# First Detection and Molecular Characterization of Apple Stem Grooving Virus, Apple Chlorotic Leaf Spot Virus, and Apple Hammerhead Viroid in Loquat in Spain

**DOI:** 10.3390/plants10112293

**Published:** 2021-10-25

**Authors:** Celia Canales, Félix Morán, Antonio Olmos, Ana Belén Ruiz-García

**Affiliations:** Center for Plant Protection and Biotechnology, Valencian Institute of Agricultural Research (IVIA), Ctra. Moncada-Náquera km 4.5, Moncada, 46113 Valencia, Spain; canales_cel@externos.gva.es (C.C.); moran_fel@gva.es (F.M.); aolmos@ivia.es (A.O.)

**Keywords:** ASGV, CTLV, ACLSV, AHVd, loquat, HTS

## Abstract

Loquat (*Eriobotrya japonica*) is an important crop in Spain. To date, only one viral species, apple stem pitting virus (ASPV), has been detected in Spanish loquat orchards. In this study, the presence of additional viruses infecting this crop in Spain was investigated. RT-PCR and high-throughput sequencing (HTS) of symptomatic loquat plants led to first-time detection and characterization of apple stem grooving virus (ASGV), also known as citrus tatter leaf virus (CTLV), and apple chlorotic leaf spot virus (ACLSV) from Spain with description of nearly complete genomic sequences. The frequency of ACLSV infection was the highest, with over 30% of the samples testing positive and were also detected as coinfections with ASGV and ASPV, although most of the samples infected were symptomless. Studies on all the full-length sequences available in the databases were performed in order to establish the phylogenetic relationships of the Spanish isolates of these two viral species. Moreover, apple hammerhead viroid (AHVd) was also detected to infect loquat, the first host different from apple reported for this viroid to date.

## 1. Introduction

Biodiversity is a key factor in the achievement of sustainable development. Agricultural biodiversity improves the resilience of production systems by reducing their vulnerability to the threats posed by pests, diseases, and climate change [1]. In this context, the diversification of crop species and varieties plays an important role for food production and agriculture. Loquat (*Eriobotrya japonica*) is an evergreen tree belonging to the family *Rosaceae* originating from China. It produces early spring sweet fruits that are highly appreciated for its organoleptic and medicinal properties [2]. Loquat is cultivated in around 30 countries in Asia and Europe, with Spain being the main European producer and exporter with an annual production of 40,000 tones [2,3]. Despite being a minor crop, loquat is very valuable in the Mediterranean region of Spain, where it represents an important economic income and contributes to increasing the genetic diversity of crop production [3,4]. 

As other crops, loquat production can be affected by pests and diseases caused by different plant pathogens. Among them, plant viruses are related to 50% of emerging diseases and produce important economic losses [5]. Loquat has been reported to be infected by four viruses: apple stem grooving virus (ASGV), apple chlorotic leaf spot virus (ACLSV), loquat virus A (LoVA), and apple stem pitting virus (ASPV) [2,3,6]. 

Apple stem grooving virus, also reported as citrus tatter leaf virus (CTLV), is a member of the family *Betaflexiviridae*, genus *Capillovirus* [7]. This virus was reported for the first time in the 1960s in the USA, as CTLV, infecting “Meyer” lemmon [8] and, as ASGV, infecting apple [9,10,11]. Since then, ASGV has been reported to infect a wide range of hosts belonging to different plant families including other citrus species, pear, apricot, cherry, lily, bamboo, soybean, fava bean, and tomato and shown to be broadly distributed worldwide [7,12,13]. Recently, ASGV has also been found to infect loquat in China, another natural host of this viral species [2]. The ASGV genome consists of a single positive-stranded RNA molecule of 6496 nt comprising two ORFs: ORF1 encoding a polyprotein containing motifs of methyltransferase, papain-like protease, the nucleotide triphosphate-binding helicase, the RNA polymerase (RdRp), and the coat protein (CP); and ORF2 that encodes the movement protein (MP) [14,15]. Although ASGV infection remains latent in many cultivars and hosts, it can cause a broad range of symptoms in sensitive cultivars, such as stem grooving, deformation on graft unions, interveinal mottling, leaf deformation, and chlorosis [12,13]. In citrus plants, it shows severe symptomatology when propagated onto trifoliate orange (*P. trifoliata*), a common rootstock in citrus-producing areas, that results in bud union incompatibility and tree decline [16]. The virus is seed and mechanically transmitted and no natural vectors have been identified to date [13,16]. 

*Apple chlorotic leaf spot virus* is the type species of the genus *Trichovirus*, in the family *Betaflexiviridae* [17,18,19]. ACLSV was first reported in England in the indicator plant *Malus platycarpa* [19]. ACLSV has a broad worldwide distribution and has been reported to infect cultivated, ornamental, and wild species belonging to the family *Rosaceae*, including apple, pear, apricot, peach, plum, almond, cherry, and quince [19,20,21]. Recently, ACLSV has also been found to infect loquat [2]. ACLSV is a positive sense single-stranded RNA virus with a genome ranging between 7545 and 7555 nt in length that comprises three ORFs: ORF1 encoding a large replication-related protein containing polymerase (RdRp), nucleotide binding helicase, and methyltransferase domains; ORF2 that encodes a movement protein (MP); and ORF3 encoding the coat protein (CP) [17,18,19]. Despite being commonly latent, ACLSV infection has been associated with different symptoms in sensitive cultivars and rootstocks including malformation and reduction in leaves, chlorotic rings, russet rings on fruits, graft incompatibility, and tree decline [18,19]. ACLSV is transmitted by grafting and vegetative propagation with no report of seed/pollen or vector-mediated transmission to date [21].

Apple hammerhead viroid is a member of the genus *Pelamoviroid*, family *Avsunviroidae* [22]. AHVd was first described by high-throughput sequencing (HTS) in apple in China as a viroid-like RNA [23] and later shown to satisfy the criteria to be considered a viroid [22]. AHVd has also been reported in apple in Canada, the USA, and Italy [24,25,26]. In addition, the detection of this viroid in apple plant material imported from Japan, New Zealand, and Spain supports its occurrence in these countries [26]. AHVd consists of a circular RNA molecule of 434 nt [22]. Although AHVd has been found in apple plants showing several symptoms, such as typical symptoms of apple scar skin disease, trunk splitting, shoot decline, and dieback, the association of this viroid to a disease remains to be clarified [22,24,26]. 

In this study, the presence of viruses and viroids in one of the main Spanish loquat-growing areas in the Mediterranean region of the country, Callosa d’en Sarrià, Alicante, was evaluated by RT-PCR and HTS. Two viruses, ASGV and ACLSV, were detected for the first time in loquat in Spain. In addition, AHVd, a viroid previously known to infect apple trees, was also identified from loquat plants. This is the first report of ASGV and ACLSV infecting loquat in Spain and the first report of loquat as a natural host of AHVd.

## 2. Results

### 2.1. Detection of Loquat Viruses by RT-PCR

In summer 2020, a random survey was carried out in one of the main Spanish loquat-growing areas, Callosa d’en Sarrià, Alicante. A total of 91 samples from different loquat cultivars were randomly collected and tested by specific RT-PCR detection methods for ASGV, ACLSV, LoVA, and ASPV [3,6,27,28,29]. Only five of the samples collected showed virus-like symptoms, in particular leaf chlorotic mottling, while the remaining plants sampled were symptomless. The results of the RT-PCR analysis (Table 1) showed the presence of three of the four viruses currently known to infect loquat, ASPV (7 out of 91 samples, 7.69%), ASGV (6 out of 91 samples, 6.59%), and ACLSV (29 out of 91 samples, 31.87%). Mixed infections were detected in nine plants that were positive for more than one virus. None of the analyzed plants tested positive for LoVA. The presence of ASPV had been found in previous studies conducted in our laboratory [3]. However, neither ASGV nor ACLSV had been previously detected in Spanish loquat orchards, thus representing the first report of these two viral species in this crop in Spain. 

To evaluate the possible effects of the presence of the viruses in loquat, symptoms were studied in these plants. All the five surveyed samples showing leaf chlorotic mottling turned out to be infected by ACLSV. However, most of the plants that tested positive for this virus were symptomless (24 out of 29 plants). Concerning ASGV and ASPV, all the samples that tested positive for these two viral species did not show any symptoms. Therefore, this analysis showed that the scarce symptomatology observed in the survey was not clearly related to the presence of any of these viruses nor to the synergism between them. 

In order to confirm the presence of ASGV and ACLSV in loquat in Spain and perform a molecular genome characterization of the Spanish isolates, HTS was conducted in two of the surveyed loquat samples, one symptomless sample that tested positive for ASGV (SL73.32) and one ACLSV-positive sample showing leaf chlorotic mottling (SL73.6).

### 2.2. First Report of ASGV in Loquat in Spain

**Figure 1:**
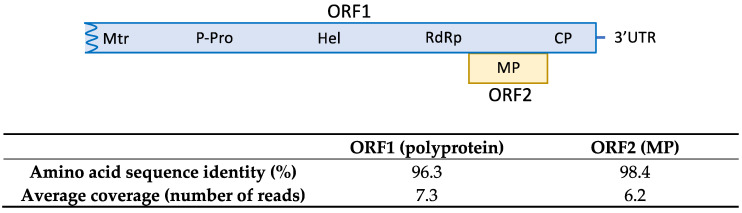
Amino acid sequence similarity of SL73.32 ORFs with the nearest ASGV isolate (LC143387). HTS average coverage for each ORF is shown.

The HTS analysis on sample SL73.32 resulted in 52,353,872 paired-end reads (average size 134.34 nt) after adapter trimming and quality control steps. Reads were mapped against the loquat genome for host genome subtraction and the 566,878 unrelated reads were subjected to de novo assembly, generating 519 contigs. Among them, 7 contigs related to ASGV ranging from 1523 nt to 310 nt were found by BLASTN/X analysis. Mapping the reads against the contigs allowed the overlapping between some of the contigs and the recovery of 3 partial sequences of 730, 1260, and 4291 nt covering 96.7 % of the genome and lacking 98 nt at the 5′ end; two small coding regions of 33 nt and 37 nt at the ORF1 region; and 48 nt at the 3′ end, with respect to the reference sequence (NC_001749). In order to cover the two ORF1 gaps, RT-PCR and Sanger sequencing were performed. Overlapping between the partial HTS sequences and the RT-PCR amplified sequences resulted in the assembly of a near full-length ASGV coding sequence of 6345 nt, named isolate SL73.32 (deposited in GenBank, accession number OK272504). SL73.32 showed the highest percentage of nucleotide identity (95.1%) with the citrus isolate FKSS2 (LC143387) from Japan and a nucleotide similarity of 83.42% with the loquat isolate L3 from China (MK599422). These results confirm the occurrence of ASGV in loquat in Spain. The SL73.32 genome organization map, HTS average coverage, and protein similarity of its ORFs with the nearest ASGV isolate (LC143387) are shown in Figure 1.

**Figure 2:**
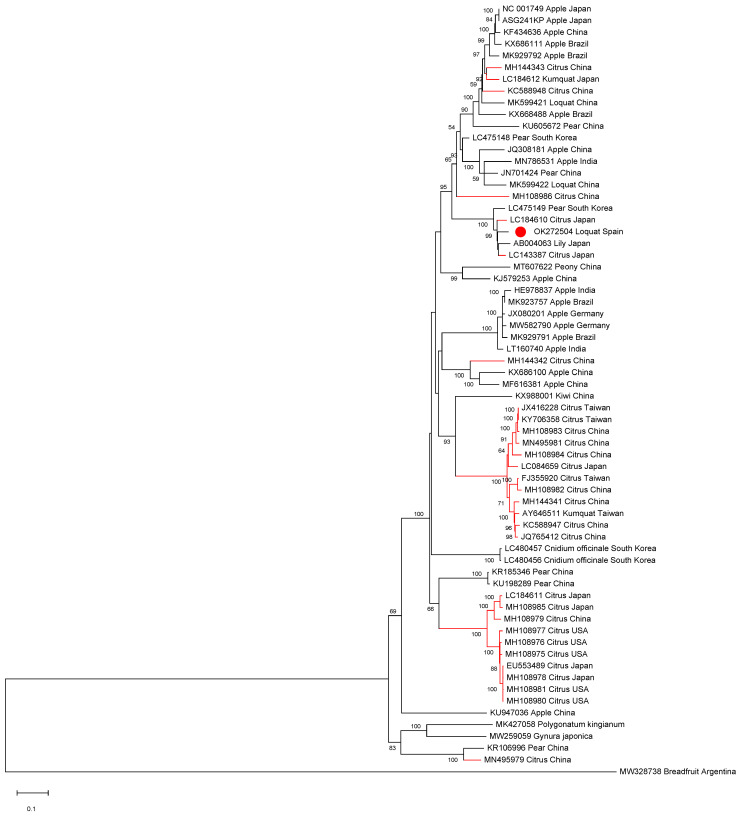
Full-length ASGV genomic sequences maximum likelihood phylogenetic tree using the GTR+G+I model. Scale bar shows genetic distance. Bootstrap percentages are indicated on the branches. ASGV isolate SL73.32, 38 ASGV full-length sequences, and 25 CTLV full-length sequences available in the databases were used. Accession numbers, host, and origin are indicated. Red branches correspond to citrus hosts, black branches correspond to non-citrus hosts. The Spanish loquat isolate is labeled with a red dot. Breadfruit capillovirus 1 (MW328738) was included in the analysis as an outgroup.

All ASGV and CTLV full-length sequences available in the databases as well as isolate SL73.6 were aligned in order to establish phylogenetic relationships. The phylogenetic analysis showed that the Spanish loquat isolate groups in a cluster containing both Rosaceous and citrus isolates and is more closely phylogenetically related to two citrus isolates: isolate FKSS2 (LC143387) and isolate N297 (LC184610), a lily isolate (AB004063), and a pear isolate (LC475149), being all grouped in a subcluster (Figure 2).

### 2.3. First Report of ACLSV in Loquat in Spain

**Figure 3:**
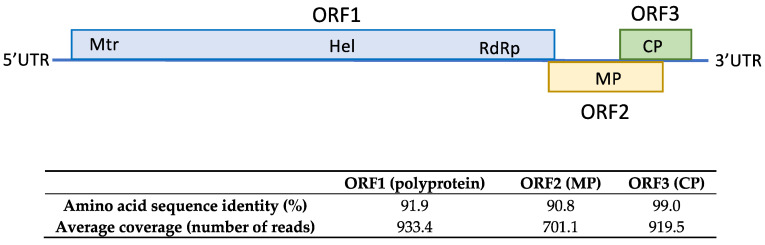
Amino acid sequence similarity of SL73.6 ORFs with the nearest ACLSV isolate (KX579123). HTS average coverage for each ORF is shown.

HTS analysis conducted on total RNA extracted from sample SL73.6 yielded 31,867,726 paired-end reads with an average size of 134.8 nt, after trimming of adapters and read quality control. Loquat genome subtraction was performed, resulting in 1,698,300 unrelated reads that were used for the assembly of 13,797 de novo contigs. Contigs related to viruses and viroids were annotated by BLASTN/X. This analysis showed 11 contigs ranging from 6511 to 281 nt related to ACLSV, confirming the presence of ACLSV in the sample. Contig extension performed by mapping the reads against the contigs allowed the recovery of a near full-length genome of 7533 nt, covered by 44,039 reads (average coverage 857.2x) named isolate SL73.6 (deposited in GenBank, accession number OK272502) that showed the highest percentage of nucleotide identity (83.7%) with the German isolate 38/85-B (KX579123) from apple. These results confirm the presence of ACLSV-infecting loquat in Spain. The SL73.6 genome organization map, HTS average coverage, and protein similarity of its ORFs with the nearest ACLSV isolate (KX579123) are shown in Figure 3.

**Figure 4:**
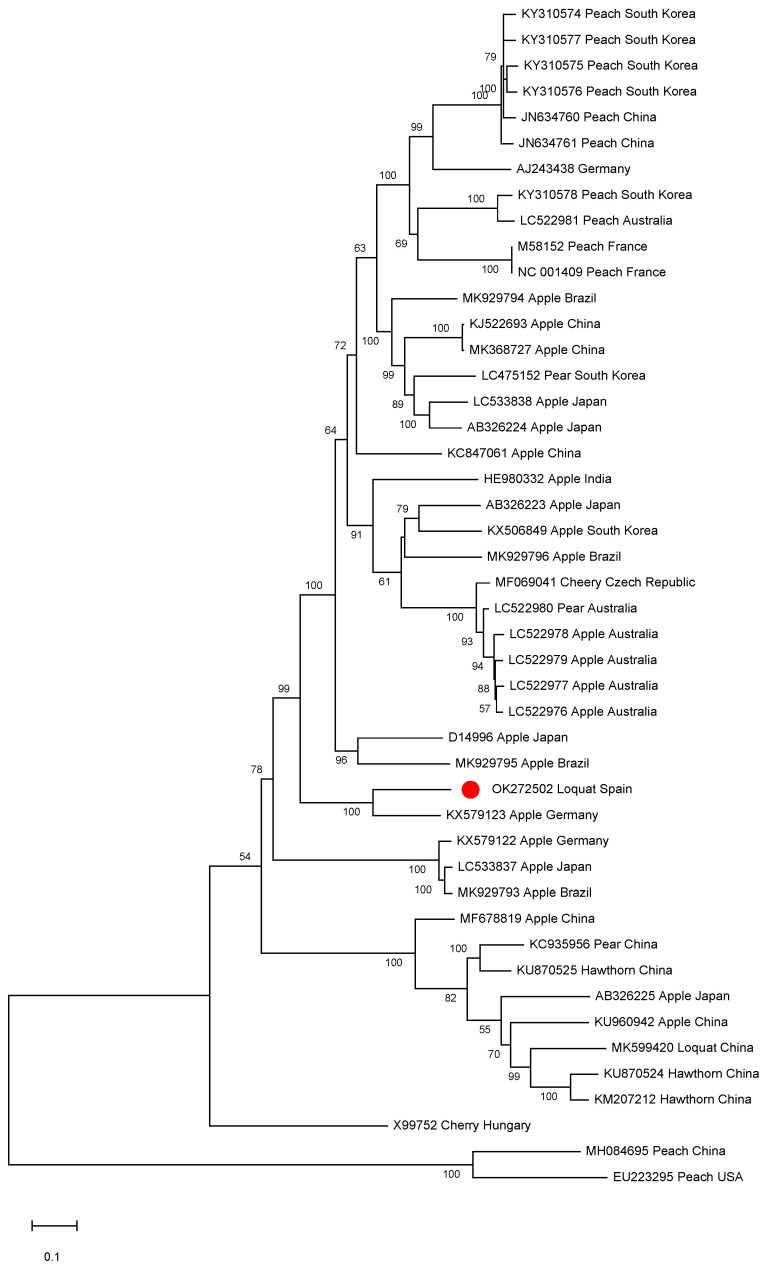
Full-length ACLSV genomic sequences maximum likelihood phylogenetic tree using the GTR+G+I model. Scale bar shows genetic distance. Bootstrap percentages are indicated on the branches. ACLSV isolate SL73.6 and 44 ACLSV full-length sequences available in the databases were used. Accession numbers, host, and origin are indicated. The Spanish loquat isolate is labeled with a red dot. Peach chlorotic leaf spot virus (MH084695) was included in the analysis as an outgroup.

A phylogenetic analysis was conducted on the ACLSV complete genomic sequences available in the databases. Isolate SL73.6 grouped into a small cluster together with the German apple isolate (Figure 4). Interestingly, a close phylogenetic relationship between isolate SL73.6 and the Chinese loquat isolate (isolate L1, MK599420) was not found, clustering the two ACLSV loquat isolates in different groups. This result is in agreement with a pairwise identity at the nucleotide level of only 75.8% between these two isolates.

### 2.4. Loquat Is a Natural Host of AHVd

**Figure 5:**
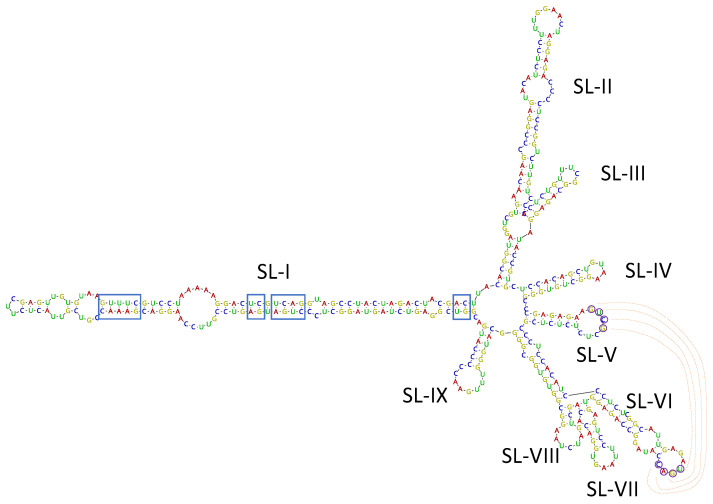
Isolate AHVd-SL73.6 predicted secondary structure by Vienna RNAfold software. Conserved nucleotides forming the hammerhead structures are marked by blue squares. Residues potentially involved in a kissing loop interaction are labelled by circles and read lines. The nine stem-loops (SL-I to SL-IX) in the multi-branched domain are indicated.

Among the virus/viroid-related contigs assembled from SL73.6 HTS data, two contigs of 489 and 376 nt related to the viroid AHVd were found. Bioinformatic analysis of these contigs allowed the recovery of a complete circular genome of 376 nt covered by 7014 reads (average coverage 2515.5x), isolate AHVd-SL73.6 (deposited in GenBank, accession number OK272503). To confirm the presence of AHVd in this sample, the complete genomic sequence of the viroid was amplified by RT-PCR using adjacent primers of opposite polarity previously reported [26] and modified for perfect matching with the HTS-recovered sequence. A 376-bp product was obtained, cloned, and Sanger sequenced, confirming 100% of the HTS-recovered sequence. These results show for the first time the presence of AHVd infecting loquat, thus identifying this crop as a new host of this viroid species. The Spanish loquat AHVd isolate showed a high nucleotide identity (93.97%) with the reference isolate (NC_028132, KR605506) from China, although the AHVd-SL73.6 genome was shorter (376 nt vs. 434 nt) due to a 56-nt deletion at the genomic position 55–110 and a 2-nt deletion at the genomic position 419–420 with respect to the reference sequence. However, the predicted secondary structure for AHVd-SL73.6 showed similar folding to the ones previously reported [23,26], resulting in a characteristic conformation of the Pelamoviroids composed of a rod-like domain containing the nucleotides forming the hammerhead structures and a multi-branched domain (Figure 5). The remaining 90 loquat plants surveyed in this study were analyzed by RT-PCR. None of these samples tested positive for AHVd. 

The HTS analysis of sample SL73.6 also revealed the presence of 1 large contig of 4922 nt covered by 4219 reads (average coverage 115.6×) that shared 66.08% nucleotide identity with the totivirus peach-associated virus 2 (isolate PaV2 BHST1, MN905503), suggesting the presence in the sample of a novel totivirus species, tentatively named loquat-associated totivirus 1 (deposited in GenBank, accession number OK318989). ASPV was also detected by HTS in the sample. 

## 3. Discussion

With the aim of studying the presence in Spain of viruses infecting loquat, a survey was conducted in one of the main Spanish loquat-growing areas in the Mediterranean region of the country. Surveyed plants were tested for the four viruses currently known to infect this crop, ASGV, ACLSV, LoVA, and ASPV, by RT-PCR using specific primers previously described in the literature [3,6,27,28,29]. The results of the study revealed the presence of ASGV, ACLSV, and ASPV but not LoVA in Spanish loquat orchards. ASPV has been already reported to infect loquat in Spain [3]. However, the presence of ASGV and ACLSV was previously unknown. Therefore, to the best of our knowledge, this is the first report of ASGV and ACLSV in loquat in Spain. The occurrence of these two viral species in loquat in Spain has been confirmed, near full-length sequences of their genomes characterized by HTS, and phylogenetic relationships studied. 

ASGV (CTLV) is a citrus pathogen fulfilling all the criteria to qualify as a European-regulated non quarantine pest (RNQP) according to the European Food Safety Authority (EFSA) [7]. Moreover, ASGV has been reported to infect citrus in Cyprus [30]. Keeping this in mind, the fact that both Chinese and Spanish loquat ASGV are phylogenetically clustered with citrus isolates might raise uncertainty about the ability of the Spanish loquat ASGV isolate to infect citrus, thus threatening Spanish citriculture. Even though no natural transmission vectors have been identified for this viral species, this issue should be taken into account in order to establish prevention measures to protect citrus infection by this pathogen. 

The ACLSV phylogenetic relationships were also studied. Spanish loquat ACLSV isolate SL73.6 groups in a small cluster together with an apple German isolate and shows a low level of sequence similarity and a far phylogenetic relationship with the Chinese loquat isolate L1. These results indicate a different origin for the loquat isolates known to date and raise questions on the putative natural spread and host transfer of this viral species, despite the fact that no natural vectors have been identified [21]. 

It is important to note that, despite the presence of chlorotic mottling symptoms in some of the loquat plants analyzed in this study, no symptomatology was clearly associated with ASGV nor ACLSV in single or mixed infections in this crop. These results are in agreement with the literature as many latent infections have been reported for both viruses. Future studies will be needed to evaluate the impact and biological significance of these viral species in loquat.

The HTS analysis performed in this study in sample SL73.6 also allowed the identification for the first time of loquat as a new natural host of AHVd, a viroid thought to be limited to apple infection to date. The complete circular genome of the Spanish loquat isolate AHVd-SL73.6 was characterized. Although the Spanish loquat AHVd isolate shows a high level of sequence similarity with the Chinese reference isolate, its genome (376 nt) is 58 nt shorter than the reference genome due to the presence of two deletions. However, the predicted secondary structure of the viroid is similar to those previously reported. Confidence on the completeness of the AHVd genome recovered in this study is given by the amplification by RT-PCR of a 376-bp product using two adjacent primers of opposite polarity. However, taking into account that the remaining analyzed plants tested negative for AHVd and therefore no more genomic sequences were characterized, whether this genomic length represents a particular variant feature more than a general loquat AHVd attribute remains to be determined.

Taken together, the results presented in this study provide valuable new knowledge on the epidemiology of these viral and viroid species that might contribute to improving the sanitary status of Spanish loquat orchards, and thus help to protect them. 

## 4. Materials and Methods

### 4.1. Plant Material and Sample Preparation

A total of 91 leaf samples from different cultivars (Andrés, Algerie, Xirlero, Juliana, Crisanto Amadeo, and Toni Tomaca) were randomly collected in loquat orchards in Callosa d’en Sarrià, one of the main loquat-producing areas in Spain. Leaf tissue from each plant sample was extracted in individual plastic bags (Bioreba, Reinach, Switzerland) containing extraction buffer (PBS supplemented with 0.2% of diethyldithiocarbamate and 2% of PVP-10) in a 1:5 ratio (*w:v*). Tissue was ground using a Homex 6 homogenizer (Bioreba, Reinach, Switzerland) and plant extract was kept on ice for subsequent processing.

### 4.2. RNA Purification

Total RNA was purified from 200 µL of plant extract using the Plant/Fungi RNA isolation kit (Norgen Biotek Corporation, Thorold, ON, Canada) following the manufacturer’s instructions. RNA concentrations were determined using a DeNovix DS-11 spectrophotometer (DeNovix Inc., Wilmington, DE, USA) and stored at −80 °C until subsequent analysis. Samples selected for HTS analysis were treated with DNase using an RNase-Free DNase I kit (Norgen Biotek Corporation, Thorold, ON, Canada) following the manufacturer’s instructions.

### 4.3. ASGV, ACLSV, LoVA, and ASPV Detection by RT-PCR 

Conventional and/or real-time RT-PCR detection of ASGV, ACLSV, LoVA, and ASPV was performed using the primers and probes described in Table 2. ASGV and ACLSV were tested by real time RT-PCR in a StepOne Plus thermal cycler (Applied Biosystems, Foster City, CA, USA). The amplification protocol consisted of one step of 45 °C for 10 min and 95 °C for 10 min, followed by 40 cycles of amplification (95 °C for 15 s, 50 °C for 15 s and 60 °C for 45 s). ASGV, LoVA, and ASPV were tested by conventional RT-PCR. The amplification protocol used consisted of one step at 45 °C for 45 min, one step at 95 °C for 10 min, and 40 cycles of amplification (95 °C for 30 s, 50 °C for 30 s, and 60 °C for 45 s) with a final step at 60 °C for 5 min. All RT-PCR reactions were carried out using AgPath One-Step RT-PCR Reagents (Applied biosystems, Foster City, CA, USA) following the manufacturer’s instructions, containing 0.4 µM of each primer, 160 nM of the probe, and 100 ng of total RNA in a total volume of 25 µL.

All PCR products were purified using a mi-PCR Purification Kit (Metabion International AG, Martinsried, Germany) following the manufacturer’s instructions and Sanger sequenced in both directions. 

### 4.4. HTS Analysis

RNA quality control, library construction, and sequencing were performed at Macrogen Inc. (Seoul, Korea) using the TruSeq Stranded Total RNA Ribo-Zero Plant Kit (Illumina, San Diego, CA, USA) and the library protocol TruSeq Stranded Total RNA Sample Prep Guide, Part #15031048 Rev. E. Libraries were sequenced (2 × 150 bp paired-end reads) on a NovaSeq 6000 platform (Illumina, San Diego, CA, USA).

Bioinformatic analysis of HTS raw data was performed by CLC Genomics Workbench v.10.1.1 (Qiagen Bioinformatics, Hilden, Germany) and Geneious Prime v2020.2.5 (Biomatters Ltd., Auckland, New Zealand). After adapter trimming and quality control, reads were mapped against *Eriobotrya japonica* genome (GWHAAZU00000000), chloroplast (NC_034639), and mitochondrion (NC_045228) for host genome subtraction using CLC Genomics Workbench software. The same software was used to perform de novo assembly of the remaining reads. Contigs over 200 nt were annotated by BLAST analysis (BLASTN/X) with a cut-off e-value of 10^−4^ against local and online virus, viroids, and nt/nr databases. For full or near full genome recovery, virus/viroid-related contigs were extended by mapping the reads against the contigs using Geneious Prime software. Two gaps in the ASGV HTS-recovered sequence were covered by conventional RT-PCR and Sanger sequencing using two sets of primers designed based on the HTS analysis, SL-ASGV-731F (5′CTGACGGTGTGGCCTCTGAAT3′, sense), and SL-ASGV-1082 (5′AGATCTCTCTTCTCCAACTGCCTC3′, antisense), targeting an amplicon of 351 bp; and SL-ASGV-1960 (5′GACAAAGGACTCTCACACGAAATG3′, sense) and SL-ASGV-2312 (5′TTGCAGAAGGCCGGATCAAAGG3′, antisense) amplifying a fragment of 352 bp.

### 4.5. AHVd Genome Amplification by RT-PCR 

The AHVd complete genome was amplified by RT-PCR using the primers L-AHVd-F (5′-GTAGCCTACTAGACTACGACTTACAC-3′) and L-AHVd-R (5′-CTGACGAGTCCTTTTTAGGACGAAACT-3′). The RT-PCR reaction was performed using a OneStep PrimeScript RT-PCR kit (Takara Bio Ink., Kasatsu, Japan) following the manufacturer’s instructions. The reaction mixture contained 1 µM of each primer and 100 ng of extracted RNA. In the first step, RNA was mixed with the primers, heated at 95 °C for 5 min, incubated at 65 °C for 5 min, and cooled on ice. The reaction mixture was then completed in a total volume of 20 µL and subjected to a second step consisting of incubation at 42 °C for 30 min, 95 °C for 10 s, and 40 cycles of amplification (95 °C for 15 s, 55 °C for 30 s, and 60 °C for 30 s). The amplicon was purified using a mi-PCR Purification Kit (Metabion International AG, Martinsried, Germany) following the manufacturer’s instructions and Sanger sequenced in both directions. 

### 4.6. Sequence Alignment and Phylogenetic Analysis

Sequence alignments were carried out using ClustalW implemented in MEGA X [31]. Phylogenetic trees were constructed with the maximum likelihood algorithm implemented in MEGA X applying the lowest BIC (Bayesian information criterion) and 500 bootstraps. For both ASGV and ACLSV, the general time reversible model [32] with rates among sites gamma distributed and with invariant sites (G+I) was used. 

### 4.7. AHVd Secondary Structure Prediction

AHVd secondary structure prediction was performed by Vienna RNAfold (v2.14.3) implemented in Geneious Prime software.

## Figures and Tables

**Table 1:** RT-PCR detection of ASGV, ACLSV, LoVA, and ASPV in the surveyed loquat samples. Plants that tested positive for one or more viruses are listed, indicating sample code, variety, symptomatology, and positive (+) or negative (−) detection result for each virus.

Sample	Variety	Symptomatology	ASGV	ACLSV	LoVA	ASPV
SL73.1	Andrés	Symptomless	−	+	−	−
SL73.2	Algerie	Symptomless	+	−	−	−
SL73.4	Xirlero	Symptomless	−	+	−	−
SL73.6	Xirlero	Chlorotic mottling	−	+	−	+
SL73.11	Juliana	Symptomless	−	+	−	−
SL73.12	Algerie	Symptomless	−	+	−	−
SL73.16	Algerie	Symptomless	−	+	−	−
SL73.18	Algerie	Symptomless	−	+	−	−
SL73.20	Algerie	Symptomless	−	+	−	−
SL73.32	Xirlero	Symptomless	+	−	−	−
SL73.33	Xirlero	Chlorotic mottling	−	+	−	−
SL73.40	Crisanto Amadeo	Symptomless	−	+	−	−
SL73.41	Crisanto Amadeo	Symptomless	−	+	−	−
SL73.42	Xirlero	Chlorotic mottling	−	+	−	−
SL73.43	Xirlero	Chlorotic mottling	−	+	−	−
SL73.54	Xirlero	Symptomless	+	+	−	+
SL73.55	Xirlero	Symptomless	−	+	−	−
SL73.56	Xirlero	Symptomless	−	+	−	−
SL73.57	Algerie	Symptomless	−	+	−	−
SL73.61	Algerie	Symptomless	−	+	−	−
SL73.69	Xirlero	Chlorotic mottling	−	+	−	−
SL73.71	Xirlero	Symptomless	−	+	−	−
SL73.72	Toni tomaca	Symptomless	−	+	−	−
SL73.76	Algerie	Symptomless	−	+	−	+
SL73.78	Algerie	Symptomless	−	+	−	+
SL73.79	Algerie	Symptomless	−	+	−	+
SL73.82	Toni tomaca	Symptomless	−	+	−	−
SL73.83	Toni tomaca	Symptomless	+	−	−	+
SL73.84	Toni tomaca	Symptomless	+	+	−	+
SL73.87	Toni tomaca	Symptomless	+	+	−	−
SL73.89	Xirlero	Symptomless	−	+	−	+

**Table 2:** Primers and probes used to detect, ASGV, ACLSV, LoVA, and ASPV.

Virus	Primer	Sequence (5′–3′)	Size (bp)	Reference
ASGV	qASG-F	GGGACTTGCCACYAACATTT	72	[27]
	qASG-R	CACCCAAGGGCTYTTTTCAA		
	ASG-P	AGAAATGGCCCAAAGC		
	ASGV-499-1	CCCGCTGTTGGATTTGATACACCTC	499	[28]
	ASGV-499-2	GGAATTTCACACGACTCCTAACCCTCC		
ACLSV	ACLSV 5F	GCCTACAAATTAGGTGAGAGGCTC	288	[29]
	ACLSV 8R	TTCCAATGGATCATGAGGTC		
	MGB26	FAM-ATTCACATCTTGAAATT-MGB		
LoVA	LoVA-F	TAATGAAGTCCAAGGAAGCACC	453	[6]
	LoVA-R	GGTCTCATTTCTGAAACCTCGA		
ASPV	qASP-FS	CGCTTTCTACGCGAAGCATGT	385	[3]
	ASPV-RS	TTGGGATCAACTTTATTAAAAGCATAA		

## Data Availability

All data supporting the results are contained within the article or Supplementary Material.

## Supplementary Materials

The following are available online at are available online at https://www.mdpi.com/article/10.3390/plants10112293/s1. FASTA and FASTAQ files containing the HTS-assembled ASGV, ACLSV, AHVd, and LaTV1 sequences as well as the reads covering them.
