# Supplementary material for: First Detection and Molecular Characterization of Apple Stem Grooving Virus, Apple Chlorotic Leaf Spot Virus, and Apple Hammerhead Viroid in Loquat in Spain
Source: Plants (Basel). 2021 Oct 25;10(11):2293. doi: 10.3390/plants10112293 (PMC8624106; doi:10.3390/plants10112293)
Supplement: Supplementary file 1 [file plants-10-02293-s001.zip › Mapping_reads_ASGV.pdf]

```
@A01056:60:HMTK7DSXY:1:2413:14714:27320 2:N:0:TCCTTAGC+ACTCGATT
GATCAATTGCTTTCCTTGACCACAGACGAGATTGAAAAGACCTTAGAAGTAACAAACCGCTTCTTCTC
TTTTTCAATCACCCCAGAAGATCAAGAATTGTTAACCAAACATGGTATAACACTTGCGCCTGTTGGAT
TCAAGTCACATTCCC
+
FFFFFFFFFFFFFFFFFFFFFFFFFFFFFFF:FF:FFFFFFFFFFFFFFFFFFFFFFFFF:FFFFFFF
FFFFFFFFFFFFFFFFFF:FFFFFFFFFFFFFFFFFFFFFFFFFFFFFFFFFFFFFFFFFF:FFFFF
FFFFFFFFFFFFFF:
@A01056:60:HMTK7DSXY:1:2413:14714:27320 1:N:0:TCCTTAGC+ACTCGATT
CAAACCGCTTCTTCTCTTTTTCAATCACCCCAGAAGATCAAGAATTGTTAACCAAACATGGTATAACA
CTTGCGCCTGTTGGATTCAAGTCACATTCCCACCCAATATCCAAAATGATAGAGAATCATCTCTTGTA
CATATGTATCCCCAG
+
FF:FFFFFFFFFFFFFF,FFFFFFFFFFFFFFFFFFFFFFFFFFFFFFFFFFFFFFFFFFFFFFFFFF
FFFFFFFFFFFFFFFFFFFFFFFFFF:FFFFFFFFFFFFFFFFFFFFFFFFFFFFFFFFFFFFFFFFF
FFFFFFFFFFFFFFF
@A01056:60:HMTK7DSXY:1:1438:3441:20040 2:N:0:TCCTTAGC+ACTCGATT
CAAGAATTGTTAACCAAACATGGTATAACACTTGCGCCTGTTGGATTCAAGTCACATTCCCACCCAAT
ATCCAAAATGATAGAGAATCATCTCTTGTACATATGTATCCCCAGTCTTCTATCTTCTTTTAGGTCAG
TTGCCTTCTTTTCCC
+
FFFFFFFFFFFFFFFFFFFFFFFFFFFFFFFFFFFFFFFFFFFFFFFFFFFFFFFFFFFFFFFFFFFF
FFFFFFFF:FFFFFFFFFFFFFFFFFFFFFF,FFFFFFFFFFFFFFFFFFFFFF,FFFF,FFFFFFFFF
FFFF:FFFFFFFFF,
@A01056:60:HMTK7DSXY:1:2647:32750:4476 2:N:0:TCCTTAGC+ACTCGATT
CCAAAATGATAGAGAATCATCTCTTGTACATATGTATCCCCAGTCTTCTATCTTCTTTTAGGTCAGTT
GCCTTCTTTTCCCTTAGAGAGAACAAAGTTGATAATTTTCTTAAAATGCATTCTGTATTTTCACATGG
AAAAATCAAACCTTT
+
FFFFFF:FFFFFFFFFFFFFFFFFFFFFFFFFFFFFFFFFFFFFFFFFFFFFFFFF,:FFFFFFFFF
FFFFFFFFFFFFFF:FFFFFFFFFFFFFFFFFFFFFFFFFFFFFF:FFFFFFFFFFFFFFFFFFFFF:FF
F:FFFFFFFFFFF,F
@A01056:60:HMTK7DSXY:1:2647:32750:4476 1:N:0:TCCTTAGC+ACTCGATT
AAAATGATAGAGAATCATCTCTTGTACATATGTATCCCCAGTCTTCTATCTTCTTTTAGGTCAGTTGC
CTTCTTTTCCCTTAGAGAGAACAAAGTTGATAATTTTCTTAAAATGCATTCTGTATTTTCACATGGAA
AAATCAAACCTTTAG
+
:FFFFFFFFF:FFFFFFFFFFFFFFFF:FFFFFFFFFFFFFFFFFFFFFFFFFFFFFFFFFFFFFFFFF
FFFFFFFFFFFFFFFFFFFFFFFFFFFFFFFFFFFFFFFFF:FFFFFFFFFFFFFFFFFFFFFFFFFFF
FFFFFFFFFFFFFFF
@A01056:60:HMTK7DSXY:1:2137:7355:19586 1:N:0:TCCTTAGC+ACTCGATT
GCTCTTCCGATCTCCCAGTCTTCTATCTTCTTTTAGGTCAGTTGCCTTCTTTTCCCTTAGAGAGAACA
AAGTTGATAATTTTCTTAAAATGCATTCTGTATTTTCACATGGAAAAATCAAATCTTTAGGAATGTAC
AATGCGATAATTGAC
+
FFFFFFFFFFFFFFFFFFFFFFFFFFFF:FFFFFFFFFFFFFFFFFFFFFFF:FFFFFFFFFFFFFFFFF
FFFFFFFFFFFFFFFFFFFFFFFFFFFFFFFFFFFFFFFFFFFFFFFFFFFFFFFFFFFFFFFFFFFF
FFFFFFFFFFFFFFF
@A01056:60:HMTK7DSXY:1:2137:7355:19586 2:N:0:TCCTTAGC+ACTCGATT
CCCAGTCTTCTATCTTCTTTTAGGTCAGTTGCCTTCTTTTCCCTTAGAGAGAACAAAGTTGATAATTT
TCTTAAAATGCATTCTGTATTTTCACATGGAAAAATCAAATCTTTAGGAATGTACAATGCGATAATTG
ACAGATCGGAAGAGC
+
FFFFFFFFFFFFFFFFFFFFFFFFFFFFFFFFFFFFFFFFFFFFFFFFFFFFFFFFFFFFFFFFFFFF
```

```
FFFFF:FFFFFFFFFFFFFFFFFFFFFFFFFFFFFFFFFFFFFFFFFFFFFFFFFFFFFFFFFFFF
FF,:FFFFFFFF:FF
@A01056:60:HMTK7DSXY:1:1438:3441:20040 1:N:0:TCCTTAGC+ACTCGATT
CCTTAGAGAGAACAAAGTTGATAATTTTCTTAAAATGCATTCTGTATTTTCACATGGAAAAATCAAAT
CTTTAGGAATGTACAATGCGATAATTGACGGGAAGGACAAGTATAGGTATGGTGAGGTCTCTTTCACC
TCCTTCAGAGATAGG
+
FFF,FFFFFFFFFFFFFFFFFFFFFFFFFFFFFFFFFFFFFFFFFFFFFFFFFFFFFFFFFFFFFFFF
FFFFFFFFFFFFFFFFFFFFFFFFFFFFFFFFFFFFFFFFFFFFFFFFFFFFFFFFFFFFFFFFFFFF
FFFFFFFFFFFFFFF
@A01056:60:HMTK7DSXY:1:1643:13331:22451 2:N:0:TCCTTAGC+ACTCGATT
GAAAAATCAAATCTTTAGGAATGTACAATGCGATAATTGACGGGAAGGACAAGTATAGGTATGGTGAG
GTCTCTTTCACCTCCTTCAGAGATAGGGTGATAGGTCTTAGAGATCAATGCCTTACGCGTAATAAATT
TCCAAAAGTGTTATT
+
FFFFFFFFFFFFFFFFFFFFFFFFFFFFFFFFFFFFFFFFFFFFFFFFFFF:FFFFFFFFFFFFFFFFF
FFFFFFFFFFFFFFFFFFFFFFFFFFFFFFFFFF:FFFFFFFFFFFFF:FFFFFFFFFFFFFFFFFFFF
FFFFFFFFFFFFFFF
@A01056:60:HMTK7DSXY:1:2344:30228:16830 2:N:0:TCCTTAGC+ACTCGATT
CTTTAGGAATGTACAATGCGATAATTGACGGGAAGGACAAGTATAGGTATGGTGAGGTCTCTTTCACC
TCCTTCAGAGATAGGGTGATAGGTCTTAGAGATCAATGCCTTACGCGTAATAAATTTCCAAAAGTGTT
ATTCCTTCATGATGA
+
FFFFFFFFFFFFFFFFFFFFFFFFFFFFFFFFFFFFFFFFFFFFFFFFFFFFFFFFFFFFFFF,FFFF
FFFFFFFFFFF:FFFFFFFFFFFFFFFFFFFFFFFFFFFFFFFFFFFFFFFFFFFFFFFFFFFFFFFF
FFFFFFFFFFFFFFF
@A01056:60:HMTK7DSXY:1:2344:30228:16830 1:N:0:TCCTTAGC+ACTCGATT
ATGTACAATGCGATAATTGACGGGAAGGACAAGTATAGGTATGGTGAGGTCTCTTTCACCTCCTTCAG
AGATAGGGTGATAGGTCTTAGAGATCAATGCCTTACGCGTAATAAATTTCCAAAAGTGTTATTCCTTC
ATGATGAACTGCACC
+
FFFFFFF,FFFFFFFFFFFFFFFFFFFFFFFFFFFFFFFFFFFFFFFFFFFFFFFF,FFFFFFFFFFFF
FFFFFFFFFFFFFFFFFFFFFFFFFFFFFFFFFFFFFFFFFFFFFFFFFFFFFFFFFFFFFFFFFFFF
FFFFFFFFFFFFFFF
@A01056:60:HMTK7DSXY:1:2552:32823:21512 2:N:0:TCCTTAGC+ACTCGATT
GTGAGGTCTCTTTCACCTCCTTCAGAGATAGGGTGATAGGTCTTAGAGATCAATGCCTTACGCGTAAT
AAATTTCCAAAAGTGTTATTCCTTCATGATGAACTGCACTTCCTTAGCCCCTTTGATATTGCTTTTTT
GTTTGAAACAATTCC
+
FFFFFFFFFFFFF:FFFFFFFFFFFFFFFFFFFFFFFF:FFFF:FFFFFFFFFFFFFFFFFFFFFFFFFF
FFFFFFFFFFFFFFFFFFFFFFFFFFFFFFFFFFFFFFF:FFFFFFFFFFFFFFFFFFFFFFFFFFFFF
FF:FFFFFFFFFFFF
@A01056:60:HMTK7DSXY:1:2552:32832:21496 2:N:0:TCCTTAGC+ACTCGATT
GTGAGGTCTCTTTCACCTCCTTCAGAGATAGGGTGATAGGTCTTAGAGATCAATGCCTTACGCGTAAT
AAATTTCCAAAAGTGTTATTCCTTCATGATGAACTGCACTTCCTTAGCCCCTTTGATATTGCTTTTTT
GTTTGAAACAATTCC
+
FFFF:FFFFFFFFFFFFFF::FFFFFFF:FFFFFFFFFFFFF:FFFFFFFFFFFFFFFFFFFFF:FFF
FFFFFFFFFFFFFFFFFFFFFFFFFFFFFFFFFFFFFFFFFFF:FFFFFF:FFFFFFFFFFFFF
FF,FFFFFFF:FFFF
@A01056:60:HMTK7DSXY:1:2459:21847:22639 2:N:0:TCCTTAGC+ACTCGATT
GTGAGGTCTCTTTCACCTCCTTCAGAGATAGGGTGATAGGTCTTAGAGATCAATGCCTTACGCGTAAT
AAATTTCCAAAAGTGTTATTCCTTCATGATGAACTGCACTTCCTTAGCCCCTTTGATATTGCTTTTTT
GTTTGAAACAATTCC
```

```
+
FFFFFFFFFFFFFFFFFFFFFFFFFFFFFFFFFFFFFFFFFFFFFFFFFFFFFFFFFFFFFFFFFFFFFF
FFFFFFFFFFFFFFFFFFFFFFFFFFFFFFFFFFFFFFFFFFFFFFFFFFFFFFFFFFFFFFFFFFFFFF
FFFFFFFFFF:FFF:
@A01056:60:HMTK7DSXY:1:1575:18069:4914 2:N:0:TCCTTAGC+ACTCGATT
GTCTCTTTCACCTCCTTCAGAGATAGGGTGATAGGTCTTAGAGATCAATGCCTTACGCGTAATAAATT
TCCAAAAGTGTTATTCCTTCATGATGAACTGCACTTCCTTAGCCCCTTTGATATTGCTTTTTTGTTTG
AAACAATTCCAGAAA
+
FFFFFFFFFFFFFFFFFFFFFFFFFFFFFFFFFFFFFFFFFFFFFFFFFFFFFFFFFFFFFFFFFFFFFF
FFFFFFFFFFFFFFFFFFFFFFFFFFFFFFFFFFFFFFFFFFFFFFFFFFFFFFFFFFFFFFFFFFFFFF
FFFFFFFFFFFF:FF
@A01056:60:HMTK7DSXY:1:1542:19461:13025 1:N:0:TCCTTAGC+ACTCGATT
AGACGTGTGCTCTTCCGATCTTAGAGATCAATGCCTTACGCGTAATAAATTTCCAAAAGTGTTATTCC
TTCATGATGAACTGCACTTCCTTAGCCCCTTTGATATTGCTTTTTTGTTTGAAACAATTCCAGAAATA
GACAGAGTTATTGCG
+
FFFFFFFFFFFFFFFFFFF,FFFFFFFFFFFFFFFFFFFFF:FFFFFFFFFFFFFFFFFFF:FFFFFF
FFFFFFFFFFFF:FFFF:FFFFFFFFFFFFFFFFFFFFFFFFFFFFFFFFFFFFFFFFFFFFFFFFFF
FFFFFFFFFFFFFFF
@A01056:60:HMTK7DSXY:1:1575:18069:4914 1:N:0:TCCTTAGC+ACTCGATT
GAGATAGGGTGATAGGTCTTAGAGATCAATGCCTTACGCGTAATAAATTTCCAAAAGTGTTATTCCTT
CATGATGAACTGCACTTCCTTAGCCCCTTTGATATTGCTTTTTTGTTTGAAACAATTCCAGAAATAGA
CAGAGTTATTGCGAC
+
FFFFFFFFFFFFFFFFFFFFFF:FFFFFFFFFFFFFFFFFFFFFFFFFFFFFFFFFFFFFFFFFFFFF
FFFFFFFFFFFFFFF::FF,FFFF:FFFFFFFFFFFFFFFF:FFFFFFFFFFFFFFFFFFFFFFFFFF
FFFFFFFFFFFFFFF
@A01056:60:HMTK7DSXY:1:1643:13331:22451 1:N:0:TCCTTAGC+ACTCGATT
GAGATAGGGTGATAGGTCTTAGAGATCAATGCCTTACGCGTAATAAATTTCCAAAAGTGTTATTCCTT
CATGATGAACTGCACTTCCTTAGCCCCTTTGATATTGCTTTTTTGTTTGAAACAATTCCAGAAATAGA
CAGAGTTATTGCGAC
+
FFFFFFFFFFFFFFFFFFFFFFFFFFFFFFFFFFFFFFFFFFFFFFFFFFFFFFFFFFFFFFFFFFFFFF
FFFFFFFFFFFFFFFFFFFFFFFFFFFFFFFFFFFFFFFFFFFFFFFFFFFFFFFFFFFFFFFFFFFFFF
FFFFFFFFFFFFFFF
@A01056:60:HMTK7DSXY:1:1542:19461:13025 2:N:0:TCCTTAGC+ACTCGATT
TAGAGATCAATGCCTTACGCGTAATAAATTTCCAAAAGTGTTATTCCTTCATGATGAACTGCACTTCC
TTAGCCCCTTTGATATTGCTTTTTTGTTTGAAACAATTCCAGAAATAGACAGAGTTATTGCGAGATCG
GAAGAGCGTCGTGTA
+
FFFFFFFFFFFFFFFFFFFFFFFFFFFFFFFFFFFFFFFFFFFFFFFFFFFFFFFFFFFFFFFFFFFFFF
FFFFFFFFFFFFFFFFFFFFFFFFFFFFFFFFFFFF:FFFFFFFFFFFFFFF:FFFFFFFFFFFFFF
FF:FFFFFFFFFF,F
@A01056:60:HMTK7DSXY:1:2552:32832:21496 1:N:0:TCCTTAGC+ACTCGATT
TCCAAAAGTGTTATTCCTTCATGATGAACTGCACTTCCTTAGCCCCTTTGATATTGCTTTTTTGTTTG
AAACAATTCCAGAAATAGACAGAGTTATTGCGACCACTATATTTCCGATTGAATTGTTATTTGGGGAC
AAGGTCTCTAAGGAG
+
FFFFFFFFFFFFF:FFFFFFFFFFFFFFFFFFFF,FFFFFFF:FFFFFFFF:FFFFFFFFFFFFFFF
FFFFFFFFFFFF:,,FF:FFFFFFFF:FFFFFFFFF:F:FFFFFFFFF:FFFFFFFFFFFFFFFF
FFFFFFFFFFFFFFF
@A01056:60:HMTK7DSXY:1:2552:32823:21512 1:N:0:TCCTTAGC+ACTCGATT
TCCAAAAGTGTTATTCCTTCATGATGAACTGCACTTCCTTAGCCCCTTTGATATTGCTTTTTTGTTTG
```

```
AAACAATTCCAGAAATAGACAGAGTTATTGCGACCACTATATTTCCGATTGAATTGTTATTTGGGGAC
AAGGTCTCTAAGGAG
+
FFFFF,FFFFF:F,FFFFFFFFFFFFFFFFFFFF:FFFFFFFFFFFFFFFFFFFFF:FFFFFFFFFFF
FFFFFF:FFFFFF,:FFFFFFFFFFFFFFFFFFFFFFFFFFFFFFFFFFFFFFFFFFFFFFFFFFFFF
FFFFFFFFFFFFFFF
@A01056:60:HMTK7DSXY:1:2459:21847:22639 1:N:0:TCCTTAGC+ACTCGATT
TCCAAAAGTGTTATTCCTTCATGATGAACTGCACTTCCTTAGCCCCTTTGATATTGCTTTTTTGTTTG
AAACAATTCCAGAAATAGACAGAGTTATTGCGACCACTATATTTCCGATTGAATTGTTATTTGGGGAC
AAGGTCTCTAAGGAG
+
FFFFFFFFFFFFFFFFFFFFFFFFFFFFFFFFFFFFFFF,FFFFFFFFFFFFFFFFFFFFFFFFFFFFF
FFFFFFFFFFFFFF:FFF,FFF:,FFFFFFFFFFFFFF:FFFFFFFFFFFFFFFFFFFFFFFFFFFF:F
FFFFFFFFFFFFFFF
@A01056:60:HMTK7DSXY:1:2675:2763:9126 2:N:0:TCCTTAGC+ACTCGATT
TGATGAACTGCACTTCCTTAGCCCCTTTGATATTGCTTTTTTGTTTGAAACAATTCCAGAAATAGACA
GAGTTATTGCGACCACTATATTTCCGATTGAATTGTTATTTGGGGACAAGGTCTCTAAGGAGCCCAGG
GTTTACACCTATAAA
+
FFFFFFFFFFFFFFFFFFFFFFFFFFFFFFFFFFFFFFFFFFFFFFFFFFFFFFFFFFFFFFFFFFFF
FFFFFFFFFFFFFFFFFFFFFFFF::FFFFFFFFFFFFFFFFF:FFFFFFF:FFFFFFFFFFFFFFFFF
FF:FFFFFFFFFFFF
@A01056:60:HMTK7DSXY:1:2675:2763:9126 1:N:0:TCCTTAGC+ACTCGATT
ATTGCTTTTTTGTTTGAAACAATTCCAGAAATAGACAGAGTTATTGCGACCACTATATTTCCGATTGA
ATTGTTATTTGGGGACAAGGTCTCTAAGGAGCCCAGGGTTTACACCTATAAAGTTCATGGATCCTCCT
TTTCATTTTACCCTG
+
FFFF:FFFFFF:FFFFFFFFFFFFFFFFFFFFFFFFFFF:FFFFFFFFFFFFFFFFFFFFFFFFFFFFF
FFFFFFFFFFFFFFFFFFFFFFFFFFFFFFFFFFFFFFFFFFFFFFFFFFFFFFFFFFFFFFFFFFFF
FFFFFFFFFFFFFFF
@A01056:60:HMTK7DSXY:1:1473:19217:10567 1:N:0:TCCTTAGC+ACTCGATT
GAGTTATTGCGACCACTATATTTCCGATTGAATCGTTATTTGGGGACAAGGTCTCTAAGGAGCCCAGG
GTTTACACCTATAAAGTTCATGGATCCTCCTTTTCATTTTACCCTGACGGTGTGGCCTCTGAATGTTA
TGAACAAAATTTGGC
+
FFFFFFFFFFFFFFFFFFFF,FFFFFFFFFFFFF:FFFFFFFFFFFFFFFFFFFFFFFFFFFFFFFFFF
FFFFFFFFFFFFFFFFFFFFFFFFFFFFFFFFFFFFF:FFFFFFFFFFFFFFFFFFFFFFFFFFFFFFF
FFFFFFFFFFFFFFF
@A01056:60:HMTK7DSXY:1:1473:19217:10567 2:N:0:TCCTTAGC+ACTCGATT
GAGTTATTGCGACCACTATATTTCCGATTGAATCGTTATTTGGGGACAAGGTCTCTAAGGAGCCCAGG
GTTTACACCTATAAAGTTCATGGATCCTCCTTTTCATTTTACCCTGACGGTGTGGCCTCTGAATGTTA
TGAACAAAATTTGGC
+
FFFFFFFFFFFFFFFFFFFFFFFFFFFFFFFFFFFFFFFFFFFFFFFFFFFFFFFFFFFFFFFFFFFF
FFFFFFFFFFFFFFFFFFFFFFFFFFFFFFFFFFFFFFFFFFFFFFFFFFFFFFFFFFFFFFFFFFFF
FFFFF:FFFFFFFFF
@A01056:60:HMTK7DSXY:1:2221:27688:21637 2:N:0:TCCTTAGC+ACTCGATT
CCACTATATTTCCGATTGAATTGTTATTTGGGGACAAGGTCTCTAAGGAGCCCAGGGTTTACACCTAT
AAAGTTCATGGATCCTCCTTTTCATTTTACCCTGACGGTGTGGCCTCTGAATGTTATGAACAAAATTT
GGCAAATTCAAAATG
+
FFFFFFFFFFFFFFFFFFFFF,FFFFFFFFFFFFFFFFFFFFFFFFFFFFFFFFFFFFFFFFFFFFFF
FFFFFFFFFFFFFFFFFFFF:FFFFF:FFFFFFFFFFFFFFFFFFFFFFFFFFFFFF,FFFFFFFFFFFF
FFFFFFFFFFFFFFF
```

```
@A01056:60:HMTK7DSXY:1:2234:12906:6746 2:N:0:TCCTTAGC+ACTCGATT
CCACTATATTTCCGATTGAATTGTTATTTGGGGACAAGGTCTCTAAGGAGCCCAGGGTTTACACCTAT
AAAGTTCATGGATCCTCCTTTTCATTTTACCCTGACGGTGTGGCCTCTGAATGTTATGAACAAAATTT
GGCAAATTCAAAATG
+
FFFFFFFFFFFFFFFFFF:FF:FFFFF:FFFFFFFFFFFFFFFFFFFFFFFFFFFFFFFFFFFFF:FF
FFFFFFFFFFFFFFFFFFFFFFFFFFFFFF:FFFFFFFFFFFFFFFFFFFFFFFFFFFFFFFFFFFFF
FFFFFFFFFFFFFF:
@A01056:60:HMTK7DSXY:1:1632:20401:8328 2:N:0:TCCTTAGC+ACTCGATT
ATTTGGGGACAAGGTCTCTAAGGAGCCCAGGGTTTACACCTATAAAGTTCATGGATCCTCCTTTTCAT
TTTACCCTGACGGTGTGGCCTCTGAATGTTATGAACAAAATTTGGCAAATTCAAAATGGCCTTTCACC
TGCAGTGGCATTCAA
+
FFFFFFFFFFFFFFFFFFFFFFFFFFFFFFFFFFFFFFFFFFFFFFFFFFFFFFFFFFFFFFFFFFFF
FFFFFFFFFFFFFFFFFFFFFFFFFFFFFFFFFFFFFFFFFFFFFFFFFFFFFFFFFFFFFFFFFFFF
FFFFFFFFFFFFFFF
@A01056:60:HMTK7DSXY:1:1632:15275:14606 1:N:0:TCCTTAGC+ACTCGATT
CTCTTCCGATCTCTAAGGAGCCCAGGGTTTACACCTATAAAGTTCATGGATCCTCCTTTTCATTTTAC
CCTGACGGTGTGGCCTCTGAATGTTATGAACAAAATTTGGCAAATTCAAAATGGCCTTTCACCTGCAG
TGGCATTCAATGGGC
+
FFFFFFFFFFFFFFFFFFFFFFFFFFFFFFFFFFFFFFFFFFFFFFFFFFFFFFFFFFFFFFFFFFFF
FFFFFFFFFFFFFFFFFFFFFFFFFFFFFFFFFFFFFFFFFFFF:FFFF:FFFFFFFFFFFFFFFFFF
FFFFFFFFFFFFFFF
@A01056:60:HMTK7DSXY:1:1632:16387:16063 1:N:0:TCCTTAGC+ACTCGATT
CTCTTCCGATCTCTAAGGAGGCCTGTGTTTACACCTATAAAGTTCATGGATCCTCCTTTTCATTTTAC
CCTGACGGTGTGGCCTCTGAATGTTATGATCATAATTTGGCAAATTCAAAATGTCCTTTCACCTGCAG
TGGCATTCAATGGGC
+
,F,FF,,FFF,F:FFFF:FF,,:,F,FF:FF,F,:FFF:F:FFF:FF,,:F:FF:,FFF:,FFFFF,F
F,FF,FFFF,FFF,FF,FF,FF,FFFFF:,,:,,:FFFF,FFFFFF:,FF:FF,F:FFFFFF:FFF:F
FFFFFFFF,FFFF:F
@A01056:60:HMTK7DSXY:1:2221:27688:21637 1:N:0:TCCTTAGC+ACTCGATT
AAGGTCTCTAAGGAGCCCAGGGTTTACACCTATAAAGTTCATGGATCCTCCTTTTCATTTTACCCTGA
CGGTGTGGCCTCTGAATGTTATGAACAAAATTTGGCAAATTCAAAATGGCCTTTCACCTGCAGTGGCA
TTCAATGGGCTAATA
+
FFFFFFFFFFFFFFFFFFFFFFFFFFFFFFFFFFFFFFFFFFFFFFFFFFFFFFFFFFFFFFFFFFFF
FFFFFFFFFFFFFFFFFFFFFFFFFFFFFFFFFFFFFFF:FFFFFFFFFFFFFFFFFFFFFFFFFFFF
FFFFFFFFFFFFFFF
@A01056:60:HMTK7DSXY:1:2234:12906:6746 1:N:0:TCCTTAGC+ACTCGATT
AAGGTCTCTAAGGAGCCCAGGGTTTACACCTATAAAGTTCATGGATCCTCCTTTTCATTTTACCCTGA
CGGTGTGGCCTCTGAATGTTATGAACAAAATTTGGCAAATTCAAAATGGCCTTTCACCTGCAGTGGCA
TTCAATGGGCTAATA
+
FFFFFFFFFFFFFFFFFFFFFFFFFFFFFFFFFFFFFFFFFFFFFFFFFFFFFFFFFFFFFFFFFFFF
FFFFFFFFFFFFFFFFFFFFFFFFFFFFF:FFFFFFFFFFFFFFF:,FFFFFFFFFFFFFFFFFFFFF
FFFFFFFFFFFFFFF
@A01056:60:HMTK7DSXY:1:2613:31521:11741 2:N:0:TCCTTAGC+ACTCGATT
GGTCTCTAAGGAGCCCAGGGTTTACACCTATAAAGTTCATGGATCCTCCTTTTCATTTTACCCTGACG
GTGTGGCCTCTGAATGTTATGAACAAAATTTGGCAAATTCAAAATGGCCTTTCACCTGCAGTGGCATT
CAATGGGCTAATAGG
+
FFFFFFF:FFFFFFFFFFFFFFFFFFFFFFFF:FFFFFFFFFFFFFFFF::FFFFFF:FFFFFFFFFF
```

```
F,FFFFFFFFFFFFFFF,FFFF:FFFFFF,FFFFFFFFFFFFFFFFFFFFFFFFFFFFFFF,FFFFFF
FFFFFFFF,FF,FFF
@A01056:60:HMTK7DSXY:1:2613:31195:12117 2:N:0:TCCTTAGC+ACTCGATT
GGTCTCTAAGGAGCCCAGGGTTTACACCTATAAAGTTCATGGATCCTCCTTTTCATTTTACCCTGACG
GTGTGGCCTCTGAATGTTATGAACAAAATTTGGCAAATTCAAAATGGCCTGTCACCTGCAGTGGCATT
CAATGGGCTAATAGG
+
:FFFFFFFF:FFFFFFFFFFFFFFFFFF,F,FFFFFFFF,FFFFFFFFFF:,FFF:,FFFFFFF:FFF
F:FFFFFF:F,FFFFF,F:F:F:FFF:F,FFFFFF,FF:FF:FFFFFFFF,FFFFFFFFFF,FFFF,F
FFFFF:FF,:F:FFF
@A01056:60:HMTK7DSXY:1:1632:20401:8328 1:N:0:TCCTTAGC+ACTCGATT
CTCTAAGGAGCCCAGGGTTTACACCTATAAAGTTCATGGATCCTCCTTTTCATTTTACCCTGACGGTG
TGGCCTCTGAATGTTATGAACAAAATTTGGCAAATTCAAAATGGCCTTTCACCTGCAGTGGCATTCAA
TGGGCTAATAGGAAG
+
FFFFFFFFFFFFFFFFF:FFFFFFFFFFFFFFFFFFFFFFFFFFFFFFFFFFFFFFFFFFFFFFFFFF
FFFFFFFFFFFFFFFFFFFFFFFFFFFFFFFFFFFFFFFFFFFFFFFFFFFFFFFFFFFFFFFFFFFF
FFFFFFFFFFFFFFF
@A01056:60:HMTK7DSXY:1:1174:22209:34006 2:N:0:TCCTTAGC+ACTCGATT
CTAAGGAGCCCAGGGTTTACACCTATAAAGTTCATGGATCCTCCTTTTCATTTTACCCTGACGGTGTG
GCCTCTGAATGTTATGAACAAAATTTGGCAAATTCAAAATGGCCTTTCACCTGCAGTGGCATTCAATG
GGCTAATAGGAAGAT
+
FFFFFFFFFFFFFFFFFFFFFFFFFFFFFFFFFFFFFFFFFFFFFFFFFFFFFFFFFFFFFFFFFFFF
FFFFFFFFFFF,FFFFFFFFFFFFFFFFFFFFFFFFFFFFFFFFFFFFFFFFFFFFFFFFFFFFFFFF
FFFF::FFFFFFFFF
@A01056:60:HMTK7DSXY:1:1632:15275:14606 2:N:0:TCCTTAGC+ACTCGATT
CTAAGGAGCCCAGGGTTTACACCTATAAAGTTCATGGATCCTCCTTTTCATTTTACCCTGACGGTGTG
GCCTCTGAATGTTATGAACAAAATTTGGCAAATTCAAAATGGCCTTTCACCTGCAGTGGCATTCAATG
GGCAGATCGGAAGAG
+
FFFFFFFFFFFFFFFFFFFFFFFFFFFFFFFFFFFFFFFFFFFFFFFFFFFFFFFFFFFFFFFFFFFF
FFFFFFFFFFFFFFFFFFFFFFFFFFFFFFFFFFFFFFFFFFFFFFFFFFFFFFFFFFFFFFFFFFFF
FFFFFFFF,FFFFFF
@A01056:60:HMTK7DSXY:1:1632:16387:16063 2:N:0:TCCTTAGC+ACTCGATT
CTAAGGAGCCCAGGGTTTACACCTATAAAGTTCATGGATCCTCCTTTTCATTTTACCCTGACGGTGTG
GCCTCTGAATGTTATGAACAAAATTTGGCAAATTCAAAATGGCCTTTCACCTGCAGTGGCATTCAATG
GGCAGATCGGAAGAG
+
FFFFFFFFFF,F:FFFF,FFF:F:F,FFF:FFFF::FFFFF:FFFF::FFFFFFFFF:FFFFF:::FF
FFFFFFFFFFFFFFFFFFFFFFFFF,F,:FFFFF:FFFFF:F:FF:FFFFF:,FFFFFFFFFFF:FFF,
FFFFFFF:FFFFFFF
@A01056:60:HMTK7DSXY:1:2268:7229:29512 2:N:0:TCCTTAGC+ACTCGATT
CTAAGGAGCCCAGGGTTTACACCTATAAAGTTCATGGATCCTCCTTTTCATTTTACCCTGACGGTGTG
GCCTCTGAATGGTATGAACAAAATTTGGCAAATTCAAAATGGCCTTTCACCTGCAGTGGCATTCAATG
GGCTAATAGGAAGAT
+
FFFFFFFFFFFFFFFFFFFFFFFFFFFFFFFFFFFFFFFFFFFFFFFFFFFFFFFFFFFFFFFFFFFF
FFFFFFFFFFF,FFFFFFFFFFFFFFFFFFFFFFFFFFFFFFFFFFFFF:FFFF,FFFFFFFFFFF::F
FFFF:F,FFFFFF,:
@A01056:60:HMTK7DSXY:1:1174:22209:34006 1:N:0:TCCTTAGC+ACTCGATT
GGAGCCCAGGGTTTACACCTATAAAGTTCATGGATCCTCCTTTTCATTTTACCCTGACGGTGTGGCCT
CTGAATGTTATGAACAAAATTTGGCAAATTCAAAATGGCCTTTCACCTGCAGTGGCATTCAATGGGCT
AATAGGAAGATAAGG
```

```
+
FFFFFFFFFFFFFFF:FFFFFFFFFFFFFFFFFFFFFFFFFFFFFFFFFFFFFFFFFFFFFFFFFFFFF
FFFFFFFFFFFFFFFFFFFFFFFFFFFFFFFFFFFFFFFFFFFFFFFFFFFFFFFFFFFFFFFFFFFFF
FFFFFFFFFFFFFFF
@A01056:60:HMTK7DSXY:1:2268:7229:29512 1:N:0:TCCTTAGC+ACTCGATT
GGAGCCCAGGGTTTACACCTATAAAGTTCATGGATCCTCCTTTTCATTTTACCCTGACGGTGTGGCCT
CTGAATGCTATGAACAAAATTTGGCAAATTCAAAATGGCCTTTCACCTGCAGTGGCATTCAATGGGCT
AATAGGAAGATAAGG
+
FFFFFFFFFFFFFFFFFFFFFFFFFFFFFFFFFFFFFFFFFFFFFFFFFFFFFFFFFFFFFFFFFFFFF
FFFFFFF,FFFFFFFFFFFFFFFFFFFFFFFFFFFFFFFFFFFFFFFFFFFFFFFFFFFFFFFFFFFFF
FFFFFFFFFFFFFFF
@A01056:60:HMTK7DSXY:1:2260:24849:14716 2:N:0:TCCTTAGC+ACTCGATT
TGTTTTTTCGTTTGACAGGGGTAGGGCCTGTAATGAGTTTAACCATTTCGACAAACCAAGTTGCCTGC
TCGCAGAGGAAATGCGTCTTTTGACAAAAAGGTTTGATTCTGCAGTCATCAATCGAAGCACAGTTTCC
TCTCTTAGCACTTAC
+
FFFFFFFFFFFFFFFFFFFFFFFFFFFFFFFFFFFFFFFFFFFFFFFFFFFFFF:FFFFFFFFFFFFF
FFFFFFFFFF:FFF:FFFFFFFFFFFFFFFF:FFFFFFFFFFFFFFFFFFFFFFFF,:FFFFFFFFFF
FFFFFFFFFFFFFFF
@A01056:60:HMTK7DSXY:1:2260:24849:14716 1:N:0:TCCTTAGC+ACTCGATT
GTAGGGCCTGTAATGAGTTTAACCATTTCGACAAACCAAGTTGCCTGCTCGCAGAGGAAATGCGTCTT
TTGACAAAAAGGTTTGATTCTGCAGTCATCAATCGAAGCACAGTTTCCTCTCTTAGCACTTACATGGC
TTGCCTCAAAACTGC
+
FFFFFFFFFFFFFFFFFFFFFFFFFFFFFFFFFF:FFFFFFFFFFFFFFFF:FFFFFFFFFFFFFFFFF
FFFFFFFFFFFFFFFFFFFFFFFFFFFFFFFFFFFFFFFFFFFFFFFFFFFFFFFFFFFFFFFFFFFFF
FFFFFFFFFFFFFFF
@A01056:60:HMTK7DSXY:1:1354:26413:10379 1:N:0:TCCTTAGC+ACTCGATT
GATCTGTTTAACCATTTCGACAAACCAAGTTGCCTGCTCGCAGAGGAAATGCGTCTTTTGACAAAAAG
GTTTGATTCTGCAGTCATCAATCGAAGCACAGTTTCCTCTCTTAGCACTTACATGGCTTGCCTCAAAA
CTGCGAATGCAGCCG
+
FFFFFFFFFFFFFF:FFFFFF:FFFFFFFF:FFFFFFFFFFFFFFFFFFFFFFFFFFFFFFFFFFFFFF
FFFFFFFFFFFFFFFFFFFFFFFFFFFFFFFFFFFFFFFFFFFFFFFFFF:FFFFFFFFFFFFFFFFFF
FFFFFFFFFFFFFFF
@A01056:60:HMTK7DSXY:1:1354:26413:10379 2:N:0:TCCTTAGC+ACTCGATT
GTTTAACCATTTCGACAAACCAAGTTGCCTGCTCGCAGAGGAAATGCGTCTTTTGACAAAAAGGTTTG
ATTCTGCAGTCATCAATCGAAGCACAGTTTCCTCTCTTAGCACTTACATGGCTTGCCTCAAAACTGCG
AATGCAGCCGAGATC
+
FFFFFFFFFFFFFFFFFFFFFFFFFFFFFFFFFFFFFFFFFF:FFFFFFFFFFFF,FFFFFFFFFFFF
F:FFFFFFFFFFFFFFFFFFFFFFFFFFFFFFFFFFFFFFFFFFFF:FFFFF:FFFFFFFFFFFFFFF
FFFFFFFFFFF:FFF
@A01056:60:HMTK7DSXY:1:2572:23945:35790 2:N:0:TCCTTAGC+ACTCGATT
TTAACCATTTCGACAAACCAAGTTGCCTGCTCGCAGAGGAAATGCGTCTTTTGACAAAAAGGTTTGAT
TCTGCAGTCATCAATCGAAGCACAGTTTCCTCTCTTAGCACTTACATGGCTTGCCTCAAAACTGCGAA
TGCAGCCTCAGCAGT
+
FFFFFFFFFFFFFFFFFFFFFFFFFFFFFFFFFFFFFFFFFFFFFFFFFFFFFFFFFFFFFFFFFFFFF,
FFFFFFFFFFFFFFFFFF,FFFFFFFFFFFFFFFFFFFFFFFFFFFFFFFFFFFFFFFFFFFFFFFFFF
FFFFFFFFFFFFFFF
@A01056:60:HMTK7DSXY:1:2572:23945:35790 1:N:0:TCCTTAGC+ACTCGATT
TGCAGTCATCAATCGAAGCACAGTTTCCTCTCTTAGCACTTACATGGCTTGCCTCAAAACTGCGAATG
```

CAGCCTCAGCAGTTGCTAAATTGAGGCAGTTGGAGAAGAGAGATCTCTATCCAGATGAGCTGAATTTT
GTTTACTCTTTCGGG
+
FFFFFFFFFFFFFFFFFFFFFFFF:FFFFFFFFFFFFFFFFFFFFFFFFFFFFFFFFFFFFFFFFFFFFF
FFFFFFFFFFFFFFFFFFFFFFFFFFFFFFFFFFFFFFFFFFFFFFFFFFFFFFFFFFFFFFFFFFFFFF
FFFFFFFFFFFFFFF
@A01056:60:HMTK7DSXY:1:2104:21269:4476 2:N:0:TCCTTAGC+ACTCGATT
GTTTCCTCTCTTAGCACTTACATGGCTTGCCTCAAAACTGCGAATGCAGCCTCAGCAGTTGCTAAATT
GAGGCAGTTGGAGAAGAGAGATCTCTATCCAGATGAGCTGAATTTTGTTTACTCTTTCGGGGAACATT
TCAAGAATTTTGGGA
+
FFFFFFFFFFFFFFFFFFFFFFFFFFFF:FFFFFFFFFFFFFFFFFFFFFFFFFFFFFFFFFFFFF:FFF
FFFFFFFFFFFFFFFFFFFFFFFFFFFFFFFFFFFFFFFFFFFFFFFFFFFFFFFFFFFFFFFFFFFFFF
FFFFFFFFFFFF:FF
@A01056:60:HMTK7DSXY:1:2104:21269:4476 1:N:0:TCCTTAGC+ACTCGATT
CTCAAAACTGCGAATGCAGCCTCAGCAGTTGCTAAATTGAGGCAGTTGGAGAAGAGAGATCTCTATCC
AGATGAGCTGAATTTTGTTTACTCTTTCGGGGAACATTTCAAGAATTTTGGGATGAGAGACGACTTTG
ATGTGTCGATTCTCC
+
FFFFF,FFFFFFFFFFFFFFFFFFFFFFFFFFFFFFFFFFFFFFFFFFFFFFFFFFFFFFFFFFFF
FFFFFFFFFFFFFFFFFFFFFFFFFFFFFFFFFFFFFFFFFFFFFFFFFFFFFFFFFFFFFFFFFFFFFF
FFFFFFFFFFFFFFF
@A01056:60:HMTK7DSXY:1:1551:30662:30107 2:N:0:TCCTTAGC+ACTCGATT
CTCAAAACTGCGAATGCAGCCTCAGCAGTTGCTAAATTGAGGCAGTTGGAGAAGAGAGATCTCTATCC
AGATGAGCTGAATTTTGTTTACTCTTTCGGGGAACATTTCAAGAATTTTGGGATGAGAGACGACTTTG
ATGTGTCGATTCTCC
+
FFFFFFFFFFFFFFFFFFFFFFFFFFFFFFFFFF:FFFFFFFFFFFFFFFFFFF:FFFFFFFFFFFFFFF
:FFFFFFFFFF:FFFFFFFFFFFFFFFFFFFFFFFFFFFFF:FFFFFFFFFFFFFFFFFFFFFFFFF:FF
F:FFFFFFFFFFFFF
@A01056:60:HMTK7DSXY:1:2613:31195:12117 1:N:0:TCCTTAGC+ACTCGATT
AACTGCGAATGCAGCCTCAGCAGTTGCTAAATTGAGGCAGTTGGAGAAGAGAGATCTCTATCCAGATG
AGCTGAATTTTGTTTACTCTTTCGGGGAACATTTCAAGAATTTTGGGATGAGAGACGACTTTGATGTG
TCGATTCTCCAATGG
+
FFFFFFFFFFFFFFFFFFFFFF:F:FFF:FFFF:FFFFFFFFFFFFFFFFFFFFFFFFFFFFFFFFFFFF
FFFFFFFFFFFFFFFFFFFFFFFFFFFFFFFFFFFFFFFFFFFFFFFFFFFFFFFFFFFFFFF:FFFFFF
FFFFFFFFFFFFFFF
@A01056:60:HMTK7DSXY:1:2613:31521:11741 1:N:0:TCCTTAGC+ACTCGATT
AACTGCGAATGCAGCCTCAGCAGTTGCTAAATTGAGGCAGTTGGAGAAGAGAGATCTCTATCCAGATG
AGCTGAATTTTGTTTACTCTTTCGGGGAACATTTCAAGAATTTTGGGATGAGAGACGACTTTGATGTG
TCGATTCTCCAATGG
+
,FFFFFFFFFFFFFFFFFFFFFFFFFFFFFFFFFFFFFFFFFFFFF:F:FFFFFFFFFFFFF:FFFFFFF
FFFFFFFFFFFFFFFFFFFFFFFFFFF,FFFFFFFFFFFFFFFFFFFFFFFFFFFFFFFFFFFFFFFFFF
FFFFFFFFFFFFFFF
@A01056:60:HMTK7DSXY:1:2428:25283:17190 2:N:0:TCCTTAGC+ACTCGATT
CAGCAGTTGCTAAATTGAGGCAGTTGGAGAAGAGAGATCTCTATCCAGATGAGCTGAATTTTGTTTAC
TCTTTCGGGGAACATTTCAAGAATTTTGGGATGAGAGACGACTTTGATGTGTCGATTCTCCAATGGGT
CAAAGACAAATTTTG
+
FFFFFFFFFFFFFFFFFFFFFFFFFFFFFFFFFFFFFFFFFFFFFFFFFFFFFFFFFFF:FFFFFFFFFF
FFFFFFFFFFFFFFFFFFFFFFFFFF:FFFFFFFFFFFFFFFFFFFFFFFFFFFFFFFFFFFFFFFFFFF
FFFFFFFF:FFF,FF

```
@A01056:60:HMTK7DSXY:1:1551:30662:30107 1:N:0:TCCTTAGC+ACTCGATT
AGATGAGCTGAATTTTGTTTACTCTTTCGGGGAACATTTCAAGAATTTTGGGATGAGAGACGACTTTG
ATGTGTCGATTCTCCAATGGGTCAAAGACAAATTTTGTCAAGTTATGCCTCACTTCATTGCTGCCAGC
TTCTTTGAGCCAACA
+
FFFFFFFFFFFFFFFFFFFF:FFFFFFFFFFFFFFFFFFFFFFF:FFFFFFFFFFFFFFFFFFFFFFFFF
FFFFFFFFFFFFFFFFFFFFFFFFFFFFFFFFFFFFFFFFFFFFFFFFFFFFFFFFFFFFFFFFFFFFFF
FFFFFFFFFFFFFFF
@A01056:60:HMTK7DSXY:1:1165:4246:1767 2:N:0:TCCTTAGC+ACTCGATT
GCTGAATTTTGTTTACTCTTTCGGGGAACATTTCAAGAATTTTGGGATGAGAGACGACTTTGATGTGT
CGATTCTCCAATGGGTCAAAGACAAATTTTGTCAAGTTATGCCTCACTTCATTGCTGCCAGCTTCTTT
GAGCCAACAGAGTTT
+
FFFFFFFFFFFFFFFFFFFFFFFFFFFFFFFFFFFFFFFFFFFFFFFFFFFFFFFFFFFFFFFFFFFFFF
FFFFFFFFFFFFFFFFFFFFFFFFFFFFFFFFFFFFFFFFFFFFFFFFFFFFFFFFFFFFFFFFFFFFFF
FFFFFFFFFFFFFFF
@A01056:60:HMTK7DSXY:1:1173:28203:23970 1:N:0:TCCTTAGC+ACTCGATT
CTATTTTGTTTACTCTTTCGGGGAACATTTCAAGAATTTTGGGATGAGAGACGACTTTGATGTGTCGA
TTCTCCAATGGGTCAAAGACAAATTTTGTCAAGTTATGCCTCACTTCATTGCTGCCAGCTTCTTTGAG
CCAACAGAGTTTCAC
+
FFFF:FFFFFFFFFFFFFFFFFFFFFFFFFFFFFFFFFFFFFFFFFFFFFFFFFFFFFFFFFFFFFFFF
FFFFFFFFFFFFFFFFFFFFFFFFFFFFFFFFFFFFFFFFFFFFFFFFFFFFFFFFFFFFFFFFFFFFFF
FFFFFFFFFFFFFFF
@A01056:60:HMTK7DSXY:1:1173:28203:23970 2:N:0:TCCTTAGC+ACTCGATT
ATTTTGTTTACTCTTTCGGGGAACATTTCAAGAATTTTGGGATGAGAGACGACTTTGATGTGTCGATT
CTCCAATGGGTCAAAGACAAATTTTGTCAAGTTATGCCTCACTTCATTGCTGCCAGCTTCTTTGAGCC
AACAGAGTTTCACAG
+
FFFFFFFFFFFFFFFFFFFFFFF:FFFFFFFFFFFFFFFFFFFFFFFFFFFFFFFFFFFFFFFFFFFFFF
FFFFFFFFFFFFFFFFFFFFFFFFFFFFFFFFFFFFFFFFFFFFFFFFFFFFFFFFFFFFFFFFFFFFFF
FFFFFFFFFFFFFF:
@A01056:60:HMTK7DSXY:1:2366:7139:15828 2:N:0:TCCTTAGC+ACTCGATT
ATTTTGTTTACTCTTTCGGGGAACATTTCAAGAATTTTGGGATGAGAGACGACTTTGATGTGTCGATT
CTCCAATGGGTCAAAGACAAATTTTGTCAAGTTATGCCTCACTTCATTGCTGCCAGCTTCTTTGAGCC
AACAGAGTTTCACCT
+
FFFFFFFFFFFFFFFFFFFFFFFFFFFFFFFFFFFFFFFFFFFFFFFFFFFFFFFFFFFFFFFFFFFFFF
FFFFFFFFFFFFFFFFFFFFFFFFFFFF:FFFFFFFFFFFFFFFFFFFFFFFFFFFFFFF,FFFFFF:F:
FFFFFFFFFFFF:FF
@A01056:60:HMTK7DSXY:1:2428:25283:17190 1:N:0:TCCTTAGC+ACTCGATT
ACTCTTTCGGGGAACATTTCAAGAATTTTGGGATGAGAGACGACTTTGATGTGTCGATTCTCCAATGG
GTCAAAGACAAATTTTGTCAAGTTATGCCTCACTTCATTGCTGCCAGCTTCTTTGAGCCAACAGAGTT
TCACCTCAACATGCG
+
FFFFFFFFFFFFFFFFFFFFFFFFFFFFFFFFFFFFFFFFFFFFFFFFFFFFFFFFFFFFFFFFFFFFFF
FFFFFFFFFFFFFFFFFFFFFFFFFFFFFFFFFFFFFFFFFFFFFFFFFFFFF:FFFFFFFFFFFFFFFF
FFFFFFFFFFFFFFF
@A01056:60:HMTK7DSXY:1:1165:4246:1767 1:N:0:TCCTTAGC+ACTCGATT
ACTCTTTCGGGGAACATTTCAAGAATTTTGGGATGAGAGACGACTTTGATGTGTCGATTCTCCAATGG
GTCAAAGACAAATTTTGTCAAGTTATGCCTCACTTCATTGCTGCCAGCTTCTTTGAGCCAACAGAGTT
TCACCTCAACATGCG
+
FFFFFFFFFFFFFFFFFFFFFFFFFFFFFFFFFFFFFFFFFFFFFFFFFFFFFFFFFFFFFFFFFFFFFF
```

FFFFFFFFFFFFFFFFFFFFFFFFFFFFFFFFFFFFFFFFFFFFFFFFFFFFFFFFFFFFFFFFFFFF
FFFFFFFFFFFFFFF
@A01056:60:HMTK7DSXY:1:1213:1669:13557 2:N:0:TCCTTAGC+ACTCGATT
CGACTTTGATGTGTCGATTCTCCAATGGGTCAAAGACAAATTTTGTCAAGTTATGCCTCACTTCATTG
CTGCCAGCTTCTTTGAGCCAACAGAGTTTCACCTCAACATGCGAAAATTATTGAATGATCTGGCCACC
AAGGGGATAGAGGTC
+
FFF:FFFFFFFFFFFFFFFFFFFFFFFFFFFFF,FFFFFFFFFFFFFFFFFFFFF:FFFFFFFFFFFF
FFFFFFFFFFFFFFFFFFFFFFFFFFFFFFF::FFFFFF,FFFFFFFFFFFF:FFFFFFFFFF,FFF:
FF:FFFFFFFFFFFF
@A01056:60:HMTK7DSXY:1:2511:26133:6856 2:N:0:TCCTTAGC+ACTCGATT
CAAATTTTGTCAAGTTATGCCTCACTTCATTGCTGCCAGCTTCTTTGAGCCAACAGAGTTTCACCTCA
ACATGCGAAAATTATTGAATGATCTGGCCACCAAGGGGATAGAGGTCCCACTTTCCGTCATTGTGTTA
GACAATGTCGATTTT
+
FFFFFFFFFFFFFFFFFFFFFFFFFFFFFFFFFFFFFFFFFFFFFFFFFFFFFFFFFFFFFFFFFFFF
FFFFFFFFFFFFFFFFFFFFFFFFFFFFFFFFFFFFFFFFFFFFFFFFF:FF:FFFFFFFFFFFFFFF
FF:FFFFFFFFFFFF
@A01056:60:HMTK7DSXY:1:1213:1669:13557 1:N:0:TCCTTAGC+ACTCGATT
GAATGATCTGGCCACCAAGGGGATAGAGGTCCCACTTTCCGTCATTGTGTTAGACAATGTCGATTTTA
TTGAAACTAGGTTCCATGCCAGGATGTTTGACATCGCGCAGGCTATTGGGGTCAACTTGAACTTGCTC
GGGCGCAGATTTGAC
+
FFFFFFFF,:FFFFFF,:F:FFFFFFFFFFFFFFFF:FFFFFFFFFFFFFFFFFFFFFFFFFFFFFFF
FFFFFFFFFFFFFFFFFFFFFFFFFFFFFFFFFFFFFFFFFFFFFFFFFFFFFFFFFFFFFFFFFFFF
FFFFFFFFFFFFFFF
@A01056:60:HMTK7DSXY:1:2366:7139:15828 1:N:0:TCCTTAGC+ACTCGATT
GGGGATAGAGGTCCCACTTTCCGTCATTGTGTTAGACAATGTCGATTTTATTGAAACTAGGTTCCATG
CCAGGATGTTTGACATCGCGCAGGCTATTGGGGTCAACTTGAACTTGCTCGGGCGCAGATTTGACTAC
GATACTGAAAGTGAG
+
FFFFFFFFFFFFFFFFFFFFFFFFFFFFFFFFFFFFFF:FFFFFFFFFFFFFF:FFFFFFF,FFFFFF
FFFFFFFF:FFFFFFFFFFFFFFFFFFFFFFFFFFFFFFFFFFFFFFFFFFFFFFFFFFFFFFFFFFF
FFFFFFFFFFFFFFF
@A01056:60:HMTK7DSXY:1:2511:26133:6856 1:N:0:TCCTTAGC+ACTCGATT
CAATGTCGATTTTATTGAAACTAGGTTCCATGCCAGGATGTTTGACATCGCGCAGGCTATTGGGGTCA
ACTTGAACTTGCTCGGGCGCAGATTTGACTACGATACTGAAAGTGAGAAATATTTCTCAGAAAATGGT
TATGTCTTCATGCCG
+
FFFFFFFFFFFFFFFFFF,FFFFFFFFFFFFFFFFFFFFFFFFFFFFFFFFFFFFFFFFFFFFFFFFF
FFFFFFFFFFFFFFFFFFFFFFFFFFFFFFFFFFFFFFFFFFFFFFFFFFFFFFFFFFFFFFFFFFFF
FFFFFFFFFFFFFFF
@A01056:60:HMTK7DSXY:1:1252:1380:15812 2:N:0:TCCTTAGC+ACTCGATT
ATTTTATTGAAACTAGGTTCCATGCCAGGATGTTTGACATCGCGCAGGCTATTGGGGTCAACTTGAAC
TTGCTCGGGCGCAGATTTGACTACGATACTGAAAGTGAGAAATATTTCTCAGAAAATGGTTATGTCTT
CATGCCTTCAAAATC
+
FFFFFFFFFF:FFFFFFFFFFFFFFFFFFFFFFFFFFFFFFFFFFFFF:F:FFFFFFFFFFFFFFFFF
FFFFFFFFFFFFFFFF:FFFFFFFFFFFFFF:,FF:FFFFFFFFFFFFFFFFFFFFFFFFFFFFFFFF
FFFFFFFFFFFFFFF
@A01056:60:HMTK7DSXY:1:2674:29451:28260 2:N:0:TCCTTAGC+ACTCGATT
CAGGATGTTTGACATCGCGCAGGCTATTGGGGTCAACTTGAACTTGCTCGGGCGCAGATTTGACTACG
ATACTGAAAGTGAGAAATATTTCTCAGAAAATGGTTATGTCTTCATGCCTTCAAAATCAAATCCAGAC
AGGAATTGGATTCTG

+
FFFFFFFFFFFFFFFFFFFFFFFFFF,FFFFFFFFFFFFFFFFFFFFFFFFFFFFFFFFFFFFFFFFFFF
FFFFFFFFFFFFFFFFFFFFFFFFFFFF:FFFFFFFFFFFFFFFFFFFFFFFFFFFFFFFFFFFFFFFFF
FFFF:FFFFFFFFFF
@A01056:60:HMTK7DSXY:1:2674:27091:23641 2:N:0:TCCTTAGC+ACTCGATT
CAGGATGTTTGACATCGCGCAGGCTATTGGGGTCAACTTGAACTTGCTCGGGCGCAGATTTGACTACG
ATACTGAAAGTGAGAAATATTTCTCAGAAAATGGTTATGTCTTCATGCCTTCAAAATCAAATCCAGAC
AGGAATTGGATTCTG
+
FFFFFFFFFFFFFFFFFFFFFFFFFFFFFFFFFFFFFFFFFFFFFFFFFFFFFFFFFFFF:FFFFFFFFF
FFFFFFFFFFFFFFFFFFFFFFFFFFFFFFFFFFFFFFFFFFFFFFFFFFFFFFFFFFFFFFFFFFFFFF
FFFFFFFFFFFFFFF
@A01056:60:HMTK7DSXY:1:2674:29451:28260 1:N:0:TCCTTAGC+ACTCGATT
TGACATCGCGCAGGCTATTGGGGTCAACTTGAACTTGCTCGGGCGCAGATTTGACTACGATACTGAAA
GTGAGAAATATTTCTCAGAAAATGGTTATGTCTTCATGCCTTCAAAATCAAATCCAGACAGGAATTGG
ATTCTGAACTCTGGC
+
FFFFFFFFFFFFFFFFFFFFFFFFFFFFFFFFFFFFFFFFFFFFFFFFFFFFFFFFFFFFFFFFFFFFFF
FFFFFFFFFFFFFFFFFFFFFFFFFFFFFFFFFFFFFFFFFFFFFFFFFFFFFFFFFFFFFFFFFFFFFF
FFFFFFFFFFFFFFF
@A01056:60:HMTK7DSXY:1:2674:27091:23641 1:N:0:TCCTTAGC+ACTCGATT
TGACATCGCGCAGGCTATTGGGGTCAACTTGAACTTGCTCGGGCGCAGATTTGACTACGATACTGAAA
GTGAGAAATATTTCTCAGAAAATGGTTATGTCTTCATGCCTTCAAAATCAAATCCAGACAGGAATTGG
ATTCTGAACTCTGGC
+
FFF:FFFFFFFFFFFFFFFFFFFFFFFFFFFFFFFFFFFFFFFFFFFFFFFFFFFFFFFFFFFFFFFFFF
FFFFFFFFFFFFFFFFFFFFFFFFFFFFFFFFFFFFFFFFFFFFFFFFFFFFFFFFFFFFFFFFFFFFFF
FFFFFFFFFFFFFFF
@A01056:60:HMTK7DSXY:1:2502:29975:16611 2:N:0:TCCTTAGC+ACTCGATT
ATTTGACTACGATACTGAAAGTGAGAAATATTTCTCAGAAAATGGTTATGTCTTCATGCCTTCAAAAT
CAAATCCAGACAGGAATTGGATTCTGAACTCTGGCTCTTTAAAGATTGACTACTTCAAATTGGTCAAA
GTCAGGAGATTCAGA
+
FFFFFFFFFFFFFFFFFFFFFFFFFFFFFFFFFFFFFFFFFFFFFFFFFFFFFFFFFFFFFFFFFFFFFF
:FFF:FFFFFFFFFFFFFFFFFFFFFFFFFFFFFFFFFFFFFFFFFFFF:FFFF:FFFFFFFFFFFFFFF
FFFFFFFFFFFFFFF
@A01056:60:HMTK7DSXY:1:2502:29975:16611 1:N:0:TCCTTAGC+ACTCGATT
ACGATACTGAAAGTGAGAAATATTTCTCAGAAAATGGTTATGTCTTCATGCCTTCAAAATCAAATCCA
GACAGGAATTGGATTCTGAACTCTGGCTCTTTAAAGATTGACTACTTCAAATTGGTCAAAGTCAGGAG
ATTCAGATTGAGGAG
+
FF,FFFFFF:FFFFFFFFFFFFFFFFFFFFFFFFFFFFFFFFFFFFFFFFFFFFFFFFFFFFFFFFFFFF
:FFFFFFFFFFFFFFFFFFFFFFFFFFFFFFFFFFFFFFFFFFFFFFFFFFFFFFFFFFFFFFFFFFFFF
FFFFFFFFFFFFFFF
@A01056:60:HMTK7DSXY:1:1252:1380:15812 1:N:0:TCCTTAGC+ACTCGATT
ATACTGAAAGTGAGAAATATTTCTCAGAAAATGGTTATGTCTTCATGCCTTCAAAATCAAATCCAGAC
AGGAATTGGATTCTGAACTCTGGCTCTTTAAAGATTGACTACTTCAAATTGGTCAAAGTCAGGAGATT
CAGATTGAGGAGGGG
+
FFFFFFFFFFFFFFFFFFFF:FFFFFFFFFFFF:FFFFFFFFFFFFFFFFF:FFFFFFFFFFFFFFFFFF
FFFFF:FFFFFFFFFFFFFFFFFFFFFFFFFFFFFFFFFFFFFFFFFFFFFFFFFFFFFFFFFFFFFFFF
FFFFFFFFFFFFFFF
@A01056:60:HMTK7DSXY:1:1661:8223:28166 2:N:0:TCCTTAGC+ACTCGATT
GTCTTCATGCCTTCAAAATCAAATCCAGACAGGAATTGGATTCTGAACTCTGGCTCTTTAAAGATTGA

CTACTTCAAATTGGTCAAAGTCAGGAGATTCAGATTGAGGAGGGATTTTCTGGATCCCATATCTAAAG
GTGATTCTCCCAGGA
+
FFFFFFFFFFFFFFFFFFFFFFFFFFFFFFFFFFFFFFFFFFFFFFFFFFFFFFFFFFFFFFFFFFFF
FFFFFFFFFFFFFFFFFFFFFFFFFFFFFFFFFFFFFFFFFFFFFFFFFFFFFFFF:FFFFFFFFFFF
FFFFFFFFF:FFFFF
@A01056:60:HMTK7DSXY:1:2254:12418:16611 2:N:0:TCCTTAGC+ACTCGATT
ATTGGATTCTGAACTCTGGCTCTTTAAAGATTGACTACTTCAAATTGGTCAAAGTCAGGAGATTCAGA
TTGAGGAGGGATTTTCTGGATCCCATATCTAAAGGTGATTCTCCCAGGAGGCAACTCTTCTTAGAATC
AGCCAGTGACCCTAG
+
FFFFFFFFFFFFFFFFFFFFFFFFFFFFFFFFFFFFFFFFFFFFFFFFFFFF:FFFFFFFFFFFFFFF
FFFFFFFFFFFFFFFFFFFFFFFFFFFFFFFFFFFFFFFFFFFFFFFFFFFFFFFFFFF:FFFFFFFF
FFFFFFFFFFFFF:F
@A01056:60:HMTK7DSXY:1:2254:12418:16611 1:N:0:TCCTTAGC+ACTCGATT
CTCTGGCTCTTTAAAGATTGACTACTTCAAATTGGTCAAAGTCAGGAGATTCAGATTGAGGAGGGATT
TTCTGGATCCCATATCTAAAGGTGATTCTCCCAGGAGGCAACTCTTCTTAGAATCAGCCAGTGACCCT
AGGTCAAACCCTGAG
+
FFFFFFFFFFFFFFFFFFFFFFFFFFFFFFFFFFFFFFFFFFFFFFFFF:FFFFFFFFFFFFFFFFFF
FFFFFFFFFFFFFFFFFFFFFFFFFFFFFFFFFFFFFFFFFFFFFFFFFFFFFFFFFFFFFFFFFFFF
FFFFFFFFFFFFFFF
@A01056:60:HMTK7DSXY:1:2205:27950:17080 2:N:0:TCCTTAGC+ACTCGATT
GACTACTTCAAATTGGTCAAAGTCAGGAGATTCAGATTGAGGAGGGATTTTCTGGATCCCATATCTAA
AGGTGATTCTCCCAGGAGGCAACTCTTCTTAGAATCAGCCAGTGACCCTAGGTCAAACCCTGAGGTTC
CCAGGAGTGGTGAAA
+
FFFFFFFFFFFFFFFFFFFFFFFFFFFFFFFFFFFFFFFFFFFFFFFFFFFFFFFFFF:FFFFFFFFF
FFFFF:FFFFFFFFFFFFFFFFFFFFFFFFFFFFFFFFFFFFFFFF:FFFFFFFFFFFFFFFFFFFFF
F:FFFFFFFFFFFFF
@A01056:60:HMTK7DSXY:1:2562:6162:19366 2:N:0:TCCTTAGC+ACTCGATT
AATTGGTCAAAGTCAGGAGATTCAGATTGAGGAGGGATTTTCTGGATCCCATATCTAAAGGTGATTCT
CCCAGGAGGCAACTCTTCTTAGAATCAGCCAGTGACCCTAGGTCAAACCCTGAGGTTCCCAGGAGTGG
TGAAATTAGAGAGGC
+
FFFFFFF:FF:FFFFFFFFFFFFFFF:FFFFFFFFFFFFFFFFFFFFFF:FFFFF:FFFFFFFFFFFF
FFFFFFFFFFFFFFFFFFFFFFFFFFFFFFFFF:FFFF:FF:FFFFFF:F:FFFFFFF,FFFFFFFFF
:FFF:F,FFFFFFFF
@A01056:60:HMTK7DSXY:1:2423:19913:15687 2:N:0:TCCTTAGC+ACTCGATT
AATTGGTCAAAGTCAGGAGATTCAGATTGAGGAGGGATTTTCTGGATCCCATATCTAAAGGTGATTCT
CCCAGGAGGCAACTCTTCTTAGAATCAGCCAGTGACCCTAGGTCAAACCCTGAGGTTCCCAGGAGTGG
TGAAATTAGAGAGGC
+
FFFFFFFFFFFFFFFFFFFFFFFFFFFFFFFFFFFFFFFFFFFFFFFFFFFFFFFFFFFFFFFFFFFF
FFFFFFFFFFFFFFFFFFFF:FFFFFFFFFFFFFFFFFFFFFFFF:FFFF:FFFFFFFFFFFFFFFFF
,FFF,F:FFFFF:FF
@A01056:60:HMTK7DSXY:1:1302:5620:18490 2:N:0:TCCTTAGC+ACTCGATT
GGAGATTCAGATTGAGGAGGGATTTTCTGGATCCCATATCTAAAGGTGATTCTCCCAGGAGGCAACTC
TTCTTAGAATCAGCCAGTGACCCTAGGTCAAACCCTGAGGTTCCCAGGAGTGGTGAAATTAGAGAGGC
AGAACCATTGGTCAA
+
FFFFFFFFFFFFFFFFFFFFFFFFFFFFFFFFFFFFFFFFFF:FF:FFFFFFFFFFFFFFFFFFFFFF
FFF:,FFFFFFFFFF:FFFFFFFFFFFFFFFFFFFFFFFFFFFFF:FFFFFFFFFFFFFFF:FFFFFF
FFFFFFFF,FFFFFF

```
@A01056:60:HMTK7DSXY:1:2205:27950:17080 1:N:0:TCCTTAGC+ACTCGATT
GAGATTCAGATTGAGGAGGGATTTTCTGGATCCCATATCTAAAGGTGATTCTCCCAGGAGGCAACTCT
TCTTAGAATCAGCCAGTGACCCTAGGTCAAACCCTGAGGTTCCCAGGAGTGGTGAAATTAGAGAGGCA
GAACCATTGGTCAAT
+
FFFFFFFFFFFFFFFFFFFFFF,FFFFFFFFFFFFFFFFFFFFFFFFFFFFFFFFFFFFFFFFFFFFF
FFFFFFFFFFFFFFFFFFFFFFFFFFFFFFFFFFFFFFFF:FFFFFFFFFFFFFFFFFFFFFFFFFFF
FFFFFFFFFFFFFFF
@A01056:60:HMTK7DSXY:1:1311:22046:5948 2:N:0:TCCTTAGC+ACTCGATT
CTGGATCCCATATCTAAAGGTGATTCTCCCAGGAGGCAACTCTTCTTAGAATCAGCCAGTGACCCTAG
GTCAAACCCTGAGGTTCCCAGGAGTGGTGAAATTAGAGAGGCAGAACCATTGGTCAATGACGAGAGCT
GCCAGTCACCAAGAG
+
FFFFFFFFFFFFFFFFFFFFFFFFFFFFFFFFFFF:FFFFFFFFFFFFFFFFFFFFFFFFFFFFFFFF
FFFFFFFFFFFFFFFFFFFFFFFFFFFFFFFFFFFFFFFFFFFFFFFFFFFFFFFFFF:FFFFFFFFFF
FFFFFFFFFFFF:FF
@A01056:60:HMTK7DSXY:1:1661:8223:28166 1:N:0:TCCTTAGC+ACTCGATT
GATCCCATATCTAAAGGTGATTCTCCCAGGAGGCAACTCTTCTTAGAATCAGCCAGTGACCCTAGGTC
AAACCCTGAGGTTCCCAGGAGTGGTGAAATTAGAGAGGCAGAACCATTGGTCAATGACGAGAGCTGCC
AGTCACCAAGAGAGC
+
FFFFFFFFFFFFFFFFFFFFF:FFFFFFFFFFFFFFFFFFFFFFFFFFFFFFFFFFFFFFFFFFFFFFF
FFFFFFFFFFFFFFFFFFFFFFFFFFFFFFFFFFFFFFFFFFFFFFFFFFFFFFFFFFFFFFFFFFFFF
FFFFFFFFFFFFFFF
@A01056:60:HMTK7DSXY:1:1302:5620:18490 1:N:0:TCCTTAGC+ACTCGATT
CCATATCTAAAGGTGATTCTCCCAGGAGGCAACTCTTCTTAGAATCAGCCAGTGACCCTAGGTCAAAC
CCTGAGGTTCCCAGGAGTGGTGAAATTAGAGAGGCAGAACCATTGGTCAATGACGAGAGCTGCCAGTC
ACCAAGAGAGCTTTG
+
FFFFFFFFF:FF:FFFFFFFFFFFFFFFFFFFFFFFFFFF:FFFFFFFFFFFF:FFFFFFFFFFFFFF
FFFFFFF:FFFFFFF,F:FFFFFFFFFFFFFFFFFFFF:FFFFFFFFFFFFFFFFFFFFFFFFFFFFFFF
FFFFFFFFFFFFFFF
@A01056:60:HMTK7DSXY:1:2423:19913:15687 1:N:0:TCCTTAGC+ACTCGATT
GAGGCAACTCTTCTTAGAATCAGCCAGTGACCCTAGGTCAAACCCTGAGGTTCCCAGGAGTGGTGAAA
TTAGAGAGGCAGAACCATTGGTCAATGACGAGAGCTGCCAGTCACCAAGAGAGCTTTGTGTGCAAATG
TCCACAGAGGACGGC
+
FFFFFFFFFFFFFFFFFFFFFFFFFFFFFFFFFFFFFFFFFFFFFFFFFFFFFFFFFFFFFFFFFFFFF
FFFFFFFFFFFFFFFFFFFFFFFFFFFFFFFFFFFFFFFFFFFFFFFFFFFFFFFFFFFFFFFFFFFFF
FFFFFFFFFFFFFFF
@A01056:60:HMTK7DSXY:1:2562:6162:19366 1:N:0:TCCTTAGC+ACTCGATT
GAGGCAACTCTTCTTAGAATCAGCCAGTGACCCTAGGTCAAACCCTGAGGTTCCCAGGAGTGGTGAAA
TTAGAGAGGCAGAACCATTGGTCAATGACGAGAGCTGCCAGTCACCAAGAGAGCTTTGTGTGCAAATG
TCCACAGAGGACGGC
+
FFFFFFFFFFF::FFFFFFFFFFFFFFFFFFFFFFFFFFFFFFFFF,FFF:FFFF:F:FF:FFFFFFFFF
:FFFFFFFFFFFFFF:FFFFFFFFFFFFFFFFFFFFFFFFFFFFFFFFFFFFFF:,FFFFFFFFFFFFFF
FFFFFFFFFFFFF:F
@A01056:60:HMTK7DSXY:1:2673:4616:26991 2:N:0:TCCTTAGC+ACTCGATT
CCTAGGTCAAACCCTGAGGTTCCCAGGAGTGGTGAAATTAGAGAGGCAGAACCATTGGTCAATGACGA
GAGCTGCCAGTCACCAAGAGAGCTTTGTGTGCAAATGTCCACAGAGGACGGCCAAGGTTTCGAAGGTT
CAATTCCAATTGATT
+
FFFFFFFFFFFFFFFFFFFFFFFFFFFFFFFFFFFFFFFF:FFFFFFFFFFFFFFFFFFFFFFFFFFF
```

FFFFFFFFFFFFFFFFFFFFFFFFFFFFFFFFFFFFFFFFFFFFFFFFFFFFFFFFFFFFFFFFFFFF
FFFFFFFFFFFFFFF
@A01056:60:HMTK7DSXY:1:1616:5141:5635 2:N:0:TCCTTAGC+ACTCGATT
GTGAAATTAGAGAGGCAGAACCATTGGTCAATGACGAGAGCTGCCAGTCACCAAGAGAGCTTTGTGTG
CAAATGTCCACAGAGGACGGCCAAGGTTTCGAAGGTTCAATTCCAATTGATTTGATCAATTGTTTTGA
ACCAGAAGAAGTCAA
+
FFFFFFFFFFFFFFFFFFFFFFFFFFFFFFFFFFFFFFFFFFFFFFFFFFFFFFFFFFFFFFFFFFFF
FFFFFFFFFFFFFFFFFFFFFFFFFFFFFFFFFFFFFFFFFFFFFFFFFFFFFFFFFFFFFFFFFFFF
FFFFFFFFFF:FFFF
@A01056:60:HMTK7DSXY:1:1103:13304:26130 2:N:0:TCCTTAGC+ACTCGATT
GTGAAATTAGAGAGGCAGAACCATTGGTCAATGACGAGAGCTGCCAGTCACCAAGAGAGCTTTGTGTG
CAAATGTCCACAGAGGACGGCCAAGGTTTCGAAGGTTCAATTCCAATTGATTTGATCAATTGTTTTGA
ACCAGAAGAAGTCAA
+
F:FF,FFFFFFFFFFFFFFFFFFFFFFFFFFFFFFFFFFFFFFFFFFFFFFFFFFFFFFFFFFFFFFF
FFFFFFFFFFFFFFFFFFFFFFFFFFFFFFFFFFFFFFFFFFFFFFFFFFFFFFFFFFFFFFFFFFFF
FFFFFFFFFFFFFFF
@A01056:60:HMTK7DSXY:1:1616:5141:5635 1:N:0:TCCTTAGC+ACTCGATT
GCTGCCAGTCACCAAGAGAGCTTTGTGTGCAAATGTCCACAGAGGACGGCCAAGGTTTCGAAGGTTCA
ATTCCAATTGATTTGATCAATTGTTTTGAACCAGAAGAAGTCAAATGCCCAAGAAGAAAGAGGAAGAA
TGATTGTGTTTTTAG
+
FFFFFFFFFFFFF:FFFFFFFFFFFFFFFFFFFFFFFFFFFFFFFFF:FF:FFFFFFFFFFFFFFFFF
FFFFFFFFFFFFFFFFFFFFFFFFFFFFFFFFFFFFFFFFFFFFFFFFFFFFFFFFFFFFFFFFFFFF
:FFFFFFFFFFFFFF
@A01056:60:HMTK7DSXY:1:1103:13304:26130 1:N:0:TCCTTAGC+ACTCGATT
GCTGCCAGTCACCAAGAGAGCTTTGTGTGCAAATGTCCACAGAGGACGGCCAAGGTTTCGAAGGTTCA
ATTCCAATTGATTTGATCAATTGTTTTGAACCAGAAGAAGTCAAATGCCCAAGAAGAAAGAGGAAGAA
TGATTGTGTTTTTAG
+
F:FF,FFFFFFFF:FFFFF::F:FFFFFFF:FFFFF:FFFFFFFFFF::FFFFFFFFFF:FF:FFFFF
FFFF::FFFF:FFFFFFFFFFFFFF:FFFFFFFFFFFFFFFFFFF:FFFFFFFFFFFFFFFFFFFFFF
FFFFFFFFFFFFFFF
@A01056:60:HMTK7DSXY:1:1311:22046:5948 1:N:0:TCCTTAGC+ACTCGATT
TGCCAGTCACCAAGAGAGCTTTGTGTGCAAATGTCCACAGAGGACGGCCAAGGTTTCGAAGGTTCAAT
TCCAATTGATTTGATCAATTGTTTTGAACCAGAAGAAGTCAAATGCCCAAGAAGAAAGAGGAAGAATG
ATTGTGTTTTTAGGG
+
FFFFFFFFFFFFFFFFFFFFFFFFFFFFFFFFFFFFFFFFFFF:FFFFFFFFFFFFFFFFFFFFFFFF
FFFFFFFFFFFFFFFFFFFFFF,FFFFFFFFFFF:FFFFFFFFFFFFFFFFFFFFFFFFFFFFFFFFF
FFFFFFFFFFFFFFF
@A01056:60:HMTK7DSXY:1:2151:5755:9142 2:N:0:TCCTTAGC+ACTCGATT
CACCAAGAGAGCTTTGTGTGCAAATGTCCACAGAGGACGGCCAAGGTTTCGAAGGTTCAATTCCAATT
GATTTGATCAATTGTTTTGAACCAGAAGAAGTCAAATGCCCAAGAAGAAAGAGGAAGAATGATTGTGT
TTTTAGGGCTATCTC
+
FFFFFFFFFFFFFFFFFFFFFFFFFFFFFFFFFFFFFFFFFFFFFFFFFFFFFFFFFFFFFFFFFFFF
FFFFFFFFFFFFFFFFFFFFFFFFFFFFFFFFFFFFFFFF:FFFFFFFFFFFFF,FFFFFFFFFFFFF
FFFFFFFFFFFFFFF
@A01056:60:HMTK7DSXY:1:2673:4616:26991 1:N:0:TCCTTAGC+ACTCGATT
CCACAGAGGACGGCCAAGGTTTCGAAGGTTCAATTCCAATTGATTTGATCAATTGTTTTGAACCAGAA
GAAGTCAAATGCCCAAGAAGAAAGAGGAAGAATGATTGTGTTTTTAGGGCTATCTCTGCACACTTGGG
AATTGAAACCCAGGA

+
FFFFFF:FFFFFFFFFF:FFFFFFFF::FFFFFFFFFFFFFFFFFFFFFFFFFFF:FFFFFFFFFFFF
FFFFFFFFFFFFFFFFFF:FFFFFFFFFFFFF:FFFFFFFFFFFFFFFFFFFFFFFFFFFFFFFFFFF
FFFFFFFFFFFFFFF
@A01056:60:HMTK7DSXY:1:2151:5755:9142 1:N:0:TCCTTAGC+ACTCGATT
CAATTGTTTTGAACCAGAAGAAGTCAAATGCCCAAGAAGAAAGAGGAAGAATGATTGTGTTTTTAGGG
CTATCTCCGCACACTTGGGAATTGAAACCCAGGACTTGTTGAATTTCCTCGTTAATGAGGATATATCT
GAAGAACTAATGGAC
+
,FFFFFFFFFFFFFFFFFFFFFFFFFF:FFFFFFFFFFFF,FFFFFFFF:FFFFFFFFFFFFFFFFFF
FFFFFFFFFFFFFFFFFFFFFFFFFFFFFFFFFFFFFFFFFFFFFFFFFFFFFFFFFFFFFFFFFFFF
FFFFFFFFFFFFFFF
@A01056:60:HMTK7DSXY:1:1269:13404:23046 2:N:0:TCCTTAGC+ACTCGATT
TGATTGTGTTTTTAGGGCTATCTCTGCACACTTGGGAATTGAAACCCAGGACTTGTTGAATTTCCTCG
TTAATGAGGATATATCTGAAGAACTAATGGACTGCATTGATGAAGACAAAGGACTCTCACACGAAATG
ATTGAAGAGGTGTTA
+
FFFFFFFFFFFFFFFFFFFFFFFFFFFFFFFFFFFFF:FFFFFFFFFFFFFFFFFFFFFFFFFFFFFF
FFFFFFFFFFFFFFFFFFFFFFFFFFFFFFFFFFFFFFFFFFFFFFFFFFFFFFFFFFFFF:FFF:FF
FFFFFFFFFFFFFFF
@A01056:60:HMTK7DSXY:1:1423:30219:19883 2:N:0:TCCTTAGC+ACTCGATT
TGAAACCCAGGACTTGTTGAATTTCCTCGTTAATGAGGATATATCTGAAGAACTAATGGACTGCATTG
ATGAAGACAAAGGACTCTCACACGAAATGATTGAAGAGGTGTTAGTCACAAAAGGTCTTTCAATGGTT
TATACTTCAGACTTC
+
FFFFFFFFFFFFFFFFFFFFFFFFFFFFFFFFFFFFFFFFFFFFFFFFFFFFFFFFFFFFFFFFFFFF
FFFFFFFF:FFFFFFFFFFFFFFFFFFFFFFFFFFFFFFFFFFFFFFFFFFFFFFFFFFFFFFFFFFF
FFFFFFFFFFFFFFF
@A01056:60:HMTK7DSXY:1:2220:6569:18004 2:N:0:TCCTTAGC+ACTCGATT
CCAGGACTTGTTGAATTTCCTCGTTAATGAGGATATATCTGAAGAACTAATGGACTGCATTGATGAAG
ACAAAGGACTCTCACACGAAATGATTGAAGAGGTGTTAGTCACAAAAGGTCTTTCAATGGTTTATACT
TCAGACTTCAAAGAG
+
FFFFFFF:FFFFFFFFFFFFFFFFFFFFFFFFFFFFFFFFFFFFFFFFFFFFFFFFFFFFFFFFF,FF
FFFFFFFFFFFFFFFFFFFFFFFFFFF:FFFFFFFFFFFFFFFFFFF:FFFFFFFFFFFFFFFFFFFF
F:F:FFFFFFFFFFF
@A01056:60:HMTK7DSXY:1:1220:9290:12571 2:N:0:TCCTTAGC+ACTCGATT
CCAGGACTTGTTGAATTTCCTCGTTAATGAGGATATATCTGAAGAACTAATGGACTGCATTGATGAAG
ACAAAGGACTCTCACACGAAATGATTGAAGAGGTGTTAGTCACAAAAGGTCTTTCAATGGTTTATACT
TCAGACTTCAAAGAG
+
FFFFFFFFFFFFFFFFFFFFFFFFF:FFFFFFFFFFFFFFFFFFFFFFFFFFFFFFFFFFFFFFFFFF
FFFFFFFFFFFFFFFFFFFFFFFF:FFFFF,FFFFFFFFFFFFFFFFFFFFFFFFFFFFFFFFFFFFF
FFFFFFFFFFFFFFF
@A01056:60:HMTK7DSXY:1:1522:6108:10410 2:N:0:TCCTTAGC+ACTCGATT
TGAATTTCCTCGTTAATGAGGATATATCTGAAGAACTAATGGACTGCATTGATGAAGACAAAGGACTC
TCACACGAAATGATTGAAGAGGTGTTAGTCACAAAAGGTCTTTCAATGGTTTATACTTCAGACTTCAA
AGAGATGGCAGTCCT
+
FFFFFFFFFFFFFFFFFFFFF:FFFFFFFFFFFFFFFFFFFFFFFFFFFFFFF:FFFFFFFFF:FFFF
FFFFFFFFFFF:FFF::FFFFFFF:FFFFFFFFF:FFF,FFFFFFF:FFF,F,F::F:F:FFFFF:,F
:,::FFFFFFFF:FF
@A01056:60:HMTK7DSXY:1:1269:13404:23046 1:N:0:TCCTTAGC+ACTCGATT
AATGAGGATATATCTGAAGAACTAATGGACTGCATTGATGAAGACAAAGGACTCTCACACGAAATGAT

TGAAGAGGTGTTAGTCACAAAAGGTCTTTCAATGGTTTATACTTCAGACTTCAAAGAGATGGCAGTCC
TCAACAGAAAATATG
+
F:FFFFFFFFFFFFFFFFFFFFFFFFFFFFFFFFFFFFFFFFFFFFFFFFFFFFFFFFFFFFFFFFFFFF
FFFFFFFFFFFFFFFFFFFFFFFFFFFFFFFFFFFFFFFFFFFFF:FFFFFFFFFFFFFFFFFFFFFFFF
FFFFFFFFFFFFFFF
@A01056:60:HMTK7DSXY:1:2453:2935:21355 2:N:0:TCCTTAGC+ACTCGATT
AAGAACTAATGGACTGCATTGATGAAGACAAAGGACTCTCACACGAAATGATTGAAGAGGTGTTAGTC
ACAAAAGGTCTTTCAATGGTTTATACTTCAGACTTCAAAGAGATGGCAGTCCTCAACAGAAAATATGG
TGTGAATGGCAAAAT
+
FFFFFFFFFFFFFFFFFFFFFFFFFFFFFFFF:FF:FFFFFFFFFFFFFFFFFFFFFFFFFFFFFFFFFF
FFFFFFFFFFFFFFFFFFFFFFFFFFFFFFFFFFFF:FFFFFFF:FF:FFFFFFFFFFFFFFFFFFFFFF
FFFFF:FFFFFF:FF
@A01056:60:HMTK7DSXY:1:1123:3848:1548 1:N:0:TCCTTAGC+ACTCGATT
TGAAGACAAAGGACTCTCACACGAAATGATTGAAGAGGTGTTAGTCACAAAAGGTCTTTCAATGGTTT
ATACTTCAGACTTCAAAGAGATGGCAGTCCTCAACAGAAAATATGGTGTGAATGGCAAAATGTACTGC
ACCATCAAAGGCAAC
+
F:FFFFFFFF:FFFFFFFFFFFFFFFFFFFFFFFFFFFFFFFFFFFFFFFFFFFFFFFFFFF:FFFFFFF
FFFFFFFFFFFFFFFFFFFFFFFFFFFFFFFFFFFFFFFFFFFFFFFFFFFFFFFFFFFFFFFFFFFFFF
FFFFFFFFFFFFFFF
@A01056:60:HMTK7DSXY:1:1123:3848:1548 2:N:0:TCCTTAGC+ACTCGATT
TGAAGACAAAGGACTCTCACACGAAATGATTGAAGAGGTGTTAGTCACAAAAGGTCTTTCAATGGTTT
ATACTTCAGACTTCAAAGAGATGGCAGTCCTCAACAGAAAATATGGTGTGAATGGCAAAATGTACTGC
ACCATCAAAGGCAAC
+
FFFFFFFFFFFFFFFFFFFFFFFFFFFFFFFFFFFFF:FFFFFFFFFFFFFFFFFFFFFFFFFFFFFFFF
FFFFFFFFFFFFFFFFFFFFFFFFFFFFFFFFFFFFFFFFFFFFFFFFFFFFFFFFFFFFFFFFFFFFFF
FFFFFFFF:FFFFFF
@A01056:60:HMTK7DSXY:1:1220:9290:12571 1:N:0:TCCTTAGC+ACTCGATT
TCTCACACGAAATGATTGAAGAGGTGTTAGTCACAAAAGGTCTTTCAATGGTTTATACTTCAGACTTC
AAAGAGATGGCAGTCCTCAACAGAAAATATGGTGTGAATGGCAAAATGTACTGCACCATCAAAGGCAA
CCACTGTGAACTGAG
+
FFFFFFFFFFFFFFFFFFFFFFFFFFFFFFFFFFFFFF:FFFFFFFF:FFFFF:FFFFFFFFFFFFFFFF
FFFFFFFFFFFFFFFFFFFFFFFFFFFFFFFFFFFFFFFFFFFFF:FFFFFFFFFFFFFFFFFFFFFFFF
FFFFFFFFFFFFFFF
@A01056:60:HMTK7DSXY:1:2220:6569:18004 1:N:0:TCCTTAGC+ACTCGATT
TCTCACACGAAATGATTGAAGAGGTGTTAGTCACAAAAGGTCTTTCAATGGTTTATACTTCAGACTTC
AAAGAGATGGCAGTCCTCAACAGAAAATATGGTGTGAATGGCAAAATGTACTGCACCATCAAAGGCAA
CCACTGTGAACTGAG
+
FFFFFFFFFFFFFFFFFFFFFFFF:FFFFFFFFFFFFFFFF:FFFF:FFFF:FFFFFFFFFFFFFFFFFF
FFFFFFFFFFFFFFFFFFFFFFFF:FFFFFFFFFFFFFFFFFFFFFFFFFFFFFFFFFFFFFFFFFFFFF
FFFFFFFFFFFFFFF
@A01056:60:HMTK7DSXY:1:1423:30219:19883 1:N:0:TCCTTAGC+ACTCGATT
TCTCACACGAAATGATTGAAGAGGTGTTAGTCACAAAAGGTCTTTCAATGGTTTATACTTCAGACTTC
AAAGAGATGGCAGTCCTCAACAGAAAATATGGTGTGAATGGCAAAATGTACTGCACCATCAAAGGCAA
CCACTGTGAACTGAG
+
FFFFFFFFFFFFFFFFFFFFFFFFFFFFFFFFFFFFFFFFFFFFFFFFFFFFFFFFFFFFFFFFFFFFFF
FFFFFFFFFFFFFFFFFFFFFFFFFFFFFFFFFFFFFFFFFFFFFFFFFFFFFFFFFFFFFFFFFFFFFF
FFFFFFFFFFFFFFF

```
@A01056:60:HMTK7DSXY:1:2453:2935:21355 1:N:0:TCCTTAGC+ACTCGATT
TCTCACACGAAATGATTGAAGAGGTGTTAGTCACAAAAGGTCTTTCAATGGTTTATACTTCAGACTTC
AAAGAGATGGCAGTCCTCAACAGAAAATATGGTGTGAATGGCAAAATGTACTGCACCATCAAAGGCAA
CCACTGTGAACTGAG
+
:FFFFFFFFFFFFFFFFFFFFFFFFFFFFF:FFFFFFFFFFFFFFFFFFFFF:FFFFFFFFFFFFFFFF
FFFFFFFFFFFFFFFFFFFFFFFFFF:FFFFFFFFFFFFFFFFFFFFFFFFFFFFFFFFFFFFFFFFFF
FFFFFFFFFFFFFFF
@A01056:60:HMTK7DSXY:1:2417:29369:11710 2:N:0:TCCTTAGC+ACTCGATT
GGTGAAGCTCAAATGTCTAATGAAAACTTAAATGCTGACTCCATGTTTGACCTTGGAAAATTTGTGCA
TAACAGGGAGAGGGCCGTTAAGTTGGCAAAATCAATGGCGAGAGGTACTACAGGTCTCTTGAATTCCT
TTGATCCGGCCTTCT
+
FFFFFFFFFFFFFFFFFFFFFFFFFFFFFFFFFFFFFFFFFFFFFFFFF:FFFFFFFFF:FFFFFFFFF
FFFFFFFFFFFFFFFFFFFFFFF:FF:FFFFFF:FFFF:FFFFFFFF:FFFFFFFF,FFFFFF,F,:FF
FFFFF:FFFFF:FFF
@A01056:60:HMTK7DSXY:1:1242:7961:32409 2:N:0:TCCTTAGC+ACTCGATT
GGTGAAGCTCAAATGTCTAATGAAAACTTAAATGCTGACTCCATGTTTGACCTTGGAAAATTTGTGCA
TAACAGGGAGAGGGCCGTTAAGTTGGCAAAATCAATGGCGAGAGGTACTACAGGTCTCTTGAATTCCT
TTGATCCGGCCTTCT
+
FFFFFFFFFFFFFFFFFFFFFFFFFFFFFFFFFFFFFFFFFFFFFFFFFFFFFFFFFFFFFFFFFFFFF
FFFFFFFFFFFFFFFFFFFFFFFFFFFFFFFFFF,FFFFFFFFFFFF:FFFFFFFF:FFF:FFFFFFFF
FFFFFFFFFFFFFFF
@A01056:60:HMTK7DSXY:1:2250:24343:20948 2:N:0:TCCTTAGC+ACTCGATT
ATTTGTGCATAACAGGGAGAGGGCCGTTAAGTTGGCAAAATCAATGGCGAGAGGTACTACAGGTCTCT
TGAATTCCTTTGATCCGGCCTTCTGCAAGAGCATGGTCACTCTTTCAGAGTTGTTCCCTGAGAACTTT
TCATCTACTGTTGGA
+
FFFFFFFFFFFFFFFFFFFFFFFFFFFFFFFFFFFFFFFFFFFFFFFFFFFFFFFFFFFFFFFFFFFFF
FFFFFFFFFFFFFFFFFFFFFFFFFFFFFFFFFFFFFFFFFFFFFFFFFFFFFFFFFFFFFFFFFFFFF
FFFFFFFFFFFFFFF
@A01056:60:HMTK7DSXY:1:1268:5231:30311 2:N:0:TCCTTAGC+ACTCGATT
ATTTGTGCATAACAGGGAGAGGGCCGTTAAGTTGGCAAAATCAATGGCGAGAGGTACTACAGGTCTCT
TGAATTCCTTTGATCCGGCCTTCTGCAAGAGCATGGTCACTCTTTCAGAGTTGTTCCCTGAGAACTTT
TCATCTACTGTTGGA
+
FFFFFFFFFFFFFFFFFFFFFFFFFFFFFFFFFFFFFFFFFFFFFFFFFFFFFFFFFFFFFFFFFF,FF
FFFFFFFFFFFFFFFFFFFFFFFFFFFFFFFFFFFFFFFFFFFFFFFFFFFFFFFFFFFFFFFFFFFFF
FFFFFFFFFFFFFFF
@A01056:60:HMTK7DSXY:1:2417:29369:11710 1:N:0:TCCTTAGC+ACTCGATT
ACAGGGAGAGGGCCGTTAAGTTGGCAAAATCAATGGCGAGAGGTACTACAGGTCTCTTGAATTCCTTT
GATCCGGCCTTCTGCAAGAGCATGGTCACTCTTTCAGAGTTGTTCCCTGAGAACTTTTCATCTACTGT
TGGACTGAGATTGGG
+
FFFFFFFFFFFFF:FFF,:FFFFF:FFFFFF:FFFFFFFFFFFFFFFF:F:FF:FFFFFFFFFFFFFF
FFFFFFF:FFFFFFFFFFFF:FFFFFFFFF:FFFFFFF:FFFFFFFFFFFFFFFFFFFFFFFFFFFFFF
FFFFFFFFFFFFFFF
@A01056:60:HMTK7DSXY:1:1242:7961:32409 1:N:0:TCCTTAGC+ACTCGATT
ACAGGGAGAGGGCCGTTAAGTTGGCAAAATCTATGGCGAGAGGTACTACAGGTCTCTTGAATTCCTTT
GATCCGGCCTTCTGCAAGAGCATGGTCACTCTTTCAGAGTTGTTCCCTGAGAACTTTTCATCTACTGT
TGGACTGAGATTGGG
+
:FFFFFFFFFFFFFFFFFFFFFFFFFFFFFFF,FFFFFFFFFFFFFFFFFFFFFFFFFFFFF:FFFFFF
```

```
FFFFFFFFFFFFFFFFFFFFFFFFFF:FFFFFFFFFFFFFFFFFFFFFFFFFFFFFFFFFFFFFFFFFFF
FFFFFFFFFFFFFFF
@A01056:60:HMTK7DSXY:1:2250:24343:20948 1:N:0:TCCTTAGC+ACTCGATT
TAAGTTGGCAAAATCAATGGCGAGAGGTACTACAGGTCTCTTGAATTCCTTTGATCCGGCCTTCTGCA
AGAGCATGGTCACTCTTTCAGAGTTGTTCCCTGAGAACTTTTCATCTACTGTTGGACTGAGATTGGGA
TTTGCTGGTTCAGGA
+
FFFFFFFFFFFFFFFFFF:FFFFFFFFFFFFFFFFFFFFFFFFFFFFFFFFFFFFFFFFFFFFFFFFFFF
FFFFFFFFFFFFFFFFFFFFFFFFFFFFFFFFFFFFFFFFFFFFFFFFFFFFFFFFFFFFFFFFFFFFFF
FFFFFFFFFFFFFFF
@A01056:60:HMTK7DSXY:1:1268:5231:30311 1:N:0:TCCTTAGC+ACTCGATT
TAAGTTGGCAAAATCAATGGCGAGAGGTACTACAGGTCTCTTGAATTCCTTTGATCCGGCCTTCTGCA
AGAGCATGGTCACTCTTTCAGAGTTGTTCCCTGAGAACTTTTCATCTACTGTTGGACTGAGATTGGGA
TTTGCTGGTTCAGGA
+
FFFFFFFFFFFFFFFFFFFFFFFFFFFFFFFFFFFFFFFFFFFFFFFFFFFFFFFFFFFFFFFFFFFFFF
FFFFFFFFFFFFFFFFFFFFFFFFFFFFFFFFFFFFFFFFFFFFFFFFFFFFFFFFFFFFFFFFFFFFFF
FFFFFFFFFFFFFFF
@A01056:60:HMTK7DSXY:1:1660:2600:31140 2:N:0:TCCTTAGC+ACTCGATT
GAGCATGGTCACTCTTTCAGAGTTGTTCCCTGAGAACTTTTCATCTACTGTTGGACTGAGATTGGGAT
TTGCTGGTTCAGGAAAAACTCACAAAGTGCTCCAATGGATCAACTACACACCGAGTGTTAAAAGAATG
TTTATAAGTCCAAGA
+
:FFFFFFFFFFFFFFFFFFFFFFFFFFFFFFFFFFFFFFFFFFFFFFFFFFFFFFFF:FFFFFFFFFFFF
FFFFFFFFFFFFFFFFFFFFFFFFFFFFFFFFFFFFFFFFFFFFFFFFFFFFF:FF:FFFFFFFFFFFFF
FFFFFFFFFFFFFFF
@A01056:60:HMTK7DSXY:1:1113:6515:28056 2:N:0:TCCTTAGC+ACTCGATT
GAGCATGGTCACTCTTTCAGAGTTGTTCCCTGAGAACTTTTCATCTACTGTTGGACTGAGATTGGGAT
TTGCTGGTTCAGGAAAAACTCACAAAGTGCTCCAATGGATCAACTACACACCGAGTGTTAAAAGAATG
TTTATAAGTCCAAGA
+
FFFFFFFFFFFFFFFFFFFFFFFFFFFFFFFFFFFFFFFFFFFFFFFFFFFFFFFFFFFFFFFFFFFFFF
FFFFFFFFFFFFFFFFFF,FFFFFFF:FFFFFFFFFFFFFF:FFFFFFFFFFFFFFFFFFFFFFFFFFFF
,F:FFFFFFFFFFFF
@A01056:60:HMTK7DSXY:1:1113:7337:28072 2:N:0:TCCTTAGC+ACTCGATT
GAGCATGGTCACTCTTTCAGAGTTGTTCCCTGAGAACTTTTCATCTACTGTTGGACTGAGATTGGGAT
TTGCTGGTTCAGGAAAAACTCACAAAGTGCTCCAATGGATCAACTACACACCGAGTGTTAAAAGAATG
TTTATAAGTCCAAGA
+
FFFFFFFFFFFFFFFFFFFFFFFFFFF:FFFFFFFFFFFFFFFFFFFFFFFFFFFFFFFFFFFFFFFFFF
FFFFFFFFFFFFFFFFFFFFFFFFFFFFFFFFFFFFFFFFFFFFFFFFFFFFFFFFFFFFFFFFFFFFFF
F:FFFFF::FFFFFF
@A01056:60:HMTK7DSXY:1:1351:24207:33708 1:N:0:TCCTTAGC+ACTCGATT
GCTCTTCCGATCTGGAAAAACTCACAAAGTGCTCCAATGGATCAACTACACACCGAGTGTTAAAAGAA
TGTTTATAAGTCCAAGAAGGATGTTAGCTGATGAAGTTGGGGTCCAGCTTAAAGGAACAGCCTGTCAA
GTGCATACTTGGGAG
+
FFFF:F:FFFFFFFFFFFFFFF:FFFFFFFFFFFFFFF:FFFFFFFFFFFFFFFFFFFFFFFFFFFFFFF
FFFFFFFFFFFFFFFFFFFFFFFFFFFFFFFFFFFFFFFFFFFFFFFFFFFFFFFFFFFFFFFFFFFFFF
FFFFFFFFFFFFFFF
@A01056:60:HMTK7DSXY:1:1351:8314:30921 1:N:0:TCCTTAGC+ACTCGATT
GCTCTTCCGATCTGGAAAAACTCACAAAGTGCTCCAATGGATCAACTACACACCGAGTGTTAAAAGAA
TGTTTATAAGTCCAAGAAGGATGTTAGCTGATGAAGTTGGGGTCCAGCTTAAAGGAACAGCCTGTCAA
GTGCATACTTGGGAG
```

+
FFFFFFFFFFFFFFFFFFFFFFFFFFFFFFFFFFFFFFFFFFFFFFFFFFFFFFFFFFFFFFFFFF:FFF
:FFFFFFFFFFFFFFFFFFFFFFFFFFFFFFFFFFFFFFFFFFFFFFFFFFFFFFFFFFFFFFFFFFFFF
FFFFFFFFFFFFFFF
@A01056:60:HMTK7DSXY:1:1351:24207:33708 2:N:0:TCCTTAGC+ACTCGATT
GGAAAAACTCACAAAGTGCTCCAATGGATCAACTACACACCGAGTGTTAAAAGAATGTTTATAAGTCC
AAGAAGGATGTTAGCTGATGAAGTTGGGGTCCAGCTTAAAGGAACAGCCTGTCAAGTGCATACTTGGG
AGAGATCGGAAGAGC
+
FFFFFFFFFFFFFFFFFFFFFFFFFFF:FFFFFFFFFFFFFFFFFFFFFFFFFFFFF,FFFFFFFFFFFF
FFFFFFFFFFFFFFFFFFFFFFFFFFFFFFFFFFFFFFFFF:FFFFFFFFFFFFFFFFFFFFFFFF:FFF
FFFFFFFFFFFFFFF
@A01056:60:HMTK7DSXY:1:1351:8314:30921 2:N:0:TCCTTAGC+ACTCGATT
GGAAAAACTCACAAAGTGCTCCAATGGATCAACTACACACCGAGTGTTAAAAGAATGTTTATAAGTCC
AAGAAGGATGTTAGCTGATGAAGTTGGGGTCCAGCTTAAAGGAACAGCCTGTCAAGTGCATACTTGGG
AGAGATCGGAAGAGC
+
FFFFFFFFFFFFFFFFFFFFFFFFFFFFFFFFFFFFFFFFFFFFFFFFFFFFFF:FFFFFFFFFFFFFFF
FFFFFFFFFFFFFFFFFFFFFFFFFFFFFFFFFFFFFFFFF:FFFFFFFFFFFFFFFFFFFF:FFFFFFF
FFFFFFFFFFFFFFF
@A01056:60:HMTK7DSXY:1:2331:2483:16689 2:N:0:TCCTTAGC+ACTCGATT
AAACTCACAAAGTGCTCCAATGGATCAACTACACACCGAGTGTTAAAAGAATGTTTATAAGTCCAAGA
AGGATGTTAGCTGATGAAGTTGGGGTCCAGCTTAAAGGAACAGCCTGTCAAGTGCATACTTGGGAGAC
CGCTTTAAAGAAGAT
+
FFFFFFFFFFFFFFFFFFFFFFFFFFFFFFFFFFFFFFFFFFFFFFFF::FFFFFFFFFFFFFFF:FFFF
FFF:FFFFFFFFFFFFFFFFFFFFFFFFFFFFFFF:FFFFFFFFFFFFFFFFFFFFFFFFFFFFFFFFFF
FFFFFFFFFFFFFFF
@A01056:60:HMTK7DSXY:1:2331:2483:16689 1:N:0:TCCTTAGC+ACTCGATT
CAAGAAGGATGTTAGCTGATGAAGTTGGGGTCCAGCTTAAAGGAACAGCCTGTCAAGTGCATACTTGG
GAGACCGCTTTAAAGAAGATTGATGGAACCTTCATGGAGGTTTTCATTGATGAAATAGGTCTGTACCC
ACCTGGGTTCCTCAC
+
FFFFFFFFFFFFFFFFFFFFFFFFFFFFFFFFFFFFFFFFFFFFFFFFFFFFFFFFFFFFFFFFFFFFFF
FFFFFFFFFFFFFFFFFFFFFFFFFFFFFFFFFFFFFFFFFFFFFFFFFFFFFFFFFFFFFFFFFFFFFF
FFFFFFFFFFFFFFF
@A01056:60:HMTK7DSXY:1:2110:21920:12868 2:N:0:TCCTTAGC+ACTCGATT
TGGGAGACCGCTTTAAAGAAGATTGATGGAACCTTCATGGAGGTTTTCATTGATGAAATAGGTCTGTA
CCCACCTGGGTTCCTCACATTGTTACAAATGTGTGCTTTTAGGAAGATTGTAAAAGGACAGATCGGAA
GAGCGTCGTGTAGGG
+
FFFFFFFFFFFFFFFFFFFFFFFF:FF:FFFFFFFFFFFFFF:FFFFFFFFFFF:FFFFFFFFFFF:FFF
FFFFFFFFFFFFFFFFFFFFFFFFFFFFFF:FFFFFFFFFFFFFFFFFFFFFFFFFFFFFFFFFFFFF:F
FFFFFFFFFF:FF:F
@A01056:60:HMTK7DSXY:1:2427:4616:22639 2:N:0:TCCTTAGC+ACTCGATT
TGGAACCTTCATGGAGGTTTTCATTGATGAAATAGGTCTGTACCCACCTGGGTTCCTCACATTGTTAC
AAATGTGTGCTTTTAGGAAGATTGTAAAAGGACAGAGTGAAAAGTTCTTGAAGGGTAAGCTCAGTGAG
CTGTCAAAAACCTGT
+
FFFFFFFFFFFFFFFFFFFFFFFFFFFFFFFFFFFFFFFFFFFFFFFFFFFFFFFFFFFFFFFFFFFFFF
FFFFFFFFFFFFFFFFFFFFFFFFFFFFFFFFFFFFFFFFFFFFFFFFFFFFFFFFFFFFFFFFFFFFFF
FFFFFFFFFFF:FFF
@A01056:60:HMTK7DSXY:1:2427:3595:21840 2:N:0:TCCTTAGC+ACTCGATT
TGGAACCTTCATGGAGGTTTTCATTGATGAAATAGGTCTGTACCCACCTGGGTTCCTCACATTGTTAC

```
AAATGTGTGCTTTTAGGAAGATTGTAAAAGGACAGAGTGAAAAGTTCTTGAAGGGTAAGCTCAGTGAG
CTGTCAAAAACCTGT
+
FFFFFFFFFFFFFFFFFFFFFFFFFFFFFFFFFFFFFFFFFFFFFFFFFFFFFFFFFFFFFFFFFFFF
FFFFFFFFF:FFFFFFFFFFFFFFFF::,FFFFFF:FFFFFFFFFFFFFFFFFFFFFFFFFFFFFFFF
FFFFFFF:FFFFFFF
@A01056:60:HMTK7DSXY:1:1440:31964:7936 1:N:0:TCCTTAGC+ACTCGATT
GATCTGTTTTCATTGATGAAATAGGTCTGTACCCACCTGGGTTCCTCACATTGTTACAAATGTGTGCT
TTTAGGAAGATTGTAAAAGGACAGAGTGAAAAGTTCTTGAAGGGTAAGCTCAGTGAGCTGTCAAAAAC
CTGTCTGAACATGAG
+
FFFFFFFFFFFFFFFFFFFFFFFFFFFFFFFF:FFFFFFFF,FFFFFFFFFFF:FFFFFFFFFFFFFF
FFFFFFFFFFFFFFF:FFFFFFFFFFFFFFFFF:FFFFFFFFFFFFFFFFFFFFFFFFFFFFFFFFFF
FFFFFFFFFFFFFFF
@A01056:60:HMTK7DSXY:1:1440:31964:7936 2:N:0:TCCTTAGC+ACTCGATT
GTTTTCATTGATGAAATAGGTCTGTACCCACCTGGGTTCCTCACATTGTTACAAATGTGTGCTTTTAG
GAAGATTGTAAAAGGACAGAGTGAAAAGTTCTTGAAGGGTAAGCTCAGTGAGCTGTCAAAAACCTGTC
TGAACATGAGAGATC
+
FFFFFFFFFFFFFFFFFFFFFFFFFFFFFFFFFFFFFFFFFFFF:FFFFFFFFFFFFFFFFFFF:FFF
FFFFFFFFFFFFFFFFFFFFFFFFFFFFFFFFFFFFFFFFFFFFFFFFFFFFFFFFFFFFFFFFFFFF
FFFFFFFFFFFFF:F
@A01056:60:HMTK7DSXY:1:2427:4616:22639 1:N:0:TCCTTAGC+ACTCGATT
TGTACCCACCTGGGTTCCTCACATTGTTACAAATGTGTGCTTTTAGGAAGATTGTAAAAGGACAGAGT
GAAAAGTTCTTGAAGGGTAAGCTCAGTGAGCTGTCAAAAACCTGTCTGAACATGAGGTGTTTTGGTGA
TCCATTACAATTGAG
+
FFFFFFFFFFFFFFFFFFFFFFFFFFFFFFFFFFFFFFFFFFFFFFFFFFFFFFFFFFFFFFFFFFFF
FFFFFFFFFFFFFFFFFFFFFFFFFFFFFFFFFFFFFFFFFFFFFFFFFFFFFFFFFFFFFFFFFFFF
FFFFFFFFFFFFFFF
@A01056:60:HMTK7DSXY:1:2427:3595:21840 1:N:0:TCCTTAGC+ACTCGATT
TGTACCCACCTGGGTTCCTCACATTGTTACAAATGTGTGCTTTTAGGAAGATTGTAAAAGGACAGAGT
GAAAAGTTCTTGAAGGGTAAGCTCAGTGAGCTGTCAAAAACCTGTCTGAACATGAGGTGTTTTGGTGA
TCCATTACAATTGAG
+
:FFFFFFFFFFFFFFFFFFFFFF:FFFFFFF:FFFFFFFFFFFFFFFF:FFFFFFFFFFFFFFFFFFF
FFFFFFFFFFFFFFFFFFFFFFFFFFFFFFFFFFFFFFFFFFFFFFFFFFFFFFFFFFFFFFFFFFFF
FFFFFFFFFFFFFFF
@A01056:60:HMTK7DSXY:1:2336:30689:9580 2:N:0:TCCTTAGC+ACTCGATT
GTGAAAAGTTCTTGAAGGGTAAGCTCAGTGAGCTGTCAAAAACCTGTCTGAACATGAGGTGTTTTGGT
GATCCATTACAATTGAGATACTACTCTGCTGAGTATACAAATCTTCTAGACAGAACACATGAGATTGA
CATTTTGATCAAGAC
+
FFFFFFFF:FFFFFFFFFFFFFFFFFFFFFFFFFFFFFFFFFFFFFFFFFFFFFFFFFFFFFFFFFFF
FFFF:FFFFFFFFFF:FFFFFFFFFFFFFFFFFFFFF:F:FF:FFFFFFFFFFFFFFFFFFFFFFFFF
FFFFFFFFFFFFFFF
@A01056:60:HMTK7DSXY:1:1566:27028:2519 2:N:0:TCCTTAGC+ACTCGATT
CTGTCAAAAACCTGTCTGAACATGAGGTGTTTTGGTGATCCATTACAATTGAGATACTACTCTGCTGA
GGATACAAATCTTCTAGACAGAACACATGAGATTGACATTTTGATCAAGACGATCAAGCACAAATACC
TTCTTCAGGGGTACA
+
FFFFFFFFFFFFFFFFFFFFFFFFFFFFFF:FFFFFFFFFFFFFFFFFFFFFFFFFFFFFFFFFFFFF
FFFFFFFFFFFFFFFFFFFFFFFFFFFFFFFFFFFFFFFFFFFFFFF:FFFFFFFFFFFFFFFFFFFF
FFFFFFFFFFFFFFF
```

```
@A01056:60:HMTK7DSXY:1:1566:27028:2519 1:N:0:TCCTTAGC+ACTCGATT
TACAATTGAGATACTACTCTGCTGAGGATACAAATCTTCTAGACAGAACACATGAGATTGACATTTTG
ATCAAGACGATCAAGCACAAATACCTTCTTCAGGGGTACAGATTCGGCCAATGGTTTCAGGAGCTCAT
AAACATGCCAACTAG
+
FFFFFFFFFFFFFFFFFFFFFFFFFFFFFFFFFFFFFFFFFFFFFFFFFFFFFFFFFFFFFFFFFFFF
FFFFFFFFFFFFFFFFFFFFFFFFF:FFFFFFFFFFFFFFFFFFFFFFFFFFFFFFFFFFFFFFFFFF
FFFFFFFFFFFFFFF
@A01056:60:HMTK7DSXY:1:2249:1108:27774 2:N:0:TCCTTAGC+ACTCGATT
ATGAGATTGACATTTTGATCAAGACGATCAAGCACAAATACCTTCTTCAGGGGTACAGATTCGGCCAA
TGGTTTCAGGAGCTCATAAACATGCCAACTAGGGTGGATGACTCAAAGTTCTCAAGAAAGTTCTTTGC
GGACATCTCCAGTGT
+
FFF:FFFFFFF:FFFFFFFFFFF::F:FFFFFFFFFFFFFF::FFFFFFFFFFFFFFFFFFFFFFFFF
FFFFFFFFF:FFFFFFFFF:F,FFFFFFFFFFFFFFFFFFF:FFFFFFFFFFFFFFFFFFFFFFFFFF
FFFFFFFFFFFFFFF
@A01056:60:HMTK7DSXY:1:2336:30689:9580 1:N:0:TCCTTAGC+ACTCGATT
GATCAAGACGATCAAGCACAAATACCTTCTTCAGGGGTACAGATTCGGCCAATGGTTTCAGGAGCTCA
TAAACATGCCAACTAGGGTGGATGACTCAAAGTTCTCAAGAAAGTTCTTTGCGGACATCTCCAGTGTG
AAGACTGAAGATTAC
+
FFFFFFFFFFFFFFFFFFFFFFFFFF:FFFFFFFFFFFFFFFFFFFFFFFFFFFFFFFFFFFFFFFFF
FFFFFFFFFFFFF:FFFFFFFFFFFFFFFF:F:FFFFF:FFFFFFFFFFFFFFFFFFFFFFFFFFFFF
FFFFFFFFFFFFFFF
@A01056:60:HMTK7DSXY:1:1432:10276:3474 2:N:0:TCCTTAGC+ACTCGATT
CGATCAAGCACAAATACCTTCTTCAGGGGTACAGATTCGGCCAATGGTTTCAGGAGCTCATAAACATG
CCAACTAGGGTGGATGACTCAAAGTTCTCAAGAAAGTTCTTTGCGGACATCTCCAGTGTGAAGACTGA
AGATTACGGGCTCAT
+
FF:FFFFFFFFFFFFFFFFFFFFFFFFFFFFFFFFFFFFFFFFFFFFFFFF,FFFFFFFFFFFFFFFF
FFFFFFFFFFFFFFFFFFFFFFFFFFFFFFFFFFFFFFFFFFFFFFFFFFFFFFFFFFFFFFFFFFFF
FFFFFFFFFFFFFFF
@A01056:60:HMTK7DSXY:1:1569:4924:33442 2:N:0:TCCTTAGC+ACTCGATT
GCACAAATACCTTCTTCAGGGGTACAGATTCGGCCAATGGTTTCAGGAGCTCATAAACATGCCAACTA
GGGTGGATGACTCAAAGTTCTCAAGAAAGTTCTTTGCGGACATCTCCAGTGTGAAGACTGAAGATTAC
GGGCTCATTCTCGTT
+
:FFFFFFFFFFFFFFFFFFFFFFFFFFFFFFFFFFFFFFFFFFFFFFFFFFFFFFFFFFFFFFFFFFF
FFFFFFFFFFFFFFFFFFFFFFFFFFFFFFFFFFFFFFFFFFFFFFFFFFFFFFFFFFFFFFFFFFFF
FFFFFFFFFFFFFFF
@A01056:60:HMTK7DSXY:1:1569:4924:33442 1:N:0:TCCTTAGC+ACTCGATT
TCTTCAGGGGTACAGATTCGGCCAATGGTTTCAGGAGCTCATAAACATGCCAACTAGGGTGGATGACT
CAAAGTTCTCAAGAAAGTTCTTTGCGGACATCTCCAGTGTGAAGACTGAAGATTACGGGCTCATTCTC
GTTGCCAAAAGGGAG
+
FFFFFFFFFFFFFFFFFFFFFFFFFFFFFFFFFFFFFFFFFFFFFFFFFFFFFFFFFFFFFFFFFFFF
FFFFFFFFFFFFFFFFFFFFFFFFFFFFFFFFFFFFFFFFFFFFFFFFFFFFFFFFFFFFFFFFFFFF
FFFFFF:FFFFFFFF
@A01056:60:HMTK7DSXY:1:1432:10276:3474 1:N:0:TCCTTAGC+ACTCGATT
GGTACAGATTCGGCCAATGGTTTCAGGAGCTCATAAACATGCCAACTAGGGTGGATGACTCAAAGTTC
TCAAGAAAGTTCTTTGCGGACATCTCCAGTGTGAAGACTGAAGATTACGGGCTCATTCTCGTTGCCAA
AAGGGAGGACAAAGG
+
FFFFFFFFFFFFFFF:FFFFFFFFFFFFFFFFFFFFFFFFFFFFFFFFFFFFFFFFFFFFFFFFFFFF
```

```
FFFFFFFFFF:FFFFFFFFFFFFFFFFFFFFFFFFFFFFFFFFFFFFFFFFFFFFFFFFFFFFFF:F
FFFFFFFFFFFFFFF
@A01056:60:HMTK7DSXY:1:2249:1108:27774 1:N:0:TCCTTAGC+ACTCGATT
GGGTGGATGACTCAAAGTTCTCAAGAAAGTTCTTTGCGGACATCTCCAGTGTGAAGACTGAAGATTAC
GGGCTCATTCTCGTTGCCAAAAGGGAGGACAAAGGTGTTTTTGCTGGGAGGGTCCCAGTGGCCACAGT
TAGCGAGTCTCAAGG
+
F:F:FFFFFFF:FFFFFFFF,FFFFFFFFF:FFFFFFFFFFFFFFFFFFFFFFFFFFFFFFFFFFFFF
FFFFFFFFFFFFFFFFFF:FFFFFFFF:FFFFFFF:FF:FFFFFFFFFFFFFFFFFFFFFFFFFFFFF
FFFFFFFFFFFFFFF
@A01056:60:HMTK7DSXY:1:2653:19515:20415 2:N:0:TCCTTAGC+ACTCGATT
GATGACTCAAAGTTCTCAAGAAAGTTCTTTGCGGACATCTCCAGTGTGAAGACTGAAGATTACGGGCT
CATTCTCGTTGCCAAAAGGGAGGACAAAGGTGTTTTTGCTGGGAGGGTCCCAGTGGCCACAGTTAGCG
AGTCTCAAGGGATGA
+
FFFFFFFFFFFFFFFFFFFFFFFFFFFFFFFFFFFFFFFFFFFFFFFFFFFFFFFFFFFFFFFFFFFF
FFFFFFFFFFFFFFFFFFFFFFFFFFFFFFFFFFFFFFFFFFFFFFFFFFFFFFFFFFFFFFFFFFFF
FFFFFFFFFFFFFFF
@A01056:60:HMTK7DSXY:1:2257:27959:5040 2:N:0:TCCTTAGC+ACTCGATT
CTCAAAGTTCTCAAGAAAGTTCTTTGCGGACATCTCCAGTGTGAAGACTGAAGATTACGGGCTCATTC
TCGTTGCCAAAAGGGAGGACAAAGGTGTTTTTGCTGGGAGGGTCCCAGTGGCCACAGTTAGCGAGTCT
CAAGGGATGACACTA
+
FFFFFFFFFFFFFFFFF:FFFFFF:FFFFFFFFFFFFFFFFFFF,FFFFFFFFFFFFFFFFFFFFFFF
FFFFFFFFFFFFFFFFFFFF,F,FFFFF:F,:FFFFFFFFFFFFFFFFFFFFFFFFFFFFFFFFFFFF
FFFFFFFFFFFF,F:
@A01056:60:HMTK7DSXY:1:1315:27868:13275 2:N:0:TCCTTAGC+ACTCGATT
CTCAAGAAAGTTCTTTGCGGACATCTCCAGTGTGAAGACTGAAGATTACGGGCTCATTCTCGTTGCCA
AAAGGGAGGACAAAGGTGTTTTTGCTGGGAGGGTCCCAGTGGCCACAGTTAGCGAGTCTCAAGGGATG
ACAATAAGCAAAAGA
+
F:::FFFF::,,FFFF:FFFF,FFFFFFFFF::,F:FF::FFF:FFFFFFF:FFFF,F,FFFFFFFF:
F,F,,FFF,F,FFF,F::FFFFFFF:FF:,F:FFFFF::FF,FFFFFFF:FFFFF,,FFFF:F,,F,,
FFFF,F:,F:FFF,F
@A01056:60:HMTK7DSXY:1:2467:29487:7091 2:N:0:TCCTTAGC+ACTCGATT
GAAGACTGAAGATTACGGGCTCATTCTCGTTGCCAAAAGGGAGGACAAAGGTGTTTTTGCTGGGAGGG
TCCCAGTGGCCACAGTTAGCGAGTCTCAAGGGATGACAATAAGCAAAAGAGTGTTGATATGTCTGGAT
CAAAACCTCTTTGCC
+
FFFFFFFFFFFFFFFFFFFFFFFFFFFFFFFFFFFFFFFFFFFFFFFFFFFFFFFFFFFFFFFFFFFF
FFFFFFFFFFFFFFFFFFFFFFFFFFFFFFFFFFFFFFFF:FFFFF:FFFFFFFFFFFFFFFFFFFFFF
FFFFFF:,FFFFFFF
@A01056:60:HMTK7DSXY:1:1461:25192:32033 2:N:0:TCCTTAGC+ACTCGATT
GAAGACTGAAGATTACGGGCTCATTCTCGTTGCCAAAAGGGAGGACAAAGGTGTTTTTGCTGGGAGGG
TCCCAGTGGCCACAGTTAGCGAGTCTCAAGGGATGACAATAAGCAAAAGAGTGTTGATATGTCTGGAT
CAAAACCTCTTTGCC
+
FFFFFFFFFFFFFFFFFFFFFFFFFFFFFFFFFFFFFF:FFFFFFFFFFFFFFFFFFFFFFFFFFFFF
FFFFFFFFFFFFFFFFFFFFFFFFFFFFFFFFFFFFFFFFFFFFFFFFFFFFFFFFFFFFFFFFFFFF
FFFFFFFFFFFFFFF
@A01056:60:HMTK7DSXY:1:1301:32570:11929 2:N:0:TCCTTAGC+ACTCGATT
GAAGACTGAAGATTACGGGCTCATTCTCGTTGCCAAAAGGGAGGACAAAGGTGTTTTTGCTGGGAGGG
TCCCAGTGGCCACAGTTAGCGAGTCTCAAGGGATGACAATAAGCAAAAGAGTGTTGATATGTCTGGAT
CAAAACCTCTTTGCC
```

```
+
FFFFFFFFFFFFFFFFFFFFFFFFFFFFFFFFFFFFFF:FFFFFFFFFFFFFFF,FFFFFFFFFFFFF
FFFF,FFFFFFFFFFFFFFFFFFFFFFFFFFFFFFFFFFFFFFFF,FFFFFFFFFFFFFFFFFFFFF,
FFFFFFFFFFFFFFF
@A01056:60:HMTK7DSXY:1:2257:27959:5040 1:N:0:TCCTTAGC+ACTCGATT
ATTACGGGCTCATTCTCGTTGCCAAAAGGGAGGACAAAGGTGTTTTTGCTGGGAGGGTCCCAGTGGCC
ACAGTTAGCGAGTCTCAAGGGATGACAATAAGCAAAAGAGTGTTGATATGTCTGGATCAAAACCTCTT
TGCCGGAGGGGCCAA
+
FFFFFFFFFFFFFFFFFFFFFFFFFFFFFFFFFFFFFFFFFFFFFFFFFFFFFFFFFFFFFFFFFFFF
FFFFFFFFFFFFFFFFFFFFFFFFFFFFFFFFFFFFFFFFFFFFFFFFFFFFFFFFFFFFFFFFFFFF
FFFFFFFFFFFFFFF
@A01056:60:HMTK7DSXY:1:1315:27868:13275 1:N:0:TCCTTAGC+ACTCGATT
AAAGGGAGGACAAAGGTGTTTTTGCTGGGAGGGTCCCAGTGGCCACAGTTAGCGAGTCTCAAGGGATG
ACAATAAGCAAAAGAGTGTTGATATGTCTGGATCAAAACCTCTTTGCCGGAGGGGCCAATGCCGCCAT
TGTTGCAATCACAAG
+
FF:FF,FF:::FFF,F:FFFF,FFFFFFFFFFFF:,:,F:F:FFFF,FFFFFFFFFFFFF:,FFFFFF
,FF:F,:F:,,FFFF:FFFF:,F,:,FFFF,:F:,FFF,FFF::FFFFF,F:,:,F::FFF::::FFF
FFFFFFFFFF:FF:,
@A01056:60:HMTK7DSXY:1:2653:19515:20415 1:N:0:TCCTTAGC+ACTCGATT
AAAGGGAGGACAAAGGTGTTTTTGCTGGGAGGGTCCCAGTGGCCACAGTTAGCGAGTCTCAAGGGATG
ACAATAAGCAAAAGAGTGTTGATATGTCTGGATCAAAACCTCTTTGCCGGAGGGGCCAATGCCGCCAT
TGTTGCAATCACAAG
+
FF:FFFFFFFFFFFFFFF,FFFFFFFFFFFFFFFFFFFFFFFFFFFFFFFFFFFFFFFFFFFFFFFFF
FFFFFFFFFFFFFFFFFFFFFFFFFFFFFFFFFFFFFFFFFFFFFFFFFF:FFFFFFFFFFFFFFFFF
FFFFFFFFFFFFFFF
@A01056:60:HMTK7DSXY:1:1301:32570:11929 1:N:0:TCCTTAGC+ACTCGATT
CACAGTTAGCGAGTCTCAAGGGATGACAATAAGCAAAAGAGTGTTGATATGTCTGGATCAAAACCTCT
TTGCCGGAGGGGCCAATGCCGCCATTGTTGCAATCACAAGGTCGAAAGTTGGATTTGACTTTATCCTC
AAAGGAAACTCACTA
+
FFFFFFFFFFFFFFFFFFFFFFFFFFFFFFFFFFFFFFFFFFFFFFFFFFFFFFFFFFFFFFFFFFFF
FFFFFFFFFFFFFFFFFFFFFFFFFFFFFFFFFFFFFFFFFFFFFFFFFFFFFFFFFFFFFFFFFFFF
FFFFFFFFFFFFFFF
@A01056:60:HMTK7DSXY:1:1461:25192:32033 1:N:0:TCCTTAGC+ACTCGATT
CACAGTTAGCGAGTCTCAAGGGATGACAATAAGCAAAAGAGTGTTGATATGTCTGGATCAAAACCTCT
TTGCCGGAGGGGCCAATGCCGCCATTGTTGCAATCACAAGGTCGAAAGTTGGATTTGACTTTATCCTC
AAAGGAAACTCACTA
+
FFFFFFFFFFFFFFFFFFFFFFFFFFFFFFFFFFFFFFFFFFFFFFFFFFFFFFFFFFFFFFFFFFFF
FFFFFFFFFFFFFFFFFFFFFFFFFFFFFFFFFFFFFFFFFFFFFFFFFFFFFFFFFFFFFFFFFFFF
FFFFFFFFFFFFFFF
@A01056:60:HMTK7DSXY:1:2467:29487:7091 1:N:0:TCCTTAGC+ACTCGATT
CACAGTTAGCGAGTCTCAAGGGATGACAATAAGCAAAAGAGTGTTGATATGTCTGGATCAAAACCTCT
TTGCCGGAGGGGCCAATGCCGCCATTGTTGCAATCACAAGGTCGAAAGTTGGATTTGACTTTATCCTC
AAAGGAAACTCACTA
+
FFFFFFFFFFFFFFFFF,FFFFFFFFFFFFFFFFFFFFFFFFFFFFFFFFFFFFFFFFFFFFFFFFFF
FFFFFFFFFFFFFFFFFFFFFFF:FFFFFFFFFFFFFFFFFFFFFFFFFFFFFFFFFFFFFFFFFFFF
FFFFFFFFFFFFFFF
@A01056:60:HMTK7DSXY:1:1178:29496:32503 1:N:0:TCCTTAGC+ACTCGATT
CGAGTCTCAAGGGATGACAATAAGCAAAAGAGTGTTGATATGTCTGGATCAAAACCTCTTTGCCGGAG
```

GGGCCAATGCCGCCATTGTTGCAATCACAAGGTCGAAAGTTGGATTTGACTTTATCCTCAAAGGAAAC
TCACTAAAGGAAGTA
+
FFFFFFFFFFFFFFFFFFFFFFFFFFFFFFFFFFFFFFFFFF:FFFFFFFFFF:FFFFFFFFFFFF
FFFFFFFFFFFFFFFFFFFFFFFFFFFFFFFFFFFFFFFFFFFFFFFFFFFFFFFFFFFFFFFF:FFFF
FFFFFFFFFFF:FF:
@A01056:60:HMTK7DSXY:1:2178:25961:22435 1:N:0:TCCTTAGC+ACTCGATT
CGAGTCTCAAGGGATGACAATAAGCAAAAGAGTGTTGATATGTCTGGATCAAAACCTCTTTGCCGGAG
GGGCCAATGCCGCCATTGTTGCAATCACAAGGTCGAAAGTTGGATTTGACTTTATCCTCAAAGGAAAC
TCACTAAAGGAAGTA
+
FFFFFFFFFFFFFFFFFFFFFFFFFFFFFFFFFFFFFFFFFFFFFFFFFFFFFFFFFFFFFFFFFFFF
FFFFFFFFFFFFFFFFFFFFFFFFFFFFFFFFFFFFFFFFFFFFFFFFFFFFFFFFFFFFFFFFFFFF
FFFFFFFFFFFFFFF
@A01056:60:HMTK7DSXY:1:2178:25961:22435 2:N:0:TCCTTAGC+ACTCGATT
CCTCAAAGGAAACTCACTAAAGGAAGTACAGAGGATGGCCCAAAAAACCATTTGGCAATTCATCATTG
AGGGTAAATCCATCCCAATGGAGAGAATAGTCAACATGAACCCTGGAGCCAGCTTTTATGAAAGCCCC
TTAGACGTTGGAAAT
+
FF,FFFF:FFFFFFFFFFFFF:FF:FFFFFFFFFFFFFFFFFFFFFFFFF:FFFFFFFF,FFFFFFFFF
FFFFFFFFFFFFFFFFFFFFFFFFFFFFFFFFFFFFFFFFFFFFFFFFFFFFFFFFFFFFFFFFFFFF
FFFFFFFFFFFFFFF
@A01056:60:HMTK7DSXY:1:1178:29496:32503 2:N:0:TCCTTAGC+ACTCGATT
CCTCAAAGGAAACTCACTAAAGGAAGTACAGAGGATGGCCCAAAAAACCATTTGGCAATTCATCATTG
AGGGTAAATCCATCCCAATGGAGAGAATAGTCAACATGAACCCTGGAGCCAGCTTTTATGAAAGCCCC
TTAGACGTTGGAAAT
+
FFF,FFFFFFF,FFFFFFFF,:FFFFF,FFFF:FFFFFFFFF:,FFFFFFFFFFFFFFFFFFFFFFFF
FFFFFFFF:FFFFFFFFFFFFFFFFFFFFFFFFFFFFFFFFFFFFFFFFFFFFFFFFF:FFFFFFFFF
FFFFFFFFFFFFFFF
@A01056:60:HMTK7DSXY:1:2374:6786:12900 2:N:0:TCCTTAGC+ACTCGATT
CCCAAAAAACCATTTGGCAATTCATCATTGAGGGTAAATCCATCCCAATGGAGAGAATAGTCAACATG
AACCCTGGAGCCAGCTTTTATGAAAGCCCCTTAGACGTTGGAAATTCGTCCATTCAGGACAAAGCTTC
TCATGACTTGTTTAT
+
FFFFFFFFFFFFFFFFFFFFFFFFFFF:FFFFFFFFFFFFFFFFFFFFFFFFFFFFFFFFFFFFFFFF
FFFFFFFFFFFFFFFFFFFFFFFFFFFFFF:FFFFFFFFFFFFFFFFFFFFF:FFFFFFFFFFFFF:F
FFFFFFFFF:FFFFF
@A01056:60:HMTK7DSXY:1:2374:6786:12900 1:N:0:TCCTTAGC+ACTCGATT
CCCAATGGAGAGAATAGTCAACATGAACCCTGGAGCCAGCTTTTATGAAAGCCCCTTAGACGTTGGAA
ATTCGTCCATTCAGGACAAAGCTTCTCATGACTTGTTTATAATGCCTTTCATAAATTTGGCAGAAGAA
GAGGTTGACCCAGAG
+
FF:F:F,FFFFFFFFFFFFF::FFFFFFFF::FFFF:F:FFFFFF,FFFFFFFFFFFFFF:FFFFFFF
FFFFFFFFFFFFFFFFFFFFFFFFFFFFFFFFFFF:FFFFFFFFFFFFFFFFFFFFFFFFFFFFFFFF
FFFFFFFFFFFFFFF
@A01056:60:HMTK7DSXY:1:1356:27028:19617 2:N:0:TCCTTAGC+ACTCGATT
CTTAGACGTTGGAAATTCGTCCATTCAGGACAAAGCTTCTCATGACTTGTTTATAATGCCTTTCATAA
ATTTGGCAGAAGAAGAGGTTGACCCAGAGGAAGTTGTTGGGGATGTGATACAACCTATTGAATGGTTT
AAATGCCACATACCT
+
FFFFFFFFFFFFFFFFFFFFFFFFFFFFFFFF,FFFFFFFFFFFFFFFFFFFFFFFFFFFFFFFFFF,F
FF:FFFFFFFFFFFFFFFFFFFFFFFFFFFFF,FFFFFFFFFFFFFFFFFFFF:FFFF:FFFFFFFFFFF
FFFFFFFFFFFF:FF

```
@A01056:60:HMTK7DSXY:1:1224:29830:21872 2:N:0:TCCTTAGC+ACTCGATT
CTTAGACGTTGGAAATTCGTCCATTCAGGACAAAGCTTCTCATGACTTGTTTATAATGCCTTTCATAA
ATTTGGCAGAAGAAGAGGTTGACCCAGAGGAAGTTGTTGGGGATGTGATACAACCTATTGAATGGTTT
AAATGCCACATACCT
+
FFFFFFFFFFFFFFFFFFFFFFFFFFFFFFFFFFFFFFFFF:FFFFFFFFFFFFFFFFFFFFFFFFFF
FFFFFFFFFFFFFFFFFFFFFFFFFFFFFFFFF:F,FFF:FFFFFFFFFF:FFFFFFF:FFFFFF:FF:F
FFFF:FFFFFFFFFF
@A01056:60:HMTK7DSXY:1:1356:27028:19617 1:N:0:TCCTTAGC+ACTCGATT
TCAGGACAAAGCTTCTCATGACTTGTTTATAATGCCTTTCATAAATTTGGCAGAAGAAGAGGTTGACC
CAGAGGAAGTTGTTGGGGATGTGATACAACCTATTGAATGGTTTAAATGCCACATACCTGTCTTCGAC
ACAGATCCAATGCTG
+
FFF,FFF:F,:FFFFFFFFFF,FFFFFFFFFFF,:FFFFF::FFFFF:FFFFFF:FFFFFFFFFFFFF
FFFFFFF:FFFFFFFFFFFFFF,FFFFFF,FFFFFFFFF,FFFFFFFFFFFFFFF:FFFFFFFFFF::
FFFF:FFFF:FFFFF
@A01056:60:HMTK7DSXY:1:1224:29830:21872 1:N:0:TCCTTAGC+ACTCGATT
TCAGGACAAAGCTTCTCATGACTTGTTTATAATGCCTTTCATAAATTTGGCAGAAGAAGAGGTTGACC
CAGAGGAAGTTGTTGGGGATGTGATACAACCTATTGAATGGTTTAAATGCCACATACCTGTCTTCGAC
ACAGATCCAATGCTG
+
FFFFFFFFFFFFFFFFFFFFFFFFFFFFFFFFFFFFFFFFFFFFFFFFFFFFFFFFFFFFFFFFFFFF
FFFFFFFFFFFFFFFFFFFFFFFFFFFFFFFFFFFFFFFFFFFFFFFFFFFFFFFFFFFFFFFFFFFF
FFFFFFFFFFFFFFF
@A01056:60:HMTK7DSXY:1:2339:13892:25019 2:N:0:TCCTTAGC+ACTCGATT
CAAAGCTTCTCATGACTTGTTTATAATGCCTTTCATAAATTTGGCAGAAGAAGAGGTTGACCCAGAGG
AAGTTGTTGGGGATGTGATACAACCTATTGAATGGTTTAAATGCCACATACCTGTCTTCGACACAGAT
CCAATGCTGGCAGAG
+
FFFFFFFFFFFFFFFFFFFFFFFFFFFFFFFFFFFFFFFFFF:FFFFFFFFFFFFFFFFFFFFFFFFFF
FFFFFFFFFFFFFFFFFFFFFFFFFFFFFFF:FFFFFFFFFFFFFFFFFFFF:FFFFFFFFFFFFFFF
FFFFFFFFFFFFFFF
@A01056:60:HMTK7DSXY:1:1539:12952:7200 2:N:0:TCCTTAGC+ACTCGATT
GCTTCTCATGACTTGTTTATAATGCCTTTCATAAATTTGGCAGAAGAAGAGGTTGACCCAGAGGAAGT
TGTTGGGGATGTTATACAACCTATTGAATGGTTTAAATGCCACATACCTGTCTTCGACACAGATCCAA
TGCTGGCAGAGATAT
+
FFFFFFFFFFFFFFFFFFFFFFFFFFFFFFFFFFFFFFFFFFFFFFFFFFFFFFFFFFFFFFFFFFFF
FFFFFFFFFFFFFFFFFFFF:FF:FFFFFFFFFFFFF:FFFFFFFFFF:FFFF:FFFFFFFFFFFFF
FFFFFFFFFFFFFFF
@A01056:60:HMTK7DSXY:1:1331:12897:20134 2:N:0:TCCTTAGC+ACTCGATT
CATGACTTGTTTATAATGCCTTTCATAAATTTGGCAGAAGAAGAGGTTGACCCAGAGGAAGTTGTTGG
GGATGTGATACAACCTATTGAATGGTTTAAATGCCACATACCTGTCTTCGACACAGATCCAATGCTGG
CAGAGATATTCGACA
+
FFFFFFFFFFFFFFFFFFFFFFFFFFFFFFFFFFFFFFFFFFFFFFFFFFFFFFFFFFFFFFFFFFFF
FFFFFFFFFFFFFFFFFFFFFFFFFFFFFFFF:FFFFFFFFFFFFFFFFFFFFFFFFFFFFFFFFFFF
FFFFFFFFFFFFFFF
@A01056:60:HMTK7DSXY:1:2339:13892:25019 1:N:0:TCCTTAGC+ACTCGATT
ATAAATTTGGCAGAAGAAGAGGTTGACCCAGAGGAAGTTGTTGGGGATGTGATACAACCTATTGAATG
GTTTAAATGCCACATACCTGTCTTCGACACAGATCCAATGCTGGCAGAGATATTCGACAAAGTAGCTG
CAAAAGAAAAGAGAG
+
FFFFFFF,FFFFFFFFFFFFFFFFFFFFFFFFFFFFFFFFFFFFFFFFFFFFFFFFFFFFFFFFFFFF
```

FFFFFFFFFFFFFFFFFFFFFFFFFFFFFFFFFFFFFFFFFFFFFFFFFF:FFFFFFFFFFFFFFFFF
FFFFFFFFFFFFFFF
@A01056:60:HMTK7DSXY:1:1539:12952:7200 1:N:0:TCCTTAGC+ACTCGATT
TAAATTTGGCAGAAGAAGAGGTTGACCCAGAGGAAGTTGTTGGGGATGTTATACAACCTATTGAATGG
TTTAAATGCCACATACCTGTCTTCGACACAGATCCAATGCTGGCAGAGATATTCGACAAAGTAGCTGC
AAAAGAAAAGAGAGA
+
FFFFFFFFFFFFFFFFFFFFFFFFFFFFFFFFFFFFFFFFFFFFFFFFFFFFFFFFFFFFFFFFFFFF
:FFFFFFFFFFFFFFFFFFFFFFFFFFFFFFFFFFFFFFFFFFFFFFFFFFFFFFFFFFFFFFFFFFF
FFFFFFFFFFFFFFF
@A01056:60:HMTK7DSXY:1:1331:12897:20134 1:N:0:TCCTTAGC+ACTCGATT
TAAATTTGGCAGAAGAAGAGGTTGACCCAGAGGAAGTTGTTGGGGATGTGATACAACCTATTGAATGG
TTTAAATGCCACATACCTGTCTTCGACACAGATCCAATGCTGGCAGAGATATTCGACAAAGTAGCTGC
AAAAGAAAAGAGAGA
+
FFFFFF:FFFFFFFFFFFFFFFFFFFFFFFFFFFFFFFFFFFFFFFFFFFFFFFFFFFFFFFFFFFFF
,FFFFFFFFFFFFFFFFFFFFFFFFFFFFFFFFFFFFFFFFFFFFFFFFFFFFFFFFFFFFFFFFFFF
FFFFFFFFFFFFFFF
@A01056:60:HMTK7DSXY:1:2613:28140:26772 1:N:0:TCCTTAGC+ACTCGATT
CTCTTCCGATCTATACAACCTATTGAATGGTTTAAATGCCACATACCTGTCTTCGACACAGATCCAAT
GCTGGCAGAGATATTCGACAAAGTAGCTGCAAAAGAAAAGAGAGAGTTTCAATCCATATTGGGTCTCT
CAAATCAATTTCTGG
+
FFF:FFFFFFFFFFFFFFFFFFFFFFFFFFFFFFFFFFFFFFFFFFFFFFFFFFFFFFFFFFFFFFFF
FFFFFFFFFFFFFFFFFFFFFFFFFFFFFFFFFFFFFFFFFFFFFFFFFFFFFFFFFFFFFFFFFFFF
FFFFFFFFFFFFFFF
@A01056:60:HMTK7DSXY:1:1334:30599:33880 2:N:0:TCCTTAGC+ACTCGATT
GATACAACCTATTGAATGGTTTAAATGCCACATACCTGTCTTCGACACAGATCCAATGCTGGCAGAGA
TATTCGACAAAGTAGCTGCAAAAGAAAAGAGAGAGTTTCAATCCATATTGGGTCTCTCAAATCAATTT
CTGGATATGGAGAAA
+
FFFFFFFFFFFFFFFFFFFFFFFFFFFFFFFFFFFFFFFFFFFFFFFFFFFFFFFFFFFFFFFFFFFF
FFFFFFFFFFFFFFFFFFFFFFFFFFFFFFFFFFFFFFFFFFFFFFFFFFFFFFFFFFFFFFFFFFFF
FFFFFFF,FFFFFFF
@A01056:60:HMTK7DSXY:1:1660:13539:6558 2:N:0:TCCTTAGC+ACTCGATT
GATACAACCTATTGAATGGTTTAAATGCCACATACCTGTCTTCGACACAGATCCAGTGCTGGCAGAGA
TATTCGACAAAGTAGCTGCAAAAGAAAAGAGAGAGTTTCAATCCATATTGGGTCTCTCAAATCAATTT
CTGGATATGGAGAAA
+
FFFFFFFFFFFFFFFFFFFFFFFFFFFFFFFFFFFFFFFFFFFFFFFFFFFFFFFFFFFFFFFFFFFF
FFFFFFFFFFFFFFFFFFFFFFFFFFFFFFFFFFFFFFFFFFFFFFFFFFFFFFFFFFFFFFFFFFFF
FFFFFFFFFFFFFFF
@A01056:60:HMTK7DSXY:1:1563:23475:8672 2:N:0:TCCTTAGC+ACTCGATT
GATACAACCTATTGAATGGTTTAAATGCCACATACCTGTCTTCGACACAGATCCAATGCTGGCAGAGA
TATTCGACAAAGTAGCTGCAAAAGAAAAGAGAGAGTTTCAATCCATATTGGGTCTCTCAAATCAATTT
CTGGATATGGAGAAA
+
FFFFFFFFFFFFFFFFFF:FFFFFFFFFFFFF:FFFFFFFFFFFFFFFFFFFFFFFFFFFF:FFFFFF
FFFFFFFFFFF:FF:FFFFFFFF:FFFFFFFFFF:F:F:FFF,:FFFFF:,FFFFFF:FFFF:FFFF:
FFFFFFFFFFFFFFF
@A01056:60:HMTK7DSXY:1:2613:28140:26772 2:N:0:TCCTTAGC+ACTCGATT
ATACAACCTATTGAATGGTTTAAATGCCACATACCTGTCTTCGACACAGATCCAATGCTGGCAGAGAT
ATTCGACAAAGTAGCTGCAAAAGAAAAGAGAGAGTTTCAATCCATATTGGGTCTCTCAAATCAATTTC
TGGAGATCGGAAGAG

```
+
FFFFFFFFFFFFFFFFFFFFFFFFFFFFFFFFFFFFFFFFFFFFFFFFFFFFFFFFFFFFFFFFFFFF
FFFFFFFFFFFFFFFFFFFFFFFFFFFFFFFFFFFFFFFFFFFFFFFF:FFFFFFFFFFFFFFFFFF:FF
FFFFFFFFFFFFFFF
@A01056:60:HMTK7DSXY:1:1334:30599:33880 1:N:0:TCCTTAGC+ACTCGATT
CCTATTGAATGGTTTAAATGCCACATACCTGTCTTCGACACAGATCCAATGCTGGCAGAGATATTCGA
CAAAGTAGCTGCAAAAGAAAAGAGAGAGTTTCAATCCATATTGGGTCTCTCAAATCAATTTCTGGATA
TGGAGAAAAACGGGT
+
FFFFFFFFFFFFFFFFFFFFFFFFFFFFFFFF:FFFFFFFFFFFFFFFFFFFFFFFFFFFFFFFFFFF
FFFFF:FFFFFFFFFFFFFFFFFFFFFFFFFFFFFFFFFFFFFFFFFFFFFFFFFFFFFFFFFFFFFF
FFFFFFFFFFFFFFF
@A01056:60:HMTK7DSXY:1:1660:13539:6558 1:N:0:TCCTTAGC+ACTCGATT
CCTATTGAATGGTTTAAATGCCACATACCTGTCTTCGACACAGATCCAGTGCTGGCAGAGATATTCGA
CAAAGTAGCTGCAAAAGAAAAGAGAGAGTTTCAATCCATATTGGGTCTCTCAAATCAATTTCTGGATA
TGGAGAAAAACGGGT
+
FFFFFFFFFFFF::FFFFFFFFFFFFFFFFFFFFFFFFFFFFFFFFFFFFFF:FFFFFFFFFFFFFFF
:FFFF,FFFFFFFFFFFFFFFFFFFFFFFFFFFFFFFFFFFFFFFFFFFFFFFFFFFFFFFFFFFFFF
FFFFFFFFFFFFFFF
@A01056:60:HMTK7DSXY:1:1563:23475:8672 1:N:0:TCCTTAGC+ACTCGATT
CCTATTGAATGGTTTAAATGCCACATACCTGTCTTCGACACAGATCCAATGCTGGCAGAGATATTCGA
CAAAGTAGCTGCAAAAGAAAAGAGAGAGTTTCAATCCATATTGGGTCTCTCAAATCAATTTCTGGATA
TGGAGAAAAACGGGT
+
,FFFFF:F:F::,:FFFFF,F:FFFFF::FFFFFFFFFFFFFFFFFFFFFFFFF,F:FFFFFFFFFFFF
FFFF:FF:FF,FFFFF:FFFFFFFFFF:FFF:FFFFFFFFFFFF:FFFFFF:FF:FF:FFFFFF:FFF
F::FFFF:FFFFFFF
@A01056:60:HMTK7DSXY:1:2564:12888:15828 2:N:0:TCCTTAGC+ACTCGATT
GAGATATTCGACAAAGTAGCTGCAAAAGAAAAGAGAGAGTTTCAATCCATATTGGGTCTCTCAAATCA
ATTTCTGGATATGGAGAAAAACGGGTGTAATATTGACATACTTCCTTTTGCTAGGCAAAATGTTTTCC
CACATCACCAATCCT
+
FFFFFFFFFFFFFFFFFFFFFFFFFFFFFFFFFFFFFFFFFFFFFFFFFFFFFFFFFFFFFFFFFFFF
:F:FFFFFFFFFFFF:FFFF,FFFFFFFFFFFF:,F,FFFFFFFFF,FF:FFFFF:FFF:FFFFFF:F
,FFFFFFFFFFFFF,
@A01056:60:HMTK7DSXY:1:2360:24162:20165 2:N:0:TCCTTAGC+ACTCGATT
GTAGCTGCAAAAGAAAAGAGAGAGTTTCAATCCATATTGGGTCTCTCAAATCAATTTCTGGATATGGA
GAAAAACGGGTGTAAGATTGACATACTTCCTTTTGCTAGGCAAAATGTTTTCCCACATCACCAATCCT
CAGATGATGTCACTT
+
FFFFFFFFFFF:FFFFFFFFFFFFFFFFFFFF:FFFFFFFFFFFFFFFFFFFFFFFFFFFFFFFFFFF
FFFFF,FFFFFFFFFF:FFFFFFFFFFF:FFFFFFFFFFFFFFFFFFFFFFFFFFFFFFF,FFFFFFF
:F:FFFFFFFFF:FF
@A01056:60:HMTK7DSXY:1:1123:4381:14622 2:N:0:TCCTTAGC+ACTCGATT
TTTCAATCCATATTGGGTCTCTCAAATCAATTTCTGGATATGGAGAAAAACGGGTGTAAGATTGACAT
ACTTCCTTTTGCTAGGCAAAATGTTTTCCCACATCACCAATCCTCAGATGATGTCACTTTCTGGGCAG
GTGTTCAAAAGAGGA
+
FFFFFFFFFFFFFFFFFFFFFFFFFFFFFFFFFFFFFFFFFFFFFFFFFFFFFFFFFFFFFFFFFFFF
FFFFFFFFFFFFFFFFFFFFFFFFFFFFFFFFFFFFFFFFFFFFFFFFFFFFFFFFFFFFFFFFFFFF
FFFFFFFFFFFFFFF
@A01056:60:HMTK7DSXY:1:1123:4381:14622 1:N:0:TCCTTAGC+ACTCGATT
TCAAATCAATTTCTGGATATGGAGAAAAACGGGTGTAAGATTGACATACTTCCTTTTGCTAGGCAAAA
```

TGTTTTCCCACATCACCAATCCTCAGATGATGTCACTTTCTGGGCAGGTGTTCAAAAGAGGATAAGAA
AGTCCAATTGGAGGC
+
:FFFFFFFFFFFFFFFFFFFFFFFFFFFFFFFFFFFFFFFFFFFFFFFFFFFFFFFFFFFFFFFFFFFF
FFFFFFFFFFFFFFFFFFFFFFFFFFFFFFFFFFFFFFFFFFFFFFFFFFFFFFFFFF:FFFFFFFFFFF
FFFFFFFFFFFFFFF
@A01056:60:HMTK7DSXY:1:2564:12888:15828 1:N:0:TCCTTAGC+ACTCGATT
TAATATTGACATACTTCCTTTTGCTAGGCAAAATGTTTTCCCACATCACCAATCCTCAGATGATGTCA
CTTTCTGGGCAGGTGTTCAAAAGAGGATAAGAAAGTCCAATTGGAGGCGTGAAAAATCGAAATTTGAA
GAATTTGAGAGCCAG
+
FFFFFFFFFFFFFFFFFFFFFFFFFFFFFFFFFFFFFFFFFFFFFFFFFFFFFFFFFFFFFFFFFFFFFF
FFFFFFFFFFFFFFFFFFFFFFFFFFFFFFFFFFFFFFF:FFFFFFFFFFFFFFFFFFFFFFFFFFFFFF
FFFFFFFFFFFFFFF
@A01056:60:HMTK7DSXY:1:2360:24162:20165 1:N:0:TCCTTAGC+ACTCGATT
CCACATCACCAATCCTCAGATGATGTCACTTTCTGGGCAGGTGTTCAAAAGAGGATAAGAAAGTCCAA
TTGGAGGCGTGAAAAATCGAAATTTGAAGAATTTGAGAGCCAGGGGAAAGAACTGCTCCACGAGTTTA
TTTCAATGTTGCCCG
+
FFFFFFFFFFFFFFFFFFFFFFFFFFFFFFFFFFFFFFFFFFFFFFFFFFFFFFFFFFFFFFFFFFFFFF
FFFFFFFFFFFFFFFFFFFFFFFFFFFFFFFFFFFFFFFFFFFFFFFFFFFFFFFFFFFFFFFFFFFFFF
FFFFFFFFFFFFFFF
@A01056:60:HMTK7DSXY:1:2465:30969:20556 2:N:0:TCCTTAGC+ACTCGATT
GTCACTTTCTGGGCAGGTGTTCAAAAGAGGATAAGAAAGTCCAATTGGAGGCGTGAAAAATCGAAATT
TGAAGAATTTGAGAGCCAGGGGAAAGAACTGCTCCACGAGTTTATTTCAATGTTGCCCTTTGAATTCA
AGGTTAACATTAAAG
+
FFFFFFFFFFFFFFFFF:FFFFFFFFFFFFFFFF,FFFFFFFFFF:FFFFFFFFFFFFFFFFFFFFFFFF
FFFFFFFFFFFFFFF:FF:FFFF,FFFFFFFFFFFFFFFFFFFFFFFFFFFFFFF:FFFFFFFFFFFFF:
FF:FFFFFF:FF,FF
@A01056:60:HMTK7DSXY:1:1421:30092:26678 2:N:0:TCCTTAGC+ACTCGATT
GTCACTTTCTGGGCAGGTGTTCAAAAGAGGATAAGAAAGTCCAATTGGAGGCGTGAAAAATCGAAATT
TGAAGAATTTGAGAGCCAGGGGAAAGAACTGCTCCACGAGTTTATTTCAATGTTGCCCTTTGAATTCA
AGGTTAACATTAAAG
+
FFFFFFFFFFFFFFFFFFFFFFFFFFFFFFFFFFFFFFFFFFFFFFFFFFFFFFFFFFFFFFFFFFFFFF
FFFFFFFFFFFFFFFFFFFFFFFFFFFFFFFFF:FFFFFFFFFFFFFFF:FFFFFFFFFFFFFFFFFFFF
FFFFFFFFFFFFFFF
@A01056:60:HMTK7DSXY:1:2465:30969:20556 1:N:0:TCCTTAGC+ACTCGATT
ACGAGTTTATTTCAATGTTGCCCTTTGAATTCAAGGTTAACATTAAAGACATTGAAGATGGAGAAAAA
AGTTTCCTGGAAAAAAGAAAATTGAAGTCAGAAAAGATGTGGGCGAATCATTCAGAAAGGTCTGACAT
CGATTGGAAGCTTG
+
FFFFFFFFFFFFFFFFFFFFFFFFFFFF:FFFFFFFFFFFFFFFFFFFFFFFFFFFFFFFFFFFFFFFFF
FFFFFFFFFFFFFFFFFFF::FFFFFFFFFFFFFFFFFFFFFFFFFFFFFFFFFFFFFFFFFFFFFFFFF
FFFFFFFFFFFFFF
@A01056:60:HMTK7DSXY:1:1421:30092:26678 1:N:0:TCCTTAGC+ACTCGATT
ACGAGTTTATTTCAATGTTGCCCTTTGAATTCAAGGTTAACATTAAAGACATTGAAGATGGAGAAAAA
AGTTTCCTGGAAAAAAGAAAATTGAAGTCAGAAAAGATGTGGGCGAATCATTCAGAAAGGTCTGACAT
CGATTGGAAGCTTG
+
FFFFFFFFFFFFFFFFFFFFFF:FFFFFFFFFFFF:FFFFFFFFFFFFFFFFFFFFFFFFFFFFFFFFFF
FFFFFFFFFFFFFFFFFFFF:FFFFFFFFFFFFFFFFFFFFFFFFFFF:FFFFFFFFFFFFFFFFFFFFF
FFFFFFFFFFFFFF

```
@A01056:60:HMTK7DSXY:1:1540:16342:13166 2:N:0:TCCTTAGC+ACTCGATT
GAAAAAAGTTTCCTGGAAAAAAGAAAATTGAAGTCAGAAAAGATGTGGGCGAATCATTCAGAAAGGTC
TGACATCGATTGGAAGCTTGACCATGTTTTCCTTTTTATGAAATCACAGTATTGCACGAAGGAAGGGA
AGATGTTTACTGAAG
+
FFFFF::FFFFFFFFFFFFF:FFFFFFF:FFFFFFFFFFFFFFFFFFFFFFFFFFFFFFFFFFFFFFF
FFFF::FFFFFFFFFFFF:FFFF,FFFFFFFFFF:FFFFFFFFFFFFFFFFFFFFFFFFFF:FFFFFF
FFFFFFFFFFFFFFF
@A01056:60:HMTK7DSXY:1:2235:28637:13949 2:N:0:TCCTTAGC+ACTCGATT
GAAAAAAGTTTCCTGGAAAAAAGAAAATTGAAGTCAGAAAAGATGTGGGCGAATCATTCAGAAAGGTC
TGACATCGATTGGAAGCTTGACCATGTTTTCCTTTTTATGAAATCACAGTATTGCACGAAGGAAGGGA
AGATGTTTACTGAAG
+
FFFFFFFFFFFFFFFFFFFFFFFFFFFFFFFFFFFFFFFFFFFFFFFFF,FFFFFFFFFFFFFFFFFF
FFFF:,FFFFFFFFF:FFF:FFFFFFFFFFFFFFFFFFFFFFFFFFFFFFFF:FFFF:FFFFFFFFFFF
FFF:FFFFFFFFF:F
@A01056:60:HMTK7DSXY:1:1458:27697:32769 2:N:0:TCCTTAGC+ACTCGATT
GAAAAAAGTTTCCTGGAAAAAAGAAAATTGAAGTCAGAAAAGATGTGGGCGAATCATTCAGAAAGGTC
TGACATCGATTGGAAGCTTGACCATGTTTTCCTTTTTATGAAATCACAGTATTGCACGAAGGAAGGGA
AGATGTTTACTGAAG
+
FFFFF:FFFFFFFFFFFFFFFFFFFFFFFFFFFFF:FFFFFFFFFFFFFFFFFFFFFFFFFFFFFFFF
FFFFFFFFFFFFFFFFFFFFFFFFFFFFFFFFFFFFFFFFFFFFFFFFFFFFFFFFFFFFFFFFFFF:
:FFFFFFFFFFFFFF
@A01056:60:HMTK7DSXY:1:1458:27697:32769 1:N:0:TCCTTAGC+ACTCGATT
TTTATGAAATCACAGTATTGCACGAAGGAAGGGAAGATGTTTACTGAAGCGAAAGCCGGCCAAACTTT
AGCCTGCTTCCAGCACATTGTCTTGTTCAGATTCGGACCCATGCTTAGAGCAATTGAAAGTGCCTTCT
TAAGGAGCTGTGGGG
+
FFFFFFFFFFFFFFFFFFFFFFFFF,FFFFFFFFFFFFFF:FFFFFFFFFFFFFFFFFFFFFF,FFFFF
FFFFFFFFFFFFFFFFFFFFFFFFFFFFFFFFFFFFFFFFFFFFFFFFFFFFFFFFFFFFFFFFFFFF
FFFFFFFFFFFFFFF
@A01056:60:HMTK7DSXY:1:1540:16342:13166 1:N:0:TCCTTAGC+ACTCGATT
TTTATGAAATCACAGTATTGCACGAAGGAAGGGAAGATGTTTACTGAAGCGAAAGCCGGCCAAACTTT
AGCCTGCTTCCAGCACATTGTCTTGTTCAGATTCGGACCCATGCTTAGAGCAATTGAAAGTGCCTTCT
TAAGGAGCTGTGGGGACTCATAC
+
FFFFFFFF,FFFFFFFF:FFF:FFFFFFFFFFFFFFFFFFF:FFFFFFFFFF,FFFFFFFFF,:FFFF
FFFFFFFFFFFFFFFFFFFFFFFFFFFFFFF:FFFFFFFFFFFFFFFFFFFFFFFFFFFFFFFFFFFF
FFFFFFFFFFFFFFF````````
@A01056:60:HMTK7DSXY:1:2235:28637:13949 1:N:0:TCCTTAGC+ACTCGATT
TTTATGAAATCACAGTATTGCACGAAGGAAGGGAAGATGTTTACTGAAGCGAAAGCCGGCCAAACTTT
AGCCTGCTTCCAGCACATTGTCTTGTTCAGATTCGGACCCATGCTTAGAGCAATTGAAAGTGCCTTCT
TAAGGAGCTGTGGGGACTCATAC
+
FFFFFFFFFFFFFFFFFFFFFFFFFFFFFFFFFFFFFFF:F:FFFFFFFFFFFFFFFFFFFFFFFF:FFFF
FFFFFFFFFFFFFFFFFFFFFFFFFFFFFFFFFFFFFFFFFFFFFFFFFFFFFFFFFFF:FFFFFFFFF
FFFFFFFFFFFFFFF````````
@A01056:60:HMTK7DSXY:1:1336:25979:7247 2:N:0:TCCTTAGC+ACTCGATT
ACTACATACACTCCGGAAAAAACTTTTTCTGCCTAGATAGCTTTGTAACAAGAAATGCACATGTTTTT
GACGGGTTCTCAATAGAGTCCGACTACACTGCTTTTGATTCGTCCCAGGACCACGTCATTTTGGCCTT
CGAGATGGCGCTATT
+
FFFFFFFFFFFFFFFFFFFFFFFFFFFFFFFFFFFFFFFFFFFFFFFFFFFFFFFFFFFFFFFFF:FFFF
```

```
FFFFFFFFFFFFFFFFFFFFFFFFFFFFFFFFFFFFFFFFFFFFFFFFFFFFFFFFFFFFFFFFFFFFFF
FFFFFFFFFFFFFFF
@A01056:60:HMTK7DSXY:1:2544:12644:27242 2:N:0:TCCTTAGC+ACTCGATT
ACTACATACACTCCGGAAAAAACTTTTTCTGCCTAGATAGCTTTGTAACAAGAAATGCACATGTTTTT
GACGGGTTCTCAATAGAGTCCGACTACACTGCTTTTGATTCGTCCCAGGACCACGTCATTTTGGCCTT
CGAGATGGCGCTATT
+
FFFFFFFFFFFFFFFFFFFFFFFFF:FFFFFFFFFFFFFFFFFFFFFFFFFFFFF,FFFF:FFFFFFFFFF
F,FFFFFFFFFFFF:FFFFFFFFFFFF:FFFFFFFFFFFFFFFFFFFFFFFF:F:FFFFFFFFFFFFFF
FFFF:FFFFFFF:FF
@A01056:60:HMTK7DSXY:1:2658:27362:4257 2:N:0:TCCTTAGC+ACTCGATT
AAAACTTTTTCTGCCTAGATAGCTTTGTAACAAGAAATGCACATGTTTTTGACGGGTTCTCAATAGAG
TCCGACTACACTGCTTTTGATTCGTCCCAGGACCACGTCATTTTGGCCTTCGAGATGGCGCTATTACA
ATATTTGGGTGTATC
+
FFFFFFFFFFFFFFFFFFFFFFFFFFFFFFFFFFFFFFFFFF:FFFFFFFFFFFFFF:FFFFFFFFFFF
FFFFFFFFFFFFFFFFFFFFFFFFFFFFFFFFFFFF:FFFFFFFF:FF:FFFFFFFFFFFFF,FFFFF
FF,FFFFFF:FFFFF
@A01056:60:HMTK7DSXY:1:1343:21386:17018 2:N:0:TCCTTAGC+ACTCGATT
AAAACTTTTTCTGCCTAGATAGCTTTGTAACAAGAAATGCACATGTTTTTGACGGGTTCTCAATAGAG
TCCGACTACACTGCTTTTGATTCGTCCCAGGACCACGTCATTTTGGCCTTCGAGATGGCGCTATTACA
ATATTTGGGTGTATC
+
FFFFFFFFFFFFFFFFFFFFFFFFFFFFFFFFFFFFFFFFFFFFFFFFFFFFFFFFFFFFFFFFFFFFFF
FFFFFFFFFFFFFFFFFFFFFFFFFFFFFFFFFFFFFFFFFFFFFFFFFFFFFFFFFFFFFFFFFFFFFF
FFFFFFFFFFFFFFF
@A01056:60:HMTK7DSXY:1:2216:16342:27414 2:N:0:TCCTTAGC+ACTCGATT
AAAACTTTTTCTGCCTAGATAGCTTTGTAACAAGAAATGCACATGTTTTTGACGGGTTCTCAATAGAG
TCCGACTACACTGCTTTTGATTCGTCCCAGGACCACGTCATTTTGGCCTTCGAGATGGCGCTATTACA
ATATTTGGGTGTATC
+
FFF,FFFFFFFFFFFFFFFFFFFFF:FFFFFFFFFFFFFFFFFFF:FFFFFF:FFFFFFFFFFFFFFFFF
FFFF:FFFFFFFFFFFFFFF,FFFFFFFFFFFFFFFFFFFFF:FF:FFFFFFFFFFFFFFFFFFFFF:FF
FFFFFFF:FFFFFFF
@A01056:60:HMTK7DSXY:1:1336:25979:7247 1:N:0:TCCTTAGC+ACTCGATT
TCTCAATAGAGTCCGACTACACTGCTTTTGATTCGTCCCAGGACCACGTCATTTTGGCCTTCGAGATG
GCGCTATTACAATATTTGGGTGTATCTAAAGAATTTCAGCTTGACTATCTGAGATTGAAGTTAACTCT
TGGTTGTCGACTTGG
+
FFFFFFFFFFFFFFFFFFFFFFFFFFFFFFFFFFFFFFFFFFFFFFFFFFFFFFFFFFFFFFFFFFFFFF
FFFFFFFFFFFFFFFFFFFFFFFFFFFFFFFFFFFFFFFFFFFFFFFFFFFFFFFFFFFFFFFFFFFFFF
FFFFFFFFFFFFFFF
@A01056:60:HMTK7DSXY:1:2544:12644:27242 1:N:0:TCCTTAGC+ACTCGATT
TCTCAATAGAGTCCGACTACACTGCTTTTGATTCGTCCCAGGACCACGTCATTTTGGCCTTCGAGATG
GCGCTATTACAATATTTGGGTGTATCTAAAGAATTTCAGCTTGACTATCTGAGATTGAAGTTAACTCT
TGGTTGTCGACTTGG
+
FF:FFFFFFFFFFFFFFFFFFFFFFFF:FFFFFFFFFFFFFFFFFFFFFFFFFFFFFFFFFFFFFFFFFF
FFFFFFFFFFFFFFFFFFFFFFFFFFFFFFFFFFFFFFFFFFFFFFFFFFFFFFFFFFFFFFFFFFFFFF
FFFFFFFFFFFFFFF
@A01056:60:HMTK7DSXY:1:2136:10601:35321 2:N:0:TCCTTAGC+ACTCGATT
CCCAGGACCACGTCATTTTGGCCTTCGAGATGGCGCTATTACAATATTTGGGTGTATCTAAAGAATTT
CAGCTTGACTATCTGAGATTGAAGTTAACTCTTGGTTGTCGACTTGGGTCATTGGCAATAATGAGATT
CACTGGGGAATTTTG
```

```
+
FFFF:FFFFFFFFFFFFFFFFFFFFFFFFFFFFFFFFFFFFFFFFFFFFFF,:FFFFFFFFFFFFFF
F:FFFF,FFFFFFFFFFFF::FFFF:F:FFFF:::FF,FFFF:F,FFFFFFFFFFFFFFFF:FF::F
FFFF::FFFFFFFF,
@A01056:60:HMTK7DSXY:1:1343:21386:17018 1:N:0:TCCTTAGC+ACTCGATT
AGGACCACGTCATTTTGGCCTTCGAGATGGCGCTATTACAATATTTGGGTGTATCTAAAGAATTTCAG
CTTGACTATCTGAGATTGAAGTTAACTCTTGGTTGTCGACTTGGGTCATTGGCAATAATGAGATTCAC
TGGGGAATTTTGCAC
+
FFFFFFFFFFFFFFFFFFFFFFFFFFFFFFFFFFFFFFFFFFFFFFFFFFFFFFFFFFFFFFFFFFFF
FFFFFFFFFFFFFFF:FFFFFFFFFF,F:FFFFFFFFFFFFFFFFFFFFFFFFFFFFFFFFFFFFFFF
FFFFFFFFFFFFFFF
@A01056:60:HMTK7DSXY:1:2658:27362:4257 1:N:0:TCCTTAGC+ACTCGATT
AGGACCACGTCATTTTGGCCTTCGAGATGGCGCTATTACAATATTTGGGTGTATCTAAAGAATTTCAG
CTTGACTATCTGAGATTGAAGTTAACTCTTGGTTGTCGACTTGGGTCATTGGCAATAATGAGATTCAC
TGGGGAATTTTGCAC
+
FFF:FFFFF:FFFFFFFFFF,FFFFFFFFFFFFFFFFFFFFFFFF:FFFFFFFFFFFFFFFFFFFFFF
FFFFFFFFFFFFFFF:FFFFFFFFFFFFFFFFFFFFFFFFFFFFFFFFFFFFFFFFFFFFFFFFFFFF
FFFFFFFFFFFFFFF
@A01056:60:HMTK7DSXY:1:2216:16342:27414 1:N:0:TCCTTAGC+ACTCGATT
AGGACCACGTCATTTTGGCCTTCGAGATGGCGCTATTACAATATTTGGGTGTATCTAAAGAATTTCAG
CTTGACTATCTGAGATTGAAGTTAACTCTTGGTTGTCGACTTGGGTCATTGGCAATAATGAGATTCAC
TGGGGAATTTTGCAC
+
FFFFFFFFF:FFFFFFFFFFFFFFFFFFFFFFFF:F,FFFFFFF:F:FFFFFFFFFFFFFFFFFFFFF
FFFFFFFFFFFFFFF:FFFFFFFFFFFFFFFF:FFFFFFF:FFFFFFFFFFFFFFFFFFFFFFFFFFF
FFFFFFFFFFFFFFF
@A01056:60:HMTK7DSXY:1:2255:28257:18051 2:N:0:TCCTTAGC+ACTCGATT
CGTCATTTTGGCCTTCGAGATGGCGCTATTACAATATTTGGGTGTATCTAAAGAATTTCAGCTTGACT
ATCTGAGATTGAAGTTAACTCTTGGTTGTCGACTTGGGTCATTGGCAATAATGAGATTCACTGGGGAA
TTTTGCACCTTTCTG
+
FFFFFFFFFFFFFFFFFFFFFFFFFFFFFFFFFFFFFFFFFFFFFFFFFFFFFFFFFFFFFFFFFFFF
FFFFFFFF:FFFFFFFFFFFFFFFFFFFFFFFFFFFFFFFFFFFFFFFFFFFFF:FFFFFFFFFFF
FFFF:FFFFFFFFFF
@A01056:60:HMTK7DSXY:1:2345:18005:3145 1:N:0:TCCTTAGC+ACTCGATT
GGTGTATCTAAAGAATTTCAGCTTGACTATCTGAGATTGAAGTTAACTCTTGGTTGTCGACTTGGGTC
ATTGGCAATAATGAGATTCACTGGGGAATTTTGCACCTTTCTGTTTAACACATTTGCCAACATGCTTT
TCACTCAGTTGAAGT
+
FFFFFFFFFFFFF:FFFFFFFFFFFFFFFFFFFFFFFFFFFFFFFFFFFFFFFF:FFFFFFFFFFFFF
FFFFFFFFFFFFFFFFFFFFFFFFFFFFFFFFFFFFFFFFFFFFFFFFFFFFFFFFFFFFFFFFFFFF
FFF:FFFFFFFFFFF
@A01056:60:HMTK7DSXY:1:1637:22598:6652 1:N:0:TCCTTAGC+ACTCGATT
GGTGTATCTAAAGAATTTCAGCTTGACTATCTGAGATTGAAGTTAACTCTTGGTTGTCGACTTGGGTC
ATTGGCAATAATGAGATTCACTGGGGAATTTTGCACCTTTCTGTTTAACACATTTGCCAACATGCTTT
TCACTCAGTTGAAGT
+
FFFFFFFF:FFFFFFFFFFFFFFFFFFFFFFFFFFFFFFFFFFFFFFF:FFFF:FFFFFFFFFFFFFF
FFFFFFFFFFFFFFFFFFFFFFFFFFFFFFFFFFFFFFFFFFFFFFFFFFFFFFFFFFFF:FFFFFFFF
FFFFFFFFFFFFFFF
@A01056:60:HMTK7DSXY:1:1152:5764:14356 2:N:0:TCCTTAGC+ACTCGATT
AAAGAATTTCAGCTTGACTATCTGAGATTGAAGTTAACTCTTGGTTGTCGACTTGGGTCATTGGCAAT
```

AATGAGATTCACTGGGGAATTTTGCACCTTTCTGTTTAACACATTTGCCAACATGCTTTTCACTCAGT
TGAAGTACAAAATAG
+
FFFFFFFFFFFFFFFFFFFFFFFFFFFFFFFFFFFFFFFFFFFFFFFFFFFFFFFFFFFFFFFFFFFFFF
FFFFFFFFFFFFFFFFFFFFFFFFFF:FFFFFFFFFFFFFFFFFFFFFFFFFFFFFFFFFFFF:FFFFFF
FFFFFFFFFF:FFFF
@A01056:60:HMTK7DSXY:1:2255:28257:18051 1:N:0:TCCTTAGC+ACTCGATT
TTTCAGCTTGACTATCTGAGATTGAAGTTAACTCTTGGTTGTCGACTTGGGTCATTGGCAATAATGAG
ATTCACTGGGGAATTTTGCACCTTTCTGTTTAACACATTTGCCAACATGCTTTTCACTCAGTTGAAGT
ACAAAATAGACCCAC
+
FFFFFFFFFFFFFFFFFFFFFFFFFFFFFF:FFFFFFFFFFFFFFFFFFFFFFFFFFFF:FFFFFFFFFF
FFFFFFFFFFFFFFFFFFFFFFFFFFFFFFFFFFFFFFFFFFFFFFFFFFFFFFFFFFFFFFFFFFFFFF
FFFFFFFFFFFFFFF
@A01056:60:HMTK7DSXY:1:1152:5764:14356 1:N:0:TCCTTAGC+ACTCGATT
TTCAGCTTGACTATCTGAGATTGAAGTTAACTCTTGGTTGTCGACTTGGGTCATTGGCAATAATGAGA
TTCACTGGGGAATTTTGCACCTTTCTGTTTAACACATTTGCCAACATGCTTTTCACTCAGTTGAAGTA
CAAAATAGACCCACG
+
FFFFFFFFFFFFFFFFFFFFFFFFFFFFFFFFFFFFFFFFF:FFFF:FFFFFFFFFFFFFFFFFFFFFFF
FFFFFFFFFFFFFFFFFFFFFFFFFFFFFFFFFFFFFFFFFFFFFFFFFFFFFFFFFFFFFFFFFFFFFF
FFFFFFFFFFFFFFF
@A01056:60:HMTK7DSXY:1:2136:10601:35321 1:N:0:TCCTTAGC+ACTCGATT
TTCAGCTTGACTATCTGAGATTGAAGTTAACTCTTGGTTGTCGACTTGGGTCATTGGCAATAATGAGA
TTCACTGGGGAATTTTGCACCTTTCTGTTTAACACATTTGCCAACATGCTTTTCACTCAGTTGAAGTA
CAAAATAGACCCACG
+
::FFF,F:,,:FFFFFF:,:FF:,FFF::,:,,:FFF:FF::F,,F,FF:FFFFFFF:F:::FFFFFF
FFFFFFF,FF,,FFFFFFF:FFFFFFFFFFF:FFFF,:FFFFF:F,FFFFFFFFFFFFFFFFFFFFFF
FFF,FFFFFFFFF:F
@A01056:60:HMTK7DSXY:1:1637:22598:6652 2:N:0:TCCTTAGC+ACTCGATT
AGATTGAAGTTAACTCTTGGTTGTCGACTTGGGTCATTGGCAATAATGAGATTCACTGGGGAATTTTG
CACCTTTCTGTTTAACACATTTGCCAACATGCTTTTCACTCAGTTGAAGTACAAAATAGACCCACGGA
AACATAGAATCTTAT
+
FFFFFFFFFFFFFFFF:FF:FFF:FFFFFFFFFFFFFFFFFFFFFFFFFFFF,FFFFFFFFFFFFFFFF
FFFFFFFF:FFFFFFFFFFFFFFFFFFFFFFFFFFFFFFF:FFFFFFFFFFFFFFFFFFFFFFFFFFFFF
FFFFFFFFFFFFFFF
@A01056:60:HMTK7DSXY:1:2345:18005:3145 2:N:0:TCCTTAGC+ACTCGATT
AGATTGAAGTTAACTCTTGGTTGTCGACTTGGGTCATTGGCAATAATGAGATTCACTGGGGAATTTTG
CACCTTTCTGTTTAACACATTTGCCAACATGCTTTTCACTCAGTTGAAGTACAAAATAGACCCACGGA
AACATAGAATCTTAT
+
FFFF,FFFFFF:FFFF::FFFFFFFFFFFFFFF:FFFFFFFFFFF:FFFFF,:FFFFFFFFFFFFFFF
FFFFFFFFFF,:FF:FFFFFFFFFFFFFFFFFFFFFFFFFFFFFFFFFFFFFFFFFFFFFFFFFFFFFFF
FFFFFFFFFFFFFFF
@A01056:60:HMTK7DSXY:1:2618:10393:1673 2:N:0:TCCTTAGC+ACTCGATT
ATTGAAGTTAACTCTTGGTTGTCGACTTGGGTCATTGGCAATAATGAGATTCACTGGGGAATTTTGCA
CCTTTCTGTTTAACACATTTGCCAACATGCTTTTCACTCAGTTGAAGTACAAAATAGACCCACGGAAA
CATAGAATCTTATTC
+
FFFFFFFFFFFFFFFFFFFFFFFFFFFFFFFFFFFFFFFFFFFFFFFFFFFFFFFFFFFFFFFFFFFFFF
:FFFFFFFFFF:FFFFFFFFFFFFFFFFFFFFFFFF:FFFFFFFF:FFFF:FFFFFFFFF,FFFFFFF,
::F:,FFF:FFFFF,

```
@A01056:60:HMTK7DSXY:1:2618:10393:1673 1:N:0:TCCTTAGC+ACTCGATT
AAGTTAACTCTTGGTTGTCGACTTGGGTCATTGGCAATAATGAGATTCACTGGGGAATTTTGCACCTT
TCTGTTTAACACATTTGCCAACATGCTTTTCACTCAGTTGAAGTACAAAATAGACCCACGGAAACATA
GAATCTTATTCGCAG
+
FF,FFFFFFFF:FFFFFFFFFF,:F,F:FFFFFFFFFFFFFFFFFFFFFFFFF:FFFFFFFFFF::FF
,FFFFFFFFFFFFFFFFFFFFFFFFF:FFFFFFFFFFFFFFFFFFFFFFFFFFFFFFFFFFFFFFFFF
FFFFFFFFFFFFFFF
@A01056:60:HMTK7DSXY:1:2508:2465:16783 2:N:0:TCCTTAGC+ACTCGATT
CGACTTGGGTCATTGGCAATAATGAGATTCACTGGGGAATTTTGCACCTTTCTGTTTAACACATTTGC
CAACATGCTTTTCACTCAGTTGAAGTACAAAATAGACCCACGGAAACATAGAATCTTATTCGCAGGGG
ATGACATGTGTTCCT
+
FFFFFFFFFFFFFFFFFFFFFFFFFFFFFFFFFFFFFFFFFFFFFFFFFFFFFFFFFFFFFFFFFFFF
FFFFFFFFFFFFFFFFFFFFFFFFFFFFFFFFFFFFFFFFFFFFFFFFFFFFFFFFFFFFFFFFFFFF
F,FFFFFFFFFFF,F
@A01056:60:HMTK7DSXY:1:2523:4047:28322 2:N:0:TCCTTAGC+ACTCGATT
CGACTTGGGTCATTGGCAATAATGAGATTCACTGGGGAATTTTGCACCTTTCTGTTTAACACATTTGC
CAACATGCTTTTCACTCAGTTGAAGTACAAAATAGACCCACGGAAACATAGAATCTTATTCGCAGGGG
ATGACATGTGTTCCT
+
FFFFFFFFFFFFFFFFFFFFFFFFFFFFFFFFFFFFF:FFFFFFFFFFFFFFFFFFFF:FFFFFFFFF
FFFFFFFFFFFFFFFFFFFFFFFFFFFFFFFFFFFFFFFFFFFFFFFFFFFFFFFFFFFFFF,,FFFF
FFFFFFFFFFFFF,F
@A01056:60:HMTK7DSXY:1:1374:14714:1423 2:N:0:TCCTTAGC+ACTCGATT
GACTTGGGTCATTGGCAATAATGAGATTCACTGGGGAATTTTGCACCTTTCTGTTTAACACATTTGCC
AACATGCTTTTCACTCAGTTGAAGTACAAAATAGACCCACGGAAACATAGAATCTTATTCGCAGGGGA
TGACATGTGTTCCTT
+
FFFFFFFFFFF:FF:FFF:FFFFFFFFFFFFFFFFFFFFFFFFFFFFFFFFFFFFFFFFFFFFFFFFF
FFFFFFFFFFFFFFFFFFFFFFFFFFFFFFFFFFF:FFFFFFFFFFFFFFFFF:FFFFF::FF,FFFF
FFFFF:FFFFFFFFF
@A01056:60:HMTK7DSXY:1:2508:2465:16783 1:N:0:TCCTTAGC+ACTCGATT
TTCACTCAGTTGAAGTACAAAATAGACCCACGGAAACATAGAATCTTATTCGCAGGGGATGACATGTG
TTCCTTGAGCTCTCTCAAGAGGAGAAGAGGAGAAAGAGCAAAAAGGCTGATGAAGAGCTTTTCCCTAA
CTGCGGTGGAGGAAG
+
FFFFF:FF,F:FFFFFFFFFFFFFFFFFFFF:FFFF:FFFFFFFFFFFFFFFFFFFFFFFFFFFFFFF
FFFFFFFFFFFFFF:FFFFFFFFFFFFFFFFFFFFFFFFFFFFFFFFFFFFFFFFFFFFFFFFFFFFF
FFFFFFFFFFFFFFF
@A01056:60:HMTK7DSXY:1:2523:4047:28322 1:N:0:TCCTTAGC+ACTCGATT
TTCACTCAGTTGAAGTACAAAATAGACCCACGGAAACATAGAATCTTATTCGCAGGGGATGACATGTG
TTCCTTGAGCTCTCTCAAGAGGAGAAGAGGAGAAAGAGCAAAAAGGCTGATGAAGAGCTTTTCCCTAA
CTGCGGTGGAGGAAG
+
FFFFFFFFFFFFFFFFFFFFFFFFFFFFFFF:FFFFFF:FFFFFFFFFFFFFFFFFFFFFFFFFFFFF
FFFFFFFFFFFFFFFFFFFFFFFFFFFFFFFFFFFFFFFFF:FFFFFFFFFFFFFFFFFF:FFFFFFF
FFFFFFFFFFFFFFF
@A01056:60:HMTK7DSXY:1:2117:26467:8093 2:N:0:TCCTTAGC+ACTCGATT
AAACATAGAATCTTATTCGCAGGGGATGACATGTGTTCCTTGAGCTCTCTCAAGAGGAGAAGAGGAGA
AAGAGCAAAAAGGCTGATGAAGAGCTTTTCCCTAACTGCGGTGGAGGAAGTGAGGAAATTCCCTATGT
TCTGTGGATGGTATT
+
FFFFFFFFFFFFFFFFFFFF:FFFFFFFFFFFFFFFFFFFFFFFFFFFFFFFFFFFFFFFFF:FFFFF
```

FFFF:FFFFFFFFFFFFFFFFFFFFFFFFFFFFFFFFFFFFFF:FFFFFFFFFFFFFFFFFFFFFFFF
FF:FFFFFFF::FFF
@A01056:60:HMTK7DSXY:1:2632:17580:26616 2:N:0:TCCTTAGC+ACTCGATT
AGAGCTTTTCCCTAACTGCGGTGGAGGAAGTGAGGAAATTCCCTATGTTCTGTGGATGGTATTTGAGT
CCATATGGAATTATTAAATCTCCAAAATTGCTATGGGCCAGAATTAAGATGATGAGTGAAAGACAGCT
CCTGAAAGAATGCGT
+
FFFFFFFFFFFFFFFFFFFFFFFFFFFFFFFFFFFFFFFFFFFFFFFFFFFFFFFFFFFFFFFFF,FF
FFFFFFFFFFFFFFFFFFFFFFFFFFFFFFFFFFFFFFFFFFFFFFFFFFFFFFFFFF:FFFFFFFFF
FFFFFFFFF:FFFFF
@A01056:60:HMTK7DSXY:1:1374:14714:1423 1:N:0:TCCTTAGC+ACTCGATT
GAGCTTTTCCCTAACTGCGGTGGAGGAAGTGAGGAAATTCCCTATGTTCTGTGGATGGTATTTGAGTC
CATATGGAATTATTAAATCTCCAAAATTGCTATGGGCCAGAATTAAGATGATGAGTGAAAGACAGCTC
CTGAAAGAATGCGTT
+
FFFFFFFFFFFFFFFFFFFFFFFFFFFFFFFFFFFFFFFFFFFFFFFFFFFFFFFFFFFF:FFFFFFF
FFFFFFFFFFFFFFFFFFFFFFFFFFFFFFFFFFFFFFFFFFFFFFFFFFFFFFFFFFFFFFFFFFFF
FFFFFFFFFFFFFFF
@A01056:60:HMTK7DSXY:1:1568:19723:8688 2:N:0:TCCTTAGC+ACTCGATT
CTAACTGCGGTGGAGGAAGTGAGGAAATTCCCTATGTTCTGTGGATGGTATTTGAGTCCATATGGAAT
TATTAAATCTCCAAAATTGCTATGGGCCAGAATTAAGATGATGAGTGAAAGACAGCTCCTGAAAGAAT
GCGTTGACAACTATC
+
FFFFFFFFFFFFFFFFFFFFFFFFFFFFFFFFFFFF:FFFFFFFFFFFFFFFFFFFFFFFFFFFFFFF
FFFFFFF:FFFFFFFFFFFFF:FFFFFFFFFFFFFFFFF:FFFFFFFFFFFFFFFFFFFFFFFFFFFF
FFFFFF:FFFFFFFF
@A01056:60:HMTK7DSXY:1:2618:26765:30467 2:N:0:TCCTTAGC+ACTCGATT
GAGGAAATTCCCTATGTTCTGTGGATGGTATTTGAGTCCATATGGAATTATTAAATCTCCAAAATTGC
TATGGGCCAGAATTAAGATGATGAGTGAAAGACAGCTCCTGAAAGAATGCGTTGACAACTATCTGTTT
GAGGCAATATTTGCC
+
FFFFFFFFFFFFFFFFFFFFFFFFFFFFF:FFFFFFFFFFFFFFFFFFFFFFF:FFFFFFFFFFFFFF
F:FFFFFFFFFFFFFF:FFFFFFFFFFFFFFFFFFFFFFFFFFFFFFFFFFFFFFFFFFFFFFFFFFF
FFFFFF:FF:FFFFF
@A01056:60:HMTK7DSXY:1:1361:24171:20964 1:N:0:TCCTTAGC+ACTCGATT
TGGATGGTATTTGAGTCCATATGGAATTATTAAATCTCCAAAATTGCTATGGGCCAGAATTAAGATGA
TGAGTGAAAGACAGCTCCTGAAAGAATGCGTTGACAACTATCTGTTTGAGGCAATATTTGCCTACAGA
TTAGGTGAGAGGCTG
+
FFFFFF:FFFFFFFFFFFFFFFFFFF:FFFFFFFFFFFFFFFFFFFFFFFFFFFFFFFFFFFFFFFFF
FFFF:FFFFFFFFFFFFFFFFFFFFFFFFFFFFFFFFFFFFFFFFFFFFFFFFFFFFFFFFFFFFFFF
FFFFFFFFFFFFFFF
@A01056:60:HMTK7DSXY:1:2633:27715:16924 2:N:0:TCCTTAGC+ACTCGATT
TGGAATTATTAAATCTCCAAAATTGCTATGGGCCAGAATTAAGATGATGAGTGAAAGACAGCTCCTGA
AAGAATGCGTTGACAACTATCTGTTTGAGGCAATATTTGCCTACAGATTAGGTGAGAGGCTGTACACA
ATTTTGAAAGAAGAA
+
FFFFFFFFFFFFFFFFFFFFFFFFFFF:F:FFFFF:FFFFFFFFFFFFFFFFFFFFFFFFFFFFFFFF
FFFFFFFFFFFFFFFFFFFFFFFFFFFFFFFFFFFFFFFFFF:FFFFFFFFFFFFFFFFF:FFFFFFF
FFFFFFFFFFFFFFF
@A01056:60:HMTK7DSXY:1:1568:19723:8688 1:N:0:TCCTTAGC+ACTCGATT
GAATTATTAAATCTCCAAAATTGCTATGGGCCAGAATTAAGATGATGAGTGAAAGACAGCTCCTGAAA
GAATGCGTTGACAACTATCTGTTTGAGGCAATATTTGCCTACAGATTAGGTGAGAGGCTGTACACAAT
TTTGAAAGAAGAAGA

+
FFFFFFFFFFFFFFFFFFFFFFFFFFFFFFFFFFFFFFFFFFFFFFFFFFFFFFFFFFFFFFFFFFFFF
FFFFFFFFFFFFFFFFFFFFFFFFFFFFFFFFFFFF:FFFFFFFFFFFFFFFFFFFFFFFFFFFFFFFF
FFFFFFFFFFFFFFF
@A01056:60:HMTK7DSXY:1:1371:32859:6856 2:N:0:TCCTTAGC+ACTCGATT
AATTATTAAATCTCCAAAATTGCTATGGGCCAGAATTAAGATGATGAGTGAAAGACAGCTCCTGAAAG
AATGCGTTGACAACTATCTGTTTGAGGCAATATTTGCCTACAGATTAGGTGAGAGGCTGTACACAATT
TTGAAAGAAGAAGAC
+
FF:FFFFFFFFFFFFFFFFFFFF:FFFFFFFFFFFFFFFFFF:FFFFFFFFFFFFFFFFF:FFFFFFFF
FFFFFFFFFFF:FFFFFFFFFFFFFFFFFFFFFFFFFFFFFFFFF,FFFFFFFFFFFFFFFFFFFFFFF
FFFFFFF:FFFFFFF
@A01056:60:HMTK7DSXY:1:1448:6162:30201 2:N:0:TCCTTAGC+ACTCGATT
TGATGAGTGAAAGACAGCTCCTGAAAGAATGCGTTGACAACTATCTGTTTGAGGCAATATTTGCCTAC
AGATTAGGTGAGAGGCTGTACACAATTTTGAAAGAAGAAGACTTTGAATATCATTATCTTGTTATAAG
ATTCTTTGTCAAGAA
+
FFFFFFFFFFFFFFFFFFFFFFFFFFFFFFFFFFFFFFFFFFFFFFFFFFFFFFFFFFFFFFFFFFFFF
FFFFFFFFFFFF:FFFFFFFFFFFFFF:FF:FFFFFF:FFFFFFFFFFFFFFFFFFFFFFFFFFFFFFF
FFFFFFFFFFFFFF:
@A01056:60:HMTK7DSXY:1:2117:26467:8093 1:N:0:TCCTTAGC+ACTCGATT
GCTCCTGAAAGAATGCGTTGACAACTATCTGTTTGAGGCAATATTTGCCTACAGATTAGGTGAGAGGC
TGTACACAATTTTGAAAGAAGAAGACTTTGAATATCATTATCTTGTTATAAGATTCTTTGTCAAGAAT
TCGAAACTACTAACG
+
FFFFF:FF:FFFFFFFFFFFFFFFFFFFFFFFFFFFFFFFF,FFFFFFFFFFFFFFFFFFFFFFFFFFF
FFFFFFFFFFFFFFFFFFFFFFFFFF:FFFFFFFFFFFFFFFFFFFFFFFFFFFFFFFFFFFFFFFFFF
FFFFFFFFFFFFFFF
@A01056:60:HMTK7DSXY:1:1371:32859:6856 1:N:0:TCCTTAGC+ACTCGATT
TCCTGAAAGAATGCGTTGACAACTATCTGTTTGAGGCAATATTTGCCTACAGATTAGGTGAGAGGCTG
TACACAATTTTGAAAGAAGAAGACTTTGAATATCATTATCTTGTTATAAGATTCTTTGTCAAGAATTC
GAAACTACTAACGGG
+
FFFFFFFFFFFFFFFFFFFFFFFFFFFFFFFFFFFFFFFFFFFFFFFFFFFFF:FFFFFFFFFFFFFFF
FFFFFFFFFFFFFFFFFFFFFFFFFFFFFFFFFFFFF:FFFFFFFFFFFFFFFF,FFFFFFFFFFFFFF
FFFFFFFFFFFFFFF
@A01056:60:HMTK7DSXY:1:2618:26765:30467 1:N:0:TCCTTAGC+ACTCGATT
TCCTGAAAGAATGCGTTGACAACTATCTGTTTGAGGCAATATTTGCCTACAGATTAGGTGAGAGGCTG
TACACAATTTTGAAAGAAGAAGACTTTGAATATCATTATCTTGTTATAAGATTCTTTGTCAAGAATTC
GAAACTACTAACGGG
+
::FFFFFFFFFFFFFFFFFFFFFFFFFFFFFFFFFFFFFFFFFFFFFFFFFFFFFFFFFFFFFFFFFFF
FFFFFFFFFFFFFFFFFFFFFFFFFFFFFFFFFFFFFFFFFFFFFFFFFFFFFFFFFFFFFFFFFFFFF
FFFFFFFFFFFFFFF
@A01056:60:HMTK7DSXY:1:2208:14742:17566 2:N:0:TCCTTAGC+ACTCGATT
ATCTGTTTGAGGCAATATTTGCCTACAGATTAGGTGAGAGGCTGTACACAATTTTGAAAGAAGAAGAC
TTTGAATATCATTATCTTGTTATAAGATTCTTTGTCAAGAATTCGAAACTACTAACGGGTTTAAGCAA
AAGCTTAATTCTTGA
+
FFFFFFFFFFFFFFFFFFFFFFFFFFFFFFFFFFFFFFFFFFFFFFFFFFFFFFFFFFFFFFFFFFFFF
FFFFFFFFFFFFFFFFFFFFFFFFF,FFF:FFFFFFF:FFFFFFFFFFFFFFFFFFFFFFFFFFF:FFF
FFFFFFFFFFFFFFF
@A01056:60:HMTK7DSXY:1:1448:6162:30201 1:N:0:TCCTTAGC+ACTCGATT
GAGGCAATATTTGCCTACAGATTAGGTGAGAGGCTGTACACAATTTTGAAAGAAGAAGACTTTGAATA

```
TCATTATCTTGTTATAAGATTCTTTGTCAAGAATTCGAAACTACTAACGGGTTTAAGCAAAAGCTTAA
TTCTTGAAATTGGAG
+
FFFFFFFFFF,FFFFFFFFFFFFFFFFFFFFFFFFFFFFFFFFFFFFFFFFFFFFFFFFFFF:::FFFFF
FFFFFFFFFFFFFFFFFFFFFFFFFFFFFFFFFFFFFFFFFFFFFFFFFFFFFFFFFFFFFFFFFFFFFF
FFFFFFFFFFFFFFF
@A01056:60:HMTK7DSXY:1:2632:17580:26616 1:N:0:TCCTTAGC+ACTCGATT
GAGGCAATATTTGCCTACAGATTAGGTGAGAGGCTGTACACAATTTTGAAAGAAGAAGACTTTGAATA
TCATTATCTTGTTATAAGATTCTTTGTCAAGAATTCGAAACTACTAACGGGTTTAAGCAAAAGCTTAA
TTCTTGAAATTGGAG
+
FFFFFF:FFF:FFFFFFFFFFFFFFFFFFFFF:FFFFFFFFFF:FFFFFFFFFFFFF:FFFFFFFFFF
FFFFFFFFFFFFFFFFFFFFFFFFFFFFFFFFFFFFFFFFFFFFFFFFFFFFFFFFFFFFFFFFFFFFFF
FFFFFFFFFFFFFFF
@A01056:60:HMTK7DSXY:1:2633:27715:16924 1:N:0:TCCTTAGC+ACTCGATT
TGCCTACAGATTAGGTGAGAGGCTGTACACAATTTTGAAAGAAGAAGACTTTGAATATCATTATCTTG
TTATAAGATTCTTTGTCAAGAATTCGAAACTACTAACGGGTTTAAGCAAAAGCTTAATTCTTGAAATT
GGAGAAGGAATTGGG
+
FFFFFFFFFFFFFFFFFFFFFF:FFFFFFFFFFFFFFFFFFFFFFFFFFFFFFFFFFFFFFFFFFFFFF
FFFFFFFFFFF:FFFFFFFFFFFFFFFFFFFFFFFFFFFFFFFFFFFFFFFFFFFFFFFFFFFFFFFFFF
FFFFFFFFFFFFFFF
@A01056:60:HMTK7DSXY:1:2208:14742:17566 1:N:0:TCCTTAGC+ACTCGATT
CTACAGATTAGGTGAGAGGCTGTACACAATTTTGAAAGAAGAAGACTTTGAATATCATTATCTTGTTA
TAAGATTCTTTGTCAAGAATTCGAAACTACTAACGGGTTTAAGCAAAAGCTTAATTCTTGAAATTGGA
GAAGGAATTGGGTCG
+
FFFFFF,:FFFF:FFF:FFFFFFFFFFFFFFFFFFFFFFFFFFFFFFFFFFFFFFFFFFFFFFFFF:FF
FFFFFFFF:FFFFFFFFFFFFFFFFFFFFFFFFFFFFFFFFFFFFFFFFFFFFFFFFFFFFFFFFFFFF
FFFFFFFFFFFFFFF
@A01056:60:HMTK7DSXY:1:2335:29414:36088 2:N:0:TCCTTAGC+ACTCGATT
GCTGTACACAATTTTGAAAGAAGAAGACTTTGAATATCATTATCTTGTTATAAGATTCTTTGTCAAGA
ATTCGAAACTACTAACGGGTTTAAGCAAAAGCTTAATTCTTGAAATTGGAGAAGGAATTGGGTCGGAA
TGGCGATCGTCAATG
+
FFFFFFFFFFFFFFFFFFFFFFFFFFFFFFFFFFFFFFFFFFF:FFFFFFFFFFFFF:F:FFFFFFFF
FF:FFFFFFFFFFFFFF:FFFFFFF:FF:FF:FFFFFFFFFFFF:FF,FFFFFFFFF:FFFFFFFFFF
FFF:FFFFFFFFFFF
@A01056:60:HMTK7DSXY:1:1351:12915:15624 2:N:0:TCCTTAGC+ACTCGATT
ATTGGAGAAGGAATTGGGTCGGAATGGCGATCGTCAATGTCAACCATTTCCTCAAGGAGGTCGAGTCA
ACAGATCTCAAGATTGACGCAATCTCATCCTCAGAACTTTACAAAGATGCAACCTTTTTCAAGCCGGA
TGTGCTCAATTGTAT
+
F::FFFFFFFFF:FFFFFFFFFFFFFFFFFFFFFFFFFFFFFFFFFFFFFFFFFFFFFFFFFFFFFFF
FFFFFFFFFFFFFF:FFFFFF:FFFFFFFFFFFFFFFFFFFFFFFFFFFFFFFFFF,FFFFFFFF:FFF
FF:FF:FFFFFFFFF
@A01056:60:HMTK7DSXY:1:1351:12915:15624 1:N:0:TCCTTAGC+ACTCGATT
ACCATTTCCTCAAGGAGGTCGAGTCAACAGATCTCAAGATTGACGCAATCTCATCCTCAGAACTTTAC
AAAGATGCAACCTTTTTCAAGCCGGATGTGCTCAATTGTATTAAAAGATTCGAGTCAAATGTCAAAGT
TTCCTCCAGGTCTGG
+
FFFFFFFFF:FFFFFFFFFFFFFFFFFFFFFFFFFFFFFFFFFFFFFFFFFFFFFFFFFFFFFFFFFF
FFFFFFFFFFFFFFFFFFFFFFFFFFFFFFFFFFFFFFFFFFFFFFFFFFFFFFFFFFFFFFFFFFFFFF
FFFFFFFFFFFFFFF
```

@A01056:60:HMTK7DSXY:1:2545:30138:27853 2:N:0:TCCTTAGC+ACTCGATT
CGCAATCTCATCCTCAGAACTTTACAAAGATGCAACCTTTTTCAAGCCGGATGTGCTCAATTGTATTA
AAAGATTCGAGTCAAATGTCAAAGTTTCCTCCAGGTCTGGTGATGGACTCGTTTTGTCAGATTTCAAG
TTGCTTGATGACACT
+
FFFFFF:FFFFFFFFFFFF:FFFFFFFFFFFFFFFFFFFFFFFFFFFFFFFFFFFF:FFF,FFFF:FF
FFFFFFFFFFFFFFFFFFFF::FF:FFFFFFFFFFFFFFFFFFFFF::,FFFFFFFFFFFFFFFFFFF
FFFFFFFFFFFFFF:
@A01056:60:HMTK7DSXY:1:1605:27254:11616 2:N:0:TCCTTAGC+ACTCGATT
ACCTTTTTCAAGCCGGATGTGCTCAATTGTATTAAAAGATTCGAGTCAAATGTCAAAGTTTCCTCCAG
GTCTGGTGATGGACTCGTTTTGTCAGATTTCAAGTTGCTTGATGACACTGAAATCGACTCAATCAGAA
AGAAGAGCAACAAGT
+
FFFFFFFFFFFFFFFFFFFFFFFFFFFFFFFF:FFFFFFFFFFFFFFFFFFFFFFFFFFFFFFFFFFF
FFFFFFFFFFFFFFFFFFFFFFFFFFFFFFFFFFFFFFFFFFFFFFFFFFFFFFFFFFFFFFFFFFFF
FFFFFFFFFFFFFFF
@A01056:60:HMTK7DSXY:1:2605:23484:24565 2:N:0:TCCTTAGC+ACTCGATT
ACCTTTTTCAAGCCGGATGTGCTCAATTGTATTAAAAGATTCGAGTCAAATGTCAAAGTTTCCTCCAG
GTCTGGTGATGGACTCGTTTTGTCAGATTTCAAGTTGCTTGATGACACTGAAATCGACTCAATCAGAA
AGAAGAGCAACAAGT
+
FFFFFFFFFFFFFFFFFFFFFFFFFFFFFFFFFFFF:FFFFFFFFFFFFFFFFFFFFFFFFFFFFFFF
FFFFFFFFFFFFFFFFFFFFFFFFFFFFFFFFFFFFFF:FF:FFFFFFFFFFFFFFFFFFFFFFFF:F
FFFF:FFFFFFFFFF
@A01056:60:HMTK7DSXY:1:1605:27254:11616 1:N:0:TCCTTAGC+ACTCGATT
CTCAATTGTATTAAAAGATTCGAGTCAAATGTCAAAGTTTCCTCCAGGTCTGGTGATGGACTCGTTTT
GTCAGATTTCAAGTTGCTTGATGACACTGAAATCGACTCAATCAGAAAGAAGAGCAACAAGTACAAGT
ACTTGCATTATGGAG
+
FFF,,FFFF:FFFF,:F:FFF,:::,F:F:::,FF,FFFFF:FFF,FFF,FF:F,FFF,F:F:F,FFF
,F,F,,FFFFF:FFFF,FFFFF,FFF,F,FF,FFFFFFFFFFFFFFFFFFFFFFFFFFFFFFFFFFFF
FFFFFFFFFFFFFFF
@A01056:60:HMTK7DSXY:1:2605:23484:24565 1:N:0:TCCTTAGC+ACTCGATT
CTCAATTGTATTAAAAGATTCGAGTCAAATGTCAAAGTTTCCTCCAGGTCTGGTGATGGACTCGTTTT
GTCAGATTTCAAGTTGCTTGATGACACTGAAATCGACTCAATCAGAAAGAAGAGCAACAAGTACAAGT
ACTTGCATTATGGAG
+
,F,F:FFFFFFFFFFF,FFFF,FFFFFF,F:F:FFFFFFFFFFFFFFFF:F:FFF,FFFFFFF,FFFF
:F:FFFFFF:FFFFFFFFF:FFFFFFFF,FF:FFFFFFFFFFFFFFFFFFFFFFFFFFFFFFFFFFFF
FFFFFFFFFFFFFFF
@A01056:60:HMTK7DSXY:1:2335:29414:36088 1:N:0:TCCTTAGC+ACTCGATT
TCAAAGTTTCCTCCAGGTCTGGTGATGGACTCGTTTTGTCAGATTTCAAGTTGCTTGATGACACTGAA
ATCGACTCAATCAGAAAGAAGAGCAACAAGTACAAGTACTTGCATTATGGAGTCATTCTGGTTGGAAT
CAAAGCAATGCTGCC
+
FFFF::FFFFFFFF,F,FFFFFFFFF:FF:F:FFFFFFFFFF:FFF,FFF:FF,FFF:FFFFFFF:FF
FFFFFFFFFFFFFFFFFFFFFFFFFFFFFFFFFFFFFFFFFFFFFFFFFFFFFFFFFFFFFFFFFFFF
FFFFFFFFFFFFFFF
@A01056:60:HMTK7DSXY:1:2148:1542:31939 2:N:0:TCCTTAGC+ACTCGATT
GATGACACTGAAATCGACTCAATCAGAAAGAAGAGCAACAAGTACAAGTACTTGCATTATGGAGTCAT
TCTGGTTGGAATCAAAGCAATGCTGCCAAACTTCAGAGGCATGGAAGGAAGGGTCATTGTGTATGATG
GAGCCTGCCTTGATC
+
FFFFFFFFFFFFFFFFFFFFFFFFFFFFFFFFFFFFFFFFFFFF:FFFFFFFFFFFFFFFFFFFFFFF

FFFFFFFFFFFFFFFFFFFFFFFFFFFFFFFFFFFFFFFFFFFFFFFFFFFFFFFFFFFFFFFFFFF:FF
FFFF:FFFFFFFFFF
@A01056:60:HMTK7DSXY:1:2545:30138:27853 1:N:0:TCCTTAGC+ACTCGATT
AATCGACTCAATCAGAAAGAAGAGCAACAAGTACAAGTACTTGCATTATGGAGTCATTCTGGTTGGAA
TCAAAGCAATGCTGCCAAACTTCAGAGGCATGGAAGGAAGGGTCATTGTGTATGATGGAGCCTGCCTT
GATCCGGAAAGAGGC
+
FFFFFFFFFFFFFFFFFFFFFFFFFFFFFFFFFFFFFFFFFFFFFFFFFFFFFFFFFFFFFFFFFFFFFF
FFFFFFFFFFFFFFFFFFFFFFFFFFFFFFFFFFFFFFFFFFFFFFFFFFFFFFFFFFFFFFFFFFFFFF
FFFFFFFFFFFFFFF
@A01056:60:HMTK7DSXY:1:2148:1542:31939 1:N:0:TCCTTAGC+ACTCGATT
GACTCAATCAGAAAGAAGAGCAACAAGTACAAGTACTTGCATTATGGAGTCATTCTGGTTGGAATCAA
AGCAATGCTGCCAAACTTCAGAGGCATGGAAGGAAGGGTCATTGTGTATGATGGAGCCTGCCTTGATC
CGGAAAGAGGCCAAT
+
FFFFFFFFFF:FFFFFF:F,FFFFFFFFFFFFFFFFFFFFFFFFFFFFFFFFFFFFFFFFFFFFFFFFFF
FFFFFF:FF:FFFFFFFFFFFFFFFFFFFFFFFFFFFF:FFFFFFF:FFFFFFFFFFFFFFFFFFFFFFF
FFFFFFFFFFFFFFF
@A01056:60:HMTK7DSXY:1:2218:13847:29168 2:N:0:TCCTTAGC+ACTCGATT
ATTCTGGTTGGAATCAAAGCAATGCTGCCAAACTTCAGAGGCATGGAAGGAAGGGTCATTGTGTATGA
TGGAGCCTGCCTTGATCCGGAAAGAGGCCATATATGCTCATATTTGTTTAAGTTTGAATCTGACTGCT
GTTACTTCGGTCTTA
+
FFFFFFFFFFFFFFFFFFFFFFFFFFFFFFFFFFFFFFFFFFFFFFFFF:FFFFFFFFFFFFFFFFFFFF
FFFFFFFFFFFFFFFFFFFFFFFFFFFFFFFFFFFFFFFFFFFFFFFFFFFFFFFFFFFFFFFFF:FFFF
FFFFFFFF::FFFFF
@A01056:60:HMTK7DSXY:1:2218:13847:29168 1:N:0:TCCTTAGC+ACTCGATT
TGCTGCCAAACTTCAGAGGCATGGAAGGAAGGGTCATTGTGTATGATGGAGCCTGCCTTGATCCGGAA
AGAGGCCATATATGCTCATATTTGTTTAAGTTTGAATCTGACTGCTGTTACTTCGGTCTTAGACCTGA
ACACTGCTTATCCAC
+
FFFFFFFFFFFFFFFFFFFFFFFFFFFFFFFFFFFFFFFFFFFFFFFFFFFFFF:FFFFFFFFFFFFFFF
FFFFFFFFFFFFFFFFFFFFFFFFFFFFFFF,FFFFFFFFFFFFFFFFFFFFFFFFFFFFFFFFFFFFFF
FFFFFFFFFFFFFFF
@A01056:60:HMTK7DSXY:1:1576:30951:13416 2:N:0:TCCTTAGC+ACTCGATT
CGGAAAGAGGCCATATATGCTCATATTTGTTTAAGTTTGAATCTGACTGCTGTTACTTCGGTCTTAGA
CCTGAACACTGCTTATCCACCACAGATGCCAACCTGGCAAAGAGATTTAGATTTCGTGTGGACTTTGA
TTGCCCACAGTATGA
+
FFFFFFFFFFFFFFFFFFFFFFFFFFFFFFFFFFFFFFFFFFFFFFFFF:FFFFFFFFFFFFFFFFFFFF
FFFFFFFFFFFFFFFFFFFFFFFFF:FFFFFFFFFFFFFFFFFFFFFFFF:FFFFF:FFFFFFFFFFFFFF
FFFFFFFFFFFFFFF
@A01056:60:HMTK7DSXY:1:1528:3965:5666 2:N:0:TCCTTAGC+ACTCGATT
CGGAAAGAGGCCATATATGCTCATATTTGTTTAAGTTTGAATCTGACTGCTGTTACTTCGGTCTTAGA
CCTGAACACTGCTTATCCACCACAGATGCCAACCTGGCAAAGAGATTTAGATTTCGTGTGGACTTTGA
TTGCCCACAGTATGA
+
FFFFFFFFFFFF:FFFFFFFFFFFFFFFFFFFFFFFFFFFF:FFFFFFFFFFFFFFFFFFFFFFFFFFFF
FFFFFFFFFFFFFFFFFFFFFFFFFFFFFFFFFFFFFFFFF:FFF:FFFFFFFFFF:F:FFFFFFFFF:
FFFFF:FFFF,FFFF
@A01056:60:HMTK7DSXY:1:2521:2636:10285 2:N:0:TCCTTAGC+ACTCGATT
GGAAAGAGGCCATATATCCTCATATTTGTTTAAGTTTGAATCTGACTGCTGTTACTTCGGTCTTAGAC
CTGAACACTGCTTATCCACCACAGATGCCAACCTGGCAAAGAGATTTAGATTTCGTGTGGACTTTGAT
TGCCCACAGTATGAA

```
+
FFFFFF,FFFFFFFFFFFFFFFFFFFFFFFFFFFFFFFFFFFFFFFFFFFFFFFFFFFFFFFFFFFFFF
FFFFFFFFFFFFFFFFFFFFFFFFFFFFFFFFFFFFFFFFFFFFFFFFFFFFFFFFFFFFFFFFFF:F
FF:FFFFFFFFFFFF
@A01056:60:HMTK7DSXY:1:2548:15266:10488 2:N:0:TCCTTAGC+ACTCGATT
GAAAGAGGCCATATATGCTCATATTTGTTTAAGTTTGAATCTGACTGCTGTTACTTCGGTCTTAGACC
TGAACACTGCTTATCCACCACAGATGCCAACCTGGCAAAGAGATTTAGATTTCGTGTGGACTTTGATT
GCCCACAGTATGAAC
+
FF:FFFFFFFFFFFFFFFFFFFFFFFFFFFFFFFFFFFFFFFFFFFFFFFFFFFFFFFFFFFFFFFFFF
FFFFFFFFFFFFFFFFFFFFFFFFFFFF:FFFFFFFFFFFFFFF,FFFFFFFFFFFFFFFFFFFFF:F
FFFFFFFFFFFFFFF
@A01056:60:HMTK7DSXY:1:1576:30951:13416 1:N:0:TCCTTAGC+ACTCGATT
AAAGAGGCCATATATGCTCATATTTGTTTAAGTTTGAATCTGACTGCTGTTACTTCGGTCTTAGACCT
GAACACTGCTTATCCACCACAGATGCCAACCTGGCAAAGAGATTTAGATTTCGTGTGGACTTTGATTG
CCCACAGTATGAACA
+
FFFFFFFFFFFFFFFFFFFFFFFFFFFFFFFFFFFFFFFFFFFFFFF:FFFFFFFFFFFFFFFFFFFFF
FFFFFFFFFFFFFFFFFFFFFFFFFFFFFFFFFFFFFFFFFFFFFFFFFFFFFFFFFFFFFFFFFFFF
FFFFFFFFFFFFFFF
@A01056:60:HMTK7DSXY:1:1528:3965:5666 1:N:0:TCCTTAGC+ACTCGATT
GAGGCCATATATGCTCATATTTGTTTAAGTTTGAATCTGACTGCTGTTACTTCGGTCTTAGACCTGAA
CACTGCTTATCCACCACAGATGCCAACCTGGCAAAGAGATTTAGATTTCGTGTGGACTTTGATTGCCC
ACAGTATGAACAGGA
+
FFFFFFFFFFFF:FFFFFFFFFFFFF:FFFFFF:FFFFFFFFFFFFFFFFFFFFFFFFFFFFFFFFFFF
FFFFFFFFFFFFFFFFFFFFFFFFFFFFFFFFFFFFFFFFFFFFFFFFFFFFFFFFFFFFFFFFFFFF
FFFFFFFFFFFFFFF
@A01056:60:HMTK7DSXY:1:2521:2636:10285 1:N:0:TCCTTAGC+ACTCGATT
AGGCCATATATCCTCATATTTGTTTAAGTTTGAATCTGACTGCTGTTACTTCGGTCTTAGACCTGAAC
ACTGCTTATCCACCACAGATGCCAACCTGGCAAAGAGATTTAGATTTCGTGTGGACTTTGATTGCCCA
CAGTATGAACAGGAC
+
FFFFFF:FFF:,FFFFFFFFFFFFFFFF:FFFFFFFFFFFFFFFFFFFFFFFFFFFFFFFFFFFFFFFF
FFFFFFFFFFFFFFFFFFFFFFFFFFFFFFFFFFFFFFFFFFFFFFFFFFFFFFFFFFFFFFFFFFFF
FFFFFFFFFFFFFFF
@A01056:60:HMTK7DSXY:1:2548:15266:10488 1:N:0:TCCTTAGC+ACTCGATT
AGGCCATATATGCTCATATTTGTTTAAGTTTGAATCTGACTGCTGTTACTTCGGTCTTAGACCTGAAC
ACTGCTTATCCACCACAGATGCCAACCTGGCAAAGAGATTTAGATTTCGTGTGGACTTTGATTGCCCA
CAGTATGAACAGGAC
+
FFFFFFF:::FFFFFFF:FFFFFFFFFFFFFFFFFFFFFFFFFFFFFFFFF:FFFFFFFFFFFFFFFFFF
FFFFFFFFFFFFFFFFFFFFFFFFFFFFFFFFFFFFFFFFFFFFFFFFFFFFFFFFFFFFFFFFFFFF
FFFFFFFFFFFFFFF
@A01056:60:HMTK7DSXY:1:2150:30391:18928 2:N:0:TCCTTAGC+ACTCGATT
CTTCGGTCTTAGACCTGAACACTGCTTATCCACCACAGATGCCAACCTGGCAAAGAGATTTAGATTTC
GTGTGGACTTTGATTGCCCACAGTATGAACAGGACACTGAGTTGTTTGCTCTTGACATTGGCGTGGCC
TACAGATGCGTCAAT
+
FFFFFFFFFFFFFFFFFFFFFFFFFFFFFFFFFFFFFFFFFFFF,FFFFFFFFFFFFFFFFFFFFFFFFF
FFFFFFFFFFFFFFFFFFFFFFFFF,FFF,F:FFFFFFFFFFF:FFFFFF:FFFFFFFFFFFFFFFFFF
FFFFFFFFFFFFFFF
@A01056:60:HMTK7DSXY:1:2414:25509:35650 2:N:0:TCCTTAGC+ACTCGATT
GAGATTTAGATTTCGTGTGGACTTTGATTGCCCACAGTATGAACAGGACACTGAGTTGTTTGCTCTTG
```

ACATTGGCGTGGCCTACAGATGCGTCAATTCAGCTCGTTTCTTGGAGACCAAGACTGGAGACTCAGGA
TGGGCCTCACAGGCT
+
FFFFFFFFFFFFFFFFFFFFFFFFF:FFFFFFFFFFFFFFFFFFFFFFFFFFFFFF,FFFFFFFFFFF:
FF:FFFFFFFF:FFFFFFFFFFFFFFFFFFFFF:F:FFFFFFFF:FFFFFFFFFF,:FFFFFFFF:,F
:FFFFF:FF:FFFF:
@A01056:60:HMTK7DSXY:1:1434:4824:36620 2:N:0:TCCTTAGC+ACTCGATT
ATTTAGATTTCGTGTGGACTTTGATTGCCCACAGTATGAACAGGACACTGAGTTGTTTGCTCTTGACA
TTGGCGTGGCCTACAGATGCGTCAATTCAGCTCGTTTCTTGGAGACCAAGACTGGAGACTCAGGATGG
GCCTCACAGGCTATT
+
FFFFFFFFFFFF:FFFFFFFFFFFFF:FFFFFFFFF:FFFF:,FFFFFFFFF:FFFF,FFFFF:FFFF
F::FFFFFF,FFFFF:FFF:FFFFFFFFFFF,,:FFFFFFF:FFF,FFF:FFFF:F:FF,FFF:FFF:
FFFFFFFF:FFFFFF
@A01056:60:HMTK7DSXY:1:2150:30391:18928 1:N:0:TCCTTAGC+ACTCGATT
ACACTGAGTTGTTTGCTCTTGACATTGGCGTGGCCTACAGATGCGTCAATTCAGCTCGTTTCTTGGAG
ACCAAGACTGGAGACTCAGGATGGGCCTCACAGGCTATTAGCGGCTGTGAAGCACTTAGATTCAATGA
AGAAATCAAAATGGC
+
FFFFFFFFFFFF:FFFFFFFFFFFFFFFFFFFFFFFFFFFFFFFFFFFFFFFFFFFFFFFFFFFFFFF
FFFF:FFFFFFFFFFFFFFFFFFFFFFFFFFFFFFFFFFFFFFFFFFFFFFFFFFFFFFFFFFFFFFF
FFFFFFFFFFFFFFF
@A01056:60:HMTK7DSXY:1:2414:25509:35650 1:N:0:TCCTTAGC+ACTCGATT
ACACTGAGTTGTTTGCTCTTGACTTTGGCGTGGCCTACAGATGCGTCAATTCAGCTCGTTTCTTGGAG
ACCAAGACTGGAGACTCAGGATGGGCCTCACAGGCTATTAGCGGCTGTGAAGCACTTAGATTCAATGA
AGAAATCAAAATGGC
+
::F:FFFFFFFFFFFFF:FFF,,,F,FFFFFFFF,:FFFFFFFF:F:F,FF:F,FFFFF:FFFFFFFF
FFFFFFF:FFF,FFFFF,FF:F:FFFFFFFFFF,:FFFF:FFFF:FFFFF,FFF:FFFF,FF:FFFFF
F:F:FFFFFFFFF:F
@A01056:60:HMTK7DSXY:1:1434:4824:36620 1:N:0:TCCTTAGC+ACTCGATT
ACACTGAGTTGTTTGCTCTTGACATTGGCGTGGCCTACAGATGCGTCAATTCAGCTCGTTTCTTGGAG
ACCAAGACTGGAGACTCAGGATGGGCCTCACAGGCTATTAGCGGCTGTGAAGCACTTAGATTCAATGA
AGAAATCAAAATGGC
+
FF,FFF:FFFFFFFFFFFFFFFF,F:FFF,FFFFFFFFFFF:FFFFFFFFFFFFFF:FFF:FFFFFF
FFF:FFF:FFFFFFFFFFFF:FFFFF:FFFFFFF:F:FF:FFFFFF:FFFFFFFFFFFFFFFF,FFFF
F:FFFFFFFFFFF,F
@A01056:60:HMTK7DSXY:1:1309:23529:1564 2:N:0:TCCTTAGC+ACTCGATT
CCAAGACTGGAGACTCAGGATGGGCCTCACAGGCTATTAGCGGCTGTGAAGCACTTAGATTCAATGAA
GAAATCAAAATGGCAATTTTGGATCACAAATCTCCGCTGTTTCTGGAGGAAGGTGTTCCAAACATGCA
TGTCGAAAAGAGACT
+
FFFFFFFFFFFFFFFFFFFFFFFFFFFFFFFFFFFFFFFFFFFFFFFFFFFFFFFFFFFFFFFFFFFF
FFFFFFFFFFFFFFF:FFFFFFFFFFFFFFFFFFFFFFFFFFFF:FFFFFFF:FFFFFFFFFFFFFFFF
FFFFFFFFFFFFFFF
@A01056:60:HMTK7DSXY:1:1309:23529:1564 1:N:0:TCCTTAGC+ACTCGATT
AATCAAAATGGCAATTTTGGATCACAAATCTCCGCTGTTTCTGGAGGAAGGTGTTCCAAACATGCATG
TCGAAAAGAGACTGTTCAGAGGTGACAAGATTAGAAGGTCACGCTCGATTTCTGCAAAAAGGGGGCCA
AACTCGAAACCGCAG
+
FFFFFFFFFF:FFFFFFFFFFFFFFFFFFFFFFFFFFFFFFFFFFFFFFFFFFFFFFFFFFFFFFFFF
FFFFFFFFFFFFFFFFFFFFFFFFFFFFFFFFFFFFFFFFFFFFFFFFFFFFFFFFFFFFFFFFFFFF
FFFFFFFFFFFFFFF

```
@A01056:60:HMTK7DSXY:1:2234:4444:24158 2:N:0:TCCTTAGC+ACTCGATT
GTTCCAAACATGCATGTCGAAAAGAGACTGTTCAGAGGTGACAAGATTAGAAGGTCACGCTCGATTTC
TGCAAAAAGGGGGCCAAACTCGAAACCGCAGGAAAAGAGAGGATTTAGGTCCCTCTCAGCTAGAATTG
AAAGATTCGGAGAAA
+
FFFFFFFFFFFFFFFFFFFFFFFFFFFFFFFFFFFFFFFFFFFFFFFFFFFFFFFFFFFFFFFFFFFF
FFFFFFFFFFFFFFFFFFFFFFFFFFFFFFFFF:FFFFFFFFFF:FFFFFFFFFFFFFFFFFFFFFFF
FFFFFFF:FFFFFFF
@A01056:60:HMTK7DSXY:1:2566:13747:5259 2:N:0:TCCTTAGC+ACTCGATT
GTCGAAAAGAGACTGTTCAGAGGTGACAAGATTAGAAGGTCACGCTCGATTTCTGCAAAAAGGGGGCC
AAACTCGAAACCGCAGGAAAAGAGAGGATTTAGGTCCCTCTCAGCTAGAATTGAAAGATTCGGAGAAA
ATGAGTTTGGAAGAC
+
FFFFFFFFFFFFFFFFFFFFFFFFFFFFFFFFFFFFFFFFFFFFFFFFFFFFFFFFFF:FFFFFFFFF
FFFFFFFFFFFFFF:FFFFFFFFFFF:FFFFFFFFFFFFFFFFFFFF:FFFFFFFFFFFF:FFFFFFF
FFFFFFFFFF:FF:F
@A01056:60:HMTK7DSXY:1:1566:13575:15233 2:N:0:TCCTTAGC+ACTCGATT
GTCGAAAAGAGACTGTTCAGAGGTGACAAGATTAGAAGGTCACGCTCGATTTCTGCAAAAAGGGGGCC
AAACTCGAAACCGCAGGAAAAGAGAGGATTTAGGTCCCTCTCAGCTAGAATTGAAAGATTCGGAGAAA
ATGAGTTTGGAAGAC
+
FFFFFFFFFFFFFFFFFFFFFFFFFFFFFFFFFFFFFFFFFFFFFFFFFFFFFFFFFFFFFFFFFFFF
FFFFFFFFFFFFFFFFFF:FFFFFFFFFFFFFFFFFFFFFFFFFFFFFFFFFFFFFFFFFFFFFFFFF
FFFFFFFFFFFFFFF
@A01056:60:HMTK7DSXY:1:1566:13657:2691 2:N:0:TCCTTAGC+ACTCGATT
GTCGAAAAGAGACTGTTCAGAGGTGACAAGATTAGAAGGTCACGCTCGATTTCTGCAAAAAGGGGGCC
AAACTCGAAACCGCAGGAAAAGAGAGGATTTAGGTCCCTCTCAGCTAGAATTGAAAGATTCGGAGAAA
ATGAGTTTGGAAGAC
+
FFFFFFFFFFFFFFFFFFFFFFFFFFFFFFFFFFFFFFFFFFFFFFFFFFFFFFFFFFFFFFFFFFFF
FFFFFFFFFFFFFFFFFFFFFFFFFFFFFFFFFFFFFFFFFFFFFFFFFFFFFFFFFFFFFFFFFFFF
FFFFFFFFFFFFFFF
@A01056:60:HMTK7DSXY:1:2234:4444:24158 1:N:0:TCCTTAGC+ACTCGATT
AAAGAGACTGTTCAGAGGTGACAAGATTAGAAGGTCACGCTCGATTTCTGCAAAAAGGGGGCCAAACT
CGAAACCGCAGGAAAAGAGAGGATTTAGGTCCCTCTCAGCTAGAATTGAAAGATTCGGAGAAAATGAG
TTTGGAAGACGTGCT
+
FFFFFFFFFFF:FFFFFFFFFFFFFFFFFF:FFFFFFFFFFFFFFFFFFFFFFFFFFFFFFFFFFF:F:
FFFFFFFFFFFFFFFFFFFFFFFFFFFFFFFFFFFFFFFF:FFFFFFFFFFFFFFFFFFFFFFFFFFF
FFFFFFFFFFFFFFF
@A01056:60:HMTK7DSXY:1:1607:26738:4398 2:N:0:TCCTTAGC+ACTCGATT
GTGACAAGATTAGAAGGTCACGCTCGATTTCTGCAAAAAGGGGGCCAAACTCGAAACCGCAGGAAAAG
AGAGGATTTAGGTCCCTCTCAGCTAGAATTGAAAGATTCGGAGAAAATGAGTTTGGAAGACGTGAGAT
CGGAAGAGCGTCGTG
+
FFFFFFFFFFFFFFFFFFFFFFFFFFFFFFFFFFFFFFFFFFFFFFFFFFFFFFFFFFFFFFFFFFFF
FFFFFFFFFFFFFFFFFFFFFFFFFFFFFFFFFFFFFFFFFFFFFFFFFFFFFFFFFFFFFFFFFFFF
FFFFFFFFFFFFFFF
@A01056:60:HMTK7DSXY:1:2566:13747:5259 1:N:0:TCCTTAGC+ACTCGATT
GATTAGAAGGTCACGCTCGATTTCTGCAAAAAGGGGGCCAAACTCGAAACCGCAGGAAAAGAGAGGAT
TTAGGTCCCTCTCAGCTAGAATTGAAAGATTCGGAGAAAATGAGTTTGGAAGACGTGCTTCAACAAGC
GAGGCGCCACCGGGT
+
FFFFFFFFFFFFFFFFFFFFFFFFFFFFF:FFFFFF:FFF::FFFFFFFFFFFFFFFFFFFFFFFFFF
```

```
FFFFFFFFFFFFFFFFFFFFFFFFFFFFFFFFFFFFFFFFFFFFFFFFFFFFFFFFFFFFFFFFFFFF
FFFFFFFFFFFFFFF
@A01056:60:HMTK7DSXY:1:1566:13575:15233 1:N:0:TCCTTAGC+ACTCGATT
GATTAGAAGGTCACGCTCGATTTCTGCAAAAAGGGGGCCAAACTCGAAACCGCAGGAAAAGAGAGGAT
TTAGGTCCCTCTCAGCTAGAATTGAAAGATTCGGAGAAAATGAGTTTGGAAGACGTGCTTCAACAAGC
GAGGCGCCACCGGGT
+
FFFFFFFFFFFFFFFFFFFFFFFFFFFFFFFFFFFFFFF,F:FFFF,FFFFFFFFFFFFFFFFFFFF
FFFFFFFFFFFFFFFFFFFFFFFFFFFFFFFFFFFFFFFFFFFFFFFFFFFFFFFFFFFFFFFFFFFF
FFFFFFFFFFFFFFF
@A01056:60:HMTK7DSXY:1:1566:13657:2691 1:N:0:TCCTTAGC+ACTCGATT
GATTAGAAGGTCACGCTCGATTTCTGCAAAAAGGGGGCCAAACTCGAAACCGCAGGAAAAGAGAGGAT
TTAGGTCCCTCTCAGCTAGAATTGAAAGATTCGGAGAAAATGAGTTTGGAAGACGTGCTTCAACAAGC
GAGGCGCCACCGGGT
+
FFFFFFFFFFFFFFFFFFFFFFFFFFFFFFFFFFFFFFF,:FFFFFFFFFFFFFFFFFFFFFFFFFFF
FFFFFFFFFFFFFFFFFFFFFFFFFFFFFFFFFFFFFFFFFFFFFFFFFFFFFFFFFFFFFFFFF:FF
FFFFFFFFFFFFFFF
@A01056:60:HMTK7DSXY:1:2275:24415:13213 2:N:0:TCCTTAGC+ACTCGATT
CTCGAAACCGCAGGAAAAGAGAGGATTTAGGTCCCTCTCAGCTAGAATTGAAAGATTCGGAGAAAATG
AGTTTGGAAGACGTGCTTCAACAAGCGAGGCGCCACCGGGTAGGAGTGTATCTTTGGAAGACGCATAT
AGACCCGGCAAAGGA
+
FFFFFFFFFFFFFFFFFF::FFFFF,FFFFFFFFFFFFFFFFFFFFFFFFFFFFFFFFFFFFFFFFFF
FFFFFFFFFF:FFFFFFFFFFFFFFFFFFFFFFFFFFFFFFFFFFFFFFFFFFFFFFFFFFFFFFFFF
FFFFFFFFFFFFFFF
@A01056:60:HMTK7DSXY:1:2275:24415:13213 1:N:0:TCCTTAGC+ACTCGATT
GTATCTTTGGAAGACGCATATAGACCCGGCAAAGGAACTTCTGACGGTTCCTCCCCCTGAAGGGTTTA
AGGAAGGTGAAAGCTTTGAAGGCAAAGAGCTTTACCTTCTTCTTTGCAACCATTATTGTAAATATTTA
TTTGGTAATATTGCT
+
F:F:FFFFFFFFFFFFFFFFFFFFFFFFFFFFFFFFF:FFFFFFFFFFFFFFFFFFFFFFFFFFF::FF
FFFFFFFFFFFFFFFFFFFFFFFFFFFFFFFFFFFFF:FFFFFFFFFFFFFFFFFFFFFFFFFFFFFF
FFFFFFFFFFFFFFF
@A01056:60:HMTK7DSXY:1:2110:21856:23156 2:N:0:TCCTTAGC+ACTCGATT
TATTGCTGTTTTCGGGTCATCTGATAAGACCCAGTTTCCTGCTGTTGGATTTGATACCCCTCCGGTTC
ATTACAATTTGACAACGATCCCGAAAGAAGGGGAAACTGAAGAGCAAAAGAAGGCCAGAGAGGGTTCG
TCTGGCGAAAAAACA
+
FFFFFFFFFFFFFFFFFFFFFFFFFFFFFFFFFFFFFFFFFFFFFFFFFFFF:FFFFFFFFFFFFFFF
FFFFFFFFFFFFFFFFFFFFF:FFFFFFFFFFFFFFFFF:FFFFFF:,FFFFFFFFFFFFFFF,FFFFF
FFFFFF:FFF:FFFF
@A01056:60:HMTK7DSXY:1:1309:4851:7420 2:N:0:TCCTTAGC+ACTCGATT
GCTGTTTTCGGGTCATCTGATAAGACCCAGTTTCCTGCTGTTGGATTTGATACCCCTCCGGTTCATTA
CAATTTGACAACGATCCCGAAAGAAGGGGAAACTGAAGAGCAAAAGAAGGCCAGAGAGGGTTCGTCTG
GCGAAAAAACAAAAA
+
F:FFFFFFFFFFFFFFFFFFFFFFFFFFFFFFFFFFFFFFFFFFFFFFFFFFFFFFFFFFFFFFFFFF
FFFFFFFFFFFFFF:FFFFFFFFFFFFFFFFFFFFFFFF:FFFFFFFFFFFFFFFFFFFFFFFFFFFFF
FFFFFFF:FFFFFFF
@A01056:60:HMTK7DSXY:1:1309:4851:7420 1:N:0:TCCTTAGC+ACTCGATT
CCTGCTGTTGGATTTGATACCCCTCCGGTTCATTACAATTTGACAACGATCCCGAAAGAAGGGGAAAC
TGAAGAGCAAAAGAAGGCCAGAGAGGGTTCGTCTGGCGAAAAAACAAAAATTTGGAGAATCGACTTGT
CAAATGTTGTACCTG
```

```
+
FFFFFFFFFFFFFFFFFFFFFFFFFF:FFFFFFFFFFFFFFFFFFFFFFFFFFFFFFFFFFFFFFFFF
FFFFFFFFFFFFFFFFFFFFFFFFFFFFFFFFFFFFFFFFFFFFFFFFFFFFFFFFFFFFFFFFFFFF
FFFFFFFFFFFFFFF
@A01056:60:HMTK7DSXY:1:1440:9661:31093 2:N:0:TCCTTAGC+ACTCGATT
CTGCTGTTGGATTTGATACCCCTCCGGTTCATTACAATTTGACAACGATCCCGAAAGAAGGGGAAACT
GAAGAGCAAAAGAAGGCCAGAGAGGGTTCGTCTGGCGAAAAAACAAAAATTTGGAGAATCGACTTGTC
AAATGTTGTACCTGA
+
:FF:,FFF,FFFFFFFFFFFFFFFFFFFFFFFFFFFFFFFFFFFF:FF:FFFFFFF:FFF:FFFFF:F
FFFFFFFFFF:FFFFFFFF:FFFFFFFFFFFFF,FFFFFFFFFFFFFFFFF:FFFFFFFFFFF:FFFF
FFF:F:F,F,::FFF
@A01056:60:HMTK7DSXY:1:1567:6008:11772 2:N:0:TCCTTAGC+ACTCGATT
GGGAAACTGAAGAGCAAAAGAAGGCCAGAGAGGGTTCGTCTGGCGAAAAAACAAAAATTTGGAGAATC
GACTTGTCAAATGTTGTACCTGAATTGAAAACCTTTGCTGCCACTTCTAGGCAGAACTCTTTGAATGA
ATGTACATTCAGAAA
+
FFF:FFF:FFFFF:FFFFFFFFFFFFFFFFF::FFFFFFFFFFFFFFFFFFFFFFFFFFFFFFFFFFF
FFFFFFFFF:,FFFFFFF,::FFF,FFFFFFFFFFFFFFF,FFFFFFFF:FFFFFFF,FFFFFFFFF:
FFFF:FF:FFFFFFF
@A01056:60:HMTK7DSXY:1:1518:27588:34303 2:N:0:TCCTTAGC+ACTCGATT
GGGAAACTGAAGAGCAAAAGAAGGCCAGAGCGGGTTCGTCTGGCGAAAAAACAAAAATTTGGAGAATC
GACTTGTCAAATGTTGTACCTGAATTGAAAACCTTTGCTGCCACTTCTAGGCAGAACTCTTTGAATGA
ATGTACATTCAGAAA
+
FFF:FFFFFFFFFFFFFFFFFF:FFFFF:F,FFFFFFFFFFFFFFFFFF:FFFFFF:F:FFFFFFFFF
FFFFFFF,FFFFFFFF,FF,:FF::F:FFFFF:FF::FFFFFFFF:F:F,F:FFFF:FFFFFF:FFFF
,F,FFFF:FF:FF:F
@A01056:60:HMTK7DSXY:1:2337:18240:21621 2:N:0:TCCTTAGC+ACTCGATT
GGGAAACTGAAGAGCAAAAGAAGGCCAGAGAGGGTTCGTCTGGCGAAAAAACAAAAATTTGGAGAATC
GACTTGTCAAATGTTGTACCTGAATTGAAAACCTTTGCTGCCACTTCTAGGCAGAACTCTTTGAATGA
ATGTACATTCAGAAA
+
FFFFFFFFFFFFFFFFFFFFFFFFFFFFFFFFFFFFFFFFFFFFFFFFFFFFFFFFF,FFFFF:FFFF
FFFFFFFFFFFFF::FFFFFFFFFF:FFFFFF,FFF:FFF:FFFFFF:FFFFFFF,FFFFFF,:FFF:
FFFFFF:FFF,FFFF
@A01056:60:HMTK7DSXY:1:1165:4707:8422 2:N:0:TCCTTAGC+ACTCGATT
TGAAGAGCAAAAGAAGGCCAGAGAGGGTTCGTCTGGCGAAAAAACAAAAATTTGGAGAATCGACTTGT
CAAATGTTGTACCTGAATTGAAAACCTTTGCTGCCACTTCTAGGCAGAACTCTTTGAATGAATGTACA
TTCAGAAAGCTTTGT
+
FFFFFFFFFFFFFFFFFFFFFFFFFFFFFFFFFF:FFFFFFFFFFFFFFFFFFFFFFFFFFFFFF::F
FFFFFFFFFFFFFFFFFFFFF:F,FFFFFFFFFFFFFFFFFFFFFFFFFFFFFFFFFF:FFFFFFFFF
FFFFFFFFFF:FFF:
@A01056:60:HMTK7DSXY:1:1165:5556:18474 2:N:0:TCCTTAGC+ACTCGATT
TGAAGAGCAAAAGAAGGCCAGAGAGGGTTCGTCTGGCGAAAAAACAAAAATTTGGAGAATCGACTTGT
CAAATGTTGTACCTGAATTGAAAACCTTTGCTGCCACTTCTAGGCAGAACTCTTTGAATGAATGTACA
TTCAGAAAGCTTTGT
+
FFFFFFFFFFFFFFFFFFFFFFFFFFFFFFFFFFFFFFFFFFFFFFF:FFFFFFFFFFFFFFFFFFFF
FFFFFFFFFFFFFFFFFFFFFF:FFFFFFF:FFFFFFFFFFF:FFF:FFFFFFFFFFFFFFFFFFFFF
FFFFFFFFFF:FFFF
@A01056:60:HMTK7DSXY:1:2478:24957:32847 2:N:0:TCCTTAGC+ACTCGATT
GTTCGTCTGGCGAAAAAACAAAAATTTGGAGAATCGACTTGTCAAATGTTGTACCTGAATTGAAAACC
```

```
TTTGCTGCCACTTCTAGGCAGAACTCTTTGAATGAATGTACATTCAGAAAGCTTTGTGAGCCATTCGC
TGATTTGGCCCGTGA
+
FFFFFFFFFFFFFFFFFFFFFFFFFFFFFFFFFFFFFFFFFFFFFFFFFFFFFFFFFFFFFFFFFFFF
FFFFFFFFFFFFFFFFFFFFFFFFFFFFFFFFFFF:FFFFFFFFFFFFFFFFFFFFFF:FFFFFFFFF
:FFF:FFFFFFF:F:
@A01056:60:HMTK7DSXY:1:1440:9661:31093 1:N:0:TCCTTAGC+ACTCGATT
AAACAAAAATTTGGAGAATCGACTTGTCAAATGTTGTACCTGAATTGAAAACCTTTGCTGCCACTTCT
AGGCAGAACTCTTTGAATGAATGTACATTCAGAAAGCTTTGTGAGCCATTCGCTGATTTGGCCCGTGA
GTTTCTTCATGAAAG
+
:FFFFFFFFFFFFFF:F:FFFFFFFFFFFFFFFFFFFFFFFFFFFFFFFFFFFFFFFFFFFFFF:FFF
FFFFFFFFFFF:FFFFFFFFFFFFFFFFFFFFFFFFFFFFFFFFFFFF,FFFFFFFFFFFFFFFFFFF
FFFFFFFFFFFFFFF
@A01056:60:HMTK7DSXY:1:2123:9688:34115 2:N:0:TCCTTAGC+ACTCGATT
CCTGAATTGAAAACCTTTGCTGCCACTTCTAGGCAGAACTCTTTGAATGAATGTACATTCAGAAAGCT
TTGTGAGCCATTCGCTGATTTGGCCCGTGAGTTTCTTCATGAAAGGTGGTCGAAAGGATTGGCCACCA
ACATTTATAAGAAAT
+
FFFFFFFFFFFFFFFFFFFFFFFFFFFFFFFFFFFFFFFFFFFFFFFFFFFFFFFFFFFFFFFFFFFF
FFFFFFFFFFFFFFFFFFFFFFFFFFFFFFFFFFFFFFFFFFFFFFFFFFFFFFFFFFFFFFFFFFFF
FFFFFFFFFFFFFFF
@A01056:60:HMTK7DSXY:1:1437:20654:12493 2:N:0:TCCTTAGC+ACTCGATT
CCTGAATTGAAAACCTTTGCTGCCACTTCTAGGCAGAACTCTTTGAATGAATGTACATTCAGAAAGCT
TTGTGAGCCATTCGCTGATTTGGCCCGTGAGTTTCTTCATGAAAGGTGGTCGAAAGGATTGGCCACCA
ACATTTATAAGAAAT
+
FFFFFFFFFFFFFFFFFFFFFFFFFFFFFFFFFFFFFFFFFFFFFFFFFFFFFFFFFFFFFFFFFFFF
FFFFFFFFFFFFFFFFFFFFFFFFFFFFFFFFFFFFFFFFFFFF:FFFFFFFFFFFFFFFFFFFFFFF
FFFF,FFFFFFFFFF
@A01056:60:HMTK7DSXY:1:2478:24957:32847 1:N:0:TCCTTAGC+ACTCGATT
GAATTGAAAACCTTTGCTGCCACTTCTAGGCAGAACTCTTTGAATGAATGTACATTCAGAAAGCTTTG
TGAGCCATTCGCTGATTTGGCCCGTGAGTTTCTTCATGAAAGGTGGTCGAAAGGATTGGCCACCAACA
TTTATAAGAAATGGC
+
FFFFFFFFFFFFF:FFFFFFFFFFFFFFFFFFFFFFFFFFFFFFFFFFFFFFFFFFFFFFFFFFFFFF
FFFFFFFFFFFFFFFFFFFFFFFFFFFFFFFFFFFFFFFFFFFFFFFFFFFFFFFFFFFFFFFFFFFF
FFFFFFFFFFFFFFF
@A01056:60:HMTK7DSXY:1:1437:20654:12493 1:N:0:TCCTTAGC+ACTCGATT
GAATTGAAAACCTTTGCTGCCACTTCTAGGCAGAACTCTTTGAATGAATGTACATTCAGAAAGCTTTG
TGAGCCATTCGCTGATTTGGCCCGTGAGTTTCTTCATGAAAGGTGGTCGAAAGGATTGGCCACCAACA
TTTATAAGAAATGGC
+
FFFFF:FFFFFFFFFFF:FFFFF:FF:FFFFFFFFFF:FFFFFFFFFFFFFFFF,FFFFFFFFFF:FF
FFFFFFFFFFFFFFFFFFFFFFFFFFFFFFFFFFFFFFFFFFFFFFFFFFFFFFFFFFFFFFFFFFFF
FFFFFFFFFFFFFFF
@A01056:60:HMTK7DSXY:1:1165:5556:18474 1:N:0:TCCTTAGC+ACTCGATT
GAATTGAAAACCTTTGCTGCCACTTCTAGGCAGAACTCTTTGAATGAATGTACATTCAGAAAGCTTTG
TGAGCCATTCGCTGATTTGGCCCGTGAGTTTCTTCATGAAAGGTGGTCGAAAGGATTGGCCACCAACA
TTTATAAGAAATGGC
+
FFFFF:FFFFFFFFFFFFFFFFFFF:FFFFFFFFFFFFFFFFFFFFFFFFFFFFFFFFFFFFFFFFFF
FFFFFFFFFFFFFFFFFFFFFFFFFFFFFFFFFFFFFFFFFFFFFFFFFFFFFFFFFFFFFFFFFFFF
FFFFFFFFFFFFFFF
```

```
@A01056:60:HMTK7DSXY:1:1165:4707:8422 1:N:0:TCCTTAGC+ACTCGATT
GAATTGAAAACCTTTGCTGCCACTTCTAGGCAGAACTCTTTGAATGAATGTACATTCAGAAAGCTTTG
TGAGCCATTCGCTGATTTGGCCCGTGAGTTTCTTCATGAAAGGTGGTCGAAAGGATTGGCCACCAACA
TTTATAAGAAATGGC
+
FFFFFFFFFFFFF:FFFFFFFFFF,FFFFFFFFFFFFFFF:FFFFFFFFFFFFFFFFFFFFFFFFF:FFF
FFFFFFFFFFFFFFFFFFFFFFFFFFFFFFFF::FFFFFFFFFFFFFFFFFFFFFFFFFFFFFFFFFFFF
FFFFFFFFFFFFF:F
@A01056:60:HMTK7DSXY:1:2123:9688:34115 1:N:0:TCCTTAGC+ACTCGATT
GAATTGAAAACCTTTGCTGCCACTTCTAGGCAGAACTCTTTGAATGAATGTACATTCAGAAAGCTTTG
TGAGCCATTCGCTGATTTGGCCCGTGAGTTTCTTCATGAAAGGTGGTCGAAAGGATTGGCCACCAACA
TTTATAAGAAATGGC
+
FFFFFFFFFFFFFFFFFFFFFFFFFFFFFFFFFFFFFFFFFFFFFFFFFFFFFFF,FFFFFFFFFF:FFF
FFFFFFFFFFFFFFFFFFFFFFFFFFFFFFFFFFFFFFFFFFFFFFFFFFFFFFFFFFFFFFFFFFFFFF
FFFFFFFFFFFFFFF
@A01056:60:HMTK7DSXY:1:2260:9489:26256 2:N:0:TCCTTAGC+ACTCGATT
CGTGAGTTTCTTCATGAAAGGTGGTCGAAAGGATTGGCCACCAACATTTATAAGAAATGGCCCAAAGC
TTTTGAAAAAAGTCCTTGGGTGGCATTTGATTTTGCCACCGGTCTAAAAATGAACCGTTTAACACCTG
ATGAAAAACAGGTGA
+
FFFFFFFFFFFFFFFFFFFFFFFFFFFFFFFFFFFFFFFFFFFFFFFFFFFFFFFFFFFFFFFFFFFFFF
FFFFFFFFFFFFFFFFFFFFFFFFFFFFFFFFFFFFFFFFFFFFFFFFFFFFFFF:FFFFFFFFFFFFF:
:FFFFFFFFFF:FFF
@A01056:60:HMTK7DSXY:1:2260:9489:26256 1:N:0:TCCTTAGC+ACTCGATT
GGTGGCATTTGATTTTGCCACCGGTCTAAAAATGAACCGTTTAACACCTGATGAAAAACAGGTGATCG
ACAGGATGACCAAAAGGCTTTTTCGTACTGAAGGACAAAAAGGGGTTTTTGAGGCAGGTTCGGAGGGT
AACCTGGAACTGGAG
+
FFFFFFFFFFFFFFFFFFFFFFFFFFFFFFFFFFFFFFFFFF,F:FFFFFFFF:FFFFFFFFFFFFFFFF
FFFFFFFFFFFFFFFFFFFFFFFFFFFFFFFFFFFFFFFF:FFFFFFFFFFFFFFFFFFFFFFFFFFFFF
FFFFFFFFFFFFFFF
@A01056:60:HMTK7DSXY:1:2171:23647:18709 2:N:0:TCCTTAGC+ACTCGATT
ATTTGATTTTGCCACCGGTCTAAAAATGAACCGTTTAACACCTGATGAAAAACAGGTGATCGACAGGA
TGACCAAAAGGCTTTTTCGTACTGAAGGACAAAAAGGGGTTTTTGAGGCAGGTTCGGAGGGTAACCTG
GAACTGGAGGGTTAG
+
FFFFFFFFFFFFFFFFFFFFFFFFFFFFFFFFFFFFFFFFFFFFFFFFFFFFFFFFFFFFFFFFFFFFFF
FFFFFFFFFFFFFFFFFFFFFFFFFFFFFFFFFFFFFFFFFFFFFFFFFFFFFFFFFFFFFFFFFFFFFF
FFFFFFFFFFF:FFF
@A01056:60:HMTK7DSXY:1:2171:23647:18709 1:N:0:TCCTTAGC+ACTCGATT
GATGAAAAACAGGTGATCGACAGGATGACCAAAAGGCTTTTTCGTACTGAAGGACAAAAAGGGGTTTT
TGAGGCAGGTTCGGAGGGTAACCTGGAACTGGAGGGTTAGGAGTCGTGTGAAATTCCGCAAAATTGGT
CGCGGTCTTGCAGGT
+
:FFFFFFFFFFFF:FFFFFFFFFFFFFFFFFFFFFFFFFFF:FFFFFFFFFFFFFFFFFFFFFFFFFFFF
FFFFFFFFFFFFFFFFFFFFFFFFFFFFFFFFFFFFFFFFFFFFFFFFFFFFFFFFFFFFFFFFFFFFFF
FFFFFFFFFFFFFFF
@A01056:60:HMTK7DSXY:1:1567:6008:11772 1:N:0:TCCTTAGC+ACTCGATT
GGACAAAAAGGGGTTTTTGAGGCAGGTTCGGAGGGTAACCTGGAACTGGAGGGTTAGGAGTCGTGTGA
AATTCCGCAAAATTGGTCGCGGTCTTGCAGGTTGACATGCCTGCCTTTATACTTAAATAAAGGGTTCA
CCCGGTTTTCTGTGC
+
FFFFF,FFFFFFFFFFFFFFFFFFFFFFFFFFFF:FFFFFFFFFFFFFFFFFFFFFFFFFF:FFFFFFFF
```

```
FFFFFFFFFFFFFFFFFFFFFF:FFFFFFFFFFFFFFFFFFFFFFFFFFFFFF:FFFFFFFFFFFFFF
FFFFFFFFFFFFFFF
@A01056:60:HMTK7DSXY:1:2337:18240:21621 1:N:0:TCCTTAGC+ACTCGATT
GGACAAAAAGGGGTTTTTGAGGCAGGTTCGGAGGGTAACCTGGAACTGGAGGGTTAGGAGTCGTGTGA
AATTCCGCAAAATTGGTCGCGGTCTTGCAGGTTGACATGCCTGCCTTTATACTTAAATAAAGGGTTCA
CCCGGTTTTCTGTGC
+
F:FFFFF,FFFFFFFF:FFFFFFFFFFFFFFFFF:FFFFFFFFF,FFFFFFFFFFFFFFFFFFFFFFF
:F:FFFFFFF:FFFFFFFFFFFFFFFFFFFFFFFFFFFFFFFFFFFFF:F:FFFFFFFFFFFFFFFFF
FFFFFFFFFFFFFFF
@A01056:60:HMTK7DSXY:1:1518:27588:34303 1:N:0:TCCTTAGC+ACTCGATT
GGACAAAAAGGGGTTTTTGAGGCAGGTTCGGAGGGTAACCTGGAACTGGAGGGTTAGGAGTCGTGTGA
AATTCCGCAAAATTGGTCGCGGTCTTGCAGGTTGACATGCCTGCCTTTATACTTAAATAAAGGGTTCA
CCCGGTTTTCTGTGC
+
,:FFFF:FFFFFF:F:FFFFFFFFFFFFFFFFFFFFFFFFFFFFFFF::FFFFFFFFFFFFFFFFFFF
FFFFFFFFFFFFFFFFFFFF:FFFFFFFFFFFFFFFFFF:FF:FFFFF:F,F:FFFFFFFFFFFFFFF
FFFFFFFFFFFF::F
```
