# Supplementary material for: First Detection and Molecular Characterization of Apple Stem Grooving Virus, Apple Chlorotic Leaf Spot Virus, and Apple Hammerhead Viroid in Loquat in Spain
Source: Plants (Basel). 2021 Oct 25;10(11):2293. doi: 10.3390/plants10112293 (PMC8624106; doi:10.3390/plants10112293)
Supplement: Supplementary file 1 [file plants-10-02293-s001.zip › Mapping_reads_LaTV1.pdf]

@A00155:342:HHGFNDSXY:1:1501:7057:3255 1:N:0:GAACCTAG+TCCGCATA
GATCACTCTATAACAGTACTCGTAACGTGTAATCTACATCTGTTACGTGTGCTATAAGCTTTTTAACA
AGCCTCGCACTACTGTCACTTATCATGCTTATTGGCGCTATTCCTACAAATCTCGCCTTCTCTATAAA
+
FFFFFFFFFFFFFFFFFFFFFFFFFFFFF:FFFFFFFFFFFFFFFFFFFFFFFFFFFFFFFFFFFFFF
FFFFFFFFFFFFFFFFFFFFFFFFFFFFFFFFFFFFFFFF,FFFFFFFFFFFFFFFFFFFFFFFFFFF
@A00155:342:HHGFNDSXY:1:2303:10926:24361 2:N:0:GAACCTAG+TCCGCATA
CTCTATAACAGTACTCGTAACGTGTAATCTACATCTGTTACGTGTGCTATAAGCTTTTTAACAAGCCT
CGCACTACTGTCACTTATCATGCTTATTGGCGCTATTCCTACAAATCTCGCCTTCTCTATAAACGGTA
+
FFF:FFFFFFFFFFFFFF::F,F:FFFFFFFFFFFFFFFFF,FFFFFF,FFFFF:FFFFFFFFFFFF:
FFFFF:FFFFFFFFFFFFFFFFFF:,FFFFFFFFFFFFFF,F:FFFFFFFFFFFFFFF:F,F:FFFF:
@A00155:342:HHGFNDSXY:1:2248:20763:35415 2:N:0:GAACCTAG+TCCGCATA
CTCTATAACAGTACTCGTAACGTGTAATCTACATCTGTTACGTGTGCTATAAGCTTTTTAACAAGCCT
CGCACTACTGTCACTTATCATGCTTATTGGCGCTATTCCTACAAATCTCGCCTTCTCTATAAACGGTA
+
FFFFFFFFFFFFFFFFFFFFFFFFFFFFFFFFFFFFFFFFFFFFFFFFFFFFFFFFFFFFFFFF:FFF
FFFFFFFFFF:FFFFFFFFFFFFFFFFFFFFFFF:FFFFFFFFFFFFFF,FFFFFFFFFFFFFFFFFF
@A00155:342:HHGFNDSXY:1:2563:19144:35618 2:N:0:GAACCTAG+TCCGCATA
CTCTATAACAGTACTCGTAACGTGTAATCTACATCTGTTACGTGTGCTATAAGCTTTTTAACAAGCCT
CGCACTACTGTCACTTATCATGCTTATTGGCGCTATTCCTACAAATCTCGCCTTCTCTATAAACGGTA
+
FFFFFFFFFFFFFFFFFFFFFFFFFFFFFFFFFFFFFFFFFFFFFFFFF:FFFFFFFFFFFFFFFFFF
FFFFFFFFFFFFFFFFFFFFFFF:FFF:FFFFFFFFFFFFFFFFFFFFFFFFFFFFFFFFFFFFFFFF
@A00155:342:HHGFNDSXY:1:2464:7030:20588 2:N:0:GAACCTAG+TCCGCATA
AACGTGTAATCTACATCTGTTACGTGTGCTATATGCTTTTTAACAAGCCTCGCACTACTGTCACTTAT
CATGCTTATTGGCGCTATTCCTACAAATCTCGCCTTCTCTATAAACGGTATGTCTACGATGTGCCGGT
+
FFFFFFFFFFFFFFFFFFFFFFFFFFFFFFFFFFFFFFFFFFFFFFFFFFFFFFFFFFFFFFFFFFFF
FFFFF:FFFFFFFFFFFFFFFFFFF:FFFFFFFFFFFFFFFFFFFFFF:FFFFFFFFFFFFFFFFFFF
@A00155:342:HHGFNDSXY:1:2464:7030:20588 1:N:0:GAACCTAG+TCCGCATA
CGTGTAATCTACATCTGTTACGTGTGCTATATGCTTTTTAACAAGCCTCGCACTACTGTCACTTATCA
TGCTTATTGGCGCTATTCCTACAAATCTCGCCTTCTCTATAAACGGTATGTCTACGATGTGCCGGTAC
+
FFFFFFFFFFFFFFFFFFFFFFFFFFFFFFFFFFFFFFFFFFFFFFFFFFFFFFFFFFFFFFFFFFFF
FFFFFFFFFFFFFFFFFFFFFFFFFFFFFFFFFFFFFFFFFFFFFFFFFFFFFFFFFFFFFFFFFFFF
@A00155:342:HHGFNDSXY:1:2248:20763:35415 1:N:0:GAACCTAG+TCCGCATA
TAATCTACATCTGTTACGTGTGCTATAAGCTTTTTAACAAGCCTCGCACTACTGTCACTTATCATGCT
TATTGGCGCTATTCCTACAAATCTCGCCTTCTCTATAAACGGTATGTCTACGATGTGCCGGTACTGCC
+
FFFFFFFFFFFFFFFFFFFFFFFFFFFFFFFFFFFFFFFFFFFFFFFFF,FFFFFFFFFFFFFFFFFF
FFFFFFFFFFFFFFFFFFFFFFFFFFFF:FFFFFFFFFFFFFFFFFFFFFFFFFFFFFFFFFFFFFFF
@A00155:342:HHGFNDSXY:1:2303:10926:24361 1:N:0:GAACCTAG+TCCGCATA
TAATCTACATCTGTTACGTGTGCTATAAGCTTTTTAACAAGCCTCGCACTACTGTCACTTATCATGCT
TATTGGCGCTATTCCTACAAATCTCGCCTTCTCTATAAACGGTATGTCTACGATGTGCCGGTACTGCC
+
FFFFF:F,FF,FFFF:FFFFFFFFFF,FFF:FFFFF,F,:FFFF:F:,,FFFFFF,FF:::F:FFF,F
FFFFF,,F:FF,FFF:F,,,FFFFFF:FFFFF:FFFFFFFFFFFFFF:F::FFFFFF:FFFFF,FFF,
@A00155:342:HHGFNDSXY:1:2563:19144:35618 1:N:0:GAACCTAG+TCCGCATA
TAATCTACATCTGTTACGTGTGCTATAAGCTTTTTAACAAGCCTCGCACTACTGTCACTTATCATGCT
TATTGGCGCTATTCCTACAAATCTCGCCTTCTCTATAAACGGTATGTCTACGATGTGCCGGTACTGCC
+
FFFFFFFFFFFFFFFFFFFFFFFFFFFFFFFFFFFFFFFFFFFFFFFFFFFFFFFFFFFFFFFF:FFF
FFFFFFFFFFFFFFFFFFFF:FFFFFFFFFFFFFFFF:FFFFFFFFFFFFFFFFFFFFFFFFFFFFFF

```
@A00155:342:HHGFNDSXY:1:1212:22408:29340 2:N:0:GAACCTAG+TCCGCATA
CGTGTGCTATAAGCTTTTTAACAAGCCTCGCACTACTGTCACTTATCATGCTTATTGGCGCTATTCCT
ACAAATCTCGCCTTCTCTATAAACGGTATGTCTACGATGTGCCGGTACTGCCTGAACAATGCCC
+
FFFFFFFFFFFFFF:FFFFFFFFF:FFFFFFFFFFFFFFFFFFFFFFFFFFFF:FFFFFFFF:FFFFF
FFFFFFFFFFFFFFFFFFFFFFFFFFFFFFFFFFFFFFFFFFFFFFFFFFFFFFFFFF:FFFFF
@A00155:342:HHGFNDSXY:1:2637:3558:28260 1:N:0:GAACCTAG+TCCGCATA
TGTGCTATAAGCTTTTTAACAAGCCTCGCACTACTGTCACTTATCATGCTTATTGGCGCTATTCCTAC
AAATCTCGCCTTCTCTATAAACGGTATGTCTACGATGTGCCGGTACTGCCTGAACAATGCCCTACCGT
+
FFFF,FFFFFFF:FFFFFFFFFFFFFFFFFFFFFFFFFFFFFFFFFFFFFFFFFFFFFFFF:FFFFFF
FFFFFFFFFFFFFFFFFFFFFFFFFFFFFFFFFFFFFFFFFFFFFFFFFFFFFFFFFFF:FFFFFFFF
@A00155:342:HHGFNDSXY:1:1212:22408:29340 1:N:0:GAACCTAG+TCCGCATA
ATAAGCTTTTTAACAAGCCTCGCACTACTGTCACTTATCATGCTTATTGGCGCTATTCCTACAAATCT
CGCCTTCTCTATAAACGGTATGTCTACGATGTGCCGGTACTGCCTGAACAATGCCCTACCGTATGCAT
+
FFFFFFFFFFFFFFFFFFFFFFFFFFFFFF:FFFFFFFFFFFFFF:FFFFFFFFF:FFFFFFFFFFFF
FFFFFFFFFFFFFF:FFFFFFFFFFFFFFFF:FFFFFFFFFFFFFFFFFFFFFFFFFFFFFFFFFFFF
@A00155:342:HHGFNDSXY:1:1369:23113:4789 1:N:0:GAACCTAG+TCCGCATA
AGCTTTTTAACAAGCCTCGCACTACTGTCACTTATCATGCTTATTGGCGCTATTCCTACAAATCTCGC
CTTCTCTATAAACGGTATGTCTACGATGTGCCGGTACTGCCTGAACAATGCCCTACCGTATGCATACC
+
FFFFFFFFFFFF:FFFFFFFFFF:FFFFFFFFFFFFFFFFFFFFFFFFFFFFFFFFFFFFFFFFFFFF
F,FFFFFFFFFFFFFFFFFFFFFFFFFFFFFFFFFFFFFFFFFFFFF:FFFFFFFFFF:FFFF,FFFF
@A00155:342:HHGFNDSXY:1:2637:3558:28260 2:N:0:GAACCTAG+TCCGCATA
AGCTTTTTAACAAGCCTCGCACTACTGTCACTTATCATGCTTATTGGCGCTATTCCTACAAATCTCGC
CTTCTCTATAAACGGTATGTCTACGATGTGCCGGTACTGCCTGAACAATGCCCTACCGTATGCATACC
+
FFFFFFFFFFFFFFFFFFFFFFFFFFFFFFFFFFFFFFFFFFFFFFFFFFFFFFFFFFFFFFFFFFFF
FFFFFFFFFFFFF:FF:FFFFFFFF:FFFFFFFFFFFFFFFFFFFFFFF:FFFFFFFF:FFFFFFFFF
@A00155:342:HHGFNDSXY:1:1501:7057:3255 2:N:0:GAACCTAG+TCCGCATA
GCTTTTTAACAAGCCTCGCACTACTGTCACTTATCATGCTTATTGGCGCTATTCCTACAAATCTCGCC
TTCTCTATAAACGGTATGTCTACGATGTGCCGGTACTGCCTGAACAATGCCCTACCGTATGCATACCT
+
FF:FFFFFFFFFFFFFFFFFFFFFFFFFFFFFFFFFFFFFFFFFFFFFFFFFFFFFFFFFFFFFFFFF
:F:F:FFFFFFFFFFFFFFFFFFFFFFFFFFFFFFFFFFFFF:FFFFFFFFFFFFFFFFFFFFFFFFF
@A00155:342:HHGFNDSXY:1:1369:23113:4789 2:N:0:GAACCTAG+TCCGCATA
AACAAGCCTCGCACTACTGTCACTTATCATGCTTATTGGCGCTATTCCTACAAATCTCGCCTTCTCTA
TAAACGGTATGTCTACGATGTGCCGGTACTGCCTGAACAATGCCCTACCGTATGCATACCTCCCTACG
+
FFFFFFFFFFFFFF:FF:FFFFF:FFFFFFFFFFFFFFFFFFFFFFFFFFFFF:FFFFFFFFFFFFF:
F:FFFFFFFFFFFFFFF:FF:FFFFFFFFFFFFFFFFFFFFFFFFFFFFFFFFFFFFFFFFFFFFFFF
@A00155:342:HHGFNDSXY:1:2146:16812:19053 1:N:0:GAACCTAG+TCCGCATA
CTTATCATGCTTATTGGCGCTATTCCTACAAATCTCGCCTTCTCTATAAACGGTATGTCTACGATGTG
CCGGTACTGCCTGAACAATGCCCTACCGTATGCATACCTCCCTACGGCCCGTACGTCTTCCACTTGCA
+
FFFFFFFFFFFFFFFFFFFFFFFFFFFFFFFFFFFFFFFFFFFFFFFFFFFFFFFFFFFFFFFFFFFF
FFFFFFFFFFFFFFFFFFFFFFFFFFFFFFFFFFFFFFFFFFFFFFFFFFFFFFFFFFFFFFFFFFFF
@A00155:342:HHGFNDSXY:1:1173:24569:26349 1:N:0:GAACCTAG+TCCGCATA
ATCATGCTTATTGGCGCTATTCCTACAAATCTCGCCTTCTCTATAAACGGTATGTCTACGATGTGCCG
GTACTGCCTGAACAATGCCCTACCGTATGCATACCTCCCTACGGCCCGTACGTCTTCCACTTGCAACC
+
FFFFFFFFFFFFFFFFFFFFFFFFFFFFFFFFFFFFFFFFFFFFFFFFFFFFFFFFFFFFFFFFFFFF
FFFFFFFFFFFFFFFFFFFFFFFFFFF:FFFFFFFFFFFFFFFFFFFFFFFFFFFFFFFFFFFFFFFF
```

```
@A00155:342:HHGFNDSXY:1:2556:14507:34006 1:N:0:GAACCTAG+TCCGCATA
GGCGCTATTCCTACAAATCTCGCCTTCTCTATAAACGGTATGTCTACGATGTGCCGGTACTGCCTGAA
CAATGCCCTACCGTATGCATACCTCCCTACGGCCCGTACGTCTTCCACTTGCAACCACGTTTTG
+
FFFFFFFFFFFFFFFFFFFFFFFFFFFFFFFFFFFFFFFFFFFFFFFFFFFFFFFFFFFFFFFFFFFF
FFFFFFFFFFFFFFFFFFFFFFFFFFFFFFFFFFFF:FFFFFFFFFFFFFFFFFFFFFF:FFFF
@A00155:342:HHGFNDSXY:1:1618:9146:3740 2:N:0:GAACCTAG+TCCGCATA
GCGCTATTCCTACAAATCTCGCCTTCTCTATAAACGGTATGTCTACGATGTGCCGGTACTGCCTGAAC
AATGCCCTACCGTATGCATACCTCCCTACGGCCCGTACGTCTTCCACTTGCAACCACG
+
FFFFFFFF:FFFFFFFFFFFFFFF:FFFFFFFFFFFFFFFFFFFFFFFFFFFFFFFFFFFFFFFFFFF
FF:FFFFFFFFFFFFFFFFFFFFFFFFF:FFFFFFFFFFFFFFFFFFFFFFFFFFFFFFF
@A00155:342:HHGFNDSXY:1:2665:13268:19429 2:N:0:GAACCTAG+TCCGCATA
GCGCTATTCCTACAAATCTCGCCTTCTCTATAAACGGTATGTCTACGATGTGCCGGTACTGCCTGAAC
AATGCCCTACCGTATGCATACCTCCCTACGGCCCGTACGTCTTCCACTTGCAACCACG
+
FFFFFFFFFFFFFFFFFFFFFFFFFFFFFFFFFFFFFFFFFFFFFFFFFFFFFFFFFFFFFFF:FFFF
FFFFFFFFFFFFFFFFFFFFFFFFFFFFFFFFFFFFFFFFFFFFFFFFFFFFFFFFFFF
@A00155:342:HHGFNDSXY:1:2451:4065:27320 2:N:0:GAACCTAG+TCCGCATA
GCTATTCCTACAAATCTCGCCTTCTCTATAAACGGTATGTCTACGATGTGCCGGTACTGCCTGAACAA
TGCCCTACCGTATGCATACCTCCCGACGGCCCGTACGTCTTCCACTTGCAACCACGTTTTGCGTGTAA
+
FFFFFF:FF:FF,FFFF,FFF:F::,FFFFFFFFF:FF:FFFFFFFFF:FFFFFFFF:FFF:FFFFF:
FFFFF:FF,FFFF,FF,FFFFFFF,,FFFFFFF:FFFFF:FFF,F,FFFFFFF:FF,F::FFFFFFFF
@A00155:342:HHGFNDSXY:1:2451:4065:27320 1:N:0:GAACCTAG+TCCGCATA
TATTCCTACAAATCTCGCCTTCTCTATAAACGGTATGTCTACGATGTGCCGGTACTGCCTGAACAATG
CCCTACCGTATGCATACCTCCCTACGGCCCGTACGTCTTCCACTTGCAACCACGTTTTGCGTGTAACT
+
FFFFFFFFFFFFFFFFFFFFFFFFFFFF:FFFFF:FFFFFFFFFFFFFFFFFFFFFFFFFFFFFFFFF
FFFFFFFFFFFFFFFFFF:FFFFFFFFFFFFFFFFFFFFFFFFFF:FFFF,FFFFFF:FFFF:FFFFFF
@A00155:342:HHGFNDSXY:1:1173:24569:26349 2:N:0:GAACCTAG+TCCGCATA
ATTCCTACAAATCTCGCCTTCTCTATAAACGGTATGTCTACGATGTGCCGGTACTGCCTGAACAATGC
CCTACCGTATGCATACCTCCCTACGGCCCGTACGTCTTCCACTTGCAACCACGTTTTGCGTGTAACTG
+
F,FFFFFFFFFFFFFFF:FFFFFFFFFFFFFFFFFFFFFFFFFFFFFFF:FFFFFFFFFFFF:FFFF
FFFFFFFFFFFFFFFFFFFFFFFFFFFFFFFFFFFFFFFFFF:FFFFFFFFFFFFFFFFFFFFFFFFF
@A00155:342:HHGFNDSXY:1:2560:6732:35634 1:N:0:GAACCTAG+TCCGCATA
ATTCCTACAAATCTCGCCTTCTCTATAAACGGTATGTCTACGATGTGCCGGTACTGCCTGAACAATGC
CCTACCGTATGCATACCTCCCTACGGCCCGTACGTCTTCCACTTGCAACCACGTTTTGCGTGTAACTG
+
FFFFFFFFFFFFF:FFFFFF:F,FFFFFFF,,FFFFFFFF:FFFFFF:FFFFFF:F:FFFFFFFFFFF
FFFFFFF:FFFFFFF:,FFFFFFF:F:FFFFFFFFFF:FFFFF,:,FF:FF,FFF,,FFFF,FFF:FF
@A00155:342:HHGFNDSXY:1:2423:18276:3176 1:N:0:GAACCTAG+TCCGCATA
TTCCTACAAATCTCGCCTTCTCTATAAACGGTATGTCTACGATGTGCCGGTACTGCCTGAACAATGCC
CTACCGTATGCATACCTCCCTACGGCCCGTACGTCTTCCACTTGCAACCACGTTTTGCGTGTAACTGC
+
FFFFFFFFFFFFFFFFFFFFFFFFFFFFFFFFFFFFFFFFFFF:FFFFFFFFFFFFFFFFFFFFFFFFF
FFFFFFFFFFFFFFFFFFFFFFFFFFFFFFFFFFFFFFFFFFFFFFFFFFFFFFFFFFFFFFFFFFFFF
@A00155:342:HHGFNDSXY:1:2572:6343:16673 1:N:0:GAACCTAG+TCCGCATA
TCCTACAAATCTCGCCTTCTCTATAAACGGTATGTCTACGATGTGCCGGTACTGCCTGAACAATGCCC
TACCGTATGCATACCTCCCTACGGCCCGTACGTCTTCCACTTGCAACCACGTTTTGCGTGTAACTGCA
+
FF:FFFFFFFFFFFFFFFFFFFFFFFFFFFFFFFFFFFFFFFF:FFFFFFFFFFFFFFFFFFFFFFFFF
F:FFFFFFFFFFFFFFF,F,FFF:FFF,FFFFFFFFF:FFFFFFFFFFF,FFFFF:,F:F:FFFFFF:
```

```
@A00155:342:HHGFNDSXY:1:1618:9146:3740 1:N:0:GAACCTAG+TCCGCATA
TCCTACAAATCTCGCCTTCTCTATAAACGGTATGTCTACGATGTGCCGGTACTGCCTGAACAATGCCC
TACCGTATGCATACCTCCCTACGGCCCGTACGTCTTCCACTTGCAACCACGTTTTGCGTGTAACTGCA
+
FFFFFFFFFFFFFFFFFFFFFFFFFFFFF:FFFFFFFFFFFFFFFFFFFFFFFFFFFF:FFFFFFFFF
FFFFFFFFFFFFFFFFFFFFFFFFFFFFFFFFFFFFFFFFFFFFFFFFFFFFFFFFFFFF:F:FFFFF
@A00155:342:HHGFNDSXY:1:1603:13024:36479 1:N:0:GAACCTAG+TCCGCATA
AAATCTCGCCTTCTCTATAAACGGTATGTCTACGATGTGCCGGTACTGCCTGAACAATGCCCTACCGT
ATGCATACCTCCCTACGGCCCGTACGTCTTCCACTTGCAACCACGTTTTGCGTGTAACTGCAAGCTGA
+
FFFFFFFFFF:FFFFFFFFFFFFFFFFFFFFFFFFFFFFFFFFFFFFFFFFFFFFFFFFFFFFFFFFF
FFFFFFFFFFFFFFFFFFFFFFFFFFFFFFFFFFFFFFFFFFFFFFFFFFFFFFFFFFFF:FFFFFFF
@A00155:342:HHGFNDSXY:1:2665:13268:19429 1:N:0:GAACCTAG+TCCGCATA
ATCTCGCCTTCTCTATAAACGGTATGTCTACGATGTGCCGGTACTGCCTGAACAATGCCCTACCGTAT
GCATACCTCCCTACGGCCCGTACGTCTTCCACTTGCAACCACGTTTTGCGTGTAACTGCAAGC
+
FFFFFFFFF:FFFFFFFFFFFFFFFFFFFFFFFFFFFFFFFFFFFFFFFFFFFFFFFFFFFFFFFFFF
FFFFFFFFFFFFFFFFFFFFFFFFFFFFFFFFFFFFFFFFFFFFFFFFFF:FFFFFFFFF:FF
@A00155:342:HHGFNDSXY:1:2277:23439:12305 2:N:0:GAACCTAG+TCCGCATA
TCTCGCCTTCTCTATAAACGGTATGTCTACGATGTGCCGGTACTGCCTGAACAATGCCCTACCGTATG
CATACCTCCCTACGGCCCGTACGTCTTCCACTTGCAACCACGTTTTGCGTGTAACTGCAAGCTGACGT
+
FFFFFFFFFFFFFFFFFFFFFFFFFFFFFFFFFFFFFFFFFFFFFFFFFFFFFFFFFFFFFFFFFFFF
FFFFFFFFFFFFFFFFFFFFFFFFFFFFFFFFFFFFFFFFFFFFFFFFFFFFFFFFFFFFFFFFFFFF
@A00155:342:HHGFNDSXY:1:2556:14507:34006 2:N:0:GAACCTAG+TCCGCATA
TTCTCTATAAACGGTATGTCTACGATGTGCCGGTACTGCCTGAACAATGCCCTACCGTATGCATACCT
CCCTACGGCCCGTACGTCTTCCACTTGCAACCACGTTTTGCGTGTAACTGCAAGCTGACGTCTAGTAG
+
FFFFF,F:FFFFFFFFFFFFFFFFFFFFFFFFFFFFFFFFFFFFFFF:FFFFFFFFFFFFFFFFFFFF
FFFF,FFFFFFFFFFFFFFFFFFFFF:FF:FFFFF:FFFFFFFFFFFFFFFFFFFFFFFFFFFFFFFF
@A00155:342:HHGFNDSXY:1:2277:23439:12305 1:N:0:GAACCTAG+TCCGCATA
CTCTATAAACGGTATGTCTACGATGTGCCGGTACTGCCTGAACAATGCCCTACCGTATGCATACCTCC
CTACGGCCCGTACGTCTTCCACTTGCAACCACGTTTTGCGTGTAACTGCAAGCTGACGTCTAGTAGCC
+
FFFFFFFFFFFFFFFFFFFFFFFFFFFFFFFF:FFFFFFFFFFFFFFFFFFFFFFFFFFFFFFFFFFF
FFFFFFFFFFFFFFFFFFFFFFFFFFFFFFFFFFFFFFFFFFFFFFFFFFFFFFFFFFFFFFFFFFFF
@A00155:342:HHGFNDSXY:1:2560:6732:35634 2:N:0:GAACCTAG+TCCGCATA
GGTATGTCTACGATGTGCCGGTACTGCCTGAACAATGCCCTACCGTATGCATACCTCCCTACGGCCCG
TACGTCTTCCACTTGCAACCACGTTTTGCGTGTAACTGCAAGCTGACGTCTAGTAGCCCTAGAGATAC
+
F,FFFFF,FFFF:FFFFF:FFFFFFF,FF:FFFFFFFF,FFFFFFFFFFFFFFFFFFFF:F:FFF:,F
FFFFFF:FFFFFF:FFFFFFFFF:FFFFF,FFF:FFFFFFFFFFFFFFFFFFFFFFFFFFFFFF,FFF
@A00155:342:HHGFNDSXY:1:2150:4001:15624 1:N:0:GAACCTAG+TCCGCATA
TATGTCTACGATGTGCCGGTACTGCCTGAACAATGCCCTACCGTATGCATACCTCCCTACGGCCCGTA
CGTCTTCCACTTGCAACCACGTTTTGCGTGTAACTGCAAGCTGACGTCTAGTAGCCCTAGAGATACTG
+
FFFFFFFFFFFFFFFFFFFFFFFFFFFFFFFFFFFFFFFFFFFFFFFFFFFFFFFFFFFFFFFFFFFF
FFFFFFFFFFFFFFFFFFFFFFFFFFFFFFFFFFFFFF:FFFFFFFFFFFFFFFFFFF::FFFFFFFF
@A00155:342:HHGFNDSXY:1:2465:8874:32049 1:N:0:GAACCTAG+TCCGCATA
TATGTCTACGATGTGCCGGTACTGCCTGAACAATGCCCTACCGTATGCATACCTCCCTACGGCCCGTA
CGTCTTCCACTTGCAACCACGTTTTGCGTGTAACTGCAAGCTGACGTCTAGTAGCCCTAGAGATACTG
+
FFFFFFFFFFFFFFFFFFFFFF,FFFFFFFFFFFFFFFFFFFFFFFFFFFFFFFFFFFFFFFFFFF:F
FFFFFFFFFFFFFFFFF:FFFF:FFFFF:FFFFF,FFFFFFFFFF,FFFFF:FFFFF::FFFFFFF:F
```

```
@A00155:342:HHGFNDSXY:1:1603:13024:36479 2:N:0:GAACCTAG+TCCGCATA
TATGTCTACGATGTGCCGGTACTGCCTGAACAATGCCCTACCGTATGCATACCTCCCTACGGCCCGTA
CGTCTTCCACTTGCAACCACGTTTTGCGTGTAACTGCAAGCTGACGTCTAGTAGCCCTAGAGATACTG
+
FFFFFFFFFFFFFFFFFFFFFFFFFFFFFFFFFFFFFFFFFFFFFFFFFFFFFFFFFFFFFFFFFF:F
FFFFFFFF:FFFFFFFFFFFFFFFFFFFFFFFFFFFFFFFFFFFFFFFFFFFFFFFFFFFFFFFFFFF
@A00155:342:HHGFNDSXY:1:1425:9010:2472 2:N:0:GAACCTAG+TCCGCATA
GTACTGCCTGAACAATGCCCTACCGTATGCATACCTCCCTACGGCCCGTACGTCTTCCACGTGCAACC
ACGTTTTGCGTGTAACTGCAAGCTGACGTCTAGTAGCCCTAGAGATACTGTCTACTATCTGCTTCCTT
+
FF:FFFF,FFFFF::F:FFFFFF:,FF:FFF,FFFF::F:FFFFFFFFFFFFFFFFFFFF,FFFFFFF
FF,:F:FFFFF:F:FFFFFFFFFFFF,FFF:FFFFFFF:F::FFFFFFFFFFFFFF:FFF:FFF::FF
@A00155:342:HHGFNDSXY:1:2150:4001:15624 2:N:0:GAACCTAG+TCCGCATA
GTACTGCCTGAACAATGCCCTACCGTATGCATACCTCCCTACGGCCCGTACGTCTTCCACTTGCAACC
ACGTTTTGCGTGTAACTGCAAGCTGACGTCTAGTAGCCCTAGAGATACTGTCTACTATCTGCTTCCTT
+
FFFFFFFFFFFFFFFFFFFF:FFFFFFFFFFFFFFFFFFFFF:FFFFFFFFFFFFFFFFF:FFFFFFF
FFFFFF:FFFFFFFFFFFFFFFFFFFFFFFFFFFFFFFFFFFFF:FFFFFFFFFFFFFFFFFFFFFFF
@A00155:342:HHGFNDSXY:1:1277:15600:22968 2:N:0:GAACCTAG+TCCGCATA
GTACTGCCTGAACAATGCCCTACCGTATGCATACCTCCCTACGGCCCGTACGTCTTCCACTTGCAACC
ACGTTTTGCGTGTAACTGCAAGCTGACGTCTAGTAGCCCTAGAGATACTGTCTACTATCTGCTTCCTT
+
FFFFFFFF:FFFFFFFFFFFFFFFFFF:FFFFFFFFFFFF:F:FFFFFFFFFFFFFFFFF,FFFFFFF
FFF:FFFFFF:FFFFFFFFFFFFFFFFFFFFFFFFFFFFFFFFFFFFFFFFFFFFFFFFFFFFFFFFF
@A00155:342:HHGFNDSXY:1:2465:8874:32049 2:N:0:GAACCTAG+TCCGCATA
GTACTGCCTGAACAATGCCCTACCGTATGCATACCTCCCTACGGCCCGTACGTCTTCCACTTGCAACC
ACGTTTTGCGTGTAACTGCAAGCTGACGTCTAGTAGCCCTAGAGATACTGTCTACTATCTGCTTCCTT
+
FFFFFFFFFFFFFFFF:FFFF:FFFFFFFFFFFFFFFFFFFFFFFFFFFFFFFFFFFFFFF:FFFFFF
FFFFFFFFFFFFFFFFFFFFFFFFFFFFFFFFFFFFFFFFFFFFFFFFFFFFFFFFFFFFFF,FFFFF
@A00155:342:HHGFNDSXY:1:1425:9010:2472 1:N:0:GAACCTAG+TCCGCATA
ACTGCCTGAACAATGCCCTACCGTATGCATACCTCCCTACGGCCCGTACGTCTTCCACTTGCAACCAC
GTTTTGCGTGTAACTGCAAGCTGACGTCTAGTAGCCCTAGAGATACTGTCTACTATCTGCTTCCTTCC
+
FF:FFFFFFFFFFFF:FFFFFFFFF:FF:FFFF:FFFFFFFFFFFFFFF,:FFFF:FFF:F:FFFFFF
F:FFFFFFFFFFFFFF:FFFFF:FF,F:FFFFF::FFFFFFFFFFFFFFFFFFF::FFFFFFF,FFF:
@A00155:342:HHGFNDSXY:1:1277:15600:22968 1:N:0:GAACCTAG+TCCGCATA
ACTGCCTGAACAATGCCCTACCGTATGCATACCTCCCTACGGCCCGTACGTCTTCCACTTGCAACCAC
GTTTTGCGTGTAACTGCAAGCTGACGTCTAGTAGCCCTAGAGATACTGTCTACTATCTGCTTCCTTCC
+
FFFFFFFFFFFFFFFFFFFFFFFFFFFFFFFFFFFFFFFFFFFFFFFFFFFFFFFFFFFFFFFFFFFF
FFFFFFFFFFFFFFFFFFFFFFFFFFFFFFFFFFFFFFFFFFFFFFFFFFFFFFFFFFFFFFFFFFF:
@A00155:342:HHGFNDSXY:1:2572:6343:16673 2:N:0:GAACCTAG+TCCGCATA
ACAATGCCCTACCGTATGCATACCTCCCTACGGCCCGTACGTCTTCCACTTGCAACCACGTTTTGCGT
GTAACTGCAAGCTGACGTCTAGTAGCCCTAGAGATACTGTCTACTATCTGCTTCCTTCCTACGTACTC
+
FFFFFFFFFF:FFFFFFFFFFFF:FFFFFFFFFFFFF,FFFFFFFFFFFFFFFFFFFFFFFFFFFFFF
FFFFFFFFFFFFFFFFFFFFFFFFFFFFFF:FFFFFFFFFFFFFFFFFFFFFFFFFFFFF:FFFFFFF
@A00155:342:HHGFNDSXY:1:2423:18276:3176 2:N:0:GAACCTAG+TCCGCATA
CCCTACCGTATGCATACCTCCCTACGGCCCGTACGTCTTCCACTTGCAACCACGTTTTGCGTGTAACT
GCAAGCTGACGTCTAGTAGCCCTAGAGATACTGTCTACTATCTGCTTCCTTCCTACGTACTCACCGTA
+
FFFFFFFFFFFFFFFFFFFFFFFFFFFFFFFFFFFFFFFFFFFF:FFFFFFFFFFFFFFFFFFFFFFF
FFFFFFFFFFFFFFFFFFFFFFF,FFF::FFFFFFFFFFFFFFFFFFFFFFFFFFFFFFFFFFFF:FF
```

```
@A00155:342:HHGFNDSXY:1:2265:20518:18584 1:N:0:GAACCTAG+TCCGCATA
TACCGTATGCATACCTCCCTACGGCCCGTACGTCTTCCACTTGCAACCACGTTTTGCGTGTAACTGCA
AGCTGACGTCTAGTAGCCCTAGAGATACTGTCTACTATCTGCTTCCTTCCTACGTACTCACCGTATTG
+
FFFFFFFFFFFFFFFFFFFFFFFFFFFFFFFFFFFFFFFFFFFFFFFFFFFFFFFFFFFFFFFFFFFF
FFFFFFFFFFFFFFFFFFFFFFFFFFFFFFFFFFFFFFFFFFFFFFFFFFFFFFFFFFFFFFFFFFFF
@A00155:342:HHGFNDSXY:1:1221:24361:31187 1:N:0:GAACCTAG+TCCGCATA
ACCGTATGCATACCTCCCTACGGCCCGTACGTCTTCCACTTGCAACCACGTTTTGCGTGTAACTGCAA
GCTGACGTCTAGTAGCCCTAGAGATACTGTCTACTATCTGCTTCCTTCCTACGTACTCACCGTATTGC
+
:FFFFFF::F:FFFFFF:FFFFF:FFFFFFFFFFFF::FFFF:FF:FFFFFF,FFFF:FFFFFFF,::
::FFFF:FFFF:FFF:FFFF:FFFFFFF:FF:FFFF:FF:,:FF,F,F:FFFFFFFFFFFFFF:::FF
@A00155:342:HHGFNDSXY:1:2265:20518:18584 2:N:0:GAACCTAG+TCCGCATA
TGCATACCTCCCTACGGCCCGTACGTCTTCCACTTGCAACCACGTTTTGCGTGTAACTGCAAGCTGAC
GTCTAGTAGCCCTAGAGATACTGTCTACTATCTGCTTCCTTCCTACGTACTCACCGTATTGCCTAACT
+
FFFFFFFFFFFFFFFFFFFFFFFFFFFFFFFFFFFFFFFFFFFFFFFFFFFFFFFFFFFFFFFFFFFF
FFF:FFFFFFFFFFFFFFFFFFFFFFFFFFFFFFFFFFFFFFFF:FFFFFFFFFFFFF:FFFFF:,F,
@A00155:342:HHGFNDSXY:1:2648:12301:15655 2:N:0:GAACCTAG+TCCGCATA
ATACCTCCCTACGGCCCGTACGTCTTCCACTTGCAACCACGTTTTGCGTGTAACTGCAAGCTGACGTC
TAGTAGCCCTAGAGATACTGTCTACTATCTGCTTCCTTCCTACGTACTCACCGTATTGCCTAACTAGC
+
FFFFF:FFFFFFFFFFFFFFFFFF,FFFFFFFFFFFFFFFFFFFFFFFFFFFFFFFFF:FFFFFFFFF
FFFFFFFFFFFFFF:FFFFFFFFFFFFFFFFFFFFFFFFFFFFFFFFFFFFFFFFFFFFFFFFFFFFF
@A00155:342:HHGFNDSXY:1:2544:24442:27508 1:N:0:GAACCTAG+TCCGCATA
ACCTCCCTACGGCCCGTACGTCTTCCACTTGCAACCACGTTTTGCGTGTAACTGCAAGCTGACGTCTA
GTAGCCCTAGAGATACTGTCTACTATCTGCTTCCTTCCTACGTACTCACCGTATTGCCTAACTAGCAC
+
FFFFFFFFFFFFFFFFFFFFFFFFFFFFFFFFFFFFFFFFFFFFFF:FFFFFFFFFFFFFFFFFFFFF
FFFFFFFFFFFFFFFFFFFFFFFFFFFFFFFFFFFFFFFFFFF:FFFFFFFFFFFFFFFFFFFFFFFF
@A00155:342:HHGFNDSXY:1:1653:6976:34835 1:N:0:GAACCTAG+TCCGCATA
ACCTCCCTACGGCCCGTACGTCTTCCACTTGCAACCACGTTTTGCGTGTAACTGCAAGCTGACGTCTA
GTAGCCCTAGAGATACTGTCTACTATCTGCTTCCTTCCTACGTACTCACCGTATTGCCTAACTAGCAC
+
FFFFFFFFFF:FFFFFFFFFFFFFFFFFFFFFFFFFFFFFFFFFFF:FF:FFFFFF,FFFFFFFFFF:
FFFFFFFFFFFFFFFFFFFFFFFFFFFFFFFFFFFFFFFFFFFFFFFFFFFFFFFFFFFFFFFF:FFF
@A00155:342:HHGFNDSXY:1:2648:12301:15655 1:N:0:GAACCTAG+TCCGCATA
CCTCCCTACGGCCCGTACGTCTTCCACTTGCAACCACGTTTTGCGTGTAACTGCAAGCTGACGTCTAG
TAGCCCTAGAGATACTGTCTACTATCTGCTTCCTTCCTACGTACTCACCGTATTGCCTAACTAGCACG
+
FFFFFFFFFFFFFFFFFFFFFFFFFFFFFFFFFFFFFFFFFFFFF:FFFFFFFFFFFFFFFFFFFFFF
FFFFFFFFFFFFFFFFFFFFFFFFFFFFFFFFFFFFFFFFFFFFFFFFFFFFFFFFFFFFFFFFFFFF
@A00155:342:HHGFNDSXY:1:1221:24361:31187 2:N:0:GAACCTAG+TCCGCATA
CCCTACGGCCCGTACGTCTTCCACTTGCAACCACGTTTTGCGTGTAACTGCAAGCTGACGTCTAGTAG
CCCTAGAGATACTGTCTACTATCTGCTTCCTTCCTACGTACTCACCGTATTGCCTAACTAGCACGTTA
+
FFF:FFFFFFF:FFFFF,FFFFFFFFFFFFFF,FFFFFFFFFFFFFFFFFFFFFFFFFFFFFFFFFFF
FF:FFFFF:F:FFFFFFFFFFFFFFFFFFFFFFFFF:FFFFF:FFFFFFFFFFFFFF:FFFFFFFFFF
@A00155:342:HHGFNDSXY:1:2672:6180:9815 2:N:0:GAACCTAG+TCCGCATA
CGGCCCGTACGTCTTCCACTTGCAACCACGTTTTGCGTGTAACTGCAAGCTGACGTCTAGTAGCCCTA
GAGATACTGTCTACTATCTGCTTCCTTCCTACGTACTCACCGTATTGCCTAACTAGCACGTTAGCAT
+
FFFFFFFFFFFFFFFFFFF:FFFFFFF,FFFFFFFFFFFFFFFFFFFFFFFFFFFFFFFFFFFFFFFF
FFFFF:FFFFFFFFFFFFFFF:FFFFFFFF,FFFFF:FFFFFFFFFFFFFF:FFFFFFFFFFFFFFF
```

```
@A00155:342:HHGFNDSXY:1:2672:6180:9815 1:N:0:GAACCTAG+TCCGCATA
ACGTCTTCCACTTGCAACCACGTTTTGCGTGTAACTGCAAGCTGACGTCTAGTAGCCCTAGAGATACT
GTCTACTATCTGCTTCCTTCCTACGTACTCACCGTATTGCCTAACTAGCACGTTAGCATAGTCATATA
+
FFFFFFFFFFFFFFFFFFFFFFFFFFFFFFFF,FFFFFFFFFFFFFFFFFFFFFFFFFFFFFFFFFFFFF
FFFFFFFFFFFFFFFFFFFFFFFFFFFFFFFFFFFFFFFFFFFFFFFFFFFFFFFF:FFFFFFFFFFFFF
@A00155:342:HHGFNDSXY:1:1220:4010:25880 1:N:0:GAACCTAG+TCCGCATA
CCTTCCTACGTACTCACCGTATTGCCTAACTAGCACGTTAGCATAGTCATATATTCCTGGTCCTAGCT
CACGTACCGATGCCCTTCTCTCTTTCTGCTCTTCATTCATTGCTTCTTCATCTTCGTATTCTATCATC
+
FFFFFFF,FFFFFFFFFFFFFFFFFFFFFFFFFFFFFFFFFFFFFFFFFFFFFFFFFFFFFFFFFFFFFF
FFFFFFFFFFFFFFFFFFFFFFFFFFFFFFFFFFFFFFFF:FF:FFFFFFFFFFFFFFFFFFFFFFFFFF
@A00155:342:HHGFNDSXY:1:1526:2709:32581 1:N:0:GAACCTAG+TCCGCATA
CCTTCCTACGTACTCACCGTATTGCCTAACTAGCACGTTAGCATAGTCATATATTCCTGGTCCTAGCT
CACGTACCGATGCCCTTCTCTCTTTCTGCTCTTCATTCATTGCTTCTTCATCTTCGTATTCTATCATC
+
FFFFFFFFFFFFFFFFFFFFFFFFFFFFFFFFFFFFFFFFFFFFFFFFFFFFFFFFFFFFFFFFFFFFFF
FFFFFFFFFFFFFFFFFFFFFFFFFFFFFFFFFFFFFFFF:FFFFFFFFFFFFFFFFFFFFFFFFFFFFF
@A00155:342:HHGFNDSXY:1:1429:28944:7999 1:N:0:GAACCTAG+TCCGCATA
CCTTCCTACGTACTCACCGTATTGCCTAACTAGCACGTTAGCATAGTCATATATTCCTGGTCCTAGCT
CACGTACCGATGCCCTTCTCTCTTTCTGCTCTTCATTCATTGCTTCTTCATCTTCGTATTCTATCATC
+
FFFFFFFFFFFFFFFFFFFFFFFFFFFFFFFFFFFFFFFFFFFFFFFFF,FFFFFFFFFFFFFFFFFFFF
FFFFFFFFFFFFFF,:FFFFFFF:FFFFFFFFFFFFFF:FFFFF:FFFFFFFFFFFFFFFFFF:FFFFFF
@A00155:342:HHGFNDSXY:1:2403:7545:24518 1:N:0:GAACCTAG+TCCGCATA
CCTTCCTACGTACTCACCGTATTGCCTAACTAGCACGTTAGCATAGTCATATATTCCTGGTCCTAGCT
CACGTACCGATGCCCTTCTCTCTTTCTGCTCTTCATTCATTGCTTCTTCATCTTCGTATTCTATCATC
+
FFF:FF:FFFFFFFFFFFFFFFFFFFFFFFFFFFFFFFFFFFFFFFFFFFFFFFFFFFFFFFF:FFFFFF
FFFFFFFF:FFFFFF,:FFFFFF:F:FF:FFFFFFFFF:FFFFF:FFF:FFFF,:,F:FFFFFFFFFFFF
@A00155:342:HHGFNDSXY:1:1429:28944:7999 2:N:0:GAACCTAG+TCCGCATA
CTTCCTACGTACTCACCGTATTGCCTAACTAGCACGTTAGCATAGTCATATATTCCTGGTCCTAGCTC
ACGTACCGATGCCCTTCTCTCTTTCTGCTCTTCATTCATTGCTTCTTCATCTTCGTATTCTATCATCT
+
FFFFFFFFFFFFFFF::FFFFFFFFFFF:F:FFFFFFFFFFFFFFF:F,FFFFFFFFF:FFFFFFFFFFF
FFFFFFFFFFFFFFFFFFFFFFFFF,:FFFFFFFFFFFF,FFFFFFFFFFFFFFFFFFFFFFFFFFFFFF
@A00155:342:HHGFNDSXY:1:2403:7545:24518 2:N:0:GAACCTAG+TCCGCATA
CTTCCTACGTACTCACCGTATTGCCTAACTAGCACGTTAGCATAGTCATATATTCCTGGTCCTAGCTC
ACGTACCGATGCCCTTCTCTCTTTCTGCTCTTCATTCATTGCTTCTTCATCTTCGTATTCTATCATCT
+
FFFFFF,::FFFFFFFF,FFFFFFFFFF:FFFFFFFFFFFFFFFFFFFFFFF:F,FFFFF:FFFFFFFFF
FFF:FFFFFFFF,FFFFFFFFFFFFFF:FFFFF:F:FFFF:FFFFFF:FFFFFFFFFFFFFFFFFFFFFF
@A00155:342:HHGFNDSXY:1:1526:2709:32581 2:N:0:GAACCTAG+TCCGCATA
TTCCTACGTACTCACCGTATTGCCTAACTAGCACGTTAGCATAGTCATATATTCCTGGTCCTAGCTCA
CGTACCGATGCCCTTCTCTCTTTCTGCTCTTCATTCATTGCTTCTTCATCTTCGTATTCTATCATCT
+
FFFFFFFFFFFFFFFFFFFFFFFFFFFFF:FFFFFF,FFFFFFFF:FFFFFFFFFFFFFFFFFFFFFFFF
FFFFFFFFFFFFFFFFFFFFFFFFFFFFFFFFFFFFFF:FFFFFFFFFFFFFFFFFFFFFFFFFFFFFF
@A00155:342:HHGFNDSXY:1:2440:1714:26130 1:N:0:GAACCTAG+TCCGCATA
CACCGTATTGCCTAACTAGCACGTTAGCATAGTCATATATTCCTGGTCCTAGCTCACGTACCGATGCC
CTTCTCTCTTTCTGCTCTTCATTCATTGCTTCTTCATCTTCGTATTCTATCATCTCTTTATACAAATT
+
FFFFFFF,FFFF::FFF:FFFFFFF:FFFFFF,FFFF:FFFFFFFFFFFFFFFFFFFFFFFFFFF:FFFF
F:FFFFFFFFFFFFFFFFF::F:F:FF:FFF,:F,FFFFF:FFFFF:FF:FFF,:FFFF,FF:FFF,FFF
```

```
@A00155:342:HHGFNDSXY:1:2111:24460:3897 1:N:0:GAACCTAG+TCCGCATA
CCGTATTGCCTAACTAGCACGTTAGCATAGTCATATATTCCTGGTCCTAGCTCACGTACCGATGCCCT
TCTCTCTTTCTGCTCTTCATTCATTGCTTCTTCATCTTCGTATTCTATCATCTCTTT
+
FFFFFFFFFF:FFFFFFFFFFFFFFFFFFFFFFFFFFFFFFFFFFFFFFFFFFFFFFFFFFFFFF:F,
FFFFFFFFFFFFFFFFFFFFFFFFFFFFFF:FFFFFFFFFFFFFFFF:FFFFFFFFF
@A00155:342:HHGFNDSXY:1:1126:23565:20885 1:N:0:GAACCTAG+TCCGCATA
CCGTATTGCCTAACTAGCACGTTAGCATAGTCATATATTCCTGGTCCTAGCTCACGTACCGATGCCCT
TCTCTCTTTCTGCTCTTCATTCATTGCTTCTTCATCTTCGTATTCTATCATCTCTTTATACAAATTAT
+
FFFFFFFFFFFFFFFFFFFFFFFFFFFFFFFFFFFFFFFF:FFFFFFFFFFFFFFFFFFFFFFFFFFF
,FFFFFFFFFFFFFFFFFFFFFFFFFFFF:FFFFFFFFFF:F:FFF,FFFFFFFFFFFF:FFFFFFFF
@A00155:342:HHGFNDSXY:1:2440:1714:26130 2:N:0:GAACCTAG+TCCGCATA
GTATTGCCTAACTAGCACGTTAGCATAGTCATATATTCCTGGTCCTAGCTCACGTACCGATGCCCTTC
TCTCTTTCTGCTCTTCATTCATTGCTTCTTCATCTTCGTATTCTATCATCTCTTTATACAAATTATCT
+
::F,::FF:F:F:FFFFF,F,F:FF,F:FF,FF,F,FFF,:F:FFFFFF:FFFF::FF:F,:FFFF:F
FFFFF:FFFFFFFFFFF:FFF,:,F:FFFFF,FFFFFFFF:FF:FFFFFFFFF,FF:FFFFF,:FFFF
@A00155:342:HHGFNDSXY:1:2544:24442:27508 2:N:0:GAACCTAG+TCCGCATA
CCTAACTAGCACGTTAGCATAGTCATATATTCCTGGTCCTAGCTCACGTACCGATGCCCTTCTCTCTT
TCTGCTCTTCATTCATTGCTTCTTCATCTTCGTATTCTATCATCTCTTTATACAAATTATCTATACAA
+
FFFFFFFFFFFFFFFFFFFFFF:FFFFFFFFFFFFFFFFFFFFFFFFFFFFFFFFFFFFFFFFFFFFF
FFFFFFFFFFF,FFFFFFFFFFFFFFFFFFFFFFFFFFFFFFFFFFFFFFFFFFFFFFFFFFFFFFFF
@A00155:342:HHGFNDSXY:1:1653:6976:34835 2:N:0:GAACCTAG+TCCGCATA
CCTAACTAGCACTTTAGCATAGTCATATATTCCTGGTCCTAGCTCACGTACCGATGCCCTTCTCTCTT
TCTGCTCTTCATTCATTGCTTCTTCATCTTCGTATTCTATCATCTCTTTATACAAATTATCTATACAA
+
F,:F,FFFFFFF,F:F,FF,FFFFFFFFFFFFFF:FFFFFFFFFFF,FFFFFF:FFFFFFFFFFFF:F
F,FF:FF:FFFFFFF,F::FFFFFFFFFF,FFFFFF:FFFFFFFF,FFFFFFFFFFFFFF:,FFFF:F
@A00155:342:HHGFNDSXY:1:2242:17318:11694 1:N:0:GAACCTAG+TCCGCATA
TAGCATAGTCATATATTCCTGGTCCTAGCTCACGTACCGATGCCCTTCTCTCTTTCTGCTCTTCATTC
ATTGCTTCTTCATCTTCGTATTCTATCATCTCTTTATACAAATTATCTATACAACCATTCCTGTCTGA
+
FFFFFFFFFFFFFFFFFFFFFFFFFFFFFFFFFFFFFFFFFFFFFFFFFFFFF:FFFFFFFFFFFFFF
FFFF:FFFFFFFFFFFFFFFFFFFFFFFFFFFFF:FFFFFFFFF:FFFFFFFFFFFFFFFFFFFFFFFF
@A00155:342:HHGFNDSXY:1:2242:13593:13291 1:N:0:GAACCTAG+TCCGCATA
TAGCATAGTCATATATTCCTGGTCCTAGCTCACGTACCGATGCCCTTCTCTCTTTCTGCTCTTCATTC
ATTGCTTCTTCATCTTCGTAGTCTATCATCTCTTTATACAAATTATCTATACAACCATTCCTGTCTGA
+
FFFFFFFFFFFFFFFFFFFFFFFFFFFFFFFFFFFFFFFFFFFFFFFFFFFFFFFFFFFFFFFFFFFF
FFFF:FFF:FFFFFFFFFFF,FFF:,FFFFFFFFFF:FFFFFFFFFFFFFFFFF:FFFFFFFFFFFFF
@A00155:342:HHGFNDSXY:1:1220:4010:25880 2:N:0:GAACCTAG+TCCGCATA
TCCTAGCTCACGTACCGATGCCCTTCTCTCTTTCTGCTCTTCATTCATTGCTTCTTCATCTTCGTATT
CTATCATCTCTTTATACAAATTATCTATACAACCATTCCTGTCTGATATCGGACCTCCGACTATGACA
+
FFFFFFFFFFFFFFFFFFFFF:FF:FFF:F:FFFFFF:FFFFFFFFF:FFFFFFFFF:FFF::FFFFF
:FFFFFFF:FFFFFFFFFFFFFFF::FFFFFFFFF:FFFFFFFFFFFFFFFFFFFFFFFFFFFFFFFFF
@A00155:342:HHGFNDSXY:1:2242:17318:11694 2:N:0:GAACCTAG+TCCGCATA
TCCTAGCTCACGTACCGATGCCCTTCTCTCTTTCTGCTCTTCATTCATTGCTTCTTCATCTTCGTATT
CTATCATCTCTTTATACAAATTATCTATACAACCATTCCTGTCTGATATCGGACCTCCGACTATGACA
+
FFFFF:F:FFFFFFFFFFFF:FFFFFFFFFFFFFFFFFFF:FFFFFFFFFFFF:FFFFFFFFFFFFFF
FFFFFFFFF:FFF:FFFFFFFFFFFFFFFFFFFFF,FFFFFFFFFFFFFFFFFFFFFFFFFFFFFFFFF
```

```
@A00155:342:HHGFNDSXY:1:2242:13593:13291 2:N:0:GAACCTAG+TCCGCATA
TCCTAGCTCACGTACCGATGCCCTTCTCTCTTTCTGCTCTTCATTCATTGCTTCTTCATCTTCGTATT
CTATCATCTCTTTATACAAATTATCTATACAACCATTCCTGTCTGATATCGGACCTCCGACTATGACA
+
FFFFFFFFFFFFFFFFFFFFFFFFFFFFFFFFFFFFFFFFFFFFFFFFFFFFFFFFF:FFFFFFFFFF
FFFFFFFFFFFFFFFFFFFFFFFFFFFFFFF:FFFFFFFFFFFFFFFFFFFFFFFFFFFFFFFFFFFF
@A00155:342:HHGFNDSXY:1:2325:20772:1391 1:N:0:GAACCTAG+TCCGCATA
ACGTACCGATGCCCTTCTCTCTTTCTGCTCTTCATTCATTGCTTCTTCATCTTCGTATTCTATCATCT
CTTTATACAAATTATCTATACAACCATTCCTGTCTGATATCGGACCTCCGACTATGACATGGGCAGCA
+
FFFFFFFFFFFFFFFFFFFFFFFFFFFFFFFFFFFFFFFFFFFFFFFF:FFFFFFFFFFFFFFFFFFF
FF:FFFFFFFFFFFFFFFFFFFFFFFFFFFFFFFFFFFFFFFFFFFFFFFFFFFFFFFFFFFFFFFFF
@A00155:342:HHGFNDSXY:1:2158:4119:31454 2:N:0:GAACCTAG+TCCGCATA
CGTACCGATGCCCTTCTCTCTTTCTGCTCTTCATTCATTGCTTCTTCATCTTCGTATTCTATCATCTC
TTTATACAAATTATCTATACAACCATTCCTGTCTGATATCGGACCTCCGACTATGACATGGGCAGCA
+
FFFFFFFFFFF:FF:FFFFFFFFFFFFFFFFFFFFFFFFFF,FFFFFFFFF,FFFFFFFF:FFFFFFF
FFFFFFFFFFFFFF:FFFFFFF:F:FF,FFFFFFFFFFF::FFFFFFFFFFFFFFFFFFFFFFFFFF
@A00155:342:HHGFNDSXY:1:1401:7247:23531 2:N:0:GAACCTAG+TCCGCATA
CCGATGCCCTTCTCTCTTTCTGCTCTTCATTCATTGCTTCTTCATCTTCGTATTCTATCATCTCTTTA
TACAAATTATCTATACAACCATTCCTGTCTGATATCGGACCTCCGACTATGACATGGGCAGCAACTAG
+
FFFF,,FFFFFFFF:FFFFF:FFFFFFFFF:FFFFFFF:FFFFFFFFFFFFFFFFFFFFF:FFFFFFF
FFFFFFFFFFFFFFF:FFFFFFFFFFFFFFFFFFFFFFFFFFFFFFFFFFFFFFFFFFFFFFFFFFFF
@A00155:342:HHGFNDSXY:1:1401:7247:23531 1:N:0:GAACCTAG+TCCGCATA
CCGATGCCCTTCTCTCTTTCTGCTCTTCATTCATTGCTTCTTCATCTTCGTATTCTATCATCTCTTTA
TACAAATTATCTATACAACCATTCCTGTCTGATATCGGACCTCCGACTATGACATGGGCAGCAACTAG
+
FFFFFFFFFFFFFFFFFFFFFFFFFFFFFFFF:FFF:F::FFFFFFFFFFFFFFFFFFFFFFFFFFFF
FFFFFFFFFFFFFFFFFFFFFFFFFFFFFFFFFFFFFFFFFFFFFFFFFFFFFFFFFFFFFFFFFFFF
@A00155:342:HHGFNDSXY:1:1569:25563:27571 1:N:0:GAACCTAG+TCCGCATA
CGATGCCCTTCTCTCTTTCTGCTCTTCATTCATTGCTTCTTCATCTTCGTATTCTATCATCTCTTTAT
ACAAATTATCTATACAACCATTCCTGTCTGATATCGGACCTCCGACTATGACATGGGCAGCAACTAGC
+
FFFFFFFFFFFFFFFFFFFFFFFFFFFFFFFFFFFFFFF,FFFF:FFFFFFFFFFFFFFFFFFFFFFF
FFFFFFFFFFFFFFFFFFFFFFFFFFFFFFFFFFFFFFFFFFFFFF:FFFFFFFFFFFFFFFFFFFFF
@A00155:342:HHGFNDSXY:1:1344:11803:8750 1:N:0:GAACCTAG+TCCGCATA
TGCTCATCATTCATTGCTTCTTCATCTTCGTATTCTATCATCTCTTTATACAAATTATCTATACAACC
ATTCCTGTCTGATATCGGACCTCCGACTATGACATGGGCAGCAACTAGCTTCCTACATACCTTCTCAT
+
FFFFF,FFFFF:F:FF:FF:FF:FFFFFFFFFFFFFFFFFFFFFFFFFFFFF:FFFFFFFFFFFFFFF
F,FFFFFFFFFFFFFFFFFFFFFFFFFFFFFFFFFFFFFFF:FFFFF,F:FFFFFFFFFFFFFFFFFF
@A00155:342:HHGFNDSXY:1:2564:14380:24831 1:N:0:GAACCTAG+TCCGCATA
TCTTCATTCATTGCTTCTTCATCTTCGTATTCTATCATCTCTTTATACAAATTATCTATACAACCATT
CCTGTCTGATATCGGACCTCCGACTATGACATGGGCAGCAACTAGCTTCCTACATACCTTCTCATCCA
+
FFFFFFFFFFFFFF,FF:FFFFFFFFFFFFFFFFFFFFFFFFFFFFFF:FFFFFFFFFFFFFFFFFFF
FFFFFFFFFFFFFFFFFFFFFFFFFFFFFFFFFFFFFFFFFFFFFFFFFFFFFFFFFFFFFFFFFFFF
@A00155:342:HHGFNDSXY:1:2564:15022:26005 1:N:0:GAACCTAG+TCCGCATA
TCTTCATTCATTGCTTCTTCATCTTCGTATTCTATCATCTCTTTATACAAATTATCTATACAACCATT
CCTGTCTGATATCGGACCTCCGACTATGACATGGGCAGCAACTAGCTTCCTACATACCTTCTCATCCA
+
FFFFFFFFF:FFF:::FFFFFFF:FFFFFFFFFFFFFFFFFFFFFFFFFFFFFFFFFFFFFFFFFFFF
FFFFFFFFFFFFFFFFFFFFFFFFFFFFFFFFFFFFFFFF,FFFFFFFFFFFFFFFFFFFFFFFFFFF
```

```
@A00155:342:HHGFNDSXY:1:2325:20772:1391 2:N:0:GAACCTAG+TCCGCATA
TTCATTCATTGCTTCTTCATCTTCGTATTCTATCATCTCTTTATACAAATTATCTATACAACCATTCC
TGTCTGATATCGGACCTCCGACTATGACATGGGCAGCAACTAGCTTCCTACATACCTTCTCATCCACA
+
FFFFFFFF:FF::FFFFFFFFFFFFFFFFFFFFFFFFFFFFFFFFFFFFFFFFFFFFFFFFFFFFF
FFFFFFF:FFFFFFFFFFFFFFF:FFFFFFFFFFFFFFFFFFFFFF:FFFFFFFFFFFFFFFFFFF
@A00155:342:HHGFNDSXY:1:2423:29830:2174 2:N:0:GAACCTAG+TCCGCATA
TTCATCTTCGTATTCTATCATCTCTTTATACAAATTATCTATACAACCATTCCTGTCTGATATCGGAC
CTCCGACTATGACATGGGCAGCAACTAGCTTCCTACATACCTTCTCATCCACACTGAACACATT
+
FFFFF,FF,FFFFF,FFFFFFFFFFFFFFFF,FFFFFFFFFFFFFFFFFFFFFFFFFFFFFF:FFF
FFFF::FFF:FFFFFFF:FFFFFFFFFFF:FF::FF:F:F:FFFFFFFFFFFFFF:FFFFFF
@A00155:342:HHGFNDSXY:1:2568:32796:33739 2:N:0:GAACCTAG+TCCGCATA
TTCATCTTCGTATTCTATCATCTCTTTATACAAATTATCTATACAACCATTCCTGTCTGATATCGGAC
CTCCGACTATGACATGGGCAGCAACTAGCTTCCTACATACCTTCTCATCCACACTGAACACATT
+
F:FF:FFFFFFFFF:FFFFFFF:FFFFFFFFFFFFF:FFFFFFFFFFFFFFFFFFFFFFF:FFF:F
FFFFFFFFFFF:FFFFFFFFFFFFFFFFF:FFFFFFFFFFFFF,FFFFFFFFFFFFFFFFFF
@A00155:342:HHGFNDSXY:1:1569:25563:27571 2:N:0:GAACCTAG+TCCGCATA
TTCATCTTCGTATTCTATCATCTCTTTATACAAATTATCTATACAACCATTCCTGTCTGATATCGGAC
CTCCGACTATGACATGGGCAGCAACTAGCTTCCTACATACCTTCTCATCCACACTGAACACATT
+
:FFFFFFFFFFFFFFFFFF:FFFFFF:FFFFFFFFFFFFF,FFFFFFF:FFFFFFFFFFFFFFFFF
FFFFFFFFFFFFFFFFFFFFFFFFFFFFFFFFFFFFFFFFFFFFFFFFFFFFFFFFFFFFFF
@A00155:342:HHGFNDSXY:1:1129:9435:27727 1:N:0:GAACCTAG+TCCGCATA
TTCATCTTCGTATTCTATCATCTCTTTATACAAATTATCTATACAACCATTCCTGTCTGATATCGGAC
CTCCGACTATGACATGGGCAGCAACTAGCTTCCTACATACCTTCTCATCCACACTGAACACATTAGAC
+
FFFFFFFFFFFFFFFFFFFFFFFFFFFFFFFFFFFFFFFFFFFFFFFFF:FFFFFFFFFFFF:FFF
FFFFFFFFFFFFFFFFFFFFFFFFFFFFFFFFFFFFFFFFFFFFFFFFFFFFFFFFFFFFFFFFFF
@A00155:342:HHGFNDSXY:1:2423:29830:2174 1:N:0:GAACCTAG+TCCGCATA
TTCATCTTCGTATTCTATCATCTCTTTATACAAATTATCTATACAACCATTCCTGTCTGATATCGGAC
CTCCGACTATGACATGGGCAGCAACTAGCTTCCTACATACCTTCTCATCCACACTGAACACATTAGAC
+
FFFFFFFFF:FFFF:FFFF:FFFFFFFFFFFFFFFFF:FFFFFFFFFFFFFFFFFFFFFFFFF::F
FFFFFFFFFFFFFF:FFFF:FF:FFFFFFFFFFFFFFFFFFFFF:FFFFF,FFFFFFFFFFFFFFF
@A00155:342:HHGFNDSXY:1:1129:8865:15593 1:N:0:GAACCTAG+TCCGCATA
TTCATCTTCGTATTCTATCATCTCTTTATACAAATTATCTATACAACCATTCCTGTCTGATATCGGAC
CTCCGACTATGACATGGGCAGCAACTAGCTTCCTACATACCTTCTCATCCACACTGAACACATTAGAC
+
FF:FFFFFFFFF:F:FFFFFFFFFFF,FFFF::FFFF:FFFFFFFFFF:F,F:F:FFFFFFFFFF:
FFFFFFFFFFFFFF:F:FFFFFF:F,FFF:FFFFFFFFFFFFFFF:FFFFFF:FFFFFFFF,F,F:
@A00155:342:HHGFNDSXY:1:2568:32796:33739 1:N:0:GAACCTAG+TCCGCATA
TTCATCTTCGTATTCTATCATCTCTTTATACAAATTATCTATACAACCATTCCTGTCTGATATCGGAC
CTCCGACTATGACATGGGCAGCAACTAGCTTCCTACATACCTTCTCATCCACACTGAACACATTAGAC
+
FFFFFFFFFFFFFFFFFFFFFFFFFFFFFFFFFFFFFFFFFFFFFFFFFFFFFFFFFFFFFFFFFF
FFFFFFFFFFFFFFFFFFFFFFFFFFFFFFFFFFFFFFFFFFFFFFFFFFFFFFFFFFFFFFFFFF
@A00155:342:HHGFNDSXY:1:2667:28176:33348 2:N:0:GAACCTAG+TCCGCATA
TTCGTATTCTATCATCTCTTTATACAAATTATCTATACAACCATTCCTGTCTGATATCGGACCTCCGA
CTATGACATGGGCAGCAACTAGCTTCCTACATACCTTCTCATCCACACTGAACACATTAGACAATCTC
+
FFFFF:FF::FFFFFF:FFFFFFFFFFFFFF:F:FFFFFFF,F:FFFFFFFFF:FFFFFFFFFFFF
FFFFF:F,FF:FFFFFFFFFFFFFFFFFFF:FFFFFFFFFFFFFFFFFFFFFFFFFFFFFFFFFFF
```

```
@A00155:342:HHGFNDSXY:1:1126:23565:20885 2:N:0:GAACCTAG+TCCGCATA
TTCGTATTCTATCATCTCTTTATACAAATTATCTATACAACCATTCCTGTCTGATATCGGACCTCCGA
CTATGACATGGGCAGCAACTAGCTTCCTACATACCTTCTCATCCACACTGAACACATTAGACAATCTC
+
FFFFFFFFFFFF:FFFF,FFFFFFFFFFFFFF::FFFFFFFFF:FFFFFF,F:FFFFFFFFFFFFFFF
,FFFFFFFFF:FFFFFFFFFFFF:FFFF:FFFFFFFFFFFFFFFFFFFFFFFFFFFFFFFFFFFFFFF
@A00155:342:HHGFNDSXY:1:2667:28176:33348 1:N:0:GAACCTAG+TCCGCATA
GTATTCTATCATCTCTTTATACAAATTATCTATACAACCATTCCTGTCTGATATCGGACCTCCGACTA
TGACATGGGCAGCAACTAGCTTCCTACATACCTTCTCATCCACACTGAACACATTAGACAATCTCACA
+
FFFFFFFFFFFFFFFFFFFFFFFFFFFFFFFFFFFFFFFFFF:FFFFFFFFFFFFFFFFFFFFFFFFF
FFFFFF:FFF:F:FFFFFFFFFFFFFFFFFFFFFFFFFFFFFFFF:FFFFFFFFFFFFFF:FFFFFFF
@A00155:342:HHGFNDSXY:1:2158:4119:31454 1:N:0:GAACCTAG+TCCGCATA
GTATTCTATCATCTCTTTATACAAATTATCTATACAACCATTCCTGTCTGATATCGGACCTCCGACTA
TGACATGGGCAGCAACTAGCTTCCTACATACCTTCTCATCCACACTGAACACATTAGACAATCTCACA
+
FFFFFFFFFFFFFFFFFFFFFFFFFFFFFFFFFFFFFFFFFFFFFFFFFFFFFF:FFFFFFFFFFFFF
FFFFFF:FFFFFFFFFFFFFFFF:FFFF:F,FFFFFFFFFFFFFFFFFFFFFFFFFFFFFFFFFFFFF
@A00155:342:HHGFNDSXY:1:2111:24460:3897 2:N:0:GAACCTAG+TCCGCATA
CTTTATACAAATTATCTATACAACCATTCCTGTCTGATATCGGACCTCCGACTATGACATGGGCAGCA
ACTAGCTTCCTACATACCTTCTCATCCACACTGAACACATTAGACAATCTCACAACTGACATGTCCAG
+
FFFFFFFFFFFFFFFFFF:FFFFFFF,FFFFFFFFFFFFFFFFFFFFFFFFFFFFFFFFFFFFFFFFF
FFFFFFFFFFFFFFFFFFFFFFFFFFFFFFFFFFFFFFFFFFFFFFFFFFFFFFFF:FFFFFFFFFFF
@A00155:342:HHGFNDSXY:1:1672:2736:21042 2:N:0:GAACCTAG+TCCGCATA
TTATACAAATTATCTATACAACCATTCCTGTCTGATATCGGACCTCCGACTATGACATGGGCAGCAAC
TAGCTTCCTACATACCTTCTCATCCACACTGAACACATTAGACAATCTCACAACTGACATGTCCAGCA
+
FFFFFFFFFFF,FFFFFFFFFFF:FFFFFFFFFFFFFF:FFF:FFFFFF:FFFFFFFFF:FFF,FFFF
FF:FFFFFFFFFFFFFFF,FFFFFFF::FFFFF:FFFFFFFF:FFFFF:F:FFF:FFF:FFF:FFFFF
@A00155:342:HHGFNDSXY:1:1129:8865:15593 2:N:0:GAACCTAG+TCCGCATA
TACAAATTATCTATACAACCATTCCTGTCTGATATCGGACCTCCGACTATGACATGGGCAGCAACTAG
CTTCCTACATACCTTCTCATCCACACTGAACACATTAGACAATCTCACAACTGACATGTCCAGCAAGG
+
F::FFFFFFFFFFFFFFF,:F,FFFF:FFFFFFFFFFFFFFFF:FFFFFFFFFFFFFFFFFFFFFFFF
FFFFFFFFFFFFFFFFFFFFFFFFFFFFFFFFFF:FFFFFFFFFFFFFFFFF:FFFFFFFFFFFFF:F
@A00155:342:HHGFNDSXY:1:1129:9435:27727 2:N:0:GAACCTAG+TCCGCATA
TACAAATTATCTATACAACCATTCCTGTCTGATATCGGACCTCCGACTATGACATGGGCAGCAACTAG
CTTCCTACATACCTTCTCATCCACACTGAACACATTAGACAATCTCACAACTGACATGTCCAGCAAGG
+
FFFFFF,FFFFFFFFFFFFFF,FFFFFFFFFFFFFFFFFFFFFFFFFFFFFFFFFF:FFFFFFFFFFF
FFFF:FFFFFFFFFFFFFFFFFFFFFFFFFFF:FFFFFFFFFFFFF:FFFFFFFFF::FFFFFFF,FF
@A00155:342:HHGFNDSXY:1:2564:14380:24831 2:N:0:GAACCTAG+TCCGCATA
AAATTATCTATACAACCATTCCTGTCTGATATCGGACCTCCGACTATGACATGGGCAGCAACTAGCTT
CCTACATACCTTCTCATCCACACTGAACACATTAGACAATCTCACAACTGACATGTCCAGCAAGGCTA
+
FFFFFFFF:FFFFFFFFF:FFFFFFFFFFFFFFFFFFFFFFFFFFF,FFFFFFFFFFFFFFFFFFFFF
FFFFFF:FFFFFFFFFFFFFFFFFFFFFFFF,FFFFFFFFFFFFFFFFFFFFFFFFFFFFFFFFFFFF
@A00155:342:HHGFNDSXY:1:2564:15022:26005 2:N:0:GAACCTAG+TCCGCATA
AAATTATCTATACAACCATTCCTGTCTGATATCGGACCTCCGACTATGACATGGGCAGCAACTAGCTT
CCTACATACCTTCTCATCCACACTGAACACATTAGACAATCTCACAACTGACATGTCCAGCAAGGCTA
+
FFF::FF::FFFFFFFFFFFFFFFFFFFFFFFFFFFFFFFFFFFFFFFFFFFFFFFFFFFFFFFFFFF
FF:FFFFFFFFFFFFFFFFFFFFFFFFFFFFFFFFFFFFFFFFFFFFFFFFFFFFFFFFFFFFFFFFF
```

```
@A00155:342:HHGFNDSXY:1:1473:15167:21966 1:N:0:GAACCTAG+TCCGCATA
TATCTATACAACCATTCCTGTCTGATATCGGACCTCCGACTATGACATGGGCAGCAACTAGCTTCCTA
CATACCTTCTCATCCACACTGAACACATTAGACAATCTCACAACTGACATGTCCAGCAAGGCTAGTAT
+
FFFFFFFFFFF:FFFFFFFFFFFFFFFFFFFFFFFFFFFFFFFFFFFFFFFFFFFFFFFFFFFFF:FF
FFFFFFFFFFFFFFFFFFFFFFFFFFFFFFFFF:FFFFFFFFFFFFFFFFFFFFFFFFFF:FF:F:FF
@A00155:342:HHGFNDSXY:1:2242:23863:23124 1:N:0:GAACCTAG+TCCGCATA
TCTATACAACCATTCCTGTCTGATATCGGACCTCCGACTATGACATGGGCAGCAACTAGCTTCCTACA
TACCTTCTCATCAACACTGAACACATTAGACAATCTCACAACTGACATGTCCAGCAAGGCTAGTATC
+
FFFFFFFFFF,FF,FFF,F::FF,FFFF,F,:F:F:FF:FFF:FFFF,,FF::FF,:FFFFF::FF,F
FFFF:F,FFFF,FFFFFF,F:FFFF:,F,FFFFFFFFFFFF:,FFFFF::FFF,,:FF,F:F,FF:F
@A00155:342:HHGFNDSXY:1:2175:2745:35086 1:N:0:GAACCTAG+TCCGCATA
ATATCGGACCTCCGACTATGACATGGGCAGCAACTAGCTTCCTACATACCTTCTCATCCACACTGAAC
ACATTAGACAATCTCACAACTGACATGTCCAGCAAGGCTAGTATCATTTCTTCTGAACCCCTGCACCT
+
FFFFFFFFFFFFF:FFFFFFFF:FFFFFFFFFFF:F:FFFFFFFFFFFFFFFFFF:FFFFFFFFFFFF
FFFFFFFFFFFFFFFFFF:FFFFFFFFFFFF:FFFFFFFFFFFFFFFFF:FFFF:FFFFFFFFF::FF
@A00155:342:HHGFNDSXY:1:1344:11803:8750 2:N:0:GAACCTAG+TCCGCATA
CTCCGACTATGACATGGGCAGCAACTAGCTTCCTACATACCTTCTCATCCACACTGAACACATTAGAC
AATCTCACAACTGACATGTCCAGCAAGGCTAGTATCATTTCTTCTGAACCCCTGCACCTAGCACCTAC
+
:F:,FF,FFFFFF,FFFFFFFFFFFFFFFFFFFFFFFFFFFFFFF:FFFFFFFFFFFFFFFFFFFFFF
FFFFFFFFFFFFFFFFFFFFFF,FFFFF,FFFFFFFFFFFFFF:FFFFFFFFFFFFFF,FFFFFFFFF
@A00155:342:HHGFNDSXY:1:2571:22480:18912 1:N:0:GAACCTAG+TCCGCATA
GGGCAGCAACTAGCTTCCTACATACCTTCTCATCCACACTGAACACATTAGACAATCTCACAACTGAC
ATGTCCAGCAAGGCTAGTATCATTTCTTCTGAACCCCTGCACCTAGCACCTACTTCCTCACATCTTGC
+
FFFFFFFFFFFFFFFFFFFFFFFFFFFFFFFFFFFFFFFFFFFFFFFFFFFFFFFFFFFFFFFFFFFF
FFFFFFFFFFFFFFFFFFFFFFFFFFFFFFFFFFFFFFFFFFFFFFFFFFFFFFFFFFFFFFFFFFFF
@A00155:342:HHGFNDSXY:1:1660:11369:17362 1:N:0:GAACCTAG+TCCGCATA
GGGCAGCAACTAGCTTCCTACATACCTTCTCATCCACACTGAACACATTAGACAATCTCACAACTGAC
ATGTCCAGCAAGGCTAGTATCATTTCTTCTGAACCCCTGCACCTAGCACCTACTTCCTCACATCTTGC
+
FFFFFFFFFFFFFFFFFFFFFFFFFFFFFFFFFFFFFFFFFFFFFFFFFFFFFFFFFFFFFFFFFFFF
:FFFFFFFFFFFFFFFFFFFFFFFFFFFFFFFFFFFFFFFFFFFFFFFFFF:F,,FFFFFFFFFFFFF
@A00155:342:HHGFNDSXY:1:1223:12599:34773 1:N:0:GAACCTAG+TCCGCATA
CTACATACCTTCTCATCCACACTGAACACATTAGACAATCTCACAACTGACATGTCCAGCAAGGCTAG
TATCATTTCTTCTGAACCCCTGCACCTAGCACCTACTTCCTCACATCTTGCCACACACGCTTTCAACG
+
FFFFFFFFFFFFFFFFFFFFFFFFFFFFFFFFFFFFFFFFFFFFFFFFFFFFFFFFFFFFFFFFFF:F
FFFFFFFFFFFFFFFFFFFFFFFFFFFFFFFFFFFFFFFFFFFF:FFFFFFF:FFFFFFFF:FFFFFF
@A00155:342:HHGFNDSXY:1:2175:2745:35086 2:N:0:GAACCTAG+TCCGCATA
CATCCACACTGAACACATTAGACAATCTCACAACTGACATGTCCAGCAAGGCTAGTATCATTTCTTCT
GAACCCCTGCACCTAGCACCTACTTCCTCACATCTTGCCACACACGCTTTCAACGCCTCTAGCAGCC
+
FF:FF:FFF,:FFF,FF,FFFF:FF:FFF,:,:F,:FFF,FF,FFF:F:FFFFF:FFFFFFFF:FFFF
FF::FFFFF:FF,F:,FF,FF::,:FF:FF,F,F,F::,FFFFFFFFFF:FFFF,FFFFFF:FFFFF
@A00155:342:HHGFNDSXY:1:1663:2582:9533 2:N:0:GAACCTAG+TCCGCATA
GAACACATTAGACAATCTCACAACTGACATGTCCAGCAAGGCTAGTATCATTTCTTCTGAACCCCTGC
ACCTAGCACCTACTTCCTCACATCTTGCCACACACGCTTTCAACGCCTCTAGCAGCCTGCTAGGTTCC
+
FFF,F:FFFFF:FFFFFFFFFFFFFFFFFFFFFFFFFF:FFF:FFFFFFFFF:FFF,FF,FFFFFFFF
FF,FFFFFFFFFFFFFFFFFFFFFFFF,FFFFFFFFFFFFFFFF,FFFFF:FFF:FFFFFFFFFFF:F
```

```
@A00155:342:HHGFNDSXY:1:2121:16179:26819 1:N:0:GAACCTAG+TCCGCATA
AACACATTAGACAATCTCACAACTGACATGTCCAGCAAGGCTAGTATCATTTCTTCTGAACCCCTGCA
CCTAGCACCTACTTCCTCACATCTTGCCACACACGCTTTCAACGCCTCTAGCAGCCTGCTAGGTTCCT
+
FFFFFFFFFFFFF:FFFFFFFFFFFFFFFFFFFFFFFFFFFFFFFFFFFFFFFFFFFFFFFFFFFFFFFF
FFFFFFFFFFFFFFFFFFFFFFFFFFFFFFFFFFFFFFFFFFFFFFFFFFFFFFFFFFFFFFF:FFFF
@A00155:342:HHGFNDSXY:1:2571:22480:18912 2:N:0:GAACCTAG+TCCGCATA
CACATTAGACAATCTCACAACTGACATGTCCAGCAAGGCTAGTATCATTTCTTCTGAACCCCTGCACC
TAGCACCTACTTCCTCACATCTTGCCACACACGCTTTCAACGCCTCTAGCAGCCTGCTAGGTTCCTGG
+
FFFFFFFFFFFFFFFFFFFFFFFFFFFFFFFFFFFFFFF:FFFFFFFFFFFFFFFFFFFFFFFFFFFFF
FFFFFFFFFF,FFFFFFF,FFFFFFFFFFFFFFFFFFFFFFFFFFFFFFFFFFFFFFFFFFFFFFFFFF
@A00155:342:HHGFNDSXY:1:1660:11369:17362 2:N:0:GAACCTAG+TCCGCATA
CACATTAGACAATCTCACAACTGACATGTCCAGCAAGGCTAGTATCATTTCTTCTGAACCCCTGCACC
TAGCACCTACTTCCTCACATCTTGCCACACACGCTTTCAACGCCTCTAGCAGCCTGCTAGGTTCCTGG
+
FFFFFFFFFFF:FFFFFFFF:FFFFFFFFFFFFFFFF:FFFFFFFF:FFFFFFFFFFFFFFFFFFFFF
FFFFFFFFFF,FFF:FFFFFFFFFFFFFFFFFFFFFFFFFFFFFFFFFFFFFFFFFFFFFFFFFFFFF
@A00155:342:HHGFNDSXY:1:1513:21667:6136 1:N:0:GAACCTAG+TCCGCATA
ATCTCACAACTGACATGTCCAGCAAGGCTAGTATCATTTCTTCTGAACCCCTGCACCTAGCACCTACT
TCCTCACATCTTGCCACACACGCTTTCAACGCCTCTAGCAGCCTGCTAGGTTCCTGGCTCTCCACCCT
+
FFF:FFFFFFFFFFFFFFF,FFFFFF:FF:FFFFFFF:FFFFFFFFFFFFFFFFFFFFFFFFFFFFFF
FFFFFFFFFFFFFFFFFFFFFFFFF,FFFFFFF:FFF,FFFFFFFF:FFFFFFFFFFFFFFFFF:F:FF
@A00155:342:HHGFNDSXY:1:2121:16179:26819 2:N:0:GAACCTAG+TCCGCATA
CACAACTGACATGTCCAGCAAGGCTAGTATCATTTCTTCTGAACCCCTGCACCTAGCACCTACTTCCT
CACATCTTGCCACACACGCTTTCAACGCCTCTAGCAGCCTGCTAGGTTCCTGGCTCTCCACCCTAGAG
+
FFFFFF:FFFFFFFFFFFF:FFFFFFFFFFFFFFFFFFFFFFFFFFFFFFFFFFFFFFFFFFFFFFFFF
FFF:FF:FFFFFFFFFFFFFFFFF:FFFFFFF:FFFFFFFFFFFFFFFFFFFFFFFFFFFF:FFFFFFF
@A00155:342:HHGFNDSXY:1:1232:1606:7498 2:N:0:GAACCTAG+TCCGCATA
CTGACATGTCCAGCAAGGCTAGTATCATTTCTTCTGAACCCCTGCACCTAGCACCTACTTCCTCACAT
CTTGCCACACACGCTTTCAACGCCTCTAGCAGCCTGCTAGGTTCCTGGCTCTCCACCCTAGAGTGCAC
+
FFFF:,F,FFFF:FFF,:FFFFFF,F:FF,FFFFFFFFFF:FFFFF:FFFFFFFFFFFFFFF:FFFFF
FFFFFFFFFFFFFF,FFFFFFFFF,FFFFFFFFF,FFFFFFFFF:F:FFFFFFFFFFFF,FFFFFFF
@A00155:342:HHGFNDSXY:1:1232:1606:7498 1:N:0:GAACCTAG+TCCGCATA
TGACATGTCCAGCAAGGCTAGTATCATTTCTTCTGAACCACTGCACCTAGCACCTACTTCCTCACATC
TTGCCACACACGCTTTCAACGCCTCTAGCAGCCTGCTAGGTTCCTGGCTCTCCACCCTAGAGTGCACT
+
FFFFFFFFFFFFFFFFFFFFFFFFFFFFFFFFFFFF::FFF,FFFFFFFFF,FF:FFFFFFFFF:FFFFF
FFFFFFFFFFFFFFFFFFFFFFFFFFFFFFFFFFFFFFFFFFFFFFFFFFFFFFFFFFFFFFFFFFFF
@A00155:342:HHGFNDSXY:1:1608:22851:11350 1:N:0:GAACCTAG+TCCGCATA
TCCAGCAAGGCTAGTATCATTTCTTCTGAACCCCTGCACCTAGCACCTACTTCCTCACATCTTGCCAC
ACACGCTTTCAACGCCTCTAGCAGCCTGCTAGGTTCCTGGCTCTCCACCCTAGAGTGCACTAGTGTCG
+
FFFFFFFFFFFFFFFFFFFF:FFFFFFFFFFFFFFFFFFFFFFFFFFFFFFFFFFFFFFFFFFFF:FFF
FFFF:FFFFFFFFF:FFFFFFFFFFFFFFFFFFFFFFFFFFFFFFFFFFFFFFFFFFFFFFFFF:FFFF
@A00155:342:HHGFNDSXY:1:2371:17065:17989 2:N:0:GAACCTAG+TCCGCATA
CAGCAAGGCTAGTATCATTTCTTCTGAACCCCTGCACCTAGCACCTACTTCCTCACATCTTGTCACAC
ACGCTTTCAACGCCTCTAGCAGCCTGCTAGGTTCCTGGCTCTCCACCCTAGAGTGCACTAGTGT
+
FFFFFFFFFFFFFFFFFFFFFFFFFFFFFFFFFFFFFFF:FFFFFFFFFFFFFFFFF:FFFFFFFFFFF
FFFFFFFFFFFFFFFFFFFFFFFFFFFFFFFFFFFFFFFFFFFFFFFFFFFFFFFFFFFFFFFF
```

@A00155:342:HHGFNDSXY:1:2146:18276:30483 1:N:0:GAACCTAG+TCCGCATA
AGGCTAGTATCATTTCTTCTGAACCCCTGCACCTAGCACCTACTTCCTCACATCTTGCCACACACGCT
TTCAACGCCTCTAGCAGCCTGCTAGGTTCCTGGCTCTCCACCCTAGAGTGCACTAGTGTCGCTACTCC
+
FFFFFFFFFFFFFFFFFFFFFFFFFFFFFFFFFFFFFFFFFFFFFFFFFFFFFFFFFFFFFFFFFFFF
FFFFFFFFFFFFFFFFFFFFFFFFFFFFFFFFFFFFFFFFFFFFFFFFFFFFFFFFFFFFFFFFFFFF
@A00155:342:HHGFNDSXY:1:2146:18276:30483 2:N:0:GAACCTAG+TCCGCATA
CTTCTGAACCCCTGCACCTAGCACCTACTTCCTCACATCTTGCCACACACGCTTTCAACGCCTCTAGC
AGCCTGCTAGGTTCCTGGCTCTCCACCCTAGAGTGCACTAGTGTCGCTACTCCTCTGGTCAAGTATTG
+
FF:FFFFFFFFFFFFFFFFFFFFFFFFFFFFFFFFF,FFFFFFFFFFFFFFFFFFFFFFFFFFFFFFF
FFFFFFFFFFFFFFFFFFFFFFFFFFFFFFFFFFFFFFFFFFFFFFFFFFFFFFFFFFFFFFFFFFFF
@A00155:342:HHGFNDSXY:1:1135:32344:11631 1:N:0:GAACCTAG+TCCGCATA
TTCTGAACCCCTGCACCTAGCACCTACTTCCTCACATCTTGCCACACACGCTTTCAACGCCTCTAGCA
GCCTGCTAGGTTCCTGGCTCTCCACCCTAGAGTGCACTAGTGTCGCTACTCCTCTGGTCAAGTATTGT
+
:FFFFFFFFFFFFF:FFFFFFFFFFFFF:FFFFFFF:FFFFFF:FFFFFFFFFFFFFFFFFFFF:FFF
FFFFFFFFFF:FFFFFFFFFFFFFFFFF:FFFFFFFFFFFFFFFFFFFFFFFFFFFFFFFFFFFFFFF
@A00155:342:HHGFNDSXY:1:1157:15420:3239 1:N:0:GAACCTAG+TCCGCATA
TTCTGAACCCCTGCACCTAGCACCTACTTCCTCACATCTTGCCACACACGCTTTCAACGCCTCTAGCA
GCCTGCTAGGTTCCTGGCTCTCCACCCTAGAGTGCACTAGTGTCGCTACTCCTCTGGTCAAGTATTGT
+
FFFFFFFFFFFFFFFFFFFFFFFFFFFFFFFFFFFF:FFFFFFFFFFFFFFFFFFFFFFFFFFFFFFF
FFFFFFFFFFFFFFFFFFFFFFFFFFFFF:FFFFFFFFFFFFFFFFFFFFFFFFFFFFF:FFFF:FFF
@A00155:342:HHGFNDSXY:1:1651:12491:33959 1:N:0:GAACCTAG+TCCGCATA
TTCTGAACCCCTGCACCTAGCACCTACTTCCTCACATCTTGCCACACACGCTTTCAACGCCTCTAGCA
GCCTGCTAGGTTCCTGGCTCTCCACCCTAGAGTGCACTAGTGTCGCTACTCCTCTGGTCAAGTATTGT
+
FFFFFFFFFFFFFFFFFFFF:FFFFFFFFFFFFFFFF:FFFFFFFFFFFFFFFFFFF:FFFFFFF:FF
:FFFFFFFFFFFFFFFFFFFFFFFFFFFFFFFFFFFFFFFFFFFFFFFFFFFFFFFFFFFFFFFFFFF
@A00155:342:HHGFNDSXY:1:1329:22444:8202 1:N:0:GAACCTAG+TCCGCATA
TCTGAACCCCTGCACCTAGCACCTACTTCCTCACATCTTGCCACACACGCTTTCAACGCCTCTAGCAG
CCTGCTAGGTTCCTGGCTCTCCACCCTAGAGTGCACTAGTGTCGCTACTCCTCTGGTCAAGTATTGTG
+
FFFFFFFFFFFFFFFFFFFFFFFFFFFFFFFFFFFFFFFF,FFFFFFFFFFFFFFFFFFFFFFFFFFF
FFFFFFFFFFFFFFFFFFF:FFFFFFFFFFFFFFFFFFFFFFFFFFFFFFFFFFFFFFFFFFFFFFFF
@A00155:342:HHGFNDSXY:1:1157:15420:3239 2:N:0:GAACCTAG+TCCGCATA
CCCCTGCACCTAGCACCTACTTCCTCACATCTTGCCACACACGCTTTCAACGCCTCTAGCAGCCTGCT
AGGTTCCTGGCTCTCCACCCTAGAGTGCACTAGTGTCGCTACTCCTCTGGTCAAGTATTGTGCACCCA
+
:FFFFFFFFFFFFFFFFFFF:FFFFFFFFFFFFF:FFFFFFFFFFFFFFFFFFFFFFFFFFF:FFFFF
FFFFFFFFFFFFFFFFFFFFFFFFFFFFFFFFFFFFFFFFFFFFFFFFFFFFFFFFFFFFFFFFFFFF
@A00155:342:HHGFNDSXY:1:1329:22444:8202 2:N:0:GAACCTAG+TCCGCATA
CCCCTGCACCTAGCACCTACTTCCTCACATCTTGCCACACACGCTTTCAACGCCTCTAGCAGCCTGCT
AGGTTCCTGGCTCTCCACCCTAGAGTGCACTAGTGTCGCTACTCCTCTGGTCAAGTATTGTGCACCCA
+
FFFFF:FFFFFFFFFFFFFFF:,FFFFFFFFFFFFFFFFF:FFFFFFFFFFFFFFFFFFFFFFFFFFF
FFFFFFFFFFFFFFFFFFFFFFFFFFFFFFFFFFFF:FFFFFFFFFFFFFFFFFFFFFFFFFFFFF:F
@A00155:342:HHGFNDSXY:1:1370:32018:4366 2:N:0:GAACCTAG+TCCGCATA
CCCTGCACCTAGCACCTACTTCCTCACATCTTGCCACACACGCTTTCAACGCCTCTAGCAGCCTGCTA
GGTTCCTGGCTCTCCACCCTAGAGTGCACTAGTGTCGCTACTCCTCTGGTCAAGTATTGTGCA
+
FFFFFFFFFFFFFFFFFFFFFFFFFFFFFFFFFFFFFFFFFFFFFFFFFFFFFFFFFFFFFFFFFFFF
FFFFFFFFFFFFFFFFFFFFFFFFFFFFFFFFFFFFFF:FFFFFFFFFFFFFFFFF::FFFFF

```
@A00155:342:HHGFNDSXY:1:1608:22851:11350 2:N:0:GAACCTAG+TCCGCATA
CCTGCACCTAGCACCTACTTCCTCACATCTTGCCACACACGCTTTCAACGCCTCTAGCAGCCTGCTAG
GTTCCTGGCTCTCCACCCTAGAGTGCACTAGTGTCGCTACTCCTCTGGTCAAGTATTGTGCACCCAGT
+
:FFFFF:FFFFF:FFFFFFFFFFFF:FFFFFFFFFFF:FFF:FFFFFFFFFFFF,FFFFFFFFFFFFF
FFFF:FFFFFFFFFFFFFF:FFFFFF:FFFFFFFFFF:FFFFFFFF:FFFFF:FFFFF:FFFFFFF,FF
@A00155:342:HHGFNDSXY:1:2523:7970:28823 1:N:0:GAACCTAG+TCCGCATA
GCACCTAGCACCTACTTCCTCACATCTTGCCACACACGCTTTCAACGCCTCTAGCAGCCTGCTAGGTT
CCTGGCTCTCCACCCTAGAGTGCACTAGTGTCGCTACTCCTCTGGTCAAGTATTGTGCACCCAGTCCT
+
FFFFFFFFFFFFFFFFFFFFFFFFFFFFFFFFFFFFFFFFFFFFFFFFFFFFFFFFFFFFFFFFFFFF
FFFFFFFFFFFFFFFFFFFFFFFFFFFFFFFFFFFFFFFFFFFFFFFF:FFFFFFFFFFFFF:FFFFF
@A00155:342:HHGFNDSXY:1:1513:21667:6136 2:N:0:GAACCTAG+TCCGCATA
TAGCACCTACTTCCTCACATCTTGCCACACACGCTTTCAACGCCTCTAGCAGCCTGCTAGGTTCCTGG
CTCTCCACCCTAGAGTGCACTAGTGTCGCTACTCCTCTGGTCAATTATTGTGCACCCAGTCCTTGTTC
+
F:FFFFFF,FFFFFFFFF,FF,FFFFF::FFFFFFFFFF,FF:FFFFFFFFFFFFF:FF:F:FFFFFF
FFFFFFFFFFFFFFFFFFFFFFFFFFFFFFFFFFFFFFFFFFF,FFFFFFFFFFFFFFFFFFFF,FF
@A00155:342:HHGFNDSXY:1:2329:5782:17895 1:N:0:GAACCTAG+TCCGCATA
ACCTACTTCCTCACATCTTGCCACACACGCTTTCAACGCCTCTAGCAGCCTGCTAGGTTCCTGGCTCT
CCACCCTAGAGTGCACTAGTGTCGCTACTCCTCTGGTCAAGTATTGTGCACCCAGTCCTTGTTCCTTA
+
FFFFFFF:FFFFFFFFFFFFFFFFFFFFF:FFF:FFFFFFFFFFFFFFFFFFFFFFFFFFFFFFFFFFF
FFF:FFFFFFFFFFFFFFFFFFFFFFFF:FF:FFFFFFFFFFFFFFFFFFFFFFFF:FF:,FFFFFFFFF
@A00155:342:HHGFNDSXY:1:1426:13006:30154 1:N:0:GAACCTAG+TCCGCATA
ACCTACTTCCTCACATCTTGCCACACACGCTTTCAACGCCTCTAGCAGCCTGCTAGGTTCCTGGCTCT
CCACCCTAGAGTGCACTAGTGTCGCTACTCCTCTGGTCAAGTATTGTGCACCCAGTCCTTGTTCCTTA
+
FFFFFFFFFFFFFFFFFFFFFFFFFFFFFFFFFFFFFFFFFFFFFFFFFFFFFFFFFFFFFFFFFFFF
FFFFFFFFFFFFFFFFFFFFFFFFFFFFFFFFFFFFFFFFFFFFFFFFFFFFFFFFFFFFFFFFFFFF
@A00155:342:HHGFNDSXY:1:1426:13105:30389 1:N:0:GAACCTAG+TCCGCATA
ACCTACTTCCTCACATCTTGCCACACACGCTTTCAACGCCTCTAGCAGCCTGCTAGGCTCCTGGCTCT
CCACCCTAGAGTGCACTAGTGTCGCTACTCCTCTGGTCAAGTATTGTGCACCCAGTCCTTGTTCCTTA
+
FFFFFFFFFFFFF:FFFFFFFFFFFFFFFFFFFFFFFFFFFFFFFFFFFFFFFFFFFFFFFFFFFFFFF
FFFFFFFFFFFFFFFFFFFFFFFFFFFFFFF:FFFFFFFFFFFFFFFFFFFFFFFFFFFFFF:FFFFFF
@A00155:342:HHGFNDSXY:1:1370:32018:4366 1:N:0:GAACCTAG+TCCGCATA
CTACTTCCTCACATCTTGCCACACACGCTTTCAACGCCTCTAGCAGCCTGCTAGGTTCCTGGCTCTCC
ACCCTAGAGTGCACTAGTGTCGCTACTCCTCTGGTCAAGTATTGTGCACCCAGTCCTTGTTCC
+
FFFFFFFFFFFFFFFFFFFFFFFFFFFFFFFFFFFFFFFFFFFFFFFFFFFFFFFFFFFFFFFFFFFF
FFFFFFF:FFFFFFFFFFFFFFFFFFFFFFFFFFFFFF:FFFFFFFFFFFFFFFFFFFFFFFFF
@A00155:342:HHGFNDSXY:1:2454:10836:35822 1:N:0:GAACCTAG+TCCGCATA
TCCTCACATCTTGCCACACACGCTTTCAACGCCTCTAGCAGCCTGCTAGGTTCCTGGCTCTCCACCCT
AGAGTGCACTAGTGTCGCTACTCCTCTGGTCAAGTATTGTGCACCCAGTCCTTGTTCCTTATCTACTT
+
F:FF:F,F:FF::F:FFFFFF:FFFF,FFFFFFFFFFFFFFFFFF:FFFF,F,,FFFF:FFFFFFFFF
FFFFFFFFF,FF::FFFFFFF,F:F:FFF,,FFF,FFFF,F,FF,FF:,F,FFF,F,F:,FFFFFF:F
@A00155:342:HHGFNDSXY:1:1205:19633:20995 2:N:0:GAACCTAG+TCCGCATA
CTCACATCTTGCCACACACGCTTTCAACGCCTCTAGCAGCCTGCTAGGTTCCTGGCTCTCCACCCTAG
AGTGCACTAGTGTCGCTACTCCTCTGGTCAAGTATTGTGCACCCAGTCCTTGTTCCTTATCTACTTT
+
FFFFFFFFFFFFFFFFFFFFFFFFFFFFFFFFFFFFFF:FFFFF:FFFFFFFFFFFFFFFFFFFF,F
FFFFFFFFFF:FFFFFFFFFFFFFFFFFFFFFFFFFFFFFFFFFF:FFFFFFF,FFFFFFFFFFFFF
```

```
@A00155:342:HHGFNDSXY:1:1341:8395:28181 2:N:0:GAACCTAG+TCCGCATA
ACATCTTGCCACACACGCTTTCAACGCCTCTAGCAGCCTGCTAGGTTCCTGGCTCTCCACCCTAGAGT
GCACTAGTGTCGCTACTCCTCTGGTCAAGTATTGTGCACCCAGTCCTTGTTCCTTATCTACTTTGTGC
+
:FFFFFFFFFFFFFFFF:F:FFFFFFFFFF,:FFFFFFFFF,FFFFFFFFFFFFFFFFFFFFFFFFFF
FFFFFFFFFFFFF:FFFFF:FFFF:FFFFFFFFFFFFFFFFFFFFFF:FFFFFFFFFFFF:FF:,FFFF
@A00155:342:HHGFNDSXY:1:1341:8395:28181 1:N:0:GAACCTAG+TCCGCATA
CTTGCCACACACGCTTTCAACGCCTCTAGCAGCCTGCTAGGTTCCTGGCTCTCCACCCTAGAGTGCAC
TAGTGTCGCTACTCCTCTGGTCAAGTATTGTGCACCCAGTCCTTGTTCCTTATCTACTTTGTGCTCGA
+
FFFFFFFFFFFFFFFFFFF:FFFFFFFFFFFFFFFFFFFFFFFFFFFFFFFFFFFFFFFFFFFFFFFF
FFFFF:FFFFFFFFFFFFFFFFFFFF:FFFFFFFFFFFFFFFFFFFFFFFFFFFFF:FFFFFFFFFFFF
@A00155:342:HHGFNDSXY:1:1205:19633:20995 1:N:0:GAACCTAG+TCCGCATA
ACACGCTTTCAACGCCTCTAGCAGCCTGCTAGGTTCCTGGCTCTCCACCCTAGAGTGCACTAGTGTCG
CTACTCCTCTGGTCAAGTATTGTGCACCCAGTCCTTGTTCCTTATCTACTTTGTGCTCGACTCTCAG
+
FFFFFFFFFFFFFFFFFFFFFFFFFFFFFFFFFFFFFFFFFFFFFFFFFFFFFFFFFFFFFFFFFFFF
FFFFFFFFFFFFFF:FFFF:FFFFFFFFFFFFFFFFFFFFFFFF:FFFFFFFFFFFFFFFFFFFFFF
@A00155:342:HHGFNDSXY:1:2508:8133:18897 2:N:0:GAACCTAG+TCCGCATA
TACGCCTCTAGCAGCCTGCTAGGTTCCTGGCTCTCCACCCTAGAGTGCACTAGTGTCGCTACTCCTCT
GGTCAAGTATTGTGCACCCAGTCCTTGTTCCTTATCTACTTTGTGCTCGACTCTCAGAAAC
+
FFFFFFFFFFFFFFFFFFFFFFFFFFFFFF:FFFFFFFFFFF:FFFFFFFFFFFFFFFFFFFFF:FFFF
FFFFFFFFFFFFFFFFFFFFFFFFFFFF:FFFF:FFFFFFFFFFFFFFFFFFFFFFFFFFFF
@A00155:342:HHGFNDSXY:1:2508:7129:18818 2:N:0:GAACCTAG+TCCGCATA
TACGCCTCTAGCAGCCTGCTAGGTTCCTGGCTCTCCACCCTAGAGTGCACTAGTGTCGCTACTCCTCT
GGTCAAGTATTGTGCACCCAGTCCTTGTTCCTTATCTACTTTGTGCTCGACTCTCAGAAAC
+
FFFFFFFFF,FFFFFFFFFFFFF::FFFFFF:FFFFFFFFFFFFFFFFFF,FFFFFFFFFFFFFFFFF
FFFFFFFFFFFFFFFFFFFFFFFFFFFF,FFFFFFFFFFF:FFFFFFFF:FFFFFFFFFFFF
@A00155:342:HHGFNDSXY:1:2624:30879:24565 1:N:0:GAACCTAG+TCCGCATA
AACGCCTCTAGCAGCCTGCTAGGTTCCTGGCTCTCCACCCTAGAGTGCACTAGTGTCGCTACTCCTCT
GGTCAAGTATTGTGCACCCAGTCCTTGTTCCTTATCTACTTTGTGCTCGACTCTCAGAAACTCACCTA
+
FFFFFFFFFFFFFFFFFFFFFFFFFFFFFFFFFFFFFFFFFFFFFFFFFFFFFFFFFFFFFFFFFFFF
FFFFFFFFFFFFFFFFFFFFFFFFFFFFFFFFF:FFFFFFFFFFFFFFFFFFFFFFFFFFFFFFFFFF
@A00155:342:HHGFNDSXY:1:1624:32235:19022 1:N:0:GAACCTAG+TCCGCATA
AACGCCTCTAGCAGCCTGCTAGGTTCCTGGCTCTCCACCCTAGAGTGCACTAGTGTCGCTACTCCTCT
GGTCAAGTATTGTGCACCCAGTCCTTGTTCCTTATCTACTTTGTGCTCGACTCTCAGAAACTCACCTA
+
FFFFFFFFFFFFFFFFFFFFFFFFFFFFFFFFFFFFFFFFFFFFFFFFFFFFFFFFFFFFFFFFFFFF
FFFFFFF:FFFFFFFFFFFFFFFFFFFFFFFFFFFFFFFFFFFFFFFFFFFFFFFFFFFFFFFFFFF:
@A00155:342:HHGFNDSXY:1:1322:24777:19413 1:N:0:GAACCTAG+TCCGCATA
AACGCCTCTAGCAGCCTGCTAGGTTCCTGGCTCTCCACCCTAGAGTGCACTAGTGTCGCTACTCCTCT
GGTCAAGTATTGTGCACCCAGTCCTTGTTCCTTATCTACTTTGTGCTCGACTCTCAGAAACTCACCTA
+
FFFFFFFFFF:FFFFFFFFFFFFFFFFFFFFFFFFFFFFFFF:FFFFFFFFFFFFFFFFFFFFFFFFFF
FFFFFFF,FFFFFFFFFFFFFFFFFFF:FFFFFFFFFFFFFFFFFFFFFFFFFFFFFFFFFFFFFFFF
@A00155:342:HHGFNDSXY:1:2454:10836:35822 2:N:0:GAACCTAG+TCCGCATA
TCTAGCAGCCTGCTAGGTTCCTGGCTCTCCACCCTAGAGTGCACTAGTGTCGCTACTCCTCTGGTCAA
GTATTGTGCACCCAGTCCTTGTTCCTTATCTACTTTGTGCTCGACTCTCAGAAACTCACCTACTGAGA
+
FFF:FFFFFF:FFFFFF,FFFFFFFFFFFFF:FF:FF,:FFFFF:FFFFFF,FFFFFFFFFFFF:FF,
FF:FFFF:F:FFF:FFFFFFFFF:FFF,F:FFFFFFFF:FFFFF:FF:FFFFFFFF:FF:FFFFFFFF
```

```
@A00155:342:HHGFNDSXY:1:2523:7970:28823 2:N:0:GAACCTAG+TCCGCATA
AGCCTGCTAGGTTCCTGGCTCTCCACCCTAGAGTGCACTAGTGTCGCTACTCCTCTGGTCAAGTATTG
TGCACCCAGTCCTTGTTCCTTATCTACTTTGTGCTCGACTCTCAGAAACTCACCTACTGAGAACACGT
+
FFFFFFFFFFFFFFFFFFFFFFFFFFFFFFFFFFFFFFFFFFFFFFFFFFFFFFFFFFFFFFFFFFFF
FFFFFFFFFFFFFFF:FFFFFFFFFFFFFFFFFFFFFFFFFFFFFFFFFFFFFFFFFFFFFFFFFFFF
@A00155:342:HHGFNDSXY:1:1651:12491:33959 2:N:0:GAACCTAG+TCCGCATA
GCCTGCTAGGTTCCTGGCTCTCCACCCTAGAGTGCACTAGTGTCGCTACTCCTCTGGTCAAGTATTGT
GCACCCAGTCCTTGTTCCTTATCTACTTTGTGCTCGACTCTCAGAAACTCACCTACTGAGAACACGTT
+
FFFFFFFFFFFFFFFFFFFFFFFFFFFFFFFFFFFFFFFFFFFFFFFFFFFFFF:FFFFFFFFFFFFF
FFFFFFFFFFFFFFFFFFFFFFFFF:FFFFFFFFFFFFFFFFFFFFFFFFFFFFF:FFFFFFFFFFFFF
@A00155:342:HHGFNDSXY:1:2508:8133:18897 1:N:0:GAACCTAG+TCCGCATA
CTGCTAGGTTCCTGGCTCTCCACCCTAGAGTGCACTAGTGTCGCTACTCCTCTGGTCAAGTATTGTGC
ACCCAGTCCTTGTTCCTTATCTACTTTGTGCTCGACTCTCAGAAACTCACCTACTGAGAAC
+
FFFFFFFFFFFFFFFFFFFFFFFFFFFFFFFFFFFFFFFFFFFFFFFFFFFFFFFFFFFF:FFFFFFF
FFFF:FFFFFFFFFFFFFFFFFFFFFFFFFFFFFFFFFFFFFFFFF:FFFF,:FFFFFFFFF
@A00155:342:HHGFNDSXY:1:2508:7129:18818 1:N:0:GAACCTAG+TCCGCATA
CTGCTAGGTTCCTGGCTCTCCACCCTAGAGTGCACTAGTGTCGCTACTCCTCTGGTCAAGTATTGTGC
ACCCAGTCCTTGTTCCTTATCTACTTTGTGCTCGACTCTCAGAAACTCACCTACTGAGAAC
+
FFFFFFFFFFFFFFFFFFFFFFFFFFFFFFFFFFFFFFFFFFFFFFFFFFFFFFFFFFFF,FFFFFFF
FFFFFFFFFFFFFFFFFFFFFFFFFFFFFFFFFFFFFFFFFFFFFFFFFFFFFFFFFFFFF
@A00155:342:HHGFNDSXY:1:1651:27109:3129 1:N:0:GAACCTAG+TCCGCATA
TTCCTGGCTCTCCACCCTAGAGTGCACTAGTGTCGCTACTCCTCTGGTCAAGTATTGTGCACCCAGTC
CTTGTTCCTTATCTACTTTGTGCTCGACTCTCAGAAACTCACCTACTGAGAACACGTTACACTTAGTG
+
FFFFFFFFFFFFFFFFFFFFFFFFFFFFFFFFFFFFFFFFFFFFFFFFFFFFFFFFFFFFFFFFFFFF
FFFF:FFFFFFFFFFF:FFFFFFFFFFFFFFFFFF:FFFFFFF:FFFFFFFFFFFFFFFFFFFFFFFF
@A00155:342:HHGFNDSXY:1:2507:15483:27023 2:N:0:GAACCTAG+TCCGCATA
CCTGGCTCTCCACCCTAGAGTGCACTAGTGTCGCTACTCCTCTGGTCAAGTATTGTGCACCCAGTCCT
TGTTCCTTATCTACTTTGTGCTCGACTCTCAGAAACTCACCTACTGAGAACACGTTACAC
+
FFFFFFFFFFFFFFFFFFFFFFFFFF,FFFFFFFFFFFFFFFFFFFFFFFFFFFFFFFF:FFFFFFFFF
FF:FFFFFFFFF:FFF:FFFFFFF:FFFFFFFFFFFFFFFFFFFFFFFFFFFFFFF,FFFF
@A00155:342:HHGFNDSXY:1:1527:17372:14074 2:N:0:GAACCTAG+TCCGCATA
CCTGGCTCTCCACCCTAGAGTGCACTAGTGTCGCTACTCCTCTGGTCAAGTATTGTGCACCCAGTCCT
TGTTCCTTATCTACTTTGTGCTCGACTCTCAGAAACTCACCTACTGAGAACACGTTACAC
+
FFFFFFFFFFFFFFFFFFFFFFFFFFFFFFFFFFFFFFFFFFFFFFFFFFFFF:FFFFFFFFFFFFFF
:F,FFFF:FFFFFFFFFFFFFFFFFFFFFFFFFFFFFFFFFFF:FFFFFFFFFFFFFFFFF
@A00155:342:HHGFNDSXY:1:1527:17083:14888 2:N:0:GAACCTAG+TCCGCATA
CCTGGCTCTCCACCCTAGAGTGCACTAGTGTCGCTACTCCTCTGGTCAAGTATTGTGCACCCAGTCCT
TGTTCCTTATCTACTTTGTGCTCGACTCTCAGAAACTCACCTACTGAGAACACGTTACAC
+
FFFFFFFF:FFFFFFF:FFFFFFFF,FFFFFFFFFFFFFFFFFFFFFFFFFFFFFFFFFFFFFFFFFF
FF,FFFFFFFFFFF:FFFFFFFFFFFFFFFFFFF:FF:FFFFF,FF:FFFFFFFFFFFFFF
@A00155:342:HHGFNDSXY:1:1527:18204:23437 2:N:0:GAACCTAG+TCCGCATA
CCTGGCTCTCCACCCTAGAGTGCACTAGTGTCGCTACTCCTCTGGTCAAGTATTGTGCACCCAGTCCT
TGTTCCTTATCTACTTTGTGCTCGACTCTCAGAAACTCACCTACTGAGAACACGTTACAC
+
FFFFFFFFFFFFFFFFFFFFFFFFFFFFFFFFFFFFFFFFFFFFFFFFFFFFFFFFFFFFFF,FFFFF
FFFFFFFF:FFF:FFFFFFFFFFFFFFFFFFFFFFFFFFFFFFFFFFFFFFFFFFFFFFFF
```

```
@A00155:342:HHGFNDSXY:1:1549:26341:20807 2:N:0:GAACCTAG+TCCGCATA
GCCCTAGAGTGCACTAGTGTCGCTACTCCTCTGGTCAAGTATTGTGCACCCAGTCCTTGTTCCTTATC
TACTTTGTGCTCGACTCTCAGAAACTCACCTACTGAGAACACGTTACACTTAGTGTGCTGCGCCCTAG
+
FFFFFFFFFFFFFF:FFFFFFFFFFFFFFFFF:FFFFFFFFFFFFFFFFFFF:FFFFFFFFFFFFFFF
,FF:FFFFFFFFFFFFFFFFFFFFFFFFFF:FFFFFFFFF:FFFFFFFFFFFFFFFFFFFFFFFFFFF
@A00155:342:HHGFNDSXY:1:1651:27109:3129 2:N:0:GAACCTAG+TCCGCATA
CCCTAGAGTGCACTAGTGTCGCTACTCCTCTGGTCAAGTATTGTGCACCCAGTCCTTGTTCCTTATCT
ACTTTGTGCTCGACTCTCAGAAACTCACCTACTGAGAACACGTTACACTTAGTGTGCTGCGCCCTAGC
+
FFFFFFFFFFFFFFFFFFFFFFFFFFFFFFFFFFFFFFFFFFFFFFFFFFFFFFFFFF:FFFFFFFFF
FFFFFFFFFFFFFFFFFFFFFFFFFFFFFFFF:FFFFFFFFF:FFFFFFF:FFFFFFFFFFFFFFFFF
@A00155:342:HHGFNDSXY:1:2602:27651:32503 1:N:0:GAACCTAG+TCCGCATA
CCCTAGAGTGCACTAGTGTCGCTACTCCTCTGGTCAAGTATTGTGCACCCAGTCCTTGTTCCTTATCT
ACTTTGTGCTCGACTCTCAGAAACTCACCTACTGAGAACACGTTACACTTAGTGTGCTGCGCCCTAGC
+
FFFFFFFFFFFFFFFFFFFFFFFFFFFFFFFFFFFFFFFFFFFFFFFFFFFFFFFFFFFF:FFFFFFF
FFFFFFFFFFFFFFFFFFFFF,FFFF:FFFFFFFFFFFFFFFFFFFFFFFFFFFFFFFFFFFF:,FFF
@A00155:342:HHGFNDSXY:1:1527:17083:14888 1:N:0:GAACCTAG+TCCGCATA
TAGAGTGCACTAGTGTCGCTACTCCTCTGGTCAAGTATTGTGCACCCAGTCCTTGTTCCTTATCTACT
TTGTGCTCGACTCTCAGAAACTCACCTACTGAGAACACGTTACACTTAGTGTGCTGCGCC
+
FFFFFFFFFFFFFFFFFFFFFFFFFFFFFFFFFFFFF:FFFFFFFFFFFFFFFFFFFFFFFFFFFFFF
FFFFFFFFFFFFFFFFFFFFFFFFFFFFFFFFFFFFFFFFFFFFFFFFFFFFFFFFFFFF
@A00155:342:HHGFNDSXY:1:1527:17372:14074 1:N:0:GAACCTAG+TCCGCATA
TAGAGTGCACTAGTGTCGCTACTCCTCTGGTCAAGTATTGTGCACCCAGTCCTTGTTCCTTATCTACT
TTGTGCTCGACTCTCAGAAACTCACCTACTGAGAACACGTTACACTTAGTGTGCTGCGCC
+
FFFFFFFFFFFFFFFFFFFFFFFFFFFFFFFFFFF,FFFFFFFFFFFFFFFFFFFFFFFFFFFFFFFF
FFFFFFFFF:FFFFFFFFFFFFFFF::FFFFFF:FFFFFFFFFFF,FFFFFFFFFFFFFF
@A00155:342:HHGFNDSXY:1:1527:18204:23437 1:N:0:GAACCTAG+TCCGCATA
TAGAGTGCACTAGTGTCGCTACTCCTCTGGTCAAGTATTGTGCACCCAGTCCTTGTTCCTTATCTACT
TTGTGCTCGACTCTCAGAAACTCACCTACTGAGAACACGTTACACTTAGTGTGCTGCGCC
+
FFFFFFFFFFFFFFFFFFFFFFFFFFFFFFFFFFF:,FFFFFFFFFFFFFFF:FF:FFFFFFFFFF::
FFFFFFFFFFFFFFFFFFFFFFF:FFFFFFFFFFFFFFFFFFFFFF:FFFFFFFFFFFFF
@A00155:342:HHGFNDSXY:1:2507:15483:27023 1:N:0:GAACCTAG+TCCGCATA
TAGAGTGCACTAGTGTCGCTACTCCTCTGGTCAAGTATTGTGCACCCAGTCCTTGTTCCTTATCTACT
TTGTGCTCGACTCTCAGAAACTCACCTACTGAGAACACGTTACACTTAGTGTGCTGCGCC
+
FFFFFFFFFFFFFFFFFFFF:FFFFFF,FFFFFFFFFFFFFFFFFFF:FFFF:FFFFF,FFFFFFFFF
,FFFFF:FFFFFFFFFFFFFFFFFFFFFFFFFFFFFFFFFFFFFFFFFFFFFFFFFFFFF
@A00155:342:HHGFNDSXY:1:2230:30002:34851 1:N:0:GAACCTAG+TCCGCATA
AGAGTGCACTAGTGTCGCTACTCCTCTGGTCAAGTATTGTGCACCCAGTCCTTGTTCCTTATCTACTT
TGTGCTCGACTCTCAGAAACTCACCTACTGAGAACACGTTACACTTAGTGTGCTGCGCCCTAGCGTGT
+
FFFFFFFFFFFFFFFFFFFFFFFFFFFFFFFFFF,FFFFFFFFFFF:FFFF:FFFFFFFFFFFFFFFF
FFFFFFFFFFFFFFFFFFFFFFFFFFFFFFFFFFFFFFFFFFFF:FFFFFFFFFFFFFFFF:FFFF,F
@A00155:342:HHGFNDSXY:1:2351:29062:24956 1:N:0:GAACCTAG+TCCGCATA
GAGTGCACTAGTGTCGCTACTCCTCTGGTCAAGTATTGTGCACCCAGTCCTTGTTCCTTATCTACTTT
GTGCTCGACTCTCAGAAACTCACCTACTGAGAACACGTTACACTTAGTGTGCTGCGCCCTAGCGGTT
+
FFF:FFFFFFFFFFFFFFFFFFFFFFFFFFFFFFFFFFFFFFFFFFFFFFFFFFFFFFFFFFFFFF,F
FFFFFFFFFFFFFFFFF:FFFFFFFFFFFFFFFFFFFFFFFFFFFFFFFFFFFF:FF:FFFFFF,FF
```

@A00155:342:HHGFNDSXY:1:1426:13006:30154 2:N:0:GAACCTAG+TCCGCATA
AGTGCACTAGTGTCGCTACTCCTCTGGTCAAGTATTGTGCACCCAGTCCTTGTTCCTTATCTACTTTG
TGCTCGACTCTCAGAAACTCACCTACTGAGAACACGTTACACTTAGTGTGCTGCGCCCTAGCGTTTAT
+
FFFFFFFFFFFFFFFFFFFFFFFFFFFFFFFFFFFFFFFFFFFFFFFFFFFFFFFFFFFFFFFFFFFF
FFFFFFFFFFFFFFFFFFFFFFFFFFFFFFFFFFFFFFFFFFFFFFFFFFFFFFFFFFFFFFFFFFFF
@A00155:342:HHGFNDSXY:1:1426:13105:30389 2:N:0:GAACCTAG+TCCGCATA
AGTGCACTAGTGTCGCTACTCCTCTGGTCAAGTATTGTGCACCCAGTCCTTGTTCCTTATCTACTTTG
TGCTCGACTCTCAGAAACTCACCTACTGAGAACACGTTACACTTAGTGTGCTGCGCCCTAGCGTTTAT
+
FFFF::,FFF:F:FFFFFFFFFFFFFFF:FFF,FFFFFFFF:FFFFFFFFFF:FF,,FFFFFFFFFFF
FFFFF:FFFFFFFFFFFFFFFFFFFFFFFFFF,FFFFFFFFFFFFFFFFFF:FFFFFFFFFFFFFFFF
@A00155:342:HHGFNDSXY:1:1549:26341:20807 1:N:0:GAACCTAG+TCCGCATA
CTAGTGTCGCTACTCCTCTGGTCAAGTATTGTGCACCCAGTCCTTGTTCCTTATCTACTTTGTGCTCG
ACTCTCAGAAACTCACCTACTGAGAACACGTTACACTTAGTGTGCTGCGCCCTAGCGTTTATCATTCT
+
FFFFFFFFFFFFFFFFFFFFFFF:FFFFFFFFFFFFFFFFFFFFFFFFFFFFFFFFFFFFFFFFFFFF
FFFFFFFFFFFFFFFFFFFFFFFFFFFFFFFFFFFFFFFFFFFFFFFFFFFFFFFFF,FFFFFFFFFF
@A00155:342:HHGFNDSXY:1:2520:20609:25598 2:N:0:GAACCTAG+TCCGCATA
TGTCGCTACTCCTCTGGTCAAGTATTGTGCACCCAGTCCTTGTTCCTTATCTACTTTGTGCTCGACTC
TCAGAAACTCACCTACTGAGAACACGTTACACTTAGTGTGCTGCGCCCTAGCGTTTATCATTCTCA
+
FFFFFFFFFFFFFFFFFFFFFFFFFFFFFFFFFFFFFFFFFF:FFFF:FFFFFFFFFFFFFFFFFFFF
FFFFFFFFFFFFF:FFFFFFFFFFFFFFFFFFFFFFFFFFFFFFFFFFFFFFFFFFFFFFFFFFFFFF
@A00155:342:HHGFNDSXY:1:2676:20925:23610 2:N:0:GAACCTAG+TCCGCATA
TGTCGCTACTCCTCTGGTCAAGTATTGTGCACCCAGTCCTTGTTCCTTATCTACTTTGTGCTCGACTC
TCAGAAACTCACCTACTGAGAACACGTTACACTTAGTGTGCTGCGCCCTAGCGTTTATCATTCTCA
+
FFFFFFFFFFFFFFFFFFFFFFFFFFFFFFFFFFFFFFFFFF:FFFFFFFFFFFFFFFFFFFFFFFFF
FFFFFFFFFFFFFFFFFFFFFFFFFFFFFFFFFFFFFFFFFFFFFFFFFFFFFFFFFFFFFFFFFF
@A00155:342:HHGFNDSXY:1:2130:3016:7435 2:N:0:GAACCTAG+TCCGCATA
GTCGCTACTCCTCTGGTCAAGTATTGTGCACCCAGTCCTTGTTCCTTATCTACTTTGTGCTCGACTCT
CAGAAACTCACCTACTGAGAACACGTTACACTTAGTGTGCTGCGCCCTAGCGTTTATCATTCTCATTT
+
FFFFFFFFFFFFFFFFFFFF,FFFFFFFFFFFFF:FFFFFF:FFFFFFFFFFFFFFFFFFFFFFFFFF
FFFFFFFFFFFFFFFFFFFFFFFFFFFFFFFFFFFFFFFFFFFFFFFFFFFF:FFFFFFFFFFFFFFF
@A00155:342:HHGFNDSXY:1:2676:20925:23610 1:N:0:GAACCTAG+TCCGCATA
GGTCAAGTATTGTGCACCCAGTCCTTGTTCCTTATCTACTTTGTGCTCGACTCTCAGAAACTCACCTA
CTGAGAACACGTTACACTTAGTGTGCTGCGCCCTAGCGTTTATCATTCTCATTTTTTCGTGTATAA
+
FFFFFFFFF:FFFFFFFFFFFFFFFFFFFFFFFFFFFFFFFFFFFFFFFFFFFFFFFFFFFFFFFFFF
FFFFFFFFFFFFFFFFFFFFFFFFFFFFFFFFFFFFFFF,:FFFFFFFFFFFFFFFFFFFFFFFFF
@A00155:342:HHGFNDSXY:1:2520:20609:25598 1:N:0:GAACCTAG+TCCGCATA
GGTCAAGTATTGTGCACCCAGTCCTTGTTCCTTATCTACTTTGTGCTCGACTCTCAGAAACTCACCTA
CTGAGAACACGTTACACTTAGTGTGCTGCGCCCTAGCGTTTATCATTCTCATTTTTTCGTGTATAA
+
FFFFFFFFF:FFFFFFFFFFFFFFFFFFFFFFFFFFFFFFFFFFFFFFFFFFFFFFFFFFFFFFFFFF
FFFFFFFFFF:FFFFFFFFFFFFFFFFFFFFF:FFFFF:FFFFFF:FFFFFFFFFFF,:FFFFFFF
@A00155:342:HHGFNDSXY:1:2605:22137:31594 1:N:0:GAACCTAG+TCCGCATA
GTCAAGTATTGTGCACCCAGTCCTTGTTCCTTATCTACTTTGTGCTCGACTCTCAGAAACTCACCTAC
TGAGAACACGTTACACTTAGTGTGCTGCGCCCTAGCGTTTATCATTCTCATTTTTTCGTGTATAACGT
+
FFFFFFFF:FFFFFFFFFFFFFFFFFFFFFFFFFFFFFFFFFFFFFFFFFFFFFFFFFFFFFFFFFFF
FFFFFFFFFFFFFFFFFFFFFFFFFFFFFFFFFFFFFF::FFF:FFFFFFFFFFFFFFFFFF:FFFFF

```
@A00155:342:HHGFNDSXY:1:2130:3016:7435 1:N:0:GAACCTAG+TCCGCATA
GTCAAGTATTGTGCACCCAGTCCTTGTTCCTTATCTACTTTGTGCTCGACTCTCAGAAACTCACCTAC
TGAGAACACGTTACACTTAGTGTGCTGCGCCCTAGCGTTTATCATTCTCATTTTTTCGTGTATAACGT
+
FFFFFFFFFFFFFFFFFFFFFFFFFFFFFFFFFFFFFFFFFFFFFFFFFFFFFFFFFFFFFFFFFFFF
FFFFFFFFFFFFFFFFFFFFFFFFFFF:FF:FFFFFFFFFFFFFFFFFFFF:FFFF::FFFFFFF:FF
@A00155:342:HHGFNDSXY:1:2331:22905:7811 1:N:0:GAACCTAG+TCCGCATA
GTCAAGTATTGTGCACCCAGTCCTTGTTCCTTATCTACTTTGTGCTCGACTCTCAGAAACTCACCTAC
TGAGAACACGTTACACTTAGTGTGCTGCGCCCTAGCGTTTATCATTCTCATTTTTTCGTGTATAACGT
+
FFF:FFFF:FFFFFFFFFFFFFFFFFFFFFFFFFFFFFFFFFFFFFFFFFFFFFFFFFFFFFFFFFFF
FFFFFFFFFFFFFFFFFFFFFFFFFFFFFFFFFFFFFFF:FFFF,FFFFFFFFFFF:FFFFFFFFFFF
@A00155:342:HHGFNDSXY:1:2477:22399:22592 1:N:0:GAACCTAG+TCCGCATA
CAAGTATTGTGCACCCAGTCCTTGTTCCTTATCTACTTTGTGCTCGACTCTCAGAAACTCACCTACTG
AGAACACGTTACACTTAGTGTGCTGCGCCCTAGCGTTTATCATTCTCATTTTTTCGTGTATAAC
+
FFFFFFFFFFFFFFFFFFFFFFFFFFFFFFFFFFFFFFFFFFFFFFFFFFFFFFFFFFFFFFFFFFFF
FFFFFFFFFFFFFFFFFFFFFFFFFFFFFFF,FFFF,FF:FFFFFFFFFFFFFFF:FFFFFFFF
@A00155:342:HHGFNDSXY:1:2266:23556:7623 2:N:0:GAACCTAG+TCCGCATA
CAAGTATTGTGCACCCAGTCCTTGTTCCTTATCTACTTTGTGCTCGACTCTCAGAAACTCACCTACTG
ATAACACGTTACACTTAGTGTGCTGCGCCCTAGCGTTTATCATTCTCATTTTTTCGTGTATAACGTTC
+
FFFFFFFFFFFFFFFFFFFFFFFFFFFFFF:FF,FFF:FFFFFFFFFFFFFFFFFFFFFFFFFFFFFF
FFFFFFFF:FFFFFFFFFFFFFFFFFFFFFFF:F:FFFFFFFFFFFF:FFFFFFFFFFFFFFFFFFF
@A00155:342:HHGFNDSXY:1:2566:31458:16986 2:N:0:GAACCTAG+TCCGCATA
CAAGTATTGTGCACCCAGTCCTTGTTCCTTATCTACTTTGTGCTCGACTCTCAGAAACTCACCTACTG
ATAACACGTTACACTTAGTGTGCTGCGCCCTAGCGTTTATCATTCTCATTTTTTCGTGTATAACGTTC
+
FFFFFFFFFFFFFFFFFFFFFFFFFFFFFFFFFFFFFFFFFFFFFFFFFFFFFFFFFFFFFFFFFFFF
FFFFFFFFFFFFFFFFFFFFFFFFFFFFFFFFFFFFFFFFFFFFFFFFFFFFFFFFFFFFFFFFFFFF
@A00155:342:HHGFNDSXY:1:2477:22399:22592 2:N:0:GAACCTAG+TCCGCATA
GTATTGTGCACCCAGTCCTTGTTCCTTATCTACTTTGTGCTCGACTCTCAGAAACTCACCTACTGAGA
ACACGTTACACTTAGTGTGCTGCGCCCTAGCGTTTATCATTCTCATTTTTTCGTGTATAACGTTCGCT
+
FFFFFFFFFFFFFFFFFFFFF,FFFFFFFFFFF:FFFFFFFFFFFFFFFFFFFFFFFFFFFFF:FFFF
FFF:FFFFFFFFFFFFFFFFFFFFFFFFFFFFFFFFFFFFFFFFFFFF:FFFFFFFFFFFFFFFFFFF
@A00155:342:HHGFNDSXY:1:2407:23520:13667 1:N:0:GAACCTAG+TCCGCATA
ATTGTGCACCCAGTCCTTGTTCCTTATCTACTTTGTGCTCGACTCTCAGAAACTCACCTACTGAGAAC
ACGTTACACTTAGTGTGCTGCGCCCTAGCGTTTATCATTCTCATTTTTTCGTGTATAACGTTCGCTGT
+
FFFFFFFFFFFFFFFFFFFFFFFFFFFFFFFFF:FF:FF:FFFFFFFFFFFFFFFFFFFFFFFFFFFF
FFFFFFFFFFFFFFFF:FFFF:FFF:FFFFFFFFFFFFFFFFFFFFFFFFFFFFFFFFFFFFFFFFFF
@A00155:342:HHGFNDSXY:1:1663:2582:9533 1:N:0:GAACCTAG+TCCGCATA
TTGTGCACCCAGTCCTTGTTCCTTATCTACTTTGTGCTCGACTCTCAGAAACTCACCTACTGAGAACA
CGTTACACTTAGTGTGCTGAGCCCTAGCGTTTATCATTCTCATTTTTTCGTGTATAACGTTCGCTGTC
+
FFFFFFFF::FFFFFFFF,FFFFFFFFFFFFFFFFFFFFFF:FFFFF:FFFFFFFFFFFFFFF:FFFF
FFFFFFFFFFFFFFFF:FF:,FFFFFF:,,FFF:F:FFFFFF:FFFFFF,FFFFFFFFFFFFFFFFFF
@A00155:342:HHGFNDSXY:1:2439:19452:25880 1:N:0:GAACCTAG+TCCGCATA
GTGCACCCAGTCCTTGTTCCTTATCTACTTTGTGCTCGACTCTCAGAAACTCACCTACTGAGAACACG
TTACACTTAGTGTGCTGCGCCATAGCGTTTATCATTCTCATTTTTTCGTGTATAACGTTCGCTGTC
+
FF:,FFF,F,:,FF:,:F:F:FF,,FF,,,,:,,:,F,FFFF,FFFFFFF:FFFF:FF:F::FF:F::
:,F,F:F,FF,,,,,F,F:,,,,FF:,,:FFF:F:F:F,F,:F,,FFFFFFFFFF,:FFFF:,,FF
```

```
@A00155:342:HHGFNDSXY:1:2439:19407:25488 1:N:0:GAACCTAG+TCCGCATA
GTGCACCCAGTCCTTGTTCCTTATCTACTTTGTGCTCGACTCTCAGAAACTCACCTACTGAGAACACG
TTACACTTAGTGTGCTGCGCCCTAGCGTTTATCATTCTCATTTTTTCGTGTATAACGTTCGCTGTCCT
+
FFFFFFFFFFFFFFFFFFFFFFFFFFFFFFFFFFFFFFFFFFFFFFFFFFFFFFFFFFFFFFFFFFFF
FFFFFFFFFFFFFFFFFFFFFFFFFFFFFF:FF:FFFFFFFFFFFFFFFFFFFFFFFFFFFFFFFFFF
@A00155:342:HHGFNDSXY:1:2439:20645:25066 1:N:0:GAACCTAG+TCCGCATA
GTGCACCCAGTCCTTGTTCCTTATCTACTTTGTGCTCGACTCTCAGAAACTCACCTACTGAGAACACG
TTACACTTAGTGTGCTGCGCCCTAGCGTTTATCATTCTCATTTTTTCGTGTATAACGTTCGCTGTCCT
+
FFFFFFFFFFFFFFFFFFFFFFFFFFFFFFFFFFFFFFFFFFFFFFFFFFFFFFFFFFFFFFFFFFFF
FFFFFFFFFFFFFFFFFFFFFFFFFFFFFFFFFFFFFFFFFFFFFFFFFFFFFFFFFFFFFFFFFFFF
@A00155:342:HHGFNDSXY:1:2631:5511:6527 1:N:0:GAACCTAG+TCCGCATA
GTGCACCCAGTCCTTGTTCCTTATCTACTTTGTGCTCGACTCTCAGAAACTCACCTACTGAGAACACG
TTACACTTAGTGTGCTGCGCCCTAGCGTTTATCATTCTCATTTTTTCGTGTATAACGTTCGCTGTCCT
+
FFFFFFFFFFFFFFFFFFFFFFFFFFFFFFFFF:FFFFFFFFFFFFFFFFFFFF:FFFFFF:FFFFFF
FFFFFFFFFFFFFFFFFFFFFFFFFFFF,:FFFFFFFFFF:FFFFFFFFFFFFFFF:FFF:FFFFFFF
@A00155:342:HHGFNDSXY:1:2407:23520:13667 2:N:0:GAACCTAG+TCCGCATA
CCAGTCCTTGTTCCTTATCTACTTTGTGCTCGACTCTCAGAAACTCACCTACTGAGAACACGTTACAC
TTAGTGTGCTGCGCCCTAGCGTTTATCATTCTCATTTTTTCGTGTATAACGTTCGCTGTCCTAATATT
+
::,F::,:FFF:F,:FFFFFFFFFFFF:,F,F:FFFFFF,F:FF:FF:::F:FF::::F:F:F:FF::
FF::FFFFF::F,F:FFF:F:FFFFFFF:FFFFFFFFFF:FFF:F::F:::FFFFF:F:FFFFFFFF:
@A00155:342:HHGFNDSXY:1:1135:32344:11631 2:N:0:GAACCTAG+TCCGCATA
TCCTTGTTCCTTATCTACTTTGTGCTCGACTCTCAGAAACTCACCTACTGAGAACACGTTACACTTAG
TGTGCTGCGCCCTAGCGTTTATCATTCTCATTTTTTCGTGTATAACGTTCGCTGTCCTAATATTGTCT
+
FFF:FF,FFFFFF,FFFF,FFFFF:FFFFFFFFFFFF:F:FFFFF:FFFFFFFFFFFFFF,FFFFFFF
FFFFFFFFFFFF::FFFFFFFFFFFFFFFFFFFFFFFFFFFFFFFFFFFFFFFFFFFF:::F:FFFF
@A00155:342:HHGFNDSXY:1:2351:29062:24956 2:N:0:GAACCTAG+TCCGCATA
TCCTTGTTCCTTATCTACTTTGTGCTCGACTCTCAGAAACTCACCTACTGAGAACACGTTACACTTAG
TGTGCTGCGCCCTAGCGTTTATCATTCTCATTTTTTCGTGTATAACGTTCGCTGTCCTAATATTGTCT
+
:FFFFF,F,FFFFFFFFFFFFFFFFFF,FFFFFFF:FFFFFFFF,FFFFFFFFFFFFFFFFFFFFFFF
FFFFFFFFFF:FFFFFFFFFFFFF:FFFFFFFF:FFFFFFFFFFFF:FFFF:,FFFFFF:FFFFFFFF
@A00155:342:HHGFNDSXY:1:1322:24777:19413 2:N:0:GAACCTAG+TCCGCATA
CTTGTTCCTTATCTACTTTGTGCTCGACTCTCAGAAACTCACCTACTGAGAACACGTTACACTTAGTG
TGCTGCGCCCTAGCGTTTATCATTCTCATTTTTTCGTGTATAACGTTCGCTGTCCTAATATTGTCTAT
+
FFFFFFFFFFFFFFFFFF:FFFFFFFFFFFFFFFFFFFFFFFFFF:FFFFFFFFFFFFFFFFFFFFFF
FFFFFFFFFFFFFFFFFFFFFFFFFFFFFFFFFFFFFFFFF:FFFFF,FFFFFFFFFFFFFFFF:FF:
@A00155:342:HHGFNDSXY:1:1624:32235:19022 2:N:0:GAACCTAG+TCCGCATA
CTTGTTCCTTATCTACTTTGTGCTCGACTCTCAGAAACTCACCTACTGAGAACACGTTACACTTAGTG
TGCTGCGCCCTAGCGTTTATCATTCTCATTTTTTCGTGTATAACGTTCGCTGTCCTAATATTGTCTAT
+
FFFF,FFFFFFFFFFFFFFFFFFFFFFFFFFFFFFFFFFFFFFFF:FFFFFFF:FFFFFFFF:FFFFF
FFF:FFFFFFFFFFFF:FFFF,FFFFFFFFFFFFFFFFFFFFFFFFFFFFFFFFFFF:FFFFFFFFFF
@A00155:342:HHGFNDSXY:1:2624:30879:24565 2:N:0:GAACCTAG+TCCGCATA
CTTGTTCCTTATCTACTTTGTGCTCGACTCTCAGAAACTCACCTACTGAGAACACGTTACACTTAGTG
TGCTGCGCCCTAGCGTTTATCATTCTCATTTTTTCGTGTATAACGTTCGCTGTCCTAATATTGTCTAT
+
F,FFF:FF:FFFFFFFFFFFFFFFFF,FFFFFFFFFFFFFFFFFFFFFFFFFFFFFFFFFFFFFFFFF
FFFFFFFFFFFFFFFFFFFFFFFFFFFFFFFFFFFFFFFFFFFFFFFFFFFFFFFFFFFFFFFFFFFF
```

```
@A00155:342:HHGFNDSXY:1:2331:22905:7811 2:N:0:GAACCTAG+TCCGCATA
TGTTCCTTATCTACTTTGTGCTCGACTCTCAGAAACTCACCTACTGAGAACACGTTACACTTAGTGTG
CTGCGCCCTAGCGTTTATCATTCTCATTTTTTCGTGTATAACGTTCGCTGTCCTAATATTGTCTATGG
+
FF,FFF::F:FFFFFFFFF::FFFFFFFFFFFFFFFFFFFFFFFFFFFFFFFFF:FFFFFFFFFFFFFFF
FFFFFFFFFFFFFF:FFFFFFFFFFFFFFFFFFFFFFFFFFFFFFFFFFFFFFFFFFFFFFFFFFFFFFF
@A00155:342:HHGFNDSXY:1:2605:22137:31594 2:N:0:GAACCTAG+TCCGCATA
TCCTTATCTACTTTGTGCTCGACTCTCAGAAACTCACCTACTGAGAACACGTTACACTTAGTGTGCTG
CGCCCTAGCGTTTATCATTCTCATTTTTTCGTGTATAACGTTCGCTGTCCTAATATTGTCTATGGAT
+
FFFFFFFFFFFFFFFFFFFFFFFFFFFFFFFFFFFFFFFFFFFFFFFFFFFFFFFFFFFFFFFFFFFFFF
FFFFFFFFFFFFFFFFFFFFFFFFFFFFFFFFFFFFFFFFF:FFFFFFFFFFFFFFFFFFFFFFFFFFF
@A00155:342:HHGFNDSXY:1:2230:30002:34851 2:N:0:GAACCTAG+TCCGCATA
TCCTTATCTACTTTGTGCTCGACTCTCAGAAACTCACCTACTGAGAACACGTTACACTTAGTGTGCTG
CGCCCTAGCGTTTATCATTCTCATTTTTTCGTGTATAACGTTCGCTGTCCTAATATTGTCTATGGAT
+
FFFFFFFFF,FF,FFFFFFFFFFFFFFFFFFFFFFFFFFF:,FFFFFFFFFFFF,FFFFFF:FFFFF:FF
FFFF:FFFFFFFFFFFFFFFFFFFFFFFFFFFFFFFFFF:FFFFFFFFFFFFF:,FF:FFFFFF:FFFF
@A00155:342:HHGFNDSXY:1:2567:27263:19586 2:N:0:GAACCTAG+TCCGCATA
TCCTTATCTACTTTGTGCTCGACTCTCAGAAACTCACCTACTGAGAACACGTTACACTTAGTGTGCTG
CGCCCTAGCGTTTATCATTCTCATTTTTTCGTGTATAACGTTCGCTGTCCTAATATTGTCTATGGATA
+
FFFFFFFFFFFFFFFFFFFFFFFFFFFFFFFF:FFFFFFFFFFFFFFFFFFFFFFFFFFFFFFFFFFFFF
FFFFFFFFFFFFFFFFF,FFFFFFFFFFFFFFFFFFFFFFFFFFFFFFFFFFFFFFFFFFFFFFFFFFFF
@A00155:342:HHGFNDSXY:1:1559:23068:8719 1:N:0:GAACCTAG+TCCGCATA
TCCTTATCTACTTTGTGCTCGACTCTCAGAAACTCACCTACTGAGAACACGTTACACTTAGTGTGCTG
CGCCCTAGCGTTTATCATTCTCATTTTTTCGTGTATAACGTTCGCTGTCCTAATATTGTCTATGGATA
+
FFFFFFFFFFFFFFFFFFFFFFFFFFFFFFFFFFFFFFFFFFFFFFFFFFFFFFFFFFFFFFFFFFFFFF
FFFFFFFFFFF:F:FFFFFFFFFFFFFFFFFFFFFFFFFFFFFFFFFFFFFFFFFFFFFFFFFFFFFFFF
@A00155:342:HHGFNDSXY:1:1559:23068:8719 2:N:0:GAACCTAG+TCCGCATA
TCCTTATCTACTTTGTGCTCGACTCTCAGAAACTCACCTACTGAGAACACGTTACACTTAGTGTGCTG
CGCCCTAGCGTTTATCATTCTCATTTTTTCGTGTATAACGTTCGCTGTCCTAATATTGTCTATGGATA
+
FFFFFFFF:FFFFFFFFFFFFFFFFFFFFFFFFFFFFFFFFFFFFFFFFFFFFFFFFFFFFFFFFFFFF
FFFFFFFFFFFFFFFFFFFFFFFFF:FFFFFFFFFFFFFFFFFFFFFFFFFFFF:FFFFFFFFFFFFF:F
@A00155:342:HHGFNDSXY:1:2266:23556:7623 1:N:0:GAACCTAG+TCCGCATA
TCCTTATCTACTTTGTGCTCGACTCTCAGAAACTCACCTACTGATAACACGTTACACTTAGTGTGCTG
CGCCCTAGCGTTTATCATTCTCATTTTTTCGTGTATAACGTTCGCTGTCCTAATATTGTCTATGGATA
+
FFFFFFFFFFFFFFFFFFFFFFFFFFFFFFFFFFFFFFFFFFFFFFFFFFFFFFFFFFFF:FFFFFFFFF
FFFFF:FFFFFFFFFFFFFFFFFFFFFFFFFFFFFFFFFFFFFFFFFFFFFFFFFFFFFFFFFF:FFFFF
@A00155:342:HHGFNDSXY:1:2566:31458:16986 1:N:0:GAACCTAG+TCCGCATA
TCCTTATCTACTTTGTGCTCGACTCTCAGAAACTCACCTACTGATAACACGTTACACTTAGTGTGCTG
CGCCCTAGCGTTTATCATTCTCATTTTTTCGTGTATAACGTTCGCTGTCCTAATATTGTCTATGGATA
+
FFFFFFFF:FFFFFFFFFFFFFFFFFFFFFFFFFFFFFFF:FFFFFFFFF:FFFFFFFFFFFFFFFFFFF
FFFFFFFFFFFFFFFFFFFFFFFFFFFFFFFFFFFF:FFFFFFFFFFFFFFFFFFFFFFFFFFFFFFF,F
@A00155:342:HHGFNDSXY:1:2518:1633:21699 1:N:0:GAACCTAG+TCCGCATA
CCTTATCTACTTTGTGCTCGACTCTCAGAAACTCACCTACTGAGAACACGTTACACTTAGTGTGCTGC
GCCCTAGCGTTTATCATTCTCATTTTTTCGTGTATAACGTTCGCTGTCCTAATATTGTCTATGGATAC
+
FFFFFFFFFFFFFFFFFFFFFFFFFFFFFF:FFFFFFFFFFFFFFFFFFFFFFFFFFFFFFFFFFFFFFF
FFFFFFFFFFFFFFFFFFFFFFFFFFFFFFFFFFFFFFFFFFFFFFFFFFFFFFF:FFFFFFFFFFFFFF
```

@A00155:342:HHGFNDSXY:1:2410:26720:26694 1:N:0:GAACCTAG+TCCGCATA
CCTTATCTACTTTGTGCTCGACTCTCAGAAACTCACCTACTGAGAACACGTTACACTTAGTGTGCTGC
GCCCTAGCGTTTATCATTCTCATTTTTTCGTGTATAACGTTCGCTGTCCTAATATTGTCTATGGATAC
+
FF:FFFFFF,FFFFFFFFFFFFFFFFFFFFFFFFFFFFFFFFFFFFFFFFFFFFFFF:FFFFFFFFFF
FFFFFFFFFFFFFFFFFFFFFFFFFFFFFFFFFFFFFFFFFFFFFFFFFFFFF:FFFFFFFFFFFFFF
@A00155:342:HHGFNDSXY:1:1442:6189:26522 1:N:0:GAACCTAG+TCCGCATA
CTTATCTACTTTATGCTCGACTCTCAGAAACTCACCTACTGAGAACACGTTACACTTAGTGTGCTGCG
CCCTAGCGTTTATCATTCTCATTTTTTCGTGTATAACGTTCGCTGTCCTAATATTGTCTATGGATACC
+
FFFFFFFFFFFFFFFFFFFFFFFFFFFFFFFFFFFFFFFFFFFFFFFFFFFFFFFFFFFFFFFFFFFF
FFFFF:FFF:FFFFF:FFFFFFFFFFFF:FFFFFFFFFFFFFFFFF:FFFFFFFFFFFFFF:FFFFFF
@A00155:342:HHGFNDSXY:1:1473:15167:21966 2:N:0:GAACCTAG+TCCGCATA
TTATCTACTTTGTGCTCGACTCTCAGAAACTCACCTACTGAGAACACGTTACACTTAGTGTGCTGCGC
CCTAGCGTTTATCATTCTCATTTTTTCGTGTATAACGTTCGCTGTCCTAATATTGTCTATGGATAC
+
FFFFFFF:FFFFFFFFFFF:F,F:FFF:FFFFF::FFFFFFFFFFFF,FFF,FFFFFFFFFF:FF:FF
FFFFFFFFFFFF:FFFFFFFFFFFFFFFFFF:FFFFFFFFFFFFFFFF:FFFFFFF:FFFFFFFFF
@A00155:342:HHGFNDSXY:1:2567:27263:19586 1:N:0:GAACCTAG+TCCGCATA
TTTGTGCTCGACTCTCAGAAACTCACCTACTGAGAACACGTTACACTTAGTGTGCTGCGCCCTAGCGT
TTATCATTCTCATTTTTTCGTGTATAACGTTCGCTGTCCTAATATTGTCTATGGATACCAA
+
FFFFFFFFFFFFFFFFFFFFFFFFFFFFFFFFFFFFFFFFFFFFFFFFFFFFFFFFFFFFFFFFFFFF
:FF:FFFFFFFFFFFFFFFFFFFFFFFFFFFFFFFFFFFFFFFFFFFFFFFFFFFFFFFFF
@A00155:342:HHGFNDSXY:1:2439:20645:25066 2:N:0:GAACCTAG+TCCGCATA
TCGACTCTCAGAAACTCACCTACTGAGAACACGTTACACTTAGTGTGCTGCGCCCTAGCGTTTATCAT
TCTCATTTTTTCGTGTATAACGTTCGCTGTCCTAATATTGTCTATGGATACCAATACGTCGTCCCCAT
+
:FFFFFFFFFFFFFFFFFFFFFFFFFFFFFFFFFFFFFFFFFFFFFFFFFFFFFF:FFFFFFFFFFF
FFFFFFFF:FFFFFFFFFFFFFFFFFFFFFFFFF:FFFFFFFFFF:FFFFFFFFFFFFFFFFFFFFFF
@A00155:342:HHGFNDSXY:1:2439:19407:25488 2:N:0:GAACCTAG+TCCGCATA
TCGACTCTCAGAAACTCACCTACTGAGAACACGTTACACTTAGTGTGCTGCGCCCTAGCGTTTATCAT
TCTCATTTTTTCGTGTATAACGTTCGCTGTCCTAATATTGTCTATGGATACCAATACGTCGTCCCCAT
+
FFFFFFFFFFFFFFFFFFFFF:FFFFFFFFFFFFFFFFFFFFFFFFFFFFFFFFFFFFFFFFFFFFFF
FFFFFFFFFFFFFFFFFFFFFFFFFFFFF:FFFFFFFFFFFFFFFFFFFFFFFFFFFFFFFFFFFFFF
@A00155:342:HHGFNDSXY:1:2439:19452:25880 2:N:0:GAACCTAG+TCCGCATA
TCGACTCTCAGAAACTCACCTACTTAGAACACGTTACACTTAGTGTGCTGCGCCCTAGCGTTTATCAT
TCTCATTTTTTCGTGTATAACGTTCGCTGTCCTAATATTGTCTATGGATACCAATACGTCGTCCCCAT
+
FFF,FF:F,FFF:,FFFF:FFFFF,:FF,FFFFFFFF::FFFFFFFFFFFFFF,:F:FFF,FFFFFFF
F:F:FFFFFFF::FFFFFF:FFFFFFFFFFFFFFFFF:FF:FFFFFF:FFF:FFFFFFFFFFFFFFFF
@A00155:342:HHGFNDSXY:1:2602:27651:32503 2:N:0:GAACCTAG+TCCGCATA
CTCTCAGAAACTCACCTACTGAGAACACGTTACACTTAGTGTGCTGCGCCCTAGCGTTTATCATTCTC
ATTTTTTCGTGTATAACGTTCGCTGTCCTAATATTGTCTATGGATACCAATACGTCGTCCCCATTATG
+
FFFFFFFFFFFFFFFFFFFFFFFFFF:FFF:FFFFFFFFFFFFFFFFFFFF:FFFFFFFFFFFFFFFF
FFFFFFFFFFFFFFFFFFFFFFFFFFFFFFFFFFFFFFFFFFFF:FFFFFFFFFFFFFFFFFFFFFFF
@A00155:342:HHGFNDSXY:1:1218:15402:1329 1:N:0:GAACCTAG+TCCGCATA
AAACTCACCTACTGAGAACACGTTACACTTAGTGTGCTGCGCCCTAGCGTTTATCATTCTCATTTTTT
CGTGTATAACGTTCGCTGTCCTAATATTGTCTATGGATACCAATACGTCGTCCCCATTATGTACGGAG
+
FFFFFFFFFFFFFFFFFFFFFFFFFFFFFFFFFFFFFFFFFFFFFFFFFFFF:FFFFFFFFFFFFFFF
FFFFFFFFFFFFFFFFFFFFFFFFFFFFFFFFFFFFFFFFFFFFFFFFFFFFFFFFFFFFFFFFFFF:

```
@A00155:342:HHGFNDSXY:1:2474:24587:22185 1:N:0:GAACCTAG+TCCGCATA
AGAACACGTTACACTTAGTGTGCTGCGCCCTAGCGTTTATCATTCTCATTTTTTCGTGTATAACGTTC
GCTGTCCTAATATTGTCTATGGATACCAATACGTCGTCCCCATTATGTACGGAGTCGTATACTCCCTT
+
FFFFFFFFFFFFFFFFFFFFFFFFFFFFFFFFFFFFFFFFFFFFFFFFFFFFFFFFFFFFFFFFFFFF
FFFFFFFFFFFFFFFFFFFFFFFFFFFFFFFFFFFFFFFFFFFFFFFFFFFFFFFFFFFFFFFFFFFF
@A00155:342:HHGFNDSXY:1:2647:20690:21825 2:N:0:GAACCTAG+TCCGCATA
TACACTTAGTGTGCTGCGCCCTAGCGTTTATCATTCTCATTTTTTCGTGTATAACGTTCGCTGTCCTA
ATATTGTCTATGGATACCAATACGTCGTCCCCATTATGTACGGAGTCGTATACTCCCTTCACATCAAA
+
FFFFFFFFFFFFFFFFFFFFFFFFFFFF,FF,FFFFFFF:FFFFFFFFFFFFFFFFFFFFFFFFFFFF
:F:FFFFFFFFFFFFFFFFFFFFFFFFFFFFFFFFFFFFFFFFFF:FFFFFFFFFFFFFFFF,FFFFF,F
@A00155:342:HHGFNDSXY:1:2329:5782:17895 2:N:0:GAACCTAG+TCCGCATA
TTAGTGTGCTGCGCCCTAGCGTTTATCATTCTCATTTTTTCGTGTATAACGTTCGCTGTCCTAATATT
GTCTATGGATACCAATACGTCGTCCCCATTATGTACGGAGTCGTATACTCCCTTCACATCAAATACCC
+
FFFFFFFFFFFFFFFFFFF:FFFFF:FFFFFFFFFFFFFFFFFFFFFFFFFFFFFFFFFFFFFFFFFF
FFFFFFFFFFFFFFFFFFFFFFFF:FFFFFFFFFFFF:FFFFFFFFFFFFFFFFFFFFFFFFFFFFFF
@A00155:342:HHGFNDSXY:1:2636:8874:8563 1:N:0:GAACCTAG+TCCGCATA
CTGCGCCCTAGCGTTTATCATTCTCATTTTTTCGTGTATAACGTTCGCTGTCCTAATATTGTCTATGG
ATACCAATACGTCGTCCCCATTATGTACGGAGTCGTATACTCCCTTCACATCAAATACCCCCGCTAAT
+
FFFFFFFFFFFFFFFFFFFFFFFFFFFFFFFFFFFFFFFFFFFFFFFFFFFFFFFFFFFFFFFFFFFF
FFFFFFFFF:FFFFFFFFFFFFFFFFFF:FFFFFFFFFFFFFFFFFFFFFFFFFFFFFF:FFFFFFFFF
@A00155:342:HHGFNDSXY:1:1218:15402:1329 2:N:0:GAACCTAG+TCCGCATA
TGCGCCCTAGCGTTTATCATTCTCATTTTTTCGTGTATAACGTTCGCTGTCCTAATATTGTCTATGGA
TACCAATACGTCGTCCCCATTATGTACGGAGTCGTATACTCCCTTCACATCAAATACCCCCGCTAATT
+
FFFFFFFFFFFF,FFF:FFFFFFFFFFFFFFFFFFFFFFFFFFFFFFFFFFF,FFFFFFFFFFF:F:FF
FFFFFFFFFFFFFFFFF:FFFFFFFF:FFFFFFFFFFFFFFFFFFFFFFFFFFFFFFFFFFFFFFF,FF
@A00155:342:HHGFNDSXY:1:2419:16803:4726 2:N:0:GAACCTAG+TCCGCATA
CTAGCGTTTATCATTCTCATTTTTTCGTGTATAACGTTCGCTGTCCTAATATTGTCTATGGATTCCAA
TACGTCGTCCCCATTATGTACGGAGTCGTATACTCCCTTCACATCAAATACCCCCGCTAATTTGAAAT
+
FFFFFFFFFFFF:FFFFFFFFFFFFFFFFFFFFFFFF:FFFFFFF:FFFFFFFFFFFFF:FFFF,FFFF
FFFFFFFFFFFFFFFFFFFFFFFFFFFFFFFFFFFFFFFFFFFFFFFFFFFFFFFFFFFFFFFFFFFF
@A00155:342:HHGFNDSXY:1:2419:16803:4726 1:N:0:GAACCTAG+TCCGCATA
TTATCATTCTCATTTTTTCGTGTATAACGTTCGCTGTCCTAATATTGTCTATGGATACCAATACGTCG
TCCCCATTATGTACGGAGTCGTATACTCCCTTCACATCAAATACCCCCGCTAATTTGAAATATA
+
FFFFFFFFFFFFFFFFFFFFFFFFFFFFFFFFFFFFFFFFFFFFFFFFFFFFFFFFF:FFFFFFFFFFF
FFFFFFFFFFFFFFFFFFFFFFFFFFFFFFFFFFFFFFFFFFF:FFFFFFFFFFFFFFFFFFF
@A00155:342:HHGFNDSXY:1:2518:1633:21699 2:N:0:GAACCTAG+TCCGCATA
CATTCTCATTTTTTCGTGTAGAACGTTCGCTGTCCTAATATTGTCTATGGATACCAATACGTCGTCCC
CATTATGTACGGAGTCGTATACTCCCTTCACATCAAATACCCCCGCTAATTTGAAATATATGTAATTC
+
FFFFFFFFFFF:FFFFFFFF,F:FF,FFFFFFFFFFFFFF,FFFFF,FFFFFFFFFFFFFFF:FF:FF
FFFFFFFFFFFFFFFFFFFFFFFFFFFFFFFFFFF,FFF:FFFFFFFFFFFFFFFFFFFFF,FFFFF:FF
@A00155:342:HHGFNDSXY:1:2410:26720:26694 2:N:0:GAACCTAG+TCCGCATA
CATTCTCATTTTTTCGTGTATAACGTTCGCTGTCCTAATATTGTCTATGGATACCAATACGTCGTCCC
CATTATGTACGGAGTCGTATACTCCCTTCACATCAAATACCCCCGCTAATTTGAAATATATGTAATTC
+
FFFFFFFFFFF:FFFFFF:FFFFFFFFFFFFFFFF:FFFF,FFFFF:FFFFF:FFFFFFFFF:FFFF:
FFFFFFFFFFFFFFFFFFFF:FFFFFFFFFFFFFFFFFFFFFFFFFFFFFFFFFFFFFFFFFFFFFFF
```

```
@A00155:342:HHGFNDSXY:1:1436:1470:20666 2:N:0:GAACCTAG+TCCGCATA
CATTCTCATTTTTTCGTGTATAACGTTCGCTGTCCTAATATTGTCTATGGATACCAATACGTCGTCCC
CATTATGTACGGAGTCGTATACTCCCTTCACATCAAATACCCCCGCTAATTTGAAATATATGTAATTC
+
FFFFFFFFF::F:FFFFFFFFFFFFFFFFFFFFFFFFF:FFFF:FFFFFFFFFFFFFFFFFFFFFFFF
FFFFFFFFFFFFFFFFFFFFFFFFFFF:FFFF,FFFFFFFFFFFFFFFF:F:FFFFFFFFFFFFFFFF
@A00155:342:HHGFNDSXY:1:1539:31602:23719 1:N:0:GAACCTAG+TCCGCATA
ATTCTCATTTTTTCGTGTATAACGTTCGCTGTCCTAATATTGTCTATGGATACCAATACGTCGTCCCC
ATTATGTACGGAGTCGTATACTCCCTTCACATCAAATACCCCCGCTAATTTGAAATATATGTAATTCA
+
FFFFFFFFFFFFFFFFFFFFFFFFFFFFFFFFFFFFFFFFFFFFFFFFFFFFFFFFFFFFFFFFFFFF
FFFFFFFFFFFFFFFFFFFFFFFFFFFFFFFFFFFFFFFFFFFFFFFFFFFFFFFFFFFFFFF:FFFF
@A00155:342:HHGFNDSXY:1:2367:7491:17033 1:N:0:GAACCTAG+TCCGCATA
TTCTCATTTTTTCGTGTATAACGTTCGCTGTCCTAATATTGTCTATGGATACCAATACGTCGTCCCCA
TTATGTACGGAGTCGTATACTCCCTTCACATCAAATACCCCCGCTAATTTGAAATATATGTAATTCAG
+
FFFFFFFFFFFFFFFFFFFFFFFFFFFFFFFFFFFFFFFFFFFFFFFFFFFFFFFFFFFFFFFFFFFF
FFFFFFFFFFFFFFFFFFFFF:FFFFFFFFFFFFFFFFFFFFFFFFFF,FFFFFFFFFFFFFFFFFFF
@A00155:342:HHGFNDSXY:1:1623:19786:21324 1:N:0:GAACCTAG+TCCGCATA
CTCATTTTTTCGTGTATAACGTTCGCTGTCCTAATATTGTCTATGGATACCAATACGTCGTCCCCATT
ATGTACGGAGTCGTATACTCCCTTCACATCAAATACCCCCGCTAATTTGAAATATATGTAATTCAGAG
+
FFFFFFFFFFFFFFFFFFFFFFFFFFFFFFFFFFFFFFFFFFFFFFFFFFFFFFFF:FFFFF:FFFFF
FFFFFF,FFFFFFFFFFFFFFFFF:FFF:FF:FFFFFFFFFFFFFFFFFFFFFFFFFFFFFFFFFFFF
@A00155:342:HHGFNDSXY:1:1114:29206:16501 1:N:0:GAACCTAG+TCCGCATA
CTCATTTTTTCGTGTATAACGTTCGCTGTCCTAATATTGTCTATGGATACCAATACGTCGTCCCCATT
ATGTACGGAGTCGTATACTCCCTTCACATCAAATACCCCCGCTAATTTGAAATATATGTAATTCAGAG
+
F:FFFFFFFFFFFFFFFFFFFFFF:FFF:FFFFFFFFFFF:FFFFFFFFFFFFFFFFFFFFFFFFFFF
FFFFFF,FFF,FFFFFFFFF,F:FFFFFFFFFFFF,FFFFFFFFFFFFFFFFFFFF,FFFFFFFFFFF
@A00155:342:HHGFNDSXY:1:2407:24948:25003 1:N:0:GAACCTAG+TCCGCATA
TCATTTTTTCGTGTATAACGTTCGCTGTCCTAATATTGTCTATGGATACCAATACGTCGTCCCCATTA
TGTACGGAGTCGTATACTCCCTTCACATCAAATACCCCCGCTAATTTGAAATATATGTAATTCAGAGC
+
F:FFFFFFFFFFFFFFFFF:FFFFF:FFFF:FFFFFFFFFFF:FFFFFFF:FFFF:FFFF,FF:FFFF
F:FF:FFFFFFFFFFFFFFF:FF:FFF:FFFFFFFFFFFFFFFF,FF:FFFFFFFFFFF:FF,F,F,F
@A00155:342:HHGFNDSXY:1:2407:24948:25003 2:N:0:GAACCTAG+TCCGCATA
TTTTTTCGTGTATAACGTTCGCTGTCCTAATATTGTCTATGGATACCAATACGTCGTCCCCATTATGT
ACGGAGTCGTATACTCCCTTCACATCAAATACCCCCGCTAATTTGAAATATATGTAAT
+
F,FFFFFFFFF:FF,FF,FFFFFFFF:F:FFFFFFFFFFF:FFFFFFFFF:FFFFFFFF,F,FFFFFF
F:::FFFFFFFFFFF:F:FFFFFFFFFFFFFFFFF:FFFF:FF:F:FFFFFFFFFFFF
@A00155:342:HHGFNDSXY:1:2367:7491:17033 2:N:0:GAACCTAG+TCCGCATA
TTCGTGTATAACGTTCGCTGTCCTAATATTGTCTATGGATACCAATACGTCGTCCCCATTATGTACGG
AGTCGTATACTCCCTTCACATCAAATACCCCCGCTAATTTGAAATATATGTAATTCAGAGCCGTATTC
+
FFFFFFFF:FFFFFFFFFFFFFFFFF::FFFF:F:FFFFFFFFFFFFFFFFFFFFFFFFFFFFFFFFF
FFFFFFFFFFFFFFF:F:FFFFFFF:FFFFFFFFFFFFFFFFFFFFFFFFFFFFFFFFFFFFFFFFFF
@A00155:342:HHGFNDSXY:1:1539:31602:23719 2:N:0:GAACCTAG+TCCGCATA
CGTGTATAACGTTCGCTGTCCTAATATTGTCTATGGATACCAATACGTCGTCCCCATTATGTACGGAG
TCGTATACTCCCTTCACATCAAATACCCCCGCTAATTTGAAATATATGTAATTCAGAGCCGTATTCAT
+
FFFFFFF::FFFFFFFF:FFFFFF:,F:FFFFFFFFFF,FFFFFFFFFFFFFFFFFF:FFFFFFFFFF
FFFFF:FFFFFFFFFFFF:FFF::FFFFFFFFFFFFFFFFFFFFFFFFFFFFFFFFFFFFFFFFFFFF
```

```
@A00155:342:HHGFNDSXY:1:2647:20690:21825 1:N:0:GAACCTAG+TCCGCATA
TGTATAACGTTCGCTGTCCTAATATTGTCTATGGATACCAATACGTCGTCCCCATTATGTACGGAGTC
GTATACTCCCTTCACATCAAATACCCCCGCTAATTTGAAATATATGTAATTCAGAGCCGTATTCATGA
+
FFF:FFFFFFFFFFFFFFFFFFFFFFFFFFFFFFFFFFFFFFFFFFFFFFFFFFFFFFFFFFFFFFFF
FFFFFFFFFFFFFFFFFFFFFFFFFFFFFFFFFFFFFFFFFFFFFFFFFFFFFFFFFFFFFFFFFFFF
@A00155:342:HHGFNDSXY:1:1513:16902:11694 1:N:0:GAACCTAG+TCCGCATA
ACGTTCGCTGTCCTAATATTGTCTATGGATACCAATACGTCGTCCCCATTATGTACGGAGTCGTATAC
TCCCTTCACATCAAATACCCCCGCTAATTTGAAATATATGTAATTCAGAGCCGTATTCATGAATGTAG
+
FFFFFFFFFFFFFFFFFFFFFFFFFFFFFFFFFFFFFFFFFFFFFFFFFFFFFFFFFFFFFFFFFFFF
FFFFFFFFFFFFFF:FFFFFFFFFFFFFFFFFFFFFFFFFFFFFFFFFFFFFFFFFFFFFFFFFFFFF
@A00155:342:HHGFNDSXY:1:2319:20139:26099 1:N:0:GAACCTAG+TCCGCATA
CGCTGTCCTAATATTGTCTATGGATACCAATACGTCGTCCCCATTATGTACGGAGTCGTATACTCCCT
TCACATCAAATACCCCCGCTAATTTGAAATATATGTAATTCAGAGCCGTATTCATGAATGTAGTAAGC
+
FFFFFFFFFFFFFFFFFFFFFFFFFFFFFFF:FFFFFFFFFFFFFFFFFFFFFFFFFFFFFFFFFFFF
FFFFFFFFFFFFFFFFFFFF:FFFFFFFFFFFFFFFFFFFFFFFFFFFF::FFFFFFFFFFFFFFFFF
@A00155:342:HHGFNDSXY:1:2657:19045:27524 2:N:0:GAACCTAG+TCCGCATA
CTGTCCTAATATTGTCTATGGATACCAATACGTCGTCCCCATTATGTACGGAGTCGTATACTCCCTTC
ACATCAAATACCCCCGCTAATTTGAAATATATGTAATTCAGAGCCGTATTCATGAATGTAGTAAGCCT
+
FFFFFFFFFFFFFFFFFFFFFFFFFFFFFFFFFFFFFFFFFFFFFFFFFFF:FFFFFFFFFFFFFFFF
FFFFFFFFFFFFFFFFFFFFFFFFFFFFFFFFFFFFFFFFFFFFFFFFFFFFFFFFFFFFFFFFFFFF
@A00155:342:HHGFNDSXY:1:2638:20039:15499 2:N:0:GAACCTAG+TCCGCATA
CTGTCCTAATATTGTCTATGGATACCAATACGTCGTCCCCATTATGTACGGAGTCGTATACTCCCTTC
ACATCAAATACCCCCGCTAATTTGAAATAGATGTAATTCAGAGCCGTATTCATGAATGTAGTAAGCCT
+
FFFFFFFFFFFFFFFFFFFFFFFFFFFFFFFFFFFFFFFFFFFFFFFFFFFFFFFFFF:FFFFFFFFF
FFFFFFFFFFFFFFFFFFFFFFFFFFFFF,FFFFFFFFFFFFFFFFFFFFFFFFFFFFFFFFFFFFFF
@A00155:342:HHGFNDSXY:1:2657:18204:20995 2:N:0:GAACCTAG+TCCGCATA
CTGTCCTAATATTGTCTATGGATACCAATACGTCGTCCCCATTATGTACGGAGTCGTATACTCCCTTC
ACATCAAATACCCCCGCTAATTTGAAATATATGTAATTCAGAGCCGTATTCATGAATGTAGTAAGCCT
+
FFFFFFF,FFFFFFFFF,FFFFFFFFFFFFFFFFFFFFFFFFFFFFFFFFFFFFFFFFF:FFFFFFFF
FFFFFFFFFFFFFFFFFFFFFFFFFFFFF:FFFFFFFFF:FFFFFFFFFFFFFFFFFFFFFFFFFFFF
@A00155:342:HHGFNDSXY:1:1442:6189:26522 2:N:0:GAACCTAG+TCCGCATA
GTCCTAATATTGTCTATGGATACCAATACGTCGTCCCCATTATGTACGGAGTCGTATACTCCCTTCAC
ATCAAATACCCCCGCTAATTTGAAATATATGTAATTCAGAGCCGTATTCATGAATGTAGTAAGCCTCC
+
FFF:FFFFFFFFFFFFFFFFFFFFFF,FFFF:F,,FFF:FFFFFFFFFFFFFFFFFFFFFFFFFFFFF
FFFFFFFFFFFFFFFFFFFFFFFFFFFF:FFFFFFFFFFFFFFFFFFFFFFFFFFFFFFFFFFFFFFF
@A00155:342:HHGFNDSXY:1:1114:29206:16501 2:N:0:GAACCTAG+TCCGCATA
CCTAATATTGTCTATGGATACCAATACGTCGTCCCCATTATGTACGGAGTCGTATACTCCCTTCACAT
CAAATACCCCCGCTAATTTGAAATATATGTAATTCAGAGCCGTATTCATGAATGTAGTAAGCCTCCAT
+
FFF:FFFFFFFF:FFFFF:FFFFFFFFFFFFFFFFFFFFFFFFFFFFFFFF:FFF:FFFFFF:FFFFFF
FFFF:FFFFFFFFFFFFFFFFFFFFFFFFFFFFFFFFFFFFFFF::FFFFFFFFFFF:FFFF:FFFF
@A00155:342:HHGFNDSXY:1:1623:19786:21324 2:N:0:GAACCTAG+TCCGCATA
CCTAATATTGTCTATGGATACCAATACGTCGTCCCCATTATGTACGGAGTCGTATACTCCCTTCACAT
CAAATACCCCCGCTAATTTGAAATATATGTAATTCAGAGCCGTATTCATGAATGTAGTAAGCCTCCAT
+
FFFFFFFFFFF::FF:FFFFFFFFFFFFFFFFFFFF:FFFFFFFFFFFFFF:FFFFFFFFFFFFFFFF
FFFF:F:FF::FFFFFFFFFFFFFFF:FFFFFFFFFFFFFFFFFFFFFFFFFFFFFFFFFFFFFFFFF
```

```
@A00155:342:HHGFNDSXY:1:2371:7401:3286 2:N:0:GAACCTAG+TCCGCATA
TAATATTGTCTATGGATACCAATACGTCGTCCCCATTATGTACGGAGTCGTATACTCCCTTCACATCA
AATACCCCCGCTAATTTGAAATATATGTAATTCAGAGCCGTATTCATGAATGTAGTAAGCCTCCATCC
+
FFFF:FFFFFFFFFFFFFFFFFFFFFFFFF,FFFFFFFFFFFF:FFFFFFFFFFFFFFFFFFFFFFFF
FFFFFFFFFFFFFFFFFFFFFFFFFFFFFFFFFFFFFFFFFFFFFFFFFFFFFFFFFFFFFF,FFFFF
@A00155:342:HHGFNDSXY:1:1513:16902:11694 2:N:0:GAACCTAG+TCCGCATA
ATATTGTCTATGGATACCAATACGTCGTCCCCATTATGTACGGAGTCGTATACTCCCTTCACATCAAA
TACCCCCGCTAATTTGAAATATATGTAATTCAGAGCCGTATTCATGAATGTAGTAAGCCTCCATCCTG
+
FFFFFFFFFFFFFFFFFFFFFFFFFFFFFFFFFFFFFFFFFFFFFFFFF:FFFFFFFFFFFFFFFFFF
FFFFFFFFFFFFFFFFFFFFFFFFFFFFFFFFFFFFFFFFFFFFFFFFFFFFFFFFFFFFFFFFFFFF
@A00155:342:HHGFNDSXY:1:2660:13810:12132 2:N:0:GAACCTAG+TCCGCATA
TTGTCTATGGATACCAATACGTCGTCCCCATTATGTACGGAGTCGTATACTCCCTTCACATCAAATAC
CCCCGCTAATTTGAAATATATGTAATTCAGAGCCGTATTCATGAATGTAGTAAGCCTCCATCCTGATA
+
FFFFFFFF:FFFFFFFFFFFFFFFFFFFFFFFFFFFFFFFFFFFFFFFFFFFFFFFFFFFFFFFFFFF
FFFFFFFFFFFFFFFFFFFFFFFFFFFFFFFFFFFFFFFFFFFFFFFFFFFFFFFFFFFFFFFFFFFF
@A00155:342:HHGFNDSXY:1:2657:18204:20995 1:N:0:GAACCTAG+TCCGCATA
GTCTATGGATACCAATACGTCGTCCCCATTATGTACGGAGTCGTATACTCCCTTCACATCAAATACCC
CCGCTAATTTGAAATATATGTAATTCAGAGCCGTATTCATGAATGTAGTAAGCCTCCATCCTGATAGT
+
FFFFFFFFFFFFFFFFFFFFFFFFFFFFFFFFFFFFFFFFFFFFFFFFFFFFFFFFFFFFFFFFFFFF
FFFFFFFFFFFFFFFFFFFFFFFFFFFFFFFFFFFFFFFFFFFFFFFFFFFFFFFFFFFFF:FF:FFF
@A00155:342:HHGFNDSXY:1:2657:19045:27524 1:N:0:GAACCTAG+TCCGCATA
GTCTATGGATACCAATACGTCGTCCCCATTATGTACGGAGTCGTATACTCCCTTCACATCAAATACCC
CCGCTAATTTGAAATATATGTAATTCAGAGCCGTATTCATGAATGTAGTAAGCCTCCATCCTGATAGT
+
FFFFFFFFFFFFFFFFFFFFFFFFFFFFFFFFFFFFFFFFFFFFFFFFFFFFFFFFFFFFFFFFFFFF
FFFFFFFFFFFFFFFFFFFFFFFFFFFFFFFFFFFFFFFFFFFFFFFFFFFFFFFFFFFFFFFFFFFF
@A00155:342:HHGFNDSXY:1:2638:20039:15499 1:N:0:GAACCTAG+TCCGCATA
GTCTATGGATACCAATACGTCGTCCCCATTATGTACGGAGTCGTATACTCCCTTCACATCAAATACCC
CCGCTAATTTGAAATAAATGTAATTCAGAGCCGTATTCATGAATGTAGTAAGCCTCCATCCTGATAGT
+
FFFFFFFFFFFFFFFFFFFFFFFFFFFFFFFFFFFFFFFFFFFFFFFFFFFFFFFFFFFFFFFFFFFF
FFFFFFFFFFFFFFFF,F:FFFFFFFFFFFFFFFFFFFFF,FFFFFFFFFFFFFFFFFFFFFFFFFFF
@A00155:342:HHGFNDSXY:1:2660:13810:12132 1:N:0:GAACCTAG+TCCGCATA
GATACCAATACGTCGTCCCCATTATGTACGGAGTCGTATACTCCCTTCACATCAAATACCCCCGCTAA
TTTGAAATATATGTAATTCAGAGCCGTATTCATGAATGTAGTAAGCCTCCATCCTGATAGTAGTGTTC
+
FFFFFFFFFFFFFFFFFFFFFFFFFFFFFFFFFFFFFFFFFFFFFFFFFFFFFFFFFF:FFFFFFFFF
FFFFFFFFFFFFFFFFFFFFFFFFFFFFFFFFFFFFFFFFFFFFF:FFFFFFFF,FFFFFFFFFFFFF
@A00155:342:HHGFNDSXY:1:1347:30761:26960 1:N:0:GAACCTAG+TCCGCATA
GATACCAATACGTCGTCCCCATTATGTACGGAGTCGTATACTCCCTTCACATCAAATACCCCCGCTAA
TTTGAAATATATGTAATTCAGAGCCGTATTCATGAATGTAGTAAGCCTCCATCCTGATAGTAGTGTTC
+
FFFFFFFFFFFFFFFFFFFFFFFFFFFFFFFFFF:FFFFFFFFFFFFFFFFF:FFFFFFFFFFFF:FF
FFFFFFFFFFFF:FF:FFFFFFFFFF:FFFFFFFFFF:FFFFFF:FFFFFFFFFFFFFFFFFFFFFFF
@A00155:342:HHGFNDSXY:1:2371:7401:3286 1:N:0:GAACCTAG+TCCGCATA
GATACCAATACGTCGTCCCCATTATGTACGGAGTCGTATACTCCCTTCACATCAAATACCCCCGCTAA
TTTGAAATATATGTAATTCAGAGCCGTATTCATGAATGTAGTAAGCCTCCATCCTGATAGTAGTGTTC
+
FFFFFFFFFFFFFFFFFFFFFFFFFFFFFFFFFFFFFFFFFFFFFFFFFFFFFFFFFFFFFFFFFFF:
FF:FFFFFFFFFFFFFFFF:FFFFFFFFFFFFFFFFFFFFFFFFFFFFFFFFFFFFFFFFFFFFFFFFFFFFFFFF:FFFFF
```

```
@A00155:342:HHGFNDSXY:1:2636:8874:8563 2:N:0:GAACCTAG+TCCGCATA
AATACGTCGTCCCCATTATGTACGGAGTCGTATACTCCCTTCACATCAAATACCCCCGCTAATTTGAA
ATATATGTAATTCAGAGCCGTATTCATGAATGTAGTAAGCCTCCATCCTGATAGTAGTGTTCCTTTCA
+
FF:FFFFFFFFF::FFFFFFFFF:,FFFF:FF:FFFFFFFFFFFFFFFF:FFFFFFF:FFFFFFFFF
FFFFFFFFFFFFFFFFF::FFFF:FFFFFFFF:FFFFFFFFFFFFFFFFFF:FFFFFFFFFFFFFFFF
@A00155:342:HHGFNDSXY:1:2427:16676:21919 1:N:0:GAACCTAG+TCCGCATA
GTCCCCATTATGTACGGAGTCGTATACTCCCTTCACATCAAATACCCCCGCTAATTTGAAATATATGT
AATTCAGAGCCGTATTCATGAATGTAGTAAGCCTCCATCCTGATAGTAGTGTTCCTTTCACTTCGTAT
+
FFFFFFFFFFFFFFFFFFFFFFFFFFFFFFFFFFFFFFFFFFFFFFFFFFF:FFFFFFFFFFFFFFFF
FFFFFFFFFFFFFFFFFFFFFFFFFFFFFFFFFFFFFFFFFFFFFFFFFFFFFFFFFFFFFFFFFFFF
@A00155:342:HHGFNDSXY:1:1569:8350:7874 2:N:0:GAACCTAG+TCCGCATA
CCATTATGTACGGAGTCGTATACTCCCTTCACATCAAATACCCCCGCTAATTTGAAATATATGTAATT
CAGAGCCGTATTCATGAATGTAGTAAGCCTCCATCCTGATAGTAGTGTTCCTTTCACTTCGTATTC
+
FFFFFFFFFFFFFFFFFFFFFFFFFFFFFFFFFFFFFFFFFFFFFFFFFFFFFFFFFFFFFFFFFFFF
FFFFFFFFFFFFFFFFFFFFFFFFFFFFFFFFFFFFFFFFFFFFFFFFFFFFFFFFFFFFFFFFFF
@A00155:342:HHGFNDSXY:1:2415:31774:15436 2:N:0:GAACCTAG+TCCGCATA
ATTATGTTCGGAGTCGTATACTCCCTTCACATCAAATACCCCCGCTAATTTGAAATATATGTAATTCA
GAGCCGTATTCATGAATGTAGTAAGCCTCCATCCTGATAGTAGTGTTCCTTTCACTTCGTATTCATCA
+
FFF:FFFFFFFFFFFFFFFFFFFFFFFFFFFF:FFFFFFFFFFFFF:FFFFFFFF:F:FFFFFFFFFF
FFFFFFFFFFFFFFFFFFFFFFFFFFFFFFFFFFFFFF,FF,F:FFFFFFFFFF:FFFF,FFF:FFF
@A00155:342:HHGFNDSXY:1:2427:16676:21919 2:N:0:GAACCTAG+TCCGCATA
ATTATGTACGGAGTCGTATACTCCCTTCACATCAAATACCCCCGCTAATTTGAAATATATGTAATTCA
GAGCCGTATTCATGAATGTAGTAAGCCTCCATCCTGATAGTAGTGTTCCTTTCACTTCGTATTCATCA
+
FFFFFFFFFFFFFFFFFFFFFFFFFFFFFFFFFFFFFFFFFFFFFFFFFFFFFFFFFFFFFFFFFFFF
FFFFFFFFFFFFFFFFFFFFFFFFFFFFFFFFFFFFFF:FFFFFFFFFFFFFFFFFFFFFFFFFFFFF
@A00155:342:HHGFNDSXY:1:2319:20139:26099 2:N:0:GAACCTAG+TCCGCATA
TTATGTACGGAGTCGTATACTCCCTTCACATCAAATACCCCCGCTAATTTGAAATATATGTAATTCAG
AGCCGTATTCATGAATGTAGTAAGCCTCCATCCTGATAGTAGTGTTCCTTTCACTTCGTATTCATCAC
+
F:FFF:FFFFFFFFFFFFFFFFFFFFFFFFFFFFFFFFFFFFFFFFFF,FFFFFFFFFFFFFFFFFFF
FFFFFFFFFFFFFFFFF,FFFFFFFFFFF:FFFFFFFFFFFFFFFFFFFFFFFFFFFFFFFFFFFFFF
@A00155:342:HHGFNDSXY:1:2415:31774:15436 1:N:0:GAACCTAG+TCCGCATA
CGGAGTCGTATACTCCCTTCACATCAAATACCCCCGCTAATTTGAAATATATGTAATTCAGAGCCGTA
TTCATGAATGTAGTAAGCCTCCATCCTGATAGTAGTGTTCCTTTCACTTCGTATTCATCACTAT
+
FFFFFFFFFF:FFFFF:FFFFFFFFFFFFFFFFFFFFFFFFFFFFFFFFFFFFFFFFFFFFFFFFFFF
FFFFFFFFFFFFFFFFFFFFFFFFFFFFFFFFFFFFFF,FFF:FFFFFFFFFFFFFFFFFFFFFFF
@A00155:342:HHGFNDSXY:1:2133:16938:24533 1:N:0:GAACCTAG+TCCGCATA
CGGAGTCGTATACTCCCTTCACATCAAATACCCCCGCTAATTTGAAATATATGTAATTCAGAGCCGTA
TTCATGAATGTAGTAAGCCTCCATCCTGATAGTAGTGTTCCTTTCACTTCGTATTCATCACTATGCTC
+
FFFFFFFFFFFFFFFFFFFFFFFFFFFFFFFFFFFFFFFF:FFFFFF:FFFFFFFFFFFFFFFFFFFFF
FFFFFFFFFFFFFFFFFFFFFFFFFFFFFFFFFFFFFFFFFFFFFFFFFFFFFFFFFFFFFFFFFFFF
@A00155:342:HHGFNDSXY:1:1552:22607:24173 1:N:0:GAACCTAG+TCCGCATA
GGAGTCGTATACTCCCTTCACATCAAATACCCCCGCTAATTTGAAATATATGTAATTCAGAGCCGTAT
TCATGAATGTAGTAAGCCTCCATCCTGATAGTAGTGTTCCTTTCACTTCGTATTCATCACTATGCTCT
+
FFFFFFFFFFFFFFFFFFFFFFFFFFFFFFFFFFFFFFFFFFFFFFFFFFFFFFFFFFFFFFFFFFFF
FFFFFFFFFFFFFFFFFFFFFFFFFFFFFFFFFFFFFFFFFFFFFFFFFFFFFFFFFFFFFFFFFFFF
```

```
@A00155:342:HHGFNDSXY:1:1569:8350:7874 1:N:0:GAACCTAG+TCCGCATA
TCGTATACTCCCTTCACATCAAATACCCCCGCTAATTTGAAATATATGTAATTCAGAGCCGTATTCAT
GAATGTAGTAAGCCTCCATCCTGATAGTAGTGTTCCTTTCACTTCGTATTCATCACTATGCTCTCC
+
FFFFFFFFFFFFFFFFFFFFFFFFFFFFFFFFFFFFFFFFF:FFFFFFFFFFFFFF:FFFFFFFFFFF
FFFFFFFFFFFFFFFFFFFFFFFFFFFFFFFFFFFFFFFFFFFFFFFFFFFFFFFFFFFFFFFFFF
@A00155:342:HHGFNDSXY:1:1221:29939:4178 1:N:0:GAACCTAG+TCCGCATA
TCGTATACTCCCTTCACATCAAATACCCCCGCTAATTTGAAATATATGTAATTCAGAGCCGTATTCAT
GAATGTAGTAAGCCTCCATCCTGATAGTAGTGTTCCTTTCACTTCGTATTCATCACTATGCTCTCCTT
+
FFFFFFFFFFFFFFFFFFFFFFFFFFFFFFFFFFFFFFFFFFFFFFFFFFFFFFFFFFFFFFFFFFFF
FFFFFFFFFFFF:FFFFFFFFFFF:FFFFFFFFFFFFFFFFFFFFFFFFFFFFFFFFFFFFFFFFFFF
@A00155:342:HHGFNDSXY:1:2551:28682:6073 1:N:0:GAACCTAG+TCCGCATA
TCGTATACTCCCTTCACATCAAATACCCCCGCTAATTTGAAATATATGTAATTCAGAGCCGTATTCAT
GAATGTAGTAAGCCTCCATCCTGATAGTAGTGTTCCTTTCACTTCGTATTCATCACTATGCTCTCCTT
+
FFFFFFFFFFFFFFFFFFFFFFFFFFFFFFFFFFFFFFFFFFFFFFFFFFFFFFFFFFFFFFFFFFFF
FFFFFFFFFFFFFFFFFFFFFFF:FFFFFFFFFFFFFFFFFFFFFFFFFFFFFFFFFFFFF,FFFFFF
@A00155:342:HHGFNDSXY:1:2502:22535:29246 1:N:0:GAACCTAG+TCCGCATA
TCGTATACTCCCTTCACATCAAATACCCCCGCTAATTTGAAATATATGTAATTCAGAGCCGTATTCAT
GAATGTAGTAAGCCTCCATCCTGATAGTAGTGTTCCTTTCACTTCGTATTCATCACTATGCTCTCCTT
+
FFFFFFFFFFFFFFFFFFFFFFFFFFFFFFFFFFFFFFFFF:FFFFFFFFFFFFFFFFFFFFFFFFFF
FFFFFFFFFFFFFFFFFFFFFFFFFFFFFFFFFFFFFFFFFFFFFFFFFFFFFFFFFFFFFFFFFFF:
@A00155:342:HHGFNDSXY:1:1207:10393:9471 1:N:0:GAACCTAG+TCCGCATA
TCGTATACTCCCTTCACATCAAATACCCCCGCTAATTTGAAATATATGTAATTCAGAGCCGTATTCAT
GAATGTAGTAAGCCTCCATCCTGATAGTAGTGTTCCTTTCACTTCGTATTCATCACTATGCTCTCCTT
+
FFFFFFFFFFFFFFFFFFFFFFFFFFFFFFFFF:FFFFFFF,FFFF:FFFFFFFFF:FFFFFFFFFFF
FFFFFFFFFFFFFFFFFFFFFFFFFFFF,FFFFFFFF,FFFFFFFFFFFFFFFFFFFFFFFFFFFFFF
@A00155:342:HHGFNDSXY:1:1207:10393:9471 2:N:0:GAACCTAG+TCCGCATA
CCCTTCACATCAAATACCCCCGCTAATTTGAAATATATGTAATTCAGAGCCGTATTCATGAATGTAGT
AAGCCTCCATCCTGATAGTAGTGTTCCTTTCACTTCGTATTCATCACTATGCTCT
+
FFFFFF:F:FFFFF,FFFFFFFFFFFFFFFFFFFFF,FF:FFF:FFFFFF:FFFFFFFFFFFFFFFFF
FFFFFFFFFFFFFFFFFFFFFFFFFFFFFFFFFFFFFFFFFFFFFFFF:FFFFF:
@A00155:342:HHGFNDSXY:1:2551:28682:6073 2:N:0:GAACCTAG+TCCGCATA
TCACATCAAATACCCCCGCTAATTTGAAATATATGTAATTCAGAGCCGTATTCATGAATGTAGTAAGC
CTCCATCCTGATAGTAGTGTTCCTTTCACTTCGTATTCATCACTATGCTCT
+
FFFFFFFFFFFFFFFFFFFFFFFFFFFFFFFFFFFFFFFFFFFFFFFFFFFFFFFFFFFFFFFFFFFF
FFFFFFFFFFF:FFFFFFFFFFFFFFFFFFFFFFFFFFFFFFFFFFFFFFF
@A00155:342:HHGFNDSXY:1:1221:29939:4178 2:N:0:GAACCTAG+TCCGCATA
TCACATCAAATACCCCCGCTAATTTGAAATATATGTAATTCAGAGCCGTATTCATGAATGTAGTAAGC
CTCCATCCTGATAGTAGTGTTCCTTTCACTTCGTATTCATCACTATGCTCT
+
FFFFFFFFFF,FFFFFFFFFFFFFFFFFFFFFFFFFFFFFFFFFFFFFFFFFFFFFFFFFFFFFFFFF
FFFFFFFFFFFFFFFFFFFFFFFFFF:FFFFFFFFFFFFFFFFFFFFFFFF
@A00155:342:HHGFNDSXY:1:2502:22535:29246 2:N:0:GAACCTAG+TCCGCATA
TCACATCAAATACCCCCGCTAATTTGAAATATATGTAATTCAGAGCCGTATTCATGAATGTAGTAAGC
CTCCATCCTGATAGTAGTGTTCCTTTCACTTCGTATTCATCACTATGCTCT
+
FFFFFFFFFFFFFFFFFFFFFFFFFFFFFFF::FFFFFFFFFFFFFFFFFFFFFFFFFFF:FFF:FFF
FFFFFFFFFFFFFFFFFFFFFFFFFFFFFFFFFFF:FFFFFFFFFFFF:FF
```

```
@A00155:342:HHGFNDSXY:1:1552:22607:24173 2:N:0:GAACCTAG+TCCGCATA
ACATCAAATACCCCCGCTAATTTGAAATATATGTAATTCAGAGCCGTATTCATGAATGTAGTAAGCCT
CCATCCTGATAGTAGTGTTCCTTTCACTTCGTATTCATCACTATGCTCT
+
FFFFFFFFFFFFFFFFFFFFFFFFFFFFFFFFFFFFFFFFFFFFFFFFFFFFFFFFFFFFFFFFFFFF
FFFFFFFFFFFFFFFFFFFFFFFFFFFFFFFFFFFFFFFFFFFFFFFFF
@A00155:342:HHGFNDSXY:1:2659:8820:14199 1:N:0:GAACCTAG+TCCGCATA
CAAATACCCCCGCTAATTTGAAATATATGTAATTCAGAGCCGTATTCATGAATGTAGTAAGCCTCCAT
CCTGATAGTAGTGTTCCTTTCACTTCGTATTCATCACTATGCTCTCCTTCATTATTGACCACGCTCAT
+
FFFFFFF:FFFFFFFFFFFFFFFFFFFFFFFFFFFFFFFFFFFFFFFFFFFFFFFFFFFFFFFFFFFF
FFFFFFFFFFFFFFFFFFFF:FFFFFFF:FFFFFFFFFFF:,FFFF,FFFF,F:FF,F,FFF:FFFFF
@A00155:342:HHGFNDSXY:1:2631:5511:6527 2:N:0:GAACCTAG+TCCGCATA
AAATACCCCCGCTAATTTGAAATATATGTAATTCAGAGCCGTATTCATGAATGTAGTAAGCCTCCATC
CTGAGAGTAGTGTTCCTTTCACTTCGTATTCATCACTATGCTCT
+
F:F,F:FF:F,::FFFFFFFFFFFFFF,FFFFFFFFFFFF:FFFFFFFFFFFFFFFF:F::FFFFFFF
FFFF,FFFFFFFFFFFFFFFFFFFFFFFFFFFFFFFF:FFFFFF
@A00155:342:HHGFNDSXY:1:2614:15691:6370 1:N:0:GAACCTAG+TCCGCATA
TATGTAATTCAGAGCCGTATTCATGAATGTAGTAAGCCTCCATCCTGATAGTAGTGTTCCTTTCACTT
CGTATTCATCACTATGCTCTCCTTCATTATTGACCACGCTCATTCTCTTCACACTCTCACATACCCAT
+
FFFFFFFFFFFFFFFFFFFFFFFFFFFFFFFFFFFFFFFFFFFFFFFFFFFFFFFFFFFFFFFFFFFF
FFFFFFFFFFFFFFFFFFFFFFFFFFFFFFFFFFFFFFFFFFFFFFFFFFFFFFFFFFFFFFFFFFFF
@A00155:342:HHGFNDSXY:1:2659:10140:15608 2:N:0:GAACCTAG+TCCGCATA
AGCCGTATTCATGAATGTAGTAAGCCTCCATCCTGATAGTAGTGTTCCTTTCACTTCGTATTCATCAC
TATGATCTCCTTCATTATTGACCACGCTCATTCTCTTCACACTCTCACATACCCATGCCATAGCTTG
+
FFF,F:FFFF,FF,FFFFF:FFFF::FFFFFFFFF,:FFF,FFFFFF,F,,:F,FF,FFFFFF:FF,:
,FFF,F,F:FFFFFFFFFFFF::F,F,,:,,F,FFFFFFF:FF,FF:F:,:F::FFF,F,F:FFFF:
@A00155:342:HHGFNDSXY:1:2659:8820:14199 2:N:0:GAACCTAG+TCCGCATA
AGCCGTATTCATGAATGTAGTAAGCCTCCATCCTGATAGTAGTGTTCCTTTCACTTCGTATTCATCAC
TATGCTCTCCTTCATTATTGACCACGCTCATTCTCTTCACACTCTCACATACCCATGCCATAGCTTGC
+
FF:F:FF:FFF:FFFFFFFFFFFFF:FFFFFFFFF:FFFFFFFFFFFFF:FFFFFFFFFFFFFFFFFF
FFF,FFFFFFFFFFFFFFFFFFFFFFFFFFFFFFFFFFFFFFFFFFFFFFFFFF,FFFFFFFFF,FFF
@A00155:342:HHGFNDSXY:1:1157:6433:34147 1:N:0:GAACCTAG+TCCGCATA
TAAGCCTCCATCCTGATAGTAGTGTTCCTTTCTCTTCGTATTCATCACTATGCTCTCCTTCATTATTG
ACCACGCTCATTCTCTTCACACTCTCACATACCCATGCCATAGCTTGCCTCTGCTCCACCTCCATCTC
+
FFFFFFFFFFFFFFFFFFFFFFFFFFFFFFFFF:FFFFFFFFFFFFF:FFFFFFFFFFFFFFFFFF:F
FFFFFFFFFF:FFFFFFFFFFFFFFFFFFFFFFFFFFFFFFFFFFFF:FFFFFFFFFFFFFFFFFFFF
@A00155:342:HHGFNDSXY:1:1157:6433:34147 2:N:0:GAACCTAG+TCCGCATA
CCTCCATCCTGATAGTAGTGTTCCTTTCTCTTCGTATTCATCACTATGCTCTCCTTCATTATTGACCA
CGCTCATTCTCTTCACACTCTCACATACCCATGCCATAGCTTGCCTCTGCTCCACCTCCATCTCGTGG
+
FFFFFFFFFFFFFFFFFFFFFF:FFFFFFFFFFFFFFFFFFFFFF,FFFFFFFFFFFFFFFFFF:FFF
FFFFFFFFFFFFFFFFFFFFFFFFFFFFFFFFFFF,FFFFFFFFFFFFFFFFFFFFFFFFFFFFFFFF
@A00155:342:HHGFNDSXY:1:2133:16938:24533 2:N:0:GAACCTAG+TCCGCATA
CTGATAGTAGTGTTCCTTTCACTTCGTATTCATCACTATGCTCTCCTTCATTATTGACCACGCTCATT
CTCTTCACACTCTCACATACCCATGCCATAGCTTGCCTCTGCTCCACCTCCATCTCGTGGTAAAACGT
+
FFFFFFFFFFFFFFFFFFFFFFFF:FFFFFFFFFFFFFFFFFFFFFFFFFFFFFFFFFFFFFFFFFFF
FFFFFFFFFFFFFFFFF,FFFF:FFFFFFFFF:FFFFFFFFFFFF:FFFFFFFFFFFFFFFFFFFFFF
```

```
@A00155:342:HHGFNDSXY:1:1259:31322:26960 1:N:0:GAACCTAG+TCCGCATA
TAGTGTTCCTTTCACTTCGTATTCATCACTATGCTCTCCTTCATTATTGACCACGCTCATTCTCTTCA
CACTCTCACATACCCATGCCATAGCTTGCCTCTGCTCCACCTCCATCTCGTGGTAAAACGTGTCGTAA
+
FFFFFFFFF:FFFFF:FFFFFFFFFFFFFFFFF:FFFFFFFFFFFFFFFFFFFFFF:FFFFF:FFFFF
FFFFFFFFFFFFFFFFFFFFFFFFFFFFFFFFFFFFFFFFFFFFFFFFFFF::FFFFFFFFFFFFFFF
@A00155:342:HHGFNDSXY:1:2620:10393:1861 1:N:0:GAACCTAG+TCCGCATA
TAGTGTTCCTTTCACTTCGTATTCATCACTATGCTCTCCTTCATTATTGACCACGCTCATTCTCTTCA
CACTCTCACATACCCATGCCATAGCTTGCCTCTGCTCCACCTCCATCTCGTGGTAAAACGTGTCGTAA
+
FFFFFFFFFFFFFFFFFFFFFFFFFFFFFFFFFF:FFFFFFFFFFFFFFFFFFFFFFFFFFFFFFFFF
:FFFFFFFFFFFFFFFFF:FFFFFFFFFFFFFFFFFFFFFFFFFFFFFFFFFFFFFFF:FFFFFFFFF
@A00155:342:HHGFNDSXY:1:2174:12156:34319 1:N:0:GAACCTAG+TCCGCATA
TAGTGTTCCTTTCACTTCGTATTCATCACTATGCTCTCCTTCATTATTGACCACGCTCATTCTCTTCA
CACTCTCACATACCCATGCCATAGCTTGCCTCTGCTCCACCTCCATCTCGTGGTAAAACGTGTCGTAA
+
FFFFFFFFFFFFFFFFFFFFFFFFFFFFFFFFFFFFFFFFFFFFFFFFFFFFFFFFFFFFFFFFFFFF
FFFFFFFFFFFFFFFFFF:FFFFFFFFFFFFFFFFFFF:FFFFFFFFFFFFFFFFFFFFFFFFFFFFF
@A00155:342:HHGFNDSXY:1:1610:2727:13949 1:N:0:GAACCTAG+TCCGCATA
GTTCCTTTCACTTCGTATTCATCACTATGCTCTCCTTCATTATTGACCACGCTCATTCTCTTCACACT
CTCACATACCCATGCCATAGCTTGCCTCTGCTCCACCTCCATCTCGTGGTAAAACGTGTCGTAATACG
+
FFFFFFFFFFFFFFFFFFFFFFFFFF:FF:F,F:FFFF,FFFFFFFFF,FFFFFFF:FF:FFFFFFFF
FF:FFF::FFFFFFFFFFFFFFFFFFFFFFFFFFFFFFFFF:FFFFFFFFFFFFFFFFFFFFFFFFFF
@A00155:342:HHGFNDSXY:1:1368:29930:7921 2:N:0:GAACCTAG+TCCGCATA
TTTCACTTCGTATTCATCACTATGCTCTCCTTCATTATTGACCACGCTCATTCTCTTCACACTCTCAC
ATACCCATGCCATAGCTTGCCTCTGCTCCACCTCCATCTCGTGGTAAAACGTGTCGGAATACGCCAGT
+
FFFFFFFFFFFFFFFFFFFFFFFFFFFFFFFFFFFF:FFFFFFFFFFFFFFFFFFFFFFFFFFFFFFF
FFFFFFFFF:FFFFFFFFFFFFFFFFFFFFFFFFFFFFFFFFFFFFFFFFF:FFFFFFFFFFFFFFFF
@A00155:342:HHGFNDSXY:1:1368:29930:7921 1:N:0:GAACCTAG+TCCGCATA
CACTTCGTATTCATCACTATGCTCTCCTTCATTATTGACCACGCTCATTCTCTTCACACTCTCACATA
CCCATGCCATAGCTTGCCTCTGCTCCACCTCCATCTCGTGGTAAAACGTGTCGGAATACGCCAGTAAA
+
FFFFFFFFFFFFFFFFFFFFFFFFFFFFFFFFFFFFFFFFFFFFFFFFFFFFFFFFFFFFFFFFFFFF
FFFFFFFFFFFFFFFFFFFFFFFFFFFFFFFFFFFFFFFFFFFFFFFFFFFFFFFFFFFFFFFFFFFF
@A00155:342:HHGFNDSXY:1:1537:19334:5415 2:N:0:GAACCTAG+TCCGCATA
CGTATTCATCACTATGCTCTCCTTCATTATTGACCACGCTCATTCTCTTCACACTCTCACATACCCAT
GCCATAGCTTGCCTCTGCTCCACCTCCATCTCGTGGTAAAACGTGTCGTAATACGCCAGTAAAACGGC
+
FFF:FFFFFFFFFFFFFFFFFFFFFFFFFFFFFFFFFFFFFFFFFFFFFFFFFFFFFFFFFFFFFFFF
FFFFFFFF:FFFFFFFFFFFFFFFFFFFFFFFFFFFFFFFFFFFFFFFFFFFFFFFFFFFFFFFFFFF
@A00155:342:HHGFNDSXY:1:2248:20862:6402 1:N:0:GAACCTAG+TCCGCATA
TATTCATCACTATGCTCTCCTTCATTATTGACCACGCTCATTCTCTTCACACTCTCACATACCCATGC
CATAGCTTGCCTCTGCTCCACCTCCATCTCGTGGTAAAACGTGTCGTAATACGCCAGTAAAACGGCAT
+
FFFFFFFFFFFFFFFFFFFFFFFFFFFFFFFFFFFFFFFFFFFFFFFFFFFFFFFFFFFFFFFFFFF:
FFFFFFFFFFFFFFFFFFFFFFFFFFFFFFFFFFFFFFFFFFFFFFFFFFFFFFFFFFFFFFFFFFFF
@A00155:342:HHGFNDSXY:1:1673:16532:36323 1:N:0:GAACCTAG+TCCGCATA
TATTCATCACTATGCTCTCCTTCATTATTGACCACGCTCATTCTCTTCACACTCTCACATACCCATGC
CATAGCTTGCCTCTGCTCCACCTCCATCTCGTGGTAAAACGTGTCGTAATACGCCAGTAAAACGGCAT
+
FFFFFFFFFFF:FF,FFFFFFFFFFFFFFFFFFFFFFFFFFFFFFFFFFFFFFFFFFFFFFFFFFFFF
FFFFFFFFFFFFFFFFFFFFFFFFFFF:FFFFFFFFFFFFFFFFFFFFFFFFFFFFFFFFFFFFFFFF
```

@A00155:342:HHGFNDSXY:1:1327:9887:5525 1:N:0:GAACCTAG+TCCGCATA
TATTCATCACTATGCTCTCCTTCATTATTGACCACGCTCATTCTCTTCACACTCTCACATACCCATGC
CATAGCTTGCCTCTGCTCCACCTCCATCTCGTGGTAAAACGTGTCGTAATACGCCAGTAAAACGGCAT
+
FFFFFFFFFFFFFFFFFFFFFFFFFFFFFFFF,F:FFFFFFFFFFFFFFFFFFFFFFFFFFF:FFFF:
FFFFFFFFFFFFFFFFFFFFFFFFFFFFFFFFFFFFFFFFFFFFFFFFFFFFFFFFFFFFFFFFFFFF
@A00155:342:HHGFNDSXY:1:1347:30761:26960 2:N:0:GAACCTAG+TCCGCATA
TATTCATCACTATGCTCTCCTTCATTATTGACCACGCTCATTCTCTTCACACTCTCACATACCCATGC
CATAGCTTGCCTCTGCTCCACCTCCATCTCGTGGTAAAACGTGTCGTAATACGCCAGTAAAACGGCAT
+
FFFFFFFFF:FFFFFFFFF:FFFFFFFFFFFFFFFFFFFFFFFFFFFFFFFFFFFFFFFFFFFFFF:F
FFFFFF:FFFFFFFFFF:FFFFFFFFFFF:FFFFFFFFFFFFFFFFFFFFFFFFFFFFFFFFFFFFFF
@A00155:342:HHGFNDSXY:1:2313:17797:36699 1:N:0:GAACCTAG+TCCGCATA
CATCACTATGCTCTCCTTCATTATTGACCACGCTCATTCTCTTCACACTCTCACATACCCATGCCATA
GCTTGCCTCTGCTCCACCTCCATCTCGTGGTAAAACGTGTCGTAATACGCCAGTAAAACGGCATGCAT
+
FFFFFFFFFFFFFFFFFFFFFFFFFFFFFFFFFFFFFFFFFFFFFFFFFFFFFFFFFFFFFFFFFFFF
FFFFFFFFFFFFFFFFFFFFFFFFFFFFFFFFFFFFFFFFFFFFFFFF:FFFFFFFFF:FFFFFFFFF
@A00155:342:HHGFNDSXY:1:1537:19334:5415 1:N:0:GAACCTAG+TCCGCATA
CATCACTATGCTCTCCTTCATTATTGACCACGCTCATTCTCTTCACACTCTCACATACCCATGCCATA
GCTTGCCTCTGCTCCACCTCCATCTCGTGGTAAAACGTGTCGTAATACGCCAGTAAAACGGCATGCAT
+
FFFFFFFFFFFFFFFFFFFFFFFFFFFFFFFFFFFFFFFFFFFFFFFFFFFFFFFFFFFFFFFFFFFF
FFFFFFFFFFFFFFFFFFFFFFFFFFFFFFFFFFFFFFFFFFFFFFFFFFFFFFFFFFFFFFFFFFFF
@A00155:342:HHGFNDSXY:1:1628:21612:19946 1:N:0:GAACCTAG+TCCGCATA
ATCACTATGCTCTCCTTCATTATTGACCACGCTCATTCTCTTCACACTCTCACATACCCATGCCATAG
CTTGCCTCTGCTCCACCTCCATCTCGTGGTAAAACGTGTCGTAATACGCCAGTAAAACGGCATGCATG
+
FFFFFFFFFFFFFFFFFFFFFFFFFFFFFFFFFFFFFFFFFFFFFFFFFFFFFFFFFFFFFFFFFFFF
FFFFFFFFFFFFFFFFFFFFFFFFFFFFFFFFFFFFFFFFFFF:FFFFFFFFFFFFFFFFFFFFFFFF
@A00155:342:HHGFNDSXY:1:2346:5466:35728 2:N:0:GAACCTAG+TCCGCATA
TCCTTCATTATTGACCACGCTCATTCTCTTCACACTCTCACATACCCATGCCATAGCTTGCCTCTGCT
CCACCTCCATCTCGTGGTAAAACGTGTCGTAATACGCCAGTAAAACGGCATGCATGGACTCAGTTGAA
+
FFFFFFFFFFFFFFFFFFFFFFFFFFFFFFFFFFFFFFFFFFFFFFFFFFFFFFFFFFFFFFFFFFFF
FFFFFFFFFFFFFFFFFFFFFFFFFFFFFFFFFFFFFFFFFFFFFFFFFFFFFFFFFFFFFFFFFFFF
@A00155:342:HHGFNDSXY:1:1424:30418:26991 1:N:0:GAACCTAG+TCCGCATA
CATTATTGACCACGCTCATTCTCTTCACACTCTCACATACCCATGCCATAGCTTGCCTCTGCTCCACC
TCCATCTCGTGGTAAAACGTGTCGTAATACGCCAGTAAAACGGCATGCATGGACTCAGTTGAATGCTG
+
FF:FFFFFFFFFFFFFFFFFFFFFFFFFFFFFFFFFFFFF:FFFFFFFFFFFFFFFFFFFFFFFFFFF
FFFFFFFFFFFFFFFFFFFFFFFFFFFFFFFFFFFFFF:FFFFFFFFFFFFFFFFFFFFFFFFFFFFF
@A00155:342:HHGFNDSXY:1:2313:17797:36699 2:N:0:GAACCTAG+TCCGCATA
ATTGACCACGCTCATTCTCTTCACACTCTCACATACCCATGCCATAGCTTGCCTCTGCTCCACCTCCA
TCTCGTGGTAAAACGTGTCGTAATACGCCAGTAAAACGGCATGCATGGACTCAGTTGAATGCTGCGCA
+
F:FFFFFFFFFFFFFFFF::FFFFFFFFFFFFF,FFFFF:FFFF,FFFFFFFFFFFFFFFFFFFFFFF
FF:FFFFFFFFFFFFFFFFFFFFFFFFFFF:FFFFFFFFFFFFFFFFFFFFFFFFFFFF:FFFFFFFF
@A00155:342:HHGFNDSXY:1:2641:29622:9017 1:N:0:GAACCTAG+TCCGCATA
TGACCACGCTCATTCTCTTCACACTCTCACATACCCATGCCATAGCTTGCCTCTGCTCCACCTCCATC
TCGTGGTAAAACGTGTCGTAATACGCCAGTAAAACGGCATGCATGGACTCAGTTGAATGCTGCGCATT
+
FFFFFFFFFFFFFFFFFFFFFFFFFFFFFFFFFFFFFFF::FFFFFFFFFFFFFFFFFFFFFFFFFFF
FFFFFFFFF:FFFFFFFFFFFFFFFFFFFFFFFFFF:FFFFFFFFFFFFFFFFFFF:FFFFFF:FFFF

```
@A00155:342:HHGFNDSXY:1:2659:26078:7075 1:N:0:GAACCTAG+TCCGCATA
GACCACGCTCATTCTCTTCACACTCTCACATACCCATGCCATAGCTTGCCTCTGCTCCACCTCCATCT
CGTGGTAAAACGTGTCGTAATACGCCAGTAAAACGGCATGCATGGACTCAGTTGAATGCTGCGCATTA
+
FFFFFFFFFFFFFFFFFFFFFFFFFFFFFFFFFFFFFFFFFFFFFFFFFFFFFFFFFFFFFFFFFFFF
FFFFFFF:FFFFFFFFFFFFFFFFFFFFFFFFFFFFFFFFFFFFFFFFFFFFFFFFFFFFFFFFFFFF
@A00155:342:HHGFNDSXY:1:2477:20907:7388 1:N:0:GAACCTAG+TCCGCATA
GACCACGCTCATTCTCTTCACACTCTCACATACCCATGCCATAGCTTGCCTCTGCTCCACCTCCATCT
CGTGGTAAAACGTGTCGTAATACGCCAGTAAAACGGCATGCATGGACTCAGTTGAATGCTGCGCATTA
+
FFFFFFFFFFFFFFFFFFFFFFFFFFFFFFFFFFFFFFFFFFFFFFFFFFFFFFFFFFFFFFFFFFFF
FFFFFFFFFFFFFFFFFFFFFFFFFFFFFFFFFFFFFFFFFFFFFFFFFFFFFFFFFFFFFFFFFFFF
@A00155:342:HHGFNDSXY:1:1624:17725:4539 1:N:0:GAACCTAG+TCCGCATA
GACCACGCTCATTCTCTTCACACTCTCACATACCCATGCCATAGCTTGCCTCTGCTCCACCTCCATCT
CGTGGTAAAACGTGTCGTAATACGCCAGTAAAACGGCATGCATGGACTCAGTTGAATGCTGCGCATTA
+
FFFFFFFFFFFFFF:FFFFFFFFFF:FFFFFFF:FFF:::FFFFF:FFFFF:FFFFFFFFFF,FF:F,
::FFFFFFFFFFFFFF:FFFFF::FFF,:FFFFF:,FF:FFFFF:FFF,FFF:FFF,:FFFFFFFF:F
@A00155:342:HHGFNDSXY:1:1624:17192:4335 1:N:0:GAACCTAG+TCCGCATA
GACCACGCTCATTCTCTTCACACTCTCACATACCCATGCCATAGCTTGCCTCTGCTCCACCTCCATCT
CGTGGTAAAACGTGTCGTAATACGCCAGTAAAACGGCATGCATGGACTCAGTTGAATGCTGCGCATTA
+
FFFFFFFFFFFFFFFFFFFFFFFFFFFFFFFFFFFFFF:FFFFFFFFFFFFFFFFFFFFFFFFFFFFF
FFFFFFFFFFFFFFFFFFFFFFFFFFFFFFFFFFFFFFFFFFFFFFFFFFFFFFFFFFFFFFFFFFFF
@A00155:342:HHGFNDSXY:1:2346:5466:35728 1:N:0:GAACCTAG+TCCGCATA
CCACGCTCATTCTCTTCACACTCTCACATACCCATGCCATAGCTTGCCTCTGCTCCACCTCCATCTCG
TGGTAAAACGTGTCGTAATACGCCAGTAAAACGGCATGCATGGACTCAGTTGAATGCTGCGCATTAAA
+
FFFFFFFFFFFFFFFFFFFFFFFFFFFFFFFFFFFFFFFFFFFFFFFFFFFFFFFFFFFFFFFFFFFF
FFFFFFF:FFFFFFFFFFFFFFFFFFFFFFFFFFFFFFFFFFFFFFFFFFFFFFFFFFFFFF:FF:FF
@A00155:342:HHGFNDSXY:1:2433:23276:2879 1:N:0:GAACCTAG+TCCGCATA
CGCTCATTCTCTTCACACTCTCACATACCCATGCCATAGCTTGCCTCTGCTCCACCTCCATCTCGTGG
TAAAACGTGTCGTAATACGCCAGTAAAACGGCATGCATGGACTCAGTTGAATGCTGCGCATTAAAGTC
+
FFFFFFFFFFF:FFFFFFFFFFFFFFFFFFFFFFFFFFFFFFFFFFFFFFFFFFFFFFFFFFFFFFFF
FFFFFFFFFFFFFFFFFFFFFFFFFFFFFFFFFFFFFFFFFFFFFFFFFFFFFFFFFFFFFFFFFFFF
@A00155:342:HHGFNDSXY:1:1352:25409:22701 1:N:0:GAACCTAG+TCCGCATA
GCTCATTCTCTTCACACTCTCACATACCCATGCCATAGCTTGCCTCTGCTCCACCTCCATCTCGTGGT
AAAACGTGTCGTAATACGCCAGTAAAACGGCATGCATGGACTCAGTTGAATGCTGCGCATTAAAGTCG
+
FFFFFFFFFFFFFFFFFFFFFFFFFFFFFFFF:FFFFFFFFFFFFFFFFFFFFFFFFFFFFFFFFFFF
:FFFFFFFFFFFFFFFFFFFFFFFFFFFFFFFFFFFFFFFFFFFFFFFFFFFFFFFFFFFFFFFFFFF
@A00155:342:HHGFNDSXY:1:1352:25427:22701 1:N:0:GAACCTAG+TCCGCATA
GCTCATTCTCTTCACACTCTCACATACCCATGCCATAGCTTGCCTCTGCTCCACCTCCATCTCGTGGT
AAAACGTGTCGTAATACGCCAGTAAAACGGCATGCATGGACTCAGTTGAATGCTGCGCATTAAAGTCG
+
FFFFFFFFFFFFFFFFFFFFFFFFFFFFFFFF:F:FFFFFFFFFFFFFFFFFFFFFFFFFFFFFFFFF
FFFFFFFFFFFFFFF:FFFFFFFFFFFFFFFFFFFFFFFFFFFFFFFFFFFFFFFFFFFFFFFFFFFF
@A00155:342:HHGFNDSXY:1:1628:21612:19946 2:N:0:GAACCTAG+TCCGCATA
GCTCATTCTCTTCACACTCTCACATACCCATGCCATAGCTTGCCTCTGCTCCACCTCCATCTCGTGGT
AAAACGTGTCGTAATACGCCAGTAAAACGGCATGCATGGACTCAGTTGAATGCTGCGCATTAAAGTCG
+
FFFFFFFFFFFFFFFFFFFFFFF:FFFFF,FFFFFFFFFFFFFFFFFFFFFFFFFFFFFFFFFFFFFF
FFFFFFFFFFFFFFFFFFFFFFFFFFFFFFFFFFFFFFFFFFFFFFFFFFFFFFFFFFFFFFFFFFFF
```

@A00155:342:HHGFNDSXY:1:1673:16532:36323 2:N:0:GAACCTAG+TCCGCATA
CATTCTCTTCACACTCTCACATACCCATGCCATAGCTTGCCTCTGCTCCACCTCCATCTCGTGGTAAA
ACGTGTCGTAATACGCCAGTAAAACGGCATGCATGGACTCAGTTGAATGCTGCGCATTAAAGTCGTC
+
FFFFFFFFFF,F,:,FFF:F:F:FFF,:FFFF,FFF:FFFFFFFFFFFFFFF,FFFFFFFFFFFFFFF
FFFFF,FFFFFFFFFFFFFFFFF:FFFFFFFFFFFFFFFFFFFFFFFF:FFFF:FFFFFFFFFFFFF
@A00155:342:HHGFNDSXY:1:1673:1072:5885 1:N:0:GAACCTAG+TCCGCATA
TCACACTCTCACATACCCATGCCATAGCTTGCCTCTGCTCCACCTCCAGCTCGTGGTAAAACGTGTCG
TAATACGCCAGTAAAACGGCATGCATGGACTCAGTTGAATGCTGCGCATTAAAGTCGTCAAAGTCATA
+
FFFF,FFFFFFF:F,F:::FF,:,F:FFF:FFFFF,FFFFFFFFFFFF,F,FF:FF,,,FFFF:FFFF
F:FF,FFFF,FFF:FFFFFF::FFFFFFFFFF,F,:FF::FF,FFFF:FF,FFFFFFFFFFFFFFFFF
@A00155:342:HHGFNDSXY:1:2160:1551:15389 1:N:0:GAACCTAG+TCCGCATA
TCACACTCTCACATACCCATGCCATAGCTTGCCTCTGCTCCACCTCCATCTCGTGGTAAAACGTGTCG
TAATACGCCAGTAAAACGGCATGCATGGACTCAGTTGAATGCTGCGCATTAAAGTCGTCAAAGTCATA
+
FFFFFFFFFFFFFFFFFFFFF::FFFFFFFFFFFFFFFFFFFFFFFFFFFFFFF:FFFF::FFF:FFF
FFFFFFFFFFFFFFFFFFFFFFFFFFFFFFFFFFFFFFF:F:FFFFFFFFF:F,FFFFFFFFFFFFFF
@A00155:342:HHGFNDSXY:1:2122:18195:19789 1:N:0:GAACCTAG+TCCGCATA
ACTCTCACATACCCATGCCATAGCTTGCCTCTGCTCCACCTCCATCTCGTGGTAAAACGTGTCGTAAT
ACGCCAGTAAAACGGCATGCATGGACTCAGTTGAATGCTGCGCATTAAAGTCGTCAAAGTCATAGCAC
+
FFFFFFFFFFFFFFFFFFFFFFFFFFFFFFFFFFFFFFFFFFFFFFFFFFFFFFFFFFFFFFFFFFFF
FFFFFFFFFFFFFFFFFFFFFFFFFFFFFFFFFFFFFFFFFFFFFFFFFFFFFFFFFFFFFFFFFFFF
@A00155:342:HHGFNDSXY:1:1424:30418:26991 2:N:0:GAACCTAG+TCCGCATA
ACATACCCATGCCATAGCTTGCCTCTGCTCCACCTCCATCTCGTGGTAAAACGTGTCGTAATACGCCA
GTAAAACGGCATGCATGGACTCAGTTGAATGCTGCGCATTAAAGTCGTCAAAGTCATAGCACAGTGAC
+
FFF:FFFF:FFFFFFFFFFFFFFFFFF::FFFFFFFF,FFFFF:FFFFFFFFFFFFFFFFFFFFFFFF
FFFFFFFFFFFFFFFFFFFFFFFFFFFFFFFFFFFFFFFFFFFFFFFFFFFFFFFFFFFFFFFFFFFF
@A00155:342:HHGFNDSXY:1:1352:25409:22701 2:N:0:GAACCTAG+TCCGCATA
CCATGCCATAGCTTGCCTCTGCTCCACCTCCATCTCGTGGTAAAACGTGTCGTAATACGCCAGTAAAA
CGGCATGCATGGACTCAGTTGAATGCTGCGCATTAAAGTCGTCAAAGTCATAGCACAGTGACTCGCAG
+
FFFFFFF:FFFF,FFFFFFFFFFFFFFFFFFFFFFFFFFFFFFFFFFFFFFFFFFFFFFFFFFFFFF
FFFFFFFFFFFFFFFFFFFFFFFFFFFFFFFFFFFFFFFFFFFFFFFFFFFFFFFFFFFFFFFFFFFF
@A00155:342:HHGFNDSXY:1:1352:25427:22701 2:N:0:GAACCTAG+TCCGCATA
CCATGCCATAGCTTGCCTCTGCTCCACCTCCATCTCGTGGTAAAACGTGTCGTAATACGCCAGTAAAA
CGGCATGCATGGACTCAGTTGAATGCTGCGCATTAAAGTCGTCAAAGTCATAGCACAGTGACTCGCAG
+
FFFFFFFFF:FF:FFFFFFFFFFFFFFFFFFFFFFFFFFFFFFFFFFFFFFFFFFF:FFFFFFFFFFF
FFFFFFFFFFFFFFFFFFFFFFFFFFFFFFFFFFFFFFFFFFFFFFFFFFFFFFFFFFFFFFFFFFFF
@A00155:342:HHGFNDSXY:1:1220:1416:35947 2:N:0:GAACCTAG+TCCGCATA
CTTGCCTCTGCTCCACCTCCATCTCGTGGTAAAACGTGTCGTAATACGCCAGTAAAACGGCATGCATG
GACTCAGTTGAATGCTGCGCATTAAAGTCGTCAAAGTCATAGCACAGTGACTCGCAGTCCTTCAACAT
+
FFFFFFFFFFFFFFFFFFFFFFFFFFFFFFFFFFFFFFFFFFFFFFFFFFFFFFFFFFFF:FFFFFFF
FFFFFFFFFFFFFFFFFFFFFFFFFFFFFFFFFFFFFFFFFFFFFFFFFFFFFFFFFFFFFFFFFFFF
@A00155:342:HHGFNDSXY:1:1106:6795:22060 2:N:0:GAACCTAG+TCCGCATA
CTTGCCTCTGCTCCACCTCCATCTCGTGGTAAAACGTGTCGTAATACGCCAGTAAAACGGCATGCATG
GACTCAGTTGAATGCTGCGCATTAAAGTCGTCAAAGTCATAGCACAGTGACTCGCAGTCCTTCAACAT
+
FFFFFFFFFFFFFFFFFFFFFFFFFFFFFFFFFFFFFFFFFFFFFFFFFFFFFFFFFFFFFFFFFFFF
FFFFFFFFFFFFFFFFFFFFFFFFFFFFFFFFFFFFFFFFFFFFFFFFFFFFFFFFFFFFFFFFFFFF

@A00155:342:HHGFNDSXY:1:2569:1678:6715 1:N:0:GAACCTAG+TCCGCATA
TTGCCTCTGCTCCACCTCCATCTCGTGGTAAAACGTGTCGTAATACGCCAGTAAAACGGCATGCATGG
ACTCAGTTGAATGCTGCGCATTAAAGTCGTCAAAGTCATAGCACAGTGACTCGCAGTCCTTCAAC
+
:FFFFFFFFFFFFFFFFFFFFFFFFFFFFFFFFFFFF:FF,F:FFFFFFFFF,,FFFFFFFFFFFFFF
:FFFF:FFFFFFFFFFFFFFFFFFFFFFFFFFFFFFFFFFFFFFFFFFFFFFFFFFFFFFFFFFF
@A00155:342:HHGFNDSXY:1:2122:18195:19789 2:N:0:GAACCTAG+TCCGCATA
TTGCCTCTGCTCCACCTCCATCTCGTGGTAAAACGTGTCGTAATACGCCAGTAAAACGGCATGCATGG
ACTCAGTTGAATGCTGCGCATTAAAGTCGTCAAAGTCATAGCACAGTGACTCGCAGTCCTTCAACATG
+
:FFFFFFFFF:FFFFFFFFFFFFFFFFFFFFFFFFFFFFFFFFFFFFFFFFFFFFFFFFFFFFFFFFF
FFFFFFFFFFFFFFFFFFFFFFFFFFFFFFFFFFFFFFFFFFFFFFFFFFFFFFFFFFFFFFFFFFFF
@A00155:342:HHGFNDSXY:1:2218:32651:6151 2:N:0:GAACCTAG+TCCGCATA
TGCCTCTGCTCCACCTCCATCTCGTGGTAAAACGTGTCGTAATACGCCAGTAAAACGGCATGCATGGA
CTCAGTTGAATGCTGCGCATTAAAGTCGTCAAAGTCATAGCACAGTGACTCGCAGTCCTTCAACATG
+
FFFFFF:FF::FF:FFFF:FFFFFFFFFFFFFFFFFFFFFFFFFFFFFFFFFF:FFFFFFFFFFFF:F
FFFFFFFFFFFFFFFFFFFFFFFFFFFFFFFFF:FFFFFFFFFFFFFFFFFFFFFFFFFFFF,FFFF
@A00155:342:HHGFNDSXY:1:2629:30752:30295 1:N:0:GAACCTAG+TCCGCATA
GCCTCTGCTCCACCTCCATCTCGTGGTAAAACGTGTCGTAATACGCCAGTAAAACGGCATGCATGGAC
TCAGTTGAATGCTGCGCATTAAAGTCGTCAAAGTCATAGCACAGTGACTCGCAGTCCTTCAACATGTA
+
FFFFFFFFFFFFFFFFFFFFFFF:FFFFFFFFFFFFFFFFFFFFFFFFFFFFFFFFFFFFFFFFFFFF
FFFFFFFFFFFF:FFFFFFFFFFFFFFFFFFFFFFFFFFFFFFFFFFFFFFFFFFFFFFFFFFFFFFF
@A00155:342:HHGFNDSXY:1:2318:26838:11772 1:N:0:GAACCTAG+TCCGCATA
GCCTCTGCTCCACCTCCATCTCGTGGTAAAACGTGTCGTAATACGCCAGTAAAACGGCATGCATGGAC
TCAGTTGAATGCTGCGCATTAAAGTCGTCAAAGTCATAGCACAGTGACTCGCAGTCCTTCAACATGTA
+
FFFFFFFFFFFFFFFFFFFFFFFFFF:FFFFFFFFFFFFF:FFFFFFFFFFFFFFFFFFFFFFFFFFF
FFFFFFFFFFFFFFFFFFFFFFFFFFFFFFF:FFFFFFFFFFFFFFFFFFFF:FFFFFFFFFFFFFFF
@A00155:342:HHGFNDSXY:1:2318:26250:11318 1:N:0:GAACCTAG+TCCGCATA
GCCTCTGCTCCACCTCCATCTCGTGGTAAAACGTGTCGTAATACGCCAGTAAAACGGCATGCATGGAC
TCAGTTGAATGCTGCGCATTAAAGTCGTCAAAGTCATAGCACAGTGACTCGCAGTCCTTCAACATGTA
+
FFF:FFFFFFFFFFFFFFFFFFFFFFFFFF:FFFFFFF:FFFFFF:FFFFFFFFFFFFF:FF:FFFFF
FFFFFFFFFFFFFFFFFFFFFFF,FFFFFFF:FFFFFFFFFFFFFFFFFFFFFFFFFFFFFFFFFFFF
@A00155:342:HHGFNDSXY:1:2433:23276:2879 2:N:0:GAACCTAG+TCCGCATA
CCTCTGCTCCACCTCCATCTCGTGGTAAAACGTGTCGTAATACGCCAGTAAAACGGCATGCATGGACT
CAGTTGAATGCTGCGCATTAAAGTCGTCAAAGTCATAGCACAGTGACTCGCAGTCCTTCAACATGTAC
+
FFFFFFFFFFFFFFFFFFFF:FFFFFFFFFFFFFFFFFFFFFFFFFFFFFFFFFFFFFFFFFFFFFFF
F:FFFFFFFFFFFFFFFFFFFFFFFFFFFFFFFFFFFFFFFFFFFFFFFFFFFFFFFFFFFFFFFFFF
@A00155:342:HHGFNDSXY:1:1374:6967:27242 1:N:0:GAACCTAG+TCCGCATA
CCTCTGCTCCACCTCCATCTCGTGGTAAAACGTGTCGTAATACGCCAGTAAAACGGCATGCATGGACT
CAGTTGAATGCTGCGCATTAAAGTCGTCAAAGTCATAGCACAGTGACTCGCAGTCCTTCAACATGTAC
+
FFFFFFFFFF:FFFFFF,F:::,FFFFFFFFFFF:FF,FF:FFFFFFFFFFFFFFFFFFFF,FFFFF:
FFF:FFFFFFF:FF:FFFFFFFF:FFFFFFFFFFFFFFFF:,FFFFFFFFFFFFFFFFFFFFFFFFFF
@A00155:342:HHGFNDSXY:1:2576:5005:22122 1:N:0:GAACCTAG+TCCGCATA
CCTCTGCTCCACCTCCATCTCGTGGTAAAACGTGTCGTAATACGCCAGTAAAACGGCATGCATGGACT
CAGTTGAATGCTGCGCATTAAAGTCGTCAAAGTCATAGCACAGTGACTCGCAGTCCTTCAACATGTAC
+
FFFFFFFFFFFFFFFFFFFFFFFFFFFFFFFF:FFFF:FFFFFFFFFFFFFFFFFFFFFFFFFFFFFF
FFFFFFFFFFFFFFFFFFFFFFFFFFFFFFFFFFFFFFFFFFFFFFFFFFF:FFFFFFFFFFFFFFFF

```
@A00155:342:HHGFNDSXY:1:1358:14886:22263 1:N:0:GAACCTAG+TCCGCATA
CCTCTGCTCCACCTCCATCTCGTGGTAAAACGTGTCGTAATACGCCAGTAAAACGGCATGCATGGACT
CAGTTGAATGCTGCGCATTAAAGTCGTCAAAGTCATAGCACAGTGACTCGCAGTCCTTCAACATGTAC
+
FFFFFFFFFFFFFFFFFFFFFFFFFFFFFFFFFFFFFF:FFFFFFFFFFFFFFFFFFFFFFFFFFFFF
FFFFFFFFFFFFFFFFF:FFFFFFFFFFFFFFFFFFFFFFF:FFFFFFFFFFFFFFF:FFFFFFFFFF
@A00155:342:HHGFNDSXY:1:1102:19144:2143 2:N:0:GAACCTAG+TCCGCATA
CTCTGCTCCACCTCCATCTCGTGGTAAAACGTGTCGTAATACGCCAGTAAAACGGCATGCATGGACTC
AGTTGAATGCTGCGCATTAAAGTCGTCAAAGTCATAGCACAGTGACTCGCAGTCCTTCAACATGTA
+
FFFFFFFFFFFFFFFFFF:FFFFFFFFFFFFFFFFFFFFFFFFFFFFFFFFFFFFFFFFFFFFFFFFF
:FFFFFFFFFFFFFFFFFFFF:FFFFFFFFFFFFFFFFFFFFFFFFFFFFFFFFFFFFFFFFFFFF
@A00155:342:HHGFNDSXY:1:1102:18756:10645 2:N:0:GAACCTAG+TCCGCATA
CTCTGCTCCACCTCCATCTCGTGGTAAAACGTGTCGTAATACGCCAGTAAAACGGCATGCATGGACTC
AGTTGAATGCTGCGCATTAAAGTCGTCAAAGTCATAGCACAGTGACTCGCAGTCCTTCAACATGTA
+
FFFFFFFFFFFFFFFFFFFFFFFFFFFFFFFFFFFFFFFFFFFFFFFFFFFFFFFFFFFFFFFFFFFF
FFFFFFFFFFFFFFFFFFFFFFFFFFFFFFFFFF:FFFFFFFFFFFFFFFFFFFFFFFFFFFFFFF
@A00155:342:HHGFNDSXY:1:2659:26078:7075 2:N:0:GAACCTAG+TCCGCATA
CTGCTCCACCTCCATCTCGTGGTAAAACGTGTCGTAATACGCCAGTAAAACGGCATGCATGGACTCAG
TTGAATGCTGCGCATTAAAGTCGTCAAAGTCATAGCACAGTGACTCGCAGTCCTTCAACATGTACCT
+
FFFFFFFFFFFFF:FFFFF,FFFFFFFFFFFFFFFFF:FFFFF:FFFFFFFFFFFFFFF:FFFFFFF:
FFFFFFFFFFFFFFFFFFFFFFFFFFF,FFFF:FFFFF:F:FFFFFFFFFFF:FFFFFFFFFFFFFF
@A00155:342:HHGFNDSXY:1:1624:17192:4335 2:N:0:GAACCTAG+TCCGCATA
CTGCTCCACCTCCATCTCGTGGTAAAACGTGTCGTAATACGCCAGTAAAACGGCATGCATGGACTCAG
TTGAATGCTGCGCATTAAAGTCGTCAAAGTCATAGCACAGTGACTCGCAGTCCTTCAACATGTACCTC
+
FFFFFFFFFFFFFFFFFFFFFFFFFF:FFFFFFFFFFFFFFFFFFFFFFFFFFFFFFFFFFFFFFFFF
FFFFFFFFFFFFFFFFFFFFFFFFFFFFFFFFFFFFFFFF:FFFFFFFFFFFFFFFFFFFFFFFFFFF
@A00155:342:HHGFNDSXY:1:1624:17725:4539 2:N:0:GAACCTAG+TCCGCATA
CTGCTCCACCTCCATCTCGTGGTAAAACGTGTCGTAATACGCCAGTAAAACGGCATGCATGGACTCAG
TTGAATGCTGCGCATTAAAGTCGTCAAAGTCATAGCACAGTGACTCGCAGTCCTTCAACATGTACCTC
+
FFFFFFFFFFFFFFFFFFFFFFFFFFFFFFFFFFFFFFFFFFFFFFFFFFFFFFFFFFFFFFFFFFFF
FFFFFFFFFFFFFFFFFFFFFFFFFFFFFFFFFFFFFFFF:FFFFFFFFFFFFFFFFFFFFFFFFFFF
@A00155:342:HHGFNDSXY:1:2477:20907:7388 2:N:0:GAACCTAG+TCCGCATA
CTGCTCCACCTCCATCTCGTGGTAAAACGTGTCGTAATACGCCAGTAAAACGGCATGCATGGACTCAG
TTGAATGCTGCGCATTAAAGTCGTCAAAGTCATAGCACAGTGACTCGCAGTCCTTCAACATGTACCTC
+
FFFFFFFFFFFFFFFFFFFFFFFFFFFFFFFFFFFFFFFFFFFFFFFFF:FFFFFFFFFFFFFFFFFF
FFFFFFFFFFFFFFFFFFFFFFFFFFFFFFFFFFFFFFFFFFFFFFFFFFFFFFFFFFFFFFFFFFFF
@A00155:342:HHGFNDSXY:1:1434:7057:29215 1:N:0:GAACCTAG+TCCGCATA
TGCTCCACCTCCATCTCGTGGTAAAACGTGTCGTAATACGCCAGTAAAACGGCATGCATGGACTCAGT
TGAATGCTGCGCATTAAAGTCGTCAAAGTCATAGCACAGTGACTCGCAGTCCTTCAACATGTACCTCA
+
FFFFFFFFFFFFFFFFFF:FFFFFFFFFFFFFFFF:FFFFFFFFFFFFFFFFFFFFFFFFFFF:FFFF
FFFFFFFFFFFF:FFFFFFFFFFFFF:FFFFFFFFFFFFFFFFFFFFFFFF::FFFFFFFFFFFFFFF
@A00155:342:HHGFNDSXY:1:1462:28546:19147 1:N:0:GAACCTAG+TCCGCATA
TGCTCCACCTCCATCTCGTGGTAAAACGTGTCGTAATACGCCAGTAAAACGGCATGCATGGACTCAGT
TGAATGCTGCGCATTAAAGTCGTCAAAGTCATAGCACAGTGACTCGCAGTCCTTCAACATGTACCTCA
+
FFFFFFFFFFFFFFFFFF:FFFFFFFFF,FFFFFFFFFFFFFFFFFFFFFFFFFFFFFFFFFFFFFFF
FFFFFFFFFFFFFFFFFFFFFFFFFFFFFFFFFFFFFFFFFFFFFFFFFFFFFFFFFFFFFFFFFFFF
```

```
@A00155:342:HHGFNDSXY:1:2218:32651:6151 1:N:0:GAACCTAG+TCCGCATA
GCTCCACCTCCATCTCGTGGTAAAACGTGTCGTAATACGCCAGTAAAACGGCATGCATGGACTCAGTT
GAATGCTGCGCATTAAAGTCGTCAAAGTCATAGCACAGTGACTCGCAGTCCTTCAACATGTACCTCAG
+
FFFFFFFFFFFFFFFFFFFFFFFFFFFFFFFFFFFFFFFFFFFFFFFFFFFFFFFFFFFFFFFFFFFF
FFFFFFFFFFFFFFF:FFFFFFFFFFFFFFFFFFFFFFFFFFFFFFFFFFFFFFFFFFFFFFFFFFFF
@A00155:342:HHGFNDSXY:1:1220:1416:35947 1:N:0:GAACCTAG+TCCGCATA
CCACCTCCATCTCGTGGTAAAACGTGTCGTAATACGCCAGTAAAACGGCATGCATGGACTCAGTTGAA
TGCTGCGCATTAAAGTCGTCAAAGTCATAGCACAGTGACTCGCAGTCCTTCAACATGTACCTCA
+
FFFFFFFFFFFFFFFFFFFFFFFFFFFFFF,FFFFF:FFFFFFFFFFFFFFFFFFFFFFFFFFFFFFF
FFFFFFFFFFFFFFFFFFFFFF,FFFFFFFFFFFFFFFFFF,FFFFFFFFFF:FFFFFFFFFF:
@A00155:342:HHGFNDSXY:1:1322:27453:28776 2:N:0:GAACCTAG+TCCGCATA
CCTCCCTCTCGTGGTAAAACGTGTCGTAATACGCCAGTAAAACGGCATGCATGGACTCAGTTGAATGC
TGCGCATTAAAGTCGTCAAAGTCATAGCACAGTGACTCGCAGTCCTTCAACATGTACCTCAGCCGTTT
+
:F:F,:FFFFFFFFFFFFFFFFFFF:FFFFFFFFFF:FFFFFFFFFFFFFFFFFFFFFFFFFFFFFFF
FFFFFFFFFFFFFFFFFFFFFFFFFFFFFFFFFFFFFFFFFFFFFFFFF:FFF,FF,FFFFFFFFFFF
@A00155:342:HHGFNDSXY:1:2323:2835:4492 2:N:0:GAACCTAG+TCCGCATA
CCTCCATCTCGTGGTAAAACGTGTCGTAATACGCCAGTAAAACGGCATGCATGGACTCAGTTGAATGC
TGCGCATTAAAGTCGTCAAAGTCATAGCACAGTGACTCGCAGTCCTTCAACATGTACCTCAGCCGTTT
+
FFFFFFFFFFFFFFFFFFFFF:FFFFFF:FFFFFFFFFFFFFFFFFFFFFFFFFFFFFFFFFFFFFFF
FFFF:FFFFFFFFFFFFFFFFFFFFF:FFFFFFFFFFFFFFFFFFFFFFFFFFFF:FFFFFFFFFFFF
@A00155:342:HHGFNDSXY:1:2234:24451:7670 2:N:0:GAACCTAG+TCCGCATA
CCTCCATCTCGTGGTAAAACGTGTCGTAATACGCCAGTAAAACGGCATGCATGGACTCAGTTGAATGC
TGCGCATTAAAGTCGTCAAAGTCATAGCACAGTGACTCGCAGTCCTTCAACATGTACCTCAGCCGTTT
+
FFFFFF,FFFFFFFFFFF:FFFFFFFFFFFFFFFFFFFFFFFFFFFFFFFFFFFFFFF,FFFFF:FFF
FFFFFF,FFFFFFFFFFFFFFFFFFFFFFFFFFFFFFFFFFFFFFFFFFFFFFFFFFFFFFFFFFFFF
@A00155:342:HHGFNDSXY:1:1102:19144:2143 1:N:0:GAACCTAG+TCCGCATA
ATCTCGTGGTAAAACGTGTCGTAATACGCCAGTAAAACGGCATGCATGGACTCAGTTGAATGCTGCGC
ATTAAAGTCGTCAAAGTCATAGCACAGTGACTCGCAGTCCTTCAACATGTACCTCAGCCGTTTGTG
+
FFFFFFFFFFFFF,FFFFFFF:FFFFFFFFFFFFFFFFFFFFFFFFFFFFFFFFFFFFFFFF:FFFFF
FFFF:FFFFFFFFF:FFFFFFFFFFFFFFFFFFFFFFFFFFFFFFFFFFFFFFFFFFFF,FFF:F
@A00155:342:HHGFNDSXY:1:1102:18756:10645 1:N:0:GAACCTAG+TCCGCATA
ATCTCGTGGTAAAACGTGTCGTAATACGCCAGTAAAACGGCATGCATGGACTCAGTTGAATGCTGCGC
ATTAAAGTCGTCAAAGTCATAGCACAGTGACTCGCAGTCCTTCAACATGTACCTCAGCCGTTTGTG
+
FFFFFFFFFFFFFFFFFFFFFFFFFFFFFFFFFFFFFFFFFFFFFFFFFFFFFFFFFFFFFFFFFFFF
FFFFFFFFFFFFFFFFFFFFFFFFFFFF:FFFFFFFFFFFFFFFFFFFFFFFFFFFFFFFFFFFFF
@A00155:342:HHGFNDSXY:1:2323:2835:4492 1:N:0:GAACCTAG+TCCGCATA
ATCTCGTGGTAAAACGTGTCGTAATACGCCAGTAAAACGGCATGCATGGACTCAGTTGAATGCTGCGC
ATTAAAGTCGTCAAAGTCATAGCACAGTGACTCGCAGTCCTTCAACATGTACCTCAGCCGTTTGTGCA
+
FFFFFFFFFFFFFFFFFFFFFFFFFFFFFFFFFFFFFFFFFFFFFFFFFFFFFFFFFFFFFFFFFFFF
FFFFFFFFFFFFFFFFFFFFFFFFFFFFFFFFFFFFFFFFFFFFFFFFFFFFFFFFFFFFFFFFFFFF
@A00155:342:HHGFNDSXY:1:1322:27453:28776 1:N:0:GAACCTAG+TCCGCATA
ATCTCGTGGTAAAACGTGTCGTAATACGCCAGTAAAACGGCATGCATGGACTCAGTTGAATGCTGCGC
ATTAAAGTCGTCAAAGTCATAGCACAGTGACTCGCAGTCCTTCAACATGTACCTCAGCCGTTTGTGCA
+
F:FFFFFFFFFFFFFFFF:FFFF::FF:FFFFFFFFF,FFFFF:FFFF:FFFFFFFFFFFFFFF:F:F
FFFFFFFFFF:FFF:FF,FFFF,,FF:FFFFFFFF,,,:F:F:FFF:F:FFFFFFF,FF,:F::FFFF
```

```
@A00155:342:HHGFNDSXY:1:2478:4580:13463 1:N:0:GAACCTAG+TCCGCATA
ATCTCGTGGTAAAACGTGTCGTAATACGCCAGTAAAACGGCATGCATGGACTCAGTTGAATGCTGCGC
ATTAAAGTCGTCAAAGTCATAGCACAGTGACTCGCAGTCCTTCAACATGTACCTCAGCCGTTTGTGCA
+
FFFFFFFFFFFFFFFFFFFFFFFFFFFFFFFFFFFFFFFFFFFFFFFFFFFFFFFFFF:FFFFFFFFF
F:FFFFFFFFFFFFFFFFFFFFFFFFFFFFFFFFFFFFFFFFFFFFFFFFFFFFFFFFFFFFFFFFFF
@A00155:342:HHGFNDSXY:1:2234:24451:7670 1:N:0:GAACCTAG+TCCGCATA
ATCTCGTGGTAAAACGTGTCGTAATACGCCAGTAAAACGGCATGCATGGACTCAGTTGAATGCTGCGC
ATTAAAGTCGTCAAAGTCATAGCACAGTGACTCGCAGTCCTTCAACATGTACCTCAGCCGTTTGTGCA
+
FFFFFFFFFFFFFFFFFFFFFFFFFFFFFFFFFFFFFFFFFFFFFFFFFFFFFFFFFFFFFF:FFFFF
FFFFFF:FFFFFFFFFFFFFFFFFFFFFFFFFFFFFFFFFFFFFFFFFFFFFFFFFFFFFFFFFFFFF
@A00155:342:HHGFNDSXY:1:2410:31620:36338 1:N:0:GAACCTAG+TCCGCATA
ATCTCGTGGTAAAACGTGTCGTAATACGCCAGTAAAACGGCATGCATGGACTCAGTTGAATGCTGCGC
ATTAAAGTCGTCAAAGTCATAGCACAGTGACTCGCAGTCCTTCAACATGTACCTCAGCCGTTTGTGCA
+
FFFFFFFFFFFFFFFFFFFFFFFFFFFFFFFFFFFFFFFFFFFFFFFFFFFFFFFFFFFFFFFFFFFF
FFFFFFFFFFFFFFFFFFFFFFFFFFFFFFFFFFFFFFFFFFFFFFFFFFFFFFFFFFFFF:FFFFFF
@A00155:342:HHGFNDSXY:1:2478:4580:13463 2:N:0:GAACCTAG+TCCGCATA
TCGTGGTAAAACGTGTCGTAATACGCCAGTAAAACGGCATGCATGGACTCAGTTGAATGCTGCGCATT
AAAGTCGTCAAAGTCATAGCACAGTGACTCGCAGTCCTTCAACATGTACCTCAGCCGTTTGTGCACTC
+
FFFF,FFFFFFFFFFFFFFFFFFFFFFFFFFFFFFFFFFFFFFFFFFFFFFFFFFFFFFFFFFFFFFF
FFFFFFFFFFFFFFFFFFFFFFFFFFFFFFFFFFFFFFFFFFFFFFFFFFFFFFFFFFFFFFFFFFFF
@A00155:342:HHGFNDSXY:1:2410:31620:36338 2:N:0:GAACCTAG+TCCGCATA
TCGTGGTAAAACGTGTCGTAATACGCCAGTAAAACGGCATGCATGGACTCAGTTGAATGCTGCGCATT
AAAGTCGTCAAAGTCATAGCACAGTGACTCGCAGTCCTTCAACATGTACCTCAGCCGTTTGTGCACTC
+
FFFFFFFFFFFFFFFF:FFFFFFFFFFFFFFF:FFFFFFFFFFFFFFFFFFFFFFF:FFFFFFFFFF,
FFFFFFFFFFFFFFFFFFFFFFFF,FFFFFFFFFFFFFFFFFFFFFFFFFFFFFFFFFFF:FFFFFFF
@A00155:342:HHGFNDSXY:1:1319:21106:33786 2:N:0:GAACCTAG+TCCGCATA
CGTGGTAAAACGTGTCGTAATACGCCAGTAAAACGGCATGCATGGACTCAGTTGAATGCTGCGCATTA
AAGTCGTCAAAGTCATAGCACAGTGACTCGCAGTCCTTCAACATGTACCTCAGCCGTTTGTGCACTCT
+
FFFFFFFFFFFFFFFFFFFFFFFFFFFFFFFFFFFF:FFFFFFFFFFFFFFFFFFFFFFFF,FFFFFF
FFFFFFFFFFFFFFFFFFFFFFFFFFFFFFFFFFFFF:FFFFFFFFFFFFFFFFFFFFFFF:FFFFFF
@A00155:342:HHGFNDSXY:1:2443:4010:26068 1:N:0:GAACCTAG+TCCGCATA
CGTGGTAAAACGTGTCGTAATACGCCAGTAAAACGGCATGCATGGACTCAGTTGAATGCTGCGCATTA
AAGTCGTCAAAGTCATAGCACAGTGACTCGCAGTCCTTCAACATGTACCTCAGCCGTTTGTGCACTCT
+
FFFFFFFFFFFFFFFFFFFFFFFFFFFFFFFFFFFFFFFFFFFFFFFFFFFFFFFFFFFFFFFFFFFF
FFFFFFFFFFFFFFFFFFFFFFFFFFFFFFFFFFFFFFFFFFFFFFFFFFFFFFFFFFFFFFFFFFFF
@A00155:342:HHGFNDSXY:1:2327:18457:33677 1:N:0:GAACCTAG+TCCGCATA
AAAACGTGTCGTAATACGCCAGTAAAACGGCATGCATGGACTCAGTTGAATGCTGCGCATTAAAGTCG
TCAAAGTCATAGCACAGTGACTCGCAGTCCTTCAACATGTACCTCAGCCGTTTGTGCACTCTCTCGGC
+
FFFFFFFFFFFFFFFFFFFFFFFFFFFFFFFFFFFFFFFFFFFFFFFFFFFFFFFFFFFFFFFFFFFF
FFFFFFFFFFFFFFFFFFFFFFFFFFFFFFFFFFFFFFFFFFFFFFFFFFFFFFFFFFFFFFFFFFFF
@A00155:342:HHGFNDSXY:1:1427:32036:14325 1:N:0:GAACCTAG+TCCGCATA
AAACGTGTCGTAATACGCCAGTAAAACGGCATGCATGGACTCAGTTGAATGCTGCGCATTAAAGTCGT
CAAAGTCATAGCACAGTGACTCGCAGTCCTTCAACATGTACCTCAGCCGTTTGTGCACTCTCTCGGCT
+
FFFFFFFFFFFFFFFFFFFFFFF:FFFFFFFFFFFFFFFFFFFFFFFFFFFFFFFFFFFFFFFFFFFF
FFFFFFFFFFFFFF:FFFFFFFFFFFFFFFFFFFFFFFFFFFFFFFFFF:FFFFFFFFFFFFFFFFFF
```

```
@A00155:342:HHGFNDSXY:1:1427:31629:15468 1:N:0:GAACCTAG+TCCGCATA
AAACGTGTCGTAATACGCCAGTAAAACGGCATGCATGGACTCAGTTGAATGCTGCGCATTAAAGTCGT
CAAAGTCATAGCACAGTGACTCGCAGTCCTTCAACATGTACCTCAGCCGTTTGTGCACTCTCTCGGCT
+
FFFFFFFFFF:FFFFFFFFFFFFFFFFFFFFFFFFFFFFFFFFFFFFFFFFF:FFFFFFFFFFFFFFF
FFFFFFFFFFFFFFFFFFFFFFFFFFFFFFFFFFFFFFFFFFFFFFFFF:FFFFFFFFFFFFFFFFFF
@A00155:342:HHGFNDSXY:1:1277:20112:26365 1:N:0:GAACCTAG+TCCGCATA
AAACGTGTCGTAATACGCCAGTAAAACGGCATGCATGGACTCAGTTGAATGCTGCGCATTAAAGTCGT
CAAAGTCATAGCACAGTGACTCGCAGTCCTTCAACATGTACCTCAGCCGTTTGTGCACTCTCTCGGCT
+
FFFFFFF:FFFFFFFFFFFFFFFFFFFFFFFFFFFFFFFFFFFFFFFFFFFFFFFFF:FFFFFFFFFF
FF:FFFFFFFFFFFFFFFFFFFFFFFFFFFFFFFFFFFFFFFFFFFFFFFFFFFFFFF:FFFFFFFFF
@A00155:342:HHGFNDSXY:1:2462:1099:23093 1:N:0:GAACCTAG+TCCGCATA
AAACGTGTCGTAATACGCCAGTAAAACGGCATGCATGGACTCAGTTGAATGCTGCGCATTAAAGTCGT
CAAAGTCATAGCACAGTGACTCGCAGTCCTTCAACATGTACCTCAGCCGTTTGTGCACTCTCTCGGCT
+
FFFFFFFFFFFFFFFFFFFFFFFFF,FFFFFFFFFFFFF:FFFFFFFFFFFFFFFFFFFFFF:FFFFF
:FFFFFFFF:FFFFFFFFFFFFFFFFFFFFF:,:FFFFFFF,FFF:FFFFFFFFF:FFFFFF:FFFFF
@A00155:342:HHGFNDSXY:1:2576:5005:22122 2:N:0:GAACCTAG+TCCGCATA
GTGTCGTAATACGCCAGTAAAACGGCATGCATGGACTCAGTTGAATGCTGCGCATTAAAGTCGTCAAA
GTCATAGCACAGTGACTCGCAGTCCTTCAACATGTACCTCAGCCGTTTGTGCACTCTCTCGGCTGATG
+
FFFFFFFFFFFFFFFFFFFFFFFFFFFFFFFFFFFFFFFFFFFFFFFFFFFFFFFFFFFFFFFFFFFF
FFFF:FFFFFFFFFFFFFFFFFFFFFFFFFFFFFFFFFFFFFFFFFFFFFFFFFFFFFFFFFFFFFFF
@A00155:342:HHGFNDSXY:1:2443:4010:26068 2:N:0:GAACCTAG+TCCGCATA
GTGTCGTAATACGCCAGTAAAACGGCATGCATGGACTCAGTTGAATGCTGCGCATTAAAGTCGTCAAA
GTCATAGCACAGTGACTCGCAGTCCTTCAACATGTACCTCAGCCGTTTGTGCACTCTCTCGGCTGATG
+
FFFFFFFFFFFFFFFFFFFF:FFFFFFFFFFFFFFFFFFFFFFFFFFFFFFF:FFFFFFFFFFFFFFF
FFFFFFFFFFFFFFFFFFFFFFFFFFFFFFFFFFFFFFF:FFFFFFFFFFFFFFFFFFFFFFFFFFFF
@A00155:342:HHGFNDSXY:1:1319:21106:33786 1:N:0:GAACCTAG+TCCGCATA
GTCGTAATACGCCAGTAAAACGGCATGCATGGACTCAGTTGAATGCTGCGCATTAAAGTCGTCAAAGT
CATAGCACAGTGACTCGCAGTCCTTCAACATGTACCTCAGCCGTTTGTGCACTCTCTCGGCTGATGCC
+
FFFFFFFFFFFFFFFFFFFFFF:FFFFF:FFFFFFFFF,FFFFFFFFFFFF:FFFFFFFFFFFFF,FF
FFFFFFFFFFFFFFFFFFFFFF::FFFFFFFFFFFFFFFF,FFFFFFFFFFFF:F:FFFFFFFFF:FF
@A00155:342:HHGFNDSXY:1:2665:24044:31548 2:N:0:GAACCTAG+TCCGCATA
CGTAATACGCCAGTAAAACGGCATGCATGGACTCAGTTGAATGCTGCGCATTAAAGTCGTCAAAGTCA
TAGCACAGTGACTCGCAGTCCTTCAACATGTACCTCAGCCGTTTGTGCACTCTCTCGGCTGATG
+
FFFFFFFFFFFFFFFFFFFFFFFFFFFFFFFFFFFFFFFFFFFFFFFFFFFFFFFFFFFFFFFFFFFF
FFFFFFFFFFFFFFFFFFFFFFFFFFFFFFFFFFFFFFFFFFFFFFFFFFFFFFFFFFFFFFFF
@A00155:342:HHGFNDSXY:1:1549:8865:34945 1:N:0:GAACCTAG+TCCGCATA
CGTAATACGCCAGTAAAACGGCATGCATGGACTCAGTTGAATGCTGCGCATTAAAGTCGTCAAAGTCA
TAGCACAGTGACTCGCAGTCCTTCAACATGTACCTCAGCCGTTTGTGCACTCTCTCGGCTGATGCCTC
+
FFFFFFFFFFFFFFF:FFFFFFFFFFFFFFFFFFFFFFFFFFFFFFFFFFFFFFFFFFFFFFFFFFFF
FFFFFFFFFFFFFFFFFFFFFFFFFFFFFFFFFFFFFFFFF:FFFFFFFFFFFFFFFFFFFFFFFFFF
@A00155:342:HHGFNDSXY:1:2318:26250:11318 2:N:0:GAACCTAG+TCCGCATA
GTAATACGCCAGTAAAACGGCATGCATGGACTCAGTTGAATGCTGCGCATTAAAGTCGTCAAAGTCAT
AGCACAGTGACTCGCAGTCCTTCAACATGTACCTCAGCCGTTTGTGCACTCTCTCGGCTGATGCCTCC
+
FFFFFFFFFFFFFFFFFFFFFFFFFFFFFFFFFFFFFFFFFFFFF::FFFFFFFFFFFFFFFFFFFFF
FFFFFFFFFFFFFFFFFFFFFFFFFFFFFFFFFFFFFFFFFFF,FFFFFFFFFFFFFFFFFFFFFFFF
```

```
@A00155:342:HHGFNDSXY:1:2318:26838:11772 2:N:0:GAACCTAG+TCCGCATA
GTAATACGCCAGTAAAACGGCATGCATGGACTCAGTTGAATGCTGCGCATTAAAGTCGTCAAAGTCAT
AGCACAGTGACTCGCAGTCCTTCAACATGTACCTCAGCCGTTTGTGCACTCTCTCGGCTGATGCCTCC
+
FFFFFFFFFFFFFFFFFFFFFF:FFFFFFFFFF:FFFFFFFFFFFFFFFFFFFFFFFFFFFFFFFFFF
FFFFFFFFFFFFFFFFFFFFFFFFFFFFFFFFFFFFFFFFFFF:FFFFFFFFFFFFFFFFFFFFFFFF
@A00155:342:HHGFNDSXY:1:2629:30752:30295 2:N:0:GAACCTAG+TCCGCATA
GTAATACGCCAGTAAAACGGCATGCATGGACTCAGTTGAATGCTGCGCATTAAAGTCGTCAAAGTCAT
AGCACAGTGACTCGCAGTCCTTCAACATGTACCTCAGCCGTTTGTGCACTCTCTCGGCTGATGCCTCC
+
FFFFFFFFFFFFFFFFFFFFFFFFFFFFFFFFFFFFFFFFFFFFF:FFFF:FFFFFFFFFFFFFFFFF
FFFFFFFFFFFFFFFFFFFFF:FFFFFFFFFFFFFFFFFFFFFF:FFFFFFFFFFFFFFFFFFFFFFF
@A00155:342:HHGFNDSXY:1:2540:17092:33505 1:N:0:GAACCTAG+TCCGCATA
TAAAACGGCATGCATGGACTCAGTTGAATGCTGCGCATTAAAGTCGTCAAAGTCATAGCACAGTGACT
CGCAGTCCTTCAACATGTACCTCAGCCGTTTGTGCACTCTCTCGGCTGATGCCTCCTCCCCTATCGGG
+
FFFFFFFFFFFFFFFFFFFFFFFFFFFFFFFFFFFFFFFFFFFFFFFFFFFFFFFFFFFFFFFFFFFF
FFFFFFFFFFFFFFFFFFFFFFFFFFFFFFFFFFFFFFFFFFFFFFFFFFFFFFFFFFFFFFFFFFFF
@A00155:342:HHGFNDSXY:1:2106:12563:2863 1:N:0:GAACCTAG+TCCGCATA
TAAAACGGCATGCATGGACTCAGTTGAATGCTGCGCATTAAAGTCGTCAAAGTCATAGCACAGTGACT
CGCAGTCCTTCAACATGTACCTCAGCCGTTTGTGCACTCTCTCGGCTGATGCCTCCTCCCCTATCGGG
+
FFFFFFFFFFFFFFFFFFFFFFFFFFFFFFFFFFFFFFFFFFFFFFFFFFFFFFFFFFFFFFFFFFFF
FFFFFFFFFFFFFFFFFFFFFFFFFFFFFFFFFFFFFFFFFFFFFFFFFFFFFFFFFFFFFFFFFFFF
@A00155:342:HHGFNDSXY:1:2665:24044:31548 1:N:0:GAACCTAG+TCCGCATA
AAACGGCATGCATGGACTCAGTTGAATGCTGCGCATTAAAGTCGTCAAAGTCATAGCACAGTGACTCG
CAGTCCTTCAACATGTACCTCAGCCGTTTGTGCACTCTCTCGGCTGATGCCTCCTCCCCTATCG
+
FF:FFFFFFFFFFFFFFFFFFFFFFFFFFFFFFFFFFFFFFFFFFFFFFFFFFFFFFFFFFFFFFFFF
FFFFFFFFFFFFFFFFFFFFFFFFFFFFFFFFFFFFFFFFFFFFFFFFFFFFFFFFFFFFFFFF
@A00155:342:HHGFNDSXY:1:1258:27073:32722 2:N:0:GAACCTAG+TCCGCATA
ACGGCATGCATGGACTCAGTTGAATGCTGCGCATTAAAGTCGTCAAAGTCATAGCACAGTGACTCGCA
GTCCTTCAACATGTACCTCAGCCGTTTGTGCACTCTCTCGGCTGATGCCTCCTCCCCTATCGGGAAT
+
,FFF,FFFF:FF:FFF:FFFF,FFFFFFFFF:F,FFFFFFFFFFFFFFF:F:FFFFFFFF,F:F,FFF
F:FFFFFFFFFF,FFFFFFFFFFFF,FFFFFFFFFFFFFFFFFFFFFFFFFFFFFFFF:FFFF:FFF
@A00155:342:HHGFNDSXY:1:1434:7057:29215 2:N:0:GAACCTAG+TCCGCATA
CGGCATGCATGGACTCAGTTGAATGCTGCGCATTAAAGTCGTCAAAGTCATAGCACAGTGACTCGCAG
TCCTTCAACATGTACCTCAGCCGTTTGTGCACTCTCTCGGCTGATGCCTCCTCCCCTATCGGGAATCT
+
FFFFFFFFFFFFFFFFFFFFFFFFFFFFFFFFFFFFFFFFFFFFFFFFFF,FFFFFFFFFFFFFFFFF
FFFFFFFFFFFFFFFFFFFFFFFFFFFFFFFFFFFFFFFFFFFFFFFFFFFFFFFFFFFFFFFFFFFF
@A00155:342:HHGFNDSXY:1:1427:32036:14325 2:N:0:GAACCTAG+TCCGCATA
CGGCATGCATGGACTCAGTTGAATGCTGCGCATTAAAGTCGTCAAAGTCATAGCACAGTGACTCGCAG
TCCTTCAACATGTACCTCAGCCGTTTGTGCACTCTCTCGGCTGATGCCTCCTCCCCTATCGGGAATCT
+
:FFF:F::FFFFFFFFFFFFF::FFF:FFFFFFFFFFF:FFFFF:F,FF:FFF:FFFFFFFFFFFFFF
FFFFFFFFFFFFFFF:FFFFFFFFFF:FFFFFFFFF,FFFFFFF:FFF,FFFFFFFFFFFFFFFFFFF
@A00155:342:HHGFNDSXY:1:1427:31629:15468 2:N:0:GAACCTAG+TCCGCATA
CGGCATGCATGGACTCAGTTGAATGCTGCGCATTAAAGTCGTCAAAGTCATAGCACAGTGACTCGCAG
TCCTTCAACATGTACCTCAGCCGTTTGTGCACTCTCTCGGCTGATGCCTCCTCCCCTATCGGGAATCT
+
FF:FFFFFFFFFFFFFFFFFFFFFFFFFFFFFFFFFFFFFFFFFFFFFFFFFFFFFFFFFFFFFFFFF
FFFFFFFFFFFFFFFFFFFFFFFFFFFFFFFFFFFFFFFFFFFFFFFFFFFFFFFFF,FFFFFFFFFF
```

```
@A00155:342:HHGFNDSXY:1:1277:20112:26365 2:N:0:GAACCTAG+TCCGCATA
CGGCATGCATGGACTCAGTTGAATGCTGCGCATTAAAGTCGTCAAAGTCATAGCACAGTGACTCGCAG
TCCTTCAACATGTACCTCAGCCGTTTGTGCACTCTCTCGGCTGATGCCTCCTCCCCTATCGGGAATCT
+
FFFFFFFFFFFFFFFFFFFFFFFFFFFFFFFF:FF:FFFFFFFFFFFFFF,FFFFFFFFFFFFFFFFF
FFFFFFFFFF:FFFFFFFFFFFFFFFFFFFFFFFFFFFFFFFFFFFFFFFFFFFFFF:FFFFFFFFFF
@A00155:342:HHGFNDSXY:1:1462:28546:19147 2:N:0:GAACCTAG+TCCGCATA
CGGCATGCATGGACTCAGTTGAATGCTGCGCATTAAAGTCGTCAAAGTCATAGCACAGTGACTCGCAG
TCCTTCAACATGTACCTCAGCCGTTTGTGCACTCTCTCGGCTGATGCCTCCTCCCCTATCGGGAATCT
+
FFFFFFFFFFFFFFFFFFFFFFFFFFFFFFFFFFFFFFFFFFFFFFFFFFFFFFFFFF:FFFFFFFFF
FFFFFFFFFFFFFFFFFFFFFFFFFFFFFFFFFFFFFFFFFFFFFFFFFFFFFFFF:FFFFFFFFFFF
@A00155:342:HHGFNDSXY:1:2174:12156:34319 2:N:0:GAACCTAG+TCCGCATA
CGGCATGCATGGACTCAGTTGAATGCTGCGCATTAAAGTCGTCAAAGTCATAGCACAGTGACTCGCAG
TCCTTCAACATGTACCTCAGCCGTTTGTGCACTCTCTCGGCTGATGCCTCCTCCCCTATCGGGAATCT
+
:FFFFFFFFFFFFFFFFFFFFFFFFFFFFFFFFFFFFFFFFFFFFFFFFFFFFFFFFFFFFFFFFFFF
FFFFFFFFFFFFFFFFFFFFFFFFFFFFFFFFFFFFFFFFFFFFFFFFFFFFFFFFF::FFFFFFFFF
@A00155:342:HHGFNDSXY:1:2248:20862:6402 2:N:0:GAACCTAG+TCCGCATA
GGCATGCATGGACTCAGTTGAATGCTGCGCATTAAAGTCGTCAAAGTCATAGCACAGTGACTCGCAGT
CCTTCAACATGTACCTCAGCCGTTTGTGCACTCTCTCGGCTGATGCCTCCTCCCCTATCGGGAATCTG
+
FFFFFFFFFFFFFFFFFFFFFFFFFFFFFFFFFFFFFFFFFFFFFFFFFFFFFFFFFFFFFFFFFFFF
FFFFFFFFFFFFFFFFFFFFFFFFFFFFFFFFFFFFFFFF:FFFFFFFFFFFFFFFFFFFFFFFFFFF
@A00155:342:HHGFNDSXY:1:2161:31828:32972 1:N:0:GAACCTAG+TCCGCATA
GCATGCATGGACTCAGTTGAATGCTGCGCATTAAAGTCGTCAAAGTCATAGCACAGTGACTCGCAGTC
CTTCAACATGTACCTCAGCCGTTTGTGCACTCTCTCGGCTGATGCCTCCTCCCCTATCGGGAATCTGT
+
FFFFFFFFFFFFFFFFFFFFFFFFFFFFFFFFFFFFFFFFFFFFFFFFFFFFFFFFFFFFFFFFFFFF
FFFFFFFFFFFFFFFFFFFFFFFFFFFFFFFFFFFFFFFFFFFFFFFFFFFFFFFFFFFFFFFFFFFF
@A00155:342:HHGFNDSXY:1:2540:17092:33505 2:N:0:GAACCTAG+TCCGCATA
ATGCATGGACTCAGTTGAATGCTGCGCATTAAAGTCGTCAAAGTCATAGCACAGTGACTCGCAGTCCT
TCAACATGTACCTCAGCCGTTTGTGCACTCTCTCGGCTGATGCCTCCTCCCCTATCGGGAATCTGTGC
+
FFFFFFFFFFFFFFFF:FFFFFF:FFFFF:FFFFFFFFFFFFFFFFFF:FFFFFFFFFF:FFFFFFFF
FFFFFFFFFFFFFFFFFFFFF:F:FFFFFFFFFFFFFFFFFFFFFFFFFFFFFFFFFFFFFFFFFFFF
@A00155:342:HHGFNDSXY:1:2641:29622:9017 2:N:0:GAACCTAG+TCCGCATA
ATGGACTCAGTTGAATGCTGCGCATTAAAGTCGTCAAAGTCATAGCACAGTGACTCGCAGTCCTTCAA
CATGTACCTCAGCCGTTTGTGCACTCTCTCGGCTGATGCCTCCTCCCCTATCGGGAATCTGTGCTTAA
+
:FFFFFF::FFF,F:FF:FFFFFFFFFFFFFFFFFFFFFFFF:F:FFFFFFFFFFF:FFFFFFFFFFF
FFFFFFFFFFFFFFFFFF:F,FFFFFFFFFFFFFFFFFFFFFFFFFFFFFFFFFFFFFFFFF:FFFFF
@A00155:342:HHGFNDSXY:1:2348:19208:16470 1:N:0:GAACCTAG+TCCGCATA
ATGGACTCAGTTGAATGCTGCGCATTAAAGTCGTCAAAGTCATAGCACAGTGACTCGCAGTCCTTCAA
CATGTACCTCAGCCGTTTGTGCACTCTCTCGGCTGATGCCTCCTCCCCTATCGGGAATCTGTGCTTAA
+
FFFFFFFFFFFFFFFFFFFFFFFFFFFFFFFFFFFFFFFFFFFFFFFFFFFFFFFFFFFFFFFFFFFF
FFFF:FFFFF:FFFFFFFFFFFFFFFFFFFFFFFFFFFFFFFFFFFFF:FFFFFFFFFFFFFFFFFFF
@A00155:342:HHGFNDSXY:1:1258:27073:32722 1:N:0:GAACCTAG+TCCGCATA
GACTCAGTTGAATGCTGCGCATTAAAGTCGTCAAAGTCATAGCACAGTGACTCGCAGTCCTTCAACAT
GTACCTCAGCCGTTTGTGCACTCTCTCGGCTGATGCCTCCTCCCCTATCGGGAATCTGTGCTTAAA
+
FF,FF::F:FFFFFFFFFFF,F,FF,FFFFFFFFFFFFF::,F:FFFFF:,:FFF:FFFFF,FFFFFF
:FFFF:,::FFFFFF:FFFFF:::FFF::,::F,:FFF:F,FFFFFFFF,,FFFFFFFFFFF,FFF
```

```
@A00155:342:HHGFNDSXY:1:2348:19208:16470 2:N:0:GAACCTAG+TCCGCATA
ACTCAGTTGAATGCTGCGCATTAAAGTCGTCAAAGTCATAGCACAGTGACTCGCAGTCCTTCAACATG
TACCTCAGCCGTTTGTGCACTCTCTCGGCTGATGCCTCCTCCCCTATCGGGAATCTGTGCTTAAACAC
+
FFFFFFFFFFFFFFFFFFFFFFFFFFFFFFFFFFFFFFFFFFFFFFFFFFFFFF:FFFF:FFFFFFFF
FFFFFFFFFFF:FFFFFFFFFFFFFFFFFFFFFFFFFFFFFFFFF:FFFFFF:FFFFFFFFFFF,FFF
@A00155:342:HHGFNDSXY:1:2614:15691:6370 2:N:0:GAACCTAG+TCCGCATA
GAATGCTGCGCATTAAAGTCGTCAAAGTCATAGCACAGTGACTCGCAGTCCTTCAACATGTACCTCAG
CCGTTTGTGCACTCTCTCGGCTGATGCCTCCTCCCCTATCGGGAATCTGTGCTTAAACACTTCCTCAC
+
FFFFFFFFFFFFFFFFFFFFFFFFFFFFFFFFFFFFFFFFFFFFFFFFFFFFFFFFFFFF:FFFFFFF
F,FFFFFFFFFFFFFFFFFFFFFFFFFFFFFFFFFFF:,FFFFFFFFFFFFFFFFFFFFFFFFFFFFF
@A00155:342:HHGFNDSXY:1:2106:12563:2863 2:N:0:GAACCTAG+TCCGCATA
GCGCATTAAAGTCGTCAAAGTCATAGCACAGTGACTCGCAGTCCTTCAACATGTACCTCAGCCGTTTG
TGCACTCTCTCGGCTGATGCCTCCTCCCCTATCGGGAATCTGTGCTTAAACACTTCCTCACACCGGTA
+
FFFFFFFFFFFFFFFFFFFFFFFFFFFFFFFFFFFFFFFFFFFFFFFFFFFFFFFFFFFFFFFFFFF:
FFFFF,FFFFFFFFFFFFFFFFFFFFFFFFFFFFFFFFFFFFFFFFFFFFFFFFFFFFFFFFFF
@A00155:342:HHGFNDSXY:1:1549:8865:34945 2:N:0:GAACCTAG+TCCGCATA
CGCATTAAAGTCGTCAAAGTCATAGCACAGTGACTCGCAGTCCTTCAACATGTACCTCAGCCGTTTGT
GCACTCTCTCGGCTGATGCCTCCTCCCCTATCGGGAATCTGTGCTTAAACACTTCCTCACACCGGTAC
+
FFFFFFFFFFFFFFFFFFF:FFFFFFFFFFFFFFFFFFFFFFFFFFFFFFFFFFFFFFFFFFFFFFFF
FFFFFFFFFFFFFFFFFFFFFFFFFFFFF:FFFFFFFFFFFFFFFFFFFFFF,FFFFFFF:FFFFFFF
@A00155:342:HHGFNDSXY:1:1547:21694:1110 2:N:0:GAACCTAG+TCCGCATA
AAGTCGTCAAAGTCATAGCACAGTGACTCGCAGTCCTTCAACATGTACCTCAGCCGTTTGTGCACTCT
CTCGGCTGATGCCTCCTCCCCTATCGGGAATCTGTGCTTAAACACTTCCTCACACCGGTACATAGCAT
+
FFFFFFFFFFFFFFFFFFFFFFF:FFFFF:FFFFFFF:FFFFFFFF,:FFFFFFFFFFFFFFFFF:FF
FFFFFFFFFFFFFFFFFFFFFFFFFFFFFFFFFFFFFFFFFFFFF:,FFFFFFFFFFFFFFFFFFFFF
@A00155:342:HHGFNDSXY:1:2327:18457:33677 2:N:0:GAACCTAG+TCCGCATA
AAGTCGTCAAAGTCATAGCACAGTGACTCGCAGTCCTTCAACATGTACCTCAGCCGTTTGTGCACTCT
CTCGGCTGATGCCTCCTCCCCTATCGGGAATCTGTGCTTAAACACTTCCTCACACCGGTACATAGCAT
+
FFFFFFFFFFFFFFFFFFFFFFFFFFFFFFFFFFFFFFFFFFFFFFFFFFFFFFFFFFFFFFFFFFFF
FFFFFFFFFFFFFFFFFFFFFF:FFFFFFFFFFFFFFFFFFFFFFFFFFFFFF:FFFF,FFFFFFFFF
@A00155:342:HHGFNDSXY:1:2175:5330:2456 2:N:0:GAACCTAG+TCCGCATA
TCAAAGTCATAGCACAGTGACTCGCAGTCCTTCAACATGTACCTCAGCCGTTTGTGCACTCTCTCGGC
TGATGCCTCCTCCCCTATCGGGAATCTGTGCTTAAACACTTCCTCACACCGGTACATAGCATAGTTGG
+
FFF,FFFFF:FFFFFF:FFFFFFFFFFFFFF:FFFFFFFFFFFFF::FFFFFFFFFFFFFFFFFFFFF
FFFFFFFFFFFFFFFF,FFF:FFFFFFFFFFFFFFFFFF:FFFFFFFFFF:FFFFFFF,FFFFFFFFF
@A00155:342:HHGFNDSXY:1:2174:4562:32722 2:N:0:GAACCTAG+TCCGCATA
TCAAAGTCATAGCACAGTGACTCGCAGTCCTTCAACATGTACCTCAGCCGTTTGTGCACTCTCTCGGC
TGATGCCTCCTCCCCTATCGGGAATCTGTGCTTAAACACTTCCTCACACCGGTACATAGCATAGTTGG
+
FFFFFFFFFFF,FFFFFFFFFFFFFFFFFFFFFFFFFFFF,:FFFFFFFFFFFFFFFFFFFFFFFFFF
FFFFF,FFFFFFFFFFFFFFFFFFFFFFFFFF:FF:FFFFF:FFFFFFFFF,FFFFFFFFFFFFFFFF
@A00155:342:HHGFNDSXY:1:2161:31828:32972 2:N:0:GAACCTAG+TCCGCATA
CAAAGTCATAGCACAGTGACTCGCAGTCCTTCAACATGTACCTCAGCCGTTTGTGCACTCTCTCGGCT
GATGCCTCCTCCCCTATCGGGAATCTGTGCTTAAACACTTCCTCACACCGGTACATAGCATAGTTGGT
+
FFFFFFFFFFFFFFFFFFFFFFFFFFFFFFFFFFFFFFFFFFFFFFFFFFFFFFFFFFFFFFFFFFFF
FFFFFFFFFFFFFFFFFFFFFFFFFFFFFFFFFFFFFFFFFFFFFFFFFFFF:FF,FFFFFFFFFFFF
```

```
@A00155:342:HHGFNDSXY:1:1547:21694:1110 1:N:0:GAACCTAG+TCCGCATA
AAGTCATAGCACAGTGACTCGCAGTCCTTCAACATGTACCTCAGCCGTTTGTGCACTCTCTCGGCTGA
TGCCTCCTCCCCTATCGGGAATCTGTGCTTAAACACTTCCTCACACCGGTACATAGCATAGTTGGTTA
+
FFFFFFFFFFFFFFFFFFFFFFFFFFFFFFFFFFFFFFFFFFFFFFFFF:F:FFFFFFFFFFFFFFFF
FF:FFFFFFFFFFFFFF,FFFF:FFFFFFFFFFFFFFFFFFFFFFFFF:FFFFFFF:FFFFFFF:FFFF
@A00155:342:HHGFNDSXY:1:1327:9887:5525 2:N:0:GAACCTAG+TCCGCATA
ATAGCACAGTGACTCGCAGTCCTTCAACATGTACCTCAGCCGTTTGTGCACTCTCTCGGCTGATGCCT
CCTCCCCTATCGGGAATCTGTGCTTAAACACTTCCTCACACCGGTACATAGCATAGTTGGTTATAACC
+
FFFFFFFFFFFFFFFFFFFFFFFFFFFFFFFFFFFFFFFFFFFFFFFFFFFFFFFFFFFFFFFFFFFF
FFFFFFFFFFFFFFFFFFFFFFFFFFFFFFFF,FFFFFFFFFFFFFFFFFFFFFFFFFFFFFFFFFFF
@A00155:342:HHGFNDSXY:1:2462:1099:23093 2:N:0:GAACCTAG+TCCGCATA
GCACAGTGACTCGCAGTCCTTCAACATGTACCTCAGCCGTTTGTGCACTCTCTCGGCTGATGCCTCCT
CCCCTATCGGGAATCTGTGCTTAAACACTTCCTCACACCGGTACATAGCATAGTTGGTTATAACCGAA
+
:FFFFF,F:F:FFFFFFFF:,FFFFFF::F,FFFFFFFF,FFF:FFF:FFFF,F:F,:FFFFFF,FFF
FFFFFF:F::F,F:F:FFFFFFFFFFF,FFF::FFFFFF,FFFFF:FF:FF:F::FF::FFFFFFFFF
@A00155:342:HHGFNDSXY:1:2410:17318:9596 1:N:0:GAACCTAG+TCCGCATA
GACTCGCAGTCCTTCAACATGTACCTCAGCCGTTTGTGCACTCTCTCGGCTGATGCCTCCTCCCCTAT
CGGGAATCTGTGCTTAAACGCTTCCTCACACCGGTACATAGCATAGTTGGTTATAACCGAACT
+
FFFFFFFFFFFFFFFFFFFFFFFFFFFFFFFFFFFFFFFFFFFFFFFFFFFFFFFFFFFFFFFFFFFF
FFFFFFFFFFFFFFFFFFFFF:FFFFFFFFFFFFFFFFFFFFFFFFFFFFFFFFFFFFFFFFF
@A00155:342:HHGFNDSXY:1:2174:4562:32722 1:N:0:GAACCTAG+TCCGCATA
GACTCGCAGTCCTTCAACATGTACCTCAGCCGTTTGTGCACTCTCTCGGCTGATGCCTCCTCCCCTAT
CGGGAATCTGTGCTTAAACACTTCCTCACACCGGTACATAGCATAGTTGGTTATAACCGAACTT
+
FFFFFFFFFFFFFFFFFFFFFFFFFFFFFFFFFFFFFFFFFFFFFFFFFFFFFFFFFFFFFFFFFFFF
FFFFFFFFFFFFFFFFFFFFFFFFFFFFFFFFFFFFFFFFF,FFFFFFFFFFFFFFFFFFF,FFF
@A00155:342:HHGFNDSXY:1:2175:5330:2456 1:N:0:GAACCTAG+TCCGCATA
GACTCGCAGTCCTTCAACATGTACCTCAGCCGTTTGTGCACTCTCTCGGCTGATGCCTCCTCCCCTAT
CGGGAATCTGTGCTTAAACACTTCCTCACACCGGTACATAGCATAGTTGGTTATAACCGAACTT
+
FFFFFFFFF:FFFFFFFFFFFFFFF,FFFFFFFFF:FFFFF:FFFF:F,FFFFF:F::FFFFFFF:FF
FFFFF,FFFFFFF:FFFFFFFFFFFFFFFFFFFF:FFFF,F::FFF,FFFFFFFFFFFFFFFFFF
@A00155:342:HHGFNDSXY:1:2160:1551:15389 2:N:0:GAACCTAG+TCCGCATA
CTTCAACATGTACCTCAGCCGTTTGTGCACTCTCTCGGCTGATGCCTCCTCCCCTATCGGGAATCTGT
GCTTAAACACTTCCTCACACCGGTACATAGCATAGTTGGTTATAACCGAACTTGTTAGATCTACTCCA
+
F:FFFFFF:FF:FFFFFFF,FFFFFFFFFFFFFFFFFFFFFFFFFFFFFFFFFFFFFFFFF,F:FFFFFF
F:FFFFFFFFFFFFFFFFFFFFFFFFFFFFFFFFFFFFFFFFFFFFFFFFFFFFFFFFFFFFFFFFFF
@A00155:342:HHGFNDSXY:1:1259:31322:26960 2:N:0:GAACCTAG+TCCGCATA
CTTCAACATGTACCTCAGCCGTTTGTGCACTCTCTCGGCTGATGCCTCCTCCCCTATCGGGAATCTGT
GCTTAAACACTTCCTCACACCGGTACATAGCATAGTTGGTTATAACCGAACTTGTTAGATCTACTCCA
+
F:FFFFFFFFF:FFFFFFFFFFFFFFFFFFFFFFFFFFFFFFFFFFFFFFFFFF:FFFFFFFFF:FF
FFFFFFFFFFFFFFFFFFFFFFFFFFFFFFFFFFFFFFFFFFFFFFFF:FFFFFFFFFFFFFFFFFFF
@A00155:342:HHGFNDSXY:1:2620:10393:1861 2:N:0:GAACCTAG+TCCGCATA
CTTCAACATGTACCTCAGCCGTTTGTGCACTCTCTCGGCTGATGCCTCCTCCCCTATCGGGAATCTGT
GCTTAAACACTTCCTCACACCGGTACATAGCATAGTTGGTTATAACCGAACTTGTTAGATCTACTCCA
+
FF:FFFFFFFFFFFFFFFFFF,FFFFFF:FFFFFFFFFFFFFFFFFFFFFFFFFFFFFFF,FF:FFFFFF
FFFFFFFFFFF:FFFFFFFFFFFFFFFFFFFFFFFFFFFFFFFFFFFFFFFFFFFFFFFFFFFFFFFFF
```

```
@A00155:342:HHGFNDSXY:1:1509:12924:18646 2:N:0:GAACCTAG+TCCGCATA
TACCTCAGCCGTTTGTGCACTCTCTCGGCTGATGCCTCCTCCCCTATCGGGAATCTGTGCTTAAACAC
TTCCTCACACCGGTACATAGCATAGTTGGTTATAACCGAACTTGTTAGATCTACTCCATATATAGCTC
+
FFFFFFFFFFFFFFFFFFFFFFFFFFFFFFFFFFFFFFFFFFFFFFFFFFF:FFFFFFFFFFFFFF
FFFFFFFFFFFFFFFFFFFFFFFFFFFFFFFFFFFFFFFF:FFFFFFF:FFFFFFFFFFFFFFFFF:
@A00155:342:HHGFNDSXY:1:1673:14968:21746 2:N:0:GAACCTAG+TCCGCATA
GTTTGTGCACTCTCTCGGCTGATGCCTCCTCCCCTATCGGGAATCTGTGCTTAAACACTTCCTCACAC
CGGTACATAGCATAGTTGGTTATAACCGAACTTGTTAGATCTACTCCATATATAGCTCTCATCTTGGC
+
FFFFFFFFFFFFFFFFFFFFFFFFFFFFFFFFFFFFFFFFFFFFFFFFFFFFFFFFF,FFFFFFFFF
F:FFFFF,FFFF:FFFFFFFFFFFFFFFFFFFFFFFFFFFFFFFFFFFFFFFFFFFFFFFFFFFFFF
@A00155:342:HHGFNDSXY:1:2251:32597:34178 1:N:0:GAACCTAG+TCCGCATA
CACTCTCTCGGCTGATGCCTCCTCCCCTATCGGGAATCTGTGCTTAAACACTTCCTCACACCGGTACA
TAGCATAGTTGGTTATAACCGAACTTGTTAGATCTACTCCATATATAGCTCTCATCTTGGCCCACTCA
+
FFFFFFFFFFFFFFFFFFFFFFFFFFF:FFF:FFFFFFFFFFFFFFFFFF:FFFFFFFF:FFF::FF
FFFF:FFFFFFFFFFFFFFFFFFFFFFFFFFFFFFF:FFFFFFFFFFFFFFFFFFFFFFFFFFFFFFF
@A00155:342:HHGFNDSXY:1:2265:5502:18474 1:N:0:GAACCTAG+TCCGCATA
CACTCTCTCGGCTGATGCCTCCTCCCCTATCGGGAATCTGTGCTTAAACACTTCCTCACACCGGTACA
TAGAATAGTTGGTTATAACCGAACTTGTTAGATCTACTCCATATATAGCTCTCATCTTGGCCCACTCA
+
F:FFFFFFF,,:F,FF,F,FFFFFFFFFF,F:::,FF:FF,,,FF:,F:FFFF,FF:F:FFFF:FF::
FFF,FF:FFFFFFFFFFFFFFFFFFFF:FFF,:F,FF,F,FFF,FFFF:FFFFF,F:FFFFFFFF,FFF
@A00155:342:HHGFNDSXY:1:1509:12924:18646 1:N:0:GAACCTAG+TCCGCATA
ACTCTCTCGGCTGATGCCTCCTCCCCTATCGGGAATCTGTGCTTAAACACTTCCTCACACCGGTACAT
AGCATAGTTGGTTATAACCGAACTTGTTAGATCTACTCCATATATAGCTCTCATCTTGGCCCACTCAT
+
FFFFFFFFFFFFFFFFFFFFFFFFFFFFFFFFFFFFFFFFFFFFFFFFFF,FFFFFFFFFFFFFFFFF
FFFFFFFFFFFFFFFFFFFFFFFFFFF:FFFFFFFFFF:FFFFF:FFFFFFFFFFFFFFFFFFFFFFF
@A00155:342:HHGFNDSXY:1:1148:22516:23578 1:N:0:GAACCTAG+TCCGCATA
ACTCTCTCGGCTGATGCCTCCTCCCCTATCGGGAATCTGTGCTTAAACACTTCCTCACACCGGTACAT
AGCATAGTTGGTTATAACCGAACTTGTTAGATCTACTCCATATATAGCTCTCATCTTGGCCCACTCAT
+
FFFFFFFFFFFFFFFFFFFFFFFFFFFFFFFFFFFFFFFFFFFFFFFFFFFFFFFFFFFFFFFFFFFF
FFFFFFFFFFFFFFFFFFFFFFFFFFFFFFFFFFFFFFFF,FFFFFFFFFFFFF:FFFFFFFFFFFF
@A00155:342:HHGFNDSXY:1:2362:8495:14199 1:N:0:GAACCTAG+TCCGCATA
ACTCTCTCGGCTGATGCCTCCTCCCCTATCGGGAATCTGTGCTTAAACACTTCCTCACACCGGTACAT
AGCATAGTTGGTTATAACCGAACTTGTTAGATCTACTCCATATATAGCTCTCATCTTGGCCCACTCAT
+
FFFFFFFFFFFFFFFFFFFFFFFFFFFFFFFFFFFFFFFFFFFFFF:FFFFFFFFFFFFFFFFFFFFF
FFFFFFFFFFFFFFFFF:FFFFFFFFFFFFFFFF:FF,F:FF:FFFFFFFFFFFFFFFFF:FFFFFFF
@A00155:342:HHGFNDSXY:1:2251:32597:34178 2:N:0:GAACCTAG+TCCGCATA
TCTCTCGGCTGATGCCTCCTCCCCTATCGGGAATCTGTGCTTAAACACTTCCTCACACCGGTACATAG
CATAGTTGGTTATAACCGAACTTGTTAGATCTACTCCATATATAGCTCTCATCTTGGCCCACTCATAC
+
FFFFFFFFFFFFFFFFFFFFFFFFFFFFFFFFF,FFFFFFFFFFFFFFFFFFFFFFFFFFFFFFFFFF
FFFFFFFFF,FFFFFFFFFFFF:FFFFFFFFFFFFFFFFFFFFFFFFFFFFF:FF:FFFFFFFFFF:F
@A00155:342:HHGFNDSXY:1:2265:5502:18474 2:N:0:GAACCTAG+TCCGCATA
CTCTCGGCTGATGCCTCCTCCCCTATCGGGAATCTGTGCTTAAACACTTCCTCACACCGGTACATAGC
ATAGTTGGTTATAACCGAACTTTTTAGATCTACTCCATATATAGCTCTCATCTTGGCCCACTCATA
+
FFFFF:F,F:FFFFFF:::F::FFFFFF:FFFFFFFFFFF,FF,FFFFFFFFFFF:FFF:FFFF:,F
FFFF::F::FF:FFFFFFFF,F,F:FFFFF:FFF,FFFFFFFF,FFF:FF:FFF,,,,,FF:FF:F
```

```
@A00155:342:HHGFNDSXY:1:2501:17128:33285 1:N:0:GAACCTAG+TCCGCATA
TGATGCCTCCTCCCCTATCGGGAATCTGTGCTTAAACACTTCCTCACACCGGTACATAGCATAGTTGG
TTATAACCGAACTTGTTAGATCTACTCCATATATAGCTCTCATCTTGGCCCACTCATACTTAACACTT
+
FFFFFFFFFFFFFFFFFFFFFFFFFFFFFFFFF:FFFFFFFFFFFFFFFF:FFF::FFFFFF:FFF
FFFFFFFFFFF:FFFFFFFFFFFFFFFFFFFFFFFFFFFFFFFFFFFFFFFFFFFFFFFFFFFFFF
@A00155:342:HHGFNDSXY:1:1374:6967:27242 2:N:0:GAACCTAG+TCCGCATA
GCCTCCTCCCCTCTCGGGAATCTGTGCTTAAACACTTCCTCACACCGGTACATAGCATAGTTGGTTAT
AACCGAACTTGTTAGATCTACTCCATATATAGCTCTCATCTTGGCCCACTCATACTTAACACTTGCCC
+
FF:FFFFFFFFF,FFFFFFFFFFFFFFFFFFFF,FFFFFFFFFF:FFFFFFFFFFFFFFFFFFF:F
FFFFFF::FFFFFFFFFFFFFFFFFFFFFFFFFF:FFFFFFF:FFFF:FFFFFFFFFFFF,FFFFF
@A00155:342:HHGFNDSXY:1:1358:14886:22263 2:N:0:GAACCTAG+TCCGCATA
GCCTCCTCCCCTATCGGGAATCTGTGCTTAAACACTTCCTCACACCGGTACATAGCATAGTTGGTTAT
AACCGAACTTGTTAGATCTACTCCATATATAGCTCTCATCTTGGCCCACTCATACTTAACACTTGCCC
+
FFFFFFF:FFFFFFFFFFFFFFFFFFFFFFFFFFFFFFFFFFFFFFFFFFFFFFF:FFF:FFFFFF
FFFFFFF:FFFFFFFFFFF:FF:FFFFFF:FFFFFFFFFFFFFFFFFFFFFFFFFFFFFFFFFFFF
@A00155:342:HHGFNDSXY:1:2362:8495:14199 2:N:0:GAACCTAG+TCCGCATA
CCCTATCGGGAATCTGTGCTTAAACACTTCCTCACACCGGTACATAGCATAGTTGGTTATAACCGAAC
TTGTTAGATCTACTCCATATATAGCTCTCATCTTGGCCCACTCATACTTAACACTTGCCCACGCCTTT
+
FFFF:F:FFFFFFFFFFFFFFFFFFFFFFFFFFFFFFFFFFFFFFFFFFFFFFFFFFFFFFF:FFF
FFFFFFFFFFFFF:FFFFFFFFFFFFFFFFFFFFFFFFFFFFFFFFFFFFFFFFFFFFFFFFFFFF
@A00155:342:HHGFNDSXY:1:1148:22516:23578 2:N:0:GAACCTAG+TCCGCATA
CCCTATCGGGAATCTGTGCTTAAACACTTCCTCACACCGGTACATAGCATAGTTGGTTATAACCGAAC
TTGTTAGATCTACTCCATATATAGCTCTCATCTTGGCCCACTCATACTTAACACTTGCCCACGCCTTT
+
FFFFFFFFFFFFFFFFFFFFFFFFFFFFFFFFFFFFFFFFFFFFFFF,FFFFFFFFFFFFFFFFFF
FFFFFFFFFFFFFFFFFFFFFFFFFFFF:FFFFFFFFF:FFFFFFFFFFFFF:FFFFFFFFFFFFF
@A00155:342:HHGFNDSXY:1:1610:2727:13949 2:N:0:GAACCTAG+TCCGCATA
CGGGAATCTGTGCTTAAACACTTCCTCACACCGGTACATAGCATAGTTGGTTATAACCGAACTTGTTA
GATCTACTCCATATATAGCTCTCATCTTGGCCCACTCATACTTAACACTTGCCCACGCCTTTATTTCT
+
F,FFFFFFFFFF:FFFFFFFFFFFFFFFFFFFFF:FFFF:FFFFFFFFFFFFFF:,FFFFFFFFFF
FFFFFFFFFFFFFFFFFFFFFFFFFFFFFFF,FFFFFFFFFFFFFFFF:FFFFFFF,FFFFFFFFF
@A00155:342:HHGFNDSXY:1:2337:19922:23469 1:N:0:GAACCTAG+TCCGCATA
GGGAATCTGTGCTTAAACACTTCCTCACACCGGTACATAGCATAGTTGGTTATAACCGAACTTGTTAG
ATCTACTCCATATATAGCTCTCATCTTGGCCCACTCATACTTAACACTTGCCCACGCCTTTATTTCTG
+
FFFFFFFFFFFFFFFFFFFFFFFFFFFFFFFFFFFFFFFFFFFFFFFFFFFFFFFFFFFFFFFFFF
FFFFFFFFFFFFFFFFFFFFFFFFFFFFFFFFFFFFFFFFFFFFFFFFFFFFFFFFF:FF:FFFFF
@A00155:342:HHGFNDSXY:1:1632:14841:19429 1:N:0:GAACCTAG+TCCGCATA
GCTTAAACACTTCCTCACACCGGTACATAGCATAGTTGGTTATAACCGAACTTGTTAGATCTACTCCA
TATATAGCTCTCATCTTGGCCCACTCATACTTAACACTTGCCCACGCCTTTATTTCTGGTTTTCTCTT
+
FFFFFFFFFFFFFFFFFFFFFFFFFFFFFFFFFFFFFFFFFFFFFFFFFFFFFFFFFFFFFFFFFF
FFFFFFFFFFFFFFFFFFFFFFFFFFFFFFFFFFFFFFFFFFFFFFFFFFFF:FFFFFFFFFFFFF
@A00155:342:HHGFNDSXY:1:2107:31792:2942 2:N:0:GAACCTAG+TCCGCATA
GCTTAAACACTTCCTCACACCGGTACATAGCATAGTTGGTTATAACCGAACTTGTTAGATCTACTCCA
TATATAGCTCTCATCTTGGCCCACTCATACTTAACACTTGCCCACGCCTTTATTTCTGGTTTTCTCTT
+
FFFFFFFFFF:FFFFF,F:FFFFFFFF:FFFFFFFFFFFFFFFFFFFF:F:FFFFFFFFFFFFFFF
F:FFFFFFFFFFFFFFFFFFFFFFFFFFFFFFFFFFFFFFFFFFFFFFFFFF:FFFFFFFFFFFFF
```

```
@A00155:342:HHGFNDSXY:1:2107:26603:2347 2:N:0:GAACCTAG+TCCGCATA
GCTTAAACACTTCCTCACCCCGGTACATAGCATAGTTGGTTATAACCGAACTTGTTAGATCTACTCCA
TATATAGCTCTCATCTTGGCCCACTCATACTTAACACTTGCCCACGCCTTTATTTCTGGTTTTCTCTT
+
FFFFFFFFFFFFFFFFFF,FFFFF:FF,FFFFFFFFFFFFFFFFFFFFFFFFFFFFFFFFFFFFFFFF
FF,FFFFFFFFFFFFFFFFFFFFFFFFF::FFF,F:F:FF:FFFFFFFFFFFFFFFFFFFF:FFFFF,
@A00155:342:HHGFNDSXY:1:1632:14841:19429 2:N:0:GAACCTAG+TCCGCATA
CTTAAACACTTCCTCACACCGGTACATAGCATAGTTGGTTATAACCGAACTTGTTAGATCTACTCCAT
ATATAGCTCTCATCTTGGCCCACTCATACTTAACACTTGCCCACGCCTTTATTTCTGGTTTTCTCTTA
+
FFFFFFFFFFFFFFFFFFFFFFFFFFFFFFFFFFFFFFFFFFFFFFFFFFFFFFFFFFFFFFFFFFFF
FFFFFFFFFFFFFFFFFFFFFFFFFFFFFFFFFFFFFFFFFFFFFFFFFFFFFFFFFFFFFFFFFFFF
@A00155:342:HHGFNDSXY:1:2648:14353:27289 1:N:0:GAACCTAG+TCCGCATA
TTAAACACTTCCTCACACCGGTACATAGCATAGTTGGTTATAACCGAACTTGTTAGATCTACTCCATA
TATAGCTCTCATCTTGGCCCACTCATACTTAACACTTGCCCACGCCTTTATTTCTGGTTTTCTCTTAA
+
FFFFFFFFFFFFFFFFFFFFFFFFFFFFFFFFFFFFFFFFFFFFFFFFF:FFFFFFFFFFFFFFFFFF
FFFFFFFFFFFFFFFFFFFFFFFFFFFFFFFFFFFFFFFFFFFFFFFFFF,FFFFFFFFFFFFFFFFF
@A00155:342:HHGFNDSXY:1:2107:31792:2942 1:N:0:GAACCTAG+TCCGCATA
CACACCGGTACATAGCATAGTTGGTTATAACCGAACTTGTTAGATCTACTCCATATATAGCTCTCATC
TTGGCCCACTCATACTTAACACTTGCCCACGCCTTTATTTCTGGTTTTCTCTTAAACATTGACGCTAT
+
FFFFFFFFFFFFFFFFFFFFFFFFFFFFFFFFFFFFFFFFFFFFFFFFFFFFFFFFFFFFFFFFFFFF
FFFFFFFFFFFFFFFFFFFFFFFFFFFF:FFFF:,,FF,FFFFFFFFFFFFFFFFFFFFFFFFFFFFF
@A00155:342:HHGFNDSXY:1:2107:26603:2347 1:N:0:GAACCTAG+TCCGCATA
CACACCGGTACATAGCATAGTTGGTTATAACCGAACTTGTTAGATCTACTCCATATATAGCTCTCATC
TTGGCCCACTCATACTTAACACTTGCCCACGCCTTTATTTCTGGTTTTCTCTTAAACATTGACGCTAT
+
FFFFFFFFFFFFFFFFFFFFFFFFFFFFFFFFFFFFFFFFFFFFFFFFFFFFFFFFFFFFFFFFFFFF
FFFFFFFFFFFFFFFFFFFFFFFFFFFFFFFFFF:FFFFFFFFFFFFFFFFFFFFFFFFFFFF:FF:F
@A00155:342:HHGFNDSXY:1:2362:15365:29951 2:N:0:GAACCTAG+TCCGCATA
CACCGGTACATAGCATAGTTGGTTATAACCGAACTTGTTAGATCTACTCCATATATAGCTCTCATCTT
GGCCCACTCATACTTAACACTTGCCCACGCCTTTATTTCTGGTTTTCTCTTAAACATTGACGCT
+
FFFFFFFFFFFFFFFFFFFFFFFFFFFFFFFFFFFFFFFFFFF:FFFFFFFF,FFFFFFFFFFFFFFF
FFFFF:FFFF:FFFFFFFFF:FFFFFFFFFFFFFFFFFFFFFFFFFFFFFFFFFFFFFF
@A00155:342:HHGFNDSXY:1:1671:19334:5885 2:N:0:GAACCTAG+TCCGCATA
CACCGGTACATAGCATAGTTGGTTATAACCGAACTTGTTAGATCTACTCCATATATAGCTCTCATCTT
GGCCCACTCATACTTAACACTTGCCCACGCCTTTATTTCTGGTTTTCTCTTAAACATTGACGCT
+
FFFFFFFFFFFFFFFFFFFFFFFFFFFFFFFFFFFFFFFFFFFFFFFFFFFFFFFFFFFFFFFFFFFF
FFFFFFFFFFFFFFFFFFFFFFFFFFFFFFFFFFFFFFFFFFFFFFFFFFFFFFFFFFFF
@A00155:342:HHGFNDSXY:1:2625:12138:19069 1:N:0:GAACCTAG+TCCGCATA
TAGCATAGTTGGTTATAACCGAACTTGTTAGATCTACTCCATATATAGCTCTCATCTTGGCCCACTCA
TACTTAACACTTGCCCACGCCTTTATTTCTGGTTTTCTCTTAAACATTGACGCTATATGTTCCTTCTT
+
FFFFFFFFFFFFFFFFFFFFFFFFFFFFFFFFFFFFFFFFFFFFFFFFFFF:FFFFFFFFFFFFFFFF
FFFFFFFFFFF:FFFFFFFF:F:FFF:FFFFFFFFFFFFFFFFFFFFFFFFFFFFF:FFFFFFFFFFF
@A00155:342:HHGFNDSXY:1:1137:16595:16360 1:N:0:GAACCTAG+TCCGCATA
TAGCATAGTTGGTTATAACCGAACTTGTTAGATCTACTCCATATATAGCTCTCATCTTGGCCCACTCA
TACTTAACACTTGCCCACGCCTTTATTTCTGGTTTTCTCTTAAACATTGACGCTATATGTTCCTTCTT
+
FFFFFFFFFFFFFFFFFFFFFFFFFFF:FFFFFFFFFFFFFFFFFFFFFFFFFFFFFFFFFFFFFFFF
FFFFFFFFFFFFFFFFFFFFF:::F:FFFFFFFFFFFFFFFFFFFFFFFFFFFFF,FFFFFFFFFFFF
```

```
@A00155:342:HHGFNDSXY:1:1671:19334:5885 1:N:0:GAACCTAG+TCCGCATA
TAGTTGGTTATAACCGAACTTGTTAGATCTACTCCATATATAGCTCTCATCTTGGCCCACTCATACTT
AACACTTGCCCACGCCTTTATTTCTGGTTTTCTCTTAAACATTGACGCTATATGTTCCTTCTTC
+
FFFFFFF:FFFFFFFFFFFFFFFFFFFFFFFFFFFFFFFFFFFFFFFFFFFFFFFFFFFFFFFF
FFFFFFFFFFFFFFF,F:FFFFFFFFFFFFFFFFFFFFFFFFFFFFFFFFFFFFFFFFFFFFFF
@A00155:342:HHGFNDSXY:1:2362:15365:29951 1:N:0:GAACCTAG+TCCGCATA
TAGTTGGTTATAACCGAACTTGTTAGATCTACTCCATATATAGCTCTCATCTTGGCCCACTCATACTT
AACACTTGCCCACGCCTTTATTTCTGGTTTTCTCTTAAACATTGACGCTATATGTTCCTTCTTC
+
FFFFFFFFFFFFFFFFFFFFFFFFFFFFFFFFFFFFFFFFFFF:FFFFFFFFFFFFFFFFFFFFFFFF
FFFFFFFFFFFFFFF:,F:FFFFFFFFFFFFFFFFFFFFFFFFFFFFFFF:FFFFFFFFFFFFF
@A00155:342:HHGFNDSXY:1:1157:13404:1720 1:N:0:GAACCTAG+TCCGCATA
AGTTGGTTATAACCGAACTTGTTAGATCTACTCCATATATAGCTCTCATCTTGGCCCACTCATACTTA
ACACTTGCCCACGCCTTTATTTCTGGTTTTCTCTTAAACATTGACGCTATATGTTCCTTCTTCATCAT
+
FFFFFFFFFFFFFFFFFFFFFFFFFFFFFFFFFFFFFFFF:FFFFFFFFFFFFFFFFFFFFFFFFFFF
FFFFF:FFFFFFFF:,FFFFFFFFFFFFFFFFFFFFFFFFFFFF,FF:FF:FFFFFFFFFFFFFFFFF
@A00155:342:HHGFNDSXY:1:2648:14353:27289 2:N:0:GAACCTAG+TCCGCATA
GTTGGTTATAACCGAACTTGTTAGATCTACTCCATATATAGCTCTCATCTTGGCCCACTCATACTTAA
CACTTGCCCACGCCTTTATTTCTGGTTTTCTCTTAAACATTGACGCTATATGTTCCTTCTTCATCATG
+
FFF:FFFFFFFFFFFFFFFFFFFFFFFFFFF:FFF,F:F,FFFFFFFFFFFFFFFFFFFFFFFFFFFF
FFFFFFFFFFFFFFFFFFFFFFFFFFFFFFFFFFFFFFFFFFFFFFFFFFFFFFFFFFFFFFFFFFFF
@A00155:342:HHGFNDSXY:1:2501:17128:33285 2:N:0:GAACCTAG+TCCGCATA
GTTATAACCGAACTTGTTAGATCTACTCCATATATAGCTCTCATCTTGGCCCACTCATACTTAACACT
TGCCCACGCCTTTATTTCTGGTTTTCTCTTAAACATTGACGCTATATGTTCCTTCTTCATCATGTTCA
+
FFFFFFFFFFFFFFFFFFFFFFFFFFFFFFFFFFFFFFFF:FF:FFFFFFFFFFFFFFFFFFFFFFFF
FFFFFFFFFFFFFFFFFFFFFFFFFFFFFFFFFFFFFFFFFFFFFFFF:FFFF:FFFFFFFFFFFFFF
@A00155:342:HHGFNDSXY:1:2337:19922:23469 2:N:0:GAACCTAG+TCCGCATA
ACTTGTTAGATCTACTCCATATATAGCTCTCATCTTGGCCCACTCATACTTAACACTTGCCCACGCCT
TTATTTCTGGTTTTCTCTTAAACATTGACGCTATATGTTCCTTCTTCATCATGTTCAATGCCACGAAC
+
FFFFFFFFFFFFFFFFFFFFFFFFFFFFFFFFFFFFFFFFFFFFFFFFFFFFFFF:FFFFFFFFFFF
FFFFF,FFFFFFFFFFFFFFFFFFFFFFFFFFFFFFF,FFFFFFFFFF,FFFFFFFFFFFFFFFFFFF
@A00155:342:HHGFNDSXY:1:1137:16595:16360 2:N:0:GAACCTAG+TCCGCATA
TAGCTCTCATCTTGGCCCACTCATACTTAACACTTGCCCACGCCTTTATTTCTGGTTTTCTCTTAAAC
ATTGACGCTATATGTTCCTTCTTCATCATGTTCAATGCCACGAACTTGGTCCTGTGCCTGTACTCTTT
+
FFFFFFFFFFFFFFFFFFFFFF:FFFFFFFFFFFFFFFFFFFFFFFFFFFFFFFFFFFFFFFFFFFFF
FFFFFFFFFFFFFFFFFFFFFFFFFFFFFFFFFFFFFFFFFFFFFFFFFFFFFFFFFFFFFFFFFFFF
@A00155:342:HHGFNDSXY:1:2625:12138:19069 2:N:0:GAACCTAG+TCCGCATA
TAGCTCTCATCTTGGCCCACTCATACTTAACACTTGCCCACGCCTTTATTTCTGGTTTTCTCTTAAAC
ATTGACGCTATATGTTCCTTCTTCATCATGTTCAATGCCACGAACTTGGTCCTGTGCCTGTACTCTTT
+
FFFFFF:FFFFFFFFFFFFFFF,FFFFFFFFFFFF::FFFFFFFFF:FFFFFF:FFFFFFFFFFFFFF
FFFFFFFFFFFFFFFFFFFFFFFFFFFFFFFFFFFFFFFFFFFFFFFFFFFFFFFFFFFFFFFFFF:F
@A00155:342:HHGFNDSXY:1:2659:12463:4194 1:N:0:GAACCTAG+TCCGCATA
CTCATCTTGGCCCACTCATACTTAACACTTGCCCACGCCTTTATTTCTGGTTTTCTCTTAAACATTGA
CGCTATATGTTCCTTCTTCATCATGTTCAATGCCACGAACTTGGTCCTGTGCCTGTACTCTTTTTTGA
+
FFFFFFFFFFFFFFFFFFFFFFFFFFFFFFFFFFFFFFFFFFFFFFFFFFFFFFFFFFFFFFF:FFFF
FFFF,F,FFFFFF:FFFFFFFFFFFFFFFFFFFFFFFFFFFFFFFFFFFFFFFFFFFFFFFFFFFFF:
```

@A00155:342:HHGFNDSXY:1:1446:11324:2096 1:N:0:GAACCTAG+TCCGCATA
TCATCTTGGCCCACTCATACTTAACACTTGCCCACGCCTTTATTTCTGGTTTTCTCTTAAACATTGAC
GCTATATGTTCCTTCTTCATCATGTTCAATGCCACGAACTTGGTCCTGTGCCTGTACTCTTTTTTGAT
+
FFFFFFFFFFFFFFFFFFFFFFFFFFFF:FFFFFFFFF::FFFFFFFFFFFFFFFFFFFFFFFFFFFF
FFFF:FFFFFFFFFFFFFFFFFFFFFF:FFFFFFFFFFFFFFFFFFFFFFFFFFFFFFFFFF:FFFFF
@A00155:342:HHGFNDSXY:1:1176:8739:22106 1:N:0:GAACCTAG+TCCGCATA
TCATCTTGGCCCACTCATACTTAACACTTGCCCACGCCTTTATTTCTGGTTTTCTCTTAAACATTGAC
GCTATATGTTCCTTCTTCATCATGTTCAATGCCACGAACTTGGTCCTGTGCCTGTACTCTTTTTTGAT
+
FFFFFFFFFFFFFFFFFFFFFFFFFFFFFFFFFFF:FFFFFFFFFFFFFFFFFFFFFFFFFFFFFFFF
F,FFFF,FFFFFFFFFFFFFFFFF:FFFFFFFFFFFFFFFFFFFFFFFFFFFFFFFFFFFFFFFFFFF
@A00155:342:HHGFNDSXY:1:1407:15402:17957 2:N:0:GAACCTAG+TCCGCATA
ACTCATACTTAACACTTGCCCACGCCTTTATTTCTGGTTTTCTCTTAAACATTGACGCTATATGTTCC
TTCTTCATCATGTTCAATGCCACGAACTTGGTCCTGTGCCTGTACTCTTTTTTGATGTACTGTCTATC
+
FFFFFFFFFFFFFFFFFFFFFFFFFFF:FFFFF,FFFF:FFFFFFFFFFF:FFFFFFF,,FFFFFFFF
FFFFFFFFFFFFFFFFFFFFFFFF:,F:FF,FFFFFFFFFFFFFFFFFFFFFFFFFFFFFFFFFFFFF
@A00155:342:HHGFNDSXY:1:2327:18285:24267 2:N:0:GAACCTAG+TCCGCATA
TCATACTTAACACTTGCCCACGCCTTTATTTCTGGTTTTCTCTTAAACATTGACGCTATATGTTCCTT
CTTCATCATGTTCAATGCCACGAACTTGGTCCTGTGCCTGTACTCTTTTTTGATGTACTGTCTATCCT
+
FFFFFFFFFFFFFFFFFFFFFFFFFFFFFFFFFFFFFFFFFFFFFFFFFFFFFFFFFFFFFFFFFFFF
FFFFFFFFFFFFFFFFFFFFFFFFFFFFFFFFFFFFFFFFFFFFFFFFFFFFFFFFFFFFFFFFFFFF
@A00155:342:HHGFNDSXY:1:1157:13404:1720 2:N:0:GAACCTAG+TCCGCATA
ACTTAACACTTGCCCACGCCTTTATTTCTGGTTTTCTCTTAAACATTGACGCTATATGTTCCTTCTTC
ATCATGTTCAATGCCACGAACTTGGTCCTGTGCCTGTACTCTTTTTTGATGTACTGTCTATCCTCTTC
+
FFFFFFF:FFFFFFFFFFFFFFFFFFFFFFFFFFFFFFFFFFFFFFFFFFFFFFFFFFFFFFFFF:FF
FFFFFFF:FFFFFFFFFFFFFFFFFFFFFFFFFFFFFFFFFFFFFFFFFFFFFF:FFFFFFFF:FFFF
@A00155:342:HHGFNDSXY:1:1407:15402:17957 1:N:0:GAACCTAG+TCCGCATA
CTTAACACTTGCCCACGCCTTTATTTCTGGTTTTCTCTTAAACATTGACGCTATATGTTCCTTCTTCA
TCATGTTCAATGCCACGAACTTGGTCCTGTGCCTGTACTCTTTTTTGATGTACTGTCTATCCTCTTCA
+
FFFFFFFFFFFFFFFFFFFFFFFFFFFFFFFFFFFFFFFFFFFFFFFFFF:FFFFFFFFFFFFFFFFF
FFFFFF:FFFFFFFFFFFFFFFFFFFFFFFFFFFFFFFFFF:FFFFFFFFFFFFFFFFFFFFFFFFFFF
@A00155:342:HHGFNDSXY:1:2327:18285:24267 1:N:0:GAACCTAG+TCCGCATA
CACGCCTTTATTTCTGGTTTTCTCTTAAACATTGACGCTATATGTTCCTTCTTCATCATGTTCAATGC
CACGAACTTGGTCCTGTGCCTGTACTCTTTTTTGATGTACTGTCTATCCTCTTCATACTGT
+
FFFFFFFFFFFFFFFFFFFFFFFFFFFFFFFFFFFFFFFFFFFFFFFFFFFFFFFFFFFFFFFFFFFF
FFFFFFFFFFFFFFFFFFFFFFFFFFFFFFFFFFFFFFFFFFFFFFFFFFFFFFFFFFFFF
@A00155:342:HHGFNDSXY:1:1446:11324:2096 2:N:0:GAACCTAG+TCCGCATA
ACGCCTTTATTTCTGGTTTTCTCTTAAACATTGACGCTATATGTTCCTTCTTCATCATGTTCAATGCC
ACGAACTTGGTCCTGTGCCTGTACTCTTTTTTGATGTACTGTCTATCCTCTTCATACTGTGAATGTAC
+
FFFF:FFFFFFFFFFFFFFFFFFFFF:FFFFFFFFFFFFFFFF:FFFFFFFFFFFFFFFFFFFFFFFF
FFFFFFFFFFFFFFFFFFFFFFFFFFFFFFFFFFFFFFFFFFFFFFFF:FFFFFFFFFFFFFFFFFFF
@A00155:342:HHGFNDSXY:1:1176:8739:22106 2:N:0:GAACCTAG+TCCGCATA
ACGCCTTTATTTCTGGTTTTCTCTTAAACATTGACGCTATATGTTCCTTCTTCATCATGTTCAATGCC
ACGAACTTGGTCCTGTGCCTGTACTCTTTTTTGATGTACTGTCTATCCTCTTCATACTGTGAATGTAC
+
FFFFFFFFFFFFFFFFFFFFFFFFFFFFFFFFFFFFFFFFFFFFFFF:FFFFFFFF:FFFFFFFFFFF
FFFFFFFFFFFFFFFFFFFFFFFFFFFFFFFFFFFFFFFFFFFFFFFFFFFFFFFFFFFFFFFFFFFF

@A00155:342:HHGFNDSXY:1:1249:12463:14246 1:N:0:GAACCTAG+TCCGCATA
TATTTCTGGTTTTCTCTTAAACATTGACGCTATATGTTCCTTCTTCATCATGTTCAATGCCACGAACT
TGGTCCTGTGCCTGTACTCTTTTTTGATGTACTGTCTATCCTCTTCATACTGTGAATGTACACTTCCT
+
FFFFFFFFFFFFFFFFFFFFFF:FFFFFFF:FFFFFFFFF,FF:FFFFFFFFFFFFFFFFF:FFFFF,
FFFF:FFFFFFF,FFF:FFFFFFFFFFF:FFF,FFFFFFFF:FFFFF,:F::F,F:FFFFFFF:F:F:
@A00155:342:HHGFNDSXY:1:1369:27434:9549 1:N:0:GAACCTAG+TCCGCATA
TGGTTTTCTCTTAAACATTGACGCTATATGTTCCTTCTTCATCATGTTCAATGCCACGAACTTGGTCC
TGTGCCTGTACTCTTTTTTGATGTACTGTCTATCCTCTTCATACTGTGAATGTACACTTCCTGATGGC
+
FFFFFFFFFFFFFFFFFFFFFFFFF,FFFFFFFFFFFFFFFFFFFFFFFFFF:F::FFFFFFFF:F:F
FFFFFFFFFFF:FFFFFFFFFFFFFFFFFFFFFFFFFFFFFFFF:FFFFF:FF:FFFFF:FFFFFFFF
@A00155:342:HHGFNDSXY:1:1369:27434:9549 2:N:0:GAACCTAG+TCCGCATA
TGGTTTTCTCTTAAACATTGACGCTATATGTTCCTTCTTCATCATGTTCAATGACACGAACTTGGTCC
TGTGCCTGTACTCTTTTTTGATGTACTGTCTATCCTCTTCATACTGTGAATGTACACTTCCTGATGGC
+
FFFFFFFFFFFFFFFFFFFFFFFFFFFFFF:FFFFFFFFFFFFFFFF:FFFFF,FFFFFFFFFFFFFF
FFFF:FFFFFFFFFFFFFFFFFFFFFF:FFFFFFFFFFFFFFFFFFFFFFFF:FFFFFFFFFFFFFFF
@A00155:342:HHGFNDSXY:1:2429:19009:21104 1:N:0:GAACCTAG+TCCGCATA
TGGTTTTCTCTTAAACATTGACGCTATATGTTCCTTCTTCATCATGTTCAATGCCACGAACTTGGTCC
TGTGCCTGTACTCTTTTTTGATGTACTGTCTATCCTCTTCATACTGTGAATGTACACTTCCTGATGGC
+
FFFFFFFFFFFFFFFFFFFFFFF:FFFFFFFFFFFFFFFFFFFFFFFFFFFFF:FFF:FFFFFFFF,F
FFFF:FFFFFFFFFFFFFFFFFFFFFFFFFFFFFFFF,FFFFFFFFFFFFFFFFFFFFFFFFFFFFFF
@A00155:342:HHGFNDSXY:1:2659:12463:4194 2:N:0:GAACCTAG+TCCGCATA
CTTAAACATTGACGCTATATGTTCCTTCTTCATCATGTTCAATGCCACGAACTTGGTCCTGTGCCTGT
ACTCTTTTTTGATGTACTGTCTATCCTCTTCATACTGTGAATGTACACTTCCTGATGGCACCCACTCC
+
FFFFFFFFFFFF::FFFFFFF:FF:FFFFFFFFFFFFFFFFFFFFFFFFFFFFFFFFFFFFFFFFFFF
FFFFFFFFFFFFFFFFFFFFFFFFFFFFFFFFFFFFFFFFFFFFFFFFFFFFFFFFFFFFFFFFFFFF
@A00155:342:HHGFNDSXY:1:2617:31485:14810 2:N:0:GAACCTAG+TCCGCATA
GGCGCTATATGTTCCTTCTTCATCATGTTCAATGCCACGAACTTGGTCCTGTGCCTGTACTCTTTTTT
GATGTACTGTCTATCCTCTTCATACTGTGAATGTACACTTCCTGATGGCACCCACTCCCAACGT
+
FFFFFFFFFFFFFFFFFFFFFFFFFFFFFFFFFFFFFFFFFFFFFF:FFFFFFFFFFFFFFFFFFFFF
FFFFFFF:F:F:FFFFFFFFFFFFFFFFFFF:FFFFFFFFFFFFFFFFFFFFFFFFFFFFFFFF
@A00155:342:HHGFNDSXY:1:1331:2419:35211 1:N:0:GAACCTAG+TCCGCATA
GACGCTATATGTTCCTTCTTCATCATGTTCAATGCCACGAACTTGGTCCTGTGCCTGTACTCTTTTTT
GATGTACTGTCTATCCTCTTCATACTGTGAATGTACACTTCCTGATGGCACCCACTCCCAACGTGAAT
+
FF:F,FFF:FFFFFFFFFFFFFF,FFFFFFFFFFF,FFFFFFFFFFFFFFFF,FFFFFFFFFFFFFFF
FFFFFFFFFFFFFFFFFFFFFFFFFFFFFFFFFFF:FFFFFFFFFF:FFFFFFFFFFFFFFFFFFF:F
@A00155:342:HHGFNDSXY:1:2617:31485:14810 1:N:0:GAACCTAG+TCCGCATA
TTCTTCATCATGTTCAATGCCACGAACTTGGTCCTGTGCCTGTACTCTTTTTTGATGTACTGTCTATC
CTCTTCATACTGTGAATGTACACTTCCTGATGGCACCCACTCCCAACGTGAATCTAAGTATTTG
+
FFFFFFFFFFFFFFFFFFFFFFFFFFFFFF:F:FFFFFFFFFF:F:FFFFFFFFFFFFFFFFF:F:FF
FFFFFFFFFFFFFFF:FFFFFFFF:FFFFFFFFFFFFF:FFF::FFFFFF::FFFFFF,:FFFF
@A00155:342:HHGFNDSXY:1:1610:25726:16110 2:N:0:GAACCTAG+TCCGCATA
CACGAACTTGGTCCTGTGCCTGTACTCTTTTTTGATGTACTGTCTATCCTCTTCATACTGTGAATGTA
CACTTCCTGATGGCACCCACTCCCAACGTGAATCTAAGTATTTGTCCAGCCTCATTTTCGGATACTTG
+
FFFFFFFFFFFFFFFFFFFFFFFFFFFFFFFFFFFFFFFFFFFFFFFFFFFFFFFFFFF:FFFFFFFF
FFFFFFFFFFFFFFFFFFFFFFFFFFFFFFFFFFFFFFFFFFFFFFFFFFFFFFFFFFFFFFFFFFFF

```
@A00155:342:HHGFNDSXY:1:1141:7184:7921 2:N:0:GAACCTAG+TCCGCATA
ACGAACTTGGTCCTGTGCCTGTACTCTTTTTTGATGTACTGTCTATCCTCTTCATACTGTGAATGTAC
ACTTCCTGATGGCACCCACTCCCAACGTGAATCTAAGTATTTGTCCAGCCTCATTTTCGGATACTTGT
+
F:FFFFFFFFFFFFFFFFFFFFF:F:FFFFFFFFF:FFFFFFFFFFF:FFFFFFFFFFF,FFFFFFFF
FFFFFFFFFFFFFFFFFFFFFFFFFF:FFFFFFFFFFFFFFFFFFFFFFFFFFFFFFFFFFFFFFFFF
@A00155:342:HHGFNDSXY:1:2624:26657:35164 1:N:0:GAACCTAG+TCCGCATA
CGAACTTGGTCCTGTGCCTGTACTCTTTTTTGATGTACTGTCTATCCTCTTCATACTGTGAATGTACA
CTTCCTGATGGCACCCACTCCCAACGTGAATCTAAGTATTTGTCCAGCCTCATTTTCGGATACTTGTA
+
FFFFFFFFFFFFFFFFFFFFFFFFFFFFFFFFFFFFFFFFFFFFFFFFFFFFFFFFFFFFFFFFFFFF
FFFFFFFFFFFFFFFFFFFFFFFFFFFFFFFFFFFFFFFFFFFFFF:FFFFFFFF,F:FFFFFFFFF
@A00155:342:HHGFNDSXY:1:1331:2419:35211 2:N:0:GAACCTAG+TCCGCATA
GTCCTGTGCCTGTACTCTTTTTTGATGTACTGTCTATCCTCTTCATACTGTGAATGTACACTTCCTGA
TGGCACCCACTCCCAACGTGAATCTAAGTATTTGTCCAGCCTCATTTTCGGATACTTGTATCCGTGAT
+
FFFFFFFFFFFFFFFFFFFFFFFFFFFFFF,FFFFFFF:FFFFFFFFFFFFFFFFFFFF:FFFFFF
FFFFFF:FFFF:FFFF:F:FFFFFFFF:FFFFFFFFFFFF:FFFFFFFFFFFFFFFFFFFFFFFFFF
@A00155:342:HHGFNDSXY:1:1322:18231:14998 1:N:0:GAACCTAG+TCCGCATA
GTGCCTGTACTCTTTTTTGATGTACTGTCTATCCTCTTCATACTGTGAATGTACACTTCCTGATGGCA
CCCACTCCCAACGTGAATCTAAGTATTTGTCCAGCCTCATTTTCGGATACTTGTATCCGTGATTCACC
+
FFFFFFFFFFFFFFFFFFFFFFFFFFFFFF:FFFFFFFFFFFFFFFFFFFFFFFFFFFFFFFFFFFFF
FFFFFFFFFFFFFFFFFFFFFFFFFFFFFFFFFFFFFFFFFFFFFFFFFFFFFFFFFFFFFFFFFFFF
@A00155:342:HHGFNDSXY:1:1141:7184:7921 1:N:0:GAACCTAG+TCCGCATA
GTGCCTGTACTCTTTTTTGATGTACTGTCTATCCTCTTCATACTGTGAATGTACACTTCCTGATGGCA
CCCACTCCCAACGTGAATCTAAGTATTTGTCCAGCCTCATTTTCGGATACTTGTATCCGTGATTCACC
+
FFFFFF:FFFFFFFFFFFFFFFFFFF,F:FFFFFFFFFFFFFFFFFFF:FFFFFFFFFFFFFFFFFFF
FFFFFFFFFF:FFFFF:FFFFFFFFFFFFF:FF:FFFFFFF:F,FFFFFF:FFFFF:FFF,FFFFF,F
@A00155:342:HHGFNDSXY:1:2440:26467:3771 1:N:0:GAACCTAG+TCCGCATA
GTGCCTGTACTCTTTTTTGATGTACTGTCTATCCTCTTCATACTGTGAATGTACACTTCCTGATGGCA
CCCACTCCCAACGTGAATCTAAGTATTTGTCCAGCCTCATTTTCGGATACTTGTATCCGTGATTCACC
+
F:FFFFFFFFFFFFFFF:FFFFFF:FFFFFFFFFFFFFF:FFF:FFF:FFFFF:::FFFFFFFFFF
FFFFFFF:FFFFFFFFFFFFFFFFFFF:FF:FF,FFFFFFFFFF:FFFFFF:,FFFFF:F,FFFFF:F
@A00155:342:HHGFNDSXY:1:2429:19009:21104 2:N:0:GAACCTAG+TCCGCATA
TACTCTTTTTTGATGTACTGTCTATCCTCTTCATACTGTGAATGTACACTTCCTGATGGCACCCACTC
CCAACGTGAATCTAAGTATTTGTCCAGCCTCATTTTCGGATACTTGTATCCGTGATTCACCCCTTCCG
+
FFFFFFF:FFFFFFFF:FFFFFFFFFFFFFFF:FFFFFFFFFFFFFFFFF,FFFFFFFFFFFFFFFFF
FFFFFFFFFFFFF:FFFFFFFFFFFFFFFFFFFFFFFFFFFFFFFFFFFFFFFFFFFFFFFFFFFFFF
@A00155:342:HHGFNDSXY:1:1249:12463:14246 2:N:0:GAACCTAG+TCCGCATA
TTTTGATGTACTGTCTATCCTCTTCATACTGTGAATGTACACTTCCTGATGGCACCCACTCCCAACGT
GAATCTAAGTATTTGTCCAGCCTCATTTTCGGATACTTGTATCCGTGATTCACCCCTTCCGAGAATAC
+
FFFFFFFFFFFFFFFFFFFFFFFFFFFFFFFFFFFFFFFFFFFFFFFFFFFFFFFFFFFFFFFFFFFF
FFFFFFFFF:FFF:FFFFFFFFFFFFFFFFFFFFFFFFFFFFFFFFFFFFFFFFFFFFFFFFFFFFFF
@A00155:342:HHGFNDSXY:1:1322:18231:14998 2:N:0:GAACCTAG+TCCGCATA
TCTATCCTCTTCATACTGTGAATGTACACTTCCTGATGGCACCCACTCCCAACGTGAATCTAAGTATT
TGTCCAGCCTCATTTTCGGATACTTGTATCCGTGATTCACCCCTTCCGAGAATACCTTTTTTGCTTCT
+
FFFFFFFFFFFFFFFFF:FFFFFFFFFFFFFFFFFFFFFFFFFFFFFFFFFFFF,FFFFFFFFFFFFFF
FFFFFFFFFFFFFFFFFFFFFFFFFFFFFFFFFFFFFFFFFFFFFFFFFFFFFFFF:FFFFFFFFFFF
```

@A00155:342:HHGFNDSXY:1:2440:26467:3771 2:N:0:GAACCTAG+TCCGCATA
TCTATCCTCTTCATACTGTGAATGTACACTTCCTGATGGCACCCACTCCCAACGTGAATCTAAGTATT
TGTCCAGCCTCATTTTCGGATACTTGTATCCGTGATTCACCCCTTCCGAGAATACCTTTTTTGCTTCT
+
FFFFFFFFFFFFF,FFFFFFFFFFFFFFFFFFFFFFFFFFFFFFFFFFFFF:FFFFFFFFFFFFFFFF
:FFFFFFFFFFFFFFFFFFFFFFFFFFFFFFFFFF,FFFFFFFFFFFFFFFFFFFFFFFFFFFFFFFF
@A00155:342:HHGFNDSXY:1:2624:26657:35164 2:N:0:GAACCTAG+TCCGCATA
TTCATACTGTGAATGTACACTTCCTGATGGCACCCACTCCCAACGTGAATCTAAGTATTTGTCCAGCC
TAATTTTCGGATACTTGTATCCGTGATTCACCCCTTCCGAGAATACCTTTTTTGCTTCTTGGTATACA
+
FFFFFFFFFFF,FFFF::FF,F:FFFF,FF:FF,F:FFF,FFF,FFFF:F,FF:FFFFFFFFFFFFF:
F,FFFFF:FFFFF:FFFFFFFFFFFF,FFFFFFFFFF:FFFFFFFFFFFF:F:FFFFFFFFFFFFFFF
@A00155:342:HHGFNDSXY:1:1263:22363:31798 1:N:0:GAACCTAG+TCCGCATA
TTCATACTGTGAATGTACACTTCCTGATGGCACCCACTCCCAACGTGAATCTAAGTATTTGTCCAGCC
TCATTTTCGGATACTTGTATCCGTGATTCACCCCTTCCGAGAATACCTTTTTTGCTTCTTGGTATACA
+
FFFFFFFFFFFFFFFFFFFFFFFFFFFFFFFFFFFFFFFFFFFFFFFFF,F:FFFFFFFFFFFFFFFF
FFFFFFFFFFFFFFFFFFFFFFFFFFFFFFFFFFFFFFFFFF,FFFFFFFFFFFFFFFFFFFFFFF:F
@A00155:342:HHGFNDSXY:1:1275:25527:35650 1:N:0:GAACCTAG+TCCGCATA
AATGTACACTTCCTGATGGCACCCACTCCCAACGTGAATCTAAGTATTTGTCCAGCCTCATTTTCGGA
TACTTGTATCCGTGATTCACCCCTTCCGAGAATACCTTTTTTGCTTCTTGGTATACATCTTGCTTTTT
+
FFFFFFFFFFFFFFFFFFFFFFFFFFFFFFFFFFFFFFFFFFFFFFFFFFFFFFFFFFFFFFFFFFFF
FFFFFFFFFFFFFF:FFFFFFFFFFFFFFFFFFFFFFFFFFFFFFFFFFFFFFFFFFFFFFFFFFFFF
@A00155:342:HHGFNDSXY:1:2263:10863:7185 1:N:0:GAACCTAG+TCCGCATA
CTGATGGCACCCACTCCCAACGTGAATCTAAGTATTTGTCCAGCCTCATTTTCGGATACTTGTATCCG
TGATTCACCCCTTCCGAGAATACCTTTTTTGCTTCTTGGTATACATCTTGCTTTTTTACCTTTATCGC
+
FFFFFFFFFFFFFFFFFFFFFFFFF:FFFFFFFFFFFFFFFFFFFFFFFFFFFFFFFFFFFFFFFFFF
FFFFFFFFFFFFFFFFFFFFFFFFFFFFFFFF:FFFFFFFFFFFF:FFFFFFFFFFFFFFF,FFFFFF
@A00155:342:HHGFNDSXY:1:1335:2501:29183 1:N:0:GAACCTAG+TCCGCATA
GTGAATCTAAGTATTTGTCCAGCCTCATTTTCGGATACTTGTATCCGTGATTCACCCCTTCCGAGAAT
ACCTTTTTTGCTTCTTGGTATACATCTTGCTTTTTTACCTTTATCGCGTCAGGTTTAATCCTGTGCTC
+
FFFFFFFFFFFFFFFFFFFFFFFFFFFFFFFFFFFFFFFFFFFFFFFFFF:FFFFFFFFFFFFFFFFF
FFFFFFFFFF:FFFFFFF,FFFFFF:FFFFFFFFFFFFFFFFFFF:FFFFFFFFFFFFFF,FFFF:FF
@A00155:342:HHGFNDSXY:1:2342:10700:11882 2:N:0:GAACCTAG+TCCGCATA
AATCTAAGTATTTGTCCAGCCTCATTTTCGGATACTTGTATCCGTGATTCACCCCTTCCGAGAATACC
TTTTTTGCTTCTTGGTATACATCTTGCTTTTTTACCTTTATCGCGTCAGGTTTAATCCTGTGCTCTTT
+
FFFFFFFFFFFFFFFFFFFFFFFFFFFFFFFFFFFFFFFFFFFFFFFFFFFFFFFF:FFFFFFFFFFF
FF:FFFFFF::FFFFFFFFFFFFFFFFFFFFFFFFFFFFFFFFFFFF,FFF:FFFFF:FFFFFFFFFF
@A00155:342:HHGFNDSXY:1:2140:29423:3724 2:N:0:GAACCTAG+TCCGCATA
AATCTAAGTATTTGTCCAGCCTCATTTTCGGATACTTGTATACGTGATTCACCCCTTCCGAGAATACC
TTTTTTGCTTCTTGGTATACATCTTGCTTTTTTACCTTTATCGCGTCAGGTTTAATCCTGTGCTCTTT
+
FFFF:FFF:FFFF:F,FFFFFFFFFFFFFFFFF:FFF:FFF,:FFFFFFFFFFFFFFFFFF:FFFFF,
FF:FFFF,:F::FF:FFF:F,FFFFFFF,FFFFFFFF:FF:,,FFFFFF:FFFFFFF:FFFF,F:FFF
@A00155:342:HHGFNDSXY:1:1275:25527:35650 2:N:0:GAACCTAG+TCCGCATA
ATCTAAGTATTTGTCCAGCCTCATTTTCGGATACTTGTATCCGTGATTCACCCCTTCCGAGAATACCT
TTTTTGCTTCTTGGTATACATCTTGCTTTTTTACCTTTATCGCGTCAGGTTTAATCCTGTGCTCTTTC
+
FFFFFFFFFFF:FFFFFFFFFFFFF:FF:FFFF,FFFFFFFFFF:FF:FFFFFFFFFFFFFFFFFFFF
FFFFFFFFFFFFFFFFFFFFFFFFFFFFFFFFF:FFFFFFFFFFFFFFFFFFFFFFFFFFFFFFFFFF

@A00155:342:HHGFNDSXY:1:2140:29423:3724 1:N:0:GAACCTAG+TCCGCATA
ATTTGTCCAGCCTCATTTTCGGATACTTGTATCCGTGATTCACCCCTTCCGAGAATACCTTTTTTGCT
TCTTGGTATACATCTTGCTTTTTTACCTTTATCGCGTCAGGTTTAATCCTGTGCTCTTTCTCTTTCT
+
FFFFFF:FFFF:F,FFFFFFF::FFFFFFFFFFFFF:FFFFFF:,FFFFFFFFF:FFFFFFFFFFFFF
:FFFFFFFFFFFFFFFFFFFFFFFFFFFFF:FFFFFFFFFFFFFFFFFFFF:FFFFFFFFF:FFFFF
@A00155:342:HHGFNDSXY:1:2342:10700:11882 1:N:0:GAACCTAG+TCCGCATA
ATTTGTCCAGCCTCATTTTCGGATACTTGTATCCGTGATTCACCCCTTCCGAGAATACCTTTTTTGCT
TCTTGGTATACATCTTGCTTTTTTACCTTTATCGCGTCAGGTTTAATCCTGTGCTCTTTCTCTTTCTG
+
FFFFFFFFFFFFFFFFFFFFFFFF:FFFFFFFFFFFFFFFFFFFFFFFFFFFFFFFFFFFFFF:FFFF
FFFFFFFFFFFFFF:FFFFFF:FFFFFFFFFFFFFF,FFFFFFFFFFFFFFFFFFFFFFFFFFFFFFF
@A00155:342:HHGFNDSXY:1:2275:21992:21042 1:N:0:GAACCTAG+TCCGCATA
TTGTCCAGCCTCATTTTCGGATACTTGTATCCGTGATTCACCCCTTCCGAGAATACCTTTTTTGCTTC
TTGGTATACATCTTGCTTTTTTACCTTTATCGCGTCAGGTTTAATCCTGTGCTCTTTCTCTTTCTGCC
+
FFFFFFFFFFFFFFFFFFFFFFFFFFFFFFFFFFFFFFFFFFFFFFFFFFFFFFFFFFF:FFFFFFFF
FFFFF,,FFFFFFFFFFFFFFFFFFFFFFFFFFFFFFFFFFFFFFFFFFFFFFFFFFFFFFFFFFFFF
@A00155:342:HHGFNDSXY:1:1347:2944:34053 1:N:0:GAACCTAG+TCCGCATA
TTGTCCAGCCTCATTTTCGGATACTTGTATCCGTGATTCACCCCTTCCGAGAATACCTTTTTTGCTTC
TTGGTATACATCTTGCTTTTTTACCTTTATCGCGTCAGGTTTAATCCTGTGCTCTTTCTCTTTCTGCC
+
FFFFFFFFFFFFFFFFFFFFFFFFFFFFFFFFFFFFFFFFFF:FFFFFFFFFFFFFFFFFFFFFFFFF
FFFFF,FFFFFFFFFFFFFFFFFF,FFFFFFFFFFFFFFFFFFFFFFFFFFFFFFFFFFFF:FFFFFF
@A00155:342:HHGFNDSXY:1:2247:13648:10661 1:N:0:GAACCTAG+TCCGCATA
CTTGTATCCGTGATTCACCCCTTCCGAGAATACCTTTTTTGCTTCTTGGTATACATCTTGCTTTTTTA
CCTTTATCGCGTCAGGTTTAATCCTGTGCTCTTTCTCTTTCTGCCAGTTTATGCTCCC
+
FFFFFFFFFFFFFFFFFFFFFFFFF:FFFFF:FFFFFFFFFFFF:FFFFFFFFFFFFFFFFFFFF::FF
FFFFFFFFFFFFFFFFFFFFFFFFFFFFFFFFFFFFFFFFFFFFFFFFFFFFFFFFFF
@A00155:342:HHGFNDSXY:1:2165:1732:5619 2:N:0:GAACCTAG+TCCGCATA
TTGTATCCGTGATTCACCCCTTCCGAGAATACCTTTTTTGCTTCTTGGTATACATCTTGCTTTTTTAC
CTTTATCGCGTCAGGTTTAATCCTGTGCTCTTTCTCTTTCTGCCAGTTTATGCTCCCCACACCTCTAT
+
:,::F,FF:F::,FFFFFFF:FFF:F:F:,FFFFFF:,:,F:FF::F::F:FFF,F,::FFFFFFFFF
FF,F,FF:FF:FFFFF:FFF:FF::::FFFFFFFFFF:FFFFFFFFF:FF,FF,FFF:,FFFFFF:,F
@A00155:342:HHGFNDSXY:1:1263:22363:31798 2:N:0:GAACCTAG+TCCGCATA
TCCGTGATTCACCCCTTCCGAGAATACCTTTTTTGCTTCTTGGTATACATCTTGCTTTTTTACCTTTA
TCGCGTCAGGTTTAATCCTGTGCTCTTTCTCTTTCTGCCAGTTTATGCTCCCCACACCTCTATTCACC
+
FFFFFFFFFFFFFFFFFFFFFFFF:F:FFFFFFFFFFFFFFF:FFFFFFFFFFFFFFFFFFFFFFFFF
FFFFFFFFFFFFFFFFFFFFFFFFFFFFFFFFFFFFFFFFFFFFFFFFFFFFFFFFFFFFFFFFFFFF
@A00155:342:HHGFNDSXY:1:2247:13648:10661 2:N:0:GAACCTAG+TCCGCATA
CCTTCCGAGAATACCTTTTTTGCTTCTTGGTATACATCTTGCTTTTTTACCTTTATCGCGTCAGGTTT
AATCCTGTGCTCTTTCTCTTTCTGCCAGTTTATGCTCCCCACACCTCTATTCACCAAGC
+
FFFFFFFFFFFFFFFFFFFFFFFFFFFFFFFFFFFFFFFFFFFFFFFFFFFFFFFFFFFFFFFFFFFF
FFFFFFFFFFFFFFFFFFFFFFFFFFFFFFFFFFFFFFFFFFFFFFFFFFFFFFFFFFF
@A00155:342:HHGFNDSXY:1:1476:4218:3568 2:N:0:GAACCTAG+TCCGCATA
GAATACCTTTTTTGCTTCTTGGTATACATCTTGCTTTTTTACCTTTATCGCGTCAGGTTTAATCCTGT
GCTCTTTCTCTTTCTGCCAGTTTATGCTCCCCACACCTCTATTCACCAAGCACATCATCTCAAACATT
+
FFFFFFFFF:FFFFFFF:FFFFFF:FFFFFF:FFFF:FF:F:FFF:FFFFFFFFFFFFF:FFFFFFFF
:FFFF::FFFFFF:FFFFFFFFFF,FFFFFFFFF:FFFFFF,FFFFFFFFFFFF,FFFFFFFFFFF:F

```
@A00155:342:HHGFNDSXY:1:2275:21992:21042 2:N:0:GAACCTAG+TCCGCATA
GAATACCTTTTTTGCTTCTTGGTATACATCTTGCTTTTTTACCTTTATCGCGTCAGGTTTAATCCTGT
GCTCTTTCTCTTTCTGCCAGTTTATGCTCCCCACACCTCTATTCACCAAGCACATCATCTCAAACATT
+
FFFFFFFFFFFFF:FFFFFFFFFFFFFFFFFFFFFFFFFFFFFFFF,FFFFFFFFFFFFFFFFFFFFF
FFFFFFFFFF,FFFFFFFFFFFFFFFFFFFFFFFFFFFFFFFF,FFFFFFFFFFFFFFFFFFFFFFFFF
@A00155:342:HHGFNDSXY:1:1347:2944:34053 2:N:0:GAACCTAG+TCCGCATA
AATACCTTTTTTGCTTCTTGGTATACATCTTGCTTTTTTACCTTTATCGCGTCAGGTTTAATCCTGTG
CTCTTTCTCTTTCTGCCAGTTTATGCTCCCCACACCTCTATTCACCAAGCACATCATCTCAAACATT
+
FFF:FF,FF,FFFFF,FFFFFFFFFFFF:FFFFF,FFFFF:FFFFFFFFFFFFFFF,FFFFFF:FFFF
FFFFF:FFFFFFFFFFFFFFFFFFFFFFFFFFFFFFFFFFFFFFF:FFFFFFFFFFFFFFFFFFFFFF
@A00155:342:HHGFNDSXY:1:2263:10863:7185 2:N:0:GAACCTAG+TCCGCATA
TACATCTTGCTTTTTTACCTTTATCGCGTCAGGTTTAATCCTGTGCTCTTTCTCTTTCTGCCAGTTTA
TGCTCCCCACACCTCTATTCACCAAGCACATCATCTCAAACATTTCTGTCAGATCTAATATCTCCTTG
+
FFF:FFFF:F:FF,,FFFFFFFFFFFFFFFFFFFFFFFFFFFFFFFFF:FFFF:FFF:FFFFFFFFFFF
FFFFFFFF:FFFFFFFF:FFFFFFFFFFFFFFFFFFFFFFFFFFFFFFFFFFFFFFFFFFFFFFFFFF
@A00155:342:HHGFNDSXY:1:2268:27398:35853 2:N:0:GAACCTAG+TCCGCATA
GCTTTTTTACCTTTATCGCGTCAGGTTTAATCCTGTGCTCTTTCTCATTCTGCCAGTTTATGCTCCCC
ACACCTCTATTCACCAAGCACATCATCTCAAACATTTCTGTCAGATCTAATATCTCCTTGTTA
+
FFFFF:FFFFFFFFFFFFFFFFFFFFF:FFFFFFFFF:FFFFFFFFFFFFFFFFFFFFFFFFFFFFF:F
FFFFFFFFFFFFFFFFFFFFFFFFFFFFFFFFFFFFFFFFFFFFFFFFFFFFFFFFFFFFFFF
@A00155:342:HHGFNDSXY:1:1414:23032:7999 2:N:0:GAACCTAG+TCCGCATA
ACCTTTATCGCGTCAGGTTTAATCCTGTGCTCTTTCTCTTTCTGCCAGTTTATGCTCCCCACACCTCT
ATTCACCAAGCACATCATCTCAAACATTTCTGTCAGATCTAATATCTCCTTGTTATGCATACTTTTCA
+
FFFFFFFFFF:FFFFFFFFFFFFFFFFFFFFFFFFFFFFFFFFFFFFFFFFFFFFF:FFFFFFF:FFF
:FFFFFFFFFFFFFFFFFFF:FFFFFFFFFFFFFFFFFFF:FFFFFFFFF,FFF:FFFFFF:,FFFFF
@A00155:342:HHGFNDSXY:1:1335:2501:29183 2:N:0:GAACCTAG+TCCGCATA
TTATCGCGTCAGGTTTAATCCTGTGCTCTTTCTCTTTCTGCCAGTTTATGCTCCCCACACCTCTATTC
ACCAAGCACATCATCTCAAACATTTCTGTCAGATCTAATATCTCCTTGTTATGCATACTTTTCAATG
+
FFFFFFFFFFFFFFFFFFFFFFFFF:FFFFFFFFFFFFF:FFFFFFFFFFFFFFFFFFFFFF:FF::F
FFF:FFFFFFFFFFFFFFFFFFFFFFFFFFFFFFFFFFFFFFFFF:FFF:FFF:FFFFFFFFFFFFF
@A00155:342:HHGFNDSXY:1:2268:27398:35853 1:N:0:GAACCTAG+TCCGCATA
TCGCGTCAGGTTTAATCCTGTGCTCTTTCTCATTCTGCCAGTTTATGCTCCCCACACCTCTATTCACC
AAGCACATCATCTCAAACATTTCTGTCAGATCTAATATCTCCTTGTTATGCATACTTTTCAAT
+
FFFFFFFFFFFFFFFFFFFFFFFFFFFFFFFFFFFFFFFF:FFFFFF,FFFFFFFFFFFFFFFFFFF
FFFFFFFFFFFFFFFFFFFFFFFFFFFFFF::FFFFFFFF:FFFFF:FFFFFFFFFFFFFFFFF
@A00155:342:HHGFNDSXY:1:1356:23918:18114 2:N:0:GAACCTAG+TCCGCATA
TCTTTCTCTTTCTGCCAGTTTATGCTCCCCACACCTCTATTCACCAAGCACATCATCTCAAACATTTC
TGTCAGATCTAATATCTCCTTGTTATGCATACTTTTCAATGGTGTGGCTAACTCCTTTAGTCTTCCTG
+
FFFFFFFFFFFFFF:FFFFFFFFFFFFFFFFFFFFFFFFF:FFFFFFFFFFFFFFFFFFFFFFFFFFFF
FFFFFFFFFFFFF:FFFFFFFFFFFFFFFFFFFFFFFFFFFFFFFFFFFFFFFFFFFFFFFFFFFFFFF
@A00155:342:HHGFNDSXY:1:2469:20130:2284 1:N:0:GAACCTAG+TCCGCATA
CTGCCAGTTTATGCTCCCCACACCTCTATTCACCAAGCACATCATCTCAAACATTTCTGTCAGATCTA
ATATCTCCTTGTTATGCATACTTTTCAATGGTGTGGCTAACTCCTTTAGTCTTCCTGCCAACTCGTCC
+
FFFFFFFFFFFF:F:FFFFFFFFFFFFFFFFFFFFFFFFFFFFFFFFFFFF:FFFFFFFFFFFFFFFF
FFFFFFFFFF::FFFFFFFFF:FFFFFFFFFFFFFFFFFF:FFFFFFFFFFFFFFFFFFFFFFFFFFFF
```

```
@A00155:342:HHGFNDSXY:1:2658:4300:12195 1:N:0:GAACCTAG+TCCGCATA
TGCCAGTTTATGCTCCCCACACCTCTATTCACCAAGCACATCATCTCAAACATTTCTGTCAGATCTAA
TATCTCCTTGTTATGCATACTTTTCAATGGTGTGGCTAACTCCTTTAGTCTTCCTGCCAACTCGTCCA
+
FFFFFFFFFFFFFFFFFFFFFF:FFFFFFFF:FFFFFFFFF,FFFFFFFFF,FFFFFFFF:FF,,:::
F,:FF,FF:FFFFFF,FFFFFFFFFFFFFFFFFF:,FFFFFFFFFFFFF:FFF,FFFFFF,FFFFFFF
@A00155:342:HHGFNDSXY:1:1356:23918:18114 1:N:0:GAACCTAG+TCCGCATA
GCCAGTTTATGCTCCCCACACCTCTATTCACCAAGCACATCATCTCAAACATTTCTGTCAGATCTAAT
ATCTCCTTGTTATGCATACTTTTCAATGGTGTGGCTAACTCCTTTAGTCTTCCTGCCAACTCGTCCAA
+
FFFFFFFFFFFFF:FFF:FFFFFFFFFFFFFFFFFFFFFFFFFFFFFFFFFFFFFFFFFFFF,F,FFF
FFFFFF:FFFFFFFFFFFFFFFFFFFFFFFFFFFFFFFFFFFFFFFFFFFFFFFFFFFFFFFFF
@A00155:342:HHGFNDSXY:1:1414:23032:7999 1:N:0:GAACCTAG+TCCGCATA
CCAGTTTATGCTCCCCACACCTCTATTCACCAAGCACATCATCTCAAACATTTCTGTCAGATCTAATA
TCTCCTTGTTATGCATACTTTTCAATGGTGTGGCTAACTCCTTTAGTCTTCCTGCCAACTCGTCCAAA
+
FFFFFFFFFFFFFFFFFFFFFFFFFFFFFFFFFFFFFFFFFFFFF:FFFFF,FFFFFFFFFFF:FFFF
FFFFFFFFFFFFFFFFFFFFFFFFFFFFFFFFFFFFFFFFFFFFFFFFFFFFFFFFFFFFFFFFFFFF
@A00155:342:HHGFNDSXY:1:1634:11939:34914 1:N:0:GAACCTAG+TCCGCATA
CCCCACACCTCTATTCACCAAGCACATCATCTCAAACATTTCTGTCAGATCTAATATCTCCTTGTTAT
GCATACTTTTCAATGGTGTGGCTAACTCCTTTAGTCTTCCTGCCAACTCGTCCAAACTCTCACACTCC
+
FFFFFF:FFFFFFFFFFFFFFFFFFFFFFFFFFFFFFF:FFFFFFFFFFFF:F:F,:FFFFFFFFF:F
FFFFFFFFFFFFF:FFFFFFFFF::FFFFFFFFFFFFFFFFFFFFFFFFFFFFFFFFFFFFFFFFFFF
@A00155:342:HHGFNDSXY:1:1401:6931:24674 1:N:0:GAACCTAG+TCCGCATA
CCCACACCTCTATTCACCAAGCACATCATCTCAAACATTTCTGTCAGATCTAATATCTCCGTGTTATG
CATACTTTTCAATGGTGTGGCTAACTCCTTTAGTCTTCCTGCCAACTCGTCCAAACTCTCACACTCC
+
FFFFF,FFFFFFFFFFFFFFFFFFFFFF:FFFFF:F:::FFFFFFFF::FF,F,FFFFFF,FFFFFFF
FFFFFFFFFFFFFFFFFFFFFFFFF:FFFFFFFFFFFFFFFFFFFFFFFFFFFFFFFFFFFFFFFFF
@A00155:342:HHGFNDSXY:1:2315:27742:22639 1:N:0:GAACCTAG+TCCGCATA
ACATCATCTCAAACATTTCTGTCAGATCTAATATCTCCTTGTTATGCATACTTTTCAATGGTGTGGCT
AACTCCTTTAGTCTTCCTGCCAACTCGTCCAAACTCTCACACTCCCACAGCCTTCTTGATGTGCAGAA
+
FFFFFFFFFFFFFFFFFFFFFFFFFF:FFFF:,FFF:FFFFFFFFFFFFFFFFFFFFFFFFFFFFFFF
FFFFFFFFFFFFFFFFFFFFFFFFFFFFFFFFFFFFFFFFFFFFFFFFFFFFFFFFFFFFFFFFFFFF
@A00155:342:HHGFNDSXY:1:1315:27380:33411 1:N:0:GAACCTAG+TCCGCATA
ACATCATCTCAAACATTTCTGTCAGATCTAATATCTCCTTGTTATGCATACTTTTCAATGGTGTGGCT
AACTCCTTTAGTCTTCCTGCCAACTCGTCCAAACTCTCACACTCCCACAGCCTTCTTGATGTGCAGAA
+
FFFFFFFFFFFFFFFFFFFFFFFFF,:::F:F:FF:FFFFFFFFFFFFFFFFFFFFFFFFFF,FFFFF
FFFFFF:FFFFFFFFFFFFFFFFFFFFFFFFFFFFFFFFFFFFFFFFFFFFFFFFFFFF,FFFFFFFF
@A00155:342:HHGFNDSXY:1:1331:22299:11584 2:N:0:GAACCTAG+TCCGCATA
ATCATCTCAAACATTTCTGTCAGATCTAATATCTCCTTGTTATGCATACTTTTCAATGGTGTGGCTAA
CTCCTTTAGTCTTCCTGCCAACTCGTCCAAACTCTCACACTCCCACAGCCTTCTTGATGTGCAGAATA
+
FFFFFFFFFFFFFFFFFFFFFF,FFFFFFFFFFFFFFFF:,FFFFFFFFFFF:FF:FFFFFFFFFFF,
FFF:FFFFFFF,FFFFFF,FFFFF:FF:FFF,FFFF:::FF,:F:FFFFFFFFF:FFFFFFFFFFFFF
@A00155:342:HHGFNDSXY:1:1621:29143:6621 2:N:0:GAACCTAG+TCCGCATA
ATCATCTCAAACATTTCTGTCAGATCTAATATCTCCTTGTTATGCATACTTTTCAATGGTGTGGCTAA
CTCCTTTAGTCTTCCTGCCAACTCGTCCAAACTCTCACACTCCCACAGCCTTCTTGATGTGCAGAATA
+
FF,FF,FF,:F:FF::FFFFFFFFFFFF:FFFFFFFFF:FFFFF,FFF:FFFFFFFFFFF:FFF:FFF
FFFFFFFFFF,FFF:FFFFF,FFFFFFFFFFFFFFFF:FFFFFF:FFF:FFFFFFFFF,FF,FFFFFF
```

```
@A00155:342:HHGFNDSXY:1:1529:26277:12712 2:N:0:GAACCTAG+TCCGCATA
ATCATCTCACACATTTCTGTCAGATCTAATATCTCCTTGTTATGCATACTTTTCAATGGTGTGGCTAA
CTCCTTTAGTCTTCCTGCCAACTCGTCCAAACTCTCACACTCCCACAGCCTTCTTGATGTGCAGAATA
+
FFFFFFF,F,FFFF:FFFFFFF:FFFFFFFFFFFFFFFFFFFF:FFFFF:FFFFFFF,FFFFFFFFFF
FFFFFFFFFFFFFFFFFFFFFFFFFFFFFFFFFFFFFFFFF:FFFFFFFFFFFFFFFFFFFFFFFFFF
@A00155:342:HHGFNDSXY:1:1634:11939:34914 2:N:0:GAACCTAG+TCCGCATA
TCAAACATTTCTGTCAGATCTAATATCTCCTTGTTATGCATACTTTTCAATGGTGTGGCTAACTCCTT
TAGTCTTCCGGCCAACTCGTCCAAACTCTCACACTCCCACAGCCTTCTTGATGTGCAGAATATGTAT
+
F,FFFFFFFFFFFFFF:FF:F,FF:FFFF:FF:FFFFF,,FFFFFFFFFFFFF:F:FFFFFFFFF,FF
FFFF:FF::,FFFFFFFF:FFFFFFFFFFFFFFFFFFFFF,FFFFF:FFF,FFFFFFFFFFFFFFFF
@A00155:342:HHGFNDSXY:1:2625:5963:33771 1:N:0:GAACCTAG+TCCGCATA
TCAAACATTTCTGTCAGATCTAATATCTCCTTGTTATGCATACTTTTCAATGGTGTGGCTAACTCCTT
TAGTCTTCCTGCCAACTCGTCCAAACTCTCACACTCCCACAGCCTTCTTGATGTGCAGAATATGTATG
+
FFFFFFFFFFFFFFFFF:,F::FFFFF,FFFFFFFFFFFFFFFFFFFFFFFFFFFFF:FFFF:FFFFF
FFFFFFFFFFFFFFFFFF,FFFFFFFFFFFFFFFFFFFFFFFFFF:F,FFFFFFF:,FFFFFFFFFFF
@A00155:342:HHGFNDSXY:1:2625:5963:33771 2:N:0:GAACCTAG+TCCGCATA
TGTCAGATCTAATATCTCCTTGTTATGCATACTTTTCAATGGTGTGGCTAACTCCTTTAGTCTTCCTG
CCAACTCGTCCAAACTCTCACACTCCCACAGCCTTCTTGATGTGCAGAATATGTATGCCACCTGTGGT
+
FFFFFFFF:FFFFFFFFFFFFFFFFFFFFFFFFFFF:FFFFFFFFFFFFFFFF::FFFFFFFFFFFF,
FFFFFFFFFFFFF,FFFFFFFFFFFFFFFF:FFFF:FFFFFFFFFFFFFFFFFFFFFFFFFFFF:FFF
@A00155:342:HHGFNDSXY:1:1170:10140:1830 2:N:0:GAACCTAG+TCCGCATA
CTCCTTGTTATGCATACTTTTCAATGGTGTGGCTAACTCCTTTAGTCTTCCTGCCAACTCGTCCAAAC
TCTCACACTCCCACAGCCTTCTTGATGTGCAGAATATGTATGCCACCTGTGGTCTGGCTAGTA
+
FFF:FFFFFFFFFFFFFF:FFFFFF:FFFFFFFFFFFFFF:FFFFFFFFFFFFFFFFFFFFFFFFFFF
FFFFFFFFFFFFFF:FFFF:FFFFFFFFFFFFFFFFFFFFFFFFFFFFFFFFFFFFFFFFFFF
@A00155:342:HHGFNDSXY:1:1170:10520:16235 2:N:0:GAACCTAG+TCCGCATA
CTCCTTGTTATGCATACTTTTCAATGGTGTGGCTAACTCCTTTAGTCTTCCTGCCAACTCGTCCAAAC
TCTCACACTCCCACAGCCTTCTTGATGTGCAGAATATGTATGCCACCTGTGGTCTGGCTAGTA
+
FFFFFFFFFFFFFF:FFFFFFFFFFFFFFFFFFFFFFFFFF:FFFFFFFFFFFFFFFFFFFFFFFFFF
FFFFFFFFFFFFFFFFFFFFFFFFFFFFFFFFFFFFFFFFFFFFFFFFFFFFFFFFFFFFFFF
@A00155:342:HHGFNDSXY:1:2469:20130:2284 2:N:0:GAACCTAG+TCCGCATA
TCCTTGTTATGCATACTTTTCAATGGTGTGGCTAACTCCTTTAGTCTTCCTGCCAACTCGTCCAAACT
CTCACACTCCCACAGCCTTCTTGATGTGCAGAATATGTATGCCACCTGTGGTCTGGCTAGTAGCGCGT
+
FFFFFFFFFFFFFFFFFFFFFFFFFFFFFFFFFFFFFFF:FFFFFFFFFFFFFFFFFFFFFFFFFFFF
FFFFFFFFFFFFFFFFFFFFFFFFFFFFFFFFFFFFFFFFFFFFFFFFFFFFFFFF:FFFFFFFFFFF
@A00155:342:HHGFNDSXY:1:1233:19271:24878 1:N:0:GAACCTAG+TCCGCATA
TGTTATGCATACTTTTCAATGGTGTGGCTAACTCCTTTAGTCTTCCTGCCAACTCGTCCAAACTCTCA
CACTCCCACAGCCTTCTTGATGTGCAGAATATGTATGCCACCTGTGGTCTGGCTAGTAGCGCGT
+
FFFFFFFFFFFFFFFFFFFFFFFFFFFFFFFFFFFFFFFFFFFFFFFF:FFFFFFFFFFFFFFFFFFF
FFFFFFFFFFFFFFFFFFFFFFFFFFFFFFFFFFFFFFFFFFFFFFFFFFFFFFFFFFFFFFF:
@A00155:342:HHGFNDSXY:1:1233:19271:24878 2:N:0:GAACCTAG+TCCGCATA
TATGCATACTTTTCAATGGTGTGGCTAACTCCTTTAGTCTTCCTGCCAACTCGTCCAAACTCTCACAC
TCCCACAGCCTTCTTGATGTGCAGAATATGTATGCCACCTGTGGTCTGGCTAGTAGCGCGTACATCAT
+
FFFFFFFFFFFFFFFFFFFFFFFFFFFFFFFFFFFFFFFFFFFFFFFFFFFFFFFFFFFFFFFFFFFF
FFFFFFFFFFFFFFFFFFFFFFFFFFFFFFFFFFFFFFFFFFFFFFFFFFFFF:FFFFFFFFFFFFFF
```

```
@A00155:342:HHGFNDSXY:1:1365:30101:32456 2:N:0:GAACCTAG+TCCGCATA
ATGCATACTTTTCAATGGTGTGGCTAACTCCTTTAGTCTTCCTGCCAACTCGTCCAAACTCTCACACT
CCCACAGCCTTCTTGATGTGCAGAATATGTATGCCACCTGTGGTCTGGCTAGTAGCGCGTACATCATG
+
FFFFFFFF:FFFF:FFFFFFFFFFFFFFFFFFFFFFFFFFFFFFFFFFFFFFFFFFFFFFFFFFFFFF
FFFFFFFFF:FFFFFFFFFFFFFFFFFFFFFFFFFFFFFFFFFFFFFFFFFFFFFFFFFFFFFFFFFF
@A00155:342:HHGFNDSXY:1:1170:10140:1830 1:N:0:GAACCTAG+TCCGCATA
AATGGTGTGGCTAACTCCTTTAGTCTTCCTGCCAACTCGTCCAAACTCTCACACTCCCACAGCCTTCT
TGATGTGCAGAATATGTATGCCACCTGTGGTCTGGCTAGTAGCGCGTACATCATGC
+
F:FFFFFFFFFFFFFFFFFFFFFFFFFFFFFFFFFFFFFFFFFFFFFFFFFFFFFFFFFFFFFFFFFF
FFFFFFFFFFFFFFFFFFFFFFFFFFFFFFFFFFFFFFFF::FFFFFFFFFFFFFF
@A00155:342:HHGFNDSXY:1:1170:10520:16235 1:N:0:GAACCTAG+TCCGCATA
AATGGTGTGGCTAACTCCTTTAGTCTTCCTGCCAACTCGTCCAAACTCTCACACTCCCACAGCCTTCT
TGATGTGCAGAATATGTATGCCACCTGTGGTCTGGCTAGTAGCGCGTACATCATGC
+
FFFFFFFFFFFFFFFFFFFFFFFFFFFFFFFFFFFFFFFFFFFFFFFFFFFFFFFFFFFFFFFFFFFF
FFFFFFFFFFFFFFFFFFFFFFFFFFFFFFFFFFFFF:FF,FFFFFFFFFFFFFFF
@A00155:342:HHGFNDSXY:1:2315:27742:22639 2:N:0:GAACCTAG+TCCGCATA
AATGGTGTGGCTAACTCCTTTAGTCTTCCTGCCAACTCGTCCAAACTCTCACACTCCCACAGCCTTCT
TGATGTGCAGAATATGTATGCCACCTGTGGTCTGGCTAGTAGCGCGTACATCATGCAAGTTGCCACAG
+
FFFFFFFFFFFFFFF:FFFFFFFFFFFFFFFFFFFFFFFFFFFFFFFFFFFFFFFFFFFFFFFFFFFF
FFFFFFFFFFFFFFFFFFFFFFFFFFFFFFFFFFFFFFFFFFFFFFFFFFFFFFFFFFF:FFFFFFFF
@A00155:342:HHGFNDSXY:1:1315:27380:33411 2:N:0:GAACCTAG+TCCGCATA
AATGGTGTGGCTAACTCCTTTAGTCTTCCTGCCAACTCGTCCAAACTCTCACACTCCCACAGCCTTCT
TGATGTGCAGAATATGTATGCCACCTGTGGTCTGGCTAGTAGCGCGTACATCATGCAAGTTGCCACAG
+
FFFFFFFFFFFFFFFFFFFFFFFFF:FFFF:FFFF:FFFFFFFFFFFFFFFFFFFFFFFFFFFFFFFF
:FFFFFFFFFFFFFFFFFFFFFFFFFFFFFFFFFFFFFFFFFFFFFFFFFFFFFFFFF:FFFFFFFFF
@A00155:342:HHGFNDSXY:1:1365:30101:32456 1:N:0:GAACCTAG+TCCGCATA
TGGTGTGGCTAACTCCTTTAGTCTTCCTGCCAACTCGTCCAAACTCTCACACTCCCACAGCCTTCTTG
ATGTGCAGAATATGTATGCCACCTGTGGTCTGGCTAGTAGCGCGTACATCATGCAAGTTGCCACAGCA
+
FFFFFFF:F:FFFFFFFFF:FFFFFFFFFFFFFFFFFFFFFF:FFFFFFFFFFFFFFFFFFFFFFFFF
FFF:FFFFF::F:FFFFFFFFFFFFFFFFF:FFF::F:FFFFFFFFFFFFFFFFFFFFFFFFFFFFFF
@A00155:342:HHGFNDSXY:1:2658:4300:12195 2:N:0:GAACCTAG+TCCGCATA
AACTCCTTTAGTCTTCCTGCCAACTCGTCCAAACTCTCACACTCCCACAGCCTTCTTGATGTGCAGAA
TATGTATGCCACCTGTGGTCTGGCTAGTAGCGCGTACATCATGCAAGTTGCCACAGCAGCCTCCGTTA
+
FFFFFFFF,FFFF,FFFFFFFFFFFFFF,FFFFFFFFFFFFFF:F:FFFF:FFFFFFFFFF:FFFFFF
FFFFFFFFFFFFFFFFFFFFFFFFFFFFFFFFF:FFFFFFFFFFFFFFFFFFFFFFFFFFFFFFFFFF
@A00155:342:HHGFNDSXY:1:2446:22064:7169 1:N:0:GAACCTAG+TCCGCATA
GTCTTCCTGCCAACTCGTCCAAACTCTCACACTCCCACAGCCTTCTTGATGTGCAGAATATGTATGCC
ACCTGTGGTCTGGCTAGTAGCGCGTACATCATGCAAGTTGCCACAGCAGCCTCCGTTATACCTCTCAT
+
FFFFFFFFFFFFFFFFFFFFFF:FFFFFFFFFFFFFFFFFFFFFFFFFFFFFFFFFFFFFFFF:FFFF
FFFFFFFFFFFFFFF:FFFFFFFFFFFFFFFFFFFFFFFFFFFFFFFFFFFFFFF:F:FF:FFFFFFF
@A00155:342:HHGFNDSXY:1:1443:5963:20776 2:N:0:GAACCTAG+TCCGCATA
CGTCCAAACTCTCACACTCCCACAGCCTTCTTGATGTGCAGAATATGTATGCCACCTGTGGTCTGGCT
AGTAGCGCGTACATCATGCAAGTTGCCACAGCAGCCTCCGTTATACCTCTCATTCTTCTCATCTCCTT
+
:FFFFFFFFFFFFFFFFFFFFFFFFFFF:FFFFFFFFF,FFFFFF,FFFFFFFFFFFFFFFFFFFFFF
FFFFFFFFFFFFFFFFFFFFFFFFFFFFFFFFFFFFFFFFFFFFFFFFFFFFFFFFFFFF:FFFFFFF
```

```
@A00155:342:HHGFNDSXY:1:1443:5963:20776 1:N:0:GAACCTAG+TCCGCATA
AACTCTCACACTCCCACAGCCTTCTTGATGTGCAGAATATGTATGCCACCTGTGGTCTGGCTAGTAGC
GCGTACATCATGCAAGTTGCCACAGCAGCCTCCGTTATACCTCTCATTCTTCTCATCTCCTTTAGTAC
+
FFFFFFFFFFFFFFFFFFFFFFFFFFFFFFFFFFFF:FFFFFFFFFFFFFFFFFFFFFF:FFFFFFFF
FFFFFFFFFFFFFFFFFFFF:FFFFFFFFFFFFFFFFFFF,::FFFFFFFFFFFFFFFF:FFFF:FFFFFF
@A00155:342:HHGFNDSXY:1:1144:27995:25927 1:N:0:GAACCTAG+TCCGCATA
ACACTCCCACAGCCTTCTTGATGTGCAGAATATGTATGCCACCTGTGGTCTGGCTAGTAGCGCGTACA
TCATGCAAGTTGCCACAGCAGCCTCCGTTATACCTCTCATTCTTCTCATCTCCTTTAGTACCAATCCT
+
FFFFFFFFFFFFFFFFFFFFFFFFFFFFFFFFFFFFFFFFFFFFFFFFFFFFFFFFFFFF:FFFFFFFF
FFFFFFFFFFFFFFFFFFFFFFFFFFFFF::::,,FFFFFFFFFFFFFFFFFFFFFFFFFFFFFFFFFFF
@A00155:342:HHGFNDSXY:1:1538:9209:5916 1:N:0:GAACCTAG+TCCGCATA
ACACTCCCACAGCCTTCTTGATGTGCAGAATATGTATGCCACCTGTGGTCTGGCTAGTAGCGCGTACA
TCATGCAAGTTGCCACAGCAGCCTCCGTTCTACCTCTCATTCTTCTCATCTCCTTTAGTACCAATCCT
+
FFFFFFFFFFFFFFFFFFFFFFFFFFF:FFFFFFFFFFFFFFFFFFFFFFFFFFFFFFFFF,FFFFFFFFF
FFFFFFFFFFFFFFFFFFFFFFFFFFFF,F,F,,,,FFF:F::::FFFFFFFFFFFFFFFFFFFFFFFFF
@A00155:342:HHGFNDSXY:1:2130:1913:7310 1:N:0:GAACCTAG+TCCGCATA
TTGATGTGCAGAATATGTATGCCACCTGTGGTCTGGCTAGTAGCGCGTACATCATGCAAGTTGCCACA
GCAGCCTCCGTTATACCTCTCATTCTTCTCATCTCCTTTAGTACCAATCCTACACATCTCTTCTGTAC
+
FFFFFFFFFFFF:FFFFFFFF:FFFFFFFFFFFFFFFF,F:,FFFFFFFF:FFFFFFF,FFFFFFFFF
FFFFFF:FFF,F,,:FFFF:FFFFFF,FFFFFFFFFF,::FFFFF,FFFFFFFFFF,F:FF:FF,FFF
@A00155:342:HHGFNDSXY:1:1240:3242:32753 1:N:0:GAACCTAG+TCCGCATA
GTGCAGAATATGTATGCCACCTGTGGTCTGGCTCGTAGCGCGTACATCATGCAAGTTGCCACAGCAGC
CTCCGTTATACCTCTCATTCTTCTCATCTCCTTTAGTACCAATCCTACACATCTCTTCTTTACGTTAA
+
FFFFFF::FFFFFFFFFFF:FF,FFFFFF:FFF,:,,FFFFFFFFFF:FFFF,FFFFFFFFFFFFFFF
FFFFF,,:,:::,FFFFF:FFFFF:FFFFF:FFFFFFF::FFFFFFFFFFFFFFFFFFFFFFF,FFFFF
@A00155:342:HHGFNDSXY:1:2603:26006:8296 1:N:0:GAACCTAG+TCCGCATA
GCAGAATATGTATGCCACCTGTGGTCTGGCTAGTAGCGCGTACATCATGCAAGTTGCCACAGCAGCCT
CCGTTATACCTCTCATTCTTCTCATCTCCTTTAGTACCAATCCTACACATCTCTTCTTTACGTTAAAT
+
,:FFFFFFFFFFFFFFFFFFFFFFFFF:FFF:FF:FFFFFFFFFFFFFFF:FF:FFFFFFFFF:F:FF:
:FFF::,:::F:FFFF:,FFFFFFFFFFFFFFF:FFFFFFFF:FF:FFFFFFF:FFFFFF,FFFFFFF
@A00155:342:HHGFNDSXY:1:1240:3242:32753 2:N:0:GAACCTAG+TCCGCATA
TGGCTAGTAGCGCGTACATCATGCAAGTTGCCACAGCAGCCTCCGTTATACCTCTCATTCTTCTCATC
TCCTTTAGTACCAATCCTACACATCTCTTCTTTACGTTAAATTTCTCCGCTACTTCCCAAATCTCCTC
+
FFFFFFFFFFFFFFFFFFFFFFFFF:FF:FFFFFFFFF:FF:FF:FFFFFFFFFFFFFFFFFFFFFFFF
FFFFFFFFFFFFFFFFFFFFFFFFFFFFFFFFFFFFFFFFFFFFFFFFFFFFFFFFFFFFFFFFF,FFF
@A00155:342:HHGFNDSXY:1:2563:23005:2785 1:N:0:GAACCTAG+TCCGCATA
GCTAGTAGCGCGTACATCATGCAAGTTGCCACAGCAGCCTCCGTTATAGCTCTCATTCTTCTCATCTC
CTTTAGTACCAATCCTACACATCTCTTCTTTACGTTAAATTTCTCCGCTACTTCCCAAATCTCCTCGG
+
FFFFFFFFFFFFFFFFFFFFFFFF,FFFFFFFFFFFFFFFFFFF,F,F,,,F:F:,,,::FFFF:FFFF
FFFFFFFFFFFFFFFFFFFFFFFFF:FFFF,FFFFFFFFFFFFFFF:FFFFFFFFFFFFFFFFFFFFFFF
@A00155:342:HHGFNDSXY:1:1538:9209:5916 2:N:0:GAACCTAG+TCCGCATA
AGCGCGTACATCATGCAAGTTGCCACAGCAGCCTCCGTTATACCTCTCATTCTTCTCATCTCCTTTAG
TACCAATCCTACACATCTCTTCTTTACGTTAAATTTCTCCGCTACTTCCCAAATCTCCTCGGGCCGAT
+
FFFFFFFFFFFFFFFFFFFFFFFFFFFFFFFFF:FFFFFFFFFFFFFFFFFFFFFFFFFFFFFFFFFFF
FFFFFFFFFFFFFFFFFFFFF:,FFFFFFFFFFFFFFFFFFFFFFFFFFFF:FFFFFFFFFFFFFFFFFF:
```

```
@A00155:342:HHGFNDSXY:1:2130:1913:7310 2:N:0:GAACCTAG+TCCGCATA
CGCGTACATCATGCAAGTTGCCACAGCAGCCTCCGTTATACCTCTCATTCTTCTCATCTCCTTTAGTA
CCAATCCGACACATCTCTTCTTTACGTTAAATTTCTCCGCTACTTCCCAAATCTCCTCGGGCCGATAG
+
FFFFFFFFFFF:FFFFF:FFFFFF:FFFFFFFFFFFFF:,FFFFFFFFF:FFFFFFF,FFFFFF:FFF:F
FFF,FFF,FF:F:FFFF,FFFFFFFFFFFFFFFF:,FFFFFF:FFFFFFF,FFFFFFFFFFFFFFF:,FF
@A00155:342:HHGFNDSXY:1:1323:13901:34554 2:N:0:GAACCTAG+TCCGCATA
CACCGTTATACCTCTCATTCTTCTCATCTCCTTTAGTACCAATCCTACACATCTCTTCTTTACGTTAA
ATTTCTCCGCTACTTCCCAAATCTCCTCGGGCCGATAGTGCGTGTGATGTTCTGTGGACACGCTTGA
+
FFFF:FFFFFFFFFFFF:FFFFFF:FFFFFFFFFFFFFFFFFFFFFFFFFFFFFFFFFFFFF:FFFFFFF
FFFFFFF:FFFFFFFFFFFFFFFFFFFFFFFFFFFFFFFFFFFFFFFFFFFFFFFFFFF:FFFFFFFFF
@A00155:342:HHGFNDSXY:1:2446:22064:7169 2:N:0:GAACCTAG+TCCGCATA
TTATACCTCTCATTCTTCTCATCTCCTTTAGTACCAATCCTACACATCTCTTCTTTACGTTAAATTTC
TCCGCTACTTCCCAAATCTCCTCGGGCCGATAGTGCGTGTGATGTTCTGTGGACACGCTTGAGCTCTC
+
FFFFFFFFFFFFFFFFFFF,,FFFFFFFFFFFFFFFFFFFF,FF::FFFFFFFFFFFFFFFFFFFFFFFF
FFFFFFFFFFFFFFF:FFFFFFFFFFFFFFFFFFFFFFFFFFFFFF:FFFFFFFFFFFFFFFFFFFFFFF
@A00155:342:HHGFNDSXY:1:1637:20283:27352 2:N:0:GAACCTAG+TCCGCATA
TACCTCTCATTCTTCTCATCTCCTTTAGTACCAATCCTACACATCTCTTCTTTACGTTAAATTTCTCC
GCTACTTCCCAAATCTCCTCGGGCCGATAGTGCGTGTGATGTTCTGTGGACACGCTTGACCTCTCAAA
+
FFFFFFFFFFFFFFFFFFFFFFFFF:FFFFFFFF:FFFFFFFFFFFFFFFFFFFF:FFF:FFFFFFFFFF
FFFFFFFFFFFFFFFFFFFFFFFFFFFFFF:FFFFFF:FFFFFFFFFFFFFFFFFFFFFFFFFFFFFFFF
@A00155:342:HHGFNDSXY:1:1144:27995:25927 2:N:0:GAACCTAG+TCCGCATA
TACCTCTCATTCTTCTCATCTCCTTTAGTACCAATCCTACACATCTCTTCTTTACGTTAAATTTCTCC
GCTACTTCCCAAATCTCCTCGGGCCGATAGTGCGTGTGATGTTCTGTGGACACGCTTGACCTCTCAAA
+
FFFFFFFFF,FFFFFFFFFFFFFFFFFFFFFFFF:FFFFFFFFFFFFFFFFFFF,FFFFFFFFFFFFFFF
FFFFFFFFFF:F:FFFFFFFFFFFFFFFFFFFFFFFFFFFFFFFFFFFFFFFFFFFFFFFFFFFFFFFF
@A00155:342:HHGFNDSXY:1:1323:13901:34554 1:N:0:GAACCTAG+TCCGCATA
CATTCTTCTCATCTCCTTTAGTACCAATCCTACACATCTCTTCTTTACGTTAAATTTCTCCGCTACTT
CCCAAATCTCCTCGGGCCGATAGTGCGTGTGATGTTCTGTGGACACGCTTGACCTCTCAAATTCACC
+
FFFFFFFFFFFFFFFFFFFFFFFFF:FF:FFFFFFFFFF:FFFFFFFFFFFFFF:FFF:FF:FFFFFFFF
F:FFFFFF::FFFFFFF,FFFFF:F:FFFFFF:FFFF:FFFFFF::FFFFFFFFFFF:FFF:F:FFF
@A00155:342:HHGFNDSXY:1:1121:26078:14622 1:N:0:GAACCTAG+TCCGCATA
CATTCTTCTCATCTCCTTTAGTACCAATCCTACACATCTCTTCTTTACGTTAAATTTCTCCGCTACTT
CCCAAATCTCCTCGGGCCGATAGTGCGTGTGATGTTCTGTGGACACGCTTGACCTCTCAAATTCACCT
+
FFFFFFFFFFFFFFFFFFFFFF:FF:FFF,FFFFFFFFFFFFFFFFFFFFF::FF,FF:::F:FF,F:
FFF:FF::::FFFFF:::FFFFFF:FFF:FFF:FFFFFFFF::FFFFFFFFFF:FFFFF:FF:F:FF
@A00155:342:HHGFNDSXY:1:2603:26006:8296 2:N:0:GAACCTAG+TCCGCATA
TTCTCATCTCCTTTAGTACCAATCCTACACATCTCTTCTTTACGTTAAATTTCTCCGCTACTTCCCAA
ATCTCCTCGGGCCGATAGTGCGTGTGATGTTCTGTGGACACGCTTGACCTCTCAAATTCACCTGATTT
+
:FFFF:FFFFFFF:FF:FFFFFFFFF:FFFFFFFFFFFFFF,FFFFFFFFFFFFFF:FFFF,FF:FF:
FFFFFFFFFFFFFFFFFFFFFFF,FFFFFFFF:FFFFFFFFFFFFFFFFFFFFFFFFFFFFF:FFFFF:F
@A00155:342:HHGFNDSXY:1:1121:26078:14622 2:N:0:GAACCTAG+TCCGCATA
TCATCTCCTTTAGTACCAATCCTACACATCTCTTCTTTACGTTAAATTTCTCCGCTACTTCCCAAATC
TCCTCGGGCCGATAGTGCGTGTGATGTTCTGTGGACACGCTTGACCTCTCAAATTCACCTGATTTAGG
+
FF:FFFFFFFFFF:FFFF:FFF:FFFF:FFFFFFF:FFFFFFFFFFFFFF:FFFFF:FFFFFFF:FFF
FFFFFFFFFFFF:FFFFFFFFFFFFFFFFFFFFFFFFFFFFFFFFFFFFFFFFFFFFFFFFFFFFFFFF
```

```
@A00155:342:HHGFNDSXY:1:2240:22851:6120 2:N:0:GAACCTAG+TCCGCATA
ATCTCTTCTTTACGTTAAATTTCTCCGCTACTTCCCAAATCTCCTCGGGCCGATAGTGCGTGTGATGT
TCTGTGGACACGCTTGACCTCTCAAATTCACCTGATTTAGGTGGGTCTACGTTGTGTGCTCTGGT
+
FFFFFFFFFFFFFFFFFFFFFFFFFFFFFFFF:FFFFFFFFFFFFFFFFFFFFFFFFFFFFFFFFFFF
FFFFFFFFFFFFFFFFFFFFFFFFF:FFFFFFFFFFFFFFFFF:FFFFFFFFFFFFFFFFFFFFFFF
@A00155:342:HHGFNDSXY:1:1627:13367:22983 2:N:0:GAACCTAG+TCCGCATA
CTTTACGTTAAATTTCTCCGCTACTTCCCAAATCTCCTCGGGCCGATAGTGCGTGTGATGTTCTGTGG
ACACGCTTGACCTCTCAAATTCACCTGATTTAGGTGGGTCTACGTTGTGTGCTCTGGTTATTCTTTCC
+
FFFFFFFFFFFFFFF:FFFFFFFFFFFFFFF,FFFFFFFFFFFFFFFFFFFFFFFFFFFFFFFFFFFF
FFFFFFFFFFFFFFFFF:FFFFFFFFFF:FFFFFFFFFFFFFFFFFFFFFFFFFFFFFFFFFFFFFFF
@A00155:342:HHGFNDSXY:1:1149:27091:33473 1:N:0:GAACCTAG+TCCGCATA
AATTTCTCCGCTACTTCCCAAATCTCCTCGGGCCGATAGTGCGTGTGATGTTCTGTGGACACGCTTGA
CCTCTCAAATTCACCTGATTTAGGTGGGTCTACGTTGTGTGCTCTGGTTATTCTTTCCCTCTCCACTC
+
FFFFFFFFFFFFFFFFFFFFFFFFFFFFFFFFFFFFFFFFFFFFFFFFFFFFFFFFFFFFFFFFFFFF
FFFFFFFFFFFFFFFFFFFFFFFFFFFFFFFFFFFFFFFFFFFFFFFFFFFFFFFFFFFFFFFFFFFF
@A00155:342:HHGFNDSXY:1:2240:22851:6120 1:N:0:GAACCTAG+TCCGCATA
TTTCTCCGCTACTTCCCAAATCTCCTCGGGCCGATAGTGCGTGTGATGTTCTGTGGACACGCTTGACC
TCTCAAATTCACCTGATTTAGGTGGGTCTACGTTGTGTGCTCTGGTTATTCTTTCCCTCTC
+
FFFFFFFFFFFFFFFFFFFFFFFFFFFFFFFFFFFFFFFFFFFFFFFFFFFFFFFFFFFFFFFFFFFF
FFFFFFFFFFFFFFFFFFFFFFFFFFFFFFFF:FFFFFFFFFFFFFFFFFFFFFFFFF:FFFF
@A00155:342:HHGFNDSXY:1:1627:13367:22983 1:N:0:GAACCTAG+TCCGCATA
CTCCGCTACTTCCCAAATCTCCTCGGGCCGATAGTGCGTGTGATGTTCTGTGGACACGCTTGACCTCT
CAAATTCACCTGATTTAGGTGGGTCTACGTTGTGTGCTCTGGTTATTCTTTCCCTCTCCACTCCGAAC
+
FFFFFFFFFFFFFFFFFFFFFFFFFFFFFFFFFFFFFFFFFFFFFFFFFFFFFFFFFFFFFFFFFFFF
FFFFFFFFFFFFF,FFFFFFFFFFFFFFFFFFFFFFFFF:FFFFFFFFFFFFFFFFFFFFFFFFFFFF
@A00155:342:HHGFNDSXY:1:2134:29234:28040 1:N:0:GAACCTAG+TCCGCATA
AATCTCCTCGGGCCGATAGTGCGTGTGATGTTCTGTGGACACGCTTGACCTCTCAAATTCACCTGATT
TAGGTGGGTCTACGTTGTGTGCTCTGGTTATTCTTTCCCTCTCCACTCCGAACCTCCTCCTCAGGTAC
+
FFFFFFFFFFFFFFFFFFFFFFFFFFFFFFFFFFFFFFFFFFFFFFFFFFFFFFFFFFFFFFFFFFFF
FFFFFFFFFFFFFFFFFFFFFFFFFFFFFFFFFFFFFFFFFFFFFFFFFFFFFFFFFFFF,FFFFFFF
@A00155:342:HHGFNDSXY:1:1169:2826:15092 1:N:0:GAACCTAG+TCCGCATA
AATCTCCTCGGGCCGATAGTGCGTGTGATGTTCTGTGGACACGCTTGACCTCTCAAATTCACCTGATT
TAGGTGGGTCTACGTTGTGTGCTCTGGTTATTCTTTCCCTCTCCACTCCGAACCTCCTCCTCAGGTAC
+
:FFFFFFFFFFFFFFFFFFFFFFFFFFFFFFFFFFFF,FFFFFFFFFFFFFFFFFFFFFFFFFFFFFF
FFFFFFFFFFFFFFFFFFFFFFFFFFFFFFFFFFFFFFFFFFFFFFFFFFF:FFFFFFF,,FFFFF,F
@A00155:342:HHGFNDSXY:1:1565:19904:7623 1:N:0:GAACCTAG+TCCGCATA
AATCTCCTCGGGCCGATAGTGCGTGTGATGTTCTGTGGACACGCTTGACCTCTCAAATTCACCTGATT
TAGGTGGGTCTACGTTGTGTGCTCTGGTTATTCTTTCCCTCTCCACTCCGAACCTCCTCCTCAGGTAC
+
FFFFFFFFFFFFFFFFFFFFFFFFFFFFFFFFFFFFFFFFFFFFFFFFFFFFFFFF:FFFFFFFFFF:
FFFFFFFFFFFFFFFFFFFFFFFFFFFFFFFFFFFFFFFFFFFFFFFFFFFFFF:FFFFFFFFF:FFF:F
@A00155:342:HHGFNDSXY:1:1543:13801:9079 1:N:0:GAACCTAG+TCCGCATA
AATCTCCTCGGGCCGATAGTGCGTGTGATGTTCTGTGGACACGCTTGACCTCTCAAATTCACCTGATT
TAGGTGGGTCTACGTTGTGTGCTCTGGTTATTCTTTCCCTCTCCACTCCGAACCTCCTCCTCAGGTAC
+
FFFFFFFFFFFFFFFFFFFFFFFFFFFFFFFFFFFFFFFFFFFFFFFFFFFFFFFFFFFFFFFFFFFF
FFFFFFFFFFFFFFFFFFFFFFFFFFFFF:FFFFFFFFFFFFFFFFFFFFFFFFFFFFFFFFFFFFFF
```

@A00155:342:HHGFNDSXY:1:2223:17852:4131 1:N:0:GAACCTAG+TCCGCATA
AATCTCCTCGGGCCGATAGTGCGTGTGATGTTCTGTGGACACGCTTGACCTCTCAAATTCACCTGATT
TAGGTGGGTCTACGTTGTGTGCTCTGGTTATTCTTTCCCTCTCCACTCCGAACCTCCTCCTCAGGTAC
+
FFFFFFFFFFFF:FFFFFFFFFFFFFFFFFFFFFFFFFFFFFFFF:FFFFFFFFFFFF:FFFFFFFFF
FF:FFFFFFFFFFFFFFFFFFFFFFFFFFFFFFFFFFFFFFFFFFFFFFFF,:FFFFFFFFFFFFFFF
@A00155:342:HHGFNDSXY:1:1604:16694:32847 1:N:0:GAACCTAG+TCCGCATA
AATCTCCTCGGGCCGATAGTGCGTGTGATGTTCTGTGGACACGCTTGACCTCTCAAATTCACCTGATT
TAGGTGGGTCTACGTTGTGTGCTCTGGTTATTCTTTCCCTCTCCACTCCGAACCTCCTCCTCAGGTAC
+
FFFFFF:FFFFFFFFFF,FFFFFFFFFFFFFFFFFFFFFFFFFFFFFFFFFFFFFFFFFFFFFFFF:F
FFFFFFFFFFFFF:FFFFFFFFFFFFFFFFFFFFFFFFFFFFFFFFFFFFFFFFFFFFFFFFFFFF:F
@A00155:342:HHGFNDSXY:1:1131:14931:24032 2:N:0:GAACCTAG+TCCGCATA
GCTCCTCGGGCCGATAGTGCGTGTGATGTTCTGTGGACACGCTTGACCTCTCAAATTCACCTGATTTA
GGTGGGTCTACGTTGTGTGCTCTGGTTATTCTTTCCCTCTCCACTCCGAACCTCCTCCTCAGGTACC
+
FFFFFFFFFFFFFF,FFFFFFFFFFFFFFFFFFFFFFFFFFFFFFFFFFFFFFFFFFFFFFFFFFFFF
FFFFFFFFFFFFFFFFFFFFFFFFFFFFFFFFFFFFFFFFFF,FFFFFFFFFFFFFFFFFFFFFFFF
@A00155:342:HHGFNDSXY:1:1330:15718:24643 1:N:0:GAACCTAG+TCCGCATA
TCTCCTCGGGCCGATAGTGCGTGTGATGTTCTGTGGACACGCTTGACCTCTCAAATTCACCTGATTTA
GGTGGGTCTACGTTGTGTGCTCTGGTTATTCTTTCCCTCTCCACTCCGAACCTCCTCCTCAGGTACCT
+
FFFFFFFFFFFFFFFFFFFFFFFFFFFFFFFFFFFFFFFFFFFFFFFFFFFFFFFFFFFFFFFFFFFF
FFFFFFFFFFFFFFFFFFFFFFFFFFFFFFFFFFFFFFFFFFFFFFFFFFFFFFFFFFFFFFFFFFFF
@A00155:342:HHGFNDSXY:1:2610:14995:7232 1:N:0:GAACCTAG+TCCGCATA
TCTCCTCGGGCCGATAGTGCGTGTGATGTTCTGTGGACACGCTTGACCTCTCAAATTCACCTGATTTA
GGTGGGTCTACGTTGTGTGCTCTGGTTATTCTTTCCCTCTCCACTCCGAACCTCCTCCTCAGGTACCT
+
FFFFFFFFFFFFFFFFFFFFFFFFFFFFFFFFFFFFFFFFFFFFFFFFFFFFFFFFFFFFFFFFFFFF
FFFFFFFFFFFFFFFFFFFFFFFFFFFFFFFFFFFFFFFFFFFFFFFFFFFFFFFFFFFF:FFFFFFF
@A00155:342:HHGFNDSXY:1:1149:27091:33473 2:N:0:GAACCTAG+TCCGCATA
GGCCGATAGTGCGTGTGATGTTCTGTGGACACGCTTGACCTCTCAAATTCACCTGATTTAGGTGGGTC
TACGTTGTGTGCTCTGGTTATTCTTTCCCTCTCCACTCCGAACCTCCTCCTCAGGTACCTAGCTCCAT
+
FFFFFF:FFFFFFFFFFFFFFF:FFFFFFFFFFFFFFFFFFFFFFFFFFFFFFFFFFFFFFFFFFFFF
FFFFFFFFFFFFFFFFFFFFFFFFFFFFFFFFFFFFFFFFFFFFFFFFFFFFFFFFFFFFFFFFFFFF
@A00155:342:HHGFNDSXY:1:1330:15718:24643 2:N:0:GAACCTAG+TCCGCATA
CCGATAGTGCGTGTGATGTTCTGTGGACACGCTTGACCTCTCAAATTCACCTGATTTAGGTGGGTCTA
CGTTGTGTGCTCTGGTTATTCTTTCCCTCTCCACTCCGAACCTCCTCCTCAGGTACCTAGCTCCATTA
+
FFFF:FFFFFFFFFFFFFFFFFFFFFFFFFFFFFFFFFFFFFFFFFFFFFFFFFFFF:FFF:FFFFFF
FFFFFFFFFFFFFFFFFFFFFFFFFFFFFFFFFFFFFFFF:FFFFFFFFFFFFFFFFFFFFFFFFFFF
@A00155:342:HHGFNDSXY:1:1575:28040:22592 2:N:0:GAACCTAG+TCCGCATA
CGATAGTGCGTGTGATGTTCTGTGGACACGCTTGACCTCTCAAATTCACCTGATTTAGGTGGGTCTAC
GTTGTGTGCTCTGGTTATTCTTTCCCTCTCCACTCCGAACCTCCTCCTCAGGTACCTAGCTCCATTAA
+
FFFFFFFFFFFFFFFFFFFFFFFFFFFFFFFFFFFFFFFFFFFFFFFFFFFFFFFFFFFFFFFFFFFF
FFFFFFFFFFFFFFFFFFFFF:FFFFFFFFFFFFFFFFFFFFFFFFFFFFFFFFFFFFFFFFFFFFFF
@A00155:342:HHGFNDSXY:1:1649:17309:28150 2:N:0:GAACCTAG+TCCGCATA
CGATAGTGCGTGTGATGTTCTGTGGACACGCTTGACCTCTCAAATTCACCTGATTTAGGTGGGTCTAC
GTTGTGTGCTCTGGTTATTCTTTCCCTCTCCACTCCGAACCTCCTCCTCAGGTACCTAGCTCCATTAA
+
FFF:FFFFFFFFFFFFFFFFFFFFFFFFFFFFFFFFFFFFFFFFFFFFFFFFFFFFFFFFFFFFFFFF
FFFFFF:FFFFFFFFFFFFFFFFFFFFFFFFFFFFFFFFFFFFFFFFFFFFFFFFFFFFFFFFFFFFF

```
@A00155:342:HHGFNDSXY:1:1649:17309:28150 1:N:0:GAACCTAG+TCCGCATA
ATAGTGCGTGTGATGTTCTGTGGACACGCTTGACCTCTCAAATTCACCTGATTTAGGTGGGTCTACGT
TGTGTGCTCTGGTTATTCTTTCCCTCTCCACTCCGAACCTCCTCCTCAGGTACCTAGCTCCATTAAAA
+
FFFFFFFFFFFFFFFFFFFFFFFFFFFFFFFFFFFFFFFFFFFFFFFFFFFFFFFFFFFFFFFFFFFF
FFFFFFFFFFFFFFFFFFFFFFFFFFFFFFFFFFFFFFFFFFFFFFFFFFFFFFFFFF:FFFFF:FF:
@A00155:342:HHGFNDSXY:1:1575:28040:22592 1:N:0:GAACCTAG+TCCGCATA
ATAGTGCGTGTGATGTTCTGTGGACACGCTTGACCTCTCAAATTCACCTGATTTAGGTGGGTCTACGT
TGTGTGCTCTGGTTATTCTTTCCCTCTCCACTCCGAACCTCCTCCTCAGGTACCTAGCTCCATTAAAA
+
FFFFFFFFFFFFFFFFFFFFFFFFFFFFFFFFFFFFFFFFFFFFFFFFFFFF:FFFFFFFFFFFFFFF
FFFFFFFFFFFFFFFFFFFFFFFFFFFFFFFFFFFFFFFFFFFFFFFFFFFFFFFFFFFFFF:FFFFF
@A00155:342:HHGFNDSXY:1:1131:14931:24032 1:N:0:GAACCTAG+TCCGCATA
AGTGCGTGTGATGTTCTGTGGACACGCTTGACCTCTCAAATTCACCTGATTTAGGTGGGTCTACGTTG
TGTGCTCTGGTTATTCTTTCCCTCTCCACTCCGAACCTCCTCCTCAGGTACCTAGCTCCATTAAAAT
+
FFFFFFFFFFFFFFFFFFFFFFFFFFFFFFFFF:FFFFFFFFFFFFFFFFFFFFFFFFFFFFFFFFFF
FFFFFFFFFFFFFFFF:FFFFFFFFFFFFFFFFF:FFFFFFFFFFFFFFFFFFFFFFFFFFF:FFFF
@A00155:342:HHGFNDSXY:1:2135:9227:23891 1:N:0:GAACCTAG+TCCGCATA
GTGCGTGTGATGTTCTGTGGACACGCTTGACCTCTCAAATTCACCTGATTTAGGTGGGTCTACGTTGT
GTGCTCTGGTTATTCTTTCCCTCTCCACTCCGAACCTCCTCCTCAGGTACCTAGCTCCATTAAAATCT
+
FFFFFFFFFFFFFFFFFFFFFFFFFFFFFFFFFFFFFFFFFFFFFFFFFFFFFFFFFFFFFFFFFFFF
FFFFFFFFFFFFFFFFFFFFFFFFFFFFFFFFFFFFFFFFFFFF:FFFFFFFFFFFFFFFFFFFFFFF
@A00155:342:HHGFNDSXY:1:1450:12111:7059 1:N:0:GAACCTAG+TCCGCATA
CGTGTGATGTTCTGTGGACACGCTTGACCTCTCAAATTCACCTGATTTAGGTGGGTCTACGTTGTGTG
CTCTGGTTATTCTTTCCCTCTCCACACCGAACCTCCTCCTCAGGTACCTAGCTCCATTAAAATCTGAA
+
FFFFFFFFF,FFFFFFFFFFFFFF:FFFF:FFFFFFFFFFFF,:FFF:FFFFFFFFFFFF:FF:FFFF
FFF:F:F:FFF:FFF::F:FFFFFF:FF,FFFF,FF,FF,FF,,FF:F:F::,FFF:,FFFF:,F:FF
@A00155:342:HHGFNDSXY:1:1628:11053:35790 1:N:0:GAACCTAG+TCCGCATA
CGTGTGATGTTCTGTGGACACGCTTGACCTCTCAAATTCACCTGATTTAGGTGGGTCTACGTTGTGTG
CTCTGGTTATTCTTTCCCTCTCCACTCCGAACCTCCTCCTCAGGTACCTAGCTCCATTAAAATCTGAA
+
FFFFFFFFFFFFFFFFFFFFFFFFFFFFFFFFFFFFFFFFFFFFFFFFFFFFFFFFFFFFFFFFFFFF
FFF:FFFFFFFFFFFFFFFFFFFFFFFFFFF:FFFF:FFFFFFFFFFFFFFFFFFFFFFFFFFFFFFF
@A00155:342:HHGFNDSXY:1:1643:3360:27571 1:N:0:GAACCTAG+TCCGCATA
TGTGATGTTCTGTGGACACGCTTGACCTCTCAAATTCACCTGATTTAGGTGGGTCTACGTTGTGTGCT
CTGGTTATTCTTTCCCTCTCCACTCCGAACCTCCTCCTCAGGTACCTAGCTCCATTAAAATCTGAAAA
+
FF:FFF,FFFFFF:FFFFFFF:FFF:FFF:FFF:F:FFFFFFFFFFF,::FFFFFFFFFFF:F,FFFF
FFF,:FFFFFFF::FFFFFF,FFFFFFF::FF:F::::FFF:FFFFF:F::FFFF,:::FFFFF::FF
@A00155:342:HHGFNDSXY:1:1433:9245:16344 1:N:0:GAACCTAG+TCCGCATA
GTGATGTTCTGTGGACACGCTTGACCTCTCAAATTCACCTGATTTAGGTGGGTCTACGTTGTGTGCTC
TGGTTATTCTTTCCCTCTCCACTCCGAACCTCCTCCTCAGGTACCTAGCTCCATTAAAATCTGAAAAA
+
FFFFFFFFFFFFFFFFFFFFFFFFFFFFFFFFFFFFFFFFFFFFFFFFFFFFFFFFFFFFFFFFFFFF
FFFFFFFFFFFFFFFFFFFFFFFFFFFFFFFFFFFFFFFFFFFFFFFFFFFFFFFFFFFFFFFFFFFF
@A00155:342:HHGFNDSXY:1:1305:4182:13401 1:N:0:GAACCTAG+TCCGCATA
TGATGTTCTGTGGACACGCTTGACCTCTCAAATTCACCTGATTTAGGTGGGTCTACGTTGTGTGCTCT
GGTTATTCTTTTCCTCTCCACTCCGAACCTCCTCCTCAGGTACCTAGCTCCATTAAAATCTGAAAAA
+
FFFFFFFFFFFFFFFFFF:FFFFFFFFF:FFFFFFFF,FFFFFFFF:FFFFF:FFFFFFFFFFFFF::
FFFFFFFFFF:FFFFFFFFFFFFFFFFF,FFFFF,FFF::FFFFFFFFFFFFFFFFFFFFFFFFFFF
```

```
@A00155:342:HHGFNDSXY:1:2135:9227:23891 2:N:0:GAACCTAG+TCCGCATA
TTCTGTGGACACGCTTGACCTCTCAAATTCACCTGATTTAGGTGGGTCTACGTTGTGTGCTCTGGTTA
TTCTTTCCCTCTCCACTCCGAACCTCCTCCTCAGGTACCTAGCTCCATTAAAATCTGAAAGATCGTAT
+
FFFFFFFFFFFFFFFFFFF:FF:FFFFFFFFFFFFFFFFFFFFFFFFFFFFFFFFFFFFFFFFFFFFF
FFFF:FFFFFFFFFFFFFFFFFFFFFFF:F:FFFFF,FFF:FFFFFFFFFFFFFFFFFFFFFFFFFFFF
@A00155:342:HHGFNDSXY:1:1433:9245:16344 2:N:0:GAACCTAG+TCCGCATA
ACACGCTTGACCTCTCAAATTCACCTGATTTAGGTGGGTCTACGTTGTGTGCTCTGGTTATTCTTTCC
CTCTCCACTCCGAACCTCCTCCTCAGGTACCTAGCTCCATTAAAATCTGAAAAATCGTATTGATCAAC
+
FFFFFFFFFFFFFFFFFFFFFFFFFFFFFFFFFFFFFFFF::FFFFFFFFFFFFFFFFFFFFF:FFF
FFFFFFFFFFFFFFFFFFFFFFFFFFFFFFFFFFFFFFFFF:FFFFFFFFFFFFFFFFFFFFFFFFFF
@A00155:342:HHGFNDSXY:1:1305:4182:13401 2:N:0:GAACCTAG+TCCGCATA
ACGCTTGACCTCTCAAATTCACCTGATTTAGGTGGGTCTACGTTGTGTGCTCTGGTTATTCTTTTCCT
CTCCACTCCGAACCTCCTCCTCAGGTACCTAGCTCCATTAAAATCTGAAAAATCGTATTGATCAACC
+
FFFFFFFFFFFFFFFFFFFFFFFFFFFFFFFFFFFFFFFFFFFFFFFFFFFFFFFFFFFFFFFFFFFF
FFFFFFFFFFFFFFFFFFFFFFFFFFFFFFFFFFFFFFFFFFFFFFFFFFFFF:FFFFFFFF:FF:F
@A00155:342:HHGFNDSXY:1:1304:2980:28197 2:N:0:GAACCTAG+TCCGCATA
ACGCTTGACCTCTCAAATTCACCTGATTTAGGTGGGTCTACGTTGTGTGCTCTGGTTATTCTTTTCCT
CTCCACTCCGAACCTCCTCCTCAGGTACCTAGCTCCATTAAAATCTGAAAAATCGTATTGATCAACC
+
FFFFFFFFFFFFFFFFFFFFFFFFFFFFFFFFFFF,FFFFFFFFFFFF:FFFFFFFFFFFFFFFFFFFF
FFF:FFF:F:FF:FFFFFFFFFFFFFFFFFFFFFFFFFFFFF:FFFFFFFFFFFFFFFFFFF:FF:FF:
@A00155:342:HHGFNDSXY:1:1249:20157:16172 1:N:0:GAACCTAG+TCCGCATA
TTGACCTCTCAAATTCACCTGATTTAGGTGGGTCTACGTTGTGTGCTCTGGTTATTCTTTCCCTCTCC
ACTCCGAACCTCCTCCTCAGGTACCTAGCTCCATTAAAATCTGAAAAATCGTATTGATCAACCAGGCT
+
FFFFFFFFFFFFFFFFFFFFFFFFFFFFFFFFFFFFFFFFFFFFFFFFFFFFFFFFFFFFFFFFFFFF
FFFFFFF,,FFFFFFFFFFFFFFFFFFFFFFFFFFFFFFFFFFFFFFFFFFFFFFFFFFF:FFFFFF
@A00155:342:HHGFNDSXY:1:2474:19000:33552 2:N:0:GAACCTAG+TCCGCATA
TTGACCTCTCAAATTCACCTGATTTAGGTGGGTCTACGTTGTGTGCTCTGGTTATTCCTTCCCTCTCC
ACTCCGAACCTCCTCCTCAGGTACCTAGCTCCATTAAAATCTGAAAAATCGTATTGATCAACCAGGCT
+
:FFFFFFFFFF,:,FFFFFFFFFFFFFFFFFFFFFFFFFFFFFFFFFFFFFFFFFFFFFFFFFFFFF
FFFFFFFFFFFFF:FFFFFFFFFFFF:FFFFFFFFFFFFFFFFFF,FFFFFFF,FFFFFFFFF:FFFF
@A00155:342:HHGFNDSXY:1:2610:14995:7232 2:N:0:GAACCTAG+TCCGCATA
CTCTCAAATTCACCTGATTTAGGTGGGTCTACGTTGTGTGCTCTGGTTATTCTTTCCCTCTCCACTCC
GAACCTCCTCCTCAGGTACCTAGCTCCATTAAAATCTGAAAAATCGTATTGATCAACCAGGCTGTACA
+
FFFFFFFF:FFFFFFFFFFFFFFFFFFFFFFFFF:FFFFFFF:FFFFFFFFFFF,FFFFFFFFFFFFF
FFFFFFFFFFFFFFFF:FFFFFFFFFFFFFFFFFFFFFFFFFFFFFFF:FFFFFFFFFFFFFFFFFFFF
@A00155:342:HHGFNDSXY:1:2561:17083:29763 1:N:0:GAACCTAG+TCCGCATA
CTGATTTAGGTGGGTCTACGTTGTGTGCTCTGGTTATTCTTTCCCTCTCCACTCCGAACCTCCTCCTC
AGGTACCTAGCTCCATTAAAATCTGAAAAATCGTATTGATCAACCAGGCTGTACATTGCAGAC
+
FFFFFFFFFFFFFFFFFFFFFFFFFFFFFFFFFFFFFFFFFFFFFFFFFFFFFFFFFF,,FFFFFFFF
FFFFFFFFFFFFFFFFFFFFFFFFFFFFFFFFFFFF:FFFFFFFFFFFFFFFFFFFFFFFFFF
@A00155:342:HHGFNDSXY:1:2662:23014:33708 1:N:0:GAACCTAG+TCCGCATA
CTGATTTAGGTGGGTCTACGTTGTGTGCTCTGGTTATTCTTTCCCTCTCCACTCCGAACCTCCTCCTC
AGGTACCTAGCTCCATTAAAATCTGAAAAATCGTATTGATCAACCAGGCTGTACATTGCAGAC
+
FFFFFFFFFFFFFFFFFFFFFFFFFFFFFFFFFFFFFFFFFFFFFFFFFFFFFFFFFF::FFFFFFFF
FFFFFFFFFFFFFFFFFFFFFFFFFFFFFFFFFFFF:FFFFFFFFFFF:FFFFFFFFFFFFF
```

```
@A00155:342:HHGFNDSXY:1:1364:19018:1830 1:N:0:GAACCTAG+TCCGCATA
TTTAGGTGGGTCTACGTTGTGTGCTCTGGTTATTCTTTCCCTCTCCACTCCGAACCTCCTCCTCAGGT
ACCTAGCTCCATTAAAATCTGAAAAATCGTATTGATCAACCAGGCTGTACATTGCAGACATCGCCCTC
+
FFFFFFF:FFFFFFFFFFFFFFFFFFFFFFFFFF:FFFFFFFFFFFFFFFFFFFFFFFFFFFFFFFFF
FFFFFFFFFFFF:FFFFFFFFFFFFFFFFFFFFFFFFFFFFFFFFFFFFFFFFFFFFFFFF:F:FFFF
@A00155:342:HHGFNDSXY:1:1169:2826:15092 2:N:0:GAACCTAG+TCCGCATA
GGTGGGTCTACGTTGTGTGCTCTGGTTATTCTTTCCCTCTCCACTCCGAACCTCCTCCTCAGGTACCT
AGCTCCATTAAAATCTGAAAAATCGTATTGATCAACCAGGCTGTACATTGCAGACATCGCCCTCCTGA
+
FFFF:FFFFFFFFFFFFFFFFFFFFFFFFFFF,FFFFFFFFFFFFFFFFFFFFFFFFFFFFFFFFFFF
FFFFFFFFFFFFFFFFFFFFFFFFFFFFFFFF:FFFFFFFFF:FF,FFFFFFFFFFFFFFFFFFFFFF
@A00155:342:HHGFNDSXY:1:2223:17852:4131 2:N:0:GAACCTAG+TCCGCATA
GTGGGTCTACGTTGTGTGCTCTGGTTATTCTTTCCCTCTCCACTCCGAACCTCCTCCTCAGGTACCTA
GCTCCATTAAAATCTGAAAAATCGTATTGATCAACCAGGCTGTACATTGCAGACATCGCCCTCCTGA
+
FFF:FFFF,FFFFFFFFFFFFFFFFFFFFFFFFFFFFFFFFFFFFFFFFFFFFFFFF:FFFFFFFFF
FFFFFFFFFFFFFFFFFF:FFFFFFFFFFFFFFFF:FFFFFFFFFFFFFFFFFFFFFFFFFFFFFF
@A00155:342:HHGFNDSXY:1:2134:29234:28040 2:N:0:GAACCTAG+TCCGCATA
GCTCTGGTTATTCTTTCCCTCTCCACTCCGAACCTCCTCCTCAGGTACCTAGCTCCATTAAAATCTGA
AAAATCGTATTGATCAACCAGGCTGTACATTGCAGACATCGCCCTCCTGACTGACGCTGGCAGCGTCC
+
FFFFFFFFFFFFFFFFFFFF:FFFFFFFFFFFFFFFFFFFFFFFFFFFFFF,FFFFFFF,FFFFFFFFF
:F:FFFFF,FFFFFFFFFFFFFFFFFFFFFFFFFFFFFFFFFFFFFFFFFFFFFFFFFFFFFFFFFFF
@A00155:342:HHGFNDSXY:1:1565:19904:7623 2:N:0:GAACCTAG+TCCGCATA
GCTCTGGTTATTCTTTCCCTCTCCACTCCGAACCTCCTCCTCAGGTACCTAGCTCCATTAAAATCTGA
AAAATCGTATTGATCAACCAGGCTGTACATTGCAGACATCGCCCTCCTGACTGACGCTGGCAGCGTCC
+
F:FFFFFFFFFFF:FFFF:FFFFFFFFFFFFFFFFFFFFFFFFFFF,FFFFFF:FFFFFFFFFFFFFF
FFFFFFFFFFFFFFFFFFFFFFFFFFF,FFFFFFFFFFFFFFFFFFFF:FFFFFFFFFFFFFFFFFFF
@A00155:342:HHGFNDSXY:1:1628:11053:35790 2:N:0:GAACCTAG+TCCGCATA
GGTTATTCTTTCCCTCTCCACTCCGAACCTCCTCCTCAGGTACCTAGCTCCATTAAAATCTGAAAAAT
CGTATTGATCAACCAGGCTGTACATTGCAGACATCGCCCTCCTGACTGACGCTGGCAGCGTCCTCGCG
+
FFFFFFFFFFFFFFFFFFFFFFFFFFFFFFFFFFFFFFFFFFFFFFFFFFFFFFFFFFFFFFFFFFFF
FF:F:FFFFFFFFFFFFFFFFFFF,FFFFFFFF:FFFFFFFFFFFFFFFFFFFFFFFFFFFFFFFFFF
@A00155:342:HHGFNDSXY:1:1450:12111:7059 2:N:0:GAACCTAG+TCCGCATA
GTTATTCTTTCCCTCTCCACTCCGAACCTCCTCCTCAGGTACCTAGCTCCATTAAAATCTGAAAAATC
GTATTGATCAACCAGGCTGTACATTGCAGACATCGCCCTCCTGACTGACGCTGGCAGCGTCCTCGCG
+
FFFFFF:FFFFFFF:FFF:FF:FFFFFFFF:FFFFFFFFFF:FFFFFF::FFFFFFFFFFFFFFFFF:
FFFFFFFFFFFFFFFFFFFFFFFFFFFFFFFFFFFFFFFFFFFFFFFFFFFFFFFFFFFFFFFFFFF
@A00155:342:HHGFNDSXY:1:1209:30906:18443 1:N:0:GAACCTAG+TCCGCATA
CTCTCCACTCCGAACCTCCTCCTCAGGTACCTAGCTCCATTAAAATCTGAAAAATCGTATTGATCAAC
CAGGCTGTACATTGCAGACATCGCCCTCCTGACTGACGCTGGCAGCGTCCTCGCGTCGGTTCGCGTGT
+
FFFFFFFFFFFFFF:FFFFFFFFFFFFFFFFFFFFFFFFFFFFFFFFFFFFFFFFFFFF:FFFFFFFFF
FFFFFFFFFFFFFFFFFFFFFFFFFFFFFFFFFFFF:FFFFFFFFFFFFFFFFFFFFFFFFFFFFFFF
@A00155:342:HHGFNDSXY:1:1504:15691:7560 1:N:0:GAACCTAG+TCCGCATA
CTCTCCACTCCGAAGCTCCTCCTCAGGTACCTAGCTCCATTAAAATCTGAAAAATCGTATTGATCAAC
CAGGCTGTACATTGCAGACATCGCCCTCCTGACTGACGCTGGCAGCGTCCTCGCGTCGGTTCGCGTGT
+
FFFFFFFFFFFFF:,FFFFFFFFFFFF:FFFFFFFFFFFFFFFFFFFFFFFFFFFFFFFFFFFFFFFFF
FF:FFFFF:FFFFFFFFFFFFFFFFFFFFFFFFFFFFFFFFFFFFFFFFFFFFFFFFFFFFFFFFFFF
```

```
@A00155:342:HHGFNDSXY:1:1554:27697:19680 1:N:0:GAACCTAG+TCCGCATA
TCTCCACTCCGAACCTCCTCCTCAGGTACCTAGCTCCATTAAAATCTGAAAAATCGTATTGATCAACC
AGGCTGTACATTGCAGACATCGCCCTCCTGACTGACGCTGGCAGCGTCCTCGCGTCGGTTCGCGTGTT
+
FFFFFFFFFFFF:FFFFFFFFFFFFFFFFFFFFFFFFFFFFFFFF,FFFFFFFFFFFFFFFFFFFFFF
FFFFFFFFFFFFFFFFFFFFFFFFFFFFFFFFFFFFFFFFFFFFFFFFFFFFFFFFFFFFFFFFFFFF
@A00155:342:HHGFNDSXY:1:2372:27887:12211 1:N:0:GAACCTAG+TCCGCATA
TCCACTCCGAACCTCCTCCTCAGGTACCTAGCTCCATTAAAATCTGAAAAATCGTATTGATCAACCAG
GCTGTACATTGCAGACATCGCCCTCCTGACTGACGCTGGCAGCGTCCTCGCGTCGGTTCGCGTGTTAA
+
FFF:FFFF,FF:FFFFFFFF:FFF:FFFFFFFFFFFFFFFFF:FFFFFFFFFFF:::,:F,FFFF:FF
FFFFF:FFFFFFF:FFF,FF,FF,FFF:FFFFFFFFFFFFF:FF,FF,FFF:FFFF,FF:FFFF,F,F
@A00155:342:HHGFNDSXY:1:1209:30906:18443 2:N:0:GAACCTAG+TCCGCATA
TCCGAACCTCCTCCTCAGGTACCTAGCTCCATTAAAATCTGAAAAATCGTATTGATCAACCAGGCTGT
ACATTGCAGACATCGCCCTCCTGACTGACGCTGGCAGCGTCCTCGCGTCGGTTCGCGTGTTAACGTAG
+
FFFFFFFFFFFFFFFFFFFFFFFFFFFFFFFFFFFFFFFFFFFFFFFFFFFFFFFFFFFFFFFFFFFF
FFFFFFFFFFFFFFFFFFFFFFFFFFFFFFFFFFFFFFFFFFFFFFFFFFFFFFFFFFFFFFFFFFFF
@A00155:342:HHGFNDSXY:1:1554:27697:19680 2:N:0:GAACCTAG+TCCGCATA
AACCTCCTCCTCAGGTACCTAGCTCCATTAAAATCTGAAAAATCGTATTGATCAACCAGGCTGTACAT
TGCAGACATCGCCCTCCTGACTGACGCTGGCAGCGTCCTCGCGTCGGTTCGCGTGTTAACGTAGTACA
+
FFFFF:FFFFFFFFFFFFFFFFFFFFFFFFFFFFFFFFFFFFFFFF:FFFFFFFFFFFFFFFF,FFFF
FFFFFFFFFFFFFFFFFFFFFFFFFFFFFFFFFFFFFFFFFFFFFFFFFFFFFFFFFFFFFFFFFFFF
@A00155:342:HHGFNDSXY:1:2323:17345:10864 2:N:0:GAACCTAG+TCCGCATA
ACCTCCTCCTCAGGTACCTAGCTCCATTAAAATCTGAAAAATCGTATTGATCAACCAGGCTGTACATT
GCAGACATCGCCCTCCTGACTGACGCTGGCAGCGTCCTCGCGTCGGTTCGCGTGTTAACGTAGTACA
+
FFFFFFFFFFF:FF,FFFFFFFFFFFFFFFFF:FFFFFFFFFFFFFFFFFFFFFFFFFFFFFFFFFF:
FF:FFFFFFFFFFFFFFFFFFFFFFF:FFF:FFFFFFFFFFFFFFFFFFFFFFFFFFFFFFFFFFFF
@A00155:342:HHGFNDSXY:1:2323:17345:10864 1:N:0:GAACCTAG+TCCGCATA
CCTCCTCAGGTACCTAGCTCCATTAAAATCTGAAAAATCGTATTGATCAACCAGGCTGTACATTGCAG
ACATCGCCCTCCTGACTGACGCTGGCAGCGTCCTCGCGTCGGTTCGCGTGTTAACGTAGTACAACCAA
+
FFFFFFFFFFF:FFFFFFFFFFFFFFFFFFFFFFFFFFFFFFFFFFFFFFFFFFFFFFFFFFFFFFFF
FFFFFFFFFFFFFFFFFFFFFFFFFF,FFFFFFFFFFFFFFFFFFFFFFFFFFFFFFFFFFFFFFFFF
@A00155:342:HHGFNDSXY:1:2521:32949:10426 2:N:0:GAACCTAG+TCCGCATA
CTCAGGTACCTAGCTCCATTAAAATCTGAAAAATCGTATTGATCAACCAGGCTGTACATTGCAGACAT
CGCCCTCCTGACTGACGCTGGCAGCGTCCTCGCGTCGGTTCGCGTGTTAACGTAGTACA
+
:FFFFFFFFFFFFFFFFFFFFF,FFFF,FFF:FFF:FFFFFFFFF:FFFFFFFFFFFFFFFF,F:FFF
F,:FFFF:FFFFFF:FF:F,FFF:FFF:FFF:F:FFFFFFFF:FFF:FFFFFFF,FF:,
@A00155:342:HHGFNDSXY:1:1526:31665:24267 2:N:0:GAACCTAG+TCCGCATA
TCCATTAAAATCTGAAAAATCGTATTGATCAACCAGGCTGTACATTGCAGACATCGCCCTCCTGACTG
ACGCTGGCAGCGTCCTCGCGTCGGTTCGCGTGTTAACGTAGTACAACCAAGCGTCCTCACACCTCACT
+
FFFFFFF:FFFFFFFFFFFFFFFFFFFFFFFFFFFFFFFFFFFF:FFFFFFFFFFFFFFFFFFFFFFF
FFFFFFFFFFFFFFFFFFFFFFFF:FFFFFFFFFFFFF:FFFFFFFFFFFFFFFFFFFFFFFFFF,FF
@A00155:342:HHGFNDSXY:1:2521:32949:10426 1:N:0:GAACCTAG+TCCGCATA
CCATTAAAATCTGAAAAATCGTATTGATCAACCAGGCTGTACATTGCAGACATCGCCCTCCTGACTGA
CGCTGGCAGCGTCCTCGCGTCGGTTCGCGTGTTAACGTAGTACAACCAAGCGTCCTCAC
+
FFFFF:FFFFFFFFFFFFFFFFFFFFFFFFFFFFFFFFFFFFFFF:FFFFFFFFFFFFFFFFFFFFFF
FFFFFFFFFFF:FFFFFFFFFFFFFFFFFFFFFFFFFFFFFFF:FFFFFF,F:FF:FFF
```

@A00155:342:HHGFNDSXY:1:2422:2248:12931 2:N:0:GAACCTAG+TCCGCATA
CATTAAAATCTGAAAAATCGTATTGATCAACCAGGCTGTACATTGCAGACATCGCCCTCCTGACTGAC
GCTGGCAGCGTCCTCGCGTCGGTTCGCGTGTTAACGTAGTACAACCAAGCGTCCTCACACCTCACTAT
+
FF:FFFFFFFF,FFFFFFFFFFFFFFFFFFFFFFFFFFFFFFFFFFFFFFFFFFFFFFFFF:FFFFFFFF
FFFFFFFFFFFFFFFFFFFFFFFFFFFFFFF:FF:FFFFFFFFFFFFFFFF,FFFFFFFFFFFFFFFFFF
@A00155:342:HHGFNDSXY:1:2249:17390:31767 1:N:0:GAACCTAG+TCCGCATA
TCTGAAAAATCGTATTGATCAACCAGGCTGTACATTGCAGACATCGCCCTCCTGACTGACGCTGGCAG
CGTCCTCGCGTCGGTTCGCGTGTTAACGTAGTACAACCAAGCGTCCTCACACCTCACTATTGTACAAT
+
FFFFFFFFFFFFFFFFFFFFFFFFFFFFFFFFFFFFFFFFFFFFFFFFFFFFFFFFFFFFFFFFFFFFFF
FFFFFFFFFFFFFFFFFFFFFFFFFFFFFFFFFFFFFFFFFFFFFFFFFFFFFFFFFFFFF,FFFFFFFF
@A00155:342:HHGFNDSXY:1:2662:23014:33708 2:N:0:GAACCTAG+TCCGCATA
CTGAAAAATCGTATTGATCAACCAGGCTGTACATTGCAGACATCGCCCTCCTGACTGACGCTGGCAGC
GTCCTCGCGTCGGTTCGCGTGTTAACGTAGTACAACCAAGCGTCCTCACACCTCACTATTGTACAATA
+
FFFFFFFFFFFFFFFFFFFFFFFFFFFFFFFFFF:FFFFFFFFFF:FFFFFFFFFFFFFFFFFFFFFFFF
FFFFFFFFFFFFFFFFFFFFFFFFFFFFFFFFFFFFFFFFFFFFFFFFFFFFFFFFFFFFFFFFFFFFFF
@A00155:342:HHGFNDSXY:1:2561:17083:29763 2:N:0:GAACCTAG+TCCGCATA
CTGAAAAATCGTATTGATCAACCAGGCTGTACATTGCAGACATCGCCCTCCTGACTGACGCTGGCAGC
GTCCTCGCGTCGGTTCGCGTGTTAACGTAGTACAACCAAGCGTCCTCACACCTCACTATTGTACAATA
+
FFFFFFFFFFFF:FFFFFFFFFFFFFFFFF:FFFFFFFFFFFFFFFFFFFFFFFFFFFFFFFFFFFFFFF
FFFFFFFFFFFFFFFFFFFFFFFFFFFFFFFFFFFFFFFFFFFFFFFFFFFFFFFFFFFFFFFFFFFFFF
@A00155:342:HHGFNDSXY:1:2103:30147:21981 1:N:0:GAACCTAG+TCCGCATA
GAAAAATCGTATTGATCAACCAGGCTGTACATTGCAGACATCGCCCTCCTGACTGACGCTGGCAGCGT
CCTCGCGTCGGTTCGCGTGTTAACGTAGTACAACCAAGCGTCCTCACACCTCACTATTGTACAATATA
+
FFFFFFFFFFFFFFFFFFFFFFFFFFFFFFFFFFFFFFFFFFFFFFFFFFFFFFFFFFFFFFFFFFFFFF
FFFFFFFFFFFFFFFFFFFFFFFFFFFFFFFFFFFFFFFFF:FFFFFFFFFFFFFFF:FFFFFFFFFF,F
@A00155:342:HHGFNDSXY:1:2108:27344:34350 1:N:0:GAACCTAG+TCCGCATA
GAAAAATCGTATTGATCAACCAGGCTGTACATTGCAGACATCGCCCTCCTGACTGACGCTGGCAGCGT
CCTCGCGTCGGTTCGCGTGTTAACGTAGTACAACCAAGCGTCCTCACACCTCACTATTGTACAATATA
+
FFFFFFFFFFFFFFFFFFFFFFFFFFFFFFFFFFFFFFFFFFFFFFFFFFFFFFFFFFFFFFFFFFFFFF
FFFFFFFFFFFFFFFFFFFFFFFFFFFFFFFFFFFFFFFFFFFFFFFFFFFFFFFF,FFFFFFFFFFFFF
@A00155:342:HHGFNDSXY:1:1526:31665:24267 1:N:0:GAACCTAG+TCCGCATA
AAAAATCGTATTGATCAACCAGGCTGTACATTGCAGACATCGCCCTCCTGACTGACGCTGGCAGCGTC
CTCGCGTCGGTTCGCGTGTTAACGTAGTACAACCAAGCGTCCTCACACCTCACTATTGTACAATATAC
+
FFFFFFFFFFFFFFFFFFFFFFFFFFFFFFFFFFFFFFFFFFFFFFFFFFFFFFFFFFFFFFFFFFFFFF
FFFFFF:FFFFFFFFFFFFFFFFFFFFFFFFFFFFFFFFFFFFFFFFFFFFFFFFFFFFF,FFFFFF:F
@A00155:342:HHGFNDSXY:1:2422:2248:12931 1:N:0:GAACCTAG+TCCGCATA
AAAAATCGTATTGATCAACCAGGCTGTACATTGCAGACATCGCCCTCCTGACTGACGCTGGCAGCGTC
CTCGCGTCGGTTCGCGTGTTAACGTAGTACAACCAAGCGTCCTCACACCTCACTATTGTACAATATAC
+
FFFFFFFFFFFFFFFFFFFFFFFFFFFFFFFFFFFFFFFFFFFFFFFFFFFFFFFFFFFFFFFFFFFFFF
FFFFFFFFFFFFFFFFFFFFFFFFFFFFFFFFFFFFFFFFFFFFFFFFFFFFFFFFFFFFFF:FFFFFFF
@A00155:342:HHGFNDSXY:1:1371:3260:34914 1:N:0:GAACCTAG+TCCGCATA
AAAAATCGTATTGATCAACCAGGCTGTACATTGCAGACATCGCCCTCCTGACTGACGCTGGCAGCGTC
CTCGCGTCGGTTCGCGTGTTAACGTAGTACAACCAAGCGTCCTCACACCTCACTATTGTACAATATAC
+
FFFFFFFFFFFFFFFFFFFFFFFFFFFFFFFFFFFF:FFFFFFFFFFFFFFFFFFFF:FFFFFFFFFFFF
FFFFFFFFFFFFFFFFFFFFFFFFFFFFFF:FFFFFFFFFFFFFFFFFFFFFFFFFFFFFFFFFFF,FFF

```
@A00155:342:HHGFNDSXY:1:1543:13801:9079 2:N:0:GAACCTAG+TCCGCATA
AATCGTATTGATCAACCAGGCTGTACATTGCAGACATCGCCCTCCTGACTGACGCTGGCAGCGTCCTC
GCGTCGGTTCGCGTGTTAACGTAGTACAACCAAGCGTCCTCACACCTCACTATTGTACAATATACCAC
+
FFF:FFFFFFFFFFFFFFFFFFFFFF:FFFFFFFFFFFFFFFFFFFFFFFFFFFFFFFFFFFFFFFFF
F:FFFFFFFFFFFFFFFFFFFFFFFF:FFFFFFFF:FFFFFFFFFFFFFFFF:FFFFFFFFFFFFFFF
@A00155:342:HHGFNDSXY:1:1604:16694:32847 2:N:0:GAACCTAG+TCCGCATA
GATCAACCAGGCTGTACATTGCAGACATCGCCCTCCTGACTGACGCTGGCAGCGTCCTCGCGTCGGTT
CGCGTGTTAACGTAGTACAACCAAGCGTCCTCACACCTCACTATTGTACAATATACCACGTCTTTTCC
+
FFFFFFFFFFFFFFFFFFFFFFFFFFF:F,F:FFFFFFFFFF:FFF:FFFFFFFFFFF,FFFF:FFFF
FFFFFFFFFFFFFFFFFFFFFFFFF:FFFFFFFFFFFFFFFFFFFFFFFFFFFFFFFFFFFFFFFFFF
@A00155:342:HHGFNDSXY:1:2307:13431:11757 1:N:0:GAACCTAG+TCCGCATA
CAGGCTGTACATTGCAGACATCGCCCTCCTGACTGACGCTGGCAGCGTCCTCGCGTCGGTTCGCGTGT
TAACGTAGTACAACCAAGCGTCCTCACACCTCACTATTGTACAATATACCACGTCTTTTCCTATCCTA
+
FFFFFFFF:FFFFFFFFFFFFFFFFF:FFFFFFFFFFFFFFFFFFFFFFFFFFFFFFFFFFFFFFFFF
FFFFFFFFF:FFFFFFFFFFFFFFFFFFFFFFFFFFFFFFFFFFFFFFFFFFFFFFFFFFFFFFFFFF
@A00155:342:HHGFNDSXY:1:1107:11858:14231 1:N:0:GAACCTAG+TCCGCATA
GCTGTACATTGCAGACATCGCCCTCCTGACTGACGCTGGCAGCGTCCTCGCGTCGGTTCGCGTGTTAA
CGTAGTACAACCAAGCGTCCTCACACCTCACTATTGTACAATATACCACGTCTTTTCCTATCCTATGC
+
FFFFFFFFFFFFFFFFFFFFFFFFFFFFFFFFFFFFFFFFFFFFFFFFFFFFFFFFFFFFFFFFFFFF
FFFFFFFFFFFFFFFFFFFFFFFFFFFFFFFF:FFFFFFFFFFFFFFFFFFFFFFFFFFFFFFFFFFF
@A00155:342:HHGFNDSXY:1:1371:3260:34914 2:N:0:GAACCTAG+TCCGCATA
ACATCGCCCTCCTGACTGACGCTGGCAGCGTCCTCGCGTCGGTTCGCGTGTTAACGTAGTACAACCAA
GCGTCCTCACACCTCACTATTGTACAATATACCACGTCTTTTCCTATCCTATGCGCTCCAAAGCCTCC
+
FFFFFFFFFFFFFFFFFFFFFFFFFFFFFFFFFFFFFFFFFFFFFFFFFFFFFFFFFFFFFFFFFFFF
FFFFFFFFFFF,FFFF,FFFFFFFFFFFFFFFFF:FF:FFFFFFFFFFFFFFFFFFFFFFFFFFFFF:
@A00155:342:HHGFNDSXY:1:2249:17390:31767 2:N:0:GAACCTAG+TCCGCATA
CATCGCCCTCCTGACTGACGCTGGCAGCGTCCTCGCGTCGGTTCGCGTGTTAACGTAGTACAACCAAG
CGTCCTCACACCTCACTATTGTACAATATACCACGTCTTTTCCTATCCTATGCGCTCCAAAGCCTCCA
+
FFFFFFFFFFFFFFFFFFF:F:FFFFF:FFF:FFFFFFF:F:FF:FFFFFFFF:FFFFFFFFFFFFFF
FFFFFFFFFFFFFFFFFFFFFFFFFFFFFFFFFFFFFFFFFFFFFFFFFFFFFFFFFFFFFFFFFFFF
@A00155:342:HHGFNDSXY:1:1504:15691:7560 2:N:0:GAACCTAG+TCCGCATA
CGCCCTCCTGACTGACGCTGGCAGCGTCCTCGCGTCGGTTCGCGTGTTAACGTAGTACAACCAAGCGT
CCTCACACCTCACTATTGTACAATATACCACGTCTTTTCCTATCCTATGCGCTCCAAAGCCTCCAACA
+
FFFFFFFFFFFFFFFFFFFFFFFFFFFFFFFFFFFFFFFFFF:FFFFFFFFFFFFFFFFFFFFFFFFF
FFFFFFFFFFFFFFFFFFFFFFF,FFFFFFFFFFF:FFFFF:FFFFFFFFFFFFFFFFFFFFFFFFF:
@A00155:342:HHGFNDSXY:1:2372:27887:12211 2:N:0:GAACCTAG+TCCGCATA
CCCTCCTGACTGACGCTGGCAGCGTCCTCGCGTCGGTTCGCGTGTTAACGTAGTACAACCAAGCGTCC
TCACACCTCACTATTGTACAATATACCACGTCTTTTCCTATCCTATGCGCTCCAAAGCCTCCAACACC
+
F:FF:FFFFFFFFFFFFFFF:FFFFFFFFFFFFF:FFFFF,FFFFFFF:F:F:FFFFFFFFFFFFFF:
FF:FFFFF,FFFFFF,FFFFFFFFFFFF:FF:FFFFFF:FFFFFFFFFFFFFFF:FF:FFFFFFFFFF
@A00155:342:HHGFNDSXY:1:1249:20157:16172 2:N:0:GAACCTAG+TCCGCATA
CCCTCCTGACTGACGCTGGCAGCGTCCTCGCGTCGGTTCGCGTGTTAACGTAGTACAACCAAGCGTCC
TCACACCTCACTATTGTACAATATACCACGTCTTTTCCTATCCTATGCGCTCCAAAGCCTCCAACACC
+
FFFFFFFFFFFFFFFFFFFFFFFFFFFFFFFFFFFFFFFFFFFFFFF:FFFFFFFFFFFFFFFFFFFF
FFFFF:FFFFF:FFFFFFFFFFF:FFF,FFFFFFFFFFFFFFFFF:FFFFFFFFFFFFFFFFFFF:FF
```

```
@A00155:342:HHGFNDSXY:1:2108:27344:34350 2:N:0:GAACCTAG+TCCGCATA
CCCTCCTGACTGACGCTGGCAGCGTCCTCGCGTCGGTTCGCGTGTTAACGTAGTACAACCAAGCGTCC
TCACACCTCACTATTGTACAATATACCACGTCTTTTCCTATCCTATGCGCTCCAAAGCCTCCAACACC
+
FFFFFFFFFFFFFFFFFFFFFFFFF:FFFFFFFFFFFFFFFFFFFFFFFFFFFFFFFFFFFFFFFFFF
FFFFFFFFFFFFFFFFFFFFFFFFFFFFFFFFF:FFFFFFFFFFFFFFFFFFFFFFFFFFFFFFFFFF
@A00155:342:HHGFNDSXY:1:2103:30147:21981 2:N:0:GAACCTAG+TCCGCATA
CCCTCCTGACTGACGCTGGCAGCGTCCTCGCGTCGGTTCGCGTGTTAACGTAGTACAACCAAGCGTCC
TCACACCTCACTATTGTACAATATACCACGTCTTTTCCTATCCTATGCGCTCCAAAGCCTCCAACACC
+
FFFFFFFFFFFFFFFFFFFFFFFFFFFFF:FFFFFFFFFFFFFFFFFFFF:FFFFFFFFFFFFFFFFF
FFFFFFFFFFFFFFFFFFFFFFFFFFFFFFFFFFF:FFFFFFFFFFFFFFFFFFFFFFFFFFFFFFFF
@A00155:342:HHGFNDSXY:1:2168:11324:10864 1:N:0:GAACCTAG+TCCGCATA
GGCAGCGTCCTCGCGTCGGTTCGCGTGTTAACGTAGTACAACCAAGCGTCCTCACACCTCACTATTGT
ACAATATACCACGTCTTTTCCTATCCTATGCGCTCCAAAGCCTCCAACACCATCTCTGTTTTCGAGGC
+
FFFFFFFFFFFFFFFFFFFFFFFFFFFFFFFFFFFFFFFFFFFFFFFFFFFFFFFFFFFFFFF,FFFF
FFFFFFFFFFFFFFFFFFFFFFFFFFFFFFFFFFFFFFFFFFFFFFFFFFFFFFF:FFFFFFFFFFFF
@A00155:342:HHGFNDSXY:1:2168:11324:10864 2:N:0:GAACCTAG+TCCGCATA
GGCAGCGTCCTCGCGTCGGTTCGCGTGTTAACGTAGTACAACCAAGCGTCCTCACACCTCACTATTGT
ACAATATACCACGTCTTTTCCTATCCTATGCGCTCCAAAGCCTCCAACACCATCTCTGTTTTCGAGGC
+
FFFFFFFFFFFFFFFFFFFFFF,FFFF:FFF:FFFFFFFFFFFFFFFFFFF:FFFFFFFFFFF::FFF
FFFFFFFFFF:FFFFFFFFFFFFFFFFFFFFFFFFFFFFFFFFFFFFFFFFF:FFFFFFFFFFFFFFF
@A00155:342:HHGFNDSXY:1:1463:9986:25864 2:N:0:GAACCTAG+TCCGCATA
GGCAGCGTCCTCGCGTCGGTTCGCGTGTTAACGTAGTACAACCAAGCGTCCTCACACCTCACTATTGT
ACAATATACCACGTCTTTTCCTATCCTATGCGCTCCAAAGCCTCCAACACCATCTCTGTTTTCGAGGC
+
FFFFFFFFFFFF:FFFFFFFFFFFFFFFFFFFFFFFFFFFFFFFFFFFFFFFFFFFFFFFFFFFFFF
FFFFFFFF:FFFFFFFFFFFFFFFFFFF:FFFFFFF:FFFFFFFFFFFF,FFFFFFFFFFFFFFFFFF
@A00155:342:HHGFNDSXY:1:1463:9986:25864 1:N:0:GAACCTAG+TCCGCATA
GGCAGCGTCCTCGCGTCGGTTCGCGTGTTAACGTAGTACAACCAAGCGTCCTCACACCTCACTATTGT
ACAATATACCACGTCTTTTCCTATCCTATGCGCTCCAAAGCCTCCAACACCATCTCTGTTTTCGAGGC
+
FFFFFFFFFFFFFFFFFFFFFFFFFFFFFFFFFFFFFFFFFFFFFFFFFFFFFFFFFFFFFFF,:FFF
FFFFFFFFFFFFFFFFFFFFFFFFFFFFFFFFFFFFFFFFFFFFFFFFFFFFFFFFFFFFFFFFFFFF
@A00155:342:HHGFNDSXY:1:1509:32452:18803 2:N:0:GAACCTAG+TCCGCATA
CGTCCTCGCGTCGGTTCGCGTGTTAACGTAGTACAACCAAGCGTCCTCACACCTCACTATTGTACAAT
ATACCACGTCTTTTCCTATCCTATGCGCTCCAAAGCCTCCAACACCATCTCTGTTTTCGAGGCTGA
+
FFFFFFFFFFFFFF:FFF:FFFFFFFFFFFFFFFFFFF,:FFFFFFFFFFFFFFFFFFFFFFFFFFFF
FFFFFFFFFFFFFFFF::FFF,:FFFFFFFFFF,FFFFFFFFFFFFFFFFFFFFFFFFFFFFFFFF
@A00155:342:HHGFNDSXY:1:1122:29071:30358 1:N:0:GAACCTAG+TCCGCATA
GCGTCGGTTCGCGTGTTAACGTAGTACAACCAAGCGTCCTCACACCTCACTATTGTACAATATACCAC
GTCTTTTCCTATCCTATGCGCTCCAAAGCCTCCAACACCATCTCTGTTTTCGAGGCTGACATGGACTA
+
FFFFFFFFFFFFFFFFFFFFFFFFFFFFFFFFF:FFFFFFFFFFFFFFFFF::FFFFFFFF:FFFFFF
FFFFFFFFFF,FFFFFFFFFFFFFFFFFFFFFFFFFFFFFFFFF:FFFFFF:FFFFFFFFFFFFFFFF
@A00155:342:HHGFNDSXY:1:1122:29071:30358 2:N:0:GAACCTAG+TCCGCATA
GCGTCGGTTCGCGTGTTAACGTAGTACAACCAAGCGTCCTCACACCTCACTATTGTACAATATACCAC
GTCTTTTCCTATCCTATGCGCTCCAAAGCCTCCAACACCATCTCTGTTTTCGAGGCTGACATGGACTA
+
FFFFF:FFF,FFFFFFFFFFFFFFFFFFFFFFFFFFFFFFFFFFFFFFFF:FF:FFFFFF:FFFFFFF
FF:FFFFFFFFFFFFFFFFFFFFFFFFFFFFFFFFFFFF,FFFFFFFFFFFFFFFF:FFFFFF:FFFF
```

```
@A00155:342:HHGFNDSXY:1:1633:22480:21543 1:N:0:GAACCTAG+TCCGCATA
GCGTCGGTTCGCGTGTTAACGTAGTACAACCAAGCGTCCTCACACCTCACTATTGTACAATATACCAC
GTCTTTTCCTATCCTATGCGCTCCAAAGCCTCCAACACCATCTCTGTTTTCGAGGCTGACATGGACTA
+
FFFFFFFFFFFFFFFFFFF:FFFFFFFFFFFFFFFFFFFFFFF:FFFFFFFFF:FFFF:F:FFFFFFFFF
FFFFFFFF:FFFFFFF:FFFFFFFFFFFFFFFFFFFFFF:F:,F:FFFFFFFFF:FFFFF:FFFFFFFFF
@A00155:342:HHGFNDSXY:1:1633:22480:21543 2:N:0:GAACCTAG+TCCGCATA
GCGTCGGTTCGCGTGTTAACGTAGTACAACCAAGCGTCCTCACACCTCACTATTGTACAATATACCAC
GTCTTTTCCTATCCTATGCGCTCCAAAGCCTCCAACACCATCTCTGTTTTCGAGGCTGACATGGACTA
+
FFFFF:FFFFFFFFFFF:FFFFFFFFFFFFFFFFFFFFFFFFFFFFFFFFFFFFFFFFFFFFFF:FFFFF
FFFFFFFFFFFFFFFFFFFFFFFFFFFFFFFFFFFFFFFFFFFFFFFFFF:FFFFFFF:FFFFFFFFFFF
@A00155:342:HHGFNDSXY:1:1509:32452:18803 1:N:0:GAACCTAG+TCCGCATA
TCGCGTGTTAACGTAGTACAACCAAGCGTCCTCACACCTCACTATTGTACAATATACCACGTCTTTTC
CTATCCTATGCGCTCCAAAGCCTCCAACACCATCTCTGTTTTCGAGGCTGACATGGACTAACACAT
+
FFFFFFFFFFFFFFFFFFFFFFFFFFFFF,FFFFFFFFFFFFFF,:FF:FFFFFFFFFFFFFFFFFFFFF
FFFFFFFFFFFFFFFFFFFFFFFFFFFFFFFFFFFFFFFFFFFFFFFF:FF,FFFFFFFFFFFF:FFF
@A00155:342:HHGFNDSXY:1:2528:21603:33739 1:N:0:GAACCTAG+TCCGCATA
CGCGTGTTAACGTAGTACAACCAAGCGTCCTCACACCTCACTATTGTACAATATACCACGTCTTTTCC
TATCCTATGCGCTCCAAAGCCTCCAACACCATCTCTGTTTTCGAGGCTGACATGGACTAACACATACA
+
FFFFFFFFFFFFFFFFFFFFFFFFFFFFFFFFFFFFFFFFFFFF:FFFFFFF:F:FFFFFFFFFFFFFFF
FFFFFFFFFFFFFFFFFFFFFFFFFFFFFFFFFFFFFFFFFFFFFFFFFFFFFFFFFFFFFFFFFFFFFF
@A00155:342:HHGFNDSXY:1:1224:5285:2628 2:N:0:GAACCTAG+TCCGCATA
GTACAACCAAGCGTCCTCACACCTCACTATTGTACAATATACCACGTCTTTTCCTATCCTATGCGCTC
CAAAGCCTCCAACACCATCTCTGTTTTCGAGGCTGACATGGACTAACACATACACCGCCTTATGTCTC
+
FFFFFFFFFFFFFFFFFFFFFFFFFFFFFF:FFFFFFF::FFFFFFFFFFFFFFFFFFFFFFFFFFFFFF
FFF:FFFFFFFFFF:FF,FFFFFFFFFFFFFFFFFFFF:FFFFFFFFFFFFFFFFFFFFFFFFFFFFFFF
@A00155:342:HHGFNDSXY:1:2528:21603:33739 2:N:0:GAACCTAG+TCCGCATA
GTACAACCAAGCGTCCTCACACCTCACTATTGTACAATATACCACGTCTTTTCCTATCCTATGCGCTC
CAAAGCCTCCAACACCATCTCTGTTTTCGAGGCTGACATGGACTAACACATACACCGCCTTATGTCTC
+
FFFFFFFFFFFFFFFFFFF:FFFFFFFFFFFFFFFFFFFFFFFFFFFFFFFFFFFFFFFFFFFFFFFFFF
FFF:FFFFFFFFFFFFFFFFFFFFFFFFFFFFFFFFFFF:FFFFFFFFFFF,FFFFFFFFFFFFFFFFFF
@A00155:342:HHGFNDSXY:1:2307:13431:11757 2:N:0:GAACCTAG+TCCGCATA
TACAACCAAGCGTCCTCACACCTCACTATTGTACAATATACCACGTCTTTTCCTATCCTATGCGCTCC
AAAGCCTCCAACACCATCTCTGTTTTCGAGGCTGACATGGACTAACACATACACCGCCTTATGTCTCG
+
:FFFFFFFFF:FFFFFFFFFFFFFFFFFFFFF,FFFFFFFFFFFFFFFFFFFFFFF,FFFFFFFFFFFFF
FF,FFFF:FFFFFFFFFFFFFFFF::FFFFFFFFFFFFFFFFFFFFF:FFFFFFFFFF:FF::FFFFFFF
@A00155:342:HHGFNDSXY:1:1224:5285:2628 1:N:0:GAACCTAG+TCCGCATA
ACAACCAAGCGTCCTCACACCTCACTATTGTACAATATACCACGTCTTTTCCTATCCTATGCGCTCCA
AAGCCTCCAACACCATCTCTGTTTTCGAGGCTGACATGGACTAACACATACACCGCCTTATGTCTCGG
+
FFFFFFFFFFFF:FFFFFFFFFFFFFFF::FFFFFF:F:FFFFFFFFFFFFFFFFFFFFFFFFFFFFFFF
FFFFFFFFFFFFFF:FFFFFFFFFFFFFFFFFFFFFFFFFFFFFFFFFFFFFFFFFFFFFF:FFFFFFFF
@A00155:342:HHGFNDSXY:1:2216:30346:9706 2:N:0:GAACCTAG+TCCGCATA
CACCTCACTATTGTACAATATACCACGTCTTTTCCTATCCTATGCGCTCCAAAGCCTCCAACACCATC
TCTGTTTTCGAGGCTGACATGGACTAACACATACACCGCCTTATGTCTCGGGACCACACTTATATTGC
+
FFFFFFFFFFFFFFFFFFFF,FFFFFFFFFF:FFFF:FFFFFFFFFFFFFFFF:FFFFFFFFFFFFFFFF
FFFFFFFFFFFFFFFFFFFFFFFFFFFFFFF,FFFFFFFFFFFFFFFFFF:FFFFFFFFFF:FFFFFFFF
```

```
@A00155:342:HHGFNDSXY:1:1107:11858:14231 2:N:0:GAACCTAG+TCCGCATA
CACCTCACTATTGTACAATATACCACGTCTTTTCCTATCCTATGCGCTCCAAAGCCTCCAACACCATC
TCTGTTTTCGAGGCTGACATGGACTAACACATACACCGCCTTATGTCTCGGGACCACACTTATATTGC
+
FFFFFFFFFFFFFFFFFF,FFFFFFFFF:F::FFFFFFFFF:FFFFF,FFFF:FFFFFFF:FFFF,FF
FFFFFFFFFFFFFFFFFFFFFFFFFFFFFF,FFFFFFFFFFFFFFFFFFFFFFFFFFFFFFFFFFFFF
@A00155:342:HHGFNDSXY:1:1541:14705:8265 2:N:0:GAACCTAG+TCCGCATA
CCTCACTATTGTACAATATACCACGTCTTTTCCTATCCTATGCGCTCCAAAGCCTCCAACACCATCTC
TGTTTTCGAGGCTGACATGGACTAACACATACACCGCCTTATGTCTCGGGACCACACTTATATT
+
FFFFFFFFFFFFFFFFFFFFFFFFFFFFFFFFFFFFFFFF,FFFFFFFFF:FFFFFFFFF:FFFFFFF
FFFFFFFFFFFFFFFF:FFFFFFFFFFFFFFF,FFFFFF:FFFFFFFFFFFFFFFFF,FFFF,F
@A00155:342:HHGFNDSXY:1:2611:8043:2080 1:N:0:GAACCTAG+TCCGCATA
TATTGTACAATATACCACGTCTTTTCCTATCCTATGCGCTCCAAAGCATCCAACACCATCTCTGTTTT
CGAGGCTGACATGGACTAACACATACACCGCCTTATGTCTCGGGACCACACTTATATTGCCATCCCGT
+
FFFFFFFFFFFFFFFF:F,FFFF:FFFFFFFFFFFF:FFF::FFFFF,FF:FFFFFFFF:FFF:FFFF
F,FFFFFFF,FF:FFFFFFFFFFFFFFFF,FFF:FFF:FFF,,FF:FFFF,,FFFFFF,F:FFF:F,:
@A00155:342:HHGFNDSXY:1:2611:7663:2644 1:N:0:GAACCTAG+TCCGCATA
TATTGTACAATATACCACGTCTTTTCCTATCCTATGCGCTCCAAAGCCTCCAACACCATCTCTGTTTT
CGAGGCTGACATGGACTAACACATACACCGCCTTATGTCTCGGGACCACACTTATATTGCCATCCCGT
+
FFFFFFFFFFFFFFFFFFFFFFFFFFFFFFFFFFFF:FF:FFFFFFF:FFFFFFFF:FFFFFF,FFFF
F,FF:FFFFFFFF:FFFFFFFFF:FFFFFFFFFFFFFFFFFFFFFFFFFFF,:FFFFFFFFFFFF,FFF
@A00155:342:HHGFNDSXY:1:1266:16821:8422 1:N:0:GAACCTAG+TCCGCATA
TATTGTACAATATACCACGTCTTTTCCTATCCTATGCGCTCCAAAGCCTCCAACACCATCTCTGTTTT
CGAGGCTGACATGGACTAACACATACACCGCCTTATGTCTCGGGACCACACTTATATTGCCATCCCTT
+
FFFFFFFFFFFFFFFFFFFFFFFFFFFFFFFFFFFFFFFFFFFFFFFFFFFFFFFFFFFFFFFFFFFF
FFFFFFFFFFF:FFFFFFFFFFFFFFFFFFFFFFFFFFFFFFFFFFFFFFFF,FFFF:FFFFFFFFF
@A00155:342:HHGFNDSXY:1:1636:10854:21073 1:N:0:GAACCTAG+TCCGCATA
TATTGTACAATATACCACGTCTTTTCCTATCCTATGCGCTCCAAAGCCTCCAACACCATCTCTGTTTT
CGAGGCTGACATGGACTAACACATACACCGCCTTATGTCTCGGGACCACACTTATATTGCCATCCCTT
+
FFFFFFFFFFFFFFFFFFFFFFFFFFFFFFFFFFFFFFFFFFFFFFFFFFF,FFFFFFFFFFFFFFFFF
FFFFFFFFFFFFFF:FFFFFFFFFFFFFFFFFFFFFFFFFFFFFFFFFFFFFFFF:FF:FFFFFF,FFF
@A00155:342:HHGFNDSXY:1:1541:14705:8265 1:N:0:GAACCTAG+TCCGCATA
TGTACAATATACCACGTCTTTTCCTATCCTATGCGCTCCAAAGCCTCCAACACCATCTCTGTTTTCGA
GGCTGACATGGACTAACACATACACCGCCTTATGTCTCGGGACCACACTTATATTGCCATCCCGTATC
+
FFF:FFFFFFFFFFFFFFFFFFFFFFFFFFFFFFFFFFFFFFFFFFFFFFFFFFFFFFFFFFFFFFFF
FFFFFFFFFFFFFFFFFFFFFFFFFFFFFFFFFFFFFFFFFFFFFFFFFFFFFFFFFFFFFFFFFFFF
@A00155:342:HHGFNDSXY:1:2216:30346:9706 1:N:0:GAACCTAG+TCCGCATA
TGTACAATATACCACGTCTTTTCCTATCCTATGCGCTCCAAAGCCTCCAACACCATCTCTGTTTTCGA
GGCTGACATGGACTAACACATACACCGCCTTATGTCTCGGGACCACACTTATATTGCCATCCCTTATC
+
FFFFFFFFFFFFFFFFFFFFFFFFFFFFFFFFFFFFFFFFFFFFFFFFFFFFFFFFFFFFFFFFFFFF
FFFFFFFFFFFFFFFFFFFFFFFFFFFFFFFFFFFFFFFFFFFFFFFFFFFFFFFFFFFFFFFFFFFF
@A00155:342:HHGFNDSXY:1:2160:31937:20134 1:N:0:GAACCTAG+TCCGCATA
ATATACCACGTCTTTTCCTATCCTATGCGCTCCAAAGCCTCCAACACCATCTCTGTTTTCGAGGCTGA
CATGGACTAACACATACACCGCCTTATGTCTCGGGACCACACTTATATTGCCATCCCGTATCGAACAC
+
FFFFFFFFFFFFFFFFFFFFFFFFFFFFFFFFFFFFFFFFFFFFFFFFFFFFFFFFFFFFFFFFFFFF
FFFFFFFFFFFFFFFFFFFFFFFFFFFFFFFFFFFFFFFFFFFFFF:FFFFFFFFFFFFFF:FFFFFFFFF
```

```
@A00155:342:HHGFNDSXY:1:1329:32353:27774 2:N:0:GAACCTAG+TCCGCATA
GTCTTTTCCTATCCTATGCGCTCCAAAGCCTCCAACACCATCTCTGTTTTCGAGGCTGACATGGACTA
ACACATACACCGCCTTATGTCTCGGGACCACACTTATATTGCCATCCCGTATCGAACACCACAC
+
FF:FFFFFFF:FFFFFFFF:FFFF,:FFFFFFFF,FFFFFFFFF:FFFFFFFFFFFFFFFFFFFFFFF
FFFFFFFFFFFFFFFFFFFF:FFFFFFFFFFFFFFFFFFFFFFFFFFFFFFFFFFFFFFFFF:F
@A00155:342:HHGFNDSXY:1:2160:31937:20134 2:N:0:GAACCTAG+TCCGCATA
TTTCCTATCCTATGCGCTCCAAAGCCTCCAACACCATCTCTGTTTTCGAGGCTGACATGGACTAACAC
ATACACCGCCTTATGTCTCGGGACCACACTTATATTGCCATCCCGTATCGAACACCACACTGGGATAT
+
F,FFFF::FFFFFFFFFFFFFF:FFFFFF:FF:FFFFFFF:FFFFFFFFFFFFFFFFFFFFFFFFFFF
F:FFFFFFFFF:F,FFFFFFFFFFFFFFFFFFFFFFFFFFFFFFFFFFFFFFFFFFFFFFFFFFFFFF
@A00155:342:HHGFNDSXY:1:1329:25084:16470 1:N:0:GAACCTAG+TCCGCATA
CCTATGCGCTCCAAAGCCTCCAACACCATCTCTGTTTTCGAGGCTGACATGGACTAACACATACACCG
CCTTATGTCTCGGGACCACACTTATATTGCCATCCCGTATCGAACACCACACTGGGATATTGTTTTTT
+
FFFFFFFFFFFFFFFFFFFFFFFFFFF:FFFFFFFFFFFFFFFFFFFF:FFFFFFFFFFFFFFFFFFF
FFFFFFFFFFFFFFFFFFFFFFF:FFFFFFFFFFFFF,FFFFFFFFFFFF:F:FFFF:FFFFFFFFFF
@A00155:342:HHGFNDSXY:1:1353:29451:36902 1:N:0:GAACCTAG+TCCGCATA
CCTATGCGCTCCAAAGCCTCCAACACCATCTCTGTTTTCGAGGCTGACATGGACTAACACATACACCG
CCTTATGTCTCGGGACCACACTTATATTGCCATCCCGTATCGAACACCACACTGGGATATTGTTTTTT
+
FFFFFF:FFFFFFFFFFFFFFFFFFFFFFFFFFFFFF:F:FFFFFFFF:FFFFFFFFFFFFFFFFFFF
FFFFFFFFFFFFFFFFFFFFF:FFFFFFFFFFFFFFFFFFF:FFFFFFFFFFFFFFF,FFFFFFFFFF
@A00155:342:HHGFNDSXY:1:1329:32353:27774 1:N:0:GAACCTAG+TCCGCATA
ATGCGCTCCAAAGCCTCCAACACCATCTCTGTTTTCGAGGCTGACATGGACTAACACATACACCGCCT
TATGTCTCGGGACCACACTTATATTGCCATCCCGTATCGAACACCACACTGGGATATTGTTTTT
+
FFFFFFFFFFFFFFFFFFFFFFFFFFFFFFFFFFFFFFFFFFFFFFFFFFFFFFFFFFFFFFFFFFFF
FFFFFFFFFFFFFFFFFFFFFFFFFFFFFFFF:FFFFFFFFFFFFFFFFFFFFFFFFFFFFFFF
@A00155:342:HHGFNDSXY:1:2341:4390:12665 1:N:0:GAACCTAG+TCCGCATA
ATGCGCTCCAAAGCCTCCAACACCATCTCTGTTTTCGAGGCTGACATGGACTAACACATACACCGCCT
TATGTCTCGGGACCACACTTATATTGCCATCCCGTATCGAACACCACACTGGGATATTGTTTTTTGTC
+
FFFFFFFFFFFFFFFFFFFFFFFFFFFFFFFFFFFFFFFFFFFFFFFFFFFFFFFFFFFFFFFFFFFF
FFFFFFFFFFFFFFFFFFF:FFFFF:FFFFFFFFFFFFF:,FFFFFF:FFFFFFFFFFFFFFFFFFFF
@A00155:342:HHGFNDSXY:1:1132:21305:22044 1:N:0:GAACCTAG+TCCGCATA
CTCCAAAGCCTCCAACACCATCTCTGTTTTCGAGGCTGACATGGACTAACACATACACCGCCTTATGT
CTCGGGACCACACTTATATTGCCATCCCGTATCGAACACCACACTGGGATATTGTTTTTTGTCAAAGC
+
FFFFFFFFFFFFFFFFFFFFFFFFFFFFFFFFFFFFFFFFFFFFFFFFFFFF:FFFFFFFFFFFFFFF
FFF:FFFFFF:F:FFFFFFFFFFFFFFFFFFFFF:FFFFFFFFFFFFFFFFFFFFFFFFFFFFFFFFF
@A00155:342:HHGFNDSXY:1:1221:5575:31501 1:N:0:GAACCTAG+TCCGCATA
CCAAAGCCTCCAACACCATCTCTGTTTTCGAGGCTGACATGGACTAACACATACACCGCCTTATGTCT
CGGGACCACACTTATATTGCCATCCCTTATCGAACACCACACTGGGATATTGTTTTTTGTCAAAGCTT
+
FFFFF,FFFFFFFFFFFFFFF,FFF:FFFFFFFFFFFF:FFFFFFFFFFFFFFFFFF:FFFFFFFFFF
F:FFFF,,FFFF:FFFFFFFFFFFFFFFFFFFFFFFFFFF::FFF:FFF,FFFFFFFFFFFFFFFFF:
@A00155:342:HHGFNDSXY:1:2311:1651:25113 2:N:0:GAACCTAG+TCCGCATA
CTCCAACACCATCTCTGTTTTCGAGGCTGACATGGACTAACACATACACCGCCTTATGTCTCGGGACC
ACACTTATATTGCCATCCCGTATCGAACACCACACTGGGATATTGTTTTTTGTCAAAGCTTCCTT
+
FFFFFFFFFFFFFFFFFFF:FFFFFFFFFFFFFFFFFFFFFFFFFFFFFFFFFFFFFFFFFFFFFFFF
FFFFFFFFF,FFFFFFFFFF:FFFFFFFFFFFFFFFFFFFFFFFFFFFFFFFFFFFFFF:FFFFF
```

```
@A00155:342:HHGFNDSXY:1:1636:10854:21073 2:N:0:GAACCTAG+TCCGCATA
CCATCTCTGTTTTCGAGGCTGACATGGACTAACACATACACCGCCTTATGTCTCGGGACCACACTTAT
ATTGCCATCCCTTATCGAACACCACACTGGGATATTGTTTTTGTCAAAGCTTCCTTAACTACTCGAC
+
FFFFFFF:FFFFFFFFFFFFFFF:FFFFF:FFFFFFFFFFFFFFFFFFFFFFFFFFFFFFFFFFFFFF
FFFFFFFFFFFFFFFFFFFFFFFFFFFFFFFFFFFFFFFF:FFFFFFFFFFFFFFFFFFFFFFFFFFF
@A00155:342:HHGFNDSXY:1:2526:11143:12367 1:N:0:GAACCTAG+TCCGCATA
TCTGTTTTCGAGGCTGACATGGACTAACACATACACCGCCTTATGTCTCGGGACCACACTTATATTGC
CATCCCGTATCGAACACCACACTGGGATATTGTTTTTGTCAAAGCTTCCTTAACTACTCGACCTCTC
+
FFFFFFFFFFFFFFFFFFFFFFFFFFFFFFFFFFFFFFFFFFFFFFFFFFFFFFFFFFFF:FFFFFFF
FFFFFFFFFFFFFFFFFFFFFFFFFFFFFFFFFFFFFFFFFFFFFFFFFFFFFFFFFFFFFFFFFFFF
@A00155:342:HHGFNDSXY:1:2311:1651:25113 1:N:0:GAACCTAG+TCCGCATA
TGTTTTCGAGGCTGACATGGACTAACACATACACCGCCTTATGTCTCGGGACCACACTTATATTGCCA
TCCCGTATCGAACACCACACTGGGATATTGTTTTTGTCAAAGCTTCCTTAACTACTCGACCTCT
+
FFFFFFFFFFFFFFFFFFFFFFFFFFFFFFFFFFFFFFFF:FFFFFFFFFFFFFFFFFF,FFFFFFFF:
FFFFFFF:FFFFFFFFFF:F,FFFF:FFFFFFFFFFFFFFFFFFFFFFFFF,FFFFFFFFFFFFFF
@A00155:342:HHGFNDSXY:1:2563:23005:2785 2:N:0:GAACCTAG+TCCGCATA
TGTTTTCGAGGCTGACATGGACTAACACATACACCGCCTTATGTCTCGGGACCACACTTATATTGCCA
TCCCGTATCGAACACCACACTGGGATATTGTTTTTGTCAAAGCTTCCTTAACTACTCGACCTCTCCC
+
FFFFFF:FFF:FFFFFF::FFFFFFFFFFFFFFFFFF,FFFF:FFFF:FFFFFFFF:FFFFFFFFFF
FFFFFFFF:FFFFFFFFFFFFFFFFFFFFFFF,FFFFFFFFF:FFFFFFF:FFFFFFFFFFFFFFFFF
@A00155:342:HHGFNDSXY:1:1132:20790:25817 2:N:0:GAACCTAG+TCCGCATA
TGTTTTCGAGGCTGACATGGACTAACACATACACCGCCTTATGTCTCGGGACCACACTTATATTGCCA
TCCCGTATCGAACACCACACTGGGATATTGTTTTTGTCAAAGCTTCATTAACTACTCGACCTCTCCC
+
FF:FFFFFF:FF:F::F:,,F,FF:F,:,F,,F:FFFFFF:FFFFFFF:FF,,F,FFFF:FF:FF,:F
F:F:FF::,FFF::F:F:,FF::FF:::F,F,FFFFFF:FFF:,FFF,FFFFFFFFFFFFF,F:FFF:
@A00155:342:HHGFNDSXY:1:1132:21305:22044 2:N:0:GAACCTAG+TCCGCATA
TGTTTTCGAGGCTGACATGGACTAACACATACACCGCCTTATGTCTCGGGACCACACTTATATTGCCA
TCCCGTATCGAACACCACACTGGGATATTGTTTTTGTCAAAGCTTCCTTAACTACTCGACCTCTCCC
+
FFFFFFFFFFFFFFFFFFFFFFFFFFFF:FFF:FFFFFFFF:FF:FFFFFFFFFFFFFFFF::FFFFF
FFFFFF:F:FFFFFFFFFFFFFFFFFFFFFFF:FFFFF:FFFFFF:FFFFFFFFFFFFFFFFFFFF:FF
@A00155:342:HHGFNDSXY:1:2341:4390:12665 2:N:0:GAACCTAG+TCCGCATA
TGTTTTCGAGGCTGACATGGACTAACACATACACCGCCTTATGTCTCGGGACCACACTTATATTGCCA
TCCCGTATCGAACACCACACTGGGATATTGTTTTTGTCAAAGCTTCCTTAACTACTCGACCTCTCCC
+
FFFFFFFFFFFFFFFFFFFFFFFFFFFFFFFFFFFFFF:FFFFFFFFFFFFFFFFFFFFFFFF:FFF,F
FFFFFFFFFFFFFFFF,FFFFFFFFFFFFFFFFFFFFFFFFFFFFFFFFFFFFFFFFFFFFFFFFFFF
@A00155:342:HHGFNDSXY:1:1320:31837:9032 2:N:0:GAACCTAG+TCCGCATA
TCGAGGCTGACATGGACTAACACATACACCGCCTTATGTCTCGGGACCACACTTATATTGCCATCCCG
TATCGAACACCACACTGGGATATTGTTTTTGTCAAAGCTTCCTTAACTACTCGACCTCTCCCTGCCG
+
FFFFFFF:FFFFFFFFFFFFFFFFFFFFFF,FF:FFFFFFF:FF:F,FFFFFF::FF,FFF:FFFFFF:
F,FF:FFFFFFFFFF::FFFF::FFF,F,FFFFFFFFFFFFFFF,FFFFF,:FFFFFF:FFFFFF:F:
@A00155:342:HHGFNDSXY:1:2611:8043:2080 2:N:0:GAACCTAG+TCCGCATA
CATGGACTAACACATACACCGCCTTATGTCTCGGGACCACACTTATATTGCCATCCCGTATCGAACAC
CACACTGGGATATTGTTTTTGTCAAAGCTTCCTTAACTACTCGACCTCTCCCTGCCGTCCTCCCTCC
+
:FFFFFFFFFFFFFFFFFFFFFFFFFFFFFFFFFFFF,FFFFFFFFFFFFFFFF,FFFFFFFF::FFFF
FFFFFFFF,FF:FFFFFFFFFFFFFFFFFFFFFFFFFFFFF:FFFF,FF:FFFFFFFFFFFFFFFFFFF
```

@A00155:342:HHGFNDSXY:1:2611:7663:2644 2:N:0:GAACCTAG+TCCGCATA
CATGGACTAACACATACACCGCCTTATGTCTCGGGACCACACTTATATTGCCATCCCGTATCGAACAA
CACACTGGGATATTGTTTTTTGTCAAAGCTTCCTTAACTACTCGACCTCTCCCTGCCGTCCTCCCTCC
+
:FFFFFFFFFFFFFFF:F:FFFFFFFFFFFFFFFFF:FFFFFFFFFFFF:FFF,FFFFFFF,F:FFF,
F::FFFF:FFFFFF:FFF:FFFFFFF::FFFFFFFFFFFF,FFF:FFFFFF,FFFFFFFF:FFFFFFF
@A00155:342:HHGFNDSXY:1:1221:5575:31501 2:N:0:GAACCTAG+TCCGCATA
ACACATACACCGCCTTATGTCTCGGGACCACACTTATATTGCCATCCCTTATCGAACACCACACTGGG
ATATTGTTTTTTGTCAAAGCTTCCTTAACTACTCGACCTCTCCCTGCCGTCCTCCCTCCACCATTCAG
+
:FFFFFFFFFFFFF,F:FFFFF:FFFFFFFFFFF,FFFFFFFFF,FFFFFFFFFFFFFFFFF:FFFFF
FFFFFFFFF:F,FFFFFFFFFFFF,FFFFFF:,:FFFFFFFFFFFFFFFFFFFFFFFFFFFFFFFFFF
@A00155:342:HHGFNDSXY:1:1329:25084:16470 2:N:0:GAACCTAG+TCCGCATA
ACCACACTTATATTGCCATCCCGTATCGAACACCACACTGGGATATTGTTTTTTGTCAAAGCTTCCTT
AACTACTCGACCTCTCCCTGCCGTCCTCCCTCCACCATTCAGGCTGGCCGGGTCACTATTCAGTAGTG
+
FFFFFFFFFFF:FFFFFFFFFFFFFFFFFFFFFF:FFFFFFFFFFFFFF:FFFFFFFFFFFF:F:FFF
FFFFFFFFFFFFFFFFFFFFFFFFFF:FFFFFF:FF:FFFFFFFFFFFFFFFFFFFFFFFFFFFFFFF
@A00155:342:HHGFNDSXY:1:1353:29451:36902 2:N:0:GAACCTAG+TCCGCATA
ACCACACTTATATTGCCATCCCGTATCGAACACCACACTGGGATATTGTTTTTTGTCAAAGCTTCCTT
AACTACTCGACCTCTCCCTGCCGTCCTCCCTCCACCATTCAGGCTGGCCGGGTCACTATTCAGTAGTG
+
FFFFFFFFFFFFFFFFFFFFFFFFFFFFFF:FFFFFFFFFFFFFFFFFFFF:FFFFFFFFFFFFFFFF
FFFFFFFFFFFFFFF:FFFFFFFFFFFFFFFFFFFFF,FFFFFFFFFFFFFFFFFFFFFFFFFFFFFF
@A00155:342:HHGFNDSXY:1:2371:8721:11929 1:N:0:GAACCTAG+TCCGCATA
ACACTGGGATATTGTTTTTTGTCAAAGCTTCCTTAACTACTCGACCTCTCCCTGCCGTCCTCCCTCCA
CCATTCAGGCTGGCCGGGTCACTATTCAGTAGTGTTATCTCTTCAGCGCTGTACTCCCCCAACGCCTG
+
FFFF:FFFF:FFFFFFFFFFFFFFFFFFFFFFFFFFFFFFFFFFFFFFFFFF:FFFFFFFFFFFFFFF
FFFFFFFFFFFFFFFFFFFFFFFFFFFFFFFFFFFFFFFFFFFFFFF:FFFFFFFFFFFFFFFFFFFF
@A00155:342:HHGFNDSXY:1:1617:29866:29982 2:N:0:GAACCTAG+TCCGCATA
ACACTGGGATATTGTTTTTTGTCAAAGCTTCCTTAACTACTCGACCTCTCCCTGCCGTCCTCCCTCCA
CCATTCAGGCTGGCCGGGTCACTATTCAGTAGTGTTATCTCTTCAGCGCTGTACTCCCCCAACGCCTG
+
FFFF:FFFFFFFFFFFFFFFFFFFFFFFFFFFFFFFFFFFFFFFFFFFFFFFFFFFFFFFFFFFFFFF
FFFFFFFFFFFFFFFFFFFFFFFFFFFFFFFFFFFFFFFFFFFFFFFFFFFFFFFFFFFFFFFFFFFF
@A00155:342:HHGFNDSXY:1:1617:29866:29982 1:N:0:GAACCTAG+TCCGCATA
GTTTTTTGTCAAAGCTTCCTTAACTACTCGACCTCTCCCTGCCGTCCTCCCTCCACCATTCAGGCTGG
CCGGGTCACTATTCAGTAGTGTTATCTCTTCAGCGCTGTACTCCCCCAACGCCTGATCCCACCATGCC
+
FFFFFFFFFFFFFFFFFFFFFFFFFFFFFFFFFFFFFFFFFFFFFFFFFFFFFFFFFFFFFFFFFFFF
FFFFFFFFFFFFFFFFFFFFFFFFFFFFFFFFFFFFFFFFFFFFFFFFFFFFFFFFFFFFFFFFFFFF
@A00155:342:HHGFNDSXY:1:1572:27299:1423 1:N:0:GAACCTAG+TCCGCATA
CGACCTCTCCCTGCCGTCCTCCCTCCACCATTCAGGCTGGCCGGGTCACTATTCAGTAGTGTTATCTC
TTCAGCGCTGTACTCCCCCAACGCCTGATCCCACCATGCCCCTACATGCCTTCCACAGGTGGGGCACC
+
FFFFFFFFFFFFF:FFFFFFFFFFFFFFFFFFFFFF:FFFFFFFFFFFFFFFFFFFFFFFFFF:FFFF
FFFFFFFFFFF:F:FFFFFFFF:FFFFFFFFFFFFFFFFFFFFFFFFFFFFFFFFFFFFFFFFFFFFF
@A00155:342:HHGFNDSXY:1:2233:4725:4820 1:N:0:GAACCTAG+TCCGCATA
TCTCCCTGCCGTCCTCCCTCCACCATTCAGGCTGGCCGGGTCACTATTCAGTAGTGTTATCTCTTCAG
CGCTGTACTCCCCCAACGCCTGATCCCACCATGCCCCTACATGCCTTCCACAGGTGGGGCACCCTGCT
+
FFFFFFFFFFFFFFFFFFFFFFFFFFFFFFFFFFFFFFFFFFFFFFFFFFFFFFFFFFFFFFFFFFFF
FFFFFFFFFFFFFFFFFFFFFFFFFFFFFFFFFFFFFFFFFFFFFFFFFFFFF,FFFFFFFFFFFFFF

```
@A00155:342:HHGFNDSXY:1:1517:13069:31548 2:N:0:GAACCTAG+TCCGCATA
CCTGCCGTCCTCCCTCCACCATTCAGGCTGGCCGGGTCACTATTCAGTAGTGTTATCTCTTCAGCGCT
GTACTCCCCCAACGCCTGATCCCACCATGCCCCTACATGCCTTCCACAGGTGGGGCACCC
+
FFFFFFFFFFFFFFFFFFFFFFFFFFFFFFFFF:FFFFFFFFFFFFFFFFFF:FFFFFFFFFFFFFFF
FFFFFFFFFFFFFFFFFFF,:FFFFFFFFFFFFFFFFFFFFFFFFFFFFFFFFFFFFFFF
@A00155:342:HHGFNDSXY:1:2526:11143:12367 2:N:0:GAACCTAG+TCCGCATA
GCCGTCCTCCCTCCACCATTCAGGCTGGCCGGGTCACTATTCAGTAGTGTTATCTCTTCAGCGCTGTA
CTCCCCCAACGCCTGATCCCACCATGCCCCTACATGCCTTCCACAGGTGGGGCACCCTGCTCCAAGTT
+
FFFFFFFFFFFFFFFFFFFFFFFFF,FFFFFFFFFFFFF:FFFF:FFFFFFFFFFFFFFFFFFFFFFF
FFFFFFF:FFFFFFFFFFFFFFFFFFFFFFFFFFFFFFFFFFFFFFFFFFFFFFFFFFFFFFFFFFFF
@A00155:342:HHGFNDSXY:1:2371:8721:11929 2:N:0:GAACCTAG+TCCGCATA
CCTCCCTCCACCATTCAGGCTGGCCGGGTCACTATTCAGTAGTGTTATCTCTTCAGCGCTGTACTCCC
CCAACGCCTGATCCCACCATGCCCCTACATGCCTTCCACAGGTGGGGCACCCTGCTCCAAGTTTGGCT
+
FFFFFFFFFFFFF:FFFFFFFFF,FFFFFFFFF:FFF,F,FFFFFFFFFFFFFFFFFFFFF:FFFFFF
FF:FFFFFFFFFFFF:FFFFFFFFFF,FFFFFFFFFFFF,FFFFFFFFFFFFFFFFFFFFFFFFFFFF
@A00155:342:HHGFNDSXY:1:1517:13069:31548 1:N:0:GAACCTAG+TCCGCATA
CCACCATTCAGGCTGGCCGGGTCACTATTCAGTAGTGTTATCTCTTCAGCGCTGTACTCCCCCAACGC
CTGATCCCACCATGCCCCTACATGCCTTCCACAGGTGGGGCACCCTGCTCCAAGTTTGGC
+
FFFFFFFFFFFFFFFFFFFFFFFFFFFFFFFFFFFFFFFFFFFFFFFFFFFFFFFFFFFFFFFFFFFF
FFFFFFFFFFFFFFFFFFFFFFFFFFFFFFFFFFFFFFFFFFFFFFFFFFFFFFFFFFFF
@A00155:342:HHGFNDSXY:1:2505:12219:26976 1:N:0:GAACCTAG+TCCGCATA
GCCGGGTCACTATTCAGTAGTGTTATCTCTTCAGCGCTGTACTCCCCCAACGCCTGATCCCACCATGC
CCCTACATGCCTTCCACAGGTGGGGCACCCTGCTCCAAGTTTGGCTGAGGGATCACGCCAGCCTGAAC
+
FFFF:FF:FFFFFFFFFFFF:F,:F,FF:FF:F:F:FF:FFFFFFFF:F:F,F:F,F:FFFFFFFFFF
F,FFFFF,::FF,FFFFFFFFFFF:FFFFFFFFFFFFF:FF::,FF:FFFFFFFFFFFFFF:FF:FFF
@A00155:342:HHGFNDSXY:1:1238:12454:23218 1:N:0:GAACCTAG+TCCGCATA
CCGGGTCACTATTCAGTAGTGTTATCTCTTCAGCGCTGTACTCCCCCAACGCCTGATCCCACCATGCC
CCTACATGCCTTCCACAGGTGGGGCACCCTGCTCCAAGTTTGGCTGAGGGATCACGCCAGCCTGAACA
+
FFFFF,::FFFFFF,FFFFFF:FFF,FFFFFF:FFFFFFFFFFFFFFFFFFFFFFFFFFFFFF:FFFF
FFFFFF:FFF,FFFFFFFF:FFFFFFFFFFFFFFFFF,FFFFFFFFFFFFFF:FFFFFFFFFFFFFFF
@A00155:342:HHGFNDSXY:1:1561:4354:21590 1:N:0:GAACCTAG+TCCGCATA
CCGGGTCACTATTCAGTAGTGTTATCTCTTCAGCGCTGTACTCCCCCAACGCCTGATCCCACCATGCC
CCTACATGCCTTCCACAGGTGGGGCACCCTGCTCCAAGTTTGGCTGAGGGATCACGCCAGCCTGAACA
+
FFFFFFFFFFFFFFFFFFFFFFF:FFFFFFFFFFFFFFFFFFFFFFF:,FFFFFFFFF:FFFFFFFFF
FFFFFFFFFFFFFFFFFFFFFFFFFFFFFFFFFFFFFFFFFFFFFFFFFFFFFFFFFFFFFFFFFFFF
@A00155:342:HHGFNDSXY:1:2428:30255:33379 1:N:0:GAACCTAG+TCCGCATA
CCGGGTCACTATTCACTAGTGTTATCTCTTCAGCGCTGTACTCCCCCAACGCCTGATCCCACCATGCC
CCTACATGCCTTCCACAGGTGGGGCACCCTGCTCCAAGTTTGGCTGAGGGATCACGCCAGCCTGAACA
+
FF,FFF:FF:F:F:FF,F,::,FFFFFF:FFF,F:F:FFF,FFFFFFFF:FFF,FFF,FFFFF,,:FF
FF,F:F:FFF:FFFF,F,FFFFFFFFFFF,FF,FFFF,FFFFFFFFFFFFFFFFF:F::,FF,FFF:F
@A00155:342:HHGFNDSXY:1:2665:28420:6997 2:N:0:GAACCTAG+TCCGCATA
TATTCAGTAGTGTTATCTCTTCAGCGCTGTACTCCCCCAACGCCTGATCCCACCATGCCCCTACATGC
CTTCCACAGGTGGGGCACCCTGCTCCAAGTTTGGCTGAGGGATCACGCCAGCCTGAACACCTGTTACA
+
FF,FFFFFFFFF:FFFFFFFF:FFFFFFF:FFFFFFFFF::FFFFF::FFFF:F:FFFFFFFFFF,FF
F:FF:,FFFFFFFFFFFFFFFFFFFFFFFFFFFF,FFFFFFFFFFFFFFFFFFFFFFFFFFFF,FFFF
```

```
@A00155:342:HHGFNDSXY:1:1572:27299:1423 2:N:0:GAACCTAG+TCCGCATA
TTCAGTAGTGTTATCTCTTCAGCGCTGTACTCCCCCAACGCCTGATCCCACCATGCCCCTACATGCCT
TCCACAGGTGGGGCACCCTGCTCCAAGTTTGGCTGAGGGATCACGCCAGCCTGAACACCTGTTACAAA
+
:FFFFFFFFFFFFFFFFFFFFF:FFFF:FFFFFFFFFFFFFFFFFFFFFFFFFFFFFFFFFFFFFFFF
FFFFFFFFFFFFFFFFFFFFFFFFFFFFFFFFFFFFFFFFFFFFFFFFFFFFFFFFFFFFFFFFFFF:
@A00155:342:HHGFNDSXY:1:2665:28420:6997 1:N:0:GAACCTAG+TCCGCATA
TAGTGTTATCTCTTCAGCGCTGTACTCCCCCAACGCCTGATCCCACCATGCCCCTACATGCCTTCCAC
AGGTGGGGCACCCTGCTCCAAGTTTGGCTGAGGGATCACGCCAGCCTGAACACCTGTTACAAACCGAA
+
FFFFFFFFFFFFFFFFFFFFF:FFFFFFFFFFFFFFFFFFFFFFFFFFFFFFFFFFFFF:FFFF:FFF
FFFFFFFFFFFFFFFFFFFFFFFFFFFFFFF:FFFFFFFFFFFFFFFFFFFFFFFFFFFFFFFFFFFF
@A00155:342:HHGFNDSXY:1:1226:7934:18427 2:N:0:GAACCTAG+TCCGCATA
TCTCTTCAGCGCTGTACTCCCCCAACGCCTGATCCCACCATGCCCCTACATGCCTTCCACAGGTGGGG
CACCCTGCTCCAAGTTTGGCTGAGGGATCACGCCAGCCTGAACACCTGTTACAAACCGAAAACCCTGC
+
FFFFFFFFFFFFFF:FFFFFFFF:FFFFFFFFFFFFFFF:FFFFFFFFFFFFFFFFFFFFFFFFFFFF
FFFFFFFFFFFFFFFFFFFFFFFFFFFFFFFFFFFFFFFFFFFFFFF:FFFFFFFFFFFFFFFFFFFF
@A00155:342:HHGFNDSXY:1:2505:12219:26976 2:N:0:GAACCTAG+TCCGCATA
TCTCTTCAGCGCTGTACTCCCCCAACGCCTGATCCCACCATGCCCCTACATGCCTTCCACAGGTGGGG
CACCCTGCTCCAAGTTTGGCTGAGGGATCACGCCAGCCTGAACACCTGTTACAAACCGAAAACCCTGC
+
FFFFFFFFF,,FFFFFFF,FFF:FFFFFFFFFF:FF:FFFFFFFFFF:FFFFFFF:FFFFFFF:FFFF
F,FFFFFFFF:FFFFFFFF:FFF:FFFFFFF:FF:FF:FFFFFFF:F:FFFFFFFFFFFFFFFFFFFF
@A00155:342:HHGFNDSXY:1:2657:13612:26036 2:N:0:GAACCTAG+TCCGCATA
TTCAGCGCTGTACTCCCCCAACGCCTGATCCCACCATGCCCCTACATGCCTTCCACAGGTGGGGCACC
CTGCTCCAAGTTTGGCTGAGGGATCACGCCAGCCTGAACACCTGTTACAAACCGAAAACCCTGCTCTT
+
FFFFFFFFFFFFFFFFFFFFFFFFFFFFFFFFFFFFFFFFFFFFFFFFFFFFFFFFFFFFFFFFFFFF
FFFFFFFFFFFFFFFFFFFFFFFFFFFFFFFFFFFFFFFFFF:FFFFFFF:FFFFFFFFFF:FFFFFF
@A00155:342:HHGFNDSXY:1:2428:30255:33379 2:N:0:GAACCTAG+TCCGCATA
TTCAGCGCTGTACTCCCCCAACGCCTGATCCCACCATGCCCCTACATGCCTTCCACAGGTGGGGCACC
CTGCTCCAAGTTTGGCTGAGGGATCACGCCAGCCTGAACACCTGTTACAAACCGAAAACCCTGCTCTT
+
FFFFFFFFFF,FFFFFFFFF:FFFFFFFFFFFFFF:FFFFFFFFF:FF:F:FFFFFFFFFFFFFFFFF
FFFFFFFFFFFFFFFFFFFFFFFFFFFFFFFFFFFFFFFFFFF:FFFFF:FFFFFFFFFFFFFFFFFF
@A00155:342:HHGFNDSXY:1:1238:12454:23218 2:N:0:GAACCTAG+TCCGCATA
TTCAGCGCTGTACTCCCCCAACGCCTGATCCCACCATGCCCCTACATGCCTTCCACAGGTGGGGCACC
CTGCTCCAAGTTTGGCTGAGGGATCACGCCAGCCTGAACACCTGTTACAAACCGAAAACCCTGCTCTT
+
FFFFFFFFFF,FFFFFFFF:FFFFF:F:F:FFFFF:FFFFFFF,FFFFFFFFFFFF,FFFFFFFFFFF
FFFFFFFFFF:FFFFFFFFFFFFFFFFFFFFFFFFFFFFFFFFFFFFFFFFFFFFFFFFFFFFFFFFF
@A00155:342:HHGFNDSXY:1:1278:11966:17989 2:N:0:GAACCTAG+TCCGCATA
TTCAGCGCTGTACTCCCCCAACGCCTGATCCCACCATGCCCCTACATGCCTTCCACAGGTGGGGCACC
CTGCTCCAAGTTTGGCTGAGGGATCACGCCAGCCTGAACACCTGTTACAAACCGAAAACCCTGCTCTT
+
FFFFFFFFFFFFFFFFFFF:FFFFFFFFFFFFFFFFFFFFFF:,F:FFFFFFFFFFFFFFFFFFFFFF
FFFFFFFFFFFF:FFFFFFFFFFFFFFFFFFFFFFFFFFFFFFFFFFFF:FFFFFFF,FFFFFFFFF:
@A00155:342:HHGFNDSXY:1:1278:11966:17989 1:N:0:GAACCTAG+TCCGCATA
CAGCGCTGTACTCCCCCAACGCCTGATCCCACCATGCCCCTACATGCCTTCCACAGGTGGGGCACCCT
GCTCCAAGTTTGGCTGAGGGATCACGCCAGCCTGAACACCTGTTACAAACCGAAAACCCTGCTCTTCT
+
FFFFFFFF:FFFFFFFFFFFFFFFF:FFFFFFFFFFFFFFFFFFFFFFFFFFFFFFFFFFFFF:FFFF
FFFFFFFFFFFFFFFFFFFFFFFFFFFFFFFFFFFFFFFFFFFFFFFFFFFFFFFFFFFFFFFFFFFF
```

```
@A00155:342:HHGFNDSXY:1:1366:4381:24706 2:N:0:GAACCTAG+TCCGCATA
GCGTTGTACTCCCCCAACGCCTGATCCCACCATGCCCCTACATGCCTTCCACAGGTGGGGCACCCTGC
TCCAAGTTTGGCTGAGGGATCACGCCAGCCTGAACACCTGTTACAAACCGAAAACCCTGCTCTT
+
FFFFFFF:FFFFFFFFFFFFFFF:FFFFFFFFFFFFFFFFF:FFFFFFFFFFFFFFFFFFFFFFFFFF
F:FF:FFFFFFFFFFFFFFFFFFFFFFFFFFFFFFFFFFFFFFFFFFFFFFFFFFFFFFF,FFF
@A00155:342:HHGFNDSXY:1:2657:13612:26036 1:N:0:GAACCTAG+TCCGCATA
CTGTACTCCCCCAACGCCTGATCCCACCATGCCCCTACATGCCTTCCACAGGTGGGGCACCCTGCTCC
AAGTTTGGCTGAGGGATCACGCCAGCCTGAACACCTGTTACAAACCGAAAACCCTGCTCTTCTCGGGT
+
FFFFFFFFFFFFFFFFFFFFFFFFFFFFFFFFFFFFFFFFFFFFFFFFFFFFFFFFFFFFFFFFFFFF
FFFFFFFFFFFFFFFFFFFFFFFFFFFFFFFFFFFFFFFFFFFFFFFFFFFFFFFFFFFFFFFFFFFF
@A00155:342:HHGFNDSXY:1:2645:29179:21715 1:N:0:GAACCTAG+TCCGCATA
GTACTCCCCCAACGCCTGATCCCACCATGCCCCTACATGCCTTCCACAGGTGGGGCACCCTGCTCCAA
GTTTGGCTGAGGGATCACGCCAGCCTGAACACCTGTTACAAACCGAAAACCCTGCTCTTCTCGGGTTA
+
FFFFFFFFFFFFFFFFFFFFFFFFFFFFFFFFFFFFFFFFFFFFFFFFFFFFFFFF:FFFFFFFFFFF
FFFFFFFFFFFFFFFFFFFFFFFFFFFFFFFFFFFFFFFFFFFFFFFFFFFFFFFFFFFFFFFFFFFF
@A00155:342:HHGFNDSXY:1:1243:27109:8234 1:N:0:GAACCTAG+TCCGCATA
CTCCCCCAACGCCTGATCCCACCATGCCCCTACATGCCTTCCACAGGTGGGGCACCCTGCTCCAAGTT
TGGCTGAGGGATCACGCCAGCCTGAACACCTGTTACAAACCGAAAACCCTGCTCTTCTCGGGTTATGT
+
FFFFFFFFFFFFFFFFFFFFFFFFFFFFFFFFFFFFFFFFFFFFFFFF:FFFFFFFFFFFFFFFFFFF
FFFFFFFFFFFFFFFFFFFFFFFFFFFFFFFFFFFFFFFFFFFFFFFFFFFFFFFFFFFFFFFF:FFF
@A00155:342:HHGFNDSXY:1:1226:7934:18427 1:N:0:GAACCTAG+TCCGCATA
CCCCCAACGCCTGATCCCACCATGCCCCTACATGCCTTCCACAGGTGGGGCACCCTGCTCCAAGTTTG
GCTGAGGGATCACGCCAGCCTGAACACCTGTTACAAACCGAAAACCCTGCTCTTCTCGGGTTAT
+
FFFFFFFFFFFFFFFFFFFFFFFF:FFFFFFFFFFFFFFFFFFFFFFFFFFFF:FFFFFFFFFFFFFFF
FFFFFFFFFFFFFFFFFFFFFFFFFF:FFFFFFFFFF:FFFFFF:FFFFFFFFFFFFFFFFF:FF
@A00155:342:HHGFNDSXY:1:2254:9118:9643 2:N:0:GAACCTAG+TCCGCATA
CCCCACCGCCTGATCCCACCATGCCCCTACATGCCTTCCACAGGTGGGGCACCCTGCTCCAAGTTTGG
CTGAGGGATCACGCCAGCCTGAACACCTGTTACAAACCGAAAACCCTGCTCTTCTCGGGTTATGTAAC
+
F,F::,:FFFFFFFF,FFFF,FFFFFFFFFFFFFF:FFFFF,FFFFF,FFFFFFFFFFFFFFFFFFF
FFFFFFFFFFFFFFFFFFFFFFFFFFFF:FFFFFFF:FFFFFF,FFFFF:FF:FFFFFFFFFFFFFFF
@A00155:342:HHGFNDSXY:1:1141:18150:28134 2:N:0:GAACCTAG+TCCGCATA
CCCCAACGCCTGATCCCACCATGCCCCTACATGCCTTCCACAGGTGGGGCACCCTGCTCCAAGTTTGG
CTGAGGGATCACGCCAGCCTGAACACCTGTTACAAACCGAAAACCCTGCTCTTCTCGGGTTATGTAAC
+
FFFF,:FFFFFFFFFFFFFFFFFFFFFFFFFFFFFFFFF:FFF:FFFFFFFFFFFFFFFFFFFFFFFFF
FFFFFFFFFFFFFFFFFFFFFFFFFFFFFFFFFFFFFFFFFFFFFFFF:F:FFFFFFFFFFFFFFFF
@A00155:342:HHGFNDSXY:1:1561:4354:21590 2:N:0:GAACCTAG+TCCGCATA
CCCAACGCCTGATCCCACCATGCCCCTACATGCCTTCCACAGGTGGGGCACCCTGCTCCAAGTTTGGC
TGAGGGATCACGCCAGCCTGAACACCTGTTACAAACCGAAAACCCTGCTCTTCTCGGGTTATGTAACC
+
FFFF,FFFFFFFFFFFFFFFFFFFFFFFFFFFFFFFFFFFFFFFFFFFFFFFFFFFFFFFFFFFFF:F
FFFFFFFFFFF:FFFFFFFFFFFFFFFFFFFFFF,FFFFFFF,FFFFFFFFFFFFFFFFFFFFFFFFFF,
@A00155:342:HHGFNDSXY:1:1366:4381:24706 1:N:0:GAACCTAG+TCCGCATA
AACGCCTGATCCCACCATGCCCCTACATGCCTTCCACAGGTGGGGCACCCTGCTCCAAGTTTGGCTGA
GGGATCACGCCAGCCTGAACACCTGTTACAAACCGAAAACCCTGCTCTTCTCGGGTTATGTAAC
+
FFFFFFFFFFFFFFFFFFFFFFFFFFFFFFFFFFFFFFF:FFFFFFFFFFFFFFFFFFFFFF:FFFFFFF
FFFFFFFFFFFFFFFFFFFFFFFFFFFFFFFFFFFFFFFF,FFFFFFFFFFFFFFFF:,FFFFFFF
```

```
@A00155:342:HHGFNDSXY:1:2226:21603:28322 2:N:0:GAACCTAG+TCCGCATA
CCTGATCCCACCATGCCCCTACATGCCTTCCACAGGTGGGGCACCCTGCTCCAAGTTTGGCTGAGGGA
TCACGCCAGCCTGAACACCTGTTACAAACCGAAAACCCTGCTCTTCTCGGGTTATGTAACC
+
FFFFFFFFFFFF,FFFFFFFFFFFFFFFFFFFF,FFFFFFFFFFFFFFFFFFFFF:FFFFFFFFFFFF
FFFFFFFFFFFFFFFFFFFFFFFFFFF:FFFF,FFFFFFFFFFFFFFFFFFFFFFFFFFFF
@A00155:342:HHGFNDSXY:1:2645:29179:21715 2:N:0:GAACCTAG+TCCGCATA
TGATCCCACCATGCCCCTACATGCCTTCCCCAGGTGGGGCACCCTGCTCCAAGTTTGGCTGAGGGATC
ACGCCAGCCTGAACACCTGTTACAAACCGAAAACCCTGCTCTTCTCGGGTTATGTAACCTCGCATCCG
+
FFF:FFF,FF:FFFFFFFFFF:FFFF:FF,FFFFFFFFFFFFFFFFFFFFFFFFFFFFFFFFFFFFFF
FFFFFFFFFFFFFFFFFFFFFFFFF:FFFF:FFFFFFFFFFFFFFFFFFFFFFFFFFFFFFFF:FFFF
@A00155:342:HHGFNDSXY:1:1630:25916:8985 1:N:0:GAACCTAG+TCCGCATA
GATCCCACCATGCCCCTACATGCCTTCCACAGGTGGGGCACCCTGCTCCAAGTTTGGCTGAGGGATCA
CGCCAGCCTGAACACCTGTTACAAACCGAAAACCCTGCTCTTCTCGGGTTATGTAACCTCGCATCCGC
+
FFFFFFFFFFFFFFFFFFFFFFFFFFFFFFFFFFFFFFFFFFFFFFFFFFFFFFFFFFFFFFFFFFFF
FFFFFFFFFFFFFFFFFFFFFFFFFFFFFFFFFFFFFFFFFFFFFFFFFFFFFFFFFFFFFFFFFFFF
@A00155:342:HHGFNDSXY:1:2558:28411:33004 1:N:0:GAACCTAG+TCCGCATA
ATCCCACCATGCCCCTACATGCCTTCCACAGGTGGGGCACCCTGCTCCAAGTTTGGCTGAGGGATCAC
GCCAGCCTGAACACCTGTTACAAACCGAAAACCCTGCTCTTCTCGGGTTATGTAACCTCGCATCCGC
+
FFFFFFFFFFFFFFFFFFFFFFFFFFFFFFFFFFFFFFFFFFFFFFFFFFFFFFFFFFFFFFFFFFFF
FFFFFFF:FF,F:FFFFFFFFFF,FFFFFFFFFFFFFFFFFFFFFFFFFFFFF:FFFFFFFF:FFFF
@A00155:342:HHGFNDSXY:1:1243:27109:8234 2:N:0:GAACCTAG+TCCGCATA
ATCCCACCATGCCCCTACATGCCTTCCACAGGTGGGGCACCCTGCTCCAAGTTTGGCTGAGGGATCAC
GCCAGCCTGAACACCTGTTACAAACCGAAAACCCTGCTCTTCTCGGGTTATGTAACCTCGCATCCGCT
+
FFFFFFFFFFFFFFFFFFFFFFFFFFFFFFFFFFFFFFFFFFFFFFFFFFFFFFFFFFFFFFFFFFFF
FFFFFFFFFFFFFFFFFFFFFFFFFFFFFFFFFFFFFFFFFFFFFFFFFFFFFFFFFFFFFFFFFFFF
@A00155:342:HHGFNDSXY:1:2254:9118:9643 1:N:0:GAACCTAG+TCCGCATA
ATCCCACCATGCCCCTACATGCCTTCCACAGGTGGGGCACCCTGCTCCAAGTTTGGCTGAGGGATCAC
GCCAGCCTGAACACCTGTTACAAACCGAAAACCCTGCTCTTCTCGGGTTATGTAACCTCGCATCCGCT
+
FFFFFFFFFFFFFFFFFFFFFFFFFFFFFFFFFFFFFFFFFFFFFFFFFFFFFFFFFFFFFFFFFFFF
FFFFFFFFFFFFFFFFFFFFFFFFFFFFFFFFFFFFFFFFFFFFFFFF,F:FFF:FFFFFFFFFFFFF
@A00155:342:HHGFNDSXY:1:1141:18150:28134 1:N:0:GAACCTAG+TCCGCATA
ATCCCACCATGCCCCTACATGCCTTCCACAGGTGGGGCACCCTGCTCCAAGTTTGGCTGAGGGATCAC
GCCAGCCTGAACACCTGTTACAAACCGAAAACCCTGCTCTTCTCGGGTTATGTAACCTCGCATCCGCT
+
FFFFFFFFFFFFFFFFFFFFFFFFFFFFFFFFFFFFFFFFFFFFFFFFFFFFFFFFFFFFFFFFFFFF
FFFFFFFFFFFFFFFFFFFFFFFFFFFFFFFFFFFFFFFFFFFFFFF,FFFFFFFFFFFFFFFFFFFF
@A00155:342:HHGFNDSXY:1:1313:10529:12994 1:N:0:GAACCTAG+TCCGCATA
ATCCCACCATGCCCCTACATGCCTTCCACAGGTGGGGCACCCTGCTCCAAGTTTGGCTGAGGGATCAC
GCCAGCCTGAACACCTGTTACAAACCGAAAACCCTGCTCTTCTCGGGTTATGTAACCTCGCATCCGCT
+
FFFFFFFFFFFFFFFFFFFFFFFFFFFFFFFFFFFFFFFFFFFFFFFFFFFFFFFFFFFFFFFFFFFF
FFFFFFFFFFFFFFFFFFFFFFFFFFFFFFFFFFFFFFFFFFFFFFFF::FFFFFFFFFFFF:FFFFF
@A00155:342:HHGFNDSXY:1:1322:5132:22404 2:N:0:GAACCTAG+TCCGCATA
CACCATGCCCCTACATGCCTTCCACAGGTGGGGCACCCTGCTCCAAGTTTGGCTGAGGGATCACGCCA
GCCTGAACACCTGTTACAAACCGAAAACCCTGCTCTTCTCGGGTTATGTAACCTCGCATCCG
+
FFFFFFFFFFFFFFFFFFFFFFFFFFFFFFFFFFFFF:FFFFFFFFFFFFFFFFFFFFFFFFFFFFFF
FFFFFFFFFFFFFFFFFFFFFFFFFFFFFFFFFFFFFFFFFFFFFFFFFFFFFFFFFFFFFF
```

```
@A00155:342:HHGFNDSXY:1:1519:17526:2284 2:N:0:GAACCTAG+TCCGCATA
CACCATGCCCCTACATGCCTTCCACAGGTGGGGCACCCTGCTCCAAGTTTGGCTGAGGGATCACGCCA
GCCTGAACACCTGTTACAAACCGAAAACCCTGCTCTTCTCGGGTTATGTAACCTCGCATCCG
+
FFFFFFFFFFFFFFFFFFFF:FFFFFFFFFFFFFFFFFFFFFFFFFFFFFFFFFFFFFFFFFFFFFFF
FFFFFFFFFFFFFFFFFFFFFFFFFFFFFFFFFFFFFFFFFFFFFFFFFFFFFFFFFFFFFF
@A00155:342:HHGFNDSXY:1:1322:5014:21386 2:N:0:GAACCTAG+TCCGCATA
CACCATGCCCCTACATGCCTTCCACAGGTGGGGCACCCTGCTCCAAGTTTGGCTGAGGGATCACGCCA
GCCTGAACACCTGTTACAAACCGAAAACCCTGCTCTTCTCGGGTTATGTAACCTCGCATCCG
+
FFFFFFFFFFF:FFFFFFFFFFFFFFFFFFFFFFFFFFFFFFFFFFFFFFFFFFFFFFFFFFFFFFFF
FFFFFFFFFFFFF,FFFFFFFFFFFFFFFFFFFFFFFFFFFFFFFFFFFFFFFFFFFFFFFFFF
@A00155:342:HHGFNDSXY:1:2607:12219:2832 1:N:0:GAACCTAG+TCCGCATA
CACCATGCCCCTACATGCCTTCCACAGGTGGGGCACCCTGCTCCAAGTTTGGCTGAGGGATCACGCCA
GCCTGAACACCTGTTACAAACCGAAAACCCTGCTCTTCTCGGGTTATGTAACCTCGCATCCGCTGCAC
+
FFFFFFFFFFFFFFFFFFFFFFFFFFFFFFFFFFFFFFFFFFFFFFFFFFFFFFFFFFFFFFFFFFFF
FFFFFF:FFFFFFFFFFFFFFFFF:FFFFFFFFFFFFFFFFFFFFFFFFFFFFFFFFFFFFFFFFFFF
@A00155:342:HHGFNDSXY:1:2607:12626:1219 1:N:0:GAACCTAG+TCCGCATA
CACCATGCCCCTACATGCCTTCCACAGGTGGGGCACCCTGCTCCAAGTTTGGCTGAGGGATCACGCCA
GCCTGAACACCTGTTACAAACCGAAAACCCTGCTCTTCTCGGGTTATGTAACCTCGCATCCGCTGCAC
+
FFFFFFFFFFFFFFFFFFFFFFFFFFFFFFFFFF:FFFFFFFFFFFFFFFFFFFFFFFFFFFFFFFFF
FFFFFFFFFFFFFFFFFFFFFFFFFF:FFFFFFFFFFFFFFFFFFFFFFFFFFFFFFFFFFFFFFFFF
@A00155:342:HHGFNDSXY:1:2607:12626:1125 1:N:0:GAACCTAG+TCCGCATA
CACCATGCCCCTACATGCCTTCCACAGGTGGGGCACCCTGCTCCAAGTTTGGCTGAGGGATCACGCCA
GCCTGAACACCTGTTACAAACCGAAAACCCTGCTCTTCTCGGGTTATGTAACCTCGCATCCGCTGCAC
+
FFFFFFFFFFFFFFFFFFFFFFFFFFFFFFFFFFFFFFFFFFFFFFFFFFFFFFFFFFFFFFFFFFFF
FFFFFFFFFFFFFFFFFFFFFFFFFFFFFFFFFFFFFFFFFFFF:FFFFFFFFFFFFFFFFFFFFFFF
@A00155:342:HHGFNDSXY:1:2433:2284:18599 1:N:0:GAACCTAG+TCCGCATA
ATGCCCCTACATGCCTTCCACAGGTGGGGCACCCTGCTCCAAGTTTGGCTGAGGGATCACGCCAGCCT
GAACACCTGTTACAAACCGAAAACCCTGCTCTTCTCGGGTTATGTAACCTCGCATCCGCTGCACTGTT
+
FFFFFFFFFFFFFFFFFFFFFFFFFFFFFFFFFFFFFFFFFFFFFFFFFFFFFFFF:FFFFFFFFFFF
FFFFFFFFFFFFFFFFFFFFFFFFFFFFFFFFFFFFFFFFFFFFFFFFFFFFFFFFFFFFFFFFFFFF
@A00155:342:HHGFNDSXY:1:1629:21133:32675 1:N:0:GAACCTAG+TCCGCATA
ATGCCCCTACATGCCTTCCACAGGTGGGGCACCCTGCTCCAAGTTTGGCTGAGGGATCACGCCAGCCT
GAACACCTGTTACAAACCGAAAACCCTGCTCTTCTCGGGTTATGTAACCTCGCATCCGCTGCACTGTT
+
:FFFFFFFFFFFFFFFFFFFFFFFFFFFFFFFFFFFFFFFFFFFFFFFFFFFFFFFFFFFFFFFFFFF
FFFFFFFFFFFFFFFFFFFFFFFFFFFFFFFFFFFFFFFFFFFF:FFFFFFFFFFFFFF:FFFFFFFF
@A00155:342:HHGFNDSXY:1:1629:21007:33270 1:N:0:GAACCTAG+TCCGCATA
ATGCCCCTACATGCCTTCCACAGGTGGGGCACCCTGCTCCAAGTTTGGCTGAGGGATCACGCCAGCCT
GAACACCTGTTACAAACCGAAAACCCTGCTCTTCTCGGGTTATGTAACCTCGCATCCGCTGCACTGTT
+
:FFFFFFFFFF:FFFFFFFFFFFFFFFFFFFFFFFFFFFFFFFFFFFFFFFFFFFFFFFFFFFFFFFF
FFFFFFFFFFFFFFFFFFFFFFFFFFFFFFFFFFF:FFFFFFFFFFFFFFF:FFFFFFFFFFFFFFFF
@A00155:342:HHGFNDSXY:1:2407:25274:26725 1:N:0:GAACCTAG+TCCGCATA
GCCCCTACATGCCTTCCACAGGTGGGGCACCCTGCTCCAAGTTTGGCTGAGGGATCACGCCAGCCTGA
ACACCTGTTACAAACCGAAAACCCTGCTCTTCTCGGGTTATGTAACCTCGCATCCGCTGCACTGTTTC
+
:FFFFFFFF:,FFF:F:FFFF,FF:FFFF:FFFFFFF:FFF:,FF:FFFFFFFFFFFFF,F:FFFFFF
FFFFFFFF:FFFF:FFFFFF,FFFFFFFFFFFFFFF:FF,FFFFFFFFFF:F:FFFF,FFFF:F,FFF
```

@A00155:342:HHGFNDSXY:1:2407:25247:26334 1:N:0:GAACCTAG+TCCGCATA
GCCCCTACATGCCTTCCACAGGTGGGGCACCCTGCTCCAAGTTTGGCTGAGGGATCACGCCAGCCTGA
ACACCTGTTACAAACCGAAAACCCTGCTCTTCTCGGGTTATGTAACCTCGCATCCGCTGCACTGTTTC
+
FFFFFFFFFFFF:FFFFFFFFFFFFFFFFFFFFFFFFFFFFFFFFFF:FFFFFFFFFFFFFFFFFFFF
FFFFFFFFFFFFFFFFFFFFFFFFFFFFF:FFFFFFFFF:FFFFFFFFFFFFFFFFFFFFFFFFFF:F
@A00155:342:HHGFNDSXY:1:2226:21603:28322 1:N:0:GAACCTAG+TCCGCATA
CCCCTACATGCCTTCCACAGGTGGGGCACCCTGCTCCAAGTTTGGCTGAGGGATCACGCCAGCCTGAA
CACCTGTTACAAACCGAAAACCCTGCTCTTCTCGGGTTATGTAACCTCGCATCCGCTGCAC
+
FFFFFFFFFFFFFFFFFFFFFFFFFFFFFFFFFFFFFFFFFFFFFFFFFFFFFFFFFFFFFFFFFFFF
FFFFFFFFFFFFFFFFFFFFFFFFFFFFFFFFFFFF:FFFFFFFFFFFFFFFFFFFFFFFF
@A00155:342:HHGFNDSXY:1:1629:21133:32675 2:N:0:GAACCTAG+TCCGCATA
CCCCTACATGCCTTCCACAGGTGGGGCACCCTGCTCCAAGTTTGGCTGAGGGATCACGCCAGCCTGAA
CACCTGTTACAAACCGAAAACCCTGCTCTTCTCGGGTTATGTAACCTCGCATCCGCTGCACTGTTTCA
+
FFFFFFFFFFFF:FFF:F:FFFFFFFFFFFFFFF:FFFFFFFFFFF:FFFFFFFFFFFFFFFFFFFFF
FFFFFFFFFFFF,FFFFF:FFFFFFFFFF,FFFFFFFFFFFFFFFFFFFFFFFFFFFFFFFFFFFFFF
@A00155:342:HHGFNDSXY:1:1629:21007:33270 2:N:0:GAACCTAG+TCCGCATA
CCCCTACATGCCTTCCACAGGTGGGGCACCCTGCTCCAAGTTTGGCTGAGGGATCACGCCAGCCTGAA
CACCTGTTACAAACCGAAAACCCTGCTCTTCTCGGGTTATGTAACCTCGCATCCGCTGCACTGTTTCA
+
FFFFFFFFFFFFFFFFFFFFFFFFFFF:FFFFFFFFFFFF:FFFFF:FFFFFFFF:FFFFFFFFFFFF
FFFFFFFFFFFFFFFFFFF:FFFFFFFF:FFFFFFFFFFFFFFFFFFFFFFFFFFFFFFFF:FFFFFF
@A00155:342:HHGFNDSXY:1:2433:2284:18599 2:N:0:GAACCTAG+TCCGCATA
CCCCTACATGCCTTCCACAGGTGGGGCACCCTGCTCCAAGTTTGGCTGAGGGATCACGCCAGCCTGAA
CACCTGTTACAAACCGAAAACCCTGCTCTTCTCGGGTTATGTAACCTCGCATCCGCTGCACTGTTTCA
+
FFFFFFFFFFFFFFFFFFFFFFFFFFFFFFFFFFFFFF:FFFFFFFFFFFFFFFFFFFFFFFFFFFFF
FFFFFFFFFFFF,FFFFFFFFFFFFFFFFFFFFFFFFFFFFFFFFFFFFFFFFFFFFFFFFFFFFFF:
@A00155:342:HHGFNDSXY:1:2503:29514:1031 1:N:0:GAACCTAG+TCCGCATA
TACATGCCTTCCACAGGTGGGGCACCCTGCTCCAAGTTTGGCTGAGGGATCACGCCAGCCTGAACACC
TGTTACAAACCGAAAACCCTGCTCTTCTCGGGTTATGTAACCTCGCATCCGCTGCACTGTTTCATC
+
FFFFFFFFFFFFFFFFFFFFFFFFFFFFFFFFFFFFFFFFFFFFFFFFFFFFFFFFFFFFFFFFFFFF
FFFFFFFFFFFFFFFFFFFFFFFFFFFFFFFFFFFFFFFFFFFFFFFFFFFFFFFFFFFFFFFFFF
@A00155:342:HHGFNDSXY:1:1322:5014:21386 1:N:0:GAACCTAG+TCCGCATA
TGCCTTCCACAGGTGGGGCACCCTGCTCCAAGTTTGGCTGAGGGATCACGCCAGCCTGAACACCTGTT
ACAAACCGAAAACCCTGCTCTTCTCGGGTTATGTAACCTCGCATCCGCTGCACTGTTTCATC
+
FFFFFFFFFFFFFFFFFFFFFFFFFFFFFFFFFFFFFFFFFFFFFFFFFFFFFFFFFFFFFFFFFFFF
FFFF:FFFFFFFFFFFFFFFFFFFFFFFFFFFFFFFFFFFFFFFFFFFFFFFFFFFFFFFF
@A00155:342:HHGFNDSXY:1:1322:5132:22404 1:N:0:GAACCTAG+TCCGCATA
TGCCTTCCACAGGTGGGGCACCCTGCTCCAAGTTTGGCTGAGGGATCACGCCAGCCTGAACACCTGTT
ACAAACCGAAAACCCTGCTCTTCTCGGGTTATGTAACCTCGCATCCGCTGCACTGTTTCATC
+
FFFFFFFFFFFFFFFFFFFFFFFFFFFFFFFFFFFFFFFFFFFFFFFFFFFFFFFFFFFFFFFFFFFF
FFFFFFFFFFFFFFFFFFFFF:FFFFFFFFFFFFFFFFFFFFFFFFF:FFFFFFFFFFFFFF
@A00155:342:HHGFNDSXY:1:1519:17526:2284 1:N:0:GAACCTAG+TCCGCATA
TGCCTTCCACAGGTGGGGCACCCTGCTCCAAGTTTGGCTGAGGGATCACGCCAGCCTGAACACCTGTT
ACAAACCGAAAACCCTGCTCTTCTCGGGTTATGTAACCTCGCATCCGCTGCACTGTTTCATC
+
FFFFFFFFFFFFFFFFFFFFFFFFFFFFFFFFFFFFFFFFFFFFFFFFFFFFFFFFFFFFFFFFFFFF
FFFFFFFFFFFFFFFFFFFFFFFFFFFFFFFFFFFFFFFFFFFFFFFFFFFFFFFFFFFFFF

```
@A00155:342:HHGFNDSXY:1:2407:25247:26334 2:N:0:GAACCTAG+TCCGCATA
TGCCTTCCACAGGTGGGGCACCCTGCTCCAAGTTTGGCTGAGGGATCACGCCAGCCTGAACACCTGTT
ACAAACCGAAAACCCTGCTCTTCTCGGGTTATGTAACCTCGCATCCGCTGCACTGTTTCATCTGTATT
+
FFFFFFFFFFFFFF:FFFFFFFFFFFFFFFFFFFFFFFFFFFFFFFF:FFFFFFFFFFFFFFFFF:F
FF:FF:FFFFFFF:FFFFFFFFFFFFFFFFFFFFFFFFFFF:FFFFFFFFFFFFFFFFFFFFFFFFFFF
@A00155:342:HHGFNDSXY:1:2606:27832:5666 1:N:0:GAACCTAG+TCCGCATA
CACAGGTGGGGCACCCTGCTCCAAGTTTGGCTGAGGGATCACGCCAGCCTGAACACCTGTTACAAACC
GAAAACCCTGCTCTTCTCGGGTTATGTAACCTCGCATCCGCTGCACTGTTTCATCTGTATTTACTGT
+
FFFFFFFFFFFFFFFFFFFFFFFFFFFFFFFFFFFFFFFFFFFFFFFFFFFFFFFFFFFFFFFFFFFF
FFFF:FFFFFFFFFFFFFFFFFFFFFFFFFFFFFFFFFFFFFFFFFFFFFFF:FFFFFFFFFFFF:
@A00155:342:HHGFNDSXY:1:2606:27832:5666 2:N:0:GAACCTAG+TCCGCATA
CACAGGTGGGGCACCCTGCTCCAAGTTTGGCTGAGGGATCACGCCAGCCTGAACACCTGTTACAAACC
GAAAACCCTGCTCTTCTCGGGTTATGTAACCTCGCATCCGCTGCACTGTTTCATCTGTATTTACTGTC
+
FFFFFFFFFFFFFFFFFFFFFFFFFFFFFFFFFFFFFFFFFFFFFFFFFFFFFFFFFFFFFFFFFFFF
FFFFFFFFFFFFFFFFFFFFFFFFFFFFFFFFFFFFFFFFFFFFFFFFFFFFFFFFFFFFFFFFFFFF
@A00155:342:HHGFNDSXY:1:2618:14769:22247 1:N:0:GAACCTAG+TCCGCATA
ACAGGTGGGGCACCCTGCTCCAAGTTTGGCTGAGGGATCACGCCAGCCTGAACACCTGTTACAAACCG
AAAACCCTGCTCTTCTCGGGTTATGTAACCTCGCATCCGCTGCACTGTTTCATCTGTATTTACTGTCA
+
FFFFFFFFFFFFFFFFFFFFFFFFFFFFFF:FFFFFFFFFFFFFFFFFFFFFFFFFFFFFFFFFFFFF
FFFFFFFFFFFFFFF:FFFFFFFFFFFFFFFFFFFFFFFFFFFFFFFFFFF,F:FF:FFFFFFFFFFFF
@A00155:342:HHGFNDSXY:1:2607:12626:1219 2:N:0:GAACCTAG+TCCGCATA
GGGGCACCCTGCTCCAAGTTTGGCTGAGGGATCACGCCAGCCTGAACACCTGTTACAAACCGAAAACC
CTGCTCTTCTCGGGTTATGTAACCTCGCATCCGCTGCACTGTTTCATCTGTATTTACTGTCACTGTTG
+
:FFFFFFFFFFFFFFFFFF:FFFFFFFFFFFFF:FFFFFFFFFFFFFFFFFFFFFFFFFFFFFFFFFF
FFFFFFFFFFFFFFFFFFFFFFFFFFFFFFFFFFFFFFFFFFFFFFFFFFFFFFFFFFFFFFFFFFFF
@A00155:342:HHGFNDSXY:1:2607:12219:2832 2:N:0:GAACCTAG+TCCGCATA
GGGGCACCCTGCTCCAAGTTTGGCTGAGGGATCACGCCAGCCTGAACACCTGTTACAAACCGAAAACC
CTGCTCTTCTCGGGTTATGTAACCTCGCATCCGCTGCACTGTTTCATCTGTATTTACTGTCACTGTTA
+
FFF:FFFFFFFFFFFFFFFFFFFFFFFFFFFFF:FFFFFFFFFFFFFF:FFFFFFFFFFFFFFF:FFF
FFFFFFFFFFFFFFFF:FFFFFFFFFFFFFFFFFFFFFFFFFFFFFFFFFFFFFFFFFFFFFFFFFF
@A00155:342:HHGFNDSXY:1:2558:28411:33004 2:N:0:GAACCTAG+TCCGCATA
GGGGCACCCTGCTCCAAGTTTGGCTGAGGGATCACGCCAGCCTGAACACCTGTTACAAACCGAAAACC
CTGCTCTTCTCGGGTTATGTAACCTCGCATCCGCTGCACTGTTTCATCTGTATTTACTGTCACTGTTA
+
FFFFFFFFFFFFFFFFFF:::FFFFFFFFFFFFFFFFFFFFFFFFFFFFFFFF:FFFF:FFFFFF:FFF
F:FFFFFFFFFFFFFFFFFFFFFFFFFF:FFFFFFFFFFFFFFFFFFFFFFFFFFFFF:FFFFFFFFFF
@A00155:342:HHGFNDSXY:1:2607:12626:1125 2:N:0:GAACCTAG+TCCGCATA
GGGGCACCCTGCTCCAAGTTTGGCTGAGGGATCACGCCAGCCTGAACACCTGTTACAAACCGAAAACC
CTGCTCTTCTCGGGTTATGTAACCTCGCATCCGCTGCACTGTTTCATCTGTATTTACTGTCACTGTTG
+
FFFFFFFFFFFFFFFFFFFFFFFFFFFFFFFFFFFFFFFFFFFFFFFFFFFFFFFFFFFFFFFFFFFF
FFFFFFFFFFFFFFFFFFFFFFFFFFFFFFFFFFFFFFFFFFFFFFFFFFFFFFFFFFFFFFFFFFFF
@A00155:342:HHGFNDSXY:1:1240:10827:30358 2:N:0:GAACCTAG+TCCGCATA
CCTGCTCCAAGTTTGGCTGAGGGATCACGCCAGCCTGAACACCTGTTACAAACCGAAAACCCTGCTCT
TCTCGGGTTATGTAACCTCGCATCCGCTGCACTGTTTCATCTGTATTTACTGTCACTGTTA
+
FFFFFFFFFFFFFFFFFFFFFFFFFFFFFFFFFFFFFFFFFFFFFFFFFFFFFFFFF::FFFFFFFFF
F:FFFFFFFFFFFFFFFFFFFFFFFFFFFFFFFFFFFFFFF:FFFFFFFFFFFFFFFFFFFF
```

@A00155:342:HHGFNDSXY:1:1240:9570:30217 2:N:0:GAACCTAG+TCCGCATA
CCTGCTCCAAGTTTGGCTGAGGGATCACGCCAGCCTGAACACCTGTTACAAACCGAAAACCCTGCTCT
TCTCGGGTTATGTAACCTCGCATCCGCTGCACTGTTTCATCTGTATTTACTGTCACTGTTA
+
FF,FFFFFFFFFFFFFFFFFFFFFFFFFFFFFFFFFFF:FFFFFFF,FFFFFFFFFFFFFFFFFFFFF
FFFFFFFFFFFF:FFFFFFFFFFFFFFFFFFFFFFFFFFFFFFFFFFFFFFFFFFFFFFFF
@A00155:342:HHGFNDSXY:1:1365:31349:20838 1:N:0:GAACCTAG+TCCGCATA
GGCTGAGGGATCACGCCAGCCTGAACACCTGTTACAAACCGAAAACCCTGCTCTTCTCGGGTTATGTA
ACCTCGCATCCGCTGCACTGTTTCATCTGTATTTACTGTCACTGTTATATCTTTGGTTATCCTTACTC
+
FFFFFFF:FFFFFFFFFFFFFFFFFFFFFFFFFFFFFFFFFFFF:FFFFFFFFFFFFFFFFF:FFF:F
FFF:FFFF::FFFFFFFFFFFFFFFFFFFFFFFFFFFFFFFFFFFFFFFFFFFFFFFFFFFFFFFF:
@A00155:342:HHGFNDSXY:1:2423:24686:16595 2:N:0:GAACCTAG+TCCGCATA
GGCTGAGGGATCACGCCAGCCTGAACACCTGTTACAAACCGAAAACCCTGCTCTTCTCGGGTTATGTA
ACCTCGCATCCGCTGCACTGTTTCATCTGTATTTACTGTCACTGTTATATCTTTGGTTATCCTTACTC
+
FFFFFFFFFFFFFFFFFFFFFFFFFFFFFFFFFFFFF:FFFFF,FFF:FFFFF,FFFFFFFFFFFFFF
FFFFFFFFFFFFFFFFFFFFFFFFFFFFFFFFFFFFFFFFFFFFFFFFF:F:FFFFFFFFFFFFFFFF
@A00155:342:HHGFNDSXY:1:1240:9570:30217 1:N:0:GAACCTAG+TCCGCATA
GCTGAGGGATCACGCCAGCCTGAACACCTGTTACAAACCGAAAACCCTGCTCTTCTCGGGTTATGTAA
CCTCGCATCCGCTGCACTGTTTCATCTGTATTTACTGTCACTGTTATATCTTTGGTTATCC
+
FFFFFFFFFFFFFFFF:FFFFFFFFFFFFFFFFFFF:FFFFFFFFFFFFFFFFFFFFFFF,FFFFFFF
FFFFFF:FFFFFFFFFFFFFFFFFFFFF:FF:FFFFFFFFF:FF,,F,FFF,FFFFFFFFF
@A00155:342:HHGFNDSXY:1:1240:10827:30358 1:N:0:GAACCTAG+TCCGCATA
GCTGAGGGATCACGCCAGCCTGAACACCTGTTACAAACCGAAAACCCTGCTCTTCTCGGGTTATGTAA
CCTCGCATCCGCTGCACTGTTTCATCTGTATTTACTGTCACTGTTATATCTTTGGTTATCC
+
FFFFFFFFFFFFFFFFFFFFFFFFFFFFFFFFFFFFFFFFFFFFFFFFFFFFFFFFFFFFFFFFFFFF
FFFFFFFFFFFFFFFFFFFFFFFFFFFFFFFFFFFFFFFFFFFFFFFFFFFFFFFFFFFFF
@A00155:342:HHGFNDSXY:1:1408:25672:1329 1:N:0:GAACCTAG+TCCGCATA
GCTGAGGGATCACGCCAGCCTGAACACCTGTTACAAACCGAAAACCCTGCTCTTCTCGGGTTATGTAA
CCTCGCATCCGCTGCACTGTTTCATCTGTATTTACTGTCACTGTTATATCTTTGGTTATCCTTACTCT
+
FFFFFFFFFFFFFFFFFFFFFFFFFFFFFFFFFFFFFFFFFFFFFFFFFFFFFFFFFFFFFFFF:FFF
FFFFFFFFFFFFFFFFFFFFFFFFFFFFFFFFFFFFFFFFFFFFFFFFFFFFF:FFFFFFFFFFFFF:
@A00155:342:HHGFNDSXY:1:1408:25355:1094 1:N:0:GAACCTAG+TCCGCATA
GCTGAGGGATCACGCCAGCCTGAACACCTGTTACAAACCGAAAACCCTGCTCTTCTCGGGTTATGTAA
CCTCGCATCCGCTGCACTGTTTCATCTGTATTTACTGTCACTGTTATATCTTTGGTTATCCTTACTCT
+
FFFFFFFFFFFFFFFFFFFFFFFFFFFFFFFFFFFFFFFFFFFFFFFFFFFFFFFFFFFFFFF,FF:F
FFFFFFFFFFFFFFFFFFFFFFFFFFFF:FFFFFFFFFFFFFFFFFFFFFFFFFFFFFFFF:FFFFFF
@A00155:342:HHGFNDSXY:1:2509:32262:23109 1:N:0:GAACCTAG+TCCGCATA
GCTGAGGGATCACGCCAGCCTGAACACCTGTTACAAACCGAAAACCCTGCTCTTCTCGGGTTATGTAA
CCTCGCATCCGCTGCACTGTTTCATCTGTATTTACTGTCACTGTTATATCTTTGGTTATCCTTACTCT
+
FFFFFFFFFFFFFFFFFFFFFFFFFFFFFFFFFFFFFFFFFFFFFFFFFFFFFFFFFFFFFFFFFFFF
FFFFFFFFFFFFFFFFFFFFFFFFFFFFFFFFFFFFFFFFFFFFFFFFFFFFFFFFFFFFFFFFFFFF
@A00155:342:HHGFNDSXY:1:2407:32063:14841 1:N:0:GAACCTAG+TCCGCATA
AGGGATCACGCCAGCCTGAACACCTGTTACAAACCGAAAACCCTGCTCTTCTCGGGTTATGTAACCTC
GCATCCGCTGCACTGTTTCATCTGTATTTACTGTCACTGTTATATCTTTGGTTATCCTTACTCTACCA
+
FFFFFFFFFFFFFFFFFFFFFFFFFFFFFFFFFFFFF:FFFFFFFFFFFFFFFFFFFFFFFFFFF:FF
FFFFFFFFFFFFFFFFFFFFFFFFFFFFFFFFFFFFFFFFFFFFFFFFFF:FFFFFFFFFFFFFFFFF

```
@A00155:342:HHGFNDSXY:1:2423:24686:16595 1:N:0:GAACCTAG+TCCGCATA
AGGGATCACGCCAGCCTGAACACCTGTTACAAACCGAAAACCCTGCTCTTCTCGGGTTATGTAACCTC
GCATCCGCTGCACTGTTTCATCTGTATTTACTGTCACTGTTATATCTTTGGTTATCCTTACTCTACCA
+
FFFFFFFFFFFFFFFFFFFFFFFFFFFFFFFFFFFFFFFFFFFFFFFFFFFFFFFFFFFFFFFFFFFF
FFFFFFFFFFFFFFFFFFFFFFFFFFFFFFFFFFFFFFFFFFFFFFFFFFFFFFFFFFFFFFFFFFFF
@A00155:342:HHGFNDSXY:1:2533:29984:34632 1:N:0:GAACCTAG+TCCGCATA
AGGGATCACGCCAGCCTGAACACCTGTTACAAACCGAAAACCCTGCTCTTCTCGGGTTATGTAACCTC
GCATCCGCTGCACTGTTTCATCTGTATTTACTGTCACTGTTATATCTTTGGTTATCCTTACTCTACCA
+
FFFFFFFFFFFFFFFFFFFFFFFFFFFFFFFFFFFFFFFFFFFFFFFFFFFFFFFFFFFFFFFFFFFF
FFFFFFFFFFFFFFFFFFFFFFFFFFFFFFFFFFFFFFFFFFFFFFFFFFFFFFFFF:FFFFFFFFFF
@A00155:342:HHGFNDSXY:1:2618:14769:22247 2:N:0:GAACCTAG+TCCGCATA
TCACGCCAGCCTGAACACCTGTTACAAACCGAAAACCCTGCTCTTCTCGGGTTATGTAACCTCGCATC
CGCTGCACTGTTACATCTGTATTTACTGTCACTGTTATATCTTTGGTTATCCTTACTCTACCACCAAA
+
FFFFF:FFFFFFFFFFFFFFFFFFFF:FFFFF:F,FFFFFFFFFFFFFFFFFFFFFFFFFFFFFFF:F
FFFFFFFFFFFF,FFFFFFFFFFFFFFFFFFFFFFFF,FFFFFFFFFFFFFFFFFFFF:FFFFFFFFF
@A00155:342:HHGFNDSXY:1:2533:29984:34632 2:N:0:GAACCTAG+TCCGCATA
GCCAGCCTGAACACCTGTTACAAACCGAAAACCCTGCTCTTCTCGGGTTATGTAACCTCGCATCCGCT
GCACTGTTTCATCTGTATTTACTGTCACTGTTATATCTTTGGTTATCCTTACTCTACCACCAAATTCT
+
FFFFFFFFFFFFFFFFFFFFFFFFFFFFFFFFFFFFFFFFFFFFFFFFFFFFFFFFFFFFFFFFFFFF
:F:FFFFFFFFFFFFFFFFFFFFFFFFFFFFFFFFFFFFFFFFFFFFFFFFFFFFFFFFFFFFFFFFF
@A00155:342:HHGFNDSXY:1:2602:25807:26334 1:N:0:GAACCTAG+TCCGCATA
CAGCCTGAACACCTGTTACAAACCGAAAACCCTGCTCTTCTCGGGTTATGTAACCTCGCATCCGCTGC
ACTGTTTCATCTGTATTTACTGTCACTGTTATATCTTTGGTTATCCTTACTCTACCACCAAATTCTGA
+
FFFFFFFFFFFFFFFFFFFFFFFFFFFFFFFFFFFFFFFFFFFFFFFFFFFFFFFFFFFFFFFFFFFF
FFFFFFFFFFFFFFFFFFFFFFFFFFFFFFFFFFFFFFFFFFFFFFFFFFFFFFFFFFFFFFFFFFFF
@A00155:342:HHGFNDSXY:1:2602:25807:26365 1:N:0:GAACCTAG+TCCGCATA
CAGCCTGAACACCTGTTACAAACCGAAAACCCTGCTCTTCTCGGGTTATGTAACCTCGCATCCGCTGC
ACTGTTTCATCTGTATTTACTGTCACTGTTATATCTTTGGTTATCCTTACTCTACCACCAAATTCTGA
+
FFFFFFFFFFFFF::FFFFFFFFFFFFFFFF:FFFFFFFFFFFFFFFFFFFFFF:FFFFFFFF:FFFF
FFFFFFFFFFFFF:FFFFFFFFFFFFFFFFFFFFFFFFFFFFFFFFFFFFFFFF:FFFFFFFFFFFFF
@A00155:342:HHGFNDSXY:1:1656:6054:21778 2:N:0:GAACCTAG+TCCGCATA
AGCCTGAACACCTGTTACAAACCGAAAACCCTGCTCTTCTCGGGTTATGTAACCTCGCATCCGCTGCA
CTGTTTCATCTGTATTTACTGTCACTGTTATATCTTTGGTTATCCTTACTCTACCACCAAATTCTGAT
+
FFFFFFFFFFFF:FFFFFFF:F:FFFF:FFFFFFFFFFFFFFF:FFFFFFFF:FFFFFFFFF,FFFFF
,FFFFFFFFFFFFFFFFFFFFFFFFFFFFFFFF:FFFFFFFFFFFFFFFFFF:F:FFFFFFFFFFFFF
@A00155:342:HHGFNDSXY:1:1630:25916:8985 2:N:0:GAACCTAG+TCCGCATA
CCTGAACACCTGTTACAAACCGAAAACCCTGCTCTTCTCGGGTTATGTAACCTCGCATCCGCTGCACT
GTTTCATCTGTATTTACTGTCACTGTTATATCTTTGGTTATCCTTACTCTACCACCAAATTCTGATAT
+
FF:FFFFFFFF,FFFFFFFF:FFFFFFFFFFFFFFFFFFFFFFFFFFFFFFFFFFFFFFFFFFFFFFF
FFFFFFFFFFFFFFFFFFFFFFFFFFFFFFFFF:FFFFFFFFF,FFFFFFFFFFFFFFFFFFFF:FFF
@A00155:342:HHGFNDSXY:1:1512:30617:10081 1:N:0:GAACCTAG+TCCGCATA
TGAACACCTGTTACAAACCGAAAACCCTGCTCTTCTCGGGTTATGTAACCTCGCATCCGCTGCACTGT
TTCATCTGTATTTACTGTCACTGTTATATCTTTGGTTATCCTTACTCTACCACCAAATTCTGATATAT
+
FFFFFFFFFFFFFFFFFFFFFFFFFFFFFFFFFFFFFFFFF,FFFF,FFFFFFFFFFFFFFFFFFFFF
FFFFFFFFFFFFFFFFFFFFFFFFF:FFFF:FFFFFF:FFFFFFFFFFFFFFFFFFFFFFFFF,:FFF
```

```
@A00155:342:HHGFNDSXY:1:2227:4761:35822 2:N:0:GAACCTAG+TCCGCATA
AACACCTGTTACAAACCGAAAACCCTGCTCTTCTCGGGTTATGTAACCTCGCATCCGCTGCACTGTTT
CATCTGTATTTACTGTCACTGTTATATCTTTGGTTATCCTTACTCTACCACCAAATTCTGAT
+
FFFFFFFFFFFFFFFFFFFF,FFFFFFFFFF:FFFFFF:FFFFF:FFFFFFFFFFFFFFFFFFFF,:FF
FFFFF,FFFFFFFFFFFFFFFFFFFF,FFFFFFFFFFFFFFFFFFFFFFFFFFFFFFFFFFF
@A00155:342:HHGFNDSXY:1:2602:25807:26365 2:N:0:GAACCTAG+TCCGCATA
ACCTGTTACAAACCGAAAACCCTGCTCTTCTCGGGTTATGTAACCTCGCATCCGCTGCACTGTTTCAT
CTGTATTTACTGTCACTGTTATATCTTTGGTTATCCTTACTCTACCACCAAATTCTGATATATCCTTC
+
FFFFFF:FFFFFFFFFF:FFFFFF:FFFF:FFF:FFFFFFF:FFFFF:FFFFF,FFFFFFFFFFFFFF
,FFFFFFFF,FFFFFFFFFFFFFFFF,FFFFFFFFF:F:FFFFFFFF,:FFFFFFFFFFFFFFFFFFF
@A00155:342:HHGFNDSXY:1:2136:16043:23923 1:N:0:GAACCTAG+TCCGCATA
ACCTGTTACAAACCGAAAACCCTGCTCTTCTCGGGTTATGTAACCTCGCATCCGCTGCACTGTTTCAT
CTGTATTTACTGTCACTGTTATATCTTTGGTTATCCTTACTCTACCACCAAATTCTGATATATCCTTC
+
FFFFFFFFFFFFFFFFFFFFFFFFFFFFFFFFFFF,FF:FFFFFFFFFFFFFFFFFFFFFFFFFFFFF
FFFFFFFFFFFFFFFFFF:FFFFFFFFFFFFFFFFFFFFFFFFFF:FFFFFFFFFFFFFFFFFFFFFF
@A00155:342:HHGFNDSXY:1:2602:25807:26334 2:N:0:GAACCTAG+TCCGCATA
ACCTGTTACAAACCGAAAACCCTGCTCTTCTCGGGTTATGTAACCTCGCATCCGCTGCACTGTTTCAT
CTGTATTTACTGTCACTGTTATATCTTTGGTTATCCTTACTCTACCACCAAATTCTGATATATCCTTC
+
FFFFFFFFFFFFFFFFFFFFFFFFFFFFFFFFFFFFFFFFFFFFFFF:FFFFFFF:FF:FFFFFFFFF
FFFFFFFFFFFFFFFFFF:FFFFFFFFFFFFFFFFFFFFFFFFFFFFFFFFFFFFFFFFFFFFFFFFF
@A00155:342:HHGFNDSXY:1:2514:14190:8124 2:N:0:GAACCTAG+TCCGCATA
CCTGTTACAAACCGAAAACCCTGCTCTTCTCGGGTTATGTAACCTCGCATCCGCTGCACTGTTTCATC
TGTATTTACTGTCACTGTTATATCTTTGGTTATCCTTACTCTACCACCAAATTCTGATATATCCTTC
+
FFFFFFFFFFFF:FFFFF:FF::FFFFFFFFFFFFFFF:F:FFFFFFFFFFFFFFFFFFFF:FFFFFF
FFFFFFFFFFFFFFFFF:FFFFFFFFFFFFFFFFFFFFFFFFFFFFFFFFFFFFFFFFFFFFFFFF
@A00155:342:HHGFNDSXY:1:1650:5602:25692 1:N:0:GAACCTAG+TCCGCATA
TGTTACAAACCGAAAACCCTGCTCTTCTCGGGTTATGTAACCTCGCATCCGCTGCACTGTTTCATCTG
TATTTACTGTCACTGTTATATCTTTGGTTATCCTTACTCTACCACCAAATTCTGATATATCCTTCCTG
+
FFFFFFFFFFFFFFFFFFFFFFFFFFFFFFFF:FF:FFFFFFFFFFFFFFFFFFFFFFFFFFFFFFFF
,FFFFFFFFFFFFFFFFFFFFFFFFFFFFFFFFFFFFFFFFFFFFFFFFFFFFFFFFFFFFFFFFFFF
@A00155:342:HHGFNDSXY:1:1234:32443:35289 1:N:0:GAACCTAG+TCCGCATA
TGTTACAAACCGAAAACCCTGCTCTTCTCGGGTTATGTAACCTCGCATCCGCTGCACTGTTTCATCTG
TATTTACTGTCACTGTTATATCTTTGGTTATCCTTACTCTACCACCAAATTCTGATATATCCTTCCTG
+
FFFFFFFFFFFFFFF:FFFFFFFFFFFFFFFFFFFFFFFFFFFFFFFFFFFFFFFFFFFF:FFF:FFF
FFFFFFFFFFFFFFF:FFFFFFFFFFFFFFFFFFFFFFFFFFFFFFFFFFFFFFF,FFFFF:FFFFFF
@A00155:342:HHGFNDSXY:1:1662:26196:33458 1:N:0:GAACCTAG+TCCGCATA
TGTTACAAACCGAAAACCCTGCTCTTCTCGGGTTATGTAACCTCGCATCCGCTGCACTGTTTCATCTG
TATTTACTGTCACTGTTATATCTTTGGTTATCCTTACTCTACCACCAAATTCTGATATATCCTTCCTG
+
FFFFFFFFFFFFFFF:FFFFFFFFFFFFFFFFFFFFFFFFFFFFFFFFFFFFFFFFFFFFFFFFFFFF
F:FFFFFFFFFFFFFFFFFFFFFFFFFFFFFFFFFFFFFFFFFFFFFFFFFFFFFFFFFFFFFFFFFF
@A00155:342:HHGFNDSXY:1:1656:6054:21778 1:N:0:GAACCTAG+TCCGCATA
TGTTACAAACCGAAAACCCTGCTCTTCTCGGGTTATGTAACCTCGCATCCGCTGCACTGTTTCATCTG
TATTTACTGTCACTGTTATATCTTTGGTTATCCTTACTCTACCACCAAATTCTGATATATCCTTCCTG
+
FFFFFFFFFFFFFFFFFFFFFFFFFFFFFFFF:FFFFF:FFFFFFFFFFFFFFFFFFFFFFFFFF::F
FFFFFFF:F,FFFFFFFFFFFFFFFFFFFFFFFFFFFFF:FFFFFFFFFFFFFFFFFFFFFFFFF:FF
```

```
@A00155:342:HHGFNDSXY:1:1118:10122:33301 1:N:0:GAACCTAG+TCCGCATA
AAACCGAAAACCCTGCTCTTCTCGGGTTATGTAACCTCGCATCCGCTGCACTGTTTCATCTGTATTTA
CTGTCACTGTTATATCTTTGGTTATCCTTACTCTACCACCAAATTCTGATATATCCTTCCTGTC
+
FFFFFFFFFFFFFFFFFFFFFFFFFFFFFFFFFFFFFFFFFFFFFFFFFFFFFFFFFFFFFFFFFFFF
FFFFFFFFFFFFFFFFFFFFFFFFFFFFFFFFFFFFFFFFFFFFFFFFFFFFFFFFFFFFFFFF
@A00155:342:HHGFNDSXY:1:1118:10963:34663 1:N:0:GAACCTAG+TCCGCATA
AAACCGAAAACCCTGCTCTTCTCGGGTTATGTAACCTCGCATCCGCTGCACTGTTTCATCTGTATTTA
CTGTCACTGTTATATCTTTGGTTATCCTTACTCTACCACCAAATTCTGATATATCCTTCCTGTC
+
FFFFFFFFFFFFFFFFFF:FFFFFFFFFF:FFFFFFFF:FFFFFFFFFFFFFFFFFFFFFFFFFFFFF
FFFFFFFFFFFFFFF:FFFFFFFFFFF:FFFFFFFFFFFFFFF:FFFFF:F:FFFF:FFFFFFF
@A00155:342:HHGFNDSXY:1:1365:31349:20838 2:N:0:GAACCTAG+TCCGCATA
ACCGAAAACCCTGCTCTTCTCGGGTTATGTAACCTCGCATCCGCTGCACTGTTTCATCTGTATTTACT
GTCACTGTTATATCTTTGGTTATCCTTACTCTACCACCAAATTCTGATATATCCTTCCTGTCTA
+
FF:FFFF:F,FFF:FFFF:F:F:FFF:FFF:,FFFFF::FFFFFFFF,FFFFFF:FFFF,FFFFFF:F
FF:,,FFFFFF:FFFFFFFFFFF:FFFFFF:FFF:,FFF:FFFFFFFFFFFFFFFFF,FFFFFF
@A00155:342:HHGFNDSXY:1:2227:4761:35822 1:N:0:GAACCTAG+TCCGCATA
CCGAAAACCCTGCTCTTCTCGGGTTATGTAACCTCGCATCCGCTGCACTGTTTCATCTGTATTTACTG
TCACTGTTATATCTTTGGTTATCCTTACTCTACCACCAAATTCTGATATATCCTTCCTGTCT
+
FFFFFF:FFF,FFFFFFFFFFFF,FFFFFFFFFFFFFFFFFFFFFFFFFFFFFFFFFFFFFFFFFFFF
FFFFFFFFFFFFFFFFFFFFFFFF:FF:F:FFFFFFFF:F:FFFFFFFFFFF:FFFFFFFFF
@A00155:342:HHGFNDSXY:1:1319:26232:2174 1:N:0:GAACCTAG+TCCGCATA
AAAACCCTGCTCTTCTCGGGTTATGTAACCTCGCATCCGCTGCACTGTTTCATCTGTATTTACTGTCA
CTGTTATATCTTTGGTTATCCTTACTCTACCACCAAATTCTGATATATCCTTCCTGTCTACATCATAC
+
FFFFF:F,::F:F:FFFF:F,FF:FFFFFFFFFFF:FFFFFFFFFFF::,,,FFF:FF:,FFFF,FFF
F:FFFFFFFFFFFFFFFFF:FFFFFFFF,,FFF:,F,F:FFFF,FF,FFFFFFF:FF:FFFFF,FFFF
@A00155:342:HHGFNDSXY:1:2514:14190:8124 1:N:0:GAACCTAG+TCCGCATA
AAACCCTGCTCTTCTCGGGTTATGTAACCTCGCATCCGCTGCACTGTTTCATCTGTATTTACTGTCAC
TGTTATATCTTTGGTTATCCTTACTCTACCACCAAATTCTGATATATCCTTCCTGTCTACATCATAC
+
FFFFFFFFFFFFFFFFFFFFFFFFFFFFFFFFFFFFFFFFFFFFFFFFFFFFFFFFFFFFFFFFFFFF
FFFFFFFFFFFFFFFFFFFFFFFFFFFFFFFFFFFFFFFFFFFFFFF:FFFF:FFFFFFFFFFFFFF
@A00155:342:HHGFNDSXY:1:1209:14913:14199 1:N:0:GAACCTAG+TCCGCATA
AAACCCTGCTCTTCTCGGGTTATGTAACCTCGCATCCGCTGCACTGTTTCATCTGTATTTACTGTCAC
TGTTATATCTTTGGTTATCCTTACTCTACCACCAAATTCTGATATATCCTTCCTGTCTACATCATACC
+
FFFFFFFFFFF,FFFFFFFFFFFFFFFFFFFFFFFFFFFFFFFFFFFF:FFFFFFF:FFFFFFFFFFF
FFFFF:FFFFFFFFFFFFFFFFFF:FFFFFFFFFFFFFFFFFFFFFFFFFFFFFFFFFFFFFFFF,FF
@A00155:342:HHGFNDSXY:1:1118:10122:33301 2:N:0:GAACCTAG+TCCGCATA
AACCCTGCTCTTCTCGGGTTATGTAACCTCGCATCCGCTGCACTGTTTCATCTGTATTTACTGTCACT
GTTATATCTTTGGTTATCCTTACTCTACCACCAAATTCTGATATATCCTTCCTGTCTACATCATACCA
+
FFFFFFFFFFFFFFFFFFFFFFFFFFFFFFFFFFFFFFFFFFFFFFFFFFFFFFFFFFFFFFFFFFFF
FFFFFFFFFFFFFFFFFFFFFFFFFFFFFFFFFFFFFFFFFFFFFFFFFFFFFFFFFFFFFFFFF:FF
@A00155:342:HHGFNDSXY:1:1118:10963:34663 2:N:0:GAACCTAG+TCCGCATA
AACCCTGCTCTTCTCGGGTTATGTAACCTCGCATCCGCTGCACTGTTTCATCTGTATTTACTGTCACT
GTTATATCTTTGGTTATCCTTACTCTACCACCAAATTCTGATATATCCTTCCTGTCTACATCATACCA
+
FFFFFFFFFFFFFFFFFF,FFFFFFFFFFFFFFFFFFFFFFFFFFF,FFFFF:FFFFFFFFFFFF:F:
FFF:FFFFFFFFFFFFFFFFFFFFFFFFFFFFFFFFFFFFFF,FFFFFFFFFFF:FFFFFFFFFFFFF
```

```
@A00155:342:HHGFNDSXY:1:2139:12527:19867 1:N:0:GAACCTAG+TCCGCATA
AACCCTGCTCTTCTCGGGTTATGTAACCTCGCATCCGCTGCACTGTTTCATCTGTATTTACTGTCACT
GTTATATCTTTGGTTATCCTTACTCTACCACCAAATTCTGATATATCCTTCCTGTCTACATCATACCA
+
FFFFFFFFFFFFFFFFFF:FFFFFFFFFFFFFF:FFFFFFFFFFFFFFFFFFFFFFFFFFFFFFFFFF
FFFFFF:FFFFFF:FFFFFFFFFFFFFFFFFFFFFF:FFFFFFFFFFFFFFFFFFFFFFFFFFFFFFF
@A00155:342:HHGFNDSXY:1:2123:19633:19398 1:N:0:GAACCTAG+TCCGCATA
ACCCTGCTCTTCTCGGGTTATGTAACCTCGCATCCGCTGCACTGTTTCATCTGTATTTACTGTCACTG
TTATATCTTTGGTTATCCTTACTCTACCACCAAATTCTGATATATCCTTCCTGTCTA
+
FFFFFFFFFFFFFFFFFFFFFFFFFFFF:FFFFFFF,FFFFFFFFFFFFF:FFFFFFFFFFFFFFFFF
FFFFFFFFFFFFFFFFFFFFFFFFFFFFFFFFFFFFFFFFFFFFFFFFFFFFFFFFF
@A00155:342:HHGFNDSXY:1:1423:5801:9001 1:N:0:GAACCTAG+TCCGCATA
ACCCTGCTCTTCTCGGGTTATGTAACCTCGCATCCGCTGCACTGTTTCATCTGTATTTACTGTCACTG
TTATATCTTTGGTTATCCTTACTCTACCACCAAATTCTGATATATCCTTCCTGTCTA
+
FF:FFFFFFFFF:FFFFFFF,FFFFFFFFFFFFFFFFFFFFFFFFFFFFFFFFFFF:FFFFFFFFFFF
FFFFFFFFFFFFFFFFFFFFFFFFFFFFFFFFFFFFFFFFFFFFFFFFFFFFFFFFF
@A00155:342:HHGFNDSXY:1:1259:9335:11459 1:N:0:GAACCTAG+TCCGCATA
ACCCTGCTCTTCTCGGGTTATGTAACCTCGCATCCGCTGCACTGTTTCATCTGTATTTACTGTCACTG
TTATATCTTTGGTTATCCTTACTCTACCACCAAATTCTGATATATCCTTCCTGTCTA
+
FFFFFFFFFFFFFF:FFFFFFFFFFFFFFFFFFFFFFFFFFFFFFFFFFFFFFFFFFFFFFFFFFFFF
FFFFFFFFFFFFFFFFFFFFFFFFFFFFFFFFFFFF:FFFFFFFFFFFFFFFFFFFF
@A00155:342:HHGFNDSXY:1:2673:1398:23516 1:N:0:GAACCTAG+TCCGCATA
ACCCTGCTCTTCTCGGGTTATGTAACCTCGCATCCGCTGCACTGTTTCATCTGTATTTACTGTCACTG
TTATATCTTTGGTTATCCTTACTCTACCACCAAATTCTGATATATCCTTCCTTTCTACATCATAC
+
FFFF:FFFFFFFFFFF,FF:FFFFFFFFFFF:F::F,FF::,F:FFFFFFFFFFFFFFFFFFF,F,F:
FFFF:FFFFFF,FFFFFFFFFFF,F:FF:,,F,:FF:F,,F:FFF:,FF,F:,F:FFF,F:FFF:
@A00155:342:HHGFNDSXY:1:1462:21956:15154 1:N:0:GAACCTAG+TCCGCATA
CCTGCTCTTCTCGGGTTATGTAACCTCGCATCCGCTGCACTGTTTCATCTGTATTTACTGTCACTGTT
ATATCTTTGGTTATCCTTACTCTACCACCAAATTCTGATATATCCTTCCTGTCTACATCATACC
+
FFFFFFFFFFFFFFFFFFFFFFFFFFFFFFFFFFFFFFFFFFFFFFFFFFFFFFFFFFFFFFFFFFFF
FFFFFFFFFFFF,FFFFFFFFFFFFFFFFFFFFFFFFFFFFFFFFFFFFFFFFFFF
@A00155:342:HHGFNDSXY:1:1320:31837:9032 1:N:0:GAACCTAG+TCCGCATA
TGCTCTTCTCGGGTTATGTAACCTCGCATCCGCTGCACTGTTTCATCTGTATTTACTGTCACTGTTAT
ATCTTTGGTTATCCTTACTCTACCACCAAATTCTGATATATCCTTCCTGTCTACATCATACCATAGAC
+
FFFFFFFFFFFF:FFF:FFFFFFFFFFFFFFFFFFFFFFFFFFFFFFFFFFFFFFF:FFFF:F::FF:
FF:FFFFFFFFFFF,::,FFFFFFFFFFFFFFFFFFFF:FFFFFFFFFFFFFFFFFFFFFFFFFFFFF
@A00155:342:HHGFNDSXY:1:1319:26232:2174 2:N:0:GAACCTAG+TCCGCATA
GGGTTATGTAACCTCGCATCCGCTGCACTGTTTCATCTGTATTTACTGTCACTGTTATATCTTTGGTT
ATCCTTACTCTACCACCAAATTCTGATATATCCTTCCTGTCTACATCATACCATAGACACACTCCCT
+
FFFFFFFFF:F,:FF::FF::FFF:F:,FFFFF:FF:FFF:FFFF:FFFFFFFFFFFF:FFFF:FFFF
FFF,:F,FFF:FFFFFFFFF:FFF,FFFF:FFFFFFFFFF,FF,FFF,F,FFFF,FFF:FFFF:FFF
@A00155:342:HHGFNDSXY:1:1662:26196:33458 2:N:0:GAACCTAG+TCCGCATA
GGGTTATGTAACCTCGCATCCGCTGCACTGTTTCATCTGTATTTACTGTCACTGTTATATCTTTGGTT
ATCCTTACTCTACCACCAAATTCTGATATATCCTTCCTGTCTACATCATACCATAGACACACTCCCTT
+
FFFFFFFFFFFFFFFFFFFFFFFFFFFFFFFFFFFFFFFFFFFFFFFFFFFFFFFFFFFFFFFFFFFF
FFFFFFFFFFF:FFFFFFFFFFFFFFFFFFFFFFFFFFFFFFFFFFFFFFFFFFFFFFFF
```

@A00155:342:HHGFNDSXY:1:2136:8250:2159 2:N:0:GAACCTAG+TCCGCATA
GGGTTATGTAACCTCGCATCCTCTGCACTGTTTCATCTGTATTTACTGTCACTGTTATATCTTTGGTT
ATCCTTACTCTACCACCAAATTCTGATATATCCTTCCTGTCTACATCATACCATAGACACACTCCCTT
+
FF:FFFF,FF:FF:FFFFFFF,FFF,F:FFFFF,FFFFFFFFFF:FFFFFFFFFFFFFFFFFFFFFFF
FFFFFFFFF:FFFFFFFFFFFFFFFFFFFFFFFFF:FFFFFFFFFFFFFFFFFFFFFF:FF,FFFFFF
@A00155:342:HHGFNDSXY:1:1423:5801:9001 2:N:0:GAACCTAG+TCCGCATA
GGTTATGTAACCTCGCATCCGCTGCACTGTTTCATCTGTATTTACTGTCACTGTTATATCTTTGGTTA
TCCTTACTCTACCACCAAATTCTGATATATCCTTCCTGTCTACATCATACCATAGACACACTCC
+
FFFF:FFFFFFFFFFFFFFFFFFFFFFFFFFFFFFFFF:FFFFFFFFFFFFFFFFFFFFFFFFFFFFF
FFFFFFFFFFFFFFFFFFFF:FFFFFFFFFFFFFFFFFFFFFFFFFFFFFFFFF,FFFFFFFFF
@A00155:342:HHGFNDSXY:1:2123:19633:19398 2:N:0:GAACCTAG+TCCGCATA
GGTTATGTAACCTCGCATCCGCTGCACTGTTTCATCTGTATTTACTGTCACTGTTATATCTTTGGTTA
TCCTTACTCTACCACCAAATTCTGATATATCCTTCCTGTCTACATCATACCATAGACACACTCC
+
FFFFFFFFFFFFFFFFFFFFFFFFFFFFFF:FFFFFFFFFFFFFFFFFFFFFF,FFFFFF:FFFFFFF
FFFFFFFFFFFFFFFFFFFFFFFFFFFFFFFFFFFFFFFFFFFFFFFFFFFFFFFFFFFFFFFF
@A00155:342:HHGFNDSXY:1:1259:9335:11459 2:N:0:GAACCTAG+TCCGCATA
GGTTATGTAACCTCGCATCCGCTGCACTGTTTCATCTGTATTTACTGTCACTGTTATATCTTTGGTTA
TCCTTACTCTACCACCAAATTCTGATATATCCTTCCTGTCTACATCATACCATAGACACACTCC
+
FFFFFFFFFFFFFFFFFFFFFFFFFFFFFFFFFFFFFFFFFFFFFFFFFFFFFFFF,FFFFFFFFFFF
FFFFFFFFFFFFFFFFFFFFFFFFFFFFFFFFFFFFFFFFFFFFFFFFFFFFFFFFFFFFFFFF
@A00155:342:HHGFNDSXY:1:2136:16043:23923 2:N:0:GAACCTAG+TCCGCATA
GTTATGTAACCTCGCATCCGCTGCACTGTTTCATCTGTATTTACTGTCACTGTTATATCTTTGGTTAT
CCTTACTCTACCACCAAATTCTGATATATCCTTCCTGTCTACATCATACCATAGACACACTCCC
+
FFFFFFFFFFFFFFFFFFFFFFFFFFFFFFFFFFFFFFFFFFFFFFFFFFFFF:FFFFFFFFFFFFFF
FFFFFFFFFFFFFFFFFFFFFFFFFFFFFFFFFFFFFFFFFFFFF:FFFFFFFFFFFFFFFFFF
@A00155:342:HHGFNDSXY:1:1304:28221:26976 1:N:0:GAACCTAG+TCCGCATA
GTTATGTAACCTCGCATCCGCTGCACTGTTTCATCTGTATTTACTGTCACTGTTATATCTTTGGTTAT
CCTTACTCTACCACCAAATTCTGATATATCCTTCCTGTCTACATCATACCATAGACACACTCCCTTAC
+
FFFFFFFFFFFFFFFFFFFFFFFFFFFFFFFFFFFFFFFFFFFFFFFFFFFFFFFFFFFFFFFFFFFF
FFFFFFFFFFFFFFFFFFFFFFFFFFFFFFFFFFFFFFFFFFFFFFFFFFFFFFFFFFFFFFFFFFFF
@A00155:342:HHGFNDSXY:1:2149:27046:37027 1:N:0:GAACCTAG+TCCGCATA
GTTATGTAACCTCGCATCCGCTGCACTGTTTCATCTGTATTTACTGTCACTGTTATATCTTTGGTTAT
CCTTACTCTACCACCAAATTCTGATATATCCTTCCTGTCTACATCATACCATAGACACACTCCCTTAC
+
F:FFFFFFFFFFFFFFFFFFFFFFFFFFFFFFFFFFFFFFFFFFFFFFFFFFFFFFFFFFFFFF:FFF
FFFFFFFFFFFFFFFFFFFFFFFFFFFFFFFFFFFFFFFFFFFFFFFFFFFFFFFFFFFFFFFFFFFF
@A00155:342:HHGFNDSXY:1:2170:16188:17347 1:N:0:GAACCTAG+TCCGCATA
GTTATGTAACCTCGCATCCGCTGCACTGTTTCATCTGTATTTACTGTCACTGTTATATCTTTGGTTAT
CCTTACTCTACCACCAAATTCTGATATATCCTTCCTGTCTACATCATACCATAGACACACTCCCTTAC
+
FFFFFFFFFFFFFFFFFFFFFFFFFFFFFFFFFFFFFFFFFFFFFFFFFFFFFFFFFFFFFFFFFFFF
FFFFFFFFFFFFFFFFFFFFFFFFFFFFFFFFFFFFFFFFFFFFFFFFFFFFFFFFFFFFFFFFFFFF
@A00155:342:HHGFNDSXY:1:2149:26973:36933 1:N:0:GAACCTAG+TCCGCATA
GTTATGTAACCTCGCATCCGCTGCACTGTTTCATCTGTATTTACTGTCACTGTTATATCTTTGGTTAT
CCTTACTCTACCACCAAATTCTGATATATCCTTCCTGTCTACATCATACCATAGACACACTCCCTTAC
+
FFFFFFF:FFFFFFFFFFFFFFFFFFFFFFFFFFFFFFFFFFFFFFFFFFFFFFFFFFFFFFFFFFFF
FFFFFFFFFFFFFFFFFFFFFFFFFFFFFFFFFFF:FFFFFFFFFFFFFFFFFFFFFFFFFFFFFFFF

@A00155:342:HHGFNDSXY:1:1265:25437:35336 1:N:0:GAACCTAG+TCCGCATA
GTTATGTAACCTCGCATCCGCTGCACTGTTTCATCTGTATTTACTGTCACTGTTATATCTTTGGTTAT
CCTTACTCTACCACCAAATTCTGATATATCCTTCCTGTCTACATCATACCATAGACACACTCCCTTAC
+
FFFFFFFFFFFFFFFFFFFFFFFFFFFFFFFFFFFFFFFFF:FFFFFFFFFFFFFFFFFFFFF,FFFF
FFFFFFFFFFFFFFFFFF:FFFFFFFFFFFFFFFFFFFFFFFFFFFFFFFFFFFFFFFFFFFFFFFFF
@A00155:342:HHGFNDSXY:1:1507:11812:5196 1:N:0:GAACCTAG+TCCGCATA
GTTATGTAACCTCGCATCCGCTGCACTGTTTCATCTGTATTTACTGTCACTGTTATATCTTTGGTTAT
CCTTACTCTACCACCAAATTCTGATATATCCTTCCTGTCTACATCATACCATAGACACACTCCCTTAC
+
FFFFFFFFFFFFFFFFFFFFFFFFFFFFFFFFFFFFFFFF:FFFFFFFFFFFFF:FFFFFFFFFFFFF
FFFFFFFFFFFFFFFFFFFFFFFFFFFFFFFFFFFFFFFFFFFFFFFFFFFFFFFFFFFFFFFFFFFF
@A00155:342:HHGFNDSXY:1:1462:21956:15154 2:N:0:GAACCTAG+TCCGCATA
TTATGTAACCTCGCATCCGCTGCACTGTTTCATCTGTATTTACTGTCACTGTTATATCTTTGGTTATC
CTTACTCTACCACCAAATTCTGATATATCCTTCCTGTCTACATCATACCATAGACACACTCCCT
+
FFFFFFFFFFFFFFFFFFFFFFFFFFF:FFFFFFFFFFFFFFFFFFFFFFFFFFFFFFFFFFFFFFFF
FFFFFFFFFFFFFFFFFFFFFFF:FFFFFFFFFFFFFFFFFFFFFFFFFFFF:FFFFFFFFFFF
@A00155:342:HHGFNDSXY:1:2149:26973:36933 2:N:0:GAACCTAG+TCCGCATA
TATGTAACCTCGCATCCGCTGCACTGTTTCATCTGTATTTACTGTCACTGTTATATCTTTGGTTATCC
TTACTCTACCACCAAATTCTGATATATCCTTCCTGTCTACATCATACCATAGACACACTCCCTTACGC
+
FFFFFFFFFFFFFFFFFFFF:F:FFFFFF:FF,FFFFFFFFFFFFF:FF:FFFFFFFFFFFFFF:FFF
,FFFFFFFF,FFFFFFFFFFFFFFFFFFFFFFFFF:FFFFFFFFFFFFFFFFF:FFFFFFFFFFFFFF
@A00155:342:HHGFNDSXY:1:2149:27046:37027 2:N:0:GAACCTAG+TCCGCATA
TATGTAACCTCGCATCCGCTGCACTGTTTCATCTGTATTTACTGTCACTGTTATATCTTTGGTTATCC
TTACTCTACCACCAAATTCTGATATATCCTTCCTGTCTACATCATACCATAGACACACTCCCTTACGC
+
FFFFFFFFFFFFFFFFF:FFFFFFFFFFFFFFFFFFFFFFFFFFFFFFFFFFFFFFFFFFFFFFFFFF
FFFFFFFF:FFFFFFFFFFFFFFFFFFFFFFFFFFF:FFFFFFFFFFFFFFFFFFFFFFFFFF:FFFF
@A00155:342:HHGFNDSXY:1:2136:8250:2159 1:N:0:GAACCTAG+TCCGCATA
GTAACCTCGCATCCGCTGCACTGTTTCATCTGTATTTACTGTCACTGTTATATCTTTGGTTATCCTTA
CTCTACCACCAAATTCTGATATATCCTTCCTGTCTACATCATACCATAGACACACTCCCTTACGCTGT
+
,FFFFFFFFFFFFFFFFFFFFFFFFFFFFFFFFFFFFFFFFFFFFFFFFFFFFFFFFFFFF,FFFFFF
FFFFFFFFFFFFFFFFFFFFFFFFFFFF::F,FFFFFFFFFFFFFFFFFF::FFFFF:,FFFFFFFFF
@A00155:342:HHGFNDSXY:1:2428:21160:36949 1:N:0:GAACCTAG+TCCGCATA
CACTGTTTCATCTGTATTTACTGTCACTGTTATATCTTTGGTTATCCTTACTCTACCACCAAATTCTG
ATATATCCTTCCTGTCTACATCATACCATAGACACACTCCCTTACGCTGTATCTGTACACTATACTCT
+
:FFFFFFFFFF:FFFFFFFFFFFFFF:FFFFFFFFFFFFFFFFFFFF::FFFFFFFFFFFF:FFFFFF
FFFFFFFFFFFFFFFFFFFFFFFFFF::FFFFF:FFFFFFF:FFF:FFFFFF:FFFFFFFFFFFFFFF
@A00155:342:HHGFNDSXY:1:1650:5602:25692 2:N:0:GAACCTAG+TCCGCATA
CACTGTTTCATCTGTATTTACTGTCACTGTTATATCTTTGGTTATCCTTACTCTACCACCAAATTCTG
ATATATCCTTCCTGTCTACATCATACCATAGACACACTCCCTTACGCTGTATCTGTACACTATACTCT
+
F:FFFFFFFFFFFFFFF:FFFFFFFFFFFFFFFFFFFF,FFFFFFFF:FFFFFFFFFFFFFFFFFFFF
FFFFFFFFF:FFF:FFFFFFFFFFFFFFFFFFFFFFFFFFFFFFFFFFFFFFFFFFFFFFFFFFFFFF
@A00155:342:HHGFNDSXY:1:1554:6162:9846 1:N:0:GAACCTAG+TCCGCATA
ACTGTTTCATCTGTATTTACTGTCACTGTTATATCTTTGGTTATCCTTACTCTACCACCAAATTCTGA
TATATCCTTCCTGTCTACATCATACCATAGACACACTCCCTTACGCTGTATCTGTACACTATACTCTA
+
FFFFFFFFFFFFFFFFFFFFFF:FFFF:,FFFFFFFF,:FFF,FFFFFFFFFFFFFFFFFF:FFFFFF
FFFFFF,FFFFF:FFFFFFFF:FFFFFFFFFFFFFFFFFF:FFFFFFFFFFFFFFFFFFFFFFFFF:F

```
@A00155:342:HHGFNDSXY:1:1209:14913:14199 2:N:0:GAACCTAG+TCCGCATA
TGTTTCATCTGTATTTACTGTCACTGTTATATCTTTGGTTATCCTTACTCTACCACCAAATTCTGATA
TATCCTTCCTGTCTACATCATACCATAGACACACTCCCTTACGCTGTATCTGTACACTATACTCTAAC
+
FFFFFFFFFFFFFFFFFFFFFFFFFFFFFFFFF:FFFFFFFFFFFFF:,FFFFFFFFFFFFFFFFFFF
FFFFFFFFFFFFFFFFFFFFFFFFFFFFFFFFFFFF:FFF:FFFFFFFFFFFFFFFFFFF:FFF::FF
@A00155:342:HHGFNDSXY:1:2570:3794:13041 1:N:0:GAACCTAG+TCCGCATA
GTTTCATCTGTATTTACTGTCACTGTTATATCTTTGGTTATCCTTACTCTACCACCAAATTCTGATAT
ATCCTTCCTGTCTACATCATACCATAGACACACTCCCTTACGCTGTATCTGTACACTATACTCTAACT
+
FFFFFFFFFFFFFFFFFFFFFFFFFFFFFFFFF,FFFFFFFFF:FFFFFFFFFFFFFFFFFFFFFFFF
FFFFFFFFFFFFFFFFFFFFFFFFFF:FFFFFFFFFFFFFFFFFFFFFFFFF:FFFFFFFFFFF:FFF
@A00155:342:HHGFNDSXY:1:1216:32777:14951 2:N:0:GAACCTAG+TCCGCATA
TTCATCTGTATTTACTGTCACTGTTATATCTTTGGTTATCCTTACTCTACCACCAAATTCTGATATAT
CCTTCCTGTCTACATCATACCATAGACACACTCCCTTCCGCTGTATCTGTACACTATACTCTAACTTC
+
:FFFF:FFFFF:FFFFFFFFFFFFFFFF:FFFFFFFFFFFFFFFFFFF,FFFFF,FFFFFFF,FFFFF
FFFFFFFF:FFFFFFFFFFFFFFFFF:FFFFFFFFFF,FFFFFFFFFFFF,FF:FF::FFFF,,F:FF
@A00155:342:HHGFNDSXY:1:1216:32533:15060 2:N:0:GAACCTAG+TCCGCATA
TTCATCTGTATTTACTGTCACTGTTATATCTTTGGTTATCCTTACTCTACCACCAAATTCTGATATAT
CCTTCCTGTCTACATCATACCATAGACACACTCCCTTACGCTGTATCTGTACACTATACTCTAACTTC
+
FFFFFFFFFFFFF:FFFFFF:FFFFFF::F:FFFFFFFFFFFFFFFFFFFFFFFFFFFFFFFFFFFFF
FFFFFFFFFFF:FFFF::FFFFFFFFFFFFFF:FF:,::FFFFFFF:FFF:FFFFFF:FFFF:,FFFF
@A00155:342:HHGFNDSXY:1:2657:13521:4022 2:N:0:GAACCTAG+TCCGCATA
TTCATCTGTATTTACTGTCACTGTTATATCTTTGGTTATCCTTACTCTACCACCAAATTCTGATATAT
CCTTCCTGTCTACATCATACCATAGACACACTCCCTTACGCTGTATCTGTACACTATACTCTAACTTC
+
FFFFFFFFFFFFFFFFFFFFFFFFFFFFF:FFFFFFFFFFFFFFFFFFFFFFFFFFFFFFFFFFFFFF
FFFFFFFFFFFFFFFFFFFFFFFFFFFFFFFFFFFFFFFFFFFFFFFFFFFFFFFFFFFFFFFFFFFF
@A00155:342:HHGFNDSXY:1:2671:4960:21919 1:N:0:GAACCTAG+TCCGCATA
TCATCTGTATTTACTGTCACTGTTATATCTTTGGTTATCCTTACTCTACCACCAAATTCTGATATATC
CTTCCTGTCTACATCATACCATAGACACACTCCCTTACGCTGTATCTGTACACTATACTCTAACTTCT
+
FFFFFFFFFFFFFFFFFFFFFFFFFFFFFFFFFFFFFFFFFFFFFFFFFFFFFFFFFFFF:FFFFFF:
FFFFFFFFFFFFFFFFFFFFFFFFFFFFFFFFFFFF:FFFFFFFFFFFFFFFFFFFFFFFFFFFFFF:
@A00155:342:HHGFNDSXY:1:2621:25138:16564 1:N:0:GAACCTAG+TCCGCATA
ATCTGTATTTACTGTCACTGTTATATCTTTGGTTATCCTTACTCTACCACCAAATTCTGATATATCCT
TCCTGTCTACATCATACCATAGACACACTCCCTTACGCTGTATCTGTACACTATACTCTAACTTCTGG
+
FFFFFFFFFFFFFFFFFFFFFFFFFFFFFFFFFFFFFFFFFFFFFFFFFFFFFFFFFFFFFFFFFFFF
FFFFFFFFFFFFFFFFFFFFFFFFFFFFFFFFFFFFFFFF:FFFFFFFFFFFFFFFFFFFFFFFFFFF
@A00155:342:HHGFNDSXY:1:1276:5828:8516 1:N:0:GAACCTAG+TCCGCATA
TCTGTATTTACTGTCACTGTTATATCTTTGGTTATCCTTACTCTACCACCAAATTCTGATATATCCTT
CCTGTCTACATCATACCATAGACACACTCCCTTACGCTGTATCTGTACACTATACTCTAACTTCTGGT
+
FFFFFFFFFFFFFFFFFFFFFFFFFF:FF,FFFFFFFFFFFFFFFFFFFFFFFFFFFFFFFFFFFFFF
FFFFFFFFFFFFFFFFFFFFFFFFFFFFFFFFFFFFFFFFFFFFFFFFFFFFFFFFFFFFFFFFFFFF
@A00155:342:HHGFNDSXY:1:2139:7139:27070 1:N:0:GAACCTAG+TCCGCATA
CTGTATTTACTGTCACTGTTATATCTTTGGTTATCCTTACTCTACCACCAAATTCTGATATATCCTTC
CTGTCTACATCATACCATAGACACACTCCCTTACGCTGTATCTGTACACTATACTCTAACTTCTGGTT
+
FFFFFFFFFFFFFFFFFFFFFFFFFFFFFFFFFFFFFFFFFFFFFFFFFFFFFFFFFFFFFFFFFFFF
FFFFFFFFFFFFFFFFFFFFFFFFFFFFFFFFFFFFFFFFFFFF:FFFFFFFFFFFFFFFFFFFFFFF
```

```
@A00155:342:HHGFNDSXY:1:2139:12527:19867 2:N:0:GAACCTAG+TCCGCATA
TGTATTTACTGTCACTGTTATATCTTTGGTTATCCTTACTCTACCACCAAATTCTGATATATCCTTCC
TGTCTACATCATACCATAGACACACTCCCTTACGCTGTATCTGTACACTATACTCTAACTTCTGGTTA
+
FFF:FFF:FFFF:F:F:FFFFFFFFFFF:FFFFFFFFFFFF:FFFFFFF:FFFFF:FFFFFFFFFFFF
FFFFFFFFFFFFFFFFFFFFFFFFFFFFFFFFFFFFFFFFFFFFFFFFFFFFFFFFFFFFFFFFFFFF
@A00155:342:HHGFNDSXY:1:2570:3794:13041 2:N:0:GAACCTAG+TCCGCATA
GTATTTACTGTCACTGTTATATCTTTGGTTATCCTTACTCTACCACCAAATTCTGATATATCCTTCCT
GTCTACATCATACCATAGACACACTCCCTTACGCTGTATCTGTACACTATACTCTAACTTCTGGTTAG
+
:FFFFFFFFFFFFF:FFFFFFFFFFFFFFFFFFFFFFFFFFFFFFFFFFFFFFFFFFFFFFFFFFFF:
FFF:FFFFFF:FFFFFFFFF:FFFFFFFFF:FFFFFFFFFFFFFFFFFFFFFF:F:FFFFFFFFFFFF
@A00155:342:HHGFNDSXY:1:1356:25807:7294 1:N:0:GAACCTAG+TCCGCATA
GTATTTACTGTCACTGTTATATCTTTGGTTATCCTTACTCTACCACCAAATTCTGATATATCCTTCCT
GTCTACATCATACCATAGACACACTCCCTTACGCTGTATCTGTACACTATACTCTAACTTCTGGTTAG
+
FFFFFFFFFFFFFFFF:FFFFFFFFFFFFFFF:FFFFFFFFFFFFFFFFFFFFFFFFFFFFFFFFFFF
FFFFFFFFFFFFFFFFFFFFFFFF:FFFFFFFFFFFFFFFFFFFFFFFF:FFFFFFFF:FFFFF:FFF
@A00155:342:HHGFNDSXY:1:1304:28221:26976 2:N:0:GAACCTAG+TCCGCATA
GTATTTACTGTCACTGTTATATCTTTGGTTATCCTTACTCTACCACCAAATTCTGATATATCCTTCCT
GTCTACATCATACCATAGACACACTCCCTTACGCTGTATCTGTACACTATACTCTAACTTCTGGTTAG
+
FFFFFFFFFFFFFFFFFFFFFFFFFFFFFFFFFFFFFFFFFFFFFFFFFFFFFFFFFFFFFFFFFFFF
FFFFFFFFFFFFFFFFFFFFFFFFFFFFFF:FFFFFFFFFFFFFFFFFFFFFFFFFFFFFFFFFFFFF
@A00155:342:HHGFNDSXY:1:2170:16188:17347 2:N:0:GAACCTAG+TCCGCATA
GTATTTACTGTCACTGTTATATCTTTGGTTATCCTTACTCTACCACCAAATTCTGATATATCCTTCCT
GTCTACATCATACCATAGACACACTCCCTTACGCTGTATCTGTACACTATACTCTAACTTCTGGTTAG
+
FFFFFFFF:FFFFFFF:F:FFFFFFFFFFFFFFFFFFFFFFFFFFFFFFFFF:FFFFFFFFFFFFFFF
FFFFFF:FFF,FFF:FFFFFFFFFFFFFFFFFFFFFFFFFFFFFFFFFFFFFFFF::F,FFFFFFFFF
@A00155:342:HHGFNDSXY:1:2657:13521:4022 1:N:0:GAACCTAG+TCCGCATA
TATTTACTGTCACTGTTATATCTTTGGTTATCCTTACTCTACCACCAAATTCTGATATATCCTTCCTG
TCTACATCATACCATAGACACACTCCCTTACGCTGTATCTGTACACTATACTCTAACTTCTGGTTAGC
+
FFFFFFFFFFFFFFFFFFFFFFFFFFFFFFFFFFFFFFFFFFFFFFFFFFFFFFFFFFFFFFFFFFFF
FFFFFFFFFFFFFFFFFFFFFFFFFFFFFFFFFFFFFFFFFFFFFFFFFFFFFFFFFFFFFFFFFFFF
@A00155:342:HHGFNDSXY:1:1216:32777:14951 1:N:0:GAACCTAG+TCCGCATA
TATTTACTGTCACTGTTATATCTTTGGTTATCCTTACTCTACCACCAAATTCTGATATATCCTTCCTG
TCTACATCATACCATAGACACACTCCCTTACGCTGTATCTGTACACTATACTCTAACTTCTGGTTAGC
+
FFFFFFFFFFFFFFFFFFFFFFFFFFFFFFFFFFFFFFFFFFFFFFFFFFFFFFFFFFFF:FFFFFFF
FFFFFFFFFFFFFFFFFFFFFFFFFFFFFFFFFFFFFFFFFFFFFFFFFFFFFFFFFFFFFFFFFFF:
@A00155:342:HHGFNDSXY:1:1216:32533:15060 1:N:0:GAACCTAG+TCCGCATA
TATTTACTGTCACTGTTATATCTTTGGTTATCCTTACTCTACCACCAAATTCTGATATATCCTTCCTG
TCTACATCATACCATAGACACACTCCCTTACGCTGTATCTGTACACTATACTCTAACTTCTGGTTAGC
+
FFFFFFFFFFFFFFFFFFFFFFFFFFFFFFFFFFFFF:FFFFFFFFFFFFFFFFFFFFFFFFFFFFFF
FFFFF:FFFFFFFFFFFFFFFFFFFFFFFFFFFFFFFFFFFFFFFFFFF:FFFFFFFFFFFFFFFFFF
@A00155:342:HHGFNDSXY:1:1670:12635:20744 2:N:0:GAACCTAG+TCCGCATA
CACTGTTATATCTTTGGTTATCCTTACTCTACCACCAAATTCTGATATATCCTTCCTGTCTACATCAT
ACCATAGACACACTCCCTTACGCTGTATCTGTACACTATACTCTAACTTCTGGTTAGCGAAG
+
FFFFFFFFFFFFFFFFFFFFFFFFFFFFFFFFFFFFFFFFFFFFFFFFFFFFFFFFFFFFFFFFFFF,
FFFFFFFFFFFFFFFFFFFFFFFFFFFFFFFFFFFFFFF:FFFFFFFFFFFFFFFFFFFFFF
```

```
@A00155:342:HHGFNDSXY:1:1550:6081:34882 2:N:0:GAACCTAG+TCCGCATA
CACTGTTATATCTTTGGTTATCCTTACTCTACCACCAAATTCTGATATATCCTTCCTGTCTACATCAT
ACCATAGACACACTCCCTTACGCTGTATCTGTACACTATACTCTAACTTCTGGTTAGCGAAG
+
FFFFFFFFFFFFFFFFFFFFFFFFFFFFFFFFFFFFFFFFFFFFFFFFFFFFFFFFFFFFFFFFFFFF
FFFFFFFFFFFFFFFFFFFFFFFFFFFFFFFFFFFFFFFF:F:FFFFFFFFFFFFFFFFFFFF
@A00155:342:HHGFNDSXY:1:2529:2483:14403 1:N:0:GAACCTAG+TCCGCATA
CTGTTATATCTTTGGTTATCCTTACTCTACCACCAAATTCTGATATATCCTTCCTGTCTACATCATAC
CATAGACACACTCCCTTACGCTGTATCTGTACACTATACTCTAACTTCTGGTTAGCGAAGAACTTATT
+
FFFFFFFFF:FFFFFFFFFFFFFFFFFFFFFFFFF,FFFFFFFFFFFFFFFFFFFFFFFFFFFFFFFF
FFFFFFFFFFFFFFFFFFFFFFFFFFFFFFFFFFFFFFFFFFFFFFFFFFFFFFFFFFFFFFFFFFFF
@A00155:342:HHGFNDSXY:1:2624:7726:9956 1:N:0:GAACCTAG+TCCGCATA
CTGTTATATCTTTGGTTATCCTTACTCTACCACCAAATTCTGATATATCCTTCCTGTCTACATCATAC
CATAGACACACTCCCTTACGCTGTATCTGTACACTATACTCTAACTTCTGGTTAGCGAAGAACTTATT
+
FF:::FFF:FFFFFFFFFFFF::FFF,FF::F,:FFF:F:FFFFFFFFFFFFF,FFFFFFFFFFFFFF
,F:FFFFF,F:FFF,FFFF,FFFFFFFFFFFFFFF,FFFFFF:FFF,FFFFFFFFF,FF:,FFFF,FFF
@A00155:342:HHGFNDSXY:1:1507:11812:5196 2:N:0:GAACCTAG+TCCGCATA
TATCTTTGGTTATCCTTACTCTACCACCAAATTCTGATATATCCTTCCTGTCTACATCATACCATAGA
CACACTCCCTTACGCTGTATCTGTACACTATACTCTAACTTCTGGTTAGCGAAGAACTTATTGTGTAA
+
FFFFFFFFFFFFFFFFFFFFFFFF:FFFFFFFFFFFFFFFFFF:FFFFFFFFFFFFFFFFFF:FFFFFF
FFFFFFFFFFFFFFFFFFFFFFFFFFFFFFF:,FFFFFFFFFFFFFFFFFFFFFFFFFFFFFFFFFFF
@A00155:342:HHGFNDSXY:1:1637:21802:11537 1:N:0:GAACCTAG+TCCGCATA
TTTGGTTATCCTTACTCTACCACCAAATTCTGATATATCCTTCCTGTCTACATCATACCATAGACACA
CTCCCTTACGCTGTATCTGTACACTATACTCTAACTTCTGGTTAGCGAAGAACTTATTGTGTAACGGC
+
F:FFFFFFFFFFFFF,FFFFFFFFFFF,FFFFFFFFFFFFF:FFFF:FFFFF,FF,FFFF,::FFFFF
FFF:FF:FFF::FFFFFFFFFFFFFF:FF:FF,FFFFF:FF:FFF:FFFF:F,,:FFFFFFFFFFF,F
@A00155:342:HHGFNDSXY:1:1649:6976:24220 2:N:0:GAACCTAG+TCCGCATA
TTGGTTATCCTTACTCTACCACCAAATTCTGATATATCCTTCCTGTCTACATCATACCATAGACACAC
TCCCTTACGCTGTATCTGTACACTATACTCTAACTTCTGGTTAGCGAAGAACTTATTGTGTAACGGCG
+
F:F:FFFFFFFFFFFFFFFFFFFF:F:FFFFFFFFFFFFFFFFFF:FFFFFFFF,,FF,:FFFFFFFF
FFFFFFFFFFFFFFFFFFFFFFFF:F:FFFFFFF:FFFFFFFFFFFFFFFFFFF,FFFFFFFFFFFFF
@A00155:342:HHGFNDSXY:1:1550:6081:34882 1:N:0:GAACCTAG+TCCGCATA
GGTTATCCTTACTCTACCACCAAATTCTGATATATCCTTCCTGTCTACATCATACCATAGACACACTC
CCTTACGCTGTATCTGTACACTATACTCTAACTTCTGGTTAGCGAAGAACTTATTGTGTAAC
+
FFFFFFFFFFFFFFFFFFFFFFFFFFFFFFFFFF:FFFFFFFFFFFFFFFFFFFFFFFFFFFFFFFFF
FFFFFFFFFFFFFFFFFFFFFFFFFFFFFF,FFF,FFFFFFFFFFFFFFF:FFFFFFFFFFFF
@A00155:342:HHGFNDSXY:1:1670:12635:20744 1:N:0:GAACCTAG+TCCGCATA
GGTTATCCTTACTCTACCACCAAATTCTGATATATCCTTCCTGTCTACATCATACCATAGACACACTC
CCTTACGCTGTATCTGTACACTATACTCTAACTTCTGGTTAGCGAAGAACTTATTGTGTAAC
+
FFFFFFFFFFFFFFFFFFFFFFFFFFFFFFFFFFFFFFFFFFFFFFFFFFFFFFFFFFFFFFFFFFFF
FFFFFFFFFFFFFFFFFFFFFFFFFFFFFFFFFFFFFFFFFFFFFF:FFFFFFFFFFFFFFFF
@A00155:342:HHGFNDSXY:1:1554:6162:9846 2:N:0:GAACCTAG+TCCGCATA
GTGATCCTTACTCTACCACCAAATTCTGATATATCCTTCCTGTCTACATCATACCATAGACACACTCC
CTTACGCTGTATCTGTACACTATACTCTAACTTCTGGTTAGCGAAGAACTTATTGTGTAACGGCGGT
+
FF,FF,FFFFFFFFFFFFF:FFFFF,FFFFFFFF:FFFF:FFFFFFFFFFFFFFFFFF:F:FFFFFF:
:FFF,F,FFFFFFF,FFFFFFFFFFFFFFFF,:FFFFFFFFFFFFFFFF:FFFF::FFFF,F,FF,F
```

```
@A00155:342:HHGFNDSXY:1:2529:2483:14403 2:N:0:GAACCTAG+TCCGCATA
ATCCTTACTCTACCACCAAATTCTGATATATCCTTCCTGTCTACATCATACCATAGACACACTCCCTT
ACGCTGTATCTGTACACTATACTCTAACTTCTGGTTAGCGAAGAACTTATTGTGTAACGGCGGTAGCT
+
FFFFFFFFFFFFFFFFFFFFFFFFF:FFFFFFFFFFFFFFFFFFFFFFF:FFFFFFFFFFFFFFFFFF
,FFFFFFFF,FFFFFFF:FFFFFFFF:FFFFFFFFFFFFFFFFFFFFFFFFFFFFFFFFFFFFFFFFF
@A00155:342:HHGFNDSXY:1:1574:1913:9909 2:N:0:GAACCTAG+TCCGCATA
CCTTACTCTACCACCAAATTCTGATATATCCTTCCTGTCTACATCATACCATAGACACACTCCCTTAC
GCTGTATCTGTACACTATACTCTAACTTCTGGTTAGCGAAGAACTTATTGTGTAACGGCG
+
FF:FFFFF::FFFFFFF,F:FFFFFF:FFFFFFFFFFFFFFFFFFFFFFFFFFFFFFFFFF:FFF:F,F
FFFFFFFFFFFFFFFFFFFFFFFF:FFFFFFFFFFFFFFFFFFFFFFF:FFF:FFFFFFF
@A00155:342:HHGFNDSXY:1:1445:26015:35556 2:N:0:GAACCTAG+TCCGCATA
CCTTACTCTACCACCAAATTCTGATATATCCTTCCTGTCTACATCATACCATAGACACACTCCCTTAC
GCTGTATCTGTACACTATACTCTAACTTCTGGTTAGCGAAGAACTTATTGTGTAACGGCGG
+
FFFFFFFFFFFFFF:FFF,FFFFFFFFFFFF:FFFFF:FF,F:FFFFFFFF,FFFFFFF:FFFFF:FF
FFFFFFFFFFFFFFFFFF:FFFFF,F,FFFFFFFFFFFFFFFFFFFFFFFFFFFFFFFFFF
@A00155:342:HHGFNDSXY:1:1150:28239:34961 2:N:0:GAACCTAG+TCCGCATA
CCTTACTCTACCACCAAATTCTGATATATCCTTCCTGTCTACATCATACCATAGACACACTCCCTTAC
GCTGTATCTGTACACTATACTCTAACTTCTGGTTAGCGAAGAACTTATTGTGTAACGGCGGTAGCTGT
+
FFFFFFFFFFFFFF,F,FFFFFFFFF,FFFF:FFFFFFFFFFFFFFFF:FFFFFFFFFFFFFFF:F,F
FFFFFFFFFFFFFFFFFFFFFFFFFFF:FFFFF:FFFFFFFFFFFFFF:FFFFFFFFFFFFFFFFFFFF
@A00155:342:HHGFNDSXY:1:1234:32443:35289 2:N:0:GAACCTAG+TCCGCATA
CCTTACTCTACCACCAAATTCTGATATATCCTTCCTGTCTACATCATACCATAGACACACTCCCTTAC
GCTGTATCTGTACACTATACTCTAACTTCTGGTTAGCGAAGAACTTATTGTGTAACGGCGGTAGCTGT
+
FFFFFFFF:FFF:FFFFFFFFFF:FFFFFFFFFFF:FFFFFFFFFFFFFF:FFFFFFF:FFFFFF:FF
FFFFFFFFFFFFFFF:F::FFFFFFFFFFFF:FFFFFFFFFFFF:FFF:F:FFFFFFFFFFFFFFFFF
@A00155:342:HHGFNDSXY:1:2278:3848:19711 2:N:0:GAACCTAG+TCCGCATA
CCTTACTCTACCACCAAATTCTGATATATCCTTCCTGTCTACATCATACCATAGACACACTCCCTTAC
GCTGTATCTGTACACTATACTCTAACTTCTGGTTAGCGAAGAACTTATTGTGTAACGGCGGTAGCTGT
+
FFFFFFFF:FFFFFFFF,FFFFFF,FF:FFFFFFFFFFFFFFFFFFFFFFFF,FFFFFF:FFFFF:FFF
FFFFFFFFFFFFFFFF:FFFFFFFFFFFFFFFFFFFFFFFFFFFFFFFFFFFFFFFFFFFFFFFFFFF
@A00155:342:HHGFNDSXY:1:1649:6976:24220 1:N:0:GAACCTAG+TCCGCATA
CTTACTCTACCACCAAATTCTGATATATCCTTCCTGTCTACATCATACCATAGACACACTCCCTTACG
CTGTATCTGTACACTATACTCTAACTTCTGGTTAGCGAAGAACTTATTGTGTAACGGCGGTAGCTGTA
+
FFFFFFFFFFFFFFFFFFFFFFFF:FFFFFFFFFFFFFFFFFFFFFFFFFFFFFFFFFFFFFFFFFFF
FFFFFFFFFFFFFFFFFFFFFFFFFFFFF:FFFFFFFFFFF:FFFFFFFFFFFFFFFFFFFFFFFFFFF
@A00155:342:HHGFNDSXY:1:1574:1913:9909 1:N:0:GAACCTAG+TCCGCATA
AAATTCTGATATATCCTTCCTGTCTACATCATACCATAGACACACTCCCTTACGCTGTATCTGTACAC
TATACTCTAACTTCTGGTTAGCGAAGAACTTATTGTGTAACGGCGGTAGCTGTATGAATA
+
FFFFFFFFFFFFFFFFFFFFFFFFFFFFFFFFFFFFFFF,FFFFFFFFFFFFFFFFFFFFFFFFFFFF
FFF:FFFFFFFFFFFFFFFFFFFFFFFF:FFFFFFFFFFFFF:FFFFFFFFFFFFFFFFFF
@A00155:342:HHGFNDSXY:1:1445:26015:35556 1:N:0:GAACCTAG+TCCGCATA
AAATTCTGATATATCCTTCCTGTCTACATCATACCATAGACACACTCCCTTACGCTGTATCTGTACAC
TATACTCTAACTTCTGGTTAGCGAAGAACTTATTGTGTAACGGCGGTAGCTGTATGAATAC
+
FFFFFFFFFFFFFFFFFFFFFFFFFFFFFFFFFFFFFFFFFFFFFFFFFFFFFFFFFFFFFFFFFFFF
FFFFFFFFFFFFFFFFFFFFFFFFFFFFFFFFFFFFFFFFFFFFFFF:FFFFFFFFFFFFFFFF
```

```
@A00155:342:HHGFNDSXY:1:2278:3848:19711 1:N:0:GAACCTAG+TCCGCATA
AAATTCTGATATATCCTTCCTGTCTACATCATACCATAGACACACTCCCTTACGCTGTATCTGTACAC
TATACTCTAACTTCTGGTTAGCGAAGAACTTATTGTGTAACGGCGGTAGCTGTATGAATACACGCGAC
+
FFFFFFFFFFFFFFFFFFFFFFFFFFFFFFFFFFFFFFFFFFFFFFFFFFFFFFFFFFFFFFFFFFFF
FFFFFFFFFFFFFFFFFFFFFFFFFFFFFFFFFFFFFFFFFFFFFFFFFFFFFFFFFFFFFFFF:FFF
@A00155:342:HHGFNDSXY:1:2672:31259:28635 1:N:0:GAACCTAG+TCCGCATA
AAATTCTGATATATCCTTCCTGTCTACATCATACCATAGACACACTCCCTTACGCTGTATCTGTACAC
TATACTCTAACTTCTGGTTAGCGAAGAACTTATTGTGTAACGGCGGTAGCTGTATGAATACACGCGAC
+
FFFFFFFFF:FFFFFFFFFFFFFFFFFFFFFFFFFFFFFFFF,FFFFFFFFFFFFFFFFFFFFFFFFF
FFFFFFFFFFFFFFFFFFFFFFFF,FF,FFFFFFFFFFFFFFFFFFFFFFFFFFFFFFFFFFFFFFFF
@A00155:342:HHGFNDSXY:1:1637:21802:11537 2:N:0:GAACCTAG+TCCGCATA
AAATTCTGATATATCCTTCCTGTCTACATCATACCATAGACACACTCCCTTACGCTGTATCTGTACAC
TATACTCTAACTTCTGGTTAGCGAAGAACTTATTGTGTAACGGCGGTAGCTGTATGAATACACGCGAC
+
FFFFFFFFFFFFFFFFFFFFFFFF:FFFFFFF:FFF,FF,FFFFF:FFFFFFFFFFFFFFFFFFFFFF
F,F:FFF:,FFFFFFFFFFFFFFFFF:FFFFFF:FFFFF:FFFFFFFFFFFFF:FFFFFFFFFFFFFF
@A00155:342:HHGFNDSXY:1:1105:7129:29559 1:N:0:GAACCTAG+TCCGCATA
AAATTCTGATATATCCTTCCTGTCTACATCATACCATAGACACACTCCCTTACGCTGTATCTGTACAC
TATACTCTAACTTCTGGTTAGCGAAGAACTTATTGTGTAACGGCGGTAGCTGTATGAATACACGCGAC
+
FFFFFFFFFFFFFFFFFFFFF,FFFFFFFFFFF,FFFFFFFFFFFFFFFFFFFFFFFFFFFFFFFFFF
FFFFFFFFFFFFFFFFFFFFFFFFFFFFFFFFFFFFFFFFFFFFF:FFFFFFFFFFFFFFFFF:FFFF
@A00155:342:HHGFNDSXY:1:1227:24994:2722 1:N:0:GAACCTAG+TCCGCATA
AAATTCTGATATATCCTTCCTGTCTACATCATACCATAGACACACTCCCTTACGCTGTATCTGTACAC
TATACTCTAACTTCTGGTTAGCGAAGAACTTATTGTGTAACGGCGGTAGCTGTATGAATACACGCGAC
+
FFFFFFFFFFFFFFFFFFFFFFFFFFFFFFFFFFFFFFFFFFFFFFFFFFFFFFFFFFFFFFFFFFFF
FFFFFFFFFFFFFFFFFFFFFFFF:FFFFFFFFFFFFFFF:FFFFFFFFFFFFFFFFFFFFFFFFFFF
@A00155:342:HHGFNDSXY:1:1150:28239:34961 1:N:0:GAACCTAG+TCCGCATA
AAATTCTGATATATCCTTCCTGTCTACATCATACCATAGACACACTCCCTTACGCTGTATCTGTACAC
TATACTCTAACTTCTGGTTAGCGAAGAACTTATTGTGTAACGGCGGTAGCTGTATGAATACACGCGAC
+
FFFFFFFFFFFFFFFFFFFF::FFFFFFFFFFF:FFFFFFF:FFFFFFFFFFFFFFFFFFFFFFFF:F
FFFFFFFFFFFFFFFFFFFFFFF,:FFFFFFFFFFFFFFFFFFFFFFFFFFFFFFFFF:FFFF:FFFF
@A00155:342:HHGFNDSXY:1:2654:10248:31704 1:N:0:GAACCTAG+TCCGCATA
AAATTCTGATATATCCTTCCTGTCTACATCATACCATAGACACACTCCCTTACGCTGTATCTGTACAC
TATACTCTAACTTCTGGTTAGCGAAGAACTTATTGTGTAACGGCGGTAGCTGTATGAATACACGCGAC
+
FFFFFFFFFFFFFFFFFFFFFFFFFFFFFFFFFFFFFFFFFFFFF:FFFFFFFFFFFFFFFFFFFFFF
FFFFFFFFFFFFF:FFFFFFFFFFFFFFFFFFFFFFFFFFFFFFFFFFFFFFFFFFFFFFFFFFFFFF
@A00155:342:HHGFNDSXY:1:1265:25437:35336 2:N:0:GAACCTAG+TCCGCATA
ATTCTGATATATCCTTCCTGTCTACATCATACCATAGACACACTCCCTTACGCTGTATCTGTACACTA
TACTCTAACTTCTGGTTAGCGAAGAACTTATTGTGTAACGGCGGTAGCTGTATGAATACACGCGACCT
+
FFFFFFFFFFFFFFFFFF:FFFFFFFFFF,:FFFFFFFF:FFFFFFFFFFFFFFFFF:FF:F:FFFFF
FFFFFF:F::FFFFFF,FFFFFFFFFFFF:,:FFFFFF:FFFFFFFFFFFFFFFFFFFFFFFFFFFFF
@A00155:342:HHGFNDSXY:1:1408:25672:1329 2:N:0:GAACCTAG+TCCGCATA
TTCTGATATATCCTTCCTGTCTACATCATACCATAGACACACTCCCTTACGCTGTATCTGTACACTAT
ACTCTAACTTCTGGTTAGCGAAGAACTTATTGTGTAACGGCGGTAGCTGTATGAATACACGCGACCTC
+
FFFFFFFFFFFFFFFFFFFFFFFFFFFF:FFFF:FFFFFFFFFFFFFFFFFFFFFFFFFFF:FFFFFF
FFFFFF:FFFFFFF:F:FFFFFFFFFFFFFFFFFFFFFFFFFFFFFFFFFFFFFFFFFFFFFFFFFFF
```

```
@A00155:342:HHGFNDSXY:1:1408:25355:1094 2:N:0:GAACCTAG+TCCGCATA
TTCTGATATATCCTTCCTGTCTACATCATACCATAGACACACTCCCTTACGCTGTATCTGTACACTAT
ACTCTAACTTCTGGTTAGCGAAGAACTTATTGTGTAACGGCGGTAGCTGTATGAATACACGCGACCTC
+
FFFFFFFF:FFFFFFFFFFFFFFFFFFF:FFFFFFFFFFFFFFFFFFFFFFFF::FFFFFFFFFFFFF:
FFFFFF:F:FFFFFFFFFFFFFFFFFFFF,FFFFFFFFFFFFFFFFFFFFFFFFFFFFFFFFFFFFFFF
@A00155:342:HHGFNDSXY:1:1622:17183:27806 1:N:0:GAACCTAG+TCCGCATA
GATATATCCTTCCTGTCTACATCATACCATAGACACACTCCCTTACGCTGTATCTGTACACTATACTC
TAACTTCTGGTTAGCGAAGAACTTATTGTGTAACGGCGGTAGCTGTATGAATACACGCGACCTCTCCG
+
FFFFFFFFFFFFFFFFFFFFFFFFFFFFFFFFFFFFFFFFFFFFFFFFFFFFFFFFFFFFFFFFFFFF
FFFF,FFFFFFFFFFF:FFFFFFFFFFFFFFFFFFFFFFFFFFFFFFFFFFFFFFFFFFFFFFFFFFF:
@A00155:342:HHGFNDSXY:1:2624:7726:9956 2:N:0:GAACCTAG+TCCGCATA
ATATCCTTCCTGTCTACATCATACCATAGACACACTCCCTTACGCTGTATCTGTACACTATACTCTAA
CTTCTGGTTAGCGAAGAACTTATTGTGTAACGGCGGTAGCTGTATGAATACACGCGACCTCTCCGTCG
+
FFFF,FFF,FFFF:FFFFF,FFF:,FF:FFFFFFFF,,,FFF:,:FFFFF:FFFFF::FF:FFF,FF:
:FF:FF,FFF:F,:F:FF:FFFFFFFFF,FFF::,FFFFFFFFFFFFFF:FFFF::FF:F:FFFFFF:
@A00155:342:HHGFNDSXY:1:1107:31521:11021 1:N:0:GAACCTAG+TCCGCATA
TATCCTTCCTGTCTACATCATACCATAGACACACTCCCTTACGCTGTATCTGTACACTATACTCTAAC
TTCTGGTTAGCGAAGAACTTATTGTGTAACGGCGGTAGCTGTATGAATACACGCGACCTCTCCGTCGT
+
FFFFFFFFFFFFFFFF:FFFFFFFFFFFFFFFFFFFFFFFFFFFF:FF:FFFFFFFFFFFFFFFFFFF
,FFFFFFFFFFFFFFF,FFFF,FFFFFFFFFFFFFFFFFFF:FFFFFFFFFFFFFFFFFFFFFFFFFF
@A00155:342:HHGFNDSXY:1:2621:25138:16564 2:N:0:GAACCTAG+TCCGCATA
TATCCTTCCTGTCTACATCATACCATAGACACACTCCCTTACGCTGTATCTGTACACTATACTCTAAC
TTCTGGTTAGCGAAGAACTTATTGTGTAACGGCGGTAGCTGTATGAATACACGCGACCTCTCCGTCGT
+
FFFFFFFFFFFFFFFFFFFFFFFFFFFFFFFFFFFFFFFF,FFFFFFFFFFFFFFFFFF,FFFFFFFF
FFFFFFFFFFFFFFFFFFFFF,FFFFFFFFFFFFFFFFFFFFFFFFFFFFFFFFFFFFFFFFFFFFFF
@A00155:342:HHGFNDSXY:1:1116:9842:1877 1:N:0:GAACCTAG+TCCGCATA
TATCCTTCCTGTCTACATCATACCATAGACACACTCCCTTACGCTGTATCTGTACACTATACTCTAAC
TTCTGGTTAGCGAAGAACTTATTGTGTAACGGCGGTAGCTGTATGAATAAACGCGACCTCTCCGTCGT
+
FFFFF:F:FFFFFFF,FFFFFFFFFFFFF:FFFFFFFFFFFFFFFFFFFFFFFFF:FFFFFFFFFFFF
FFFF,FFFFFFF,FFFF,FFFFFFFFFFFFFFFFFFF,,FFFFFFFFFF,FFF:,FFFFFFFFFFFFF
@A00155:342:HHGFNDSXY:1:1512:30617:10081 2:N:0:GAACCTAG+TCCGCATA
CCTTCCTGTCTACATCATACCATAGACACACTCCCTTACGCTGTATCTGTACACTATACTCTAACTTC
TGGTTAGCGAAGAACTTATTGTGTAACGGCGGTAGCTGTATGAATACACGCGACCTCTCCGTCGTAGA
+
FFFFFFFFFFFFFFFFFFFFFFFFFFFFFFFFFFFFF,,FFFFFFFFFFFFFFFFFF:FFFFFFFF:FF
FF:FFFFFFFFFF:FFFFF:F,FFFFFFFFFFFFFFFFFFFFFFFFFFFFFFFFFFFFFFF:FFFFFF
@A00155:342:HHGFNDSXY:1:1276:5828:8516 2:N:0:GAACCTAG+TCCGCATA
TCCTGTCTACATCATACCATAGACACACTCCCTTACGCTGTATCTGTACACTATACTCTAACTTCTGG
TTAGCGAAGAACTTATTGTGTAACGGCGGTAGCTGTATGAATACACGCGACCTCTCCGTCGTAGACGT
+
FFFFFFFFFFFFFFFFFFFFFFFFFFFFFFFFFFFFFFFFFFFFFFFFF:FFFFFFFFF:F:FF:FF
FFFFFFFFFFFFF:FFFFFFFFFFFFFFFFFFFFFFFFFFFFFFFFFFFFFFF:FFFFFFFFFFFFF
@A00155:342:HHGFNDSXY:1:1226:32940:7122 1:N:0:GAACCTAG+TCCGCATA
CCTGTCTACATCATACCATAGACACACTCCCTTACGCTGTATCTGTACACTATACTCTAACTTCTGGT
TAGCGAAGAACTTATTGTGTAACGGCGGTAGCTGTATGAATACACGCGACCTCTCCGTCGTAGACGTG
+
FFFFFFFFFFFFFFFFFFFFFFFFFFFFFFFFFFFFFFFFF:FFFFFFFFFFFFFFFFFFFFFFFFFF
::FFFFFFFFFFF:FFF:FFFFFFFFFFFFFFFFFFFFFFFFFFFFFFFFFFFFFFFFFFFFFFFFFF
```

```
@A00155:342:HHGFNDSXY:1:1313:10529:12994 2:N:0:GAACCTAG+TCCGCATA
TGTCTACATCATACCATAGACACACTCCCTTACGCTGTATCTGTACACTATACTCTAACTTCTGGTTA
GCGAAGAACTTATTGTGTAACGGCGGTAGCTGTATGAATACACGCGACCTCTCCGTCGTAGACGTGAT
+
FFFFF:FF:FF:FF:FFFFFFFFFFFFFFF::FFFF:F:FFFFFFFFFFFF,FFFFFFF,:FFFFFFF
FFFFFFFFFFFFFFFFFFFFFFFFFFFFFFFFFFFFFFFFFFFFFFFFFFFFFFFFFFFFFFFFFFFF
@A00155:342:HHGFNDSXY:1:1512:30436:8703 1:N:0:GAACCTAG+TCCGCATA
TGTCTACATCATACCATAGACACACTCCCTTACGCTGTATCTGTACACTATACTCTAACTTCTGGTTA
GCGAAGAACTTATTGTGTAACGGCGGTAGCTGTATGAATACACGCGACCTCTCCGTCGTAGACGTGAT
+
FFFFFFFFFFFFFFFFFFFFFFFFFFFFFFFFFFFFFFF:FFFFFFFFFFFFFFFFFFFFFFFFFFFF
F:FFFFFFFFFFFFFFFFFFFFFFFFFFFFFFFFFFF,FFFFFFFFFFFFFFFFFFFFFFFFFFFFFF
@A00155:342:HHGFNDSXY:1:1220:5972:21668 1:N:0:GAACCTAG+TCCGCATA
ACATCATACCATAGACACACTCCCTTACGCTGTATCTGTACACTATACTCTAACTTCTGGTTAGCGAA
GAACTTATTGTGTAACGGCGGTAGCTGTATGAATACACGCGACCTCTCCGTCGTAGACGTGATGGTCA
+
FFFFFFFFFFFFFFFFFFFFFFFFFFFFFFFFFFFFFFFFFF:FFFFFFFFFFFFFFFFFFFFFFFFF
FFFFFFFFFFFFFFFFFFFFFFFFFFFFFFFFFFFFFFFF:FFFFFFFFFFFFFFFFFFFFFFFFFFF
@A00155:342:HHGFNDSXY:1:1508:17517:9846 1:N:0:GAACCTAG+TCCGCATA
CATCATACCATAGACACACTCCCTTACGCTGTATCTGTACACTATACTCTAACTTCTGGTTAGCGAAG
AACTTATTGTGTAACGGCGGTAGCTGTATGAATACACGCGACCTCTCCGTCGTAGACGTGATGGTCAT
+
FFFFFFFFFFFFFFFFFFFFFFFFFFFFFFFF:FFFFFFFFFFFFFFFFFFFFFFFFFFFFFFFFFFF
FFFFFFFFFFFFFFFFFFFFFFFFFFFFFFFFFFFFFFFFFFFFFFFFFFFFFFFFFFFFFFFFFFFF
@A00155:342:HHGFNDSXY:1:1216:22489:27759 2:N:0:GAACCTAG+TCCGCATA
ACCATAGACACACTCCCTTACGCTGTATCTGTACACTATACTCTAACTTCTGGTTAGCGAAGAACTTA
TTGTGTAACGGCGGTAGCTGTATGAATACACGCGACCTCTCCGTCGTAGACGTGATGGTCATGTCTTC
+
FFFF:FFFFFFFFFFF:FFFFFFFFFFFFFFFFFFF,:F,FFFF:FFFFFFFFFFFFFFFFFFFFFFF
F:FFFFFFFFFFFFFFFFFFFFFFFFFFFFFFFFFFFFFFFFFFFF:FFFFFFFF:FFFFFFFFFFFF
@A00155:342:HHGFNDSXY:1:1471:10538:8531 1:N:0:GAACCTAG+TCCGCATA
CCATAGACACACTCCCTTACGCTGTATCTGTACACTATACTCTAACTTCTGGTTAGCGAAGAACTTAT
TGTGTAACGGCGGTAGCTGTATGAATACACGCGACCTCTCCGTCGTAGACGTGATGGTCATGTCTTCA
+
FFFFFFFFFFFFFFFFFFFFFFFFFFFFFFFFFFF:FFFFFFFFFFFFFFFFFFFFFFFFFFFFFFFF
FFFFFFFFFFFFFFFFFFFFFFFFFFFFFFFFFFFFFFFFFFFFFFFFFFFFFFFFFFFFFFFFFFFF
@A00155:342:HHGFNDSXY:1:1216:22489:27759 1:N:0:GAACCTAG+TCCGCATA
CCATAGACACACTCCCTTACGCTGTATCTGTACACTATACTCTAACTTCTGGTTAGCGAAGAACTTAT
TGTGTAACGGCGGTAGCTGTATGAATACACGCGACCTCTCCGTCGTAGACGTGATGGTCATGTCTTCA
+
FF:FFFFFFFFFFFFFFFFFFFFFFFFFFFFFFFFFFFFFFFFFFFFFFFFF:FFFFFF:FFFFFFF:
FFFFFFFFFFFFFFFFFFFFFFFFFFFFFFFFFFFFFFFFFFFFFFFFFFFFFFFFFFFFFFFFFFFF
@A00155:342:HHGFNDSXY:1:1231:7383:24580 1:N:0:GAACCTAG+TCCGCATA
CCATAGACACACTCCCTTACGCTGTATCTGTACACTATACTCTAACTTCTGGTTAGCGAAGAACTTAT
TGTGTAACGGCGGTAGCTGTATGAATACACGCGACCTCTCCGTCGTAGACGTGATGGTCATGTCTTCA
+
FFFFFFFFFFFFFFFFFFFFFFFFFFFFFFFFFFFFFFFFFFFFFFFF:FFFFFFFFFFFFFFFFFFF
FFFFFFFFFFFFFFFFFFFFFFFFFFFFFFFFFFFFFFFFFFFFFFFFFFFFFFFFFFFFFFFFFFFF
@A00155:342:HHGFNDSXY:1:1246:4752:22467 1:N:0:GAACCTAG+TCCGCATA
CCATAGACACACTCCCTTACGCTGTATCTGTACACTATACTCTAACTTCTGGTTAGCGAAGAACTTAT
TGTGTAACGGCGGTAGCTGTATGAATACACGCGACCTCTCCGTCGTAGACGTGATGGTCATGTCTTCA
+
FFFFFFFFFFFFFFFFFFFF,,FFFFFFFFFF,FFFFFFFFFFFFFFFFFFFFFFFFFFFFFFFFFFF
FFFFFFFFFFFFFFFFFFFFFFFFFFFFFFFFFFFFFFFFF,FFFFFF:FFFFFFFFFFFFFFFFFFF
```

```
@A00155:342:HHGFNDSXY:1:1109:19416:1579 1:N:0:GAACCTAG+TCCGCATA
CATAGACACACTCCCTTACGCTGTATCTGTACACTATACTCTAACTTCTGGTTAGCGAAGAACTTATT
GTGTAACGGCGGTAGCTGTATGAATACACGCGACCTCTCCGTCGTAGACGTGATGGTCATG
+
FF:FFF:FF:FF,,FF:F,,,F:FF,:F,:FF,,,,,FF,FFF:,:FF,:FFF::FF,FFF,:F,FF,
FF,:FF,FF,FF,F,:,:FFFFF,,FFFFFF,FFF:FFFFFFFF,:FFF,,FF,:F,:,FF
@A00155:342:HHGFNDSXY:1:2422:10881:16110 1:N:0:GAACCTAG+TCCGCATA
CACACTCCCTTACGCTGTATCTGTACACTATACTCTAACTTCTGGTTAGCGAAGAACTTATTGTGTAA
CGGCGGTAGCTGTATGAATACACGCGACCTCTCCGTCGTAGACGTGATGGTCATGTCT
+
FFFFFFFFFFFFFFFFFFFFFFFFFFFFFFFFFFFFFFFFFFFFFFFFFFFF:F:FFFFFFFFFFFFF
FFFFFFFFFFFFFFFFFFFFFFFFFFFFFFFFFFFFFFFFFFFFFFFFFFFFFFFFFF
@A00155:342:HHGFNDSXY:1:2422:11469:18349 1:N:0:GAACCTAG+TCCGCATA
CACACTCCCTTACGCTGTATCTGTACACTATACTCTAACTTCTGGTTAGCGAAGAACTTATTGTGTAA
CGGCGGTAGCTGTATGAATACACGCGACCTCTCCGTCGTAGACGTGATGGTCATGTCT
+
FFFFFFFFFFFFFFFFFFFFFFFFFFFFFFFFFFFFFFFFFFFFFFFFFFFFFFFFFFFFFFFFFFFF
FFFFFFFFFFFFFFFFFFFFFFFFFFFFFFFFFFFFFFFFFFFFFFFFFFFFFFFFFF
@A00155:342:HHGFNDSXY:1:1508:17517:9846 2:N:0:GAACCTAG+TCCGCATA
CACTCCCTTACGCTGTATCTGTACACTATACTCTAACTTCTGGTTAGCGAAGAACTTATTGTGTAACG
GCGGTAGCTGTATGAATACACGCGACCTCTCCGTCGTAGACGTGATGGTCATGTCTTCATCTTGCTCT
+
FFFFFF:FFFFFFFFFFFFFFFFFFFFFF:FFFF,FFFFF:FFFFFFFFFFFFFFFFF:FFFFFFFFF
FFFFFFFFFFFFFFFFFFFFFFFFFFFFFFFFFFFFFFFFFFFFFFFFFFFFFFFFFFFFFFFFFFFF
@A00155:342:HHGFNDSXY:1:2469:15835:21371 2:N:0:GAACCTAG+TCCGCATA
CCCTTACGCTGTATCTGTACACTATACTCTAACTTCTGGTTAGCGAAGAACTTATTGTGTAACGGCGG
TAGCTGTATGAATACACGCGACCTCTCCGTCGTAGACGTGATGGTCATGTCTTCATCTTGCTCTTCAG
+
FFFFFFFFFFFFFFFFFFFFFFFFFFFFFFF:FFFFFFFFFFFFFFFFFFF:FFFFFFFFFFFFFFFF
FFFFFFFFFFF:FFFFFFFFFFFFFFFFFFFFFFFFFFFFFFFFFFFFFFFFFFFFFFFFFFFFFF:F
@A00155:342:HHGFNDSXY:1:2139:7139:27070 2:N:0:GAACCTAG+TCCGCATA
CCCTTACGCTGTATCTGTACACTATACTCTAACTTCTGGTTAGCGAAGAACTTATTGTGTAACGGCGG
TAGCTGTATGAATACACGCGACCTCTCCGTCGTAGACGTGATGGTCATGTCTTCATCTTGCTCTTCAG
+
FFFFFFFFFFFFFFFFFFFF:FF,FFFFFF:FFFFFFFFFFFFFFFFFFFFFFFFFFFFFF:FFFFFF
F:FFFFFFFF:FFFFFFFFF:FFFFFFFFFFFFFFFFFFFFFFFFFFFFFFFFFFFFFFFFFFFFFFF
@A00155:342:HHGFNDSXY:1:1512:30436:8703 2:N:0:GAACCTAG+TCCGCATA
CTTACGCTGTATCTGTACACTATACTCTAACTTCTGGTTAGCGAAGAACTTATTGTGTAACGGCGGTA
GCTGTATGAATACACGCGACCTCTCCGTCGTAGACGTGATGGTCATGTCTTCATCTTGCTCTTCAGGT
+
FFFFFFFFFFFFFFFFFFFFFFFFFFFFF,FFFFFFFFFFFFFFFFFFFFFFFFFFFFFFFFFFFFFF
FFFFFFFFFFF:FFFFFFFFFFFFFFFFFFFFFFFFFFFFFFFFFFFFFFFFFFF:FFFFFFFFFFFF
@A00155:342:HHGFNDSXY:1:1622:17183:27806 2:N:0:GAACCTAG+TCCGCATA
CTTACGCTGTATCTGTACACTATACTCTAACTTCTGGTTAGCGAAGAACTTATTGTGTAACGGCGGTA
GCTGTATGAATACACGCGACCTCTCCGTCGTAGACGTGATGGTCATGTCTTCATCTTGCTCTTCAGGT
+
FFF,FFFFFFF:FFFFFFFFF,FFFFFF:FFFFFFFFFFFFFFFFFFFFFFFFFFFFFFFFFFFFFFF
FFFFFFFFFFFFFFFFFFFFFFFFFFFFFFFFFFFFFFFFFFFFFFFFFFFFFFFFFFFF:FFF:FFF
@A00155:342:HHGFNDSXY:1:2411:12789:17315 1:N:0:GAACCTAG+TCCGCATA
TACGCTGTATCTGTACACTATACTCTAACTTCTGGTTAGCGAAGAACTTATTGTGTAACGGCGGTAGC
TGTATGAATACACGCGACCTCTCCGTCGTAGACGTGATGGTCATGTCTTCATCTTGCTCTTCAGGTGC
+
FFFFFFFFFFFFFFFFFFFFFFFFFFFFFFFFFFFFFFFFFFFFFFFFFFFFFFFFFFFFFFFFFFFF
FFFFFFFFFFFFFFFFFFFFFFFFFFFFFFFFFFFFFFFFFFFFFFF:FFFFFFFFFFFFFFFFFFFF
```

```
@A00155:342:HHGFNDSXY:1:2671:4960:21919 2:N:0:GAACCTAG+TCCGCATA
CTGTATCTGTACACTATACTCTAACTTCTGGTTAGCGAAGAACTTATTGTGTAACGGCGGTAGCTGTA
TGAATACACGCGACCTCTCCGTCGTAGACGTGATGGTCATGTCTTCATCTTGCTCTTCAGGTGCATTT
+
FFFFF:FFFFFFFFFFFFFFF,FFFFF,FFFFF:FFFFFFFFFFFFFF:FFFF:FFFFFFFFFFFFFF
FFFF:FFFFFFFFFFFFFFFFFFFFFFFFFFFFFFFFFFFFFFFFFFF:FFFFFFFFFFFFFFFFFFF
@A00155:342:HHGFNDSXY:1:2654:10248:31704 2:N:0:GAACCTAG+TCCGCATA
ATCTGTACACTATACTCTAACTTCTGGTTAGCGAAGAACTTATTGTGTAACGGCGGTAGCTGTATGAA
TACACGCGACCTCTCCGTCGTAGACGTGATGGTCATGTCTTCATCTTGCTCTTCAGGTGCATTTAACA
+
FFFFFFFFFFFFFFFFFFF:FFFFFFFFFFF:FFFFFFFFFFFFFFFF:FFFFFFFFFFFFFFFFFFF
FFFFFFFFFFFFFFFFFFFFFFFFFFFFFFFFFFFFFFFFFFFFFFFFF,FFFFFFFFFFF:FFFFFF
@A00155:342:HHGFNDSXY:1:1105:7129:29559 2:N:0:GAACCTAG+TCCGCATA
ATCTGTACACTATACTCTAACTTCTGGTTAGCGAAGAACTTATTGTGTAACGGCGGTAGCTGTATGAA
TACACGCGACCTCTCCGTCGTAGACGTGATGGTCATGTCTTCATCTTGCTCTTCAGGTGCATTTAACA
+
FFFFFFFFFFFFFFFFFFFFF,FF:FFFFFFFFFFFFFFFFFFFFFFFFFFFFFFFFFFFFFFFFFFF
FFFFFFFFFFFFFFFFFFFFFFFFFFFFFFFFFFFFFFFFFFFFFFFFFFFFFFFFFFFFFFFFFFFF
@A00155:342:HHGFNDSXY:1:1227:24994:2722 2:N:0:GAACCTAG+TCCGCATA
ATCTGTACACTATACTCTAACTTCTGGTTAGCGAAGAACTTATTGTGTAACGGCGGTAGCTGTATGAA
TACACGCGACCTCTCCGTCGTAGACGTGATGGTCATGTCTTCATCTTGCTCTTCAGGTGCATTTAACA
+
FF:FFFFFFFFFFFFFF:FFFFFFFFFFFFFFFFFFFFFFFFFFFFF:FFF:FF:F:FFFFFFFFFFF
FFFFFFFFFFFFFFFFFFFFFFFF:FFFFFFFFFFFFFFFFFFFFFFFFFFFFFFFFFFF:FFFFFFF
@A00155:342:HHGFNDSXY:1:2361:30011:36871 1:N:0:GAACCTAG+TCCGCATA
TCTGTACACTATACTCTAACTTCTGGTTAGCGAAGAACTTATTGTGTAACGGCGGTAGCTGTATGAAT
ACACGCGACCTCTCCGTCGTAGACGTGATGGTCATGTCTTCATCTTGCTCTTCAGGTGCATTTAACAC
+
FFFFFFFFFFFFFFFFFFFFF:FFFFFFFFFFFFFFFFFFFFFFFFFFFF:FFFFFFFFFFFFFFFFF
FFFFFFFFFFFFFFFFFFFFFFFFFFFFFF:FFFFFFFFFFFFFFFFF:FFFFFFFFFFFFFFFFFFF
@A00155:342:HHGFNDSXY:1:1143:13901:13322 1:N:0:GAACCTAG+TCCGCATA
TCTGTACACTATACTCTAACTTCTGGTTAGCGAAGAACTTATTGTGTAACGGCGGTAGCTGTATGAAT
ACACGCGACCTCTCCGTCGTAGACGTGATGGTCATGTCTTCATCTTGCTCTTCAGGTGCATTTAACAC
+
FFFFFFFFFFFFFFFFFFFFFFFFFFFFFFFFFFFFFFFFFFFFFFFFFFFFFFFFFFFFFFFFFFFF
FFFFFFFFFFFFFFFFFFFFFFFFFFFFFFFFFFFFFFFFFFFFFFFFFFFFFFFFFFFFFFFFFFFF
@A00155:342:HHGFNDSXY:1:2469:15835:21371 1:N:0:GAACCTAG+TCCGCATA
TCTGTACACTATACTCTAACTTCTGGTTAGCGAAGAACTTATTGTGTAACGGCGGTAGCTGTATGAAT
ACACGCGACCTCTCCGTCGTAGACGTGATGGTCATGTCTTCATCTTGCTCTTCAGGTGCATTTAACAC
+
FFFFFFFFFFFFFFFFFFFFFFFFFFFFFFFFFFFFFF:FFFFFFFFFFFFFFFFFFFFFFFFFFFFF
FFFFFFFFFFFFFFFFFFFFFFFFFFFFFFFFFFFFFFFFFFFFFFFFFFFFFFFFFFFFFFFFFFFF
@A00155:342:HHGFNDSXY:1:2562:28221:19429 1:N:0:GAACCTAG+TCCGCATA
TCTGTACACTATACTCTAACTTCTGGTTAGCGAAGAACTTATTGTGTAACGGCGGTAGCTGTATGAAT
ACACGCGACCTCTCCGTCGTAGACGTGATGGTCATGTCTTCATCTTGCTCTTCAGGTGCATTTAACAC
+
FFFFFFFFFFFFFFFFFFFFFFFFFFFFFFFFFFFFFFFFFFFFFFFFFFFFFFFFFFFFFFFFFFFF
FFFFFFFFFFFFFFFFFFFFFFFFFFFFFFFFFFFFFFFFFFFFFFFFFFFFFFFFFFFFFFFFFFFF
@A00155:342:HHGFNDSXY:1:2533:30644:12727 1:N:0:GAACCTAG+TCCGCATA
TCTGTACACTATACTCTAACTTCTGGTTAGCGAAGAACTTATTGTGTAACGGCGGTAGCTGTATGAAT
ACACGCGACCTCTCCGTCGTAGACGTGATGGTCATGTCTTCATCTTGCTCTTCAGGTGCATTTAACAC
+
FFFFFFFFFFFFFFFFFFFFFFFFFFFFFFFFFFFFFFFFFFFFFFFFFFFFFFFFFFFFFFFFFF,F
FFFFFFFFFFFFFFFFFFFFFFFFFFFFFFFFFFFFFFFFFFFFFFFFFFFFFFFFFFFFFFFFFFFF
```

```
@A00155:342:HHGFNDSXY:1:1226:32940:7122 2:N:0:GAACCTAG+TCCGCATA
CTGTACACTATACTCTAACTTCTGGTTAGCGAAGAACTTATTGTGTAACGGCGGTAGCTGTATGAATA
CACGCGACCTCTCCGTCGTAGACGTGATGGTCATGTCTTCATCTTGCTCTTCAGGTGCATTTAACACT
+
FFF:F:FFFFFFFFF:::FFFFFFFFFFFF:FFFFFFFF:FFFFFFFFFFFFFFF:FFFFFFFFF,,F
FFFFFF:FF:FFFFFFFFFFFFFFFFFFFFFFFFFFFFFFFFF:FFFFFFFFFFFFFFFFF:FFFFF,
@A00155:342:HHGFNDSXY:1:2413:26512:27430 1:N:0:GAACCTAG+TCCGCATA
TGTACACTATACTCTAACTTCTGGTTAGCGAAGAACTTATTGTGTAACGGCGGTAGCTGTATGAATAC
ACGCGACCTCTCCGTCGTAGACGTGATGGTCATGTCTTCATCTTGCTCTTCAGGTGCATTTAACACTG
+
FFFFFFFFFFFFFFFFFFFFFFFFFFFFFFFFFFFF:FFFFFFFFFFFFFFFFFFFFFFFFFFFFFFF
FFFFFFFFFFFF:FFFFFFFFFFFFFFFFFFFFFFFFFFFFFFFFFFFFFFFFFFFFFFFFFFFFFFF
@A00155:342:HHGFNDSXY:1:2413:25988:30123 1:N:0:GAACCTAG+TCCGCATA
TGTACACTATACTCTAACTTCTGGTTAGCGAAGAACTTATTGTGTAACGGCGGTAGCTGTATGAATAC
ACGCGACCTCTCCGTCGTAGACGTGATGGTCATGTCTTCATCTTGCTCTTCAGGTGCATTTAACACTG
+
FFFFFFFFFFFFFFFFFFFFFFFFFFFFFFF:FFFFFFFFFFFFFFFFFFFFFFFFFFFFFFFFFFFF
FFFFFFFFFFFFFFFFFFFFFFFFFFFFFFFFFFFFFFFFFFFFF:FFFFFFFFFFFFFFFFFFFFFF
@A00155:342:HHGFNDSXY:1:1432:30038:12868 1:N:0:GAACCTAG+TCCGCATA
TACACTATACTCTAACTTCTGGTTAGCGAAGAACTTATTGTGTAACGGCGGTAGCTGTATGAATACAC
GCGACCTCTCCGTCGTAGACGTGATGGTCATGTCTTCATCTTGCTCTTCAGGTGCATTTAACACTGGC
+
FFFFF:FFFFFFFFFFFFFFFFFFFFFFFFFFFFFFFFFFFFFFFFFFFFFFFFFFFFFFFFFFFFFF
FFFFFFFFFFFFFFFFFFFFFFFFFFFFFFFFFFFFFFFFFFFFFFFFFFFFFFFFFFFFFFFFFFFF
@A00155:342:HHGFNDSXY:1:1439:29080:21512 1:N:0:GAACCTAG+TCCGCATA
TACACTATACTCTAACTTCTGGTTAGCGAAGAACTTATTGTGTAACGGCGGTAGCTGTATGAATACAC
GCGACCTCTCCGTCGTAGACGTGATGGTCATGTCTTCATCTTGCTCTTCAGGTGCATTTAACACTGGC
+
FFFFFFFFFFFFFFF:FFFFFFFFFFFFFF:FFFFFFFFFFFFFFFFFFFFFFFFFFFFFFFFFFFFF
FFFFFFFFFFFFFFFFFFFFFFFFFFFFFFFFFFF:FFFFFFFFF:FFFFF:FFFFFF:FFFFFFFFF
@A00155:342:HHGFNDSXY:1:1428:15817:9189 2:N:0:GAACCTAG+TCCGCATA
TACACTATACTCTAACTTCTGGTTAGCGAAGAACTTATTGTGTAACGGCGGTAGCTGTATGAATACAC
GCGACCTCTCCGTCGTAGACGTGATGGTCATGTCTTCATCTTGCTCTTCAGGTGCATTTAACACTGGC
+
FFFFFFFFFFFFFFFFFFFFFFFF:FF:FFFFFFFFFFFFFFFFFFFFFFFFFFFFFFFFFFFFFFFF
FFFFFFFFFFFFFFFFFFFFFFFFFFFF:FFFFFFFFFFFFF:FFFFFFFFFFFFFFFFFFFFFFFFF
@A00155:342:HHGFNDSXY:1:1116:9842:1877 2:N:0:GAACCTAG+TCCGCATA
CACTATACTCTAACTTCTGGTTAGCGAAGAACTTATTGTTTAACGGCGGTAGCTGTATGAATACACGC
GACCTCTCCGTCGTAGACGTGATGGTCATGTCTTCATCTTGCTCTTCAGGTGCATTTAACACTGGCCA
+
FFFFFFFFFFFFFFFFFF:,:FFFFFFFFFFFFFFFFFF,F:FFFFFFFFFFFFFFFFFFFFF,FFFF
FFFFFFFFFF:FF:FFFFFFFFFFF:FFFF:FF:FFFFFFFFFFFFFFFFFFFFFFFFFFFFF:FFFF
@A00155:342:HHGFNDSXY:1:1428:15817:9189 1:N:0:GAACCTAG+TCCGCATA
CTCTAACTTCTGGTTAGCGAAGAACTTATTGTGTAACGGCGGTAGCTGTATGAATACACGCGACCTCT
CCGTCGTAGACGTGATGGTCATGTCTTCATCTTGCTCTTCAGGTGCATTTAACACTGGCCACACCATA
+
FFFFFF:::FFFFFFFFFFFFFFFFFF:FFFFFFFFF:FFFFFFFFFF:FFFFFFFFFFFFFFFFFFF
FFFFFFFFFFFFFFFFF:FFFFFFFFFFFFFFFFFFFFFFFFFFFFFFFFFFFFFFFF:FFFFFFF:F
@A00155:342:HHGFNDSXY:1:1421:32606:27430 1:N:0:GAACCTAG+TCCGCATA
ACTTCTGGTTAGCGAAGAACTTATTGTGTAACGGCGGTAGCTGTATGAATACACGCGACCTCTCCGTC
GTAGACGTGATGGTCATGTCTTCATCTTGCTCTTCAGGTGCATTTAACACTGGCCACACCATATTGAC
+
FFFFFFFFFFFFFFFFFF:FFFFFFFFF:FFFFFFFFF:FFFFFFFFF,FFFFFFFFFFFFFFFFFFF
FFFFFFFFFFFFFFFFFFFFFFFF:FFFFF:FFFFFFFFFFFF:FFFFFFFFFFF:FFFFFF,FFFFF
```

```
@A00155:342:HHGFNDSXY:1:2411:12789:17315 2:N:0:GAACCTAG+TCCGCATA
CTTCTGGTTAGCGAAGAACTTATTGTGTAACGGCGGTAGCTGTATGAATACACGCGACCTCTCCGTCG
TAGACGTGATGGTCATGTCTTCATCTTGCTCTTCAGGTGCATTTAACACTGGCCACACCATATTGACG
+
FFFFFFFFFFFFFFFFFFFFFF:FFFFFFFFFFFFFFFFFFFFFFFFFFFFFFFFFFFFFFFFFFFFF
FFFFFFFFFFFFFFFFFFFFFFFFFFFFFFFFFFFFFFFFFFFFFFFFFFFFFFFFFFFFFFFFFFFF
@A00155:342:HHGFNDSXY:1:1424:12373:11929 1:N:0:GAACCTAG+TCCGCATA
TTCTGGTTAGCGAAGAACTTATTGTGTAACGGCGGTAGCTGTATGAATACACGCGACCTCTCCGTCGT
AGACGTGATGGTCATGTCTTCATCTTGCTCTTCAGGTGCATTTAACACTGGCCACACCAGATTGACGT
+
FFFFFFFFFFFFFFFFFFFFFFFFFFFFFFFFFFFFFFFFFFFFFFFFFFFFFFFFFFFFFFFFFFFF
FFFFFFFFFFFFFFFFFFFFFFFFFFFFFFFFFFFFFFFFFFFFFFFFFFFFFFFFFFF,FFFFFFFF
@A00155:342:HHGFNDSXY:1:1659:8874:33019 1:N:0:GAACCTAG+TCCGCATA
TGGTTAGCGAAGAACTTATTGTGTAACGGCGGTAGCTGTATGAATACACGCGACCTCTCCGTCGTAGA
CGTGATGGTCATGTCTTCATCTTGCTCTTCAGGTGCATTTAACACTGGCCACACCATATTGACGTCGT
+
FFFFFFFFFFFFFFFFFFFFFFFFFFFFFFFFFFFFFFFFF,FFFFFFFFFFFFFFFFFFFFFFFFFF
FFFFFFFFFFFFFFF:FFFFFFFFFFFFFFFFFFFFFFFFFFFFFFFFFFFFFFFFFFFFFFFFFFFF
@A00155:342:HHGFNDSXY:1:1615:27679:9784 1:N:0:GAACCTAG+TCCGCATA
TGGTTAGCGAAGAACTTATTGTGTAACGGCGGTAGCTGTATGAATACACGCGACCTCTCCGTCGTAGA
CGTGATGGTCATGTCTTCATCTTGCTCTTCAGGTGCATTTAACACTGGCCACACCATATTGACGTCGT
+
FFFFFFFFFFFFFFFFFFFFFFFFFFFFFFFFFFFFFFFFFFF:FFFFFFFFFFFFFFFFFFFFFFFF
FFFFFFFFFFFFFFF:FFFFFFFFFFFFFFFFFFFFFFFFFFFFFFFFFFFFFFFFFFFFFFFFFFFF
@A00155:342:HHGFNDSXY:1:1357:7337:29418 2:N:0:GAACCTAG+TCCGCATA
GGTTAGCGAAGAACTTATTGTGTAACGGCGGTAGCTGTATGAATACACGCGACCTCTCCGTCGTAGAC
GTGATGGTCATGTCTTCATCTTGCTCTTCAGGTGCATTTAACACTGGCCACACCATATTG
+
FFFFFFFFFFFFFFFFFFFFFFFFFFFFFFFFF:FFFF:FFFFFFFFFFFFFFFFFFFFFFFFFFFFF
FFFFFFFFFFFFFFF:FFFFFFFFFFFFFFFFFFF::FFFFFFFFFFFFFFFFFFFFFFF
@A00155:342:HHGFNDSXY:1:2672:31259:28635 2:N:0:GAACCTAG+TCCGCATA
GGTTAGCGAAGAACTTATTGTGTAACGGCGGTAGCTGTATGAATACACGCGACCTCTCCGTCGTAGAC
GTGATGGTCATGTCTTCATCTTGCTCTTCAGGTGCATTTAACACTGGCCACACCATATTGACGTCGTT
+
FFFFFF:F:FFFFFFFF,FFFFFFFFFFFFF,FFFFFFFFFF::FFFFFFFFFFFFFFFFFFFFFFFF
FFFFFFFFFFFFFFFFFFFF:FFFFFFFFFFFFFFFFFFF,FFF,FFFFFF:FFFFFFFF,FFFFFFF
@A00155:342:HHGFNDSXY:1:1421:32606:27430 2:N:0:GAACCTAG+TCCGCATA
TTAGCGAAGAACTTATTGTGTAACGGCGGTAGCTGTATGAATACACGCGACCGCTCCGTCGTAGACGT
GATGGTCATGTCTTCATCTTGCTCTTCAGGTGCATTTAACACTGGCCACACCATATTGACGTCGTTCG
+
F:FFFFFFFFFFFF,FFFFFFFFFFFFFF:FFFFFFFFFFF,FF:FFFFFF:,FFFFFFFFFFFFFFF
FF,FFFFFFFFFFFFFFFFFFFFFFFFF,FFFFFFFFFFFF,F,FFFF,FFFFFF,FFFFFFFFFFFF
@A00155:342:HHGFNDSXY:1:1220:5972:21668 2:N:0:GAACCTAG+TCCGCATA
TTAGCGAAGAACTTATTGTGTAACGGCGGTAGCTGTATGAATACACGCGACCTCTCCGTCGTAGACGT
GATGGTCATGTCTTCATCTTGCTCTTCAGGTGCATTTAACACTGGCCACACCATATTGACGTCGTTCG
+
F:FFFFFFFFFFFFFFFFFFFFFFFF:FFFFFFFFFFFFFFFFFFFFFFFFFFFFFFFFFFFFFFFFF
FFFFFFFFFFFFFFFFFFFFFFFFFFFFFFFFFFFFFFFFFFFFFFFFFFFFFFFFFFFFFFF:FFFF
@A00155:342:HHGFNDSXY:1:1424:12373:11929 2:N:0:GAACCTAG+TCCGCATA
TAGCGAAGAACTTATTGTGTAACGGCGGTAGCTGTATGAATACACGCGACCTCTCCGTCGTAGACGTG
ATGGTCATGTCTTCATCTTGCTCTTCAGGTGCATTTACCACTGGCCACACCATATTGACGTCGTTCGG
+
FFFFFF:FFFFF:FFF:FFFFFFFFFFFFFFFFFFFFFFFFF:FFFFFFFFFFFFFFFFFFFFFFFFF
FFFFFFFFFFFFFFFFFFFFFFFFFFFFFFFFFFFFF,FFFFFFFF,FFFFFFFFFFFFF:FFFFFFF
```

@A00155:342:HHGFNDSXY:1:1520:20103:24565 1:N:0:GAACCTAG+TCCGCATA
AGCGAAGAACTTATTGTGTAACGGCGGTAGCTGTATGAATACACGCGACCTCTCCGTCGTAGACGTGA
TGGTCATGTCTTCATCTTGCTCTTCAGGTGCATTTAACACTGGCCACACCATATTGACGTCGTTCGGC
+
FFFFFFFFFFFFFFFFFFFFFFFFFFFFFFFFFFFFFFFFFFFFFFFFFFFFFFF:FFFFFFFFFFFF
FFFFFFFFFFFFFF,FFFFFFFFFFFFFFFFFFFFFFFFFFFFFFFFFFFFFFFFFFFFFFFFFFFFF
@A00155:342:HHGFNDSXY:1:1356:25807:7294 2:N:0:GAACCTAG+TCCGCATA
AGCGAAGAACTTATTGTGTAACGGCGGTAGCTGTATGAATACACGCGACCTCTCCGTCGTAGACGTGA
TGGTCATGTCTTCATCTTGCTCTTCAGGTGCATTTAACACTGGCCACACCATATTGACGTCGTTCGGC
+
FFFFFFFFFFFFFFFFFFFFFFFFFFF:FFFFFFFFFFFFFFFFFFFFFFFFFFFFFFFFFFFFFFFF
FFFFFFFFFFFFFFFFFFFFFFFFFFFFFFFFFFFFFF:FFFFFFFFFFFFFFFFFFFFFFFFFFFFF
@A00155:342:HHGFNDSXY:1:2509:32262:23109 2:N:0:GAACCTAG+TCCGCATA
GCGAAGAACTTATTGTGTAACGGCGGTAGCTGTATGAATACACGCGACCTCTCCGTCGTAGACGTGAT
GGTCATGTCTTCATCTTGCTCTTCAGGTGCATTTAACACTGGCCACACCATATTGACGTCGTTCGGCG
+
FFFFFFFFFFFFFFFFFFFFFFFFFFFFFFFF:FFFFFFFFFFFFFFFFFFFFFFFFFFFFFFFFFFF
FFFFFFFFFFFFFFFFFFFFFFFFFFFFFFFFFFFFF,FFFFFFFFFFFF:FFFFFFFFFFFFFFFFF
@A00155:342:HHGFNDSXY:1:2407:9824:26365 1:N:0:GAACCTAG+TCCGCATA
CGAAGAACTTATTGTGTAACGGCGGTAGCTGTATGAATACACGCGACCTCTCCGTCGTAGACGTGATG
GTCATGTCTTCATCTTGCTCTTCAGGTGCATTTAACACTGGCCACACCATATTGACGTCGTTCGGCGA
+
FF:FFFFFFFFFFFFFFFFFFFFFFFFFFFFFFFFFFFFFFFFFFFFFFFFFFFFFFFFFFFFFFFFF
FFFFFFFFFFFFFFFFFFFFFFFFFFFFFFFFFFFFFFFFFFFFFFFFFFFFFFFFFFFFFFFFF,FF
@A00155:342:HHGFNDSXY:1:1573:19967:21668 2:N:0:GAACCTAG+TCCGCATA
GACTTATTGTGTAACGGCGGTAGCTGTATGAATACACGCGACCTCTCCGTCGTAGACGTGATGGTCAT
GTCTTCATCTTGCTCTTCAGGTGCATTTAACACTGGCCACACCATATTGACGTCGTTCGG
+
FFFFFF,FFFFFFFFFFFFFFFFFFFFFFFFFFFFFFFFFFFFFFFFFFFFFFFFFFFFFFFFFFFFF
FFFFFFFFFFFFFFFFFFFFFFFFFFFFFFFFFFFFFFFFFFFFFFFFF:FF,FFFFFFF
@A00155:342:HHGFNDSXY:1:2630:13340:29011 2:N:0:GAACCTAG+TCCGCATA
GACTTATTGTGTAACGGCGGTAGCTGTATGAATACACGCGACCTCTCCGTCGTAGACGTGATGGTCAT
GTCTTCATCTTGCTCTTCAGGTGCATTTAACACTGGCCACACCATATTGACGTCGTTCGG
+
FFFFFFFFFFFFFFFFF,FFFFFFFFFFFFFFFF:FFFFFFFFFFFFF:F:FFFFFFFFF,FFFFFFF
FFFFFFFFF,FFFFFFFFFFFFFFFFFFFFFFF:FFFFFFFFFFFF:FFFF:FFFFFFFF
@A00155:342:HHGFNDSXY:1:1357:7337:29418 1:N:0:GAACCTAG+TCCGCATA
TATTGTGTAACGGCGGTAGCTGTATGAATACACGCGACCTCTCCGTCGTAGACGTGATGGTCATGTCT
TCATCTTGCTCTTCAGGTGCATTTAACACTGGCCACACCATATTGACGTCGTTCGGCGAG
+
FFFFFFFFFFFFFFFFFFFFFFF:FFFFFFFFFFFFFFFFFFFFFFFF:FFFFFFFFFFFFFFFFFFF
FFFFFFFFFFFFFFFFFFFFFFFFFFFFFFFFFFFFFFFFFFFFFFFFFFFFFFFFFFFF
@A00155:342:HHGFNDSXY:1:2228:18701:28087 1:N:0:GAACCTAG+TCCGCATA
ATTGTGTAACGGCGGTAGCTGTATGAATACACGCGACCTCTCCGTCGTAGACGTGATGGTCATGTCTT
CATCTTGCTCTTCAGGTGCATTTAACACTGGCCACACCATATTGACGTCGTTCGGCGAGAAGAATGCC
+
FFFFFFFFFFFFFFFFFFFFFFFFFFFFFFFFFFFFFFFFFFFFFFFFFFFFFFFFFFFFFFFFFFFF
FFFFFFFFFFFFFFFFFFFFFFFFFFFFFFFFFFFFFFFFFFFFFFFFFFFFFFFFFFFFFFFFFFFF
@A00155:342:HHGFNDSXY:1:1246:4752:22467 2:N:0:GAACCTAG+TCCGCATA
TTGTGTAACGGCGGTAGCTGTATGAATACACGCGACCTCTCCGTCGTAGACGTGATGGTCATGTCTTC
ATCTTGCTCTTCAGGTGCATTTAACACTGGCCACACCATATTGACGTCGTTCGGCGAGAAGAATGCCG
+
FFFFFFFFFFF:F:FFFFFFFFFFF:FFFFFFFFFFFFFFFFFFFFFFFFFFFFFFFFFFFFFFFFFF
FFFFFFFFFFFFFFFFFFFF:FFFFFFFFFFFFFFFFFFFFFFFFF:FFFFFFFFFFFFFFFFFFFFF

```
@A00155:342:HHGFNDSXY:1:1615:27679:9784 2:N:0:GAACCTAG+TCCGCATA
TTGTGTAACGGCGGTAGCTGTATGAATACACGCGACCTCTCCGTCGTAGACGTGATGGTCATGTCTTC
ATCTTGCTCTTCAGGTGCATTTAACACTGGCCACACCATATTGACGTCGTTCGGCGAGAAGAATGCCG
+
FFFFFFFFFFFFFFFFFFFFFFFFFFFFFFFFFF:FFFFFFFFFFFFFFFFFFFFFFFFFFFFFFFFF
FFFFFFFFFFFFFFFFFFFFFFFFFFFFFFFFFFFFFFFFFFFFFFFFFFFFFFFFFFFFFFFFFFFF
@A00155:342:HHGFNDSXY:1:1471:10538:8531 2:N:0:GAACCTAG+TCCGCATA
TTGTGTAACGGCGGTAGCTGTATGAATACACGCGACCTCTCCGTCGTAGACGTGATGGTCATGTCTTC
ATCTTGCTCTTCAGGTGCATTTAACACTGGCCACACCATATTGACGTCGTTCGGCGAGAAGAATGCCG
+
FFFFFFFFFFFFFFFFFFFFFFFFFFFFFFFFFFFFFFFFFFFFFFFFFFFFFFFFFFFFFFFFFFFF
FFFFFFFFFFFFFFFFF,FFFFFFFFFFFFFFFFFFFFFFFFFFFFFFFFFFFFFFFFFFFFFFFFFF
@A00155:342:HHGFNDSXY:1:1231:7383:24580 2:N:0:GAACCTAG+TCCGCATA
TTGTGTAACGGCGGTAGCTGTATGAATACACGCGACCTCTCCGTCGTAGACGTGATGGTCATGTCTTC
ATCTTGCTCTTCAGGTGCATTTAACACTGGCCACACCATATTGACGTCGTTCGGCGAGAAGAATGCCG
+
FFFFFFFFFFFFFFFFFFFFFFFFFFFFFFFFFFFFFFFFFFFFFFFFFFFFFFFFFFFFFFFFFFFF
FFFFFFFFFFFFFFFFFFFFFFFFFFFFFFFFFFFFFFFFFFFFFFFFFFFFFFFFFFFFFFFFFFFF
@A00155:342:HHGFNDSXY:1:2228:18701:28087 2:N:0:GAACCTAG+TCCGCATA
GTAACGGCGGTAGCTGTATGAATACACGCGACCTCTCCGTCGTAGACGTGATGGTCATGTCTTCATCT
TGCTCTTCAGGTGCATTTAACACTGGCCACACCATATTGACGTCGTTCGGCGAGAAGAATGCCGTTGG
+
FFFFFFFFFFFFFFFFFFFFFFFFFFFFFFFFFFFFFFFFFFFFFFFFFFFFFFFFFFFFFFFFFFFF
FFFFFFFFFFFFFFFFFFFFFFFFFFFFFFFFFFFFFFFFFFFFFFFFFFFFFFFFFFFFFFFFFFFF
@A00155:342:HHGFNDSXY:1:2533:30644:12727 2:N:0:GAACCTAG+TCCGCATA
GTAACGGCGGTAGCTGTATGAATACACGCGACCTCTCCGTCGTAGACGTGATGGTCATGTCTTCATCT
TGCTCTTCAGGTGCATTTAACACTGGCCACACCATATTGACGTCGTTCGGCGAGAAGAATGCCGTTGG
+
FFFFFFFFFFFFFFFFFFFFFFFFFFFFFFFFFFFFFFFFFFFFFFFFFFFFFFFFFFFFFFFFFFFF
FFFFFFFFFFFFFFFFFFFFFFFFFFFFFF:FFFFFFFFFFFFFFFFFFFFFFFFFFFFFFFFFFFFF
@A00155:342:HHGFNDSXY:1:2361:30011:36871 2:N:0:GAACCTAG+TCCGCATA
GTAACGGCGGTAGCTGTATGAATACACGCGACCTCTCCGTCGTAGACGTGATGGTCATGTCTTCATCT
TGCTCTTCAGGTGCATTTAACACTGGCCACACCATATTGACGTCGTTCGGCGAGAAGAATGCCGTTGG
+
:FFFFF:FFFFFFFFFFFFFFFFFFFFFFFFFFFFFFFFFFFFFFFFFFFFFFFFFFFFFFFFFFFFF
FFFFFFFFFFFFFFFFFFFFFFFFFFFFFFFFFFFFFFFFFFFFFFFFFFFFFFFFFFFFFFFFFFFF
@A00155:342:HHGFNDSXY:1:2308:1624:30107 2:N:0:GAACCTAG+TCCGCATA
AACGGCGGTAGCTGTATGAATACACGCGACCTCTCCGTCGTAGACGTGATGGTCATGTCTTCATCTTG
CTCTTCAGGTGCATTTAACACTGGCCACACCATATTGACGTCGTTCGGCGAGAAGAATGCCGTTGG
+
FFFFFFFF:FFFFFFFFFF:FFFFFFFF:FFFFFFFFFFFFFFFFFFFF:FFFFFFFFFFFFFFFFFF
FFF::FFFFFFF:FFF:FF,FFFFFFFF:FF:FF:FF:FF:FFFFFFFFF:FFFFFFFFFFF,FFF
@A00155:342:HHGFNDSXY:1:1205:1443:25848 2:N:0:GAACCTAG+TCCGCATA
ACGGCGGTAGCTGTATGAATACACGCGACCTCTCCGTCGTAGACGTGATGGTCATGTCTTCATCTTGC
TCTTCAGGTGCATTTAACACTGGCCACACCATATTGACGTCGTTCGGCGAGAAGAATGCCGTTGGCCC
+
FFFFFFFFFFFFFFFFFFFFFF:FFFFFFFFFFFFFFFFF:F:FFFFFFFFFFFFFFFFFFFFFFFFF
FFFFFFFFFFFFFFF:FFFFFFFFFFFFFFFFFFFFF:FFFFFFFFFFF:FF:FFFFFFFFFFFFFFF
@A00155:342:HHGFNDSXY:1:1205:2094:26443 2:N:0:GAACCTAG+TCCGCATA
ACGGCGGTAGCTGTATGAATACACGCGACCTCTCCGTCGTAGACGTGATGGTCATGTCTTCATCTTGC
TCTTCAGGTGCATTTAACACTGGCCACACCATATTGACGTCGTTCGGCGAGAAGAATGCCGTTGGCCC
+
FFFFFFFFFFFFFFFFFFFFFFFFFFFFFFFFFFFFFFFFFFFFFFFFFFFFFFFFFFFFF:FFFFFFFFFF
FFFFFFFFFFFFFFFFFFFFFFFFFFFFFFFFFFFFFFFFFFFFFFFFFF:FFFFFFFFFFFFFFF:F
```

```
@A00155:342:HHGFNDSXY:1:1676:12952:17253 2:N:0:GAACCTAG+TCCGCATA
GGCGGTAGCTGTATGAATACACGCGACCTCTCCGTCGTAGACGTGATGGTCATGTCTTCATCTTGCTC
TTCAGGTGCATTTAACACTGGCCACACCATATTGACGTCGTTCGGCGAGAAGAATGCCGT
+
FFFFFFFFFFFFFFFFFFFFFFFFFFFFFFFFFF:FFFFFFFFFFFFFFFFFFFFF,FFFFFFFFFFF
FFF:FFFFFFFFFFFFFFFFFFFFFFFFFF:,FFFF::FFFFFFFFFFFFFFFFFFFFFF
@A00155:342:HHGFNDSXY:1:1676:15194:14090 2:N:0:GAACCTAG+TCCGCATA
GGCGGTAGCTGTATGAATACACGCGACCTCTCCGTCGTAGACGTGATGGTCATGTCTTCATCTTGCTC
TTCAGGTGCATTTAACACTGGCCACACCATATTGACGTCGTTCGGCGAGAAGAATGCCGT
+
FFFFFFFFFFFFFFFFFFFFFFFFFFFFFFFFFFFFFFFFFFFFFFFFFFFFFFFFFFFFFFFFFFFF
FFFFFFFFFFFFFFFFFFFFFFFFFFFFFFFF:FFFFFFFFFFFFFFFFFFFFFFFFFFF
@A00155:342:HHGFNDSXY:1:1573:19967:21668 1:N:0:GAACCTAG+TCCGCATA
GGCGGTAGCTGTATGAATACACGCGACCTCTCCGTCGTAGACGTGATGGTCATGTCTTCATCTTGCTC
TTCAGGTGCATTTAACACTGGCCACACCATATTGACGTCGTTCGGCGAGAAGAATGCCGT
+
FFFFFFFFFFFFFFFFFFFFFFFFFFFFFFFFFFFFFFFFFFFFFFFFFFFFFFFFFFFFFFFFFFFF
FFFFFFFFFFFFFFFFFFFFFFFFF:FFFFFFFFFFFFFFFFFFFFFFFFFFF:FFFFFF
@A00155:342:HHGFNDSXY:1:2630:13340:29011 1:N:0:GAACCTAG+TCCGCATA
GGCGGTAGCTGTATGAATACACGCGACCTCTCCGTCGTAGACGTGATGGTCATGTCTTCATCTTGCTC
TTCAGGTGCATTTAACACTGGCCACACCATATTGACGTCGTTCGGCGAGAAGAATGCCGT
+
FF:FFFFFFFFFFFFFFFFFFFFFFFFFF,FFFFFF:FFFFFFFFFFFFFFFFFFFFFFFFFFFFFFF
FFFF,FFF:FF:FFFFFFFFFFFFFFF:F:F:FFFFFFFFFF:FFFFFFF:FFFFFFFFF
@A00155:342:HHGFNDSXY:1:2362:10357:3396 2:N:0:GAACCTAG+TCCGCATA
CTGTATGAATACACGCGACCTCTCCGTCGTAGACGTGATGGTCATGTCTTCATCTTGCTCTTCAGGTG
CATTTAACACTGGCCACACCATATTGACGTCGTTCGGCGAGAAGAATGCCGTTGGCCCCGCCAATTCG
+
:FFFFFFFFFFFFFFFFFFFFFFFFFFFFFFFFFFFFFFFFFFFFFFFFFFFFFFFFFFFFFFFFFF
FFFFFFFFFFFFFFFFFFFFFFFFFFFFFFFFFFFFFFFFFFFFFFFFFFFFFFFFFFFFFFFFFFFF
@A00155:342:HHGFNDSXY:1:2425:32642:11866 1:N:0:GAACCTAG+TCCGCATA
ATGAATACACGCGACCTCTCCGTCGTAGACGTGATGGTCATGTCTTCATCTTGCTCTTCAGGTGCATT
TAACACTGGCCACACCATATTGACGTCGTTCGGCGAGAAGAATGCCGTTGGCCCCGCCAATTCG
+
FFFFFFFFFFFFFFFFFFFFFFFFFFFFFFFFFFFFFFFF:FFFFFFFFF:FFFFF:FFFFFFFFF:F
FFFFFFFFFFFFFFFFF:FFFFFFFFFFFFFFFFFFFFFFFFFFFFFFFFFFFFFFFFFFF:
@A00155:342:HHGFNDSXY:1:2426:21486:16939 1:N:0:GAACCTAG+TCCGCATA
ATGAATACACGCGACCTCTCCGTCGTAGACGTGATGGTCATGTCTTCATCTTGCTCTTCAGGTGCATT
TAACACTGGCCACACCATATTGACGTCGTTCGGCGAGAAGAATGCCGTTGGCCCCGCCAATTCG
+
FFFFFFFFFFFFFFFFFFFFFFFFFFFFFFFFFFFFFFFFFFFFFFFFFFFFFFFFFFFFFFFFFFFF
FFFFFFFFFFFFFFFFFFFFFFFFFFFFFFFFFFFFFFFFFFFFFFFFFFFFFFFFFFFFFFFF
@A00155:342:HHGFNDSXY:1:2308:1624:30107 1:N:0:GAACCTAG+TCCGCATA
ATGAATACACGCGACCTCTCCGTCGTAGACGTGATGGTCATGTCTTCATCTTGCTCTTCAGGTGCATT
TAACACTGGCCACACCATATTGACGTCGTTCGGCGAGAAGAATGCCGTTGGCCCCGCCAATTCGGC
+
FFFFFFFFFFFFFFFFFFFFFF:FFFFFFFFFFFFFFFFFFFFFFFFF:FF:FFFFF:FFFFFFFF,F
:FFFFFFFFFFFFFFFFF:::FFFFFFFFFFFFFFFFF:FFFFFFFFFFFFFFFFFFFFFFFFFFF
@A00155:342:HHGFNDSXY:1:2604:25690:13416 1:N:0:GAACCTAG+TCCGCATA
TGAATACACGCGACCTCTCCGTCGTAGACGTGATGGTCATGTCTTCATCTTGCTCTTCAGGTGCATTT
AACACTGGCCACACCATATTGACGTCGTTCGGCGAGAAGAATGCCGTTGGCCCCGCCAATTCGGCCCC
+
FFFFFFFFFFFFFFFFFFFFFFFFFFFFFFFFFFFFFFFFFFFFFFFFFFFFFFFFFFFFFFFFFFFF
FFFFFFFFFFFFFFFFFFFFFFFFFFFFFFFFFFFFFFFFFFFFFFFFFFFFFFFFFFFFFFFFFFFF
```

```
@A00155:342:HHGFNDSXY:1:2232:22516:30436 1:N:0:GAACCTAG+TCCGCATA
GAATACACGCGACCTCTCCGTCGTAGACGTGATGGTCATGTCTTCATCTTGCTCTTCAGGTGCATTTA
ACACTGGCCACACCATATTGACGTCGTTCGGCGAGAAGAATGCCGTTGGCCCCGCCAATTCGGC
+
F:FFFFFFFFFFFFFFFFFFFFFFFFFFFFFFFFFFFFFFFFFFFFFFFFFFFFFFFFFFFFFFFFFF
FFFFFFFFFFFFFFFFFFFFFFFFFFFFFFFFFFFFFFFFFFFFFFFFFFFFFFFFFFFFFFFF
@A00155:342:HHGFNDSXY:1:1676:15194:14090 1:N:0:GAACCTAG+TCCGCATA
AATACACGCGACCTCTCCGTCGTAGACGTGATGGTCATGTCTTCATCTTGCTCTTCAGGTGCATTTAA
CACTGGCCACACCATATTGACGTCGTTCGGCGAGAAGAATGCCGTTGGCCCCGCCAATTC
+
FFFFFFFFFFFFFFFFFFFFFFFFFFFFFFFFFFFFFFFFFFFFFFFFFFFFFFFFFFFFFFFFFFFF
FFFFFFFFFFFFFFFF,FFFFF:FFFFFFFFFFFFFFFFFFFFFFFFFFFFFFFFFFFFFF
@A00155:342:HHGFNDSXY:1:1676:12952:17253 1:N:0:GAACCTAG+TCCGCATA
AATACACGCGACCTCTCCGTCGTAGACGTGATGGTCATGTCTTCATCTTGCTCTTCAGGTGCATTTAA
CACTGGCCACACCATATTGACGTCGTTCGGCGAGAAGAATGCCGTTGGCCCCGCCAATTC
+
F,:FFFFF:FFFFFF:FFFFFF:F:FFFFFFFFFFFFFFFFFFFF:FF:FFFFFFFFFFFFFF:FF:F
FFFFFFFFFFFFF,:,FFFFFFFFFFFFFFFFFFFFFFFF:FFFFF:FFFFFFFFFFFF:F
@A00155:342:HHGFNDSXY:1:2356:1118:17143 1:N:0:GAACCTAG+TCCGCATA
AATACACGCGACCTCTCCGTCGTAGACGTGATGGTCATGTCTTCATCTTGCTCTTCAGGTGCATTTAA
CACTGGCCACACCATATTGACGTCGTTCGGCGAGAAGAATGCCGTTGGCCCCGCCAATTCGGCCCCTG
+
:FFFFFFFFFFFFFFFFFFFFF:FFFFFFFFFFFF::FFFFFFFFFFFFFFFFFFFFFFFFFFFFFFF
:F,FFFFFFF:F:FFFFF::F:FFFFF:FFF,FFFFF:FFFFFFFFFFFFFFFFFFFFFFFFF:,F:F:
@A00155:342:HHGFNDSXY:1:2664:25654:16141 1:N:0:GAACCTAG+TCCGCATA
AATACACGCGACCTCTCCGTCGTAGACGTGATGGTCATGTCTTCATCTTGCTCTTCAGGTGCATTTAA
CACTGGCCACACCATATTGACGTCGTTCGGCGAGAAGAATGCCGTTGGCCCCGCCAATTCGGCCCCTG
+
FFFFFFFFFFFFFFFFFFFFFFFFFFFFFFFFFFFFFFFFFFFFFFFFFFFFFFFFFFFFFFFFFFFF
FFFFFFFFFFFFFFFFFFFFFFFFFFFFFFFFFFFFFFFFFFFFFFFFFFFFFFFFFFFFFFFFFFFF
@A00155:342:HHGFNDSXY:1:2362:10357:3396 1:N:0:GAACCTAG+TCCGCATA
ATACACGCGACCTCTCCGTCGTAGACGTGATGGTCATGTCTTCATCTTGCTCTTCAGGTGCATTTAAC
ACTGGCCACACCATATTGACGTCGTTCGGCGAGAAGAATGCCGTTGGCCCCGCCAATTCGGCCCCTGT
+
FFFFFFFFFFFF:F:FFFFFFFF:FFFFFFFFFFFFFFFFFFFFFFFFFFFFF:FFFFFFFFFFFFFF
FFFFFFFFFFFFFFFFFFFFFFFFFFFFFFFFFFFFFFFFFFFFFFFFFFFFFF,FFFFF:FFFFFFF
@A00155:342:HHGFNDSXY:1:2606:10809:20055 2:N:0:GAACCTAG+TCCGCATA
CACGCGACCTCTCCGTCGTAGACGTGATGGTCATGTCTTCATCTTGCTCTTCAGGTGCATTTAACACT
GGCCACACCATATTGACGTCGTTCGGCGAGAAGAATGCCGTTGGCCCCGCCAATTCGGCCCCTGTTCT
+
FFFFFFFFFFFFFFFFFFFFFFFFFFFFFFFFFFFFFFFFFFFFFFFFFFFFFFFFFFF:FFFFFFFF
FFFFFFFFFFFFFFFFFFFFFFFFFFFFFFFFFFFFFFFFFFFFFFFFFFFFFFFFFFFFFFFFFFFF
@A00155:342:HHGFNDSXY:1:2155:19063:19789 1:N:0:GAACCTAG+TCCGCATA
CGACCTCTCCGTCGTAGACGTGATGGTCATGTCTTCATCTTGCTCTTCAGGTGCATTTAACACTGGCC
ACACCATATTGACGTCGTTCGGCGAGAAGAATGCCGTTGGCCCCGCCAATTCGGCCCCTGTTCTGAAG
+
FFFFFFFFFFFFFFFFFFFFFFFFFFF:FFFFFFFFFFFFFFFFFFFFFFFFF:FFFFFFFFFFFFFFF
FFFFFFFFFFFF:FFFFFFFFFFFFFFFFFFFFFFFFFFFFF:FFFF:F:FFFFFFF:FFFF:FFFFFFF
@A00155:342:HHGFNDSXY:1:2413:25988:30123 2:N:0:GAACCTAG+TCCGCATA
GACCTCTCCGTCGTAGACGTGATGGTCATGTCTTCATCTTGCTCTTCAGGTGCATTTAACACTGGCCA
CACCATATTGACGTCGTTCGGCGAGAAGAATGCCGTTGGCCCCGCCAATTCGGCCCCTGTTCTGAAGT
+
FFFFFFFF:FFFFFFFFF:FFFF,FFFFFFFF:F::FFFFFFFFFF:FFFFFFFFFFFFFFFFFFF:FF
FFFFFFFFFFFFFFFFFFFFFFFFFFFFFFFFFFFFFFFFFFFFFFFFFFFFFFFFFFFFFFFFFFFF
```

```
@A00155:342:HHGFNDSXY:1:2413:26512:27430 2:N:0:GAACCTAG+TCCGCATA
GACCTCTCCGTCGTAGACGTGATGGTCATGTCTTCATCTTGCTCTTCAGGTGCATTTAACACTGGCCA
CACCATATTGACGTCGTTCGGCGAGAAGAATGCCGTTGGCCCCGCCAATTCGGCCCCTGTTCTGAAGT
+
FFFFFFFFFFFFFFFFFFFFFFFFFFFFFFFFFFFFFFFFFFFFFFFFFFFFFFFFFFFFFFFFFFFFFF
FFFFFFFFFFFFFFFFFFFFFF:FFFFFFFFFFFFFFFFFFFFFFFFFFFFFFFFFFFFFFFFFFFFFFF
@A00155:342:HHGFNDSXY:1:2606:10809:20055 1:N:0:GAACCTAG+TCCGCATA
ACCTCTCCGTCGTAGACGTGATGGTCATGTCTTCATCTTGCTCTTCAGGTGCATTTAACACTGGCCAC
ACCATATTGACGTCGTTCGGCGAGAAGAATGCCGTTGGCCCCGCCAATTCGGCCCCTGTTCTGAAGTC
+
FFFFFFFFFFFFFFFFFFFFFFFFFFFFFFFFFFFFFFFFFFFFFFFFFFFFFFFFFFFFFFFFFFFFFF
FFFFF,FFFFFFFFFFFFFFFFFFFFFFFFFFFFFFFFFFFFFFFFFFFFFFFFFFFFFFFFFFFF:FFF
@A00155:342:HHGFNDSXY:1:1110:19018:26757 2:N:0:GAACCTAG+TCCGCATA
CTCTCCGTCGTAGACGTGATGGTCATGTCTTCATCTTGCTCTTCAGGTGCATTTAACACTGGCCACAC
CATATTGACGTCGTTCGGCGAGAAGAATGCCGTTGGCCCCGCCAATTCGGCCCCTGTTCTGAAGTCTA
+
FFFFFFFFFFFFFFFFFFFFFFFFFFFFFFFFFFFFFFFFFFFFFFFFFFFF,FFFFFFFFFFFFFFFFF
FFFFFFFFFFFFFFFFFFFFFFFFFFFFFFFFFFFFFFFFFFFFFFFFFFFFFFFFFFFFFFFFFFFFFF
@A00155:342:HHGFNDSXY:1:1143:13901:13322 2:N:0:GAACCTAG+TCCGCATA
GTCGTAGACGTGATGGTCATGTCTTCATCTTGCTCTTCAGGTGCATTTAACACTGGCCACACCATATT
GACGTCGTTCGGCGAGAAGAATGCCGTTGGCCCCGCCAATTCGGCCCCTGTTCTGAAGTCTATATCGT
+
FFFFFFFFFFFFFFFFFFFFFFFFFFFFFFFFFFFFFFFFFFFFFFFFFFFFFFFFFFFFFFFFFFFFFF
FFFFFFFFFFFFFFFFFFFFFFFFFFFFFFFFFFFFFFFFFFFFFFFFFFFF:FFFFFFFFFFF:FFFFF
@A00155:342:HHGFNDSXY:1:2407:9824:26365 2:N:0:GAACCTAG+TCCGCATA
CGTGATGGTCATGTCTTCATCTTGCTCTTCAGGTGCATTTAACACTGGCCACACCATATTGACGTCGT
TCGGCGAGAAGAATGCCGTTGGCCCCGCCAATTCGGCCCCTGTTCTGAAGTCTATATCGTAACCACA
+
FFFFFFFFFFFFFFFFFFFFFFFFFFFFFFFFFFFFFFFFFFFFFFFFFFFFFFFFFFFFFFFFFFFFFF
FFFFFFFFFFFFFFFFFFFFFFFFFFFFFFFFFFFFFFFFFFFFFFFFFFFFFFFFFFFFFFFFFFFFF
@A00155:342:HHGFNDSXY:1:1439:29080:21512 2:N:0:GAACCTAG+TCCGCATA
GATGGTCATGTCTTCATCTTGCTCTTCAGGTGCATTTAACACTGGCCACACCATATTGACGTCGTTCG
GCGAGAAGAATGCCGTTGGCCCCGCCAATTCGGCCCCTGTTCTGAAGTCTATATCGTAACCACACAGC
+
FFFFFFFFFFFF:FFFFFFFFFFFFFFFFFFFFFFFFFFFFFF:FFFFFFFFFFFFFFFFFFFFFF:FFF
FFFFFFFFFFFFFFFFFFFFFFFFFFFFF,FFFFFF,FFFFF:FFFFFFFFFFFFFFFFFFFF:FFFFFF
@A00155:342:HHGFNDSXY:1:1646:28483:30592 2:N:0:GAACCTAG+TCCGCATA
GATGGTCATGTCTTCATCTTGCTCTTCAGGTGCATTTAACACTGGCCACACCATATTGACGTCGTTCG
GCGAGAAGAATGCCGTTGGCCCCGCCAATTCGGCCCCTGTTCTGAAGTCTATATCGTAACCACACAGC
+
FFFFFFFFFFFFFFFFFFFFFFFFFFFFFFFFFFFFFFFFFFFFFFFFFFFFFFFFFFFF:FFFFFFFFF
FFFFFFFFFFFFFFFFFFFFFFFFFFFFFFFF:FFFFF,F:FFFFFFFFFFF::FFFFFFFFFFFFFFFF
@A00155:342:HHGFNDSXY:1:1107:31521:11021 2:N:0:GAACCTAG+TCCGCATA
GATGGTCATGTCTTCATCTTGCTCTTCAGGTGCATTTAACACTGGCCACACCATATTGACGTCGTTCG
GCGAGAAGAATGCCGTTGGCCCCGCCAATTCGGCCCCTGTTCTGAAGTCTATATCGTAACCACACAGC
+
FFFFFFFFF:FFFFFFFF:FFFFFFFFFFFFFFFFFFFFFFFFFFFFFFFFF:FFFFFFFFFFFFFFFFF
FFFFFFFFFFFFFFFFFFFFFFFFFFFFFFF:FFFFFFFFFFFFFFFFFFFFFFFFFFFFFFFFFFFFFF
@A00155:342:HHGFNDSXY:1:2452:1353:1517 1:N:0:GAACCTAG+TCCGCATA
GGTCATGTCTTCATCTTGCTCTTCAGGTGCATTTAACACTGGCCACACCATATTGACGTCGTTCGGCG
AGAAGAATGCCGTTGGCCCCGCCAATTCGGCCCCTGTTCTGAAGTCTATATCGTAACCACACAGCCTC
+
FFFFFFFFFFFFFFFFFFFFFFFFFF:FFFF,:FFFFFF,FFFFFFFF:FFFFFFFF:FFFFFFFFFFFF
FF:FFFFFFFFFFFFFFFFFFFFFFFFFFFFFF::FFFFFFFF,:FF,F:F:F,FF,:,:FF:F,F::FF
```

```
@A00155:342:HHGFNDSXY:1:2176:25907:12790 2:N:0:GAACCTAG+TCCGCATA
GTCATGTCTTCATCTTGCTCTTCAGGTGCATTTAACACTGGCCACACCATATTGACGTCGTTCGGCGA
GAAGAATGCCGTTGGCCCCGCCAATTCGGCCCCTGTTCTGAAGTCTATATCGTAACCACACGGC
+
FFFFFFFFFFFFFFFFFFFFFFFFFFFFFFFFFFFFFFFFFFFFFFFFFFFFFFFFFFFFFFFFFFFF
FFFFFFFFFFFFFFFFFFFFFFFFFFFFFFFFFFFFFFFFFFFFFFFFFFFFFFFFFFFFFFFF
@A00155:342:HHGFNDSXY:1:1139:27760:22921 2:N:0:GAACCTAG+TCCGCATA
GTCTTCATCTTGCTCTTCAGGTGCATTTAACACTGGCCACACCATATTGACGTCGTTCGGCGAGAAGA
ATGCCGTTGGCCCCGCCAATTCGGCCCCTGTTCTGAAGTCTATATCGTAACCACACAGC
+
FFFFFF,FFFFFFFFFFFFFFFFFFFFFFFFFFFFFFFFFFFFFF:,FFFFF:FFFFFFFFFFFFFFF
FFFFFFFFFFFFFFFFFFFFFFFFFFFFFFFFFFFFFFFFFFFFFFFFFFFF:FFFFFF
@A00155:342:HHGFNDSXY:1:2452:1353:1517 2:N:0:GAACCTAG+TCCGCATA
GTCTTCATCTTGCTCTTCAGGTGCATTTAACACTGGCCACACCATATTGACGTCGTTCGGCGAGAAGA
ATGCCGTTGGCCCCGCCAATACGGCCCCTGTTCTGAAGTCTATATCGTAACCACACAGCCTCGCCAA
+
F,FF,FF,F:FFF,F,:FF:F,FFFF,FFFFFF,FFFFFFFF:FFFFFFFF,:F::,FFFFFFFFF:F
F:FFF:FF:FFFFF::FFFF,FFFFFFF:FF:FFFFFF:FFFFFFF:FFFFFFFFFFFFFFF,FF:F
@A00155:342:HHGFNDSXY:1:1432:30038:12868 2:N:0:GAACCTAG+TCCGCATA
GTCTTCATCTTGCTCTTCAGGTGCATTTAACACTGGCCACACCATATTGACGTCGTTCGGCGAGAAGA
ATGCCGTTGGCCCCGCCAATTCGGCCCCTGTTCTGAAGTCTATATCGTAACCACACAGCCTCGCCAAA
+
FFFFFFFFFFFFFFFFFFFFFFFF:FFFFFFFFFFFFFFFFFFFFFFFFFFFFFFFFFFFFFFFFFFF
FFFFFFFFFFFFFFFFFFFFFFFFFFFFFFFFFFFFFFFFFFFFFFFFFFFFFFFFFFFFFFFFFFFF
@A00155:342:HHGFNDSXY:1:1101:13467:2206 1:N:0:GAACCTAG+TCCGCATA
CATCTTGCTCTTCAGGTGCATTTAACACTGGCCACACCATATTGACGTCGTTCGGCGAGAAGAATGCC
GTTGGCCCCGCCAATTCGGCCCCTGTTCTGAAGTCTATATCGTAACCACACAGCCTCGCCAAATTCGC
+
FFFFFFFFFFFFFFFFFFFFFFFFFFFFFFFFFFFFFFFFFFFFFFFFFFFFFFFFFFFFFFFFFFFF
F,FFFFFFFFFFFFFFFFFFFFFFFFFFFFF:FFFFFFFFFFFFFFFFFFFFFFF:FFFFFFFFFFFF
@A00155:342:HHGFNDSXY:1:1446:7455:4758 2:N:0:GAACCTAG+TCCGCATA
CTCTTCAGGTGCATTTAACACTGGCCACACCATATTGACGTCGTTCGGCGAGAAGAATGCCGTTGGCC
CCGCCAATTCGGCCCCTGTTCTGAAGTCTATATCGTAACCACACAGCCTCGCCAAATTCGCGACCACC
+
FFF:FFFFFFFFF:FFFFFFFFFFFFFFFFFFFFFFFFFFFFFFFFFFFFFFFFFFFFFFFFFFFFFF
FFFFFFFFFFFFFFFFFFFFFFFFFFFFFFFFFFF:FFFFFFFFFFFFFFFFF:FFFFFFFFFFFFFF
@A00155:342:HHGFNDSXY:1:2176:25907:12790 1:N:0:GAACCTAG+TCCGCATA
CTTCAGGTGCATTTAACACTGGCCACACCATATTGACGTCGTTCGGCGAGAAGAATGCCGTTGGCCCC
GCCAATTCGGCCCCTGTTCTGAAGTCTATATCGTAACCACACGGCCTCGCCAAATTCGCG
+
FFFFFFFFFFFFFFFFFFFFFFFFFFFFFFFFFFFFFFFFFFFFFFFFFFFFFFFFFFFFFFFFFFFF
FFFFFFFFFFFFFFFFFFFFFFFFFFFFFF:FFFFFFFFFFFFFFFFFFFFFFFFFFFFF
@A00155:342:HHGFNDSXY:1:1535:3803:22608 1:N:0:GAACCTAG+TCCGCATA
CTTCAGGTGCATTTAACACTGGCCACACCATATTGACGTCGTTCGGCGAGAAGAATGCCGTTGGCCCC
GCCAATTCGGCCCCTGTTCTGAAGTCTATATCGTAACCACACAGCCTCGCCAAATTCGCGACCA
+
FFFFFFFFFFFFFFFFFFFFFFFFFFFFF:FFF:FFFFFFFFFFFFFFFFFFFFFFFFFFFFFFFFFF
FFFFFFFFFFFFFFFFFFFFFFFFFFFFFFFFFFFFFFFFFFFFFFFFFFFF:FFFFFFFFFFF
@A00155:342:HHGFNDSXY:1:2567:6289:11757 2:N:0:GAACCTAG+TCCGCATA
TTCAGGTGCATTTAACACTGGCCACACCATATTGACGTCGTTCGGCGAGAAGAATGCCGTTGGCCCCG
CCAATTCGGCCCCTGTTCTGAAGTCTATATCGTAACCACACAGCCTCGCCAAATTCGCGACCACCCAC
+
FFFFFFFFFF:FFFFFFFFF,F:FFFFFFFFFFFFFFFFFFFFFFFFFFFFFFFFFFFFFFFFFFFFF
FFFFFFFFFFFFFFFFFFFFFFFFFFFFFFFFFFFFFFFFFFFFFFFFFFFFFFFFFFFFFFFFFFFF
```

```
@A00155:342:HHGFNDSXY:1:2476:21124:13369 2:N:0:GAACCTAG+TCCGCATA
TTCAGGTGCATTTAACACTGGCCACACCATATTGACGTCGTTCGGCGAGAAGAATGCCGTTGGCCCCG
CCAATTCGGCCCCTGTTCTGAAGTCTATATCGTAACCACACAGCCTCGCCAAATTCGCGACCACCCAC
+
FFFFFFFFFFFFFFFFFFFFFFFFFFFFFFFFFFFFFFFFFFFFFFFFFFFFFFFFFFFFFFFFFFFF
FFFFFFFFFFFFFFFFFFFFFFFFFFFFFFFFFFFFFFFFFFFFFFFFFFFFFFFFFFFFFFFFFFFF
@A00155:342:HHGFNDSXY:1:2403:30897:4022 2:N:0:GAACCTAG+TCCGCATA
TTCAGGTGCATTTAACACTGGCCACACCATATTGACGTCGTTCGGCGAGAAGAATGCCGTTGGCCCCG
CCAATTCGGCCCCTGTTCTGAAGTCTATATCGTAACCACACAGCCTCGCCAAATTCGCGACCACCCAC
+
FFFFFFF:FF:FFFFFFFFFFFFFFFFFFFFFFFFFFFFFFFFFFFFFFFFFFFFFFFFFFFFFFFFF
FFFF:FFFFFFFFFFFFFFFFFFFFFFFFFFF:FFFFFFFFFFFFFFFFFFFFFFFFFFFFF:FFF:F
@A00155:342:HHGFNDSXY:1:2664:25654:16141 2:N:0:GAACCTAG+TCCGCATA
CAGGTGCATTTAACACTGGCCACACCATATTGACGTCGTTCGGCGAGAAGAATGCCGTTGGCCCCGCC
AATTCGGCCCCTGTTCTGAAGTCTATATCGTAACCACACAGCCTCGCCAAATTCGCGACCACCCACGC
+
FFFFFFFFFFFFFFFFFFFFFFFFFFFFFFFFFFFFFFFFFFFFFFFFFFFFFFFFFFFFFFFFFFFF
FFFFFFFFFFFFFFFFFFFFFFFFFFFFFFFFFFFFFFFFFFFFFFFFFFFFFFFFFFFFFFFFFFFF
@A00155:342:HHGFNDSXY:1:2356:1118:17143 2:N:0:GAACCTAG+TCCGCATA
CAGGTTCATTTAACACTGGCCACACCATATTGACGTCGTTCGGCGAGAAGAATGCCGTTGGCCCCGCC
AATTCGGCCCCTGTTCTGAAGTCTATATCGTAACCACACAGCCTCGCCAAATTCGCGACCACCCACGC
+
FF:F:,FF:FFFFFFFFFF:FFFFFFFFFF,FFF,,F:FFFFFFFFFFFFFFFFFFFFFFFFFFFFFF
FFFFFFFFFFF:FF,FFFFF:F:FFFF,FFFFF:FFF:FFF:FFF:FF,:FFFFFFFFFFFF:FFF,F
@A00155:342:HHGFNDSXY:1:1263:29152:13589 2:N:0:GAACCTAG+TCCGCATA
GGTGCATTTAACACTGGCCACACCATATTGACGTCGTTCGGCGAGAAGAATGCCGTTGGCCCCGCCAA
TTCGGCCCCTGTTCTGAAGTCTATATCATAACCACACAGCCTCGCCAAATTCGCGACCACCCACGCGT
+
FFFFFFFFF:FFFF:FFFFFFFFFFFFFFFFF:FFFFFFFFFFFFFFFFFFFFFFFFFFFFFFFFFFF
:FFFFFFFFFFFFFFFFFFFFFFFFFFFFFFFFFFFFFFFFFFFFFFFFFFFFFFFFFFFFFFFFFFF
@A00155:342:HHGFNDSXY:1:1334:14814:36573 2:N:0:GAACCTAG+TCCGCATA
GTGCATTTAACACTGGCCACACCATATTGACGTCGTTCGGCGAGAAGAATGCCGTTGGCCCCGCCAAT
TCGGCCCCTGTTCTGAAGTCTATATCGTAACCACACAGCCTCGCCAAATTCGCGACCACCC
+
FFFFFFFFFFFFFFFFFFFFFFFFFFFFFFFFFFFFFFFFFFFF:FFFFFFFFFFFFFFFFFFFFFF,
F:FFFFFFFFFFFFFFFFFFFFFFFFFFFFFFFFFFFFFFFFFFFFFFFFFFFFFFFFFFF
@A00155:342:HHGFNDSXY:1:1329:8124:31845 2:N:0:GAACCTAG+TCCGCATA
TGCATTTAACACTGGCCACACCATATTGACGTCGTTCGGCGAGAAGAATGCCGTTGGCCCCGCCAATT
CGGCCCCTGTTCTGAAGTCTATATCGTAACCACACAGCCTCGCCAAATTCGCGACCACCCACGCGTCC
+
FFFFFFFFFFFFFFFFFFFFFFFFFFFFFFFFFFFFFFFFFFFFFFFFFFFFFFFFFFFFFFFFFFFF
FFFFFFFFFFFFFFFFFFFFFFFFFFFFFFFFFFFFFFFFFFFFFFFFFFFFFFFFFFFFFFFFFFFF
@A00155:342:HHGFNDSXY:1:1168:21151:28009 2:N:0:GAACCTAG+TCCGCATA
ATTTAACACTGGCCACACCATATTGACGTCGTTCGGCGAGAAGAATGCCGTTGGCCCCGCCAATTCGG
CCCCTGTTCTGAAGTCTATATCGTAACCACACAGCCTCGCCAAATTCGCGACCACCCACGCGTCCTTA
+
FF:FFFFFFFFFFFFFFFFFFFFFFFFFFFFF:FFFFFFFFFFFFFFFFFFFFFFFFFFFFFFFFFFF
FFFFFFFFFFFFFFFFFFFFFFFFFFFFFFFFFFFFFFFFF:FFFFFFFFFFFFFFFFFFFFFFFFFF
@A00155:342:HHGFNDSXY:1:1659:8874:33019 2:N:0:GAACCTAG+TCCGCATA
ATTTAACACTGGCCACACCATATTGACGTCGTTCGGCGAGAAGAATGCCGTTGGCCCCGCCAATTCGG
CCCCTGTTCTGAAGTCTATATCGTAACCACACAGCCTCGCCAAATTCGCGACCACCCACGCGTCCTTA
+
FFFFFFFFFF:FFFFFFFFFFFFFFFFFFFFFFFFFFFFFFFFFFFFFFFFFFFFFFFFFFFFFFF:F
FFFFFFFFFFFFFFFFFFFFFFFFFFFFFFFFFFFFFFFFFFFFFFFFFFFFFFFFFFFFFFFFFFFF
```

```
@A00155:342:HHGFNDSXY:1:1101:13467:2206 2:N:0:GAACCTAG+TCCGCATA
ATTTAACACTGGCCACACCATATTGACGTCGTTCGGCGAGAAGAATGCCGTTGGCCCCGCCAATTCGG
CCCCTGTTCTGAAGTCTATATCGTAACCACACAGCCTCGCCAAATTCGCGACCACCCACGCGTCCTTA
+
FFFFFFFFFFFFFFFFFFFFFFF:FFFFFFFFFFFFFFFFFFFFFFFFFFFFFFFFFFFFFFFFFFFF
FFFFFFFFFFFFFFFFFFFFFFFFFFFFFFFFFFFFFFFFFFFFFFFFFFFFFFFFFFFFFFFFFFFF
@A00155:342:HHGFNDSXY:1:1446:7455:4758 1:N:0:GAACCTAG+TCCGCATA
TTTAACACTGGCCACACCATATTGACGTCGTTCGGCGAGAAGAATGCCGTTGGCCCCGCCAATTCGGC
CCCTGTTCTGAAGTCTATATCGTAACCACACAGCCTCGCCAAATTCGCGACCACCCACGCGTCC
+
FFFFFFFFFFFFFFFFFF:FFFFFFFFFFFFFFFFFFFFFFFFFFFFFFFFFFFFFFFFFFFFFFFFF
FFFFF:FFFFFFFFFFFFFFFFFFFFFFFFFFFFFFFFFFFFFFFFFFFFFFFFFFFFFFFFFF
@A00155:342:HHGFNDSXY:1:1625:4490:25488 1:N:0:GAACCTAG+TCCGCATA
TTAACACTGGCCACACCATATTGACGTCGTTCGGCGAGAAGAATGCCGTTGGCCCCGCCAATTCGGCC
CCTGTTCTGAAGTCTATATCGTAACCACACAGCCTCGCCAAATTTGCGACCACCCACGCGTCCTTA
+
FFFFFFFFFFFFFFFFFFFFFFFFFFFFFFFFFFFFFFFFFFFFFFFFFFFFFFFFFFFFFFFFFFFF
FFFFFFFFFFFFFFFF,FFFFFFF:FFFFFFFFFFFFFFFFFFFFFFFFFFFFFFFFFFFFFFFFF
@A00155:342:HHGFNDSXY:1:2625:32705:4586 1:N:0:GAACCTAG+TCCGCATA
TTAACACTGGCCACACCATATTGACGTCGTTCGGCGAGAAGAATGCCGTTGGCCCCGCCAATTCGGCC
CCTGTTCTGAAGTCTATATCGTAACCACACAGCCTCGCCAAATTCGCGACCACCCACGCGTCCTTAGC
+
FFFFFFFFFFFFFFFFFFFFFFFFFFFFFFFFFFFFFFFFFFFFFFFFFFFFFFFFFFFFFFFFFFFF
FFFFFFFFFFFFFFFFFFFFFFFFFFFFFFFFFFFFFFFFFFFFFFFFFF:FFFFFFFFFFFFFFFF:
@A00155:342:HHGFNDSXY:1:1560:32786:4914 1:N:0:GAACCTAG+TCCGCATA
TTAACACTGGCCACACCATATTGACGTCGTTCGGCGAGAAGAATGCCGTTGGCCCCGCCAATTCGGCC
CCTGTTCTGAAGTCTATATCGTAACCACACAGCCTCGCCAAATTCGCGACCACCCACGCGTCCTTAGC
+
FFFFFFFFFFFFFFFFFF:FFFFFFFFFFFFFFFFFFFFFFFFFFFFFFFFFFFFFFFFFFFFFFFFF
FFFFFFFFFFFFFFFFFFFFFFFFFFFFFFFFFFFFFFFFFFFFFFFFFFFFFFFFFFFFFFFFFFFF
@A00155:342:HHGFNDSXY:1:2403:30897:4022 1:N:0:GAACCTAG+TCCGCATA
TTAACACTGGCCACACCATATTGACGTCGTTCGGCGAGAAGAATGCCGTTGGCCCCGCCAATTCGGCC
CCTGTTCTGAAGTCTATATCGTAACCACACAGCCTCGCCAAATTCGCGACCACCCACGCGTCCTTAGC
+
FFFFFFFFFFFFFFFFFFFFFFFFFFFFFFFFFFFFFFFFFFFFFFFFFFFFFFFFFFFFFFFFFFFF
FFFFFFFFFFFFFFFFFFFFFFFFFFFFFFFFFFFFFFFFFFFFFFFFFFFFFFFFFFFFFFFFFFFF
@A00155:342:HHGFNDSXY:1:2660:7654:24893 1:N:0:GAACCTAG+TCCGCATA
TTAACACTGGCCACACCATATTGACGTCGTTCGGCGAGAAGAATGCCGTTGGCCCCGCCAATTCGGCC
CCTGTTCTGAAGTCTATATCGTAACCACACAGCCTCGCCAAATTCGCGACCCCCCACGCGTCCTTAGC
+
FFFFFFFFFFFFFFFFFFFFFFFFFFF:FFFFFFFFFFFFFFFFFFFFFFF:FFFFFFFFFFFFF:F,
FFFFFFFF:FFFFFFFFFFFFFFFFFFFFFFFFFFFFFFFFFFFFFF,FF:FFFFFFFFFFFFFFFF
@A00155:342:HHGFNDSXY:1:1139:27760:22921 1:N:0:GAACCTAG+TCCGCATA
TAACACTGGCCACACCATATTGACGTCGTTCGGCGAGAAGAATGCCGTTGGCCCCGCCAATTCGGCCC
CTGTTCTGAAGTCTATATCGTAACCACACAGCCTCGCCAAATTCGCG
+
FFFFFFFFFFFFFFFFFFFFFFFFFFFFFFFFFFFFFFFFFFFFFFFFFFFFFFFFFFFFFFFFFFFF
FFFFFFFFFFFFFFFFFFFFFFFFFFFFFFFFFFFFFFFFFFFFFFF
@A00155:342:HHGFNDSXY:1:1401:32036:18646 1:N:0:GAACCTAG+TCCGCATA
TAACACTGGCCACACCATATTGACGTCGTTCGGCGAGAAGAATGCCGTTGGCCCCGCCAATTCGGCCC
CTGTTCTGAAGTCTATATCGTAACCACACAGCCTCGCCAAATTCGCGACCACCCAC
+
FFFFFFFFFFFFFFFFFFFFFFFFFFFFFFFFFFFFFFFFFFFFFFFFFFFFFFFFFFFFFFFFFFFF
FFFFFFFFFFFFF:FFFFFFFFFFFFFFFFFFFFFFFFFFFFFFFFFFFFFFFFFF
```

```
@A00155:342:HHGFNDSXY:1:1263:29152:13589 1:N:0:GAACCTAG+TCCGCATA
TAACACTGGCCACACCATATTGACGTCGTTCGGCGAGAAGAATGCCGTTGGCCCCGCCAATTCGGCCC
CTGTTCTGAAGTCTATATCATAACCACACAGCCTCGCCAAATTCGCGACCACCCAC
+
FFFFFFFFFFFFFFFFFFFFFFFFFFFFFFFFFFFFFFFFFFFFFFFFFFFFFFFFFFFFFFFFFFFF
FFFFFFFFFFFFFFFFFFFFFFFFFFFFFFFFFFFFFFFFFFFFFFFFFFFFFFFF
@A00155:342:HHGFNDSXY:1:2476:21124:13369 1:N:0:GAACCTAG+TCCGCATA
TAACACTGGCCACACCATATTGACGTCGTTCGGCGAGAAGAATGCCGTTGGCCCCGCCAATTCGGCCC
CTGTTCTGAAGTCTATATCGTAACCACACAGCCTCGCCAAATTCGCGACCACCCAC
+
FFFFFFFFFFFFFFFFFFFFFFFFFFFFFFFFFFFFFFFFFFFFFFFFFFFFFFFFFFFFFFFFFFFF
FFFFFFFFFFFFFFFFFFFFFFFFFFFFFFFFFFFFFFFFFFFFFFFFFFFFFFFF
@A00155:342:HHGFNDSXY:1:2567:6289:11757 1:N:0:GAACCTAG+TCCGCATA
TAACACTGGCCACACCATATTGACGTCGTTCGGCGAGAAGAATGCCGTTGGCCCCGCCAATTCGGCCC
CTGTTCTGAAGTCTATATCGTAACCACACAGCCTCGCCAAATTCGCGACCACCCAC
+
FFFFFFFFFFFFFFFFFFFFFFFFFFFFFFFFFFFFFFFFFFFFFFFFFFFFFFFFFFFF:FFFFFFF
FFFFFFFFFFF:FFFFFFFFF::FFFFFFFFFFFFFFFFFFFFFFFFFFFFFFFFF
@A00155:342:HHGFNDSXY:1:1524:12888:11350 1:N:0:GAACCTAG+TCCGCATA
TAACACTGGCCACACCATATTGACGTCGTTCGGCGAGAAGAATGCCGTTGGCCCCGCCAATTCGGCCC
CTGTTCTGAAGTCTATATCGTAACCACACAGCCTCGCCAAATTCGCGACCACCCAC
+
FFFFFFFFFFFFFFFFFFFFFFFFFFFFFFFFFFFFFFFFFFFFFFFFFFFFFFFF:FFFFFFFFFFF
FFFFFFFFFFFFFFFFFFFFFFFFFFFFFFFFFFFFFFFFFFFFFFFFFFFFFFFF
@A00155:342:HHGFNDSXY:1:1471:29152:2691 1:N:0:GAACCTAG+TCCGCATA
TAACACTGGCCACACCATATTGACGTCGTTCGGCGAGAAGAATGCCGTTGGCCCCGCCAATTCGGCCC
CTGTTCTGAAGTCTATATCGTAACCACACAGCCTCGCCAAATTCGCGACCACCCACG
+
FFFFFFFF,FFF:FFFF:FF,,F,FFFF:FF,,FFF::F:FF:F,F,FF:FFF:F:,FFFFFFFFF::
FF:FF:,:FFF:F:FFFFF,,FF::FFFFFFF,FF:FFFFFF,F::,F,FF,FFFF:
@A00155:342:HHGFNDSXY:1:2666:4291:35008 1:N:0:GAACCTAG+TCCGCATA
TAACACTGGCCACACCATATTGACGTCGTTCGGCGAGAAGAATGCCGTTGGCCCCGCCAATTCGGCCC
CTGTTCTGAAGTCTATATCGTAACCACACAGCCTCGCCAAATTCGCGACCACCCACGC
+
FFFFFFF:FFFFFFFFFFFFFFFFFFFFFFFFFFFFFFFFFFFFFFFFFFFFFFFFFF:FFFFFFFFF
FFFFFFFF:FFFFFFFFFF:F:::FFFFFFFFFFFFFFFFFFFF,FFFFFFFFFFFFF
@A00155:342:HHGFNDSXY:1:1168:21151:28009 1:N:0:GAACCTAG+TCCGCATA
TAACACTGGCCACACCATATTGACGTCGTTCGGCGAGAAGAATGCCGTTGGCCCCGCCAATTCGGCCC
CTGTTCTGAAGTCTATATCGTAACCACACAGCCTCGCCAAATTCGCGACCACCCACGCGTCCTT
+
FFFFFFFFFFFFFFFFFFFFFFFFFFFFFFFFFFFFFFFFFFFFFFFFFFFFFFFFFFFFFFFFFFFF
FFFFFFFFFFFFFFFFFFFFFFFFFFFFFFFFFFFFFFFFFFFFFFFFFFFFFFFFFFFFFFFF
@A00155:342:HHGFNDSXY:1:2447:2944:21590 1:N:0:GAACCTAG+TCCGCATA
TAACACTGGCCACACCATATTGACGTCGTTCGGCGAGAAGAATGCCGTTGGCCCCGCCAATTCGGCCC
CTGTTCTGAAGTCTATATCGTAACCACACAGCCTCGCCAAATTCGCGACCACCCACGCGTCCTTAGCT
+
FFFFFFFFFFFFFFFFFFFFFFFFFFFFFFFFFFFFFFFFFFFFFFFFFFFFFFFFFFFFFFFFFFFF
FFFFFFFFFFFFFFFFFFFFFFFFFFFFFFFFFFFFFFFFFFFFFFFFFFFFFFFFFFFFFFFFFFFF
@A00155:342:HHGFNDSXY:1:1466:22661:30906 1:N:0:GAACCTAG+TCCGCATA
TAACACTGGCCACACCATATTGACGTCGTTCGGCGAGAAGAATGCCGTTGGCCCCGCCAATTCGGCCC
CTGTTCTGAAGTCTATATCGTAACCACACAGCCTCGCCAAATTCGCGACCACCCACGCGTCCTTAGCT
+
FFFFFFFFFFFFFF:FFFFFFFFFFFFFFFFFFFFFFFFFFFFFFFFFFFFFFFFFFFFFFFFFFFFF
FFFFFFFFFFFFFF:F:FFFFFFFFFFFFFFFFFFFFFFFFFFFFFFFFFFFFFFFFFFFFFFFFFFF
```

```
@A00155:342:HHGFNDSXY:1:1554:14904:6574 1:N:0:GAACCTAG+TCCGCATA
TAACACTGGCCACACCATATTGACGTCGTTCGGCGAGAAGAATGCCGTTGGCCCCGCCAATTCGGCCC
CTGTTCTGAAGTCTATATCGTAACCACACAGCCTCGCCAAATTCGCGACCACCCACGCGTCCTTAGCT
+
FFFFFFFFFFFFFFFFFFFFFFFFFFFFFFFFFFFFFFFFFFFFFFFFFFFFFFFFFFFFFFFFFFFF
FFFFFFFFFFFFF:FFFFFFFFFFFFFFFFFFFFFFFFFFFFFFFFFFFFFFFFFFFFFFFFFFFFFF
@A00155:342:HHGFNDSXY:1:2124:21423:19398 2:N:0:GAACCTAG+TCCGCATA
AACACTGGCCACACCATATTGACGTCGTTCGGCGAGAAGAATGCCGTTGGCCCCGCCAATTCGGCCCC
TGTTCTGAAGTCTATATCGTAACCACACAGCCTCGCCAAATTCGCGACCACCCACGCGTCCTTAGCT
+
FFFFFFFFFFFFFFFFFFFFFFFFFFFFFFFFFFFFFFFFFFFFFFFFFFFFFFFFFFFFFFFFFFFF
FFFFFFFFFFFFFF::FFFFFFFFFFFFFFFFFFFFFFFFFFFFFFFFFFFF:FFFFFFFFFFFFFF
@A00155:342:HHGFNDSXY:1:2604:25690:13416 2:N:0:GAACCTAG+TCCGCATA
ACTGGCCACACCATATTGACGTCGTTCGGCGAGAAGAATGCCGTTGGCCCCGCCAATTCGGCCCCTGT
TCTGAAGTCTATATCGTAACCACACAGCCTCGCCAAATTCGCGACCACCCACGCGTCCTTAGCTTTCA
+
FFFFFFFFFFFFFFFFFFFFFFFFFFFF:FFFFFFFFFFFFFFFFFFFFFFFFFFFFFFFFFFFFFFF
FFFFF:FFF,F,FFFFFFFFFFFFFFFFFFFFFF:F:FFFFFFFFFFFFF,FFFFFFFFFFFFFFFF:
@A00155:342:HHGFNDSXY:1:1329:8124:31845 1:N:0:GAACCTAG+TCCGCATA
TGGCCACACCATATTGACGTCGTTCGGCGAGAAGAATGCCGTTGGCCCCGCCAATTCGGCCCCTGTTC
TGAAGTCTATATCGTAACCACACAGCCTCGCCAAATTCGCGACCACCCACGCGTCCTTAGCTTTCATC
+
FFFFFFFFFFFFFFFFFFFFFFFFFFFFFFFFFFFFFFFFFFFFFFFFFFFFFFFFFFFFFFFFFFFF
FFFFFFFFFFFFFFFFFFFFFFFFFFFFFFFF:FFFFFFFFFFFFFFFFFFFFFFFFFFFFFFFFFFF
@A00155:342:HHGFNDSXY:1:2446:23484:30107 1:N:0:GAACCTAG+TCCGCATA
GGCCACACCATATTGACGTCGTTCGGCGAGAAGAATGCCGTTGGCCCCGCCAATTCGGCCCCTGTTCT
GAAGTCTATATCGTAACCACACAGCCTCGCCAAATTCGCGACCACCCACGCGTCCTTAGCTTTCATCT
+
FFFFFFFFFFFFFFFFFFFFFFFFFFFFFFFFFFFFFFFFFFFFFFFFFFFFFFFFFFFFFFFFFFFF
FFFFFFFFFFFFFFFFFFFFFFFFFFFFFFFFFFFFFFFFFFFFFFFFFFFFFFFFFFFFFFFFFFFF
@A00155:342:HHGFNDSXY:1:1334:14814:36573 1:N:0:GAACCTAG+TCCGCATA
GCCACACCATATTGACGTCGTTCGGCGAGAAGAATGCCGTTGGCCCCGCCAATTCGGCCCCTGTTCTG
AAGTCTATATCGTAACCACACAGCCTCGCCAAATTCGCGACCACCCACGCGTCCTTAGCTT
+
FFFFFFFFFFFFFFFFFFFFFFFFFFFFFFFFF:FFFFFFFFFFFFFFFFFFFFFFFFFFFFFFFFFF
FFFFF:FFFFFFFFFFFFFFFFFFFFFFFFFFFFFFFFFFFFFFFFFFFFFFFFFFFFFFF
@A00155:342:HHGFNDSXY:1:1519:17589:30545 1:N:0:GAACCTAG+TCCGCATA
GCCACACCATATTGACGTCGTTCGGCGAGAAGAATGCCGTTGGCCCCGCCAATTCGGCCCCTGTTCTG
AAGTCTATATCGTAACCACACAGCCTCGCCAAATTCGCGACCACCCACGCGTCCTTAGCTTTCATCT
+
FFFFFFFFFFFFFFFFFFFFFFFFFFFFFFFFFFFFFFFFFFFFFFFFFFFFFFFFFFFFFFFFFFFF
FFFFFFFFFFFFFFFFFFFFFFFFFFFFFFFFFFF:FFFFFFFFFFFFFFFFFFFFFFFFFFFFFFF
@A00155:342:HHGFNDSXY:1:1115:29460:10676 1:N:0:GAACCTAG+TCCGCATA
GCCACACCATATTGACGTCGTTCGGCGAGAAGAATGCCGTTGGCCCCGCCAATTCGGCCCCTGTTCTG
AAGTCTATATCGTAACCACACAGCCTCGCCAAATTCGCGACCACCCACGCGTCCTTAGCTTTCATCTT
+
FFFFFFFFFFFFFFFFFFFFFFFFFFFFFFFFFFFFFFFFFFFFFFFFFFFFFFFFFFFFFFFFFFFF
FFFFFFFFFFFFFFFFFFFFFFFFFFFFFFF:FFFFFFFFFFFFFFFFFFFFFFFFFFFFFFFFFFFF
@A00155:342:HHGFNDSXY:1:1355:13548:24862 1:N:0:GAACCTAG+TCCGCATA
GCCACACCATATTGACGTCGTTCGGCGAGAAGAATGCCGTTGGCCCCGCCAATTCGGCCCCTGTTCTG
AAGTCTATATCGTAACCACACAGCCTCGCCAAATTCGCGACCACCCACGCGTCCTTAGCTTTCATCTT
+
F:FFFFFFFFFFFF:FF:::FFFF:FFFFFFFFF:FF:FFFFFFFFF,FFFFFF:F,F:FFFFFFFFF
F:FFF:F:FF:FFFFF:F:FFF:FFFFFFFFFFFFF:FFF::FFF,F,FFF,F:FFFFFFFFFF:F::
```

```
@A00155:342:HHGFNDSXY:1:1355:13548:24862 2:N:0:GAACCTAG+TCCGCATA
ACACCATATTGACGTCGTTCGGCGAGAAGAATGCCGTTGGCCCCGCCAATTCGGCCCCTGTTCTGAAG
TCTATATCGTAACCACACAGCCTCGCCAAATTCGCGACCACCCACGCGTCCTTAGCTTTCATCTTAAA
+
:FF:FFFFFFFFFF:FF:FFFFFFFF:FFFFFFF:FFFFFFFF,FF:FFF,:F,FFF:F,F:FFF:FF
F,FFFFFFFFFFFFFFF,F:FF:FFFFFFFFFFF:FFF:FFF::FFFFFF:FFF,FFFF:FFFF:FFF
@A00155:342:HHGFNDSXY:1:2540:7455:14403 1:N:0:GAACCTAG+TCCGCATA
CACCATATTGACGTCGTTCGGCGAGAAGAATGCCGTTGGCCCCGCCAATTCGGCCCCTGTTCTGAAGT
CTATATCGTAACCACACAGCCTCGCCAAATTCGCGACCACCCACGCGTCCTTAGCTTTCATCTTAAAC
+
FFFFFFFFFFFFFFFFFFFFFFFFFFFFFFFFFFFFFFFFFFFFFFFFFFFFFFFFFFFFFFFFFFFF
FFFFFFFFFFFFFFFFFFFFFFF:FFFFFFF:FFFFFFFFFFFFFFFFFFFFFFFF:FFF:FFFFFFF
@A00155:342:HHGFNDSXY:1:1625:4490:25488 2:N:0:GAACCTAG+TCCGCATA
CCATATTGACGTCGTTCGGCGAGAAGAATGCCGTTGGCCCCGCCAATTCGGCCCCTGTTCTGAAGTCT
ATATCGTAACCACACAGCCTCGCCAAATTTGCGACCACCCACGCGTCCTTAGCTTTCATCTTAAAC
+
FFFFFFFFFFFFFFFFF,FFFFFFFFFFFFFFFFFFFFFFFFFFFFFFFFFFFFFFFFFFFF,FFFF
F:FFFFFFFFFFFFFFFF:FFFFFFFFFFFFFFFFFFFFFFFFFFFF:FFFFFFFFFFFFFFF:FF
@A00155:342:HHGFNDSXY:1:2660:7654:24893 2:N:0:GAACCTAG+TCCGCATA
CATATTGACGTCGTTCGGCGAGAAGAATGCCGTTGGCCCCGCCAATTCGGCCCCTGTTCTGAAGTCTA
TATCGTAACCACACAGCCTCGCCAAATTCGCGACCACCCACGCGTCCTTAGCTTTCATCTTAAACCCC
+
FFFFF:FFFFFFFFFFFF:FFFFFFFFFFFFFFFFFFFFFFFFFFFFFFFFFF:FFFFFFFFFFF:FF
FFFFFFFFFFFFFFFF:FFFFFFFFFFFFFFFFFFFFFFFFFFFFFFFFFFFFFFFFFFFFFFFFFFF
@A00155:342:HHGFNDSXY:1:2124:21423:19398 1:N:0:GAACCTAG+TCCGCATA
ATATTGACGTCGTTCGGCGAGAAGAATGCCGTTGGCCCCGCCAATTCGGCCCCTGTTCTGAAGTCTAT
ATCGTAACCACACAGCCTCGCCAAATTCGCGACCACCCACGCGTCCTTAGCTTTCATCTTAAACCCC
+
FFFFFFFFFFFFFFFFFFFFFFFFFFFFFFFFFFFFFFFFFFFFFFFFFFFFFFFFFFFFFFFFFFFF
FFFFFFFFFFFFFFFFFFFFFFFFFFFFFFFFFFFFFFFFFFFFFFFFFFFFFFFFFFFFFFFFFFF
@A00155:342:HHGFNDSXY:1:1470:19126:21590 1:N:0:GAACCTAG+TCCGCATA
ATTGACGTCGTTCGGCGAGAAGAATGCCGTTGGCCCCGCCAATTCGGCCCCTGTTCTGAAGTCTATAT
CGTAACCACACAGCCTCGCCAAATTCGCGACCACCCACGCGTCCTTAGCTTTCATCTTAAACCCCTTG
+
FFFFF:FF:FFFFFFFFFFFFFFFFFFFFFFFFFFFFFFF,FFFFFF:FFFFFFFFFFFF:FFFFFFF
FFFFF:FFFFFFFFFFFFFFFFFFFFFFFFFFFFFFFFFFFFFFFFFFFFFFFFFFFFFFFFFFF,FF
@A00155:342:HHGFNDSXY:1:1115:29460:10676 2:N:0:GAACCTAG+TCCGCATA
ATTGACGTCGTTCGGCGAGAAGAATGCCGTTGGCCCCGCCAATTCGGCCCCTGTTCTGAAGTCTATAT
CGTAACCACACAGCCTCGCCAAATTCGCGACCACCCACGCGTCCTTAGCTTTCATCTTAAACCCCTTG
+
FFFFFFFFFFFFFFFFFFFFFFFFFFFFFFFFFFFFFFFFFFFFFFFFFFFFFFFFFFFFFFFFFFFF
FFFFFFFFFFFFFFFFFFFFFFFFFFFFFFFFFFFFFFFFFFFFFFFFFFFFFFFFFFFFFFFFFFFF
@A00155:342:HHGFNDSXY:1:1211:8648:34131 1:N:0:GAACCTAG+TCCGCATA
ACGTCGTTCGGCGAGAAGAATGCCGTTGGCCCCGCCAATTCGGCCCCTGTTCTGAAGTCTATATCGTA
ACCACACAGCCTCGCCAAATTCGCGACCACCCACGCGTCCTTAGCTTTCATCTTAAACCCCTTGCGTG
+
FFFFFFFFFFFFFFFFFFFFFFFFFFFFFFFFFFFFFFFFFFFFFFFFF:FFFFF:FFFFFFFFFFFF
FFFFFFFFFFFFFFFFFFFFFFFFFFFFFFFFFFFFFFFFFFFFFFFFFFFFFFFFFFFFFFFFFFFF
@A00155:342:HHGFNDSXY:1:2359:5556:24862 1:N:0:GAACCTAG+TCCGCATA
ACGTCGTTCGGCGAGAAGAATGCCGTTGGCCCCGCCAATTCGGCCCCTGTTCTGAAGTCTATATCGTA
ACCACACAGCCTCGCCAAATTCGCGACCACCCACGCGTCCTTAGCTTTCATCTTAAACCCCTTGCGTG
+
FFFFFFFFFFFFFFFF:FFFFFFFFFFFFFFFFFFFFFFF:FFFFFFFFFFFFFF:FFFFFFFFFFFF
FFFFFFFFFFFFFFFF:FFFFFFFFF:FFFFFFFFFFFFFFFFFFFFFFFFFFFFFFFFFFFFFFF:F
```

```
@A00155:342:HHGFNDSXY:1:2548:28122:11209 1:N:0:GAACCTAG+TCCGCATA
CGTCGTTCGGCGAGAAGAATGCCGTTGGCCCCGCCAATTCGGCCCCTGTTCTGAAGTCTATATCGTAA
CCACACAGCCTCGCCAAATTCGCGACCACCCACGCGTCCTTAGCTTTCATCTTAAACCCCTTGCGTGT
+
FFFFFFFFFFFFFFF:FFFFFFFFFFFFFFFFFFFFFFFFFFFFFFFFFFFFFFFFFFFFFFFFFFF:
FFFFFFFFFFFFFFFFFFFFFFFFFFFFFFFFFFFFFFFFFFFFFFFFFFFFFFFFFFFFFFFFFFFF
@A00155:342:HHGFNDSXY:1:1644:18982:36558 1:N:0:GAACCTAG+TCCGCATA
CGTCGTTCGGCGAGAAGAATGCCGTTGGCCCCGCCAATTCGGCCCCTGTTCTGAAGTCTATATCGTAA
CCACACAGCCTCGCCAAATTCGCGACCACCCACGCGTCCTTAGCTTTCATCTTAAACCCCTTGCGTGT
+
FFFFFFFFFFFFFFFFFFFFFFFFFFFFFFFFFFFFFFFFFFFFFFFFFFFFFFFFFFFFFFFFFFFF
FFFFFFFFFFFFFFFFFFFFFFFFFFFFFFFFFFFFFFFFFFFFFFFFFFFFFFFFFFFFFFFFFFFF
@A00155:342:HHGFNDSXY:1:1424:16125:36401 1:N:0:GAACCTAG+TCCGCATA
CGTCGTTCGGCGAGAAGAATGCCGTTGGCCCCGCCAATTCGGCCCCTGTTCTGAAGTCTATATCGTAA
CCACACAGCCTCGCCAAATTCGCGACCACCCACGCGTCCTTAGCTTTCATCTTAAACCCCTTGCGTGT
+
FFFFFFFFFFFFFFFFFFFFFFFFFFFFFFFFFFFFFFFFFFFFFFFFFFFFFFFFFFFFFFFFFFFF
FFFFFFFFFFFFFFFFFFFFFFFFFFFFFFFFFFFFFFFFFFFFF:FFFFFFFFF:F:FFFFFFFFFF
@A00155:342:HHGFNDSXY:1:1472:7374:31015 1:N:0:GAACCTAG+TCCGCATA
CGTCGTTCGGCGAGAAGAATGCCGTTGGCCCCGCCAATTCGGCCCCTGTTCTGAAGTCTATATCGTAA
CCACACAGCCTCGCCAAATTCGCGACCACCCACGCGTCCTTAGCTTTCATCTTAAACCCCTTGCGTGT
+
FFFF:FFFFFFFFFFFFFFFFF,F::FFF::FFFFFFFFFFFFFFFFFFFFFF,FFFFFFFFFFFFFF
FFFFFFFFFFFFFFFFFFFFFFFFFFFFFFFFF:FFFFFFFF:FFFFFFFFFFFFFFFFFFFF:FFFF
@A00155:342:HHGFNDSXY:1:1163:25590:33191 1:N:0:GAACCTAG+TCCGCATA
CGTCGTTCGGCGAGAAGAATGCCGTTGGCCCCGCCAATTCGGCCCCTGTTCTGAAGTCTATATCGTAA
CCACACAGCCTCGCCAAATTCGCGACCACCCACGCGTCCTTAGCTTTCATCTTAAACCCCTTGCGTGT
+
FFFFFFFFFFFF:FFFFFFFFFFFFFFFFFFFFFFFFFFFFFFFFFFFFFFFFF:FFFFFFFFFFFFF
FFFFFFFFFFFFFFFFFFFFFFFFFFFFFFFFFFFFFFFFFFFFFFFFFFFFFFFFFFFFFFFFFFFF
@A00155:342:HHGFNDSXY:1:1218:15745:27132 1:N:0:GAACCTAG+TCCGCATA
CGTCGTTCGGCGAGAAGAATGCCGTTGGCCCCGCCAATTCGGCCCCTGTTCTGAAGTCTATATCGTAA
CCACACAGCCTCGCCAAATTCGCGACCACCCACGCGTCCTTAGCTTTCATCTTAAACCCCTTGCGTGT
+
FFFFFFFF:FF:FFFFFFFFFFFFFFFFFFFFFFFFF:FFFFFFFF:FFFF:FFFFFFFFFFFFFFFF
FFFFFFFFFFFFFFFFFFFFF:FFFFFFFFFFFFF:FFFFFFFFFFFFFFFFFFF:F:FFFF:FFFFF
@A00155:342:HHGFNDSXY:1:1424:16125:36401 2:N:0:GAACCTAG+TCCGCATA
TCGTTCGGCGAGAAGAATGCCGTTGGCCCCGCCAATTCGGCCCCTGTTCTGAAGTCTATATCGTAACC
ACACAGCCTCGCCAAATTCGCGACCACCCACGCGTCCTTAGCTTTCATCTTAAACCCCTTGCGTGTTA
+
FFFFFFFF:FFFFFFFFFFFFFFFFFFFFFFFFFFFFFFFFFFFFFFFFFFFFFFFFFF:FFFFFFFF
FFFFFFFFFFFFFFFFFFFFFFFFFFF:FFFFFFFFFFFFFFFFFFFFFF:FFFFFFFFFFFF:FFFF
@A00155:342:HHGFNDSXY:1:2447:2944:21590 2:N:0:GAACCTAG+TCCGCATA
TCGTTCGGCGAGAAGAATGCCGTTGGCCCCGCCAATTCGGCCCCTGTTCTGAAGTCTATATCGTAACC
ACACAGCCTCGCCAAATTCGCGACCACCCACGCGTCCTTAGCTTTCATCTTAAACCCCTTGCGTGTTA
+
FFFFFFFF:FFFFF:FFFF:FFFFFFFFFFFFFFFFFFFFFFFFFFFFFFFFFFFFFFFFFFFFFFFF
FFFFFFFFFFFFFFFFFFFFFFFFFFFFF:F:FFFFFFFFFFFFFFFFFFFFFFFFFFF:FFFFFFFF
@A00155:342:HHGFNDSXY:1:1536:28483:25363 1:N:0:GAACCTAG+TCCGCATA
GTTCGGCGAGAAGAATGCCGTTGGCCCCGCCAATTCGGCCCCTGTTCTGAAGTCTATATCGTAACCAC
ACAGCCTCGCCAAATTCGCGACCACCCACGCGTCCTTAGCTTTCATCTTAAACCCCTTGCGTGTTAGC
+
FFFFFFFFFFFFFFFFFFFFFFFFFFFFFFFFFFFFFFFFFFFFFFFFFFFFFFFFF:FFFFFFFFFF
FF::FFFFFFFFFFFFFFFF:FFFFFFFFFFFFFFFFFFFFFFFFFFFFF:FFFFFFFFFFFFFFFFF
```

```
@A00155:342:HHGFNDSXY:1:1470:19126:21590 2:N:0:GAACCTAG+TCCGCATA
TTCGGCGAGAAGAATGCCGTTGGCCCCGCCAATTCGGCCCCTGTTCTGAAGTCTATATCGTAACCACA
CAGCCTCGCCAAATTCGCGACCACCCACGCGTCCTTAGCTTTCATCTTAAACCCCTTGCGTGTTAGCT
+
FF:FFFFFF:FFFFFFF:FFFFFFFFF:FFFFFFFFF:FF:FFFFFF:FFFFFFFFFFFFFFFFFFFF
FFFFFF:FFFFFFFFFFFFFFFFFFFFFFFFFFFFFFFFFFFFFFFFFFFFFF:F:FFFFFFFFFFFFF
@A00155:342:HHGFNDSXY:1:1520:20103:24565 2:N:0:GAACCTAG+TCCGCATA
AGAAGAATGCCGTTGGCCCCGCCAATTCGGCCCCTGTTCTGAAGTCTATATCGTAACCACACAGCCTC
GCCAAATTCGCGACCACCCACGCGTCCTTAGCTTTCATCTTAAACCCCTTGCGTGTTAGCTCTCCCAT
+
FFFFFFFFFFFFFFFFFFFFFFFF:FFFFFFFFFFFFFFFFFFFFFFF:FFFFFFFFFFFFFFFFFFF
FFFFFFFFFFFFFFFFFFFFFFFFFFFFFFFFFFFFFFFFFFFFFFFFFFFFFFFFFFFFFFFFFFF:
@A00155:342:HHGFNDSXY:1:2666:4291:35008 2:N:0:GAACCTAG+TCCGCATA
GAAGAATGCCGTTGGCCCCGCCAATTCGGCCCCTGTTCTGAAGTCTATATCGTAACCACACAGCCTCG
CCAAATTCGCGACCACCCACGCGTCCTTAGCTTTCATCTTAAAACCCTTGCGTGTTAGCTCTCCCATT
+
F:FF,FFFF,FFFFF:FFFFFF:FFFFFFFF:FFFFF:FFFFFFFFF,FF,,F:F:FFFFFFF:FFF:
FF:FFFFFFFFF,FFFFFF,FFF::FF,FFFFFF:FFFFFFFF,F,FF::FF:FFF::FFFFFFFF,F
@A00155:342:HHGFNDSXY:1:2562:28221:19429 2:N:0:GAACCTAG+TCCGCATA
GAAGAATGCCGTTGGCCCCGCCAATTCGGCCCCTGTTCTGAAGTCTATATCGTAACCACACAGCCTCG
CCAAATTCGCGACCACCCACGCGTCCTTAGCTTTCATCTTAAACCCCTTGCGTGTTAGCTCTCCCATT
+
FFFFFFFFFFF,FFFFFFFFFFFF:FFFFFFFFFFFFFFFFFFFFFFF:FFFFFFF:FFFFFFFFFFF
FFFFFFFFFFFFFFFFFFFFFFFFFFFFFFFFFFFFF:FFFFFFFFFF:FFFFFFFFFFFFFFFFFFFF
@A00155:342:HHGFNDSXY:1:1163:25590:33191 2:N:0:GAACCTAG+TCCGCATA
GAAGAATGCCGTTGGCCCCGCCAATTCGGCCCCTGTTCTGAAGTCTATATCGTAACCACACAGCCTCG
CCAAATTCGCGACCACCCACGCGTCCTTAGCTTTCATCTTAAACCCCTTGCGTGTTAGCTCTCCCATT
+
FFFFFFFFFFFFFFFFFFFFFFFFFFFFFFFFFFFFFFFFFFFFFFFFFFFFFFFFFFFFFFFFFFFF
FFFFFFFFFFFFFFFFFFFFFFFFFFFFFFFFFFFFFFFFFFFFFFFFFFFFFFFFFFFFFFFFFFFF
@A00155:342:HHGFNDSXY:1:1560:32786:4914 2:N:0:GAACCTAG+TCCGCATA
AAGAATGCCGTTGGCCCCGCCAATTCGGCCCCTGTTCTGAAGTCTATATCGTAACCACACAGCCTCGC
CAAATTCGCGACCACCCACGCGTCCTTAGCTTTCATCTTAAACCCCTTGCGTGTTAGCTCTCCCATTC
+
FFFFFFFFFFFFFFFFFFFFFFFFFFFFFFFFFFFFFFFFFFFFFFFFFFFFFFFF:FFFFFF:FFFF
FFFFFFFFFFFFFFFFFFFFFFFFFFFFFFFFFFFFFFFFFFFFFFFFFFFFFFFFFFFFFFFFFFFF
@A00155:342:HHGFNDSXY:1:2625:32705:4586 2:N:0:GAACCTAG+TCCGCATA
AAGAATGCCGTTGGCCCCGCCAATTCGGCCCCTGTTCTGAAGTCTATATCGTAACCACACAGCCTCGC
CAAATTCGCGACCACCCACGCGTCCTTAGCTTTCATCTTAAACCCCTTGCGTGTTAGCTCTCCCATTC
+
F:F:FFFFFFFFFFFFFFFFFFFFFFFFFFFFFFFFFFFFFFFFFFFFFFFFFFFFFFFFFFFFFFFF
FFFFFFFFFFFFFFFFFFFFFFFFFFFFFFFFFFFFFFFFFFFFFFFFFFFFFFFFFFFFFFFFFFFF
@A00155:342:HHGFNDSXY:1:1535:3803:22608 2:N:0:GAACCTAG+TCCGCATA
CCGTTGGCCCCGCCAATTCGGCCCCTGTTCTGAAGTCTATATCGTAACCACACAGCCTCGCCAAATTC
GCGACCACCCACGCGTCCTTAGCTTTCATCTTAAACCCCTTGCGTGTTAGCTCTCCCATTCCTGGGTC
+
FFFFFFFFFFFFFFFFFFFFFFFFFFFFFFFFFFFFFFFF:FFFFFFFFFFFFFFFFFFFFFFFFFFF
FFFFFFFFFFFFFFFFFFFFFF:FFFFFFFFFFFFFFFFFFFFFFFFFFFFFFFFFFFFFFFFFFFF:F
@A00155:342:HHGFNDSXY:1:2348:28754:30405 1:N:0:GAACCTAG+TCCGCATA
TTGGCCCCGCCAATTCGGCCCCTGTTCTGAAGTCTATATCGTAACCACACAGCCTCGCCAAATTCGCG
ACCACCCACGCGTCCTTAGCTTTCGTCTTAAACCCCTTGCGTGTTAGCTCTCCCATTCCTGGGTCAAT
+
FFFFFFFFFFFFFFFFFFFFFFFFFFFFFFFFFFFFFFFFFFFFFFFFFFFFFFFFFFFFFFFFFFFF
FFFFFFFFFFFFFFFFFFFFFFFFFFFFFFFFFFFFFFFFFFFFFFFFFFFF:FFFFFFFFFFFFFF:
```

```
@A00155:342:HHGFNDSXY:1:2426:21486:16939 2:N:0:GAACCTAG+TCCGCATA
CCCCGCCAATTCGGCCCCTGTTCTGAAGTCTATATCGTAACCACACAGCCTCGCCAAATTCACGACCA
CCCACGCGTCCTTAGCTTTCATCTTAAACCCCTTGCGTGTTAGCTCTCCCATTCCTGGGTCAATCACG
+
FFFFFFFFF:FFFFFFFFFFFFFFFFFFFFF,FFFFFFFFFFFFFFFFFFFFFFFFFFFFFFFFFFFF
FFFFFFFFFFFF:FFFFFFFFFFFFF:FFFFFFFFFFFFFFFFFFFFFFFF,FFFFFFFFFFFFFFFF
@A00155:342:HHGFNDSXY:1:1536:28483:25363 2:N:0:GAACCTAG+TCCGCATA
CCCCGCCAATTCGGCCCCTGTTCTGAAGTCTATATCGTAACCACACAGCCTCGCCAAATTCGCGACCA
CCCACGCGTCCTTAGCTTTCATCTTAAACCCCTTGCGTGTTAGCTCTCCCATTCCTGGGTCAATCACG
+
FFFFFFFFF::FFFFFFFFFFFFFFFFFFFFFFFF:FFFFFFFFFFFFFF:FFFFFFFFFFFFFFFFF
FFFFFFFFFFFFF:FFFFFFFFFFFFF:FFFF::FFFFFFFFFF:FFFFFFFFFFFFFFFFFFFFFFF
@A00155:342:HHGFNDSXY:1:1554:14904:6574 2:N:0:GAACCTAG+TCCGCATA
CGCCAATTCGGCCCCTGTTCTGAAGTCTATATCGTAACCACACAGCCTCGCCAAATTCGCGACCACCC
ACGCGTCCTTAGCTTTCATCTTAAACCCCTTGCGTGTTAGCTCTCCCATTCCTGGGTCAATCACGCCT
+
FFFFFFFFFFFFFFFFFFFFFFFFFFFFFF:FFFFFFFFFFFFFFFFFFFFFFFFFFFFFFFFFFFFF
FFFFFFFFFFFFFFFFFFFFFFFFFFFFFFFFFFFFFFFFFFFFFFFF:FFFFFFFFFFFFFFFFFFF
@A00155:342:HHGFNDSXY:1:1466:22661:30906 2:N:0:GAACCTAG+TCCGCATA
CAATTCGGCCCCTGTTCTGAAGTCTATATCGTAACCACACAGCCTCGCCAAATTCGCGACCACCCACG
CGTCCTTAGCTTTCATCTTAAACCCCTTGCGTGTTAGCTCTCCCATTCCTGGGTCAATCACGCC
+
FFF:FFFFFFFFFFFFFFF::FFFFFFFFFFFFFFFFFFFFFFFFFFFFFFF:FFFFFFFFFFF:FFF
FFFFFFFFFFFFFFFFFFFFFF:FFFFFFFFFFFFFFFFFFFFFFFFFFFFFFFFFFFF,FFFF
@A00155:342:HHGFNDSXY:1:2540:7455:14403 2:N:0:GAACCTAG+TCCGCATA
CAATTCGGCCCCTGTTCTGAAGTCTATATCGTAACCACACAGCCTCGCCAAATTCGCGACCACCCACG
CGTCCTTAGCTTTCATCTTAAACCCCTTGCGTGTTAGCTCTCCCATTCCTGGGTCAATCACGCC
+
FFF:FFFFF:FFFFFFFFFFF:FFFFFFFFFF:FFFF:FFFFFFFFFFFFFFFFFFFFFFFFFFFFFF
FFFFFFFFF:FFFFFF,FFFFFFFFFFFFFF:FFFF:FFFFFFFFFFFFFFFFFFFFFFFFFFF
@A00155:342:HHGFNDSXY:1:1218:15745:27132 2:N:0:GAACCTAG+TCCGCATA
CAATTCGGCCCCTGTTCTGAAGTCTATATCGTAACCACACAGCCTCGCCAAATTCGCGACCACCCACG
CGTCCTTAGCTTTCATCTTAAACCCCTTGCGTGTTAGCTCTCCCATTCCTGGGTCAATCACGCC
+
FFFFFFFFFFFFFFFFFFFFFFFFFFF,FFFFF,FFFFFFFFFFFFFFFFFFFFFF:FFFFFFFF:FF
FFFFFFFFF:FFFFFFFFFF:FFFFFFFFFFFFFFFFFFFF,FF,:FF:FFFFFFFFFFFFFFF
@A00155:342:HHGFNDSXY:1:2125:5050:20635 2:N:0:GAACCTAG+TCCGCATA
CGGCCCCTGTTCTGAAGTCTATATCGTAACCACACAGCCTCGCCAAATTCGCGACCACCCACGCGTCC
TTAGCTTTCATCTTAAACCCCTTGCGTGTTAGCTCTCCCATTCCTGGGTCAATCACGCCTTTCAGGTT
+
FFFFFFFFFFFFFFFFFF,FFFFFFFFFF:FFFFFFFFFFFFFF:FFFFFFFFFFFFF:FFFFFFFFF
FFFFF:FFFFFFFFFFFFFFFFFFFFFFFFFFFFFFFFFF:FFFFFFFFFFFFFFFFFFFFFFFFFFF
@A00155:342:HHGFNDSXY:1:1524:12888:11350 2:N:0:GAACCTAG+TCCGCATA
CGGCCCCTGTTCTGAAGTCTATATCGTAACCACACAGCCTCGCCAAATTCGCGACCACCCACGCGTCC
TTAGCTTTCATCTTAAACCCCTTGCGTGTTAGCTCTCCCATTCCTGGGTCAATCACGCCTTTCAGGTT
+
FFFFFFFFFFFFFFFFFFFFFFFFFFFFFFFFFFFFFFFFFFFFFFFFFFFFFFFFFFFFFFFFFFFF
FFFFFFFFFFFFFFFFFFFFFFFFFFFFFFFFFFFFFFFFFFFFFFFFFFFFFFFFFFFFFFFFFFFF
@A00155:342:HHGFNDSXY:1:1262:14579:27054 1:N:0:GAACCTAG+TCCGCATA
CCCCTGTTCTGAAGTCTATATCGTAACCACACAGCCTCGCCAAATTCGCGACCACCCACGCGTCCTTA
GCTTTCATCTTAAACCCCTTGCGTGTTAGCTCTCCCATTCCTGGGTCAATCACGCCTTTCAGGTTAAA
+
F:FFFFFFFFFFFFFFFFFFFFFFFFFFFFFFFFFFFFFFFFFFFFFFFFFFFFFFFFFFFFFFFFFF
FFFFFFFFFFFFFFFFFF:FFFFFFFFFFFFFFFFF:FFFFFFFFFFFFFFFFFFFFFFFFFFF:FFF
```

```
@A00155:342:HHGFNDSXY:1:1504:8250:29778 1:N:0:GAACCTAG+TCCGCATA
CCCCTGTTCTGAAGTCTATATCGTAACCACACAGCCTCGCCAAATTCGCGACCACCCACGCGTCCTTA
GCTTTCATCTTAAACCCCTTGCGTGTTAGCTCTCCCATTCCTGGGTCAATCACGCCTTTCAGGTTAAA
+
FFFFFFFFFFFFFFFFFFFFFFFFFFFFF,FFFFFFFFFFFFFFFFFFFFFFFFFFFFFFFFFFFFFF
FFFFFFFFFFFFFFFFFFFFFFFFFFFF:::FFF:FFFFFF:FFFFFFFFFFFFFFFFFFFFFFFFFFF
@A00155:342:HHGFNDSXY:1:1504:7545:28588 1:N:0:GAACCTAG+TCCGCATA
CCCCTGTTCTGAAGTCTATATCGTAACCACACAGCCTCGCCAAATTCGCGACCACCCACGCGTCCTTA
GCTTTCATCTTAAACCCCTTGCGTGTTAGCTCTCCCATTCCTGGGTCAATCACGCCTTTCAGGTTAAA
+
FFFFFFFFFFFFFFFFFFFFFFFFFFFFFFFFFFFFFFFFFFFFFFFFFFFFFFFFFFFFFFFFFFFF
FFFFFFFFFFFFF:FFFFFFFFFFFFFFFFFFFFFFFFFFFFFFFFFFFFFFFFFFFFFFFFFFFFFF
@A00155:342:HHGFNDSXY:1:1407:19633:19586 1:N:0:GAACCTAG+TCCGCATA
CCTGTTCTGAAGTCTATATCGTAACCACACAGCCTCGCCAAATTCGCGACCACCCACGCGTCCTTAGC
TTTCATCTTAAACCCCTTGCGTGTTAGCTCTCCCATTCCTGGGTCAATCACGCCTTTCAGGTTAAAAG
+
FFFF:FFFFFFFFFFFFFFFFFFFFFFFFFFFFFFFFFFFFFFFF:FFFFFFFFFFFFFFFFFFFFFFF
FFFFFFFFFFFFFFFFFFFFFFFFFFFFFFFFFFFFFFFFFFF:FFFFFFFFFFFFFFFFFF:FFFF:FF
@A00155:342:HHGFNDSXY:1:2338:17490:21480 2:N:0:GAACCTAG+TCCGCATA
GTTCTGAAGTCTATATCGTAACCACACAGCCTCGCCAAATTCGCGACCACCCACGCGTCCTTAGCTTT
CATCTTAAACCCCTTGCGTGTTAGCTCTCCCATTCCTGGGTCAATCACGCCTTTCAGGT
+
FFFFFFFFFF:FFFFFFFFFFFFFFFFFFFFFFFFFFFFFFFFFFFFFFFFFF:FFFFFFFFFFFFFFF
FFFFFFFFFFFFFF:FFFFFFFFFFFFFFFFFFFFFFFFFFFFFFFFFFFFFFFFFFF
@A00155:342:HHGFNDSXY:1:1429:12762:21089 2:N:0:GAACCTAG+TCCGCATA
GTCTATATCGTAACCACACAGCCTCGCCAAATTCGCGACCACCCACGCGTCCTTAGCTTTCATCTTAA
ACCCCTTGCGTGTTAGCTCTCCCATTCCTGGGTCAATCACGCCTTTCAGGTTAAAAGGTGACGCATAT
+
FFFFFF:FFFFFFFF:FFF:FFFFFFFFFFFFFFFFFFFFFFFFFFFFFFFFFFFFFFFFFFFFFFFF
FFFFFFFFFFFFF:FFFFFFFFFF:FFF,FFFFFFFFFFFFFFFFFFFFFFFFFFFFFFFFFFFFFFF
@A00155:342:HHGFNDSXY:1:1610:20347:8328 1:N:0:GAACCTAG+TCCGCATA
GTCTATATCGTAACCACACAGCCTCGCCAAATTCGCGACCACCCACGCGTCCTTAGCTTTCATCTTAA
ACCCCTTGCGTGTTAGCTCTCCCATTCCTGGGTCAATCACGCCTTTCAGGTTAAAAGGTGACGCATAT
+
FFFFFFFFFFFFFFFFFFFFFFFFFFFFFFFFFFFFFFFFFFFFFFFFFFFFFFFFFFFFFFFFFFFF
FFFFFFFFFFFFFFFFFFFFFFFFFFFFFFFFFFFFFFFFFFFFFFFFFF,FFFFFF:FFFFFFFF,F
@A00155:342:HHGFNDSXY:1:2557:6135:35947 1:N:0:GAACCTAG+TCCGCATA
GTCTATATCGTAACCACACAGCCTCGCCAAATTCGCGACCACCCACGCGTCCTTAGCTTTCATCTTAA
ACCCCTTGCGTGTTAGCTCTCCCATTCCTGGGTCAATCACGCCTTTCAGGTTAAAAGGTGACGCATAT
+
FFFFF,FFFFFFFFFFFFFFFFFFFFFF,FFFFF:FFFFFFFFFFFFFFFFFFFFFFFFFFF:FFFFFFF
FF:FFFFFFFFFFFFFFFFFFFFFFFFFFFFFFFFFF:FFFFFFFFFFFFFFFFFFFF:FFFFFFFFFFF
@A00155:342:HHGFNDSXY:1:2446:23484:30107 2:N:0:GAACCTAG+TCCGCATA
GTCTATATCGTAACCACACAGCCTCGCCAAATTCGCGACCACCCACGCGTCCTTAGCTTTCATCTTAA
ACCCCTTGCGTGTTAGCTCTCCCATTCCTGGGTCAATCACGCCTTTCAGGTTAAAAGGTGACGCATAT
+
FFFFFF:FFFFFFFFFFFFFFFFFFFFFFFFFFFFFF:FF,FFFFFFFFFFFF:FFFFF:FFFFFFFF
FFFFFFFFFFFFFFFFFFFFFFFFFFFFFFFFFFFFFFFFFFFFFFFFFFFFFFFFFFFFFFFFFFFF
@A00155:342:HHGFNDSXY:1:2348:28754:30405 2:N:0:GAACCTAG+TCCGCATA
GTCTATATCGTAACCACACAGCCTCGCCAAATTCGCGACCACCCACGCGTCCTTAGCTTTCGTCTTAA
ACCCCTTGCGTGTTAGCTCTCCCATTCCTGGGTCAATCACGCCTTTCAGGTTAAAAGGTGACGCATAT
+
FFFFFFFFFFFFFFFFFFFFFFFFF:FFFFFF,FFFFFFFFFFFFFFFFFFFFFFFFFFF:FFFFFFFF
FFFFFFFFFFFFFFFFFFFFFF,FFFFFFFFFFFFFFFFFFFFFFFFFFFFFFFFFFFFFFFFFFFFF
```

```
@A00155:342:HHGFNDSXY:1:2338:17490:21480 1:N:0:GAACCTAG+TCCGCATA
TCGTAACCACACAGCCTCGCCAAATTCGCGACCACCCACGCGTCCTTAGCTTTCATCTTAAACCCCTT
GCGTGTTAGCTCTCCCATTCCTGGGTCAATCACGCCTTTCAGGTTAAAAGGTGAGGCAT
+
FFFFFFFFFFFFFFFFFFFFFFFFFFFFFFFFFFFFFFFFFFFFFFFFFFFFFFFFFFFFFFFFFFFF
FFFFFFFFFFFFFFFFF,FFF,FFFFFFFFFFFFFFFFFFFFFFFFFFFFFFFFFFFFF
@A00155:342:HHGFNDSXY:1:1503:2980:9189 1:N:0:GAACCTAG+TCCGCATA
TAACCACACAGCCTCGCCAAATTCGCGACCACCCACGCGTCCTTAGCTTTCATCTTAAACCCCTTGCG
TGTTAGCTCTCCCATTCCTGGGTCAATCACGCCTTTCAGGTTAAAAGGTGACGCATATGGGAATGGAT
+
FFFFFFFFFFFFFFFFFFFFFFFFFFFFFFFFFFFFFFFFFFFFFFFFFFFFFFFF:FFFFFFFFFFF
F:FFFFFFFFFFFFFFFFFFFFFFFFFFFFFFFFFFFFFFFFFFFFFFFFFFFFFFFFFFFF:FFFFF
@A00155:342:HHGFNDSXY:1:1429:12762:21089 1:N:0:GAACCTAG+TCCGCATA
TAACCACACAGCCTCGCCAAATTCGCGACCACCCACGCGTCCTTAGCTTTCATCTTAAACCCCTTGCG
TGTTAGCTCTCCCATTCCTGGGTCAATCACGCCTTTCAGGTTAAAAGGTGACGCATATGGGAATGGAT
+
FFFFFFFFFFFFFFFFFFFFFFFFFFFFFFFFFFFFFFFFFFFFFFFFFFFFFFFFFFFFFFFFFFFF
FFFFFFFFFFFFFF,FFFFFFFFFFFFFFFFFFFFFFFFFFF,FFFFFFFFFFFF:F:FFFF:FFFFF
@A00155:342:HHGFNDSXY:1:2602:30951:24471 2:N:0:GAACCTAG+TCCGCATA
CACAGCCTCGCCAAATTCGCGACCACCCACGCGTCCTTAGCTTTCATCTTAAACCCCTTGCGTGTTAG
CTCTCCCATTCCTGGGTCAATCACGCCTTTCAGGTTAAAAGGTGACGCATATGGGAAAGGATTTAAGG
+
FFFFFFFFFFFFFFFFFFFFFFFFFFFFFFFFFFFFF,FFFFFFFFFFFFFFFFFFF:FFFFFFF:FF
FFFFFFFFFFFFFFFFFFFFFFFFFFFFFFFFFFFFFFFFFFFFFFFFFFFFFFFFFFFFFFFFFFFF
@A00155:342:HHGFNDSXY:1:1471:29152:2691 2:N:0:GAACCTAG+TCCGCATA
CACAGCCTCGCCAAATTCGCGACCACCCACGCGTCCTTAGCTTTCATCTTAAACCCCTTGCGTGTTAG
CTCTCCCATTCCTGGGTCAATCACGCCTTTCAGGTTAAAAGGTGACGCATATGGGAATGGATTTAAGG
+
F:FF:FFF,FF:,FF:FF:FFF:FFFF:FFFFFFFFFF:,FFFFF:F:FFFFFF::FFFFFFFFFFFF
FFFFFF:F::F:FFFFF:,FFF,F:FFFFFFFFFFFFFF:FFFFFFFFFFFFFF,FFFFFFFFFF:F:
@A00155:342:HHGFNDSXY:1:1610:20347:8328 2:N:0:GAACCTAG+TCCGCATA
CACAGCCTCGCCAAATTCGCGACCACCCACGCGTCCTTAGCTTTCATCTTAAACCCCTTGCGTGTTAG
CTCTCCCATTCCTGGGTCAATCACGCCTTTCAGGTTAAAAGGTGACGCATATGGGAATGGATTTAAGG
+
FFFF:FFFFFFFFFFFFFFFFFFFFFFFFFFFFFFFFF::FFF,FF,FFFFFFFFFF:FFFFFFFFFF
FFFFFFFFF,FFFFFFFFFFFFFFFFFFFFFFFFFFFFFFFFFFFFFFFFFFFFFFFFFFFFFFFFFF
@A00155:342:HHGFNDSXY:1:1472:7374:31015 2:N:0:GAACCTAG+TCCGCATA
ACAGCCTCGCCAAATTCGCGACCACCCACGCGTCCTTAGCTTTCATCTTAAACCCCTTGCGTGTTAGC
TCTCCCATTCCTGGGTCAATCACGCCTTTCAGGTTAAAAGGTGACGCATATGGGAATGGATTTAAGGG
+
FFFFFFFFFFFFFF:FFFFFFFFFFFF:FFFFFFFFFFFFFFFFFFFFFFFFFFFFFFFFFF:FFFFF
FFFFFFF,FFFFFFFFFFFFF:FFFFFFFFFFFFFFFFFFFFFFFF:FFFFFFFFFFFFFFFFFFFFF
@A00155:342:HHGFNDSXY:1:2425:32642:11866 2:N:0:GAACCTAG+TCCGCATA
ACAGCCTCGCCAAATTCGCGACCACCCACGCGTCCTTAGCTTTCATCTTAAACCCCTTGCGTGTTAGC
TCTCCCATTCCTGGGTCAATCACGCCTTTCAGGTTAAAAGGTGACGCATATGGGAATGGATTTAAGGG
+
F::FFFF:FFFFFFFFFFFFFFFFFFFFFFFFFFFFF:FFFFFF:FFFFFFFFFF:FFFFF:FFFFFF
FFFFFFF:FFFFFFFFFFFFF,FFFFFFFFFFFFFFFFFFFFFFFFFFFFFFFFFFFFFFFFFFFFFF
@A00155:342:HHGFNDSXY:1:1354:29044:16939 2:N:0:GAACCTAG+TCCGCATA
ACAGCCTCGCCAAATTCGCGACCACCCACGCGTCCTTAGCTTTCATCTTAAACCCCTTGCGTGTTAGC
TCTCCCATTCCTGGGTCAATCACGCCTTTCAGGTTAAAAGGTGACGCATATGGGACTGGATTTAAGGG
+
FFFFFFFFFFFFFFFFFFFFFFFFFFFFFFFFFFFFFFFFFFFFFFFFFFFFFFFFFFFFF,F,FFFF
FFFFFFFFFFFFFFFFFF,FFFFFFFF:FFFFF:F,:FFFFFFFFFF:FFFFFFF,FFFF:FFFFFFF
```

```
@A00155:342:HHGFNDSXY:1:1519:17589:30545 2:N:0:GAACCTAG+TCCGCATA
ACAGCCTCGCCAAATTCGCGACCACCCACGCGTCCTTAGCTTTCATCTTAAACCCCTTGCGTGTTAGC
TCTCCCATTCCTGGGTCAATCACGCCTTTCAGGTTAAAAGGTGACGCATATGGGAATGGATTTAAGGG
+
FFFFFFFFFFFFFFFFFFFFFFFFFF:FFFFFFFFF:FF:FFFFF::FFFFFFFFFFFFFFFFFFFFF
FFFFFFF:FFF:FFFFFFFFFFFFFFFFFFFFFFFFFFFFFFFFFFFFFFFFFFFFFFFFFFFFFFFF
@A00155:342:HHGFNDSXY:1:1354:29044:16939 1:N:0:GAACCTAG+TCCGCATA
CAGCCTCGCCAAATTCGCGACCACCCACGCGTCCTTAGCTTTCATCTTAAACCCCTTGCGTGTTAGCT
CTCCCATTCCTGGGTCAATCACGCCTTTCAGGTTAAAAGGTGACGCATATGGGAATGGATTTAAGGGC
+
FFFFFFFFFFFFF:FFFFFFFFFFFF:FFFFFFFF:FFFFFFFFFFFFFF:FFFFFFFFFFFFFFFFF
FFFFF::FFFFFFFFFF:FF:FFF,FFFFFFFFFFFFFFFFFFFFFFFFFFFFFFFFFFFFFFFFFFF
@A00155:342:HHGFNDSXY:1:2116:31864:27868 1:N:0:GAACCTAG+TCCGCATA
CCTCGCCAAATTCGCGACCACCCACGCGTCCTTAGCTTTCATCTTAAACCCCTTGCGTGTTAGCTCTC
CCATTCCTGGGTCAATCACGCCTTTCAGGTTAAAAGGTGACGCATATGGGAATGGATTTAAGGGCATG
+
FFFFFFFFFFFFFFFFFFFFFFFFFFFFFFFFFFFFFFFFFFFFFFFFFFFFFFFFFFFFFFFFFFFF
FFF,FFF:FFFFFFFFF:FFFFFFFFFFF,:FFFFFFFFFFFFF,FFFFFFF:FFFFFFFFFFFFF:F
@A00155:342:HHGFNDSXY:1:2262:32560:21339 1:N:0:GAACCTAG+TCCGCATA
CTCGCCAAATTCGCGACCACCCACGCGTCCTTAGCTTTCATCTTAAACCCCTTGCGTGTTAGCTCTCC
CATTCCTGGGTCAATCACGCCTTTCAGGTTAAAAGGTGACGCATATGGGAATGGATTTAAGGGCATG
+
FFFFFFFFFFFFFFFFFFFFFFFFFFFFFF,FFFFF,FF:FF:FFFFFFFF:FFFF,F,::FFFFFFF
F:F:FF,FFFFFF:FFFFFFFF,:FFFFF:FFFFFFFFFFFFFFFFFFFF:FFFF:F:FFFFFF,:F
@A00155:342:HHGFNDSXY:1:1659:14190:18834 2:N:0:GAACCTAG+TCCGCATA
CGCCAAATTCGCGACCACCCACGCGTCCTTAGCTTTCATCTTAAACCCCTTGCGTGTTAGCTCTCCCA
TTCCTGGGTCAATCACGCCTTTCAGGTTAAAAGGTGACGCATATGGGAATGGATTTAAGG
+
FFFFFFFFFFFFFFFFFFFFFFFFFFFFFFFFFFFFFFFFFFFFFFFFFFFFF:FFFFFFFFFFFFF
,FFFFFFFFFFFF:,FFFFFFFFFFFFFFFFFFFFFFFFFFFFFFFFFFFFFFFFFFFFF
@A00155:342:HHGFNDSXY:1:2463:6262:20447 2:N:0:GAACCTAG+TCCGCATA
ATTCGCGACCACCCACGCGTCCTTAGCTTTCATCTTAAACCCCTTGCGTGTTAGCTCTCCCATTCCTG
GGTCAATCACGCCTTTCAGGTTAAAAGGTGACGCATATGGGAATGGATTTAAGGGCATGGCCAGTGTC
+
FFFFF,FFFFFFFF:FFFFFFFFFF,F:FFFFFFFFFFFFFFF,F:FFFF:FF::FFFFFFF:FFFFF
FFFFFF:FFFFFFFFFFFF,FFFFFFFFFFFFFFFFFF:FF:FFFFFFFFFFFFFFFFFFFFFFFFFF
@A00155:342:HHGFNDSXY:1:1503:2980:9189 2:N:0:GAACCTAG+TCCGCATA
ATTCGCGACCACCCACGCGTCCTTAGCTTTCATCTTAAACCCCTTGCGTGTTAGCTCTCCCATTCCTG
GGTCAATCACGCCTTTCAGGTTAAAAGGTGACGCATATGGGAATGGATTTAAGGGCATGGCCAGTGTC
+
FFFFFFFFFFFFFFFF:FFFFFF:FFFFFFFFFFFFFFFFFFFFFFFFFFFFFFFFFFFFFFFFFFFF
FFFFFFFFFFFFFFFFFFFFFFFFFFFFFFFFFFFFFFFFFFFFFFFFFFFFFFFFFFFFFFFFFFFF
@A00155:342:HHGFNDSXY:1:2359:5556:24862 2:N:0:GAACCTAG+TCCGCATA
TTCGCGACCACCCACGCGTCCTTAGCTTTCATCTTAAACCCCTTGCGTGTTAGCTCTCCCATTCCTGG
GTCAATCACGCCTTTCAGGTTAAAAGGTGACGCATATGGGAATGGATTTAAGGGCATGGCCAGTGTCG
+
FFFFFFFFFFFFFFFFFFFFFFFFFFFFFFFFFFFFFFFFFFFFFFFFFFFFFFFFFF:FFFFFFFFF
FFFFFFFFFFFFFFFFFF,FFFFFFFFFFFFFFFFFFFFFFFFFFFFFFFFFFFFFFFFFFFFFFFFF
@A00155:342:HHGFNDSXY:1:1211:8648:34131 2:N:0:GAACCTAG+TCCGCATA
TTCGCGACCACCCACGCGTCCTTAGCTTTCATCTTAAACCCCTTGCGTGTTAGCTCTCCCATTCCTGG
GTCAATCACGCCTTTCAGGTTAAAAGGTGACGCATATGGGAATGGATTTAAGGGCATGGCCAGTGTCG
+
FFFFFFFFFFFFFFFFFFFFFFFFFFFFFFFFFFFFFFFFFFFFFFFFFFFFFFFFFFFFF:FFFFFF
FFFFFFFFFFFFFFFFFFFFFFFFFFFFFFFFFFFFFFFFFFFFFFFFFFFFFFFFFFFFFFFFFFFF
```

```
@A00155:342:HHGFNDSXY:1:2602:30951:24471 1:N:0:GAACCTAG+TCCGCATA
TTCGCGACCACCCACGCGTCCTTAGCTTTCATCTTAAACCCCTTGCGTGTTAGCTCTCCCATTCCTGG
GTCAATCACGCCTTTCAGGTTAAAAGGTGACGCATATGGGAAAGGATTTAAGGGCATGGCCAGTGTCG
+
FFFFFFFFFFFFFFFFFFFFFFFFFFFFFFFFFFFFFFFFFFFFFFFFFFFFFFFFFFFFFFFFFFFF
FFFFFFFFFFFFFFFFFFFFFFFFFFFFFFFFFF,F:FFFFFFFFFFFFFFFFFF:FFFFFFFFFFFF
@A00155:342:HHGFNDSXY:1:2548:5891:20337 1:N:0:GAACCTAG+TCCGCATA
TTCGCGACCACCCACGCGTCCTTAGCTTTCATCTTAAACCCCTTGCGTGTTAGCTCTCCCATTCCTGG
GTCAATCACGCCTTTCAGGTTAAAAGGTGACGCATATGGGAAAGGATTTAAGGGCATGGCCAGTGTCG
+
FFFFFFFFFFFFFFFFFFFFFFFFFFFFFFFFFFFFF:FFFFFFFFFFFFFFFFFFFFFFF:FFFFFF
FFFFFFFFFFFFFF,FFFFFFFFFFFFFFFFFF,FF,FFFF,:FFF,FFFFFFFF,FFFF:,F:FFFF
@A00155:342:HHGFNDSXY:1:2463:6262:20447 1:N:0:GAACCTAG+TCCGCATA
CGACCACCCACGCGTCCTTAGCTTTCATCTTAAACCCCTTGCGTGTTAGCTCTCCCATTCCTGGGTCA
ATCACGCCTTTCAGGTTAAAAGGTGACGCATATGGGAATGGATTTAAGGGCATGGCCAGTGTCGGTAC
+
FFFFFFFFFFFFFFFFFFFFFFFFFFFFFFFFF:FFFFFFFFFFFF:FF:FFFFFFFFFFFFF:FFFF
FFFF:FFFFFFFFFFFFFF:FFFFFFFFFFFF:FFFF,FFFFFFFFFFFF,FFF:FFFFFFFF:FFFF
@A00155:342:HHGFNDSXY:1:1659:14190:18834 1:N:0:GAACCTAG+TCCGCATA
CACCCACGCGTCCTTAGCTTTCATCTTAAACCCCTTGCGTGTTAGCTCTCCCATTCCTGGGTCAATCA
CGCCTTTCAGGTTAAAAGGTGACGCATATGGGAATGGATTTAAGGGCATGGCCAGTGTCG
+
FFFFFFFFFFFFFFFFFFFFFFFFFFFFFFFFFFFFFFFFFFFFFFFFFFFFFFFFFFF:FFFFFFFF
FFFFFFFF:FFFFFFFFFFFFFFFFFFFFFFFFFFFFFFFFFFFFFFFFFFFFFFFFFF
@A00155:342:HHGFNDSXY:1:1210:6822:17190 1:N:0:GAACCTAG+TCCGCATA
ACCCACGCGTCCTTAGCTTTCATCTTAAACCCCTTGCGTGTTAGCTCTCCCATTCCTGGGTCAATCAC
GCCTTTCAGGTTAAAAGGTGACGCATATGGGAATGGATTTAAGGGCATGGCCAGTGTCGGTACACCCG
+
FFFFFFFFFFFFF:FFFF:FFFFFFFFFFFFFFFFFFF,FFFFFFFFFFFFFFFFFFFFFFFFFFFFF
FFFFFFFFFFFFFFFFFFFFFFFFF:FFFFFFFFFFFFFFFFFFFFFFFFFFFFFFFFFFFFFFFFFF
@A00155:342:HHGFNDSXY:1:2547:5104:29559 1:N:0:GAACCTAG+TCCGCATA
CGCGTCCTTAGCTTTCATCTTAAACCCCTTGCGTGTTAGCTCTCCCATTCCTGGGTCAATCACGCCTT
TCAGGTTAAAAGGTGACGCATATGGGAATGGATTTAAGGGCATGGCCAGTGTCGGTACACCCGCAAAC
+
FFFFF:FFFF:FFFFFFF,FFFFFFFFFFFFFFFF,FFFFFFFFFFF:FFF:FF::FFFFFF,FFFFF
FFFF:FFFFFFFFFFF:FFF,FFFFFFFFFFFFFF::FFF:FF,F:FF:FFFFFFF:FF:FFFFF,FF
@A00155:342:HHGFNDSXY:1:1401:32036:18646 2:N:0:GAACCTAG+TCCGCATA
CGCGTCCTTAGCTTTCATCTTAAACCCCTTGCGTGTTAGCTCTCCCATTCCTGGGTCAATCACGCCTT
TCAGGTTAAAAGGTGACGCATATGGGAATGGATTTAAGGGCATGGCCAGTGTCGGTACACCCGCAAAC
+
FFFFFFFFFFFFFFFFFFFFFFFFFFFFFFFFFFFFFFFFFFFFFFFFFFFFFFFFFFFFFFFFFFFF
FFFFFFFFFFFFFFFFFFFFFFFFFFFFFFFFFFFFFFFFFFFFFFFFFFFFFFFFFFFFFFFFFFFF
@A00155:342:HHGFNDSXY:1:1504:8250:29778 2:N:0:GAACCTAG+TCCGCATA
CGCGTCCTTAGCTTTCATCTTAAACCCCTTGCGTGTTAGCTCTCCCATTCCTGGGTCAATCACGCCTT
TCAGGTTAAAAGGTGACGCATATGGGAATGGATTTAAGGGCATGGCCAGTGTCGGTACACCCGCAAAC
+
FFFFFFF,F:FFF:FFFFFFFFFF:FFFFFFFFFFFFFF:FFFFFFFFFFFFFFFFFFFFFFFFFFFF
:FFFFFFFFFFFFFFFFFFFFFFFFFFFFFFFFFFFFFFFFFFFF,FFFFFFFFFFFFFFFFFFFF,F
@A00155:342:HHGFNDSXY:1:1504:7545:28588 2:N:0:GAACCTAG+TCCGCATA
CGCGTCCTTAGCTTTCATCTTAAACCCCTTGCGTGTTAGCTCTCCCATTCCTGGGTCAATCACGCCTT
TCAGGTTAAAAGGTGACGCATATGGGAATGGATTTAAGGGCATGGCCAGTGTCGGTACACCCGCAAAC
+
:FFFFFF,FFFFFFFF:FFFFFFFFFFF,FFFFFFFFFFFFFFFFFFFFFFFFFFFFFFFFFFFFFFF
FFFFFFFFFFFFFFFFFFFFFFFFFFFFFFFFFFFFFFFFFFFFFFFFFFFFFFFFFFF:FFFFFFFF
```

```
@A00155:342:HHGFNDSXY:1:1262:14579:27054 2:N:0:GAACCTAG+TCCGCATA
CGCGTCCTTAGCTTTCATCTTAAACCCCTTGCGTGTTAGCTCTCCCATTCCTGGGTCAATCACGCCTT
TCAGGTTAAAAGGTGACGCATATGGGAATGGATTTAAGGGCATGGCCAGTGTCGGTACACCCGCAAAC
+
FFFFFFFFF:FFFFFFFFF:FFFFFFFFFFFFFFFFFFFFFFFFFFFFFFFFFFFFFF,FFFFFFFFF
FFFFFFFFFFFFFFFFFFFFFFFFFFFF:FFFFFFFFFFFFFFFFFFFFFFFFFFFFFFFFFFFFFFF
@A00155:342:HHGFNDSXY:1:2155:19063:19789 2:N:0:GAACCTAG+TCCGCATA
GCGTCCTTAGCTTTCATCTTAAACCCCTTGCGTGTTAGCTCTCCCATTCCTGGGTCAATCACGCCTTT
CAGGTTAAAAGGTGACGCATATGGGAATGGATTTAAGGGCATGGCCAGTGTCGGTACACCCGCAAACG
+
FFFFF:,FF:FFFFF:,FFFFFFFF:FFFF,::,FFFFFFFFFF,FFFFFFF,FFF:FFF,FFFFFFF
FFFFFFFFFFFFFFFF,FFF:FF:F:FFF,FFFFFF:FF,FFFF:FFFFFFF:,FF:F:F:FF:F,:F
@A00155:342:HHGFNDSXY:1:2557:6135:35947 2:N:0:GAACCTAG+TCCGCATA
AAACCCCTTGCGTGTTAGCTCTCCCATTCCTGGGTCAATCACGCCTTTCAGGTTAAAAGGTGACGCAT
ATGGGAATGGATTTAAGGGCATGGCCAGTGTCGGTACACCCGCAAACGGTATCCATGTTAATCTCACC
+
FF,FFFF,FFFFFFFFFFFF:FFFFFFFFFFFFFFF,FFFFFF:FFFFFFFFFFF:FFFFFFFFFFFF
FFFFFFF:FFFF:FFFFFFFFFF,FFFFFFFFFFFFFFF:FFFFFFF,FFFFFFFFFFFFFF:FFFFF
@A00155:342:HHGFNDSXY:1:2365:30038:17127 1:N:0:GAACCTAG+TCCGCATA
CATTCCTGGGTCAATCACGCCTTTCAGGTTAAAAGGTGACGCATATGGGAATGGATTTAAGGGCATGG
CCAGTGTCGGTACACCCGCAAACGGTATCCATGTTAATCTCACCGCTAGCTTCTCTTCTCCCTCTTCT
+
FF:FFFFFFFFFFFFFFFFFFFFFFFFFFFFFFFFFFFFFFFFFFFFFFFFFFFFFFFFFFFFFFFFF
FFFFFFFFFFFFFFFFFFFFFFFFFF:FFFFFFFFFFFFFFFFFFFFFFFFFFFFFFFFFFFFFFFFF
@A00155:342:HHGFNDSXY:1:2569:26449:7028 1:N:0:GAACCTAG+TCCGCATA
CATTCCTGGGTCAATCACGCCTTTCAGGTTAAAAGGTGACGCATATGGGAATGGATTTAAGGGCATGG
CCAGTGTCGGTACACCCGCAAACGGTATCCATGTTAATCTCACCGCTAGCTTCTCTTCTCCCTCTTCT
+
FFFFFFFFFFFFFFFFFFFFFFFFFFFFFFFFFFFFFFFFFFFFFFFFFFFFFFFFFFFFFFFFFFFF
FFFFFFFFFFFFFFFFFFFF:FFFFFFFFFFFFFFFFFFFFFFFFFFFFFFFFFFFFFFFFFFFFFF
@A00155:342:HHGFNDSXY:1:2548:28122:11209 2:N:0:GAACCTAG+TCCGCATA
CACGCCTTTCAGGTTAAAAGGTGACGCATATGGGAATGGATTTAAGGGCATGGCCAGTGTCGGTACAC
CCGCAAACGGTATCCATGTTAATCTCACCGCTAGCTTCTCTTCTCCCTCTTCTATCTCCTGGTCCTCC
+
FFFFFFFFFFFFFFFFFFFFFFFFFFFFFFFFFFFFFFFFFFFFFFFFFFFFFFFFFFFFFFFFFFFF
FFFFFFFFFFFFFFFFFFFFFFFFF:FFFFFFFFF,FFFF:FFFFFFFFFFFFFFFFFFFFFFFFFFF
@A00155:342:HHGFNDSXY:1:1644:18982:36558 2:N:0:GAACCTAG+TCCGCATA
CACGCCTTTCAGGTTAAAAGGTGACGCATATGGGAATGGATTTAAGGGCATGGCCAGTGTCGGTACAC
CCGCAAACGGTATCCATGTTAATCTCACCGCTAGCTTCTCTTCTCCCTCTTCTATCTCCTGCTCCTCC
+
FFFFFFFFFFFFFFFFFFFFFFFFFFFFFF:F:FF:FFFFFFFFFFFFFFFFFFFFFFFFFFFFFFFF
FFFFFF,FFFFFFFFFFFF:FFFFFFFFFF:FFFFFFFFFFFFFFFFFFFFFFFFFFFFFFFFFFFFF
@A00155:342:HHGFNDSXY:1:2422:10881:16110 2:N:0:GAACCTAG+TCCGCATA
CGCCTTTCAGGTTAAAAGGTGACGCATATGGGAATGGATTTAAGGGCATGGCCAGTGTCGGTACACCC
GCAAACGGTATCCATGTTAATCTCACCGCTAGCTTCTCTTCTCCCTCTTCTATCTCCTGCTCCTCCAG
+
:FFFFFFFFFFFFFF,FFFFFFF,FFFFFFFF:FFFFFFFFFFFFFFFFFFFFFFFFFFFFFFFFFFF
FFFFFFFFFFFFFFFFFFF:FF:FFFFFFFFFFFFFFF:FFFFFFFFFFFFFFFFFFFFFFFFFFFFF
@A00155:342:HHGFNDSXY:1:2422:11469:18349 2:N:0:GAACCTAG+TCCGCATA
CGCCTTTCAGGTTAAAAGGTGACGCATATGGGAATGGATTTAAGGGCATGGCCAGTGTCGGTACACCC
GCAAACGGTATCCATGTTAATCTCACCGCTAGCTTCTCTTCTCCCTCTTCTATCTCCTGCTCCTCCAG
+
FF,FFFFFFFF:FFFFFFFFFFFFF:FFFFFFFFF:FFFFFFFFFFFFFFFFFFFFFFFFFFFFFFFF
:FFFFFFFFFFFFFFFFFFFFFFFFFF:FFFFFFFFF:FFFFFFFFFFFFFFFFFFFFFFFFFFFFFF
```

@A00155:342:HHGFNDSXY:1:2262:32560:21339 2:N:0:GAACCTAG+TCCGCATA
CTTTCAGGTTAAAAGGTGACGCATATGGGAATGGATTTAAGGGCATGGCCAGTGTCGGTACACCCGCA
AACGGTATCCATGTTAATCTCACCGCTAGCTTCTCTTCTCCCTCTTCTATCTCCTGCTCCTCCAGTAT
+
FFFFFFFFFFFFFFF:FFFFFFFFFF:FFFF,FFFFF::FFFFFFFFFFFFFFFFFFFFFFFFFFFFF
:FFFFFFFFFFFFFFFFFFFFFFF:FFFFF,FFFF:FFFFFFFFFFF:FFFFFFFFFFFFFFFFFFF:
@A00155:342:HHGFNDSXY:1:2116:31864:27868 2:N:0:GAACCTAG+TCCGCATA
CTTTCAGGTTAAAAGGTGACGCATATGGGAATGGATTTAAGGGCATGGCCAGTGTCGGTACACCCGCA
AACGGTATCCATGTTAATCTCACCGCTAGCTTCTCTTCTCCCTCTTCTATCTCCTGCTCCTCCAGTAT
+
FFFFFFFFFFFFFFFFFFFF:FFFFFFF:F:F:FFFFFFFFFFFFFFFFFFFFFFFFFFFFFFFFFF:
FFFFFFFFFFFFFFFFFFFFFFFFFFFFFF:FFFFFFFFFFFFFFFFFFFFFFFFFF:FFFFF:FFF:
@A00155:342:HHGFNDSXY:1:1210:6822:17190 2:N:0:GAACCTAG+TCCGCATA
TTTCAGGTTAAAAGGTGACGCATATGGGAATGGATTTAAGGGCATGGCCAGTGTCGGTACACCCGCAA
ACGGTATCCATGTTAATCTCACCGCTAGCTTCTCTTCTCCCTCTTCTATCTCCTGCTCCTCCAGTATT
+
FFFFFFFFFFFFFFFFFFFFFFF:FFFFFFF:FFFFFFFFFF:FFFFFFFFFFFFFFFFFFFFFFFFF
FFFFFFFFFFFFFFFFFFFFFFFFFFFFFFFFFF:FFFFFF:FFFF:FFFFFFFFFFFF:FF:FFFFF
@A00155:342:HHGFNDSXY:1:1233:13395:17268 1:N:0:GAACCTAG+TCCGCATA
AAGGTGACGCATATGGGAATGGATTTAAGGGCATGGCCAGTGTCGGTACACCCGCAAACGGTATCCAT
GTTAATCTCACCGCTAGCTTCTCTTCTCCCTCTTCTATCTCCTGCTCCTCCAGTATTGCATCTTCTCT
+
FFFFFFFFFFFFFFFFFFFFFFFFFFFF:FFFFFFFFFFFFFFFFFFFFFFFF:FFFFFFFFFFFFFF
FFFFFFFFFFFFFFFFFFFFFFFFFFFFFFFFFFFFFFFFFFFFFFFFFFFFFFFFFFFFFFFFFFFF
@A00155:342:HHGFNDSXY:1:1311:30653:34945 1:N:0:GAACCTAG+TCCGCATA
AAGGTGACGCATATGGGAATGGATTTAAGGGCATGGCCAGTGTCGGTACACCCGCAAACGGTATCCAT
GTTAATCTCACCGCTAGCTTCTCTTCTCCCTCTTCTATCTCCTGCTCCTCCAGTATTGCATCTTCTCT
+
FFFFFFFFFFFFFFFFFFFFFFFFFFFFFFFFFFFFFF:FFFFFFFFFFFFFFFFFFFFFFFFFFFFF
FFFFFFFFFFFFFFFFFFFFFFFFFFFFFFFFFFFFFFFFFFFFFFFFFFFFFFFFFFFFFFFFFFFF
@A00155:342:HHGFNDSXY:1:2365:30038:17127 2:N:0:GAACCTAG+TCCGCATA
CATATGGGAATGGATTTAAGGGCATGGCCAGTGTCGGTACACCCGCAAACGGTATCCATGTTAATCTC
ACCGCTAGCTTCTCTTCTCCCTCTTCTATCTCCTGCTCCTCCAGTATTGCATCTTCTCTAGCTGCCAT
+
FFFFFFFFFFFFFFFF:F,FFFFFFFFFFFFFFFFFFFFFFFFFFFFFFFFFFFFFFFFFFFFFFFFF
FFFFF,FFF,FFFFFFFFFFFF:FFFFF:FFFFFFFFFF:FFFFFF:FFF,FFFFFFFFFFFFFFFFF
@A00155:342:HHGFNDSXY:1:2569:26449:7028 2:N:0:GAACCTAG+TCCGCATA
CATATGGGAATGGATTTAAGGGCATGGCCAGTGTCGGTACACCCGCAAACGGTATCCATGTTAATCTC
ACCGCTAGCTTCTCTTCTCCCTCTTCTATCTCCTGCTCCTCCAGTATTGCATCTTCTCTAGCTGCCAT
+
F:F:FFFFFFFFFFFFFFFFFFFFFFFFFFFFFFFFFFFFFFF:FFF:FFFFFFFFFFFFFFFFFFFF
FFFFFFFFFFFFFFFFFFFFFFFFFFF,FFFFFFFFFFFFFFFFFFFFFFFFF,FFFFFFFFFFFFFF
@A00155:342:HHGFNDSXY:1:1358:28465:9392 2:N:0:GAACCTAG+TCCGCATA
GGAATGGATTTAAGGGCATGGCCAGTGTCGGTACACCCGCAAACGGTATCCATGTTAATCTCACCGCT
AGCTTCTCTTCTCCCTCTTCTATCTCCTGCTCCTCCAGTATTGCATCTTCTCTAGCTGCCATCTCGTC
+
FFFFFFFFFFFFFFFFFFFFFFFFFFFFFFFFFFFFFFFFFFFFFFFFFFFFFFFFFFFFFFFFFFFF
FFFFFFFFFFFFFFFFFFFFFFFFFFFFFFFFFFFFFFFFFFFFFFFFFFFFFFFFFFFFFFFFFFFF
@A00155:342:HHGFNDSXY:1:1251:3839:29027 2:N:0:GAACCTAG+TCCGCATA
GGAATGGATTTAAGGGCATGGCCAGTGTCGGTACACCCGCAAACGGTATCCATGTTAATCTCACCGCT
AGCTTCTCTTCTCCCTCTTCTATCTCCTGCTCCTCCAGTATTGCATCTTCTCTAGCTGCCATCTCGTC
+
FFF:FFFFFFFF:FFFFFF:FFFFFFFFFFFFFFFFFFFFFFFFFFFFFFFFFFFFFFFFFFFFFFFF
FFFFFFFF:FFFFFFFFFF:FFFFFFFFFFFFFFFFFFFFFFFFFFFFFFFFFFFFFFFFFFFFFFFF

@A00155:342:HHGFNDSXY:1:2628:11532:27665 2:N:0:GAACCTAG+TCCGCATA
GGAATGGATTTAAGGGCATGGCCAGTGTCGGTACACCCGCAAACGGTATCCATGTTAATCTCACCGCT
AGCTTCTCTTCTCCCTCTTCTATCTCCTGCTCCTCCAGTATTGCATCTTCTCTAGCTGCCATCTCGTC
+
FFFFFFFFFFFFFFFFFFFFFFFFFFFFFFFFFFFFFFFFFFFFFFFFFFFFF:FFFFFFFFFFFFFF
FFFFFFFFFFFFFFFFFFFFFFFFFFFFFFFFFFFFFFFFFFFFFFFFFFFFFFFFFFFFFFFFFFFF
@A00155:342:HHGFNDSXY:1:1357:13277:11240 2:N:0:GAACCTAG+TCCGCATA
GGAATGGATTTAAGGGCATGGCCAGTGTCGGTACACCCGCAAACGGTATCCATGTTAATCTCACCGCT
AGCTTCTCTTCTCCCTCTTCTATCTCCTGCTCCTCCAGTATTGCATCTTCTCTAGCTGCCATCTCGTC
+
FFFFFFFFFFFFFFFFFFFFFFFFF:FFFFFFFFFFFFFFFFFFFFFFFFFF:FFFFFFFFFFFFFFF
FFFFFFFFFFFFFFFFFFFFF:FFFFFFFFFFFFFFFFFFFFFFFFFFFFFFFFFFFFFFFFFFFFFF
@A00155:342:HHGFNDSXY:1:1233:13395:17268 2:N:0:GAACCTAG+TCCGCATA
AATGGATTTAAGGGCATGGCCAGTGTCGGTACACCCGCAAACGGTATCCATGTTAATCTCACCGCTAG
CTTCTCTTCTCCCTCTTCTATCTCCTGCTCCTCCAGTATTGCATCTTCTCTAGCTGCCATCTCGTCGC
+
FFFFFFFFFFFFFFFFFFFFFFFFFFFFFFFFFFFFFFFFFFFFFFFFFFFFFFFFFFFFFFFFFFFF
F,FFFFFFFFFFFFFFFFFFFFFFFFFFFFFFFFFFFFFFFFFFFFFFFFFFFFFFFFFFFFFFFFFF
@A00155:342:HHGFNDSXY:1:1311:30653:34945 2:N:0:GAACCTAG+TCCGCATA
AATGGATTTAAGGGCATGGCCAGTGTCGGTACACCCGCAAACGGTATCCATGTTAATCTCACCGCTAG
CTTCTCTTCTCCCTCTTCTATCTCCTGCTCCTCCAGTATTGCATCTTCTCTAGCTGCCATCTCGTCGC
+
:FFFFFFFFFFFFFFFFFFFFFFFFFFFFFFFFFFFFFFFFFFFFFFFFFFFFFFFFFFFFFFFFFFF
FFFFFFFFFFFFFFFFFFFFFFFFFFFFFFFF:FFFFFFFFFFFFFFFFFFFFFFF,FFFFF:FFFFF
@A00155:342:HHGFNDSXY:1:1357:13277:11240 1:N:0:GAACCTAG+TCCGCATA
ATGGATTTAAGGGCATGGCCAGTGTCGGTACACCCGCAAACGGTATCCATGTTAATCTCACCGCTAGC
TTCTCTTCTCCCTCTTCTATCTCCTGCTCCTCCAGTATTGCATCTTCTCTAGCTGCCATCTCGTCGCC
+
:FFFFFFFFFFFFF,FFFFFFFFFFFFFFFFFFFFFFFFFFFFFFFFFFFFFFFFFFFFFFFFFFFFF
FFFFFFFFFFFFFFFFFFFFFFFFFFFFFFFFFFFFFFFFFFFFFFFFFFFFFFFFFFFFFFFFFFFF
@A00155:342:HHGFNDSXY:1:2628:11532:27665 1:N:0:GAACCTAG+TCCGCATA
GGATTTAAGGGCATGGCCAGTGTCGGTACACCCGCAAACGGTATCCATGTTAATCTCACCGCTAGCTT
CTCTTCTCCCTCTTCTATCTCCTGCTCCTCCAGTATTGCATCTTCTCTAGCTGCCATCTCGTCGCCGT
+
FFFFFFFFFFFFFFFFFFFFFFFFFFFFFFFFFFFFFFFFFFFFFFFFFFFFFFFFFFFFFFFFFFFF
FFFFFFFFFFFFFFFFFFFFFFFFFFFFFFFFFFFFFFFFFFFFFFFFFFFFFFFFFFFFFFFFFFFF
@A00155:342:HHGFNDSXY:1:2520:15700:35540 1:N:0:GAACCTAG+TCCGCATA
TTAAGGGCATGGCCAGTGTCGGTACACCCGCAAACGGTATCCATGTTAATCTCACCGCTAGCTTCTCT
TCTCCCTCTTCTATCTCCTGCTCCTCCAGTATTGCATCTTCTCTAGCTGCCATCTCGTCGCCGTTAAG
+
FFFFFFFFFFFFFFFFFFFFFFFFFFFFFFFFFFFFFFFFFFFFFFFFFFFFFFFFFFFFF:,FFF:F
FFFFFFFFFFFFFFFFFFFFFFFFFFFFFFFFFFFFFFFFFFFFFFFFFFFFFFFFFFFFFFFFFFFF
@A00155:342:HHGFNDSXY:1:1251:3839:29027 1:N:0:GAACCTAG+TCCGCATA
GGGCATGGCCAGTGTCGGTACACCCGCAAACGGTATCCATGTTAATCTCACCGCTAGCTTCTCTTCTC
CCTCTTCTATCTCCTGCTCCTCCAGTATTGCATCTTCTCTAGCTGCCATCTCGTCGCCGTTAAGTCTA
+
FFFF:FFFFFFFFFFFFFFFFFFFFFFFFFF:FFFFFFFFFFFFFFFFFFFFFFFFFFFFFFFFFFFF
:FFFFF:FFFFFFFFFFFFFFFFFFFFFFFFFFFFFFFFFFFFFFFFFFFFFFFFFFFFFFFFFFFFF
@A00155:342:HHGFNDSXY:1:1358:28465:9392 1:N:0:GAACCTAG+TCCGCATA
GGGCATGGCCAGTGTCGGTACACCCGCAAACGGTATCCATGTTAATCTCACCGCTAGCTTCTCTTCTC
CCTCTTCTATCTCCTGCTCCTCCAGTATTGCATCTTCTCTAGCTGCCATCTCGTCGCCGTTAAGTCTA
+
FFFF:FFFFFFFFFFFFFFFFFFFFFFF:FFFFFFFFFFFFFFFFFFFFFFFFFFFFFFFFFFFFFFF
FFFFF:FFFFFFFFFFFFFFFFFFFFFFFFFFFFFFFFFFFFFFFFFFFFFFFFF:FFFFFFFFFFFF

```
@A00155:342:HHGFNDSXY:1:2341:11776:18505 1:N:0:GAACCTAG+TCCGCATA
TGGCCAGTGTCGGTACACCCGCAAACGGTATCCATGTTAATCTCACCGCTAGCTTCTCTTCTCCCTCT
TCTATCTCCTGCTCCTCCAGTATTGCATCTTCTCTAGCTGCCATCTCGTCGCCGTTAAGTCTAACCGG
+
FFFFFFFFFFFFFFFFFFFFFFFFFFFFFFFFFFFFFFFFFFFFFFFFFFFFF,FFFFFFFFFFFFFF
FFFFFFFFFFFFFFFFFFFFFFFFFFFFFFFFFFFFFFFFFFFFFFFFFFFFFFFFFFFFFFFFFFFF
@A00155:342:HHGFNDSXY:1:2621:27986:11945 1:N:0:GAACCTAG+TCCGCATA
TGGCCAGTGTCGGTACACCCGCAAACGGTATCCATGTTAATCTCACCGCTAGCTTCTCTTCTCCCTCT
TCTATCTCCTGCTCCTCCAGTATTGCATCTTCTCTAGCTGCCATCTCGTCGCCGTTAAGTCTAACCGG
+
FFFFFFFFFFFF:FFFFFFFFFFFFFFFF:FFFFFFFFFFFFFFFFFFFFFFFFFFFF,F:FFFFFFF
FFF:FFFFFFFFFFFFFFFFFFFFFFFFFFFFFFFFFFFFFFFFFFFFFFFFFFFFFFFFFF::FFFF
@A00155:342:HHGFNDSXY:1:2547:5104:29559 2:N:0:GAACCTAG+TCCGCATA
GTACACACGCAAACGGTATCCATGTTAATCTCACCGCTAGCTTCTCTTCTCCCTCTTCTATCTCCTGC
TCCTCCAGTATTGCATCTTCTCTAGCTGCCATCTCGTCGCCGTTAAGTCTAACCGGCCGCTCGTACTT
+
::F,FF,FF,,FFFFFFF::FF:FFFFF:FFFFF,FFFFFFFFFFFF:FFFF:FFFFFFFFFFFFF:F
FFFFFFFFFFFFF:F:FFFFFFF,FFFFFFFFFFFFFFF:FFFFFFFFFFF,FF:FFFF:FFFF:FFF
@A00155:342:HHGFNDSXY:1:1610:1407:31548 1:N:0:GAACCTAG+TCCGCATA
ACCCGCAAACGGTATCCATGTTAATCTCACCGCTAGGTTCTCTTCTCCCTCTTCTATCTCCTGCTCCT
CCAGTATTGCATCTTCTCTAGCTGCCATCTCGTCGCCGTTAAGTCTAACCGGCCGCTCGTACTTCTCC
+
FFFFFFFFFFFFFFFFFFFFFFFFFFFFFFFFFF,F,:F:F,,FFFFFFFFFFFFFFFFFFFFFFFFF
FFFFFFFFFFFFFFFFFFFFFFFFFFFFFFFFFFFFFFFFFFFFFF:F:FFFFFFFFFFFFFFFFFFF
@A00155:342:HHGFNDSXY:1:2108:27299:4492 1:N:0:GAACCTAG+TCCGCATA
CGCAAACGGTATCCATGTTAATCTCACCGCTAGCTTCTCTTCTCCCTCTTCTATCTCCTGCTCCTCCA
GTATTGCATCTTCTCTAGCTGCCATCTCGTCGCCGTTAAGTCTAACCGGCCGCTCGTACTTCTCCTTT
+
FFFFFFFFFFFFF:FFFFFFFFFFFFFFFFFFF:FFFFFFFFFFFFFFFFFFFFFFFFFFFFFFFFFF
FFFFFFFFFFFFFFFFFFFFFFFFFFFFFFFFFFFFFFFFFFF:FFFFFFFFFFFFFF:FFFFFFFFF
@A00155:342:HHGFNDSXY:1:1661:16098:16845 2:N:0:GAACCTAG+TCCGCATA
CGCAAACGGTATCCATGTTAATCTCACCGCTAGCTTCTCTTCTCCCTCTTCTATCTCCTGCTCCTCCA
GTATTGCATCTTCTCTAGCTGCCATCTCGTCGCCGTTAAGTCTAACCGGCCGCTCGTACTTCTCCTTT
+
FFFFFFFFFFFFFFFFFFFFFFFFFFFFFFFFFFFFFFFFFFFFFFFFFFFFFFFFFFFFFFFFFFFF
FFFFFFFFFF,FFFFFFFFFFFFFF:FFFFFFFFFFFFFFFFFFFFFFFFFFFFFFFFF,FFFFFF:F
@A00155:342:HHGFNDSXY:1:2108:28971:2440 1:N:0:GAACCTAG+TCCGCATA
CGCAAACGGTATCCATGTTAATCTCACCGCTAGCTTCTCTTCTCCCTCTTCTATCTCCTGCTCCTCCA
GTATTGCATCTTCTCTAGCTGCCATCTCGTCGCCGTTAAGTCTAACCGGCCGCTCGTACTTCTCCTTT
+
FFFFFFFFFF:,FFFFFFFF:FFFFFFFFFFFF:F:FF:,FFFFFFFFFFFFFFFFFFFFFFFFFFFF
FFFFFFFFFFFFFFFFFFFFFFFFFFFFFFFFFFFFFFFFFFFFFFFFFFFFFFFFFFFFFF:FFFFF
@A00155:342:HHGFNDSXY:1:2621:27986:11945 2:N:0:GAACCTAG+TCCGCATA
CGCAAACGGTATCCATGTTAATCTCACCGCTAGCTTCTCTTCTCCCTCTTCTATCTCCTGCTCCTCCA
GTATTGCATCTTCTCTAGCTGCCATCTCGTCGCCGTTAAGTCTAACCGGCCGCTCGTACTTCTCCTTT
+
FFFFFFFFF:F:FFFF:F,FFFFFFFFF:F:FFF,F:FF:FFFFFFFF,FFFFFFFFFFFFFFFFF:F
,:F:FFFFFFFFFFFFFFFFFFFFFFFFFFFFFF:F:FFFFFFFFFFFFFF:FFFFFFFFFFFFF,:F
@A00155:342:HHGFNDSXY:1:1406:11767:12571 1:N:0:GAACCTAG+TCCGCATA
AACGGTATCCATGTTAATCTCACCGCTAGCTTCTCTTCTCCCTCTTCTATCTCCTGCTCCTCCAGTAT
TGCATCTTCTCTAGCTGCCATCTCGTCGCCGTTAAGTCTAACCGGCCGCTCGTACTTCTCCTTTACAG
+
FF:F,FFFFFFFFFFF:FF:FFFF,FF,F:,FF,F,FFFFF:FF:FFFF,F,FFFF:FFFFFFFF:FF
FFFF::FFF:FFFFFFFFFF:FF,,:FF:FF,FFF,FFFFF::FFFFFF:FFFF:,FFFFFFF:FFF:
```

```
@A00155:342:HHGFNDSXY:1:1661:16098:16845 1:N:0:GAACCTAG+TCCGCATA
CGGTATCCATGTTAATCTCACCGCTAGCTTCTCTTCTCCCTCTTCTATCTCCTGCTCCTCCAGTATTG
CATCTTCTCTAGCTGCCATCTCGTCGCCGTTAAGTCTAACCGGCCGCTCGTACTTCTCCTTTAC
+
FFFF,FFFFFF:FFFFFFFFFFFFFFFF:FF::,FFFFFFFFFFFFFFFFFFFFF:FF:FFFFFFFFF
FFFFFFFFFFFFFFFFFFFFFFFFFFFFFFFFFFFF,FFFFFFFFF,FFFFFFFFFFFFFFFF
@A00155:342:HHGFNDSXY:1:1674:14877:1110 1:N:0:GAACCTAG+TCCGCATA
CATGTTAATCTCACCGCTAGCTTCTCTTCTCCCTCTTCTATCTCCTGCTCCTCCAGTATTGCATCTTC
TCTAGCTGCCATCTCGTCGCCGTTAAGTCTAACCGGCCGCTCGTACTTCTCCTTTACAGATATAATTT
+
FFFFFFFFFFFFFFFFFFFFF,FFFFFFFFFFFFFFFFFFFFFFFFFFFFFFFFFFFFFFFFFFF:FFF
FFFFFFFFFFFFFFFFFFFFFFFFFFFFFFF:FFFFFFFFFFFFFFFFFFFFF::FFFFFFFFFFFFFF
@A00155:342:HHGFNDSXY:1:1610:1407:31548 2:N:0:GAACCTAG+TCCGCATA
ACCGCTAGCTTCTCTTCTCCCTCTTCTATCTCCTGCTCCTCCAGTATTGCATCTTCTCTAGCTGCCAT
CTCGTCGCCGTTAAGTCTAACCGGCCGCTCGTACTTCTCCTTTACAGATATAATTTCCTTATGATATT
+
FFFF:FFFFFFFFFFFFFFFFFFFFF,:FFFF:FF:FFFFFFFFFFFFFFFFFFFFFFFFFFFFFFFF
FFFFFFFFFFFFFFFFFFFFFFFFFFFFFFFFFFFFFFFFFF:FFFFFFFFFFFFFFFFFFFFFFFFF
@A00155:342:HHGFNDSXY:1:1466:24415:19288 2:N:0:GAACCTAG+TCCGCATA
GCTAGCTTCTCTTCTCCCTCTTCTATCTCCTGCTCCTCCAGTATTGCATCTTCTCTAGCTGCCATCTC
GTCGCCGTTAAGTCTAACCGGCCGCTCGTACTTCTCCTTTACAGATATAATTTCCTTATG
+
FFFFFFFFFFFFFFFFFFFFFFFFFFFFFFFFFFFFFFFFFFFFFF,FFFFFFFFFFFFFFFFFFFFF
FFFFFFFFFFFFFFFFFFFFFFFFFFFFFFFFFFFFFFFFFF,FFFFFFFFFFFFFFFFFFF
@A00155:342:HHGFNDSXY:1:2155:25400:22623 1:N:0:GAACCTAG+TCCGCATA
GCTAGCTTCTCTTCTCCCTCTTCTATCTCCTGCTCCTCCAGTATTGCATCTTCTCTAGCTGCCATCTC
GTCGCCGTTAAGTCTAACCGGCCGCTCGTACTTCTCCTTTACAGATATAATTTCCTTATGATATTTTA
+
FFFFF:FFFFFFFFFFFFFFFFFFFFFFFFFFFFFFFFFFFFFFFFFFFFFFFFFFFFFFFFFFFFFF
FFFFFFFFFFFFFFFFFFFFFFFFFFFFFFFFFFFFFFFFFFFFFFFFFFFFFFFFFFFFFFFFFFFF
@A00155:342:HHGFNDSXY:1:2520:32606:30248 1:N:0:GAACCTAG+TCCGCATA
AGCTTCTCTTCTCCCTCTTCTATCTCCTGCTCCTCCAGTATTGCATCTTCTCTAGCTGCCATCTCGTC
GCCGTTAAGTCTAACCGGCCGCTCGTACTTCTCCTTTACAGATATAATTTCCTTATGATATTTTAAC
+
FFF,:FFFFFFFFFFFFFFFFFF:FFFFFFF:FFFFFFF:FFF,FF:FF,FFFF:FF:FFFF:FFFFF
FFFF:FFFFFFFFFF:FFFFFFFFFFFFFFFFF,FFFF,FFF:FFF::FFFFFFFFFFF::FFFFFF
@A00155:342:HHGFNDSXY:1:1266:22272:1642 1:N:0:GAACCTAG+TCCGCATA
TTCTCCCTCTTCTATCTCCTGCTCCTCCAGTATTGCATCTTCTCTAGCTGCCATCTCGTCGCCGTTAA
GTCTAACCGGCCGCTCGTACTTCTCCTTTACAGATATAATTTCCTTATGATATTTTAACTTCTTTACC
+
FFFFFFF,FFFFF::FF::FF::FFFFFF::FF,F:,FFFFFF::FFF:F:,:F:F:F::F::FF::F
F::F:FF,,F:FFF:FFFFFFFFFF:F,F:F:F:FFF:FF:F,FFF:FFFF:FFFFFFF,FFFFFF,:
@A00155:342:HHGFNDSXY:1:1466:24415:19288 1:N:0:GAACCTAG+TCCGCATA
CCCTCTTCTATCTCCTGCTCCTCCAGTATTGCATCTTCTCTAGCTGCCATCTCGTCGCCGTTAAGTCT
AACCGGCCGCTCGTACTTCTCCTTTACAGATATAATTTCCTTATGATATTTTAACTTCTT
+
FFFFFFFFFFFFFFFFFFFFFFFFFFF:FFFFFFFF:FFFFFFFFFF:FFFFFFFFFFFFFFFFFFFF
FFFFFFFFFFFFFFFFFF:FFFFFFFFFFFFFFFFFFFFFFFFFFFFFFFFFFFFFFFFFFFF
@A00155:342:HHGFNDSXY:1:2108:27299:4492 2:N:0:GAACCTAG+TCCGCATA
CCCTCTTCTATCTCCTGCTCCTCCAGTATTGCATCTTCTCTAGCTGCCATCTCGTCGCCGTTAAGTCT
AACCGGCCGCTCGTACTTCTCCTTTACAGATATAATTTCCTTATGATATTTTAACTTCTTTACCATTT
+
FFFFFFFFFFFFFFFFFFFFFFFFFFFFFF,FF,FFFFFFFFFFFFFFFFFFFFFFFFFFFFFFFFFF
FFFFFFFFFFFFFFFFFFFFFFFFFFFFFFFFFFFFFFFFFFFFFFFFFFFFFFFFFFFFFFFFFFFF
```

```
@A00155:342:HHGFNDSXY:1:2108:28971:2440 2:N:0:GAACCTAG+TCCGCATA
CCCTCTTCTATCTCCTGCTCCTCCAGTATTGCATCTTCTCTAGCTGCCATCTCGTCGCCGTTAAGTCT
AACCGGCCGCTCGTACTTCTCCTTTACAGATATAATTTCCTTATGATATTTTAACTTCTTTACCATTT
+
FFFFFFFF:F:F:FFFFFFFF:FFFFFFFFFFF:FFFFFF:FFFFFFFFFF:FFFFFFFFFFFFFFF:
FFFFFFFFFF:FFFFFFFF:FFFFFFFFFFFF:FFFFFFFFFFFFF:FFFFFFFFFFFFFFFFFFFFF
@A00155:342:HHGFNDSXY:1:2520:15700:35540 2:N:0:GAACCTAG+TCCGCATA
CTTCTATCTCCTGCTCCTCCAGTATTGCATCTTCTCTAGCTGCCATCTCGTCGCCGTTAAGTCTAACC
GGCCGCTCGTACTTCTCCTTTACAGATATAATTTCCTTATGATATTTTAACTTCTTTACCATTTCTGC
+
FFFFFFFFFFFFFFFFFFFFFFFFFFFFFFFFFFF,FFFF:FFFFFFFFFFFFFFFFFFFFF:FFFF
FFFF:FFFFFFFFFFFFFF:FFFFFFFFFFFFFFFFFFFFFFFFFFFFF:FFFFFFFFFFFFFFFFFFFF
@A00155:342:HHGFNDSXY:1:1407:19633:19586 2:N:0:GAACCTAG+TCCGCATA
TTCTATCTCCTGCTCCTCCAGTATTGCATCTTCTCTAGCTGCCATCTCGTCGCCGTTAAGTCTAACCG
GCCGCTCGTACTTCTCCTTTACAGATATAATTTCCTTATGATATTTTAACTTCTTTACCATTTCTGCA
+
FFFF:FFFF:FF,FF:FFFFFF,F:FF,FFFFFFFFFFFF:FFFFFF,FFF,FFFFFFFFFF:FFFF,
F:FF:F,FF:F:FFFF:FFFFFFF,FFFFFFF:FFFFFFF:FFFFFFFF:FF:FFF:FFFFFFFFFFF
@A00155:342:HHGFNDSXY:1:1563:4020:15843 1:N:0:GAACCTAG+TCCGCATA
CTGCTCCTCCAGTATTGCATCTTCTCTAGCTGCCATCTCGTCGCCGTTAAGTCTAACCGGCCGCTCGT
ACTTCTCCTTTACAGATATAATTTCCTTATGATATTTTAACTTCTTTACCATTTCTGCATGCTGTTCT
+
FFFFFFFFFFFFFFFFFFFFFFFF:F:FFFFFFFFFFFFFFFFFFFF:FFFFFFFF,FFFFF:FFFFF
FF,FFFFFF:FFFFFFFFF,F:FF:FF:FFFFFFF:,FFFFFFF:FFF::F:FFFF,:F::,FFFFF:
@A00155:342:HHGFNDSXY:1:2555:18502:32252 1:N:0:GAACCTAG+TCCGCATA
CTGCTCCTCCAGTATTGCATCTTCTCTAGCTGCCATCTCGTCGCCGTTAAGTCTAACCGGCCGCTCGT
ACTTCTCCTTTACAGATATAATTTCCTTATGATATTTTAACTTCTTTACCATTTCTGCATGCTGTTCT
+
FFFFFFFFFFFFFFFFFFFFFFFFFFFF:FFFFF::FF,FFFFFFFFFFFFFFFFFFFFFFFFFFFFF
F:FFFFFFFFFFFFFFFFFFF,FFFFFFFFFFF:FFFFF:FFFFFFFFFFFFFFFFFF:FFFFFFFFF
@A00155:342:HHGFNDSXY:1:2203:23430:18396 1:N:0:GAACCTAG+TCCGCATA
TGCTCCTCCAGTATTGCATCTTCTCTAGCTGCCATCTCGTCGCCGTTAAGTCTAACCGGCCGCTCGTA
CTTCTCCTTTACAGATATAATTTCCTTATGATATTTTAACTTCTTTACCATTTCTGCATGCTGTTCTG
+
FFFFFFFFFFFFFFFFFFFFFFFFFFFFFFFFFFFFFFFFFFFFFFFFFFFFFFFFFFFFFFFFFFFF
,FFFFFFFFFFFF,FFFFFFFFFFF,FF:FF:FFFFFFFFFFFFFFF:FFFFFFF:FFFFFF::FFFF
@A00155:342:HHGFNDSXY:1:2520:32606:30248 2:N:0:GAACCTAG+TCCGCATA
CCTCCAGTATTGCATCTTCTCTAGCTGCCATCTCGTCGCCGTTAAGTCTAACCGGCCGCTCGTACTTC
TCCTTTACAGATATAATTTCCTTATGATATTTTAACTTCTTTACCATTTCTGCATGCTGTTCTGTTCC
+
FFFFFFFFF:FFFFFFFFFFFFFFFFFFFFFFFFFFFFFFFFFFFFFFF:FFFFFFFFFFFFFFFFFF
FFFFFFFFFFFFFFFFFFFFFFFFFFFFFFFFFFFFFFFFFFF:FF,FFFFFFFFFFF,FFFFFFFFFF
@A00155:342:HHGFNDSXY:1:2517:7979:9267 2:N:0:GAACCTAG+TCCGCATA
TCCAGTATTGCATCTTCTCTAGCTGCCATCTCGTCGCCGTTAAGTCTAACCGGCCGCTCGTACTTCTC
CTTTACAGATATAATTTCCTTATGATATTTTAACTTCTTTACCATTTCTGCATGCTGTTCTGTTCCTA
+
FFFFFFFFFFFF:FFFFFF,FFFFFFFFFFFFFFFFFFFFFFFFFFFFF:FFFFFFFFFFFFFFFFFF
F:FFFFFFF:F:F:FF:FFFFF,FF,FFFFFFFFFFF:FFFFFFF:FFFFFFFFFFFFFF,FF:FF:,
@A00155:342:HHGFNDSXY:1:2204:8015:24236 1:N:0:GAACCTAG+TCCGCATA
TATTGCATCTTCTCTAGCTGCCATCTCGTCGCCGTTAAGTCTAACCGGCCGCTCGTACTTCTCCTTTA
CAGATATAATTTCCTTATGATATTTTAACTTCTTTACCATTTCTGCATGCTGTTCTGTTCCTACATAC
+
FFFFFFFFFFFFFFFFFFFFFFFFFFFFFFFFFFFFFFFFFFFFFFFFFFFF:FFFFFFFFFFFFFFF
FFFFFFFFFFFFFFFFF:FFFFFFFFFF:FF:,FFFFFFFFFFF:FFFFFFFFFFFFFFFFFFFFF:F
```

@A00155:342:HHGFNDSXY:1:2442:29839:8077 2:N:0:GAACCTAG+TCCGCATA
TATTGCATCTTCTCTAGCTGCCATCTCGTCGCCGTTAAGTCTAACCGGCCGCTCGTACTTCTCCTTTA
CAGATATAATTTCCTTATGATATTTTAACTTCTTTACCATTTCTGCATGCTGTTCTGTTCCTACATAC
+
FF:FFFFFFFFFFFFFFFFFFFFFFFFFFFFFFF:FFFFFFFFFFFFFFFFFFFFFFFFF:FFFFFF:FF
FFFFFFFFFF::FFFFFFFFFFFFFFFFFFFFFFFFFFFFFF:FFFFFFFFF,FFFFFF:FFF:FFFFFF
@A00155:342:HHGFNDSXY:1:2442:29839:8077 1:N:0:GAACCTAG+TCCGCATA
TTGCATCTTCTCTAGCTGCCATCTCGTCGCCGTTAAGTCTAACCGGCCGCTCGTACTTCTCCTTTACA
GATATAATTTCCTTATGATATTTTAACTTCTTTACCATTTCTGCATGCTGTTCTG
+
FFFFFFFFFFFFFFFFFFFFFFFFFFF:FFFFFFFFFFFFFFFFFFFFFFFFFFFFFFFFFFFFFFFFFF
FFFFFFF:FFFFFFFFFFFFFFFFFF::FFFFFFFFFFFF:FFFFFF:FFFFFFF
@A00155:342:HHGFNDSXY:1:2203:23701:17613 1:N:0:GAACCTAG+TCCGCATA
TTGCATCTTCTCTAGCTGCCATCTCGTCGCCGTTAAGTCTAACCGGCCGCTCGTACTTCTCCTTTACA
GATATAATTTCCTTATGATATTTTAACTTCTTTACCATTTCTGCATGCTGTTCTG
+
FFFFFFFFFFFFFFFFFFFFFFFFFFFFFFFFFFFFFFFFFF:FFFFFFFFFFFFFFFFFFFFF:FFFFF
:FFFFFFFFFFF:FFFFFFFFFFFFFFFFFFFFFFFFFFFFFFFFFFFFFFFFFF
@A00155:342:HHGFNDSXY:1:2539:9381:4586 1:N:0:GAACCTAG+TCCGCATA
TTGCATCTTCTCTAGCTGCCATCTCGTCGCCGTTAAGTCTAACCGGCCGCTCGTACTTCTCCTTTACA
GATATAATTTCCTTATGATATTTTAACTTCTTTACCATTTCTGCATGCTGTTCTGTT
+
FFFFFFFFFFFFFFF:FFFFFFFFFFFFFFFFFFFFFFFFFFFFFFFFFFFFFFFFFFFFFFFFFFFFFF
FFFFFFFFFFFFFFFFFFFFFFFFFFFFFFFFFFFFFFFFFFFFFFFFFFFFFFFFF
@A00155:342:HHGFNDSXY:1:2132:18502:1063 2:N:0:GAACCTAG+TCCGCATA
TTGCATCTTCTCTAGCTGCCATCTCGTCGCCGTTAAGTCTAACCGGCCGCTCGTACTTCTCCTTTACA
GATATAATTTCCTTATGATATTTTAACTTCTTTACCATTTCTGCATGCTGTTCTGTTCCTACATACTC
+
F:FFFFF:FF:FFFFFFFFFFFFFFFFFFFFFFFFFFFFFFFFFFFFFFFFFFFFFFFFFFFFFFFFFFF
FFFFFFFFFFFFFFFFFFFFFFFFFFFFFF:FFFFFFFFFFFFFFFFFFF:FFFFFFFFFFFFFFFFFFF
@A00155:342:HHGFNDSXY:1:2341:11776:18505 2:N:0:GAACCTAG+TCCGCATA
TGCATCTTCTCTAGCTGCCATCTCGTCGCCGTTAAGTCTAACCGGCCGCTCGTACTTCTCCTTTACAG
ATATAATTTCCTTATGATATTTTAACTTCTTTACCATTTCTGCATGCTGTTCTGTTCCTACATACTCG
+
FFFFFFFFFFFFFFFFFFFFFFFFFFFFFFFFFFFFFFFFFFFFFFFFFFFFFFFFFFFFFFF:FFFFFF
FFFFFFFFFFF:FFFFFFFFFFFFFFFFFF:FFFFFFFFFFFFFFFFFFF:FFFF,FFFFFFFFFFFFFF
@A00155:342:HHGFNDSXY:1:1524:29776:28823 2:N:0:GAACCTAG+TCCGCATA
ATCTTCTCTAGCTGCCATCTCGTCGCCGTTAAGTCTAACCGGCCGCTCGTACTTCTCCTTTACAGATA
TAATTTCCTTATGATATTTTAACTTCTTTACCATTTCTGCATGCTGTTCTGTTCCTACATACTCGCTT
+
FFFFFFFFFFFFFFFFFFFFFFFFFFFFFFFFFFFFFFFFFFFFFFFFFFFFFFFFFFFFFFFFFFFFFF
FFFFFFFFFFFFFFFFFFFFFFFFFFFFFFFFFFFFFFFFFFFFFFFFFFFFFFFFFFFFFFFFFFFFFF
@A00155:342:HHGFNDSXY:1:2517:7979:9267 1:N:0:GAACCTAG+TCCGCATA
ATCTTCTCTAGCTGCCATCTCGTCGCCGTTAAGTCTAACCGGCCGCTCGTACTTCTCCTTTACAGATA
TAATTTCCTTATGATATTTTAACTTCTTTACCATTTCTGCATGCTGTTCTGTTCCTACATACTCGCTT
+
FFFFFFFFFFFFFFFFFFFFFFFFFFFFFFFFFFFFFFFFFFFFFFFFFFFFFFFFFFFFFFFFFFFFFF
FFFF:FFFFFFFFFFFF:FFFFFFFFFFFFFFFFFFFFF,FFFFFFFFFFFFFFFFFF::FFFFFFFFFF
@A00155:342:HHGFNDSXY:1:1645:9353:18912 1:N:0:GAACCTAG+TCCGCATA
CTGCCATCTCGTCGCCGTTAAGTCTAACCGGCCGCTCGTACTTCTCCTTTACAGATATAATTTCCTTA
TGATATTTTAACTTCTTTACCATTTCTGCATGCTGTTCTGTTCCTACATACTCGCTTATC
+
FFFFFFFFFFFFFFFFFFFFFFFFFFFF:FFF:FFFFFFFFFFFFFFFFFFFFFFFFFFFFFFFFF:FFF
FFFFFFFFFFF,FFFFFFF:FFFFFFFF:FFFFFFFFFFFFFFFFFFFFFFFFFFFFFF

@A00155:342:HHGFNDSXY:1:1524:29776:28823 1:N:0:GAACCTAG+TCCGCATA
CTGCCATCTCGTCGCCGTTAAGTCTAACCGGCCGCTCGTACTTCTCCTTTACAGATATAATTTCCTTA
TGATATTTTAACTTCTTTACCATTTCTGCATGCTGTTCTGTTCCTACATACTCGCTTATC
+
FFFFFFFFFFFFFFFFFFFFFFFFFFFFFFFFFFFFFFFFFFFFFFFFF:FFFFFFFFFFFFFF:FFFF
FFFFF:F:FFF:F,FFFFF:FF:FFFFFFFFF:FFFFFFFFFFFFFFFFFFFFFFFFFFF
@A00155:342:HHGFNDSXY:1:2208:31729:11537 1:N:0:GAACCTAG+TCCGCATA
CTGCCATCTCGTCGCCGTTAAGTCTAACCGGCCGCTCGTACTTCTCCTTTACAGATATAATTTCCTTA
TGATATTTTAACTTCTTTACCATTTCTGCATGCTGTTCTGTTCCTACATACTCGCTTATC
+
FFFFFFFFFFFFFFFFFFFFFFFFFFFFFFFFFFFFF:FFFFFFFFFFFFFFFFFFF:FFFFFF:
FFF,FFFFFFF::FFFFFFFF:F:FFFFFFFFFFF,FFFFFFFFFFFFFFFFFFFFFFFF
@A00155:342:HHGFNDSXY:1:2507:15646:1720 1:N:0:GAACCTAG+TCCGCATA
CTGCCATCTCGTCGCCGTTAAGTCTAACCGGCCGCTCGTACTTCTCCTTTACAGATATAATTTCCTTA
TGATATTTTAACTTCTTTACCATTTCTGCATGCTGTTCTGTTCCTACATACTCGCTTATC
+
FFFFFF:FFFFFFFFFFFFFFFFFFFFFFF,FFFFFFFFFFFFFFFFF:FFFFFFFFFFFFFFFFFFF
FF:FFFFFFFFFFFFF:FFFFFFFF:FFFFFFFFFFFFFFFF:FFFFFFFFFFFFFFFFF
@A00155:342:HHGFNDSXY:1:1627:9055:2832 1:N:0:GAACCTAG+TCCGCATA
GCCATCTCGTCGCCGTTAAGTCTAACCGGCCGCTCGTACTTCTCCTTTACAGATATAATTTCCTTATG
ATATTTTAACTTCTTTACCATTTCTGCATGCTGTTCTGTTCCTACATACTCGCTTATCGGTACCCTGA
+
FFFFFF:FFFFFFFFFFFFFFFFFFFFFFFFFFFFFFFFFFFFFFFFFFFFFFFFF:FFFFFFFFFF
FFFFFFFFFFFFFFFFFFFFFFFFFFFFFFFFFFFFFFFFFFFFFFFFFFFFFFFFFFFFFFFFFFFF
@A00155:342:HHGFNDSXY:1:2614:25346:34867 1:N:0:GAACCTAG+TCCGCATA
GCCATCTCGTCGCCGTTAAGTCTAACCGGCCGCTCGTACTTCTCCTTTACAGATATAATTTCCTTATG
ATATTTTAACTTCTTTACCATTTCTGCATGCTGTTCTGTTCCTACATACTCGCTTATCGGTACCCTGA
+
FFFFFFFFFFFFFFFFFFFFFFFFFFFFFFFFFFFFFFFFFFFFFFFFFFFFFFFFFFFFFFFFFFF:
FFFFFFFFFF:FFFFFFFFF::F,F,FFFFFFFFFFFF,FFFFFFFFFFF:FFFFFFFFFFF:FFFFF
@A00155:342:HHGFNDSXY:1:1604:4047:4836 2:N:0:GAACCTAG+TCCGCATA
GCCGTTAAGTCTAACCGGCCGCTCGTACTTCTCCTTTACAGATATAATTTCCTTATGATATTTTAACT
TCTTTACCATTTCTGCATGCTGTTCTGTTCCTACATACTCGCTTATCGGTACCCTGACCATCTGCTTT
+
FFFFFF:FFFFFFFFFFFFFFFFFFFFFF,FFF,F:FFFFFFFFFFFFFFFFFFFF:FFFFFFFFFF:F
FFFFFFFFFFF:FFFFFFFFFFFFFFFF:FFF:,FFFFFFFFFFFFFFFFFFFFFFFFFFFFFFFFFF
@A00155:342:HHGFNDSXY:1:2155:25400:22623 2:N:0:GAACCTAG+TCCGCATA
GCCGTTAAGTCTAACCGGCCGCTCGTACTTCTCCTTTACAGATATAATTTCCTTATGATATTTTAACT
TCTTTACCATTTCTGCATGCTGTTCTGTTCCTACATACTCGCTTATCGGTACCCTGACCATCTGCTTT
+
FFFFFFFFFFF:FFFFFFFFFFFFFFFFFFFFFFF:FFFFFF:FFFFFFFFFFF:FFFFFFFFFFFFF
FFFFFFFFFF:FFFFFFFFFFF::FFF:FFFFFFFFFFFFFFFFFFFFFFFFFFFFFFFFFFFFFFFF
@A00155:342:HHGFNDSXY:1:2203:23701:17613 2:N:0:GAACCTAG+TCCGCATA
CCGTTAAGTCTAACCGGCCGCTCGTACTTCTCCTTTACAGATATAATTTCCTTATGATATTTTAACTT
CTTTACCATTTCTGCATGCTGTTCTGTTCCTACATACTCGCTTATCGGTACCCTGACCATCTGCTTTG
+
FFFFFFFFFF:F:FFFFFFFFFFFFFF,FFFFF,FF:FFFFFFF:FFFFFFF:FFFFFFFFFFFF:F
FFFFFFFFF:FFFFFFFFF:F,:FFFFFFFFFFFFFFFFFFFFFFFFFFFFFFFFFFFFFFFFFFFF
@A00155:342:HHGFNDSXY:1:2203:23430:18396 2:N:0:GAACCTAG+TCCGCATA
CCGTTAAGTCTAACCGGCCGCTCGTACTTCTCCTTTACAGATATAATTTCCTTATGATATTTTAACTT
CTTTACCATTTCTGCATGCTGTTCTGTTCCTACATACTCGCTTATCGGTACCCTGACCATCTGCTTTG
+
FFFFFFFFFFFF,FFFFFFFF:FFFFFFFFFFF:FFFFFFFFF:FFFFFFFFF,FFFFFF:FFFFFFF
FFFFFFFFF,FFFFFFFFF,FF:FFFFFFF:FFFFFFFFFFFFFFFFFFFFFFFFFFFFFFFFFFFFF

```
@A00155:342:HHGFNDSXY:1:1674:14877:1110 2:N:0:GAACCTAG+TCCGCATA
CGTTAAGTCTAACCGGCCGCTCGTACTTCTCCTTTACAGATATAATTTCCTTATGATATTTTAACTTC
TTTACAATTTCTGCATGCTGTTCTGTTCCTACATACTCGCTTATCGGTACCCTGACCATCTGCTTTGA
+
FFFFFFFFFFFFFFFFFFFFFFF,FF,F:FFFFFFFFFFFFFFFFF,FFFFFFFFFFFFFF:FFFFFF
FFFFFFFFFFFFFFFFFFFFFFF:FFFFFFFFF:FFFFFFFFFFFFFFFFFF:FFFFFFFFFFFFFFF
@A00155:342:HHGFNDSXY:1:2311:3115:1971 1:N:0:GAACCTAG+TCCGCATA
CGTTAAGTCTAACCGGCCGCTCGTACTTCTCCTTTACAGATATAATTTCCTTATGATATTTTAACTTC
TTTACCATTTCTGCATGCTGTTCTGTTCCTACATACTCGCTTATCGGTACCCTGACCATCTGCTTTGA
+
FFFFFFFFFFFFFFFFFFFFFFFFFFFFF,FFFFFFFF:FFFFFFFFFFF,FFFFFFF:,FFFFFFFF
FFFFFFF:FF::FFFFF:FFFFFFFFFFF,FFFFFFFFFFFFFFF:FFFFFFFFFFF:F:F,,,FFFF
@A00155:342:HHGFNDSXY:1:1366:17164:20917 1:N:0:GAACCTAG+TCCGCATA
GTTAAGTCTAACCGGCCGCTCGTACTTCTCCTTTACAGATATAATTTCCTTATGATATTTTAACTTCT
TTACCATTTCTGCATGCTGTTCTGTTCCTACATACTCGCTTATCGGTACCCTGACCATCTGCTTTGAA
+
FFFFFFFFFFFFFFFFFFFFFFFFFFFFFFFFFFFFFFFFFFFF:FFFF,FFFFFFFFFFFFFFFFFF
FFFFFFFFFFFFFFFFFFFFFFFFFFFFFFFFFFFFFFFFFFFFFFFFFFFFFFFFFFFFFFFFFFFF
@A00155:342:HHGFNDSXY:1:1373:21269:28995 1:N:0:GAACCTAG+TCCGCATA
GTTAAGTCTAACCGGCCGCTCGTACTTCTCCTTTACAGATATAATTTCCTTATGATATTTTAACTTCT
TTACCATTTCTGCATGCTGTTCTGTTCCTACATACTCGCTTATCGGTACCCTGACCATCTGCTTTGAA
+
FFFFFFFF:FFFFFFFFFFFFF:FFFFF:FFFFFFFFFFFFFF:FFFFFFFFFFFFF:FFFFFFFFFF
FFFFFFFFFFFFFFFFFFFFFFFFFFFFFFFFFFFFFFFFFFFFFFFFFFFFFFFFFFFFFFFFFF:F
@A00155:342:HHGFNDSXY:1:2672:13693:10551 1:N:0:GAACCTAG+TCCGCATA
TTAAGTCTAACCGGCCGCTCGTACTTCTCCTTTACAGATATAATTTCCTTATGATATTTTAACTTCTT
TACCATTTCTGCATGCTGTTCTGTTCCTACATACTCGCTTATCGGTACCCTGACCATCTGCTTTGAAG
+
FFFFFFFFFFFFFFFFFFFFFFFFFFFFFFFFFFFFFFFFFFFFFFFFFFFFFFFFFFFFFFFFFFFF
FFFFFFFFFFFFFFFFFFFFFFFFFFFFFFFFFFFFFFFFFFFFFFFFFFFFFFFFFFFFFFFFFFFF
@A00155:342:HHGFNDSXY:1:2539:9381:4586 2:N:0:GAACCTAG+TCCGCATA
TAAGTCTAACCGGCCGCTCGTACTTCTCCTTTACAGATATAATTTCCTTATGATATTTTAACTTCTTT
ACCATTTCTGCATGCTGTTCTGTTCCTACATACTCGCTTATCGGTACCCTGACCATCTGCTTTGAAGT
+
FFFF:FFFFFFFFFFFFFFFFFFFFFFFFFFFFFFFFFFFFFFFFFFFFFFFFFFFFFFFFFFFFFFF
FFFFFFFFFFFFFFFF:F,FFFFFFFFFFFFFFFFFFFFFFF:FFFFFF,FFFFFFFFFFFFFFFFFF
@A00155:342:HHGFNDSXY:1:1413:18945:7404 1:N:0:GAACCTAG+TCCGCATA
TAAGTCTAACCGGCCGCTCGTACTTCTCCTTTACAGATATAATTTCCTTATGATATTTTAACTTCTTT
ACCATTTCTGCATGCTGTTCTGTTCCTACATACTCGCTTATCGGTAACCTGACCATCTGCTTTGAAGT
+
FFFFFFFFF,F:::,:FF,:FFF,,,F,:,F,F:::FFFFF:FFF:,,:F,,F,FF,F,FF,,:F,:F
FFF:FFF,::,F,,::,,FFFF::F,:FFF:F,:FFF:FF,F,FFF,F:F,:F:FF:F,:FFFFFFFF
@A00155:342:HHGFNDSXY:1:2127:1461:25034 2:N:0:GAACCTAG+TCCGCATA
ACCGGCCGCTCGTACTTCTCCTTTACAGATATAATTTCCTTATGATATTTTAACTTCTTTACCATTTC
TGCATGCTGTTCTGTTCCTACATACTCGCTTATCGGTACCCTGACCATCTGCTTTGAAGTGGACGTTA
+
FFF:,,F::FF,:FFFFFF:F,:FFFF,FFFFFFFF:,FFFFFFFFF:FFFFFFFFF:FFFFFFFFFF
:,FFFFFFFFFFFFFFFFFFFFFFFFFFFFFFFFFFFFFFFFFFFFF:FFFFFFFFFFFFFFFFFFFF
@A00155:342:HHGFNDSXY:1:1366:17164:20917 2:N:0:GAACCTAG+TCCGCATA
ACCGGCCGCTCGTACTTCTCCTTTACAGATATAATTTCCTTATGATATTTTAACTTCTTTACCATTTC
TGCATGCTGTTCTGTTCCTACATACTCGCTTATCGGTACCCTGACCATCTGCTTTGAAGTGGACGTTA
+
:FFFFFFFFFFFFFFFFFFFF::FFFFFFFFFFFFFFFFFF:FFFFFFFFFFFFFFF,FFFFFFFFFF
FFFF,FF,F,FFFF:FFF,:FFFFFFFFFFFFFFFFFFFFFFFFFFFFFFFFFFFFFFFFFFFFFFFF
```

```
@A00155:342:HHGFNDSXY:1:1106:22571:19664 2:N:0:GAACCTAG+TCCGCATA
GCCGCTCGTACTTCTCCTTTACAGATATAATTTCCTTATGATATTTTAACTTCTTTACCATTTCTGCA
TGCTGTTCTGTTCCTACATACTCGCTTATCGGTACCCTGACCATCTGCTTTGAAGTGGACGTTATTTC
+
FFFFFFFFFFFFFFFFFFFFFFFFFFFF:F,F:FFFFF:FFFFFFFFFFFFFFF:FFFFFFFFFFFFFFF
FFF:FFFFFF,FFFFFFFFFFFFFFFFFFFFFFFFFF:F,FFFFFFF,FFFFFFFFFFFFFFFFFFFFFF
@A00155:342:HHGFNDSXY:1:1645:9353:18912 2:N:0:GAACCTAG+TCCGCATA
CCGCTCGTACTTCTCCTTTACAGATATAATTTCCTTATGATATTTTAACTTCTTTACCATTTCTGCAT
GCTGTTCTGTTCCTACATACTCGCTTATCGGTACCCTGACCATCTGCTTTGAAGTGGACGTTATTTCG
+
FFFFFFFFFFFF:FFFFFFFFF:FFFFFFFFFFFFFFFFFFFFFFFFFFFF:FFF:FFFFFFFFFFFFFF
FF:FFFFFFFFFFFFFFFFFFFFFFFFFFFFFFFFFFFFFFFFFFFFFFFFFFFFFFFFFFFFFFFFFFF
@A00155:342:HHGFNDSXY:1:1122:16857:7169 1:N:0:GAACCTAG+TCCGCATA
TCCTTTACAGATATAATTTCCTTATGATATTTTAACTTCTTTACCATTTCTGCATGCTGTTCTGTTCC
TACATACTCGCTTATCGGTACCCTGACCATCTGCTTTGAAGTGGACGTTATTTCGACCACTTCAGTGT
+
FFFFFFFFFFFFFFF:FFFFF:FF:FFFF:FFFFFFFFFFFFFFFFFFFFFFF:FFF:FFFFFFFFFFFF
FFFFFFFFFFFF,FFFFFFFFFFFFFFFFFFFFFFFF:FFFFFFFFF,FFFFFFFFFFF,FFFF:F:FFF
@A00155:342:HHGFNDSXY:1:1106:22571:19664 1:N:0:GAACCTAG+TCCGCATA
CCTTTACAGATATAATTTCCTTATGATATTTTAACTTCTTTACCATTTCTGCATGCTGTTCTGTTCCT
ACATACTCGCTTATCGGTACCCTGACCATCTGCTTTGAAGTGGACGTTATTTCGACCACTTCAGTGTC
+
FFFFFFFF:FFFFFFFFFFFF:,FFFFFFFFFFFFFFFFFFFFFFFFFFFFFF,FFFFFFFFFFFFFFFF
FFFFFFFFFFFFFFFFFFFF:FFFFFFFFFFFF,FFFFFFFFFFFFFFFFFFFFFFFFFFFFFFFFFFFF
@A00155:342:HHGFNDSXY:1:2205:25509:9283 1:N:0:GAACCTAG+TCCGCATA
TTTACAGATATAATTTCCTTATGATATTTTAACTTCTTTACCATTTCTGCATGCTGTTCTGTTCCTAC
ATACTCGCTTATCGGTACCCTGACCATCTGCTTTGAAGTGGACGTTATTTCGACCACTTCAGTGTCGT
+
FFFF:FFFFFFFFFFFFFFFFFFFFFFFFFFFFFFFFF:FFFFFFFFFFFFF:FFFFFFFFFFFFFFFFF
FFFFFF:FFFFFF:FFFFFF:FFFFFFFF:FFFFFFFFFFFFFFFFFFFFFFFFF:FFFFFFF:FFFFFF
@A00155:342:HHGFNDSXY:1:2208:31729:11537 2:N:0:GAACCTAG+TCCGCATA
TACAGATATAATTTCCTTATGATATTTTAACTTCTTTACCATTTCTGCATGCTGTTCTGTTCCTACAT
ACTCGCTTATCGGTACCCTGACCATCTGCTTTGAAGTGGACGTTATTTCGACCACTTCAGTGTCGTA
+
:FFFFFFFFFFFFFFFFFFFFFFFFFFFFFFFFFFFF::FFFFFFFFFFFFFFFFFFFFFFFFFFFFFFF
FFFFFFFFFFFF:FFFFFFFFFFFFFFFFFFF,FFFFFFFFFFFFF:FFFF:FFFFFFFFFFFFFFFFF
@A00155:342:HHGFNDSXY:1:1455:5285:7764 1:N:0:GAACCTAG+TCCGCATA
TACAGATATAATTTCCTTATGATATTTTAACTTCTTTACCATTTCTGCATGCTGTTCTGTTCCTACAT
ACTCGCTTATCGGTACCCTGACCATCTGCTTTGAAGTGGACGTTATTTCGACCACTTCAGTGTCGTAA
+
FFFFFFFFFFFFFFFFFFFFFFFFFFFFFFFFFFF:FFFF:FFFFFFFFFFFFFFFFFFFFFFF:FFFF:
FFFFFFFFFFFFF:FFFFFFFFFFFFFFFFFFFFFFFFFFFFFFFFFFFFFFFFFFFFFFFFFFFFFFFF
@A00155:342:HHGFNDSXY:1:1342:14416:13933 1:N:0:GAACCTAG+TCCGCATA
TACAGATATAATTTCCTTATGATATTTTAACTTCTTTACCATTTCTGCATGCTGTTCTGTTCCTACAT
ACTCGCTTATCGGTACCCTGACCATCTGCTTTGAAGTGGACGTTATTTCGACCACTTCAGTGTCGTAA
+
:FFFFFFFFFFFFFFFFFFFFFFFFFFFFFFFFFFFFFFFF:FFFFFFFFFFFFFFFFFFFFFFFFFFFF
FFFFFFFFFFFFFFFFFFFFFFFFFFFFFFFFFFFFFFFFFFFFFFFFFFFFFFFFFFFFFFFFFFFFFF
@A00155:342:HHGFNDSXY:1:1413:18945:7404 2:N:0:GAACCTAG+TCCGCATA
TACAGATATAATTTCCTTATTATATTTTAACTTCTTTACCATTTCTGCATGCTGTTCTGTTCCTACAT
ACTCGCTTATCGGTACCCTGACCATCTGCTTTGAAGTGGACGTTATTTCGACCACTTCAGTGTCGTAA
+
::,F,FFFFFFFFFF:F,:F,:FFFFFFFF,FF,,FF:,:::FFFF,,FF,FFF:FFFF:FFF:F,,F
FFF,F,FF,FF,:F,F::FF,FFFF:F,:,FFF,FFF,:FFFFF::FFFF,,,,FFFFFFF:,::,F
```

```
@A00155:342:HHGFNDSXY:1:2133:8431:31062 2:N:0:GAACCTAG+TCCGCATA
ATATAATTTCCTTATGATATTTTAACTTCTTTACCATTTCTGCATGCTGGTCTGTTCCTACATACTCG
CTTATCGGTACCCTGACCATCTGCTTTGAAGTGGACGTTATTTCGACCACTTCAGTGTCGTAACCTAC
+
FFFFF:FFFFFFFFFFFFFFFFFFFFFFF:F:FFFF:FFFFFFFFFF,F,FFFFFF:FFFF:FFFFFF
FFFFFF:FF:FFFFF,F:F:F,FFFFFFFFFFFFFFFFFFFFFFFFFFFFFFFFFFFFFFFFFFFFFF
@A00155:342:HHGFNDSXY:1:2519:3893:34194 2:N:0:GAACCTAG+TCCGCATA
ATAATTTCCTTATGATATTTTAACTTCTTTACCATTTCTGCATGCTGTTCTGTTCCTACATACTCGCT
TATCGGTACCCTGACCATCTGCTTTGAAGTGGACGTTATTTCGACCACTTCAGTGTCGTAACCTAC
+
:FFFFFFF,FFFFFFFFFFFFFFFFF:FFFFFFFFFFFFFFFFFFFFFFFFFFFFF:FFFFFFF,FFF
FFFF:F:FFFF:FF:FFFFFFFFFFFF:FFFFFFFFF:FFF:FFFFFFF:FFFFFFFFFFFFFFFF
@A00155:342:HHGFNDSXY:1:1154:24451:18818 2:N:0:GAACCTAG+TCCGCATA
ATAATTTCCTTATGATATTTTAACTTCTTTACCATTTCTGCATGCTGTTCTGTTCCTACATACTCGCT
TATCGGTACCCTGACCATCTGCTTTGAAGTGGACGTTATTTCGACCACTTCAGTGTCGTAACCTAC
+
FFFFFFFFFFFFFFFFFFFFFFFFFFF:FFFFFFFFFFFFFF:FF:FFFFFF,FFFFFFF:FFFFFFF
FFFFFFFFF::,FFFFFFFFFFFFFFFFFFFFFFFFFFFFFFFFFFFFF:FFFFFFFFFFFFFFFF
@A00155:342:HHGFNDSXY:1:1455:5285:7764 2:N:0:GAACCTAG+TCCGCATA
TTATGATATTTTAACTTCTTTACCATTTCTGCATGCTGTTCTGTTCCTACATACTCGCTTATCGGTAC
CCTGACCATCTGCTTTGAAGTGGACGTTATTTCGACCACTTCAGTGTCGTAACC
+
FFFFFFFFF:FFFFFFFFFFFFFFFFFFFFFFFFFFFFFFFFFFFFFFFFFFFFFFFFFFFFFFFFFF
FFFFFFFFFFFFFFFFFFFFFFFFFF:FFFFFFFFFFFFFFFFFFFFFFFFFFF
@A00155:342:HHGFNDSXY:1:1342:14416:13933 2:N:0:GAACCTAG+TCCGCATA
TTATGATATTTTAACTTCTTTACCATTTCTGCATGCTGTTCTGTTCCTACATACTCGCTTATCGGTAC
CCTGACCATCTGCTTTGAAGTGGACGTTATTTCGACCACTTCAGTGTCGTAACC
+
FFFFFFFFFFFFFFFFFFFFFFFFFFFFFFFFFFFFFFFFFFFFFFFFFFFFFFFFFFFFFF:FFFFF
FFFFFFFFFFFFFFFFF:FFFFFFFFFFFFFFFFFFF:FFFFFFFFFFFFFFFF
@A00155:342:HHGFNDSXY:1:2204:8015:24236 2:N:0:GAACCTAG+TCCGCATA
TTATGATATTTTAACTTCTTTACCATTTCTGCATGCTGTTCTGTTCCTACATACTCGCTTATCGGTAC
CCTGACCATCTGCTTTGAAGTGGACGTTATTTCGACCACTTCAGTGTCGTAACCTACTAGTTCTACGG
+
FFFFF:FFFFFFFFFFFFFFFFFFFFFFFFFFFFFFFF,FFFFFFFFFFFF:FFFFFFFFFFFFFFFF
FFFFFFFFFFF,FFFFFFFFFFFFFFFFFFFFFFFFFFFFFFFFFFFFFFFFFFFFFFFFFFFFFFFF
@A00155:342:HHGFNDSXY:1:1266:22272:1642 2:N:0:GAACCTAG+TCCGCATA
TTATGATATTTTAACTTCTTTACCATTTCTGCATGCTGTTCTGTTCCTACATACTCGCTTATCGGTAC
CCTGACCATCTGCTTTGAAGTGGACGTTATTTCGACCACTTCAGTGTCGTAACCTACTAGTTCTACGG
+
FFFFFFF:FFFFFFFFFFFFFFFFFF,FFFFFF:FFFFFFF:F:FFFFFFF:FFFFFFFFFFFFFFFF
FFFFFFFFFFFFFFFFFFFFFFFFFFFFFFFFFFFFFFFFFFFFFFFFF:FFFFFFFFFFF,FFFFFF
@A00155:342:HHGFNDSXY:1:2555:18502:32252 2:N:0:GAACCTAG+TCCGCATA
GATATTTTAACTTCTTTACCATTTCTGCATGCTGTTCTGTTCCTACATACTCGCTTATCGGTACCCTG
ACCATCTGCTTTGAAGTGGACGTTATTTCGACCACTTCAGTGTCGTAACCTACTAGTTCTACGGTGTT
+
FFFFFFFFFFFFFFFFFFFFFF:FFFFFFFFF,FFFFFF,FFF:FFFF:FFFFFFFFFFFFFFFFFFF
FFFFFFFFFFFFFFFFFFFFFFFFFFFFFFFFFFFFFFFFFFFFFFFFFFFFFFFFFFFFFFFFFFFF
@A00155:342:HHGFNDSXY:1:1563:4020:15843 2:N:0:GAACCTAG+TCCGCATA
GATATTTTAACTTCTTTACCATTTCTGCATGCTGTTCTGTTCCTACATACTAGCTTATCGGTACCCTG
ACCATCTGCTTTGAAGTGGACGTTATTTCGACCACTTCAGTGTCGTAACCTACTAGTTCTACGGTGTT
+
FFFFFFFFFFFFFF,:FFF:FFFF,FF:FF:FF:FF,FFFFFFFFFFFFFF,FFFFFFF:,FFFF,FF
FF,FFFFF:FFFFFFFFF:FFFFFFFFFFFFF:FFFFF:FFFF:,F,FFFFFF,FFFFFFF:,FFFFF
```

```
@A00155:342:HHGFNDSXY:1:2519:3893:34194 1:N:0:GAACCTAG+TCCGCATA
TATTTTAACTTCTTTAACATTTCTGCATGCTGTTCTGTTCCTACATACTCGCTTATCGGTACCCTGAC
CATCTGCTTTGAAGTGGACGTTATTTCGACCACTTCAGTGTCGTAACCTACTAGTTCTACGGTGTT
+
FFFFFFFFFFFFFFFF,FFFFFFFFFFFFFFFFFFFFFFFFFFFFFFFFFFFFFFFFF:FFF:FFFFF
FFFFFF,F:FFFF,FFF:FFFFFF:FFFFFFFFFFFFFFFFFFFFFFF,FFFFF:FFFFFFF,F:F
@A00155:342:HHGFNDSXY:1:1154:24451:18818 1:N:0:GAACCTAG+TCCGCATA
TATTTTAACTTCTTTACCATTTCTGCATGCTGTTCTGTTCCTACATACTCGCTTATCGGTACCCTGAC
CATCTGCTTTGAAGTGGACGTTATTTCGACCACTTCAGTGTCGTAACCTACTAGTTCTACGGTGTT
+
FFFF:FFF:F:F:FFFFFFFFFFFFFFFFFFFFFFFFFFFFFFFFFFFFFFF:FFFFFFFFFFFFFFF
FFFFFFFFFFFFFFFFFFF:FFFFFFFFFFFFFFFFFFFFFFFFFFFFFFFFFFFFFFF,FFF,:F
@A00155:342:HHGFNDSXY:1:1652:31006:31172 1:N:0:GAACCTAG+TCCGCATA
CTTCTTTACCATTTCTGCATGCTGTTCTGTTCCTACATACTCGCTTATCGGTACCCTGACCATCTGCT
TTGAAGTGGACGTTATTTCGACCACTTCAGTGTCGTAACCTACTAGTTCTACGGTGTTGTCATTCATC
+
FFFFFFFFFF:FFFFFFFFFFFFFFFFFFFFFFFFFFFFFFFFFFFF::FFFF:FFF:FFFFFFFFFF
FFFFFFF:FFFFFFFFFFF,FFFFFFFFFFFFFFFFFFFFFFFFFFFFFFFFFFFFF:FFFFFF:FFF
@A00155:342:HHGFNDSXY:1:2108:17056:24549 1:N:0:GAACCTAG+TCCGCATA
CTTTACCATTTCTGCATGCTGTTCTGTTCCTACATACTCGCTTATCGGTACCCTGACCATCTGCTTTG
AAGTGGACGTTATTTCGACCACTTCAGTGTCGTAACCTACTAGTTCTACGGTGTTGTCATTCATCGTA
+
F:FFFFFF:F,FFFFFFFFFFFFF,:F:FFFFFF,FFFFFF::FF:FFFFFFFFFFFFF:F,:::,FF
FFF:FFFF:,::F:,FFFFFFF,,FF:,FFFFFFFFFF:FFF::::FFF::FF::F:FFF:FF:FFFF
@A00155:342:HHGFNDSXY:1:2334:21350:14794 1:N:0:GAACCTAG+TCCGCATA
TTTACCATTTCTGCATGCTGTTCTGTTCCTACATACTCGCTTATCGGTACCCTGACCATCTGCTTTGA
AGTGGACGTTATTTCGACCACTTCAGTGTCGTAACCTACTAGTTCTACGGTGTTGTCATTCATCGTAA
+
FFFFFFFFFFFFFFFFFFFFFFFFFFFFFFFF:FFFFFFFFFFFFFFFFFFFFFFFFFFFFFFFFFFF
FFFFFFFFFFFFFFFFFFFFFFFFFFFFFFFFFFFF:FFFFFFFFFFFFFFFFFFFFFFFFFFF:FFF
@A00155:342:HHGFNDSXY:1:2614:25346:34867 2:N:0:GAACCTAG+TCCGCATA
CATTTCTGCATGCTGTTCTGTTCCTACATACTCGCTTATCGGTACCCTGACCATCTGCTTTGAAGTGG
ACGTTATTTCGACCACTTCAGTGTCGTAACCTACTAGTTCTACGGTGTTGTCATTCATCGTAATTATC
+
FFFF:FFFFFFFFFF:FFFFFFFFFFFFFFFFFFFFFFFF:F:FFFFFFFF:FFFFFFFFFFFFFFFF
F:FFFFFFF:FFFFFFFFF:FFFFFFFFFFFFFFFFFFFFF:FFFFFFFFFFFFFFFFFFFFFFFFFF
@A00155:342:HHGFNDSXY:1:1627:9055:2832 2:N:0:GAACCTAG+TCCGCATA
CATTTCTGCATGCTGTTCTGTTCCTACATACTCGCTTATCGGTACCCTGACCATCTGCTTTGAAGTGG
ACGTTATTTCGACCACTTCAGTGTCGTAACCTACTAGTTCTACGGTGTTGTCATTCATCGTAATTATC
+
FFFFFFFFFFFFFFFFFFFFFFFFFFFF:FFFFF,:FFFF:F:FFFFFFFFFFFFFFFFFFFFFFFFF
FFFFFFFFFFFFFFFFFFFFFFFFFFFFFFFFFFFFFFFFFFFFFFFF:FFFF:FFFFFFFFFFF,FF
@A00155:342:HHGFNDSXY:1:1360:21658:22060 1:N:0:GAACCTAG+TCCGCATA
GCATGCTGTTCTGTTCCTACATACTCGCTTATCGGTACCCTGACCATCTGCTTTGAAGTGGACGTTAT
TTCGACCACTTCAGTGTCGTAACCTACTAGTTCTACGGTGTTGTCATTCATCGTAATTATCTTGAAGT
+
FFFFFFFFFFFFFFFFFFFFFFFFFFFFFFFFFFFFFFFFFFFFFFFFFFFFFFFFFFFFFFFFFFFF
FFFFFFFFFFFFFFFFFFFFFFFFFFFFF::FFFFFFFFFFFFFFFFFFFFFFFFFFFFFFFFFFFFF
@A00155:342:HHGFNDSXY:1:1360:21866:21167 1:N:0:GAACCTAG+TCCGCATA
GCATGCTGTTCTGTTCCTACATACTCGCTTATCGGTACCCTGACCATCTGCTTTGAAGTGGACGTTAT
TTCGACCACTTCAGTGTCGTAACCTACTAGTTCTACGGTGTTGTCATTCATCGTAATTATCTTGAAGT
+
FFFFFFFFFFFFFFFFFFFFFFFFFFFFFFFFFFFFFFFFFFFFFFFFFFFF:FFFFFFFFFFFFFFF
FF:FFFFFFFFFFF,FFFFFFFFFFFFFFFFFFFFF:FFFFFFFFFFFFFFFFFFF::FF:FFFFFFF
```

```
@A00155:342:HHGFNDSXY:1:2666:16233:26005 2:N:0:GAACCTAG+TCCGCATA
ATGCGGTTCTGTTCCTACATACTCGCTTATCGGTACCCTGACCATCTGCTTTGAAGTGGACGTTATTT
CGACCACTTCAGTGTCGTAACCTACTAGTTCTACGGTGTTGTCATTCATCGTAATTATCTTGAAGTCG
+
FF,F,:,FFFFFFFFF:FFFFFFFFFFFFFFFFFFFF,FFFFFFFFFFFFFFFFFFFFFFF,FFFFFF
F:FFFFFFFFFFFFFFF,FFF:F,FFFFFF,FFF:FF:FFFFFF,FFFFFFF,F:F:FFF:FFFFFFF
@A00155:342:HHGFNDSXY:1:1642:1588:7686 1:N:0:GAACCTAG+TCCGCATA
ATGCTGTTCTGTTCCTACATACTCGCTTATCGGTACCCTGACCATCTGCTTTGAAGTGGACGTTATTT
CGACCACTTCAGTGTCGTAACCTACTAGTTCTACGGTGTTGTCATTCATCGTAATTATCTTGAAGTCG
+
FFFFFFFFFFFFFFFFFFFF:FFFFFFFFFFFFFFF:FFF::FFFFFFFFFFF:FFF:FFFFFFFFFF
FFFFF:FFFFFFFFFFFFFFFFFFFFFFFFFFFFFFFFFFFFFFFFFFFFFFFFFFFFFFF:FFF:F
@A00155:342:HHGFNDSXY:1:1642:1886:11428 1:N:0:GAACCTAG+TCCGCATA
ATGCTGTTCTGTTCCTACATACTCGCTTATCGGTACCCTGACCATCTGCTTTGAAGTGGACGTTATTT
CGACCACTTCAGTGTCGTAACCTACTAGTTCTACGGTGTTGTCATTCATCGTAATTATCTTGAAGTCG
+
FFFFFFFFFFFFFFFFFFFFFFFFFFFFFFFFFFFFFFFFFFFFFFFFFFFFFFFFFFFFFFFFFFFF
FFFFFFFFFFFFFFFFFFFFFFFFFFFFFFFFFFFFFFFFFFFFFFFFFFFFFFFFFFFFF:FFFFFF
@A00155:342:HHGFNDSXY:1:1622:21034:32127 1:N:0:GAACCTAG+TCCGCATA
ATGCTGTTCTGTTCCTACATACTCGCTTATCGGTACCCTGACCATCTGCTTTGAAGTGGACGTTATTT
CGACCACTTCAGTGTCGTAACCTACTAGTTCTACGGTGTTGTCATTCATCGTAATTATCTTGAAGTCG
+
FFFFFFFFFFFFFFFFFFFFFFFFFFFFFFFFFFFFFFFFFFFFFFFFFFFFFFFFFFFFFFFFFFFF
FFFFFFFFFFFFFFFFFFFFFFFFFFFFFFFFFFFFFFFFFFFFFFFFFFFFFFFFFFFFFFFFFF:F
@A00155:342:HHGFNDSXY:1:2512:5719:32722 2:N:0:GAACCTAG+TCCGCATA
TGCTGTTCTGTTCCTACATACTCGCTTATCGGTACCCTGACCATCTGCTTTGAAGTGGACGTTATTTC
GACCACTTCAGTGTCGTAACCTACTAGTTCTACGGTGTTGTCATTCATCGTAATTATCTTGAAGTCGT
+
FFFFFFFFFF:FFFFFFFFFFF,FFFFFFF:FFFFFFFFFFFFF:F:,FFFFFFFFFFFFFFFFFFFF
FF:FFFFFFFFFF:FF,FFFFF:FFFFFFFFFFFFFFFFFFFFFFFFFFFFFFFFFFFFFFFFFFF:F
@A00155:342:HHGFNDSXY:1:1363:8467:16877 1:N:0:GAACCTAG+TCCGCATA
TGTTCTGTTCCTACATACTCGCTTATCGGTACCCTGACCATCTGCTTTGAAGTGGACGTTATTTCGAC
CACTTCAGTGTCGTAACCTACTAGTTCTACGGTGTTGTCATTCATCGTAATTATCTTGAAGTCGTGTG
+
FFFFFFFFFFFFFFFFFFFF:FFFFFFFFFFFFFFFFFFFF:FFFFFFFFFFFFFFFFFFFFFFFFFF
FFFFFFFFFFFFFFFFFFFFFFFFFFFFFFFFFFFFFFFFFFFFFFFFFFFFFFFFFF:F,F:FFFFF
@A00155:342:HHGFNDSXY:1:1332:18213:13526 1:N:0:GAACCTAG+TCCGCATA
TGTTCTGTTCCTACATACTCGCTTATCGGTACCCTGACCATCTGCTTTGAAGTGGACGTTATTTCGAC
CACTTCAGTGTCGTAACCTACTAGTTCTACGGTGTTGTCATTCATCGTAATTATCTTGAAGTCGTGTG
+
FFFFFFFFFFFFFFFFFFFFFFFFFFFFFFFFFFFFFFFFFFFFFFFFFFFFFFFFFFFFFFFFFFFF
FFFFFFFFFFFFFFFFFFFFFFFFFFFFFF:FFFFFFFFFFFFFFFFFFFF:FFFFFFFFFFFFFFFF
@A00155:342:HHGFNDSXY:1:1332:17598:13369 1:N:0:GAACCTAG+TCCGCATA
TGTTCTGTTCCTACATACTCGCTTATCGGTACCCTGACCATCTGCTTTGAAGTGGACGTTATTTCGAC
CACTTCAGTGTCGTAACCTACTAGTTCTACGGTGTTGTCATTCATCGTAATTATCTTGAAGTCGTGTG
+
FFFFFFFFFFFFFFFFFFFFFFFFFFFFFFFFFF:FFFFFFFFFFF:F:FFFFFFFFFFFFFFFFFFF
FFFFFFFF:FFF:FFFFF:FFFFFFFFFFFFFFFFFFFFFFFFF:FFFFFFFFFFFFFFFFFFFFFFF
@A00155:342:HHGFNDSXY:1:1654:6659:19946 1:N:0:GAACCTAG+TCCGCATA
TGTTCTGTTCCTACATACTCGCTTATCGGTACCCTGACCATCTGCTTTGAAGTGGACGTTATTTCGAC
CACTTCAGTGTCGTAACCTACTAGTTCTACGGTGTTGTCATTCATCGTAATTATCTTGAAGTCGTGTG
+
FFFFFFFFFFFFFFFFFFFFFFFFFFFFFFFFFFFFFFFFFFFFFFFFFFFFFFFFFFFFFFFFFFFF
FFFFFFFFFFFFFFFFFFFFFFFFFFFFFFFFFFFFFFFFFFFFF:FFFFFFFFFFFFFFFFFFFFFF
```

```
@A00155:342:HHGFNDSXY:1:1430:18982:20619 2:N:0:GAACCTAG+TCCGCATA
GTTCTGTTCCTACATACTCGCTTATCGGTACCCTGACCATCTGCTTTGAAGTGGACGTTATTTCGACC
ACTTCAGTGTCGTAACCTACTAGTTCTACGGTGTTGTCATTCATCGTAATTATCTTGAAGTCG
+
FFFFFFFFFFFFFFFFFFFFFFFFFFFF:FFFFFFFFF:FFFFFFFFFFFFFFFFFFFFFFFFFFFFF
FFFFFFFFFFFFFFFFFFFFFFFFFFFFFFFFFFFFFFFFFFFFFFFFFFFFFFFFFFF:FFF
@A00155:342:HHGFNDSXY:1:2108:17056:24549 2:N:0:GAACCTAG+TCCGCATA
GTTCTGTTCCTACATACTCGCTTATCGGTACCCTGACCATCTGCTTTGAAGTGGACGTTATTTCGACC
ACTTCAGTGTCGTAACCTACTAGTTCTACGGTGTTGTCATTCATCGTAATTATCTTGAAGTCGTGTG
+
FFFFFFFFFFFFFF:FFFFFFFFFFF:FFFFFFFFFFFFFFFFFFFFFFFFFFFFFFFFFFFFFFFFF
FFFFFFFFFFFFFFFFFFFFFFFFFFFFFFFFFFFFFFFFFFFFFFFFFFFFFFFFFFFFFFFFFFF
@A00155:342:HHGFNDSXY:1:2132:18502:1063 1:N:0:GAACCTAG+TCCGCATA
GTTCTGTTCCTACATACTCGCTTATCGGTACCCTGACCATCTGCTTTGAAGTGGACGTTATTTCGACC
ACTTCAGTGTCGTAACCTACTAGTTCTACGGTGTTGTCATTCATCGTAATTATCTTGAAGTCGTGTGG
+
F:FFFF,FFFFFFFFFFFFFFFFFFFFFFFFFFFFFFFFFFFFFFFFFFFFFFFFFFFFFFFFFFFFF
FFFFFFFFFFFFFFFFFFFFFFFFFFFFFFFFFFFFFFFFFFFFFFFFFFFFFFFFFFFFFFFFFFFF
@A00155:342:HHGFNDSXY:1:2250:24207:28479 2:N:0:GAACCTAG+TCCGCATA
TTCTGTTCCTACATACTCGCTTATCGGTACCCTGACCATCTGCTTTGAAGTGGACGTTATTTCGACCA
CTTCAGTGTCGTAACCTACTAGTTCTACGGTGTTGTCATTCATCGTAATTATCTTGAAGTCGTGTGGG
+
FFFFFFFFFFFFFFFFFFFFF:FFFFFFFFFFFFFFFFFFFFFFFFFFFFFFFFFFFFFFFFFFFFFF
FFFFFFFFFFFFFFFFFFFFFFFFFFFFFFFFFFFFFFFFFFFFFFFFFFFFFFFFFFFFFFFFFFFF
@A00155:342:HHGFNDSXY:1:1240:14027:3771 1:N:0:GAACCTAG+TCCGCATA
TTCTGTTCCTACATACTCGCTTATCGGTACCCTGACCATCTGCTTTGAAGTGGACGTTATTTCGACCA
CTTCAGTGTCGTAACCTACTAGTTCTACGGTGTTGTCATTCATCGTAATTATCTTGAAGTCGTGTGGG
+
FFFFFFFFFFFFFFFFFFFFFFFFFFFFFFFFFFFFFFFFFFFFFFFFFFFFFFFFFFFFFFFFFFFF
FFFFFFFFFFFFFFFFFFFFFFFFFFFFFFFFFFFFFFFFFFFFFFFFFFFFFFFFF:FFFFFFFFFF
@A00155:342:HHGFNDSXY:1:2538:9652:13260 1:N:0:GAACCTAG+TCCGCATA
TTCTGTTCCTACATACTCGCTTATCGGTACCCTGACCATCTGCTTTGAAGTGGACGTTATTTCGACCA
CTTCAGTGTCGTAACCTACTAGTTCTACGGTGTTGTCATTCATCGTAATTATCTTGAAGTCGTGTGGG
+
FFFFFFFFFFFFFFFFFFFFFFFFFFFFFFFFFFFFFFFFFFFFFFFFFFFFFFFFFFFFFFFFFFFF
FFFFFFFFFFFFFFFFFFFFFFFFFFFFFFFFFFFFFFFFFFFFFFFFFFFFFFFFFFFFFFFFFFFF
@A00155:342:HHGFNDSXY:1:2250:24207:28479 1:N:0:GAACCTAG+TCCGCATA
TCTGTTCCTACATACTCGCTTATCGGTACCCTGACCATCTGCTTTGAAGTGGACGTTATTTCGACCAC
TTCAGTGTCGTAACCTACTAGTTCTACGGTGTTGTCATTCATCGTAATTATCTTGAAGTCGTGTGGG
+
FFFFFFFFFFFFFFFFFFFFFFFFFFFFFFFFFFFFFFFFFFFFFFFFFFFFFFFFFFFFFFFFFFFF
FFFFFFFFFFFFFFFFFFFFFFFFFFFFFFFFFFFFFFFFFFFFFFFFFFFFFFFFFFFFFFFFFFF
@A00155:342:HHGFNDSXY:1:2229:3622:11678 2:N:0:GAACCTAG+TCCGCATA
TTCCTACATACTCGCTTATCGGTACCCTGACCATCTGCTTTGAAGTGGACGTTATTTCGACCACTTCA
GTGTCGTAACCTACTAGTTCTACGGTGTTGTCATTCATCGTAATTATCTTGAAGTCGTGTGGGTCGTC
+
FFFFFFFFFFFFFFFFFFFFFF,FFFFFFFFFFFFFFFFFFFFFFFFFFFFFFFFFFFFFFFFFFFFF
FFFFFFFFFFFFFFFFFFFFFFFFFFFFFFFFFFFFFFFFFFFFFFFFFFFFFFFFFFFFFFFFFFFF
@A00155:342:HHGFNDSXY:1:1566:15935:14465 2:N:0:GAACCTAG+TCCGCATA
TTCCTACATACTCGCTTATCGGTACCCTGACCATCTGCTTTGAAGTGGACGTTATTTCGACCACTTCA
GTGTCGTAACCTACTAGTTCTACGGTGTTGTCATTCATCGTAATTATCTTGAAGTCGTGTGGGTCGTC
+
:FFFFFFFFFFFFFFFFFFFFFFFFFFFFFFFFFFFFFFFFFFFFFFFFFFFFFFFFFFFFFFFFFFF
FFFFFFFFFFFF,F:,FFFFF,FFFFF:FFFFFFFFFFFFFFFFFFFFFFFFFFFFFFFFFFFFFFFF
```

```
@A00155:342:HHGFNDSXY:1:2671:27787:6151 2:N:0:GAACCTAG+TCCGCATA
TCCTACATACTCGCTTATCGGTACCCTGACCATCTGCTTTGTAGTGGACGTTATTTCGACCACTTCAG
TGTCGTAACCTACTAGTTCTACGGTGTTGTCATTCATCGTAATTATCTTGAAGTCGTGTGGGTCGT
+
:,FF:F:FFFF,FFFFF:F:FFF:,FFF:FFFFFFFF,FFF,FFFFFFFFFFFFFFF:::FF,:FFFF
FFFFF,F:F,:FF,,FFF:F:FFFFFFFFFFFFFFFF::FF:FFFF:FFF:,F::FFFFF:FFFFF
@A00155:342:HHGFNDSXY:1:1206:30436:28933 2:N:0:GAACCTAG+TCCGCATA
TCCTACATACTCGCTTATCGGTACCCTGACCATCTGCTTTGAAGTGGACGTTATTTCGACCACTTCAG
TGTCGTAACCTACTAGTTCTACGGTGTTGTCATTCATCGTAATTATCTTGAAGTCGTGTGGGTCGTC
+
FFFFFFFFFFFFF,FFFFFFFFFFFFFFF:FFFFFFF:FFFFFFFFFFFFFF,FF::FFFFFFFFFFF
FFFFFFFFFFFFFFFFFFFFFFFFFFFFFFFFFFFFFFFFFFFFFFF:FFF:FFFFFFFFFFFFFFF
@A00155:342:HHGFNDSXY:1:2512:5719:32722 1:N:0:GAACCTAG+TCCGCATA
TCCTACATACTCGCTTATCGGTACCCTGACCATCTGCTTTGAAGTGGACGTTATTTCGACCACTTCAG
TGTCGTAACCTACTAGTTCTACGGTGTTGTCATTCATCGTAATTATCTTGAAGTCGTGTGGGTCGTCG
+
FFFFFFFFFFFFFFFFFF:FFFFFFFFFFFFFFFFFFFFFF:FFFFFFFFFFFFFFFFFFFFFFFFF:
FFFFFFFFFFFFFFFFFFFFFFFFFFFFFFFFFF:FFFFFFFFFFFFFFFFFFFFFFF:FFFFF:FFF
@A00155:342:HHGFNDSXY:1:1376:23339:9189 1:N:0:GAACCTAG+TCCGCATA
CCTACATACTCGCTTATCGGTACCCTGACCATCTGCTTTGAAGTGGACGTTATTTCGACCACTTCAGT
GTCGTAACCTACTAGTTCTACGGTGTTGTCATTCATCGTAATTATCTTGAAGTCGTGTGGGTCGTCGA
+
FFFFFFFFFFFFFFFFFFFFFFFFFFFFFFFFFFFFFFFFFFFFFFFFFFFFFFFFFFFFFF:FFFFF
FFFFFFFFFFFFFFFFFFFFFFFFFFFFFFFFFFFFFFFFFFFFFFFFFF:FFFFFFFFFFFFFFFFF
@A00155:342:HHGFNDSXY:1:2274:14886:31782 1:N:0:GAACCTAG+TCCGCATA
CCTACATACTCGCTTATCGGTACCCTGACCATCTGCTTTGAAGTGGACGTTATTTCGACCACTTCAGT
GTCGTAACCTACTAGTTCTACGGTGTTGTCATTCATCGTAATTATCTTGAAGTCGTGTGGGTCGTCGA
+
FFFFFFFFFFFFFFFFFFFFFFFFF:FFFFFFFF:FFFFFFFFFFFFFFFFFFFFFFFFFFFFFFFFF
FFFFFFFFFFFFFFFFFFFFFFFFFFFFFFFFFFFFFFFFFFFFFFFFFFFFFF:FFFFFFFFFFFFF
@A00155:342:HHGFNDSXY:1:1530:14109:35289 1:N:0:GAACCTAG+TCCGCATA
CCTACATACTCGCTTATCGGTACCCTGACCATCTGCTTTGAAGTGGACGTTATTTCGACCACTTCAGT
GTCGTAACCTACTAGTTCTACGGTGTTGTCATTCATCGTAATTATCTTGAAGTCGTGTGGGTCGTCGA
+
FFFFFFFFFFFFFFFFFFFFFFFFFFFFFFFFFFFFFFFFFFFFFFFFFFFFFFFFFFFFFFFFFFFF
FFFFFFFFFFFFFFFFFFFFFFFFFFFFFFFFFFFFFFFFFFFFFFFFFFFFFFFFFFFFFFFFFFFF
@A00155:342:HHGFNDSXY:1:2229:3622:11678 1:N:0:GAACCTAG+TCCGCATA
CCTACATACTCGCTTATCGGTACCCTGACCATCTGCTTTGAAGTGGACGTTATTTCGACCACTTCAGT
GTCGTAACCTACTAGTTCTACGGTGTTGTCATTCATCGTAATTATCTTGAAGTCGTGTGGGTCGTCGA
+
FFFFFFFFFFFFFFFFFFFFFFFFFFFFFFFFFFFFFFFFFFFFFFFFFFFFFFFFFFFFFFFFFFFF
FFFFFFFFFFFFFFFFFFFFFFFFFFFFFFFFFFFFFFFFFFFFFFFFFFFFFFFFFFFFFFFFFFFF
@A00155:342:HHGFNDSXY:1:2310:18819:11537 1:N:0:GAACCTAG+TCCGCATA
CTACATACTCGCTTATCGGTACCCTGACCATCTGCTTTGAAGTGGACGTTATTTCGACCACTTCAGTG
TCGTAACCTACTAGTTCTACGGTGTTGTCATTCATCGTAATTATCTTGAAGTCGTGTGGGTCGTCGAA
+
FFFFFFFFFFFFFFFFFFFFFFFFFFFFFFFFFFFFFFFFFFFFFFFFFFFFFFFFFFFFFFFFFFFF
FFFFFFFFFFFFFFF:FFFFFFFFFFFFFFFFFFFFFFFFFFFFFFFFFFFFF:FFFF:FFFFFFFFF
@A00155:342:HHGFNDSXY:1:2108:30391:28322 1:N:0:GAACCTAG+TCCGCATA
TACATACTCGCTTATCGGTACCCTGACCATCTGCTTTGAAGTGGACGTTATTTCGACCACTTCAGTGT
CGTAACCTACTAGTTCTACGGTGTTGTCATTCATCGTAATTATCTTGAAGTCGTGTGGGTCGTCGAAC
+
FFFFFFFFFFFFFFFFFFF:FFFFFFFFFFFFFFFFFFFFFFF:FFFFFFFFFF,FFFFFFFFFFFFF
FFFFFFFFFFFFFFFFFFFFFFFFFFFFFFFFFFFFFFFFFFFFFFFFFFFFFFFFFF:FF:FFFFFF
```

```
@A00155:342:HHGFNDSXY:1:2178:17779:24298 1:N:0:GAACCTAG+TCCGCATA
TACATACTCGCTTATCGGTACCCTGACCATCTGCTTTGAAGTGGACGTTATTTCGACCACTTCAGTGT
CGTAACCTACTAGTTCTACGGTGTTGTCATTCATCGTAATTATCTTGAAGTCGTGTGGGTCGTCGAAC
+
FFFFFFFFFFFFFFFFFFFFFFFFFFFFFFFFFFFFFFFFFFFFFFFFFFFFFFFFFFFFFFFFFFFF
FFFFFFFFFFFFFFFFFFFFFFFFFFFFFFFFFFFFFFFFFFFFFFFFFFFFFFFFFFFFFFFFFFFF
@A00155:342:HHGFNDSXY:1:1351:8088:26303 2:N:0:GAACCTAG+TCCGCATA
CATACTCGCTTATCGGTACCCTTACCATCTGCTTTGAAGTGGACGTTATTTCGACCACTTCAGTGTCG
TAACCTACTAGTTCTACGGTGTTGTCATTCATCGTAATTATCTTGAAGTCGTGTGGGTCGTCGA
+
FFFFFFFFFFFFFFFFFF,F::,FFFFFFFFF:FF,FFFFFFFFFFFFFF:FFFFFFFFFFFFFFFFF
FFFFFF:FFFFFFFFFFFFFFFFFFFFF:FFFFFFFFFFF:FFFFFFFFFFFFFFFFFFFFFFF
@A00155:342:HHGFNDSXY:1:2153:20247:32393 1:N:0:GAACCTAG+TCCGCATA
CATACTCGCTTATCGGTACCCTGACCATCTGCTTTGAAGTGGACGTTATTTCGACCACTTCAGTGTCG
TAACCTACTAGTTCTACGGTGTTGTCATTCATCGTAATTATCTTGAAGTCGTGTGGGTCGTCGAACCT
+
FFFFFFFFFFFFFFFFFFFFFFFFFFFFFFFF:FFFFFFFFFFFFFFFFFFFFFFFFF:FFFFFFFFF
FFFFFFFFFFFFFFFFFFFFFFFFFFFFFFFFFFFFFFFFF:FFFFFFFFFFFFFFFFFFFFFFFFFF
@A00155:342:HHGFNDSXY:1:1351:8088:26303 1:N:0:GAACCTAG+TCCGCATA
ATACTCGCTTATCGGTACCCTGACCATCTGCTTTGAAGTGGACGTTATTTCGACCACTTCAGTGTCGT
AACCTACTAGTTCTACGGTGTTGTCATTCATCGTAATTATCTTGAAGTCGTGTGGGTCGTC
+
FFFFFFFFFFFFFFFFFFFFFFFFFFFFFFF,FFFFFFFFFFFFFF:FFFFFFFFFFFFFFFFFFFFF
FFFFFFFFFFFFFFF,FFFFFFFFFFFFFFFFFFFFFFFFFFFFFFFFFFFFFFFFFFFFF
@A00155:342:HHGFNDSXY:1:1635:24135:14857 2:N:0:GAACCTAG+TCCGCATA
ATACTCGCTTATCGGTACCCTGACCATCTGCTTTGAAGTGGACGTTATTTCGACCACTTCAGTGTCGT
AACCTACTAGTTCTACGGTGTTGTCATTCATCGTAATTATCTTGAAGTCGTGTGGGTCGTCGAACCTC
+
FFFFFFFFFFFFFFFFFFFFFFFFFFFFFFFFFFFFFFFFFFFFFFFFFFFFFFFFFFFFFFFFFFFF
FFFFFFFFFFFFFFFFFFFFFFFFFFFFFFFFFFFFFFFFFFFFFFFFFFFFFFFFFFFFFFFFFFFF
@A00155:342:HHGFNDSXY:1:2607:24686:2754 2:N:0:GAACCTAG+TCCGCATA
ATACTCGCTTATCGGTACCCTGACCATCTGCTTTGAAGTGGACGTTATTTCGACCACTTCAGTGTCGT
AACCTACTAGTTCTACGGTGTTGTCATTCATCGTAATTATCTTGAAGTCGTGTGGGTCGTCGAACCTC
+
FFFFFFFFF:FFFFF:FFFFFFFFFFFFFFFFFFFFFFFFFFFFFFFFFFFFFFFFF::FFFFFFFF:
FFFFFFFFFFFFFFFFFFFFFFFFFFFFFFFFF:FFFFFFFFFFFFFFFFFFFFFFFFFFFFFFFFFF
@A00155:342:HHGFNDSXY:1:2644:32551:21856 1:N:0:GAACCTAG+TCCGCATA
ATACTCGCTTATCGGTACCCTGACCATCTGCTTTGAAGTGGACGTTATTTCGACCACTTCAGTGTCGT
AACCTACTAGTTCTACGGTGTTGTCATTCATCGTAATTATCTTGAAGTCGTGTGGGTCGTCGAACCTC
+
FFFFFFFFFFFFFFFF:FFFFFFFFFFFFFFFFFFFFFFFF:FFFFFFFFFFFFFFFFFFFFFF:FFF
FFFFFFFFFFFFFFFFFFFFFFFFFFFFFFFFFFFFFFFFFFFFFFFFFFFFFFFFFFFFF:FFFFFF
@A00155:342:HHGFNDSXY:1:1430:18982:20619 1:N:0:GAACCTAG+TCCGCATA
ACTCGCTTATCGGTACCCTGACCATCTGCTTTGAAGTGGACGTTATTTCGACCACTTCAGTGTCGTAA
CCTACTAGTTCTACGGTGTTGTCATTCATCGTAATTATCTTGAAGTCGTGTGGGTCGTCGAAC
+
FFFFFFFFFFFFFFFFFFFFFFFFFFFFF:FFFFFFFFFFFFFFFFFFFFFFFFFFFFFFFFFFFFFF
FFFFFFFF:FFFFFFFFFFFFFFFFF:FFFFFFFFFFFFFFFFFFFFFFFFFFFFFFFFFFFF
@A00155:342:HHGFNDSXY:1:1174:7627:1141 1:N:0:GAACCTAG+TCCGCATA
ACTCGCTTATCGGTACCCTGACCATCTGCTTTGAAGTGGACGTTATTTCGACCACTTCAGTGTCGTAA
CCTACTAGTTCTACGGTGTTGTCATTCATCGTAATTATCTTGAAGTCGTGTGGGTCGTCGAACCTCAC
+
FFFFFFFFFFFFFFFFFFFFFFFFFFFFFF:FFFF:FFFFFFFFFFFFFFFFFFFFFFFFFFFFFFFF
FFFFFFFFFFFFFFFFFFFFFFFFFFFFFFFFFFFFFFFFFFFFFFFFFFFFFFFFFFFFFFFFFFFF
```

@A00155:342:HHGFNDSXY:1:2422:25292:19366 1:N:0:GAACCTAG+TCCGCATA
ACTCGCTTATCGGTACCCTGACCATCTGCTTTGAAGTGGACGTTATTTCGACCACTTCAGTGTCGTAA
CCTACTAGTTCTACGGTGTTGTCATTCATCGTAATTATCTTGAAGTCGTGTGGGTCGTCGAACCTCAC
+
FFFFFFFFFFFFFFFFFFFFFFFFFFFFFFFFFFFFFFFFFFFFFFFFFFFFFFFFFFFFFFFFFFFF
FFFFFFFFFFFFFFFFFFFFFFFFFFFF:FFFFFFFFFFFFFFFFFFFFFFFFFFFFFFFFFFFFFFF
@A00155:342:HHGFNDSXY:1:2338:30951:29794 1:N:0:GAACCTAG+TCCGCATA
ACTCGCTTATCGGTACCCTGACCATCTGCTTTGAAGTGGACGTTATTTCGACCACTTCAGTGTCGTAA
CCTACTAGTTCTACGGTGTTGTCATTCATCGTAATTATCTTGAAGTCGTGTGGGTCGTCGAACCTCAC
+
FFFFFFFFFFFFFFFFFFFFFFFFFFFFFFFFFFFFFFFFFFFFFFFFFFFF:FFFFFFFFFFFFFFF
FFFFFFFFFFFFFFFFFFFFFFFF:FFFFFFFFFFFFFFFFFFFFFFFFFFFFFFFFFFFFFFFFFFF
@A00155:342:HHGFNDSXY:1:2571:11595:19570 1:N:0:GAACCTAG+TCCGCATA
ACTCGCTTATCGGTACCCTGACCATCTGCTTTGAAGTGGACGTTATTTCGACCACTTCAGTGTCGTAA
CCTACTAGTTCTACGGTGTTGTCATTCATCGTAATTATCTTGAAGTCGTGTGGGTCGTCGAACCTCAC
+
FFFFFFFFFFFFFFFFFFFFFFFFFFFFFFFFFFFFFFFFFFFFFFFFFFFFFFFFFFFFFFFFFFFF
FFFFFFFFFFFFFFFFFFFFFFFFFFFFFFFFFFFFFFFFFFFFFFFFFFFFFFFFFFFFFFFFFFFF
@A00155:342:HHGFNDSXY:1:2574:5918:16814 1:N:0:GAACCTAG+TCCGCATA
ACTCGCTTATCGGTACCCTGACCATCTGCTTTGAAGTGGACGTTATTTCGACCACTTCAGTGTCGTAA
CCTACTAGTTCTACGGTGTTGTCATTCATCGTAATTATCTTGAAGTCGTGTGGGTCGTCGAACCTCAC
+
FFFFFFFFFFFFFFFFFFFFFFF:FFFFFFFFFFFFFFFFFFFFFFFFFFFFFFF:FFFFFFFFFFFF
FFFFFFFF:FFFFFFFFFFFFFFFFFFFFFFFFFFFFFFFFF:FFFF,FFFFFFFF,,FFFFFFFFFF
@A00155:342:HHGFNDSXY:1:1245:11650:5415 2:N:0:GAACCTAG+TCCGCATA
CTCGCTTATCGGTACCCTGACCATCTGCTTTGAAGTGGACGTTATTTCGACCACTTCAGTGTCGTAAC
CTACTAGTTCTACGGTGTTGTCATTCATCGTAATTATCTTGAAGTCGTGTGGGTCGTCGAACCTCACA
+
FFFFFFFFFFFFFFFFFFFFFFFFFFFFFFFFFFFFFFFFFFFFFFFFFFFFFFFFFFFFFFFFFFFF
FF:FFFF:FFFFFFFFFFFFFF:FFFFFFFFFFFFFFFFFFFFFFFFFFF:FFFFFFFFFFFFFFFFF
@A00155:342:HHGFNDSXY:1:2333:16080:14998 2:N:0:GAACCTAG+TCCGCATA
TCGCTTATCGGTAACCTGACCATCTGCTTTGAAGTGGACGTTATTTCGACCACTTCAGTGTCGTAACC
TACTAGTTCTACGGTGTTGTCATTCATCGTAATTATCTTGAAGTCGTGTGGGTCGTCGAACCTCACAC
+
FFFFFFFFFFFFFFFFFFFFFFFFFFFFFFFFFFFFFFFFFFFFFFFFFFFFFFFFFFFFFFFFFFFF
FFFFFFFFFFFFFFFFFFFFFFFFFFFFFFFFFFFFFFFFFFFFFFFFFFFFFFFFFFFFFFFFFFFF
@A00155:342:HHGFNDSXY:1:1206:30436:28933 1:N:0:GAACCTAG+TCCGCATA
CTTATCGGTACCCTGACCATCTGCTTTGAAGTGGACGTTATTTCGACCACTTCAGTGTCGTAACCTAC
TAGTTCTACGGTGTTGTCATTCATCGTAATTATCTTGAAGTCGTGTGGGTCGTCGAACCTCACACCGC
+
FFFFFFFFFFFFFFFFFFFFFFFFFFFFFFFFFFFFFFFFFFFFFFFFFFFFFFFFFFFFFFFFFFFF
FFFFFFFFFFFFFFFFFFFFFFFFFFFF:FFF:FFFFFFFFFFFFFFFFFFFFFFF:FFFFFFFFFFF
@A00155:342:HHGFNDSXY:1:1566:15935:14465 1:N:0:GAACCTAG+TCCGCATA
CTTATCGGTACCCTGACCATCTGCTTTGAAGTGGACGTTATTTCGACCACTTCAGTGTCGTAACCTAC
TAGTTCTACGGTGTTGTCATTCATCGTAATTATCTTGAAGTCGTGTGGGTCGTCGAACCTCACATCGC
+
FFFFFFFFF,FFFFFFFFFFFFFFFFFFFFFFFFFFFFFFFFFFFFFFFFFFFFFFFFFFFFFFFFFF
F:FFFFFFFFFFFFFFFFFFFFFFFFFFFFFFFF:FFFFFFFFFFFFFFFFFFFFFFFFFFFFFFFFF
@A00155:342:HHGFNDSXY:1:1312:22724:34585 1:N:0:GAACCTAG+TCCGCATA
CTTATCGGTACCCTGACCATCTGCTTTGAAGTGGACGTTATTTCGACCACTTCAGTGTCGTAACCTAC
TAGTTCTACGGTGTTGTCATTCATCGTAATTATCTTGAAGTCGTGTGGGTCGTCGAACCTCACACCGC
+
FFFFFFFFFFFFFFFFFFFFFFFFFFFFFFFFFFFF,F:FFFFFFFFFFFFFFFFFFFFFFFFF::FF
FFFFFFFFFFFFFFFFFFFFFFFFFFFFFFFFFFFFFFFFFFFFFFFFFFFFFFFFFFFFFFFFFF,F

```
@A00155:342:HHGFNDSXY:1:2108:30391:28322 2:N:0:GAACCTAG+TCCGCATA
TTATCGGTACCCTGACCATCTGCTTTGAAGTGGACGTTATTTCGACCACTTCAGTGTCGTAACCTACT
AGTTCTACGGTGTTGTCATTCATCGTAATTATCTTGAAGTCGTGTGGGTCGTCGAACCTCACACCGCC
+
FFFFFFFFFFFFFFFFFFFFFF:FFFFFFFFFFFFFFFF:FFFFFFF,FFFFFFFFFFFFFFFFFFFF
::FFFFFFFFFFFFFFFFFFFFFFFFFFFFFFFFFFFFFFFFFFFFFFFFFFFFFFFFFFFFFFFFFF
@A00155:342:HHGFNDSXY:1:2178:17779:24298 2:N:0:GAACCTAG+TCCGCATA
TTATCGGTACCCTGACCATCTGCTTTGAAGTGGACGTTATTTCGACCACTTCAGTGTCGTAACCTACT
AGTTCTACGGTGTTGTCATTCATCGTAATTATCTTGAAGTCGTGTGGGTCGTCGAACCTCACACCGCC
+
FFFFFFFFFFFFFFFFFFFFFFFFFFFFFFFFFFFFFFFFFFFFFFFFFFFFFFFFFFFFFFFFFFFF
FFFFFFFFFFFFFFFFFFFFFFFFFFFFFFFFFFFFFFFFFFFFFFFFFFFFFFFFFFFFFFFFFFFF
@A00155:342:HHGFNDSXY:1:1635:24135:14857 1:N:0:GAACCTAG+TCCGCATA
ATCGGTACCCTGACCATCTGCTTTGAAGTGGACGTTATTTCGACCACTTCAGTGTCGTAACCTACTAG
TTCTACGGTGTTGTCATTCATCGTAATTATCTTGAAGTCGTGTGGGTCGTCGAACCTCACACCGCCAT
+
FFFFFFFFFFFFFFFFFFFFFFFFFFFFFFFFFFFFFFFFFFFFFFFFFFFFFFFFFFFFFFFFFFFF
FFFFFFFFFFFFFFFFFFFFFFFFFFFFFFFFFFFFFFFFFFFFFFFFFFFFFFFFFFFFFFFFFFFF
@A00155:342:HHGFNDSXY:1:1654:6659:19946 2:N:0:GAACCTAG+TCCGCATA
ATCGGTACCCTGACCATCTGCTTTGAAGTGGACGTTATTTCGACCACTTCAGTGTCGTAACCTACTAG
TTCTACGGTGTTGTCATTCATCGTAATTATCTTGAAGTCGTGTGGGTCGTCGAACCTCACACCGCCAT
+
FFFFF,FFFFFFFFFFFFFFFFFFFFFFFFFFFFFFFFFFFFFFFFFFFFFFFFFFF,FFFFFFFFFF
FFFFFFFFFFFFFFFFFFFFFFFFFFFFFFFFFFFFFFFFFFFFFFFFFFFFFFFFFFFFFFFFFFFF
@A00155:342:HHGFNDSXY:1:1363:8467:16877 2:N:0:GAACCTAG+TCCGCATA
ATCGGTACCCTGACCATCTGCTTTGAAGTGGACGTTATTTCGACCACTTCAGTGTCGTAACCTACTAG
TTCTACGGTGTTGTCATTCATCGTAATTATCTTGAAGTCGTGTGGGTCGTCGAACCTCACACCGCCAT
+
FFFFFFFFFFFFFFFFFFF:F:FFFFFFFFFFFFFFFFFFFFFFFFFFFFFFFFFFFFFFFFFFFFFF
FFFFFFFFFFFFFFFFFFFFFFFFFFFFFFFFFFFFFFFFFFFFFFFFFFFFFFFFFFFFFFFFFFFF
@A00155:342:HHGFNDSXY:1:1332:17598:13369 2:N:0:GAACCTAG+TCCGCATA
ATCGGTACCCTGACCATCTGCTTTGAAGTGGACGTTATTTCGACCACTTCAGTGTCGTAACCTACTAG
TTCTACGGTGTTGTCATTCATCGTAATTATCTTGAAGTCGTGTGGGTCGTCGAACCTCACACCGCCAT
+
FFFFFFFFFFFFFFFFF,FFFFFFFF:FFFFFFFFFFFFFFFFFF:FFFFFFFFFFFFFFFFFFFFFF
FFFFFFFFFFFFFFFFFFFFFFFFFFFF:FFFFF:FFFFFFFFFFF:FFFFFFFFFFFFFFFFFFFFF
@A00155:342:HHGFNDSXY:1:1259:4309:25081 1:N:0:GAACCTAG+TCCGCATA
ATCGGTACCCTGACCATCTGCTTTGAAGTGGACGTTATTTCGACCACTTCAGTGTCGTAACCTACTAG
TTCTACGGTGTTGTCATTCATCGTAATTATCTTGAAGTCGTGTGGGTCGTCGAACCTCACACCGCCAT
+
FFFFFFFFFFFFFFFFFFFFFFFFFFFFFFFFFFFFFFFFFFFFFFFFFFFFFFFFFFFFFFFFFFFF
FFFFFFFFFFFFFFFFFFFFFFFFFFFFFFF,FF,:FFFFFFFFFFFFFFFFFF:FFFFFFFFFFFFF
@A00155:342:HHGFNDSXY:1:2121:31620:28260 1:N:0:GAACCTAG+TCCGCATA
ATCGGTACCCTGACCATCTGCTTTGAAGTGGACGTTATTTCGACCACTTCAGTGTCGTAACCTACTAG
TTCTACGGTGTTGTCATTCATCGTAATTATCTTGAAGTCGTGTGGGTCGTCGAACCTCACACCGCCAT
+
FFFFFFFFFFFFFFFFFFFFF:FFFFFFFFFFFFFFFFFFFFFFFFFFFFFFFFFFFFFFFFFFFFFF
FFFFFFFFFFFFFFFFFFFFFFFFF:FFFFFFFFFFFFFFFFFFFFFFFFFFFFFFFFFFFFFFFF:F
@A00155:342:HHGFNDSXY:1:1259:5041:14325 1:N:0:GAACCTAG+TCCGCATA
ATCGGTACCCTGACCATCTGCTTTGAAGTGGACGTTATTTCGACCACTTCAGTGTCGTAACCTACTAG
TTCTACGGTGTTGTCATTCATCGTAATTATCTTGAAGTCGTGTGGGTCGTCGAACCTCACACCGCCAT
+
FF,FFFFFFFFFFF:FFFFF::FFFFFFFFF:FFFFFFFF:FF:FFFFFFFFFFFFFFFFFFFFFFFF
FF,FFFFFFFFFF,FFFFFFFFFFFFFFFFFFFFFFFF:FFFFF::F,FFFFFFFFFFFFFFFFFFFF
```

```
@A00155:342:HHGFNDSXY:1:2607:24686:2754 1:N:0:GAACCTAG+TCCGCATA
ATCGGTACCCTGACCATCTGCTTTGAAGTGGACGTTATTTCGACCACTTCAGTGTCGTAACCTACTAG
TTCTACGGTGTTGTCATTCATCGTAATTATCTTGAAGTCGTGTGGGTCGTCGAACCTCACACCGCCAT
+
FFFFFFFFFFFFFFFF:FFFFFFFFFFFFFFFFFFFFFFFFFFFFFFFFFFFFFFFFFFFFFFFFFFF
FFFFFFFFFFFFFFFFFFFFFFFFFFFFFFFFFFFFFFFFFFFFFFFFFFFFFFFFFFFFFFFFFFFF
@A00155:342:HHGFNDSXY:1:1332:18213:13526 2:N:0:GAACCTAG+TCCGCATA
ATCGGTACCCTGACCATCTGCTTTGAAGTGGACGTTATTTCGACCACTTCAGTGTCGTAACCTACTAG
TTCTACGGTGTTGTCATTCATCGTAATTATCTTGAAGTCGTGTGGGTCGTCGAACCTCACACCGCCAT
+
FFFFFFFFFFFFFFFFFFFFFFFFFFFFFFFFFFFFFFFFFFFFFFFFFFFFFFFFFFFFFF:FFF:F
FFFFFFFFFFFFFFFF:FFFFFFF:FFFFFFFFFFFFFFFFFFFFFFFFFFFFFFFFFFFFFFFFFFF
@A00155:342:HHGFNDSXY:1:1556:3929:36730 1:N:0:GAACCTAG+TCCGCATA
TCGGTACCCTGACCATCTGCTTTGAAGTGGACGTTATTTCGACCACTTCAGTGTCGTAACCTACTAGT
TCTACGGTGTTGTCATTCATCGTAATTATCTTGAAGTCGTGTGGGTCGTCGAACCTCACACCGCCATC
+
FFFFFFFFFFFFFFFFFFFFFFFFFFFFFFFFFFFFFFFFFFFFFFFFFFFFFFFFFFFFFFFFFFFF
FFFFFFFFFFFFFFFFFFFFFFFFFFFFFFFFFFFFF:FFFFFFFFFFFFFFFFFFFFFFFFFFFFFF
@A00155:342:HHGFNDSXY:1:1245:11650:5415 1:N:0:GAACCTAG+TCCGCATA
CGGTACCCTGACCATCTGCTTTGAAGTGGACGTTATTTCGACCACTTCAGTGTCGTAACCTACTAGTT
CTACGGTGTTGTCATTCATCGTAATTATCTTGAAGTCGTGTGGGTCGTCGAACCTCACACCGCC
+
FFFFFFFFFFFFFFFFFFFFFFFFFFFFFFFFFFFFFFFFFFFFFFFFFFFFFFFFFFFFFFFFFFFF
,FFFFFFFFFFFFFFFFFFFFFFFFFFFFFFFFFFFFFF:FFFFFFFFFFFFFFFFFFFFFFFF
@A00155:342:HHGFNDSXY:1:2334:21350:14794 2:N:0:GAACCTAG+TCCGCATA
CGGTACCCTGACCATCTGCTTTGAAGTGGACGTTATTTCGACCACTTCAGTGTCGTAACCTACTAGTT
CTACGGTGTTGTCATTCATCGTAATTATCTTGAAGTCGTGTGGGTCGTCGAACCTCACACCGCC
+
FFFFFFFFFFFFFFFFFFFF:FFFFFFFFFFFFFFFFFFFFFFFFFFFFFFFFFFFFFFFFFFFFFFF
FFFFFFFFFFFFFFFFFFFFFFFFFFFFFFFFFFFFFFFFFFFFFFFFFFFFFFFFFFFFFFFF
@A00155:342:HHGFNDSXY:1:2412:6027:30185 1:N:0:GAACCTAG+TCCGCATA
CGGTACCCTGACCATCTGCTTTGAAGTGGACGTTATTTCGACCACTTCAGTGTCGTAACCTACTAGTT
CTACGGTGTTGTCATTCATCGTAATTATCTTGAAGTCGTGTGGGTCGTCGAACCTCACACCGCCATCA
+
FFFFFFFFFFFFFFF:FFFF:FFFFFFFFFFFFFFF:FFFFFFFFFFFFF:FFFFFFFFF:FFFFFFF
FFFF:FFFFFFFFFFFFFFFFFFFFFFFFFFFFFFF:FFFFFFFFF,,FFFFFFFFFFFFFFFFFFFF
@A00155:342:HHGFNDSXY:1:2333:16080:14998 1:N:0:GAACCTAG+TCCGCATA
CGGTAACCTGACCATCTGCTTTGAAGTGGACGTTATTTCGACCACTTCAGTGTCGTAACCTACTAGTT
CTACGGTGTTGTCATTCATCGTAATTATCTTGAAGTCGTGTGGGTCGTCGAACCTCACACCGCCATCA
+
FFFFFFFFFFFFFFFFFFFFFFFFFFFFFFFFFFFFFFFFFFFFFFFFFFFFFFFFFFFFFFFFFFFF
FFFFFFFFFFFFFFFFFFFF:FFFFFFFFFFFFFFFFFFFFFFFFFFFF:FFFFFFFFFFFFFFFFFF
@A00155:342:HHGFNDSXY:1:1259:5041:14325 2:N:0:GAACCTAG+TCCGCATA
GGTACCCTGACCATCTGCTTTGAAGTGGACGTTATTTCGACCACTTCAGTGTCGTAACCTACTAGTTC
TACGGTGTTGTCATTCATCGTAATTATCTTGAAGTCGGGTGGGTCGTCGAACCTCACACCGCCATCAA
+
:FFFFFFFFFF:FFFFFFFFFFFFFFFFFFFFFFFFFFFFFFFFFFFFFFFFFFF:FFFFFFFFFFFF
FFFFFFF:FFFFFFFF:FFFFFFFFFFFFFFFF:FFF,FFFFFFFFFFFFFFFFFFFFFFFFFFFFF:
@A00155:342:HHGFNDSXY:1:1259:4309:25081 2:N:0:GAACCTAG+TCCGCATA
GGTACCCTGACCATCTGCTTTGAAGTGGACGTTATTTCGACCACTTCAGTGTCGTAACCTACTAGTTC
TACGGTGTTGTCATTCATCGTAATTATCTTGAAGTCGTGTGGGTCGTCGAACCTCACACCGCCATCAA
+
:FFFFFFFFFFFFFFFFFFFFFFFFFFFFFFFFFFFFFFFFFFFFFFFFFFFFFFFFFFFFFFFFFFF
FFFFFFFFFFFFFFFFFFFFFFFFFFFFFFFFFFFFF:FFFFFFFFFFFFFFFFFFFFFF:FFFFFFF
```

@A00155:342:HHGFNDSXY:1:1376:23339:9189 2:N:0:GAACCTAG+TCCGCATA
CCTGACCATCTGCTTTGAAGTGGACGTTATTTCGACCACTTCAGTGTCGTAACCTACTAGTTCTACGG
TGTTGTCATTCATCGTAATTATCTTGAAGTCGTGTGGGTCGTCGAACCTCACACCGCCATCAAGATAT
+
FFFF:FFFFFFFFFFFFFFFFFFFFFFFFFFFFFFFFFFFFFFFFFFFFFFFFF:,FFFF:FFF:FFF
FFFFFFFFFFFFFFFFFFFFFFFFFFFFFFFFFFFFFFFFFFFF,FFFFFFFFFFFFFFFFFFFFFFF
@A00155:342:HHGFNDSXY:1:2546:17815:21167 2:N:0:GAACCTAG+TCCGCATA
CCTGACCATCTGCTTTGAAGTGGACGTTATTTCGACCACTTCAGTGTCGTAACCTACTAGTTCTACGG
TGTTGTCATTCATCGTAATTATCTTGAAGTCGTGTGGGTCGTCGAACCTCACACCGCCATCAAGATAT
+
FFFFFFFFFFFFFFFFFFFFFFFFFFFFFFFFFFFFFFFFFFFFFFFFFFFFFFF,FF:FFFFFFFFF
FFFFFFFFFFFFFFFFFFFFFFFFFFFFFFFFFFFFFFFFFFFFFFFFFFFFFFFFFF:FFFFFFFFF
@A00155:342:HHGFNDSXY:1:2334:5791:32910 1:N:0:GAACCTAG+TCCGCATA
CCTGACCATCTGCTTTGAAGTGGACGTTATTTCGACCACTTCAGTGTCGTAACCTACTAGTTCTACGG
TGTTGTCATTCATCGTAATTATCTTGAAGTCGTGTGGGTCGTCGAACCTCACACCGCCATCAAGATAT
+
FFFFFFFFFFFFFFFFFFFFFFFFFFFFFFFFFFFFFFFFFFFFFFFFFFFFFFFFFFFFFFFFFFFF
FFFFFFFFFFFFFFFFFFFFFFFFFFFFFFFFFFFFFFFFFFFF:FFFFFFFFFFFFFFFFFFFFFFF
@A00155:342:HHGFNDSXY:1:1571:13150:29935 1:N:0:GAACCTAG+TCCGCATA
CCTGACCATCTGCTTTGAAGTGGACGTTATTTCGACCACTTCAGTGTCGTAACCTACTAGTTCTACGG
TGTTGTCATTCATCGTAATTATCTTGAAGTCGTGTGGGTCGTCGAACCTCACACCGCCATCAAGATAT
+
FFFFFFFFFFFFFFFFFFFFFFFFFFFFFFFFFFFFFFFFFFFFFFFFFFFFFFFFFFFFFFFFFFFF
FFFFFFFFFFFFFFFFFFFFFFFFFF:FFFFFFFFFFFFFFFF:FFFFFFFFFFFFFFFFFFFFFFFF
@A00155:342:HHGFNDSXY:1:2274:14886:31782 2:N:0:GAACCTAG+TCCGCATA
CCTGACCATCTGCTTTGAAGTGGACGTTATTTCGACCACTTCAGTGTCGTAACCTACTAGTTCTACGG
TGTTGTCATTCATCGTAATTATCTTGAAGTCGTGTGGGTCGTCGAACCTCACACCGCCATCAAGATAT
+
FFFFFFF::FFFFFFFFFFFFFFFFFFF:F:FFFFFFFFFFFFFFFFFFFFFFFF:FFF,FFFFFFFF
FFFFFFFFFFFFFFFFFFFFFFFF:FFFFFFFFFFFFFFFFFFFFFFFFFFFFFFFFFFFFFFFFFF
@A00155:342:HHGFNDSXY:1:1621:18530:26788 1:N:0:GAACCTAG+TCCGCATA
CCTGACCATCTGCTTTGAAGTGGACGTTATTTCGACCACTTCAGTGTCGTAACCTACTAGTTCTACGG
TGTTGTCATTCATCGTAATTATCTTGAAGTCGTGTGGGTCGTCGAACCTCACACCGCCATCAAGATAT
+
FFFFFFFFFFFFFFFFFFFFFFFFFFFFFFFFFFFFF:FFFFFFFFFFFFFFFFFFFFFFFFFFFF:F
FFFFFFFFFFFFFFFFFFFFFFFFFFFFFFF:FFFFFF:FFFFFFFFFFFFFFFFFFFFFFFFFFFFF
@A00155:342:HHGFNDSXY:1:2119:22923:34428 2:N:0:GAACCTAG+TCCGCATA
CCTGACCATCTGCTTTGAAGTGGACGTTATTTCGACCACTTCAGTGTCGTAACCTACTAGTTCTACGG
TGTTGTCATTCATCGTAATTATCTTGAAGTCGTGTGGGTCGTCGAACCTCACACCGCCATCAAGATAT
+
FFFFFFFFFFFFFFFFFFFFFFFFFFFFFFFFFFFFFFFFFFFFFFFFFFFFFFFFFFFFFFFFFFFF
FFFFFFFFFFFFFFFFFFFFFFFFFFFFFFFFFFFFFFFFFFFFFFFFFFFFFFFFFFFFFFFFFFFF
@A00155:342:HHGFNDSXY:1:1122:16857:7169 2:N:0:GAACCTAG+TCCGCATA
CTGACCATCTGCTTTGAAGTGGACGTTATTTCGACCACTTCAGTGTCGTAACCTACTAGTTCTACGGT
GTTGTCATTCATCGTAATTATCTTGAAGTCGTGTGGGTCGTCGAACCTCACACCGCCATCAAGATATA
+
FFFFFFFFFFFFFFFFFFFFFFFFFFFFFFFFFFFFFFFFFFFFFFFFFFFFFFFFFFFFFFFFFFFF
FFFFFFFF,F:FFFFFFFFFFF:FFFFFFFFFFFFFFFFFFFFFFFFFFFFFFFFFFF:FFFFFFFFF
@A00155:342:HHGFNDSXY:1:1323:10348:11553 2:N:0:GAACCTAG+TCCGCATA
CTGACCATCTGCTTTGAAGTGGACGTTATTTCGACCACTTCAGTGTCGTAACCTACTAGTTCTACGGT
GTTGTCATTCATCGTAATTATCTTGAAGTCGTGTGGGTCGTCGAACCTCACACCGCCATCAAGATATA
+
FFF:FFFFFFFFFFFFFFFFFFFFFFFFFFFFFFFFFFF:FFFFFFFFFFFFFFFFFFFFFFFFFFFF
FFFF:FFFFFFFFFFF,FFF:FFFFFFFFFFFFFFFFFFFFFF:FFFFFFFFFFFFFFFFFFFFF:FF

```
@A00155:342:HHGFNDSXY:1:1323:10348:11553 1:N:0:GAACCTAG+TCCGCATA
TGACCATCTGCTTTGAAGTGGACGTTATTTCGACCACTTCAGTGTCGTAACCTACTAGTTCTACGGTG
TTGTCATTCATCGTAATTATCTTGAAGTCGTGTGGGTCGTCGAACCTCACACCGCCATCAAGATATAT
+
FFFFFFFFFFFFFFFFFFFFFFFFF:FFFFFFFFFFFFFFFFFFFFFFFFFFFFFF:FFFFFFFFFF
FFFFFFFFFFFFFFFFFFFFFFFFFFFFFFFFFFFFFFFFFFFFFFFFFFFFFFFFFFF:FFFFFFF
@A00155:342:HHGFNDSXY:1:2538:9652:13260 2:N:0:GAACCTAG+TCCGCATA
GACCATCTGCTTTGAAGTGGACGTTATTTCGACCACTTCAGTGTCGTAACCTACTAGTTCTACGGTGT
TGTCATTCATCGTAATTATCTTGAAGTCGTGTGGGTCGTCGAACCTCACACCGCCATCAAGATATATG
+
FFFFFFFFFFFFFFFFFFFFFFFFFFFFFFFFFFFFFFFFFFFFFFFFFFFFFFFFFFFFFFFFFFFF
FFFFFFFFFFFFFFFFFFFFFFFFFFFFFFFFFFFFFFFFFFFFFFFFFFFFFFFFFFFFFFFFFFFF
@A00155:342:HHGFNDSXY:1:2121:31620:28260 2:N:0:GAACCTAG+TCCGCATA
GACCATCTGCTTTGAAGTGGACGTTATTTCGACCACTTCAGTGTCGTAACCTACTAGTTCTACGGTGT
TGTCATTCATCGTAATTATCTTGAAGTCGTGTGGGTCGTCGAACCTCACACCGCCATCAAGATATATG
+
FFFFFFFFFFFFFFFFFFFFFFF:FF,FF:FFFFFFFFFFFFFFFFFFFFFFFFFFFFFFFFFFFFF:
FFFFFFFFFFFFFFFFFFFFFFFFFFFFFFFFFFFFFFFFFFFFFFFFFFFFFFFFFFFFFFFFFFFF
@A00155:342:HHGFNDSXY:1:1510:6027:21449 1:N:0:GAACCTAG+TCCGCATA
ACCATCTGCTTTGAAGTGGACGTTATTTCGACCACTTCAGTGTCGTAACCTACTAGTTCTACGGTGTT
GTCATTCATCGTAATTATCTTGAAGTCGTGTGGGTCGTCGAACCTCACACCGCCATCAAGATAT
+
FFFFFFFFFFFFFFFFF:FF,FFFFFFFF:FFFFFFFFFFFFFFFFFFFFFFFFFFFFFFFFFFFFFF
FFFFFFFFFFFFFFFFFFFFFF,FFFFFFFFFFF,FFFFFFFFFFFFFFFFFFFFFFFFFFFFF:F
@A00155:342:HHGFNDSXY:1:2620:2989:24737 1:N:0:GAACCTAG+TCCGCATA
ACCATCTGCTTTGAAGTGGACGTTATTTCGACCACTTCAGTGTCGTAACCTACTAGTTCTACGGTGTT
GTCATTCATCGTAATTATCTTGAAGTCGTGTGGGTCGTCGAACCTCACACCGCCATCAAGATAT
+
FFFFFFFFFFFFFFFFFFFFFFFFFFFFFFFFFFFFFFFFFFFFFFFFFFFFFFFFFFFFFFFFFFFF
FFFFFFFFFFFFFFFFFFFFFFFFFFFFFFFFFFFFFFFFFFFFFFFFFFFFFFFFFFFFFFFF
@A00155:342:HHGFNDSXY:1:1117:24722:25801 1:N:0:GAACCTAG+TCCGCATA
ACCATCTGCTTTGAAGTGGACGTTATTTCGACCACTTCAGTGTCGTAACCTACTAGTTCTACGGTGTT
GTCATTCATCGTAATTATCTTGAAGTCGTGTGGGTCGTCGAACCTCACACCGCCATCAAGATATATGC
+
FFFFFFFFFFFFFFFFFFFFFFFFFFFFFFFFFFFFFFFFFFFFFFFFFFFFFFFF:FFFFFFFFFF
FFFFFFFFFFFFFFFFFFFFFFFFFFFFFFFFF:FFFFFF,FFFFFFFFFFFFFF:FFFFFFFFFFF
@A00155:342:HHGFNDSXY:1:1240:14027:3771 2:N:0:GAACCTAG+TCCGCATA
CTGCTTTGAAGTGGACGTTATTTCGACCACTTCAGTGTCGTAACCTACTAGTTCTACGGTGTTGTCAT
TCATCGTAATTATCTTGAAGTCGTGTGGGTCGTCGAACCTCACACCGCCATCAAGATATATGCCTGAC
+
FFFFFFFFFFFFFFFFFFFFFFFFFFFFFFFFFFFFFFFFFFFFFFFFFFFFFFFFFFFFFFFFFFFF
FFFFFFFFFFFFFFFFFFFFFFFFFFFFFFFFFFFFFFFFFFFFFFFFFFFFFFFFFFFFFFFFFFFF
@A00155:342:HHGFNDSXY:1:1652:31006:31172 2:N:0:GAACCTAG+TCCGCATA
CTTTGAAGTGGACGTTATTTCGACCACTTCAGTGTCGTAACCTACTAGTTCTACGGTGTTGTCATTCA
TCGTAATTATCTTGAAGTCGTGTGGGTCGTCGAACCTCACACCGCCATCAAGATATATGCCTGACATC
+
FFFFFFFFFFFFFFFFFFFF:F:FFFFFFFFFFFFFFFFFFFFFFFFFFFFFFFFFFFFFFFF:FFF
F:FFFFFFFFFFFFFFFFFFFFFFFFFFF:FFFFFFFFFF,FFFFFFFFFFFFFFFFFFFFFFFFFFF
@A00155:342:HHGFNDSXY:1:1510:6027:21449 2:N:0:GAACCTAG+TCCGCATA
CTTTGAAGTGGACGTTATTTCGACCACTTCAGTGTCGTAACCTACTAGTTCTACGGTGTTGTCATTCA
TCGTAATTATCTTGAAGTCGTGTGGGTCGTCGAACCTCACACCGCCATCAAGATATATGCCTGACATC
+
:FFFFFFFF,FFF,FFFFFFFFFFFFFFFFFFF:FFFFFFFFFF:FFFFFFFFFFFFFFFFFFFFFFF
FFFFFFFFFFFFFFFFFFFFFFFFFFFFFFFFFFFFFFFFFFFFFFFFFFFFFFFFFFFFFFFFFFFF
```

```
@A00155:342:HHGFNDSXY:1:1644:20943:8735 1:N:0:GAACCTAG+TCCGCATA
TTGAAGTGGACGTTATTTCGACCACTTCAGTGTCGTAACCTACTAGTTCTACGGTGTTGTCATTCATC
GTAATTATCTTGAAGTCGTGTGGGTCGTCGAACCTCACACCGCCATCAAGATATATGCCTGACATCCC
+
FFFFFFFFFFFFFFFFFFFFFFFFF:FFFFFFFFFFFFFFFFFFFFFFFFFFFFFFFFFFFFFFFFFF
FFFFFFFFFFFFFFFFFFFFFFFFFFFFFFFFFFFFFFFFFFFFFFFFFFFFFFFFFFFFFFFFFFFF
@A00155:342:HHGFNDSXY:1:2546:17815:21167 1:N:0:GAACCTAG+TCCGCATA
TGAAGTGGACGTTATTTCGACCACTTCAGTGTCGTAACCTACTAGTTCTACGGTGTTGTCATTCATCG
TAATTATCTTGAAGTCGTGTGGGTCGTCGAACCTCACACCGCCATCAAGATATATGCCTGACATCCCC
+
FFFFFFFFFFFFFFFFFFFFFFFFFFFFFFFFFFFFFFFFFFFFFFFFFFFFFFFFFFFFFFFFFFFF
FFFFFFFFFFFFFFFFFFFFFFFFFFFFFFFFFFFFFFFFFFFFFFFFFFFFFFFFFFFFFFFFFFFF
@A00155:342:HHGFNDSXY:1:2119:22923:34428 1:N:0:GAACCTAG+TCCGCATA
TGAAGTGGACGTTATTTCGACCACTTCAGTGTCGTAACCTACTAGTTCTACGGTGTTGTCATTCATCG
TAATTATCTTGAAGTCGTGTGGGTCGTCGAACCTCACACCGCCATCAAGATATATGCCTGACATCCCC
+
FFFFFFFFFFFFFFFFFFFFFFFFFFFFFFFFFFFFFFFFFFFFFFFFFFFFFFFFFFFFFFFFFFFF
FFFFFFFFFFFFFFFFFFFFFFFFFFFFFFFFFFFFFFFFFFFFFFFFFFFFFFFFFFFFFFFFFFFF
@A00155:342:HHGFNDSXY:1:1249:31837:4116 1:N:0:GAACCTAG+TCCGCATA
TGAAGTGGACGTTATTTCGACCACTTCAGTGTCGTAACCTACTAGTTCTACGGTGTTGTCATTCATCG
TAATTATCTTGAAGTCGTGTGGGTCGTCGAACCTCACACCGCCATCAAGATATATGCCTGACATCCCC
+
FFF:FFFFFFFFFFFFFFFFFFFFFFFFFFF,FFFFFFFFFFFFF:FFFFFFFFFFFFFFFFFFFFFF
FFFFF:FFFFFFFFF:FFFFFFFFFFFFFFFFFFFFFFF:FFFFFFFFFFFFFFFF:FFFFFFF:FFF
@A00155:342:HHGFNDSXY:1:1246:21468:30029 1:N:0:GAACCTAG+TCCGCATA
TGAAGTGGACGTTATTTCGACCACTTCAGTGTCGTAACCTACTAGTTCTACGGTGTTGTCATTCATCG
TAATTATCTTGAAGTCGTGTGGGTCGTCGAACCTCACACCGCCATCAAGATATATGCCTGACATCCCC
+
FFFFFFFFFFFFFFFFFFFFFFFFFFFFFFFFFFFFFFFFFFFFFFFFFFFFFFFFFFFFFFFFFFFF
FFFFFFFFFFFFFFFFFFFFFFFFFFFFFFFFFFFFFFFFFFFFFFFFFFFFF:FFFFFFFFFF:FFF
@A00155:342:HHGFNDSXY:1:1420:7690:10802 1:N:0:GAACCTAG+TCCGCATA
TGAAGTGGACGTTATTTCGACCACTTCAGTGTCGTAACCTACTAGTTCTACGGTGTTGTCATTCATCG
TAATTATCTTGAAGTCGTGTGGGTCGTCGAACCTCACACCGCCATCAAGATATATGCCTGACATCCCC
+
:FFFFFFFFFFFFFFFFFFFFFFFFFFFFFFFFFFFFFFFFFFFFFFFFFFFFFFFFFFFFFFFFFFF
FFFFFFFFFFFFFFFFFFFFFFFFFFFFFFFFFFFFFFF:FFFFF,FF,FFFFFFFFFFFFFFFFFFF
@A00155:342:HHGFNDSXY:1:1420:8070:13275 1:N:0:GAACCTAG+TCCGCATA
TGAAGTGGACGTTATTTCGACCACTTCAGTGTCGTAACCTACTAGTTCTACGGTGTTGTCATTCATCG
TAATTATCTTGAAGTCGTGTGGGTCGTCGAACCTCACACCGCCATCAAGATATATGCCTGACATCCCC
+
FFFFFFFFFFFFFFFFF:FFFFFFFFFFFF:FFFFFFFFFFFFFFFFFFFFFFFFFFFFFFFFFFFFF
FF:FFFFFFFFFFFFFFFFFFFFFFFFFFFFFFFFFFFFFFFFFFFFFFFFFFFFFFFFFFFFFFFFF
@A00155:342:HHGFNDSXY:1:2516:3224:18724 2:N:0:GAACCTAG+TCCGCATA
GAAGTGGACGTTATTTCGACCACTTCAGTGTCGTAACCTACTAGTTCTACGGTGTTGTCATTCATCGT
AATTATCTTGAAGTCGTGTGGGTCGTCGAACCTCACACCGCCATCAAGATATATGCCTGACATCCCCG
+
FFFFFFFFFFF:FFFF:FFFFFFFFFFFFFF,FFFFFFFFFFFFFFFFFFFFFFFFFFFFFFFFFFFF
FFFFFFFFFFFFFFFFFFFFFFFFFFFFFFFFFFFFFFFFFFFFFFFFFFFFFFFFFFFFFFFFFFFF
@A00155:342:HHGFNDSXY:1:2412:6027:30185 2:N:0:GAACCTAG+TCCGCATA
GAAGTGGACGTTATTTCGACCACTTCAGTGTCGTAACCTACTAGTTCTACGGTGTTGTCATTCATCGT
AATTATCTTGAAGTCGTGTGGGTCGTCGAACCTCACACCGCCATCAAGATATATGCCTGACATCCCCG
+
FFFFFFFFFFFFFFFFFFFFFFFFFFFFFFFFFFFF,FF:,FFF,FFFFFFFF:FFFFFFFFFF:FFF
FFFFFFFFFFFFF:FFFFF:FFFFFFFFFFFFFFFF,FFFFFFFFFFFFFFFFFFFFF:FFFFFFFFF
```

```
@A00155:342:HHGFNDSXY:1:2311:5529:8375 1:N:0:GAACCTAG+TCCGCATA
AAGTGGACGTTATTTCGACCACTTCAGTGTCGTAACCTACTAGTTCTACGGTGTTGTCATTCATCGTA
ATTATCTTGAAGTCGTGTGGGTCGTCGAACCTCACACCGCCATCAAGATATATGCCTGACAACCCCG
+
FFFFF:FFF,:FFFFFFFFFFFFFFFF:FF:FFFFFFFFFFFFFFFFFF:F:FFFFFFFFFFF:F,:F
FFFFFFFFFFFFFFFFFFFF,F:FFFFFFFFF,FFFFF,FFFFFFFFFFFFF:FF,:FFFF,FFFFF
@A00155:342:HHGFNDSXY:1:1373:21269:28995 2:N:0:GAACCTAG+TCCGCATA
AAGTGGACGTTATTTCGACCACTTCAGTGTCGTAACCTACTAGTTCTACGGTGTTGTCATTCATCGTA
ATTATCTTGAAGTCGTGTGGGTCGTCGAACCTCACACCGCCATCAAGATATATGCCTGACATCCCCGG
+
FFF:FFFFFFFF:FF:FFFFFFFFFFFFFFFFFFFFFFFFFFF:FFFFFFFFFFFFFFFFFFFFFFFF
FF:FFFFFFFFFFFFFFFFFFFFFFFFFFFFFFFFFFFFFFFFFFFFFFFFFFFFFFFFFFFFFFFFF
@A00155:342:HHGFNDSXY:1:2516:3224:18724 1:N:0:GAACCTAG+TCCGCATA
AAGTGGACGTTATTTCGACCACTTCAGTGTCGTAACCTACTAGTTCTACGGTGTTGTCATTCATCGTA
ATTATCTTGAAGTCGTGTGGGTCGTCGAACCTCACACCGCCATCAAGATATATGCCTGACATCCCCGG
+
FFFFFFFFFFFFFFFFFFFFFFFFFFFFFFFFFFFFFFFFFFFFFFFFFFFFFFFFFFFFFFFFFFFF
FFFFFFFFFFF:FFFFFFFFFFFFFFFFFFFFFFFFFFFFFFFFFFFFFFFFF:FFFFFFFFFF:F::
@A00155:342:HHGFNDSXY:1:1154:7039:26490 1:N:0:GAACCTAG+TCCGCATA
AGTGGACGTTATTTCGACCACTTCAGTGTCGTAACCTACTAGTTCTACGGTGTTGTCATTCATCGTAA
TTATCTTGAAGTCGTGTGGGTCGTCGAACCTCACACCGCCATCAAGATATATGCCTGACATCCCCGGC
+
FFFFFFFFFFFFFFFFFFFFFFFFFFFFFFFFFFFFFFFFFFFFFFFFFFFFFFFFFFFFFFFFFFFF
FFFFFFFFFFFFFFFFFFFFFFFFFFFFFFFFFFFFFFFFFFFFFFFFF:FFF:FFFFFFFFFFFFFF
@A00155:342:HHGFNDSXY:1:2575:23683:23563 1:N:0:GAACCTAG+TCCGCATA
AGTGGACGTTATTTCGACCACTTCAGTGTCGTAACCTACTAGTTCTACGGTGTTGTCATTCATCGTAA
TTATCTTGAAGTCGTGTGGGTCGTCGAACCTCACACCGCCATCAAGATATATGCCTGACATCCCCGGC
+
FFFFFFFFFFFFFFFFFFFFFFFFFFFFFFFFFFFFFFFFFFFFFFFFFFFFFFFFFFFFFFFFFFFF
FFFFFFFFFFFFFFFFFFFFFFFFFFFFFFFFFFFFFFFFFFFFFFFFFFFFFFFFFFFFFF:FFFFF
@A00155:342:HHGFNDSXY:1:2422:25292:19366 2:N:0:GAACCTAG+TCCGCATA
GTGGACGTTATTTCGACCACTTCAGTGTCGTAACCTACTAGTTCTACGGTGTTGTCATTCATCGTAAT
TATCTTGAAGTCGTGTGGGTCGTCGAACCTCACACCGCCATCAAGATATATGCCTGACATCCCCGGCA
+
FFFFFFFFFFFFFFFFFFFFFFFFFFFFFFFFFFFFFFF,FFFFFFFFFFFFFFFFFFFFFFFFFFFF
F::FFFFFFFFFFFFFFFFFFFFFFFFFFFFFFFFFFFFFFFFFFFFFFFFFFF:FFFFFFFFFFFFF
@A00155:342:HHGFNDSXY:1:1562:3242:7232 2:N:0:GAACCTAG+TCCGCATA
GACGTTATTTCGACCACTTCAGTGTCGTAACCTACTAGTTCTACGGTGTTGTCATTCATCGTAATTAT
CTTGAAGTCGTGTGGGTCGTCGAACCTCACACCGCCATCAAGATATATGCCTGACATCCCCGGC
+
FF:FFFFFFFFFFFFFFFFFFFFFFFFFFFFFFFFFFFFFFFFFFFFFFFFFFFFFFFFFFFFFFFFF
FFFFFFFFFFFFFFFFFFFFFFFFFFFFFFFFFFFFFFFFFFFFFFFFFFFFFFFFFFFFFFFF
@A00155:342:HHGFNDSXY:1:2620:2989:24737 2:N:0:GAACCTAG+TCCGCATA
GTTATTTCGACCACTTCAGTGTCGTAACCTACTAGTTCTACGGTGTTGTCATTCATCGTAATTATCTT
GAAGTCGTGTGGGTCGTCGAACCTCACACCGCCATCAAGATATATGCCTGACATCCCCGGCATCGGG
+
FFFFFFFFFFFFF:FFFFFFFF:FFFFFFFFFFFFFFFFFFFFFFFFFFFFFFFFFFFFFFFFFFFFF
FFFFFFF:FFFFFFFFFFFFFFFFFFFFFFFFFFFFFFFFFFFFFFFFFFFFFFFFFFFFFFFFFFF
@A00155:342:HHGFNDSXY:1:1422:10465:15233 1:N:0:GAACCTAG+TCCGCATA
TTATTTCGACCACTTCAGTGTCGTAACCTACTAGTTCTACGGTGTTGTCATTCATCGTAATTATCTTG
AAGTCGTGTGGGTCGTCGAACCTCACACCGCCATCAAGATATATGCCTGACATCCCCGGCATCG
+
FFFFFFFFFFFFFFFFFFFFFFFFFFFFFFFFFFFFFFFFFFFFFFFFFFFFFFFFFFFFFFFFFFFF
FFFFFFFFFFFFFFFFFFFFFFFFFFFFFFFFFFFFFFFFFFFFFFFFFFFFFFFFFFFFFFFF
```

```
@A00155:342:HHGFNDSXY:1:1560:14624:33896 2:N:0:GAACCTAG+TCCGCATA
TATTTCGACCACTTCAGTGTCGTAACCTACTAGTTCTACGGTGTTGTCATTCATCGTAATTATCTTGA
AGTCGTGTGGGTCGTCGAACCTCACACCGCCATCAAGATATATGCCTGACATCCCCGGCATCGGGAAC
+
FF:::FFFFFF:,:FFFFFFFFFFFF:FFF:F:FFFFFFFFFF:FFF:FFFFFFFFFFFFFF:FFFFF
FFFFFFFF:FFF:FFFFFFFFFFFFFFFFFFFFFFFFFF:FFF:FFFFFFFFFFFFFFFF:FFFFFFF
@A00155:342:HHGFNDSXY:1:1420:7690:10802 2:N:0:GAACCTAG+TCCGCATA
TATTTCGACCACTTCAGTGTCGTAACCTACTAGTTCTACGGTGTTGTCATTCATCGTAATTATCTTGA
AGTCGTGTGGGTCGTCGAACCTCACACCGCCATCAAGATATATGCCTGACATCCCCGGCATCGGGAAC
+
FFFFFFFFFFFFFFFFFFFFFFFFFFFFFFFFFFFFFFFFFFFFFFFFFFFFFFFFFFFFFFFFFFFF
FFFFFFFFFFFFFFFFFFFFFFFFFFFFFFFFFFFFFFFFFFFFFFFFFFFFFFFFFFFFFFFFFFFF
@A00155:342:HHGFNDSXY:1:1420:8070:13275 2:N:0:GAACCTAG+TCCGCATA
TATTTCGACCACTTCAGTGTCGTAACCTACTAGTTCTACGGTGTTGTCATTCATCGTAATTATCTTGA
AGTCGTGTGGGTCGTCGAACCTCACACCGCCATCAAGATATATGCCTGACATCCCCGGCATCGGGAAC
+
FFFFFFFFFFFFFFFFFFFFFFFFFFF:FFFFFFFFFFFFFFFFFFFFFFFFFFFFFFFFFF:FFFFF
FFFFFFFFFFFFFFFFFFFFFFFFFFFFFFFFFFFFFFFFFFFFFFFFFFFFFFFFFFFF:FFFFFFF
@A00155:342:HHGFNDSXY:1:1556:3929:36730 2:N:0:GAACCTAG+TCCGCATA
CGACCACTTCAGTGTCGTAACCTACTAGTTCTACGGTGTTGTCATTCATCGTAATTATCTTGAAGTCG
TGTGGGTCGTCGAACCTCACACCGCCATCAAGATATATGCCTGACATCCCCGGCATCGGGAACTCAGC
+
FFFFFFFFFF:F:FFFFFFFFFFFFFFFFFFFFFFFFFFFFFFFFFFFFFFFFFFFFFFFFFFFFFF
FFFFFFFFFFFFFFFFFF:FFFFFFFFFFFFFFFFFFFFFFFFFFF:FFFFFFFFFFFFFFFFFFFFF
@A00155:342:HHGFNDSXY:1:2153:20247:32393 2:N:0:GAACCTAG+TCCGCATA
CCACTTCAGTGTCGTAACCTACTAGTTCTACGGTGTTGTCATTCATCGTAATTATCTTGAAGTCGTGT
GGGTCGTCGAACCTCACACCGCCATCAAGATATATGCCTGACATCCCCGGCATCGGGAACTCAGCCCT
+
:FFFFFF:FFFFFFFFFFFFFFFFFFFFFFFFFFFFFFFFFFFFFFF:FFF:FFFFFFFFFFFFFFFF
FFFFFFFFFFFFFFFFFFFFFFFFFFFF:FFFFFF:FFFFFFFFFFFFFFFFFFFFFFFFFFFFFFFF
@A00155:342:HHGFNDSXY:1:2644:32551:21856 2:N:0:GAACCTAG+TCCGCATA
CCACTTCAGTGTCGTAACCTACTAGTTCTACGGTGTTGTCATTCATCGTAATTATCTTGAAGTCGTGT
GGGTCGTCGAACCTCACACCGCCATCAAGATATATGCCTGACATCCCCGGCATCGGGAACTCAGCCCT
+
FFFFF:FFFFFFF::FFFF:FF:FFFFFFFFF:FF:FFFFF:FFFFF:::FFFFFFFFFFFFFFFFFF
F:FFFFFFFFFFFFFFFFFFFFFFFFFF:FFFFFF:FFFFFFFFFFFFFFFFFFFFFFFFFFFFFFFF
@A00155:342:HHGFNDSXY:1:1562:3242:7232 1:N:0:GAACCTAG+TCCGCATA
ACTTCAGTGTCGTAACCTACTAGTTCTACGGTGTTGTCATTCATCGTAATTATCTTGAAGTCGTGTGG
GTCGTCGAACCTCACACCGCCATCAAGATATATGCCTGACATCCCCGGCATCGGGAACTCAGCC
+
FFFFFFFFFFFFFFFFFFFFFF:FFFFFFFFFFFFFFFFFFFFFFFFFFFFFFFFFFFFFFFFFFFFF
FFFFFFFFFFFFFFFFFFFFFFFFFFFFFFFFFFFFFFFFFFFFFFFFFFFFFFFF:FFFFFFF
@A00155:342:HHGFNDSXY:1:1622:21034:32127 2:N:0:GAACCTAG+TCCGCATA
TTCAGTGTCGTAACCTACTAGTTCTACGGTGTTGTCATTCATCGTAATTATCTTGAAGTCGTGTGGGT
CGTCGAACCTCACACCGCCATCAAGATATATGCCTGACATCCCCGGCATCGGGAACTCAGCCCTAATC
+
FFFFFFFFFFFFFFFFFFFFFFFFFFFFFFFF:FFFFFFFFFFFFFFFFFFFFFFFFFFFFFFFFFFF
FFFFFFFFFFFFF:FFFFFFFFFFFFFFFFFFFFFFFFFFFFFFFFFFFFFFFFFFFFFFFFFFFFFF
@A00155:342:HHGFNDSXY:1:1642:1886:11428 2:N:0:GAACCTAG+TCCGCATA
TTCAGTGTCGTAACCTACTAGTTCTACGGTGTTGTCATTCATCGTAATTATCTTGAAGTCGTGTGGGT
CGTCGAACCTCACACCGCCATCAAGATATATGCCTGACATCCCCGGCATCGGGAACTCAGCCCTAATC
+
FFFFFFFFFFFFFF:FFFFFFF,FFFFFFFFFFFFFFF:FFF:FFFFFFF:FFFFFFFFFFFFFFFFF
:FFFFFFFFFF:FFFFFFFFFFFFFFF::FFFFFFFFFFFFFFFFFFFFFFFFFFFFFFFFFFFF:FF
```

```
@A00155:342:HHGFNDSXY:1:1642:1588:7686 2:N:0:GAACCTAG+TCCGCATA
TTCAGTGTCGTAACCTACTAGTTCTACGGTGTTGTCATTCATCGTAATTATCTTGAAGTCGTGTGGGT
CGTCGAACCTCACACCGCCATCAAGATATATGCCTGACATCCCCGGCATCGGGAACTCAGCCCTAATC
+
F:FFFFFFFFF:FFFFFFFFFFFFFFFFFFFF,F::FF,FF:FFFFFFFF,FFFFFFFFFFFFFFFF:
FFFFFFFFFFFFFFFFFFFFFFFFFFFFF:FFFFFFFFFFFFFFFFFFFFFFFFF:FFFFFFFFFFFF:F
@A00155:342:HHGFNDSXY:1:2632:7672:32910 2:N:0:GAACCTAG+TCCGCATA
AGTGTCGTAACCTACTAGGTCTACGGTGTTGTCATTCATCGTAATTATCTTGAAGTCGTGTGGGTCGT
CGAACCTCACACCGCCATCAAGATATATGCCTGACATCCCCGGCATCGGGAACTCAGCCCTAATCGCT
+
:FFFFFF::F,:FFFF,:,F,FFFFF:FFFFFFFFF,FF:FF:FFFFFFFFFFFFF,FFFFFFF:FFF
FFF:FFFFFFFFFFF:FF:F:FF:FFFFFFFFFFFFF,FFFFF:FFF,FFFFFFF:FFFFFFFF,F:F
@A00155:342:HHGFNDSXY:1:1507:30409:34679 2:N:0:GAACCTAG+TCCGCATA
GTCGTAACCTACTAGTTCTACGGTGTTGTCATTCATCGTAATTATCTTGAAGTCGTGTGGGTCGTCGA
ACCTCACACCGCCATCAAGATATATGCCTGACATCCCCGGCATCGGGAACTCAGCCCTAATCGC
+
FFFFFFFFFFFFFFFFFFFFFFFFFFFFFFFF:FFFFFFFFFFFFFFFFFFFFFFFFFFFFFFFFFFF
FFFFFFFFFFFFFFFFFFFFF:FFFFFFFFFFFFFFFFFFFFFFFFFFFFFFFFFFFFFFFFFFF
@A00155:342:HHGFNDSXY:1:2642:14018:7420 2:N:0:GAACCTAG+TCCGCATA
GTCGTAACCTACTAGTTCTACGGTGTTGTCATTCATCGTAATTATCTTGAAGTCGTGTGGGTCGTCGA
ACCTCACACCGCCATCAAGATATATGCCTGACATCCCCGGCATCGGGAACTCAGCCCTAATCGCT
+
FFFFFFFFFF,F:FFFFFFFFFFFFFFFFFFFFFFFFFFFFFFFFF:FFFFFFFFFFFFFFFFFFFFFFF
FFFFFFF:FFFFFFFFFFFFFFFFFFFFFFFFFFF:FFFFFFFFFFFFF:FFFFFFFFFFFFFFFF:
@A00155:342:HHGFNDSXY:1:1602:23411:32456 2:N:0:GAACCTAG+TCCGCATA
GTCGTAACCTACTAGTTCTACGGTGTTGTCATTCATCGTAATTATCTTGAAGTCGTGTGGGTCGTCGA
ACCTCACACCGCCATCAAGATATATGCCTGACATCCCCGGCATCGGGAACTCAGCCCTAATCGCTT
+
FFFFFFFFFFFF:FFFFFFFFFFFFFFF:FFFFFFFFF:FFFFFFFFFFFFFFFFFFFFFFFFFFFFFF
FFFFFFFFFFFFFFFFFFFFFFFFFFFFFFFFFFFFFFFFFFFFFFFFFFFF:FFFFFFFFFFFFF
@A00155:342:HHGFNDSXY:1:1444:22806:32158 1:N:0:GAACCTAG+TCCGCATA
GTAACCTACTAGTTCTACGGTGTTGTCATTCATCGTAATTATCTTGAAGTCGTGTGGGTCGTCGAACC
TCACACCGCCATCAAGATATATGCCTGACATCCCCGGCATCGGGAACTCAGCCCTAATCGCTTCGCTC
+
FFFFFFFFFFFFFFFFFFFFFFFFFFFFFFFFFFFFFFFFFFFFFFFFFFFFFFFFFFFFFFFFFFFF
FFFFFFFFFFFFFFFFFFFFFFFFFFFFFFFFF:FFFFFF:FFFFFFFFFF:FFFFFFFFFFFFFFFF
@A00155:342:HHGFNDSXY:1:1508:16649:11600 2:N:0:GAACCTAG+TCCGCATA
GAACCTACTAGTTCTACGGTGTTGTCATTCATCGTAATTATCTTGAAGTCGTGTGGGTCGTCGAACCT
CACACCGCCATCAAGATATATGCCTGACATCCCCGGCATCGGGAACTCAGCCCTAATCGCTTCGCT
+
FFFFFFFFFF:FFFFFFFFFF:FFFFFFFFFFFFFFFFFFFFFFFFFFFFFFFFFFFFFFFFFFFFFF
FFFFFFFFFF:FFFFFFFFFFFFFFFFFFFFFFFFFFFFFFFFFFFFFFFFFFFFFFFFFFFFF
@A00155:342:HHGFNDSXY:1:2175:1488:11115 2:N:0:GAACCTAG+TCCGCATA
CCTACTAGTTCTACGGTGTTGTCATTCATCGTAATTATCTTGAAGTCGTGTGGGTCGTCGAACCTCAC
ACCGCCATCAAGATATATGCCTGACATCCCCGGCATCGGGAACTCAGCCCTAATCGCTTCGCTCACTG
+
FFFFFFF,F:FFFFFF:FF,F::FF:FF,FF,FF,FFFFF,,FFFFFFFF:FFFF:F:FFFFFFFFFF
FFFFFFFFFFFFFFFFFFF:FFFFFFFFFFFFFFFFFFFFFFFF:FFFF::FFF:FFFFFFFFF:FFFF
@A00155:342:HHGFNDSXY:1:1360:21658:22060 2:N:0:GAACCTAG+TCCGCATA
CTACTAGTTCTACGGTGTTGTCATTCATCGTAATTATCTTGAAGTCGTGTGGGTCGTCGAACCTCACA
CCGCCATCAAGATATATGCCTGACATCCCCGGCATCGGGAACTCAGCCCTAATCGCTTCGCTCACTGC
+
FFFFFFFFFFFFFFFFFFFFFFFFFFFFFFFFFFF:FFFF:FFFFFFFFFFFFFFFFFFFFFFFFFFFF
FFFFFFFFFFFFFFFFFFFFFFF:FFFFFFFFFFFFFFFFFFFFFFFFFFFFFFFFFFFFFFFFF,FFFF
```

```
@A00155:342:HHGFNDSXY:1:1360:21866:21167 2:N:0:GAACCTAG+TCCGCATA
CTACTAGTTCTACGGTGTTGTCATTCATCGTAATTATCTTGAAGTCGTGTGGGTCGTCGAACCTCACA
CCGCCATCAAGATATATGCCTGACATCCCCGGCATCGGGAACTCAGCCCTAATCGCTTCGCTCACTGC
+
FFFFFFFFFFFFFFFFFFFFFFFFFFFFFFFF:FFFFFFFFFFFFFFFFFFFFFFFFFFFFFFFFFFF
FF,FFFFFFFFFFFFFFFFFFFFFFFFFFFFFFFFFFFFFFFFFFFFFFFFFFFFFFFFFFFFFFFFF
@A00155:342:HHGFNDSXY:1:2311:5529:8375 2:N:0:GAACCTAG+TCCGCATA
ACTAGTTCTACGGTGTTGTCATTCATCGTAATTATCTTGAAGTCGTGTGGGTCGTCGAACCTCACACC
GCCATCAAGATATATGCCTGACATCCCCGGCATCGGGAACTCAGCCCTAATCGCTTCGCTCACTGCCG
+
FFFFFF:F,FFFFFFFFFFFFF::FFFFFF:FFFFF:FFFFFFFFFFFFFFFFFFFFFFFFFFFFFFF
F:FFFFFFFFFFFFFFFFFFFFFFFFF:FFFFFFFFF:FFFFFFFFFFFFFFFFFFFFFFFFFFFFFF
@A00155:342:HHGFNDSXY:1:1444:10004:14528 2:N:0:GAACCTAG+TCCGCATA
GTTCTACGGTGTTGTCATTCATCGTAATTATCTTGAAGTCGTGTGGGTCGTCGAACCTCACACCGCCA
TCAAGATATATGCCTGACATCCCCGGCATCGGGAACTCAGCCCTAATCGCTTCGCTCACT
+
FFFFFFFFFFFFFFFFFFFFFFFFFFFFFFFFF:FFFFFFFFFFFFFFFFFFFFFFFFFFFFFFFFFF
FFFFFFFFFFFFFFFFFFFFFFFFFFFFFFFFFFFFFFFFFFFFF:FFFF:FFFFFFFFF
@A00155:342:HHGFNDSXY:1:1331:16966:4883 2:N:0:GAACCTAG+TCCGCATA
GTTCTACGGTGTTGTCATTCATCGTAATTATCTTGAAGTCGTGTGGGTCGTCGAACCTCACACCGCCA
TCAAGATATATGCCTGACATCCCCGGCATCGGGAACTCAGCCCTAATCGCTTCGCTCACT
+
FFFFFFFFFFFFFFFFFFFFFFFFFFFFFFFFFFFFFFFFFFFFFFFFFFFFFFFFFFFFFFFFFFFF
FFFFFFFFFFFFFFFFFFFFFFFFFFFFFFFFFFFFFFFFFFFF,:FFFFFFFFFFFFFF
@A00155:342:HHGFNDSXY:1:2520:16920:8625 2:N:0:GAACCTAG+TCCGCATA
GTTCTACGGTGTTGTCATTCATCGTAATTATCTTGAAGTCGTGTGGGTCGTCGAACCTCACACCGCCA
TCAAGATATATGCCTGACATCCCCGGCATCGGGAACTCAGCCCTAATCGCTTCGCTCACT
+
FFFFFFFFFFFFFFFFFFFFFFFFFFFFFFFFFFFFFFFFFFFFFFFFFFFFF:FFFFFFFFFFFFFF
:FFFFFFFFFFFFFFF,FFFFFFFFFFFFFFFFFFFFFFFFFFFFFFFFFFFFFFFFFFF
@A00155:342:HHGFNDSXY:1:2417:11225:7467 2:N:0:GAACCTAG+TCCGCATA
GTTCTACGGTGTTGTCATTCATCGTAATTATCTTGAAGTCGTGTGGGTCGTCGAACCTCACACCGCCA
TCAAGATATATGCCTGACATCCCCGGCATCGGGAACTCAGCCCTAATCGCTTCGCTCACT
+
FFFFFFFFFFFFFFFFFFFFFFFFFFFFFFFFFFFFFFFFFFFFFFF:FFFFFFFFFFFFFFFFFFFF
FFFFFFFFFFFFFFFFFFFFFFFFFFFFFFFFFFFFFF:FFFFFFFFFFFFFFFFFFFFF
@A00155:342:HHGFNDSXY:1:1507:30409:34679 1:N:0:GAACCTAG+TCCGCATA
TTCTACGGTGTTGTCATTCATCGTAATTATCTTGAAGTCGTGTGGGTCGTCGAACCTCACACCGCCAT
CAAGATATATGCCTGACATCCCCGGCATCGGGAACTCAGCCCTAATCGCTTCGCTCACTGCCGCT
+
FFFFFFFFFFFFFFFFFFFFFFFFFFFFFFFFFFFFFFFFFFFFFFFFFFFFFFFFFFFFFFFFFFFF
FFFFFFFFFFFFFFFFFFFF:FFFFFFFFFFFF,FFFFFFFFFFFFFFF,FFFFFFFF:FFFFFF
@A00155:342:HHGFNDSXY:1:2642:14018:7420 1:N:0:GAACCTAG+TCCGCATA
TTCTACGGTGTTGTCATTCATCGTAATTATCTTGAAGTCGTGTGGGTCGTCGAACCTCACACCGCCAT
CAAGATATATGCCTGACATCCCCGGCATCGGGAACTCAGCCCTAATCGCTTCGCTCACTGCCGCT
+
FFF:F:FFF::FFFFFFFFFFF:FFFF:FFFFFFFFFFFFFFFF:FFFFFFFFFFFFFFFFFFFFFF:
FFFFFFFF,FFFFFFFFFF:FFFF:F:FFFFFFFFF,FFFFFFFF,F,F:F:FFFFFFFFFFFFF
@A00155:342:HHGFNDSXY:1:1602:23411:32456 1:N:0:GAACCTAG+TCCGCATA
TTCTACGGTGTTGTCATTCATCGTAATTATCTTGAAGTCGTGTGGGTCGTCGAACCTCACACCGCCAT
CAAGATATATGCCTGACATCCCCGGCATCGGGAACTCAGCCCTAATCGCTTCGCTCACTGCCGCTG
+
FFFFFFFFFFFFFFFFFFFFFFFFFFFFFFFFFFFFFFFFFFFFFFFFFFFFFFFFFFFFFFFFFFFF
FFFFFFFFFFFFFFFFFFFFFFFFFFFFFFFFFFFFFFFFFFFFFFFFFFFFFFFFFFFFFFFFFF
```

```
@A00155:342:HHGFNDSXY:1:2632:28664:8422 1:N:0:GAACCTAG+TCCGCATA
TTCTACGGTGTTGTCATTCATCGTAATTATCTTGAAGTCGTGTGGGTCGTCGAACCTCACACCGCCAT
CAAGATATATGCCTGACATCCCCGGCATCGGGAACTCAGCCCTAATCGCTTCGCTCACTGCCGCTGA
+
FFFFFFFFFFFFFFFFFFFFFFFFFFFFFFFFFFFFFFFFFFFFFFF:FFFFFF:FFFFF:FFF:FFF
FFFFFFFFFF:FFFFFFFFFFFFFFFFFFFFF:FFFFFFF,F:F:FF,FFFFFFFFF,FFF:FFFF:
@A00155:342:HHGFNDSXY:1:1548:29577:15170 1:N:0:GAACCTAG+TCCGCATA
TTCTACGGTGTTGTCATTCATCGTAATTATCTTGAAGTCGTGTGGGTCGTCGAACCTCACACCGCCAT
CAAGATATATGCCTGACATCCCCGGCATCGGGAACTCAGCCCTAATCGCTTCGCTCACTGCCGCTGAT
+
FFFFFFFFFFFFFFFFFFFFFFFFFFFFFFFFFFFFFFFFFFFFFFFFFFFFFFFFFFFFFFFFFFFF
FFFFFFFFFFFFFFFFFFFFFFFFFFFFFFFFF,FFFFFFFFFFFFFFFFFFFFFFFFFFFFFFFFFF
@A00155:342:HHGFNDSXY:1:2213:9824:28025 1:N:0:GAACCTAG+TCCGCATA
TTCTACGGTGTTGTCATTCATCGTAATTATCTTGAAGTCGTGTGGGTCGTCGAACCTCACACCGCCAT
CAAGATATATGCCTGACATCCCCGGCATCGGGAACTCAGCCCTAATCGCTTCGCTCACTGCCGCTGAT
+
FFFFFFFFFFFFFFFFFFFFFFFFFFFFFFFFFF:FFFFFFFFFFFFFFFFFFFFFFFF,FFFFFFFF
FFFFFFFFFFFFFFFFFFFFFFFFF:FFFFFFFFFFFF:FFFFF:FFFFFFFFFFFFFFFFFFFFFFF
@A00155:342:HHGFNDSXY:1:1209:18611:20322 1:N:0:GAACCTAG+TCCGCATA
TTCTACGGTGTTGTCATTCATCGTAATTATCTTGAAGTCGTGTGGGTCGTCGAACCTCACACCGCCAT
CAAGATATATGCCTGACATCCCCGGCATCGGGAACTCAGCCCTAATCGCTTCGCTCACTGCCGCTGAT
+
FFFFFFFFFFFFFFFFFFFFFFFFFFFFFFFFFFFFFFFFFFFFFFFFFFFFF:FFFFFFFFFFFFFF
FFFFFFFFFFF:FFFFFFFFFFFFFFFFFFFFFFFFFFFFFFFFFFFFFFFFFFFFFFFFFFFFFFFF
@A00155:342:HHGFNDSXY:1:2632:28845:8797 1:N:0:GAACCTAG+TCCGCATA
TTCTACGGTGTTGTCATTCATCGTAATTATCTTGAAGTCGTGTGGGTCGTCGAACCTCACACCGCCAT
CAAGATATATGCCTGACATCCCCGGCATCGGGAACTCAGCCCTAATCGCTTCGCTCACTGCCGCTGAT
+
FFFFFFFFFFFFFFFFFFFFFFFFFFFFFFFFFFFFFFFF:FFFFFFFFFFFFFF:FFFF:FFFFFFF
FFFFFFFFFFFF:FFFFFFFFFFFFFFFFFFFF:FFF,FFFFFFFFF,FFFFFFFFFFF:FFFFF::F
@A00155:342:HHGFNDSXY:1:1407:25455:18270 1:N:0:GAACCTAG+TCCGCATA
TTCTACGGTGTTGTCATTCATCGTAATTATCTTGAAGTCGTGTGGGTCGTCGAACCTCACACCGCCAT
CAAGATATATGCCTGACATCCCCGGCATCGGGAACTCAGCCCTAATCGCTTCGCTCACTGCCGCTGAT
+
FFFFFFFFFFFFFFFFFFFFFFFFFFFFFFFFFFFFFFFFFFFFFFFFFFFFFFFFFFFFFFFFFFFF
FFFFFFFFFFF:FFFFFFFFFFFFFFFFFFFFFFFFFFFFFFFFFFFFFFFFFFFFFFFFFFFFFFFF
@A00155:342:HHGFNDSXY:1:2233:23511:28025 1:N:0:GAACCTAG+TCCGCATA
TTCTACGGTGTTGTCATTCATCGTAATTATCTTGAAGTCGTGTGGGTCGTCGAACCTCACACCGCCAT
CAAGATATATGCCTGACATCCCCGGCATCGGGAACTCAGCCCTAATCGCTTCGCTCACTGCCGCTGAT
+
FFFFFFFFFFFFFFFFFFFFFFFFFFFFFFFFFFFFFFFFFFFFFFFFFFFFFFFFFFFFFFFFFFFF
FFFFFFFFFFFFFFFFFFFFFFFFFFFFFFFFFFFFFFFFFFFFFFFFFFFFFFFFFFFFFFFFFFFF
@A00155:342:HHGFNDSXY:1:2233:23466:28510 1:N:0:GAACCTAG+TCCGCATA
TTCTACGGTGTTGTCATTCATCGTAATTATCTTGAAGTCGTGTGGGTCGTCGAACCTCACACCGCCAT
CAAGATATATGCCTGACATCCCCGGCATCGGGAACTCAGCCCTAATCGCTTCGCTCACTGCCGCTGAT
+
FFFFFFFFFFFFFFFFFFFFFFFFFFFFFFFFFFFFFFFFFFFFFFFFFFFFFFFFFFFFFFFFFFFF
FFFFFFFFFFFFFFFFFFFFFFFFFFFFFFFFFFFFFFFFFFFFFFFFFFFFFFFFFFFFFFFFFFFF
@A00155:342:HHGFNDSXY:1:2604:15121:7795 1:N:0:GAACCTAG+TCCGCATA
TTCTACGGTGTTGTCATTCATCGTAATTATCTTGAAGTCGTGTGGGTCGTCGAACCTCACACCGCCAT
CAAGATATATGCCTGACATCCCCGGCATCGGGAACTCAGCCCTAATCGCTTCGCTCACTGCCGCTGAT
+
FFFFFFFFFFFFFFFFFFFFFFFFFFFFFFFFFFFFFFFFFFFFFFFFFFFFFFFFFFFFFFFFFFFF
FFFFFFFFFFFFFFFFFFFFFFFFFFFFFFFFFFFFFFFFFFFFFFFFFFFFFFFFFFFFFFFFFFFF
```

@A00155:342:HHGFNDSXY:1:1621:18530:26788 2:N:0:GAACCTAG+TCCGCATA
TACGGTGTTGTCATTCATCGTAATTATCTTGAAGTCGTGTGGGTCGTCGAACCTCACACCGCCATCAA
GATATATGCCTGACATCCCCGGCATCGGGAACTCAGCCCTAATCGCTTCGCTCACTGCCGCTGATGCG
+
FFFFFFFFFFFFF:FFFFFFFFFFFFFFFFFFFFFFFFFFFFFFFFFFFFFFFFFFFFFFFFFFFFFF
FFFFFFFFFFFFFFF:FFFFFFFFFFFFFFFFFFFFFFFFF:FFFF:FFFFFFFFFFFFFFFFFFFFF
@A00155:342:HHGFNDSXY:1:1508:16649:11600 1:N:0:GAACCTAG+TCCGCATA
ACGGTGTTGTCATTCATCGTAATTATCTTGAAGTCGTGTGGGTCGTCGAACCTCACACCGCCATCAAG
ATATATGCCTGACATCCCCGGCATCGGGAACTCAGCCCTAATCGCTTCGCTCACTGCCGCTGATGC
+
FFFFFFFFFFFFFFFF:FFFFFFFFFFFFFFFFFFFFFFFFFFFFFFFFFFFFFFFFFFFFFFFF:FF
FF,FFFFFFFFFFFFFFFFFFF:FFFFFFFFFFFFFFFFFFFFFFFFFFFFFFFFFFFFFFFFFFF
@A00155:342:HHGFNDSXY:1:1110:24261:28698 2:N:0:GAACCTAG+TCCGCATA
ACGGTTTTGTCATTCATCGTAATTATCTTGAAGTCGTGTGGGTCGTCGAACCTCACACCGCCATCAAG
ATATATGCCTGACATCCCCGGCATCGGGAACTCAGCCCTAATCGCTTCGCTCACTGCCGCTGATGCGA
+
FFFF:FFF:FFFFFFFFFFFFFFFFFFFFFFFFFFFFFFFFFFFFFFFFFFFFFF:F::FFFFFFFFF
FFFFFFF:FFFFFFFFFFFFFFFFF,FFFFFFFFFF:FFFFFFFF:FFFFFFFFFFFF,F,FFFF:FF
@A00155:342:HHGFNDSXY:1:2457:29188:34945 1:N:0:GAACCTAG+TCCGCATA
GTGTTGTCATTCATCGTAATTATCTTGAAGTCGTGTGGGTCGTCGAACCTCACACCGCCATCAAGATA
TATGCCTGACATCCCCGGCATCGGGAACTCAGCCCTAATCGCTTCGCTCACTGCCGCTGATGCGAAGT
+
FFF:FFFFFFFFFFFFFFFFFFFFFFFFFFFFFFFFFFFFFFFFFFFFFFFFFFFFFFFFFFFFFFFF
FFFFFFFFFFFFFFFFFFFFFFFFFFFFFFFFFFFFFFFFFFFFF:FFFFFFFFFFFFFFFFFFFFFF
@A00155:342:HHGFNDSXY:1:1225:7726:28870 1:N:0:GAACCTAG+TCCGCATA
GTGTTGTCATTCATCGTAATTATCTTGAAGTCGTGTGGGTCGTCGAACCTCACACCGCCATCAAGATA
TATGCCTGACATCCCCGGCATCGGGAACTCAGCCCTAATCGCTTCGCTCACTGCCGCTGATGCGAAGT
+
FFFFFFFFFFFFFFFFFFFFFFFFFFFFFFFFFF:FFFFFFFFFFFFF:FFFFFFFFFFFFFFFFFFF
FFFFFFFFFFFFFFFFFFFFFFFFFF:FFFFFFFFFFFFFFFFFFFFFFFFFF:FFFFFFFFFFFFFF
@A00155:342:HHGFNDSXY:1:1601:26612:24659 2:N:0:GAACCTAG+TCCGCATA
TGTTGTCATTCATCGTAATTATCTTGAAGTCGTGTGGGTCGTCGAACCTCACACCGCCATCAAGATAT
ATGCCTGACATCCCCGGCATCGGGAACTCAGCCCTAATCGCTTCGCTCACTGCCGCTGATGCGAAGTT
+
FFFFFFF,FFFFFFFFFF:FFF:FFFFFFF:FFFF:F,:FFF,F:FF:FFFF::FFFFFFFF,FFFFF
:FF:F:FFFFFFFFFFFFFFFFFFF,FF:FFFFFFFFF:FFF:FF:FFFF,,,FF:FFFFFFFF:F:F
@A00155:342:HHGFNDSXY:1:1245:7952:30921 1:N:0:GAACCTAG+TCCGCATA
TGTTGTCATTCATCGTAATTATCTTGAAGTCGTGTGGGTCGTCGAACCTCACACCGCCATCAAGATAT
ATGCATGACATCCCCGGCATCGGGAACTCAGCCCTAATCGCTTCGCTCACTGCCGCTGATGCGAAGTT
+
FFFFFFFFFFFFFFFFFFFFFFFFFFFFFFFFFFFFFFFFFFFFFFFFFFFFFFFFFFFFFFFFFFFF
FFFF,FFFFFFFFFFFFFFFFFFFFFFFFFFFFFFFFFFFFFFFFFFFFFFFFFFFFFFFFFFFFFFF
@A00155:342:HHGFNDSXY:1:2142:2763:33646 2:N:0:GAACCTAG+TCCGCATA
GTTGTCATTCATCGTAATTATCTTGAAGTCGTGTGGGTCGTCGAACCTCACACCGCCATCAAGATATA
TGCCTGACATCCCCGGCATCGGGAACTCAGCCCTAATCGCTTCGCTCACTGCCGCTGATGCGAAGTTT
+
FFFFFFFFFFFFFFFFFFFFFFFFFFFFFFFFFFFFFFFFFFFFFFFFFFFFFFFFFFFFFFFFFFFF
FFFFFFFFFFFFFFFFFFFFFFFFFFFFFFFFFFFFFFFFFFFFFFFFFFFFFFFFFFFFFFFFFFFF
@A00155:342:HHGFNDSXY:1:1319:5972:24267 1:N:0:GAACCTAG+TCCGCATA
TTGTCATTCATCGTAATTATCTTGAAGTCGTGTGGGTCGTCGAACCTCACACCGCCATCAAGATATAT
GCCTGACATCCCCGGCATCGGGAACTCAGCCCTAATCGCTTCGCTCACTGCCGCTGATGCGAAGTTTG
+
FFFFFFFFFFFFFFFFFFFFFFFFFF:FFFFFFFFFFFFFFFFFFFFFFFFFFFFFFFFFFFFFFFFF
FFFFFFFFFFFFFFFFFFFFFFF:FFFFFFFFFFFFFFFFFFFFFFFFFFFFFFFFFFFFFFFFFFFF

```
@A00155:342:HHGFNDSXY:1:2570:21251:22263 2:N:0:GAACCTAG+TCCGCATA
TTGTCATTCATCGTAATTATCTTGAAGTCGTGTGGGTCGTCGAACCTCACACCGCCATCAAGATATAT
GCCTGACATCCCCGGCATCGGGAACTCAGCCCTAATCGCTTCGCTCACTGCCGCTGATGCGAAGTTTG
+
FFFFFFFFF:FFFFFFFFF,FFFFFFFFFFFFFFFFFFFFFFFFFFFFFFFFFFFFFFFFFFFFFFFF
FFFFFFFFFFFFFFFFFFFFFFFFFFFFFFFFFFFFFFFFFFFFFFFFFFFFFFFFFFFFFFFFFFFF
@A00155:342:HHGFNDSXY:1:2401:9561:17550 1:N:0:GAACCTAG+TCCGCATA
TTGTCATTCATCGTAATTATCTTGAAGTCGTGTGGGTCGTCGAACCTCACACCGCCATCAAGATATAT
GCCTGACATCCCCGGCATCGGGAACTCAGCCCTAATCGCTTCGCTCACTGCCGCTGATGCGAAGTTTG
+
FFFFFFFFFFFFFFFFFFFFFFFFFFFFFFFFFFFFFFFFFFFFFFFFFFFFFFFFFFFFFFFFFFFF
FFFFFFFFFFFFFFFFFFFFFFF,FFFFFFFFFFFFFFFF:FFFFFFFFFFFFFFFFFFFFFFFFFFF
@A00155:342:HHGFNDSXY:1:1634:14299:28823 1:N:0:GAACCTAG+TCCGCATA
TTGTCATTCATCGTAATTATCTTGAAGTCGTGTGGGTCGTCGAACCTCACACCGCCATCAAGATATAT
GCCTGACATCCCCGGCATCGGGAACTCAGCCCTAATCGCTTCGCTCACTGCCGCTGATGCGAAGTTTG
+
F:FFFF:FFFFF:FFFFFFFFFFFFF,F,FFF:F:,FFFFFFFFFFFFFFFFFFFFFFFFFFFFFFFF
::FFFFF:FFFFFFFFFFFFFFFFFFFFFFFFFFF,FFFFF:FFFFFFFFFFFFFFFF:FFFF:F:FF
@A00155:342:HHGFNDSXY:1:2401:9561:17550 2:N:0:GAACCTAG+TCCGCATA
TTGTCATTCATCGTAATTATCTTGAAGTCGTGTGGGTCGTCGAACCTCACACCGCCATCAAGATATAT
GCCTGACATCCCCGGCATCGGGAACTCAGCCCTAATCGCTTCGCTCACTGCCGCTGATGCGAAGTTTG
+
FFFFFFFFFFFFFFFFFFFFFFFFFFFFFFFFFFFFFFFFFFFFFFFFFF:FFFFFFFFFFFFFFFFF
FFFFFFFFFFFFFFFFFFFFFFFFFFFFFFFFFFFFFFFFFFFFFFFFFFFFFFFFFFFFFFFFFFFF
@A00155:342:HHGFNDSXY:1:2570:21251:22263 1:N:0:GAACCTAG+TCCGCATA
TTGTCATTCATCGTAATTATCTTGAAGTCGTGTGGGTCGTCGAACCTCACACCGCCATCAAGATATAT
GCCTGACATCCCCGGCATCGGGAACTCAGCCCTAATCGCTTCGCTCACTGCCGCTGATGCGAAGTTTG
+
FFFFFFFFFFFFFFFFFFFFFFFFFFFFFFFFFFFFFFFFFFFFFFFFFFFFFFFFFFFFFFFFFFFF
FFFFFFFFFFFFFFFFFFFFFFFFFFFFFFFFFFFFFFFFFFFFFFFFFFFFFFFFFFFFFFFFFFFF
@A00155:342:HHGFNDSXY:1:1634:14299:28823 2:N:0:GAACCTAG+TCCGCATA
TTGTCATTCATCGTAATTATCTTGAAGTCGTGTGGGTCGTCGAACCTCACACCGCCATCAAGATATAT
GCCTGACATCCCCGGCATCGGGAACTCAGCCCTAATCGCTTCGCTCACTGCCGCTGATGCGAAGTTTG
+
FFFFFFFFFFFFF:FFFFFFF:FFFFFFFFFFFFFFFFFFFFFFFFFFFFFFFFFFFFFFFFFFFFFF
FFFFFFFFFFFFFFFFFFFFFFFFFFFFFFFFFF,FFFF,FFFFFFFFFFFFFFFFFFFFFFFFFFFF
@A00155:342:HHGFNDSXY:1:1260:11035:26146 2:N:0:GAACCTAG+TCCGCATA
TTGTCATTCATCGTAATTATCTTGAAGTCGTGTGGGTCGTCGAACCTCACACCGCCATCAAGATATAT
GCCTGACATCCCCGGCATCGGGAACTCAGCCCTAATCGCTTCGCTCACTGCCGCTGATGCGAAGTTTG
+
:FFFFFFFFFFFFFFFFFFFFFFFFFFFFFFFFFFFFFFFFFFFFFFFFFFFFFFFFFFFFFF:FFFF
FFFFFFFFFFFFFFFFFFFFFFFFFFFFFFFFFFFFFFFFFFFFFFFFFFFFFFFFFFFFFFFFFFFF
@A00155:342:HHGFNDSXY:1:1276:19678:29340 1:N:0:GAACCTAG+TCCGCATA
TTGTCATTCATCGTAATTATCTTGAAGTCGTGTGGGTCGTCGAACCTCACACCGCCATCAAGATATAT
GCCTGACATCCCCGGCATCGGGAACTCAGCCCTAATCGCTTCGCTCACTGCCGCTGATGCGAAGTTTG
+
FFFFFFFFFF:FFFFFF,FFFF:FFF:FFF:F:FFFFF,FFFFFFFF,FFFFFFFFFFFFFFFFFFFF
:FFFFFFFFFFFFFFFFFFFFFFFFFFFFFFFFFFFFFFFFFFFFFFFFFFFFFFFFFFFFFF:FFF
@A00155:342:HHGFNDSXY:1:2672:13693:10551 2:N:0:GAACCTAG+TCCGCATA
TTGTCATTCATCGTAATTATCTTGAAGTCGTGTGGGTCGTCGAACCTCACACCGCCATCAAGATATAT
GCCTGACATCCCCGGCATCGGGAACTCAGCCCTAATCGCTTCGCTCACTGCCGCTGATGCGAAGTTTG
+
FFFFF:F:FFFFFFFFFFFFFFFFFFFFFFFFFFFFFFFFFFFFFFFFFFFFFFFFFFFFFFFFFFFF
FFFFFFFFFFFFFFFFFFFFFFFFFFFFFFFFFF:FFFFFFFFFFFFFFFFFFFFFFFFFFFFFFFFF
```

```
@A00155:342:HHGFNDSXY:1:2142:2763:33646 1:N:0:GAACCTAG+TCCGCATA
TTGTCATTCATCGTAATTATCTTGAAGTCGTGTGGGTCGTCGAACCTCACACCGCCATCAAGATATAT
GCCTGACATCCCCGGCATCGGGAACTCAGCCCTAATCGCTTCGCTCACTGCCGCTGATGCGAAGTTTG
+
FFFFFFFFFFFFFFFFFFFFFFFFFFFFFFFFFFFFFFFFFFFFFFFFFFFFFFFFFFFFFFFFFFFF
FFFFFFFFFFFFFFFFFFFFFFF:FFFFFFFFFFFFFFFFFFFFFFFFFFFFFFFFFFFFFFFFFFFF
@A00155:342:HHGFNDSXY:1:2574:26069:5400 1:N:0:GAACCTAG+TCCGCATA
TTGTCATTCATCGTAATTATCTTGAAGTCGTGTGGGTCGTCGAACCTCACACCGCCATCAAGATATAT
GCCTGACATCCCCGGCATCGGGAACTCAGCCCTAATCGCTTCGCTCACTGCCGCTGATGCGAAGTTTG
+
FFFFFFFFFFFFFFFFFFFFFFFFFFFFFFFFFFFFFFFFFFFFFFFFFFFFFFFFFFFFFFFFFFFF
FFFFFFFFFFFFFFFFFFFFFFFFFFFFFFFFFFFFFFFFFFFFFFFFFFFFFFFFFFFFFFFFF:FF
@A00155:342:HHGFNDSXY:1:2372:11921:22952 1:N:0:GAACCTAG+TCCGCATA
TTGTCATTCATCGTAATTATCTTGAAGTCGTGTGGGTCGTCGAACCTCACACCGCCATCAAGATATAT
GCCTGACATCCCCGGCATCGGGAACTCAGCCCTAATCGCTTCGCTCACTGCCGCTGATGCGAAGCTTG
+
FFFFFFFFFFFFFFFFFFFFFFFFFFFFFFFFFFFFFFFFFFFFFFFFFFFFFFFFFFFFFFFFFFFF
FFFFFFFFFFFFFFFFFFFFFFFFFFFFFFFFFFFFFFFFFFFFFFFFFFFFFFFFFFFFFFFFFFFF
@A00155:342:HHGFNDSXY:1:1444:22806:32158 2:N:0:GAACCTAG+TCCGCATA
TTGTCATTCATCGTAATTATCTTGAAGTCGTGTGGGTCGTCGAACCTCACACCGCCATCAAGATATAT
GCCTGACATCCCCGGCATCGGGAACTCAGCCCTAATCGCTTCGCTCACTGCCGCTGATGCGAAGTTTG
+
FFFFFFFFFFFFFFFFFFF:FFFF:FFFFFFFFFFFFFFFFFFFFFFFFFFFFFFFFFFFFFFFFFFF
FFFFFFFFFFFFFFFFFFFFFFFFFFFFFFFFFFFFFFFFFFFFFFFFFFFFFFFFFFFFFFFFFFFF
@A00155:342:HHGFNDSXY:1:1260:11035:26146 1:N:0:GAACCTAG+TCCGCATA
TTGTCATTCATCGTAATTATCTTGAAGTCGTGTGGGTCGTCGAACCTCACACCGCCATCAAGATATAT
GCCTGACATCCCCGGCATCGGGAACTCAGCCCTAATCGCTTCGCTCACTGCCGCTGATGCGAAGTTTG
+
FFFFFFF:FFFFFFFFFFFFFFFFFFFFFFFFFFFFFFFFFFFFFFFFFFFFFFFFFFFFFFFFFFFF
FFFFFFFFFFFFFFFF:FFFFFF:FFFFFFFFFFFFFFFFFFFFFFFFFFFFFFFFFFFFFFFFFFFF
@A00155:342:HHGFNDSXY:1:1425:7672:7451 1:N:0:GAACCTAG+TCCGCATA
TGTCATTCATCGTAATTATCTTGAAGTCGTGTGGGTCGTCGAACCTCACACCGCCATCAAGATATATG
CCTGACATCCCCGGCATCGGGAACTCAGCCCTAATCGCTTCGCTCACTGCCGCTGATGCGAAGTTTG
+
FFFFFFFFFFFFFFFFFFFFFFFFFFFFFFFFFFFFFFFFFFFFFFFFFFFFFFFFFF:FFFFFFFFF
FFFFFFFFFFFFFFFFFFFFFF:FFFFFFFFFFFFFFFFFFFFFFFFFFFFFFFFFFFFFFFFFFFF
@A00155:342:HHGFNDSXY:1:2601:27941:18818 1:N:0:GAACCTAG+TCCGCATA
TGTCATTCATCGTAATTATCTTGAAGTCGTGTGGGTCGTCGAACCTCACACCGCCATCAAGATATATG
CCTGACATCCCCGGCATCGGGAACTCAGCCCTAATCGCTTCGCTCACTGCCGCTGATGCGAAGTTTGA
+
FFFFFFFFFFFFFFFFFFFFFFFFFFFFFFFFFF,FFFFFFFFFFFFFFFF:FFFFFFFFFFFFFFFF
:FFFFFFFFFFFFF:FFFFFFFFFFFFF:FFFFFFFFFFFFFFFFFFFFFFFFFFFFFFFFFFFFFFF
@A00155:342:HHGFNDSXY:1:2601:28131:17926 1:N:0:GAACCTAG+TCCGCATA
TGTCATTCATCGTAATTATCTTGAAGTCGTGTGGGTCGTCGAACCTCACACCGCCATCAAGATATATG
CCTGACATCCCCGGCATCGGGAACTCAGCCCTAATCGCTTCGCTCACTGCCGCTGATGCGAAGTTTGA
+
FFFFFFFFFFFFFFFFFFFFFFFFFFFFFFFFFFFFFFFFFFFFFFFFFFFFFFFFFFFFFFFFFFFF
FFFFFFFFFFFFFFFFFFFFFF:FFFFFFFFFFFFFFFFFFFFFFFFFFFFFFFFFFFFFFFFFFFFF
@A00155:342:HHGFNDSXY:1:1460:26973:17300 1:N:0:GAACCTAG+TCCGCATA
TGTCATTCATCGTAATTATCTTGAAGTCGTGTGGGTCGTCGAACCTCACACCGCCATCAAGATATATG
CCTGACATCCCCGGCATCGGGAACTCAGCCCTAATCGCTTCGCTCACTGCCGCTGATGCGAAGTTTGA
+
FFFFFFFFFFFFFFFFFFFFFFFFFFFFFFFFFFFFFFFFFFFFFFFFFFFFFFFFFFFFFFFFFFF:
FFFFFFFFFFFFFFFFFFFFFFFFFFFFFFFFFFF:FFFFFFFFFFFFFFFFFFFFFFFFF:F:FFF:
```

```
@A00155:342:HHGFNDSXY:1:2470:6063:16752 1:N:0:GAACCTAG+TCCGCATA
TGTCATTCATCGTAATTATCTTGAAGTCGTGTGGGTCGTCGAACCTCACACCGCCATCAAGATATATG
CCTGACATCCCCGGCATCGGGAACTCAGCCCTAATCGCTTCGCTCACTGCCGCTGATGCGAAGTTTGA
+
FFFFFFFFFFFFFFFFFFFFFFFFFFFFFFFFFFFFFFFFFFFFFFFFFFFFFFFFFFFFFFFFFFF:
FFFFFFFFFFFFFFFFFFFFFFFFFFFFFFFFFFFFFFFFFFFFFFFFFFFFFFF:FFFFFFFFFFFF
@A00155:342:HHGFNDSXY:1:2601:27941:18818 2:N:0:GAACCTAG+TCCGCATA
TGTCATTCATCGTAATTATCTTGAAGTCGTGTGGGTCGTCGAACCTCACACCGCCATCAAGATATATG
CCTGACATCCCCGGCATCGGGAACTCAGCCCTAATCGCTTCGCTCACTGCCGCTGATGCGAAGTTTGA
+
FFFFFFFFFFFFFFFFFFFFFFFFFFFFFFFFFFFFFFFFFFFFFFFFFFFFFFFFFFF:FFFFFFFF
FFFFFFFFFFFFFFFFFFFFFFFFFFFFFFFFFFFFFFFFFFFF:FFFFFFFFFFFFFFFFFFFFFFF
@A00155:342:HHGFNDSXY:1:1543:5339:12524 1:N:0:GAACCTAG+TCCGCATA
TGTCATTCATCGTAATTATCTTGAAGTCGTGTGGGTCGTCGAACCTCACACCGCCATCAAGATATATG
CCTGACATCCCCGGCATCGGGAACTCAGCCCTAATCGCTTCGCTCACTGCCGCTGATGCGAAGTTTGA
+
FFFFFFFFFFFFFFFFFFFFFFFFFFFFFFFFFFFFFFFFFFFFFFFF,FF:FFFFFFFFFFFFFFFF
FFFFFFFFFFFFFFFFFFFFFFFFFFFFFFFFFFFFFFFFFFFFFFFFFFFFF:FFFFFFFFFFFFFF
@A00155:342:HHGFNDSXY:1:2601:28131:17926 2:N:0:GAACCTAG+TCCGCATA
TGTCATTCATCGTAATTATCTTGAAGTCGTGTGGGTCGTCGAACCTCACACCGCCATCAAGATATATG
CCTGACATCCCCGGCATCGGGAACTCAGCCCTAATCGCTTCGCTCACTGCCGCTGATGCGAAGTTTGA
+
FFFFF:FFFFFFFFFFFFFFFFFFFFFFFFFFFFFFFFFFFFFFFFFFFFFFFFFFFFFFFFFFFFFF
FFFFFFFFFFFFFFFFFFFFFFFFFFFFFFFFFFFFFFFFFFFFFFFFFFFFFFFFFFFFFFFFFFFF
@A00155:342:HHGFNDSXY:1:2601:28085:18192 1:N:0:GAACCTAG+TCCGCATA
TGTCATTCATCGTAATTATCTTGAAGTCGTGTGGGTCGTCGAACCTCACACCGCCATCAAGATATATG
CCTGACATCCCCGGCATCGGGAACTCAGCCCTAATCGCTTCGCTCACTGCCGCTGATGCGAAGTTTGA
+
FFFFFFFFFFFFFFFFFFFFFFFFFFFFFFFFFFFFFFFFFFFFFFFFFFFFFFFFFFFFFFFFFFFF
FFFFFFFFFFFFFFFFFFFFFFFFFFFFFFFF:FFFFFFFFFFFFFFFFFFFFFFFFFFFF:FFFFFF
@A00155:342:HHGFNDSXY:1:1638:23900:13260 1:N:0:GAACCTAG+TCCGCATA
TGTCATTCATCGTAATTATCTTGAAGTCGTGTGGGTCGTCGAACCTCACACCGCCATCAAGATATATG
CCTGACATCCCCGGCATCGGGAACTCAGCCCTAATCGCTTCGCTCACTGCCGCTGATGCGAAGTTTGA
+
FFFFFFFFFFFFFFFFFFFFFFFFFFFFFFFFFFFFFFFFFFFFFFFFFF:FFFFFFFFFFFFFFFFF
FFFFFFFFFFFFFFFFFFFFFFFFFFFFFFFFFFFF:FFFFFFFFFFFFFFFFFFFFFFFFFFFFFFF
@A00155:342:HHGFNDSXY:1:2601:28085:18192 2:N:0:GAACCTAG+TCCGCATA
TGTCATTCATCGTAATTATCTTGAAGTCGTGTGGGTCGTCGAACCTCACACCGCCATCAAGATATATG
CCTGACATCCCCGGCATCGGGAACTCAGCCCTAATCGCTTCGCTCACTGCCGCTGATGCGAAGTTTGA
+
FFFFFFF:FF:FF:FFFFFFFFFFFFFFFFFFFFFFFFFFFFFFFFFFF:FFFFFFFFFFFFFFFFFF
FFFFFFFFFFFFFFFFFFFFFFFFFFFFFFFF::FFFFFFFFFFFFFFFFFFFFFFFFFFFFFFFFFF
@A00155:342:HHGFNDSXY:1:1225:7726:28870 2:N:0:GAACCTAG+TCCGCATA
GTCATTCATCGTAATTATCTTGAAGTCGTGTGGGTCGTCGAACCTCACACCGCCATCAAGATATATGC
CTGACATCCCCGGCATCGGGAACTCAGCCCTAATCGCTTCGCTCACTGCCGCTGATGCGAAGTTTGAT
+
F:FFFFFFFFFFFFFFFFFFFFFFFFFFFFFFFFFFFFFFFFFFFFFFFFFFFFFFFFFFFFFFFFFF
FFFFFFFFFFFFFFFFFFFFFFFFFFFFFFFFFFFFFFFFFFFFFFFFFFFFFFFFFFFFFFFFFFFF
@A00155:342:HHGFNDSXY:1:1319:5972:24267 2:N:0:GAACCTAG+TCCGCATA
TCATTCATCGTAATTATCTTGAAGTCGTGTGGGTCGTCGAACCTCACACCGCCATCAAGATATATGCC
TGACATCCCCGGCATCGGGAACTCAGCCCTAATCGCTTCGCTCACTGCCGCTGATGCGAAGTTTGATG
+
FFFFFFFFFFFFFFFFFFFFFFFFFFFFFFFFFFFFFFFFFFFFFFF:FFFFFFFFFFFFFFFFFFFF
FFFFFFFFFFFFFFFFFFFFFFFFFFFFFF::FFFFFFFFF:FFFFFFFFFFFFFFFFFFFFFFFFFF
```

@A00155:342:HHGFNDSXY:1:1639:7238:15123 2:N:0:GAACCTAG+TCCGCATA
TCATTCATCGTAATTATCTTGAAGTCGTGTGGGTCGTCGAACCTCACACCGCCATCAAGATATATGCC
TGACATCCCCGGCATCGGGAACTCAGCCCTAATCGCTTCGCTCACTGCCGCTGATGCGAAGTTTGATG
+
FFFFFFFFFFFFFFFFFFFFFFFFFFFFFFFFFFFFFFFFFFFFFFFFFFFFFFFFFFFFFFFFFFFF
FFFF:FFFFFFFFFFFFFFFFFFFFFFFFFFFFFFFFFFFFFFFFFFFFFFFFFFFFFFFFFFFFFFF
@A00155:342:HHGFNDSXY:1:2601:26982:17597 2:N:0:GAACCTAG+TCCGCATA
TCATTCATCGTAATTATCTTGAAGTCGTGTGGGTCGTCGAACCTCACACCGCCATCAAGATATATGCC
TGACATCCCCGGCATCGGGAACTCAGCCCTAATCGCTTCGCTCACTGCCGCTGATGCGAAGTTTGATG
+
FFFFFFFFFFFFFFFFFFFFFFFFFFFFFFFFFFFFFFFFFFFFFFFFFFFFFFFFFFFFFFFFFFFF
FFFFFFFFFFFFFFFFFFFFFFFFFFFFFFFFFFFFFFFFFFFFFFFFFFFFFFFFFFFFFFFFFFFF
@A00155:342:HHGFNDSXY:1:2575:23683:23563 2:N:0:GAACCTAG+TCCGCATA
TCATTCATCGTAATTATCTTGAAGTCGTGTGGGTCGTCGAACCTCACACCGCCATCAAGATATATGCC
TGACATCCCCGGCATCGGGAACTCAGCCCTAATCGCTTCGCTCACTGCCGCTGATGCGAAGTTTGATG
+
FFFF:FFFFFFFFFFFFFFFFFFFFFFFFFFFFFFFFFFFFFFFFFFFFFFFFFFFFFFFFFF:FFFF
FFFFFFFFFFFFFFFFFFFFFFFFFFFFFFF,FFFF,FFFFFFFFFFFFFFFFFFFFFFFFFFFFFFF
@A00155:342:HHGFNDSXY:1:1331:16966:4883 1:N:0:GAACCTAG+TCCGCATA
CATTCATCGTAATTATCTTGAAGTCGTGTGGGTCGTCGAACCTCACACCGCCATCAAGATATATGCCT
GACATCCCCGGCATCGGGAACTCAGCCCTAATCGCTTCGCTCACTGCCGCTGATGCGAAG
+
FFFFFFFFFFFFFFFFFFFFFFFFFFFFFFFFFFFFFFFFFFFFFFFFFFFFFFFFFFFFFFFFFFFF
FFFFFFFFFFFFFFFFFFFFFFFFFFFFFFFFFFFFFFFFFFFFFFFFFFFFFFFFFFFF
@A00155:342:HHGFNDSXY:1:1444:10004:14528 1:N:0:GAACCTAG+TCCGCATA
CATTCATCGTAATTATCTTGAAGTCGTGTGGGTCGTCGAACCTCACACCGCCATCAAGATATATGCCT
GACATCCCCGGCATCGGGAACTCAGCCCTAATCGCTTCGCTCACTGCCGCTGATGCGAAG
+
FFFFFFFFFFFFFFFFFFFFFFFFFFFFFFFFFFFFFFFFFFFFFFFFF:FFFFFFFFFFFFFFFFFF
FFFFFFFFFFFFFFFFFFFF:FFFFFFFFFFFFFFFFFFFFFFFFFFFFFFFFFFFFFFF
@A00155:342:HHGFNDSXY:1:2520:16920:8625 1:N:0:GAACCTAG+TCCGCATA
CATTCATCGTAATTATCTTGAAGTCGTGTGGGTCGTCGAACCTCACACCGCCATCAAGATATATGCCT
GACATCCCCGGCATCGGGAACTCAGCCCTAATCGCTTCGCTCACTGCCGCTGATGCGAAG
+
FFFFFFFFFFFFFFFFFFFFFFFFFFFFFFFFFFFFFFFFFFFFFFFFFFFFFFFFFFFFFFFFFFFF
FFFFFFFFFFFFFFFFFFFF:FFFFFFFFF:FFFFFFFFFFFFFFFFFFFFFFFFFFFFF
@A00155:342:HHGFNDSXY:1:2417:11225:7467 1:N:0:GAACCTAG+TCCGCATA
CATTCATCGTAATTATCTTGAAGTCGTGTGGGTCGTCGAACCTCACACCGCCATCAAGATATATGCCT
GACATCCCCGGCATCGGGAACTCAGCCCTAATCGCTTCGCTCACTGCCGCTGATGCGAAG
+
FFFFFFFFFFFFFFFFFFFFFFFFFFFFFFFFFFFFFFFFFFFFFFFFFFFFFFFFFFFFFFFFFFFF
FFFFFFFFFFFFFFFFFFFFFFFFFFFFFFFFFFFFFFFFFFFFFFFFFFFFFFFFFFFF
@A00155:342:HHGFNDSXY:1:1153:7328:15092 1:N:0:GAACCTAG+TCCGCATA
CATTCATCGTAATTATCTTGAAGTCGTGTGGGTCGTCGAACCTCACACCGCCATCAAGATATATGCCT
GACATCCCCGGCATCGGGAACTCAGCCCTAATCGCTTCGCTCACTGCCGCTGATGCGAAGTTTGATGC
+
FFFFFFFFFFFFFFFFFFFFFFFFFFFFFFFFFFFFFFFFFFFFFFFFFFFFFFFFFFFFFFFFFFFF
FFFFFFFFFFFFFFFFFFFFFFFFFFFFFFFFFFFFFFFFFFFFFFFFFFFFFFFFFFFFFFFFFFFF
@A00155:342:HHGFNDSXY:1:2334:5791:32910 2:N:0:GAACCTAG+TCCGCATA
CATTCATCGTAATTATCTTGAAGTCGTGTGGGTCGTCGAACCTCACACCGCCATCAAGATATATGCCT
GACATCCCCGGCATCGGGAACTCAGCCCTAATCGCTTCGCTCACTGCCGCTGATGCGAAGTTTGATGC
+
FFF:FFF:F:F:FFFF:F:FFFFFFFFFFFFFFFFFFFFFFFFFFFFFFFFFFFFFFFFFFFFFFFFF
FFFFFFFFFFFFFFFFFFFFFFFFFFFFFFFFFFFFFFFFFFFFFFFFFFFFFFFFFFFFFFFFFFFF

```
@A00155:342:HHGFNDSXY:1:1543:5339:12524 2:N:0:GAACCTAG+TCCGCATA
CATTCATCGTAATTATCTTGAAGTCGTGTGGGTCGTCGAACCTCACACCGCCATCAAGATATATGCCT
GACATCCCCGGCATCGGGAACTCAGCCCTAATCGCTTCGCTCACTGCCGCTGATGCGAAGTTTGATGC
+
FFFFFFFFFFFFFFF:FFFFFFFFFFFFFFFFFFFFFFFFFFFFFFFFFFFFFFFFFFFFFFFFFFFF
FFFFFFFFFFFFFFFFFFFFFFFFFFFFFFFFFFFFFFFFFFFFFFFFFFFFFFFFFFFFFFFFFFFF
@A00155:342:HHGFNDSXY:1:2443:1362:30686 1:N:0:GAACCTAG+TCCGCATA
CATTCATCGTAATTATCTTGAAGTCGTGTGGGTCGTCGAACCTCACACCGCCATCAAGATATATGCCT
GACATCCCCGGCATCGGGAACTCAGCCCTAATCGCTTCGCTCACTGCCGCTGATGCGAAGTTTGATGC
+
FFFFFFFFFFFFFFFFFFFF:FFFFFFFFFFFFFFFFF:FF:F:FFFF:FFFFFFFFFFFFFFFFFFF
FFFFFFFFFFFFFFFFFFFF:FFF:F:FFFFFFF,FF,FFF:FFFF,:FFFFFF,FF:F:FFF,:F:F
@A00155:342:HHGFNDSXY:1:2265:5330:11130 1:N:0:GAACCTAG+TCCGCATA
ATTCATCGTAATTATCTTGAAGTCGTGTGGGTCGTCGAACCTCACACCGCCATCAAGATATATGCCTG
ACATCCCCGGCATCGGGAACTCAGCCCTAATCGCTTCGCTCACTGCCGCTGATGCGAAGTTTGATGCA
+
FFFFFFFFFFFFFFFFFFFFFFFFFFFFF:FFFFFFFFFF:FFFFFFFFFFFFFFFFFFFFFFFFFFF
FF:FFFF:FFFFFFFF:FFFFFFFFFFFFFFFFFFFFFFFFFFFF,FFFFFFF,FFFFFFFFFFFFFF
@A00155:342:HHGFNDSXY:1:1639:7238:15123 1:N:0:GAACCTAG+TCCGCATA
TCATCGTAATTATCTTGAAGTCGTGTGGGTCGTCGAACCTCACACCGCCATCAAGATATATGCCTGAC
ATCCCCGGCATCGGGAACTCAGCCCTAATCGCTTCGCTCACTGCCGCTGATGCGAAGTTTGATGCAGA
+
FFFFFFFFFFFFFFFFFFFFFFFFFFFFFFFFFFFFFFFFFFFFFFFFFFFFFFFFFFFFFFFFFFFF
FFFFFFFFFFFFFFFFFFFFFFFFFFFFFFFFFFFFFFFFFFFFFFFFFFFFFFFFFFFFFFFFFFFF
@A00155:342:HHGFNDSXY:1:2507:15646:1720 2:N:0:GAACCTAG+TCCGCATA
TCATCGTAATTATCTTGAAGTCGTGTGGGTCGTCGAACCTCACACCGCCATCAAGATATATGCCTGAC
ATCCCCGGCATCGGGAACTCAGCCCTAATCGCTTCGCTCACTGCCGCTGATGCGAAGTTTGATGCAGA
+
FFFFFFFFFFFF,FFFFFFFFFFFFFFFF:FFFFF:FFFFFFFFFFFFFFFFFFFFFFFFFFFFFFFF
,FFFF:FFFFFFFFFFFFFFF:FFFFFFFFFFFFFFFFFFFFFFFFFFFFFFFFFFFFFFFFFFFFFF
@A00155:342:HHGFNDSXY:1:2601:26982:17597 1:N:0:GAACCTAG+TCCGCATA
TCATCGTAATTATCTTGAAGTCGTGTGGGTCGTCGAACCTCACACCGCCATCAAGATATATGCCTGAC
ATCCCCGGCATCGGGAACTCAGCCCTAATCGCTTCGCTCACTGCCGCTGATGCGAAGTTTGATGCAGA
+
FFFFFFFFFFFFFFFFFFFFFFFFFFFFFFFFFFFFFFFFFFFFFFFFFFFFFFFFFFFFFFFFFFFF
FFFFFFFFFFFFFFFFFFFFFFFFFFFFFFFFFFFFFFFFFFFFFFFFFFFFFFFFFFFFFFFF:FFF
@A00155:342:HHGFNDSXY:1:1464:30987:30389 1:N:0:GAACCTAG+TCCGCATA
TCATCGTAATTATCTTGAAGTCGTGTGGGTCGTCGAACCTCACACCGCCATCAAGATATATGCCTGAC
ATCCCCGGCATCGGGAACTCAGCCCTAATCGCTTCGCTCACTGCCGCTGATGCGAAGTTTGATGCAGA
+
FFFFFFFFFFFFFFFFFFFFFFFFFFFFFFFFFFF:FFFFFFFFFFFFFFFFFFFFFFFFFFFFFFFF
FFFFFFFFFFFFFFFFFFFFFFFFFFFFFFFFFFF:FFFFFFFFFFFFFFFFFFFFFFFFFFFFFFFF
@A00155:342:HHGFNDSXY:1:2575:16721:10097 1:N:0:GAACCTAG+TCCGCATA
TCGTAATTATCTTGAAGTCGTGTGGGTCGTCGAACCTCACACCGCCATCAAGATATATGCCTGACATC
CCCGGCATCGGGAACTCAGCCCTAATCGCTTCGCTCACTGCCGCTGATGCGAAGTTTGATGCAGAGTA
+
FFFFFFFFFFFFFFFFFFFFFFFFFFFFFFFFFFFFFFFFFFFFFFFFFFFFFFFFFFFFFFFFFFFF
FFFFFFFFFFFFFFFFFFFFFFFFFFFFFFFFFFFFFFFFFFFFFFFFFFFFFFFFFFFFFFFFFFFF
@A00155:342:HHGFNDSXY:1:2108:29686:25473 1:N:0:GAACCTAG+TCCGCATA
TCGTAATTATCTTGAAGTCGTGTGGGTCGTCGAACCTCACACCGCCATCAAGATATATGCCTGACATC
CCCGGCATCGGGAACTCAGCCCTAATCGCTTCGCTCACTGCCGCTGATGCGAAGTTTGATGCAGAGTA
+
FFFFFFFFFFFF:FFFFFFFFFFFFFFFFFFFFFFFFFFFFFFFFFFFFFF:F:FFF:FFFFFFFFFF
FFFFFFFFFFFFFFF:FFFFFFFFFFFFFF:FFFFFFFFFFFFFFFFFFFFFFFFFFFFFFFFFFFFF
```

```
@A00155:342:HHGFNDSXY:1:2233:23466:28510 2:N:0:GAACCTAG+TCCGCATA
CGTAATTATCTTGAAGTCGTGTGGGTCGTCGAACCTCACACCGCCATCAAGATATATGCCTGACATCC
CCGGCATCGGGAACTCAGCCCTAATCGCTTCGCTCACTGCCGCTGATGCGAAGTTTGATGCAGAGTAA
+
F:FFFFFFFFFFFFFFFFFFFFFFFFFFFFFFFFFFFFFFFFFFFFFFFFFFFFFFFFFFFFFFFFFF
FFFFFFFFFFFFFFFFFFF,FFFFFFFFFFFFFFFFFFFFFFFFFFFFFFFFFFFFFFFFFFFFFFFF
@A00155:342:HHGFNDSXY:1:2233:23511:28025 2:N:0:GAACCTAG+TCCGCATA
CGTAATTATCTTGAAGTCGTGTGGGTCGTCGAACCTCACACCGCCATCAAGATATATGCCTGACATCC
CCGGCATCGGGAACTCAGCCCTAATCGCTTCGCTCACTGCCGCTGATGCGAAGTTTGATGCAGAGTAA
+
:FFFF:FFFFFFFFFFFFFFFFFFFFFFFFFFFFFFFFFFFFFFFFFFFFFFFFFFFFFFFFFFF:FF
FFFFFFFFFFFFFFFFFFFFFFFFFFFFFFFFFFFFFFFFFFFFFFFFFFFFFFFFFFFFFFFFFFFF
@A00155:342:HHGFNDSXY:1:2153:25138:13651 2:N:0:GAACCTAG+TCCGCATA
CGTAATTATCTTGAAGTCGTGTGGGTCGTCGAACCTCACACCGCCATCAAGATATATGCCTGACATCC
CCGGCATCGGGAACTCAGCCCTAATCGCTTCGCTCACTGCCGCTGATGCGAAGTTTGATGCAGAGTAA
+
FFFFFFF:FFFFFFFFFFFFFFFFFFFFFFFFFFFFFFFFFFFFFFFFFFFFFFFFFFFFFFFFFFFF
FFFFFFFFFFFFFFFFFFFFFFF:FFFFFFFFFFFFFFFFFFFFFFFFFFFFFFFFFFFFFFFFFFFF
@A00155:342:HHGFNDSXY:1:2457:29188:34945 2:N:0:GAACCTAG+TCCGCATA
AATTATCTTGAAGTCGTGTGGGTCGTCGAACCTCACACCGCCATCAAGATATATGCCTGACATCCCCG
GCATCGGGAACTCAGCCCTAATCGCTTCGCTCACTGCCGCTGATGCGAAGTTTGATGCAGAGTAAAAA
+
FFFFFFFFFFFFFFFFFFFFFFFFFFFFFFFFFFFFFFFFFFFFFFFFFFF:F:FFFFFFFF:FFFFF
FFFFFFFFFFFFFFFFFFFFFFFFFFFFFFFFFFFFFFFFFFFFFFFFFFFFFFF:FFFFFFFFFFFF
@A00155:342:HHGFNDSXY:1:1217:9742:35837 1:N:0:GAACCTAG+TCCGCATA
AATTATCTTGAAGTCGTGTGGGTCGTCGAACCTCACACCGCCATCAAGATATATGCCTGACATCCCCG
GCATCGGGAACTCAGCCCTAATCGCTTCGCTCACTGCCGCTGATGCGAAGTTTGATGCAGAGTAAAAA
+
FFFFFFFFFFFFF:FFFFFFFFFFFFFFFFFFFFFFFFFFFFFFFFFFFFFFFFFFFFFFFFFFFFFF
FFFFFFFFFFFFFFFFFFFFFFFFFFFFFFFFFFFFFFFFFFFFFFFFFFF:FFFFFFFFFFFFFFFF
@A00155:342:HHGFNDSXY:1:1667:10239:30436 1:N:0:GAACCTAG+TCCGCATA
AATTATCTTGAAGTCGTGTGGGTCGTCGAACCTCACACCGCCATCAAGATATATGCCTGACATCCCCG
GCATCGGGAACTCAGCCCTAATCGCTTCGCTCACTGCCGCTGATGCGAAGTTTGATGCAGAGTAAAAA
+
FFFFFFFFFFFFFFFFFFFFFFFFFFFFFFF:FFF:FFFFFFFFFFFFFFFFFFFFFFFFFFFFFFFF
FFFFFFFFFFFFFFFFFFFFFFFFFFFFFFFFFFFFFFFFFFFFFFFFFFFFFFFFFFFFFFFFFFF:
@A00155:342:HHGFNDSXY:1:1364:18159:1438 1:N:0:GAACCTAG+TCCGCATA
AATTATCTTGAAGTCGTGTGGGTCGTCGAACCTCACACCGCCATCAAGATATATGCCTGACATCCCCG
GCATCGGGAACTCAGCCCTAATCGCTTCGCTCACTGCCGCTGATGCGAAGTTTGATGCAGAGTAAAAA
+
F,F:FFFFFFFFFFFFFFFFFF,FFFFFFFFFFFFFFF:FFFFFFFFFFFFFFFFFFFFFFFFFFFFF
FFFFFFFFFFFFFFFFFF:FFFFFFFFF:FFFFFFFFFFFFFFFFFFFFFFF:FFFFFFFFFFFFFFF
@A00155:342:HHGFNDSXY:1:2409:32660:1626 1:N:0:GAACCTAG+TCCGCATA
AATTATCTTGAAGTCGTGTGGGTCGTCGAACCTCACACCGCCATCAAGATATATGCCTGACATCCCCG
GCATCGGGAACTCAGCCCTAATCGCTTCGCTCACTGCCGCTGATGCGAAGTTTGATGCAGAGTAAAAA
+
FFFFFFFFFFFFFFFFFFFFFFF:FFFFFFFFFFFFFFFFFFF:FFFFFFFFFFFFFFFFFFFFFFFF
FFFFFFFFFFFFFFFFFFFFFFFFFFFFFFFFFFFFFFFFFFFFFFFFFFFFFFFFFFFFFFFFFFFF
@A00155:342:HHGFNDSXY:1:1557:23014:11256 1:N:0:GAACCTAG+TCCGCATA
ATTATCTTGAAGTCGTGTGGGTCGTCGAACCTCACACCGCCATCAAGATATATGCCTGACATCCCCGG
CATCGGGAACTCAGCCCTAATCGCTTCGCTCACTGCCGCTGATGCGAAGTTTGATGCAGAGTAAAAA
+
FFFFFFFFFFF:FF:FFFF,FF::F:FFFFFF:FFFFF:FFFFFFFFF:FFFFFFFFFF:FFFF,FFF
FFFF:F:FFFFFF,FFF,FFFFFFFF:FFFFF,FFF:,,FFFF:F:FF:FFF:FF,:F:FFFFFFFF
```

```
@A00155:342:HHGFNDSXY:1:2310:18819:11537 2:N:0:GAACCTAG+TCCGCATA
CTTGAAGTCGTGTGGGTCGTCGAACCTCACACCGCCATCAAGATATATGCCTGACATCCCCGGCATCG
GGAACTCAGCCCTAATCGCTTCGCTCACTGCCGCTGATGCGAAGTTTGATGCAGAGTAAAAATC
+
FFFFFFFFFFFFFFFFFFFFFFFFFFFFFFFFFFFFFFFFFFFFFF,FFFFFFFFF:FFFFFFFFFFF
FFFFFFFFFFFFFF,FFFFFFFFFFFFFFFFFFFFFFFFFFFFFFFFFFFFFFFFFFFFFFFFF
@A00155:342:HHGFNDSXY:1:1174:7627:1141 2:N:0:GAACCTAG+TCCGCATA
CTTGAAGTCGTGTGGGTCGTCGAACCTCACACCGCCATCAAGATATATGCCTGACATCCCCGGCATCG
GGAACTCAGCCCTAATCGCTTCGCTCACTGCCGCTGATGCGAAGTTTGATGCAGAGTAAAAAAC
+
FFFFFFFFFFFFFFFFFFFFFFFFFFFFFFFFFFFFFFFFFFFFFFFFFFFFFFFFFFFFFFFFFFFF
FFFFFFFFFFFFFF:FFFFFFFFFFFFFFFFFFFFFFFFFFFFFFFFFFFFFFFFFFFFFFFFF
@A00155:342:HHGFNDSXY:1:1312:22724:34585 2:N:0:GAACCTAG+TCCGCATA
CTTGAAGTCGTGTGGGTCGTCGAACCTCACACCGCCATCAAGATATATGCCTGACATCCCCGGCATCG
GGAACTCAGCCCTAATCGCTTCGCTCACTGCCGCTGATGCGAAGTTTGATGCAGAGTAAAAATC
+
FFFFFFFFF:FFFF:FFF:FFFFFFFF:FF::F,:FFFF:FFFFF:FFFF,FFFFFFF:F,FFFFFFF
FFFFFF:F:FFFFFFF:FF:FFFFFFFFFFFF:FFFFFFFFFFFF:FFFFFFFFFFFFFFFF:F
@A00155:342:HHGFNDSXY:1:2153:25138:13651 1:N:0:GAACCTAG+TCCGCATA
CTTGAAGTCGTGTGGGTCGTCGAACCTCACACCGCCATCAAGATATATGCCTGACATCCCCGGCATCG
GGAACTCAGCCCTAATCGCTTCGCTCACTGCCGCTGATGCGAAGTTTGATGCAGAGTAAAAATCCCCC
+
FFFFFFFFFFFFFFFFFFFFFFFFFFFFFFFFFFFFFFFFFFFFFFFFFFFFFFFFFFFFFFFFFFFF
FFFFFFFFFFFFFFFFFFFFFFFFFFFFFFFFFFFFFFFFFFFF:FFFFFFFFFFFFFFFFFFFFFFF
@A00155:342:HHGFNDSXY:1:2562:30680:27164 1:N:0:GAACCTAG+TCCGCATA
TTGAAGTCGTGTGGGTCGTCGAACCTCACACCGCCATCAAGATATATGCCTGACATCCCCGGCATCGG
GAACTCAGCCCTAATCGCTTCGCTCACTGCCGCTGATGCGAAGTTTGATGCAGAGTAAAAATCC
+
FFFFFFFFFFFFFFFFFFFFFFFFFFFFFFFFFFFFFFFFFFFFFFFFFFFFFFFFFFFFFFFFFFFF
FFFFFFFFFFFFFFFFFFFFFFFFFFFFFFFFFFFFFFFFFFFFFFFFFFFFFFFFFFFFFFFF
@A00155:342:HHGFNDSXY:1:1571:13150:29935 2:N:0:GAACCTAG+TCCGCATA
TGAAGTCGTGTGGGTCGTCGAACCTCACACCGCCATCAAGATATATGCCTGACATCCCCGGCATCGGG
AACTCAGCCCTAATCGCTTCGCTCACTGCCGCTGATGCGAAGTTTGATGCAGAGTAAAAATCCCCCTG
+
FFFFFF,FFFFFFFFFFFFFFFFFFFFFF:FFFFFFFFFFFFFFFFFFFFFFFFFFFFFFFFFFFFFF
:FFFFFFFFFF:FFFFFFFFFFFFFFFFFFFFFFFFFFFFFFFFFFFFFFFFFFFFFFFFFFFFFFFF
@A00155:342:HHGFNDSXY:1:1210:27868:33379 1:N:0:GAACCTAG+TCCGCATA
AAGTCGTGTGGGTCGTCGAACCTCACACCGCCATCAAGATATATGCCTGACATCCCCGGCATCGGGAA
CTCAGCCCTAATCGCTTCGCTCACTGCCGCTGATGCGAAGTTTGATGCAGAGTAAAAATCCCCCTGTG
+
FFFFFFFFFFFFFFFFFFFFFFFFFFFFFFFFFFFFFFFFFFFFFFFFFFFFFFFFFFFFFFFFFFFF
FFFFFFFFFFFFFFFFFFFFFFFFFFFFFFFFFFFFFFFFF:FFFFFFFFFFFFFFFFFFFFFFFFFF
@A00155:342:HHGFNDSXY:1:2110:31222:18082 1:N:0:GAACCTAG+TCCGCATA
AAGTCGTGTGGGTCGTCGAACCTCACACCGCCATCAAGATATATGCCTGACATCCCCGGCATCGGGAA
CTCAGCCCTAATCGCTTCGCTCACTGCCGCTGATGCGAAGTTTGATGCAGAGTAAAAATCCCCCTGTG
+
FFFFFFFFFFFFFFFFFFFFFFFFFFFFFFFFFFFFFFFFFFFFFFFFFFFFFFFFFFFFFFFFFFFF
FFFFFFFFF:FFFFFFFFFFFFFFFFFFFFFFF:FFFFFFFF:FFFFFFFFFFFFFFFFFFFFFFFFF
@A00155:342:HHGFNDSXY:1:1210:26341:31234 1:N:0:GAACCTAG+TCCGCATA
AAGTCGTGTGGGTCGTCGAACCTCACACCGCCATCAAGATATATGCCTGACATCCCCGGCATCGGGAA
CTCAGCCCTAATCGCTTCGCTCACTGCCGCTGATGCGAAGTTTGATGCAGAGTAAAAATCCCCCTGTG
+
FFFFFFFFFFFFFFFFFFFFFFFFFFFFFFFFFFFFFFFF:F:FFFFFFFFFFFFFFFFFFFFFFFFF
FFFFFFFFFFFFFFFFFFFFFFFFFFF,FF:F:FFFFF:FFFFFFFFFFFFF:FFFFFFFFFFFFFFF
```

```
@A00155:342:HHGFNDSXY:1:1249:31837:4116 2:N:0:GAACCTAG+TCCGCATA
AGTCGTGTGGGTCGTCGAACCTCACACCGCCATCAAGATATATGCCTGACATCCCCGGCATCGGGAAC
TCAGCCCTAATCGCTTCGCTCACTGCCGCTGATGCGAAGTTTGATGCAGAGTAAAAATCCCCCTGTGT
+
FFFFF,FFFFFFFFFFFFFFFFFFFFFFFFFFFFFFFFFFFFFFFFFFFFFFFFFFFFFFFFFFFFFF
FFFFFFFFF:FFFF:FFFFFFFFFFFFFFFFFFFFFFFFFFFFFFFFFFFFFFFFFFFFFFFFFFFFF
@A00155:342:HHGFNDSXY:1:1246:21468:30029 2:N:0:GAACCTAG+TCCGCATA
AGTCGTGTGGGTCGTCGAACCTCACACCGCCATCAAGATATATGCCTGACATCCCCGGCATCGGGAAC
TCAGCCCTAATCGCTTCGCTCACTGCCGCTGATGCGAAGTTTGATGCAGAGTAAAAATCCCCCTGTGT
+
FFFFFFFFFFFFFFFFFFFFFFFFFFFFFFFFFFFFFFFFFFFFFFFFFF:FFFFFFFFFFFFFFFFF
FFFFFFFFF,FFFFFFFFFFFFFFFFFFFFFFFFFFFFFFFFFFFFFFFFFFFFFFFFFFFFFFFFFF
@A00155:342:HHGFNDSXY:1:1154:7039:26490 2:N:0:GAACCTAG+TCCGCATA
AGTCGTGTGGGTCGTCGAACCTCACACCGCCATCAAGATATATGCCTGACATCCCCGGCATCGGGAAC
TCAGCCCTAATCGCTTCGCTCACTGCCGCTGATGCGAAGTTTGATGCAGAGTAAAAATCCCCCTGTGT
+
FFFFFFFFFFFFFFFFFFFFFFFFFFFFFFFFFFFFFFFFFFFFFFFFFFFFFFFFFFFFFFFFFFFF
FFFFFFFFFFFFFF:FFFFFFFFFFFFFFFFFFFFFFFFFFFFFFFFFFFFFFFFFFFFFFFFFFFFF
@A00155:342:HHGFNDSXY:1:2575:16721:10097 2:N:0:GAACCTAG+TCCGCATA
AGTCGTGTGGGTCGTCGAACCTCACACCGCCATCAAGATATATGCCTGACATCCCCGGCATCGGGAAC
TCAGCCCTAATCGCTTCGCTCACTGCCGCTGATGCGAAGTTTGATGCAGAGTAAAAATCCCCCTGTGT
+
FFFFFFFFFFFFFFFFFFFFFFFFFFFFFFFFFFFFFFFFFFFFFFFFFFFFFFFFFFFFFFFFFFFF
FFFFFFFFFFFFFFFFFFFFFFFFFFFFFFFFFFFFFFFFFFFFFFFFFFFFFFFFFFFFFFFFFFFF
@A00155:342:HHGFNDSXY:1:1542:10411:5118 2:N:0:GAACCTAG+TCCGCATA
TCGTGTGGGTCGTCGAACCTCACACCGCCATCAAGATATATGCCTGACATCCCCGGCATCGGGAACTC
AGCCCTAATCGCTTCGCTCACTGCCGCTGATGCGAAGTTTGATGCAGAGTAAAAATCCCCCTGTGTGG
+
FFF:FFFFFFFFFFFFFFFFFFFFFFFFFFFFFFFFFFFFFFFFFFFFFFFFFFFFFFFFFFFF,FFF
:FFFFFFFFFFFFFFFFFFFFFF,FFFFFF:FFF:FFFFFFFFFFFFFFFFF,FFFFFFFFFFFFFFF
@A00155:342:HHGFNDSXY:1:1654:20952:23218 1:N:0:GAACCTAG+TCCGCATA
TCGTGTGGGTCGTCGAACCTCACACCGCCATCAAGATATATGCCTGACATCCCCGGCATCGGGAACTC
AGCCCTAATCGCTTCGCTCACTGCCGCTGATGCGAAGTTTGATGCAGAGTAAAAATCCCCCTGTGTGG
+
FFFFFFFFFFFFFFFFFFFFFFFFFFFFFFFFFFFFFFFFFFFFFFFFFFFFFFFFFFFFFFFFFFFF
FFFFFFF:FFFFFFFFFFFFFFFFFFFFFF:FFFFFFFFFFFFFFFFFFFFFFFFFFFFFFFFFFFFF
@A00155:342:HHGFNDSXY:1:2372:11921:22952 2:N:0:GAACCTAG+TCCGCATA
TCGTGTGGGTCGTCGAACCTCACACCGCCATCAAGATATATGCCTGACATCCCCGGCATCGGGAACTC
AGCCCTAATCGCTTCGCTCACTGCCGCTGATGCGAAGCTTGATGCAGAGTAAAAATCCCCCTGTGTGG
+
FFFFFFFFFFFFFFFFFFFFFFFFFFFFFFFFFFFFFFFFFFFFFFFFFFFFFFFFFFFFFFFFFFFF
FFFFFFFFFFFFFFFFFFFFFFFFFFFFFFFFFFFFF:FFFFFFFFFFFFFFFFFFFFFFFFFFFFFF
@A00155:342:HHGFNDSXY:1:2245:14850:35540 2:N:0:GAACCTAG+TCCGCATA
TCGTGTGGGTCGTCGAACCTCACACCGCCATCAAGATATATGCCTGACATCCCCGGCATCGGGAACTC
AGCCCTAATCGCTTCGCTCACTGCCGCTGATGCGAAGTTTGATGCAGAGTAAAAATCCCCCTGTGTGG
+
FFFFFFFFFFFFFFFFFFFFFFFFFFFFFFFFFFFFFFFFFFFFFFFFFFFFFFFFFFFFFFFFFFFF
FFFFFFFFFFFFFFFFFFFFFFFFFFFFFFFFFFFFFFFFFFFFFFFFFFFFFFFFFFFFFFFFFFFF
@A00155:342:HHGFNDSXY:1:2571:11595:19570 2:N:0:GAACCTAG+TCCGCATA
TCGTGTGGGTCGTCGAACCTCACACCGCCATCAAGATATATGCCTGACATCCCCGGCATCGGGAACTC
AGCCCTAATCGCTTCGCTCACTGCCGCTGATGCGAAGTTTGATGCAGAGTAAAAATCCCCCTGTGTGG
+
FFFFFFFFFFFFFFFFFFFFFFFFFFFFFFFFFFFFFFFFFFFFFFFFFFFFFFFFFFFFFFFFFFFF
FFFFFFF,FFFFFFFFFFFFFFFFFFFFFFFFFFFFFFFFFFFFFFFFFFFFFFFFFFFFFFFFFFFF
```

```
@A00155:342:HHGFNDSXY:1:2245:14850:35540 1:N:0:GAACCTAG+TCCGCATA
TCGTGTGGGTCGTCGAACCTCACACCGCCATCAAGATATATGCCTGACATCCCCGGCATCGGGAACTC
AGCCCTAATCGCTTCGCTCACTGCCGCTGATGCGAAGTTTGATGCAGAGTAAAAATCCCCCTGTGTGG
+
FFFFFFFFFFFFFFFFFFFFFFFFFFFFFFFFFFFFFFFFFFFFFFFFFFFFFFFFFFFFFFFFFFFF
FFFFFFFFFFFFFFFFFFFFFFFFFFFFFFFFFFFF:FFFFFFFFFFFFFFFFFFFFFFFFFFFFFFF
@A00155:342:HHGFNDSXY:1:1138:14977:36354 1:N:0:GAACCTAG+TCCGCATA
CGTGTGGGTCGTCGAACCTCACACCGCCATCAAGATATATGCCTGACATCCCCGGCATCGGGAACTCA
GCCCTAATCGCTTCGCTCACTGCCGCTGATGCGAAGTTTGATGCAGAGTAAAAATCCCCCTGTGTGGA
+
FFFFFFFFFFFFFFFFFFFFFFFFFFFFFFFFFFFFFFFFFFFFFFFFFFFFFFFFFFFFFFF:FFFF
FFFFFFFFFFFFFFFFFFFF:FFFFFFFFFFFFF:FFFFFFFFFFFFFFFFFFFFFFFFFFF,FFFF:
@A00155:342:HHGFNDSXY:1:1138:14977:36354 2:N:0:GAACCTAG+TCCGCATA
GGGTCGTCGAACCTCACACCGCCATCAAGATATATGCCTGACATCCCCGGCATCGGGAACTCAGCCCT
AATCGCTTCGCTCACTGCCGCTGATGCGAAGTTTGATGCAGAGTAAAAATCCCCCTGTGTGGAGTAC
+
FFFFFFFFFFFFFFFFFFFFFFFFFFFFFFFFFFFFFFF:FFFFFFFFFFFFFFFFFFFFFFFFFFFF
::FFFFFFFFFFFFFFFFFFF:FFFFFFFFFFFFFFFFFFFFFFFFFFFFFFFFFFF,F:FFFFFFFFF
@A00155:342:HHGFNDSXY:1:1276:19678:29340 2:N:0:GAACCTAG+TCCGCATA
GGGTCGTCGAACCTCACACCGCCATCAAGATATATGCCTGACATCCCCGGCATCGGGAACTCAGCCCT
AATCGCTTCGCTCACTGCCGCTGATGCGAAGTTTGATGCAGAGTAAAAATCCCCCTGTGTGGAGTACA
+
FFFFFFFFF:FFFFFFFFFFFFFFFFFFFFFFFFFFF:FFFFFFFFFFFFFF:FFFFFFFFFFFFFFF,
FFFFFFFFFFFFF,FFFFFFFFFFFFFF,FFFFFFFFFFFFFFFFFFFFFFFFFFFFFFFFFFFFFFFF
@A00155:342:HHGFNDSXY:1:1407:25455:18270 2:N:0:GAACCTAG+TCCGCATA
GTCGAACCTCACACCGCCATCAAGATATATGCCTGACATCCCCGGCATCGGGAACTCAGCCCTAATCG
CTTCGCTCACTGCCGCTGATGCGAAGTTTGATGCAGAGTAAAAATCCCCCTGTGTGGAGTACAAGTCA
+
FFFFFFFFFFFFFFFFFFFFFFFFFFFFFFFFFFFFFF:FFFFFFFFFFF:FFFFFFFFFFFFFFFFF
FFFFFFFF:FFFFFFFFFFFFFFFFFFFFFFFFFFFFFFFFFFFFFFFFFFFFFFFFFFFFFFFFFFF
@A00155:342:HHGFNDSXY:1:1548:29577:15170 2:N:0:GAACCTAG+TCCGCATA
GTCGAACCTCACACCGCCATCAAGATATATGCCTGACATCCCCGGCATCGGGAACTCAGCCCTAATCG
CTTCGCTCACTGCCGCTGATGCGAAGTTTGATGCAGAGTAAAAATCCCCCTGTGTGGAGTACAAGTCA
+
FFFFFF,FFFFFFFFFFFFFFFFFFFFFFFFFFFFFFF,FFFFFFFFFFFFFFFFFFFFFFFFFFFFF
FFFFFFFFFFFFFFFFFFFFFFFFFFFFFFFFFFFFFFFFFFFFFFFFFFFFFFFFFFFFFFFFFFFF
@A00155:342:HHGFNDSXY:1:1260:30888:24925 2:N:0:GAACCTAG+TCCGCATA
CGAACCTCACACCGCCATCAAGATATATGCCTGACATCCCCGGCATCGGGAACTCAGCCCTAATCGCT
TCGCTCACTGCCGCTGATGCGAAGTTTGATGCAGAGTAAAAATCCCCCTGTGTGGAGTACAAGTCATG
+
FFFFFFFF,F,FFFFFFFFFFFFFFFFFFFFFFFFF,FFFFFFFFFFFFFFFFFFFFFFFFFFFFFFF
FFFFFFFFFFFFFFFFFFFFFFFFFFFFFFFFFFFFFFFFFFFFFFFFFFFFFFFFFFFFFFFFFFFF
@A00155:342:HHGFNDSXY:1:2261:31114:8844 2:N:0:GAACCTAG+TCCGCATA
CGAACCTCCCACCGCCATCAAGATATATGCCTGACATCCCCGGCATCGGGAACTCAGCCCTAATCGCT
TCGCTCACTGCCGCTGATGCGAAGTTTGATGCAGAGTAAAAATCCCCCTGTGTGGAGTACAAGTCATG
+
FFFFFFFF,FFFFFFFFFFF:FFF,FF,FFFFFFF,FFFFFFFFFFFFFFF,FFFFFF:FFF::FFFF
FFFFFFFFFFFFFFFFFFFFFFFFFFFFFFFFFFFFFFFFFFFFFFFFFFFFFFFFFFFFFFFFFFFF
@A00155:342:HHGFNDSXY:1:1644:20943:8735 2:N:0:GAACCTAG+TCCGCATA
CGAACCTCACACCGCCATCAAGATATATGCCTGACATCCCCGGCATCGGGAACTCAGCCCTAATCGCT
TCGCTCACTGCCGCTGATGCGAAGTTTGATGCAGAGTAAAAATCCCCCTGTGTGGAGTACAAGTCATG
+
FFFFFFFFFFFFFFFFFFFFFFFFFFFFFFFFFFFFFFFFFFFFFFFFFFFFFFFFFFFFFFFFFFFF
FFFFFFFFFFFFFFFFFFFFFFFFFFFFFFFFF:FFFFFFFFFFFFFFFFFFFFFFFFFFFFFFFFFF
```

```
@A00155:342:HHGFNDSXY:1:2142:7292:17033 2:N:0:GAACCTAG+TCCGCATA
AACCTCACACCGCCATCAAGATATATGCCTGACATCCCCGGCATCGGGAACTCAGCCCTAATCGCTTC
GCTCACTGCCGCTGATGCGAAGTTTGATGCAGAGTAAAAATCCCCCTGTGTGGAGTACAAGTCATGAG
+
FFFFFFFFFFFFFFFFF:FFFFFFFFFFFFFFFFFFFFFFFFFFFFFFFFFFFFFFFFFFFFFFFFFF
FFFFFFFFFFFFFFFFFFFFFFFFFFFFFFFFFFFFFFFFFFFFFFFFFFFFFFFFFFFFFFFFFFFF
@A00155:342:HHGFNDSXY:1:2205:25509:9283 2:N:0:GAACCTAG+TCCGCATA
AACCTCACACCGCCATCAAGATATATGCCTGACATCCCCGGCATCGGGAACTCAGCCCTAATCGCTTC
GCTCACTGCCGCTGATGCGAAGTTTGATGCAGAGTAAAAATCCCCCTGTGTGGAGTACAAGTCATGAG
+
FFFFFFFFFFFFFFFFFFFFFFFFFFFFFFFFFFFFFFFFFFFFFF:FFFFFFFFFFFFFFFFFFFFF
FFFFFFFFFFFFFFFFFFFFFFFFFFFFFFFFFFFFFFFFFFFFFFFFFFFFFFFFFFFFFFFFFFFF
@A00155:342:HHGFNDSXY:1:2170:14904:6511 2:N:0:GAACCTAG+TCCGCATA
CTCACACCGCCATCAAGATATATGCCTGACATCCCCGGCATCGGGAACTCAGCCCTAATCGCTTCGCT
CACTGCCGCTGATGCGAAGTTTGATGCAGAGTAAAAATCCCCCTGTGTGGAGTACAAGTCA
+
FFFFFFFFFFF:FFFFFFFFFFFFFFFFFFFFFFFFFFFFFFFFFFFFFFFF:FFFFFF:F:FFFFFF
FFFFFFFFFFFFFFFFFFFFFFFFFFFFFFFFFFFFFFFFFFFFFFFFFFFFF:FFFFFFF
@A00155:342:HHGFNDSXY:1:2574:26069:5400 2:N:0:GAACCTAG+TCCGCATA
CTCACACCGCCATCAAGATATATGCCTGACATCCCCGGCATCGGGAACTCAGCCCTAATCGCTTCGCT
CACTGCCGCTGATGCGAAGTTTGATGCAGAGTAAAAATCCCCCTGTGTGGAGTACAAGTCATGAGGGT
+
FFFFFFFFFFFFFFFFFFFFFFFFFFFFFFFFFFFFFFFFFFFFFFFFFFFFFFFFFFFFFFFFFFFF
FFFFFFFFFFFFFFFFFFFFFFFFFFFFFFFFFFFFFFFFFFFFFFFFFFFFFFFFFFFFFFFFFFFF
@A00155:342:HHGFNDSXY:1:1260:30888:24925 1:N:0:GAACCTAG+TCCGCATA
GCCATCAAGATATATGCCTGACATCCCCGGCATCGGGAACTCAGCCCTAATCGCTTCGCTCACTGCCG
CTGATGCGAAGTTTGATGCAGAGTAAAAATCCCCCTGTGTGGAGTACAAGTCATGAGGGTCCACTTCG
+
FFFFFFFFFFFFF:FFFFFFFFFFFFFFFFFFFFFFFFFFFFFFFFFFFFFFFFFFFFFFFFFFFFFF
FFFFFFFFFFFFFFFFFFFFFFFFF:FFFFFFFFFFFFFFFFFFFFFFFFFFFFFFFFFFFFFFFFFF
@A00155:342:HHGFNDSXY:1:2569:11351:10504 2:N:0:GAACCTAG+TCCGCATA
GCCATCAAGATATATGCCTGACATCCCCGGCATCGGGAACTCAGCCCTAATCGCTTCGCTCACTGCCG
CTGATGCGAAGTTTGATGCAGAGTAAAAATCCCCCTGTGTGGAGTACAAGTCATGAGGGTCCACTTCG
+
FFFFFFFFFFFFFFFFFFFFFFFFFFFFFFFFFFFFFFFFFFFFFFFFFFFFFFFFFFFFFFFFFFFF
FFFFFFFFFFFFFFFFFFFFFFF:FFFFFFFFFFFFFFFFFFFFFFFFFFFFFFFFFFFFFFFFFFFF
@A00155:342:HHGFNDSXY:1:2261:31114:8844 1:N:0:GAACCTAG+TCCGCATA
GCCATCAAGATATATGCCTGACATCCCCGGCATCGGGAACTCAGCCCTAATCGCTTCGCTCACTGCCG
CTGATGCGAAGTTTGATGCAGAGTAAAAATCCCCCTGTGTGGAGTACAAGTCATGAGGGTCCACTTCG
+
FFFF,FFFFFFFFFF:::FF:FFFFFFFFFF:FFFFFFFFFFFF:FFFFFFFFFFFFFFFFFFF:FFF
FFFFFFFFF,F:,FFFFFFFFFFFFFFFF,FF::FF:FF:F:FFFFFFFFFFF:FFF:F,FFFF:FFF
@A00155:342:HHGFNDSXY:1:2569:11351:10504 1:N:0:GAACCTAG+TCCGCATA
ATCAAGATATATGCCTGACATCCCCGGCATCGGGAACTCAGCCCTAATCGCTTCGCTCACTGCCGCTG
ATGCGAAGTTTGATGCAGAGTAAAAATCCCCCTGTGTGGAGTACAAGTCATGAGGGTCCACTTCGAGC
+
FFFFFFFF:FFFFFFFFFFFFFFFFFFFFFFFFFFFFFFFFFFFFFFFFFFFFFFFFFFFFFFFFFFF
FFFFFFFFFFFFFFFFFFFFFFFFFFFFFFFFFFFFFFFFFFFFFFFFFFFFFFFFFFFFFFFFFFFF
@A00155:342:HHGFNDSXY:1:2604:15121:7795 2:N:0:GAACCTAG+TCCGCATA
ATCAAGATATATGCCTGACATCCCCGGCATCGGGAACTCAGCCCTAATCGCTTCGCTCACTGCCGCTG
ATGCGAAGTTTGATGCAGAGTAAAAATCCCCCTGTGTGGAGTACAAGTCATGAGGGTCCACTTCGAGC
+
FFFFFFFFFFFFFFFFFFFFFFFFFFFFFFFFFFFFFFFFFFFFFF:FFFFFFFFFFFFFFFF:FFFF
FFFFFFFFFFFFFFFFFFFFFFFFFFFFFFFFF:FFFFFFFFFFFFFFFFFFFFFFFFFFFFFFFFFF
```

```
@A00155:342:HHGFNDSXY:1:1422:10465:15233 2:N:0:GAACCTAG+TCCGCATA
ATCAAGATATATGCCTGACATCCCCGGCATCGGGAACTCAGCCCTAATCGCTTCGCTCACTGCCGCTG
ATGCGAAGTTTGATGCAGAGCAAAAATCCCCCTGTGTGGAGTACAAGTCATGAGGGTCCACTTCGAGC
+
FFFFFFFFFFFFFFFFFFFFFFFFFFFFFFFFFFFFFFFFFFFFFFFFFFFFFFFFFFFFFFFFFFFF
FFFFFFFFFFFFFFFFFFFFFFFFFFFFFFFFFFFFFFFFFFFFFFFFFFFFFFFFFFFFF:FFFFFF
@A00155:342:HHGFNDSXY:1:2508:8947:4617 1:N:0:GAACCTAG+TCCGCATA
AAGATATATGCCTGACATCCCCGGCATCGGGAACTCAGCCCTAATCGCTTCGCTCACTGCCGCTGATG
CGAAGTTTGATGCAGAGTAAAAATCCCCCTGTGTGGAGTACAAGTCATGAGGGTCCACTTCGAGCCCC
+
FFFFFFFFFFFF,FFFFFFFFFFFFFFFFFFFFFFFFFFFFFFFFFFFF:FFF:FFFFFFFFFFFFFF
FFFFF:FFFFFFFFFFFF,F:FFFFFFFFFF:F:::FFFFFFF:FFFF:FFFFFFFFF:FFFFFFFFF
@A00155:342:HHGFNDSXY:1:2170:14904:6511 1:N:0:GAACCTAG+TCCGCATA
AGATATATGCCTGACATCCCCGGCATCGGGAACTCAGCCCTAATCGCTTCGCTCACTGCCGCTGATGC
GAAGTTTGATGCAGAGTAAAAATCCCCCTGTGTGGAGTACAAGTCATGAGGGTCCACTTCG
+
FFFFFFFFFFFFFFFFFFFFFFFFFFFFFFFFFFFFFFFF:FFFFFFFFFFFFFFFFFFFFFFFFFFF
FFFFFFFFFFFFFFFFFFFFFFFFFFFFFFFFFFFFFFFFFFFFFFFFFFFFFFFFFFFFF
@A00155:342:HHGFNDSXY:1:1571:22435:15546 1:N:0:GAACCTAG+TCCGCATA
TATGCCTGACATCCCCGGCATCGGGAACTCAGCCCTAATCGCTTCGCTCACTGCCGCTGATGCGAAGT
TTGATGCAGAGTAAAAATCCCCCTGTGTGGAGTACAAGTCATGAGGGTCCACTTCGAGCCCCATTCTG
+
FFFFFFFFFFFFFFFFFFFFFFFFFFF:FFFFFFFFFFFFFFFFFFFFFFFFFFFFFFFFFFFFFFFF
FFFFFFFFFFFFFFFFFFFFFFFFFFFFFFFFFF:FFF:FFFFFFFFFFFFFFFFFFFFFFFFFFFFF
@A00155:342:HHGFNDSXY:1:2121:5195:6198 1:N:0:GAACCTAG+TCCGCATA
ATGCCTGACATCCCCGGCATCGGGAACTCAGCCCTAATCGCTTCGCTCACTGCCGCTGATGCGAAGTT
TGATGCAGAGTAAAAATCCCCCTGTGTGGAGTACAAGTCATGAGGGTCCACTTCGAGCCCCATTCTGT
+
FFFFFFFFFFFFFFFFFFFFFFFFFFFFFFFFFFFFFFFFFFFFFFFFFFFFFFFFFFFFFFFFFFFF
FFFFFF,FFFFFFF:FFF:FFFFFFFFFFFFFFFFFFFFFFFFFFFFFFFFFFFFFFFFFFFFFFFFF
@A00155:342:HHGFNDSXY:1:2551:5873:15765 1:N:0:GAACCTAG+TCCGCATA
ATGCCTGACATCCCCGGCATCGGGAACTCAGCCCTAATCGCTTCGCTCACTGCCGCTGATGCGAAGTT
TGATGCAGAGTAAAAATCCCCCTGTGTGGAGTACAAGTCATGAGGGTCCACTTCGAGCCCCATTCTGT
+
FFFFFFFFFFFFFFFFFFFFFFFFFFFFFFFFFFFFFFFFFFFFFFFFFFFFFFFFFFFFFFFFFFFF
FFFFFFFFFFFFFF:FFFFFFFFFFFFFFFFFFFFFFFFFFFFFFFFFFFFFFFFFFFFFFFFFFFFF
@A00155:342:HHGFNDSXY:1:2669:15808:16125 1:N:0:GAACCTAG+TCCGCATA
ATGCCTGACATCCCCGGCATCGGGAACTCAGCCCTAATCGCTTCGCTCACTGCCGCTGATGCGAAGTT
TGATGCAGAGTAAAAATCCCCCTGTGTGGAGTACAAGTCATGAGGGTCCACTTCGAGCCCCATTCTGT
+
FFFFFFFFFFFFFFFFFFFFFFFFFFFFFFFFFFFFFFFFFFFFFFFFFFFFFFFFFFFFFFFFFFFF
FFFFFFFFFFFFFFFFFFFFFFFFFFFFFFFFFFFFFFFFFFFFFFFFFFFFFFFFFFFFFFFFFFFF
@A00155:342:HHGFNDSXY:1:1555:24297:17268 1:N:0:GAACCTAG+TCCGCATA
TGCCTGACATCCCCGGCATCGGGAACTCAGCCCTAATCGCTTCGCTCACTGCCGCTGATGCGAAGTTT
GATGCAGAGTAAAAATCCCCCTGTGTGGAGTACAAGTCATGAGGGTCCACTTCGAGCCCCATTCTGTT
+
FFFFFFFFFFFFFFFFFFFFFFFFFFFFFFFFFF:FFFFFFFFFFFFFFFFFFFFFFFFFFFFFFF:F
FFFFFFFFFFFFFFFFFFFFFFFFFFFFFFFFFFFFFFFFFFFFFFFFFFFFFFFFFFFFFFFFFFFF
@A00155:342:HHGFNDSXY:1:1641:11035:26741 1:N:0:GAACCTAG+TCCGCATA
GCCTGACATCCCCGGCATCGGGAACTCAGCCCTAATCGCTTCGCTCACTGCCGCTGATGCGAAGTTTG
ATGCAGAGTAAAAATCCCCCTGTGTGGAGTACAAGTCATGAGGGTCCACTTCGAGCCCCATTCTGTTA
+
FFFFFFFFFFFFFFFFFFFFFFF:FFFFFFFFFFFFFFFFFFFFFFFFFFFFFFFFFFFFFFFFFFFF
FFFFFFFFFFFFFFFFFFFFFFFFFFFFFFFFFFF:FFFFFFFFFFFFFFFFFFFFFFFFFFFFFFFF
```

```
@A00155:342:HHGFNDSXY:1:2150:13548:32565 1:N:0:GAACCTAG+TCCGCATA
GCCTGACATCCCCGGCATCGGGAACTCAGCCCTAATCGCTTCGCTCACTGCCGCTGATGCGAAGTTTG
ATGCAGAGTAAAAATCCCCCTGTGTGGAGTACAAGTCATGAGGGTCCACTTCGAGCCCCATTCTGTTA
+
FFFFFFFFFFFFFFFFFFFFFFFFFFFFFFFFFFFFFFFFFFFFFFFFFFFFFFFFFFFFFFFFFFFF
FFFFFFFFFFFFFFFFFFFFFFFFFFFFFFFFFFFFFFFFFFFFFFFFFFFFFFFFFFFFFFFFFFFF
@A00155:342:HHGFNDSXY:1:1544:30969:23124 1:N:0:GAACCTAG+TCCGCATA
GCCTGACATCCCCGGCATCGGGAACTCAGCCCTAATCGCTTCGCTCACTGCCGCTGATGCGAAGTTTG
ATGCAGAGTAAAAATCCCCCTGTGTGGAGTACAAGTCATGAGGGTCCACTTCGAGCCCCATTCTGTTA
+
FFFFFFFFFFFFFFFFFFFFFFFFFFFFFFFFFFFFFFFFFFFFFFFFFFFFFFFFFFFFFFFFF:FF
FFFFFFFFFFFFFFFFFFFFFFFFFFFFFFFFFFFFFFFFFFFFFFFFFFFFFFFFFFFFFFFFFFFF
@A00155:342:HHGFNDSXY:1:1234:30626:4805 2:N:0:GAACCTAG+TCCGCATA
CTGACATCCCCGGCATCGGGAACTCAGCCCTAATCGCTTCGCTCACTGCCGCTGATGCGAAGTTTGAT
GCAGAGTAAAAATCCCCCTGTGTGGAGTACAAGTCATGAGGGTCCACTTCGAGCCCCAT
+
FFFFFFFFFFFFFFFFFFFFFFFFFFFFFFFF,FFFFFFFFFFFFFFFFFFFFFFFFFFFFFFFFFFF
FFFFFFFFFFFFFFFFFFFFFFFFFFFFFFFFFFFFFFFFFFFFFFFFFFFFFFFFFFF
@A00155:342:HHGFNDSXY:1:1126:20193:13166 1:N:0:GAACCTAG+TCCGCATA
CTGACATCCCCGGCATCGGGAACTCAGCCCTAATCGCTTCGCTCACTGCCGCTGATGCGAAGTTTGAT
GCAGAGTAAAAATCCCCCTGTGTGGAGTACAAGTCATGAGGGTCCACTTCGAGCCCCATTCTGTTAGA
+
FFFFFFFFFFFFFFFFFFFFF:FFFFFFFFFFFFFFFFFFFFFFFFFFFFFFFFFFFFFFFFFFFFFF
FFFFFFF:FFFFFFFFFFFFFFFFFFFFFFFFFFFFFFFFFFFFFFFFFFFFFFFFFFFFFFFFFFFF
@A00155:342:HHGFNDSXY:1:2375:16089:19805 1:N:0:GAACCTAG+TCCGCATA
TGACATCCCCGGCATCGGGAACTCAGCCCTAATCGCTTCGCTCACTGCCGCTGATGCGAAGTTTGATG
CAGAGTAAAAATCCCCCTGTGTGGAGTACAAGTCATGAGGGTCCACTTCGAGCCCCATTCTGTTAGAC
+
FFFFFFFFFFFFFFFFFFFFFFFFFFFFFFFFFFFFFFFFFFFFFFFFFFFFFFFFFFFFFFFFFFFF
FFFFFFFFFFFFFFFFFFFFFFFFFFFFFFFFFFFFFFFFFFFFFFFFFFFFFFFFFFFFFFFFFFFF
@A00155:342:HHGFNDSXY:1:2646:14570:28573 1:N:0:GAACCTAG+TCCGCATA
GACATCCCCGGCATCGGGAACTCAGCCCTAATCGCTTCGCTCACTGCCGCTGATGCGAAGTTTGATGC
AGAGTAAAAATCCCCCTGTGTGGAGTACAAGTCATGAGGGTCCACTTCGAGCCCCATTCTGTTAGAC
+
FFFFFFFFFFFFFFF:FFFFFFFFFFFFFFFFFF:FFF:FFFFFFFFFFF:FFFFFFFFFFFFF:FFF
:FFFFFF:FFFFFFFFFF::FFF:FFFF,FFFFF:FFFFFFFF,FFFFFFFFFFF,FFFFF:F:FFF
@A00155:342:HHGFNDSXY:1:2174:32154:12649 1:N:0:GAACCTAG+TCCGCATA
GACATCCCCGGCATCGGGAACTCAGCCCTAATCGCTTCGCTCACTGCCGCTGATGCGAAGTTTGATGC
AGAGTAAAAATCCCCCTGTGTGGAGTACAAGTCATGAGGGTCCACTTCGAGCCCCATTCTGTTAGACC
+
FFFFFFFFFFFFFFFFFFFFFFFFFFFFFFFFFFFFFFFFFFFFFFFFFFFFFF:FFFFFFFFFFFFF
FFFFFFFFFFFFFFFFFFFFFFFFFFFFFFFFFFFFFFFFFFFFFFFFFFFFFFFFFFFFFFFFFFF:
@A00155:342:HHGFNDSXY:1:1542:10411:5118 1:N:0:GAACCTAG+TCCGCATA
ACATCCCCGGCATCGGGAACTCAGCCCTAATCGCTTCGCTCACTGCCGCTGATGCGAAGTTTGATGCA
GAGTAAAAATCCCCCTGTGTGGAGTACAAGTCATGAGGGTCCACTTCGAGCCCCATTCTGTTAGACCT
+
FFFFFFFFF:FFFFFFFFFFFFFFFFFFFF:FFFFFFFFFFFF:FFFFFFFFFFFFFFF:FFFFFFFF
FF::FFFFFFFFFFFFFFFFFFFFFFFFFFFFFFFFFFFFFFFFFFFFFFFFFFFF:FFFFFFFFFFF
@A00155:342:HHGFNDSXY:1:2409:32660:1626 2:N:0:GAACCTAG+TCCGCATA
CATCCCCGGCATCGGGAACTCAGCCCTAATCGCTTCGCTCACTGCCGCTGATGCGAAGTTTGATGCAG
AGTAAAAATCCCCCTGTGTGGAGTACAAGTCATGAGGGTCCACTTCGAGCCCCATTCTGTTAGACCT
+
FF:FF:FF,FFFFFFFF:FFFFFFFFFFFFFFFFFF:FFF:FFFFF:FF:FFFFFFFFFFFFFFFFFF
FFFFFF:FFF,FFFFFFFF:FFFFFFFFFF:FFFFFFFFFFFFFFFFFFFFFFFFFFFFFF:FFF:F
```

```
@A00155:342:HHGFNDSXY:1:1217:9742:35837 2:N:0:GAACCTAG+TCCGCATA
CATCCCCGGCATCGGGAACTCAGCCCTAATCGCTTCGCTCACTGCCGCTGATGCGAAGTTTGATGCAG
AGTAAAAATCCCCCTGTGTGGAGTACAAGTCATGAGGGTCCACTTCGAGCCCCATTCTGTTAGACCTT
+
FFFFFFFFFFFFFFFFFFFFFFFFFFFFFFFFF,FFFFFFFFFFFFFFFFFFFFFFFFFFFFFFFFFF
FFFFFFFFFFFFFF:FFFFFFFFFFFFFFFFF:FFFFFFFFFFFFFFFFFFFFFF:FFFFFFFFFFFF
@A00155:342:HHGFNDSXY:1:1654:20952:23218 2:N:0:GAACCTAG+TCCGCATA
CATCCCCGGCATCGGGAACTCAGCCCTAATCGCTTCGCTCACTGCCGCTGATGCGAAGTTTGATGCAG
AGTAAAAATCCCCCTGTGTGGAGTACAAGTCATGAGGGTCCACTTCGAGCCCCATTCTGTTAGACCTT
+
FFFFFFFFFFFFFFFFFFF:FFFFFFFFFFFFF,FFFFFFFFFFFFFFFFFFFFFFFFFFFFFFFFFF
FFFFFFFFFFFFFF:FFFFFFFFFFFFFFFFFFFFFFFFFFFFFFFFFF::FFFFFFFFFFFFFFFFF
@A00155:342:HHGFNDSXY:1:2265:25898:21136 2:N:0:GAACCTAG+TCCGCATA
CATCCCCGGCATCGGGAACTCAGCCCTAATCGCTTCGCTCACTGCCGCTGATGCGAAGTTTGATGCAG
AGTAAAAATCCCCCTGTGTGGAGTACAAGTCATGAGGGTCCACTTCGAGCCCCATTCTGTTAGACCTT
+
FFFF:FFFFFFFFFFFFFFFFFFFFFFF:FFFFFFFFFFFFFFFFFFFFFFFFFFFFFFFFFFFFFFF
FFFFFFFFFFFFFFFFFFFFFFFFFFFFFFFFFFFFFFFFFFFFFFFFFFFFFFFFFFFFFFFFFFFF
@A00155:342:HHGFNDSXY:1:2368:7853:28463 2:N:0:GAACCTAG+TCCGCATA
CATCCCCGGCATCGGGAACTCAGCCCTAATCGCTTCGCTCACTGCCGCTGATGCGAAGTTTGATGCAG
AGTAAAAATCCCCCTGTGTGGAGTACAAGTCATGAGGGTCCACTTCGAGCCCCATTCTGTTAGACCTT
+
FFFFFFFFFFFFFFFFFFFFFFFFFFFFFFFFFFFFFFFFFFFFFFFFFFFFFFFFFFFFFFFFFFFF
FFFFFFFFFFFFFFFFFFFFFFFFFFFFFFFFFFFFFFFFFFFFFFFFFFFFFFFFFFFFFFFFFFFF
@A00155:342:HHGFNDSXY:1:2549:11939:15060 2:N:0:GAACCTAG+TCCGCATA
CATCCCCGGCATCGGGAACTCAGCCCTAATCGCTTCGCTCACTGCCTCTGATGCGAAGTTTGATGCAG
AGTAAAAATCCCCCTGTGTGGAGTACAAGTCATGAGGGTCCACTTCGAGCCCCATTCTGTTAGACCTT
+
FFFFFFFFFFFFFFFF:FFFFFFFFFFFFFFFF:FFFFFFFFFFFF,F:FFFF:FFFF,FFFFFFFFF
FF:FFFF:FFFFFFFFFFFFFFFF:FF:FFFFFFFFFFFFFFFFFFFFFFFFFFFFFFFFFFFFFFFF
@A00155:342:HHGFNDSXY:1:2265:25301:25113 2:N:0:GAACCTAG+TCCGCATA
CATCCCCGGCATCGGGAACTCAGCCCTAATCGCTTCGCTCACTGCCGCTGATGCGAAGTTTGATGCAG
AGTAAAAATCCCCCTGTGTGGAGTACAAGTCATGAGGGTCCACTTCGAGCCCCATTCTGTTAGACCTT
+
FFFFFFFFFFFFFFFFFFFFFFFFFFFFFFFFFFFFFFFFFFFFFFFFFFFFFFFFFFFFFFFFFFFF
FFFFFFFFFFFFFFFFFFFFFFFFFFFFFFFFFFFFFFFFFFFFFFFFFFFFFFFFFFFFFFFFFFFF
@A00155:342:HHGFNDSXY:1:2470:6063:16752 2:N:0:GAACCTAG+TCCGCATA
ATCCCCGGCATCGGGAACTCAGCCCTAATCGCTTCGCTCACTGCCGCTGATGCGAAGTTTGATGCAGA
GTAAAAATCCCCCTGTGTGGAGTACAAGTCATGAGGGTCCACTTCGAGCCCCATTCTGTTAGACCTTA
+
F:FFF,FFFFFFFFFFFF:FFFFF:FFFFFFFFFFFFFFFFFFFFF:FFFFFFFFFF:FFFFFFFFFF
FFFFFFFFFFFFFFFFFFFFFFFFFFFFFFFFFFFFFFFFFFFFFFFFFFFFFFFFFFFFFFFFFFFF
@A00155:342:HHGFNDSXY:1:1557:23014:11256 2:N:0:GAACCTAG+TCCGCATA
ATCCCCGGCATCGGGAACTCAGCCCTAATCGCTTCGCTCACTGCCGCTGATGCGAAGTTTGATGCAGA
GTAAAAATCCCCCTGTGTGGAGTACAAGTCATGAGGGTCCACTTCGAGCCCCATTCTGTTAGACCTTA
+
FFFFFFFF:FFFFFFFFFFFFFFF:FF:FFF:FF:FFFFFF,FFFF:FFFFFFFFFFFFFFFFFFF:F
FFFFFFFFFFFFFFFFFFFFFFFFFF:FFFFFFFFFFFFFFFFFFFF,F:FFFF::FFFFFFFFFF:F
@A00155:342:HHGFNDSXY:1:1425:7672:7451 2:N:0:GAACCTAG+TCCGCATA
ATCCCCGGCATCGGGAACTCAGCCCTAATCGCTTCGCTCACTGCCGCTGATGCGAAGTTTGATGCAGA
GTAAAAATCCCCCTGTGTGGAGTACAAGTCATGAGGGTCCACTTCGAGCCCCATTCTGTTAGACCTTA
+
FFFFFFFFFFFFFFFFFF:FFFFFFFFFFFFFFFFFFFFFFFFFFFFFFFFFF:FFFFFFFFFFFFFF
FFFFFFFFFF:FFFFFFFFFFFFFFFFFFFFFFFFFFFFFFFFFFFFFFFFFFFFFFFFFFFFFFFFF
```

```
@A00155:342:HHGFNDSXY:1:1364:18159:1438 2:N:0:GAACCTAG+TCCGCATA
ATCCCCGGCATCGGGAACTCAGCCCTAATCGCTTCGCTCACTGCCGCTGATGCGAAGTTTGATGCAGA
GTAAAAATCCCCCTGTGTGGAGTACAAGTCATGAGGGTCCACTTCGAGCCCCATTCTGTTAGACCTTA
+
FFFFFFFFFFFFFFFFFFFFFFFFFFF::FFFF:FFFFFFFFFFFFFFFFFF:FFFF:FFFFFFFFFF
FFFFFFFFFFFFFFFFFFFFFFFFFFFFFFFFFFFFFFFFFFFFFFFFF:FFFFFFFFF:FFFFFF:F
@A00155:342:HHGFNDSXY:1:2174:32154:12649 2:N:0:GAACCTAG+TCCGCATA
TCCCCGGCATCGGGAACTCAGCCCTAATCGCTTCGCTCACTGCCGCTGATGCGAAGTTTGATGCAGAG
TAAAAATCCCCCTGTGTGGAGTACAAGTCATGAGGGTCCACTTCGAGCCCCATTCTGTTAGACCTTA
+
FFFFFFFFFFFFFFFFFFFFFFFFFFFF:FF:FFFFFFFFFFFFFFFFFFFFFFFFFFFFFFFFFFFF
FFFFFFFFFFFFFFFFFFFFFFFFFFFFFFFFFFFFFFFFFFFFFFFFFFFFFFFFFFFFFFF:,:F
@A00155:342:HHGFNDSXY:1:1110:24261:28698 1:N:0:GAACCTAG+TCCGCATA
CCGGCATCGGGAACTCAGCCCTAATCGCTTCGCTCACTGCCGCTGATGCGAAGTTTGATGCAGAGTAA
AAATCCCCCTGTGTGGAGTACAAGTCATGAGGGTCCACTTCGAGCCCCATTCTGTTAGA
+
FFFFFFFFFFFFFFFFFFFFFFF:FFFFFFFFFFFFFFFFFF:FF::FFFFFFFFFFFFFFFFFFFFF
FFFFFFFFFFFFFFFFFFFFFFFFFFFFFFFFFFFFFFFFFFFFFFFFFFFFFFFFFFF
@A00155:342:HHGFNDSXY:1:2154:5665:31501 2:N:0:GAACCTAG+TCCGCATA
CCGGCATCGGGAACTCAGCCCTAATCGCTTCGCTCACTGCCGCTGATGCGAAGTTTGATGCAGAGTAA
AAATCCCCCTGTGTGGAGTACAAGTCATGAGGGTCCACTTCGAGCCCCATTCTGTTAGACCTT
+
FFFFFFFFFFFFFFFFFFFFFFFFFFF::FFFFFFFFFF,FFFFFFFFFFFFFFFFFFFFFFFFFFFF
FFFFFFF:FFFFF:FFFFF:FFFFFFFFFFFFFFFFFFFFFFFFFFFFFFF:FFFFFFFFFFF
@A00155:342:HHGFNDSXY:1:2549:23511:23829 2:N:0:GAACCTAG+TCCGCATA
CTGGCATCGGGAACTCAGCCCTAATCGCTTCGCTCACTGCCGCTGATGCGAAGTTTGATGCAGAGTAA
AAATCCCCCTGTGTGGAGTACAAGTCATGAGGGTCCACTTCGAGCCCCATTCTGTTAGACCTTA
+
FFFFFFFFFFFFFFFFFFFFFFFFFFFFFFFFFFFFFFFFFFFFFFFF:FFFF:FFFFFFFFFFFFFF
FFFFFFFFFFFFFFFFFFFFFFFFFFFFFFFFFFFFFFFFFFFFFFFFFFFFFFFFFFFFFFFF
@A00155:342:HHGFNDSXY:1:1332:31241:26287 2:N:0:GAACCTAG+TCCGCATA
GGCATCGGGAACTCAGCCCTAATCGCTTCGCTCACTGCCGCTGATGCGAAGTTTGATGCAGAGTAAAA
ATCCCCCTGTGTGGAGTACAAGTCATGAGGGTCCACTTCGAGCCCCATTCTGTTAGACCTTACCGCAC
+
FFFFFF,FFFFFFFFFFFFFFFFFFFFFFFFFFFFFFFFFFFFFFFFFFFFFFFFFFFFFFFFFFFFF
FFFFFFFFFFFFFFFFFFFFFFFFFFFFFFFFFFFFFFFFFFFFFFFFFFFFFFFFFFFFFFFFFFFF
@A00155:342:HHGFNDSXY:1:1621:29360:36307 1:N:0:GAACCTAG+TCCGCATA
GCATCGGGAACTCAGCCCTAATCGCTTCGCTCACTGCCGCTGATGCGAAGTTTGATGCAGAGTAAAAA
TCCCCCTGTGTGGAGTACAAGTCATGAGGGTCCACTTCGAGCCCCATTCTGTTAGACCTTACCGCACA
+
FFFFFFFFFFFFFFFFFFFFFFFFFFFFFFFFFFFFFFFF:FFFFFFFFFFFFFFFFFFFFFFFFFFF
FFFFFFFFFFFFFFFFFFFFFFFFFFFFFFFFFFF:FFFFFFFFFFFFFFFFFFFFFFFFFFFFFFFF
@A00155:342:HHGFNDSXY:1:1621:29541:36119 1:N:0:GAACCTAG+TCCGCATA
GCATCGGGAACTCAGCCCTAATCGCTTCGCTCACTGCCGCTGATGCGAAGTTTGATGCAGAGTAAAAA
TCCCCCTGTGTGGAGTACAAGTCATGAGGGTCCACTTCGAGCCCCATTCTGTTAGACCTTACCGCACA
+
FFFFFFFFFFFFFFFFFFFFFFFFFFFFFFFFFFFFFFFF:FFFFFFFFFFF:FFFFFFFFFFFFFFF
FFFFFFFFFFFFFFFFFFFFFFFF:FFFFFFFFFF:FFFFFFFFFFFFFFFFFFFFFFFFFFFFFFFF
@A00155:342:HHGFNDSXY:1:1126:20193:13166 2:N:0:GAACCTAG+TCCGCATA
CATCGGGAACTCAGCCCTAATCGCTTCGCTCACTGCCGCTGATGCGAAGTTTGATGCAGAGTAAAAAT
CCCCCTGTGTGGAGTACAAGTCATGAGGGTCCACTTCGAGCCCCATTCTGTTAGACCTTACCGC
+
FFFFFFFFFFFFFFFFFF,,FFFFFFFFFFFFF:FFFFFFFFFFFFFFFFFFFFFFFFFFFFFFFFFF
FFFFFFFFFFFFFF:FFFFFFFFFFFFFF:FFFFFFFFFFFFFFF,FFFFFFFFFFFFFFFFFF
```

```
@A00155:342:HHGFNDSXY:1:2562:30680:27164 2:N:0:GAACCTAG+TCCGCATA
CATCGGGAACTCAGCCCTAATCGCTTCGCTCACTGCCGCTGATGCGAAGTTTGATGCAGAGTAAAAAT
CCCCCTGTGTGGAGTACAAGTCATGAGGGTCCACTTCGAGCCCCATTCTGTTAGACCTTACCGC
+
FFFFF:FFFFFFFFFFFFFFFFFFFFF:FFFFFFFFFFFFFFFFFFFFFFFFFFFFFFFFFFFFFFFF
FFFFFFFFFFFFFFFFFFFFFFFFFFFFFFFFFFFFFFFFFFFFFFFFFFFFFFFFFFFFFFFF
@A00155:342:HHGFNDSXY:1:1224:4137:26725 1:N:0:GAACCTAG+TCCGCATA
CATCGGGAACTCAGCCCTAATCGCTTCGCTCACTGCCGCTGATGCGAAGTTTGATGCAGAGTAAAAAT
CCCCCTGTGTGGAGTACAAGTCATGAGGGTCCACTTCGAGCCCCATTCTGTTAGACCTTACCGCACAA
+
FFFFFFFFFFFFFFFFFFFFFFFFFFFFFFFFFFFF:FFFFFF:FFFFFFF:FFFFFFFFF:FFFFFF
FFFFFFFFFFFFFFFFFFFFFFFFF:FFFFFFFFFFFFFFFFFFFFFFFFFFFFFFFFFFF:FFFFFF
@A00155:342:HHGFNDSXY:1:1617:26558:7341 1:N:0:GAACCTAG+TCCGCATA
CATCGGGAACTCAGCCCTAATCGCTTCGCTCACTGCCGCTGATGCGAAGTTTGATGCAGAGTAAAAAT
CCCCCTGTGTGGAGTACAAGTCATGAGGGTCCACTTCGAGCCCCATTCTGTTAGACCTTACCGCACAA
+
FFFFFFFFFFFFFFFFFF:FFFFFFFFFFFFFFFFFFFFFFFFFFFFFFFFFFFFFFFFFFFFFFFFF
FFFFFFFFFFFFFFFFFFFFFFFFFFFFFFFFFFFFFFFFFFFFFFFFFFFFFFFFFFFFFFFFFFFF
@A00155:342:HHGFNDSXY:1:2320:28122:29810 1:N:0:GAACCTAG+TCCGCATA
ATCGGGAACTCAGCCCTAATCGCTTCGCTCACTGCCGCTGATGCGAAGTTTGATGCAGAGTAAAAATC
CCCCTGTGTGGAGTACAAGTCATGAGGGTCCACTTCGAGCCCCATTCTGTTAGACCTTACCGCACAA
+
:FF:FFFFFF:FFFFFFFFFFFFF:FFFFFFFFFF:F:FFFFF,FFF:FF::FFFFF,FFFF:FFFF:
FF,FFFF,FFFFFF:FFF,FFF:FFF,FFF:FF,FF:FFFF:FFF:F:FFFF:FFFF:FFFFFFFF:
@A00155:342:HHGFNDSXY:1:1234:30626:4805 1:N:0:GAACCTAG+TCCGCATA
TCGGGAACTCAGCCCTAATCGCTTCGCTCACTGCCGCTGATGCGAAGTTTGATGCAGAGTAAAAATCC
CCCTGTGTGGAGTACAAGTCATGAGGGTCCACTTCGAGCCCCATTCTGTTAGACCTTAC
+
FFFFFFFFFFFFFFF:FFFFFFFFFFFFFFFFFFFFFFF:FFFF:FFFFFFFFFFFFFF:FFFFFFFF
FFFFFFFFFFFFFFFFFFFFFFFFFFFFFFFFFFFFFFFFFFFFFFFFFFFFFFFFFFF
@A00155:342:HHGFNDSXY:1:2549:11939:15060 1:N:0:GAACCTAG+TCCGCATA
TCGGGAACTCAGCCCTAATCGCTTCGCTCACTGCCGCTGATGCGAAGTTTGATGCAGAGTAAAAATCC
CCCTGTGTGGAGTACAAGTCATGAGGGTCCACTTCGAGCCCCATTCTGTTAGACCTTACCGCACAACC
+
FFFFFFFFFFFFFFFFFFFFFFFFFFFFFFFFFFF,FFFFFFFFFFFFFFFFFFFFF:FFF:FFFFFF
FFFFFFFFFFFFFFFFFFFFFFFFFFFFFFFFFFFFFFFFFFFFFFFFFFFFFF:,FFFFFFFFFFFF
@A00155:342:HHGFNDSXY:1:1332:31241:26287 1:N:0:GAACCTAG+TCCGCATA
TCGGGAACTCAGCCCTAATCGCTTCGCTCACTGCCGCTGATGCGAAGTTTGATGCAGAGTAAAAATCC
CCCTGTGTGGAGTACAAGTCATGAGGGTCCACTTCGAGCCCCATTCTGTTAGACCTTACCGCACAACC
+
FFFFFFFFFFFFFFF,FFFFFFFFFFFFFFFFFFFFFFFFFFFFFFFFFFFFFFFFFFFFFFFFFFFF
FFFFFFFFFFFFFFFFFFFFFF:FFFFFF:FFFFFFFFFFFFF:FFFFFFFFFFFFFFFFFFFFFFFF
@A00155:342:HHGFNDSXY:1:2265:25898:21136 1:N:0:GAACCTAG+TCCGCATA
TCGGGAACTCAGCCCTAATCGCTTCGCTCACTGCCGCTGATGCGAAGTTTGATGCAGAGTAAAAATCC
CCCTGTGTGGAGTACAAGTCATGAGGGTCCACTTCGAGCCCCATTCTGTTAGACCTTACCGCACAACC
+
FFFFFFFFFFFFFFFFFF:FFFFFFFFFFFFFFFFFFFFFFFFFFFFFF:FFFFFFFFFFFFFFFFFF
FFFFFFFFFFFFFFFFFFFFFFFFFFFFFFFFFFFFFFFFFFFFFFFFFFFFFFFF:FFFFFFFFFFF
@A00155:342:HHGFNDSXY:1:2108:29686:25473 2:N:0:GAACCTAG+TCCGCATA
TCGGGAACTCAGCCCTAATCGCTTCGCTCACTGCCGCTGATGCGAAGTTTGATGCAGAGTAAAAATCC
CCCTGTGTGGAGTACAAGTCATGAGGGTCCACTTCGAGCCCCATTCTGTTAGACCTTACCGCACAACC
+
FFFFFFFFFFFFFFFFFFFFFFFFFFFFFFFFFFFFFFFFFFFFFFFFFFFFFFFFFFFFFFFFFFFF
FFFFFFFFF:FFFFFFFFFFFFFFFFFFFFFFFFFFFFFFFFFFFFFFF:FFFFFFFFFFFFFFFFFF
```

```
@A00155:342:HHGFNDSXY:1:2265:25301:25113 1:N:0:GAACCTAG+TCCGCATA
TCGGGAACTCAGCCCTAATCGCTTCGCTCACTGCCGCTGATGCGAAGTTTGATGCAGAGTAAAAATCC
CCCTGTGTGGAGTACAAGTCATGAGGGTCCACTTCGAGCCCCATTCTGTTAGACCTTACCGCACAACC
+
FFFFFFFFFFFFFFFFFFFFFFFFFFFFFFFFFFFFFFFFFFFFFFFFFFFFFFFFFFFFFFFFFFFF
FFFFFFFFFFFFFFFFFFFFF:FFFFFFFFFFFFFFFFFFFFFFFFFF:FFFFFFFFFFFFFFFFFFF
@A00155:342:HHGFNDSXY:1:2108:30327:27242 2:N:0:GAACCTAG+TCCGCATA
TCGGGAACTCAGCCCTAATCGCTTCGCTCACTGCCGCTGATGCGAAGTTTGATGCAGAGTAAAAATCC
CCCTGTGTGGAGTACAAGTCATGAGGGTCCACTTCGAGCCCCATTCTGTTAGACCTTACCGCACAACC
+
FFFFFFFFFFFFFFF:,FFFFF:FFFFFFFF:FFFFFFFFFFFFFFFFFFFFFF:FFFFFFFFFFFFF
:FFFFFFFFFFFFFFF:FFFFFFFFF,FFFFFFFF:F:FFFFFFFFFFFFFFFFFFFFFFFFFFFFFF
@A00155:342:HHGFNDSXY:1:2368:7853:28463 1:N:0:GAACCTAG+TCCGCATA
TCGGGAACTCAGCCCTAATCGCTTCGCTCACTGCCGCTGATGCGAAGTTTGATGCAGAGTAAAAATCC
CCCTGTGTGGAGTACAAGTCATGAGGGTCCACTTCGAGCCCCATTCTGTTAGACCTTACCGCACAACC
+
FFFFFF:FFFFFFFFFFFFFFFFFFFFFFFFFFFFFFFFFFFFFF:FFFFFFFFFFFFFF:FFFFFFF
FFFFFFFFFFFFFFFFFFFFFFFFFFFFFFFF:FFFFFFFFFF,FFFFFFFFFF:F:FFFFFFF:FFF
@A00155:342:HHGFNDSXY:1:1153:7328:15092 2:N:0:GAACCTAG+TCCGCATA
CGGGAACTCAGCCCTAATCGCTTCGCTCACTGCCGCTGATGCGAAGTTTGATGCAGAGTAAAAATCCC
CCTGTGTGGAGTACAAGTCATGAGGGTCCACTTCGAGCCCCATTCTGTTAGACCTTACCGCACAACCT
+
:FFFFFFFFFF:FFFFFFFFF,FFFFFFFFFFFFFFFFF:FFFFFFFFFFFFFFFFFFFFFFFFFFFF
FFFFFFFFFFFFFFFFFFFFFFFFFFFFFFFFFFFFFFFFFF:FFFFFFFFFFFFFFFFFFFFFFFFF
@A00155:342:HHGFNDSXY:1:2443:1362:30686 2:N:0:GAACCTAG+TCCGCATA
AACACAGCCCTAATCGCTTCGCTCACTGCCGCTGATGCGAAGTTTGATGCAGAGTAAAAATCCCCCTG
TGTGGAGTACAAGTCATGAGGGTCCACTTCGAGCCCCATTCTGTTAGACCTTACCGCACAACCT
+
FFF,FF:F,FFFFF:FFFFFF:,FFF:FFFFF,FF,F:FFF:,:FFFF:FF:F::FFFFF:FFFFF::
::FFF::,FFFF:,FF:FFFFFFFFFFFFFFFFFFFFFFFF:F:FFFFFF:FFFF:FFFFFFFF
@A00155:342:HHGFNDSXY:1:1460:26973:17300 2:N:0:GAACCTAG+TCCGCATA
CTCAGCCCTAATCGCTTCGCTCACTGCCGCTGATGCGAAGTTTGATGCAGAGTAAAAATCCCCCTGTG
TGGAGTACAAGTCATGAGGGTCCACTTCGAGCCCCATTCTGTTAGACCTTACCGCACAACCTGTCTGG
+
FFFFFFFFFFFFFFFFFFFFFFFFFFFFFFFFFFFFFFFFFFFFFFFFFFFFFFFFFFFFFFFFFFFF
FFFFFFFFFFFFFFFFFFFFFFFFFFFFFFFFFFFFFFFFFFFFFFFFFFFFFFFFFFFFFFFFFFFF
@A00155:342:HHGFNDSXY:1:1638:23900:13260 2:N:0:GAACCTAG+TCCGCATA
CTCAGCCCTAATCGCTTCGCTCACTGCCGCTGATGCGAAGTTTGATGCAGAGTAAAAATCCCCCTGTG
TGGAGTACAAGTCATGAGGGTCCACTTCGAGCCCCATTCTGTTAGACCTTACCGCACAACCTGTCTGG
+
FFFFFFFFFF:FFFFFFFFFFFFFFFFFFFFFFF:FFFFFFFFFFFFFFFFFFFFFFFFFFFFFFFFF
FFFFFFFFFFFFFFFFFFFFFFFFFFFFFFFFFFFFFFFFFFFFFFFFFFFFFFFFFFFFFFFFFFFF
@A00155:342:HHGFNDSXY:1:2154:5665:31501 1:N:0:GAACCTAG+TCCGCATA
CAGCCCTAATCGCTTCGCTCACTGCCGCTGATGCGAAGTTTGATGCAGAGTAAAAATCCCCCTGTGTG
GAGTACAAGTCATGAGGGTCCACTTCGAGCCCCATTCTGTTAGACCTTACCGCACAACCTGTC
+
FFFFFFFFFFFFFFFFFFFFFFFFFFFFFFFFFFFF:FFFF:FFFFFFFFFFFFFFFFFFFFFFFFFF
FFFFFFFFFFFFFFFFFFFFFFFFFFFFFFFFFFFFFFFFFFFFFFFFFFFFFFFFFFFFFFF
@A00155:342:HHGFNDSXY:1:2549:23511:23829 1:N:0:GAACCTAG+TCCGCATA
CAGCCCTAATCGCTTCGCTCACTGCCGCTGATGCGAAGTTTGATGCAGAGTAAAAATCCCCCTGTGTG
GAGTACAAGTCATGAGGGTCCACTTCGAGCCCCATTCTGTTAGACCTTACCGCACAACCTGTCT
+
FFFFFFFFFFFFFFFFFFFFFFFFFFFFFFF,FFFFFFFFFFFFFFFFFF:FFFFFFFFFFFFFFFFF
FFFFFFFFFFFFFFFFFFFFFFFFFFFFFFFFFF:FFFFFFFFFFFFFFFFFFFFFFFF:FFFF
```

```
@A00155:342:HHGFNDSXY:1:1123:5936:16501 1:N:0:GAACCTAG+TCCGCATA
CAGCCCTAATCGCTTCGCTCACTGCCGCTGATGCGAAGTTTGATGCAGAGTAAAAATCCCCCTGTGTG
GAGTACAAGTCATGAGGGTCCACTTCGAGCCCCATTCTGTTAGACCTTACCGCACAACCTGTCTGGAA
+
FFFFFFFFFFFFFFFFFFFFFFFFFFFFFFFFFFFFFFF:FFFFFFFFFFFFFFFFFFFFFFFFFFFF
FFFFFFFFFFF:FFFFFFFFFFFFFFFFFFFFFFFFFFFFFFFFFFFFFFFFFFFFFFFFFFFFFFFF
@A00155:342:HHGFNDSXY:1:1328:16486:4398 2:N:0:GAACCTAG+TCCGCATA
CAGCCCTAATCGCTTCGCTCACTGCCGCTGATGCGAAGTTTGATGCAGAGTAAAAATCCCCCTGTGTG
GAGTACAAGTCATGAGGGTCCACTTCGAGCCCCATTCTGTTAGACCTTACCGCACAACCTGTCTGGAA
+
FFFFFFFFFFFFFFFFFFFFFFFFFFFFFFFFFFFFFFFFFFFFFFFFFFFFFFFFFFFFFFFFFFFF
FFFFFFFFFFFFFFFFFFFFFFFFFFFFFFFFFFFFFFFFFFFFFFFFFFFFFFFFFFFFFFFFFFFF
@A00155:342:HHGFNDSXY:1:2127:7943:1877 2:N:0:GAACCTAG+TCCGCATA
CTAATCGCTTCGCTCACTGCCGCTGATGCGAAGTTTGATGCAGAGTAAAAATCCCCCTGTGTGGAGTA
CAAGTCATGAGGGTCCACTTCGAGCCCCATTCTGTTAGACCTTACCGCACAACCTGTCTGGAAACACT
+
FFFFFFFFFFFFFFFFFFFFFFFFFFFFFFFFFFFFFFFFFFFFFFFFFFFFFFFFFFFFFFFFFFFF
FF:FFFFFFFFFFFFFFFFFFFFFFFFFFFFFFF:FFFFFFFFFFFFFFFFFFFFFFFFFFFFFFFFF
@A00155:342:HHGFNDSXY:1:2126:8540:37043 2:N:0:GAACCTAG+TCCGCATA
CTAATCGCTTCGCTCACTGCCGCTGATGCGAAGTTTGATGCAGAGTAAAAATCCCCCTGTGTGGAGTA
CAAGTCATGAGGGTCCACTTCGAGCCCCATTCTGTTAGACCTTACCGCACAACCTGTCTGGAAACACT
+
FFFFFFFFFFFFF:FFFFFFFFFFFFFFFFFFFFFFFFFFFFFFFFFFFFFFFFFFFFFFFFFFFFFF
FFFFFFFFFFFFFFFFFFFFFFFFFFFFFFFFFFFFFFFFFFFFFFFFFFFFFFFFFFFFFFFFFFFF
@A00155:342:HHGFNDSXY:1:2127:7771:1861 2:N:0:GAACCTAG+TCCGCATA
CTAATCGCTTCGCTCACTGCCGCTGATGCGAAGTTTGATGCAGAGTAAAAATCCCCCTGTGTGGAGTA
CAAGTCATGAGGGTCCACTTCGAGCCCCATTCTGTTAGACCTTACCGCACAACCTGTCTGGAAACACT
+
FF:,:FFFFFFFFF:FFFFFFFFFFFFF:FFFFFF:FFFFFFFFFFFFFFFFFF:FFFFFFFFFFFFF
FFFFFFFFFFFFFFFFFFFF:FFFFFFFFFFFFF:FFFFFF:FFFFFFFFFFFFFFFFFFF:FFFFFF
@A00155:342:HHGFNDSXY:1:2435:28736:28275 1:N:0:GAACCTAG+TCCGCATA
AATCGCTTCGCTCACTGCCGCTGATGCGAAGTTTGATGCAGAGTAAAAATCCCCCTGTGTGGAGTACA
AGTCATGAGGGTCCACTTCGAGCCCCATTCTGTTAGACCTTACCGCACAACCTGTCTGGAAACACTGC
+
FFFFFFFFFFFFFFFFFFFFFFFFFFFFFFF:FFFFFFFFFFFFFFFFFFFFFFFFFFFFFFFFFFFF
FFF:FFFFFFFFFFFFFFFFFFFFFFFFFFFFFFFFFFFFFFFFFFFFFFFFFFFFFFFFFFFFFFFF
@A00155:342:HHGFNDSXY:1:1667:10239:30436 2:N:0:GAACCTAG+TCCGCATA
ATCGCTTCGCTCACTGCCGCTGATGCGAAGTTTGATGCAGAGTAAAAATCCCCCTGTGTGGAGTACAA
GTCATGAGGGTCCACTTCGAGCCCCATTCTGTTAGACCTTACCGCACAACCTGTCTGGAAACACTGCG
+
FFFFFFFFFFFFFFF:FFFFFFFFFFFFFFFFFFFFFFFFFFFFFFFFFFFFFFFFFFFFFFFFFFFF
FFFFFFFFFFFFFFFFFFFFFFFFFFFFFFFFFFFFFFFFFFFFFFFFFFFF:FFFFFFFFFFFFFFF
@A00155:342:HHGFNDSXY:1:1328:16486:4398 1:N:0:GAACCTAG+TCCGCATA
TTCGCTCACTGCCGCTGATGCGAAGTTTGATGCAGAGTAAAAATCCCCCTGTGTGGAGTACAAGTCAT
GAGGGTCCACTTCGAGCCCCATTCTGTTAGACCTTACCGCACAACCTGTCTGGAAACACTGCGCTATC
+
FFFFFFFFFFFFFFFFFFFFFFFFFFFFFFFFFFFFFFFFFFFFFFFFFFFFFFFFFFFFFFFFFFFF
FFFFFFFFFFFFFFFFFFFFFFFFFFFFFFFFFFFFFFFFFFFFFFFFFFFFFFFFFFFFFFFFFFFF
@A00155:342:HHGFNDSXY:1:2551:5873:15765 2:N:0:GAACCTAG+TCCGCATA
TCGCTCACTGCCGCTGATGCGAAGTTTGATGCAGAGTAAAAATCCCCCTGTGTGGAGTACAAGTCATG
AGGGTCCACTTCGAGCCCCATTCTGTTAGACCTTACCGCACAACCTGTCTGGAAACACTGCGCTATCA
+
FFFFFFFFFFFFFFFFFFFFFFFFFFFFFFFFFFFFFFFFFFFFFFFFFFFFFFFFFFFFFFFFFFFF
FFFFFFFFFFFFFFFFFFFFFFFFFFFFFFFFFFFFFFFFFFFFFFFFFFFFFFFFFFFFFFFFF:FF
```

```
@A00155:342:HHGFNDSXY:1:2121:5195:6198 2:N:0:GAACCTAG+TCCGCATA
TCGCTCACTGCCGCTGATGCGAAGTTTGATGCAGAGTAAAAATCCCCCTGTGTGGAGTACAAGTCATG
AGGGTCCACTTCGAGCCCCATTCTGTTAGACCTTACCGCACAACCTGTCTGGAAACACTGCGCTATCA
+
FFFFFFFFFFFFFFFFFFFFFFFFFFFFFFFFFFFFFFFFFFFFFFFFFFFFFFFFFFFFFFFFFFFFFF
FFFFFFFFFFFFFFFFFFFFFFFFFFFFFFFFFFFFFFFFFFFFFFFFFFFFFFFFFFFFFFFFFFFFFF
@A00155:342:HHGFNDSXY:1:2429:6705:14418 1:N:0:GAACCTAG+TCCGCATA
CGCTCACTGCCGCTGATGCGAAGTTTGATGCAGAGTAAAAATCCCCCTGTGTGGAGTACAAGTCATGA
GGGTCCACTTCGAGCCCCATTCTGTTAGACCTTACCGCACAACCTGTCTGGAAACACTGCGCTATCAG
+
FFFFFFFFFFFFFFFFFFFFFFFFFFFFFFFFFFFFF:FFFFFFF:FFFFFFFFFFFFFFFFFFFFFFFF
FFFFFFFFFFFFFFFFFFFFFFFFFFFFFFFFFFFFFFFFFFFFFFFFFFFFFFFFFFFFFFFFFFFFFF
@A00155:342:HHGFNDSXY:1:2139:21251:15906 1:N:0:GAACCTAG+TCCGCATA
CTCACTGCCGCTGATGCGAAGTTTGATGCAGAGTAAAAATCCCCCTGTGTGGAGTACAAGTCATGAGG
GTCCACTTCGAGCCCCATTCTGTTAGACCTTACCGCACAACCTGTCTGGAAACACTGCGCTATCAGTA
+
FFFFFFFFFFFFFFFFFFFFFFFFFFFFFFFFFFFFFFFFFFFFFFFFFFFFFFFFFFFFFFFFFFFFFF
FFFFFFFFFFFFFFFFFFFFFFFFFFFFFFFFFFFFFFFFFFFFFFFFFFFFFFFFFFFFFFFFFFFFFF
@A00155:342:HHGFNDSXY:1:2126:8540:37043 1:N:0:GAACCTAG+TCCGCATA
TCACTGCCGCTGATGCGAAGTTTGATGCAGAGTAAAAATCCCCCTGTGTGGAGTACAAGTCATGAGGG
TCCACTTCGAGCCCCATTCTGTTAGACCTTACCGCACAACCTGTCTGGAAACACTGCGCTATCAGTAG
+
FFFFFFFFFFFFFFFFFFFFFFFFFFFFFFFFFFFFFFFFFFFFFFFFFFFFFFFFFFFFFFFFFFFFFF
FFFFFFFFFFFFFFFFFFFFFFFFF:FFFFFFFFFFFFFFFFFFFFFFFFFFFFFFFFFFFFFFFFF:FF
@A00155:342:HHGFNDSXY:1:2127:7771:1861 1:N:0:GAACCTAG+TCCGCATA
TCACTGCCGCTGATGCGAAGTTTGATGCAGAGTAAAAATCCCCCTGTGTGGAGTACAAGTCATGAGGG
TCCACTTCGAGCCCCATTCTGTTAGACCTTACCGCACAACCTGTCTGGAAACACTGCGCTATCAGTAG
+
FFFFFFFFFFFFFFFFFFFFF:FFFFFFFFFFFFFFFFFFFFFFFFFFFFFFFFFFFFFFFFFFFFFFFF
FFFFFFFFFFFFFFFFFFFFFFFFFFFFFFFF:FFFFFFFFFFFFFFFFFFFFFFFFFFFFFFFFFFFFF
@A00155:342:HHGFNDSXY:1:2127:7943:1877 1:N:0:GAACCTAG+TCCGCATA
TCACTGCCGCTGATGCGAAGTTTGATGCAGAGTAAAAATCCCCCTGTGTGGAGTACAAGTCATGAGGG
TCCACTTCGAGCCCCATTCTGTTAGACCTTACCGCACAACCTGTCTGGAAACACTGCGCTATCAGTAG
+
FFFFFFFFFFFFFFFFFFFFFFFFFFFFFFFFFF:FFFFFFFFFFFFFFFFFFFFFFFFFFFFFFFFFFF
FFFFFFFFFFFFFFFFFFFFFFFFF:FFFFFFFFFFFFFFFFFFFFFFF:FFFFFFFFFFFFFFFFFFFF
@A00155:342:HHGFNDSXY:1:2631:27100:27884 1:N:0:GAACCTAG+TCCGCATA
CACTGCCGCTGATGCGAAGTTTGATGCAGAGTAAAAATCCCCCTGTGTGGAGTACAAGTCATGAGGGT
CCACTTCGAGCCCCATTCTGTTAGACCTTACCGCACAACCTGTCTGGAAACACTGCGCTATCAGTAGG
+
FFFFFFFFFFFFFFFFFFFFFFFFFFFFFFFFFFFFFFFFFFFFFFFFFFFFFFFFFFFFFFFFFFFFFF
FFFFF,FFFFFFFFFFFFFFFFFFFFFFFFFFFFFFFFFFFFFFFFFFFFFFFFFFFFFFF,FFFFFFFF
@A00155:342:HHGFNDSXY:1:2435:28736:28275 2:N:0:GAACCTAG+TCCGCATA
CACTGCCGCTGATGCGAAGTTTGATGCAGAGTAAAAATCCCCCTGTGTGGAGTACAAGTCATGAGGGT
CCACTTCGAGCCCCATTCTGTTAGACCTTACCGCACAACCTGTCTGGAAACACTGCGCTATCAGTAGG
+
FFFFFFFFFFFFFFFFFFFFFFFFFFFFFFFFFFFFFFFFFFFFFFFFFFFFFFFFFFFFFFFFFFFFFF
FFFFFFFFFFFFFFFFFFFFFFFFFFFFFFFFFFFFFFFFFFFFFFFFFFFFFFFFFFFFFFFFFFFFF:
@A00155:342:HHGFNDSXY:1:2637:11939:23985 2:N:0:GAACCTAG+TCCGCATA
CACTGCCGCTGATGCGAAGTTTGATGCAGAGTAAAAATCCCCCTGTGTGGAGTACAAGTCATGAGGGT
CCACTTCGAGCCCCATTCTGTTAGACCTTACCGCACAACCTGTCTGGAAACACTGCGCTATCAGTAGG
+
FFFF,FFFFFFFFFFFFFFFFF:FFFFFFFFFFFF:FFFFFFFFFFFFF:FFFFFFFFFFFFFFFFFFFF
FFFFFFFFFFFFFFFFFFFFF:FFFFFFFFFFFFFFFFFFFFFFFFFFFF:FFFFFFF:FFFFF:FF:::
```

```
@A00155:342:HHGFNDSXY:1:2454:23656:29496 1:N:0:GAACCTAG+TCCGCATA
ACTGCCGCTGATGCGAAGTTTGATGCAGAGTAAAAATCCCCCTGTGTGGAGTACAAGTCATGAGGGTC
CACTTCGAGCCCCATTCTGTTAGACCTTACCGCACAACCTGTCTGGAAACACTGCGCTATCAGTAGGC
+
FFFFFFFFFFFFFFFFFFFFFFFFFFFFFFFFFFFFFFFFFFFFFFFFFFFFFFFFFFFFFFFFFFFF
FFFFFFFFFFFFFFFFFFFFFFFFFFFFFFFFFFFFFFFFFFFFFFFFFFFFFFFFFFFFFFFFFFFF
@A00155:342:HHGFNDSXY:1:2401:14389:19680 2:N:0:GAACCTAG+TCCGCATA
GCCGCTGATGCGAAGTTTGATGCAGAGTAAAAATCCCCCTGTGTGGAGTACAAGTCATGAGGGTCCAC
TTCGAGCCCCATTCTGTTAGACCTTACCGCACAACCTGTCTGGAAACACTGCGCTATCAGTAGGCATA
+
FFFFFFFFFF:FFFFFFFFFFFFFFFFFFFFFFFFF:FFFFFFFFFFFFFFFFFFFFFFFFFFFFFFF
FFFFFFF:FFFFFFFFFFFFFFF:FFFFFFFFFFFFFFFFFFFFFFFFFFFFFFFFFFFFF:FFFFFF
@A00155:342:HHGFNDSXY:1:1673:14262:24659 2:N:0:GAACCTAG+TCCGCATA
GCCGCTGATGCGAAGTTTGATGCAGAGTAAAAATCCCCCTGTGTGGAGTACAAGTCATGAGGGTCCAC
TTCGAGCCCCATTCTGTTAGACCTTACCGCACAACCTGTCTGGAAACACTGCGCTATCAGTAGGCATA
+
FFFFFFFFFFFFF:FFFFFFFFFFFFFFFFFFFFFFFFFFFFFFFFFFFFFFFFFFFFFFFFFFFFFF
FFFFFFFFFFF:FFFFFFFFFFFF:FFFFFFFF:FFF:FFFFFFFFFFFFFFFFFFFFFFFFFFFFFF
@A00155:342:HHGFNDSXY:1:1530:14109:35289 2:N:0:GAACCTAG+TCCGCATA
CCGCTGATGCGAAGTTTGATGCAGAGTAAAAATCCCCCTGTGTGGAGTACAAGTCATGAGGGTCCACT
TCGAGCCCCATTCTGTTAGACCTTACCGCACAACCTGTCTGGAAACACTGCGCTATCAGTAGGCATAT
+
FFFFFFFFFFFFFF:FFF:FFFFFFFFFFFFFFFFFFFFFFFFFFFFFFFFFF:FFFFFFFFFFFFFF
FFFFFFFFFFFFFFFFFFFFFFF:FFFFFFFFFFFFFFFF:FFFFFFFFFFFFFFFFFFFFFFFFFFF
@A00155:342:HHGFNDSXY:1:1617:26558:7341 2:N:0:GAACCTAG+TCCGCATA
CCGCTGATGCGAAGTTTGATGCAGAGTAAAAATCCCCCTGTGTGGAGTACAAGTCATGAGGGTCCACT
TCGAGCCCCATTCTGTTAGACCTTACCGCACAACCTGTCTGGAAACACTGCGCTATCAGTAGGCATAT
+
FF:F:FFF,FFFFFFFFFFF:FFFF:FFFFFFFFFFFFFFFFFFFFFFFFFFFFFFFFF:FFFFFFFF
FFFFFFFFFFFFFFFFFFFFFFFFFFFFFFFFFFFFFFFFFFFFFFFFFFFFFFFFFFFFFFFFFFFF
@A00155:342:HHGFNDSXY:1:2320:28122:29810 2:N:0:GAACCTAG+TCCGCATA
CGCTGATGCGAAGTTTGATGCAGAGTAAAAATCCCCCTGTGTGGAGTACAAGTCATGAGGGTCCACTT
CGAGCCCCATTCTGTTAGACCTTACCGCACAACCTGTCTGGAAACACTGCGCTATCAGTAGGCATAT
+
F,:F:FF:F,FF:FFFFFF,:,FFFFFF:FFFF,FFFFFFFFF:FFFFF:F:F,:FFFFFFFFF,FFF
F:F,:F:FFFF,FFFF,,FFFF:FFF::F:FF:FF:FFF,:,FF:FFFFF,FF,FFFFF,:F:FFFF
@A00155:342:HHGFNDSXY:1:1566:7609:14951 1:N:0:GAACCTAG+TCCGCATA
CGCTGATGCGAAGTTTGATGCAGAGTAAAAATCCCCCTGTGTGGAGTACAAGTCATGAGGGTCCACTT
CGAGCCCCATTCTGTTAGACCTTACCGCACAACCTGTCTGGAAACACTGCGCTATCAGTAGGCATATT
+
FFFFFFFFFFFFFFFFFFFFFFFFFFFFFFFFFFFFFFFFF:FFFFFFFFFFFFFFFFFFFFFFFFFF
FFFFFFFFFFFFFFFFFFFFFFFFFFFFFFFFFFFF:FFFF:FFFFFFFFFFFFFFFFFFFFFFFFFF
@A00155:342:HHGFNDSXY:1:2246:8612:27837 2:N:0:GAACCTAG+TCCGCATA
CGCTGATGCGAATTTTGATGCAGAGTAAAAATCCCCCTGTGTGGAGTACAAGTCATGAGGGTCCACTT
CGAGCCCCATTCTGTTAGACCTTACCGCACAACCTGTCTGGAAACACTGCGCTATCAGTAGGCATATT
+
FFFFF:FFFFFF,FFFFFFFFFFF:FFFF:FFFFFFF::FFFFFFFFFF:F,FFFFFFFFFFFFFFFF
FFFF:FFFFFFFFFFFFFF,FFFFFFFFF:FFFFFFFFFF:FFF::FFF:FFFFFFFFF:F::FFFFF
@A00155:342:HHGFNDSXY:1:1357:29577:9064 1:N:0:GAACCTAG+TCCGCATA
GCTGATGCGAAGTTTGATGCAGAGTAAAAATCCCCCTGTGTGGAGTACAAGTCATGAGGGTCCACTTC
GAGCCCCATTCTGTTAGACCTTACCGCACAACCTGTCTGGAAACACTGCGCTATCAGTAGGCATATTA
+
FFFFFFFFFFFFFFFFFFFFFFFFFFFFFFFFFFFFFFFFFFFFFFFFFFFFFFFFFFFFFFFFFFFF
FFFFFFFFFFFFFFFFFFFFFFFFFFFFFFFFFFFFFFFFFFFFFFFFFFFFFFFFFFFFFFFFFFFF
```

```
@A00155:342:HHGFNDSXY:1:1433:9824:5854 1:N:0:GAACCTAG+TCCGCATA
GCTGATGCGAAGTTTGATGCAGAGTAAAAATCCCCCTGTGTGGAGTACAAGTCATGAGGGTCCACTTC
GAGCCCCATTCTGTTAGACCTTACCGCACAACCTGTCTGGAAACACTGCGCTATCAGTAGGCATATTA
+
FF:FFFFFFFFFFFFFFFFFFFFFFFFFFFFFFFFFFFFFFFFFFFFFFFFFFFFFFFFFFFFFFFFF
FFFFFFFFFFFFFFFFFFFFFFFFFFFFFFFFFFFFFFFFFFFFFFFFFFFFFFFFFFFFFFFFFFFF
@A00155:342:HHGFNDSXY:1:2123:32723:28103 1:N:0:GAACCTAG+TCCGCATA
GCTGATGCGAAGTTTGATGCAGAGTAAAAATCCCCCTGTGTGGAGTACAAGTCATGAGGGTCCACTTC
GAGCCCCATTCTGTTAGACCTTACCGCACAACCTGTCTGGAAACACTGCGCTATCAGTAGGCATATTA
+
FFFFFFFFFFFFFFFFFFFFFFFFFFFFFFFFFFFFFFFFFFFFFFFFFFFFFFFFFFFFFFFFFFFF
FFFFFFFFFFFFFFFFF:FFFFFFF:FFFFFFFFFFFFFFFFFFFFFFFFFFFFFFFFFFFFFFFFFF
@A00155:342:HHGFNDSXY:1:1673:14262:24659 1:N:0:GAACCTAG+TCCGCATA
GCTGATGCGAAGTTTGATGCAGAGTAAAAATCCCCCTGTGTGGAGTACAAGTCATGAGGGTCCACTTC
GAGCCCCATTCTGTTAGACCTTACCGCACAACCTGTCTGGAAACACTGCGCTATCAGTAGGCATATTA
+
FFFFFFFFFFFFF:FFFFFFFFFFFFFFFFFFFFFFFFF:FFFFFFFFFFFFFFFFFFFFFFFFFFFF
FFFFFFFFFFFFFFFFFFFFFFFFFFFFFFFFFFFFF:F,FFFFFFFFFFFFFFFFFFFFFFFFFFF:F
@A00155:342:HHGFNDSXY:1:2551:25048:19194 1:N:0:GAACCTAG+TCCGCATA
GCTGATGCGAAGTTTGATGCAGAGTAAAAATCCCCCTGTGTGGAGTACAAGTCATGAGGGTCCACTTC
GAGCCCCATTCTGTTAGACCTTACCGCACAACCTGTCTGGAAACACTGCGCTATCAGTAGGCATATTA
+
FFFFFFFFFFFFFFFFFFFFFFFFFFFFFFFFFFFFFFFFFFFFFFFFFFFFFFFFFFFFFFFFFF:F
FFFFFFF:FFFFF:FFFFFFFFFFFFFFFFFFFFFFFFFFFFFFFFFFFFFFFFF:FFFFFFFF:FF:
@A00155:342:HHGFNDSXY:1:2637:11939:23985 1:N:0:GAACCTAG+TCCGCATA
CTGATGCGAAGTTTGATGCAGAGTAAAAATCCCCCTGTGTGGAGTACAAGTCATGAGGGTCCACTTCG
AGCCCCATTCTGTTAGACCTTACCGCACAACCTGTCTGGAAACACTGCGCTATCAGTAGGCATATTA
+
FFFFFFF:FFFFFFFF,FFFFF:FFFFFFFFFFFF:FFFFFFF:FFFFF:F:FF:FF:FFFFFF:FF:
FFFFFFF,:FFFF:F:FFF:FFFFFFFFFFFFF:FFFF:F,FFFFF:,FFFFFFFFFF:,FF,FFFF
@A00155:342:HHGFNDSXY:1:2610:4833:24392 1:N:0:GAACCTAG+TCCGCATA
CTGATGCGAAGTTTGATGCAGAGTAAAAATCCCCCTGTGTGGAGTACAAGTCATGAGGGTCCACTTCG
AGCCCCATTCTGTTAGACCTTACCGCACAACCTGTCTGGAAACCCTGCGCTATCAGTAGGCATATTA
+
FFFFFF,FFFFF:F,FFFFFFFFFFFFFF,,FFF,FFF:FFFFF,FFFFFFFF:FF:FF,FFF,FFF:
FFFF,FFFF,FFFFF,FFFF:FF,,:FFFF:FFFFFFF:F,FF,FF:FFFF:FFF,,F,,:FF:FFF
@A00155:342:HHGFNDSXY:1:2401:14389:19680 1:N:0:GAACCTAG+TCCGCATA
CTGATGCGAAGTTTGATGCAGAGTAAAAATCCCCCTGTGTGGAGTACAAGTCATGAGGGTCCACTTCG
AGCCCCATTCTGTTAGACCTTACCGCACAACCTGTCTGGAAACACTGCGCTATCAGTAGGCATATTAA
+
FFFFFFFFFFFFFFFFFFFFFFFFFFFFFFFFFFFFFFFFFFFFFFFFFFFFFFFFFFFFFFFFFFFF
FFFFFFFFFFFFFFFFFFFFFFFFFFFFFFFFFFFFFFFFFFFFFFFFFFFFFFFFFFFFFF,F:FFFF
@A00155:342:HHGFNDSXY:1:2653:2184:29450 1:N:0:GAACCTAG+TCCGCATA
CTGATGCGAAGTTTGATGCAGAGTAAAAATCCCCCTGTGTGGAGTACAAGTCATGAGGGTCCACTTCG
AGCCCCATTCTGTTAGACCTTACCGCACAACCTGTCTGGAAACACTGCGCTATCAGTAGGCATATTAA
+
FFFFFFFFFFFFFFFFFFFFFFFFFFFFFFFFFFFFFFFFFFFFFFFFFF:FFFFFFFFFFFFFFFFFF
FFFFFFFFFFFFFFFFFFFFFFFFFFFFFFFFFFFFFFFF,FFFFFFFFFFFFFF:FFFFFFFF,FFFF:
@A00155:342:HHGFNDSXY:1:1307:20609:22905 1:N:0:GAACCTAG+TCCGCATA
CTGATGCGAAGTTTGATGCAGAGTAAAAATCCCCCTGTGTGGAGTACAAGTCATGAGGGTCCACTTCG
AGCCCCATTCTGTTAGACCTTACCGCACAACCTGTCTGGAAACACTGCGCTATCAGTAGGCATATTAA
+
FFFFFFFFFF:FF,FF,FFFFFF:FFFFFF,FFFF:FFFFFFFFFFFFF::F:FFFFFFFFFFFF:FF
FFFFFFF,FFF:,FFF:FFF:FFFFFFFFFFFFF,FF,:F:FFFF:FFFFF,F:,F:FF,F,,F::FF
```

```
@A00155:342:HHGFNDSXY:1:2471:28836:36401 1:N:0:GAACCTAG+TCCGCATA
TGATGCGAAGTTTGATGCAGAGTAAAAATCCCCCTGTGTGGAGTACAAGTCATGAGGGTCCACTTCGA
GCCCCATTCTGTTAGACCTTACCGCACAACCTGTCTGGAAACACTGCGCTATCAGTAGGCATATTAAG
+
FFFFFFFFFFFFFFFFFFFFFFFFFFFFFFFFFFFFFFFFFFFFFFFFFFFFFFFFFFFFFFFFFFFF
FFFFFFFFFFFF:FFFFFFFFFFFFFFFFFFFFFFFFFFFFFFFFFFFFFFFFFFFFFFFFFFFFFF:
@A00155:342:HHGFNDSXY:1:1655:13865:14262 1:N:0:GAACCTAG+TCCGCATA
ATGCGAAGTTTGATGCAGAGTAAAAATCCCCCTGTGTGGAGTACAAGTCATGAGGGTCCACTTCGAGC
CCCATTCTGTTAGACCTTACCGCACAACCTGTCTGGAAACACTGCGCTATCAGTAGGCATATTAAGTT
+
FFFFFFFFFFFFFFFFFFFFFFFFFFFFFFFFFFFFFFFFFFFFFFFFFFFFFFFFFFFFFFFFFFFF
FFFFFFFFFFFFFFFFFFFFFFFFFFFFFFFFFFFFFFFFFFFFFFFFFFFFFFFFFFFFFFFFFFFF
@A00155:342:HHGFNDSXY:1:2245:22562:29731 1:N:0:GAACCTAG+TCCGCATA
TGCGAAGTTTGATGCAGAGTAAAAATCCCCCTGTGTGGAGTACAAGTCATGAGGGTCCACTTCGAGCC
CCATTCTGTTAGACCTTACCGCACAACCTGTCTGGAAACACTGCGCTATCAGTAGGCATATTAAGTTT
+
FFFFFFFFFFFFFFFFFFFFFFFFFFFFFFFFFFFFFFFFFFFFFFFFFFFFFFFFFFFFFFFFFFFF
FFFFFFFFFFFFFFFFFFFFFFFFFFFFFFFFFFFFFFFFFFFFFFFFFFFFFFFF:FFFFFFFFFF
@A00155:342:HHGFNDSXY:1:2454:23656:29496 2:N:0:GAACCTAG+TCCGCATA
CGAAGTTTGATGCAGAGTAAAAATCCCCCTGTGTGGAGTACAAGTCATGAGGGTCCACTTCGAGCCCC
ATTCTGTTAGACCTTACCGCACAACCTGTCTGGAAACACTGCGCTATCAGTAGGCATATTAAGTTTAC
+
F:FFFFFFFFFFFFFFFFFFFFFFFFFFFFFFFFFFFFFFFFFFFFFFFFFFFFFFFFFFFFFFFFFF
FFFFFFFFFFFFFFFFFFFFFFFFFFFFFFFFFFFFFFFFFFFFFFFFFFFFFFFFFFFFFFFFFFFF
@A00155:342:HHGFNDSXY:1:1621:29360:36307 2:N:0:GAACCTAG+TCCGCATA
GTTTGATGCAGAGTAAAAATCCCCCTGTGTGGAGTACAAGTCATGAGGGTCCACTTCGAGCCCCATTC
TGTTAGACCTTACCGCACAACCTGTCTGGAAACACTGCGCTATCAGTAGGCATATTAAGTTTACTTTC
+
FFFFFFFFFFFFFFFFFFFFFF:FFFFFFFFFFFFFFFFFFFFFFFFFFFFFFFFFFFFFFFFFFFFF
FFFFFFFFFFFFFFFFFFFFFFFFFFFFFFFFFFFFFFFFFFFFFFFFFFFFFFFFFFFFFFFFFFFF
@A00155:342:HHGFNDSXY:1:1621:29541:36119 2:N:0:GAACCTAG+TCCGCATA
GTTTGATGCAGAGTAAAAATCCCCCTGTGTGGAGTACAAGTCATGAGGGTCCACTTCGAGCCCCATTC
TGTTAGACCTTACCGCACAACCTGTCTGGAAACACTGCGCTATCAGTAGGCATATTAAGTTTACTTTC
+
FFFFFFFFFFFFFFFFFFFFFFF::FFFFFFFFFFFFF:FF::FFFFFFFFFFFFFFFFFFFFFFFFF
FFFFFFFFFFFFFFFFFFFFFFFFFFFFFFFFFFFFFFFF,FFFFFFFFFFFFFFFFFFFFFFFFFF,
@A00155:342:HHGFNDSXY:1:2327:7048:18082 1:N:0:GAACCTAG+TCCGCATA
TTTGATGCAGAGTAAAAATCCCCCTGTGTGGAGTACAAGTCATGAGGGTCCACTTCGAGCCCCATTCT
GTTAGACCTTACCGCACAACCTGTCTGGAAACACTGCGCTATCAGTAGGCATATTAAGTTTACTTTCT
+
FFFFFFFFFFFFFFFFFFFFFFFFFFFFFFFFFFFFFFFFFFFFFFFFFFFFFFFFFFFFFFFFFFFF
FFFFFFFFFFFFFFFFFFFFFFFFFFFFFF:FFFFFFFFFFFFFFFFFFFFFFFFFFFFFFFFFFFFF
@A00155:342:HHGFNDSXY:1:2132:3269:18396 1:N:0:GAACCTAG+TCCGCATA
TTTGATGCAGAGTAAAAATCCCCCTGTGTGGAGTACAAGTCATGAGGGTCCACTTCGAGCCCCATTCT
GTTAGACCTTACCGCACAACCTGTCTGGAAACACTGCGCTATCAGTAGGCATATTAAGTTTACTTTCT
+
FFFFFFFFFFFFFFFFFFFF,FF:FFFFFFFFFFFFFFFFFFFFFF,FFFFFFFFFFFFFFFFFFFFF
FFFF:FFFFFFFFFF:FFFFFFFFFFFFFFFFFFFFFFFFFFFFFFFFFFFF:FFFFFFFFFFF:FFF
@A00155:342:HHGFNDSXY:1:2631:27100:27884 2:N:0:GAACCTAG+TCCGCATA
TTGATGCAGAGTAAAAATCCCCCTGTGTGGAGTACAAGTCATGAGGGTCCACTTCGAGCCCCATTCTG
TTAGACCTTACCGCACAACCTGTCTGGAAACACTGCGCTATCAGTAGGCATATTAAGTTTACTTTCTC
+
FFFFFFFFFFFFF:FFF,FFFFFFFFFFFFFFFFFFFFFFFFFFFFFFFFFFFFFFFFFFFFFFF:FF
FFFFFFFFFFFFFFFFFFFF:FFFFFFFFFFFFFFFFFFFFFFF:FFFFFFFFFFFFF:FFFFFFFFF
```

```
@A00155:342:HHGFNDSXY:1:2110:31222:18082 2:N:0:GAACCTAG+TCCGCATA
TTGATGCAGAGTAAAAATCCCCCTGTGTGGAGTACAAGTCATGAGGGTCCACTTCGAGCCCCATTCTG
TTAGACCTTACCGCACAACCTGTCTGGAAACACTGCGCTATCAGTAGGCATATTAAGTTTACTTTCTC
+
:F:FF:FFFFFFFFF,F:FFFFFFFFFFFFFFFFFFFFFFFFFFFFF:FFF:FFF:FFFFFFFFFFFF
FFFFFFFF:FFFFFFFFFFFFFFFFFFFFFFFFFFF:FFFFFFFFFFFFFFFFFFFFF:FFFFFFFFF
@A00155:342:HHGFNDSXY:1:2574:5918:16814 2:N:0:GAACCTAG+TCCGCATA
TGATGCAGAGTAAAAATCCCCCTGTGTGGAGTACAAGTCATGAGGGTCCACTTCGAGCCCCATTCTGT
TAGACCTTACCGCACAACCTGTCTGGAAACACTGCGCTATCAGTAGGCATATTAAGTTTACTTTCTCC
+
:FFFFFF::FFF:FFFFFFFFFFF,F:FFFFFFFFFFF:FFFFF:FF,FFFFFFFF,:FFFFFFFFFF
FFFF,FFFFFF,FFF:FF:,FFFFFFF:FFF,F,FFFFFFFFFFFFFFFFFFFFFFFFFFFFFFF,FF
@A00155:342:HHGFNDSXY:1:2338:30951:29794 2:N:0:GAACCTAG+TCCGCATA
TGATGCAGAGTAAAAATCCCCCTGTGTGGAGTACAAGTCATGAGGGTCCACTTCGAGCCCCATTCTGT
TAGACCTTACCGCACAACCTGTCTGGAAACACTGCGCTATCAGTAGGCATATTAAGTTTACTTTCTCC
+
FFFF:FF:FFFFFFFF,F,FFFF,::F:F:FFFFFFF,FFFFFFFF:FF:FFFFFFFFFFFF:FFFFF
FFFFFFFFFFFFFFFF:FFFFFFFFFFFFFFFFFFFFFFFFFFFFFFF:FFFF:FFFFFFF:FFFFFF
@A00155:342:HHGFNDSXY:1:2157:2645:17973 1:N:0:GAACCTAG+TCCGCATA
TGATGCAGAGTAAAAATCCCCCTGTGTGGAGTACAAGTCATGAGGGTCCACTTCGAGCCCCATTCTGT
TAGACCTTACCGCACAACCTGTCTGGAAACACTGCGCTATCAGTAGGCATATTAAGTTTACTTTCTCC
+
FFFFFFFFFFFFFFFFFFFFFFFFFFFFFFFFFFFFFFFFF:FFFFFFFFFFFFFFFFFFFFFFFFFF
FFFFFFFFFFFFFFFFFFFFFFFFFFFFFFFFFFFFFF:F:FFFFFFFFFFFFFFFFFFFFF:FFFFF
@A00155:342:HHGFNDSXY:1:1555:24297:17268 2:N:0:GAACCTAG+TCCGCATA
GCAGAGTAAAAATCCCCCTGTGTGGAGTACAAGTCATGAGGGTCCACTTCGAGCCCCATTCTGTTAGA
CCTTACCGCACAACCTGTCTGGAAACACTGCGCTATCAGTAGGCATATTAAGTTTACTTTCTCCAATC
+
FFF:FFFFFFFFF:FFFFFFFFFFFFFFFFFFFFFFFFFFFFFFFFFFFFFFFFFFFFFFFFFFFFFF
FFFFFFFFFFFFFFFFFFFFFFF:FFFFFFFFFFFFFFFFFFFFFFFFFFFFFFFFFFFFFFFFFFFF
@A00155:342:HHGFNDSXY:1:2669:15808:16125 2:N:0:GAACCTAG+TCCGCATA
GCAGAGTAAAAATCCCCCTGTGTGGAGTACAAGTCATGAGGGTCCACTTCGAGCCCCATTCTGTTAGA
CCTTACCGCACAACCTGTCTGGAAACACTGCGCTATCAGTAGGCATATTAAGTTTACTTTCTCCAATC
+
FFFFFFFFFFFFFFFFFFFFFFFFFFFFFFFFFFFFFFFFFFFFFFFFFFFFFF,FFFFFFFFFFFFF
FFFFFFFFFFFFFFFFFFFFFFFFFFFFFFFFFFFFFFFFFFFFFFFFFFFFFFF:FFFFFFFFFFFF
@A00155:342:HHGFNDSXY:1:2245:22562:29731 2:N:0:GAACCTAG+TCCGCATA
GCAGAGTAAAAATCCCCCTGTGTGGAGTACAAGTCATGAGGGTCCACTTCGAGCCCCATTCTGTTAGA
CCTTACCGCACAACCTGTCTGGAAACACTGCGCTATCAGTAGGCATATTAAGTTTACTTTCTCCAATC
+
FFFFFFFFFFFFFFFFFFFF:FFFFFFFFFFFFFFFFFFFFFFFFFFFFFFFFFFFFFFFFFFFFFFF
FFFFFFFFFFFFFFFFFFFFFFFFFFFFFFFFFFFFFFFFFFFFFFFFFFFFFFFFFFFFFFFFFFFF
@A00155:342:HHGFNDSXY:1:1307:20609:22905 2:N:0:GAACCTAG+TCCGCATA
GCAGAGTAAAAATCCCCCTGTGTGGAGTACAAGTCATGAGGGTCCACTTCGAGCCCCATTCTGTTAGA
CCTTACCGCACAACCTGTCTGGAAACACTGCGCTATCAGTAGGCATATTAAGTTTACTTTCTCCAATC
+
:FFFFFFFFFFFFF:FFFFFFF:FFFFFFFFFFFF::FFFF::FFFFFFFFFFFFFFF:FFFFFFF:F
FFFFFFFFFFF:FFF:FFFFFFFFFFFFF:FFFFFFFFFF:F:FFFFFFFFFFFFFFFFFFFFFFFFF
@A00155:342:HHGFNDSXY:1:2375:16089:19805 2:N:0:GAACCTAG+TCCGCATA
GCAGAGTAAAAATCCCCCTGTGTGGAGTACAAGTCATGAGGGTCCACTTCGAGCCCCATTCTGTTAGA
CCTTACCGCACAACCTGTCTGGAAACACTGCGCTATCAGTAGGCATATTAAGTTTACTTTCTCCAATC
+
FFFFFFFFFFFFFFFFFFFFFFFFFFFFFFFFFFFFFFFFFFFFFFFFFFFFFFFFFFFFFFFFFFFF
FFFFFFFFFFFFFFFFFFFFFFFFFFFFFFFFFFFFFFFFFFFFFFFFFFFFFFFFFFFFFFFFFFFF
```

```
@A00155:342:HHGFNDSXY:1:2630:22028:15029 2:N:0:GAACCTAG+TCCGCATA
GCAGAGTAAAAATCCCCCTGTGTGGAGTACAAGTCATGAGGGTCCACTTCGAGCCCCATTCTGTTAGA
CCTTACCGCACAACCTGTCTGGAAACACTGCGCTATCAGTAGGCATATTAAGTTTACTTTCTCCAATC
+
FFFFFFFFFFFFFFFFFFFFFFFFFFFFFFF:FFFFFFFFFFFFFFFFFFFFFFFFFFFFFFFFFFFF
FFFFFFFFFFFFFFFFFFFFFFFFFFFFFFFFFFFFFFFFFFFFFFFFFFFFFFFFFFFFFFFFFFFF
@A00155:342:HHGFNDSXY:1:1571:22435:15546 2:N:0:GAACCTAG+TCCGCATA
GCAGAGTAAAAATCCCCCTGTGTGGAGTACAAGTCATGAGGGTCCACTTCGAGCCCCATTCTGTTAGA
CCTTACCGCACAACCTGTCTGGAAACACTGCGCTATCAGTAGGCATATTAAGTTTACTTTCTCCAATC
+
FFFFFFF:FFFF,FFF:FFFFFF:FFFFFFFFFFFFFFFF,FFFFFFFF:FFFFFFFFFFFFFFFFFF
FFF:FFFFFFFFFFFFFFFFFFFFFFFFFFFFFFFFFFFFFFFFFFFFFFFFFFFFFFFFFFFFF:FF
@A00155:342:HHGFNDSXY:1:2508:8947:4617 2:N:0:GAACCTAG+TCCGCATA
GCAGAGTAAAAATCCCCCTGTGTGGAGTACAAGTCATGAGGGTCCACTTCGAGCCCCATTCTGTTAGA
CCTTACCGCACAACCTGTCTGGAAACACTGCGCTATCAGTAGGCATATTAAGTTTACTTTCTCCAATC
+
FFFFFFFFFFFFF:FFFFFFFFFFFFFFFF:FFFFFFFFFFFFFFFFFFFFFFFFF,FFFFFFFFFFF
FFFFFFFFFFFFFFFFFFFFFFFFFFFFFFFFFFFFFFFFFFFFFFFFFFFFFFFFFFF:FFFFFFFF
@A00155:342:HHGFNDSXY:1:2653:2184:29450 2:N:0:GAACCTAG+TCCGCATA
CAGAGTAAAAATCCCCCTGTGTGGAGTACAAGTCATGAGGGTCCACTTCGAGCCCCATTCTGTTAGAC
CTTACCGCACAACCTGTCTGGAAACACTGCGCTATCAGTAGGCATATTAAGTTTACTTTCTCCAATC
+
FFFFFFFFFFFFFFFFFFFFFF:FFFFFFFFFFFFFFFF:FF:FFFFFFFFF:FFFFFFFFFFFFFFF
FFFFFF:FFFFFFFFFFFFF:FFFFFFFFF,FFFFFFFFFFFFFFFFFFFFFFFFFFFFFFFFFFFF
@A00155:342:HHGFNDSXY:1:2132:3269:18396 2:N:0:GAACCTAG+TCCGCATA
CAGAGTAAAAATCCCCCTGTGTGGAGTACAAGTCATGAGGGTCCACTTCGAGCCCCATTCTGTTAGAC
CTTACCGCACAACCTGTCTGGAAACACTGCGCTATCAGTAGGCATATTAAGTTTACTTTCTCCAATC
+
FFFFFFFFFFFFFFF:FFFFFFFFFFFFFFF:FFFFFFFFFFFFFFFF:FFF:FFFFFFFFFFFFFFF
FFFFF:F:FFFF,FFFFFFFFFFFFF:FFFFFFFFFFFFFFF:FFFFFFFFFFFFFFF:FFF:FFFF
@A00155:342:HHGFNDSXY:1:2213:9824:28025 2:N:0:GAACCTAG+TCCGCATA
AGTAAAAATCCCCCTGTGTGGAGTACAAGTCATGAGGGTCCACTTCGAGCCCCATTCTGTTAGACCTT
ACCGCACAACCTGTCTGGAAACACTGCGCTATCAGTAGGCATATTAAGTTTACTTTCTCCAATCTCCC
+
FFF:FFFFFFFFFFFFFFFFF:FFFFFFFFFFFFFFFFFFFFFFFFFFFFFFFFFFFFFFFFFFFFFF
FFFFFFFFFFFFFFFFFFFFFFFFFFFFFFFFFFFFFFFFFFFFFFFFFFFFFFFFFFFFFFFFFFFF
@A00155:342:HHGFNDSXY:1:1224:4137:26725 2:N:0:GAACCTAG+TCCGCATA
CCCCCTGTGTGGAGTACAAGTCATGAGGGTCCACTTCGAGCCCCATTCTGTTAGACCTTACCGCACAA
CCTGTCTGGAAACACTGCGCTATCAGTAGGCATATTAAGTTTACTTTCTCCAATCTCCCATTGATATA
+
FFFFFFFFFFFFF:FFFFFFFFFFFFF,FFFFF,F::FFFFFFFFFF:FFFFFFFFFFFFF:FFFFFF
:F::FF:FFFFFFFFFFFFFFFFFFFFFFFFFFFFFFFFFFFFFFFF,:FFFFFFFFF,FFFFFFFFF
@A00155:342:HHGFNDSXY:1:2610:4833:24392 2:N:0:GAACCTAG+TCCGCATA
CCCCCTGTGTGGAGTACAAGTCATGAGGGTCCACTTCGAGCCCCATTCTGTTAGACCTTACCGCACAA
CCTGTCTGGAAACACTGCGCGATCAGTAGGCATATTAAGTTTACTTTCTCCAATCTCCCATTGATATA
+
FFFF,:,:FF,FFFFF,,:F:FFFFFF:F:FFFFFFFFFFFFF,FFFFFF:FFFFFFF:FF:FFFFFF
FFF:FFFF:FFFFFFF,F,:,FFF:FF::FFFFFFF,FFFFF:,FFFFFFFFFFFFFFF:FFFFFFFF
@A00155:342:HHGFNDSXY:1:1539:18557:12305 1:N:0:GAACCTAG+TCCGCATA
CCCCCTGTGTGGAGTACAAGTCATGAGGGTCCACTTCGAGCCCCATTCTGTTAGACCTTACCGCACAA
CCTGTCTGGAAACACTGCGCTATCAGTAGGCATATTAAGTTTACTTTCTCCAATCTCCCATTGATATA
+
FFFFFFFFFFFFFFFFFFFFFFFFFF:FFFFFFFFFFFFFFFFFF:FFFFFFFFFFFFFFFFFFFFFF
FFFFFFFFFF:FF:F:FFFFFFFFFF:FFFFFFFFFFFFFFFFFFFFFFFFFFFFFFFFF,FFFFFFF
```

@A00155:342:HHGFNDSXY:1:2630:22028:15029 1:N:0:GAACCTAG+TCCGCATA
CCCCCTGTGTGGAGTACAAGTCATGAGGGTCCACTTCGAGCCCCATTCTGTTAGACCTTACCGCACAA
CCTGTCTGGAAACACTGCGCTATCAGTAGGCATATTAAGTTTACTTTCTCCAATCTCCCATTGATATA
+
FFFFFFFFFFFFFFFFFFFFFFF:FFFFFFFFFFFFFFFFFFFFFFFFFFFFFFFFFFFFFFFFFFFF
FFFFFFFFFFFFFFFFFFFFF:FFFFFFFFFF:FFFFFFFFFFFFFFFFFFFFFFFFFFFFFFFFFFF
@A00155:342:HHGFNDSXY:1:1433:9824:5854 2:N:0:GAACCTAG+TCCGCATA
CCCCCTGTGTGGAGTACAAGTCATGAGGGTCCACTTCGAGCCCCATTCTGTTAGACCTTACCGCACAA
CCTGTCTGGAAACACTGCGCTATCAGTAGGCATATTAAGTTTACTTTCTCCAATCTCCCATTGATATA
+
FFFFFFFFFFFFFFFFFFFFFFFFFFFFFFFFFFFFFFFFFFFFFF:FFFFFF,FF:FFFFFFFFFFF
FFFFFFFFFFFFFFFFFFFFFFFFFFFFFFFFFFFFFFFFFFFFFFFFFFFFFFFFFFFFFFFFFFFF
@A00155:342:HHGFNDSXY:1:2343:10312:20165 1:N:0:GAACCTAG+TCCGCATA
CCCCCTGTGTGGAGTACAAGTCATGAGGGTCCACTTCGAGCCCCATTCTGTTAGACCTTACCGCACAA
CCTGTCTGGAAACACTGCGCTATCAGTAGGCATATTAAGTTTACTTTCTCCAATCTCCCATTGATATA
+
FFFFFFFFFFFFFFFFFFFFFFFFFFFFFFFFFFFFFFFFFFFFFFFFFF:FFFFFFFFFFFFFFFFF
FFFFFF:FFFFFFFFFFFFFFFFFFFFFFFFFFFFFFFFFFFFFFFFFFFFFFFFFFFFFFFFFFFFF
@A00155:342:HHGFNDSXY:1:2551:25048:19194 2:N:0:GAACCTAG+TCCGCATA
CCCCCTGTGTGGAGTACAAGTCATGAGGGTCCACTTCGAGCCCCATTCTGTTAGACCTTACCGCACAA
CCTGTCTGGAAACACTGCGCTATCAGTAGGCATATTAAGTTTACTTTCTCCAATCTCCCATTGATATA
+
FFFFFFFFFFFFFFFFFFFFFF:FFFFFFFFFFFFFFFFFFFFFF:FFFFFFFFFFFFFFFFFFFFFF
FFFFFFFFFFFFFFFFFFFFFFFFFF:FFFFFFFFFFFFFFFFFFFFFFFFFFFFFFFFFFFFFFF,F
@A00155:342:HHGFNDSXY:1:2378:12472:20118 1:N:0:GAACCTAG+TCCGCATA
CCCCCTGTGTGGAGTACAAGTCATGAGGGTCCACTTCGAGCCCCATTCTGTTAGACCTTACCGCACAA
CCTGTCTGGAAACACTGCGCTATCAGTAGGCATATTAAGTTTACTTTCTCCAATCTCCCATTGATATA
+
FFFFFFFFFFFFFFFFFFFFFFFFFFFFFFFFFFFFFFFFFFFFFFFFFFFFFFFFFFFFFFFFFFFF
FFFFFFFFFFFFFFFFFFFFFFFFFFFFFFFFFF:FFFFFFFFFFFFFFFFFFFFFFFFFFFFFFFFF
@A00155:342:HHGFNDSXY:1:2631:31765:34929 2:N:0:GAACCTAG+TCCGCATA
CCCCTGTGTGGAGTACAAGTCATGAGGGTCCACTTCGAGCCCCATTCTGTTAGACCTTACCGCACAAC
CTGTCTGGAAACACTGCGCTATCAGTAGGCATATTAAGTTTACTTTCTCCAATCTCCCATTGATATA
+
FFFFFFFFFFFFFFFFFFFFFFFFFFFF:FFFFFFFFFFFFFFFFFFFFFFFFFFFFFFFFFFFFFFF
FFFFFFFFFFFFFFFFFFFFFFFFFFFFFFFFFFFFFFFFFFFFFFF:FFFFFFFFFFFFFFFFFFF
@A00155:342:HHGFNDSXY:1:2645:29577:24627 2:N:0:GAACCTAG+TCCGCATA
CCCTGTGTGGAGTACAAGTCATGAGGGTCCACTTCGAGCCCCATTCTGTTAGACCTTACCGCACAACC
TGTCTGGAAACACTGCGCTATCAGTAGGCATATTAAGTTTACTTTCTCCAATCTCCCATTGAT
+
FFF,FFFFFFFFFFFFFFFFFFFFFFFFFFFFFFFFF:FFFFFFFFFFFFFFFFFFF:FFFFFFFFFF
FFFFFFFFFFFFFFFFFF:FFFFFFFFFFFFFFFFFFFFFFFFFFFFF:FFFFFFFFFFFFFF
@A00155:342:HHGFNDSXY:1:1358:13792:31422 2:N:0:GAACCTAG+TCCGCATA
CCCTGTGTGGAGTACAAGTCATGAGGGTCCACTTCGAGCCCCATTCTGTTAGACCTTACCGCACAACC
TGTCTGGAAACACTGCGCTATCAGTAGGCATATTAAGTTTACTTTCTCCAATCTCCCATTGAT
+
FFFFFFFFFFFFFFFFFFFFFFFFFFFFFFFFFFFFFFFFFFFFFFFFFFFFFFFFFFFFFFFFFFFF
FFFFFFFFFFFFFFFFFFFF:FFFFFFFFFFFFFFFFFFFFFFFFFFFFFFFFFFFF:FFFFF
@A00155:342:HHGFNDSXY:1:1274:1868:17440 2:N:0:GAACCTAG+TCCGCATA
CCCTGTGTGGAGTACAAGTCATGAGGGTCCACTTCGAGCCCCATTCTGTTAGACCTTACCGCACAACC
TGTCTGGAAACACTGCGCTATCAGTAGGCATATTAAGTTTACTTTCTCCAATCTCCCATTGATATA
+
FFF,F,FFFFFFFFFFFFFFFFFFFFFFFFFFFFFFFFFFFFFF::FFFFFFFF:FFFF:FFFFFFFF
FFFFFFFFFFFFFFFFFFFFFFFF:FFFFFFFFFFFFFFFFFFF:FFFFFFFFFFFFFFFFFFFFF

```
@A00155:342:HHGFNDSXY:1:2157:2645:17973 2:N:0:GAACCTAG+TCCGCATA
CCCTGTGTGGAGTACAAGTCATGAGGGTCCACTTCGAGCCCCATTCTGTTAGACCTTACCGCACAACC
TGTCTGGAAACACTGCGCTATCAGTAGGCATATTAAGTTTACTTTCTCCAATCTCCCATTGATATAAC
+
F:FFFFFFFFFFFFFFFFFFFF:FF:FFFFFFFFFFFFFFFFFFFFFFFFFFF:FF:FFFFFFFFFFF
FFFFFFFFFFFF:FFFFFFFFFFFF:FFFFFFFF:FFFFFFFFFFFFFFFFFFFFFFFFFFFFFFFFF
@A00155:342:HHGFNDSXY:1:1306:1009:36620 2:N:0:GAACCTAG+TCCGCATA
CCTGTGTGGAGTACAAGTCATGAGGGTCCACTTCGAGCCCCATTCTGTTAGACCTTACCGCACAACCT
GTCTGGAAACACTGCGCTATCAGTAGGCATATTAAGTTTACTTTCTCCAATCTCCCATTGATA
+
FFFFFFFFFFFFFFFFFFFFFFFFFFFFFFFFFFFFFFFFFFFFF:FFFFFFFF,FFFFFFFFFFFFF
:FFFFFFFFFFF:FFF,,FFFFF:FFFF:FFFFFFF::FFFF:FFFFFFFFFFFFFFF:FF:F
@A00155:342:HHGFNDSXY:1:1117:24722:25801 2:N:0:GAACCTAG+TCCGCATA
CCTGTGTGGAGTACAAGTCATGAGGGTCCACTTCGAGCCCCATTCTGTTAGACCTTACCGCACAACCT
GTCTGGAAACACTGCGCTATCAGTAGGCATATTAAGTTTACTTTCTCCAATCTCCCATTGATATAACC
+
FFFFFFFFFFFFFFFFFFFFFFFFFFFFFFFFFFFFFFFFFFFFFFFFFFFFF,FFFFFFFFF:FFFF
FFFFFFFFFFFFFFFF:FFFFFFFFFFFFFFFFFFFFFFFFFFFFFFFFFFFFFFFFFFFFFFF:FFF
@A00155:342:HHGFNDSXY:1:1511:9869:32142 2:N:0:GAACCTAG+TCCGCATA
CCTGTGTGGAGTACAAGTCATGAGGGTCCACTTCGAGCCCCATTCTGTTAGACCTTACCGCACAACCT
GTCTGGAAACACTGCGCTATCAGTAGGCATATTAAGTTTACTTTCTCCAATCTCCCATTGATATAACC
+
FFFFFFFFFFFFFFFFFFFFFFFFFFFFFFFFFFFFFFFFFFFFFFFFFFFFFFFFFFFFFF:FFFFF
FFFFF:FFFFFFFFFFFFFFFFFFFFFFFFFFFFFFFFFFFFFFFFFFFFFFFFFFFFFFFFFF
@A00155:342:HHGFNDSXY:1:2603:17924:1313 2:N:0:GAACCTAG+TCCGCATA
CCTGTGTGGAGTACAAGTCATGAGGGTCCACTTCGAGCCCCATTCTGTTAGACCTTACCGCACAACCT
GTCTGGAAACACTGCGCTATCAGTAGGCATATTAAGTTTACTTTCTCCAATCTCCCATTGATATAACC
+
FFFF,FFFFFFFFFFFFFF:FFFFFFFFFFFFFFFFFFFFF:FFFFFFFFF:F:FFFF:FFFFFFFFF
FFFFFFFFFFFFFFFFFFF:FFFFFFFFFFFFFFFFFFFFFFFF:FFFFFFFFFFFFFFFFFFFFFFF
@A00155:342:HHGFNDSXY:1:2429:6705:14418 2:N:0:GAACCTAG+TCCGCATA
CCTGTGTGGAGTACAAGTCATGAGGGTCCACTTCGAGCCCCATTCTGTTAGACCTTACCGCACAACCT
GTCTGGAAACACTGCGCTATCAGTAGGCATATTAAGTTTACTTTCTCCAATCTCCCATTGATATAACC
+
FFFF:FFFFFF:::FFFFFFFFFFFFFFFFFFF:FFFFF:FFFFFFFFFFFFFFFFFF:FFFFFFFFF
FFFF:FFFFFF:FFFFF:FFFF,FFFFFFFFFFFFF:,FFFFFFFFFFFFFFFFFFFFFFFFFFFFFF
@A00155:342:HHGFNDSXY:1:2469:23990:20087 2:N:0:GAACCTAG+TCCGCATA
CCTGTGTGGAGTACAAGTCATGAGGGTCCACTTCGAGCCCCATTCTGTTAGACCTTACCGCACAACCT
GTCTGGAAACACTGCGCTATCAGTAGGCATATTAAGTTTACTTTCTCCAATCTCCCATTGATATAACC
+
FFFFFFFFFFFFFFFFFFFFFFFF:FFFFFFFFF:FFFFFFFFFFFF:FFFFFFFFFFFFFFFFFFFF
FFFFFFFFFFFFFFFFFFFFFFFF:FFFFFFFFFFFFFFFFFFFFFFFFFFFFF:FFFFFFFFFFFFF
@A00155:342:HHGFNDSXY:1:1511:9869:32142 1:N:0:GAACCTAG+TCCGCATA
GTACAAGTCATGAGGGTCCACTTCGAGCCCCATTCTGTTAGACCTTACCGCACAACCTGTCTGGAAAC
ACTGCGCTATCAGTAGGCATATTAAGTTTACTTTCTCCAATCTCCCATTGATATAACCCCACTCATTA
+
FFFFFFFFFFFFFFFFFFFFFFFFFFFFFFFFFFFFFFFFF:FFFFFF:FFFFFFFFFFFFFFFFFFF
FFFFFFFFFFFFFFFFFFFFFFFFFFFFFFFFFFFFFFFFFFFFFFFFFFFFFFFFFFFFFFFFFFFF
@A00155:342:HHGFNDSXY:1:2469:23990:20087 1:N:0:GAACCTAG+TCCGCATA
GTACAAGTCATGAGGGTCCACTTCGAGCCCCATTCTGTTAGACCTTACCGCACAACCTGTCTGGAAAC
ACTGCGCTATCAGTAGGCATATTAAGTTTACTTTCTCCAATCTCCCATTGATATAACCCCACTCATTA
+
FFFFFFF:FFFFFFFFFFFFFFFFFFFFFFFFFFFFFFFFFFFFFFFFFFFFFFFFF:FFF:FFFFFF
FFFFFFFFFFFFFFFFFFFFFF:FFFFFFFFFF:FFFFFFFFFFFF:F:FFFFFFF:FFFFFFF:F:F
```

```
@A00155:342:HHGFNDSXY:1:1269:12066:32127 1:N:0:GAACCTAG+TCCGCATA
TACAAGTCATGAGGGTCCACTTCGAGCCCCATTCTGTTAGACCTTACCGCACAACCTGTCTGGAAACA
CTGCGCTATCAGTAGGCATATTAAGTTTACTTTCTCCAATCTCCCATTGATATAACCCCACTCATTAA
+
FFFFFFFFFFFFFFFFFFFFFFFFFFFFFFFFFFFFFFFFFFFFFFFFFFFFFFFFFFFFFFFFFFFF
FFFFFFFFFFFFFFFFFFFFFFFFFFFFFFFFFFFFFFFFFFFFFFFFFFFFFFFFFFFF:FFFFFFF
@A00155:342:HHGFNDSXY:1:1429:32841:34757 1:N:0:GAACCTAG+TCCGCATA
TACAAGTCATGAGGGTCCACTTCGAGCCCCATTCTGTTAGACCTTACCGCACAACCTGTCTGGAAACA
CTGCGCTATCAGTAGGCATATTAAGTTTACTTTCTCCAATCTCCCATTGATATAACCCCACTCATTAA
+
FFFFFFFFFFFFFFFFFFFFFFFFFFFFFFFFFFFFFFFFFFFFFFFFFFFFFFFFFFFFFFFFFFFF
FFFFFFFFFFFFFFFFFFFFFFFFFFFFFFFFFFFFFFFFFFFFFFFFFFFFFFFFFFF:F:F,FFF:
@A00155:342:HHGFNDSXY:1:1178:4978:13964 1:N:0:GAACCTAG+TCCGCATA
ACAAGTCATGAGGGTCCACTTCGAGCCCCATTCTGTTAGACCTTACCGCACAACCTGTCTGGAAACAC
TGCGCTATCAGTAGGCATATTAAGTTTACTTTCTCCAATCTCCCATTGATATAACCCCACTCATTAAT
+
FF:FFFFFFFFFFFFFFFFFFFFFFFFFFFFFFFFFFFFFFFFFFFFFFFFFFFFFFFFFFFFFFFFF
FFFFFFFFFFFF:FFFFFFFFFFFFFFFFFFFFFFFFFFFFFFFFFFFFFFFFFFFFFF,FFFF,FF
@A00155:342:HHGFNDSXY:1:2631:31765:34929 1:N:0:GAACCTAG+TCCGCATA
CAAGTCATGAGGGTCCACTTCGAGCCCCATTCTGTTAGACCTTACCGCACAACCTGTCTGGAAACACT
GCGCTATCAGTAGGCATATTAAGTTTACTTTCTCCAATCTCCCATTGATATAACCCCACTCATTAAT
+
FFFFFFFFFFFFFFFFFFFFFFFFFFFFFFFFFF:FFFFFFFFFFFFF,FFFFFFFFF:FFFFFFFF:
FFFFF:FFFFFFFFFFFF:FFFFFFFFFFFFFFFFFF:FFFFF:FFFF,F:FFFFFFFF:FFFFF:F
@A00155:342:HHGFNDSXY:1:1405:31295:21308 1:N:0:GAACCTAG+TCCGCATA
CAAGTCATGAGGGTCCACTTCGAGCCCCATTCTGTTAGACCTTACCGCACAACCTGTCTGGAAACACT
GCGCTATCAGTAGGCATATTAAGTTTACTTTCTCCAATCTCCCATTGATATAACCCCACTCATTAATA
+
FFFFFFFFFFFFFFFFFFFFFFFFFFFFF,FFFFFFFFFFFFFFFFFFFFFFFFFF:F:FFFFFFFF:
FFFFFFFFFFFFFFFFFFFFFFFFFFFFFFFFFFFFFFFFFFFFFFFFFFFFFFFFFFFFFFFFFFFF
@A00155:342:HHGFNDSXY:1:1322:8440:35180 1:N:0:GAACCTAG+TCCGCATA
CAAGTCATGAGGGTCCACTTCGAGCCCCATTCTGTTAGACCTTACCGCACAACCTGTCTGGAAACACT
GCGCTATCAGTAGGCATATTAAGTTTACTTTCTCCAATCTCCCATTGATATAACCCCACTCATTAATA
+
FFFFFFFFFFFFFFFFFFFFFFFFFFFFFFFFFFFFFFFFFFFFFFFFFFFFFFFFFFFFFFFFFFFF
FFFFFFFFFFFFFFFFFFFFFFFFFFFFFFFFFFFFFFFFFFFFFFFFFFFFFFFFFFFFFFFFFFFF
@A00155:342:HHGFNDSXY:1:2645:29577:24627 1:N:0:GAACCTAG+TCCGCATA
AAGTCATGAGGGTCCACTTCGAGCCCCATTCTGTTAGACCTTACCGCACAACCTGTCTGGAAACACTG
CGCTATCAGTAGGCATATTAAGTTTACTTTCTCCAATCTCCCATTGATATAACCCCACTCATT
+
FFFFFFFFFFFFFFFFFFFFFFFFFFFFFFFFFFFFFFFFFFFFFFF:FFFF:FFFFFFFFFFFFFFF
FFFFFFFFFFFFFFFFFFFFFFFFFFFFFFFFFFFFFFFF:F:,FF:FFFFFFFFF:FFFF,F
@A00155:342:HHGFNDSXY:1:1358:13792:31422 1:N:0:GAACCTAG+TCCGCATA
AAGTCATGAGGGTCCACTTCGAGCCCCATTCTGTTAGACCTTACCGCACAACCTGTCTGGAAACACTG
CGCTATCAGTAGGCATATTAAGTTTACTTTCTCCAATCTCCCATTGATATAACCCCACTCATT
+
FFFFFFFFFFFFFFFFFFFFFFFFFFFFFFFFFFFFFFFFFFFFFFFFFFFFFFFFFFFFFFFFFFFF
FFFFFFFFFFFFFFFFFFFFFFFFFFFFFFFFFFFFFFFFFFFFFFFFFFFFFFFFFFFFFFF
@A00155:342:HHGFNDSXY:1:1274:1868:17440 1:N:0:GAACCTAG+TCCGCATA
AAGTCATGAGGGTCCACTTCGAGCCCCATTCTGTTAGACCTTACCGCACAACCTGTCTGGAAACACTG
CGCTATCAGTAGGCATATTAAGTTTACTTTCTCCAATCTCCCATTGATATAACCCCACTCATTAAT
+
FFFFF:FFFFFFFFFFFFFFFFFFFFFF:FFFF::FFFFFFFFFFFFFFFFFFF:F:FFFFFFFFFFF
FFFF:FFFFFFFFFFFFFFFFFFFFFFFFFFFFFFFFFFF:FFFFFFFFFFFF:FFFFFFFFFF,F
```

```
@A00155:342:HHGFNDSXY:1:1306:1009:36620 1:N:0:GAACCTAG+TCCGCATA
AGTCATGAGGGTCCACTTCGAGCCCCATTCTGTTAGACCTGACCGCACAACCTGTCTGGAAACACTGC
GCTATCAGTAGGCATATTAAGTTTACTTTCTCCAATCTCCCATTGATATAACCCCACTCATTA
+
FFF::FFFFFFFFFFFF,FFFFFFFF:F,F,F:FFF:FFF,FFFFFFF,,FFFF,F,FF::FFFF:FF
FFF:FF:FF,FFF:F::F:FF:FF,FFFFFFFF:F,FFFFF:,FFF,FFFFFFFFFFFFFFFF
@A00155:342:HHGFNDSXY:1:2364:32931:29997 1:N:0:GAACCTAG+TCCGCATA
AGTCATGAGGGTCCACTTCGAGCCCCATTCTGTTAGACCTTACCGCACAACCTGTCTGGAAACACTGC
GCTATCAGTAGGCATATTAAGTTTACTTTCTCCAATCTCCCATTGATATAACCCCACTCATTAATAGC
+
FFFFFFFFFFFFFFFFFFFFFFFFFFFFF:FFFFFFFFFFFFFFFFFFFFFFFFFFFFFFFF:FFFFF
FFFFFFFFFFFFFFFFFFFFFFFFFFFFFFFFFFFFFFFFFFFFFFFFFFF:FFF:FFFFFFF:FFFF
@A00155:342:HHGFNDSXY:1:1545:5963:31735 1:N:0:GAACCTAG+TCCGCATA
AGTCATGAGGGTCCACTTCGAGCCCCATTCTGTTAGACCTTACCGCACAACCTGTCTGGAAACACTGC
GCTATCAGTAGGCATATTAAGTTTACTTTCTCCAATCTCCCATTGATATAACCCCACTCATTAATAGC
+
FFFF:FFFFFFFFFFFFFFFFFFFFFFFFFFFFFFFFFFFFFFFFFFFFFFFFFFFFFFFFFFFFFFF
FFFFFFFFFFFFFFFFFFFFFFFFFFFFFFFFFFFFFFFFFFFFFFFFFFFFFFFFFFFFFF:FFFFF
@A00155:342:HHGFNDSXY:1:2619:12671:24972 1:N:0:GAACCTAG+TCCGCATA
AGTCATGAGGGTCCACTTCGAGCCCCATTCTGTTAGACCTTACCGCACAACCTGTCTGGAAACACTGC
GCTATCAGTAGGCATATTAAGTTTACTTTCTCCAATCTCCCATTGATATAACCCCACTCATTAATAGC
+
FFFFFFFFFFFFFFFFFFFFFFFFFFFFFFFFFFFFFFFFFFFFFFFFF:FFFFFFFFFFFFFFFFFF
FFFFFFFFFFFFFFFFFFFFFFFFFFFFFFFFFFFFFFFFFFFFFFFFFFFFFFFFFFFFFFFFFFFF
@A00155:342:HHGFNDSXY:1:2442:19994:12759 1:N:0:GAACCTAG+TCCGCATA
AGTCATGAGGGTCCACTTCGAGCCCCATTCTGTTAGACCTTACCGCACAACCTGTCTGGAAACACTGC
GCTATCAGTAGGCATATTAAGTTTACTTTCTCCAATCTCCCATTGATATAACCCCACTCATTAATAGC
+
FFFFFFFFFFFFFFFFFFFFFFFFFFFFFFFFFFFFFFFFFFFFFFFFFFFFFFFFFFFFFFFFFFFF
FFFFFFFF:FFFFFFFFFFFFFFFFFFFFFFFFFFFFFFFF:F:FFFFFFFFFFFFFFFFFFFFFFFF
@A00155:342:HHGFNDSXY:1:2151:20799:25895 1:N:0:GAACCTAG+TCCGCATA
AGTCATGAGGGTCCACTTCGAGCCCCATTCTGTTAGACCTTACCGCACAACCTGTCTGGAAACACTGC
GCTATCAGTAGGCATATTAAGTTTACTTTCTCCAATCTCCCATTGATATAACCCCACTCATTAATAGC
+
FFFFFFFFFFFFFFFFFFFFFFFFFFFFFFFFFFFFFFFFFFFFFFFFFFFFFF:FFFFFFFFFF:FF
FFFFFFFFFFFFFFF:FFFFFFFFFFFFFFFFFFFFFFFFFFFFFFFFFFFFFFFFFFFFFFFFFFFF
@A00155:342:HHGFNDSXY:1:2303:29062:23015 1:N:0:GAACCTAG+TCCGCATA
AGTCATGAGGGTCCACTTCGAGCCCCATTCTGTTAGACCTTACCGCACAACCTGTCTGGAAACACTGC
GCTATCAGTAGGCATATTAAGTTTACTTTCTCCAATCTCCCATTGATATAACCCCACTCATTAATAGC
+
FFFFFFFFFFFFFFFFFFFFFFFFFFFFFFFFFFFFFFFFFFFFFFFFFFFFFFFFFFFFFFFFFFFF
FFFFFFFFFFFFFFFFFFFFFFFFFFFFFFFFFFFFFFFFFFFFFFFFFFFF:FFFFFFF:FFFFFFF
@A00155:342:HHGFNDSXY:1:2603:17924:1313 1:N:0:GAACCTAG+TCCGCATA
AGTCATGAGGGTCCACTTCGAGCCCCATTCTGTTAGACCTTACCGCACAACCTGTCTGGAAACACTGC
GCTATCAGTAGGCATATTAAGTTTACTTTCTCCAATCTCCCATTGATATAACCCCACTCATTAATAGC
+
FFFFFFFFFFFFFFFFFFFFFFFFFFFFFFFFFFFFFFFFFFFFFFFFFFFFFF,FFFFFFFFFFFFF
FFFFFFFFFFFFFF:FFFFFFFFFFFFFFFFFFFFFFFFFFFFFFF:FFFFFFFF:FFFFF:FFFFFF
@A00155:342:HHGFNDSXY:1:2378:12472:20118 2:N:0:GAACCTAG+TCCGCATA
ATGAGGGTCCACTTCGAGCCCCATTCTGTTAGACCTTACCGCACAACCTGTCTGGAAACACTGCGCTA
TCAGTAGGCATATTAAGTTTACTTTCTCCAATCTCCCATTGATATAACCCCACTCATTAATAGCTCTG
+
FFFFFFFFFFFFFFFFFFFFFFFFFFFFFFFFFFFFFFFFFFFFFFFF:FFFFFFFFFFFFFFFFFFF
FFFFFFFFFFFFFFFFFFFFFFFFFFFFFFFFFFFFFFFFFFFFFFFFFFFFFFFFFFFFFFFFFFFF
```

```
@A00155:342:HHGFNDSXY:1:2317:7572:3302 1:N:0:GAACCTAG+TCCGCATA
TGAGGGTCCACTTCGAGCCCCATTCTGTTAGACCTTACCGCACAACCTGTCTGGAAACACTGCGCTAT
CAGTAGGCATATTAAGTTTACTTTCTCCAATCTCCCATTGATATAACCCCACTCATTAATAGCTCTGT
+
FFFFFFFFFFFFFFFFFFFFFFFFFFFFFFFFFFFFFFFFFFFFFFFFFFFFFFFFFFFFFFFFFFFFFF
FFFFFFFFFFFFFFFFFFFFFFFFFFFFFFFFFFFFFFFFFFFFFFFFFFFFFFFFFFFFFFFFFFFFFF
@A00155:342:HHGFNDSXY:1:2263:16532:33473 1:N:0:GAACCTAG+TCCGCATA
TGAGGGTCCACTTCGAGCCCCATTCTGTTAGACCTTACCGCACAACCTGTCTGGAAACACTGCGCTAT
CAGTAGGCATATTAAGTTTACTTTCTCCAATCTCCCATTGATATAACCCCACTCATTAATAGCTCTGT
+
FFFFFFFFFFFFFFFFFFFFFFFFFFFFFFFFFFFFFFFFFFFFFFFFFFFFFFFFFFFFFFFFFFFFFF
FFFFFFFFFFFFFFFFFFFFFFFFFFFFFFFFFFFFFFFFFFFFFFFFFFFFFFFFF:FFFFFFFFFFFF
@A00155:342:HHGFNDSXY:1:1328:4300:29543 1:N:0:GAACCTAG+TCCGCATA
AGGGTCCACTTCGAGCCCCATTCTGTTAGACCTTACCGCACAACCTGTCTGGAAACACTGCGCTATCA
GTAGGCATATTAAGTTTACTTTCTCCAATCTCCCATTGATATAACCCCACTCATTAATAGCTCT
+
FFFFFFFFFFFFFFFFFFFFFFFFFFFFFF,FF:FFF:FFFFFFFF:FFF::FFFFFFFFFFFFFFFFFF
FFFFFFFFFFFFFFFFFF:FFFFFF:FF:FFFFFFFFFFFFFFFFFFFFFFFFFFFFFFFFFFFFF
@A00155:342:HHGFNDSXY:1:1613:22110:18490 1:N:0:GAACCTAG+TCCGCATA
AGGGTCCACTTCGAGCCCCATTCTGTTAGACCTTACCGCACAACCTGTCTGGAAACACTGCGCTATCA
GTAGGCATATTAAGTTTACTTTCTCCAATCTCCCATTGATATAACCCCACTCATTAATAGCTCT
+
FFFFFFFFFFFFFFFFFFFFFFFFFFFFFFFFFFFFFFFFFFFFFFFFFFFFFFFFFFFFFFFFFFFFFF
FFFFFFFFFFFFFFFFFFFFFFFFFFFFF,FFFFFFFFFFFF::FFFFFFFFFFFFFFFF:FFFFF
@A00155:342:HHGFNDSXY:1:2470:27190:5024 1:N:0:GAACCTAG+TCCGCATA
AGGGTCCACTTCGAGCCCCATTCTGTTAGACCTTACCGCACAACCTGTCTGGAAACACTGCGCTATCA
GTAGGCATATTAAGTTTACTTTCTCCAATCTCCCATTGATATAACCCCACTCATTAATAGCTCT
+
FFFFFFFFFFFFFFFFFFFFFFFFFFFFFFFFFFFFFFFFFFFFFFFFFFFFFFFFFFFF:FFFFFFFFF
FFFFFFFFFFFFFFFFFF:FFFFFFFFFFFFFFFFFFFFFFFFFFFFFFFFFFF:FFFFFFFFFF
@A00155:342:HHGFNDSXY:1:1432:8178:12179 1:N:0:GAACCTAG+TCCGCATA
AGGGTCCACTTCGAGCCCCATTCTGTTAGACCTTACCGCACAACCTGTCTGGAAACACTGCGCTATCA
GTAGGCATATTAAGTTTACTTTCTCCAATCTCCCATTGATATAACCCCACTCATTAATAGCTCT
+
FFFFFFFFFFFFFFFFFFFFFFFFFFFFFFFFFFFFFFFFFFFFFFFFFFFFFFFFFFFFFFFFFFFFFF
FFFFFFFFFFFFFFFFFFFFFFFFFFFFFFFFFFFFFFFFFFFFFFFFFFFFFFFFFFFFFFFFFF
@A00155:342:HHGFNDSXY:1:2303:29062:23015 2:N:0:GAACCTAG+TCCGCATA
AGGGTCCACTTCGAGCCCCATTCTGTTAGACCTGACCGCACAACCTGTCTGGAAACACTGCGCTATCA
GTAGGCATATTAAGTTTACTTTCTCCAATCTCCCATTGATATAACCCCACTCATTAATAGCTCTGTGG
+
FFFFFFFFFF:FF:FFFFFFFFFFFFFF:FFFFF,FFFFFFFF:FFFFFF:FFFFFFFFFFFFFFFF:FF
FFFFFFFFFFFFFFF,FFFFFFFFFFFF:FFFFFFFFFFFFFFFFFFFFFFFFF,FFFFFFFF,FFFFFF
@A00155:342:HHGFNDSXY:1:1123:5936:16501 2:N:0:GAACCTAG+TCCGCATA
AGGGTCCACTTCGAGCCCCATTCTGTTAGACCTTACCGCACAACCTGTCTGGAAACACTGCGCTATCA
GTAGGCATATTAAGTTTACTTTCTCCAATCTCCCATTGATATAACCCCACTCATTAATAGCTCTGTGG
+
FFFFFFFFFFF:FFFFFFFFFFFFFFFFFFFFFFFFFFFF:FFFFFFFFFFFFFFFFFFFFFFFFFF:FF
FFFFFFFFFFFFFFFFFFFFFFFFFFFFFFFFFFFFFFFFFFFFFFF:FFFFFFFFF,FFFFFFFFFFFF
@A00155:342:HHGFNDSXY:1:1245:7952:30921 2:N:0:GAACCTAG+TCCGCATA
AGGGTCCACTTCGAGCCCCATTCTGTTAGACCTTACCGCACAACCTGTCTGGAAACACTGCGCTATCA
GTAGGCATATTAAGTTTACTTTCTCCAATCTCCCATTGATATAACCCCACTCATTAATAGCTCTGTGG
+
:FFFFFF,FFFFFFF:FFF:FFFFFFFFFFFFFFFFFFFFFFFFFFFFFFFFFFFFF:FFFFFFFFFFFF
FFFFFFFFFFFFFFFFFFFFFFFFFFFFFFFF:FFFFFF:FFFFFFFFFFF:FFFFFFFF:,FFFFFFFF
```

```
@A00155:342:HHGFNDSXY:1:1566:7609:14951 2:N:0:GAACCTAG+TCCGCATA
GGTCCACTTCGAGCCCCATTCTGTTAGACCTTACCGCACAACCTGTCTGGAAACACTGCGCTATCAGT
AGGCATATTAAGTTTACTTTCTCCAATCTCCCATTGATATAACCCCACTCATTAATAGCTCTGTGGCT
+
FFFFFFFFFFFFFFFFFFFFFFFFFFFFFF:FFFFFFFFFFFFFF:FFFFFFFFFFFFFFFFFFFFFF
FFFFFFFFFFFFFFFFFFFFFFFFFFFF:FFF:FFFFFFFFFFFFFFFFF:FFFFFFFFFFFFFFFFF
@A00155:342:HHGFNDSXY:1:2103:12943:18145 2:N:0:GAACCTAG+TCCGCATA
GTCCACTTCGAGCCCCATTCTGTTAGACCTTACCGCACAACCTGTCTGGAAACACTGCGCTATCAGTA
GGCATATTAAGTTTACTTTCTCCAATCTCCCATTGATATAACCCCACTCATTAATAGCTCTGT
+
FFFFFFFFFFFFFFFFFFFFFFFFFFFFFFFFFFFFFFFFFFFFFFFFFFFFFFFFFFFFFFFFFFFF
FFFFFFFFFFFFFFFFFFFFFFFFFFFFFFF:::FFFFFFFFFFFFFFFFFFFFFFFFFFFFF
@A00155:342:HHGFNDSXY:1:1432:8178:12179 2:N:0:GAACCTAG+TCCGCATA
CCACTTCGAGCCCCATTCTGTTAGACCTTACCGCACAACCTGTCTGGAAACACTGCGCTATCAGTAGG
CATATTAAGTTTACTTTCTCCAATCTCCCATTGATATAACCCCACTCATTAATAGCTCTGTGGCTTAC
+
FFFFFFFFFFFFFFFFFFFFFFFFFFFF,FFFFFFFFFFFFFFFFFFFFFFFFFFFFFFFFFFFFFFF
FFFFFFFFFFFFFFFFFFFFFF,FFFFFFFFFFFFFFFFFFFFFFFFFFFFFFFFFFFFFFFFF:FFF
@A00155:342:HHGFNDSXY:1:1545:5963:31735 2:N:0:GAACCTAG+TCCGCATA
CCACTTCGAGCCCCATTCTGTTAGACCTTACCGCACAACCTGTCTGGAAACACTGCGCTATCAGTAGG
CATATTAAGTTTACTTTCTCCAATCTCCCATTGATATAACCCCACTCATTAATAGCTCTGTGGCTTAC
+
FFFFF:FFFFFFFFF:FFFF:FFFFFFFFFFFFFFFFFFFFFFFFFFFFFFFFFFFFFFFFFFFFFFF
:FFFFFFFFFFFFFFFFFFFFFFFFFFFFFFFFFFFFF:FFFF:FFFFFFFFFFFFFFFFFFFFFFFF
@A00155:342:HHGFNDSXY:1:1365:19479:13463 1:N:0:GAACCTAG+TCCGCATA
CGAGCCCCATTCTGTTAGACCTTACCGCACAACCTGTCTGGAAACACTGCGCTATCAGTAGGCATATT
AAGTTTACTTTCTCCAATCTCCCATTGATATAACCCCACTCATTAATAGCTCTGTGGCTTACTAGAGC
+
FFFFFFFFF,FFFFFFFFFFFFFFFFFFFFFFFFFFFFFFFFFFFFFFFFFFFFFFFFFFFFFFFFFF
FFFFFFFFFFFFFFFFFFFFFFFFFFFFFFFFFFFFFFFFFFFFFFFFFFFFFFFFFFFFFFFFFFFF
@A00155:342:HHGFNDSXY:1:1323:11153:28009 1:N:0:GAACCTAG+TCCGCATA
AGCCCCATTCTGTTAGACCTTACCGCACAACCTGTCTGGAAACACTGCGCTATCAGTAGGCATATTAA
GTTTACTTTCTCCAATCTCCCATTGATATAACCCCACTCATTAATAGCTCTGTGGCTTACTAGA
+
FFFFFFFFFFFFFFFFFFFFFFFFFFFFFFFFFFFFFFFFFFFFFFFFFFFFFFFFFFFFFFFFFFFF
FFFFFFFFFFFFFFFFFFFFFFFFFFFFFFF:FFFFFFFFFFFFFFFFFFFFFFFFFFFFFFFF
@A00155:342:HHGFNDSXY:1:1323:11514:29324 1:N:0:GAACCTAG+TCCGCATA
AGCCCCATTCTGTTAGACCTTACCGCACAACCTGTCTGGAAACACTGCGCTATCAGTAGGCATATTAA
GTTTACTTTCTCCAATCTCCCATTGATATAACCCCACTCATTAATAGCTCTGTGGCTTACTAGA
+
FFFFFFFFFFFFFFFFFFFFFFFFFFFFFFFFFFFFFFFFFFFFFFFFFFFFFFFFFFFFFFFFFFFF
FFFFFFFFFFFFFFFFFFFFFF:FFFFFFFFFFFFFF:FFFFFFFFFFFFFFFFFFFFFFFFFF
@A00155:342:HHGFNDSXY:1:1609:7699:21997 1:N:0:GAACCTAG+TCCGCATA
AGCCCCATTCTGTTAGACCTTACCGCACAACCTGTCTGGAAACACTGCGCTATCAGTAGGCATATTAA
GTTTACTTTCTCCAATCTCCCATTGATATAACCCCACTCATTAATAGCTCTGTGGCTTACTAGA
+
FFFFFFFFFFFFFFFFFFFFFFFFFFFFFFFFFF:FFFFFFFFFFFFFFFFFFFFFF:FFFFFFFFFF
FFFFFFFFFFF:FFFFFFFFF:FFFFFFFFFFFFFFFFFFFFFFFFFFFFFFFFFFFFFFFF:F
@A00155:342:HHGFNDSXY:1:1347:21938:35884 1:N:0:GAACCTAG+TCCGCATA
AGCCCCATTCTGTTAGACCTTACCGCACAACCTGTCTGGAAACACTGCGCTATCAGTAGGCATATTAA
GTTTACTTTCTCCAATCTCCCATTGATATAACCCCACTCATTAATAGCTCTGTGGCTTACTAGA
+
FFFFFFFFFFFFFFFFFFFFFFFFFFFFFFFFFFFFFFF:,FFFFFFFFFFFFFFFFFFFFFFFFFFF
FFFFFFFFFFFFFFFFFFFFFFFFFFFFFFFFFFFFFFFFFFFFFFFFFFFFFFFFFFFFFFFF
```

@A00155:342:HHGFNDSXY:1:2604:6994:9032 2:N:0:GAACCTAG+TCCGCATA
GCCCCATTCTGTTAGACCTTACCGCACAACCTGTCTGGAAACACTGCGCTATCAGTAGGCATATTAAG
TTTACTTTCTCCAATCTCCCATTGATATAACCCCACTCATTAATAGCTCTGTGGCTTAC
+
FF:FFF:FFFFF:FFFFF:FFFFFFFFFFFFFFFFFFFF:FFFFFFFFFFFFFFFFFFFFFFFFFFFF
FFFFFFFFFFFFF,FFFFFFFFFFFFFFFFF:FF,FFFFF:FFFFFFFFFFFFFFFFFF
@A00155:342:HHGFNDSXY:1:2571:12201:18239 2:N:0:GAACCTAG+TCCGCATA
CCCCATTCTGTTAGACCTTACCGCACAACCTGTCTGGAAACACTGCGCTATCAGTAGGCATATTAAGT
TTACTTTCTCCAATCTCCCATTGATATAACCCCACTCATTAATAGCTCTGTGGCTTACTAGAGCCGCT
+
FF:FFFFFFFFFFFFFFFFFFF:F:FFFFFFFFFFFFFFFF:FFFFFFFFFFFFFF:FFFFFFFFFFF
FFFFFFFFFF:FFFFFFFF:FFFFFFFFFFF:FFFFFFFFFFFFFFFFFFFFFFFFFFFFFFFF:FFF
@A00155:342:HHGFNDSXY:1:1347:21938:35884 2:N:0:GAACCTAG+TCCGCATA
CCCCATTCTGTTAGACCTTACCGCACAACCTGTCTGGAAACACTGCGCTATCAGTAGGCATATTAAGT
TTACTTTCTCCAATCTCCCATTGATATAACCCCACTCATTAATAGCTCTGTGGCTTACTAGAGCCGCT
+
FFFFF:FFFFFFFFFFFFFFFFFFFFFFFFFFFFFFFFFFFFFFFFF:FFFFFFFFFFFFFFFFFFFF
:FFFFFFFFFF,FFFFFFFFFFFFFFFF:FFFFFFFFFFFFFFFFFFFFFFFFFFFFFFFFFFFFFFF
@A00155:342:HHGFNDSXY:1:1655:13865:14262 2:N:0:GAACCTAG+TCCGCATA
CCCCATTCTGTTAGACCTTACCGCACAACCTGTCTGGAAACACTGCGCTATCAGTAGGCATATTAAGT
TTACTTTCTCCAATCTCCCATTGATATAACCCCACTCATTAATAGCTCTGTGGCTTACTAGAGCCGCT
+
FFFFFFFFFF:FFFFFFFFFFFFF:FFFFFFFFFFFFFFFFFFFFFFFFFFFFFFFFFFFFFFFFFF:
FFFFFFFFFFFFFFFFFFFFFFFFFFFFFFFFFFFFFFFFFFFFFFFFFFFFFFFFFFFFFFFFFFFF
@A00155:342:HHGFNDSXY:1:1609:7699:21997 2:N:0:GAACCTAG+TCCGCATA
CCCCATTCTGTTAGACCTTACCGCACAACCTGTCTGGAAACACTGCGCTATCAGTAGGCATATTAAGT
TTACTTTCTCCAATCTCCCATTGATATAACCCCACTCATTAATAGCTCTGTGGCTTACTAGAGCCGCT
+
FFFFFFFFFFFFFFF:FFFFFFFFFFFFFFFFFFFFFFFFFFFFFFFFFFFFFFFFF:FFFFFFFFFF
FFFFFFFFFFFFFFFFF:FFFFFFFFFFFFFFFFFFFFFFFFFFFFFFFFFFFFFFFFFFFFFFFFFF
@A00155:342:HHGFNDSXY:1:2537:18096:7780 2:N:0:GAACCTAG+TCCGCATA
CCATTCTGTTAGACCTTACCGCACAACCTGTCTGGAAACACTGCGCTATCAGTAGGCATATTAAGTTT
ACTTTCTCCAATCTCCCATTGATATAACCCCACTCATTAATAGCTCTGTGGCTTACTAGAGCCG
+
:FFFFFFFFFFFFFFFFFFFFFFFFFFFFFFFF,FFFFFFFFFFFFFF:FFFFFFF:FFFFFFFFFFF
FFFFFFFFFFFFFFFF:FFFFFFFFFFFFFFFF:FFFFFFFFFFFFFFFFFFFFFFFFFFFFFF
@A00155:342:HHGFNDSXY:1:1357:29577:9064 2:N:0:GAACCTAG+TCCGCATA
CCATTCTGTTAGACCTTACCGCACAACCTGTCTGGAAACACTGCGCTATCAGTAGGCATATTAAGTTT
ACTTTCTCCAATCTCCCATTGATATAACCCCACTCATTAATAGCTCTGTGGCTTACTAGAGCCG
+
FFF,F:FFFFFFFFFFFFFFFFFFFFFFFFFFFFFFFFFFFFFFFFFFFFFFFFFFFFFFFFFFFFFF
FFFFFFFFFFFFFFF:FFFFFFFFFFFFFFFFFFFFFFFFFFFFFFFFFFFFFFFFFFFFFFFF
@A00155:342:HHGFNDSXY:1:2103:12943:18145 1:N:0:GAACCTAG+TCCGCATA
CATTCTGTTAGACCTTACCGCACAACCTGTCTGGAAACACTGCGCTATCAGTAGGCATATTAAGTTTA
CTTTCTCCAATCTCCCATTGATATAACCCCACTCATTAATAGCTCTGTGGCTTACTAGAGCCG
+
FFFFFFFFFFFFFFFFFFFFFFFFFFFFFFFFFFFFFFFFFFFFFFFFFFFFFFFFFF:FFFFFFFFF
FFFFFFFFFFFFFFFFFFFFFFFFFFFFFFFF:FFFFFFFFFFFFFFFFFFFFFFFFFFFFFF
@A00155:342:HHGFNDSXY:1:2571:12201:18239 1:N:0:GAACCTAG+TCCGCATA
CATTCTGTTAGACCTTACCGCACAACCTGTCTGGAAACACTGCGCTATCAGTAGGCATATTAAGTTTA
CTTTCTCCAATCTCCCATTGATATAACCCCACTCATTAATAGCTCTGTGGCTTACTAGAGCCGCTTCA
+
FFFFFFF:FFFFFFFFFFFFFFFFFFFFF,FFFFFFFFFFFFFFFFFFFFFFFFFFFFF:FFF:FFFF
FFFFFFFFFFFFFFFFF,F,FFFFFFFFF:FFFFFFFF:FFFFFFFFFF,F:FFFFFFFFFFFFFFFF

```
@A00155:342:HHGFNDSXY:1:2348:28275:18677 1:N:0:GAACCTAG+TCCGCATA
ATTCTGTTAGACCTTACCGCACAACCTGTCTGGAAACACTGCGCTATCAGTAGGCATATTAAGTTTAC
TTTCTCCAATCTCCCATTGATATAACCCCACTCATTAATAGCTCTGTGGCTTACTAGAGCCGCTTCAC
+
FFFFFFFFFFFFFFFFFFFFFFFFFFFFFFFFFFFFFFFFFFFFFFFFFFFFFFFFFFFFFFFFFFFF
FFFFFFFF:FFFFFFFFFFFFFFFFFFFFFFFFFFFFFFFFFFFFFFFFFFFFFFFFFFFFFFFFFFF
@A00155:342:HHGFNDSXY:1:2537:30996:4789 1:N:0:GAACCTAG+TCCGCATA
TTCTGTTAGACCTTACCGCACAACCTGTCTGGAAACACTGCGCTATCAGTAGGCATATTAAGTTTACT
TTCTCCAATCTCCCATTGATATAACCCCACTCATTAATAGCTCTGTGGCTTACTAGAGCCGCTTCACC
+
FFFFFFFFFFFFFFFFFFFFFFFFFFFFF:FFFFFFFFFFFFFFFFFFFFFFFFFF:FFFFFFFFFFF
FFFFFFFFFFFFFFFFFFFFFFFFFFFFFFFFFFFF:FFFFFFFFFFFFFF:FFFFFFFFFFFFFFFFF
@A00155:342:HHGFNDSXY:1:2151:20799:25895 2:N:0:GAACCTAG+TCCGCATA
TCTGTTAGACCTTACCGCACAACCTGTCTGGAAACACTGCGCTATCAGTAGGCATATTAAGTTTACTT
TCTCCAATCTCCCATTGATATAACCCCACTCATTAATAGCTCTGTGGCTTACTAGAGCCGCTTCACCA
+
FFFFFFFFFFFFFFFFFFFF:FFFFFFFFFFFFFFFFFFFFFFFFFFFFFFFFFFFFFFFFFFFFFFF
FFFFFFFFFFFFFFFFFFFFFFFFFFFFFFFFFFFFFFFFFFFF:FFF:FFFFFFFFFFFFFFFFFFF
@A00155:342:HHGFNDSXY:1:1178:4978:13964 2:N:0:GAACCTAG+TCCGCATA
CTGTTAGACCTTACCGCACAACCTGTCTGGAAACACTGCGCTATCAGTAGGCATATTAAGTTTACTTT
CTCCAATCTCCCATTGATATAACCCCACTCATTAATAGCTCTGTGGCTTACTAGAGCCGCTTCACCAT
+
FFFFFFFFFF:FFFFFFFFF:FFFFF:FFF:FFFFFFFFFF:FFFFFFFFFFFFFFFFFFFFFFFFFF
FFFFFFFFFFFFFFFFFFFFFFFFFF,FF,FFFFFFFFF:FFFFFFFFFFFFFFFFFF:FFFFFFFFFF
@A00155:342:HHGFNDSXY:1:1464:30987:30389 2:N:0:GAACCTAG+TCCGCATA
GTTAGACCTTACCGCACAACCTGTCTGGAAACACTGCGCTATCAGTAGGCATATTAAGTTTACTTTCT
CCAATCTCCCATTGATATAACCCCACTCATTAATAGCTCTGTGGCTTACTAGAGCCGCTTCACCATCA
+
FFFFFF,FFFFFFFFFFFFF:FFFFFFF:FF:FFFFF:FFF::FFFFF:FFFFFFFFFFFFFFFFFFF
FFFFF:FFF:FFFF:FFFFFF,FFFFFFFFFFFFFF:FFFFFFFFFFFFFFFFFFFFFFFFFFFFFFF
@A00155:342:HHGFNDSXY:1:2317:7572:3302 2:N:0:GAACCTAG+TCCGCATA
TTAGACCTTACCGCACAACCTGTCTGGAAACACTGCGCTATCAGTAGGCATATTAAGTTTACTTTCTC
CAATCTCCCATTGATATAACCCCACTCATTAATAGCTCTGTGGCTTACTAGAGCCGCTTCACCATCAT
+
FFFFFF:FFFFFFFFFFFFF:FFFFFFFFFFFFFFFF:::FFFFFFFFFFFFFFFFF:FFFFFF:FFF
FFFFFFFFF,FFFFFFFFFFFFFFFFFF:FFFFFFF:FFFFFFFFFFFFFFFFFFFFFFFFFFF,FFFF
@A00155:342:HHGFNDSXY:1:1323:11514:29324 2:N:0:GAACCTAG+TCCGCATA
TAGACCTTACCGCACAACCTGTCTGGAAACACTGCGCTATCAGTAGGCATATTAAGTTTACTTTCTCC
AATCTCCCATTGATATAACCCCACTCATTAATAGCTCTGTGGCTTACTAGAGCCGCTTCACCATCATT
+
FFFFFF:FF:FFFFFFFFFFFFFFFFFFFFFFFFFF:FFFFFFFFFFFFFFFFFFFFFFFFFFFFFFF
FFFFFFFF::FFFFFFFFFFFFFFF:FFFFFFFFFFFFFFFFFFFFFFFFFFFFFFFFFFFFFFFFF:F
@A00155:342:HHGFNDSXY:1:1323:11153:28009 2:N:0:GAACCTAG+TCCGCATA
TAGACCTTACCGCACAACCTGTCTGGAAACACTGCGCTATCAGTAGGCATATTAAGTTTACTTTCTCC
AATCTCCCATTGATATAACCCCACTCATTAATAGCTCTGTGGCTTACTAGAGCCGCTTCACCATCATT
+
FFFFFFFFFF:FFFFFFFFFFFFFFFFFFFFFFFFFFFFFFFFFFFFFFFFFFFFFFFFFFF:FFFFF
F,FFFFFFFFFFFFFFFFFFFFFFF:FFFFFFFFFFFFFFFFFFFFFFFFFFFFFFFFF:FFFFFFFF:FF
@A00155:342:HHGFNDSXY:1:1365:19479:13463 2:N:0:GAACCTAG+TCCGCATA
GACCTTACCGCACAACCTGTCTGGAAACACTGCGCTATCAGTAGGCATATTAAGTTTACTTTCTCCAA
TCTCCCATTGATATAACCCCACTCATTAATAGCTCTGTGGCTTACTAGAGCCGCTTCACCATCATTTA
+
FFFFFF:FFFFFFFFFFFFFFFFFFFFFFFFFFFFFFFFFFFFFFFFFFFFFFFFFFFFFFFFFFFFF
FFFFFFFFFFFFFFF:FFFF:FFFFFFFFFFFFFFFFFFFFFFFFFFFFFFFFFFFFFFFFFFFFFFF
```

```
@A00155:342:HHGFNDSXY:1:2604:6994:9032 1:N:0:GAACCTAG+TCCGCATA
ACCTTACCGCACAACCTGTCTGGAAACACTGCGCTATCAGTAGGCATATTAAGTTTACTTTCTCCAAT
CTCCCATTGATATAACCCCACTCATTAATAGCTCTGTGGCTTACTAGAGCCGCTTCACC
+
FFFFFFFFFF:FFFFFFFFFFFFFFFFFFFFFFFFFFFFFFFFFFFFFFFFFFFFFFFFFFFFFF
FFFFFFFFFFFFFFFF:FFFFFFFFFFFFFFFFFFFFFFFFFFFFFFFFFFFFFFF,FF
@A00155:342:HHGFNDSXY:1:2607:25156:29559 1:N:0:GAACCTAG+TCCGCATA
ACCTTACCGCACAACCTGTCTGGAAACACTGCGCTATCAGTAGGCATATTAAGTTTACTTTCTCCAAT
CTCCCATTGATATAACCCCACTCATTAATAGCTCTGTGGCTTACTAGAGCCGCTTCACCATCATTTAA
+
FFFFFFFFFFFFFFFFFFFFFFFFFFFFFFFFFFFFFFFFFFFFFFFFFFFFFFFFFFFFFFFFFFFF
FFFFFFFFFFFFFFFFFFFFFFFFFFFFFFFFFFFFFF:FFFFFFFFFFFFFFFFFFFFFFFFFFFFF
@A00155:342:HHGFNDSXY:1:1172:11388:3646 1:N:0:GAACCTAG+TCCGCATA
ACCTTACCGCACAACCTGTCTGGAAACACTGCGCTATCAGTAGGCATATTAAGTTTACTTTCTCCAAT
CTCCCATTGATATAACCCCACTCATTAATAGCTCTGTGGCTTACTAGAGCCGCTTCACCATCATTTAA
+
FFFFFFFFFFFFFFFFFFFFFFFFFFFFFFFFFFFFFFFFFFFFF:FFFFFFFFFFFFFFFFFFFFFF
FFFFFFFFFFFFFFFFFFFFFFFFFFFFFFFFFFFFFFFFFFFFFFFFF:FFFFFFFFFFFF:FF:FF
@A00155:342:HHGFNDSXY:1:2348:28275:18677 2:N:0:GAACCTAG+TCCGCATA
ACCTTACCGCACAACCTGTCTGGAAACACTGCGCTATCATTAGGCATATTAAGTTTACTTTCTCCAAT
CTCCCATTGATATAACCCCACTCATTAATAGCTCTGTGGCTTACTAGAGCCGCTTCACCATCATTTAA
+
:FFFFFFFFFFFFFFFFFFFFFFFFFFFFFFF:FFFFFF,FFFFFFFFFF,FFFFFFFFFFFFFFFFF
FFFFFFFFFFFFFFFFFFFFFFF:FFFFFFFFFFFFFFFFFFFFFFFFFFFFFFFFFFFFFFFFFFFF
@A00155:342:HHGFNDSXY:1:2378:30978:22075 1:N:0:GAACCTAG+TCCGCATA
ACCTTACCGCACAACCTGTCTGGAAACACTGCGCTATCAGTAGGCATATTAAGTTTACTTTCTCCAAT
CTCCCATTGATATAACCCCACTCATTAATAGCTCTGTGGCTTACTAGAGCCGCTTCACCATCATTTAA
+
FFFFFFFFFFFFF:FFFFFFFFFFFFFFFFFFFFFFFFFFFFFFFFFFFFFFFFFFFFFFFFFFFFFF
FFFFFFFFFFFFFFFFFFFFFFFFFFFFFFFFFFFFFFFFFFFFFF,FFFFFFFFFFFFFFFFFFFFF
@A00155:342:HHGFNDSXY:1:1422:14841:10974 1:N:0:GAACCTAG+TCCGCATA
ACCTTACCGCACAACCTGTCTGGAAACACTGCGCTATCAGTAGGCATATTAAGTTTACTTTCTCCAAT
CTCCCATTGATATAACCCCACTCATTAATAGCTCTGTGGCTTACTAGAGCCGCTTCACCATCATTTAA
+
FFFFFFFFFFFFFFFFFFFFFFFFFFFFFFFFFFFFFFF:FFFFFFFFFFFFFFFFFFFFFFFFF:FF
FFFFFFFFFFFFFFFFFFFFFFFFFFFFFFF:FFFFFFFFFFFFFFFFFFFFFFFFFFFFFFF:FFFF
@A00155:342:HHGFNDSXY:1:1328:4300:29543 2:N:0:GAACCTAG+TCCGCATA
ACCTTACCGCACAACCTGTCTGGAAACACTGCGCTATCAGTAGGCATATTAAGTTTACTTTCTCCAAT
CTCCCATTGATATAACCCCACTCATTAATAGCTCTGTGGCTTACTAGAGCCGCTTCACCATCATTTAA
+
FFFFFFFFFFFFFFFFFFFFFFFFFFFFFFFFFFFFFFFFFFFFFFFFFFFFFFFFFFFFFFFFFFFF
FFFFFFFFFFFFFFFFFFFFFFFFFFFFFFFFFFFFFFFFFFFFFFFFFFFFFFFFFFF:FFFFFFFF
@A00155:342:HHGFNDSXY:1:1511:16947:13683 1:N:0:GAACCTAG+TCCGCATA
CCTTACCGCACAACCTGTCTGGAAACACTGCGCTATCAGTAGGCATATTAAGTTTACTTTCTCCAATC
TCCCATTGATATAACCCCACTCATTAATAGCTCTGTGGCTTACTAGAGCCGCTTCACCATCATTTAAC
+
FFFFFFFFFFFFFFFFFFFFFFFFFFFFFFFFFFFFFFFFFFFFFFFFFFFFFFFFFFFFFFFFFFFF
FFFFFFFFFFFFFFFFFFFFFFFFFFFFFFFFFFFFFFFFFFFFFFFFFFFFFFFFFFFFFFFFFFFF
@A00155:342:HHGFNDSXY:1:2108:16938:21371 1:N:0:GAACCTAG+TCCGCATA
CCTTACCGCACAACCTGTCTGGAAACACTGCGCTATCAGTAGGCATATTAAGTTTACTTTCTCCAATC
TCCCATTGATATAACCCCACTCATTAATAGCTCTGTGGCTTACTAGAGCCGCTTCACCATCATTTAAC
+
FF,FFFFFFFFFFFFFFFFFF:F:FFFFFFFFFFFFFF,,FFFFFFFFFFFFF:FFFFFFFFFFFFFF
FF,FF:,FFFF:FF:FF,FFFFF::FFFF::FFFFFFFFFFFFFFFFFFF:FFFFFFFF:FFF,FFFF
```

@A00155:342:HHGFNDSXY:1:2231:12156:26897 1:N:0:GAACCTAG+TCCGCATA
CCTTACCGCACAACCTGTCTGGAAACACTGCGCTATCAGTAGGCATATTAAGTTTACTTTCTCCAATC
TCCCATTGATATAACCCCACTCATTAATAGCTCTGTGGCTTACTAGAGCCGCTTCACCATCATTTAAC
+
FFFFFFFFFFFFFFFFFFFFFFFFFFFFFFFFFFFFFFFFFFFF:FF:FFFFFFFFFFFFFFFFFFFF
FFFFFFFFFFFFFFFFFFFFFFFFFFFFFFFFFFFFFFFFFFFFFFFFFFFFFFFFFFFFFFFFFFFF
@A00155:342:HHGFNDSXY:1:2231:14091:23296 1:N:0:GAACCTAG+TCCGCATA
CCTTACCGCACAACCTGTCTGGAAACACTGCGCTATCAGTAGGCATATTAAGTTTACTTTCTCCAATC
TCCCATTGATATAACCCCACTCATTAATAGCTCTGTGGCTTACTAGAGCCGCTTCACCATCATTTAAC
+
FFFFFFFFFFFFFFFFFFFFFFFFFFFFFFFFFFFFFFFFFFFFFFFFFFFFFFFFFFFFFFFFFFFF
FFFFFFFFFFFFFFFFFFFFFFFFFFFFFFFFFFFFFFFFFFFFFFFFFFFFFFFFFFFFFFF:FFFF
@A00155:342:HHGFNDSXY:1:2571:25156:22545 1:N:0:GAACCTAG+TCCGCATA
CCTTACCGCACAACCTGTCTGGAAACACTGCGCTATCAGTAGGCATATTAAGTTTACTTTCTCCAATC
TCCCATTGATATAACCCCACTCATTAATAGCTCTGTGGCTTACTAGAGCCGCTTCACCATCATTTAAC
+
FFFFFFFFFFFFFFFFFFFFFFFFFFFFFFFFFFFFFFFFFFFFFFF,FFFFFFFFFFFFFFFFFFFF
FFFFFFFFFFFFFFFFFFFFFFFFFFFFFFFFFFFFFFFFFFFFFFFFFFFFFFFFFFFFFFFFFFFF
@A00155:342:HHGFNDSXY:1:2537:18096:7780 1:N:0:GAACCTAG+TCCGCATA
CCTTACCGCACAACCTGTCTGGAAACACTGCGCTATCAGTAGGCATATTAAGTTTACTTTCTCCAATC
TCCCATTGATATAACCCCACTCATTAATAGCTCTGTGGCTTACTAGAGCCGCTTCACCATCATTTAAC
+
FFFFFFFFFFFFFFFFFFFFF:FFFFFFFFFFFFFFFFFFFFFFFFFFFFFFFFFFFFFFFFFFFFFF
FF:FFFFFFFFFFFFFFFFFFFFFFFFFFFFFFFFFFFFFFFFFFFFF:FFFFFFFFFFFFFFFFFF:
@A00155:342:HHGFNDSXY:1:2140:1542:8077 1:N:0:GAACCTAG+TCCGCATA
CCTTACCGCACAACCTGTCTGGAAACACTGCGCTATCAGTAGGCATATTAAGTTTACTTTCTCCAATC
TCCCATTGATATAACCCCACTCATTAATAGCTCTGTGGCTTACTAGAGCCGCTTCACCATCATTTAAC
+
FFFFFFFFFFFFFFFFFFFFFFFFFFFFFFFFFFFFFFFFFFFFFFFFFFFFFFFFFFFFFFFFF:FF
FFFFFFFFFFFFFFFFFFFFFFFFFF:FFFFFFFFFFFFFFF:F:FFFF:FFFFF,FFFFFFFFFF:F
@A00155:342:HHGFNDSXY:1:1155:29071:31485 1:N:0:GAACCTAG+TCCGCATA
TACCGCACAACCTGTCTGGAAACACTGCGCTATCAGTAGGCATATTAAGTTTACTTTCTCCAATCTCC
CATTGATATAACCCCACTCATTAATAGCTCTGTGGCTTACTAGAGCCGCTTCACCATCATTTAACACA
+
FFFFFFFFFFFFFFFFFFFFFFFFFFFFFFFFFFFFFFFFFFFFFFFFFFFFFFFFFFFFFFFFFFFF
FF,FFFFFFFFFFFFFFFFFFFFFFFFFFFFFFFFFFFFFFFFFFFFFFFFFFFFF:FFFFFFFFFF
@A00155:342:HHGFNDSXY:1:2205:17499:33708 2:N:0:GAACCTAG+TCCGCATA
CGCACAACCTGTCTGGAAACACTGCGCTATCAGTAGGCATATTAAGTTTACTTTCTCCAATCTCCCAT
TGATATAACCCCACTCATTAATAGCTCTGTGGCTTACTAGAGCCGCTTCACCATCATTTAA
+
FFFFFFFFFFFFFFFFFFFFFFFFFFFFFFFFFFFFFFFFFFFFFFFFFFFFFFFFFFFFFFFFFFFF
FFFFFFFFFFFFFFFFFFFFFFFFFFFFFFFFFFFFFFFFFFFFFFFFFFFFFFFFFFFFF
@A00155:342:HHGFNDSXY:1:2267:16432:3583 2:N:0:GAACCTAG+TCCGCATA
GCACAACCTGTCTGGAAACACTGCGCTATCAGTAGGCATATTAAGTTTACTTTCTCCAATCTCCCATT
GATATAACCCCACTCATTAATAGCTCTGTGGCTTACTAGAGCCGCTTCACCATCATTTAA
+
,FFFFFFFFFFFFFFFFFFFFFFFFFFFFFFFFFFF:FFFFFFFFFFFFFFFFFFFFFFFFF:FF:FF
FFFFFFFFFFFFFFFFFFFFFFFFFFFF:FFFFFFFFFFFFFFFFFFFFFFFFFFFFFFF
@A00155:342:HHGFNDSXY:1:1242:20645:12633 2:N:0:GAACCTAG+TCCGCATA
GCACAACCTGTCTGGAAACACTGCGCTATCAGTAGGCATATTAAGTTTACTTTCTCCAATCTCCCATT
GATATAACCCCACTCATTAATAGCTCTGTGGCTTACTAGAGCCGCTTCACCATCATTTAA
+
FFFFFFFFFFFFFFFFFFFFFFFFFFFFFFFFFFFFFFFFFF:FFFFFFFFFFFFFFFFFFFFFFFFF
FFFFFFFFFFFFFFF:FFFF:FFFFFFFFFFFFFFFFFFFFFFF:FFFFFFFFFFFFFFF

```
@A00155:342:HHGFNDSXY:1:1613:22110:18490 2:N:0:GAACCTAG+TCCGCATA
ACAACCTGTCTGGAAACACTGCGCTATCAGTAGGCATATTAAGTTTACTTTCTCCAATCTCCCATTGA
TATAACCCCACTCATTAATAGCTCTGTGGCTTACTAGAGCCGCTTCACCATCATTTAACACATCGTAT
+
:FFFFFFFFFFFFFFFFFFFFFFFFFFFFF:FFFFFFFFFFFFFFFFFFFFFFFFF,:FFFFFFFFFF
FFFF:FFFFFFFFFFF:FFFFFFFFFFFFFFFFFFFFFFFFFFFFF:FFFFFFFFFFFFFFF:FFFFFF
@A00155:342:HHGFNDSXY:1:2469:28637:28729 1:N:0:GAACCTAG+TCCGCATA
TGTCTGGAAACACTGCGCTATCAGTAGGCATATTAAGTTTACTTTCTCCAATCTCCCATTGATATAAC
CCCACTCATTAATAGCTCTGTGGCTTACTAGAGCCGCTTCACCATCATTTAACACATCGTATCTTCCT
+
FFFFFFFFFFFFFFFFFFFFFFFFFFFFFFFFFFFFFFFFFFFFFFFFFFFFFFFFFFFFFFFFFFFF
FFFFFFFFFFFFFFFF:FFFFFFFFFFFFF:FFFFFFFFFFFFFFFFFFFFFFFFFFFFFF:FFF:FFF
@A00155:342:HHGFNDSXY:1:1177:18611:23578 1:N:0:GAACCTAG+TCCGCATA
TGTCTGGAAACACTGCGCTATCAGTAGGCATATTAAGTTTACTTTCTCCAATCTCCCATTGATATAAC
CCCACTCATTAATAGCTCTGTGGCTTACTAGAGTCGCTTCACCATCATTTAACACATCGTATCTTCCT
+
FFFFFFFFFFFFFFFFFFFFFFFFFFFFFFFFFFFFFFFFFFFFFFFFFFFFFFFFFFFFFFFFFFFF
FFFFFFFFFFFFFFFFFFFFFFFFFFFFFFFFFFFFF,FFFFFF:FFFFFFF:FFFFFFFF:FFFFFF
@A00155:342:HHGFNDSXY:1:1444:18114:24565 1:N:0:GAACCTAG+TCCGCATA
TCTGGAAACACTGCGCTATCAGTAGGCATATTAAGTTTACTTTCTCCAATCTCCCATTGATATAACCC
CACTCATTAATAGCTCTGTGGCTTACTAGAGCCGCTTCACCATCATTTAACACATCGTATCTTCCTCT
+
FFFFFFFFFFFFFFFFFFFFFFFFFFFFFFFFFFFFFFFFFFFFFFFFFFFFFFFFFFFFFFFFFFFF
FFFFFFFFFFFFFFFFFFFFFFFFFFFFF:F:FFFFFFFFFFFFFFFFFFFFFFFFFFFFFFFFFFFFF
@A00155:342:HHGFNDSXY:1:1410:31819:28385 1:N:0:GAACCTAG+TCCGCATA
CTGGAAACACTGCGCTATCAGTAGGCATATTAAGTTTACTTTCTCCAATCT
+
FFFFFFFFFFFFFFFFFFFFFFFFFFFFFFFFFFFFFFFFFFFFFFFFFFF
@A00155:342:HHGFNDSXY:1:1528:10565:15436 1:N:0:GAACCTAG+TCCGCATA
CTGGAAACACTGCGCTATCAGTAGGCATATTAAGTTTACTTTCTCCAATCTCCCATTGATATAACCCC
ACTCATTAATAGCTCTGTGGCTTACTAGAGCCGCTTCACCATCATTTAACACATCGTATCTTCCTCTT
+
FFFFFFFFFFFFFFFFFFFFFFFFFFFFFFFFFFFFFFFFFFFFFFFFFFFFFFFFFFFFFFFFFFFF
,FFFFFFFFFFFFFFFFFFFFFFFFF:FFFFFFFFFFFFFFFFF:FFFFFFFFFFFFFF:FFFFFFFF
@A00155:342:HHGFNDSXY:1:1641:11035:26741 2:N:0:GAACCTAG+TCCGCATA
TGGAAACACTGCGCTATCAGTAGGCATATTAAGTTTACTTTCTCCAATCTCCCATTGATATAACCCCA
CTCATTAATAGCTCTGTGGCTTACTAGAGCCGCTTCACCATCATTTAACACATCGTATCTTCCTCTTA
+
FFFFFFFFFFF:FFFFFFFFF:FFFFFFFFFFFFFFFFFFFFFFFFFFFFFFFFFFFFFFFFFFFFFF
FFFFFFFF:FFFF:FFF:FFFFFFFFFFFFFFFFFFFFFFFFFFFFFFFFFFFFFFFFFFFFFFFFFF:
@A00155:342:HHGFNDSXY:1:2554:7591:27352 2:N:0:GAACCTAG+TCCGCATA
TGGAAACACTGCGCTATCAGTAGGCATATTAAGTTTACTTTCTCCAATCTCCCATTGATATAACCCCA
CTCATTAATAGCTCTGAGGCTTACTAGAGCCGCTTCACCATCATTTAACACATCGTATCTTCCTCTTA
+
FFFFFFFFFFFFF:FF:FFFF:FFFFFFFFFF,FF:FFFFFFFFFFFFFFFFFFFFFFFFFFFFFFFF
FFFFFFFFFFFFFFFF,FFFFFFFFFFFFFFFFFFFFFFFFFFFFFFFFFFFFFFFFFFFFFFFFFFF
@A00155:342:HHGFNDSXY:1:1155:29071:31485 2:N:0:GAACCTAG+TCCGCATA
TGGAAACACTGCGCTATCAGTAGGCATATTAAGTTTACTTTCTCCAATCTCCCATTGATATAACCCCA
CTCATTAATAGCTCTGTGGCTTACTAGAGCCGCTTCACCATCATTTAACACATCGTATCTTCCTCTTA
+
FFFFFFFFFFF,FFFFFFFFFFFFFFFFFFFFFFFFFFFFFFFFFFFFFFFFFFFFFFFFFFFFFFF:
FFFFFFFFFFFFFFFFFFFFFFFFFFFFFFFFFFFFFFFFFFF:FFFFFFFF:FFFFFFFFFFFFFFF
@A00155:342:HHGFNDSXY:1:2150:13548:32565 2:N:0:GAACCTAG+TCCGCATA
TGGAAACACTGCGCTATCAGTAGGCATATTAAGTTTACTTTCTCCAATCTCCCATTGATATAACCCCA
```

```
CTCATTAATAGCTCTGTGGCTTACTAGAGCCGCTTCACCATCATTTAACACATCGTATCTTCCTCTTA
+
FFFF:FFFFFFF:FFFFFFFFFFFFFFFFFFFFFFFFFFFFFFFFFFFFFFFFFFFFFFFFFFFFFF:
FFF:FFFFFFFFFF:FFFFFFFFFFFFFFFFFFFFFFFFF:FFFFFFFFFFFFFFFFFFFFFFFF:FF
@A00155:342:HHGFNDSXY:1:1249:29261:31344 2:N:0:GAACCTAG+TCCGCATA
TGGAAACACTGCGCTATCAGTAGGCATATTAAGTTTACTTTCTCCAATCTCCCATTGATATAACCCCA
CTCATTAATAGCTCTGTGGCTTACTAGAGCCGCTTCACCATCATTTAACACATCGTATCTTCCTCTTA
+
F:FFFF:FFFFFFFFFFFFFFF:FFFFFFFFFFFFFFFFFFFFFFFFF,FFFFFFFFFFF,FFFFF::
FFFFFFFFFFFFFFFFFF:FFFF:FFFFFFFFFFFFFFFFFFFF::FFFFFF:FFFFFFFFFFF:FFF
@A00155:342:HHGFNDSXY:1:1210:27868:33379 2:N:0:GAACCTAG+TCCGCATA
GGAAACACTGCGCTATCAGTAGGCATATTAAGTTTACTTTCTCCAATCTCCCATTGATATAACCCCAC
TCATTAATAGCTCTGTGGCTTACTAGAGCCGCTTCACCATCATTTAACACATCGTATCTTCCTCTTAT
+
FFFFFFFFFFFFFFFFFFFFFFFFFFFFFFFFFFFFFFFFFFFFFFFFFFFFFF:FFFFFF:FFFFF:
FFFFFFFFFFFFFFFFFFFFFFFFFFFFFFFFFFFFFFFFFFFFFFFFFFFFFFFFFFFFFFFFFFFF
@A00155:342:HHGFNDSXY:1:1507:26259:28432 2:N:0:GAACCTAG+TCCGCATA
GGAAACACTGCGCTATCAGTAGGCATATTAAGTTTACTTTCTCCAATCTCCCATTGATATAACCCCAC
TCATTAATAGCTCTGTGGCTTACTAGAGCCGCTTCACCATCATTTAACACATCGTATCTTCCTCTTAT
+
FFFFFFFFFFFFFFFFFFFFFFFFFFFFFFFFFF:FFFFFFFFFF:FFFFFFFFFFF:FFFFFFFFFF
FFFF:FFFFFFFFFFFFFFFFFFFFFFFFFFF:FFFFFFFFFFFFFFFFFFFFFFFFFFFFFFFFFFF
@A00155:342:HHGFNDSXY:1:2442:19994:12759 2:N:0:GAACCTAG+TCCGCATA
GGAAACACTGCGCTATCAGTAGGCATATTAAGTTTACTTTCTCCAATCTCCCATTGATATAACCCCAC
TCATTAATAGCTCTGTGGCTTACTAGAGCCGCTTCACCATCATTTAACACATCGTATCTTCCTCTTAT
+
FFFFFFFFF:FFFFFFFFFFFFFFFFFF,FFFFFFFFFFFFFFFFFFFFFFFFFFFFFFFF:FFFF,F
FFFFFFFFFFFFFFFFFFFFFFFFFFFFFFFFFFFFFFFFFFFFFFFFFFFFFFFFFFFFFFFFFFFF
@A00155:342:HHGFNDSXY:1:1210:26341:31234 2:N:0:GAACCTAG+TCCGCATA
GGAAACACTGCGCTATCAGTAGGCATATTAAGTTTACTTTCTCCAATCTCCCATTGATATAACCCCAC
TCATTAATAGCTCTGTGGCTTACTAGAGCCGCTTCACCATCATTTAACACATCGTATCTTCCTCTTAT
+
FFFFFFFFFF:FFFF:F:FFFFFFFFFFFFFFF:FFFF,FFFFFF::FFFFFFF:FFFFF::FFFFFF
FFFFFFF:FFFFFFF:FFFFFFFFFFFFFFFFFFFFFFFFFFF:FFFFFFFFFFFFFF,FFFFF,FFF
@A00155:342:HHGFNDSXY:1:1351:29206:20823 2:N:0:GAACCTAG+TCCGCATA
GGAAACACTGCGCTATCAGTAGGCATATTAAGTTTACTTTCTCCAATCTCCCATTGATATAACCCCAC
TCATTAATAGCTCTGTGGCTTACTAGAGCCGCTTCACCATCATTTAACACATCGTATCTTCCTCTTAT
+
FFFFFFFFFFFFFFFFFFFFFFFFFFFFFFFFFFFFFFFFFFFFFFFFFFFFFFFFFFFFFFFFFFFF
FFFFFFFFFFFFFFFFFFFFFFFFFFFFFFFFFFFFFFFFFFFFFFFFFFFFFFFFFFFFFFFFFFFF
@A00155:342:HHGFNDSXY:1:2205:17499:33708 1:N:0:GAACCTAG+TCCGCATA
GAAACACTGCGCTATCAGTAGGCATATTAAGTTTACTTTCTCCAATCTCCCATTGATATAACCCCACT
CATTAATAGCTCTGTGGCTTACTAGAGCCGCTTCACCATCATTTAACACATCGTATCTTCC
+
FFFFFFFFFFFFFFFFFFFFFFFFFFFFFFFFFFFFFFFFFFFFFFFFFFFF:FFFFFFFFFFFFFFF
FFFFFFFFFFFFFFFFFFFFFFFF,FFFFFFFFFFFFFFFFFF:FFF:FFFFFFFFFFFFF
@A00155:342:HHGFNDSXY:1:1351:28058:22310 2:N:0:GAACCTAG+TCCGCATA
GAAACACTGCGCTATCAGTAGGCATATTAAGTTTACTTTCTCCAATCTCCCATTGATATAACCCCACT
CATTAATAGCTCTGTGGCTTACTAGAGCCGCTTCACCATCATTTAACACATCGTATCTTCCTCTTAT
+
FFFFFFFFFFFFFFFFFFFFFFFFFFFFFFFFFFFFFFFFFFFFFFFFFFFFFFFFFFFFFFFFFFFF
FFFFFFFFFFFFFFFFFFFFFFFFFFFFFFFFFFFFFFFFFFFFFFFFFFFFFFFFFFFFFFFFFFF
@A00155:342:HHGFNDSXY:1:1574:1642:26913 1:N:0:GAACCTAG+TCCGCATA
GAAACACTGCGCTATCAGTAGGCATATTAAGTTTACTTTCTCCAATCTCCCATTGATATAACCCCACT
```

```
CATTAATAGCTCTGTGGCTTACTAGAGCCGCTTCACCATCATTTAACACATCGTATCTTCCTCTTATC
+
FFFFFFFFFFFFFFFFFFFFFFFFFFFFFFFFFFFFFFFFFFFFFFFFFFFFFFFFFFFFFFFFFFFF
FFFFF:FFFFFFFFFFFFFFFFFFF:FFFFFFFFFFFFFFFFFFFFFFFFFFFFFFFFFFFFFFFFFF
@A00155:342:HHGFNDSXY:1:1242:20645:12633 1:N:0:GAACCTAG+TCCGCATA
AAACACTGCGCTATCAGTAGGCATATTAAGTTTACTTTCTCCAATCTCCCATTGATATAACCCCACTC
ATTAATAGCTCTGTTGCTTACTAGAGCCGCTTCACCATCATTTAACACATCGTATCTTCC
+
FFF:FFFFF:FFF,:FFFFFFFF:FFFFF:FFFFF:FF:F,FFFFF::FFF:FFF,F,FF,FFFF:,F
F,FFF,F:F:,:,F,FFFFFF,F:F,FF,F,F,FFF:FFF:FFFF:F,F,:,FF,,:FFF
@A00155:342:HHGFNDSXY:1:2267:16432:3583 1:N:0:GAACCTAG+TCCGCATA
AAACACTGCGCTATCAGTAGGCATATTAAGTTTACTTTCTCCAATCTCCCATTGATATAACCCCACTC
ATTAATAGCTCTGTGGCTTACTAGAGCCGCTTCACCATCATTTAACACATCGTATCTTAC
+
FFF,FF:FFFFFFF:F,FF:FFFFFFFFF:FFFF:FFFFFFFFFF:FFFFFF,:FFF:FFFFFFFF:F
FFFFF:F:FFF:FF,FF:FFF:F,FFFFFFFF:F:FFF,FFFFFF,F:F::FFF,,,F,F
@A00155:342:HHGFNDSXY:1:1405:31295:21308 2:N:0:GAACCTAG+TCCGCATA
AAACACTGCGCTATCAGTAGGCATATTAAGTTTACTTTCTCCAATCTCCCATTGATATAACCCCACTC
ATTAATAGCTCTGTGGCTTACTAGAGCCGCTTCACCATCATTTAACACATCGTATCTTCCTCTTATCG
+
FFFFFFFFFFFFFFFFFFFFFFFFFFFFFFFFFFFFFFFFFF:FFFFFFFFFFFFFFFFFFFFFFFFF
FFFFFFFFFFFFFFFFFFFFFFFFFFFFFFFFFFFFFFF:FFFFFFFFFFFFFFFFFFFFFFFFFFFF
@A00155:342:HHGFNDSXY:1:2550:4788:18928 2:N:0:GAACCTAG+TCCGCATA
CACTGCGCTATCAGTAGGCATATTAAGTTTACTTTCTCCAATCTCCCATTGATATAACCCCACTCATT
AATAGCTCTGTGGCTTACTAGAGCCGCTTCACCATCATTTAACACATCGTATCTTCCTCTTA
+
F:FFFFFF:FFFFFFFFFFF,FFF:FFFFFFF:FFFFFF:FFFFFFFF:FFFFF:FFFFFFFFFFFFF
FFFFFFFFFFFFFFFFFFFFFFFFFFFFFFFFFFFFFFFFFFFFFFFFF:FFFFFFFFFF,F
@A00155:342:HHGFNDSXY:1:2231:12156:26897 2:N:0:GAACCTAG+TCCGCATA
CACTGCGCTATCAGTAGGCATATTAAGTTTACTTTCTCCAATCTCCCATTGATATAACCCCACTCATT
AATAGCTCTGTGGCTTACTAGAGCCGCTTCACCATCATTTAACACATCGTATCTTCCTCTTATCGATG
+
FFF,F:FFFFFFFFFFFFFFFFFFFF:FF:FFFFFFFFFF:FF:FFFFFFFFFFFFFFFFFFFFFFFF
FFFFFFFFFFFFFFFFFFFFFFFFFFFFFFFFFF:FFFFFFFFFF:FFF,FFFFFFFFF,FF:FFFFF
@A00155:342:HHGFNDSXY:1:2231:14091:23296 2:N:0:GAACCTAG+TCCGCATA
CACTGCGCTATCAGTAGGCATATTAAGTTTACTTTCTCCAATCTCCCATTGATATAACCCCACTCATT
AATAGCTCTGTGGCTTACTAGAGCCGCTTCACCATCATTTAACACATCGTATCTTCCTCTTATCGATG
+
FFFFFFFFFFFFFFFFFFFFFFFFFFFFFFFFFFFFFFFFFFFFFFFFFFFFFFFFFFFFFFFFFFFF
FFFFFFFFFFFFFFFFFFFFFFFFFFFFFFFFFFFFFFFFFFFFFFFFFFFFFFFFFFFFFFFFFFFF
@A00155:342:HHGFNDSXY:1:2327:7048:18082 2:N:0:GAACCTAG+TCCGCATA
ACTGCGCTATCAGTAGGCATATTAAGTTTACTTTCTCCAATCTCCCATTGATATAACCCCACTCATTA
ATAGCTCTGTGGCTTACTAGAGCCGCTTCACCATCATTTAACACATCGTATCTTCCTCTTATCG
+
FFFFFFFFFFFFFFFFFFFFFFFFFF,:FFFFFFFFFFFFFFFFFFFFFFFF:FFFFFFFFFFFFFFF
F:FFFFFF:FFFFFFFFFFFFFFFFFFFFFFFF,FFFFFFFFFFFFFFF:F:FFFFFFFFFFFF
@A00155:342:HHGFNDSXY:1:2123:32723:28103 2:N:0:GAACCTAG+TCCGCATA
CTGCGCTATCAGTAGGCATATTAAGTTTACTTTCTCCAATCTCCCATTGATATAACCCCACTCATTAA
TAGCTGTGTGGCTTACTAGAGCCGCTTCACCATCATTTAACACATCGTATCTTCCTCTTATCGATGTG
+
:FFFFFFFFFFFFFFFFFFF:FF:F:FFFFFFFFFFF::FFFFFFFFFFFFFFFFFFFFFFFF:FFFF
FFFFFFFFFFFFFFFFFFFFFFFFFFFFFFFFFFFFFFFFFFFF:FFFFFF:FFFFFFFFFFFFFFFF
@A00155:342:HHGFNDSXY:1:2537:30996:4789 2:N:0:GAACCTAG+TCCGCATA
CTGCGCTATCAGTAGGCATATTAAGTTTACTTTCTCCAATCTCCCATTGATATAACCCCACTCATTAA
```

TAGCTCTGTGGCTTACTAGAGCCGCTTCACCATCATTTAACACATCGTATCTTCCTCTTATCGATGTG
+
FFFFFFFFFFFF,FFFFFFF:FFFFF,:FFFFFFFFFFFFFFFFFF:FFFFF,F,FFFF,FFFFFFFF
FFFFFFFFFFFFFFFFFFFFFFFFF:FFFFF:FFFFFFFFFFFFFFFFFFFFFFFFFFF,FFF:FFFF
@A00155:342:HHGFNDSXY:1:1574:1642:26913 2:N:0:GAACCTAG+TCCGCATA
GCGCTATCAGTAGGCATATTAAGTTTACTTTCTCCAATCTCCCATTGATATAACCCCACTCATTAATA
GCTCTGTGGCTTACTAGAGCCGCTTCACCATCATTTAACACATCGTATCTTCCTCTTATCGATGTGAA
+
FFFFFFFFFFFFFFFFFFFFFFFFFFFFF:FFFFFFFFFFFFFFFFFFFFFF:FFFFF,FF,FFFFF
FFFFFF:FFFFFFFFFFFFFFFFFFFFFFFFFFF:FFFFFFFF:FFFF:FF:FFFFFFFFFFFFF,FFF
@A00155:342:HHGFNDSXY:1:2571:25156:22545 2:N:0:GAACCTAG+TCCGCATA
GCGCTATCAGTAGGCATATTAAGTTTACTTTCTCCAATCTCCCATTGATATAACCCCACTCATTAATA
GCTCTGTGGCTTACTAGAGCCGCTTCACCATCATTTAACACATCGTATCTTCCTCTTATCGATGTGAA
+
FFFFFFFFFFFFFFFFFFFFFFF:FFFFFFFFFFFF:FFFFFFFFFFFFFFFFFFFFFFFFFFFFFFF
FFFFFFFFFFFFFFFFFFFFFFFFFFFFFFFFFFFFFFFFFFFFFFFFFFFFFFFFFFFFFFFFFFFF
@A00155:342:HHGFNDSXY:1:2554:7591:27352 1:N:0:GAACCTAG+TCCGCATA
GCGCTATCAGTAGGCATATTAAGTTTACTTTCTCCAATCTCCCATTGATATAACCCCACTCATTAATA
GCTCTGAGGCTTACTAGAGCCGCTTCACCATCATTTAACACATCGTATCTTCCTCTTATCGATGTGAA
+
FFFFFFFFFFFFFFFFFFFFFFFFFFFFFFFFFFFFFFFFFFFFFFFFFFFFFFFFFFFFFFFFFFFF
FFFFFF,FFFFFFFFFFFFFFFFFFFFFFFFFFFFFFFFFFFFF:FFFFFFFFFFFFFF:FFF:FFFF
@A00155:342:HHGFNDSXY:1:1512:16740:34898 1:N:0:GAACCTAG+TCCGCATA
CGCTATCAGTAGGCATATTAAGTTTACTTTCTCCAATCTCCCATTGATATAACCCCACTCATTAATAG
CTCTGTGGCTTACTAGAGCCGCTTCACCATCATTTAACACATCGTATCTTCCTCTTATCGATGTGAAT
+
FFFFFFFFFFFFFFFFFFFFFFFFFFFFFFFFFFFFFFFFFFFFFFFFFFFFFFFFFF,FF:FFFFFF
F:FFFFFFFFFFFFFFFFFFFFFFFFF:FFF:FFFFFFFF:FFF:FFFFFFFFF:FFFFFFFFFFFFF
@A00155:342:HHGFNDSXY:1:1642:10565:36479 2:N:0:GAACCTAG+TCCGCATA
CGCTATCAGTAGGCATATTAAGTTTACTTTCTCCAATCTCCCATTGATATAACCCCACTCATTAATAG
CTCTGTGGCTTACTAGAGCCGCTTCACCATCATTTAACACATCGTATCTTCCTCTTATCGATGTGAAT
+
FFFFFFFFFFFFFFFFFFFFFFFFFFFFFFFFFFFFFFFFFFFFFFFFFFFFFFFFFFFFFFFFFFFF
FFFFFFFFFFFFFFFFFFFFFFFFFFFF:FFFFFFFFFFFFFFFFFFFFFFFFFFFFFFFFFFFFFFF
@A00155:342:HHGFNDSXY:1:2323:6976:10755 1:N:0:GAACCTAG+TCCGCATA
GCTATCAGTAGGCATATTAAGTTTACTTTCTCCAATCTCCCATTGATATAACCCCACTCATTAATAGC
TCTGTGGCTTACTAGAGCCGCTTCACCATCATTTAACACATCGTATCTTCCTCTTATCGATGTGAATT
+
FFFFFFFFFFFFFFFFFFFFFFFFFFFFF:FFFFFFFFFFFFFFFFFFFFFFFFFFFFFFFFFFFFFF
FFFFFFFFF:FFFF:FFFFFFFFFFFFFFFFFFFFFFFFFFF:FFFFFFFFFFFFFFFFFFFFFFFFF
@A00155:342:HHGFNDSXY:1:1528:10565:15436 2:N:0:GAACCTAG+TCCGCATA
GCTATCAGTAGGCATATTAAGTTTACTTTCTCCAATCTCCCATTGATATAACCCCACTCATTAATAGC
TCTGTGGCTTACTAGAGCCGCTTCACCATCATTTAACACATCGTATCTTCCTCTTATCGATGTGAATT
+
FFFFFFFFFFF:FFFFFF,FFFFFFFFFFFFFFF:FFFFFFFF:FFFFFFFFFFFFFFF:F:FFFFF
FFFFFFFFFFFFFFFFFFFFFFFFFFFF:FFF:FFFFFFFFFFFFFFFFFFF:FFFFFFFFFFFFFFF
@A00155:342:HHGFNDSXY:1:1642:10565:36479 1:N:0:GAACCTAG+TCCGCATA
GCTATCAGTAGGCATATTAAGTTTACTTTCTCCAATCTCCCATTGATATAACCCCACTCATTAATAGC
TCTGTGGCTTACTAGAGCCGCTTCACCATCATTTAACACATCGTATCTTCCTCTTATCGATGTGAATT
+
FFFFFFFFFFFFFFFFFFFFFFFFFFFFFFFFFFFFFFFFFFFFFFFFFFFFFFFFFFFFFFFFFFFF
FFFFFFFFFFFFFFFFFFFFFFFFFFFFFFFFFFFFFFFFFFFFFFFFFFFFFFFFFFFFFFFFFFFF
@A00155:342:HHGFNDSXY:1:1507:26259:28432 1:N:0:GAACCTAG+TCCGCATA
TATCAGTAGGCATATTAAGTTTACTTTCTCCAATCTCCCATTGATATAACCCCACTCATTAATAGCTC

```
TGTGGCTTACTAGAGCCGCTTCACCATCATTTAACACATCGTATCTTCCTCTTATCGATGTGAATTGA
+
FFFFFFFFFFFFFFFFFFFFFFFFFFFFFFFFFFFFFFFFFFFFFFFFFFF:FFFFFFFFFFFFFFFF
FF:FFFFFFFFFFFFFFFFFFFFFFFFFFFFFFFFFFFFFFFFFFFFFFFFFFFFFFFFFFFF,FFFF
@A00155:342:HHGFNDSXY:1:2218:21386:36933 1:N:0:GAACCTAG+TCCGCATA
TATCAGTAGGCATATTAAGTTTACTTTCTCCAATCTCCCATTGATATAACCCCACTCATTAATAGCTC
TGTGGCTTACTAGAGCCGCTTCACCATCATTTAACACATCGTATCTTCCTCTTATCGATGTGAATTGA
+
FFFFFFFFFFFFFFFFFFFFFFFFFFFFFFFFFFFFFFFFFFFFFFFFFFFFFFFFFFF:FFFFFFFF
FFFFFFFFFFFFFFFFFFFFFFF:FFFFFFFFFFFFFFFFFFFFFFFFFFFFFFFFFFFFFFFFFFFF
@A00155:342:HHGFNDSXY:1:2556:6497:19257 1:N:0:GAACCTAG+TCCGCATA
TATCAGTAGGCATATTAAGTTTACTTTCTCCAATCTCCCATTGATATAACCCCACTCATTAATAGCTC
TGTGGCTTACTAGAGCCGCTTCACCATCATTTAACACATCGTATCTTCCTCTTATCGATGTGAATTGA
+
F:FFFFFFFFFFFF:F:FFFFFFFFFFFFFFFFFFFFFFFFFFFFFFFFFFFF:FFFFFFFFFFFFFF
FFFFFFFFFF:FFFFF:FFFFFF,:F:FFFFFFFFFFFFFFFFFF:FFFFFF:FFFFFFFFFF,:FFF
@A00155:342:HHGFNDSXY:1:2420:32850:2988 1:N:0:GAACCTAG+TCCGCATA
TATCAGTAGGCATATTAAGTTTACTTTCTCCAATCTCCCATTGATATAACCCCACTCATTAATAGCTC
TGTGGCTTACTAGAGCCGCTTCACCATCATTTAACACATCGTATCTTCCTCTTATCGATGTGAATTGA
+
FFF:FFFFFFFFFFFFFFFFFFFFFFFFFFFF:F:FFFFFFFFFFFFFFFFFFF:FFFFFFFFFFFFF
FFFFFFFFFFFFF:FFFFFFFFFF:FFFFFFFF,FFFFFFFF:FFFFFFFFFFFFFF:FFFFFFFFFF
@A00155:342:HHGFNDSXY:1:1351:29206:20823 1:N:0:GAACCTAG+TCCGCATA
ATCAGTAGGCATATTAAGTTTACTTTCTCCAATCTCCCATTGATATAACCCCACTCATTAATAGCTCT
GTGGCTTACTAGAGCCGCTTCACCATCATTTAACACATCGTATCTTCCTCTTATCGATGTGAATTGAG
+
FFFFFFFFFFFFFFFFFFFFFFFFFFFFFFFFFFFFFFFFFFFFFFFFFFFFFFFFFFFFFFFFFFFF
FFFFFFFFFFFFFFFFFFFFFFFFFFFFFFFFFFFFFFFFFFFFFFFFFFFFFF,FFFFFFFFFFFFF
@A00155:342:HHGFNDSXY:1:1351:28058:22310 1:N:0:GAACCTAG+TCCGCATA
ATCAGTAGGCATATTAAGTTTACTTTCTCCAATCTCCCATTGATATAACCCCACTCATTAATAGCTCT
GTGGCTTACTAGAGCCGCTTCACCATCATTTAACACATCGTATCTTCCTCTTATCGATGTGAATTGAG
+
FFFFFFFFFFF:FFFFFFFFFFFFFFFFFFFFFFFFFFFFFFFFFFFFFFFFFFFFFFFFFFFFFFFF
FFFFFFFFFFFFFFFFFFFFFFFFFFFFFFFFFFFFFFFFFFFFFFF:FFFFFFFFF:FFFFFFFFFF
@A00155:342:HHGFNDSXY:1:2619:12671:24972 2:N:0:GAACCTAG+TCCGCATA
ATCAGTAGGCATATTAAGTTTACTTTCTCCAATCTCCCATTGATATAACCCCACTCATTAATAGCTCT
GTGGCTTACTAGAGCCGCTTCACCATCATTTAACACATCGTATCTTCCTCTTATCGATGTGAATTGAG
+
FFFFF:FFFFFF:FFFFFFFFFFFFFFFFFFFFFFFFFFFFFFF:FFFFFFFFFFFFFFFFFFFF:FF
FFFFFFFFFFFFFFFFFFFFFFFFFFFFFFFFFFFFFFFFFFFFFFFFFFFFFFFFFFFFFFFFFFFF
@A00155:342:HHGFNDSXY:1:2218:21386:36933 2:N:0:GAACCTAG+TCCGCATA
ATCAGTAGGCATATTAAGTTTACTTTCTCCAATCTCCCATTGATATAACCCCACTCATTAATAGCTCT
GTGGCTTACTAGAGCCGCTTCACCATCATTTAACACATCGTATCTTCCTCTTATCGATGTGAATTGAG
+
FFFFF:FF:FF:FFFFF:FF:FFFF:FFFFFFFFFFFFFFFFFFFFFFFFFFFFFFFF:FF:FFFFFF
FFFFF:F:F:FFFFFFFF:FFFFFFFFFFF:FFFFFFFFF:FFF:FFFFFFFFFFFF:F:FFFF:FFF
@A00155:342:HHGFNDSXY:1:1128:9453:9126 2:N:0:GAACCTAG+TCCGCATA
AGTAGGCATATTAAGTTTACTTTCTCCAATCTCCCATTGATATAACCCCACTCATTAATAGCTCTGTG
GCTTACTAGAGCCGCTTCACCATCATTTAACACATCGTATCTTCCTCTTATCGATGTGAATTGAGGAT
+
FFFFFFFFFFFFFFF,FFFFFFFFFF:FFFFFF:F,FFFFFFFFFFFFFFFFFF:FFFFFFF:FFFFF
FFFFFFFFFFFFFFFFFFFFFFFFFFFFFFFFFFFFFFFFFFFFFFFFFFFFFFFFFFFFFFFFFFFF
@A00155:342:HHGFNDSXY:1:1539:18557:12305 2:N:0:GAACCTAG+TCCGCATA
AGTAGGCATATTAAGTTTACTTTCTCCAATCTCCCATTGATATAACCCCACTCATTAATAGCTCTGTG
```

```
GCTTACTAGAGCCGCTTCACCATCATTTAACACATCGTATCTTCCTCTTATCGATGTGAATTGAGGAT
+
FFFFFFFFFFFFFFFF:FFFFFFFFFFFFFFFFFFFFFFFFFFFFFFFFFFFFFFFFFFFFFFFFFFF
FFFFFFFFFFFFFFFFFFFFFFFFFFFFFFFFFFFFFFFFFFFFFFFFFFFFFFFFFFFFFFFFFFFF
@A00155:342:HHGFNDSXY:1:2469:28637:28729 2:N:0:GAACCTAG+TCCGCATA
TAGGCATATTAAGTTTACTTTCTCCAATCTCCCATTGATATAACCCCACTCATTAATAGCTCTGTGGC
TTACTAGAGCCGCTTCACCATCATTTAACACATCGTATCTTCCTCTTATCGATGTGAATTGAGGATAC
+
FFFFFFFFFFFFFFFFFFFFFFFFF:FFFFFFFFFFFFFFFFFFFFFFFFF:FFFFFFFFFFFFFFFF
FFFFFFFFFFFFFFFFFFFFFFFFFFFFFFFFFFFFFFFFFFFFFFFFFFFFFFFFFFFFFFFFFFFF
@A00155:342:HHGFNDSXY:1:2550:4788:18928 1:N:0:GAACCTAG+TCCGCATA
AGGCATATTAAGTTTACTTTCTCCAATCTCCCATTGATATAACCCCACTCATTAATAGCTCTGTGGCT
TACTAGAGCCGCTTCACCATCATTTAACACATCGTATCTTCCTCTTATCGATGTGAATTGAG
+
FFFF:FFFFFFFFFFFFFFFFFFFFFFFFFFFFFFFFFFFFFFFFFFFFFFFFFFFFFFFFFFFFFFF
FFFFFFFFFFFFFFFFFFFFFFFFFFFFFFFFFFFFFFFFFFFFFF:FFFFF:,FFF::FFFF
@A00155:342:HHGFNDSXY:1:1220:26847:2018 2:N:0:GAACCTAG+TCCGCATA
GGCATATTAAGTTTACTTTCTCCAATCTCCCATTGATATAACCCCACTCATTAATAGCTCTGTGGCTT
ACTAGAGCCGCTTCACCATCATTTAACACATCGTATCTTCCTCTTATCGATGTGAATTGA
+
FFFFF:FFFFFFFFFFFFFFFFFFFFFFFFFFFFFFFFFFFFFFFFFFFFFFFFFFFFFFFFFFFFFF
FFFFFFFFFF:,FFFFFFFFFFFFFFFFFFFFFFFFFFFFFFFF:FFFFFFFFFFFFFFFF
@A00155:342:HHGFNDSXY:1:2343:10312:20165 2:N:0:GAACCTAG+TCCGCATA
GCATATTAAGTTTACTTTCTCCAATCTCCCATTGATATAACCCCACTCATTAATAGCTCTGTGGCTTA
CTAGAGCCGCTTCACCATCATTTAACACATCGTATCTTCCTCTTATCGATGTGAATTGAGGATACACC
+
FF:FFFFFFFFFFFFFFFFFFFFFFFFFFFFFFFFFFFFFFFFFFFFFFFFFFFFFFFFFFFFFFFFF
FFFFFFFFFFF:FFFFFFFFFFFFFFFFFFFFFFFFFFFFFFFFFFFFFFFFFFFFFF:FFFFFFFFF
@A00155:342:HHGFNDSXY:1:1641:1524:4038 1:N:0:GAACCTAG+TCCGCATA
CATATTAAGTTTACTTTCTCCAATCTCCCATTGATATAACCCCACTCATTAATAGCTCTGTGGCTTAC
TAGAGCCGCTTCACCATCATTTAACACATCGTATCTCCCTCTTATCGATGTGAATTGAGGATACA
+
FFFFFFFFFFFFFFFFFFFFFFFFFFFFFFFFFFFFFFFFFFFFFFFFFFFFFFFFFFFFFFFFFFFF
FFFFFFFFFFFFFFFFFFFFFFFFF:FFFFF:FFFFFFFFFFFFFFFFFFFFFFFFFFF:FFFFF:F
@A00155:342:HHGFNDSXY:1:2619:11270:29309 1:N:0:GAACCTAG+TCCGCATA
CATATTAAGTTTACTTTCTCCAATCTCCCATTGATATAACCCCACTCATTAATAGCTCTGTGGCTTAC
TAGAGCCGCTTCACCATCATTTAACACATCGTATCTTCCTCTTATCGATGTGAATTGAGGATACACCA
+
FFFFFFFFFFFFFF:F:FFFFFFFFFFFFFFFF:FFFFFFFFFFFFFFFFFFFFFFFFFFF:FFFFFFF
:FFFFFFFFFFFF,FFFFF:FFFF:FFFF:FFFFFFFFFFFFFFFFFFF,F:FFFFF:FFFFFFFFFFF
@A00155:342:HHGFNDSXY:1:1128:9453:9126 1:N:0:GAACCTAG+TCCGCATA
ATATTAAGTTTACTTTCTCCAATCTCCCATTGATATAACCCCACTCATTAATAGCTCTGTGGCTTACT
AGAGCCGCTTCACCATCATTTAACACATCGTATCTTCCTCTTATCGATGTGAATTGAGGATACACCAC
+
FFFFFFFFFFFFFFFFFFFFFFFFFFFFFFFFFFFFFFFFFFFFFFFFFFFFFFFFFFFFFFFFFFFF
FFFFFFFFFFFFFFFFFFFFFFFFFFFFFFFFFFFFFFFFFFFFFFFFFFFFFFFFFFFFFFFFFFFF
@A00155:342:HHGFNDSXY:1:2368:29414:11725 2:N:0:GAACCTAG+TCCGCATA
TATTAAGTTTACTTTCTCCAATCTCCCATTGATATAACCCCACTCATTAATAGCTCTGTGGCTTACTA
GAGCCGCTTCACCATCATTTAACACATCGTATCTTCCTCTTATCGATGTGAATTGAGGATACACCACC
+
FFFFFF:FFFFF:FFFFFF:FFFFFFFFFFFFFFFFFFFFFFFFFFFFFFFFFFFFFFFFFFFFFFFF
FFFFFF:FFFFFFFFFFFFFFFFFFFFFFFFFFFFFFFFFFFFFFFFFFFFFFFFFFFFFFFFFFFFF
@A00155:342:HHGFNDSXY:1:1234:9037:14387 2:N:0:GAACCTAG+TCCGCATA
TATTAAGTTTACTTTCTCCAATCTCCCATTGATATAACCCCACTCATTAATAGCTCTGTGGCTTACTA
```

GAGCCGCTTCACCATCATTTAACACATCGTATCTTCCTCTTATCGATGTGAATTGAGGATACACCACC
+
FFFFFFFFFFFFFFFFFFFFFFFFFFFFFFFFFFFFFFFF,FFFFFFFFFF:FFFFFFFFFFFFFFFFF
FFFFFFFFFFFFFFFFFFFFFFFFFFFFFFFFFFFFFFFFFF:FFFFFFFFFFFFFFFFFFFFFFFFFF
@A00155:342:HHGFNDSXY:1:2263:20654:1939 2:N:0:GAACCTAG+TCCGCATA
TATTAAGTTTACTTTCTCCAATCTCCCATTGATATAACCCCACTCATTAATAGCTCTGTGGCTTACTA
GAGCCGCTTCACCATCATTTAACACATCGTATCTTCCTCTTATCGATGTGAATTGAGGATACACCACC
+
FFFFFFFFFFFFFFFFFFFF:FFFFFFFFFFFFFFF:FFFFF,FFFFFF,::FFFFFFFFFFF:FFFF:
FFFFF:F,FFFFF,FFFFFFFFFFF:FFFFFFF:,FFFF,:FFFFFFFFFFFF:FFFFFFFFFFFFFF
@A00155:342:HHGFNDSXY:1:2646:14570:28573 2:N:0:GAACCTAG+TCCGCATA
TTAAGTTTACTTTCTCCAATCTCCCATTGATATAACCCCACTCATTAATAGCTCTGTGGCTTACTAGA
GCCGCTTCACCATCATTTAACACATCGTATCTTCCTCTTATCGATGTGAATTGAGGATACACCACCTT
+
FFFFFF,FFFFFFFF:FFFFFFFFFFFF::FF:FFFFFFFFFFFFFFFFFFFFFFFFFFFFFFFFFFFF
FFFFFFFFFFFFFFFFFFFFFFFFFFFFFF,FFFFFFFFF::FFFFFFFFFFFFFFFFFFFFFFF:FFF:
@A00155:342:HHGFNDSXY:1:1444:18114:24565 2:N:0:GAACCTAG+TCCGCATA
TAAGTTTACTTTCTCCAATCTCCCATTGATATAACCCCACTCATTAATAGCTCTGTGGCTTACTAGAG
CCGCTTCACCATCATTTAACACATCGTATCTTCCTCTTATCGATGTGAATTGAGGATACACCACCTTC
+
:FFFFF:FF:F:FFFFFFFF:FFFFFFFFFFFFFFFFFFFFFFFFFFFFF:FFFFFFF:FFFFFFFFFF
FFFFFFF:FFFFFFFF:FFFFFFFFFFFFFFFFFFFFFFFFFFFFFFFFFFFFFFFFFFFFFFFFFFFF
@A00155:342:HHGFNDSXY:1:1512:16740:34898 2:N:0:GAACCTAG+TCCGCATA
TTACTTTCTCCAATCTCCCATTGATATAACCCCACTCATTAATAGCTCTGTGGCTTACTAGAGCCGCT
TCACCATCATTTAACACATCGTATCTTCCTCTTATCGATGTGAATTGAGGATACACCACCTTCC
+
FFFFFF:FFF:FFFFFFFFFFFFFFFFFFFFF,FFFFFFFFFF:FFFFFF:F:FFF:FFFFFFFFFFFF
FF:FFFFFFFFFFFFFFFFFFFFFFFFFFFFFFFFFFFFFFFFF:F:FFFFFFFFFFFFFFFFFFFF
@A00155:342:HHGFNDSXY:1:2101:26006:13996 2:N:0:GAACCTAG+TCCGCATA
ACTTTCTCCAATCTCCCATTGATATAACCCCACTCATTAATAGCTCTGTGGCTTACTAGAGCCGCTTC
ACCATCATTTAACACATCGTATCTTCCTCTTATCGATGTGAATTGAGGATACACCACCT
+
FFF:FFFFF:FFFFFFFFFFFFFFFFFFFFFFFFFFFFFFF:FFFFFFFFF:FFFFFFFFFFFFFFFFF
FFFFFFFFFFFFFFFFFFFFFFFFFFFFFFFFFFFFFFFFFFFFFFFFFFFFFFFFFFF
@A00155:342:HHGFNDSXY:1:2623:3414:24345 1:N:0:GAACCTAG+TCCGCATA
ACTTTCTCCAATCTCCCATTGATATAACCCCACTCATTAATAGCTCTGTGGCTTACTAGAGCCGCTTC
ACCATCATTTAACACATCGTATCTTCCTCTTATCGATGTGAATTGAGGATACACCACCTTCCAATCCA
+
FFFFFFFFFFFFFFFFFFFFFFFFFFFFFFFFFFFFFFFFFFFFFFFFFFFFFFFFFFFFFFFFFFFFF
FFFFFFFFFFFFFFFFFFFFFFFFFFFFFFFFFFF:FFFFFFF:FFFF,FFFFFFFFFFFFFFFFF,F
@A00155:342:HHGFNDSXY:1:1325:18186:6433 2:N:0:GAACCTAG+TCCGCATA
CTTTCTCCAATCTCCCATTGATATAACCCCACTCATTAATAGCTCTGTGGCTTACTAGAGCCGCTTCA
CCATCATTTAACACATCGTATCTTCCTCTTATCGATGTGAATTGAGGATACACCACCTT
+
FFFFFFFFF,FFFFFF:FFFFFFF:FFFFFFFFFFFFFFFFFFFFFFFFFFFFFFFFFFFFFFFFFFFF
FFFFFFFF:FFFFFFFFFFFFFFFFFFFFFFFFFF:FFFFFFFFFFFFFFFFFFFFFFFFF
@A00155:342:HHGFNDSXY:1:1455:3134:7639 2:N:0:GAACCTAG+TCCGCATA
CTTTCTCCAATCTCCCATTGATATAACCCCACTCATTAATAGCTCTGTGGCTTACTAGAGCCGCTTCA
CCATCATTTAACACATCGTATCTTCCTCTTATCGATGTGAATTGAGGATACACCACCTT
+
FFFFFF:FFF:FFFFFFFFFFFFFFFFFFFFF:FF:FFFFFFFFFFFFFFFFFFFFFFFFFFFFFFFFF
FFFFFFFFFFFFFFFFFFFFFFFFFFFFF:FFFFFFF:FFFFFFFFFFFFFFFFFFFFFFFF
@A00155:342:HHGFNDSXY:1:1220:26847:2018 1:N:0:GAACCTAG+TCCGCATA
CTTTCTCCAATCTCCCATTGATATAACCCCACTCATTAATAGCTCTGTGGCTTACTAGAGCCGCTTCA

```
CCATCATTTAACACATCGTATCTTCCTCTTATCGATGTGAATTGAGGATACACCACCTTC
+
FFFFFFFFFFFFFFFFFFFFFFFFFFFFFFFFFFFFFFFFFFFFFFFFFFFFFFFFFFFFFFFFFFF
FFFFFFF:FFFFFFFFFFFFFFFFFFFF:FFFFFFFFFFF,FFFFFFF,FFFFFFFFFFF
@A00155:342:HHGFNDSXY:1:2263:20654:1939 1:N:0:GAACCTAG+TCCGCATA
CTTTCTCCAATCTCCCATTGATATAACCCCACTCATTAATAGCTCTGTGGCTTACTAGAGCCGCTTCA
CCATCATTTAACACATCGTATCTTCCTCTTATCGATGTGAATTGAGGATACACCACCTTCCAATCCAT
+
FFFFFFFFFFFFFFFFFFFFFFFFFFFFFFFFFFFFFFFFFFFFFFFFFFFFFFFFFFFFFFFFFFFF
FFFFFFFFFFFFFFFFFFFFFFFFFFFFFFFFFFFFFFFF:FFFFFFFFFFFFFFFFFFFFFFFFFFF
@A00155:342:HHGFNDSXY:1:2409:5086:37043 1:N:0:GAACCTAG+TCCGCATA
CTTTCTCCAATCTCCCATTGATATAACCCCACTCATTAATAGCTCTGTGGCTTACTAGAGCCGCTTCA
CAATCATTTAACACATCGTATCTTCCTCTTATCGATGTGAATTGAGGATACACCACCTTCCAATCCAT
+
FFFFFFFFFFFFFFFFFFFFFFFFFFFFFFFFFF:FFFFFFFFFFFFFFFFFFFFFFFFFFFFFFFFF
F,FFFFFFFFFFFFFFF,FFFFFFFFFFFFFFFFFFFF,FFFF,FFFFFF::,FF,FF:FFFFFFFFF
@A00155:342:HHGFNDSXY:1:2410:5493:1047 1:N:0:GAACCTAG+TCCGCATA
CTTTCTCCAATCTCCCATTGATATAACCCCACTCATTAATAGCTCTGTGGCTTACTAGAGCAGCTTCA
CCATCATTTAACACATCGTATCTTCCTCTTATCGATGTGAATTGAGGATACACCACCTTCCAATCCAT
+
FFFFFFF:FFFFF,FFF,FFFFFFFFFF,:FF::FFFFFFFFFFFF:::FFFFFFFFFF:F,,FFFFF
FFFFFF:F,FFFFFFFFFFFFF:FF,:FFFFF:,FFFFF::FFFFFFFFFFFFFFF:FFF:FFFF,FF
@A00155:342:HHGFNDSXY:1:1463:5746:14606 1:N:0:GAACCTAG+TCCGCATA
CTTTCTCCAATCTCCCATTGATATAACCCCACTCATTAATAGCTCTGTGGCTTACTAGAGCCGCTTCA
CCATCATTTAACACATCGTATCTTCCTCTTATCGATGTGAATTGAGGATACACCACCTTCCAATCCAT
+
FFFFFFFFFFFFFFFFFFFFFFFFFFFFFFFFFFFFF,FFFFFFFFFFFFFFFFFFFFFFFFFFFFFF
FFFFFFFFFFFFFFFFFFFFFFFFFFFFFFFFFFFFF:FFFFFFFFFFFFFFFFFFFFFFFFFFFFFF
@A00155:342:HHGFNDSXY:1:1650:14253:29935 1:N:0:GAACCTAG+TCCGCATA
CTTTCTCCAATCTCCCATTGATATAACCCCACTCATTAATAGCTCTGTGGCTTACTAGAGCCGCTTCA
CCATCATTTAACACATCGTATCTTCCTCTTATCGATGTGAATTGAGGATACACCACCTTCCAATCCAT
+
FFFFFFFFFFFFFFFFFFFFFFFFFFFFFFFFFFFFFFFFFFFFFFFFFFFFFFFFFFFFFFFFFFFF
FFFFFFF:FFFFFFFFFFFFFFFFFFFFFFFFFFFFFFFFF:FFFFFFFFFFFFFFFFFFFFFFFFFF
@A00155:342:HHGFNDSXY:1:1234:9037:14387 1:N:0:GAACCTAG+TCCGCATA
CTTTCTCCAATCTCCCATTGATATAACCCCACTCATTAATAGCTCTGTGGCTTACTAGAGCCGCTTCA
CCATCATTTAACACATCGTATCTTCCTCTTATCGATGTGAATTGAGGATACACCACCTTCCAATCCAT
+
FFFFFFFFFFFFFFFFFFFFFFFFFFFFFFFFFFFF:FFFFFFFFFFFFFFFFFFFFFFFFFFFFFFF
FFFFFFFFFFFFFFFFFFFFFFFFFFFFFFFFFFFFFFFFFFFFFFFFFFFFF:FFFFFFFFFFFFFF
@A00155:342:HHGFNDSXY:1:2565:25238:31015 1:N:0:GAACCTAG+TCCGCATA
CTTTCTCCAATCTCCCATTGATATAACCCCACTCATTAATAGCTCTGTGGCTTACTAGAGCCGCTTCA
CCATCATTTAACACATCGTATCTTCCTCTTATCGATGTGAATTGAGGATACACCACCTTCCAATCCAT
+
FFFFFFFFFFFFFFFFFFFFFFFFFFFFFFFFFFFFFFFFFFFFFF:FFFFFFFFFFFFFFFFFFFFF
FFFFFFFFFFFFFFFFFFFFFFFFFFFFF:FFFFFFFFFFFFFFFFFFFFFFFFFFFFFFFFFFFFFF
@A00155:342:HHGFNDSXY:1:1331:6822:3474 1:N:0:GAACCTAG+TCCGCATA
CTTTCTCCAATCTCCCATTGATATAACCCCACTCATTAATAGCTCTGTGGCTTACTAGAGCCGCTTCA
CCATCATTTAACACATCGTATCTTCCTCTTATCGATGTGAATTGAGGATACACCACCTTCCAATCCAT
+
FFFFFFFFFFFFFFFFFFFFFFFFFFFFFFFFFFFFFFFFFFFFFFFFFFFFFFFF:FF,FFFFFFFFF
FFFFFFFFFFFFFFFFFFFFFFFFF:FFF:FFFFFFFFFFFFFFFFFFFFFFFFFFFFFFFFFFF:FFF
@A00155:342:HHGFNDSXY:1:2368:29414:11725 1:N:0:GAACCTAG+TCCGCATA
CTTTCTCCAATCTCCCATTGATATAACCCCACTCATTAATAGCTCTGTGGCTTACTAGAGCCGCTTCA
```

```
CCATCATTTAACACATCGTATCTTCCTCTTATCGATGTGAATTGAGGATACACCACCTTCCAATCCAT
+
FFFFFFFFFFFFFFFFFFFFFFFFFFFFFFFFFFFFFFFFFFFFFFFFFFFFFFFFFFF:FFFFFFFF
FFFFFFFFFFFFFFFFFFFFFFFFFFFFFFFFFFFFFFFFFFFFFFFFFFFFFFFFFFFFFFFFFFFF
@A00155:342:HHGFNDSXY:1:2240:31141:28526 1:N:0:GAACCTAG+TCCGCATA
CTTTCTCCAATCTCCCATTGATATAACCCCACTCATTAATAGCTCTGTGGCTTACTAGAGCCGCTTCA
CCATCATTTAACACATCGTATCTTCCTCTTATCGATGTGAATTGAGGATACACCACCTTCCAATCCAT
+
FFFFFFFFFFFFFFFFFFFFFFFFFFFFFFFFFFF:FFFFFFFFFFFFFFFFFFFFFFFFF:FFFFFF
FFF:FFFFFFFFFFFFFFFFFFFFFFFFFFFFFFFFFFFFFFFFFFFFFFFFFFFFFF:FFFFFFFFF
@A00155:342:HHGFNDSXY:1:2108:22128:6464 1:N:0:GAACCTAG+TCCGCATA
CTTTCTCCAATCTCCCATTGATATAACCCCACTCATTAATAGCTCTGTGGCTTACTAGAGCCGCTTCA
CCATCATTTAACACATCGTATCTTCCTCTTATCGATGTGAATTGAGGATACACCACCTTCCAATCCAT
+
FFFFFFFFFFFFFFFFFFFFFFFFFFFFFFFFFFFFFFFFFFFFFFFFFFF:FFFFFFFFFFFFFFFF
FFFFFFFFFFFFFFFFFFF,F:FFFF:FFFFFFFFFFFFFFFFFFFFFFFFFFFFFFFFFFFFFFFF:
@A00155:342:HHGFNDSXY:1:2221:18900:35978 1:N:0:GAACCTAG+TCCGCATA
TTTCTCCAATCTCCCATTGATATAACCCCACTCATTAATAGCTCTGTGGCTTACTAGAGCCGCTTCAC
CATCATTTAACACATCGTATCTTCCTCTTATCGATGTGAATTGAGGATACACCACCTTCCAATCCATC
+
FFFFFFFFFFF,FFFFFF:FFFFFFFFF:FFFFFFFFFFF:FFFFFFF:FFFF:FF:FFFFFFFFFFF
FF:FFF,FFFFFFFF:FFFFFFFFFFFFFFF:FFF:F:FF:FFFFFFFFFFFFFF:FFFFFFFFFFF:
@A00155:342:HHGFNDSXY:1:1422:14841:10974 2:N:0:GAACCTAG+TCCGCATA
TTCTCCAATCTCCCATTGATATAACCCCACTCATTAATAGCTCTGTGGCTTACTAGAGCCGCTTCACC
ATCATTTAACACATCGTATCTTCCTCTTATCGATGTGAATTGAGGATACACCACCTTCCAATCCATCT
+
FFFF::FFFFFF:FFFFFFFFFFFFFFF:FFFFFFFFFFFFFFFFFFFFFFFFFFFFFFFFFFFFFFF
FFFFFFFFFFFF,FFFFFFFFFFFFFFF:FFFFFFFFFFFFFFFF::F:FFFFFFFFFFFFFFFFFFF
@A00155:342:HHGFNDSXY:1:2508:13548:2472 1:N:0:GAACCTAG+TCCGCATA
TTCTCCAATCTCCCATTGATATAACCCCACTCATTAATAGCTCTGTGGCTTACTAGAGCCGCTTCACC
ATCATTTAACACATCGTATCTTCCTCTTATCGATGTGAATTGAGGATACACCACCTTCCAATCCATCT
+
FFFFFFFFFFFFFFFFFFFFFFFFFFFFFFFFFFFFFFFFFFFFFFFFFFFFFFFFFFFFFFFFFFFF
FFFFFFFFFFFFFFFFFFFFFFFFFFFFFFFFFFFFFFFFFFFFFFFFFFFFFFFFFFFFFFFFFFFF
@A00155:342:HHGFNDSXY:1:2516:32868:28040 1:N:0:GAACCTAG+TCCGCATA
TCTCCAATCTCCCATTGATATAACCCCACTCATTAATAGCTCTGTGGCTTACTAGAGCCGCTTCACCA
TCATTTAACACATCGTATCTTCCTCTTATCGATGTGAATTGAGGATACACCACCTTCCAATCCATCTG
+
FFFFFFFFFFFFFFFFFFFFFFF:FFFFFF:F:FFFFF:FFFFFFFFFFFFFFF:FFFFFFFFFFFFF
FFFFFFFFFFFFFFFFFFFFFFFFFFFFFFFFFFF:FFFFFFFFFFFFFFFFFFFFFFFFF,FFFFFF
@A00155:342:HHGFNDSXY:1:1338:11342:14497 2:N:0:GAACCTAG+TCCGCATA
CTCCAATCTCCCATTGATATAACCCCACTCATTAATAGCTCTGTGGCTTACTAGAGCCGCTTCACCAT
CATTTAACACATCGTATCTTCCTCTTATCGATGTGAATTGAGGATACACCACCTTCCAATCCATCTG
+
FF,FFFFFFFF:FFFFFFFFFFFFFFFFFFFFFFFFFFFFFFFFFFFFFFFFFFFFFFFFFFFFFFFF
FFFFFFFFFFFFFFFFFFFFFFFFF:FF:FFFFFFFFFFFFFFFFFFFFFFFFFFFFFFFFFFFFFF
@A00155:342:HHGFNDSXY:1:2607:25156:29559 2:N:0:GAACCTAG+TCCGCATA
CTCCAATCTCCCATTGATATAACCCCACTCATTAATAGCTCTGTGGCTTACTAGAGCCGCTTCACCAT
CATTTAACACATCGTATCTTCCTCTTATCGATGTGAATTGAGGATACACCACCTTCCAATCCATCTGC
+
:FFFF:FFFFFFFFFFFFF:FFFFFFFFFFFF:FFFFFFFFFFFFFFFFFFFFFFFFFFFFFFFFFFF
FFFFFFFFFFFFFFFF:FFFFFFFFFFFFFFFFFFFFFFFFFFFFFFFFFFFFFFFFFFFFFFFFFFF
@A00155:342:HHGFNDSXY:1:2240:31141:28526 2:N:0:GAACCTAG+TCCGCATA
CTCCAATCTCCCATTGATATAACCCCACTCATTAATAGCTCTGTGGCTTACTAGAGCCGCTTCACCAT
```

```
CATTTAACACATCGTATCTTCCTCTTATCGATGTGAATTGAGGATACACCACCTTCCAATCCATCTGC
+
FFFFFFFFFF:FFFFFFFFF:FFFFFFFFFFFF:FFFFFFFFFFFFFFFFFFFFFFFFFFFFFFFFFFFF
FFFFFFFFFFFFFFFFFFFFFFFFF::FFFFFFFFFFFFFFFF:F:FFFFFFFFFFFFFFFFFFFFFFFF
@A00155:342:HHGFNDSXY:1:2269:2998:30044 1:N:0:GAACCTAG+TCCGCATA
TCCAATCTCCCATTGATATAACCCCACTCATTAATAGCTCTGTGGCTTACTAGAGCCGCTTCACCATC
ATTTAACACATCGTATCTTCCTCTTATCGATGTGAATTGAGGATACACCACCTTCCAATCCATCTGCA
+
FFFFFFF:FFFFFFFFFFFFF:FFFFFFFFFFFFF:FFFFFFFFFFFFFFFFFFFFFFFFFFFFFFFF:F
FFFFFFFFFFFFFFFFFFFFFFFFFFFFFFFFFFFFFFFFFFFFFFFFFFFFFFFFFFFFFFFFFFFFFF
@A00155:342:HHGFNDSXY:1:1167:16324:8500 2:N:0:GAACCTAG+TCCGCATA
CAATCTCCCATTGATATAACCCCACTCATTAATAGCTCTGTGGCTTACTAGAGCCGCTTCACCATCAT
TTAACACATCGTATCTTCCTCTTATCGATGTGAATTGAGGATACACCACCTTCCAATCC
+
FFFFFFFFFF:FFFFFFFFFFFF:FFFFFFFFFFFFFFFFFFFFFFFFFFFFFFF:F:FFFFFFFFFFFF
FFFFFFFFFFFFFFFFFFFFFFF:FFFFFFFFFFFFFFFFFFFFFFFFFFF:FFFFFFFF
@A00155:342:HHGFNDSXY:1:2653:8585:5807 1:N:0:GAACCTAG+TCCGCATA
TCTCCCATTGATATAACCCCACTCATTAATAGCTCTGTGGCTTACTAGAGCCGCTTCACCATCATTTA
ACACATCGTATCTTCCTCTTATCGATGTGAATTGAGGATACACCACCTTCCAATCCATCTGCAGCCAC
+
FFFFFFFFFFFFFFFFFFFFFFFFFFFFFFFFFFFFFFFFFFFFFFFFFFFFFFFFFFFFFFFFFFFFFF
FFFFFFFFFFFFFFFFFFFFFFFFF:F,FFFFFFFFFFFFFFFFFFFFFFFFFFFFFFFFFFFFFFF:FF
@A00155:342:HHGFNDSXY:1:2223:20500:8124 2:N:0:GAACCTAG+TCCGCATA
TCTCCCATTGATATAACCCCACTCATTAATAGCTCTGTGGCTTACTAGAGCCGCTTCACCATCATTTA
ACACATCGTATCTTCCTCTTATCGATGTGAATTGAGGATACACCACCTTCCAATCCATCTGCAGCCAC
+
FFFFFFFFFFFF:FFFFFFFFFFFFF:FF:FFFF:FFFFFFFFFFFFFFFFFFFF,FFFFFFFFFFFFFF
FFFF,F::FFFFFFFFFFF,FFFF:FFFFFFFFFFFFFFFFFFFFFFFFFFFFFFFFF:FFFFFFFFF,F
@A00155:342:HHGFNDSXY:1:2101:26006:13996 1:N:0:GAACCTAG+TCCGCATA
CCATTGATATAACCCCACTCATTAATAGCTCTGTGGCTTACTAGAGCCGCTTCACCATCATTTAACAC
ATCGTATCTTCCTCTTATCGATGTGAATTGAGGATACACCACCTTCCAATCCATCTGCA
+
FFFFFFFFFFF:FFFFFFFFFFFFFFFFFFFFFFFFFFFFFFFFFFFFFFFFFFFFFFFFFFFFFFFFFF
FFFFFFFFFFFFFFFFFFFFFFFFFFFFFFFFFFFFFFFFFFFFFFFFFFFFFFFFFFFF
@A00155:342:HHGFNDSXY:1:1325:18186:6433 1:N:0:GAACCTAG+TCCGCATA
CATTGATATAACCCCACTCATTAATAGCTCTGTGGCTTACTAGAGCCGCTTCACCATCATTTAACACA
TCGTATCTTCCTCTTATCGATGTGAATTGAGGATACACCACCTTCCAATCCATCTGCAG
+
FFFFFFFFFFFFFFFFFFFFFFFFFFFFFFFFFFFFFFFFFFFFFFFFFFFFFFFFFFFF::FFFF:FFF
FFFFFFFFFFFFFFFFFFFFFFFFFFFFFFFFFFFFFFFFFFFFFFFFFFFFFFFFFFFF
@A00155:342:HHGFNDSXY:1:1455:3134:7639 1:N:0:GAACCTAG+TCCGCATA
CATTGATATAACCCCACTCATTAATAGCTCTGTGGCTTACTAGAGCCGCTTCACCATCATTTAACACA
TCGTATCTTCCTCTTATCGATGTGAATTGAGGATACACCACCTTCCAATCCATCTGCAG
+
FFFFFFFFFFFFFFFFFFFFFFFFFFFFFFFFFFFFFFFFFF:FFFFFFFFFFFFFFFFFFFFFF:FFFF
FFFFFFFFFFFFFFFFFFFFFFF:FFFFFFFFFFFFFFFFFFFFFFFFFFFFFFFFFF:F
@A00155:342:HHGFNDSXY:1:1602:5737:31563 1:N:0:GAACCTAG+TCCGCATA
CATTGATATAACCCCACTCATTAATAGCTCTGTGGCTTACTAGAGCCGCTTCACCATCATTTAACACA
TCGTATCTTCCTCTTATCGATGTGAATTGAGGATACACCACCTTCCAATCCATCTGCAGCCACACGCA
+
FFFFFFFFFFFFFFFFFFFF:FFFFF,FFFFFFFFFFF:FF,FFFFFFFFFFFFFFF:FFF:FFFFFFFF
FFFFFFFFFFFFF:FF:FFFFFFFFFFFFFFFFFFFFFF:FF:FFFFFFFFFFFFFFFFFFFF:FFFFFF
@A00155:342:HHGFNDSXY:1:2604:27281:8625 1:N:0:GAACCTAG+TCCGCATA
CATTGATATAACCCCACTCATTAATAGCTCTGTGGCTTACTAGAGCCGCTTCACCATCATTTAACACA
```

```
TCGTATCTTCCTCTTATCGATGTGAATTGAGGATACACCACCTTCCAATCCATCTGCAGCCACACGCA
+
FFFFFFFFFFFFFFFFFFFFFFFFFFFFFFFFFFFFFFFFFFFFFFFFFFFFFFFFFFFFFFFFFFFF
FFFFFFFFFFFFFFFFFFFFFFFFFFFFFFFFFFFFFFFFFFFFFFFFFFFFFFFFFFFFFF:FFFFFF
@A00155:342:HHGFNDSXY:1:1338:11342:14497 1:N:0:GAACCTAG+TCCGCATA
GATATAACCCCACTCATTAATAGCTCTGTGGCTTACTAGAGCCGCTTCACCATCATTTAACACATCGT
ATCTTCCTCTTATCGATGTGAATTGAGGATACACCACCTTCCAATCCATCTGCAGCCACACGCATGC
+
FFFFFFFFFFFFFFFFFFFFFFFFFFFFFFFFFFFFFFFFFF:FFFFFFFF:FFFFFFFFFFFF
FFFFFFFFF,FFFFFFFFFFFFFFFFFFF:FFFFFFFFFFFFFFFFFFFFFFFFFF,FFFFFFFFF
@A00155:342:HHGFNDSXY:1:2374:2510:28573 1:N:0:GAACCTAG+TCCGCATA
GATATAACCCCACTCATTAATAGCTCTGTGGCTTACTAGAGCCGCTTCACCATCATTTAACACATCGT
ATCTTCCTCTTATCGATGTGAATTGAGGATACACCACCTTCCAATCCATCTGCAGCCACACGCATGCT
+
F,FFFFFFFFFFFFFFFFF:FF,,FFFFFFFFFFFFFFFFFFFFFFFFFFFFFFFFF:FFFFFF::FF
FFFF,FFFFF,FFFFFF:,FFFFF:FFFFFFFF,FFFFFFF:F:F,FFFFFFFFFFFFFFF::F::FF
@A00155:342:HHGFNDSXY:1:1118:1497:26256 1:N:0:GAACCTAG+TCCGCATA
GATATAACCCCACTCATTAATAGCTCTGTGGCTTACTAGAGCCGCTTCACCATCATTTAACACATCGT
ATCTTCCTCTTATCGATGTGAATTGAGGATACACCACCTTCCAATCCATCTGCAGCCACACGCATGCT
+
F,FFFFFFFFFFFF:FFFFFFF:FFFFFF,FFFFFFFF:F:F,F,FFFF:,FFF:FFFFFFFFFFFF
FFFFFF,FFFFFFF:FFFFF:FFFFFFFFFF:FF,FFFFFFF:FFFFFF:FFFFFFFFFFFFF:FFFF
@A00155:342:HHGFNDSXY:1:1467:1551:28980 1:N:0:GAACCTAG+TCCGCATA
GATATAACCCCACTCATTAATAGCTCTGTGGCTTACTAGAGCCGCTTCACCATCATTTAACACATCGT
ATCTTCCTCTTATCGATGTGAATTGAGGATACACCACCTTCCAATCCATCTGCAGCCACACGCATGCT
+
F:FFF:FFFFFFFFFFFFFFFFF,FFFFFFFFFFFFFFFFFFFFFFFFFFF,FF:FFFFF:FFFFFFFF
FFFFFFFFFFFFFFFFFFFFFF,FFFFFFFFFF:FFFFFFFFFF:FFFFFFFFFF:FFFFFFF:FFFF
@A00155:342:HHGFNDSXY:1:1569:24867:9580 1:N:0:GAACCTAG+TCCGCATA
GATATAACCCCACTCATTAATAGCTCTGTGGCTTACTAGAGCCGCTTCACCATCATTTAACACATCGT
ATCTTCCTCTTATCGATGTGAATTGAGGATACACCACCTTCCAATCCATCTGCAGCCACACGCATGCT
+
FFFFFFFFFFFFFFFFFFFFFFFFFFFFFFFFFFFFFFFFFFFFFFFFFFFFFFFFFFFFFFFFFFFF
FFFFFFFFFFFFFFFFFFFFFFFFFFFFFFFFFFFFFFFFFFFFFFFFFFFFFFFFFFFFFFFFFFFF
@A00155:342:HHGFNDSXY:1:2223:20500:8124 1:N:0:GAACCTAG+TCCGCATA
GATATAACCCCACTCATTAATAGCTCTGTGGCTTACTAGAGCCGCTTCACCATCATTTAACACATCGT
ATCTTCCTCTTATCGATGTGAATTGAGGATACACCACCTTCCAATCCATCTGCAGCCACACGCATGCT
+
FFFFFFFFFFFFFFFFFFFFFFFFF:FFFFFFFFFFFFFFFFFFFFFFFFFFFFFFFFFFFFFFFFFF
,FFFFFFFFFFFFFFFFFFFFFFFFFFFFFFFFFFFFFFFFFFFF:FFFFFFFFFFFFFFFFFFFFFF
@A00155:342:HHGFNDSXY:1:1632:32560:11882 1:N:0:GAACCTAG+TCCGCATA
GATATAACCCCACTCATTAATAGCTCTGTGGCTTACTAGAGCCGCTTCACCATCATTTAACACATCGT
ATCTTCCTCTTATCGATGTGAATTGAGGATACACCACCTTCCAATCCATCTGCAGCCACACGCATGCT
+
FFFFFFFFFFFFFFFFFFFFF:FFFFFFFFFFFFFFFFFFFFFFFFFFFFFFFFFF:FFFFFFFFFFFF
:FFFFFFFFF:FFFFFFFFFFFFFFFFFFFFFFFFFFFFFFFFFFFFFFFFFFFFFFFFFFFFFFF:F
@A00155:342:HHGFNDSXY:1:2215:24397:22200 1:N:0:GAACCTAG+TCCGCATA
GATATAACCCCACTCATTAATAGCTCTGTGGCTTACTAGAGCCGCTTCACCATCATTTAACACATCGT
ATCTTCCTCTTATCGATGTGAATTGAGGATACACCACCTTCCAATCCATCTGCAGCCACACGCATGCT
+
FFFFFFFFFFFFFFFFFFFFFFFFFFFFFFFFFFFFFFFFFFFFFFFFFFFFFFFFFFFFFFFFFFFF
FFFFFFFFFFFFFFFFFFFFFFFFFFFFFFFFFFFFFFFFFFFFFFFFFFFFFFFFFFFFFFFFFFFF
@A00155:342:HHGFNDSXY:1:2348:13494:20917 1:N:0:GAACCTAG+TCCGCATA
GATATAACCCCACTCATTAATAGCTCTGTGGCTTACTAGAGCCGCTTCACCATCATTTAACACATCGT
```

```
ATCTTCCTCTTATCGATGTGAATTGAGGATACACCACCTTCCAATCCATCTGCAGCCACACGCATGCT
+
FFFF:FFF:FFFFF:FFFFFFFFFFFFFFFFFFFFFFFFFFFF:FFFFFFFFFFFFFFFFFF,F:FFF
FFFFFFF:F:FFF,FFFFFFFFFFFFFFFFFF:FFFFF,FFFFF,FF:FFF:F:FFFFFFFFFFFFFF
@A00155:342:HHGFNDSXY:1:1577:11912:27320 1:N:0:GAACCTAG+TCCGCATA
ATATAACCCCACTCATTAATAGCTCTGTGGCTTACTAGAGCCGCTTCACCATCATTTAACACATCGTA
TCTTCCTCTTATCGATGTGAATTGAGGATACACCACCTTCCAATCCATCTGCAGCCACACGCATGCT
+
FFF,FF:FFFF:FFF:FFFFFFFFFFFFFFFFFFFFFFFFFFFFFFFFFFFF:FFFF,FFFFFFFFFF
FFFFFFFFF:F:F,FFFFFFFFFFFFFFFFFFFFFF,F:FFFFFFFFFFFFFFFFFFFFFFFF:FFF
@A00155:342:HHGFNDSXY:1:1577:11912:27320 2:N:0:GAACCTAG+TCCGCATA
TATAACCCCACTCATTAATAGCTCTGTGGCTTACTAGAGCCGCTTCACCATCATTTAACACATCGTAT
CTTCCTCTTATCGATGTGAATTGAGGATACACCACCTTCCAATCCATCTGCAGCCACACGCATGCTTC
+
FFFFFFF,FF,FFF,FFFFF:FF:F:F:FFFFFFFFFFFFF:,FFFFFFF,FFFFFFF:FF::FFFFF
:FF,FFFFF:FFFFFFFFFF,FFFFFFFFFFFFFFFFFFFFFF:FF:FFFFFFFF,:FFFFFFFFFFF
@A00155:342:HHGFNDSXY:1:1167:16324:8500 1:N:0:GAACCTAG+TCCGCATA
ATAACCCCACTCATTAATAGCTCTGTGGCTTACTAGAGCCGCTTCACCATCATTTAACACATCGTATC
TTCCTCTTATCGATGTGAATTGAGGATACACCACCTTCCAATCCATCTGCAGCCACACG
+
FFFFFFFFFFFFF:FFFFFFFFFFFFFFF,FFFFFFFFFFFFFFFFFFFFFFFFFFFFFFFFFFFFFF
FFFFFFFFFFFFFFFFFFFFFFFFFFF:FFFFFFFFFFFFFFFFFFFFFFFFFFFFFFF
@A00155:342:HHGFNDSXY:1:1544:30969:23124 2:N:0:GAACCTAG+TCCGCATA
ACCCCACTCATTAATAGCTCTGTGGCTTACTAGAGCCGCTTCACCATCATTTAACACATCGTATCTTC
CTCTTATCGATGTGAATTGAGGATACACCACCTTCCAATCCATCTGCAGCCACACGCATGCTTCCGCC
+
FFFFFFFFFFFFFFFFFFFFFFFFFFFFFFFFFFFFFF:FFFFFFFFFFF:FFFFFFFF:FFFFF:FF
FFFF::FFFFFFFFFFFFFFF:FFFFFFF,FF:FFFFFFFFFFFFFF:FFFFFFFFFFFFFFF:FFFF
@A00155:342:HHGFNDSXY:1:1331:6822:3474 2:N:0:GAACCTAG+TCCGCATA
ACCCCACTCATTAATAGCTCTGTGGCTTACTAGAGCCGCTTCACCATCATTTAACACATCGTATCTTC
CTCTTATCGATGTGAATTGAGGATACACCACCTTCCAATCCATCTGCAGCCACACGCATGCTTCCGCC
+
FFFFFFFFFFFFFFFFFFFFFFFFFFFFFFFFFFFFFFFFFFFFFFFFFFFFFFFFFFFFFFFFFFFF
FFFFFFFFFFFFFFFFFFFFFFFFFFFFFFFF:FFFF:FFFFFFFFFFFFFFFFFFFFFFFFFFFFFF
@A00155:342:HHGFNDSXY:1:2210:16812:27320 1:N:0:GAACCTAG+TCCGCATA
CCCCACTCATTAATAGCTCTGTGGCTTACTAGAGCCGCTTCACCATCATTTAACACATCGTATCTTCC
TCTTATCGATGTGAATTGAGGATACACCACCTTCCAATCCATCTGCAGCCACACGCATGCTTCCGCCG
+
FFFFFFFFFFFFFFFFFFFFFFFFFFFFFFFFFFFFFFFFFFFFFFF:FFFFFFFFFFFFFFFFFFFF
FFFFFFFFFFFFFFFFFFFFFFFFFFFFFFFFFFFFFFFFFFFFFFFFFFFFFFFFFFFFFFFFFFFF
@A00155:342:HHGFNDSXY:1:2210:17924:27273 1:N:0:GAACCTAG+TCCGCATA
CCCCACTCATTAATAGCTCTGTGGCTTACTAGAGCCGCTTCACCATCATTTAACACATCGTATCTTCC
TCTTATCGATGTGAATTGAGGATACACCACCTTCCAATCCATCTGCAGCCACACGCATGCTTCCGCCG
+
FFFFFFFFFFFFFFFFFFFFFFFFFFFFFFFFFFFFFFFFFFFFFFFFF:FFFFFF:FFFFFFFFFFF
FFFFFFFFFFFFFFFFFFFFFFFFFFFFFFFFFFFFFFFFFFFFFFFFFFFFFFFFFFFFFFFFFFFF
@A00155:342:HHGFNDSXY:1:2210:19732:28526 1:N:0:GAACCTAG+TCCGCATA
CCCCACTCATTAATAGCTCTGTGGCTTACTAGAGCCGCTTCACCATCATTTAACACATCGTATCTTCC
TCTTATCGATGTGAATTGAGGATACACCACCTTCCAATCCATCTGCAGCCACACGCATGCTTCCGCCG
+
FFFFFFFFFFFFFFFFFFFFFFFFFFFFFFFFFFFFFFFFFFFFFFFFFFFFFFFFFFFFFFFFFFFF
FFFFFFFFFFFFFFF:FFFFFFFFFFFFFFFFFFFFFFFFFFFFFFFFFFFFFFFFFFFFFFFFFFFF
@A00155:342:HHGFNDSXY:1:1557:2790:10238 1:N:0:GAACCTAG+TCCGCATA
CCCCACTCATTAATAGCTCTGTGGCTTACTAGAGCCGCTTCACCATCATTTAACACATCGTATCTTCC
```

```
TCTTATCGATGTGAATTGAGGATACACCACCTTCCAATCCATCTGCAGCCACACGCATGCTTCCGCCG
+
FFFFFFFFFFFFFFFFFFFFFFFFFFFFFFFFFFFFFFFFFFFFFFFFFFFFFFFFFFFFFFFFFFFF
FFFFFFFFFFFFFFFFFFFFFFFFFFFFFFFFFFFFFF:FFFFFFFFFFFFFFFFFFFFFFFFFFFFF
@A00155:342:HHGFNDSXY:1:2210:17969:26663 1:N:0:GAACCTAG+TCCGCATA
CCCCACTCATTAATAGCTCTGTGGCTTACTAGAGCCGCTTCACCATCATTTAACACATCGTATCTTCC
TCTTATCGATGTGAATTGAGGATACACCACCTTCCAATCCATCTGCAGCCACACGCATGCTTCCGCCG
+
FFFFFFFFFFFFFFFFFFFFFFFFFFFFFFFFFFFFFFFFFFFFFFFFFFFFFFFFFFFFFFFFFFFF
FFFFFFFFFFFFFFFFFFFFFFFFFFFFFFFFFFFFFFFFFFFFFFFFFFFFFFFFFFFF:FFFFFFF
@A00155:342:HHGFNDSXY:1:1141:22110:5368 1:N:0:GAACCTAG+TCCGCATA
CCCCACTCATTAATAGCTCTGTGGCTTACTAGAGCCGCTTCACCATCATTTAACACATCGTATCTTCC
TCTTATCGATGTGAATTGAGGATACACCACCTTCCAATCCATCTGCAGCCACACGCATGCTTCCGCCG
+
FFFFFFFFFFFFFFFFFFFFFFFFFFFFFFFFFFFFFFFFFFFFFFFFFFFFFFFFFFFFFFFFFFFF
FFFFF:FFFFFFFFFFFFFFFFFFFFFFFFFFFFFFFFFFFFFFFFFFFFFFFFFFFFFFFFFFFFFF
@A00155:342:HHGFNDSXY:1:2268:19759:31015 2:N:0:GAACCTAG+TCCGCATA
CCCACTCATTAATAGCTCTGTGGCTTACTAGAGCCGCTTCACCATCATTTAACACATCGTATCTTCCT
CTTATCGATGTGAATTGAGGATACACCACCTTCCAATCCATCTGCAGCCACACGCATGCTTCCGCC
+
FFFFF,FFFFFFFFFFFFFFFFFFFFFFFFFFFFFFFFFFFFFFFFFFFFFFFFFFFFFFFFFFFFF:
FFFFFFFFFFFFFFFFFFFFFFFFFFFFFFFFFFFFFFFFFF:FFFFFF:FFFFFFFFFFFFFFFF
@A00155:342:HHGFNDSXY:1:2653:8585:5807 2:N:0:GAACCTAG+TCCGCATA
CCCACTCATTAATAGCTCTGTGGCTTACTAGAGCCGCTTCACCATCATTTAACACATCGTATCTTCCT
CTTATCGATGTGAATTGAGGATACACCACCTTCCAATCCATCTGCAGCCACACGCATGCTTCCGCCGT
+
F:FFFFFFFFFFFFFFFFFFFFFFFFFFFFFFFFFFFFFFFF,FFFFFFFFFFFFFFFFFFFFFFFFF
FFF:FFFFFFFFFFFFFFFFFFFFFFF:FFFFFFF,FFFFFFFFFFFFFFFFF:FFFFFFFFFFFFFF
@A00155:342:HHGFNDSXY:1:1478:32976:34334 1:N:0:GAACCTAG+TCCGCATA
CCACTCATTAATAGCTCTGTGGCTTACTAGAGCCGCTTCACCATCATTTAACACATCGTATCTTCCTC
TTATCGATGTGAATTGAGGATACACCACCTTCCAATCCATCTGCAGCCACACGCATGCTTCCGCCGTT
+
FFFFFFFFFFFFFFFFFFFFFFFFFFFFFFFFFFFFFFFFFFFFFFFF:FFFFFFFFFFFFFFFFFFF
FFFFFFFFFFFFFFFFFFFFFFFFFFFFFFFFFFF:FFFFFFFFFFFFFFFFFFFFFFFFFFFFFFFF
@A00155:342:HHGFNDSXY:1:2604:27281:8625 2:N:0:GAACCTAG+TCCGCATA
CACTCATTAATAGCTCTGTGGCTTACTAGAGCCGCTTCACCATCATTTAACACATCGTATCTTCCTCT
TATCGATGTGAATTGAGGATACACCACCTTCCAATCCATCTGCAGCCACACGCATGCTTCCGCCGTTG
+
FFFFFFFFFFFFFFFFFFFFFFFFFFFFFFFFFFF:FFFFFFFFFFFFFFFFFFFFFFFFF:FFFFFF
FFFFFFFFFFFFFFFFFFFFFFFFFFFFFFFFFFFFFFFFFFFFFFF:FFFFFFFFFFFFFFFFFFFF
@A00155:342:HHGFNDSXY:1:1467:1551:28980 2:N:0:GAACCTAG+TCCGCATA
CACTCATTAATAGCTCTGTGGCTTACTAGAGCCGCTTCACCATCATTTAACACATCGTATCTTCCTCT
TATCGATGTGAATTGAGGATACACCACCTTCCAATCCATCTGCAGCCACACGCATGCTTCCGCCGTTG
+
FFFF,F,FFFFFFF:FF::FFFFFFFFF:FFFFF:FFFFFFF,FFFFFFFFFFF:FFFFFFF:FFFFF
FFFFFFFFFFFFFFFFFFFFFFFFFFFFFFFFFFFFFFFFFFFFFFFFFFFFFFFF:F:FFFFFFFFF
@A00155:342:HHGFNDSXY:1:2263:16532:33473 2:N:0:GAACCTAG+TCCGCATA
CACTCATTAATAGCTCTGTGGCTTACTAGAGCCGCTTCACCATCATTTAACACATCGTATCTTCCTCT
TATCGATGTGAATTGAGGATACACCACCTTCCAATCCATCTGCAGCCACACGCATGCTTCCGCCGTTG
+
FFFFFFF,FF:FFF:FFFFFFF:FFF,FFFFFFFFFFFFFFFFFFFFFFFFFFFFFFFFFFFFFFFF:
F:FFF,FFFFF:FFFFFFFFFFFFFFFF,FFFFFFFFFFFFF:FFFFF,FFFFFFFFFFFFFFFFFFF
@A00155:342:HHGFNDSXY:1:2658:25482:29747 1:N:0:GAACCTAG+TCCGCATA
ACTCATTAATAGCTCTGTGGCTTACTAGAGCCGCTTCACCATCATTTAACACATCGTATCTTCCTCTT
```

ATCGATGTGAATTGAGGATACACCACCTTCCAATCCATCTGCAGCCACACGCATGCTTCCGCCGTTGA
+
FFFFFFFFFFFFFFFFFFFFFFFFFFFFFFFFFFFFFFFFFFFFFFFFFFFFFFFFFFFFFFFFFFFF
FFFFFFFFFFFFFFFFFFFFFFFFFFFFFFFFFFFFFFFFFFFFFFFFFFFFFFFFFFFFFFFFFFFF
@A00155:342:HHGFNDSXY:1:1477:27561:16313 1:N:0:GAACCTAG+TCCGCATA
ACTCATTAATAGCTCTGTGGCTTACTAGAGCCGCTTCACCATCATTTAACACATCGTATCTTCCTCTT
ATCGATGTGAATTGAGGATACACCACCTTCCAATCCATCTGCAGCCACACGCATGCTTCCGCCGTTGA
+
FFFFFFFFFFFFFFFFFFFFFFFFFFF:FF:FFFF:FFFFFFFFFFFFFFFFFFFFFFFFFFFFFFFF
FFFFFFFFFFFFFFFFFFFFFFFFFFFFFFFFFFFFFFFFFFFFFFFFFFFFFFFFFFFFFFFFFFFF
@A00155:342:HHGFNDSXY:1:1405:14136:14606 1:N:0:GAACCTAG+TCCGCATA
ACTCATTAATAGCTCTGTGGCTTACTAGAGCCGCTTCACCATCATTTAACACATCGTATCTTCCTCTT
ATCGATGTGAATTGAGGATACACCACCTTCCAATCCATCTGCAGCCACACGCATGCTTCCGCCGTTGA
+
FFFFFFFFFFFFFFFFFFFFFFFFFFFFFFFFFFFFFFFFFFFFFFFFFFFFFFFFFFFFFFFFF:FF
FFF:FFFFFFFFFFFFFFFFFFFFFFFFFFFF:FFFFFFF:FFFFFFFFFFFFFFFFFFFFFFFFFFF
@A00155:342:HHGFNDSXY:1:2645:19388:1939 1:N:0:GAACCTAG+TCCGCATA
ACTCATTAATAGCTCTGTGGCTTACTAGAGCCGCTTCACCATCATTTAACACATCGTATCTTCCTCTT
ATCGATGTGAATTGAGGATACACCACCTTCCAATCCATCTGCAGCCACACGCATGCTTCCGCCGTTGA
+
FFFFFFFFFFFFFFFFFFFFFFFFFFFFFFFFFFFFFFFFFFFFFFFFFFFFFFFFFFFFFFFFFFFF
FFFFFFFFFFFFFFFFFFFFFFFFFFFFFFFFFFFFFFFFFFFFFFFFFFFFFFFFFFFFFFFFFFFF
@A00155:342:HHGFNDSXY:1:1370:4788:14105 1:N:0:GAACCTAG+TCCGCATA
CTCATTAATAGCTCTGTGGCTTACTAGAGCCGCTTCACCATCATTTAACACATCGTATCTTCCTCTTA
TCGATGTGAATTGAGGATACACCACCTTCCAATCCATCTGCAGCCACACGCATGCTTCCGCCGTTGAT
+
FFFF:FFFFFFFFFFFFFFFFFFFFFFFFFFFFFFFFFFFFFFFFFFFFFFFFFFFFFFFFFFFFFFF
FFFFFFFFFFFFFFFFFFFFFF:FFFFFFFFFFFFFFFFFFFFFFFF:FFFFFFFFFF:FFFFFF:FF
@A00155:342:HHGFNDSXY:1:1624:20419:11992 2:N:0:GAACCTAG+TCCGCATA
CATTAATAGCTCTGTGGCTTACTAGAGCCGCTTCACCATCATTTAACACATCGTATCTTCCTCTTATC
GATGTGAATTGAGGATACACCACCTTCCAATCCATCTGCAGCCACACGCATGCTTCCGCC
+
FFFFFFFFFFFFFFFFFFFFFFFFFFFFFFFFFFFFFFFFFFFFFFFFFFFFFFFFFFFFFFFFFFFF
FFFFFFFFFFFFFFFFFFFFFFFFFFFFFFFFFFFFFFFFFFFFFFFFFFFFFFFFFFFF
@A00155:342:HHGFNDSXY:1:1477:27561:16313 2:N:0:GAACCTAG+TCCGCATA
ATTAATAGCTCTGTGGCTTACTAGAGCCGCTTCACCATCATTTAACACATCGTATCTTCCTCTTATCG
ATGTGAATTGAGGATACACCACCTTCCAATCCATCTGCAGCCACACGCATGCTTCCGCCGTTGATGG
+
FFFFF:FFFFFFFFFFFFFFFFFFFFFFFFFFFFFF,FF:FFFFFFFFFFFFFFFF:FFFFF:FFFFF
FFFFFF:FFFFFFFFFFFFFFFF:FFFFFFFFFFFFFFFFFFFFFFFFFFFFFFFFFFFFFFFFFFF
@A00155:342:HHGFNDSXY:1:1557:2790:10238 2:N:0:GAACCTAG+TCCGCATA
ATTAATAGCTCTGTGGCTTACTAGAGCCGCTTCACCATCATTTAACACATCGTATCTTCCTCTTATCG
ATGTGAATTGAGGATACACCACCTTCCAATCCATCTGCAGCCACACGCATGCTTCCGCCGTTGATGGC
+
FFFFFFFFFFFFFFFFFFFFFFFFFFFFFFFFFFFFFFFFF,FFFFFFFFFFFFFFFFFFFFFFFF:F
FFFFFFFFFFFFFFFFFFFFFFFFFFFFFFFFFFFFFFFFFFFFFFFFFFFFFFFFFFFFFFFFFFFF
@A00155:342:HHGFNDSXY:1:1511:16947:13683 2:N:0:GAACCTAG+TCCGCATA
ATTAATAGCTCTGTGGCTTACTAGAGCCGCTTCACCATCATTTAACACATCGTATCTTCCTCTTATCG
ATGTGAATTGAGGATACACCACCTTCCAATCCATCTGCAGCCACACGCATGCTTCCGCCGTTGATGGC
+
F:FFFFFFFFFFFFFFFFFFFFFFFFFFFFFFFFFFFFFFFFFFFFFFFFFFFFFFFFFFFFFFFFFF
FFFFFFFFFFFFFFFFFFFFFFF:FFFFFFFFFFFFFFFFFF:F:FFFFFFF:FFFFFFFFFFFFFFF
@A00155:342:HHGFNDSXY:1:2541:2700:17253 1:N:0:GAACCTAG+TCCGCATA
AGCTCTGTGGCTTACTAGAGCCGCTTCACCATCATTTAACACATCGTATCTTCCTCTTATCGATGTGA

```
ATTGAGGATACACCACCTTCCAATCCATCTGCAGCCACACGCATGCTTCCGCCGTTGATGGCACG
+
FFFF:FFF:FFFFFFFFFFFFFF:FFFFFFFFFFF:FFFFFF,FFFFFF:FFFFF,FF:FFFFFFFFF
FFFFFFFFFFFFF:FFF:FFF:FFFFFFFFFFFFFFFFFFFF:FFFFFFFFFFF:FFFFF:FFF:
@A00155:342:HHGFNDSXY:1:2541:2736:17190 1:N:0:GAACCTAG+TCCGCATA
AGCTCTGTGGCTTACTAGAGCCGCTTCACCATCATTTAACACATCGTGTCTTCCTCTTATCGATGTGA
ATTGAGGATACACCACCTTCCAATCCATCTGCAGCCACACGCATGCTTCCGCCGTTGATGGCACGAAC
+
FFFFFFFFFFFFFFFFFFFFFFFFFFFFFFFFFFFFFFFFFFFFFFF,FFFFFFFFFFFFFFFFFFFF
FFFFFFFFFFFFFFFFFFFFFFFFFFFFFFFFFFFFFFFFFFFFFFFFFFFFFFFFFFFFFFFFFFFF
@A00155:342:HHGFNDSXY:1:2133:30237:10300 1:N:0:GAACCTAG+TCCGCATA
GCTCTGTGGCTTACTAGAGCCGCTTCACCATCATTTAACACATCGTATCTTCCTCTTATCGATGTGAA
TTGAGGATACACCACCTTCCAATCCATCTGCAGCCACACGCATGCTTCCGCCGTTGATGGCACGAACT
+
FFFFFFFFFFFFFFFFFFFFFFFFFFFFFFFFFFFFFFFF:FFFFFFFFFFFFFFFFFFFFFFFFFFF
FFFFFFFFFFFFFFFFFFFFFFFFFF:FFFFFFFFFFFFFFFFFFFFF:FFFFFFFFFFFFFFFFFFF
@A00155:342:HHGFNDSXY:1:2243:27037:27367 1:N:0:GAACCTAG+TCCGCATA
GCTCTGTGGCTTACTAGAGCCGCTTCACCATCATTTAACACATCGTATCTTCCTCTTATCGATGTGAA
TTGAGGATACACCACCTTCCAATCCATCTGCAGCCACACGCATGCTTCCGCCGTTGATGGCACGAACT
+
FFFFFFFFFFFFFFFFFFFFFFFFFFFFFFFFFFFFFFFFFFF:FFFFFFFFFFFFFFFFFFFFFFFF
FFFFFFFFFFFFFFFFFFFFFFF:FFFFFFFFFFFFFFFFFFFFFFFFFFFFFFFFFFF,FFFFFFFF
@A00155:342:HHGFNDSXY:1:2105:6415:5087 1:N:0:GAACCTAG+TCCGCATA
GCTCTGTGGCTTACTAGAGCCGCTTCACCATCATTTAACACATCGTATCTTCCTCTTATCGATGTGAA
TTGAGGATACACCACCTTCCAATCCATCTGCAGCCACACGCATGCTTCCGCCGTTGATGGCACGAACT
+
FFFFFFFFFFFFFFFFFFFFFFFFFFFFFFFFFFFFFFFFFF:FFFFFF:F:F:FFFFFFFFF,FFFF
FFFF:FF:FFFFFFFF,FFFFFFFFFFFFFFFFFFFFFFFFF:FFFFFFFFFFF:FFFFFFFFFFFFF
@A00155:342:HHGFNDSXY:1:1552:17797:19820 1:N:0:GAACCTAG+TCCGCATA
GCTCTGTGGCTTACTAGAGCCGCTTCACCATCATTTAACACATCGTATCTTCCTCTTATCGATGTGAA
TTGAGGATACACCACCTTCCAATCCATCTGCAGCCACACGCATGCTTCCGCCGTTGATGGCACGAACT
+
FFFFFFFFFFFFFFFFFFFFFFFFFFF:FFFFFFFFFFFFFFFFFFFFFFFFFFFFFFFFFFFFFFFF
FFFFFFFFFFFFFFFFFFFFFFFFFFFFFFFFFFFFFFFFFFFFFFFFFFFFFFFFFFF:FFFFFFFF
@A00155:342:HHGFNDSXY:1:2268:19759:31015 1:N:0:GAACCTAG+TCCGCATA
CTCTGTGGCTTACTAGAGCCGCTTCACCATCATTTAACACATCGTATCTTCCTCTTATCGATGTGAAT
TGAGGATACACCACCTTCCAATCCATCTGCAGCCACACGCATGCTTCCGCCGTTGATGGCACGAAC
+
FFFFFFFFFFFFFFF:FFFFFFFFF:FFFFFFFFFFFFFFFFFFFFFFFFFFFFFFFFFFFFFFFFFF
FFFFFFFFFFFFFFFFFFFFFFFFFFFFFFFFFFFFFFFFFFFFFFFFFFFFFFFFFFFFFFFFFF
@A00155:342:HHGFNDSXY:1:1152:17824:30326 1:N:0:GAACCTAG+TCCGCATA
CTCTGTGGCTTACTAGAGCCGCTTCACCATCATTTAACACATCGTATCTTCCTCTTATCGATGTGAAT
TGAGGATACACCACCTTCCAATCCATCTGCAGCCACACGCATGCTTCCGCCGTTGATGGCACGAACTG
+
FFFFFFFFFFFFFF,FFFFFFFFFFFFFFFFFFFFFFFFFFFFFFFFFFFFFFFFFFFFFFFFFFFFF
FFFFFFFFFFFFFFFFFFFFFFFFFFFFFFFFFF,FFFFFFFFFFFFFFFFFFFFFFFFFFFFFF:FFF
@A00155:342:HHGFNDSXY:1:1247:18276:15796 2:N:0:GAACCTAG+TCCGCATA
CTCTGTGGCTTACTAGAGCCGCTTCACCATCATTTAACACATCGTATCTTCCTCTTATCGATGTGAAT
TGAGGATACACCACCTTCCAATCCATCTGCAGCCACACGCATGCTTCCGCCGTTGATGGCACGAACTG
+
FFFFFFFFFFFFFF:FFFFFFFFFFFFFF::FFFFFFFFFFFF:FFFF:FFFFFFFFFFFFFFFFFFF
FFFFFFFFFFFFFFFFFFFFFFFFFFFFFFFFFFFFFFFFFFFFFFFFFFFFFFFFFFFFFFFFFFFF
@A00155:342:HHGFNDSXY:1:2244:26323:30170 1:N:0:GAACCTAG+TCCGCATA
CTCTGTGGCTTACTAGAGCCGCTTCACCATCATTTAACACATCGTATCTTCCTCTTATCGATGTGAAT
```

```
TGAGGATACACCACCTTCCAATCCATCTGCAGCCACACGCATGCTTCCGCCGTTGATGGCACGAACTG
+
FFFFFFFFFFFFFFFFFFFFFFFFFFFFFFFFFFFFFFFFFFFFFFFFFFFFFFFFFFFFFFFFFFFF
FFFFFFFFFFFFFFFFFFFFFFFFFFFFFFFFFFFFFFFFFFFFFFFFFFFFFFFFFFFFFFFFFFFF
@A00155:342:HHGFNDSXY:1:1659:27100:3302 1:N:0:GAACCTAG+TCCGCATA
CTGTGGCTTACTAGAGCCGCTTCACCATCATTTAACACATCGTATCTTCCTCTTATCGATGTGAATTG
AGGATACACCACCTTCCAATCCATCTGCAGCCACACGCATGCTTCCGCCGTTGATGGCACGAACTGGT
+
FFFFF:FFFFFFFFF,FFFFFFFFFFFFFFFFFFFFFFFF:FFFFFFFFFFFFFFFFFF:FFFFFFF:
FFF::FFFFFFFFFFFFFF:FFFFFFFFFF:FFFFF:FFFFF,:FF:FF:F:FFFFFFFFFFFF::FF
@A00155:342:HHGFNDSXY:1:1659:26350:4382 1:N:0:GAACCTAG+TCCGCATA
CTGTGGCTTACTAGAGCCGCTTCACCATCATTTAACACATCGTATCTTCCTCTTATCGATGTGAATTG
AGGATACACCACCTTCCAATCCATCTGCAGCCACACGCATGCTTCCGCCGTTGATGGCACGAACTGGT
+
FFFFFFFFFFFFFFFFFFFFFFFFFFFFFFFFFFFFFFFFFFFFFFFFFFFF:FFFFFFFFFFFFFFF
FFFFFFFFFFFFFFFFFFFFFFFFFFFFFFFFFFFFFFFFFFFFFFFFFFFFFFFFFFFFFFFFFFFF
@A00155:342:HHGFNDSXY:1:2658:25482:29747 2:N:0:GAACCTAG+TCCGCATA
TGTGGCTTACTAGAGCCGCTTCACCATCATTTAACACATCGTATCTTCCTCTTATCGATGTGAATTGA
GGATACACCACCTTCCAATCCATCTGCAGCCACACGCATGCTTCCGCCGTTGATGGCACGAACTGGTA
+
FFFFFFFFFFFFFFFFFFFFFFFFFFFFFFFFFFFFFFFFFFFFFFFFFFFFFFFFFFFFFFFFFFFF
FFFFFFFFFFFFFFFFFFFFFFFFFFFFFFFFFFFFFFFFFF:FFFFFFFFFFFFFFFFFFFFFFFFF
@A00155:342:HHGFNDSXY:1:1430:17607:28510 1:N:0:GAACCTAG+TCCGCATA
TGTGGCTTACTAGAGCCGCTTCACCATCATTTAACACATCGTATCTTCCTCTTATCGATGTGAATTGA
GGATACACCACCTTCCAATCCATCTGCAGCCACACGCATGCTTCCGCCGTTGATGGCACGAACTGGTA
+
FFFFFF:FFFFFFFFFFFFFFFFFFFFFFFFFFFFFFFFFFFFFFFFFFFFFFFFFFFFFFFF:FFFF
FFFFFFFFFFFFFFFFFFFFFF:FFFFFFFFFFFFFFFFFFFFFFFFFFFFFFFFFFFFFFFFFFFFF
@A00155:342:HHGFNDSXY:1:1225:24062:3333 1:N:0:GAACCTAG+TCCGCATA
TGTGGCTTACTAGAGCCGCTTCACCATCATTTAACACATCGTATCTTCCTCTTATCGATGTGAATTGA
GGATACACCACCTTCCAATCCATCTGCAGCCACACGCATGCTTCCGCCGTTGATGGCACGAACTGGTA
+
FFFFFFF:FFFFFFFFFFFFFFFFFFFFFFFFFFFFFFFFFFFFFFFFFFFFFFFFFFFFFFFFFFFF
FFFFFFFFFFFFFFFFFFF:FFFFFFFFFFFFFFFFFFFFFFFFFFFFFFFFFFFFFFFFFFFFFFFF
@A00155:342:HHGFNDSXY:1:2221:18900:35978 2:N:0:GAACCTAG+TCCGCATA
TGTGGCTTACTAGAGCCGCTTCACCATCATTTAACACATCGTATCTTCCTCTTATCGATGTGAATTGA
GGATACACCACCTTCCAATCCATCTGCAGCCACACGCATGCTTCCGCCGTTGATGGCACGAACTGGTA
+
FFFFF::FFFFFFFFFFF,FFFFF,FFFFFFFFFFFFFFFFFFFFFFFFFFF,FFF::FFFFFFFFFF
FFF,FFFFFFFFFFFF:FFFFFFFFFFFFFFFFFFF:FFFF:FFFFFFFFFFFFF:FFFFFF:FFFFF
@A00155:342:HHGFNDSXY:1:1405:14136:14606 2:N:0:GAACCTAG+TCCGCATA
TGTGGCTTACTAGAGCCGCTTCACCATCATTTAACACATCGTATCTTCCTCTTATCGATGTGAATTGA
GGATACACCACCTTCCAATCCATCTGCAGCCACACGCATGCTTCCGCCGTTGATGGCACGAACTGGTA
+
FFFFFFFFFFFFFFFFFFFFFFFFF,FFFFFFFFFFFFFFFFFFFFFFFFFFFFFFF:FFFFFFFFFF
FFFFFFFFFFFFFFFFF:FFFFFFFFF:FFFFFFFFFFFFFFFFFFFFFFFFFFFFFFFFFFFFFFFF
@A00155:342:HHGFNDSXY:1:1569:24867:9580 2:N:0:GAACCTAG+TCCGCATA
TGTGGCTTACTAGAGCCGCTTCACCATCATTTAACACATCGTATCTTCCTCTTATCGATGTGAATTGA
GGATACACCACCTTCCAATCCATCTGCAGCCACACGCATGCTTCCGCCGTTGATGGCACGAACTGGTA
+
FFFFFF,FFFFFFFFFFFFFFFFFFFFFFFFFFFFFFFFFFFFFFFFFFFFFFFFFFFFFFFFFFFFF
FFFFFFFFFFFFFFFFFFFFFFFFFFFFFFFFFFFFFFFFF,FFFFFFFFFFFFFFFFFFFFFFFFFF
@A00155:342:HHGFNDSXY:1:2565:25238:31015 2:N:0:GAACCTAG+TCCGCATA
GTGGCTTACTAGAGCCGCTTCACCATCATTTAACACATCGTATCTTCCTCTTATCGATGTGAATTGAG
```

GATACACCACCTTCCAATCCATCTGCAGCCACACGCATGCTTCCGCCGTTGATGGCACGAACTGGTAT
+
FFFFFFFFFFFFFFFFFFFFFFFFFFFFFFFFFF:FFFFF:FFFFFFFFFFFFFFF:FFFFFFFFFFF
FFFFFFFFFFFFFFFFFFFFFFFFFFFFFF:FFFFFFFFF:FFFFFFFFFFFFFFFFFFFFFFFFFFF
@A00155:342:HHGFNDSXY:1:1430:17607:28510 2:N:0:GAACCTAG+TCCGCATA
GTGGCTTACTAGAGCCGCTTCACCATCATTTAACACATCGTATCTTCCTCTTATCGATGTGAATTGAG
GATACACCACCTTCCAATCCATCTGCAGCCACACGCATGCTTCCGCCGTTGATGGCACGAACTGGTAT
+
FFFFFF:FFFFFFFFFF,FF,F,FFFFFFFFFFFFFFFFFFFFFFFFFFFFFFFFFFFFFFFFFFFFF
FFFFFFFFFFFFFFFFFFFFFFFFFFF:FFFFFFFFFFFFFFFFFFFFFFFFFFFFFFFFFFFFFFFF
@A00155:342:HHGFNDSXY:1:2619:11270:29309 2:N:0:GAACCTAG+TCCGCATA
GTGGCTTACTAGAGCCGCTTCACCATCATTTAACACATCGTATCTTCCTCTTATCGATGTGAATTGAG
GATACACCACCTTCCAATCCATCTGCAGCCACACGCATGCTTCCGCCGTTGATGGCACGAACTGGTAT
+
FFFFFFFFFFFFFFFFF::FFFFFFFFFFFFFFFF:FFFFF:FFFFFFFFFFFFFFFFFFFFFFFFFF
FF:FFFFFFFFFFFFFFFFFFFFFFFFFFFFFFFFFFFFFFFFFFFFFFFFFFFFFFFFFFFFFFFFF
@A00155:342:HHGFNDSXY:1:1225:24062:3333 2:N:0:GAACCTAG+TCCGCATA
GTGGCTTACTAGAGCCGCTTCACCATCATTTAACACATCGTATCTTCCTCTTATCGATGTGAATTGAG
GATACACCACCTTCCAATCCATCTGCAGCCACACGCATGCTTCCGCCGTTGATGGCACGAACTGGTAT
+
FFFFFFFFFFFFFFFFFFFFFFFFFFFFFFFFFFFFFFFFFFFFFFFFFFFFFFFFFFFFFFFFFFFF
FFFFFFFFFFFFFFFFFFFFFFFFFFFFFF:FFFFFFFFF,FFFFFFFFFFFFFFFFFFFFFFFFFFF
@A00155:342:HHGFNDSXY:1:1247:18276:15796 1:N:0:GAACCTAG+TCCGCATA
GTGGCTTACTAGAGCCGCTTCACCATCATTTAACACATCGTATCTTCCTCTTATCGATGTGAATTGAG
GATACACCACCTTCCAATCCATCTGCAGCCACACGCATGCTTCCGCCGTTGATGGCACGAACTGGTAT
+
FFFFFF:FFFFFFFFFFFFFFFFFFFFFF:FFFFFFFFFFFFFFFFFFFFFFFFFFFFFFFFFFFFFF
FFFFFFFFFFFFFFFFFFFFFFFFFFFFFFFF,FFFFFFFFFFF:FFFFFFF:FFFFFFFFF:F:FFF
@A00155:342:HHGFNDSXY:1:1624:20419:11992 1:N:0:GAACCTAG+TCCGCATA
GGCTTACTAGAGCCGCTTCACCATCATTTAACACATCGTATCTTCCTCTTATCGATGTGAATTGAGGA
TACACCACCTTCCAATCCATCTGCAGCCACACGCATGCTTCCGCCGTTGATGGCACGAAC
+
FFFFFFFFFFFFFFFFFFFFFFFFFFFFFFFFFFFFFFFFFFFFFFFFFFFFFFFFFFFFFFFFFFFF
FFFFFFFFFFFFFFFFFFFFFFFFFFFFFFFFFFFFFFFFFFFFFFFFFFFFFFFFFFFF
@A00155:342:HHGFNDSXY:1:1330:3495:36980 1:N:0:GAACCTAG+TCCGCATA
CTTACTAGAGCCGCTTCACCATCATTTAACACATCGTATCTTCCTCTTATCGATGTGAATTGAGGATA
CACCACCTTCCAATCCATCTGCAGCCACACGCATGCTTCCGCCGTTGATGGCACGAACTGG
+
FFFFFFFFFFFFFFFFFFFFFFF:FFFFFFFFFF:FFFFFFF:FFFFFFFFFFFFFFFFFFFFFFFFF
FFFFFFF:FFFFFFFFFFFFFFFFFFFFFFFFFFFFFFFFFFFFFFFFFFFFFFFFFFFFF
@A00155:342:HHGFNDSXY:1:1608:20211:24690 2:N:0:GAACCTAG+TCCGCATA
TACTAGAGCCGCTTCACCATCATTTAACACATCGTATCTTCCTCTTATCGATGTGAATTGAGGATACA
CCACCTTCCAATCCATCTGCAGCCACACGCATGCTTCCGCCGTTGATGGCACGAACTGGTATGCCGCA
+
FFFFFFFFFFFF:FFFFFFFF,FFFFF:,F:FFFFFFFFFFFFFFFFFFFFFFFFFFFFFFFFF:FFF
FFFFFFFFFFFFFFFFFFFFFFFFFFFFFFFFFF:FFFFFFFFFFFFFFFFFFFFFFFFFFFFFFFFF
@A00155:342:HHGFNDSXY:1:1502:16676:33473 2:N:0:GAACCTAG+TCCGCATA
TACTAGAGCCGCTTCACCATCATTTAACACATCGTATCTTCCTCTTATCGATGTGAATTGAGGATACA
CCACCTTCCAATCCATCTGCAGCCACACGCATGCTTCCGCCGTTGATGGCACGAACTGGTATGCCGCA
+
FFFFFFFFFF:FFF:F,FFFFFFFFFFFFFFFFFFFFFFFF,FFFFFFFFFFFFFFFFFFFFFFFFFF
FFFFFFFFFFFFFFFFFFFFFF:FFFFFF:FFFFFFFFFFFFFFFFFFFFFFFFFFFFFFFFFFFFFF
@A00155:342:HHGFNDSXY:1:1322:8440:35180 2:N:0:GAACCTAG+TCCGCATA
ACTAGAGCCGCTTCACCATCATTTAACACATCGTATCTTCCTCTTATCGATGTGAATTGAGGATACAC

```
CACCTTCCAATCCATCTGCAGCCACACGCATGCTTCCGCCGTTGATGGCACGAACTGGTATGCCGCAA
+
FFFFFFFFF,FFFFFFFFFFFFFFFFFFFFF:FFFFFFFFFF:FFFFFFFFFFFFFFFFFFFFFFFFF
FFFFFFFFFFFFFFFFFFFF,FFFFFFFFFFFFFFFFFF:FFFFFFFFFFFFFFFFFFF:FFFFFFFF
@A00155:342:HHGFNDSXY:1:2508:13548:2472 2:N:0:GAACCTAG+TCCGCATA
ACTAGAGCCGCTTCACCATCATTTAACACATCGTATCTTCCTCTTATCGATGTGAATTGAGGATACAC
CACCTTCCAATCCATCTGCAGCCACACGCATGCTTCCGCCGTTGATGGCACGAACTGGTATGCCGCAA
+
FFFFFFFFFFFFFFFFFFFFFFFFFFFFFFFFFFFFFFFFFFF:FFFFFFFFFFFFFFFFFFFF:FFF
FFFFFFFFFFFFFFFFFFFFFFFFFFFFFFFFFFFFFFFFFFFFFFFFFFFFFFFFFFFFFFFFFFFF
@A00155:342:HHGFNDSXY:1:1659:27100:3302 2:N:0:GAACCTAG+TCCGCATA
CTAGAGCCGCTTCACCATCATTTAACACATCGTATCTTCCTCTTATCGATGTGAATTGAGGATACACC
ACCTTCCAATCCATCTGCAGCCACACGCATGCTTCCGCCGTTGATGGCACGAACTGGTATGCCGCAAA
+
FFFFFFFFFFFFFFFFFFFFFFFFFFFFFFFFFFFFFFFFFFFFFFFFFFFFFFFFFFFFFFFFFFFF
:FFFFFFFFFFFFFFFFFFFFF:FFFFFFFFF:FFFFFFFF:FFFFFFFFFFFFFFFFFFF:FFFFFF
@A00155:342:HHGFNDSXY:1:1659:26350:4382 2:N:0:GAACCTAG+TCCGCATA
CTAGAGCCGCTTCACCATCATTTAACACATCGTATCTTCCTCTTATCGATGTGAATTGAGGATACACC
ACCTTCCAATCCATCTGCAGCCACACGCATGCTTCCGCCGTTGATGGCACGAACTGGTATGCCGCAAA
+
FFFFFFFFFFFFFF:FFFFFFFFFFFFFFFFFFF:FFFFFFFFF:FFFFFFFFFFFFFFFFFFFFFFF
FFFFFFFFFFFFFFFFFFFFFFFFFFFFFFFFFFFFFFFFFFFFFFFFFFFFFFFFFFFFFFFFFFFF
@A00155:342:HHGFNDSXY:1:2471:28836:36401 2:N:0:GAACCTAG+TCCGCATA
CTAGAGCCGCTTCACCATCATTTAACACATCGTATCTTCCTCTTATCGATGTGAATTGAGGATACACC
ACCTTCCAATCCATCTGCAGCCACACGCATGCTTCCGCCGTTGATGGCACGAACTGGTATGCCGCAAA
+
FFFFFFFFFFFFFFFFFFFFFFFFFFFFFFFFFFFFFFFFF,FFFFFFFFFFFFFFFFFFFFFFFFFF
FFFFFFFFFFFFFFFFFFFFFFFFFFFFFFFFFFFFFFFFFFFFFFFFFFFFFFFFFFFFFFFFFFFF
@A00155:342:HHGFNDSXY:1:2123:22535:32534 2:N:0:GAACCTAG+TCCGCATA
AGCCGCTTCACCATCATTTAACACATCGTATCTTCCTCTTATCGATGTGAATTGAGGATACACCACCT
TCCAATCCATCTGCAGCCACACGCATGCTTCCGCCGTTGATGGCACGAACTGGTATGCC
+
FFFFFFFFFFFFFFFFFFFFFFFFFFFFFFFFFFFFFFFFFFFFFFFFFFFFFFFFFF:FFFFFFFFF
FFFFF:FFFFFFFFFFFFFFFFFFFFFFFFFFFFFFFFFFFFFFFFFFFFFFFFFFFFF
@A00155:342:HHGFNDSXY:1:2244:26323:30170 2:N:0:GAACCTAG+TCCGCATA
CGCTTCACCATCATTTAACACATCGTATCTTCCTCTTATCGATGTGAATTGAGGATACACCACCTTCC
AATCCATCTGCAGCCACACGCATGCTTCCGCCGTTGATGGCACGAACTGGTATGCCGCAAAACCTATG
+
F:F:FFFFFFFFFFFFFFFFFFFFF,FFFFFFFFFFFFFFFFFFFFFFFFFFFFFFFFFFFFFFFFFF
FF,FFFFFFFFFFFFFFFFFFFFFFFFFFFFFFFFFFFFFFFFFFFFFFFFFFFFFFFFFFFFFFFFF
@A00155:342:HHGFNDSXY:1:1502:16676:33473 1:N:0:GAACCTAG+TCCGCATA
CGCTTCACCATCATTTAACACATCGTATCTTCCTCTTATCGATGTGAATTGAGGATACACCACCTTCC
AATCCATCTGCAGCCACACGCATGCTTCCGCCGTTGATGGCACGAACTGGTATGCCGCAAAACCTATG
+
FFFFFFFFFFFFFFFFFFFFFFFFFFFFFFFFFFFFFFFFFFFFFFFFFFFF:FFFFFFFFFFFFFFF
FFFFFFFFFFFFFFFFFFFFFF:FFFFFFFFFFFFFFFFFFFFFFFFFFFFFFFFFFFF:FFFFFFFF
@A00155:342:HHGFNDSXY:1:1429:32841:34757 2:N:0:GAACCTAG+TCCGCATA
CGCTTCACCATCATTTAACACATCGTATCTTCCTCTTATCGATGTGAATTGAGGATACACCACCTTCC
AATCCATCTGCAGCCACACGCATGCTTCCGCCGTTGATGGCACGAACTGGTATGCCGCAAAACCTATG
+
FFFFFFFFFFFFFFFF:FFFF:FFFFFFF:FF:FFFFFFFFFFFFFF:FFFFFFFFFFFFF:FFFF:F
FFFFFFFFFFFFFFFFFFFFFFFF:,FFF:FFFFFFFFFFFFFFFFFFFFFFFFFFFFFFFFFFFFFF
@A00155:342:HHGFNDSXY:1:1269:12066:32127 2:N:0:GAACCTAG+TCCGCATA
CGCTTCACCATCATTTAACACATCGTATCTTCCTCTTATCGATGTGAATTGAGGATACACCACCTTCC
```

```
AATCCATCTGCAGCCACACGCATGCTTCCGCCGTTGATGGCACGAACTGGTATGCCGCAAAACCTATG
+
FFFFFFFFFFFFFFF:FFFFFFFFFFFFFFFFFFFFFF:FFF:FFFFF:FFFFFFFFFFFFFFFFFFFF
FF:FFFFFF:FFFFFFFFFFFFFFFFFFFFFFFFFFFFFFFFFFFFFFFFFFFFFFFFFF:FFFFFFF
@A00155:342:HHGFNDSXY:1:1608:20211:24690 1:N:0:GAACCTAG+TCCGCATA
CGCTTCACCATCATTTAACACATCGTATCTTCCTCTTATCGATGTGAATTGAGGATACACCACCTTCC
AATCCATCTGCAGCCACACGCATGCTTCCGCCGTTGATGGCACGAACTGGTATGCCGCAAAACCTATG
+
FFFFFFFFFFFFFF:FFFFFF,FFFFFFFFFFFFFF:FFFFFFFFFFFFFFFFFFFFFFFFFFFFFFF
FFFFFFFFFFFFFFFFFFFFFFFFFFFFFFFFFFFFFFFFFFFFFFFFFF:FFFFFFFFF,FFFFFF
@A00155:342:HHGFNDSXY:1:2364:32931:29997 2:N:0:GAACCTAG+TCCGCATA
ACCATCATTTAACACATCGTATCTTCCTCTTATCGATGTGAATTGAGGATACACCACCTTCCAATCCA
TCTGCAGCCACACGCATGCTTCCGCCGTTGATGGCACGAACTGGTATGCCGCAAAACCTATGATGTAC
+
FFFFFFFFFFFFFFFFFFFFFFFFFFFFFFFFFFFFFFFFFFFFFFFFFFFFFFFFFFFFFFFFFFFF
FFFFFFFFF:FFFFFFFFFFFFFFFFFFFFFFFFFFFFFFFFFFFFFFFFFFFFFFFFFFFFFFFFFF
@A00155:342:HHGFNDSXY:1:2139:21251:15906 2:N:0:GAACCTAG+TCCGCATA
CATCATTTAACACATCGTATCTTCCTCTTATCGATGTGAATTGAGGATACACCACCTTCCAATCCATC
TGCAGCCACACGCATGCTTCCGCCGTTGATGGCACGAACTGGTATGCCGCAAAACCTATGATGTACAG
+
FFFFFFFFFFFFFFFFF,FFFFFFFFFF,FFF,FFFFFF:FFFFFFFFFFFFFFFFFFFFF,FFFFFF
FFFFFFFFFFFFFFFFF:FFFFFFFFF:FFFFFFFFFFFFFFFFFFFFFFFFFFFFFFFFFFFFFFFF
@A00155:342:HHGFNDSXY:1:1406:9959:26287 2:N:0:GAACCTAG+TCCGCATA
ATCATTTAACACATCGTATCTTCCTCTTATCGATGTGAATTGAGGATACACCACCTTCCAATCCATCT
GCAGCCACACGCATGCTTCCGCCGTTGATGGCACGAACTGGTATGCCGCAAAACCTATG
+
FFFFFFFFFFFF:FFFFFFFFFFFFFFFFFFF:FFFFFFFFFFFFFFFFFFFFFFFFFFFFFFFFFFF
FFFFFFFFFFFFFFFFFFFFFFFFFFFFFFFFFFFFFFFFFFFFFFFFFFFFFFFFFFF
@A00155:342:HHGFNDSXY:1:1156:14579:36542 2:N:0:GAACCTAG+TCCGCATA
ATCATTTAACACATCGTATCTTCCTCTTATCGATGTGAATTGAGGATACACCACCTTCCAATCCATCT
GCAGCCACACGCATGCTTCCGCCGTTGATGGCACGAACTGGTATGCCGCAAAACCTATGATGTACAGG
+
FFFFF:FFFFFF:FFFFFFFFFFFFFFFFFFFFFFFFFFFFFFFFFFFFFFF:FFFFFFFFFFFFFFF
FFFFFF:F,FFFFFFF:FFFFFFFFFFFFFFFFFFFFFFFFFFFFFFFFFFFFFFFFFFFFFFFFFFF
@A00155:342:HHGFNDSXY:1:1330:3495:36980 2:N:0:GAACCTAG+TCCGCATA
TCATTTAACACATCGTATCTTCCTCTTATCGATGTGAATTGAGGATACACCACCTTCCAATCCATCTG
CAGCCACACGCATGCTTCCGCCGTTGATGGCACGAACTGGTATGCCGCAAAACCTATGATGTACAGGG
+
FFFFFFFFFFFFFFFFFFFFFFFFFFFFFFF,FFFFFFFFFFFFFFFFFFFFFFFFFFFFFFFFFF:F
FFFFF,FFFFFFFFFFFFFFFFFFFFFFFFFFFFFFFFFFFFFFFFFFFFFFF:FFFFFFFFFFFF
@A00155:342:HHGFNDSXY:1:2374:2510:28573 2:N:0:GAACCTAG+TCCGCATA
TCATTTAACACATCGTATCTTCCTCTTATCGATGTGAATTGAGGATACACCACCTTCCAATCCATCTG
CAGCCACACGCATGCTTCCGACGTTGATGGCACGAACTGGTATGCCGCAAAACCTATGATGTACAGGG
+
F,FFFFFFFFFFFF,FF,:FFFFFFFFFF:FFFFFFFFFFFFFFFFFFFFFFF:FFF:FFFFFFF:FFF
FFFF::FFF,FFFFFFFF,,,FFFF,:FF:F,FFFFFF,FFFFFFFFFFFFFFFFFFF,FFFFFFFFF
@A00155:342:HHGFNDSXY:1:1177:18611:23578 2:N:0:GAACCTAG+TCCGCATA
TCATTTAACACATCGTATCTTCCTCTTATCGATGTGAATTGAGGATACACCACCTTCCAATCCATCTG
CAGCCACACGCATGCTTCCGCCGTTGATGGCACGAACTGGTATGCCGCAAAACCTATGATGTACAGGG
+
FFFFFFFFFFFFFFFFFFFFFFFFFFFFFFFFFFFFFFFFF:FF:FFFFFF,FFFFFFFFFFFFFFF
FFFFFFFFFFFFFF::FFFFFFFFFFFFFFFFFFFFFFFFFFFFFFFFFFFFFFFFFFFFFFFFFFFF
@A00155:342:HHGFNDSXY:1:2147:28420:28040 2:N:0:GAACCTAG+TCCGCATA
CATTTAACACATCGTATCTTCCTCTTATCGATGTGAATTGAGGATACACCACCTTCCAATCCATCTGC
```

```
AGCCACACGCATGCTTCCGCCGTTGATGGCACGAACTGGTATGCCGCAAAACCTATGATGTACAGGGC
+
FFFFFFFFFFFFFF:FFF:FFFFFFF:FFFFFFFFFFFFFFFFFFFFFFFFFFFFFFFFFFFFFFFFF
FFFFFFFFFFFFFF:FFFFFFFFFFFFFFFFFFFFFFFFFFFFFFFFFFFFFFFFFFFFFFFFFFFFF
@A00155:342:HHGFNDSXY:1:1427:31602:28729 2:N:0:GAACCTAG+TCCGCATA
CATTTAACACATCGTATCTTCCTCTTATCGATGTGAATTGAGGATACACCACCTTCCAATCCATCTGC
AGCCACACGCATGCTTCCGCCGTTGATGGCACGAACTGGTATGCCGCAAAACCTATGATGTACAGGGC
+
FFFFFFFFF::FFF:FFFFFFFFFFFFFFFFFFFFFFFFFFFFFFFFFFFFFFFFFFFFFFFFFFFFF
FFFF,FFFFFFFFFFFFFFFFFFFFFFFFFFFFFFFFFFFFFFFFFFFFFFFFF,FFFFFFFFFFFFF
@A00155:342:HHGFNDSXY:1:2607:19741:17143 2:N:0:GAACCTAG+TCCGCATA
CATTTAACACATCGTATCTTCCTCTTATCGATGTGAATTGAGGATACACCACCTTCCAATCCATCTGC
AGCCACACGCATGCTTCCGCCGTTGATGGCACGAACTGGTATGCCGCAAAACCTATGATGTACAGGGC
+
FFFFFFFFFFFFFFFFFFFFFFFFFF:FFFFFF:FFFFFFFFFFFFFFFFFFFFFFFFFFFFFFFFFF
FFFFFFFFFFFFFFFFFFFFFFFFFFFFFFFFFFFFFFFFFFFFFFFFFFFFFFF:FFFFFFFFFFFF
@A00155:342:HHGFNDSXY:1:1325:31729:18427 2:N:0:GAACCTAG+TCCGCATA
CATTTAACACATCGTATCTTCCTCTTATCGATGTGAATTGAGGATACACCACCTTCCAATCCATCTGC
AGCCACACGCATGCTTCCGCCGTTGATGGCACGAACTGGTATGCCGCAAAACCTATGATGTACAGGGC
+
FFFFFFFFFFFFFF:FFFFFFFFFFFFFFFFFFFFFFFFFFFFFFFFFFFFFFFFFFFFFFFFFFFFF
FFFFFFFFFFFFFF:FFFFFFFFFFFFFFFFFFFFFFFFFFFFFFFFFFFFFFFF:FFFFFFFFFFFF
@A00155:342:HHGFNDSXY:1:2123:23041:32972 1:N:0:GAACCTAG+TCCGCATA
ATTTAACACATCGTATCTTCCTCTTATCGATGTGAATTGAGGATACACCACCTTCCAATCCATCTGCA
GCCACACGCATGCTTCCGCCGTTGATGGCACGAACTGGTATGCCGCAAAACCTATGATG
+
FFFFFFFFFFFFFFFFFFFFFFFFFFFFFFFFFFFFFFFFFFFFFFFFFFFFFFFFFFFFFFFFFFFF
FFFFFFFFFFFFFFFFFFFFFFFFFFFFFFFFFFFFFFFFFFFFFFFFFFFFFFFFFFF
@A00155:342:HHGFNDSXY:1:2123:22535:32534 1:N:0:GAACCTAG+TCCGCATA
ATTTAACACATCGTATCTTCCTCTTATCGATGTGAATTGAGGATACACCACCTTCCAATCCATCTGCA
GCCACACGCATGCTTCCGCCGTTGATGGCACGAACTGGTATGCCGCAAAACCTATGATG
+
FFFFFFFFFFFFFFFFFFFFFFF:FFFFFFFFFFFFFFFFFFFFFFFFFFFFFFFFFFFFFFFFFFFF
FFFFFFFFFFFFFFFFFFFFFFFFFFFFFFFFF,FFFFFFFFFFFFFFFFFFFFFFFFF
@A00155:342:HHGFNDSXY:1:1316:29053:34147 1:N:0:GAACCTAG+TCCGCATA
ATTTAACACATCGTATCTTCCTCTTATCGATGTGAATTGAGGATACACCACCTTCCAATCCATCTGCA
GCCACACGCATGCTTCCGCCGTTGATGGCACGAACTGGTATGCCGCAAAACCTATGATGTACAGGGCT
+
FFFFFFFFFFFFFFFFFFFFFFF,:FFFFFFFFFFFFFFFFFFFFFFFFFFFFFFFFFF:FFFFFFFF
FFFFFFFFFFFFFFFFFFFFFF:FFFFFFFFFFFF:FFFFFFFFFFFFFFFFFFFFFFFFFFFFF:FF
@A00155:342:HHGFNDSXY:1:2312:27290:5040 1:N:0:GAACCTAG+TCCGCATA
ATTTAACACATCGTATCTTCCTCTTATCGATGTGAATTGAGGATACACCACCTTCCAATCCATCTGCA
GCCACACGCATGCTTCCGCCGTTGATGGCACGAACTGGTATGCCGCAAAACCTATGATGTACAGGGCT
+
FFFFFFFFFFFFFFFFFFFFFFFFFFFFFFFFFFFFFFFFFFFFFFFFFFFFFFFFFFFFFFFFFFFF
FFFFFFFFFFFFFFFFFFFFFFFFFFFFFFFFFFFFFFFFFFFFFFFFFFFFFFFFFFFFFFFFFFFF
@A00155:342:HHGFNDSXY:1:2210:26205:20760 1:N:0:GAACCTAG+TCCGCATA
ATTTAACACATCGTATCTTCCTCTTATCGATGTGAATTGAGGATACACCACCTTCCAATCCATCTGCA
GCCACACGCATGCTTCCGCCGTTGATGGCACGAACTGGTATGCCGCAAAACCTATGATGTACAGGGCT
+
FFFFFFFFFFFFFFFFFFFFFFFFFFFFFFFFFFFFFFFFFFFFFFFFFFFFFFFFFFFFFFFFFFFF
FFFFFFFFFFFFFFFFFFFFFF:FFF:FFFFFFFFFFFFFFFFFFFFFFFFF:FFFFFFFF:FFFFFF
@A00155:342:HHGFNDSXY:1:2526:28501:29434 1:N:0:GAACCTAG+TCCGCATA
ATTTAACACATCGTATCTTCCTCTTATCGATGTGAATTGAGGATACACCACCTTCCAATCCATCTGCA
```

GCCACACGCATGCTTCCGCCGTTGATGGCACGAACTGGTATGCCGCAAAACCTATGATGTACAGGGCT
+
FFFFFFFFFFFFFFFFFFFFFFFFFFFFFFFFFFFFFFFFFFFFFFFFFFFFFFFFFFFFFFFFFFFF
FFFFFFFFFFFFFFFFFFFFFFFFFFFFFFFFFFFFFFFFFFFFFFFFFFFFFFFFFFFF:FFFFFFF
@A00155:342:HHGFNDSXY:1:2607:19741:17143 1:N:0:GAACCTAG+TCCGCATA
ATTTAACACATCGTATCTTCCTCTTATCGATGTGAATTGAGGATACACCACCTTCCAATCCATCTGCA
GCCACACGCATGCTTCCGCCGTTGATGGCACGAACTGGTATGCCGCAAAACCTATGATGTACAGGGCT
+
FFFFFFFFFFFFFFFFFFFFFFFFFFFFFFFFFFFFFFFFFFFFFFFFFFFFFFFFFFFFFFFFFFFF
FFFFFFFFFF:FFFFFFFFFFFFFFFFFFFFFFFFFFFFFFFFFFFFFFFFFFFFFFFFFFFFFFFFF
@A00155:342:HHGFNDSXY:1:2172:12418:24815 1:N:0:GAACCTAG+TCCGCATA
TTTAACACATCGTATCTTCCTCTTATCGATGTGAATTGAGGATACACCACCTTCCAATCCATCTGCAG
CCACACGCATGCTTCCGCCGTTGATGGCACGAACTGGTATGCCGCAAAACCTATGATGTACAGGGCTG
+
FFFFFFFFFFFFFFFFFFFFFFFFFFFFFFFFFFF:FFFFFFFFFFFFFFFFFFFFFFFFFFFFFFFF
FFFFFFFFFFFFFFFFFFFFFFFFFFFFFFFFFFFFFFFFFFFFFFFFFFFF:FFFFFFFFFFFFFFF
@A00155:342:HHGFNDSXY:1:1325:31729:18427 1:N:0:GAACCTAG+TCCGCATA
TTTAACACATCGTATCTTCCTCTTATCGATGTGAATTGAGGATACACCACCTTCCAATCCATCTGCAG
CCACACGCATGCTTCCGCCGTTGATGGCACGAACTGGTATGCCGCAAAACCTATGATGTACAGGGCTG
+
FFFFFFFFFFFFF:FFFFFFFF:FFFFFFFFFFF,FFFFF,FFFFFFFFFFFFFFFFFFFFFFFFFFF
FF,FFFFFFFFFFFFFFFFFFFFFFFFFF:FFFF:FFF,FFFFFFFFFFFFFFFFFFFFFFFFF:FFF
@A00155:342:HHGFNDSXY:1:1334:1542:4006 1:N:0:GAACCTAG+TCCGCATA
TTTAACACATCGTATCTTCCTCTTATCGATGTGAATTGAGGATACACCACCTTCCAATCCATCTGCAG
CCACACGCATGCTTCCGCCGTTGATGGCACGAACTGGTATGCCGCAAAACCTATGATGTACAGGGCTG
+
FFFF:F:FFFFFFFFFFFFFFFFFFFFFFFFFFFFFFF,F:FFFFFFFFFFFF:FFFFFFFFFFFFFF
FFFF:FFF:FFFFFFFFFFFFFFFFFFF::F:FFFFFF,FFF:F:FFF,FFFFFFFFFFFFFFFFFFF
@A00155:342:HHGFNDSXY:1:1156:1280:25880 1:N:0:GAACCTAG+TCCGCATA
TTTAACACATCGTATCTTCCTCTTATCGATGTGAATTGAGGATACACCACCTTCCAATCCATCTGCAG
CCACACGCATGCTTCCGCCGTTGATGGCACGAACTGGTATGCCGCAAAACCTATGATGTACAGGGCTG
+
FFFFFFFFFFFFFFFFFFFF:FFFFFFFFFFFFF,FFFF:FFFFFFFFFFF,FFFF:FFFFFF:FFFF
F:FFFFFF:FFFFFFFFFFFFFFFFF:FFF::FFFF:,FFFFFFFF,FFFFFFFFFFFFFFFFFFFF:
@A00155:342:HHGFNDSXY:1:1427:31602:28729 1:N:0:GAACCTAG+TCCGCATA
TTTAACACATCGTATCTTCCTCTTATCGATGTGAATTGAGGATACACCACCTTCCAATCCATCTGCAG
CCACACGCATGCTTCCGCCGTTGATGGCACGAACTGGTATGCCGCAAAACCTATGATGTACAGGGCTG
+
FFFFFFFFFFFFFFFFFFFFFFFF:FFFFFFFFFFFFFFFFFFFFFFFFFFFFFFFFFFFFFFFFFFF
FFFFFFFFFFFFFFFFFFFFFFFFFFFFFFFFFFFFFFFFFFFFFFFFFFFF:FFFFFFFFFFFFFFF
@A00155:342:HHGFNDSXY:1:2147:28420:28040 1:N:0:GAACCTAG+TCCGCATA
TAACACATCGTATCTTCCTCTTATCGATGTGAATTGAGGATACACCACCTTCCAATCCATCTGCAGCC
ACACGCATGCTTCCGCCGTTGATGGCACGAACTGGTATGCCGCAAAACCTATGATGTACAGGGCTGCT
+
FFFFFFFFFFFFFFFFFFFFFFFFFFFFFFFFFFFFFFFFFFFFFFFFFFFFFFFFFFFFFFFFFFFF
FFFFFFFFFFFFFFFFFFFFFFFFFFFFFFFFFFFFFFFFFFFFFFFFF:FFFFFFFFFFFFFFFFFF
@A00155:342:HHGFNDSXY:1:2516:27407:14074 1:N:0:GAACCTAG+TCCGCATA
TAACACATCGTATCTTCCTCTTATCGATGTGAATTGAGGATACACCACCTTCCAATCCATCTGCAGCC
ACACGCATGCTTCCGCCGTTGATGGCACGAACTGGTATGCCGCAAAACCTATGATGTACAGGGCTGCT
+
FFFFFFFFFFFFFFFFFFFFFFFFFFFFFFFFFFFFFFFFFFFFFFFFFFFFFFFFFFFFFFFFFFFF
FFFFFFFFFFFFFFFFFFFFFFFFFFFFFFFFFFFFFFFFFFFFFFFFFFFFFFFFFFFFFFFFFFFF
@A00155:342:HHGFNDSXY:1:2366:18936:35321 1:N:0:GAACCTAG+TCCGCATA
CATCGTATCTTCCTCTTATCGATGTGAATTGAGGATACACCACCTTCCAATCCATCTGCAGCCACACG

```
CATGCTTCCGCCGTTGATGGCACGAACTGGTATGCCGCAAAACCTATGATGTACAGGGCTGCTGAAAA
+
FFFFFFFFFFFFFFFFFFFFFFFFFFFFFFFFFFFFFFFFFFFFFFFFFFFFFFFFFFFFFFFFFFFF
FFFFFFFFFFFFFFFFFFFFFFFFFFFFFFFFFFFFFFFFFFFFFF,FFFFFFFFFFFFFFFFFFFFF
@A00155:342:HHGFNDSXY:1:2142:2067:24048 2:N:0:GAACCTAG+TCCGCATA
ATCGTATCTTCCTCTTATCGATGTGAATTGAGGATACACCACCTTCCAATCCATCTGCAGCCACACGC
ATGCTTCCGCCGTTGATGGCACGAACTGGTATGCCGCAAAACCTATGATGTACAGGGCTGCTGAAAAC
+
:FFFFFFFFFFFFFFFFFFFFFFF:FFFFFFFFFFFFFFFFFFFFFFFFFFFFFFFFFFFFFFFFF:F
FFFFFFFFFFFFFFFFFFFFFFFFFFFFFFFFFFFFFFFFFFFFFFFFFFFFFFFFFFFFFFFFFFFF
@A00155:342:HHGFNDSXY:1:2526:28501:29434 2:N:0:GAACCTAG+TCCGCATA
TCGTATCTTCCTCTTATCGATGTGAATTGAGGATACACCACCTTCCAATCCATCTGCAGCCACACGCA
TGCTTCCGCCGTTGATGGCACGAACTGGTATGCCGCAAAACCTATGATGTACAGGGCTGCTGAAAACT
+
FFFFFFFFFFFFFFFFFFFFF:FFFFFFFFFFFFFFFFFFFFFFFFFFFFFFFFFFFFFFF:FFFFFF
FFF:FFFFFFFFFFFFFFFFFFFFFFFFFFFFFFFFFFFFFFFF:FFFFFFFFFFFFFFFFFFFFFFF
@A00155:342:HHGFNDSXY:1:2471:13367:10019 1:N:0:GAACCTAG+TCCGCATA
TCGTATCTTCCTCTTATCGATGTGAATTGAGGATACACCACCTTCCAATCCATCTGCAGCCACACGCA
TGCTTCCGCCGTTGATGGCACGAACTGGTATGCCGCAAAACCTATGATGTACAGGGCTGCTGAAAACT
+
FFF:FFFFFFFFFFFFFFFFFFFFFFFFFFFFFFFFFFFFFFFFFFFFFFFFFFFFFFFFFFFFFFFF
FFFFFFFFFFFFFFFFFFFFFFFFFFFFFFFFFFFFFFFFFFFF,FFFFF:FFFFFFFFFFFF:FFFF
@A00155:342:HHGFNDSXY:1:1138:18050:34413 1:N:0:GAACCTAG+TCCGCATA
TCGTATCTTCCTCTTATCGATGTGAATTGAGGATACACCACCTTCCAATCCATCTGCAGCCACACGCA
TGCTTCCGCCGTTGATGGCACGAACTGGTATGCCGCAAAACCTATGATGTACAGGGCTGCTGAAAACT
+
FFFFFFFFFFFFFFFFFFFFFFFFFFFFFFFFFFFFFFFFFFFFFFFFFFFFFFFFFFFFFFFFFFFF
FFFFFFFFFFFFFFFFFFFFFFFFFFFFFFFFFFFFFFFFFFFFFFFFFFFFFFFFFFFFFFFF:FFF
@A00155:342:HHGFNDSXY:1:2646:28393:24017 1:N:0:GAACCTAG+TCCGCATA
CGTATCTTCCTCTTATCGATGTGAATTGAGGATACACCACCTTCCAATCCATCTGCAGCCACACGCAT
GCTTCCGCCGTTGATGGCACGAACTGGTATGCCGCAAAACCTATGATGTACAGGGCTGCTGAAAACTG
+
FFF:FFFFFFFFFFFFFFFFFFFFFFFFFFFFFFFFFFFFFFFFFFFFFFFFFFFFFFFFFFFFFFFF
FFFFFFFFFFFFFFFFFFFFFFFFFFFF:FFFFFFFFFFFFFFFFFFFFFFFFFFFFFFFFFFFFF:F
@A00155:342:HHGFNDSXY:1:1406:9959:26287 1:N:0:GAACCTAG+TCCGCATA
GTATCTTCCTCTTATCGATGTGAATTGAGGATACACCACCTTCCAATCCATCTGCAGCCACACGCATG
CTTCCGCCGTTGATGGCACGAACTGGTATGCCGCAAAACCTATGATGTACAGGGCTGCT
+
FFFFFFFFFFF,FFFFFFFFFFFFFFFFFFFF:FFFFFFFFFFFFFFFFFFFFFFFFFFFFFFFFFFF
FFFFFFFFFFFFFFFFFFFFFFFFFFFFFFFFFFFFFF:FFFFFFFFFFFFFFFFFFFF
@A00155:342:HHGFNDSXY:1:1156:14579:36542 1:N:0:GAACCTAG+TCCGCATA
GTATCTTCCTCTTATCGATGTGAATTGAGGATACACCACCTTCCAATCCATCTGCAGCCACACGCATG
CTTCCGCCGTTGATGGCACGAACTGGTATGCCGCAAAACCTATGATGTACAGGGCTGCTGAAAACTGG
+
FFFFFFFFFFFFFFFFFFFFFFFFFFFFFFFFFFFFFFFFFFFFFFFFFFFFFFFFFFFFFFFFFFFF
FFFFFFFFFFFFFFFFFFFFFFFFFFFFFFFFFFFFFFFF:FFFFFFFF:FFFFFFFFFFFFFFFFFF
@A00155:342:HHGFNDSXY:1:2314:23873:23766 1:N:0:GAACCTAG+TCCGCATA
GTATCTTCCTCTTATCGATGTGAATTGAGGATACACCACCTTCCAATCCATCTGCAGCCACACGCATG
CTTCCGCCGTTGATGGCACGAACTGGTATGCCGCAAAACCTATGATGTACAGGGCTGCTGAAAACTGG
+
FFFFFFFFFFFFFFFFFFFFFFFFFFFFFFFFFFFFFFFFFFFFFFFFFFFFFFFFFFFFFFFFFFFF
FFFFFFFFFFFFFFFFFFFFFFFFFFFFFFFFFFFFFFFFFFFFFFFFFFFFFFFFFFFFFFFFFFFF
@A00155:342:HHGFNDSXY:1:2274:23963:16595 1:N:0:GAACCTAG+TCCGCATA
GTATCTTCCTCTTATCGATGTGAATTGAGGATACACCACCTTCCAATCCATCTGCAGCCACACGCATG
```

```
CTTCCGCCGTTGATGGCACGAACTGGTATGCCGCAAAACCTATGATGTACAGGGCTGCTGAAAACTGG
+
FFFFFFFFFFF,FFFFFFFFFFFFFFFFFFFFFFF:FFF,FFFFFFFFFFFF,FFFFFFFFFFFFFFF
FF:FFFFFFF,FFFFFFFFFFFF:F,FFFFFFFFFFFFFFFFF:FFFFFFF,FFFFFFFFFFFFF:FF
@A00155:342:HHGFNDSXY:1:1206:31403:18803 1:N:0:GAACCTAG+TCCGCATA
GTATCTTCCTCTTATCGATGTGAATTGAGGATACACCACCTTCCAATCCATCTGCAGCCACACGCATG
CTTCCGCCGTTGATGGCACGAACTGGTATGCCGCAAAACCTATGATGTACAGGGCTGCTGAAAACTGG
+
FFFFFFFFFFFFFF:FFFFF,FF:FFFFFFFFFFFFFFFFFFFFFFFFFFFFFFFFFFFFFFFFFFFF
FFFFFFFFFFFFFFFFFFFFFFFFFFFFFFFFFFFFFFFFFFFFFFFFFFFFFFFFFFFFF,FFFFFF
@A00155:342:HHGFNDSXY:1:2229:20410:32299 1:N:0:GAACCTAG+TCCGCATA
GTATCTTCCTCTTATCGATGTGAATTGAGGATACACCACCTTCCAATCCATCTGCAGCCACACGCATG
CTTCCGCCGTTGATGGCACGAACTGGTATGCCGCAAAACCTATGATGTACAGGGCTGCTGAAAACTGG
+
FFF:FFF:FFFFFFFFF:FFFF:F:FFFFFFFFF:FFFFFFFFFFFFFF:FFFFFFFFFFFFFFFF:F
FFFFFF:FFFFFFFFFFFFFFFFFFFFFFFFFFFFFFFFFFFFFFFFF:FF:FFFFFFF:FFFFFFF:
@A00155:342:HHGFNDSXY:1:1107:14570:29230 2:N:0:GAACCTAG+TCCGCATA
TCTTCCTCTTATCGATGTGAATTGAGGATACACCACCTTCCAATCCATCTGCAGCCACACGCATGCTT
CCGCCGTTGATGGCACGAACTGGTATGCCGCAAAACCTATGATGTACAGGGCTGCTGAAAACTGGTCA
+
FFFFFFF:FFFFFFFFFFFFFFFFFFFFFFFFFFFFFFFFFFFFFFFFFFFFFFFFFF:FFFFFFF:F
FFFFFFFFFFFFFFFFFFFF:FFFFFFFFFFFFFFFFFFFFFFFFFFFFFFFFFFFFFFFFFFFFFF
@A00155:342:HHGFNDSXY:1:1650:14253:29935 2:N:0:GAACCTAG+TCCGCATA
TCTTCCTCTTATCGATGTGAATTGAGGATACACCACCTTCCAATCCATCTGCAGCCACACGCATGCTT
CCGCCGTTGATGGCACGAACTGGTATGCCGCAAAACCTATGATGTACAGGGCTGCTGAAAACTGGTCA
+
FFFFFFFFFFFFFFFFF:FFFFFFFFFFFFFFFFFFFFFFFF:FFFFFFFFFFFFFFFFFFFFFFFFF
FFFFFFFFFFFFFFFFFFFFFFFFFFFFFFFFFFFFFFFFFFFFFFFFFFF:FFFFFFFFFFFFFFFF
@A00155:342:HHGFNDSXY:1:1107:15203:29857 2:N:0:GAACCTAG+TCCGCATA
TCTTCCTCTTATCGATGTGAATTGAGGATACACCACCTTCCAATCCATCTGCAGCCACACGCATGCTT
CCGCCGTTGATGGCACGAACTGGTATGCCGCAAAACCTATGATGTACAGGGCTGCTGAAAACTGGTCA
+
:FFFFFFFFFFF:FFFFFFFFFFFFFFFFFFFFFFFF:FFFFFFFFFFFFFFFFFFFFFFFFFFFFFF
FFFFFFFFFFFFFFFFFFFFFFF:FFFFFFFFFFFFFFFFFFFFFFFFFFFFFFFFFFFFFFFFFFFF
@A00155:342:HHGFNDSXY:1:2345:21441:14732 2:N:0:GAACCTAG+TCCGCATA
CTTCCTCTTATCGATGTGAATTGAGGATACACCACCTTCCAATCCATCTGCAGCCACACGCATGCTTC
CGCCGTTGATGGCACGAACTGGTATGCCGCAAAACCTATGATGTACAGGGCTGCTGAAA
+
FFFFFFF:FFFFFFFFFFFFFFFFFFFFFFFFFFFF:FFFFFFFFFFFFFFFFFFFF:FFFFFFFFFF
FFFFFFFFFFFFFFFFFFFFFFFFFFFFFFFFFFFFFFFFFFFFFFFFFFFFFFFFFFF
@A00155:342:HHGFNDSXY:1:2108:16938:21371 2:N:0:GAACCTAG+TCCGCATA
CTTCCTCTTATCGATGTGAATTGAGGATACACCACCTTCCAATCCATCTGCAGCCACACGCATGCTTC
CGCCGTTGATGGCACGAACTGGTATGCCGCAAAACCTATGATGTACAGGGCTGCTGAAAACTGGTCAT
+
FFFF:FFF,FFFFFFFFFFFFFFFFFFF:FFFFFFFFFFFFF:FFFFFF:FFFFF,:FFFFFFF::FF
FF:F:FFFFFFFFFFFFFFF:FF:FFFFFFFF::F:FFFFFFFFFFFFFF:FFFFFFFFFFFFFFFFF
@A00155:342:HHGFNDSXY:1:2516:27407:14074 2:N:0:GAACCTAG+TCCGCATA
CTTCCTCTTATCGATGTGAATTGAGGATACACCACCTTCCAATCCATCTGCAGCCACACGCATGCTTC
CGCCGTTGATGGCACGAACTGGTATGCCGCAAAACCTATGATGTACAGGGCTGCTGAAAACTGGTCAT
+
F:FFFFFF:FFFFFF:FFFFFFFFFFFFFFFFFFF,:FFFFFFFFFFFFFFFFFF:FFFFFFFFFFFF
FFFFFFFFFFFFFFFFFFFFFFFFFFFFFFFFFFFFFFFFFFFFFFFFFFFFFFFFFFFFF:FFFFFF
@A00155:342:HHGFNDSXY:1:1370:4788:14105 2:N:0:GAACCTAG+TCCGCATA
CTTCCTCTTATCGATGTGAATTGAGGATACACCACCTTCCAATCCATCTGCAGCCACACGCATGCTTC
```

```
CGCCGTTGATGGCACGAACTGGTATGCCGCAAAACCTATGATGTACAGGGCTGCTGAAAACTGGTCAT
+
FFFFF:FFFFFFFFFFFFFFFFFF:FFFFFFFFFFFFFFFFFFFFFFFFFFFFFFFFFFFFFFFFFFF
FFFFFFFFFFFFFFFFFFFFFFFFFFFFFFFFFFFFFF:FFFFFFFFFFFFFFFFFFFFFFFFFFFFF
@A00155:342:HHGFNDSXY:1:2623:3414:24345 2:N:0:GAACCTAG+TCCGCATA
TTCCTCTTATCGATGTGAATTGAGGATACACCACCTTCCAATCCATCTGCAGCCACACGCATGCTTCC
GCCGTTGATGGCACGAACTGGTATGCCGCAAAACCTATGATGTACAGGGCTGCTGAAAACTGGTCAT
+
FFFFFFFF,FFFFFFFFFFFFFFFFFFFFFFFFFFFFFF:FFFFFF:FFFFFFFFFFFFFFFFFFFFF
FFFFFFFFFF,FFFFFFFFFFFFFFFFFFFFFFFFFFFFFFFFFFFFFFFFFFFFFFFFFFFFFFFF
@A00155:342:HHGFNDSXY:1:2238:18982:7717 2:N:0:GAACCTAG+TCCGCATA
CTCTTATCGATGTGAATTGAGGATACACCACCTTCCAATCCATCTGCAGCCACACGCATGCTTCCGCC
GTTGATGGCACGAACTGGTATGCCGCAAAACCTATGATGTACAGGGCTGCTGAAAACTGGTCA
+
FFFFF,FFFFFFFFFFFFFFFFFFFFFFFFFF:FFFFFFFFFFFFFFFFFFFFFFFFFFFFFFFFFFF
FFFFFFFFFFFFFFFFFFFFFFF:FFFFFFFFF:FFFFFFFFFFFFFFFFFFFFFFFFFFFFF
@A00155:342:HHGFNDSXY:1:1312:16947:19413 2:N:0:GAACCTAG+TCCGCATA
CTCTTATCGATGTGAATTGAGGATACACCACCTTCCAATCCATCTGCAGCCACACGCATGCTTCCGCC
GTTGATGGCACGAACTGGTATGCCGCAAAACCTATGATGTACAGGGCTGCTGAAAACTGGTCA
+
,FF:FFFFFFFFFFFFFFFFFFFFFFFFFFFFFFFFFFFFFFFFFFFFFFFFFFFFFFFF:FFFFFF
FFFFFFFFFFFFFFFFFFFFFFFFFFFFFFFFFFFFFFFFFFFFFFFFFFFFFFFFFFFFFF:
@A00155:342:HHGFNDSXY:1:1674:26711:31720 2:N:0:GAACCTAG+TCCGCATA
CTCTTATCGATGTGAATTGAGGATACACCACCTTCCAATCCATCTGCAGCCACACGCATGCTTCCGCC
GTTGATGGCACGAACTGGTATGCCGCAAAACCTATGATGTACAGGGCTGCTGAAAACTGGTCAT
+
FFFFFFFFFFFFFFFFFFFFFFFFFFFFFFFF:FFFF:FFFFFFFFFFFFFFFFFFFFFFF:FFFFFF
FFFFFFFFFFFFFFFFFFFFFFFFFFFFFFFFFFFFFFFFFFFFFFFFFFFFFFFFFFFFFFFF
@A00155:342:HHGFNDSXY:1:2133:30237:10300 2:N:0:GAACCTAG+TCCGCATA
CTCTTATCGATGTGAATTGAGGATACACCACCTTCCAATCCATCTGCAGCCACACGCATGCTTCCGCC
GTTGATGGCACGAACTGGTATGCCGCAAAACCTATGATGTACAGGGCTGCTGAAAACTGGTCATAAAG
+
FFFFFFFFFFFFFFFFFFFFFFFFFFFFFFFF:FFFFFFFFFFFFFFFFFFFFFF:FFFFF:FFFFFF
FFFFF:FFFFFFFFFFFFFFFFFFFFFFF:FFFFFFFFFFFFFFFFFFFFFFFFFFFFFFFFFFFFFF
@A00155:342:HHGFNDSXY:1:2243:27037:27367 2:N:0:GAACCTAG+TCCGCATA
CTCTTATCGATGTGAATTGAGGATACACCACCTTCCAATCCATCTGCAGCCACACGCATGCTTCCGCC
GTTGATGGCACGAACTGGTATGCCGCAAAACCTATGATGTACAGGGCTGCTGAAAACTGGTCATAAAG
+
FFFFFFFFFFFFFFFFFFFFFFFFFFFFFFFF:FFFFFFFFFFFFFFFFFFFFFF:FFFFFFFFFFFF
:FFFFFFFF:FFFFFFFFF:FFFFFFFFFFFFFFFFFFFFFFFFFFFFFFFFFFFFFFFFFFFFFFFF
@A00155:342:HHGFNDSXY:1:2131:3803:2503 2:N:0:GAACCTAG+TCCGCATA
TCTTATCGATGTGAATTGAGGATACACCACCTTCCAATCCATCTGCAGCCACACGCATGCTTCCGCCG
TTGATGGCACGAACTGGTATGCCGCAAAACCTATGATGTACAGGGCTGCTGAAAACTGGTCATAAAGC
+
FF:FFFFFFFF,FFFFFFFFFFFFF,FFFFFFFFFFFFFFFFFFFFFFFFFFFFFFFFFFFFFFFFFF
FFFFFFFF:FFFFFFFFFFFFFFFFFFFFFFFFFFFF:FFFFFFFFFFFFFFF,:FFFFFFFFF:FFF
@A00155:342:HHGFNDSXY:1:2131:4643:2832 2:N:0:GAACCTAG+TCCGCATA
TCTTATCGATGTGAATTGAGGATACACCACCTTCCAATCCATCTGCAGCCACACGCATGCTTCCGCCG
TTGATGGCACGAACTGGTATGCCGCAAAACCTATGATGTACAGGGCTGCTGAAAACTGGTCATAAAGC
+
FFFFFFFFFFFFFF:FFFFFFFFFFFFFFFFFFFFFFFFFFFFFFFFFFFFFFFFFFFFFFFFFFFFF
FFFFFFFFFFFFFFFFFFFFFFFF:FFFFFFFFFFFFFFFFFFFFFFFFFFF,FF:FFFFFFFFFFFF
@A00155:342:HHGFNDSXY:1:1325:30391:30953 2:N:0:GAACCTAG+TCCGCATA
TCTTATCGATGTGAATTGAGGATACACCACCTTCCAATCCATCTGCAGCCACACGCATGCTTCCGCCG
```

```
TTGATGGCACGAACTGGTATGCCGCAAAACCTATGATGTACAGGGCTGCTGAAAACTGGTCATAAAGC
+
FFFFFFFFFFFFFFFFFFFFFFFFFFFFFFFFFFFFFFFFFFFFFFFFFFFFFFFFFFFFFFFFFFFF
FFFFFFFFFFFFFFFFFF:FFFFFFFFFFFFFFFFFFFFFFFFFFFFFFFFFFFFFFFFFF,FFFFFF
@A00155:342:HHGFNDSXY:1:2541:16785:23202 1:N:0:GAACCTAG+TCCGCATA
TATCGATGTGAATTGAGGATACACCACCTTCCAATCCATCTGCAGCCACACGCATGCTTCCGCCGTTG
ATGGCACGAACTGGTATGCCGCAAAACCTATGATGTACAGGGCTGCTGAAAACTGGTCATAAAGCCGG
+
FFFFFFFFFFFFFFFFFFFFFFFFFFFFFFFFFFFFFFFFFFFFFFFFFFFFFFFFFFFFFFFFFFFF
FFFFFFFFFFFFFFFFFFFFFFFFFFFFFFFFFFFFFFFFFFFFFFFFFFFFFFFFFFFFFFFFFFFF
@A00155:342:HHGFNDSXY:1:2142:2067:24048 1:N:0:GAACCTAG+TCCGCATA
TATCGATGTGAATTGAGGATACACCACCTTCCAATCCATCTGCAGCCACACGCATGCTTCCGCCGTTG
ATGGCACGAACTGGTATGCCGCAAAACCTATGATGTACAGGGCTGCTGAAAACTGGTCATAAAGCCGG
+
FFFFFFFFFFFFFFFFFFFFFFFFFFFFFFFFFFFFFFFFFFFFFFFFFFFFFFFFFFFFFFFFFFFF
FFFFFFFFFFFFFFFFFFFFFFFFFFFFFFFFFFFFFFFFFFFFFFFFFFFFFFFFFFFFFFFFF:FF
@A00155:342:HHGFNDSXY:1:1107:14570:29230 1:N:0:GAACCTAG+TCCGCATA
TATCGATGTGAATTGAGGATACACCACCTTCCAATCCATCTGCAGCCACACGCATGCTTCCGCCGTTG
ATGGCACGAACTGGTATGCCGCAAAACCTATGATGTACAGGGCTGCTGAAAACTGGTCATAAAGCCGG
+
FFFFFFFFFFFF:FFFFFFFFFFFFFFFFFFFFFFFFFFFFFFFFFFFFFFFFFFFFFFFFFFFFFFF
:FFFFFFFFFFFFFFFFFFFFFFFFFFFFFFFFFFFFFFFFFFFFFFFFFFFFFFFFFFFFFFFFFFF
@A00155:342:HHGFNDSXY:1:1107:15203:29857 1:N:0:GAACCTAG+TCCGCATA
TATCGATGTGAATTGAGGATACACCACCTTCCAATCCATCTGCAGCCACACGCATGCTTCCGCCGTTG
ATGGCACGAACTGGTATGCCGCAAAACCTATGATGTACAGGGCTGCTGAAAACTGGTCATAAAGCCGG
+
FFFFFFFFFFFFFFFFFFFFFFFFFFFFFFFFFFFFFFFFFFFFFFFFFFFFFFFFFFFFFFFFFFFF
FFFFFFFFFFFFFFFFFFFFFFFFFFFFFFFFFFFFFFFFFFFFFFFFFFFFFFFFFFFFFFFFFFFF
@A00155:342:HHGFNDSXY:1:1205:1651:34695 1:N:0:GAACCTAG+TCCGCATA
TCGATGTGAATTGAGGATACACCACCTTCCAATCCATCTGCAGCCACACGCATGCTTCCGCCGTTGAT
GGCACGAACTGGTATGCCGCAAAACCTATGATGTACAGGGCTGCTGAAAACTGGTCATAAAGCCGGTT
+
FFFFFFFFFFFFFFFFFFFF:F,::FFF,FFFFFF:FFFF:F:FFFFFF,FFFFFFFFFFFFFFFFFF
FFFFFF:FFFFFFFFF:FFF:FFFFFFFFFFFFFFFFF:FFFFFFF::F,FFFFFFFF:F,FFFFFFF
@A00155:342:HHGFNDSXY:1:2345:21441:14732 1:N:0:GAACCTAG+TCCGCATA
GTGAATTGAGGATACACCACCTTCCAATCCATCTGCAGCCACACGCATGCTTCCGCCGTTGATGGCAC
GAACTGGTATGCCGCAAAACCTATGATGTACAGGGCTGCTGAAAACTGGTCATAAAGCC
+
FFFFFFFFFFFFFFFFFFFFFFFFFFFFFFFFFFFFFFFFFFFFFFFFFFFFFFFFFFFFFFFFFFFF
FFFFFFFF,FFFFFFFFFFFFFF:FFFFFFFFFFFFFFFFFFFFFFFFFFFFFFFFFFF
@A00155:342:HHGFNDSXY:1:2312:28763:16642 1:N:0:GAACCTAG+TCCGCATA
GTGAATTGAGGATACACCACCTTCCAATCCATCTGCAGGCACACGCATGCTTCCGCCGTTGATGGCAC
GAACTGGTATGCCGCAAAACCTATGATGTACAGGGCTGCTGAAAACTGGTCATAAAGCCGGTTTTGCC
+
FFFFFFFFFFFF:FFFFFFFFFFFFFFFFFFFFFFFFFFFFFFFFFFFFFFFFFFFFFFFFFFFFFFF
FFFFFFFFFFFFFFFFFFFFFFFFFFFFFFFFFFFFFFFF:FFFFFFFFFFFFFFFFFFFFFFFFF,F
@A00155:342:HHGFNDSXY:1:1316:29053:34147 2:N:0:GAACCTAG+TCCGCATA
GAATTGAGGATACACCACCTTCCAATCCATCTGCAGCCACACGCATGCTTCCGCCGTTGATGGCACGA
ACTGGTATGCCGCAAAACCTATGATGTACAGGGCTGCTGAAAACTGGTCATAAAGCCGGTTTTGCCCT
+
FFFFFFFFFFFFFFFFFFFFFFFFFFFFFFF:FFFFFFFFFFFFFF:FFFFFFFFFFFFFFFFFFFFF
FFFFFFFFFFFFFFFFFFFF,FFFFFFFFFFFFFFFFFFFFFFFFFFFFFFF,FFFFFFFFFFFFFFF
@A00155:342:HHGFNDSXY:1:2312:27290:5040 2:N:0:GAACCTAG+TCCGCATA
GAATTGAGGATACACCACCTTCCAATCCATCTGCAGCCACACGCATGCTTCCGCCGTTGATGGCACGA
```

ACTGGTATGCCGCAAAACCTATGATGTACAGGGCTGCTGAAAACTGGTCATAAAGCCGGTTTTGCCCT
+
FFFFFFFFFFFFFFFFFFFFFFFFFFFFFFFFFFFFFFFFFFFFFFFFFFFFFFFFFFFFFFFFFFFF
FFFFFFFFFFFFFFFFFFFFFFFFFFFFFFFFFFFFFFFFFFFFFFFFFFFF:FFFFFFFFFFFFFF:
@A00155:342:HHGFNDSXY:1:2210:26205:20760 2:N:0:GAACCTAG+TCCGCATA
GAATTGAGGATACACCACCTTCCAATCCATCTGCAGCCACACGCATGCTTCCGCCGTTGATGGCACGA
ACTGGTATGCCGCAAAACCTATGATGTACAGGGCTGCTGAAAACTGGTCATAAAGCCGGTTTTGCCCT
+
FFFFFFFFFFFFFFFFFFFFFFFFFFFFFFFFFFF:FFFFFFF:FFFFFFFFFFFFFFFFFFFF,FFF
FFFFFFFFFFFFFFFFFFFFF::FFFFFFFFFFFFFFFFFFFFFFFFFFFFFFFFFFFFFFFFFFFFF
@A00155:342:HHGFNDSXY:1:1312:16947:19413 1:N:0:GAACCTAG+TCCGCATA
ATTGAGGATACACCACCTTCCAATCCATCTGCAGCCACACGCATGCTTCCGCCGTTGATGGCACGAAC
TGGTATGCCGCAAAACCTATGATGTACAGGGCTGCTGAAAACTGGTCATAAAGCCGGTTTTGC
+
FFFFFFFFFFFFFFFFFFFFFFFFFFFFFFFFFFFFFFFFFFFFFFFFFFFFFFFFFFFFFFFFFFFF
FFFFFFFFFFFF:FFFFFFFFFFFFFFFFFFFFFFFFFFFFFFFFFFFFFFFFFFFFFFFFFF
@A00155:342:HHGFNDSXY:1:2238:18982:7717 1:N:0:GAACCTAG+TCCGCATA
ATTGAGGATACACCACCTTCCAATCCATCTGCAGCCACACGCATGCTTCCGCCGTTGATGGCACGAAC
TGGTATGCCGCAAAACCTATGATGTACAGGGCTGCTGAAAACTGGTCATAAAGCCGGTTTTGC
+
FFFFFFFFFFFFFFFFFFFFFFFFFFFFFFFFFFFFFFFFFFFFFFFFFFFFFFFFFFFFFFFFFFFF
FFFFFFFFFFFFFFFFFF:FFFFFF:FFFFFFFFFFFFF,FFFFFFFFFFFFFFFFFFFFFFF
@A00155:342:HHGFNDSXY:1:1674:26711:31720 1:N:0:GAACCTAG+TCCGCATA
ATTGAGGATACACCACCTTCCAATCCATCTGCAGCCACACGCATGCTTCCGCCGTTGATGGCACGAAC
TGGTATGCCGCAAAACCTATGATGTACAGGGCTGCTGAAAACTGGTCATAAAGCCGGTTTTGCC
+
FFFFFFFFFFFFFFFFFFFFFFFFFFFFFFFFFFFFFFFFFFFFFFFFFFFFFFFFFFFFFFFFFFFF
FFFFFFFFFFFFFFFFFFFFFFFFFFFFFFFFFFFFFF,FFFFFFFFFFFFFFFFFFFFFFFFF
@A00155:342:HHGFNDSXY:1:2410:1479:23343 1:N:0:GAACCTAG+TCCGCATA
ATTGAGGATACACCACCTTCCAATCCATCTGCAGCCACACGCATGCTTCCGCCGTTGATGGCACGAAC
TGGTATGCCGCAAAACCTATGATGTACAGGGCTGCTGAAAACTGGTCATAAAGCCGGTTTTGCCCTAC
+
FFFFFFFFFFFFFFF,FFFFFFFFFFFFFFFFFFFFFFFFFFFFFFFFFFFFFFFFFFFFFFFFFFFF
FFFFFFFFFFFFFF:FFFFF:,FFFFFFFFFFFFFFF,FF:FFFFF:FFFF:FF:FFFFFFFF:FF:F
@A00155:342:HHGFNDSXY:1:2447:1579:25927 1:N:0:GAACCTAG+TCCGCATA
ATTGAGGATACACCACCTTCCAATCCATCTGCAGCCACACGCATGCTTCCGCCGTTGATGGCACGAAC
TGGTATGCCGCAAAACCTATGATGTACAGGGCTGCTGAAAACTGGTCATAAAGCCGGTTTTGCCCTAC
+
FFFFFFFFFFFFFFFFFFFFFFFFFFFFFFFFFFFFFFFFFFFFFFFFFFFFFFFFFFFFFFFFFFFF
FFFFFFFFFFFFFFFFFFFFFFFFFFFFFFFFFFFFFFFFFFFFFFFFFFFFFFFFFFFFFF,::FFF
@A00155:342:HHGFNDSXY:1:1570:29017:30953 1:N:0:GAACCTAG+TCCGCATA
ATTGAGGATACACCACCTTCCAATCCATCTGCAGCCACACGCATGCTTCCGCCGTTGATGGCACGAAC
TGGTATGCCGCAAAACCTATGATGTACAGGGCTGCTGAAAACTGGTCATAAAGCCGGTTTTGCCCTAC
+
FFFFFFFFFFFFFFFFFFFFFFFFFFFFFFFFFFFFFFFFFFFFFF:FFFFFFFFFFFFFFFFFFFFF
FFFFFFFFFFFFFF:FFFFFFFFFFFFFFFFFFFFFF:FFFFFFFFFF:FFFFFFFFFFFFFFFFFFF
@A00155:342:HHGFNDSXY:1:2516:32868:28040 2:N:0:GAACCTAG+TCCGCATA
TGAGGATACACCACCTTCCAATCCATCTGCAGCCACACGCATGCTTCCGCCGTTGATGGCACGAACTG
GTATGCCGCAAAACCTATGATGTACAGGGCTGCTGAAAACTGGTCATAAAGCCGGTTTTG
+
:FFFF:FFFFFFFFFFF,FFFFFFFFFF:FF::FFFFFFFFFFFFFFFFFF:F:FFFFF:F,FFFFFF
F,FFFFFFFFFFFFFFFFFFFFFFFFF:FFFFFFF:FFFFF:FF:FFFFFFFFF:F:FFF
@A00155:342:HHGFNDSXY:1:1552:17797:19820 2:N:0:GAACCTAG+TCCGCATA
GGATACACCACCTTCCAATCCATCTGCAGCCACACGCATGCTTCCGCCGTTGATGGCACGAACTGGTA

TGCCGCAAAACCTATGATGTACAGGGCTGCTGAAAACTGGTCATAAAGCCGGTTTTGCCCTACATATG
+
:F:FFF,F,::FF:FFFFF:FFFFFFF:FFFFFFFFFFFFFF:FFFFFFFFFFFFFFFFFFFFFFFFFF
FFFFFFFFFFFFFFFFFFFFFFFFFFFFFFFFFFFFFFFFFFFFFFFFFFFFFFFF:FFFFFFFFFFFFF
@A00155:342:HHGFNDSXY:1:2410:1479:23343 2:N:0:GAACCTAG+TCCGCATA
GATACACCACCTTCCAATCCATCTGCAGCCACACGCATGCTTCCGCCGTTGATGGCACGAACTGGTAT
GCCGCAAAACCTATGATGTACAGGGCTGCTGAAAACTGGTCATAAAGCCGGTTTTGCCCTACATATG
+
FFFFFFFF:,FFF:FFFFFFFFFF:FFFFFFFFFFFFFFFFFFFFFFFFFF:FFFFFFFFFF:FFFFFFF
FFFFFFFFFFFFFFFFFFFFFF:FFFFFFF:FFFFFFFFFFFFFFFFFFFFFFFFF:FFFFFFFFFFFF
@A00155:342:HHGFNDSXY:1:1205:1651:34695 2:N:0:GAACCTAG+TCCGCATA
CACCACCTTCCAATCCATCTGCAGCCACACGCATGCTTCCGCCGTTGATGGCACGAACTGGTATGCCG
CAAAACCTATGATGTACAGGGCTGCTGAAAACTGGTCATAAAGCCGGTTTTGCCCTACATATGCCCTC
+
FF:FFFF:FFFFFFFFF:FFFFFFFFFFFFFFFFFFFFF,FFF:FFFFFFF:FFFFFFF:FFFFF:FFFF
FFFFFFFFFFFFFFFFFFFFFFFFFFFFFFFFFFFF:FFFFFFFFFFFF:,FFFFF:FFFF:FFFFFF,F
@A00155:342:HHGFNDSXY:1:1141:22110:5368 2:N:0:GAACCTAG+TCCGCATA
CACCACCTTCCAATCCATCTGCAGCCACACGCATGCTTCCGCCGTTGATGGCACGAACTGGTATGCCG
CAAAACCTATGATGTACAGGGCTGCTGAAAACTGGTCATAAAGCCGGTTTTGCCCTACATATGCCCTC
+
FF:FFFFFFFFFFFFF:F:FFF:FFFFFFFFFFFFFF:FFFFFFF:F:FFFFFFFFFFFFFFFFFFFFFF
FFFFF:FFFFFFFFFFFFFFFFFFFFFFFFFFFFFFFFFFFFFFFFFFFFFFFFFFFFFFFFFFFFFFFF
@A00155:342:HHGFNDSXY:1:2470:27190:5024 2:N:0:GAACCTAG+TCCGCATA
CCACCTTCCAATCCATCTGCAGCCACACGCATGCTTCCGCCGTTGATGGCACGAACTGGTATGCCGCA
AAACCTATGATGTACAGGGCTGCTGAAAACTGGTCATAAAGCCGGTTTTGCCCTACATATACCCTCAA
+
FFFFFFF,FF:FFFFFFFFFFFFFFFFFFF,FFFF:FFFFFFFFFFFFFFFFFFFFFFFF,FFFFF,FFF
FFFFFFFFFFFFFFFFFF,FFFFFFFFFFFFFFFFFFFFFF:FF:FFFFFFFFF:FFFFFFFFFFFFFFF
@A00155:342:HHGFNDSXY:1:2351:6054:7498 2:N:0:GAACCTAG+TCCGCATA
CACCTTCCAATCCATCTGCAGCCACACGCATGCTTCCGCCGTTGATGGCACGAACTGGTATGCCGCAA
AACCTATGATGTACAGGGCTGCTGAAAACTGGTCATAAAGCCGGTTTTGCCCTACATATGCCCTCAA
+
FFFFFFFFFFFFFFFFFFFFFFFFFFFFFFFFFFFFFFFF,FFFFFFFFFFFFFF,FFFFFFFFFFFFFF
FFFFFFFFFFFFFFFFFF:FFFFFFFFFFFFFFFFFFFFFFFFFFFFFFFFFFFFF:FFFF:FFFFFFF
@A00155:342:HHGFNDSXY:1:1365:22173:22075 2:N:0:GAACCTAG+TCCGCATA
ACCTTCCAATCCATCTGCAGCCACACGCATGCTTCCGCCGTTGATGGCACGAACTGGTATGCCGCAAA
ACCTATGATGTACAGGGCTGCTGAAAACTGGTCATAAAGCCGGTTTTGCCCTACATATGCCCTCAACG
+
FFFFFFFF,FFFFFFFFFFFFFFFF:FFFFFFF:,FFFFFFFFFFFFFFFFFFFFFFFFFFFFFFFFFFF
FFFFFFFFFFFFFFFFFFFFFF:FFFFFFFFFFFFFFFFFFFFFF:FFFFFFFFF:FFFFFFFFFFFFFF
@A00155:342:HHGFNDSXY:1:2645:19388:1939 2:N:0:GAACCTAG+TCCGCATA
CCTTCCAATCCATCTGCAGCCACACGCATGCTTCCGCCGTTGATGGCACGAACTGGTATGCCGCAAAA
CCTATGATGTACAGGGCTGCTGAAAACTGGTCATAAAGCCGGTTTTGCCCTACATATGCCCTCAACGC
+
FFFFFFFFFFFFFFFFFFFFFFFFFF:FFFFFFFFFFFFFFFFFFFFF:FFFFFFFFFF:FFFFFFFFFF
FFFFFFFFF:FFFFFFFFFFFFFFFFFFFFFFFFFFFFFFFFFFFFFFFFFFFFFFFFFFF:FFFFFFFF
@A00155:342:HHGFNDSXY:1:1260:33003:32064 2:N:0:GAACCTAG+TCCGCATA
CTTCCACTCCATCTGCAGCCACACGCATGCTTCCGCCGTTGATGGCACGAACTGGTATGCCGCAAAAC
CTATGATGTACAGGGCTGCTGAAAACTGGTCATAAAGCCGGTTTTGCCCTACATATGCCCTC
+
F::FFF,:FFF,FFFFFFFFF::FF::FFF,FFFFFFFFFF:FF:F:F,FFFFFF:FFF:FFFFFF:,FF
F,FFFF:F:FFFFFFFF:FF:FF:FFF:FFFFFFFFF:F:FFFFF,:FFFFFFFFF:,FFF:FF
@A00155:342:HHGFNDSXY:1:1246:22064:22138 2:N:0:GAACCTAG+TCCGCATA
CTTCCAATCCATCTGCAGCCACACGCATGCTTCCGCCGTTGATGGCACGAACTGGTATGCCGCAAAAC

```
CTATGATGTACAGGGCTGCTGAAAACTGGTCATAAAGCCGGTTTTGCCCTACATATGCCCTC
+
FFFFFFFFFFFFFFFFFFFFFF,FFFFFFF,FFFFFFFFFFFFFFFFFFF,FFFF:FFFFFFFFFFFF
FFFFFFFFFFFFFFFFFFFFFFFFFFFFFFFF:FFF:FFFFFF:F:FFFFF:FFFFFFFFFFF
@A00155:342:HHGFNDSXY:1:1263:19388:34256 2:N:0:GAACCTAG+TCCGCATA
CTTCCAATCCATCTGCAGCCACACGCATGCTTCCGCCGTTGATGGCACGAACTGGTATGCCGCAAAAC
CTATGATGTACAGGGCTGCTGAAAACTGGTCATAAAGCCGGTTTTGCCCTACATATGCCCTCA
+
FF,FFF:FFFFFFFFFFFFFFFFFFFFFF:,FFFFFFFFFFFFFFFFFFF,FFFFFFFFFFFFFFFFF
FFFFFFFFF,FFFFFFFFFFFFFFFFFFFFFFFFFFFFFFFF,FFFFFFFFFFFFFFFFFFFFF
@A00155:342:HHGFNDSXY:1:2228:26142:5494 2:N:0:GAACCTAG+TCCGCATA
TTCCAATCCATCTGCAGCCACACGCATGCTTCCGCCGTTGATGGCACGAACTGGTATGCCGCAAAACC
TATGATGTACAGGGCTGCTGAAAACTGGTCATAAAGCCGGTTTTGCCCTACATATGCCCT
+
FFFFFFFFFFFFFFFFFFFFFFFFFFFFFF:FFFFFFFFFFFFFFFFFFFFFFFFFFFFFFFFFFFFF
FFFFF:FFFFFFFFFFFFFFFFFFFF:FFFFFFFFFFFFFFFFF:FFFFFFFFFFFFFFFFF
@A00155:342:HHGFNDSXY:1:2228:25798:5588 2:N:0:GAACCTAG+TCCGCATA
TTCCAATCCATCTGCAGCCACACGCATGCTTCCGCCGTTGATGGCACGAACTGGTATGCCGCAAAACC
TATGATGTACAGGGCTGCTGAAAACTGGTCATAAAGCCGGTTTTGCCCTACATATGCCCT
+
FFFFFFFFFFFFFFFFFFFFFFFFFFFFFFFFFFFFFFFFFFFFFFFFFFFFFFFFFFFFFFFFFFFF
FF:FFFFFFFFFFFFFFFFFFFFFFFFFFFFFFFFFFFFFFFFFFFFFFFFFFFFFF:FFFFF
@A00155:342:HHGFNDSXY:1:2105:6415:5087 2:N:0:GAACCTAG+TCCGCATA
CAATCCATCTGCAGCCACACGCATGCTTCCGCCGTTGATGGCACGAACTGGTATGCCGCAAAACCTAT
GATGTACAGGGCTGCTGAAAACTGGTCATAAAGCCGGTTTTGCCCTACATATGCCCTCAACGCAGACC
+
:FFFFFFF,FFFFFFF,FF:FFFF,FFFFFFFFFFFFFFFFFFFFFFFFFFFFFFFFFFFFFFFFFFF
FFFFFFFFFFFFFFFFFFF,FFFFFFFF:FFFFFFFFFFFFFFFFFFFFFFFFFFFFFFFFFFFFFFF
@A00155:342:HHGFNDSXY:1:2229:20410:32299 2:N:0:GAACCTAG+TCCGCATA
CCATCTGCAGCCACACGCATGCTTCCGCCGTTGATGGCACGAACTGGTATGCCGCAAAACCTATGATG
TACAGGGCTGCTGAAAACTGGTCATAAAGCCGGTTTTGCCCTACATATGCCCTCAACGCAGACCATAT
+
FF:FFFFFFFFF:FFFFFFFFF,FFFFFFFFFFFFFFFFFFFFFFFFFFFFFFFFFFFFFFF,FFFFF
FFFFFFFFFFFFFFFFFF:FFFFFFFF,FFFFFFFF,FFFFFFFFFFFFFFFFFFFFFFFFFFFFFFF
@A00155:342:HHGFNDSXY:1:1206:31403:18803 2:N:0:GAACCTAG+TCCGCATA
CCATCTGCAGCCACACGCATGCTTCCGCCGTTGATGGCACGAACTGGTATGCCGCAAAACCTATGATG
TACAGGGCTGCTGAAAACTGGTCATAAAGCCGGTTTTGCCCTACATATGCCCTCAACGCAGACCATAT
+
FFFFFFFFFFFFFFFFFFFFFF:FFFFFFFFFFFFFFFFFFFFFFFFFFFFFFFFFFFFFFFFFFFFF
FFFFFFFFFFFFFFFFFFFFFFFFFFFFFFFF:FFFFFFFFFFFFFFFFFFFFFFFFFFFFFFFFFFF:
@A00155:342:HHGFNDSXY:1:2172:12418:24815 2:N:0:GAACCTAG+TCCGCATA
CCATCTGCAGCCACACGCATGCTTCCGCCGTTGATGGCACGAACTGGTATGCCGCAAAACCTATGATG
TACAGGGCTGCTGAAAACTGGTCATAAAGCCGGTTTTGCCCTACATATGCCCTCAACGCAGACCATAT
+
FFFFFFFFFFFFFFF:FFFFFFFFFFFFFFF:FFFFFFFFFFFFFFFFFFFFFFFFFFFFFFFFFFFF
FFFFFFFFFFFFFFFFFFFFFFFFFFFFFFFFFFFFFFFFFFFFFFFFFFFFFFFFFFFFFFFFFFFF
@A00155:342:HHGFNDSXY:1:2314:23873:23766 2:N:0:GAACCTAG+TCCGCATA
CCATCTGCAGCCACACGCATGCTTCCGCCGTTGATGGCACGAACTGGTATGCCGCAAAACCTATGATG
TACAGGGCTGCTGAAAACTGGTCATAAAGCCGGTTTTGCCCTACATATGCCCTCAACGCAGACCATAT
+
FFFFFFFFFFFFFF,FFFFF:F,FFFFFFFFFFFFFFFFFFFFFFFFFFFFFFFFFFFFFF:FFFFFF
FFFFFFFFFFFFFFFFFFFFFFFFFFFFFFFFFFFFFFFFFFFFFFFFFFFFFFFFFFFFFFFFFFFF
@A00155:342:HHGFNDSXY:1:2312:28763:16642 2:N:0:GAACCTAG+TCCGCATA
CATCTGCAGGCACACGCATGCTTCCGCCGTTGATGGCACGAACTGGTATGCCGCAAAACCTATGATGT
```

ACAGGGCTGCTGAAAACTGGTCATAAAGCCGGTTTTGCCCTACATATGCCCTCAACGCAGACCATATC
+
F,FFF:FFFFFFFFFFFFFFFFFFFFFFFFFFFFFFFFFFFFFFFFFFFFFFFFFF:FFFF::FFFFF
FFFFFFFFFFFFFFFFFFFFFFF,FFFFFFFFFFFFFFFFFFFFFFFFFFFFFFFFFFFFFFFFF:FF
@A00155:342:HHGFNDSXY:1:2269:2998:30044 2:N:0:GAACCTAG+TCCGCATA
ATCTGCAGCCACACGCATGCTTCCGCCGTTGATGGCACGAACTGGTATGCCGCAAAACCTATGATGTA
CAGGGCTGCTGAAAACTGGTCATAAAGCCGGTTTTGCCCTACATATGCCCTCAACGCAGACCATATCT
+
FFFFFFFFFFFFFFF:FFFFFFFFFFFFFFFFFFFFFFFFFFFFFFFFFFFFFFFFFFFFFFFFFFFF
FFFFFFFFFFFFFFFFFFFFFFFFFFFFFFFF:FFFFFFFFFFFFF:FFFFFFFFFFFFFFFFFFFFF
@A00155:342:HHGFNDSXY:1:1112:12500:1344 1:N:0:GAACCTAG+TCCGCATA
ATCTGCAGCCACACGCATGCTTCCGCCGTTGTTGGCACGAACTGGTATGCCGCAAAACCTATGATGTA
CAGGGCTGCTGAAAACTGGTCATAAAGCCGGTTTTGCCCTACATATGCCCTCAACGCAGACCATATCT
+
FFFFFFFFFFFFFFFFFFFFFFFFFFFFFF:,FFFFFFF:FFFFFFFFFFFFFFFFFFFFFFFFFFFF
FFFFFFFFFFFFFFFFFFFFFFFFFFFFFFFFFFFFFFFF,FFFFFF:FFFFFFFFFFFFFFFFFFFF
@A00155:342:HHGFNDSXY:1:2215:3197:7874 1:N:0:GAACCTAG+TCCGCATA
TCTGCAGCCACACGCATGCTTCCGCCGTTGATGGCACGAACTGGTATGCCGCAAAACCTATGATGTAC
AGGGCTGCTGAAAACTGGTCATAAAGCCGGTTTTGCCCTACATATGCCCTCAACGCAGACAATATCT
+
F:FFFFFFFFFFFFFFFFFFFFFFFFFFFFFFFFFFFFFFFFFF:FFFFFFFFFFFFFFFFFFFFFFF
FFFFFFFFFFFFFFFFFFFFFFFFFFFFFFFFFFFFFFF:F:FFF,FFFFFFFFFFFFFFFF,:FF:FF
@A00155:342:HHGFNDSXY:1:2140:14534:18521 1:N:0:GAACCTAG+TCCGCATA
TCTGCAGCCACACGCATGCTTCCGCCGTTGATGGCACGAACTGGTATGCCGCAAAACCTATGATGTAC
AGGGCTGCTGAAAACTGGTCATAAAGCCGGTTTTGCCCTACATATGCCCTCAACGCAGACCATATCTG
+
FFFFFFFFFFFFFFFFFFFFFFFFFFFFFFFFFFFFFFFFFFFFFFFFFFFFFFFFFFFFFFFFFFFF
FFFFFFFFFFFFFFFFFFFFFFFFFFFFFFFFFFFFFFFFFFF:FFFFFFFFFFFFFFFFFFFFFFFF
@A00155:342:HHGFNDSXY:1:2451:24325:6198 1:N:0:GAACCTAG+TCCGCATA
TCTGCAGCCACACGCATGCTTCCGCCGTTGATGGCACGAACTGGTATGCCGCAAAACCTATGATGTAC
AGGGCTGCTGAAAACTGGTCATAAAGCCGGTTTTGCCCTACATATGCCCTCAACGCAGACCATATCTG
+
FFFFFFFFFFFFFFFFFFFFFFFFFFFFFFFFFFFFFFFFFFFFFFFFFFFFFFFFFFFFFFFFFFFF
FFFFFFFFFFFFFFFFFFFFFFFFFFFFFFFFFFFFFFFFFFFF,FFFFFFFFFFFFFFFFF:FFFFFF
@A00155:342:HHGFNDSXY:1:1274:30861:31641 1:N:0:GAACCTAG+TCCGCATA
CTGCAGCCACACGCATGCTTCCGCCGTTGATGGCACGAACTGGTATGCCGCAAAACCTAAGATGTACA
GGGCTGCTGAAAACTGGTCATAAAGCCGGTTTTGCCCTACATATGCCCTCAACGCAGACCATATCTGT
+
FFFFFFFFFFFFFFFFFFFFFFF:FFF,FFFFFFFFFFFFFFFFFFFFFFFFFFFFFFF,F:FFF:FFFF
FFFFFFFFFFFFFFFFFFFFFFFFF:F,FF::FFFFFFFFFFFFFFFFFFFF:FFFFFFFFFFFFFFFF
@A00155:342:HHGFNDSXY:1:1324:22833:31892 1:N:0:GAACCTAG+TCCGCATA
CTGCAGCCACACGCATGCTTCCGCCGTTGATGGCACGAACTGGTATGCCGCAAAACCTATGATGTACA
GGGCTGCTGAAAACTGGTCATAAAGCCGGTTTTGCCCTACATATGCCCTCAACGCAGACCATATCTGT
+
FFFFFFFFFFFFFFFFFFFFFFFFFFFFFFFFFFFFFFFFFFFFFFFFFFFFFFFFFFFFFFFFFFFF
FFFFFFFFFFFFFFFFFFFFFFFFFFFFFFFFFFFFFFFFFF,FFFFFFFFFFFFFFFFFFFFFF:FFFF
@A00155:342:HHGFNDSXY:1:2113:26467:4773 1:N:0:GAACCTAG+TCCGCATA
CTGCAGCCACACGCATGCTTCCGCCGTTGATGGCACGAACTGGTATGCCGCAAAACCTATGATGTACA
GGGCTGCTGAAAACTGGTCATAAAGCCGGTTTTGCCCTACATATGCCCTCAACGCAGACCATATCTGT
+
FFFFFFFFFFFFFFFFFFFFFFFFFFFFFFFFFFFFFFFFFFFFFFFFFFFFFFFFFFFFFFFFFFFF
FFFFFFFFFFFFFFFFFFFFFFFFFFFFFFFFFFFFFFFFFFFFFFFFFFFFFFFFFFFFFFFFFFFF
@A00155:342:HHGFNDSXY:1:2677:1154:34460 1:N:0:GAACCTAG+TCCGCATA
CTGCAGCCACACGCATGCTTCCGCCGTTGATGGCACGAACTGGTATGCCGCAAAACCTATGATGTACA

```
GGGCTGCTGAAAACTGGTCATAAAGCCGGTTTTGCCCTACATATGCCCTCAACGCAGACCATATCTGT
+
FFFFFFFFFF:FFFFFFFFFFFFFFFFFF:FFFFFFF,:FFFFFFFFFFFFFF:FFFFFFFFFFFFFF
FFFFFFFFFFFFFFFFFFFFFFFFF:FFFFFFFFFFF:FF:FFFFFFFFFFFFFFF:FF:FFFF:F:FFF
@A00155:342:HHGFNDSXY:1:1365:22173:22075 1:N:0:GAACCTAG+TCCGCATA
TGCAGCCACACGCATGCTTCCGCCGTTGATGGCACGAACTGGTATGCCGCAAAACCTATGATGTACAG
GGCTGCGGAAAACTGGTCATAAAGCCGGTTTTGCCCTACATATGCCCTCAACGCAGACCATATCTGTG
+
FFFFFFFFFFFFFFFFFFFFFFFFFFFFFFFFF:FFFFFFFFFFFFFFFFFFFFFFFFFFFFFFFFFF
FFFFFF,FFFFFFFFFFFFFFFFFFFFFFFFFFFFFFFFFFFFFFFFFFFFFFFFFFFFFFFFFFFFFF
@A00155:342:HHGFNDSXY:1:1516:5168:26913 1:N:0:GAACCTAG+TCCGCATA
TGCAGCCACACGCATGCTTCCGCCGTTGATGGCACGAACTGGTATGCCGCAAAACCTATGATGTACAG
GGCTGCTGAAAACTGGTCATAAAGCCGGTTTTGCCCTACATATGCCCTCAACGCAGACCATATCTGTG
+
FFFFFFFFFFFFFFFFFFFFFFFFFFFFFFFFFFFFFFFFFFFFFFFFFFFFFFFFFFFFFFFFFFFF
FFFFFFFFFFFFFFFFFFFFFFFFFF,FFFFFFFFFFFFFFFFFFFFFFFFFFFFFFFFFF:FFFFFFF
@A00155:342:HHGFNDSXY:1:2338:13892:3599 1:N:0:GAACCTAG+TCCGCATA
TGCAGCCACACGCATGCTTCCGCCGTTGATGGCACGAACTGGTATGCCGCAAAACCTATGATGTACAG
GGCTGCTGAAAACTGGTCATAAAGCCGGTTTTGCCCTACATATGCCCTCAACGCAGACCATATCTGTG
+
FFFFFFFFFFFFFFFFFFFFFFFFFFFFFFFFFFFFFFFFFFFFFFFFFFFFFFFFFFFFFFFFFFFF
FFFFFFFFFFFFFFFFFFFFFFFFFFFFFFFFFFFFFFFFFFFFFFFFFFFFFFFFFFFFFFFFFFFF
@A00155:342:HHGFNDSXY:1:1128:6307:26725 1:N:0:GAACCTAG+TCCGCATA
TGCAGCCACACGCATGCTTCCGCCGTTGATGGCACGAACTGGTATGCCGCAAAACCTATGATGTACAG
GGCTGCTGAAAACTGGTCATAAAGCCGGTTTTGCCCTACATATGCCCTCAACGCAGACCATATCTGTG
+
FFFFFFFFFFFFFFFFFFFFFFFFFF:FFFFFFFFFF:FFFFFFFFF:FFFFFFFFFFFFFFFFFFFF:
FFFFFFFFFFFFFFFFFFFFFFFFFF:FFFFFFFFFF:FFFFFFFFFFFFFFFFFFFFFFFFFFFFFFF
@A00155:342:HHGFNDSXY:1:1128:5728:26819 1:N:0:GAACCTAG+TCCGCATA
TGCAGCCACACGCATGCTTCCGCCGTTGATGGCACGAACTGGTATGCCGCAAAACCTATGATGTACAG
GGCTGCTGAAAACTGGTCATAAAGCCGGTTTTGCCCTACATATGCCCTCAACGCAGACCATATCTGTG
+
FFFFFFFFFFFFFFFFFFFFFF,FFFFFFFFFFFFFFF:FFFFFFFFFFFFFFFFFFFFFFFFFFFFFF
FFFFFFFFFFFFFFFFFFFFFFFFFFFFFFFFFFFFFFFFFFFFFFFFFFFFFFFFFFFFFF:FFFFFF
@A00155:342:HHGFNDSXY:1:1510:23249:2362 1:N:0:GAACCTAG+TCCGCATA
AGCCACACGCATGCTTCCGCCGTTGATGGCACGAACTGGTATGCCGCAAAACCTATGATGTACAGGGC
TGCTGAAAACTGGTCATAAAGCCGGTTTTGCCCTACATATGCCCTCAACGCAGACCATATCTGTGCCG
+
FFFFFFFFFFFFFFFFFFFFFFFFFFFFFFFFFFFFFFFFFFFFFFFFFFFFFFFFFFFFFFFFFFFF
FF:FFFFFFFFFFFFFFFFFFFFFFFFFFFFFFFFFFFFFF,FFFFFFFFFFFFFFFFFFFFFFFFFFF
@A00155:342:HHGFNDSXY:1:1263:20473:2628 1:N:0:GAACCTAG+TCCGCATA
AGCCACACGCATGCTTCCGCCGTTGATGGCACGAACTGGTATGCCGCAAAACCTATGATGTACAGGGC
TGCTGAAAACTGGTCATAAAGCCGGTTTTGCCCTACATATGCCCTCAACGCAGACCATATCTGTGCCG
+
FFFFFFFFFFFFFFFFFFFFFFFFFF,FFFFFF:FFFFFFFFFFFFFFFF,FFFFF,FFFFFFFFFFFF
FFFFFFF,FFFFFFF::FFFFFFFFFFFFFFFFF:FFFF:FFFFFF:FFFFFFFFFFFFFFFF::FFF
@A00155:342:HHGFNDSXY:1:2646:28393:24017 2:N:0:GAACCTAG+TCCGCATA
GCCACACGCATGCTTCCGCCGTTGATGGCACGAACTGGTATGCCGCAAAACCTATGATGTACAGGGCT
GCTGAAAACTGGTCATAAAGCCGGTTTTGCCCTACATATGCCCTCAACGCAGACCATATCTGTGCCGA
+
FFFFFFFFFFFFFFFFFFFFFFFFFFFFFFFFFFFFFFFFFFFFFFFFFFFF:FFFFFFFFFFFFFFF
FFFFFFFFFFFFFFFFFFFFFFFFFFFFFFFFFFFFFFFFFFFFFFFFFFFFFFFFF:FFFFFFFFF
@A00155:342:HHGFNDSXY:1:1634:23149:24674 1:N:0:GAACCTAG+TCCGCATA
CCACACGCATGCTTCCGCCGTTGATGGCACGAACTGGTATGCCGCAAAACCTATGATGTACAGGGCTG
```

```
CTGAAAACTGGTCATAAAGCCGGTTTTGCCCTACATATGCCCTCAACGCAGACCATATCTGTGCCGAG
+
FFFFFFFFFFFFFFFFFFFFFFFFFFFFFFFF:FFFFFFFFFFFFFFF:FFFFFFFFFFFFFFFFFFF
FFFFFFFFFFFFFFFFFFFFFFFFFFFFFFFFFFFFFFF:FFFF:FFFFFFFFFFFFFFFFFFFFFFF
@A00155:342:HHGFNDSXY:1:1507:32072:31641 1:N:0:GAACCTAG+TCCGCATA
CACGCATGCTTCCGCCGTTGATGGCACGAACTGGTATGCCGCAAAACCTATGATGTACAGGGCTGCTG
AAAACTGGTCATAAAGCCGGTTTTGCCCTACATATGCCCTCAACGCAGACCATATCTGTGCCGAGTTG
+
FFFFFFFFFFFFFFFFFFFFFFFFFFFFFFFFFFFFFFFFFFFFFFFFFFFFFFFFFFFFFFFFFFFF
FFFFFFFFFFFFFFFFFFFFFFFFFFFFFFFF:FF:FFFFFFFFFFFFFFFFFFFFFFFFFFFFFFFF
@A00155:342:HHGFNDSXY:1:1607:1389:31673 1:N:0:GAACCTAG+TCCGCATA
CACGCATGCTTCCGCCGTTGATGGCACGAACTGGTATGCCGCAAAACCTATGATGTACAGGGCTGCTG
AAAACTGGTCATAAAGCCGGTTTTGCCCTACATATGCCCTCAACGCAGACCATATCTGTGCCGAGTTG
+
FFFFFFFFFFFFFFFFFFFFFFFFF:FF::FF:FFFFF,FFF,FF:FFFFFFFFFFFFFFFFFFFFFF
FF,,FFFFFFFFFF,:F,FFFFFFFF:FF:FF,:F,:F,FF:FFFF:FFF,,FFFFFF:FFFFFFFFF
@A00155:342:HHGFNDSXY:1:1139:5584:11193 1:N:0:GAACCTAG+TCCGCATA
CGCATGCTTCCGCCGTTGATGGCACGAACTGGTATGCCGCAAAACCTATGATGTACAGGGCTGCTGAA
AACTGGTCATAAAGCCGGTTTTGCCCTACATATGCCCTCAACGCAGACCATATCTGTGCCGAGTTGGG
+
FFFFFFFFFFFFFFFFFFFFFFFFFFFFFFFFF:FFFFFFFFFFFFFFFFFFFFFFFFFFFFFFFF:F
F:FFFFFFFFFFFFFFFFFFFFFFFFFFFFFFFFFFFFFFFFFFFFFFFF:FFFFFFFFFFF:FFFF:
@A00155:342:HHGFNDSXY:1:1246:22064:22138 1:N:0:GAACCTAG+TCCGCATA
CATGCTTCCGCCGTTGATGGCACGAACTGGTATGCCGCAAAACCTATGATGTACAGGGCTGCTGAAAA
CTGGTCATAAAGCCGGTTTTGCCCTACATATGCCCTCAACGCAGACCATATC
+
FFFFFFFFFFFFFFFFFFFFFFFFFFFFFFFFFFFFFFFFFFFFF:FFFFFFFFFFFFFFFFFFFFFF
FFFFFFFFFFFFFFFFFFFFFFFFFFFFFFFFFFFFFFFFFFFFFFFFFFFF
@A00155:342:HHGFNDSXY:1:1260:33003:32064 1:N:0:GAACCTAG+TCCGCATA
CATGCTTCCGCCGTTGATGGCACGAACTGGTATGCCGCAAAACCTATGATGTACAGGGCTGCTGAAAA
CTGGTCATAAAGCCGGTTTTGCCCTACATATGCCCTCAACGCAGACCATATC
+
FFFFF,FFFFFFF:FFF:FFFFFFFFF:FFF,FFFF:F:FFFFFF,FFFFF:FFFFFFFFFFFFF:F:
FFFFFF:FFFFFFFF:,FFFFFFFFFF,F,F:FFFFFFFFFF:FFFF,F:FF
@A00155:342:HHGFNDSXY:1:1263:19388:34256 1:N:0:GAACCTAG+TCCGCATA
CATGCTTCCGCCGTTGATGGCACGAACTGGTATGCCGCAAAACCTATGATGTACAGGGCTGCTGAAAA
CTGGTCATAAAGCCGGTTTTGCCCTACATATGCCCTCAACGCAGACCATATCT
+
FFFFFFFFFFFFFFFFFFFFFFFFFFFFFFFFFFFFFFFFFFFFFFFFFFFFFFFFFFFFFFFFFFFF
FFFFFFFFFFFFFFFFFFFFFFFF:FFFFFFFFFFFFFFFFFFFFFFFF:FFF
@A00155:342:HHGFNDSXY:1:1363:20518:32988 1:N:0:GAACCTAG+TCCGCATA
TGCTTCCGCCGTTGATGGCACGAACTGGTATGCCGCAAAACCTATGATGTACAGGGCTGCTGAAAACT
GGTCATAAAGCCGGTTTTGCCCTACATATGCCCTCAACGCAGACCATATCTGTGCCGAGTTGGGTAGG
+
FFFFFFFFFFFF,FF,FFFFFFFFFFFFFFFFFFFFFFFFFFFFFFFFFFFFFFFF,FFFFFFFFFFF
FFFFFFFFFFF,FFFF,FFFFFFFFFFFFFFFFFFFFFFFFFFFFFFFFFFFFFFF:FFFFFFFFFF
@A00155:342:HHGFNDSXY:1:2274:23963:16595 2:N:0:GAACCTAG+TCCGCATA
TGCTTCCGCCGTTGATGGCACGAACTGGTATGCCGCAAAACCTATGATGTACAGGGCTGCTGAAAACT
GGTCATAAAGCCGGTTTTGCCCTACATATGCCCTCAACGCAGACCATATCTGTGCCGAGTTGGGTAGG
+
FFFFFFFF:F:FFFFFFFFFFFFFFFFFFFFFF:FF:FFFFFFFFFFFFFFFFFFFFFFFFFFFFFFF
FFFFFFF,:FFFF:FFFFFFF:FF:FFFFFF:FFFFFFFFF,FFFF:FFFFFFFFFFFFFFFFFFFFF
@A00155:342:HHGFNDSXY:1:2236:27724:19006 1:N:0:GAACCTAG+TCCGCATA
CTTCCGCCGTTGATGGCACGAACTGGTATGCCGCAAAACCTATGATGTACAGGGCTGCTGAAAACTGG
```

```
TCATAAAGCCGGTTTTGCCCTACATATGCCCTCAACGCAGACCATATCTGTGCCGAGTTGGGTA
+
FFFFFFFFFFFFFFFFFFFFFFFFFFFFFFFFFFFFFFFFFFFFFFFFFFFFFFFFFFFFFFFFFFFF
FFFFFFFFFFFFFFFFFFFFFFFF:FFFFFFFFFFFFFFFFFFF:FFFFFFFFFFFFFFFFFFF
@A00155:342:HHGFNDSXY:1:2573:7283:18333 1:N:0:GAACCTAG+TCCGCATA
CTTCCGCCGTTGATGGCACGAACTGGTATGCCGCAAAACCTATGATGTACAGGGCTGCTGAAAACTGG
TCATAAAGCCGGTTTTGCCCTACATATGCCCTCAACGCAGACCATATCTGTGCCGAGTTGGGTA
+
FFFFFFFFFFFFFFFFFFFFFFFFFFFFFFFFFFFF:FFFFFFFFFFFFFFFFFFFFFFFFFFFFFFF
FFFFFFFFFFFFFFF:FFFFFFFFFFFF:FFFFFFFFFFFFFFFFFFFFFFFF,FFFFFFFFFFF
@A00155:342:HHGFNDSXY:1:2431:25256:14231 1:N:0:GAACCTAG+TCCGCATA
CTTCCGCCGTTGATGGCACGAACTGGTATGCCGCAAAACCTATGATGTACAGGGCTGCTGAAAACTGG
TCATAAAGCCGGTTTTGCCCTACATATGCCCTCAACGCAGACCATATCTGTGCCGAGTTGGGTA
+
FFFFFFFFFFFFFFF:FFFFFFF:FFFFF:F:FFFFFFF:FFF:FFFFFFFF:FFFFF:FFFFFFFFF
FFFFFFF:FFFFFFFFFFFFFFFFFFFFFFFFFFFFFFFFFFFFFFFFFFFFFFFFFFFFFFF:FF
@A00155:342:HHGFNDSXY:1:2236:27932:19272 1:N:0:GAACCTAG+TCCGCATA
CTTCCGCCGTTGATGGCACGAACTGGTATGCCGCAAAACCTATGATGTACAGGGCTGCTGAAAACTGG
TCATAAAGCCGGTTTTGCCCTACATATGCCCTCAACGCAGACCATATCTGTGCCGAGTTGGGTA
+
FFFFFFFFFFFFFFFFFFFFFFFFFFFFFFFFFFFFFFFFFFFFFFFFFFFFFFFFFFFFFFF:FFFF
FFFFFFFF,FFFFFFFFFFFFFFFFFFFFFFFFFFFFFFFFFFF,FFFFFFFFFFFFFFFFFFF
@A00155:342:HHGFNDSXY:1:1324:22833:31892 2:N:0:GAACCTAG+TCCGCATA
CTTCCGCCGTTGATGGCACGAACTGGTATGCCGCAAAACCTATGATGTACAGGGCTGCTGAAAACTGG
TCATAAAGCCGGTTTTGCCCTACATATGCCCTCAACGCAGACCATATCTGTGCCGAGTTGGGTAGGTC
+
F,FFFFFFFFFF:FFFFFFFFFFFFFFFFFFFFFFFFFFFFF:FFFFFFFFFFFFFFFFFFFFFFFFFF
FFFFFFFFFFFFFFFFFFFFFFFFFFFFFFFFFFFFFFFFFFFFFFFFFFFFFFFFFFFFFFFFFFFF
@A00155:342:HHGFNDSXY:1:2131:3803:2503 1:N:0:GAACCTAG+TCCGCATA
CTTCCGCCGTTGATGGCACGAACTGGTATGCCGCAAAACCTATGATGTACAGGGCTGCTGAAAACTGG
TCATAAAGCCGGTTTTGCCCTACATATGCCCTCAACGCAGACCATATCTGTGCCGAGTTGGGTAGGTC
+
F:FFFFFFFFFFFFFFFFFFFFFFFF,:FFFFFFF,FFFFF,FFFFF,FFFFFFFFFFFFFFFFF:FF
FFFFF:FFFFFFFFFFFFFFFFFFFFFFFFFFFFFFFFFFFFFFFFFFFFFFFFFFFFFFFFFFFFFF
@A00155:342:HHGFNDSXY:1:2131:4643:2832 1:N:0:GAACCTAG+TCCGCATA
CTTCCGCCGTTGATGGCACGAACTGGTATGCCGCAAAACCTAGGATGTACAGGGCTGCTGAAAACTGG
TCATAAAGCCGGTTTTGCCCTACATATGCCCTCAACGCAGACCATATCTGTGCCGAGTTGGGTAGGTC
+
FFFFFFFF:FFFF:FFFFFFFF:FFF:F,FFFFFFFF:F:F,,FFFF,FFFFFFFFFFFFFFFFFFFF
,FFFFF,FFFFFFF:FFFFF::FFFFFFFFFFFFFFFFFFFFFFFFFFFFFFFFFFFFFFFFFFFFFF
@A00155:342:HHGFNDSXY:1:1325:30391:30953 1:N:0:GAACCTAG+TCCGCATA
CTTCCGCCGTTGATGGCACGAACTGGTATGCCGCAAAACCTATGATGTACAGGGCTGCTGAAAACTGG
TCATAAAGCCGGTTTTGCCCTACATATGCCCTCAACGCAGACCATATCTGTGCCGAGTTGGGTAGGTC
+
F:FFFFFFFFFFFFFFFFFFFFFFFFFF:FFFFFFFFFFFFFFFFFFFFFFFFFFFFFFFFFFFFFFFF
FFFFFFFFFFFFFFFFFFFFFFFFFFFFFFFFFFFFFFFFFFFF:FFFFFFFFFFFFFFFFFFFFFFFF
@A00155:342:HHGFNDSXY:1:2541:2700:17253 2:N:0:GAACCTAG+TCCGCATA
TCCGCCGTTGATGGCACGAACTGGTATGCCGCAAAACCTATGATGTACAGGGCTGCTGAAAACTGGTC
ATAAAGCCGGTTTTGCCCTACATATGCCCTCAACGCAGACCATATCTGTGCCGAGTTGGGTAGGTCA
+
FFFFF:FFFFFFFFFF,FFFFFFFFFFFFFFFFFFF:FF:,:FFF:FFFFFFFFF,FFFFFFFFFFF
FF:F:FFFFFF::FFFFFF:F::F:F,FF,FFF:FFFFFFFFFF:FFF:FFFFFFFFFFFFFFF,FF
@A00155:342:HHGFNDSXY:1:2541:2736:17190 2:N:0:GAACCTAG+TCCGCATA
TCCGCCGTTGATGGCACGAACTGGTATGCCGCAAAACCTATGATGTACAGGGCTGCTGAAAACTGGTC
```

ATAAAGCCGGTTTTGCCCTACATATGCCCTCAACGCAGACCATATCTGTGCCGAGTTGGGTAGGTCA
+
FFFFFFFFFFFFFFFFFFFFFFFFFFFFFFFFFFFFFF:FFF:FFFFFFFFFFFFFFFFFFFFFFFFF
FFFFFFFFFFFFFFFFFFFFFFFFFFFFFFFF:FFFFFFFFF,FFFFFFFFFFFFFFFFFFFFFFFF
@A00155:342:HHGFNDSXY:1:2167:10818:1282 1:N:0:GAACCTAG+TCCGCATA
TCCGCCGTTGATGGCACGAACTGGTATGCCGCAAAACCTATGATGTACAGGGCTGCTGAAAACTGGTC
ATAAAGCCGGTTTTGCCCTACATATGCCCTCAACGCAGACCATATCTGTGCCGAGTTGGGTAGGTCAG
+
FFFFFFFFFFFFFFFFFFFFFFFFFFFFFFFFFFFFFFFFFFFFFFFFFFFFFFFFFFFFFFFFFFFF
FFFFFFFFFFFF:FFFFFFFFF,FFFFFFFFFFFFFFFFFFFFFFFFFFFFFFFFFFFFFFFFFFFFF
@A00155:342:HHGFNDSXY:1:2167:12626:5071 1:N:0:GAACCTAG+TCCGCATA
TCCGCCGTTGATGGCACGAACTGGTATGCCGCAAAACCTATGATGTACAGGGCTGCTGAAAACTGGTC
ATAAAGCCGGTTTTGCCCTACATATGCCCTCAACGCAGACCATATCTGTGCCGAGTTGGGTAGGTCAG
+
FFFFFFFFFFFFFFFFFFFFFFFFFFFFFFFFFFFFFFFFFFFFFFFFFFFFFFFFFFFFFFFFFFFF
FFFFFFFFFFFFFFFFFFFFFFFFFFFFFFFFFFFFFFFFF:FFFFFFFFFFFFFFFFFFFFFFFFFF
@A00155:342:HHGFNDSXY:1:1153:24261:29418 1:N:0:GAACCTAG+TCCGCATA
CCGCCGTTGATGGCACGAACTGGTATGCCGCAAAACCTATGATGTACAGGGCTGCTGAAAACTGGTCA
TAAAGCCGGTTTTGCCCTACATATGCCCTCAACGCAGACCATATCTGTGCCGAGTTGGGTAGGTCAGG
+
FFFFFFFFFFFFFFFFFFFFFFFFFFFFFFFFFFFFFFFFFFFFFFFFFFFFFFFFFFFFFFFFFFFF
FFFFFFFFFFFFFFFFFFFFFFFFFFFFFFFFFFFFFFFF:FFFFFFFFFFFFFFFFFFFFFFFFFFF
@A00155:342:HHGFNDSXY:1:2123:4101:36620 1:N:0:GAACCTAG+TCCGCATA
CCGCCGTTGATGGCACGAACTGGTATGCCGCAAAACCTATGATGTACAGGGCTGCTGAAAACTGGTCA
TAAAGCCGGTTTTGCCCTACATATGCCCTCAACGCAGACCATATCTGTGCCGAGTTGGGTAGGTCAGG
+
FFFFFFFFFFFFFFFFFFFFFFFF:FFFFFFFFFFFFFFFFFFFFFFFFFFFFFFFFFFFFFFFFFFF
FFFFFFFFFFFFFFFFFFFFFFFFFFFFFFFFFFFFFFFFFFFFFFFFFFFFFFFFFFFFFFFFFFFF
@A00155:342:HHGFNDSXY:1:2415:27317:34303 1:N:0:GAACCTAG+TCCGCATA
CCGCCGTTGATGGCACGAACTGGTATGCCGCAAAACCTATGATGTACAGGGCTGCTGAAAACTGGTCA
TAAAGCCGGTTTTGCCCTACATATGCCCTCAACGCAGACCATATCTGTGCCGAGTTGGGTAGGTCAGG
+
FFFFFFFFFFFFFFFFFFFFFFFFFFFFFFFFFFFFFFFFFFFFFFFFFFFFFFFFFFFFFFFF:FFF
FFF:FFFFFFFFFFFFFFFFFFFFFFFFFFFFFFFFFFFFFFFFFFFFFFFFFFFFFFFFFFFFFFFF
@A00155:342:HHGFNDSXY:1:2274:31421:26600 1:N:0:GAACCTAG+TCCGCATA
CCGCCGTTGATGGCACGAACTGGTATGCCGCAAAACCTATGATGTACAGGGCTGCTGAAAACTGGTCA
TAAAGCCGGTTTTGCCCTACATATGCCCTCAACGCAGACCATATCTGTGCCGAGTTGGGTAGGTCAGG
+
FFFFFFFFFFFFFFFFFFFFFFFFFFFFFFFFFFFFFFFFFFFFFFFFFFFFFFFFFFFFFFFFFFFF
FFFFFFFFFFFFFFFFF:FFF:FFFFFFFFFFFFFFFFFFF,FFFFF:FFFFFFFFFFFFFFFFFFFF
@A00155:342:HHGFNDSXY:1:2139:17137:18865 1:N:0:GAACCTAG+TCCGCATA
CCGCCGTTGATGGCACGAACTGGTATGCCGCAAAACCTATGATGTACAGGGCTGCTGAAAACTGGTCA
TAAAGCCGGTTTTGCCCTACATATGCCCTCAACGCAGACCATATCTGTGCCGAGTTGGGTAGGTCAGG
+
FFFFFFFFFFFFFFFFFFFFFFFFFFFFFFFFFF:FFFFFFFFFFFFFFFFFFFFFFFFFFFFFFFFF
FFFFFFFFFFFFFFFFFFFFFFFFFFFFFFFFFFFFFFFFF,FFFFFFFFFFFFFFFFFFFFFFF:FF
@A00155:342:HHGFNDSXY:1:2305:12753:32565 1:N:0:GAACCTAG+TCCGCATA
CGCCGTTGATGGCACGAACTGGTATGCCGCAAAACCTATGATGTACAGGGCTGCTGAAAACTGGTCAT
AAAGCCGGTTTTGCCCTACATATGCCCTCAACGCAGACCATATCTGTGCCGAGTTGGGTAGGTCAGGT
+
FFFFFFFFFFFFFFFFFFFFFFFFFFFFFFFFFFFFFFFFFFFFFFFFFFFFFFFFFFFFFFFFFFFF
FFFFFFFFFFFFFFFFFFFFFFFFFFFFFFFFFFFFFFFFFFFFFFFFFFFFFFFFFFFFFFFFFFFF
@A00155:342:HHGFNDSXY:1:1463:5746:14606 2:N:0:GAACCTAG+TCCGCATA
CGCCGTTGATGGCACGAACTGGTATGCCGCAAAACCTATGATGTACAGGGCTGCTGAAAACTGGTCAT

```
AAAGCCGGTTTTGCCCTACATATGCCCTCAACGCAGACCATATCTGTGCCGAGTTGGGTAGGTCAGGT
+
:FF,F:FFFFF:FFFFFFFFFFFFFFF,FFFFFFFFFFFFFFFFFFFFFFFFFFFFFFFFFFFFFFFF
FFFFFFFFFFFFFFF:FFFFFFFFFF:F:FFFFFFFFFFFF:FFFFFFFFFFFFFFFFFFFFFFFFFF
@A00155:342:HHGFNDSXY:1:2462:21847:28369 1:N:0:GAACCTAG+TCCGCATA
GCCGTTGATGGCACGAACTGGTATGCCGCAAAACCTATGATGTACAGGGCTGCTGAAAACTGGTCATA
AAGCCGGTTTTGCCCTACATATGCCCTCAACGCAGACCATATCTGTGCCGAGTTGGGTAGGTCAGGTG
+
FFFFFFFFFFFFFFFFFFFFFFFFFFFFFFFFFFFFFFFFFFFFFFFFFFFFFFFFFFFFFFFFFFFF
FFFFFFFFFFFFFFFFFFFFFFFFFFFFFFFFFFFFFFFFFFFFFFFFFFFFFFFFFFFFFFFFFFFF
@A00155:342:HHGFNDSXY:1:2347:15429:2127 1:N:0:GAACCTAG+TCCGCATA
GCCGTTGATGGCACGAACTGGTATGCCGCAAAACCTATGATGTACAGGGCTGCTGAAAACTGGTCATA
AAGCCGGTTTTGCCCTACATATGCCCTCAACGCAGACCATATCTGTGCCGAGTTGGGTAGGTCAGGTG
+
FFFFFFFFFFFFFFFFFFFFFFFFFFFFFFFFFFFFFFFFFFFFFFFFFFFFFFFFFFFFFFFFFFFF
F:FFFFFFFFFFFFFFFFFFFFFFFFFFFFFFFFFF:FFFFFFFFFFFFFFFFFFFFFF:FFFFFFFFF
@A00155:342:HHGFNDSXY:1:2341:4255:8547 1:N:0:GAACCTAG+TCCGCATA
TTGATGGCACGAACTGGTATGCCGCAAAACCTATGATGTACAGGGCTGCTGAAAACTGGTCATAAAGC
CGGTTTTGCCCTACATATGCCCTCAACGCAGACCATATCTGTGCCGAGTTGGGTAGGTCAGGTGCCCT
+
FFFFFFFFFFFFF:FFFFFFFFFFFFFFFFFFFFFFFFFFFFFFFFFFFFFFFFFFFFFFFFFFFFFF
FFFFFFFFFFFFFFFFFFFFFFFFFFFFFFFFFFFFFFF:FFFFFFFFFFFFFFFFFFFFFFFFFFFF
@A00155:342:HHGFNDSXY:1:1274:30861:31641 2:N:0:GAACCTAG+TCCGCATA
TTGATGGCACGAACTGGTATGCCGCAAAACCTATGATGTACAGGGCTGCTGAAAACTGGTCATAAAGC
CGGTTTTGCCCTACATATGCCCTCAACGCAGACCATATCTGTGCCGAGTTGGGTAGGTCAGGTGCCCT
+
FFFFFFFFFFFFFFFFFFFFFFFFFF:F:F:F:FFFFFFFFFFFFFFFFFFFFFFFFFFFFFFFFFFF
FFFFFFFFF,FFFFFFFFFFFFFFFFFFFFFFFFFFFFFFFFFFFFFFFFFFFFFFFFFFFFFFFFFF
@A00155:342:HHGFNDSXY:1:1263:20473:2628 2:N:0:GAACCTAG+TCCGCATA
TTGATGGCACGAACTGGTATGCCGCAAAACCTATGATGTACAGGGCTGCTGAAAACTGGTCATAAAGC
CGGTTTTGCCCTACATATGCCCTCAACGCAGACCATATCTGTGCCGAGTTGGGTAGGTCAGGTGCCCT
+
FFFFFFFFFFFFFFFFFFFFFFFFFFFFFFFFFFFF:FFFFFFFFFFFFFFFF:FFFFFFFFFFFFFF
FFFFFFFFFFFFFFFFFFFFFFFFFFFFFFFFFFF,FFFFFFFFFFFFFFFFFFF:FFFFFFFFFFFF:
@A00155:342:HHGFNDSXY:1:2323:6976:10755 2:N:0:GAACCTAG+TCCGCATA
TTGATGGCACGAACTGGTATGCCGCAAAACCTATGATGTACAGGGCTGCTGAAAACTGGTCATAAAGC
CGGTTTTGCCCTACATATGCCCTCAACGCAGACCATATCTGTGCCGAGTTGGGTAGGTCAGGTGCCCT
+
FFFFFFFFFFFFFFFFFFFFFFFFFFFFFFFFFF,FFFFFFFFFFFFFFFFFFFFFFFFFFFFFFFFF
:FF:FFFFFFFFFFFFFFFFFFFFFFFFFFFFFFFF:FFFFFFFFFFFFFFFFFFFFFFFFFFFFFFF
@A00155:342:HHGFNDSXY:1:2123:4101:36620 2:N:0:GAACCTAG+TCCGCATA
TTGATGGCACGAACTGGTATGCCGCAAAACCTATGATGTACAGGGCTGCTGAAAACTGGTCATAAAGC
CGGTTTTGCCCTACATATGCCCTCAACGCAGACCATATCTGTGCCGAGTTGGGTAGGTCAGGTGCCCT
+
FFFFFFFFFFFFFFFFFF:FFFFFFFF,FFFF::FFFFFFFFFFFFFFFFFFFFFFFFFFFFFFFFFF
FFFFFFFFFFFFFFFFFFFFFFFFFFFFFFFFFFFFFFFFFFFFFFFFFFFFFFFFFFFFFFFFFFFF
@A00155:342:HHGFNDSXY:1:2541:16785:23202 2:N:0:GAACCTAG+TCCGCATA
TTGATGGCACGAACTGGTATGCCGCAAAACCTATGATGTACAGGGCTGCTGAAAACTGGTCATAAAGC
CGGTTTTGCCCTACATATGCCCTCAACGCAGACCATATCTGTGCCGAGTTGGGTAGGTCAGGTGCCCT
+
FFFFFFFFF:FFFFFFFFFFFFFFFFFFF:F:F,FFFFFFF:FFFFFFFFFFFFFFFFFF:FFFF:FF
FFFFFFFFFFFFFFFFF:FFFFFFFFFFFFFFFFF,FFFFFFFFFFFFFFFFFFFFFFFFFFFFFFFF
@A00155:342:HHGFNDSXY:1:1538:4038:10019 1:N:0:GAACCTAG+TCCGCATA
TTGATGGCACGAACTGGTATGCCGCAAAACCTATGATGTACAGGGCTGCTGAAAACTGGTCATAAAGC
```

```
CGGTTTTGCCCTACATATGCCCTCAACGCAGACCATATCTGTGCCGAGTTGGGTAGGTCAGGTGCCCT
+
FFFFFFFFFFFFFFFFFFFFFFFFFFFFFFFFFFFFFFFFFFFFFFFFFFFFFFFFFFFFFFFFFFFF
FFFFFFFFFFFFFFFFFFFFFFFFFFFFFFFFFFF,FFFFFFFFFFFFFFFFFFFFFFF:FFFFFFFF
@A00155:342:HHGFNDSXY:1:1478:32976:34334 2:N:0:GAACCTAG+TCCGCATA
TGATGGCACGAACTGGTATGCCGCAAAACCTATGATGTACAGGGCTGCTGAAAACTGGTCATAAAGCC
GGTTTTGCCCTACATATGCCCTCAACGCAGACCATATCTGTGCCGAGTTGGGTAGGTCAGGTGCCCTA
+
FF,FFFFFFFFFFFFFFFFFFFFFFFFFFF,FFFFFFFFFFFFFFFFFFFFF,FFFFFFFFF,FFFFF
FFFFFFFFFFFFFFFFFFFFFFFFFFFFFFFFFF::FFFFFFFFFFFFFFFFFFFFFFFFF:FFFFFF
@A00155:342:HHGFNDSXY:1:1152:17824:30326 2:N:0:GAACCTAG+TCCGCATA
TGGCACGAACTGGTATGCCGCAAAACCTATGATGTACAGGGCTGCTGAAAACTGGTCATAAAGCCGGT
TTTGCCCTACATATGCCCTCAACGCAGACCATATCTGTGCCGAGTTGGGTAGGTCAGGTGCCCTAG
+
FFFFFFFFFFF:FFFFFFFFFFFFFFFFFFFFFFFFFF:FFFFFFFFFFFFFF:FFFFFF::,FFFFFF
FFFFFFFFFFFFFFFFFFFFFF,FFFFFFFF:FFFFFFFFFFFFFFFFFFFFFFFFFFFFFFFFF,FF
@A00155:342:HHGFNDSXY:1:2113:26467:4773 2:N:0:GAACCTAG+TCCGCATA
CACGAACTGGTATGCCGCAAAACCTATGATGTACAGGGCTGCTGAAAACTGGTCATAAAGCCGGTTTT
GCCCTACATATGCCCTCAACGCAGACCATATCTGTGCCGAGTTGGGTAGGTCAGGTGCCCTAGCACCT
+
FFFFFFFFFFFFFFFFFFFFFFFFFFFFFFFFFFFFFFFFFFFFFFFFFFFFFFFFFFFFFFFFFFFF
FFFFFFFFFFFFFFFFFFFFFFFFFFFFFFFFFFFFFFFFFFFFFFFFFFFFFFFFFFFFFFFFFFF:
@A00155:342:HHGFNDSXY:1:2677:1154:34460 2:N:0:GAACCTAG+TCCGCATA
CACGAACTGGTATGCCGCAAAACCTATGATGTACAGGGCTGCTGAAAACTGGTCATAAAGCCGGTTTT
GCCCTACATATGCCCTCAACGCAGACCATATCTGTGCCGAGTTGGGTAGGTCAGGTGCCCTAGCACCT
+
FFFFFFFFF,,FFFFFFFFFFFFFFFF:F:F:FFF::FF:FFFFFFFFFFF::FF,FFFFFFF:F,F:
FFFFFFFFFFFFFFF:FFFFFFFFFFFFFFFFF::FFFFFF:FFFFFFFFFFFFFFFFFFFF:FFFFF
@A00155:342:HHGFNDSXY:1:1334:28429:27367 1:N:0:GAACCTAG+TCCGCATA
CGAACTGGTATGCCGCAAAACCTATGATGTACAGGGCTGCTGAAAACTGGTCATAAAGCCGGTTTTGC
CCTACATATGCCCTCAACGCAGACCATATCTGTGCCGAGTTGGGTAGGTCAGGTGCCCTAGCACCTTC
+
FFFFFFFFFFFFFFFFFFFFFFFFFFFFFFFFFFFFFFFFFFFFFFFFFFFFFFFFFFFFFFFFFFFF
FFFFFFFFFFFFFFFFFFFFFFFFFFFFFFFFFFFFFFFFFFFF:FFFFFFFFFFFFFFFFFFFF,FF
@A00155:342:HHGFNDSXY:1:2542:19831:9596 1:N:0:GAACCTAG+TCCGCATA
CGAACTGGTATGCCGCAAAACCTATGATGTACAGGGCTGCTGAAAACTGGTCATAAAGCCGGTTTTGC
CCTACATATGCCCTCAACGCAGACCATATCTGTGCCGAGTTGGGTAGGTCAGGTGCCCTAGCACCTTC
+
FFFFFFFFFFFFFFFFFFFFFFFFFFFFFFFFFFFFFFFFFFFFFFFFFFFFFFFFFFFFFFFFFFFF
FFFFFFFFFFFFFFFFFFFFF,FFFFFFFFFFFFFFFFFFFFFFFFFFFFFFFFFFFFFF:FF:F:,:F
@A00155:342:HHGFNDSXY:1:2118:30337:13166 1:N:0:GAACCTAG+TCCGCATA
AACTGGTATGCCGCAAAACCTATGATGTACAGGGCTGCTGAAAACTGGTCATAAAGCCGGTTTTGCCC
TACATATGCCCTCAACGCAGACCATATCTGTGCCGAGTTGGGTAGGTCAGGTGCCCTAGCACCTTCCT
+
FFFFFFFFFFFFFFFFFFFFFFFFFFFFFFFFFFFFFFFFFFFFFFFFFFFFFFFFFFFFFFFFFFFF
FFFFFFFFFFFFFFFFFFFFFFFFFFFFFFFFFFFFFFFFFFFFFFFFFFFFFFFFFFFFFFFF:FF:
@A00155:342:HHGFNDSXY:1:2215:24397:22200 2:N:0:GAACCTAG+TCCGCATA
AACTGGTATGCCGCAAAACCTATGATGTACAGGGCTGCTGAAAACTGGTCATAAAGCCGGTTTTGCCC
TACATATGCCCTCAACGCAGACCATATCTGTGCCGAGTTGGGTAGGTCAGGTGCCCTAGCACCTTCCT
+
FFFFFFFFFFFFFFFFFFFFFFFFFFFFFFFFFFFF:FFFFFFFFFFFFFFFFFFFFFFFFFFFFFFF
FFFFFFFFFFFFFFFFFFFFFFFFFFFFFFF:FFFFFFFFFFFFFFFFFFFFFFFFFFFFFFFFFFFF
@A00155:342:HHGFNDSXY:1:2140:14534:18521 2:N:0:GAACCTAG+TCCGCATA
ACTGGTATGCCGCAAAACCTATGATGTACAGGGCTGCTGAAAACTGGTCATAAAGCCGGTTTTGCCCT
```

ACATATGCCCTCAACGCAGACCATATCTGTGCCGAGTTGGGTAGGTCAGGTGCCCTAGCACCTTCCTC
+
FFFFFFFFFFFFFFF:F,FFF:FFFFFFFFFFFFFFFFFFFFFFFFFFFFFFFFFFFFFFFFFFFFFF
FF,FFFFFFFFFFFFFFFFFFFFFFFFFFFFFFFFFFFFFFFFFFFFFFF,FFFFFFFFFFFFFFFFF
@A00155:342:HHGFNDSXY:1:1262:15338:13714 1:N:0:GAACCTAG+TCCGCATA
ACTGGTATGCCGCAAAACCTATGATGTACAGGGCTGCTGAAAACTGGTCATAAAGCCGGTTTTGCCCT
ACATATGCCCTCAACGCAGACCATATCTGTGCCGAGTTGGGTAGGTCAGGTGCCCTAGCACCTTCCTC
+
FFFFFFFFFFFFFFFFFFFFFFFFFFFFFFFFFFFFFFFFFFFFFFFFFFFFFFFFFFFFFFFFFFFF
FFFFFFFFFFFFFFFFFFFFFFFFFFFFFFFFFFFFFFFFFFFFFFFFFFFFFFFFFFFFFFFFFFFF
@A00155:342:HHGFNDSXY:1:1628:10890:28933 1:N:0:GAACCTAG+TCCGCATA
CTGGTATGCCGCAAAACCTATGATGTACAGGGCTGCTGAAAACTGGTCATAAAGCCGGTTTTGCCCTA
CATATGCCCTCAACGCAGACCATATCTGTGCCGAGTTGGGTAGGTCAGGTGCCCTAGCACCTTCCTCC
+
FFFFFFFFFFFFFFFFFFFFFFFFFFFFFFFFFFFFFFFFF,FFFFFFFFFF:FFFFFF:FFFFFFFF
FFFFFFFFFFFFFFFFFFFFFF:FFFFFFFFFFFFFFFFFFFFFFFFFFFFFFFFFFFFFFFFFF,FF
@A00155:342:HHGFNDSXY:1:2523:12816:19179 1:N:0:GAACCTAG+TCCGCATA
CTGGTATGCCGCAAAACCTATGATGTACAGGGCTGCTGAAAACTGGTCATAAAGCCGGTTTTGCCCTA
CATATGCCCTCAACGCAGACCATATCTGTGCCGAGTTGGGTAGGTCAGGTGCCCTAGCACCTTCCTCC
+
FFFFFFFFFFFFFFFFFFFFFFFFFFFFFFFFFFFFFFFFFFFFFFFFFFFFFFFFFFFFFFFFFFFF
FFFFFFFFFFFFFFFFFFFFFFF:FFFFFFFFFFFFFFFFFFFFFFFFFFFFFFFFFFFFFFFFFFFF
@A00155:342:HHGFNDSXY:1:1628:10972:28980 1:N:0:GAACCTAG+TCCGCATA
CTGGTATGCCGCAAAACCTATGATGTACAGGGCTGCTGAAAACTGGTCATAAAGCCGGTTTTGCCCTA
CATATGCCCTCAACGCAGACCATATCTGTGCCGAGTTGGGTAGGTCAGGTGCCCTAGCACCTTCCTCC
+
FFFFFFFFFFFFFFFFFFFFFF:FFFFFFFFFFFFFFFFFFFFFFFFFFFFFFFFFFFFFFFFFFFFF
FFFFFFFFFFFFFFFFFFFFFF:FFFFFFFFFFFFFFFFFFFFFFFFFFFFFFFFFFFFFFF,FFFFF
@A00155:342:HHGFNDSXY:1:1138:18050:34413 2:N:0:GAACCTAG+TCCGCATA
CTGGTATGCCGCAAAACCTATGATGTACAGGGCTGCTGAAAACTGGTCATAAAGCCGGTTTTGCCCTA
CATATGCCCTCAACGCAGACCATATCTGTGCCGAGTTGGGTAGGTCAGGTGCCCTAGCACCTTCCTCC
+
FFFFFFFFFFFFFF:FFFFFFFFFFFFFFFFFFFFFFFFFFFFFFFFFFFFFFFFFFFFFFFFFFFFF
FFFFFFFFFFFFFFFFFFFFFFFFFFFFFFFFFFFFFFFFFFFFFFFFFFFFF:FFFFFFFFFFFFFF
@A00155:342:HHGFNDSXY:1:1510:23249:2362 2:N:0:GAACCTAG+TCCGCATA
CTGGTATGCCGCAAAACCTATGATGTACAGGGCTGCTGAAAACTGGTCATAAAGCCGGTTTTGCCCTA
CATATGCCCTCAACGCAGACCATATCTGTGCCGAGTTGGGTAGGTCAGGTGCCCTAGCACCTTCCTCC
+
FFFFFFFFFFFFFFFFFFFFFF:FFFFFFF:FFFFFFFFFFFFFFFFFFFFFFFFFFFFFFFFFFFFF
FFFFFFFFFFFFFFFFFFFFFF:FFFFFFFFFFFFFFFFFFFFFFFFFFFFFFFFFFFFFFFFFFFFF
@A00155:342:HHGFNDSXY:1:2253:15917:27524 1:N:0:GAACCTAG+TCCGCATA
CTGGTATGCCGCAAAACCTATGATGTACAGGGCTGCTGAAAACTGGTCATAAAGCCGGTTTTGCCCTA
CATATGCCCTCAACGCAGACCATATCTGTGCCGAGTTGGGTAGGTCAGGTGCCCTAGCACCTTCCTCC
+
F:FFFFFFFFFFFFFFFFFFFFFFFFFFFFFFFFFFFFFFFFFFFFFFFFFFFFFFFFFFFFFFFFFF
FFFFFFFFFFFFFFFFFFFFFFFFFFFFFFFFFFFFFFFFFFFFFFFFFFFFFFFFFFFFFFFFF:FF
@A00155:342:HHGFNDSXY:1:2210:17969:26663 2:N:0:GAACCTAG+TCCGCATA
TGGTATGCCGCAAAACCTATGATGTACAGGGCTGCTGAAAACTGGTCATAAAGCCGGTTTTGCCCTAC
ATATGCCCTCAACGCAGACCATATCTGTGCCGAGTTGGGTAGGTCAGGTGCCCTAGCACCTTCCTCCC
+
FFFFFFFFFFFFFFFFFFF:FFFFFFF:FFFFFFFFFFFF:FFFFFFFFFF:FFFFFFFFFFFFFFFF
FFFFFFFFFFFFFFFFFFFFFF,FFFFFFFFFFFFFFFFFFFFFFFFFFFFFFFFFFFFFFFFFFFFF
@A00155:342:HHGFNDSXY:1:2210:19732:28526 2:N:0:GAACCTAG+TCCGCATA
TGGTATGCCGCAAAACCTATGATGTACAGGGCTGCTGAAAACTGGTCATAAAGCCGGTTTTGCCCTAC

ATATGCCCTCAACGCAGACCATATCTGTGCCGAGTTGGGTAGGTCAGGTGCCCTAGCACCTTCCTCCC
+
FFFFFFFFFFFFFFFFFF:FFFFFFFFFFFFFFFFFFFFFFFFFF,FFFFFFFFFFFFFFFFFFFFFFF:FF
FFFFFFFFFFFFFFFFFFFFFFFFFFFFFFFFFFFFFFFFFFFFFFFFFFFFFFFFFFFFFF:FFFFFFF
@A00155:342:HHGFNDSXY:1:2210:17924:27273 2:N:0:GAACCTAG+TCCGCATA
TGGTATGCCGCAAAACCTCTGATGTACAGGGCTGCTGAAAACTGGTCATAAAGCCGGTTTTGCCCTAC
ATATGCCCTCAACGCAGACCATATCTGTGCCGAGTTGGGTAGGTCAGGTGCCCTAGCACCTTCCTCCC
+
FFFFF:FF,FFFFFFFFF,FFFFFFFFFFFFFFFFFFFFF:FFFFFFFFFFFF:F:FFF,FFFFFFFF
FFFFF:FFFFF:FFFFFFFFFF:FFFFFFFFFFFFFFFF:FFFFFFFFFFFFFFFFFF:FFFFFFFFF
@A00155:342:HHGFNDSXY:1:1525:2347:26381 1:N:0:GAACCTAG+TCCGCATA
TGGTATGCCGCAAAACCTATGATGTACAGGGCTGCTGAAAACTGGTCATAAAGCCGGTTTTGCCCTAC
ATATGCCCTCAACGCAGACCATATCTGTGCCGAGTTGGGTAGGTCAGGTGCCCTAGCACCTTCCTCCC
+
FFFFFF:FFFFFFFFFFFFFFFFFFFFFFFFFFFFFFFFFFFFFFFFFFFFFFFFFFFFFFF:FFFFFF
FFFFFFFFFFFFFF,FFFFFFFFFFFFFFFFFFFFFFF:FFFFFFFFFFFFFFFFFFFFFF:FFF:FFFF
@A00155:342:HHGFNDSXY:1:2258:18719:15593 2:N:0:GAACCTAG+TCCGCATA
TGGTATGCCGCAAAACCTATGATGTACAGGGCTGCTGAAAACTGGTCATAAAGCCGGTTTTGCCCTAC
ATATGCCCTCAACGCAGACCATATCTGTGCCGAGTTGGGTAGGTCAGGTGCCCTAGCACCTTCCTCCC
+
FFFFFFFFFFFFFFFFFF,FFFFFFFFFFFFFFFFFFFFFFFFFFFFFFFFFFFFFFFFFFFFFFFFFF
FFFFFFFFFFFFFFFFFFF:FFFFFFFFFFFFFFFFFFFFFFFFFFFFFFFFFFFFFFFFFF:FFFFFFF
@A00155:342:HHGFNDSXY:1:2210:16812:27320 2:N:0:GAACCTAG+TCCGCATA
TGGTATGCCGCAAAACCTATGATGTACAGGGCTGCTGAAAACTGGTCATAAAGCCGGTTTTGCCCTAC
ATATGCCCTCAACGCAGACCATATCTGTGCCGAGTTGGGTAGGTCAGGTGCCCTAGCACCTTCCTCCC
+
FFFFFFFFFFFFFFFFFF:FFFFFFFFFFFFFFFFFFFFFFFFFFFFFFFFF:FFFFFFFFFFFFFFFF
FFFFFFFFFFFFFFFFFFFFFFFFFFFFFFFFFFFFFFFFFFFFFFFFFFFFFFFFFFFFFFFFFFFFF
@A00155:342:HHGFNDSXY:1:2118:30337:13166 2:N:0:GAACCTAG+TCCGCATA
ATGCCGCAAAACCTATGATGTACAGGGCTGCTGAAAACTGGTCATAAAGCCGGTTTTGCCCTACATAT
GCCCTCAACGCAGACCATATCTGTGCCGAGTTGGGTAGGTCAGGTGCCCTAGCACCTTCCTCCCCTTT
+
FF:F:FFFFFFFFF,FFFFF:FFFFFFFFFFFFFFFFFFFFFFFFFF:FFFFFFFFFFFFFFFFFFFFF
FFFFFFFFFFFFFFFFFFFFFFFFFFFFFFFFFFFFFFFFFFFFF:FFFFFFFFFFF:FFFFFFFFFF
@A00155:342:HHGFNDSXY:1:1665:17833:1031 2:N:0:GAACCTAG+TCCGCATA
TGCCGCAAAACCTATGATGTACAGGGCTGCTGAAAACTGGTCATAAAGCCGGTTTTGCCCTACATATG
CCCTCAACGCAGACCATATCTGTGCCGAGTTGGGTAGGTCAGGTGCCCTAGCACCTTCCT
+
FFFFFFFFFFFFFFFFFFFFFFFFFFFFFFFFFFFFFFFFFFFFFFFFFFFFFFFFFF:FFFFFFFFF
FFFFFFFFFFFFFFFFFFFFFFFFFFFFFFFFFFFFFFFFFFFFFFFFFFFFFFFFFFFF
@A00155:342:HHGFNDSXY:1:2218:29613:7279 2:N:0:GAACCTAG+TCCGCATA
GCCGCAAAACCTATGATGTACAGGGCTGCTGAAAACTGGTCATAAAGCCGGTTTTGCCCTACATATGC
CCTCAACGCAGACCATATCTGTGCCGAGTTGGGTAGGTCAGGTGCCCTAGCACCTTCCTCCCC
+
FFFFFFFFFFFFFFFFFFFFFFFFFFFFFFFFFFFFFFFF:FFFFFFFFFF:FFFFFFFFFFFFFFFF
FFFFFFFFFFFF:FFFFFFFFFFFFFFFFFFFFFFFFFFFFFFFFFFFFFFFFFFFFF:FFFF
@A00155:342:HHGFNDSXY:1:2218:27679:7874 2:N:0:GAACCTAG+TCCGCATA
GCCGCAAAACCTATGATGTACAGGGCTGCTGAAAACTGGTCATAAAGCCGGTTTTGCCCTACATATGC
CCTCAACGCAGACCATATCTGTGCCGAGTTGGGTAGGTCAGGTGCCCTAGCACCTTCCTCCCC
+
FFFFFFFFFF:FFFFFF,:FFFF,FFFFFFF:FFFFFFFF:FFFFFF,FFFFFFFFFFFFFFFFF:FF
FFF:FF,FFFFFFFFFFFFFFFFF,FFFFFFFFF:FFFFFFFFFFFFFFFFFFF:FFFFFFFF
@A00155:342:HHGFNDSXY:1:2258:18719:15593 1:N:0:GAACCTAG+TCCGCATA
CCGCAAAACCTATGATGTACAGGGCTGCTGAAAACTGGTCATAAAGCCGGTTTTGCCCTACATATGCC

```
CTCAACGCAGACCATATCTGTGCCGAGTTGGGTAGGTCAGGTGCCCTAGCACCTTCCTCCCCTTTCAA
+
FFFFFFF,FFFFFFFFFFFFFFFFFFFFFFFFF:FFFFFFFFFFFFFFFFFFFFFFFFFFFFFFFFFF
FFFFFFFFFFFFFFFFFFFFFFFFFFFFFFFFFFFFFFFFFFFFFFFFFFFFFFF:FFFFFFFFFFFFF
@A00155:342:HHGFNDSXY:1:1672:20844:26725 2:N:0:GAACCTAG+TCCGCATA
CGCAAAACCTATGATGTACAGGGCTGCTGAAAACTGGTCATAAAGCCGGTTTTGCCCTACATATGCCC
TCAACGCAGACCATATCTGTGCCGAGTTGGGTAGGTCAGGTGCCCTAGCACCTTCCTCCCCTTTCAAC
+
FFFFFFFFFFFFFFFFFFFFFFFFFFFFFFFF:FFFFFFFFFFFFFFFFFFFFFFFFFFFFFFFFFFF
FFFFFFFFFFFFFFFFFFFFFFFFFFFFFFFFFFFFFFFFFFFFFFFFFFFFFFFFFFFFFFFFFFFF
@A00155:342:HHGFNDSXY:1:2305:12753:32565 2:N:0:GAACCTAG+TCCGCATA
GCAAAACCTATGATGTACAGGGCTGCTGAAAACTGGTCATAAAGCCGGTTTTGCCCTACATATGCCCT
CAACGCAGACCATATCTGTGCCGAGTTGGGTAGGTCAGGTGCCCTAGCACCTTCCTCCCCTTTCAACC
+
FFFFFFFFFFFFFFFFFFFFFFFFFFFFFFFFFFFFFFFFFFFFFFF:FFFFFFFFFFFFFFFFFFFF
FFFFFFFFFFFFFFFFFFFFFFFFFFFFFFFFFFFFFFFFFFFFFFFFFFFF:FFFFFFFFFFFFFFFF
@A00155:342:HHGFNDSXY:1:2638:32380:17581 1:N:0:GAACCTAG+TCCGCATA
CAAAACCTATGATGTACAGGGCTGCTGAAAACTGGTCATAAAGCCGGTTTTGCCCTACATATGCCCTC
AACGCAGACCATATCTGTGCCGAGTTGGGTAGGTCAGGTGCCCTAGCACCTTCCTCCCCTTTCAACCA
+
FFFFFFFFFFFFFFFFFFFFFFFFFFFFFFFFFFFFF,FFFFFFFFFFFFFFFFFFFFFFFFFFFFFF
FFFFFFFFFFFF:FFFFFFFFFFF:FFFFFFFFFFFFFFFFFFFFFFFFFFF:FFFFFFFFF:FFFFFFF
@A00155:342:HHGFNDSXY:1:2507:12084:22420 1:N:0:GAACCTAG+TCCGCATA
CAAAACCTATGATGTACAGGGCTGCTGAAAACTGGTCATAAAGCCGGTTTTGCCCTACATATGCCCTC
AACGCAGACCATATCTGTGCCGAGTTGGGTAGGTCAGGTGCCCTAGCACCTTCCTCCCCTTTCAACCA
+
FFFFFFFFFFFFFFFFFFFFFFFFFFFFFFFFFFFFFFFFFFFFFFFFFFFFFFFFFFFFFFFFFFFF
FFFFFFFFFFFFFFFFFFFFFFFFFFFFFFFFFFFFFFFFFFFFFFFFFFFFFFFFFFFFFFFFFFF:
@A00155:342:HHGFNDSXY:1:2215:3197:7874 2:N:0:GAACCTAG+TCCGCATA
AACCTATGATGTACAGGGCTGCTGAAAACTGGTCATAAAGCCGGTTTTGCCCTACATATGCCCTCAAC
GCAGACCATATCTGTGCCGAGTTGGGTAGGTCAGGTGCCCTAGCACCTTCCTCCCCTTTCAACCATGC
+
F:FFFFFFFFFFFFFFFFFFFFFFFFFFFFFFFFFFFF,FFFFFFFFFFFFFFFFFFFFFFFFFFFFF
FFF:FFFFFFFFFFFFFFFFFFFFFFFF:FFFFFFFFFFFFFFFFFFF:FFFFFFFFFFFFFFFFFFFF
@A00155:342:HHGFNDSXY:1:2410:5493:1047 2:N:0:GAACCTAG+TCCGCATA
CCTATGATGTACAGGGCTGCTGAAAACTGGTCATAAAGCCGGTTTTGCCCTACATATGCCCTCAACGC
AGACCATATCTGTGCCGAGTTGGGTAGGTCAGGTGCCCTAGCACCTTCCTCCCCTTTCAACCATGCGT
+
F,FFFFFFFF,F,FFFFFFFF::FFFFFFFFFFFFFFFF,FFFFFFF,FFFFFFFFFFFFFFFFFFF:
F:FFFFFFFFFFFFFF:FFF:F:FF:FFFFFFFFFFFFFFFFFFFF:FFFFFF:FFFFFF:FFFFFFFF
@A00155:342:HHGFNDSXY:1:2409:5086:37043 2:N:0:GAACCTAG+TCCGCATA
CCTATGATGTACAGGGCTGCTGAAAACTGGTCATAAAGCCGGTTTTGCCCTACATATGCCCTCAACGC
AGACCATATCTGTGCCGAGTTGGGTAGGTCAGGTGCCCTAGCACCTTCCTCCCCTTTCAACCATGCGT
+
FF,FFFFFFFFFFFFFFFFFFFFFFFF,:FFFFFFFFFFFFFFFFFFF,FFFFF:FF:FFF,FFFFFFFF
FFFFFFFFFFFFFFFFF:FFFFFFFFFFFFFF:FFFFFFFFFFFF,FFFFFFFFFFFFFFFFFFFFFF
@A00155:342:HHGFNDSXY:1:1665:17833:1031 1:N:0:GAACCTAG+TCCGCATA
GATGTACAGGGCTGCTGAAAACTGGTCATAAAGCCGGTTTTGCCCTACATATGCCCTCAACGCAGACC
ATATCTGTGCCGAGTTGGGTAGGTCAGGTGCCCTAGCACCTTCCTCCCCTTTCAACCATGC
+
FFFFFFFFFFFFFFFFFFFFFFFFFFFFFFFFFFFFFFFFFFFFFFFFFFFFFFFFFFFFFFFFFFFF
FFFFFFFFFFFFFFFFFFFFFFFFFFFFFFFFFFFFFFFFFF,:FF:FFFFFFFFFFFFFFFF
@A00155:342:HHGFNDSXY:1:1303:22073:21684 2:N:0:GAACCTAG+TCCGCATA
GATGTACAGGGCTGCTGAAAACTGGTCATAAAGCCGGTTTTGCCCTACATATGCCCTCAACGCAGACC
```

ATATCTGTGCCGAGTTGGGTAGGTCAGGTGCCCTAGCACCTTCCTCCCCTTTCAACCATGCGTC
+
FFFFFFFFFFFFFFFFFF:FFFFFFFFFFFFFFFFFFFFFFFFFFF:F:FFFFFFFFFFFFFFFFFFF
FFFFFFFFFFFFFFFFFFFFFFFFFFFFFFFFFFFFFFFF:FFFFFFFFFFFFFFFF:FFFFFF
@A00155:342:HHGFNDSXY:1:1127:1949:7341 2:N:0:GAACCTAG+TCCGCATA
GATGTACAGGGCTGCTGAAAACTGGTCATAAAGCCGGTTTTGCCCTACATATGCCCTCAACGCAGACC
ATATCTGTGCCGAGTTGGGTAGGTCAGGTGCCCTAGCACCTTCCTCCCCTTTCAACCATGCGTC
+
F:FFFFFFFFFFFFFFFFFFFFFFFFFFFFFFFFFFFFFFFFFFFFFF,FFFFFFFFFFFFFFFFFFF
FFFFFFFFFFFFFFFFFFFFFFFFFFFFFFF:FFFFFFFFFFFFFFFFFFFFFFFFFFFFFFFF
@A00155:342:HHGFNDSXY:1:1303:21305:6856 2:N:0:GAACCTAG+TCCGCATA
GATGTACAGGGCTGCTGAAAACTGGTCATAAAGCCGGTTTTGCCCTACATATGCCCTCAACGCAGACC
ATATCTGTGCCGAGTTGGGTAGGTCAGGTGCCCTAGCACCTTCCTCCCCTTTCAACCATGCGTC
+
FFFFFFFFFFFFFFFFFFFFFFFFFFFFFFFFFFFFFFFFFFFFFFFFFFFFFFFFFFFFFFFFFFFF
FFFFFFFFFFFFFFFFF:FFFFF:FFFFFFF:FFFFFFFF:FFFFFFFFFFF:FFFFFFFFFFF
@A00155:342:HHGFNDSXY:1:2167:12626:5071 2:N:0:GAACCTAG+TCCGCATA
GATGTACAGGGCTGCTGAAAACTGGTCATAAAGCCGGTTTTGCCCTACATATGCCCTCAACGCAGACC
ATATCTGTGCCGAGTTGGGTAGGTCAGGTGCCCTAGCACCTTCCTCCCCTTTCAACCATGCGTC
+
F,FFFFFFF:FFFFFFFFFFFFFFFFFFFFFFFFFFFFFFFFFFFFFFF:FFFFFFFFFFFFFFFFFF
FFFFFFFFFFFFFFFFFFFFFFFFFFFFFFFFFFFFFFFFFFFFFFFFFFFFFFFFFFFFFFFF
@A00155:342:HHGFNDSXY:1:2167:10818:1282 2:N:0:GAACCTAG+TCCGCATA
GATGTACAGGGCTGCTGAAAACTGGTCATAAAGCCGGTTTTGCCCTACATATGCCCTCAACGCAGACC
ATATCTGTGCCGAGTTGGGTAGGTCAGGTGCCCTAGCACCTTCCTCCCCTTTCAACCATGCGTC
+
FFFFFFFF:FFFFFFFFFFFFFFFFFFF:FFFFFFFFFF,FFFFFFFFFF:FFFFFFF:FFFFFFFFF
FFFFFFFFFFFFFFFFFFFFFFFFFFFFFFFFFFFFFFFFFFFFFFFFFFFFFFFFFFFFFFFF
@A00155:342:HHGFNDSXY:1:2645:15465:36761 2:N:0:GAACCTAG+TCCGCATA
GATGTACAGGGCTGCTGAAAACTGGTCATAAAGCCGGTTTTGCCCTACATATGCCCTCAACGCAGACC
ATATCTGTGCCGAGTTGGGTAGGTCAGGTGCCCTAGCACCTTCCTCCCCTTTCAACCATGCGTC
+
FFFFFFFFF:FFFFFFFFFFF:FFFFFFFF:FF::FFFFFFF,FFFFFFFFFFFFFFFFFF:FFFFFF
FFFF:FFFFFFFFFFFFFFFFFFFFFF,FF:,FFFFFFFF:FFFFFFFFFFFFFFFFFFFFFFF
@A00155:342:HHGFNDSXY:1:2366:18936:35321 2:N:0:GAACCTAG+TCCGCATA
GATGTACAGGGCTGCTGAAAACTGGTCATAAAGCCGGTTTTGCCCTACATATGCCCTCAACGCAGACC
ATATCTGTGCCGAGTTGGGTAGGTCAGGTGCCCTAGCACCTTCCTCCCCTTTCAACCATGCGTC
+
FFFFFFFFFFFFFFFFFFFFFFFFFFFFFFF:FFFFFFFFFFFFFFFFFFFFFFFFFFFFFFFFFFFF
FFFFFFFFFFFFFFFFFFFFFFFFFFFFFFFFFFFFFFFFFFFFFFFFFFFFFFFFFFFFFFFF
@A00155:342:HHGFNDSXY:1:2347:15429:2127 2:N:0:GAACCTAG+TCCGCATA
GATGTACAGGGCTGCTGAAAACTGGTCATAAAGCCGGTTTTGCCCTACATATGCCCTCAACGCAGACC
ATATCTGTGCCGAGTTGGGTAGGTCAGGTGCCCTAGCACCTTCCTCCCCTTTCAACCATGCGTC
+
FFFFFFFFFFFFFFFFFFFFFFFFFFFFFFFFFFFFFFFFFFFFFFFFFFFFFFFFFFFFFFFFFFFF
:FFFFFFFFFFFFFFFFFFFFFFFFFFFFFFFFFFFFFFFFFFFFFFFFFFFFFFFFFFFFFFF
@A00155:342:HHGFNDSXY:1:2218:29613:7279 1:N:0:GAACCTAG+TCCGCATA
ATGTACAGGGCTGCTGAAAACTGGTCATAAAGCCGGTTTTGCCCTACATATGCCCTCAACGCAGACCA
TATCTGTGCCGAGTTGGGTAGGTCAGGTGCCCTAGCACCTTCCTCCCCTTTCAACCATGCGTC
+
FFFFFFFFFFFFFFFFFFFFFFFFFFFFFFFFFFFFFFFFFFFFFFFFFFFFFFFFFFFFFFFFFFFF
FFFFFFFFFFFFFFFF:FFFFFFFFFFFFFFFFFFFFFFFFFFFFFFFFFFFFFFFFFFFFF
@A00155:342:HHGFNDSXY:1:2343:24975:9079 1:N:0:GAACCTAG+TCCGCATA
ATGTACAGGGCTGCTGAAAACTGGTCATAAAGCCGGTTTTGCCCTACATATGCCCTCAACGCAGACCA

```
TATCTGTGCCGAGTTGGGTAGGTCAGGTGCCCTAGCACCTTCCTCCCCTTTCAACCATGCGTCCACCC
+
FFFFFFFFFFFFFFFFFFFFFFFFFFFFFFFFFFFFFFFFFFFFFFFFFFFFFFFFFFFFFFFFFFFFFF
FFFFFFFFFFFFFFFFFFFFFFFFF:FFFFFFFFFFFFFFF,FFFFFFFFFFFFFFFFFFFFFFFFFFFF
@A00155:342:HHGFNDSXY:1:1127:1949:7341 1:N:0:GAACCTAG+TCCGCATA
ATGTACAGGGCTGCTGAAAACTGGTCATAAAGCCGGTTTTGCCCTACATATGCCCTCAACGCAGACCA
TATCTGTGCCGAGTTGGGTAGGTCAGGTGCCCTAGCACCTTCCTCCCCTTTCAACCATGCGTCCACCC
+
FFFFF:FFFFFFFFF:FFFFFFF,F,FFFFFF,FFFFF:FFFFFFFFFFFFFF,FFFFFFFFFFFFFF
FFFFFFFFFFFFFFFF:FFFFFFFFFFFFFFFFFFFFFFFFFFFFF:FFFFFF:FFFFFFF:FF:FFF
@A00155:342:HHGFNDSXY:1:1131:1669:21668 1:N:0:GAACCTAG+TCCGCATA
ATGTACAGGGCTGCTGAAAACTGGTCATAAAGCCGGTTTTGCCCTACATATGCCCTCAACGCAGACCA
TATCTGTGCCGAGTTGGGTAGGTCAGGTGCCCTAGCACCTTCCTCCCCTTTCAACCATGCGTCCACCC
+
FFFFFFFFFFFFFFFFFFFFFFFFFFFF:FFFFFFFFFFFFFFFFFFFFFFFFFFFFFF:FFFFFFF
FFFFFFFF:FFFFFFFFFFFFFFFFF:FFFFFFFFFFFFFF:FFFFFF:FFFFFFFFFFFFFFFFFFF
@A00155:342:HHGFNDSXY:1:1632:11514:6370 1:N:0:GAACCTAG+TCCGCATA
ATGTACAGGGCTGCTGAAAACTGGTCATAAAGCCGGTTTTGCCCTACATATGCCCTCAACGCAGACCA
TATCTGTGCCGAGTTGGGTAGGTCAGGTGCCCTAGCACCTTCCTCCCCTTTCAACCATGCGTCCACCC
+
FFFFFFFFFFFFFFFFFFFFFFFFFFFFFFFFFFFFFFFFFFFFFFFFFFFFFFFFFFFFFFFF:FFF
FFFFFFFFFFFFFFFFFFFFFFFFFFFFFFFFFFFFFFFF:FFFFFFFFFFFFFFFFFFFFFFFFFFF
@A00155:342:HHGFNDSXY:1:1625:5421:15734 1:N:0:GAACCTAG+TCCGCATA
ATGTACAGGGCTGCTGAAAACTGGTCATAAAGCCGGTTTTGCCCTACATATGCCCTCAACGCAGACCA
TATCTGTGCCGAGTTGGGTAGGTCAGGTGCCCTAGCACCTTCCTCCCCTTTCAACCATGCGTCCACCC
+
FFFFFFFFFFFFFFFFFFFFFFFFFFFFFFF:FFFFFFFF:FFFFFFFFFFFFFFFFFFFFFFF:FFF
:FFFFFFFFFFFFFF:FFFFFFFFFFFFFFFFFFFFFFF:FFFFFFFFFFFFFFFFFFFFFF:FFFFFF
@A00155:342:HHGFNDSXY:1:1302:5683:9987 1:N:0:GAACCTAG+TCCGCATA
ATGTACAGGGCTGCTGAAAACTGGTCATAAAGCCGGTTTTGCCCTACATATGCCCTCAACGCAGACCA
TATCTGTGCCGAGTTGGGTAGGTCAGGTGCCCTAGCACCTTCCTCCCCTTTCAACCATGCGTCCACCC
+
FFFFFFFFFFFFFFFFFFFFFFFFF:F:FFFF:::FFFF:FF:FFFFFF:FFFFF:FFFFFFFFFFFF
FFFFFFFFFFF:FFFFFFFFFFFFFFFFFFFFFFFFFFFF:,FFFFFFFFFFFFFFFFFFFFFFFFFFF
@A00155:342:HHGFNDSXY:1:2218:27679:7874 1:N:0:GAACCTAG+TCCGCATA
TGTACAGGGCTGCTGAAAACTGGTCATAAAGCCGGTTTTGCCCTACATATGCCCTCAACGCAGACCAT
ATCTGTGCCGAGTTGGGTAGGTCAGGTGCCCTAGCACCTTCCTCCCCTTTCAACCATGCGTC
+
FFFFFFFFFFFFFF:FF:FFFFFFFF:FFFFF:FF::FFFFFFFFFFFF:FFFF:FFFF:F,FFFFF
F:FFFF,F:FFFFFFFFFF:FFFF:,FF,F::FFFFFFFF:F:FFFFF:FFFFFFF:FFFFF
@A00155:342:HHGFNDSXY:1:2624:27236:22263 1:N:0:GAACCTAG+TCCGCATA
TGTACAGGGCTGCTGAAAACTGGTCATAAAGCCGGTTTTGCCCTACATATGCCCTCAACGCAGACCAT
ATCTGTGCCGAGTTGGGTAGGTCAGGTGCCCTAGCACCTTCCTCCCCTTTCAACCATGCGTCCACCCC
+
FFFFFFFFFFFFFFFFFFFFFFFFFFFFFFFFFFFFFFFFFF:FFFFFFFFFFFFFFFFFFFFFFFFF
FFFFFFFFFFFFFFFFFFFFFFFF:FFFFFFFFFFF:FFFFF,FFFFFF,FFFFFFFFFFFF,:FFFF
@A00155:342:HHGFNDSXY:1:2624:29134:17942 1:N:0:GAACCTAG+TCCGCATA
TGTACAGGGCTGCTGAAAACTGGTCATAAAGCCGGTTTTGCCCTACATATGCCCTCAACGCAGACCAT
ATCTGTGCCGAGTTGGGTAGGTCAGGTGCCCTAGCACCTTCCTCCCCTTTCAACCATGCGTCCACCCC
+
FFFFFFFFFFFFFFFFFFFFFFFFFFFFFFFFFFFFFFFFFFFFFFFFFFFFFFFFFFFFFFFFFFFF
FFFFFFFFFFFFFFFFFFFFFFFFFFFFFFFFFFFFFFFFFFFFFFFFFFFFFF:FFFFFFFFFFFFF
@A00155:342:HHGFNDSXY:1:2568:3775:2738 1:N:0:GAACCTAG+TCCGCATA
TGTACAGGGCTGCTGAAAACTGGTCATAAAGCCGGTTTTGCCCTACATATGCCCTCAACGCAGACCAT
```

ATCTGTGCCGAGTTGGGTAGGTCAGGTGCCCTAGCACCTTCCTCCCCTTTCAACCATGCGTCCACCCC
+
FFFFFFFFFFFFFFFFFFFFFFFFFFFF:FFFFFFFFFFFFFFFFFFFFFFFFFFFFFFFFFFFFFFFF
FFFFFFFFFFFFFFFFFFFFFFFF:FFFFFFFFFFFFFFFFFFFFFFFFFFFFFFFFFFFFFFFFFFFF
@A00155:342:HHGFNDSXY:1:1375:25527:14074 1:N:0:GAACCTAG+TCCGCATA
TGTACAGGGCTGCTGAAAACTGGTCATAAAGCCGGTTTTGCCCTACATATGCCCTCAACGCAGACCAT
ATCTGTGCCGAGTTGGGTAGGTCAGGTGCCCTAGCACCTTCCTCCCCTTTCAACCATGCGTCCACCCC
+
FFFFFFFFFFFFFFFFFFFF:FFFFFFFFFFFFFFFFFFFFFFFFFFFFFFFFFFFFFFFFFFFFFFFF
FFFFFFFFFFFFFFFFFFFFFFFFFFFFFFFFFFFFFF:,FFFFFFFFFFFFFFFFFFFFFFFFFFFFF
@A00155:342:HHGFNDSXY:1:2142:21685:15029 1:N:0:GAACCTAG+TCCGCATA
TGTACAGGGCTGCTGAAAACTGGTCATAAAGCCGGTTTTGCCCTACATATGCCCTCAACGCAGACCAT
ATCTGTGCCGAGTTGGGTAGGTCAGGTGCCCTAGCACCTTCCTCCCCTTTCAACCATGCGTCCACCCC
+
FFFFFFFFFFFFFFFFFFFFFFFFFFFFFFFFFFFFFFFFFFFFFFFFFFFFFFFFFFFFFFFFFFFFF
FFFFFFFFFFFFFFFFFFFFFFFFFFFFFFFFFFFFFFFFFFFFFFFFFFFFFFFFFFFFFFFFFFFFF
@A00155:342:HHGFNDSXY:1:2578:3088:29731 1:N:0:GAACCTAG+TCCGCATA
TGTACAGGGCTGCTGAAAACTGGTCATAAAGCCGGTTTTGCCCTACATATGCCCTCAACGCAGACCAT
ATCTGTGCCGAGTTGGGTAGGTCAGGTGCCCTAGCACCTTCCTCCCCTTTCAACCATGCGTCCACCCC
+
FFFFFFFFFFFFFFFFFF:FFFFFFFFFFFFFFFFFFFFFF:FFFFFFFFFFFFFFFFFFFFFFFFFFFF
FFFFFFFFFFFFFFFFFFFFFFFFFF:FFF:FFFFFFFFFFFFFFFFFFFFFFFFFFFFFFFFFFFFFF
@A00155:342:HHGFNDSXY:1:1115:11505:14403 1:N:0:GAACCTAG+TCCGCATA
GTACAGGGCTGCTGAAAACTGGTCATAAAGCCGGTTTTGCCCTACATATGCCCTCAACGCAGACCATA
TCTGTGCCGAGTTGGGTAGGTCAGGTGCCCTAGCACCTTCCTCCCCTTTCAACCATGCGTCCACCCCG
+
FFFFFFFFFFFFFFFFFFFFFFFFFFFFFF:FFFFFFFF:F:FFFFFFFFFFFFFFFFFFFFFFF,FF
FFFFFFFFFFFFFFFFFFFFFFFFFFFFF,:FFF:FFFF:FFFFFFFFFFFFFFFFFFFFFFFFFFFFF
@A00155:342:HHGFNDSXY:1:1377:19334:10269 1:N:0:GAACCTAG+TCCGCATA
GTACAGGGCTGCTGAAAACTGGTCATAAAGCCGGTTTTGCCCTACATATGCCCTCAACGCAGACCATA
TCTGTGCCGAGTTGGGTAGGTCAGGTGCCCTAGCACCTTCCTCCCCTTTCAACCATGCGTCCACCCCG
+
FFFFFFFFFFFFFF:FF:FFFFFFFFFFFFFFFFFFFFFFFFFFFFFFFFFFFFFFFFFFFFFFFFFFF
FFFF,FFFFFFFFFFFFFFFFFFFFFFFFFFFF:FFF:FFFFFFFFFFFFFFFFFFFFFFFFFFFFFFF
@A00155:342:HHGFNDSXY:1:2210:4227:7686 1:N:0:GAACCTAG+TCCGCATA
GTACAGGGCTGCTGAAAACTGGTCATAAAGCCGGTTTTGCCCTACATATGCCCTCAACGCAGACCATA
TCTGTGCCGAGTTGGGTAGGTCAGGTGCCCTAGCACCTTCCTCCCCTTTCAACCATGCGTCCACCCCG
+
FFFFFFFFFFFFFFFFFFFFFFFFFFFFFFFFFFF:FFFFFFFFFF:FFFFFFF:FFFFFFFFFFF,
FF,:FFFFFFF,FFFFFFFF:FFFFFFFFFF,FFFFFFFFFFFFFFFFFF::FF::FFFFFFFFFFFF
@A00155:342:HHGFNDSXY:1:1372:32362:15890 1:N:0:GAACCTAG+TCCGCATA
GTACAGGGCTGCTGAAAACTGGTCATAAAGCCGGTTTTGCCCTACATATGCCCTCAACGCAGACCATA
TCTGTGCCGAGTTGGGTAGGTCAGGTGCCCTAGCACCTTCCTCCCCTTTCAACCATGCGTCCACCCCG
+
FFFFFFFFFFFFFFFFFFFFFFFFFFFFF:FFFF,FF,FFFFFFFF,FFFFFFF:FFFFFFFF:FF:F
FFFF:FFFFFFFFFFF:FFFF:FFFFFF:FFFFF:F::FFFFFFFFFFFFF:FFFFFFFFF:FFFFFF
@A00155:342:HHGFNDSXY:1:2210:4128:7827 1:N:0:GAACCTAG+TCCGCATA
GTACAGGGCTGCTGAAAACTGGTCATAAAGCCGGTTTTGCCCTACATATGCCCTCAACGCAGACCATA
TCTGTGCCGAGTTGGGTAGGTCAGGTGCCCTAGCACCTTCCTCCCCTTTCAACCATGCGTCCACCCCG
+
FFFFFFFFFFFFFFFFFFFFFFFFFFFFFFFFFFFFFFFFFFFFFFFF:FFFFFFFFFFFFFFFFFFFF
FFFFFFFFFFFFFFFFFFFFF:FFFFFFFFFF:FFFFFF:FFFFFFFFFFFFFFF,FFFFFFFFFFFFF
@A00155:342:HHGFNDSXY:1:1413:21007:11099 1:N:0:GAACCTAG+TCCGCATA
GTACAGGGCTGCTGAAAACTGGTCATAAAGCCGGTTTTGCCCTACATATGCCCTCAACGCAGACCATA

TCTGTGCCGAGTTGGGTAGGTCAGGTGCCCTAGCACCTTCCTCCCCTTTCAACCATGCGTCCACCCCG
+
FFFFFFFFFF,F,FFFFFF:FFFFF,,FFFFFF::FFFF,F,:FFFFFF:FF:FFFFFF,:,FFFFFF
:F:FF,FF:FFFFFFFFFFFFF:FFFFF:FFFF:FF:F:,FFF:,FF::FF:F:F,FFF:F:FFFFFF
@A00155:342:HHGFNDSXY:1:2137:27037:28307 1:N:0:GAACCTAG+TCCGCATA
GTACAGGGCTGCTGAAAACTGGTCATAAAGCCGGTTTTGCCCTACATATGCCCTCAACGCAGACCATA
TCTGTGCCGAGTTGGGTAGGTCAGGTGCCCTAGCACCTTCCTCCCCTTTCAACCATGCGTCCACCCCG
+
FFFFFFFFFFFFFFFFFFFFFFFFFFFFFFFFFFFFFFFFFFFFFFFFFFFFFFFFFFFFFFFFFFFF
FFFFFFFFFFFFFFFFFFFFFFFFFFF:FFFFFFFFF,FFFFFFFFFFFFFFFFFFFFFFF:F:FFFF
@A00155:342:HHGFNDSXY:1:1446:9218:33364 1:N:0:GAACCTAG+TCCGCATA
TACAGGGCTGCTGAAAACTGGTCATAAAGCCGGTTTTGCCCTACATATGCCCTCAACGCAGACCATAT
CTGTGCCGAGTTGGGTAGGTCAGGTGCCCTAGCACCTTCCTCCCCTTTCAACCATGCGTCCACCCCGG
+
FFFFFFFFFFFFFFFFFFFFFFFFFFFFFFFFFFFFFFF,FFFFFFFFF,FFFFFFFFFFFFFFFFFF
FFFFFFFFFFFFFFFFFFFFFFFFFFFFFFFFFFFFFFFFFFFFFFFFFFFFFFFFFFFFFFFFFFFF
@A00155:342:HHGFNDSXY:1:1220:18909:19053 1:N:0:GAACCTAG+TCCGCATA
TACAGGGCTGCTGAAAACTGGTCATAAAGCCGGTTTTGCCCTACATATGCCCTCAACGCAGACCATAT
CTGTGCCGAGTTGGGTAGGTCAGGTGCCCTAGCACCTTCCTCCCCTTTCAACCATGCGTCCACCCCGG
+
FFFFFFF:FFFFFFFFFFFFFFFFFFFFFFFFFFFFFFFF:FFFFFFFFFFFFFFFFFFFFFFFF:FF
FFFFF:FFFFFFFFFFFFFFFFFFFFFFFFFF:FFFF:FF:FFFFFF:FFFFFFFFFF:FFFF:FFFF
@A00155:342:HHGFNDSXY:1:2638:32380:17581 2:N:0:GAACCTAG+TCCGCATA
ACAGGGCTGCTGAAAACTGGTCATAAAGCCGGTTTTGCCCTACATATGCCCTCAACGCAGACCATATC
TGTGCCGAGTTGGGTAGGTCAGGTGCCCTAGCACCTTCCTCCCCTTTCAACCATGCGTCCACCCCGGT
+
FF,FFFFFFFFFFFFFFFFFFFFFF:FFFFFFFFF:FFFFFFFFFF:FFFFFFF:FFFFFFFFF:FFF
FFFFFFFFFFFFFFFFFFFFFFFFFFFF:FFFFFF:FFFFFFFFFFFFFFFFFFFFFFFFFFFFFFFF
@A00155:342:HHGFNDSXY:1:2542:19831:9596 2:N:0:GAACCTAG+TCCGCATA
ACAGGGCTGCTGAAAACTGGTCATAAAGCCGGTTTTGCCCTACATATGCCCTCAACGCAGACCATATC
TGTGCCGAGTTGGGTAGGTCAGGTGCCCTAGCACCTTCCTCCCCTTTCAACCATGCGTCCACCCCGGT
+
FFFFFF:FFFFFFFFFFFF:FFFFFF:FFFFF,FFFFFFFFFFFFFFFFFFFFFFFFFFFFF:FFFF:
FFFFFFFFFFFFFFFFFFFFFFFFFFFFFFFFFFFFFFFFFFFFFFFFF,FFFFFFFFFFFFFFFFFF
@A00155:342:HHGFNDSXY:1:1377:19334:10269 2:N:0:GAACCTAG+TCCGCATA
AGGGCTGCTGAAAACTGGTCATAAAGCCGGTTTTGCCCTACATATGCCCTCAACGCAGACCATATCTG
TGCCGAGTTGGGTAGGTCAGGTGCCCTAGCACCTTCCTCCCCTTTCAACCATGCGTCCACCCCG
+
:FFFFFFFFFFFFFFFFFFFFFFFFFFFFFFFFFFFFFFFFFF,FFFFFFFF,FFFFFFFFFFF::FF
FFFFFFFFFFFFFFFFFFFFF:FF:FFFFFFFFFFFFFFFFFFFFF:FFFFFFFFFFFFFFFFF
@A00155:342:HHGFNDSXY:1:2578:3088:29731 2:N:0:GAACCTAG+TCCGCATA
GGGCTGCTGAAAACTGGTCATAAAGCCGGTTTTGCCCTACATATGCCCTCAACGCAGACCATATCTGT
GCCGAGTTGGGTAGGTCAGGTGCCCTAGCACCTTCCTCCCCTTTCAACCATGCGTCCACCCCG
+
FFFFFFF:FFFFFFFF:FFFFFFFFFFFFF:FFFFFFFFFFF,FFFFFFFFFFFFFFFFFFFFF:FFF
FFFFFFFFFFFFFFFFFFFFFFFFFFFFFFFFFFFFFFFFFFFFFFFFFFFFFFFFFFFFFFFF
@A00155:342:HHGFNDSXY:1:1672:20844:26725 1:N:0:GAACCTAG+TCCGCATA
GGGCTGCTGAAAACTGGTCATAAAGCCGGTTTTGCCCTACATATGCCCTCAACGCAGACCATATCTGT
GCCGAGTTGGGTAGGTCAGGTGCCCTAGCACCTTCCTCCCCTTTCAACCATGCGTCCACCCCGGTTAA
+
FFFFFFFFFFFFFFFFFFFFFFF:FFFFFFFF:FFFFFFFFFFFFFFFFFF:FFFFFFFFFFFFFFFF
FFFFFFF:FFFFFFFFFFFFFFFFFFFFFFFFFFFFFFFFFFFFFFFFFFFFFFFFFFFFFFFFFFFF
@A00155:342:HHGFNDSXY:1:2415:27317:34303 2:N:0:GAACCTAG+TCCGCATA
GGCTGCTGAAAACTGGTCATAAAGCCGGTTTTGCCCTACATATGCCCTCAACGCAGACCATATCTGTG

CCGAGTTGGGTAGGTCAGGTGCCCTAGCACCTTCCTCCCCTTTCAACCATGCGTCCACCCCGGTTAAT
+
FFFFFFFFFF:FFFFFFFFFFFFFFFFFFFFFFFFFFFF:FFF:FFFFFF:FFFFFFFF:FFFFFFFF
FFFFFFFFFFFFFFFFFFF:FFFFFFFFFFFFFFFFFFFFFFFFFFFFF:FFFFFFFFFFFFFFFFFF
@A00155:342:HHGFNDSXY:1:2461:17092:33567 1:N:0:GAACCTAG+TCCGCATA
CTGCTGAAAACTGGTCATAAAGCCGGTTTTGCCCTACATATGCCCTCAACGCAGACCATATCTGTGCC
GAGTTGGGTAGGTCAGGTGCCCTAGCACCTTCCTCCCCTTTCAACCATGCGTCCACCCCGGTTAATTT
+
FFFFFFFFFFFFFFFFFFFFFFFFFFFFFFFFFFFFFFFFFFFFFFFFFFFFFFFFFFFFFFFFFFFF
FFFFFFFFFFFFFFFFFFFFFFFFFFFFF::FFFFFFFFFFFFFFFFFFFFFFFFFFFFFFFFFFFFF
@A00155:342:HHGFNDSXY:1:2163:3667:36338 1:N:0:GAACCTAG+TCCGCATA
CTGCTGAAAACTGGTCATAAAGCCGGTTTTGCCCTACATATGCCCTCAACGCAGACCATATCTGTGCC
GAGTTGGGTAGGTCAGGTGCCCTAGCACCTTCCTCCCCTTTCAACCATGCGTCCACCCCGGTTAATTT
+
FFFFFFFFFFFFFFFFFFFFFFFFFFFFFFFFFFFFFFFFFFFFFFFFFFFFFFFFFFFFFFFFFFFF
FFFFFFFFFFFFFFFFFFFFFFF:FFFFF:FFFFFFFFFFFFFFFFFF:FFFFFFFFFFFFFFFFFFF
@A00155:342:HHGFNDSXY:1:2430:23222:16188 1:N:0:GAACCTAG+TCCGCATA
CTGCTGAAAACTGGTCATAAAGCCGGTTTTGCCCTACATATGCCCTCAACGCAGACCATATCTGTGCC
GAGTTGGGTAGGTCAGGTGCCCTAGCACCTTCCTCCCCTTTCAACCATGCGTCCACCCCGGTTAATTT
+
FFFFFFFFFFFFFFFFFFFFFFFFFFFFFFFFFFFFFFFFFFFFFFFFFFFFFFFFF:F:FFFFFFFF
FFFFFFFFFFFFFFFFFFFFFFFFFFFFFF,FFFFFFFFFFFFFFFFFFFFFFFFFFFFFFFFFFFFF
@A00155:342:HHGFNDSXY:1:2239:17381:8234 1:N:0:GAACCTAG+TCCGCATA
CTGCTGAAAACTGGTCATAAAGCCGGTTTTGCCCTACATATGCCCTCAACGCAGACCATATCTGTGCC
GAGTTGGGTAGGTCAGGTGCCCTAGCACCTTCCTCCCCTTTCAACCATGCGTCCACCCCGGTTAATTT
+
FFFFFFFFFFFFFFFFFFFFFFFFFFFFFFFFFFFFFFFFFFFFFFFFFFFFFFFFFFFFFFFFFFFF
FFFFFFFFFFFFFFFFFFFFFFFFFFFF::FFFFFFFFFFFFFFFFFFFFFFFFFFFFFFFFFFFFFF
@A00155:342:HHGFNDSXY:1:1413:21007:11099 2:N:0:GAACCTAG+TCCGCATA
GCTGAAAACTGGTCATAAAGCCGGTTTTGCCCTACATATGCCCTCAACGCAGACCATATCTGTGCCGA
GTTGGGTAGGTCAGGTGCCCTAGCACCTTCCTCCCCTTTCAACCATGCGTCCACCCCGGTTAATTTTG
+
FFF:F,FFF:FFF:FFF:FFF:FFF:FF,:FF:FF,FFFF,FFFF,,:FFFFFF,F:,FFF,FF:FF:
FF,FFFFFFFF:,F,FF,FFFF,FFF,FF:FF,F,FFFF,FF,FF:FFFFFFFFF:,FFFFFF,FFF:
@A00155:342:HHGFNDSXY:1:2343:24975:9079 2:N:0:GAACCTAG+TCCGCATA
GCTGAAAACTGGTCATAAAGCCGGTTTTGCCCTACATATGCCCTCAACGCAGACCATATCTGTGCCGA
GTTGGGTAGGTCAGGTGCCCTAGCACCTTCCTCCCCTTTCAACCATGCGTCCACCCCGGTTAATTTTG
+
FFFFFFFFFFFFFF:FFFFFFFFFFFFFFF:FFF:FF:FFFFFFFFFFFFFFFFFFFFFFFFFFFFFF
FFFFFFFFFFFFFFFFFFFFFFFFF:FFFFFFFFFFFFFFFFFFFFFFFFFFFFFFFFFFFFFFFFFF
@A00155:342:HHGFNDSXY:1:2645:15465:36761 1:N:0:GAACCTAG+TCCGCATA
GCTGAAAACTGGTCATAAAGCCGGTTTTGCCCTACATATGCCCTCAACGCAGACCATATCTGTGCCGA
GTTGGGTAGGTCAGGTGCCCTAGCACCTTCCTCCCCTTTCAACCATGCGTCCACCCCGGTTAATTTTG
+
FFFFFFFFFFF:FFFFFFFFF:FFFFFFFFFFFFFFFFF,FFFF:FFFFFFFFFFFFFFFFFFFFFFF
FFFFFFFFFFF:FFF:FFFFFFFFFFFFFFFFFFFFFFFFFFFFFFFFFFFFFFFFFFFFFFFFFF:F
@A00155:342:HHGFNDSXY:1:1303:22073:21684 1:N:0:GAACCTAG+TCCGCATA
GCTGAAAACTGGTCATAAAGCCGGTTTTGCCCTACATATGCCCTCAACGCAGACCATATCTGTGCCGA
GTTGGGTAGGTCAGGTGCCCTAGCACCTTCCTCCCCTTTCAACCATGCGTCCACCCCGGTTAATTTTG
+
FFFFFFF:FFFFFFFFFFFFFFFFFFFFFFFFFFFFFFFFFFFFFFFFFFFFF:FFFFFFFFFFFFFF
FFFFFFFFFFFFFFFFFFFFFFFFFFF:FFFFFFFFFFFFFFFFFFFFFFFFFFFFFFFFFFFF:FFF
@A00155:342:HHGFNDSXY:1:1303:21305:6856 1:N:0:GAACCTAG+TCCGCATA
GCTGAAAACTGGTCATAAAGCCGGTTTTGCCCTACATATGCCCTCAACGCAGACCATATCTGTGCCGA

```
GTTGGGTAGGTCAGGTGCCCTAGCACCTTCCTCCCCTTTCAACCATGCGTCCACCCCGGTTAATTTTG
+
FFFFFFFFFFFFFFFFFFFFFFFFFFFFFFFFFFFFFFFFFFFFFFFFFFFFFFFFFFFFFFFFFFFF
FFFFFFFFFFFFFFFFFFFFFFFFFFFFFFFFFFFFFFFFFFFFFFFFFFFFFFFFFFFFFFFFFFFF
@A00155:342:HHGFNDSXY:1:2378:12328:11569 1:N:0:GAACCTAG+TCCGCATA
CTGAAAACTGGTCATAAAGCCGGTTTTGCCCTACATATGCCCTCAACGCAGACCATATCTGTGCCGAG
TTGGGTAGGTCAGGTGCCCTAGCACCTTCCTCCCCTTTCAACCATGCGTCCACCCCGGTTAATTTTGA
+
FFFFFFFFFFFFFFFFFFFFFFFFFFFFFFFFFFFFFFFFFFFFFFFFFFFFFFFFFFFFFFFFFFFF
FFFFFFFFFFFFFFFFFFFFFFFFFF,FFFFFFFFFFFFFFFFFFFFFFFFFFFFFFFFFFFFFFFFF
@A00155:342:HHGFNDSXY:1:1602:5737:31563 2:N:0:GAACCTAG+TCCGCATA
TGAAAACTGGTCATAAAGCCGGTTTTGCCCTACATATGCCCTCAACGCAGACCATATCTGTGCCGAGT
TGGGTAGGTCAGGTGCCCTAGCACCTTCCTCCCCTTTCAACCATGCGTCCACCCCGGTTAATTTTGA
+
F:FFFFFFFFFFFFF,FFFFFFF:FFF:FFFFFF,:FFF::FFFFFFFFFFFF:FFFFFFFFFFFFFF
FFFFFFFFFFFFFFFFFF,FFFFFFF:FFFF:FFFF:FFFFFFFFFFFFFFF:FFFFFFFFFFFFF:F
@A00155:342:HHGFNDSXY:1:1632:11514:6370 2:N:0:GAACCTAG+TCCGCATA
GAAAACTGGTCATAAAGCCGGTTTTGCCCTACATATGCCCTCAACGCAGACCATATCTGTGCCGAGTT
GGGTAGGTCAGGTGCCCTAGCACCTTCCTCCCCTTTCAACCATGCGTCCACCCCGGTTAATTTTGACC
+
FFFFFFFFFFFFFFFFFFFFFFFFFFFFFFFFFFFFFFFFFFFFFFFFFFFFFFFFFFFFFFFFFFFF
FFFFFFFFFFFFFFFFFFFFFFFFFFFFFFFFFFFFFFFFFFFFFFFFFFFFFFFFFFFFFFFFFFFF
@A00155:342:HHGFNDSXY:1:2378:12328:11569 2:N:0:GAACCTAG+TCCGCATA
AACTGGTCATAAAGCCGGTTTTGCCCTACATATGCCCTCAACGCAGACCATATCTGTGCCGAGTTGGG
TAGGTCAGGTGCCCTAGCACCTTCCTCCCCTTTCAACCATGCGTCCACCCCGGTTAATTTTGACCCGT
+
FFFFFFFFFFFFFFF,:FFFFFFFFFFFFFFFFFFFFFFFF,FFFFFFFFFFFFFFFFFF:FFFFFFFF
FFFFFFFFFFFFFFFFFFFFF:FFFFFFFFFFFFFFFFFFFFFFFFFFFFFFFFFFFFFFFF:FFFFF
@A00155:342:HHGFNDSXY:1:2414:12527:6809 1:N:0:GAACCTAG+TCCGCATA
AACTGGTCATAAAGCCGGTTTTGCCCTACATATGCCCTCAACGCAGACCATATCTGTGCCGAGTTGGG
TAGGTCAGGTGCCCTAGCACCTTCCTCCCCTTTCAACCATGCGTCCACCCCGGTTAATTTTGACCCGT
+
FFFFFFFFFFFFFFFFFFFFFFFFFFFFF:F:FFFFFFFFFFFFFFFFFFFFFFFFFFFFFFFFFFFF
FFFFFFFFFFFFFFFFFFFFF,FFFFFFFFFFFFFFFFFFFFFFFFFFFFFFFFFFFFFFFFFFFFFF
@A00155:342:HHGFNDSXY:1:1570:29017:30953 2:N:0:GAACCTAG+TCCGCATA
ACTGGTCATAAAGCCGGTTTTGCCCTACATATGCCCTCAACGCAGACCATATCTGTGCCGAGTTGGGT
AGGTCAGGTGCCCTAGCACCTTCCTCCCCTTTCAACCATGCGTCCACCCCGGTTAATTTTGACCCGTG
+
FFFFFFFFF:FFFFFFFFFFFFFFFFFFFFFFFFFFFFFFFFFF:FFFFFFFF:FFFFFFFFFFFFFF
FFFFFFFFFFFFFFFFFFFF,FFFFFFFFFFFFFFFFFFFFFFFFFFFFFFFFFFFFFFFFFFFFFFF
@A00155:342:HHGFNDSXY:1:1152:3766:9424 2:N:0:GAACCTAG+TCCGCATA
TGGTCATAAAGCCGGTTTTGCCCTACATATGCCCTCAACGCAGACCATATCTGTGCCGAGTTGGGTAG
GTCAGGTGCCCTAGCACCTTCCTCCCCTTTCAACCATGCGTCCACCCCGGTTAATTTTGACCCGTGAC
+
FFF:FFFFFFFFFFFFFFFFFFFFFFFFFFFFFFFFFFF,F:FFFFFFFFFFFFF,FFFFFFFFFFFFF
FFFFFFFFFFFF,FFFFFFFFFFFFFFFFFFFFFFF:FFFFFFFFFFFFFFFFFFFFFFFFFFFFFFF
@A00155:342:HHGFNDSXY:1:2350:5602:21966 2:N:0:GAACCTAG+TCCGCATA
TGGTCATAAAGCCGGTTTTGCCCTACATATGCCCTCAACGCAGACCATATCTGTGCCGAGTTGGGTAG
GTCAGGTGCCCTAGCACCTTCCTCCCCTTTCAACCATGCGTCCACCCCGGTTAATTTTGACCCGTGAC
+
FF::FFFF:FFFFFFFFFFFFF:FFFFFFFFFFFFFFFFFFFFFFFFFF:FFFFFFFFFFFFFFFFFF
FFFFFFFFFFFFFFFF:F:FFFFFF:,FFF:F:FF:FFFFFFFFFFFFFFFFFF,FFFFFFFFFFFFF
@A00155:342:HHGFNDSXY:1:2431:25256:14231 2:N:0:GAACCTAG+TCCGCATA
TGGTCATAAAGCCGGTTTTGCCCTACATATGCCCTCAACGCAGACCATATCTGTGCCGAGTTGGGTAG
```

```
GTCAGGTGCCCTAGCACCTTCCTCCCCTTTCAACCATGCGTCCACCCCGTTTAATTTTGACCCGTGAC
+
FF,FFFF::FFFFFFFFFFFFFFFF,FFFFF:FFFFFFFFFFF:,FFFFFF:FF:FFF:FFFFFFFFF
,FFF,FFFF,FFF,FFFFFFFFFF:F,FFFFFF,FFFFF,F,F:FFFFF,FFF:F:FFFFFFF:F::F
@A00155:342:HHGFNDSXY:1:2151:3477:25394 2:N:0:GAACCTAG+TCCGCATA
TGGTCATAAAGCCGGTTTTGCCCTACATATGCCCTCAACGCAGACCATATCTGTGCCGAGTTGGGTAG
GTCAGGTGCCCTAGCACCTTCCTCCCCTTTCAACCATGCGTCCACCCCGGTTAATTTTGACCCGTGAC
+
FFFFFFFFFFFFFFFFFFFFFFFFFFFFFF:FFFFFF:FFFFFFFFF:FFFFFFFFFFFFFFFFF:FF
FFFFFFFFFFFFFFFFFFFFFFFFFFFFF:FFFFFFFFFFFFFFFFFFFFFFFFFFFFFFFFFFFFFF
@A00155:342:HHGFNDSXY:1:2163:3667:36338 2:N:0:GAACCTAG+TCCGCATA
GGTCATAAAGCCGGTTTTGCCCTACATATGCCCTCAACGCAGACCATATCTGTGCCGAGTTGGGTAGG
TCAGGTGCCCTAGCACCTTCCTCCCCTTTCAACCATGCGTCCACCCCGGTTAATTTTGACCCGTGACG
+
FFFFFFFFFFFF:FFFFFFF:FFFFFFFFFFFF:FFFFFFFFFFFFFFFFFFFFFFFFFFFFFFFFFF
FFFFFFFFFFFF:FFFFFFFFFF:FFFFFFFFFF:FFFFFFFFFFFFFFFFFFFFFFFFFFFFFFFFF
@A00155:342:HHGFNDSXY:1:2430:23222:16188 2:N:0:GAACCTAG+TCCGCATA
GGTCATAAAGCCGGTTTTGCCCTACATATGCCCTCAACGCAGACCATATCTGTGCCGAGTTGGGTAGG
TCAGGTGCCCTAGCACCTTCCTCCCCTTTCAACCATGCGTCCACCCCGGTTAATTTTGACCCGTGACG
+
FFFFFFF:FFFFFFFFFFFFFFFFFFFF:FFFFFFFFFFFFFFFF:F:FFFFFFFFFFFFFFFFFFFF
FFFFFFFFFFFFFFFFF,FFFFFFFFFFFFFFFF:FFFFFFFFFFFFFFFFFFFFFFFFFFFFFFFFF
@A00155:342:HHGFNDSXY:1:1565:22110:15546 1:N:0:GAACCTAG+TCCGCATA
GTCATAAAGCCGGTTTTGCCCTACATATGCCCTCAACGCAGACCATATCTGTGCCGAGTTGGGTAGGT
CAGGTGCCCTAGCACCTTCCTCCCCTTTCAACCATGCGTCCACCCCGGTTAATTTTGACCCGTGACGG
+
FFFFFFFFFFFFFFFFFFFFFF:FFFFFFFFFFFFFFFFF,FFFFFFFFFFFFFFFFFFFFFFFFFFF
FFFFFFFFFFFFFFFFFFFFFFFFFFFFFFFFFFFFFFFFFFFFFFFFFFFFFFFFFFFFFFFFFFFF
@A00155:342:HHGFNDSXY:1:1565:22209:16157 1:N:0:GAACCTAG+TCCGCATA
GTCATAAAGCCGGTTTTGCCCTACATATGCCCTCAACGCAGACCATATCTGTGCCGAGTTGGGTAGGT
CAGGTGCCCTAGCACCTTCCTCCCCTTTCAACCATGCGTCCACCCCGGTTAATTTTGACCCGTGACGG
+
FFFFFFFFFFFFFFFFFFFFFFFFFFFFFFFFFFFFFFFFFFFFFFFFFFFFFFFFFFFFFFFFFFFF
FFFFFFFFFFFFFFFFF,FFFFFFFFFFFFFFFFFFFFFFFFFFFFFFFFFFFFFFFFFFFFFFFFFF
@A00155:342:HHGFNDSXY:1:1473:13395:1799 1:N:0:GAACCTAG+TCCGCATA
GTCATAAAGCCGGTTTTGCCCTACATATGCCCTCAACGCAGACCATATCTGTGCCGAGTTGGGTAGGT
CAGGTGCCCTAGCACCTTCCTCCCCTTTCAACCATGCGTCCACCCCGGTTAATTTTGACCCGTGACGG
+
FFFFFFFFFFFFFFFFFFFFFFFFFFFFFFFFFFFFFFFFFFFFFFFFFFFFFFFFFFFFFFFFFFFF
FFFFFFFFFFFFFFFFFFFFFFFFFFFFFFFFFFFFFFFFFFFFFFFFFFFFFFFFFFFFFFFFFF:F
@A00155:342:HHGFNDSXY:1:1523:3278:10457 1:N:0:GAACCTAG+TCCGCATA
GTCATAAAGCCGGTTTTGCCCTACATATGCCCTCAACGCAGACCATATCTGTGCCGAGTTGGGTAGGT
CAGGTGCCCTAGCACCTTCCTCCCCTTTCAACCATGCGTCCACCCCGGTTAATTTTGACCCGTGACGG
+
FFFFFFFFFFFFFFFFFFFFFFFFFFFFFFFFFFFFF,FFFFFFFFFFFFFFFFFFFFFFFFFFFFFF
FFFFFFFFFFFFF:FFFFFFFFFFFF,FFFFFFFFFFFFFFFFFFFFFFFFFFFFFFFFFFFFFFFFF
@A00155:342:HHGFNDSXY:1:2615:32253:15107 1:N:0:GAACCTAG+TCCGCATA
GTCATAAAGCCGGTTTTGCCCTACATATGCCCTCAACGCAGACCATATCTGTGCCGAGTTGGGTAGGT
CAGGTGCCCTAGCACCTTCCTCCCCTTTCAACCATGCGTCCACCCCGGTTAATTTTGACCCGTGACGG
+
FFFFFFFFFFFFFFFFFFFFFFFFFFF:FFFFFFFFFFFFFFFFFFFFFFFFFFFFFFFFFFFFFFFF
FFFFFFFFFFFFFFFFF:FFFFFFFFFFFFFFFFFFFFFFFFFFFFFFFFFFFFFFFFFFFFFFFFFF
@A00155:342:HHGFNDSXY:1:2112:18304:11052 1:N:0:GAACCTAG+TCCGCATA
GTCATAAAGCCGGTTTTGCCCTACATATGCCCTCAACGCAGACCATATCTGTGCCGAGTTGGGTAGGT
```

```
CAGGTGCCCTAGCACCTTCCTCCCCTTTCAACCATGCGTCCACCCCGGTTAATTTTGACCCGTGACGG
+
FFFFFFFFFFFFFFFFFFFFFFFFFFFFFFFFFFFFFFFFFFFFFFFFFFFFFFFFFFFFFFFFFFFF
FFFFFFFFFFFFFFFF:FF:FFFFFFFFFFFFFFFFFFFFFFFFFFFFFFFFFFFFFFFFFFFFFFFF
@A00155:342:HHGFNDSXY:1:2112:18295:11068 1:N:0:GAACCTAG+TCCGCATA
GTCATAAAGCCGGTTTTGCCCTACATATGCCCTCAACGCAGACCATATCTGTGCCGAGTTGGGTAGGT
CAGGTGCCCTAGCACCTTCCTCCCCTTTCAACCATGCGTCCACCCCGGTTAATTTTGACCCGTGACGG
+
FFFFFFFFFFFFFFFFFFFFFFFFFFFFFFFFFFFFFFFFFFFFFFFFFFFFFFFFFFFFFFFFFFFF
FFFFFFFFFFFFFFFF:FFFFFFFFFFFFFFFFFFFFFFFFFFFFFFFFFFFFFFFFFFFFFFFFFF
@A00155:342:HHGFNDSXY:1:2644:4327:19539 1:N:0:GAACCTAG+TCCGCATA
TCATAAAGCCGGTTTTGCCCTACATATGCCCTCAACGCAGACCATATCTGTGCCGAGTTGGGTAGGTC
AGGTGCCCTAGCACCTTCCTCCCCTTTCAACCATGCGTCCACCCCGGTTAATTTTGACCCGTGACGGT
+
FFFFFFFFFFFFFFFFFFFFFFFFFF:FFFFFFFFFFFFFFFFFFFFFFFFFFFFFFFFFFFFFFFFF
FFFFFFFFFFFFFFFFFFFFFFFFFFFFFFFFFFFFFFFFFFFFFFFFFFFFFFFFFFFFFFFFFFFF
@A00155:342:HHGFNDSXY:1:2333:6117:21010 1:N:0:GAACCTAG+TCCGCATA
TCATAAAGCCGGTTTTGCCCTACATATGCCCTCAACGCAGACCATATCTGTGCCGAGTTGGGTAGGTC
AGGTGCCCTAGCACCTTCCTCCCCTTTCAACCATGCGTCCACCCCGGTTAATTTTGACCCGTGACGGT
+
FFFFFFFFFFFFFFFFFFFFFFFFFFFFFFFFFFFFFFFFFFFF:FFFFFFFFFFFFFFFFFFFFFFF
FFFFFFFFFFFFFFFFFFFFFFFFFFFFFFFFFFFFFFFFFFFFFFF:FFF:FFFFF:FFFFFFFFFF
@A00155:342:HHGFNDSXY:1:1634:23149:24674 2:N:0:GAACCTAG+TCCGCATA
ATAAAGCCGGTTTTGCCCTACATATGCCCTCAACGCAGACCATATCTGTGCCGAGTTGGGTAGGTCAG
GTGCCCTAGCACCTTCCTCCCCTTTCAACCATGCGTCCACCCCGGTTAATTTTGACCCGTGACGGTAA
+
:FFFFFFFFF:FFFFFFFF,FFFFFFFFFFFF:FFFFFFFFFF,FFFFFFFFFFFFFFFFFFFFFFFF
FFFFFFFFFFFFFFFFFFFFFFFFF:FFFFFFFFFFFFFFF:FFFFFFFFFFFFFFFFFFFFFFFFFF
@A00155:342:HHGFNDSXY:1:1152:3766:9424 1:N:0:GAACCTAG+TCCGCATA
TAAAGCCGGTTTTGCCCTACATATGCCCTCAACGCAGACCATATCTGTGCCGAGTTGGGTAGGTCAGG
TGCCCTAGCACCTTCCTCCCCTTTCAACCATGCGTCCACCCCGGTTAATTTTGACCCGTGACGGTAAT
+
FFFFFFFFFFFFFFFFFFFFFFFFFFFFFFFFFFFFFFFFFFFF:FFFFFFFFFFFFFFF,FFFFFFF
FFFFFF,FF:FFF:FFFFFFFFFFFFFFFFFFFFFFFFFFFFFFFFFFFFFFFFFFFFFFFFFFFFFF
@A00155:342:HHGFNDSXY:1:2151:3477:25394 1:N:0:GAACCTAG+TCCGCATA
TAAAGCCGGTTTTGCCCTACATATGCCCTCAACGCAGACCATATCTGTGCCGAGTTGGGTAGGTCAGG
TGCCCTAGCACCTTCCTCCCCTTTCAACCATGCGTCCACCCCGGTTAATTTTGACCCGTGACGGTAAT
+
FFFFFFFFF:FFFFFFFFFFFFFFFFFFFFFFFFFFF:FFFFFFFFFFFFFFFFFFFFFFFFFFFFFF
FFFFFFFFFF:FF,FFFFFFFFFFFFFFFFFFFFFFFFFFF:FFFFFFFFFFFFFFFFF:F:FFFFF:
@A00155:342:HHGFNDSXY:1:2142:21685:15029 2:N:0:GAACCTAG+TCCGCATA
AGCCGGTTTTGCCCTACATATGCCCTCAACGCAGACCATATCTGTGCCGAGTTGGGTAGGTCAGGTGC
CCTAGCACCTTCCTCCCCTTTCAACCATGCGTCCACCCCGGTTAATTTTGACCCGTGACGGTAATACA
+
FFFFFF:FFFFFFFFFF:FF:FF:FFFFFFFFFFFFFF:FFFFFFFFFFFFFFFFFFFFFFFFFFFFF
FFFFFFFFFFFFFFFFFFFFFFFFFFFFF:FFFFFFFF:FFFFFFFFFFFFFFFFFFFFFFFFFFFFF
@A00155:342:HHGFNDSXY:1:1220:18909:19053 2:N:0:GAACCTAG+TCCGCATA
AGCCGGTTTTGCCCTACATATGCCCTCAACGCAGACCATATCTGTGCCGAGTTGGGTAGGTCAGGTGC
CCTAGCACCTTCCTCCCCTTTCAACCATGCGTCCACCCCGGTTAATTTTGACCCGTGACGGTAATACA
+
FFFFFFFFFFF:::FFFFFFFFFFFFFFFFFFFFFFFFFFFFFFFFF:FFFFF:FFFFFFFFFFFFFF
FFFFFFFFF:FFFFFFFFFFFFFF,FFF:FFFFF,FFFFFFFF,FFFF:FFFFFFFFFFFFF:FFFFF
@A00155:342:HHGFNDSXY:1:2414:12527:6809 2:N:0:GAACCTAG+TCCGCATA
GGTTTTGCCCTACATATGCCCTCAACGCAGACCATATCTGTGCCGAGTTGGGTAGGTCAGGTGCCCTA
```

```
GCACCTTCCTCCCCTTTCAACCATGCGTCCACCCCGGTTAATTTTGACCCGTGACGGTAATACAATAG
+
FFF,,FFFFFFFFFFFFFFFFFFFFFFFFFFFFFFFFFFFFFFFFFFFFFFFFFFFFFFFFFFFFFFFF
FFFFFFFFFFFFFFFFFFFFFFFFFFFFFFFFFFFFFFFFFFFFFFFFFFFFFFFFFFFFFFF:FFFF
@A00155:342:HHGFNDSXY:1:2447:1579:25927 2:N:0:GAACCTAG+TCCGCATA
GGTTTTGCCCTACATATGCCCTCAACGCAGACCATATCTGTGCCGAGTTGGGTAGGTCAGGTGCCCTA
GCACCTTCCTCCCCTTTCAACCATGCGTCCACCCCGGTTAATTTTGACCCGTGACGGTAATACAATAG
+
FFFFFFFFFFFFFFFFFFFFFFFFFF:FFFFFFFFFFFFF:FFFFFFFFFFFFFFF:FFFFFFFFFFF
FFFFFFFFFFFFFFFFFFFFF,FFFFFFFFFFFFFFFFFFFFFFFFFFFFFFFFFFFFFFFFFFFFFF
@A00155:342:HHGFNDSXY:1:1131:1669:21668 2:N:0:GAACCTAG+TCCGCATA
GGTTTTGCCCTACATATGCCCTCAACGCAGACCATATCTGTGCCGAGTTGGGTAGGTCAGGTGCCCTA
GCACCTTCCTCCCCTTTCAACCATGCGTCCACCCCGGTTAATTTTGACCCGTGACGGTAATACAATAG
+
FF:FFFFFFFFFFFFFFFFFFFFFFFFFFFFFFFFFFF:FFFFFFFFFFFFFFFFFFFFFFFFFFFFF
FFFFFFFFFFFFFFFFFFFFFFFFFFFFFFFFFFFFFFFFFF:F:FFFFFFFFFFFFFFFFFFFFFFFF
@A00155:342:HHGFNDSXY:1:2321:18276:33270 2:N:0:GAACCTAG+TCCGCATA
TTGCCCTACATATGCCCTCAACGCAGACCATATCTGTGCCGAGTTGGGTAGGTCAGGTGCCCTAGCAC
CTTCCTCCCCTTTCAACCATGCGTCCACCCCGGTTAATTTTGACCCGTGACGGTAATACAATAGCTCG
+
FFFFFFFFFFFFFFFFFFFFFFFFFFFFFFFFFFFFFFFFFFFFFFFFFFFFFFFFF,FFFFFFFFFF
FFFFFFFFFFFFFFFFFFFFFFFFFFFFFFFFFFFFFFFFFFFFFFFFFFFFFFFFFFFFFFFFFFFF
@A00155:342:HHGFNDSXY:1:1544:29206:26490 1:N:0:GAACCTAG+TCCGCATA
GCCCTACATATGCCCTCAACGCAGACCATATCTGTGCCGAGTTGGGTAGGTCAGGTGCCCTAGCACCT
TCCTCCCCTTTCAACCATGCGTCCACCCCGGTTAATTTTGACCCGTGACGGTAATACAATAGCTCGGT
+
FFFFFFFFFFFFFFFFFFFFFFFFFFFFFFFFFFFFFFFFFFFFFFFFFFFFFFFFFFFFFFFFFFFF
FFFFFFFFFFFFFFFFFFFFFFFFFFFFFFFFFFFFFFFFFFFFFFFFFFFFFFFFFFFFFFFFFFFF
@A00155:342:HHGFNDSXY:1:2554:16288:31485 2:N:0:GAACCTAG+TCCGCATA
TACATATGCCCTCAACACAGACCATATCTGTGCCGAGTTGGGTAGGTCAGGTGCCCTAGCACCTTCCT
CCCCTTTCAACCATGCGTCCACCCCGGTTAATTTTGACCCGTGACGGTAATACAATAGCTCGGTAAGC
+
FFF:F:FFFFF,FF,FFFFFFFFF:FFFFFFFFFFFFFFFFFFFFFFFFFFFFFFFFFFFFFFFFFFF
FFFFFFFFFFFFF:FFFFFFFFFFFFFFFFFFFFFFFFFFFFFFFFFFFFFFFF:FFFFFFFFFFFFFF
@A00155:342:HHGFNDSXY:1:2523:12816:19179 2:N:0:GAACCTAG+TCCGCATA
ACATATGCCCTCAACGCAGACCATATCTGTGCCGAGTTGGGTAGGTCAGGTGCCCTAGCACCTTCCTC
CCCTTTCAACCATGCGTCCACCCCGGTTAATTTTGACCCGTGACGGTAATACAATAGCTCGGTAAGCT
+
FFFFFFFFFFFFFFFFFFFFFFFF:FFFFFFFFFFFFFFFFFFFFFFFFFFFFFFFFFFFFFFFFFFF
FFFFFFFFFFFFFFFFFFFFFFFFFFFFFFFFFFFFFFFFFFFFFFFFFFFFFFFFFFFFFFFFFF:F
@A00155:342:HHGFNDSXY:1:1628:10972:28980 2:N:0:GAACCTAG+TCCGCATA
ACATATGCCCTCAACGCAGACCATATCTGTGCCGAGTTGGGTAGGTCAGGTGCCCTAGCACCTTCCTC
CCCTTTCAACCATGCGTCCACCCCGGTTAATTTTGACCCGTGACGGTAATACAATAGCTCGGTAAGCT
+
FFFFFFFFFFFFFFFFFFFFFFFF:FFFFFFFFFFFF,FFFFFFFFFFFFFFFFFFFFFFFFFFFFFF
FFFFFFFFFFFFFFFFFFFFFFFFFFFFFFFFFFFFFFFFFFFFFFFFFFFFFFFFFFFFFFFFFFFF
@A00155:342:HHGFNDSXY:1:2471:13367:10019 2:N:0:GAACCTAG+TCCGCATA
ACATATGCCCTCAACGCAGACCATATCTGTGCCGAGTTGGGTAGGTCAGGTGCCCTAGCACCTTCCTC
CCCTTTCAACCATGCGTCCACCCCGGTTAATTTTGACCCGTGACGGTAATACAATAGCTCGGTAAGCT
+
FFFF::FFFFFFFFFFFFFFFFFF:FFFFFFFFFFFFFFFFFFFFFFFFFFFFFFFF:FFFFFFFFFFF
FFFFFFFFFFFFFFFFFFFFFFFFFFFFFFFFFFFFFFFFFFFFFFFFFFFFFFF:FFFFFF:FFFFF
@A00155:342:HHGFNDSXY:1:1516:5168:26913 2:N:0:GAACCTAG+TCCGCATA
ACATATGCCCTCAACGCAGACCATATCTGTGCCGAGTTGGGTAGGTCAGGTGCCCTAGCACCTTCCTC
```

CCCTTTCAACCATGCGTCCACCCCGGTTAATTTTGACCCGTGACGGTAATACAATAGCTCGGTAAGCT
+
FFFFFFFFFFF:FFFFFFFFFFF,FFFFFFFFF:FFFFFFFFFFFFFFFF,FFFFFFFFFF:FF:FFF
FFFFFFFFFFFFFFF:FFFFFFFFFFFFFFF:FFFFFFFFFFFF:FFFFFFFFFFFF:FFF:FFFFFF
@A00155:342:HHGFNDSXY:1:2333:6117:21010 2:N:0:GAACCTAG+TCCGCATA
ACATATGCCCTCAACGCAGACCATATCTGTGCCGAGTTGGGTAGGTCAGGTGCCCTAGCACCTTCCTC
CCCTTTCAACCATGCGTCCACCCCGGTTAATTTTGACCCGTGACGGTAATACAATAGCTCGGTAAGCT
+
FF,FFFFFFFFFFFFFFFFFF::FFFFFFFFFFFFFFFFFF::FFFFFFF:FFFF:FFFFFFFFFFF,
FFF:FFFFFFF:FF:FFFFFFFFFFFFFFFFFFFFFFFFFFFFFFFFFFFFFFF:FFFFFFFFFFFFF
@A00155:342:HHGFNDSXY:1:1628:10890:28933 2:N:0:GAACCTAG+TCCGCATA
ACATATGCCCTCAACGCAGACCATATCTGTGCCGAGTTGGGTAGGTCAGGTGCCCTAGCACCTTCCTC
CCCTTTCAACCATGCGTCCACCCCGGTTAATTTTGACCCGTGACGGTAATACAATAGCTCGGTAAGCT
+
FFFFFFFFFF:FFFFFFFFFFFFFFFFFFFFF:FFFFFFFF::FFFFFFFFFFFFFFFFFFF,FFFFF
FFFFFFFF,FFFFFFFFFFFFFFFFFFFF::FFFFFFFFFFFFFFFFFFFFFFF,FFFFFFFFFFFFF
@A00155:342:HHGFNDSXY:1:1507:32072:31641 2:N:0:GAACCTAG+TCCGCATA
ACATATGCCCTCAACGCAGACCATATCTGTGCCGAGTTGGGTAGGTCAGGTGCCCTAGCACCTTCCTC
CCCTTTCAACCATGCGTCCACCCCGGTTAATTTTGACCCGTGACGGTAATACAATAGCTCGGTAAGCT
+
FFFFFFFFFFFFF,FFFFFFFFFFFFFFFFFFFFFF:FFFFFFFFFFFFFFFFFFF:FFFFFFFFFFF
FFFFFFF,FFF:FFFFFFF,FFFFFFFFFFFF:FFFFFFFFFFFFFFFFFFFFFFFFFFFFFFFFFFF
@A00155:342:HHGFNDSXY:1:2253:15917:27524 2:N:0:GAACCTAG+TCCGCATA
ACATATGCCCTCAACGCAGACCATATCTGTGCCGAGTTGGGTAGGTCAGGTGCCCTAGCACCTTCCTC
CCCTTTCAACCATGCGTCCACCCCGGTTAATTTTGACCCGTGACGGTAATACAATAGCTCGGTAAGCT
+
FFFFFFFFFFFFFFFFFFFFFFFFFFFFFFFFFFFFFFFFFFFFFFFFFFFFFFFFFFFFFFFFFFFF
FFFFFFFFFFFFFFFFFFFFFFFFFFFFFFFFFFFFFFFFFFFFFFFFFFFFFFFFFFFFFFFFFFFF
@A00155:342:HHGFNDSXY:1:1644:21206:16485 2:N:0:GAACCTAG+TCCGCATA
ACATATGCCCTCAACGCAGACCATATCTGTGCCGAGTTGGGTAGGTCAGGTGCCCTAGCACCTTCCTC
CCCTTTCAACCATGCGTCCACCCCGGTTAATTTTGACCCGTGACGGTAATACAATAGCTCGGTAAGCT
+
FFF:FFFFFFFFF:FFFFFFFFFFFFFFFFFFFFFFFFFFFFFFFFFFFF:FFFFFFFFFFFFFFF:F
FFFFFFFF:FFFFFFFFFFFFFFFFFFFFFFFFFFFFFFFFFFFFFFFFFFFFF,FFFFF
@A00155:342:HHGFNDSXY:1:2643:8829:28244 2:N:0:GAACCTAG+TCCGCATA
ACATATGCCCTCAACGCAGACCATATCTGTGCCGAGTTGGGTAGGTCAGGTGCCCTAGCACCTTCCTC
CCCTTTCAACCATGCGTCCACCCCGGTTAATTTTGACCCGTGACGGTAATACAATAGCTCGGTAAGCT
+
FFFF:FFFFFFFFFFFFFFFFF:FFFFFFFFFFFFFFFFFFFFFFFFFFFFFFFFFFFFFFFFFFFFF
FFFFFFFFFFFFFFFFFFFFFFFFFFFFFFFFFFFFFFFFFFFFFFFFFFFFFFFFFFFFFFFFFFFF
@A00155:342:HHGFNDSXY:1:1607:1389:31673 2:N:0:GAACCTAG+TCCGCATA
ACATATGCCCTCAACGCAGACCATATCTGTGCCGAGTTTGGTAGGTCAGGTGCCCTAGCACCTTCCTC
CCCTTTCAACCATGCGTCCACCCCGGTTAATTTTGACCCGTGACGGTAATACAATAGCTCGGTAAGCT
+
FFFFFFFFFFFFFFFFFFF::FFFFFFF:FFFFFFFFF::F,F:FFFFFFFFF::FF:FFFF:FF::FF:
::FFFFFFF:FFFF:F:FFFFFFFFFFFFF,:,FF,,FFFFFFFFFF,FFFFF:,FFFF,,FFFF:F:
@A00155:342:HHGFNDSXY:1:1224:31033:24768 2:N:0:GAACCTAG+TCCGCATA
GCATATGCCCTCAACGCAGACCATATCTGTGCCGAGTTGGGTAGGTCAGGTGCCCTAGCACCTTCCTC
CCCTTTCAACCATGCGTCCACCCCGGTTAATTTTGACCCGTGACGGTAATACAATAGCTCGGTAAGCT
+
FFFFFFFFFFFFF:FFFFFFFFFFFFFFFFFFFFFFFFFFFFFFFFFFFFFFFFFFFFFFFFFFFFFFF
FFFFFFFFFFF:FFFFFFFFFFFFFFFFFFFFFFF:FFFFFFFFFFFFFFFFFFF:FFFFFFFFFFFF
@A00155:342:HHGFNDSXY:1:1578:4264:33708 1:N:0:GAACCTAG+TCCGCATA
CATATGCCCTCAACGCAGACCATATCTGTGCCGAGTTGGGTAGGTCAGGTGCCCTAGCACCTTCCTCC

```
CCTTTCAACCATGCGTCCACCCCGGTTAATTTTGACCCGTGACGGTAATACAATAGCTCGGTAAGCTT
+
FFFFFFFFFFFFFFFFFFFFFF:FFFFF:FFFFFFFFFF:FFFFFFFFF,FFFFFFFFFFFFFFFFFF
FFFFFFF:FFFFFFFFFFFFFFFFFFFFFFFFFFFFFFFFFFFFFFFFFFFFFFFFFFFFFFFFFFFF
@A00155:342:HHGFNDSXY:1:2566:7392:35493 1:N:0:GAACCTAG+TCCGCATA
CATATGCCCTCAACGCAGACCATATCTGTGCCGAGTTGGGTAGGTCAGGTGCCCTAGCACCTTCCTCC
CCTTTCAACCATGCGTCCACCCCGGTTAATTTTGACCCGTGACGGTAATACAATAGCTCGGTAAGCTT
+
FFFFFFFFFFFFFFFFFFFFFFFFFFFFFFFFFFFFFFFFFFFFFFFFFFFFFF,FFFFFFFFFFFFF
FFFFFFFFFFFFFFFFFFFFFFFFF:FFFFF,FFFFFFFFFFFFFFFFFFFFFFFFF:FFFFFFFFFF
@A00155:342:HHGFNDSXY:1:2308:28266:12524 1:N:0:GAACCTAG+TCCGCATA
CATATGCCCTCAACGCAGACCATATCTGTGCCGAGTTGGGTAGGTCAGGTGCCCTAGCACCTTCCTCC
CCTTTCAACCATGCGTCCACCCCGGTTAATTTTGACCCGTGACGGTAATACAATAGCTCGGTAAGCTT
+
FFFFFFFFFFFFFFFFFFFFFFFFFFFFFFFFFFFFFFFFFFFFF:FFFFFFFFFFFFFFFFFFFFFF
FFFFFFFFFFFFFFFFFFFFFFFFFFFFFFFFFFFFFFFFFFFFF,FFFFFFFFFFFFFFFFFFFFFF
@A00155:342:HHGFNDSXY:1:2321:18276:33270 1:N:0:GAACCTAG+TCCGCATA
CATATGCCCTCAACGCAGACCATATCTGTGCCGAGTTGGGTAGGTCAGGTGCCCTAGCACCTTCCTCC
CCTTTCAACCATGCGTCCACCCCGGTTAATTTTGACCCGTGACGGTAATACAATAGCTCGGTAAGCTT
+
FFFFFFFFFFFFFFFFFFFFFFFFFFFFFFFFFFFFFFFFFFFFFFFFFFFFFFFFFFFFFFFFFFFF
FFFFFFFFFFFFFFFFFFFFFFFFFFFFFFFFFFFFFFFFFFFFFFFFFFFFFFFFFFFFFFF:FFFF
@A00155:342:HHGFNDSXY:1:1172:11388:3646 2:N:0:GAACCTAG+TCCGCATA
CATATGCCCTCAACGCAGACCATATCTGTGCCGAGTTGGGTAGGTCAGGTGCCCTAGCACCTTCCTCC
CCTTTCAACCATGCGTCCACCCCGGTTAATTTTGACCCGTGACGGTAATACAATAGCTCGGTAAGCTT
+
FFFFFFFFFFFFFFFFFFFFFFFFFFFFFFFFFFFFFFFFFFFFFFFFFFFFFFFFFFFFFFFFFFFF
FFFFFFFFFFFFFFFFFFFFFFFFFF:FFFFFFFFFFFFFFFFFFFFFFFFFF:F:FFFFFFFFFFFF
@A00155:342:HHGFNDSXY:1:2446:20871:22388 1:N:0:GAACCTAG+TCCGCATA
CATATGCCCTCAACGCAGACCATATCTGTGCCGAGTTGGGTAGGTCAGGTGCCCTAGCACCTTCCTCC
CCTTTCAACCATGCGTCCACCCCGGTTAATTTTGACCCGTGACGGTAATACAATAGCTCGGTAAGCTT
+
FF,FFFFFFFFFFFFFFFFFFFFFFFFFFFFFFFFFFFFFFFFFFFFFFFFFFFFFFFFFFFFFFFFF
FFFFFFF:FFFFFFFFFFFFFFFFFFFFFFFFFFFFFFFFFFFFFFFFFFFFFFFFFFFFFFFFFFFF
@A00155:342:HHGFNDSXY:1:1339:12093:12759 1:N:0:GAACCTAG+TCCGCATA
CATATGCCCTCAACGCAGACCATATCTGTGCCGAGTTGGGTAGGTCAGGTGCCCTAGCACCTTCCTCC
CCTTTCAACCATGCGTCCACCCCGGTTAATTTTGACCCGTGACGGTAATACAATAGCTCGGTAAGCTT
+
FFFFFFFFFFFFFFFFFFFFFFFFFFFFFFFFFFFFFFFFFFFFFFFFFFFFFFFFFFFFF::FFFFF
FFFFFFFFFFFFFFFFFFFFFFFFFFFFFFFF:FFFFFFFFFFFF::FFFFFFFFFFFFFFFFFFFFF
@A00155:342:HHGFNDSXY:1:1132:25165:13009 1:N:0:GAACCTAG+TCCGCATA
CATATGCCCTCAACGCAGACCATATCTGTGCCGAGTTGGGTAGGTCAGGTGCCCTAGCACCTTCCTCC
CCTTTCAACCATGCGTCCACCCCGGTTAATTTTGACCCGTGACGGTAATACAATAGCTCGGTAAGCTT
+
FFFFFFFFFFFFFFFFFFFFFFFFFFFFFFFFFFFFFFFFFFFFFFFFFFFFFFFFFFFFFFFFFFFF
FFFFFFF:FFFFFFFFFFFFFFFFFFFFFFFFFFFFFFFFFFFFFFFFFFFFFFFFFF,FFF:F
@A00155:342:HHGFNDSXY:1:2226:14841:17206 1:N:0:GAACCTAG+TCCGCATA
CATATGCCCTCAACGCAGACCATATCTGTGCCGAGTTGGGTAGGTCAGGTGCCCTAGCACCTTCCTCC
CCTTTCAACCATGCGTCCACCCCGGTTAATTTTGACCCGTGACGGTAATACAATAGCTCGGTAAGCTT
+
FFFFFFFFFFFFFFFFFFFFFFFFFFFFFFFFFFFFFFFFFFFFFFFFFFFFFFFFFFFFFFFFFFFF
FFFFFFFFFFFFFFFFFFFFFFFFFFFFFFFFFFFFFFFFFFFFFFFFFFFFFFFFFFFFFFFFFFFF
@A00155:342:HHGFNDSXY:1:2378:30978:22075 2:N:0:GAACCTAG+TCCGCATA
ATATGCCCTCAACGCAGACCATATCTGTGCCGAGTTGGGTAGGTCAGGTGCCCTAGCACCTTCCTCCC
```

```
CTTTCAACCATGCGTCCACCCCGGTTAATTTTGACCCGTGACGGTAATACAATAGCTCGGTAAGCTT
+
FFFFFFFFFFF:FFFF:F,F:F,FFFFFFF:FFFFFFFFF,FFF,FFFFF:FF:FFFFFFFFFFFFF:
FFFFFFF::FFFFFFFFF:F:FFFFF,FFFFFFFFFFFFFFFFFFFFFFFFFFFFFF:FFFFFFFF
@A00155:342:HHGFNDSXY:1:1165:29776:15013 2:N:0:GAACCTAG+TCCGCATA
TATGCCCTCAACGCAGACCATATCTGTGCCGAGTTGGGTAGGTCAGGTGCCCTAGCACCTTCCTCCCC
TTTCAACCATGCGTCCACCCCGGTTAATTTTGACCCGTGACGGTAATACAATAGCTCGGTAAGCTTGG
+
F:FFFFFFFFFFF:FFF,FFFFFFFFF,FFFFFFFFFFFFFFFFFFFF,FFFFFFFFFFFFFFFFF
FFFFFFFFFFFFFF:FFF:FF,FFFFFFFFFFFFFFFFFFFFFFFFFFFFFFFFFFFFFFFFFFFFF
@A00155:342:HHGFNDSXY:1:2373:4616:10113 2:N:0:GAACCTAG+TCCGCATA
ATGCCCTCAACGCAGACCATATCTGTGCCGAGTTGGGTAGGTCAGGTGCCCTAGCACCTTCCTCCCCT
TTCAACCATGCGTCCACCCCGGTTAATTTTGACCCGTGACGGTAATACAATAGCTCGGTAAGCTTGGG
+
FFFFFFFFF,FFFFFFFF:FFFFFFFFFFF:FFFFFFFFFFFFFFFFFFFFFFFFFFFFFFFFFFF:F
FFFFFFFFFFFFFFFFFFFFFFFFFFFFFFFFFFFFFFFFFFFFFFFFFFFFFFFFFFFFFFFFFFFF
@A00155:342:HHGFNDSXY:1:1118:1497:26256 2:N:0:GAACCTAG+TCCGCATA
ATGCCCTCAACGCAGACCATATCTGTGCCGAGTTGGGTAGGTCAGGTGCCCTAGCACCTTCCTCCCCT
TTCAACCATGCGTCCACCCCGGTTAATTTTGACCCGTGACGGTAATACAATAGCTCGGTAAGCTTGGG
+
FFFFF:FFFFF,FFFFFFFFFFFFF:F,FFFFFFF,:FF:F:FFF:FFFF:FF:FFFFFFFFFF,FFF
FFFFFFFFFFFF:FFFFFFF:,,FFFFFFF,F:FF:FFFFF,FFFFFFFFFFFFFFFFFFFFFFFF::
@A00155:342:HHGFNDSXY:1:1632:32560:11882 2:N:0:GAACCTAG+TCCGCATA
ATGCCCTCAACGCAGACCATATCTGTGCCGAGTTGGGTAGGTCAGGTGCCCTAGCACCTTCCTCCCCT
TTCAACCATGCGTCCACCCCGGTTAATTTTGACCCGTGACGGTAATACAATAGCTCGGTAAGCTTGGG
+
FFFFFFFFF,FFFFFFFF::FFFFFFFFF:FF:FFFFFFFFFFFFFFFF:::FFFFFFFFFFFFFF:
FFF:FFF,FFFFFFF:FFFFFFFFF,FFF,F,FFFFFFFFFFFFF:FFF:,FFFFFFFFFFFFFFFF
@A00155:342:HHGFNDSXY:1:2348:13494:20917 2:N:0:GAACCTAG+TCCGCATA
ATGCCCTCAACGCAGACCATATCTGTGCCGAGTTGGGTAGGTCAGGTGCCCTAGCACCTTCCTCCCCT
TTCAACCATGCGTCCACCCCGGTTAATTTTGACCCGTGACGGTAATACAATAGCTCGGTAAGCTTGGG
+
FFFFFFFFFFFFFFF:FF,FFFFFFFFFFFFFFF,FFFFF,,:FFFFFFFFFFFFFFF:FFF::FFFF
FFF,FFFFFFFFFF:FFF:,,F:FFFFFFFFFFFFFFFFFFFFFFFFFFFFFFFF:FFFFFFFFFFF
@A00155:342:HHGFNDSXY:1:1467:19018:15734 2:N:0:GAACCTAG+TCCGCATA
ATGCCCTCAACGCAGACCATATCTGTGCCGAGTTGGGTAGGTCAGGTGCCCTAGCACCTTCCTCCCCT
TTCAACCATGCGTCCACCCCGGTTAATTTTGACCCGTGACGGTAATACAATAGCTCGGTAAGCTTGGG
+
FFFFFFFFFFFFFFFFFFFFFFFFFFFFFFFFFFFFFFFFFFFFFFFFFFFFFFFFFFF:FFFFFFFF
FFFFFFFF,FFFFFFFFFFFFFFFFFFFFFFFFFFFFFFFFFFFFFFFFFFFFFFFFFFFFFF:FFFF
@A00155:342:HHGFNDSXY:1:2568:3775:2738 2:N:0:GAACCTAG+TCCGCATA
CTCAACGCAGACCATATCTGTGCCGAGTTGGGTAGGTCAGGTGCCCTAGCACCTTCCTCCCCTTTCAA
CCATGCGTCCACCCCGGTTAATTTTGACCCGTGACGGTAATACAATAGCTCGGTAAGCTTGGGTGAGT
+
FFFFF,FFFFFF:FFFFFFFFFFFFFFFFFFFFFFFFFFFFFFFFFFFFFFFFFFFFFFFFFFFFFFF
FFFFFFFFFFFFFFFFFFFFFF:FFFFFFFFFFFFF:FFFFFFFFFFF:FFFFFF:FFFFFFFFFFFFF
@A00155:342:HHGFNDSXY:1:2462:6189:23453 1:N:0:GAACCTAG+TCCGCATA
CTCAACGCAGACCATATCTGTGCCGAGTTGGGTAGGTCAGGTGCCCTAGCACCTTCCTCCCCTTTCAA
CCATGCGTCCACCCCGGTTAATTTTGACCCGTGACGGTAATACAATAGCTCGGTAAGCTTGGGTGAGT
+
FFFFFFFFFFFFFFFFFFFFFFFFFFFFFFFFFFFFFFFFFFFFFFFFFFFFFFFFFFFFFFFFFFFF
FFFFFFFFFFFFFFFFFFFFFFFFFFFFFFFFFFFFFFFFF:FFFFFFFFFFFFFFFFF:FFFFFFFFFF
@A00155:342:HHGFNDSXY:1:1617:7301:18803 1:N:0:GAACCTAG+TCCGCATA
TCAACGCAGACCATATCTGTGCCGAGTTGGGTAGGTCAGGTGCCCTAGCACCTTCCTCCCCTTTCAAC
```

CATGCGTCCACCCCGGTTAATTTTGACCCGTGACGGTAATACAATAGCTCGGTAAGCTTGGGTGAGTC
+
FFFFFFFFFFFFFFFFFFFFFFFFFFFFFFFFFFFFFFFFFFFFFFFFFFFFFFFFFFFFFFFFFFFF
FFFFFFFFFFFFFFFFFFFFFFFFFFFFFFFFFFFFF,,FFFFFFFFF:FFFFFFFFFFFFFFFFFFF
@A00155:342:HHGFNDSXY:1:1224:31033:24768 1:N:0:GAACCTAG+TCCGCATA
TCAACGCAGACCATATCTGTGCCGAGTTGGGTAGGTCAGGTGCCCTAGCACCTTCCTCCCCTTTCAAC
CATGCGTCCACCCCGGTTAATTTTGACCCGTGACGGTAATACAATAGCTCGGTAAGCTTGGGTGAGTC
+
FFFFFFFFFFFFFFFFFFFFFFFFFFFFFFFFFFFFFFFFFFFFFFFFFFFFF:FFFFFFFFFFFFFF
FFFFFFFFFFFFFFFFFFFFFFFFFFFFFFFFFFFFFF,FFFFFFFFFFFFFFFFFFFFFFFFFFFFF
@A00155:342:HHGFNDSXY:1:1343:10502:35305 1:N:0:GAACCTAG+TCCGCATA
TCAACGCAGACCATATCTGTGCCGAGTTGGGTAGGTCAGGTGCCCTAGCACCTTCCTCCCCTTTCAAC
CATGCGTCCACCCCGGTTAATTTTGACCCGTGACGGTAATACAATAGCTCGGTAAGCTTGGGTGAGTC
+
FFFFFFFFFFFFFFFFFFFFFFFFFF:FFFFFFFFFFFFFFFFFFFFFFFF:FFFFFFFFFFFFFFFF
FFFFFFFFFFFFFFFFFFFFFFFFFFFFFFFFFFFFFFFFFFFFFFFFFFFFFFFFFFFFFFFFFFFF
@A00155:342:HHGFNDSXY:1:2373:4616:10113 1:N:0:GAACCTAG+TCCGCATA
TCAACGCAGACCATATCTGTGCCGAGTTGGGTAGGTCAGGTGCCCTAGCACCTTCCTCCCCTTTCAAC
CATGCGTCCACCCCGGTTAATTTTGACCCGTGACGGTAATACAATAGCTCGGTAAGCTTGGGTGAGTC
+
FFFFFFFFFFFFFFFFFFF:FFFFFFFFFFFFFFFFFFFFFFFFFFFFFFFFFFFFFFFFFFFFFFFF
FFFFFFFFFFFFFFFF::FFFFFFFFFFFFFFFFFFFFFFFFFFFFFFFFFFFFFFFFFFFFFFFFFF
@A00155:342:HHGFNDSXY:1:1114:3513:11490 1:N:0:GAACCTAG+TCCGCATA
TCAACGCAGACCATATCTGTGCCGAGTTGGGTAGGTCAGGTGCCCTAGCACCTTCCTCCCCTTTCAAC
CATGCGTCCACCCCGGTTAATTTTGACCCGTGACGGTAATACAATAGCTCGGTAAGCTTGGGTGAGTC
+
FFFFF:FFFFFFFFFFFFFF:F,FFFFFFFFFFF:FF,,FF,FFFFF,:F,,:F:F::FFFFFFFFFF
:FFFFF,FFFFFFFFFFFFFFFFFFF,FFFFFF,FFFFF,F:FFFFFFFFFFFFFFF::FFFFFF:FF
@A00155:342:HHGFNDSXY:1:2554:16288:31485 1:N:0:GAACCTAG+TCCGCATA
TCAACACAGACCATATCTGTGCCGAGTTGGGTAGGTCAGGTGCCCTAGCACCTTCCTCCCCTTTCAAC
CATGCGTCCACCCCGGTTAATTTTGACCCGTGACGGTAATACAATAGCTCGGTAAGCTTGGGTGAGTC
+
FFFFFFFFFFFFFFFFFFFFFFFFFFFFFFFFFFFFFFFFFFFFFFFFFFFF:FFFFFFFFFFFFFFF
FFFFFFFFFFFFFFFFFFFFFFFFFFFFFFFFFFFFF:FFFFFFF:FFFFFFFFFFF:FFFFFFFFFF
@A00155:342:HHGFNDSXY:1:1214:4309:27211 1:N:0:GAACCTAG+TCCGCATA
TCAACGCAGACCATATCTGTGCCGAGTTGGGTAGGTCAGGTGCCCTAGCACCTTCCTCCCCTTTCAAC
CATGCGTCCACCCCGGTTAATTTTGACCCGTGACGGTAATACAATAGCTCGGTAAGCTTGGGTGAGTC
+
FFFFFFFFFFFFFFFFFFFFFFFFFFFFFFFFFFFFFFFFFFFFFFFFFFFF:FFFFFFFFFFFFFFF
FFFFFFFFFFFFFFFFFFFFFFFF:FFFFFFFFFFFFFFFFFFFFFFFFFFFFFFFFFFFFFFFFFFF
@A00155:342:HHGFNDSXY:1:2675:25129:19335 1:N:0:GAACCTAG+TCCGCATA
TCAACGCAGACCATATCTGTGCCGAGTTGGGTAGGTCAGGTGCCCTAGCACCTTCCTCCCCTTTCAAC
CATGCGTCCACCCCGGTTAATTTTGACCCGTGACGGTAATACAATAGCTCGGTAAGCTTGGGTGAGTC
+
FFFFFFFFFFFFFFFFFFFFFFFFFFFFFFFFFFFFFFFFFFFFFFFFFFFFFFFFFFFFFFFFFFFF
FFFFFFFFFFFFFFFFFFFFFFFFFFFFFFFFFFFFF:FFFFFFFFFFFFFFF:FFFFFFFFFFFFFF
@A00155:342:HHGFNDSXY:1:1343:10502:35305 2:N:0:GAACCTAG+TCCGCATA
TCAACGCAGACCATATCTGTGCCGAGTTGGGTAGGTCAGGTGCCCTAGCACCTTCCTCCCCTTTCAAC
CATGCGTCCACCCCGGTTAATTTTGACCCGTGACGGTAATACAATAGCTCGGTAAGCTTGGGTGAGTC
+
FFFFFFFFFFFFFF:FFFFFFFFFFFFFFFFFFFFFFFFFFFFFFFFFFFFFFFFFFFFFFFFFFF:F
FFFFFFFFFFFFFFFFFFFFFFFFFFFFFFFFFFFFFFFF:FFFFFFFFFFFFFFFFFFFFFFFFFFF
@A00155:342:HHGFNDSXY:1:2460:11532:25316 1:N:0:GAACCTAG+TCCGCATA
TCAACGCAGACCATATCTGTGCCGAGTTGGGTAGGTCAGGTGCCCTAGCACCTTCCTCCCCTTTCAAC

```
CATGCGTCCACCCCGGTTAATTTTGACCCGTGACGGTAATACAATAGCTCGGTAAGCTTGGGTGAGTC
+
FFFFFFFFFFFFFFFFFFFFFFFFFFFFFFFFFFFFFFFFFFFFFFFF,:F:FFFFFFFFFFFFFFF
FFFFFFFFFFFFFFFFFFFFFFFFFFFFFFFFFFFFFFFFFFFFFFFFFFFFFFFFFFFFFFFFFFFF
@A00155:342:HHGFNDSXY:1:1472:6506:3709 1:N:0:GAACCTAG+TCCGCATA
TCAACGCAGACCATATCTGTGCCGAGTTGGGTAGGTCAGGTGCCCTAGCACCTTCCTCCCCTTTCAAC
CATGCGTCCACCCCGGTTAATTTTGACCCGTGACGGTAATACAATAGCTCGGTAAGCTTGGGTGAGTC
+
FFFFFFFFFFFFFFF:FFF:FFFFFFFFFFFFFFFFFFFFFFFFFFFFF:FF::FFFFFFFFFFFFF
FFFFFFFFFFFFFFFFFFFFFFFFFFFFFFFFFFFFF:FFFFFFFFFFFFFFFFFFFFFFFFFFFFFF
@A00155:342:HHGFNDSXY:1:1114:4833:12931 1:N:0:GAACCTAG+TCCGCATA
TCAACGCAGACCATATCTGTGCCGAGTTGGGTAGGTCATGTGCCCTAGCACCTTCCTCCCCTTTCAAC
CATGCGTCCACCCCGGTTAATTTTGACCCGTGACGGTAATACAATAGCTCGGTAAGCTTGGGTGAGTC
+
FFFFFFFFFFFFFFFF,FFF,,FFFFFF:,FFF,FFFF,,FFFF:FF:FFFF:FFFFFFFFFFFFFF
FFFFFFFFFFFFFFFFFFFFFFFFFFFFFFFFFFF:,FFFFFFFFFFFF:FF:F:FFFFFFFFFFFF
@A00155:342:HHGFNDSXY:1:1617:6325:19554 1:N:0:GAACCTAG+TCCGCATA
TCAACGCAGACCATATCTGTGCCGAGTTGGGTAGGTCAGGTGCCCTAGCACCTTCCTCCCCTTTCAAC
CATGCGTCCACCCCGGTTAATTTTGACCCGTGACGGTAATACAATAGCTCGGTAAGCTTGGGTGAGTC
+
FFFFFFFFFFFFFFFFFFFFFFFFFF:FFFFFFF,FFFFFFFFFFFFFFFFFFF:FFFFFFFFFFFFF
FFFFFFFFFFFFFFFFFFFFFFFFFFFFFFFFFFFFFFFFFFFFFFFFFFFFFF:FFF:FFFFFFFFF
@A00155:342:HHGFNDSXY:1:2360:2763:22999 1:N:0:GAACCTAG+TCCGCATA
TCAACGCAGACCATATCTGTGCCGAGTTGGGTAGGTCAGGTGCCCTAGCACCTTCCTCCCCTTTCAAC
CATGCGTCCACCCCGGTTAATTTTGACCCGTGACGGTAATACAATAGCTCGGTAAGCTTGGGTGAGTC
+
FFFFFFFFFFFFFFFFFFFFFFFFFFFFFFFFFFFFFFFFFFFFFFFFFFF:FF:FFFFFFFFFFFFF
FFFFFFFFFFFFFFFFFFFFFFFFFF:FFFFFFFFFF:FFFFFFFFFFFFFFFFFFFF,FFFFFFFFF
@A00155:342:HHGFNDSXY:1:1167:7328:15562 1:N:0:GAACCTAG+TCCGCATA
TCAACGCAGACCATATCTGTGCCGAGTTGGGTAGGTCAGGTGCCCTAGCACCTTCCTCCCCTTTCAAC
CATGCGTCCACCCCGGTTAATTTTGACCCGTGACGGTAATACAATAGCTCGGTAAGCTTGGGTGAGTC
+
FFFFFFFFFFFFFFFFFFFFFFFFFFFFFFFFFFFFFFFFFFFFFFF:FF,FFFFFFFFFFFFFFFFF
FFFFFFFFFFFFFFFFFFFFFFFFF:FFFFFFFFFFFFFFFFFFFFFFFF:FFFFFFFFFFFFFFFFFF
@A00155:342:HHGFNDSXY:1:1557:30662:16892 1:N:0:GAACCTAG+TCCGCATA
TCAACGCAGACCATATCTGTGCCGAGTTGGGTAGGTCAGGTGCCCTAGCACCTTCCTCCCCTTTCAAC
CATGCGTCCACCCCGGTTAATTTTGACCCGTGACGGTAATACAATAGCTCGGTAAGCTTGGGTGAGTC
+
FFFFFFFFFFFFFFFFFFFFFFFFFFFFFFFFFFFFFFFFFFFFFFFFFFFF:FFFFFFFFFFFFFF
FFFFFFFFFFFFFFFFFFFFFFFFFFFFFFFFFFFFFFFFFFFFFFFFFFFFFFFFFFFFFFFFFFFF
@A00155:342:HHGFNDSXY:1:2147:18566:11099 1:N:0:GAACCTAG+TCCGCATA
CAACGCAGACCATATCTGTGCCGAGTTGGGTAGGTCAGGTGCCCTAGCACCTTCCTCCCCTTTCAACC
ATGCGTCCACCCCGGTTAATTTTGACCCGTGACGGTAATACAATAGCTCGGTAAGCTTGGGTGAGTCA
+
FFFFFFFFFFFFFFFFFF:FFFFFFFFFFFFFFF:FFFFF:FFFFFFF,:F:FFFFFFFFFFFFFFFF
FFFFF:,FFFFFFFFFFFF:,::FFFFFFFFFFFFF::,FFFFF:FF,FFFFF:FFFFFFFFFFF:FF
@A00155:342:HHGFNDSXY:1:1644:21206:16485 1:N:0:GAACCTAG+TCCGCATA
CAACGCAGACCATATCTGTGCCGAGTTGGGTAGGTCAGGTGCCCTAGCACCTTCCTCCCCTTTCAACC
ATGCGTCCACCCCGGTTAATTTTGACCCGTGACGGTAATACAATAGCTCGGTAAGCTTGGGTGAGTCA
+
FFFFFFFFFFFFFFFFFFFFFFFFFFFFFFFFFFFFFFFFFFFFFFFFFFFFFFFFFFFFFFFFFFFF
FFFFFFFFFFFFFFFFFFFFFFFFFFFFFFFFFFFFFFFFFF,FFFFFFFFFFFFFFFFFFFFFFFFF
@A00155:342:HHGFNDSXY:1:2213:29369:21825 1:N:0:GAACCTAG+TCCGCATA
CAACGCAGACCATATCTGTGCCGAGTTGGGTAGGTCAGGTGCCCTAGCACCTTCCTCCCCTTTCAACC
```

ATGCGTCCACCCCGGTTAATTTTGACCCGTGACGGTAATACAATAGCTCGGTAAGCTTGGGTGAGTCA
+
FFFFFFFFFFFFF:FFFFFFFFFFFFFFFFFFFFFFFFFFFFFFFFFFFFFFFFFFFFFFFFFFFFFFFFF
FFFFFFFFFFFFFFFFFFFFFFFFFFFFFFFFFFFFFFFFFFFFFFFFFFFFFFFFFFFFFFFFFFFFFFF
@A00155:342:HHGFNDSXY:1:1254:19063:19664 1:N:0:GAACCTAG+TCCGCATA
CAACGCAGACCATATCTGTGCCGAATTGGGTAGGTCAGGTGCCCTAGCACCTTCCTCCCCTTTCAACC
ATGCGTCCACCCCGGTTAATTTTGACCCGTGACGGTAATACAATAGCTCGGTAAGCTTGGGTGAGTCA
+
FFFFFFFFFFFFFFFFFFFFFFFFFFFFFFFFFFFFFFFFFFFFFFFFFFFFFFFFFFFFFFFFFFFFFFF
FFFFFFFFFFFFFFFFFFFFFFFFFFFFFFFFFFFFFFFFFFFFFFFFFFFFFFFF:FFFFFF,FFFF
@A00155:342:HHGFNDSXY:1:2643:8829:28244 1:N:0:GAACCTAG+TCCGCATA
AACGCAGACCATATCTGTGCCGAGTTGGGTAGGTCAGGTGCCCTAGCACCTTCCTCCCCTTTCAACCA
TGCGTCCACCCCGGTTAATTTTGACCCGTGACGGTAATACAATAGCTCGGTAAGCTTGGGTGAGTCAA
+
FFFFFFFFFFFFFFFFFFFFFFFFFFFFFFFFFFFFFFFFFFFFFFFFFFFFFFFFFFFFFFFFFFFFFFF
FFFFFFFFFFFFFFFFFFFFFFFFFFFFFFFFFFFFFFFFFFFFFFFFFFFFFFFFFFFFFFFFFFFFFFF
@A00155:342:HHGFNDSXY:1:1672:10248:15608 1:N:0:GAACCTAG+TCCGCATA
AACGCAGACCATATCTGTGCCGAGTTGGGTAGGTCAGGTGCCCTAGCACCTTCCTCCCCTTTCAACCA
TGCGTCCACCCCGGTTAATTTTGACCCGTGACGGTAATACAATAGCTCGGTAAGCTTGGGTGAGTCAA
+
FFFFFFFFFFFFFFFFFFFFFFFFFFFFFFFFFFFFFFFFFFFFFFFFFFFFFFFFFFFFFFFFFFFFFFF
FFF:FFFFFFFFFFFFFFFFFFFFFFFFFFFFFFFFFFFFFFFFFFFFFFFFFFFFFFFFFFFFFFFFFFF
@A00155:342:HHGFNDSXY:1:1329:17282:36213 1:N:0:GAACCTAG+TCCGCATA
AACGCAGACCATATCTGTGCCGAGTTGGGTAGGTCAGGTGCCCTAGCACCTTCCTCCCCTTTCAACCA
TGCGTCCACCCCGGTTAATTTTGACCCGTGACGGTAATACAATAGCTCGGTAAGCTTGGGTGAGTCAA
+
FFFFFFFFFFFFFFFFFFFFFFFFFFFFFFFFFFFFFFFFFFFFFFFFFFF,FF:FFFFFFFFFFFFF
FFFFFFFFFFFFFFFFFFFFFFFFFFFFFFFFFFFFFFFFFFFFFFFFFFFFFFFFFFFFFFFFFFFFFFF
@A00155:342:HHGFNDSXY:1:1625:4327:9392 1:N:0:GAACCTAG+TCCGCATA
AACGCAGACCATATCTGTGCCGAGTTGGGTAGGTCAGGTGCCCTAGCACCTTCCTCCCCTTTCAACCA
TGCGTCCACCCCGGTTAATTTTGACCCGTGACGGTAATACAATAGCTCGGTAAGCTTGGGTGAGTCAA
+
FFFFFFFFFFFFFFFFFFFFFFFFFFFFFFFFFFFFFFFFFFFF:FFFFFFFF,FFFFFFFFFFFFF
FFFFFFFFFFFFFFFFFFFFFFFF::FFFFFFF:FFFFFFFFFFFFFFFF:FFFFFFFFFFFFFFFFFF
@A00155:342:HHGFNDSXY:1:2305:14968:19335 1:N:0:GAACCTAG+TCCGCATA
AACGCAGACCATATCTGTGCCGAGTTGGGTAGGTCAGGTGCCCTAGCACCTTCCTCCCCTTTCAACCA
TGCGTCCACCCCGGTTAATTTTGACCCGTGACGGTAATACAATAGCTCGGTAAGCTTGGGTGAGTCAA
+
FFFFFFFFFFFFFFFFFFFFFFFFFFFFFFFFFFFFFFFFFFFFFFFFFFFFFFFFFFFFFFFFFFFFFFF
FFFFFFFFFFFFFFFFFFF:FFFFFFFFFFFFFFFFFFFFFFFFFFFFFFFFFFFFFFFFFFFFF:FFFF
@A00155:342:HHGFNDSXY:1:1207:3242:7169 1:N:0:GAACCTAG+TCCGCATA
AACGCAGACCATATCTGTGCCGAGTTGGGTAGGTCAGGTGCCCTAGCACCTTCCTCCCCTTTCAACCA
TGCGTCCACCCCGGTTAATTTTGACCCGTGACGGTAATACAATAGCTCGGTAAGCTTGGGTGAGTCAA
+
FFF:FFFFF,FFFFFFFFF::FFFFFF:,:FFF:F,FFFFFFFFFFFF:F:FFFFFFFFFFFFFFFFF
FF:FFFFFFFFFFFFFFFFFFFFFFFFFFFFFFFFF:FFF,,FFFFFFFF:FFFFFFFFFFFFFFFFFF
@A00155:342:HHGFNDSXY:1:2165:4915:23594 1:N:0:GAACCTAG+TCCGCATA
AACGCAGACCATATCTGTGCCGAGTTGGGTAGGTCAGGTGCCCTAGCACCTTCCTCCCCTTTCAACCA
TGCGTCCACCCCGGTTAATTTTGACCCGTGACGGTAATACAATAGCTCGGTAAGCTTGGGTGAGTCAA
+
FFFFFFFFFFF:FFFFFFFFFFFFFFFFF:FFFFFFFFFFFFFFFFFFFFFFFFFFFFFFFFFFFFFFFF
FFFFF:FFFFFFFFFFFFFFFFFFFFFFFFFFFFFFFFFFFFFFFFFFFFFFF:FFFFFFFFFFFFFFFF
@A00155:342:HHGFNDSXY:1:2458:22860:27430 1:N:0:GAACCTAG+TCCGCATA
AACGCAGACCATATCTGTGCCGAGTTGGGTAGGTCAGGTGCCCTAGCACCTTCCTCCCCTTTCAACCA

```
TGCGTCCACCCCGGTTAATTTTGACCCGTGACGGTAATACAATAGCTCGGTAAGCTTGGGTGAGTCAA
+
FFFFFFFFFFFFFFFFFFFFFFFFFFFFFFFFFFFFFFFFFFFFFFF::FFFFFFFFFFFFFFFFFFF
FFFFFFFFFFFFFFFFFFFFFFFFFFFFFFFFFFFFFFFFFFFFFFFFFFFFFFFFFFFFFFFFFFFF
@A00155:342:HHGFNDSXY:1:2511:8169:35243 1:N:0:GAACCTAG+TCCGCATA
AACGCAGACCATATCTGTGCCGAGTTGGGTAGGTCAGGTGCCCTAGCACCTTCCTCCCCTTTCAACCA
TGCGTCCACCCCGGTTAATTTTGACCCGTGACGGTAATACAATAGCTCGGTAAGCTTGGGTGAGTCAA
+
FFFFFF:FFFFFFFFFFFF:FFFFFFFFFFFFFFFFFFFFFFFFFFFFFFFFFFFFFFFFFFFFFFFF
FFFFFFFFFFFFFFFFFFFFFFF:FFFFFFFFFFFFFFFFFFFFFFFFFFFFFFFFFFFFFFFFFFFF
@A00155:342:HHGFNDSXY:1:1609:9598:36370 1:N:0:GAACCTAG+TCCGCATA
AACGCAGACCATATCTGTGCCGAGTTGGGTAGGTCAGGTGCCCTAGCACCTTCCTCCCCTTTCAACCA
TGCGTCCACCCCGGTTAATTTTGACCCGTGACGGTAATACAATAGCTCGGTAAGCTTGGGTGAGTCAA
+
FFFFFFFFFFFFFFFFFFFFFFFFFFFFFFFFFFFFFFFFFFFFFFFFFFFFFFFFFFFFFFFFFFFF
FFFFFFFFFFFFFFFFFFFFFFFFFFFFFFFF:FFFF:FFFFFFFFFFFFFFFFFFFFFFFFFFFFFF
@A00155:342:HHGFNDSXY:1:1547:8449:11365 1:N:0:GAACCTAG+TCCGCATA
AACGCAGACCATATCTGTGCCGAGTTGGGTAGGTCAGGTGCCCTAGCACCTTCCTCCCCTTTCAACCA
TGCGTCCACCCCGGTTAATTTTGACCCGTGACGGTAATACAATAGCTCGGTAAGCTTGGGTGAGTCAA
+
FFFFFFFFFFFFFFFFFFFFFFFFFFFFFFFFFFFFFFFFFFFFFFFFF:,FFFFFFFFFFFFFFFFF
FFFFFFFFFFFFFFFFFFFFFFF:FFFFFFFFFFFFFFFFFFFFFFFFFFFFFFF:FFFFFFFFFFFF
@A00155:342:HHGFNDSXY:1:1226:22046:20165 1:N:0:GAACCTAG+TCCGCATA
AACGCAGACCATATCTGTGCCGAGTTGGGTAGGTCAGGTGCCCTAGCACCTTCCTCCCCTTTCAACCA
TGCGTCCACCCCGGTTAATTTTGACCCGTGACGGTAATACAATAGCTCGGTAAGCTTGGGTGAGTCAA
+
FFFFFFFFFFFFFFFFFFFFFFFFFFFFF:FFFFFFFFFFFFFFFFF:FF:FF:FFFFFFFFFFFFFF
:FFFFFFFFFFFFFFFFFF,FFFFFFFFFFFFFFFFFFFFFFFFFFFFFFFF:FFF:,FFFFFFFFFF
@A00155:342:HHGFNDSXY:1:2178:10041:9424 1:N:0:GAACCTAG+TCCGCATA
ACGCAGACCATATCTGTGCCGAGTTGGGTAGGTCAGGTGCCCTAGCACCTTCCTCCCCTTTCAACCAT
GCGTCCACCCCGGTTAATTTTGACCCGTGACGGTAATACAATAGCTCGGTAAGCTTGGGTGAGTCAAA
+
FFFFFFFFFFFFFFFFFFFFFFFFFFFFFFFFFFFFFFFFFFFFFFFFFFFFFFFFFFFFFFFFFFFF
FFFFFFFFFFFFFFFFFFFFFFFFFFFFFFFFFF:FFFFFFFFFFFFFFFFFFFFFFFFFFFFFFFFF
@A00155:342:HHGFNDSXY:1:1446:9218:33364 2:N:0:GAACCTAG+TCCGCATA
GCAGACCATATCTGTGCCGAGTTGGGTAGGTCAGGTGCCCTAGCACCTTCCTCCCCTTTCAACCATGC
GTCCACCCCGGTTAATTTTGACCCGTGACGGTAATACAATAGCTCGGTAAGCTTGGGTGAGTCAAAAT
+
FFFFFFFFFFFFFFFFFFFFFFFFFFFF:FFFFFFFFFFFFFFFFFFFFFFFFFFFFFFFFFFFFFFF
FFFFFFFFFFFFFFFFFFFFFFFFFFFFF:FFFFFFFFFFF:FFFFFFFFFFFFFFFFFFFFFFFF:F
@A00155:342:HHGFNDSXY:1:1668:10673:35509 2:N:0:GAACCTAG+TCCGCATA
CAGACCATATCTGTGCCGAGTTGGGTAGGTCAGGTGCCCTAGCACCTTCCTCCCCTTTCAACCATGCG
TCCACCCCGGTTAATTTTGACCCGTGACGGTAATACAATAGCTCGGTAAGCTTGGGTGAGTCAAAATC
+
F:FFFFF,FF,FFFF:FF,FFFF,FFFFF:FFFF:FF:F:FFF:F::FFFFFF:FFFFFFF,FFFFFF
FFFFFFF:FFFFFFFFFFFFFFFFFFFF:FFFFFFF:F:F:FFFFF:FFF:FFFFFFFFFFFFFF,FF
@A00155:342:HHGFNDSXY:1:1668:12029:27837 2:N:0:GAACCTAG+TCCGCATA
CAGACCATATCTGTGCCGAGTTGGGTAGGTCAGGTGCCCTAGCACCTTCCTCCCCTTTCAACCATGCG
TCCACCCCGGTTAATTTTGACCCGTGACGGTAATACAATAGCTCGGTAAGCTTGGGTGAGTCAAAATC
+
F,FF:FFF:FFFFF:FFF:FFFFFFFFFFFF,FF:F,FFFFF,FFFFFFFFF:F,FFFFFFFFFFFFF
FFFFF::FFFFFF,FFF:FFF:FFFFF:FFFFFF:FFFFFFFFFFFFFF,FFFFF,:FFFFFFFFFFF
@A00155:342:HHGFNDSXY:1:2147:18566:11099 2:N:0:GAACCTAG+TCCGCATA
CAGACCATATCTGTGCCGAGTTGGGTAGGTCAGGTGCCCTAGCACCTTCCTCCCCTTTCAACCATGCG
```

```
TCCACCCCGGTTAATTTTGACCCGTGACGGTAATACAATAGCTCGGTAAGCTTGGGTGAGTCAAAATC
+
FFFFFFFFFFFFFFFFFF:FFFF:FFFFFFFFFFFFFF:FFFFFFFFFFFFFFFFFFFFFFFFFFFFF:F
FFFFF:FFFFFFFFFFFFFFFFFFFFFFFFFFFFFFFFF::FFFFFFFFFFFFFFFFFFFFFFFFFFFFF
@A00155:342:HHGFNDSXY:1:2327:9037:32831 2:N:0:GAACCTAG+TCCGCATA
AGACCATATCTGTGCCGAGTTGGGTAGGTCAGGTGCCCTAGCACCTTCCTCCCCTTTCAACCATGCGT
CCACCCCGGTTAATTTTGACCCGTGACGGTAATACAATAGCTCGGTAAGCTTGGGTGAGTCAAAATCT
+
F:FF:FFFFF:F:FFFFFFFFFFFFFF,FFFFFFFFFFF:FF:FFFF::FFFFFF,FFFFFFFFF:FF:F
FFFFFFFFFFFFFFFFFFFFFFFFFFFFFF:FFFFFFFFFFFFFFFFFFFFFFFFFFFFFFF:FFFFFFF
@A00155:342:HHGFNDSXY:1:1241:20021:8609 2:N:0:GAACCTAG+TCCGCATA
GACCATATCTGTGCCGAGTTGGGTAGGTCAGGTGCCCTAGCACCTTCCTCCCCTTTCAACCATGCGTC
CACCCCGGTTAATTTTGACCCGTGACGGTAATACAATAGCTCGGTAAGCTTGGGTGAGTCAAAATCTA
+
FFFFFF,FFFFFFFFFFFF:FFFFFFFFFFFFFFFFFF:FFFFFF:FFF:FFFFFFFFFFFFFFFFFFFF
FFFFFFFFFFFFFFFFFFFFFFFFFFFFFFFFFFFFFFFFFFFFFFFFFFFFFFFFFFFF:FFFFFFFFF
@A00155:342:HHGFNDSXY:1:2234:10411:16611 1:N:0:GAACCTAG+TCCGCATA
ACCATATCTGTGCCGAGTTGGGTAGGTCAGGTGCCCTAGCACCTTCCTCCCCTTTCAACCATGCGTCC
ACCCCGGTTAATTTTGACCCGTGACGGTAATACAATAGCTCGGTAAGCTTGGGTGAGTCAAAATCTAG
+
F:FFFFFF:FFFFFFFFFFFFF:FFFFFFFF:FF:FFFFFFF,,FFFFF:FF:FFFFFFFFFF:FFF:FF
FFFF:FFFFFFFF:F:FFF,FFFF:F::FFFFFFFFFFFFFFFF:FFFF:FF:FFFFFFFFFF:FFFFFF
@A00155:342:HHGFNDSXY:1:1467:19018:15734 1:N:0:GAACCTAG+TCCGCATA
ACCATATCTGTGCCGAGTTGGGTAGGTCAGGTGCCCTAGCACCTTCCTCCCCTTTCAACCATGCGTCC
ACCCCGGTTAATTTTGACCCGTGACGGTAATACAATAGCTCGGTAAGCTTGGGTGAGTCAAAATCTAG
+
FFFFFFFFFFFFFFFFFFFFFFFFFFFFFFFFFFFFFFFFFFFFFFFFFFFFFFFFFFFFFFFFFFFFFF
FFFFFFFFFFFFFFFFFFFFFFFFFFFFFF:FFFFFFFFFFFFFFFFFFFFFFFFFFFFFFFFFFFFFFF
@A00155:342:HHGFNDSXY:1:2460:11532:25316 2:N:0:GAACCTAG+TCCGCATA
CCATATCTGTGCCGAGTTGGGTAGGTCAGGTGCCCTAGCACCTTCCTCCCCTTTCAACCATGCGTCCA
CCCCGGTTAATTTTGACCCGTGACGGTAATACAATAGCTCGGTAAGCTTGGGTGAGTCAAAATCTAGT
+
FFFFFFFFFFFFFFFFFFFFFFFFFFFFFFFFFFFFFFFFFFFFFFFFFFFFFF:FFFFFFFFFFFFFFF
FFFFFFFFFFFFFFFFFFFFFFFFFFFFFFFFFFFFFFFFFFFFFFFFFFFFFF:FFFFFFFFFFFFFFF
@A00155:342:HHGFNDSXY:1:2360:2763:22999 2:N:0:GAACCTAG+TCCGCATA
CCATATCTGTGCCGAGTTGGGTAGGTCAGGTGCCCTAGCACCTTCCTCCCCTTTCAACCATGCGTCCA
CCCCGGTTAATTTTGACCCGTGACGGTAATACAATAGCTCGGTAAGCTTGGGTGAGTCAAAATCTAGT
+
FFFFFFFFFFFFFFFFFFFFFFFFFFFFFFFFF,FFFF:FFFF:FFFFFFFFFFFFFFFFFFFFFFFFFF
FFFFFFFFFFFFFFFFFFFFFFFFFFFFFFFFFFFFFFFFFFFFFFFFFFFFFFFFFFFFFFFFFFFFFF
@A00155:342:HHGFNDSXY:1:2117:1434:11459 1:N:0:GAACCTAG+TCCGCATA
CATATCTGTGCCGAGTTGGGTAGGTCAGGTGCCCTAGCACCTTCCTCCCCTTTCAACCATGCGTCCAC
CCCGGTTAATTTTGACCCGTGACGGTAATACAATAGCTCGGTAAGCTTGGGTGAGTCAAAATCTAGTC
+
FFFFFFFFFFFFFFFFFFFFFFFFFFFFFFFFFFFFFFFFFFF:FFFFFFFFFFFFF:FFFFFFFFFFFF
FFFFFFFFFFFFFFFFFFFFFFFFFFFFFFFFFFFFFFFFFFFFFFF:FFFFFFFFFFF:F::F:FFFFF
@A00155:342:HHGFNDSXY:1:1315:11993:27273 1:N:0:GAACCTAG+TCCGCATA
CATATCTGTGCCGAGTTGGGTAGGTCAGGTGCCCTAGCACCTTCCTCCCCTTTCAACCATGCGTCCAC
CCCGGTTAATTTTGACCCGTGACGGTAATACAATAGCTCGGTAAGCTTGGGTGAGTCAAAATCTAGTC
+
FFFFFFFFFFFFFFFFFFFFFFFFFFFFFFFFFFFFFFFFF:FFFF,FFFFFFFFFFFFFFFFF:FFFFF
FFFF:FFFFFFFFFFFF,FF:FFFFFFFFFFFFFFFFFFFF:FFFFFFF:F:FFFFFFFFFFFFFFFFFF
@A00155:342:HHGFNDSXY:1:2425:7301:12226 1:N:0:GAACCTAG+TCCGCATA
CATATCTGTGCCGAGTTGGGTAGGTCAGGTGCCCTAGCACCTTCCTCCCCTTTCAACCATGCGTCCAC
```

```
CCCGGTTAATTTTGACCCGTGACGGTAATACAATAGCTCGGTAAGCTTGGGTGAGTCAAAATCTAGTC
+
FFFFFFFF:FFFFFFFFFFFFFFFFFFFF:FFFFF:FFFFFF:FFFFF:FFFFFFFFFFFFFFFFFFF
FFFFFFFFFFFFFFFFFFFFFFFFF:,FFF,FFFFFFFFF:FFFFFFFFFFFFFFFFFFFFFFFFFFF
@A00155:342:HHGFNDSXY:1:1241:20021:8609 1:N:0:GAACCTAG+TCCGCATA
CATATCTGTGCCGAGTTGGGTAGGTCAGGTGCCCTAGCACCTTCCTCCCCTTTCAACCATGCGTCCAC
CCCGGTTAATTTTGACCCGTGACGGTAATACAATAGCTCGGTAAGCTTGGGTGAGTCAAAATCTAGTC
+
FFFFFFFFFFFFFFFFFFFFFFFFFFFFFFFFFFFFFFFFF,:FF:FFFFFFFFFFFFFFFFFFFFFF
FFFFFFFFFFFFFFFFFFFFFFFFFFF,FFFFFFFFF:FFFF:FFFFFFFFFFFFFFFF:FFFF:F:F
@A00155:342:HHGFNDSXY:1:1146:1470:33567 1:N:0:GAACCTAG+TCCGCATA
CATATCTGTGCCGAGTTGGGTAGGTCAGGTGCCCTAGCACCTTCCTCCCCTTTCAACCATGCGTCCAC
CCCGGTTAATTTTGACCCGTGACGGTAATACAATAGCTCGGTAAGCTTGGGTGAGTCAAAATCTAGTC
+
FFFFFFFFFFFFFFFFFFFFFFF,FFFFFFFFFFFFFFFFFFFFFFFFFFFFFFFFFFFFFFFFFFFF
FFFFFFFFFFFFFFFFFFFFFFFFFFFFFFFFFFFFFFFFF:FFFFFFFFFFFFFFFFFFFFFFFFFF
@A00155:342:HHGFNDSXY:1:1138:12798:11851 2:N:0:GAACCTAG+TCCGCATA
ATATCTGTGCCGAGTTGGGTAGGTCAGGTGCCCTAGCACCTTCCTCCCCTTTCAACCATGCGTCCACC
CCGGTTAATTTTGACCCGTGACGGTAATACAATAGCTCGGTAAGCTTGGGTGAGTCAAAATCTAGTCT
+
FFFFFFFFFFFFFFFFFFFFFFFFFFFFFFFFFFFFFFFFFFFFFFFFFFFFFFFFFFFFFFFFFFFF
FFFFFFFF:FFFFFFFFFFFFFFFFFFFFFFFFFFFFFFFFFFFFFFFFF:FFFFFFFFFFFFFFFFF
@A00155:342:HHGFNDSXY:1:2507:12084:22420 2:N:0:GAACCTAG+TCCGCATA
ATATCTGTGCCGAGTTGGGTAGGTCAGGTGCCCTAGCACCTTCCTCCCCTTTCAACCATGCGTCCACC
CCGGTTAATTTTGACCCGTGACGGTAATACAATAGCTCGGTAAGCTTGGGTGAGTCAAAATCTAGTCT
+
FFFFFFFFFFFFFFFFFFFFFFFFFFFFFFFFFFFFFFFFFFFFFFFFFFFFFFFFFFFFFFFFFFFF
FFFFFFF:FFFFFFFFFFFFFFFFFFFFFFFFFFFFFFFFFFFFFFFFFFFFFFFFFFFFFFFFFFFF
@A00155:342:HHGFNDSXY:1:2458:22860:27430 2:N:0:GAACCTAG+TCCGCATA
ATATCTGTGCCGAGTTGGGTAGGTCAGGTGCCCTAGCACCTTCCTCCCCTTTCAACCATGCGTCCACC
CCGGTTAATTTTGACCCGTGACGGTAATACAATAGCTCGGTAAGCTTGGGTGAGTCAAAATCTAGTCT
+
FFFFFFFFFFFFFFFFFFFFFFFFFFFFFFFFFF:FFFFF:FFFFFFFFFFFFFFFFFFFFFFFFFFF
FFFFFFFFFFFFFFFFFFFFFFFFFFFFFFFFFF:FFFFFFFFFFFFFFF:FFFFFFFFFFFFFFFFF
@A00155:342:HHGFNDSXY:1:1226:5367:24627 1:N:0:GAACCTAG+TCCGCATA
ATATCTGTGCCGAGTTGGGTAGGTCAGGTGCCCTAGCACCTTCCTCCCCTTTCAACCATGCGTCCACC
CCGGTTAATTTTGACCCGTGACGGTAATACAATAGCTCGGTAAGCTTGGGTGAGTCAAAATCTAGTCT
+
FFFFFFFFFFFFFFFFFFFFFFFFFFFFFFFFFF:FFF:FFFFFFFFFFFFFFFFFFFFFFFFFFFFF
FFFFFFFFFFFFFFFFFFFFFFFFFFFFFFFFFFFFFFFFFF:FFFFFFFFFFFFFFFFFFFFFFFFF
@A00155:342:HHGFNDSXY:1:2649:14534:14418 2:N:0:GAACCTAG+TCCGCATA
ATATCTGTGCCGAGTTGGGTAGGTCAGGTGCCCTAGCACCTTCCTCCCCTTTCAACCATGCGTCCACC
CCGGTTAATTTTGACCCGTGACGGTAATACAATAGCTCGGTAAGCTTGGGTGAGTCAAAATCTAGTCT
+
F:FFFFFFFFFFFFFFFFFFFFFFFFFFFFFFFFFFFFFF:FFFFFFFFFFFFFFFFFFFFFFFFFFF
FFFFFFFFFFFFFFFFFFFFFFFFFFFFFF:FFFFFFFFF:FFFFFFFFFFFFFFFFFFFFFFFFFFF
@A00155:342:HHGFNDSXY:1:1138:12798:11851 1:N:0:GAACCTAG+TCCGCATA
TATCTGTGCCGAGTTGGGTAGGTCAGGTGCCCTAGCACCTTCCTCCCCTTTCAACCATGCGTCCACCC
CGGTTAATTTTGACCCGTGACGGTAATACAATAGCTCGGTAAGCTTGGGTGAGTCAAAATCTAGTCTC
+
FFFFFFFFFFFFFFFFFFFFFFFFFFFFFFFFFFFFFFFFFFF:FFFFFFFFFFFFFFFFFFFFFFFF
FFFFFFFFFFFFFFFFFFFFFFFF:FFFFFFFFFFFFFFFFFFFFFFFFFFFFFFFFFFFFFFFFFF
@A00155:342:HHGFNDSXY:1:1427:24514:29982 1:N:0:GAACCTAG+TCCGCATA
TATCTGTGCCGAGTTGGGTAGGTCAGGTGCCCTAGCACCTTCCTCCCCTTTCAACCATGCGTCCACCC
```

```
CGGTTAATTTTGACCCGTGACGGTAATACAATAGCTCGGTAAGCTTGGGTGAGTCAAAATCTAGTCTC
+
FFFFFFFFFFFFFFFFFFFFFFFFFFFFFFFFFFFFFFF,FFFFFFFFFFFFFFFFFFFFFFFFFFFF
FFFFFFFFFFFFFFFFFFFFFFFF::FFFFFFFFFFFFFFFFFFFFFFFFFFFFFFFFFFF:FFFFFF
@A00155:342:HHGFNDSXY:1:2451:24325:6198 2:N:0:GAACCTAG+TCCGCATA
CTGTGCCGAGTTGGGTAGGTCAGGTGCCCTAGCACCTTCCTCCCCTTTCAACCATGCGTCCACCCCGG
TTAATTTTGACCCGTGACGGTAATACAATAGCTCGGTAAGCTTGGGTGAGTCAAAATCTAGTCT
+
FFFFFFFFFFFFFFFFFFFF:FFFFFFFF:FFFFFFFFFFFFFFFFFFFFFFFFFFFFFFFFFFFFFF
FFFFFFFFFFFFFFFFFFFFFFFFFFF:F:FFFFFFFFFFFFFFFFFFFFF:FFFFFFFFFFFF
@A00155:342:HHGFNDSXY:1:2644:4327:19539 2:N:0:GAACCTAG+TCCGCATA
CTGTGCCGAGTTGGGTAGGTCAGGTGCCCTAGCACCTTCCTCCCCTTTCAACCATGCGTCCACCCCGG
TTAATTTTGACCCGTGACGGTAATACAATAGCTCGGTAAGCTTGGGTGAGTCAAAATCTAGTCT
+
FFFF:FFFFFFFFFFFFFFFFFFFFFFFFF::FFFFFF:FFFFFFFFFFF:FF,FFFFFFFFFFFF:F
F:FFFFFFFFFFFFFFFFFFFFFFFFFFFFFF:FFFFFFFFFFFFFFFFFFFFFFFFFFFF:FF
@A00155:342:HHGFNDSXY:1:2462:21847:28369 2:N:0:GAACCTAG+TCCGCATA
CTGTGCCGAGTTGGGTAGGTCAGGTGCCCTAGCACCTTCCTCCCCTTTCAACCATGCGTCCACCCCGG
TTAATTTTGACCCGTGACGGTAATACAATAGCTCGGTAAGCTTGGGTGAGTCAAAATCTAGTCT
+
FFFFFFFFFFFFFFFFFFFFFFFFFFFFFF,FFFFF,FFF:FFFFFFFFF:FF:FFFFFFFFFFFF:F
FFFFFFFFFFFFFFFFFFFFFFFFFFFF:FFFFFFFFFFFFFFFFFFFFFFFFF,FFFFFFFFF
@A00155:342:HHGFNDSXY:1:2446:20871:22388 2:N:0:GAACCTAG+TCCGCATA
CTGTGCCGAGTTGGGTAGGTCAGGTGCCCTAGCACCTTCCTCCCCTTTCAACCATGCGTCCACCCCGG
TTAATTTTGACCCGTGACGGTAATACAATAGCTCGGTAAGCTTGGGTGAGTCAAAATCTAGTCT
+
FFFFFFFFFFFFFFFFFFFFFFFFFFFFFFFFFFFFFFFFFFFFFFFFF:FFFFFFFFFFFFFFFFFF
FFFFF:FFFFFFFFFFFFFFFFF,FF:FFFFFFFFFFFFFFFFFFFFFFFFFFFFFFFFFFFFF
@A00155:342:HHGFNDSXY:1:1334:28429:27367 2:N:0:GAACCTAG+TCCGCATA
CTGTGCCGAGTTGGGTAGGTCAGGTGCCCTAGCACCTTCCTCCCCTTTCAACCATGCGTCCACCCCGG
TTAATTTTGACCCGTGACGGTAATACAATAGCTCGGTAAGCTTGGGTGAGTCAAAATCTAGTCT
+
FFFFFFFFFFFFFFFFFFFFFFFFFFFFFFFFFFFFFFFFFFFFFFFFFFFFFFFFFFFFFFFFFFFF
FFFFFFFFFFFFFFFFFFFFFFFFFFFFFFFFFFFFFFFFFFFFFFFFFFFFFFFFFFFFFFFF
@A00155:342:HHGFNDSXY:1:2649:14534:14418 1:N:0:GAACCTAG+TCCGCATA
CTGTGCCGAGTTGGGTAGGTCAGGTGCCCTAGCACCTTCCTCCCCTTTCAACCATGCGTCCACCCCGG
TTAATTTTGACCCGTGACGGTAATACAATAGCTCGGTAAGCTTGGGTGAGTCAAAATCTAGTCTCGCT
+
FFFFFFFFFFFFFFFFFFFFFFFFFFFFFFFFFFFFFFFFFFFFFFFFFFFFFFFFFFFFFFFFFFFF
FFFFFFFFF:FFFFFFFFFFF:FFFFFFFFFFFFFFF:FFF,FFFFFFFFFFFFFFFFFFFFFFFFFF
@A00155:342:HHGFNDSXY:1:2426:5122:19570 1:N:0:GAACCTAG+TCCGCATA
CTGTGCCGAGTTGGGTAGGTCAGGTGCCCTAGCACCTTCCTCCCCTTTCAACCATGCGTCCACCCCGG
TTAATTTTGACCCGTGACGGTAATACAATAGCTCGGTAAGCTTGGGTGAGTCAAAATCTAGTCTCGCT
+
FFFFFFFFFFFFFFFFFFFFFFFFFFFFFFFFFFFFFFFFFFFFFFFFFFFFFFFFFFFFFFFFFFFF
FFFFFF:FFFFFFFFFFFFFFFFFFFFFFFFFFFFFFFFFFFFFFFFFFFFFFFFFFFFFFFFF
@A00155:342:HHGFNDSXY:1:2327:9037:32831 1:N:0:GAACCTAG+TCCGCATA
CTGTGCCGAGTTGGGTAGGTCAGGTGCCCTAGCACCTTCCTCCCCTTTCAACCATGCGTCCACCCCGG
TTAATTTTGACCCGTGACGGTAATACAATAGCTCGGTAAGCTTGGGTGAGTCAAAATCTAGTCTCGCT
+
FFFFFFFFFFFFF:FF::F:FFFFFFFFFFFFFFFFFFFFFFFF,FFFFFFF:FFF:FF:FFFFFFFF
FFFFFF:FFFFFF:FFFFF,FFFFFFFFFFFFFF:F:FFFFF:F:FFFFFFFFFFFFFFFFFFFF::F
@A00155:342:HHGFNDSXY:1:2239:17381:8234 2:N:0:GAACCTAG+TCCGCATA
TGTGCCGAGTTGGGTAGGTCAGGTGCCCTAGCACCTTCCTCCCCTTTCAACCATGCGTCCACCCCGGT
```

TAATTTTGACCCGTGACGGTAATACAATAGCTCGGTAAGCTTGGGTGAGTCAAAATCTAGTCTCGCTC
+
FFFFFFFFFFFFFFFFFFFFFFFFFFFFFFFFFFF,FFFFFFFFFFFFFFFFFFFFFFFFFFFFFFFF
FFFFFFFFFFFFFFFFFFFFFFFFFFFFFFFFFFFFFFFFFFFFFFFFFFFFFF:FFFFFFFFFFFFF
@A00155:342:HHGFNDSXY:1:2615:32253:15107 2:N:0:GAACCTAG+TCCGCATA
TGTGCCGAGTTGGGTAGGTCAGGTGCCCTAGCACCTTCCTCCCCTTTCAACCATGCGTCCACCCCGGT
TAATTTTGACCCGTGACGGTAATACAATAGCTCGGTAAGCTTGGGTGAGTCAAAATCTAGTCTCGCTC
+
FFFFFFFFFFFFFFFFFFFFFFFFFFFFFFFFFFF,FFFFFFFFFFFFFFFFFFFFFFFFFFFFF:FF
FF:FFFFFFFFFFFFFFFFFFFFFFFFF:FFFFFFFFFFFFFFFFFFFFFFFFFFFFFFFFFFFFFFF
@A00155:342:HHGFNDSXY:1:1613:20292:7952 2:N:0:GAACCTAG+TCCGCATA
TGTGCCGAGTTGGGTAGGTCAGGTGCCCTAGCACCTTCCTCCCCTTTCAACCATGCGTCCACCCCGGT
TAATTTTGACCCGTGACGGTAATACAATAGCTCGGTAAGCTTGGGTGAGTCAAAATCTAGTCTCGCTC
+
FFFFFFFFFFF,FFFFFFF:FFF:FF:FF,,F::FFF,F,F:FFFFF:F:FFFFFFFFFFFFFFFF:F
FFFFFFFFFFFFFFFF,FFFFFF,FFFF,F:,FFFFFF:FFFF,FF:FFF:FFFFFFFFFFFFFFFFF
@A00155:342:HHGFNDSXY:1:1214:4309:27211 2:N:0:GAACCTAG+TCCGCATA
TGTGCCGAGTTGGGTAGGTCAGGTGCCCTAGCACCTTCCTCCCCTTTCAACCATGCGTCCACCCCGGT
TAATTTTGACCCGTGACGGTAATACAATAGCTCGGTAAGCTTGGGTGAGTCAAAATCTAGTCTCGCTC
+
FFFFFFFFFFFFFFFFFFFFFFFFFFFFFFFFFFF:FFFFFFFFFFFFFFFFFFFFFFFFFFFFFFFF
FFFFFFFFFFFFFFFFF:FFFFFFFFFFFFFFFFF,FFFFFF:FFFFFFFFFFFFF:FFF,FFFFFFF
@A00155:342:HHGFNDSXY:1:2305:14968:19335 2:N:0:GAACCTAG+TCCGCATA
TGTGCCGAGTTGGGTAGGTCAGGTGCCCTAGCACCTTCCTCCCCTTTCAACCATGCGTCCACCCCGGT
TAATTTTGACCCGTGACGGTAATACAATAGCTCGGTAAGCTTGGGTGAGTCAAAATCTAGTCTCGCTC
+
:F:F:F:FFFFFFF:FFF:F:FFFFFFF:F:F:FF::FF:FFF::F:FFFFFFFFFF:FF:FFFFFF:
:FFFFF:F:FFFF:F:FFF:FF:FFFF:FFF:FFF:FFFF::FFFFFFF:FFFFF:F:FF:FFFFFFF
@A00155:342:HHGFNDSXY:1:1372:32362:15890 2:N:0:GAACCTAG+TCCGCATA
TGTGCCGAGTTGGGTAGGTCAGGTGCCCTAGCACCTTCCTCCCCTTTCAACCATGCGTCCACCCCGGT
TAATTTTGACCCGTGACGGTAATACAATAGCTCGGTAAGCTTGGGTGAGTCAAAATCTAGTCTCGCTC
+
FFFF:FFFFFFFFFFFFFFFFFFFFFFF:FFFFFF:FFFFFFFFFFFFFFFF:FFFF,FFFFFFFFFF
FFFFFFF:FFFFFFFFFFF:FFF:FF::F:F:FFFFFFF:FFFFF:FFFFFF:F:F:FFFFFFFFFFF
@A00155:342:HHGFNDSXY:1:2137:27037:28307 2:N:0:GAACCTAG+TCCGCATA
TGTGCCGAGTTGGGTAGGTCAGGTGCCCTAGCACCTTCCTCCCCTTTCAACCATGCGTCCACCCCGGT
TAATTTTGACCCGTGACGGTAATACAATAGCTCGGTAAGCTTGGGTGAGTCAAAATCTAGTCTCGCTC
+
FFFFFFFFFFFFFFFFFFFFFFFFFFFF:FFFFFF:FFFFFFFFFFFFFFFFFFFFFFFFFFF:FFFF
FFFFFFFFFFFFFFFFFFFFFFFFFFFFFFFFFFFFFFFFFFFFFFFFFFFFFFFFFFFFFFFFFFFF
@A00155:342:HHGFNDSXY:1:2675:25129:19335 2:N:0:GAACCTAG+TCCGCATA
TGTGCCGAGTTGGGTAGGTCAGGTGCCCTAGCACCTTCCTCCCCTTTCAACCATGCGTCCACCCCGGT
TAATTTTGACCCGTGACGGTAATACAATAGCTCGGTAAGCTTGGGTGAGTCAAAATCTAGTCTCGCTC
+
FFFFFFFFFFFFFFFFFFFFFFFFFFFFFFFFFFFFFFFFFFFFFFFFFFFFFFFFFFFFFFFFFFFF
FFFFFFFFFFFFFFFFFFFFFFFFFFFFFFFFFFFFFFFFFFFFFFFFFFFFFFFFFFFFFFFFFFFF
@A00155:342:HHGFNDSXY:1:1238:27832:7482 2:N:0:GAACCTAG+TCCGCATA
GTGCCGAGTTGGGTAGGTCAGGTGCCCTAGCACCTTCCTCCCCTTTCAACCATGCGTCCACCCCGGTT
AATTTTGACCCGTGACGGTAATACAATAGCTCGGTAAGCTTGGGTGAGTCAAAATCTAGTCT
+
FFFFFFFFFFF:FFFFFFFFFFFFFFFFFFFF:FFFFFFFFFFFFFFFFFFFFFFFFFFFFFFFFFFF
FFFFFFFFFFFFFFFFFFFFFFFFFFFFFFFFFFFFFFFFFFFFFFFFFFFFFFFFFFFFF:
@A00155:342:HHGFNDSXY:1:2439:26576:15233 2:N:0:GAACCTAG+TCCGCATA
GTGCCGAGTTGGGTAGGTCAGGTGCCCTAGCACCTTCCTCCCCTTTCAACCATGCGTCCACCCCGGTT

```
AATTTTGACCCGTGACAGTAATACAATAGCTCGGTAAGCTTGGGTGAGTCAAAATCTAGTCTCGCTCT
+
FFFFFFFFFFFFF:FF:FFFFFFFF:FFFFFFFF,FFFFFFFFF:FF,FFFFFFFFFF:FFFFFFFFF
FFFF:FFFFFFFFFFF:FFFFFFFFFFFFF,FFFFFFFF:FFFFFFFFFFFFFFFFFFFFFFFFFFFF
@A00155:342:HHGFNDSXY:1:2228:26503:23907 2:N:0:GAACCTAG+TCCGCATA
GTGCCGAGTTGGGTAGGTCAGGTGCCCTAGCACCTTCCTCCCCTTTCAACCATGCGTCCACCCCGGTT
AATTTTGACCCGTGACGGTAATACAATAGCTCGGTAAGCTTGGGTGAGTCAAAATCTAGTCTCGCTCT
+
FFFFFFFFFFFFFFFF:F,FFFFFF,FFF::FFF,FFFFFFFFFFFFFFFF:F,F,FF,FF:FF,FFF
FFFFFFFFFFFFFFFF:FFFFFFFF:FFFFFFFFFFFFFFFFFFFFFFFFFF:FFFFFFFFF:FFFFF
@A00155:342:HHGFNDSXY:1:1355:18593:31845 2:N:0:GAACCTAG+TCCGCATA
CGCCGAGTTGGGTAGGTCAGGTGCCCTAGCACCTTCCTCCCCTTTCAACCATGCGTCCACCCCGGTTA
ATTTTGACCCGTGACGGTAATACAATAGCTCGGTAAGCTTGGGTGAGTCAAAATCTAGTCTCG
+
FFFFFFFFFFFFFFFFFFFFF:FFFFFFFFFFF,FFFFFFFFFFFFFFFFFFFFFFFF:FFFFFFFFF
FFFFFFFFFFFFFFFFFFFFFFFFF:FFF:FFFFFFF:FFFFFFFFFFFFFFFFFFFFFF:FF
@A00155:342:HHGFNDSXY:1:1546:8269:24549 2:N:0:GAACCTAG+TCCGCATA
CCGAGTTGGGTAGGTCAGGTGCCCTAGCACCTTCCTCCCCTTTCAACCATGCGTCCACCCCGGTTAAT
TTTGACCCGTGACGGTAATACAATAGCTCGGTAAGCTTGGGTGAGTCAAAATCTAGTCTCGCTCTAGA
+
FFFFFFFFFFFFFFFFFFFF:FFFFFFFFFFFFFFFF:FFFFFFFFFFFFFFFFFFFFFFFFF:FFFF
F:FFFFFFFFFFF:FFFFFFFFFFFFFFFFFFFFFFFFFFFFFFFFFFFF:FFFFFFFFFFFFFFFFF
@A00155:342:HHGFNDSXY:1:1139:5584:11193 2:N:0:GAACCTAG+TCCGCATA
CCGAGTTGGGTAGGTCAGGTGCCCTAGCACCTTCCTCCCCTTTCAACCATGCGTCCACCCCGGTTAAT
TTTGACCCGTGACGGTAATACAATAGCTCGGTAAGCTTGGGTGAGTCAAAATCTAGTCTCGCTCTAGA
+
FFFFFFFFFFFFFFFFFFFFFFFFFFFFFFFFFFFFFFFFFFFFFFFFFFF:FFFFFFFFFFFFFFFF
FFFF:FFFFFFFFFFFFF,FFFFFFFFFFFFFFFFFFFFFF:FFFFFFFFFFFFFFFFFFFFF:FFFF
@A00155:342:HHGFNDSXY:1:1115:11505:14403 2:N:0:GAACCTAG+TCCGCATA
CCGAGTTGGGTAGGTCAGGTGCCCTAGCACCTTCCTCCCCTTTCAACCATGCGTCCACCCCGGTTAAT
TTTGACCCGTGACGGTAATACAATAGCTCGGTAAGCTTGGGTGAGTCAAAATCTAGTCTCGCTCTAGA
+
FFFFFFFFFFFFFFFFFFFFFFFFF:FFFFFFFFFFFFFFFFFFFFFFFFFFF:FFFFFFFFF:FFFF
FFFFFFFFFFFFFFFFFFFFFFFFFFFFFFFFFFFFFFFFFFFFFFFFFFFFFFFFFFFFFFFFFFFF
@A00155:342:HHGFNDSXY:1:1330:2446:28557 2:N:0:GAACCTAG+TCCGCATA
CGAGTTGGGTAGGTCAGGTGCCCTAGCACCTTCCTCCCCTTTCAACCATGCGTCCACCCCGGTTAATT
TTGACCCGTGACGGTAATACTATAGCTCGGTAAGCTTGGGTGAGTCAAAATCTAGTCTCGCTCTAGAC
+
FFFF:FFFFFFFFFFFFFFFFFFFFFF:FFFFFFFF,FFFF:FFFFFF:FFFFFFFFFFFFFFFFFFF
FFFFFFFFFFFFFFFFFFFFFFFFF:FFFFF,F,FFFFFFFFFFFFFFFFFFFFFFFFFFFF:FF,:F
@A00155:342:HHGFNDSXY:1:1330:2944:26944 2:N:0:GAACCTAG+TCCGCATA
CGAGTTGGGTAGGTCAGGTGCCCTAGCACCTTCCTCCCCTTTCAACCATGCGTCCACCCCGGTTAATT
TTGACCCGTGACGGTAATACTATAGCTCGGTAAGCTTGGGTGAGTCAAAATCTAGTCTCGCTCTAGAC
+
FFFFFFFFFFFFFFFFFFFFFFFFFFFFFFFFFFFFFFFFFFFF:FFFFFF:FFFFFFFFFFFFFFFF
FFFFFFFFFFFFFFFF:FFFFFFFFFFFFFFFFFFFFFFFFFFFFFF:FFF:FFFFFFFFFFFFFFFF
@A00155:342:HHGFNDSXY:1:1116:29550:23484 1:N:0:GAACCTAG+TCCGCATA
GAGTTGGGTAGGTCAGGTGCCCTAGCACCTTCCTCCCCTTTCAACCATGCGTCCACCCCGGTTAATTT
TGACCCGTGACGGTAATACAATAGCTCGGTAAGCTTGGGTGAGTCAAAATCTAGTCTCGCTCTAGACC
+
FFFFFFFFFFFFFFFFFFFFFFFFFFFFFFFFFFFFFFFFFFFFFFFFFFFFFFFFFFFFFFFFFFFF
FFFFFFFFFFFFFFFFFFFFFFFFFFFFFFF:FFF:FFFFFFFFFFFFFFFFFFFF:FFFFFFFFFFF
@A00155:342:HHGFNDSXY:1:2573:7283:18333 2:N:0:GAACCTAG+TCCGCATA
AGTTGGGTAGGTCAGGTGCCCTAGCACCTTCCTCCCCTTTCAACCATGCGTCCACCCCGGTTAATTTT
```

```
GACCCGTGACGGTAATACAATAGCTCGGTAAGCTTGGGTGAGTCAAAATCTAGTCTCGCTCTAGACCT
+
FFFFF,FFFFFFFFFF:FFF,FFFFFFFFFFFFFFFFFFF,FFFFFFFFFFFFFFF:FFF:FFF:F:F
FFF:FFFFFFFFFFFFFFF:FFFFFFFFFFFFFFFFFF:FFFFFFFFFFFFFFFFFFFFFFFFFFF:F
@A00155:342:HHGFNDSXY:1:1226:5367:24627 2:N:0:GAACCTAG+TCCGCATA
AGTTGGGTAGGTCAGGTGCCCTAGCACCTTCCTCCCCTTTCAACCATGCGTCCACCCCGGTTAATTTT
GACCCGTGACGGTAATACAATAGCTCGGTAAGCTTGGGTGAGTCAAAATCTAGTCTCGCTCTAGACCT
+
FFFFF:FFFFFFFFFFFFFFFFFFFFFF,FFFFFF:FFFFFFFFFFFFFFFFFFFFFFFFFFFFFFFF
F:FFFFFFFFFFFFFFFFFFFFFFFFFFFFFFFFFFFF:FFFFFFFFFFFFFFFFFFFFFFFFFFFFF
@A00155:342:HHGFNDSXY:1:2236:27724:19006 2:N:0:GAACCTAG+TCCGCATA
AGTTGGGTAGGTCAGGTGCCCTAGCACCTTCCTCCCCTTTCAACCATGCGTCCACCCCGGTTAATTTT
GACCCGTGACGGTAATACAAAAGCTCGGTAAGCTTGGGTGAGTCAAAATCTAGTCTCGCTCTAGACCT
+
F,FFFF:FFFFFFFFFFFFFFFF:FFFF:F:FFFFFFFFFFFFFFFFFFFFFFFFFFFFFFFFFFFFF
FFFFFFFFFFFFFFFFFFFFFFFFFFFFFFFFFFFFFF,FFFFFFFFFFFFFFFFFFFFFFFFFFFFF
@A00155:342:HHGFNDSXY:1:1519:21468:28307 2:N:0:GAACCTAG+TCCGCATA
AGTTGGGTAGGTCAGGTGCCCTAGCACCTTCCTCCCCTTTCAACCATGCGTCCACCCCGGTTAATTTT
GACCCGTGACGGTAATACAATAGCTCGGTAAGCTTGGGTGAGTCAAAATCTAGTCTCGCTCTAGACCT
+
FF:FFFFFFFFFFFFFFFFFFFFFFF:FFFFFFFFFFFFFFF:FFFFFFFFFFFFFFFFFFFFFFFFF
FFFFFFFFFFFFFFFFFFFFF,FFFFFFFFFFF:FFFFFFFFFFFFFFFFFFFFFFFFFFFFFFFFFF
@A00155:342:HHGFNDSXY:1:2236:27932:19272 2:N:0:GAACCTAG+TCCGCATA
AGTTGGGTAGGTCAGGTGCCCTAGCACCTTCCTCCCCTTTCAACCATGCGTCCACCCCGGTTAATTTT
GACCCGTGACGGTAATACAAAAGCTCGGTAAGCTTGGGTGAGTCAAAATCTAGTCTCGCTCTAGACCT
+
FFFFFFFFFFFFFFFFFF:FFFFFFFFF:FF,FF:FFFFFFFFF:FFFF:FFFFFFFF,FFFFFF:FF
,::FFFFFF,:FFFFFF:FFFFF,F:,:FFFFFFFFFFFFFFFFFFFFFFF::FFFFFFFFFFFF:FF
@A00155:342:HHGFNDSXY:1:1519:21468:28307 1:N:0:GAACCTAG+TCCGCATA
AGTTGGGTAGGTCAGGTGCCCTAGCACCTTCCTCCCCTTTCAACCATGCGTCCACCCCGGTTAATTTT
GACCCGTGACGGTAATACAATAGCTCGGTAAGCTTGGGTGAGTCAAAATCTAGTCTCGCTCTAGACCT
+
FFFFFFFF:FFFFFFFFFFFFFFFFFFFFFFFFFFFFFFFFFFFFFFFFFFFFFFFFFFFFF:FFFFF
F:FFFFFFFFFFFFFFFFFFFFFFFFFFFFFFFFFFFFFFFFFFFFFFFFFFF:FFFFFFFFFFFFFF
@A00155:342:HHGFNDSXY:1:2369:30789:9345 2:N:0:GAACCTAG+TCCGCATA
AGTTGGGTAGGTCAGGTGCCCTAGCACCTTCCTCCCCTTTCAACCATGCGTCCACCCCGGTTAATTTT
GACCCGTGACGGTAATACAATAGCTCGGTAAGCTTGGGTGAGTCAAAATCTAGTCTCGCTCTAGACCT
+
FFFFFFFFFFFFFFFFFFFFFFFFFFFFFFFFFFFFFFFFF:FFFFFFFFFFFFFFFFFF:FFFFFFF
FFFFFFFFFFFFFFFFFFFFFFFFFFFFFFFFFFFFFFFFFFFFFFFFFFFFFFFFFFFFFFFFFFFF
@A00155:342:HHGFNDSXY:1:1658:16324:4742 1:N:0:GAACCTAG+TCCGCATA
AGTTGGGTAGGTCAGGTGCCCTAGCACCTTCCTCCCCTTTCAACCATGCGTCCACCCCGGTTAATTTT
GACCCGTGACGGTAATACAATAGCTCGGTAAGCTTGGGTGAGTCAAAATCTAGTCTCGCTCTAGACCT
+
FFFFFFFFFFFFFFFFFFFFFFFFFFFF,FFFFFFFFFFFFFFFFFFFFFFFFFFFFFFFFFFFFFFF
FFFFFFFFFFFFFF:FFFFFFFFFFFFFFFFFFFFFFFFFFFFFFFFFFFFFFFFFFFFFFFFFFFFF
@A00155:342:HHGFNDSXY:1:1226:22046:20165 2:N:0:GAACCTAG+TCCGCATA
TGGGTAGGTCAGGTGCCCTAGCACCTTCCTCCCCTTTCAACCATGCGTCCACCCCGGTTAATTTTGAC
CCGTGACGGTAATACAATAGCTCGGTAAGCTTGGGTGAGTCAAAATCTAGTCTCGCTCTAGACCTTCT
+
FFFFFFFFFFFFFFFFFF,FFFFFFFFFFFFFFFFFFFFFFF,FFFFFFFFFFFFFFFF:FFFFFFFF
FFFFFFFFFFF:FFF::F:FFFFFFFFFFFFFFFFFFFFFFFF::FFFFFFFFFFFFFFFFFFFFFFF
@A00155:342:HHGFNDSXY:1:1427:24514:29982 2:N:0:GAACCTAG+TCCGCATA
TAGGTCAGGTGCCCTAGCACCTTCCTCCCCTTTCAACCATGCGTCCACCCCGGTTAATTTTGACCCGT
```

```
GACGGTAATACAATAGCTCGGTAAGCTTGGGTGAGTCAAAATCTAGTCTCGCTCTAGACCTTCTTGAC
+
FFFFFFFFFFFFFFFFFFFFF,FFFFFFFFFFFFFFFF,FFFFFFFFF,FFFFFFF:FFFFFFFFFFF
FFFFFFFFFFFF:FFFFFFFFFFFFFFFFFFFFFFFFFFFFFFFFFFFFFFFFFFFFFFFFFFFFFFF
@A00155:342:HHGFNDSXY:1:2540:2456:18239 1:N:0:GAACCTAG+TCCGCATA
TAGGTCAGGTGCCCTAGCACCTTCCTCCCCTTTCAACCATGCGTCCACCCCGGTTAATTTTGACCCGT
GACGGTAATACAATAGCTCGGTAAGCTTGGGTGAGTCAAAATCTAGTCTCGCTCTAGACCTTCTTGAC
+
FFFFFFFFFFFFFFFFFFFFFFFFFFFFFFFFFFFFFFFFFFFFFFFFFFFFFFFFFFFFFFFFFFFF
FFFFFFFFFFFFFFFFFFFFFFFFFFFFFFFFFFFFFFFF:FFFFFFFFFFFFFFFFFFFFFFFFFFF
@A00155:342:HHGFNDSXY:1:1238:27832:7482 1:N:0:GAACCTAG+TCCGCATA
GGTCAGGTGCCCTAGCACCTTCCTCCCCTTTCAACCATGCGTCCACCCCGGTTAATTTTGACCCGTGA
CGGTAATACAATAGCTCGGTAAGCTTGGGTGAGTCAAAATCTAGTCTCGCTCTAGACCTTCT
+
FFFFFFFFFFFFFFFFFFFFFFFFFFFFFFFFFFFFFFFFFFFFFFFFFFFFFFFFFFFFFFFFFFFF
FFFF:FFFFFFFFFFFFFFFFFFF:FF:FFFFFFFFFFFFFFFFFFFFFFFFFFFFFFFFFF
@A00155:342:HHGFNDSXY:1:2410:31602:13949 1:N:0:GAACCTAG+TCCGCATA
GGTCAGGTGCCCTAGCACCTTCCTCCCCTTTCAACCATGCGTCCACCCCGGTTAATTTTGACCCGTGA
CGGTAATACAATAGCTCGGTAAGCTTGGGTGAGTCAAAATCTAGTCTCGCTCTAGACCTTCTTGACCA
+
FFFFFFFFFFFFFFFFFFF:FFFFFFFFFFFFFFFFF:FFFFFFFFFFFFFFFFFFFFFFFFFFFFFF
FFFF::FFFFFFFFFFFFFFFFFFFFFFFFFFFFFFFFFFFFFFFF:FFF:FFFFFFF:FFFFFFFFF
@A00155:342:HHGFNDSXY:1:2228:26503:23907 1:N:0:GAACCTAG+TCCGCATA
GGTCAGGTGCCCTAGCACCTTCCTCCCCTTTCAACCATGCGTCCACCCCGGTTAATTTTGACCCGTGA
CGGTAATACAATAGCTCGGTAAGCTTGGGTGAGTCAAAATCTAGTCTCGCTCTAGACCTTCTTGACCA
+
FFFFFFFF:FF:FF:FFF:::F,FFFFFFFFFFF::F:FF:FFFFFFFF:F,FFFFF:FFF:F:FFFF
FFF:FFFFFFFFF:::FF:FFFFF:FFF,FFF:F:FFFFF:FF,F:F:F,FF:F:FFFFFFFF:F,FF
@A00155:342:HHGFNDSXY:1:1658:16324:4742 2:N:0:GAACCTAG+TCCGCATA
GGTCAGGTGCCCTAGCACCTTCCTCCCCTTTCAACCATGCGTCCACCCCGGTTAATTTTGACCCGTGA
CGGTAATACAATAGCTCGGTAAGCTTGGGTGAGTCAAAATCTAGTCTCGCTCTAGACCTTCTTGACCA
+
FFFFFFFFFFFF:FFFFFF:FFFFFFFFFFFFFFFFFFFFFFFFFFFFFFFFFFFFFFFFFFFFFFFF
FFFFFFFFFF,FFFFFFFFFFFFFFFFFF:FFFFFFFFFFFFFFFFFFFFFFFFFFFFFFFFFFFFFF
@A00155:342:HHGFNDSXY:1:1310:17879:26819 1:N:0:GAACCTAG+TCCGCATA
GGTCAGGTGCCCTAGCACCTTCCTCCCCTTTCAACCATGCGTCCACCCCGGTTAATTTTGACCCGTGA
CGGTAATACAATAGCTCGGTAAGCTTGGGTGAGTCAAAATCTAGTCTCGCTCTAGACCTTCTTGACCA
+
FFFFFFFFFFFFFFFFFFFFFFFFFFFFFFFFFFFFFFFFFFFFFFFFFFFFFFFFFFFFFFFFFFFF
FFFFFFFFFFFFFFFFFFFFFFFFFFFFFFFFFFFFFFFFFFFFFFFFFFFFFFFFFFFFFFFFFFFF
@A00155:342:HHGFNDSXY:1:2439:26576:15233 1:N:0:GAACCTAG+TCCGCATA
GGTCAGGTGCCCTAGCACCTTCCTCCCCTTTCAACCATGCGTCCACCCCGGTTAATTTTGACCCGTGA
CAGTAATACAATAGCTCGGTAAGCTTGGGTGAGTCAAAATCTAGTCTCGCTCTAGACCTTCTTGACCA
+
FFFFFFFFFFFFFFFFFFF,:FFFFFFFFFFFFFFFFFFFFFFFFFFFFFFFFFFFFFFFFFFFFFFF
FFFFFFFFFFFFFFFFFFFFFFFFFFFFFFFFFFFFFFFFFFFFFFFFFFFFFFFFFFFFFFFFFFFF
@A00155:342:HHGFNDSXY:1:2275:29993:12195 1:N:0:GAACCTAG+TCCGCATA
GGTCAGGTGCCCTAGCACCTTCCTCCCCTTTCAACCATGCGTCCACCCCGGTTAATTTTGACCCGTGA
CGGTAATACAATAGCTCGGTAAGCTTGGGTGAGTCAAAATCTAGTCTCGCTCTAGACCTTCTTGACCA
+
FFFFFFFFFFFFFFFFFFFFFFFFFFFFFFFFFFFFFFFFFFFFFFFFFFFFFFFFFFFFFFFFFFFF
FFFFFFFFFFFFFFFFFFFFFFFFFFFFFFFFFFFFFFFFFFFFFFFFFFFFFFFFFFFFFFFFFFFF
@A00155:342:HHGFNDSXY:1:2566:13096:16814 1:N:0:GAACCTAG+TCCGCATA
GGTCAGGTGCCCTAGCACCTTCCTCCCCTTTCAACCATGCGTCCACCCCGGTTAATTTTGACCCGTGA
```

```
CGGTAATACAATAGCTCGGTAAGCTTGGGTGAGTCAAAATCTAGTCTCGCTCTAGACCTTCTTGACCA
+
FFFFFFFFFFFFFFFFFFFFFFFFFFFFFFFFFFFFFFFFFFFFFFFFFFFFFFFFFFFFFFFFFFFF
FFFFFFFFFFFFFFFFFFFFFFFFFFFFFFFFFFFFFFFFFFFFFFFFFFFFFFF:FFFFFFFFFFFFF
@A00155:342:HHGFNDSXY:1:2461:11912:18834 1:N:0:GAACCTAG+TCCGCATA
GGTCAGGTGCCCTAGCACCTTCCTCCCCTTTCAACCATGCGTCCACCCCGGTTAATTTTGACCCGTGA
CGGTAATACAATAGCTCGGTAAGCTTGGGTGAGTCAAAATCTAGTCTCGCTCTAGACCTTCTTGACCA
+
FFFFFFFFFFFFFFFFFFFFFFFFFFFFFFFFFFFFFFFFFFFFFFFFFFFFFFFFFFFFFFFFFFFF
,FFFFFFFFFFFFFFFFFFFFFFFFFFFFFFFFFFFFFFFF,FFFFFFFFFFFFFFFF:FFFFFFFF
@A00155:342:HHGFNDSXY:1:2461:11803:19022 1:N:0:GAACCTAG+TCCGCATA
GGTCAGGTGCCCTAGCACCTTCCTCCCCTTTCAACCATGCGTCCACCCCGGTTAATTTTGACCCGTGA
CGGTAATACAATAGCTCGGTAAGCTTGGGTGAGTCAAAATCTAGTCTCGCTCTAGACCTTCTTGACCA
+
FFFFFFFFFFFFFFFFFFFFFFFFFFFFFFFFF:FFFFFFFFFFFFFFFFFFFFFFFFFFFFFFFFFF
FFFFFFFFFFFFFFFFFFFFFFFFF,FFFFFFFFFFFFFFFFFFFFFFFFFFFFFFFFFFFFFFFFFFF
@A00155:342:HHGFNDSXY:1:1252:13955:8281 1:N:0:GAACCTAG+TCCGCATA
GGTCAGGTGCCCTAGCACCTTCCTCCCCTTTCAACCATGCGTCCACCCCGGTTAATTTTGACCCGTGA
CGGTAATACAATAGCTCGGTAAGCTTGGGTGAGTCAAAATCTAGTCTCGCTCTAGACCTTCTTGACCA
+
FFFF:FFFFFFFFFFFFFFFFFFFFFFFFFFFFFFFFFFFFFFFFFFFFFFFFFFFFFFFFFFFFFFF
FFFFFFFFFFFFFFFFFFFFFFFFFFFFFFFFFFFF,FFFFFFFFFFFFFFFFFFFFFFFFFFFFFFF
@A00155:342:HHGFNDSXY:1:1355:18593:31845 1:N:0:GAACCTAG+TCCGCATA
GTCAGGTGCCCTAGCACCTTCCTCCCCTTTCAACCATGCGTCCACCCCGGTTAATTTTGACCCGTGAC
GGTAATACAATAGCTCGGTAAGCTTGGGTGAGTCAAAATCTAGTCTCGCTCTAGACCTTCTTG
+
FFFFFFFFFFFFFFF:FF,FFFFFFFFFFFFFFFF,FFFFFFFFFFFFFFFFFFFFFFFFFFFFFFFF
FFFFFFFFFFFFFFFFFFFFFFFFFFFFFFFFFFFFF:FFFFFF:FFFFFFFFFFFFFFFFFFFF
@A00155:342:HHGFNDSXY:1:1507:2935:1720 1:N:0:GAACCTAG+TCCGCATA
GTCAGGTGCCCTAGCACCTTCCTCCCCTTTCAACCATGCGTCCACCCCGGTTAATTTTGACCCGTGAC
GGTAATACAATAGCTCGGTAAGCTTGGGTGAGTCAAAATCTAGTCTCGCTCTAGACCTTCTTGACCA
+
FFFFFFFFFFFFF,FFFFF:,FFFFFFFFFFFF::FFF,,FFFFF::FF,FFFFFFFFFFFF:FFFFF
FFF:FFFFFFFFFFFF:FFF:FFFFFFFFFFFFFFFFFFFFFFFFFFFFFFFFFFFFFFFFFFFFFF
@A00155:342:HHGFNDSXY:1:1550:31286:18129 1:N:0:GAACCTAG+TCCGCATA
GTCAGGTGCCCTAGCACCTTCCTCCCCTTTCAACCATGCGTCCACCCCGGTTAATTTTGACCCGTGAC
GGTAATACAATAGCTCGGTAAGCTTGGGTGAGTCAAAATCTAGTCTCGCTCTAGACCTTCTTGACCAT
+
FFFFFFFFFFFFFFFFFFFFFFFFFFFFFFFFFFFFFFFFFFFFFFFFFFF:FFFFFFFFFFFFFFFF
FFFFFFFFFFFFFFFFFFFFFFFF:,FFFFFFFFFFFFFFFFFFFFFFFFFFFFFFFF:FFFF:FFFFFF
@A00155:342:HHGFNDSXY:1:1546:8269:24549 1:N:0:GAACCTAG+TCCGCATA
GTCAGGTGCCCTAGCACCTTCCTCCCCTTTCAACCATGCGTCCACCCCGGTTAATTTTGACCCGTGAC
GGTAATACAATAGCTCGGTAAGCTTGGGTGAGTCAAAATCTAGTCTCGCTCTAGACCTTCTTGACCAT
+
FFFFFFFFFFFF:FFF:F,FFFFFFFFFFFFFFFFFFFFFFFFFFFFFFFFFFFFFFFFFFFFFFFFF
FFFFFFFFFFF:FFFFFFFFFFF:FFFFFFFFFFFFFFFFFFFFFFFFFFFFFFFFFFFFFFFFFFFF
@A00155:342:HHGFNDSXY:1:1507:2944:1892 1:N:0:GAACCTAG+TCCGCATA
GTCAGGTGCCCTAGCACCTTCCTCCCCTTTCAACCATGCGTCCACCCCGGTTAATTTTGACCCGTGAC
GGTAATACAATAGCTCGGTAAGCTTGGGTGAGTCAAAATCTAGTCTCGCTCTAGACCTTCTTGACCAT
+
FFFFFFFFFFFF:FFFFFFFFF,FFFFF:FFF:FFFFFFF:FF,FFFFFFFFFFFFFFFFFFFFFFFFF
FFFFFFFFFFFFFFFFFFFFFFFF:FFFFFFFFFFF:FFFFFFFFFFFFFFFFFFFFFFFFFFFFFFFF
@A00155:342:HHGFNDSXY:1:1550:28339:12211 1:N:0:GAACCTAG+TCCGCATA
GTCAGGTGCCCTAGCACCTTCCTCCCCTTTCAACCATGCGTCCACCCCGGTTAATTTTGACCCGTGAC
```

```
GGTAATACAATAGCTCGGTAAGCTTGGGTGAGTCAAAATCTAGTCTCGCTCTAGACCTTCTTGACCAT
+
FFFFFFFFFFFFFFFFFFFFFFFFFFFFFFFFFFFFFFFFFFFFFFFFFFFFFFFFFFFFFFFFFFFF
FF,FFFFFFFFFFFFFFFFFFFFFFFFFFFFFFFFFFFFFFFFFFFFFFFFFFFFFFFFFFFFFFFFF
@A00155:342:HHGFNDSXY:1:2369:30789:9345 1:N:0:GAACCTAG+TCCGCATA
TCAGGTGCCCTAGCACCTTCCTCCCCTTTCAACCATGCGTCCACCCCGGTTAATTTTGACCCGTGACG
GTAATACAATAGCTCGGTAAGCTTGGGTGAGTCAAAATCTAGTCTCGCTCTAGACCTTCTTGACCATC
+
FFFFFFFFFFFFFFFFFFFFFFFFFFFFFFFFFFFFFFFFFFFF:FFFFFFFFFFFFFFFFFFFFFFF
FFFFFFFFFFFFFFFFFFFFFFFF:FFFFFFFFFFFFFFFFFFFFFFFFFFF:FFFFFFFFFFFFFFF
@A00155:342:HHGFNDSXY:1:2569:29460:5165 1:N:0:GAACCTAG+TCCGCATA
TCAGGTGCCCTAGCACCTTCCTCCCCTTTCAACCATGCGTCCACCCCGGTTAATTTTGACCCGTGACG
GTAATACAATAGCTCGGTAAGCTTGGGTGAGTCAAAATCTAGTCTCGCTCTAGACCTTCTTGACCATC
+
FFFFFFFFFFFFFFFFFFFFFFFFFFFFFFFFFFFFFFFFFFFFFFFFFFFFFFFFFFFFFFFFFFFF
FFFFFFFF:FFFFFFFFFFFFFFFFFFFFFFFFFFFFFFFFFFFFFFFFFFFFFFF:FFFFFFFFFF
@A00155:342:HHGFNDSXY:1:1514:24876:4899 1:N:0:GAACCTAG+TCCGCATA
TCAGGTGCCCTAGCACCTTCCTCCCCTTTCAACCATGCGTCCACCCCGGTTAATTTTGACCCGTGACG
GTAATACAATAGCTCGGTAAGCTTGGGTGAGTCAAAATCTAGTCTCGCTCTAGACCTTCTTGACCATC
+
FFFFFFFFFFFFFFFFFFFFFFFFFFFFF:FFFFFFFFFFFFFFFFFFFFFFFFFFFFFFFFFFFFFF
FFF:FFFFFFFFFFFFFFFFFFF:FFFFFFFFF:FFFFFFFFFFFFFFFFFFFFFFFFFFFFFFFF,F
@A00155:342:HHGFNDSXY:1:2465:14977:36166 1:N:0:GAACCTAG+TCCGCATA
TCAGGTGCCCTAGCACCTTCCTCCCCTTTCAACCATGCGTCCACCCCGGTTAATTTTGACCCGTGACG
GTAATACAATAGCTCGGTAAGCTTGGGTGAGTCAAAATCTAGTCTCGCTCTAGACCTTCTTGACCATC
+
FFFFFFFFFFFFFFFFFFFFFFFFFFFFFFFFFFFFFFFFFFFFFFFFFFFFFFFFFFFFFFFFFFFF
FFFFFFFFFFFFFFFFFFFFFFFFFFFFFFFFFFFFFFFFFFFFFFFFFFFFFFF:FFFFFFFFFFFF
@A00155:342:HHGFNDSXY:1:1169:9480:30749 1:N:0:GAACCTAG+TCCGCATA
TCAGGTGCCCTAGCACCTTCCTCCCCTTTCAACCATGCGTCCACCCCGGTTAATTTTGACCCGTGACG
GTAATACAATAGCTCGGTAAGCTTGGGTGAGTCAAAATCTAGTCTCGCTCTAGACCTTCTTGACCATC
+
FFFFFFFFFFFFFF:FFFFFFFFFFFFFFFFFF:FFFFFFFFFFFFFFFFFFFFFFFFFFFFFFFFFF
FFFFFFFFFFFFF:FFFFFFFFFFFFFFFFFFFFFFFFFFFFFFFFFFFFFFFFFFFFFFFFFFFFFF
@A00155:342:HHGFNDSXY:1:2556:6497:19257 2:N:0:GAACCTAG+TCCGCATA
TGCCCTAGCACCTTCCTCCCCTTTCAACCATGCGTCCACCCCGGTTAATTTTGACCCGTGACGGTAAT
ACAATAGCTCGGTAAGCTTGGGTGAGTCAAAATCTAGTCTCGCTCTAGACCTTCTTGACCATCTGG
+
FFFF:FFFF:FFFFFFFFFFF,F,FFFFFFFFFFFFFFFF:F,FFFFFFFFFFFFFFFFF,FFFFFFF
FFFFF:FFFFFFFFFF,FFFFF:FFFFFFFFFFFFFFFFF:FFFFFFFFFFFFFF,FFFFFFFFFF
@A00155:342:HHGFNDSXY:1:1565:22209:16157 2:N:0:GAACCTAG+TCCGCATA
TGCCCTAGCACCTTCCTCCCCTTTCAACCATGCGTCCACCCCGGTTAATTTTGACCCGTGACGGTAAT
ACAATAGCTCGGTAAGCTTGGGTGAGTCAAAATCTAGTCTCGCTCTAGACCTTCTTGACCATCTGGCA
+
FFFFFFFFFFFF:FFFFFFFFFFFFFFFFFFFFFFFFFFFFFFFFFFFFF:FFFFFFFFFFFFFFFFF
FFFF::FFFFFFFFFFFFFFFFFFFFFFFFFFFFFFFFFFFFFFFFFFFFFFFFFFFFFFFFFFFFFF
@A00155:342:HHGFNDSXY:1:2420:32850:2988 2:N:0:GAACCTAG+TCCGCATA
TGCCCTAGCACCTTCCTCCCCTTTCAACCATGCGTCCACCCCGGTTAATTTTGACCCGTGACGGTAAT
ACAATAGCTCGGTAAGCTTGGGTGAGTCAAAATCTAGTCTCGCTCTAGACCTTCTTGACCATCTGGCA
+
FFFFFFF:FF::FF:FFFFFFFFFFFFFFFFFFFF,FFFFFF:F:FFF::FFFFFFFF:FFFFFFFFF
FF:F:F:FFFF:FFFFFFFF:F:FF:FFFFFFFFFFFFFFF:FF,FFFFFFF:FFFFFFFFFFFFFFF
@A00155:342:HHGFNDSXY:1:1565:22110:15546 2:N:0:GAACCTAG+TCCGCATA
GCCCTAGCACCTTCCTCCCCTTTCAACCATGCGTCCACCCCGGTTAATTTTGACCCGTGACGGTAATA
```

CAATAGCTCGGTAAGCTTGGGTGAGTCAAAATCTAGTCTCGCTCTAGACCTTCTTGACCATCTGGCA
+
FFFF,FFFFFFFFFFFFFFFFFFFFFFF::FFFFFFFFFFFFFFFFFFFFFFFFFFFFFFFFFFFFFF
FFFFFFFFFFF:FFFFFFFFFFFFFFFFFF:FFFFFFFFFFFFFFFFFFFFFFFFFFFFFFFFFFFF
@A00155:342:HHGFNDSXY:1:1375:25527:14074 2:N:0:GAACCTAG+TCCGCATA
CCCTAGCACCTTCCTCCCCTTTCAACCATGCGTCCACCCCGGTTAATTTTGACCCGTGACGGTAATAC
AATAGCTCGGTAAGCTTGGGTGAGTCAAAATCTAGTCTCGCTCTAGACCTTCTTGACCATCTGGCAGT
+
FFFFFFFFFF:FFFFFFFFFFFFFFFFFFFFFFFFFFFFFFFFFFFFFFFFFFFFFFFFFFFFFFFFF
FFFFFFFFFFFFFFFFFFFFFFFFFFFFFFFFFFFFFFFFFFFFFFFFFFFFF:FFFFFFFFFFFFFF
@A00155:342:HHGFNDSXY:1:2140:1542:8077 2:N:0:GAACCTAG+TCCGCATA
CCCTAGCACCTTCCTCCCCTTTCAACCATGCGTCCACCCCGGTTAATTTTGACCCGTGACGGTAATAC
AATAGCTCGGTAAGCTTGGGTGAGTCAAAATCTAGTCTCGCTCTAGACCTTCTTGACCATCTGGCAGT
+
FF:FF:FF:FFF,FFFF:FFFFFFFFFFFFFFFFFFF:FFF::FFF,,FFFFFFFFFFF,FFFFFFFF
FFFFFFFFFFFFFFFFFFFF,FFF:FFFFFFFFFFFFFFFF:FFFFFFFFFFFFFFFFFFFFFFFFFF
@A00155:342:HHGFNDSXY:1:2117:1434:11459 2:N:0:GAACCTAG+TCCGCATA
CCCTAGCACCTTCCTCCCCTTTCAACCATGCGTCCACCCCGGTTAATTTTGACCCGTGACGGTAATAC
AATAGCTCGGTAAGCTTGGGTGAGTCAAAATCTAGTCTCGCTCTAGACCTTCTTGACCATCTGGCAGT
+
FFFFF:FFFFFFFFFFFF:FF,FF:FFFF::F:FFFFFFFF::FFFF:,FFFFFFFFFFFF:FFF:FF
FFFFFF:FF:F:FFFFFFFF:FFF:FFFFFFFFF:FFFFFFFFFFFFFFF:FFFFFFFFFFFFFFF:F
@A00155:342:HHGFNDSXY:1:2234:10411:16611 2:N:0:GAACCTAG+TCCGCATA
CCCTAGCACCTTCCTCCCCTTTCAACCATGCGTCCACCCCGGTTAATTTTGACCCGTGACGGTAATAC
AATAGCTCGGTAAGCTTGGGTGAGTCAAAATCTAGTCTCGCTCTAGACCTTCTTGACCATCTGGCAGT
+
FFFFFFFFFFFFFFF:F:FFFFFFFFFFFFFFFFFFFFFFFFFFFFFFFFFFFFFFFFFFFFFFFFFF
,FFFFFFFFFFFFFFFFF:FFFFFFFFFFFFFFFFFFFFFFFFFFFFFFFFFFFFFFFFFFFFFFFFF
@A00155:342:HHGFNDSXY:1:1167:7328:15562 2:N:0:GAACCTAG+TCCGCATA
CCTAGCACCTTCCTCCCCTTTCAACCATGCGTCCACCCCGGTTAATTTTGACCCGTGACGGTAATACA
ATAGCTCGGTAAGCTTGGGTGAGTCAAAATCTAGTCTCGCTCTAGACCTTCTTGACCATCTGGCAGTC
+
FFFFFFFFFFFFFFF:FFFFFFFFFFFFFFFFFFFFFFFF:FFFFFFFFFFFFFFFFFFFFFFFFFFF
FFFFFFFFFFFFFFFFFFFFFFFFFFFFFFFFFFFFFFFFF:FFFFFFFFFFFFFFFFFFFFFFFFFF
@A00155:342:HHGFNDSXY:1:1128:6307:26725 2:N:0:GAACCTAG+TCCGCATA
CTAGCACCTTCCTCCCCTTTCAACCATGCGTCCACCCCGGTTAATTTTGACCCGTGACGGTAATACAA
TAGCTCGGTAAGCTTGGGTGAGTCAAAATCTAGTCTCGCTCTAGACCTTCTTGACCATCTGGCAGTCA
+
FFFF:FF:FFFFFFFFFFFFFFF:FFFFFFFFFFF:FFFFFFFF,:FFFFFFFFFFFFFFFFFFFFFF
FFFFFF:FFFFFFFFF:FFFFFFFFFFFFFFFFFFFFFFFFFFFFFFFFFFFFFFFFFFFFFFFFFFF
@A00155:342:HHGFNDSXY:1:1128:5728:26819 2:N:0:GAACCTAG+TCCGCATA
CTAGCACCTTCCTCCCCTTTCAACCATGCGTCCACCCCGGTTAATTTTGACCCGTGACGGTAATACAA
TAGCTCGGTAAGCTTGGGTGAGTCAAAATCTAGTCTCGCTCTAGACCTTCTTGACCATCTGGCAGTCA
+
FFFFFFFFFFFFFFFFFFFFFF:FFFFFFFFFFFFFFFFFFFFFFFF,FFFF:FFFFFFFFFFFFFFF
FFFFFFFFFFFFFFFFFFFFFFFFFFFFFFFFFFFFFFF,FFFFFFFFFFFFFFFFF:FFFFFFFFFF
@A00155:342:HHGFNDSXY:1:1225:9019:12602 2:N:0:GAACCTAG+TCCGCATA
CTAGCACCTTCCTCCCCTTTCAACCATGCGTCCACCCCGGTTAATTTTGACCCGTGACGGTAATACAA
TAGCTCGGTAAGCTTGGGTGAGTCAAAATCTAGTCTCGCTCTAGACCTTCTTGACCATCTGGCAGTCA
+
FFFFFFFF:FFFFFFFFF::FFFFFFFFFFFFFFFFFFFFFFFF:F,:F:FFFFFFFFFFFFF:FFFF
::FFFFFFFFFFF,FFFFFFFFFFFFFFFF,FFFFFFFFFF,FFFFFFFFFFFFFFFFFFFFFFFFFF
@A00155:342:HHGFNDSXY:1:2441:23294:20572 2:N:0:GAACCTAG+TCCGCATA
CTAGCACCTTCCTCCCCTTTCAACCATGCGTCCACCCCGGTTAATTTTGACCCGTGACGGTAATACAA

TAGCTCGGTAAGCTTGGGTGAGTCAAAATCTAGTCTCGCTCTAGACCTTCTTGACCATCTGGCAGTCA
+
FFFFFFFFFFFFFFF,FFFFFFF:FFF:FFFFFFFF,FF,F,FFFF:FFFF:F,FFFFF:FFFFFFF:
FFFFFF:FFFFFF:FFFFFFFFFFFFFFFFFFFFFFFFFF,FFFFFFF::FFFFFFFFFF:FFFFFFF
@A00155:342:HHGFNDSXY:1:1523:3278:10457 2:N:0:GAACCTAG+TCCGCATA
CTAGCACCTTCCTCCCCTTTCAACCATGCGTCCACCCCGGTTAATTTTGACCCGTGACGGTAATACAA
TAGCTCGGTAAGCTTGGGTGAGTCAAAATCTAGTCTCGCTCTAGACCTTCTTGACCATCTGGCAGTCA
+
FFFFFFFF,FFFFFFFFFFFFFFFFFFFFFFFFFFFFFFFFFFFF:FFFFFFFFFFFFFFFFFFFF:F
FFFFFFFFFFFFFFFFFFFFFFFFFF::FFFFFFFFFFFFFFFFFFFFFFFFFFFFFFFFFFFFFFFF
@A00155:342:HHGFNDSXY:1:1473:13395:1799 2:N:0:GAACCTAG+TCCGCATA
CTAGCACCTTCCTCCCCTTTCAACCATGCGTCCACCCCGGTTAATTTTGACCCGTGACGGTAATACAA
TAGCTCGGTAAGCTTGGGTGAGTCAAAATCTAGTCTCGCTCTAGACCTTCTTGACCATCTGGCAGTCA
+
FFFFFFFFFFFFFFFFF:FFFFFFF:FFFFFFFFFFFFFFFFFFFFFFFFFFFFFFF:FFFFFFFFFF
FFFFFFFFFFFFFFFFFFFFFFFFFFFFF:FFF,FFFFFFFFFFFFFFFFFFFFFFFFFFFFFFFFFF
@A00155:342:HHGFNDSXY:1:2338:13892:3599 2:N:0:GAACCTAG+TCCGCATA
CTAGCACCTTCCTCCCCTTTCAACCATGCGTCCACCCCGGTTAATTTTGACCCGTGACGGTAATACAA
TAGCTCGGTAAGCTTGGGTGAGTCAAAATCTAGTCTCGCTCTAGACCTTCTTGACCATCTGGCAGTCA
+
F:FFFFFFFFFFFFFFFF:FF:FFFFFFFFFFFFFFFFFFFFFFFFFFFFFFFFFFFFFFFFFFFFFF
,FFFFFFFFFFFFFFFFFFFFFFFFFFFFFFFFFFFFFFFFFFFFFFFFFFFFFFFFFFFFFFFFFFF
@A00155:342:HHGFNDSXY:1:1525:2347:26381 2:N:0:GAACCTAG+TCCGCATA
CTAGCACCTTCCTCCCCTTTCAACCATGCGTCCACCCCGGTTAATTTTGACCCGTGACGGTAATACAA
TAGCTCGGTAAGCTTGGGTGAGTCAAAATCTAGTCTCGCTCTAGACCTTCTTGACCATCTGGCAGTCA
+
FF:FFFFFFFFFFFFF,FF,:FFF:FFFFFFFFFFFFFF:FFFFFFFFFFFFFFFFFFFFFFFFFFFF
FFFFFFFFFFF,FFFFFF:FFFFFFFFFFFFFFFFFF:FFFFFFFFFFFFFFFFFFF,F:FFFFFFFF
@A00155:342:HHGFNDSXY:1:1112:12500:1344 2:N:0:GAACCTAG+TCCGCATA
GCACCTTCCTCCCCTTTCAACCATGCGTCCACCCCGGTTAATTTTGACCCGTGACGGTAATACAATAG
CTCGGTAAGCTTGGGTGAGTCAAAATCTAGTCTCGCTCTAGACCTTCTTGACCATCTGGCAGTCAGCA
+
FFFFFFFFF:FFFFFFFFFFFFFFFFFFFFFFFFF:FFFFFFFF:F:FFFFFFFFFFFFFFFFFFFFF
FFFFFFFFFFFFFFFFFFFFFFFFFFFFFFFFFFFFFFFFFFFFFFFFFFFFF::FFFFFFFFFFFFF
@A00155:342:HHGFNDSXY:1:1544:29206:26490 2:N:0:GAACCTAG+TCCGCATA
GCACCTTCCTCCCCTTTCAACCATGCGTCCACCCCGGTTAATTTTGACCCGTGACGGTAATACAATAG
CTCGGTAAGCTTGGGTGAGTCAAAATCTAGTCTCGCTCTAGACCTTCTTGACCATCTGGCAGTCAGCA
+
FFFFFFFFFFFFFFFFFFFFFFFFFFFFFFFFFFFFFFFFFFFFFFFFFFFFFFFFFFFFFFFFFFFF
FFFFFFFFFFFFFFFFFFFFFFFFFFFFFFFFFFFFFFFFFFFFFFFFFFFFFFFFFFFFFFFFFFFF
@A00155:342:HHGFNDSXY:1:1330:2944:26944 1:N:0:GAACCTAG+TCCGCATA
CTTCCTCCCCTTTCAACCATGCGTCCACCCCGGTTAATTTTGACCCGTGACGGTAATACTATAGCTCG
GTAAGCTTGGGTGAGTCAAAATCTAGTCTCGCTCTAGACCTTCTTGACCATCTGGCAGTCAGCATAAG
+
FFFFFFFFFFFFFFFFFF:FFFFFFFFFFF:FFFFFFFFFFFFFFFFFFFFFFFFFFFFFFFFFFFFF
FFFFFFFFFFF:FFFFFFFFFFFFFFFFFFFFFFFFFFFFFFFFFFFFFFFFFFFFFFFFFFFFFFFF
@A00155:342:HHGFNDSXY:1:1330:2446:28557 1:N:0:GAACCTAG+TCCGCATA
CTTCCTCCCCTTTCAACCATGCGTCCACCCCGGTTAATTTTGACCCGTGACGGTAATACTATAGCTCG
GTAAGCTTGGGTGAGTCAAAATCTAGTCTCGCTCTAGACCTTCTTGACCATCTGGCAGTCAGCATAAG
+
FFFFFF,F:,FFF:FFF,FFFF:FFF:FFFF,:FFFFF:F,FFFFFFFFFFF,,F,FFFF,FFFFFFF
FF,FFFFFFF:FFF,F,FFFFFFFFFF,FFFFFFFFFFF,FFFFFFFF:FFFF,F,FFFFF::FFFFF
@A00155:342:HHGFNDSXY:1:2554:20799:25081 2:N:0:GAACCTAG+TCCGCATA
CTCCCCTTTCAACCATGCGTCCACCCCGGTTAATTTTGACCCGTGACGGTAATACAATAGCTCGGTAA

```
GCTTGGGTGAGTCAAAATCTAGTCTCGCTCTAGACCTTCTTGACCATCTGGCAGTCAGCATA
+
FFFFFFFFFFFFFF:FFFFFFFFFFFFFFFFFFF:FFFFFFFFFFFFFFFFFFFFFFFFFFFFFFFF
FFFFFFFFFFFFFFFFFFFFFFFFFFFFFFFFFFFFFFFFFFFFFFFFFFFFFFFFFFFFFF
@A00155:342:HHGFNDSXY:1:1252:13955:8281 2:N:0:GAACCTAG+TCCGCATA
CTCCCCTTTCAACCATGCGTCCACCCCGGTTAATTTTGACCCGTGACGGTAATACAATAGCTCGGTAA
GCTTGGGTGAGTCAAAATCTAGTCTCGCTCTAGACCTTCTTGACCATCTGGCAGTCAGCATAAGGACG
+
FFFFFFFFFFFFFFFFFFFFFFFFFFFFFFFFFFFFFFFFFFFFFFFFFFFFFFFFF,FFFFFFFFFF
FFFFFFFFFFFFFFFFFFFFFFFFFFFFF:FFFFFFFFFFF:FFFFFFFFFFFFFFFFFFFFF:FFFFF
@A00155:342:HHGFNDSXY:1:2222:28700:1752 2:N:0:GAACCTAG+TCCGCATA
CCCCTTTCAACCATGCGTCCACCCCGGTTAATTTTGACCCGTGACGGTAATACAATAGCTCGGTAAGC
TTGGGTGAGTCAAAATCTAGTCTCGCTCTAGACCTTCTTGACCATCTGGCAGTCAGCATA
+
FFFFFFFFF,FF:FFFFFFFFFFFFFFFFFFFFF:FFFFFFFFFFFFFFFF:F:F,FFFF:FFFFFFF
FFFF:FFFFFF:::FFFFFFFFFFFFFFFFFFFFFFFFFFFFFFFFFFFFFFFFFF:FFFFFF
@A00155:342:HHGFNDSXY:1:2674:6334:11146 2:N:0:GAACCTAG+TCCGCATA
CCCCTTTCAACCATGCGTCCACCCCGGTTACTTTTGACCCGTGACGGTAATACAATAGCTCGGTAAGC
TTGGGTGAGTCAAAATCTAGTCTCGCTCTAGACCTTCTTGACCATCTGGCAGTCAGCATA
+
FFFFF:FFF,FF,,FFFFFFFFFFFFFF:F,::FFFFFFFFFFFFFFFFFFF:F::F:FFFFFFFF:F
F:FFF:FFFFFFFFFF:FFFFFFF:FFFFF:FFFFFFFFFFFF:FFF:FFFFFFFFFFFF
@A00155:342:HHGFNDSXY:1:1644:14931:3270 2:N:0:GAACCTAG+TCCGCATA
CCTCTTTCAACCATGCGTCCACCCCGGTTAATTTTGACCCGTGACGGTAATACAATAGCTCGGTAAGC
TTGGGTGAGTCAAAATCTAGTCTCGCTCTAGACCTTCTTGACCATCTGGCAGTCAGCATA
+
:FF,FFFF,,,F:F,FFFFFF:FF,:FFF:FFFFFFFFFF:FFF:FFFFFF,FF,,FFFF::,FFFFF
FFF,FFFFFFFF,F,FFF:FF:FFFFF,FFFFF:FFFFFFFFFFFFFFF:FFFFFFFFF,
@A00155:342:HHGFNDSXY:1:1132:25165:13009 2:N:0:GAACCTAG+TCCGCATA
CCCCTTTCAACCATGCGTCCACCCCGGTTAATTTTGACCCGTGACGGTAATACAATAGCTCGGTAAGC
TTGGGTGAGTCAAAATCTAGTCTCGCTCTAGACCTTCTTGACCATCTGGCAGTCAGCATAAGGA
+
FFFFFFFFFFFFFFFFFFFFFFFFFFFFFFFFFFFFFFFFFFFFFFFFFFFFFFFFFFFFFFFFFFFF
FFFFFFFFFFFFFFFFFFFFFFFFFFFFFFFFFFFFFFFFFFFFFFFFFFFFFFFFFFFFFFFF
@A00155:342:HHGFNDSXY:1:2341:4255:8547 2:N:0:GAACCTAG+TCCGCATA
CCTTTCAACCATGCGTCCACCCCGGTTAATTTTGACCCGTGACGGTAATACAATAGCTCGGTAAGCTT
GGGTGAGTCAAAATCTAGTCTCGCTCTAGACCTTCTTGACCATCTGGCAGTCAGCATAAGGACGATAG
+
FFFFFFFFFFFFFFFFFFFFFFFFFFFFFFFFFFFFFFFFFFFFFFFFFFFF:FFFFFFFFFFFFFFF
FFFFFFFFFFFFFFF,FFFFFFFFFFFFFFFFFFFFFFFFFFFF:FFFFFFFFFFF:FFFFFFFFFFF
@A00155:342:HHGFNDSXY:1:1538:4038:10019 2:N:0:GAACCTAG+TCCGCATA
CCTTTCAACCATGCGTCCACCCCGGTTAATTTTGACCCGTGACGGTAATACAATAGCTCGGTAAGCTT
GGGTGAGTCAAAATCTAGTCTCGCTCTAGACCTTCTTGACCATCTGGCAGTCAGCATAAGGACGATAG
+
FFFFFFFFFFF:FFFFFFFFF:F,FFFFF:F,FFFFFFFFFFFFFFFFFFFF:FFFFFFFF:FFFFFF
FFFFFFFFFFFF:FFFFFFFFFFFFFFFF:FFFFFFF:FFFFFFFFFFFFFFFFFFFFFFFFFFFFFF
@A00155:342:HHGFNDSXY:1:1114:4833:12931 2:N:0:GAACCTAG+TCCGCATA
CTTTCAACCATGCGTCCACCCCGGTTAATTTTGACCCGTGACGGTAATACAATAGCTCGGTAAGCTTG
GGTGAGTCAAAATCTAGTCTCGCTCTAGACCTTCTTGACCATCTGGCAGTCAGCATAAGGACGATAGT
+
FFFFFFFFFFFFFFFFFFFFFF,FFFFFFFFFFFFFFFFFFFFFFFFFFFFF:FFFFFFFFFFFFFFF
FF,FFFFFFFFFFFFFFFFFFFFFFFFFFFFFFFFFFFFFFFFFFFFFFF:FFFFFFFFFFFFFFFFFF
@A00155:342:HHGFNDSXY:1:1114:3513:11490 2:N:0:GAACCTAG+TCCGCATA
TTTCAACCATGCGTCCACCCCGGTTAATTTTGACCCGTGACGGTAATACAATAGCTCGGTAAGCTTGG
```

```
GTGAGTCAAAATCTAGTCTCGCTCTAGACCTTCTTGACCATCTGGCAGTCAGCATAAGGACGATAGT
+
FF:FF::F,FF,FFF:FFFFFFFFFFFFFFFFFFFFFFFFFFF:FFFFFFFF:,FFFFFFFFFFFFFF
FFFFF:FFFFFFFFFFFFFFFFFFFFFFFFFFFFFFFFF,FFFFFFFFF:FFFFFFFFFFF:FFFFF
@A00155:342:HHGFNDSXY:1:2139:17137:18865 2:N:0:GAACCTAG+TCCGCATA
TTTCAACCATGCGTCCACCCCGGTTAATTTTGACCCGTGACGGTAATACAATAGCTCGGTAAGCTTGG
GTGAGTCAAAATCTAGTCTCGCTCTAGACCTTCTTGACCATCTGGCAGTCAGCATAAGGACGATAGTT
+
FFFFFFFFFFFFFFFFFFFFFFFFFFF:FFFFFFFFFFFFFFFFFF,FFFFF:FFFFFFFFFFF:FFF
FFFFFFFF:FFFFFFFFFFFFFFFFFFFFFFFFFFFFFFFFFFFFFFFFFFFFFFFFFFFFFFFFFF
@A00155:342:HHGFNDSXY:1:1153:24261:29418 2:N:0:GAACCTAG+TCCGCATA
TTTCAACCATGCGTCCACCCCGGTTAATTTTGACCCGTGACGGTAATACAATAGCTCGGTAAGCTTGG
GTGAGTCAAAATCTAGTCTCGCTCTAGACCTTCTTGACCATCTGGCAGTCAGCATAAGGACGATAGTT
+
FFFFFFFF,FFFFFFFFFFFFFFFFFFFFFFFFFFFFFFFFFFFFFFFFFFFFFFFFFFFFFFFFFFF
FFFFFFFFFFFFFFFFFFFFFFFFFFF:FFFFFFFFFFFF:FFF,FFFFFFFFFFFFFFFFFFFFFFF
@A00155:342:HHGFNDSXY:1:2274:31421:26600 2:N:0:GAACCTAG+TCCGCATA
TTTCAACCATGCGTCCACCCCGGTTAATTTTGACCCGTGACGGTAATACAATAGCTCGGTAAGCTTGG
GTGAGTCAAAATCTAGTCTCGCTCTAGACCTTCTTGACCATCTGGCAGTCAGCATAAGGACGATAGTT
+
FFFFFFFFFFFFFFFFFFFFFFFFFFFFFFFFFFFFFFF:FFF:FFFFFFF,FFFFFFFFFFFFFFFF
FFFFFFFFFFFFFFFFFFFFFFFFFFFFFFFFFFFFFFFFFFFFFFFFFFFFFFFFFFFFFFFFFFFF
@A00155:342:HHGFNDSXY:1:2425:7301:12226 2:N:0:GAACCTAG+TCCGCATA
CAACCATGCGTCCACCCCGGTTAATTTTGACCCGTGACGGTAATACAATAGCTCGGTAAGCTTGGGTG
AGTCAAAATCTAGTCTCGCTCTAGACCTTCTTGACCATCTGGCAGTCAGCATAAGGACGATAGTTGC
+
F:FF:FFFFFFFFFFFFF:FFFFFFF:FFFFFFFFFFFFFFFF:FFFF,:FFFFFFF:FFFFFFFFFF
FFFFFFFFFFFFFFFFFFFFF:FFFFFFFFFFFFFFFFFFF:FFFFFFFFFFFFFFFFFF:FF:FFF
@A00155:342:HHGFNDSXY:1:1138:31485:7639 2:N:0:GAACCTAG+TCCGCATA
CAACCATGCGTCCACCCCGGTTAATTTTGACCCGTGACGGTAATACAATAGCTCGGTAAGCTTGGGTG
AGTCAAAATCTAGTCTCGCTCTAGACCTTCTTGACCATCTGGCAGTCAGCATAAGGACGATAGTTGCC
+
FFFFFFFFFFFFFFFFFFFFFFFFFFFFFFFFFFFFFFFFFFF,FFFF:FFFFFFFFFFFF,FFFFFF
FF:FFFFFFFFFFFFFFF:FF:FFFFFFFFFFFFFFF::FFFFFFFFFFFFFFFFFFFF:FF:FFFFF
@A00155:342:HHGFNDSXY:1:2557:26955:8124 2:N:0:GAACCTAG+TCCGCATA
CAACCATGCGTCCACCCCGGTTAATTTTGACCCGTGACGGTAATACAATAGCTCGGTAAGCTTGGGTG
AGTCAAAATCTAGTCTCGCTCTAGACCTTCTTGACCATCTGGCAGTCAGCATAAGGACGATAGTTGCC
+
FFFFFF:FFFFFFFFFFFFFFFFFFFFFFFFF:FFFFF:FFFFFFFFFFFFFFFFFFFFFFFFFFFFF
FFFFFFFFFFFFFFFFFFFFFF,FFFFFFFFFFFFFFFFFFFFFFFFFFFFFFFFFFFFFFFF:FFFF
@A00155:342:HHGFNDSXY:1:1633:9534:8766 1:N:0:GAACCTAG+TCCGCATA
AACCATGCGTCCACCCCGGTTAATTTTGACCCGTGACGGTAATACAATAGCTCGGTAAGCTTGGGTGA
GTCAAAATCTAGTCTCGCTCTAGACCTTCTTGACCATCTGGCAGTCAGCATAAGGACGATAGTTGCCT
+
FFFFFFFFFFFFFFFFFFFFFFFFFFFFFFFFFFFFFFFFFFFFFFFFFFFFFFFFFFFFFFFFFFFF
FFFFFFFFFFFFFFFFFFFFFFFFFFFFFFFFFFFFFFFFFFFFFFFFFFFFFFFFFFFFFFFFFFFF
@A00155:342:HHGFNDSXY:1:1633:9688:17957 1:N:0:GAACCTAG+TCCGCATA
AACCATGCGTCCACCCCGGTTAATTTTGACCCGTGACGGTAATACAATAGCTCGGTAAGCTTGGGTGA
GTCAAAATCTAGTCTCGCTCTAGACCTTCTTGACCATCTGGCAGTCAGCATAAGGACGATAGTTGCCT
+
FFFFFFFFFFFFFFFFFFFFFFFFFFFFFFFFFFFFFFFFFFFFFFFFFFFFFFFFFFFFFFFFFFFF
FFFFFFFFFFFFFFFFFFFFFFFFFFFFFFFFFFFFFFFFFFFFFFFFFFFFFFFFFFFFFFFFFFFF
@A00155:342:HHGFNDSXY:1:1416:6262:35383 2:N:0:GAACCTAG+TCCGCATA
ACCATGCGTCCACCCCGGTTAATTTTGACCCGTGACGGTAATACAATAGCTCGGTAAGCTTGGGTGAG
```

```
TCAAAATCTAGTCTCGCTCTAGACCTTCTTGACCATCTGGCAGTCAGCATAAGGACGATAGTTGC
+
FFF,FFFFF,FFFFFF,:FFFFF:FFFFFFFFFFFFFFFFFFF:FFFFF:FFFFFFFF,FFFFFFFFF
FFFF:FFFFFFFFFFFFFFFFFFF:F:FFFFFFFFFFFF,FFFFFFFFFFFFFFFFFFFFFFFFF
@A00155:342:HHGFNDSXY:1:1112:3233:34992 2:N:0:GAACCTAG+TCCGCATA
CATGCGTCCACCCCGGTTAATTTTGACCCGTGACGGTAATACAATAGCTCGGTAAGCTTGGGTGAGTC
AAAATCTAGTCTCGCTCTAGACCTTCTTGACCATCTGGCAGTCAGCATAAGGACGATAGTTGCCTCCA
+
FFFFFFFFFFFFFFFFF:FF:F:F::FFFFFFFF:FFFFFFF,FFFFFFFFFF:FFFFFFFFFFFFFF
FFFFFFFFFFFFFFFFFFFFFFFFFFFF:FFFFFFFFFFFFFFFFFFFF:FFFFFFFFFFFFFFFFFF
@A00155:342:HHGFNDSXY:1:1234:24162:19069 1:N:0:GAACCTAG+TCCGCATA
ATGCGTCCACCCCGGTTAATTTTGACCCGTGACGGTAATACAATAGCTCGGTAAGCTTGGGTGAGTCA
AAATCTAGTCTCGCTCTAGACCTTCTTGACCATCTGGCAGTCAGCATAAGGACGATAGTTGCCTCCAT
+
FFFFFFFFFFFFFFFFFFFFFFFFFFFFFFFFFFFFF,FFFFFFFFFFFFFFFFFF:FFFFFFFFFFF
FFFFFFFFFFFFFFFFFFFFFFFFF:FFFFFFFFFFFFFFFFFFFFFFFFFFFFFFFFFFFFFFFFFF
@A00155:342:HHGFNDSXY:1:2554:20799:25081 1:N:0:GAACCTAG+TCCGCATA
TGCGTCCACCCCGGTTAATTTTGACCCGTGACGGTAATACAATAGCTCGGTAAGCTTGGGTGAGTCAA
AATCTAGTCTCGCTCTAGACCTTCTTGACCATCTGGCAGTCAGCATAAGGACGATAGTTGCC
+
FFFFFFFFFFFFFFFFFFFFFFFFFFFFFFFFFFFFFFFFFFFFFF:FFFFFFFFFFFFFFFFFFFFF
FFFFFFFFFFFFFFFFFFFFFFFFFFFFFFFFFFFFFFFFFFFFFFFFFFFFFFF:FFFFFF
@A00155:342:HHGFNDSXY:1:1613:3432:32612 1:N:0:GAACCTAG+TCCGCATA
TGCGTCCACCCCGGTTAATTTTGACCCGTGACGGTAATACAATAGCTCGGTAAGCTTGGGTGAGTCAA
AATCTAGTCTCGCTCTAGACCTTCTTGACCATCTGGCAGTCAGCATAAGGACGATAGTTGCCTCCATG
+
FFFFFFFFFFFFFFFFFFFFFFFFFFFFFFFFFFFFF:FFF,FFFFFFFFFF:FFF,FFFFFFFFFFF
FFFFFFFFFFFFFFFFFFFFF:FFFFFFFFFFFFFFFFFFFFFFFFFFFFFFFFFFFFFFFFFFFFFF
@A00155:342:HHGFNDSXY:1:2557:26955:8124 1:N:0:GAACCTAG+TCCGCATA
GCGTCCACCCCGGTTAATTTTGACCCGTGACGGTAATACAATAGCTCGGTAAGCTTGGGTGAGTCAAA
ATCTAGTCTCGCTCTAGACCTTCTTGACCATCTGGCAGTCAGCATAAGGACGATAGTTGCCTCCATGA
+
FFFFFFFFFFFFFFFFFFFFFFFFFFFFFFFFFFF:FFFFFFFFFFFFFFF,FFFFFFFFFFFFFFFF
FFFFFFFFFFFFFFFFFFFFFFFFFFFFFFFFF:FFFFFFFFFFFFFFFFFFFF,FFFFFFFFFFFFF
@A00155:342:HHGFNDSXY:1:1137:22724:25629 2:N:0:GAACCTAG+TCCGCATA
GCGTCCACCCCGGTTAATTTTGACCCGTGACGGTAATACAATAGCTCGGTAAGCTTGGGTGAGTCAAA
ATCTAGTCTCGCTCTAGACCTTCTTGACCATCTGGCAGTCAGCATAAGGACGATAGTTGCCTCCATGA
+
F:FFFFFFFFFFFFFFF:FFFFFFFFFFFFFFFFFFFFFFFFFFFFFFFFFFFFFFFFFFFFFFFFFF
FFFFFFFFFFFFFFFFFFFFFFFFFFFFFFFFFFFFFFFFFFFFFFFFFFFFFFFFFFFFFFFFFFFF
@A00155:342:HHGFNDSXY:1:2524:22860:32972 1:N:0:GAACCTAG+TCCGCATA
GCGTCCACCCCGGTTAATTTTGACCCGTGACGGTAATACAATAGCTCGGTAAGCTTGGGTGAGTCAAA
ATCTAGTCTCGCTCTAGACCTTCTTGACCATCTGGCAGTCAGCATAAGGACGATAGTTGCCTCCATGA
+
FFFFFFFFFFFFFFFFFFFFFFFFFFFFFFFFFFFFFFFFFFFFFFFFFFFFFFF:FFFFFFFFFFFF
FFFFFFFFFFFFFFFFFFFFFFFFFFFFFF:FFFFFFFFFFFFFF,FFFFFF:FFF,FFFFFFFF:FF
@A00155:342:HHGFNDSXY:1:2222:28700:1752 1:N:0:GAACCTAG+TCCGCATA
CGTCCACCCCGGTTAATTTTGACCCGTGACGGTAATACAATAGCTCGGTAAGCTTGGGTGAGTCAAAA
TCTAGTCTCGCTCTAGACCTTCTTGACCATCTGGCAGTCAGCATAAGGACGATAGTTGCC
+
FFFFFFFFFFFFFFFFFFFFFFFFFFFFFFFF:FFFFFFFFFFF:FF:FFFFFFFFFF:FFFFFFFFF
FFFFFFFFFFFFFFFFFF:FFFFFFFFFFFFFFFFFFFFFFFFFFFFFFFF:FFFFFFFF
@A00155:342:HHGFNDSXY:1:2674:6334:11146 1:N:0:GAACCTAG+TCCGCATA
CGTCCACCCCGGTTAATTTTGACCCGTGACGGTAATACAATAGCTCGGTAAGCTTGGGTGAGTCAAAA
```

```
TCTAGTCTCGCTCTAGACCTTCTTGACCATCTGGCAGTCAGCATAAGGACGATAGTTGCC
+
FFFFFFFFFFFFFFFFFFFFFFFFFFFF:FFFFFFFFFFFFFFFFFF:FFFF:FFFFFFFFFFFFFFF
F,FF:FFFFFFFFFFFFF,FFFFFF,FFFFFFFFF:FFFFFFFFF:FFFF:FFFF:FFFF
@A00155:342:HHGFNDSXY:1:1335:13340:27508 1:N:0:GAACCTAG+TCCGCATA
CGTCCACCCCGGTTAATTTTGACCCGTGACGGTAATACAATAGCTCGGTAAGCTTGGGTGAGTCAAAA
TCTAGTCTCGCTCTAGACCTTCTTGACCATCTGGCAGTCAGCATAAGGACGATAGTTGCCTCCATGA
+
FFFFFFFFFFFFFFFFFFFFFFFFFFFFFFFFFFFFFFFFFFFFFFFFFFFFFFFFFFFFFFFFFFFF
FFFFFFFFFFFFFFFFFFFF:FFFFFFFFFFFFFFFFFFFFFFFFFFFFFFFFFFFFFFFFFFFFFFF
@A00155:342:HHGFNDSXY:1:2527:10836:16219 1:N:0:GAACCTAG+TCCGCATA
CGTCCACCCCGGTTAATTTTGACCCGTGACGGTAATACAATAGCTCGGTAAGCTTGGGTGAGTCAAAA
TCTAGTCTCGCTCTAGACCTTCTTGACCATCTGGCAGTCAGCATAAGGACGATAGTTGCCTCCATGA
+
FF:F:FFFFFFF:FFF:FFFF,FFFFFFFFF,,FFFFFFFFFFF,:FFFFFFFFFFFFFF:FFFFFF:
FFFFFFFFFFF:,FFFF::F:FFFF:F:F,FFFF,FFFFF::FFFFFFF:,F:FFFFF:,FFFFFFF
@A00155:342:HHGFNDSXY:1:1532:17237:27649 1:N:0:GAACCTAG+TCCGCATA
CGTCCACCCCGGTTAATTTTGACCCGTGACGGTAATACAATAGCTCGGTAAGCTTGGGTGAGTCAAAA
TCTAGTCTCGCTCTAGACCTTCTTGACCATCTGGCAGTCAGCATAAGGACGATAGTTGCCTCCATGAC
+
FFFFFFFFFFFFFFFFFFFFFFFFFFFFFFFFFFFF:FFFFF:F::FFFF:FF,,FFFFFFFF:FFFF
FFFFFFFFF:FFFFFFF,FFF:FFFFFFFFFFFFFFFFFFFFFFFFFFFFFFFFFF::FFFFFFFFFF
@A00155:342:HHGFNDSXY:1:2310:7139:12790 1:N:0:GAACCTAG+TCCGCATA
GTCCACCCCGGTTAATTTTGACCCGTGACGGTAATACAATAGCTCGGTAAGCTTGGGTGAGTCAAAAT
CTAGTCTCGCTCTAGACCTTCTTGACCATCTGGCAGTCAGCATAAGGACGATAGTTGCCTCCATGACG
+
FFFFFFFFFFFFFFFFFFFFFFFFFFFFFFF:FFFFFFFFFFFFFFFFFFFF,FFFFFFFF:FFFFFF
FFFFFFFFFF:FFFFFFF:FFFFFFFFFFFFFFFFFFFFFFFFFF:FFFFFFFFFFFFFFFFFFFFFF
@A00155:342:HHGFNDSXY:1:1302:5683:9987 2:N:0:GAACCTAG+TCCGCATA
GTCCACCCCGGTTAATTTTGACCCGTGACGGTAATACAATAGCTCGGTAAGCTTGGGTGAGTCAAAAT
CTAGTCTCGCTCTAGACCTTCTTGACCATCTGGCAGTCAGCATAAGGACGATAGTTGCCTCCATGACG
+
FFFFFFFFFFFFFFFFFFFFFFFFFFFFFF,FFFFFF:FFFFFFFFFFFFFFFFFFFFFFFFFFFFFF
FFFFFFFFFFFFFFFFF:FFFFFFFFFFFFFFFFFFFFFFFFFFFFFFFFFFFFFFFFFFFFFFFF:F
@A00155:342:HHGFNDSXY:1:1625:5421:15734 2:N:0:GAACCTAG+TCCGCATA
GTCCACCCCGGTTAATTTTGACCCGTGACGGTAATACAATAGCTCGGTAAGCTTGGGTGAGTCAAAAT
CTAGTCTCGCTCTAGACCTTCTTGACCATCTGGCAGTCAGCATAAGGACGATAGTTGCCTCCATGACG
+
FF,FFFFFF,,FFFFFF:FFFFFFFFFFFFFFFFF:FFFFFFFFFF:FFFF,FF:FFF,FFFFFFFFF
,FF,F:FFFFFFFFFFFFFFFFFFFFFFFFFFFFFFFFFF,FFFFFFFF:,FFFFFF,FF:,,FFFFF
@A00155:342:HHGFNDSXY:1:1633:9534:8766 2:N:0:GAACCTAG+TCCGCATA
CCACCCCGGTTAATTTTGACCCGTGACGGTAATACAATAGCTCGGTAAGCTTGGGTGAGTCAAAATCT
AGTCTCGCTCTAGACCTTCTTGACCATCTGGCAGTCAGCATAAGGACGATAGTTGCCTCCATGACGTT
+
FFFFFFFFFFFFFFFFFFFFFFFFFFFFFFFFFFFFFFFFFFFFFFFFFFFFFFFFFFFFFFFFFFFF
FFFFFFFFFFFFFFFFFFFFFFFFFFFFFFFFFFFFFFFFFFFFFFFFFFFFFFFFFFFFFFFFFFFF
@A00155:342:HHGFNDSXY:1:1633:9688:17957 2:N:0:GAACCTAG+TCCGCATA
CCACCCCGGTTAATTTTGACCCGTGACGGTAATACAATAGCTCGGTAAGCTTGGGTGAGTCAAAATCT
AGTCTCGCTCTAGACCTTCTTGACCATCTGGCAGTCAGCATAAGGACGATAGTTGCCTCCATGACGTT
+
FFFFFFFFFFFFFFFFFFFFFFFFFFFFFFFFFFFFFFFFFFFFFFFFFFFFFFFFFFFFFFFFFFFF
FFFFFFFFFFFFFFF:FFFFFFFFFFFFFFFFFFFFFFFF:FFFFFFF:FFFFFFFFFFF:FFFFFFF
@A00155:342:HHGFNDSXY:1:2263:2338:32252 1:N:0:GAACCTAG+TCCGCATA
CCGGTTAATTTTGACCCGTGACGGTAATACAATAGCTCGGTAAGCTTGGGT
```

+
FFFFFFFFFFFFFFFFFFFFFFFFFFFFFFFFFFFFFFFFFFFFFFFFFFF
@A00155:342:HHGFNDSXY:1:2163:32922:31516 1:N:0:GAACCTAG+TCCGCATA
CCGGTTAATTTTGACCCGTGACGGTAATACAATAGCTCGGTAAGCTTGGGT
+
FFFFFFFFFFFFFFFFFFFFFFFFFFFFFFFFFFFFFFFFFFFFFFFFFFF
@A00155:342:HHGFNDSXY:1:1322:22932:34287 2:N:0:GAACCTAG+TCCGCATA
CCGGTTAATTTTGACCCGTGACGGTAATACAATAGCTCGGTAAGCTTGGGTGAGTCAAAATCTAGTCT
CGCTCTAGACCTTCTTGACCATCTGGCAGTCAGCATAAGGACGATAGTTGCCTCCATGACGTTTA
+
FFFFFFFFFFFFF:FFFFFFFFFFFFFFFFFFFFFFFFFFFFFFFFFFFFFFFFFFFFFFFFFFFFFF
FFFFFFFFFFFFFFFFFFFFFFFFFFFFFFFFFFFFFFFFFFFFFFF:FFFFFFF:FFFFFFFFF
@A00155:342:HHGFNDSXY:1:1416:6262:35383 1:N:0:GAACCTAG+TCCGCATA
CGGTTAATTTTGACCCGTGACGGTAATACAATAGCTCGGTAAGCTTGGGTGAGTCAAAATCTAGTCTC
GCTCTAGACCTTCTTGACCATCTGGCAGTCAGCATAAGGACGATAGTTGCCTCCATGACGTTTAG
+
FFFFFFFFFFFFFFFFFFFFFFFFFFFFFFFFFFFFFFFFFFFFFFFFFFFFFFFFFFFFFFF:FFFF
FFFFFFFFFFFFFFFFFFFFFFFFFFFFFFFFFFFFFFFFFFFFFFFFFFFFFFFFFFFFF,FFF
@A00155:342:HHGFNDSXY:1:2473:6985:11553 1:N:0:GAACCTAG+TCCGCATA
GGTTAATTTTGACCCGTGACGGTAATACAATAGCTCGGTAAGCTTGGGTGAGTCAAAATCTAGTCTCG
CTCTAGACCTTCTTGACCATCTGGCAGTCAGCATAAGGACGATAGTTGCCTCCATGACGTTTAGCCCC
+
FFFFFFFFFFFFFFFFFFFFFFFFFFFFFFFFFFFFFFFFFFFFFFFFFFFFFFFFFFFFFFFFFFFF
FFFFFFFFFFFFFFFFFFFFFFFFFFFFFFFFFFFFFFFFFFFFFFFFFFFFFFFF:FFFFFFFFFFF
@A00155:342:HHGFNDSXY:1:2149:6180:31610 1:N:0:GAACCTAG+TCCGCATA
GTTAATTTTGACCCGTGACGGTAATACAATAGCTCGGTAAGCTTGGGTGAGTCAAAATCTAGTCTCGC
TCTAGACCTTCTTGACCATCTGGCAGTCAGCATAAGGACGATAGTTGCCTCCATGACGTTTAGCCCCC
+
FFFFFFFFFFFFFFFFFFFFFFFFFFFFFFFFFFFFFFFFFF:FFFFFFFFFFFFFFFFFFFFFFFFF
FFFFF:FFFFFFFFFFFFFFFFFFFFFF:FFFFFFFFFFFFFFFFFFFFFFFFFFFFFFFFFFFFFFF
@A00155:342:HHGFNDSXY:1:2633:13087:20306 1:N:0:GAACCTAG+TCCGCATA
GTTAATTTTGACCCGTGACGGTAATACAATAGCTCGGTAAGCTTGGGTGAGTCAAAATCTAGTCTCGC
TCTAGACCTTCTTGACCATCTGGCAGTCGGCATAAGGACGATAGTTGCCTCCATGACGTTTAGCCCCC
+
FFFFFFFFFFFFFFFFFFFFFFF:FFFFFFFFFFFFFFFFFFFFFFFFFFFFFFFFFFFFFFFFFFFF
FFFFFFFFFFFFFFFFFFFFFFFFFFFF,FFFFFFFFFFFFFFFFFF:FFFFFFFFFFFFFFFFFFFF
@A00155:342:HHGFNDSXY:1:1639:20166:29747 1:N:0:GAACCTAG+TCCGCATA
GTTAATTTTGACCCGTGACGGTAATACAATAGCTCGGTAAGCTTGGGTGAGTCAAAATCTAGTCTCGC
TCTAGACCTTCTTGACCATCTGGCAGTCAGCATAAGGACGATAGTTGCCTCCATGACGTTTAGCCCCC
+
FFFFFFFFFFFFFFFFFFFFFFFFFFFFFFFFFFFFFFFFFFFFFFFFFFFFFFFFFFFFFFFFFFFF
FFFFFFFFFFFFFFFFFFFF:FFFFFFFFFFFFFFFFFFFFF,FFFFFFFFF:FFFFFFFFFFFFFFF
@A00155:342:HHGFNDSXY:1:1107:2320:6386 1:N:0:GAACCTAG+TCCGCATA
GTTAATTTTGACCCGTGACGGTAATACAATAGATCGGTAAGCTTGGGTGAGTCAAAATCTAGTCTCGC
TCTAGACCTTCTTGACCATCTGGCAGTCAGCATAAGGACGATAGTTGCCTCCATGACGTTTAGCCCCC
+
FFFFFFFFFFFFF:FFF:FFFFFFFFFFFFF:,FFFFFFFF,:FFFFFFFFFFFFFFFFFFFFFFFFF
FFFFFFFFFFFFF:FFFFFFFFFFFFFFF:FFFFFFFF,FFFFFFFFF:FFFFFFFFFFFFFFFFFFF
@A00155:342:HHGFNDSXY:1:1424:19018:22028 1:N:0:GAACCTAG+TCCGCATA
GTTAATTTTGACCCGTGACGGTAATACAATAGCTCGGTAAGCTTGGGTGAGTCAAAATCTAGTCTCGC
TCTAGACCTTCTTGACCATCTGGCAGTCAGCATAAGGACGATAGTTGCCTCCATGACGTTTAGCCCCC
+
FFFFFFFFFFFFFFFFFFFFFFF,FFFFFFFFFFFFFFFFFFFFFFFFFFFFFFFFFFFFFFFF:FFF
FFF:FFFF,FFFFFFFFFFFFFFFFFFFFFF,,FFFFFFFF,FFFFFFFFFFFFFFFFFFFFFFFFFF

```
@A00155:342:HHGFNDSXY:1:1340:12536:20290 1:N:0:GAACCTAG+TCCGCATA
GTTAATTTTGACCCGTGACGGTAATACAATAGCTCGGTAAGCTTGGGTGAGTCAAAATCTAGTCTCGC
TCTAGACCTTCTTGACCATCTGGCAGTCAGCATAAGGACGATAGTTGCCTCCATGACGTTTAGCCCCC
+
FFFFFFFFFFFFFFFFFFFFFF,FFFFFFFFFFFFFFFF:FFFFFFFFFFFFFFFFFFFFFFFFFFFF
FFFFFFFFFFFFFFFFFFFFFFFFFFFFFFFFFFFFFFFFFFFF:FFFFFFFFFFFFFFFFFFFFFFF
@A00155:342:HHGFNDSXY:1:2320:30825:18490 1:N:0:GAACCTAG+TCCGCATA
GTTAATTTTGACCCGTGACGGTAATACAATAGCTCGGTAAGCTTGGGTGAGTCAAAATCTAGTCTCGC
TCTAGACCTTCTTGACCATCTGGCAGTCAGCATAAGGACGATAGTTGCCTCCATGACGTTTAGCCCCC
+
FFFFFFFFFFFFFFFFFFFFFFFFFFFFFFFFFFFFFFFFFFFFFFFFFFFFFFFFFFFFFFFFFFFF
FFFFFFFFF:FFFFFFFF:FFFFFFFFFFFFFFFFFFFFFFFFFFFFFFFFFFFFFFFFFFFFFFFFF
@A00155:342:HHGFNDSXY:1:1137:22724:25629 1:N:0:GAACCTAG+TCCGCATA
GTTAATTTTGACCCGTGACGGTAATACAATAGCTCGGTAAGCTTGGGTGAGTCAAAATCTAGTCTCGC
TCTAGACCTTCTTGACCATCTGGCAGTCAGCATAAGGACGATAGTTGCCTCCATGACGTTTAGCCCCC
+
FFFFFFFFFFFFFFFFFFFFFFFFFFFFFFFFFFFFFFFFFFFFFFFFFFFFFFFFF:FF:FFFFFFF
FFFFFFFF:FFFFFFFFFFFFFFFFFFFFFFFFFFFFFFFFFFFFFFFFFFFFFFFFF:FFFFFFFF
@A00155:342:HHGFNDSXY:1:1601:30996:23390 1:N:0:GAACCTAG+TCCGCATA
GTTAATTTTGACCCGTGACGGTAATACAATAGCTCGGTAAGCTTGGGTGAGTCAAAATCTAGTCTCGC
TCTAGACCTTCTTGACCATCTGGCAGTCAGCATAAGGACGATAGTTGCCTCCATGACGTTTAGCCCCC
+
,FFFFFFFF,FFFFF,:FF,:FFF:FFFFFFFFFFFFFF,,FFF::FFFF::FFFFFF,:FF:F:F::
F:FFFFFFF:FF,FFF,FFF:FFFF::FF:FFFFFFFFFFFFFFF,FFFFFFFFFFF,FFFF,:FFFF
@A00155:342:HHGFNDSXY:1:2621:5764:34021 1:N:0:GAACCTAG+TCCGCATA
GTTAATTTTGACCCGTGACGGTAATACAATAGCTCGGTAAGCTTGGGTGAGTCAAAATCTAGTCTCGC
TCTAGACCTTCTTGACCATCTGGCAGTCAGCATAAGGACGATAGTTGCCTCCATGACGTTTAGCCCCC
+
FFFFFFFFFFFFFFFFFFFFFFFFFFFFFFFFFFFFFFFFFFFFFFFFFFFFFFFFFFFFFFFFFFFF
FFFFFFFFFFFFFFFFFF:FFF:FFFFFFFF:FFFFFFFFFFFFFFFFFFFFFFFFFFFFFFFFFFFF
@A00155:342:HHGFNDSXY:1:1340:11053:21104 1:N:0:GAACCTAG+TCCGCATA
GTTAATTTTGACCCGTGACGGTAATACAATAGCTCGGTAAGCTTGGGTGAGTCAAAATCTAGTCTCGC
TCTAGACCTTCTTGACCATCTGGCAGTCAGCATAAGGACGATAGTTGCCTCCATGACGTTTAGCCCCC
+
FFFFFFFFFFFFFFFFFFFFFFFFFFFFFFFFFFFFFFF:FFFFFFFFFFFFFFFFFFFFFF,FFFFF
FFFFFFFF,FFFFFFFFFFF:FFFFFFFFFFF:FFFFFFFF:FFFFFFFFFFFFFFFFFFFFFFFFFF
@A00155:342:HHGFNDSXY:1:1237:14814:21292 1:N:0:GAACCTAG+TCCGCATA
GTTAATTTTGACCCGTGACGGTAATACAATAGCTCGGTAAGCTTGGGTGAGTCAAAATCTAGTCTCGC
TCTAGACCTTCTTGACCATCTGGCAGTCAGCATAAGGACGATAGTTGCCTCCATGACGTTTAGCCCCC
+
FFFFFFFFFFFFFFFFFFFFFFFFFFFFFFFFFFFFFFFFFFFFFFFFFFFFFFFFFFFFFFFFFFFF
FFFFFFFFFFFFFFFFFFFFFFFFFFFFFFFFFFFFFFFF:FFFFFFFFFFFFFFFF:FFFFFFFFFF
@A00155:342:HHGFNDSXY:1:2409:15763:11945 1:N:0:GAACCTAG+TCCGCATA
TTAATTTTGACCCGTGACGGTAATACAATAGCTCGGTAAGCTTGGGTGAGTCAAAATCTAGTCTCGCT
CTAGACCTTCTTGACCATCTGGCAGTCAGCATAAGGACGATAGTTGCCTCCATGACGTTTAGCCCCC
+
::FFFFF:FF:FFFFF:FF:F,,FFFFFFFFF:FFF:FFFFFF::FFFFFF:FF:F,F:FFFFF:FFF
FFFFF:,,:F:F:F:FF:FF,FFFF::FFFFF,F,FFFFF:FF,,,FFFFFF,FFF:F,FFF,FF:F
@A00155:342:HHGFNDSXY:1:1557:30662:16892 2:N:0:GAACCTAG+TCCGCATA
TTAATTTTGACCCGTGACGGTAATACAATAGCTCGGTAAGCTTGGGTGAGTCAAAATCTAGTCTCGCT
CTAGACCTTCTTGACCATCTGGCAGTCAGCATAAGGACGATAGTTGCCTCCATGACGTTTAGCCCCCG
+
FFFFFFFFFFFFFFFFFFFFFFFFFFFFFFFFFFFFFFFFFFFFFF,FFFFFFFFFFFFFFFFFFFFF
FFFFFFFFFFFFFFFFFFFFFFFFFFFFFFFFFFFFFFFFFFFFFFFFFFFFFFFFFFFFFFFFFFFF
```

```
@A00155:342:HHGFNDSXY:1:1109:7572:9126 2:N:0:GAACCTAG+TCCGCATA
TAATTTTGACCCGTGACGGTAATACAATAGCTCTGTAAGCTTGGGTGAGTCAAAATCTAGTCTCGCTC
TAGACCTTCTTGACCATCTGGCAGTCAGCATAAGGACGATAGTTGCCTCCATGACGTTTAGCCCCCGC
+
FFFFFFFFFFF:FFFFFFFFFFFFFFFF:FFFF,FF,FF,,FF,FFFFFFFF,FFFFFFFFFFFFFFF
FFF:FFFFFFFFFF:FFFF:F,FFFFFFFFFFFFFF,FFFFFFF,,:FF::FF:F:FFFFFFFFFFFF
@A00155:342:HHGFNDSXY:1:2624:27236:22263 2:N:0:GAACCTAG+TCCGCATA
TAATTTTGACCCGTGACGGTAATACACTAGCTCGGTAAGCTTGGGTGAGTCAAAATCTAGTCTCGCTC
TAGACCTTCTTGACCATCTGGCAGTCAGCATAAGGACGATAGTTGCCTCCATGACGTTTAGCCCCCGC
+
FFFFFFFFFFFFFFFFFFFFFFFFFF,F:FFFFFFFFFFFFFFFF,FFFFFF,F:FF:FFFFFFFFFF
FFFFFFFFFFFFFFFFFFFFFFFFFFFFFFFFFFFFFFFFFF:FFFF,FFFFFFFFFFFF:FFFFFFF
@A00155:342:HHGFNDSXY:1:1266:1461:6308 1:N:0:GAACCTAG+TCCGCATA
TAATTTTGACCCGTGACGGTAATACAATAGCTCGGTAAGCTTGGGTGAGTCAAAATCTAGTCTCGCTC
TAGACCTTCTTGACCATCTGGCAGTCAGCATAAGGACGATAGTTGCCTCCATGACGTTTAGCCCCCGC
+
FFFFFFFFFFFFFFFFFFFFFFFFFFFFFFFFFFFFFFFFFF:FFFFFFFFFFFFFFFF:FFFFFFFF
FFFFFFFFFFFFFF::FFFFFFFFFFFFFFF:FFFFFFFFFFFFFFFFFFFFF,FFFFFFFFFFFFFF
@A00155:342:HHGFNDSXY:1:2624:29134:17942 2:N:0:GAACCTAG+TCCGCATA
TAATTTTGACCCGTGACGGTAATACAATAGCTCGGTAAGCTTGGGTGAGTCAAAATCTAGTCTCGCTC
TAGACCTTCTTGACCATCTGGCAGTCAGCATAAGGACGATAGTTGCCTCCATGACGTTTAGCCCCCGC
+
FFFFFFFF:FFFFFFFFFFFFFFFFFFFFFFFFFFFFFF:FFFFFFFFFFFFFFFFFFFFFFFFFFF:
FFFFFFFFFFFFFFFFFFFFFFFFFFFFFFFFFFFFFFFFFFFFFFFFFFFFFFFFFFFFFFFFFFFF
@A00155:342:HHGFNDSXY:1:2459:3161:11976 1:N:0:GAACCTAG+TCCGCATA
TAATTTTGACCCGTGACGGTAATACAATAGCTCGGTAAGCTTGGGTGAGTCAAAATCTAGTCTCGCTC
TAGACCTTCTTGACCATCTGGCAGTCAGCATAAGGACGATAGTTGCCTCCATGACGTTTAGCCCCCGC
+
FFFFFFFFFFFFFFFFFFFFFFFFFFFFFFFFFFFFFFFFFFFFFFFFFFFFFFFFFFFFFFFFFFFF
FFFFFFFFFFFFFFFFFFFFFFFFFFFF:FFFFFFFFFFFFFFFFFFFFFFFFFFFFFFFF:FFFFFF
@A00155:342:HHGFNDSXY:1:1438:5981:2362 1:N:0:GAACCTAG+TCCGCATA
TTTTGACCCGTGACGGTAATACAATAGCTCGGTAAGCTTGGGTGAGTCAAAATCTAGTCTCGCTCTAG
ACCTTCTTGACCATCTGGCAGTCAGCATAAGGACGATAGTTGCCTCCATGACGTTTAGCCCCCGCAGG
+
FFFFFFFFFFFFFFFFFF:FFFFFFFFFFFFFFFFFFFFFFFFFFFFFFFFFFFFFFFFFF:FFFFFF
FFFFFFFFFFFFFFFFFFFFFFFFFFFFFFFFFFFFFFFFFFFFFFFFFFFFFFFFFFFFFFFFFFFF
@A00155:342:HHGFNDSXY:1:2461:17092:33567 2:N:0:GAACCTAG+TCCGCATA
TTTTGACCCGTGACGGTAATACAATAGCTCGGTAAGCTTGGGTGAGTCAAAATCTAGTCTCGCTCTAG
ACCTTCTTGACCATCTGGCAGTCAGCATAAGGACGATAGTTGCCTCCATGACGTTTAGCCCCCGCAGG
+
FFFFFFFFFFFFFFFFFFFFFFFF,:FFFFFFFFFFFFFFFF::FFFFFFF:FFFFF:FFFFFFFFFF
FFFFFFFFFFFFFFFFFFFFFFFFFFFFFFFFFFFFFFF:FFFFFFFFFFFFFFFFFFFFFFFFFFFF
@A00155:342:HHGFNDSXY:1:2650:19162:28541 1:N:0:GAACCTAG+TCCGCATA
TTTTGACCCGTGACGGTAATACAATAGCTCGGTAAGCTTGGGTGAGTCAAAATCTAGTCTCGCTCTAG
ACCTTCTTGACCATCTGGCAGTCAGCATAAGGACGATAGTTGCCTCCATGACGTTTAGCCCCCGCAGG
+
F:FFFFFFFFFFFFFFFFFFFFFFFFFFFFFFFFFFFFFFFFFFFFFFFFFF:FFFFFFFFFFFFFFF
FFFFFFFFFFFFFFFFFFFFFFFFFFFFFFFFFFFFFFFFFFFFFFFFFFFFFFFFFFFFFFFFFFFF
@A00155:342:HHGFNDSXY:1:1672:10248:15608 2:N:0:GAACCTAG+TCCGCATA
TTTGACCCGTGACGGTAATACAATAGCTCGGTAAGCTTGGGTGAGTCAAAATCTAGTCTCGCTCTAGA
CCTTCTTGACCATCTGGCAGTCAGCATAAGGACGATAGTTGCCTCCATGACGTTTAGCCCCCGCAGGT
+
F:FFF:FFFFFFFFFFFF:FFFFFFFFFFFFFFFFFFFFFF,FFFFFFFFFFFFFFFFFFFFFFFFFF
FFFFFFFFFFFFFFFFFFFFFFFFFFFFFFFFFFFFFFFFFFFFFFF,FFFFFFFFFFFFFFFFFFFF
```

```
@A00155:342:HHGFNDSXY:1:1107:2320:6386 2:N:0:GAACCTAG+TCCGCATA
TTTGACCCGTGACGGTAATACAATAGCTCGGTAAGCTTGGGTGAGTCAAAATCTAGTCTCGCTCTAGA
CCTTCTTGACCATCTGGCAGTCAGCATAAGGACGATAGTTGCCTCCATGACGTTTAGCCCCCGCAGGT
+
FFFFF:FFFFFFFFFFFFFFFFF:F:FFFFFFFF,,FFFFF:FFFFFF:FFFFFFF:FF:FFF:FFFF
FFFFFFFFFFFFFFFF:FFFFFF::FFFFF:FFFFFF:FFFF,FF:FFFF:FFFFFF,FFFFFFFFFF
@A00155:342:HHGFNDSXY:1:1601:30996:23390 2:N:0:GAACCTAG+TCCGCATA
TTTGACCCGTGACGGTAATACAATAGCTCGGTAAGCTTGGGTGAGTCAAAATCTAGTCTCGCTCTAGA
CCTTCTTGACCATCTGGCAGTCAGCATAAGGACGATAGTTGCCTCCATGACGTTTAGCCCCCGCAGGT
+
FFFF:F:FFFFFFFF:FFFF:F:F:FFFFFFFF:FF:FFF,FF,FFFFFFF,FFFFFFFFFFF:FFFF:
FFFF,::F::F:F:FFF,FFFFFFFF,FFFFFFFFF:,FFFFFFFFF,F:FFF:F:,FFFF,FFFFFF
@A00155:342:HHGFNDSXY:1:1625:4327:9392 2:N:0:GAACCTAG+TCCGCATA
TTTGACCCGTGACGGTAATACAATAGCTCGGTAAGCTTGGGTGAGTCAAAATCTAGTCTCGCTCTAGA
CCTTCTTGACCATCTGGCAGTCAGCATAAGGACGATAGTTGCCTCCATGACGTTTAGCCCCCGCAGGT
+
FF:FFFFFFFFFFFFFFFFF,F:FFFFFFFFFFFF:FFFFFFFFF:FFFFFFFFFFFFFFFFFFFFF
FFFFFFFFFFFF:FFFF:FFFFFFFFFFFFFFFFF:FFFFFFFFFFFFFFFFFFFFFFFFFFFFFFFF
@A00155:342:HHGFNDSXY:1:2428:16450:16360 1:N:0:GAACCTAG+TCCGCATA
TTTGACCCGTGACGGTAATACAATAGCTCGGTAAGCTTGGGTGAGTCAAAATCTAGTCTCGCTCTAGA
CCTTCTTGACCATCTGGCAGTCAGCATAAGGACGATAGTTGCCTCCATGACGTTTAGCCCCCGCAGGT
+
FFFF:FFFFFFFFFFF,FFFFFFFFFFFFFFFFFFFFFFFFFFFFFFFFFFFFFFFFFFFFFFFFFFFF
FFFFFFFFF:FFFFFF:FFFFFFFFFFFFFFFFFFFFF:FFFFFFFFFF,FFF,FFFFFFFFFFFFFF
@A00155:342:HHGFNDSXY:1:2162:19452:8187 1:N:0:GAACCTAG+TCCGCATA
TTGACCCGTGACGGTAATACAATAGCTCGGTAAGCTTGGGTGAGTCAAAATCTAGTCTCGCTCTAGAC
CTTCTTGACCATCTGGCAGTCAGCATAAGGACGATAGTTGCCTCCATGACGTTTAGCCCCCGCAGGTC
+
FFFFFFFFFFFFFF:FFFFFFFFFFFFFFFFFFFFFFFFFFFFFFFFFFFFFFFFFFFFFFFFFFFFF
FFFFFFFFFFFFFFFFFFFFFFFFFFFFFFFFFFFFFFFFFFFFFFFFFFFFFFFFFFFFFFFFFFFF
@A00155:342:HHGFNDSXY:1:2631:20943:19695 1:N:0:GAACCTAG+TCCGCATA
TTGACCCGTGACGGTAATACAATAGCTCGGTAAGCTTGGGTGAGTCAAAATCTAGTCTCGCTCTAGAC
CTTCTTGACCATCTGGCAGTCAGCATAAGGACGATAGTTGCCTCCATGACGTTTAGCCCCCGCAGGTC
+
FFFFFFFFFFFFFFFFFFFFFFFFFFFFFFFF:FF:FFFFFFFFFFFFFF:FFFFFFFFFFFFFFFFF
F:FFFFFFFFF:FFFFFFFFFFFFFFFFFFFFFFFFFFFFFFFFFF:FFFFFFFFFFFFFFFFFFFFF
@A00155:342:HHGFNDSXY:1:1472:6506:3709 2:N:0:GAACCTAG+TCCGCATA
TGACCCGTGACGGTAATACAATAGCTCGGTAAGCTTGGGTGAGTCAAAATCTAGTCTCGCTCTAGACC
TTCTTGACCATCTGGCAGTCAGCATAAGGACGATAGTTGCCTCCATGACGTTTAGCCCCCGCAGGTCC
+
FFFFFFFFFFFFF:FFFFFF:FFFFFFFFFFFFFFFFFF,FFFFFFFFFFF:FFFFFFFFFFFFFFFF
FFFFFFFFFFFFFFFFFFFFFFFFFFFFFFFFFFFFFFFFFF,FF:FFFFFFFFFFFFFFFFFFFF
@A00155:342:HHGFNDSXY:1:2557:12355:10113 1:N:0:GAACCTAG+TCCGCATA
TGACCCGTGACGGTAATACAATAGCTCGGTAAGCTTGGGTGAGTCAAAATCTAGTCTCGCTCTAGACC
TTCTTGACCATCTGGCAGTCAGCATAAGGACGATAGTTGCCTCCATGACGTTTAGCCCCCGCAGGTCC
+
FFFFFFFFFFFFFF:FFFFFFFFFFFFFFFFFFFFFFFFFFFFFFFFFFFFFF:FFFFFFFFFFFFFFF
FFFFFFFFFFFFFFFFFFFFFFFFFFFFFFFFFFFFFFFFFFFFFFFFFF:FFFFFFFFFFFFFFFF
@A00155:342:HHGFNDSXY:1:1144:6307:10316 1:N:0:GAACCTAG+TCCGCATA
TGACCCGTGACGGTAATACAATAGCTCGGTAAGCTTGGGTGAGTCAAAATCTAGTCTCGCTCTAGACC
TTCTTGACCATCTGGCAGTCAGCATAAGGACGATAGTTGCCTCCATGACGTTTAGCCCCCGCAGGTCC
+
FFFFFFFFFFFFFFFFFFFFFFFFFFFFFFFFFFFFFFFFFFFFFF:F:FFFFFFFFFF:F:FFFFFFF
FFFFFFFFFFFFFFFFFFFFFFFFFFFFFFFFFFFFFF:FFFFFFFFFFFFF,FFFFFFFFFFFFFFFF
```

```
@A00155:342:HHGFNDSXY:1:2557:12454:10911 1:N:0:GAACCTAG+TCCGCATA
TGACCCGTGACGGTAATACAATAGCTCGGTAAGCTTGGGTGAGTCAAAATCTAGTCTCGCTCTAGACC
TTCTTGACCATCTGGCAGTCAGCATAAGGACGATAGTTGCCTCCATGACGTTTAGCCCCCGCAGGTCC
+
FFFFFFFFFFFFFFF:FFFFFFFFFFFFFFFFFF:FFFFFFFFFFFFFFFFFFFFFFFFFFFFFFF:F
FFFFFFFFFFFFFFFFFFFFFFFFF,FFFFFFFF:FFFFFFFFFFFFFFF:FFFFFFFFFFFFFFFFF
@A00155:342:HHGFNDSXY:1:2205:13666:17832 1:N:0:GAACCTAG+TCCGCATA
GACCCGTGACGGTAATACAATAGCTCGGTAAGCTTGGGTGAGTCAAAATCTAGTCTCGCTCTAGACCT
TCTTGACCATCTGGCAGTCAGCATAAGGACGATAGTTGCCTCCATGACGTTTAGCCCCCGCAGGTCCA
+
FFFFFFFFFFFFF:FFFFFFFFFFFFFFFFFFFFFFFFFFFFFFFFFFFFFFFFFFFFFFFFFFFFFF
FFFFFFFF:FFFFFFFFFFFFFFFFFFFFFFFFFFFFFFFFFFFFFFFFFFFFFFFFFFFFFFFFFFF
@A00155:342:HHGFNDSXY:1:2633:13087:20306 2:N:0:GAACCTAG+TCCGCATA
ACCCGTGACGGTAATACAATAGCTCGGTAAGCTTGGGTGAGTCAAAATCTAGTCTCGCTCTAGACCTT
CTTGACCATCTGGCAGTCGGCATAAGGACGATAGTTGCCTCCATGACGTTTAGCCCCCGCAGGTCCAC
+
FFFFFFFFFFFFFFFFFFFFFFFFFFFFFFFFFFFFFFFFFFFFFFFFFFFFFFFFFFFFFFFFFFFF
FFFFFFFFFFFFFFFFFF,FFFFFFFFFFFFFFFFFFFFFFFFFFFFFFFFFFFFFFFFFFFFFFFFF
@A00155:342:HHGFNDSXY:1:1678:7482:30107 1:N:0:GAACCTAG+TCCGCATA
ACCCGTGACGGTAATACAATAGCTCGGTAAGCTTGGGTGAGTCAAAATCTAGTCTCGCTCTAGACCTT
CTTGACCATCTGGCAGTCAGCATAAGGACGATAGTTGCCTCCATGACGTTTAGCCCCCGCAGGTCCAC
+
FFFFFFFFFFFFFFF:FFFFFF:FFFFF:FFFFFFF,FFFF:F:FFFFFFFFFFFFFFFFFFFFFFFF
FFF,FFFFFFFFFFFFFFFFFFFFFFFFFFFFFFFFFFFF:FFF:FF,FFFFFFFFFFFFFFFFFFFF
@A00155:342:HHGFNDSXY:1:1339:12093:12759 2:N:0:GAACCTAG+TCCGCATA
ACCCGTGACGGTAATACAATAGCTCGGTAAGCTTGGGAGAGTCAAAATCTAGTCTCGCTCTAGACCTT
CTTGACCATCTGGCAGTCAGCATAAGGACGATAGTTGCCTCCATGACGTTTAGCCCCCGCAGGTCCAC
+
:FFFFFFFFFFFFFFFFFF:FFFFFFFFFFF:FFFFF,FFFFFFFFFFFFFFFFF:FFFFFFFFFFFF
FFFFFFFFFFFFFFFFFFFFFFFFFFFFFFFFFFFFFFFFFFFFFFFFFFFFFFFFFFFFFFFFFFFF
@A00155:342:HHGFNDSXY:1:2463:2383:18740 2:N:0:GAACCTAG+TCCGCATA
ACCCGTGACGGTAATACAATAGCTCGGTAAGCTTGGGAGAGTCAAAATCTAGTCTCGCTCTAGACCTT
CTTGACCATCTGGCAGTCAGCATAAGGACGATAGTTGCCTCCATGACGTTTAGCCCCCGCAGGTCCAC
+
FFFFF:FFFFFFFFFFFFFFFFFFFFFFFFFFFFFFF,FFFFFFFFFFFFFFFFFFFFFFFFFFFFF:
FFFFFFFFFFFFFFFFFFFFFFFFFFFFFFFFFFFFFFFFFFFFFFFFFFFFFFFFFFFFFFFFFFFF
@A00155:342:HHGFNDSXY:1:2354:9815:5744 1:N:0:GAACCTAG+TCCGCATA
ACCCGTGACGGTAATACAATAGCTCGGTAAGCTTGGGTGAGTCAAAATCTAGTCTCGCTCTAGACCTT
CTTGACCATCTGGCAGTCAGCATAAGGACGATAGTTGCCTCCATGACGTTTAGCCCCCGCAGGTCCAC
+
FFFFFFFFFFFFFFFFFFFFFFFFFFFFFFFFFFFFFFFFFFFFFFFFFFFFFFFFFFFFFFFFFFFF
FFFFFFFFFFFFFFFFFFFFFFF:FFFFFFFFFFFFFFFFFFFFFFFFFFFFFFFFFFFFFFFFFFFF
@A00155:342:HHGFNDSXY:1:2378:20283:4648 1:N:0:GAACCTAG+TCCGCATA
ACCCGTGACGGTAATACAATAGCTCGGTAAGCTTGGGTGAGTCAAAATCTAGTCTCGCTCTAGACCTT
CTTGACCATCTGGCAGTCAGCATAAGGACGATAGTTGCCTCCATGACGTTTAGCCCCCGCAGGTCCAC
+
FFFFFFFFFFFFFFFFFFFFFFFFFFF:FFFFFFFFFFFFFFFFFFFFFFFFFFFFFFFFFFFFFFFF
FFFFFFFFFFFFFFFFFFFFFFFFFFFFFFFFFFFFFFFFFFFFFFFFFFFFFFFFFFFFFFFFFFFF
@A00155:342:HHGFNDSXY:1:1222:3070:7498 2:N:0:GAACCTAG+TCCGCATA
ACCCGTGACGGTAATACAATAGCTCGGTAAGCTTGGGTGAGTCAAAATCTAGTCTCGCTCTAGACCTT
CTTGACCTTCTGGCAGTCAGCATAAGGACGATAGTTGCCTCCATGACGTTTAGCCCCCGCAGGTCCAC
+
FFFFFFFFFFFFFFFFFFFFFFFFFFFFFFFFFFFFFFFFFFFFFFFFFF:FFFFFFFFFFFFFFFFF
FFFFF:F:,FFFFFFFFFFFFFFFFFFFFF:FFFFFFFFFFFFFFFFFFFFFFFFFFFFFFFFFFF:F
```

```
@A00155:342:HHGFNDSXY:1:1477:16821:28995 1:N:0:GAACCTAG+TCCGCATA
ACCCGTGACGGTAATACAATAGCTCGGTAAGCTTGGGTGAGTCAAAATCTAGTCTCGCTCTAGACCTT
CTTGACCATCTGGCAGTCAGCATAAGGACGATAGTTGCCTCCATGACGTTTAGCCCCCGCAGGTCCAC
+
FFFFFFFFFFFFFFFFFFFFFFFFFFFF:FFF::FFFFFFFFFFFFFFFFFFFFFFFFFFFFFFFFFF
FFFFFFFFFFFFFFFFFFFFFFFFFF:FFFF,F:FFFFFFFFFFFFFFFFFFFFFFFFFFFFF:FFFF
@A00155:342:HHGFNDSXY:1:1327:14714:28385 1:N:0:GAACCTAG+TCCGCATA
ACCCGTGACGGTAATACAATAGCTCGGTAAGCTTGGGTGAGTCAAAATCTAGTCTCGCTCTAGACCTT
CTTGACCATCTGGCAGTCAGCATAAGGACGATAGTTGCCTCCATGACGTTTAGCCCCCGCAGGTCCAC
+
FFFFFFFFFFFFFFFFFFFFFFFFFFFFFFFFFFFFFFFFFFFFFFFFFFFFFFFFFFFFFFFFFFFF
FFFFFFFFFFFFFFFFFFFFF:FFFFFFFFFFFFFFFFFFFFFFFFFF:FFFFFFFFFFFFFFFFFFF
@A00155:342:HHGFNDSXY:1:1234:24162:19069 2:N:0:GAACCTAG+TCCGCATA
ACCCGTGACGGTAATACAATAGCTCGGTAAGCTTGGGTGAGTCAAAATCTAGTCTCGCTCTAGACCTT
CTTGACCATCTGGCAGTCAGCATAAGGACGATAGTTGCCTCCATGACGTTTAGCCCCCGCAGGTCCAC
+
FFFFFFFFFFFFFFFFFFFFFFFFFFFFFFF:FFFFF,FFFFFFF:FFFFFFFFFFFFFFFFFFFFFF
FFFFFFFFFFFFFFFFFFFFFFFFFFFFFFFFFFFFFFFFFFFFFFFFFFFFFFFFFFFFFFFFFFF:
@A00155:342:HHGFNDSXY:1:1478:23773:21840 1:N:0:GAACCTAG+TCCGCATA
ACCCGTGACGGTAATACAATAGCTCGGTAAGCTTGGGTGAGTCAAAATCTAGTCTCGCTCTAGACCTT
CTTGACCATCTGGCAGTCAGCATAAGGACGATAGTTGCCTCCATGACGTTTAGCCCCCGCAGGTCCAC
+
FFFFFFFFFFFFFFFFFFF:FFFFFFFFFFFFFFFFFFF:FFFFFFFFFF:FFFFFFFFFFF,FFFFF
FFFFFFFFFFFFFFFFFFFFFFFFFFFFFFFFFF,FFFFFFFFFFFF:FFFFFFFFFFFFFFFFFFFF
@A00155:342:HHGFNDSXY:1:2233:20889:28056 1:N:0:GAACCTAG+TCCGCATA
CCCGTGACGGTAATACAATAGCTCGGTAAGCTTGGGTGAGTCAAAATCTAGTCTCGCTCTAGACCTTC
TTGACCATCTGGCAGTCAGCATAAGGACGATAGTTGCCTCCATGACGTTTAGCCCCCGCAGGTCCACG
+
FFFFFFFFFFFFFFFFFFFFFFFFFFFFFFFFFFFFFFFFFFFFFF,FFFFFFFFFFFFFFFFFFFFF
FFFFFFFFFFFFFFFFFFFFFFFFFFFFFFFFF:FFFFFFFFFFFFFFFFFFFFFFFFFFFFFFFFFF
@A00155:342:HHGFNDSXY:1:2438:11252:3787 1:N:0:GAACCTAG+TCCGCATA
CCCGTGACGGTAATACAATAGCTCGGTAAGCTTGGGTGAGTCAAAATCTAGTCTCGCTCTAGACCTTC
TTGACCATCTGGCAGTCAGCATAAGGACGATAGTTGCCTCCATGACGTTTAGCCCCCGCAGGTCCACG
+
FFFFFFFFFFFFFFFFFFFFFFFFFFFFFFFFFFFFFFFFFFFFFFFFFFFFFFFFFFFFFFFFFFFF
FFFFFFFFFFFFFFFFFFFFFFFFFFFFFFFFFFFFFFFFFFFFFFFFFFFFFFFFFFFFFFFFFFFF
@A00155:342:HHGFNDSXY:1:2314:31304:21073 1:N:0:GAACCTAG+TCCGCATA
CCCGTGACGGTAATACAATAGCTCGGTAAGCTTGGGTGAGTCAAAATCTAGTCTCGCTCTAGACCTTC
TTGACCATCTGGCAGTCAGCATAAGGACGATAGTTGCCTCCATGACGTTTAGCCCCCGCAGGTCCACG
+
FFFFFFFFFF:FFFFFFFFFFFFFFFFFFFFFFFFFFFFFFFFFFFFFFFF:FFFFFFFFFFFFFFFF
FFF:FFF:FFFFFFFFF:FF:FFFFFFFFFFFFFFFFFFFFFFFFFFFFFFFFFFFFFFFFFFFFFF
@A00155:342:HHGFNDSXY:1:1322:22932:34287 1:N:0:GAACCTAG+TCCGCATA
CCGTGACGGTAATACAATAGCTCGGTAAGCTTGGGTGAGTCAAAATCTAGTCTCGCTCTAGACCTTCT
TGACCATCTGGCAGTCAGCATAAGGACGATAGTTGCCTCCATGACGTTTAGCCCCCGCAGGTCCA
+
FFFFFFFFFFFFFFFFFFFFFFFFFFFFFFFFFFFFFFFFFFFFFFFFFFFFFFFFFFFFFFFFFFFF
FF:FFFFFFFFFFFFFFFFFFFFFFFFFFFFFFFFFFFFFFFF:FFFFFFFFFFFFFFFFFFFFF
@A00155:342:HHGFNDSXY:1:2307:32687:16110 1:N:0:GAACCTAG+TCCGCATA
CCGTGACGGTAATACAATAGCTCGGTAAGCTTGGGTGAGTCAAAATCTAGTCTCGCTCTAGACCTTCT
TGACCATCTGGCAGTCAGCATAAGGACGATAGTTGCCTCCATGACGTTTAGCCCCCGCAGGTCCACGG
+
FFFFFFFFFFFFFFFFFFFFFFFFFFFFFFFFFFFFFFFFFFFFFFFFFFFFFFFFFFFFFFFF:FFF
FFFFFFFFFFFFFFFFFFFFFFFFFFFFFFFFFFFFFFFFFFFFFFFFFFFFFFFFFFFFFFFFFFFF
```

```
@A00155:342:HHGFNDSXY:1:1511:23005:13495 1:N:0:GAACCTAG+TCCGCATA
CCGTGACGGTAATACAATAGCTCGGTAAGCTTGGGTGAGTCAAAATCTAGTCTCGCTCTAGACCTTCT
TGACCATCTGGCAGTCAGCATAAGGACGATAGTTGCCTCCATGACGTTTAGCCCCCGCAGGTCCACGG
+
FFFFFFFFF:FFFFFFFFFFF:FFFFFFFF:FFFFFFFFFFFFFFFFFFFFFFFFFFFFFFFFF:FFF
FFFFFF:FFFFFFFFFFFFFFFFFFFFFFFFFFFFF:FFFFFFFFFFFFFFFFFFFFFFFFFFFFFFF
@A00155:342:HHGFNDSXY:1:1610:7229:1517 1:N:0:GAACCTAG+TCCGCATA
CCGTGACGGTAATACAATAGCTCGGTAAGCTTGGGTGAGTCAAAATCTAGTCTCGCTCTAGACCTTCT
TGACCATCTGGCAGTCAGCATAAGGACGATAGTTGCCTCCATGACGTTTAGCCCCCGCAGGTCCACGG
+
FFFFFFFFFFFFFFFFFFF:FFFFFFF:F,FFFFFFFFF:FFFFFFFFF:FFFFFFFFFFFFFFFFFF
FFFFFFFFFFFFFFFFFFFFFFFF:FFFFFFFFFF:FFFFFFFFFFFFFFFFFFFFFFFFFFFFFFFF
@A00155:342:HHGFNDSXY:1:1212:20961:26929 1:N:0:GAACCTAG+TCCGCATA
CCGTGACGGTAATACAATAGCTCGGTAAGCTTGGGTGAGTCAAAATCTAGTCTCGCTCTAGACCTTCT
TGACCATCTGGCAGTCAGCATAAGGACGATAGTTGCCTCCATGACGTTTAGCCCCCGCAGGTCCACGG
+
FFFFFFFFFFFFFFFFFFFFFFFFFFFFFFFFFFFFFFFFFFFFFFFFFFFFFFFFFFF:FFFFFFFF
FFFFFFFFFFFFFFFFFFFFFFFFFFFFFFFFFFFFFFFFFFFFFFFFFFFFFFFFFFFFFFFFFFFF
@A00155:342:HHGFNDSXY:1:2304:3558:14105 1:N:0:GAACCTAG+TCCGCATA
CCGTGACGGTAATACAATAGCTCGGTAAGCTTGGGTGAGTCAAAATCTAGTCTCGCTCTAGACCTTCT
TGACCATCTGGCAGTCAGCATAAGGACGATAGTTGCCTCCATGACGTTTAGCCCCCGCAGGTCCACGG
+
F,:,FF:F,F:FFFFFFF::FFF,FFFF:FFFF,F,FFF:FFF:,:,,:,FFFFFFFFFFFFFFF,:,
FFFFFF:FFF,,FFFFF:,FFFFF,FF,F:F,F,,F,FFFF,:FF:FFFFFF,FFF,:,,:FFFFF:F
@A00155:342:HHGFNDSXY:1:1547:8449:11365 2:N:0:GAACCTAG+TCCGCATA
CGTGACGGTAATACAATAGCTCGGTAAGCTTGGGTGAGTCAAAATCTAGTCTCGCTCTAGACCTTCTT
GACCATCTGGCAGTCAGCATATGGACGATAGTTGCCTCCATGACGTTTAGCCCCCGCAGGTCCACGGC
+
FFFFFFFFFFFFFFFFFFFFFFFF:FFFFFFFFFFFFFFFFFFFFFFFFFFFFFFFFFFFFFFFFFFF
FFFFFFFFFFFFFFFFFFFFF,FFFFFFFFFFFFFFFFFFFFFFFFF:FFFFFFFFFFFFFFFFFFFF
@A00155:342:HHGFNDSXY:1:1267:24424:6840 1:N:0:GAACCTAG+TCCGCATA
CGTGACGGTAATACAATAGCTCGGTAAGCTTGGGTGAGTCAAAATCTAGTCTCGCTCTAGACCTTCTT
GACCATCTGGCAGTCAGCATAAGGACGATAGTTGCCTCCATGACGTTTAGCCCCCGCAGGTCCACGGC
+
FFFFFFFFFFFFFFFFFFFFFFFFFFFFF:FFFFFFFFFFFFFFFFFFFFFFFFFFFFFFFFFFFFFF
F:FFFFFFFFFFFFFFFFFFFFFFFFFFFFFFFFFFFFFF:FFFFFFFF:FFFFFFFFFFFFFFFFFF
@A00155:342:HHGFNDSXY:1:1549:19633:11631 1:N:0:GAACCTAG+TCCGCATA
CGTGACGGTAATACAATAGCTCGGTAAGCTTGGGTGAGTCAAAATCTAGTCTCGCTCTAGACCTTCTT
GACCATCTGGCAGTCAGCATAAGGACGATAGTTGCCTCCATGACGTTTAGCCCCCGCAGGTCCACGGC
+
FFFFFFFFFFFFFF:FFFFFFFFFFFFFFF:FFFFFFFFFFFFFFFFFFFFFFFFFFFFFFFF:FF:F
FFFFFFFFFFFFFFFFFFFFFFFFFFFFFFFFFFFFFFFFFFFFFFFFFFFFFFFFFFFFFFFFFFFF
@A00155:342:HHGFNDSXY:1:1448:15031:19946 1:N:0:GAACCTAG+TCCGCATA
GTGACGGTAATACAATAGCTCGGTAAGCTTGGGTGAGTCAAAATCTAGTCTCGCTCTAGACCTTCTTG
ACCATCTGGCAGTCAGCATAAGGACGATAGTTGCCTCCATGACGTTTAGCCCCCGCAGGTCCACGGCC
+
FFFFFFFFFFFFFFFFFFFFFFFFFFFFFFFFFFFFFFFFFFFFFFFFFFFFFFFFFFFFFFFFFFFF
FFFFFFFFFFFFFFFFFFFFFFFFFFFFFFFFFFFFFFFFFFFFF:FFFFFFFFFFFFFFFFFFFFFF
@A00155:342:HHGFNDSXY:1:1564:26612:33301 1:N:0:GAACCTAG+TCCGCATA
GTGACGGTAATACAATAGCTCGGTAAGCTTGGGTGAGTCAAAATCTAGTCTCGCTCTAGACCTTCTTG
ACCATCTGGCAGTCAGCATAAGGACGATAGTTGCCTCCATGACGTTTAGCCCCCGCAGGTCCACGGCC
+
FFFFFFFFFFFFFFFFFFFFFFFFFFFFFFFFFFFFFFFFFFFFFFFFFFFFFFFFFFFFFFFFFFFF
FFFFFFFFFFFFFF:FFFFFFFFFFFFFFFFFFFFFFFFFFFFFFFFFFFFFFFFFFFFFFFFFFFFF
```

```
@A00155:342:HHGFNDSXY:1:2463:2383:18740 1:N:0:GAACCTAG+TCCGCATA
TGACGGTAATACAATAGCTCGGTAAGCTTGGGTGAGTCAAAATCTAGTCTCGCTCTAGACCTTCTTGA
CCATCTGGCAGTCAGCATAAGGACGATAGTTGCCTCCATGACGTTTAGCCCCCGCAGGTCCACGGCCG
+
FFFFFFFFFFFFFFFFFFFFFFFFFFFFFFFFFFFFFFFFFFFFFFFFFFFFFFFFFFFFFFFFFFFF
FFFFFFFFF:FFFFFFFFFFFFFFFFFFFFFFFFFFFFFFFFFFFFFFFF:FFFFFFFFFFFFFFFFF
@A00155:342:HHGFNDSXY:1:2628:21070:35164 2:N:0:GAACCTAG+TCCGCATA
TGACGGTAATACAATAGCTCGGTAAGCTTGGGTGAGTCAAAATCTAGTCTCGCTCTAGACCTTCTTGA
CCATCTGGCAGTCAGCATAAGGACGATAGTTGCCTCCATGACGTTTAGCCCCCGCAGGTCCACGGCCG
+
FFFFFF:FF:FFFF,FFFFFFFFFFFFFFFFFFFF:FFFFFFFF:FFFFFFFFFFFFFFFFFFFFFFF
FFFFFFFFFFFFFFFFFFFFFFFFFFFFFFFFFFFFFFFFFFF:FFFFFFFFFFFFFFFFFFFFFFFF
@A00155:342:HHGFNDSXY:1:1530:10194:35430 1:N:0:GAACCTAG+TCCGCATA
TGACGGTAATACAATAGCTCGGTAAGCTTGGGTGAGTCAAAATCTAGTCTCGCTCTAGACCTTCTTGA
CCATCTGGCAGTCAGCATAAGGACGATAGTTGCCTCCATGACGTTTAGCCCCCGCAGGTCCACGGCCG
+
FFFFFFFFFFFFFFFFFFFFFFFF:FFFFFFFFFFFFFFFFFFFFFFFFFFFFFFFFFFFFFFFFFFF
FFF:FFFFFFFFFFFFFFFFFFFFFFFFFFFFFFFFFFFFFFFFFFFFFFFFFFFFFFFFFFFFFFFF
@A00155:342:HHGFNDSXY:1:2628:21070:35164 1:N:0:GAACCTAG+TCCGCATA
TGACGGTAATACAATAGCTCGGTAAGCTTGGGTGAGTCAAAATCTAGTCTCGCTCTAGACCTTCTTGA
CCATCTGGCAGTCAGCATAAGGACGATAGTTGCCTCCATGACGTTTAGCCCCCGCAGGTCCACGGCCG
+
FFFFFFF:FFFFFFFFFFFFFFFFFFFFFFFFFFFFFF:FFFFFFFFFFFFFFFFFFFFFFFFFFFFF
FFFFFFFFFFFFFFFFFFFFFFFFFFFFFFFFFFFFFFFFFFFFFFFFFFFFFFFFFFFFFFFFFFFF
@A00155:342:HHGFNDSXY:1:1421:24514:1454 1:N:0:GAACCTAG+TCCGCATA
TGACGGTAATACAATAGCTCGGTAAGCTTGGGTGAGTCAAAATCTAGTCTCGCTCTAGACCTTCTTGA
CCATCTGGCAGTCAGCATAAGGACGATAGTTGCCTCCATGACGTTTAGCCCCCGCAGGTCCACGGCCG
+
FFFFFFFFFFFFFFFFFFFFFFFFFFFFFFF:FFFFFFFFFFFFFFFFFFFFFFFFFFFFFFFFFFF:
FFFFFFFFFFFFFFFFFFFFFFFFFFFFFFFFFFFFFFFFFFF,FFFFFFFFFFFFFF:FFFFFFFFF
@A00155:342:HHGFNDSXY:1:2622:23294:21825 1:N:0:GAACCTAG+TCCGCATA
TGACGGTAATACAATAGCTCGGTAAGCTTGGGTGAGTCAAAATCTAGTCTCGCTCTAGACCTTCTTGA
CCATCTGGCAGTCAGCATAAGGACGATAGTTGCCTCCATGACGTTTAGCCCCCGCAGGTCCACGGCCG
+
FFFFFFFFFFFFFFFFFFFFFFFFFFFFFFFFFFFFFFFFF:FFFFFFFFFFFFFFFFFFFFFFFFFF
FFFFFFFFFFFFFFFFFFFFFFFFFFFFFFFFFFFFFFFFFFFFFFFFFFFFFFFFFFFFFFFFFFFF
@A00155:342:HHGFNDSXY:1:1559:21983:9408 1:N:0:GAACCTAG+TCCGCATA
TGACGGTAATACAATAGCTCGGTAAGCTTGGGTGAGTCAAAATCTAGTCTCGCTCTAGACCTTCTTGA
CCATCTGGCAGTCAGCATAAGGACGATAGTTGCCTCCATGACGTTTAGCCCCCGCAGGTCCACGGCCG
+
:FFFF:FFFFFFFFFFFFFFFFFFFFFFFFFFFFFFFFFFFFFFF:FFFFFFFFFFFFFFFF:FFFFF
FFFFFFFFFFFFFFFFFFFFFFFFFFFFFFF,FFFFFFFFFFF,FFFF:FFFFFFFFFFFFFFFFFFF
@A00155:342:HHGFNDSXY:1:2377:13756:1579 2:N:0:GAACCTAG+TCCGCATA
GACGGTAATACAATAGCTCGGTAAGCTTGGGTGAGTCAAAATCTAGTCTCGCTCTAGACCTTCTTGAC
CATCTGGCAGTCAGCATAAGGACGATAGTTGCCTCCATGACGTTTAGCCCCCGCAGGTCCAC
+
FFFFFFFFFFFFFFFFFFFFFFFFFFFFFFFFFFFFFFFFFFFFFFFFFFFFFFFFFFFFFFFFFFFF
FFFFFFFFFFFFFFFFFFFFFFFFFFFFFFFFFFFFFFFFFFFFFFFFFFFFFFFFFFFFFF
@A00155:342:HHGFNDSXY:1:1212:20961:26929 2:N:0:GAACCTAG+TCCGCATA
GTAATACAATAGCTCGGTAAGCTTGGGTGAGTCAAAATCTAGTCTCGCTCTAGACCTTCTTGACCATC
TGGCAGTCAGCATAAGGACGATAGTTGCCTCCATGACGTTTAGCCCCCGCAGGTCCACGGCCGGGTGC
+
F:F:FFF,,F,FFF:FFFFFFFFFFFF:F,FFFFFF,FFFFFFFFFFFFFFFFFFFFFFFFF,FFFFF
FFFFFFFFFFFFFFFFFFFFFFFFFFFFFFFFF:FFFFFFFFFFFFFFFFFFFFFF:FFFFFFFFFFF
```

```
@A00155:342:HHGFNDSXY:1:1134:24415:23202 2:N:0:GAACCTAG+TCCGCATA
TAATACAATAGCTCGGTAAGCTTGGGTGAGTCAAAATCTAGTCTCGCTCTAGACCTTCTTGACCATCT
GGCAGTCAGCATAAGGACGATAGTTGCCTCCATGACGTTTAGCCCCCGCAGGTCCACGGCCGGGTGC
+
FFFFFFFFFFFFFFFFFFFFFFFFFF:FFFFFF:FFFFFFFFFFFFFFFFFFFFFFFFFFFFFFFFFF
FFFFFFFFFFFFFFFFFFFFFFFFFFFFFFFFFFFFFFFFFFFFFFFFFFFFFFFFFFFFFFFFFFF
@A00155:342:HHGFNDSXY:1:1134:24442:23156 2:N:0:GAACCTAG+TCCGCATA
TAATACAATAGCTCGGTAAGCTTGGGTGAGTCAAAATCTAGTCTCGCTCTAGACCTTCTTGACCATCT
GGCAGTCAGCATAAGGACGATAGTTGCCTCCATGACGTTTAGCCCCCGCAGGTCCACGGCCGGGTGC
+
FFFFFFF,FFFFFFFFFFFFFFFFFFFFFFFFFFF,FFF,FFFFFFFFFFFFFFFFFFFFFFFFFFFF
FFFFFFFFFFFFFFFFFFFFFFF:FFFFFFFFFFFFFFFFFFFFFFFFFFFFFFFFFFFFFFFFFFF
@A00155:342:HHGFNDSXY:1:2622:23294:21825 2:N:0:GAACCTAG+TCCGCATA
AATACAATAGCTCGGTAAGCTTGGGTGAGTCAAAATCTAGTCTCGCTCTAGACCTTCTTGACCATCTG
GCAGTCAGCATAAGGACGATAGTTGCCTCCATGACGTTTAGCCCCCGCAGGTCCACGGCCGGGTGCTC
+
FFFFFFF,FFFFFFFFFFFFFFFFFFFFFFFFFFFFFFFFFFFFFFFFFFFFFFFFFFFFFFFFFFFF
FFFFFFFFFFFFFFFFFFFFFFFFFFFFFFFFFFFFFFFFFFFFFFFFFFFFFFFFFFFFFFFFFFFF
@A00155:342:HHGFNDSXY:1:2566:13096:16814 2:N:0:GAACCTAG+TCCGCATA
ATACAATAGCTCGGTAAGCTTGGGTGAGTCAAAATCTAGTCTCGCTCTAGACCTTCTTGACCATCTGG
CAGTCAGCATAAGGACGATAGTTGCCTCCATGACGTTTAGCCCCCGCAGGTCCACGGCCGGGTGCTCC
+
FFFFFF:FFFFFFFFFFFFFFFFFFFFFFFFFFFFFFFFFFFFFFFFFFFFFFFFFFFFFF:FFFFF
FFFFFFFFFFFFFFFFFFFFFFFFFFFFFFFFFFFFFFFFFFFFFFFFFFFFFFFFFFFFFFFFFFFF
@A00155:342:HHGFNDSXY:1:2468:10556:14168 2:N:0:GAACCTAG+TCCGCATA
ATACAATAGCTCGGTAAGCTTGGGTGAGTCAAAATCTAGTCTCGCTCTAGACCTTCTTGACCATCTGG
CAGTCAGCATAAGGACGATAGTTGCCTCCATGACGTTTAGCCCCCGCAGGTCCACGGCCGGGTGCTCC
+
FFFF:FFFFFFFFFFFFFFFFFFF:FFFFFFFFFFFFFFFFFFFFFFFFFFFFFFFFFFFFFFFFFFF
FFFFFFFFFFFFFFFFFFFFFFFFFFFFFFFFFFFFFFFFFFFFFFFFFFFFFFFFFFFFFFFFFFFF
@A00155:342:HHGFNDSXY:1:2461:11803:19022 2:N:0:GAACCTAG+TCCGCATA
ATACAATAGCTCGGTAAGCTTGGGTGAGTCAAAATCTAGTCTCGCTCTAGACCTTCTTGACCATCTGG
CAGTCAGCATAAGGACGATAGTTGCCTCCATGACGTTTAGCCCCCGCAGGTCCACGGCCGGGTGCTCC
+
FFFFFFFFFFFFFFFFFFFFFFFF:FFFFF:FFFFFFFFFFFFFFFFFFFFFFFFFFFFFFFFFFFFF
FFFFFFFF:FFFFFFFFFFFFFFFFFFFFFFFFFFFFFFFFFFFFFFFFFFFFFFFFFFFFFFFFFF
@A00155:342:HHGFNDSXY:1:2461:11912:18834 2:N:0:GAACCTAG+TCCGCATA
ATACAATAGCTCGGTAAGCTTGGGTGAGTCAAAATCTAGTCTCGCTCTAGACCTTCTTGACCATCTGG
CAGTCAGCATAAGGACGATAGTTGCCTCCATGACGTTTAGCCCCCGCAGGTCCACGGCCGGGTGCTCC
+
FFFFFFFFFFFFFFFFFFFFFFFFFFFFFFFFFFFFFFFFFFFFFFFFFFFFFFFFFFFFFFFFFFFF
FFFFFFFFFFFFFFFFFFFFFFFFFFFFFFFFFFFFFFFFFFFFFFFFFFFFFFFFFFFFFFFFFFFF
@A00155:342:HHGFNDSXY:1:1654:19506:30483 2:N:0:GAACCTAG+TCCGCATA
ATACAATAGCTCGGTAAGCTTGGGTGAGTCAAAATCTAGTCTCGCTCTAGACCTTCTTGACCATCTGG
CAGTCAGCATAAGGACGATAGTTGCCTCCATGACGTTTAGCCCCCGCAGGTCCACGGCCGGGTGCTCC
+
FFFF:F:FFFFFF:FFFFF:FFFF:FFFFFFFFFFFFFFFFFFFFF:FFFFFFFFFFFFFFFFFFFFF
FFFFFFFFFFFFFF:FFFFFF:FFFFFFFFFFFFFFFFFFFFFFFFFFFFFFFFFFFFFFFFFFFFFF
@A00155:342:HHGFNDSXY:1:2275:29993:12195 2:N:0:GAACCTAG+TCCGCATA
ATACAATAGCTCGGTAAGCTTGGGTGAGTCAAAATCTAGTCTCGCTCTAGACCTTCTTGACCATCTGG
CAGTCAGCATAAGGACGATAGTTGCCTCCATGACGTTTAGCCCCCGCAGGTCCACGGCCGGGTGCTCC
+
FFFFFFFFFFFFFFFFFFFFFFFFFFFFFFFFFFFFFFFFFFFFFFFFFFFFFFFFFFFFFFFFFFFF
FFFFFFFFFFFFFFFFFFFFFFFFFFFFFFFFFFFFFFFFFFFFFFFFFFFFFFFFFFFFFFFFFFFF
```

```
@A00155:342:HHGFNDSXY:1:1639:20166:29747 2:N:0:GAACCTAG+TCCGCATA
CAATAGCTCGGTAAGCTTGGGTGAGTCAAAATCTAGTCTCGCTCTAGACCTTCTTGACCATCTGGCAG
TCAGCATAAGGACGATAGTTGCCTCCATGACGTTTAGCCCCCGCAGGTCCACGGCCGGGTGCTCCAGC
+
FFF,FFFFFFFFFFFF:FFFFFFFFFFFFFFFF:FFFFFFFFFFFFFFFFFFFFFFFFFFFFFFFFFF
FFFFFFFFFFFFFFFFFFFFFFFFFFFFFFFFFFFFFFFFFFFFFFFFFFFFFFFFFFFFFFFFFFFF
@A00155:342:HHGFNDSXY:1:1166:12689:33802 1:N:0:GAACCTAG+TCCGCATA
CAATAGCTCGGTAAGCTTGGGTGAGTCAAAATCTAGTCTCGCTCTAGACCTTCTTGACCATCTGGCAG
TCAGCATAAGGACGATAGTTGCCTCCATGACGTTTAGCCCCCGCAGGTCCACGGCCGGGTGCTCCAGC
+
FFFFFFFFFFFFFFFFFFFFFFFFFFFFFFFFFFFFFFFFFFFFFFFFFFFFFFFFFFFFFFFFFFFF
FFFFFFFFFFFFFFF:FFFFFFFFFFFFFFFFF:FFFFFFFFFFFFFFFFFFFFFFFFFFFFFFFFFF
@A00155:342:HHGFNDSXY:1:1606:12237:25817 1:N:0:GAACCTAG+TCCGCATA
CAATAGCTCGGTAAGCTTGGGTGAGTCAAAATCTAGTCTCGCTCTAGACCTTCTTGACCATCTGGCAG
TCAGCATAAGGACGATAGTTGCCTCCATGACGTTTAGCCCCCGCAGGTCCACGGCCGGGTGCTCCAGC
+
FFFFFFFFFFFFFFFFFFFFFFFFFFFFFFFFFFFFFFFFFFFFFFFFF:FFFFFF:FFFFFFFFFFF
FFFFFFFFFFFFFFFFFFFFFFFFFFFFFFFFFFFFFFFFFFFFFFFFFFFFFFFFFFFFFFFFFFFF
@A00155:342:HHGFNDSXY:1:1166:13376:34334 1:N:0:GAACCTAG+TCCGCATA
CAATAGCTCGGTAAGCTTGGGTGAGTCAAAATCTAGTCTCGCTCTAGACCTTCTTGACCATCTGGCAG
TCAGCATAAGGACGATAGTTGCCTCCATGACGTTTAGCCCCCGCAGGTCCACGGCCGGGTGCTCCAGC
+
FFFFFFFFFFFFF:FFFFFFFFFFFFFFFFFFFFFFFFFFFFFFFFFFFF:FFFFFFFFFFFFFFFFF
FFFFFFFFFFFFFFFFFFFFFFFFFFFFFFFFFFFFFFFFFFFFFFFFFFFFFFFFFFFFFFFFFFFF
@A00155:342:HHGFNDSXY:1:2569:29460:5165 2:N:0:GAACCTAG+TCCGCATA
AATAGCTCGGTAAGCTTGGGTGAGTCAAAATCTAGTCTCGCTCTAGACCTTCTTGACCATCTGGCAGT
CAGCATAAGGACGATAGTTGCCTCCATGACGTTTAGCCCCCGCAGGTCCACGGCCGGGTGCTCCAGCG
+
F:FFFFFFFFFFFFFFFFFF:FFFFFFF,FFFF:FFFFFFFFFFFFFF:FFFFFFFFFFFFFFFFFFF
FFFFFFFFFFFFFFFFFFFFFFFFFFFFFFFFFFFFFFFFFFFFFFFFFFFFFFFFFFFFFFFFFFFF
@A00155:342:HHGFNDSXY:1:1147:14407:19085 1:N:0:GAACCTAG+TCCGCATA
ATAGCTCGGTAAGCTTGGGTGAGTCAAAATCTAGTCTCGCTCTAGACCTTCTTGACCATCTGGCAGTC
AGCATAAGGACGATAGTTGCCTCCATGACGTTTAGCCCCCGCAGGTCCACGGCCGGGTGCTCCAGCGC
+
FFFFFFFFFFFFFFFFFFFFFFFFFFFFFFFFFFFFFFFFFFFFFFFF:FFFFFFFFFFFFFFFFFFF
FFFFFFFFFFFFFFFFFFFFFFFFFFFFFFFFFFFFFFFFFFFFFFFFFFFFFFFFFFFFFFFFFFFF
@A00155:342:HHGFNDSXY:1:2349:1579:35383 1:N:0:GAACCTAG+TCCGCATA
ATAGCTCGGTAAGCTTGGGTGAGTCAAAATCTAGTCTCGCTCTAGACCTTCTTGACCATCTGGCAGTC
AGCATAAGGACGATAGTTGCCTCCATGACGTTTAGCCCCCGCAGGTCCACGGCCGGGTGCTCCAGCGC
+
FFFFFFFFFFFFFFFFFFFFFFFFFFFFFFFFFFFFFFFFFFFFFFFFFFFFFFFFFFFFFFFFFFFF
FFFFFFFFFFFFFFFFFFFFFFF:FFFFFFFFFFFFFFFF,FFFFFFFFFFFFFFFFFFFFFFFFFF:
@A00155:342:HHGFNDSXY:1:1651:13910:31125 1:N:0:GAACCTAG+TCCGCATA
ATAGCTCGGTAAGCTTGGGTGAATCAAAATCTAGTCTCGCTCTAGACCTTCTTGACCATCTGGCAGTC
AGCATAAGGACGATAGTTGCCTCCATGACGTTTAGCCCCCGCAGGTCCACGGCCGGGTGCTCCAGCGC
+
FFFFFFFFFFFFFFFFFFFFFFFFFFFFFFFFFFFFFFFFFFFFFFFFFFFFFFFFFFFFFFFFFFFF
FFFFFFFFFFFFFFFFFFFFFFFFFFFFFFFFFFFFFFFFFFFFFFFFFFFFFFFFFFFFFFF:FFFF
@A00155:342:HHGFNDSXY:1:2551:21621:24627 1:N:0:GAACCTAG+TCCGCATA
ATAGCTCGGTAAGCTTGGGTGAGTCAAAATCTAGTCTCGCTCTAGACCTTCTTGACCATCTGGCAGTC
AGCATAAGGACGATAGTTGCCTCCATGACGTTTAGCCCCCGCAGGTCCACGGCCGGGTGCTCCAGCGC
+
FFFFFFFFFFFFFFFFFFFFFFFFFFFFFFFFFFFFFFFFFFFFFFFFFFFFFFFFFFFFFFFFFFFF
FFFFFFFFFFFFFFFFFFFFFFFFFFFFFFFFFFFFFFFFFFFFFFFFFFFFF:FFFFFFFFFFFFFF
```

```
@A00155:342:HHGFNDSXY:1:1542:29704:12821 1:N:0:GAACCTAG+TCCGCATA
ATAGCTCGGTAAGCTTGGGTGAGTCAAAATCTAGTCTCGCTCTAGACCTTCTTGACCATCTGGCAGTC
AGCATAAGGACGATAGTTGCCTCCATGACGTTTAGCCCCCGCAGGTCCACGGCCGGGTGCTCCAGCGC
+
FFFFFFFFFFFFFFFFFFFFFFFFFFFFFFFFFFFFFFFFFFFFFFFFFFFFFFFFFF:FFFFFFFFF
FFFFFFFFFFFFFFFFFFFFFFFFFF,FFFFFFFFF:FFFFFFFFFFFFFFFFFFFFFFFFFFFFFFF
@A00155:342:HHGFNDSXY:1:1537:28203:21277 1:N:0:GAACCTAG+TCCGCATA
TAGCTCGGTAAGCTTGGGTGAGTCAAAATCTAGTCTCGCTCTAGACCTTCTTGACCATCTGGCAGTCA
GCATAAGGACGATAGTTGCCTCCATGACGTTTAGCCCCCGCAGGTCCACGGCCGGGAGCTCCAGCGC
+
:F:,FFFFFFFFF:::,,F:FFFFFFFFFFFF:FFFF:F,FFF,F,FFF,,FFFFFFFF,FF:FFFFF
FFF:FFF,F:FF:FF:::,FFFFFF:,FF:F,FFFFFFF:FFFFFFFFF:FFFF,F,FFFFFFFFFF
@A00155:342:HHGFNDSXY:1:1234:13783:14278 1:N:0:GAACCTAG+TCCGCATA
TAGCTCGGTAAGCTTGGGTGAGTCAAAATCTAGTCTCGCTCTAGACCTTCTTGACCATCTGGCAGTCA
GCATAAGGACGATAGTTGCCTCCATGACGTTTAGCCCCCGCAGGTCCACGGCCGGGTGCTCCAGCGCT
+
FFFFFFFFFFFFFFFFFFFFFFFF:FFFFFFFFFFFFFFFFFFFFFFFFFFFFFFFFFFFFFFFFFFF
FFFFFFFFFFFFFFFFFFFFFFFFFFFFFFFFFFFFFFFFFFFFFFFFFFFFFFFFFFFFFFFFFFFF
@A00155:342:HHGFNDSXY:1:1471:11134:9502 1:N:0:GAACCTAG+TCCGCATA
TAGCTCGGTAAGCTTGGGTGAGTCAAAATCTAGTCTCGCTCTAGACCTTCTTGACCATCTGGCAGTCA
GCATAAGGACGATAGTTGCCTCCATGACGTTTAGCCCCCGCAGGTCCACGGCCGGGTGCTCCAGCGCT
+
FFFFFFFFFFFFFFFFFFFFF:FFFFFFFFFFFFFFFFFFFFFFFFFFFFFFFFFFFFFFFFFFFFFF
FFFFFFFFFFFFFFFFFFFFFFFFFFFFFFFFFFFFFFFFFFFFFFFFF:FFFFFFFFFFFFFFFFFF
@A00155:342:HHGFNDSXY:1:1158:6533:34632 1:N:0:GAACCTAG+TCCGCATA
TAGCTCGTTAAGCTTGGGTGAGTCAAAATCTAGTCTCGCTCTAGACCTTCTTGACCATCTGGCAGTCA
GCATAAGGACGATAGTTGCCTCCATGACGTTTAGCCCCCGCAGGTCCACGGCCGGGTGCTCCAGCGCT
+
FFFFFFF,FFFFF:FFF::FFFFFFF:F,FFF,FFFFFFFFFF,FF:::F::FFF:FFF,FFFF:FFF
FFF:FFFFFFFFFFFFFFFFFFFFFFFFF:FFFF,FFFFFFFF:FFFFFFFFFFFFFFFFFFFFFFFF
@A00155:342:HHGFNDSXY:1:2264:31665:36166 1:N:0:GAACCTAG+TCCGCATA
TAGCTCGGTAAGCTTGGGTGAGTCAAAATCTAGTCTCGCTCTAGACCTTCTTGACCATCTGGCAGTCA
GCATAAGGACGATAGTTGCCTCCATGACGTTTAGCCCCCGCAGGTCCACGGCCGGGTGCTCCAGCGCT
+
FFFFFFFFFFFFFFFFFFFFFFFFFFFFFFFFFFFFFFFFFFFFFFFFFFFFFFFFFFFFFFFFFFFF
FFFFFFFFFFFFFFFFFFFFFFFFFFFFFFFFFFFFFFFFFFFFFFFFFFFFFFFFFFFFFFFFFFFF
@A00155:342:HHGFNDSXY:1:1656:26494:3662 1:N:0:GAACCTAG+TCCGCATA
TAGCTCGGTAAGCTTGGGTGAGTCAAAATCTAGTCTCGCTCTAGACCTTCTTGACCATCTGGCAGTCA
GCATAAGGACGATAGTTGCCTCCATGACGTTTAGCCCCCGCAGGTCCACGGCCGGGTGCTCCAGCGCT
+
:FFFFFFFFFFFFFFFFFFFFFFFFFFFFF:FFFFFFFFFFFFFFFFFFFFFFFFFFFFFFFFFFFFF
FFFFFFFFFFFFFFFFFFFFFFFFFFFFFFFFFFFFFFFFFFFFFFFFFFFFFFFFFFFFFFFFFFFF
@A00155:342:HHGFNDSXY:1:2106:1235:3443 1:N:0:GAACCTAG+TCCGCATA
TAGCTCGGTAAGCTTGGGTGAGTCAAAATCTAGTCTCGCTCTAGACCTTCTTGACAATCTGGCAGTCA
GCATAAGGACGATAGTTGCCTCCATGACGTTTAGCCCCCGCAGGTCCACGGCCGGGTGCTCCAGCGCT
+
FFF:F:FFFFFFFFFFFFFFFFFFFF:FF:FFFFFFFF,FFFF:FFFFFF,FFFF,F,,:FF:FFFFF
FFF::F:F::FFFFFFFFFFFF:FFFFFFFFF,,FFFFFFFFF:FFFF,FFF:FFFFF:FFFFFFFFF
@A00155:342:HHGFNDSXY:1:2225:24804:36933 1:N:0:GAACCTAG+TCCGCATA
TAGCTCGGTAAGCTTGGGTGAGTCAAAATCTAGTCTCGCTCTAGACCTTCTTGACCATCTGGCAGTCA
GCATAAGGACGATAGTTGCCTCCATGACGTTTAGCCCCCGCAGGTCCACGGCCGGGTGCTCCAGCGCT
+
FFFFFFFFFFFFFFFFFFFFFFFFFFFFFFFFFFFFFFFFFFFFFFFFFFFFFFFFFFFFFFFFFFFF
FFFFFFFFFFFFFFFFFFFFFFFFFFFFFFFFFFFFFFFFFFFFFFFFFFFFFFFFFFFFFFFFFFFF
```

```
@A00155:342:HHGFNDSXY:1:1151:29731:21136 1:N:0:GAACCTAG+TCCGCATA
TAGCTCGGTAAGCTTGGGTGAGTCAAAATCTAGTCTCGCTCTAGACCTTCTTGACCATCTGGCAGTCA
GCATAAGGACGATAGTTGCCTCCATGACGTTTAGCCCCCGCAGGTCCACGGCCGGGTGCTCCAGCGCT
+
FF:FFFFFFFFFFFFFFFFFFFFFFFFFFFFFFFFFFFFFFFFFFFFFFFFFFFFFFFF:FFFFFFF:F
FFFFFFFFFFFFFFFFFFFFFFFFFFFFFFFFFFFFFFFFFFFFFFFFFFFFFFFFFFFFFFFFFFFFFF
@A00155:342:HHGFNDSXY:1:2315:3224:10645 1:N:0:GAACCTAG+TCCGCATA
AGCTCGGTAAGCTTGGGTGAGTCAAAATCTAGTCTCGCTCTAGACCTTCTTGACCATCTGGCAGTCAG
CATAAGGACGATAGTTGCCTCCATGACGTTTAGCCCCCGCAGGTCCACGGCCGGGTGCTCCAGCGCT
+
FF,FFFF:F,F,:FF::FF:FFF:FFF,FFF:FFF:FF:FFFFFFFFFF:FF,FFFFFFFF,:FFF,F,,
FFFFF::F:FF:FF:FFFFF:FFFFFFF,FFFFFFFFFFFFF,FFFFFFFFFFFFF:FFF:FF:FF,FF
@A00155:342:HHGFNDSXY:1:2377:13756:1579 1:N:0:GAACCTAG+TCCGCATA
GCTCGGTAAGCTTGGGTGAGTCAAAATCTAGTCTCGCTCTAGACCTTCTTGACCATCTGGCAGTCAGC
ATAAGGACGATAGTTGCCTCCATGACGTTTAGCCCCCGCAGGTCCACGGCCGGGTGCTCCAG
+
FFFFFFFFFFFFFFFFFFFFFFFFFFFFFFFFFFFFFFFFFFFFFFFFFFFFFFFFFFFFFFFFFFFFFF
FFFFFFFFFFFFFFFFFFFFFFFFFFFFFFFFFFFFFFFFFFFFFFFFFFFFFFFFFFFFFFFF
@A00155:342:HHGFNDSXY:1:1411:28275:29481 1:N:0:GAACCTAG+TCCGCATA
CTCGGTAAGCTTGGGTGAGTCAAAATCTAGTCTCGCTCTAGACCTTCTTGACCATCTGGCAGTCAGCA
TAAGGACGATAGTTGCCTCCATGACGTTTAGCCCCCGCAGGTCCACGGCCGGGTGCTCCAGCGCTGGC
+
FFFFFFFFFFFFFF:FFFFFFFFFFFFFFFFFFFFFFFFFFFFF:FFFFFFFFFFFFFFFFFFFFFFFFF
FFFFFFFFFFFFFFFFFFFFFFFFFFFFFFFFFFFFFFFFFFFFFFFFFFFFFFFFFFFFFFFFFFFFFF
@A00155:342:HHGFNDSXY:1:1501:6994:6026 1:N:0:GAACCTAG+TCCGCATA
TCGGTAAGCTTGGGTGAGTCAAAATCTAGTCTCGCTCTAGACCTTCTTGACCATCTGGCAGTCAGCAT
AAGGACGATAGTTGCCTCCATGACGTTTAGCCCCCGCAGGTCCACGGCCGGGTGCTCCAGCGCTGGCG
+
FFFFFFFFFFFFFFFFFFFFFFFFFFFFFFFFFFFFFFF:FFF:FFF:FFFFFF:FFFFFFFFFFFFFFF
FFFFFFFFFFFFFFFFFFFFFFFFFF,FFFFFFFFFFFFFFF,:FFFFFFFFFFFFFFFFFFFFFFFFFF
@A00155:342:HHGFNDSXY:1:2468:10556:14168 1:N:0:GAACCTAG+TCCGCATA
TCGGTAAGCTTGGGTGAGTCAAAATCTAGTCTCGCTCTAGACCTTCTTGACCATCTGGCAGTCAGCAT
AAGGACGATAGTTGCCTCCATGACGTTTAGCCCCCGCAGGTCCACGGCCGGGTGCTCCAGCGCTGGCG
+
FFFFFFFFFFFFFFFFFFFFFFFFFFFFFFFFFFFFFFFFFFFFFFFFFFFFFFFFFFFFFFFFFFFFFF
FFFFFFFFFFFFFFFFFFFFFFFFFFFFFFFFFFFFFFFFFFFFFFFFFFFFFFFFFFFFFFFFFFFFFF
@A00155:342:HHGFNDSXY:1:1561:4155:1736 1:N:0:GAACCTAG+TCCGCATA
TCGGTAAGCTTGGGTGAGTCAAAATCTAGTCTCGCTCTAGACCTTCTTGACCATCTGGCAGTCAGCAT
AAGGACGATAGTTGCCTCCATGACGTTTAGCCCCCGCAGGTCCACGGCCGGGTGCTCCAGCGCTGGCG
+
FFFFFFFFFFFFFFFFFFFFFFFFFFFFFFFFF,FFFFFFFFFFFFFFF:FFFFFFFFFFFFFFFFFFFF
FFFFFFFFFFFFFFFFFFFFFFFFFFFFFFFFF:FFFFFFFF,F,FF:FFFFFFFFFFFFFFFFFFFFFF
@A00155:342:HHGFNDSXY:1:2443:16957:8719 1:N:0:GAACCTAG+TCCGCATA
TCGGTAAGCTTGGGTGAGTCAAAATCTAGTCTCGCTCTAGACCTTCTTGACCATCTGGCAGTCAGCAT
AAGGACGATAGTTGCCTCCATGACGTTTAGCCCCCGCAGGTCCACGGCCGGGTGCTCCAGCGCTGGCG
+
FFFFFFFFFFFFFFFFFFFFFFFFFFFFFFFFFFFFFFFFFFFFFFFFFFFFFFFFFFFFFFFFFFFFFF
FFFFFFFFFFFFFFFFFFFFFFFFFFFFFFFFFFFFFFFFFFFFFFFFFFFFFFFFFFFFF:FFFFFFFF
@A00155:342:HHGFNDSXY:1:2311:24306:36699 1:N:0:GAACCTAG+TCCGCATA
TCGGTAAGCTTGGGTGAGTCAAAATCTAGTCTCGCTCTAGACCTTCTTGACCATCTGGCAGTCAGCAT
AAGGACGATAGTTGCCTCCATGACGTTTAGCCCCCGCAGGTCCACGGCCGGGTGCTCCAGCGCTGGCG
+
FFFFFFFFFFFFFFFFFFFFFFFFFFFFFFFFFFFFFFFFFFFFFFFFFFFFFFFFFFFFFFFFFFFFFF
FFFFFFFFFFFFFFFFFFFFFFFFFFFFFFFFFFFFFFFFFFFFFFFFFFFFFFFFFFFFFFFFFFFFFF
```

```
@A00155:342:HHGFNDSXY:1:2162:19452:8187 2:N:0:GAACCTAG+TCCGCATA
TCGGTAAGCTTGGGTGAGTCAAAATCTAGTCTCGCTCTAGACCTTCTTGACCATCTGGCAGTCAGCAT
AAGGACGATAGTTGCCTCCATGACGTTTAGCCCCCGCAGGTCCACGGCCGGGTGCTCCAGCGCTGGCG
+
FFFFFFFF:FFFFFFFFFFFFFFFFFFFFFFFFFFFFFFFFFFFFFFFFFFFFFFFFFFFFFFFFFFF
FFFFFFFFFFFFFFFFFFFF:FFFFFFFFFFFFFFFFFFFFFFFFFFFFFFFFFFFFFFFFFFFFFFF
@A00155:342:HHGFNDSXY:1:2126:4978:25864 1:N:0:GAACCTAG+TCCGCATA
TCGGTAAGCTTGGGTGAGTCAAAATCTAGTCTCGCTCTAGACCTTCTTGACCATCTGGCAGTCAGCAT
AAGGACGATAGTTGCCTCCATGACGTTTAGCCCCCGCAGGTCCACGGCCGGGTGCTCCAGCGCTGGCG
+
FFFFFFFFFFFFFFFFFFFFFFFFFFFFFFFFFFFFFFFFFFFFFFFFFFFFFFFFFFFFFFFFFFFF
FFFFFFFF:FFFFFFFFFFFFFFFFFFFFFFFFFFFFFFFFFFFFFFFFFFFFFFFFFFFFFFFFFFF
@A00155:342:HHGFNDSXY:1:2442:15718:19445 1:N:0:GAACCTAG+TCCGCATA
TCGGTAAGCTTGGGTGAGTCAAAATCTAGTCTCGCTCTAGACCTTCTTGACCATCTGGCAGTCAGCAT
AAGGACGATAGTTGACTCCATGACGTTTAGCCCCCGCAGGTCCACGGCCGGGTGCTCCAGCGCTGGCG
+
:::FFFFFF::,:F,FFF,FFFFFF,FF:FF:F:FFF,F:FFFF,FFFFFFFFFFFFFFF:FFF,FFF
FF:FFFFFFFF:FF,FFFFF:FFFFFFFFFFF,F,:FF:FFFFF,,F,FFF,FFFFFFF,FF,,FFFF
@A00155:342:HHGFNDSXY:1:2658:17372:13542 1:N:0:GAACCTAG+TCCGCATA
TCGGTAAGCTTGGGTGAGTCAAAATCTAGTCTCGCTCTAGACCTTCTTGACCATCTGGCAGTCAGCAT
AAGGACGATAGTTGCCTCCATGACGTTTAGCCCCCGCAGGTCCACGGCCGGGTGCTCCAGCGCTGGCG
+
FFFFFFFFFFFFFFFFFFFFFFFFFFFFFFFFFFFFFFFFFFFFFFFFFFFFFFFFFFFFFFFFFFFF
FFFFFFFFFFFFFFFFFFFFFFFFF:FFFFFFFFFFFFFFFFFFFFFFFFFFFFFFFF,:FFFFFFFF
@A00155:342:HHGFNDSXY:1:1654:19506:30483 1:N:0:GAACCTAG+TCCGCATA
TCGGTAAGCTTGGGTGAGTCAAAATCTAGTCTCGCTCTAGACCTTCTTGACCATCTGGCAGTCAGCAT
AAGGACGATAGTTGCCTCCATGACGTTTAGCCCCCGCAGGTCCACGGCCGGGTGCTCCAGCGCTGGCG
+
FFFFFFFFF:FFFFFFFFFFFFFFFFFFFFFFFFFFFFFFFFFFFFFFFFFFFFFFF:FFFFFFFFFF
FFFFFFFFFFFFFFFFFFFFFFFFFFFFFFFFFFFFFFFFFFFFFFFFFFFFFFFFFFFFFFFFFFFF
@A00155:342:HHGFNDSXY:1:2631:20943:19695 2:N:0:GAACCTAG+TCCGCATA
TCGGTAAGCTTGGGTGAGTCAAAATCTAGTCTCGCTCTAGACCTTCTTGACCATCTGGCAGTCAGCAT
AAGGACGATAGTTGCCTCCATGACGTTTAGCCCCCGCAGGTCCACGGCCGGGTGCTCCAGCGCTGGCG
+
FFFFFFFFFFFFFF:FFFFFFFFF:FFFFFFFFFFFFFFFFFFFFFFFFFFFFFFFFFFFFFFFFFF
FFFFFFFFFFFFFFFFFFFFFFFFFFFF:FFFFFFFFFFFFFFFFFFFFFFFFFFFFFFFFFFFFFFF
@A00155:342:HHGFNDSXY:1:2658:17210:13730 1:N:0:GAACCTAG+TCCGCATA
TCGGTAAGCTTGGGTGAGTCAAAATCTAGTCTCGCTCTAGACCTTCTTGACCATCTGGCAGTCAGCAT
AAGGACGATAGTTGCCTCCATGACGTTTAGCCCCCGCAGGTCCACGGCCGGGTGCTCCAGCGCTGGCG
+
FF,F,FF,FFFF:F,FFF,FFFFFFFFFF::FFFFFF:FFFFF:F,F:,FFFFFFFFFFFFFFFFF,F
FFF,FF,FFFFFF,FFFF:FFFFFFFFFFFFFFF,FFFFFFFFFFFFFFF:FF::FFFFFFFFFFFFF
@A00155:342:HHGFNDSXY:1:1615:8567:35963 1:N:0:GAACCTAG+TCCGCATA
CGGTAAGCTTGGGTGAGTCAAAATCTAGTCTCGCTCTAGACCTTCTTGACCATCTGGCAGTCAGCATA
AGGACGATAGTTGCCTCCATGACGTTTAGCCCCCGCAGGTCCACGGCCGGGTGCTCCAGCGCTGGCG
+
FFFFFFFFFFFFFFFFFFFFFFFFFFFFFFFFFFFFFFFFFFFFFFFFFFFFFFFFFFFFFFFFFFFF
FFFFFFFFF:FFFFFFFFFFFFFFFFFFFFFFFFFFFFFFFFFFFFFFFFFFFFFFFFFFFFFFF
@A00155:342:HHGFNDSXY:1:2360:31693:13980 1:N:0:GAACCTAG+TCCGCATA
CGGTAAGCTTGGGTGAGTCAAAATCTAGTCTCGCTCTAGACCTTCTTGACCATCTGGCAGTCAGCATA
AGGACGATAGTTGCCTCCATGACGTTTAGCCCCCGCAGGTCCACGGCCGGGTGCTCCAGCGCTGGCG
+
FFFFFFFF:FFFFFFFFFFFFFFFFFFFFFFFFFFFFFFFFFFFFFFFFFFFFFFFFFFFFFFFFFFF
FFFFFFFFFFFFFFFFFFFFFFFFFFFFFFFFFFFFFFFFFFFFFFFFFFFFFFFFFFFFFFFFFFF
```

```
@A00155:342:HHGFNDSXY:1:2645:16631:8970 2:N:0:GAACCTAG+TCCGCATA
CGGTAAGCTTGGGTGAGTCAAAATCTAGTCTCGCTCTAGACCTTCTTGACCATCTGGCAGTCAGCATA
AGGACGATAGTTGCCTCCATGACGTTTAGCCCCCGCAGGTCCACGGCCGGGTGCTCCAGCGCTGGCGT
+
FFFFFFFFFFFFF:FFF:FFFFFFFFFFFFFFFFFFFFFFFFFFFFFFFFFFFFFFFFFFFFFFFFF
FFFFFFFFFFFFFFFFFFF:FFFFFFFFFFFFFFFFFFFFFFFFFFFFFFFFFFFFFFFFFFFFFFF
@A00155:342:HHGFNDSXY:1:1620:9679:4351 1:N:0:GAACCTAG+TCCGCATA
GGTAAGCTTGGGTGAGTCAAAATCTAGTCTCGCTCTAGACCTTCTTGACCATCTGGCAGTCAGCATAA
GGACGATAGTTGCCTCCATGACGTTTAGCCCCCGCAGGTCCACGGCCGGGTGCTCCAGCGCTGGCGTG
+
FFFFFFFFFFFFFFFFFFFFFFFFFFFFFFFFFFFFFFFFFFFFFFFFFFFFFFFFFFFFFFFFFFF
FFFFFFFFFFFFFFFFFFFFFFFFFFFFFFFFFFFFFFFFFFFFFFFFFFFFFFF:FFFFFFFFFFF
@A00155:342:HHGFNDSXY:1:1134:24442:23156 1:N:0:GAACCTAG+TCCGCATA
GTAAGCTTGGGTGAGTCAAAATCTAGTCTCGCTCTAGACCTTCTTGACCATCTGGCAGTCAGCATAAG
GACGATAGTTGCCTCCATGACGTTTAGCCCCCGCAGGTCCACGGCCGGGTGCTCCAGCGCTGGCGTG
+
FFFFFFFFFFFFFFFFFFFFFFFFFFFFFFFFFFFFFFFFFFFFFFFFFFFFFFFFFFFFFFFFFFF
FFFFFFFFFFFFFFFFFFFFFFFFFFFFFFFFFFF:FFF:FFFFFFFFFFFFFFFFFFFFFFFFFF
@A00155:342:HHGFNDSXY:1:1134:24415:23202 1:N:0:GAACCTAG+TCCGCATA
GTAAGCTTGGGTGAGTCAAAATCTAGTCTCGCTCTAGACCTTCTTGACCATCTGGCAGTCAGCATAAG
GACGATAGTTGCCTCCATGACGTTTAGCCCCCGCAGGTCCACGGCCGGGTGCTCCAGCGCTGGCGTG
+
FFFFFFFFFFFFFFFFFFFFFFFFFFFFFFFFFFFFFFFFFFFFFFFFFFFFFFFFFFFFFFFFFFF
FFFFFFFFFFFFFFFFFFFFFFFFFFFFFFFFFFFFFF::FFFFFFFFFFFFFFFFFFFFFFFFFF
@A00155:342:HHGFNDSXY:1:1146:1470:33567 2:N:0:GAACCTAG+TCCGCATA
TAAGCTTGGGTGAGTCAAAATCTAGTCTCGCTCTAGACCTTCTTGACCATCTGGCAGTCAGCATAAGG
ACGATAGTTGCCTCCATGACGTTTAGCCCCCGCAGGTCCACGGCCGGGTGCTCCAGCGCTGGCGTGGT
+
FFFF:FFFFF:FFFFFFF,:FF:F::FFFFFFFFFFFFFFFFFFFFFFFFFFFFFFFFFFFFFFFFF
FFFFFFFFFFFFFFF:FFFFFFFFFFFFFFFFFFFFFFFFF,FFFFFFFFFFFFFFFFFFFFFFFFF
@A00155:342:HHGFNDSXY:1:1530:10194:35430 2:N:0:GAACCTAG+TCCGCATA
TAAGCTTGGGTGAGTCAAAATCTAGTCTCGCTCTAGACCTTCTTGACCATCTGGCAGTCAGCATAAGG
ACGATAGTTGCCTCCATGACGTTTAGCCCCCGCAGGTCCACGGCCGGGTGCTCCAGCGCTGGCGTGGT
+
FFFFFFFFFF,FFFFFFFFFFFFFFFFFFFFFFFFFFFFFF,FFFFFFFFFFFFFFFFFFFFFFFFF
FFFFFFFFFFFFFFFFFFFFFFFFFFFFFFFFFFFFFFFFFFFFFFFFFFFFFFFFFFFFFFFFFFF
@A00155:342:HHGFNDSXY:1:1158:6533:34632 2:N:0:GAACCTAG+TCCGCATA
TAAGCTTGGGTGAGTCAAAATCTAGTCTCGCTCTAGACCTTCTTGACCATCTGGCAGTCAGCATAAGG
ACGATAGTTGCCTCCATGACGTTTAGCCCCCGCAGGTCCACGGCCGGGTGCTCCAGCGCTGGCGTGGT
+
FFFFFFF:FFFFFFFFFFF,F,:FFFFFFFFFFFFFFFFFFF,FFFFFFFFFFFFFFFFFFFFFFFF
FFFF,:F,FFFFFFFFFFFFFFFFFFFFFFFFFFFFFFFFFFFFFFFFFFF,FFFFFF:FFFFF,FF
@A00155:342:HHGFNDSXY:1:2527:10836:16219 2:N:0:GAACCTAG+TCCGCATA
TAAGCTTGGGTGAGTCAAAATCTAGTCTCGCTCTAGACCTTCTTGACCATCTGGCAGTCAGCATAAGG
ACGATAGTTGCCTCCATGACGTTTAGCCCCCGCAGGTCCACGGCCGGGTGCTCCAGCGCTGGCGTGGT
+
FFFFFFFFFF:F:FFF:FF,FFFFFFFFFFF:FFFF:FFFFFFFFFFFFFFFFF,FFFFF:FFFFFF
F:FFFFF:FFFFFFFFFF:FFFFFFFF:FFF,F,FF:FFFFFF,:F,FFFFF:FFFF:FFFFFFFFF
@A00155:342:HHGFNDSXY:1:1514:24876:4899 2:N:0:GAACCTAG+TCCGCATA
TAAGCTTGGGTGAGTCAAAATCTAGTCTCGCTCTAGACCTTCTTGACCATCTGGCAGTCAGCATAAGG
ACGATAGTTGCCTCCATGACGTTTAGCCCCCGCAGGTCCACGGCCGGGTGCTCCAGCGCTGGCGTGGT
+
FFFFFFFFFFFFFFFFFFFFFF:FFFFFF:FFFF:FFFFFFFFFFFFFFFFFFFFFFFFFFFFFFFF
FFFFFFFFFFFFFFFF:FFFFF:,FFFFFFFFFFFF:FFFFFFFFFFFFFFF:FFFFFFFFFFFFFF
```

@A00155:342:HHGFNDSXY:1:1617:7301:18803 2:N:0:GAACCTAG+TCCGCATA
TAAGCTTGGGTGAGTCAAAATCTAGTCTCGCTCTAGACCTTCTTGACCATCTGGCAGTCAGCATAAGG
ACGATAGTTGCCTCCATGACGTTTAGCCCCCGCAGGTCCACGGCCGGGTGCTCCAGCGCTGGCGTGGT
+
FFFFFFFFFFFFFFFFFFF,FFFFFFFFFFFFFFFFFFFFFFFFFFFFFFFFFFFFFFFFFFFFFFFF
FFFFFFFFFFFFFFFFFFFFF:FFFFFFFFFFFFFFFFFFFFFFFFFFFFFFFFFFFFFFFFFFFFFF
@A00155:342:HHGFNDSXY:1:1617:6325:19554 2:N:0:GAACCTAG+TCCGCATA
TAAGCTTGGGTGAGTCAAAATCTAGTCTCGCTCTAGACCTTCTTGACCATCTGGCAGTCAGCATAAGG
ACGATAGTTGCCTCCATGACGTTTAGCCCCCGCAGGTCCACGGCCGGGTGCTCCAGCGCTGGCGTGGT
+
:F,FF,F::FFFF:FFFFFFF:FFFF,FFFFFFFFFFFFF:FFFFFFFFFF:FFFFFFFFFFFFFFFF
FFFFFFFFFFFFFFFF:FFFFFFFFF:F:FFFFFFFFFFFFFFFFFFFFFFFFFFFFFFFFFFFFFFF
@A00155:342:HHGFNDSXY:1:1620:9679:4351 2:N:0:GAACCTAG+TCCGCATA
TAAGCTTGGGTGAGTCAAAATCTAGTCTCGCTCTAGACCTTCTTGACCATCTGGCAGTCAGCATAAGG
ACGATAGTTGCCTCCATGACGTTTAGCCCCCGCAGGTCCACGGCCGGGTGCTCCAGCGCTGGCGTGGT
+
FFFFFFFFFF:FFFFFFFFFFFFFFFFFFFFFFFFFFFFFFFFFFFFFFFFFFFFFFFFFFFFFFFFF
FFFFFFFFFFFFFFFFFFFFFFFFFFFFFFFFFFFFFFFFFFFFFFFFFFFFFFFFFFFFFFFFFFFF
@A00155:342:HHGFNDSXY:1:2611:9326:3740 2:N:0:GAACCTAG+TCCGCATA
TAAGCTTGGGTGAGTCAAAATCTAGTCTCGCTCTAGACCTCCTTGACCATCTGGCAGTCAGCATAAGG
ACGATAGTTGCCTCCATGACGTTTAGCCCCCGCAGGTCCACGGCCGGGTGCTCCAGCGCTGGCGTGGT
+
FFFFFFFFFFFFFFFFFFFFFFFFFFFFFFFFFFFFFFFFFFFFFFFFFFFFFFFFFFFFFFFFFFFF
FFFFFFFFFFFFFFFFFFFFFFFFFFFFFFFFFFFFFFFFFFFFFFFFFFFFFFFFFFFFFFFFFFFF
@A00155:342:HHGFNDSXY:1:1471:11134:9502 2:N:0:GAACCTAG+TCCGCATA
TAAGCTTGGGTGAGTCAAAATCTAGTCTCGCTCTAGACCTTCTTGACCATCTGGCAGTCAGCATAAGG
ACGATAGTTGCCTCCATGACGTTTAGCCCCCGCAGGTCCACGGCCGGGTGCTCCAGCGCTGGCGTGGT
+
FFFFFFFFFFFFFFFFFFFFFFFFFFFFFFFFFFFFFFFFFFFFFFFFFFFFFFFFFFFFFFFFFFFF
FFFFFFFFFFFFFFFFFFFFFFFFFFFF:F::FFFFFFFFFFFFFFFFFFFFFFFFFFFFFFFFFFFF
@A00155:342:HHGFNDSXY:1:1651:13910:31125 2:N:0:GAACCTAG+TCCGCATA
TAAGCTTGGGTGAATCAAAATCTAGTCTCGCTCTAGACCTTCTTGACCATCTGGCAGTCAGCATAAGG
ACGATAGTTGCCTCCATGACGTTTAGCCCCCGCAGGTCCACGGCCGGGTGCTCCAGCGCTGGCGTGGT
+
FFFFFFFFFFFFFFFFFFFFF:FFF:F:FFFFFFFFFFFFFFF,FFFFFFFFFF:FFFFFFFFFFFFF
FFFFFFFFFFFFFFFFFFFFFFFFFFFFFFFFFFFFFFFFFFFFFFFFFFFFFFFFFFFFFFFFFFFF
@A00155:342:HHGFNDSXY:1:2378:20283:4648 2:N:0:GAACCTAG+TCCGCATA
TAAGCTTGGGTGAGTCAAAATCTAGTCTCGCTCTAGACCTTCTTGACCATCTGGCAGTCAGCATAAGG
ACGATAGTTGCCTCCATGACGTTTAGCCCCCGCAGGTCCACGGCCGGGTGCTCCAGCGCTGGCGTGGT
+
FFFFFFFFFF:FFFFFFFF,FFFFFFFFFFFFFFFFFFFFFFFFFFFFFFFFFFFFFFFFFFFFFFFF
FFFFF:FFFFFFFFFFFFFFFFFFFFFFFFFFFFFFFFFFFFFFFFFFFFFFFFFFFFFFFFFFFFFF
@A00155:342:HHGFNDSXY:1:2106:1235:3443 2:N:0:GAACCTAG+TCCGCATA
TAAGCTTGTGTGAGTCAAAATCTAGTCTCGCTCTAGACCTTCTTGACCATCTGGCAGTCAGCATAAGG
ACGATAGTTGCCTCCATGACGTTTAGCCCCCGCAGGTCCACGGCCGGGTGCTCCAGCGCTGGCGTGGT
+
FFFFFFFF,F,FFF,FFFFF::FF,:FF,F,:FFFFFFFFFFFFF:FFF:FFFFFFFFFFFFFFFFFF
FFF:FFFFF,FF:FFFFFFFFFFFFFFFFFFFF:FF:FFFFFFFFF:,FFFF:F:F:FFFFFFFFFF:
@A00155:342:HHGFNDSXY:1:1411:28275:29481 2:N:0:GAACCTAG+TCCGCATA
TAAGCTTGGGTGAGTCAAAATCTAGTCTCGCTCTAGACCTTCTTGACCATCTGGCAGTCAGCATAAGG
ACGATAGTTGCCTCCATGACGTTTAGCCCCCGCAGGTCCACGGCCGGGTGCTCCAGCGCTGGCGTGGT
+
FFFFFFFFFFFFFFFFFFFFFFFFFFFFFFFFFFFFFFFFFFFFFFFFFFF:FFFFFFFFFFFFFFFF
FFFFFFFF,FFFFFFF:FFFFFFFFFFFFFFFFFFFFFFFFFFFFFFFFFFFFF,FFFFFFFFFFFFF

```
@A00155:342:HHGFNDSXY:1:2274:16884:21903 1:N:0:GAACCTAG+TCCGCATA
TAAGCTTGGGTGAGTCAAAATCTAGTCTCGCTCTAGACCTTCTTGACCATCTGGCAGTCAGCATAAGG
ACGATAGTTGCCTCCATGACGTTTAGCCCCCGCAGGTCCACGGCCGGGTGCTCCAGCGCTGGCGTGGT
+
FF:F:F::F:,FF:FFFFFFFFFFFFFFFFFFFFFFFFF:FF,F:FFFFFFF,FFFFFFFFFF:FFFF
FFFF,FFFFFF,FFFF,FFFF:,FFFFFF::F:FFF,FFFF:FFFFFFFFFFFF:FFFFFF:F::F::
@A00155:342:HHGFNDSXY:1:2213:29369:21825 2:N:0:GAACCTAG+TCCGCATA
TAAGCTTGGGTGAGTCAAAATCTAGTCTCGCTCTAGACCTTCTTGACCATCTGGCAGTCAGCATAAGG
ACGATAGTTGCCTCCATGACGTTTAGCCCCCGCAGGTCCACGGCCGGGTGCTCCAGCGCTGGCGTGGT
+
FFFFF,FFFF,FFFFFFFFFFFFFFFFFFFFFFFFFFFFFFFFFFFFFFFFFFFFFFFFFFFFFFFFF
FFFFFFF:FFFFFFF,FFFFFFFFFFFFFFFFFFFFFFFFFFFFFFFFFFFFFFFFFF,FFFFFFFF
@A00155:342:HHGFNDSXY:1:1167:32497:7607 2:N:0:GAACCTAG+TCCGCATA
GCTTGGGTGAGTCAAAATCTAGTCTCGCTCTAGACCTTCTTGACCATCTGGCAGTCAGCATAAGGACG
ATAGTTGCCTCCATGACGTTTAGCCCCCGCAGGTCCACGGCCGGGTGCTCCAGCGCTGGCGTGGTT
+
FFF:FFF,FFFFFFFFFFFFFFFFFFFFFF:FFFFFFFFFFF,FFFFFFFFFFFFFFFFFFF:FFFFF
FFFFFFFFFFFFFFFFFFFFFFFFFFFFFFFFFFFFFFFFFFFFFFFFFFFFFFFF:FFFFFFFFFF
@A00155:342:HHGFNDSXY:1:1221:28854:7373 2:N:0:GAACCTAG+TCCGCATA
CTTGGGTGAGTCAAAATCTAGTCTCGCTCTAGACCTTCTTGACCATCTGGCAGTCAGCATAAGGACGA
TAGTTGCCTCCATGACGTTTAGCCCCCGCAGGTCCACGGCCGGGTGCTCCAGCGCTGGCGTGGTTGTA
+
FFFFFFFFFFFFFF::FFFFFFFFFFFFF,FFFFFFFFFFFFFFFFFFFFFFFF,FFFFF:FFFFFFFF
FFF:FFFFFFFFFFFFFFFFFFFFFFFFFFFFFFFFFFFFFFFFFFFF:FFFFFFFFFFFFFFFFFFFF
@A00155:342:HHGFNDSXY:1:1501:6994:6026 2:N:0:GAACCTAG+TCCGCATA
GGGTGAGTCAAAATCTAGTCTCGCTCTAGACCTTCTTGACCATCTGGCAGTCAGCATAAGGACGATAG
TTGCCTCCATGACGTTTAGCCCCCGCAGGTCCACGGCCGGGTGCTCCAGCGCTGGCGTGGTTGTAGTC
+
FFF,FFF:FFF:FFFFFFFFFFFFFFFFFFFFF:FFFFFFFFFFFFFFFFFFFFFFFFFFFFFFFFFFF
FFFFFFFFFFFFFFFFFFFFFFFFFFFFFFFFFFFF:FFFFFFFFFFFFFFF,FFFFFFFFFFFFFF:
@A00155:342:HHGFNDSXY:1:2645:16631:8970 1:N:0:GAACCTAG+TCCGCATA
GGTGAGTCAAAATCTAGTCTCGCTCTAGACCTTCTTGACCATCTGGCAGTCAGCATAAGGACGATAGT
TGCCTCCATGACGTTTAGCCCCCGCAGGTCCACGGCCGGGTGCTCCAGCGCTGGCGTGGTTGTAGTCA
+
FFFFFFFFFFFFFFFFFFFFFFFFFFFFFFFFFFFFFFFFFFFFFFFFFFFFFFFFFFFFFFFFF:FF
FFFFFFFFFFFFFFFFFFFFFFFFFFFFFFFFFFFFFFFFFFFFFFFFFFFFFFFF:FFFFFFFFFF
@A00155:342:HHGFNDSXY:1:1345:6551:13056 1:N:0:GAACCTAG+TCCGCATA
GGTGAGTCAAAATCTAGTCTCGCTCTAGACCTTCTTGACCATCTGGCAGTCAGCATAAGGACGATAGT
TGCCTCCATGACGTTTAGCCCCCGCAGGTCCACGGCCGGGTGCTCCAGCGCTGGCGTGGTTGTAGTCA
+
FFFFFFFFFFFFFFFFFFFFFFFFFFFFFFFFFFFFFFFFFFFFFFFFFFFFFFFFF:F:FFFFFFFF
FFFFFFFFFFFFFFFFFFFFFFFFFFFFFFFFFFFF,FFFFFFFFFF:FFFFFFFFFFFFFFFFFFFF
@A00155:342:HHGFNDSXY:1:2239:19334:12524 1:N:0:GAACCTAG+TCCGCATA
GTGAGTCAAAATCTAGTCTCGCTCTAGACCTTCTTGACCATCTGGCAGTCAGCATAAGGACGATAGTT
GCCTCCATGACGTTTAGCCCCCGCAGGTCCACGGCCGGGTGCTCCAGCGCTGGCGTGGTTGTAGTCAA
+
:,FFF:FFF:FFFFF,FFFFFFFFFFF:FF,FFFFF,FF:,F,F,FFFFFFF:F,FF:FFFFFFF:FF
FFFFF,FFFFFFFF:FFFF,FFFF,:::FF,FFFFF,FFF:FFF:FFF:F:F:FFFFF,FFFFFFFFF
@A00155:342:HHGFNDSXY:1:1221:28854:7373 1:N:0:GAACCTAG+TCCGCATA
GTGAGTCAAAATCTAGTCTCGCTCTAGACCTTCTTGACCATCTGGCAGTCAGCATAAGGACGATAGTT
GCCTCCATGACGTTTAGCCCCCGCAGGTCCACGGCCGGGTGCTCCAGCGCTGGCGTGGTTGTAGTCAA
+
FFFFFFFFFFFFFFFFFFFFFFFFFFFFFFF:FFFFFFFFFFFFFFFFFFFFFFFFFFFFFFFFFFFF
FFFFFFFFFFFFFFFFFFFFFFFFFFFFFFF:FFFFFFFFFFFFFFFFFFFFFFFFFFFFFFFFFFFF
```

```
@A00155:342:HHGFNDSXY:1:1613:3884:31172 1:N:0:GAACCTAG+TCCGCATA
GTGAGTCAAAATCTAGTCTCGCTCTAGACCTTCTTGACCATCTGGCAGTCAGCATAAGGACGATAGTT
GCCTCCATGACGTTTAGCCCCCGCAGGTCCACGGCCGGGTGCTCCAGCGCTGGCGTGGTTGTAGTCAA
+
FFFFFFFFFFFFFFFFFFFFFFFFF,FFFFFFFFFFF:::FFFFFFFFF:FFFFFFFFFFFFFFFFFF
FFFFFFFFFFFFFFFFFFFFFFFFFFFFFFFFFFFFFFFFFFFFFFFFFFFFFFFFF:FFFFFFFF:
@A00155:342:HHGFNDSXY:1:1267:24424:6840 2:N:0:GAACCTAG+TCCGCATA
TGAGTCAAAATCTAGTCTCGCTCTAGACCTTCTTGACCATCTGGCAGTCAGCATAAGGACGATAGTTG
CCTCCATGACGTTTAGCCCCCGCAGGTCCACGGCCGGGTGCTCCAGCGCTGGCGTGGTTGTAGTCAAA
+
FFFFFFFF:FF:FFFFFF:F,F,FFFFFFFFFFFFFFFFFFFFFFFFFFF:FFFFFFFFFFFF:FFFF
FFFFFFFFFFFFFFFFFFFFFFFFFFFFFFFFFFFFFFFFFFFFFFFFFFFFFFFFFFFFFFFFFFFF
@A00155:342:HHGFNDSXY:1:1607:25138:20917 1:N:0:GAACCTAG+TCCGCATA
TGAGTCAAAATCTAGTCTCGCTCTAGACCTTCTTGACCATCTGGCAGTCAGCATAAGGACGATAGTTG
CCTCCATGACGTTTAGCCCCCGCAGGTCCACGGCCGGGTGCTCCAGCGCTGGCGTGGTTGTAGTCAAA
+
FFFFFFFFFF:FF:FFFFFFFFFFFFFFFFFFFFFFFFFFFFFFFFFFFFFFFFFFFFFFFFFFFFFF
FFFFFFFFFFFFFFFFFFFFFFFFFFFFFFFFFFFFFFFFFFFFFFFFFFFFFFFFF,FFFFFFFFFF
@A00155:342:HHGFNDSXY:1:2321:30978:33755 1:N:0:GAACCTAG+TCCGCATA
AGTCAAAATCTAGTCTCGCTCTAGACCTTCTTGACCATCTGGCAGTCAGCATAAGGACGATAGTTGCC
TCCATGACGTTTAGCCCCCGCAGGTCCACGGCCGGGTGCTCCAGCGCTGGCGTGGTTGTAGTCAAATG
+
FFFFFFFFFFFFFFFFFFFFFFFFFFFFFFFFFFFFFFFFFFFFFFFFFFFFFFFFFFFFFFFFFFFF
FFFFFFFFFFFFFFFFFFFFFFFFFFFFFFFFFFFFFFFFFFFFFFFFFFFFFFF:FFFFFFFFFFFF
@A00155:342:HHGFNDSXY:1:1473:18141:21887 1:N:0:GAACCTAG+TCCGCATA
AGTCAAAATCTAGTCTCGCTCTAGACCTTCTTGACCATCTGGCAGTCAGCATAAGGACGATAGTTGCC
TCCATGACGTTTAGCCCCCGCAGGTCCACGGCCGGGTGCTCCAGCGCTGGCGTGGTTGTAGTCAAATG
+
FFFFFFFFFFFFFFFFFFFFFFFFFFFFFFFFFFFFFFFFFFFFFFFFFFFFFFFFFFFFFFFFFFFF
FFFFF:FFFFFFFFFFFFFFFFFFFFFFFFFFFFFFFFFFFFFFFFFFFFFFFFFFFFFFFFFFFFFF
@A00155:342:HHGFNDSXY:1:2514:1533:20525 2:N:0:GAACCTAG+TCCGCATA
GTCAAAATCTAGTCTCGCTCTAGACCTTCTTGACCATCTGGCAGTCAGCATAAGGACGATAGTTGCCT
CCATGACGTTTAGCCCCCGCAGGTCCACGGCCGGGTGCTCCAGCGCTGGCGTGGTTGTAGTCAAATGT
+
F:FFFFFFFFFFFFFFFFFFFFFFFFF:FFFFFFFFFFF:FFFF:FFFF:FFFFFFFFFFFFFFFFF:
FFFFFFFFFFFFFFFFFFFFFFFFFFFFFFFFFFFFFFFFFFFFFFFFFFFFFFFFFFFFFFFFFFFF
@A00155:342:HHGFNDSXY:1:1609:9598:36370 2:N:0:GAACCTAG+TCCGCATA
TCAAAATCTAGTCTCGCTCTAGACCTTCTTGACCATCTGGCAGTCAGCATAAGGACGATAGTTGCCTC
CATGACGTTTAGCCCCCGCAGGTCCACGGCCGGGTGCTCCAGCGCTGGCGTGGTTGTAGTCAAATGTT
+
FFFFFFFFFFFFFFFFFFFFFFFFFFFFFFFFFFFFFFFFFFFF:FFFFFFFFFFFF:FFF:FFFF
FFFFFFFFFFFFFFFFFFFF:FFFFFFFFFFFF:FFFF:FFFFFFFFFFFFFFFFF:FFFFFFF,FFF
@A00155:342:HHGFNDSXY:1:2370:4336:22529 1:N:0:GAACCTAG+TCCGCATA
TCAAAATCTAGTCTCGCTCTAGACCTTCTTGACCATCTGGCAGTCAGCATAAGGACGATAGTTGCCTC
CATGACGTTTAGCCCCCGCAGGTCCACGGCCGGGTGCTCCAGCGCTGGCGTGGTTGTAGTCAAATGTT
+
:FFFFFFFFF:FFFF,FFFFFFFFFFFFFFFFFFFFFFFFFFFFF,,,FFFF::FFFFFFF,F,FF:F
FFFFFF:FFFFFF:FF,:FFF,:,FFF,::F,FF,FFFFFFFFFFFFF:F:FFF,:FF:FFFFFFF,F
@A00155:342:HHGFNDSXY:1:1169:9480:30749 2:N:0:GAACCTAG+TCCGCATA
TCAAAATCTAGTCTCGCTCTAGACCTTCTTGACCATCTGGCAGTCAGCATAAGGACGATAGTTGCCTC
CATGACGTTTAGCCCCCGCAGGTCCACGGCCGGGTGCTCCAGCGCTGGCGTGGTTGTAGTCAAATGTT
+
FFFFFFFFFFFFFFFFF:FFFFFFFFFFFFFFFFFFFFFFFFFFFFFFFFFFFFFFFFFFFFFFFFFF
FFFFFFFFFFFFFFFFFFFFFFFFFFFFFFFFFFFFFFFFFFFFFFFFFFFFFFFFFFFFFFFFFFFF
```

```
@A00155:342:HHGFNDSXY:1:2465:14977:36166 2:N:0:GAACCTAG+TCCGCATA
TCAAAATCTAGTCTCGCTCTAGACCTTCTTGACCATCTGGCAGTCAGCATAAGGACGATAGTTGCCTC
CATGACGTTTAGCCCCCGCAGGTCCACGGCCGGGTGCTCCAGCGCTGGCGTGGTTGTAGTCAAATGTT
+
FFFFF,FFFFFFFFFFFFFFFFFFF:FFFFFFFFFFFFFFFFFFFFFFFFFFFFFFFFFFFFFFFFFF
FFFFFFFFF:FFFFFFFFFFFFFFFFFFFFFFFFFFFFFFFFFFFFFFFFFFFFFFFFFFFFFFFFFF
@A00155:342:HHGFNDSXY:1:1678:7482:30107 2:N:0:GAACCTAG+TCCGCATA
TCAAAATCTAGTCTCGCTCTAGACCTTCTTGACCATCTGGCAGTCAGCATAAGGACGATAGTTGCCTC
CATGACGTTTAGCCCCCGCAGGTCCACGGCCGGGTGCTCCAGCGCTGGCGTGGTTGTAGTCAAATGTT
+
FFFFFFFFFFFFFFFFFFFFFFFFFFFF:FFFFFFFFF:FFFFFFFFFFFFFFFFFFFFFF,FFFFFF
FFFFFFFFFFFFFFFFFFFFFFFFFFFFFFFFFFFFFFFFFFFFFFFFFFFFFFFFFFFFFFFFFFFF
@A00155:342:HHGFNDSXY:1:1334:1542:4006 2:N:0:GAACCTAG+TCCGCATA
CAAAATCTAGTCTCGCTCTAGACCTTCTTGACCATCTGGCAGTCAGCATAAGGACGATAGTTGCCTCC
ATGACGTTTAGCCCCCGCAGGTCCACGGCCGGGTGCTCCAGCGCTGGCGTGGTTGTAGTCAAATGTT
+
FFFFF:FFFFFFF:FF:F:FFFFFF,F,FFFFFFFFFFFFFFFFFFFFFFFFFFFFFFFFFFFFFFFF
FFFFFFFFFFFFFFFFFFF:FFFFFF:FFFFFFFF,FF:FFFFF:FFFFFFFFFFFFF,FFFFFFFF
@A00155:342:HHGFNDSXY:1:1156:1280:25880 2:N:0:GAACCTAG+TCCGCATA
CAAAATCTAGTCTCGCTCTAGACCTTCTTGACCATCTGGCAGTCAGCATAAGGACGATAGTTGCCTCC
ATGACGTTTAGCCCCCGCAGGTCCACGGCCGGGTGCTCCAGCGCTGGCGTGGTTGTAGTCAAATGTTG
+
FF,,FFFFFF,FFFFFFFFFFFFFFFFFFFFFF:FFFFFFFFFFFFFFFFFFFFFF,FFFFFFFFFFF
:FFFFFFFF:FFF:FFFF:FF:FFFFFFFFFFFFFFFFFFFFFF:FFFFFFFFFFFFF:FFF:FFFFF
@A00155:342:HHGFNDSXY:1:1561:4155:1736 2:N:0:GAACCTAG+TCCGCATA
CAAAATCTAGTCTCGCTCTAGACCTTCTTGACCATCTGGCAGTCAGCATAAGGACGATAGTTGCCTCC
ATGACGTTTAGCCCCCGCAGGTCCACGGCCGGGTGCTCCAGCGCTGGCGTGGTTGTAGTCAAATGTTG
+
FFFFFFF:FFFFFFFFFFFFFFFFF:FFFFFFFFFFFFFFFFFFFFFFFFFFFFFFFFFFFFFFFFFF
FFFFFFFFFFFFFFFFFFFFFFFFFFFFFFFFFFFFFFFFFFFFFFFFFFFFFFFFFFFFFFFFFFFF
@A00155:342:HHGFNDSXY:1:2443:16957:8719 2:N:0:GAACCTAG+TCCGCATA
CAAAATCTAGTCTCGCTCTAGACCTTCTTGACCATCTGGCAGTCAGCATAAGGACGATAGTTGCCTCC
ATGACGTTTAGCCCCCGCAGGTCCACGGCCGGGTGCTCCAGCGCTGGCGTGGTTGTAGTCAAATGTTG
+
FFFFFF:FFFFFFFFFFFFFFFFFFFFFFFFFFFFFFFFFFFFFFFFFFFFFFFFFFFFFFFFFFFFF
FFFFFFFFFFFFFFFFFFFFFFFFFFFFFFFFFFFFFFFFFFFFFFFFFFFFFFFFFFFFFFFFFFFF
@A00155:342:HHGFNDSXY:1:2442:15718:19445 2:N:0:GAACCTAG+TCCGCATA
CAAAATCTAGTCTCGCTCTAGACCTTCTTGACCATCTGGCAGTCAGCATAAGGACGATAGTTGCCTCC
ATGACGTTTAGCCCCCGCAGGTCCACGGCCGGGTGCTCCAGCGCTGGCGTGGTTGTAGTCAAATGTTG
+
FFFFFFFFFFFFFFFFFFFFFF:FFFFFFFFFFFFFFFFFFFFFFFFFFFFFFFFFFFFFFFFFFFFF
FFFFFFFFFFFF:FFFFFFFFFFFFFFFFFFFFFFFFFFFFFFFFFFFFFFFFFFFFFFFFFFFFFFF
@A00155:342:HHGFNDSXY:1:2658:17372:13542 2:N:0:GAACCTAG+TCCGCATA
AAAATCTAGTCTCGCTCTAGACCTTCTTGACCATCTGGCAGTCAGCATAAGGACGATAGTTGCCTCCA
TGACGTTTAGCCCCCGCAGGTCCACGGCCGGGTGCTCCAGCGCTGGCGTGGTTGTAGTCAAATGTTGG
+
FFFFFF:FFFFFFFFFFF:F:FFFFFFFFFFFFFFFFFFFFFFFFFFFFFF:FF:F,:FFFFFFFFFF
FFF:FFFFFFFFFFFFFFFFFFF:FFFFFFFFFFFFFF,FFFFFFFFFFFFFFFFFF,FFFFFFFFFF
@A00155:342:HHGFNDSXY:1:2310:7139:12790 2:N:0:GAACCTAG+TCCGCATA
AAAATCTAGTCTCGCTCTAGACCTTCTTGACCATCTGGCAGTCAGCATAAGGACGATAGTTGCCTCCA
TGACGTTTAGCCCCCGCAGGTCCACGGCCGGGTGCTCCAGCGCTGGCGTGGTTGTAGTCAAATGTTGG
+
FF:FFFFFFFFFFFFFFFFFFFFFFFFFFFFFFFFFFFFFFFFFFFFFFFFFFFFFFFFFFFFFFFFF
FFFFFFFFFFFFFFFFFFFFFFFFFFFFFFFFFFFFFFFFFFFFFFFFFFFFFFFFFFFFFFFFFFFF
```

```
@A00155:342:HHGFNDSXY:1:2165:4915:23594 2:N:0:GAACCTAG+TCCGCATA
AAAATCTAGTCTCGCTCTAGACCTTCTTGACCATCTGGCAGTCAGCATAAGGACGATAGTTGCCTCCA
TGACGTTTAGCCCCCGCAGGTCCACGGCCGGGTGCTCCAGCGCTGGCGTGGTTGTAGTCAAATGTTGG
+
:FF:FFFF:FFFFFFFFFFFFFFFFF:FFF:FFFFFFFFFFFFFFFFFFFFFFFFFFFFFFF,FFFFFFF
FFFFF,FFFFFFFFFFFFFFFFFFFFFFFFFFFFFFFFFFFFFF:FFFFFFFFFFFFFFFFFFFFFFFFF
@A00155:342:HHGFNDSXY:1:2658:17210:13730 2:N:0:GAACCTAG+TCCGCATA
AAAATCTAGTCTCGCTCTAGACCTTCTTGACCATCTGGCAGTCAGCATAAGGACGATAGTTGCCTCCA
TGACGTTTAGCCCCCGCAGGTCCACGGCCGGGTGCTCCAGCGCTGGCGTGGTTGTAGTCAAATGTTGG
+
FFF:FF,FFFFFFFFFFFFFFFFFFF:FFFFFFFFFFFFFFFFFFFFFFFF:FFFFFFFFFFFFFFF:F:
FFFFFFFFFFFFFFFFFFFFFFFFFFFFFFFFFFFFFFFFFFFFFFFFFFFFFFFFFFFFFFFFFFFFFF
@A00155:342:HHGFNDSXY:1:2122:17833:12430 2:N:0:GAACCTAG+TCCGCATA
AAATCTAGTCTCGCTCTAGACCTTCTTGACCATCTGGCAGTCAGCATAAGGACGATAGTTGCCTCCAT
GACGTTTAGCCCCCGCAGGTCCACGGCCGGGTGCTCCAGCGCTGGCGTGGTTGTAGTCAAATGTTGGT
+
FFFFFFFFFFFFFFFFFFFFFFFFFFFFF:FFFFFFFFFFFFFFFFFFF:FFFFFFFFFFFFFFFFFFFF
FFFFFFFFFFFFFFFFFFFFFFFFFFFFFFFFFFFFFFFFFFFFFFFFFFFFFFFFFFFFFFFFFFFFFF
@A00155:342:HHGFNDSXY:1:2426:5122:19570 2:N:0:GAACCTAG+TCCGCATA
AAATCTAGTCTCGCTCTAGACCTTCTTGACCATCTGGCAGTCAGCATAAGGACGATAGTTGCCTCCAT
GACGTTTAGCCCCCGCAGGTCCACGGCCGGGTGCTCCAGCGCTGGCGTGGTTGTAGTCAAATGTTGGT
+
FFFF:FFFFFFFFFFFFFFFFFFFFFFFFFFFFFFFFFFFFFFFFFFFFFFFFFFFFFF:FFFFFFF:F
FFFFFFFFFFFFFFFFFFFFFFFFFFFFFFFFFFFFFFFFFFFFFFFFFFFFFFFFFFFFFFFFFFFFFF
@A00155:342:HHGFNDSXY:1:2540:2456:18239 2:N:0:GAACCTAG+TCCGCATA
AAATCTAGTCTCGCTCTAGACCTTCTTGACCATCTGGCAGTCAGCATAAGGACGATAGTTGCCTCCAT
GACGTTTAGCCCCCGCAGGTCCACGGCCGGGTGCTCCAGCGCTGGCGTGGTTGTAGTCAAATGTTGGT
+
FFFFFFFFFFFFFFFFFFFF:FFFFFFFFFFFFFFFFFFFFFFFFFFFFFFFFFFFFFFFFFFFFFFFFF
FFFFFFFFFFFFFFFFFFFFFFFFFFFFFFFFFFFFFFFFFFFFFFFFFFFFFFFFFFFFFFFFFFFFFF
@A00155:342:HHGFNDSXY:1:1559:21983:9408 2:N:0:GAACCTAG+TCCGCATA
AATCTAGTCTCGCTCTAGACCTTCTTGACCATCTGGCAGTCAGCATAAGGACGATAGTTGCCTCCATG
ACGTTTAGCCCCCGCAGGTCCACGGCCGGGTGCTCCAGCGCTGGCGTGGTTGTAGTCAAATGTTGGTA
+
F:FFFFFFFFFFFFFFFFFFF:FF,FFFFFFFFFFFFFFFFFFFFFFFFFFFFFFFFFFFFFFFFFFFFF
FFFFFFFFFFFFFFFFFFFFFFFFFFFFFFFFFFFFFFFFFF,FFFFFFFFFFFFFFFFFFFFFFFFFFF
@A00155:342:HHGFNDSXY:1:2551:21621:24627 2:N:0:GAACCTAG+TCCGCATA
ATCTAGTCTCGCTCTAGACCTTCTTGACCATCTGGCAGTCAGCATAAGGACGATAGTTGCCTCCATGA
CGTTTAGCCCCCGCAGGTCCACGGCCGGGTGCTCCAGCGCTGGCGTGGTTGTAGTCAAATGTTGGTAG
+
FFFFFFFFFFFFFFFFFFFFFFFFFFFFFFFFFFFFFFFFFFFFFFFFFFFFFFFFFF:FFFFFFFFFFF
FFFFFFFFFFFFFFFFFFFFFFFFFFFFFFFFFFFFFFFFFFFFFFFFFFFFFFFFFFFFFFFFFFFFFF
@A00155:342:HHGFNDSXY:1:1335:13340:27508 2:N:0:GAACCTAG+TCCGCATA
ATCTAGTCTCGCTCTAGACCTTCTTGACCATCTGGCAGTCAGCATAAGGACGATAGTTGCCTCCATGA
CGTTTAGCCCCCGCAGGTCCACGGCCGGGTGCTCCAGCGCTGGCGTGGTTGTAGTCAAATGTTGGTAG
+
FFFFFFFFFFFFFFFFFFFFFFFFF:FFFFFFFFF:FFFFFFFFFFFFFFFFFFFFFFFFFFFFFFFFFF
FF:FFFFFFFFFFFFFFFFFFFFFFFFFFFFFFFFFFFFFFFFFFFFFFFFFFFFFFFFFFFFFFFFFFF
@A00155:342:HHGFNDSXY:1:2264:31665:36166 2:N:0:GAACCTAG+TCCGCATA
ATCTAGTCTCGCTCTAGACCTTCTTGACCATCTGGCAGTCAGCATAAGGACGATAGTTGCCTCCATGA
CGTTTAGCCCCCGCAGGTCCACGGCCGGGTGCTCCAGCGCTGGCGTGGTTGTAGTCAAATGTTGGTAG
+
FFFF,FFFFFFFFFFFFFFFFFFF:FFFFFFFFFFFFFFFFFFFFFF:FFFFFF:FFFFFFFFFFFFFFF
FFFFFFFFFFFFFFFFFFFFFFFFFFFFFFFFFFFFFFFFFFFFFFFFFFFFFFFFFFFFFFFFFFFFFF
```

```
@A00155:342:HHGFNDSXY:1:2307:32687:16110 2:N:0:GAACCTAG+TCCGCATA
ATCTAGTCTCGCTCTAGACCTTCTTGACCATCTGGCAGTCAGCATAAGGACGATAGTTGCCTCCATGA
CGTTTAGCCCCCGCAGGTCCACGGCCGGGTGCTCCAGCGCTGGCGTGGTTGTAGTCAAATGTTGGTAG
+
FFFFFFFFFFFFFFFFFFFFFFFFFFFFFFFFFFFFFFFFFFFFFFFFFFFFFFFFFFFFFFFFFFFFFF
FFFFFFFFFFFFFFFFFFFFFFFFFFFFFFFFFFFFFFFFFFFFFFFFFFFFFFFFFFFFFFFFFFFFFF
@A00155:342:HHGFNDSXY:1:2654:22408:8453 2:N:0:GAACCTAG+TCCGCATA
ATCTAGTCTCGCTCTAGACCTTCTTGACCATCTGGCAGTCAGCATAAGGACGATAGTTGCCTCCATGA
CGTTTAGCCCCCGCAGGTCCACGGCCGGGTGCTCCAGCGCTGGCGTGGTTGTAGTCAAATGTTGGTAG
+
:FFFFFFFFFFFFFFFFFFFFFFFFFFFFFFFFFFFFFFFFFFFFFFFFFFFFFFFFFFFFFFFFFFFFF
FFFFFFFFFFFFFFFFFFFF:FFFFFFFFFFF:FF:FFFFFFFFFFFFFFFFFFFFFFFFFFFFFFFFFF
@A00155:342:HHGFNDSXY:1:1310:17879:26819 2:N:0:GAACCTAG+TCCGCATA
ATCTAGTCTCGCTCTAGACCTTCTTGACCATCTGGCAGTCAGCATAAGGACGATAGTTGCCTCCATGA
CGTTTAGCCCCCGCAGGTCCACGGCCGGGTGCTCCAGCGCTGGCGTGGTTGTAGTCAAATGTTGGTAG
+
FFFFFFFFFFFFFFFFFFFFFFFFFFFFFFFFFFFFFFFFFFFFFFFFFFFFFFFFFFFFFFFFFF:FFF
FFFFFFFFFFFFFFFFFFFFFFFFFFFFFFFFFFFFFFFFFFFFFFFFFFFFFFFFFFFFFFFFFFFFFF
@A00155:342:HHGFNDSXY:1:1237:14814:21292 2:N:0:GAACCTAG+TCCGCATA
TCTAGTCTCGCTCTAGACCTTCTTGACCATCTGGCAGTCAGCATAAGGACGATAGTTGCCTCCATGAC
GTTTAGCCCCCGCAGGTCCACGGCCGGGTGCTCCAGCGCTGGCGTGGTTGTAGTCAAATGTTGGTAGT
+
F:FFFFFFFFFFFFFFFFFFFFFF:FFFFFFFFFFFFFFFFFFFFFFFFFFFFFFFFFFFFFFFFFFFFF
FFFFFFFFFFFFFFFFFFFFFFFFFFFFFFFFFFFFFFFFFFFFFFFFFFFFFFFFFFF:FFFFFF:FFF
@A00155:342:HHGFNDSXY:1:1166:13376:34334 2:N:0:GAACCTAG+TCCGCATA
TCTAGTCTCGCTCTAGACCTTCTTGACCATCTGGCAGTCAGCATAAGGACGATAGTTGCCTCCATGAC
GTTTAGCCCCCGCAGGTCCACGGCCGGGTGCTCCAGCGCTGGCGTGGTTGTAGTCAAATGTTGGTAGT
+
FFFFFFFFFFFFFFFFFFFF:FFFFFFFFFFF:FFFFFFFFFFFFFFFFFFFFFFFFFFFFFFFFFFFFF
FFFFFFFFFFFFFFFFFFFFFFFFFFFFFFFFFFFFFFFFFFFFFFFFFF:FFFFFFFF:FFFFFFFFFF
@A00155:342:HHGFNDSXY:1:1574:8784:10441 2:N:0:GAACCTAG+TCCGCATA
TCTAGTCTCGCTCTAGACCTTCTTGACCATCTGGCAGTCAGCATAAGGACGATAGTTGCCTCCATGAC
GTTTAGCCCCCGCAGGTCCACGGCCGGGTGCTCCAGCGCTGGCGTGGTTGTAGTCAAATGTTGGTAGT
+
FF:FFFFFFFFFFFFFFFFFFFFFFFFFFFFFF:FFFFFFFFFFFFFFFFFFFFFFFFFFFFFFFFFFFF
FFFFFFFFFFFFFFFFFFFFFFFFFFFFFFFFFFFFFFFFFFFFFFFFF:FFFFFFFFFFFFFFFFFFFF
@A00155:342:HHGFNDSXY:1:1166:12689:33802 2:N:0:GAACCTAG+TCCGCATA
TCTAGTCTCGCTCTAGACCTTCTTGACCATCTGGCAGTCAGCATAAGGACGATAGTTGCCTCCATGAC
GTTTAGCCCCCGCAGGTCCACGGCCGGGTGCTCCAGCGCTGGCGTGGTTGTAGTCAAATGTTGGTAGT
+
FFFFFFFFFFFFFFFFFFFFFFFFFFFFFFFFFFFFFFFFFFFFFFFFFFFFFFFFFFFFFFFFF:FFFF
FFFFFFFFFFFFFFFFFFFFFFFFFFFFFFFFFFFFFFFF:FFFFFFFFFFFFFFFFFFFFFF:FFFFFF
@A00155:342:HHGFNDSXY:1:1606:12237:25817 2:N:0:GAACCTAG+TCCGCATA
TCTAGTCTCGCTCTAGACCTTCTTGACCATCTGGCAGTCAGCATAAGGACGATAGTTGCCTCCATGAC
GTTTAGCCCCCGCAGGTCCACGGCCGGGTGCTCCAGCGCTGGCGTGGTTGTAGTCAAATGTTGGTAGT
+
FFFFFFFFFFFFFFFFFFFFFFFFFFFFFFFFFFFFFFFFFFFFFFFFFFFFFFFFFFFFFFFFFFFFFF
FFFFFFFFFFFFFFFFFFFFFFFFFFFFFFFFFFFFFFFFFFFFFFFFFFFFFFFFFFFFFFFFFFFFFF
@A00155:342:HHGFNDSXY:1:2514:1533:20525 1:N:0:GAACCTAG+TCCGCATA
TAGTCTCGCTCTAGACCTTCTTGACCATCTGGCAGTCAGCATAAGGACGATAGTTGCCTCCATGACGT
TTAGCCCCCGCAGGTCCACGGCCGGGTGCTCCAGCGCTGGCGTGGTTGTAGTCAAATGTTGGTAGTC
+
FFFFFFFFFFFFFFFFFFFFFFFFFFFFFFFFFFFFFFFFFFFFFFFFFFFFFFFFFFFFFFFFFFFFFF
FFFFFFFFFFFFFFFFFFFFFFFFFFFFFFFFFFFFFFFFFFFFFFFFFFFFFFFFF,FFFF,FFFFFF
```

```
@A00155:342:HHGFNDSXY:1:2122:17833:12430 1:N:0:GAACCTAG+TCCGCATA
TAGTCTCGCTCTAGACCTTCTTGACCATCTGGCAGTCAGCATAAGGACGATAGTTGCCTCCATGACGT
TTAGCCCCCGCAGGTCCACGGCCGGGTGCTCCAGCGCTGGCGTGGTTGTAGTCAAATGTTGGTAGTCA
+
FFFFFFFFFFFFFFFFFFFFFFFFFFFFFFFFFFFFFFFFFFFFFFFFFFFFFFFFFFFFFFFFFFFF
FFFFFFFFFFFFFFFFFFFFFFFFFFFFFFFFFFFFFFFFFF:FFFFFFFFFFFFF:FFFFFFFFFFF
@A00155:342:HHGFNDSXY:1:1167:32497:7607 1:N:0:GAACCTAG+TCCGCATA
AGTCTCGCTCTAGACCTTCTTGACCATCTGGCAGTCAGCATAAGGACGATAGTTGCCTCCATGACGTT
TAGCCCCCGCAGGTCCACGGCCGGGTGCTCCAGCGCTGGCGTGGTTGTAGTCAAATGTTGG
+
FFFFFFFFFFFFFFFFFFFFFFFFFFFFFFFFFFFFFFFFFFFFFFFFFFFFFFFFFFFFFFFFFFFF
FFFFFFFFFFFFFFFFFFFFFFFFFFFFFFF:FFFF:FFFF:FFFFFFFFFFFFFFFF:FF
@A00155:342:HHGFNDSXY:1:1574:8784:10441 1:N:0:GAACCTAG+TCCGCATA
AGTCTCGCTCTAGACCTTCTTGACCATCTGGCAGTCAGCATAAGGACGATAGTTGCCTCCATGACGTT
TAGCCCCCGCAGGTCCACGGCCGGGTGCTCCAGCGCTGGCGTGGTTGTAGTCAAATGTTGGTA
+
FFFFFFFFFFFFFFFFFFFFFFFFFFFFFFFFFFFFFFFFFFFFFFFFFFFFFFFFFFFFFFFFFFFF
FFFFFFFFFFFFFFFFFFFFFFFFFFFFFFFFFFFFFFFFFFFF:,FFFFFFFFFFFFFFFFF
@A00155:342:HHGFNDSXY:1:1667:14190:6339 1:N:0:GAACCTAG+TCCGCATA
AGTCTCGCTCTAGACCTTCTTGACCATCTGGCAGTCAGCATAAGGACGATAGTTGCCTCCATGACGTT
TAGCCCCCGCAGGTCCACGGCCGGGTGCTCCAGCGCTGGCGTGGTTGTAGTCAAATGTTGGTA
+
FFFFFFFFFFFFFFFFFFFFFFFFFFFFFFFFFFFFFFFFFFFFFFFFFFFFFFFFFFFFFFFFFFFF
FFFFFFFFFFFFFFFFFFFFFFFFFFFFFFFFFFFFFFFFFFFF:FFFFFFFFF:FFF:FFFF
@A00155:342:HHGFNDSXY:1:2654:22408:8453 1:N:0:GAACCTAG+TCCGCATA
AGTCTCGCTCTAGACCTTCTTGACCATCTGGCAGTCAGCATAAGGACGATAGTTGCCTCCATGACGTT
TAGCCCCCGCAGGTCCACGGCCGGGTGCTCCAGCGCTGGCGTGGTTGTAGTCAAATGTTGGTA
+
FFFFFFFFFFFFFFFFFFFFFFFFFFFF:FFFFFFFFFFFFFFFFFFFFFFFFFFFFFFFFFFFFFFF
FFFFFFFFFFFF:FFFFFFFFFFFFFFFFFFFFFFFFFFFFFFFFFFFFFFFFFFFFFFFFF
@A00155:342:HHGFNDSXY:1:1371:24306:33536 1:N:0:GAACCTAG+TCCGCATA
AGTCTCGCTCTAGACCTTCTTGACCATCTGGCAGTCAGCATAAGGACGATAGTTGCCTCCATGACGTT
TAGCCCCCGCAGGTCCACGGCCGGGTGCTCCAGCGCTGGCGTGGTTGTAGTCAAATGTTGGTAG
+
FFFFFFFFFFFFFFFFFFFF:FFFFFFFFFFFFFFFFFFFFFFFFFF:FFFFFFFF,FFFFFFFFFFF
FFFFFFFFFFFFFFFFFFFFFFFFFFFFFFFF,FFFFFFFFFFFFFFFFFFFFFFF,,:FFFFF
@A00155:342:HHGFNDSXY:1:1371:24288:33786 1:N:0:GAACCTAG+TCCGCATA
AGTCTCGCTCTAGACCTTCTTGACCATCTGGCAGTCAGCATAAGGACGATAGTTGCCTCCATGACGTT
TAGCCCCCGCAGGTCCACGGCCGGGTGCTCCAGCGCTGGCGTGGTTGTAGTCAAATGTTGGTAG
+
FFFFFFFFFFFFFFFFFFFFFFFFFFFFFFFFFFFFFFFFFFFFFFFFFFF:FFFFFFFFFFFFFFFF
FFFFFFFFFFFFFFFFFFFFFFFFFFFFFFFFFFFFFFFFFFFFFFFFFFFFFFFFFFFFFFFF
@A00155:342:HHGFNDSXY:1:2503:6153:8547 2:N:0:GAACCTAG+TCCGCATA
AGTCTCGCTCTAGACCTTCTTGACCATCTGGCAGTCAGCATAAGGACGATAGTTGCCTCCATGACGTT
TAGCCCCCGCAGGTCCACGGCCGGGTGCTCCAGCGCTGGCGTGGTTGTAGTCAAATGTTGGTAG
+
FFFFFFFFF,FFFFFFFF,FFFFFF:FFFFFFFFFFFFFFFFFFFFFFFFFFFFFFFFFFFFFFF:FF
FFFFFFFFFFFFFFFFFF:FF,FFFFFFFFF:FFFFFFFFFFFFFFFFFFFFFF:F:FFF,FFF
@A00155:342:HHGFNDSXY:1:1314:30861:18176 1:N:0:GAACCTAG+TCCGCATA
AGTCTCGCTCTAGACCTTCTTGACCATCTGGCAGTCAGCATAAGGACGATAGTTGCCTCCATGACGTT
TAGCCCCCGCAGGTCCACGGCCGGGTGCTCCAGCGCTGGCGTGGTTGTAGTCAAATGTTGGTAGTCAG
+
FFFFFFFFFFFFFFFFFFFFFFFFFFFFFFFFFFFFFFFFFFFFFFFFFFFFFFFFFFFFFFFFFFFF
FFFFFFFFFFFFFFFFFFFFFFFFFFFFFFFFFFFFFFFFFFFFFFFFFFFFFFFFFFFFFFF:FFFFF
```

```
@A00155:342:HHGFNDSXY:1:1244:21911:35086 1:N:0:GAACCTAG+TCCGCATA
AGTCTCGCTCTAGACCTTCTTGACCATCTGGCAGTCAGCATAAGGACGATAGTTGCCTCCATGACGTT
TAGCCCCCGCAGGTCCACGGCCGGGTGCTCCAGCGCTGGCGTGGTTGTAGTCAAATGTTGGTAGTCAG
+
FFFFFFFFFFFFFFFFFFFFFFFFFFFFFFFFFFFFFFFFFFFFFFFFFFFFFFFFFFFFFFFFFFFF
FFFFFFFFFFFFFFFFFFFFFFFF:FFFFFFFFFFFFFFFFFFFFFFFFFFFFFFFFFFFFFFFFFFF
@A00155:342:HHGFNDSXY:1:1127:20500:24596 1:N:0:GAACCTAG+TCCGCATA
AGTCTCGCTCTAGACCTTCTTGACCATCTGGCAGTCAGCATAAGGACGATAGTTGCCTCCATGACGTT
TAGCCCCCGCAGGTCCACGGCCGGGTGCTCCAGCGCTGGCGTGGTTGTAGTCAAATGTTGGTAGTCAG
+
FFFFFFFFFFFF:FFFFFFFFFFFFFFFFFFFFFFFFFFFFFFFFFFFFFFFFFFFFFFFFFFFFFFF
FFFFFFFFFFFFFFFF:FFFFFFFFFFFFFFFFFFFFFFFFFFFFFFFFFFFFFFFFFFFFFFFFFFF
@A00155:342:HHGFNDSXY:1:1115:9742:2801 1:N:0:GAACCTAG+TCCGCATA
AGTCTCGCTCTAGACCTTCTTGACCATCTGGCAGTCAGCATAAGGACGATAGTTGCCTCCATGACGTT
TAGCCCCCGCAGGTCCACGGCCGGGTGCTCCAGCGCTGGCGTGGTTGTAGTCAAATGTTGGTAGTCAG
+
FFFFFFFFFFFFFFF,FFFFFFF:FFFFFFFFFFFFF:FFFFF:FFFFFFFF,FFFFFFFFFF:FFFF
FFFFFFFFFF:FFFFF,FFFFFFFFFFFFFFFFFFF:FF:F,FFF:FFFFFFFFFFFFFFFFFFFFFF
@A00155:342:HHGFNDSXY:1:2638:21621:19993 1:N:0:GAACCTAG+TCCGCATA
GTCTCGCTCTAGACCTTCTTGACCATCTGGCAGTCAGCATAAGGACGATAGTTGCCTCCATGACGTTT
AGCCCCCGCAGGTCCACGGCCGGGTGCTCCAGCGCTGGCGTGGTTGTAGTCAAATGTTGGTAGTCAGG
+
FFFFFFFFFFFFFFFFFFFFFFFFFFFFFFFFFFFFFFFFFFFFFFFF:FFFFFFFFFFFFFFFFF,F
FFFFFFFFFFFFFFFFFFFFFFFFFFFFFFFFFFFFFFFFFFF:FFFFFFFFFFFFFFFFFFFFFFFF
@A00155:342:HHGFNDSXY:1:1424:19018:22028 2:N:0:GAACCTAG+TCCGCATA
GTCTCGCTCTAGACCTTCTTGACCATCTGGCAGTCAGCATAAGGACGATAGTTGCCTCCATGACGTTT
AGCCCCCGCAGGTCCACGGCCGGGTGCTCCAGCGCTGGCGTGGTTGTAGTCAAATGTTGGTAGTCAGG
+
FFF,FFFFFFFFFFFFFFFFFFFFFFFFFFFFFFF:FFFFFFFFFFFFFFFFFFFFFFFF:FFFFFF:
FFFFFFFFF:FFFFFFFFFFFFFFFFFFFF:FFFFFFFFFFFFFFF:FFFFFFFFFFFFFFFFFFFFF
@A00155:342:HHGFNDSXY:1:1613:3884:31172 2:N:0:GAACCTAG+TCCGCATA
GTCTCGCTCTAGACCTTCTTGACCATCTGGCAGTCAGCATAAGGACGATAGTTGCCTCCATGACGTTT
AGCCCCCGCAGGTCCACGGCCGGGTGCTCCAGCGCTGGCGTGGTTGTAGTCAAATGTTGGTAGTCAGG
+
FFFFFFFFFFFFFFFFFFFFFFFFFFFFFFFFFFFFFF:FFFFFFFFFFFF,FFFFFFFFFFFFFFFF
FFFFFFFFFFFFF:FFFFFFFFFFFFFFFFFFFFFFFFFFFFFFFFFFFFFFFFFFFFFFFFFFFFFF
@A00155:342:HHGFNDSXY:1:2314:31304:21073 2:N:0:GAACCTAG+TCCGCATA
GTCTCGCTCTAGACCTTCTTGACCATCTGGCAGTCAGCATAAGGACGATAGTTGCCTCCATGACGTTT
AGCCCCCGCAGGTCCACGGCCGGGTGCTCCAGCGCTGGCGTGGTTGTAGTCAAATGTTGGTAGTCAGG
+
F:F:F:F:F:F:FFF::F::FFFFF:F:FFFFF:FF:FF,FFFF:F:F,FF::FFF:FFF:FFFF:::
F:FFFFF:FFFF:FFFFFFFFFFF:FF,FFF:F:F:FFFF:FF::F:FF:FFFF:F::FF:F::FF::
@A00155:342:HHGFNDSXY:1:1553:21992:14152 2:N:0:GAACCTAG+TCCGCATA
CTCGCTCTAGACCTTCTTGACCATCTGGCAGTCAGCATAAGGACGATAGTTGCCTCCATGACGTTTAG
CCCCCGCAGGTCCACGGCCGGGTGCTCCAGCGCTGGCGTGGTTGTAGTCAAATGTTGGT
+
FFFFFFF,FF:FFFFFF:FFF:,FFF:FFFFFFFFFFFFFF:FFFF,FFFFFFFFFFFFFFFFFFFFF
:FF,FFFFFF,FFF:FFFFFFFFF:FFFFF:F:FFFFFFFFFFFFFFFFFFFFFFFFFF
@A00155:342:HHGFNDSXY:1:1649:17842:13823 2:N:0:GAACCTAG+TCCGCATA
CTCGCTCTAGACCTTCTTGACCATCTGGCAGTCAGCATAAGGACGATAGTTGCCTCCATGACGTTTAG
CCCCCGCAGGTCCACGGCCGGGTGCTCCAGCGCTGGCGTGGTTGTAGTCAAATGTTGGT
+
FFFFFFFFFFFFFFFFFFFFFFF:FFFFFFFFFFFFF:FFFFFFFFFFFFFFFFFFFF:FFFFFFFFF
FFFFFFFFFFFFFFFFFFFFFFFFFFFFFFFFFFFFFFFFFFFFFFFFFFFFFFFFFFF
```

```
@A00155:342:HHGFNDSXY:1:2650:19162:28541 2:N:0:GAACCTAG+TCCGCATA
CTCGCTCTAGACCTTCTTGACCATCTGGCAGTCAGCATAAGGACGATAGTTGCCTCCATGACGTTTAG
CCCCCGCAGGTCCACGGCCGGGTGCTCCAGCGCTGGCGTGGTTGTAGTCAAATGTTGGTAGTCAGGGT
+
FFFFFFFFFFFFFFFFFFFFFF,FFFFFFFFFFFFFFFFFFFFFFFFFFFFFFFFFFFFFFFF:FFFF
FFFFFFFFFFFFFFFFFFFFFFFFFFFFFFFFFFFFFFFFFFFFFFFFFFFFFFFFFFFFFFFFFFFF
@A00155:342:HHGFNDSXY:1:1315:11993:27273 2:N:0:GAACCTAG+TCCGCATA
CTCGCTCTAGACCTTCTTGACCATCTGGCAGTCAGCATAAGGACGATAGTTGCCTCCATGACGTTTAG
CCCCCGCAGGTCCACGGCCGGGTGCTCCAGCGCTGGCGTGGTTGTAGTCAAATGTTGGTAGTCAGGGT
+
FF:FFFFFFFF,FFFFFFFFF,FFFF,FFF,::F:FFFFFFFF,:,FFF,FF:FFF,FFFFFFFFFF:
:F,F,FFFF:F,FF,,F,F:FFFFFF::FF:FFFFF:FFFFFFFFFFFFFFFFFFFF:FFFFFF:FFFF
@A00155:342:HHGFNDSXY:1:2473:6985:11553 2:N:0:GAACCTAG+TCCGCATA
CGCTCTAGACCTTCTTGACCATCTGGCAGTCAGCATAAGGACGATAGTTGCCTCCATGACGTTTAGCC
CCCGCAGGTCCACGGCCGGGTGCTCCAGCGCTGGCGTGGTTGTAGTCAAATGTTGGTAGTCAGGGTAG
+
FFFFFFFFFFFFFFFFFFFFFFFFFFFFFFFFFFFFFFFFFFFFFFFFFFFFFFFFFFFFFFFFFFFF
FFFFFFFFFFFFFFFFFFFFFFFFFFFFFFFFFFFFFFFFFFFFFFFFFFFFFFFFFFFFFFFFFFFF
@A00155:342:HHGFNDSXY:1:2178:10041:9424 2:N:0:GAACCTAG+TCCGCATA
CGCTCTAGACCTTCTTGACCATCTGGCAGTCAGCATAAGGACGATAGTTGCCTCCATGACGTTTAGCC
CCCGCAGGTCCACGGCCGGGTGCTCCAGCGCTGGCGTGGTTGTAGTCAAATGTTGGTAGTCAGGGTAG
+
FFFFFFFFFFFFFFFFFFFFFFFFFFFFFFFFFFFFFFFFFFFFFFFFFFFFFFFFFFFFFFFFFFFF
FFFFFFFFFFFFFFFFFFFFFFFFFFFFFFFFFFFFFFFFFFFFFFFFFFFFFFFFFFFFFFFFFFFF
@A00155:342:HHGFNDSXY:1:1252:30427:18176 2:N:0:GAACCTAG+TCCGCATA
CGCTCTAGACCTTCTTGACCATCTGGCAGTCAGCATAAGGACGATAGTTGCCTCCATGACGTTTAGCC
CCCGCAGGTCCACGGCCGGGTGCTCCAGCGCTGGCGTGGTTGTAGTCAAATGTTGGTAGTCAGGGTAG
+
FFFFFFFFFFFFFFFFFFFF:FFFFFFFFFFFFFFFFFFFFFFFFFFF:FFFFFFFFFFFFFFFFFFFF
FFFFFFFFFFFFFFFFFFFFFFF:FFFFFFFFFFFFFFFFFFFFFFFFFFFFFFFFFFFFFFFFFFFF
@A00155:342:HHGFNDSXY:1:1252:30129:28620 2:N:0:GAACCTAG+TCCGCATA
CGCTCTAGACCTTCTTGACCATCTGGCAGTCAGCATAAGGACGATAGTTGCCTCCATGACGTTTAGCC
CCCGCAGGTCCACGGCCGGGTGCTCCAGCGCTGGCGTGGTTGTAGTCAAATGTTGGTAGTCAGGGTAG
+
FFFFFFFFFFFFFF:FFFFFFFFFFFFFFFFFFFFFFF:FFFFFFFFFFFFFFFFFFFFFFFFFFFFF
FFFFFFFFFFFFFFFFFFFFFFFFFFFFFFFFFFFFFFFFFFFFFFFFFFFFFFFFFFFFFFFFFFFF
@A00155:342:HHGFNDSXY:1:2621:5764:34021 2:N:0:GAACCTAG+TCCGCATA
GCTCTAGACCTTCTTGACCATCTGGCAGTCAGCATAAGGACGATAGTTGCCTCCATGACGTTTAGCCC
CCGCAGGTCCACGGCCGGGTGCTCCAGCGCTGGCGTGGTTGTAGTCAAATGTTGGTAGTCAGGGTAGT
+
FF:FFFFFFFFFFFFFFFFFFFFFFFFF:FFFFF:FFFFFFFFFFFFFFFFFFFFFFFFFFFFFFFFF
FFFFFFFFFFFFFFFFFFFFFFFFFFFFFFFFFFFFFFFFFFFFFFFFFFFFFFFFFFFFFFFFFFF:
@A00155:342:HHGFNDSXY:1:1262:27010:36683 1:N:0:GAACCTAG+TCCGCATA
CTAGACCTTCTTGACCATCTGGCAGTCAGCATAAGGACGATAGTTGCCTCCATGACGTTTAGCCCCCG
CAGGTCCACGGCCGGGTGCTCCAGCGCTGGCGTGGTTGTAGTCAAATGTTGGTAGTCAGGG
+
FFFFFFFFFFFFFFFFFFFFFFFFFFFFFFFFFFFFFFFFFFFFFFFFFFFFFFFFFFFFFFFFFFFF
FFFFF:FFFFFFFFFF:FFFFFFFFFFFFFFF:FFFFFFFFFFFFFFFFFFFFFFFFFFFF
@A00155:342:HHGFNDSXY:1:1669:13693:11177 1:N:0:GAACCTAG+TCCGCATA
CTAGACCTTCTTGACCATCTGGCAGTCAGCATAAGGACGATAGTTGCCTCCATGACGTTTAGCCCCCG
CAGGTCCACGGCCGGGTGCTCCAGCGCTGGCGTGGTTGTAGTCAAATGTTGGTAGTCAGGGTAG
+
FFFFFFFFFFFFFFFFFFFFFFFFFFFFFFFFFFFFFFFFFFFFFFFFFFFFFFFFFFFFFFFFFFFF
F:FFFFFFFFFFFFFFFFF:FFFFFFFFFFFFFFF:FFFFFFFFFFFFFFFFFFFFFFFFFFFF
```

```
@A00155:342:HHGFNDSXY:1:2615:14217:9987 1:N:0:GAACCTAG+TCCGCATA
CTAGACCTTCTTGACCATCTGGCAGTCAGCATAAGGACGATAGTTGCCTCCATGACGTTTAGCCCCCG
CAGGTCCACGGCCGGGTGCTCCAGCGCTGGCGTGGTTGTAGTCAAATGTTGGTAGTCAGGGTAG
+
FFFFFFFFFFFFFFFFFFFFFFFFFFFFFFFFFFFFFFFFFFFFFFFFFFFFFFFFFFFFFFFFFFFF
FFFFFFFFFFFFFFFFFFFFFFFFFFFFFFFFFFF:FFFFFFFFFFFFFFFF:FFFFFFFFF:F
@A00155:342:HHGFNDSXY:1:2335:22281:7795 1:N:0:GAACCTAG+TCCGCATA
CTAGACCTTCTTGACCATCTGGCAGTCAGCATAAGGACGATAGTTGCCTCCATGACGTTTAGCCCCCG
CAGGTCCACGGCCGGGTGCTCCAGCGCTGGCGTGGTTGTAGTCAAATGTTGGTAGTCAGGGTAG
+
:FFFF,FFFFFFFFFFF:FFFFFF:FFF:F:FFFFFFFFF,FFFFFFFFFFFFFF:FFFFFFFFFFFF
FFFFFFFFFFFFFFFFFFF:FFFFFFF:FFF,F:FFFF,FF:FFFFFFFFFFFFFFFFFFFFFF
@A00155:342:HHGFNDSXY:1:1340:21386:14387 1:N:0:GAACCTAG+TCCGCATA
CTAGACCTTCTTGACCATCTGGCAGTCAGCATAAGGACGATAGTTGCCTCCATGACGTTTAGCCCCCG
CAGGTCCACGGCCGGGTGCTCCAGCGCTGGCGTGGTTGTAGTCAAATGTTGGTAGTCAGGGTAG
+
FFFFFFFFFFFFFFFFFFFFFFFFFFFFFFFFFF:FFFFFFFFFFFFFFFFFFFFFFFFFF:FFFFFF
:FFFFFFFFFFFFFFF:FFFF:FFFFFFFFFF:FFFFFFFFFFFFFFFFFFFFFFFFFFFFFFF
@A00155:342:HHGFNDSXY:1:1578:4264:33708 2:N:0:GAACCTAG+TCCGCATA
CTAGACCTTCTTGACCATCTGGCAGTCAGCATAAGGACGATAGTTGCCTCCATGACGTTTAGCCCCCG
CAGGTCCACGGCCGGGTGCTCCAGCGCTGGCGTGGTTGTAGTCAAATGTTGGTAGTCAGGGTAGTTTT
+
FFFFFFFFF:FFFFFFFFFFFFFFFFFFFFFFFFFFFFFFFFFFFFFFFFFF:FFFF,FFFFFFFFFF
FFFFFFFFFFFFFFFFFFFFFFFFFFFFFFFFFFFFFFFFFFFFFFFFFFF:FFFFFFFFFFFFFFFF
@A00155:342:HHGFNDSXY:1:2566:7392:35493 2:N:0:GAACCTAG+TCCGCATA
CTAGACCTTCTTGACCATCTGGCAGTCAGCATAAGGACGATAGTTGCCTCCATGACGTTTAGCCCCCG
CAGGTCCACGGCCGGGTGCTCCAGCGCTGGCGTGGTTGTAGTCAAATGTTGGTAGTCAGGGTAGTTTT
+
FFFFFFFFFF::FF,F:FFF:FFFFFFFFFFFF:FFFFFFF,FFFFFFFFF:FFFFF,,FFFFFFFFF
FFFFFFFFFFFFFFFFFFFFFFFFFFFFFFFFFFFF:FFFFFFFFFFFFFFFFFF:FFFFFFFFFF:F
@A00155:342:HHGFNDSXY:1:2226:14841:17206 2:N:0:GAACCTAG+TCCGCATA
CTAGACCTTCTTGACCATCTGGCAGTCAGCATAAGGACGATAGTTGCCTCCATGACGTTTAGCCCCCG
CAGGTCCACGGCCGGGTGCTCCAGCGCTGGCGTGGTTGTAGTCAAATGTTGGTAGTCAGGGTAGTTTT
+
FFFFFFFFFFFFFFFFFFFFFFFFFFFFFFFFFFFFFFFFFFFFFFFFFFFFFFFFFFFFFFFFFFFF
FFFFFFFFFFFFFFFFFFFFFFFFFFFFFFFFFFFFFFFFFFFFFFFFFFFFFFFFFFFFF:FFFFFF
@A00155:342:HHGFNDSXY:1:2308:28266:12524 2:N:0:GAACCTAG+TCCGCATA
CTAGACCTTCTTGACCATCTGGCAGTCAGCATAAGGACGATAGTTGCCTCCATGACGTTTAGCCCCCG
CAGGTCCACGGCCGGGTGCTCCAGCGCTGGCGTGGTTGTAGTCAAATGTTGGTAGTCAGGGTAGTTTT
+
FFFFFF:FFFFFFFFF:FFFFFFFFFFFFFFFFFFFFFFFFFFFFFFFFFFFFFFFFFFFFFFFFFFF
FFFFFFFFFFFFFFFFFFFFFFFFFFFFFFFFFFFFFFFFFFFFFFFFFFFFFFFFFFFFFFFF:FFF
@A00155:342:HHGFNDSXY:1:1262:27010:36683 2:N:0:GAACCTAG+TCCGCATA
TAGACCTTCTTGACCATCTGGCAGTCAGCATAAGGACGATAGTTGCCTCCATGACGTTTAGCCCCCGC
AGGTCCACGGCCGGGTGCTCCAGCGCTGGCGTGGTTGTAGTCAAATGTTGGTAGTCAGGGTAGTTTTC
+
FFFFF:FFFFFFFFFFFFFFFFFFFFFFFFFFFFFFFFFFFFFFFFFFFFFFFFFFFFFFFFFFFFFF
FFFFF:FFFFFFFFFFFFFFFFFFFFFFFFFFFFFFFFFFFFFFFFFFFFFFFFFFFFFFFFFFFFFF
@A00155:342:HHGFNDSXY:1:1314:30861:18176 2:N:0:GAACCTAG+TCCGCATA
TAGACCTTCTTGACCATCTGGCAGTCAGCATAAGGACGATAGTTGCCTCCATGACGTTTAGCCCCCGC
AGGTCCACGGCCGGGTGCTCCAGCGCTGGCGTGGTTGTAGTCAAATGTTGGTAGTCAGGGTAGTTTTC
+
FFFFFFFFFFFFFFFFFFFFFFFFFFFFFFFFFFFFFFF,FFFFFFFFFFFFFFFFFFFFFFFFFFFF
FFFFFFFFFFFFFFFFFFFFFFFFFF:FFFFFFFFFFFFFFFFFFFFFFFFFFFFFFFFFFFFFFFFF
```

```
@A00155:342:HHGFNDSXY:1:2311:24306:36699 2:N:0:GAACCTAG+TCCGCATA
TAGACCTTCTTGACCATCTGGCAGTCAGCATAAGGACGATAGTTGCCTCCATGACGTTTAGCCCCCGC
AGGTCCACGGCCGGGTGCTCCAGCGCTGGCGTGGTTGTAGTCAAATGTTGGTAGTCAGGGTAGTTTTC
+
FFFFFFFFFFFFFFFFFFFFFFFFFFFFFFFFFFFFFFFFFFFFFFFFFFFFFFFFFFFFFFFFFFFF
FFFFFFFFFFFFFFFFFFFFFFFFFFFFFFFFFFFFF:FFFFFFFFFFFFFFFFFFFFFFFFFFFFFF
@A00155:342:HHGFNDSXY:1:1115:9742:2801 2:N:0:GAACCTAG+TCCGCATA
TAGACCTTCTTGACCATCTGGCAGTCAGCATAAGGACGATAGTTGCCTCCATGACGTTTAGCCCCCGC
AGGTCCACGGCCGGGTGCTCCAGCGCTGGCGTGGTTGTAGTCAAATGTTGGTAGTCAGGGTAGTTTTC
+
FFFFFFFFFFFF:FFFFFFFFFFFFFFFFFFFFFFFFFFFFFFFFFFFFFFFFFFF:FFF:FFF:FFF
FF:FFFFFFFFFFFFFFFFFFFFFFFFFFFFFFFFFFFFFFFFFFFFFFFFFFFFFF,FFFFFFFF
@A00155:342:HHGFNDSXY:1:2354:9815:5744 2:N:0:GAACCTAG+TCCGCATA
ACCTTCTTGACCATCTGGCAGTCAGCATAAGGACGATAGTTGCCTCCATGACGTTTAGCCCCCGCAGG
TCCACGGCCGGGTGCTCCAGCGCTGGCGTGGTTGTAGTCAAATGTTGGTAGTCAGGGTAGTTTTCCT
+
F:FFFFFFFFFF:F,FFFFFFF:FFF:FFFFFF:FF,FFFFFFFFFFF:F,FFFF,FFFFFFFF,FF
FFFF:FFFFFF:,FF:FF:FF:,FFF:FFF,F:FF::,:FFFF:FFF:FFF:F,F:,F:,FF:FFFF
@A00155:342:HHGFNDSXY:1:1549:19633:11631 2:N:0:GAACCTAG+TCCGCATA
CTTCTTGACCATCTGGCAGTCAGCATAAGGACGATAGTTGCCTCCATGACGTTTAGCCCCCGCAGGTC
CACGGCCGGGTGCTCCAGCGCTGGCGTGGTTGTAGTCAAATGTTGGTAGTCAGGGTAGTTTTCCTCCC
+
FFFFFFFFFFFFF,FFFFFFFFFFFFFFFFFFFFFFF:FFFFFFFFFFFFFFF:FFFFFFFFFFFFFF
FFFFFFFFFFFFFFFFFFFFFFFFFFF,FFFFFFFF:FFFFFFFFFFFFFFFFFFFFFFFFFFFFFFF
@A00155:342:HHGFNDSXY:1:2369:7961:30874 1:N:0:GAACCTAG+TCCGCATA
TTCTTGACCATCTGGCAGTCAGCATAAGGACGATAGTTGCCTCCATGACGTTTAGCCCCCGCAGGTCC
ACGGCCGGGTGCTCCAGCGCTGGCGTGGTTGTAGTCAAATGTTGGTAGTCAGGGTAGTTTTCCTCCCC
+
FFFFFFFFFFFFFFFFFFFFFFFFFFFFFFFFFFFFFFFFFFFFFFFFFFFFFFFFFFFFFFFFFFFF
FFFFFFFFFFFFFFFFFFFFFFFFFFFFFFFFFFFFFFFFFFFFFFFFFFFFFFFFFFFFFFFFFFFF
@A00155:342:HHGFNDSXY:1:2319:26603:28682 1:N:0:GAACCTAG+TCCGCATA
TTCTTGACCATCTGGCAGTCAGCATAAGGACGATAGTTGCCTCCATGACGTTTAGCCCCCGCAGGTCC
ACGGCCGGGTGCTCCAGCGCTGGCGTGGTTGTAGTCAAATGTTGGTAGTCAGGGTAGTTTTCCTCCCC
+
FFFF,FFFFFF,FFF:F::FF,FFFFFF:FF,FFF,:FFFF:FFFFFFFFFFFFFFF::::FFFFFF:
FFFFFF::F:FFFFFF,:FFF,FFF,FFFFFF:FFFFFFF:F:F:FFFFFFFFFF,,:FFF:FF,FFF
@A00155:342:HHGFNDSXY:1:2650:12364:19961 1:N:0:GAACCTAG+TCCGCATA
TTCTTGACCATCTGGCAGTCAGCATAAGGACGATAGTTGCCTCCATGACGTTTAGCCCCCGCAGGTCC
ACGGCCGGGTGCTCCAGCGCTGGCGTGGTTGTAGTCAAATGTTGGTAGTCAGGGTAGTTTTCCTCCCC
+
FFFFFFFFFFFFFFFFFFFFFFFFFFFFFFFFFFFFFFFFFFFFFFFFFFFFFFFFFFFFFFFFFFFF
FFFFFFFFFFFFFFFFFFFFFFFFFFFFFFFFFFFFFF:FFFFFFFFFFFFFFFFFFFFFFFFFFFFF
@A00155:342:HHGFNDSXY:1:1462:23647:8437 1:N:0:GAACCTAG+TCCGCATA
TTCTTGACCATCTGGCAGTCAGCATAAGGACGATAGTTGCCTCCATGACGTTTAGCCCCCGCAGGTCC
ACGGCCGGGTGCTCCAGCGCTGGCGTGGTTGTAGTCAAATGTTGGTAGTCAGGGTAGTTTTCCTCCCC
+
FFFFFF:FFFFFFFFFFFFFFFFFFFFFFFFFFFFFFFFFFFFFFFFFFFFFFFFFFFFFFFFFFFFF
FFFFFFFFFFFFFFFFFFFFFFFFFFFFFFFFFFFFFFFFFFFFFFFFFFFFFFFFFFFFFFFFFFFF
@A00155:342:HHGFNDSXY:1:1512:1687:14873 1:N:0:GAACCTAG+TCCGCATA
TTCTTGACCATCTGGCAGTCAGCATAAGGACGATAGTTGCCTCCATGACGTTTAGCCCCCGCAGGTCC
ACGGCCGGGTGCTCCAGCGCTGGCGTGGTTGTAGTCAAATGTTGGTAGTCAGGGTAGTTTTCCTCCCC
+
FFFFFFFFFFFFFFFFFFFF:F,FFFFF:FFFFFFFFFFFFFFFFFFFFFFFFFFFFFFFFFFFFFFF
FFFFFFFFFFFFFFFFFFFFFFFFFFFFFFFFFFFFFFFFF,FFFFFFFFFFFFFFFFFFFFFFFFFF
```

```
@A00155:342:HHGFNDSXY:1:1525:3269:12821 1:N:0:GAACCTAG+TCCGCATA
TCTTGACCATCTGGCAGTCAGCATAAGGACGATAGTTGCCTCCATGACGTTTAGCCCCCGCAGGTCCA
CGGCCGGGTGCTCCAGCGCTGGCGTGGTTGTAGTCAAATGTTGGTAGTCAGGGTAGTTTTCCTCCCCT
+
FFFFFFFFFFFFFFFFFFFFFFFFFFFFFFFFFFFFFFFFFFFFFFFFFFFFFFFFFFFFFFFFFFFF
FFFFFFFFFFFFFFFFFFFFFFFFFFFFFFFFFFFFFFFFFFFFFFFFFFFFF:FFFFFFFFFFFFFF
@A00155:342:HHGFNDSXY:1:1378:7509:9768 1:N:0:GAACCTAG+TCCGCATA
TCTTGACCATCTGGCAGTCAGCATAAGGACGATAGTTGCCTCCATGACGTTTAGCCCCCGCAGCTCCA
CGGCCGGGTGCTCCAGCGCTGGCGTGGTTGTAGTCAAATGTTGGTAGTCAGGGTAGTTTTCCTCCCCT
+
FFFFFFFFFFFFFFFFF:FFFFFFFFFFFFFFFFFFFFFFFFFFFFFFFF,FF,FFFFFFFFFFFFFF
FFFFFFFFFFFFFFFFFFFFFFFFFFFFFFFFFFFFFFFFFFFFFFFFFFFFFFFFFFFFFFFFFFFF
@A00155:342:HHGFNDSXY:1:1252:30129:28620 1:N:0:GAACCTAG+TCCGCATA
TCTTGACCATCTGGCAGTCAGCATAAGGACGATAGTTGCCTCCATGACGTTTAGCCCCCGCAGGTCCA
CGGCCGGGTGCTCCAGCGCTGGCGTGGTTGTAGTCAAATGTTGGTAGTCAGGGTAGTTTTCCTCCCCT
+
FFFF:FFFFFFFFFFFFFFFFFFFFFFFFFFFFFFFFFFFFFFFFFFFFFFFFFFFFFFFFFFFFFF:
FFFFFFFFFFFFFFFFFFFFFFFF:FFFFF,FFFFFFF,FFFFFFFFFFFFFFFFFFFFFFFFFFFFF
@A00155:342:HHGFNDSXY:1:1266:1461:6308 2:N:0:GAACCTAG+TCCGCATA
TCTTGACCATCTGGCAGTCAGCATAAGGACGATAGTTGCCTCCATGACGTTTAGCCCCCGCAGGTCCA
CGGCCGGGTGCTCCAGCGCTGGCGTGGTTGTAGTCAAATGTTGGTAGTCAGGGTAGTTTTCCTCCCCT
+
FFFFFFFFFFFFFFFFFFFFFFF:FFFFFFFFFFFFFFFFFFFFFFFFFFFFFFFFFFFFFFFFFFFF
FFFFFFFFFFFFFFFFFFFFFFFFFFFFFFFFFFFFFFFFFFFFFFFFFFFFFFFFFFFFFFFFFFFF
@A00155:342:HHGFNDSXY:1:2531:19886:34460 1:N:0:GAACCTAG+TCCGCATA
TCTTGACCATCTGGCAGTCAGCATAAGGACGATAGTTGCCTCCATGACGTTTAGCCCCCGCAGGTCCA
CGGCCGGGTGCTCCAGCGCTGGCGTGGTTGTAGTCAAATGTTGGTAGTCAGGGTAGTTTTCCTCCCCT
+
FFFFFFFFFFFFFFFFFFFFFFFFFFFFFFFFFFFFFFFFFFFFFFFFFFFFFFFFFFFFFFFFFFFF
FFFFFFFFFFFFFFFFFFFFFFFFFFFFFFFFFFFFFFFFFFFFFFFFFFFFFFFFFFFFFFFFFFFF
@A00155:342:HHGFNDSXY:1:1252:30427:18176 1:N:0:GAACCTAG+TCCGCATA
TCTTGACCATCTGGCAGTCAGCATAAGGACGATAGTTGCCTCCATGACGTTTAGCCCCCGCAGGTCCA
CGGCCGGGTGCTCCAGCGCTGGCGTGGTTGTAGTCAAATGTTGGTAGTCAGGGTAGTTTTCCTCCCCT
+
FFFFFFFFFFFFFFFFFFFFFFFFFFFFFFFFFFFFFFFFF,FFFFFFFFFFFFFFFFFFFFFFFFFF
FFFFFFFFFFFFFFFFFFFFFFFFFFFFFF:FFFFFFF:FFFFFFFFFFFFFFFFFFFFFFFFFFFFF
@A00155:342:HHGFNDSXY:1:1462:23647:8437 2:N:0:GAACCTAG+TCCGCATA
TCTTGACCATCTGGCAGTCAGCATAAGGACGATAGTTGCCTCCATGACGTTTAGCCCCCGCAGGTCCA
CGGCCGGGTGCTCCAGCGCTGGCGTGGTTGTAGTCAAATGTTGGTAGTCAGGGTAGTTTTCCTCCCCT
+
FF:FFFFFFFFFFFFFFFFFFFFFFFFF:FFFFFFFFFFFFFF,FFFFFFFFFFFFFFFFFFFFFFFF
FFFFFFFFFFFFFFFFFFFFFFFFFFFFFF:FFFFFFFFFFFFFFFFFFFFFFFFFFF:FFFFFFFFF
@A00155:342:HHGFNDSXY:1:2459:3161:11976 2:N:0:GAACCTAG+TCCGCATA
TCTTGACCATCTGGCAGTCAGCATAAGGACGATAGTTGCCTCCATGACGTTTAGCCCCCGCAGGTCCA
CGGCCGGGTGCTCCAGCGCTGGCGTGGTTGTAGTCAAATGTTGGTAGTCAGGGTAGTTTTCCTCCCCT
+
FFFFFFFFFFF:FFFFFFFFFFFFFFFFFFFFFFFFFFFFFFFFFFFFF:FFFFFFFFFFFFFFFFFF
FFFFFFFFFFFFFFFFFFFFFFFFFFFFFFFFFFFFFFFFFFFFFFFFFFFFFFFFFF:FFF:FFFFF
@A00155:342:HHGFNDSXY:1:2218:20455:19445 1:N:0:GAACCTAG+TCCGCATA
TCTTGACCATCTGGCAGTCAGCATAAGGACGATAGTTGCCTCCATGACGTTTAGCCCCCGCAGGTCCA
CGGCCGGGTGCTCCAGCGCTGGCGTGGTTGTAGTCAAATGTTGGTAGTCAGGGTAGTTTTCCTCCCCT
+
FFFFFFFFFFFFFFFFFFFFFFFFFFFFFFFFFFFFFFFFFFFFFFFFFFFFFFFFFFFFFFFFFFFF
FFFFFFFFFFFFFFFFFFFFFFFFFFF:FFFFFFFFFFFFFFFFFFFFFFFFFFFFFFFFFFFFFFFF
```

```
@A00155:342:HHGFNDSXY:1:1649:17842:13823 1:N:0:GAACCTAG+TCCGCATA
CTTGACCATCTGGCAGTCAGCATAAGGACGATAGTTGCCTCCATGACGTTTAGCCCCCGCAGGTCCAC
GGCCGGGTGCTCCAGCGCTGGCGTGGTTGTAGTCAAATGTTGGTAGTCAGGGTAGTTTT
+
FFFFFFFFF:FFFFFFFFFFFFF,FFFFFFFFFFFFFFFFFFFFFFFFFFFFFFFFFFFFFFFFFFFF
FFFFFFFFFFFFFFFFFFFFFFFFFFFFF:FFFFFFFFFF,FFF:FFFFFFFFFFFFFFF
@A00155:342:HHGFNDSXY:1:2354:22453:6590 1:N:0:GAACCTAG+TCCGCATA
CTTGACCATCTGGCAGTCAGCATAAGGACGATAGTTGCCTCCATGACGTTTAGCCCCCGCAGGTCCAC
GGCCGGGTGCTCCAGCGCTGGCGTGGTTGTAGTCAAATGTTGGTAGTCAGGGTAGTTTTCCTCCCCTG
+
FFFFFFFFFFFFFFFFFFFFFFFFFFFFFFFFFFFFFFFFFFFFFFFFFFFFFFFFFFFFFFFFFF:F
FFFFFFFFFFFFFFFFFFFFFFFFFFFFFFFFFFFFFFFFFFFFFFFFFFFF:FFFFFFFFFFFFFFF
@A00155:342:HHGFNDSXY:1:2354:24885:4507 1:N:0:GAACCTAG+TCCGCATA
CTTGACCATCTGGCAGTCAGCATAAGGACGATAGTTGCCTCCATGACGTTTAGCCCCCGCAGGTCCAC
GGCCGGGTGCTCCAGCGCTGGCGTGGTTGTAGTCAAATGTTGGTAGTCAGGGTAGTTTTCCTCCCCTG
+
FFFFFFFFFFFFFFFFFFFFFFFFFFFFFFFFFFFFFFFFFFFFFFFFFFFFFFFFFFFFFFFFFFFF
FFFFFFFFFFFFFFFFFFFFFFFFFFFFFFF:FFFFFFFFFFFFFFFFFFFFFFFFFFFFFFFFFFFF
@A00155:342:HHGFNDSXY:1:2205:13666:17832 2:N:0:GAACCTAG+TCCGCATA
TGACCATCTGGCAGTCAGCATAAGGACGATAGTTGCCTCCATGACGTTTAGCCCCCGCAGGTCCACGG
CCGGGTGCTCCAGCGCTGGCGTGGTTGTAGTCAAATGTTGGTAGTCAGGGTAGTTTTCCTCCCCTGCT
+
FFFFFFFFFFFFFFFFFFFFFFFFFFFFFFFFFFFFFFFFFFFFFFFFFFFFFFFFFFF:FFFFFFFF
FFFFFFFFFFFFFFFFFFFFFFFFFFFFFFFFFFFFFFFFFFFFFFFFFFFFFFFFFFFFFFFFFFFF
@A00155:342:HHGFNDSXY:1:2233:20889:28056 2:N:0:GAACCTAG+TCCGCATA
TGACCATCTGGCAGTCAGCATAAGGACGATAGTTGCCTCCATGACGTTTAGCCCCCGCAGGTCCACGG
CCGGGTGCTCCAGCGCTGGCGTGGTTGTAGTCAAATGTTGGTAGTCAGGGTAGTTTTCCTCCCCTGCT
+
FFFFFFFFFFFFFFFFFFFF:FFFFFFFFFFF,FFFFFFFFFFFFFFF,:FFFFFFFFFFFFFFFFFF
FFFFFFFFFFFFFFFFFFFFFFFFFFFFFFFFFFFFFFFFFFFFFFFFFFFFFFFFFFFFFFFFFFFF
@A00155:342:HHGFNDSXY:1:2259:29649:34053 1:N:0:GAACCTAG+TCCGCATA
TGACCATCTGGCAGTCAGCATAAGGACGATAGTTGCCTCCATGACGTTTAGCCCCCGCAGGTCCACGG
CCGGGTGCTCCAGCGCTGGCGTGGTTGTAGTCAAATGTTGGTAGTCAGGGTAGTTTTCCTCCCCTGCT
+
FFFFFFFFFFFFFFFFFFFFFFFFFFFFFFFFFFFFFFFFFFF:FFFFFFFF:FFFFFFFFFFFFFFF
FFFFFFFFFFFFFFFFFFFFFFFFFFFFFFFFFF:F:FFFFFFFFFFFFFFF:FFFFFFFFFFFFFFF
@A00155:342:HHGFNDSXY:1:2335:22281:7795 2:N:0:GAACCTAG+TCCGCATA
TGACCATCTGGCAGTCAGCATAAGGACGATAGTTGCCTCCATGACGTTTAGCCCCCGCAGGTCCACGG
CCGGGTGCTCCAGCGCTGGCGTGGTTGTAGTCAAATGTTGGTAGTCAGGGTAGTTTTCCTCCCCTGCT
+
:FFF:FFFFF:F:FFFFFF:,FFFFFFFFFFFF:FFFFF:FFFFFFFF:FFFFFFFFFFFFFFFFFFF
FFFFFFFFFFFFFFFFFFFFFFFFFFFFFFFFFFFFFFFFFFFFFFFFFFFFFFFFFFFFFFFFFFFF
@A00155:342:HHGFNDSXY:1:1553:1732:28009 2:N:0:GAACCTAG+TCCGCATA
TGACCATCTGGCAGTCAGCATAAGGACGATAGTTGCCTCCATGACGTTTAGCCCCCGCAGGTCCACGG
CCGGGTGCTCCAGCGCTGGCGTGGTTGTAGTCAAATGTTGGTAGTCAGGGTAGTTTTCCTCCCCTGCT
+
:FFFFFFFFFFFFF:FF:FFFFFFFFFFFFFFFFFFFFFFF,FFFFFFFFFFFFFFFFFFFFFFFFFF
FFFFFFFFFFFFFFFFFFFFFFFFFFFFFF:FFFFFFFFFFFFFFF:FFFFFFFFFFFFFF:FFFFFF
@A00155:342:HHGFNDSXY:1:2428:16450:16360 2:N:0:GAACCTAG+TCCGCATA
TGACCATCTGGCAGTCAGCATAAGGACGATAGTTGCCTCCATGACGTTTAGCCCCCGCAGGTCCACGG
CCGGGTGCTCCAGCGCTGGCGTGGTTGTAGTCAAATGTTGGTAGTCAGGGTAGTTTTCCTCCCCTGCT
+
FFFFFFFFFFFFFFFFFFFFF,FFFFFFFFFFFFFFFFFFFFFFFFFFFFFFFFFFFFFFFFFFFFFF
FFFFFFFFFFFFFFFFFFFFFFFFFFFFFFFFFFFF:FFFFFFFFFFFFFFFFFFFFFFFFFF:FFFF
```

@A00155:342:HHGFNDSXY:1:2127:5647:2597 1:N:0:GAACCTAG+TCCGCATA
GACCATCTGGCAGTCAGCATAAGGACGATAGTTGCCTCCATGACGTTTAGCCCCCGCAGGTCCACGGC
CGGGTGCTCCAGCGCTGGCGTGGTTGTAGTCAAATGTTGGTAGTCAGGGTAGTTTTCCTCCCCTGCTC
+
FFFFFFFFFFFFFFFFFFFFFFFFFFFFFFFFFFFFFFFFFF:FFFFFFFFFFFFFFFFFFFFFFFFF
FFFFFFFFFFFFFFFFFFFFFFFFFFFFFFFFFFFFFFFFFFFFFFFFFFFFFFFFF:FFFFFFFFFF
@A00155:342:HHGFNDSXY:1:2514:31087:10645 2:N:0:GAACCTAG+TCCGCATA
GCCATCTGGCAGTCAGCATAAGGACGATAGTTGCCTCCATGACGTTTAGCCCCCGCAGGTCCACGGCC
GGGTGCTCCAGCGCTGGCGTGGTTGTAGTCAAATGTTGGTAGTCAGGGTAGTTTTCCTCC
+
FFFF:FFFFFFFFFFFFFFFFFFFFFFFFFFF:FFFFFFF::FFFFFFFFFFFFFFFFFFFFFFFFFFF
FFFFFFFFFFFFFFFFFFFFFFFFFFFFFFFFFFFFFFFFFFFFFFFFFFFFF:FFFFFF
@A00155:342:HHGFNDSXY:1:2578:7328:25113 2:N:0:GAACCTAG+TCCGCATA
ACCATCTGGCAGTCAGCATAAGGACGATAGTTGCCTCCATGACGTTTAGCCCCCGCAGGTCCACGGCC
GGGTGCTCCAGCGCTGGCGTGGTTGTAGTCAAATGTTGGTAGTCAGGGTAGTTTTCCTCCCCTGCTCC
+
FFFFFFFFFFF:FFFFFFFFFFFFFFFFFFFFFFFFFFFFFFFFFFFF,FFFFFFFFFFFFFFFFFFF
FFFFFFFFFFFFFFFFFFFFFFFFFFFFFFFFFFFFFFFFFFFFFFFFFFF:FFFF:FF,FFFF,FFFF
@A00155:342:HHGFNDSXY:1:1210:20500:15515 1:N:0:GAACCTAG+TCCGCATA
CATCTGGCAGTCAGCATAAGGACGATAGTTGCCTCCATGACGTTTAGCCCCCGCAGGTCCACGGCCGG
GTGCTCCAGCGCTGGCGTGGTTGTAGTCAAATGTTGGTAGTCAGGGTAGTTTTCCTCCCCTGCTCCAC
+
FFFFFFFFFFFFFFFFFFFFFFFFFFFFFFFFFFFFFFFFFFFFFFFFFFFFFFFFFFFFFFFFFFFF
FFFFFFFFFFFFFFFFFFFFFFFFFFFFFFFFFFFFFFFFFFFFFFFFFFFFFFFFFFFFFFFFFFFF
@A00155:342:HHGFNDSXY:1:2254:1479:32957 1:N:0:GAACCTAG+TCCGCATA
TCTGGCAGTCAGCATAAGGACGATAGTTGCCTCCATGACGTTTAGCCCCCGCAGGTCCACGGCCGGGT
GCTCCAGCGCTGGCGTGGTTGTAGTCAAATGTTGGTAGTCAGGGTAGTTTTCCTCCCCTGC
+
FFFFFFFFFFF:FFFFFFFFFFFFFFFFFFFFFFFFFF:FFFFFFFFFFFFFFFFFF:FFFFFFFFFFF
FFFFFFFFFFFFFFFFFFFFFFFFFFFFFFFF:FFFFFFFFFFFFFFFFFF:FFFF:F:FF
@A00155:342:HHGFNDSXY:1:1363:20518:32988 2:N:0:GAACCTAG+TCCGCATA
CTGGCAGTCAGCATAAGGACGATAGTTGCCTCCATGACGTTTAGCCCCCGCAGGTCCACGGCCGGGTG
CTCCAGCGCTGGCGTGGTTGTAGTCAAATGTTGGTAGTCAGGGTAGTTTTCCTCCCCTGCTCCACCT
+
F,FFFFFFFFFFFF,FFFFF,FFFF,FFFFFFF:FFFFFFFFFFFFFFFFFFF:FF,FFFFFFFF:FFF
FFFF:F,F,FFFF:F:FFFF,FFFF:F:FFFFFFFFFFFFFFFFFFFFFFFFFF:F:FFFFFFF,FFF
@A00155:342:HHGFNDSXY:1:1339:24966:27070 2:N:0:GAACCTAG+TCCGCATA
CTGGCAGTCAGCATAAGGACGATAGTTGCCTCCATGACGTTTAGCCCCCGCAGGTCCACGGCCGGGTG
CTCCAGCGCTGGCGTGGTTGTAGTCAAATGTTGGTAGTCAGGGTAGTTTTCCTCCCCTGCTCCACCTG
+
FFFF,FFF:FFFFF:FFFFFFF:FFFFF,FFFFFFFFFFFFFFF,FFFFFFFFFFF:FFFFFFFFFFF
FFFFFFFFFFFFFFFFFFFF::FF:F:FFFFFFFFFFFFFFF:FFFFFFFFFFFFF:FFFFFFFFFF:
@A00155:342:HHGFNDSXY:1:2315:3224:10645 2:N:0:GAACCTAG+TCCGCATA
CTGGCAGTCAGCATAAGGACGATAGTTGCCTCCATGACGTTTAGCCCCCGCAGGTCCACGGCCGGGTG
CTCCAGCGCTGGCGTGGTTGTAGTCAAATGTTGGTAGTCAGGGTAGTTTTCCTCCCCTGCTCCACCTG
+
FFF,F,,::F:F:FF:,,:FFFFF:,FFF:FFFF,FFFFF:F:FF:F:FFFFFFFFFFF,:FFFFFFF
FFFF,FF:FFFFFFFF,:FFFFFFFF,,FFFF,FFFFFFFFFFFFF,F,:FFF:FFFFFFF:FFF,FF
@A00155:342:HHGFNDSXY:1:1262:15338:13714 2:N:0:GAACCTAG+TCCGCATA
TGGCAGTCAGCATAAGGACGATAGTTGCCTCCATGACGTTTAGCCCCCGCAGGTCCACGGCCGGGTGC
TCCAGCGCTGGCGTGGTTGTAGTCAAATGTTGGTAGTCAGGGTAGTTTTCCTCCCCTGCTCCACCTGG
+
FFFFFFFFFFFFFFFFFFFFFFFFFFFFFFFFFFFF:FFFFFFFFFFFFFFFF:FFFFFFFFFFF:FF
FFFFFFFFFF:FFFFFFFF:FFFFFFFFFFFFFFFFFFFF:FFFFFFFFFFFFFFFFFFFFFFFFFFF

```
@A00155:342:HHGFNDSXY:1:1248:25690:28917 2:N:0:GAACCTAG+TCCGCATA
CAGTCAGCATAAGGACGATAGTTGCCTCCATGACGTTTAGCCCCCGCAGGTCCACGGCCGGGTGCTCC
AGCGCTGGCGTGGTTGTAGTCAAATGTTGGTAGTCAGGGTAGTTTTCCTCCCCTGCTCCACCTGGCCA
+
FFFFFFFFF:FFFFFFFFFFF:FFFFFFFFFFFFFFFFFFFFFFFFFFFFFFFFFFFFFFFFFFFF:F
FFFFFFFFFFFFFFFFFFFFFFFFFFFFFFFFFFFFFFFFFFFFFFFFFFFFFFFFFFFFFFFFFFFF
@A00155:342:HHGFNDSXY:1:1345:6551:13056 2:N:0:GAACCTAG+TCCGCATA
CAGTCAGCATAAGGACGATAGTTGCCTCCATGACGTTTAGCCCCCGCAGGTCCACGGCCGGGTGCTCC
AGCGCTGGCGTGGTTGTAGTCAAATGTTGGTAGTCAGGGTAGTTTTCCTCCCCTGCTCCACCTGGCCA
+
FFFFFFFFFFFFFFFFFFFFFFFFFFFFFFFFFFFFFFFFFF:FFFFFFFFFFFFFFFFFFFFFFFFF
FFFFFFFFFFFFFFFFFFFFFFF:FFFFFFFFFFFFFFFFFFFF:FFFFFFFFFFFFFFFFFFFFFFF
@A00155:342:HHGFNDSXY:1:2331:22444:29371 1:N:0:GAACCTAG+TCCGCATA
CAGTCAGCATAAGGACGATAGGTGCCTCCATGACGTTTAGCCCCCGCAGGTCCACGGCCGGGTGCTCC
AGCGCTGGCGTGGTTGTAGTCAAATGTTGGTAGTCAGGGTAGTTTTCCTCCCCTGCTCCACCTGGCCA
+
F:FFFFF:FFFFFFFFFFFFF,FFF:FFFFFFFFFFFF:FFFFFFFF:FF:FFFFF::FFFFFFFFFF
FF:FFFFFFFFFFFFFFFFFF::FFFFFFFFFFFFFFFFFFFFFFFFFFFF:FFFFFFFFF:FFFFFF
@A00155:342:HHGFNDSXY:1:1352:13322:17425 2:N:0:GAACCTAG+TCCGCATA
GTCAGCATAAGGACGATAGTTGCCTCCATGACGTTTAGCCCCCGCAGGTCCACGGCCGGGTGCTCCAG
CGCTGGCGTGGTTGTAGTCAAATGTTGGTAGTCAGGGTAGTTTTCCTCCCCTGCTCCACCTGGCC
+
FFFFFFFFFFFFFFFFFFFFFFFFFFFFFFFFFFFFFFFFFFFFFFFFFFFFFFFFFFFFFFFFFFFF
FFFFFFFFFFFFFFFFFFFFFFFFFFFFFFFFFFFFFFFFFFFFFFFFFFFFFFFFFFFFFFFFF
@A00155:342:HHGFNDSXY:1:2410:31602:13949 2:N:0:GAACCTAG+TCCGCATA
CAGCATAAGGACGATAGTTGCCTCCATGACGTTTAGCCCCCGCAGGTCCACGGCCGGGTGCTCCAGCG
CTGGCGTGGTTGTAGTCAAATGTTGGTAGTCAGGGTAGTTTTCCTCCCCTGCTCCACCTGGCCACGAG
+
FFFFFFFFFFFFFF,FFF:FFFFFFF:FFFFFF:FFFFFFFFFFFFFFFFFFFFF:FFFFFFFF:FFF
FFFFFFFFFFFFFFFFFFFFFFFFFFFFFFFFFFF,FFFF:FFFFFFFFFFFFFFFFFFFFFFFFFFF
@A00155:342:HHGFNDSXY:1:2514:31087:10645 1:N:0:GAACCTAG+TCCGCATA
GCATAAGGACGATAGTTGCCTCCATGACGTTTAGCCCCCGCAGGTCCACGGCCGGGTGCTCCAGCGCT
GGCGTGGTTGTAGTCAAATGTTGGTAGTCAGGGTAGTTTTCCTCCCCTGCTCCACCTGGC
+
FFFFF:FFFFFFFFFFFFFFFFFFFFFFFFFFFFFFFFFFFFFFFFF:FFFFFFFFFFFFFFFFFFFF
FFFFFFF,FFFFFFFFFFFFFFFFFFFFFFFFFFFFFFFFFFFF:FFFFFFFFFFFFFFF
@A00155:342:HHGFNDSXY:1:1553:1732:28009 1:N:0:GAACCTAG+TCCGCATA
GCATAAGGACGATAGTTGCCTCCATGACGTTTAGCCCCCGCAGGTCCACGGCCGGGTGCTCCAGCGCT
GGCGTGGTTGTAGTCAAATGTTGGTAGTCAGGGTAGTTTTCCTCCCCTGCTCCACCTGGCC
+
FFFFFFFFFFFFFFFFFFFFFFFFFFFFFFFFFFFFFFFFFFFFFFFFFFFFFFFFFFF:FFFFFFFF
FFFFFFFFFFFF:FFFFFFFF:FFFFFFFFFFFFFFFFFFFFFFFFFFFFFFFFFFFFFFF
@A00155:342:HHGFNDSXY:1:2331:22444:29371 2:N:0:GAACCTAG+TCCGCATA
GCATAAGGACGATAGTTGCCTCCATGACGTTTAGCCCCCGCAGGTCCACGGCCGGGTGCTCCAGCGCT
GGCGTGGTTGTAGTCAAATGTTGGTAGTCAGGGTAGTTTTCCTCCCCTGCTCCACCTGGCCACG
+
:FFFFFFFFFFF:FFFFFFF:FFFFFFFFF:,FFFFFFFFF,FF:FFFFFFFFFFF:FFFFF,FFFFF
FFFF:FFF,F:FFFFFFFFFFFFFFFFFFFF:FFFFFFFFFFFF,FFFF:FFFFFFF:FFFFFF
@A00155:342:HHGFNDSXY:1:1210:20500:15515 2:N:0:GAACCTAG+TCCGCATA
GCATAAGGACGATAGTTGCCTCCATGACGTTTAGCCCCCGCAGGTCCACGGCCGGGTGCTCCAGCGCT
GGCGTGGTTGTAGTCAAATGTTGGTAGTCAGGGTAGTTTTCCTCCCCTGCTCCACCTGGCCACGAGTC
+
FFFF::FFFFFFFFF,FFFFFFFFFFFFFFFFFFFFFFFFFFFFFFFFFFFFFFFFFFFFFFFFFFFF
FFFFFFFFFF:FFFFFFFFFFFFFFFFFFFFFFFFFFFFFFFFFFFFFFFFFFFFFFFFFFFFFFFFF
```

@A00155:342:HHGFNDSXY:1:2218:20455:19445 2:N:0:GAACCTAG+TCCGCATA
GCATAAGGACGATAGTTGCCTCCATGACGTTTAGCCCCCGCAGGTCCACGGCCGGGTGCTCCAGCGCT
GGCGTGGTTGTAGTCAAATGTTGGTAGTCAGGGTAGTTTTCCTCCCCTGCTCCACCTGGCCACGAGTC
+
FFF:FFFFFFFFFFFFFFFFFFFFFFFFFFF:FFFFFFFFFFFFFFFFFFFFFFFFFFFFFFFFFFFFFF
FFFFFFFFFFFFFFFFFFFFFFFFFFFFFFFFFFFFFFFFFFFFFFFFFFFFFFFFFFFFFFFFFFFFFF
@A00155:342:HHGFNDSXY:1:2531:19886:34460 2:N:0:GAACCTAG+TCCGCATA
GCATAAGGACGATAGTTGCCTCCATGACGTTTAGCCCCCGCAGGTCCACGGCCGGGTGCTCCAGCGCT
GGCGTGGTTGTAGTCAAATGTTGGTAGTCAGGGTAGTTTTCCTCCCCTGCTCCACCTGGCCACGAGTC
+
FF:FFFFFFFFFFFFFFFFFFFFFFFFFFFFFFFFFFFFFF:FFFFFFFFFFFFFFFFFFFFFFFFFFFF
FFFFFFFFFF:FFFFFFFFFFFFFFFFFFFFFFFFFFF:FFFFFFFFFFFFFFFFFFFFFFFFFFFFFFF
@A00155:342:HHGFNDSXY:1:2575:14498:20494 1:N:0:GAACCTAG+TCCGCATA
GCATAAGGACGATAGTTGCCTCCATGACGTTTAGCCCCCGCAGGTCCACGGCCGGGTGCTCCAGCGCT
GGCGTGGTTGTAGTCAAATGTTGGTAGTCAGGGTAGTTTTCCTCCCCTGCTCCACCTGGCCACGAGTC
+
FFFFFFFFFFFFFFFFFFFFFFFFFFFFFFFFFFFFFFFFFFFFFFFFFFFFFFFFFFFFFFFFFFFFFF
FFFFFFFFFFFFFFFFFFFFFFFFFFFFFFFFFFFFFFFFFFFFFFFFFFFFFFFFFFFFFFFFFFFFFF
@A00155:342:HHGFNDSXY:1:2511:8169:35243 2:N:0:GAACCTAG+TCCGCATA
CATAAGGACGATAGTTGCCTCCATGACGTTTAGCCCCCGCAGGTCCACGGCCGGGTGCTCCAGCGCTG
GCGTGGTTGTAGTCAAATGTTGGTAGTCAGGGTAGTTTTCCTCCCCTGCTCCACCTGGCCACGAGTCA
+
FFFFFFFFFFFFFFFFFFFFFFFFFFF:FFFF::FFFFFFFFFFFFFFFFFF:F,FFFFFFFFFFFFFFF
FFFFFFFFFFFFFFFFFFFFFFFFFFFFFFFFF:FFFFFFFFFFF:FFFFFFFFFFFFFFFFFFFFFFFF
@A00155:342:HHGFNDSXY:1:2638:21621:19993 2:N:0:GAACCTAG+TCCGCATA
CATAAGGACGATAGTTGCCTCCATGACGTTTAGCCCCCGCAGGTCCACGGCCGGGTGCTCCAGCGCTG
GCGTGGTTGTAGTCAAATGTTGGTAGTCAGGGTAGTTTTCCTCCCCTGCTCCACCTGGCCACGAGTCA
+
FFFFFFFFFFFFFFFFFFFFFFFFFFFFFFFFFFFFFFFFFFFFFFFFFFFFFFFFFFFFFFFFFFFFFF
F:FFFFFFFFFFFFFFF,FFFFFFFF:FFFFFFFFFFFFFFFF:FFFFFFFFFFFFFFFFFFFFFFFFFF
@A00155:342:HHGFNDSXY:1:2369:7961:30874 2:N:0:GAACCTAG+TCCGCATA
CATAAGGACGATAGTTGCCTCCATGACGTTTAGCCCCCGCAGGTCCACGGCCGGGTGCTCCAGCGCTG
GCGTGGTTGTAGTCAAATGTTGGTAGTCAGGGTAGTTTTCCTCCCCTGCTCCACCTGGCCACGAGTCA
+
FFFFFFFFFFFFFF,FFFFFFFFFFFFFFFFFFFFFFFFFFFFFFFFFFFFFFFFFFFFFFFFFFFFFFF
FFFFFFFFFFFFFFFFFFFFFFFFFFFFFFFFFFF:FFFFFFFFFFFFFFFFFFFFFFFFFFFFFFFFFF
@A00155:342:HHGFNDSXY:1:1329:17282:36213 2:N:0:GAACCTAG+TCCGCATA
CATAAGGACGATAGTTGCCTCCATGACGTTTAGCCCCCGCAGGTCCACGGCCGGGTGCTCCAGCGCTG
GCGTGGTTGTAGTCAAATGTTGGTAGTCAGGGTAGTTTTCCTCCCCTGCTCCACCTGGCCACGAGTCA
+
FFFFFFFFFFFFFFFFFFFFFF:FFFFFFFFFFFFFFFFFFFFFFFFFFFFFFFFFFFFFFFFFFFFFFF
FFFFFFFFFFFFFFFFFFFFFFFFFFFFFFFFFFF:FFFFFFFFFFFFFFFFFFFFFFFFFFFFFFFFFF
@A00155:342:HHGFNDSXY:1:1339:24966:27070 1:N:0:GAACCTAG+TCCGCATA
CATAAGGACGATAGTTGCCTCCATGACGTTTAGCCCCCGCAGGTCCACGTCCGGGTGCTCCAGCGCTG
GCGTGGTTGTAGTCAAATGTTGGTAGTCAGGGTAGTTTTCCTCCCCTGCTCCACCTGGCCACGAGTCA
+
FFFFFF:FF,FFFFFFFFFFFFFFFFF,FFFFF::FFF:F:F,FF:FFFF,FFFFFF::FF:FFFFFFFF
FFFFFF::F:FFFFFFFF,FFFFFFFFFFFFFFF:FFF,FFFFFFFFFF:FFFFFFFFF:FFFFFF:FFF
@A00155:342:HHGFNDSXY:1:2255:8712:11036 2:N:0:GAACCTAG+TCCGCATA
AAGGACGATAGTTGCCTCCATGACGTTTAGCCCCCGCAGGTCCACGGCCGGGTGCTCCAGCGCTGGCG
TGGTTGTAGTCAAATGTTGGTAGTCAGGGTAGTTTTCCTCCCCTGCTCCACCTGGCCACGAGTCAAAA
+
FFFF:FFFF:FFFFFFFFFFFFFFFFFFFFFFFFFFFFFFFFFFFFFF:FFFFF:FFFFFFFFFFFFFFF
FFFFFFFFFFF::FFFFF,FFFF:FFFFFFFFFFFFFFFFFFFFFFFFFFFFFFFFFFFFFFFFFFF:FF

```
@A00155:342:HHGFNDSXY:1:1176:30327:33411 2:N:0:GAACCTAG+TCCGCATA
AAGGACGATAGTTGCCTCCATGACGTTTAGCCCCCGCAGGTCCACGGCCGGGTGCTCCAGCGCTGGCG
TGGTTGTAGTCAAATGTTGGTAGTCAGGGTAGTTTTCCTCCCCTGCTCCACCTGGCCACGAGTCAAAA
+
FFFFFFFFFFFF:FFFFFFFFFFFFFFFFFFFFFFFFFFFFFFFFFFFFFFFFFFFFFFFFFFFFFFF
FFFFFF::FF:FFFFFFFFFFFFFFFFFFFFFFF:FFFFFFFF:FFFFFFFFFFFFFFFFFFFFFFFF
@A00155:342:HHGFNDSXY:1:1248:25690:28917 1:N:0:GAACCTAG+TCCGCATA
AAGGACGATAGTTGCCTCCATGACGTTTAGCCCCCGCAGGTCCACGGCCGGGTGCTCCAGCGCTGGCG
TGGTTGTAGTCAAATGTTGGTAGTCAGGGTAGTTTTCCTCCCCTGCTCCACCTGGCCACGAGTCAAAA
+
FFFFFFFFFFFFFFFFFFFFFFFFFFFFFFFFFFFFFF:FFFFFFFFFFFFFFFFFFFFFFFFFFFFF
FFFFFFFFFFFFFFFFF:FFFFFFFFF:FFFFFFFFFFFF:FFFFFFFFFFFFFFF:FFFFFFFFFFF
@A00155:342:HHGFNDSXY:1:2420:22290:15201 1:N:0:GAACCTAG+TCCGCATA
AGGACGATAGTTGCCTCCATGACGTTTAGCCCCCGCAGGTCCACGGCCGGGTGCTCCAGCGCTGGCGT
GGTTGTAGTCAAATGTTGGTAGTCAGGGTAGTTTTCCTCCCCTGCTCCACCTGGCCACGAGTCAAAAC
+
FFFFFFFFFFFFFFFFFFFFFFFFFFFFFFFF:FFFFFFFFFFFF:FFFFFFFFFFFF:FFFFFFF:F
FFFFFFFFFFFFFF:FF:FFFFFFFFFFF:FFFF:FFFFFFFFFFFFFFFFFFFFFFF:FFFFFFFFF
@A00155:342:HHGFNDSXY:1:2432:25102:25958 1:N:0:GAACCTAG+TCCGCATA
AGGACGATAGTTGCCTCCATGACGTTTAGCCCCCGCAGGTCCACGGCCGGGTGCTCCAGCGCTGGCGT
GGTTGTAGTCAAATGTTGGTAGTCAGGGTAGTTTTCCTCCCCTGCTCCACCTGGCCACGAGTCAAAAC
+
FFFFFFFFFFFFFFFFFFFFFFFFFFFFFFFFFFFFFFFFFFFFFFFFFFFFFFFFFFFFFFFFFFFF
FFF:FFFFFFFFFFFFFFFFFFFFFFFFFFFFFFFFFFFFFFFFFFFFFFFFFFFFFFFFFFFFFFFF
@A00155:342:HHGFNDSXY:1:2420:22146:15702 1:N:0:GAACCTAG+TCCGCATA
AGGACGATAGTTGCCTCCATGACGTTTAGCCCCCGCAGGTCCACGGCCGGGTGCTCCAGCGCTGGCGT
GGTTGTAGTCAAATGTTGGTAGTCAGGGTAGTTTTCCTCCCCTGCTCCACCTGGCCACGAGTCAAAAC
+
FFFFFFFFFFFFFFFFFFFFF:FFFFFFFFFFFFFFFFFFFFFFFFFFFFF:FFFFFFFFFFFFFFFF
FFFFFFFFFFFFFFFFFFFFFFFFFFFFFFFFFFFFFFFFFFFFFFFFFFFFFFFFFFFFFFFFFF:FF
@A00155:342:HHGFNDSXY:1:1358:23800:36793 1:N:0:GAACCTAG+TCCGCATA
AGGACGATAGTTGCCTCCATGACGTTTAGCCCCCGCAGGTCCACGGCCGGGTGCTCCAGCGCTGGCGT
GGTTGTAGTCAAATGTTGGTAGTCAGGGTAGTTTTCCTCCCCTGCTCCACCTGGCCACGAGTCAAAAC
+
FFFFFFFFFFFFFFFFFFFFFFFFFFFFFFFFFFFFFFFFFFFFFFFFFFFFFFFFFFFFFFFFFFFF
FFFFFFFFFFFFFFFFFFFFFFFFFFFFFFFFFFFFFFFFFFFFFFFFFFFFFFFFFFF:FFFFFFFF
@A00155:342:HHGFNDSXY:1:1609:23095:11052 1:N:0:GAACCTAG+TCCGCATA
GGACGATAGTTGCCTCCATGACGTTTAGCCCCCGCAGGTCCACGGCCGGGTGCTCCAGCGCTGGCGTG
GTTGTAGTCAAATGTTGGTAGTCAGGGTAGTTTTCCTCCCCTGCTCCACCTGGCCACGAGTCAAAACT
+
FFFFFFFFFFFFFFFFFFFFFFFFFFFFFFFFFFFFFFFFF:FFFFFFFFFFFFFFFFFFFFFFFFFF
FFFFFFFFFFF:FFFFFFFFFFFFFFFFFFFFFFFFFFFFFFFFFFFFFFFFFFFFFFFFFFFFFFFF
@A00155:342:HHGFNDSXY:1:2642:7554:26224 1:N:0:GAACCTAG+TCCGCATA
GACGATAGTTGCCTCCATGACGTTTAGCCCCCGCAGGTCCACGGCCGGGTGCTCCAGCGCTGGCGTGG
TTGTAGTCAAATGTTGGTAGTCAGGGTAGTTTTCCTCCCCTGCTCCACCTGGCCACGAGTCAAAACTA
+
FFFFFFFFFFFFFFFFFFFFFFFFFFFFFFFFFFFFFFFFFFFFFFFFFFFFFFFFF:FFFFFFFFFF
FFFFFFFFFFFFFFFFFFFFFFFFFFFFFFFFFFFFFFFFFFFFFFFFFF:FFFFFFFFFFFFF:FFF
@A00155:342:HHGFNDSXY:1:2319:26603:28682 2:N:0:GAACCTAG+TCCGCATA
GACGATAGTTGCCTCCATGACGTTTAGCCCCCGCAGGTCCACGGCCGGGTGCTCCAGCGCTGGCGTGG
TTGTAGTCAAATGTTGGTAGTCAGGGTAGTTTTCCTCCCCTGCTCCACCTGGCCACGAGTCAAAACTA
+
FFFFFFFFFFFFFFFFFFFFFFFFFFFFFFFFFFFFFFFFFFFFFFFFFFFFFFFFFFFFFFFFFFFF
FFFFFFFFFFFFFFFFF:FFFFFFFFFFF:FFF:F:FFFFFFFFFFFFFFFFFFFFFFFFFFFFFF:F
```

```
@A00155:342:HHGFNDSXY:1:2610:27425:29951 2:N:0:GAACCTAG+TCCGCATA
GACGATAGTTGCCTCCATGACGTTTAGCCCCCGCAGGTCCACGGCCGGGTGCTCCAGCGCTGGCGTGG
TTGTAGTCAAATGTTGGTAGTCAGGGTAGTTTTCCTCCCCTGCTCCACCTGGCCACGAGTCAAAACTA
+
FFFFFFFFFFFFFFFFFFFFFFFFFFF,FF,FFFFFFFFFFFFFFFFFFFFFFFFFFFFFF:FFFFFF
FFF:FFFFFF,:FFFF:FFFFFFFFFFFF:FFF:FFFFFFFFFFF:FFFFFFFFFFFFFFFFFFFFFF
@A00155:342:HHGFNDSXY:1:1669:13693:11177 2:N:0:GAACCTAG+TCCGCATA
ATAGTTGCCTCCATGACGTTTAGCCCCCGCAGGTCCACGGCCGGGTGCTCCAGCGCTGGCGTGGTTGT
AGTCAAATGTTGGTAGTCAGGGTAGTTTTCCTCCCCTGCTCCACCTGGCCACGAGTCAAAACTAGCCT
+
F,FFFFFFFFFF,FFFFFFFFFF:FFFFFFFFFFFFFFFFFFFFFFFFFFFFFFFF:FFFFFFFFFFF
FFFFFFFFFFFFFFFFFFFFFFFFFFFFFFFFFFFFFFFFFFFFFFFFFFFFFFFFFFFFFFFFFFFF
@A00155:342:HHGFNDSXY:1:2631:14714:35931 2:N:0:GAACCTAG+TCCGCATA
TAGTTGCCTCCATGACGTTTAGCCCCCGCAGGTCCACGGCCGGGTGCTCCAGCGCTGGCGTGGTTGTA
GTCAAATGTTGGTAGTCAGGGTAGTTTTCCTCCCCTGCTCCACCTGGCCACGAGTCAAAACTAGCCTC
+
FFFFFFFFFFFFFFFFF:FFFFFFFFFFFFFFFFFFFFFFFFFFFFFFFFFFFFFFFFFFFFFFF:FF
FFFFFFFFFFFFFFFFFFFFFFFFFFFFFFFFFFFFFFFFFFFFFFFFFFFFFFFFFFFFFFFFFFFF
@A00155:342:HHGFNDSXY:1:1610:7229:1517 2:N:0:GAACCTAG+TCCGCATA
TAGTTGCCTCCATGACGTTTAGCCCCCGCAGGTCCACGGCCGGGTGCTCCAGCGCTGGCGTGGTTGTA
GTCAAATGTTGGTAGTCAGGGTAGTTTTTCTCCCCTGCTCCACCTGGCCACGAGTCAAAACTAGCCTC
+
FFFFFFFFFFFFFFFFFF:FFFFFFFFFFFFFFFFFFFFFFFFFFFFFFFFFFFFFFFFFFFFFFFFF
FFFFFFFFFFFFFFFFFFFFFFFFFFFFFFFFFFFFFFFFFFFFFFFFFFFFFFFFFFFFFFFFFFFF
@A00155:342:HHGFNDSXY:1:1564:26612:33301 2:N:0:GAACCTAG+TCCGCATA
GTTGCCTCCATGACGTTTAGCCCCCGCAGGTCCACGGCCGGGTGCTCCAGCGCTGGCGTGGTTGTAGT
CAAATGTTGGTAGTCAGGGTAGTTTTCCTCCCCTGCTCCACCTGGCCACGAGTCAAAACTAGCCTCTT
+
F:FFFFFFFF:FFFFFF:FFFFFFFFFFFFFFFFFFFFFFFFFFFFFFFFFFFFFFFFFFFFFFFFFF
FFFFFFFFFFFFFFFFFFFFFFFFFFFFFFFFFFFFFFFFFFFFFFFFFFFFFFFFFFFFFFFFFFFF
@A00155:342:HHGFNDSXY:1:2631:14714:35931 1:N:0:GAACCTAG+TCCGCATA
GTTGCCTCCATGACGTTTAGCCCCCGCAGGTCCACGGCCGGGTGCTCCAGCGCTGGCGTGGTTGTAGT
CAAATGTTGGTAGTCAGGGTAGTTTTCCTCCCCTGCTCCACCTGGCCACGAGTCAAAACTAGCCTCTT
+
FFFFFFFFFFFFFFFFFFFFFFFFFFFFFFFFFFFFFFFFFFFFFFFFFFFFFFFFFFFFFFFFFFFF
FFFFFFFFFFFFFFFFFFFFFFFFFFFFFFFFFFFFFFFFFFFFFFFFFFFFFFFFFFFFFFFFFFFF
@A00155:342:HHGFNDSXY:1:1121:19551:20917 2:N:0:GAACCTAG+TCCGCATA
GTTGCCTCCATGACGTTTAGCCCCCGCAGGTCCACGGCCGGGTGCTCCAGCGCTGGCGTGGTTGTAGT
CAAATGTTGGTAGTCAGGGTAGTTTTCCTCCCCTGCTCCACCTGGCCACGAGTCAAAACTAGCCTCTT
+
FFFFFFFFF:FFFFFFFFFFFFFFFFFFFFFFFFFFFFFFFFFFFFFFFFFFF,FFFFFFFFFFFFFF
FFFFFFFFFFFFFFFFFFFFFFFFFFFFFFFFFFFFFFFFFFFFFFFFFFFFFFFFFFF:FFFFFFFF
@A00155:342:HHGFNDSXY:1:2110:24415:22200 2:N:0:GAACCTAG+TCCGCATA
GTTGCCTCCATGACGTTTAGCCCCCGCAGGTCCACGGCCGGGTGCTCCAGCGCTGGCGTGGTTGTAGT
CAAATGTTGGTAGTCAGGGTAGTTTTCCTCCCCTGCTCCACCTGGCCACGAGTCAAAACTAGCCTCTT
+
F,FFFFFFFFFFFFFFFFFFFFFFFFFFFFFFFFFFFFFFFFFFFFFFFFFFFFFFFFFFFFFFFFFF
FFFFFFFFFF:FFFFFFFFFFFFF,FFFFFFFFFFFFFFFFFFFFFFFFFFFFFFFFFFFFFFFFFFF
@A00155:342:HHGFNDSXY:1:1448:15031:19946 2:N:0:GAACCTAG+TCCGCATA
GTTGCCTCCATGACGTTTAGCCCCCGCAGGTCCACGGCCGGGTGCTCCAGCGCTGGCGTGGTTGTAGT
CAAATGTTGGTAGTCAGGGTAGTTTTCCTCCCCTGCTCCACCTGGCCACGAGTCAAAACTAGCCTCTT
+
FF,FFFFFFFFFFFFFFFFFFFFFFFFFFFFFFFFFFFFFFFFFFFFFFFFFFFFFFFFFFFFF:FFF
FFFFFFFFFFFFFFFFFFFFFFFFFFFFFFFFFFFFFFFFFFFFFFFFFFFFFF:FFFFFFFFFFFFF
```

```
@A00155:342:HHGFNDSXY:1:1358:23800:36793 2:N:0:GAACCTAG+TCCGCATA
GTTGCCTCCATGACGTTTAGCCCCCGCAGGTCCACGGCCGGGTGCTCCAGCGCTGGCGTGGTTGTAGT
CAAATGTTGGTAGTCAGGGTAGTTTTCCTCCCCTGCTCCACCTGGCCACGAGTCAAAACTAGCCTCTT
+
FFFFFFFFFFFFFFFFFFFFFFFFFFFFFFFFFFFFFFFFFFFFFFFFFFFFFFFFFFFFFFFFFFFF
FF:FFFFFFFFFFFFFFFFFFFFFFFFFFFFFFFFFFFFFFFFFFFFFFFFFFFFFFFFFFFFFFFFF
@A00155:342:HHGFNDSXY:1:1116:29550:23484 2:N:0:GAACCTAG+TCCGCATA
GTTGCCTCCATGACGTTTAGCCCCCGCAGGTCCACGGCCGGGTGCTCCAGCGCTGGCGTGGTTGTAGT
CAAATGTTGGTAGTCAGGGTAGTTTTCCTCCCCTGCTCCACCTGGCCACGAGTCAAAACTAGCCTCTT
+
FFFFFFFFFFFFFFF:FFFFFF:FFFFFFFFFFFFFFFFFFFFFFFFFFFFFFFFFFFFFFFFFFFFF
FFFFFFFFFFFFFFFFFFFFFF:FFFFFFF:FFFFFFFFFFFFFFFF:FFFFFFFFFFFFFF:FFFFF
@A00155:342:HHGFNDSXY:1:2642:7554:26224 2:N:0:GAACCTAG+TCCGCATA
GTTGCCTCCATGACGTTTAGCCCCCGCAGGTCCACGGCCGGGTGCTCCAGCGCTGGCGTGGTTGTAGT
CAAATGTTGGTAGTCAGGGTAGTTTTCCTCCCCTGCTCCACCTGGCCACGAGTCAAAACTAGCCTCTT
+
FFFFFFFFFFFFFFFFFFFFFFFFFFFFFFFFFFFFFFFFFFFFFFFFFFFFFFFFFFFFFFFFFFFF
FFFFFFFFFFFFFFFFFFFFFFFF:FFFFFFFFFFFFFFFFFFFFFFFFFFFFFFFFFFFFFFFFFFF
@A00155:342:HHGFNDSXY:1:1532:17237:27649 2:N:0:GAACCTAG+TCCGCATA
GTTGCCTCCATGACGTTTAGCCCCCGCAGGTCCACGGCCGGGTGCTCCAGCGCTGGCGTGGTTGTAGT
CAAATGTTGGTAGTCAGGGTAGTTTTCCTCCCCTGCTCCACCTGGCCACGAGTCAAAACTAGCCTCTT
+
F:FF:FFFFFFFFFFFFFFF:FFF:,FFFFFFFFFFFFFF,:FF:FFFFFFFF:FFFFFF:FFFFFFF
F:FFFFFFFFFF:FFFFF,FFFFFF:FF:F:FFF,FFFFFFFFFFFFFFFFFFF:FF:F:FFFFF,FF
@A00155:342:HHGFNDSXY:1:2255:8712:11036 1:N:0:GAACCTAG+TCCGCATA
GTTGCCTCCATGACGTTTAGCCCCCGCAGGTCCACGGCCGGGTGCTCCAGCGCTGGCGTGGTTGTAGT
CAAATGTTGGTAGTCAGGGTAGTTTTCCTCCCCTGCTCCACCTGGCCACGAGTCAAAACTAGCCTCTT
+
FFFFFFFFFFFFFFFFFFFFFFFFFFFFFFFFFFFFFFFFFFFFFFFFFFFFFFFFFFFFFFFFFFFF
FFFFFF:FFFFFF:FFFFFFFFFFFF:FFFFFFFFFFFFFFFFFFFFFF:FFFFFFFFFFFFFFFFFF
@A00155:342:HHGFNDSXY:1:1421:24514:1454 2:N:0:GAACCTAG+TCCGCATA
GTTGCCTCCATGACGTTTAGCCCCCGCAGGTACACGGCCGGGTGCTCCAGCGCTGGCGTGGTTGTAGT
CAAATGTTGGTAGTCAGGGTAGTTTTCCTCCCCTGCTCCACCTGGCCACGAGTCAAAACTAGCCTCTT
+
FFFFFFFF:FFFFFFFFFFFFF,FF:FFFFF,FF:FFFFFFFFFF:FF:FFFFFFFF,FFF:FFFF:F
FFFFFFFFF:FFFFFFF:FFFFFFFFFFFFF,FFFFFFFFFFFFFFF:FFFFFFFFFFFFFFFFFFFF
@A00155:342:HHGFNDSXY:1:1667:14190:6339 2:N:0:GAACCTAG+TCCGCATA
GTTGCCTCCATGACGTTTAGCCCCCGCAGGTCCACGGCCGGGTGCTCCAGCGCTGGCGTGGTTGTAGT
CAAATGTTGGTAGTCAGGGTAGTTTTCCTCCCCTGCTCCACCTGGCCACGAGTCAAAACTAGCCTCTT
+
FFFFFFFFFFFFF:FFFFFFFFFFFFFFFFFFFFFFFFFF:FFFFFFFFFFFFFFFFFFFFFFFFFFF
FFFFFFFFFFFFFFFFFFFFFFFFFFFFFFFFFFFFFFFFFFFFFFFFFFFFFFFFFFFFFFFFFFFF
@A00155:342:HHGFNDSXY:1:1255:2989:11835 1:N:0:GAACCTAG+TCCGCATA
TTGCCTCCATGACGTTTAGCCCCCGCAGGTCCACGGCCGGGTGCTCCAGCGCTGGCGTGGTTGTAGTC
AAATGTTGGTAGTCAGGGTAGTTTTCCTCCCCTGCTCCACCTGGCCACGAGTCAAAACTAG
+
FFFFFF,FFF,FF,FF:F:FFF:FF:FFFFFFF:,FFFFFFF:FFF,FFFFFFFFFFFFFFFFFFFF,
FFFFFF:FFFFFFFFF,:FFFFFFFFFF:FF:FFFFFFFFFFFFFFFFFFFFFFF:F,FFF
@A00155:342:HHGFNDSXY:1:2631:13602:23954 1:N:0:GAACCTAG+TCCGCATA
TTGCCTCCATGACGTTTAGCCCCCGCAGGTCCACGGCCGGGTGCTCCAGCGCTGGCGTGGTTGTAGTC
AAATGTTGGTAGTCAGGGTAGTTTTCCTCCCCTGCTCCACCTGGCCACGAGTCAAAACTAG
+
FFFFFFFFFFFFFFFFFFFFFFFFFFFFFFFFFFFFFFFFFFFFFFFFFFFFFFFFFFFFFFFFFFFF
FFFFFFFFFFFFFFFFFFFFFFFFFFFFFFFFFFFFFFFFFFFFFFFFFFFFFFFFFFFFF
```

```
@A00155:342:HHGFNDSXY:1:1352:13322:17425 1:N:0:GAACCTAG+TCCGCATA
TTGCCTCCATGACGTTTAGCCCCCGCAGGTCCACGGCCGGGTGCTCCAGCGCTGGCGTGGTTGTAGTC
AAATGTTGGTAGTCAGGGTAGTTTTCCTCCCCTGCTCCACCTGGCCACGAGTCAAAACTAG
+
FFFFFFFFFFFFFFFFFFFFFFFFFFFFFFFFFFFFFFFFFFFFFFFFFFFFFFFFFFFFFFFFFFFF
FFFFFFFFFFFFFFFFFFFFFFFFFFFFFFFFFFFFFFFFFFFFFFFFFFFFFFFFFFFFF
@A00155:342:HHGFNDSXY:1:1358:3052:6464 1:N:0:GAACCTAG+TCCGCATA
TTGCCTCCATGACGTTTAGCCCCCGCAGGTCCACGGCCGGGTGCTCCAGCGCTGGCGTGGTTGTAGTC
AAATGTTGGTAGTCAGGGTAGTTTTCCTCCCCTGCTCCACCTGGCCACGAGTCAAAACTAGCC
+
FFFFFFFFFFFFFFFFFFFFFFFFFFFFFFFFFFFFFFFFFFFFFFFFFFFFFFFFFFFFFFFFFFFF
FFFFFFFFFFFFFFFFFFFFFFFFFFFFFFFFFFFFFFFFFFFFFFFFFFFFFFF:FFFFFFF
@A00155:342:HHGFNDSXY:1:2646:21902:22670 1:N:0:GAACCTAG+TCCGCATA
TTGCCTCCATGACGTTTAGCCCCCGCAGGTCCACGGCCGGGTGCTCCAGCGCTGGCGTGGTTGTAGTC
AAATGTTGGTAGTCAGGGTAGTTTTCCTCCCCTGCTCCACCTGGCCACGAGTCAAAACTAGCC
+
FFFFF,FFFFFFFFFFFFFFFFFFFFFFFFFFFFFFFFFFFFFFFFFFFFFFFFFF:FFFFFFF
FFFFFFFFFFFFFFFFFFFFFFFFFFFFFFFFFFFFFFFFFFFFFFFFFFFFFFFFFFFFFFF
@A00155:342:HHGFNDSXY:1:2110:31105:36010 1:N:0:GAACCTAG+TCCGCATA
TTGCCTCCATGACGTTTAGCCCCCGCAGGTCCACGGCCGGGTGCTCCAGCGCTGGCGTGGTTGTAGTC
AAATGTTGGTAGTCAGGGTAGTTTTCCTCCCCTGCTCCACCTGGCCACGAGTCAAAACTAGCCT
+
FFFFFFFFFFFFFFFFFFFFFFFFFFFFFFFFFFFFFFFFFFFFFFFFFFFFFFFFFFFFFFFFFFFF
FFFFFFFFFFFFFFFFFFFFFFFFFFFFFFFFFFFFFFFFFFFFFFFFFFFFFFFFFFFFFFFF
@A00155:342:HHGFNDSXY:1:1121:19551:20917 1:N:0:GAACCTAG+TCCGCATA
TTGCCTCCATGACGTTTAGCCCCCGCAGGTCCACGGCCGGGTGCTCCAGCGCTGGCGTGGTTGTAGTC
AAATGTTGGTAGTCAGGGTAGTTTTCCTCCCCTGCTCCACCTGGCCACGAGTCAAAACTAGCCT
+
FFFFFFFFFFFFFFFFFFFFFFFFFF:FFFFF:FFFFFFFFFFFFFFFFFFFFFFFFFFFFFFFFFFF
FFFFFFFFFFFFFFFFFFFFFFFFFFFFFFFFFFFFFFFFFFFFFFFFFFFFFFFFFFFFFFFF
@A00155:342:HHGFNDSXY:1:1514:9254:1141 1:N:0:GAACCTAG+TCCGCATA
TTGCCTCCATGACGTTTAGCCCCCGCAGGTCCACGGCCGGGTGCTCCAGCGCTGGCGTGGTTGTAGTC
AAATGTTGGTAGTCAGGGTAGTTTTCCTCCCCTGCTCCACCTGGCCACGAGTCAAAACTAGCCT
+
FFFFFFFFFFFFFFFFFFFFFFFFFFFFFFFFFFFFFFFFFFFFFFFFFFFFFFFFFFFFFFFFFF:F
FFFFFFFFFFFFFFFFFFFFFFFFFFFFFFFFFFFFFFFFFFFFFFFFFFFFFFFFFFFFFFFF
@A00155:342:HHGFNDSXY:1:1176:30327:33411 1:N:0:GAACCTAG+TCCGCATA
TTGCCTCCATGACGTTTAGCCCCCGCAGGTCCACGGCCGGGTGCTCCAGCGCTGGCGTGGTTGTAGTC
AAATGTTGGTAGTCAGGGTAGTTTTCCTCCCCTGCTCCACCTGGCCACGAGTCAAAACTAGCCT
+
FFFFFFFFFFFFFFFFFFFFFFFFFFFFFFFFFFFFFFFFFFFFFFFFFFFFFFFFFFFFFFFFFFFF
FFFFFFFFF:FFFFFFFFFFFFFFFFFFFFFFFFFFFFFFFFFFFFFFFF::FFFF
@A00155:342:HHGFNDSXY:1:1401:5981:26569 1:N:0:GAACCTAG+TCCGCATA
TTGCCTCCATGACGTTTAGCCCCCGCAGGTCCACGGCCGGGTGCTCCAGCGCTGGCGTGGTTGTAGTC
AAATGTTGGTAGTCAGGGTAGTTTTCCTCCCCTGCTCCACCTGGCCACGAGTCAAAACTAGCCT
+
FFFFFFFFFFFFFFFFFF:FFFFFFFFFFFFFFFFFFFFFFFFFFFFFFFFFFFFFFFFFFFFFFFFF
,FFFFFFFFFFFFFFFFFFFFFFFFFFFFFFFFFFFFFFFFFFFFFFFFFFFFFFFFFFFFFFF
@A00155:342:HHGFNDSXY:1:2225:24804:36933 2:N:0:GAACCTAG+TCCGCATA
TGCCTCCATGACGTTTAGCCCCCGCAGGTCCACGGCCGGGTGCTCCAGCGCTGGCGTGGTTGTAGTCA
AATGTTGGTAGTCAGGGTAGTTTTCCTCCCCTGCTCCACCTGGCCACGAGTCAAAACTAGCCTCTTCT
+
FFFFFFFFFFFFFFFFFFFFFFFFFFFFFFFFFFFFFFFFFFFFFFFFFFFFFFFFFFFFFFFFFFFF
FFFFFFFFFFFFFFFFFFFFFF:FFFFFFFFFFFFFFFFFFFFFFFFFFFFFFFFFFFFFFFFFFFFF
```

```
@A00155:342:HHGFNDSXY:1:2409:15763:11945 2:N:0:GAACCTAG+TCCGCATA
TGCCTCCATGACGTTTAGCCCCCGCAGGTCCACGGCCGGGTGCTCCAGCGCTGGCGTGGTTGTAGTCA
AATGTTGGTAGTCAGGGTAGTTTTCCTCCCCTGCTCCACCTGGCCACGAGTCAAAACTAGCCTCTTCT
+
F:FFFFFFFFFFFF:FFFF,F,F,,F,:F,F,F,F,FF,FF:,:::FFF,FF:FFFFFFFFF:FFFF:
FFFFFFF::FFF,F:FFFFFFF:FFFFFF,FF,FFF,FF,FF::F,F,,FF:FF:FFF:FF::FFFF:
@A00155:342:HHGFNDSXY:1:2149:6180:31610 2:N:0:GAACCTAG+TCCGCATA
TGCCTCCATGACGTTTAGCCCCCGCAGGTCCACGGCCGGGTGCTCCAGCGCTGGCGTGGTTGTAGTCA
AATGTTGGTAGTCAGGGTAGTTTTCCTCCCCTGCTCCACCTGGCCACGAGTCAAAACTAGCCTCTTCT
+
:FFFFFF:FFFFFFFFFFFFFFFF:FFFFFF:FFFFFFFFFFFFFF:FFFFFFFFFFFFFFFFFFFF:
FFFFFFFFFFFFFFFFFFFFFFFFFFFFFFFFFFFFFFFFFFFFFFFFFFFFFFF:FF:FFFFFFFF:
@A00155:342:HHGFNDSXY:1:2620:11532:7498 1:N:0:GAACCTAG+TCCGCATA
TGCCTCCATGACGTTTAGCCCCCGCAGGTCCACGGCCGGGTGCTCCAGCGCTGGCGTGGTTGTAGTCA
AATGTTGGTAGTCAGGGTAGTTTTCCTCCCCTGCTCCACCTGGCCACGAGTCAAAACTAGCCTCTTCT
+
FFFFFFFFFFFFFFFFFFFFFFFFFFFFFFFFFFFFFFFFFFFFFF,FFFFFFFFFFFFFFFFFFFFF
FFFFFFFFFFFFFFFFFFFFFFFFFFFFFFFFFFFFFFFFFFFFFFFFFFFFFFFFFFF:FFFFFFFF
@A00155:342:HHGFNDSXY:1:1151:29731:21136 2:N:0:GAACCTAG+TCCGCATA
TGCCTCCATGACGTTTAGCCCCCGCAGGTCCACGGCCGGGTGCTCCAGCGCTGGCGTGGTTGTAGTCA
AATGTTGGTAGTCAGGGTAGTTTTCCTCCCCTGCTCCACCTGGCCACGAGTCAAAACTAGCCTCTTCT
+
:FFFFFFFFFFFFFFFFFFFFFFFFFFFFFFFFFFFFFFFFFFFFFFFFFFFFFFFFFFFFFFFFFFF
FFFFFFFFFFFFFFFFFFFFFFFFFFFFFFFFFFFFFFFFFFFFFFFFFFFFFFFFFFFFFFFFFFFF
@A00155:342:HHGFNDSXY:1:2225:2483:19319 2:N:0:GAACCTAG+TCCGCATA
GCCTCCATGACGTTTAGCCCCCGCAGGTCCACGGCCGGGTGCTCCAGCGCTGGCGTGGTTGTAGTCAA
ATGTTGGTAGTCAGGGTAGTTTTCCTCCCCTGCTCCACCTGGCCACGAGTCAAAACTAGCCTCTT
+
FFFFFF:FFFFFFFFFFFFFFFFFFFFFFFFFFFFFFFFFFFFFFFFFFF:FFFFFFFFFFFFFFFFF
FFFFFFFFFFFFFF:F:FFFF:FFFFFFFFFFFFFFFFFFFFFFFFFFFFFFFFFFFFFFFFFFF
@A00155:342:HHGFNDSXY:1:2225:17553:16423 2:N:0:GAACCTAG+TCCGCATA
CTCCATGACGTTTAGCCCCCGCAGGTCCACGGCCGGGTGCTCCAGCGCTGGCGTGGTTGTAGTCAAAT
GTTGGTAGTCAGGGTAGTTTTCCTCCCCTGCTCCACCTGGCCACGAGTCAAAACTAGCCTCTT
+
FFFFFFFFFFFFFFFFFFFFFFFFFFFFFFFFFFFFFFFFFFFFFFFFFFFFFFFFFFFFFFFFFFFF
FFFFFFFFFFFFFFFFFFFFFFFFFFFFFFFFFFFFFFFFFFFFFFFFF:FFFFFFFFFFFFF
@A00155:342:HHGFNDSXY:1:2620:11532:7498 2:N:0:GAACCTAG+TCCGCATA
CTCCATGACGTTTAGCCCCCGCAGGTCCACGGCCGGGTGCTCCAGCGCTGGCGTGGTTGTAGTCAAAT
GTTGGTAGTCAGGGTAGTTTTCCTCCCCTGCTCCACCTGGCCACGAGTCAAAACTAGCCTCTTCTTCA
+
FFFFFFFFFF:FFFFFFFFFFFFFFFFFFFF:FFFFFFFFFFFFFFFFFFFFFFFFFFFFFFFFFFFF
F:FFFFFFFFFFFF:FF:FFFFFFFFFFFFFFFFFFFFFFFFFFFFFFFFFFFFFFFFFFFFFF:FFF
@A00155:342:HHGFNDSXY:1:2222:29478:16971 1:N:0:GAACCTAG+TCCGCATA
CCATGACGTTTAGCCCCCGCAGGTCCACGGCCGGGTGCTCCAGCGCTGGCGTGGTTGTAGTCAAATGT
TGGTAGTCAGGGTAGTTTTCCTCCCCTGCTCCACCTGGCCACGAGTCAAAACTAGCCTCTTCTTCATA
+
FFFFFFFFFFFFFFFFFFFFFFFFFFFFFFFFFFFFFFFFFFFFFFFFFFFFFFFFFFFFFFFFFFFF
FFFFFFFFFFFFFFFFFFFFFFFFFFFFFFFFFFFFFFFFFFFFFFFFFFFFFFFFFFFFFFFFFFFF
@A00155:342:HHGFNDSXY:1:1406:8015:24017 1:N:0:GAACCTAG+TCCGCATA
CCATGACGTTTAGCCCCCGCAGGTCCACGGCCGGGTGCTCCAGCGCTGGCGTGGTTGTAGTCAAATGT
TGGTAGTCAGGGTAGTTTTCCTCCCCTGCTCCACCTGGCCACGAGTCAAAACTAGCCTCTTCTTCATA
+
FFFFFFFFF:FFFFFFFFFFFFFFFFFFF,FFFF:FFFFFF:FFFFFFFFFFFFFFFFFFFF:FFFFF
FFFFFFFFFF,FFFFFFFFFFFFFFFFFFFFFFFFFFFFFFFF:FFFFFFFFFFFFFFFFFFFFFFFF
```

```
@A00155:342:HHGFNDSXY:1:1314:16731:6637 1:N:0:GAACCTAG+TCCGCATA
CCATGACGTTTAGCCCCCGCAGGTCCACGGCCGGGTGCTCCAGCGCTGGCGTGGTTGTAGTCAAATGT
TGGTAGTCAGGGTAGTTTTCCTCCCCTGCTCCACCTGGCCACGAGTCAAAACTAGCCTCTTCTTCATA
+
FFFFFFFFFFFFFFFFFFFFFFFFFFFFFFFFFFFFFFFFFFFFFFFFFFF:FFFFF:FFFFFFFFFF
FFFFFFFFFFFFFFFFFFFFFFFFFFFFFFFFFFFFFFFFFFFFFFFFFFFFFFFFFFFFFFFFFFFF
@A00155:342:HHGFNDSXY:1:1140:23303:15608 1:N:0:GAACCTAG+TCCGCATA
CATGACGTTTAGCCCCCGCAGGTCCACGGCCGGGTGCTCCAGCGCTGGCGTGGTTGTAGTCAAATGTT
GGTAGTCAGGGTAGTTTTCCTCCCCTGCTCCACCTGGCCACGAGTCAAAACTAGCCTCTTCTTCATAT
+
FFFF:FFFFFFFFFFFFFFFFFFFFFFFFFFFFFFFFFFFFFFFFFFFFF:FFFFFFFFFFFFFFFFF
FFFFFFFFFFFFFFFFFFFFFFFFFFFFFFFFFFFFFFFFFFFFFFFFFFFFFFFFFFFFFFFFFFFF
@A00155:342:HHGFNDSXY:1:2126:4978:25864 2:N:0:GAACCTAG+TCCGCATA
CATGACGTTTAGCCCCCGCAGGTCCACGGCCGGGTGCTCCAGCGCTGGCGTGGTTGTAGTCAAATGTT
GGTAGTCAGGGTAGTTTTCCTCCCCTGCTCCACCTGGCCACGAGTCAAAACTAGCCTCTTCTTCATAT
+
FFFFFFFFF,FFFFFFFFFFFFFFFFFFFFFFFFFFFFFFFFFFFFFFFFFFFFFFFFFFFFFFFFFF
FFFFFFFFFFFFFFFFFFFFFFFFFFFFFFFFFFFFFFFFFFFFFFFFFFFFFFFFFFFFFFFFFFFF
@A00155:342:HHGFNDSXY:1:1348:27254:2284 2:N:0:GAACCTAG+TCCGCATA
ATGACGTTTAGCCCCCGCAGGTCCACGGCCGGGTGCTCCAGCGCTGGCGTGGTTGTAGTCAAATGTTG
GTAGTCAGGGTAGTTTTCCTCCCCTGCTCCACCTGGCCACGAGTCAAAACTAGCCTCTT
+
FFFFFFFFFFFFFFFFFFFFFFFFFFFFFFFFFFFFFFFFFFFF:FFFFFFFFFFFFFFFFFFFFFFF
FF:FFFFFFFFFFFFFFFFFFFFFF:FFFFFFFFFFFFFFFFFFFFFFFFFFFFFFFFF
@A00155:342:HHGFNDSXY:1:2667:20003:19758 1:N:0:GAACCTAG+TCCGCATA
ATGACGTTTAGCCCCCGCAGGTCCACGGCCGGGTGCTCCAGCGCTGGCGTGGTTGTAGTCAAATGTTG
GTAGTCAGGGTAGTTTTCCTCCCCTGCTCCACCTGGCCACGAGTCAAAACTAGCCTCTTCTTCATATC
+
FFFFFFFFFFFFFFFFFFFFFFFFFFFFFFFFFFFFFFFFFFFFFFFFFFFFFFFFFFFFFFFFFFFF
FFFFFFFFFFFFFFFFFFFFFFFFFFFFFFFFFFFFFFFFFFFFFFFFFFFFFFFFFFFFFFFFFF:F
@A00155:342:HHGFNDSXY:1:2342:7066:13197 1:N:0:GAACCTAG+TCCGCATA
ATGACGTTTAGCCCCCGCAGGTCCACGGCCGGGTGCTCCAGCGCTGGCGTGGTTGTAGTCAAATGTTG
GTAGTCAGGGTAGTTTTCCTCCCCTGCTCCACCTGGCCACGAGTCAAAACTAGCCTCTTCTTCATATC
+
FFFFFFFFFFFFFFFFFFFFFFFFFFFFFFFFFFFFFFFFFFFFFFFFFFFFFFFFFFFFFFFFFFFF
FFFFFFFFFFFFFF:FFFFFFFFFFFFFFFFFFFFFFFFFFFFFFFFF:FFFFFFFFFFFFFFFFFFF
@A00155:342:HHGFNDSXY:1:1305:31213:30655 1:N:0:GAACCTAG+TCCGCATA
TTTAGCCCCCGCAGGTCCACGGCCGGGTGCTCCAGCGCTGGCGTGGTTGTAGTCAAATGTTGGTAGTC
AGGGTAGTTTTCCTCCCCTGCTCCACCTGGCCACGAGTCAAAACTAGCCTCTTCTTCATATCTCTCCG
+
FFFFFFFFFFFFFFFFFFFF:FFFFFFFFFFFFFFFFFFFFFFFFFFFFFFFFFFFFFFFFFFFFFFF
FFFFFFFFFFFFF:FFFFFFFFFFFFFFFFFFFFFFFFFFFFFFFFFFFFFFFFFFFFFFFFFFFFFF
@A00155:342:HHGFNDSXY:1:2575:14498:20494 2:N:0:GAACCTAG+TCCGCATA
TTTAGCCCCCGCAGGTCCACGGCCGGGTGCTCCAGCGCTGGCGTGGTTGTAGTCAAATGTTGGTAGTC
AGGGTAGTTTTCCTCCCCTGCTCCACCTGGCCACGAGTCAAAACTAGCCTCTTCTTCATATCTCTCCG
+
FFF:FFFFFFFFFFFFFFFFFFFFFFFFFFFFFFFFFFFFFFFFFFFFF,FFFFFFFFFFFFFFFFFF
FFFFFFFFFFFFFFFFFFFFFFFFFFFFFFFFFFFFFFFFFFFFFFFFFFFFFFFFFFFFFFFFFFFF
@A00155:342:HHGFNDSXY:1:1221:24171:21308 1:N:0:GAACCTAG+TCCGCATA
TAGCCCCCGCAGGTCCACGGCCGGGTGCTCCAGCGCTGGCGTGGTTGTAGTCAAATGTTGGTAGTCAG
GGTAGTTTTCCTCCCCTGCTCCACCTGGCCACGAGTCAAAACTAGCCTCTTCTTCATATCTCTCCGGC
+
FFFFFFFFFFFFFFFF:FFFFFFFF:FFFFFFFFFFFFFFF:FF:FFF:FFF:FFFFFFFFFFFFFFF
FFF:FFFFFFFFFFFFFFFFFFFFFFFFFFFFFFFFFFFFFFF:FFFFF,FFFFFFFFFFFFFFFFFF
```

```
@A00155:342:HHGFNDSXY:1:1470:18475:2550 1:N:0:GAACCTAG+TCCGCATA
TAGCCCCCGCAGGTCCACGGCCGGGTGCTCCAGCGCTGGCGTGGTTGTAGTCAAATGTTGGTAGTCAG
GGTAGTTTTCCTCCCCTGCTCCACCTGGCCACGAGTCAAAACTAGCCTCTTCTTCATATCTCTCCGGC
+
FFFFFFFFFFFFFFFF,FFFFFFFFFFFFFFFFFFFFFFFFFFFFFFFFFFFFFF::FFFFFFFFFFF
FFFFFFFFFFFFFFFFFFFFFFFFFFFFFFFFFFFFFFFF:FFFFFFFFFFFFFFFFFFFFFFFFFFF
@A00155:342:HHGFNDSXY:1:2225:2483:19319 1:N:0:GAACCTAG+TCCGCATA
AGCCCCCGCAGGTCCACGGCCGGGTGCTCCAGCGCTGGCGTGGTTGTAGTCAAATGTTGGTAGTCAGG
GTAGTTTTCCTCCCCTGCTCCACCTGGCCACGAGTCAAAACTAGCCTCTTCTTCATATCTCTCCG
+
FFFFFFFFFFFFFFFFFFFFFFFFFFFFFFFFFFFFFFFFFFFFFFFFFFFFFFFFFFFFFFFFFFFF
FFFFFFFFFFFFFFFFFFFFFFFFFFFFFFFFFFFFFFFFFFFFF,FFFFFFFFFFFFFFFFFFF
@A00155:342:HHGFNDSXY:1:1405:32497:35102 1:N:0:GAACCTAG+TCCGCATA
AGCCCCCGCAGGTCCACGGCCGGGTGCTCCAGCGCTGGCGTGGTTGTAGTCAAATGTTGGTAGTCAGG
GTAGTTTTCCTCCCCTGCTCCACCTGGCCACGAGTCAAAACTAGCCTCTTCTTCATATCTCTCCGGCC
+
FFFFFFFFFFFFFFFFFFFFFFFFFFFFFFFFFFFFFFFFFFFFFF:F:FFFFFFFFFFFFFFFFFFF
FFFFFFFFFFFFFFFFFF:FFFFFFFFFFFFFFFFFFFFFFFFFFFFFFFFF:FFFFFFFFFFFFFFF
@A00155:342:HHGFNDSXY:1:1101:6289:30545 1:N:0:GAACCTAG+TCCGCATA
AGCCCCCGCAGGTCCACGGCCGGGTGCTCCAGCGCTGGCGTGGTTGTAGTCAAATGTTGGTAGTCAGG
GTAGTTTTCCTCCCCTGCTCCACCTGGCCACGAGTCAAAACTAGCCTCTTCTTCATATCTCTCCGGCC
+
FFFFF:FFFFFFFFFFF,FFFFF:FFFF:FFFFFFFFFFF:,FFFFF,FFFFFFFFFFFFFFFFFFFF
F:FFFFFFFFFFFFFFFFFFFFFFFFFFFFFFFFFFFFFFFFFFFFFFFFFFFFFFFFFFFFFFFFFF
@A00155:342:HHGFNDSXY:1:2631:13602:23954 2:N:0:GAACCTAG+TCCGCATA
AGCCCCCGCAGGTCCACGGCCGGGTGCTCCAGCGCTGGCGTGGTTGTAGTCAAATGTTGGTAGTCAGG
GTAGTTTTCCTCCCCTGCTCCACCTGGCCACGAGTCAAAACTAGCCTCTTCTTCATATCTCTCCGGCC
+
FFFFFFFFFFFFFFFFFFFFFFFFFFFFFFFFFFFFFFFFFFFFFFFFFFFFFFFFFFFFFFFFFFFF
FFFFFFFFFFFFFFFFFFFFFFFFFFFFFFFFFFFFFFFFFFFFFFFFFFFFFFFFFFFFFFFFFFFF
@A00155:342:HHGFNDSXY:1:1313:15619:17362 1:N:0:GAACCTAG+TCCGCATA
AGCCCCCGCAGGTCCACGGCCGGGTGCTCCAGCGCTGGCGTGGTTGTAGTCAAATGTTGGTAGTCAGG
GTAGTTTTCCTCCCCTGCTCCACCTGGCCACGAGTCAAAACTAGCCTCTTCTTCATATCTCTCCGGCC
+
FFFFFFFFFFFFFFFFFFFFFFFFFFFFFFFFFFFFFFFFFFFFFF::F,FFFFFF,FFFFFFFFFFF
FFFFFFFFFFFFFFFFFF,FFFFFFFFFFFFFFF,FFFFFFFFFFFFFF:F,FFFFF,FFFFFFFFFF
@A00155:342:HHGFNDSXY:1:1162:25545:19147 1:N:0:GAACCTAG+TCCGCATA
AGCCCCCGCAGGTCCACGGCCGGGTGCTCCAGCGCTGGCGTGGTTGTAGTCAAATGTTGGTAGTCAGG
GTAGTTTTCCTCCCCTGCTCCACCTGGCCACGAGTCAAAACTAGCCTCTTCTTCATATCTCTCCGGCC
+
FFFFFFFFFFFFFFFFFFFFFFFFFFFFFFFFFFFFFFFFFFFFFFFFFFFFFFFFFFFFFFFFFFFF
FFFFFFFFFFFFFFFFFFFFFFFFFFFFFFFFFFFFF:FFFFFFFFFFFFFFFFFFFFFFFFFFFFFF
@A00155:342:HHGFNDSXY:1:2507:4960:31657 1:N:0:GAACCTAG+TCCGCATA
AGCCCCCGCAGGTCCACGGCCGGGTGCTCCAGCGCTGGCGTGGTTGTAGTCAAATGTTGGTAGTCAGG
ATAGTTTTCCTCCCCTGCTCCACCTGGCCACGAGTCAAAACTAGCCTCTTCTTCATATCTCTCCGGCC
+
FFFF:FFFFFFFFFFFFFFFFFFFFFFFFF:FFFF:FFFF:FF,,:,FF:F:FFFF::FFFFFFFFFF
FFFFFFFFFFFFFF:FFFFFFF:FFFFFFFFFFF:FFFFFFF:FFFFF:,F,:FFF:FF:FFFFFFFF
@A00155:342:HHGFNDSXY:1:2123:20066:16861 1:N:0:GAACCTAG+TCCGCATA
AGCCCCCGCAGGTCCACGGCCGGGTGCTCCAGAGCTGGCGTGGTTGTAGTCAAATGTTGGTAGTCAGG
GTAGTTTTCCTCCCCTGCTCCACCTGGCCACGAGTCAAAACTAGCCTCTTCTTCATATCTCTCCGGCC
+
FFFFFFFFFFFFFFFFFFFFFFFFFFFFFFFFFFFFFFFFFFF,FFFFFFFFFFFFFFFFFFFFF:FF
FFFFFFFFFFFFFFFFFFFFFFFFFFFFFFFFFFFFFFFFFFFFFFFFFFFFFFFFFFFFFFFFFFFF
```

```
@A00155:342:HHGFNDSXY:1:1211:12527:27007 1:N:0:GAACCTAG+TCCGCATA
AGCCCCCGCAGGTCCACGGCCGGGTGCTCCAGAGCTGGCGTGGTTGTAGTCAAATGTTGGTAGTCAGG
GTAGTTTTCCTCCCCTGCTCCACCTGGCCACGAGTCAAAACTAGCCTCTTCTTCATATCTCTCCGGCC
+
FFFFFFFFFFFFFFFFFFFFFFFFFFFFFFFFFFFFFFFFFFFFFFFFFFFFFFFFFFFFFFFFFFFF
FFFFFFFFFFFFFFFFFFFFFFFFFFFFFFFFFFFFFFFFFFFFFFFFFFF:FFFFFFFFFFFFFFFF
@A00155:342:HHGFNDSXY:1:2363:8721:17221 1:N:0:GAACCTAG+TCCGCATA
AGCCCCCGCAGGTCCACGGCCGGGTGCTCCAGCGCTGGCGTGGTTGTAGTCAAATGTTGGTAGTCAGG
GTAGTTTTCCTCCCCTGCTCCACCTGGCCACGAGTCAAAACTAGCCTCTTCTTCATATCTCTCCGGCC
+
FFFFFFFFFFFFFFF:FFFFFFFFFFFFFFFFFFFFFFFFFFFFFFFFFFFFFFFFFFFFFFFFFFFF
FFFFFFFFFFFFFFFFFFFFFFFFFFFFFFFFFFFFFFFFFFFFFFFFFFFFFFFFFFFFFFFFFFFF
@A00155:342:HHGFNDSXY:1:2174:8648:8578 1:N:0:GAACCTAG+TCCGCATA
AGCCCCCGCAGGTCCACGGCCGGGTGCTCCAGCGCTGGCGTGGTTGTAGTCAAATGTTGGTAGTCAGG
GTAGTTTTCCTCCCCTGCTCCACCTGGCCACGAGTCAAAACTAGCCTCTTCTTCATATCTCTCCGGCC
+
FFFFFFFFFFFFFFFFFFFFFFFFFFFFFFFFFFFFFFFFFFFFFFFFFFFFFFFFFFFFFFFFFFFF
FFFFFFFFFFFFFFFFFFFFFFFFFFFFFFFFFFFFFFF:FFFFFFFFFFFFFFFFFFFFFFFFFFFF
@A00155:342:HHGFNDSXY:1:1412:13530:31939 1:N:0:GAACCTAG+TCCGCATA
AGCCCCCGCAGGTCCACGGCCGGGTGCTCCAGCGCTGGCGTGGTTGTAGTCAAATGTTGGTAGTCAGG
GTAGTTTTCCTCCCCTGCTCCACCTGGCCACGAGTCAAAACTAGCCTCTTCTTCATATCTCTCCGGCC
+
FFFFFFFFFFFFFFFFFFFFFFFFFFFFFFFFFFFFFFFFFFFFFFFFFFFFFFFFFFFFFFFFFFFF
FFFFFFFFFFFFFFFFFFFFFFFFFFFFFFFF:FFFFFFFFFF:FFFFF,FFFFF:,FF,F:::,,F:
@A00155:342:HHGFNDSXY:1:1101:5674:30013 1:N:0:GAACCTAG+TCCGCATA
AGCCCCCGCAGGTCCACGGCCGGGTGCTCCAGCGCTGGCGTGGTTGTAGTCAAATGTTGGTAGTCAGG
GTAGTTTTCCTCCCCTGCTCCACCTGGCCACGAGTCAAAACTAGCCTCTTCTTCATATCTCTCCGGCC
+
FFFF:FFFFFFFFFF:FFFFFFF,FFFFFF:FF:FFFFFF:FFFFF:FFFFF:FFFFFFFFFFFFFFF
FFFFFFFFFFFFFFFFFFFFFFFFFFFFFFFFFF,FFFFFFFFFFFFFFFFFFFFFF:FFFFFFFFFF
@A00155:342:HHGFNDSXY:1:2472:12427:12868 1:N:0:GAACCTAG+TCCGCATA
AGCCCCCGCAGGTCCACGGCCGGGTGCTCCAGCGCTGGCGTGGTTGTAGTCAAATGTTGGTAGTCAGG
GTAGTTTTCCTCCCCTGCTCCACCTGGCCACGAGTCAAAACTAGCCTCTTCTTCATATCTCTCCGGCC
+
FFFFFFFFFFFFFFFFFFFFFFFFFFFFFF:FFFFFFFFFFFFFFFFFFFFFFFFFFFFFFFFFFFFF
FFFFFFFFFFFFFFFFFFFFFFFFFFFFFFFFFFFFFFFFFFFFF:FFFFFFFFFFFFFFFFFFFFFF
@A00155:342:HHGFNDSXY:1:2253:2546:1266 1:N:0:GAACCTAG+TCCGCATA
AGCCCCCGCAGGTCCACGGCCGGGTGCTCCAGCGCTGGCGTGGTTGTAGTCAAATGTTGGTAGTCAGG
GTAGTTTTCCTCCCCTGCTCCACCTGGCCACGAGTCAAAACTAGCCTCTTCTTCATATCTCTCCGGCC
+
FFFFFFFFFFFFFFFFFFFFFFFFFFFFFFFFFFFFFFFFFFF:FFFFFFFFFFFFFFFFFFFFFFFF
FFFFFFFFFFFFFFFFFFFFFFFFFFFFFFFFFFFFFFFFFFFFFFFFFFFFFFFFFFFFFFFFFFFF
@A00155:342:HHGFNDSXY:1:2247:27335:29356 1:N:0:GAACCTAG+TCCGCATA
AGCCCCCGCAGGTCCACGGCCGGGTGCTCCAGCGCTGGCGTGGTTGTAGTCAAATGTTGGTAGTCAGG
GTAGTTTTCCTCCCCTGCTCCACCTGGCCACGAGTCAAAACTAGCCTCTTCTTCATATCTCTCCGGCC
+
FFFFFFFFFFFFFFFFFFFFFFFFFFFFFFFFFFFFFFFFFFFFFFF:FFFFFFFFFFFFFFFFFFFF
FFFFFFF:FFFFFFFFFFFFFFFFFFFFFFFFFFFFFFFFFFFFFFFFFFFFFFFFFFFFFFFFFFFF
@A00155:342:HHGFNDSXY:1:1431:13856:4789 1:N:0:GAACCTAG+TCCGCATA
AGCCCCCGCAGGTCCACGGCCGGGTGCTCCAGCGCTGGCGTGGTTGTAGTCAAATGTTGGTAGTCAGG
GTAGTTTTCCTCCCCTGCTCCACCTGGCCACGAGTCAAAACTAGCCTCTTCTTCATATCTCTCCGGCC
+
F:F:FFF:FFFFFFFFFFFFFFF::FFFFFFFFFFFFFFFFFFFFFFFFFFFFFFFF:FFFFFFFFFF
FFFFFFFFFFFFFFFFFFF:FFFFFFFFFFFF:FFFFFFFFFFFFFFF,FFFFFFFFFFFFFFF:FFF
```

```
@A00155:342:HHGFNDSXY:1:2306:6587:28902 1:N:0:GAACCTAG+TCCGCATA
GCCCCCGCAGGTCCACGGCCGGGTGCTCCAGCGCTGGCGTGGTTGTAGTCAAATGTTGGTAGTCAGGG
TAGTTTTCCTCCCCTGCTCCACCTGGCCACGAGTCAAAACTAGCCTCTTCTTCATATCTCTCCGGCC
+
FFFFFFFFFFFFFFFFFFFFFFFFFFFFFFFFFFFFFFFFFFFFF:FFFFFF:FFFFFFFFFFFFFFF
FFFFFFFFFFFFFFFFFFFFFFFFFFFFFFFFFFFFFFFFFFFFFFFFFFFFFFFFFFFFFFFFFFF
@A00155:342:HHGFNDSXY:1:1319:14615:11459 1:N:0:GAACCTAG+TCCGCATA
GCCCCCGCAGGTCCACGGCCGGGTGCTCCAGCGCTGGCGTGGTTGTAGTCAAATGTTGGTAGTCAGGG
TAGTTTTCCTCCCCTGCTCCACCTGGCCACGAGTCAAAACTAGCCTCTTCTTCATATCTCTCCGGCCA
+
FFFFFFFFFFFFFF:FFFFFFFFFFFFFFFFFFFFFFFFFFFFFF:FFFFFF:FFFFFFFFFFFFFFF
FFFFFFFFFFFFFFFFFFFFFFFFFFFFFFFFFFFFFFFFFFFFFFFFFF:FFFFFFFFFFFFFFFFF
@A00155:342:HHGFNDSXY:1:2225:17553:16423 1:N:0:GAACCTAG+TCCGCATA
CCCCCGCAGGTCCACGGCCGGGTGCTCCAGCGCTGGCGTGGTTGTAGTCAAATGTTGGTAGTCAGGGT
AGTTTTCCTCCCCTGCTCCACCTGGCCACGAGTCAAAACTAGCCTCTTCTTCATATCTCTCCG
+
FFFFFFFFFFFFFFFFFFFFFFFFFFFFFFFFFFFFFFFFFFFF,:FFFFFFFFFFFFFFFFFFFFFF
FFFFFFFFFFFFFFFFFFFFFFFFFFFFFFFFFFFFFFFFFFFFFFFFFFFFFFFFF:FFFFF
@A00155:342:HHGFNDSXY:1:2544:12048:24298 2:N:0:GAACCTAG+TCCGCATA
CCTCCGCAGGTCCACGGCCGGGTGCTCCAGCGCTGGCGTGGTTGTAGTCAAATGTTGGTAGTCAGGGT
AGTTTTCCTCCCCTGCTCCACCTGGCCACGAGTCAAAACTAGCCTCTTCTTCATATCTCTCCGGCCAC
+
FFFFFFFFFFFFFFFFFFFFFFFFFFFFFFFFFFFFFFFFFFFFFFFFFFFFFFFFFFFFFFFFFFFF
FFFFFFFFFFFFFFFFFFFFFFFFFFFFFFFFFFFFFFFFFFFFFFFFFFFFFFFFFFFFFFFFFFFF
@A00155:342:HHGFNDSXY:1:2526:5213:20760 1:N:0:GAACCTAG+TCCGCATA
CCCCCGCAGGTCCACGGCCGGGTGCTCCAGCGCTGGCGTGGTTGTAGTCAAATGTTGGTAGTCAGGGT
AGTTTTCCTCCCCTGCTCCACCTGGCCACGAGTCAAAACTAGCCTCTTCTTCATATCTCTCCGGCCAC
+
FFFFFFFFFFFFFFFFFFFFFFFFFFFFFFFFFFFFFFFFF:FFFFFFFFFFFFFFFFFFFFFFFFFF
FFFFFFFFFFFFFFFFFFFFFFFFFFFFFFFFFFFFFFFFFFF:FF:FFFFF,FF::FFFFFFFFF:F
@A00155:342:HHGFNDSXY:1:1213:12165:12132 1:N:0:GAACCTAG+TCCGCATA
CCCCCGCAGGTCCACGGCCGGGTGCTCCAGCGCTGGCGTGGTTGTAGTCAAATGTTGGTAGTCAGGGT
AGTTTTCCTCCCCTGCTCCACCTGGCCACGAGTCAAAACTAGCCTCTTCTTCATATCTCTCCGGCCAC
+
FFFFFFFFFFFFFFFFFFFFFFFFFFFFFFFFFFFFFFFFFFFFFFFFFFFFFFFFFFFFFFFFFFFF
FFFFFFFFFFFFFFFFFFFFFFFFFFFFFFFFFFFFFFFFFFFFFFFFFFFFFFFFFFFFFFFFFF:F
@A00155:342:HHGFNDSXY:1:1508:19099:23578 1:N:0:GAACCTAG+TCCGCATA
CCCCCGCAGGTCCACGGCCGGGTGCTCCAGCGCTGGCGTGGTTGTAGTCAAATGTTGGTAGTCAGGGT
AGTTTTCCTCCCCTGCTCCACCTGGCCACGAGTCAAAACTAGCCTCTTCTTCATATCTCTCCGGCCAC
+
FFFFFFFFFFFFFFFFFFFFFFFFFFFFFFFFFFFFFFFFFFFFFFFFFFFFFFFFFFFFFFFFFFFF
FFFFFFFFFFFFFFFFFFFFFFFFFFFFFFFFFFFFFFFFFFFFFFFFFFFFFFFFFFFFFFFFFFFF
@A00155:342:HHGFNDSXY:1:1173:32470:36839 1:N:0:GAACCTAG+TCCGCATA
CCCCCGCAGGTCCACGGCCGGGTGCTCCAGCGCTGGCGTGGTTGTAGTCAAATGTTGGTAGTCAGGGT
AGTTTTCCTCCCCTGCTCCACCTGGCCACGAGTCAAAACTAGCCTCTTCTTCATATCTCTCCGGCCAC
+
FFFFFFF:FFFFFFFFFFFFFFFFFFFFFFFFFFFFFFFFFFFF:FFFF:FFFFFFFFFFFFFFFFFF
FFFFFFFFFFFFFFFF:FFFFFFFFFFFFFFFFFFFF,FFFFFFFFFFFFFFFFFFFFFFFFFFFFFF
@A00155:342:HHGFNDSXY:1:1258:13096:10457 1:N:0:GAACCTAG+TCCGCATA
CCCCCGCAGGTCCACGGCCGGGTGCTCCAGCGCTGGCGTGGTTGTAGTCAAATGTTGGTAGTCAGGGT
AGTTTTCCTCCCCTGCTCCACCTGGCCACGAGTCAAAACTAGCCTCTTCTTCATATCTCTCCGGCCAC
+
FFFFFFFFFFFFFFFFFFFFFFFFFFFFFFFFFFFFFFFFF:,FFFFFFFFFFFFFFFFFFFFFFFFF
FFFFFFFFFFFFFFFFFFFFFFFFFFFFFFFFFFFFFFFFFFFFFFFFFFFFFFFFFFFFFFFFFFFF
```

```
@A00155:342:HHGFNDSXY:1:1458:11134:15828 2:N:0:GAACCTAG+TCCGCATA
CCCGCAGGTCCACGGCCGGGTGCTCCAGCGCTGGCGTGGTTGTAGTCAAATGTTGGTAGTCAGGGTAG
TTTTCCTCCCCTGCTCCACCTGGCCACGAGTCAAAACTAGCCTCTTCTTCATATCTCTCCGGCCACCA
+
FFFF:FFFFFFFFFFFFFFFFFFFFFFFFFFFFF:FFFFF:FFFFFFFFFFFFFFFFFFFFFFFFFFF
F::FFFFFFFFFFFFFFFFFFFFFFFFFFFFFFFFFFFFFFFFFFFFFFFFFFFFFFFFFFFFFFFFF
@A00155:342:HHGFNDSXY:1:2521:21820:8656 1:N:0:GAACCTAG+TCCGCATA
CCCGCAGGTCCACGGCCGGGTGCTCCAGCGCTGGCGTGGTTGTAGTCAAATGTTGGTAGTCAGGGTAG
TTTTCCTCCCCTGCTCCACCTGGCCACGAGTCAAAACTAGCCTCTTCTTCATATCTCTCCGGCCACCA
+
FFFFFFFFFFFFFFFFFFFFFFFFFFFFFFFFFFFFFFFFFFFFFFFFFFFFFFFFFFFFFFFFFF:F
FFFFFFFFFFFFFFFFFFFFFFFFFFFFFFFFFFFFFFFFFFFFFFFFFFFFFFFFFFFFFFFFFFFF
@A00155:342:HHGFNDSXY:1:2456:4101:16548 1:N:0:GAACCTAG+TCCGCATA
CCCGCAGGTCCACGGCCGGGTGCTCCAGCGCTGGCGTGGTTGTAGTCAAATGTTGGTAGTCAGGGTAG
TTTTCCTCCCCTGCTCCACCTGGCCACGAGTCAAAACTAGCCTCTTCTTCATATCTCTCCGGCCACCA
+
FFFFFFFFFFFFFFFFFFFFFFFFFF:FFFFFFFFFFFFFFFFFFFFFFFFFFFFFFFFFFFFFFFFF
FFFFFFFFFFFFFFFFFFFFFFF,FFFFFFFFFFFFFFFFFFFFFFFFFFFFFFFFFFFFFFFFFFFF
@A00155:342:HHGFNDSXY:1:1370:28411:4038 2:N:0:GAACCTAG+TCCGCATA
CCGCAGGTCCACGGCCGGGTGCTCCAGCGCTGGCGTGGTTGTAGTCAAATGTTGGTAGTCAGGGTAGT
TTTCCTCCCCTGCTCCACCTGGCCACGAGTCAAAACTAGCCTCTTCTTCATATCTCTCCGG
+
FFFFFFFFFF:FFFFFFFFFFFFFFFFFFFFFFFFFFFFFFFFFFFFFFFFFFFFFFFFFFFFFFFFF
FFFFFFFFFFFFFFFFFFFFFFFFFFFFFFFFFFFFFFFFFFFFFFFFFFFFFFFFFFFFF
@A00155:342:HHGFNDSXY:1:1314:18584:6089 1:N:0:GAACCTAG+TCCGCATA
CCGCAGGTCCACGGCCGGGTGCTCCAGCGCTGGCGTGGTTGTAGTCAAATGTTGGTAGTCAGGGTAGT
TTTCCTCCCCTGCTCCACCTGGCCACGAGTCAAAACTAGCCTCTTCTTCATATCTCTCCGGCCACCAC
+
FFFFFFFFFF,FFFFFFFFFFFFFFFFFFFFFFFFFFFFFFFFFFFFFFFFFFFFFFFFFFFFF:FFF
FFFFFFFFFFFFFFFFFFFFFFFFFFFFFFFFFFFFFFFFFFFFFFFFFFFFFFFFFFFFFFF,FFFF
@A00155:342:HHGFNDSXY:1:1348:27254:2284 1:N:0:GAACCTAG+TCCGCATA
CGCAGGTCCACGGCCGGGTGCTCCAGCGCTGGCGTGGTTGTAGTCAAATGTTGGTAGTCAGGGTAGTT
TTCCTCCCCTGCTCCACCTGGCCACGAGTCAAAACTAGCCTCTTCTTCATATCTCTCCG
+
FFFFFFFFFF:FFFFFFFFFFF:F:FFFFFFFFFFFFFFFFFFFFFF,FFFFFFFFFFFFFF:FFFFF
:FFFF:FFFFFFFFFF:FFFFFFFFFFFFFFFFFFFFFFFFFFFFFFFFFFFFFFFFFF
@A00155:342:HHGFNDSXY:1:2371:19958:26600 1:N:0:GAACCTAG+TCCGCATA
CGCAGGTCCACGGCCGGGTGCTCCAGCGCTGGCGTGGTTGTAGTCAAATGTTGGTAGTCAGGGTAGTT
TTCCTCCCCTGCTCCACCTGGCCACGAGTCAAAACTAGCCTCTTCTTCATATCTCTCCGGCCACCAC
+
FFFFFFFFFFFFFFFFFFFFFFFFFFFFFFFFFFFFFFFFFFFFFFFFFFFFFFFFFFFFFFFFFFFF
FFFFFFFFFFFFFFFFFFFFFFFFFFFFFFFFFFFFFFFFFFFFFFFFFFFFFFFFFFFFFFFFFFF
@A00155:342:HHGFNDSXY:1:2161:13169:9862 1:N:0:GAACCTAG+TCCGCATA
CGCAGGTCCACGGCCGGGTGCTCCAGCGCTGGCGTGGTTGTAGTCAAATGTTGGTAGTCAGGGTAGTT
TTCCTCCCCTGCTCCACCTGGCCACGAGTCAAAACTAGCCTCTTCTTCATATCTCTCCGGCCACCAC
+
FFFFFFFFFFFFFFFFFFFFFFFFFFFFFFFFFFFFFFFFFFFFFFFFFFFFF:FFFFFFFFFFFFFF
FFFFFFFFFFFFFFFFFFFFFFFFFFFFFFFF:FFFFFFFFFFFFFFFFFFFFFFFFFFFFFFFFFF
@A00155:342:HHGFNDSXY:1:1632:18087:19758 1:N:0:GAACCTAG+TCCGCATA
CGCAGGTCCACGGCCGGGTGCTCCAGCGCTGGCGTGGTTGTAGTCAAATGTTGGTAGTCAGGGTAGTT
TTCCTCCCCTGCTCCACCTGGCCACGAGTCAAAACTAGCCTCTTCTTCATATCTCTCCGGCCACCACT
+
FFFFFFFFFFFFFFFFFFFFFFFFFFFFFFFFFF:FFFFFFFFFFFFFFFFFFFFFFFFFFFFF,FFF
FFFFFFFFFFFFFFFFFFFFFFFFFFFFFFFFFFFFFFFFFFFFFFFFFFFFFFFFF:FFFFFFFFFF
```

```
@A00155:342:HHGFNDSXY:1:1352:15908:25254 1:N:0:GAACCTAG+TCCGCATA
CGCAGGTCCACGGCCGGGTGCTCCAGCGCTGGCGTGGTTGTAGTCAAATGTTGGTAGTCAGGGTAGTT
TTCCTCCCCTGCTCCACCTGGCCACGAGTCAAAACTAGCCTCTTCTTCATATCTCTCCGGCCACCACT
+
FFFFFFFFFFFFFFFFFFFFFFFFFFFFFFFFFFFFF:FFFFFFFFF:FFFFFFFFFFFFFFFFFFFF
FFFFFFFFFFFFFFFFFFFFFFFFFFFFFFFFFFFFFFFFFFFFF:FFFFFFFFFFFFFFFF,FFFFF
@A00155:342:HHGFNDSXY:1:1608:31376:3223 1:N:0:GAACCTAG+TCCGCATA
CGCAGGTCCACGGCCGGGTGCTCCAGCGCTGGCGTGGTTGTAGTCAAATGTTGGTAGTCAGGGTAGTT
TTCCTCCCCTGCTCCACCTGGCCACGAGTCAAAACTAGCCTCTTCTTCATATCTCTCCGGCCACCACT
+
FFFFFFFFFFFFFFFFFFFFFFFFFFFFFFFFFFFFF:FFFFFFFFFFFFFFFFFFFFFFFFFFFFFF
FFFFFFFFFFFFFFFFFFFFFFFFFFFFFFFFFFFFFFFFFFFFFFFFFFFF:FFFFFFFFFFFFFFF
@A00155:342:HHGFNDSXY:1:1458:11134:15828 1:N:0:GAACCTAG+TCCGCATA
CGCAGGTCCACGGCCGGGTGCTCCAGCGCTGGCGTGGTTGTAGTCAAATGTTGGTAGTCAGGGTAGTT
TTCCTCCCCTGCTCCACCTGGCCACGAGTCAAAACTAGCCTCTTCTTCATATCTCTCCGGCCACCACT
+
FFFFFFFFFFFFFFFFFFFFFFFFFFFFFFFFFFFFFFFFFFFF:FFFFFFFFFFFFFFFFFFFFFFF
FFFFFFFFFFFFFF:FFFFFFFFFFFFFFFFFFFFFFFFFFFFFFFFFFFFFF:FFFFFFFF:FFFF:
@A00155:342:HHGFNDSXY:1:1570:12707:25629 1:N:0:GAACCTAG+TCCGCATA
CGCAGGTCCACGGCCGGGTGCTCCAGCGCTGGCGTGGTTGTAGTCAAATGTTGGTAGTCAGGGTAGTT
TTCCTCCCCTGCTCCACCTGGCCACGAGTCAAAACTAGCCTCTTCTTCATATCTCTCCGGCCACCACT
+
FFFFFFFFFFFFFFFFFFFFFFFFFFFFFFFFFFFFFF:FF:FFFFF:FFFFFFFFFFFFFFFFFFFF
FFFFFFFFFFFFFFFFFFFFFFFFFFFFFFFFFFFFFFFFFFFFFFFFF:FFFFFFFFFFFFFFFFFF
@A00155:342:HHGFNDSXY:1:2636:29957:28103 1:N:0:GAACCTAG+TCCGCATA
CGCAGGTCCACGGCCGGGTGCTCCAGCGCTGGCGTGGTTGTAGTCAAATGTTGGTAGTCAGGGTAGTT
TTCCTCCCCTGCTCCACCTGGCCACGAGTCAAAACTAGCCTCTTCTTCATATCTCTCCGGCCACCACT
+
FFFFFF,FFFFFFFFFFFF:FFFFF:FFFFF,::FFF,,:FF:FFFFFFFFFFFFFFF:FF:FF:F:F
FFFFFFF:::F,FFFFFF:FF:FFF:FF::FFFFFFFF:FFFFFFFFFFFFFFF:FF:FFFF:FFFFF
@A00155:342:HHGFNDSXY:1:2161:12653:9502 1:N:0:GAACCTAG+TCCGCATA
CGCAGGTCCACGGCCGGGTGCTCCAGCGCTGGCGTGGTTGTAGTCAAATGTTGGTAGTCAGGGTAGTT
TTCCTCCCCTGCTCCACCTGGCCACGAGTCAAAACTAGCCTCTTCTTCATATCTCTCCGGCCACCACT
+
FFFFFFFFFFFFFFFFFFFFFFFFFFFFFFFFFFFFFFFF:FFFFFFFFFFFFFFFFFFFFFFFFFFF
FFFFFFFFFFFFFFFFFFFFFFFFFFFFFFFFFFFFFFFFFFFFFFFFFFFFFFFFFFFFFFFFFFFF
@A00155:342:HHGFNDSXY:1:2139:18059:1767 1:N:0:GAACCTAG+TCCGCATA
CGCAGGTCCACGGCCGGGTGCTCCAGCGCTGGCGTGGTTGTAGTCAAATGTTGGTAGTCAGGGTAGTT
TTCCTCCCCTGCTCCACCTGGCCACGAGTCAAAACTAGCCTCTTCTTCATATCTCTCCGGCCACCACT
+
FFFFFFFFFFFFFFFFFFFFFFFFFFFFFFFFFF:FF,FFFFFFFFFFFFFFFFFFFFFFFFFF:FFF
FFFFFFFFFFFFFFFFFFFFFFFFFFFFFFFFFFFFFFFFFFFFFFFFFFFFFFFFFFFFFFFFFFFF
@A00155:342:HHGFNDSXY:1:2677:7563:19319 1:N:0:GAACCTAG+TCCGCATA
GCAGGTCCACGGCCGGGTGCTCCAGCGCTGGCGTGGTTGTAGTCAAATGTTGGTAGTCAGGGTAGTTT
TCCTCCCCTGCTCCACCTGGCCACGAGTCAAAACTAGCCTCTTCTTCATATCTCTCCGGCCACCACTG
+
FFFFFFFFFFFFFFFFFFFFFFFFFFFFFFFFFFFFFFFFFFFFFF:FFFFFFFFFFFFFFFFFFFFF
FFFFFFFFFFFFFFFFFFFFFFFFFFFFFFFFFFFFFFFFFFFFFFFFFFFFFFFFFFFFF:FFFFFF
@A00155:342:HHGFNDSXY:1:1213:12165:12132 2:N:0:GAACCTAG+TCCGCATA
GCAGGTCCACGGCCGGGTGCTCCAGCGCTGGCGTGGTTGTAGTCAAATGTTGGTAGTCAGGGTAGTTT
TCCTCCCCTGCTCCACCTGGCCACGAGTCAAAACTAGCCTCTTCTTCATATCTCTCCGGCCACCACTG
+
FFFFFFFFFFFFFFFFFFFFFFFFFFFFFFFFFFFFFFF:FFFFFFFFFFFFFFFFFFFFFFFFFFFF
FFFFFFFFFFFFFFFFFFFFFFFFFFFFFFFFFFFFFFFFFFFFFFFFFFFFFFFFFFFFFFFFFFFF
```

```
@A00155:342:HHGFNDSXY:1:1526:29378:15327 2:N:0:GAACCTAG+TCCGCATA
CAGGTCCACGGCCGGGTGCTCCAGCGCTGGCGTGGTTGTAGTCAAATGTTGGTAGTCAGGGTAGTTTT
CCTCCCCTGCTCCACCTGGCCACGAGTCAAAACTAGCCTCTTCTTCATATCTCTCCGGCCACCACTGG
+
FFFFFFFFFFFFFFFFFFFFFFFFFFFFFFF,F,FFFFFFFFFFFFFFFFFFFFFFFFFFFFFFFFFF
FFFFFFFFFFFFFFFFFFFFFFFFFFFFFFFFFFFFFFFFFFFFFFF:FFFFFFFFFFF,FF:FFF:FF
@A00155:342:HHGFNDSXY:1:2667:11568:34773 1:N:0:GAACCTAG+TCCGCATA
CAGGTCCACGGCCGGGTGCTCCAGCGCTGGCGTGGTTGTAGTCAAATGTTGGTAGTCAGGGTAGTTTT
CCTCCCCTGCTCCACCTGGCCACGAGTCAAAACTAGCCTCTTCTTCATATCTCTCCGGCCACCACTGG
+
FFFFFFFFFFFFFFFFFFFFFFFFFFFFFFFFFFF:FFFFFFFFFFFFFFFFFFFFFFFFFFF:FFFFF
FFFFFFFFFFFFFFFFFFFFFFFFFFFFFFFFFFFFFFFFFFFFFFFFFFF:FFFFFFFFFFFFFFFF
@A00155:342:HHGFNDSXY:1:2306:6587:28902 2:N:0:GAACCTAG+TCCGCATA
AGGTCCACGGCCGGGTGCTCCAGCGCTGGCGTGGTTGTAGTCAAATGTTGGTAGTCAGGGTAGTTTTC
CTCCCCTGCTCCACCTGGCCACGAGTCAAAACTAGCCTCTTCTTCATATCTCTCCGGCCACCACTGGT
+
FFFFFFFFFFFFFFFFFFFFFFFFFFFFFFFFFFF:FFFFFF:FFFFFFFFFFFFFFFFFFFFFFFFFF
FFFFFFFFFFFFFFFFFFFFFFFFFFFFFFFFFFFFFFFFFFFFFFFFFFFFFFFFFFFFFFFFFFFFF
@A00155:342:HHGFNDSXY:1:1314:18584:6089 2:N:0:GAACCTAG+TCCGCATA
AGGTCCACGGCCGGGTGCTCCAGCGCTGGCGTGGTTGTAGTCAAATGTTGGTAGTCAGGGTAGTTTTC
CTCCCCTGCTCCACCTGGCCACGAGTCAAAACTAGCCTCTTCTTCATATCTCTCCGGCCACCACTGGT
+
FFFFFF:FFFFFFFFFFFFFFFFFFFFFFFFFFFFFFF,FFFFFFFFFFFFFFFFFF:FFFF:FFFFFF
FFFFFFFFFFFFFFFFFFFFFFFFFFFFFFFFFFFFFFFFFFF:FFFFFFFFFFFFFFFFFFFFFFFFFF
@A00155:342:HHGFNDSXY:1:1140:23303:15608 2:N:0:GAACCTAG+TCCGCATA
GGTCCACGGCCGGGTGCTCCAGCGCTGGCGTGGTTGTAGTCAAATGTTGGTAGTCAGGGTAGTTTTCC
TCCCCTGCTCCACCTGGCCACGAGTCAAAACTAGCCTCTTCTTCATATCTCTCCGGCCACCACTGGTC
+
FFFFFFFFFFFFFFFFFFFFFFFFFFFFFFFFFFFFFFFFFFFFFFFFFFFFFFFFFFFFFFFFFFFFF
FFFFF:FFFFFFFFFFFFFFFFF:FFFFFFFFFFFFFFFFFFFFFFFFFFFFFFFFFFFFFFFFFFFFF
@A00155:342:HHGFNDSXY:1:1162:25545:19147 2:N:0:GAACCTAG+TCCGCATA
GGTCCACGGCCGGGTGCTCCAGCGCTGGCGTGGTTGTAGTCAAATGTTGGTAGTCAGGGTAGTTTTCC
TCCCCTGCTCCACCTGGCCACGAGTCAAAACTAGCCTCTTCTTCATATCTCTCCGGCCACCACTGGTC
+
FFFFFFFFFFFFFFFFFFFFFFFFFFFFFFFFFFFFFFFFFFFFFFFFFFFFFFFFFFFFFFFFFFFFF
FFFFFFFFFFFFFFFFFFFFFFFFFFF:FFFFFFFFFFFFFFFFFFFFFFFFFFFFFFFFFFFFFFFFF
@A00155:342:HHGFNDSXY:1:1414:6777:6590 2:N:0:GAACCTAG+TCCGCATA
GGTCCACGGCCGGGTGCTCCAGCGCTGGCGTGGTTGTAGTCAAATGTTGGTAGTCAGGGTAGTTTTCC
TCCCCTGCTCCACCTGGCCACGAGTCAAAACTAGCCTCTTCTTCATATCTCTCCGGCCACCACTGGTC
+
FF,FF:FFFFFFFFFFFFFFFFFFFFFFFFFFFFFFF:FFFFFFFFFF:FFF:FFFFFFFFFFFFFFF
FFFFFFFFFFFFFFFFFFFFFF:FFFFFFFFFFFFFFFFFF:FFFFFFFFFFFFFFFFFFFFF:FFFFFF
@A00155:342:HHGFNDSXY:1:2127:5647:2597 2:N:0:GAACCTAG+TCCGCATA
GGTCCACGGCCGGGTGCTCCAGCGCTGGCGTGGTTGTAGTCAAATGTTGGTAGTCAGGGTAGTTTTCC
TCCCCTGCTCCACCTGGCCACGAGTCAAAACTAGCCTCTTCTTCATATCTCTCCGGCCACCACTGGTC
+
FFFFFFFFFFFFFFFFFFFFFFFFFFFFFFFFF:F:F:FF:FFFFFF:FFFFFFFFFFFFFFFFFFFFF
FFFFFFFFF:FFFFFFF:FFFFFFFF:FFFFFFFFFFFFFFFFFFFFFFFFFFFFFFFFFFFFFFFFF,
@A00155:342:HHGFNDSXY:1:1537:28203:21277 2:N:0:GAACCTAG+TCCGCATA
GTCCACGGCCGGGTGCTCCAGCGCTGGCGTGGTTGTAGTCAAATGTTGGTAGTCAGGGTAGTTTTCCT
CCCCTGCTCCACCTGGCCACGAGTCAAAACTAGCCTCTTCTTCATATCTCTCCGGCCACCACTGGTC
+
FF,:F:FFF,:FFF:F::F,FFFFFF,,FF,FFFF:FFFF:FFFFF:FFF:FFF:FF::FFFF,FFFF
:F:FFFFF:FFF:FF:FFF:F,FFFFFFFFFFFFFF:FFFFF:FF,FFFFFF:FFFF,,FF::FFF:
```

@A00155:342:HHGFNDSXY:1:2667:20003:19758 2:N:0:GAACCTAG+TCCGCATA
GTCCACGGCCGGGTGCTCCAGCGCTGGCGTGGTTGTAGTCAAATGTTGGTAGTCAGGGTAGTTTTCCT
CCCCTGCTCCACCTGGCCACGAGTCAAAACTAGCCTCTTCTTCATATCTCTCCGGCCACCACTGGTCC
+
FFFFFFFFFFFFFFFFFFFFFFFFFFFFFFFFFFFFFFFFFFFFFFFFFFFFFFFFFFFFFFF:FFFF
FFFFFFFFFFFFFFFFFFFFFFFFFFFFFFFFFFFFFF:FFFFFFFFFFFFFFFFFFFFFFFFFFFFF
@A00155:342:HHGFNDSXY:1:2274:16884:21903 2:N:0:GAACCTAG+TCCGCATA
GTCCACGGCCGGGTGCTCCAGCGCTGGCGTGGTTGTAGTCAAATGTTGGTAGTCAGGGTAGTTTTCCT
CCCCTGCTCCACCTGGCCACGAGTCAAAACTCGCCTCTTCTTCATATCTCTCCGGCCACCACTGGTCC
+
FFFF,FFF,FFFFFFF:FFFFFFFFF:FFFFFFFFFFFFFF::FF,FFFFFFFFFFFFFFFFF,:FFF
FF:FFFFFFF,FFFF:FFFFFF:F:FFFFFF,FFFF:,FFFF:FFFFFFF:FFFF::FFFFFFFFFFF
@A00155:342:HHGFNDSXY:1:2342:7066:13197 2:N:0:GAACCTAG+TCCGCATA
GTCCACGGCCGGGTGCTCCAGCGCTGGCGTGGTTGTAGTCAAATGTTGGTAGTCAGGGTAGTTTTCCT
CCCCTGCTCCACCTGGCCACGAGTCAAAACTAGCCTCTTCTTCATATCTCTCCGGCCACCACTGGTCC
+
FFFFFFFFFFFFFFFFFFFFFFFFFFFFFFFFFFFFFFFFFFFFFFFFFFFFFFFFFFFFFFFFFFFF
FFFFFFFFFFFFFFFFFFFFFFFFFFFFFFFFFFFFFFFFFFFFFFFFFFFFFFFFFFFFFF:FFFFF
@A00155:342:HHGFNDSXY:1:1405:32497:35102 2:N:0:GAACCTAG+TCCGCATA
GTCCACGGCCGGGTGCTCCAGCGCTGGCGTGGTTGTAGTCAAATGTTGGTAGTCAGGGTAGTTTTCCT
CCCCTGCTCCACCTGGCCACGAGTCAAAACTAGCCTCTTCTTCATATCTCTCCGGCCACCACTGGTCC
+
FFFFFFFFFFFFFFFFFFFFFFFFFFFFFFFFFFFFFFFFFF:FFFFFFFFFFFFFFFFFFFFFFFFF
FFFFFFFFFFFFFFFFFFFFFFFFFFFFFF:FFFFFFFFFFFFFFFFFFFFFFFFFFFFFFFFFF:FF
@A00155:342:HHGFNDSXY:1:1514:9254:1141 2:N:0:GAACCTAG+TCCGCATA
GTCCACGGCCGGGTGCTCCAGCGCTGGCGTGGTTGTAGTCAAATGTTGGTAGTCAGGGTAGTTTTCCT
CCCCTGCTCCACCTGGCCACGAGTCAAAACTAGCCTCTTCTTCATATCTCTCCGGCCACCACTGGTCC
+
F:FFFFFFFFFFFFFFFFFFFFFFFFFFFFFFFFF:FFFFFFFFFFFFFFFF:FFFFFFFF,FFFFFF
FFFFFFFFFFFFFFFFFFFFFFFFFFFFFFFFFFFFFFFFFFFFFFFFFFFFFFFFFFFFFFFFFFFF
@A00155:342:HHGFNDSXY:1:2259:29649:34053 2:N:0:GAACCTAG+TCCGCATA
GTCCACGGCCGGGTGCTCCAGCGCTGGCGTGGTTGTAGTCAAATGTTGGTAGTCAGGGTAGTTTTCCT
CCCCTGCTCCACCTGGCCACGAGTCAAAACTAGCCTCTTCTTCATATCTCTCCGGCCACCACTGGTCC
+
FFFFFFFFFFFFFFFFFFFFFFFFFFFFFFFFFFFFFFFFFFFFFFFF::FFFFFFFFFFFFFFFFFF
FFFF,FF:FFFFFFFFFF,FFFFFFFFFFFFFFFFFFFFF:FFF:FFF:F:FFFFFFFFFFFFFFFFF
@A00155:342:HHGFNDSXY:1:2615:14217:9987 2:N:0:GAACCTAG+TCCGCATA
GTCCACGGCCGGGTGCTCCAGCGCTGGCGTGGTTGTAGTCAAATGTTGGTAGTCAGGGTAGTTTTCCT
CCCCTGCTCCACCTGGCCACGAGTCAAAACTAGCCTCTTCTTCATATCTCTCCGGCCACCACTGGTCC
+
FFFFFFFFFFFFFFFFFFFFFFFFFFFFFFFFFFFFFFFFFFFFFFFFFFFFFFFFFFFFF:FFFFFF
FFFFFFFFFFFFFFFFFFFFFFFFFFFFFFFFFFFFFFFFFFFFFFFFFFFFFFFFFFFFFFFFFFFF
@A00155:342:HHGFNDSXY:1:2420:22290:15201 2:N:0:GAACCTAG+TCCGCATA
GTCCACGGCCGGGTGCTCCAGCGCTGGCGTGGTTGTAGTCAAATGTTGGTAGTCAGGGTAGTTTTCCT
CCCCTGCTCCACCTGGCCACGAGTCAAAACTAGCCTCTTCTTCATATCTCTCCGGCCACCACTGGTCC
+
FFFF:FFFF:FFFFFFFFFFFFFFFFFFFFFFFFFFFFFFFFFFFFFFFFFFFFFFFF:FFFFFFFFF
FFFFFFFFFFFFFFFFFFFFFFFFFFFFFFFFFFFFFFFFFFFFFFFFFFFFFFFF:FFFFFFFFFFF
@A00155:342:HHGFNDSXY:1:2420:22146:15702 2:N:0:GAACCTAG+TCCGCATA
GTCCACGGCCGGGTGCTCCAGCGCTGGCGTGGTTGTAGTCAAATGTTGGTAGTCAGGGTAGTTTTCCT
CCCCTGCTCCACCTGGCCACGAGTCAAAACTAGCCTCTTCTTCATATCTCTCCGGCCACCACTGGTCC
+
FFFF,FFFFFFFFFFFFFF:FFFFFFFFFF:FFFFFFFFFFFFFFFFFFFFFFFFFFF:FFFFFFFF,
FFFFFFFFFFFFFFFFFFFFFFFFFFFFFFFFFFFFFFFFFFFFFFFF,FFFFFFFFFFFFFFFFFFF

```
@A00155:342:HHGFNDSXY:1:2667:11568:34773 2:N:0:GAACCTAG+TCCGCATA
GTCCACGGCCGGGTGCTCCAGCGCTGGCGTGGTTGTAGTCAAATGTTGGTAGTCAGGGTAGTTTTCCT
CCCCTGCTCCACCTGGCCACGAGTCAAAACTAGCCTCTTCTTCATATCTCTCCGGCCACCACTGGTCC
+
FFFF:FFFFFFFFFFFFF,FFFFF:FFFFFFFFFFFFF:FFFFFFFFFFFFFFFFFFFFFFFFFFFFF
FFFFFFFFFFFFFFFFFFFFFFFFFFFFFFFFFFFFFFFFFFFFFFFFFFFFFFFFFFFFFFFFFFFF
@A00155:342:HHGFNDSXY:1:1101:6289:30545 2:N:0:GAACCTAG+TCCGCATA
GTCCACGGCCGGGTGCTCCAGCGCTGGCGTGGTTGTAGTCAAATGTTGGTAGTCAGGGTAGTTTTCCT
CCCCTGCTCCACCTGGCCACGAGTCAAAACTAGCCTCTTCTTCATATCTCTCCGGCCACCACTGGTCC
+
FFFFFFFFFFFFFFFFFFFFFFFFFFFFFFFFFFFFFFFFFFFFFFFFF:FFFFFFFFFFFFFFFFFF
FFF:FFF:FFFFFFFFFFFFFFFFFFFFFFFFFFFFF:FFFFFFFFFFFFFFFFFFFFFFFFFFFFFF
@A00155:342:HHGFNDSXY:1:1101:5674:30013 2:N:0:GAACCTAG+TCCGCATA
GTCCACGGCCGGGTGCTCCAGCGCTGGCGTGGTTGTAGTCAAATGTTGGTAGTCAGGGTAGTTTTCCT
CCCCTGCTCCACCTGGCCACGAGTCAAAACTAGCCTCTTCTTCATATCTCTCCGGCCACCACTGGTCC
+
FFFFFFFFFFFFFFFFFFFFFFFFFFFFFFFFFFF:FFFFFFFFFFFFFFFFFFFFFFFFFFF:FFFF
FFFFFFFFFFFFFFFFFFFFFFFFFFFFFFFFFFFFFFFFFFFFFFFFFFFFFFFFFFFFFFFFFFFF
@A00155:342:HHGFNDSXY:1:1305:31213:30655 2:N:0:GAACCTAG+TCCGCATA
TCCACGGCCGGGTGCTCCAGCGCTGGCGTGGTTGTAGTCAAATGTTGGTAGTCAGGGTAGTTTTCCTC
CCCTGCTCCACCTGGCCACGAGTCAAAACTAGCCTCTTCTTCATATCTCTCCGGCCACCACTGGTCCA
+
FFFFFFFFFFFFFFFFFFFFFFFFFFFFFFFFFFFFFFFFFFFFFFFFFFFFFFFFFFFFFFFFFFFF
FFFFFFFFFFFFFFFFFFFFFFFFFFFFFF:FFFFFFFFFFFFFFFFFFFFFFFFFFFFFFFFFFFF:
@A00155:342:HHGFNDSXY:1:1438:5981:2362 2:N:0:GAACCTAG+TCCGCATA
CCACGGCCGGGTGCTCCAGCGCTGGCGTGGTTGTAGTCAAATGTTGGTAGTCAGGGTAGTTTTCCTCC
CCTGCTCCACCTGGCCACGAGTCAAAACTAGCCTCTTCTTCATATCTCTCCGGCCACCACTGGTCCAT
+
FFFFFFFFFFF:FFFFFFFFFFFFFFFFFFFFFFFFFFFFFFFFFFFFFFFFFFFFFFFFFFFFFFFF
FFFFFFFFFFFFFFFFFFFFFFFFFFFFFFFFFFFFFFFFFFFFFFFFFFFFFFFFFFFFFFFFFFFF
@A00155:342:HHGFNDSXY:1:1358:3052:6464 2:N:0:GAACCTAG+TCCGCATA
CCACGGCCGGGTGCTCCAGCGCTGGCGTGGTTGTAGTCAAATGTTGGTAGTCAGGGTAGTTTTCCTCC
CCTGCTCCACCTGGCCACGAGTCAAAACTAGCCTCTTCTTCATATCTCTCCGGCCACCACTGGTCCAT
+
FFFFFFFFFFFFFFFFFFFFFFFFFFFFFFFFFFFFFFFFFFFFFFFFFFFFFFFFFFF:FFFFFFFF
FFFFFFFFFFFFFFFFFFFFFFFFFFFFFFFFFFFF:FFFFFFFFFFFFFFFFFFFFFFFFFFFFFFF
@A00155:342:HHGFNDSXY:1:2646:21902:22670 2:N:0:GAACCTAG+TCCGCATA
CCACGGCCGGGTGCTCCAGCGCTGGCGTGGTTGTAGTCAAATGTTGGTAGTCAGGGTAGTTTTCCTCC
CCTGCTCCACCTGGCCACGAGTCAAAACTAGCCTCTTCTTCATATCTCTCCGGCCACCACTGGTCCAT
+
FFFFFFFFFFFFFFFFFFFFFFFFF:FFFFFFFFFFFFFFFFFFFFFFFFFFFFFFFFFFFFFFFFFF
FFFFFFFFFFFFFFFFFFFFFFFFFFFFFFFFFFFFFFFFFFFFFFFFFFFFFFFFFFFFFFFFFFFF
@A00155:342:HHGFNDSXY:1:2658:16197:26882 2:N:0:GAACCTAG+TCCGCATA
CACGGCCGGGTGCTCCAGCGCTGGCGTGGTTGTAGTCAAATGTTGGTAGTCAGGGTAGTTTTCCTCCC
CTGCTCCACCTGGCCACGAGTCAAAACTAGCCTCTTCTTCATATCTCTCCGGCCACCACTG
+
FFFFFFFFFFFFFFFFFFFFF:FFFF:FFFFFFFFFFFFFFFFFFFFFFFFFFFFFFFFFFFFFFFFF
FFFFFFFFFFFFFFFFFFFFFFFFFFFFFFFFFFFFFFFFFFFFFFFFFFFFFFFFFFFFF
@A00155:342:HHGFNDSXY:1:1169:6045:32064 2:N:0:GAACCTAG+TCCGCATA
CACGGCCGGGTGCTCCAGCGCTGGCGTGGTTGTAGTCAAATGTTGGTAGTCAGGGTAGTTTTCCTCCC
CTGCTCCACCTGGCCACGAGTCAAAACTAGCCTCTTCTTCATATCTCTCCGGCCACCACTGGTC
+
FFFFFFFFFFFFFFFFFFFFFFFFFFFFFFFFFFFFFFFFFFFFFFFFFFFFFFFFFFFFFFFFFFFF
FFFFFFFFFFFFFFFFFFFFFFFFFFFFFFFFFFFFFFFFFFFFFFFFFFFFFFFFFFFFFFFF
```

```
@A00155:342:HHGFNDSXY:1:1169:5791:31751 2:N:0:GAACCTAG+TCCGCATA
CACGGCCGGGTGCTCCAGCGCTGGCGTGGTTGTAGTCAAATGTTGGTAGTCAGGGTAGTTTTCCTCCC
CTGCTCCACCTGGCCACGAGTCAAAACTAGCCTCTTCTTCATATCTCTCCGGCCACCACTGGTC
+
F:FFF:FFFFFFFFFFFFFFFFFFFFFFFFFFFFFFFFFFFFFFFFFFFFFFFFFFFFFFFFFFFFFF
FFFFF:FFFFFFFFFFFFFFFFFFFFFFFFFFFFFFFFFFFFFFFFFFF:FFFFFFFFFFFFFF
@A00155:342:HHGFNDSXY:1:2153:14950:5682 1:N:0:GAACCTAG+TCCGCATA
ACGGCCGGGTGCTCCAGCGCTGGCGTGGTTGTAGTCAAATGTTGGTAGTCAGGGTAGTTTTCCTCCCC
TGCTCCACCTGGCCACGAGTCAAAACTAGCCTCTTCTTCATATCTCTCCGGCCACCACTGGTCCATAT
+
,FFFFFFFFFFFFFFFFFFFFFFFFFFFFFFFFFFFFFFFFFFFFFFFFFFFFFFFFFFFFFFFFFFF
FFFFFFFFFFFFFFFFFFFFFFFFFFFFFFFFFFFFFFFFFFFFFFFFFFFFFFFFFFFFFFFFFFFF
@A00155:342:HHGFNDSXY:1:2672:31665:27649 1:N:0:GAACCTAG+TCCGCATA
ACGGCCGGGTGCTCCAGCGCTGGCGTGGTTGTAGTCAAATGTTGGTAGTCAGGGTAGTTTTCCTCCCC
TGCTCCACCTGGCCACGAGTCAAAACTAGCCTCTTCTTCATATCTCTCCGGCCACCACTGGTCCATAT
+
FFFFFFFFFFFFFFFFFFFFFFFFFFFFFFFFFFFFFFFFFFFFFFFFFFFFFFF,FFFFFFFFFFFF
FFFFFFFFFFFFFFFFFFFFFFFFFFFFFFFFFFFFFFFFFFFFFFFFFFFFFFFFFFFFFFFFFFFF
@A00155:342:HHGFNDSXY:1:1313:5791:12962 1:N:0:GAACCTAG+TCCGCATA
ACGGCCGGGTGCTCCAGCGCTGGCGTGGTTGTAGTCAAATGTTGGTAGTCAGGGTAGTTTTCCTCCCC
TGCTCCACCTGGCCACGAGTCAAAACTAGCCTCTTCTTCATATCTCTCCGGCCACCACTGGTCCATAT
+
FFFFFFFFFFFFFFFFFFFFFFFF::FFFFFFF:FFFFFF::FFFFFFFFFFFFFFFFFFFFFFFFFF
FFFFFFFFFFFFFFFFFFFFFFFFFFFFFFFFFFFFFFFF:FFFFFFFFFFFF:FFFFFFFFFFFFFF
@A00155:342:HHGFNDSXY:1:1677:30960:4852 1:N:0:GAACCTAG+TCCGCATA
ACGGCCGGGTGCTCCAGCGCTGGCGTGGTTGTAGTCAAATGTTGGTAGTCAGGGTAGTTTTCCTCCCC
TGCTCCACCTGGCCACGAGTCAAAACTAGCCTCTTCTTCATATCTCTCCGGCCACCACTGGTCCATAT
+
FFFFFFFFFFFFFFFFFFFFFFFFFFFFFFFFFFFFFFFFFFFFFFFFFFFFFFFFFFFFFFFFFFFF
FFFFFFFFFFFFFFFFFFFFFFFFFFFFFF:FFFFFFFFFFFFFFFFFFFFFFFFFFFFFFFFFFFFF
@A00155:342:HHGFNDSXY:1:2544:12048:24298 1:N:0:GAACCTAG+TCCGCATA
CGGCCGGGTGCTCCAGCGCTGGCGTGGTTGTAGTCAAATGTTGGTAGTCAGGGTAGTTTTCCTCCCCT
GCTCCACCTGGCCACGAGTCAAAACTAGCCTCTTCTTCATATCTCTCCGGCCACCACTGGTCCATATC
+
FFFFFFFFFFFFFFFFFFFFFFFFFFFFFFFFFFFFFFFFFFFFFFFFFFFFFFFFFFFFFFFFFFFF
FFFFFFFFFFFFFFFFFFFFFFFFFFFFFFFFFFFFFFFFFFF:FFFFFFFFFFFFFFFFFFFFFFFF
@A00155:342:HHGFNDSXY:1:1525:3269:12821 2:N:0:GAACCTAG+TCCGCATA
CGGCCGGGTGCTCCAGCGCTGGCGTGGTTGTAGTCAAATGTTGGTAGTCAGGGTAGTTTTCCTCCCCT
GCTCCACCTGGCCACGAGTCAAAACTAGCCTCTTCTTCATATCTCTCCGGCCACCACTGGTCCATATC
+
FFFFFFFFFFFFFFFFFFFFFFFFFFFFFFFFFFFF:FFFFFFFFFFFFFFFFFFFFFFFFFFFFFFF
FFFFFFFFFFFFFFFFFFFFFFFFFFFFFFFFFFFFFFFFFFFFFFFFFFFFFFFFFF:FFFFFFFF:
@A00155:342:HHGFNDSXY:1:2269:16731:28870 1:N:0:GAACCTAG+TCCGCATA
CGGCCGGGTGCTCCAGCGCTGGCGTGGTTGTAGTCAAATGTTGGTAGTCAGGGTAGTTTTCCTCCCCT
GCTCCACCTGGCCACGAGTCAAAACTAGCCTCTTCTTCATATCTCTCCGGCCACCACTGGTCCATATC
+
FFFFFFFFFFFFFFFFFFFFFFFFFFFFFFFFFFFFFFFFFFFFFFFFFFFFFFFFFFFFFFFFFFFF
FFFFFFFFFFFFFFFFFFFFFFFFFFFFFFFFFFFFFFFFFFFFFFFFFFFFFFFFFFFFFFFFFFFF
@A00155:342:HHGFNDSXY:1:1144:6307:10316 2:N:0:GAACCTAG+TCCGCATA
CGGCCGGGTGCTCCAGCGCTGGCGTGGTTGTAGTCAAATGTTGGTAGTCAGGGTAGTTTTCCTCCCCT
GCTCCACCTGGCCACGAGTCAAAACTAGCCTCTTCTTCATATCTCTCCGGCCACCACTGGTCCATATC
+
FFFFFFFFFFFFFFFF:FFFFFFFFFFFFFFFF:FFFFFFFFFFF:FFFFFFF::FFFFFFFFFFF:F
FFFFFFFFFFFFFFFFFFFFF:FFFFFFFFFFFFFFFFFFFFFFFFFFFFFFFFFFFFFFFFFFFFFF
```

@A00155:342:HHGFNDSXY:1:2557:12454:10911 2:N:0:GAACCTAG+TCCGCATA
CGGCCGGGTGCTCCAGCGCTGGCGTGGTTGTAGTCAAATGTTGGTAGTCAGGGTAGTTTTCCTCCCCT
GCTCCACCTGGCCACGAGTCAAAACTAGCCTCTTCTTCATATCTCTCCGGCCACCACTGGTCCATATC
+
FFFFFFFFFFFFFFFFFFFFFFFFFFFFFF:FFFFFFFFFFFFF:FFFFFFFFFFF::FFFFFFFFFF
FFFFFFFFFFFFFFFFFFFFFFFFFFFFFFFFFFFF:FFF:FFF:FFFFFFFFFFFFFFFFFFFFFFFF
@A00155:342:HHGFNDSXY:1:2557:12355:10113 2:N:0:GAACCTAG+TCCGCATA
CGGCCGGGTGCTCCAGCGCTGGCGTGGTTGTAGTCAAATGTTGGTAGTCAGGGTAGTTTTCCTCCCCT
GCTCCACCTGGCCACGAGTCAAAACTAGCCTCTTCTTCATATCTCTCCGGCCACCACTGGTCCATATC
+
FFFFFFFFFFFFFFFFFFFFFF:FFFFFFF:FFFFFFFFFFFFFFFF:FFFFFFFFFFFFFFFFFFFF
FFFFFFFFFFFFFFFFFFFFFFF:FFFFFFFFFFFFFFFFFFFFFFFFFFFFFFFFFFFFFFFFF:FF
@A00155:342:HHGFNDSXY:1:1567:26404:9298 1:N:0:GAACCTAG+TCCGCATA
CGGCCGGGTGCTCCAGCGCTGGCGTGGTTGTAGTCAAATGTTGGTAGTCAGGGTAGTTTTCCTCCCCT
GCTCCACCTGGCCACGAGTCAAAACTAGCCTCTTCTTCATATCTCTCCGGCCACCACTGGTCCATATC
+
FFFFFFFFFFFFFFFFFFFFFFFFFFFFFFFFFFFFFFFFFFFFFFFFFFFFFFFFFFFFFFFFFFFF
FFFFFFFFFFFFFFFFFFFFFFFFFFFFFFFFFFFFFFFFFFFFFFFFFFFFFFFFFFFFFFFFFFFF
@A00155:342:HHGFNDSXY:1:2456:4101:16548 2:N:0:GAACCTAG+TCCGCATA
GGCCGGGTGCTCCAGCGCTGGCGTGGTTGTAGTCAAATGTTGGTAGTCAGGGTAGTTTTCCTCCCCTG
CTCCACCTGGCCACGAGTCAAAACTAGCCTCTTCTTCATATCTCTCCGGCCACCACTGGTCCATATCG
+
:FFFFFFFFFFFFFFFFFFFFFFFFFFFFF:FFFFFFFFF:FF:FFFFFFFFFFFFFFFFFFFFFFFFF
F:FFFFF,FFFFFFFFFFFFF:FFFFFFFFFFFFFFFFFFFFFFFFFFFFFFFFFFFFFFFFFFFFFF
@A00155:342:HHGFNDSXY:1:2607:22932:19789 1:N:0:GAACCTAG+TCCGCATA
GGCCGGGTGCTCCAGCGCTGGCGTGGTTGTAGTCAAATGTTGGTAGTCAGGGTAGTTTTCCTCCCCTG
CTCCACCTGGCCACGAGTCAAAACTAGCCTCTTCTTCATATCTCTCCGGCCACCACTGGTCCATATCG
+
FFFFFFFFFFFFFFFFFFFFFFFFFFFFFFFFFFFFFFFFFFFFFFFFFFFFFFFFFFFFFFFFFFFF
FFFFFFFFFFFFFFFFFFFFFF:FFFFFFFF:FFFFFFFFFFFFFFFFFFFFFFFFFFFFFFF:FFFF
@A00155:342:HHGFNDSXY:1:1526:29378:15327 1:N:0:GAACCTAG+TCCGCATA
GCCGGGTGCTCCAGCGCTGGCGTGGTTGTAGTCAAATGTTGGTAGTCAGGGTAGTTTTCCTCCCCTGC
TCCACCTGGCCACGAGTCAAAACTAGCCTCTTCTTCATATCTCTCCGGCCACCACTGGTCCATATCGA
+
FFFFFFFFFFFFFFFFFFFFFFFFFFFFF:FFFFFFFFFFFFFFFFFFFFFFFFFFFFFFFFFFFFFF
FFFFFFFFFFFFFFFFFFFFFFFFFFFFFFFFFFFFFFFFFFFFFF,FFFFFFFFFFFFF:F,FFFFF
@A00155:342:HHGFNDSXY:1:1161:14244:26381 1:N:0:GAACCTAG+TCCGCATA
GCCGGGTGCTCCAGCGCTGGCGTGGTTGTAGTCAAATGTTGGTAGTCAGGGTAGTTTTCCTCCCCTGC
TCCACCTGGCCACGAGTCAAAACTAGCCTCTTCTTCATATCTCTCCGGCCACCACTGGTCCATATCGA
+
FFFFFFFFFFFFFFFFFFFFFF:FFFFFFFFF:FF,FFFFFFFFFFFFFFFFFFFFFFFFFFFFFFFF
FFFFFFFFFFFFF:FFFFFFFFFFFF:FFFF:FFFFFF:FFFFFFFFFFFFFF:F,FFFFFFF:FFFF
@A00155:342:HHGFNDSXY:1:1475:13141:31454 1:N:0:GAACCTAG+TCCGCATA
GCCGGGTGCTCCAGCGCTGGCGTGGTTGTAGTCAAATGTTGGTAGTCAGGGTAGTTTTCCTCCCCTGC
TCCACCTGGCCACGAGTCAAAACTAGCCTCTTCTTCATATCTCTCCGGCCACCACTGGTCCATATCGA
+
FFFFFFFFFFFFFFFFFFFFFFFFFFFFFFFFFFFFFFFFFFFFF:FFFFFFFFFFFFFFFFFFFFFF
FFFFFFFFFFFFFFFFFFFFFFFFFFFFFFFFFFFFFFFFFFFFFFFFFFFFFFF:FFFFFFFFFFFF
@A00155:342:HHGFNDSXY:1:2575:17535:23657 1:N:0:GAACCTAG+TCCGCATA
GCCGGGTGCTCCAGCGCTGGCGTGGTTGTAGTCAAATGTTGGTAGTCAGGGTAGTTTTCCTCCCCTGC
TCCACCTGGCCACGAGTCAAAACTAGCCTCTTCTTCATATCTCTCCGGCCACCACTGGTCCATATCGA
+
FFFF:FFFFFFFFFF,FFFFFF,FFF:F:FFFF:FFFF,F,FFFFFFFFF,FF:FFFF:FFFFFF:F,
FFFFFFFFFFFFF,F:FFFFFFFFF:FFF,,FFFFFFFFFFFFFF:FFFFFFFFFFFFFFFFF,FFF

```
@A00155:342:HHGFNDSXY:1:2178:24243:19711 1:N:0:GAACCTAG+TCCGCATA
CCGGGTGCTCCAGCGCTGGCGTGGTTGTAGTCAAATGTTGGTAGTCAGGGTAGTTTTCCTCCCCTGCT
CCACCTGGCCACGAGTCAAAACTAGCCTCTTCTTCATATCTCTCCGGCCACCACTGGTCCATATCGA
+
F:F:,FFF:FFFFFFF,FFFF,FFFFF,FFFFFFFFFFFFFF::,FFFF:FFFFFF:FFFFF:FF,FF
FFFFF:FFFFFFFF:FFF:::FFFFFFFF:FF:FFFFFFFFFFFF:FFFFFFFFFFFFFF:F:FFFF
@A00155:342:HHGFNDSXY:1:2354:24885:4507 2:N:0:GAACCTAG+TCCGCATA
CCGGGTGCTCCAGCGCTGGCGTGGTTGTAGTCAAATGTTGGTAGTCAGGGTAGTTTTCCTCCCCTGCT
CCACCTGGCCACGAGTCAAAACTAGCCTCTTCTTCATATCTCTCCGGCCACCACTGGTCCATATCGAT
+
FFFFFFFFFFFFFFFFFFFFFFFFFFFFFFFFFFFFFFFFFFFFFFFFFFFFFFF,FFFFFFFFFFFF
FFFFFFFFFFFFFFFFFFFFFFFFFFFFFFFFFFFFFFFFFFFFFFFFFFFFFFFFFFFFFFFFFFFF
@A00155:342:HHGFNDSXY:1:2328:32208:36448 1:N:0:GAACCTAG+TCCGCATA
CCGGGTGCTCCAGCGCTGGCGTGGTTGTAGTCAAATGTTGGTAGTCAGGGTAGTTTTCCTCCCCTGCT
CCACCTGGCCACGAGTCAAAACTAGCCTCTTCTTCATATCTCTCCGGCCACCACTGGTCCATATCGAT
+
FFFFFFFFFFFFFFFFFFFFFFFFFFF:FFFFFFFFFFFFFFFFFFFFFFFFFFFFFFFFFFFFFFFF
FFFFFFFFFFFFFFFFFFFFFFFFF:FFFFF:FFFFFFFFFFFFFFFFFFFFFFFFF:FFFFFFFFFF
@A00155:342:HHGFNDSXY:1:2465:12861:24925 1:N:0:GAACCTAG+TCCGCATA
CCGGGTGCTCCAGCGCTGGCGTGGTTGTAGTCAAATGTTGGTAGTCAGGGTAGTTTTCCTCCCCTGCT
CCACCTGGCCACGAGTCAAAACTAGCCTCTTCTTCATATCTCTCCGGCCACCACTGGTCCATATCGAT
+
FFFFFFFFFFF:FFFFFFFFFFFFFFFFFFFFFFFFFFFFFF:FFFFFFFFFFFFFFFFFFFFFFFFF
FFFFFFFFFFFFFFFFFFFFFFFFFFFFFFFFFFFFFFFFFFFFFFFFFFFFFFFFFFFFFF:FFFFF
@A00155:342:HHGFNDSXY:1:1417:5493:5274 1:N:0:GAACCTAG+TCCGCATA
CCGGGTGCTCCAGCGCTGGCGTGGTTGTAGTCAAATGTTGGTAGTCAGGGTAGTTTTCCTCCCCTGCT
CCACCTGGCCACGAGTCAAAACTAGCCTCTTCTTCATATCTCTCCGGCCACCACTGGTCCATATCGAT
+
FFFFFFFFFFFFFFFFFFFFF:FFFFFFFFFFFFFFFFFFFFF,FFFFFFFFFFFFFFFFFFFFFFFF
FFFFFFFFFFFFFFFFFFFFFFFFFFFFFFFFFFFFFFFFFFFFFFFFFFFFFFFFFFFFFFFFFFFF
@A00155:342:HHGFNDSXY:1:1611:12563:7185 1:N:0:GAACCTAG+TCCGCATA
CCGGGTGCTCCAGCGCTGGCGTGGTTGTAGTCAAATGTTGGTAGTCAGGGTAGTTTTCCTCCCCTGCT
CCACCTGGCCACGAGTCAAAACTAGCCTCTTCTTCATATCTCTCCGGCCACCACTGGTCCATATCGAT
+
FFFFFFFFFFFFFFFFFFFFFFFFFFFFFFFFFFFFFFFFFFFFFFFFFFFFFFFFFFFFFFFFFFFF
FFFFFFFFFFFFFFFFFFFFFFFFFFFFFFFFFFFFFFFFFFFFFFFFFFFFFFFFFFFFFFFFFFFF
@A00155:342:HHGFNDSXY:1:1414:6777:6590 1:N:0:GAACCTAG+TCCGCATA
CCGGGTGCTCCAGCGCTGGCGTGGTTGTAGTCAAATGTTGGTAGTCAGGGTAGTTTTCCTCCCCTGCT
CCACCTGGCCACGAGTCAAAACTAGCCTCTTCTTCATATCTCTCCGGCCACCACTGGTCCATATCGAT
+
FFFFFF:FFFFFFF:FFF:FFFFFFFFFFF:F:FF:FFFFFFFF:FFFFFF,FFFFFFFFFFFFFFFF
FFFFFFF:FFFFFFF:FFFFFFF:F:FFF:FF,F:F:F,F:FF:F:FFFFFFFFF:::F:,F,FFF,F
@A00155:342:HHGFNDSXY:1:2354:22453:6590 2:N:0:GAACCTAG+TCCGCATA
CCGGGTGCTCCAGCGCTGGCGTGGTTGTAGTCAAATGTTGGTAGTCAGGGTAGTTTTCCTCCCCTGCT
CCACCTGGCCACGAGTCAAAACTAGCCTCTTCTTCATATCTCTCCGGCCACCACTGGTCCATATCGAT
+
FFFFFFF:FFFFFFFFFFFFFFFFFFF:FFFFFFFFFFFFFFFFFFFFFFFFFFFFFFFFFFFFFFFF
FFFFFFFFFFFFFFFFFFFFFFFFFFFFFFFFFFFFFFFFFFFFFFFFFFFFFFFFFFFFFFFF:FFF
@A00155:342:HHGFNDSXY:1:1370:28411:4038 1:N:0:GAACCTAG+TCCGCATA
CGGGTGCTCCAGCGCTGGCGTGGTTGTAGTCAAATGTTGGTAGTCAGGGTAGTTTTCCTCCCCTGCTC
CACCTGGCCACGAGTCAAAACTAGCCTCTTCTTCATATCTCTCCGGCCACCACTGGTCCAT
+
FFFFFFFFFFFFFFFFFFFFFFFFFFFFFFFFFFFFFFFFFFFFFFFFFFFFFFFFFFFFFFFFFFFF
FFFFFFFFFFFFFFFFFFFFFFFFFFFFFFFFFFFFFFFFFFFFFFFFFFFFFFFFFFFFF
```

```
@A00155:342:HHGFNDSXY:1:1508:19099:23578 2:N:0:GAACCTAG+TCCGCATA
CGGGTGCTCCAGCGCTGGCGTGGTTGTAGTCAAATGTTGGTAGTCAGGGTAGTTTTCCTCCCCTGCTC
CACCTGGCCACGAGTCAAAACTAGCCTCTTCTTCATATCTCTCCGGCCACCACTGGTCCATATCGATA
+
FFFFFFFFFFFFF:FFFFFFFFFFFFFFFFFF:FFFFFFFFFFFFFFFFFFFFF:FFFFFFFF:FFFF
FFFFFFFFFF:FFFFFFFF:FFFFFFFFFFFFF::FFFFFFFFFFFFFFFFFFFFFFFFFFFFFF,FF
@A00155:342:HHGFNDSXY:1:1260:24876:23030 1:N:0:GAACCTAG+TCCGCATA
CGGGTGCTCCAGCGCTGGCGTGGTTGTAGTCAAATGTTGGTAGTCAGGGTAGTTTTCCTCCCCTGCTC
CACCTGGCCACGAGTCAAAACTAGCCTCTTCTTCATATCTCTCCGGCCACCACTGGTCCATATCGATA
+
FFFFFFFFFFFFFFFFFFFFFFFFFFFFFFFFFFFFFFFFFFFFFFFFFFFFFFFFFFFFFFFFFFFF
FFFFFFFFFFFFFFFFFFFFFFFFFFFFFFFFFFFFFFFFFFFFFF:FFFFFFFFFFFFFFFFFFFFF
@A00155:342:HHGFNDSXY:1:2608:26992:13823 1:N:0:GAACCTAG+TCCGCATA
CGGGTGCTCCAGCGCTGGCGTGGTTGTAGTCAAATGTTGGTAGTCAGGGTAGTTTTCCTCCCCTGCTC
TACCTGGCCACGAGTCAAAACTAGCCTCTTCTTCATATCTCTCCGGCCACCACTGGTCCATATCGATA
+
FFFFF:FFFFF:F:FFFFFF,FF,FFFFFF:FFFFFFFFFFFFFFFFFFFFFFFFF:F,:FFF,FFFF
,FFFFFFFFFFFFFFFFFFFFF::FFFF:FFFFF:FFF:F:F:FFFFF:FFFF,FF:FF:,FFF,:FF
@A00155:342:HHGFNDSXY:1:1363:3830:6621 1:N:0:GAACCTAG+TCCGCATA
CGGGTGCTCCAGCGCTGGCGTGGTTGTAGTCAAATGTTGGTAGTCAGGGTAGTTTTCCTCCCCTGCTC
CACCTGGCCACGAGTCAAAACTAGCCTCTTCTTCATATCTCTCCGGCCACCACTGGTCCATATCGATA
+
FFFFFFFFFFFFFFFFFFFFF,FFFFFFFFFFFFFFFFFFFFFFFFFFFFFFFFFFFFFFFFFFFFFF
FFFFFFFFFFFFFFFFFFFFFF:FFFFFFFFFFFFF:FF:FFFF:FFFFFFFF:FFFFFFFFFFFFFF
@A00155:342:HHGFNDSXY:1:1337:30110:32158 1:N:0:GAACCTAG+TCCGCATA
GGGTGCTCCAGCGCTGGCGTGGTTGTAGTCAAATGTTGGTAGTCAGGGTAGTTTTCCTCCCCTGCTCC
ACCTGGCCACGAGTCAAAACTAGCCTCTTCTTCATATCTCTCCGGCCACCACTGGTCCATATCGATAC
+
FFFFFFFFFFFFFFFFFFFFFFFFFFFFFFFFFFFFFFFFFFFFFFFFFFFFFFFFFFFFFFFFFFFF
FFFFFFFFFFFFFFFFFFFFFFFFFFF:FFFFFFFFFFFFFFFFFFFFFFFFFFFFFFFFFFFFFFFF
@A00155:342:HHGFNDSXY:1:1656:26494:3662 2:N:0:GAACCTAG+TCCGCATA
GTGCTCCAGCGCTGGCGTGGTTGTAGTCAAATGTTGGTAGTCAGGGTAGTTTTCCTCCCCTGCTCCAC
CTGGCCACGAGTCAAAACTAGCCTCTTCTTCATATCTCTCCGGCCACCACTGGTCCATATCGATACTC
+
FF,FFFFFFFFFFFFFFFFFFFFFFFFFFF:FFFFFF:F:F:FFFFFFFFFFFFFF:FFF:FFFFFFF
FFFFFFFFFFFFFFF:FFFFFFFFFFFFFF:FFFFFFF:FFFFFFFFFFFFFFFFFFFFFFFFFFFFF
@A00155:342:HHGFNDSXY:1:2321:30978:33755 2:N:0:GAACCTAG+TCCGCATA
GTGCTCCAGCGCTGGCGTGGTTGTAGTCAAATGTTGGTAGTCAGGGTAGTTTTCCTCCCCTGCTCCAC
CTGGCCACGAGTCAAAACTAGCCTCTTCTTCATATCTCTCCGGCCACCACTGGTCCATATCGATACTC
+
FFFFFFF,FFFFFFFFFF:FFFFFFFFFFF,FFFFFFFFFFFFFFFFFFFFFFFFFFFFFFFFF:FFF
:FFFFFFFFFFFFFFFFFFFFFFFFFFFFFFFFFFFFFFFFFFFFFFFFFFFFFFFFFFFFFF::FFF
@A00155:342:HHGFNDSXY:1:2371:19958:26600 2:N:0:GAACCTAG+TCCGCATA
GTGCTCCAGCGCTGGCGTGGTTGTAGTCAAATGTTGGTAGTCAGGGTAGTTTTCCTCCCCTGCTCCAC
CTGGCCACGAGTCAAAACTAGCCTCTTCTTCATATCTCTCCGGCCACCACTGGTCCATATCGATACTC
+
FFFFFFFFFFFFFFFFFFFFFFFFFFFFFFFFFFFFFFFFFFFFFFFFF:FFFFFFFFFFFFFFFFFF
FFFFFFFFFFFFFFFFFFFFFFFFFFFFFFFFFFFFFFFFFFFFFFFFFFFFFFFFFFFFFFFFFFFF
@A00155:342:HHGFNDSXY:1:1352:15908:25254 2:N:0:GAACCTAG+TCCGCATA
GTGCTCCAGCGCTGGCGTGGTTGTAGTCAAATGTTGGTAGTCAGGGTAGTTTTCCTCCCCTGCTCCAC
CTGGCCACGAGTCAAAACTAGCCTCTTCTTCATATCTCTCCGGCCACCACTGGTCCATATCGATACTC
+
FFFFFFFFFFFFFFFFFFFFFFFFFFFFFFFFFFFFFFFFFFFFFFFFFFFFFFFFFFFFFFFFFFFF
FFFFFFFFFFFFFFFFFFFFFFFFFFFFFFFFFFFFFFFFFFFFFFFFFFFFFFFFFFFFFFFFFFFF
```

```
@A00155:342:HHGFNDSXY:1:2465:12861:24925 2:N:0:GAACCTAG+TCCGCATA
GTGCTCCAGCGCTGGCGTGGTTGTAGTCAAATGTTGGTAGTCAGGGTAGTTTTCCTCCCCTGCTCCAC
CTGGCCACGAGTCAAAACTAGCCTCTTCTTCATATCTCTCCGGCCACCACTGGTCCATATCGATACTC
+
FF:FFF:FFFFFFFFFFFFF::F:FFFFFFFFFFFFF:FFFFFFFFFFFFFFFFFFFFFFFFFFFFFF
FFFFFFFFFFFFFFFFFFFFFFFFFFFFFFF:FFFFFFFFFFFFFFFFFFFFFFFF:FFFFFFFFFFF
@A00155:342:HHGFNDSXY:1:2432:25102:25958 2:N:0:GAACCTAG+TCCGCATA
GTGCTCCAGCGCTGGCGTGGTTGTAGTCAAATGTTGGTAGTCAGGGTAGTTTTCCTCCCCTGCTCCAC
CTGGCCACGAGTCAAAACTAGCCTCTTCTTCATATCTCTCCGGCCACCACTGGTCCATATCGATACTC
+
FFFFFFFFFFFFFFFFFFFFFFF:FFFFFFFFFFFFFFFFFFFFFFFFFFF,FFFFFFFFFFFFFFFF
FFFFFFFFFFFFFFFFFFFFFFFFFFFFFFFFFFFFFFFFFFFFFFFFFFFFFFFFFFFFFFFFFFFF
@A00155:342:HHGFNDSXY:1:1234:13783:14278 2:N:0:GAACCTAG+TCCGCATA
GTGCTCCAGCGCTGGCGTGGTTGTAGTCAAATGTTGGTAGTCAGGGTAGTTTTCCTCCCCTGCTCCAC
CTGGCCACGAGTCAAAACTAGCCTCTTCTTCATATCTCTCCGGCCACCACTGGTCCATATCGATACTC
+
FFFFFFFFFFFFFFFF::FFFFFFFF:FFF,FFFFFFFFFFFFFFFFFFFFFFFF:FFFFFFFFFFFF
FFFFFFFFFFFFFFFFFF::FFFFF,FFFFFFFFFFFF:FFFFFFFFFFFFFFFFFFFFFFFFFFFFF
@A00155:342:HHGFNDSXY:1:1570:12707:25629 2:N:0:GAACCTAG+TCCGCATA
GTGCTCCAGCGCTGGCGTGGTTGTAGTCAAATGTTGGTAGTCAGGGTAGTTTTCCTCCCCTGCTCCAC
CTGGCCACGAGTCAAAACTAGCCTCTTCTTCATATCTCTCCGGCCACCACTGGTCCATATCGATACTC
+
FFFFFFFFFFFFFFFFFFFFFFFFFFFFFFFFFFFFFFFFFFFFFFFFFFFFFF:FFFFFFFFFFFFF
FFFFFFFFFFFFFFFFFFFFFFFF:FF:FFFFFFFFFFFFFFFFFFFFFFFFFFFFFFFFFFFFFFFF
@A00155:342:HHGFNDSXY:1:1511:23005:13495 2:N:0:GAACCTAG+TCCGCATA
GTGCTCCAGCGCTGGCGTGGTTGTAGTCAAATGTTGGTAGTCAGGGTAGTTTTCCTCCCCTGCTCCAC
CTGGCCACGAGTCAAAACTAGCCTCTTCTTCATATCTCTCCGGCCACCACTGGTCCATATCGATACTC
+
FFFFFFFFFFFF:FFFFFFFFFF:FFFFFFFFFFFFFFFFFFFFFFFFFFFFFFFFFFFFFFFFFFFF
FFFFFFFFFFFFFFFF:FFFFFFFFFFFFFFFFFFFFFFFFFFFFF::FFF:FFFF:FFFFFFFFFFF
@A00155:342:HHGFNDSXY:1:2328:32208:36448 2:N:0:GAACCTAG+TCCGCATA
GTGCTCCAGCGCTGGCGTGGTTGTAGTCAAATGTTGGTAGTCAGGGTAGTTTTCCTCCCCTGCTCCAC
CTGGCCACGAGTCAAAACTAGCCTCTTCTTCATATCTCTCCGGCCACCACTGGTCCATATCGATACTC
+
FFFFFFFFFFFFFFFFFFFFFFF:FFFFFFFFFF,FFFFFFFFFFF:FFFFFFFFFFFFFFFFFFFFF
FFFFFFFFFFFFFFFFFFFFFFFFFFFFFFFFFFFFFFFFFFFFFFFFFFFFFFFFFFFFFFFFFFFF
@A00155:342:HHGFNDSXY:1:1117:31006:8281 2:N:0:GAACCTAG+TCCGCATA
GTGCTCCAGCGCTGGCGTGGTTGTAGTCAAATGTTGGTAGTCAGGGTAGTTTTCCTCCCCTGCTCCAC
CTGGCCACGAGTCAAAACTAGCCTCTTCTTCATATCTCTCCGGCCACCACTGGTCCATATCGATACTC
+
FFFFFFFFFFFFFFFFFFFFFFFFFFFFFF:FFFFFFFFFFFFFFFFFFFFFFFFFFFFF:FFFFFFF
FFFFFFFFFFFFF:FFFFFFFFFFFFFFFFFFFFFFFFFFFFFFFFFFFFFFFFFFFFFFFFFFFFFF
@A00155:342:HHGFNDSXY:1:2426:11659:8061 1:N:0:GAACCTAG+TCCGCATA
CTCCAGCGCTGGCGTGGTTGTAGTCAAATGTTGGTAGTCAGGGTAGTTTTCCTCCCCTGCTCCACCTG
GCCACGAGTCAAAACTAGCCTCTTCTTCATATCTCTCCGGCCACCACTGGTCCATATCGATACTCACG
+
FFFFFFFFFFFFFFFFFFFFFFFFFFFFFFFFFFFFFFFFFFFFFFFFFFFFFFFFFFFFFFFFFFFF
FFFFFFFFFFFFF:FFFFFFFFFFFFF:FFFFFFFFFFFFFFFFFFFFFFFFFFFFF:FFFFFFFFFF
@A00155:342:HHGFNDSXY:1:2467:21748:18521 1:N:0:GAACCTAG+TCCGCATA
CTCCAGCGCTGGCGTGGTTGTAGTCAAATGTTGGTAGTCAGGGTAGTTTTCCTCCCCTGCTCCACCTG
GCCACGAGTCAAAACTAGCCTCTTCTTCATATCTCTCCGGCCACCACTGGTCCATATCGATACTCACG
+
FFFFFFFFFFFFFFFFFFFFFFFFFFFFFFFFFFFFFFFFFFFFFFFFFFFFFFFFFFFFFFFFFFFF
FFFFFFFFFFFFFFFFFFFFFFFFF:FFFFFFF:FFFFFFFFFFFFFFFFFFFFFFFFFFFFFFFFF:
```

```
@A00155:342:HHGFNDSXY:1:1374:26702:8688 1:N:0:GAACCTAG+TCCGCATA
CTCCAGCGCTGGCGTGGTTGTAGTCAAATGTTGGTAGTCAGGGTAGTTTTCCTCCCCTGCTCCACCTG
GCCACGAGTCAAAACTAGCCTCTTCTTCATATCTCTCCGGCCACCACTGGTCCATATCGATACTCACG
+
FFFFFFFFFFFFFFFFFFFFFFFFFFFFFFF:FFFFFFFFFFFFFFFFFFFFFFFFFFFFFFFFFFFF
FFFFFFFFFFFFFFFFFFFFFFFFFFF:FFFFFFFFFFFFFFFFFFFFFFFFF:FFFFFFFFFFFFFF
@A00155:342:HHGFNDSXY:1:2573:24542:35603 1:N:0:GAACCTAG+TCCGCATA
CTCCAGCGCTGGCGTGGTTGTAGTCAAATGTTGGTAGTCAGGGTAGTTTTCCTCCCCTGCTCCACCTG
GCCACGAGTCAAAACTAGCCTCTTCTTCATATCTCTCCGGCCACCACTGGTCCATATCGATACTCACG
+
FFFFFFFFFFFFFFFFFFFFFFF,FFFFFFFFFFFFFFFFFFFFFFFFFFFFFFFFFFFFFFFFFFFF
FFFFFFFFFFFFFFFFFFFFFFFFFFFFFFFFF:FFFFFFFFFFFFFFFFFFFFFFFFFFFFFFFFF:
@A00155:342:HHGFNDSXY:1:2161:12653:9502 2:N:0:GAACCTAG+TCCGCATA
TCCAGCGCTGGCGTGGTTGTAGTCAAATGTTGGTAGTCAGGGTAGTTTTCCTCCCCTGCTCCACCTGG
CCACGAGTCAAAACTAGCCTCTTCTTCATATCTCTCCGGCCACCACTGGTCCATATCGATACTCACGT
+
FFFFFFFFFFFFFFFFFFF:FFFFFFFFFFFFFFFFFFFFFFFFFFFFFFFFFFFFFFFFFFFFFFFF
FFFFFFFFFFFFFFFFFFFFFFFFFFFFFFFFFFFFFFFFFFFFFFFFFFFF:FFFFFFFFFFFFFFF
@A00155:342:HHGFNDSXY:1:2161:13169:9862 2:N:0:GAACCTAG+TCCGCATA
TCCAGCGCTGGCGTGGTTGTAGTCAAATGTTGGTAGTCAGGGTAGTTTTCCTCCCCTGCTCCACCTGG
CCACGAGTCAAAACTAGCCTCTTCTTCATATCTCTCCGGCCACCACTGGTCCATATCGATACTCACGT
+
FFFFFFFFFFFFFFFFFF:FFFFFFFFFFFFFFFFFFFFFFFFFFFFFFFFFFFFFFFFFFFFFFFFF
FFFFFFFFFFFFFFFFFFFFFFFFFFFFFFFFFFFFFFFFFFFFFFFFFFFFFFFFFFFFFFFFFFFF
@A00155:342:HHGFNDSXY:1:1314:16731:6637 2:N:0:GAACCTAG+TCCGCATA
TCCAGCGCTGGCGTGGTTGTAGTCAAATGTTGGTAGTCAGGGTAGTTTTCCTCCCCTGCTCCACCTGG
CCACGAGTCAAAACTAGCCTCTTCTTCATATCTCTCCGGCCACCACTGGTCCATATCGATACTCACGT
+
FFFFFFFFFFFFFFFFFFFFFFFFFFFFFFFFFFFFFFFFFFFFFFFF:FFFFFFFFFFFFFFFF:FF
FFFFFFFFFFFFFFFFFFFFFFFF:FFFFFFFFFFFFFFFFFFFFFFFFFFFFFFFFFFFFFFFFFFF
@A00155:342:HHGFNDSXY:1:1173:32470:36839 2:N:0:GAACCTAG+TCCGCATA
TCCAGCGCTGGCGTGGTTGTAGTCAAATGTTGGTAGTCAGGGTAGTTTTCCTCCCCTGCTCCACCTGG
CCACGAGTCAAAACTAGCCTCTTCTTCATATCTCTCCGGCCACCACTGGTCCATATCGATACTCACGT
+
FFFFFFFFFFFFFFFFFFFFFFFFFF:FFFFFFFFFFFFFFFFFFFFFFFFFFFFFFFFFFFFFFFFF
FFFFFFFF:FFFFFFFFFFFFFFFFFFFFFFFFFFFFFFFFFFFFFFFFFFFFFFFFFFFFFFFFFFF
@A00155:342:HHGFNDSXY:1:1406:8015:24017 2:N:0:GAACCTAG+TCCGCATA
TCCAGCGCTGGCGTGGTTGTAGTCAAATGTTGGTAGTCAGGGTAGTTTTCCTCCCCTGCTCCACCTGG
CCACGAGTCAAAACTAGCCTCTTCTTCATATCTCTCCGGCCACCACTGGTCCATATCGATACTCACGT
+
FFFFFFFFFFFFFFFFFFFFFFFFFFFFFF:FF:FFFFFFFFF:FFFFFFFFFFF:FFFFFFFF:FFF
FFFFFFFFFFFFFFFFFFFFFFFFF:FFFFFFFFFFFFFFFFFFFFFFFFFFFFFFFFFFFFFFFFFF
@A00155:342:HHGFNDSXY:1:2658:16197:26882 1:N:0:GAACCTAG+TCCGCATA
CAGCGCTGGCGTGGTTGTAGTCAAATGTTGGTAGTCAGGGTAGTTTTCCTCCCCTGCTCCACCTGGCC
ACGAGTCAAAACTAGCCTCTTCTTCATATCTCTCCGGCCACCACTGGTCCATATCGATACT
+
FFFFFFFFFFFFFFFFFFFFFFFFFFFFFFFFFFFFFFFFFFFFFFFFFFFFFFFFFFFFFFFFFFFF
FFFFFFFFFFFFFFFFFFFFFFFFFFFFFFFFFFFFFFFFFFFFFFFFFFFFFFFFFFFFF
@A00155:342:HHGFNDSXY:1:1169:5791:31751 1:N:0:GAACCTAG+TCCGCATA
CAGCGCTGGCGTGGTTGTAGTCAAATGTTGGTAGTCAGGGTAGTTTTCCTCCCCTGCTCCACCTGGCC
ACGAGTCAAAACTAGCCTCTTCTTCATATCTCTCCGGCCACCACTGGTCCATATCGATACTCAC
+
,FFFFFFFFFFFFF:F:FFF,FFFFFFF:FFFFFFFFF,FF,,FFFFFFF:FFFFFFFFFFFFFFFFF
FFFFFFF:FFFFFFFFFFFFFFF:FFFFFF:FFFFF:FF,F,FFFFFFFFFFFFFFFFFFFFFF
```

```
@A00155:342:HHGFNDSXY:1:1169:6045:32064 1:N:0:GAACCTAG+TCCGCATA
CAGCGCTGGCGTGGTTGTAGTCAAATGTTGGTAGTCAGGGTAGTTTTCCTCCCCTGCTCCACCTGGCC
ACGAGTCAAAACTAGCCTCTTCTTCATATCTCTCCGGCCACCACTGGTCCATATCGATACTCAC
+
FFFFFFFFFFFFFFFFFFFF:FFFFFFFFFFFFFFFFFFFFF:FFFFFFFFFFFFFFFFFFFFFFFFFF
FFFFFFF:FFFFFFFFFFFFFFFFFFFFFFFFFFFFFFFFFFFFFFFFFFFFFFFFF
@A00155:342:HHGFNDSXY:1:2467:21748:18521 2:N:0:GAACCTAG+TCCGCATA
GCGCTGGCGTGGTTGTAGTCAAATGTTGGTAGTCAGGGTAGTTTTCCTCCCCTGCTCCACCTGGCCAC
GAGTCAAAACTAGCCTCTTCTTCATATCTCTCCGGCCACCACTGGTCCATATCGATACTCACGTGTCC
+
FFFFFFFFFFFFFFFFFFFFFFFFFFFFFFFFFFFFFFFFFF:FFFFFFFFFFFFFFFFFFFFFFFFFF
FFFFFFFFFFFFFFFFFFF:FFF:FFFFFFFFFFFFFFFFFFFFFFFFFFFFFFFFFFFFFFFFFFFFF
@A00155:342:HHGFNDSXY:1:1229:12075:25191 1:N:0:GAACCTAG+TCCGCATA
CGCTGGCGTGGTTGTAGTCAAATGTTGGTAGTCAGGGTAGTTTTCCTCCCCTGCTCCACCTGGCCACG
AGTCAAAACTAGCCTCTTCTTCATATCTCTCCGGCCACCACTGGTCCATATCGATACTCACGTGTCCG
+
FFFFFFFFFFFFFFFFFFFFFFF:FF:FFFFFFFFFFFFF:FFFFFFFF:FFFFFFFFFFFFFFFFFFF
FFFFFFFFFFFFFFFFFFFFFFFF:FFFFFFFF:FFF:FFFF:FFFFFFF:FFFFFFFFFFFFFFFFFF
@A00155:342:HHGFNDSXY:1:1431:13856:4789 2:N:0:GAACCTAG+TCCGCATA
CGCTGGCGTGGTTGTAGTCAAATGTTGGTAGTCAGGGTAGTTTTCCTCCCCTGCTCCACCTGGCCACG
AGTCAAAACTAGCCTCTTCTTCATATCTCTCCGGCCACCACTGGTCCATATCGATACTCACGTGTCCG
+
FFFFFF:FFFFFFFFFFFFFFFFF:FFFF:FFFFFFFFFFF:FFFF:FFFFFFFFFFFFFFFFFFFFFF
FFFFFFF:FFFFFFFFFFFFFFFFFFFFFFFFFFFFFFFFFFF:FFFFFF,FFFFFFFFFFFFFFFFFF
@A00155:342:HHGFNDSXY:1:2475:22001:4523 1:N:0:GAACCTAG+TCCGCATA
GCTGGCGTGGTTGTAGTCAAATGTTGGTAGTCAGGGTAGTTTTCCTCCCCTGCTCCACCTGGCCACGA
GTCAAAACTAGCCTCTTCTTCATATCTCTCCGGCCACCACTGGTCCATATCGATACTCACGTGTCCG
+
:FF:FFFF,FFFFFFFFFFFFF:,,FFFF:FFF:F:FFF:F,F:,FFFFFFF,FFFFFFF:,FFFF,F
FFFFFFFFFFFFFF,FF:F,:F:FFFFFFFF:,F,FFFFFFF,FFFFF:F,FFF,F:FFFFF,,FFFF
@A00155:342:HHGFNDSXY:1:1340:21386:14387 2:N:0:GAACCTAG+TCCGCATA
GCTGGCGTGGTTGTAGTCAAATGTTGGTAGTCAGGGTAGTTTTCCTCCCCTGCTCCACCTGGCCACGA
GTCAAAACTAGCCTCTTCTTCATATCTCTCCGGCCACCACTGGTCCATATCGATACTCACGTGTCCGT
+
F::FFFFFFFFFFFF:FFF:FFFFFFFFFFFFFFFFFFFFFFFFFFF:F:F,FFFF:FFFFFFFFFFFF
FFFFF:FFF:FFFF:FFFFFFFFFFFFFFFFFFFFFFFFFF:FFF,FFFFFFFFFFFFFFFFFFFFFFF
@A00155:342:HHGFNDSXY:1:1609:23095:11052 2:N:0:GAACCTAG+TCCGCATA
GCTGGCGTGGTTGTAGTCAAATGTTGGTAGTCAGGGTAGTTTTCCTCCCCTGCTCCACCTGGCCACGA
GTCAAAACTAGCCTCTTCTTCATATCTCTCCGGCCACCACTGGTCCATATCGATACTCACGTGTCCGT
+
FFFFFFFFFFFFFFFFFFFFFFF,FFFFFFFFFFFFF:FFFFFFFFFFFFFFFFFFFFFFFFFFFFFFF
FFFFFFFFFFFFFFFFFFFFFFFFFFFFFFFFFFFFFFFFFFFFFFFFFFFFFF,FFFFFFFFFFFFFF
@A00155:342:HHGFNDSXY:1:1340:12536:20290 2:N:0:GAACCTAG+TCCGCATA
GCTGGCGTGGTTGTAGTCAAATGTTGGTAGTCAGGGTAGTTTTCCTCCCCTGCTCCACCTGGCCACGA
GTCAAAACTAGCCTCTTCTTCATATCTCTCCGGCCACCACTGGTCCATATCGATACTCACGTGTCCGT
+
FFFFFFFFFFFFFFFFFFFFFFFFFFFFFFFFFFFFFFFFFFFFFF:FFFFFFFFFFFFFFFFFFFFFF
FFFFFFFFFFFFFFFFFFFFFFFFFFFFFFFFFFFFFFFFFFFFFFFFFFFFF,FFFFFFFFFFFFFFF
@A00155:342:HHGFNDSXY:1:1340:11053:21104 2:N:0:GAACCTAG+TCCGCATA
GCTGGCGTGGTTGTAGTCAAATGTTGGTAGTCAGGGTAGTTTTCCTCCCCTGCTCCACCTGGCCACGA
GTCAAAACTAGCCTCTTCTTCATATCTCTCCGGCCACCACTGGTCCATATCGATACTCACGTGTCCGT
+
FFFFFFFFFFFFFFFFFFFFFFFFFFFFFFFFFFFFFFFFFFFFFFFFFFFFFFFFFFFFFFFFFFFFF
FFFFFFFFFFFFFFFFFFFFFFFFF:FFFFFFFFFFFFFFF:FFFFF:FFFFFFFFFFFFFF,FFFFFF
```

```
@A00155:342:HHGFNDSXY:1:1313:5791:12962 2:N:0:GAACCTAG+TCCGCATA
CTGGCGTGGTTGTAGTCAAATGTTGGTAGTCAGGGTAGTTTTCCTCCCCTGCTCCACCTGGCCACGAG
TCAAAACTAGCCTCTTCTTCATATCTCTCCGGCCACCACTGGTCCATATCGATACTCACGGGTCCGTC
+
FFFFFFFFFFFFFFFFFFFFFFFFF:FFFFFFFFFFFF:FFFFFFFFFFFFFF:FFFFFFFFFFFFFF
FFFFFFFFFF,FFFFFFFFFFFFFFFFFFFFFF:FFFFFFF:FFFFFFF:FFFF:FFFFF,FFFFFFF
@A00155:342:HHGFNDSXY:1:2349:1579:35383 2:N:0:GAACCTAG+TCCGCATA
TGGCGTGGTTGTAGTCAAATGTTGGTAGTCAGGGTAGTTTTCCTCCCCTGCTCCACCTGGCCACGAGT
CAAAACTAGCCTCTTCTTCATATCTCTCCGGCCACCACTGGTCCATATCGATACTCACGTGTCCGTCA
+
FFFFFFFFFFFFFFF,F,FFFFFFFF:FFFFFFFFFF:FFFFFFFFFFFFFFFFFFFFFFFFFFFFFF
FFFFFFFFFFFFFFFFFFF:FFFFFFFFFFFFFFFFFF:FFFFFFFFFFFFFFFFFFFFFFFFFFFFF
@A00155:342:HHGFNDSXY:1:2247:27335:29356 2:N:0:GAACCTAG+TCCGCATA
TGGCGTGGTTGTAGTCAAATGTTGGTAGTCAGGGTAGTTTTCCTCCCCTGCTCCACCTGGCCACGAGT
CAAAACTAGCCTCTTCTTCATATCTCTCCGGCCACCACTGGTCCATATCGATACTCACGTGTCCGTCA
+
FFFFFFFFFFFFFFFFFFFFFFFFFFFFFFFFFF:FFF:FFFFFFFFFFFFFFFFFFFFFFFFFFFFF
:FFFFFFFFFFFFFFFFFFFFFFFFFFFFFFFFF:FFFFFFFFFFFFFFFFF:FFFFFF:F:FFFFFF
@A00155:342:HHGFNDSXY:1:1671:8024:2988 2:N:0:GAACCTAG+TCCGCATA
GGCGTGGTTGTAGTCAAATGTTGGTAGTCAGGGTAGTTTTCCTCCCCTGCTCCACCTGGCCACGAGTC
AAAACTAGCCTCTTCTTCATATCTCTCCGGCCACCACTGGTCCATATCGATACTCACGTGTCCGT
+
FFFFF:FFFFFFFFFFFFFFF:FFFFFFFFF:FFFF,FFFFFFFFFFFFFFFFFFFFFF:FFFFFFF,
FFFFFF,FFFFFFFFFFFFFFFFFFFFFFFF:FFFFFF:FFFFFFFFF:FFFFFFFFFFFFFFFF
@A00155:342:HHGFNDSXY:1:1117:31006:8281 1:N:0:GAACCTAG+TCCGCATA
GCGTGGTTGTAGTCAAATGTTGGTAGTCAGGGTAGTTTTCCTCCCCTGCTCCACCTGGCCACGAGTCA
AAACTAGCCTCTTCTTCATATCTCTCCGGCCACCACTGGTCCATATCGATACTCACGTGTCCGTCAGA
+
FFFFFFFFFFFFFFFFFFFFFFF:FFFFFFFFFFFFFFFFFFFFFFFFFFFFFFFFFFFFFFFFFFFF
FF:FFFFFFFFFFFFFFFFFFFFFFFFFFFFFFFFFFFFFFFFFFFFFFFFFFFFFF:FFFFFFFFFF
@A00155:342:HHGFNDSXY:1:2516:10981:27712 1:N:0:GAACCTAG+TCCGCATA
GCGTGGTTGTAGTCAAATGTTGGTAGTCAGGGTAGTTTTCCTCCCCTGCTCCACCTGGCCACGAGTCA
AAACTAGCCTCTTCTTCATATCTCTCCGGCCACCACTGGTCCATATCGATACTCACGTGTCCGTCAGA
+
FFFFFFFFFFFFFFFFFFFFFFFFFFFFFFFFFFFFFFFFFFFFFFFFFFFFFFFFFFFFFFFFFFFF
FFFFFF:FFFFFFFFFFFFFFF:FFFFFFFFFFFFFFFFFFFFFFFFFFFFFFFFFFFFFFFFFF:FF
@A00155:342:HHGFNDSXY:1:1258:13096:10457 2:N:0:GAACCTAG+TCCGCATA
TGGTTGTAGTCAAATGTTGGTAGTCAGGGTAGTTTTCCTCCCCTGCTCCACCTGGCCACGAGTCAAAA
CTAGCCTCTTCTTCATATCTCTCCGGCCACCACTGGTCCATATCGATACTCACGTGTCCGTCAGAGTA
+
F:FFFFFFFFFFFFFFFFFFFFFFFFFFFFFFFF:FF::FFFFFFFFFFFFFFFFFFFFFFFFFFFFF
FFFFFFFFFFFFFFFFFFFFFFFFFFFFFFFFFFFFFFFFFFFFFFFFFF,FFFFFFFFFFFFFFFF
@A00155:342:HHGFNDSXY:1:2655:14850:16376 1:N:0:GAACCTAG+TCCGCATA
GGTTGTAGTCAAATGTTGGTAGTCAGGGTAGTTTTCCTCCCCTGCTCCACCTGGCCACGAGTCAAAAC
TAGCCTCTTCTTCATATCTCTCCGGCCACCACTGGTCCATATCGATACTCACGTGTCCGTCAGAGTAT
+
FF:FFFFFFFFFFFFFFFFF:FFFFFFFFFFFFFFFFFFFFFFFFFFFFFFFFFFFFFFFFFFFFFFF
FFFFFFFFFFFFFFFFFFFFFFFFFFFFFFFFFFFFFFFFFFFFFFFFFFFFFFFFFFFFFFFFFFFF
@A00155:342:HHGFNDSXY:1:2565:29903:21527 1:N:0:GAACCTAG+TCCGCATA
GGTTGTAGTCAAATGTTGGTAGTCAGGGTAGTTTTCCTCCCCTGCTCCACCTGGCCACGAGTCAAAAC
TAGCCTCTTCTTCATATCTCTCCGGCCACCACTGGTCCATATCGATACTCACGTGTCCGTCAGAGTAT
+
FFFFFFFFFFFFFFFFFFFFFFFFFFFFFFFFFFFFFFFFFFFFFFFFFFFFFFFFFFFFFFFFFFFF
FFFFFFFFFFFFFFFFFFFFFFFFFFFFFFFFFFFFFFFFFFFFFFFFFFFFFFFFFFFFF,FFFFFF
```

```
@A00155:342:HHGFNDSXY:1:2540:27344:1470 1:N:0:GAACCTAG+TCCGCATA
GGTTGTAGTCAAATGTTGGTAGTCAGGGTAGTTTTCCTCCCCTGCTCCACCTGGCCACGAGTCAAAAC
TAGCCTCTTCTTCATATCTCTCCGGCCACCACTGGTCCATATCGATACTCACGTGTCCGTCAGAGTAT
+
FFFFFFFFFFFFFFFFFFFFFFFFFFFFFFFFFFFFFFFFFFFFFFFFFFFFFFFFFFFFFFFFFFFF
FFFFFFFFFFFFFFFFFFFFFFFFFFFFFFFFFFFFFFFFFFFFFFFFFFFFFFFFFFFFFFFFF::F
@A00155:342:HHGFNDSXY:1:1449:7518:10911 1:N:0:GAACCTAG+TCCGCATA
GGTTGTAGTCAAATGTTGGTAGTCAGGGTAGTTTTCCTCCCCTGCTCCACCTGGCCACGAGTCAAAAC
TAGCCTCTTCTTCATATCTCTCCGGCCACCACTGGTCCATATCGATACTCACGTGTCCGTCAGAGTAT
+
FFFFFFFFFFFFFFFFFFFF,FFFFFFFFFFFFFFFFFFFFFFFFFFFFFFFFFFFFFFFFFFFFFFF
FFFFFFFFFFFFFFFFFF:FFFFFFFFFFFFFFFFFFFFFFFFFFFFFFFFF:FFFFFFFFFFFFF:F
@A00155:342:HHGFNDSXY:1:2668:26874:35978 1:N:0:GAACCTAG+TCCGCATA
GGTTGTAGTCAAATGTTGGTAGTCAGGGTAGTTTTCCTCCCCTGCTCCACCTGGCCACGAGTCAAAAA
TAGCCTCTTCTTCATATCTCTCCGGCCACCACTGGTCCATATCGATACTCACGTGTCCGTCAGAGTAT
+
FF:F:FF:FFFFFFFF,,:FFF:FFFFFFF:FFF:FFFFFFF:FFF:FFFFFFFFFFFFFFFFFFFF,
:FFFFFFFFFFFFFFFFFFF,FF,FFFFF,FF:::FF:FFFFFFFFFF:FFFFF:FFFF,F::FF,FF
@A00155:342:HHGFNDSXY:1:2650:12364:19961 2:N:0:GAACCTAG+TCCGCATA
GTAGTCAAATGTTGGTAGTCAGGGTAGTTTTCCTCCCCTGCTCCACCTGGCCACGAGTCAAAACTAGC
CTCTTCTTCATATCTCTCCGGCCACCACTGGTCCATATCGATACTCACGTGTCCGTCAGAGTATGTTC
+
F:FFFFFFFF:FFFFFFFFF,FFFFFFFFFFFFFFFFFFFFFFF:FFFFFFFFFFFFFFFFFFFFFFF
FFFFFFFFFFFFFFFFFFFFFFFFFFFFFFFFFF:FFFFFFFFFFFFFFFFFFFFFFFFFFFFFF,FF
@A00155:342:HHGFNDSXY:1:1512:1687:14873 2:N:0:GAACCTAG+TCCGCATA
TAGTCAAATGTTGGTAGTCAGGGTAGTTTTCCTCCCCTGCTCCACCTGGCCACGAGTCAAAACTAGCC
TCTTCTTCATATCTCTCCGGCCACCACTGGTCCATATCGATACTCACGTGTCCGTCAGAGTATGTTCC
+
FFF:FFFFFFFFFFFFFFFFFFFFFFFFFFFFFF:FFFFFFFFFFFFFFFFFFFFFFFFFFFFFFFFF
FFFFFFFFFFFFFFFFFFFFFFFFFFFFFFFFFFFF:FFFFFFFFFF:FFFFFFFFFFFFFFFFFFFF
@A00155:342:HHGFNDSXY:1:2516:10981:27712 2:N:0:GAACCTAG+TCCGCATA
TAGTCAAATGTTGGTAGTCAGGGTAGTTTTCCTCCCCTGCTCCACCTGGCCACGAGTCAAAACTAGCC
TCTTCTTCATATCTCTCCGGCCACCACTGGTCCATATCGATACTCACGTGTCCGTCAGAGTATGTTCC
+
FFFFFF:FFFFFFFFFFFFFFFFFFFFFFFFFFF:FFFFFFFFFFFFFFFFFFFFFFFFFFFFFFFFF
FFFFFFFFFFFFFFFFFFFFFFFFFFFFFFFFF:FFFFFFFFFFF:FF:FFFFFFF:FFFFFFFFFFF
@A00155:342:HHGFNDSXY:1:2406:23439:23672 1:N:0:GAACCTAG+TCCGCATA
TAGTCAAATGTTGGTAGTCAGGGTAGTTTTCCTCCCCTGCTCCACCTGGCCACGAGTCAAAACTAGCC
TCTTCTTCATATCTCTCCGGCCACCACTGGTCCATATCGATACTCACGTGTCCGTCAGAGTATGTTCC
+
FFFFFFFFFFFFFFFFFFFFFFFFFFFFFFFFFFFFFFFFFFFFFFFFFFFFFFFFFFFFF:FFFFFF
FFFFFFFFFFFFFFFFFFFFFFFFFFFFFFFFFFF,FFFFFFFFFFFF,FFFFFFFFFFFFFFFFFFF
@A00155:342:HHGFNDSXY:1:2210:6641:33943 1:N:0:GAACCTAG+TCCGCATA
AGTCAAATGTTGGTAGTCAGGGTAGTTTTCCTCCCCTGCTCCACCTGGCCACGAGTCAAAACTAGCCT
CTTCTTCATATCTCTCCGGCCACCACTGGTCCATATCGATACTCACGTGTCCGTCAGAGTATGTTCCC
+
FFFFFFFFFFFFFFFFFF:,FFFFFFFFFFF:FFFFFFFFFFFFFFFFFFFFFFFFFFFFFFFFFFFF
FFFFFFFFFFFFFFFFFFF:FFFFFF:FFFFF:FFFFFFFFFFFFFFFFFFFFFFFFFFFFFFFFFFF
@A00155:342:HHGFNDSXY:1:2607:20708:16219 1:N:0:GAACCTAG+TCCGCATA
AGTCAAATGTTGGTAGTCAGGGTAGTTTTCCTCCCCTGCTCCACCTGGCCACGAGTCAAAACTAGCCT
CTTCTTCATATCTCTCCGGCCACCACTGGTCCATATCGATACTCACGTGTCCGTCAGAGTATGTTCCC
+
FFFFFFFFFFFFFFFFFFFFFFFFFFFFFFFFFFFFFFFFFFFFFFFFFFFFFFFFFFFFFFFFFFFF
FFFFFFFFFFFFFFFFFFFFFFFFFFFFFFFFFFFFFFFFFFFFFFFFFFFFFFFFFFFFFFFFFFFF
```

```
@A00155:342:HHGFNDSXY:1:2672:31665:27649 2:N:0:GAACCTAG+TCCGCATA
AGTCAAATGTTGGTAGTCAGGGTAGTTTTCCTCCCCTGCTCCACCTGGCCACGAGTCAAAACTAGCCT
CTTCTTCATATCTCTCCGGCCACCACTGGTCCATATCGATACTCACGTGTCCGTCAGAGTATGTTCCC
+
FFFFFFFFFFFFFFFFFFFFFFFFFFFFFFFFFFFFFFFFFFFFFFFFFFFFFFFFFFFFFFFFFFFF
FFFFFFFFFFFFFFFFFFFFFFFFFFFFFFFFF:FFFFF:FFFFFFFFFFFFFFFFFFFFFF:FFFF
@A00155:342:HHGFNDSXY:1:2505:19569:4163 1:N:0:GAACCTAG+TCCGCATA
AGTCAAATGTTGGTAGTCAGGGTAGTTTTCCTCCCCTGCTCCACCTGGCCACGAGTCAAAACTAGCCT
CTTCTTCATATCTCTCCGGCCACCACTGGTCCATATCGATACTCACGTGTCCGTCAGAGTATGTTCCC
+
FFFFFFFFFFFFFFFFFFFFFFFFFFFFFFFFFFFFFFFFFFFFFFFFFFFF:FFFFFFFFFFFFFF:
FFFFFFFFFFFFFFFFFFFFFFFFFFFFFFFFFFFFFFFFFFFFFFFFFFFFFFFFFFFFFFFFFFFF
@A00155:342:HHGFNDSXY:1:1222:3070:7498 1:N:0:GAACCTAG+TCCGCATA
AGTCAAATGTTGGTAGTCAGGGTAGTTTTCCTCCCCTGCTCCACCTGGCCACGAGTCAAAACTAGCCT
CTTCTTCATATCTCTCCGGCCACCACTGGTCCATATCGATACTCACGTGTCCGTCAGAGTATGTTCCC
+
FFF:FFFFFFFFFFFFFFFFFFFF:FFFFFFFFFFFFFFFFFFFFFFFFFFFFFFFFFFFFFFFFFFF
FFFFFFFFFFFFFFFFFFFFFF:FFFFFFFFFFFFFFFFFFFFFFFFFFFFFFFFFFFFFFFFFFFFF
@A00155:342:HHGFNDSXY:1:1374:26702:8688 2:N:0:GAACCTAG+TCCGCATA
AGTCAAATGTTGGTAGTCAGGGTAGTTTTCCTCCCCTGCTCCACCTGGCCACGAGTCAAAACTAGCCT
CTTCTTCATATCTCTCCGGCCACCACTGGTCCATATCGATACTCACGTGTCCGTCAGAGTATGTTCCC
+
FF:FFFFFFFFFFFFFFFFFFFFFFFF,FFFFFFFFFFFFFFFFFFFFFFFFFFFFFFFFFFFFFFFF
FFFF:FFFFFFFFFFFFFFFFFFF:FFFFFFFFFFFFFFFFFFFFFFFFFFFFFFFFFFFFF:FFFF
@A00155:342:HHGFNDSXY:1:2546:7798:32158 1:N:0:GAACCTAG+TCCGCATA
GTCAAATGTTGGTAGTCAGGGTAGTTTTCCTCCCCTGCTCCACCTGGCCACGAGTCAAAACTAGCCTC
TTCTTCATATCTCTCCGGCCACCACTGGTCCATATCGATACTCACGTGTCCGTCAGAGTATGTTCCCA
+
FFFFFFFFF:FFFFFFFFFFFFFFFFFFFFFFFFFFFFFFFFFFFFFFFFFFFFFFFFFFFFFFFFFF
FFFFFFFFFFF:FFFFFFFFFFFFFFFFFFFFFFFFFFFFFFFFFFFFFFFF:FFFFFFFFFFFFFFF
@A00155:342:HHGFNDSXY:1:1658:12816:31454 1:N:0:GAACCTAG+TCCGCATA
GTCAAATGTTGGTAGTCAGGGTAGTTTTCCTCCCCTGCTCCACCTGGCCACGAGTCAAAACTAGCCTC
TTCTTCATATCTCTCCGGCCACCACTGGTCCATATCGATACTCACGTGTCCGTCAGAGTATGTTCCCA
+
FFFFFFFFFFFFFFFFFFFFFFFFFFFFFFFFFFFFFFFFFFFFFFFFFFFFFFFFFFFFFFFFFFFF
FFFFFFFFFFFFFFFFFFFFFFFFF:FFFFFFFFFFFFFFFFFFFFFFFFFFFF:FFFFFFF:FFFFF
@A00155:342:HHGFNDSXY:1:2269:29857:7764 1:N:0:GAACCTAG+TCCGCATA
GTCAAATGTTGGTAGTCAGGGTAGTTTTCCTCCCCTGCTCCACCTGGCCACGAGTCAAAACTAGCCTC
TTCTTCATATCTCTCCGGCCACCACTGGTCCATATCGATACTCACGTGTCCGTCAGAGTATGTTCCCA
+
FFFFFFFFFFFFFFFFFFFFFFFFFFFFFFFFFFFFFFFFFFFFFFFFFFFFFFFFFFFFFFFFFFFF
FFFFFFFFFFFFFFFFFFFFFFFFFFFFFFFFFFFFFFFFFFFFFFFFFFFFFFFFFFFFFFFFFFFF
@A00155:342:HHGFNDSXY:1:2567:23240:24799 1:N:0:GAACCTAG+TCCGCATA
GTCAAATGTTGGTAGTCAGGGTAGTTTTCCTCCCCTGCTCCACCTGGCCACGAGTCAAAACTAGCCTC
TTCTTCATATCTCTCCGGCCACCACTGGTCCATATCGATACTCACGTGTCCGTCAGAGTATGTTCCCA
+
FFFFFFFFFFFFFFFFFFFFFFFFFFFFFFFFFFFFFFFFFFFFFFFFFFFFFFFFFFFFFFFFFFFF
FFFFFFFFFFFFFFFFFFFFFFFFFFFFFFFFFFFFFFFFFFFFFFFFFFFFFFFFFFFFFFFFFFFF
@A00155:342:HHGFNDSXY:1:1128:24804:18051 1:N:0:GAACCTAG+TCCGCATA
GTCAAATGTTGGTAGTCAGGGTAGTTTTCCTCCCCTGCTCCACCTGGCCACGAGTCAAAACTAGCCTC
TTCTTCATATCTCTCCGGCCACCACTGGTCCATATCGATACTCACGTGTCCGTCAGAGTATGTTCCCA
+
FFFFFFFFFFFFFFFFFFFFFFFFFFFFFFFFFFFFFFFFFFFFFFFFFFFFFFFFFFFFFFFFFFFF
FFFFFFFFFFFFFFFFFFFFFFFFFFFFFFFFFFFFFFF,FFFFFFFFFFFFFFFFFFF:FFFFFFFF
```

```
@A00155:342:HHGFNDSXY:1:2319:25843:32941 1:N:0:GAACCTAG+TCCGCATA
TCAAATGTTGGTAGTCAGGGTAGTTTTCCTCCCCTGCTCCACCTGGCCACGAGTCAAAACTAGCCTCT
TCTTCATATCTCTCCGGCCACCACTGGTCCATATCGATACTCACGTGTCCGTCAGAGTATGTTCCCAG
+
FFFFFFFFFFFFFFFFFFFFFFFFFFFFFFFFFFFFFFFFFFFFFFFFFF:FFFFFFFFFFFFFFFF,
FFFFFFFFFFFFFFFFFFFFFFFFFFFFFFFFFFFFFFFFFFFFFFFFFFFFFFFFFFFFFFFFFF:F
@A00155:342:HHGFNDSXY:1:2503:6153:8547 1:N:0:GAACCTAG+TCCGCATA
CAAATGTTGGTAGTCAGGGTAGTTTTCCTCCCCTGCTCCACCTGGCCACGAGTCAAAACTAGCCTCTT
CTTCATATCTCTCCGGCCACCACTGGTCCATATCGATACTCACGTGTCCGTCAGAGTATGTTCCCAGC
+
::FFFFFFFFFFFFFFFFFFFFFFFFFFFFFFFFFFFFFFFFFFFFFFFFFFFFFFFFFFFFFFFFFF
FFFFFFFFFFFFFFFFFFFFFFFFFFFFFFFFFFFFFFFFF:FFFFFFFFFFFFFFFFFFFFFFFFFF
@A00155:342:HHGFNDSXY:1:2524:22860:32972 2:N:0:GAACCTAG+TCCGCATA
CAAATGTTGGTAGTCAGGGTAGTTTTCCTCCCCTGCTCCACCTGGCCACGAGTCAAAACTAGCCTCTT
CTTCATATCTCTCCGGCCACCACTGGTCCATATCGATACTCACGTGTCCGTCAGAGTATGTTCCCAGC
+
FF:F:FF:FF,FFFFFFFFFFFF,FFFFFFFFFFFFFFFFFFFFFF,:F,FFFFFFFFFFFFFF,FFF
FFFFF,FFF:FFF,FFFFFFF:FFFFFFFF:F,FFFFFFFF:FF,FFFFF:FFFFFFFFF,FFFFFFF
@A00155:342:HHGFNDSXY:1:1671:8024:2988 1:N:0:GAACCTAG+TCCGCATA
AAATGTTGGTAGTCAGGGTAGTTTTCCTCCCCTGCTCCACCTGGCCACGAGTCAAAACTAGCCTCTTC
TTCATATCTCTCCGGCCACCACTGGTCCATATCGATACTCACGTGTCCGTCAGAGTATGTTCCCA
+
FFFFFF:FFFFFFFFFFFFFF:FFFFFFFFFFF:FF:FFF:FF:FFFF,FFFFFFFFFFFFFFFFFFF
FFFFFFFF:FFFFFFFFFFFFFFFFFF:FFFFFFFF:FFFFF:FFFFFFFFFFFFFFFFFFFFFF
@A00155:342:HHGFNDSXY:1:2578:7328:25113 1:N:0:GAACCTAG+TCCGCATA
AAATGTTGGTAGTCAGGGTAGTTTTCCTCCCCTGCTCCACCTGGCCACGAGTCAAAACTAGCCTCTTC
TTCATATCTCTCCGGCCACCACTGGTCCATATCGATACTCACGTGTCCGTCAGAGTATGTTCCCAGCT
+
FFFFFFFFFFFFFFFFFFFFFFFFF,FFFFFFFFFFFFFFFFF:FFFFFFFFFFFFFFFFFFFFFF:F
FF:FFFFFFFF:FFFF:FFFFFFFFFFFFFFFFFFFF:FFFFFFF:FFFFFFFFFFFFF:FFFFFFFF
@A00155:342:HHGFNDSXY:1:2319:25843:32941 2:N:0:GAACCTAG+TCCGCATA
AAATGTTGGTAGTCAGGGTAGTTTTCCTCCCCTGCTCCACCTGGCCACGAGTCAAAACTAGCCTCTTC
TTCATATCTCTCCGGCCACCACTGGTCCATATCGATACTCACGTGTCCGTCAGAGTATGTTCCCAGCT
+
FFFFFFFFFFFFFFFFFFFFF:FFFFFFFFFFFFFFFFFFFFFFFFFFFFFFFFFFFFFFFFFFFFFF
FFF,FFFFFFFFFFFFFFFFFFFFFFFFFFFFFFFFFFFFFFF:FFFFFFFFFFFFFFF,FF:FFFFF
@A00155:342:HHGFNDSXY:1:2210:6641:33943 2:N:0:GAACCTAG+TCCGCATA
ATGTTGGTAGTCAGGGTAGTTTTCCTCCCCTGCTCCACCTGGCCACGAGTCAAAACTAGCCTCTTCTT
CATATCTCTCCGGCCACCACTGGTCCATATCGATACTCACGTGTCCGTCAGAGTATGTTCCCAGCTCA
+
FFFF:FFFFFFFFF:FFFFFFFFFFFFFFFFFFFFFFFFFFFFFFFFFFFFFFFFFFFF:FFFFFFFF
:FFFF:FFFFFFFFFFFFFFFFFFFFF,FFFFFFFFFFFFF,FFF:FFFFFFFFFFFFFF:FFFFFFF
@A00155:342:HHGFNDSXY:1:2450:10529:16532 1:N:0:GAACCTAG+TCCGCATA
ATGTTGGTAGTCAGGGTAGTTTTCCTCCCCTGCTCCACCTGGCCACGAGTCAAAACTAGCCTCTTCTT
CATATCTCTCCGGCCACCACTGGTCCATATCGATACTCACGTGTCCGTCAGAGTATGTTCCCAGCTCA
+
FFFFFFFFFFFFFFFFFFFFFFFFFFFFFFFFFFFFFFFFFFFFFFFFFFFFFFFFFFFFFFFFFFFF
FFFFFF:FFFFFFFFFFFFFFFFFFFFFFFFFFF:FFFFFFFFFFFFFFFFFFFFFFFFFFFFFFFFF
@A00155:342:HHGFNDSXY:1:2607:20708:16219 2:N:0:GAACCTAG+TCCGCATA
ATGTTGGTAGTCAGGGTAGTTTTCCTCCCCTGCTCCACCTGGCCACGAGTCAAAACTAGCCTCTTCTT
CATATCTCTCCGGCCACCACTGGTCCATATCGATACTCACGTGTCCGTCAGAGTATGTTCCCAGCTCA
+
FFFFFFFFFFFFFFFFFFFFFFFFFFFFFFFFFFFFFFFFFF:FFFFFFFFFFFFFFFFFFFFFFFFF
FFFFFFFFFFFFFFFFFFFF:FFFFF:FFFFFFFFFFFFFFFFFFFFFFFFFFFFFFFF:FFFFFFFF
```

```
@A00155:342:HHGFNDSXY:1:1637:16911:11616 1:N:0:GAACCTAG+TCCGCATA
TGTTGGTAGTCAGGGTAGTTTTCCTCCCCTGCTCCACCTGGCCACGAGTCAAAACTAGCCTCTTCTTC
ATATCTCTCCGGCCACCACTGGTCCATATCGATACTCACGTGTCCGTCAGAGTATGTTCCCAGCTCAC
+
FFFFFFFFFFFFFFFFFFFFFFFFFFFFFFFFFFFFFFFFFFFFFFFFFFFFFFFFFF:FFFFFFFFF
FFFFFFFFFFFFFFFFFFFFFFFFFFFFFFFFFFFFFFFFFFFFFFFFFFFFFFFFFFF:FFF:FFFF
@A00155:342:HHGFNDSXY:1:2575:17535:23657 2:N:0:GAACCTAG+TCCGCATA
GTTGGTAGTCAGGGTAGTTTTCCTCCCCTGCTCCACCTGGCCACGAGTCAAAACTAGCCTCTTCTTCA
TATCTCTCCGGCCACCACTGGTCCATATCGATACTCACGTGTCCGTCAGAGTATGTTCCCAGCTCACC
+
FFFFFFFFFFFFFFFFFFFFFF:FFFFFFFFFFFFFFFFFFFFFFFFFFF:F:FFFFFFFFFFF,FF:
F:FFFFFFFFFFFFFFFFFFFFFF:F,:FFFFFFFFFFFFFFFFFFFFFFFFFFFFFFFFFFFFFFFF
@A00155:342:HHGFNDSXY:1:2153:8983:32456 1:N:0:GAACCTAG+TCCGCATA
TGGTAGTCAGGGTAGTTTTCCTCCCCTGCTCCACCTGGCCACGAGTCAAAACTAGCCTCTTCTTCATA
TCTCTCCGGCCACCACTGGTCCATATCGATACTCACGTGTCCGTCAGAGTATGTTCCCAGCTCACCTT
+
:FFFFFFFFFFFFFFFFFFFFFFFFFFFFFFFFFFFFFFFFFFFFFFFFFFFFFFFFFFFFFFFFFFF
FFFFFFFFFFFFFFFFFFFFFFFFFFFFFFFFFFFFFFFFFFFFFFFFFFFFFFFFFFFFFFFFFFFF
@A00155:342:HHGFNDSXY:1:1507:2944:1892 2:N:0:GAACCTAG+TCCGCATA
GGTAGTCAGGGTAGTTTTCCTCCCCTGCTCCACCTGGCCACGAGTCAAAACTAGCCTCTTCTTCATAT
CTCTCCGGCCACCACTGGTCCATATCGATACTCACGTGTCCGTCAGAGTATGTTCCCAGCTCACCTTT
+
FF:FFFFFFFFFFFFF,FFFFFFFFFFFFFFFFFFFFFF,FFFFFFFFFFFFFFFFFFFFFFFFFFFF
FFFFFFF:FFFFFFFFFFF,FFFFFFFFFFFFFF,FFF:FFFFFFFFFFFFFFFFFFFFFFF:FFF:F
@A00155:342:HHGFNDSXY:1:1507:2935:1720 2:N:0:GAACCTAG+TCCGCATA
GGTAGTCAGGGTAGTTTTCCTCCCCTGCTCCACCTGGCCACGAGTCAAAACTAGCCTCTTCTTCATAT
CTCTCCGGCCACCACTGGTCCATATCGATACTCACGTGTCCGTCAGAGTATGTTCCCAGCTCACCTTT
+
F:FFFFF::FFFFFFF,FFFFFFFFFFFFFFFFFFFF:FFFFFFFFFFFFFFFF:FFFFFFFFFFFFF
FFF:FFFFFFFFFFFFFFFFF::FF,FFF:FFF,:F:FF,FFFFFFFFFFFFFFFFFFFFFFFFFFFF
@A00155:342:HHGFNDSXY:1:2567:23240:24799 2:N:0:GAACCTAG+TCCGCATA
GGTAGTCAGGGTAGTTTTCCTCCCCTGCTCCACCTGGCCACGAGTCAAAACTAGCCTCTTCTTCATAT
CTCTCCGGCCACCACTGGTCCATATCGATACTCACGTGTCCGTCAGAGTATGTTCCCAGCTCACCTTT
+
FFFFFFFFFFFFFFFFFFFFFFFFFFFFFFFFFFFFFFFFFFFFFFFFFFFFFFFFFFFFFFFFFFFF
FFFFFFFFFFFFFFFFFFFFFFF:FFFFFFFFFFFF:FFFFFFFFFFFFFF:FFFFFFFFFFFFFFFF
@A00155:342:HHGFNDSXY:1:1550:31286:18129 2:N:0:GAACCTAG+TCCGCATA
GGTAGTCAGGGTAGTTTTCCTCCCCTGCTCCACCTGGCCACGAGTCAAAACTAGCCTCTTCTTCATAT
CTCTCCGGCCACCACTGGTCCATATCGATACTCACGTGTCCGTCAGAGTATGTTCCCAGCTCACCTTT
+
:FFFFFFFFFF:FFFF:FFFFFFFF:FFFFFF:FFFFFFFFFFFFFFFFF:FFFFFFFFFFFFFF:F,
FFFFFFFFFFFFFF:FFFFFF,,FFFFFFFFFFFFFFFFFF:FFFFFFFFFFFFFFF:FFFFFFFFFF
@A00155:342:HHGFNDSXY:1:1550:28339:12211 2:N:0:GAACCTAG+TCCGCATA
GTAGTCAGGGTAGTTTTCCTCCCCTGCTCCACCTGGCCACGAGTCAAAACTAGCCTCTTCTTCATATC
TCTCCGGCCACCACTGGTCCATATCGATACTCACGTGTCCGTCAGAGTATGTTCCCAGCTCACCTTT
+
FFFFFFFFFFFFFFFFFFFF:F:FFFFFF,F:FF:F:FFFFFFF,FFFFFFFF,:FFFFFFFFFFFFF
FFFFFF::FFFFFFFFFFFFFFFFF,FFFFFFFFFFFFFFFFFF:FFFFFFFFFFFF:FFFFFFFFF
@A00155:342:HHGFNDSXY:1:2163:8938:7263 1:N:0:GAACCTAG+TCCGCATA
TAGTCAGGGTAGTTTTCCTCCCCTGCTCCACCTGGCCACGAGTCAAAACTAGCCTCTTCTTCATATCT
CTCCGGCCACCACTGGTCCATATCGATACTCACGTGTCCGTCAGAGTATGTTCCCAGCTCACCTT
+
:FFFFFFF:FFFFFF,F,F:FFFFFFFF:FFF:FF:,FFF:F,FF:FFFFFFFF:F::FFFFFFF:F,
FFFFFFFFF:FFFF,F:,FF:FFFFFFFFFFFF::FFFFFFFFFFF,FF:F:F:FFFFFFFFFFF
```

@A00155:342:HHGFNDSXY:1:1148:5837:12195 1:N:0:GAACCTAG+TCCGCATA
TAGTCAGGGTAGTTTTCCTCCCCTGCTCCACCTGGCCACGAGTCAAAACTAGCCTCTTCTTCATATCT
CTCCGGCCACCACTGGTCCATATCGATACTCACGTGTCCGTCAGAGTATGTTCCCAGCTCACCTTTC
+
FFFFFFFFFF:FFFFFFFFFFFFF:FFFFFFFFFFFFFFFF:FFFFFFFF,FF,FFFFFFFFFFFFFF
FFFFFFFFFFFFFFFFFFF:FFFF:FFFFFFFFFFFFFFFFF:FFFF,FF:FF:FFFFFF:FFF:FF
@A00155:342:HHGFNDSXY:1:1475:13141:31454 2:N:0:GAACCTAG+TCCGCATA
GTCAGGGTAGTTTTCCTCCCCTGCTCCACCTGGCCACGAGTCAAAACTAGCCTCTTCTTCATATCTCT
CCGGCCACCACTGGTCCATATCGATACTCACGTGTCCGTCAGAGTATGTTCCCAGCTCACCTTT
+
FFF:FFFFFFFFFFFFFFFFF,FFFFFFFFFFFFFFFFFFFFFFFFF:FFFFFFFFFFFFFFFFFFFF
FFFFFFFFFFFFFFFFFFFFFFFFFFFFFFFFFFFFFFFFFFFFFFF:FFFFFFFFFFFFFFFF
@A00155:342:HHGFNDSXY:1:2268:31919:23202 1:N:0:GAACCTAG+TCCGCATA
CAGGGTAGTTTTCCTCCCCTGCTCCACCTGGCCACGAGTCAAAACTAGCCTCTTCTTCATATCTCTCC
GGCCACCACTGGTCCATATCGATACTCACGTGTCCGTCAGAGTATGTTCCCAGCTCACCTTTCAGCGA
+
FFFFFFFFFFFFFFFFFFFFFFFFFFFFFFFFFFFFFFFFFFFFFFFFFFFFFFFFFFFFFFFFFFFF
FFFFFFFFFFFFFFFFFFFFFFFFF:FFFFFFFFFFFFFFF:FFF:FFFFFFFFFFFFFFFFFFFFFF
@A00155:342:HHGFNDSXY:1:2526:5213:20760 2:N:0:GAACCTAG+TCCGCATA
AGGGTAGTTTTCCTCCCCTGCTCCACCTGGCCACGAGTCAAAACTAGCCTCTTCTTCATATCTCTCCG
GCCACCACTGGTCCATATCGATACTCACGTGTCCGTCAGAGTATGTTCCCAGCTCACCTTTCAGCGAT
+
FFFFFFFF,,FFFFFFFF:FFFFFFFFFFFFFFFFFFFFFFFFFFFFFFFFFFFFFFFFFFFFFFFFF
FFFFFFFFFFFFFFFFFFFFFFFFFFFFFFFFFFFFFFFFFFFFFFFFFFFFFFFFFFFFFFFFFFFF
@A00155:342:HHGFNDSXY:1:2324:5782:10942 1:N:0:GAACCTAG+TCCGCATA
GGTAGTTTTCCTCCCCTGCTCCACCTGGCCACGAGTCAAAACTAGCCTCTTCTTCATATCTCTCCGGC
CACCACTGGTCCATATCGATACTCACGTGTCCGTCAGAGTATGTTCCCAGCTCACCTTTCAGCG
+
FFFFFFFFFFFFFFFFFFFFFFFFFFFFFFFFFFFFFFFFFFFFFFFFFFFFFFFFFFFFFFFFFFFF
FFFFFFFFFFFFFF:FFFFFFFFFFFFFFFFFFFFFFFF,FFFFFFFFFFFFFFFFFFFFFFFF
@A00155:342:HHGFNDSXY:1:2250:28357:25833 1:N:0:GAACCTAG+TCCGCATA
GGTAGTTTTCCTCCCCTGCTCCACCTGGCCACGAGTCAAAACTAGCCTCTTCTTCATATCTCTCCGGC
CACCACTGGTCCATATCGATACTCACGTGTCCGTCAGAGTATGTTCCCAGCTCACCTTTCAGCG
+
FFFFFFFFFFFFFFFFFFFFFFFFFFFFFFFFFFFFFFFF:F:FFFFFFFFFFFFFFFFFFFFFFFFF
FFFFFFFFFFFFFFFFFFFFFFFFFFFFFFFFFFFFFFFFFFF:FFFFFFFFFFFFFFFFFFFF
@A00155:342:HHGFNDSXY:1:1363:3830:6621 2:N:0:GAACCTAG+TCCGCATA
GGTAGTTTTCCTCCCCTGCTCCACCTGGCCACGAGTCAAAACTAGCCTCTTCTTCATATCTCTCCGGC
CACCACTGGTCCATATCGATACTCACGTGTCCGTCAGAGTATGTTCCCAGCTCACCTTTCAGCGATCC
+
FFFFF:FFFFFFFFFFFFFFFFFFFFFFFFFFFFFFFFFFFFFFFFFFFFFFFFFFFFFFFFFFFFFF
FFFFFFFFFFFFFFFFFFFFFFFFFFF,FFFFFFFFFFFFFFFFFFFFFFFFFFFFFFFFFFFFFFFF
@A00155:342:HHGFNDSXY:1:2112:18295:11068 2:N:0:GAACCTAG+TCCGCATA
GGTAGTTTTCCTCCCCTGCTCCACCTGGCCACGAGTCAAAACTAGCCTCTTCTTCATATCTCTCCGGC
CACCACTGGTCCATATCGATACTCACGTGTCCGTCAGAGTATGTTCCCAGCTCACCTTTCAGCGATCC
+
FF:FFF:FFFFFFFFFFFFFFFFFFFFFFF:FFFFFFFFFFFFFFFFFFFF:FFF,FFFFFFFF:FFF
FFFFFFFFFFFFFFFFFFFFFFFFFFFFFFFFFFFFFFFFFFFFFFFFFFFFFFFFFFFFFFFFFFF:
@A00155:342:HHGFNDSXY:1:2112:18304:11052 2:N:0:GAACCTAG+TCCGCATA
GGTAGTTTTCCTCCCCTGCTCCACCTGGCCACGAGTCAAAACTAGCCTCTTCTTCATATCTCTCCGGC
CACCACTGGTCCATATCGATACTCACGTGTCCGTCAGAGTATGTTCCCAGCTCACCTTTCAGCGATCC
+
FFFFFFFFFFFFFFFFFFFFFFFFFFFFFFFFFFFFFFFFFFFFFFFFFFFFFFFFFFFFFFFFFFFF
FFFFFFFFFFFFFFFFFFFFFFFFFFF:FFFFFFFFFFFFFFFFFF:FFFFFF:FFFFFFFF::FFFF

```
@A00155:342:HHGFNDSXY:1:1473:18141:21887 2:N:0:GAACCTAG+TCCGCATA
GGTAGTTTTCCTCCCCTGCTCCACCTGGCCACGAGTCAAAACTAGCCTCTTCTTCATATCTCTCCGGC
CACCACTGGTCCATATCGATACTCACGTGTCCGTCAGAGTATGTTCCCAGCTCACCTTTCAGCGATCC
+
FFFFF:FFFFFFFF:FFFFFFFFFFFFFFFFFFFFFFFFF:F:FFFFFFF:FFFFFFFFFFFFFFFFF
FFFFFFFFFFFFFF:FFFFFFFFFFFFFFFFFFFFFFFFFFFFFFFFFFFFFFFFF:FFFFFFFFFFF
@A00155:342:HHGFNDSXY:1:1260:24876:23030 2:N:0:GAACCTAG+TCCGCATA
GGTAGTTTTCCTCCCCTGCTCCACCTGGCCACGAGTCAAAACTAGCCTCTTCTTCATATCTCTCCGGC
CACCACTGGTCCATATCGATACTCACGTGTCCGTCAGAGTATGTTCCCAGCTCACCTTTCAGCGATCC
+
FFFFFFFFFFFFFFFFFFFFFFFFFFFFFFFFFFFFFFFFFFFFFFFFFFFFFFFFFFFFFFFFFFFF
FFFFFFFFFFFFFFFFFFFFFFFFFFFFFFFFFFFFFFFF:FF:FFFFFF:FFFFFFFFFFFFFFFFF
@A00155:342:HHGFNDSXY:1:1412:13530:31939 2:N:0:GAACCTAG+TCCGCATA
GTAGTTTTCCTCCCCTGCTCCACCTGGCCACGAGTCAAAACTAGCCTCTTCTTCATATCTCTCCGGCC
ACCACTGGTCCATATCGATACTCACGTGTCCGTCAGAGTATGTTCCCAGCTCACCTTTCAGCGATCC
+
FFFFF:FFFFFFFFFFFFFF::FFFFFF:,FFFFFFFF:,F:FFFFFFFFFFFFFFFFFFFFF:FFFF
F:FF,FFFFFFFFFFFFFF:FFF::FFFF:FFFFFFFFF:FFFFFFF:FFF::::FFF::FFF,F,,
@A00155:342:HHGFNDSXY:1:2507:4960:31657 2:N:0:GAACCTAG+TCCGCATA
ATAGTTTTCCTCCCCTGCTCCACCTGGCCACGAGTCAAAACTAGCCTCTTCTTCATATCTCTCCGGCC
ACCACTGGTCCATATCGATACTCACGTGTCCGTCAGAGTATGTTCCCAGCTCACCTTTCAGCGATCCC
+
FF:FF:FFFFFFF:FFFFFFFFFFFFFF::FF,FFFF:FF,FFFFFFFFFFFFFFFFFFFFFFFFFFF
FFFFFFFFFFF:F,FFFFFFFFFFFFFFFFFFFF:FFFFFFFFFFFFFFFFFFFFFFFFFFFFF:FFF
@A00155:342:HHGFNDSXY:1:1319:14615:11459 2:N:0:GAACCTAG+TCCGCATA
GTAGTTTTCCTCCCCTGCTCCACCTGGCCACGAGTCAAAACTAGCCTCTTCTTCATATCTCTCCGGCC
ACCACTGGTCCATATCGATACTCACGTGTCCGTCAGAGTATGTTCCCAGCTCACCTTTCAGCGATCCC
+
FFFFFFFFFFFFFFFFFFFFFFFFFFFFFFFFF,FFFF:FFFFFFFFFFFFFFFFF:FFFFFFFFFFF
FFFFFFFFFFF:FFFFFFFFFFFFFFFFFFFFFFFFFFFFFF:FFFFFFFFFFFFFFFFFFFFFFFFF
@A00155:342:HHGFNDSXY:1:2254:1479:32957 2:N:0:GAACCTAG+TCCGCATA
AGTTTTCCTCCCCTGCTCCACCTGGCCACGAGTCAAAACTAGCCTCTTCTTCATATCTCTCCGGCCAC
CACTGGTCCATATCGATACTCACGTGTCCGTCAGAGTATGTTCCCAGCTCACCTTTCAGCGATCCCTC
+
:FF:FFFFFFFFFFF::FFFFFFFF:F:FFF:F:FFFFFFFFFF,FF:FFFFFFFFFF:FFF:FFFFF
FFFFF:FFF:FFF:FF:FF:FFFFFFFFFFFFFF:FFFFFFFFF:F::FFFFFFFFFFFFFFFFFFFF
@A00155:342:HHGFNDSXY:1:2565:29903:21527 2:N:0:GAACCTAG+TCCGCATA
AGTTTTCCTCCCCTGCTCCACCTGGCCACGAGTCAAAACTAGCCTCTTCTTCATATCTCTCCGGCCAC
CACTGGTCCATATCGATACTCACGTGTCCGTCAGAGTATGTTCCCAGCTCACCTTTCAGCGATCCCTC
+
FFF:FFFFFFFFFFFFFFFFFFFFFFFF:FFFFFFFFFFFFFFFFFFFFFFFFFFFFFFFFFFFFFFF
FFFFFFFFFFFFFFFF:FFFFFFFFFFFFFFFFFFFFFFFFFFFFFFFFFFFFFFFFFFFFFFFFFFF
@A00155:342:HHGFNDSXY:1:2222:29478:16971 2:N:0:GAACCTAG+TCCGCATA
TTTTCCTCCCCTGCTCCACCTGGCCACGAGTCAAAACTAGCCTCTTCTTCATATCTCTCCGGCCACCA
CTGGTCCATATCGATACTCACGTGTCCGTCAGAGTATGTTCCCAGCTCACCTTTCAGCGATCCCTCAT
+
FFFFFFFFFFFFFFFFFFFFFFFFFFFFFFFFFFFFFFFFFFFFFFFFFFFFFFFFFFFFFFFFFFFF
FF:FFFF:FFFFFFFFFFFFFFFFFFFFFFFFFFFFFFFFFFFFFFFFFFFFFFFFFFFFFFFFFFFF
@A00155:342:HHGFNDSXY:1:2546:7798:32158 2:N:0:GAACCTAG+TCCGCATA
TTCCTCCCCTGCTCCACCTGGCCACGAGTCAAAACTAGCCTCTTCTTCATATCTCTCCGGCCACCACT
GGTCCATATCGATACTCACGTGTCCGTCAGAGTATGTTCCCAGCTCACCTTTCAGCGATCCCTCATTA
+
FFFFFFFFFFFFFFFFFFFFFFFFFFFFFFFFFFFFFFFFFFFFFFFFFFFFFFFFFFFFFFFFFFFF
FFFFF:FFFFFFFFFFFFFFFFFFFFFFFFFFFFFFFFFFFFFFFFFFFFFFFFFFFFFFFFFF::FF
```

```
@A00155:342:HHGFNDSXY:1:1608:31376:3223 2:N:0:GAACCTAG+TCCGCATA
TTCCTCCCCTGCTCCACCTGGCCACGAGTCAAAACTAGCCTCTTCTTCATATCTCTCCGGCCACCACT
GGTCCATATCGATACTCACGTGTCCGTCAGAGTATGTTCCCAGCTCACCTTTCAGCGATCCCTCATTA
+
:FFF,FF,FFFFFFFFFFFFFFF:FFFFFFFFFFFFFFFFFFFFF:FFFFFFFFFFFFFFFFFFF:FF
FFFFFFFFFFFFFFFFFFFFFFFFFFFFFFFFFFFFFFFFFFFFFFFFFFFFFFFFFFFFFFFF:FFF
@A00155:342:HHGFNDSXY:1:2608:26992:13823 2:N:0:GAACCTAG+TCCGCATA
TTCCTCCCCTGCTCTACCTGGCCACGAGTCAAAACTAGCCTCTTCTTCATATCTCTCCGGCCACCACT
GGTCCATATCGATACTCACGTGTCCGTCAGAGTATGTTCCCAGCTCACCTTTCAGCGATCCCTCATTA
+
FFFFFFFFFFFFF:F:FFFFFFFFFFF,,FFFFFFFFFFFFFFFF:FF:FFFFFFFFFFFFFFFFFFF
FFFFFFFF:FFFFFFF:FFFFFFFFFFFFFFFF:FFFFFFFFFFFFFFFF,FFFFFFFFFFFFFFFFF
@A00155:342:HHGFNDSXY:1:1658:12816:31454 2:N:0:GAACCTAG+TCCGCATA
TTCCTCCCCTGCTCCACCTGGCCACGAGTCAAAACTAGCCTCTTCTTCATATCTCTCCGGCCACCACT
GGTCCATATCGATACTCACGTGTCCGTCAGAGTATGTTCCCAGCTCACCTTTCAGCGATCCCTCATTA
+
FFFFFFFFFFFFFFFFFFFFFFFFFFFFFFFFFFFFFFFFFFFFFFFFFFFFFFFFFFFFFFFFFFF:
FFFFFFFFFFFFFFFFFFFF:FFFFFFFFFFFFFFFFFFFFFFFFFFFFFFFFFFFFFFFFFFFFFF
@A00155:342:HHGFNDSXY:1:2139:18059:1767 2:N:0:GAACCTAG+TCCGCATA
TTCCTCCCCTGCTCCACCTGGCCACGAGTCAAAACTAGCCTCTTCTTCATATCTCTCCGGCCACCACT
GGTCCATATCGATACTCACGTGTCCGTCAGAGTATGTTCCCAGCTCACCTTTCAGCGATCCCTCATTA
+
FFFFFFFFF:FFFFFFFFFFFFFFFFFFFFFFFFFFFFFFFFFFFFFFFFFFFFFFFFFFFFFFFF:F
FFFFFFFFFFFFFFFFFFFFFFFFFFFFFFFFFFFFFFFFF:FFFFFFFFFF:FFFFFFFFFFFFF:F
@A00155:342:HHGFNDSXY:1:2560:17626:36620 2:N:0:GAACCTAG+TCCGCATA
GCCTCCCCTGCTCCACCTGGCCACGAGTCAAAACTAGCCTCTTCTTCATATCTCTCCGGCCACCACTG
GTCCATATCGATACTCACGTGTCCGTCAGAGTATGTTCCCAGCTCACCTTTCAGCGATCC
+
FFFFFFFF:F:FFFFFFFFFFFFFFFFFFFFFFFFFFFFFFFFFFF:FFFFFFFFFF:FFFFFFFFFF
F:F:FFFF:FFFFFFFFFF,FFFFFFFFFFFFFFFFFFFFFFFFFF,F:F:F:FFFFFFF
@A00155:342:HHGFNDSXY:1:1607:25138:20917 2:N:0:GAACCTAG+TCCGCATA
TCCTCCCCTGCTCCACCTGGCCACGAGTCAAAACTAGCCTCTTCTTCATATCTCTCCGGCCACCACTG
GTCCATATCGATACTCACGTGTCCGTCAGAGTATGTTCCCAGCTCACCTTTCAGCGATCCCTCATTAG
+
FFFFFFFFFFFFFFFFFFFFFFFFFFFFFFFFFFFFFFFF,FFFFFFFFFFFFFFFFFFFFFFFFFFF
FFFF:FFF:FFFFFFFFFF,FFFFFFFFFFFFFFF:FFFFFFF,FFF:FFFFFFFFFF:FFFFFF:FF
@A00155:342:HHGFNDSXY:1:1449:7518:10911 2:N:0:GAACCTAG+TCCGCATA
TCCTCCCCTGCTCCACCTGGCCACGAGTCAAAACTAGCCTCTTCTTCATATCTCTCCGGCCACCACTG
GTCCATATCGATACTCACGTGTCCGTCAGAGTATGTTCCCAGCTCACCTTTCAGCGATCCCTCATTAG
+
:FFFFFFFFFFFFFFFFFFFFFFFFFFFFFFFFFFFFFFFFFFFFFFFFFFFFFFFFFFFFFFFFFF
FFFFFFFFFFFFFFFFFFFFFFFFFFFFFFFFFFFFFFFFFFFFFFFFFFFFFFFFFFFFFFFFFFFF
@A00155:342:HHGFNDSXY:1:1632:18087:19758 2:N:0:GAACCTAG+TCCGCATA
TCCTCCCCTGCTCCACCTGGCCACGAGTCAAAACTAGCCTCTTCTTCATATCTCTCCGGCCACCACTG
GTCCATATCGATACTCACGTGTCCGTCAGAGTATGTTCCCAGCTCACCTTTCAGCGATCCCTCATTAG
+
FFFFFFFFFFFFFFFFFFFFFFFFFFFFFFFFFFFFFFF,FFFFFFFFFFFFFFFFFFFFFFFFFFFF
FFFF::FFFFFFFFFFFFFFFFFFFFFFFFFFFF:FFFFFF:FFFFFFFFFFFFFFFFFFFFFFF:FF
@A00155:342:HHGFNDSXY:1:2677:7563:19319 2:N:0:GAACCTAG+TCCGCATA
TCCTCCCCTGCTCCACCTGGCCACGAGTCAAAACTAGCCTCTTCTTCATATCTCTCCGGCCACCACTG
GTCCATATCGATACTCACGTGTCCGTCAGAGTATGTTCCCAGCTCACCTTTCAGCGATCCCTCATTAG
+
F:FFFFFFFFFFFFFFFFFFFFFFFFFFFFFFFFFFFFFFFFFFFFFFFFFFFFFFFFFFFFFFFFFF
FFFFFF,FFFFFFFFFFFFFFFFFF:FFFFFFFFFFFFFFFFFFFFFF:FFFFFFFFFF,FFFFFFF,
```

```
@A00155:342:HHGFNDSXY:1:1613:3432:32612 2:N:0:GAACCTAG+TCCGCATA
TCCTCCCCTGCTCCACCTGGCCACGAGTCAAAACTAGCCTCTTCTTCATATCTCTCCGGCCACCACTG
GTCCATATCGATACTCACGTGTCCGTCAGAGTATGTTCCCAGCTCACCTTTCAGCGATCCCTCATTAG
+
FFFF,FFFF,FFFFFFFFFFFFFFFFFFFFFFFFFFFFFFFFFFFFFFFFFFFFFFFFFFFFFFFFFF
FFFFFFFFFFFFFFFFFFFFFFFFFFFFFFFFFFFFFFFFFFFFFFFFFFFFFFFFFF,FFFFFFFFF
@A00155:342:HHGFNDSXY:1:2655:14850:16376 2:N:0:GAACCTAG+TCCGCATA
TCCTCCCCTGCTCCACCTGGCCACGAGTCAAAACTAGCCTCTTCTTCATATCTCTCCGGCCACCACTG
GTCCATATCGATACTCACGTGTCCGTCAGAGTATGTTCCCAGCTCACCTTTCAGCGATCCCTCATTAG
+
FFFFFFFFFFFFFFFFFFF:FFFFFFFFFFF,FFFFFFFFFFFFFFFFFFFFFFFFFFFFFFFFFF:F
FFFF:FFFFFFFFFFFFFFFFFFFFFFFFFFFFFF:FFFFFFFFFFFF:FFFFFFFFFFFFFF::FFF
@A00155:342:HHGFNDSXY:1:1378:7509:9768 2:N:0:GAACCTAG+TCCGCATA
TCCTCCCCTGCTCCACCTGGCCACGAGTCAAAACTAGCCTCTTCTTCATATCTCTCCGGCCACCACTG
GTCCATATCGATACTCACGTGTCCGTCAGAGTATGTTCCCAGCTCACCTTTCAGCGATCCCTCATTAG
+
F:FFFF:FFFFFFFFFFFFF:FFFFFFFFFFFFFF:FFFFFFF:FFFFFFFFFFFFFFF,FFFFFFFF
FFFFFFFFFFFFFFF::FFFFFFFFFFFFFFF:FF,FFFFFFFFFFFFFFFFFFFFFFFFFFFFFFFF
@A00155:342:HHGFNDSXY:1:2540:27344:1470 2:N:0:GAACCTAG+TCCGCATA
TCCTCCCCTGCTCCACCTGGCCACGAGTCAAAACTAGCCTCTTCTTCATATCTCTCCGGCCACCACTG
GTCCATATCGATACTCACGTGTCCGTCAGAGTATGTTCCCAGCTCACCTTTCAGCGATCCCTCATTAG
+
FFFFFFFFFFFFFFFFFFFFFFFFFFFFFFF:FF:FFFF:FFFFFFFFFFFFFFFFFFFFFFFFFFFF
FFFF:FFFFFFFFFF:FFF:FFFFFFFFFFFFFFF:FFFFFFFFFFFFFFFFFFFFFFFFFFFFFFFF
@A00155:342:HHGFNDSXY:1:1229:12075:25191 2:N:0:GAACCTAG+TCCGCATA
CTCCCCTGCTCCACCTGGCCACGAGTCAAAACTAGCCTCTTCTTCATATCTCTCCGGCCACCACTGGT
CCATATCGATACTCACGTGTCCGTCAGAGTATGTTCCCAGCTCACCTTTCAGCGATCCCTCATTAGAT
+
FFFFFFFFFFFFFF:FFFFFF,FF,F,FF:FFFFFFFFFFFFFFFFFFFF:FFFFFFFFFFFF:FFFF
FFFFFFFFF:F:FFFFFFFFFFFF,F:FFFFFF:F:::FFFFFFF:FFFFFFFFFFFFFFFFFFF:FF
@A00155:342:HHGFNDSXY:1:2370:4336:22529 2:N:0:GAACCTAG+TCCGCATA
CCCTGCTCCACCTGGCCACGAGTCAAAACTAGCCTCTTCTTCATATCTCTCCGGCCTCCACTGGTCCA
TATCGATACTCACGTGTCCGTCAGAGTATGTTCCCAGCTCACCTTTCAGCGATCCCTCATTAGATTCT
+
:::F:FFFF,,FFFFFFF:FFFF,:FFFFF:FFFFFFF,FFFFFFFFFFFFF:FFF,FF,FF:FF,FF
FFFFFFFFFF:F:,,FF,FFFFFFF:FFF,FFF:FFFFFFFFFFFF,:,FFFFF:FFF:F:FFFFF,F
@A00155:342:HHGFNDSXY:1:2544:16938:36808 2:N:0:GAACCTAG+TCCGCATA
CCTGCTCCACCTGGCCACGAGTCAAAACTAGCCTCTTCTTCATATCTCTCCGGCCACCACTGGTCCAT
ATCGATACTCACGTGTCCGTCAGAGTATGTTCCCAGCTCACCTTTCAGCGATCCCTCATTAGATTCTT
+
FFFFFFFFFFFFFFFFFF,FFFFFFFFFF:FFF:F,:FFFFFFFFFFFFFFFFFF:FF:FFFF:FF,F
FFFFFFFFFFFFFFFFFFFFFFFFFF,FFFFFFF::FF:FFFFFFFFFFFFFFFFFFFFFFFFFFFFF
@A00155:342:HHGFNDSXY:1:2651:26883:30608 2:N:0:GAACCTAG+TCCGCATA
CTGCTCCACCTGGCCACGAGTCAAAACTAGCCTCTTCTTCATATCTCTCCGGCCACCACTGGTCCATA
TCGATACTCACGTGTCCGTCAGAGTATGTTCCCAGCTCACCTTTCAGCGATCCCTCATTAGATTCTT
+
F::FFFFF::,FF,F:F,F:FFFFFFFFF:FF:F,FF:FFFFF:,:FFFF,,FFFFFFF:F:FFF:,F
FF:F:FF,FF:F,,FF,FFFFFFF:FF,FFF:FFF:FFFFFFF,FFFFFFFFF::FFFFFFFFF,FF
@A00155:342:HHGFNDSXY:1:2450:10529:16532 2:N:0:GAACCTAG+TCCGCATA
CACCTGGCCACGAGTCAAAACTAGCCTCTTCTTCATATCTCTCCGGCCACCACTGGTCCATATCGATA
CTCACGTGTCCGTCAGAGTATGTTCCCAGCTCACCTTTCAGCGATCCCTCATTAGATTCTTGCATCTT
+
FFFFFFFFFFFFFFFFFFFFFFFFFFFFFFFFFF:FFFFFFFFFFFFFFFFFF:FFFFFFFFFFFFFF
FFFFFFFFFFFFFFFFFFFFFFFFFFFFFFFFFFFFFFFFFFFFFFFFFFFFFFFFFFFFFFFFFFFF
```

@A00155:342:HHGFNDSXY:1:1673:6090:6277 2:N:0:GAACCTAG+TCCGCATA
ACCTGGCCACGAGTCAAAACTAGCCTCTTCTTCATATCTCTCCGGCCACCACTGGTCCATATCGATAC
TCACGTGTCCGTCAGAGTATGTTCCCAGCTCACCTTTCAGCGATCCCTCATTAGATTCTTGCATCTTC
+
FFFFF:FF:FFFFFFF:F,F,FFFFFFFFFF:FFFFFFFFFFFF:F:FFFFFFFFFFFFFFFFFFFF
FFFFFFFFFFFFFFFFFFFFF:FFFFFFFFFFFFFFFF:FFFFFFFFFFFFFFFF:FF:FFFFFFFFF
@A00155:342:HHGFNDSXY:1:2560:17626:36620 1:N:0:GAACCTAG+TCCGCATA
CCTGGCCACGAGTCAAAACTAGCCTCTTCTTCATATCTCTCCGGCCACCACTGGTCCATATCGATACT
CACGTGTCCGTCAGAGTATGTTCCCAGCTCACCTTTCAGCGATCCCTCATTAGATTCTTG
+
FFFFFFFFFFFFFFFFFFFFFFFFFFFFFFFFFFFFFFFF:FFFFFFFFFFFFFFFFFFFFFFF,FF
FFFFFF:FFFFFFFFFFFFFFFFFF:FFFFFFFFFF:FFFFFFFFFFFF:F:FFF:F:FFF
@A00155:342:HHGFNDSXY:1:2451:29288:1642 1:N:0:GAACCTAG+TCCGCATA
CTGGCCACGAGTCAAAACTAGCCTCTTCTTCATATCTCTCCGGCCACCACTGGTCCATATCGATACTC
ACGTGTCCGTCAGAGTATGTTCCCAGCTCACCTTTCAGCGATCCCTCATTAGATTCTTGCATCTTCAT
+
FFFFFFFFFFFFFFFFFFFFFFFFFFFFFFFFFFFFFFFFFFFFFFFFFFFFFFFFFFFFFFFFFFFF
FFFFFFFFFFFFFFFFFFFFFFFFFFFFFFFFFFFFFFFFFFF:FFFFFFFFFFFFFFFFFF,FFFFFFF
@A00155:342:HHGFNDSXY:1:2324:5782:10942 2:N:0:GAACCTAG+TCCGCATA
CTGGCCACGAGTCAAAACTAGCCTCTTCTTCATATCTCTCCGGCCACCACTGGTCCATATCGATACTC
ACGTGTCCGTCAGAGTATGTTCCCAGCTCACCTTTCAGCGATCCCTCATTAGATTCTTGCATCTTCAT
+
FFFFFFFFFF:FFFFFFFFFFFFFF:FFFFF:FFFFFFFFFFFFFFFFFFFFFFFFFFFF:FFFFFFFF
FFFFFFFFFFFFFFFFFFFFFFFFFFFFFFFFFFFFFFFFFFFFFFFFFFF:FFFFFFFFFFFFFFFFF
@A00155:342:HHGFNDSXY:1:1274:29559:20744 1:N:0:GAACCTAG+TCCGCATA
TGGCCACGAGTCAAAACTAGCCTCTTCTTCATATCTCTCCGGCCACCACTGGTCCATATCGATACTCA
CGTGTCCGTCAGAGTATGTTCCCAGCTCACCTTTCAGCGATCCCTCATTAGATTCTTGCATCTTCATC
+
FFFFFFFFFFFFFFFFFFFFFFFFFFFFFFFFFFFFFFFF:FFFFFFFFFFFFFFFFFFFFFFFFFFFF
FFFFFFFFFFFF:FFFFFFFFFFFFFFFFFFFFFFFFFFFFFFFFFFFFFFFFFFFFFF:F:FFFFFF
@A00155:342:HHGFNDSXY:1:2677:19768:20823 1:N:0:GAACCTAG+TCCGCATA
TGGCCACGAGTCAAAACTAGCCTCTTCTTCATATCTCTCCGGCCACCACTGGTCCATATCGATACTCA
CGTGTCCGTCAGAGTATGTTCCCAGCTCACCTTTCAGCGATCCCTCATTAGATTCTTGCATCTTCATC
+
FFFFFFFFFFFFFFFFFFFFFFFFFFFFFFFFFFFFFFFFFFFFFFFFFFFFFFFFFFFFFFFFFFFF
FFFFFFFFFFFFFFFFFF,FFFFFFFFFFFFFFFFFFFFF,FFFFFFFFFFF:FFFFFF:FFF,FFFF
@A00155:342:HHGFNDSXY:1:2567:8196:15123 2:N:0:GAACCTAG+TCCGCATA
GGCCACGAGTCAAAACTAGCCTCTTCTTCATATCTCTCCGGCCACCACTGGTCCATATCGATACTCAC
GTGTCCGTCAGAGTATGTTCCCAGCTCACCTTTCAGCGATCCCTCATTAGATTCTTGCATCTTCATCA
+
FFFFFFFF:FFFFFFFFFFFFFFF,FFFFFFFFF:FFFF:FFFFFFFF:FFFFFFF:,FF:FFF,FFF
:FFFF:FFFFFFFFFFFFFFFFFFFFF:FFFF:FFFFFFFFFFFFF:FFFFFFFFFFFF,FFFF:FFF
@A00155:342:HHGFNDSXY:1:2475:22001:4523 2:N:0:GAACCTAG+TCCGCATA
GGCCACGAGTCAAAACTAGCCTCTTCTTCATATCTCTCCGGCCACCACTGGTCCATATCGATACTCAC
GTGTCCGTCAGAGTATGTTCACAGCTCACCTTTCAGCGATCCCTCATTAGATTCTTGCATCTTCATCA
+
::FFFFFFFFFF::,FFFF::FFFFFFFFFFFFFFFF,FFFFF,,FFF,FFF,::,FFF:FFFFF:F:
:FFFFF:F:F::FF:FFFFF,FF:FF:::F::FF,FFF:F:,:,,,F:F:FFFFFFF:FF:FFFFFFF
@A00155:342:HHGFNDSXY:1:2152:31304:5948 2:N:0:GAACCTAG+TCCGCATA
GCCATGAGTCAAAACTAGCCTCTTCTTCATATCTCTCCGGCCACCACTGGTCCATATCGATACTCACG
TGTCCGTCAGAGTATGTTCCCAGCTCACCTTTCAGCGATCCCTCATTAGATTCTTGCATCTTCAT
+
FFFFFFFF:FFF:FFFFFFFFFFFFFFFFF:FFFFFFFFFFFFFFFFFFFFFFFFFFFFFFFFFF:FF
:FFFFFFFFFFFFF:F:FFFFFFFFFFFFFFFFFFFFF:FFFFFF:FFFFFFFFFFFFFFFFFF:F

```
@A00155:342:HHGFNDSXY:1:2246:25111:14888 2:N:0:GAACCTAG+TCCGCATA
CCACGAGTCAAAACTAGCCTCTTCTTCATATCTCTCCGGCCACCACTGGTCCATATCGATACTCACGT
GTCCGTCAGAGTATGTTCCCAGCTCACCTTTCAGCGATCCCTCATTAGATTCTTGCATCTTCAT
+
FFFFFFFFFFFFFFFFFFFFFFFFFFFFFFFFFFFFFFFFFFFFFFFFFFFFFF:FFFFFFFFFFFFF
FFFFFFFFFFFF:FFFFFFF:FFFFFFFFFFFFFFFFFFFFFFFFFFFFFFFFFFFFFFFFFFF
@A00155:342:HHGFNDSXY:1:1548:12906:9095 2:N:0:GAACCTAG+TCCGCATA
CCACGAGTCAAAACTAGCCTCTTCTTCATATCTCTCCGGCCACCACTGGTCCATATCGATACTCACGT
GTCCGTCAGAGTATGTTCCCAGCTCACCTTTCAGCGATCCCTCATTAGATTCTTGCATCTTCATC
+
FFFFFFFFFFFFFFFFFFFFFFFFFFFFFFFFFFFF:FFFFFFFFF:FFFFFFFFFFFFFFFFFFFFF
FFFFFFFFFFFFFFF:FFFFFFFFFFFF:F,FFFFFFFFFFFFFFFFFFFFF,FFFFFFFFFFFF
@A00155:342:HHGFNDSXY:1:2153:14950:5682 2:N:0:GAACCTAG+TCCGCATA
CCACGAGTCAAAACTAGCCTCTTCTTCATATCTCTCCGGCCACCACTGGTCCATATCGATACTCACGT
GTCCGTCAGAGTATGTTCCCAGCTCACCTTTCAGCGATCCCTCATTAGATTCTTGCATCTTCATCAGC
+
FFFFFFFF:FFFFFFFFFFFFFFFFFFFFFFFFFFFFFFFFFFFFFFFFFFFFFFFFFFFFFF:FFF
FFFFFFFFFFFFFFF:FFFFFFFFFFFFFFFFFFFFFFFFFFFF:FFFFFFFFFFFFFFFFFFFFFFF
@A00155:342:HHGFNDSXY:1:1139:32678:11428 1:N:0:GAACCTAG+TCCGCATA
CCACGAGTCAAAACTAGCCTCTTCTTCATATCTCTCCGGCCACCACTGGTCCATATCGATACTCACGT
GTCCGTCAGAGTATGTTCCCAGCTCACCTTTCAGCGATCCCTCATTAGATTCTTGCATCGTCATCAGC
+
FFFFFFFFFFFFFFFFFFFFF::F:FFFFFFFFFFFFFFFFFFFFFFFFFFF:FFFFFFFFFFFFFFF
FFFFFFFFFFFFF:FF:FFFFFFFFFFFF:FF:FFF:,FFF:FFFFFFFFFFFFFF::F,FFF,FFFF
@A00155:342:HHGFNDSXY:1:2450:32524:7216 2:N:0:GAACCTAG+TCCGCATA
CCACGAGTCAAAACTAGCCTCTTCTTCATATCTCTCCGGCCACCACTGGTCCATATCGATACTCACGT
GTCCGTCAGAGTATGTTCCCAGCTCACCTTTCAGCGATCCCTCATTAGATTCTTGCATCTTCATCCGC
+
FFFFFFFFFFFFFF,,FFF,FFFFFFFF:,FFFFFFFFFF:FFFFF,FFFFFFFFFFFF,FFFFFFF,
FFFFFFFFFFF:F,F,FFFFFFF,F:FFF:FF:FFFFFFFF:FFF,FFF:FF:FFFFF:,FF,FF,FF
@A00155:342:HHGFNDSXY:1:2161:12825:3630 2:N:0:GAACCTAG+TCCGCATA
CCACGAGTCAAAACTAGCCTCTTCTTCATATCTCTCCGGCCACCACTGGTCCATATCGATACTCACGT
GTCCGTCAGAGTATGTTCCCAGCTCACCTTTCAGCGATCCCTCATTAGATTCTTGCATCTTCATCAGC
+
FFFFFFFFFFFFFFFFFFFFFFFFFFFFFFFFFFFFFFFFFFFFFFFFFFFFFFFFFFFFFFFFFFF:
FFFFFFFFFFFFFFFFFFFFFFFFFFFFFFFFFFFFFFFFFFFFFFFFFFFFFFFFFFFFFFFFFFFF
@A00155:342:HHGFNDSXY:1:2264:11496:31015 1:N:0:GAACCTAG+TCCGCATA
CCACGAGTCAAAACTAGCCTCTTCTTCATATCTCTCCGGCCACCACTGGTCCATATCGATACTCACGT
GTCCGTCAGAGTATGTTCCCAGCTCACCTTTCAGCGATCCCTCATTAGATTCTTGCATCTTCATCAGC
+
FFFFFFFFFFFF:FFFFFFFFFFFFFFFFFFFFFFFFFFFFFFFFFFFFFFFFFFFFFFFFFFFFFF:
FFFFFFFFFFFFFFFFFFFFFFFFFFFFFFFFFFFF,FFFFFFFFFFFFFFFFFF::FF,:FFFF,FF
@A00155:342:HHGFNDSXY:1:2153:21685:3662 2:N:0:GAACCTAG+TCCGCATA
CCACGAGTCAAAACTAGCCTCTTCTTCATATCTCTCCGGCCACCACTGGTCCATATCGATACTCACGT
GTCCGTCAGAGTATGTTCCCAGCTCACCTTTCAGCGATCCCTCATTAGATTCTTGCATCTTCATCAGC
+
FFFFF,FFFFFFFFFFFFFFFFFFFFFFF:FFFFFFFFFFFFFFF:FFFFFFF:FFFFFFFFFF:FFF
FFFFFFFFFFFFFFFFFFFFFFFFFFFFFFFFFFFFFFFFF,F:FFFFFFFFFFFFFFFFFFFFFFFF
@A00155:342:HHGFNDSXY:1:1156:13720:33771 2:N:0:GAACCTAG+TCCGCATA
CCACGAGTCAAAACTAGCCTCTTCTTCATATCTCTCCGGCCACCACTGGTCCATATCGATACTCACGT
GTCCGTCAGAGTATGTTCCCAGCTCACCTTTCAGCGATCCCTCATTAGATTCTTGCATCTTCATCAGC
+
F::FF,FFFFFFFFFFFFF:FFFFFFFFFF:FFFFFFFFFFFFFFFFFFFFFFFF:FFFFFFFFFFFF
FFFFFFFFFFFFFFF,FFFFFFFFFFFF,FFFFFFFFFFFFFFFFFFFFFFFFFFFFFFFFFFFFFFF
```

```
@A00155:342:HHGFNDSXY:1:2533:7129:1595 2:N:0:GAACCTAG+TCCGCATA
CCACGAGTCAAAACTAGCCTCTTCTTCATATCTCTCCGGCCACCACTGGTCCATATCGATACTCACGT
GTCCGTCAGAGTATGTTCCCAGCTCACCTTTCAGCGATCCCTCATTAGATTCTTGCATCTTCATCAGC
+
FFFFFFFFFFFFFFFFFFFFFFFFFFFFFFFFFFFFFFFFFFFFFFFF:FFFFFFFFFFFFFFFFFFF
FFFFFFFFFFFFFFFFFFFFFFFFFFFFFFFFF:FFFFFFFFFF:FFFFFFFFFFFFFFFFFFFFFFF
@A00155:342:HHGFNDSXY:1:2472:24478:2237 2:N:0:GAACCTAG+TCCGCATA
CCACGAGTCAAAACTAGCCTCTTCTTCATATCTCTCCGGCCACCACTGGTCCATATCGATACTCACGT
GTCCGTCAGAGTATGTTCCCAGCTCACCTTTCAGCGATCCCTCATTAGATTCTTGCATCTTCATCAGC
+
FFFFFF:FFFFFFF:FFFFFFFFFFFFFFFFFFFFFFFFFFFFFFFFFFFFFFFFFFFFFFFFFFFFF
FFFFFFFFFFFFFFFFFFFFFFFFFFFFFFFFFFFFFFFFFFFFFFFFFFFFFFFFFFFFFFFFFFFF
@A00155:342:HHGFNDSXY:1:1677:30960:4852 2:N:0:GAACCTAG+TCCGCATA
CCACGAGTCAAAACTAGCCTCTTCTTCATATCTCTCCGGCCACCACTGGTCCATATCGATACTCACGT
GTCCGTCAGAGTATGTTCCCAGCTCACCTTTCAGCGATCCCTCATTAGATTCTTGCATCTTCATCAGC
+
FFF:FFFFFFFFFFFFFFFFFFFFF:FFFFFFFFFFFFFFFFFFFFFFFFFFF:FFFFFFFFFFFFFF
FFFFFFFFFFFFFFFFFFFF:FFFFFFFFFFFFFFFFFFFFF:F:FF:FFFFFFFFFFFFFFFFFFFF
@A00155:342:HHGFNDSXY:1:2172:16034:5024 2:N:0:GAACCTAG+TCCGCATA
CCACGAGTCAAAACTAGCCTCTTCTTCATATCTCTCCGGCCACCACTGGTCCATATCGATACTCACGT
GTCCGTCAGAGTATGTTCCCAGCTCACCTTTCAGCGATCCCTCATTAGATTCTTGCATCTTCATCAGC
+
FFF:FFFFFFFFF:,FFFFFFFFFFFFFFFFFFFFFFFFFFFFFFFFFFFFFFFFFFFFFFFFFFF:F
FFFFFFFFFFFFFFFFFF:FFFFFFFFFFFFFFFFFFFFFFFFFFF:FFFFFFFFFFFFFFFFFFFFF
@A00155:342:HHGFNDSXY:1:1312:3016:24940 1:N:0:GAACCTAG+TCCGCATA
CCACGAGTCAAAACTAGCCTCTTCTTCATATCTCTCCGGCCACCACTGGTCCATATCGATACTCACGT
GTCCGTCAGAGTATGTTCCCAGCTCACCTTTCAGCGATCACTCATTAGATTCTTGCATCTTCATCAGC
+
FFFFFFFFFFFFFFFFFF:FFFFFFFFFFFFF:FFFFFFFF::FFFFFF:FFFFFFF,FFFFFFFFF:
FFFFFFFFFFFFFFFFFFF:FFFFFFFFFFFFFFFF:,,,FFFFFFFFFFFFFF::FFFFFFFFFFFF
@A00155:342:HHGFNDSXY:1:2472:24478:2237 1:N:0:GAACCTAG+TCCGCATA
CCACGAGTCAAAACTAGCCTCTTCTTCATATCTCTCCGGCCACCACTGGTCCATATCGATACTCACGT
GTCCGTCAGAGTATGTTCCCAGCTCACCTTTCAGCGATCCCTCATTAGATTCTTGCATCTTCATCAGC
+
FFFFFFFFFFFFFFFFFFFFFFFFFFFFFFFFFFFFFFFFFFFFFFFFFFFFFFFFFFFFFFFFFFFF
FFFFFFFFFFFFFFFFFFFFFFFFFFFFFFFF:FFFFFFFFFFFFFFFFFFFFFFFFFFFFFFFFFFF
@A00155:342:HHGFNDSXY:1:1542:29704:12821 2:N:0:GAACCTAG+TCCGCATA
CCACGAGTCAAAACTAGCCTCTTCTTCATATCTCTCCGGCCACCACTGGTCCATATCGATACTCACGT
GTCCGTCAGAGTATGTTCCCAGCTCACCTTTCAGCGATCCCTCATTAGATTCTTGCATCTTCATCAGC
+
FFFFFFFFFFFFFFF:FFFFFFFFFFFFFFFFFFFFFFFFFFFFFFFFFFFFFFFFFFFFFFFFFFFF
FFFFFFFFFFFFFFFFF:FFF:FFFFFFFFFFFFFFFFFF:FFFFFFFFFFFFFFFFF:FFFFFFFFF
@A00155:342:HHGFNDSXY:1:2567:8196:15123 1:N:0:GAACCTAG+TCCGCATA
CCACGAGTCAAAACTAGCCTCTTCTTCATATCTCTCCGGCCACCACTGGTCCATATCGATACTCACGT
GTCCGTCAGAGTATGTTCCCAGCTCACCTTTCAGCGATCCCTCATTAGATTCTTGCATCTTCATCAGC
+
FFFFFFFFFFFF,FFFFFFFFFFFFFFFFFFFFFFFFFFFFFFFFFFFFFFFFFFFFFFFFFFFFFFF
FFFFFFFFFFFFFFFFFFFFFFFFFFFFFFFFFFFFFFFFFFFFFFFFFFFFFFFFFFFFFFFFFFF:
@A00155:342:HHGFNDSXY:1:1147:14407:19085 2:N:0:GAACCTAG+TCCGCATA
CCACGAGTCAAAACTAGCCTCTTCTTCATATCTCTCCGGCCACCACTGGTCCATATCGATACTCACGT
GTCCGTCAGAGTATGTTCCCAGCTCACCTTTCAGCGATCCCTCATTAGATTCTTGCATCTTCATCAGC
+
FFF:FFFFFFFFF,FFFFFFF,:FFFFFFFFFFFFFFFFFFFFFFFF,:FFFFFFFFFFFFFFFFFFF
FFF:FFFFFFFFFFF:FFFFFFFFFFFFFFFFFFFFFFFFFFFFF:FFFF:FFFFFFFFFFFFFFFFF
```

@A00155:342:HHGFNDSXY:1:2268:31919:23202 2:N:0:GAACCTAG+TCCGCATA
CCACGAGTCAAAACTAGCCTCTTCTTCATATCTCTCCGGCCACCACTGGTCCATATCGATACTCACGT
GTCCGTCAGAGTATGTTCCCAGCTCACCTTTCAGCGATCCCTCATTAGATTCTTGCATCTTCATCAGC
+
FFFFFFFFFFF:FFF:FFFFFFFFFFFFFFFFFFFFFFFFFFFFFFFFFFFFFFFFFFFFFFFFFFFF
FFFFFFFFFFFFFFFFFFFFFFFFFFFFFFFFFFFFFFFFFFFFFFFFFFFFFFFFFFFFFFFFFFFF
@A00155:342:HHGFNDSXY:1:1156:13720:33771 1:N:0:GAACCTAG+TCCGCATA
CCACGAGTCAAAACTAGCCTCTTCTTCATATCTCTCCGGCCACCACTGGTCCATATCGATACTCACGT
GTCCGTCAGAGTATGTTCCCAGCTCACCTTTCAGCGATCCCTCATTAGATTCTTGCATCTTCATCAGC
+
FFFFFFFFFFFFFFFFFFFFFFFFFFFFFFFFFFFFFFFFFFFFFFFFFFFFFFFFFFFFFFFFFFFF
FFFFFFFFFFF:FFFFFFFFFFFFFFFFFFFFFFFFFFFFFFFFFFFFFFFFFFFFFFFFFFFFFFFF
@A00155:342:HHGFNDSXY:1:1303:8133:33019 2:N:0:GAACCTAG+TCCGCATA
CACGAGTCAAAACTAGCCTCTTCTTCATATCTCTCCGGCCACCACTGGTCCATATCGATACTCACGTG
TCCGTCAGAGTATGTTCCCAGCTCACCTTTCAGCGATCCCTCATTAGATTCTTGCATCTTCATC
+
FFFFFF:FFFFFFFFFFFFFFFFFFFFF,FFFFFFFFFFFFFFFFFFFFFFFFFFFFFFFFFFFFFFF
FFF:FFFFFFFFFFFFFFFFFFFFFFFFFFFFFFFFFFFFF:FFFFFFFFFFFFFFFFFFFFF
@A00155:342:HHGFNDSXY:1:1303:4408:14481 2:N:0:GAACCTAG+TCCGCATA
CACGAGTCAAAACTAGCCTCTTCTTCATATCTCTCCGGCCACCACTGGTCCATATCGATACTCACGTG
TCCGTCAGAGTATGTTCCCAGCTCACCTTTCAGCGATCCCTCATTAGATTCTTGCATCTTCATCAGC
+
FFFFFFFFFF:FFFFFFF,:FFFF,FFFFFFFFFFFFF:FFFFFFF:,FFFFFFFFFFFFFFFFFFFF
FFFFFFFFFF:FFFFFFFFFFFFFFFFFFFFFFFFFFFFFFFFFFFF:FFFFFFFFF:FFF:F:FFFF
@A00155:342:HHGFNDSXY:1:2373:11216:7952 2:N:0:GAACCTAG+TCCGCATA
CACGAGTCAAAACTAGCCTCTTCTTCATATCTCTCCGGCCACCACTGGTCCATATCGATACTCACGTG
TCCGTCAGAGTATGTTCCCAGCTCACCTTTCAGCGATCCCTCATTAGATTCTTGCATCTTCATCAGC
+
FFFFFFFFFFFFFFFFFFFFFFFFFFFFFFFFFFFFFFFFFFFFFFFFFFFFFFFFFFFFFFFFFFFF
FFFFFFFFFFFFFFFFFFFFFFFFFFFFFFFFFFFFFFFFFFFFFFFFFFFFFFFFFFFFFFFFFFF
@A00155:342:HHGFNDSXY:1:1537:7500:32330 1:N:0:GAACCTAG+TCCGCATA
ACGAGTCAAAACTAGCCTCTTCTTCATATCTCTCCGGCCACCACTCGTCCATATCGATACTCACGTGT
CCGTCAGAGTATGTTCCCAGCTCACCTTTCAGCGATCCCTCATTAGATTCTTGCATCTTCATCAGCAT
+
FFFFFFF:FFFFFFFFFFFFFFFFFFFFFFFFFFFFFFFFFFFFFFFFFFFFFFFFFFFFFFFFFFFF
FFFFFFFFFFFFF,FFFFFFFFF,FF:FFF:FFF,FF:FFFFFFFF:FFFFFFF,,:,:F,FF:FF:F
@A00155:342:HHGFNDSXY:1:2334:15212:19194 1:N:0:GAACCTAG+TCCGCATA
ACGAGTCAAAACTAGCCTCTTCTTCATATCTCTCCGGCCACCACTGGTCCATATCGATACTCACGTGT
CCGTCAGAGTATGTTCCCAGCTCACCTTTCAGCGATCCCTCATTAGATTCTTGCATCTTCATCAGCAT
+
FFFFFFFFFF:FFFFFFFFFFFFFFFFFFFFFFFFFFFFFFFFFFFFFFFFFFFFFFFFFFFFFFFFF
FFFFF:FFFF:FF:FFFFFFFFFFFFFFFFFFFFFFFFFF:FFFFFFFFFFFFFF:FFFFFFFFFFFF
@A00155:342:HHGFNDSXY:1:1677:20302:7185 2:N:0:GAACCTAG+TCCGCATA
CGAGTCAAAACTAGCCTCTTCTTCATATCTCTCCGGCCACCACTGGTCCATATCGATACTCACGTGTC
CGTCAGAGTATGTTCCCAGCTCACCTTTCAGCGATCCCTCATTAGATTCTTGCATCTTCATCAGC
+
FFFFFFFFFFFF,FFFFFFFFFFFFFFFFF:FFFFFFFFFFF:FFFFFFFFF:FFFFFFFFFFFFF:F
FFFFFFFFFFFF:FFFFFFFFFFFF:FFFFFFFFFFFFFFFFFFFFFFFFFFFFFFFFFFFFFFFF
@A00155:342:HHGFNDSXY:1:2660:18023:21997 1:N:0:GAACCTAG+TCCGCATA
CGAGTCAAAACTAGCCTCTTCTTCATATCTCTCCGGCCACCACTGGTCCATATCGATACTCACGTGTC
CGTCAGAGTATGTTCCCAGCTCACCTTTCAGCGATCCCTCATTAGATTCTTGCATCTTCATCAGCATG
+
FFFFFFFFFFFFFFFFFFFFFFFFFFFFFFFFFFFFFFFFFFFFFFFFFFFFFFFFFFFFFFFFFFFF
FFFFFFFFFFFFFFFFFFFFFFFFFFFFFFFFFF,,FFFFFFFFFFFFFFFFFFFFFFFFFFFFFFFFF

```
@A00155:342:HHGFNDSXY:1:2617:22426:27461 1:N:0:GAACCTAG+TCCGCATA
GAGTCAAAACTAGCCTCTTCTTCATATCTCTCCGGCCACCACTGGTCCATATCGATACTCACGTGTCC
GTCAGAGTATGTTCCCAGCTCACCTTTCAGCGATCCCTCATTAGATTCTTGCATCTTCATCAGCATGC
+
FFFFFFFFFFFFFFFFFFFFFFFFFFFFFFFFFFFFFFFFFFFFFFFFFFFFFFFFFFFFFFFFFFFF
FFFFFFFFFFFFFFFFFFFFFFFFFFFFFFFFFFFFFFFFF:FFFFFFFFFFFFFFFFFFFFFFF,FF
@A00155:342:HHGFNDSXY:1:2533:7129:1595 1:N:0:GAACCTAG+TCCGCATA
GAGTCAAAACTAGCCTCTTCTTCATATCTCTCCGGCCACCACTGGTCCATATCGATACTCACGTGTCC
GTCAGAGTATGTTCCCAGCTCACCTTTCAGCGATCCCTCATTAGATTCTTGCATCTTCATCAGCATGC
+
FFFFFFFFFFFFFFFFFFFFFFFFFFFFFFFFFFFFFFFFFFFFFFFFFFFFFFFFFFFFFFFFFFFF
FFFFFFFFFFFFFFFFFFFFFFFFFFFFFFFFFFFFFFFFFFFFFFFFFFFFFFFFFFFFFFFFFFF:
@A00155:342:HHGFNDSXY:1:2362:8748:10692 1:N:0:GAACCTAG+TCCGCATA
GAGTCAAAACTAGCCTCTTCTTCATATCTCTCCGGCCACCACTGGTCCATATCGATACTCACGTGTCC
GTCAGAGTATGTTCCCAGCTCACCTTTCAGCGATCCCTCATTAGATTCTTGCATCTTCATCCGCATGC
+
FFFFFFFFFFFFF:FFF:FFFFFFFFFFFF:FFFFFFFFFFFFFFFFFFF:FFFFFFFFFFFFFFFFF
FFFFFFFFFFFFFFFFFFFFFFFFFFFFFFFFFFFFFFFFFFFFFFFFFFFFF:FFFFFFF,:FFFFF
@A00155:342:HHGFNDSXY:1:1673:6090:6277 1:N:0:GAACCTAG+TCCGCATA
GAGTCAAAACTAGCCTCTTCTTCATATCTCTCCGGCCACCACTGGTCCATATCGATACTCACGTGTCC
GTCAGAGTATGTTCCCAGCTCACCTTTCAGCGATCCCTCATTAGATTCTTGCATCTTCATCAGCATGC
+
FFFFFFFFFFFFF,FFFFFFFFFFFFFF:FFF,FFFFFFFFFFFFFFFFFFFFFFFFFF,FFFFFFFF
FFFFFFFFFFFFFFFFFF,FFF:F:FFFFF,FFFFFFFFFF:FFF:FFFFFFFFFFFFFFF,FFFFFF
@A00155:342:HHGFNDSXY:1:2242:7482:19586 1:N:0:GAACCTAG+TCCGCATA
GAGTCAAAACTAGCCTCTTCTTCATATCTCTCCGGCCACCACTGGTCCATATCGATACTCACGTGTCC
GTCAGAGTATGTTCCCAGCTCACCTTTCAGCGATCCCTCATTAGATTCTTGCATCTTCATCAGCATGC
+
FFFFFFFFFFFFFFFFFFFFFFFFFFFFFFFFFFFFFFFFFFFFFFFFFFFFFFFFFFFFFFFFFFFF
FFFFFFFFFFFFFFFFFFFFFFFFFFFFFFFFFFFFFFFFFFFFFFFFFFFFFFFFFFFFFFFFFFFF
@A00155:342:HHGFNDSXY:1:1678:27715:24001 1:N:0:GAACCTAG+TCCGCATA
GAGTCAAAACTAGCCTCTTCTTCATATCTCTCCGGCCACCACTGGTCCATATCGATACTCACGTGTCC
GTCAGAGTATGTTCCCAGCTCACCTTTCAGCGATCCCTCATTAGATTCTTGCATCTTCATCAGCATGC
+
FFFFFFF,FFFF:F:FFFFFFFFFFFFFFFFFFFFFFFFFFFFFFFFFFFFFFFFFFFFFFFF:FFFF
FFFFFFFFF:FFFFFFFFFFFFFF,FFFFFFF:FFFFFFF:FFFFFFFFFFF:FFFFFFFF:FFFFFF
@A00155:342:HHGFNDSXY:1:1549:14091:28682 1:N:0:GAACCTAG+TCCGCATA
AGTCAAAACTAGCCTCTTCTTCATATCTCTCCGGCCACCACTGGTCCATATCGATACTCACGTGTCCG
TCAGAGTATGTTCCCAGCTCACCTTTCAGCGATCCCTCATTAGATTCTTGCATCTTCATCAGCATGCA
+
FFFFFFFFFFFFFFFFFFFFFFFFFFFFFFFFFFFFFFFFFFFFFFFFFFFFFFFFFFFFFFFFFFFF
FFFFFFF:FFFFFFFFFFFFFFFFFFFFFFFFFFFFFFFFFFFFFFFFFFFFFFFFFFFFFFFFFFFF
@A00155:342:HHGFNDSXY:1:1460:18530:10755 1:N:0:GAACCTAG+TCCGCATA
AGTCAAAACTAGCCTCTTCTTCATATCTCTCCGGCCACCACTGGTCCATATCGATACTCACGTGTCCG
TCAGAGTATGTTCCCAGCTCACCTTTCAGCGATCCCTCATTAGATTCTTGCATCTTCATCAGCATGCA
+
FFFFFFFFFFFFFFFFFFFFFFFF:FFFFFFFFFFFFFFFFFFFFFFFFFFFFFFFFFFFFFFFFFFF
FFFFFFFFFFFFFFFFFFFFFFFFF:FFFFFFFFFFFFFFFFFFFFFFFFFFFFFFFFFF:FFFFFFF
@A00155:342:HHGFNDSXY:1:2269:29857:7764 2:N:0:GAACCTAG+TCCGCATA
AGTCAAAACTAGCCTCTTCTTCATATCTCTCCGGCCACCACTGGTCCATATCGATACTCACGTGTCCG
TCAGAGTATGTTCCCAGCTCACCTTTCAGCGATCCCTCATTAGATTCTTGCATCTTCATCAGCATGCA
+
FFFFFFFFFFFFFFFFFFFFFFFFFFFFFFFFFFFFFFFFFFFFFFFFFFFFFFFFFFFFFFFFFFFF
FFFFFFFFFFFFFFFFFFFFFFFFFFFFFFFFFFFFFFFFFFFFFFFFFFFFFFFFFFFFFFFFFFFF
```

```
@A00155:342:HHGFNDSXY:1:1664:15040:29168 1:N:0:GAACCTAG+TCCGCATA
TCAAAACTAGCCTCTTCTTCATATCTCTCCGGCCACCACTGGTCCATATCGATACTCACGTGTCCGTC
AGAGTATGTTCCCAGCTCACCTTTCAGCGATCCCTCATTAGATTCTTGCATCTTCATCAGCATGCAAA
+
FFFFF:FFFFFFFFF:FFFFFFFFFFFFFFFFFFFFFFFFFFFFFFFFFFFFFFFFFFFFFFFFFFFF
FFFFFFFFFFFFFFFFFFFFFFFFFFFFF,:FFFFFFFFFFF,FFFFFFFFFFFFFFFFFFFFFFFF:
@A00155:342:HHGFNDSXY:1:2450:32524:7216 1:N:0:GAACCTAG+TCCGCATA
CAAAACTAGCCTCTTCTTCATATCTCTCCGGCCACCACTGGTCCATATCGATACTCACGTGTCCGTCA
GAGTATGTTCCCAGCTCACCTTTCAGCGATCCCTCATTAGATTCTTGCATCTTCATCAGCATGCAAAA
+
FFF:FFF:FFFFF,FFFFFF:FFFFFFFFFFFF:FFFF:FFFFF,,:,FF:F:FFFFFFFFFFFFFFF
FFF,,FF,,FFFFFF:F,FFF,:FFFFFFFFFFFFF,,FFF,:FF:FF,,F,FFFFF,FFFFFF,:FF
@A00155:342:HHGFNDSXY:1:2242:14154:15640 2:N:0:GAACCTAG+TCCGCATA
CAAAACTAGCCTCTTCTTCATATCTCTCCGGCCACCACTGGTCCATATCGATACTCACGTGTCCGTCA
GAGTATGTTCCCAGCTCACCTTTCAGCGATCCCTCATTAGATTCTTGCATCTTCATCAGCATGCAAAA
+
FFFFFFFFFFFFFFFFFFF:FFFFFFFFFFFFFFFFFFFFFFFF:FFFFFFFFFFFFFFFFFFFFFFF
FFFFFFFFFFFF:FFFFFFFFFFFFFFFFFFFFFFFFF:FFFFFFFFFFFFFFFFFFFFFFFFFFFFF
@A00155:342:HHGFNDSXY:1:1125:8422:10504 1:N:0:GAACCTAG+TCCGCATA
CAAAACTAGCCTCTTCTTCATATCTCTCCGGCCACCACTGGTCCATATCGATACTCACGTGTCCGTCA
GAGTATGTTCCCAGCTCACCTTTCAGCGATCCCTCATTAGATTCTTGCATCTTCATCAGCATGCAAAA
+
FFFFF:FF:FF:FFFFFFFFFFFFFFFFFFFFF:FFFFFFFFFFFFFFFFFFFFFF:FFFFFFFFF:F
F:FFFFF,FFFFFFFFFFFFFFF:FFFF:,F:FFFFFFFFFFFFFFF:FFFFFFFFFFFFFFFFF,FF
@A00155:342:HHGFNDSXY:1:2153:21685:3662 1:N:0:GAACCTAG+TCCGCATA
CAAAACTAGCCTCTTCTTCATATCTCTCCGGCCACCACTGGTCCATATCGATACTCACGTGTCCGTCA
GAGTATGTTCCCAGCTCACCTTTCAGCGATCCCTCATTAGATTCTTGCATCTTCATCAGCATGCAAAA
+
FFFFFFFFFFFFFFFFFFFFFFFFFFFFFFFFFFFFFFFFFFFFFFFFFFFFFFFFFFFFFFFFFFFF
F:FFFFFFFFFFFFFFFFFFFFFFFFFFFFFFFFFFFFFFFFFFFFFFFFFFFFFFFFFFFFFFF,FF
@A00155:342:HHGFNDSXY:1:1549:21721:7420 1:N:0:GAACCTAG+TCCGCATA
CAAAACTAGCCTCTTCTTCATATCTCTCCGGCCACCACTGGTCCATATCGATACTCACGTGTCCGTCA
GAGTATGTTCCCAGCTCACCTTTCAGCGATCCCTCATTAGATTCTTGCATCTTCATCCGCATGCAAAA
+
FFFFFFFFFFFFFFFFFFFFFFFFFFFFFFFFFFFFFFFFFFFF:FFFFFFFFFFFFFFFFFFFFFFF
FFFFFFFF:FFFFFFFFF:FFFFFFFFFF,FFFFFFFFFFFFFFFFFFF:FFFFF:F,FFFFFFFFFF
@A00155:342:HHGFNDSXY:1:2216:13250:33614 2:N:0:GAACCTAG+TCCGCATA
AAAACTAGCCTCTTCTTCATATCTCTCCGGCCACCACTGGTCCATATCGATACTCACGTGTCCGTCAG
AGTATGTTCCCAGCTCACCTTTCAGCGATCCCTCATTAGATTCTTGCATCTTCATCAGCATGCAAAAT
+
FFFF,,FFFFFFFFFFFFFFFFFFFFFFFFFFFFFFF:FFFFF:FFFFFFFFFFFFFFFFFFFFFFFF
FFFFFFFFFFFFFFFFFFFFFFFFFFFFFFFF:FF:FFFFFFFFFFFFFFFFFFFFFFFFFFFFFFF:
@A00155:342:HHGFNDSXY:1:2261:28013:29058 1:N:0:GAACCTAG+TCCGCATA
AAACTAGCCTCTTCTTCATATCTCTCCGGCCACCACTGGTCCATATCGATACTCACGTGTCCGTCAGA
GTATGTTCCCAGCTCACCTTTCAGCGATCCCTCATTAGATTCTTGCATCTTCATCAGCATGCAAAATC
+
FFFFFFFFFFFFFFFFFFFFFFFFFFFFFFFFFFFFFFFFFFFFFFFFFFFFFFFFFFFFFFFFFFFF
FFFFFFFFFFFFFFFFFFFFFFFFFFFFFFFFFFFFFFFFFFFFFFFFFFFFFFFFFFFFFFFFFFFF
@A00155:342:HHGFNDSXY:1:2261:28013:29058 2:N:0:GAACCTAG+TCCGCATA
AACTAGCCTCTTCTTCATATCTCTCCGGCCACCACTGGTCCATATCGATACTCACGTGTCCGTCAGAG
TATGTTCCCAGCTCACCTTTCAGCGATCCCTCATTAGATTCTTGCATCTTCATCAGCATGCAAA
+
FFFFFFFFFFFFFFFFFFFFFFFFFFFFFFFFFFFFFFFFFF,FFFFFFFFFFFFFFFFFFFFFFFFF
FFFFFFFFFFFFFFFFFFFFFFFFFFFFFFFFFFFFFFFFFFFFFFFFFFFFFFFFFFFFFFFF
```

@A00155:342:HHGFNDSXY:1:2255:12445:33724 1:N:0:GAACCTAG+TCCGCATA
AACTAGCCTCTTCTTCATATCTCTCCGGCCACCACTGGTCCATATCGATACTCACGTGTCCGTCAGAG
TATGTTCCCAGCTCACCTTTCAGCGATCCCTCATTAGATTCTTGCATCTTCATCAGCATGCAAAATCT
+
FFFFFFFFFFFFFFFFFFFFFFFFFFFFFFFFFFFFFFFFFFFFFFFFFFFFFFFFFFFFFFFFFFFF
FFFFFFFFFFFFFFFFFF:FFFFFFFFFFFFFFFFFFFFFFFFFFFFFFFFFFFFFFFFFFFFFFFFF
@A00155:342:HHGFNDSXY:1:1525:8133:33176 1:N:0:GAACCTAG+TCCGCATA
AACTAGCCTCTTCTTCATATCTCTCCGGCCACCACTGGTCCATATCGATACTCACGTGTCCGTCAGAG
TATGTTCCCAGCTCACCTTTCAGCGATCCCTCATTAGATTCTTGCATCTTCATCAGGATGCAAAATCT
+
F:FFFFFFFFFFFFFFFFFFFFFFFFFFFFFFFFFFFFFFFFFFFFFFFFFFFFFFFFFFFFFFFFFF
FFFFFFFFFFFFFFFFFF:FFFFFFF:FFFFFFFFFFFFFFFFFFFFFFFFFFFFFFFFFFF:F:FFF
@A00155:342:HHGFNDSXY:1:1669:19768:19539 1:N:0:GAACCTAG+TCCGCATA
AACTAGCCTCTTCTTCATATCTCTCCGGCCACCACTGGTCCATATCGATACTCACGTGTCCGTCAGAG
TATGTTCCCAGCTCACCTTTCAGCGATCCCTCATTAGATTCTTGCATCTTCATCAGGATGCAAAATCT
+
FFFFFFFFFFFFFFFFFFFFFFFFFFFFFFFFFFFFFFFFFFFFFFFFFFFFFFFFFFFFFFFFFFFF
FFFFFFFFFFFFFFFFFFFFFFFFF:FFFFFFFFFFFFFFFFFFFFFFFFFFFFFFFF:FFFFF,FFF
@A00155:342:HHGFNDSXY:1:2610:27425:29951 1:N:0:GAACCTAG+TCCGCATA
CTAGCCTGTTCTTCATATCTCTCCGGCCACCACTGGTCCATATCGATACTCACGTGTCCGTCAGAGTA
TGTTCCCAGCTCACCTTTCAGCGATCCCTCATTAGATTCTTGCATCTTCATCAGCATGCAAAATCTT
+
FFFFFFF,FFF:FFFF,FFF:FFFFF:,:F:,FFFFFF,F:FFFFFFFFF,FF:FF:FF,FF,,F,F:
F,::::,F:FFF,F:FFFFFFFFFF:::F,:FFFFFFFFFFFFFFFFFFFF:FFF,FFFFFF:F,FF
@A00155:342:HHGFNDSXY:1:1128:24804:18051 2:N:0:GAACCTAG+TCCGCATA
TAGCCTCTTCTTCATATCTCTCCGGCCACCACTGGTCCATATCGATACTCACGTGTCCGTCAGAGTAT
GTTCCCAGCTCACCTTTCAGCGATCCCTCATTAGATTCTTGCATCTTCATCAGCATGCAAA
+
FFFFFFFFFFFFFFFFFFFFFFFFFFFFFFFFFFFFFFFFFFFFFFFFFFFFFFFFFFFFFFFFFFFF
FFFFFFFFFFFFFFFFFFFFFFFFFFFFFFFFFFFFFFFFFFFFFFFFFFFFFFFFFFFFF
@A00155:342:HHGFNDSXY:1:2152:31304:5948 1:N:0:GAACCTAG+TCCGCATA
TAGCCTCTTCTTCATATCTCTCCGGCCACCACTGGTCCATATCGATACTCACGTGTCCGTCAGAGTAT
GTTCCCAGCTCACCTTTCAGCGATCCCTCATTAGATTCTTGCATCTTCATCAGCATGCAAAATCT
+
FFFFFFFFFFFFFFFFFFFFFFFFFFFFFFFFFFFFFFFF:FFFFF:FFFFFFFFFFFFFFFFFFFFF
FFFFFFFFFFFFFFFFFFFFFFFFFFFFFFFFFFFFFFFFFFFFFFFFFFFFFFFFFFF,FFFFF
@A00155:342:HHGFNDSXY:1:2472:11957:3255 1:N:0:GAACCTAG+TCCGCATA
TAGCCTCTTCTTCATATCTCTCCGGCCACCACTGGTCCATATCGATACTCACGTGTCCGTCAGAGTAT
GTTCCCAGCTCACCTTTCAGCGATCCCTCATTAGATTCTTGCATCTTCATCAGCATGCAAAATCTTA
+
FFFFF,FF,FFFFFFF:FFFFFFFFFF,FF,FFFFFFFFFFFFFF:FF:F:FFFFFFFFFFFFFFFFF
FFFFFFFFFFFFFFF::FFFFF:,FFFF:FF:FFFFFFFFF:F:F:FF:,F:FFFF:FFF:FFFF,F
@A00155:342:HHGFNDSXY:1:2503:6027:22357 1:N:0:GAACCTAG+TCCGCATA
TAGCCTCTTCTTCATATCTCTCCGGCCACCACTGGTCCATATCGATACTCACGTGTCCGTCAGAGTAT
GTTCCCAGCTCACCTTTCAGCGATCCCTCATTAGATTCTTGCATCTTCATCAGCATGCAAAATCTTAA
+
FFFFFFFFFFFFFFFFFFFFFFFFFFFFFFFFFFFFFFFFFFFFFFFFFFFFFFFFFFFFFF:FFF:F
F:FFFFFFFFFFFFFFFFFFFFFFF:FFFFFFFFFFFFFFFFFFFFFFFFFFFFF:FFFFFFFFFFFF
@A00155:342:HHGFNDSXY:1:1622:15492:28103 1:N:0:GAACCTAG+TCCGCATA
TAGCCTCTTCTTCATATCTCTCCGGCCACCACTGGTCCATATCGATACTCACGTGTCCGTCAGAGTAT
GTTCCCAGCTCACCTTTCAGCGATCCCTCATTAGATTCTTGCATCTTCATCAGCATGCAAAATCTTAA
+
FFFFFFF,FFFFFFFFFFFFFFFFFFFFFFFFFFFFFFFFFFFFFFFFFFFFFFFFFFFFFFFFFF:F
FFFFFFFFFFFFFFFFFFFFFFFFFFFFFFFFFFFFFFFFF:F::FFFFFFFFFFFFFFFFFFFFFFF

```
@A00155:342:HHGFNDSXY:1:2242:14154:15640 1:N:0:GAACCTAG+TCCGCATA
TAGCCTCTTCTTCATATCTCTCCGGCCACCACTGGTCCATATCGATACTCACGTGTCCGTCAGAGTAT
GTTCCCAGCTCACCTTTCAGCGATCCCTCATTAGATTCTTGCATCTTCATCAGCATGCAAAATCTTAA
+
FFFFFFFFFFFFFFFFFFFFFFFFFFFFFFFFFFFFFFFFFFFFFFFFFFFFFFFFFFFFFFFFFFFF
FFFFFFFFFFFFFFFFFFFFFFFFFFFFFFFFFFFFFFFFFFFFFFFFFFF:FFFFFFF:FFFFFFFF
@A00155:342:HHGFNDSXY:1:1335:7012:18928 1:N:0:GAACCTAG+TCCGCATA
TAGCCTCTTCTTCATATCTCTCCGGCCACCACTGGTCCATATCGATACTCACGTGTCCGTCAGAGTAT
GTTCCCAGCTCACCTTTCAGCGATCCCTCATTAGATTCTTGCATCTTCATCAGCATGCAAAATCTTAA
+
FFFFFFFFFFF:FFFFFFFFFFFFFFFFFFFFFFF:FFFFFFFFFFFFFFFFFFFFFFFFFFFFFFFF
FFFFFFFFFFFFFFFFFFFFFF,FFFFFFFFFFFFFFFFFFF,,:FFF:FFFFFFFFFFFFFFFFFFF
@A00155:342:HHGFNDSXY:1:2161:12825:3630 1:N:0:GAACCTAG+TCCGCATA
TAGCCTCTTCTTCATATCTCTCCGGCCACCACTGGTCCATATCGATACTCACGTGTCCGTCAGAGTAT
GTTCCCAGCTCACCTTTCAGCGATCCCTCATTAGATTCTTGCATCTTCATCAGCATGCAAAATCTTAA
+
FFFFFFFFFFFFFFFFFFFFFFFFFFFFFFFFFFFFFFFFFFFFFFFFFFFFFFFFFFFFFFFFFFFF
FFFFFFFFFFFFFFFFFFFFFFFFFFFFFFFFFFFFFFFFFFFFFFFFFFFFFFFFFFFFFFFFFFFF
@A00155:342:HHGFNDSXY:1:2172:16034:5024 1:N:0:GAACCTAG+TCCGCATA
TAGCCTCTTCTTCATATCTCTCCGGCCACCACTGGTCCATATCGATACTCACGTGTCCGTCAGAGTAT
GTTCCCAGCTCACCTTTCAGCGATCCCTCATTAGATTCTTGCATCTTCATCAGCATGCAAAATCTTAA
+
FFFF:FFFF:FFFFFFFF:FFF:FFFFFFFFFF:FFFFFFFFF:FFFFF:F,FFFFFF:FFFFFFFFF
FFFFFFFF:FFFFF:FFFFFFFFFFFFFFFFFF:F:FFFFFFFFFF:FFFFF:FFFFFFFFFFFFFFF
@A00155:342:HHGFNDSXY:1:2216:13250:33614 1:N:0:GAACCTAG+TCCGCATA
TAGCCTCTTCTTCATATCTCTCCGGCCACCACTGGTCCATATCGATACTCACGTGTCCGTCAGAGTAT
GTTCCCAGCTCACCTTTCAGCGATCCCTCATTAGATTCTTGCATCTTCATCAGCATGCAAAATCTTAA
+
FFFFFFFFFFFFFFFFFFFFFFFFFFFFFFFFFFFFFFFFFFFFFFFFFFFFFFFFFFFFFFFFFFFF
FFFFFFFFFFFFFFFFFFFFFFFFFFFFFFFFFFFFFFFFFFFFFFFFFFFFFFFFFFFFFFFFF:FF
@A00155:342:HHGFNDSXY:1:1378:29930:27743 1:N:0:GAACCTAG+TCCGCATA
TAGCCTCTTCTTCATATCTCTCCGGCCACCACTGGTCCATATCGATACTCACGTGTCCGTCAGAGTAT
GTTCCCAGCTCACCTTTCAGCGATCCCTCATTAGATTCTTGCATCTTCATCAGCATGCAAAATCTTAA
+
FFFFFFFFFFFFFFFFFF:FFFFFFFFFFFFFFFFFFFFFFFFFFFFFFFFFFFFFFFFFFFFFFFFF
FFFFFFFFFFFFFFFFFFFFFFFFFFFFFFFFFFFFFFFFFFFFFFFFFFFFFFFFFFFFFFFFFFFF
@A00155:342:HHGFNDSXY:1:2220:24379:14184 1:N:0:GAACCTAG+TCCGCATA
TAGCCTCTTCTTCATATCTCTCCGGCCACCACTGGTCCATATCGATACTCACGTGTCCGTCAGAGTAT
GTTCCCAGCTCACCTTTCAGCGATCCCTCATTAGATTCTTGCATCTTCATCAGCATGCAAAATCTTAA
+
FFFFFFFFFFFFFFFFFFFFFFFFFFFFFFFFFFFFFFFFFFFFFFFFFFFFFFFFFFFFFFFFFFFF
FFFFFFFFFFFFFFFFFFFFFF:FFFFFFFFFFFFFFFFFFFFFF:FFFFFFFFFFFFFFFFFFF:FF
@A00155:342:HHGFNDSXY:1:2204:16604:25958 1:N:0:GAACCTAG+TCCGCATA
TAGCCTCTTCTTCATATCTCTCCGGCCACCACTGGTCCATATCGATACTCACGTGTCCGTCAGAGTAT
GTTCCCAGCTCACCTTTCAGCGATCCCTCATTAGATTCTTGCATCTTCATCAGCATGCAAAATCTTAA
+
FFFFFFFFFFFFFFFFFFFFFFFFFFFFFFFFFFFFFFFFFFFFFFFFFFFFFFFFFFFFFFFFFFFF
F:FFFFFFFFFFFFFFFFFFFFF:FFFFFFFFFFFFFFFFFFFFFFFFF,:,FFF:FFF:FFF:FFFF
@A00155:342:HHGFNDSXY:1:1678:27715:24001 2:N:0:GAACCTAG+TCCGCATA
AGCCTCTTCTTCATATCTCTCCGGCCACCACTGGTCCATATCGATACTCACGTGTCCGTCAGAGTATG
TTCCCAGCTCACCTTTCAGCGATCCCTCATTAGATTCTTGCATCTTCATCAGCATGCAAA
+
FFFFFFFFFFFFFFFFFFFFFFFFFFF:FFFFFFFFFFFFFFFFFFFFFFFFFFFFFFFFFFFFFFFF
FFFFFFFFFFFFFFFFFFFFFFFFFFFFFFFFFFFFFFFFFFFFFFFFFFFFFFFFFFFF
```

```
@A00155:342:HHGFNDSXY:1:2246:25111:14888 1:N:0:GAACCTAG+TCCGCATA
AGCCTCTTCTTCATATCTCTCCGGCCACCACTGGTCCATATCGATACTCACGTGTCCGTCAGAGTATG
TTCCCAGCTCACCTTTCAGCGATCCCTCATTAGATTCTTGCATCTTCATCAGCATGCAAAATCT
+
FFFFFFFFFFFFFFFFFFFFFFFFFFFFFFFFFFFFFFFFFFFFFFFFFFFFFFFFFFFFFFFFFFFF
FFFFF:FFFFFFFFFFFFFFFFFFFFFFFFFFFFFFFFFFFFFF:FFFFFFFFFFFFF:FFFFF
@A00155:342:HHGFNDSXY:1:1548:12906:9095 1:N:0:GAACCTAG+TCCGCATA
AGCCTCTTCTTCATATCTCTCCGGCCACCACTGGTCCATATCGATACTCACGTGTCCGTCAGAGTATG
TTCCCAGCTCACCTTTCAGCGATCCCTCATTAGATTCTTGCATCTTCATCAGCATGCAAAATCTT
+
:FFFFFFFFFFFFFFFFFFFFFFFFFFFFFFFFFFFFFFFFFFFFFFFFFFFFFFFFFFF:FFFFFFF
FFFFFFFFFFFFFFF:FFFFFFFFFF:FFFFFFFFFFFFFFFFFFFFFFFF:F:FFFFFFFFFFF
@A00155:342:HHGFNDSXY:1:2624:17210:30420 1:N:0:GAACCTAG+TCCGCATA
AGCCTCTTCTTCATATCTCTCCGGCCACCACTGGTCCATATCGATACTCACGTGTCCGTCAGAGTATG
TTCCCAGCTCACCTTTCAGCGATCCCTCATTAGATTCTTGCATCTTCATCCGCATGCAAAATCTTAAC
+
FFFFFFFFFFFFFFFFFFFFFFFFFFFFFFFFFFFFFFFFFFFFFFFFFFFFFFFFFFFFFFFFFFFF
FFFFFFFFFFFFFFFFFFFFF:FFFFFFFFFFFFFFFFFFFFFFFFFFFF,FFFFFFFFFFFFFFFFF
@A00155:342:HHGFNDSXY:1:1604:26413:10473 2:N:0:GAACCTAG+TCCGCATA
GCCTCTTCTTCATATCTCTCCGGCCACCACTGGTCCATATCGATACTCACGTGTCCGTCAGAGTATGT
TCCCAGCTCACCTTTCAGCGATCCCTCATTAGATTCTTGCATCTTCATCAGCATGCAAA
+
FF:FF:FFFFFFFFFFFFFFFFFFFFFFFFFFFFFF:FFFFFFFFFFFFFFFFFFFFFFFFFFFFFF:
FFFFFFFFFFFFFFFFFFFFFFFFFFFFFFFFFFFFFFFFFFFFFFFFFFFFFFFFFFF
@A00155:342:HHGFNDSXY:1:1314:8350:8688 2:N:0:GAACCTAG+TCCGCATA
GCCTCTTCTTCATATCTCTCCGGCCACCACTGGTCCATATCGATACTCACGTGTCCGTCAGAGTATGT
TCCCAGCTCACCTTTCAGCGATCCCTCATTAGATTCTTGCATCTTCATCAGCATGCAAAA
+
FFFFFFFFFFFFFFFFFF:FFFFFF:FFFFFFFFFFFFFFFFF:FFFFFFFFFFFFFFFFFFFFFFF:
FFFFFFFFFFFFF:FFFFFFFFFFFFFFFFFFFFFF:FFFFFFFFFFFFFFFFFFFFFFF
@A00155:342:HHGFNDSXY:1:1303:8133:33019 1:N:0:GAACCTAG+TCCGCATA
GCCTCTTCTTCATATCTCTCCGGCCACCACTGGTCCATCTCGATACTCACGTGTCCGTCAGAGTATGT
TCCCAGCTCACCTTTCAGCGATCCCTCATTAGATTCTTGCATCTTCATCAGCATGCAAAATCTT
+
FFFFF:FFFFFFFFFFFFFF:FFFFFFFFFFFFFFFF,,FFFFFFFFFFFFFFFFFFFFFFFFFFFFF
FFFFFFFFFFFFFFFFFFFFFFFF:FFFFFFFFFFFFFFFFFFFFFFFFFFFFFFFFFFFFFFF
@A00155:342:HHGFNDSXY:1:1371:24596:10457 1:N:0:GAACCTAG+TCCGCATA
GCCTCTTCTTCATATCTCTCCGGCCACCACTGGTCCATATCGATACTCACGTGTCCGTCAGAGTATGT
TCCCAGCTCACCTTTCAGCGATCCCTCATTAGATTCTTGCATCTTCATCAGCATGCAAAATCTTAA
+
FFFFF::F,FFF:F,FFFFFFFF:FFFFFFFFFFFFFFFFFFFF:FFF:F:FFFFFFFFFFFFF:FFF
FF::FF,,FFFFF,FFFF:FFFF::FFFFFFFF:F:FF:FFFFFFFFFFFFFF:FFFFFF:FFFFF
@A00155:342:HHGFNDSXY:1:2373:11216:7952 1:N:0:GAACCTAG+TCCGCATA
GCCTCTTCTTCATATCTCTCCGGCCACCACTGGTCCATATCGATACTCACGTGTCCGTCAGAGTATGT
TCCCAGCTCACCTTTCAGCGATCCCTCATTAGATTCTTGCATCTTCATCAGCATGCAAAATCTTAAC
+
FFFFFFFFFFFFFFFFFFFFFFFFFFFFFFFFFFFFFFFFFFFFFFFFFFFFFFFFFFFFFFFFFFFF
FFFFFFFFFFFFFFFFFFFFFFFFFFFFFFFFFFFFFFFFFFFFFFFFFFFFFFFFFFFFFFFFFFF
@A00155:342:HHGFNDSXY:1:1303:4408:14481 1:N:0:GAACCTAG+TCCGCATA
GCCTCTTCTTCATATCTCTCCGGCCACCACTGGTCCATATCGATACTCACGTGTCCGTCAGAGTATGT
TCCCAGCTCACCTTTCAGCGATCCCTCATTAGATTCTTGCATCTTCATCAGCATGCAAAATCTTAAC
+
FFFFFFFFFFFFFFFFFFF::FFFFFFFFFF,,F:FFFFFF,FFFFF:FFF:FF,FFFFFFFFFFFFF
FFFFFFFFFFFFFFFFFFFFFFF:::FFFFFFFFFFFF,FFFFFFFFFFFFFFFFFFFFFFFFFFFF
```

```
@A00155:342:HHGFNDSXY:1:1444:8097:18239 1:N:0:GAACCTAG+TCCGCATA
GCCTCTTCTTCATATCTCTCCGGCCACCACTGGTCCATATCGATACTCACGTGTCCGTCAGAGTATGT
TCCCAGCTCACCTTTCAGCGATCCCTCATTAGATTCTTGCATCTTCATCAGCATGCAAAATCTTAAC
+
FFFFFFFFFFFFFFFFFFFFFFFFFFFFFFFFFFFFFFFFF::FFFFFFFFFFFFFFFFFFFFFFFFF
FFFFFFFFFFFFFFFFFFFFFFFFFFFFFFFFFFFFFFFFFFFFFFFFFFFFFFFFF:FFFFFFFFF
@A00155:342:HHGFNDSXY:1:1405:10782:33912 1:N:0:GAACCTAG+TCCGCATA
GCCTCTTCTTCATATCTCTCCGGCCACCACTGGTCCATATCGATACTCACGTGTCCGTCAGAGTATGT
TCCCAGCTCACCTTTCAGCGATCCCTCATTAGATTCTTGCATCTTCATCAGCATGCAAAATCTTAAC
+
FFFFFF:FFFFFFFFFFFFFFFFFFFFFFFFFFFFFFFFFFFFFFFFFFFFFFFFFFFFFFFFFFFF:
FFFFFFFFFFFFFFFF:FFFFFFFFFFFFFFFFFFFFFFF,FF:FFFFFFFF:FFFFFFFFFFFFFF
@A00155:342:HHGFNDSXY:1:2207:19678:15029 1:N:0:GAACCTAG+TCCGCATA
GCCTCTTCTTCATATCTCTCCGGCCACCACTGGTCCATATCGATACTCACGTGTCCGTCAGAGTATGT
TCCCAGCTCACCTTTCAGCGATCCCTCATTAGATTCTTGCATCTTCATCAGCATGCAAAATCTTAACA
+
FFFFFFFFFFFF:FFFFFFFFFFFFFFFFFFF:FFFFFFFFFFFFF,FFFFFFFFFFFFFFFF:,FFF
FFFFFFFFFFFF:,FFFFFFFF,FFFFFFFFFFFFFFFFF,F:F:F,FFFFFFFFFFFFFFFF:FFF:
@A00155:342:HHGFNDSXY:1:2543:5484:1376 1:N:0:GAACCTAG+TCCGCATA
GCCTCTTCTTCATATCTCTCCGGCCACCACTGGTCCATATCGATACTCACGTGTCCGTCAGAGTATGT
TCCCAGCTCACCTTTCAGCGATCCCTCATTAGATTCTTGCATCTTCATCAGCATGCAAAATCTTAACA
+
FFFFFFFFFFFFFFFFFFFFFFFFFFFFFFF:FFFFFFFF:FFFFFFFFFFF:FFFFFFF:FFFFFFF
FFFFF,FFFF,FF:FFFFF::FFFFFFFFFFFFFFFFFFF:FFFFFFFFFFFFFFFFFFFFFFFFFFF
@A00155:342:HHGFNDSXY:1:1124:2220:31203 1:N:0:GAACCTAG+TCCGCATA
GCCTCTTCTTCATATCTCTCCGGCCACCACTGGTCCATATCGATACTCACGTGTCCGTCAGAGTATGT
TCCCAGCTCACCTTTCAGCGATCCCTCATTAGATTCTTGCATCTTCATCAGCATGCAAAATCTTAACA
+
FFFFFFFFFFFFFFFFFFFFFFFFFFFFFFF:FFFFFFFFFFFFFFFFFFFFFFFFFFFFFFFFFFFF
F:FFFFFFFFFFFFFFFFFF:FFFFFFFFFFFFFFFFFF:FFFFFFFFFFFFF,FFFFFFFFFFFFF:
@A00155:342:HHGFNDSXY:1:2231:16929:28964 1:N:0:GAACCTAG+TCCGCATA
GCCTCTTCTTCATATCTCTCCGGCCACCACTGGTCCATATCGATACTCACGTGTCCGTCAGAGTATGT
TCCCAGCTCACCTTTCAGCGATCCCTCATTAGATTCTTGCATCTTCATCAGCATGCAAAATCTTAACA
+
FFFFFFFFFFFFFFFFFFF:FFFFFFFFFFFFFFFFFFFFFFFFFFFFFFFFFFFFFFFFFFFFFFFF
FFFFFFFFFFFFFFFFFFFF::FFF:FFFFFFFFFFF:FF:FFFFFF,FFFFFFFFF:FFFFFFFFF:
@A00155:342:HHGFNDSXY:1:2362:26820:22482 1:N:0:GAACCTAG+TCCGCATA
GCCTCTTCTTCATATCTCTCCGGCCACCACTGGTCCATATCGATACTCACGTGTCCGTCAGAGTATGT
TCCCAGCTCACCTTTCAGCGATCCCTCATTAGATTCTTGCATCTTCATCAGCATGCAAAATCTTAACA
+
FFFFFFFFFFFFFFFFFFFFFFFF:FFFFFFFFFFFFFFFFFFFFFFFFFFFFFFFFFFFFFFFFFFF
FFFFFFFFFFFFFFFFFFFFF:FFFFFF:FFFFFFFFFFFFFFFFFFFFFFFFFFFFFFFF:FFFFF:
@A00155:342:HHGFNDSXY:1:2640:21251:25394 2:N:0:GAACCTAG+TCCGCATA
CTCTTCTTCATATCTCTCCGGCCACCACTGGTCCATATCGATACTCACGTGTCCGTCAGAGTATGTTC
CCAGCTCACCTTTCAGCGATCCCTCATTAGATTCTTGCATCTTCATCAGCATGCAAA
+
FFFFFFFFFFFFFFFFFFFFFFFFFFFFFFFFFFFFFFFFFFFFFFFFFFFFF:FFFFFFFFFFF:FF
FFFFFFFFFFFFFFFFFFFFFFFFFFFFFFFFFFFFFFFFFFFFFFFFFFFFFFFFF
@A00155:342:HHGFNDSXY:1:1549:14091:28682 2:N:0:GAACCTAG+TCCGCATA
CTCTTCTTCATATCTCTCCGGCCACCACTGGTCCATATCGATACTCACGTGTCCGTCAGAGTATGTTC
CCAGCTCACCTTTCAGCGATCCCTCATTAGATTCTTGCATCTTCATCAGCATGCAAA
+
FFFFFFFFFFFFFFFFFFFFFFFFFFFFFFFFFFFFFFFFFFFFFFFFFFFFFFFFFFFFFFFFFFFF
FFFFFFFFFFFFFFFFFFFFFFFF:FFFFFFFFFFFFFFFFFFFFFFFFFFFFFFFF
```

```
@A00155:342:HHGFNDSXY:1:1460:18530:10755 2:N:0:GAACCTAG+TCCGCATA
CTCTTCTTCATATCTCTCCGGCCACCACTGGTCCATATCGATACTCACGTGTCCGTCAGAGTATGTTC
CCAGCTCACCTTTCAGCGATCCCTCATTAGATTCTTGCATCTTCATCAGCATGCAAA
+
FFFFF:FFFFFFFFFFFFFFFFFFFFFFFFFFFFFFFFFFFF:FFFFFFFFFFFFFFFFFFFFFFFFF
FFFFFFFFFFFFFFFFFFFFFFFFF:FFFFFFFFFFFFFFFFFFFFFFFFFFFFFFF
@A00155:342:HHGFNDSXY:1:2231:16929:28964 2:N:0:GAACCTAG+TCCGCATA
CTTCTTCATATCTCTCCGGCCACCACTGGTCCATATCGATACTCACGTGTCCGTCAGAGTATGTTCCC
AGCTCACCTTTCAGCGATCCCTCATTAGATTCTTGCATCTTCATCAGCATGCAAA
+
FFFFFFFFFFFFFFFFFFFFFFFFFFFFFFFFFFFFFFFFFFF:FFFFFFFFF:FFFFFFFFF:FFFF
FFFFFFFFFFFFFFF:FFFFFFFFF:FFFFFF:FFFF:F:FFFFFFFFFFFFFFF
@A00155:342:HHGFNDSXY:1:1622:15492:28103 2:N:0:GAACCTAG+TCCGCATA
TCTTCATATCTCTCCGGCCACCACTGGTCCATATCGATACTCACGTGTCCGTCAGAGTATGTTCCCAG
CTCACCTTTCAGCGATCCCTCATTAGATTCTTGCATCTTCATCAGCATGCAAAATCTTAACATATTAA
+
FFF:FFFFFFFFFFFFFFFFFF:FFFFFFFFF:FFFFFFFFFFFFFFFFFFFFFFFFFFFFFFFFFFF
FFFFFFFFFF:FFFFFFFFFFFFFFFFF:FFFFFFFFFFFFFFFFFFFFFFFFF:FFFFFFFFFFFFF
@A00155:342:HHGFNDSXY:1:2634:4029:23030 2:N:0:GAACCTAG+TCCGCATA
TCTTCATATCTCTCCGGCCACCACTGGTCCATATCGATACTCACGTGTCCGTCAGAGTATGTTCCCAG
CTCACCTTTCAGCGATCCCTCATTAGATTCTTGCATCTTCATCAGCATGCAAAATCTTAACATATTAA
+
FFFFFFFFF:FFFFFFFFFF:FF:F:F:FFFFFFFFFFFFFFFFFFFFFFFFFFFFFFFFFFFFFFFF
FFFFFFFFFFFFFFFFFFFFFFFFFFFFFFFFFFFFFFFFFFFFFFFFFFFFFFFFFFFFFFFFFFFF
@A00155:342:HHGFNDSXY:1:1611:6705:19460 2:N:0:GAACCTAG+TCCGCATA
CTTCATATCTCTCCGGCCACCACTGGTCCATATCGATACTCACGTGTCCGTCAGAGTATGTTCCCAGC
TCACCTTTCAGCGATCCCTCATTAGATTCTTGCATCTTCATCAGCATGCAAAATCTTAACATATTAA
+
FFFFFFFFFFF:FF:FFFFFFFFFFFFFFFFFF:FFFFFFFFFFFFFFFFFFFFFFFFFFFFFFFFFF
FFFFFFFF:FF:FFFFFFF:FFFFFFFFFFFF:FFFFFFFFFF:FFFF:FFFFFFFFFFFFFFFF::
@A00155:342:HHGFNDSXY:1:1611:6424:19664 2:N:0:GAACCTAG+TCCGCATA
CTTCATATCTCTCCGGCCACCACTGGTCCATATCGATACTCACGTGTCCGTCAGAGTATGTTCCCAGC
TCACCTTTCAGCGATCCCTCATTAGATTCTTGCATCTTCATCAGCATGCAAAATCTTAACATATTAAC
+
FFFFFFFFFFFFFFFF:FFFFFFFFFFFF:FFFFFFFFFFFFFFF:FFFFFFF,FFFFFFFFFFFFFF
FFFFFFFFFFF,FFFFFFFFFFFFFFFFFFFFFFFFFFFFFFFFFFFFFFFFFFFFFFFFFFFFFFFF
@A00155:342:HHGFNDSXY:1:1611:6406:19570 2:N:0:GAACCTAG+TCCGCATA
CTTCATATCTCTCCGGCCACCACTGGTCCATATCGATACTCACGTGTCCGTCAGAGTATGTTCCCAGC
TCACCTTTCAGCGATCCCTCATTAGATTCTTGCATCTTCATCAGCATGCAAAATCTTAACATATTAAC
+
FFFFFFFFFFFFFFFFFFFFFFFFFFFFFFFFFFFFFFFFFFFFFFFFFFFFFFFFFF:FFFFFFFFF
FFFFFFFFFFFFFFFFFFFFFFFFFFFFFFFFFFFFFFFFFFFFFFFFFFFFFFFFFFFFFFFFFF::
@A00155:342:HHGFNDSXY:1:1417:5493:5274 2:N:0:GAACCTAG+TCCGCATA
TTCATATCTCTCCGGCCACCACTGGTCCATATCGATACTCACGTGTCCGTCAGAGTATGTTCCCAGCT
CACCTTTCAGCGATCCCTCATTAGATTCTTGCATCTTCATCAGCATGCAAAATCTTAACATATTAACA
+
FFFFFFFFFFFF,FFFFFFFFFF:FFFFFF:FFFFFFFF:FFFFFFFFFFFFFFFFFFFFFFFFFFFF
FFFFFFFFFFFFFFFFFF,FF:FFFFFF:FFFFFFFFFFFFFFFFFFFFFFFFFFFFFFFF,FFF:FF
@A00155:342:HHGFNDSXY:1:1677:20302:7185 1:N:0:GAACCTAG+TCCGCATA
TCATATCTCTCCGGCCACCACTGGTCCATATCGATACTCACGTGTCCGTCAGAGTATGTTCCCAGCTC
ACCTTTCAGCGATCCCTCATTAGATTCTTGCATCTTCATCAGCATGCAAAATCTTAAC
+
FFFFFFFFFFFFFFFFFFFFFFFFFFFFFFFFFFFFFFFFFFFFFFFFFFFFFFFFFF:FFFFFFFFF
FFFFFFFFFFFFFFFFFFFFFF,FFFFFFFFFFFFFFFFFFFFFFFFF:FFFFFFFFF
```

```
@A00155:342:HHGFNDSXY:1:1655:32796:14168 1:N:0:GAACCTAG+TCCGCATA
TCATATCTCTCCGGCCACCACTGGTCCATATCGATACTCACGTGTCCGTCAGAGTATGTTCCCAGCTC
ACCTTTCAGCGATCCCTCATTAGATTCTTGCATCTTCATCAGCATGCAAAATCTTAACATATTA
+
FFFFFFFFFFFFFFFFFFFFFFFFFFFFFFFFFFFFFFFFFFFFFFFFFFFFFFFFFFFFFFFFFFFF
FFFFFFFFFFFFFFFFFFFFFFFFFFFFFFFFFFFFFFFFFFFFFFFFFFFFFFFFFF:FFFFF
@A00155:342:HHGFNDSXY:1:2271:2275:26412 1:N:0:GAACCTAG+TCCGCATA
TCATATCTCTCCGGCCACCACTGGTCCATATCGATACTCACGTGTCCGTCAGAGTATGTTCCCAGCTC
ACCTTTCAGCGATCCCTCATTAGATTCTTGCATCTTCATCAGCATGCAAAATCTTAACATATTA
+
FFFFFFFFFFFFFFFFFFFFFFF,:FFFFFFFFFFFFFF:FFFFFF:FFFFF:FFFFFFFFFF:FFFF
F:FFFFFFF:FFFFFFFFF:FF,:FFFFFF,:F:FF:FFFFF::FF,FFFFFFFFFFF:FFFFF
@A00155:342:HHGFNDSXY:1:1659:13386:26115 2:N:0:GAACCTAG+TCCGCATA
TCATATCTCTCCGGCCACCACTGGTCCATATCGATACTCACGTGTCCGTCAGAGTATGTTCCCAGCTC
ACCTTTCAGCGATCCCTCATTAGATTCTTGCATCTTCATCAGCATGCAAAATCTTAACATATTA
+
FFFFFFF:FFFFFFFFFFFFFFFFFFFFFFFFFFFFFFFFFFFFFFFFFFFFFFFFFFFFFFFFFFFF
FFFFFFFFFFFFFFFFFFFFFFFFFFFFFFFFFFFFFFFFFFFF:FFFFFFFFF,F:F:FFFFF
@A00155:342:HHGFNDSXY:1:1611:12563:7185 2:N:0:GAACCTAG+TCCGCATA
TCATATCTCTCCGGCCACCACTGGTCCATATCGATACTCACGTGTCCGTCAGAGTATGTTCCCAGCTC
ACCTTTCAGCGATCCCTCATTAGATTCTTGCATCTTCATCAGCATGCAAAATCTTAACATATTAACAA
+
FFFFFFFFFFFFFFFFFFFFFFFFFFFFFFFFFFFFFFFFFFFFFFFFFFFFFFFFFFFFFFFFFFFF
FF:FFFFFFFFFFFFFFFFFFFFFFFFFFFFFFFFFFFFFFFFFFFFFFFFFF:FFFFFFFFFFFFFF
@A00155:342:HHGFNDSXY:1:1568:4779:13714 2:N:0:GAACCTAG+TCCGCATA
CATATCTCTCCGGCCACCACTGGTCCATATCGATACTCACGTGTCCGTCAGAGTATGTTCCCAGCTCA
CCTTTCAGCGATCCCTCATTAGATTCTTGCATCTTCATCAGCATGCAAAATCTTAACATATTAACAAG
+
FFF:FFFFFFFFFFFFFFFFFFFFFFFFFFFFFFFFFFFFF,FFFFFFFFFFFFFFF:FFFFFFFFFF
FFFFFFFFFFFFFFFFFFFFFFFFF,FFFFFFFFFFFFFFFFFFFF:FFF,FFFFFFFFFFFFFFFFF
@A00155:342:HHGFNDSXY:1:1568:2871:12446 2:N:0:GAACCTAG+TCCGCATA
CATATCTCTCCGGCCACCACTGGTCCATATCGATACTCACGTGTCCGTCAGAGTATGTTCCCAGCTCA
CCTTTCAGCGATCCCTCATTAGATTCTTGCATCTTCATCAGCATGCAAAATCTTAACATATTAACAAG
+
FFFFFFFFFFFFFFFFFFFFFFFFFFFFFFFFFFFFFFFFFFFFFFFFFFFFFFFFF:FFFFFFFFFF
FF:FFFFFFFFFFFFFFFFFFFFFF:FFFFFFFFFFFFFFFFFFFF:FFF:FFF:FFFFFFFFFFFFF
@A00155:342:HHGFNDSXY:1:2636:29957:28103 2:N:0:GAACCTAG+TCCGCATA
ATCTCTCCGGCCACCACTGGTCCATATCGATACTCACGTGTCCGTCAGAGTATGTTCCCAGCTCACCT
TTCAGCGATCCCTCATTAGATTCTTGCATCTTCATCAGCATGCAAAATCTTAACATATTAACAAGCAG
+
FFFFFF:FF:FFF:FF:F::F:F,FFFF:FFFFFFF,FFFFF:FFFFF,FFFFF,FFF:FF,F:FFFF
FFFFFFFFFFFFFF:FFF:FFFFFFFFFF:FFFFFF::FFFF:FFFF:FFFF:FFFFFF:FFFF:FFF
@A00155:342:HHGFNDSXY:1:2521:21820:8656 2:N:0:GAACCTAG+TCCGCATA
ATCTCTCCGGCCACCACTGGTCCATATCGATACTCACGTGTCCGTCAGAGTATGTTCCCAGCTCACCT
TTCAGCGATCCCTCATTAGATTCTTGCATCTTCATCAGCATGCAAAATCTTAACATATTAACAAGCAG
+
FFFFFFFFFFFFFFFFFFFFFFFFFFFFFFFFF:FFFFFFFFFFFFFFFFFFFFFFFFFFFFFFFFFF
FFFFFFFFFFFFFFFFFFFFFFFFFFFFFF:FFFFFFFFFFFFFFFFFFFFFFFFFFFFFFFFFFFFF
@A00155:342:HHGFNDSXY:1:1344:22227:14215 1:N:0:GAACCTAG+TCCGCATA
TCTCTCCGGCCACCACTGGTCCATATCGATACTCACGTGTCCGTCAGAGTATGTTCCCAGCTCACCTT
TCAGCGATCCCTCATTAGATTCTTGCATCTTCATCAGCATGCAAAATCTTAACATATTAACAAGCAGT
+
FFFF:FFFF:FFFFFF:FFFFFFFFFFFFFFFFFFF:FFFFFFFFFF:FFFF:FFFFFFFFFFFFFFF
FFFFFFF::FFFFFFFFFFFFFFFFFFFFFF,FFF,F:FFFFFFFFFFFFFFF::FFFFFFFF,FFFF
```

```
@A00155:342:HHGFNDSXY:1:1314:8350:8688 1:N:0:GAACCTAG+TCCGCATA
CTCTCCGGCCACCACTGGTCCATATCGATACTCACGTGTCCGTCAGAGTATGTTCCCAGCTCACCTTT
CAGCGATCCCTCATTAGATTCTTGCATCTTCATCAGCATGCAAAATCTTAACATATTAAC
+
FFFFFFFFFFFFFFFFFFFFFFFFFFFFFFFFFFFFFFFFFFFFFFFFFFFFFFFFFFFFFFFFFFFF
FFFFFFFFFFFFFFFFFFFFFFFFFFFFFFFFFFFFFFFFFFFFFFFFFFFFFFFFFFFF
@A00155:342:HHGFNDSXY:1:1604:26413:10473 1:N:0:GAACCTAG+TCCGCATA
CTCTCCGGCCACCACTGGTCCATATCGATACTCACGTGTCCGTCAGAGTATGTTCCCAGCTCACCTTT
CAGCGATCCCTCATTAGATTCTTGCATCTTCATCAGCATGCAAAATCTTAACATATTAACAAGCAG
+
FFFFFFFFFFFFFFFFFFFFFFFFFFFFFFFFFFFFFFFFFFFFFFFFFFFFFFFFFFFFFFFFFFFF
FFFFFFFFFFFFFFFFFFFFFFFF::FFFFF:FF,FFFFFFFFFFFFFFFFFFFFFFF:FFFFFFF
@A00155:342:HHGFNDSXY:1:2472:12427:12868 2:N:0:GAACCTAG+TCCGCATA
CTCTCCGGCCACCACTGGTCCATATCGATACTCACGTGTCCGTCAGAGTATGTTCCCAGCTCACCTTT
CAGCGATCCCTCATTAGATTCTTGCATCTTCATCAGCATGCAAAATCTTAACATATTAACAAGCAGTG
+
FFFFFFFFFFFFFFFFFFFFFFFFFFFFFFFFFFFFFFFFFF:FFFFFFFFFFFFFFFFFFFFFFFFF
FFFFFFFFFFFFFFFFFFFF:FFFFFFFFFFFFFFFFFFFFFFFFFFFFFFFFFFFFFFF,FFFFFFF
@A00155:342:HHGFNDSXY:1:2304:6551:17472 1:N:0:GAACCTAG+TCCGCATA
CTCTCCGGCCACCACTGGTCCATATCGATACTCACGTGTCCGTCAGAGTATGTTCCCAGCTCACCTTT
CAGCGATCCCTCATTAGATTCTTGCATCTTCATCAGCATGCAAAATCTTAACATATTAACAAGCAGTG
+
FFFFFFFFFFFFFFFFFFFFF:FFFF:FFF,FFFF,FFFFFFFFFFFFFFFF:FFFFFFFFFFFFF:F
FFFF:::FFF:FF::FFFF:,F:FFFFF,FFF:F,F:,FFFF,:FFFFFF:FFFF:F::FFF,FFFFF
@A00155:342:HHGFNDSXY:1:1432:28890:33207 2:N:0:GAACCTAG+TCCGCATA
CTCTCCGGCCACCACTGGTCCATATCGATACTCACGTGTCCGTCAGAGTATGTTCCCAGCTCACCTTT
CAGCGATCCCTCATTAGATTCTTGCATCTTCATCAGCATGCAAAATCTTAACATATTAACACGCAGTG
+
:FFFFFFFFFFFFFFFFFFFFFFFFFFFFF:FF:F:FF:FFFFF:FFFFFFFFFFFF:FFFF,FFFFF
FFFF:FFF,FFFFF:FFF:FF:FFFFFFFF,FF,FFFFFFFFFFFFFFFFFFFF:FF,FF,,FFFFFF
@A00155:342:HHGFNDSXY:1:2174:8648:8578 2:N:0:GAACCTAG+TCCGCATA
CTCTCCGGCCACCACTGGTCCATATCGATACTCACGTGTCCGTCAGAGTATGTTCCCAGCTCACCTTT
CAGCGATCCCTCATTAGATTCTTGCATCTTCATCAGCATGCAAAATCTTAACATATTAACAAGCAGTG
+
FFFFFFFFFFFFFFFFFFFFFFFFFFFFFFFFFFFFFFFFFFFFFFFFFFFFFFFFF:FFFFFFFFFF
FFFFFFFFFFFFFFFFFFFFFFFF:FFFFFFFFFFFFFFFFFFFFFFFFFFFFFF:F:FFFFFFF::F
@A00155:342:HHGFNDSXY:1:2239:10899:17300 1:N:0:GAACCTAG+TCCGCATA
CTCTCCGGCCACCACTGGTCCATATCGATACTCACGTGTCCGTCAGAGTATGTTCCCAGCTCACCTTT
CAGCGATCCCTCATTAGATTCTTGCATCTTCATCAGCATGCAAAATCTTAACATATTAACAAGCAGTG
+
FFFFFFFFFFFFFFFFFFFFFFFFFFFFFFFFFFFFFFFFFFFFFFFFFFFFFFFFFFFFFFFFFFFF
FFFFF:,FFFFFFFFFFFFFFFFFFFFFFFFFFFFFFFFFFFFFFFFFFFF:FFFFF:FFFFFFFFF
@A00155:342:HHGFNDSXY:1:2153:8983:32456 2:N:0:GAACCTAG+TCCGCATA
CTCTCCGGCCACCACTGGTCCATATCGATACTCACGTGTCCGTCAGAGTATGTTCCCAGCTCACCTTT
CAGCGATCCCTCATTAGATTCTTGCATCTTCATCAGCATGCAAAATCTTAACATATTAACAAGCAGTG
+
FFFFFFFFFFFFFFFFFFFFFFFFFFFFFFFFFFFFFFFFFFFFFFFFFFFFFFFFFFFFFFFFFFFF
FFFFFFFFFFFFFFFFFFFFFFFFFFFFFFFFFFFFFFFFFFFFF:FFFFFFFFFFFFFF::FFFFFF
@A00155:342:HHGFNDSXY:1:2224:7464:8907 1:N:0:GAACCTAG+TCCGCATA
CTCTCCGGCCACCACTGGTCCATATCGATACTCACGTGTCCGTCAGAGTATGTTCCCAGCTCACCTTT
CAGCGATCCCTCATTAGATTCTTGCATCTTCATCAGCATGCAAAATCTTAACATATTAACAAGCAGTG
+
FFFFFFFFFFFFFFFFFFFFFFFFFFFFFFFFFFFFFFFFFFFFFFFFFFFFFFFFFFFFFFFF:FFF
FFFFFFFFFFFFFFFFFFFFFFFFFFFFFFFFFFFFFFFFFFFFFFF:F:FF:FFFFFF,FFFFFFF:
```

```
@A00155:342:HHGFNDSXY:1:1611:6406:19570 1:N:0:GAACCTAG+TCCGCATA
CTCTCCGGCCACCACTGGTCCATATCGATACTCACGTGTCCGTCAGAGTATGTTCCCAGCTCACCTTT
CAGCGATCCCTCATTAGATTCTTGCATCTTCATCAGCATGCAAAATCTTAACATATTAACAAGCAGTG
+
FFFFFFFFFFFFFFFFFFFFFFFFFFFFFFFFFFFFFF:FFFFFFFFFFFFFFFFFFFFFFFFFFFFF
FFFFFFFFFFFFFFFFFFFFFFFF,,F:FFFFFF,FFFFFFFFFFFFF,FFFFFFFFFFFFFF,FFFF
@A00155:342:HHGFNDSXY:1:1611:6424:19664 1:N:0:GAACCTAG+TCCGCATA
CTCTCCGGCCACCACTGGTCCATATCGATACTCACGTGTCCGTCAGAGTATGTTCCCAGCTCACCTTT
CAGCGATCCCTCATTAGATTCTTGCATCTTCATCAGCATGCAAAATCTTAACATATTAACAAGCAGTG
+
FFFF:FFFFFFFFFFFFFFFFFFFFFFFFFFFFFF,FFFFFFFFFFFFFFFFFFFFFFF,FFFFFFFF
FFFF::,FFFFFFFFFFFFFFFFFF:FFFFF::FFFFFFFFFFFFFFFFFFFFFFFFFFFFFFFFFFF
@A00155:342:HHGFNDSXY:1:1142:24370:16924 1:N:0:GAACCTAG+TCCGCATA
CTCTCCGGCCACCACTGGTCCATATCGATACTCACGTGTCCGTCAGAGTATGTTCCCAGCTCACCTTT
CAGCGATCCCTCATTAGATTCTTGCATCTTCATCAGCATGCAAAATCTTAACATATTAACAAGCAGTG
+
FFFFFFFFFFFFFFFFFFFFFFFFFFFFFFFFFFFFFFFFFFFFFFFFFFFFFFFFFFFFFFFFFFFF
FFFFFFFF:FFFFFFFFFFFFFFFF:FFFFFFFFFFFFFFFFFFFFFFFFFF:FFFFFFFFF:FFFFF
@A00155:342:HHGFNDSXY:1:1221:1307:14215 2:N:0:GAACCTAG+TCCGCATA
CTCTCCGGCCACCACTGGTCCATATCGATACTCACGTGTCCGTCAGAGTATGTTCCCAGCTCACCTTT
CAGCGATCCCTCATTAGATTCTTGCATCTTCATCAGCATGCAAAATCTTAACATATTAACACGCAGTG
+
FFF:FFFFFFFFFFFFFFFFFFFFFFFFFFFFF:F::FFFFFFFFFFFFFFF,FFFFFFFFFFFF:FF
FFFFFFFFFFFFFFFFFFFFFFFFFFFF:FFFFFFFFFFFFFFFFFFF:FFFF:FF:F,FF,FFFFFF
@A00155:342:HHGFNDSXY:1:1622:3658:12493 1:N:0:GAACCTAG+TCCGCATA
CTCTCCGGCCACCACTGGTCCATATCGATACTCACGTGTCCGTCAGAGTATGTTCCCAGCTCACCTTT
CAGCGATCCCTCATTAGATTCTTGCATCTTCATCAGCATGCAAAATCTTAACATATTAACAAGCAGTG
+
FFFFFFFFFF:FFFFFFFFFFFFFFFFFFFFFFFFFFFFFFFFFFFFFFFFFF:FFFFFFFFFFFFFF
FFFFF,FFFFFFFFFFFFFFFFFFFFFFFFFFFFFFFFFFF:FFFFFFFF:F:FFFFFFFFFFF:FFF
@A00155:342:HHGFNDSXY:1:1157:32588:25050 1:N:0:GAACCTAG+TCCGCATA
CTCTCCGGCCACCACTGGTCCATATCGATACTCACGTGTCCGTCAGAGTATGTTCCCAGCTCACCTTT
CAGCGATCCCTCATTAGATTCTTGCATCTTCATCAGCATGCAAAATCTTAACATATTAACAAGCAGTG
+
FFFFFFFFFFFFFFFFFFFFFFFFFFFFF:FFFFFFFFFFFFFFFFFFFFFFFFFFFFFFFFFFFFFF
FFFFF::FFFFFFFFFFFFFFFFFF,:FFFFFFF,FFFFFFFFFFFFFFFFF:FFFFFFFFFFFFFFF
@A00155:342:HHGFNDSXY:1:1157:32588:25050 2:N:0:GAACCTAG+TCCGCATA
CTCTCCGGCCACCACTGGTCCATATCGATACTCACGTGTCCGTCAGAGTATGGTCCCAGCTCACCTTT
CAGCGATCCCTCATTAGATTCTTGCATCTTCATCAGCATGCAAAATCTTAACATATTAACAAGCAGTG
+
FFFFFFFFFFFFFFFFFFFFFFFF:FFFFFFFFFFF:F:FFFFFFFFFF:FF,FFFFFFFFFFFFFFF
FFFFFFFFFFFFFFFFFFFFFFFFFFFFFFFFFFFFFFFFFFFFFFFFFFFFFFFFF,FF,FFF,FFF
@A00155:342:HHGFNDSXY:1:1673:22625:16000 1:N:0:GAACCTAG+TCCGCATA
CTCTCCGGCCACCACTGGTCCATATCGATACTCACGTGTCCGTCAGAGTATGTTCCCAGCTCACCTTT
CAGCGATCCCTCATTAGATTCTTGCATCTTCATCAGCATGCAAAATCTTAACATATTAACAAGCAGTG
+
FFFFFFFFFFFFFFFFFFFFFFFFFFFFFFFFFFFFFFFFFFFFFFFFFFFFFFFFFFFFFFFFFFFF
FFFFFFFFFFFFFFFFFFFFFFFFFFFFFFFFFFFFFFF:FFFFFFFFFF,FFFFFFFFFFFFFFFFF
@A00155:342:HHGFNDSXY:1:1211:21160:25081 1:N:0:GAACCTAG+TCCGCATA
CTCTCCGGCCACCACTGGTCCATATCGATACTCACGTGTCCGTCAGAGTATGTTCCCAGCTCACCTTT
CAGCGATCCCTCATTAGATTCTTGCATCTTCATCAGCATGCAAAATCTTAACATATTAACAAGCAGTG
+
FFFFFFFFFFFFFFFFFFFFFFFFFFFFFFFFFFFFFFFFFFFFFFFFFFFFF:FFFFFFFFFFFFFF
FF::FFFFFFFFFFFFFFFFFFFF:,F:FFF:FF,FFF:FFFFFF,FFFFFFFFFFFFFFFFFFFF:F
```

```
@A00155:342:HHGFNDSXY:1:2634:4029:23030 1:N:0:GAACCTAG+TCCGCATA
CTCTCCGGCCACCACTGGTCCATATCGATACTCACGTGTCCGTCAGAGTATGTTCCCAGCTCACCTTT
CAGCGATCCCTCATTAGATTCTTGCATCTTCATCAGCATGCAAAATCTTAACATATTAACAAGCAGTG
+
FFFF:FFFFFFFFFFFFFFFFFFFFFFFFFFFFFFFFFFFFFFFFFFFFFFFFFFFFFFFFFFFFFF:
FF:FFFFFFF:FFFFFFFFFFFFF:FF:FFFFFF:FFFFFFFFFF,FFF:FFFFFFFFFFFFF,FF,F
@A00155:342:HHGFNDSXY:1:1221:1307:14215 1:N:0:GAACCTAG+TCCGCATA
CTCTCCGGCCACCACTGGTCCATATCGATACTCACGTGTCCGTCAGAGTATGTTCCCAGCTCACCTTT
CAGCGATCCCTCATTAGATTCTTGCATCTTCATCCGCATGCAAAATCTTAACATATTAACAAGCAGTG
+
FFFFFFFFFFFFFFFFF:FFFFFFFFFFF:FFFFFFFF:FFFFFFFFFFFFFFFFFFFFFFF:FF:F:
FFFFF,FFF:FF:F:FFF:F:FFF,:FF,,F,FF,FF,FFFF:FFFFFFFFF:FF,FFFFFFFF:FFF
@A00155:342:HHGFNDSXY:1:1268:29939:18834 2:N:0:GAACCTAG+TCCGCATA
CTCCGGCCACCACTGGTCCATATCGATACTCACGTGTCCGTCAGAGTATGTTCCCAGCTCACCTTTCA
GCGATCCCTCATTAGATTCTTGCATCTTCATCAGCATGCAAAATCTTAACATATTAACAAGCAG
+
FFFFFFFFFFFFFFFFFFFFFFFFFFFFFFF:FF:FFFFFFFFFFFFFFF,FFFFFFFFFFFFFFFF:
F:FFFFFFFFFFFFFFFFFFFFFFFFFFFFFFFFFFFFFFFFF:F:FFFFFFFFFFFFFFFFFF
@A00155:342:HHGFNDSXY:1:2640:21251:25394 1:N:0:GAACCTAG+TCCGCATA
CTCCGGCCACCACTGGTCCATATCGATACTCACGTGTCCGTCAGAGTATGTTCCCAGCTCACCTTTCA
GCGATCCCTCATTAGATTCTTGCATCTTCATCAGCATGCAAAATCTTAACATATTAACAAGCAG
+
FFFFFFFFFFFFFFFFFFFFFFFFFFFFFFFFFFFFFFFFFFFFFFFFFFFFFFFFFFFFFFFFFFFF
FFFFFFFFFFFFFFFFFFFFFFF:FFFFFFFFFFFFFFFFFFFFFFFFFF:FFFFFFFFFFFFF
@A00155:342:HHGFNDSXY:1:1327:14714:28385 2:N:0:GAACCTAG+TCCGCATA
CTCCGGCCACCACTGGTCCATATCGATACTCACGTGTCCGTCAGAGTATGTTCCCAGCTCACCTTTCA
GCGATCCCTCATTAGATTCTTGCATCTTCATCAGCATGCAAAATCTTAACATATTAACAAGCAG
+
FFFFFFFF:FFFFFFFFFFFFFF:FFFFFFFF:FFFFFFFFFFFFFFFFF:FFFFFFFF:F:FFFFFF
FFFFFFFFFFFFFFFFFFFFFFFFFFFFFFFFFFFFFFF:FFFFFFFFFFFFFFFFFFFFF:F:
@A00155:342:HHGFNDSXY:1:1478:23773:21840 2:N:0:GAACCTAG+TCCGCATA
CTCCGGCCACCACTGGTCCATATCGATACTCACGTGTCCGTCAGAGTATGTTCCCAGCTCACCTTTCA
GCGATCCCTCATTAGATTCTTGCATCTTCATCAGCATGCAAAATCTTAACATATTAACAAGCAG
+
FFFFFFFFFF:FFFFFFFFFFF:FF,FFFFFFFFFFFFFFFF:FFFFFFFFF:FFFFFFFFFF:FFFF
FFFFFFFFFFFFFFFFFFFFFFFFF:FFFFFF:FFFFFFFFFFFFFFFFFFFFFFFFFFFFFFF
@A00155:342:HHGFNDSXY:1:1567:26404:9298 2:N:0:GAACCTAG+TCCGCATA
CTCCGGCCACCACTGGTCCATATCGATACTCACGTGTCCGTCAGAGTATGTTCCCAGCTCACCTTTCA
GCGATCCCTCATTAGATTCTTGCATCTTCATCAGCATGCAAAATCTTAACATATTAACAAGCAG
+
FFFFFFFFFFFFFFFFFFFFFFFFFFFFFFFFFFFFFFFF:FFFFFFFFF:FFFFFFFFFFFFFFFFF
FFFFFFFFFFFFFFFFFFFFFFFFFFFFFFFFFFFFFFFFFFFFFFFFFFFFFFFFFFFFFF:F
@A00155:342:HHGFNDSXY:1:1221:24171:21308 2:N:0:GAACCTAG+TCCGCATA
CTCCGGCCACCACTGGTCCATATCGATACTCACGTGTCCGTCAGAGTATGTTCCCAGCTCACCATTCA
GCGATCCCTCATTAGATTCTTGCATCTTCATCAGCATGCAAAATCTTAACATATTAACAAGCAG
+
FFFFFFFFFFFFFFFFFFFFF:FFFFFFFFFFFFFFFFFFFFFFFFFFFFFFF:FFF:FFFFFFFFFF
FFFFFFF:FFF:FFFFF:FFF:FFFFFFFFFFFFFFFFFFFF,FFFFFFFFFFFFF:F:FFFFF
@A00155:342:HHGFNDSXY:1:2269:16731:28870 2:N:0:GAACCTAG+TCCGCATA
CTCCGGCCACCACTGGTCCATATCGATACTCACGTGTCCGTCAGAGTATGTTCCCAGCTCACCTTTCA
GCGATCCCTCATTAGATTCTTGCATCTTCATCAGCATGCAAAATCTTAACATATTAACAAGCAG
+
FFFFFFFFF:FFFFFFFFFFFFFFFFFFFFFFFFFFFFFFFFFFFFFFFF:FFFFFFFFFF:FFFFFF
FFFFFFFFFFFFFFFFFFFFFFFFFFFFFFFFFFFFFFFFFFFFFFFFFFFFFFFFFFFFFFFF
```

```
@A00155:342:HHGFNDSXY:1:1211:21160:25081 2:N:0:GAACCTAG+TCCGCATA
CTCCGGCCACCACTGGTCCATATCGATACTCACGTGTCCGTCAGAGTATGTTCCCAGCTCACCTTTCA
GCGATCCCTCATTAGATTCTTGCATCTTCATCAGCATGCAAAATCTTAACATATTAACAAGCAG
+
FFFFFFFFFFFFFFFFFFFFFFFFFFFFFFFFFFFFFFFFFFFFFFFFFFFFFFFFFFFFFFF:FFFF
FFFFFFFFFFFFFFFFFFFFFFFFFFFFFFFFFFFFFFFFFFF:FFFFFFFFFFFFFF::FFFF
@A00155:342:HHGFNDSXY:1:2451:29288:1642 2:N:0:GAACCTAG+TCCGCATA
CTCCGGCCACCACTGGTCCATATCGATACTCACGTGTCCGTCAGAGTATGTTCCCAGCTCACCTTTCA
GCGATCCCTCATTAGATTCTTGCATCTTCATCAGCATGCAAAATCTTAACATATTAACAAGCAG
+
FFFFFFFFFFFFF:FFFFFFFFFFFFFFFFFFFFFFFFFFFFFFFFFFFFFF,FFFFFFFFFFFFFFF:
FFFFFFFFFFFFFFFFFFFFFFFFFFFFFFFFFFFFFFFFFFFFFFFFFF:FFFFFF:FFFFF
@A00155:342:HHGFNDSXY:1:2661:32072:12759 2:N:0:GAACCTAG+TCCGCATA
CTCCGGCCACCACTGGTCCATATCGATACTCACGTGTCCGTCAGAGTATGTTCCCAGCTCACCTTTCA
GCGATCCCTCATTAGATTCTTGCATCTTCATCAGCATGCAAAATCTTAACATATTAACAAGCAG
+
FFFFFFFFFFFFFFFFFF:F,FFFFFFFFFFFFFFFFFFFFFFFFFFFF:FFFFFFFFFFFFFFFF:
FFFFFFFFFFFFFFFFFFFFFFFFFFF:FFFFFFFFFFFFFFFFFFFFFFFFFFFFFF,F:FFFFF
@A00155:342:HHGFNDSXY:1:2255:12445:33724 2:N:0:GAACCTAG+TCCGCATA
CTCCGGCCACCACTGGTCCATATCGATACTCACGTGTCCGTCAGAGTATGTTCCCAGCTCACCTTTCA
GCGATCCCTCATTAGATTCTTGCATCTTCATCAGCATGCAAAATCTTAACATATTAACAAGCAG
+
FFFFFFFFFFFFFFFFFFFFFFFFFF,FFFFFFFFFFFFFFFFFFFFFFFFFFFFFFFFFFFFFFFFF
FFFFFFFFFFFFFFFFFFFFFFFFFFFFFFFFFFFFFFFFFFFFFFFFFFFFFFFFFFFFFFFF
@A00155:342:HHGFNDSXY:1:1470:18475:2550 2:N:0:GAACCTAG+TCCGCATA
CTCCGGCCACCACTGGTCCATATCGATACTCACGTGTCCGTCAGAGTATGTTCCCAGCTCACCTTTCA
GCGATCCCTCATTAGATTCTTGCATCTTCATCAGCATGCAAAATCTTAACATATTAACAAGCAG
+
FFFFFFFFFFFFFF:FFFFFFFFFFFFFFFF,FFFFFF:FFFFFFFFFFFFFFFFFFFFFFFFFFFFF
FFFFFFFFFFFFFFFFFFFFFF,FFFFFFFFF:FFFFFFFFFFFFFFFFFF:FFFFFFFFFFFF
@A00155:342:HHGFNDSXY:1:1477:16821:28995 2:N:0:GAACCTAG+TCCGCATA
CTCCGGCCACCACTGGTCCATATCGATACTCACGTGTCCGTCAGAGTATGTTCCCAGCTCACCTTTCA
GCGATCCCTCATTAGATTCTTGCATCTTCATCAGCATGCAAAATCTTAACATCTTAACAAGCAG
+
FFFFFFFFF:FFFFFFFFFFFFFFFFFFFF:FFFFF:FFFF:,FFFFFFF:FFFFFFFFFFFFFFFFF
FFFFFFFFFFF,FFF:FFFFFFFFFFFFFFFFFFFFFFFFFFF:FFFFFFFFFF,F:,FFFFFFFF
@A00155:342:HHGFNDSXY:1:2472:11957:3255 2:N:0:GAACCTAG+TCCGCATA
CTCCGGCCACCACTGGTCCATATCGATACTCACGTGTCCGTCAGAGTATGTTCCCAGCTCACCTTTCA
GCGATCCCTCATTAGATTCTTGCATCTTCATCAGCATGCAAAATCTTAACATATTAACAAGCAG
+
FFFFFFFFFFFFFFFFFFFFFFFFFF,FFFFFFFFFFFFFFFFFFFFFFFF,FFFFFFFFFFFFFFFF
FFFFFFFFFFF:FFFFFFFFFFFFFFFFFFFFFFFFFFFFFFFFFFFFFFFFFFFF:FF::FF:
@A00155:342:HHGFNDSXY:1:1523:31593:14591 1:N:0:GAACCTAG+TCCGCATA
CTCCGGCCACCACTGGTCCATATCGATACTCACGTGTCCGTCAGAGTATGTTCCCAGCTCACCTTTCA
GCGATCCCTCATTAGATTCTTGCATCTTCATCAGCATGAAAAATCTTAACATATTAACAAGCAGTGAG
+
FFFFFFFFFFFFFFFFFFF:FFFFFFFFFFFFFFFFFFFFFFFFFFFFFFFFF:FFFFFFFFFFFFFFF
FFFFFFFFFFFFFFFFFFFFFFFFFFFFFFFFFFFF:FFFFFFFFFF:FF:FFFFFF:FFF:FFFFFF
@A00155:342:HHGFNDSXY:1:2513:26078:6136 1:N:0:GAACCTAG+TCCGCATA
CTCCGGCCACCACTGGTCCATATCGATACTCACGTGTCCGTCAGAGTATGTTCCCAGCTCACCTTTCA
GCGATCCCTCATTAGATTCTTGCATCTTCATCAGCATGCAAAATCTTAACATATTAACAAGCAGTGAG
+
FFFFFFFFFFFFFFFFFFFFFFFFFFFFFFFFFFFFFFFFFFFFFFFFFFFFFFFFFFFFFFFFFFFF
FFF:,FFFFFFFFFFFFFFFFFFFFFFFFFFFFFFFFFFFFFFFFFFFFF:FFFFFF:FFFFFFFFFF
```

```
@A00155:342:HHGFNDSXY:1:1107:13630:13448 1:N:0:GAACCTAG+TCCGCATA
CTCCGGCCACCACTGGTCCATATCGATACTCACGTGTCCGTCAGAGTATGTTCCCAGCTCACCTTTCA
GCGATCCCTCATTAGATTCTTGCATCTTCATCAGCATGCAAAATCTTAACATATTAACAAGCAGTGAG
+
FFFFFFFFFFFFFFFFFFFFFFFFFFFFFFFFFFFFFFFFFFFFFFFFFFFFFFFFFFFFFFFFFFFF
FFF,FFFFFFFFFFFFFFFFFFF,FF:FF:FFFFFFFFFFFFFFFF:FFF:FFFFFFFFFFFFFFFFF
@A00155:342:HHGFNDSXY:1:2271:6858:18067 1:N:0:GAACCTAG+TCCGCATA
CTCCGGCCACCACTGGTCCATATCGATACTCACGTGTCCGTCAGAGTATGTTCCCAGCTCACCTTTCA
GCGATCCCTCATTAGATTCTTGCATCTTCATCAGCATGCAAAATCTTAACATATTAACAAGCAGTGAG
+
FFFFFFFFFFFFFFFFFFFFFFFFFFFFFFFFFFFFFFFFFFFFFFFFFFFFFFFFFFFF:FFFFFFF
FFF:FFFFFFFFFFFFFFFFFFFFFFF:FFFFFFFFFFFF:FFFFFFFFFFFFFFFFFFF,FFFFFFF
@A00155:342:HHGFNDSXY:1:1239:1823:15107 1:N:0:GAACCTAG+TCCGCATA
CTCCGGCCACCACTGGTCCATATCGATACTCACGTGTCCGTCAGAGTATGTTCCCAGCTCACCTTTCA
GCGATCCCTCATTAGATTCTTGCATCTTCATCAGCATGCAAAATCTTAACATATTAACAAGCAGTGAG
+
FFFFFFFFFFFFFFFFFFFFFFFFFFFFFFFFFFFFFF:FFFFFFFFFFFFFFFFFFFFFFFFF:FFF
FFFFFFFFFFFFFFFFFFFFFFFFFFFFFFFFFFFFF:FFFFFFFFFFFF:FFFFFFFF,FFFFFF:F
@A00155:342:HHGFNDSXY:1:1239:1533:11537 1:N:0:GAACCTAG+TCCGCATA
CTCCGGCCACCACTGGTCCATATCGATACTCACGTGTCCGTCAGAGTATGTTCCCAGCTCACCTTTCA
GCGATCCCTCATTAGATTCTTGCATCTTCATCAGCATGCAAAATCTTAACATATTAACAAGCAGTGAG
+
FFFFFFFFFFFFFFFFFFFFFFFFF:FFFFFFFFFFFFFFFFFFFFFFFFFFFFFFFFFFFFFFFFFF
F:F:::FFFFFFFFFFFFFFFFFFF:FFFFFFFFFFF:FFFFFF:FFFFFFFFFFFFFFFFFFFFFFF
@A00155:342:HHGFNDSXY:1:1110:10230:30389 1:N:0:GAACCTAG+TCCGCATA
CTCCGGCCACCACTGGTCCATATCGATACTCACGTGTCCGTCAGAGTATGTTCCCAGCTCACCTTTCA
GCGATCCCTCATTAGATTCTTGCATCTTCATCAGCATGCAAAATCTTAACATATTAACAAGCAGTGAG
+
FFFFFFFFFFFFFFFFFFFFFFFFFFFFFFFFFFFFFF:FFFFFFFF:FFFFFFFFFFFFFFFFFFFF
FFF,,FFF:FFFFFFFFFFFFFFFFFFFFFFFFFFFFFFFFFF:FF,F:F:FFFFFFFFF:FFFFFFF
@A00155:342:HHGFNDSXY:1:2661:32072:12759 1:N:0:GAACCTAG+TCCGCATA
CTCCGGCCACCACTGGTCCATATCGATACTCACGTGTCCGTCAGAGTATGTTCCCAGCTCACCTTTCA
GCGATCCCTCATTAGATTCTTGCATCTTCATCAGCATGCAAAATCTTAACATATTAACAAGCAGTGAG
+
FFFFFFFFFFFFFFFF:FFFFFFFFFFFFFFFFFFFFFFFFFFFFFF:FFFFFFFFFFFFFFFF:FFF
FFF:FFFFFFFFFFFFFFFFFFF:FFFFF:FF:FFFFFFFF:FFFFFFFFFFFFFF:FFFFFFFFFFF
@A00155:342:HHGFNDSXY:1:1630:12933:24017 1:N:0:GAACCTAG+TCCGCATA
CTCCGGCCACCACTGGTCCATATCGATACTCACGTGTCCGTCAGAGTATGTTCCCAGCTCACCTTTCA
GCGATCCCTCATTAGATTCTTGCATCTTCATCAGCATGCAAAATCTTAACATATTAACAAGCAGTGAG
+
FFFFFFFFFFFFFFFFFFFF:FFFFFFFFFFFF,:FFFFFFFFFFFF:FFF:FFF:FFFFFFFF:FFF
FFFFFFFFFFFFF::FFFFFFFF,FF,:FF,F:FF:FFF:FF:,FFFFFF:FFFFFFFFFFFF:FFFF
@A00155:342:HHGFNDSXY:1:2437:31367:13698 1:N:0:GAACCTAG+TCCGCATA
CTCCGGCCACCACAGGTCCATATCGATACTCACGTGTCCGTCAGAGTATGTTCCCAGCTCACCTTTCA
GCGATCCCTCATTAGATTCTTGCATCTTCATCAGCATGCAAAATCTTAACATATTAACAAGCAGTGAG
+
FFFFFFFFFFFFFFFFFFFFFFFFFFF:FFFFFFFFFFFFFFFFFFFFFFFFFFFFFFFFFFFFFFFF
FFF:,FFFFFFFFFFFFFFFFFF::FFFFF,FFFFFFFFFFFFFFFFFFFFFFFFFFFFFF:FFFFFF
@A00155:342:HHGFNDSXY:1:1315:9579:5744 1:N:0:GAACCTAG+TCCGCATA
CTCCGGCCACCACTGGTCCATATCGATACTCACGTGTCCGTCAGAGTATGTTCCCAGCTCACCTTTCA
GCGATCCCTCATTAGATTCTTGCATCTTCATCAGCATGCAAAATCTTAACATATTAACAAGCAGTGAG
+
FFFFFFFFFFFFFFFFFFFFFFFFFFFFFFFFFFFF:FFFFFFFFFFFFFFFFFFFFFFFFFFFFFF:
FFF::F,FFFFFFFFFFFFFFFFFFFFFFFFFFFFFFF:FFF:FFFFFFFFFFFFFFFFFFFFFFFFF
```

@A00155:342:HHGFNDSXY:1:2370:12102:18850 1:N:0:GAACCTAG+TCCGCATA
TCCGGCCACCACTGGTCCATATCGATACTCACGTGTCCGTCAGAGTATGTTCCCAGCTCACCTTTCAG
CGATCCCTCATTAGATTCTTGCATCTTCATCAGCATGCAAAATCTTAACATATTAACAAGCAGTGA
+
FFF:,FFFFFFF,::FFFF:FFFFF,F:FFF:FF:FFFFFFFFFFF,:,FFFFFFFFFFFF::FFFFF
F::FFFFFFF,FFF:FFF,:FFF::FFFFFFFFFFFFF,FF,F:FFFFFFFFFFFFFFFFFFF,FF
@A00155:342:HHGFNDSXY:1:1537:7500:32330 2:N:0:GAACCTAG+TCCGCATA
TCCGGCCACCACTCGTCCATATCGATACTCACGTGTCCGTCAGAGTATGTTCCCAGCTCACCTTTCAG
CGATCCCTCATTAGATTCTTGCATCTTCATCAGCATGCAAAATCTTAACATATTAACAAGCAGTGAGA
+
FFFFFFFFFFFF:FFFFFFFFFFFFFFFFF:FF,FFFFFFFFFFFFFFFFFFFF,FFFFFFFF:FFFF
FFFFFFFFFFFFFFFFFFFFFFFFF:FFFFFFFFFFFFFFFF:FFFFFFFFFF:F:FF:FFFFFFFFF
@A00155:342:HHGFNDSXY:1:2239:19334:12524 2:N:0:GAACCTAG+TCCGCATA
TCCGGCCACCACTGGTCCATATCGATACTCACGTGTCCGTCAGAGTATGTTCCCAGCTCACCTTTCAG
CGATCCCTCATTAGATTCTTGCATCTTCATCAGCATGCAAAATCTTAACATATTAACAAGCAGTGAGA
+
FFFFF,F:FFFFFFFFFFF:FFFFF,FFFF:FFFFFFFFFFFFFFFFFFFFF:FFFFF:F:FFFFFFF
FFFFFFFFF:FFFFFFFFFFF,FF:FFFFFFFF:FFFFFFFFFFFFFFFFFFFFFFFFFFF,FFFFFF
@A00155:342:HHGFNDSXY:1:2363:8721:17221 2:N:0:GAACCTAG+TCCGCATA
TCCGGCCACCACTGGTCCATATCGATACTCACGTGTCCGTCAGAGTATGTTCCCAGCTCACCTTTCAG
CGATCCCTCATTAGATTCTTGCATCTTCATCAGCATGCAAAATCTTAACATATTAACAAGCAGTGAGA
+
FFFFFFFFFFFFFFFFFFFF:FFFFFFFFFFFF,FFFFFFFFFFFF:FFFFFFFFFFFFFFFFFFFFF
FFFFFFFFFF:FFFFFFFFFFFFFFFFFFFFFFFFFFFFFFFFFFFFFFFFFFFFF:FFFFFFFFFFF
@A00155:342:HHGFNDSXY:1:2334:15212:19194 2:N:0:GAACCTAG+TCCGCATA
TCCGGCCACCACTGGTCCATATCGATACTCACGTGTCCGTCAGAGTATGTTCCCAGCTCACCTTTCAG
CGATCCCTCATTAGATTCTTGCATCTTCATCAGCATGCAAAATCTTAACATATTAACAAGCAGTGAGA
+
FFFFFFFFFFFFFFFF:FFFFFFFFFFFFFFFFFFFFFFFFFFFFFFFFF:FFFFFFFFFFFFFFFFF
FFFFFFFFFFFFFFFFFFFFFFFFFFFFFFFFFFFFFFFFFF:FFFFFFFFFFFFFFFFFFFFFFFFF
@A00155:342:HHGFNDSXY:1:1124:2220:31203 2:N:0:GAACCTAG+TCCGCATA
TCCGGCCACCACTGGTCCATATCGATACTCACGTGTCCGTCAGAGTATGTTCCCAGCTCACCTTTCAG
CGATCCCTCATTAGATTCTTGCATCTTCATCAGCATGCAAAATCTTAACATATTAACAAGCAGTGAGA
+
FFFFF:FFFFFF:FFFFFFFFF:FFFFFFFFFFFFFFFFFFFFFFFFFFFFFFFFFFFFFFFFFFFF:
FFFFFFFFFFFFFFFFFFFFFFF:FFFFFFFFFFFFFFFFFFFFFFFFFFFFFFFFFFF:FFFFFFFF
@A00155:342:HHGFNDSXY:1:1568:11858:8437 2:N:0:GAACCTAG+TCCGCATA
CAGGCCACCACTGGTCCATATCGATACTCACGTGTCCGTCAGAGTATGTTCCCAGCTCACCTTTCAGC
GATCCCTCATTAGATTCTTGCATCTTCATCAGCATGCAAAATCTTAACATATTAACAAGCAG
+
FFFF:FFFFFFFFFFFFFFFFFFFFFFFFFFF:FFFFFFFFFFFFFFF:FFFFFFFFF:FF::FFFFF
FFFFFFFFFFFFFFFFF,FFFFFFFFFFFFFFFFFFFFFFFFFFFFFF,FF,F:FF,:FFFF
@A00155:342:HHGFNDSXY:1:2528:8305:20353 1:N:0:GAACCTAG+TCCGCATA
CCGGCCACCACTGGTCCATATCGATACTCACGTGTCCGTCAGAGTATGTTCCCAGCTCACCTTTCAGC
GATCCCTCATTAGATTCTTGCATCTTCATCAGCATGCAAAATCTTAACATATTAACAAGCAGTGAGA
+
FFFFFFFFFFFFFFFFFFFFFFFFFFFFFFFFFFFFFFFFFFFFFFFFFFFFFFFFFFFFFFFFFFFF
FFFFFFFFFFFFFFFFFFFFFFFFF,FFFFF:FF:FFFFFFFFFFFF:FFFFFFFFFFFFFF:FFFF
@A00155:342:HHGFNDSXY:1:1630:12933:24017 2:N:0:GAACCTAG+TCCGCATA
CCGGCCACCACTGGTCCATATCGATACTCACGTGTCCGTCAGAGTATGTTCCCAGCTCACCTTTCAGC
GATCCCTCATTAGATTCTTGCATCTTCATCAGCATGCAAAATCTTAACATATTAACAAGCAGTGAGAC
+
FFFFFFFF:,F::FFFFFF,FFFFFFFFFFFF,FFFFFFFFFFFFFFFFFFFFFFFFFFFFFFFFFFF
FFFFFFFFFFFFFFFFFFFFFFFFFFFF:FFFFFFFFFFFFFFFFFFFFFFFFFFFFFFFFFFFFFFF

```
@A00155:342:HHGFNDSXY:1:1568:4779:13714 1:N:0:GAACCTAG+TCCGCATA
CGGCCACCACTGGTCCATATCGATACTCACGTGTCCGTCAGAGTATGTTCCCAGCTCACCTTTCAGCG
ATCCCTCATTAGATTCTTGCATCTTCATCAGCATGCAAAATCTTAACATATTAACAAGCAGTGAGACA
+
FFFFFFFFFFFFFFFFFFFFFFFFFFFFFFFFFFFFFFFFFFFFFFFFFFFFFFFFFFFFFFFFFFF
::FFFFFFFFFFFFFFFFFFFFF:FF:FFFFFFFFFFF:FFFFFFFFFFFFFFFFFFFFFFFFFFFF
@A00155:342:HHGFNDSXY:1:1568:2871:12446 1:N:0:GAACCTAG+TCCGCATA
CGGCCACCACTGGTCCATATCGATACTCACGTGTCCGTCAGAGTATGTTCCCAGCTCACCTTTCAGCG
ATCCCTCATTAGATTCTTGCATCTTCATCAGCATGCAAAATCTTAACATATTAACAAGCAGTGAGACA
+
FFFFFFFFFFFFFFFFFFFFFFFFFFFFF,FFF:FF:FFFFFFFFFFFFFFFFFFFFFFFFFFFF:F
:FFFFFFFFFFFFFFFFFFFFFFFFFFFF:FFFFFFFFF:FFFFFFFFFFFFFFFFFFFFFFFFFFF
@A00155:342:HHGFNDSXY:1:1525:8133:33176 2:N:0:GAACCTAG+TCCGCATA
GGCCACCACTGGTCCATATCGATACTCACGTGTCCGTCAGAGTATGTTCCCAGCTCACCTTTCAGCGA
TCCCTCATTAGATTCTTGCATCTTCATCAGGATGCAAAATCTTAACATATTAACAAGCAGTGAGACAT
+
FFFFFFFFFFFFFFFFFFFFFFFFFFFFFFFF:FFFFFFFFFFFFFFFFFFFFFFFFFFFFFFFFFF
FFFFFFFFFFFFFFFFFFFFFFFFFFFFFFFFFFFFFFFFFFFFFFFFFFF,F:FFFFFFFFFFFFF
@A00155:342:HHGFNDSXY:1:1437:5358:24236 2:N:0:GAACCTAG+TCCGCATA
GGCCACCACTGGTCCATATCGATACTCACGTGTCCGTCAGAGTATGTTCCCAGCTCACCTTTCAGCGA
TCCCTCATTAGATTCTTGCATCTTCATCAGCATGCAAAATCTTAACATATTAACAAGCAGTGAGACAT
+
::FFFFFFFFFFFFFFFFFFFFFFFFFFFFFFFFFFFFFFFFFFFFFFFFFFFFFFFFFFFFFFFFF
FFFFFFFFFFFFFFFFFFFFFFF:FFFF:FFFFFFFFFFFFF:FFFFFFFFFFFFFFFF:FFFFFFF
@A00155:342:HHGFNDSXY:1:2377:29261:20259 2:N:0:GAACCTAG+TCCGCATA
GGCCACCACTGGTCCATATCGATACTCACGTGTCCGTCAGAGTATGTTCCCAGCTCACCTTTCAGCGA
TCCCTCATTAGATTCTTGCATCTTCATCAGCATGCAAAATCTTAACATATTAACAAGCAGTGAGACAT
+
FFFFFFFFFFFFFFFFFFFFFFFFFFFFFFFFFFFFFFFFFFFFFFFFFFFFFFFFFFFFFFFFFFF
FFFFFFFFFFFFFFFFFFFFFFFFFFFFFFFFFFFFFFFFFFFFFFFFFFF,F,,FFFFFFFFFFFF
@A00155:342:HHGFNDSXY:1:1669:19768:19539 2:N:0:GAACCTAG+TCCGCATA
GGCCACCACTGGTCCATATCGATACTCACGTGTCCGTCAGAGTATGTTCCCAGCTCACCTTTCAGCGA
TCCCTCATTAGATTCTTGCATCTTCATCAGGATGCAAAATCTTAACATATTAACAAGCAGTGAGACAT
+
FFFFFFFFFFFFFFFFFFFFFFFFFFFFFFFFFFFFFFFFFFFFFFFFFFFFFFFFFFFFFFFFFFF
FFFFFFFFFFFFFFFFFFFFFFFFFFFFFFFFFFFFFFFFFFFFFFFFFFFFFFFFFFFFFFFFFFF
@A00155:342:HHGFNDSXY:1:1242:8458:29543 2:N:0:GAACCTAG+TCCGCATA
GCCACCACTGGTCCATATCGATACTCACGTGTCCGTCAGAGTATGTTCCCAGCTCACCTTTCAGCGAT
CCCTCATTAGATTCTTGCATCTTCATCAGCATGCAAAATCTTAACATATTAACAAGCAGT
+
FFFFFFFFFFFFFFFFFFFFFFFFFFFFFFFFFFFFFFFFFFFFFFFFFFFFFFFFFFFFFFFFFFF
FFFFFFFFFFFFFFFFFF,FFFFFFFFFFFFFFFFFFFFFFFFFFFFFFFFFFFFFFFFF
@A00155:342:HHGFNDSXY:1:1664:15040:29168 2:N:0:GAACCTAG+TCCGCATA
CCACCACTGGTCCATATCGATACTCACGTGTCCGTCAGAGTATGTTCCCAGCTCACCTTTCAGCGATC
CCTCATTAGATTCTTGCATCTTCATCAGCATGCAAAATCTTAACATATTAACAAGCAGTGAGAC
+
FFFFFFFFFFFFFFFFFFFFFFF:F:FFFFFFFFFFFFFFFFFF:FFFFFFFFFFFFFFFFFFFF,F
FFFFF:FFFFFFF,FFFFFFFFF,FFFFFFFFFFFFF:FFFFFF:FFFFFFFF,FFFFFFFFFF
@A00155:342:HHGFNDSXY:1:2220:24379:14184 2:N:0:GAACCTAG+TCCGCATA
CCACTGGTCCATATCGATACTCACGTGTCCGTCAGAGTATGTTCCCAGCTCACCTTTCAGCGATCCCT
CATTAGATTCTTGCATCTTCATCAGCATGCAAAATCTTAACATATTAACAAGCAGTGAGACATGGTTA
+
FFFFFFFFFFFFFFFFFFFFFFFFFFFFFFFFFFFFFFFFFFFFFFFFFFFFFFFFFFF:FFFFFFF
FFF:FFFFFFFFFFFFFFFFFFFFFFFFFFFFFFFFFFFFFFFFFFF:FFFFFFFFFFFFFFFFFFF
```

```
@A00155:342:HHGFNDSXY:1:1313:15619:17362 2:N:0:GAACCTAG+TCCGCATA
CACTGGTCCATATCGATACTCACGTGTCCGTCAGAGTATGTTCCCAGCTCACCTTTCAGCGATCCCTC
ATTAGATTCTTGCATCTTCATCAGCATGCAAAATCTTAACATATTAACAAGCAGTGAGACATGGTTA
+
FFFFFFFFF:FF,FFFFFFFF:FFFFFFFFFFFFFFFFFF,FFFF:FFFFFFFFFFFFFFFFFFFF:F
FFFFFFF:FFFFFFFFFFFFFFFFF:FFFF:FFFFFFF:FFF:FF,,FF,FFFFFFF:,FFFFFFFF
@A00155:342:HHGFNDSXY:1:2406:23439:23672 2:N:0:GAACCTAG+TCCGCATA
CACTGGTCCATATCGATACTCACGTGTCCGTCAGAGTATGTTCCCAGCTCACCTTTCAGCGATCCCTC
ATTAGATTCTTGCATCTTCATCAGCATGCAAAATCTTAACATATTAACAAGCAGTGAGACATGGTTAT
+
FFFFFFFFFFFFFFFFFFFFFFFF:FFFFFFF:FFFFFFFFFFFF:F:F:FFFFFFFFFFFFFFFFFF
FFFFFFFFFFFF:FFFFFFFFFFFFFFFFFFFF:FFFFFFFF:FFF,FF:FFFFFFFFFFFFFFFFFF
@A00155:342:HHGFNDSXY:1:2617:22426:27461 2:N:0:GAACCTAG+TCCGCATA
GGTCCATATCGATACTCACGTGTCCGTCAGAGTATGTTCCCAGCTCACCTTTCAGCGATCCCTCATTA
GATTCTTGCATCTTCATCAGCATGCAAAATCTTAACATATTAACAAGCAGTGAGACATGGTTATCTGA
+
FFFFFFFFFFFF:FFFFFFFFFFFFFFFFFFFFFFFFFFFFFFFFFFFFFFFFFFFFFFFFFFFFF:F
FFFFFFFFFFFFFFFFFFFFFFFFFFFFFFFFFFFFF:FFF,:FFFFFFFFFFFFFFFFFFFFFFFFF
@A00155:342:HHGFNDSXY:1:1268:29939:18834 1:N:0:GAACCTAG+TCCGCATA
GTCCATATCGATACTCACGTGTCCGTCAGAGTATGTTCCCAGCTCACCTTTCAGCGATCCCTCATTAG
ATTCTTGCATCTTCATCAGCATGCAAAATCTTAACATATTAACAAGCAGTGAGACATG
+
FFFFFFFFFFFFFFFFFFFFFFFFFFFFFFFFFFFFFFFFFFF:FFFFFF:FFFFF,FFFF:FFFFFF
FFFFFFFF,F:FFF::F,FFFFFFFFFF:FFFFFFF,FFFFFFFFFFFFFFFFFF:FF
@A00155:342:HHGFNDSXY:1:1437:5358:24236 1:N:0:GAACCTAG+TCCGCATA
GTCCATATCGATACTCACGTGTCCGTCAGAGTATGTTCCCAGCTCACCTTTCAGCGATCCCTCATTAG
ATTCTTGCATCTTCATCAGCATGCAAAATCTTAACATATTAACAAGCAGTGAGACATGGTTATCTGAC
+
FFFFFFFFFFFFFFFFFFFFFFFFFFFFFFFFFFFFFFFFFFF:FFFFFFFFFFFFF:FFFFFFFFFF
FFFFFFFF:FFFFFFFFFFF:FFFFFFFF:FFFFFFFFFF:FFFFFFFFFFF:FFFFFFFFFFFFFFF
@A00155:342:HHGFNDSXY:1:2377:29261:20259 1:N:0:GAACCTAG+TCCGCATA
GTCCATATCGATACTCACGTGTCCGTCAGAGTATGTTCCCAGCTCACCTTTCAGCGATCCCTCATTAG
ATTCTTGCATCTTCATCAGCATGCAAAATCTTAACATATTAACAAGCAGTGAGACATGGTTATCTGAC
+
FFFF:FFFFFFFFFFFFFFFFFFFFFFFFFFF:FF,FFFFFFFFFFFFFFFFFFFF:FFFFFFFFFFF
FFFFFFFFFFF,FF:FFFFFFFFFFFF:FFFFFFFFFFFFFFFFFFFFFFFFFFFF:FFFFFFFFFFF
@A00155:342:HHGFNDSXY:1:1568:11858:8437 1:N:0:GAACCTAG+TCCGCATA
CCATATCGATACTCACGTGTCCGTCAGAGTATGTTCCCAGCTCACCTTTCAGCGATCCCTCATTAGAT
TCTTGCATCTTCATCAGCATGCAAAATCTTAACATATTAACAAGCAGTGAGACATGGTTATCTGAC
+
FFFFFFFFFFFFFFFFFFFFFFFFFFFFFFFFFFFFFFFFFFFFFFFFFFFFFF,FFFFFFFFFFFFF
FFFFFFF:FFFF,FF:FFFFFFFFFFFFFFFFF:FFFFFFFFFFFFF:FFFFFFFFFFFFFFFFFF
@A00155:342:HHGFNDSXY:1:2577:8793:23891 1:N:0:GAACCTAG+TCCGCATA
CCATATCGATACTCACGTGTCCGTCAGAGTATGTTCCCAGCTCACCTTTCAGCGATCCCTCATTAGAT
TCTTGCATCTTCATCAGCATGCAAAATCTTAACATATTAACAAGCAGTGAGACATGGTTATCTGACCA
+
FFFFFFFFFFFFFFFFFFFFFFFFFFFFFFFFFFFFFFFFFFFFFFFFFFFFFFFFFFFFFFFFFFFF
FFFFFF,FFFFFFFFFFF:FFFFFFFFFFFFFF:FFFFFFFFFFFFFFFFFFFFFFFFFFFFFFFFFF
@A00155:342:HHGFNDSXY:1:1278:29984:27774 1:N:0:GAACCTAG+TCCGCATA
CCATATCGATACTCACGTGTCCGTCAGAGTATGTTCCCAGCTCACCTTTCAGCGATCCCTCATTAGAT
TCTTGCATCTTCATCAGCATGCAAAATCTTAACATATTAACAAGCAGTGAGACATGGTTATCTGACCA
+
FFFFFFFFFFFFFFFFFFFFFFFFFFFFFFFF:FFFFFFFFFFFFF,:FFFFFFF,FFFFFFFFFFFF
FFFFFF::FFFFFFF:FFFFFFFFFFFFF:F:FFFFFFFFFFF,FFFFFFFFFFFFFFFFFFFFFFFF
```

@A00155:342:HHGFNDSXY:1:2178:24243:19711 2:N:0:GAACCTAG+TCCGCATA
CCATATCGATACTCACGTGTCCGTCAGAGTATGTTCCCAGCTCACCTTTCAGCGATCCCTCATTAGAT
TCTTGCATCTTCATCAGCATGCAAAATCTTAACATATTAACAAGCAGTGAGACATGGTTATCTGACCA
+
FFFF,FFFF,FFFFFFFFFFFFFFFFFFFFFFF,FFFFFFFFFFFFFFFF,FFFFFFFFFFFFFFFFF
FFFFFFFFF:FFFFFFFFF:FFF:FFFFFFFFFFF,FFF,F,,FFFFFFFFFFFFFFFFFFFFFFFFF
@A00155:342:HHGFNDSXY:1:2437:31367:13698 2:N:0:GAACCTAG+TCCGCATA
CATATCGATACTCACGTGTCCGTCAGAGTATGTTCCCAGCTCACCTTTCAGCGATCCCTCATTAGATT
CTTGCATCTTCATCAGCATGCAAAATCTTAACATATTAACAAGCAGTGAGACATGGTTATCTGACCAT
+
FF:FFFFF:FFFF,FF:FFFFFFFFFFFFFF:,FFFF:FFFFFFFFFFFFFFFFFFFFFFFF,FFFFF
FFFFFFFFF:FFFFFFFFFFFFFFFFFFFFFF:FFF:F:FFFFFFF:FFFFFFFFFFFFFFFF:FFFF
@A00155:342:HHGFNDSXY:1:1242:8458:29543 1:N:0:GAACCTAG+TCCGCATA
TATCGATACTCACGTGTCCGTCAGAGTATGTTCCCAGCTCACCTTTCAGCGATCCCTCATTAGATTCT
TGCATCTTCATCAGCATGCAAAATCTTAACATATTAACAAGCAGTGAGACATGGTTATCT
+
FFFFFFFFFFFFFFFFFFFFFFFFFFF:FFFFFFFFFFFFFFFFFFFFF,F,FFFFFFFFFFFFFFFF
FFF,F:FFFFFFFFFFFFFFFFFFFFFFFFFFFFFFF:FFFFFFFFFFFFF:FFFFFFFF
@A00155:342:HHGFNDSXY:1:1549:21721:7420 2:N:0:GAACCTAG+TCCGCATA
TATCGATACTCACGTGTCCGTCAGAGTATGGTCCCAGCTCACCTTTCAGCGATCCCTCATTAGATTCT
TGCATCTTCATCAGCATGCAAAATCTTAACATATTAACAAGCAGTGAGACATGGTTATCTGACCATGA
+
FF:FFFF:FFF:FFFF:FFFFFFFFF:FFF,FFFFFFFFFFFFFFFFFFFFFFFFFFFFFFFF:FFFF
FFFFFFF:FFFFFFFFFFFFFFF:FFFFFFFFFF:FFF,FFFFFFFFFFFFFFF:FFFFFFFFFFFFF
@A00155:342:HHGFNDSXY:1:2250:28357:25833 2:N:0:GAACCTAG+TCCGCATA
TATCGATACTCACGTGTCCGTCAGAGTATGTTCCCAGCTCACCTTTCAGCGATCCCTCATTAGATTCT
TGCATCTTCATCAGCATGCAAAATCTTAACATATTAACAAGCAGTGAGACATGGTTATCTGACCATGA
+
FFFFFF:FFFFFFFFFFFFFFFFFFFFFFF,FFFFFFFFFFFFFFFFFFFFFFFFFFFFFFFFF:FF:
FFFFFF,FFFFFFFFFFFFFFFFFFFFFFFFFFFFF:FF:FFFFFFFFFFFFFFFFFFFFFFFFFFFF
@A00155:342:HHGFNDSXY:1:1211:12527:27007 2:N:0:GAACCTAG+TCCGCATA
ATCGATACTCACGTGTCCGTCAGAGTATGTTCCCAGCTCACCTTTCAGCGATCCCTCATTAGATTCTT
GCATCTTCATCAGCATGCAAAATCTTAACATATTAACAAGCAGTGAGACATGGTTATCTGACCATGAC
+
FFFFFFFFFFFFFFFFFFFFFFFFFFFFFFFFFFFFFFFFFFFFFFFFFFFFFFFFFFFFFFFFFFFF
FFFFFFFFFFFFFFFFFFFFFFFFFFFFFFFFFFFFFFFFFFFFFFFFFFFFFFFFFFFFFFFFFFFF
@A00155:342:HHGFNDSXY:1:2123:20066:16861 2:N:0:GAACCTAG+TCCGCATA
ATCGATACTCACGTGTCCGTCAGAGTATGTTCCCAGCTCACCTTTCAGCGATCCCTCATTAGATTCTT
GCATCTTCATCAGCATGCAAAATCTTAACATATTAACAAGCAGTGAGACATGGTTATCTGACCATGAC
+
FFFFFFFFFFFFF:FFFFFFFFFFFFFFFFFF:FFFFFFFFFFFFFF:FFFFFFFFFF:FFFFFFFFF
FFFFFFFFFFFFFFFFFFFFFF:F:FF:FFFFFFFFFFFFFFFFFFFFFFFFFFFFFFFFFFFFFFFF
@A00155:342:HHGFNDSXY:1:1407:26865:25942 1:N:0:GAACCTAG+TCCGCATA
ATCGATACTCACGTGTCCGTCAGAGTATGTTCCCAGCTCACCTTTCAGCGATCCCTCATTAGATTCTT
GCATCTTCATCAGCATGCAAAATCTTAACATATTAACAAGCAGTGAGACATGGTTATCTGACCATGAC
+
FFFFFFFFFFFFFFFFFFFFFFFFFFFFFFFFFFFFFFFFFFFFFFFFFFFFFFFFFFFFFFFFFFFF
FFFFFFFFFFF:FFFFFFF:FF,FFFFFFFFFFFFFFFFFFFFFFFFFFFFFFFFFFFFFFFFFFFFF
@A00155:342:HHGFNDSXY:1:1278:29984:27774 2:N:0:GAACCTAG+TCCGCATA
ATCGATACTCACGTGTCCGTCAGAGTATGTTCCCAGCTCACCTTTCAGCGATCCCTCATTAGATTCTT
GCATCTTCATCAGCATGCAAAATCTTAACATATTAACAAGCAGTGAGACATGGTTATCTGACCATGAC
+
FF:FFFFFFFFFFFFFFFFFFFFFFFFFFFFFFFFFFFFFFFFFFF:FFFFFFFFFFFFFFFFFFFFF
FFFFFFFFFFFFFFFFFFFFFFFFFFFFFFF:FFFFFFFFFF:FF:FFFFFFFFFFFFFFFFFFFFFF

@A00155:342:HHGFNDSXY:1:1315:2935:19570 2:N:0:GAACCTAG+TCCGCATA
ATCGATACTCACGTGTCCGTCAGAGTATGTTCCCAGCTCACCTTTCAGCGATCCCTCATTAGATTCTT
GCATCTTCATCAGCATGCAAAATCTTAACATATTAACAAGCAGTGAGACATGGTTATCTGACCATGAC
+
FFFFFFFFFFFFFFFFFFFFFFFFFFFFFFFF:FFFFFFFFFFFFFFFFFFFFFFFFFFFFFFFFFFF
FFFFFFFFFFFFFFFFFFFFFFFFFFFFFFFFFFFFFFF:FFFFFFFFFFFFFFFFFFFFFFFFFFFF
@A00155:342:HHGFNDSXY:1:2577:8793:23891 2:N:0:GAACCTAG+TCCGCATA
TCGATACTCACGTGTCCGTCAGAGTATGTTCCCAGCTCACCTTTCAGCGATCCCTCATTAGATTCTTG
CATCTTCATCAGCATGCAAAATCTTAACATATTAACAAGCAGTGAGACATGGTTATCTGACCATGACC
+
FFFFFFFFFFFFFFFFFFFFFFFF:FFFFFFFFFFF,FFFFFFFFFFFFFFFFF:FFFFFFFF:FFFF
FF:FFFFFFFFFFFFFFFFFFFF:FFFFFFFFF:,FF:FFFFFFFFFFFFFFFFFFFFFFFFFFFFFF
@A00155:342:HHGFNDSXY:1:2436:16233:17112 1:N:0:GAACCTAG+TCCGCATA
TCGATACTCACGTGTCCGTCAGAGTATGTTCCCAGCTCACCTTTCAGCGATCCCTCATTAGATTCTTG
CATCTTCATCAGCATGCAAAATCTTAACATATTAACAAGCAGTGAGACATGGTTATCTGACCATGACC
+
FFFFFFFFFFFFFFFFFFFFFFFFFFFFFFFFFFFFFFFFFFFFFFFFFF:FFFFFFFFFFFFFFFFF
F,F:FFFF:FFFFFFFFFFFF:FFFFFFFFFFFFFFFFFFFFFFFFFFFFFFFFFFFFFFFFFFFFFF
@A00155:342:HHGFNDSXY:1:2513:26078:6136 2:N:0:GAACCTAG+TCCGCATA
TCGATACTCACGTGTCCGTCAGAGTATGTTCCCAGCTCACCTTTCAGCGATCCCTCATTAGATTCTTG
CATCTTCATCAGCATGCAAAATCTTAACATATTAACAAGCAGTGAGACATGGTTATCTGACCATGACC
+
FFF,,FFFFFFFFFFFFFFFFFFFFFFFFFFFF:FFFFFFFFFFFFFFFFFFFFFFFF:FFFFF:FFF
FFFF:FFFFFFFFFFFFFFFFFFFFFFFFFFFFFFFFFFFFFFFFFFFFFFFFFFFFFFFFF:FFFF
@A00155:342:HHGFNDSXY:1:2677:19768:20823 2:N:0:GAACCTAG+TCCGCATA
TCGATACTCACGTGTCCGTCAGAGTATGTTCCCAGCTCACCTTTCAGCGATCCCTCATTAGATTCTTG
CATCTTCATCAGCATGCAAAATCTTAACATATTAACAAGCAGTGAGACATGGTTATCTGACCATGACC
+
F:FFFF:FFFFFFFFF:FFFFFFFFFFFFFFFFFFFFFFFFFFFFFFFFFFFFFFFF:F:FFFFFFFF
FF:FFFFFFFFFFFFFFFFFFFFFFFFFFFFFFFFF,FFFFFFFFFFFFFFFFFFFFFFFFFFFFFFF
@A00155:342:HHGFNDSXY:1:2618:6189:16564 1:N:0:GAACCTAG+TCCGCATA
GATACTCACGTGTCCGTCAGAGTATGTTCCCAGCTCACCTTTCAGCGATCCCTCATTAGATTCTTGCA
TCTTCATCAGCATGCAAAATCTTAACATATTAACAAGCAGTGAGACATGGTTATCTGACCATGACCAC
+
FFFFFFFFF:FFFFFFFFFFFFFFFFFFFFFFFFFFFFFFFFFFFFFFFFFFFFFFFFFFFF:F:FFF
FFFFFFFFFFFFFFFFFFFFFFFFFFFFFFFFFFFFFFFFFFFFFFFFF:FFFFFFFF,FFFFF:FFF
@A00155:342:HHGFNDSXY:1:1635:12400:11099 2:N:0:GAACCTAG+TCCGCATA
ATACTCACGTGTCCGTCAGAGTATGTTCCCAGCTCACCTTTCAGCGATCCCTCATTAGATTCTTGCAT
CTTCATCAGCATGCAAAATCTTAACATATTAACAAGCAGTGAGACATGGTTATCTGACCATGACCA
+
FFFF,FFFFFFFFFFFFFFFFFFFFFFFFFFFFFFFFFFFFFFFFFFFFFFFFFFFFFFFFFFFFFFF
FFFFFFFFFFFFFFFFFFF:FFFFFFFFFFFFFFFFFFFFFFFFF,FFFFFFFFFFFFFF:FFFF:
@A00155:342:HHGFNDSXY:1:1366:7175:30890 1:N:0:GAACCTAG+TCCGCATA
ATACTCACGTGTCCGTCAGAGTATGTTCCCAGCTCACCTTTCAGCGATCCCTCATTAGATTCTTGCAT
CTTCATCAGCATGCAAAATCTTAACATATTAACAAGCAGTGAGACATGGTTATCTGACCATGACCAC
+
FFFFFFFFFFFFFFFFFFFFFFFFFFFFFFF:FFFFFFF:FFFFFFFFFFFFFFFFFFFFFFFFFFFF
FFFFFFFFFFFFFFFFFFFFFFFFFFFF:FFFFFFFFFFFFFFFFFFFFFFFFFFFFFFFFFFFFFF
@A00155:342:HHGFNDSXY:1:1337:30110:32158 2:N:0:GAACCTAG+TCCGCATA
ATACTCACGTGTCCGTCAGAGTATGTTCCCAGCTCACCTTTCAGCGATCCCTCATTAGATTCTTGCAT
CTTCATCAGCATGCAAAATCTTAACATATTAACAAGCAGTGAGACATGGTTATCTGACCATGACCACC
+
FFFFFFFFFFFFFFF:FFFFFFFFF:FFFF:FFFFFFFF,FFFFFFFFFFFFFFF:FFFFFF:FFFFF
FFFFFFFFFFFFFFFFFFFFFFFFFFF:F,FFFF,FFFFFFFF,FFFFF:FFFF,FFFFF:FFFFFFF

```
@A00155:342:HHGFNDSXY:1:1242:27498:14606 1:N:0:GAACCTAG+TCCGCATA
ATACTCACGTGTCCGTCAGAGTATGTTCCCAGCTCACCTTTCAGCGATCCCTCATTAGATTCTTGCAT
CTTCATCAGCATGCAAAATCTTAACATATTAACAAGCAGTGAGACATGGTTATCTGACCATGACCACC
+
FFFFFFFFFFFFFFFFFFFFFFFFFFFFFFFFFFFFFFFFFFFFFFFF,FFFFFFFFFFFFFFFFFFFFF
FFFFFFFFFFFFFFFFFFFFFFFFFFFFFFFFFFF:,,FFFFFFFFFFFFFFFFFFFFFFF:FFFFFFFF
@A00155:342:HHGFNDSXY:1:1637:16911:11616 2:N:0:GAACCTAG+TCCGCATA
TACTCACGTGTCCGTCAGAGTATGTTCCCAGCTCACCTTTCAGCGATCCCTCATTAGATTCTTGCATC
TTCATCAGCATGCAAAATCTTAACATATTAACAAGCAGTGAGACATGGTTATCTGACCATGACCACCT
+
FFFFFFFFFFFFFFFFFFFFFFFFF:FFFFFFFFFF:FFFFFFFFFFFFFFFFFFFFFFFFFFFFFFFFF
FFFFFFFFFFFFFFFFFFFFFFFFFFFFFFFFFFFFFFFFFFFFFFFFFFFFFFFFFFFFFFFFFFFFFF
@A00155:342:HHGFNDSXY:1:2520:30544:34444 2:N:0:GAACCTAG+TCCGCATA
ACTCACGTGTCCGTCAGAGTATGTTCCCAGCTCACCTTTCAGCGATCCCTCATTAGATTCTTGCATCT
TCATCAGCATGCAAAATCTTAACATATTAACAAGCAGTGAGACATGGTTATCTGACCATGACCACCTC
+
FFFFFFFFFFFFFFFFFFFFFFFF:FFFF:FFFFFFFFFFFFFFFFFFFFFFFF:FFFFFFFFFFFFFFF
FFFFFFFFFFFFFFFFFFFFFFFFFFFFFFFF:FFFFFFFFFFFFFFFFFFFFFFFFFFFFFFFFFFFFF
@A00155:342:HHGFNDSXY:1:2321:10691:28401 1:N:0:GAACCTAG+TCCGCATA
TCACGTGTCCGTCAGAGTATGTTCCCAGCTCACCTTTCAGCGATCCCTCATTAGATTCTTGCATCTTC
ATCAGCATGCAAAATCTTAACATATTAACAAGCAGTGAGACATGGTTATCTGACCATGACCACCTCTG
+
FFFFFFFFFFFFFFFFFFFFFFFFFFFFFFFFFFFFFFFFF:FF:FFFFFFFFFFFFFFFFFFFFFFFFF
FFFFFFFFFFFFFFFFFFFFFF:FFFFFFFFFFFFFFFFFFFFFFFFFFFFFFFFFFFFFFFFFFFFFFF
@A00155:342:HHGFNDSXY:1:2559:5113:31203 1:N:0:GAACCTAG+TCCGCATA
CACGTGTCCGTCAGAGTATGTTCCCAGCTCACCTTTCAGCGATCCCTCATTAGATTCTTGCATCTTCA
TCAGCATGCAAAATCTTAACATATTAACAAGCAGTGAGACATGGTTATCTGACCATGACCACCTCTGC
+
FFFFFFFF:FFFFFFFFFFFFFFFFFFFFFFFFFFFFFFFFF:FFFFFFFFFFFFFFFFFFFFFFFFFFF
FFFFFFFFFFFFFFFFFFFFF:FFFFF,FFFFFFFFFFFFFFFFF::FFF:F:FF,FFF:FFFFFFF:FF
@A00155:342:HHGFNDSXY:1:2269:14543:18129 1:N:0:GAACCTAG+TCCGCATA
CACGTGTCCGTCAGAGTATGTTCCCAGCTCACCTTTCAGCGATCCCTCATTAGATTCTTGCATCTTCA
TCAGCATGCAAAATCTTAACATATTAACAAGCAGTGAGACATGGTTATCTGACCATGACCACCTCTGC
+
FFFFFFFFFFFFFFFFFFFFFFFFFFFFFFFFFFFFFFFFFFF:FFFFFFFFFFFFFFFFFFF,F:FFFF
,FFFFFFFFFFFFFFF:FFF:FFFFFFFFFFF:FFFFFFFFFFFFFFFFFFFFFFFFFFFFFFFFFFFFF
@A00155:342:HHGFNDSXY:1:1157:22318:20196 1:N:0:GAACCTAG+TCCGCATA
CACGTGTCCGTCAGAGTATGTTCCCAGCTCACCTTTCAGCGATCCCTCATTAGATTCTTGCATCTTCA
TCAGCATGCAAAATCTTAACATATTAACAAGCAGTGAGACATGGTTATCTGACCATGACCACCTCTGC
+
FFFFFFFFFFFFFFFFFFFFFFFFFF:FFFFFFFFFFFFFFFF,FFFFFFFFF:FFFFFFFFFFFFFFFF
FFFFFFFFFFFFFFFFFFFFFFFFFFFFFFFFFFFFFFFFFFFFFFFF:FFFFFFFFFFFFFFFFFFFFF
@A00155:342:HHGFNDSXY:1:2468:3007:27398 1:N:0:GAACCTAG+TCCGCATA
CACGTGTCCGTCAGAGTATGTTCCCAGCTCACCTTTCAGCGATCCCTCATTAGATTCTTGCATCTTCA
TCAGCATGCAAAATCTTAACATATTAACAAGCAGTGATACATGGTTATCTGACCATGACCACCTCTGC
+
FF,FF:F:F:FFF:FFFFF:FFFFFFF:FFFFFFFFFFFF:F:FF,FFF:FFFFF:,:F:F::FFFFFFF
FF,F:F:FFFFFFFF:FF::FFFFFFFFFFFF::F,,FF,FFFFF:FFF:FFF,F,FF:F:,F,FF:FF:
@A00155:342:HHGFNDSXY:1:1203:6017:11945 1:N:0:GAACCTAG+TCCGCATA
CACGTGTCCGTCAGAGTATGTTCCCAGCTCACCTTTCAGCGATCCCTCATTAGATTCTTGCATCTTCA
TCAGCATGCAAAATCTTAACATATTAACAAGCAGTGAGACATGGTTATCTGACCATGACCACCTCTGC
+
FFFFFFFFFFFFFFF:FFFFFF:FFFFFF:F:FFF:FFF::F,:FFFFFFF:FFFFFFFFFFF,FFF:F:
:F:FFFFFFFFFF,FFFFFFFFFFFFFFFFFFF:FFFF:FFFF:FFFFFF:FFFFFFFFF:FFFFF,,FF
```

```
@A00155:342:HHGFNDSXY:1:2264:11496:31015 2:N:0:GAACCTAG+TCCGCATA
ACGTGTCCGTCAGAGTATGTTCCCAGCTCACCTTTCAGCGATCCCTCATTAGATTCTTGCATCTTCAT
CAGCCTGCAAAATCTTAACATATTAACAAGCAGTGAGACATGGTTATCTGACCATGACCACCTCTGCA
+
FFFFFFFFFFFFFFFFFFFFFFFFFFFFFFFFFFFFFFFFFFFFF:FFFFFFFFFFFFFFFFFFFFFF
FFFF,FFFFFFFFFFFFFFFFFFFFFFFFFFFFFFFFFFFFFFFFFFFFFFFFFFFFFFFF:FFFFF
@A00155:342:HHGFNDSXY:1:2207:19678:15029 2:N:0:GAACCTAG+TCCGCATA
ACGTGTCCGTCAGAGTATGTTCCCAGCTCACCTTTCAGCGATCCCTCATTAGATTCTTGCATCTTCAT
CAGCATGCAAAATCTTAACATATTAACAAGCAGTGAGACATGGTTATCTGACCATGACCACCTCTGCA
+
FFFFFFFFFFFFFFFFFFF,FFFFFFFFFFFFFFFFFFFFFFFFFFFFFFFFFFFFFFFFFFFFFFFF
FFFFFFFFFFF:FFFFFFF,FFFFF,FFFFFFFFFFFFFFFFFFFFFF:FFFFFFFFFFFFFFFFFFF
@A00155:342:HHGFNDSXY:1:2520:30544:34444 1:N:0:GAACCTAG+TCCGCATA
ACGTGTCCGTCAGAGTATGTTCCCAGCTCACCTTTCAGCGATCCCTCATTAGATTCTTGCATCTTCAT
CAGCATGCAAAATCTTAACATATTAACAAGCAGTGAGACATGGTTATCTGACCATGACCACCTCTGCA
+
FFFFFFFFFFFFFFFF:FFFFFFFFFFFFFFFFFFFFFFFF:FFFFFFFFFFFFFFFFFFF:FFFFFF
FFFFFFFFFFFFFFFFFFFFFFFFFFFFFFFFFFFFFFFFFFFFFFFFFFFFFFFFFFFFFFFFFFFF
@A00155:342:HHGFNDSXY:1:2530:2320:30906 2:N:0:GAACCTAG+TCCGCATA
CGTGTCCGTCAGAGTATG
+
F:F:FFFFFFFFFFFFFF
@A00155:342:HHGFNDSXY:1:2607:25265:8547 1:N:0:GAACCTAG+TCCGCATA
GTGTCCGTCAGAGTATGTTCCCAGCTCACCTTTCAGCGATCCCTCATTAGATTCTTGCATCTTCATCA
GCATGCAAAATCTTAACATATTAACAAGCAGTGAGACATGGTTATCTGACCATGACCACCTCTGCATA
+
FFFFFFFFFFFFFFFFFFFFFFFFFFFFFFFFFFFFFF:FFFFFFFFFFFFFFFFFFF:F:FFFF:F,
FFFFFFFFFFFFFFFFF:FFFFFFFFFFFFFFFFFFFFFFFFFFFFFFFFFFFFFFFFFFFFFFFFFF
@A00155:342:HHGFNDSXY:1:2607:25201:8437 1:N:0:GAACCTAG+TCCGCATA
GTGTCCGTCAGAGTATGTTCCCAGCTCACCTTTCAGCGATCCCTCATTAGATTCTTGCATCTTCATCA
GCATGCAAAATCTTAACATATTAACAAGCAGTGAGACATGGTTATCTGACCATGACCACCTCTGCATA
+
FFFFFFFFFFFFFFFFFFFFFFFFFFFFFFF:FFFFFFFFFFFFFFFFFFFFFFFFFF:FFFFFFFFF
FFFFFFFFFFFFFFFFF:FFFFFFFFFFFFFFFFFFFFFFFFFFFFFFFFFFF:FFFFFFFFFFFFFF
@A00155:342:HHGFNDSXY:1:2370:12102:18850 2:N:0:GAACCTAG+TCCGCATA
TGTCCGTCAGAGTATGTTCCCAGCTCACCTTTCAGCGATCCCTCATTAGATTCTTGCATCTTCATCAG
CATGCAAAATCTTAACATATTAACAAGCAGTGAGACATGGTTATCTGACCATGACCACCTCTGCATAG
+
:FFFFF:FFFFFFFFFFFFFFFFFFFFFFFFFFFF,FFFF,:FFFF,FFFFF,,F:,FFFFFFFF:F,
FFFFFFF,FFFFFFFFFFFFFF,FFF:FFFFFF,FFF:FFFFFF:F:F:FFFFF:FFFFF:FFFFFFF
@A00155:342:HHGFNDSXY:1:1161:14244:26381 2:N:0:GAACCTAG+TCCGCATA
TGTCCGTCAGAGTATGTTCCCAGCTCACCTTTCAGCGATCCCTCATTAGATTCTTGCATCTTCATCAG
CATGCAAAATCTTAACATATTAACAAGCAGTGAGACATGGTTATCTGACCATGACCACCTCTGCATAG
+
FFF:FFFFFFF:,FFF,FFFF:FFFFFFFFFFFFFFFFFFFFFFFFFFFFFFFFFFFFFFFFFFFF:F
FFFFFFFF:FFFFFFFFF:FFFFFF,FFFFFFFFFFFFFFFF,FFFFFFFFFFFFFFFFFFFFFFFFF
@A00155:342:HHGFNDSXY:1:1378:24180:16658 1:N:0:GAACCTAG+TCCGCATA
TGTCCGTCAGAGTATGTTCCCAGCTCACCTTTCAGCGATCCCTCATTAGATTCTTGCATCTTCATCAG
CATGCAAAATCTTAACATATTAACAAGCAGTGAGACATGGTTATCTGACCATGACCACCTCTGCATAG
+
FFFFFFFFFFFFFFFFFFFFFFFFFFFFFFFFFFFFFFFFFFFFFFFFFFFFFFFFFFFFFFFFFFFF
FFFFFFFFFFFFFFFFFFFFFFFFFFFFFFFFFFFFFFFFFFFFFFFFFFFFFFFFFFFFFFFFFFFF
@A00155:342:HHGFNDSXY:1:1315:2935:19570 1:N:0:GAACCTAG+TCCGCATA
TGTCCGTCAGAGTATGTTCCCAGCTCACCTTTCAGCGATCCCTCATTAGATTCTTGCATCTTCATCAG
```

```
CATGCAAAATCTTAACATATTAACAAGCAGTGAGACATGGTTATCTGACCATGACCACCTCTGCATAG
+
FFFFFFFFFFFFFFFFFFFFFFFFFFFFFF:FFFF:F:FFFFFFFFFFFFFFFFFF:,FFFF:FFF:F
FFFFFFF:FFFFFFFFFFFFFFFFFFFFFFFFFFFFFFFFFFFFFFFFFFFFFFFFFFFFFFFFFFFF
@A00155:342:HHGFNDSXY:1:2539:29658:1376 1:N:0:GAACCTAG+TCCGCATA
TGTCCGTCAGAGTATGTTCCCAGCTCACCTTTCAGCGATCCCTCATTAGATTCTTGCATCTTCATCAG
CATGCAAAATCTTAACATATTAACAAGCAGTGAGACATGGTTATCTGACCATGACCACCTCTGCATAG
+
FFFFFFFFFFFFFFFFFFFFFFFFF:FFFFFFFFFF,:FFFFFFFFFFFFFFFFF:FFFFF:FF:F
FFFFFF:FFFFFF:FFFFFFFFFFFFFFFFFFFFFFFFFFFFFFFFFFFFFFFFFFFFFFFFFFFFFF
@A00155:342:HHGFNDSXY:1:1107:13630:13448 2:N:0:GAACCTAG+TCCGCATA
TGTCCGTCAGAGTATGTTCCCAGCTCACCTTTCAGCGATCCCTCATTAGATTCTTGCATCTTCATCAG
CATGCAAAATCTTAACATATTAACAAGCAGTGAGACATGGTTATCTGACCATGACCACCTCTGCATAG
+
FF,FFFFFFFFFFFF:FFFFFFFFFFFFFFFFFFFFFFFFF:FF::FFFFFFFFFFF:FFFFFFFFF
FFFFFFFF,:FFFFFF:FFFFF:FF::FFFFFFFFFF:FFFFFFFFFFFFFFFFFFFFFFFFFFFFFF
@A00155:342:HHGFNDSXY:1:2242:7482:19586 2:N:0:GAACCTAG+TCCGCATA
GTCCGTCAGAGTATGTTCCCAGCTCACCTTTCAGCGATCCCTCATTAGATTCTTGCATCTTCATCAGC
ATGCAAAATCTTAACATATTAACAAGCAGTGAGACATGGTTATCTGACCATGACCACCTCTGCATAGC
+
FFFFFFFFFFFFFFF:FFFFFFFFFFFFFFFFFFFFFFFFFFFFFFFFFFFF:FFFFFFFFFFFFFFFF
FFFFFFFFFFFFFF:FFF:FFFFF:FFFF:FFFFFFFFFFFFFFFFFFFFFFFFFFFFFFF:FFFFFFF
@A00155:342:HHGFNDSXY:1:1410:30454:18380 1:N:0:GAACCTAG+TCCGCATA
TCCGTCAGAGTATGTTCCCAGCTCACCTTTCAGCGATCCCTCATTAGATTCTTGCATCTTCATCAGCA
TGCAAAATCTTAACATATTAACAAGCAGTGAGACATGGTTATCTGACCATGACCACCTCTGCATAGCG
+
FFFFFFFFFFFFFFFFFFFFFFFFFFFFFFFFFFFF:FFFFFFFFFFFFFFFFFFFFFFFFFFFF,FFF
FFFFFFFFFFFFFFFFFFFFFFFFFFFFFFFFFFFFFFFFFFFFFFFFFFFFFFFFFFFFFFFFFFFF
@A00155:342:HHGFNDSXY:1:2365:21685:14215 1:N:0:GAACCTAG+TCCGCATA
TCCGTCAGAGTATGTTCCCAGCTCACCTTTCAGCGATCCCTCATTAGATTCTTGCATCTTCATCAGCA
TGCAAAATCTTAACATATTAACAAGCAGTGAGACATGGTTATCTGACCATGACCACCTCTGCATAGCG
+
FFFFFFFFFFFFFFFFFFFFFFFFFFFFFFFFFFFFF:FFFFFFFFFFFFFFFFFFFFFFFFFFFFFFF
FFFFF:FFFFFFFF:FFFFFFFFFFFFFFFFFFFFFFFFFFFFFFFFF:FFFFFFFFFFFFFFFFFFF
@A00155:342:HHGFNDSXY:1:2176:16586:3349 1:N:0:GAACCTAG+TCCGCATA
CCGTCAGAGTATGTTCCCAGCTCGCCTTTCAGCGATCCCTCATTAGATTCTTGCATCTTCATCAGCAT
GCAAAATCTTAACATATTAACAAGCAGTGAGACATGGTTATCTGACCATGACCACCTCTGCATAGCGT
+
FFFFFFFFFFFFFFFFFFFFFFFFFFFFF:FFFF,,:FFFFFFFFFF,FFFFFFFFFFFFFFFFFF
FFFFFFFFFFFFF:FFFFFFFFFFFFFFFFF:FFFFFFFFFFFFFFFFFFFFFFFFFFFFFFFFFFFF
@A00155:342:HHGFNDSXY:1:2626:4969:27132 1:N:0:GAACCTAG+TCCGCATA
CCGTCAGAGTATGTTCCCAGCTCACCTTTCAGCGATCCCTCATTAGATTCTTGCATCTTCATCAGCAT
GCAAAATCTTAACATATTAACAAGCAGTGAGACATGGTTATCTGACCATGACCACCTCTGCATAGCGT
+
FFFFFFFFFF,FFFFFFFFFFFFFFFFFFFFFFFF:FFFFFFFFF:FFFFFFFFFFFFFFFFFFFFF:
FFFFFFF:FFFFFFFFFFFFFFFFFFF:FFFFFFFFFFFFFFFFFFFF,FFFFF,FFFFFFF:FFF:F
@A00155:342:HHGFNDSXY:1:2331:29423:34162 2:N:0:GAACCTAG+TCCGCATA
CGTCAGAGTATGTTCCCAGCTCACCTTTCAGCGATCCCTCATTAGATTCTTGCATCTTCATCAGCATG
CAAAATCTTAACATATTAACAAGCAGTGAGACATGGTTATCTGACCATGACCACCTCTGCA
+
FFFFFFFFFFFF:FFFFFFFFFFFFF,FF:FFFFFFFFFFFFFFFF:FFF,FFFFFFFFFFF:FFFFF
FFF,FFFFFFFF,F::,:FF,,FF:FFFFFFFFFFFFFFFFFFFFFFF:FFFFFFFFFFFFF
@A00155:342:HHGFNDSXY:1:2347:10538:17675 2:N:0:GAACCTAG+TCCGCATA
CGTCAGAGTATGTTCCCAGCTCACCTTTCAGCGATCCCTCATTAGATTCTTGCATCTTCATCAGCATG
```

CAAAATCTTAACATATTAACAAGCAGTGAGACATGGTTATCTGACCATGACCACCTCTGCATA
+
,,F:FF:FF,FFFFF::F,FF,:::FFF:F:FFFF,,FFFFFF:FFFF:FF:F:FFFFFFF:F:,:FF
:,,,:FFFFFF,:FFFF:FFF:F,:FFF:F,,FFFFFFFFFFFFF:FFF,FFFFFFFFFFFFF
@A00155:342:HHGFNDSXY:1:2628:3414:12508 2:N:0:GAACCTAG+TCCGCATA
GTCAGAGTATGTTCCCAGCTCACCTTTCAGCGATCCCTCATTAGATTCTTGCATCTTCATCAGCATGC
AAAATCTTAACATATTAACAAGCAGTGAGACATGGTTATCTGACCATGACCACCTCTGC
+
:,FFFFFFFFFFFFFFFFFFFFFFFF:FFFFFFFFFFFFFFFFFFFFFFFF:FFFFFFFFFFFFFFFF
FFFFFFFFFFFFFFFFFFFFFFFFFFFFFFF:FFFFFFFFFFFFFFFFFFFFFFFFFFF
@A00155:342:HHGFNDSXY:1:2622:30544:17190 2:N:0:GAACCTAG+TCCGCATA
GTCAGAGTATGTTCCCAGCTCACCTTTCAGCGATCCCTCATTAGATTCTTGCATCTTCATCAGCATGC
AAAATCTTAACATATTAACAAGCAGTGAGACATGGTTATCTGACCATGACCACCTCTGCA
+
FFFFFFFFFFF:FFFFFFFFFFFFFFFFFFFFFFFFFFFF,FFFF,FFFFFFFFFFFFFFF:FFFFFF
FFFFFFFFFFF:FFFFF,F,:FF:FFFFFFFFFFFFFFFFFFFFFFFFFFFFFFFFFFFF
@A00155:342:HHGFNDSXY:1:1323:15374:32941 2:N:0:GAACCTAG+TCCGCATA
GTCAGAGTATGTTCCCAGCTCACCTTTCAGCGATCCCTCATTAGATTCTTGCATCTTCATCAGCATGC
AAAATCTTAACATATTAACAAGCAGTGAGACATGGTTATCTGACCATGACCACCTCTGCA
+
FFFFFFFFFFF,FFFFFFFFFFFFFFFFFF:FFFFFFFF:FFF:FFFFFF,FFF:FFFFFFFFFFFFF
FFFFFFFFFFFFFFFFFFFFFFFFFFFFFFFFF:FFFFFFFFFFFFFFFFFFFFFFFFFF
@A00155:342:HHGFNDSXY:1:2525:16396:12790 2:N:0:GAACCTAG+TCCGCATA
GTCAGAGTATGTTCCCAGCTCACCTTTCAGCGATCCCTCATTAGATTCTTGCATCTTCATCAGCATGC
AAAATCTTAACATATTAACAAGCAGTGAGACATGGTTATCTGACCATGACCACCTCTGCA
+
FFFFFFFFFFFFFFFFFFFFFFFFFFFFFF:FFFFFF:FFFFFFFFFFFFFFFFFFFFFFFFFFFFFF
FFFFFFFFFFFFFFFFFFF:FFFFFFFFFFF:FFFFFFFF:FFFFFFFFFFFFFFFFFFF
@A00155:342:HHGFNDSXY:1:1319:3721:27383 2:N:0:GAACCTAG+TCCGCATA
GTCAGAGTATGTTCCCAGCTCACCTTTCAGCGATCCCTCATTAGATTCTTGCATCTTCATCAGCATGC
AAAATCTTAACATATTAACAAGCAGTGAGACATGGTTATCTGACCATGACCACCTCTGCATAG
+
FFFFFFFFFFF:FFFFFFFFFFFFFFFFFFFFFFFFFFFFFFFFFFFFFFFFFFFFFFFFFFFFFFFF
FFFFFFFFFFFFF,F:F:F:FFFFFFFFFFFFFFFFFFFFFFFFFFFFFFFFFFFFFFFFFFFF
@A00155:342:HHGFNDSXY:1:1124:14940:12085 2:N:0:GAACCTAG+TCCGCATA
GTCAGAGTATGTTCCCAGCTCACCTTTCAGCGATCCCTCATTAGATTCTTGCATCTTCATCAGCATGC
AAAATCTTAACATATTAACAAGCAGTGAGACATGGTTATCTGACCATGACCACCTCTGCATAGCGTTC
+
FFFFFFFFFFFFFFFFFFFFFFFFFFFFFF:FFFFFFFFFFFFFFFFFFFFFFFFFFFFFFFFFFFFF
FFFFFFFFFFFFFF:FFFFF,:FFFFFFFFFFFFFFFFFFFFFFFFFFFFFFFFFFFFFFFFFFFFFF
@A00155:342:HHGFNDSXY:1:1657:31693:21245 2:N:0:GAACCTAG+TCCGCATA
GTCAGAGTATGTTCCCAGCTCACCTTTCAGCGATCCCTCATTAGATTCTTGCATCTTCATCAGCATGC
AAAATCTTAACATATTAACAAGCAGTGAGACATGGTTATCTGACCATGACCACCTCTGCATAGCGTTC
+
FFFFFFFFFFFFFFFFFFFFFFFFFFFFFFFFFFFFFFFFFFFFFFFFFFFFFFFFFFFFFFFFFFFF
FFFFFFFFFFFFFFFFF:FFFFFFFFF:FFFFFFFFFFFFFFFFFFFFFFFFFFFFFFFFFFFFFFFF
@A00155:342:HHGFNDSXY:1:2275:31015:35321 1:N:0:GAACCTAG+TCCGCATA
GTCAGAGTATGTTCCCAGCTCACCTTTCAGCGATCCCTCATTAGATTCTTGCATCTTCATCAGCATGC
AAAATCTTAACATATTAACAAGCAGTGAGACATGGTTATCTGACCATGACCACCTCTGCATAGCGTTC
+
FFFFFFFFFFF:FFFFFFFFFFFFFFFFFFFF:,FFFFFFFFFFFFFFFFFFFFFFFFFFFFFFFFFF
FFFF:FFFF:F:FFFF:FFFFFFFFFFFFFFFFFFFFFFFFFFFFFFFFFFFFFFFFFFFFFFFFFFF
@A00155:342:HHGFNDSXY:1:2375:7762:29152 2:N:0:GAACCTAG+TCCGCATA
CAGAGTATGTTCCCAGCTCACCTTTCAGCGATCCCTCATTAGATTCTTGCATCTTCATCAGCATGCAA

```
AATCTTAACATATTAACAAGCAGTGAGACATGGTTATCTGACCATGACCACCTCTGCATAGCGT
+
FFFFFFF:FFFFFFFFFFFFFF:FFFFFFFFFFFF:FF,FFFFFF:FFFFF:FFFFFFFFFFFFFFF
FFFF:FFFFF:FFFFFFF:FF:F:FFFFFFFFFFFFFFFFFFFFFFFFFFFF:FFFFFFFFFFF
@A00155:342:HHGFNDSXY:1:2660:18023:21997 2:N:0:GAACCTAG+TCCGCATA
CAGAGTATGTTCCCAGCTCACCTTTCAGCGATCCCTCATTAGATTCTTGCATCTTCATCAGCATGCAA
AATCTTAACATATTAACAAGCAGTGAGACATGGTTATCTGACCATGACCACCTCTGCATAGCGT
+
F:FFFFF:F:FFFF:FFFFFFFFFFF:F:FFFFFFFFFFFFFFFFFFFFFFFFFFFFFFFFFFFFFF
FFFFFFFFFFFFFFFFF::FFFFFFFFFFFFFFFFFFFFFFFFFFFFFFFFFFFFFFFFFFFFF
@A00155:342:HHGFNDSXY:1:2375:6488:28260 2:N:0:GAACCTAG+TCCGCATA
CAGAGTATGTTCCCAGCTCACCTTTCAGCGATCCCTCATTAGATTCTTGCATCTTCATCAGCATGCAA
AATCTTAACATATTAACAAGCAGTGAGACATGGTTATCTGACCATGACCACCTCTGCATAGCGT
+
FFFFFFFFFFFFFFFFFFFFFFFFFFFFFFFFFFFFFFFFFFFFFFFFFFFF:FFFFFFFFFFFFFF
FF,FFFFFFFFFFFFFF:FFF:FFFFFFFFFFFFFFFFFFFFFFFFFFFFFFFF:FFFFFFFFF
@A00155:342:HHGFNDSXY:1:1114:23502:36996 2:N:0:GAACCTAG+TCCGCATA
AGAGTATGTTCCCAGCTCACCTTTCAGCGATCCCTCATTAGATTCTTGCATCTTCATCAGCATGCAAA
ATCTTAACATATTAACAAGCAGTGAGACATGGTTATCTGACCATGACCACCTCTGCATAGCGTTC
+
FFFFFFFFFFFFFFFFFFFFFFFFFFFFFFFFFFFFFFFFFFFFFFFFFFFFFFFFFFFFFFFFFFF
FFFFFFFFFFFFFFFFFFFFFFFFFFFFFFFFF:FFFFFFFFFFFFFF:FFFFF:FFFFFFFFFFF
@A00155:342:HHGFNDSXY:1:1139:32678:11428 2:N:0:GAACCTAG+TCCGCATA
AGAGTATGTTCCCAGCTCACCTTTCAGCGATCCCTCATTAGATTCTTGCATCTTCATCAGCATGCAAA
ATCTTAACATATTAACAAGCAGTGAGACATAGTTATCTGACCATGACCACCTCTGCATAGCGTTCACG
+
FFFFFFFF:FFFFFFFFFFFFFFFFFFFFFFFFFFFFFFFFFFFFFF:FFFFFF:FFFFFFFFFFFFFF
FFFFFFFFFFFFFF:F,,:FFFFFFFFFFFFFFFFFFFFFFFFFFFFFFFFFFFFFFFFFFFFFFFF
@A00155:342:HHGFNDSXY:1:2530:2320:30906 1:N:0:GAACCTAG+TCCGCATA
GAGTATGTTCCCAGCTCACCTTTCAGCGATCCCTCATTAGATTCTTGCATC
+
FFFFFFFFFFFFFFFFFFFFFFFFFFFF,FFFFFFFFFFFFFF:FFFFFFF
@A00155:342:HHGFNDSXY:1:2345:28528:1172 1:N:0:GAACCTAG+TCCGCATA
GAGTATGTTCCCAGCTCACCTTTCAGCGATCCCTCATTAGATTCTTGCATCTTCATCAGCATGCAAAA
TCTTAACATATTAACAAGCAGTGAGACATGGTTATCTGACCATGACCACCTCTGCATAGCGTTCACGT
+
FFFFFFFFFFFFFFFFFFFFFFFFFFFF,FFFFFFFFFFFFFFFFFFFFFFFFFFFF:FFFFFFFFFFF
FFFFFFFFFFFFFFFFFFFFFFFFFFFFFFFFFFFFFFFF:,FFFFFFFFFFFFFFFFFFFFFFFFFF
@A00155:342:HHGFNDSXY:1:1672:14018:34381 1:N:0:GAACCTAG+TCCGCATA
GAGTATGTTCCCAGCTCACCTTTCAGCGATCCCTCATTAGATTCTTGCATCTTCATCAGCATGCAAAA
TCTTAACATATTAACAAGCAGTGAGACATGGTTATCTGACCATGACCACCTCTGCATAGCGTTCACGT
+
FFFFFFFFFFFFFFFFFFFFFFFFFFFFFFFFFFFFFFFFFFFFFFFFFFFFFFFFFFFFFFFFFFFF
FFFFFFF:FFFFFFFFFFFFFFFFFFFFFFFFFFFFFFFFFFFFFFFFFFFFFFFFFFFFFFFFFFFF
@A00155:342:HHGFNDSXY:1:2613:21567:14888 1:N:0:GAACCTAG+TCCGCATA
GTATGTTCCCAGCTCACCTTTCAGCGATCCCTCATTAGATTCTTGCATCTTCATCAGCATGCAAAATC
TTAACATATTAACAAGCAGTGAGACATGGTTATCTGACCATGACCACCTCTGCATAGCGTTCACGTGT
+
FFFFF:FFFFFFFFFFFFFFFFFFFFFFFFFFFFFFFFFFFFFFFFFFFFFFFFFFFFFFFFFFFFFF
FF:FFFFFFFFFFFFFFFFFFFFFFFFFFFFFFFFFFFFFFFFFFFFFFFFFFFFF:FFFFF,FFFFF
@A00155:342:HHGFNDSXY:1:1673:19117:23453 1:N:0:GAACCTAG+TCCGCATA
GTATGTTCCCAGCTCACCTTTCAGCGATCCCTCATTAGATTCTTGCATCTTCATCAGCATGCAAAATC
TTAACATATTAACAAGCAGTGAGACATGGTTATCTGACCATGACCACCTCTGCATAGCGTTCACGTGT
+
```

FFFFFFFFFFFFFFFFFFFFFFFFFFFFFFFFFFFFFFFFFFFF:FFFFFF:FFFFFFFFFFFFFFFFF
FFFFFFFFFFFFFFFFFFFFFFFFFFFFFFFFFFFFFFFFFFFFFFFFFFFFFFFF:FFFFFFFFFFFF
@A00155:342:HHGFNDSXY:1:2375:6488:28260 1:N:0:GAACCTAG+TCCGCATA
ATGTTCCCAGCTCACCTTTCAGCGATCCCTCATTAGATTCTTGCATCTTCATCAGCATGCAAAATCTT
AACATATTAACAAGCAGTGAGACATGGTTATCTGACCATGACCACCTCTGCATAGCGTTCACGTGTGT
+
FFFFFFFFFFFFFFFFFFFFFFFFFFFFFFFFFFFFFFFFFFFF:FFFFFF:FFFFFFFFFFFFFFFF:
FFF:FFFFFFFFFFFFFFFFFFFFFFFFFFFFFFFFFFFFFFFFFFFFFFFF:FFFF,FFFF,FFFFF
@A00155:342:HHGFNDSXY:1:2375:7762:29152 1:N:0:GAACCTAG+TCCGCATA
ATGTTCCCAGCTCACCTTTCAGCGATCCCTCATTAGATTCTTGCATCTTCATCAGCATGCAAAATCTT
AACATATTAACAAGCAGTGAGACATGGTTATCTGACCATGACCACCTCTGCATAGCGTTCACGTGTGT
+
FFFFFFFFFFFFFFFFFFFFFFFF,FFFFFFFFFFFFFFFFFFFFFFFFFFFFFFFFFFFFFFFFFFFF
FFF:FFFFFFFFFFFFFFFFFFFFFFFFFFFFFFFFFFFFFFFFFFFFFFF,FFFFFFFF:FFFFFFFF
@A00155:342:HHGFNDSXY:1:1127:20500:24596 2:N:0:GAACCTAG+TCCGCATA
GTTCCCAGCTCACCTTTCAGCGATCCCTCATTAGATTCTTGCATCTTCATCAGCATGCAAAATCTTAA
CATATTAACAAGCAGTGAGACATGGTTATCTGACCATGACCACCTCTGCATAGCGTTCACGTGTGTAG
+
FFFFFFFFFF:FFFFFFFFFFFFFFFFFFFFFFFFFFFFFFFFFFFF,FF,FFFFFFFFF:FFFFFFFF
FFFFFFF:FFFFFFFFFFFFFFFFFFFFFFFFFFFFFFFFFFFFFFFFFFFFFFFFFFFFFFFFFFFFF
@A00155:342:HHGFNDSXY:1:1657:31693:21245 1:N:0:GAACCTAG+TCCGCATA
TTCCCAGCTCACCTTTCAGCGATCCCTCATTAGATTCTTGCATCTTCATCAGCATGCAAAATCTTAAC
ATATTAACAAGCAGTGAGACATGGTTATCTGACCATGACCACCTCTGCATAGCGTTCACGTGTGTAGA
+
FFFFFFFFFFFFF:FFFFFFFFFFFFFFFFFFFFFFFFFF:FFFFFFFFFFFFFFFFFFF:FFFFFFFFF
FFFFFFFFFFFFFFFFFFFFFFFFFFFFFFFFFFFFFFFFFFFFFFFFFFF:FFFF:FFFFFFFFFF
@A00155:342:HHGFNDSXY:1:1601:8278:16799 1:N:0:GAACCTAG+TCCGCATA
TTCCCAGCTCACCTTTCAGCGATCCCTCATTAGATTCTTGCATCTTCATCAGCATGCAAAATCTTAAC
ATATTAACAAGCAGTGAGACATGGTTATCTGACCATGACCACCTCTGCATAGCGTTCACGTGTGTAGA
+
FFFFFFFFFFFFFFFFFFFFFF,FFFFFFFFFFFFFFFF,FFFFFFF,FFFFFFFFFFFFFFFFFFFF
::FFFFFFFFFFFFFFFFFFFFFFFFFFFFFF:FFF:FFFFFFFFFFFFFFFFFFFFFFFFFFFFFFF
@A00155:342:HHGFNDSXY:1:2319:18557:12587 1:N:0:GAACCTAG+TCCGCATA
TTCCCAGCTCACCTTTCAGCGATCCCTCATTAGATTCTTGCATCTTCATCAGCATGCAAAATCTTAAC
ATATTAACAAGCAGTGAGACATGGTTATCTGACCATGACCACCTCTGCATAGCGTTCACGTGTGTAGA
+
FFFFFFFFFFFFFFFFFFFFFFFFFFFFFFFFFFFFFF:FFFFFFFFFFFF:FFFFFFFFFFFFFFFF
:FFFFFFFFFFFFFFFFFFFFFFFFFFFFFFFFFFFFFFFFFFFFFFFFFFFFFFFFFFFFFFFFFFF
@A00155:342:HHGFNDSXY:1:1546:19696:27085 1:N:0:GAACCTAG+TCCGCATA
TTCCCAGCTCACCTTTCAGCGATCCCTCATTAGATTCTTGCATCTTCATCAGCATGCAAAATCTTAAC
ATATTAACAAGCAGTGAGACATGGTTATCTGACCATGACCACCTCTGCATAGCGTTCACGTGTGTAGA
+
FFFFFFFFFFFFF:FFFFFF::FFFFFFFFFFFFFFFFFFFFFFFFFFFFFFFFFFFFFFFFFFFFFF
FFFFFFFFFFFFFFFFFFFFFFFFF::FFFFF:FFFFFFFFFFFFFFFFFFFFFFFFFFFFFFFFFFFF
@A00155:342:HHGFNDSXY:1:1632:23601:2503 1:N:0:GAACCTAG+TCCGCATA
TTCCCAGCTCACCTTTCAGCGATCCCTCATTAGATTCTTGCATCTTCATCAGCATGCAAAATCTTAAC
ATATTAACAAGCAGTGAGACATGGTTATCTGACCATGACCACCTCTGCATAGCGTTCACGTGTGTAGA
+
FFFFFFFFFFFFFFFFFFFFF,FFFFFFFFFFFFFFFFFFFFFFFFFF:FFFFFFFFFFFF::FFFFFF
:FFFFFFF:FFFFFFFFFFFFFFFFFFFFFFFFFFFFFFFFFFFFFFFFFFFFFFFFFFFFFFFFFFF
@A00155:342:HHGFNDSXY:1:2241:30092:30718 1:N:0:GAACCTAG+TCCGCATA
TTCCCAGCTCACCTTTCAGCGATCCCTCATTAGATTCTTGCATCTTCATCAGCATGCAAAATCTTAAC
ATATTAACAAGCAGTGAGACATGGTTATCTGACCATGACCACCTCTGCATAGCGTTCACGTGTGTAGA
+

```
FFFFFFFFFFFFFFFFFFFFF:FFF,FFFFFFFFFFFFFFFFFFFFFFFFFFFFFFFFFFFFFFFFFF
FFFFFFFFFF,FFFFFFFFFFFFFFFFFFFFFFFFFF:FFFFFFF:FFFFFFFFFFFFFFFFFFFF:F
@A00155:342:HHGFNDSXY:1:2358:22742:7654 1:N:0:GAACCTAG+TCCGCATA
TTCCCAGCTCACCTTTCAGCGATCCCTCATTAGATTCTTGCATCTTCATCAGCATGCAAAATCTTAAC
ATATTAACAAGCAGTGAGACATGGTTATCTGACCATGACCACCTCTGCATAGCGTTCACGTGTGTAGA
+
FFFFFFFFFFFFFFFFFFFFF:FFFFFFFFFFFFFFFFFFFFFFFFFFFFFFF:FFFFFFFFFFFFFF
FFFFFFFFFFFFFFFFFFFFFFFFFFFFFFFFFFFFFFFFFFFFFFFFFFFFFFFFFFFFFFFFFFFF
@A00155:342:HHGFNDSXY:1:1540:10990:18176 1:N:0:GAACCTAG+TCCGCATA
TTCCCAGCTCACCTTTCAGCGAGCCCTCATTAGATTCTTGCATCTTCATCAGCATGCAAAATCTTAAC
ATATTAACAAGCAGTGAGACATGGTTATCTGACCATGACCACCTCTGCATAGCGTTCACGTGTGTAGA
+
FFFFFFFFFFFFFFFFFFFFFF,:FFFFFFFFFFFFFFFFFFFFFFFFFFFFFFFFFFFFFFFFFFFF
FFFFFFFFFFFFFFFFF:FFFFFFFFF:FFFFFF:FFFFFFFFFFFFFFFFFFFFFFFFFFFFFFFFF
@A00155:342:HHGFNDSXY:1:1560:10999:13369 1:N:0:GAACCTAG+TCCGCATA
TTCCCAGCTCACCTTTCAGCGATCCCTCATTAGATTCTTGCATCTTCATCAGCATGCAAAATCTTAAC
ATATTAACAAGCAGTGAGACATGGTTATCTGACCATGACCACCTCTGCATAGCGTTCACGTGTGTAGA
+
FFFFFFFFFFFFFFFFFFFFFFFFFFFFFFFFFF:FFFFFFFFFF,FFFFFFFFFFFFFFFFFFFFFF
:FFFFFFFFFFFFFFFFFFFFFFFFFFFFFFFFFFFFFFFFFFFFFFFFFFFFFFFFFFF,FFFFFFF
@A00155:342:HHGFNDSXY:1:1667:23665:12289 1:N:0:GAACCTAG+TCCGCATA
TTCCCAGCTCACCTTTCAGCGATCCCTCATTAGATTCTTGCATCTTCATCAGCATGCAAAATCTTAAC
ATATTAACAAGCAGTGAGACATGGTTATCTGACCATGACCACCTCTGCATAGCGTTCACGTGTGTAGA
+
FFFFFFFFFFFFFFFFFFFFFF,FFFFFFFFFFFFFFFFFFFFFFFFF:FFFFFFFFFFFFFFFFFFF
:FFFFFFFFFFFFFFFFFFFFFFFFFFFFFFFFFFFFFFFFFFFFFFFFFFFFFFFFFFFFFFFFFFF
@A00155:342:HHGFNDSXY:1:2116:28537:30123 1:N:0:GAACCTAG+TCCGCATA
TTCCCAGCTCACCTTTCAGCGATCCCTCATTAGATTCTTGCATCTTCATCAGCATGCAAAATCTTAAC
ATATTAACAAGCAGTGAGACATGGTTATCTGACCATGACCACCTCTGCATAGCGTTCACGTGTGTAGA
+
FFFFFFFFFFFFFFFFFFF:F:FFFFFFFFFFFFFFFFFFFFFFFFFFFFFFFFFFFFFFFFFFFFFF
:FFFFFFFFFFFFFFFFFFFFFFFFFFFFFFFFFFFFFFFFFFFFFFFFFFFFFFFFFFFFFFFFFFF
@A00155:342:HHGFNDSXY:1:1622:14461:18709 1:N:0:GAACCTAG+TCCGCATA
TTCCCAGCTCACCTTTCAGCGATCCCTCATTAGATTCTTGCATCTTCATCAGCATGCAAAATCTTAAC
ATATTAACAAGCAGTGAGACATGGTTATCTGACCATGACCACCTCTGCATAGCGTTCACGTGTGTAGA
+
FFFFFFFFFFFFFFFFFFFFFFFFFFFFFFFFFFFFFFFFF:FFFFFFFFFFFFFFFF:FFFFFF:FF
FFFFFFFFFFFFFFFFFFFFFFFFFFFFFFFFFFFFFFFFFFFFFFFFFFFFFFFFFFFFFFFF:FFF
@A00155:342:HHGFNDSXY:1:1613:7274:16250 1:N:0:GAACCTAG+TCCGCATA
TTCCCAGCTCACCTTTCAGCGATCCCTCATTAGATTCTTGCATCTTCATCAGCATGCAAAATCTTAAC
ATATTAACAAGCAGTGAGACATGGTTATCTGACCATGACCACCTCTGCATAGCGTTCACGTGTGTAGA
+
FF:FFFFFFFFFFFFF:FFFF,:FFFFFFFFFFF:FFFFFFFFFFFFFFFFFFFFFFFFFFFFFFFFF
:FFFFFFFFFFFFFFFFFFFFF:FFFFFFFFFFFFFFFFFFFFFFFFFFFF,FFFFFFFFF:FFFFFF
@A00155:342:HHGFNDSXY:1:1108:16749:19601 1:N:0:GAACCTAG+TCCGCATA
TTCCCAGCTCACCTTTCAGCGATCCCTCATTAGATTCTTGCATCTTCATCAGCATGCAAAATCTTAAC
ATATTAACAAGCAGTGAGACATGGTTATCTGACCATGACCACCTCTGCATAGCGTTCACGTGTGTAGA
+
FFFFF:FFFFFFFFF:FFFFF,FFFFFFFFFFFFFFFFFFFFFFFFFFFF,FFFFFFFFFFFFFFFFF
:FFFFFFFFFFFFFFFFFFFFF:FFFFFFFFFFFFFFFFFFFFFFFFFFFFFFFFFFFFFFF:FFF:F
@A00155:342:HHGFNDSXY:1:2345:11098:17550 2:N:0:GAACCTAG+TCCGCATA
TCCCAGCTCACCTTTCAGCGATCCCTCATTAGATTCTTGCATCTTCATCAGCATGCAAAATCTTAACA
TATTAACAAGCAGTGAGACATGGTTATCTGACCATGACCACCTCTGCATAGCGTTCACGTGTGTAGAC
+
```

```
FFFFFFFFFFFFFFFFFF:FFFFFFFFFFFFFFFFFFFFFFFFFFFFFFFFFFFFFFFFFFFFFFFFFF
FFFFF:FF,FFFFFFFFFFFFFFFFFFFFFFFFFFFFFFFFFFFFFFFFFFFFFFFFFFFFFFFFFFFF
@A00155:342:HHGFNDSXY:1:1274:29559:20744 2:N:0:GAACCTAG+TCCGCATA
TCCCAGCTCACCTTTCAGCGATCCCTCATTAGATTCTTGCATCTTCATCAGCATGCAAAATCTTAACA
TATTAACAAGCAGTGAGACATGGTTATCTGACCATGACCACCTCTGCATAGCGTTCACGTGTGTAGAC
+
FFFFFFFFFFFFFFFFFF:FFFFFFFFFFFFFFFFFFFFFFFFFFFFFFFFFFFFFFFFFFFFFFFFFF
F,FF,:F,FFFFFFFFFFFFFFFFFFFFFFFFFFFFFF:FFFFFFFFFFFFFFFFFFFFFFFFFFFFFFF
@A00155:342:HHGFNDSXY:1:1255:2989:11835 2:N:0:GAACCTAG+TCCGCATA
TCCCAGCTCACCTTTCAGCGATCCCTCATTAGATTCTTGCATCTTAATCAGCATGCAAAATCTTAACA
TATTAACAAGCAGTGAGACATGGTTATCTGACCATGACCACCTCTGCATAGCGTTCACGTGTGTAGAC
+
:,:FF::FFFFFFFFFFF:FFFFFFFFFFFF:FFFFF:F,FFFFF,FF,FFFFFFFFFFF,FFFFFFF
FFFFFFFFFFFF,FFFFFFFFF:FFFFFFFFFFFF,FFFFFFFFFFFFFFFFFFFFFF:F:FFFF,F:
@A00155:342:HHGFNDSXY:1:1124:14940:12085 1:N:0:GAACCTAG+TCCGCATA
CCCAGCTCACCTTTCAGCGATCCCTCATTAGATTCTTGCATCTTCATCAGCATGCAAAATCTTAACAT
ATTAACAAGCAGTGAGACATGGTTATCTGACCATGACCACCTCTGCATAGCGTTCACGTGTGTA
+
FFFFFFFFFFFFFFFFFFF::FFFFFFFFFFFFFFFFFFFFFFFFFF:FFFFFFFFF:FFFFFFFFF:F
FFFFFFFFFFFFFFFFFFFFFFFFF:FFFFFFFFFFFFFFFFFFFFFFFFFFFFFFFFFFF:FFFFF
@A00155:342:HHGFNDSXY:1:2661:3739:22623 1:N:0:GAACCTAG+TCCGCATA
CCCAGCTCACCTTTCAGCGATCCCTCATTAGATTCTTGCATCTTCATCAGCATGCAAAATCTTAACAT
ATTAACAAGCAGTGAGACATGGTTATCTGACCATGACCACCTCTGCATAGCGTTCACGTGTGTA
+
FF:FFFFFFFFFFFFFFFF::F,FFFFFFF,FFFFFFFFFFFFFFFFFF:FFFFFFF::FFFFFFFFF
FFFFFFFFFFF:FFFFFFFFFFFFFF:FFFFFFF:FFFFF:FF::FFFFFFFFFFFFFFF:FFF
@A00155:342:HHGFNDSXY:1:2331:29423:34162 1:N:0:GAACCTAG+TCCGCATA
CCAGCTCACCTTTCAGCGATCCCTCATTAGATTCTTGCATCTTCATCAGCATGCAAAATCTTAACATA
TTAACAAGCAGTGAGACATGGTTATCTGACCATGACCACCTCTGCATAGCGTTCACGTGTG
+
FFFFFFFFFFFFFFFFFFFFFFFFFFFFFFFFFFFFFFFFFFFFFFFFFFFFFFFFF,FFFFFFFFFFF
:FFFFFFF:FFFFFFFFFFFFFFFF:FFFFF:FFFFFFFFFFFFFFFFFFFFFFFFF:FFFF
@A00155:342:HHGFNDSXY:1:2347:10538:17675 1:N:0:GAACCTAG+TCCGCATA
CCAGCTCACCTTTCAGCGATCCCTCATTAGATTCTTGCATCTTCATCAGCATGCAAAATCTTAACATA
TTAACAAGCAGTGAGACATGGTTATCTGACCATGACCACCTCTGCATAGCGTTCACGTGTGTA
+
F:FFFFFFFFFF:FFFFF::FFFFF:FFFFFFFFFF,FF,:FFFFFF,FFF::FFFFFF::,FF:::F
:,FFFFFFFFF:FFFFFFFF::FFFFFFFF:FFFFF::FFFF:FFFFFF,F:FFF::,::FFF
@A00155:342:HHGFNDSXY:1:1668:20166:34726 1:N:0:GAACCTAG+TCCGCATA
CCAGCTCACCTTTCAGCGATCCCTCATTAGATTCTTGCATCTTCATCAGCATGCAAAATCTTAACATA
TTAACAAGCAGTGAGACATGGTTATCTGACCATGACCACCTCTGCATAGCGTTCACGTGTGTAGACCA
+
FFFFFFFFFFFFFFFFFF:,FFFFFFFFFFFFFFFFFFFF:FFFF:FFFFFFFFF:FFFFFFFFFFF
FFFFFFFFFFFFFFFFFFFFFFFFFFFFFFFFFFFFFFFFFFFFFFFFFFFF:FFFFFFFFFFFFFFF
@A00155:342:HHGFNDSXY:1:2214:15447:7889 1:N:0:GAACCTAG+TCCGCATA
CCAGCTCACCTTTCAGCGATCCCTCATTAGATTCTTGCATCTTCATCAGCATGCAAAATCTTAACATA
TTAACAAGCAGTGAGACATGGTTATCTGACCATGACCACCTCTGCATAGCGTTCACGTGTGTAGACCA
+
FFFFFFFFFFFFFFFFFF:FFFFFFFFFFFFFFFFFFFFFFFFFFFFFFFFFFFFFFFFF:FFFFFFF:FF
FFFFFFFFFFFFFFFFFFFFFFFFFFFFFFFFFFFFFFFFFFFFFFFFFFFFFFFFFFFFFFFFFFFFF:
@A00155:342:HHGFNDSXY:1:1620:21079:25347 1:N:0:GAACCTAG+TCCGCATA
CCAGCTCACCTTTCAGCGATCCCTCATTAGATTCTTGCATCTTCATCAGCATGCAAAATCTTAACATA
TTAACAAGCAGTGAGACATGGTTATCTGACCATGACCACCTCTGCATAGCGTTCACGTGTGTAGACCA
+
```

```
FFFFFFFFFFFFFFFFFF,FFFFFFFFFFFFFFFFFFF,FFFFFFFF,FFFFFFFFFFFFFFFFFFFF
FFFFFFFFFFFFFFFFFFFFFFFF:FFFFFFFFFFFFFFFFFFFFFFFFFFFFFFFFFFFFFFFFFFF
@A00155:342:HHGFNDSXY:1:1376:3839:23046 1:N:0:GAACCTAG+TCCGCATA
CCAGCTCACCTTTCAGCGATCCCTCATTAGATTCTTGCATCTTCATCAGCATGCAAAATCTTAACATA
TTAACAAGCAGTGAGACATGGTTATCTGACCATGACCACCTCTGCATAGCGTTCACGTGTGTAGACCA
+
FFFFFFFFFFFFFFFFFFFFFFFFFFFFFFFFFFF:FFFFFFFFFFFFFFFFFFFFFFFFFFFFFFFF
FFFFFFFFFFFFFFFFFFFFFFFFFFFFFFFFFFFFFFFFFFFFFFFFFFFFF:FFFFFFFFFFFFFF
@A00155:342:HHGFNDSXY:1:1611:10375:12383 1:N:0:GAACCTAG+TCCGCATA
CCAGCTCACCTTTCAGCGATCCCTCATTAGATTCTTGCATCTTCATCAGCATGCAAAATCTTAACATA
TTAACAAGCAGTGAGACATGGTTATCTGACCATGACCACCTCTGCATAGCGTTCACGTGTGTAGACCA
+
FFFFFFFFFFFFFFFFFFF:,F:,FFF,FFFFFFFF,::,FFFFFFF,FF,FFFFF:F:,FFFF:F:F
F,FFFFF,FFFFFF:::F:FF,FF,F:FF,:FFF,FFFFFFFF:F:FFF:FFFFFFF:FFFF,FF:FF
@A00155:342:HHGFNDSXY:1:1605:28610:14434 1:N:0:GAACCTAG+TCCGCATA
CCAGCTCACCTTTCAGCGATCCCTCATTAGATTCTTGCATCTTCATCAGCATGCAAAATCTTAACATA
TTAACAAGCAGTGAGACATGGTTATCTGACCATGACCACCTCTGCATAGCGTTCACGTGTGTAGACCA
+
FFFFFFFFFFFFFFFFFF:FFFFFFFFFFFFFFFFFFFFFFFFFFFFFFFFFFFFFFFFFFFFFF:FF
FFFFFFFFFFFFFFFFFFFFFFFFFFFFFFFFFFFFFFFF:FFFFFFFFFFFFFFFFFFFFFFFFFFF
@A00155:342:HHGFNDSXY:1:2345:11098:17550 1:N:0:GAACCTAG+TCCGCATA
CCAGCTCACCTTTCAGCGATCCCTCATTAGATTCTTGCATCTTCATCAGCATGCAAAATCTTAACATA
TTAACAAGCAGTGAGACATGGTTATCTGACCATGACCACCTCTGCATAGCGTTCACGTGTGTAGACCA
+
FFFFFFFFFFFFFFFFFF,FFFFFFFFFFFFFFFFFFFFFFFFFFFFFFFFFFFFFF:FFFFFFF:FF
FFFFFFFFFFFFFFFFFFFFFFFFFFFFFF:FFFFFFFFFFFFFFFFFFFFFFFFFFFFFFFFFFFFF
@A00155:342:HHGFNDSXY:1:2216:25916:9173 1:N:0:GAACCTAG+TCCGCATA
CCAGCTCACCTTTCAGCGATCCCTCATTAGATTCTTGCATCTTCATCAGCATGCAAAATCTTAACATA
TTAACAAGCAGTGAGACATGGTTATCTGACCATGACCACCTCTGCATAGCGTTCACGTGTGTAGACCA
+
FFFFFFFFFFFFFFFFFF:FFFFFFFFFFFFFFFFFF:FFFFFFFFFFFFFFFFFFFFFFFFFFFFFF
FFFFFFFFFFFFFFFFFFFFFFFFFFFFFF:FFFFFFFF:FFFFFFFFFFFFFFFFFFFFFFF:FFFF
@A00155:342:HHGFNDSXY:1:1306:18439:6809 1:N:0:GAACCTAG+TCCGCATA
CCAGCTCACCTTTCAGCGATCCCTCATTAGATTCTTGCATCTTCATCAGCATGCAAAATCTTAACATA
TTAACAAGCAGTGAGACATGGTTATCTGACCATGACCACCTCTGCATAGCGTTCACGTGTGTAGACCA
+
FFFFFFFFFFFFFFFFFFFFFFFFFFFFFFFFFFFFFF:FFFFFFFFFFFFFFFFFFFFFFFFFF:FF
FFFFFFFFFFFFFFFFFFFFFFFFFFFFFFFFFFFFFFFFFFFFFFFFFFFFFFFFFFFFFFFFFFFF
@A00155:342:HHGFNDSXY:1:2668:26874:35978 2:N:0:GAACCTAG+TCCGCATA
CAGCTCACCTTTCAGCGATCCCTCATTAGATTCTTGCATCTTCATCAGCATGCAAAATCTTAACATAT
TAACAAGCAGTGAGACATGGTTATCTGACCATGACCACCTCTGCATAGCGTTCACGTGTGTAGACCA
+
FF:,FFFFFFFFFFFF,FFFFF,F,F:FFFFF:F,:FFFF,FFFFFFFFFFFFFFF:FFFFFFFFFFF
FF:F:FFFF,FFFFFFFF:FFFFFFFFFFFFFFFFFFFFFFFFFFFFFF,F:FFFFFFFFFFF:FFF
@A00155:342:HHGFNDSXY:1:1659:13386:26115 1:N:0:GAACCTAG+TCCGCATA
CAGCTCACCTTTCAGCGATCCCTCATTAGATTCTTGCATCTTCATCAGCATGCAAAATCTTAACATAT
TAACAAGCAGTGAGACATGGTTATCTGACCATGACCACCTCTGCATAGCGTTCACGTGTGTAGACCAC
+
FFFFFFFFFFFFFFFFFFFFFFFFFFFFFFFFFFFFFFFFFFFFFFFFFFFFFFFFFFFFFFFFFFFF
FFFFFFFFFFFFFFFFFFFFFFFFFFFFFFFFFFFFFFFFFFFFFFFFFFFFFFFFFFFFFFFFFFFF
@A00155:342:HHGFNDSXY:1:1673:19117:23453 2:N:0:GAACCTAG+TCCGCATA
CAGCTCACCTTTCAGCGATCCCTCATTAGATTCTTGCATCTTCATCAGCATGCAAAATCTTAACATAT
TAACAAGCAGTGAGACATGGTTATCTGACCATGACCACCTCTGCATAGCGTTCACGTGTGTAGACCAC
+
```

```
FFFFFFFF:FF:FFFFFFFFFFF:FFFFFFF:FFFFFFFFFFFFFFFFFFFFFFFFF:FFFFFFFFFF
F:FFFFFFFFFFFFFFFFFFFFFFFFFFFFFFFFFFFFFFFFFFFFFFFFFFFFFFFFFFFFFFFFFF
@A00155:342:HHGFNDSXY:1:1407:26865:25942 2:N:0:GAACCTAG+TCCGCATA
CAGCTCACCTTTCAGCGATCCCTCATTAGATTCTTGCATCTTCATCAGCATGCAAAATCTTAACATAT
TAACAAGCAGTGAGACATGGTTATCTGACCATGACCACCTCTGCATAGCGTTCACGTGTGTAGACCAC
+
FFFFFFFFFFFFFFFFFFFFFFFFFF,FFFFFF,FFFFFFFFFFFFFFFFFFFFFFFFFFFFFFFFFF
FFFFFFFF:FFFFFFFFFFFFFFFFFFFFFFFFFFFFFFFFFFFFFFFFFFFFFFFFFFFFFFFFFFF
@A00155:342:HHGFNDSXY:1:1523:31593:14591 2:N:0:GAACCTAG+TCCGCATA
CAGCTCACCTTTCAGCGATCCCTCATTAGATTCTTGCATCTTCATCAGCATGAAAAATCTTAACATAT
TAACAAGCAGTGAGACATGGTTATCTGACCATGACCACCTCTGCATAGCGTTCACGTGTGTAGACCAC
+
FFFFF:FFFFFFFFF:FFFFFFFF:FFFFF:FFFFFFFFF:FFFFFFFFFFFFFFFFFFF,FFFFFFF
F,:F:,FFFFFFFFFFFFFFFFFFFFFFFFFFFFFFFFFFFFFFFFFFFFFFFFF:FFFFFFFFFFFF
@A00155:342:HHGFNDSXY:1:2358:22742:7654 2:N:0:GAACCTAG+TCCGCATA
GCTCACCTTTCAGCGATCCCTCATTAGATTCTTGCATCTTCATCAGCATGCAAAATCTTAACATATTA
ACAAGCAGTGAGACATGGTTATCTGACCATGACCACCTCTGCATAGCGTTCACGTGTGTAGACCACAC
+
FFFFFFFFFFFFFFFFFFFFFFF:FFFFFFFFFFFFFFFFFFFFFFFFFFFFFFFFFFFFFFFFFFFF
,F:FFFFFFFFFFFFFFFFFFFFFFFFFFFFFFFFFFFFFFFFFFFFFFFFFFFFFFFFFFFFFFFFF
@A00155:342:HHGFNDSXY:1:1611:20907:20729 1:N:0:GAACCTAG+TCCGCATA
GCTCACCTTTCAGCGATCCCTCATTAGATTCTTGCATCTTCATCAGCATGCAAAATCTTAACATATTA
ACAAGCAGTGAGACATGGTTATCTGACCATGACCACCTCTGCATAGCGTTCACGTGTGTAGACCACAC
+
F:FFFFFFFFFFF:F:FF:F:FF:FFFFFFFFF:FFF:FFFFFFF:FFF:FFFFFFFFFFFFFFFFFF
FFFF:FFFFFFFFFFFFFFFFFF:FFFFFFFFFFFF:FFF:FFFFFFF:FFF:FFFFFFFFFFFFFF:
@A00155:342:HHGFNDSXY:1:1526:10113:17816 1:N:0:GAACCTAG+TCCGCATA
GCTCACCTTTCAGCGATCCCTCATTAGATTCTTGCATCTTCATCAGCATGCAAAATCTTAACATATTA
ACAAGCAGTGAGACATGGTTATCTGACCATGACCACCTCTGCATAGCGTTCACGTGTGTAGACCACAC
+
FFFFFFFFFFFFFFFF:FFFFFFFFFFFFFFFFFFFFFFFFFFFFFFF,FFFFFFFFFFFFF:FFFFF
FFFFFFFFFFFFFFFFFFFFFFFFFFF:FFFFFFFFFFFFFFFFFFFFFFFFFFFFFFFFFFFFFFFF
@A00155:342:HHGFNDSXY:1:1148:5837:12195 2:N:0:GAACCTAG+TCCGCATA
GCTCACCTTTCAGCGATCCCTCATTAGATTCTTGCATCTTCATCAGCATGCAAAATCTTAACATATTA
ACAAGCAGTGAGACATGGTTATCTGACCATGACCACCTCTGCATAGCGTTCACGTGTGTAGACCACAC
+
FFFFFFFFFFFFFFFFFF:FFFFFFFFFFFF,FFFFFFFFFFFFFFFFFFFFFFFFFFFFFFFFFFFF
FFF,FFFFFFFFFFFFFF:FFFFFFFFFFFFFFFFFFFFFFFFFFF:FFFFFFFFFFFFFFFFFFFFF
@A00155:342:HHGFNDSXY:1:2607:25201:8437 2:N:0:GAACCTAG+TCCGCATA
GCTCACCTTTCAGCGATCCCTCATTAGATTCTTGCATCTTCATCAGCATGCAAAATCTTAACATATTA
ACAAGCAGTGAGACATGGTTATCTGACCATGACCACCTCTGCATAGCGTTCACGTGTGTAGACCACAC
+
FFFFFFF:FFFFFFFFFFFFFFFF:FFFFFFFFFFFFFFFFFFFFFFFFFFFFFFFFFFFFF:FFF:F
FFFFFFFFFFFFFFFFFFFFFFFFFFFFFFFFFFFFFFFFFFFFFFFFFFFFFFFFFFFFFFFFFFFF
@A00155:342:HHGFNDSXY:1:2607:25265:8547 2:N:0:GAACCTAG+TCCGCATA
GCTCACCTTTCAGCGATCCCTCATTAGATTCTTGCATCTTCATCAGCATGCAAAATCTTAACATATTA
ACAAGCAGTGAGACATGGTTATCTGACCATGACCACCTCTGCATAGCGTTCACGTGTGTAGACCACAC
+
FFFFFFFFFFFFFFFFFFFFFFFFFFFFFFFFFFFFFFFFFFFFFFFFFFFFFFFFFFFFFFFFFFFF
FFFFFFFFFFFFFFFFFFFFFFFFFFFFFFFFFFFFFFFFFFFFFFFFFFFFFFFFFFFFFFFFFFFF
@A00155:342:HHGFNDSXY:1:1526:10791:19022 1:N:0:GAACCTAG+TCCGCATA
GCTCACCTTTCAGCGAGCCCTCATTAGATTCTTGCATCTTCATCAGCATGCAAAATCTTAACATATTA
ACAAGCAGTGAGACATGGTTATCTGACCATGACCACCTCTGCATAGCGTTCACGTGTGTAGACCACAC
+
```

FFFFFFFFFFFFFFFF,FFFFFFFFFFFFFFFFFFFFF:FFFFF:FFF:FFFF:FFFFFFFF,FFFFF
:FFFFFFFFFFFFFFFFFFFFFFFFFFFFFFFFFFFFFFFFFFFFFFFFFFFFFFFFFFFFFFFFFFF
@A00155:342:HHGFNDSXY:1:2271:2275:26412 2:N:0:GAACCTAG+TCCGCATA
CTCACCTTTCAGCGATCCCTCATTAGATTCTTGCATCTTCATCAGCATGCAAAATCTTAACATATTAA
CAAGCAGTGAGACATGGTTATCTGACCATGACCACCTCTGCATAGCGTTCACGTGTGTAGACCACAC
+
FFFF:FFFFFFF,FFFFFF:,FFFF:FFFFFFFFF:,F:FF:FF,FFFFF:FFF,FFF:FFFFFFFFF
FFF,FF,FFFFFFFF:FFFF:FFFFFFFFFFFFFFF::FFF:FFFF:FFFFFFFFFF:FFFFFFFF:
@A00155:342:HHGFNDSXY:1:2468:3007:27398 2:N:0:GAACCTAG+TCCGCATA
CTCACCTTTCAGCGATCCCTCATTAGATTCTTGCATCTTCATCAGCATGCAAAATCTTAACATATTAA
CAAGCAGTGAGACATGGTTATCTGACCATGACCACCTCTGCATAGCGTTCACGTGTGTAGACCACACG
+
FFFFF,:FFFF,FFFFFFFF:FFFFFFFFFF,FF:F:F,FFF:FF:FFF,FFFFFFFFFF:FFFF:FF
F:::FF:FFF:FFFFFFFFFFFFFF:FFF:FFFFFFFFFF:FFF,FFFFFFFFFFFFF::FFFFF::F
@A00155:342:HHGFNDSXY:1:2628:3414:12508 1:N:0:GAACCTAG+TCCGCATA
TCACCTTTCAGCGATCCCTCATTAGATTCTTGCATCTTCATCAGCATGCAAAATCTTAACATATTAAC
AAGCAGTGAGACATGGTTATCTGACCATTACCACCTCTGCATAGCGTTCACGTGT
+
FFFFFFFFF:FFF,,FFFFFFFFFFFFFFFFFFFF:FF:FF:,FFFF,FFFFFFFFFFFFFFFFFFFF
FF,FFF:FFFFFFFFFFFFFFFFFF:FF,FF:FFFFFFFFFFFFFF:FFFFFFFF
@A00155:342:HHGFNDSXY:1:2525:16396:12790 1:N:0:GAACCTAG+TCCGCATA
TCACCTTTCAGCGATCCCTCATTAGATTCTTGCATCTTCATCAGCATGCAAAATCTTAACATATTAAC
AAGCAGTGAGACATGGTTATCTGACCATGACCACCTCTGCATAGCGTTCACGTGTG
+
FFFFFFFFFFFFF,:FFFFFFFFFFFFFFFFFFFFFFFFFFFFFFFFFFFFFFFFFFF:FFFFFFFFF
FFFFFFFFFFFFFFFFFFFFFFFFFFFFFFFFFFFFFFFFFFFF:FFFFFFFFFFF
@A00155:342:HHGFNDSXY:1:2622:30544:17190 1:N:0:GAACCTAG+TCCGCATA
TCACCTTTCAGCGATCCCTCATTAGATTCTTGCATCTTCATCAGCATGCAAAATCTTAACATATTAAC
AAGCAGTGAGACATGGTTATCTGACCATGACCACCTCTGCATAGCGTTCACGTGTG
+
FFFFFFFFFFFFFFFFFFFFFFFFFFFFFFFFFFFFFFFFFF,FFFFFFFFFFFFFFFFFFFFFFFFF
FFFFFFFFFFFFFFFFFFFFFFFFFFFFFFFFFFFFFFFFFFF,FFFFFFFFFFFF
@A00155:342:HHGFNDSXY:1:1319:3721:27383 1:N:0:GAACCTAG+TCCGCATA
TCACCTTTCAGCGATCCCTCATTAGATTCTTGCATCTTCATCAGCATGCAAAATCTTAACATATTAAC
AAGCAGTGAGACATGGTTATCTGACCATGACCACCTCTGCATAGCGTTCACGTGTGTAG
+
FFFFFFFFFFFFF:FFFFFFFFFFFFFFFFFFFFF:FFFFFF,FF:FFFF:FFFFFFFFFFFFFFFFF
FFFF:FFFFFFFFF:FFFFFFFFFFFFFFF:FFFFFFFFFFFFFFFFFFFFF,FFF,:F
@A00155:342:HHGFNDSXY:1:2475:5674:36088 1:N:0:GAACCTAG+TCCGCATA
TCACCTTTCAGCGATCCCTCATTAGATTCTTGCATCTTCATCAGCATGCAAAATCTTAACATATTAAC
AAGCAGTGAGACATGGTTATCTGACCATGACCACCTCTGCATAGCGTTCACGTGTGTAGACCAC
+
FFFFFFFFF:FFF:FFFFFFFFFFFFF:FFFF::FFFFFFFFFFFFFFFFF:FFFFFFFF:FFFFFFF
FFFFFFFFFFFFFFFFFFFFFFFFFFFFFFF:FFFFFFFFFFF:FFFFFFFFFFFFFFF:FFFF
@A00155:342:HHGFNDSXY:1:1345:25391:21449 1:N:0:GAACCTAG+TCCGCATA
TCACCTTTCAGCGATCCCTCATTAGATTCTTGCATCTTCATCAGCATGCAAAATCTTAACATATTAAC
AAGCAGTGAGACATGGTTATCTGACCATGACCACCTCTGCATAGCGTTCACGTGTGTAGACCAC
+
FFFFFFFFFFFFF:FFFFFFFFFFF:FF:FFFF:FFFFFFFF,FF:FFFFFFFFFFFFFF:FFFFFFF
FFFFFFFFFFFFFF:FFFFFFFFFFFFFFFFFFFFFFFFFFFFFFFFFFFFFFFFFFFFFFFFF
@A00155:342:HHGFNDSXY:1:1278:13892:18912 1:N:0:GAACCTAG+TCCGCATA
TCACCTTTCAGCGATCCCTCATTAGATTCTTGCATCTTCATCAGCATGCAAAATCTTAACATATTAAC
AAGCAGTGAGACATGGTTATCTGACCATGACCACCTCTGCATAGCGTTCACGTGTGTAGACCAC
+

```
FFFFFFFFFFFFFF,FFFFFFFFFFFFFFFFFF:FFFFFFFFFFFFFFFFFFFFFFFFFFF:FFFFFF
FFFFFFFFFFFFFFFFFFFFFFFFFFFFFFFFFFFFFF:FFFFFFFFFFFFFFFFFFFFFFFFFFF
@A00155:342:HHGFNDSXY:1:1154:19660:12618 1:N:0:GAACCTAG+TCCGCATA
TCACCTTTCAGCGATCCCTCATTAGATTCTTGCATCTTCATCAGCATGCAAAATCTTAACATATTAAC
AAGCAGTGAGACATGGTTATCTGACCATGACCACCTCTGCATAGCGTTCACGTGTGTAGACCAC
+
FFFFFFFFFFFFFFFFFFFFFFFFFFFFFFFFFFFFFFFFFFFFFFFFFFFFFFFFFFFFF:FFFFFFF
FFFFFFFFFFFFFFFF:FFFFFFFFFFFFFFFFFFFFFFFFFFFFFFFFFF,FFFFFFF:FFFFFF
@A00155:342:HHGFNDSXY:1:1238:21251:17848 1:N:0:GAACCTAG+TCCGCATA
TCACCTTTCAGCGATCCCTCATTAGATTCTTGCATCTTCATCAGCATGCAAAATCTTAACATATTAAC
AAGCAGTGAGACATGGTTATCTGACCATGACCACCTCTGCATAGCGTTCACGTGTGTAGACCAC
+
FFFFFFFFFFFFF:FFFFFFFFFFFFFFFFFFFFFFFFFFFFFFFFFFFFFFFFFFFFFFFFFFFFFFF
FFFFFFFFFFFFFFFFFFFFFFFFFFFFFFFFFFFFFFFFFFFFFFFFFFFFFFFFFFFFF:FFFF
@A00155:342:HHGFNDSXY:1:2326:25364:31829 1:N:0:GAACCTAG+TCCGCATA
TCACCTTTCAGCGATCCCTCATTAGATTCTTGCATCTTCATCAGCATGCAAAATCTTAACATATTAAC
AAGCAGTGAGACATGGTTATCTGACCATGACCACCTCTGCATAGCGTTCACGTGTGTAGACCAC
+
FFFFFFFFFFFFFFFFFFFFFFFFFFF,:FFFFFF,,:,:FF,F:FFFFFFFF,:FF::FFFFFFFFF
FF,FFFFFF,FFFFFFFFFFFF,FFFFF::F,FFFFFFF,FFFF:FFF:F,FFFFFFFFF,:,F
@A00155:342:HHGFNDSXY:1:1154:20157:12665 1:N:0:GAACCTAG+TCCGCATA
TCACCTTTCAGCGATCCCTCATTAGATTCTTGCATCTTCATCAGCATGCAAAATCTTAACATATTAAC
AAGCAGTGAGACATGGTTATCTGACCATGACCACCTCTGCATAGCGTTCACGTGTGTAGACCAC
+
FFFFFFFFF:FFF,:FFFFFFFFFFFFFFFFFFFFFFFFF:FFFFFFFFFFFFFFFFFFFFFFFFFF,FF
FFFFFFFFFFFFFFFFFFFFFFFFFFFFFFFFFFFFFFFFFFFFFFFFFFFFFFFFFFFFFF:FFFFF
@A00155:342:HHGFNDSXY:1:2269:14543:18129 2:N:0:GAACCTAG+TCCGCATA
TCACCTTTCAGCGATCCCTCATTAGATTCTTGCATCTTCATCAGCATGCAAAATCTTAACATATTAAC
AAGCAGTGAGACATGGTTATCTGACCATGACCACCTCTGCATAGCGTTCACGTGTGTAGACCACACGT
+
FFFFFFFFFFFFFFFFFFFFFFFFFFFFFFFFFFFFFFFFFFFFFFFFFFFFFFFFFFFFFFFF:FF:FF
FFFFFFFFFFFFFFFFFFFFFFFFFFFFFFFFFFFFFFFFFFFFFFFFFFFFFFFFFFFFFFFFFFFFFF
@A00155:342:HHGFNDSXY:1:2436:16233:17112 2:N:0:GAACCTAG+TCCGCATA
TCACCTTTCAGCGATCCCTCATTAGATTCTTGCATCTTCATCAGCATGCAAAATCTTAACATATTAAC
AAGCAGTGAGACATGGTTATCTGACCATGACCACCTCTGCATAGCGTTCACGTGTGTAGACCACACGT
+
FFFFFFFFFFFFFFFFFFFFFFFFFFFFF:FFFFFFFFFFFFFFFFFFFFFFFFFF:FFFFFFFFFFFFF
:FFFFFFFFFFFFFFF:FFFFFFFFFFFFFFFFFFFFFFFFFFFFFFFFFFFFFFFFFFFFFFFFFFFF
@A00155:342:HHGFNDSXY:1:1419:22724:7341 2:N:0:GAACCTAG+TCCGCATA
TCACCTTTCAGCGATCCCTCATTAGATTCTTGCATCTTCATCAGCATGCAAAATCTTAACATATTAAC
AAGCAGTGAGACATGGTTATCTGACCATGACCACCTCTGCATAGCGTTCACGTGTGTAGACCACACGT
+
FFFFFFFFFFFFFFFFFFFFFFFFFFFFFFFFFFFFFFFFFFFFFFFFFFFFFFFFFFFFFFFFFFFFFF
FFFFFFFFFFFFFFFF,FFFFFFFFFFFFFFFFFFFFFFFFFF:FFFFFFFFFFFFFFFFFF,FFFFF
@A00155:342:HHGFNDSXY:1:1203:6017:11945 2:N:0:GAACCTAG+TCCGCATA
TCACCTTTCAGCGATCCCTCATTAGATTCTTGCATCTTCATCAGCATGCAAAATCTTAACATATTAAC
AAGCAGTGAGACATGGTTATCTGACCATGACCACCTCTGCATAGCGTTCACGTGTGTAGACCACACGT
+
FFFFFFFFFFF:FFFFFFF:FFFF:FFFFFFFFFFFFFFFFFFFFFF:FFFFFFFFFFFFFFFFFFFF
F:FFFFFFFFF:FFF:FFFFFFFFFFFFFFFFFFFFFFFFFFFFFFFFFFFFFFFFFFFFF:FFFFFF
@A00155:342:HHGFNDSXY:1:1335:7012:18928 2:N:0:GAACCTAG+TCCGCATA
CACCTTTCAGCGATCCCTCATTAGATTCTTGCATCTTCATCAGCATGCAAAATCTTAACATATTAACA
AGCAGTGAGACATGGTTATCTGACCATGACCACCTCTGCATAGCGTTCACGTGTGTAGACCACACGT
+
```

```
FFFFFFFFFFFFFF:FFFFFFFFFFFFFFFFFFFFFFFFFFFFFFFFFFFFFFFFFFFFFFFFFF,FF
FFFFFFFFFFFFFFFFFFFFFFFFFFFFFFFFFFFFFFFFFFFFFFFF:FFFFFFFFFFFFFFFFFFF
@A00155:342:HHGFNDSXY:1:1655:32796:14168 2:N:0:GAACCTAG+TCCGCATA
CACCTTTCAGCGATCCCTCATTAGATTCTTGCATCTTCATCAGCATGCAAAATCTTAACATATTAACA
AGCAGTGAGACATGGTTATCTGACCATGACCACCTCTGCATAGCGTTCACGTGTGTAGACCACACGTG
+
FFFFFFFF,FFFFFFFFFFFF:FFFFFF:FFFFFFFFFFFFFFFFFFFFFFFFFFFFFFFFFFFF:F:
,FF:FFFFFFFFFFFFFFFFFFFFFFFFFFFFFFFFFFFFFFFFFFFFFFFFFFFFFFFFFFFF:FFFF
@A00155:342:HHGFNDSXY:1:2163:8938:7263 2:N:0:GAACCTAG+TCCGCATA
CACCTTTCAGCGATCCCTCATTAGATTCTTGCATCTTCATCAGCATGCAAAATCTTAACATATTAACA
AGCAGTGAGACATGGTTATCTGACCATGACCACCTCTGCATAGCGTTCACGTGTGTAGACCACACGTG
+
FFFF:F:FFFF::,,FFFF:FF::FFFFF::FFFFFFFFFFFF::FF:FFFFFFFFFFFFFFFF:FFF
FFF:FFFFF:FFFFFFFFF:FFF,,:F:FFFFFFFF,:FFF:FFFFF:FFFFFFFFFFFFFFFF:FFF
@A00155:342:HHGFNDSXY:1:2607:22932:19789 2:N:0:GAACCTAG+TCCGCATA
CACCTTTCAGCGATCCCTCATTAGATTCTTGCATCTTCATCAGCATGCAAAATCTTAACATATTAACA
AGCAGTGAGACATGGTTATCTGACCATGACCACCTCTGCATAGCGTTCACGTGTGTAGACCACACGTG
+
FF:FF,FFFF:FFFFFFFFFF:FFFFFF,FFFFFFFFFFFFFFFFFFFFFFFFFFFFFFFFFFFF:FF
FFFFFFFFFFFFFFFFFFFFFFFFFFFFFFFFFFFF:FFFFFFFFFFFFFFFFFFFFFFFFFFFFFFF
@A00155:342:HHGFNDSXY:1:2162:11794:9017 2:N:0:GAACCTAG+TCCGCATA
ACCTTTCAGCGATCCCTCATTAGATTCTTGCATCTTCATCAGCATGCAAAATCTTAACATATTAACAA
GCAGTGAGACATGGTTATCTGACCATGACCACCTCTGCATAGCGTTCACGTGTGTAGACCACACGTGA
+
FFFFFFFFFFFFFFFFFFFFFFFFFFFFFFFFFFFFFFFFFFFFFFFFFFFFFFFFFFFFFFFF:FFF
FFFFFFFFFFFFFFFFFFFFFFFFFFFFFFFFFFFFFFFFFFFFFFFFFFFFFFFFFFFFFFFFF:FF
@A00155:342:HHGFNDSXY:1:1278:13892:18912 2:N:0:GAACCTAG+TCCGCATA
ACCTTTCAGCGATCCCTCATTAGATTCTTGCATCTTCATCAGCATGCAAAATCTTAACATATTAACAA
GCAGTGAGACATGGTTATCTGACCATGACCACCTCTGCATAGCGTTCACGTGTGTAGACCACACGTGA
+
FFFFFFFFFFFFFFFFFFFFFFFFFFFFFFFFFFFFFFFFFFFFFFFFFFFFFFFFFFFFFFFFFFFF
FFFFFFFFFFFFFFFFFFFFFFFFFFFFFFFFFFFFFFFFFFFFFFFFFFFFFFFFFFFFFFFFFFFF
@A00155:342:HHGFNDSXY:1:1405:10782:33912 2:N:0:GAACCTAG+TCCGCATA
ACCTTTCAGCGATCCCTCATTAGATTCTTGCATCTTCATCAGCATGCAAAATCTTAACATATTCACAA
GCAGTGAGACATGGTTATCTGACCATGACCACCTCTGCATAGCGTTCACGTGTGTAGACCACACGTGA
+
FFFF:FFF::FFFFFFFFF:FFFF:FFFFFFFFFFFFFFFFFFFFFFFFFFFFFFFFFFFFF,,:FF:
FFFFFFFFFFFFFFF:FFFFFFFFFFFFFFFFFFFFFFF:FFFFFFFFFFFFFFFFFFFFFF:FF:FF
@A00155:342:HHGFNDSXY:1:2162:12427:7827 2:N:0:GAACCTAG+TCCGCATA
ACCTTTCAGCGATCCCTCATTAGATTCTTGCATCTTCATCAGCATGCAAAATCTTAACATATTAACAA
GCAGTGAGACATGGTTATCTGACCATGACCACCTCTGCATAGCGTTCACGTGTGTAGACCACACGTGA
+
FFFFFFFFFFFFFFFFFFFFFFFFFFFFFFFFFFFFFFFFFFFFFFFFFFFFFFFFF:FFFFFFFFFF
F:FFFFFFFFFFFFFFFFFFFFFFFFFFFFFFFFFFFFFFFFFFFFFFFFFFFFFFFFFF:FFFFFF:
@A00155:342:HHGFNDSXY:1:2204:16604:25958 2:N:0:GAACCTAG+TCCGCATA
ACCTTTCAGCGATCCCTCATTAGATTCTTGCATCTTCATCAGCATGCAAAATCTTAACATATTAACAA
GCAGTGAGACATGGTTATCTGACCATGACCACCTCTGCATAGCGTTCACGTGTGTAGACCACACGTGA
+
FFFF,FFFFFFFFFFFFFFFFFFFFFF:FFFFFFFFF:FFFFFFFFFFFFFFFFFFFFFFFFF,FFF,
FFFFFFFFFFFFFF:FFFFFFFFFF:FFFFFFFFFFFFFFFFFFFFFFFFFFFFFFFFFFFFFFFFFF
@A00155:342:HHGFNDSXY:1:2162:11749:9064 2:N:0:GAACCTAG+TCCGCATA
ACCTTTCAGCGATCCCTCATTAGATTCTTGCATCTTCATCAGCATGCAAAATCTTAACATATTAACAA
GCAGTGAGACATGGTTATCTGACCATGACCACCTCTGCATAGCGTTCACGTGTGTAGACCACACGTGA
+
```

```
FFFFFFFFFFFFFFFFFFFFFFFFFFFFFFFFFFFFFFFFFF:FFFFFFFFFFFFFFFFFFFFFFFFF
FFFFFFFFFF:FFFFFFFFFFFFFFFFFFFFFFFFFFFF:FFFFFFFFFFFF:FFFFFFFFFFFFFFF
@A00155:342:HHGFNDSXY:1:1238:21251:17848 2:N:0:GAACCTAG+TCCGCATA
CCTTTCAGCGATCCCTCATTAGATTCTTGCATCTTCATCAGCATGCAAAATCTTAACATATTAACAAG
CAGTGAGACATGGTTATCTGACCATGACCACCTCTGCATAGCGTTCACGTGTGTAGACCACACGTGAG
+
:FFFFFFFFFFFFFFFFFFFFFFFFFFFFFFFFFFFFFFFFFFF:FFFFFFFFFFFFFFFFFFFFFFF
FFF:FFFFFFFFFFFFFFFFFFFFFFFFFFFFFFFFFFFFFFFFFFFFFFFFFFFFFFFFFFFFFFFF
@A00155:342:HHGFNDSXY:1:2519:5050:20885 2:N:0:GAACCTAG+TCCGCATA
TTTCAGCGATCCCTCATTAGATTCTTGCATCTTCATCAGCATGCAAAATCTTAACATATTAACAAGCA
GTGAGACATGGTTATCTGACCATGACCACCTCTGCATAGCGTTCACGTGTGTAGACCACACGTGAGTC
+
FFFFFFFFFF:F:FFFFFFFFFFFFFFFF:FFFFF:FFFFFF:,FFFFFFFFFFFFFFFFFFFFFFFF
FFFFFFF:FF:FFFFFFFFFFFFFFFFFFFFFFFFFFFFFFF:FFFFFFFFFFFFFFFFFFFFFFFFF
@A00155:342:HHGFNDSXY:1:1323:15374:32941 1:N:0:GAACCTAG+TCCGCATA
TTCAGCGATCCCTCATTAGATTCTTGCATCTTCATCAGCATGCAAAATCTTAACATATTAACAAGCAG
TGAGACATGGTTATCTGACCATGACCACCTCTGCATAGCGTTCACGTGTG
+
:,F:FF::,FFF::F:FF:FFFF:FFF:FF::FF:F,:FFFFFFF:,FFFFFF::FFFFFF:FF,FFF
FFFF:FFF::FFFF,FFF,FFF,FFF:F,F::FFFFF:FFFF,,FF,F,F
@A00155:342:HHGFNDSXY:1:1114:23502:36996 1:N:0:GAACCTAG+TCCGCATA
TTCAGCGATCCCTCATTAGATTCTTGCATCTTCATCAGCATGCAAAATCTTAACATATTAACAAGCAG
TGAGACATGGTTATCTGACCATGACCACCTCTGCATAGCGTTCACGTGTGTAGACCAC
+
FFFFFFF:FFFFFF:FFFFFFFFFFFF::FF:FFFF:FFFFFFFFFFFFFFFFF:FFFFFFFFFFFFF
FFFFFFFFFFFFFFFFFFFFFFFFFFFFFFFFFFFF:FFFFFFFFFFFFFFFFFFF:F
@A00155:342:HHGFNDSXY:1:2343:18376:37012 1:N:0:GAACCTAG+TCCGCATA
TTCAGCGATCCCTCATTAGATTCTTGCATCTTCATCAGCATGCAAAATCTTAACATATTAACAAGCAG
TGAGACATGGTTATCTGACCATGACCACCTCTGCATAGCGTTCACGTGTGTAGACCACACGTGAGTC
+
FFFFFFF,FFFFFFFFFFFF,FF:FFF,FFFFFF:F:,FFFFFFFFFFFFFFFFFFFFFFFFFF,FFF
:FF,FFFF,FFFFF,F:FFFFFFFFFFFF:FF:FF:FF:FFF,FFF,FFFFFFFFFFFFFFFFF:FF
@A00155:342:HHGFNDSXY:1:2131:1054:11960 1:N:0:GAACCTAG+TCCGCATA
TTCAGCGATCCCTCATTAGATTCTTGCATCTTCATCAGCATGCAAAATCTTAACATATTAACAAGCAG
TGAGACATGGTTATCTGACCATGACCACCTCTGCATAGCGTTCACGTGTGTAGACCACACGTGAGTCT
+
FFFFFFFFFFFFFFF:FFFFFFFFFFFFFFFFFFFFFFFFFFFFF:FFFFFF:FFFFFFFFFFFFFFF
:FFFFFFF:FF:FFFFF:FFF:FFFFFFFFF:FFFFFFFF:FFF,FFFFFFFFF:FFFFF:FFFFF:F
@A00155:342:HHGFNDSXY:1:1371:26955:32362 1:N:0:GAACCTAG+TCCGCATA
TTCAGCGATCCCTCATTAGATTCTTGCATCTTCATCAGCATGCAAAATCTTAACATATTAACAAGCAG
TGAGACATGGTTATCTGACCATGACCACCTCTGCATAGCGTTCACGTGTGTAGACCACACGTGAGTCT
+
FFFFFFFFFFFFFFFFFFFFFFFFFFFFFFFFFFFFFFFFFF:FFFFFFFFFFFFFFFFFFFFFFFFF
FFFFFFFFFFFFFFFFFFFFFFFFFFFFFFFFFFFFF,FFFFFFFFFFFFFFFFFFFFFFFFFFFFFF
@A00155:342:HHGFNDSXY:1:2314:27760:31876 1:N:0:GAACCTAG+TCCGCATA
TCAGCGATCCCTCATTAGATTCTTGCATCTTCATCAGCATGCAAAATCTTAACATATTAACAAGCAGT
GAGACATGGTTATCTGACCATGACCACCTCTGCATAGCGTTCACGTGTGTAGACCACACGTGAGTCTG
+
FFFFFFF,F:FFFFFFFFFFFFFFFFFFFFFFFFFFFFFF:FFFFFF:FFFFFFFFFFFFFFFF,FFF
FF:FFFFF,FFFFFFFFF,F:FFFFFFFFFFFFFFFF,FFFFFF:FFFFFFF:FFFFFF:FFFFFFFF
@A00155:342:HHGFNDSXY:1:2275:16152:33786 1:N:0:GAACCTAG+TCCGCATA
CAGCGATCCCTCATTAGATTCTTGCATCTTCATCAGCATGCAAAATCTTAACATATTAACAAGCAGTG
AGACATGGTTATCTGACCATGACCACCTCTGCATAGCGTTCACGTGTGTAGACCACACGTGAGTCTG
+
```

F::FFF:::FFFFF,FFFF:FFFFFFFFF:,FFF:F:FFFFFFFFFFF:FFFF,FFFFFFFFFFF:FF
FFFFFF,FFFFFFF,FF:FF:FF:FFF,F:FFFFF:FFFFFFFFFFFFFFFFFFFFFF:FFFFFFFF
@A00155:342:HHGFNDSXY:1:2245:23710:10989 1:N:0:GAACCTAG+TCCGCATA
CAGCGATCCCTCATTAGATTCTTGCATCTTCATCAGCATGCAAAATCTTAACATATTAACAAGCAGTG
AGACATGGTTATCTGACCATGACCACCTCTGCATAGCGTTCACGTGTGTAGACCACACGTGAGTCTGT
+
FFFFF:,FFFFFFFFFFFFFFFFFFFFFFFFFFFFFFFFFFFFFFFFFFFFFFFFFFFFFFFFFFFFF
FFFFFFFFFFFFFFFFFFFFFFFFFFFFFFFFFFFFFFFFFFFFFFFFFF:FFFFFFFFFFFFFFFFF
@A00155:342:HHGFNDSXY:1:1446:9534:25895 1:N:0:GAACCTAG+TCCGCATA
CAGCGATCCCTCATTAGATTCTTGCATCTTCATCAGCATGCAAAATCTTAACATATTAACAAGCAGTG
AGACATGGTTATCTGACCATGACCACCTCTGCATAGCGTTCACGTGTGTAGACCACACGTGAGTCTGT
+
FFFFF:FFFFFFFFFFFFFFFFFFFFFFFFFFFF:FFFFFFFFFFFFFFFFF:FFFFFFFFFFFFFFF
FFFFFFFFFFFFFFFFFFFFFFFFFFFFFFFFFFF:FFFFFFFFFFFFFFFFFFFFFFFFFFFFFFFF
@A00155:342:HHGFNDSXY:1:1630:8169:22216 1:N:0:GAACCTAG+TCCGCATA
CAGCGATCCCTCATTAGATTCTTGCATCTTCATCAGCATGCAAAATCTTAACATATTAACAAGCAGTG
AGACATGGTTATCTGACCATGACCACCTCTGCATAGCGTTCACGTGTGTAGACCACACGTGAGTCTGT
+
FFFFF:FFFFFFFFFFFFFFFFFFF:FFFFFFFFFFFFFFFFFFFFFFFFFFFFFFFFFFFFFFFFFF
FFFFFFFFFFFFFFFFFFFFFFFFFFFFFFFFFFFFFFFFFFFFFFFFFFFFFFFFFFFFFFFFFFFF
@A00155:342:HHGFNDSXY:1:2539:29658:1376 2:N:0:GAACCTAG+TCCGCATA
CAGCGATCCCTCATTAGATTCTTGCATCTTCATCAGCATGCAAAATCTTAACATATTAACAAGCAGTG
AGACATGGTTATCTGACCATGACCACCTCTGCATAGCGTTCACGTGTGTAGACCACACGTGAGTCTGT
+
FFF,FFFFFFFFFFFFFFFFFFFFFFFFFFFFFFFFFFFFFFFFFFFFFFFFFFFFFFFFFFFFFFFF
FFFFFFFFFFFFFFFFFFFFFFFFFFFFFFFFFFFFFFFFFFFFFFFFFFFFFFFFFFFFFFFFFFFF
@A00155:342:HHGFNDSXY:1:2304:6551:17472 2:N:0:GAACCTAG+TCCGCATA
CAGCGATCCCTCATTAGATTCTTGCATCTTCATCAGCATGCAAAATCTTAACATATTAACAAGCAGTG
AGACATGGTTATCTGACCATGACCACCTCTGCATAGCGTTCACGTGTGTAGACCACACGTGAGTCTGT
+
FFFFFFFFFFFF:FFFF:FFFFF:FFFFF:F,FFFFFFFFFFFFFFFFFFFFF,:F:F,F:FFFFF:F
FFFFF:FFF,FFFFFFFFFFFFFFFFFFFFFFFFFFFFFFFFF:FFFFFFFFFFFFFFFFFFFFFF:F
@A00155:342:HHGFNDSXY:1:1608:24026:30984 1:N:0:GAACCTAG+TCCGCATA
CAGCGATCCCTCATTAGATTCTTGCATCTTCATCAGCATGCAAAATCTTAACATATTAACAAGCAGTG
AGACATGGTTATCTGACCATGACCACCTCTGCATAGCGTTCACGTGTGTAGACCACACGTGAGTCTGT
+
FFFFFFFFFF:FFFFFFFFFFFFFFFFFFFFFFFFFFFFFFFFF:FFFFFFFFFFFFFFFFFFFFFFF
FFFFFFFFFFFFFFFFFFFFFFFFFFFFFFFFFFFFFFFFFFFFFFFFFFFFFFFFFFFFFFFFF:FF
@A00155:342:HHGFNDSXY:1:2140:31358:11334 1:N:0:GAACCTAG+TCCGCATA
AGCGATCCCTCATTAGATTCTTGCATCTTCATCAGCATGCAAAATCTTAACATATTAACAAGCAGTGA
GACATGGTTATCTGACCATGACCACCTCTGCATAGCGTTCACGTGTGTAGACCACACGTGAGTCTGTA
+
FFFF:,FFFFFFFFFFFFFFFFFFFFFFFFFFFFFFFFFFF:FFFFFFFFF:FFFFFFFFFFFFFFFF
FFF:FFFFFFFFFFFFFFFFFFFFFFFFFFFFF:FFFFFFFFFFFFFF:FFFFF:FFFFFFFFFFFFF
@A00155:342:HHGFNDSXY:1:2344:9245:36573 1:N:0:GAACCTAG+TCCGCATA
AGCGATCCCTCATTAGATTCTTGCATCTTCATCAGCATGCAAAATCTTAACATATTAACAAGCAGTGA
GACATGGTTATCTGACCATGACCGCCTCTGCATAGCGTTCACGTGTGTAGACCACACGTGAGTCTGTA
+
FFFF:,FFFFFFFFFFFFFFFFFFFFFFFFFFFFFFFFFFFFFFFFFFFFFFFFFFFFFFFFFFFFFF
FFFFFFFFFFFFFFFFFFFFFFFFFFFFFFFFFFFFFFFFFFFFFFFFF,FFFFFFFFFF:FFFFFFF
@A00155:342:HHGFNDSXY:1:1641:31222:4429 1:N:0:GAACCTAG+TCCGCATA
AGCGATCCCTCATTAGATTCTTGCATCTTCATCAGCATGCAAAATCTTAACATATTAACAAGCAGTGA
GACATGGTTATCTGACCATGACCACCTCTGCATAGCGTTCACGTGTGTAGACCACACGTGAGTCTGTA
+

FFFFFFFFFFFFFFFFFFFFFFFFFFFFFFFF:FFFFFFFFFFFFFFF:FFFFFFFFFFFF:FFFFFF
FFFFFFFFFFFFFFFFFFFFFFFFFF:FFFFFFFFFFFFFFFFFFFF,FFFFF,FFFF,FFFFFFFFF
@A00155:342:HHGFNDSXY:1:2166:3070:26944 1:N:0:GAACCTAG+TCCGCATA
AGCGATCCCTCATTAGATTCTTGCATCTTCATCAGCATGCAAAATCTTAACATATTAACAAGCAGTGA
GACATGGTTATCTGACCATGACCACCTCTGCATAGCGTTCACGTGTGTAGACCACACGTGAGTCTGTA
+
FFFFFFFFFFFFFFFFFFFFFF:FFFFFFF:FFFFFFFFFFFFFFFFFFFF:FFFFFFFFFFFFFFFF
FFFFFFFFFFFFFFFFFFFFFFFFFFFFFFFFFFFFFFFFFFFFFFFFF:FFFFFFFFFFFFFFFFFF
@A00155:342:HHGFNDSXY:1:2138:24370:16673 1:N:0:GAACCTAG+TCCGCATA
AGGGATCCCTCATTAGATTCTTGCATCTTCATCAGCATGCAAAATCTTAACATATTAACAAGCAGTGA
GACATGGTTATCTGACCATGACCACCTCTGCATAGCGTTCACGTGTGTAGACCACACGTGAGTCTGTA
+
FFFF,FFFFFFFFFFFFFFFFFFFFFFFFFFFFFFFFFFFFFFFFFFFFFF:FFFFFFFFFFFFFFFF
FFFFFFFFFFFFFFFFFFFFFF:FFFFFFFFFFFFFFFFFFFFFFFFFFFFFFFFFFFFFFFFFFFFF
@A00155:342:HHGFNDSXY:1:2314:10719:9846 1:N:0:GAACCTAG+TCCGCATA
AGCGATCCCTCATTAGATTCTTGCATCTTCATCAGCATGCAAAATCTTAACATATTAACAAGCAGTGA
GACATGGTTATCTGACCATGACCACCTCTGCATAGCGTTCACGTGTGTAGACCACACGTGAGTCTGTA
+
FFFFF,FFFFFFFFFFFFFFFFFFFFFFFFFFFFFFFFFFFFFFFFFFFFF:FFFFFFFFFFFFFFFF
FFFFFFFFFFFFFFFFFFFFFFFFFFFFFFFFFFFFFFFFFFFFFFFFFFFFFFFFFFFFFFFFFFFF
@A00155:342:HHGFNDSXY:1:1571:17101:24721 1:N:0:GAACCTAG+TCCGCATA
AGCGATCCCTCATTAGATTCTTGCATCTTCATCAGCATGCAAAATCTTAACATATTAACAAGCAGTGA
GACATGGTTATCTGACCATGACCACCTCTGCATAGCGTTCACGTGTGTAGACCACACGTGAGTCTGTA
+
FF:F,FFF,FFF,:FFFFFFFFFFFFFFFFFFFF:FFFFFF:FFFFFFFFFFFF:FFFFFF:FF:FFF
FFFF:FFFFFFFFFFFFFFFFFFFFFFFFFFFFFF:FFFFFFFFFFFFFFF:FFFFFFF:FFFFFFFF
@A00155:342:HHGFNDSXY:1:1336:15338:30561 1:N:0:GAACCTAG+TCCGCATA
AGCGATCCCTCATTAGATTCTTGCATCTTCATCAGCATGCAAAATCTTAACATATTAACAAGCAGTGA
GACATGGTTATCTGACCATGACCACCTCTGCATAGCGTTCACGTGTGTAGACCACACGTGAGTCTGTA
+
FFFFFFF:FFFFFFFFFFFFFFFFFFFFFFFFFFFFFFFFFFFFFFFFFFFFFFFFFFFFFFFFFFFF
FFFFFFFFFFFFFFFFFFFFFFFFFFFFFFFFFFFFFFFFFFFFFFFFFFFFFFFFFFFFFFFFFFFF
@A00155:342:HHGFNDSXY:1:1441:17282:36714 1:N:0:GAACCTAG+TCCGCATA
AGCGATCCCTCATTAGATTCTTGCATCTTCATCAGCATGCAAAATCTTAACATATTAACAAGCAGTGA
GACATGGTTATCTGACCATGACCACCTCTGCATAGCGTTCACGTGTGTAGACCACACGTGAGTCTGTA
+
FFFFFFFFFFFFFFFFFFFFFFFFFFFFFFFFFFFFF:FFFFFFFFFFFFFFFFFFFFFFFFFFFFFF
FFFFFFFFFFFFFFFFFFFFFFFFFFFFFFFFFFFFFFFFFFFFFFFFF,FFFFFFF:FFFF:FFFFF
@A00155:342:HHGFNDSXY:1:2116:25211:27305 1:N:0:GAACCTAG+TCCGCATA
AGCGATCCCTCATTAGATTCTTGCATCTTCATCAGCATGCAAAATCTTAACATATTAACAAGCAGTGA
GACATGGTTATCTGACCATGACCACCTCTGCATAGCGTTCACGTGTGTAGACCACACGTGAGTCTGTA
+
FFFF:FFFFFFFFFFFFFFFFFFFFFFFFFFFFFFFFFFFFFFFFFFFFFFFFFFFFFFFFFFFFFFF
FFFFFFFFFFFFFFFFFFFFFFFFFFFFFFFFFFFFFFFFFFFFFFFFFFFFFFFFFFFFFFFFFFFF
@A00155:342:HHGFNDSXY:1:1538:20283:32612 1:N:0:GAACCTAG+TCCGCATA
AGCGATCCCTCATTAGATTCTTGCATCTTCATCAGCATGCAAAATCTTAACATATTAACAAGCAGTGA
GACATGGTTATCTGACCATGACCACCTCTGCATAGCGTTCACGTGTGTAGACCACACGTGAGTCTGTA
+
FFFF,FFFFFFFFFFFFFFFFFFFFFFFFFFFFFFFFFFFFFFFFFFFFFFFFFFFFFFFFFFFFFFF
FFFFFFFFFFFFFFFFFFFFFFFFFFFFFFFFFFFFFFFFFFFFFFFFFFFFFFFFFFFFFFFFFFFF
@A00155:342:HHGFNDSXY:1:1530:17996:16125 1:N:0:GAACCTAG+TCCGCATA
AGCGATCCCTCATTAGATTCTTGCATCTTCATCAGCATGCAAAATCTTAACATATTAACAAGCAGTGA
GACATGGTTATCTGACCATGACCACCTCTGCATAGCGTTCACGTGTGTAGACCACACGTGAGTCTGTA
+

FFFF:FFFFFFFFFFFFFFFFFFFFFFF:FFFF:FFFFFFFFFFFFFFFFF:FFFFFFFFFFFFFFFF
FFFFFFFFFFFFFFFFFFFFFFFFFFFFFFFFFF:FFFFFFFFFFFFFFFFFFFFFFFFFFFFFFFFF
@A00155:342:HHGFNDSXY:1:1525:30156:26412 1:N:0:GAACCTAG+TCCGCATA
AGCGATCCCTCATTAGATTCTTGCATCTTCATCAGCATGCAAAATCTTAACATATTAACAAGCAGTGA
GACATGGTTATCTGACCATGACCACCTCTGCATAGCGTTCACGTGTGTAGACCACACGTGAGTCTGTA
+
FFF::FFFFFFFFFFFFFF:FFFFFF:FFFFFFF,FFFFFFFFFFFFFFFF:FFFFFFFFFF,FFFFF
FFFFFFFFFFFFFFFFFFFFFFFFFFFF:FFFFFFF,FFF:::F,F::FFFFFFFFFFFFFFFFFFFF
@A00155:342:HHGFNDSXY:1:2163:3188:24486 1:N:0:GAACCTAG+TCCGCATA
AGCGATCCCTCATTAGATTCTTGCATCTTCATCAGCATGCAAAATCTTAACATATTAACAAGCAGTGA
GACATGGTTATCTGACCATGACCACCTCTGCATAGCGTTCACGTGTGTAGACCACACGTGAGTCTGTA
+
FFFF,FFFFFFFFFFFFFFFFFFFFFFFFF:FF:FFFFFFFFF:FFFFFFFFFFFFFFFFFFFFFFFF
FFFFFFFFFFFFFFFFFFFFFFFFFFFFFFFFFFFFFFFFFFFFFFFFFFFFFFFFFFFFFFFFFFFF
@A00155:342:HHGFNDSXY:1:1668:20166:34726 2:N:0:GAACCTAG+TCCGCATA
GCGATCCCTCATTAGATTCTTGCATCTTCATCAGCATGCAAAATCTTAACATATTAACAAGCAGTGAG
ACATGGTTATCTGACCATGACCACCTCTGCATAGCGTTCACGTGTGTAGACCACACGTGAGTCTGTAA
+
FFFFFFFFFF:FFFFF:FFFFFFFFFFFFFFFFFFFFFFFFFF:FFFFFF:FFFF:FF,F:FFFFFFF
FFFFFFFFFFFFFFFFFFFFFFFFFFFFFFFFFFFFFFFFFFFFFFFFFFFFFFFFFFFFFFFFFFFF
@A00155:342:HHGFNDSXY:1:2216:25916:9173 2:N:0:GAACCTAG+TCCGCATA
GCGATCCCTCATTAGATTCTTGCATCTTCATCAGCATGCAAAATCTTAACATATTAACAAGCAGTGAG
ACATGGTTATCTGACCATGACCACCTCTGCATAGCGTTCACGTGTGTAGACCACACGTGAGTCTGTAA
+
F:FFFFFFFFFFFFFFFFFFFFFFFFFFFF:FFFFFFFFFFFFFFFFFFFFFFFFFFFFFFFFFFFFF
FFFFFFFFFFFFFFFFFFFFFFFFFFFFFFFFFFFFFFFFFFFFFFFFFFFFFFFFFFFFFFFFFFFF
@A00155:342:HHGFNDSXY:1:1171:7392:8594 1:N:0:GAACCTAG+TCCGCATA
GATCCCTCATTAGATTCTTGCATCTTCATCAGCATGCAAAATCTTAACATATTAACAAGCAGTGAGAC
ATGGTTATCTGACCATGACCACCTCTGCATAGCGTTCACGTGTGTAGACCACACGTGAGTCTGTAATC
+
FFFFFFFFFFFFFFFFFFFFFFFFFFFFFFFFFFFFFF:FFFFFFFFF:FFFFFFFFFFFFFFFFFFF
FFFFFFFFFFFFFFFFFFFFFFFFFFFFFFFFFFFFFFF,FFFFFFFFFFFFFFFFFFFFFFFFFFFF
@A00155:342:HHGFNDSXY:1:2608:30327:2065 1:N:0:GAACCTAG+TCCGCATA
GATCCCTCATTAGATTCTTGCATCTTCATCAGCATGCAAAATCTTAACATATTAACAAGCAGTGAGAC
ATGGTTATCTGACCATGACCACCTCTGCATAGCGTTCACGTGTGTAGACCACACGTGAGTCTGTAATC
+
FFFFFFFFFFFFFFFFFFFFFFFFFFFFFFFFFFFFFFFFFFFFFFFFFFFFFFFFFFFFFFFFFFFF
FFFFFFFFFFF:FFFFFFFFFFFFFFFFFFF:FFFFFFFFFFFFFFFFFFFFFFFFFFFFFFFFFFFF
@A00155:342:HHGFNDSXY:1:2503:32362:7748 1:N:0:GAACCTAG+TCCGCATA
GATCCCTCATTAGATTCTTGCATCTTCATCAGCATGCAAAATCTTAACATATTAACAAGCAGTGAGAC
ATGGTTATCTGACCATGACCACCTCTGCATAGCGTTCACGTGTGTAGACCACACGTGAGTCTGTAATC
+
FF:FFFFFFFFFFFFFFFFFFFFFFFFFFF:FFFFFF::FF:FFFFFFFFFFFFFFFFFF,FFFFFFF
FFFFFFFFFFFFFFFFFFFFFFFFFFFFFF:FFFFFFFFFFFFFFFFFFF:FFFFFFFFFFFFFFFFF
@A00155:342:HHGFNDSXY:1:2473:27434:2315 2:N:0:GAACCTAG+TCCGCATA
GATCCCTCATTAGATTCTTGCATCTTCATCAGCATGCAAAATCTTAACATATTAACAAGCAGTGAGAC
ATGGTTATCTGACCATGACCACCTCTGCATAGCGTTCACGTGTGTAGACCACACGTGAGTCTGTAATC
+
FFFFFFFFFFFFFFFFFFFFFFFFFFFFFFFFFFFFFFFFF:FFFFFFFFFFFFFFF:FFFFFFFFFF
FFFFFFFFFFFFFFFFFFFFFFFFFFFFFFFFFFFFFFFFFFFFFFFFFFFFFFFFFFFFFFFFFFFF
@A00155:342:HHGFNDSXY:1:2608:30038:1846 1:N:0:GAACCTAG+TCCGCATA
GATCCCTCATTAGATTCTTGCATCTTCATCAGCATGCAAAATCTTAACATATTAACAAGCAGTGAGAC
ATGGTTATCTGACCATGACCACCTCTGCATAGCGTTCACGTGTGTAGACCACACGTGAGTCTGTAATC
+

FFFFFFFFFFFFFFFFFFFFFFFF:FFFFF:F:FFFFFFFFFFFFFF:FFFFFFFFFFFFFFFFFFF
FFFFFFFFFFFFFFFFFFFFFFFFFF:FFFFFFFFFFFFFFFFFFFFFFFFFFFFFFFFFFFFFFFFFF
@A00155:342:HHGFNDSXY:1:2247:32515:18818 1:N:0:GAACCTAG+TCCGCATA
ATCCCTCATTAGATTCTTGCATCTTCATCAGCATGCAAAATCTTAACATATTAACAAGCAGTGAGACA
TGGTTATCTGACCATGACCACCTCTGCATAGCGTTCACGTGTGTAGACCACACGTGAGTCTGTAATC
+
:,FFFFFFFFFFF:FFFFFF,FF:FFF:FFFF:FFF::FFFFF,FFFFFF:FFFFFFFFFFFFFFFF:
FFFFFFFFFFFFFFFFFFFFFFFFFFFFFFFFFFFFFFFFFFF:FFFFFFFFFFFFFF:FFFFFF:FFF
@A00155:342:HHGFNDSXY:1:2528:8305:20353 2:N:0:GAACCTAG+TCCGCATA
ATCCCTCATTAGATTCTTGCATCTTCATCAGCATGCAAAATCTTAACATATTAACAAGCAGTGAGACA
TGGTTATCTGACCATGACCACCTCTGCATAGCGTTCACGTGTGTAGACCACACGTGAGTCTGTAATCC
+
FFFFFFFF:FFFFFFFFFFFFF,FFFFFFFFFF:FFFFFFF,FFFFFFFF:FFFFFFFFFFFFFFFF
FFFFFFFFFFFFFFFFFFFFFFFFFFFFFFFFFFFFFFFFFFFFFFFFFFFFFFFFFFFFFFFFFFFF
@A00155:342:HHGFNDSXY:1:1635:12400:11099 1:N:0:GAACCTAG+TCCGCATA
ATCCCTCATTAGATTCTTGCATCTTCATCAGCATGCAAAATCTTAACATATTAACAAGCAGTGAGACA
TGGTTATCTGACCATGACCACCTCTGCATAGCGTTCACGTGTGTAGACCACACGTGAGTCTGTAATCC
+
FFFFF:FFF:FFFFF:FFFFFFFF:FFFFFFFFFFFFFFFFFFFFFFFFFFF:FFFFFFF:FFFFFFF
FFFFFFFFFFFFFFFFFFFFFFFFFFFFFFFFFFFFFFFFFFFFFFFFFFFFFFFFFFFFFFFFFFFF
@A00155:342:HHGFNDSXY:1:2519:5050:20885 1:N:0:GAACCTAG+TCCGCATA
TCCCTCATTAGATTCTTGCATCTTCATCAGCATGCAAAATCTTAACATATTAACAAGCAGTGAGAAAT
GGTTATCTGACCATGACCACCTCTGCATAGCGTTCACGTGTGTAGACCACACGTGAGTCTGTAATCC
+
FFFFFFFFFFFFFFFFFFFFFFFFFFFFFFFFFFFFFFFF:FFFFFF:FFFFFF:FFF:FFFFF:F,FF
,FFFFF,FF:F,FF:FF:FFFFFFFFFFFFFF:FF:FFFFFFFFFFFFFFFF,F:F::FF,FFFFFF
@A00155:342:HHGFNDSXY:1:1165:9046:27868 1:N:0:GAACCTAG+TCCGCATA
TCCCTCATTAGATTCTTGCATCTTCATCAGCATGCAAAATCTTAACATATTAACAAGCAGTGAGACAT
GGTTATCTGACCATGACCACCTCTGCATAGCGTTCACGTGTGTAGACCACACGTGAGTCTGTAATCCT
+
FFFFFFFFFFFFFFFFFFFFFFFF,FFFF::FFFF:FF:FFFFFFFF:FFFFFFFFFFFFFFFFFFFFF
FFFFFFFFFFFFFFFFFFFFFFFFFFFFFFFFFF:FFF:FFFFF::FFFFFFFFFFFFFFFFFFFFFFF
@A00155:342:HHGFNDSXY:1:1110:10230:30389 2:N:0:GAACCTAG+TCCGCATA
TCCCTCATTAGATTCTTGCATCTTCATCAGCATGCAAAATCTTAACATATTAACAAGCAGTGAGACAT
GGTTATCTGACCATGACCACCTCTGCATAGCGTTCACGTGTGTAGACCACACGTGAGTCTGTAATCCT
+
FFFFFFFFFFFFFFFFFFFFFF:FFFFFFFFFFFFFFFFFFFFFFFFFFFFFFFFFFFF:FFFFFFFFFFF
FFFFFFFFFF,FFFFFFFFFFFFFFFFFFFFFFFFFFFFFFFF:FFFFFFFFFFFFFFFFFFFFFFFFFF
@A00155:342:HHGFNDSXY:1:1355:18222:19460 1:N:0:GAACCTAG+TCCGCATA
TCCCTCATTAGATTCTTGCATCTTCATCAGCATGCAAAATCTTAACATATTAACAAGCAGTGAGACAT
GGTTATCTGACCATGACCACCTCTGCATAGCGTTCACGTGTGTAGACCACACGTGAGTCTGTAATCCT
+
FFFFFFFFFFFFFFFFFFFFFFFFFFFFF:FFFFFFF:FFFFFFFFFFFFFFFFFFFFFFFFFFFFFFF
:FFFFFFFFFFFFFFFFFFFFFFFFFFFFFFFFFFFFFFFFFFFFFFFFFFFFFFFFFFFFFFFFFFFF
@A00155:342:HHGFNDSXY:1:2239:6370:21543 1:N:0:GAACCTAG+TCCGCATA
TCCCTCATTAGATTCTTGCATCTTCATCAGCATGCAAAATCTTAACATATTAACAAGCAGTGAGACAT
GGTTATCTGACCATGACCACCTCTGCATAGCGTTCACGTGTGTAGACCACACGTGAGTCTGTAATCCT
+
FFFFFFFFFFFFFFFFFFFFFFFFFFFFFFFFFFF:FFFFFFFFFF:FFFFFFFFFFFFFFFFFFFFF
:FFFFFFFFFFFF:FFF,FFFFFFFFFFFF:F:FFFFF:F:FFFFFFF,FFFFFFFFFFFFFFFF,FF
@A00155:342:HHGFNDSXY:1:2473:27434:2315 1:N:0:GAACCTAG+TCCGCATA
TCCCTCATTAGATTCTTGCATCTTCATCAGCATGCAAAATCTTAACATATTAACAAGCAGTGAGACAT
GGTTATCTGACCATGACCACCTCTGCATAGCGTTCACGTGTGTAGACCACACGTGAGTCTGTAATCCT
+

```
,FFFFFFFFFFFFFFFFFFFFFFFFFFFFFFFFFFFFFFFFF:FFFFFFFFFFFFFFFFFFFFFFFFF
FFFFFFFFFFFFFFFFFFFFFFFFFFFFFFFFFFFFFFFFFFFFFFFFFFFFFFFFFFFFFFFFFFFF
@A00155:342:HHGFNDSXY:1:2656:15194:31908 2:N:0:GAACCTAG+TCCGCATA
CCCTCATTAGATTCTTGCATCTTCATCAGCATGCAAAATCTTAACATATTAACAAGCAGTGAGACATG
GTTATCTGACCATGACCACCTCTGCATAGCGTTCACGTGTGTAGACCACACGTGAGTCTGTAATCCTG
+
FFFFFFFFFFFFFFFFFFFFFFFFFFFFFFFFFF:FFF:FFFFFFFFFFFFFFF:FFF,FFFFFFFFF
FFFFFFFFFFFFFFFFFFFFFFFFFF:FFFF:FFFFFFFFFFFFFFFFFFFFFFFFFFFFFFFFFFFF
@A00155:342:HHGFNDSXY:1:2475:5674:36088 2:N:0:GAACCTAG+TCCGCATA
CCTCATTAGATTCTTGCATCTTCATCAGCATGCAAAATCTTAACATATTAACAAGCAGTGAGACATGG
TTATCTGACCATGACCACCTCTGCATAGCGTTCACGTGTGTAGACCACACGTGAGTCTGTAATCCTGC
+
FFFF:FFFFF,FF,FFFF:FFFFFFFFFFFFFFFFFFFFFFFFFF:FFF,FFFFFFFFFFFFFFFFFF
FFFFFFFFFFFFFFFFFFFFFFFFFFFFFFFFFFFFFFFFFFFFFFFFFFFFFFFFFFFFFFFFFFFF
@A00155:342:HHGFNDSXY:1:1529:13268:18521 1:N:0:GAACCTAG+TCCGCATA
CCTCATTAGATTCTTGCATCTTCATCAGCATGCAAAATCTTAACATATTAACAAGCAGTGAGACATGG
TTATCTGACCATGACCACCTCTGCATAGCGTTCACGTGTGTAGACCACACGTGAGTCTGTAATCCTGC
+
FFFFFFFFFFFFFFFFFFFFFFFF:FFFFFFFFFFFFFFFFFFF:FFFFFFFFFFFFFFFFFFFFFFF
FFFFFFFFFFFFFFFFFFFFFFFFFFFFFFFFFFFFFFFFFFFFFF:FFFFFFFFFFFFFFFFFFFFF
@A00155:342:HHGFNDSXY:1:1111:10384:32690 2:N:0:GAACCTAG+TCCGCATA
CTCATTAGATTCTTGCATCTTCATCAGCATGCAAAATCTTAACATATTAACAAGCAGTGAGACATGGT
TATCTGACCATGACCACCTCTGCATAGCGTTCACGTGTGTAGACCACACGTGAGTCTGTAATCCTGCT
+
FFF,FFFFFFF:FFFFF:FFFFFFFFFFFFFFFFFFFFFFFFFF,FFF:FFFFFFFFFF:FFFFFFFF
FFFFFFFFFFFFFFFFFFFFFFFFFFFFFFFFFFFFFFFFFFFFFFFFFFFFFFFFFFFFFFFFFFF:
@A00155:342:HHGFNDSXY:1:1623:18394:4319 1:N:0:GAACCTAG+TCCGCATA
TCATTAGATTCTTGCATCTTCATCAGCATGCAAAATCTTAACATATTAACAAGCAGTGAGACATGGTT
ATCTGACCATGACCACCTCTGCATAGCGTTCACGTGTGTAGACCACACGTGAGTCTGTAA
+
FFFFFFF,FFF:FFFFFFFFFFFF:FFFFFFF:FFFFFFFFF,FFFFFFFFFFFFFFFF:FFFFFFFF
FFFFFFF:FFFFFFFFFFF:FFFF:FFFFFFF,FFFFFFFFFF,F:FFFFFFFFFFFFFF
@A00155:342:HHGFNDSXY:1:2656:15194:31908 1:N:0:GAACCTAG+TCCGCATA
TCATTAGATTCTTGCATCTTCATCAGCATGCAAAATCTTAACATATTAACAAGCAGTGAGACATGGTT
ATCTGACCATGACCACCTCTGCATAGCGTTCACGTGTGTAGACCACACGTGAGTCTGTAA
+
FFF::F:FFFFFFFFFFFFFFFFF:FFFFFFF:FFFF:FFFF::FFFFFFFFF:F:FFFFF::F:::F
F:FFFFFFF:FFFFF:F:FFFFF,F:FF,FFFF,FFFFFFFFF:FFFF,F:FFFF::FFF
@A00155:342:HHGFNDSXY:1:2428:27127:29622 1:N:0:GAACCTAG+TCCGCATA
TCATTAGATTCTTGCATCTTCATCAGCATGCAAAATCTTAACATATTAACAAGCAGTGAGACATGGTT
ATCTGACCATGACCACCTCTGCATAGCGTTCACGTGTGTAGACCACACGTGAGTCTGTAA
+
FFFFFFFFFFFFFFFFFFFFFFFFFFFFFFFFFFFFFFFFFFFFFFFFFFFFFFFFFFFFFFFFFFFF
:FFFFFF:FFFFFFFFFFFFFFFFFFFFFFFFFFFFFFFFFFF:FFFFFFFFFFFFFFFF
@A00155:342:HHGFNDSXY:1:1239:1533:11537 2:N:0:GAACCTAG+TCCGCATA
TCATTAGATTCTTGCATCTTCATCAGCATGCAAAATCTTAACATATTAACAAGCAGTGAGACATGGTT
ATCTGACCATGACCACCTCTGCATAGCGTTCACGTGTGTAGACCACACGTGAGTCTGTAATCCTGCTG
+
FFFFFF:FFFF,FF:F,FFFFFFFFFFF::FFFFFFFFFFFFFFFFF,FFF::FF:FFFFFFFFFFFF
FFFFFFFFFFFFFFFFF,FFFFFFFFFFFFFFFF,FF:FFFFFFFFFFF:FFF:FFFFFFFFFFFFFF
@A00155:342:HHGFNDSXY:1:1239:1823:15107 2:N:0:GAACCTAG+TCCGCATA
TCATTAGATTCTTGCATCTTCATCAGCATGCAAAATCTTAACATATTAACAAGCAGTGAGACATGGTT
ATCTGACCATGACCACCTCTGCATAGCGTTCACGTGTGTAGACCACACGTGAGTCTGTAATCCTGCTG
+
```

```
FF::FFFFFFFFFFFF:FFF,FFFFFFFFFFFFFFFFFFFFFFFFFFFFFF:FFFF:FFFFFFFFFFF
FFFFFFFFFFFFFFFFFFFFFFFFFFFFFFFFFFFFFFFFFFFFFFFFFFFFFFFFFFFFFFFFFFFF
@A00155:342:HHGFNDSXY:1:2416:15899:34757 2:N:0:GAACCTAG+TCCGCATA
CATGAGATTCTTGCATCTTCATCAGCATGCAAAATCTTAACATATTAACAAGCAGTGAGACATGGTTA
TCTGACCATGACCACCTCTGCATAGCGTTCACGTGTGTAGACCACACGTGAGTCTGTAATCCTGCTG
+
FFF,FFFFFF,FFFFFFFFF:FFFFF,FFFFFFFFFFF:FFFFFFF:,F:,FFFFFFFFFF:FFFFFF
FFFFFFFF:FFFFFFFFFFFFFFFFFFFFFFFFFFFFFFFFFFFFFFFFFFFFFFFFFFFFF:FFFF
@A00155:342:HHGFNDSXY:1:2357:6831:10755 2:N:0:GAACCTAG+TCCGCATA
CATTAGATTCTTGCATCTTCATCAGCATGCAAAATCTTAACATATTAACAAGCAGTGAGACATGGTTA
TCTGACCATGACCACCTCTGCATAGCGTTCACGTGTGTAGACCACACGTGAGTCTGTAATCCTGCTGT
+
FFFFFFFFFFF:FFF:FFFFF:FF::FFFFFFFFFF:FFFFFFFFFF:FF,:FFFFFFFFFFFFFFFF
FFFFFFFFFFFFFFFFFFFFFFFFFFFFFFFFFFFFFFFFFFFFFFFFFFFFFFFFFFFFFFFFFFFF
@A00155:342:HHGFNDSXY:1:1157:22318:20196 2:N:0:GAACCTAG+TCCGCATA
CATTAGATTCTTGCATCTTCATCAGCATGCAAAATCTTAACATATTAACAAGCAGTGAGACATGGTTA
TCTGACCATGACCACCTCTGCATAGCGTTCACGTGTGTAGACCACACGTGAGTCTGTAATCCTGCTGT
+
FFFFFFFFFFFFFFFFFFFFFFFFFFFFFFFFFFFFFFFFFFF:FFFFFF:FFFFFFFFFFFFFFFFF
FFFFFFFFFFFFFFFFFFFFFFFFFFFFFFFFFFFFFFFFFFFFFFFFFFFFFFFFFFFFFFFFFFFF
@A00155:342:HHGFNDSXY:1:1357:6126:14575 2:N:0:GAACCTAG+TCCGCATA
CATTAGATTCTTGCATCTTCATCAGCATGCAAAATCTTAACATATTAACAAGCAGTGAGACATGGTTA
TCTGACCATGACCACCTCTGCATAGCGTTCACGTGTGTAGACCACACGTGAGTCTGTAATCCTGCTGT
+
FFFFFFFFFFFFFFFFFFFFFF:FFFFFFFFFFFFFFFFFFFFFF,,F,FF::,F:FFFFFFFFFFFFFF
FFFFFFFFFFFFFFFFFFFFFFFFFF:FFFFFFFFFFFFFFFFFFFFFFFFFFFFFFFFFFFFFFFFFF
@A00155:342:HHGFNDSXY:1:2371:5122:3223 1:N:0:GAACCTAG+TCCGCATA
CATTAGATTCTTGCATCTTCATCAGCATGCAAAATCTTAACATATTAACAAGCAGTGAGACATGGTTA
TCTGACCATGACCACCTCTGCATAGCGTTCACGTGTGTAGACCACACGTGAGTCTGTAATCCTGCTGT
+
FFFFFFFFFFFFFFFFFFFFFF:FFFFFFFFFF:FFFFFFFFF:FFFFFFFFF:FFFFFFFFFFFFFFF
FFF:FFFF:FFFFFFFFFFF:FFFFF,FFFFFFFFFFF:FFF:FFFFFF:FFFF,FFFF:F:FFFFFF
@A00155:342:HHGFNDSXY:1:2131:1054:11960 2:N:0:GAACCTAG+TCCGCATA
CAATAGATTCTTGCATCTTCATCAGCATGCAAAATCTTAACATATTAACAAGCAGTGAGACATGGTTA
TCTGACCATGACCACCTCTGCATAGCGTTCACGTGTGTAGACCACACGTGAGTCTGTAATCCTGCTGT
+
FF,FF:FFFFF,:FFFFF,FF,FF:FF:FFFFFFF,FFFFFFFF::FFF:F,FFF,FFFFFFFFFFFF
:FF:FFFFFFFFFFFF,F:FF:FFFFFFFFFFFFFFFFFFFFFFFFFFF:FFFFFFFFFFFFF:FFFFF
@A00155:342:HHGFNDSXY:1:1371:26955:32362 2:N:0:GAACCTAG+TCCGCATA
CATTAGATTCTTGCATCTTCATCAGCATGCAAAATCTTAACATATTAACAAGCAGTGAGACATGGTTA
TCTGACCATGACCACCTCTGCATAGCGTTCACGTGTGTAGACCACACGTGAGTCTGTAATCCTGCTGT
+
FF:F::F,FFFFFFFF::F:FFFFFFFFFFFFFF:FFFFFFFFFFFFFFFFFFFFFFFFFFFFFFFFFF
FFFFFFFFFFFFFFFFFFFFFFF:FFFFFFFFFFFFFFFFFFFFFFFFFFFFFFFFFFFFFFFFFFFF:
@A00155:342:HHGFNDSXY:1:2609:6298:29027 2:N:0:GAACCTAG+TCCGCATA
ATTAGATTCTTGCATCTTCATCAGCATGCAAAATCTTAACATATTAACAAGCAGTGAGACATGGTTAT
CTGACCATGACCACCTCTGCATAGCGTTCACGTGTGTATACCACACGTGAGTCTGTAATCCT
+
FFFFFFFFFFF:,,FFFF,FF:F:F:FF::FFFFFFFFF:FF:FFFFF,,,FFFFFFF::FF:FFFF:
FFFF::,F,FFFF:FF:F::FFFFF:FFFF:FFFF:FF,FF:F:,:FFFFFF:FFFFFFFFF
@A00155:342:HHGFNDSXY:1:1530:17996:16125 2:N:0:GAACCTAG+TCCGCATA
AGATTCTTGCATCTTCATCAGCATGCAAAATCTTAACATATTAACAAGCAGTGAGACATGGTTATCTG
ACCATGACCACCTCTGCATAGCGTTCACGTGTGTAGACCACACGTGAGTCTGTAATCCTGCTGTCTGC
+
```

```
FFFFFF:FFFFFFFFFFFFFFFFFFFFFFFFFFFFFFFFFFFFFFFFFFFFFFFFFFFFFFFFFFFFFF
F:FFFFFFFFFFFFFFFFFFFFFFFFFFFFFFFFFFFFFFFFFFFFFFFFFFFFFFFFFFFFFFFFFFF
@A00155:342:HHGFNDSXY:1:1571:17101:24721 2:N:0:GAACCTAG+TCCGCATA
AGATTCTTGCATCTTCATCAGCATGCAAAATCTTAACATATTAACAAGCAGTGAGACATGGTTATCTG
ACCATGACCACCTCTGCATAGCGTTCACGTGTGTAGACCACACGTGAGTCTGTAATCCTGCTGTCTGC
+
FFFFFFFFF:FF:FFFFFFFF:FFFFFFFFFFFFFFFFFFFFFFFFF:FFFFFFFFFFFFFFFFFFFF
FFFFFFFFFF:FFFFFFF:FFFFFFFFFF:F:FFFFFFFFFFFFFFFFFFFFFFFFFFFFFFFFFFFF
@A00155:342:HHGFNDSXY:1:2543:31946:8437 2:N:0:GAACCTAG+TCCGCATA
TTCTTGCATCTTCATCAGCATGCAAAATCTTAACATATTAACAAGCAGTGAGACATGGTTATCTGACC
ATGACCACCTCTGCATAGCGTTCACGTGTGTAGACCACACGTGAGTCTGTAATCCTGCTGTCTGCTTA
+
FFF:FFFFFF:FFFFFFFFFFFFFFFF:FFFFFFFF:FFFFF:,FFFFFFFFFFFFFFFFFFFFFFFF
FFFFFF,FFFFFFFF:FFFFFFFFFFFFFFFFF:FF:FFFFFFFFFFFFFFFFFFFFF:FFFFFFFFF
@A00155:342:HHGFNDSXY:1:2367:13386:22169 1:N:0:GAACCTAG+TCCGCATA
CTTGCATCTTCATCAGCATGCAAAATCTTAACATATTAACAAGCAGTGAGACATGGTTATCTGACCAT
GACCACCTCTGCATAGCGTTCACGTGTGTAGACCACACGTGAGTCTGTAATCCTGCTGTCTGCTTAAG
+
FFFFFFFFFFFFFFFFFFFFFFFFFFFFFFFFFFFFFFFFFFFFFFFFFFFFFFFFFFFFFFFFFFFF
FFFFFFFFFFFFFFFFFFFFFFFFFFFFFFFFFFFFFFFFFFFFFFFFFFFFFFFFFFFFFFFFFFFF
@A00155:342:HHGFNDSXY:1:2552:10917:19085 1:N:0:GAACCTAG+TCCGCATA
CTTGCATCTTCATCAGCATGCAAAATCTTAACATATTAACAAGCAGTGAGACATGGTTATCTGACCAT
GACCACCTCTGCATAGCGTTCACGTGTGTAGACCACACGTGAGTCTGTAATCCTGCTGTCTGCTTAAG
+
FFFFFFFFFFFFFFFFFFFFFFFFF:FFFFFFFFFFFFFFFFFFFFFFFFFFFFFFFFFFFFFFFFFF
FFFFFFFFFFFFFFFFFFFFFFFFFFFFFFFFFFFFFFFFFFFFFFFFFFFFFFFFFFFFFFFFFFFF
@A00155:342:HHGFNDSXY:1:1533:6117:26804 1:N:0:GAACCTAG+TCCGCATA
TTGCATCTTCATCAGCATGCAAAATCTTAACATATTAACAAGCAGTGAGACATGGTTATCTGACCATG
ACCACCTCTGCATAGCGTTCACGTGTGTAGACCACACGTGAGTCTGTAATCCTGCTGTCTGCTTAAGA
+
FFFFFFFFFFFFFFFFFFFFFFFFFFFFFFFFFFF:FFFFFFF:FFFFFFFFFFFFFFFFFFFFFFFF
FFFFFFFFFFFFFFFFFFFFFF:FFFFFFFFFFFFFFFFFFFFFFFFFFFFFFFFFFFFFFFFFFFFF
@A00155:342:HHGFNDSXY:1:2533:6705:17926 1:N:0:GAACCTAG+TCCGCATA
TTGCATCTTCATCAGCATGCAAAATCTTAACATATTAACAAGCAGTGAGACATGGTTATCTGACCATG
ACCACCTCTGCATAGCGTTCACGTGTGTAGACCACACGTGAGTCTGTAATCCTGCTGTCTGCTTAAGA
+
FFFFFFFFFFFFFFFFFFFFFFFFFFFFFFFFFFFF:FFFFFFFFFFFFFFFFFFFFFFFFFFFFFFF
FFFFFFFFFFFFFFFFFFFFFFFFFFFFFFFFFF:FFFFFFFFFFFFFF,FFFFFFFFFFFFFFFFFF
@A00155:342:HHGFNDSXY:1:1268:14733:16799 1:N:0:GAACCTAG+TCCGCATA
TTGCATCTTCATCAGCATGCAAAATCTTAACATATTAACAAGCAGTGAGACATGGTTATCTGACCATG
ACCACCTCTGCATAGCGTTCACGTGTGTAGACCACACGTGAGTCTGTAATCCTGCTGTCTGCTTAAGA
+
FFFFFFFFFFFFFFFFFFFFFFFF:FFFFFFFFFFFFFFFFFFFFFFFFFFFFFFFFFFFFFFFFFFF
FFFFFFFFFFFFFFFFFFFFFFFFFFFFFFFFFFF,FFFFFFFFFFFFFFFFFFFFFFFFFFFFFFFF:
@A00155:342:HHGFNDSXY:1:2453:25852:24909 1:N:0:GAACCTAG+TCCGCATA
TGCATCTTCATCAGCATGCAAAATCTTAACATATTAACAAGCAGTGAGACATGGTTATCTGACCATGA
CCACCTCTGCATAGCGTTCACGTGTGTAGACCACACGTGAGTCTGTAATCCTGCTGTCTGCTTAAGAG
+
FFFFFFFFFFFFFFFFFFFFFFFFFFFFFFFFFFFFFFFFFFFFFFFFFFFFFFFFFFFFFFFFFFFF
FFFFFFFFFFFF:FFFFFFFFF:FFFFFFFFFFFFFFFFFFFFFFFFFFFFFFFFFFFFFFFFFFFFF
@A00155:342:HHGFNDSXY:1:2246:13865:2362 1:N:0:GAACCTAG+TCCGCATA
TGCATCTTCATCAGCATGCAAAATCTTAACATATTAACAAGCAGTGAGACATGGTTATCTGACCATGA
CCACCTCTGCATAGCGTTCACGTGTGTAGACCACACGTGAGTCTGTAATCCTGCTGTCTGCTTAAGAG
+
```

```
FFFFFFFFFFFFFFFFFFFFFFFFFFFFFFFFFFFFFFFFFFFFFFFFFFFFFFFFFFFFFFFFFFFF
FFFFFFFFFFFFFFFFFFFFFFFFFFFFFFFF:FFFFFFFFFFFFFFFFFFFFFFFFFFFFFFFFFFF
@A00155:342:HHGFNDSXY:1:2246:14507:5259 1:N:0:GAACCTAG+TCCGCATA
TGCATCTTCATCAGCATGCAAAATCTTAACATATTAACAAGCAGTGAGACATGGTTATCTGACCATGA
CCACCTCTGCATAGCGTTCACGTGTGTAGACCACACGTGAGTCTGTAATCCTGCTGTCTGCTTAAGAG
+
FFF:FFFFFFFFFFFFFFFFFFFFFFFFFFFFFFFFFFFFFFFFFFFFFFFFFFFFFFFFFFFFFFFF
FFFFFFFFFFFFFFFFFFFFFFFFFFFFFFFF:FFFFFFFFFFFFFFFFFFFFFFFFFFFFFFFFFFF
@A00155:342:HHGFNDSXY:1:2609:17463:26443 1:N:0:GAACCTAG+TCCGCATA
TGCATCTTCATCAGCATGCAAAATCTTAACATATTAACAAGCAGTGAGACATGGTTATCTGACCATGA
CCACCTCTGCATAGCGTTCACGTGTGTAGACCACACGTGAGTCTGTAATCCTGCTGTCTGCTTAAGAG
+
FFFFFFFFFFFFFFFFFFFFFF:FFFFFFFFFFFFFFFFFFFFFFFFFFFFFFFFFFFFFFFFFFFF:
FFFFFFFFFFFFF:FFFFFFFFFFFFFFFFFFFFFFFFFFFFFFFFFFFFFFFFFFFFFFFFFFFFFF
@A00155:342:HHGFNDSXY:1:2578:26069:28479 1:N:0:GAACCTAG+TCCGCATA
TGCATCTTCATCAGCATGCAAAATCTTAACATATTAACAAGCAGTGAGACATGGTTATCTGACCATGA
CCACCTCTGCATAGCGTTCACGTGTGTAGACCACACGTGAGTCTGTAATCCTGCTGTCTGCTTAAGAG
+
FFFFFFFFFFFFFFFFFFFFFFFFFFFFFF:FFFFFFFFFFFFFFF:FFFFFF:FFFFFFFFFFFFFF
FFFFFFFFFFFFFFFFFFFFFFFFFFFFFFFFFFFFFFFFFF:FFFFFFFFFFFFFFFFFFFFFFFFF
@A00155:342:HHGFNDSXY:1:1441:17644:1485 1:N:0:GAACCTAG+TCCGCATA
TGCATCTTCATCAGCATGCAAAATCTTAACATATTAACAAGCAGTGAGACATGGTTATCTGACCATGA
CCACCTCTGCATAGCGTTCACGTGTGTAGACCACACGTGAGTCTGTAATCCTGCTGTCTGCTTAAGAG
+
FFF:FFFFF,FF,FFFFFFFFFFFFFFFFF:FFFFFFFFFFFFFFFFFFFFFFFFFFFFFFFFFFFFF
FFFFFFFFFFFFFFFFFFFFFFFFFFFFFFFFFF:FF:FFFFFFFFFFFFFFFFFFFFFFFFFFFFFF
@A00155:342:HHGFNDSXY:1:1678:23484:34053 1:N:0:GAACCTAG+TCCGCATA
GCATCTTCATCAGCATGCAAAATCTTAACATATTAACAAGCAGTGAGACATGGTTATCTGACCATGAC
CACCTCTGCATAGCGTTCACGTGTGTAGACCACACGTGAGTCTGTAATCCTGCTGTCTGCTTAAGAGC
+
FF:FFFFFFFF,FFF:FFFF:F,FFFFFF:FFFFFFFFFFFFFFFFFFFFFFF,FFFFFFFFFFFFFF
FFFFFFFFFFFFFFF:FFFFFFFFFFF:FFFFFFFFFFFFFFFFFFFFFFFFFFFFFFFFFFFFFFFF
@A00155:342:HHGFNDSXY:1:2543:31946:8437 1:N:0:GAACCTAG+TCCGCATA
GCATCTTCATCAGCATGCAAAATCTTAACATATTAACAAGCAGTGAGACATGGTTATCTGACCATGAC
CACCTCTGCATAGCGTTCACGTGTGTAGACCACACGTGAGTCTGTAATCCTGCTGTCTGCTTAAGAGC
+
FF:,FF:FF,F,FFFFFFFFF:FFFFFFF:FFFFFFFFFFF,FFFFFFF:FFFFFFFFFFFFFFFFFF
FFFFFFFFFFFFFFFFFFFFFFF:FFFFFFFFFFFFFFFFFFFFFFFFFFFFFFFFFF,FFFFFFFFF
@A00155:342:HHGFNDSXY:1:2112:25771:14497 1:N:0:GAACCTAG+TCCGCATA
GCATCTTCATCAGCATGCAAAATCTTAACATATTAACAAGCAGTGAGACATGGTTATCTGACCATGAC
CACCTCTGCATAGCGTTCACGTGTGTAGACCACACGTGAGTCTGTAATCCTGCTGTCTGCTTAAGAGC
+
FFFFFFFFFFFFFFFFFFFFFFFFFFFFFFFFFFFFFFFFFFFFFFFFFFFFFFFFFFFFFFFFFFFF
FFFFFFFFFFFFFFFFFFFFF:FFF:FFFFFFFFFFFFFFFFFFFFFFFFFFFFFFFFFFFFFFFFFF
@A00155:342:HHGFNDSXY:1:2171:20428:27195 1:N:0:GAACCTAG+TCCGCATA
GCATCTTCATCAGCATGCAAAATCTTAACATATTAACAAGCAGTGAGACATGGTTATCTGACCATGAC
CACCTCTGCATAGCGTTCACGTGTGTAGACCACACGTGAGTCTGTAATCCTGCTGTCTGCTTAAGAGC
+
FFFFFFFFFFFFFFFFFFFFFFFFFFFFFFFFFFFFFFFFFFFFFFFFFFFFFFFFFFFFFFFFFFFF
FFFFFFFFFFFFFFFFFFFFFFFFFFFFFFF,FFFFFFFFFFFFFFFFFFFFFFFFFFFFFFFFFFFF
@A00155:342:HHGFNDSXY:1:1111:10384:32690 1:N:0:GAACCTAG+TCCGCATA
GCATCTTCATCAGCATGCAAAATCTTAACATATTAACAAGCAGTGAGACATGGTTATCTGACCATGAC
CACCTCTGCATAGCGTTCACGTGTGTAGACCACACGTGAGTCTGTAATCCTGCTGTCTGCTTAAGAGC
+
```

```
FFFFFFFFFFF:FFFFFFFFFFF:FFFF,FFFFF:FFFF,F::FFFF:FFF:FFFFFFFFFFF,FF:F
FFFF,F:FFFFFFFFFFFFFFFFFFFFFFFFFFFFFFFFFF:,FFFFF:FFF:FFFFFFFF:F:FFFF
@A00155:342:HHGFNDSXY:1:2430:26178:30577 1:N:0:GAACCTAG+TCCGCATA
GCATCTTCATCAGCATGCAAAATCTTAACATATTAACAAGCAGTGAGACATGGTTATCTGACCATGAC
CACCTCTGCATAGCGTTCACGTGTGTAGACCACACGTGAGTCTGTAATCCTGCTGTCTGCTTAAGAGC
+
FFFFFFFFFFF,FFFFFFFFFFFFFFFFF,FFFFFFFFFFFFFFFFFFFFFFFFFFFFFFFFFFFFFF
FFFFFFFFFFFFFFFFFFFFFFFFFFFFF:FFFFFFFFFF:FFFFFFFFFFFFFFFFFFFFFF:FFFFFFF
@A00155:342:HHGFNDSXY:1:1103:12518:1344 1:N:0:GAACCTAG+TCCGCATA
GCATCTTCATCAGCATGCAAAATCTTAACATATTAACAAGCAGTGAGACATGGTTATCTGACCATGAC
CACCTCTGCATAGCGTTCACGTGTGTAGACCACACGTGAGTCTGTAATCCTGCTGTCTGCTTAAGAGC
+
FF::F:FFF:F,FFFFFFFFFFFFFFFFFFFFFFFFFFF:FFFFFFFFFFFFFFF,FFFFFFFFFFFF
FFFFFFFFFFFFFFFFFFFFFFFFFFFFFFFF:FFFFFFFFFFFFFFFFFFFFF:FFFFFFFFFFFFFF
@A00155:342:HHGFNDSXY:1:1606:8115:20807 1:N:0:GAACCTAG+TCCGCATA
GCATCTTCATCAGCATGCAAAATCTTAACATATTAACAAGCAGTGAGACATGGTTATCTGACCATGAC
CACCTCTGCATAGCGTTCACGTGTGTAGACCACACGTGAGTCTGTAATCCTGCTGTCTGCTTAAGAGC
+
FFFFFFFFFFFFFFFFF,FFFFF:FFFF:FFFFFFFFFFFFFFF:FFFFFFFFFFF:FFFFF,FFFFF
FFFFFFFFFFFFFFFFFFFFFFFF:,FFF:FFFFFFFF:FFFFFFFFFFFFFFFFFFFFFFFFFFFFFF
@A00155:342:HHGFNDSXY:1:2450:4761:19413 1:N:0:GAACCTAG+TCCGCATA
GCATCTTCATCAGCATGCAAAATCTTAACATATTAACAAGCAGTGAGACATGGTTATCTGACCATGAC
CACCTCTGCATAGCGTTCACGTGTGTAGACCACACGTGAGTCTGTAATCCTGCTGTCTGCTTAAGAGC
+
FFFFFFFFFFFFFFFFFFFF:FFFFFFF,FFFFFFFFFFFFFFFFFFFFFFFFFFFFFFFFFFFFFFF
FFFFFFFFFFFFFFFFFFFFFFFFFFFFFFFFFFFFF:FF:FFFFFFFFFF:FFFFFFFFFFFFFFFF
@A00155:342:HHGFNDSXY:1:2637:24198:8140 2:N:0:GAACCTAG+TCCGCATA
GCATCTTCATCAGCATGCAAAATCTTAACATATTAACAAGCAGTGAGACATGGTTATCTGACCATGAC
CACCTCTGCATAGCGTTCACGTGTGTAGACCACACGTGAGTCTGTAATCCTGCTGTCTGCTTAAGAGC
+
FFFFFFFFFFFFFFFFFFFFFFFFFFFFFFFFFFFFFFFFFFFFFFFFFF:FFFFFFFFFFFFFFFFFF
FFFFFFFFFFFFFFFFFFFFFFFFFFFFFFFFFFFFF:FFFFF:FFFFFFFFFFFFFFFFFF:FFFFFF
@A00155:342:HHGFNDSXY:1:1535:16405:8703 1:N:0:GAACCTAG+TCCGCATA
GCATCTTCATCAGCATGCAAAATCTTAACATATTAACAAGCAGTGAGACATGGTTATCTGACCATGAC
CACCTCTGCATAGCGTTCACGTGTGTAGACCACACGTGAGTCTGTAATCCTGCTGTCTGCTTAAGAGC
+
FFFFFF,FFFFFFFFFFFFFFFFFFFFFF,FFFFFFFFF:FFFFFFFFFFFFFFFFFFFFFFFF:FFF
FFFFFFFF:FFFFFFFFFFFFFFFFFFFFFF:FFFFFFFFFFF:FFFFFFFFFFFFFFFFFFFFFFFF
@A00155:342:HHGFNDSXY:1:2503:7120:9878 1:N:0:GAACCTAG+TCCGCATA
GCATCTTCATCAGCATGCAAAATCTTAACATATTAACAAGCAGTGAGACATGGTTATCTGACCATGAC
CACCTCTGCATAGCGTTCACGTGTGTAGACCACACGTGAGTCTGTAATCCTGCTGTCTGCTTAAGAGC
+
FF,FFFFFFFFFFFFFFFFFFF:,FFFFF:FFF,F:FF:FFFFFFFFFFFF:F,FFFFFFFF:FFFFF
FFFFFF:F:FFF:FFFFFF:FF:FFF:FFFFF:::FF,F:FFFFF:FFF:FF,FFFFF:FFF:FFFFF
@A00155:342:HHGFNDSXY:1:2571:26277:27117 1:N:0:GAACCTAG+TCCGCATA
CATCTTCATCAGCATGCAAAATCTTAACATATTAACAAGCAGTGAGACATGGTTATCTGACCATGACC
ACCTCTGCATAGCGTTCACGTGTGTAGACCACACGTGAGTCTGTAATCCTGCTGTCTGCTTAAGAGCA
+
FFFFFFFFFF:FFFFFFFFFFFFFFFFFFFFFFFFFFFFFFFFFFFFFFFFFFFFFFFFFFFFFFFFFF
FFFFFFFFFFFFFFFFFFFFFFFFFFFFFFFFFFFFFFFFFFFFFFFFFFFFFFFFFFFFFFFFFFFFF
@A00155:342:HHGFNDSXY:1:2163:3188:24486 2:N:0:GAACCTAG+TCCGCATA
CATCTTCATCAGCATGCAAAATCTTAACATATTAACAAGCAGTGAGACATGGTTATCTGACCATGACC
ACCTCTGCATAGCGTTCACGTGTGTAGACCACACGTGAGTCTGTAATCCTGCTGTCTGCTTAAGAGCA
+
```

```
FFFFFFFFFFFFFF:FFFFFF:FFFFFFFFFFFFFFFFFFFFFFFFFFFFFFFFFFFFFFFFFFFFFF
FFFFFFFFFFFFFFFFFFFFFFFFFFFFFFFFFFFFFFFFFFFFFFFFFFFFFFFFFFFFFFFFFFFF
@A00155:342:HHGFNDSXY:1:1441:17282:36714 2:N:0:GAACCTAG+TCCGCATA
CATCTTCATCAGCATGCAAAATCTTAACATATTAACAAGCAGTGAGACATGGTTATCTGACCATGACC
ACCTCTGCATAGCGTTCACGTGTGTAGACCACACGTGAGTCTGTAATCCTGCTGTCTGCTTAAGAGCA
+
FFFFFFF,FFFFFFFFFFFFFFFFFFFFFFFFFFFF::FFF:FFFFFFFFFFFFFFFFFFFFFFFFFF
FFFFFFFFFFFFFFFFFF:FFFFFFFFFFFFFFFFFFFFFFFFFFFFFFFFFFFFFFFFFFFFFFFFF
@A00155:342:HHGFNDSXY:1:1254:9136:22795 1:N:0:GAACCTAG+TCCGCATA
CATCTTCATCAGCATGCAAAATCTTAACATATTAACAAGCAGTGAGACATGGTTATCTGACCATGACC
ACCTCTGCATAGCGTTCACGTGTGTAGACCACACGTGAGTCTGTAATCCTGCTGTCTGCTTAAGAGCA
+
FFFFFFFFFFFFFFFFFFFFFFFF:FFFFFFFFFFFFFFFFFFFFFFFFFFF:FFFFFFFFFFFFFFF
FFFFFFFFFFFFFFFFFFFFFFFFFFFFFFFFFFFFFFFFFFFFFFFFFFFFFFFFFFFFFFFFFFFF
@A00155:342:HHGFNDSXY:1:1357:6126:14575 1:N:0:GAACCTAG+TCCGCATA
CATCTTCATCAGCATGCAAAATCTTAACATATTAACAAGCAGTGAGACATGGTTATCTGACCATGACC
ACCTCTGCATAGCGTTCACGTGTGTAGACCACACGTGAGTCTGTAATCCTGCTGTCTGCTTAAGAGCA
+
F:FFFFFF,F:FFFFFFFFFFFFFFFFFFFFFFFFFFFFFFFFFFFF:FFFFFFFFFFFFFFFFFFFF
FFFFFFFFFFFFFFFFFFFFFFFFFFFFFFFFFFFFFFF:FFFFFFFFFFFFFFFFFFFFFFFFFFFF
@A00155:342:HHGFNDSXY:1:1138:6271:32330 1:N:0:GAACCTAG+TCCGCATA
CATCTTCATCAGCATGCAAAATCTTAACATATTAACAAGCAGTGAGACATGGTTATCTGACCATGACC
ACCTCTGCATAGCGTTCACGTGTGTAGACCACACGTGAGTCTGTAATCCTGCTGTCTGCTTAAGAGCA
+
FFFFFFFFFF:FFFFFFFFFFFFFFFFFFFFFFFFFFFFFFFFFFFFFFFFFFFFFFFFFFFFFFFFF
FFFFFFFFFFFFFFFFFFFFFFFFFFFF:FFFFFFFFFFFFFFFFFFFFFFFFFFFFFFFFFFFFFF:
@A00155:342:HHGFNDSXY:1:1354:27724:26772 1:N:0:GAACCTAG+TCCGCATA
CATCTTCATCAGCATGCAAAATCTTAACATATTAACAAGCAGTGAGACATGGTTATCTGACCATGACC
ACCTCTGCATAGCGTTCACGTGTGTAGACCACACGTGAGTCTGTAATCCTGCTGTCTGCTTAAGAGCA
+
FFFFFFFFFFFFFFFFFFFFFFFFFFFF:FFFFFFFFFFFFFFFFFFF:FFFFF,FFFFFFFFFFFFF
:FFFFFFFFFFFFFFFFFFFFFFFFFFFFFFFFFFFFFFFF:FFFFFFFFFFFFFFFFFFFFFF:FFF
@A00155:342:HHGFNDSXY:1:2624:17210:30420 2:N:0:GAACCTAG+TCCGCATA
CATCTTCATCAGCATGCAAAATCTTAACATATTAACAAGCAGTGAGACATGGTTATCTGACCATGACC
ACCTCTGCATAGCGTTCACGTGTGTAGACCACACGTGAGTCTGTAATCCTGCTGTCTGCTTAAGAGCA
+
FFFFFFF:FFFFFFFFFFFF:FFFFFFFFFF:F,FF,F:FFFFFFFFFFFFFFFFFFFFFFFFFFFFF
FFFFFFFFFFFFFFFFFFFF:FFFFFFFFFFFFFFFFFFFFFFFFFFFFFFFFFFFFFFFFFFFFFFF
@A00155:342:HHGFNDSXY:1:1639:25021:9596 1:N:0:GAACCTAG+TCCGCATA
CATCTTCATCAGCATGCAAAATCTTAACATATTAACAAGCAGTGAGACATGGTTATCTGACCATGACC
ACCTCTGCATAGCGTTCACGTGTGTAGACCACACGTGAGTCTGTAATCCTGCTGTCTGCTTAAGAGCA
+
FFFFFFFFFFFFFFFFFFFFFFFF,FFFFFFFFFFFFFFFFFFFFFFF:FFF:FFFFFFFFFFFFFFF
FFFFFFFFFFFFFFFFFFFFFFFFFFFFFFFFFFFFFFFFFFFFFFFFFFFF:FFFFFFFFFFFFFFF
@A00155:342:HHGFNDSXY:1:2357:6831:10755 1:N:0:GAACCTAG+TCCGCATA
CATCTTCATCAGCATGCAAAATCTTAACATATTAACAAGCAGTGAGACATGGTTATCTGACCATGACC
ACCTCTGCATAGCGTTCACGTGTGTAGACCACACGTGAGTCTGTAATCCTGCTGTCTGCTTAAGAGCA
+
FFFFFFFFFFFFFFFFFF:FFFFFFFFFFFF:FFFFFFFFFFFFFFFFFFFFFFFFFFFFF,FFFFF:
FFFFFFFFFFFFFFF:FFFFFFFFFF,FFFFFFFFFFFFFFFFFF:FFFFFFFFFFFFFFFFFFFF,F
@A00155:342:HHGFNDSXY:1:2115:5014:21418 1:N:0:GAACCTAG+TCCGCATA
CATCTTCATCAGCATGCAAAATCTTAACATATTAACAAGCAGTGAGACATGGTTATCTGACCATGACC
ACCTCTGCATAGCGTTCACGTGTGTAGACCACACGTGAGTCTGTAATCCTGCTGTCTGCTTAAGAGCA
+
```

FFFFFFFFFFFFFFFFFFFFF:FFFFFF:FFFFFFFFFFF:FFFFFFFFFFFFFFFFFFFFFFFFFFF
FFFFFFFFFFFFFFFFFFFFFFFFFFFFFFFFFFFFFFFFFFFFFFFFFFFFFFFFFFFFFFFFFFF:
@A00155:342:HHGFNDSXY:1:2115:5412:30311 1:N:0:GAACCTAG+TCCGCATA
CATCTTCATCAGCATGCAAAATCTTAACATATTAACAAGCAGTGAGACATGGTTATCTGACCATGACC
ACCTCTGCATAGCGTTCACGTGTGTAGACCACACGTGAGTCTGTAATCCTGCTGTCTGCTTAAGAGCA
+
FFFFFFF:FFFFFFFFFFFFFFFFFFFFFFFFFFFFFF,:F,FFFFFFFFFFFFFFFFFFFFFFFFF:
FFFFFFFFFFF:FFFF:FFFFFFFFFFFFFFFFFFFFFFFFFFFFFFFFFFFFFFFFFFFFFFFFFFF
@A00155:342:HHGFNDSXY:1:1615:5710:2613 1:N:0:GAACCTAG+TCCGCATA
ATCTTCATCAGCATGCAAAATCTTAACATATTAACAAGCAGTGAGACATGGTTATCTGACCATGACCA
CCTCTGCATAGCGTTCACGTGTGTAGACCACACGTGAGTCTGTAATCCTGCTGTCTGCTTAAGAGCAT
+
FFFFFFFFF,FFFF:FFFFFFF:FFFFFFFFFFFFFFFFFFFFFFFF:FFFFFFFFFFFFFFFFFFFF
FFFFFFFFFFFFFFFFFFFFFFFFF:FFFFFFFFFFFFFFFFFFFFFFFFFFFFFFFFFFFFFFFFFF
@A00155:342:HHGFNDSXY:1:2134:30246:24126 1:N:0:GAACCTAG+TCCGCATA
ATCTTCATCAGCATGCAAAATCTTAACATATTAACAAGCAGTGAGACATGGTTATCTGACCATGACCA
CCTCTGCATAGCGTTCACGTGTGTAGACCACACGTGAGTCTGTAATCCTGCTGTCTGCTTAAGAGCAT
+
FFFFFFFFFFFFFFFFFFFFFFFFFFFFFFFFFFFFFFFFFFFFFFFFFFFFFFFFFFFFFFFFFFFF
FFFFFFFFFFFFFFFFFFFFFFFFFFFFFFFFFFFFFFFFFFFFFFFFFFFFFFFFFFFFFFFFFFFF
@A00155:342:HHGFNDSXY:1:2416:15899:34757 1:N:0:GAACCTAG+TCCGCATA
TCTTCATCAGCATGCAAAATCTTAACATATTAACAAGCAGTGAGACATGGTTATCTGACCATGACCAC
CTCTGCATAGCGTTCACGTGTGTAGACCACACGTGAGTCTGTAATCCTGCTGTCTGCTTAAGAGCAT
+
FFFFFFFFFFF:FFFFFFFFFFFFFF:FFFFFFFFFFFFFFFFFFFFFFFFFFFFFFFFFFFFFFFFF
FFFFFF:FFFFFFFFFFFFFFFFFFFFFFFFFFFFFFFFFFFFFFFFFFFFFFFFFFFFFFFFFFFF
@A00155:342:HHGFNDSXY:1:1551:6298:6324 2:N:0:GAACCTAG+TCCGCATA
TCTTCATCAGCATGCAAAATCTTAACATATTAACAAGCAGTGAGACATGGTTATCTGACCATGACCAC
CTCTGCATAGCGTTCACGTGTGTAGACCACACGTGAGTCTGTAATCCTGCTGTCTGCTTAAGAGCATC
+
:FFFFFFFFFFFFFFFFFFFFFFFFFFF:FFFFFFFFFFFFFFFFFFF:FFFFFFFF,FFFFF:FF:FF
:FFFFFFFFFFFFFFFFFFF:FFFFFFFFFFFF:,FF::FFFFFFFF:FFF:FFFF,FFFFF:F::FFF
@A00155:342:HHGFNDSXY:1:1366:7175:30890 2:N:0:GAACCTAG+TCCGCATA
TCTTCATCAGCATGCAAAATCTTAACATATTAACAAGCAGTGAGACATGGTTATCTGACCATGACCAC
CTCTGCATAGCGTTCACGTGTGTAGACCACACGTGAGTCTGTAATCCTGCTGTCTGCTTAAGAGCATC
+
FFFFFFFFFFFFFFFFFFFFFFFFFFFFFFF:FF:FFFFF:FFFFFFFFF:FFFFFFF:FFFFFFFFF
FFFFFFFFFFFFFFFFFF:FFFFFFFFFFFFFFFFFFFFFFFFFFFFFFFFFFFFF,FFFFFFFFF:F
@A00155:342:HHGFNDSXY:1:2417:17327:16658 1:N:0:GAACCTAG+TCCGCATA
TCTTCATCAGCATGCAAAATCTTAACATATTAACAAGCAGTGAGACATGGTTATCTGACCATGACCAC
CTCTGCATAGCGTTCACGTGTGTAGACCACACGTGAGTCTGTAATCCTGCTGTCTGCTTAAGAGCATC
+
FFFFFFFFFFFFFFFFFFFFFFFFFFFFFFFFFFFFFFFFFFFFFFFFFFFFFFFFFFFFFFFFFFFF
FFFFFFFFFFFFFFFFFFFFFFFFFFFFFFFFFFFFFFFFFFFFFFFFFFFFFFFFFFFFFFFFFFFF
@A00155:342:HHGFNDSXY:1:2609:6298:29027 1:N:0:GAACCTAG+TCCGCATA
CTTCATCAGCATGCAAAATCTTAACATATTAACAAGCAGTGAGACATGGTTATCTGACCATGACCACC
TCTGCATAGCGTTCACGTGTGTAGACCACACGTGAGTCTGTAATCCTGCTGTCTGCTTAAGA
+
F:FFFFF,FFF:FFFFFFFFFFF:,FFFF:FFFFFFFF:,FFFFFFFFFFFFFFFFFFFFFFFF,FFF
:FFFFFFFFFFFF:FFFFFFFFFFFFFFFFFFFFFFF,FFFFF:F:FFFFF,FFFFFF:FFFF
@A00155:342:HHGFNDSXY:1:2253:2546:1266 2:N:0:GAACCTAG+TCCGCATA
CTTCATCAGCATGCAAAATCTTAACATATGAACAAGCAGTGAGACATGGTTATCTGACCATGACCACC
TCTGCATAGCGTTCACGTGTGTAGACCACACGTGAGTCTGTAATCCTGCTGTCTGCTTAAGAGCATC
+

```
FFFFFFFFFFFFFFFF:FFFFFFFF:FF,,FFF,FFF::FFFFFFFFFFFFFFFFFFFFFFFFFF:FF
FFFFFFFFFFFFFFFFFFFFFFFFFFFFFFFFFFFFFFFFFFFFFFFFF:FFFFFFFFFFFFFFF
@A00155:342:HHGFNDSXY:1:2445:16495:33317 1:N:0:GAACCTAG+TCCGCATA
CTTCATCAGCATGCAAAATCTTAACATATTAACAAGCAGTGAGACATGGTTATCTGACCATGACCACC
TCTGCATAGCGTTCACGTGTGTAGACCACACGTGAGTCTGTAATCCTGCTGTCTGCTTAAGAGCATCT
+
FFFFF:F:FFFFFFFFF::FFFF,F:FFFFFFFFFFFFFFFFFFFFFFFFFFFF,:FFFFFFFFFFF
FFFFFFFFFFFFFFFFFFFFFFFFF:FFFFFFFFFFFFFFFFFFFFFFFFFFFFFFFFFFFFFFFFFF
@A00155:342:HHGFNDSXY:1:1604:4689:25300 2:N:0:GAACCTAG+TCCGCATA
CTTCATCAGCATGCAAAATCTTAACATATTAACAAGCAGTGAGACATGGTTATCTGACCATGACCACC
TCTGCATAGCGTTCACGTGTGTAGACCACACGTGAGTCTGTAATCCTGCTGTCTGCTTAAGAGCATCT
+
FFFFFFFFFFFFFFFFFFFFFFFFFFFFFFFF:F,FFFFFFFFF:FFFFFFFFFFFFFFFFFFFFFFF
FFFFFFFFFFFFFFFFFFFFFFFFFFFFFFFFFFFFFFFFFFFFFFFFFFF:FFFFFFFFF,FF
@A00155:342:HHGFNDSXY:1:2171:22254:12571 1:N:0:GAACCTAG+TCCGCATA
CTTCATCAGCATGCAAAATCTTAACATATTAACAAGCAGTGAGACATGGTTATCTGACCATGACCACC
TCTGCATAGCGTTCACGTGTGTAGACCACACGTGAGTCTGTAATCCTGCTGTCTGCTTAAGAGCATCT
+
:FFF::FF:FFF,FFFFFF:F:FFFFFFFFFFFF::F:,:F,FFF,:FFF:FF:F:,FFFFFF,FFF
:FFFFF,F,F:F::FFFF,,:,FFFFFF,F,FFFF::F,FFFFFF:F,,F,FFF:,FFFF:FFFFFFF
@A00155:342:HHGFNDSXY:1:1103:12518:1344 2:N:0:GAACCTAG+TCCGCATA
CTTCATCAGCATGCAAAATCTTAACATATTAACAAGCAGTGAGACATGGTTATCTGACCATGACCACC
TCTGCATAGCGTTCACGTGTGTAGACCACACGTGAGTCTGTAATCCTGCTGTCTGCTTAAGAGCATCT
+
FFF:FFFFFFFFFFFFFFFFFFFFFFFFFF,:FFFFFF:FFFFFFFFFFFFFFFFFFFFFFFFFFFFF
FFFFFFFFFFFFFFFFFFFFFFFFFFFFFFFFFFFFFFFFFFFFFFFFFFFFFFFFFFFFFFFFFFFF
@A00155:342:HHGFNDSXY:1:1372:8513:1423 1:N:0:GAACCTAG+TCCGCATA
CTTCATCAGCATGCAAAATCTTAACATATTAACAAGCAGTGAGACATGGTTATCTGACCATGACCACC
TCTGCATAGCGTTCACGTGTGTAGACCACACGTGAGTCTGTAATCCTGCTGTCTGCTTAAGAGCATCT
+
FFFFFFFFFFFFFFFFFFFFFFFFFFFFFFFFF:FF,FFFFFFFFFFFFF,FFFFFFFFFFFFFFFFF
FFFFFFFFFFFFFF:FFFFFFFFFFFFFFFFFFFFFFFFFFFFFFFFFFFFFFF:FFFFFFFFFFFFF
@A00155:342:HHGFNDSXY:1:1604:4689:25300 1:N:0:GAACCTAG+TCCGCATA
CTTCATCAGCATGCAAAATCTTAACATATTAACAAGCAGTGAGACATGGTTATCTGACCATGACCACC
TCTGCATAGCGTTCACGTGTGTAGACCACACGTGAGTCTGTAATCCTGCTGTCTGCTTAAGAGCATCT
+
FFFFFFFFFFFFFFFFFFFFFFFFF:FFFFFF:FFF:F,FFFFFFFFF::FFFFFFFFFFFFFFFFFF
FFFFFFFFFFFFFFFFFFFFFFFF:FFFFFFFFFFFFFFFFFFF:FFFFFFFFFFFFFFFFFFFF:FF
@A00155:342:HHGFNDSXY:1:2362:26820:22482 2:N:0:GAACCTAG+TCCGCATA
CTTCATCAGCATGCAAAATCTTAACATATTAACAAGCAGTGAGACATGGTTATCTGACCATGACCACC
TCTGCATAGCGTTCACGTGTGTAGACCACACGTGAGTCTGTAATCCTGCTGTCTGCTTAAGAGCATCT
+
FFF:FFFFFFFFFFFFFFF:FFFFFFFFFFFFFFFFF:F:FFFFFFFFFFFFFFF:FFFFF,FFFFFF
FFFFFFFF:FFFFFFFFFFFFFFFFFFFFFFFFFFFFFFFFFFFFFFF:FFFFFFFFFFFFFFFFFFFF
@A00155:342:HHGFNDSXY:1:2216:5665:1846 1:N:0:GAACCTAG+TCCGCATA
CTTCATCAGCATGCAAAATCTTAACATATTAACAAGCAGTGAGACATGGTTATCTGACCATGACCACC
TCTGCATAGCGTTCACGTGTGTAGACCACACGTGAGTCTGTAATCCTGCTGTCTGCTTAAGAGCATCT
+
FFFFFFFFFFFFFFFFFFFFFFFF:F:FFFFFFFFFFFFFFFFF:FFFFFFF:FFFFFFFFFFFFFFF
FFFFFFFFFFF:F:FF,:FFFFFFF:FFF::FFFFFF:FFFFFFF:FFFFFFFFF:FFFFFFFFFFFF
@A00155:342:HHGFNDSXY:1:1639:25021:9596 2:N:0:GAACCTAG+TCCGCATA
CTTCATCAGCATGCAAAATCTTAACATATTAACAAGCAGTGAGACATGGTTATCTGACCATGACCACC
TCTGCATAGCGTTCACGTGTGTAGACCACACGTGAGTCTGTAATCCTGCTGTCTGCTTAAGAGCATCT
+
```

F,FF:FF:FFFFFFFFFFFFFFFFFFFFFFFFFF,FFFFFFFFFFFFFFFFFFFFFFFFFFFFFFFFF
:FFFFFFFFFFFFFFFFFFFFFFFFFFFFFFFFFFFFFFFFFFFFFFFFFFFFFFFFFFFFFFFFFF:
@A00155:342:HHGFNDSXY:1:1622:14461:18709 2:N:0:GAACCTAG+TCCGCATA
CTTCATCAGCATGCAAAATCTTAACATATTAACAAGCAGTGAGACATGGTTATCTGACCATGACCACC
TCTGCATAGCGTTCACGTGTGTAGACCACACGTGAGTCTGTAATCCTGCTGTCTGCTTAAGAGCATCT
+
FFFFFFF:FFFFFFFFFFFFFFFFFFFFFF:FF:FFFFFFFFFFFFFFFFFFFFFFFFFFFFFFFFFF
FFFFFFFFFFFFFFFFFFFFFFFFFFFFFFFFFFFFFFFFFFFFFFFFFFFFFFFFFFFFFFFFFFFF
@A00155:342:HHGFNDSXY:1:2618:6189:16564 2:N:0:GAACCTAG+TCCGCATA
CTTCATCAGCATGCAAAATCTTAACATATTAACAAGCAGTGAGACATGGTTATCTGACCATGACCACC
TCTGCATAGCGTTCACGTGTGTAGACCACACGTGAGTCTGTAATCCTGCTGTCTGCTTAAGAGCATCT
+
FFFFFFFFF,FFF:FFF:F:FFFFFF,FF,,:F:::FFFFFFFFFF:FFFFFFFFFFF:FFFFFFFFF
FF:FFFFF::FFFFFFFFFFFFFFFFFFFFFF,FFFFFF,:FFFFFFFFFFFFFFFFFF:FFF:FFFF
@A00155:342:HHGFNDSXY:1:2224:7464:8907 2:N:0:GAACCTAG+TCCGCATA
TCATCAGCATGCAAAATCTTAACATATTAACAAGCAGTGAGACATGGTTATCTGACCATGACCACCTC
TGCATAGCGTTCACGTGTGTAGACCACACGTGAGTCTGTAATCCTGCTGTCTGCTTAAGAGCATCTGC
+
FFFFFFFF:FFFFFFFFFFFFFFFFFFFF::FF::FF:FFFFFFFFFFFFFFFFFFFFFFFFFFFFFF
FFFFFFFFFFFFFFFFFFFFFFFFFFFFFF:FFFFFFFFF:FFFFFFFFFFF:FFFFFFFFFFFFFFF
@A00155:342:HHGFNDSXY:1:2362:8748:10692 2:N:0:GAACCTAG+TCCGCATA
TCATCAGCATGCAAAATCTTAACATATTAACAAGCAGTGAGACATGGTTATCTGACCATGACCACCTC
TGCATAGCGTTCACGTGTGTAGACCACACGTGAGTCTGTAATCCTGCTGTCTGCTTAAGAGCATCTGC
+
:FFFFFFFFFF:FFFFFFFFFFFFFFFFFFFFFFFFFFFFFFFFFFFFFFFFFFFFFFFFFFFFFFFF
FFFFF:FFFFFFFFFFFFFFFFFFFFFFFFFFFFFFFFFFFFFFFFFFFFFFFFFFFFFFFFFFFFFF
@A00155:342:HHGFNDSXY:1:1410:30454:18380 2:N:0:GAACCTAG+TCCGCATA
TCATCAGCATGCAAAATCTTAACATATTAACAAGCAGTGAGACATGGTTATCTGACCATGACCACCTC
TGCATAGCGTTCACGTGTGTAGACCACACGTGAGTCTGTAATCCTGCTGTCTGCTTAAGAGCATCTGC
+
FFFFFFFFFFFFFFF:FFFFFFFFFFFF:FF:FFFFFFFFFFFFFFFFFFFFFFFFFFFFFFFFFFFF
FFFFF:FFFFFFFFFFFFFFFFFFFFFFFFFFFFFFFFFFFFFFFFFFFFFFFFFFFFFFFFFFFFFF
@A00155:342:HHGFNDSXY:1:1560:10999:13369 2:N:0:GAACCTAG+TCCGCATA
TCATCAGCATGCAAAATCTTAACATATTAACAAGCAGTGAGACATGGTTATCTGACCATGACCACCTC
TGCATAGCGTTCACGTGTGTAGACCACACGTGAGTCTGTAATCCTGCTGTCTGCTTAAGAGCATCTGC
+
FFFFFF:FFFFFFFFFFFFFFFFFFFFFFFF,FFFFFFFFFFFFFFFFFFFFFFFFFFFFFFFFFFFF
FFFFFFFFFFFFFFFFFFFFFFFFFFFFFFFFFFFFFFFFFFFFFFFFFFFFFFFFFFFFFFFFFFFF
@A00155:342:HHGFNDSXY:1:1378:29930:27743 2:N:0:GAACCTAG+TCCGCATA
CATCAGCATGCAAAATCTTAACATATTAACAAGCAGTGAGACATGGTTATCTGACCATGACCACCTCT
GCATAGCGTTCACGTGTGTAGACCACACGTGAGTCTGTAATCCTGCTGTCTGCTTAAGAGCATCTGCC
+
FFFFFFFFFFF:FFFFFFFFFFFFFFFFFFFFFFFFFFFFFFFFFFFFFFFFFFFFFFFFFFFFFFFF
FFFFFFFFFFFFFFFFFFFFFFFFFFFFFFFFFFFFFFFFFFFFFFFFFFFFFFFFFFFFFFFFFFFF
@A00155:342:HHGFNDSXY:1:1354:27724:26772 2:N:0:GAACCTAG+TCCGCATA
ATCAGCATGCAAAATCTTAACATATTAACAAGCAGTGAGACATGGTTATCTGACCATGACCACCTCTG
CATAGCGTTCACGTGTGTAGACCACACGTGAGTCTGTAATCCTGCTGTCTGCTTAAGAGCATCTGCCA
+
:FFFFFFFFFFFFFFFFFFFFFF,FF::F::FFFF:FFF:FFFFFFFFFFFFFFFFFFFFFFFFFFFF
FFFFFFFFFFFFFFFFFFFFFFFFFFFFFFFFFFFFFFFFFFFFFFFFFFFFFFFFFFFFFFFFFFFF
@A00155:342:HHGFNDSXY:1:1313:31964:8563 1:N:0:GAACCTAG+TCCGCATA
ATCAGCATGCAAAATCTTAACATATTAACAAGCAGTGAGACATGGTTATCTGACCATGACCACCTCTG
CATAGCGTTCACGTGTGTAGACCACACGTGAGTCTGTAATCCTGCTGTCTGCTTAAGAGCATCTGCCA
+

```
FFFFFFFFFFFFFFFFFFFFFFF:FFFFFFFFFFFFFFFFFFFFFFF:FFFFFFFFFFFFFFFFFFFF
FFFFF:FFFFFFFFFFFFF:FFFFFFFFFFFFFFFFFFFFFFFFFFFFFFFFFFFFFFFFFFFFF:FF
@A00155:342:HHGFNDSXY:1:1138:6271:32330 2:N:0:GAACCTAG+TCCGCATA
ATCAGCATGCAAAATCTTAACATATTAACAAGCAGTGAGACATGGTTATCTGACCATGACCACCTCTG
CATAGCGTTCACGTGTGTAGACCACACGTGAGTCTGTAATCCTGCTGTCTGCTTAAGAGCATCTGCCA
+
FFFFFFFFFFFFFF:FFFFFFFFFFFF:F:F:FFFFFFFFFFFFFFFFFFFFFFFFFFFFFFFFFFFF
FFFFFFFFFFFFFFFFFFFFFFFFFFFFFFFFFFFFFFFFFFFFFFFFFFFFFFFFFFFFFFFFFFFF
@A00155:342:HHGFNDSXY:1:2138:24370:16673 2:N:0:GAACCTAG+TCCGCATA
TCAGCATGCAAAATCTTAACATATTAACAAGCAGTGAGACATGGTTATCTGACCATGACCACCTCTGC
ATAGCGTTCACGTGTGTAGACCACACGTGAGTCTGTAATCCTGCTGTCTGCTTAAGAGCATCTGCCAG
+
:FFFFFFFFFFFFFFF:FFFFFFFFFFFFFFFFFFFFFFFF,FFFFFFFFFFFFFFFFFFFFFFFFFF
FFFFFFFFFFFFFFFFFFFFFFFFFFFFFFFFFFFFFFFFFFFFFFFFFFFFFFFFFFFFFFFFFFFF
@A00155:342:HHGFNDSXY:1:1312:3016:24940 2:N:0:GAACCTAG+TCCGCATA
TCAGCATGCAAAATCTTAACATATTAACAAGCAGTGAGACATGGTTATCTGACCATGACCACCTCTGC
ATAGCGTTCACGTGTGTAGACCACACGTGAGTCTGTAATCCTGCTGTCTGCTTAAGAGCATCTGCCAG
+
FFFFFFFFFFFFF:FFFFFFFFFF:FFF,FFFFFFFFFFFFF:FFFFFFFFFFFFFFFFFFFFFF,FF
FFFF::FFFFFFFFFFFFFFFFFFFFFFFFFFFFFFFFFFFFFFFFFF:FFFFFFFF,,FFFFFFFFF
@A00155:342:HHGFNDSXY:1:1125:8422:10504 2:N:0:GAACCTAG+TCCGCATA
TCAGCATGCAAAATCTTAACATATTAACAAGCAGTGAGACATGGTTATCTGACCATGACCACCTCTGC
ATAGCGTTCACGTGTGTAGACCACACGTGAGTCTGTAATCCTGCTGTCTGCTTAAGAGCATCTGCCAG
+
FFFFFFFFF:FFFFFFFFFFFFFFFF:FFFFFFFFFFFFFFFFFF:FFFF:FFF:FFFFFFFFFFFFF
FFFFFFFFFFFFFFFFFFFFFFFFFFFFFFFFFFFFF:FFFFFFFFFFFFFFFFFFFFFFFFFFFFFF
@A00155:342:HHGFNDSXY:1:2271:6858:18067 2:N:0:GAACCTAG+TCCGCATA
TCAGCATGCAAAATCTTAACATATTAACAAGCAGTGAGACATGGTTATCTGACCATGACCACCTCTGC
ATAGCGTTCACGTGTGTAGACCACACGTGAGTCTGTAATCCTGCTGTCTGCTTAAGAGCATCTGCCAG
+
FFFFF:FFFFFFFFFFFFFF:FFF,:FF,FFFFFFFFFFFFFFFFFFFFFFFFFFFF::FFFFFF:FF
FFFFFFFFFFFFFFFFFFFFFFFFFFFFFFFFFFFFFFFFFFFFFFFFFFFFFFFFFFFFFFFFFFFF
@A00155:342:HHGFNDSXY:1:2166:3070:26944 2:N:0:GAACCTAG+TCCGCATA
CAGCATGCAAAATCTTAACATATTAACAAGCAGTGAGACATGGTTATCTGACCATGACCACCTCTGCA
TAGCGTTCACGTGTGTAGACCACACGTGAGTCTGTAATCCTGCTGTCTGCTTAAGAGCATCTGCC
+
FFFFFF,FFFFF,FFFFFFFFF:FFFFFFFFFF:FFFFFFFFFFFFF:FFFFFFFFFFFFF:FFFFFF
FFFFFFFFFFFFFFFFFFFFFFFFFF:FFFFFFFFFFF,FFFFFFFFFFFFFFFFFFFFF:FFFF
@A00155:342:HHGFNDSXY:1:1608:24026:30984 2:N:0:GAACCTAG+TCCGCATA
CAGCATGCAAAATCTTAACATATTAACAAGCAGTGAGACATGGTTATCTGACCATGACCACCTCTGCA
TAGCGTTCACGTGTGTAGACCACACGTGAGTCTGTAATCCTGCTGTCTGCTTAAGAGCATCTGCCAG
+
FFFFF:FFFFF:FFFFFFFFFFFFF,FFF:FFFFFFFFFFFFFF:FFFFFFFFFFFFFF:FFFFFFFF
FFFFFFFFFFFFFFFFFFFFFFFFFFFFFFFFFFFFFFFFFFFFFFFFFFFFFFFFFFFFFFFFF:F
@A00155:342:HHGFNDSXY:1:2465:1362:11271 1:N:0:GAACCTAG+TCCGCATA
CAGCATGCAAAATCTTAACATATTAACAAGCAGTGAGACATGGTTATCTGACCATGACCACCTCTGCA
TAGCGTTCACGTGTGTAGACCACACGTGAGTCTGTAATCCTGCTGTCTGCTTAAGAGCATCTGCCAG
+
FFFF:FFFFFFFFFFFF,,:F:FF:FFF:FFFF:FFFFFFFFFFFFFFFFFFFFFF,:,:F:FFFFFF
FFF:FFFF:FFFFFFFFFFF,FF:FFFF,FF:FFF:FFFFFF,FFF,FFFFF:F:,FFFF:FF::FF
@A00155:342:HHGFNDSXY:1:1376:3839:23046 2:N:0:GAACCTAG+TCCGCATA
CAGCATGCAAAATCTTAACATATTAACAAGCAGTGAGACATGGTTATCTGACCATGACCACCTCTGCA
TAGCGTTCACGTGTGTAGACCACACGTGAGTCTGTAATCCTGCTGTCTGCTTAAGAGCATCTGCCAGG
+
```

```
FFFFFFFFFFF:FFFFFFFFFFFFF,FFFFFFFFFFFFFFFFFFFFFFFFFFFFFFFFFFFFFFFF
FFFFFFFFFFFFFFFFFFFFFFFFFFFFFFFFFFFFFFFFFFFFFFFFFFFFFFFFFFF:FFFFFFFF
@A00155:342:HHGFNDSXY:1:2669:28320:35383 2:N:0:GAACCTAG+TCCGCATA
CAGCATGCAAAATCTTAACATATTAACAAGCAGTGAGACATGGTTATCTGACCATGACCACCTCTGCA
TAGCGTTCACGTGTGTAGACCACACGTGAGTCTGTAATCCTGCTGTCTGCTTAAGAGCATCTGCCAGG
+
:FFF:FFFFFFFFFFFFF:FFFF:,:FF:FFFFFFFFFFFF:FF:FFFFF:FFFFF,FFFFFF,FFF
FFF:FFFFFFFF:FFFFFFFFFFFFFFFFFFFFF::FFF:FFFFFFFFFFFFFFFF:FFFF:FF:FFF
@A00155:342:HHGFNDSXY:1:2115:5412:30311 2:N:0:GAACCTAG+TCCGCATA
CAGCATGCAAAATCTTAACATATTAACAAGCAGTGAGACATGGTTATCTGACCATGACCACCTCTGCA
TAGCGTTCACGTGTGTAGACCACACGTGAGTCTGTAATCCTGCTGTCTGCTTAAGAGCATCTGCCAGG
+
FFFFFF,FFFF:FFFFFFF:FFFF:FF:FFFFFFFFFFFFFFFFFFFFFFFFFFFFFFFFFFFFFFFF
FFFFFFFFFFFFF:FFFFFFF:FFFF:FFFFFFFFFFFFFFFFFF:FFFFFFFFFFFFFFFFFFFFFF
@A00155:342:HHGFNDSXY:1:2115:5014:21418 2:N:0:GAACCTAG+TCCGCATA
CAGCATGCAAAATCTTAACATATTAACCAGCAGTGAGACATGGTTATCTGACCATGACCACCTCTGCA
TAGCGTTCACGTGTGTAGACCACACGTGAGTCTGTAATCCTGCTGTCTGCTTAAGAGCATCTGCCAGG
+
FFFF,FFFFFFFFFF:FFF:F:FF,FF,FFF:FFFFFFF,FFFFFFFFFFFFFFFFFF:FFFFFFFFF
FFFFFF:FFFF:FFFF,FFFFFFFFFFFFFFFFFFFFFFFFFFFFFFFFFFFFFFFFFFFFFFFFFFF
@A00155:342:HHGFNDSXY:1:1673:22625:16000 2:N:0:GAACCTAG+TCCGCATA
CAGCATGCAAAATCTTAACATATTAACAAGCAGTGAGACATGGTTATCTGACCATGACCACCTCTGCA
TAGCGTTCACGTGTGTAGACCACACGTGAGTCTGTAATCCTGCTGTCTGCTTAAGAGCATCTGCCAGG
+
FFFFFFFFFFFFFFFFFFF:FFFFFFF:FFFFF:FFFFFFFFFFFFFFFFFFFFFFFFFFFFFFFFFF
FFFFFFFFFFFFFFFFFFFFFFFFFFFFFFFFFFFFFFFFFFF,FFFFFFFFFFFFFFFFFFFFFFFF
@A00155:342:HHGFNDSXY:1:2319:18557:12587 2:N:0:GAACCTAG+TCCGCATA
CAGCATGCAAAATCTTAACATATTAACAAGCAGTGAGACATGGTTATCTGACCATGACCACCTCTGCA
TAGCGTTCACGTGTGTAGACCACACGTGAGTCTGTAATCCTGCTGTCTGCTTAAGAGCATCTGCCAGG
+
FFFFFFFFFFFFFFFFFFFFFFFF:FFFFFFFFFFFFFFFFFF:FFFFFFFFFFFFFFFFF:FFFFFFF
FFFFFFFFFF:FFF,FFFFFFFFFFF:FFFFFFFFFFFFFFFF:FFFFFFFFFFFFFFFFFFFFFFFF
@A00155:342:HHGFNDSXY:1:2239:10899:17300 2:N:0:GAACCTAG+TCCGCATA
CAGCATGCAAAATCTTAACATATTAACAAGCAGTGAGACATGGTTATCTGACCATGACCACCTCTGCA
TAGCGTTCACGTGTGTAGACCACACGTGAGTCTGTAATCCTGCTGTCTGCTTAAGAGCATCTGCCAGG
+
FFFFFFFFFFFF:FFFFFFFFFFFFFFFFFFFFFFFFFFFFFFFFFFFFFFFFFFFFFFFF:FFFFF
FFFFFFFFFFFFFFFFFFFFFFFFFFF:FFFFFFFFFFFFFFFFFFFFFFFFFFFFFFFFFFFFFFFF
@A00155:342:HHGFNDSXY:1:1336:15338:30561 2:N:0:GAACCTAG+TCCGCATA
CAGCATGCAAAATCTTAACATATTAACAAGCAGTGAGACATGGTTATCTGACCATGACCACCTCTGCA
TAGCGTTCACGTGTGTAGACCACACGTGAGTCTGTAATCCTGCTGTCTGCTTAAGAGCATCTGCCAGG
+
FFFFFF:FFFFFFFFFFFFFFFFFFFFF:FFFFFFFFFFF:FFFFFFFFFFFFFFFFFFFFFFFFFFF
FFFFFFFFFFFFFFFFFFFFFFFFFFFFFFFFFFFFFFFFFFFFFFFFFFFFFFFFFFFFFFFFFFFF
@A00155:342:HHGFNDSXY:1:1630:8169:22216 2:N:0:GAACCTAG+TCCGCATA
CAGCATGCAAAATCTTAACATATTAACAAGCAGTGAGACATGGTTATCTGACCATGACCACCTCTGCA
TAGCGTTCACGTGTGTAGACCACACGTGAGTCTGTAATCCTGCTGTCTGCTTAAGAGCATCTGCCAGG
+
FFFFFFFFFFFFFFFFFFFFF:FFF,FFFFFFFFFFFFFFFFFFFFFFFFFFFFF,FFFFFFFFFFFFF
FFFFFFFFFFFFFFFFFFFFFFFFFFFFFFFFFFFFFFFFFFFFFFFFFFFFFFFFFFFFFFFFFFFF
@A00155:342:HHGFNDSXY:1:2134:30246:24126 2:N:0:GAACCTAG+TCCGCATA
CAGCATGCAAAATCTTAACATATTAACAAGCAGTGAGACATGGTTATCTGACCATGACCACCTCTGCA
TAGCGTTCACGTGTGTAGACCACACGTGAGTCTGTAATCCTGCTGTCTGCTTAAGAGCATCTGCCAGG
+
```

```
FFFFFFFFFFFFFFFFFFFFF,FFFFF,FFFFFFFFFFF:FFFFFFFFFFFFFFFFFFFFFFFFFFFF
FFFFFFFFFFFFFFFFFFFFFF:FFFFFFFFFFFFFFFFFFF:FFFFFFFFFFFFFFFFFFFFFFFFFFFF
@A00155:342:HHGFNDSXY:1:2330:2049:26209 2:N:0:GAACCTAG+TCCGCATA
AGCATGCAAAATCTTAACATATTAACAAGCAGTGAGACATGGTTATCTGACCATGACCACCTCTGCAT
AGCGTTCACGTGTGTAGACCACACGTGAGTCTGTAATCCTGCTGTCTGCTTAAGAGCATCTGCCAGG
+
FFFFFFFFFFFFFFFFFFFFFF,:FFFFFFFF:FFFFFF:FFFFFFFFFFFFFFFFFFFFFFFFFFFF
FFFFFFFFFFFFFFFFFFFFFF:FFFFFFFFFFFFFFFFFFFFFFFFFFFFFFFFFFFFFFFFFFFFF
@A00155:342:HHGFNDSXY:1:1446:9534:25895 2:N:0:GAACCTAG+TCCGCATA
AGCATGCAAAATCTTAACATATTAACAAGCAGTGAGACATGGTTATCTGACCATGACCACCTCTGCAT
AGCGTTCACGTGTGTAGACCACACGTGAGTCTGTAATCCTGCTGTCTGCTTAAGAGCATCTGCCAGGT
+
FFF,F,FFFFFFFFFFFFFFFF,F:F:FFF:FFF,FFFFFFFFFFFFFFFFFFFFFFFFFFFFFFFFF
FFFFFFFFFFFFFFFFFFFFFFFFFFFFFFFFFFFFFFFFFFFFFFFFFFFFFFFFFFFFFFFFFFFF
@A00155:342:HHGFNDSXY:1:2667:21947:6527 2:N:0:GAACCTAG+TCCGCATA
AGCATGCAAAATCTTAACATATTAACAAGCAGTGAGACATGGTTATCTGACCATGACCACCTCTGCAT
AGCGTTCACGTGTGTAGACCACACGTGAGTCTGTAATCCTGCTGTCTGCTTAAGAGCATCTGCCAGGT
+
FFFFFFFFFFFFFFFFFFFFF:F:FFFFFF:FFFFFFFFFFFFFFFFFFFFFFFFFFFFFFF:FFFFFF
FFFFFFFFFFFFFFFFFFFFFF,FFFFFFFFFFFFFFFFFFFFFFFFFFFFFFFFFFFFFFFFFFFFF
@A00155:342:HHGFNDSXY:1:2361:16143:30953 2:N:0:GAACCTAG+TCCGCATA
CATGCAAAATCTTAACATATTAACAAGCAGTGAGACATGGTTATCTGACCATGACCACCTCTGCATAG
CGTTCACGTGTGTAGACCACACGTGAGTCTGTAATCCTGCTGTCTGCTTAAGAGCATCTGCCAGG
+
FFFFFFFFFFFFFFFFFFFFFFFFFFFFFFFFFFFFFFFFFFFFFFFFFFFFFFFFFFFFFFFFFFFF
FFFFFFFFFFFFFFFFFFFFFFFFFFFFFFFFFFFFFFFFFFFFFFFFFFFFFFFFFFFFFFFFF
@A00155:342:HHGFNDSXY:1:2608:30327:2065 2:N:0:GAACCTAG+TCCGCATA
ATGCAAAATCTTAACATATTAACAAGCAGTGAGACATGGTTATCTGACCATGACCACCTCTGCATAGC
GTTCACGTGTGTAGACCACACGTGAGTCTGTAATCCTGCTGTCTGCTTAAGAGCATATGCCAGGTTAG
+
FFFFFFFFF:FFFFFFFFFF,FF,FFFFFFFFFFFFFFFFFFFFFFFFFFFFFFFFFFFFFFFFF:FF
FFFFFFFFFFFFFFFFFFFFFFFFFFFFFFFFFFFFFFFFFFFFFFFFFFFFFFFFFFFFFFFFFFFF
@A00155:342:HHGFNDSXY:1:2503:6027:22357 2:N:0:GAACCTAG+TCCGCATA
ATGCAAAATCTTAACATATTAACAAGCAGTGAGACATGGTTATCTGACCATGACCACCTCTGCATAGC
GTTCACGTGTGTAGACCACACGTGAGTCTGTAATCCTGCTGTCTGCTTAAGAGCATCTGCCAGGTTAG
+
FFFFFFFFFFFFFFFFFFFF,FF:FFFFFFFFFFFFFFFFFFFFFFFFFFFFFFFFFFF,FFFFFFFFF
FFFFFFFFFFFFFFFFFFFFFFFFFFFFFFFFFFFFFFFFFFFFFFFFFFFFFFFFFFFFFFF:FFFFF
@A00155:342:HHGFNDSXY:1:2608:30038:1846 2:N:0:GAACCTAG+TCCGCATA
ATGCAAAATCTTAACATATTAACAAGCAGTGAGACATGGTTATCTGACCATGACCACCTCTGCATAGC
GTTCACGTGTGTAGACCACACGTGAGTCTGTAATCCTGCTGTCTGCTTAAGAGCATATGCCAGGTTAG
+
FFFFFFFFFFFFFFFFFFF:FFF:FFFFFFFFFFFFFFFFFFFFFFFFFFFFFFFFFFFFFFFFFFFF
FFFFFFFFFFFFFFFFFFFFFFFFFFFFFFFFFFFFFFFFFFFFFFFFFFFFFFFFFFFFFFFFFFFF
@A00155:342:HHGFNDSXY:1:2637:24198:8140 1:N:0:GAACCTAG+TCCGCATA
ATGCAAAATCTTAACATATTAACAAGCAGTGAGACATGGTTATCTGACCATGACCACCTCTGCATAGC
GTTCACGTGTGTAGACCACACGTGAGTCTGTAATCCTGCTGTCTGCTTAAGAGCATCTGCCAGGTTAG
+
FFFFFFFFFFFFFFFFFFFFFFFF:FFFFFF:FFFFFFFFFFFFFFFFFFFFFFFFFFFFFFFFFFFF
FFFFFFFFFFFFFFFFFFFFFFFFFFFFFFFFFFFFFFFFFFFFFFFFFFFFFFFFFFFFFFFFFFFF
@A00155:342:HHGFNDSXY:1:1344:22227:14215 2:N:0:GAACCTAG+TCCGCATA
ATGCAAAATCTTAACATATTAACAAGCAGTGAGACATGGTTATCTGACCATGACCACCTCTGCATAGC
GTTCACGTGTGTAGACCACACGTGAGTCTGTAATCCTGCTGTCTGCTTAAGAGCATCTGCCAGGTTAG
+
```

```
:FFFF:FFF:FFFFFFFFFF:,F:,FFFFFFFFFFFFFFF:FFFFFFFFFFFFFFFFFFFFFFFFFFF
FFFFFFFFF:FFFFFFFFFFFFFFFFFFFFFFFFFFFFFFFFFFFF:FFFFFFFFFFFFFFFFFFF:F
@A00155:342:HHGFNDSXY:1:1108:16749:19601 2:N:0:GAACCTAG+TCCGCATA
TGCAAAATCTTAACATATTAACAAGCAGTGAGACATGGTTATCTGACCATGACCACCTCTGCATAGCG
TTCACGTGTGTAGACCACACGTGAGTCTGTAATCCTGCTGTCTGCTTAAGAGCATCTGCCAGGTTAGT
+
FFFFF:FFFFFFFFFFF,F:FF,,FFFFFFFFFFFFFFFFFFFFFFFFFFFFFFFFFFFFF:FFFFFF
FFFFFFFFFFFFFFFF:FFFFFFFFF:FFFFFFFF,FFFFFFFFFFFFFFFFFFFFFFFFFFFFFFFF
@A00155:342:HHGFNDSXY:1:2275:16152:33786 2:N:0:GAACCTAG+TCCGCATA
TGCAAAATCTTAACATATTAACAAGCAGTGAGACATGGTTATCTGACCATGACCACCTCTGCATAGCG
TTCACGTGTGTAGACCACACGTGAGTCTGTAATCCTGCTGTCTGCTTAAGAGCATCTGCCAGGTTAGT
+
FFFFFFFFFFF:F:FFFFFFFFFFF:FFFFFFFFFFFFFFFFFFFFFFFFF:FF:FFFFFFFFFFF:F
FFFFFFFFFFFFF:FFFFFF:FFFFFFFFFFFFFFFFFFFFFFFFFFFFFFFFFFFFF,FFFFFFFFF
@A00155:342:HHGFNDSXY:1:1441:17644:1485 2:N:0:GAACCTAG+TCCGCATA
TGCAAAATCTTAACATATTAACAAGCAGTGAGACATGGTTATCTGACCATGACCACCTCTGCATAGCG
TTCACGTGTGTAGACCACACGTGAGTCTGTAATCCTGCTGTCTGCTTAAGAGCATCTGCCAGGTTAGT
+
FFFF:FF:FFFFFF,FFFFFFFF,FFFFFFFFFFF:FFFFFFFFFFFFFFFFFFFFFFFFFFFFFFFF
,FFFFFFFFFFFFFFFFFFFFFFFFFFFFFFFFFFFFFFFFFFFFFFFFFFFFFFFFFFFFFFFFFFF
@A00155:342:HHGFNDSXY:1:2559:29966:32064 2:N:0:GAACCTAG+TCCGCATA
TGCAAAATCTTAACATATTAACAAGCAGTGAGACATGGTTATCTGACCATGACCACCTCTGCATAGCG
TTCACGTGTGTAGACCACACGTGAGTCTGTAATCCTGCTGTCTGCTTAAGAGCATCTGCCAGGTTAGT
+
:FFFFFFFFFFFFFF:FF:FFFF:FFFFFFFFFFFFFFFFFFFFFFFFFFFFFFFFF:FFFFFFFFFF
FFFFFFFFFFFFFFFFFF:FFFFFFFFFFFFFFFFFFFFFFFFFFFFFFFFFFFFFFFFFFFFFFF:F
@A00155:342:HHGFNDSXY:1:1615:5710:2613 2:N:0:GAACCTAG+TCCGCATA
TGCAAAATCTTAACATATTAACAAGCAGTGAGACATGGTTATCTGACCATGACCACCTCTGCATAGCG
TTCACGTGTGTAGACCACACGTGAGTCTGTAATCCTGCTGTCTGCTTAAGAGCATCTGCCAGGTTAGT
+
FFFFFFFFFF:FFFFFFFFF,F,F,FFFFFFFFFFFFF:FFFFFFFFFFFFFFFFFFFFFFFFFFFFF
FFFFFFFFFFFFFFFFFFFFFFFFFFFFFFFFFFFFFFFFFFFFFFFFFFFFFFFFFFFFFFFFFFFF
@A00155:342:HHGFNDSXY:1:2559:29966:32064 1:N:0:GAACCTAG+TCCGCATA
GCAAAATCTTAACATATTAACAAGCAGTGAGACATGGTTATCTGACCATGACCACCTCTGCATAGCGT
TCACGTGTGTAGACCACACGTGAGTCTGTAATCCTGCTGTCTGCTTAAGAGCATCTGCCAGGTT
+
FFFFF:FFFFFFF:FFFFFFFFFFFFFFFFFFFFFFFFFFFFFFFFFFFFFFFFFFFFFFFFFFFFFF
FFFFFFFFFFFFFFFFFFFFFFFFFFFFFF:F:FFFFFFFFFFFFFFFFFFFFFFFFFFFFFFF
@A00155:342:HHGFNDSXY:1:2171:22254:12571 2:N:0:GAACCTAG+TCCGCATA
GCAAAATCTTAACATATTAACAAGCAGTGAGACATGGTTATCTGACCATGACCACCTCTGCATAGCGT
TCACGTGTGTAGACCACACGTGAGTCTGTAATCCTGCTGTCTGCTTAAGAGCATCTGCCAGGTT
+
F,:FF,FFF:,,FFFFFFFFFF:F:F,FF:FFFFFFF::FFFFFFF::FF:FF,FFF:FFFFFF:FFF
F:,FFFFFFFFF::FF,FFFFFFF:FFF,FFFFFFFFFF:FFFFFFFFF,F:FFFFFFFF:FF
@A00155:342:HHGFNDSXY:1:1526:10113:17816 2:N:0:GAACCTAG+TCCGCATA
GCAAAATCTTAACATATTAACAAGCAGTGAGACATGGTTATCTGACCATGACCACCTCTGCATAGCGT
TCACGTGTGTAGACCACACGTGAGTCTGTAATCCTGCTGTCTGCTTAAGAGCATCTGCCAGGTTAGTT
+
FFFFF:FFFFFFF:FFFFFFF:FFFFFFFFFFFFFFFFFFFFFFFFFFFFFFFFFFFFFFFFFFFFFF
FFFFF:FFFFFFFFFFFFFFFFFFFFFFFFFFFFFFFFFFFFFFFFFFFFFFFFFFFFFFFFFFFFFF
@A00155:342:HHGFNDSXY:1:1526:10791:19022 2:N:0:GAACCTAG+TCCGCATA
GCAAAATCTTAACATATTAACAAGCAGTGAGACATGGTTATCTGACCATGACCACCTCTGCATAGCGT
TCACGTGTGTAGACCACACGTGAGTCTGTAATCCTGCTGTCTGCTTAAGAGCATCTGCCAGGTTAGTT
+
```

```
FFFFF:FFFFFF:,FFFF,,FF:FFFFFF:FFFFFFF:FFFFFFFFF,FFFFFFFFFFFFF:FFFFFF
FFFFFFFFFFFFFFFFFF:FFFFFFFFFFFFFFFFFFF:FFFFFFFFFFFFFFFFFFFFFFFFFFFFFFF
@A00155:342:HHGFNDSXY:1:1620:21079:25347 2:N:0:GAACCTAG+TCCGCATA
GCAAAATCTTAACATATTAACAAGCAGTGAGACATGGTTATCTGACCATGACCACCTCTGCATAGCGT
TCACGTGTGTAGACCACACGTGAGTCTGTAATCCTGCTGTCTGCTTAAGAGCATCTGCCAGGTTAGTT
+
FFFFFFFFFFFFFFFFFFFFFF:FFFFFFFFFFFFFFFFFFFFFFFFFFFFFFFFFFFFFFFFFFFFF
FFFFFFFFFFFFFFFFFFFFFFFFFFFFFFFFFFFFFFFFFFFFFFFFFFFFFFFFFFFFFFFFFFFF
@A00155:342:HHGFNDSXY:1:1611:20907:20729 2:N:0:GAACCTAG+TCCGCATA
GCAAAATCTTAACATATTAACAAGCAGTGAGACATGGTTATCTGACCATGACCACCTCTGCATAGCGT
TCACGTGTGTAGACCACACGTGAGTCTGTAATCCTGCTGTCTGCTTAAGAGCATCTGCCAGGTTAGTT
+
FFFFFFF:FFFFFFFFFFFFFFFFFFFFFFFFFFFFFFFFFFFFFFFFF:FFFF:FFFFFFFFFFFF
FFFFFFFFFFFFFFFFFFFFFFFFFFFFFFFFFFFFFFFFFFFFFFFFFFFFFFFFF:FFFFFFFFF
@A00155:342:HHGFNDSXY:1:1345:25391:21449 2:N:0:GAACCTAG+TCCGCATA
GCAAAATCTTAACATATTAACAAGCAGTGAGACATGGTTATCTGACCATGACCACCTCTGCATAGCGT
TCACGTGTGTAGACCACACGTGAGTCTGTAATCCTGCTGTCTGCTTAAGAGCATCTGCCAGGTTAGTT
+
FFF:FFFFFFFFF:FFFFF:FFFFFFFFFFFFFFFFFFFFFFFFFFFFFFFFFFFFFFFFFFFFFFF
FFFFFFFFFFFFFFFFFFFFFFFFFFFFFFFFFFFFFFFFFFFFFFFFFFF:FFFFFFFFFFFFFFFFF
@A00155:342:HHGFNDSXY:1:2667:21947:6527 1:N:0:GAACCTAG+TCCGCATA
ATCTTAACATATTAACAAGCAGTGAGACATGGTTATCTGACCATGACCACCTCTGCATAGCGTTCACG
TGTGTAGACCACACGTGAGTCTGTAATCCTGCTGTCTGCTTAAGAGCATCTGCCAGGTTAGTTA
+
FFFFFFFFFFFFFFFFFFF:FFFFFFFFFFFFFFFFFF:FFFFFFFF:FFFFFFFFFFFFF:FFFFFF
FFFFFFFFFFFFFFFFFFFFFFFFFFFFFFFFFFFFFFFFFFFFFFFFFFFFFFFFFFFFFFFF
@A00155:342:HHGFNDSXY:1:2162:12427:7827 1:N:0:GAACCTAG+TCCGCATA
ATCTTAACATATTAACAAGCAGTGAGACATGGTTATCTGACCATGACCACCTCTGCATAGCGTTCACG
TGTGTAGACCACACGTGAGTCTGTAATCCTGCTGTCTGCTTAAGAGCATCTGCCAGGTTAGTTATGTT
+
FFFFFFFFFFFFF:FFFFFFFFFFFFFFFFFFFFFFFFFFFFFFFFFFFFFFFFFFFFFFFF:FFFF
FFFFFFFFFFFFFFFFFFFFFFFFFFFFFFFFFFFFFFFFFFFFFFFFFFFFFFFFFFFFFFFFFFFF
@A00155:342:HHGFNDSXY:1:1622:3631:2362 2:N:0:GAACCTAG+TCCGCATA
ATCTTAACATATTAACAAGCAGTGAGACATGGTTATCTGACCATGACCACCTCTGCATAGCGTTCACG
TGTGTAGACCACACGTGAGTCTGTAATCCTGCTGTCTGCTTAAGAGCATCTGCCAGGTTAGTTATGTT
+
FFFFFFFFFFFFFFFFFFFFFFFFFFFFFFFFFFFFFFFFFFFFFFFFFFFFFFFFFFFFFFFFFFFF
FFFFFFFFFFFFFFFFFFFFFFFFFFFFFFFFFFFFFFFFFFFFFFFFFFFFFFFFFFFFFFFFFF:F
@A00155:342:HHGFNDSXY:1:2162:11794:9017 1:N:0:GAACCTAG+TCCGCATA
ATCTTAACATATTAACAAGCAGTGAGACATGGTTATCTGACCATGACCACCTCTGCATAGCGTTCACG
TGTGTAGACCACACGTGAGTCTGTAATCCTGCTGTCTGCTTAAGAGCATCTGCCAGGTTAGTTATGTT
+
FFFFFFFFFFFFFFFFFFFFFFFFFFFFFFFFFFFFFFFFFFFFFFFFFFFFFFFFFFFFFFFFFFF:
FFFFFFFFFFFFFFFFFFF:FFFFFFFFFFFFFFFFFFFFFFFFFFFFFFFFFFFFFFFFFFFFFFFF
@A00155:342:HHGFNDSXY:1:2162:11749:9064 1:N:0:GAACCTAG+TCCGCATA
ATCTTAACATATTAACAAGCAGTGAGACATGGTTATCTGACCATGACCACCTCTGCATAGCGTTCACG
TGTGTAGACCACACGTGAGTCTGTAATCCTGCTGTCTGCTTAAGAGCATCTGCCAGGTTAGTTATGTT
+
FFFFFFFFF:FFFFFF,FFFFFFFFFFFFFFFFFFFFFFFFFFFFFFFFFFFFFFFFFFFFFFFFFFF
FFFFFFFFFFFFFFFFFFFFFFFFFFFFFFFFFFFFFFFFFFFFFFFFFFFFFFFFFFFFFFFFFF:F
@A00155:342:HHGFNDSXY:1:2417:17327:16658 2:N:0:GAACCTAG+TCCGCATA
ATCTTAACATATTAACAAGCAGTGAGACATGGTTATCTGACCATGACCACCTCTGCATAGCGTTCACG
TGTGTAGACCACACGTGAGTCTGTAATCCTGCTGTCTGCTTAAGAGCATCTGCCAGGTTAGTTATGTT
+
```

```
F:FFFFFFFFFFFFFFFFFFFFFFFFFFFFFFFFFFFFFFFFFFFFFFFFFFFFFFFFFFFFFFFFFF
FFFFFFFFFFFFFFFFFFFFFFFFFFFFFFFFFFFFFFFFFFFFFFFFFFF:FFFFFFFF:FFFFFFF
@A00155:342:HHGFNDSXY:1:1652:8531:25379 2:N:0:GAACCTAG+TCCGCATA
TCTTAACATATTAACAAGCAGTGAGACATGGTTATCTGACCATGACCACCTCTGCATAGCGTTCACGT
GTGTAGACCACACGTGAGTCTGTAATCCTGCTGTCTGCTTAAGAGCATCTGCCAGGTTAGTTATGTTT
+
FF:FFFF:FFFFFFFFFFFFFFFFFFFFFFFFFFFFF:FFFFFFFFFFFFFFFFFFFFFFFFFFFFFF
FFFF:FFFF,FFFFFFFFFFFFFFFFFFFFFFFFFFFFFFFFFFFFFFFFFFFFFFFFFF:FFFFFFF
@A00155:342:HHGFNDSXY:1:2430:9706:21621 1:N:0:GAACCTAG+TCCGCATA
TCTTAACATATTAACAAGCAGTGAGACATGGTTATCTGACCATGACCACCTCTGCATAGCGTTCACGT
GTGTAGACCACACGTGAGTCTGTAATCCTGCTGTCTGCTTAAGAGCATCTGCCAGGTTAGTTATGTTT
+
FFFF:FF:FFFFFFFF:FFFFFFFFFFFFFFFFFFFFFF:FF:FFF:F,FFFFFFFFFFFFFFFFFFF
FFFFFFF:FFFFFFFFFFFFFFFFFFF:FFFFFFFFFFFFFFFFFFFFF:F,FFFF:FFFF,FFFFFF
@A00155:342:HHGFNDSXY:1:2669:28320:35383 1:N:0:GAACCTAG+TCCGCATA
TCTTAACATATTAACAAGCAGTGAGACATGGTTATCTGACCATGACCACCTCTGCATAGCGTTCACGT
GTGTAGACCACACGTGAGTCTGTAATCCTGCTGTCTGCTTAAGAGCATCTGCCAGGTTAGTTATGTTT
+
:F:FF,F:FFFFFFFFFF,FFFFFFFFFFF:F,FF,FFFFFF,,FFFFF,FFFFFFFF:FFFF:F:FF
FFFFF:FFF:FF,:,FFF,FF,FFFFF,FFFF:FFF:FFFFFFFFFF:FFFFFFFF,,FF,FFFFFFF
@A00155:342:HHGFNDSXY:1:1635:8513:21590 1:N:0:GAACCTAG+TCCGCATA
TTAACATATTAACAAGCAGTGAGACATGGTTATCTGACCATGACCACCTCTGCATAGCGTTCACGTGT
GTAGACCACACGTGAGTCTGTAATCCTGCTGTCTGCTTAAGAGCATCTGCCAGGTTAGTTATGTTTAC
+
FFFFF:FFFFFFF:FFFFFFFFFFFFFFF,FF:FFFFFFFFFFFFFFFFF:FFFFFFFFFFFFF:FFF
F:FFFFFFFFFFFFFFFFFFFFFFFFFFFFFFFFFFFFFFFFFFFFFFFFFF,FF,FFFFFFFFFFFF
@A00155:342:HHGFNDSXY:1:2278:13431:32988 1:N:0:GAACCTAG+TCCGCATA
TAACATATTAACAAGCAGTGAGACATGGTTATCTGACCATGACCACCTCTGCATAGCGTTCACGTGTG
TAGACCACACGTGAGTCTGTAATCCTGCTGTCTGCTTAAGAGCATCTGCCAGGTTAGTTATGTTTACC
+
FFFFFFFFFFFFFFFFFFFFFFFFFFFFFFFFFFFFFFFFFFFFFFFFFFFFFFFFFFFFFFFFFFFF
FFFFFFFFFFFFFFFFFFFFFFFFFFFFFFFFFFFFFFFFFFF:FFFFFFFFF:FFFFFFFFFFFFFF
@A00155:342:HHGFNDSXY:1:2412:4047:30232 1:N:0:GAACCTAG+TCCGCATA
TAACATATTAACAAGCAGTGAGACATGGTTATCTGACCATGACCACCTCTGCATAGCGTTCACGTGTG
TAGACCACACGTGAGTCTGTAATCCTGCTGTCTGCTTAAGAGCATCTGCCAGGTTAGTTATGTTTACC
+
FFFFFFFFFFFFFFFFFFFFFFFFFFFFFFFFFFFFFFFFFFFFFFFFFFFFFFFFFFF:FFFFFFFF
FFFFFFFFFFFFFFFFFFFFFFFFFFFFFFFFFFFFFFFFFFFF:FFFFF:FF,F:FFFFFFFFFFFF
@A00155:342:HHGFNDSXY:1:2412:4246:30295 1:N:0:GAACCTAG+TCCGCATA
TAACATATTAACAAGCAGTGAGACATGGTTATCTGACCATGACCACCTCTGCATAGCGTTCACGTGTG
TAGACCACACGTGAGTCTGTAATCCTGCTGTCTGCTTAAGAGCATCTGCCAGGTTAGTTATGTTTACC
+
FFFFFFFFFFFFFFFFFFFFFFFFFFF:F:FFFFFFFFFFFFFFFFFFFFFFFFFF::FFFFFFFFFF
FFFFFF:FFFF:FFFFFFFFFFFFFFFFFFFFFFFFFFFFFFFF:FFFFFFFF,FFFFFF,FFFFFFF
@A00155:342:HHGFNDSXY:1:2330:2049:26209 1:N:0:GAACCTAG+TCCGCATA
AACATATTAACAAGCAGTGAGACATGGTTATCTGACCATGACCACCTCTGCATAGCGTTCACGTGTGT
AGACCACACGTGAGTCTGTAATCCTGCTGTCTGCTTAAGAGCATCTGCCAGGTTAGTTATGTTTACC
+
FFFFFFFFFFFFFFFFFFFFFFFFFFFFFFFFFFFFFFFFFFFFFFFFFFFFFFFFFFFFFFFFFFFF
FFFFFFFFFFFFFFFFFFFFFFFFFFFFFFFFFFFFFFFFFFFFFFFFFFFF,FFFFFFFFFFFFFF
@A00155:342:HHGFNDSXY:1:1622:3631:2362 1:N:0:GAACCTAG+TCCGCATA
AACATATTAACAAGCAGTGAGACATGGTTATCTGACCATGACCACCTCTGCATAGCGTTCACGTGTGT
AGACCACACGTGAGTCTGTAATCCTGCTGTCTGCTTAAGAGCATCTGCCAGGTTAGTTATGTTTACCG
+
```

```
F,FFFFFFFFFFFFFFFFFFFFFFFFFFFFFFFFFFFFFFFFFF:FFFFFFFFFFFFFFFFFFFFFFF
FFFFFFFFFFFFFFFFFFFFFFFFFFFFFFFFFFFFFFFFFFFFFFFFFFFFF,FF:FFFFFFFFFF:
@A00155:342:HHGFNDSXY:1:2275:31015:35321 2:N:0:GAACCTAG+TCCGCATA
CATATTAACAAGCAGTGAGACATGGTTATCTGACCATGACCACCTCTGCATAGCGTTCACGTGTGTAG
ACCACACGTGAGTCTGTAATCCTGCTGTCTGCTTAAGAGCATCTGCCAGGTTAGTTATGTTTAC
+
FFFFFF:FFF,FFFFFFFFFFFFFFFFFFFFFFFFFFFFFFFFFFFFFFFFFFFFFFFFFFFFFFFF
FFFFFFFFFFFFFFFFFFFFFFFFFFFFFFFFFFFFFFFFFFFFFFFFFFFFFFFFFFFFF:FF
@A00155:342:HHGFNDSXY:1:2361:16143:30953 1:N:0:GAACCTAG+TCCGCATA
CATATTAACAAGCAGTGAGACATGGTTATCTGACCATGACCACCTCTGCATAGCGTTCACGTGTGTAG
ACCACACGTGAGTCTGTAATCCTGCTGTCTGCTTAAGAGCATCTGCCAGGTTAGTTATGTTTACC
+
FFFFFFFFFFFFFFFFFFFFFFFFFFFFFFFFFFFFFFFFFFFFFFFFFFFFFFFFFFFFFFFFFFFF
FFFFFFFFFFFFFFFFFFFFFFFFFFFFFFFFFFFFFFFFFFFFFFFFFFFFFFFFFFFFFFFFF
@A00155:342:HHGFNDSXY:1:1631:8675:30796 1:N:0:GAACCTAG+TCCGCATA
ATTAACAAGCAGTGAGACATGGTTATCTGACCATGACCACCTCTGCATAGCGTTCACGTGTGTAGACC
ACACGTGAGTCTGTAATCCTGCTGTCTGCTTAAGAGCATCTGCCAGGTTAGTTATGTTTACCGTCCCC
+
FFFFFFFFFFFFFFFFFFFFFFFFFFFFFFFF:FFFFFFFFFFFFFFFFFFFFFFFFFFFFFFFFFFF
,FFFFFFFFFFFFFFFFFFFFFFFFFFFFFFFFFFFFFFFFFFFFFF:::FFFFFFFFFFFFFFFFFF
@A00155:342:HHGFNDSXY:1:1641:31222:4429 2:N:0:GAACCTAG+TCCGCATA
ATTAACAAGCAGTGAGACATGGTTATCTGACCATGACCACCTCTGCATAGCGTTCACGTGTGTAGACC
ACACGTGAGTCTGTAATCCTGCTGTCTGCTTAAGAGCATCTGCCAGGTTAGTTATGTTTACCGTCCCC
+
FFFFFF::FFFFFFFFFFFFFFFFFFFFFFFFFFFFFFFFFFFFFFFFFFFFFFFFFFFFFFFFFFFF
:FFFFFFFFFFFFFFFFFFFFFFFFFFFFFF:FFFFFFFFFFFFFFFFFFFFFFFFFFFFFFFFFFFF
@A00155:342:HHGFNDSXY:1:2202:15790:9643 1:N:0:GAACCTAG+TCCGCATA
TTAACAAGCAGTGAGACATGGTTATCTGACCATGACCACCTCTGCATAGCGTTCACGTGTGTAGACCA
CACGTGAGTCTGTAATCCTGCTGTCTGCTTAAGAGCATCTGCCAGGTTAGTTATGTTTACCGTCCCCT
+
FFFFFFF::FFFFFFFF::FF,FFFFFFFFFF,FFFFF:FFFFFFFFF,F:FFFFFFF::F:F:FFFF
FFF:F:F,,FF,FF:F:FFFF,FFF:FFFFFFFFFFFFF:FF,FFF,,,FFFFFFFF:FFFFFF:FFF
@A00155:342:HHGFNDSXY:1:1148:30255:21887 1:N:0:GAACCTAG+TCCGCATA
TTAACAAGCAGTGAGACATGGTTATCTGACCATGACCACCTCTGCATAGCGTTCACGTGTGTAGACCA
CACGTGAGTCTGTAATCCTGCTGTCTGCTTAAGAGCATCTGCCAGGTTAGTTATGTTTACCGTCCCCT
+
FFFFFFFFFFFFFFFFFFFFFFFFFFFFFFFFFFFFFFFFFFFFFFFFFFFFFFFFFFFFFFFFFFFF
FFFF:FFFFFFFFFFFFFFFFFFFFFFFFFFFFFFF:FFFFFFFFFFFFFFFFFFFFFFFFFFFFFFF
@A00155:342:HHGFNDSXY:1:1538:3052:29074 1:N:0:GAACCTAG+TCCGCATA
TTAACAAGCAGTGAGACATGGTTATCTGACCATGACCACCTCTGCATAGCGTTCACGTGTGTAGACCA
CACGTGAGTCTGTAATCCTGCTGTCTGCTTAAGAGCATCTGCCAGGTTAGTTATGTTTACCGTCCCCT
+
FFFFFFFFFFFFFFFFFFFFFFF:FFFFFFFFFFF:FFFFFFFFFFFFFFFFFFFFFFFFFFFFFFFF
FFFFFFFF:FFFFFFFFFFFFFFFFFFFFFFFFFFFFFFFFF:FFF:FFF:FFFFFFFF:FFFFFFFF
@A00155:342:HHGFNDSXY:1:1268:14733:16799 2:N:0:GAACCTAG+TCCGCATA
TAACAAGCAGTGAGACATGGTTATCTGACCATGACCACCTCTGCATAGCGTTCACGTGTGTAGACCAC
ACGTGAGTCTGTAATCCTGCTGTCTGCTTAAGAGCATCTGCCAGGTTAGTTATGTTTACCGTCCCCT
+
F:FFF:FFFFFFFFFFFFFFFFFFFFFFFFFFFFFFFFFFFFFFFFFFFFFFFFFFFFFFFFFFFFFF
FFFFFFFFFFFFFFFFFFFFFFFFFFFFFFFFFFFFFFFFFFFFFFFFFFFFFFFFFFFFFFFFFFF
@A00155:342:HHGFNDSXY:1:2428:27127:29622 2:N:0:GAACCTAG+TCCGCATA
AACAAGCAGTGAGACATGGTTATCTGACCATGACCACCTCTGCATAGCGTTCACGTGTGTAGACCACA
CGTGAGTCTGTAATCCTGCTGTCTGCTTAAGAGCATCTGCCAGGTTAGTTATGTTTACCGTCCCCTCA
+
```

FFF:FFF,FFFFFFFFFFFFFFFFFFFFFFFFFFFFFFFFF:FFFFFFFFFFFFFFFFFFFFFFFFFF
FFFFFFFFFFFFF:FFFFFFFFFFFFFFFFFFFFFFFFFFFFFFFFFFFFFFFFFFFFFFFFFFFFFF
@A00155:342:HHGFNDSXY:1:2453:25852:24909 2:N:0:GAACCTAG+TCCGCATA
AACAAGCAGTGAGACATGGTTATCTGACCATGACCACCTCTGCATAGCGTTCACGTGTGTAGACCACA
CGTGAGTCTGTAATCCTGCTGTCTGCTTAAGAGCATCTGCCAGGTTAGTTATGTTTACCGTCCCCTCA
+
FFF,FFFFFFFFFFFFFFFFFFFFFFFFFFFFFFFFFFFFFFFFFFFFFFFFFFFFFFFFFFFFFFFF
FF:FFFFFFFFFFFFFFFFFFFFFFFFFFFFFFFFFFFFFFFFFFFFFFFFFFFFFFFFFFFFFFFFF
@A00155:342:HHGFNDSXY:1:1623:18394:4319 2:N:0:GAACCTAG+TCCGCATA
AACAAGCAGTGAGACATGGTTATCTGACCATGACCACCTCTGCATAGCGTTCACGTGTGTAGACCACA
CGTGAGTCTGTAATCCTGCTGTCTGCTTAAGAGCATCTGCCAGGTTAGTTATGTTTACCGTCCCCTCA
+
FFFF,FFFFFFFF:FFFFFFFFFFFFFFFFFFFFFFFFFFFFF:FFFFF,FFFFFFFFFFFFFFFFFF
FF:FFFFFFFFFFFFFFFFFFFFFFFFFFFFFFFFFFFFFFFFFFFFFFFFFFFFFFFFFFFFFFFFF
@A00155:342:HHGFNDSXY:1:2345:28528:1172 2:N:0:GAACCTAG+TCCGCATA
CAAGCAGTGAGACATGGTTATCTGACCATGACCACCTCTGCATAGCGTTCACGTGTGTAGACCACACG
TGAGTCTGTAATCCTGCTGTCTGCTTAAGAGCATCTGCCAGGTTAGTTATGTTTACCGTCCCCTCAGG
+
F:FFFFFFFFFFFFFFFFFFFFFFFFFFFFFFFFFFFF:FFF:FFFFFFFFFFFFFFFFFFFFFFFFF
FFFFFFFFFFFFFFFFFFFFFFFFFFFFFFFFFFFFFFFFFFFFFFFFFFFFFFFFFFFFFFFFFFFF
@A00155:342:HHGFNDSXY:1:2344:9245:36573 2:N:0:GAACCTAG+TCCGCATA
CAAGCAGTGAGACATGGTTATCTGACCATGACCGCCTCTGCATAGCGTTCACGTGTGTAGACCACACG
TGAGTCTGTAATCCTGCTGTCTGCTTAAGAGCATCTGCCAGGTTAGTTATGTTTACCGTCCCCTCAGG
+
:FFFFFFFFFFFF,FFFFFFFFFFFFFFFFFFFFFFFFFF:FFFFFFFFFFF,FFF:FFFF,F:FFFF
FFFFFFFFF:FFF:F:FFFF:FFFFFFFFFFFFFFFFFF:FFFFFFFFFFFFFFFFF,FFFF:FFFFF
@A00155:342:HHGFNDSXY:1:2116:25211:27305 2:N:0:GAACCTAG+TCCGCATA
AAGCAGTGAGACATGGTTATCTGACCATGACCACCTCTGCATAGCGTTCACGTGTGTAGACCACACGT
GAGTCTGTAATCCTGCTGTCTGCTTAAGAGCATCTGCCAGGTTAGTTATGTTTACCGTCCCCTCAGGG
+
FFFFFFFFFFFFFFFFFFFFFFFFFFFFFFFFFFFFFFFFF:FFFFFFFFFFFFFFFFF:FFFFFFFF
FFFFF:FFFFFFFFFFFFFFFFFFFFFFFFFFFFFFFFFFFFFFFFFFFFFFFFFFFFFFFFFFFFFF
@A00155:342:HHGFNDSXY:1:1538:20283:32612 2:N:0:GAACCTAG+TCCGCATA
AAGCAGTGAGACATGGTTATCTGACCATGACCACCTCTGCATAGCGTTCACGTGTGTAGACCACACGT
GAGTCTGTAATCCTGCTGTCTGCTTAAGAGCATCTGCCAGGTTAGTTATGTTTACCGTCCCCTCAGGG
+
:FFFFFFFFFFFFFFFFFFFFFFFFFFFFFFFFFFFF:FFFFFFFFFFFFFFFFFFFFFFFFFFFFFF
FFFFFFFFFFFFFFFFFFFFFFFFFFFFFFFFFFFFFFFFFFFFFFFFFFFFFFFFFFFFFFFFFFFF
@A00155:342:HHGFNDSXY:1:2112:25771:14497 2:N:0:GAACCTAG+TCCGCATA
AGCAGTGAGACATGGTTATCTGACCATGACCACCTCTGCATAGCGTTCACGTGTGTAGACCACACGTG
AGTCTGTAATCCTGCTGTCTGCTTAAGAGCATCTGCCAGGTTAGTTATGTTTACCGTCCCCTCAGGGG
+
FFFFFFFFFFFFFFFFFFFFFFFF:FFFFFFFFFFFFFFFFFF:FFFFFFFFFFFFFFFFFFFFFFFF
FFFF:FFFFFFF:FFFFFFFFFFFFFFFFFFFFFFFFFFFFFFFFFFFFFFFFFF:FFFFFFFFFFFF
@A00155:342:HHGFNDSXY:1:2247:32515:18818 2:N:0:GAACCTAG+TCCGCATA
CAGTGAGACATGGTTATCTGACCATGACCACCTCTGCATAGCGTTCACGTGTGTAGACCACACGTGAG
TCTGTAATCCTGCTGTCTGCTTAAGAGCATCTGCCAGGTTAGTTATGTTTACCGTCCCCTCAGGGGTC
+
FFFFFFFFFFFFFFFFFF::FFFFFFFFF:FFFFFFFFFFFFFFFFFFFFFFFFFFFFFFF:FFFFFF
:::FFF:FFFFFFFFFFFFFFFF,FFFFFFFF:FFFFFFF:FFFFFF::,FFFFFFFFFFF,FFFFFF
@A00155:342:HHGFNDSXY:1:1419:22724:7341 1:N:0:GAACCTAG+TCCGCATA
CAGTGAGACATGGTTATCTGACCATGACCACCTCTGCATAGCGTTCACGTGTGTAGACCACACGTGAG
TCTGTAATCCTGCTGTCTGCTTAAGAGCATCTGCCAGGTTAGTTATGTTTACCGTCCCCTCAGGGGTC
+

FFFFFFFFFFFFFFFFFFFFFFFFFFFFFFFFFFFFFFFFFFF,FFFFFFFFFFFFFFFF:FFFFFFF
FFFFFFFFFFFFFFFFFFFFFFFFFFFFFFFFFFFFFFFFFFFFFFFFFFFFFFFFFFFFFFFFFFFF
@A00155:342:HHGFNDSXY:1:1171:7392:8594 2:N:0:GAACCTAG+TCCGCATA
CAGTGAGACATGGTTATCTGACCATGACCACCTCTGCATAGCGTTCACGTGTGTAGACCACACGTGAG
TCTGTAATCCTGCTGTCTGCTTAAGAGCATCTGCCAGGTTAGTTATGTTTACCGTCCCCTCAGGGGTC
+
FFFFFFFF:F:FFFFFFFFFFFFFFFFFFFFFF:FFFFFF:FFFFFFFFFFFFFFFFFFFFFFFFFFF
FFFFFFFFFFFFFFFFFFFFFFFFFFFFFFF:FFFFFFFFFFFFFFFFFFFFFFFFFFFFFFFFFFFF
@A00155:342:HHGFNDSXY:1:2365:21685:14215 2:N:0:GAACCTAG+TCCGCATA
CAGTGAGACATGGTTATCTGACCATGACCACCTCTGCATAGCGTTCACGTGTGTAGACCACACGTGAG
TCTGTAATCCTGCTGTCTGCTTAAGAGCATCTGCCAGGTTAGTTATGTTTACCGTCCCCTCAGGGGTC
+
FFFFFFFFFFF,FFFFFFFFFFFFFFFFFFFFFFFFFFFFFFF:FFFFFFFFFFFFFFFFFF:FFFFF
FFFFFFFFFFFFFFFFFFFFFFFFFFFFFFFFFFFFFFFFFFFFFFFFFFFFFFFFFFFFFFFFFFFF
@A00155:342:HHGFNDSXY:1:2314:10719:9846 2:N:0:GAACCTAG+TCCGCATA
CAGTGAGACATGGTTATCTGACCATGACCACCTCTGCATAGCGTTCACGTGTGTAGACCACACGTGAG
TCTGTAATCCTGCTGTCTGCTTAAGAGCATCTGCCAGGTTAGTTATGTTTACCGTCCCCTCAGGGGTC
+
FFFFFFFFFFFFFFFFFFFFFFFFFFFFFFFFFFFFFFFFFFFFFFFFFFFFFFFFFFFFF:FFFFFF
FFFFFFFFFFFFFFFFFFFFFFFFFFFFFFFFFFFFFFFFFFFFFFFFFFFFF:FFFFFFFFFFFFFF
@A00155:342:HHGFNDSXY:1:1605:28610:14434 2:N:0:GAACCTAG+TCCGCATA
CAGTGAGACATGGTTATCTGACCATGACCACCTCTGCATAGCGTTCACGTGTGTAGACCACACGTGAG
TCTGTAATCCTGCTGTCTGCTTAAGAGCATCTGCCAGGTTAGTTATGTTTACCGTCCCCTCAGGGGTC
+
FFFFFFFFFFFFFFF:FFFFFFFF:FFFFFFFFFFFFFFFFFFFFFFFFFFFFFFFFFFFFFFFFFFF
FFFFFFFFFFFFFFFFFFFFFFFFFFFFFFFFFFFFFFFFFFFFFFFFFFFFFFFFFFFFFFFFFFFF
@A00155:342:HHGFNDSXY:1:1615:8567:35963 2:N:0:GAACCTAG+TCCGCATA
AGTGAGACATGGTTATCTGACCATGACCACCTCTGCATAGCGTTCACGTGTGTAGACCACACGTGAGT
CTGTAATCCTGCTGTCTGCTTAAGAGCATCTGCCAGGTTAGTTATGTTTACCGTCCCCTCAGGGGTCA
+
FFFFFFFFFFFFFFFF::FFFFFFFFFFFFFFFFFFFFFFFF:FFFFFFFFFFFFFFFFFFFFFFFFF
FFFFFFFFFFFFFFFF:FFFFFFFFFFFFFFFFFFFFFFFFFFFFFFFFFFFFFFFFF:FFFFFFFFF
@A00155:342:HHGFNDSXY:1:2626:4969:27132 2:N:0:GAACCTAG+TCCGCATA
AGTGAGACATGGTTATCTGACCATGACCACCTCTGCATAGCGTTCACGTGTGTAGACCACACGTGAGT
CTGTAATCCTGCTGTCTGCTTAAGAGCATCTGCCAGGTTAGTTATGTTTACCGTCCCCTCAGGGGTCA
+
FFFFFFFFFFFFFFFFFFFF:FFFFFFFF:FFFFFFFFFFFFFFFF:FFFFFFFFFFFFFFFFFFFFF
FFFFFFFFFFFFFFFFFFFFFFFFFFFFF,FFFFFFFFFFFFFFFF,FFFFFFFFFFFFFFF:FFFFF
@A00155:342:HHGFNDSXY:1:2176:16586:3349 2:N:0:GAACCTAG+TCCGCATA
AGTGAGACATGGTTATCTGACCATGACCACCTCTGCATAGCGTTCACGTGTGTAGACCACACGTGAGT
CTGTAATCCTGCTGTCTGCTTAAGAGCATCTGCCAGGTTAGTTATGTTTACCGGCCCCTCAGGGGTCA
+
:FFFFFFFFFFFF:F:FFFFF:FF:FFFF:F:FFFFFFFFFFFFFFFFFFFFFFFFFFFF,FFFFFFF
FFFFFFFFF,:FFFFFFFFFFFFFFFF:FFFF:FF:FFFFFFFFFFFFFFFFF,FFFFFFFFFFFFF,
@A00155:342:HHGFNDSXY:1:2360:31693:13980 2:N:0:GAACCTAG+TCCGCATA
AGTGAGACATGGTTATCTGACCATGACCACCTCTGCATAGCGTTCACGTGTGTAGACCACACGTGAGT
CTGTAATCCTGCTGTCTGCTTAAGAGCATCTGCCAGGTTAGTTATGTTTACCGTCCCCTCAGGGGTCA
+
FFFFFFFFFFFFF:FFFFFFFF:FFFFFFFFFFFF:FFFFFFFFFFFFFFFFFFFFFFFF:FFFFFFF
FFFFFFFFFFFFFFFFFFFFFFFFFFFFFFFFFFFFFFFFFFFFFFFFFFFFFFFFFFFF::FFFFFF
@A00155:342:HHGFNDSXY:1:1551:6298:6324 1:N:0:GAACCTAG+TCCGCATA
GTGAGACATGGTTATCTGACCATGACCACCTCTGCATAGCGTTCACGTGTGTAGACCACACGTGAGTC
TGTAATCCTGCTGTCTGCTTAAGAGCATCTGCCAGGTTAGTTATGTTTACCGTCCCCTCAGGGGTCA
+

```
FFFFF:FFFFFFFFF:FFFF:FFFFFFF:FFFFFFFFFF:FFFFFFFFFFF::FFFFFFFFFFFFFFF
F:FFFFFFFFFFFFFFFFFFFFFFFFF,:FFF:FFFFFFFFFFFF:FFFFFFFFFFFFFFFFFFFFFFF
@A00155:342:HHGFNDSXY:1:2116:28537:30123 2:N:0:GAACCTAG+TCCGCATA
GAGACATGGTTATCTGACCATGACCACCTCTGCATAGCGTTCACGTGTGTAGACCACACGTGAGTCTG
TAATCCTGCTGTCTGCTTAAGAGCATCTGCCAGGTTAGTTATGTTTACCGTCCCCTCAGGGGTCAAGA
+
FFFFF,FF:FFFFFFFFFFFFFFFF:FFFFFFFFF:FFFFFFFFFFFFFFFFFFFFFFFFFFFFFFFF
FF,FFFFFFFFFFFFFFFFFFFFFFFFFFFFFFFFF:FF:FFFFFFFFFFFFFFFF,FFFFFFFFFF
@A00155:342:HHGFNDSXY:1:2445:16495:33317 2:N:0:GAACCTAG+TCCGCATA
AGACATGGTTATCTGACCATGACCACCTCTGCATAGCGTTCACGTGTGTAGACCACACGTGAGTCTGT
AATCCTGCTGTCTGCTTAAGAGCATCTGCCAGGTTAGTTATGTTTACCGTCCCCTCAGGGGTCAAGA
+
FFFFFFFFFFFFFFFFFFFFFFFFFFFFF,FFFFFFFFFFFFFFFFFFFFFFFF:FFFFFFFFFFFFF
FFFFF:FFFFFFFFFFFFFFFFFFF:FFFFFFFFFFFFF:FFF:FFFFFFFFFF:FFFFFFFFFFFF
@A00155:342:HHGFNDSXY:1:1652:8531:25379 1:N:0:GAACCTAG+TCCGCATA
GACATGGTTATCTGACCATGACCACCTCTGCATAGCGTTCACGTGTGTAGACCACACGTGAGTCTGTA
ATCCTGCTGTCTGCTTAAGAGCATCTGCCAGGTTAGTTATGTTTACCGTCCCCTCAGGGGTCAAGAAT
+
FFFFFFFFFFFFFFFFFFFFFFFFFFFFF:FFF:FF:FFFFFFFFFFFFFFFFFFFFFFFFFFFF:FF
FFFFFFFFFFFFFFFFFFFFFFFFFFF:FFFFFFFFFFFFFFFFFFFFFFFFFFFFF:FFFFFF:FFFFF
@A00155:342:HHGFNDSXY:1:2367:13386:22169 2:N:0:GAACCTAG+TCCGCATA
ATGGTTATCTGACCATGACCACCTCTGCATAGCGTTCACGTGTGTAGACCACACGTGAGTCTGTAATC
CTGCTGTCTGCTTAAGAGCATCTGCCAGGTTAGTTATGTTTACCGTCCCCTCAGGGGTCAAGAATTGC
+
:FFFFFFFFFFFFFFFFFFFFFFFFFFFFFFFFFFFFFFFFFFFFFFFFFFFFFFFFFFFFFFFFFFF
FFFFFFFFFFFFFFFFFFFFFFFFFFFFFFFFFFFFFFFFFFFFFFFFFFFFFFFFFFF:FFFFFFFF
@A00155:342:HHGFNDSXY:1:2214:15447:7889 2:N:0:GAACCTAG+TCCGCATA
GGTTATCTGACCATGACCACCTCTGCATAGCGTTCACGTGTGTAGACCACACGTGAGTCTGTAATCCT
GCTGTCTGCTTAAGAGCATCTGCCAGGTTAGTTATGTTTACCGTCCCCTCAGGGGTCAAGAATT
+
FFFFFFFFFFFFFFFFFFFFFFFFFFFFFFFFFFFFFFFFFFFFFFFFFFFFFFFFFFFFFFFFFFFF
FFFFFFFFFFFFFFFFFFFFFFFFFFFFFFFFFFFFFFFFFFFFFFFFFFFFFFFFF:FFFFFF
@A00155:342:HHGFNDSXY:1:1242:27498:14606 2:N:0:GAACCTAG+TCCGCATA
GTTATCTGACCATGACCACCTCTGCATAGCGTTCACGTGTGTAGACCACACGTGAGTCTGTAATCCTG
CTGTCTGCTTAAGAGCATCTGCCAGGTTAGTTATGTTTACCGTCCCCTCAGGGGTCAAGAATTGCTTT
+
FFFFFFFFFFFFFFFFFFFFFFFFFFFFFFFFFFF:FFFFFFFFFFFFFFFFFFFFFFFFFFFFFFFF
FFFFFFFFFF:FFFFFFFFFFFFFFFFFFFFFFFFFFFFFF:FFFFFFFFFFFFFFFFFFFFFFFFFFF
@A00155:342:HHGFNDSXY:1:2552:10917:19085 2:N:0:GAACCTAG+TCCGCATA
ATCTGACCATGACCACCTCTGCATAGCGTTCACGTGTGTAGACCACACGTGAGTCTGTAATCCTGCTG
TCTGCTTAAGAGCATCTGCCAGGTTAGTTATGTTTACCGTCCCCTCAGGGGTCAAGAATTGCTTTGAG
+
FFFFFFFFFFFFFFFFF:FFFFFFFFFFFFFFFFFFFFFFFFFFFFFFFFFFFFFFFFFFFFFFFFFF
FFFFFFFFFFFFFFFFFFFFFFFFFFFFFFFFFFFFFFFFFFFFFFFFFFFFFFFFFFFFFFFFFFFF
@A00155:342:HHGFNDSXY:1:2430:9706:21621 2:N:0:GAACCTAG+TCCGCATA
TGACCATGACCACCTCTGCATAGCGTTCACGTGTGTAGACCACACGTGAGTCTGTAATCCTGCTGTCT
GCTTAAGAGCATCTGCCAGGTTAGTTATGTTTACCGTCCCCTCAGGGGTCAAGAATTGCTTTGA
+
FFFFFFFFFFFFFFFFFFFFFFFFFFFFFFFFFFFFFFFFFFFFFFFFFFFFFFFFFFFFFFFFFFFF
FFFFFFFFFFF:FFFFFF:FFFFFFFFFFFFFFFFFFFFFFFFFFF:FFFFFFFFFFFFFFFFF:
@A00155:342:HHGFNDSXY:1:2245:23710:10989 2:N:0:GAACCTAG+TCCGCATA
GACCATGACCACCTCTGCATAGCGTTCACGTGTGTAGACCACACGTGAGTCTGTAATCCTGCTGTCTG
CTTAAGAGCATCTGCCAGGTTAGTTATGTTTACCGTCCCCTCAGGGGTCAAGAATTGCTTTGAGAGTC
+
```

```
FF:FFFFFFFFFFFFFFFFFFFFFFFFFFFFFFFFFFFFF:FFFFFFFFF:FFFFFFFFFFFFFFFFF
FFFFFFFFFFFFFFFF:FFFFFFFF:FFFFFFFFFFF:FFFFFFFF:FFFFFFFFFFF:,FFFFFFFF
@A00155:342:HHGFNDSXY:1:2616:7690:17973 2:N:0:GAACCTAG+TCCGCATA
CATGACCACCTCTGCATAGCGTTCACGTGTGTAGACCACACGTGAGTCTGTAATCCTGCTGTCTGCTT
AAGAGCATCTGCCAGGTTAGTTATGTTTACCGTCCCCTCAGGGGTCAAGAATTGCTTTGAGAGTC
+
FFFFF:,,,FF::FFF,FF:FFF:F:F,,:FFF,FFFFFF:F,FFFFFFF:F,FF:F:FFFF,FF:FF
FFFFFFF:FFFF,:FFFF:FFFFFFFFFFFF:FFFF,:,FF:FFFF,FFF::FF:FFF:FFFFFF
@A00155:342:HHGFNDSXY:1:1532:25852:23657 2:N:0:GAACCTAG+TCCGCATA
ATGACCACCTCTGCATAGCGTTCACGTGTGTAGACCACACGTGAGTCTGTAATCCTGCTGTCTGCTTA
AGAGCATCTGCCAGGTTAGTTATGTTTACCGTCCCCTCAGGGGTCAAGAATTGCTTTGAGAGTCCATC
+
FFFFFFFFFFFFFFFFFFFF:FFFFFFFFFFFFFFF,FFFFFFFFFFFFFFFFFFFFF:FFFF:FFFF
FFFF:,FFF:FFFF:,F:FFF,:FFFFFFFFFFFFFFFFFFFFF:::F,FFFFF:FF:FFFFFFFFFF
@A00155:342:HHGFNDSXY:1:2511:6225:12618 2:N:0:GAACCTAG+TCCGCATA
ACCACCTCTGCATAGCGTTCACGTGTGTAGACCACACGTGAGTCTGTAATCCTGCTGTCTGCTTAAGA
GCATCTGCCAGGTTAGTTATGTTTACCGTCCCCTCAGGGGTCAAGAATTGCTTTGAGAGTCCAT
+
FFFFFFFFFFFFF:FFFFFFFFFFFFFFFFFFF,FFFFFFFFFFFFFFFFFFFF:FFFFFFFFFFFFF
FFFFFFFFFFFFFFFFFFFFFFFFFFFFFFFFFFF:FFFFFFFFFF:FFFFFFFFFFFFFFFFF
@A00155:342:HHGFNDSXY:1:2241:30092:30718 2:N:0:GAACCTAG+TCCGCATA
ACCACCTCTGCATAGCGTTCACGTGTGTAGACCACACGTGAGTCTGTAATCCTGCTGTCTGCTTAAGA
GCATCTGCCAGGTTAGTTATGTTTACCGTCCCCTCAGGGGTCAAGAATTGCTTTGAGAGTCCATCAAG
+
FFFFFF,FFFFFFFFFFFF::F:FFFFFFF:FFFFFFFFFFFFFFFFFFFFFFFFFFFFFFFFFFFFF
FFFFFFFFFFFFFFFFFFFFF:F:FFFFFFFFFFFFFFFFFFFFFFF,FFFFFFFFFFFFFFFFFFFF
@A00155:342:HHGFNDSXY:1:1372:8513:1423 2:N:0:GAACCTAG+TCCGCATA
ACCACCTCTGCATAGCGTTCACGTGTGTAGACCACACGTGAGTCTGTAATCCTGCTGTCTGCTTAAGA
GCATCTGCCAGGTTAGTTATGTTTACCGTCCCCTCAGGGGTCAAGAATTGCTTTGAGAGTCCATCAAG
+
FFFFFFFFFFFFFFFFFFFFFFFFFFFFFFFFFFFFFF,:FF:FFFFFFFFFFFFFFFFFFFFFFFFF
FFFFFFFFFFFFFFFFFFFFF:FFFFFFFFFFFFFFFFFFFFFFFFFFFFFFFFFFFFFFFFFFFFFF
@A00155:342:HHGFNDSXY:1:2352:13205:26835 2:N:0:GAACCTAG+TCCGCATA
CCCACCTCTGCATAGCGTTCACGTGTGTAGACCACACGTGAGTCTGTAATCCTGCTGTCTGCTTAAGA
GCATCTGCCAGGTTAGTTATGTTTACCGTCCCCTCAGGGGTCAAGAATTGCTTTGAGAGTCCATCAAG
+
FFFFFFFFFFFFFFFFFFFFFFFFFFFFFFFFFFFFFFFFFFFFFFFFFFFFFFFFFFFFFFFFFFFF
FFFFFFFFFFFFFFFFFFFFFFFFFFFFFFFFFFFFFFFFFF:FFFFFFFFFFFFFFFFFFFFFFFFF
@A00155:342:HHGFNDSXY:1:2578:26069:28479 2:N:0:GAACCTAG+TCCGCATA
ACCACCTCTGCATAGCGTTCACGTGTGTAGACCACACGTGAGTCTGTAATCCTGCTGTCTGCTTAAGA
GCATCTGCCAGGTTAGTTATGTTTACCGTCCCCTCAGGGGTCAAGAATTGCTTTGAGAGTCCATCAAG
+
FFFFFFFFFFFFFFFFFFFFFFFFFFFFFFFFFFFFFFFFFFFFFFFFFFFFFFFFFFFFFFFFFFFF
FFFFFFFFFFFFFFFFFFFFFFFFFFFFFFFFFFFFFFFFF:FFFF:FFFFFFFFFFFFFFFFFFFFF
@A00155:342:HHGFNDSXY:1:1601:8278:16799 2:N:0:GAACCTAG+TCCGCATA
ACCACCTCTGCATAGCGTTCACGTGTGTAGACCACACGTGAGTCTGTAATCCTGCTGTCTGCTTAAGA
GCATCTGCCAGGTTAGTTATGTTTACCGTCCCCTCAGGGGTCAAGAATTGCTTTGAGAGTCCATCAAG
+
FFFFFFFFFFFFFFFFFF:FFFFFFFFF:FFFFFFFFFFFFFF:FFFFFFFFFFFFFFFFFFFFFFFF
FFFFFFFFFFFFFFFFFFFFFFFFFFFFFFFFFFF:FFFFFFFFFFFFFFFFFFFFFFFFFFFFFFFF
@A00155:342:HHGFNDSXY:1:1568:19560:7279 2:N:0:GAACCTAG+TCCGCATA
ACCTCCTCTGCATAGCGTTCACGTGTGTAGACCACACGTGAGTCTGTAATCCTGCTGTCTGCTTAAGA
GCATCTGCCAGGTTAGTTATGTTTACCGTCCCCTCAGGGGTCAAGAATTGCTTTGAGAGTCCATCAAG
+
```

FFFFFFFFFFFFFFFFFFFFFFFFFFFFFFFFFFFFFFFFFFFFFFFFFFFFFFFFFFFFFFFFFFFF
FFFFFFFFFFFFFFFFFFFFFFFFFFFFFFFFFFFFFFFFFFFFFFFFFFFFFFFFFFFFFFFFFFFF
@A00155:342:HHGFNDSXY:1:1306:18439:6809 2:N:0:GAACCTAG+TCCGCATA
ACCACCTCTGCATAGCGTTCACGTGTGTAGACCACACGTGAGTCTGTAATCCTGCTGTCTGCTTAAGA
GCATCTGCCAGGTTAGTTATGTTTACCGTCCCCTCAGGGGTCAAGAATTGCTTTGAGAGTCCATCAAG
+
FFFFFFFFFFFFFFFFFFFFFFFFFFFFFFFFFFFFFFFFFFFFFFFFFFFFFFFFFFFFFFFFFFFF
FFFFFFFFFFFFFFFFFFFFFFFFFFFFFFFFFFFFFFFFFF:FFFFFFFFFFFFFFFFFFFFFFFFF
@A00155:342:HHGFNDSXY:1:1349:4842:2300 2:N:0:GAACCTAG+TCCGCATA
CCACCTCTGCATAGCGTTCACGTGTGTAGACCACACGTGAGTCTGTAATCCTGCTGTCTGCTTAAGAG
CATCTGCCAGGTTAGTTATGTTTACCGTCCCCTCAGGGGTCAAGAATTGCTTTGA
+
FFFFFFFFFFFFFFFFFFFFFFFFFFFFFFFFFFFFFFFFFFFFFFFFFFFFFFFFFFFFFFFFFFFF
FFFFFFFFFFFFFFFFFFFFFFFFFFFFFFFFFFFFFFFFFF:FFFFFFFFFFFF
@A00155:342:HHGFNDSXY:1:2623:2302:23954 2:N:0:GAACCTAG+TCCGCATA
CCACCTCTGCATAGCGTTCACGTGTGTAGACCACACGTGAGTCTGTAATCCTGCTGTCTGCTTAAGAG
CATCTGCCAGGTTAGTTATGTTTACCGTCCCCTCAGGGGTCAAGAATTGCTTTGA
+
FFFFF:FFFFFFF::FFFFFFFFFF:FFFFFFFF:FF,FFFFFFFFFFF:FFFFFF:FF,F:FFFFFF
FFFFFFFFFFFFFFFFFFFF:FFFFFFFFFFF:FF,FFFFF:FFFFFFFF:FFFF
@A00155:342:HHGFNDSXY:1:1312:17381:22983 2:N:0:GAACCTAG+TCCGCATA
ACCTCTGCATAGCGTTCACGTGTGTAGACCACACGTGAGTCTGTAATCCTGCTGTCTGCTTAAGAGCA
TCTGCCAGGTTAGTTATGTTTACCGTCCCCTCAGGGGTCAAGAATTGCTTTGAGAGTCCATCAAG
+
FFFFFFFFF,FFFFFFFFFFFFFFFFFFFFFFFFFFFFFFFFFFFFFFFFFFFFFFFFFFFFFFFFFF
FFFFFFFFFFFFFFFFFFFFFFFFFFFFFFFFFFFFFFFFFFFFFFFFFFFFFFFFFFFFFFFFF
@A00155:342:HHGFNDSXY:1:2351:27633:23202 2:N:0:GAACCTAG+TCCGCATA
ACCTCTGCATAGCGTTCACGTGTGTAGACCACACGTGAGTCTGTAATCCTGCTGTCTGCTTAAGAGCA
TCTGCCAGGTTAGTTATGTTTACCGTCCCCTCAGGGGTCAAGAATTGCTTTGAGAGTCCATCAAG
+
FFFFF,:FFF:FFFFFFFFFFFFFFFFFFFFFFFFFFFFFFFFFFFFFFFFFFFFFFFFFFFFFFFF:
FFFFFFFFFFFFFFFFFFFFF:FFFFFFFFFF:FFFFFFFFFFF:FFFFF:FFFFFFFFFFFFFF
@A00155:342:HHGFNDSXY:1:1527:17390:2112 2:N:0:GAACCTAG+TCCGCATA
ACCTCTGCATAGCGTTCACGTGTGTAGACCACACGTGAGTCTGTAATCCTGCTGTCTGCTTAAGAGCA
TCTGCCAGGTTAGTTATGTTTACCGTCCCCTCAGGGGTCAAGAATTGCTTTGAGAGTCCATCAAGTGA
+
FFFFFFFFF:FFFFFFFFFFFFFFFFFFFFFFFFFFFFFFFFFFFFFFFFFFFFFFFFFFFFFFFFF:
FFFFFFFFFFFFFFFFFFFFFFFF:FFFFFFFFFFFFFF:FFFF,FFFF:FFFFFFFFFFFFFFFFFF
@A00155:342:HHGFNDSXY:1:1122:27597:4413 2:N:0:GAACCTAG+TCCGCATA
CTCTGCATAGCGTTCACGTGTGTAGACCACACGTGAGTCTGTAATCCTGCTGTCTGCTTAAGAGCATC
TGCCAGGTTAGTTATGTTTACCGTCCCCTCAGGGGTCAAGAATTGCTTTGAGAGTCCATCAAG
+
FFFFFFFFFFFFFFFFFFFFFFFFFFFFFFFFFFFFFFFFFFFFFFFFFFFFFFFFFFFFFFFFFF:F
FFFFFFFFFFFFFFFFFFFFFFFFFFFFFF:FFFFFFFFFF:FFFF:FFFFFFFFFFFFFFFF
@A00155:342:HHGFNDSXY:1:2314:27760:31876 2:N:0:GAACCTAG+TCCGCATA
TCTGCATAGCGTTCACGTGTGTAGACCACACGTGAGTCTGTAATCCTGCTGTCTGCTTAAGAGCATCT
GCCAGGTTAGTTATGTTTACCGTCCCCTCAGGGGTCAAGAATTGCTTTGAGAGTCCATCAAGTGATGA
+
FFFFFFFFFF:FF::FFFFFFFFF::FFFFF:FFFFFFFFFFF:FFFFFFFFFF:FFF:FFFFFFFFF
FFFFF:FFF:FFFFFFFFFFFFFFFFFF:FFFFFFF,FFFFFFFFFFFFFFFFFFFFFFFFFFFFFFF
@A00155:342:HHGFNDSXY:1:1367:23330:24862 1:N:0:GAACCTAG+TCCGCATA
TCTGCATAGCGTTCACGTGTGTAGACCACACGTGAGTCTGTAATCCTGCTGTCTGCTTAAGAGCATCT
GCCAGGTTAGTTATGTTTACCGTCCCCTCAGGGGTCAAGAATTGCTTTGAGAGTCCATCAAGTGATGA
+

```
FFFFFFFFFFFFFFFFFFF:FFFFFFF:FFFFFFFFFFFFFFFFFFFFFFFFFFFFFFFFFFFFFFFF
FFFFFFFFFFFFFFFFFFFFFFFFFFFFFFFFFFFFFFFFF:FFFFFFFFFFFFFFFFFFFFFFFFFF
@A00155:342:HHGFNDSXY:1:2571:26277:27117 2:N:0:GAACCTAG+TCCGCATA
TCTGCATAGCGTTCACGTGTGTAGACCACACGTGAGTCTGTAATCCTGCTGTCTGCTTAAGAGCATCT
GCCAGGTTAGTTATGTTTACCGTCCCCTCAGGGGTCAAGAATTGCTTTGAGAGTCCATCAAGTGATGA
+
FFFFFF:FFFFFFFFFFFFFFFFFFFFFFFFFFFFFFFFFFFFFFFFFFFFFFFFFFFFFFFFFFFFF
FFFFFFFFFFFFFFFFFFFFFFFFFFFFF:FFFFFFFFFFFFFFFFFFFFFFFFFFFFFFFFFFFFFF
@A00155:342:HHGFNDSXY:1:1635:8513:21590 2:N:0:GAACCTAG+TCCGCATA
TCTGCATAGCGTTCACGTGTGTAGACCACACGTGAGTCTGTAATCCTGCTGTCTGCTTAAGAGCATCT
GCCAGGTTAGTTATGTTTACCGTCCCCTCAGGGGTCAAGAATTGCTTTGAGAGTCCATCAAGTGATGA
+
FFFF:FFFFFFFFFFFFFFFFFFFFFFFFFFF,FFFFFFFFFFFFFFFFFFFFFFFFFFFFFFFFFFF
FFFFFFFFFFFFFFFFFFFFFFFFFFFFFFFFFFFFFFFFFFFFFFFFF:FFFFFFFFFFFFFFFFFF
@A00155:342:HHGFNDSXY:1:1529:13268:18521 2:N:0:GAACCTAG+TCCGCATA
TCTGCATAGCGTTCACGTGTGTAGACCACACGTGAGTCTGTAATCCTGCTGTCTGCTTAAGAGCATCT
GCCAGGTTAGTTATGTTTACCGTCCCCTCAGGGGTCAAGAATTGCTTTGAGAGTCCATCAAGTGATGA
+
FFFFFFFFFFFFFF:FFFFFFFFFFFFFFFFFFFFFFFFFFFFFFFFFFFFFFFFFFF:FFFFFFFFF
FFFFFFFFFFFFFFFFFFFFFFFFFFF:F,FFFFFFFFFFFFFFFFFFFFFFFFFFFFFFFFFFFFFF
@A00155:342:HHGFNDSXY:1:2456:11921:12273 1:N:0:GAACCTAG+TCCGCATA
TGCATAGCGTTCACGTGTGTAGACCACACGTGAGTCTGTAATCCTGCTGTCTGCTTAAGAGCATCTGC
CAGGTTAGTTATGTTTACCGTCCCCTCAGGGGTCAAGAATTGCTTTGAGAGTCCATCAAGTGAT
+
FFFFFFFFFF:FFFFFFFFFFFFFF,FFFFFFFFFFFFFFFFFFFFFFFFFFFFFFFFFFFFFFFFFF
FFFF,FFFFFFFFFFFFFFFFFFFFFFFFFFFFFFFFFFFFFFFFFFFFFFFFFFFFFFFFFF
@A00155:342:HHGFNDSXY:1:1428:1371:12571 1:N:0:GAACCTAG+TCCGCATA
GCATAGCGTTCACGTGTGTAGACCACACGTGAGTCTGTAATCCTGCTGTCTGCTTAAGAGCATCTGCC
AGGTTAGTTATGTTTACCGTCCCCTCAGGGGTCAAGAATTGCTTTGAGAGTCCATCAAGTGATGACGA
+
FFF:F:FFF:F:FFFFFFFF:FFFFF:FFFFFFFFFFFFFFFFFFFFFFFFFFFF:FFFFFF,FFFFF
:FFF,:F,:FFF,FF:FFFFFFFFFFFFFFFFFF:F:FFFFFFFFFFFFFFFFFFF:FFFFFFF,FFF
@A00155:342:HHGFNDSXY:1:2336:24867:28338 1:N:0:GAACCTAG+TCCGCATA
GCATAGCGTTCACGTGTGTAGACCACACGTGAGTCTGTAATCCTGCTGTCTGCTTAAGAGCATCTGCC
AGGTTAGTTATGTTTACCGTCCCCTCAGGGGTCAAGAATTGCTTTGAGAGTCCATCAAGTGATGACGA
+
FFFFFFFFFFFFFFFFFFFFFFFFFFFFF:FFFFFFFFFFFFFFFFFFFFFFFFFFFFFFFFFFFFFF
FFFFF:FFFF,FFFFFFFFFFFFFFFFFFFFFFFFFFFFFFFFFFFFFFFFFFFFFFFFFFFFFFFF
@A00155:342:HHGFNDSXY:1:1201:25880:5666 1:N:0:GAACCTAG+TCCGCATA
GCATAGCGTTCACGTGTGTAGACCACACGTGAGTCTGTAATCCTGCTGTCTGCTTAAGAGCATCTGCC
AGGTTAGTTATGTTTACCGTCCCCTCAGGGGTCAAGAATTGCTTTGAGAGTCCATCAAGTGATGACGA
+
FFFFFFFFFFFFFFFFFFFFFFFFFFFFFFFFFFFFFFFFFFFFFFFFFFFFFFFFFFFFFFFFFFFF
FFFFFFF:FFFFFFFFFFFFFFFFFFFFFFFFFFFFFFFFFFFFFFFFFFFFFFFFFFFFFFFF:FF:
@A00155:342:HHGFNDSXY:1:1510:25391:19946 1:N:0:GAACCTAG+TCCGCATA
GCATAGCGTTCACGTGTGTAGACCACACGTGAGTCTGTAATCCTGCTGTCTGCTTAAGAGCATCTGCC
AGGTTAGTTATGTTTACCGTCCCCTCAGGGGTCAAGAATTGCTTTGAGAGTCCATCAAGTGATGACGA
+
FFFFFFFFFFFFFFFFFFFFFFFFFFFFF:FFFFF:FFFFFFF:FFFFFFFF:FFFFFFFF:FFFFFF
:FF:FF:FFFFFFFF:FFFFFFFFFF:FFFFFFF:FFFFFFFFFFFFFFFFFFFFF:FFFFFFFFF:F
@A00155:342:HHGFNDSXY:1:2403:31882:24017 1:N:0:GAACCTAG+TCCGCATA
CTTAGCGTTCACGTGTGTAGACCACACGTGAGTCTGTAATCCTGCTGTCTGCTTAAGAGCATCTGCCA
GGTTAGTTATGTTTACCGTCCCCTCAGGGGTCAAGAATTGCTTTGAGAGTCCATCAAGTGATGACGAA
+
```

```
F,FFFFFFFFFFF:FFFFFFFFFFFFFFFFFFFFFFFF:FFFFFFFFFFFFFFFFFFFFFFFFFFFF:
FF:FFF:F,FFFFFFFFFFFFFFFFFFFFFFFFFFFFFFFF:FFFFFFFFFFFFFFFFFFF:FFFFFF
@A00155:342:HHGFNDSXY:1:2154:26883:18928 2:N:0:GAACCTAG+TCCGCATA
ATAGCGTTCACGTGTGTAGACCACACGTGAGTCTGTAATCCTGCTGTCTGCTTAAGAGCATCTGCCAG
GTTAGTTATGTTTACCGTCCCCTCAGGGGTCAAGAATTGCTTTGAGAGTCCATCAAGTGATGA
+
F:FFFFFFFFFFFFFFFFFFFFFFFFFFFFFFFFFFFFFFFFFF:FFFFFFFFFFFFFFFFFFFFFFF
FFFFFFFFFFFFFFFFFFFFFFFFFFFFFFFFFFFFFFFFFFFFFFFFFFFFFFFFFFFFFFF
@A00155:342:HHGFNDSXY:1:2651:26883:30608 1:N:0:GAACCTAG+TCCGCATA
AGCGTTCACGTGTGTAGACCACACGTGAGTCTGTAATCCTGCTGTCTGATTAAGAGCATCTGCCAGGT
TAGTTATGTTTACCGTCCCCTCAGGGGTCAAGAATTGCTTTGAGAGTCCATCAAGTGATGACGAACTC
+
FF,FFFF,,FFFFFF:FF,F::FFFFFF:F:FFFFFFF,FF,FFF,F,,FF,,:F:,FF,,FF,,F,F
F::FFFFFFFF:,,FFF:::FF:F:FFF:,,FF:FFF,F:FFFFFFFFFF:FFFF:,,FFFFF:F,F:
@A00155:342:HHGFNDSXY:1:2544:16938:36808 1:N:0:GAACCTAG+TCCGCATA
AGCGTTCACGTGTGTAGACCACACGTGAGTCTGTAATCCTGCTGTCTGCTTAAGAGCATCTGCCTGGT
TAGTTATGTTTACCGTCCCCTCAGGGGTCAAGAATTGCTTTGAGAGTCCATCAAGTGATGACGAACTC
+
FF:FFF:FF:,F:FFFFF:FF:,FFF,FFFFFFFFFFFFFF::FFFF::FFFFF:F,FF,FF:,,F,F
FF,FF:F,FFFFFF:,FFF:FFFFFFFF::FF:,FFF:FFFFFFFFF:FF,FFFFFF:F:F,FFFFFF
@A00155:342:HHGFNDSXY:1:1441:16640:20509 1:N:0:GAACCTAG+TCCGCATA
GCGTTCACGTGTGTAGACCACACGTGAGTCTGTAATCCTGCTGTCTGCTTAAGAGCATCTGCCAGGTT
AGTTATGTTTACCGTCCCCTCAGGGGTCAAGAATTGCTTTGAGAGTCCATCAAGTGATGACGAACTCC
+
FFFFFFFFFFFFFFFFFFFFFFFFFFFFFFFFFFFFFFFFFFFFFFFFFFFFFFFFFFFFFFFFFFFF
,F,FFFFFFF:FFFFFFFFFFFFFFFFFF:FFFFFFFF:FFFFFFFFFFFFFFFFFFFFFFFFFFFFF
@A00155:342:HHGFNDSXY:1:1441:18629:19351 1:N:0:GAACCTAG+TCCGCATA
GCGTTCACGTGTGTAGACCACACGTGAGTCTGTAATCCTGCTGTCTGCTTAAGAGCATCTGCCAGGTT
AGTTATGTTTACCGTCCCCTCAGGGGTCAAGAATTGCTTTGAGAGTCCATCAAGTGATGACGAACTCC
+
FFFFFFFFFFFFF:FFFFF:FFFF:FFFFFFFFFFFFFFFF:FFFFFFFFFFFFFFFFFFFFFFFFFF
FFFFFFFFFFFFF:FFFFFFFFFFFFFFFF:FFFFFFFFFFFFFF:FFFFFFFFFFFFFFFFFFFF,F
@A00155:342:HHGFNDSXY:1:1444:11812:6073 1:N:0:GAACCTAG+TCCGCATA
GCGTTCACGTGTGTAGACCACACGTGAGTCTGTAATCCTGCTGTCTGCTTAAGAGCATCTGCCAGGTT
AGTTATGTTTACCGTCCCCTCAGGGGTCAAGAATTGCTTTGAGAGTCCATCAAGTGATGACGAACTCC
+
FFFFFFFFFFFFFFFFFFF,FFFFFFFFFFFFFFFFFFFFFFFFFFFFFFFFFFFFFFFFFFFFFFFF
FFFFFFFFFFFFFFFFFFFFFFFFFFFFFFF:FFFFFFFFFFFFFFFFFFFFFFFF:FFFFFFFFFFF
@A00155:342:HHGFNDSXY:1:2548:4634:20509 1:N:0:GAACCTAG+TCCGCATA
GCGTTCACGTGTGTAGACCACACGTGAGTCTGTAATCCTGCTGTCTGCTTAAGAGCATCTGCCAGGTT
AGTTATGTTTACCGTCCCCTCAGGGGTCAAGAATTGCTTTGAGAGTCCATCAAGTGATGACGAACTCC
+
FFFFFFFFFFFFFFFFFFFFFFFFFFFFFFFFFFFFFFFFFFFFFFFFFFFFFFFFFFFFFFFFFFFF
:FFFFFFFFFFFF:FFFFFFFFFFFFFFFFFFFFFFFFFFFFFFFFFFFFFFFFFFFFFFFF:FFFFF
@A00155:342:HHGFNDSXY:1:1509:20835:21355 1:N:0:GAACCTAG+TCCGCATA
GCGTTCACGTGTGTAGACCACACGTGAGTCTGTAATCCTGCTGTCTGCTTAAGAGCATCTGCCAGGTT
AGTTATGTTTACCGTCCCCTCAGGGGTCAAGAATTGCTTTGAGAGTCCATCAAGTGATGACGAACTCC
+
FFFFFFFFFFFFFFFFFFFFFFFFFFFFFFFFFFFFFF:FFFFFFFFFFFFFFFFF,FFFFFFFFFF:
FFFFF,FFFFFFFFFFFFFFFFFFFFFF:FFFFFFFFFFFFFFFFFFFFFFFFFFFFFFFFFFFFFFF
@A00155:342:HHGFNDSXY:1:1140:18105:30530 1:N:0:GAACCTAG+TCCGCATA
GCGTTCACGTGTGTAGACCACACGTGAGTCTGTAATCCTGCTGTCTGCTTAAGAGCATCTGCCAGGTT
AGTTATGTTTACCGTCCCCTCAGGGGTCAAGAATTGCTTTGAGAGTCCATCAAGTGATGACGAACTCC
+
```

FFFFFFFFF:FFFFFFFFF:FFFFFFFFF:FFFFFFFFFFF,FFFFFFFFFFFFF::FF:FFFFF::F
FFF:FFFFFFFFFF:FFFFFFFFFFFFFFF:FFFFFF,FFFFFFFFFFF,FFFF,FFFFF,FFFFFFF
@A00155:342:HHGFNDSXY:1:2511:6225:12618 1:N:0:GAACCTAG+TCCGCATA
CGTTCACGTGTGTAGACCACACGTGAGTCTGTAATCCTGCTGTCTGCTTAAGAGCATCTGCCAGGTTA
GTTATGTTTACCGTCCCCTCAGGGGTCAAGAATTGCTTTGAGAGTCCATCAAGTGATGACGAAC
+
FFFFFFFFFFFFFFFFFFFFFFFFFFFFF:FFFFFFFFFFFFFFFFFFFFFFFFFFFFFFFFFFF:FF
FFFFFFFFFFFF:,FFFFFFFFFF:FFFFFFF,FFFFFFFFFF:FFFFFFF:FFFFFFFF:FFF
@A00155:342:HHGFNDSXY:1:1622:19940:15139 1:N:0:GAACCTAG+TCCGCATA
CGTTCACGTGTGTAGACCACACGTGAGTCTGTAATCCTGCTGTCTGCTTAAGAGCATCTGCCAGGTTA
GTTATGTTTACCGTCCCCTCAGGGGTCAAGAATTGCTTTGAGAGTCCATCAAGTGATGACGAACTCCC
+
FFFFFFFFFFFFFFFFFFFFFFFFFFFFFFFFFFFFFFFFFFFFFFFFFFFFFFFFFFFFFFFFF,FF
FFFF:FFFFFFFFFFFFFFFFFFFFFFFFFFFFFFFFFFFFFFFFFFFFFFFFFFFFFFFFFFFFFF
@A00155:342:HHGFNDSXY:1:1277:11858:15702 1:N:0:GAACCTAG+TCCGCATA
CGTTCACGTGTGTAGACCACACGTGAGTCTGTAATCCTGCTGTCTGCTTAAGAGCATCTGCCAGGTTA
GTTATGTTTACCGTCCCCTCAGGGGTCAAGAATTGCTTTGAGAGTCCATCAAGTGATGACGAACTCCC
+
FFFFFFFFFFFFFFFFFFFFFFFFFFFFFFFFFFFFFFFFFFFFFFFFF:FFFFFFFFFFFFFFFFFF
FFFFFFFFFFFFFFFFFFFFFFFFFFFFFFFFFFFFFFFFFFFFFFFFFFFFFFFFFFFFFFFFFFFF
@A00155:342:HHGFNDSXY:1:2352:13205:26835 1:N:0:GAACCTAG+TCCGCATA
CGTTCACGTGTGTAGACCACACGTGAGTCTGTAATCCTGCTGTCTGCTTAAGAGCATCTGCCAGGTTA
GTTATGTTTACCGTCCCCTCAGGGGTCAAGAATTGCTTTGAGAGTCCATCAAGTGATGACGAACTCCC
+
FFFFFFFFFFFFFFFFFFFFFFFFFFFFFFFFFFFFFFFFFFFFFFFFFFFFFFFFFFFFFFFFFFFF
F:FFFFF,FFFFFFFFFFFFFFFFFFFFFFFFFFFFFFFFFFFFFFFFFFFFFFFFFFFFFFFFFFFF
@A00155:342:HHGFNDSXY:1:1568:19560:7279 1:N:0:GAACCTAG+TCCGCATA
CGTTCACGTGTGTAGACCACACGTGAGTCTGTAATCCTGCTGTCTGCTTAAGAGCATCTGCCAGGTTA
GTTATGTTTACCGTCCCCTCAGGGGTCAAGAATTGCTTTGAGAGTCCATCAAGTGATGACGAACTCCC
+
FFFFFFFFFFFFFFFFFFFFFFFFFFFFFFFFFFFFFFFFFFFFFFFFFFFFFFFFFFFFFFFFFFFF
FFFFFFFFFFFFFFFFFFFFFFFFFFFFFF:FFFFFFFFFFFFFFFFFFFFF:FFFFFFFFFFFFFFF
@A00155:342:HHGFNDSXY:1:1124:15429:23390 1:N:0:GAACCTAG+TCCGCATA
CGTTCACGTGTGTAGACCACACGTGAGTCTGTAATCCTGCTGTCTGCTTAAGAGCATCTGCCAGGTTA
GTTATGTTTACCGTCCCCTCAGGGGTCAAGAATTGCTTTGAGAGTCCATCAAGTGATGACGAACTCCC
+
FFFFFFFFFFFFFFFFFFFFFFFFFFFFFFFFFFFFFFFFFFFFFFFFFFFFFFFFFFFFFFFFFFFF
FFFFFFFFFFFFFFFFFFFFFFFFFFFFFFFFFFFFFFFFF:FFFFFFFFFFFFFFFFFFFFFFFFFF
@A00155:342:HHGFNDSXY:1:2518:25762:5932 1:N:0:GAACCTAG+TCCGCATA
CGTTCACGTGTGTAGACCACACGTGAGTCTGTAATCCTGCTGTCTGCTTAAGAGCATCTGCCAGGTTA
GTTATGTTTACCGTCCCCTCAGGGGTCAAGAATTGCTTTGAGAGTCCATCAAGTGATGACGAACTCCC
+
FFFFFFFFFFFFFFFFFFFFFFFFFFFFFFFFFFFFFFFFFFFFFFFFFFFFFFFFFFFFFFFFFFFF
FFFFFFFFFFFFFFFFFFFFFFFFFFFFFFFFFFFF:FFFFFFFFFFFFFFFFFFFFFFFFFFFFFFF
@A00155:342:HHGFNDSXY:1:1527:17390:2112 1:N:0:GAACCTAG+TCCGCATA
CGTTCACGTGTGTAGACCACACGTGAGTCTGTAATCCTGCTGTCTGCTTAAGAGCATCTGCCAGGTTA
GTTATGTTTACCGTCCCCTCAGGGGTCAAGAATTGCTTTGAGAGTCCATCAAGTGATGACGAACTCCC
+
FFFFFFFFFFFFFFFFFF:FFFFFFFFFFFFFFFFFFFFFFFFFFFFFFFFFFFF,FFFFFFFFFFFF
FFF:FF:FFFFFF:FFFFFFFFFFFFFF:FFFFFFFFFFFFFFFFFFFFF,FFFFFFFFFFFFFFFFF
@A00155:342:HHGFNDSXY:1:1554:13033:26475 1:N:0:GAACCTAG+TCCGCATA
CGTTCACGTGTGTAGACCACACGTGAGTCTGTAATCCTGCTGTCTGCTTAAGAGCATCTGCCAGGTTA
GTTATGTTTACCGTCCCCTCAGGGGTCAAGAATTGCTTTGAGAGTCCATCAAGTGATGACGAACTCCC
+

FFFFFFFFFFFFFFFFFFFFFFF:FFFFFFFFFFFFFFFFFFFFFFFFFFFFFFFFFFFFFFFFFFFF
FF,FFFFFFFFFFFFFFFFFFFFFFFFFFFFFFFFFFFFFF:FFFFFFFFFFFFFF:FFFFFFFFF:F
@A00155:342:HHGFNDSXY:1:2125:24578:4695 1:N:0:GAACCTAG+TCCGCATA
CGTTCACGTGTGTAGACCACACGTGAGTCTGTAATCCTGCTGTCTGCTTAAGAGCATCTGCCAGGTTA
GTTATGTTTACCGTCCCCTCAGGGGTCAAGAATTGCTTTGAGAGTCCATCAAGTGATGACGAACTCCC
+
FFFFFFFFFFFFFFFFFF:FFFFFFFFFFFFFFFFFFFFFFFFFFFFFFFFFFFFFFFFFFFFFFFFF
FFFFFFFFFFFFFFFFFFFFFFFFFFFFFFFFFFFFFFFFFFFFFFFFFFFFFFFFFFFFFFFFFFFF
@A00155:342:HHGFNDSXY:1:1349:4842:2300 1:N:0:GAACCTAG+TCCGCATA
GTTCACGTGTGTAGACCACACGTGAGTCTGTAATCCTGCTGTCTGCTTAAGAGCATCTGCCAGGTTAG
TTATGTTTACCGTCCCCTCAGGGGTCAAGAATTGCTTTGAGAGTCCATCAAGTGATGAC
+
F:FFFFFFFFFFFFFFF:FFFFFFFFFFFFFFFFFF:FF:FFFFFFFFFFFFFFFFFFFFFFFFFFFF
:FFFFFFFFFFFFFFFFFF:F:FFFFFFFFFFFFFFFFFFFFFFFFFFFFFFFFFFFFF
@A00155:342:HHGFNDSXY:1:2623:2302:23954 1:N:0:GAACCTAG+TCCGCATA
GTTCACGTGTGTAGACCACACGTGAGTCTGTAATCCTGCTGTCTGCTTAAGAGCATCTGCCAGGTTAG
TTATGTTTACCGTCCCCTCAGGGGTCAAGAATTGCTTTGAGAGTCCATCAAGTGATGAC
+
FFFFFFFFFFFFFFFFFFFFFFFFFFFFFFFFFFFFFFFF:FFFFFFFFF:F,FFFFFFFFFFFFFFF
FFFFFFFFFFFFFFFFFFFF:FFFFFFFFFFFFFFFFFF:FFFFF,FFFFFFFFFFFFF
@A00155:342:HHGFNDSXY:1:2217:15058:29857 1:N:0:GAACCTAG+TCCGCATA
GTTCACGTGTGTAGACCACACGTGAGTCTGTAATCCTGCTGTCTGCTTAAGAGCATCTGCCAGGTTAG
TTATGTTTACCGTCCCCTCAGGGGTCAAGAATTGCTTTGAGAGTCCATCAAGTGATGACGAACTCCC
+
:FFF:FFFFFFFFF:FFFFFFFFFFFFFFFFFF:FFFF:FFFFFFFFFFFFFFFFFFFFFFFFFFF:F
FFFFFFFFFFFFFF,FFFF:FFFFFFFFFFFFFFFFFFFFFFFFFFF::FFFFFFFF:F:FF:FFFF
@A00155:342:HHGFNDSXY:1:1135:10963:5102 1:N:0:GAACCTAG+TCCGCATA
GTTCACGTGTGTAGACCACACGTGAGTCTGTAATCCTGCTGTCTGCTTAAGAGCATCTGCCAGGTTAG
TTATGTTTACCGTCCCCTCAGGGGTCAAGAATTGCTTTGAGAGTCCATCAAGTGATGACGAACTCCCT
+
FFFFFFFFFFFFFFFFFFFFFFFFFFFFFFFFFFFFFFFFFFFFFFFFFFFFFFFFFFFFFFFFF:FF
:FFFFFFFFFFFFFFFFFFFFFFFFFFFFFFFFFFFFFFFFFFFFFFFFFFFFFFFFFFFFFFFFFFF
@A00155:342:HHGFNDSXY:1:1413:5385:3208 1:N:0:GAACCTAG+TCCGCATA
GTTCACGTGTGTAGACCACACGTGAGTCTGTAATCCTGCTGTCTGCTTAAGAGCATCTGCCAGGTTAG
TTATGTTTACCGTCCCCTCAGGGGTCAAGAATTGCTTTGAGAGTCCATCAAGTGATGACGAACTCCCT
+
FFFFFFFFFFFFFFFFF,F:FFFFFFFF:FFFFF:FFFFFFFFFFFFFFF:FFFFFFFFFFFFFFF:F
F:FFFFFFFFFFFFFFFFFFFFFFFF,FFFFFFFF,FFFFFFFFFFFFFFFFFFFFF:FFFFFFFFFF
@A00155:342:HHGFNDSXY:1:2126:27001:36323 1:N:0:GAACCTAG+TCCGCATA
TTCACGTGTGTAGACCACACGTGAGTCTGTAATCCTGCTGTCTGCTTAAGAGCATCTGCCAGGTTAGT
TATGTTTACCGTCCCCTCAGGGGTCAAGAATTGCTTTGAGAGTCCATCAAGTGATGACGAACTCC
+
FFFFFFFFFFF:FFFFFFFF,FFFFFFFFFFFFFFFF:FFFFFFFFFFFFF:FFFFF,FFFFF,FFFF
FF:FFF,F:FFFFFFFFFFFFFFFFFFFFF:FFFFFFFFF:FFFFFFF:,FFF,FFFFFFFFFFF
@A00155:342:HHGFNDSXY:1:1437:25572:22388 1:N:0:GAACCTAG+TCCGCATA
TTCACGTGTGTAGACCACACGTGAGTCTGTAATCCTGCTGTCTGCTTAAGAGCATCTGCCAGGTTAGT
TATGTTTACCGTCCCCTCAGGGGTCAAGAATTGCTTTGAGAGTCCATCAAGTGATGACGAACTCCCTT
+
FFFFFFFFFFF::FFFFFFFFFFFF,FFFFFFFFFFFFFFFFFFFFFFFFF:FFFFFFFFF:FFFFFF
FFFFFFFFFFFFFFFFFFFF:FFFFFFFFFFFFF:FFFFFFFFFFFFFFFFFFFFFFFFFFFFFFFFF
@A00155:342:HHGFNDSXY:1:1446:16911:31031 2:N:0:GAACCTAG+TCCGCATA
TTCACGTGTGTAGACCACACGTGAGTCTGTAATCCTGCTGTCTGCTTAAGAGCATCTGCCAGGTTAGT
TATGTTTACCGTCCCCTCAGGGGTCAAGAATTGCTTTGAGAGTCCATCAAGTGATGACGAACTCCCTT
+

```
FFFFFFFFFFFFFFFFFF:FFFFFFFFFFFFFFFFFFFFFFFFFFFFFFFFFFFFFFFFFFFFFFFFF
FFFFFFF:FFFFFFFFFFFFFFFFFFFFFFFFFFF:FFFFFFFFFFFFFFFF:FFFFFFFFFFFFFFF
@A00155:342:HHGFNDSXY:1:2252:6912:1658 2:N:0:GAACCTAG+TCCGCATA
TTCACGTGTGTAGACCACACGTGAGTCTGTAATCCTGCTGTCTGCTTAAGAGCATCTGCCAGGTTAGT
TATGTTTACCGTCCCCTCAGGGGTCAAGAATTGCTTTGAGAGTCCATCAAGTGATGACGAACTCCCTT
+
FFFFFFFFFFFFFFFFFFF:FFFFFFFFFFFFFFFFFFFFFFFFFFFFFFFFFFFFFFFFFFFFFFFF
FFFFFFFFFFFFFFFFFFFFFFFF:FFFFFFFFFFFFFFFFFFFFFFFFFFFFFFFFFFFFFFFFFF
@A00155:342:HHGFNDSXY:1:1526:10303:15389 1:N:0:GAACCTAG+TCCGCATA
TTCACGTGTGTAGACCACACGTGAGTCTGTAATCCTGCTGTCTGCTTAAGAGCATCTGCCAGGTTAGT
TATGTTTACCGTCCCCTCAGGGGTCAAGAATTGCTTTGAGAGTCCATCAAGTGATGACGAACTCCCTT
+
FFFFFFFF:FFFFFFF:FFFFFFFFFFFFFFFFFFFFFFFFFFFFFFFFFFFFFFFFFFFFFFFFFF:
FFFFFFFFFFFFFFFFFFFFFFFFFFFF:FFFFFFFFFFFFFFFFFFFFFFFFFFFFFFFFFFFFFF:
@A00155:342:HHGFNDSXY:1:1644:27787:14732 1:N:0:GAACCTAG+TCCGCATA
TTCACGTGTGTAGACCACACGTGAGTCTGTAATCCTGCTGTCTGCTTAAGAGCATCTGCCAGGTTAGT
TATGTTTACCGTCCCCTCAGGGGTCAAGAATTGCTTTGAGAGTCCATCAAGTGATGACGAACTCCCTT
+
FFFFFFFFFFF:FFFFFFFFFFFFFFFFFFFFFFFFFFFFFFFFFFF:FFFFFFFFFFFFFFF:FFFF
:FFFFFFFFFFFFFFFFFFFFFFFFF:FFFFFFFF:FFFFFFFFFFFFFFFFFFFFFFF:FFFFF:FF
@A00155:342:HHGFNDSXY:1:1169:9525:33489 1:N:0:GAACCTAG+TCCGCATA
TTCACGTGTGTAGACCACACGTGAGTCTGTAATCCTGCTGTCTGCTTAAGAGCATCTGCCAGGTTAGT
TATGTTTACCGTCCCCTCAGGGGTCAAGAATTGCTTTGAGAGTCCATCAAGTGATGACGAACTCCCTT
+
FFFFFFFFFFFFFFFFFFFFFFFFFFFFFFFFFFFF:FFF:FFFFFFFFFFFF:FFFFFFFFFFFFFF
FF:F:FFFFFFFFFFFFF:FFF,FFFFFFFFFFFFFFFFFFFFFFFFFFFFFFFF,FFFFFF:F:FFF
@A00155:342:HHGFNDSXY:1:2209:3161:34491 1:N:0:GAACCTAG+TCCGCATA
TTCACGTGTGTAGACCACACGTGAGTCTGTAATCCTGCTGTCTGCTTAAGAGCATCTGCCAGGTTAGT
TACGTTTACCGTCCCCTCAGGGGTCAAGAATTGCTTTGAGAGTCCATCAAGTGATGACGAACTCCCTT
+
FFFFFF:FFFFFFFFFFFFFFFFFFFFFFFFFFFFFFFFFFFFFFFFFFFFFFFFFFF:FFFFFFFFF
FFFF,FFFFFFFFFFFFFFFFFFFFFFF,FFFFFFFFFFFFFFFFFFFFFFFFFFFFFFFFFF,FF,F
@A00155:342:HHGFNDSXY:1:1312:17381:22983 1:N:0:GAACCTAG+TCCGCATA
TCACGTGTGTAGACCACACGTGAGTCTGTAATCCTGCTGTCTGCTTAAGAGCATCTGCCAGGTTAGTT
ATGTTTACCGTCCCCTCAGGGGTCAAGAATTGCTTTGAGAGTCCATCAAGTGATGACGAACTCCC
+
FFFFFFFFFFFFFFFFFFFFFFFFFFFFFF:FFFFFFFFFFFFFFFFFFFFFFFFFFFFFFFF:FFF:
FFFFFFFFFFFFFFFFFFFFFFFFFFFFFFFFF,FFFFFFFFFFFFFFFFFFFFFFFFFFFFFFF
@A00155:342:HHGFNDSXY:1:2351:27633:23202 1:N:0:GAACCTAG+TCCGCATA
TCACGTGTGTAGACCACACGTGAGTCTGTAATCCTGCTGTCTGCTTAAGAGCATCTGCCAGGTTAGTT
ATGTTTACCGTCCCCTCAGGGGTCAAGAATTGCTTTGAGAGTCCATCAAGTGATGACGAACTCCC
+
FFFFFFFF:FFFF:FFFFFFFFF::FFFFFFFFFFFFFFFFFFFFFFFFFFFFFFFFFFFFFFFFFFF
F:FF:FFFFFFF:FFFFFFFFFFFFFFFFFFFFFFFFFFFFFFFFFFFFFF:F:FFFFFF,F,F:
@A00155:342:HHGFNDSXY:1:2239:6370:21543 2:N:0:GAACCTAG+TCCGCATA
TCACGTGTGTAGACCACACGTGAGTCTGTAATCCTGCTGTCTGCTTAAGAGCATCTGCCAGGTTAGTT
ATGTTTACCGTCCCCTCAGGGGTCAAGAATTGCTTTGAGAGTCCATCAAGTGATGACGAACTCCCTTG
+
FFF:FFFFFFFFFFFFFFFFFFFFFFFF:FFFFFFFF,FFFFF,FFFFFFFFFFFFFFFFFFFFFFFF
FFFFFFFFFFFFFFFFFFFFFFFF:FFFFFFFF,FFFFFFFFFFF:FFFFFFFFFFFFFFFFFFFFF
@A00155:342:HHGFNDSXY:1:2454:26639:20134 1:N:0:GAACCTAG+TCCGCATA
TCACGTGTGTAGACCACACGTGAGTCTGTAATCCTGCTGTCTGATTAAGAGCATCTGCCAGGTTAGTT
ATGTTTACCGTCCCCTCAGGGGTCAAGAATTGCTTTGAGAGTCCATCAAGTGATGACGAACTCCCTTG
+
```

FFFFFFFFFFFFFFFFFFFFFFFFFFFFFFFFFFFFFFFFFFFFFFFFFFFFFFFFFFFFFF:FFF:F
FFFFFFFFFFFFFFFFFFFFFFFFFFFFFFFFFFFFFFFFFFFFFFFFFFFFFFFFFFFFFFF:FFFF
@A00155:342:HHGFNDSXY:1:1355:18222:19460 2:N:0:GAACCTAG+TCCGCATA
TCACGTGTGTAGACCACACGTGAGTCTGTAATCCTGCTGTCTGCTTAAGAGCATCTGCCAGGTTAGTT
ATGTTTACCGTCCCCTCAGGGGTCAAGAATTGCTTTGAGAGTCCATCAAGTGATGACGAACTCCCTTG
+
FFFFFFFFFFFFFFFFFFFF,FFFFFFFFFFFFFFFFFFFFFFFFFFFFFFFFFFFFFFFFFFFFFFF
FFFFFFFFFFFFFFFFFFFFFFFFFFFFFFFFFFFFFFFFFFFFFFFFFFFFFFFFFFFFFFFFFFFF
@A00155:342:HHGFNDSXY:1:2504:2672:27633 1:N:0:GAACCTAG+TCCGCATA
TCACGTGTGTAGACCACACGTGAGTCTGTAATCCTGCTGTCTGCTTAAGAGCATCTGCCAGGTTAGTT
ATGTTTACCGTCCCCTCAGGGGTCAAGAATTGCTTTGAGAGTCCATCAAGTGATGACGAACTCCCTTG
+
FFFFFF,FFFFFFFFFFFFFFFFFFFF:FFFFFFFFFFFFFFFFFFFFFFFFFFFFFFFFFFFFFFFF
F:FFFFFFFFFFFFFFFFFFFFFFFFFFFFFF:FFFFFFFFFFFFFFFFFFFFFFFFFFFFFFFF:FF
@A00155:342:HHGFNDSXY:1:2371:5122:3223 2:N:0:GAACCTAG+TCCGCATA
TCACGTGTGTAGACCACACGTGAGTCTGTAATCCTGCTGTCTGCTTAAGAGCATCTGCCAGGTTAGTT
ATGTTTACCGTCCCCTCAGGGGTCAAGAATTGCTTTGAGAGTCCATCAAGTGATGACGAACTCCCTTG
+
FFF:FFFFFFFFFFFFFFFFFFFFFFFF:FFFFFFFF:FFFFF:FF:FFFFFF:FFFFF:FFFF:FFF
FFFFFFFFFFFFFFFFFFFFFFFF:FFFFFFFFFFFFFFFFFFFFFFFFFFFFFFFFFFFF:FFFFFF
@A00155:342:HHGFNDSXY:1:1154:19660:12618 2:N:0:GAACCTAG+TCCGCATA
CACGTGTGTAGACCACACGTGAGTCTGTAATCCTGCTGTCTGCTTAAGAGCATCTGCCAGGTTAGTTA
TGTTTACCGTCCCCTCAGGGGTCAAGAATTGCTTTGAGAGTCCATCAAGTGATGACGAACTCCCTTGA
+
:FF:FFFFFFFFFF,FFFFFFFFFFFFFFFFFFFFFFFFFFFFFFFFFFFFFFFFFFFFFFFFFFFFF
FFF:FFFFFFFFFFFFFFFFF:F:FFFFFFFFFFFFFFFFFFFFFFFFFFFFFFFFFFFFFFFFFFFF
@A00155:342:HHGFNDSXY:1:1154:20157:12665 2:N:0:GAACCTAG+TCCGCATA
CACGTGTGTAGACCACACGTGAGTCTGTAATCCTGCTGTCTGCTTAAGAGCATCTGCCAGGTTAGTTA
TGTTTACCGTCCCCTCAGGGGTCAAGAATTGCTTTGAGAGTCCATCAAGTGATGACGAACTCCCTTGA
+
:FFFFFFFFFFFFFFFFFF:FFFF:FFFFFFFFFFFFFFFFF,FFFFFFFFFFFFFFFFFFFFFFFF:
FF:FFFF:FFFF:FFFFFFF:F:FFFFFFF,F,FFFFFFFFFFFFFF,:FFFFFFFFFFFFFFFFFFF
@A00155:342:HHGFNDSXY:1:2662:23619:36918 1:N:0:GAACCTAG+TCCGCATA
CACGTGTGTAGACCACACGTGAGTCTGTAATCCTGCTGTCTGCTTAAGAGCATCTGCCAGGTTAGTTA
TGTTTACCGTCCCCTCAGGGGTCAAGAATTGCTTTGAGAGTCCATCAAGTGATGACGAACTCCCTTGA
+
FFFFFFFFFFFFFFFFFFFFFFFFF:FFFFFFFFFFFFFFFFFFFFFFFFFFFFFFFFFFFF,FFFFF
:FFFFFFFFFFFFFFFFFFFFFFFFFFFFFFFFFFFFFFFFFFFFFFFFFFFFFFFFFFFFFFFFFFF
@A00155:342:HHGFNDSXY:1:2412:4047:30232 2:N:0:GAACCTAG+TCCGCATA
CACGTGTGTAGACCACACGTGAGTCTGTAATCCTGCTGTCTGCTTAAGAGCATCTGCCAGGTTAGTTA
TGTTTACCGTCCCCTCAGGGGTCAAGAATTGCTTTGAGAGTCCATCAAGTGATGACGAACTCCCTTGA
+
FFFFFFFFFFFFFFFFFFFFFFFFFFFFFFFFFFFFFFFFFFFFFFFFFFFFFFFFFFFFFFFFFFFF
FFFFFFFFFFFFF:FFFFFFFFFFFFFFFFFFFFFFF:FFFFFFFFFFFFFFFFFFFFFFFFFFF:FF
@A00155:342:HHGFNDSXY:1:2412:4246:30295 2:N:0:GAACCTAG+TCCGCATA
CACGTGTGTAGACCACACGTGAGTCTGTAATCCTGCTGTCTGCTTAAGAGCATCTGCCAGGTTAGTTA
TGTTTACCGTCCCCTCAGGGGTCAAGAATTGCTTTGAGAGTCCATCAAGTGATGACGAACTCCCTTGA
+
FFFFFFF:FFFFFFFFFFFFFFFFFFF:FFFFFF:FFFFFFFFFFFFFFFFFFFFFFFFFFFFFFFFF
FFFFFFFFFFFFFFFFFFFFFFF:FFFFFFFFFFFFFFFFF:FFFFFFFFFFFFFFFFFFFFFFFFFF
@A00155:342:HHGFNDSXY:1:2430:26178:30577 2:N:0:GAACCTAG+TCCGCATA
CACGTGTGTAGACCACACGTGAGTCTGTAATCCTGCTGTCTGCTTAAGAGCATCTGCCAGGTTAGTTA
TGTTTACCGTCCCCTCAGGGGTCAAGAATTGCTTTGAGAGTCCATCAAGTGATGACGAACTCCCTTGA
+

```
FFFFFFFFFFFFFFFFFFFFFFFFFFFFFFFFFFFFFFFFFFFFFFFFFFFFFFFFFFFFFFFFFFFF
FFFFFFFFFFFFFFFF:FFFFFFFFFFFFFFFFFFFFFFFFFFFFFFFFFFFFFFFFFFFFFF,FFF
@A00155:342:HHGFNDSXY:1:1510:25391:19946 2:N:0:GAACCTAG+TCCGCATA
CACGTGTGTAGACCACACGTGAGTCTGTAATCCTGCTGTCTGCTTAAGAGCATCTGCCAGGTTAGTTA
TGTTTACCGTCCCCTCAGGGGTCAAGAATTGCTTTGAGAGTCCATCAAGTGATGACGAACTCCCTTGA
+
FFFFFFFFFFFFFFFFFFFFFFFFFFFFFF:FFFFFFFFFFFFFFFFFFFFFFFFFFFFFFFFFFFFF
FFFF:FFFFFFFFFFF:FF:FFF:FFFFFFFFFFFFFFF:FFFFFFFFFFFFFFFFFFFF:FFFFFF:
@A00155:342:HHGFNDSXY:1:1144:24297:36746 1:N:0:GAACCTAG+TCCGCATA
CACGTGTGTAGACCACACGTGAGTCTGTAATCCTGCTGTCTGCTTAAGAGCATCTGCCAGGTTAGTTA
TGTTTACCGTCCCCTCAGGGGTCAAGAATTGCTTTGAGAGTCCATCAAGTGATGACGAACTCCCTTGA
+
FFFFFFFFFFFFFFFFFFFFFFFFFFFFFFFFFFFFFFFFFFFFFFFFFFFFFFFFFFFFFFFFF,FF
FFFFFFFFFFFFFFFFFFFFFFFFFFFFFFFFFFFFFFFFFFFFFFFFFFFFFFFFFFFFFFFFFFFF
@A00155:342:HHGFNDSXY:1:2662:23619:36918 2:N:0:GAACCTAG+TCCGCATA
CACGTGTGTAGACCACACGTGAGTCTGTAATCCTGCTGTCTGCTTAAGAGCATCTGCCAGGTTAGTTA
TGTTTACCGTCCCCTCAGGGGTCAAGAATTGCTTTGAGAGTCCATCAAGTGATGACGAACTCCCTTGA
+
FFFFFFFFFFFFFFFFFFFFFFFFFFFFFFFFFFFFFFFFFFFFFFFFFFFFFFFFFFFFFFFFFFFF
FFFF,FF:FFFFFFFFFFFFFFF:FF:FFFFFFF:FFFFF:FFFFFFFFFFFFFFFFFFFFFFFFFFF
@A00155:342:HHGFNDSXY:1:1423:21350:18677 1:N:0:GAACCTAG+TCCGCATA
CACGTGTGTAGACCACACGTGAGTCTGTAATCCTGCTGTCTGCTTAAGAGCATCTGCCAGGTTAGTTA
TGTTTACCGTCCCCTCAGGGGTCAAGAATTGCTTTGAGAGTCCATCAAGTGATGACGAACTCCCTTGA
+
FFFFFFFFFFFFFFFFFFFFFFFFFFFFFFFFFFFFFFFFFFFFFFFFFFFFFFFFFFFFF:FFFFFF
FFFFFFFFFFFFFFFFFFFFFFFFFFFFFFFFFFFFFFFFFFFFFFFFFFFFFFFFFFFFFFFFFFFF
@A00155:342:HHGFNDSXY:1:1361:17662:12665 2:N:0:GAACCTAG+TCCGCATA
CACGTGTGTAGACCACACGTGAGTCTGTAATCCTGCTGTCTGCTTAAGAGCATCTGCCAGGTTAGTTA
TGTTTACCGTCCCCTCAGGGGTCAAGAATTGCTTTGAGAGTCCATCAAGTGATGACGAACTCCCTTGA
+
FFFFFFFFFFFFFFFFFFFFFFFFFFFFFFFFFFFFFFFFFFFFFFFFFFFFFFFFFFFFFFFFFFFF
FFFFFFFFFFFFFFFFFFFFFFF:FFFFFFFFFFFFFFFFFFFFFFF:FFFFFFFFFFFFFFFFFFFF
@A00155:342:HHGFNDSXY:1:2240:5321:4194 2:N:0:GAACCTAG+TCCGCATA
CACGTGTGTAGACCACACGTGAGTCTGTAATCCTGCTGTCTGCTTAAGAGCATCTGCCAGGTTAGTTA
TGTTTACCGTCCCCTCAGGGGTCAAGAATTGCTTTGAGAGTCCATCAAGTGATGACGAACTCCCTTGA
+
F:FFFFFFFFFFF:FFFFFFFFFF:FFFFFFFFFFFFFFFFFFF:FFFFFFFFFFFFFFFFFFFFFFF
FFFFFFF,FFFFFFFFFFFFFFF:FFFFFFFFFFFFFFFFFFFFFFFFFFFFFFFFFFFFFFFFFFFF
@A00155:342:HHGFNDSXY:1:2278:13431:32988 2:N:0:GAACCTAG+TCCGCATA
CACGTGTGTAGACCACACGTGAGTCTGTAATCCTGCTGTCTGCTTAAGAGCATCTGCCAGGTTAGTTA
TGTTTACCGTCCCCTCAGGGGTCAAGAATTGCTTTGAGAGTCCATCAAGTGATGACGAACTCCCTTGA
+
FFFFFFFFFFFFFFFFFFFFFFFFFFFFFFFFFFFFFFFFFFFFFFFFFFFFFFFFFFFFFFFFFFFF
FFFFF:FFFFFFFFFFFFFFFFF:FFFFFFFFFFFFFFFFFFFFFFFFFFFFFFFFFFFFFFFF,FFF
@A00155:342:HHGFNDSXY:1:1122:27597:4413 1:N:0:GAACCTAG+TCCGCATA
ACGTGTGTAGACCACACGTGAGTCTGTAATCCTGCTGTCTGCTTAAGAGCATCTGCCAGGTTAGTTAT
GTTTACCGTCCCCTCAGGGGTCAAGAATTGCTTTGAGAGTCCATCAAGTGATGACGAACTCCC
+
FFFFFFFFFFFFF:FFFFFFFFFFFFFFFFFFFFFFFFFFFFFFFFFFFFFFFFFFFFFF:FFF,F:F
FFFFFFFFFFFFFFFFFFFFFF:FFFFFFFFFFFFFFFFFFFFFFFFFFFFFFFFFFFFFFFF
@A00155:342:HHGFNDSXY:1:2448:30680:21057 1:N:0:GAACCTAG+TCCGCATA
ACGTGTGTAGACCACACGTGAGTCTGTAATCCTGCTGTCTGCTTAAGAGCATCTGCCAGGTTAGTTAT
GTTTACCGTCCCCTCAGGGGTCAAGAATTGCTTTGAGAGTCCATCAAGTGATGACGAACTCCCTTGAA
+
```

FFFFFFFFFFFFFFFFFFFFFFFFFFFFFFFFFFFFFFFFFFFFFFFFFFFFFFFFFFFFFFFF,FFF
FFFFFFFFFFFFFFFFFFFFFFFFFFFFFFFFFFFFFFFFFFFFFFFFFFFFF:FFFFFFFFFFFFFF
@A00155:342:HHGFNDSXY:1:2106:26169:21386 1:N:0:GAACCTAG+TCCGCATA
ACGTGTGTAGACCACACGTGAGTCTGTAATCCTGCTGTCTGCTTAAGAGCATCTGCCAGGTTAGTTAT
GTTTACCGTCCCCTCAGGGGTCAAGAATTGCTTTGAGAGTCCATCAAGTGATGACGAACTCCCTTGAA
+
FFFFFFFFFFFFFFFFFFFFFFFFFFFFFFFFFFFFFFFFFFFFFFFFFFFFFFFFF:FF:FFFFFFF
FF:FFFFFFFFFFFFFFFFFFFFFFFFFFFFFFFFFFFFFFFFFFFFFFFFFFFFFFFFFFFFF:FFF
@A00155:342:HHGFNDSXY:1:1446:16911:31031 1:N:0:GAACCTAG+TCCGCATA
ACGTGTGTAGACCACACGTGAGTCTGTAATCCTGCTGTCTGCTTAAGAGCATCTGCCAGGTTAGTTAT
GTTTACCGTCCCCTCAGGGGTCAAGAATTGCTTTGAGAGTCCATCAAGTGATGACGAACTCCCTTGAA
+
FFFFFFFFFFFFFFFFFFFFFFFFFFFFFFFFFFFFFFFFFFFFFFFFFF:FFFFFFFFFF:FF,FF:
FFFFFFFFFFFFFFFFFFFFFFF:FFFFFFFFFFFFFFFFFFFFFFFFFFFFFFFFFFFFFFFFFFFF
@A00155:342:HHGFNDSXY:1:1154:31991:22388 1:N:0:GAACCTAG+TCCGCATA
ACGTGTGTAGACCACACGTGAGTCTGTAATCCTGCTGTCTGCTTAAGAGCATCTGCCAGGTTAGTTAT
GTTTACCGTCCCCTCAGGGGTCAAGAATTGCTTTGAGAGTCCATCAAGTGATGACGAACTCCCTTGAA
+
FFFFF,FFFFFFFFF:FFFFFFFFFFFFFFFFFFFFFFFFFFFFFFFFFFFFF:FFFFFFFFFFFFFF
FFFFFFFFFFFFFF:FFFFFFFFFFFFFFFFFFFFF:FFFFFF:F:FFFFFFFFFFFFFFFFFFFFFF
@A00155:342:HHGFNDSXY:1:1449:6759:20306 1:N:0:GAACCTAG+TCCGCATA
ACGTGTGTAGACCACACGTGAGTCTGTAATCCTGCTGTCTGCTTAAGAGCATCTGCCAGGTTAGTTAT
GTTTACCGTCCCCTCAGGGGTCAAGAATTGCTTTGAGAGTCCATCAAGTGATGACGAACTCCCTTGAA
+
FFFFFFFFFFFFFFFFFFFFFF:FFFFFFFFFFFFFFFFFFFFFFFFFFFFFFFFFFFFF:F,F,FFF
FFFFFFFFFFFFFFFFFFFFFFFFFFFFFFFFFFFFFFFFFFFFFFFFFFFFFFFFFFFFFFFFFFFF
@A00155:342:HHGFNDSXY:1:1361:17662:12665 1:N:0:GAACCTAG+TCCGCATA
ACGTGTGTAGACCACACGTGAGTCTGTAATCCTGCTGTCTGCTTAAGAGCATCTGCCAGGTTAGTTAT
GTTTACCGTCCCCTCAGGGGTCAAGAATTGCTTTGAGAGTCCATCAAGTGATGACGAACTCCCTTGAA
+
FFFFFFFFFFFFFFFFFFFFFFFFFFFFFFFFFFFFFFFFFFFFFFFFFFFFFFFFFFFF,FFFFFFF
FFF,FFFFFFFFFFFFFFFFFFFFFFFFFFFFFFFFFFFFFFFFFFFFFFFFFFFFFF:FFFFFFFFF
@A00155:342:HHGFNDSXY:1:2213:10800:8328 1:N:0:GAACCTAG+TCCGCATA
ACGTGTGTAGACCACACGTGAGTCTGTAATCCTGCTGTCTGCTTAAGAGCATCTGCCAGGTTAGTTAT
GTTTACCGTCCCCTCAGGGGTCAAGAATTGCTTTGAGAGTCCATCAAGTGATGACGAACTCCCTTGAA
+
FFFFFFFFFFFFFFFFFFFFFFFFFFFFFFFFFFFFFFFFFFFFFFFFFFFFFFFFFFFFFFFFFFFF
FFFFFFFFFFFFFFFFFFFFFFFFFFFFFFFFFFFFFFFFFFFFFFFFFFFFFFF:FFFFFFFFFFF:
@A00155:342:HHGFNDSXY:1:1158:21992:9737 1:N:0:GAACCTAG+TCCGCATA
ACGTGTGTAGACCACACGTGAGTCTGTAATCCTGCTGTCTGCTTAAGAGCATCTGCCAGGTTAGTTAT
GTTTACCGTCCCCTCAGGGGTCAAGAATTGCTTTGAGAGTCCATCAAGTGATGACGAACTCCCTTGAA
+
FFFFFFFFFFFFFFFFFFFFFFFFFFFFFFFFFFFFFFFFFFFFFFFFFFFFFFFFFFFFFFFF:FFF
FFFFFFFFFFFFFFFFFFFFFFFFFFFFFFFFFFFFFFFFFFFFFFFFFFFFFFFFFFFFFFFFFFFF
@A00155:342:HHGFNDSXY:1:1529:18954:34256 1:N:0:GAACCTAG+TCCGCATA
ACGTGTGTAGACCACACGTGAGTCTGTAATCCTGCTGTCTGCTTAAGAGCATCTGCCAGGTTAGTTAT
GTTTACCGTCCCCTCAGGGGTCAAGAATTGCTTTGAGAGTCCATCAAGTGATGACGAACTCCCTTGAA
+
FFFFFFFFFFFFFFFFFFFFFFFFFFFFFFFFFFFFFFFFFFFFFFFFFFFFFFFFFFFFFF,FFFFF
FFFFFFFFFFFFFFFFFFFFFFFFFFFFFFFFFFFFFFFFFFFFFFFFFFFFFFFFFFFFFFFFFFFF
@A00155:342:HHGFNDSXY:1:1158:21486:8077 1:N:0:GAACCTAG+TCCGCATA
ACGTGTGTAGACCACACGTGAGTCTGTAATCCTGCTGTCTGCTTAAGAGCATCTGCCAGGTTAGTTAT
GTTTACCGTCCCCTCAGGGGTCAAGAATTGCTTTGAGAGTCCATCAAGTGATGACGAACTCCCTTGAA
+

```
FFF:FFFFFFFFFFFFFFFFFFFFFFFFFFFFFFFFFFFFFFFFFFFFFFFFFFFFFFFFFFFFF:FF
FFFFFFFFFFFFFFF:FFFFFFFFFFFFFFFFFFFFFFFFFFFFFFFFFFFFFFFFFFFFFFF:FFFF
@A00155:342:HHGFNDSXY:1:2448:32515:27524 1:N:0:GAACCTAG+TCCGCATA
ACGTGTGTAGACCACACGTGAGTCTGTAATCCTGCTGTCTGCTTAAGAGCATCTGCCAGGTTAGTTAT
GTTTACCGTCCCCTCAGGGGTCAAGAATTGCTTTGAGAGTCCATCAAGTGATGACGAACTCCCTTGAA
+
FFFFFFFF,FFFF:FFFFFFFFFFFFFFFFFFFFFFFFFFFFF:FFFFFFFFFFFFFFFFF,:F::F:
FFFFFFFFFFFFFFFFFFFFFFFFFFFFFFF,FFFFFFFFFFFF:F:F:FF:FFFF:FFFFFF,FFF:
@A00155:342:HHGFNDSXY:1:2223:12102:26929 1:N:0:GAACCTAG+TCCGCATA
ACGTGTGTAGACCACACGTGAGTCTGTAATCCTGCTGTCTGCTTAAGAGCATCTGCCAGGTTAGTTAT
GTTTACCGTCCCCTCAGGGGTCAAGAATTGCTTTGAGAGTCCATCAAGTGATGACGAACTCCCTTGAA
+
FFFFFFFFFFFFFFFFFFFFFFFFFFFFFFFFFFFFFFFFFFFFFFFFFFFFFFFFFFFFFFFFFFFF
FFFFFFFFFFFFFFFFFFFFFFFFFFFFFFFFFFFFFFFFFFFFFFFFFFFFFFFFFFFFFFFFFFFF
@A00155:342:HHGFNDSXY:1:2650:30508:16658 1:N:0:GAACCTAG+TCCGCATA
ACGTGTGTAGACCACACGTGAGTCTGTAATCCTGCTGTCTGCTTAAGAGCATCTGCCAGGTTAGGTAT
GTTTACCGTCCCCTCAGGGGTCAAGAATTGCTTTGAGAGTCCATCAAGTGATGACGAACTCCCTTGAA
+
FFF:FFFFFFFFF,FFFFF:FF,FF,:FFFFFFFFFFFFFFFFFFFFFF:F:FFFFF:,F:,FF,,FF
F:,FFFFF:FFFFFFF:FFF,FFFFFF,FFFF::,FFFFFFFF:FFF:FFF,F:FFFFFFF,FFF:FF
@A00155:342:HHGFNDSXY:1:1471:13919:3020 1:N:0:GAACCTAG+TCCGCATA
ACGTGTGTAGACCACACGTGAGTCTGTAATCCTGCTGTCTGCTTAAGAGCATCTGCCAGGTTAGGTAT
GTTTACCGTCCCCTCAGGGGTCAAGAATTGCTTTGAGAGTCCATCAAGTGATGACGAACTCCCTTGAA
+
FFFFFFFFFFFFF:FFFFFFFFFFFFFFFFFFFFFFFFFFFFFFFFFFFFFFFFFFFFFFFFFF,FF:
FFFFFFFFFFFFFFFFFFFFFFFFFFFFFFFFFFFFFFFFFFFFFFFFFFFFFFFFFFFFFFF:FFFF
@A00155:342:HHGFNDSXY:1:1106:26440:28682 1:N:0:GAACCTAG+TCCGCATA
ACGTGTGTAGACCACACGTGAGTCTGTAATCCTGCTGTCTGCTTAAGAGCATCTGCCAGGTTAGTTAT
GTTTACCGTCCCCTCAGGGGTCAAGAAATGCTTTGAGAGTCCATCAAGTGATGACGAACTCCCTTGAA
+
FFFF,,:FFFFFF,FF:F,FFFF:FFF:FFFFFF,FF:FFFFF:FFFFFF:::FFFF::F,FF,F:FF
FFFFF:FFFFFFFFFF::FFFFF:FFF,:FFFFF,FFF:FF,FFFF,:FFFF:FFFFF,F,,FF::FF
@A00155:342:HHGFNDSXY:1:1223:31304:10144 1:N:0:GAACCTAG+TCCGCATA
CGTGTGTAGACCACACGTGAGTCTGTAATCCTGCTGTCTGCTTAAGAGCATCTGCCAGGTTAGTTATG
TTTACCGTCCCCTCAGGGGTCAAGAATTGCTTTGAGAGTCCATCAAGTGATGACGAACTCCCTTGAAG
+
FFFFFFFFFFFF:FFFFFFFFFFFFFFFFFFFFFFFFFFFFFFFFFFFFFFFFFFFFFFFFFF,:F:F
FFFFFFFFFFFFFFFFFFFFFFFFFFFFFFFFFFFFFFF:FFFFFFFFFFFFFFFFFFFFFFFFFFFF
@A00155:342:HHGFNDSXY:1:1631:8675:30796 2:N:0:GAACCTAG+TCCGCATA
CGTGTGTAGACCACACGTGAGTCTGTAATCCTGCTGTCTGCTTAAGAGCATCTGCCAGGTTAGTTATG
TTTACCGTCCCCTCAGGGGTCAAGAATTGCTTTGAGAGTCCATCAAGTGATGACGAACTCCCTTGAAG
+
FFFFFFFFFFFFFFFFFFFFFFFFFFFFF:FFFFFFFFFFFFFFFFFFFFFFFFFFFFFF,FFFFFFF
FFFFFFFFFFFFFFFFFFFFF:FFFFFFFFFFFFFFFFFFFFFFFFFFFFFFFFFFFFFFFFFFFFFF
@A00155:342:HHGFNDSXY:1:2625:12246:3818 2:N:0:GAACCTAG+TCCGCATA
GTGTGTAGACCACACGTGAGTCTGTAATCCTGCTGTCTGCTTAAGAGCATCTGCCAGGTTAGTTATGT
TTACCGTCCCCTCAGGGGTCAAGAATTGCTTTGAGAGTCCATCAAGTGATGACGAACTCCCTTGAAGC
+
FFFFFFFFFFFFFFFF:FFFFFFFFFFFFFFFFFFFFFFFFFFFFFFFFFFFFFFFFFFFFFFFFFFF
FFFFFFFFFFFFFFFFFFFFFFFFFFFFFFFFFFFFFFFFFFFFFFFFFFFFFFFFFFFFFFFFFFFF
@A00155:342:HHGFNDSXY:1:1144:24297:36746 2:N:0:GAACCTAG+TCCGCATA
TGTGTAGACCACACGTGAGTCTGTAATCCTGCTGTCTGCTTAAGAGCATCTGCCAGGTTAGTTATGTT
TACCGTCCCCTCAGGGGTCAAGAATTGCTTTGAGAGTCCATCAAGTGATGACGAACTCCCTTGAAGCG
+
```

```
FFFFFFFFFFF:FFFFFFFFFFFFFFFFFFFFFFFFFFFFFFFFFFFFFFFFFFFFFFFFFFFFFFFF
F:FFFFFFFFFFFFFFFFFFFFFFFFFFFFFFFFFFFFFFFFFFFFFFFFFFFF:FFFFF:FFFFFFF
@A00155:342:HHGFNDSXY:1:1140:18105:30530 2:N:0:GAACCTAG+TCCGCATA
TGTGTAGACCACACGTGAGTCTGTAATCCTGCTGTCTGCTTAAGAGCATCTGCCAGGTTAGTTATGTT
TACCGTCCCCTCAGGGGTCAAGAATTGCTTTGAGAGTCCATCAAGTGATGACGAACTCCCTTGAAGCG
+
FFFFFFFFFFFF,FFFFFFFFFFFFFFFFFF:FFFFFFFFFFFFFFFFFFFFFFF:FFFFFFFFFFFF
FFFFFFFFFFFFFFFFFFF:FFFFFFFFFFFFFFFFFFFFF:FFFFFFFFFFFFFFFFFFFFFF:FFF
@A00155:342:HHGFNDSXY:1:1444:11812:6073 2:N:0:GAACCTAG+TCCGCATA
TGTGTAGACCACACGTGAGTCTGTAATCCTGCTGTCTGCTTAAGAGCATCTGCCAGGTTAGTTATGTT
TACCGTCCCCTCAGGGGTCAAGAATTGCTTTGAGAGTCCATCAAGTGATGACGAACTCCCTTGAAGCG
+
FF:FFFFFFFFFFFFFFFFFFFFFFFFFFFFFFFFFFFFFFFFFFFFFFFFFFFFFFFFFFFFFFF:F
FFFFFFFFFFFFFFFFFFF:FFFFFFFFFFFFFFFFFFFFFFFFFFFFFFFFFFFFFFFF:FFFFFFF
@A00155:342:HHGFNDSXY:1:1509:20835:21355 2:N:0:GAACCTAG+TCCGCATA
TGTGTAGACCACACGTGAGTCTGTAATCCTGCTGTCTGCTTAAGAGCATCTGCCAGGTTAGTTATGTT
TACCGTCCCCTCAGGGGTCAAGAATTGCTTTGAGAGTCCATCAAGTGATGACGAACTCCCTTGAAGCG
+
FFFFFFFFFFFFFFFF:FFFFFFFFFFFFFFFFFFFFFFFFFFFFFFFFFFFFFFFFFFFFFFFFFF
FFFFFFFFFFFFFFFFFFFFFFFFFFFFFFFFFFFFFFFFFFFFFFFFFFFFFFFFFFFFFFFF:FFF
@A00155:342:HHGFNDSXY:1:2252:6912:1658 1:N:0:GAACCTAG+TCCGCATA
TGTGTAGACCACACGTGAGTCTGTAATCCTGCTGTCTGCTTAAGAGCATCTGCCAGGTTAGTTATGTT
TACCGTCCCCTCAGGGGTCAAGAATTGCTTTGAGAGTCCATCAAGTGATGACGAACTCCCTTGAAGCG
+
FFFFFFFFFFFFFFFFFFFFFFFFFFFFFFFFFFFFFFFFFFFFFFFFFFFFFFFFFFFFF::FFFFF
FFFFFFF:FFFFFFFFFFFFFFFFFFFFFFFFFFFFFF:FFFFFFFFFF,FFFFF:FFFFFF,FFFFF
@A00155:342:HHGFNDSXY:1:1219:22408:31876 2:N:0:GAACCTAG+TCCGCATA
GTGTAGACCACACGTGAGTCTGTAATCCTGCTGTCTGCTTAAGAGCATCTGCCAGGTTAGTTATGTTT
ACCGTCCCCTCAGGGGTCAAGAATTGCTTTGAGAGTCCATCAAGTGATGACGAACTCCCTTGAAGCGA
+
FFFFFFFFFFFFFFFFFFFFFFFFFFFF:FF,FFFFFFFFFFFFFFFFF,FFFFFFFFFFFFFFFFFF
FFFFFFFFFFFFFFF:FFFFFFFFFFFFFFFFFFFFFFFFFFFFFFFFFFFFFFFFFFFFFFFFFF:F
@A00155:342:HHGFNDSXY:1:2559:5113:31203 2:N:0:GAACCTAG+TCCGCATA
GTGTAGACCACACGTGAGTCTGTAATCCTGCTGTCTGCTTAAGAGCATCTGCCAGGTTAGTTATGTTT
ACCGTCCCCTCAGGGGTCAAGAATTGCTTTGAGAGTCCATCAAGTGATGACGAACTCCCTTGAAGCGA
+
FFFFFFFFFFFF:FF:FFFFFFFFFFFFFFFFFFFFFFFFFFFFFF:FFFFF:FFFFFFFFFFFFFFF
FFFFFFFFFFFFFFFFF::FFFFFF:FFFFFFFFFFFFFFFFFFFFFFFFFFFFFFFFF:FFFFFFFF
@A00155:342:HHGFNDSXY:1:2240:5321:4194 1:N:0:GAACCTAG+TCCGCATA
GTGTAGACCACACGTGAGTCTGTAATCCTGCTGTCTGCTTAAGAGCATCTGCCAGGTTAGTTATGTTT
ACCGTCCCCTCAGGGGTCAAGAATTGCTTTGAGAGTCCATCAAGTGATGACGAACTCCCTTGAAGCGA
+
FFFFFFFFFFFFFFFFFFFFFFFFFFFFFFFFFFFFFFFFFFFFFFFFFFFFFFFFFFFFFFFFFFFF
FFFFFFFFFFFFFFFFFFFFF:FFFFFFFFFFFFFFFFFFFFFFFFFFFFFFFFFFFFFFFFFFFFFF
@A00155:342:HHGFNDSXY:1:2503:7120:9878 2:N:0:GAACCTAG+TCCGCATA
GTGTAGACCACACGTGAGTCTGTAATCCTGCTGTCTGCTTAAGAGCATCTGCCAGGTTAGTTATGTTT
ACCGTCCCCTCAGGGTTCAAGAATTGCTTTGAGAGTCCATCAAGTGATGACGAACTCCCTTGAAGCGA
+
F:FFFFFFF:F,FFFFFFF:F,F,F:FFFFF:FFFFFFFF,FFFFFFF:FFF,FFF:F:FFF:FFFFF
FFF:FFF:FF::FFF,FFFFFF:FFFFFFFFF:FFF:FFF:FF,FF,FFFFFFF:FFFFF:FFFFFFF
@A00155:342:HHGFNDSXY:1:2503:32362:7748 2:N:0:GAACCTAG+TCCGCATA
GTGTAGACCACACGTGAGTCTGGAATCCTGCTGTCTGCTTAAGAGCATCTGCCAGGTTAGTTATGTTT
ACCGTCCCCTCAGGGGTCAAGAATTGCTTTGAGAGTCCATCAAGTGATGACGAACTCCCTTGAAGCGA
+
```

FFFFFF,FFFFFFFFFFFFFFF,:FFFFFFFFFFFFFF:FFFFFFFFFFFFFFFFFFFFF:F::FFF,
FFFFFFFFFFF,FFFFFFFFFFF,FFFFFFFFFFFFFFFFF:FFFFFFFFFFF:FFFFF,,FFFFFFF
@A00155:342:HHGFNDSXY:1:1532:25852:23657 1:N:0:GAACCTAG+TCCGCATA
GTGTAGACCACACGTGAGTCTGTAATCCTGCTGTCTGCTTAAGAGCATCTGCCAGGTTAGTTATGTTT
ACCGTCCCCTCAGGGGTCAAGAATTGCTTTGAGAGTCCATCAAGTGATGACGAACTCCCTTGAAGCGA
+
FFFFFFFFF,FF:FFFFFFFFFFFFFFFFFFFFFF:FFFFFFFFF::FFFFFFFFFFFFFFF,FFF,,
FF,FFFFFFFF,FFFFFFFFFFFFF:FFFFFFFF:FFFFFFFFF:FFFFFFFFFFFF,FFFFFFFFFF
@A00155:342:HHGFNDSXY:1:2154:26883:18928 1:N:0:GAACCTAG+TCCGCATA
GTAGACCACACGTGAGTCTGTAATCCTGCTGTCTGCTTAAGAGCATCTGCCAGGTTAGTTATGTTTAC
CGTCCCCTCAGGGGTCAAGAATTGCTTTGAGAGTCCATCAAGTGATGACGAACTCCCTTGAAG
+
FFFFFFFFFFFFFFFFFFFFFFFFFFFFFFFFFFFFFF:FFFFFFFFFFFFFF:FFF,FFFFFFFFF
FFFFFFFFFFFFFFFFFFFFFFFFFFFFFFFFFFFFFFFFFFFFFFFFFFFFFFFFFFFFFFF
@A00155:342:HHGFNDSXY:1:1650:30264:25316 1:N:0:GAACCTAG+TCCGCATA
GTAGACCACACGTGAGTCTGTAATCCTGCTGTCTGCTTAAGAGCATCTGCCAGGTTAGTTATGTTTAC
CGTCCCCTCAGGGGTCAAGAATTGCTTTGAGAGTCCATCAAGTGATGACGAACTCCCTTGAAGCGACG
+
FFFFFFF,FFFFFFFF:FFFFFFFFFFFFFFFFFFFFFFFFFFFFFFF,FFFFFFFFFFFFFFFFFFF
FFFFFFF:FF:FFFF:FFFFF:FFFFFFFFF:FFFFFFFFFF,:FFFFFFFF:FFFFFFFFFFF:FFF
@A00155:342:HHGFNDSXY:1:2625:12246:3818 1:N:0:GAACCTAG+TCCGCATA
GTAGACCACACGTGAGTCTGTAATCCTGCTGTCTGCTTAAGAGCATCTGCCAGGTTAGTTATGTTTAC
CGTCCCCTCAGGGGTCAAGAATTGCTTTGAGAGTCCATCAAGTGATGACGAACTCCCTTGAAGCGACG
+
FFFFFFFFFFFFFFFFFFFFFFFFFFFFFFFFFFFFFFFFFFFFFFFFFFFF::FFF:FFFFFFFF
FFFFFFFFFFFFFFFFFFFFFFFFFFFFFFFFFFFFFFFFFFFFFFFFFFFFFFFFFFFFFFFFFFFF
@A00155:342:HHGFNDSXY:1:2206:15383:23813 1:N:0:GAACCTAG+TCCGCATA
GACCACACGTGAGTCTGTAATCCTGCTGTCTGCTTAAGAGCATCTGCCAGGTTAGTTATGTTTACCGT
CCCCTCAGGGGTCAAGAATTGCTTTGAGAGTCCATCAAGTGATGACGAACTCCCTTGAAGCGACGCCT
+
FFFFFFFFFFFFFFFFFFFFFFFFFFFFFFFFFFFFFFFFFFFFFFFFFFFFFFFFFFFFFFFFFFFF
FFFFFFFFFFFFFFFFFFFFFFFFFFFFFFFFFFFFFFFFFFFFFFFFFFFFFFFFFFFFFFFFFFFF
@A00155:342:HHGFNDSXY:1:2212:19976:19805 2:N:0:GAACCTAG+TCCGCATA
GACCACACGTGAGTCTGTAATCCTGCTGTCTGCTTAAGAGCATCTGCCAGGTTAGTTATGTTTACCGT
CCCCTCAGGGGTCAAGAATTGCTTTGAGAGTCCATCAAGTGATGACGAACTCCCTTGAAGCGACGCCT
+
FFFFFFFFF,FFFFFFFFFFFFFFFFFFFFF,FFFFFFFFFFFFFFFFFFFFFFFFFFFFFFFFFFFF
FFFFFFFFFFFFFFFFFF:FFFFFFFFFFFFFFFFFFFFFFFFFFFFFFFFFFFFFFFFFFFFFFFFF
@A00155:342:HHGFNDSXY:1:1622:19940:15139 2:N:0:GAACCTAG+TCCGCATA
CACACGTGAGTCTGTAATCCTGCTGTCTGCTTAAGAGCATCTGCCAGGTTAGTTATGTTTACCGTCCC
CTCAGGGGTCAAGAATTGCTTTGAGAGTCCATCAAGTGATGACGAACTCCCTTGAAGCGACGCCTGAA
+
FFFFF:FFFFFFFFFFFFFFFFFFFFFFFFFFFFFFFFFFFFFFFFF:FFFFFFFFFFFFFFFFFFFF
FFFFFFFFFF:FFFFFFFFFFFFFFFFFFF:FFFFFFFFF:FFFFFFFFFFFFFFFFFFFFF:FFFFFF
@A00155:342:HHGFNDSXY:1:1554:13033:26475 2:N:0:GAACCTAG+TCCGCATA
CACACGTGAGTCTGTAATCCTGCTGTCTGCTTAAGAGCATCTGCCAGGTTAGTTATGTTTACCGTCCC
CTCAGGGGTCAAGAATTGCTTTGAGAGTCCATCAAGTGATGACGAACTCCCTTGAAGCGACGCCTGAA
+
FFF:FFFFFFFFFFF:FFFFFFFFFFFFFFFFFFFFFFFFFFFFFFFFFFFFFFFFFFFFFFFFFFFF
FFFFFFFFFF:FFFFFFFFFFFFFFFFFFFFFFFFFFFFFFFFFFFFFFFFFF,FFFFFFFFFFFFFFFF
@A00155:342:HHGFNDSXY:1:1678:23484:34053 2:N:0:GAACCTAG+TCCGCATA
CACACGTGAGTCTGTAATCCTGCTGTCTGCTTAAGAGCATCTGCCAGGTTAGTTATGTTTACCGTCCC
CTCAGGGGTCAAGAATTGCTTTGAGAGTCCATCAAGTGATGACGAACTCCCTTGAAGCGACGCCTGAA
+

```
FFFFFF:FFFFFFFFFFFFFFFFFFFFFFFFFFFFFFFFFFFFFFFFFFFFFFF::FFFFFFFF,FFF
FFFFFFFFFF:FFFFFFFFFFFFFFFFFFFFFFFFFFFFFFFFFFFFFFFFFFFFFFFFFFFFFFFFF
@A00155:342:HHGFNDSXY:1:2356:12391:27868 2:N:0:GAACCTAG+TCCGCATA
CACGTGAGTCTGTAATCCTGCTGTCTGCTTAAGAGCATCTGCCAGGTTAGTTATGTTTACCGTCCCCT
CAGGGGTCAAGAATTGCTTTGAGGGTCCATCAAGTGATGACGAACTCCCTTGAAGCGACGCCTGAAAT
+
FFFFFFFFFFFFFFFFFFFFFFFFFFFFFFFFFFFFFFFFFFFFFFFF:FFFFFFFFFFFFFFFFFFF
FFFFFFFFFFFFFFFFFFFFFFFFFFFFFFFFFFFFFFFFFFFFFFF:FFFFFFFFFFFFFFFFFFFF
@A00155:342:HHGFNDSXY:1:2622:20961:32315 1:N:0:GAACCTAG+TCCGCATA
CGTGAGTCTGTAATCCTGCTGTCTGCTTAAGAGCATCTGCCAGGTTAGTTATGTTTACCGTCCCCTCA
GGGGTCAAGAATTGCTTTGAGAGTCCATCAAGTGATGACGAACTCCCTTGAAGCGACGCCTGAAATTT
+
FFFFFFFFFFFFFFFFFFFFFFFFFFFFFFFFFFFFFFFFFFFFFFFFFFF:FFFFFFFFFFFFFFFF
FFFFFFFFFFFFFFFFFFFFFFFFFFFFFFFFFFFFFFFFFFFFFFFFFFFFFFFFFFFFFFFFFFF:
@A00155:342:HHGFNDSXY:1:1201:25880:5666 2:N:0:GAACCTAG+TCCGCATA
GTGAGTCTGTAATCCTGCTGTCTGCTTAAGAGCATCTGCCAGGTTAGTTATGTTTACCGTCCCCTCAG
GGGTCAAGAATTGCTTTGAGAGTCCATCAAGTGATGACGAACTCCCTTGAAGCGACGCCTGAAATTTC
+
FFFFFFFFFFFFFFFF:FFFFFFFF,FFFFFFFFFFFFFFFFFFFFFFFFFFFFFFFFFFFFFFFF:F
FFFFFFFFFFFFFFFFFFFFFFFFFFFFFFFFFFFFFFFFFFFFFFFFFFFFFFFFFFFFFFFFF:FF
@A00155:342:HHGFNDSXY:1:2118:3875:11992 2:N:0:GAACCTAG+TCCGCATA
GTGAGTCTGTAATCCTGCTGTCTGCTTAAGAGCATCTGCCAGGTTAGTTATGTTTACCGTCCCCTCAG
GGGTCAAGAATTGCTTTGAGAGTCCATCAAGTGATGACGAACTCCCTTGAAGCGACGCCTGAAATTTC
+
FFFFFFFFFFFFFFFFFFFFFFFFF:FFFFF:FFFFFFF:FFFFFFFFFFFFF,:FFFFFFFFFFFFF
FFFFF,FFFFFFFFFFFFFFFF,FF:FFFFFFFFFFFFFFFF:F:FFFFFFFF:FFFFFFFFFFF:FF
@A00155:342:HHGFNDSXY:1:2354:22887:11506 2:N:0:GAACCTAG+TCCGCATA
GTGAGTCTGTAATCCTGCTGTCTGCTTAAGAGCATCTGCCAGGTTAGTTATGTTTACCGTCCCCTCAG
GGGTCAAGAATTGCTTTGAGAGTCCATCAAGTGATGACGAACTCCCTTGAAGCGACGCCTGAAATTTC
+
F:FFFFFFFFFFFFFFF:FFFFFFFFFFFFF:FFFFFFFFF:FFFFFFFFFFFF:F:F:FF:FFFFFF
FFFFF:FFFFFFF:FFFFFFFF:FF:FFFFFFFFFFFFFF:FF,F:FFFFFF:FF:FFFFFF:FFFFF
@A00155:342:HHGFNDSXY:1:2209:3125:34710 2:N:0:GAACCTAG+TCCGCATA
TGAGTCTGTAATCCTGCTGTCTGCTTAAGAGCATCTGCCAGGTTAGTTACGTTTACCGTCCCCTCAGG
GGTCAAGAATTGCTTTGAGAGTCCATCAAGTGATGACGAACTCCCTTGAAGCGACGCCTGAAATTTCC
+
::FFFFFFFFFFFFFFFFFF:F:FFFFFFFFFFFFFFFFFFFFFFFFFFFFFFFFFFFFFFFFFFFFF
FFFFFFFFFFFFFFFFFFFFFFFFFFFFFFFFFFFFFFFFFFFFFFFFFF,FFFFFFFFFFFFFFFFF
@A00155:342:HHGFNDSXY:1:2125:24578:4695 2:N:0:GAACCTAG+TCCGCATA
TGAGTCTGTAATCCTGCTGTCTGCTTAAGAGCATCTGCCAGGTTAGTTATGTTTACCGTCCCCTCAGG
GGTCAAGAATTGCTTTGAGAGTCCATCAAGTGATGACGAACTCCCTTGAAGCGACGCCTGAAATTTCC
+
FFFFFFFFFFFFFFFFFFFFFFFFFFFFFFFFFFFFF:FFFFFFFFFFFFFFFFFFFFFFFFFFFFFF
FFFFFFFFF:FFFFFFFFFFFFFFFFFFFFFFFFFFFFFFFFFFFFFFFFFFFFFFFFFFFFFFFFFF
@A00155:342:HHGFNDSXY:1:2209:3423:31688 2:N:0:GAACCTAG+TCCGCATA
TGAGTCTGTAATCCTGCTGTCTGCTTAAGAGCATCTGCCAGGTTAGTTACGTTTACCGTCCCCTCAGG
GGTCAAGAATTGCTTTGAGAGTCCATCAAGTGATGACGAACTCCCTTGAAGCGACGCCTGAAATTTCC
+
FFFFFFFFFFFFFFF,FFFFFFFFFFFFFFFFFFFFFFFFFFFFFFFFFFFF:FFFFFFFFFFFFFF,
FFFFFFFFFFFFFFFFFFFFFFFFFFFFFFFFFFFFFFFFFFFFFFFFFFFFFFFFFFFFFFFF:FFF
@A00155:342:HHGFNDSXY:1:2660:8124:36793 2:N:0:GAACCTAG+TCCGCATA
GAGTCTGTAATCCTGCTGTCTGCTTAAGAGCATCTGCCAGGTTAGTTATGTTTACCGTCCCCTCAGGG
GTCAAGAATTGCTTTGAGAGTCCATCAAGTGATGACGAACTCCCTTGAAGCGACGCCTGAAATTTCCC
+
```

```
FFFFFFFFFFFFF:FFFFFFFFFFFFFFFFFFFFFF:FFFFFFFFFFFFFFFFFFFFFFFFFFFFFFF
FFFFFFFFFFFF:FFFFFFFFFFFFFFFFFFFFFFF:FFFFF,FFFFFFFFFFFFFFFFFFFFFFFFF
@A00155:342:HHGFNDSXY:1:2622:20961:32315 2:N:0:GAACCTAG+TCCGCATA
GAGTCTGTAATCCTGCTGTCTGCTTAAGAGCATCTGCCAGGTTAGTTATGTTTACCGTCCCCTCAGGG
GTCAAGAATTGCTTTGAGAGTCCATCAAGTGATGACGAACTCCCTTGAAGCGACGCCTGAAATTTCCC
+
FFFFFFFFFFFFFFFFFFFFFFFFFFFFFFFFFFFFFFFFFFFFFFFFF:F:FFFFFFFFFFFFFFFF
FFF:FFFFFFFFFFFFFFFF:FFFFFFFFFFFFFFFFFFFFFFFFFFFFFFFFF:FFFFFFFFFFFFF
@A00155:342:HHGFNDSXY:1:1632:23601:2503 2:N:0:GAACCTAG+TCCGCATA
AGTCTGTAATCCTGCTGTCTGCTTAAGAGCATCTGCCAGGTTAGTTATGTTTACCGTCCCCTCAGGGG
TCAAGAATTGCTTTGAGAGTCCATCAAGTGATGACGAACTCCCTTGAAGCGACGCCTGAAATTTCCCC
+
FFFFFFFF,FFFFFFFFFFFFFFFFFFFFFFFFFFFFFFFFFFFFFFFFFFFFFFFFFFFFFF:FFF
FFFFFFFFFF:FFFFFFFFFFFFFFFFFFFFFFFFFFFFFFFFFFFFFFFFFFFFFF:FFFFFFFFFF
@A00155:342:HHGFNDSXY:1:1428:1371:12571 2:N:0:GAACCTAG+TCCGCATA
AGTCTGTAATCCTGCTGTCTGCTTAAGAGCATCTGCCAGGTTAGTTATGTTTACCGTCCCCTCAGGGG
TCAAGAATTGCTTTGAGAGTCCATCAAGTGATGACGAACTCCCTTGAAGCGACGCCTGAAATTTCCCC
+
FFFFFFFFFFFFFFFFFFFFFFFFFFFFFFFF:FFFFFFFFF::FFFFFFFFFFFFFFFFFFFFFFFF
,F:FFFFFFFFFFFFFFFFFFFFFFFFFFFFFFFFFFFF:FFFFFFFFFFFFFFFFFFFFFF,:FFFF
@A00155:342:HHGFNDSXY:1:1667:23665:12289 2:N:0:GAACCTAG+TCCGCATA
AGTCTGTAATCCTGCTGTCTGCTTAAGAGCATCTGCCAGGTTAGTTATGTTTACCGTCCCCTCAGGGG
TCAAGAATTGCTTTGAGAGTCCATCAAGTGATGACGAACTCCCTTGAAGCGACGCCTGAAATTTCCCC
+
FFFFF:F:FFF,FFF:FFFFFFFFFFFFFFFF:FFFFFFFFFFFFFFFFFFFFFFFFFFFFFFFFFF:
FF:FFFF:FFFFFFFFFFFFFFFFFFFFFFFFFFFFFFFFFFFFFFFFFFFFFFFF:FFFFFFFFFFF
@A00155:342:HHGFNDSXY:1:2573:24542:35603 2:N:0:GAACCTAG+TCCGCATA
AGTCTGTAATCCTGCTGTCTGCTTAAGAGCATCTGCCAGGTTAGTTATGTTTACCGTCCCCTCAGGGG
TCAAGAATTGCTTTGAGAGTCCATCAAGTGATGACGAACTCCCTTGAAGCGACGCCTGAAATTTCCCC
+
FF:FFFFFFFFFF:FFFFFF,::F:FF:FFFFFFF::F:FFFFFFFFFFFFFF,FFFFF:FFFFFFFF
FF:FF,F,FF,FFFFFFFFFFFFFFFFFFFFF,FFFFFFFFFFFF,FFFFFFFFFFFFFFFFFFFFFF
@A00155:342:HHGFNDSXY:1:2426:11659:8061 2:N:0:GAACCTAG+TCCGCATA
AGTCTGTAATCCTGCTGTCTGCTTAAGAGCATCTGCCAGGTTAGTTATGTTTACCGTCCCCTCAGGGG
TCAAGAATTGCTTTGAGAGTCCATCAAGTGATGACGAACTCCCTTGAAGCGACGCCTGAAATTTCCCC
+
FFFF:FFFFFFFFFFFFFFFF:FFF:FFFFFFFFFFFFFFFFF,FFFFFFFFFFFFFF:FFFFFFFFF
FF:FFFFFFFFFFFFFFFFFFFFFFFFFFFFFFFFFFFFFFFFFFFFFFFFFFFFFFFFFFF:FFFFF
@A00155:342:HHGFNDSXY:1:1219:22498:8797 2:N:0:GAACCTAG+TCCGCATA
AGTCTGTAATCCTGCTGTCTGCTTAAGAGCATCTGCCAGGTTAGTTATGTTTACCGTCCCCTCAGGGG
TCAAGAATTGCTTTGAGAGTCCATCAAGTGATGACGAACTCCCTTGAAGCGACGCCTGAAATTTCCCC
+
FFFFFFFFFFFFFFFFFFFFFFFFFFFFFFFFFFFFFFFFFFFFFFFFFFFFFFFFFFFFFFFFFFFF
FF:FFFFFFFFFFFFFFFFFFFFFFFFFFFFFFFFFFFFFFFFFFFFFFFFFFFFFFFFFFF:FFFFF
@A00155:342:HHGFNDSXY:1:1169:14434:22294 2:N:0:GAACCTAG+TCCGCATA
AGTCTGTAATCCTGCTGTCTGCTTAAGAGCATCTGCCAGGTTAGTTATGTTTACCGTCCCCTCAGGGG
TCAAGAATTGCTTTGAGAGTCCATCAAGTGATGACGAACTCCCTTGAAGCGACGCCTGAAATTTCCCC
+
FF:FFFFFFFFFFFF,FFFFFFFFF:FFFFFFFFFFFFFFFFF:FFFFFFFFFFFFFFFFFFF:FFFF
FF,FFFFFFFFFFFFF:FFFFFFFFFFFFFFFFFFFFFFFFFFFFFFFFFFFFFFFFFFFFF:FFFFF
@A00155:342:HHGFNDSXY:1:1205:6822:26678 2:N:0:GAACCTAG+TCCGCATA
GTCTGTAATCCTGCTGTCTGCTTAAGAGCATCTGCCAGGTTAGTTATGTTTACCGTCCCCTCAGGGGT
CAAGAATTGCTTTGAGAGTCCATCAAGTGATGACGAACTCCCTTGAAGCGACGCCTGAAATTTC
+
```

```
FF,FFF:,F:FFFFF:FFFFFFFFFFFFFFFFFFFFFF:F:FFFF,FFF,FFFFFFFFFFFFFFFFFF
F:FFFF,FFFFFFFFFFFFFFFFFFFFF,,FFFFFFFFFFF,FFFF,FF:F,FFFFF:FFF:F,
@A00155:342:HHGFNDSXY:1:1378:24180:16658 2:N:0:GAACCTAG+TCCGCATA
GTCTGTAATCCTGCTGTCTGCTTAAGAGCATCTGCCAGGTTAGTTATGTTTACCGTCCCCTCAGGGGT
CAAGAATTGCTTTGAGAGTCCATCAAGTGATGACGAACTCCCTTGAAGCGACGCCTGAAATTTCCCC
+
FFFFFFFFFFFFFFFFFFFFFFFFFFFFFFFF:FFFFFFFFFFFFFFFFFFFFFFFFFFFFFFFFFFF
FFFFFFFFFFFFFFFFFFFFFFFFFFFFFFFF:FFFFFFFF:FFFFFFFFFFFFFFFFFFFFFFFFF
@A00155:342:HHGFNDSXY:1:1449:6759:20306 2:N:0:GAACCTAG+TCCGCATA
GTCTGTAATCCTGCTGTCTGCTTAAGAGCATCTGCCAGGTTAGTTATGTTTACCGTCCCCTCAGGGGT
CAAGAATTGCTTTGAGAGTCCATCAAGTGATGACGAACTCCCTTGAAGCGACGCCTGAAATTTCCCCA
+
:FFFFFFFFFF:FFFFFFFFFFF:FFFFFF,FFFFFFFFFF:FFFFFFFFFFFFFFFFFFFFFFFFFF
F:FFFFFFFFFFFFFFFFFFFFFFFFFFFFFFFFFFFFFFFFFFFFFFFFFF:FFFFFFFFFFFFFFF
@A00155:342:HHGFNDSXY:1:1133:26295:3850 2:N:0:GAACCTAG+TCCGCATA
GTCTGTAATCCTGCTGTCTGCTTAAGAGCATCTGCCAGGTTAGTTATGTTTACCGTCCCCTCAGGGGT
CAAGAATTGCTTTGAGAGTCCATCAAGTGATGACGAACTCCCTTGAAGCGACGCCTGAAATTTCCCCA
+
FFFFFFFF:FFFFFFFFFFF:FFFFFFFFF:FF,FFFFFFFFFFFFF:FFFFFFFFF:,FFFFFFFFF
F:FFFFFFFFFFFFF,FFF,FFFFFFFFFFF:FFFFFFFFFFFFFFFF,FFFF:,F:FFFF:F,FF:F
@A00155:342:HHGFNDSXY:1:2126:27001:36323 2:N:0:GAACCTAG+TCCGCATA
GTCTGTAATCCTGCTGTCTGCTTAAGAGCATCTGCCAGGTTAGTTATGTTTACCGTCCCCTCAGGGGT
CAAGAATTGCTTTGAGAGTCCATCAAGTGATGACGAACTCCCTTGAAGCGACGCCTGAAATTTCCCCA
+
F:FFFFFFFFFFFFFFFFFFFFFFFFFFFF:FFFFFFFFFFF:FFFFFF:FFFFFFFFFFFFFFFFFF
:FFFFF:FFFFFFFFFFFFFFFFFFFFFFFFFFFFFFFFFFFFFFFFFFFFFFFFFFFFF,:FFFFFF
@A00155:342:HHGFNDSXY:1:2454:26639:20134 2:N:0:GAACCTAG+TCCGCATA
GTCTGTAATCCTGCTGTCTGATTAAGAGCATCTGCCAGGTTAGTTATGTTTACCGTCCCCTCAGGGGT
CAAGAATTGCTTTGAGAGTCCATCAAGTGATGACGAACTCCCTTGAAGCGACGCCTGAAATTTCCCCA
+
:FFF:FFFFFFFFFFFFFFFFFFFFFFFFFFFFFFFF:FFFFFFFFFF:FFFFFFFFFFFFFFFFFFF
F:FFFFFFFFFFFFFFFFFFFFFFFFFFFFFFFFFFFFFFFFFFFFFFFFFFFFFFFFFFFFFFFFFF
@A00155:342:HHGFNDSXY:1:2609:17463:26443 2:N:0:GAACCTAG+TCCGCATA
GTCTGTAATCCTGCTGTCTGCTTAAGAGCATCTGCCAGGTTAGTTATGTTTACCGTCCCCTCAGGGGT
CAAGAATTGCTTTGAGAGTCCATCAAGTGATGACGAACTCCCTTGAAGCGACGCCTGAAATTTCCCCA
+
FFFFFFFFFFFFFF:FFFF:FFFFFFFFFFFFFFFFFFFFFFF:FFFFFFFFFFFFFFFFFFFFFFFF
FFFFFFFFFFFFFFFFFFFFFFFFFFFFFFFFFFFFFFFFFFFFFFFFFFFFFFFFF:FFF:FFFFFF
@A00155:342:HHGFNDSXY:1:1441:18629:19351 2:N:0:GAACCTAG+TCCGCATA
GTCTGTAATCCTGCTGTCTGCTTAAGAGCATCTGCCAGGTTAGTTATGTTTACCGTCCCCTCAGGGGT
CAAGAATTGCTTTGAGAGTCCATCAAGTGATGACGAACTCCCTTGAAGCGACGCCTGAAATTTCCCCA
+
FFFFFFFFF,FFFFFF:FF:FFF:FF:FFF:FF:FFFFFFF::FFFFFF:FFFFFFFFFFFFFFFFFF
F:F:FFFFFFFFFFF:FFFFFFFFFFFFFFFFFFF:FFFFFFFFFFFFFFFFFFFFFFFFFFFFFFFF
@A00155:342:HHGFNDSXY:1:1441:16640:20509 2:N:0:GAACCTAG+TCCGCATA
GTCTGTAATCCTGCTGTCTGCTTAAGAGCATCTGCCAGGTTAGTTATGTTTACCGTCCCCTCAGGGGT
CAAGAATTGCTTTGAGAGTCCATCAAGTGATGACGAACTCCCTTGAAGCGACGCCTGAAATTTCCCCA
+
FFFFF,FFFFFFFFFFFFFFFFFFFFFFFFFFFFF:F::FFFFF:FFFFFFFFFFFFFF,FFFFFFFF
:,FFFFFFFF,FFFFFFFF:FFFFFFFFFFFFF:FFFFFFFFFFFFFFFFFFFFFFFFFFF::FFFF,
@A00155:342:HHGFNDSXY:1:1645:8314:25786 2:N:0:GAACCTAG+TCCGCATA
TGTAATCCTGCTGTCTGCTTAAGAGCATCTGCCAGGTTAGTTATGTTTACCGTCCCCTCAGGGGTCAA
GAATTGCTTTGAGAGTCCATCAAGTGATGACGAACTCCCTTGAAGCGACGCCTGAAATTTC
+
```

```
FFFFFF:FF,FFFFFFF,FFFFF:F,:F:FFFF:FFFFF:FFFFFFFF:FFFFFF::FF:FFFFFFFF
FFFFFFFFFF,FFFFFF,FFF:FFF:FFF::FF:FFF:FFFFFFFFFFF:FF:FFF:F:F,
@A00155:342:HHGFNDSXY:1:1131:14714:7999 2:N:0:GAACCTAG+TCCGCATA
GTAATCCTGCTGTCTGCTTAAGAGCATCTGCCAGGTTAGTTATGTTTACCGTCCCCTCAGGGGTCAAG
AATTGCTTTGAGAGTCCATCAAGTGATGACGAACTCCCTTGAAGCGACGCCTGAAATTTCCCCACTGC
+
FFFFFFFFFFFFFFFFFFFFFFFFFFFFFFFFFFFFFFFFFFFFFFFFFFFFFFFFFFFFFFFFFFFFFF
FFFFFFFFFFFFFFFFFFFFFFFFFFFFFFFFFFFFFFFFFFFFFFFFFFFFFFFFFFFFFFFFFFFFFF
@A00155:342:HHGFNDSXY:1:1420:10294:3850 1:N:0:GAACCTAG+TCCGCATA
AATCCTGCTGTCTGCTTAAGAGCATCTGCCAGGTTAGTTATGTTTACCGTCCCCTCAGGGGTCAAGAA
TTGCTTTGAGAGTCCATCAAGTGATGACGAACTCCCTTGAAGCGACGCCTGAAATTTCCCCACTGCAG
+
FFFFFFFFFFFFFFFFFFFFFFFFFFFFFFFFF,FFFFF:FFFFFFFFFFFFFFFFFFFFFFFFFFFFFF
FFFFFFF:FFFFFFFFFFFFFFFFFFF:FFFFFFFFFFFFFFFFFFFFFFFFFFFFFFFFFFFFFFFFFF
@A00155:342:HHGFNDSXY:1:1169:14434:22294 1:N:0:GAACCTAG+TCCGCATA
AATCCTGCTGTCTGCTTAAGAGCATCTGCCAGGTTAGTTATGTTTACCGTCCCCTCAGGGGTCAAGAA
TTGCTTTGAGAGTCCATCAAGTGATGACGAACTCCCTTGAAGCGACGCCTGAAATTTCCCCACTGCAG
+
FFFFFFFFFFFFFFFFFFFFFFFFFFFFFFFFFFFF:FFFFFFFFFFFFFFFFFFFFFFFFFFFFFFFFF
FFFFFFFFFFFFFFFFFFFFFFFFFFFFFFFFFFFFFFFFFFFFFFFFFFFFFFFFFFFFFFFFFFFFFF
@A00155:342:HHGFNDSXY:1:2217:15058:29857 2:N:0:GAACCTAG+TCCGCATA
CCTGCTGTCTGCTTAAGAGCATCTGCCAGGTTAGTTATGTTTACCGTCCCCTCAGGGGTCAAGAATTG
CTTTGAGAGTCCATCAAGTGATGACGAACTCCCTTGAAGCGACGCCTGAAATTTCCCCACTGCAGTCA
+
:F:FFFFFFFFFFFFFFFFFFFFF:FFFFFFF:FFFF,FFFFFF:FF:FFFFFFFFFFFFFFFFFFFF,F
FFFFFFFFFFFFFFFF,FFFFFF:FFFF:FFF:FFFFFFFFFFFFFFFFFFFFFFFFFFFFFFFFF::F:
@A00155:342:HHGFNDSXY:1:1219:22498:8797 1:N:0:GAACCTAG+TCCGCATA
CTGCTGTCTGCTTAAGAGCATCTGCCAGGTTAGTTATGTTTACCGTCCCCTCAGGGGTCAAGAATTGC
TTTGAGAGTCCATCAAGTGATGACGAACTCCCTTGAAGCGACGCCTGAAATTTCCCCACTGCAGTCAT
+
FFFFFFFFFFFFFFFFFFFFFFFFFFFFFFFFFFFF,FFFFFFFFFFFFFFFFFFFFFFFFFFFFFFFFF
FFFFF:FFFFFFFFFFFFFFFFFFFFFFFFFFFFFFFFFFFF:FFFFFFFFFFFFF:FF:FFFFFFFFFF
@A00155:342:HHGFNDSXY:1:2547:30843:24533 1:N:0:GAACCTAG+TCCGCATA
CTGCTGTCTGCTTAAGAGCATCTGCCAGGTTAGTTATGTTTACCGTCCCCTCAGGGGTCAAGAATTGC
TTTGAGAGTCCATCAAGTGATGACGAACTCCCATGAAGCGACGCCTGAAATTTCCCCACTGCAGTCAT
+
FFFFFFFFFFFFFFFFFFFFFFFFFFFFF:FFFFFFFFFFFFFFFFFFFFFFFFFFFFFFFFFFFFFFFF
FFFFFFFFFFFFFFFFFFFFFFFFFFFFFFFF,FFFFFFFFFFFFFFFFFFFFFFFFFFFFFFFFFFFFF
@A00155:342:HHGFNDSXY:1:1338:12933:26083 1:N:0:GAACCTAG+TCCGCATA
TGCTGTCTGCTTAAGAGCATCTGCCAGGTTAGTTATGTTTACCGTCCCCTCAGGGGTCAAGAATTGCT
TTGAGAGTCCATCAAGTGATGACGAACTCCCTTGAAGCGACGCCTGAAATTTCCCCACTGCAGTCATC
+
FFFFFFFFFFFFFFFFFFFFFFFFFFFFFFFFFFFFFFFFFFFFFFFFFFFFFFFFFFFFFFFFFFFFFF
FFFFFFFFFFFFFFFFFFFFFFFFFFFFFFFFFFFFFFFFFFFFFFFFFFFFFFFF:FFFFFFFFFFFFF
@A00155:342:HHGFNDSXY:1:1524:30563:11021 1:N:0:GAACCTAG+TCCGCATA
TGCTGTCTGCTTAAGAGCATCTGCCAGGTTAGTTATGTTTACCGTCCCCTCAGGGGTCAAGAATTGCT
TTGAGAGTCCATCAAGTGATGACGAACTCCCTTGAAGCGACGCCTGAAATTTCCCCACTGCAGTCATC
+
FFFFFFFFFFFFFFFFFFFFFFFFFFFFFFFF,:FFFFFFFFFFFFFFFFFFFFFFFFFFFFFFFFFFFF
FFFFFFFFFFFFFFFFFFFFFFFFFFFFFFFFFFFFFFFFFFFFFFFFFFFFFFFFFFFFFFFFFFFFFF
@A00155:342:HHGFNDSXY:1:2118:3875:11992 1:N:0:GAACCTAG+TCCGCATA
TGCTGTCTGCTTAAGAGCATCTGCCAGGTTAGTTATGTTTACCGTCCCCTCAGGGGTCAAGAATTGCT
TTGAGAGTCCATCAAGTGATGACGAACTCCCTTGAAGCGACGCCTGAAATTTCCCCACTGCAGTCATC
+
```

```
FFFFFFFFFFFFFFFFFFFFFFFFFFFFFFFFFFFFFFFFFFFFFFFFFFFFFFFFFFFFFFFFFFFFFFFFFFFFFFFFFFFFFFFFFFFFFFFFFFFFFFFF
FFFFFFFFFFFFFFFFFFFFFFFFFFFFFFFFFFFFFFFFFFFFFFFFFFFFFFFFFFFFFFFFFFFFFFFFFFFFFFFFFFFFFFFFFFFFFFFFFFFFFFF:
@A00155:342:HHGFNDSXY:1:2250:14552:30639 1:N:0:GAACCTAG+TCCGCATA
TGCTGTCTGCTTAAGAGCATCTGCCAGGTTAGTTATGTTTACCGTCCCCTCAGGGGTCAAGAATTGCT
TTGAGAGTCCATCAAGTGATGACGAACTCCCTTGAAGCGACGCCTGAAATTTCCCCACTGCAGTCATC
+
FFFFFFFFFFFFFFFFFFFFFFFFFFFFFFFFFFFFFFFFFFFFFFFFFFFFFFFFFFFFFFFFFFFF
FFFFFFFFFFFFFFFFFFFFFFF:FFFFFFFFFFF::FFFFFFFFFFFFFFFFFFFFFFFFFFFFFFF
@A00155:342:HHGFNDSXY:1:2250:14705:30436 1:N:0:GAACCTAG+TCCGCATA
TGCTGTCTGCTTAAGAGCATCTGCCAGGTTAGTTATGTTTACCGTCCCCTCAGGGGTCAAGAATTGCT
TTGAGAGTCCATCAAGTGATGACGAACTCCCTTGAAGCGACGCCTGAAATTTCCCCACTGCAGTCATC
+
FFFFFFFFFFFFFFFFFFFFFFFFFFFFFFFFF:FFFF:FFFFFFFFFFFFFFFFFFFFFFFFFFFFF
FFFFFFFFFFFFFFFFFFFFFFFFFFFFFFFFFFFFFFFFFFFFFFFFFFFFFFFFFFFFFFFFF,FF
@A00155:342:HHGFNDSXY:1:2254:18783:23844 1:N:0:GAACCTAG+TCCGCATA
TGCTGTCTGCTTAAGAGCATCTGCCAGGTTAGTTATGTTTACCGTCCCCTCAGGGGTCAAGAATTGCT
TTGAGAGTCCATCAAGTGATGACGAACTCCCTTGAAGCGACGCCTGAAATTTCCCCACTGCAGTCATC
+
FFFFFFFFFFFFFFFFFFFFFFFFFFFFFFFFFFFFFFFFFFFFFFFFFFFFFFFFFFFFFFFFFFFF
FFFFFFFFFFFFFFFFFFFFFFFFFFFFFFF:FFFFFFFFFFFFFFFFFFFFFFFFFFFFFFFFFFFF
@A00155:342:HHGFNDSXY:1:2605:20103:30295 1:N:0:GAACCTAG+TCCGCATA
TGCTGTCTGCTTAAGAGCATCTGCCAGGTTAGTTATGTTTACCGTCCCCTCAGGGGTCAAGAATTGCT
TTGAGAGTCCATCAAGTGATGACGAACTCCCTTGAAGCGACGCCTGAAATTTCCCCACTGCAGTCATC
+
FFFFFFFFFFFFFFFFFFFFFFFFFFFFFFFFFFFFF:FFFFFFFFFFFFFFFFFFFFFFFFFFFFFF
FFFFFFFFFFFFFFFFFFFFFFFFFFFFFFFFFFFFFFFFFFFFFFFFFFFFFFFFFFFFFFFFFFFF
@A00155:342:HHGFNDSXY:1:2143:11053:24799 1:N:0:GAACCTAG+TCCGCATA
TGCTGTCTGCTTAAGAGCATCTGCCAGGTTAGTTATGTTTACCGTCCCCTCAGGGGTCAAGAATTGCT
TTGAGAGTCCATCAAGTGATGACGAACTCCCTTGAAGCGACGCCTGAAATTTCCCCACTGCAGTCATC
+
FFFFFFFFFFFFFFFFFFFFFFFFFFFFFFFFF:FFF,FFFFFFFFFFFFFFFFFFFFFFFFFFFFFF
FFFFFFFFFFFFFFFFFFFFFFFFFFFFFFFFFFFFFFFFFFFFFFFFFFFFFFFFFFFFFFFFFFFF
@A00155:342:HHGFNDSXY:1:2639:10276:14121 1:N:0:GAACCTAG+TCCGCATA
TGCTGTCTGCTTAAGAGCATCTGCCAGGTTAGTTATGTTTACCGTCCCCTCAGGGGTCAAGAATTGCT
TTGAGAGTCCATCAAGTGATGACGAACTCCCTTGAAGCGACGCCTGAAATTTCCCCACTGCAGTCATC
+
FFFFFFFFFFFFFFFFFFFFFFFFFFFFFFFFFFFFFFFFFFFFFFFFFFFFFFFFFFFFFFFFFFFF
FFFFFFFFFFFFFFFFFFFFFFFFFFFFFFFFFFFFFFFFFFFFFFFFFFFFFFFFFFFFFFFFFFFF
@A00155:342:HHGFNDSXY:1:2250:14705:30436 2:N:0:GAACCTAG+TCCGCATA
GCTGTCTGCTTAAGAGCATCTGCCAGGTTAGTTATGTTTACCGTCCCCTCAGGGGTCAAGAATTGCTT
TGAGAGTCCATCAAGTGATGACGAACTCCCTTGAAGCGACGCCTGAAATTTCCCCACTGCAGTCATCT
+
FF,:FF:FFFFFFFFFFFFFFFFFFFFFFFFFFF,FF,:F,FFF:FFFFFFFFFFFFF,FFF:,FFFF
FFFFFFFFFFFFFFFFFFFFFFFFFFFFFFFFFFFFFFFFFFFF,FFFFFFFFFFF,FF:FFFFFF:FF
@A00155:342:HHGFNDSXY:1:2250:14552:30639 2:N:0:GAACCTAG+TCCGCATA
GCTGTCTGCTTAAGAGCATCTGCCAGGTTAGTTATGTTTACCGTCCCCTCAGGGGTCAAGAATTGCTT
TGAGAGTCCATCAAGTGATGACGAACTCCCTTGAAGCGACGCCTGAAATTTCCCCACTGCAGTCATCT
+
FFFFFFFFFFFFFFFFFFFFFFF:FFFFFFFFFFFFFFFFFFFFF:FFFFFFFFFFFFFFFFFFFFFF
FFFFFFFFFFFFFFFFFFFFFFFFFFFFFFFF:FFFFFFFFFFFFFFFFFFFFFFFFFFFFFFFFF:FF
@A00155:342:HHGFNDSXY:1:2450:4761:19413 2:N:0:GAACCTAG+TCCGCATA
GCTGTCTTCTTAAGAGCATCTGCCAGGTTAGTTATGTTTACCGTCCCCTCAGGGGTCAAGAATTGCTT
TGAGAGTCCATCAAGTGATGACGAACTCCCTTGAAGCGACGCCTGAAATTTCCCCACTGCAGTCATCT
+
```

```
F,:,:FF,:FF:FF,:FF:FFFF:FF,FFFFF,FFF,,F:F:FFFFFF:F:FF,F:F:FF,F::FFF:
,FFFF:,FFF:FFFF,:FFF:FFFFF:FFFF:F:,FFFFFFFFFFF::FFFFFF:FFFF,F:FF,F,F
@A00155:342:HHGFNDSXY:1:2660:8124:36793 1:N:0:GAACCTAG+TCCGCATA
GCTGTCTGCTTAAGAGCATCTGCCAGGTTAGTTATGTTTACCGTCCCCTCAGGGGTCAAGAATTGCTT
TGAGAGTCCATCAAGTGATGACGAACTCCCTTGAAGCGACGCCTGAAATTTCCCCACTGCAGTCATCT
+
FFFFFFFFFFFFFFFFFFFFFFFFFFFFFFFFFFFFFFFFFFFFFFFFFFFFFFFF:FFFFFFFFFFF
FFFFFFFFFFFFFFFFFFFFFFFFFFFFFFFFFFFFFFFFFFFFFFFFFFFFFFF,FFFFFFFFFFFF
@A00155:342:HHGFNDSXY:1:1367:23330:24862 2:N:0:GAACCTAG+TCCGCATA
GCTGTCTGCTTAAGAGCATCTGCCAGGTTAGTTATGTTTACCGTCCCCTCAGGGGTCAAGAATTGCTT
TGAGAGTCCATCAAGTGATGACGAACTCCCTTGAAGCGACGCCTGAAATTTCCCCACTGCAGTCATCT
+
FF:FFFFFFFFFFFFFFFFFFFFFFFFFFFFFFFFFFFFFFFFFFFFFFFFFFFFFFFFFFFFFFFFF
FFFFFFFFFFFFFFFFFFFFFFFFFFFFFFF:FFFFFFFFFF:FFFFFFFFFFFF:FFFFFFFFFFFF
@A00155:342:HHGFNDSXY:1:1133:26295:3850 1:N:0:GAACCTAG+TCCGCATA
GCTGTCTGCTTAAGAGCATCTGCCAGGTTAGTTATGTTTACCGTCCCCTCAGGGGTCAAGAATTGCTT
TGAGAGTCCATCAAGTGATGACGAACTCCCTTGAAGCGACGCCTGAAATTTCCCCACTGCAGTCATCT
+
FFFF:FFF:FFFFFF:FF:FFFFFFFF,FFFFFFFFF,FFFF:F::FFFFFFFFFFFFF:FFFFF:F,
FFFFFF:FFFFFFFFFFFF:FFFFFFF,F:FFFFF:,FF::FFFFFFFFFFFF,FFFFFFFFF,FFFF
@A00155:342:HHGFNDSXY:1:1124:15429:23390 2:N:0:GAACCTAG+TCCGCATA
GCTGTCTGCTTAAGAGCATCTGCCAGGTTAGTTATGTTTACCGTCCCCTCAGGGGTCAAGAATTGCTT
TGAGAGTCCATCAAGTGATGACGAACTCCCTTGAAGCGACGCCTGAAATTTCCCCACTGCAGTCATCT
+
FFFFFFFFFFFFFFFFFFFF:FFFFFFFFFFFFF,FFFFFFFF:FFFF:FFFFFFFFFFFFF:FFF:F
FFFFFFFFFFFFFFFFFFFFFFFFFFFFFFFFFFFFFFFFFFFFFFFFFFFFFFFFFFFFFFFFF:F,
@A00155:342:HHGFNDSXY:1:1224:24424:8656 2:N:0:GAACCTAG+TCCGCATA
GCTGTCTGCTTAAGAGCATCTGCCAGGTTAGTTATGTTTACCGTCCCCTCAGGGGTCAAGAATTGCTT
TGAGAGTCCATCAAGTGATGACGAACTCCCTTGAAGCGACGCCTGAAATTTCCCCACTGCAGTCATCT
+
FFFF:FFFF:F,FFFFFFFF:FFFFFFFFFFFFFFF,FFFFFFFFFFF,FFF:FFFFFFFFF,FFF:F
FFFFFFFFFFFFFFFFFFFFFFFFFFFFFFFFF:FFFFFFFFF:F:FFFFFFFFF:FFF:FF,FF,F:
@A00155:342:HHGFNDSXY:1:2216:19126:9095 2:N:0:GAACCTAG+TCCGCATA
GCTGTCTGCTTAAGAGCATCTGCCAGGTTAGTTATGTTTACCGTCCCCTCAGGGGTCAAGAATTGCTT
TGAGAGTCCATCAAGTGATGACGAACTCCCTTGAAGCGACGCCTGAAATTTCCCCACTGCAGTCATCT
+
FFFFFFFFFFFFFFFFFFFFFFFFFFFFFFFFFFFFFFFFFFFFFFFFFFFFFFFFF:FFFFFFFFFF
FFFFFFFFFFFFFFFFFFFFFFFFFFFFFFFFFFFFFFFFFFFFFFFFFFFFFFFFFFFFFFFFFFFF
@A00155:342:HHGFNDSXY:1:1444:8097:18239 2:N:0:GAACCTAG+TCCGCATA
CTGTCTGCTTAAGAGCATCTGCCAGGTTAGTTATGTTTACCGTCCCCTCAGGGGTCAAGAATTGCTTT
GAGAGTCCATCAAGTGATGACGAACTCCCTTGAAGCGACGCCTGAAATTTCCCCACTGCAGTCATCT
+
:,F:FFFFFFFFFF:FF:FFFFFFFFFFFFFF:,,,::FFFF,FFFFFFF:FF:FFFFF,::,FF,:F
F:FFFFFF::F,,F,F::FFF:F:FFFFF:F:FFFF:FFFFF,FFFF,FFFFF,F:F,FFF,FF:FF
@A00155:342:HHGFNDSXY:1:1254:9136:22795 2:N:0:GAACCTAG+TCCGCATA
CTGTCTGCTTAAGAGCATCTGCCAGGTTAGTTATGTTTACCGTCCCCTCAGGGGTCAAGAATTGCTTT
GAGAGTCCATCAAGTGATGACGAACTCCCTTGAAGCGACGCCTGAAATTTCCCCACTGCAGTCATCTT
+
FFFFFFFFFFFFF:FFFFFFFFFFF:F:FFFFFFFFFFFFFFFFFFFFF,FFFFFFFFFFFF:FF:FF
FFFFFFFFF:FFFF,FFFFFFFFFFFFFFFFFFFFFFFFF:F:FFFFFFFFFFFFFFFFFFF:FFF,F
@A00155:342:HHGFNDSXY:1:2140:31358:11334 2:N:0:GAACCTAG+TCCGCATA
TGTCTGCTTAAGAGCATCTGCCAGGTTAGTTATGTTTACCGTCCCCTCAGGGGTCAAGAATTGCTTTG
AGAGTCCATCAAGTGATGACGAACTCCCTTGAAGCGACGCCTGAAATTTCCCCACTGCAGTCATCTTC
+
```

```
FFFFFFFFFFFFFFFFFFFFFFFFFFFFFFFFFFFF:FFFFFFFFFFFFFFFFFFFFFFF,FFFFFFF
FFFFFFFFFFFFFFFFFFFFFFFFFFFFFFFFFFFFFFFFFFFFFFFF::FFFF:FFFFFFFFFFFFFF
@A00155:342:HHGFNDSXY:1:1205:6822:26678 1:N:0:GAACCTAG+TCCGCATA
GTCTGCTTAAGAGCATCTGCCAGGTTAGTTATGTTTACCGTCCCCTCAGGGGTCAAGAATTGCTTTGA
GAGTCCATCAAGTGATGACGAACTCCCTTGAAGCGACGCCTGAAATTTCCCCACTGCAGTCATC
+
FFFFFFFFFFFFFFFFFFFFFFFF:FFF::FFFFFFFFFFFFFFFFFFFFFFFFFFFFFFFFFFFFFF
FFFFFFFFFFFFFFFFFFFFFFFFFFFFFFFFFFFFFFFFFFFFFFFF:FFFF:FFFFFFFFFFF
@A00155:342:HHGFNDSXY:1:2321:10691:28401 2:N:0:GAACCTAG+TCCGCATA
GTCTGCTTAAGAGCATCTGCCAGGTTAGTTATGTTTACCGTCCCCTCAGGGGTCAAGAATTGCTTTGA
GAGTCCATCAAGTGATGACGAACTCCCTTGAAGCGACGCCTGAAATTTCCCCACTGCAGTCATCTTCT
+
FFFFFFFFFF:FFFFFF:FFFFFFFFFFF:FFFFF:,FFF,FFFFFFF:,FF,FFFFFF,FFF,FFFF
FFFFFF::F:,:,:,:FFFFFFFFFFF,FFF,FFF,FFFF,F,FF:F:FFFF,FFFFFF,FF,FF:FF
@A00155:342:HHGFNDSXY:1:2461:10890:17660 1:N:0:GAACCTAG+TCCGCATA
GTCTGCTTAAGAGCATCTGCCAGGTTAGTTATGTTTACCGTCCCCTCAGGGGTCAAGAATTGCTTTGA
GAGTCCATCAAGTGATGACGAACTCCCTTGAAGCGACGCCTGAAATTTCCCCACTGCAGTCATCTTCT
+
FFFFFFFFFFFFFF:FFFFFFFFFFFFFFFFFFFFFFFFFFFFFFFFFFFFFFFFFFFFFFFF:FFFF
FFFFFFFFFFFFFFFFFFFFFFFFFFFFFFFF:FFFFFFFFFFFFFFFFFFFFFFFFFFFFFFFFFFF
@A00155:342:HHGFNDSXY:1:2206:15383:23813 2:N:0:GAACCTAG+TCCGCATA
TCTGCTTAAGAGCATCTGCCAGGTTAGTTATGTTTACCGTCCCCTCAGGGGTCAAGAATTGCTTTGAG
AGTCCATCAAGTGATGACGAACTCCCTTGAAGCGACGCCTGAAATTTCCCCACTGCAGTCATCTTCTG
+
FFFFFFFFFFFFFFFFFFFFFFFFFFFFFFFFF:FFFFFFFFFFFFFFFFFFFFFFFFFFFFFFFFFF
FFFFFFFFFFFFFFFFFFFFFFFFFFFFFFFFFFFFFFFFFFFFFFFFFFFFFFFFFFFF::FFFFFF
@A00155:342:HHGFNDSXY:1:2336:24867:28338 2:N:0:GAACCTAG+TCCGCATA
TCTGCTTAAGAGCATCTGCCAGGTTAGTTATGTTTACCGTCCCCTCAGGGGTCAAGAATTGCTTTGAG
AGTCCATCAAGTGATGACGAACTCCCTTGAAGCGACGCCTGAAATTTCCCCACTGCAGTCATCTTCTG
+
FFFFFFFFFFFFF:FFFFFFFFFFFFFFFFFFFFFF:FFFFFFFFFFFFFFFFFFFFFF,FFFFFFFFF
FFFFF:FFFFFFFFFFFFFFFFFFFFF:,FFFFFFFFFFF,FFFFFFFFFFFFF:FFFFFF::F,FFFF
@A00155:342:HHGFNDSXY:1:1524:30563:11021 2:N:0:GAACCTAG+TCCGCATA
CTGCTTAAGAGCATCTGCCAGGTTAGCTATGTTTACCGTCCCCTCAGGGGTCAAGAATTGCTTTGAGA
GTCCATCAAGTGATGACGAACTCCCTTGAAGCGACGCCTGAAATTTCCCCACTGCAGTCATCTTCTGC
+
FFFFFFFFF:FFF,FFFFFFFFF:FF,FF:FFF,FFFFFFFFFFFFFFFFFFFF:FFFFFF,FFFFFF
FFFFFFFF:FFFFFFFFFFFFFFFFF:F,FFFFFFFFFFFFF:FFFFFFFFFFFFFFFFFFFFFFFFF
@A00155:342:HHGFNDSXY:1:1141:16712:28776 1:N:0:GAACCTAG+TCCGCATA
CTGCTTAAGAGCATCTGCCAGGTTAGTTATGTTTACCGTCCCCTCAGGGGTCAAGAATTGCTTTGAGA
GTCCATCAAGTGATGACGAACTCCCTTGAAGCGACGCCTGAAATTTCCCCACTGCAGTCATCTTCTGC
+
FFFFFFFFFFFFFFFFFFFFFFF:,FFFFFFFFFFFFFFFFFFFFFFFFFFFFFFFFFFFFFFFFFFF
FFFFFFFFFFFFFFFFFFFFFFF:FF:FFFFFFFFFFFFFFFFFFFFFFFFFFFFFFFFFFFFFFFFF
@A00155:342:HHGFNDSXY:1:1224:24424:8656 1:N:0:GAACCTAG+TCCGCATA
CTGCTTAAGAGCATCTGCCAGGTTAGTTATGTTTACCGTCCCCTCAGGGGTCAAGAATTGCTTTGAGA
GTCCATCAAGTGATGACGAACTCCCTTGAAGCGACGCCTGAAATTTCCCCACTGCAGTCATCTTCTGC
+
FFFFFF,FFFFFFFFFFFFFFF::FFFFFFFFFFFFFFFFFFFFFFFFFFFFFFFFFFFFFFFFFFFF
FFFFFFFFFFFFFFFFFFFFFFFFFFFFFFFFFFFFFFFFFFFFFFF:FFFFFFFFFFFFFFFFFFFF
@A00155:342:HHGFNDSXY:1:2605:20103:30295 2:N:0:GAACCTAG+TCCGCATA
CTGCTTAAGAGCATCTGCCAGGTTAGTTATGTTTACCGTCCCCTCAGGGGTCAAGAATTGCTTTGAGA
GTCCATCAAGTGATGACGAACTCCCTTGAAGCGACGCCTGAAATTTCCCCACTGCAGTCATCTTCTGC
+
```

FFFFFFFFFFFFFFFFFFFFFFFFFFFFFFFFFFFFFF:FFFFFFFFFFFFFFFFFFFFFFFFFFFFF
FFFFFFFFFFFFFFFFFFFFF:FFFFFFFFFFFFFFFFFFFFFFFF:FFFFFFFFFFFFFFF::FFFF
@A00155:342:HHGFNDSXY:1:1546:19696:27085 2:N:0:GAACCTAG+TCCGCATA
CTGCTTAAGAGCATCTGCCAGGTTAGTTATGTTTACCGTCCCCTCAGGGGTCAAGAATTGCTTTGAGA
GTCCATCAAGTGATGACGAACTCCCTTGAAGCGACGCCTGAAATTTCCCCACTGCAGTCATCTTCTGC
+
F:FFFFFFFFFFF:FFFFF,FFF,FFF:FFFFF:FFFFFFFFFFF::FFFFFFFFF:F:FFFFFFFFF
FFFFFFFFFFFFF:FF:FF:FFFFFF,FFFFFFFFFFF,FFFF,,FFFFFFFFFFFFFFF:FFFFFFF
@A00155:342:HHGFNDSXY:1:1518:23068:35493 1:N:0:GAACCTAG+TCCGCATA
CTGCTTAAGAGCATCTGCCAGGTTAGTTATGTTTACCGTCCCCTCAGGGGTCAAGAATTGCTTTGAGA
GTCCATCAAGTGATGACGAACTCCCTTGAAGCGACGCCTGAAATTTCCCCACTGCAGTCATCTTCTGC
+
FFFFFFFFFFFF:FFFFFFFFFFF,FFFF,FFFFFFFFFFFFFFFFFFFFFFFFFFFFFFFFFFFFFF
FFFFFFFFFFFFFFFFFFFFFFFFFFFFFFFFFFFFFFFFFFFFFFFFFFFFFFFFFFFFFFFFFFFF
@A00155:342:HHGFNDSXY:1:1645:8314:25786 1:N:0:GAACCTAG+TCCGCATA
TGCTTAAGAGCATCTGCCAGGTTAGTTATGTTTACCGTCCCCTCAGGGGTCAAGAATTGCTTTGAGAG
TCCATCAAGTGATGACGAACTCCATTGAAGCGACGCCTGAAATTTCCCCACTGCAGTCATC
+
FFFFF,:FFFFFFFFFFFFFF:,:FF:FF:FFFFFF:F:FFFFFFF,F,,FFF:,FFF::,FFFF,::
:,FFFFFFFF::,FF::FF,F:F,F,,FFF,::F,,FFFF::FFFF:,FFFF:F:F:FFF:
@A00155:342:HHGFNDSXY:1:2171:20428:27195 2:N:0:GAACCTAG+TCCGCATA
GCTTAAGAGCATCTGCCAGGTTAGTTATGTTTACCGTCCCCTCAGGGGTCAAGACTTGCTTTGAGAGT
CCATCAAGTGATGACGAACTCCCTTGAAGCGACGCCTGAAATTTCCCCACTGCAGTCATCTTCTGCAC
+
FFFFF:FFFFFFFFFFFFFFFFFFFFFFFF:FFFFFFFFFF:F:FFFFFFFFFF,,FFF:FFFFFFFF
FFFFFFFFFFFFFFFF:FFFFFF:F:FFFFFFFFFFFFFFF::FFFFFFFFFFFFFFF:F,FF,FFFF
@A00155:342:HHGFNDSXY:1:2223:12102:26929 2:N:0:GAACCTAG+TCCGCATA
GCTTAAGAGCATCTGCCAGGTTAGTTATGTTTACCGTCCCCTCAGGGGTCAAGAATTGCTTTGAGAGT
CCATCAAGTGATGACGAACTCCCTTGAAGCGACGCCTGAAATTTCCCCACTGCAGTCATCTTCTGCAC
+
FFFFFFFFFFFFFFFFFFFFFFFFFFFFFFFFFFFFFFFFFFF:FFFFFFFFFFFF:FFFF,FFFFFF
FFFFFFF:FFFFFFFFFFFFFFF:FFFFFFFFFFFFFFFFFFFFFFFFFF:FFFFFFF:FFFFFFFFF
@A00155:342:HHGFNDSXY:1:1613:7274:16250 2:N:0:GAACCTAG+TCCGCATA
GCTTAAGAGCATCTGCCAGGTTAGTTATGTTTACCGTCCCCTCAGGGGTCAAGAATTGCTTTGAGAGT
CCATCAAGTGATGACGAACTCCCTTGAAGCGACGCCTGAAATTTCCCCACTGCAGTCATCTTCTGCAC
+
FFFFFFFFFFFFFFFFFFFFFFFFFFFFFFFFF:FFFFF:FFFFFFFFFFFFFF:F,FFFFFFFFFFF
FFF:FFF:FFFFFFFFFFFFFFF::FFFFFFFFFFFFFFFFFFFFFFFFFFFFFF:F,FFFFFFFFFF
@A00155:342:HHGFNDSXY:1:2639:10276:14121 2:N:0:GAACCTAG+TCCGCATA
TTAAGAGCATCTGCCAGGTTAGTTATGTTTACCGTCCCCTCAGGGGTCAAGAATTGCTTTGAGAGTCC
ATCAAGTGATGACGAACTCCCTTGAAGCGACGCCTGAAATTTCCCCACTGCAGTCATCTTCTGCACCG
+
FFFFFFFFFFFFFFFFFFFFFFFFFFFFFFFFFFFFFFFFFFFFFFFFFFFFFFFFFFFFFFFFFFFF
FFFFFFFFFFFFFFFFFFFFFFFFFFFFFFFFFFFFFFFFFFFFFFFFFFFFFFFFFFFFFFFFFFFF
@A00155:342:HHGFNDSXY:1:2356:12391:27868 1:N:0:GAACCTAG+TCCGCATA
TAAGAGCATCTGCCAGGTTAGTTATGTTTACCGTCCCCTCAGGGGTCAAGAATTGCTTTGAGGGTCCA
TCAAGTGATGACGAACTCCCTTGAAGCGACGCCTGAAATTTCCCCACTGCAGTCATCTTCTGCACCG
+
FFFFFFFFFFFFFFFFFFFFFFFFFFF:FFFFFFFFFFFFFFFFFFFFFFFF,FFFFFFFFFFFFFFF
FFFFFFFFFFFFFFFFFFFFFFFFF:F:FFFFFFFFFFF,FFFFFFFFFFFFFFFFFFF:FFFFFFF
@A00155:342:HHGFNDSXY:1:1537:30544:11209 1:N:0:GAACCTAG+TCCGCATA
AAGAGCATCTGCCAGGTTAGTTATGTTTACCGTCCCCTCAGGGGTCAAGAATTGCTTTGAGAGTCCAT
CAAGTGATGACGAACTCCCTTGAAGCGACGCCTGAAATTTCCCCACTGCAGTCATCTTCTGCACCGAG
+

```
FFFFFFFFFFFFFFFFFF,FFFF,FFFFF:FFFFFFFFFFFFFFFFFFFFFFFFFFFFFFFFFFFFFF
FFFFFFFFFFFFFFFFFFFFFFFFF:FFFFFFFFFFFFFFFFFFFFFFFFFFFFFFFFFFFFFFFFFF
@A00155:342:HHGFNDSXY:1:2216:19126:9095 1:N:0:GAACCTAG+TCCGCATA
GAGCATCTGCCAGGTTAGTTATGTTTACCGTCCCCTCAGGGGTCAAGAATTGCTTTGAGAGTCCATCA
AGTGATGACGAACTCCCTTGAAGCGACGCCTGAAATTTCCCCACTGCAGTCATCTTCTGCACCGAGAA
+
FFFFFFFFFFFFFF:FFFFFFFFFFFFFFFFFFFFFFFFFFFFFFFF:FFFFFFFFFFFFFFFFFFFF
FFFFFFFFFFFFFFFFFFFFFFF:FFFFFFFFFFFFFFFFFFFFFFFFFFFFFFFFFFFFFFFFFFFF
@A00155:342:HHGFNDSXY:1:1370:19804:24987 1:N:0:GAACCTAG+TCCGCATA
GAGCATCTGCCAGGTTAGTTATGTTTACCGTCCCCTCAGGGGTCAAGAATTGCTTTGAGAGTCCATCA
AGTGATGACGAACTCCCTTGAAGCGACGCCTGAAATTTCCCCACTGCAGTCATCTTCTGCACCGAGAA
+
FFFF,FF:FFFFFF:,:F:FFFFFFFFFFFFFFFFFFFFFFFFFFFFFFFFFFFFFFFFFFFFFFFFF
FFFFFFFFFFFFFFFFFFFF:FFFFFFFFFFFFFFFFFFFFFFFFFFFFFFFFF:FFFFFFFFFFFFF
@A00155:342:HHGFNDSXY:1:2650:30508:16658 2:N:0:GAACCTAG+TCCGCATA
AGCATCTGCCAGGTTAGTTATGTTTACCGTCCCCTCAGGGGTCAAGAATTGCTTTGAGAGTCCATCAA
GTGATGACGAACTCCCTTGAAGCGACGCCTGAAATTTCCCCACTGCAGTCATCTTCTGCACCGAGAAA
+
FFFF:FF:FFFFFFFFFFFFFF,F,:FFFFFFFFFFFFFFFFFFFF:FF,FFF:FFFFFFFFFFFF:F
FFFFFFFFFF,FFFFF:FFFFFFFFFFFFFFFFFFFFF:FFFF::FFFFF::FFFFFFFFFFFFFFFF
@A00155:342:HHGFNDSXY:1:1277:11858:15702 2:N:0:GAACCTAG+TCCGCATA
AGCATCTGCCAGGTTAGTTATGTTTACCGTCCCCTCAGGGGTCAAGAATTGCTTTGAGAGTCCATCAA
GTGATGACGAACTCCCTTGAAGCGACGCCTGAAATTTCCCCACTGCAGTCATCTTCTGCACCGAGAAA
+
FFFFFFFFFF:FFFFFFFFFFFFF:,FFFFFFFFFFFFFF:FFF::FFF:FFFF,FFFFFFFFFFFFF
:FFFFFF:FF:FFFFFFFFFFFFFFFFFFFFFF,FFFFF:F,,F:FFFFFFFF:FFFFFFFFFFFFF
@A00155:342:HHGFNDSXY:1:1420:10294:3850 2:N:0:GAACCTAG+TCCGCATA
CATCTGCCAGGTTAGTTATGTTTACCGTCCCCTCAGGGGTCAAGAATTGCTTTGAGAGTCCATCAAGT
GATGACGAACTCCCTTGAAGCGACGCCTGAAATTTCCCCACTGCAGTCATCTTCTGCACCGAGAAATA
+
FFFFFFFFFFFFFFFFFFFFFFFFFFFFFFFFFFFFFFFFFFFFFFFFFFFFFFFFFFFFFFFFFFFF
FFFFFFFFFFFFFFFFFFFFFFFFFFFFFFFFFFFFFFFFFFFFFFFFFFFFFFFFFFFFFFFFFFFF
@A00155:342:HHGFNDSXY:1:2144:23999:4633 1:N:0:GAACCTAG+TCCGCATA
TCTGCCAGGTTAGTTATGTTTACCGTCCCCTCAGGGGTCAAGAATTGCTTTGAGAGTCCATCAAGTGA
TGACGAACTCCCTTGAAGCGACGCCTGAAATTTCCCCACTGCAGTCATCTTCTGCACCGAGAAATAAT
+
FFFFFFFFFFFFFFFFFFFFFFFFFFFFFFFFFFFFFFFFFFFFFFFFFFFFFFFFFFFFFFFFFFFF
FFFFFFFFFFFFFFFFFFFFFFFFFFFFFFFFFFFFFFFFFFFFFFFFFFFFFFFFFFFFFFFFFFFF
@A00155:342:HHGFNDSXY:1:1556:7527:36417 1:N:0:GAACCTAG+TCCGCATA
TCTGCCAGGTTAGTTATGTTTACAGTCCCCTCAGGGGTCAAGAATTGCTTTGAGAGTCCATCAAGTGA
TGACGAACTCCCTTGAAGCGACGCCTGAAATTTCCCCACTGCAGTCATCTTCTGCACCGAGAAATAAT
+
FFFF:FFFF:,FFF,:FFFFFFF,FFFFFF:FF,F:F,,FFFFFFF:FF:F,F:FFFFFFFFFFFFFF
FFFFFFFFFFF:FFFFFFFFFFFFF:FFFFFFFFFFF,:FFFFFFFFFFFFFFFFFFF:FFFFFFFFF
@A00155:342:HHGFNDSXY:1:1436:22535:6073 1:N:0:GAACCTAG+TCCGCATA
TCTGCCAGGTTAGTTATGTTTACCGTCCCCTCAGGGGTCAAGAATTGCTTTGAGAGTCCATCAAGTGA
TGACGAACTCCCTTGAAGCGACGCCTGAAATTTCCCCACTGCAGTCATCTTCTGCACCGAGAAATAAT
+
FFFFFFFFFFFFFF:FFFFFFFFFFFFFFFFFFFFFFFFFFFFFFFFFFFFFFFFFFF:FFFFFFFFF
FFFFFFFFFFFFFFFFFFFFFFFFFFFFFFFFFFFFFFFFFFFFFFFFFFFFFFFFFFFFFFFFFFFF
@A00155:342:HHGFNDSXY:1:2461:10890:17660 2:N:0:GAACCTAG+TCCGCATA
CTGCCAGGTTAGTTATGTGTACCGTCCCCTCAGGGGTCAAGAATTGCTTTGAGAGTCCATCAAGTGAT
GACGAACTCCCTTGAAGCGACGCCTGAAATTTCCCCACTGCAGTCATCTTCTGCACCGAGAAATAATT
+
```

```
FFFFFFFFFFFFFFF:F,,FFFFFFFFFFFFFFFFFFFFFFFFF:FF::FFFFF:FFFFFFFFFFFFF
FFFFFFFFFFFF,FFFFFFFFFFFFFFFFFFFFFFFF:FFFFFF:FF:FFFFFFFFFFFFFFFFFFFFF
@A00155:342:HHGFNDSXY:1:2661:3739:22623 2:N:0:GAACCTAG+TCCGCATA
TGCCAGGTTAGTTATGTTTACCGTCCCCTCAGGGGTCAAGAATTGCTTTGAGAGTCCATCAAGTGATG
ACGAACTCCCTTGAAGCGACGCCTGAAATTTCCCCACTGCAGTCATCTTCTGCACCGAGAAATAATT
+
:FFFFF,FFFFFFFF,:FFFFFF:F,F::FF:,,:FF:F,FF:F,FFFF:F:F:FFFF:FFFFFFFFF
FFFFFFFFFF:F:FFF:FF::F,:FFFFFFFFF,,F:F:FFFFFFFFFFFF:FFF,FFFFFFFFFFF
@A00155:342:HHGFNDSXY:1:1423:21350:18677 2:N:0:GAACCTAG+TCCGCATA
CCAGGTTAGTTATGTTTACCGTCCCCTCAGGGGTCAAGAATTGCTTTGAGAGTCCATCAAGTGATGAC
GAACTCCCTTGAAGCGACGCCTGAAATTTCCCCACTGCAGTCATCTTCTGCACCGAGAAATAATTCCT
+
FFFFFFFFFFFFFFFFFFFFFFFFFFFFFFFFFFFFFFFFFFFFF:FFFFFFFF:FFFFFFFFFFFFF
FFFFFFFFFFFFFFFFFFFFFFFFFFFFFFFFFFFFFFFFFFFFFFFFFFFFFFFFFFFFFFFFFFFF
@A00155:342:HHGFNDSXY:1:1440:13196:11851 2:N:0:GAACCTAG+TCCGCATA
GGTTAGTTATGTTTACCGTCCCCTCAGGGGTCAAGAATTGCTTTGAGAGTCCATCAAGTGATGACGAA
CTCCCTTGAAGCGACGCCTGAAATTTCCCCACTGCAGTCATCTTCTGCACCGAGAAATAATTCCTAGT
+
FFFFFFFFFFFFFFFFFFFFFFF:,FFFFF:F:,FFF,FFF,F,F:FF,FFFFFF,:FFFF,F:FFFF
FFFFF,:FFFFFFFFFFF:FFFF::FFF:FFF,FFFF,F:,FFFFFFFFFFFFFFFFFFF:FFF:FF:
@A00155:342:HHGFNDSXY:1:1518:23068:35493 2:N:0:GAACCTAG+TCCGCATA
GGTTAGTTATGTTTACCGTCCCCTCAGGGGTCAAGAATTGCTTTGAGAGTCCATCAAGTGATGACGAA
CTCCCTTGAAGCGACGCCTGAAATTTCCCCACTGCAGTCATCTTCTGCACCGAGAAATAATTCCTAGT
+
FFFFFFFFFFFFFFFFFFFFFFFFF:FFFFFFFFFFFFFFFFFFFFFFFFFFFFFFFFFFFFFFFFFF
FFFFFFFFFFFFFFFFFFFFFFFFFFFFFFFFFFFFFFFFFFFFFFFFFFFFFFFFFFFFFFFFFFFF
@A00155:342:HHGFNDSXY:1:1529:21269:31407 2:N:0:GAACCTAG+TCCGCATA
GGTTAGTTATGTTTACCGTCCCCTCAGGGGTCAAGAATTGCTTTGAGAGTCCATCAAGTGATGACGAA
CTCCCTTGAAGCGACGCCTGAAATTTCCCCACTGCAGTCATCTTCTGCACCGAGAAATAATTCCTAGT
+
FFFFFFFFFFFFFFFFFFFFFFFFFFF:FFFFFFFFFFFFFFF,FFFFFFFFFFFFFFFFFFFFFFFF
FFFFFFFFFFFFFFFFFFFFFFFFFFFFFFFFFFFFFFFFFFFFFFFFFFFFFFFFFFFFFFFFFFF:
@A00155:342:HHGFNDSXY:1:2547:30843:24533 2:N:0:GAACCTAG+TCCGCATA
GTTAGTTATGTTTACCGTCCCCTCAGGGGTCAAGAATTGCTTTGAGAGTCCATCAAGTGATGACGAAC
TCCCATGAAGCGACGCCTGAAATTTCCCCACTGCAGTCATCTTCTGCACCGAGAAATAATTCCTAGTC
+
FFFFFFFFFFF,FFFFFFFFFF:F:FFFFFFF:F,F:FFFFF,FFFFFFFFFFFF:FFFFFF:FFF,F
FFFF,FFF,FFFFFFFFFFFFFF:FFFFFFFFFFFFFF::FFFF:FFFFFFFFF:FFFFFFFFF,FFF
@A00155:342:HHGFNDSXY:1:2144:23999:4633 2:N:0:GAACCTAG+TCCGCATA
GTTAGTTATGTTTACCGTCCCCTCAGGGGTCAAGAATTGCTTTGAGAGTCCATCAAGTGATGACGAAC
TCCCTTGAAGCGACGCCTGAAATTTCCCCACTGCAGTCATCTTCTGCACCGAGAAATAATTCCTAGTC
+
FFFFFFFFFFFF,FFFFFFFFFFFFFFFFFFFFFFFF:FFFF,FFFFFFFFFFFFFFFFFFFFFFFFFF
FFFFFFFFFFFFFFFFFFFFFFFFFFFFFF:FFFFFF:F:FF:FFFFFFFFFFFFFFFFFFFFFFFFFF
@A00155:342:HHGFNDSXY:1:1551:13765:25019 2:N:0:GAACCTAG+TCCGCATA
GTTAGTTATGTTTACCGTCCCCTCAGGGGTCAAGAATTGCTTTGAGAGTCCATCAAGTGATGACGAAC
TCCCTTGAAGCGACGCCTGAAATTTCCCCACTGCAGTCATCTTCTGCACCGAGAAATAATTCCTAGTC
+
:FFF:FFFFF,F,FFFFFFFFFFF,FFFFFFFFFFFF:FF,FFFFFFFFFFF:FFF:FFFFFFFFFFF
FFFF::FFFFFFFFFFFF,FFF:FFFFFFFFF:FFF:F,:FFFF:FFFFFFFFFFFFFFFFFFFFFFFF
@A00155:342:HHGFNDSXY:1:1141:16712:28776 2:N:0:GAACCTAG+TCCGCATA
GTTAGTTATGTTTACCGTCCCCTCAGGGGTCAAGAATTGCTTTGAGAGTCCATCAAGTGATGACGAAC
TCCCTTGAAGCGACGCCTGAAATTTCCCCACTGCAGTCATCTTCTGCACCGAGAAATAATTCCTAGTC
+
```

```
F:FFFF::,F:F,FFFF:FFFFFF,F:FF:FFFFFFF:FF:F:FFFFFFFFFFFFFFFFFFF:FFFFF
FFFF,,FF:FFFFFFFFFFFF::,FFFFFFFF:FFFFFFFFFFFFFFFFFFFFFFFFFFFFFFF:FFF
@A00155:342:HHGFNDSXY:1:1644:23592:24596 1:N:0:GAACCTAG+TCCGCATA
TTAGTTATGTTTACCGTCCCCTCAGGGGTCAAGAATTGCTTTGAGAGTCCATCAAGTGATGACGAACT
CCCTTGAAGCGACGCCTGAAATTTCCCCACTGCAGTCATCTTCTGCACCGAGAAATAATTC
+
FFFF:FF:FFFFFFFFFFFFFFFFF:F:FFFFFFFFFFFFFFFFFFFFF:FFFFFFFFFFFFFFFFFF
FFFFFFFFFFFFFFFFFFFFFFFF,FFFFFFFFFFFFFFFFFFFFFFFFFFFFFFFFFFFF
@A00155:342:HHGFNDSXY:1:2535:18801:26005 1:N:0:GAACCTAG+TCCGCATA
TTAGTTATGTTTACCGTCCCCTCAGGGGTCAAGAATTGCTTTGAGAGTCCATCAAGTGATGACGAACT
CCCTTGAAGCGACGCCTGAAATTTCCCCACTGCAGTCATCTTCTGCACCGAGAAATAATTC
+
FFFFFF:FFFFFFFFFFFFFFFFFFFFFFFFFFFFFFFFFFFFFFFFFFFFFFFFFFFFFFFFFFFFF
FFFFFFFFFFFFFFFFFFFFFFFFFFFFFFFFFFFFFFFFFFFFFFFFFFFFFFFFFFFFF
@A00155:342:HHGFNDSXY:1:2535:16495:18067 1:N:0:GAACCTAG+TCCGCATA
TTAGTTATGTTTACCGTCCCCTCAGGGGTCAAGAATTGCTTTGAGAGTCCATCAAGTGATGACGAACT
CCCTTGAAGCGACGCCTGAAATTTCCCCACTGCAGTCATCTTCTGCACCGAGAAATAATTC
+
F:FFFFFFFFFFFFFFFFFFFFFFFFFFFFFFF:FFFFFFFFFFFFFFFFFFFFFFFFFFFFFFFFFF
FFFFFFFFFFFFFFFFFFFFFFFFFFFFFFFFFFFFFFFFFFFFFFFFFFFFFFFFFFFFF
@A00155:342:HHGFNDSXY:1:1158:21992:9737 2:N:0:GAACCTAG+TCCGCATA
TAGTTATGTTTACCGTCCCCTCAGGGGTCAAGAATTGCTTTGAGAGTCCATCAAGTGATGACGAACTC
CCTTGAAGCGACGCCTGAAATTTCCCCACTGCAGTCATCTTCTGCACCGAGAAATAATTCCTAGTCGT
+
FFFFFFFFFFFFFFFFFFFFFFFFFFFFFFFFF:FFFFFFFFFFFFFFFFFF:F:FFFFFFFFFFFFF
FF:FFFFFFFFFFFFFFFFFF:FFFFF:FF,FFF,F:FFFFFFFFFFFFFFFFFFFFFFFFFFFFFFF
@A00155:342:HHGFNDSXY:1:1158:21486:8077 2:N:0:GAACCTAG+TCCGCATA
TAGTTATGTTTACCGTCCCCTCAGGGGTCAAGAATTGCTTTGAGAGTCCATCAAGTGATGACGAACTC
CCTTGAAGCGACGCCTGAAATTTCCCCACTGCAGTCATCTTCTGCACCGAGAAATAATTCCTAGTCGT
+
FFFF,FFFF:FFFFF:FFFF,F:FFF:FFFFFFFFFFF:F:FFFFFFFF,:F:,FFF,FFFFFFFFFF
FF::FF:FFFFFFFFFFF:FFFFFFFFFFF:FFF:F:FFF:F:FFFFFFF:FFFFFF,FFFFFFFFFF
@A00155:342:HHGFNDSXY:1:2448:30680:21057 2:N:0:GAACCTAG+TCCGCATA
TAGTTATGTTTACCGTCCCCTCAGGGGTCAAGAATTGCTTTGAGAGTCCATCAAGTGATGACGAACTC
CCTTGAAGCGACGCCTGAAATTTCCCCACTGCAGTCATCTTCTGCACCGAGAAATAATTCCTAGTCGT
+
FFFFF,FFFFFFFFFFFFFFFFFFFFFFFF,FFF,FFF,FFFFFFFFFFFFF:FFFF:FFFFFFFFFF
:F:FFFFFFFFFFFFFFFFFFFFFFFF:FFFFFFFFF:FFFFFFFFFFFF:F,FFFFFFFFFFF:FFF
@A00155:342:HHGFNDSXY:1:2657:13485:12383 2:N:0:GAACCTAG+TCCGCATA
AGTTATGTTTACCGTCCCCTCAGGGGTCAAGAATTGCTTTGAGAGTCCATCAAGTGATGACGAACTCC
CTTGAAGCGACGCCTGAAATTTCCCCACTGCAGTCATCTTCTGCACCGAGAAATAATTCCTAGTCGTG
+
FFFFF,FFFFFFFFFFFFFF:FFFFFFFFFFFF,FFFFF:FFFFFFFFFFFF::FFFFFFFFFFFFFF
F,,FFFFFFFFFFFFFFFFF:FFFFFFFFFFFFFFFFFFFFFFFFFFFFFFFFFFFFFFFFFFFFFFF
@A00155:342:HHGFNDSXY:1:2657:13485:12383 1:N:0:GAACCTAG+TCCGCATA
GTTATGTTTACCGTCCCCTCAGGGGTCAAGAATTGCTTTGAGAGTCCATCAAGTGATGACGAACTCCC
TTGAAGCGACGCCTGAAATTTCCCCACTGCAGTCATCTTCTGCACCGAGAAATAATTCCTAGTCGTGG
+
FFFFFFFFFFFFFFFFFFFFFFFFFFFFFFFFF:FFFFFFFFFFFFFFFFFFFFFFFFFFFF:FFFFF
FFFFFFFFFFFFFFFFFFFFFFFFFFFFFFFFFFFFFFFFFFFFFFFFFFFFFFFFFFFFFFFFFFFF
@A00155:342:HHGFNDSXY:1:1622:3658:12493 2:N:0:GAACCTAG+TCCGCATA
GTTATGTTTACCGTCCCCTCAGGGGTCAAGAATTGCTTTGAGAGTCCATCAAGTGATGACGAACTCCC
TTGAAGCGACGCCTGAAATTTCCCCACTGCAGTCATCTTCTGCACCGAGAAATAATTCCTAGTCGTGG
+
```

```
FFFFFFFFFFFFF,FF:F:F::FFFFFFFFF:F:,F:F:FFFFFFFF:FFFF:F::FFFF:FFFFF:F
F:FF::FF::FFF:FFFFFFFFFFFFFFFFF:,F:FFFFFFFFFFFFF:FFFFFFFFFFFFFFFFFFF
@A00155:342:HHGFNDSXY:1:2147:18521:23766 1:N:0:GAACCTAG+TCCGCATA
TGTTTACCGTCCCCTCAGGGGTCAAGAATTGCTTTGAGAGTCCATCAAGTGATGACGAACTCCCTTGA
AGCGACGCCTGAAATTTCCCCACTGCAGTCATCTTCTGCACCGAGAAATAATTCCTAGTCGTGGTGAT
+
FFFFFFFFFFFFFFFFFFFFFFFFFFFFFFFFFFFFFFFFFFFFFFFFFFFFFFFFFFFFFFFFFFFF
FFFFFFFFFFFFFFFFFFFFFFFFFFFFFFFFFFFFFFFFFFFFFFFFFFFFFFFFFFFFFFFFFFFF
@A00155:342:HHGFNDSXY:1:1165:15519:29277 2:N:0:GAACCTAG+TCCGCATA
TGTTTACCGTCCCCTCAGGGGTCAAGAATTGCTTTGAGAGTCCATCAAGTGATGACGAACTCCCTTGA
AGCGACGCCTGAAATTTCCCCACTGCAGTCATCTTCTGCACCGAGAAATAATTCCTAGTCGTGGTGAT
+
FF:,FFFFF:FFFF,FF:F::FFFF:FFFFFF,:FFFFFFFFFFFFFF:FF:FFFFFFFFFFFF:FF:
FFFFFFFFF:FFFFFFFFFFFFFFFF:FFF:,FFFF:FFFFFFFFFFFFFFFFFFFFFFFFFFFFFFF
@A00155:342:HHGFNDSXY:1:2431:6542:9502 2:N:0:GAACCTAG+TCCGCATA
GTTTACCGTCCCCTCAGGGGTCAAGAATTGCTTTGAGAGTCCATCAAGTGACGACGAACTCCCTTGAA
GCGACGCCTGAAATTTCCCCACTGCAGTCATCTTCTGCACCGAGAAATAATTCCTAGTC
+
FFFFFFFFFFFFFFF:FFFFFFFFFFFFFFFF:FFFFFFFFFF:FF:F:FFFFFFFFFFFFFFF:FFF
FFFFFFFFFFFFFFFFFFFF,F,,FFFFFF:FFFFFFFFFFFFFFFFFFFFFFFFFFFF
@A00155:342:HHGFNDSXY:1:2123:19036:2268 1:N:0:GAACCTAG+TCCGCATA
GTTTACCGTCCCCTCAGGGGTCAAGAATTGCTTTGAGAGTCCATCAAGTGATGACGAACTCCCTTGAA
GCGACGCCTGAAATTTCCCCACTGCAGTCATCTTCTGCACCGAGAAATAATTCCTAGTCGTGGTGATA
+
FFFFFFFF,FF:FFFFFFFFFFFFFFFFFFFFFFFFFFFFFFFFFFFFFFFFFFFFFFFFFFFFFFFF
FFFFFFFFFFFFFFFFFFFFFFFFFFFFFFFFFFFFFFFFFFFFFFFFFFFFFFFFFFFFFFFFFFFF
@A00155:342:HHGFNDSXY:1:1413:11360:4194 1:N:0:GAACCTAG+TCCGCATA
GTTTACCGTCCCCTCAGGGGTCAAGAATTGCTTTGAGAGTCCATCAAGTGATGACGAACTCCCTTGAA
GCGACGCCTGAAATTTCCCCACTGCAGTCATCTTCTGCACCGAGAAATAATTCCTAGTCGTGGTGATA
+
FFFFFFFFFFFFFFFFFFFFFFFFFFFFFFFFFFFFFFFFFFFF:FFFFFFFFFFFFFFFFFFFFFFF
FFFFFFFFFFFFFFFFFFFFFFFFFFFFFFFFFFFFFFFFFFFFFFFFFFFFFFFFFFFF:FFFFFFF
@A00155:342:HHGFNDSXY:1:2476:18403:26287 2:N:0:GAACCTAG+TCCGCATA
GTTTACCGTCCCCTCAGGGGTCAAGAATTGCTTTGAGAGTCCATCAAGTGATGACGAACTCCCTTGAA
GCGACGCCTGAAATTTCCCCACTGCAGTCATCTTCTGCACCGAGAAATAATTCCTAGTCGTGGTGATA
+
FFFFFFFFFFFFFFF,FFFF:FFFFFFFFFFFFFFFFFFFFFFFFFFFFFFFFFFFFFFFFFF,:FFF
FFFFFFFF:FFF:F,FFFFFFFFFFFFFFFFFFFFFFFFFFFFFFFFFFFFFFFFFFFFFFFFFFFFF
@A00155:342:HHGFNDSXY:1:2509:25672:20024 1:N:0:GAACCTAG+TCCGCATA
GTTTACCGTCCCCTCAGGGGTCAAGAATTGCTTTGAGAGTCCATCAAGTGATGACGAACTCCCTTGAA
GCGACGCCTGAAATTTCCCCACTGCAGTCATCTTCTGCACCGAGAAATAATTCCTAGTCGTGGTGATA
+
FF:FFFFFFFFFFFFFFFFFFFFFFFFFFFFFFFFFFFFF::FFFFFFFFFFFFFFFFFFFFFFFFFF
FFFFFFFFFFFFFFFFFFFFFFFFFFFFFFFFFFFFFFFFFFFFFFFFFFFFFFFFFFFFF:FFFFFF
@A00155:342:HHGFNDSXY:1:1113:28131:27821 2:N:0:GAACCTAG+TCCGCATA
GTTTACCGTCCCCTCAGGGGTCAAGAATTGCTTTGAGAGTCCATCAAGTGATGACGAACTCCCTTGAA
GCGACGCCTGAAATTTCCCCACTGCAGTCATCTTCTGCACCGAGAAATAATTCCTAGTCGTGGTGATA
+
FFFFFFFFFFFFF,FFFFF:FFFFFFFFFFF,FFFFFFFFFFF:FFFFFFFFFFFFFFFFFFFF:FFF
FFFFFFFFFFFFFFFFFFFF,FFFFF:FFF,FFF::FFFFFFFFFFFFFFFFFFFFFFFFFFFFFFFF
@A00155:342:HHGFNDSXY:1:1278:32054:36464 1:N:0:GAACCTAG+TCCGCATA
GTTTACCGTCCCCTCAGGGGTCAAGAATTGCTTTGAGAGTCCATCAAGTGATGACGAACTCCCTTGAA
GCGACGCCTGAAATTTCCCCACTGCAGTCATCTTCTGCACCGAGAAATAATTCCTAGTCGTGGTGATA
+
```

```
FFFFFFFFFFFFFFFFFFFFFFFFF:FFFFFFFFFFFFFFFFFFFFFFFFFFFFFFFFFFFFFFFFFF
FFFFFFFFFFFFFFFFFFFFFFFFFFFFFFFFFFFFFFFFFFFFFFFFFFFFFFFFFFFFFFFFFFFF
@A00155:342:HHGFNDSXY:1:2614:6271:30452 1:N:0:GAACCTAG+TCCGCATA
GTTTACCGTCCCCTCAGGGGTCAAGAATTGCTTTGAGAGTCCATCAAGTGATGACGAACTCCCTTGAA
GCGACGCCTGAAATTTCCCCACTGCAGTCATCTTCTGCACCGAGAAATAATTCCTAGTCGTGGTGATA
+
FFFFFFFFFFFFFFFFFFFFFFFFFFFFFFFFFFFFFFFFFFFFFFF:FFFFFFFFFFFFFFFFFFFF
:FFFFFFFFFFFFFFFFFF:FFFFFFFFFFFFFFFF:FFFFFFFFFFFF:FFFFFFFFFFFFFFFFFF
@A00155:342:HHGFNDSXY:1:2418:6822:33411 1:N:0:GAACCTAG+TCCGCATA
TTTACCGTCCCCTCAGGGGTCAAGAATTGCTTTGAGAGTCCATCAAGTGATGACGAACTCCCTTGAAG
CGACGCCTGAAATTTCCCCACTGCAGTCATCTTCTGCACCGAGAAATAATTCATAGTCGTGGTGATAT
+
FFFFFFFFFFFFFFFFFFFFFFFFFFFFFFFFFFFFFFFFFFFFFFFFFFFF,FFFFFFFFFFFFF:FF
FFFFFFFFFFFFFFF:FFFFFFFFFFFFFFFFFFFF:FFFFFFFFFFFFFFF,F::F:FFFFFFFFFF
@A00155:342:HHGFNDSXY:1:2415:32868:22153 1:N:0:GAACCTAG+TCCGCATA
TTTACCGTCCCCTCAGGGGTCAAGAATTGCTTTGAGAGTCCATCAAGTGATGACGAACTCCCTTGAAG
CGACGCCTGAAATTTCCCCACTGCAGTCATCTTCTGCACCGAGAAATAATTCCTAGTCGTGGTGATAT
+
FFFFFFFFFFFFFFFFFFFFFF:FFFFFFFFFFFFF:FFFFFFFFFFFFFFFFFFFFFFFFFFFFFFF
FFFFFFFFFFFFFFFFFFF:F:FFFFFFFFFFFFFFFFFFFFF:FFFFFFFFFFFFFFFFFFFFFFFF
@A00155:342:HHGFNDSXY:1:1440:13196:11851 1:N:0:GAACCTAG+TCCGCATA
TTTACCGTCCCCTCAGGGGTCAAGAATTGCTTTGAGAGTCCATCAAGTGATGACGAACTCCCTTGAAG
CGACGCCTGAAATTTCCCCACTGCAGTCATCTTCTGCACCGAGAAATAATTCCTAGTCGTGGTGATAT
+
FFFFFFF:FFFFFFFFFFFFFFFFFFFF:FFFFFFFFFF:FFFFFFFFFFFFFFFFFFFFFFFFFF:F
FFFFFFFFFFFFFFF:FF:FFFFFFFFFFFFFFFFFFFFFFFFFFFFFFFFFFFF:FFFFFFFFFFFF
@A00155:342:HHGFNDSXY:1:2204:31159:17816 1:N:0:GAACCTAG+TCCGCATA
TTTACCGTCCCCTCAGGGGTCAAGAATTGCTTTGAGAGTCCATCAAGTGATGACGAACTCCCTTGAAG
CGACGCCTGAAATTTCCCCACTGCAGTCATCTTCTGCACCGAGAAATAATTCCTAGTCGTGGTGATAT
+
FFFFFFFFFFFFFFFFFFFFFFFFFFFFFFFFFFFFFFFFFFFFFFFFFFFFFFFFFFFFFFFFFFFF
FFFFFFFFFFFFFFF:FFFFFFFFFFFFFFFFFFFF:FFFFFFFFFFFFFFFFF:FF:FFFFFFFF:F
@A00155:342:HHGFNDSXY:1:2476:18403:26287 1:N:0:GAACCTAG+TCCGCATA
TTTACCGTCCCCTCAGGGGTCAAGAATTGCTTTGAGAGTCCATCAAGTGATGACGAACTCCCTTGAAG
CGACGCCTGAAATTTCCCCACTGCAGTCATCTTCTGCACCGAGAAATAATTCCTAGTCGTGGTGATAT
+
FFFFFFFFFFFFFFFFFFFFFFFFFFFFFFFFFFFFFFFFFFFFFFFFFFFFFFFFFFFFFFFFFFFF
FFFFFFFFFFFFFFFFFFFFFFFFFFFFFFFFFFFFFFFFFFFFFFFFFFFFFFFFFFFFFFFFFFFF
@A00155:342:HHGFNDSXY:1:2510:12382:10786 1:N:0:GAACCTAG+TCCGCATA
TTTACCGTCCCCTCAGGGGTCAAGAATTGCTTTGAGAGTCCATCAAGTGATGACGAACTCCCTTGAAG
CGACGCCTGAAATTTCCCCACTGCAGTCATCTTCTGCACCGAGAAATAATTCCTAGTCGTGGTGATAT
+
FFFFFFFFFFFFFFFFFFFFFFFFFFFFFFFF:FFFFFFFFFFFFFFFFFFFFFFFFFFFFFFFFF:FF
FFFFFFFFFFFFFFF:FFFFFFFFFFFFFFFFFFFFFFFFFFFFFFFFFFFF:FFFFFFFFFFFFFFF
@A00155:342:HHGFNDSXY:1:1609:7997:25238 1:N:0:GAACCTAG+TCCGCATA
TTTACCGTCCCCTCAGGGGTCAAGAATTGCTTTGAGAGTCCATCAAGTGATGACGAACTCCCTTGAAG
CGACGCCTGAAATTTCCCCACTGCAGTCATCTTCTGCACCGAGAAATAATTCCTAGTCGTGGTGATAT
+
FFFFFFFFFFFFFFFFFFFFFFFFFFFFF,FFFFFFFFFFFFFFFFFFFFFFFFFFFFFFFFFFFFF:
FFFFFFFFFFFFFFFFFFFFFFFFFFFFFFFFFFFFF:FFFFFFFFFFFFFFFFFFFFFFFFFFFFFF
@A00155:342:HHGFNDSXY:1:1131:16306:3239 1:N:0:GAACCTAG+TCCGCATA
TTTACCGTCCCCTCAGGGGTCAAGAATTGCTTTGAGAGTCCATCAAGTGATGACGAACTCCCTTGAAG
CGACGCCTGAAATTTCCCCACTGCAGTCATCTTCTGCACCGAGAAATAATTCCTAGTCGTGGTGATAT
+
```

```
FFFFFFFFFFFFFFFFFFFFFFFFFFFFFFFFFFFFFFFFFFFFFFFFFFFFFFFFFFFFFFFFFFFF
FFFFFFFFFFFFFFFFFFFFFFFFFFFFFFFFFFFFFFFFFFFFFFFFFFFFFFFFFFFFFFFFFFFF
@A00155:342:HHGFNDSXY:1:1461:30138:36025 1:N:0:GAACCTAG+TCCGCATA
TTTACCGTCCCCTCAGGGGTCAAGAATTGCTTTGAGAGTCCATCAAGTGATGACGAACTCCCTTGAAG
CGACGCCTGAAATTTCCCCACTGCAGTCATCTTCTGCACCGAGAAATAATTCCTAGTCGTGGTGATAT
+
FFF:FFFFFFFFFFFFFFFFFFFFFFFFFFFFFFFFFFFFFFF:FFFFFFFFFFFFFFFFFFFFFFFF
FFFFFFFFFFFFFFFFFFFFFFFFFFFFFFFFFFFFFFFFFFFFFFFFFFFFFFFFFFFFFFFFFFFF
@A00155:342:HHGFNDSXY:1:1551:13765:25019 1:N:0:GAACCTAG+TCCGCATA
TTTACCGTCCCCTCAGGGGTCAAGAATTGCTTTGAGAGTCCATCAAGTGATGACGAACTCCCTTGAAG
CGACGCCTGAAATTTCCCCACTGCAGTCATCTTCTGCACCGAGAAATAATTCCTAGTCGTGGTGATAT
+
FFFFFFFFFFFFFFFFFFFFFFFFFFFFFFFFFFFFFFFFFFFFFFFFFFFFFFFFFFFFFFFFFFFF
FFFFFFFFFFFFFFF:FFFFFFFFFFFFFFFFFFFF:FFFFFFFFFFF:FFFFFFFFFFFFFFFFFFF
@A00155:342:HHGFNDSXY:1:1458:32253:11287 1:N:0:GAACCTAG+TCCGCATA
TTTACCGTCCCCTCAGGGGTCAAGAATTGCTTTGAGAGTCCATCAAGTGATGACGAACTCCCTTGAAG
CGACGCCTGAAATTTCCCCACTGCAGTCATCTTCTGCACCGAGAAATAATTCCTAGTCGTGGTGATAT
+
FFFFFFFFFFFFFFFFFFFFFFFFFFFFFFFFFFFFFFFFFFFFFFFFFFFFFFFFFFFFFFFFFFFF
FFFFFFFFFFFFFFFFFFFFFFFFFFFFFFFFFFFFFFFFFFFFFFFFFFFFFFFFFFFFFF,FFFFF
@A00155:342:HHGFNDSXY:1:1237:5367:14512 1:N:0:GAACCTAG+TCCGCATA
TTTACCGTCCCCTCAGGGGTCAAGAATTGCTTTGAGAGTCCATCAAGTGATGACGAACTCCCTTGAAG
CGACGCCTGAAATTTCCCCACTGCAGTCATCTTCTGCACCGAGAAATAATTCCTAGTCGTGGTGATAT
+
FFFFFFFFFFFFFFFFFFFFFFFFFFFFFFFFFFFFFFFFFFFFFFFFFFFFFFFFFFFFFFFFFFFF
FFFFFFFFFFFFFFFFFFFFFFFFFFFFFFFFFFFFFFFFFFFFFFFFFFFFFFFFFFFF:FFFFFFF
@A00155:342:HHGFNDSXY:1:1529:21269:31407 1:N:0:GAACCTAG+TCCGCATA
TTTACCGTCCCCTCAGGGGTCAAGAATTGCTTTGAGAGTCCATCAAGTGATGACGAACTCCCTTGAAG
CGACGCCTGAAATTTCCCCACTGCAGTCATCTTCTGCACCGAGAAATAATTCCTAGTCGTGGTGATAT
+
FFFFFFFFFFFFFFFFFFFFFFFFFFFFFFFFFFFFFFFFFFFFFFFFFFFFFFFFFFFFFFFFFFFF
FFFFFFFFFFFFFFF:FFFFFFFFFFFFFFFFFFFFFFFFFFFFFFFFFFFFFFFFFFFFFFFFFFFF
@A00155:342:HHGFNDSXY:1:1234:21169:6245 1:N:0:GAACCTAG+TCCGCATA
TTTACCGTCCCCTCAGGGGTCAAGAATTGCTTTGAGAGTCCATCAAGTGATGACGAACTCCCTTGAAG
CGACGCCTGAAATTTCCCCACTGCAGTCATCTTCTGCACCGAGAAATAATTCCTAGTCGTGGTGATAT
+
FFFFFFFFFFFFFFFFFFFFFFFFFFFFFFFFFFFFFFFFFFFF:FFFFFFFFFFFFFFFFFFFFFFF
FFFFFFFFFFFFFFFFFFFFFFFFFFFFFFFFFFFFFFFFFFFFFFFFFFFFFFFFFFFFFFFFFF:F
@A00155:342:HHGFNDSXY:1:2263:31060:24940 1:N:0:GAACCTAG+TCCGCATA
TTACCGTCCCCTCAGGGGTCAAGAATTGCTTTGAGAGTCCATCAAGTGATGACGAACTCCCTTGAAGC
GACGCCTGAAATTTCCCCACTGCAGTCATCTTCTGCACCGAGAAATAATTCCTAGTCGTGGTGATATT
+
FFFFFFFFFFFFFFFFFFFFFFFFFF:FFFFFFFFFFFFFFFFFFFFFFFFFFFF:FFFFFFFFFFFF
FFFFFFFFFFFFFFFFFFFFFFFFFFFFFFFFFFF,FFFFF:FFFFFFFFFFFFFF:FFFFFFFFFFF
@A00155:342:HHGFNDSXY:1:1131:16306:3239 2:N:0:GAACCTAG+TCCGCATA
TTACCGTCCCCTCAGGGGTCAAGAATTGCTTTGAGAGTCCATCAAGTGATGACGAACTCCCTTGAAGC
GACGCCTGAAATTTCCCCACTGCAGTCATCTTCTGCACCGAGAAATAATTCCTAGTCGTGGTGATATT
+
FF:FFFFFFFFFFFFFF:,FFFFFFFFFF:F:FFFFFFFFFFFF:FFFF:FFFFFFFFFFF:FFFFFF
FFFFFFFFFFFFFFFFFF:FF:FFFFF:FFFFFFFFFFFFFFFFFFFFFFFFFFFFFFFFFFFFFFFF
@A00155:342:HHGFNDSXY:1:1542:2465:29465 1:N:0:GAACCTAG+TCCGCATA
TTACCGTCCCCTCAGGGGTCAAGAATTGCTTTGAGAGTCCATCAAGTGATGACGAACTCCCTTGAAGC
GACGCCTGAAATTTCCCCACTGCAGTCATCTTCTGCACCGAGAAATAATTCCTAGTCGTGGTGATATT
+
```

FFFFFFFFFFFFFFFFFFFFFFFFFFFFFFFFFFFFFFFFFFFFFFFFFFFFFFFFFFFFFFFF:FFF
FFFFFFFFFFFFFFFFFFFFFFFFFFFFFFFFFFFFFFFFFFFFFFFFFFFFFF,FFFFF::FF:FFF
@A00155:342:HHGFNDSXY:1:2417:30364:33567 1:N:0:GAACCTAG+TCCGCATA
TTACCGCCCCCTCAGGGGTCAAGAATTGCTTTGAGAGTCCATCAAGTGATGACGAACTCCCTTGAAGC
GACGCCTGAAATTTCCCCACTGCAGTCATCTTCTGCACCGAGAAATAATTCCTAGTCGTGGTGATATT
+
FFFFFFFFFFFFFFFFFFFFFFFFFFFFFFFFFFFFFFFFFFFFFFFFFFFFFFFFFFFFFFFFFFFF
FFFFFFFFFFFFFFFFFFFFFFFFFFFFFFFFFFFFFFFFFFFFFFFFFFFFFFFFFFFFFFFFFFFF
@A00155:342:HHGFNDSXY:1:2263:31087:24956 1:N:0:GAACCTAG+TCCGCATA
TTACCGTCCCCTCAGGGGTCAAGAATTGCTTTGAGAGTCCATCAAGTGATGACGAACTCCCTTGAAGC
GACGCCTGAAATTTCCCCACTGCAGTCATCTTCTGCACCGAGAAATAATTCCTAGTCGTGGTGATATT
+
FFFFFFFFFFFFFFFFFFFFFFFFFFFFFFFFFFFFFFFFFFFFFFFFFFFFFFFFFFFFFFFFFFFF
FFFFFFFFFFFFFFFFFFFFFFFFFFFFFFFFFFFFFFFFFFFFFF:FFFFFFFFFFFFFFFFFFF:F
@A00155:342:HHGFNDSXY:1:1224:9724:24471 1:N:0:GAACCTAG+TCCGCATA
TTACCGTCCCCTCAGGGGTCAAGAATTGCTTTGAGAGTCCATCAAGTGATGACGAACTCCCTTGAAGC
GACGCCTGAAATTTCCCCACTGCAGTCATCTTCTGCACCGAGAAATAATTCCTAGTCGTGGTGATATT
+
FFFFFFFFFFFFFFFFFFFFFFFFFFFFFFFFFFFFFFFFFFFFFFFFFFFFFFFFFFFFFFFFFFFF
FFFFFFFFFFFFFFFFFFFFFFFFFFFFFFFFFFFFFFFFFFFFFFFFFFFFFFFFFFFFFFFFFFFF
@A00155:342:HHGFNDSXY:1:1315:9579:5744 2:N:0:GAACCTAG+TCCGCATA
TACCGTCCCCTCAGGGGTCAAGAATTGCTTTGAGAGTCCATCAAGTGATGACGAACTCCCTTGAAGCG
ACGCCTGAAATTTCCCCACTGCAGTCATCTTCTGCACCGAGAAATAATTCCTAGTCGTGGTGATATTG
+
F:F::FFFFFFFF::,:FFFF:F,,::F:,F:FFFFFFF:FFF:::FFFFFFFF:FFFFF,::FF::F
FFFFF,FFFFFFFFFFFFFF:F:,FFF:FFFFFFFF,FF:,FFFFFFFFFFFFFFFFFFFFFFFFFFF
@A00155:342:HHGFNDSXY:1:2207:31042:27821 1:N:0:GAACCTAG+TCCGCATA
TACCGTCCCCTCAGGGGTCAAGAATTGCTTTGAGAGTCCATCAAGTGATGACGAACTCCCTTGAAGCG
ACGCCTGAAATTTCCCCACTGCAGTCATCTTCTGCACCGAGAAATAATTCCTAGTCGTGGTGATATTG
+
FFFFFFFFFFFFFFFFFFFFFFFFFFFFFFFFFFFFF:FFFFFFFFFFFFFFFFFFFFFFFFFFFFFF
FFFFFFFFFFFFFFFFFFFFFFFFFFFFFFFFFFFFFFFFFFFF:FFFFFFF:FFFFFFFFFFFFFFF
@A00155:342:HHGFNDSXY:1:1113:28131:27821 1:N:0:GAACCTAG+TCCGCATA
TACCGTCCCCTCAGGGGTCAAGAATTGCTTTGAGAGTCCATCAAGTGATGACGAACTCCCTTGAAGCG
ACGCCTGAAATTTCCCCACTGCAGTCATCTTCTGCACCGAGAAATAATTCCTAGTCGTGGTGATATTG
+
FFFFFFFFFFFFFFFFFFFFFFFFFFFFFFFFFFFFFFFFFFFFFFFFFFFFFFFFFFFFFFFFFFFF
FF,FFFFFFFFFFF:FFFFF,FFFF:FFFFFFFF,FFFFFFFFFFFFFFFFFFFFFFFFFFFFF:FF:
@A00155:342:HHGFNDSXY:1:1143:24424:22122 1:N:0:GAACCTAG+TCCGCATA
TACCGTCCCCTCAGGGGTCAAGAATTGCTTTGAGAGTCCATCAAGTGATGACGAACTCCCTTGAAGCG
ACGCCTGAAATTTCCCCACTGCAGTCATCTTCTGCACCGAGAAATAATTCCTAGTCGTGGTGATATTG
+
FFFFFFFFFFFFFFFFFFFFFFFFFFFFFFFFFFFFFFFFFFFFFFFFFFFFFFFFFFFFFFFFFFFF
FFFFFFFFFFFFFFFFFFFFFFFFFFFFFFFFFFFFFFFFFFFFFFFFFFFFFFFFFFFFFFFFFFFF
@A00155:342:HHGFNDSXY:1:1605:9661:17879 1:N:0:GAACCTAG+TCCGCATA
TACCGTCCCCTCAGGGGTCAAGAATTGCTTTGAGAGTCCATCAAGTGATGACGAACTCCCTTGAAGCG
ACGCCTGAAATTTCCCCACTGCAGTCATCTTCTGCACCGAGAAATAATTCCTAGTCGTGGTGATATTG
+
FFFFFFFFFFFFFFFFFFFFFFFFFFFFFFFFFFFFFFFFFFFFFFFFF:FFFFFFFFFFFFFFFFFF
FFFFFFFFFFFFFFFFFFFFFFFFFFFFFFFFFFFFFFFFFFFFFFFFFFFFFFFFFFFFFFFFFFFF
@A00155:342:HHGFNDSXY:1:1615:29234:12853 1:N:0:GAACCTAG+TCCGCATA
TACCGTCCCCTCAGGGGTCAAGAATTGCTTTGAGAGTCCATCAAGTGATGACGAACTCCCTTGAAGCG
ACGCCTGAAATTTCCCCACTGCAGTCATCTTCTGCACCGAGAAATAATTCCTAGTCGTGGTGATATTG
+

```
FFFFFFFFFFFFFFFFFFFFFFFFFFFFFFFFFFFFFFFFFFFFFFFFFFFFFFFFFFFFFFFFFFFF
FFFFFFFFFFFFFFFFFFFFFFFFFFFFFFFFFFFFFFFFFFFFFFFFFFFFFFFFFFFFFFFFFFFF
@A00155:342:HHGFNDSXY:1:1125:5719:11992 2:N:0:GAACCTAG+TCCGCATA
CCGTCCCCTCAGGGGTCAAGAATTGCTTTGAGAGTCCATCAAGTGATGACGAACTCCCTTGAAGCGAC
GCCTGAAATTTCCCCACTGCAGTCATCTTCTGCACCGAGAAATAATTCCTAGTCGTGGTGATAT
+
FFFFFFFFFFFFFFFFFFFFFFFFFFFFFFFFFFFFFFFFFFFFFFFFFFFFFFFFFFFFFFFFFFFF
FFFFFFFFFFFFFFFFFFFFFFFFFFFFFFFFFFFFFFFFFFFFFFFFFFFFFFFFFFFFFFFF
@A00155:342:HHGFNDSXY:1:1165:15519:29277 1:N:0:GAACCTAG+TCCGCATA
CCGTCCCCTCAGGGGTCAAGAATTGCTTTGAGAGTCCATCAAGTGATGACGAACTCCCTTGAAGCGAC
GCCTGAAATTTCCCCACTGCAGTCATCTTCTGCACCGAGAAATAATTCCTAGTCGTGGTGATATTGCT
+
FFFFFFFFFFFFFFFFFFFFFFFFFFFFFFFFFFFFFFFFFFFFFFFFFFFFFFFFFFFFFFFFFFFF
FFFFFFFFFFFFFFFFFFFFFFFFFFFF,FFFFFFFFFFFFFFFFFFFFFFFFFFFFFFFFFFFFFFF
@A00155:342:HHGFNDSXY:1:1557:21034:22388 1:N:0:GAACCTAG+TCCGCATA
CCGTCCCCTCAGGGGTCAAGAATTGCTTTGAGAGTCCATCAAGTGATGACGAACTCCCTTGAAGCGAC
GCCTGAAATTTCCCCACTGCAGTCATCTTCTGCACCGAGAAATAATTCCTAGTCGTGGTGATATTGCT
+
FFFFFFFFFFFFFFFFFFFFFFFFFFFFFFFFFFFFFFFFFFFFFFFFFFFFFFFFFFFFFFF:FFFF
FFFFFFFFFFFFFFFFFFFFFFFFFFFFFFFFFFFFFFFFFFFF:FFFFFFFFFFFFFFFFFFFFF:F
@A00155:342:HHGFNDSXY:1:2337:29017:22310 2:N:0:GAACCTAG+TCCGCATA
CCGTCCCCTCAGGGGTCAAGAATTGCTTTGAGAGTCCATCAAGTGATGACGAACTCCCTTGAAGCGAC
GCCTGAAATTTCCCCACTGCAGTCATCTTCTGCACCGAGAAATAATTCCTAGTCGTGGTGATATTGCT
+
FFFFFFFFFFFFFFFFFFF:F:FFFFFFFFFFFFFFFFFFFFFFFFFFFFFFF:FFFFFF:FFFFFFF
:FFFFFFFFFFF:FF:FFF:FFFFFF,:FFFF:FFFFFFFF:FFFFFF,FF::F,FF,FFFFFFFFFF
@A00155:342:HHGFNDSXY:1:1323:23547:8860 1:N:0:GAACCTAG+TCCGCATA
CGTCCCCTCAGGGGTCAAGAATTGCTTTGAGAGTCCATCAAGTGATGACGAACTCCCTTGAAGCGACG
CCTGAAATTTCCCCACTGCAGTCATCTTCTGCACCGAGAAATAATTCCTAGTCGTGGTGATATTGCTG
+
FFFFFFFFFFFFFFFFFFFFFFFFFFFFFFFFFFFFFFFFFFFFFFFFFFFFFFFFFFFFFFFFFFFF
FFFFFFFFFFFFFFFFFFFFFFFFFFFFFFFFFFFFFFFFFFFFFFFFFFFFFFFFFFFFFFFFFFFF
@A00155:342:HHGFNDSXY:1:1112:6786:29496 1:N:0:GAACCTAG+TCCGCATA
CGTCCCCTCAGGGGTCAAGAATTGCTTTGAGAGTCCATCAAGTGATGACGAACTCCCTTGAAGCGACG
CCTGAAATTTCCCCACTGCAGTCATCTTCTGCACCGAGAAATAATTCCTAGTCGTGGTGATATTGCTG
+
FFFFF,FFFFFFFFFFFF:F:FF,FFFFFFFFFFFFFFF:FFFFFFFF,FFFFFFF:FFFFFFFFFFF
FFFFFFFFFFFFFF:FFFFFFFFFFFFFFFFFFFFFFFFFFFF:FFF:FF,FFFFFFFFFF,FFFFFF
@A00155:342:HHGFNDSXY:1:1227:10917:21746 1:N:0:GAACCTAG+TCCGCATA
CGTCCCCTCAGGGGTCAAGAATTGCTTTGAGAGTCCATCAAGTGATGACGAACTCCCTTGAAGCGACG
CCTGAAATTTCCCCACTGCAGTCATCTTCTGCACCGAGAAATAATTCCTAGTCGTGGTGATATTGCTG
+
FFFFFFFFFFFFFFFFF:FFFFFFFFFFFFF:FFFFFFFFFFFFFFFFFFFFFFFFFFFFFFFFFFFF
FFFFFFFFFFFFFFFFFFFFFFFFFFFFFFFFFFFFFFFFFFFFFFFFFFFFFFFFFFFFFFFFFFFF
@A00155:342:HHGFNDSXY:1:1131:14714:7999 1:N:0:GAACCTAG+TCCGCATA
TCCCCTCAGGGGTCAAGAATTGCTTTGAGAGTCCATCAAGTGATGACGAACTCCCTTGAAGCGACGCC
TGAAATTTCCCCACTGCAGTCATCTTCTGCACCGAGAAATAATTCCTAGTCGTGGTGATATTGCTGTC
+
FFFFFFF:FFFFFFFFFFFFFFFFFFFFFFFFFFFFFFFFFFFFFFFFFFFFFFFFFFFFFFFFFFFF
FFFFFFFFFFFFFFFFFFFFFFFFFFFFFFFFFFFFFFFFFFFFFFF,FFFFFFFFFFFFFFFFFFFF
@A00155:342:HHGFNDSXY:1:2661:29559:9439 2:N:0:GAACCTAG+TCCGCATA
CCCCTCAGGGGTCAAGAATTGCTTTGAGAGTCCATCAAGTGATGACGAACTCCCTTGAAGCGACGCCT
GAAATTTCCCCACTGCAGTCATCTTCTGCACCGAGAAATAATTCCTAGTCGTGGTGATATTGCTGTC
+
```

FFFFFFFFFFFFFFFFFFFFFFFFFFFFFFFFFFFFFFFFFFFFFFFFFFFFFFFFFFFFFFFFFFFF
FFFFFFFFFFFFFFFFFF,F,FFFFFFFFFFFFFFFFFFFF:FFFFFFFFFFFFFFFFFFFFFFFFF
@A00155:342:HHGFNDSXY:1:1542:2465:29465 2:N:0:GAACCTAG+TCCGCATA
CCCCTCAGGGGTCAAGAATTGCTTTGAGAGTCCATCAAGTGATGACGAACTCCCTTGAAGCGACGCCT
GAAATTTCCCCACTGCAGTCATCTTCTGCACCGAGAAATAATTCCTAGTCGTGGTGATATTGCTGTCA
+
FFFFFF,FFF:FFFFFFFF:FF:FFFFFFFFFFFFFF:F:FFFFFFFFFFFFFFFFFFFFFFFFFFFF
FFFFF:FFFFFFF::FFFFF:FFFFFFFFFFFFFFFFFFFFFFFFFFFFFFFFFFFFFFFFFFFF:FF
@A00155:342:HHGFNDSXY:1:1672:16586:12493 1:N:0:GAACCTAG+TCCGCATA
CCCTCAGGGGTCAAGAATTGCTTTGAGAGTCCATCAAGTGATGACGAACTCCCTTGAAGCGACGCCTG
AAATTTCCCCACTGCAGTCATCTTCTGCACCGAGAAATAATTCCTAGTCGTGGTGATATTGCTGTCAT
+
FFFFFFFFFFFFFFFFFFFFFFFFFFFFFFFFFFFFFFFFFFFFFFFFFFFFFFFFFFFFFFFFFFFF
FFFFFFFFFFFFFFF:FFFFFFFFFFFFFFFFFFFF:FFFFFFFFFFFFFFFFFFF,FFFFFFFFFFF
@A00155:342:HHGFNDSXY:1:1645:9435:4304 1:N:0:GAACCTAG+TCCGCATA
CCCTCAGGGGTCAAGAATTGCTTTGAGAGTCCATCAAGTGATGACGAACTCCCTTGAAGCGACGCCTG
AAATTTCCCCACTGCAGTCATCTTCTGCACCGAGAAATAATTCCTAGTCGTGGTGATATTGCTGTCAT
+
FFFFFFFFFFFFFFFFFFFFFFFFFFFFFFFFFFFFFFFFFFFFFFFFFFFFFFFFFFFFFFFFFFFF
FFFFFFFFFFFFFFFFFFFFFFFFFFFFFFFFFFFF,FFFFFFFFFFFFFFFFFFFFFFFFFFFFFFF
@A00155:342:HHGFNDSXY:1:1418:27950:17957 1:N:0:GAACCTAG+TCCGCATA
CCCTCAGGGGTCAAGAATTGCTTTGAGAGTCCATCAAGTGATGACGAACTCCCTTGAAGCGACGCCTG
AAATTTCCCCACTGCAGTCATCTTCTGCACCGAGAAATAATTCCTAGTCGTGGTGATATTGCTGTCAT
+
FFFFFFFFFFFFFFFFFFFFFFFFFFFFFFFFFFFFFFFFFFFFFFFFFFFFFFFFFFFFFFFFFFFF
FFFFFFFFFFFFFFFFFFFFFFFFFFFFFFFFFFFFFFFFFFFFFFFFFFFFFFFF,F:FFFFFFFFF
@A00155:342:HHGFNDSXY:1:1464:26268:5274 2:N:0:GAACCTAG+TCCGCATA
CTCAGGGGTCAAGAATTGCTTTGAGAGTCCATCAAGTGATGACGAACTCCCTTGAAGCGACGCCTGAA
ATTTCCCCACTGCAGTCATCTTCTGCACCGAGAAATAATTCCTAGTCGTGGTGATATTGCT
+
FFFF:FFFFFFFFFF:FFFFFFFFFFFFFFFFFF,:FFFFFFFFFFFFFFFFFFF:FFFFFFFFFFFF
F,FFFFFFFFFFFFF,FF:FFFFFFFFFFFFFFFFFFFFFFFFFFFFFFFFFFFFFFFFFF
@A00155:342:HHGFNDSXY:1:1338:26359:26412 2:N:0:GAACCTAG+TCCGCATA
CTCAGGGGTCAAGAATTGCTTTGAGAGTCCATCAAGTGATGACGAACTCCCTTGAAGCGACGCCTGAA
ATTTCCCCACTGCAGTCATCTTCTGCACCGAGAAATAATTCCTAGTCGTGGTGATATTGCTGTCAT
+
FFFFFFFFFFFFFFFF:FFFFFFFFFFFFFFFFFFFFFFFFFFFFFFFFFFFFFFFFFFFFFFFFFFF
FFFFFFFFFF:FFFFFFFFFFFFFFFFFFFFFFFFFFFFFFFFFFFFFFFFFFFFFFF
@A00155:342:HHGFNDSXY:1:2213:10800:8328 2:N:0:GAACCTAG+TCCGCATA
TCAGGGGTCAAGAATTGCTTTGAGAGTCCATCAAGTGATGACGAACTCCCTTGAAGCGACGCCTGAAA
TTTCCCCACTGCAGTCATCTTCTGCACCGAGAAATAATTCCTAGTCGTGGTGATATTGCTGTCATTCA
+
:F,FFFFFFFFFF:FFFF:F,FFFFFFFF:FF::F:FFFFFFF:FF,FFF::FF,FFFFFFFFFFFFF
F,FFFFFFFFFFFFFF,FFFFFFFFFFFF:FFFFFFFFFFFFFFFFFFFFFFFFFFFFFFFFFFFFFF
@A00155:342:HHGFNDSXY:1:1537:30544:11209 2:N:0:GAACCTAG+TCCGCATA
TCAGGGGTCAAGAATTGCTTTGAGAGTCCATCAAGTGATGACGAACTCCCTTGAAGCGACGCCTGAAA
TTTCCCCACTGCAGTCATCTTCTGCACCGAGAAATAATTCCTAGTCGTGGTGATATTGCTGTCATTCA
+
FFFFFFFFFFFFFFFFFFFFFFFFFFFFFFFF:FFFFFFFFFFFFFFFFFFFFFFFFFFFFFFFFFFF
FFFFFFFFF:F:FFFFF:F,FFFFFFFFFFFFFFFFF,FFFFFFFFFFFFFFFFFFFFFFFFFFFFFF
@A00155:342:HHGFNDSXY:1:1206:14751:8312 2:N:0:GAACCTAG+TCCGCATA
CAGGGGTCAAGAAGTGCTTTGAGAGTCCATCAAGTGATGACGAACTCCCTTGAAGCGACGCCTGAAAT
TTCCCCACTGCAGTCATCTTCTGCACCGAGAAATAATTCCTAGTCGTGGTGATATTGCTGTCATTCAC
+

```
F,FFFFFFFFFFF,FFFFFFFFFFFFFFF:FF:FFFFFFFFF:,FFFFF::FFFFFFFFFF:FFFFF
:FFFFFFFFFFFFFFFFF:FFFFFFFFFFFFFFFFFFFFFFFFFFFFFFFFFFFFFFFFFFFFFFFFF
@A00155:342:HHGFNDSXY:1:1557:21034:22388 2:N:0:GAACCTAG+TCCGCATA
CAGGGGTCAAGAATTGCTTTGAGAGTCCATCAAGTGATGACGAACTCCCTTGAAGCGACGCCTGAAAT
TTCCCCACTGCAGTCATCTTCTGCACCGAGAAATAATTCCTAGTCGTGGTGATATTGCTGTCATTCAC
+
FFFFFFFFFFFFFFF:F:FFFFFFFFFFFFFFFFFFFFFFFFFFFFFFFFFFFFFFFF:FF::FFFFF
:FFFFFFF:F:FF::FFFFFFFFFFFFFFFFFFFFFFFFFFFFFFFFFFFFFFF:FFFF:FFF:FFFF
@A00155:342:HHGFNDSXY:1:1272:20229:1360 2:N:0:GAACCTAG+TCCGCATA
CAGGGGTCAAGAATTGCTTTGAGAGTCCATCAAGTGATGACGAACTCCCTTGAAGCGACGCCTGAAAT
TTCCCCACTGCAGTCATCTTCTGCACCGAGAAATAATTCCTAGTCGTGGTGATATTGCTGTCATTCAC
+
FF,:FFFFF,FFFFFFFF::FFFFFFFFFFFF:,F:F:FFFFFFFFFFF:FF:FFFFFFFF,FFF:F
,FFFFFFFF:FFFFFFF:FFF:FFFFFFFFFFFFFFFFFFFFFFFFFFFFFFFFFFFFFFFF:FFFF
@A00155:342:HHGFNDSXY:1:2431:6542:9502 1:N:0:GAACCTAG+TCCGCATA
AGGGGTCAAGAATTGCTTTGAGAGTCCATCAAGTGACGACGAACTCCCTTGAAGCGACGCCTGAAATT
TCCCCACTGCAGTCATCTTCTGCACCGAGAAATAATTCCTAGTCGTGGTGATATTGCTG
+
FFFFFFFFFFFFFFFFFFFFFFFFFFFFFFFFFFFFFFFFFFFFFFF:FFFFFFFFFFFFFFFFFF
FFFFFFFFFFFFFFFFFFFFFFFFFFFFFFFF:FFFFFFFFFFFFFFFFFFFFFFFFFFFF
@A00155:342:HHGFNDSXY:1:2256:30183:35258 1:N:0:GAACCTAG+TCCGCATA
AGGGGTCAAGAATTGCTTTGAGAGTCCATCAAGTGATGACGAACTCCCTTGAAGCGACGCCTGAAATT
TCCCCACTGCAGTCATCTTCTGCACCGAGAAATAATTCCTAGTCGTGGTGATATTGCTGTCATTCACT
+
FFFFFFFFFFFFFFFFFFFFFFFFFFFFFFFFFFFFFFFFFFFFFFFFFFFFFFFFFFFFFFFFFFFF
FFFFFFFFFFFFFFFFFFFFFFFFFFFFFFFFFFFFFFFFFFFFFFFFFFF,,FFFFFFFFFFFFFFF
@A00155:342:HHGFNDSXY:1:2403:31882:24017 2:N:0:GAACCTAG+TCCGCATA
AGGGGTCAAGAATTGCTTTGAGAGTCCATCAAGTGATGACGAACTCCCTTGAAGCGACGCCTGAAATT
TCCCCACTGCAGTCATCTTCTGCACCGAGAAATAATTCCTAGTCGTGGTGATATTGCTGTCATTCACT
+
:FFFFFFFFFF:,FFFF:FF:FFFFFF,FFF,FFFF,FFFFF:FFFFF:FFF:FFFFFFFFFFF,:F,
FFFFF:F,FFFFFF::F:FFFFFFFFF:F:FFFFFFFFFFFFFFFFFFFFFFFFFFFFFFFF::FFFFF
@A00155:342:HHGFNDSXY:1:2417:30364:33567 2:N:0:GAACCTAG+TCCGCATA
AGGGGTCAAGAATTGCTTTGAGAGTCCATCAAGTGATGACGAACTCCCTTGAAGCGACGCCTGAAATT
TCCCCACTGCAGTCATCTTCTGCACCGAGAAATAATTCCTAGTCGTGGTGATATTGCTGTCATTCACT
+
FFFFFFFFFFF:FFFFFFFFFFFFFFFFFFFFFFFFFFFFFFFFFFFF:FFFFFFFFFFFFFFFFFF:
FFFFF,FFFFFFFF,FFFFFFFFFFFFFFFFFFFFFFFFFFFFFFFFFFFFFFFFFFFFFFFFFFFF
@A00155:342:HHGFNDSXY:1:2422:18295:31297 2:N:0:GAACCTAG+TCCGCATA
AGGGGTCAAGAATTGCTTTGAGAGTCCATCAAGTGATGACGAACTCCCTTGAAGCGACGCCTGAAATT
TCCCCACTGCAGTCATCTTCTGCACCGAGAAATAATTCCTAGTCGTGGTGATATTGCTGTCATTCACT
+
FFFFFFFFFFFFFFFFFFFFFFFFFFFFFFFFFFF:FFFFFFFFFFFF:FFFFF:FFFFFFFFFFF,F
F:FFFFFFFFFFFF:FFFFFFFFFFFFFFFFFFFFFFFFFFFFFFFFFFFFFFFFFFFFFFF:FFFFF
@A00155:342:HHGFNDSXY:1:1224:9724:24471 2:N:0:GAACCTAG+TCCGCATA
AGGGGTCAAGAATTGCTTTGAGAGTCCATCAAGTGATGACGAACTCCCTTGAAGCGACGCCTGAAATT
TCCCCACTGCAGTCATCTTCTGCACCGAGAAATAATTCCTAGTCGTGGTGATATTGCTGTCATTCACT
+
FFFFFFFFFFF:FFFFF:FFFF:FFFFFFFF,:FFFFFFFFFFFFFFFF:F,FFFFFFFFF:FFFF:F
FFFFFFFFFFFFFFF,F:FFFFFFFFFFFF,:FFFFFFFFFFFFFFFFFFFFFFFFFFFFFFFFFFFFF
@A00155:342:HHGFNDSXY:1:2334:19479:23610 1:N:0:GAACCTAG+TCCGCATA
AGGGGTCAAGAATTGCTTTGAGAGTCCATCAAGTGATGACGAACTCCCTTGAAGCGACGCCTGAAATT
TCCCCACTGCAGTCATCTTCTGCACCGAGAAATAATTCCTAGTCGTGGTGATATTGCTGTCATTCACT
+
```

FFFFFFFFFFFFFFFFFFFFFFFFFFFFFFFFFFFF:FFFFFFFFFFFFFFFFFFFFFFFFFFFFFFF
FFFFFFFFFF:FFFFFFFFFFFFFFFFFFF:FFFFFFF:FFFFFFFFFFFF,FFF:FFFFFFFFFFFF
@A00155:342:HHGFNDSXY:1:2154:1172:16360 1:N:0:GAACCTAG+TCCGCATA
AGGGGTCAAGAATTGCTTTGAGAGTCCATCAAGTGATGACGAACTCCCTTGAAGCGACGCCTGAAATT
TCCCCACTGCAGTCATCTTCTGCACCGAGAAATAATTCCTAGTCGTGGTGATATTGCTGTCATTCACT
+
FFFFFFFFFFFFFFFFFFFFFFFFFFFFFFFFFFFFFFFFFFFFFFFFFFFFFF:FFFFFFFFFFFFF
FF::FFFFFFFF:FFFFFFFFF,FFFFFFFFFFFF:FF::FFFFFFFFFFFFFFFFFFFFFFFF,FFF
@A00155:342:HHGFNDSXY:1:1525:30156:26412 2:N:0:GAACCTAG+TCCGCATA
GGGGTCAAGAATTGCTTTGAGAGTCCATCAAGTGATGACGAACTCCCTTGAAGCGACGCCTGAAATTT
CCCCACTGCAGTCATCTTCTGCACCGAGAAATAATTCCTAGTCGTGGTGATATTGCTGTCATTCACT
+
FFFFFFFFF,:::,F,F:FFFFFFFF:FFFF:FFFFFFFFFFFF,FF,FFFFFFFFFFFF,::FFFFF
FFF,FF::FFFF:FFFFFFFF,F:FFFFFFFFFFFFFFFFFFFFFFFFFFF:FFFFFFFFFFFFF:F
@A00155:342:HHGFNDSXY:1:2411:6379:17112 2:N:0:GAACCTAG+TCCGCATA
GGGGTCAAGAATTGCTTTGAGAGTCCATCAAGTGATGACGAACTCCCTTGAAGCGACGCCTGAAATTT
CCCCACTGCAGTCATCTTCTGCACCGAGAAATAATTCCTAGTCGTGGTGATATTGCTGTCATTCACT
+
FFFFFFFFF,F,:FFFFFFFFFFFFFFFFFFF:FFFFFFFFFFFFFFFFFFFFFFFFFFFFFFFF:F,
FFFFFFFFFFF:FFFFFFF,FFFFFFFFFFFFFFFFFFFFFFFFFFFFFFFFFFFFFFFFFFFFFFF
@A00155:342:HHGFNDSXY:1:1615:29234:12853 2:N:0:GAACCTAG+TCCGCATA
GGGGTCAAGAATTGCTTTGAGAGTCCATCAAGTGATGACGAACTCCCTTGAAGCGACGCCTGAAATTT
CCCCACTGCAGTCATCTTCTGCACCGAGAAATAATTCCTAGTCGTGGTGATATTGCTGTCATTCACT
+
FFFFFFFFFF:FFFFFFFFFFFFFFFFFFFFFFFFFFFFFFFFFFFF::F,FFFF:FFFF:FFFFFFF
FFFFFFFFFFF:FF:F:FF:FFFFFFFFFFFFFFFFFFFFFFFFFFFFFFFFFFFFFFFFF:FFFFF
@A00155:342:HHGFNDSXY:1:1112:6786:29496 2:N:0:GAACCTAG+TCCGCATA
GGGGTCAAGAATTGCTTTGAGAGTCCATCAAGTGATGACGAACTCCCTTGAAGCGACGCCTGAAATTT
CCCCACTGCAGTCATCTTCTGCACCGAGAAATAATTCCTAGTCGTGGTGATATTGCTGTCATTCACTC
+
FFF:FFFFFFFFFFF,FFFFFFFFFF,FFFFFFFFFFFFFFFFFFFFFFFFFFFF:FFFFFFFFFFFF
FFFFFFFFFFF:F:FFFFFFFFFFFF:FFFFFFFFF:FFFFFFFFFFFFFFFFFFFFFFFF:FFFFFF
@A00155:342:HHGFNDSXY:1:1338:12933:26083 2:N:0:GAACCTAG+TCCGCATA
GGGGTCAAGAATTGCTTTGAGAGTCCATCAAGTGATGACGAACTCCCTTGAAGCGACGCCTGAAATTT
CCCCACTGCAGTCATCTTCTGCACCGAGAAATAATTCCTAGTCGTGGTGATATTGCTGTCATTCACTC
+
FFFFFFFFFFFFFFFFF:FFFFFFFFFFFFFFFFFFFFFFFFFFFFFFFF:FFFFFFFFFFFFFFFFF
FFFF:FFFFFF:FFFF::FFFFFFFFFFFFFFFFFFFFFFFFFFFFFFFFFFFFFFFFFFFFFFFFFF
@A00155:342:HHGFNDSXY:1:1201:8440:10755 2:N:0:GAACCTAG+TCCGCATA
GGGTCAAGAATTGCTTTGAGAGTCCATCAAGTGATGACGAACTCCCTTGAAGCGACGCCTGAAATTTC
CCCACTGCAGTCATCTTCTGCACCGAGAAATAATTCCTAGTCGTGGTGATATTGCTGTCATTCACTCG
+
FFFFFFFFFFFFFFFFFFFFFFFFFFFFFFF:FFFFFF:FFFFFFFFFFFFFFFFFFFFFFFFFF:FF
FFFFFFFFFFFFF:FFFFFFFFFFFFFFFFFFFFFFFFFFFFFFFFFFFFFFFFFFFFFFFFFFFFFF
@A00155:342:HHGFNDSXY:1:1609:7997:25238 2:N:0:GAACCTAG+TCCGCATA
GGGTCAAGAATTGCTTTGAGAGTCCATCAAGTGATGACGAACTCCCTTGAAGCGACGCCTGAAATTTC
CCCACTGCAGTCATCTTCTGCACCGAGAAATAATTCCTAGTCGTGGTGATATTGCTGTCATTCACTCG
+
FFFFFFFFFF,FFF:FFFFFFFFFFFFFFFFFFFFFFFFFFFFFFFFFFFFFFFFFFFFFFFF:FFFF
FFFFFFFFFF,FFFFFFFFFFFFFFFFFFFFFFFFFFFFFFFFFFFFFFFFFFFF:FFFFFFFFFFFF
@A00155:342:HHGFNDSXY:1:2533:6705:17926 2:N:0:GAACCTAG+TCCGCATA
GGTCAAGAATTGCTTTGAGAGTCCATCAAGTGATGACGAACTCCCTTGAAGCGACGCCTGAAATTTCC
CCACTGCAGTCATCTTCTGCACCGAGAAATAATTCCTAGTCGTGGTGATATTGCTGTCATTCACTCGC
+

```
FF:FFFFFFFFFFF::FFFFFFFFFFF:FFFFFFFFFFFFFFFFF,,FF:FFFFFFFFFFFF:FFFFF
FF,FFFFFFFFF:FFFF:FFFFFFFFFFFFFFFFFFFFFFFFFFFFFFFFFFFFFFFFFFFFFFFFFF
@A00155:342:HHGFNDSXY:1:2622:17029:16736 2:N:0:GAACCTAG+TCCGCATA
GGTCAAGAATTGCTTTGAGAGTCCATCAAGTGATGACGAACTCCCTTGAAGCGACGCCTGAAATTTCC
CCACTGCAGTCATCTTCTGCACCGAGAAATAATTCCTAGTCGTGGTGATATTGCTGTCATTCACTCGC
+
FFFFFFFFFFFFFFFFFFFFFFFFFFFFFFFFFFFFFFFFFFFFF,FFFFFFFFFFFFFFFFF,FFFF
FFFFFFFFFFFFF:FFFFFFFFFFFFFFFFFFFFFFFFFFFFFFFFFFFFFFFFFFFFF:FFFFFFFF
@A00155:342:HHGFNDSXY:1:1533:6117:26804 2:N:0:GAACCTAG+TCCGCATA
GGTCAAGAAGTGCTTTGAGAGTCCATCAAGTGATGACGAACTCCCTTGAAGCGACGCCTGAAATTTCC
CCACTGCAGTCATCTTCTGCACCGAGAAATAATTCCTAGTCGTGGTGATATTGCTGTCATTCACTCGC
+
FFFFFFFFF,FFF:FFFFFFFFFFFFFFFFFFFFFFFFFF:F:FFFF::FFFFFFFFFFFFFFFFFFF
FFFFFFFFF:FFFFFFF:FFFFFFFFFFFFFFFFFFFFFFFFFFFFFFFFFFFFFFFFFF:FFFFFFF
@A00155:342:HHGFNDSXY:1:2147:3613:21245 2:N:0:GAACCTAG+TCCGCATA
GTCAAGAATTGCTTTGAGAGTCCATCAAGTGATGACGAACTCCCTTGAAGCGACGCCTGAAATTTCCC
CACTGCAGTCATCTTCTGCACCGAGAAATAATTCCTAGTCGTGGTGATATTGCTGTCATTCACTCG
+
FFFFF,FFFFF:FFFFF:FFFFFFFFFFFFFFFFFFFFFFFFFFFFFFFFFFFFFFFFFFFFFFFFFF
FFF:FFFF,F:FFFFFFFFFFFFFFFFFFFFFFFFFFFFFFFFFFFFFFFFFFFFFFFFFFFFFFFF
@A00155:342:HHGFNDSXY:1:2547:10330:25864 1:N:0:GAACCTAG+TCCGCATA
GTCAAGAATTGCTTTGAGAGTCCATCAAGTGATGACGAACTCCCTTGAAGCGACGCCTGAAATTTCCC
CACTGCAGTCATCTTCTGCACCGAGAAATAATTCCTAGTCGTGGTGATATTGCTGTCATTCACTCGCA
+
FFFFFFFFFFFFFFFFFFFFFFFFFFFFFFFFFFFFFFFFFFFFFFFFFFFFFFFFFFFFFFFFFFFF
FFFFFFFFFFFFFFFFFFFFFFFFFFFFFFFFFFFFFFFFFFFFFFFFFFFFFFFFFFFFFFFFFFFF
@A00155:342:HHGFNDSXY:1:1423:30508:7921 1:N:0:GAACCTAG+TCCGCATA
GTCAAGAATTGCTTTGAGAGTCCATCAAGTGATGACGAACTCCCTTGAAGCGACGCCTGAAATTTCCC
CACTGCAGTCATCTTCTGCACCGAGAAATAATTCCTAGTCGTGGTGATATTGCTGTCATTCACTCGCA
+
FFFFFFFFFFFFFFFFFFFFFFFFFFFFFFFFFFFFFFFFF:FFFF:FFFFFFFFFFFFFFFFFFFFF
FFFFFFFFFFFFFFFFFFFFFFFFFFFFFFFFFFFFFFFFFFFFFFFFFFFFFFFFFFFFFFFFFFFF
@A00155:342:HHGFNDSXY:1:1555:20112:16814 1:N:0:GAACCTAG+TCCGCATA
GTCAAGAATTGCTTTGAGAGTCCATCAAGTGATGACGAACTCCCTTGAAGCGACGCCTGAAATTTCCC
CACTGCAGTCATCTTCTGCACCGAGAAATAATTCCTAGTCGTGGTGATATTGCTGTCATTCACTCGCA
+
FFFFFFFFFFFFFFFFFFFFFFFFFFFFFFFFFFFFFFFFFFFFFFFFFFFFFFFFFFFFFFFFFFFF
FFFFFFFFFFFFFFFFFFFFFFFFFFFFFFFFFFFFFFFFFFFFFFFFFFFFFFFFFFFFFFFFFFF:
@A00155:342:HHGFNDSXY:1:1272:20229:1360 1:N:0:GAACCTAG+TCCGCATA
GTCAAGAATTGCTTTGAGAGTCCATCAAGTGATGACGAACTCCCTTGAAGCGACGCCTGAAATTTCCC
CACTGCAGTCATCTTCTGCACCGAGAAATAATTCCTAGTCGTGGTGATATTGCTGTCATTCACTCGCA
+
FFFFFFFFFFFFFFFFFFFFFFFFFFFFFFFFFFFFFFFFFFFFFFFFFFFFFFFFFFFFFFFFFFFF
FFFFFFFFFFFFFFFFFFFFFFFFFFFFFFFFFFFFFFFFFFFFFFFF,FFFFFFFFFFFFFFFF:FFF
@A00155:342:HHGFNDSXY:1:1235:24198:33567 1:N:0:GAACCTAG+TCCGCATA
GTCAAGAATTGCTTTGAGAGTCCATCAAGTGATGACGAACTCCCTTGAAGCGACGCCTGAAATTTCCC
CACTGCAGTCATCTTCTGCACCGAGAAATAATTCCTAGTCGTGGTGATATTGCTGTCATTCACTCGCA
+
FFFFFFFFFFFFFFFFFFFFFFFFFFFFFFFFFFFFFFFFFFFFFFFFFFFFFFFFFFFFFFFFFFFF
FFFFFFFFF:FFFFFFFFFFFFFFFFFFFFFFFFFFFFFFFFFFFFFF,FFFFFFFFFFFFFFFFFFFF
@A00155:342:HHGFNDSXY:1:2510:12382:10786 2:N:0:GAACCTAG+TCCGCATA
GTCAAGAATTGCTTTGAGAGTCCATCAAGTGATGACGAACTCCCTTGAAGCGACGCCTGAAATTTCCC
CACTGCAGTCATCTTCTGCACCGAGAAATAATTCCTAGTCGTGGTGATATTGCTGTCATTCACTCGCA
+
```

```
FFF:FFFF:FFF:::FFFFFFFFF,FFFFFFFFFFFF:FFFFFFFFFFFFFFFFFFF:FFFF:FFFFF
FFFFFFFFFFFFFFFFFFFFFFFFFFFFFFFFFFFFFFFFFFFFFFFFFFFFFFFFFFFFFFFFFFFF
@A00155:342:HHGFNDSXY:1:1314:32624:18505 1:N:0:GAACCTAG+TCCGCATA
GTCAAGAATTGCTTTGAGAGTCCATCAAGTGATGACGAACTCCCTTGAAGCGACGCCTGAAATTTCCC
CACTGCAGTCATCTTCTGCACCGAGAAATAATTCCTAGTCGTGGTGATATTGCTGTCATTCACTCGCA
+
FFFFFFFFFFFFF:FFFFFFFFFFFFFFFFFFFFFFF:FF,FFF:FFFFFFFFFFFFFFFFFFFFFFF
FFFFFFFFFFFFFFFFFFFFFFFFFFFFFFFFFFFF:FFF:FFFFF,,FFFFFFFFFFFFFFFFFFFF
@A00155:342:HHGFNDSXY:1:1219:17201:35102 1:N:0:GAACCTAG+TCCGCATA
GTCAAGAATTGCTTTGAGAGTCCATCAAGTGATGACGAACTCCCTTGAAGCGACGCCTGAAATTTCCC
CACTGCAGTCATCTTCTGCACCGAGAAATAATTCCTAGTCGTGGTGATATTGCTGTCATTCACTCGCA
+
FFFFFFFFFFFFFFFFFFFFFFFFFFFFFFFFFFFFFFFFFFFFFFFFFFFFFFFFFFFFFFFFFF:F
FFFFFFFFFFFFFFFFFFFFFFFFFFFFFFFFFFFFFFFFFFFFFFFFFFFFFFFFFF:FFFFFFFFF
@A00155:342:HHGFNDSXY:1:1555:21151:17237 1:N:0:GAACCTAG+TCCGCATA
GTCAAGAATTGCTTTGAGAGTCCATCAAGTGATGACGAACTCCCTTGAAGCGACGCCTGAAATTTCCC
CACTGCAGTCATCTTCTGCACCGAGAAATAATTCCTAGTCGTGGTGATATTGCTGTCATTCACTCGCA
+
FFFFFFFFFFFFFFFFFFFFFFFFFFFFFFFFFFFFFFFFFFFFFFFFFFFFFFFFFFFFFFFFFFFF
FFFFFFFFFFFFFFFFFFFFFFFFFFFFFFFFFFFFFFFFFFFFFFFF:FFFFFFFFFFFFFFFFFFF
@A00155:342:HHGFNDSXY:1:1125:5719:11992 1:N:0:GAACCTAG+TCCGCATA
TCAAGAATTGCTTTGAGAGTCCATCAAGTGATGACGAACTCCCTTGAAGCGACGCCTGAAATTTCCCC
ACTGCAGTCATCTTCTGCACCGAGAAATAATTCCTAGTCGTGGTGATATTGCTGTCATTCACTC
+
FFFF:FFFFFFFFFFFFFFFFFF:FFFFF,FFFFFFFF:FFFFFFFFFFFFFFFFFFFFFFFFFFFFF
FFFFFFFFFFFFFFFFFFFFFFFFFFFFFFFFFFFFFFFFFFFFF,FF:FFF:FFFFFFFFFFF
@A00155:342:HHGFNDSXY:1:2276:17644:4899 1:N:0:GAACCTAG+TCCGCATA
TCAAGAATTGCTTTGAGAGTCCATCAAGTGATGACGAACTCCCTTGAAGCGACGCCTGAAATTTCCCC
ACTGCAGTCATCTTCTGCACCGAGAAATAATTCCTAGTCGTGGTGATATTGCTGTCATTCACTCGCAG
+
FFFFFFFFFFFFFFFFFFFFFFFFFFFFFFFFFFFFFFFFFFFFFFFFFFFFFFFFFFFFFFFFFFFF
FFFFFFFFFFFFFFFFFFFFFFFFFFFFFFFFFFFFFFFFFFFFFFFFFFFFFFFFFFFF:FFFFFFF
@A00155:342:HHGFNDSXY:1:1130:7934:21966 1:N:0:GAACCTAG+TCCGCATA
TCAAGAATTGCTTTGAGAGTCCATCAAGTGATGACGAACTCCCTTGAAGCGACGCCTGAAATTTCCCC
ACTGCAGTCATCTTCTGCACCGAGAAATAATTCCTAGTCGTGGTGATATTGCTGTCATTCACTCGCAG
+
FFFFFFFFFFFFFFFFFFFF:FFFFFFFFFFFFFFFFFFFF:FFFFFFFFFFFFFFFFFFFFFFFF:F
FFFFFFFFFFFFFF:FF:FFFFFFFF:FFFFFFFFFFFFFFFFF:FFFFFFFFFFFFFF:FFFFFFFFF
@A00155:342:HHGFNDSXY:1:2411:6379:17112 1:N:0:GAACCTAG+TCCGCATA
TCAAGAATTGCTTTGAGAGTCCATCAAGTGATGACGAACTCCCTTGAAGCGACGCCTGAAATTTCCCC
ACTGCAGTCATCTTCTGCACCGAGAAATAATTCCTAGTCGTGGTGATATTGCTGTCATTCACTCGCAG
+
FFFFFFFFFFFFFFFFFFFFFFFFFFFFFFF:FFFFFFFFFFF:FFFFFFFFFFFFFFFFFFFFFFFF
FFFFFFFFFFFFFFFFFFFFFFFFFFFFFFFFFFFFFFFFFFFFFF:FFFFFFFFFFFFFFFFFFFFF
@A00155:342:HHGFNDSXY:1:1566:27073:9518 1:N:0:GAACCTAG+TCCGCATA
TCAAGAATTGCTTTGAGAGTCCATCAAGTGATGACGAACTCCCTTGAAGCGACGCCTGAAATTTCCCC
ACTGCAGTCATCTTCTGCACCGAGAAATAATTCCTAGTCGTGGTGATATTGCTGTCATTCACTCGCAG
+
FFFFFFFFFFFFFFFFFFFFFFFFFFFFFFFFFFFFFFFFFFFFFFFFFFFFFFFFFFFFFFFFFFFF
FFFFFFFFFFFFFFFFFFFFFFFFFFFFFFFFFFFFFFFFFFFFFFFFFFFFFFFFFFFFFFFFFFFF
@A00155:342:HHGFNDSXY:1:1528:30915:10191 1:N:0:GAACCTAG+TCCGCATA
CAAGAATTGCTTTGAGAGTCCATCAAGTGATGACGAACTCCCTTGAAGCGACGCCTGAAATTTCCCCA
CTGCAGTCATCTTCTGCACCGAGAAATAATTCCTAGTCGTGGTGATATTGCTGTCATTCACTCGCAGC
+
```

```
FFFFFFFFFFFFFFFFFFFFFFFFFFFFFF:FFFFFFFFFFFFFFFFFFFFFFFFFFFFFFFFFFFFF
FFFFFFFFFFFFFFFFFFFFFFFFFFFFFFFFFFFFFFFFFFFF:FF:FFFFFFFFFFFFFFFFFFFF
@A00155:342:HHGFNDSXY:1:2243:12906:6277 1:N:0:GAACCTAG+TCCGCATA
CAAGAATTGCTTTGAGAGTCCATCAAGTGATGACGAACTCCCTTGAAGCGACGCCTGAAATTTCCCCA
CTGCAGTCATCTTCTGCACCGAGAAATAATTCCTAGTCGTGGTGATATTGCTGTCATTCACTCGCAGC
+
FFFFFFFFFFFFFFFFFFFFFFFFFFFFFFFFFFFFFFFFFFFFFFFFFFFFFFFFFFFFFFFFFFFF
FFFFFFFFFFFFFFFFFFFFFFFFFFFF:F:FF,FFFFFFFFFFF:F:FF::FFFFFFFFFFFFFFFF
@A00155:342:HHGFNDSXY:1:2243:11758:4914 1:N:0:GAACCTAG+TCCGCATA
CAAGAATTGCTTTGAGAGTCCATCAAGTGATGACGAACTCCCTTGAAGCGACGCCTGAAATTTCCCCA
CTGCAGTCATCTTCTGCACCGAGAAATAATTCCTAGTCGTGGTGATATTGCTGTCATTCACTCGCAGC
+
FFFFFFFFFFFFFFFFFFFFFFFFFFFFFFFFFFFFFFFFFFFFFFFFFFFFFFFFF:FFFFFFFFFF
FFFFFFFFFFFFFFFFF:FFFFFFFFFFFFFFFFFFFFFFFFFFFFFFFFFFFFFFFFFFFFFFFFFF
@A00155:342:HHGFNDSXY:1:1533:1036:35884 1:N:0:GAACCTAG+TCCGCATA
CAAGAATTGCTTTGAGAGTCCATCAAGTGATGACGAACTCCCTTGAAGCGACGCCTGAAATTTCCCCA
CTGCAGTCATCTTCTGCACCGAGAAATAATTCCTAGTCGTGGTGATATTGCTGTCATTCACTCGCAGC
+
FFFFFFFFFF,,FFFFFFFFFF,F:FFFF:FFFFFFFFFFFF,:FFFFFFFFFFFFFFFFF:FFFFFF
FFFF:FFFF:F:FF:FF,FFFFFF:FF,F::FF:FFFFFFFF,FFF,FFFFF::F:F:FFFFFFFFFF
@A00155:342:HHGFNDSXY:1:2301:5303:19977 1:N:0:GAACCTAG+TCCGCATA
CAAGAATTGCTTTGAGAGTCCATCAAGTGATGACGAACTCCCTTGAAGCGACGCCTGAAATTTCCCCA
CTGCAGTCATCTTCTGCACCGAGAAATAATTCCTAGTCGTGGTGATATTGCTGTCATTCACTCGCAGC
+
FF:FFFFFFFFFFFFFFFFFFFFFFFFFFFFFFFFFFFFFFFFFFFFFFFFFFFFFFFFFFFFFFFF
FFFFFFFFFFFFFFFFFFFFFFFFFFFFFFFFFFFFFFFFFFFFFFFFFFFFFFFFFFFFFFFFFFFF
@A00155:342:HHGFNDSXY:1:2416:13539:6715 2:N:0:GAACCTAG+TCCGCATA
CAAGAATTGCTTTGAGAGTCCATCAAGTGATGACGAACTCCCTTGAAGCGACGCCTGAAATTTCCCCA
CTGCAGTCATCTTCTGCACCGAGAAATAATTCCTAGTCGTGGTGATATTGCTGTCATTCACTCGCAGC
+
FFFFFF:FFFFFFFFFFFFFFFFFFFFFFFFFFFFFFFFFFFFFFFFFFFFFFFFFF:FFFFFFFF:
FFFFFFFFFFF:FFFFFFFFFFFFFFFFFFFFFFFFFFFFFFFFFFFFFFFFFFFFFFFFFFFFFFFF
@A00155:342:HHGFNDSXY:1:2243:12906:6277 2:N:0:GAACCTAG+TCCGCATA
CAAGAATTGCTTTGAGAGTCCATCAAGTGATGACGAACTCCCTTGAAGCGACGCCTGAAATTTCCCCA
CTGCAGTCATCTTCTGCACCGAGAAATAATTCCTAGTCGTGGTGATATTGCTGTCATTCACTCGCAGC
+
FFFFFF,FFF:F:FFFFFFFFFFFFFFFFFFFFFFFFFFFFFF:FFFFFFFFFFFFFFFFFFFFFFF:
FFFFFF,F:FFFFFFFFFFFFFFFFFFFFFFFFFFFFFFFFFFFFFFFFFFFFFFFFFFFFFFFFFFF
@A00155:342:HHGFNDSXY:1:2243:11758:4914 2:N:0:GAACCTAG+TCCGCATA
CAAGAATTGCTTTGAGAGTCCATCAAGTGATGACGAACTCCCTTGAAGCGACGCCTGAAATTTCCCCA
CTGCAGTCATCTTCTGCACCGAGAAATAATTCCTAGTCGTGGTGATATTGCTGTCATTCACTCGCAGC
+
FFFFFFFFFFFFFFFFFFFFFFFFFF:FFFFFFFFFFFFFFF,,FFFFFFFFFFFFFFFFFFFFFFFF
F:FFFFFFFFFFFFFFFFFFFFFFFFFFFFFFFFFFFFFFFFFFFFFFFFFFFFF:FFFFFFFFFFF
@A00155:342:HHGFNDSXY:1:1509:11505:27524 1:N:0:GAACCTAG+TCCGCATA
CAAGAATTGCTTTGAGAGTCCATCAAGTGATGACGAACTCCCTTGAAGCGACGCCTGAAATTTCCCCA
CTGCAGTCATCTTCTGCACCGAGAAATAATTCCTAGTCGTGGTGATATTGCTGTCATTCACTCGCAGC
+
FFFFFFFFFFFFFFFFFFFFFFFFFFFFFFFFFFFFFFFFFFFFFFFFFFFFFFFFFFFFFFFFFFFF
FFFFFFFFFFFFFFFFFFFFFFFFFFFFFFFFFFFF:FFFFF:FFF:FF::FFFFFFFFFFFFFFFFF
@A00155:342:HHGFNDSXY:1:2108:17499:11443 1:N:0:GAACCTAG+TCCGCATA
CAAGAATTGCTTTGAGAGTCCATCAAGTGATGACGAACTCCCTTGAAGCGACGCCTGAAATTTCCCCA
CTGCAGTCATCTTCTGCACCGAGAAATAATTCCTAGTCGTGGTGATATTGCTGTCATTCACTCGCAGC
+
```

```
FFFFFFFFFFFFFFFFFFFFFFFFFFFFFFFFFFFFFFFFFFFFFFFFFFFFFFFFFFFFFFFFFFFF
FFFFFFFFFFFFFFFFFFFFFFFFFFFFFFFFFFFFFFFFFFFFFFFFFFFFFFFFFFFFFFFFFFFF
@A00155:342:HHGFNDSXY:1:1357:1796:3004 1:N:0:GAACCTAG+TCCGCATA
AAGAATTGCTTTGAGAGTCCATCAAGTGATGACGAACTCCCTTGAAGCGACGCCTGAAATTTCCCCAC
TGCAGTCATCTTCTGCACCGAGAAATAATTCCTAGTCGTGGTGATATTGCTGTCATTCACTCGCAGCA
+
FFFFFFFFFFFFFFFFFFFFFFFFFFFFFFFFFFFFFFFF:FFFFFFFFFFFFFFFFFFFFFFFFF:F
FFFFFFFFFFFFFFFFFFFFFFFFFFFFFFFFFFFFFFFFFFFFFFFFFFFFFFFFFFFFFFFFFF:F
@A00155:342:HHGFNDSXY:1:1321:10926:36479 1:N:0:GAACCTAG+TCCGCATA
AAGAATTGCTTTGAGAGTCCATCAAGTGATGACGAACTCCCTTGAAGCGACGCCTGAAATTTCCCCAC
TGCAGTCATCTTCTGCACCGAGAAATAATTCCTAGTCGTGGTGATATTGCTGTCATTCACTCGCAGCA
+
F:FFFFFF:FFFFFFFFFFFFFFFFFFFFFFFFFFFFFFFFFFFFFFFFFFFFFFFFFFFFFFFFFFF
FFFFFFFFFFFFFFFFFFFFFFFFFFFFFFFFFFFFFFFFFFFFFFFFFFFFFFFFFFFFFFFFFFFF
@A00155:342:HHGFNDSXY:1:1315:19940:35650 1:N:0:GAACCTAG+TCCGCATA
AAGAATTGCTTTGAGAGTCCATCAAGTGATGACGAACTCCCTTGAAGCGACGCCTGAAATTTCCCCAC
TGCAGTCATCTTCTGCACCGAGAAATAATTCCTAGTCGTGGTGATATTGCTGTCATTCACTCGCAGCA
+
FFFFFFFFFFFFFFFFFFFFFFFFFFFFFFFFFFFFFFFFFFFFFFFFFFFFFFFFFFFFFFFFFFFF
FFFFFFFFFFFFFFFFFFFFFFFFFFFFFFFF:FFFFFFFFFFFFF:FFFFFFF:FFFFFFFFFFFFF
@A00155:342:HHGFNDSXY:1:1229:30400:8296 1:N:0:GAACCTAG+TCCGCATA
AAGAATTGCTTTGAGAGTCCATCAAGTGATGACGAACTCCCTTGAAGCGACGCCTGAAATTTCCCCAC
TGCAGTCATCTTCTGCACCGAGAAATAATTCCTAGTCGTGGTGATATTGCTGTCATTCACTCGCAGCA
+
FFFFFFFFFFFFFFFFFFFFFFFFFFFFFFFFFFFFFFFFFFFFF:FFFFFFFFFFFFFFFFFFFFFF
FFFFFFFFFFFFFFFFFFFFFFFFFF:FFFFFFFFFFFFFF:FF:FFFFFFFFFFFFFFFFFFFFFFF
@A00155:342:HHGFNDSXY:1:1206:16667:21339 1:N:0:GAACCTAG+TCCGCATA
AAGAATTGCTTTGAGAGTCCATCAAGTGATGACGAACTCCCTTGAAGCGACGCCTGAAATTTCCCCAC
TGCAGTCATCTTCTGCACCGAGAAATAATTCCTAGTCGTGGTGATATTGCTGTCATTCACTCGCAGCA
+
FFFFFF:FFFFFFFFFFFFFFFFFFFFFFFFFFFFFFFFFFFFFFFFFFFFFFFFFFFFFFFFFFFFF
FFFFFFFFFFFFFFFFFFFFFFF:FFFFFFFFFFFFFFFFFFFFFFFFFFFFFFFFFFFFFFFFFFFF
@A00155:342:HHGFNDSXY:1:1341:18358:15718 1:N:0:GAACCTAG+TCCGCATA
AAGAATTGCTTTGAGAGTCCATCAAGTGATGACGAACTCCCTTGAAGCGACGCCTGAAATTTCCCCAC
TGCAGTCATCTTCTGCACCGAGAAATAATTCCTAGTCGTGGTGATATTGCTGTCATTCACTCGCAGCA
+
FFFFFFFFFF:FFFFFFFFFFFFFFFFFFFFFFFFFFFFFFFFFFFFFFFFFFFFFFFFFFFFFFFFF
FFFFFFFFFFFFFFFFFFFFFFFFFFFFFFFFFFFFFFFFFFFFFF:FFFFF,FFFFFFFFFFFFFFF
@A00155:342:HHGFNDSXY:1:2218:10122:35243 2:N:0:GAACCTAG+TCCGCATA
AAGAATTGCTTTGAGAGTCCATCAAGTGATGACGAACTCCCTTGAAGCGACGCCTGAAATTTCCCCAC
TGCAGTCATCTTCTGCACCGAGAAATAATTCCTAGTCGTGGTGATATTGCTGTCATTCACTCGCAGCA
+
FFFFFFFFFFFFFFFFFFFFFFFFFFFFFFFFFFFFFFFFFFFF:FFFFFFFFFFFFFFFFFFF:FFF
FFFFFFFFFFFFFFFFFFFFFFFFFFFFFFFFFFFFF:FFFFFFF:FFFFF:FFFFFFFFFF,FFFF:
@A00155:342:HHGFNDSXY:1:2416:13539:6715 1:N:0:GAACCTAG+TCCGCATA
AAGAATTGCTTTGAGAGTCCATCAAGTGATGACGAACTCCCTTGAAGCGACGCCTGAAATTTCCCCAC
TGCAGTCATCTTCTGCACCGAGAAATAATTCCTAGTCGTGGTGATATTGCTGTCATTCACTCGCAGCA
+
FFFFFFFFFFFFFFFFFFFFFFFFFFFFFFFFFFF:FFFFFFFFFFFFFFFFFFFFFFFFFFFFFFFF
FFFFFFFFFFFFFFFFFFFFFFFFFFFFFFFFFFFFFFFFFFFFFFFFFFFFFFFFFFFFFFFFFFFF
@A00155:342:HHGFNDSXY:1:1206:16233:16736 1:N:0:GAACCTAG+TCCGCATA
AAGAATTGCTTTGAGAGTCCATCAAGTGATGACGAACTCCCTTGAAGCGACGCCTGAAATTTCCCCAC
TGCAGTCATCTTCTGCACCGAGAAATAATTCCTAGTCGTGGTGATATTGCTGTCATTCACTCGCAGCA
+
```

```
FFFFF:FFFFFFFFFFFFFFFFFFFFFFFFFFFFFFFFFFFFFFFFFFFFFFFFFFFFFFFFFF
FFFFFFFFFFFFFFFFFFFFFFFFFFFFFFFFFFFFF:FFFFFFFFFFFFFFFFFFFFFFFFFFFFFF
@A00155:342:HHGFNDSXY:1:1206:15176:16595 1:N:0:GAACCTAG+TCCGCATA
AAGAATTGCTTTGAGAGTCCATCAAGTGATGACGAACTCCCTTGAAGCGACGCCTGAAATTTCCCCAC
TGCAGTCATCTTCTGCACCGAGAAATAATTCCTAGTCGTGGTGATATTGCTGTCATTCACTCGCAGCA
+
FFFFFFFFFFFFFFFFFFFFFFFFFFFFFFFFFFFFFFFFFFFFFFFFFFFFFFFFFFFFFFFFFFFF
FFFFFFFFFFFFFFFFFFFFFFFFFFFFFFFFFFFFFFFFFFFFFFFFFFFFFFFFFF:FFFFFFFFF
@A00155:342:HHGFNDSXY:1:2464:15393:35070 1:N:0:GAACCTAG+TCCGCATA
AAGAATTGCTTTGAGAGTCCATCAAGTGATGACGAACTCCCTTGAAGCGACGCCTGAAATTTCCCCAC
TGCAGTCATCTTCTGCACCGAGAAATAATTCCTAGTCGTGGTGATATTGCTGTCATTCACTCGCAGCA
+
F:FFFFFFFFFFFFFFFFFFFFFFFFFFFFFFFFFFFFFFFFFFFFFFFFFFFFFFFFFFFFFFFFFF
FFFFFFFFFFFFFFFFFFFFFFF::FFFFFFFFFFFFFFFFFFF:FFFFFFFFFFFFFFFFFFFFFFF
@A00155:342:HHGFNDSXY:1:1206:16107:16517 1:N:0:GAACCTAG+TCCGCATA
AAGAATTGCTTTGAGAGTCCATCAAGTGATGACGAACTCCCTTGAAGCGACGCCTGAAATTTCCCCAC
TGCAGTCATCTTCTGCACCGAGAAATAATTCCTAGTCGTGGTGATATTGCTGTCATTCACTCGCAGCA
+
FFFFFFFFFFFFFFFFFFFFFFFFFFFFFFFFFFFFFFFFFFFFFFFFFFFFFFFFFFFFFFFFFFFF
FFFFFFFFFFFFFFFFFFFFFFFFFFFFFFFFFFFFFFFFFFFFFFFFFFFFFFFFFFFFFFFFFFFF
@A00155:342:HHGFNDSXY:1:2437:8106:33411 1:N:0:GAACCTAG+TCCGCATA
AAGAATTGCTTTGAGAGTCCATCAAGTGATGACGAACTCCCTTGAAGCGACGCCTGAAATTTCCCCAC
TGCAGTCATCTTCTGCACCGAGAAATAATTCCTAGTCGTGGTGATATTGCTGTCATTCACTCGCAGCA
+
FFFFFFFFFFFFFFFFFFFFFFFFFFFFFFFFFFFFFFFFFFFFFFFFFFFFFFFFFFFFFFFFFFFF
FFFFFFFFFFFFFFFFFFFFFFFFFFFFFFFFFFFFFFFFFFFFF:FFFFFFFFFFFFFFFFFFFFFF
@A00155:342:HHGFNDSXY:1:1172:24469:14716 1:N:0:GAACCTAG+TCCGCATA
AAGAATTGCTTTGAGAGTCCATCAAGTGATGACGAACTCCCTTGAAGCGACGCCTGAAATTTCCCCAC
TGCAGTCATCTTCTGCACCGAGAAATAATTCCTAGTCGTGGTGATATTGCTGTCATTCACTCGCAGCA
+
FFFFFFFFFFFFFFFFFFFFFFFFFFFFFFFFFFFFFFFFF:FFFFFFFFFFFFFFFFFFFFFFFFFF
FFFFFFFFFFFFFFFFFFFFFFFFFFFFFFFFFFFFFFFFFFFFFFFFFFFFFFFFFFFFFFFFFFFF
@A00155:342:HHGFNDSXY:1:2262:8386:6183 1:N:0:GAACCTAG+TCCGCATA
AAGAATTGCTTTGAGAGTCCATCAAGTGATGACGAACTCCCTTGAAGCGACGCCTGAAATTTCCCCAC
TGCAGTCATCTTCTGCACCGAGAAATAATTCCTAGTCGTGGTGATATTGCTGTCATTCACTCGCAGCA
+
FFFFFFFFFFFFFFFF,FFFF,FFFFFFFFFFFFFFFFFF:,,FFFFFFFFFFFFFFFFF:FFFFFFF
FFFFFFFFFFFFFFFFFFFFFFF:FFFFFFF:FFFFFFFFFFFF:F:FFFFFFFFFFF:FFFFF:FFF
@A00155:342:HHGFNDSXY:1:1563:22932:30248 1:N:0:GAACCTAG+TCCGCATA
AAGAATTGCTTTGAGAGTCCATCAAGTGATGACGAACTCCCTTGAAGCGACGCCTGAAATTTCCCCAC
TGCAGTCATCTTCTGCACCGAGAAATAATTCCTAGTCGTGGTGATATTGCTGTCATTCACTCGCAGCA
+
FFFFFFFFFFFFFFFFFFFFFFFFFFFFFFFFFFFFFFFFFFFFFFFFFFFFFFFFFFFFFFFFFFFF
FFFFFFFFFFFFFFFFFFFFFFFFFFFFFFFFFFFFFFFFFFFFFFFFFFFFFFFFFFFFFFFFFFFF
@A00155:342:HHGFNDSXY:1:2422:18295:31297 1:N:0:GAACCTAG+TCCGCATA
AAGAATTGCTTTGAGAGTCCATCAAGTGATGACGAACTCCCTTGAAGCGACGCCTGAAATTTCCCCAC
TGCAGTCATCTTCTGCACCGAGAAATAATTCCTAGTCGTGGTGATATTGCTGTCATTCACTCGCAGCA
+
FFFFFFFFFFFFFFFFFFFFFFFFFFFFFFFFFFFFFFFFFFFFFFFFFFFFFFFFFFFFFFFFFFFF
FFFFFFFFFFFFFFFFFFFFFFFFFFFFFFFFFFFFFFFFFFFF:FFFFFFFFFFF:FFFFFFFFFFF
@A00155:342:HHGFNDSXY:1:1214:11451:26490 1:N:0:GAACCTAG+TCCGCATA
AAGAATTGCTTTGAGAGTCCATCAAGTGATGACGAACTCCCTTGAAGCGACGCCTGAAATTTCCCCAC
TGCAGTCATCTTCTGCACCGAGAAATAATTCCTAGTCGTGGTGATATTGCTGTCATTCACTCGCAGCA
+
```

```
FFFFFFFFFFFFFFFFFFFFFFFFFFFFFFFFFFFFFFFFF:FFFFFFFFFFFFFFFFFFFFFFFFFF
FFFFFFF:FFFFFFFFFFFFFFFFFFFFFFFFFFFFFFFFFFFFFFFFFFFFFFFFFFFFFFFFFFFF
@A00155:342:HHGFNDSXY:1:1206:14751:8312 1:N:0:GAACCTAG+TCCGCATA
AAGAATTGCTTTGAGAGTCCATCAAGTGATGACGAACTCCCTTGAAGCGACGCCTGAAATTTCCCCAC
TGCAGTCATCTTCTGCACCGAGAAATAATTCCTAGTCGTGGTGATATTGCTGTCATTCACTCGCAGCA
+
FFFFFFFFFFFFFFFFFFFFFFFFFFFFFFFFFFFFFFFFFFFFFFFFFFFFFFFFFFFFFFFFFFFF
FFFFFFFFFFFFFFFFFFFFFFFFFFFFFFFFFFFFFFFFFFFFFFFFFFFFFFFFFFFFFFFFFFFF
@A00155:342:HHGFNDSXY:1:1647:7961:14246 1:N:0:GAACCTAG+TCCGCATA
AAGAATTGCTTTGAGAGTCCATCAAGTGATGACGAACTCCCTTGAAGCGACGCCTGAAATTTCCCCAC
TGCAGTCATCTTCTGCACCGAGAAATAATTCCTAGTCGTGGTGATATTGCTGTCATTCACTCGCAGCA
+
FFFFFFFFFFFFFFFFFFFFFFFFFFFFFFFFF:FFFFFFFFFFFFFFFFFFFFFFFFFFFFFFFFFF
FFFFFFFFFFFFFFFFFFFFFFFFFFFF:FFFFFFFFFFFFFFFFFFFFFFFFFFFFFFFFFFFFFFF
@A00155:342:HHGFNDSXY:1:1154:29532:3630 1:N:0:GAACCTAG+TCCGCATA
AAGAATTGCTTTGAGAGTCCATCAAGTGATGACGAACTCCCTTGAAGCGACGCCTGAAATTTCCCCAC
TGCAGTCATCTTCTGCACCGAGAAATAATTCCTAGTCGTGGTGATATTGCTGTCATTCACTCGCAGCA
+
F,:FFF:FFFFFFF:FFFFFF:FFFF,F,FFFFFFFFFFFF,:FFFF,FFFFFF:FFFFF:FFFFFFF
FFF:FFFFFFF:FF,FFFFFFFF,FF:FFFF:,F:FF:FF:FFFFFF:,FFFFFFFFFFFFFFFF:FF
@A00155:342:HHGFNDSXY:1:2566:13847:28855 1:N:0:GAACCTAG+TCCGCATA
AAGAATTGCTTTGAGAGTCCATCAAGTGATGACGAACTCCCTTGAAGCGACGCCTGAAATTTCCCCAC
TGCAGTCATCTTCTGCACCGAGAAATAATTCCTAGTCGTGGTGATATTGCTGTCATTCACTCGCAGCA
+
FFFFFFFFFFFFFFFFFFFFFFFFFFFFFFFFFFFFFFFFFFFFFFFFFFFFFFFFFFFFFFFFFFFF
FFFFFFFFFFFFFFFFFFFFFFFFFFFFFFFFFFFFFFFFFFFFFFFFFFFFFFFFFFFFFFFFFFFF
@A00155:342:HHGFNDSXY:1:2534:25961:10066 1:N:0:GAACCTAG+TCCGCATA
AAGAATTGCTTTGAGAGTCCATCAAGTGATGACGAACTCCCTTGAAGCGACGCCTGAAATTTCCCCAC
TGCAGTCATCTTCTGCACCGAGAAATAATTCCTAGTCGTGGTGATATTGCTGTCATTCACTCGCAGCA
+
FFFFFFFFFFFFFFFFFFFFFFFFFFFFFFFFFFFFFFFFFFFFFFFFFFFFFFFFFFFFFFF,FFFF
FFFFFFFFFFFFFFFFFFFFFFFFFFFFFFFFFFFFFFFFFFFF:FFFFFFFFFFFFFFFFFFFFFFF
@A00155:342:HHGFNDSXY:1:2661:29559:9439 1:N:0:GAACCTAG+TCCGCATA
AAGAATTGCTTTGAGAGTCCATCAAGTGATGACGAACTCCCTTGAAGCGACGCCTGAAATTTCCCCAC
TGCAGTCATCTTCTGCACCGAGAAATAATTCCTAGTCGTGGTGATATTGCTGTCATTCACTCGCAGCA
+
FFFFFFFFFFFFFFFFFFFFFFFFFFFFFFFFFFFFFFFFF:FFFFFFFFFFFFFFFFFFFFFFFFFF
FFFFFFFFFFFFFFFFFFFFFFFF:FFFFFFFFFFFFFFFFFFFF::FFFFFFFFFFFFFFFFFFFFF
@A00155:342:HHGFNDSXY:1:2518:25762:5932 2:N:0:GAACCTAG+TCCGCATA
AGAATTGCTTTGAGAGTCCATCAAGTGATGACGAACTCCCTTGAAGCGACGCCTGAAATTTCCCCACT
GCAGTCATCTTCTGCACCGAGAAATAATTCCTAGTCGTGGTGATATTGCTGTCATTCACTCGCAGCAC
+
FFFFF,FF,FFFFFFFFFFFFFFFFFFFFFFFFF:FFFF:FFF:,FFFFFFFF:FFFFFFFFFFFF:F
FF:FFFF:FF:FFFFFFFF:FFFFFFFFFFFFFFFFFFFFFFFFFF:FFF:FFF::FFFFFFFFFFFF
@A00155:342:HHGFNDSXY:1:2334:19479:23610 2:N:0:GAACCTAG+TCCGCATA
GAATTGCTTTGAGAGTCCATCAAGTGATGACGAACTCCCTTGAAGCGACGCCTGAAATTTCCCCACTG
CAGTCATCTTCTGCACCGAGAAATAATTCCTAGTCGTGGTGATATTGCTGTCATTCACTCGCAGCACC
+
:FFFFFFFF:FFFFFFFFFFFFFFFFFFFFFFFFFFFFFFFFFFFFFFFFFFFFF:FFFFFF::FFF
FFFFFF,FFFFFFFFFFFF,FFFFFFFFFFFFFFFFFFFFFFFFFFFFFFFFFFFFFFFFFFFFFFFF
@A00155:342:HHGFNDSXY:1:2622:17029:16736 1:N:0:GAACCTAG+TCCGCATA
GAATTGCTTTGAGAGTCCATCAAGTGATGACGAACTCCCTTGAAGCGACGCCTGAAATTTCCCCACTG
CAGTCATCTTCTGCACCGAGAAATAATTCCTAGTCGTGGTGATATTGCTGTCATTCACTCGCAGCACC
+
```

```
FFFFFFFFFFFFFFFFFFFFFFFFFFFFFFFFFFFFFFF:FFFFFFFFFFFFFFFFFFFFFFFFFFFF
FFFFFFFFFFFFFFFFFFFFFFFFFFFFFFFFFFFFFFFFFFFF:FFFFFFFFFFFFFFF,FFFFFFF
@A00155:342:HHGFNDSXY:1:1538:3052:29074 2:N:0:GAACCTAG+TCCGCATA
GAATTGCTTTGAGAGTCCATCAAGTGATGACGAACTCCCTTGAAGCGACGCTTGAAATTTCCCCACTG
CAGTCATCTTCTGCACCGAGAAATAATTCCTAGTCGTGGTGATATTGCTGTCATTCACTCGCAGCACC
+
FFF,F:FFFFFFFFFFF:F,F:FFF,F:FFF,FFFFFFF::FFFFFFFFFF:FFFFF:FFFFFFFFFF
FFFFFFFFFFFFFFF:FFF,FFFFFFFFFFFFFFFFFFFFFFFFFFFFFFFFFFFFFFFFFFFFFFFF
@A00155:342:HHGFNDSXY:1:2467:20573:2018 1:N:0:GAACCTAG+TCCGCATA
GAATTGCTTTGAGAGTCCATCAAGTGATGACGAACTCCCTTGAAGCGACGCCTGAAATTTCCCCACTG
CAGTCATCTTCTGCACCGAGAAATAATTCCTAGTCGTGGTGATATTGCTGTCATTCACTCGCAGCACC
+
FFFFFFFFFFFFFFFFFFFFFFFFFFFFFFFFFFFFFFFFFFFFFFFFFFFFFFFFFFFFFFFFFFFF
FFFFFFFFFFFFFFFFFFFFF:FFFFFFFFFFFFFFFFFFFFFFFFFFFFFFFFFFFFFFFFFFFFFF
@A00155:342:HHGFNDSXY:1:1148:30255:21887 2:N:0:GAACCTAG+TCCGCATA
GAATTGCTTTGAGAGTCCATCAAGTGATGACGAACTCCCTTGAAGCGACGCTTGAAATTTCCCCACTG
CAGTCATCTTCTGCACCGAGAAATAATTCCTAGTCGTGGTGATATTGCTGTCATTCACTCGCAGCACC
+
F:FFFFFFFFFFFF:FFFFFFFFF,FFFFFFFFFFFFFFF:FFFFFFF:FFFFFFFFF:FFFFFFFFF
FFFFFFFFFFFFFFFFFFFFFFFFFFFFFFFFFFFFFFFFFFFF:FFFFFFFFFFFFFFFFFFFFFFF
@A00155:342:HHGFNDSXY:1:2346:30581:8202 1:N:0:GAACCTAG+TCCGCATA
GAATTGCTTTAAGAGTCCATCAAGTGATGACGAACTCCCTTGAAGCGACGCCTGAAATTTCCCCACTG
CAGTCATCTTCTGCACCGAGAAATAATTCCTAGTCGTGGTGATATTGCTGTCATTCACTCGCAGCACC
+
FFFFFFFFFFFFFFFFFFFFFFFFFFFFFFFFFFFFFFFFFFFFFFFFFFFFFFFFFFFFFFFFFFFF
FFFFFFFFFFFFFFFFFFFFFFFFFFFFFFFFFFFFFFF,FFFFFFFFFFFFFFFFFFFFFFFFFFFF
@A00155:342:HHGFNDSXY:1:2256:30183:35258 2:N:0:GAACCTAG+TCCGCATA
GAATTGCTTTGAGAGTCCATCAAGTGATGACGAACTCCCTTGAAGCGACGCCTGAAATTTCCCCACTG
CAGTCATCTTCTGCACCGAGAAATAATTCCTAGTCGTGGTGATATTGCTGTCATTCACTCGCAGCACC
+
FF::FFFF:FFFFFFFFFFFFFF::FFFFFFFF,FFFFF::FF:FFF:FFFF:F::F,::FFFFFF:F
FFFFFFFFFFF,FFFFFFFFF:FFFFFFFFFFFFFFFFFFFFF:FFFFFFFFFFFFFFFFFFFFFFFF
@A00155:342:HHGFNDSXY:1:1126:6153:17190 1:N:0:GAACCTAG+TCCGCATA
GAATTGCTTTGAGAGTCCATCAAGTGATGACGAACTCCCTTGAAGCGACGCCTGAAATTTCCCCACTG
CAGTCATCTTCTGCACCGAGAAATAATTCCTAGTCGTGGTGATATTGCTGTCATTCACTCGCAGCACC
+
FFFF:FFFFFFFFFFFFFFFFFFFFFFFFFFFFFF:FFFFFFFFFFFFFFFFFFFFFFFFFFFFFFFF
FFFFFFFFFFFFFFFFFFFFFFFFF:FFFFFFFFFF:FFFFFFFFFFFFFFF,FFFFFFFFFFFFFFF
@A00155:342:HHGFNDSXY:1:1345:13259:12618 2:N:0:GAACCTAG+TCCGCATA
GAATTGCTTTGAGAGTCCATCAAGTGATGACGAACTCCCTTGAAGCGACGCCTGAAATTTCCCCACTG
CAGTCATCTTCTGCACCGAGAAATAATTCCTAGTCGTGGTGATATTGCTGTCATTCACTCGCAGCACC
+
FFFFFFFFFFFFFFFFFFFFFFFFFFFFFFFFFFFFFFFFFFFFFFFFFFFFFFFFFFFFFFFFFFFF
FFFF:FFFFFFFFFFFFFFFFFFFFFFFFFFFFFFFFFFFFFFFFFFFFFFFFFFFFFFFFFFFFFFF
@A00155:342:HHGFNDSXY:1:2216:5665:1846 2:N:0:GAACCTAG+TCCGCATA
AATTGCTTTGAGAGTCCATCAAGTGATGACGAACTCCCTGGAAGCGACGCCTGAAATTTCCCCACTGC
AGTCATCTTCTGCACCGAGAAATAATTCCTAGTCGTGGTGATATTGCTGTCATTCACTCGCAGCACCC
+
FF,:F:,:FFFFFFFFFFFFF,:FFFFFFFFFFFFFFFF,:FFFFFFFFFFFFFFFFFFFFFFFF:FF
FFFF,:F:FFFFFFFFFFFFFFFFFFFFFFFFFFFFFFFFFFFFFFFFFFFFFFFFFFFFFFFFFFFF
@A00155:342:HHGFNDSXY:1:1278:32054:36464 2:N:0:GAACCTAG+TCCGCATA
AATTGCTTTGAGAGTCCATCACGTGATGACGAACTCCCTTGAAGCGACGCCTGAAATTTCCCCACTGC
AGTCATCTTCTGCACCGAGAAATAATTCCTAGTCGTGGTGATATTGCTGTCATTCACTCGCAGCACCC
+
```

```
F,FFFFF:FFFFFFFFFFFFF,F,:FFFFFFF:FFFFFFFFFFFFFFFFFF:FFF:::FFFFFFFF,F
FFFF:FF,FFFFF:FFFFF:FFFFFFFFFFFFFFFFFFFFFFFFFFFFFFFF:FFFFFFFFFFFFFF
@A00155:342:HHGFNDSXY:1:2348:22218:33990 1:N:0:GAACCTAG+TCCGCATA
ATTGCTTTGAGAGTCCATCAAGTGATGACGAACTCCCTTGAAGCGACGCCTGAAATTTCCCCACTGCA
GTCATCTTCTGCACCGAGAAATAATTCCTAGTCGTGGTGATATTGCTGTCATTCACTCGCAGCACCCC
+
FFFFFFFFFFFFFFFFFFFFFFFFFFFFFFFFFFFFF,FFFFFFFFFFFFFFFFFFFFFFFFFFFFFF
FFFFFFFFFFFFFFFFFFFF:FFFFFFFFFFFFFFFFFFFFFFFFFFFFFFFFFFFFFFFFFFFFFFF
@A00155:342:HHGFNDSXY:1:2337:29017:22310 1:N:0:GAACCTAG+TCCGCATA
ATTGCTTTGAGAGTCCATCAAGTGATGACGAACTCCCTTGAAGCGACGCCTGAAATTTCCCCACTGCA
GTCATCTTCTGCACCGAGAAATAATTCCTAGTCGTGGTGATATTGCTGTCATTCACTCGCAGCACCCC
+
FFFFFFFFFFFF,FFFFFFFFFFFFFFFF:FFFFF:F:FFFFFFFFF:FFFFFFFFFFFFFFFFFFFF
F:F:::FFF:FFFFFFFFFFFFFFFFFF:F:FFFFFFFFFFFFFFFFFFFFFFFFFFFFFFFFFFFFF
@A00155:342:HHGFNDSXY:1:2204:31159:17816 2:N:0:GAACCTAG+TCCGCATA
ATTGCTTTGAGAGTCCATCAAGTGATGACGAACTCCCTTGAAGCGACGCCTGAAATTTCCCCACTGCA
GTCATCTTCTGCACCGAGAAATAATTCCTAGTCGTGGTGATATTGCTGTCATTCACTCGCAGCACCCC
+
F,FFFFFFFFFFFFFFFFFF:FFFFFFFFFF:FFFFFFFFFFFFFFFFFFFFFFFFFFFFFFFFFFFF
FFFFFFFFFFFFFFFFFFFFFFFFFFFFFFFFFFFFFFFFFFFFFFFFFFF:FFFFFFFFFFFFFFFF
@A00155:342:HHGFNDSXY:1:1464:26268:5274 1:N:0:GAACCTAG+TCCGCATA
TTGCTTTGAGAGTCCATCAAGTGATGACGAACTCCCTTGAAGCGACGCCTGAAATTTCCCCACTGCAG
TCATCTTCTGCACCGAGAAATAATTCCTAGTCGTGGTGATATTGCTGTCATTCACTCGCAG
+
FFFFFFFFFFFFFFFFFFFFFFFFFFFFFFFFFFFFFFFFFFFFFFFFFFFFFFFFFFFFFFFFFFFF
FFFFFFFFFFFFFFFFFFFFFFFFFF:FFFFFFFFFFFFF,FFFFFFFFFFFFFFFFFFFFF
@A00155:342:HHGFNDSXY:1:1338:26359:26412 1:N:0:GAACCTAG+TCCGCATA
TTGCTTTGAGAGTCCATCAAGTGATGACGAACTCCCTTGAAGCGACGCCTGAAATTTCCCCACTGCAG
TCATCTTCTGCACCGAGAAATAATTCCTAGTCGTGGTGATATTGCTGTCATTCACTCGCAGCACCC
+
FFFFFFFFFFFFFFFFFFFFFFFFFFFFFFFFFFFFFFFFFFFFFFFFFFFFFFFFFFFFFFFFFFFF
FFFFFFFFFFFFFFFFFFFFFFFFFFFFFFFFFFFFFFFFFFFFFFFFFFFFFFFFFFFFFFFFFF
@A00155:342:HHGFNDSXY:1:1662:1271:24956 1:N:0:GAACCTAG+TCCGCATA
TTGCTTTGAGAGTCCATCAAGTGATGACGAACTCCCTTGAAGCGACGCCTGAAATTTCCCCACTGCAG
TCATCTTCTGCACTGAGAAATAATTCCTAGTCGTGGTGATATTGCTGTCATTCACTCGCAGCACCCC
+
FFFF:FFFFFFFF::FFFFFFFFFFFFF,FF:FFF,F:FFFF:FFFFFFFFF:FFFFFFFFFFFF,F
FFFFFFFFFFFFF:,FFFFFF,FF:FFFFFFF:FF:FFF,:FFFFFFFFF,FFFFFFFF:FFFFFFF
@A00155:342:HHGFNDSXY:1:1352:31611:18004 1:N:0:GAACCTAG+TCCGCATA
TTGCTTTGAGAGTCCATCAAGTGATGACGAACTCCCTTGAAGCGACGCCTGAAATTTCCCCACTGCAG
TCATCTTCTGCACCGAGAAATAATTCCTAGTCGTGGTGATATTGCTGTCATTCACTCGCAGCACCCCA
+
FFFFFFFFFFFFFFFFFFFFFFFFFFFFFFFFFFFFFFFFFFFFFFFFFFF:FFFFFFFFFFFFFFFF
FFFFFFFFFFFFFFFFFFFFFFFFFFFFF,FFFFFFFFFFFFFFFFFFFFF:FFFFFFFFFFFFFFFF
@A00155:342:HHGFNDSXY:1:1201:8440:10755 1:N:0:GAACCTAG+TCCGCATA
TTGCTTTGAGAGTCCATCAAGTGATGACGAACTCCCTTGAAGCGACGCCTGAAATTTCCCCACTGCAG
TCATCTTCTGCACCGAGAAATAATTCCTAGTCGTGGTGATATTGCTGTCATTCACTCGCAGCACCCCA
+
FFFFFFFFFFFFFFFFFFFFFFFFFFFFFFF:FFFFFFFFFFFFFFFFFFFFFFFFFFFFFFFFFFFF
FFFFFFFFFFFFFFFFFFFFFFFFFFFFFFFFFFFFFFFFFF:FFFFFFFFFFFFFFFFFFFFFFFFF
@A00155:342:HHGFNDSXY:1:1104:25925:20087 1:N:0:GAACCTAG+TCCGCATA
TTGCTTTGAGAGTCCATCAAGTGATGACGAACTCCCTTGAAGCGACGCCTGAAATTTCCCCACTGCAG
TCATCTTCTGCACTGAGAAATAATTCCTAGTCGTGGTGATATTGCTGTCATTCACTCGCAGCACCCCA
+
```

```
:F,FFFFFFFFFFFFFFFFFFFFFFFFF:FFFFFFF,F,FF:F:FFFFFF::FFFFF:F,FFF:,:FF
F:FFFF,FFFFFF:FFFFFFFFFFFF,FFFFFF:F::FF:FF:FFFFFFF,FFFF:FFFF::F:FFFF
@A00155:342:HHGFNDSXY:1:2104:27010:15546 1:N:0:GAACCTAG+TCCGCATA
TTGCTTTGAGAGTCCATCAAGTGATGACGAACTCCCTTGAAGCGACGCCTGAAATTTCCCCACTGCAG
TCATCTTCTGCACTGAGAAATAATTCCTAGTCGTGGTGATATTGCTGTCATTCACTCGCAGCACCCCA
+
FFFFFFFFFFFFF:FFFFFFFFFFFFFFFFFFFFFFFFFFFFFFFFFFFFFFFFFFFFFFF:FFFFFF
FFFFFFFFFFFFFFFFFF:FFFFFFFFFFFFFFFFFFFF,FFFFFFFFFFFFFFFFFFFFFFFFFF:F
@A00155:342:HHGFNDSXY:1:2330:13521:35712 1:N:0:GAACCTAG+TCCGCATA
TTGCTTTGAGAGTCCATCAAGTGATGACGAACTCCCTTGAAGCGACGCCTGAAATTTCCCCACTGCAG
TCATCTTCTGCACCGAGAAATAATTCCTAGTCGTGGTGATATTGCTGTCATTCACTCGCAGCACCCCA
+
FFFFFFFFFFFFFFFFFFFFFFFFFFFFFFFFFFFFFFFFFFFFFFFFFFFFFFFFFFFFFFFFFFFF
FFFFFFFFFFFFFFFFFFFFFFFFFFFFFFFFFFFFFFFFFFFFFFFFFFFFFFFFFFFFFFFFFFFF
@A00155:342:HHGFNDSXY:1:2514:29008:3255 1:N:0:GAACCTAG+TCCGCATA
TTGCTTTGAGAGTCCATCAAGTGATGACGAACTCCCTTGAAGCGACGCCTGAAATTTCCCCACTGCAG
TCATCTTCTGCACCGAGAAATAATTCCTAGTCGTGGTGATATTGCTGTCATTCACTCGCAGCACCCCA
+
FFFFFFFFFFFFF:FFFFFFFFFFFFFFFFFFFF,FFFFFFFFFFFFFFFFFFFFFFFFFFFFFFFFF
FFFFFFFFFFFFFFFFFFFFFFFFFFFFFFFFFFFFFFFFFFFFFFFFFFFFFFFFFFFFFFFFFFFF
@A00155:342:HHGFNDSXY:1:2131:12798:15671 1:N:0:GAACCTAG+TCCGCATA
TTGCTTTGAGAGTCCATCAAGTGATGACGAACTCCCTTGAAGCGACGCCTGAAATTTCCCCACTGCAG
TCATCTTCTGCACCGAGAAATAATTCCTAGTCGTGGTGATATTGCTGTCATTCACTCGCAGCACCCCA
+
FFFFFFFFFFFFFFFFFFFFFFFFFFFFFFFFFFFFFFFFFFFFFFFFFFFFF:FFFFFFFFFFFFFF
FFFFFFFFFFFFFFFFFFFFFFFFFFFFFFFFFFFFFFFFFFFFFFFFFFFFFFFFF:FFFFFFFFFF
@A00155:342:HHGFNDSXY:1:1345:13259:12618 1:N:0:GAACCTAG+TCCGCATA
TTGCTTTGAGAGTCCATCAAGTGATGACGAACTCCCTTGAAGCGACGCCTGAAATTTCCCCACTGCAG
TCATCTTCTGCACCGAGAAATAATTCCTAGTCGTGGTGATATTGCTGTCATTCACTCGCAGCACCCCA
+
F:FFFFFFFFFFFFFFFFFFFFFF:FFFFFFFFFFFFFFFFFFFFFFFFFFFFFFFFFF:FFF:FFFF
FFFFFFFFFFFFFFFFFFFFFFF:F:FFFFFFFFFFF:FFFFFFFF:FFFFFFFFFFF:FFFFFFFFF
@A00155:342:HHGFNDSXY:1:2457:24966:15953 1:N:0:GAACCTAG+TCCGCATA
TTGCTTTGAGAGTCCATCAAGTGATGACGAACTCCCTTGAAGCGACGCCTGAAATTTCCCCACTGCAG
TCATCTTCTGCACCGAGAAATAATTCCTAGTCGTGGTGATATTGCTGTCATTCACTCGCAGCACCCCA
+
FFFFFFFFFFFFFFFFFFFFFFFFFFFFFFFFFFFFFFFFFFFFFFFFFFFFFFFFFFFFFFFFFFFF
FFFFFFFFFFFFFFFFFFFFFFFF:FFFFFFFFFFFFFFFFFFFFFFFFFFFFFFFFFFFFFFFFFF
@A00155:342:HHGFNDSXY:1:1402:1759:6605 1:N:0:GAACCTAG+TCCGCATA
TTGCTTTGAGAGTCCATCAAGTGATGACGAACTCCCTTGAAGCGACGCCTGAAATTTCCCCACTGCAG
TCATCTTCTGCACCGAGAAATAATTCCTAGTCGTGGTGATATTGCTGTCATTCACTCGCAGCACCCCA
+
FFFFFFFFFFFFFFFFFFFFFFFFFFFF:FFFFFFFFFFFF,F:FFFFFFFFFFFFF:FFFFFFFFFF
FFFFFFFFFFFFFFFFFFFFFFFFFFFFFFFFFFFFF:FFFFFFFFFFFFFFFFFFF:FFFF,FFFFF
@A00155:342:HHGFNDSXY:1:2469:20419:11553 1:N:0:GAACCTAG+TCCGCATA
TTGCTTTGAGAGTCCATCAAGTGATGACGAACTCCCTTGAAGCGACGCCTGAAATTTCCCCACTGCAG
TCATCTTCTGCACCGAGAAATAATTCCTAGTCGTGGTGATATTGCTGTCATTCACTCGCAGCACCCCA
+
FFFFFFFFFFFFFFFFFFFFFFFFFFFFFFFFFFFFFFFFFFFFFFFFFFFFFFFFFFFFFFFFFFFF
FFFFF:FFFFFFFFFFFFFFFFFFFFFFF,:FFFFFFFFFFFFFFFFFFFFFFFFFFFFFFFFFFFFF
@A00155:342:HHGFNDSXY:1:1263:31421:7404 1:N:0:GAACCTAG+TCCGCATA
TTGCTTTGAGAGTCCATCAAGTGATGACGAACTCCCTTGAAGCGACGCCTGAAATTTCCCCACTGCAG
TCATCTTCTGCACCGAGAAATAATTCCTAGTCGTGGTGATATTGCTGTCATTCACTCGCAGCACCCCA
+
```

```
FFFFFFFFFFFFFFFFFFFFFFFFFFFFFFFFFFFFFFFFFFFFFFFFFFFFFFFFFFFFFFFFFFFF
FFFFFFFFFFFFFFFFFFFFFFFFFFFFFFFFFFFF:FFFFFFFFFFFFFFFFFFFFFFFFFFFFFFF
@A00155:342:HHGFNDSXY:1:1370:19804:24987 2:N:0:GAACCTAG+TCCGCATA
TGCTTTGAGAGTCCATCAAGTGATGACGAACTCCCTTGAAGCGACGCCTGAAATTTCCCCACTGCAGT
CATCGTCTGCACCGAGAAATAATTCCTAGTCGTGGTGATATTGCTGTCATTCACTCGCAGCACCCCAC
+
FFFFFFFFFFFFFFFFFFF:,FFFFFFFF,F,FFFF:FF,FFFFFFFF:FFF:FFFFF:FFFFFFFFF
FFFF,FFFFFFFFFFFFFFFFFFFFFFFFFFFFFFFFFFFFFFFFFFFF:FFFFFFFFFFFFFFFFF
@A00155:342:HHGFNDSXY:1:2144:6958:8093 2:N:0:GAACCTAG+TCCGCATA
CTTTGAGAGTCCATCAAGTGATGACGAACTCACTTGAAGCGACGCCTGAAATTTCCCCACTGCAGTCA
TCTTCTGCACCGAGAAATAATTCCTAGTCGTGGTGATATTGCTGTCATTCACTCGCAGCACCCC
+
FFFFFFFFFFFFFFFFFFFFFFFFFFFFF:FFFFFFFFFFFFFFFFFFFFFFFFFFFFFFFFFFFFFF
FFFFFFFFFFFFFFFFFFFFFFFFFFFFFFFFFFFFFFFFFFFFFFFFFFFFFFFFFFFFFFFF
@A00155:342:HHGFNDSXY:1:2247:11831:17253 2:N:0:GAACCTAG+TCCGCATA
CTTTGAGAGTCCATCAAGTGATGACGAACTCACTTGAAGCGACGCCTGAAATTTCCCCACTGCAGTCA
TCTTCTGCACCGAGAAATAATTCCTAGTCGTGGTGATATTGCTGTCATTCACTCGCAGCACCCC
+
FFFFFFFFFFFFFFFFFFFFFFFFFFFFFFFFFFFFFFFFFFFFFFFFFFFFFFFFFFFFFFFFFFFF
FFFFFFFFFFFFFFFFFFFFFFFFFFFFFFFFFFFFFFFFFFFFFFFFFFFFFFFFFFFFFFFF
@A00155:342:HHGFNDSXY:1:2247:11541:17722 2:N:0:GAACCTAG+TCCGCATA
CTTTGAGAGTCCATCAAGTGATGACGAACTCACTTGAAGCGACGCCTGAAATTTCCCCACTGCAGTCA
TCTTCTGCACCGAGAAATAATTCCTAGTCGTGGTGATATTGCTGTCATTCACTCGCAGCACCCC
+
FFFFFFFFFFFFFFFFFFFFFFFFFFFFFFFFFFFFFFFFFFFFFFFFFFFFFFFFFFFFFFFFFFFF
FFFFFFFFFFFFFFFFFFFFFFFFFFFFFFFFFFFFFFFFFFFFFFF:FFFFFFFFFFFFFFFF
@A00155:342:HHGFNDSXY:1:2146:28673:27258 2:N:0:GAACCTAG+TCCGCATA
TTGAGAGTCCATCAAGTGATGACGAACTCCCTTGAAGCGACGCCTGAAATTTCCCCACTGCAGTCATC
TTCTGCACCGAGAAATAATTCCTAGTCGTGGTGATATTGCTGTCATTCACTCGCAGCACCCCACCAT
+
FFFFFFFFFFFFFFFF,FF:FFFFFFFF:::FFFFFF,:FFFFFFFFFFF:F,FFFFFFFFFFF:FF:
F:FFF:F:::FFFFFFFFFFFFFFFFFFFF:FFF::FFF:FF:FFFF:F,FF,FF:FFF:FFF:FFF
@A00155:342:HHGFNDSXY:1:1555:21151:17237 2:N:0:GAACCTAG+TCCGCATA
TGAGAGTCCATCAAGTGATGACGAACTCCCTTGAAGCGACGCCTGAAATTTCCCCACTGCAGTCATCT
TCTGCACCGAGAAATAATTCCTAGTCGTGGTGATATTGCTGTCATTCACTCGCAGCACCCCACCATGG
+
FFFFFFFFFF:FFFFFFFFFFFFFFFFFFFF:FFFFFFFFFFF:FFFFF,FFFF:FFF,FFF:FFFFF
FFFFFFFFFFFFFFFFFFFFFFFFFFFFFFFFFFFFFFFFFFFFFFFFFFFFFFFFFFFFFFFFFFFF
@A00155:342:HHGFNDSXY:1:1555:20112:16814 2:N:0:GAACCTAG+TCCGCATA
TGAGAGTCCATCAAGTGATGACGAACTCCCTTGAAGCGACGCCTGAAATTTCCCCACTGCAGTCATCT
TCTGCACCGAGAAATAATTCCTAGTCGTGGTGATATTGCTGTCATTCACTCGCAGCACCCCACCATGG
+
FFFFFFFFF:FFFFF,FFFFFFFFFFFFFF::FFFFFFFFFFFFFFF:FFFFFFFFF:FFF:FF:FFF
FFFFFFFFFFFFFFFFFFFFFFFFFFFFFFFFFFF:FFFFFFFF,FFFFFFFF:FFFFFFFFFFFFFF
@A00155:342:HHGFNDSXY:1:2147:3613:21245 1:N:0:GAACCTAG+TCCGCATA
GTCCATCAAGTGATGACGAACTCCCTTGAAGCGACGCCTGAAATTTCCCCACTGCAGTCATCTTCTGC
ACCGAGAAATAATTCCTAGTCGTGGTGATATTGCTGTCATTCACTCGCAGCACCCCACCATG
+
FFFFFFFFF:FFFFFFFF:FFFFFFFFFFFFFFFFF:FFFFFFFFFFFFFFFFFFFFFFFFFFFFFFF
FFFFFFFFFFFFFFFFFFFFFFFFFFFFFFFFFFFFFFFFFFFFFFFFFFFFFFFFFFFF:F
@A00155:342:HHGFNDSXY:1:2263:31060:24940 2:N:0:GAACCTAG+TCCGCATA
CCATCAAGTGATGACGAACTCCCTTGAAGCGACGCCTGAAATTTCCCCACTGCAGTCATCTTCTGCAC
CGAGAAATAATTCCTAGTCGTGGTGATATTGCTGTCATTCACTCGCAGCACCCCACCATGGTACGTGC
+
```

FFFFFFFFFFFFFFFFFFFFFFFFFFFFFFFFFFFFFFFFFFF:FFFFFFFFFFFFFFFFFFFFFFFF
FFFFFFFFFFF:FFFFFFFFFFFFFFFFFFFFFFFFFFFFFFFFFFFFFFFFFFFFFFFFFFFFFFFF
@A00155:342:HHGFNDSXY:1:2263:31087:24956 2:N:0:GAACCTAG+TCCGCATA
CCATCAAGTGATGACGAACTCCCTTGAAGCGACGCCTGAAATTTCCCCACTGCAGTCATCTTCTGCAC
CGAGAAATAATTCCTAGTCGTGGTGATATTGCTGTCATTCACTCGCAGCACCCCACCATGGTACGTGC
+
FFFFFFFFFFF:FFFFFFFFFFFFFFFFFFFFFFFFFFFF:F:FFFFFFFFFFFFFFFFFFFFFFFFF
FFFFFFFFFFFFFFFFFFFFFFFFFFFFFFFFFFFFFFFFFFFFFF:FFFFFFFFFFFFFFFFFFFFF
@A00155:342:HHGFNDSXY:1:2415:32868:22153 2:N:0:GAACCTAG+TCCGCATA
CCATCAAGTGATGACGAACTCCCTTGAAGCGACGCCTGAAATTTCCCCACTGCAGTCATCTTCTGCAC
CGAGAAATAATTCCTAGTCGTGGTGATATTGCTGTCATTCACTCGCAGCACCCCACCATGGTACGTGC
+
FFFFFFF::FFFFFFFFFFFFFFFFFFFFFFFFFFFFFFFFFFFFFFFFF:FFFFFFFFFFFFFFFFF
FFF:FFFFFFF,FFFFFFFFFFFFFFFFF:FFFFFFFF:FFFFFFFFFFFFFFFFFFFFFFFFFF:FF
@A00155:342:HHGFNDSXY:1:1169:9525:33489 2:N:0:GAACCTAG+TCCGCATA
CATCAAGTGATGACGAACTCCCTTGAAGCGACGCCTGAAATTTCCCCACTGCAGTCATCTTCTGCACC
GAGAAATAATTCCTAGTCGTGGTGATATTGCTGTCATTCACTCGCAGCACCCCACCATGGTACGTGCT
+
FFFF:F::FF:FFFFFFFFFF::FFFFFFFFFFFFFFFFFFFFFFF:FFF:F:,:,:FFFFFFFFFFF
FFFFFFFFFFFFFFF:FFFFFFFFFFFFFFFFF::FFF:FFFFFFFFFFFFFFFFFFFFFFFFFFFFF
@A00155:342:HHGFNDSXY:1:1142:24370:16924 2:N:0:GAACCTAG+TCCGCATA
CATCAAGTGATGACGAACTCCCTTGAAGCGACGCCTGAAATTTCCCCACTGCAGTCATCTTCTGCACC
GAGAAATAATTCCTAGTCGTGGTGATATTGCTGTCATTCACTCGCAGCACCCCACCATGGTACGTGCT
+
F,FF:FFFFF,F,:FFFFFFFFF::FFFFFFF:FFFFFFFFFFFFFFF:,:F:,,,FF:FFFFFFFF:
FFFFFFFFFFFFFFFFFFFFFFFFFFFFFFFFFFFFFFFFFFFFFFFFFFFFFFFFFFFFFFFFFF:F
@A00155:342:HHGFNDSXY:1:2246:14507:5259 2:N:0:GAACCTAG+TCCGCATA
CATCAAGTGAGGACGAACTCCCTTGAAGCGACGCCTGAAATTTCCCCACTGCAGTCATCTTCTGCACC
GAGAAATAATTCCTAGTCGTGGTGATATTGCTGTCATTCACTCGCAGCACCCCACCATGGTACGTGCT
+
F:FFF,F:FF,FFFFF,FFFFF:,FFFFFFFFFFF,FFFF,,FF:FF,FFFFFFFF,:F:FFFFFFFF
F,FFFFFFFFFFFFFFFFFFFFFFFFFFFFFFFFFFFFFFF:FFFFFFFFFFFFFFFFFFFFFFFFFF
@A00155:342:HHGFNDSXY:1:2246:13865:2362 2:N:0:GAACCTAG+TCCGCATA
CATCAAGTGATGACGAACTCCCTTGAAGCGACGCCTGAAATTTCCCCACTGCAGTCATCTTCTGCACC
GAGAAATAATTCCTAGTCGTGGTGATATTGCTGTCATTCACTCGCAGCACCCCACCATGGTACGTGCT
+
FF,FFFFFFFFFFFFFFFFFFFFFFFFFFFFFFFFFFFFFFFFFFFFFFFFFFFFF,:FFFFFFFFFF
FFFFFFFFFFFFFFFFFFFFFFFFFFFFFFFFFFFFFFFFFFFFFFFFFFFFFFFFFFFFFFFFFFFF
@A00155:342:HHGFNDSXY:1:1317:1877:28072 2:N:0:GAACCTAG+TCCGCATA
TCAAGTGATGACGAACTCCCTTGAAGCGACGCCTGAAATTTCCCCACTGCAGTCATCTTCTGCACCGA
GAAATAATTCCTAGTCGTGGTGATATTGCTGTCATTCACTCGCAGCACCCCACCATGGTACGTGCTAT
+
FFFFFFFFFFFFFFFFFFFFFFFFFFFFFFFFFFFFFFFFFFFFF:FFFFFFFFFFFFFFFFFFFFFF
FFFFFFFFFFF,:FFFFFFFFFFF:FFFFFFFFFFFFFFFFFFFFFFFFFFFFF:FFFFFFFFFFFFF
@A00155:342:HHGFNDSXY:1:2436:13575:16360 2:N:0:GAACCTAG+TCCGCATA
TCAAGTGATGACGAACTCCCTTGAAGCGACGCCTGAAATTTCCCCACTGCAGTCATCTTCTGCACCGA
GAAATAATTCCTAGTCGTGGTGATATTGCTGTCATTCACTCGCAGCACCCCACCATGGTACGTGCTAT
+
FFFFFFFFFFFFFFFFFFFFFFFFFFFFFFFFFFFFFFFFFFFFFFFFFFFFFFFFFFFFFFFFFFFF
FFFFFFFFFFFF:FFFFFFFFFFFFFFFFFFFFFFFFFFFFFFFFFFFFFFFFF:FFFFFFFFFFFF
@A00155:342:HHGFNDSXY:1:2247:11831:17253 1:N:0:GAACCTAG+TCCGCATA
AAGTGATGACGAACTCACTTGAAGCGACGCCTGAAATTTCCCCACTGCAGTCATCTTCTGCACCGAGA
AATAATTCCTAGTCGTGGTGATATTGCTGTCATTCACTCGCAGCACCCCACCATGGTACGTGCT
+

```
FFFFFFFFF:FFFFFFFFFFFFFFFFFFFFFFFFFFFFFFFFFFFFFFFFFFFFFFFFFFFFFFF
:FFFFFFFFFFFFFFFFFFFFFFFFFFFFFFFFFFFFFFFFFFFFFFFFFFFFFFFFFFFFFFF
@A00155:342:HHGFNDSXY:1:2144:6958:8093 1:N:0:GAACCTAG+TCCGCATA
AAGTGATGACGAACTCACTTGAAGCGACGCCTGAAATTTCCCCACTGCAGTCATCTTCTGCACCGAGA
AATAATTCCTAGTCGTGGTGATATTGCTGTCATTCACTCGCAGCACCCCACCATGGTACGTGCT
+
FFFFFFFFFFFFFFFFFFFFFFFFFFFFFFFFFFFFFFFFFFFFFFFFFFFFFFFFFFFFFFFFFFFF
:FFFFFFFFFFFFFFFFFFFFFF:FFFFFFFFFFFFFFFFFFFFFFFFFFFFFFFFFFFFFFFF
@A00155:342:HHGFNDSXY:1:2247:11541:17722 1:N:0:GAACCTAG+TCCGCATA
AAGTGATGACGAACTCACTTGAAGCGACGCCTGAAATTTCCCCACTGCAGTCATCTTCTGCACCGAGA
AATAATTCCTAGTCGTGGTGATATTGCTGTCATTCACTCGCAGCACCCCACCATGGTACGTGCT
+
FFFFFFFFFFFFFFFFFFFFFFFFFFFFFFFFFFFFFFFFFFFFFFFFFFFF,FFFFFFFFFFFFFFF
FFFFFFFFFFFFFFFFFF:FFFFFFFFFFFFFFFF:FFFFFFFFFFFFFFFFFFFFFFFFFFFFF
@A00155:342:HHGFNDSXY:1:1461:30138:36025 2:N:0:GAACCTAG+TCCGCATA
AAGTGATGACGAACTCCCTTGAAGCGACGCCTGAAATTTCCCCACTGCAGTCATCTTCTGCACCGAGA
AATAATTCCTAGTCGTGGTGATATTGCTGTCATTCACTCGCAGCACCCCACCATGGTACGTGCTATGC
+
FFFFFFFFFFFFFFFFFF:,FFFF:FFF:FFFFFFFFFFFFFF,FFFFFFFFFF:FFFFFFFFFFFF
FFFFFFFFFFFFFFFFFFFFFFFFFFFFFFFFFFFFFFFFFFFFFFFFFFFFFFFFFFFFFFFFFFFF
@A00155:342:HHGFNDSXY:1:1317:1877:28072 1:N:0:GAACCTAG+TCCGCATA
TGATGACGAACTCCCTTGAAGCGACGCCTGAAATTTCCCCACTGCAGTCATCTTCTGCACCGAGAAAT
AATTCCTAGTCGTGGTGATATTGCTGTCATTCACTCGCAGCACCCCACCATGGTACGTGCTATGCCTT
+
FFFFFFFFFFFFFFFFFFFFFFFFFFFFFFFFFFFFFFFF:FFFFFF:FFFFFFFFFFFFFFFFFFFF
FFFFFFFFFFFFFFFFFF:FFFFFFFF:FFF:FFFFFFFFFFFF:FFFFFFFFFFFFFFFFFFFFFFF
@A00155:342:HHGFNDSXY:1:1471:13919:3020 2:N:0:GAACCTAG+TCCGCATA
TGATGACGAACTCCCTTGAAGCGACGCCTGAAATTTCCCCACTGCAGTCATCTTCTGCACCGAGAAAT
AATTCCTAGTCGTGGTGATATTGCTGTCATTCACTCGCAGCACCCCACCATGGTACGTGCTATGCCTT
+
FFFFFFFFFFFFFFFFFFFFFFFFFFFFFFFFFFFFFFFFFFF::FFF:FFFFFFFFFFFFFFFFFFFF
:FFFFFFFFFFFFFFFFFFFFFFFFFFFFFFFFFFFFFFFFFFFFFFFFFFFFFFFFFFFFFFFFFFF
@A00155:342:HHGFNDSXY:1:2436:13575:16360 1:N:0:GAACCTAG+TCCGCATA
TGATGACGAACTCCCTTGAAGCGACGCCTGAAATTTCCCCACTGCAGTCATCTTCTGCACCGAGAAAT
AATTCCTAGTCGTGGTGATATTGCTGTCATTCACTCGCAGCACCCCACCATGGTACGTGCTATGCCTT
+
FFFFFFFFFFFFFFFFFFFFFFFFFFFFFFFFFFFFFFFFFFFFFFFFFFFFFFFFFFFFFFFFFFFF
FFFFFFF:FFFFFFFFFFFFFFFFFFFFFFFFFFFFFFFFFFFFFFFFFFFFFFFFFFFFFFFFFFFF
@A00155:342:HHGFNDSXY:1:1323:23547:8860 2:N:0:GAACCTAG+TCCGCATA
TGATGACGAACTCCCTTGAAGCGACGCCTGAAATTTCCCCACTGCAGTCATCTTCTGCACCGAGAAAT
AATTCCTAGTCGTGGTGATATTGCTGTCATTCACTCGCAGCACCCCACCATGGTACGTGCTATGCCTT
+
:FFFFFFFFFFFFFFFFFFFFFFFFFFFFFFFFFFFFFFFF,FFFFFF:FFFFFFFFFFFFFFFFFFFF
FFFFFFFFFFFFFFFFFFFFFFFFFFFFFFFFFFFFFFFFFFFFFFFFFFFFFFFFFFFFFFFFFFFF
@A00155:342:HHGFNDSXY:1:1154:29532:3630 2:N:0:GAACCTAG+TCCGCATA
TGATGACGAACTCCCTTGAAGCGACGCCTGAAATTTCCCCACTGCAGTCATCTTCTGCACCGAGAAAT
AATTCCTAGTCGTGGTGATATTGCTGTCATTCACTCGCAGCACCCCACCATGGTACGTGCTATGCCTT
+
F,:FFFFFFFFFFFF,FFFFFFFF:FFFFF:F:FFFFFFF:FFFF,FFFFFFFFFFFFFFFFFFFFFF
F,FF:FFFFF:FFFFF:FFFFF:FFFFFFFFF,FFFFF:FFFFFFFFFFFFFFFFFFFFFFFFFFFFF
@A00155:342:HHGFNDSXY:1:1413:5385:3208 2:N:0:GAACCTAG+TCCGCATA
ACGAACTCCCTTGAAGCGACGCCTGAAATTTCCCCACTGCAGTCATCTTCTGCACCGAGAAATAATTC
CTAGTCGTGGTGATATTGCTGTCATTCACTCGCAGCACCCCACCATGGTACGTGCTATGCCTTAAATT
+
```

FFFFFFFFFFFFFFFFFFFFFFFFFFFFFFFFF:FFFFFFFFF:FFFFFFFFFFFFFFFFFFFFFFFF
FFFFFFFFFFFFFFFFFFFFFFFF:FFFFFFFFFFFFFFFFFF:FFFFFFFF:FFFFFFFFFFFFFFF
@A00155:342:HHGFNDSXY:1:2131:12798:15671 2:N:0:GAACCTAG+TCCGCATA
CGAACTCCCTTGAAGCGACGCCTGAAATTTCCCCACTGCAGTCATCTTCTGCACCGAGAAATAATTCC
TAGTCGTGGTGATATTGCTGTCATTCACTCGCAGCACCCCACCATGGTACGTGCTATGCCTTAAATTA
+
:FFFFFFFFFFFFFF:FF:FFFFFFFFFFF:FFF,FFFFFFFFFFFFFFFFFFF:FFFFFFFFFFFFF
FFFFFFF:FF:FFFFFFFF:FFFFFFFFFFFFFFFFFFFFFFFFFFFFFFFFFFFFFFFFFFFF:FF:
@A00155:342:HHGFNDSXY:1:1104:25925:20087 2:N:0:GAACCTAG+TCCGCATA
CGAACTCCCTTGAAGCGACGCCTGAAATTTCCCCACTGCAGTCATCTTCTGCACTGAGAAATAATTCC
TAGTCGTGGTGATATTGCTGTCATTCACTCGCAGCACCCCACCATGGTACGTGCTATGCCTTAAATTA
+
FF,,FFFF:FFF:FF,:F:FFFFFFFFFFF:F:FFFFF,FFF:FFFFFFFFFF:FFFFFF:FFFFFFF
FFFFFFF:FF,FFFFFFFFFFFFFFFF:FFF,F:::FF:F:FFFFFFFFFFF::FFFF:FFFF:FFFF
@A00155:342:HHGFNDSXY:1:2104:27010:15546 2:N:0:GAACCTAG+TCCGCATA
CGAACTCCCTTGAAGCGACGCCTGAAATTTCCCCACTGCAGTCATCTTCTGCACTGAGAAATAATTCC
TAGTCGTGGTGATATTGCTGTCATTCACTCGCAGCACCCCACCATGGTACGTGCTATGCCTTAAATTA
+
FFFFFFFFFFFFFFFFFFFFFFFFFFFFFFFFFFFFFFFFFFFFFFFFFFFFFFFFFFF,FFFF:FFF
FFFFFFFFFF:FFFFFFFFFFFFFFFFFFFFFFFFFFFFFFFFFFFFFFFFFFFFFFFFFFFFFFFFF
@A00155:342:HHGFNDSXY:1:2348:22218:33990 2:N:0:GAACCTAG+TCCGCATA
GAACTCCCTTGAAGCGACGCCTGAAATTTCCCCACTGCAGTCATCTTCTGCACCGAGAAATAATTCCT
AGTCGTGGTGATATTGCTGTCATTCACTCGCAGCACCCCACCATGGTACGTGCTATGCCTTAAATTA
+
FFFFFFFFFFFFFFFFFFFFFFFFFFFFFFFFFFFFFFFFFFFFFFFFFFFFFFFFFFFFFFFFFFFF
FFFFFFFFFFFFFFFFFFFFFF:FFFFFFFFFFFFFFFFFFFFFFFFFFFFFFFFFFFFFFFFFFFF
@A00155:342:HHGFNDSXY:1:1219:17201:35102 2:N:0:GAACCTAG+TCCGCATA
GAACTCCCTTGAAGCGACGCCTGAAATTTCCCCACTGCAGTCATCTTCTGCACCGAGAAATAATTCCT
AGTCGTGGTGATATTGCTGTCATTCACTCGCAGCACCCCACCATGGTACGTGCTATGCCTTAAATTAC
+
FFFFFFFFFFFFFFFFFFFFFFFFFFFFFFFF:FFFFFFFFFFFF,FFFFFFFFFFFFFFFFFFFFFF
FFFFFFFF::FFFFFFFFF:FFFFF:FFFFFFFFFFFFFFFFFFFFFFFFFFFFFFFFFFFFFFF::F
@A00155:342:HHGFNDSXY:1:2514:29008:3255 2:N:0:GAACCTAG+TCCGCATA
GAACTCCCTTGAAGCGACGCCTGAAATTTCCCCACTGCAGTCATCTTCTGCACCGAGAAATAATTCCT
AGTCGTGGTGATATTGCTGTCATTCACTCGCAGCACCCCACCATGGTACGTGCTATGCCTTAAATTAC
+
FFFFFFFFFFFFFFFFFFFFFFFFFFF:FFFF:F,FFFFFFFFFFFFFFFFF:FFFFFFFFFF:FFFF
FFFF:FF,FFFFF:FFFF:FFFFFFFFFFFFFFFFFFFFF:FFFF:FFFFFFFFFFFFFFFFFFFFFF
@A00155:342:HHGFNDSXY:1:1436:22535:6073 2:N:0:GAACCTAG+TCCGCATA
GAACTCCCTTGAAGCGACGCCTGAAATTTCCCCACTGCAGTCATCTTCTGCACCGAGAAATAATTCCT
AGTCGTGGTGATATTGCTGTCATTCACTCGCAGCACCCCACCATGGTACGTGCTATGCCTTAAATTAC
+
FFFFFFFFF:FFFFFFFFFFFFFFFFFFFFFFFF:FFFFFFFFFFFFFFFFFFFFFFFFF:FFFFFFF
FFFFFFFFFFFFFFFFFFFFFF:FFFFFF:FFFFFFFFFFFFFFFFFFF:FFFFFFFFFFFFFFFFFF
@A00155:342:HHGFNDSXY:1:2202:15790:9643 2:N:0:GAACCTAG+TCCGCATA
ACTCCCTTGAAGCGACGCCTGAAATTTCCCCACTGCAGTCATCTTCTGCACCGAGAAATAATTCCTAG
TCGTGGTGATATTGCTGTCATTCACTCGCAGCACCCCACCATGGTACGTGCTATGCCTTAAATTACC
+
FF:F:F:FFFFFFF,FFF:FFFF:F:FFFFFFFF:FFFFFFF:FFF,FFFFFFFFFFFFFFFFF:FFF
FFFF:FFFFFFFFFF:FFFFFFF,FFFFF:FFFFFFFFFFFFFFFF:FFF:F:FF:FFFFFFFFFFF
@A00155:342:HHGFNDSXY:1:1606:28501:10426 2:N:0:GAACCTAG+TCCGCATA
CTCCCTTGAAGCGACGCCTGAAATTTCCCCACTGCAGTCATCTTCTGCACCGAGAAATAATTCCTAGT
CGTGGTGATATTGCTGTCATTCACTCGCAGCACCCCACCATGGTACGTGCTATGCCTTAAATTA
+

```
FFFFFFFFF:FFFFFFFFFFFFFFFFFF:F,FF:FFFFFFFFFFFFFFFFFFFFFFFFFF,FFFFFFF
FFFFFFFFFFFFFFFFFFFFFFFFFFFFF:FFFFFFFF:FFFFFFFFFFFFFFFFFFFFFFF:F
@A00155:342:HHGFNDSXY:1:1206:16233:16736 2:N:0:GAACCTAG+TCCGCATA
TCCCTTGAAGCGACGCCTGAAATTTCCCCACTGCAGTCATCTTCTGCACCGAGAAATAATTCCTAGTC
GTGGTGATATTGCTGTCATTCACTCGCAGCACCCCACCATGGTACGTGCTATGCCTTAAATTACCATC
+
FFFFFFFFFFFFFFFFF::FFFFFFFFFFFFF,FFFFFFFFFFFFFFFFFFFFFFFFFF:FFFFFFFF
FF:FFFFFF,FFFFFFFFFFFFFFFFFFFFFFFFF:FFFFFFFFFFFFFFFFFFFFFFFFFFFFF,FF
@A00155:342:HHGFNDSXY:1:1206:16667:21339 2:N:0:GAACCTAG+TCCGCATA
TCCCTTGAAGCGACGCCTGAAATTTCCCCACTGCAGTCATCTTCTGCACCGAGAAATAATTCCTAGTC
GTGGTGATATTGCTGTCATTCACTCGCAGCACCCCACCATGGTACGTGCTATGCCTTAAATTACCATC
+
FFFFFFFFFFFFFFFFFFFFFFFFFFFFFFFFF,FFFFFFFFFFFFFFFFFFFFFFFFFFFFFFFFFF
FFFFFFFFFFFFFFFFFFFFFFFFFFFFFFFFFFF:FFFFFFFFFFFFFFFFFFFFFFFFFF:FFFFF
@A00155:342:HHGFNDSXY:1:1206:16107:16517 2:N:0:GAACCTAG+TCCGCATA
TCCCTTGAAGCGACGCCTGAAATTTCCCCACTGCAGTCATCTTCTGCACCGAGAAATAATTCCTAGTC
GTGGTGATATTGCTGTCATTCACTCGCAGCACCCCACCATGGTACGTGCTATGCCTTAAATTACCATC
+
FFFFFFFFFFFFFFFFFFFFF:FFFFFF:FFFFFFF:FF:FFFFFFFFFFFFFFFFFFF:FFFFFFFF
FFFFFFFFFFFFFFFFFFFFFFFFFFFFFFFFFFFFFFFFFFFFFFFFFFFFFFFFFFFFFFFFF:FF
@A00155:342:HHGFNDSXY:1:1206:15176:16595 2:N:0:GAACCTAG+TCCGCATA
TCCCTTGAAGCGACGCCTGAAATTTCCCCACTGCAGTCATCTTCTGCACCGAGAAATAATTCCTAGTC
GTGGTGATATTGCTGTCATTCACTCGCAGCACCCCACCATGGTACGTGCTATGCCTTAAATTACCATC
+
FFFF:FFFFF:FFFFFFFFFFFF,:FFFF,FF:F:F,F:FF:FFFFFFFFFFFFFFFFFFFFFFFFFF
FFFFFFFFFFFFFFFFFFFFFFFFFFFFFFFFFFFFFFFFFFFFFFFFFFFFFFFFFFFFFFFFF,FF
@A00155:342:HHGFNDSXY:1:2149:25391:28588 2:N:0:GAACCTAG+TCCGCATA
TCCCTTGAAGCGACGCCTGAAATTTCCCCACTGCAGTCATCTTCTGCACCGAGAAATAATTCCTAGTC
GTGGTGATATTGCTGTCATTCACTCGCAGCACCCCACCATGGTACGTGCTATGCCTTAAATTACCATC
+
FF:F:,FF:FFFFFFFFFFF,FFFF,,F,:::,FFFFFFFFFFFFFFFF,FF,FFFFFFF:,:FFFF:
F:,F:FFFFFF:F:FFFF:F,FFFFF:FFFFF,F:F:FF,:FFF:FF,,FFFFF,,F:F:FF:F:FFF
@A00155:342:HHGFNDSXY:1:2613:21567:14888 2:N:0:GAACCTAG+TCCGCATA
TCCCTTGAAGCGACGCCTGAAATTTCCCCACTGCAGTCATCTTCTGCACCGAGAAATAATTCCTAGTC
GTGGTGATATTGCTGTCATTCACTCGCAGCACCCCACCATGGTACGTGCTATGCCTTAAATTACCATC
+
FFFF,,FFFFFFFFFFFFFFFFF,FFF:FFFFFFFFFFFFF:FFFFFFFFFFFFFF:FFFFFFFFFFF
FFFF:FFFFFFFFFFFFFFFFFFFFFFFFFFFFFFFFFFFFFFFFFFFFFFFFFFFFFFFFFFFFFFF
@A00155:342:HHGFNDSXY:1:1143:24424:22122 2:N:0:GAACCTAG+TCCGCATA
TCCCTTGAAGCGACGCCTGAAATTTCCCCACTGCAGTCATCTTCTGCACCGAGAAATAATTCCTAGTC
GTGGTGATATTGCTGTCATTCACTCGCAGCACCCCACCATGGTACGTGCTATGCCTTAAATTACCATC
+
FFF::FFFFFFFFFFFFF:FFFFFFFFFF:FF,:FFFFF,FFFFFFFFFFFFFFFFFFFFFF:F:FFF
FFFFFFFFFFFFFFFFFF:FFFFFFFFFFFFFF,FFFFFFFFFFFFFFFFFFFFFFFFFFF:FFFFFF
@A00155:342:HHGFNDSXY:1:1605:9661:17879 2:N:0:GAACCTAG+TCCGCATA
TCCCTTGAAGCGACGCCTGAAATTTCCCCACTGCAGTCATCTTCTGCACCGAGAAATAATTCCTAGTC
GTGGTGATATTGCTGTCATTCACTCGCAGCACCCCACCATGGTACGTGCTATGCCTTAAATTACCATC
+
F:FFFFFFFFFFFFFFFFFFFFF,FFFFFFFFFFFFFFFFFFFFFFFFFFFFFFFFF:FFFFFFFFFF
FFFFFFFFFFFFFFFFFFFFFFFFFFFFFFFFFFFFFFFFFFFFFFFFFFFFFFFFFFFFFF:FFFFF
@A00155:342:HHGFNDSXY:1:2276:17644:4899 2:N:0:GAACCTAG+TCCGCATA
TCCCTTGAAGCGACGCCTGAAATTTCCCCACTGCAGTCATCTTCTGCACCGAGAAATAATTCCTAGTC
GTGGTGATATTGCTGTCATTCACTCGCAGCACCCCACCATGGTACGTGCTATGCCTTAAATTACCATC
+
```

```
FFFFFFFFFFFFFFFFFFFFFFFFFFFFFFFFFFFFFFFFFFFFFFFFFFFFFFFFFFFFFFFFFFFF
FFFFFFFFFFFFFFFFFFFFFFFFFFFFFFFFFFFFFFFFFFFFFFFFFFFFFFFFFFFFFFFFFFFF
@A00155:342:HHGFNDSXY:1:2650:25473:5556 2:N:0:GAACCTAG+TCCGCATA
CCTTGAAGCGACGCCTGAAATTTCCCCACTGCAGTCATCTTCTGCACCGAGAAATAATTCCTAGTCGT
GGTGATATTGCTGTCATTCACTCGCAGCACCCCACCATGGTACGTGCTATGCCTTAAATT
+
FFFFFFFF:FF:FFFFFFFFF,FFFFFFF:FFFFFFF:FFFF:,FFFFFFFFFFFFFFFFFFFFFFFF
FFFFFFFFFFFFFFFFFFFFFFFFFFFFFFFFF:FFFFFFFFFFFFFFFFFFFFFFFFFF
@A00155:342:HHGFNDSXY:1:1237:5367:14512 2:N:0:GAACCTAG+TCCGCATA
CCTTGAAGCGACGCCTGAAATTTCCCCACTGCAGTCATCTTCTGCACCGAGAAATAATTCCTAGTCGT
GGTGATATTGCTGTCATTCACTCGCAGCACCCCACCATGGTACGTGCTATGCCTTAAATTACCATCGA
+
FFFFFFFF:FFFFFFFFFFFFFFFFFFFFFFFFFFFFFFF::FFFFF:FFFFFFFFFFFFFFF:FFFF
FFF:FFFFFFFFFFFF:FFFFFFFFFFFFFFFFFFFFFFFFFFFF::FFFFFF,FFFFFFFFFFFFFF
@A00155:342:HHGFNDSXY:1:1235:24198:33567 2:N:0:GAACCTAG+TCCGCATA
CCTTGAAGCGACGCCTGAAATTTCCCCACTGCAGTCATCTTCTGCACCGAGAAATAATTCCTAGTCGT
GGTGATATTGCTGTCATTCACTCGCAGCACCCCACCATGGTACGTGCTATGCCTTAAATTACCATCGA
+
FFFFFF:FFFFFFFFFFFFFFFFFFFFFFFFFFFFFF:FFFFFFFFFFFFFFF:FFFFFFFFFFFFFF
FFFFFFFFFFFFFFFF:FFFFFFFFFFFFFFFFFFFFFFFFFFFFFFFFFFFFFFFFFFFFFFFFFFF
@A00155:342:HHGFNDSXY:1:2141:17644:15828 2:N:0:GAACCTAG+TCCGCATA
CTTGAAGCGACGCCTGAAATTTCCCCACTGCAGTCATCTTCTGCACCGAGAAATAATTCCTAGTCGTG
GTGATATTGCTGTCATTCACTCGCAGCACCCCACCATGGTACGTGCTATGCCTTAAATTACCA
+
FFFFFFFFFFFFFFFFFFFFFFFFFFFFFFFFFFF,FFFFFFFFFFFFFFF:FFFF:FFFFFFFFFFF
FFFFFFFFFFFFF:F:FFFFFFFFFFFFFFFFFFFFFFFFFFFFFFFFFFFFFFFFFFFFFF:
@A00155:342:HHGFNDSXY:1:1316:25979:16705 2:N:0:GAACCTAG+TCCGCATA
CTTGAAGCGACGCCTGAAATTTCCCCACTGCAGTCATCTTCTGCACCGAGAAATAATTCCTAGTCGTG
GTGATATTGCTGTCATTCACTCGCAGCACCCCACCATGGTACGTGCTATGCCTTAAATTACCA
+
FFFFFFFFFFFFFFFFFFFFFFFFFFFFFFFFFFFFFFFFFFFFFFFFFFFFFFFFFFFFFFFFFFFF
FFFFFFFFFFFFFFFFFFFFFFFFFFFFFFFFFFFFFFFFFFFFFFFFFFFFFFFFFFFFFF:
@A00155:342:HHGFNDSXY:1:1106:17228:13917 1:N:0:GAACCTAG+TCCGCATA
CTTGAAGCGACGCCTGAAATTTCCCCACTGCAGTCATCTTCTGCACCGAGAAATAATTCCTAGTCGTG
GTGATATTGCTGTCATTCACTCGCAGCACCCCACCATGGTACGTGCTATGCCTTAAATTACCATCGAA
+
FFFFFFFFFFFFFFFFFFFFFFFFFFFFFFFFFFFFFFFFFFFFFFFFFFFFFFFFFFFFFFFFFFFF
FFFFFFFFFFFFFFFFFFFFFFFFFFFFFFFFFFFFFFFFFFFFFFFFFFFFFFFFFFFFFFFFFFFF
@A00155:342:HHGFNDSXY:1:2456:11921:12273 2:N:0:GAACCTAG+TCCGCATA
TTGAAGCGACGCCTGAAATTTCCCCACTGCAGTCATCTTCTGCACCGAGAAATAATTCCTAGTCGTGG
TGATATTGCTGTCATTCACTCGCAGCACCCCACCATGGTACGTGCTATGCCTTAAATTACCATCGAAG
+
::FFFFFFFFF:FFFFF:FFF:FF:FFFFFFFFFFFFFFFFFFFF:FFFFFFFFFFFFFFFFFFFFFF
FF:FFFFFFFFFFFFFFFFFFFFFFFFFFFFFFFFFFFFFFFFFFFFFFFFFFFFFFFFFFFFFFFFF
@A00155:342:HHGFNDSXY:1:2638:22227:34162 2:N:0:GAACCTAG+TCCGCATA
TTGAAGCGACGCCTGAAATTTCCCCACTGCAGTCATCTTCTGCACCGAGAAATAATTCCTAGTCGTGG
TGATATTGCTGTCATTCACTCGCAGCACCCCACCATGGTACGTGCTATGCCTTAAATTACCATCGAAG
+
FFFFFFFFFFFFFFFFFFF,FFFFFFF,FFFFFFFFFFFF:FFF:F:FFFFFFFFFFFFFFFFFFFFF
FFFFFFFFF:FFFF:FFFFFFFFFFFFFFFFFFFFFFFFFFFFFFFF:FFFFFFFF,::FFFFFFFFF
@A00155:342:HHGFNDSXY:1:2207:31042:27821 2:N:0:GAACCTAG+TCCGCATA
TGAAGCGACGCCTGAAATTTCCCCACTGCAGTCATCTTCTGCACCGAGAAATAATTCCTAGTCGTGGT
GATATTGCTGTCATTCACTCGCAGCACCCCACCATGGTACGTGCTATGCCTTAAATTACCATCGAAGT
+
```

```
FFFFFFFFFFFFFFFFFF:FFFFFFFFFFFFFFFFFFFFFFFFFFFFFFFFFFFFFFF:FFFFFFFFF
FFFFFFFFFFFFF:FFFFFFFFFFFFFFFFFFFFFFFF:FFFFFFFFFFFFFFFFFFFFFFFF:FFFF
@A00155:342:HHGFNDSXY:1:1103:30752:28447 2:N:0:GAACCTAG+TCCGCATA
TGAAGCGACGCCTGAAATTTCCCCACTGCAGTCATCTTCTGCACCGAGAAATAATTCCTAGTCGTGGT
GATATTGCTGTCATTCACTCGCAGCACCCCACCATGGTACGTGCTATGCCTTAAATTACCATCGAAGT
+
FFFFFFFFFFFFFF,FFFFFFFFFFFFFFFFFFFF:FFFFFFFFFFFFFFFFFFFFFFFFFFFFFFFF
FFFFFFFFFFFFFFFFFFFFFFFFFFFFFFFFFFFFFFFFFFFFFFFFFFFFFFFF:FFFFFFFFFFF
@A00155:342:HHGFNDSXY:1:2237:1750:16579 2:N:0:GAACCTAG+TCCGCATA
TGAAGCGACGCCTGAAATGTCCCCACTGCAGTCATCTTCTGCACCGAGAAATAATTCCTAGTCGTGGT
GATATTGCTGTCATTCACTCGCAGCACCCCACCATGGTACGTGCTATGCCTTAAATTACCATCGAAGT
+
FF,,FFF:FFFFFFFF,F,FFFFF:FFFFFF,F,FF,FF:FFFFFFFFFF:FFF,FFF,FFFFF:FFF
FFFF:,:F,:FFF,FFFFFFFFFFFFFFFF,FFFFFFFFFFFFFFFFFFF:FFF:,,FFF:FFFF:FF
@A00155:342:HHGFNDSXY:1:2652:30047:21997 2:N:0:GAACCTAG+TCCGCATA
TGAAGCGACGCCTGAAATTTCCCCACTGCAGTCATCTTCTGCACCGAGAAATAATTCCTAGTCGTGGT
GATATTGCTGTCATTCACTCGCAGCACCCCACCATGGTACGTGCTATGCCTTAAATTACCATCGAAGT
+
FFFFFFF:FFFFFF:FF:FFFFFF:FFFFFFFFF:FFFFFFFFFFFFFFFFFFFFFFFFFFFFFFFFF
FFFFFFFFFFFFFFFFFFFFFFFFFFFFFFFFFFFFFFFFFFFFFFFFFFFFFFFF:FFFFFFFFFFF
@A00155:342:HHGFNDSXY:1:2323:2202:24439 2:N:0:GAACCTAG+TCCGCATA
GAAGCGACGCCTGAAATTTCCCCACTGCAGTCATCTTCTGCACCGAGAAATAATTCCTAGTCGTGGTG
ATATTGCTGTCATTCACTCGCAGCACCCCACCATGGTACGTGCTATGCCTTAAATTACCATCGAAGT
+
:FFFFFFFFFF:FF,FF,F:FFFFFF,FFFFFFFFFFF:FFFFFFFFFFFFFFFFFFFFFFFFFFFFF
F:FFFFFFF:FF,FF:FFFFFFFFFFFFFFFFFFFFFFFF,FFFFFFFFFFFFFFFFFFFFFFFFFF
@A00155:342:HHGFNDSXY:1:1342:26395:28573 1:N:0:GAACCTAG+TCCGCATA
GAAGCGACGCCTGAAATTTCCCCACTGCAGTCATCTTCTGCACCGAGAAATAATTCCTAGTCGTGGTG
ATATTGCTGTCATTCACTCGCAGCACCCCACCATGGTACGTGCTATGCCTTAAATTACCATCGAAGTT
+
FFFFFFFFFFFFFFFFFFFFFFFFFFFFFFFFFFFFFFFFFFFFFFFFFFFF:FFFFFFFFFFFFFFF
FFFFFFFFFFFF:FFFFFFFFFFFFFFFFFFFFFFFFFFF:FFFFFFFFFFFF:FFFFFFFFFFFFFF
@A00155:342:HHGFNDSXY:1:2465:1362:11271 2:N:0:GAACCTAG+TCCGCATA
AAGCGACGCCTGAAATTTCCCCACTGCAGTCATCTTCTGCACCGAGAAATAATTCCTAGTCGTGGTGA
TATTGCTGTCATTCACTCGCAGCACCCCACCATGGTACGTGCTATGCCTTAAATTACCATCGAAGTT
+
FFFF:FFFFF,,FFF,FF,F:FFFF,FFF,:F:,:FF,:FFFFFF:FFF:FFFFFFF,:,,FF:FFFF
:FF:FFFFF:FF:FFF,FF:F,FFFFFFF,FFFFFFFFF,,F::FFFFF:FFFF:FFFFFFFFF:FF
@A00155:342:HHGFNDSXY:1:2509:25672:20024 2:N:0:GAACCTAG+TCCGCATA
AGCGACGCCTGAAATTTCCCCACTGCAGTCATCTTCTGCACCGAGAAATAATTCCTAGTCGTGGTGAT
ATTGCTGTCATTCACTCGCAGCACCCCACCATGGTACGTGCTATGCCTTAAATTACCATCGAAGTTGA
+
FFFFFFFFFFF:FFFFFFFFFFFFFFFFFFFFFFFFFFFFFFFFFFFFFFFFFFFFFFFFFFFFFFFF
FFFFFFFFFFFFFFFFFFFFFFFFFFFFFFFFFFFFFFFFFFFFFFFFFFFFFFFFFFFF:FFFFFFF
@A00155:342:HHGFNDSXY:1:2149:25391:28588 1:N:0:GAACCTAG+TCCGCATA
AGCGACGCCTGAAATTTCCCCACTGCAGTCATCTTCTGCACCGAGAAATAATTCCTAGTCGTGGTGAT
ATTGCTGTCATTCACTCGCAGCACCCCACCATGGTACGTGCTATGCCTTAAATTACCATCGAAGTTGA
+
FFFFFFFFFF,FF:,FFFFFFFFFF,FF:FFFFF,FFF:FF:FFFFFFFFFFF,F:FFF,:F:,,:FF
FFFFFFF:FFF:FFF,FF,FFFFFFF:FF,FF,FFFFF,F,:FFF:F,:FFF:,F,FFFFFFFFFF,F
@A00155:342:HHGFNDSXY:1:1503:23927:34256 1:N:0:GAACCTAG+TCCGCATA
CGACGCCTGAAATTTCCCCACTGCAGTCATCTTCTGCACCGAGAAATAATTCCTATTCGTGGTGATAT
TGCTGTCATTCACTCGCAGCACCCCACCATGGTACGTGCTATGCCTTAAATTACCATCGAAGTTGAAT
+
```

FFFFFFFFFFFFFFFFFFF:FFFFFFFFFFFFFFFFFFFFFFFFFFFFFFFFFFFFFFFFFFFFFFFF
FFFFFFFFFFFFFFFFFFFFFFFFFFFFFFFFFFFFFFFFFFFFFFFFFFF:FFFFFFFFFFFFFFFF
@A00155:342:HHGFNDSXY:1:2262:31286:21230 2:N:0:GAACCTAG+TCCGCATA
CGACGCCTGAAATTTCCCCACTGCAGTCATCTTCTGCACCGAGAAATAATTCCTAGTCGTGGTGATAT
TGCTGTCATTCACTCGCAGCACCCCACCATGGTACGTGCTATGCCTTAAATTACCATCGAAGTTGAAT
+
FFFFFFFFFFFFFFFFFFFFFFFFFFFFFFFFFFFFFFFFFFFF::FFFFFFFFFFF:FFFF:F:FFF
FFF:FFFFFF:FFFFF:FFFFFFFFFFFFFFFFFFFFFFFFFFFFFFFFFFF:FFF,FFFFFFFF:FF
@A00155:342:HHGFNDSXY:1:1214:19732:8516 2:N:0:GAACCTAG+TCCGCATA
CGACGCCTGAAATTTCCCCACTGCAGTCATCTTCTGCACCGAGAAATAATTCCTAGTCGTGGTGATAT
TGCTGTCATTCACTCGCAGCACCCCACCATGGTACGTGCTATGCCTTAAATTACCATCGAAGTTGAAT
+
FFFF,FFFFFFF:FFFFFFFFFFF,F:FFFFFFF:FFFFF:FFFF:FFF::FFFFFFF::F,FFFFFF
FFFFFFFFFFF:FFF,FFFFF:F:FFF:FFFFFFFFFFF:FFFFFFF:FFF:FF:FFF:FFF:F:FFF
@A00155:342:HHGFNDSXY:1:1223:31304:10144 2:N:0:GAACCTAG+TCCGCATA
ACGCCTGAAATTTCCCCACTGCAGTCATCTTCTGCACCGAGAAATAATTCCTAGTCGTGGTGATATTG
CTGTCATTCACTCGCAGCACCCCACCATGGTACGTGCTATGCCTTAAATTACCATCGAAGTTGAATTC
+
FFFFFFFFFF:FFFFFF:FFFFFFFFF:FFFFFFFFFFFFFFFFFFFFFFFFFFFFFFFFFFFFFFFF
FFFFFFFFFFFFFFFFFFFFFFFFFFFFFFFFFFFFFFFFFFFFFFFFFFFFFFFFFFFFFFFFFFFF
@A00155:342:HHGFNDSXY:1:2262:8386:6183 2:N:0:GAACCTAG+TCCGCATA
CGCCTGAAATTTCCCCACTGCAGTCATCTTCTGCACCGAGAAATAATTCCTAGTCGTGGTGATATTGC
TGTCATTCACTCGCAGCACCCCACCATGGTACGTGCTATGCCTTAAATTACCATCGAAGTTGAATTCG
+
FFFF:FFFF,FFFFFFFFFFFFFFF,,F:FFFFFFFFFFFFF:FF::F,FFF:FFFFFFFFFF,FFFF
FFFFFFFFFFFFFFFFF:FFFFFFF:FFFF:FFFFFFFFFFFFF:F::FF:F,FFF:::FFF:FFFFF
@A00155:342:HHGFNDSXY:1:1172:24469:14716 2:N:0:GAACCTAG+TCCGCATA
CGCCTGAAATTTCCCCACTGCAGTCATCTTCTGCACCGAGAAATAATTCCTAGTCGTGGTGATATTGC
TGTCATTCACTCGCAGCACCCCACCATGGTACGTGCTATGCCTTAAATTACCATCGAAGTTGAATTCG
+
FFFFFFFFFF:FFF:FFFFFFFFF:FFFFFFFFFFFFFFFFFFFFFFFFFFFFFFFFFFFFFFFFFF
FFFFF:FFFFFFFFFFFFFFFFFFFFFFFFFFFFFFFFFFFFFFFFFF,FFFFFFFFFFFFFFFFFFF
@A00155:342:HHGFNDSXY:1:2505:19569:4163 2:N:0:GAACCTAG+TCCGCATA
CGCCTGAAATTTCCCCACTGCAGTCATCTTCTGCACCGAGAAATAATTCCTAGTCGTGGTGATATTGC
TGTCATTCACTCGCAGCACCCCACCATGGTACGTGCTATGCCTTAAATTACCATCGAAGTTGAATTCG
+
FFFFFFFFF:F:FFFF:FF,FFFFF::F,FFFFFFFFF::FFF:FFFFFFFFFFFFFFFFFFFF:FFF
,FFFFFFFFFFFFFFFFFFFFFFFFFFFFFFFFFFFFFFFFFFFFFFFFFFFFFFFF:FFFFFFFFFF
@A00155:342:HHGFNDSXY:1:1512:5819:28291 1:N:0:GAACCTAG+TCCGCATA
CGCCTGAAATTTCCCCACTGCAGTCATCTTCTGCACCGAGAAATAATTCCTAGTCGTGGTGATATTGC
TGTCATTCACTCGCAGCACCCCACCATGGTACGTGCTATGCCTTAAATTACCATCGAAGTTGAATTCG
+
FFFFFFFFFFFFFFF:FFFFFFFFFFFFFFFFFFFFF:FFFFFFFFFFFFFFFFFFFF:FFFFFFFFF
FFFFFFFFFFFFFFFFFFFFFFFFFFFFFFFFFFFFFFFFFFFFFF:FFFFFFFFFFFFFFFFFFFFF
@A00155:342:HHGFNDSXY:1:1207:20672:34695 1:N:0:GAACCTAG+TCCGCATA
CGCCTGAAATTTCCCCACTGCAGTCATCTTCTGCACCGAGAAATAATTCCTAGTCGTGGTGATATTGC
TGTCATTCACTCGCAGCACCCCACCATGGTACGTGCTATGCCTTAAATTACCATCGAAGTTGAATTCG
+
FFFF,FFFFFFFFFFFFFFFFFFF::FFFFFFFFFFFFFFFFFFFFF:FFFFFFFFFF:FFFFFFFFF
FFFFF:FFFFFFFFFFFFFFFFFFFFFFFFFFFFFFFFFFF:FFFF:FFFF:FFFFFFFFFFF:F,FF
@A00155:342:HHGFNDSXY:1:1313:31964:8563 2:N:0:GAACCTAG+TCCGCATA
CGCCTGAAATTTCCCCACTGCAGTCATCTTCTGCACCGAGAAATAATTCCTAGTCGTGGTGATATTGC
TGTCATTCACTCGCAGCACCCCACCATGGTACGTGCTATGCCTTAAATTACCATCGAAGTTGAATTCG
+

```
FFFF:FFFFF,FFFFF,FFFFFF::FFFFFF:FFFFFF:FF,FFFFFFFFFFFFFFFFFFFFFFFFFF
FFFFF:FFFFFFFFFFFFFFFFFFFFFFFFFFFFFFF:F:FFFFF,,:FFFFF:FFFFFFFFFFFFFFF
@A00155:342:HHGFNDSXY:1:1606:28501:10426 1:N:0:GAACCTAG+TCCGCATA
GCCTGAAATTTCCCCACTGCAGTCATCTTCTGCACCGAGAAATAATTCCTAGTCGTGGTGATATTGCT
GTCATTCACTCGCAGCACCCCACCATGGTACGTGCTATGCCTTAAATTACCATCGAAGTTGAAT
+
FFFFFFFFFFFFFFFFFFFFFFFFFFFF:FFFFFFFFFFFFFFFFFFF::FFFFFFFFFFFFFFFF:F
FFFFFFFFFFFFFFFFFFFFFFFFFFFFFFFFFFFFFFFFFFFFFFFFF::FFFFFFFFFFF:F
@A00155:342:HHGFNDSXY:1:2323:2202:24439 1:N:0:GAACCTAG+TCCGCATA
GCCTGAAATTTCCCCACTGCAGTCATCTTCTGCACCGAGAAATAATTCCTAGTCGTGGTGATATTGCT
GTCATTCACTCGCAGCACCCCACCATGGTACGTGCTATGCCTTAAATTACCATCGAAGTTGAATTCGG
+
FFFFFFFFFFFFFFFFFFFFFFFFFFFFFFFFFFFFFFFFFFFFFFFFFFFFFFFFFFFFFFFFFFFF
FFFFFFFFFFFFFFFFFFFFFFFFFFFFFFFFFFFFFFFFFFFFFFFFFFFF,FFFFFFFFFFFFFF
@A00155:342:HHGFNDSXY:1:2528:3812:30107 1:N:0:GAACCTAG+TCCGCATA
GCCTGAAATTTCCCCACTGCAGTCATCTTCTGCACCGAGAAATAATTCCTAGTCGTGGTGATATTGCT
GTCATTCACTCGCAGCACCCCACCATGGTACGTGCTATGCCTTAAATTACCATCGAAGTTGAATTCGG
+
FFFFFFFFFFFFFFFFFFFFFFFFFFFFFFFFFFFFFFFFFFFFFFFFFFFF:FFFFFFFFFFFFFFF
FFFFFFFFFFFFFFFFFFFFFFFFF:FFFFFFFFFFFFFFFFFFFFFFFFFFF,FFFFFFFFFFFFFFFF
@A00155:342:HHGFNDSXY:1:2237:1750:16579 1:N:0:GAACCTAG+TCCGCATA
GCCTGAAATTTCCCCACTGCAGTCATCTTCTGCACCGAGAAATAATTCCTAGTCGTGGTGATATTGCT
GTCATTCACTCGCAGCACCCCACCATGGTACGTGCTATGCCTTAAATTACCATCGAAGTTGAATTCGG
+
FFFFFFFFFFFFFFF:FFFF:FFFFFFFFFFFFFFFFFFFFFFF:,:FFFFFF,:FFFF,FF,FFFFFF
FFF:FFFFFFFFFFFFFFFFF,FFFFFFF,FFFFFF:FFFF:,:FF:F:FF:FFFFFFFFFF::FFFF
@A00155:342:HHGFNDSXY:1:2372:1425:5118 1:N:0:GAACCTAG+TCCGCATA
GCCTGAAATTTCCCCACTGCAGTCATCTTCTGCACCGAGAAATAATTCCTAGTCGTGGTGATATTGCT
GTCATTCACTCGCAGCACCCCACCATGGTACGTGCTATGCCTTAAATTACCATCGAAGTTGAATTCGG
+
FFFFFFFFFF:F:FF:FFFFFFFFF:FFFFF,FF:F,FFFFFFFFFFFFFFFFFF:FFFFFFFFFF:F
FF:FFFFFFF,,FFFF,F:FFFFFFFFFFFFFFF:FFFF:F:FFF:FFFFFFFFF:FFFFFF:FF:FF
@A00155:342:HHGFNDSXY:1:1635:2293:14826 1:N:0:GAACCTAG+TCCGCATA
GCCTGAAATTTCCCCACTGCAGTCATCTTCTGCACCGAGAAATAATTCCTAGTCGTGGTGATATTGCT
GTCATTCACTCGCAGCACCCCACCATGGTACGTGCTATGCCTTAAATTACCATCGAAGTTGAATTCGG
+
FFFFFFFFFFFFFFFFFFFFFFFFFFFFFFFF:,F:FFFFFFFFFFFF:FFFFFFFFFFFFFFFFF:FF
FF:FFFFFFFFFFFFF,FFFFF:FFF:FFFFFF,FFFFFFFFFFFFFFFFFFFFFFF:FFFFFFFFFFF
@A00155:342:HHGFNDSXY:1:2430:20157:9596 1:N:0:GAACCTAG+TCCGCATA
GCCTGAAATTTCCCCACTGCAGTCATCTTCTGCACCGAGAAATAATTCCTAGTCGTGGTGATATTGCT
GTCATTCACTCGCAGCACCCCACCATGGTACGTGCTATGCCTTAAATTACCATCGAAGTTGAATTCGG
+
FF:FFFFFFFFFFFFFFFFFFFFFFFFFFFFFFFFFFFFFFFFFF:FFFFFFFFFFFFFFFFFFFFFF
FFFFFFFFFFFFFFFFFFFFFFFFFFFFFFFFFFFFFF:FFF:F:FFFF:FFFFFFFFFF:FFFFFFFFF
@A00155:342:HHGFNDSXY:1:1473:10321:29293 1:N:0:GAACCTAG+TCCGCATA
CCTGAAATTTCCCCACTGCAGTCATCTTCTGCACCGAGAAATAATTCCTAG
+
FFFFFFFFFFFFFFFFFFFFFFFFFFFFFFFFFFFFFFFFFFFFFFFFFFF
@A00155:342:HHGFNDSXY:1:2469:20419:11553 2:N:0:GAACCTAG+TCCGCATA
CCTGAAATTTCCCCACTGCAGTCATCTTCTGCACCGAGAAATAATTCCTAGTCGTGGTGATATTGCTG
TCATTCACTCGCAGCACCCCACCATGGTACGTGCTATGCCTTAAATTACCATCGAAGTTGAATTCGGC
+
FFFFFFFFFFFFFFFFFFFFFFFFFF:FFFFFFFFFFFF,FFFFFFFFFFFFFFFFFFFFFFFFFFFFF
FFFFFFFFFFFFFFFFFFFFFFFFFFFFFFFFFFFFFFFFFFFFFF,FFFFFFFFFFFFFFFFF:FFFFF
```

```
@A00155:342:HHGFNDSXY:1:2658:2302:27336 1:N:0:GAACCTAG+TCCGCATA
CCTGAAATTTCCCCACTGCAGTCATCTTCTGCACCGAGAAATAATTCCTAGTCGTGGTGATATTGCTG
TCATTCACTCGCAGCACCCCACCATGATACGTGCTATGCCTTAAATTACCATCGAAGTTGAATTCGGC
+
FF:FFFFFFFFFFFFFFFFFFFFFFFFFFFFFFFFFFFF:FFFFFFFFFFFFFFFFFFFFFFFFF,FF
FFFFFFFFFF:FFFFFFFFFFFFFFFFFFFFFFFFFFFFFFFFFFFFFFFFFFFFFF:FFFF:FFF:F
@A00155:342:HHGFNDSXY:1:1315:19940:35650 2:N:0:GAACCTAG+TCCGCATA
CCTGAAATTTCCCCACTGCAGTCATCTTCTGCACCGAGAAATAATTCCTAGTCGTGGTGATATTGCTG
TCATTCACTCGCAGCACCCCACCATGGTACGTGCTATGCCTTAAATTACCATCGAAGTTGAATTCGGC
+
FFFFFFFFFFFFFFFFF:FFF:FFFFFFFFFFFFFFFFFFFFFFFFFFFFFFFFFFFFFFFFFFFFFF
FFFFFFFFFFFFFFFFFFFFFFFFFFF,FFFFFFFFFFFFFFFFFFF:FFFFFFFFFFFFFFFFFFFF
@A00155:342:HHGFNDSXY:1:1460:13856:30812 2:N:0:GAACCTAG+TCCGCATA
CTGAAATTTCCCCACTGCAGTCATCTTCTGCACCGAGAAATAATTCCTAGTCGTGGTGATATTGCTGT
CATTCACTCGCAGCACCCCACCATGGTACGTGCTATGCCTTAAATTACCATCGAAGTTGAATTCG
+
FFFFFFF,FFFFFFFFFFF:FFF,FFFF,FFFFFFFFF:FFFFFFFFF:FFFFFFFFFFFFFFFFFFF
FFFFFFFFFFFFFFFFFFFFFFFFFF:FFFFFFFFFFFFFFFFFF:FFFFFFFFFFFFFFFFFFF
@A00155:342:HHGFNDSXY:1:2650:25473:5556 1:N:0:GAACCTAG+TCCGCATA
TGAAATTTCCCCACTGCAGTCATCTTCTGCACCGAGAAATAATTCCTAGTCGTGGTGATATTGCTGTC
ATTCACTCGCAGCACCCCACCATGGTACGTGCTATGCCTTAAATTACCATCGAAGTTGAAT
+
FFFFFFFFFFFFFFFFFFFFF:FFFFFFFFFFFFFFFFFFFFFFFFFFFFFFFFFFFFFFFFFFFFFF
FFFFFFFFFFFFFFFFFFFFFFFFFFFFFFFFFFFFFFFFFFFFFFFFFFFFFFFFFFFF:
@A00155:342:HHGFNDSXY:1:1521:7916:28385 1:N:0:GAACCTAG+TCCGCATA
TGAAATTTCCCCACTGCAGTCATCTTCTGCACCGAGAAATAATTCCTAGTCGTGGTGATATTGCTGTC
ATTCACTCGCAGCACCCCACCATGGTACGTGCTATGCCTTAAATTACCATCGAAGTTGAATTCGGCTC
+
FFFFFFFFFFFFFFFFFFFFFFFFFFFFFFFFFFFFFFFFFFFFFFFFFFFFFFFFFFFFFFFF,FFF
FFFFFFFFFFFFFFFFFFFFFFFFFFFFFFFFFF:FFF:FFFFF:FF:FFFFFFFFFFFFFFFFFFFF
@A00155:342:HHGFNDSXY:1:1624:13331:10050 1:N:0:GAACCTAG+TCCGCATA
TGAAATTTCCCCACTGCAGTCATCTTCTGCACCGAGAAATAATTCCTAGTCGTGGTGATATTGCTGTC
ATTCACTCGCAGCACCCCACCATGGTACGTGCTATGCCTTAAATTACCATCGAAGTTGAATTCGGCTC
+
FFFFFFFFFFFFFFFFFFFFFFFFFFFFFFFFFFFFFFFFFFFFFFFFFFFFFFFFFFFFFFFFFFFF
FFFFFFFFFFFFFFFFFFFFFFFFFFFFFFFFFFFFFFFFFFFFFFFFFFFFFFFFFFFFFFFF:FFF
@A00155:342:HHGFNDSXY:1:1521:8106:28056 1:N:0:GAACCTAG+TCCGCATA
TGAAATTTCCCCACTGCAGTCATCTTCTGCACCGAGAAATAATTCCTAGTCGTGGTGATATTGCTGTC
ATTCACTCGCAGCACCCCACCATGGTACGTGCTATGCCTTAAATTACCATCGAAGTTGAATTCGGCTC
+
FFFFFFFFFFFFFFFFFFFFFFFFFFFFFFFFFFFFFFFFFFFFFFFFFFFFFFFFFFFFFFFFFFFF
FFFFFFFFFFFFFFFFFFFFFFFFFFFFFFFFFFFFFFFFFFFFFFFFFFFFFFFFFFFFFFFFFFFF
@A00155:342:HHGFNDSXY:1:1645:9435:4304 2:N:0:GAACCTAG+TCCGCATA
TGAAATTTCCCCACTGCAGTCATCTTCTGCACCGAGAAATAATTCCTAGTCGTGGTGATATTGCTGTC
ATTCACTCGCAGCACCCCACCATGGTACGTGCTATGCCTTAAATTACCATCGAAGTTGAATTCGGCTC
+
FFFFFFFFFFFFFFFF:FFFFFFF:FFFFFFFFFFFFFFFFFFFFFFFFFFFFFFFFFFFFFFFFFFF
FFFFFFFFFFFFFFFFFFFFFFFFFFFFFFFFFFFFFFFFFFFFFFFFFFFFFFFFFFFFFFFFFFFF
@A00155:342:HHGFNDSXY:1:2114:22101:15029 1:N:0:GAACCTAG+TCCGCATA
TGAAATTTCCCCACTGCAGTCATCTTCTGCACCGAGAAATAATTCCTAGTCGTGGTGATATTGCTGTC
ATTCACTCGCAGCACCCCACCATGGTACGTGCTATGCCTTAAATTACCATCGAAGTTGAATTCGGCTC
+
FFFFFFF:FFFFFFFFFFFFFFF:FFFFFFFFFFFFFFFFFFFFFFFFFFFFFFFFFFF:FF:FFFFF
FFFFFFFF:FFFFFFFFFFFFFF:FFFFFFFFF,FFFFFFFFF:FFFFFFFFFFFFFFFFFFF:FFFF
```

```
@A00155:342:HHGFNDSXY:1:2534:13946:22701 2:N:0:GAACCTAG+TCCGCATA
AAATTTCCCCACTGCAGTCATCTTCTGCACCGAGAAATAATTCCTAGTCGTGGTGATATTGCTGTCAT
TCACTCGCAGCACCCCACCATGGTACGTGCTATGCCTTAAATTACCATCGAAGTTGAATTCGGCTCCT
+
FFFFFFFFFFFFF,FFFFFFFFFFFFFFFFFFFFFFFFFFFFFFFFFFFFF:FFFFFFFFFFFFFF
FFFFFFFFFFFFFFFFFFFFFFFFFF:FFFFFFFFFFFFFFFFFFFFFFFFFFFFFFFFFFFFFFF
@A00155:342:HHGFNDSXY:1:2535:18801:26005 2:N:0:GAACCTAG+TCCGCATA
AATTTCCCCACTGCAGTCATCTTCTGCACCGAGAAATAATTCCTAGTCGTGGTGATATTGCTGTCATT
CACTCGCAGCACCCCACCATGGTACGTGCTATGCCTTAAATTACCATCGAAGTTGAATTCGGCTCCTG
+
FF,:FFFFFFF:FFFF:FF:FFFFFFFFFFFFFFFFFFFFF:FFFFFFFFFFFFFFFFFFFFFFFF
FFFFFFFFFFFFFFFFFFFFFFFFFFFFFFFFFFFFFFFFFFF:FFFFFFFFFFFFFFFFFFFFFF
@A00155:342:HHGFNDSXY:1:1644:23592:24596 2:N:0:GAACCTAG+TCCGCATA
AATTTCCCCACTGCAGTCATCTTCTGCACCGAGAAATAATTCCTAGTCGTGGTGATATTGCTGTCATT
CACTCGCAGCACCCCACCATGGTACGTGCTATGCCTTAAATTACCATCGAAGTTGAATTCGGCTCCTG
+
F,F,FFFFFFFFFFF:FFFF:FFFFFFFFFF:FFFFFFFFFFFF:FFFFFFFFFFFFFFFFFF,FF
FFFFFFFFFFFFFFFFFFFFFFFFFFFFFFFFFFFFFFFFFFFFFFFFFFFFFFFFFFFFFFFFFF
@A00155:342:HHGFNDSXY:1:2535:16495:18067 2:N:0:GAACCTAG+TCCGCATA
AATTTCCCCACTGCAGTCATCTTCTGCACCGAGAAATAATTCCTAGTCGTGGTGATATTGCTGTCATT
CACTCGCAGCACCCCACCATGGTACGTGCTATGCCTTAAATTACCATCGAAGTTGAATTCGGCTCCTG
+
FF:FFFFFF:FFFFFFFFFFFFFFFFFFFFFFFFFFF:FFFFFFFFFFFFFFFFFFFFFFF:FFFF
FFFFFFFFFFFFFFFFFFFFFFFFFFFFFFFFFFFF:FFFFFFFFFFFFFFFFFFFFFFFFFFFFF
@A00155:342:HHGFNDSXY:1:2644:31684:21825 2:N:0:GAACCTAG+TCCGCATA
AATTTCCCCACTGCAGTCATCTTCTGCACCGAGAAATAATTCCTAGTCGTGGTGATATTGCTGTCATT
CACTCGCAGCACCCCACCATGGTACGTGCTATGCCTTAAATTACCATCGAAGTTGAATTCGGCTCCTG
+
FFFFFFFFFFFFFFFFFFFFFFFFFFFFFFFFFFFFFFFFFFFF:FFFFFFFFF:FFFFFFFFFFF
FFFFFFFFFFFFFFFFFFFFFFFFFFFFFFFFFFFFFFFFFFFF:FFFFFFFFFFFFFFFFFFFFF
@A00155:342:HHGFNDSXY:1:1341:18358:15718 2:N:0:GAACCTAG+TCCGCATA
AATTTCCCCACTGCAGTCATCTTCTGCACCGAGAAATAATTCCTAGTCGTGGTGATATTGCTGTCATT
CACTCGCAGCACCCCACCATGGTACGTGCTATGCCTTAAAGTACCATCGAAGTTGAATTCGGCTCCTG
+
F,F:FFFFFFFFFFFF:FF:FFFFFFFFFF::F:FFFF:FFFFFFFFFFFFFFFFF:FFFFFFF:F
FFFFFFF:FFFFFFFFFFFFFFFFFFFF:FFFFF,FFF,F,FF:FFFFF,FFF:F,FFFFFFFFFF
@A00155:342:HHGFNDSXY:1:2538:32940:30702 1:N:0:GAACCTAG+TCCGCATA
AATTTCCCCACTGCAGTCATCTTCTGCACCGAGAAATAATTCCTAGTCGTGGTGATATTGCTGTCATT
CACTCGCAGCACCCCACCATGGTACGTGCTATGCCTTAAATTACCATCGAAGTTGAATTCGGCTCCTG
+
FFF,FFFFFFFFFFFFFF:FFFFFFFFFFFFF:FFFFFFFFFFFFFFFFFFFFFFFFF:FFFF:FFFF
FFFFFFFFFFFFFFFFFFFFFFFFFFF:FFFFFFFFFFFFFFFFFFFFFFFFFFFFFFFFFFFFFFFF
@A00155:342:HHGFNDSXY:1:1316:25979:16705 1:N:0:GAACCTAG+TCCGCATA
TTTCCCCACTGCAGTCATCTTCTGCACCGAGAAATAATTCCTAGTCGTGGTGATATTGCTGTCATTCA
CTCGCAGCACCCCACCATGGTACGTGCTATGCCTTAAATTACCATCGAAGTTGAATTCG
+
FFFFFFFFFFFFFFFFFFFFFFFFFFFFFFFFFFFFFFFFFFFFFFFFFFFFFFFFFFFFFFFFFF
FFFFFFFFFFFFFFFFFFFFFFFFFFF:FFFFFFFFFFFFFFFFFFFFFFFFFFFFF
@A00155:342:HHGFNDSXY:1:2141:17644:15828 1:N:0:GAACCTAG+TCCGCATA
TTTCCCCACTGCAGTCATCTTCTGCACCGAGAAATAATTCCTAGTCGTGGTGATATTGCTGTCATTCA
CTCGCAGCACCCCACCATGGTACGTGCTATGCCTTAAATTACCATCGAAGTTGAATTCG
+
FFFFFFFFFFFFFFFFFFFFFFFFFFFFFFFFFFFFFFFFFFFFFFFF,FFFFFFFFFFFFFFFFF
FFFFFFFFFFFFFFFFFFFFFFFFFFF,,FFFF:FFFFFFFFFFFFFFFFFFF:FFF
```

```
@A00155:342:HHGFNDSXY:1:2331:5855:15201 1:N:0:GAACCTAG+TCCGCATA
TTTCCCCACTGCAGTCATCTTCTGCACCGAGAAATAATTCCTAGTCGTGGTGATATTGCTGTCATTCA
CTCGCAGCACCCCACCATGGTACGTGCTATGCCTTAAATTACCATCGAAGTTGAATTCGGCTCC
+
FFFFFFFF,FFFFFFFFFFFFFFFFFF:FFFFFFFFFFFFFFFFFFFFF:FFFFFFFFFF:::FFFFF
FFFFFFFF:FFFFFFFFFF:FFFF:,FFFFFFFFFFFFFFFFFFFFFFFFFFFFFFFFFFFFFF
@A00155:342:HHGFNDSXY:1:2426:3441:9862 1:N:0:GAACCTAG+TCCGCATA
TTTCCCCACTGCAGTCATCTTCTGCACCGAGAAATAATTCCTAGTCGTGGTGATATTGCTGTCATTCA
CTCGCAGCACCCCACCATGGTACGTGCTATGCCTTAAATTACCATCGAAGTTGAATTCGGCTCC
+
FFFFFFFFFFFFFFFFFFFFFFFFFFFFFFFFFFFF:FFFFFFFFFFFFFFFFFF:FFF:FFFFFFFF
FFFFFFFFFFFFFFFFFFFFFFFFFFFFFFFFFFFFF:FFFFFFFFFFFFFFFFFFFF:FFFFF
@A00155:342:HHGFNDSXY:1:1103:30752:28447 1:N:0:GAACCTAG+TCCGCATA
TTTCCCCACTGCAGTCATCTTCTGCACCGAGAAATAATTCCTAGTCGTGGTGATATTGCTGTCATTCA
CTCGCAGCACCCCACCATGGTACGTGCTATGCCTTAAATTACCATCGAAGTTGAATTCGGCTCC
+
FFFFFFFFFFFFFFFFFFFFFFFFFFFFFFFFFFFFFFFFFFFFFFFFFFFFFFFFFFFFFFFFFFFF
FFFFFFFFFFFFFFFFFFFFFFFFFFFFFFFFFFFFFFFFFFFFFFFFFFFFFFFFFFFFFFFF
@A00155:342:HHGFNDSXY:1:2652:30047:21997 1:N:0:GAACCTAG+TCCGCATA
TTTCCCCACTGCAGTCATCTTCTGCACCGAGAAATAATTCCTAGTCGTGGTGATATTGCTGTCATTCA
CTCGCAGCACCCCACCATGGTACGTGCTATGCCTTAAATTACCATCGAAGTTGAATTCGGCTCC
+
FFFFFFFFFFFFFFFFFFFFFFFFFFFFFFFFFFF:FFFFF:FFFFFFFFFFF:FFFFFFFFFFFFFF
FFFFFFFFFFFFFFFFFFFFFFFFFFFFFFFFFF:FFFFFFFFFFFFFFFFFFFFFFFFFFFFF
@A00155:342:HHGFNDSXY:1:2534:13946:22701 1:N:0:GAACCTAG+TCCGCATA
TTTCCCCACTGCAGTCATCTTCTGCACCGAGAAATAATTCCTAGTCGTGGTGATATTGCTGTCATTCA
CTCGCAGCACCCCACCATGGTACGTGCTATGCCTTAAATTACCATCGAAGTTGAATTCGGCTCC
+
FFFFFFFFFF,FFFFFFFFFFFFFFFFFFFFFFFFFFFFFFFFFFFFFFFFFFFFFFFFFFFFFFFFF
FFFFFFFFFFFFFFFFFFFFFFFFFFFFFFFFFFFFFFFFFFFFFFFFFFFFFFFFFFFFFFFF
@A00155:342:HHGFNDSXY:1:2638:22227:34162 1:N:0:GAACCTAG+TCCGCATA
TTTCCCCACTGCAGTCATCTTCTGCACCGAGAAATAATTCCTAGTCGTGGTGATATTGCTGTCATTCA
CTCGCAGCACCCCACCATGGTACGTGCTATGCCTTAAATTACCATCGAAGTTGAATTCGGCTCC
+
FFFFFFFFF:FFFFFFFFFFFFFFFFFFFF:FF:FFFFFFFFFFFFFFFFFFFFFFFFF:FFFFF:FF
FFF:FF::FFFFFFFFFFFFF:FF:FFFFFFFFFFFFFFF:FFFFFFF:FF:FFF:FFFFFFF:
@A00155:342:HHGFNDSXY:1:1314:28167:12289 1:N:0:GAACCTAG+TCCGCATA
TTTCCCCACTGCAGTCATCTTCTGCACCGAGAAATAATTCCTAGTCGTGGTGATATTGCTGTCATTCA
CTCGCAGCACCCCACCATGGTACGTGCTATGCCTTAAATTACCATCGAAGTTGAATTCGGCTCC
+
FFFFFFFFFFFFFFFFFFFFFFFFFFFFFFFF:FFF,FFFFFFFFFFF:FFFF:FFFFFFFFFFFFFF
FFFFFFFFFFFFFFFFFFFFFFFFFFFFFFFFFFFFFFFFFFFFFFFF,FFFFFFFFFF:FFFF
@A00155:342:HHGFNDSXY:1:1574:18114:8907 1:N:0:GAACCTAG+TCCGCATA
TTTCCCCACTGCAGTCATCTTCTGCACCGAGAAATAATTCCTAGTCGTGGTGATATTGCTGTCATTCA
CTCGCAGCACCCCACCATGGTACGTGCTATGCCTTAAATTACCATCGAAGTTGAATTCGGCTCC
+
FFFFFFFFFFFFFFFFFFFFFFFFFFFFFFFFFFFFFFFFFFFFFFFFFFFFFF,FFFFFFFFFFFFF
FFFFFFFFFFFFFFFFFFFFFFFFFFFFFFFFFFFFFFFFFFFFFFFFFFFFFFFFFFFFFFFF
@A00155:342:HHGFNDSXY:1:2267:5945:20525 1:N:0:GAACCTAG+TCCGCATA
TTTCCCCACTGCAGTCATCTTCTGCACCGAGAAATAATTCCTAGTCGTGGTGATATTGCTGTCATTCA
CTCGCAGCACCCCACCATGGTACGTGCTATGCCTTAAATTACCATCGAAGTTGAATTCGGCTCC
+
FFFFFFFFFFFFFFFFFFFFFFFFFFFFFFFFFFFFFFFFFFFFFFFFFFFFFFFFFFFFFFFFFFFF
FFFFF:FFFFFFFFFFFFFFFFFFFFFFFFFFFFFFFFFFFFFFFFFFFFFFFFFFFFFFFFFF
```

```
@A00155:342:HHGFNDSXY:1:2331:5728:15076 1:N:0:GAACCTAG+TCCGCATA
TTTCCCCACTGCAGTCATCTTCTGCACCGAGAAATAATTCCTAGTCGTGGTGATATTGCTGTCATTCA
CTCGCAGCACCCCACCATGGTACGTGCTATGCCTTAAATTACCATCGAAGTTGAATTCGGCTCC
+
FFFFFFFFFFFFFFFFFF:FFFFFFF,FFFFFFFFFFFFFFFFFFFFFFFFFF:FFFFFFFFFFFFFF
FFFFFFFFFFFFFFFFFFFFFFFF:FFFFFFFFFFFF,FFFFFFFFFFFFFFFFFFFFFFFFF:
@A00155:342:HHGFNDSXY:1:2331:6008:14967 1:N:0:GAACCTAG+TCCGCATA
TTTCCCCACTGCAGTCATCTTCTGCACCGAGAAATAATTCCTAGTCGTGGTGATATTGCTGTCATTCA
CTCGCAGCACCCCACCATGGTACGTGCTATGCCTTAAATTACCATCGAAGTTGAATTCGGCTCC
+
FFFFFFFFFFFFFFFFFFFFFFFFFFFFFFFFFFFFFFFFFFFFFFFFFFFFFFFFFFFFFFFFFFFF
FFFFFFFFFFFFFFFFFFFFFFFFFFFFFFFFFF:FFFFF:FFFFFFFFFFFFFFFFFFFFFFF
@A00155:342:HHGFNDSXY:1:1367:11831:15906 1:N:0:GAACCTAG+TCCGCATA
TTTCCCCACTGCAGTCATCTTCTGCACCGAGAAATAATTCCTAGTCGTGGTGATATTGCTGTCATTCA
CTCGCAGCACCCCACCATGGTACGTGCTATGCCTTAAATTACCATCGAAGTTGAATTCGGCTCC
+
FFFFFFFFFFFFFFFFFFFFFFFFFFFFFFFFFFFFFFFFFFFFFFFFFFFFFFFFFFFFFFFFFFFF
FFFFFFFFFFFFFFFFFFFFFFFFFFF:FFFFFFFFFFFFFFFFFFFFFFFFFFFFFFFFFFFF
@A00155:342:HHGFNDSXY:1:1471:16459:2534 1:N:0:GAACCTAG+TCCGCATA
TTTCCCCACTGCAGTCATCTTCTGCACCGAGAAATAATTCCTAGTCGTGGTGATATTGCTGTCATTCA
CTCGCAGCACCCCACCATGGTACGTGCTATGCCTTAAATTACCATCGAAGTTGAATTCGGCTCC
+
FFFFFFFFFFFFFFFFFFFFFFFFFFFFFFFFFFFFFFFFFFFFFFFFFF:FFFFFFFFFFFFFFFFF
FFFFFFFFFFFFFFFFFFFFFFFFFFF:FFFFFFFFFFFFFFFFFFFFFFFFFFFFFFFFFFFF
@A00155:342:HHGFNDSXY:1:2475:24044:28855 2:N:0:GAACCTAG+TCCGCATA
TGTCCCCACTGCAGTCATCTTCTGCACCGAGAAATAATTCCTAGTCGTGGTGATATTGCTGTCATTCA
CTCGCAGCACCCCACCATGGTACGTGCTATGCCTTAAATTACCATCGAAGTTGAATTCGGCTCCTGGT
+
F,FFFFFFFFFFFFFFFFFFFFFFFFFFFFFFFFFFFFFFFFFFFFFFFFFFFFFFFFFFFFFFFFFF
FFFFFFFFFFFFFFFFFFFFFFFFFFFFFFFFFFFFFFFFFFFFFFFFFFFFFFFFFFFFFFFFFFFF
@A00155:342:HHGFNDSXY:1:2126:8187:14043 2:N:0:GAACCTAG+TCCGCATA
CCCCACTGCAGTCATCTTCTGCACCGAGAAATAATTCCTAGTCGTGGTGATATTGCTGTCATTCACTC
GCAGCACCCCACCATGGTACGTGCTATGCCTTAAATTACCATCGAAGTTGAATTCGGCTCCTGG
+
FFFFFFFFFF:FFFFFFFF:FFFFFFFFFFFFFFFFFFFFFFFFFFFFFFFFFFFFFFFFFFFFFFFF
FFFFFFFFFFFFFFFFFFFFF:FFFFFFFFFFFFFFFFFFFFFFFFFFFFFFFFFFFFFFFFFF
@A00155:342:HHGFNDSXY:1:1126:6153:17190 2:N:0:GAACCTAG+TCCGCATA
CCCCACTGCAGTCATCTTCTGCACCGAGAAATAATTCCTAGTCGTGGTGATATTGCTGTCATTCACTC
GCAGCACCCCACCATGGTACGTGCTATGCCTTAAATTACCATCGAAGTTGAATTCGGCTCCTGGTGCC
+
FFFF:FF:F:::F,FFFFFFFFFFF:F:FFFF:FFFFFFFFFFFFFFFFFF,,FFFFFFFFFFFFFFF
FFFFFFFFFFFFFFFFFF:FFFFFFFFFFFFFFFFFFFFFFFFFFFFFFFFFFFFFFF:FFFFF:FFF
@A00155:342:HHGFNDSXY:1:2669:7238:12587 2:N:0:GAACCTAG+TCCGCATA
CCACTGCAGTCATCTTCTGCACCGAGAAATAATTCCTAGTCGTGGTGATATTGCTGTCATTCACTCGC
AGCACCCCACCATGGTACGTGCTATGCCTTAAATTACCATCGAAGTTGAATTCGGCTCCTGGTGCCTC
+
FFFFFFFFFFFFFFFFFFFFFFFFFFFFFFFFFFFFFFFFFFFFFFFFFFFFFFFFFF:FFFFFFFFF
FFFFFFFFFFFFFFFFFFFFFFFFFFFFFFFFFFFFFFFFFFFFFFFFFFFFFFFFFFFFFFFFFFFF
@A00155:342:HHGFNDSXY:1:1106:26440:28682 2:N:0:GAACCTAG+TCCGCATA
CACTGCAGTCATCTTCTGCTCCGAGAAATAATTCCTAGTCGTGGTGATATTGCTGTCATTCACTCGCA
GCACCCCACCATGGTACGTGCTATGCCTTAAATTACCATCGAAGTTGAATTCGGCTCCTGGTGCCTC
+
::FFFFFFF,,::,FFFFF,F:FFFFFFFFFFFFFFFFFFFFFFFFFFFFF:FFFFFFFFFFFFFFFF
FFFFFFF,:FFFFF:FFFFFFFFFFFFFFFFF:FFFFFFFF,:FF,FFFF:FFFFFFFFF:FFFFF
```

```
@A00155:342:HHGFNDSXY:1:2106:26169:21386 2:N:0:GAACCTAG+TCCGCATA
CACTGCAGTCATCTTCTGCACCGAGAAATAATTCCTAGTCGTGGTGATATTGCTGTCATTCACTCGCA
GCACCCCACCATGGTACGTGCTATGCCTTAAATTACCATCGAAGTTGAATTCGGCTCCTGGTGCCTCG
+
FFFFFF:FFFFFFFFFFFFFFFFFFFFFFFFFFFFFFFFFFFFFFFFFFFFFFFFFFFFFFFFFFFFF
FFFFFFFFFFFFFFFFFFFFFFFFFFFFFFFFFF:FFFFFFFFFFFFFFFFFFFFFFFFFFFFFFFFF
@A00155:342:HHGFNDSXY:1:1676:12292:28948 1:N:0:GAACCTAG+TCCGCATA
ACTGCAGTCATCTTCTGCACCGAGAAATAATTCCTAGTCGTGGTGATATTGCTGTCATTCACTCGCAG
CACCCCACCATGGTACGTGCTATGCCTTAAATTACCATCGAAGTTGAATTCGGCTCCTGGTGCCTCGT
+
FFFFFFFFFFFFFFFFFFFFFFFFFFFFFFFFFFFFFFFFFFFFFFFFFFFFFFFFFFFFFFFFFFFF
FFFFFFFFFFFFFFFFFFFFFFFFFFFFFFF,FFFFFFFFFFFFFFFFFFFFFFFFFFFFFFFFFFFF
@A00155:342:HHGFNDSXY:1:1556:7527:36417 2:N:0:GAACCTAG+TCCGCATA
CTGCAGTCATCTTCTGCACCGAGAAATAATTCCTAGTCGTGGTGATATTGCTGTCATTCACTCGCAGC
ACCCCACCATGGTACGTGCTATGCCTTAAATTACCATCGAAGTTGAATTCGGCTCCTGGTGCCTCGTG
+
FFFFFFFFFFF,FFFFFFFFFFFFFFFFF,F,FFFFF:FFFFFFFFFFFFFFFFF,FFFFFFFFFFF:
FFFFFFFFFFFFFFFFFFF:FFFF:FF,FFFFFFFF::FFFFF:FFFFFFFFFFFFF,FFFFFFFFFF
@A00155:342:HHGFNDSXY:1:1460:13856:30812 1:N:0:GAACCTAG+TCCGCATA
TGCAGTCATCTTCTGCACCGAGAAATAATTCCTAGTCGTGGTGATATTGCTGTCATTCACTCGCAGCA
CCCCACCATGGTACGTGCTATGCCTTAAATTACCATCGAAGTTGAATTCGGCTCCTGGTGCCTCG
+
FFFFFFFFFFFFFFFFFFFFFFF:FFFFFFFFFFFFFFFFFFFFFFFFFFFFFFFFFFFFFFFFFFFF
FFFFFFFFFFFFFFFFFFFFFFFFFFFFFFFFFFFFFFFFFFFFFFFFFFFFFFFFFFFFFFFFF
@A00155:342:HHGFNDSXY:1:1603:24840:29356 2:N:0:GAACCTAG+TCCGCATA
TGCAGTCATCTTCTGCACCGAGAAATAATTCCTAGTCGTGGTGATATTGCTGTCATTCACTCGCAGCA
CCCCACCATGGTACGTGCTATGCCTTATATTACCATCGAAGTTGAATTCGGCTCCTGGTGCCTCGTGA
+
FFFFFFFFFFFFFFFFFFFFFFFFFFFFFFFFFFFFFFFFFFFFFFFFFFFFFFFFFFFFFFFFFFFF
FFFFFFFFFF:FFFFFFFFFFFFFFFFFFFFFFFFFFFFFFFFFFFFFFFFFFF:FF:FFFFFFFFFF
@A00155:342:HHGFNDSXY:1:2425:15275:34303 1:N:0:GAACCTAG+TCCGCATA
TGCAGTCATCTTCTGCACCGAGAAATAATTCCTAGTCGTGGTGATATTGCTGTCATTCACTCGCAGCA
CCCCACCATGGTACGTGCTATGCCTTAAATTACCATCGAAGTTGAATTCGGCTCCTGGTGCCTCGTGA
+
FFFFFFFFFFFFFFFFFFFFFFFFFFFFFFFFFFFFFFFFFFFFFFFFFFFFFFFFFFFFFFFFFFFF
FFFFFFFFFFFFFFFFFFFFFFFFFFFFFFFFFFFFFFFFFFFFFFFFFFF:FFFFFFFFFFFFFFF:
@A00155:342:HHGFNDSXY:1:2669:7238:12587 1:N:0:GAACCTAG+TCCGCATA
TGCAGTCATCTTCTGCACCGAGAAATAATTCCTAGTCGTGGTGATATTGCTGTCATTCACTCGCAGCA
CCCCACCATGGTACGTGCTATGCCTTAAATTACCATCGAAGTTGAATTCGGCTCCTGGTGCCTCGTGA
+
FFFFFFFFFFFFFFFFFFFFFFFFFFFFFFFFFFFFFFFFFFFFFFFFFFFFFFFFFFFFFFFFFFFF
FFFFFFFFFFFFFFFFFFF:FFFFFFFFFFFFFFFFFFFFFFFFFFFFFFFFFFFFFFFFFFFFFFFF
@A00155:342:HHGFNDSXY:1:2538:32940:30702 2:N:0:GAACCTAG+TCCGCATA
TGCAGTCATCTTCTGCACCGAGAAATAATTCCTAGTCGTGGTGATATTGCTGTCATTCACTCGCAGCA
CCCCACCATGGTACGTGCTATGCCTTAAATTACCATCGAAGTTGAATTCGGCTCCTGGTGCCTCGTGA
+
FFFFFFFFFFFFFFFFFFFFFF:FFFFFFFF,FFFFFFFFFFFFFFFFFFFFFFFFFFFFFFFFFFFF
FFFFFFFFFFFFFFF:FFFFFFFFFFFFFFFFFFFFFF:FFFFFFFFF:FFFFFFFFFFFFFFFFFF:
@A00155:342:HHGFNDSXY:1:2165:26431:22028 1:N:0:GAACCTAG+TCCGCATA
TGCAGTCATCTTCTGCACCGAGAAATAATTCCTAGTCGTGGTGATATTGCTGTCATTCACTCGCAGCA
CCCCACCATGGTACGTGCTATGCCTTAAATTACCATCGAAGTTGAATTCGGCTCCTGGTGCCTCGTGA
+
FFFFFFFFFFFFFFFFFFFFFFFFFFFFFFFFFFFFFFFFFFFFFFFFFF:FFFFFFFFFFFFFFFFF
FFFFFFFFFFFFFFFFFFFFFFFFFFF:FFFFFFFFFFFFFFFFFFFFFFFFFFFFFFFFFFFFFFFF
```

@A00155:342:HHGFNDSXY:1:2543:8241:9095 2:N:0:GAACCTAG+TCCGCATA
TGCAGTCATCTTCTGCACCGAGAAATAATTCCTAGTCGTGGTGATATTGCTGTCATTCACTCGCAGCA
CCCCACCATGGTACGTGCTATGCCTTATATTACCATCGAAGTTGAATTCGGCTCCTGGTGCCTCGTGA
+
FFFFFFFFFFFFFFFFFFFFFFFFFFFFFFFFFFFFFFFFFFFFFFFFFFFFFFFFFFFFFFFFFFFF
FFFFFFFFFFFFFFFFF:FFFFFFFFFFFFFFFFFFFFFFFFFFFFFFF:FFFFFFFFFFFFFFFFFF
@A00155:342:HHGFNDSXY:1:1352:31611:18004 2:N:0:GAACCTAG+TCCGCATA
TGCAGTCATCTTCTGCACCGAGAAATAATTCCTAGTCGTGGTGATATTGCTGTCATTCACTCGCAGCA
CCCCACCATGGTACGTGCTATGCCTTAAATTACCATCGAAGTTGAATTCGGCTCCTGGTGCCTCGTGA
+
FFFFF:FFFF:FFFFFFFFFFFFFFF,FFFFFFFFFFFFFFFFFFFFFFFFFFFF:FFFFFFFFFFFF
FFFF:FFFFFF:FFF:FFFF:FFFF:FF:,FFFFFFFFFFFFFFFFFFFFFFFFFFFF:FFFFFFFFF
@A00155:342:HHGFNDSXY:1:2475:24044:28855 1:N:0:GAACCTAG+TCCGCATA
TGCAGTCATCTTCTGCACCGAGAAATAATTCCTAGTCGTGGTGATATTGCTGTCATTCACTCGCAGCA
CCCCACCATGGTACGTGCTATGCCTTAAATTACCATCGAAGTTGAATTCGGCTCCTGGTGCCTCGTGA
+
FFF:FFFFFFFFFFFFFFFFFFFFFFFFFFFFFFFFFFFFFFFFFFFFFFFFFFFFFFFFFFFFFFFF
FFFFFFFFFFFFFFFFFFFFFFFFF:FFFFFFFFFFFFFFFFFFFFFFFFFFFFFFFFFFFFFFFFFF
@A00155:342:HHGFNDSXY:1:1215:7536:16047 1:N:0:GAACCTAG+TCCGCATA
TGCAGTCATCTTCTGCACCGAGAAATAATTCCTAGTCGTGGTGATATTGCTGTCATTCACTCGCAGCA
CCCCACCATGGTACGTGCTATGCCTTAAATTACCATCGAAGTTGAATTCGGCTCCTGGTGCCTCGTGA
+
FFFFFFFFFFFFFFFFFFFFFFF:FFFFFFFFFFFFFFFFF,FFFFFFFFFFFFFFFFFFFFFFFFFF
FFFFFFFFFFFFFFFFFFF:FFFFFFFFFFFFFFFFFFFFFFFFFFFFFFFFFFFFFFFFFFFFFFFF
@A00155:342:HHGFNDSXY:1:2614:6271:30452 2:N:0:GAACCTAG+TCCGCATA
GCAGTCATCTTCTGCACCGAGAAATAATTCCTAGTCGTGGTGATATTGCTGTCATTCACTCGCAGCAC
CCCACCATGGTACGTGCTATGCCTTAAATTACCATCGAAGTTGAATTCGGCTCCTGGTGCCTCGTGAT
+
FFFFFFFFFFFFFF:FFFFFFFFFFFFFFFFFFFFFFFFFFFFFFFFFFFFFFF:FFFFFFFFFFFFF
FFFFFFFFFF:FFFFFFFFFFFFFFFF:FFFFFFFFFFFFFFFF:FFFFFFFFFFFFFFFFFFFFFFF
@A00155:342:HHGFNDSXY:1:1413:11360:4194 2:N:0:GAACCTAG+TCCGCATA
GCAGTCATCTTCTGCACCGAGAAATAATTCCTAGTCGTGGTGATATTGCTGTCATTCACTCGCAGCAC
CCCACCATGGTACGTGCTATGCCTTAAATTACCATCGAAGTTGAATTCGGCTCCTGGTGCCTCGTGAT
+
FFFFFFFFFFFFFFFFFFFFFFFFFFFFFFFFFFFFFFFFFFFFFFFFFFFFFFFFFFFFFFFFFFFF
FFFFFFFFFFFFFFFFFFFFFFFFFFFFFFFFFFFFFFFFFFFFFFFFFFFFFFFFFFFFFFFFFFFF
@A00155:342:HHGFNDSXY:1:1635:2293:14826 2:N:0:GAACCTAG+TCCGCATA
GCAGTCATCTTCTGCACCGAGAAATAATTCCTAGTCGTGGTGATATTGCTGTCATTCACTCGCAGCAC
CCCACCATGGTACGTGCTATGCCTTAAATTACCATCGAAGTTGAATTCGGCTCCTGGTGCCTCGTGAT
+
FFF:,FFFFFFFFFFFFFFF:FFFFFF:FFFFFFFFFFF,FFFFFFFFFFFFFF,FFFFFFFF,FFFF
FFFFFFFFFFFFFFFFFFFFFFFFF:FFFFFFFF:FFFFFFFFF:FFFFFFFFFFFFFFFFFFFFFFF
@A00155:342:HHGFNDSXY:1:2528:3812:30107 2:N:0:GAACCTAG+TCCGCATA
GCAGTCATCTTCTGCACCGAGAAATAATTCCTAGTCGTGGTGATATTGCTGTCATTCACTCGCAGCAC
CCCACCATGGTACGTGCTATGCCTTAAATTACCATCGAAGTTGAATTCGGCTCCTGGTGCCTCGTGAT
+
FFFFFFFFFFFFFFFFFFFFFFFFFFFFFFFFFFFFFFFFFFFFFFFFFFFFFFFFFFFFFFFFFFFF
FFFFFFFFFFFFFFFFFFFFFFFFFFF:FFFFFFFFFFFFFFFFFFFFFFFFFFFFFFFFFFFFFFFF
@A00155:342:HHGFNDSXY:1:1367:11831:15906 2:N:0:GAACCTAG+TCCGCATA
CAGTCATCTTCTGCACCGAGAAATAATTCCTAGTCGTGGTGATATTGCTGTCATTCACTCGCAGCACC
CCACCATGGTACGTGCTATGCCTTAAATTACCATCGAAGTTGAATTCGGCTCCTGGTGCCT
+
FFF,F::FFFFFFFFFFFFFFF:FFFFFFFF,FFFFFFFFFFFFFFFFFFFFFFFFFFFFFFFFFFFF
FFFFFFFFFFFFFFFF:FFFFFFFFFFFFFFFFFFFFFFFFFFFFFFFFFFFFFFFFFFF

```
@A00155:342:HHGFNDSXY:1:2442:19795:15076 2:N:0:GAACCTAG+TCCGCATA
CAGTCATCTTCTGCACCGAGAAATAATTCCTAGTCGTGGTGATATTGCTGTCATTCACTCGCAGCACC
CCACCATGGTACGTGCTATGCCTTAAATTACCATCGAAGTTGAATTCGGCTCCTGGTGCCTC
+
FFFFFFFF:FFFFFFFFFFFFF:FFFFFFFF,F:FFFFFFFFFFFFFFFFFFF,FFFFFFFFFFFFFF
FFFFFFFFFFFFF:FF:FFFFFFFF:FFF:FF::FFFFFFFFFFFFFFFFFFFFFFFFFFFF
@A00155:342:HHGFNDSXY:1:2221:31675:13197 2:N:0:GAACCTAG+TCCGCATA
GTCATCTTCTGCACCGAGAAATAATTCCTAGTCGTGGTGATATTGCTGTCATTCACTCGCAGCACCCC
ACCATGGTACGTGCTATGCCTTAAATTACCATCGAAGTTGAATTCGGCTCCTGGTGCCTCGT
+
FFF:FFFFFFFFFFFF:FFFFFF:F:FF:FFFFFFFFFFFFFFFFFFFFFFFFFFFFFFFFFFFFFFF
FFFFFFFFFFFFFFFFFFFF:FFF:FF,FFFFFFF:FFFFFFFFFFFFFFFFFFFFFFFFFF
@A00155:342:HHGFNDSXY:1:2555:30689:5729 2:N:0:GAACCTAG+TCCGCATA
GTCATCTTCTGCACCGAGAAATAATTCCTAGTCGTGGTGATATTGCTGTCATTCACTCGCAGCACCCC
ACCATGGTACGTGCTATGCCTTAAATTACCATCGAAGTTGAATTCGGCTCCTGGTGCCTCGTGA
+
FFFFFFFFFFFFFFFFFFFFFFFFFFFF:FFFFFFFFFFFFFFFFFFFFFFFFFFFFFFFFFFFFFFF
FFFFFFFFFFFFFFFFFFFFFFF:FFFFFF:FFFFFFFFFFFFFFFFFFFFFFFFFFFFFFFFF
@A00155:342:HHGFNDSXY:1:2441:7048:13792 1:N:0:GAACCTAG+TCCGCATA
GTCATCTTCTGCACCGAGAAATAATTCCTAGTCGTGGTGATATTGCTGTCATTCACTCGCAGCACCCC
ACCATGGTACGTGCTATGCCTTAAATTACCATCGAAGTTGAATTCGGCTCCTGGTGCCTCGTGATTCG
+
FFFFFFFFFFFFFFFFFFFFFFFFFFFFFFFFFFFFFFFF::FFFFFFFFFFFFFFFFFFFFFFFFFF
FFFFFFFF:FFFFFFFFFFFFFFFF:FFFFFFFFFFFFFF:FFF:FFFFFFFFFFFFFFFFFFF:FFF
@A00155:342:HHGFNDSXY:1:1229:30400:8296 2:N:0:GAACCTAG+TCCGCATA
GTCATCTTCTGCACCGAGAAATAATTCCTAGTCGTGGTGATATTGCTGTCATTCACTCGCAGCACCCC
ACCATGGTACGTGCTATGCCTTAAATTACCATCGAAGTTGAATTCGGCTCCTGGTGCCTCGTGATTCG
+
F:FFFFFFF:FFFFFFFFFFFFFF:FFFFFFFFFFFFFFFFFFFFFFFFFFFFFFFFFFFFFFFFFFF
FFFFFFFFFFFFFFFFFFFFFFFFFFFFFF:FFFFFFFFFFFFFFFFFFFFFFFFFFFFFFFFFFFFF
@A00155:342:HHGFNDSXY:1:2126:8187:14043 1:N:0:GAACCTAG+TCCGCATA
CTTCTGCACCGAGAAATAATTCCTAGTCGTGGTGATATTGCTGTCATTCACTCGCAGCACCCCACCAT
GGTACGTGCTATGCCTTAAATTACCATCGAAGTTGAATTCGGCTCCTGGTGCCTCGTGATTCGG
+
FFFFFFFFFFFFFFFFFFFFFFFFFFFFFFFFFFFFFFFFFFFFFFFFFFFFFFFFFFFFFFFFFFFF
FFFFFFFFFFFFFFFFFFFFFFFFFFFFFFFFFFFFFFFFFFFFFFFFFFFFFFFFFFFFFFFF
@A00155:342:HHGFNDSXY:1:1512:5819:28291 2:N:0:GAACCTAG+TCCGCATA
TTCTGCACCGAGAAATAATTCCTAGTCGTGGTGATATTGCTGTCATTCACTCGCAGCACCCCACCATG
GTACGTGCTATGCCTTAAATTACCATCGAAGTTGAATTCGGCTCCTGGTGCCTCGTGATTCGGGAAGA
+
FFFFFF:FFFFFFFFFFFFFFFFFFFFFF:FFFFFF,FFFFFFFF,FFFFFFFFFFFFFFFF:FFFFF
F,FFFFFFFFFFFFFFFF:F,FFF:FFFFFFF:FFFFFFFFFFFFFFFFFFFFFFFFFFFFFFFF:FF
@A00155:342:HHGFNDSXY:1:2258:1705:25300 1:N:0:GAACCTAG+TCCGCATA
TTCTGCACCGAGAAATAATTCCTAGTCGTGGTGATATTGCTGTCATTCACTCGCAGCACCCCACCATG
GTACGTGCTATGCCCTAAATTACCATCGAAGTTGAATTCGGCTCCTGGTGCCTCGTGATTCGGGAAGA
+
FFFFFFFFFFFFFFFFFF,:FFF,FFFFFFFFFFFF,FFFFFFFF,FF:FFFFF::FFFFFFFFFFFF
F::FFF:F,F:FFFFF:,,FFFFF:FFFFFFFF:FF:FFFFFFFFFFFFFFFFFFFFF:FFFFFFFFF
@A00155:342:HHGFNDSXY:1:2441:7048:13792 2:N:0:GAACCTAG+TCCGCATA
TTCTGCACCGAGAAATAATTCCTAGTCGTGGTGATATTGCTGTCATTCACTCGCAGCACCCCACCATG
GTACGTGCTATGCCTTAAATTACCATCGAAGTTGAATTCGGCTCCTGGTGCCTCGTGATTCGGGAAGA
+
FFFFFFFF:FFFFFFFFFFFFFF,FFFFFFFFFF:FFFFFFFFFFFFFFFFFFFFFFFFFFFFFFFFF
FFFFFFFFFFFFFF:FFFFFFFFFFFFFFFFFFFFFFFFFFFFFFFFFFFFFFFFFFFFFFFFFFFFF
```

@A00155:342:HHGFNDSXY:1:2627:4227:27477 1:N:0:GAACCTAG+TCCGCATA
CTGCACCGAGAAATAATTCCTAGTCGTGGTTATATTGCTGTCATTCACTCGCAGCACCCCACCATGGT
ACGTGCTATGCCTTAAATTACCATCGAAGTTGAATTCGGCTCCTGGTGCCTCGTGATTCGGGAAGA
+
FF:F:F,,FF,F,FFFFFF,FFFFFFF:FF,F:FFFFFFFFFFFFF,:FF::FFFFFFF:F:F:FFFF
F:FFF:F,FF:,FFFFFFFFFFFF:F,F:FFFF:FF,FFFF:FFFFFF:FFFFFFFFF:F:FFFFF
@A00155:342:HHGFNDSXY:1:2426:3441:9862 2:N:0:GAACCTAG+TCCGCATA
TGCACCGAGAAATAATTCCTAGTCGTGGTGATATTGCTGTCATTCACTCGCAGCACCCCACCATGGTA
CGTGCTATGCCTTAAATTACCATCGAAGTTGAATTCGGCTCCTGGTGCCTCGTGATTCGGGAAGAACC
+
FFFFFFF,FFFF:FFFFFFFFFFFFFFFFFFFFFFFFFFFFFFFFFFFFFFFFFFFFFFFFFFFFFF
FF:FFFFFFFFFFFFF:,FFFFFFFFFFFFFFFFFFFFFFFFFFFFFFFFFFFFFFFFFFFFFFFFFF
@A00155:342:HHGFNDSXY:1:2254:18783:23844 2:N:0:GAACCTAG+TCCGCATA
TGCACCGAGAAATAATTCCTAGTCGTGGTGATATTGCTGTCATTCACTCGCAGCACCCCACCATGGTA
CGTGCTATGCCTTAAATTACCATCGAAGTTGAATTCGGCTCCTGGTGCCTCGTGATTCGGGAAGAACC
+
FFFFFFFF,FFFFFFFF:FFFFF:FFFFFFFFFFFFFFFFFFFFFFFFFFFFFFFFFFFFFFFFFFFF
FFFFFFFFFFF,FFFFFFFFF:FFFFFFFFFF:FFFFFFFFFFFFFFFFFFFFFFFFFFFFFFFFFFF
@A00155:342:HHGFNDSXY:1:1314:28167:12289 2:N:0:GAACCTAG+TCCGCATA
TGCACCGAGAAATAATTCCTAGTCGTGGTGATATTGCTGTCATTCACTCGCAGCACCCCACCATGGTA
CGTGCTATGCCTTAAATTACCATCGAAGTTGAATTCGGCTCCTGGTGCCTCGTGATTCGGGAAGAACC
+
FFFFFFFF:FFFFFFFFFFFFFFFFFFFFFFFFFFFFFF:FFFFFFFFFFFFFFFFFF:FFFFFFFFF
FFFFFFFF:FFFFFFFFFFFFFFFFFFFFFFFFFFFFFFFFFFFFFFFFFFFFFFFFF:FFFFFFFFF
@A00155:342:HHGFNDSXY:1:2143:11053:24799 2:N:0:GAACCTAG+TCCGCATA
GCACCGAGAAATAATTCCTAGTCGTGGTGATATTGCTGTCATTCACTCGCAGCACCCCACCATGGTAC
GTGCTATGCCTTAAATTACCATCGAAGTTGAATTCGGCTCCTGGTGCCTCGTGATTCGGGAAGAACCT
+
FFFFFFFFFFFFFFFFFFFFFFFFFFFFFFFFFFFFFFFFFFFFFFFFFFFFFFFFFFFFFFFFFFFF
FFFFFFFFFFFFFFFFFFFFFFFFFFFFFFFFFFFFFFFFFFFFFFFFFFFFFFFFFFFFFFFFFFFF
@A00155:342:HHGFNDSXY:1:1505:31051:14403 2:N:0:GAACCTAG+TCCGCATA
GCACCGAGAAATAATTCCTAGTCGTGGTGATATTGCTGTCATTCACTCGCAGCACCCCACCATGGTAC
GTGCTATGCCTTAAATTACCATCGAAGTTGAATTCGGCTCCTGGTGCCTCGTGATTCGGGAAGAACCT
+
FFFFFFF,FFFF:FFFFFFFFFFF,,FFFFFFFFFFFFFFFFFFFFFFFFFFFFFFFFFFFFFFFFFF
FFFFFFFFFFFFFFFFFF:FFFFFFF:FFF:FFFFFFFFFFFF:F:FFFFFFFF:FFF:FFFFFFFFF
@A00155:342:HHGFNDSXY:1:1428:1561:16658 2:N:0:GAACCTAG+TCCGCATA
CACCGAGAAATAATTCCTAGTCGTGGTGATATTGCTGTCATTCACTCGCAGCACCCCACCATGGTACG
TGCTATGCCTTAAATTACCATCGAAGTTGAATTCGGCTCCTGGTGCCTCGTGATTCGGGAAGAACCT
+
FFFF,F,FFFFFF:FFFFF:FF:FFFFFFFFF:,F:FFFFF,FFFFFFFF:FFFFFFFFFFFFF,FFF
,FF:FFFFF:FFFFFFF:FFFFFFF,F:FFF::FFFFFFFFF:FFFFFF:FFFFFFFFFFFFFFFF:
@A00155:342:HHGFNDSXY:1:2267:5945:20525 2:N:0:GAACCTAG+TCCGCATA
CACCGAGAAATAATTCCTAGTCGTGGTGATATTGCTGTCATTCACTCGCAGCACCCCACCATGGTACG
TGCTATGCCTTAAATTACCATCGAAGTTGAATTCGGCTCCTGGTGCCTCGTGATTCGGGAAGAACCTC
+
FFF,FFFFFFFFFFF,FFFFF,FFFFFFFFFFFFFFFFFFFFFFFFFFFFFFFFFFFFFFFFF:FFFF
FFFFFFFFFFFFFFFFFFFFFFFFFFFFFFFFFFFFFFFFFFFFFFFFFFFFFFFFFFFFFFFFFFFF
@A00155:342:HHGFNDSXY:1:2577:32506:26819 2:N:0:GAACCTAG+TCCGCATA
CACCGAGAAAGAATTCCTAGTCGTGGTGATATTGCTGTCATTCACTCGCAGCACCCCACCATGGTACG
TGCTATGCCTTAAATTACCATCGAAGTTGAATTCGGCTCCTGGTGCCTCGTGATTCGGGAAGAACCTC
+
FFFFFFF:F:,FF,:FF:,F,FFFFFFFF,F:FFFF:FFF,FFFFFFFF::FFFFFF,F:F:FF,FFF
FF:FF:FFFF:,F:,,FFFFFFFFFFF,FF,,FFFFFFFF,FF,FFFFFFF:F:FFFF:FFFF,FF:F

@A00155:342:HHGFNDSXY:1:2301:5303:19977 2:N:0:GAACCTAG+TCCGCATA
CACCGAGAAATAATTCCTAGTCGTGGTGATATTGCTGTCATTCACTCGCAGCACCCCACCATGGTACG
TGCTATGCCTTAAATTACCATCGAAGTTGAATTCGGCTCCTGGTGCCTCGTGATTCGGGAAGAACCTC
+
FFF,FFFFFFFFF:FFFFFFFFFFFFFFFFFFFFFFFFF,FFFFFFFFFFFFFFFF,FFFFFFFFFF
FFF:FFFFFFFFF,FF:FFFFFFF,FFFFFF,FFFFFFFFFFFFFFFFFF:FFFFFFFFFFFFFFFF
@A00155:342:HHGFNDSXY:1:1214:11451:26490 2:N:0:GAACCTAG+TCCGCATA
CCGAGAAATAATTCCTAGTCGTGGTGATATTGCTGTCATTCACTCGCAGCACCCCACCATGGTACGTG
CTATGCCTTAAATTACCATCGAAGTTGAATTCGGCTCCTGGTGCCTCGTGATTCGGGAAGAACCTCTT
+
FFFFFFFFFFF:FFFFFFFFFFFFFFFF:FFFFFFFFFFFFFFFFFFFFFFFFFFFFFFFFFFFFFF:F
FFFFFFFFFFFFFFFFFFFFFFFFFFFFFFFFFFFFFFFFFFFFFFFFFFFFFFFFFFFFFFFFFFFF
@A00155:342:HHGFNDSXY:1:2146:28673:27258 1:N:0:GAACCTAG+TCCGCATA
CCGAGAAATAATTCCTAGTCGTGGTGATATTGCTGTCATTCACTCGCAGCACCCCACCATGGTACGTG
CTATGCCTTAAATTACCATCGAAGTTGAATTCGGCTCCTGGTGCCTCGTGATTCGGGAAGAACCTCTT
+
FFFF,FFFF:FFF:FFFFFFFFFFFFFFFFF:FFFFFF:FFFFFFFFFFFFFFFFFFFFFFFFFFFFF
FFFFF:FFF,FFF:FFF:FFF::FFFFFFFFFFF:FFFFFFFF:FFFFFFFFFFFFFFFFFFFFFFFF
@A00155:342:HHGFNDSXY:1:1317:4408:26381 2:N:0:GAACCTAG+TCCGCATA
CGAGAAATAATTCCTAGTCGTGGTGATATTGCTGTCATTCACTCGCAGCACCCCACCATGGTACGTGC
TATGCCTTAAATTACCATCGAAGTTGAATTCGGCTCCTGGTGCCTCGTGATTCGGGAAGAACCT
+
FFFFFFFFFFFFFFFFFF:FFFFFFFFFFFFFFFFFFFFFFFFFFFFFFFFFFFFFFFFFFFFFFFFF
FFFFFFFFFFFFFFFFFFFFFFFFFFFFFFFFFFFFF,FFFFFFFFFFFFFFFFFFFFFFFFFF
@A00155:342:HHGFNDSXY:1:2327:1289:20572 2:N:0:GAACCTAG+TCCGCATA
GAGAAATAATTCCTACTCGTGGTGATATTGCTGTCATTCACTCGCAGCACCCCACCATGGTACGTGCT
ATGCCTTAAATTACCATCGAAGTTGAATTCGGCTCCTGGTGCCTCGTGATTCGGGAAGA
+
,F,FFF:FF,FFF,F,,F,::,FFF,FF,FF:,,:FF:FFFFF:FF:FFFFFFFFFFFF,:FFF,F:F
F,FFFF:FFFF:FFFF,FFFF,,:FFF::FF:F:FF:,FF:FF,F:,FF:FFF,:FF:F
@A00155:342:HHGFNDSXY:1:2577:32506:26819 1:N:0:GAACCTAG+TCCGCATA
GAGAAATAATTCCTAGTCGTGGTGATATTGCTGTCATTCACTCGCAGCACCCCACCATGGTACGTGCT
ATGCCTTAAATTACCATCGAAGTTGAATTCGGCTCCTGGTGCCTCGTGAGTCGGGAAGAACCTCTTAA
+
FFFFF:FFFF:FFFFFFFFFFF:F,:FFFFFFF:FFFFFFFFFFF,FFFFFFFFFF:FFF::FFFFF:
FFFFFFFFFF:F:FF,FFFF,FFFFF::FFFFFFFFFFF:FFF,FFFFF,FFFFF,FFF:FF:F:FFF
@A00155:342:HHGFNDSXY:1:2543:8241:9095 1:N:0:GAACCTAG+TCCGCATA
AGAAATAATTCCTAGTCGTGGTGATATTGCTGTCATTCACTCGCAGCACCCCACCATGGTACGTGCTA
TGCCTTATATTACCATCGAAGTTGAATTCGGCTCCTGGTGCCTCGTGATTCGGGAAGAACCTCTTAAC
+
FFFFFFFFFFFFFFFFFFFFFFFFFFFFFFFFFFFFFFFFFFFFFFFFFFFFFFFFFFFFFFFFFFFF
FFFFFFFFFFFFFFFFFFFFFFFFFFFFFFFFFFFFFFFFFFFFFFFFFFFFFFFFFFFFF:FFFFFF
@A00155:342:HHGFNDSXY:1:2547:10330:25864 2:N:0:GAACCTAG+TCCGCATA
AGAAATAATTCCTAGTCGTGGTGATATTGCTGTCATTCACTCGCAGCACCCCACCATGGTACGTGCTA
TGCCTTAAATTACCATCGAAGTTGAATTCGGCTCCTGGTGCCTCGTGATTCGGGAAGAACCTCTTAAC
+
FFFFFFFFFFF,FFFFFFFFFFFFFFFFFFFFFFFFFFFFFFFFFFFFF:FFFFFFFFFFF:FFFF
FFFFFFFFFFFFFFFFFFFFFFFFFFFFFFFFFFFFFFFFFFFFFFFFFFF:FFFFFFFFFFFFFFFF
@A00155:342:HHGFNDSXY:1:1563:22932:30248 2:N:0:GAACCTAG+TCCGCATA
AGAAATAATTCCTAGTCGTGGTGATATTGCTGTCATTCACTCGCAGCACCCCACCATGGTACGTGCTA
TGCCTTAAATTACCATCGAAGTTGAATTCGGCTCCTGGTGCCTCGTGATTCGGGAAGAACCTCTTAAC
+
FFFFFFFFFFFFFFFFFFFFFFFFFFFFFFFFFFFFF:FFFFFFFFFFFFFFFFFFFFFFFFFFFFFFFF
FFFFFFFFFFFFFFFFFFFFFFFFFFFFFFFFFFFFF:FFFFFFFFFFFFFFFFFFFFFFFFFFFFFFFF

```
@A00155:342:HHGFNDSXY:1:1603:24840:29356 1:N:0:GAACCTAG+TCCGCATA
AGAAATAATTCCTAGTCGTGGTGATATTGCTGTCATTCACTCGCAGCACCCCACCATGGTACGTGCTA
TGCCTTATATTACCATCGAAGTTGAATTCGGCTCCTGGTGCCTCGTGATTCGGGAAGAACCTCTTAAC
+
FFFFFFFFFFFFFFFFFFFFFFFFFFFFFFFFFFFFFFFFFFFFFFFFFFFFFFFFFFFFFFFFFFFF
FFFFFFFFFFFFFFFF:FFFFFFFFFFFFFFFFFFFFFFFFFFFFFFFFFFFFFFFFFFFFFFFFFF
@A00155:342:HHGFNDSXY:1:2364:23475:8422 1:N:0:GAACCTAG+TCCGCATA
GAAATAATTCCTAGTCGTGGTGATATTGCTGTCATTCACTCGCAGCACCCCACCATGGTACGTGCTAT
GCCTTAAATTACCATCGAAGTTGAATTCGGCTCCTGGTGCCTCGTGATTCGGGAAGAACCTCTTAACG
+
FFFFFFFFFFFFFFFFFFFF:FFFFFFFFFFFFFFFFFFFFFFFFFFFFFFFFFFFFFFFFFFFFFFF
FFFFFFFFFFFFFFFFFFFFFFFFFFFFFFFFFFFFFFFFFFFFFFF:FFFFFFFFFFFFFFFFFFFF
@A00155:342:HHGFNDSXY:1:2442:19795:15076 1:N:0:GAACCTAG+TCCGCATA
AATAATTCCTAGTCGTGGTGATATTGCTGTCATTCACTCGCAGCACCCCACCATGGTACGTGCTATGC
CTTAAATTACCATCGAAGTTGAATTCGGCTCCTGGTGCCTCGTGATTCGGGAAGAA
+
FFFFFFFFFFFFFFFFFFFFF:FFFFFFFFFFFFFFFFFFFFFF:FFFFFFFFFFFFFFFFFFFFFFF
FFFFFFFFFFFFFFFFFFFFFFFFFFFFFFFFFFFFFFFFFFFFFFFFFFFFFFFF
@A00155:342:HHGFNDSXY:1:2221:31675:13197 1:N:0:GAACCTAG+TCCGCATA
AATAATTCCTAGTCGTGGTGATATTGCTGTCATTCACTCGCAGCACCCCACCATGGTACGTGCTATGC
CTTAAATTACCATCGAAGTTGAATTCGGCTCCTGGTGCCTCGTGATTCGGGAAGAACC
+
FFFFFFFFF:FFFFFFFFFFFFFFFFFFFFFFFFFFFFFFFFFFFFFFFFFFFFFFFFFFFFFFFFFF
FFF:FFFFFFFFFFFFFFFFFFF,FFFFFFFFFFFFFFFFFFFFFF:FFFFFFFFFFF
@A00155:342:HHGFNDSXY:1:1534:13449:10222 1:N:0:GAACCTAG+TCCGCATA
AATAATTCCTAGTCGTGGTGATATTGCTGTCATTCACTCGCAGCACCCCACCATGGTACGTGCTATGC
CTTAAATTACCATCGAAGTTGAATTCGGCTCCTGGTGCCTCGTGATTCGGGAAGAACCT
+
FFFFFFFFFFFFFFFF:FFFFFFF:F,F,:FFFFFF:FFF:FFFF,:FFFFF:F,F:FFFFF:F,FF:
FFFFFF,FFFFFF::FFFFFFF:F:F:FFFFFF,,F,FF,F,FFFF:F:FFFFF,:,FF
@A00155:342:HHGFNDSXY:1:2555:30689:5729 1:N:0:GAACCTAG+TCCGCATA
AATAATTCCTAGTCGTGGTGATATTGCTGTCATTCACTCGCAGCACCCCACCATGGTACGTGCTATGC
CTTAAATTACCATCGAAGTTGAATTCGGCTCCTGGTGCCTCGTGATTCGGGAAGAACCTC
+
FFFFFFFFFFFFFFFFFFFFFFFFFFFFFFFFFFFFFFFFFFFFFFFFFFFFFFFFFFFFFFFFFFFF
FF:FFFFFFFFFFFFFFFFFFFFFFFFFFFFFFFFFFFFFFFFFFFFFFFFFFFFFFFFF
@A00155:342:HHGFNDSXY:1:2644:32099:31626 1:N:0:GAACCTAG+TCCGCATA
AATAATTCCTAGTCGTGGTGATATTGCTGTCATTCACTCGCAGCACCCCACCATGGTACGTGCTATGC
CTTAAATTACCATCGAAGTTGAATTCGGCTCCTGGTGCCTCGTGATTCGGGAAGAACCTCTT
+
FFFFFFFFFFFFFFFFFFFFFFFFFFFFFFFFFFFFFFFFFFFFFFFFFFFFFFFFFFFFFFFFFFFF
FFFFFFFFFFFFFFFFFFFFFFFFFFFFFFFFFFFFFFFFFFFFFFF,FFFFFFFFFFFFF
@A00155:342:HHGFNDSXY:1:1474:17924:10833 1:N:0:GAACCTAG+TCCGCATA
AATAATTCCTAGTCGTGGTGATATTGCTGTCATTCACTCGCAGCACCCCACCATGGTACGTGCTATGC
CTTAAATTACCATCGAAGTTGAATTCGGCTCCTGGTGCCTCGTGATTCGGGAAGAACCTCTT
+
FFFFFFFFFFFFFFFFFFFFFFFFFFFFFFFFFFFFFFFFFFFFFFFFFFFFFFFFFFFFFFFFFFFF
FFFFFFFFFFFFFFFFFFFFFFFFFFFFFFFFFF:FFFFFFFFFFFFFFFFFFFFFFFFFF
@A00155:342:HHGFNDSXY:1:1474:18114:12038 1:N:0:GAACCTAG+TCCGCATA
AATAATTCCTAGTCGTGGTGATATTGCTGTCATTCACTCGCAGCACCCCACCATGGTACGTGCTATGC
CTTAAATTACCATCGAAGTTGAATTCGGCTCCTGGTGCCTCGTGATTCGGGAAGAACCTCTT
+
FFFFF,FFFFFFFFFFFFFFFFFFFFFFFFF:FFFFFFFFFFFFFFFFFFFFFFFF:FFFFFFFFFFF
FFFFFFFFFFFFFFFF,FFFFFFFFFFFFFFFFFFFFFFFFFFFFFFFFFFFFFFFFFFF
```

```
@A00155:342:HHGFNDSXY:1:1635:7437:23046 1:N:0:GAACCTAG+TCCGCATA
AATAATTCCTAGTCGTGGTGATATTGCTGTCATTCACTCGCAGCACCCCACCATGGTACGTGCTATGC
CTTAAATTACCATCGAAGTTGAATTCGGCTCCTGGTGCCTCGTGATTCGGGAAGAACCTCTTAA
+
FFFFFFFFFFFFFFFFFFFFFFFFFFFFFFFF:FFFFFFFFFFFFFFFFFFFFFFF:FFFFFF:FFFF
FF:FFFFFFFFFFFFFFFFFFFFFFFFFFFFFFFFFFFFFFFFFF:FFFFFFFFFFFFFFFFFF
@A00155:342:HHGFNDSXY:1:1361:5104:32064 1:N:0:GAACCTAG+TCCGCATA
AATAATTCCTAGTCGTGGTGATATTGCTGTCATTCACTCGCAGCACCCCACCATGGTACGTGCTATGC
CTTAAATTACCATCGAAGTTGAATTCGGCTCCTGGTGCCTCGTGATTCGGGAAGAACCTCTTAA
+
FFFFFFFFFFFFFFFFFF,FFFFFFFFFFFFFFFFFFFFFFFFFFFFFFFFFFFFFFFFFFFFFFFFF
FFFFFFFFFFFFFFFFFFFFFFFFFFFFFFFFFFFFFFFFFFFFFFFFFFF:FFFFFFFFFFF
@A00155:342:HHGFNDSXY:1:2430:17381:15562 1:N:0:GAACCTAG+TCCGCATA
AATAATTCCTAGTCGTGGTGATATTGCTGTCATTCACTCGCAGCACCCCACCATGGTACGTGCTATGC
CTTAAATTACCATCGAAGTTGAATTCGGCTCCTGGTGCCTCGTGATTCGGGAAGAACCTCTTAAC
+
FFFFFFFFFFFFFFFFFFFFFFFFFFFFFFFFFFFFFFFFFFFFFFFFFFFFFFFFFFFFFFFFFFFF
FFFFFFFFFFFFFFFFFFFFFFFFFFFFFFFFFFFFFFFFFFFFFFFFFFFFFFFFFFFFFFFFF
@A00155:342:HHGFNDSXY:1:1467:5575:15499 2:N:0:GAACCTAG+TCCGCATA
AATAATTCCTAGTCGTGGTGATATTGCTGTCATTCACTCGCAGCACCCCACCATGGTACGTGCTATGC
CTTAAATTACCATCGAAGTTGAATTCGGCTCCTGGTGCCTCGTGATTCGGGAAGAACCTCTTAACGAA
+
FFFFFFFFFFFFFFFFFFFFFFFFFFFFF:FF:FFFFFFFFFFFFFFFFFFFFFFFFFFFFFFFFFFF
FFFFFFF:FFFF:FFFFFFFFFF:FFFFFFFFFFFFFFFFFFFFFFFFFFFFFFFFFFFFF,F:FFFF
@A00155:342:HHGFNDSXY:1:2644:31684:21825 1:N:0:GAACCTAG+TCCGCATA
AATAATTCCTAGTCGTGGTGATATTGCTGTCATTCACTCGCAGCACCCCACCATGGTACGTGCTATGC
CTTAAATTACCATCGAAGTTGAATTCGGCTCCTGGTGCCTCGTGATTCGGGAAGAACCTCTTAACGAA
+
FFFFF:FFFFFFFFFFFFFFFFFFFFFFFFFFFFFFFFFFFFFFFFFFFFFFFFFFFFFFFFFFFFFF
FFFFFFFFFFFFFFFFFFFFFFFFFFFFFFFFFFFFFFFFFFFFFFFFFFFFFFFFFFFFFFFFFFFF
@A00155:342:HHGFNDSXY:1:1650:30264:25316 2:N:0:GAACCTAG+TCCGCATA
ATAATTCCTAGTCGTGGTGATATTGCTGTCATTCACTCGCAGCACCCCACCATGGTACGTGCTATGCC
TTAAATTACCATCGAAGTTGAATTCGGCTCCTGGTGCCTCGTGATTCGGGAAGAACCTCTTAACGAAA
+
FFF:FFF:FFFFFFF:FFFFFF,FFFFFFFFFFFFFF:F:F:FFFFFFFFFFFFFFFFF:F:FFFFFF
FFFFFFFFFFFF:FFFFFFFFFFFFFFFFFFFFFF:F:FFFFFFFFFFFFFFFFF:FFFFFFFFFFFF
@A00155:342:HHGFNDSXY:1:2223:6063:35697 1:N:0:GAACCTAG+TCCGCATA
AATTCCTAGTCGTGGTGATATTGCTGTCATTCACTCGCAGCACCCCACCATGGTACGTGCTATGCCTT
AAATTACCATCGAAGTTGAATTCGGCTCCTGGTGCCTCGTGATTCGGGAAGAACCTCTTAACGA
+
FFFFFFFFFFFFFFFFFFFFFFFFFFFFFFFFFFFFFFFFFFFFFFFFFFFFFFFFFFFFF,FFFFFF
FFFFFFFFFFFFFFFFFFFFFFFFFFFFFFFFFFFFFFFFFFF::FFFFFF:,FFFFFFFFF,F
@A00155:342:HHGFNDSXY:1:2462:23800:36417 1:N:0:GAACCTAG+TCCGCATA
AATTCCTAGTCGTGGTGATATTGCTGTCATTCACTCGCAGCACCCCACCATGGTACGTGCTATGCCTT
AAATTACCATCGAAGTTGAATTCGGCTCCTGGTGCCTCGTGATTCGGGAAGAACCTCTTAACGA
+
FFFFFFFFFFFFFFFFFFFFFFFFFFFFFFFFFFFFFFFFFFFFFFFFFFFFFFFFFFFFFFFFFFFF
FFFFFFFFFFFFFFFFFFFFFFFFFFFFFFFFFFFFFFFFFFFFFFFFFFFFFFFFFFFFFFFF
@A00155:342:HHGFNDSXY:1:2430:20157:9596 2:N:0:GAACCTAG+TCCGCATA
AATTCCTAGTCGTGGTGATATTGCTGTCATTCACTCGCAGCACCCCACCATGGTACGTGCTATGCCTT
AAATTACCATCGAAGTTGAATTCGGCTCCTGGTGCCTCGTGATTCGGGAAGAACCTCTTAACGAAACT
+
:FFFFFFFFFFFFFFFFFFFFFFFFFFFF,FFFFFFFFFFFFFFFFFFF:FFFFFFFFFFFFFFFFFF
FFFFFFFFFFFFFFFFFFFFFFFFFFFFFFFFFFFFFFFFFFFFFFFFFFFFFFFFFFFFFFFFFFFF
```

```
@A00155:342:HHGFNDSXY:1:1130:7934:21966 2:N:0:GAACCTAG+TCCGCATA
AATTCCTAGTCGTGGTGATATTGCTGTCATTCACTCGCAGCACCCCACCATGGTACGTGCTATGCCTT
AAATTACCATCGAAGTTGAATTCGGCTCCTGGTGCCTCGTGATTCGGGAAGAACCTCTTAACGAAACT
+
FFFF,FFFFFFFFF:FFFF:FFFFFFFFFFFFFFFFFFFFFFFFFFFFFF:FFFFFFFFFFFFFFFFF
FFFF:FFFFFFFFFFFFFFFFFFFFFFFFFFFFFFFFFFFFFFFFFF:FFFFFFFFFFFFFFFFFFFF
@A00155:342:HHGFNDSXY:1:1423:30508:7921 2:N:0:GAACCTAG+TCCGCATA
AATTCCTAGTCGTGGTGATATTGCTGTCATTCACTCGCAGCACCCCACCATGGTACGTGCTATGCCTT
AAATTACCATCGAAGTTGAATTCGGCTCCTGGTGCCTCGTGATTCGGGAAGAACCTCTTAACGAAACT
+
FFFFFFFFFFFFFFFFFFFFFFFFFFFFFFFFFFFFFFFFFFFFFFFFFFFFFFFFFFFFFFFFFF::
FFF:FFFFFFFFFFFFFFFFFFFFFFFFFFFFFFFFFFFFFFFFFFFFFFFFFFFFFFFFFFFFFFFF
@A00155:342:HHGFNDSXY:1:1471:16459:2534 2:N:0:GAACCTAG+TCCGCATA
AATTCCTAGTCGTGGTGATATTGCTGTCATTCACTCGCAGCACCCCACCATGGTACGTGCTATGCCTT
AAATTACCATCGAAGTTGAATTCGGCTCCTGGTGCCTCGTGATTCGGGAAGAACCTCTTAACGAAACT
+
FFFFFFFFFFFFFFFFFFFFFFFFFFFFFFFFFF:FFFFFFFFFFFFFFFFFFFFFFFF:FFFFFFFF
FF:FFFFFFFFFFFFFFFFFFFFFFFFFFFFF:FFFFFFFFFFFFFFFFFFFFFFFFFFFFFFFFFFF
@A00155:342:HHGFNDSXY:1:2108:17499:11443 2:N:0:GAACCTAG+TCCGCATA
AATTCCTAGTCGTGGTGATATTGCTGTCATTCACTCGCAGCACCCCACCATGGTACGTGCTATGCCTT
AAATTACCATCGAAGTTGAATTCGGCTCCTGGTGCCTCGTGATTCGGGAAGAACCTCTTAACGAAACT
+
FFFFFFFFFFFFFFFFFFFFFFFFFFFFFFFFFFFFFFFFFFFFFFFFFFFFFFFFFFFFFFFFFFFF
FFFFFFFFFFFFFFFFFFFFFFFFFFFFFFFFFFFFFFFFFFFFFFFFFFFFFFFFFFFFFFFFFFFF
@A00155:342:HHGFNDSXY:1:2627:4227:27477 2:N:0:GAACCTAG+TCCGCATA
ATTCCTAGTCGTGGTGATATTGCTGTCATTCACTCGCAGCACCCCACCATGGTACGTGCTATGCCTTA
AATTACCATCGAAGTTGAATTCGGCTCCTGGTGCCTCGTGATTCGGGAAGAACCTCTTAACGAAAC
+
FFFFFFFFFFFF:FFFFFFFFFFF:FFFFFFFFFFFFFFFFFFFFFFFFFFFFFFFFFFFFFFFFFFF
FFFFFFFFFFFFFFFFFFFFF:FFFFFFFFFFFFFFFFFFF,FF:F:FFF,FFFFFFFFFF:FFFF
@A00155:342:HHGFNDSXY:1:1676:12292:28948 2:N:0:GAACCTAG+TCCGCATA
ATTCCTAGTCGTGGTGATATTGCTGTCATTCACTCGCAGCACCCCACCATGGTACGTGCTATGCCTTA
AATTACCATCGAAGTTGAATTCGGCTCCTGGTGCCTCGTGATTCGGGAAGAACCTCTTAACGAAACTA
+
FFFFFFFFFFFFFFFFFFFFFFF:FFFFFFFFFFFFFFFFFFFFFFFFFFFFFFFFFFFFFFFFFFFF
FFFFFFFFFFFFFFFFFF:FFFFFFFFFFFFFFFFFFFFFFFFFFFFFFFFFFFFFFFFFFFFFFFFF
@A00155:342:HHGFNDSXY:1:1233:30011:6715 2:N:0:GAACCTAG+TCCGCATA
TCCTAGTCGTGGTGATATTGCTGTCATTCACTCGCAGCACCCCACCATGGTACGTGCTATGCCTTAAA
TTACCATCGAAGTTGAATTCGGCTCCTGGTGCCTCGTGATTCGGGAAGAACCTCTTAACGAAACT
+
FFFFFFFFFFFFFFF:FFFFF:FFFFFFFFFFFFFFFFFFFFFFFFFFFFFFFF:FFFFFFFF:FFFF
FFFFFFFFFFFFFFFFFFFFFFFFFFFFFFFFFFFFFFFFFFFFFFFFFFFFFFFFFFFFFFFFF
@A00155:342:HHGFNDSXY:1:2115:5303:30561 1:N:0:GAACCTAG+TCCGCATA
TCCTAGTCGTGGTGATATTGCTGTCATTCACTCGCAGCACCCCACCATGGTACGTGCTATGCCTTAAA
TTACCATCGAAGTTGAATTCGGCTCCTGGTGCCTCGTGATTCGGGAAGAACCTCTTAACGAAACTATC
+
FFFFFFFFFFFFFFFFFFFFFFFFFFFFFFFFFFFFFFFFFFFFFFFFFFFFFFFFFFFFFFFFFFFF
FFFFFFFFFFFFFFFFFFFFFFFFFFFFFFFFFFFFFFFFFFFFFFFFFFFFFFFFFFFFFF,FFFFF
@A00155:342:HHGFNDSXY:1:1335:10710:21605 1:N:0:GAACCTAG+TCCGCATA
TCCTAGTCGTGGTGATATTGCTGTCATTCACTCGCAGCACCCCACCATGGTACGTGCTATGCCTTAAA
TTACCATCGAAGTTGAATTCGGCTCCTGGTGCCTCGTGATTCGGGAAGAACCTCTTAACGAAACTATC
+
,FF::F,FFFFF:FF,F::FF:FFFF::FFFFFFFFFFFFFFFFFFFFFFFFFFFFF,FFFFFFFFFF
FFFFFFFFFFFFFFFFFFFFFFFFFFFFFFFFFFFFFFF,FFFFFFFFFFFFFF:FFFFFFFFFF,FF
```

```
@A00155:342:HHGFNDSXY:1:1467:5575:15499 1:N:0:GAACCTAG+TCCGCATA
TCCTAGTCGTGGTGATATTGCTGTCATTCACTCGCAGCACCCCACCATGGTACGTGCTATGCCTTAAA
TTACCATCGAAGTTGAATTCGGCTCCTGGTGCCTCGTGATTCGGGAAGAACCTCTTAACGAAACTATC
+
FFFFFFFFFFFFFFFFFF:FFFFFFF:FFFFFFFFFFFFFFFFFFFFFFFFFFFFFFFFFFFFFFFFF
FFFFFFFFFF:FFFFFFFFFFFFFFFFFFFFFFFFFFFFFFFFFFFFFFFFFFFFFFFFFFFFFFFFF
@A00155:342:HHGFNDSXY:1:1458:29875:34006 1:N:0:GAACCTAG+TCCGCATA
CCTAGTCGTGGTGATATTGCTGTCATTCACTCGCAGCACCCCACCATGGTACGTGCTATGCCTTAAAT
TACCATCGAAGTTGAATTCGGCTCCTGGTGCCTCGTGATTCGGGAAGAACCTCTTAACGAAACTATCC
+
FFFFFFFFFFFFFFFFFFFFFFFFFFFFFFFFFFFFFFFFFFFFFFFFFFFFFFFFFFFFFFFFFFFF
FFFFFFFFFFFFFFFFFFFFFFFFFFFFFFFFFFFFFFFFFFFFFFFFFFFFFFFFFF:FFFFFFF:F
@A00155:342:HHGFNDSXY:1:2361:8314:14544 2:N:0:GAACCTAG+TCCGCATA
CTAGTCGTGGTGATATTGCTGTCATTCACTCGCAGCACCCCACCATGGTACGTGCTATGCCTTAAATT
ACCATCGAAGTTGAATTCGGCTCCTGGTGCCTCGTGATTCGGGAAGAACCTCTTAACGAAACT
+
F,:F:FFFFFFFFFF,FFFFFFFFFFFFFFFFFFFFFFFFFFFFF,FF,FFFFFF,FFF,F,FFF,FF
FFFFFFFFFFF:FFFFFFFFFFFF,FF:FFFFFF:FFFFFFFFFFFFFFFFFFFFFFFFFFFF
@A00155:342:HHGFNDSXY:1:1357:1796:3004 2:N:0:GAACCTAG+TCCGCATA
CTAGTCGTGGTGATATTGCTGTCATTCACTCGCAGCACCCCACCATGGTACGTGCTATGCCTTAAATT
ACCATCGAAGTTGAATTCGGCTCCTGGTGCCTCGTGATTCGGGAAGAACCTCTTAACGAAACTATCCA
+
FFFFFFFFFF:FFFFFFFFFFFFFFFFFFFFFFFFFFFFFFFFFFFFFFFFF:FFFFFFFFFFFF:::
FFFFFFFFFFFFFFFFFFFFFFFFFFFFFFFFFFFFFFFFFFFFFFFFFFFFFFFFFFFFFFFFFFFF
@A00155:342:HHGFNDSXY:1:1106:17228:13917 2:N:0:GAACCTAG+TCCGCATA
CTAGTCGTGGTGATATTGCTGTCATTCACTCGCAGCACCCCACCATGGTACGTGCTATGCCTTAAATT
ACCATCGAAGTTGAATTCGGCTCCTGGTGCCTCGTGATTCGGGAAGAACCTCTTAACGAAACTATCCA
+
FFFFFFFFFFFFFFF:FFFFFFFF:FFFFFFFF:FFFFFFFFFFFFFF,FFFFFFFFFFFFFFFFF,F
FFFF,FFFFFFFFFFFFFFFFFFFFFFFFFFFFFFFFFFFFFFFFFFFFFF:FFFFFFF:FFFFFFF
@A00155:342:HHGFNDSXY:1:1207:20672:34695 2:N:0:GAACCTAG+TCCGCATA
CTAGTCGTGGTGATATTGCTGTCATTCACTCGCAGCACCCCACCATGGTACGTGCTATGCCTTAAATT
ACCATCGAAGTTGAATTCGGCTCCTGGTGCCTCGTGATTCGGGAAGAACCTCTTAACGAAACTATCCA
+
FFFFFFFFFFFFFFFFFFFFFFFFFFFFFF:FFFFFFFFFF,FFFFFFFFFF:FFFFFFFFFFFFFF:
FFFFFFFFFFFFFFFFFFFFFFFFFFFFFFFFFFFFFFFFFFFFFFFFFFFFFF,FFFFFFFFFFFFF
@A00155:342:HHGFNDSXY:1:2217:10520:31109 2:N:0:GAACCTAG+TCCGCATA
CTAGTCGTGGTGATATTGCTGTCATTCACTCGCAGCACCCCACCATGGTACGTGCTATGCCTTAAATT
ACCATCGAAGTTGAATTCGGCTCCTGGTGCCTCGTGATTCGGGAAGAACCTCTTAACGAAACTATCCA
+
FFFFFFFFFFFFFFFFFFFFFFFFFFFFFFFFFFFFFFFFFFFFFFFFFFFFFFFFFFFFFFFFFFFF
:FFFFFFFFFFFFFFFFFFFF:FFFFFFFFFFFFFFFFFFFFFFFFFFFFFFFFFFFFFFFFFFFFFF
@A00155:342:HHGFNDSXY:1:1321:10926:36479 2:N:0:GAACCTAG+TCCGCATA
TAGTCGTGGTGATATTGCTGTCATTCACTCGCAGCACCCCACCATGGTACGTGCTATGCCTTAAATTA
CCATCGAAGTTGAATTCGGCTCCTGGTGCCTCGTGATTCGGGAAGAACCTCTTAACGAAACTATCCAT
+
FFFFFFFFFFFFFF:FFFFFFFFFFFFFFFFFFFFFFFFFFFFFFFFFFFFFFFFFFFFFFFFFF:FF
FFFFFFFFFFFFFFFFFFFFFFFFFFFFFFFFFFFFFFFFFFFFFFFFFFFFFFFFFFFFFFFFFFFF
@A00155:342:HHGFNDSXY:1:2651:4942:19163 1:N:0:GAACCTAG+TCCGCATA
TAGTCGTGGTGATATTGCTGTCATTCACTCGCAGCACCCCACCATGGTACGTACTATGCCTTAAATTA
CCATCGAAGTTGAATTCGGCTCCTGGTGCCTCGTGATTCGGGAAGAACCTCTTAACGAAACTATCCAT
+
FFFFFFFFFFFFFFFFFFFFFFFFFFFFFFFFFFFFFFFFFFFFFFFFFFFFFFFFFFFFFFFFFFF:
FFFFFFFFFFFFFF:FFFFFFFFFFFFFFFFFFFFFFFFFFFFFFFFFFFFFFFFFFFFFFFF:FFFF
```

```
@A00155:342:HHGFNDSXY:1:2113:24442:18114 1:N:0:GAACCTAG+TCCGCATA
TAGTCGTGGTGATATTGCTGTCATTCACTCGCAGCACCCCACCATGGTACGTGCTATGCCTTAAATTA
CCATCGAAGTTGAATTCGGCTCCTGGTGCCTCGTGATTCGGGAAGAACCTCTTAACGAAACTATCCAT
+
FFFFFFFFFFFFFFFFFFFFFFFFFFFFFFFFFFFFFFFFFFFFFFFFFFFFFFFFFFFFFFFFFFFFFF
FFFFFFFFFFFFFFFFFFFFFFFFFFFFFFFFFFFFFFFFFFFFFFFFFFFFFFFFFFFFFFFFFFFFFF
@A00155:342:HHGFNDSXY:1:2168:30572:29888 1:N:0:GAACCTAG+TCCGCATA
TAGTCGTGGTGATATTGCTGTCATTCACTCGCAGCACCCCACCATGGTACGTGCTATGCCTTAAATTA
CCATCGAAGTTGAATTCGGCTCCTGGTGCCTCGTGATTCGGGAAGAACCTCTTAACGAAACTATCCAT
+
FFFFFFFFFFFFFFFFFFFFFFFFFFF:FFFFFFFFFFFFFFFFFFFFFFFFFFFFFFFFFFFFFFF:FF
FFFFFFFFFFFFFFFFFFFFFFFFFFFFFFFFFF:FFFFFFFFFFFF:FF,FFFFFFFFFFFFFFFFFFF
@A00155:342:HHGFNDSXY:1:2123:19036:2268 2:N:0:GAACCTAG+TCCGCATA
TAGTCGTGGTGATATTGCTGTCATTCACTCGCAGCACCCCACCATGGTACGTGCTATGCCTTAAATTA
CCATCGAAGTTGAATTCGGCTCCTGGTGCCTCGTGATTCGGGAAGAACCTCTTAACGAAACTATCCAT
+
FFFFFFFFFFFFFFFFFFFFFFFFFFFFFFFFFFFFFFFFFFFFFFFFFFFFFFFFFFFFFFFFFFFFFF
:FFFFFFFFFFFFFFFFF:FFFFFFFFFFFFFFFFFFFFFFFFFFFFFFFFFFFFFFFFFFFFFF:FFFF
@A00155:342:HHGFNDSXY:1:1269:9561:29105 1:N:0:GAACCTAG+TCCGCATA
TAGTCGTGGTGATATTGCTGTCATTCACTCGCAGCACCCCACCATGGTACGTGCTATGCCTTAAATTA
CCATCGAAGTTGAATTCGGCTCCTGGTGCCTCGTGATTCGGGAAGAACCTCTTAACGAAACTATCCAT
+
FFFFFFFFFFFFFFFFFFFFFFF:FFFFFF,FFFFFFFFFFFFFFFFFFFFFFFFFFFFFFFFFFFFFFF
,FFFFFFFFF:FFFFFFFFFFFFFFFFFFFFFFFFFFFFFFFFFFFFFFFFFFFF:FFFFF:FFFFFFFF
@A00155:342:HHGFNDSXY:1:2252:10963:10958 1:N:0:GAACCTAG+TCCGCATA
TAGTCGTGGTGATATTGCTGTCATTCACTCGCAGCACCCCACCATGGTACGTGCTATGCCTTAAATTA
CCATCGAAGTTGAATTCGGCTCCTGGTGCCTCGTGATTCGGGAAGAACCTCTTAACGAAACTATCCAT
+
FFFFFFFFFFFFFFFFFFFFFFFFFFFFFFFFFFFFFFFFFFFFFFFFFFFFFFFFFFFFFFFFFFFFFF
FFFFFFFFFFFFFFFFFFFFFFFFFFFFFFFFFFFFFFFFFFFFFFFFFFFFFFFFFFFFFFFFFFFFFF
@A00155:342:HHGFNDSXY:1:1604:2799:4460 1:N:0:GAACCTAG+TCCGCATA
TAGTCGTGGTGATATTGCTGTCATTCACTAGCAGCACCCCACCATGGTACGTGCTATGCCTTAAATTA
CCATCGAAGTTGAATTCGGCTCCTGGTGCCTCGTGATTCGGGAAGAACCTCTTAACGAAACTATCCAT
+
FFFFFFFFFF,FFFFFFFFFFFFFF,F,:,FFFFFFFFFFFFF:FFFFFFFFFFFFFFFFFFFFFFFFFF
FFFFFFFFFFFFFFFFFFFFFFFFFFFFF:FFFFF,FFFFF,FFFFFFFFFFFFFFFFFFFF:FFFFF,F
@A00155:342:HHGFNDSXY:1:2609:26286:2080 1:N:0:GAACCTAG+TCCGCATA
TAGTCGTGGTGATATTGCTGTCATTCACTCGCAGCACCCCACCATGGTACGTGCTATGCCTTAAATTA
CCATCGAAGTTGAATTCGGCTCCTGGTGCCTCGTGATTCGGGAAGAACCTCTTAACGAAACTATCCAT
+
FFFFFFFFFFFFFFFFFFFFFFFFFFFFFFFFFFFFFFFFFFFFFFFF:FFFFFFFFFFFFFFFFFFFFF
FFFFFFFFFFFFFFFFFFFFFFFFFFFFFFFFFFFFFFFFFFFF:FFFFFFFFFFFFFFFFF::FFFFFF
@A00155:342:HHGFNDSXY:1:2415:31928:36558 1:N:0:GAACCTAG+TCCGCATA
TAGTCGTGGTGATATTGCTGTCATTCACTCGCAGCACCCCACCATGGTACGTGCTATGCCTTAAATTA
CCATCGAAGTTGAATTCGGCTCCTGGTGCCTCGTGATTCGGGAAGAACCTCTTAACGAAACTATCCAT
+
FFFFFFFFF,FFFFFFFFFFFFFFFFFFFFFFFFFFFFFFFFFFFFFFFFFFFFFFFFFFFFFFFFFFFF
FFFFFFFFFFFFFFFFFFFFFFFFFFFFFFFFFFFFFFFFFFFFFFFFFFFFFFFFFFFFFFFFFFFFFF
@A00155:342:HHGFNDSXY:1:1366:12048:13557 1:N:0:GAACCTAG+TCCGCATA
TAGTCGTGGTGATATTGCTGTCATTCACTCGCAGCACCCCACCATGGTACGTGCTATGCCTTAAATTA
CCATCGAAGTTGAATTCGGCTCCTGGTGCCTCGTGATTCGGGAAGAACCTCTTAACGAAACTATCCAT
+
FFF:FFFFF:FFFFFFF:FFFFFFFFFFFFFFF,FFFFFFFFFFFFFFFFFFFFFFFFFFFFFFFF:FFF
FFFFFFFFF::FFFFFF:FFFFFFFFFFFFFFFFFFFF:FFFFFFFF:FFFFF:FFFFFFF:FFFFFFFF
```

```
@A00155:342:HHGFNDSXY:1:2331:6008:14967 2:N:0:GAACCTAG+TCCGCATA
TAGTCGTGGTGATATTGCTGTCATTCACTCGCAGCACCCCACCATGGTACGTGCTATGCCTTAAATTA
CCATCGAAGTTGAATTCGGCTCCTGGTGCCTCGTGATTCGGGAAGAACCTCTTAACGAAACTATCCAT
+
FFFFFFFFFFFFFFFFFFFFFFFFFFFFFFFFFFFFFFFFFFFFFFFFFFFFFFFFFFF:FFFFFFF:
FFFFFFF:FFFFFFFFFFFFFFFFFFFFFFFFFFFFFFFFFFFFF:FF:FFFFFF:FFFFFFFFFFFF
@A00155:342:HHGFNDSXY:1:2331:5728:15076 2:N:0:GAACCTAG+TCCGCATA
TAGTCGTGGTGATATTGCTGTCATTCACTCGCAGCACCCCACCATGGTACGTGCTATGCCTTAAATTA
CCATCGAAGTTGAATTCGGCTCCTGGTGCCTCGTGATTCGGGAAGAACCTCTTAACGAAACTATCCAT
+
FFFFFFFFF:FFFFFFFFFFFFFFFFFFFFFFFFFFFFFFFFFFFFFFFFFFFFFFFFFFFFFFFFFF
FFFFFFFFFFFFFFFFFFFFFFFFFFFFFFFFFFFFFFFFFFFFFFFFFFFFFFFFFFFFFFFFFFFF
@A00155:342:HHGFNDSXY:1:2331:5855:15201 2:N:0:GAACCTAG+TCCGCATA
TAGTCGTGGTGATATTGCTGTCATTCACTCGCAGCACCCCACCATGGTACGTGCTATGCCTTAAATTA
CCATCGAAGTTGAATTCGGCTCCTGGTGCCTCGTGATTCGGGAAGAACCTCTTAACGAAACTATCCAT
+
FFFF:F,,FFFFFFFF:FFFFFF:FFFFFFF:F:FFFFFFFF,FFFFFFFFFFFFFFFFF:FFFF:FF
FFFFF,FFFFFFF,FFFFFFFFFFFFFFFFFFFFFFFFFFFFFFFFFFFFFFFFFFFFFFFFFFFFFF
@A00155:342:HHGFNDSXY:1:2139:2157:35978 1:N:0:GAACCTAG+TCCGCATA
TAGTCGTGGTGATATTGCTGTCATTCACTCGCAGCACCCCACCATGGTACGTGCTATGCCTTAAATTA
CCATCGAAGTTGAATTCGGCTCCTGGTGCCTCGTGATTCGGGAAGAACCTCTTAACGAAACTATCCAT
+
FFFFFFFFFFFFFFFFFFFFFFFFFFFFFFFFFFFFFFFFFFFFFFFFFFFFFFFFFFFFFFFFFFFF
:FFFFFFFFFFFFFFFFFFFFFFFFFFFFFFFFFFFFFFFFFF:FF:FFFFFFF:F:FFFFF:FF:FF
@A00155:342:HHGFNDSXY:1:1333:30427:32236 1:N:0:GAACCTAG+TCCGCATA
TAGTCGTGGTGATATTGCTGTCATTCACTCGCAGCACCCCACCATGGTACGTGCTATGCCTTAAATTA
CCATCGAAGTTGAATTCGGCTCCTGGTGCCTCGTGATTCGGGAAGAACCTCTTAACGAAACTATCCAT
+
FFFFFF:FFFFFFFFFFFFFFFFFFFFFFFFF:FFFFFFFFFFFFFFFFFFFFFFFFFFFFFFFFFFF
FFFFFFFFFFFFFFFFFFFFFFFFFFFFFFFFFFFFFFFFFFFFFFFFFFFFFFFFFFFFFFFFFFFF
@A00155:342:HHGFNDSXY:1:1317:4408:26381 1:N:0:GAACCTAG+TCCGCATA
AGTCGTGGTGATATTGCTGTCATTCACTCGCAGCACCCCACCATGGTACGTGCTATGCCTTAAATTAC
CATCGAAGTTGAATTCGGCTCCTGGTGCCTCGTGATTCGGGAAGAACCTCTTAACGAAACTATC
+
FFFFFFFFFFFFFFFFFFFFFFFFFFFFFFFFFFFFFFFFFFFFFFFFFFFFFFFFFFFFFFFFFFFF
FFFFFFFFFFFFFFFFFFFFFFFFFFFFFFFFFFFFFFFFFF:FF:FFFFFF:FFFFFFFFFFF
@A00155:342:HHGFNDSXY:1:2113:24442:18114 2:N:0:GAACCTAG+TCCGCATA
AGTCGTGGTGATATTGCTGTCATTCACTCGCAGCACCCCACCATGGTACGTGCTATGCCTTAAATTAC
CATCGAAGTTGAATTCGGCTCCTGGTGCCTCGTGATTCGGGAAGAACCTCTTAACGAAACTATCCAT
+
FFFFFFFFFFFFF,FFFFFFFFFFFFFFFFFFFFFFFFF,FFFFFFFFFFFFFFFFFFFFFFFFFFFF
FFFFFFFFFFFFFFFFFFFFFFFFFFFFFFFFFFFFFFFFFFFFFFFFFFFFFFFFFFFFFFFFFF
@A00155:342:HHGFNDSXY:1:2346:30581:8202 2:N:0:GAACCTAG+TCCGCATA
AGTCGTGGTGATATTGCTGTCATTCACTCGCAGCACCCCACCATGGTACGTGCTATGCCTTAAATTAC
CATCGAAGTTGAATTCGGCTCCTGGTGCCTCGTGATTCGGGAAGAACCTCTTAACGAAACTATCCAT
+
:FFFFF:FFFF:FFF:FFFFFFFFFFF,FFFFFFFFFFF,FFFFFFFFFFFFFFF:FFFFFFFFFFFF
F,FFFFFFFFFFFFFFFFFFFFFFFFFFFFFFFFFFFFFFFFFFFFFFFFF:FFFFFFFFFFFFFF
@A00155:342:HHGFNDSXY:1:1672:16586:12493 2:N:0:GAACCTAG+TCCGCATA
AGTCGTGGTGATATTGCTGTCATTCACTCGCAGCACCCCACCATGGTACGTGCTATGCCTTAAATTAC
CATCGAAGTTGAATTCGGCTCCTGGTGCCTCGTGATTCGGGAAGAACCTCTTAACGAAACTATCCATT
+
FFFFFFFF:FFFFF:FFFFFFFFFFFFFFFFFFFFFFFFFFFFFFFFFFF:FFFFFFFFFFFFFF:FF
FFFFFFFFFFFFFFFFFFFFFFFFFFFFFFFFFFFFFFFFFFFFFFFFFFFFFFFFFFFFFFFFFFFF
```

@A00155:342:HHGFNDSXY:1:2327:1289:20572 1:N:0:GAACCTAG+TCCGCATA
GTCGTGGTGATATTGCTGTCATTCACTCGCAGCACCCCACCATGGTACGTGCTATGCCTTAAATTACC
ATCGAAGTTGAATTCGGCTCCTGGTGCCTCGTGATTCGGGAAGAACCTCTTAACGAAAC
+
FFFFFFFFF:FFFFF:FFFFFFFF:FF::FF:F,F,FF:F:FFFFFFFFFF:FFFFF,FF,:,FF:,F
:F,FF,FFFF:FFF:,F,F:,FFFFF::FFFFFFFF:FFF,FF,:::F:FF,:,F,,,,
@A00155:342:HHGFNDSXY:1:1427:7554:36276 2:N:0:GAACCTAG+TCCGCATA
GTCGTGGTGATATTGCTGTCATTCACTCGCAGCACCCCACCATGGTACGTGCTATGCCTTAAATTACC
ATCGAAGTTGAATTCGGCTCCTGGTGCCTCGTGATTCGGGAAGAACCTCTTAACGAAACTATCCAT
+
FFFFFFFFFFFFFFFFFFFFFFFFFFFFFFFFFFFFFFFFFFFFFFFFFFFFFFFFFFFFFFFFFFFF
FFFFFFFFFFFFFFFFFFFFFFFFFFFFFFFFFFFFFFFFFFFFFFFFFFFFFFFFFFFFFFFFFF
@A00155:342:HHGFNDSXY:1:2603:21721:29058 2:N:0:GAACCTAG+TCCGCATA
GTCGTGGTGATATTGCTGTCATTCACTCGCAGCACCCCACCATGGTACGTGCTATGCCTTAAATTACC
ATCGAAGTTGAATTCGGCTCCTGGTGCCTCGTGATTCGGGAAGAACCTCTTAACGAAACTATCCAT
+
FFFFFFFFFFFFFFFFFFF::FFFFFFFF:FFFFFFFFFFFFFFFFFF:FFFFFFFFF:FFFF:FFFF
,FFFFFFFFFF:FFFFFFFFF:FFFFFFFFFFFFFFFFFFFFFFFFFFFFFFFFFFFFFFFFFFF
@A00155:342:HHGFNDSXY:1:1529:18954:34256 2:N:0:GAACCTAG+TCCGCATA
TCGTGGTGATATTGCTGTCATTCACTCGCAGCACCCCACCATGGTACGTGCTATGCCTTAAATTACCA
TCGAAGTTGAATTCGGCTCCTGGTGCCTCGTGATTCGGGAAGAACCTCTTAACGAAACTATCCATTAC
+
FFFFFFFFFFFFFFFFFFFFFFFFFFFFFFFFFFFFFFFFFFFFFFFFFFFFFFFFFFFFFFFFFFFF
FFFFFFFFFFF:FFFFFFFFFFFFF:FFFFFFFFFFFFFFFFFFFFFFFFFFFFFFFFFFFFFF,,FF
@A00155:342:HHGFNDSXY:1:2356:6343:35086 2:N:0:GAACCTAG+TCCGCATA
TCGTGGTGATAGTGCTGTCATTCACTCGCAGCACCCCACCATGGTACGTGCTATGCCTTAAATTACCA
TCGAAGTTGAATTCGGCTCCTGGTGCCTCGTGATTCGGGAAGAACCTCTTAACGAAACTATCCATTAC
+
FFFFFFFFFFF,FFFFFFFF,FFFFFFFFFFFFFFFF,FFFFFFFFFFFFFFFFFFFFFF::FFFFFF
FFFFFFFFFF:FFFFFFFFFFFFFFFFFFFFFFFFFFFFFFFFFFFFFFFFFFFFFFFFFFFFFFFFF
@A00155:342:HHGFNDSXY:1:1154:31991:22388 2:N:0:GAACCTAG+TCCGCATA
TCGTGGTGATATTGCTGTCATTCACTCGCAGCACCCCACCATGGTACGTGCTATGCCTTAAATTACCA
TCGAAGTTGAATTCGGCTCCTGGTGCCTCGTGATTCGGGAAGAACCTCTTAACGAAACTATCCATTAC
+
FFFFFFFFF:FFFFFFFFFF:FFFFFFFFFFFFFFFFFFFFFFFFFFFFFFFFFFFFFFFFFFFFFFF
FFFFFFFFFF:FFFFFFFFFFFFFFFFFFFFFFFFFFFFFFFFFFFFFFFFFFFFFFFFFFFFFFFFF
@A00155:342:HHGFNDSXY:1:2316:32597:34835 2:N:0:GAACCTAG+TCCGCATA
GTGGTGATATTGCTGTCATTCACTCGCAGCACCCCACCATGGTACGTGCTATGCCTTAAATTACCATC
GAAGTTGAATTCGGCTCCTGGTGCCTCGTGATTCGGGAAGAACCTCTTAACGAAACTATCCATTACTC
+
FFFFF:FFFFFFFFFF:FFFFFFFFFFFFFFFFFFFFFFFFFFFFFFFFFFFFFFF:FF,FF:FFFF:
FFFFFFFF:FFFFFFFFFFFFFFFFFFFFFFFFFFFFFFFFFFFFFFFFFFFFFFFFFFFFFFFFFF
@A00155:342:HHGFNDSXY:1:1548:28140:7701 2:N:0:GAACCTAG+TCCGCATA
GTGGTGATATTGCTGTCATTCACTCGCAGCACCCCACCATGGTACGTGCTATGCCTTAAATTACCATC
GAAGTTGAATTCGGCTCCTGGTGCCTCGTGATTCGGGAAGAACCTCTTAACGAAACTATCCATTACTC
+
FF,FF,:F:FF,:FFF,FFFF:FF,F::FFFF:FFFF,FF,FF:FFF:,FFFFF,FFFFFFFFFFFFF
FFFFFFFFFFFFFF:FFFFFFFFFFF:FFFFFFFFF:FFFFFFFF:FFFFFFFFF:FFFFFFFFFFFF
@A00155:342:HHGFNDSXY:1:2430:17381:15562 2:N:0:GAACCTAG+TCCGCATA
TGGTGATATTGCTGTCATTCACTCGCAGCACCCCACCATGGTACGTGCTATGCCTTAAATTACCATCG
AAGTTGAATTCGGCTCCTGGTGCCTCGTGATTCGGGAAGAACCTCTTAACGAAACTATCCATTAC
+
FFFFFFFFFFFFFFFFFFFFFFFFFFFFFFFFFFFFFFFFFFFFFFFFFFFFFFFFFFF:FFFFFFFF
FFFFFFFFFFFFFFFFFFFFFFFFFFFFF::FFFFFF,FFFFFFFFFFFFFFFFFFFFFFFFFFF

```
@A00155:342:HHGFNDSXY:1:2108:15374:22075 2:N:0:GAACCTAG+TCCGCATA
TGGTGATATTGCTGTCATTCACTCGCAGCACCCCACCATGGTACGTGCTATGCCTTAAATTACCATCG
AAGTTGAATTCGGCTCCTGGTGCCTCGTGATTCGGGAAGAACCTCTTAACGAAACTATCCATTACTC
+
FFFFFF:FFFFFFFFFFFFFFFFFFFFFFFFFFFFFFFFFF:FFFFFFFFFFFFFFFFF:FFFFFFFF
FFFFFFFFFFFFFFFFFFFFFFFFFFFFFFFFFFFFFFFFFFFFFFFFFFFFFFFFFFFFFFFFFFF
@A00155:342:HHGFNDSXY:1:2457:24966:15953 2:N:0:GAACCTAG+TCCGCATA
TGGTGATATTGCTGTCATTCACTCGCAGCACCCCACCATGGTACGTGCTATGCCTTAAATTACCATCG
AAGTTGAATTCGGCTCCTGGTGCCTCGTGATTCGGGAAGAACCTCTTAACGAAACTATCCATTACTCT
+
FFFF:FFFFFFFFFF:FFFFFFFFFFFFFFFFFF:FFFFFFFFFFFFFFFFFFFFFFFFF,FFF:FFF
FFFFFFFFFFFFFFFFFFFFFFFFFFFFFFFFFFFFFFFFFFFFFFFF:FFFFFFFFFFFFFFFFFFF
@A00155:342:HHGFNDSXY:1:1624:13331:10050 2:N:0:GAACCTAG+TCCGCATA
TGGTGATATTGCTGTCATTCACTCGCAGCACCCCACCATGGTACGTGCTATGCCTTAAATTACCATCG
AAGTTGAATTCGGCTCCTGGTGCCTCGTGATTCGGGAAGAACCTCTTAACGAAACTATCCATTACTCT
+
:FFFFFFFFFFFFFFFFFFFFFFFFFFFFFFFFFFFFFFFFFFFF:FFFFFFFFFFF:FFFFFFFFFF
FFFFFFFF:FFFFFFFFFFFFFFFFFFFFFFFFFFFFFFFFFFFFFFFFFFFFFFFFFFFFFFF,FF
@A00155:342:HHGFNDSXY:1:1566:27073:9518 2:N:0:GAACCTAG+TCCGCATA
TGGTGATATTGCTGTCATTCACTCGCAGCACCCCACCATGGTACGTGCTATGCCTTAAATTACCATCG
AAGTTGAATTCGGCTCCTGGTGCCTCGTGATTCGGGAAGAACCTCTTAACGAAACTATCCATTACTCT
+
FFFFFFFFFFF:FFFFF:FFFFFFFFFFFFFFFF:FFFFFFFFFFFFFFFFFFFFFFFFFFFFFFFFF
FFF:FFFFFF:FFFFFF:FFFFFFFFFFFFFFFFFFFFFFFFFFFFFFFFFFFFFFFFFFFFFFFFFF
@A00155:342:HHGFNDSXY:1:2425:15275:34303 2:N:0:GAACCTAG+TCCGCATA
TGGTGATATTGCTGTCATTCACTCGCAGCACCCCACCATGGTACGTGCTATGCCTTAAATTACCATCG
AAGTTGAATTCGGCTCCTGGTGCCTCGTGATTCGGGAAGAACCTCTTAACGAAACTATCCATTACTCT
+
F:FFFFFFFFFFFFFFFFFFFFFFFFFFFFFFFFFFFFFFFFFFFFFFFFFFFFFFFFFFFFFFFFFF
FFFFFFFFFFFFFFFFFFFFFFFFFFFFFFFFFFFFFFFFFFFFFFFFFFFFFFFFFFFFFFFFFFFF
@A00155:342:HHGFNDSXY:1:2330:13521:35712 2:N:0:GAACCTAG+TCCGCATA
TGGTGATATTGCTGTCATTCACTCGCAGCACCCCACCATGGTACGTGCTATGCCTTAAATTACCATCG
AAGTTGAATTCGGCTCCTGGTGCCTCGTGATTCGGGAAGAACCTCTTAACGAAACTATCCATTACTCT
+
FFFFFFFFFFFFFFFFFFFFFFFFFFFFFFFFFFFFFFF:FFFFFFFFFFFFFFFFFFFFFFFFFFFF
FFFFFFFFFFFFFFFFFFFFFFFFFFFFFFFFFFFFFFFFFFFFFFFFFFFFFFFFFFFFFFFFFFFF
@A00155:342:HHGFNDSXY:1:2165:26431:22028 2:N:0:GAACCTAG+TCCGCATA
TGGTGATATTGCTGTCATTCACTCGCAGCACCCCACCATGGTACGTGCTATGCCTTAAATTACCATCG
AAGTTGAATTCGGCTCCTGGTGCCTCGTGATTCGGGAAGAACCTCTTAACGAAACTATCCATTACTCT
+
FFFFFFFFFFFFFFFFFFFFFFFFFFFFFFFFFF:FFFFFFFFFFFFFFFFFFFFFFFFFFFFFFFFF
FFFFFFFFFFFFFFFFFFFFFFFFFFFFFFFFFFFFFFFFFFFFFFFFFFFFFFFFFFFFFFFFFFFF
@A00155:342:HHGFNDSXY:1:1509:5972:19382 2:N:0:GAACCTAG+TCCGCATA
GTGATATTGCTGTCATTCACTCGCAGCACCCCACCATGGTACGTGCTATGCCTTAAATTACCATCGAA
GTTGAATTCGGCTCCTGGTGCCTCGTGATTCGGGAAGAACCTCTTAACGAAACTATCCAT
+
FFF:FFFFFFFFFFFFFFFFFFFFFFFFFFFFFFFFFFFFFFFFFFFF,FFFFFFFFF:FFF,FFFFF
FFFFFFFFFFFFFFFFFFFFFFFFFFFFFFFFFFFFFFFFFFFFFFFFFFFFFFFFFFFF
@A00155:342:HHGFNDSXY:1:1534:30608:21371 2:N:0:GAACCTAG+TCCGCATA
GTGATATTGCTGTCATTCACTCGCAGCACCCCACCATGGTACGTGCTATGCCTTAAATTACCATCGAA
GTTGAATTCGGCTCCTGGTGCCTCGTGATTCGGGAAGAACCTCTTAACGAAACTATCCAT
+
FF:FFF:FFF:FFFFFFFFFFFFFFFFFFFFFFFFFFFFFF:FF:FFFFFFFFF:F::FFFFFFFFFF
FFFFF,FFFFFFFFFFFFFFFFFFFFFFFFFFFFFFFFFFFFFFF:FFFFFFFFFFFFF:
```

```
@A00155:342:HHGFNDSXY:1:1214:20247:8594 2:N:0:GAACCTAG+TCCGCATA
GTGATATTGCTGTCATTCACTCGCAGCACCCCACCATGGTACGTGCTATGCCTTAAATTACCATCGAA
GTTGAATTCGGCTCCTGGTGCCTCGTGATTCGGGAAGAACCTCTTAACGAAACTATCCAT
+
FFFFFFFFFFFFFFFFFFFFFFFFFFFFFFFF:FFFFFF:FFFFFFFFFFFFFFFFFFFFFFFFFFFF
FFFFFFFFFFFFFFFFFFFFFFFFFFFFFFFFFFFFFFFFFFFFF:FFFFFFFFFFFFFF
@A00155:342:HHGFNDSXY:1:1223:1416:11052 2:N:0:GAACCTAG+TCCGCATA
GTGATATTGCTGTCATTCACTCGCAGCACCCCACCATGGTACGTGCTATGCCTTAAATTACCATCGAA
GTTGAATTCGGCTCCTGGTGCCTCGTGATTCGGGAAGAACCTCTTAACGAAACTATCCATTACTCTGA
+
FF:FFFFFFFFF,FFFFFFF:FFFF:F:FFFFFF:FFFFFFFFFF::FFFFFFFF::FFFFFFFFFF:
FFFFFFFFFFFFFFFFFFFFFFFFFFFFFFFFFFFFFFFFFFFFFFFFFFFFFFFFFFFFFFFFFFFF
@A00155:342:HHGFNDSXY:1:1233:30011:6715 1:N:0:GAACCTAG+TCCGCATA
TATTGCTGTCATTCACTCGCAGCACCCCACCATGGTACGTGCTATGCCTTAAATTACCATCGAAGTTG
AATTCGGCTCCTGGTGCCTCGTGATTCGGGAAGAACCTCTTAACGAAACTATCCATTACTCTGAG
+
FFFFFFFFFFFFFFFFFFFFFFFFFFFFFFFFFFFFFFFFFFF,FF:FFFFFFFFFFFFFFFFFFFFF
FFFFFFFFFFFFF:FFFFFFFFFFFFFFFFFFFFFFFFFFFFFFFFFFFFFFFFFFFFFFFFFFF
@A00155:342:HHGFNDSXY:1:1355:18692:3834 1:N:0:GAACCTAG+TCCGCATA
TATTGCTGTCATTCACTCGCAGCACCCCACCATGGTACGTGCTATGCCTTAAATTACCATCGAAGTTG
AATTCGGCTCCTGGTGCCTCGTGATTCGGGAAGAACCTCTTAACGAAACTATCCATTACTCTGAGTTT
+
FFFFFFFFFFFFFFFFFFFFFFFFFFFFFFFFFFFFFFFFFFFFFFFFFFFFFFFFFFFFFFFFFFFF
FFFFFFFFFFFFFFFFFFFFFFFFFFFFFFFFFFFFFFFFFFFFFFFFFFFFFFFFFFFFFFF:FFFF
@A00155:342:HHGFNDSXY:1:1377:12635:12602 1:N:0:GAACCTAG+TCCGCATA
ATTGCTGTCATTCACTCGCAGCACCCCACCATGGTACGTGCTATGCCTTAAATTACCATCGAAGTTGA
ATTCGGCTCCTGGTGCCTCGTGATTCGGGAAGAACCTCTTAACGAAACTATCCATTACTCTGAGTTTG
+
FFFFFFFFFFFFFFFFFFFFFFFFFFFFFFFFFFFFFFFFFFFFFFFFFFFFFFFFFFFFFFFFFFFF
FFFFFFFFFFFFFFFFFFFFFFFFFFFFFFFFFFFFFFFFFFFFFFFFFFFFFFFFFFFFFFFFFFFF
@A00155:342:HHGFNDSXY:1:2511:2401:30733 1:N:0:GAACCTAG+TCCGCATA
ATTGCTGTCATTCACTCGCAGCACCCCACCATGGTACGTGCTATGCCTTAAATTACCATCGAAGTTGA
ATTCGGCTCCTGGTGCCTCGTGATTCGGGAAGAACCTCTTAACGAAACTATCCATTACTCTGAGTTTG
+
FFFFFFFFFFFFFFFFFFFF:FFFFFFFF:FFF:FFFFFFFFFFFF:FFFFFFFFFFFFFFFFFFFFF
FFFF,FFFFFFFFFF:FFFF:FFFFFFF:FF:FFFFFFF:FFFFFFFFFFF:FF:FFFFFFFFFFFFFF
@A00155:342:HHGFNDSXY:1:2316:19831:33990 1:N:0:GAACCTAG+TCCGCATA
ATTGCTGTCATTCACTCGCAGCACCCCACCATGGTACGTGCTATGCCTTAAATTACCATCGAAGTTGA
ATTCGGCTCCTGGTGCCTCGTGATTCGGGAAGAACCTCTTAACGAAACTATCCATTACTCTGAGTTTG
+
FFFFFFFFFFFFFFFFFFFFFFFFFFFFFFFFFFFFFFFFFFFFFFFFFFFFFFFFFFFFFFFFFFFF
FFFFFFFFFFFFFFFFFFFFFFFFFFFFFFFFFFFFFFFFFFFFFFFFFFFFF:FFFFFFFFFFFFFF
@A00155:342:HHGFNDSXY:1:1420:29423:21512 1:N:0:GAACCTAG+TCCGCATA
ATTGCTGTCATTCACTCGCAGCACCCCACCATGGTACGTGCTATGCCTTAAATTACCATCGAAGTTGA
ATTCGGCTCCTGGTGCCTCGTGATTCGGGAAGAACCTCTTAACGAAACTATCCATTACTCTGAGTTTG
+
FFFFFFFFFFFFFFFFFFFFFFFFFFFFFFFFFFFFFFFFFFFFFFFFFFFFFFFFFFFFFFFFFFFF
FFFFFFFFFFFFFFFFFFFFFFFFFFFFFFFFFFFFFFFFFFFFFFFFFFFFFFFFFFFFFFFFFFFF
@A00155:342:HHGFNDSXY:1:2459:22128:22874 1:N:0:GAACCTAG+TCCGCATA
ATTGCTGTCATTCACTCGCAGCACCCCACCATGGTACGTGCTATGCCTTAAATTACCATCGAAGTTGA
ATTCGGCTCCTGGTGCCTCGTGATTCGGGAAGAACCTCTTAACGAAACTATCCATTACTCTGAGTTTG
+
FFFFFFFFFFFFFFFFFFFFFFFFFFFFFFFFFFFFFFFFFFFFFFFFFFFFFFFFFFFFFFFFFFFF
FFFFFFFFFFFFFFFFFFFFFFFFFFFFFFFFFFFFFFFFFFFFFFFFFFFFFFFFFFFFFFFFFFFF
```

```
@A00155:342:HHGFNDSXY:1:2459:26666:23657 1:N:0:GAACCTAG+TCCGCATA
ATTGCTGTCATTCACTCGCAGCACCCCACCATGGTACGTGCTATGCCTTAAATTACCATCGAAGTTGA
ATTCGGCTCCTGGTGCCTCGTGATTCGGGAAGAACCTCTTAACGAAACTATCCATTACTCTGAGTTTG
+
FFFFFFFFFFFFFFFFFFFFFFFFFFFFFFFFFFFFFFFFFFFFFFFFFFFFFFFFFFFFFFFFFFFF
FFFFFFFFFFFFFFFFFFFFFFFFFFFFFFFFFFFFFFFFFFFFFFFFFFFFFFFFFFFFFFFFFFFF
@A00155:342:HHGFNDSXY:1:1460:16197:24377 1:N:0:GAACCTAG+TCCGCATA
ATTGCTGTCATTCACTCGCAGCACCCCACCATGGTACGTGCTATGCCTTAAATTACCATCGAAGTTGA
ATTCGGCTCCTTGTGCCTCGTGATTCGGGAAGAACCTCTTAACGAAACTATCCATTACTCTGAGTTTG
+
FFFFFFFFFFFFFFFFFF:FFFFFFFFFFFFFFFFFFFFFFFFFFFFFFFFFFFFFFFFFFFFFFFFF
F:FFFFFFFFFFFFFFFFFFFFFFFFFFFF,FFFFFFFFFFFFFFFFFFFFFFFFFFFFFFFFFFFFF
@A00155:342:HHGFNDSXY:1:2217:10520:31109 1:N:0:GAACCTAG+TCCGCATA
ATTGCTGTCATTCACTCGCAGCACCCCACCATGGTACGTGCTATGCCTTAAATTACCATCGAAGTTGA
ATTCGGCTCCTGGTGCCTCGTGATTCGGGAAGAACCTCTTAACGAAACTATCCATTACTCTGAGTTTG
+
FFFFFFFFFFFFFFFFFFFFFFFFFFFFFFFFFFFFFFFFFFFFFFFFFFFFFFFFFFFFFFFFFFFF
FFFFFFFFFFFFFFFFFFFFFFFFFFFFFFFFFFFFFFFFFFFFFFFFFFFFFFFFFFFFFFFF:FFF
@A00155:342:HHGFNDSXY:1:2361:8314:14544 1:N:0:GAACCTAG+TCCGCATA
TTGCTGTCATTCACTCGCAGCACCCCACCATGGTACGTGCTATTCCTTAAATTACCATCGAAGTTGAA
TTCGGCTCCTGGTGCCTCGTGATTCGGGAAGAACCTCTTAACGAAACTATCCATTACTCTGAG
+
FFFFFFFFFFFFFFFFFFFFFFFFFFFFFFFFFFFFFFFFFFF,FFFFFF:FFFF:FFFFFFFFFFFF
FFFF:FFFFFFFFFFFFFFFFFFFFFF:F,FFFFF,FFFFFFFFFFF,FFFFFFFFFFF:FFF
@A00155:342:HHGFNDSXY:1:2117:1362:15123 1:N:0:GAACCTAG+TCCGCATA
TTGCTGTCATTCACTCGCAGCACCCCACCATGGTACGTGCTATGCCTTAAATTACCATCGAAGTTGAA
TTCGGCTCCTGGTGCCTCGTGATTCGGGAAGAACCTCTTAACGAAACTATCCATTACTCTGAGTTTG
+
FFFFFFFFFFFFFFFFFFFFF:FF:FFFF:FFFFFFFFFFFFFFFFFFFF:FF:FFFFFFFFFFFF:F
FFFFFFFFFFFFFFFFFFFFFFFFF,FFFFF,FF,FFFFF:FF:FFFFFFFFFFFF:F:FF:FFFFF
@A00155:342:HHGFNDSXY:1:1223:1416:11052 1:N:0:GAACCTAG+TCCGCATA
TTGCTGTCATTCACTCGCAGCACCCCACCATGGTACGTGCTATGCCTTAAATTACCATCGAAGTTGAA
TTCGGCTCCTGGTGCCTCGTGATTCGGGAAGAACCTCTTAACGAAAATATCCATTACTCTGAGTTTGC
+
FFFFFFFFFFFFFFFFFFFFFFFFFFFFFFFFFFFF:FFFF:FFFFFFFFFFFFFFFFFFFFFFFFFF
FFFFFFFFFFFFFFFFFFFFFFFFFFFFFFFFFFFFFFFFF:FFFF,FFFFFFFFF,FFFF:FFFFF:
@A00155:342:HHGFNDSXY:1:2644:18132:35587 1:N:0:GAACCTAG+TCCGCATA
TTGCTGTCATTCACTCGCAGCACCCCACCATGGTACGTGCTATGCCTTAAATTACCATCGAAGTTGAA
TTCGGCTCCTGGTGCCTCGTGATTCGGGAAGAACCTCTTAACGAAACTATCCATTACTCTGAGTTTGC
+
FFFFFFFFFFFFFFFFFFFFFFFFFFFFFFFFFFFFFFFFFFFFFFFFFFFFFFFFFFFFFFFFFFFF
FFFFFFFFFFFFFFFFFFFFFFFFFFFFFFFFFFFFFFFFFFFFFFFFFFFFFFFFFFFFF:FFFFFF
@A00155:342:HHGFNDSXY:1:1658:32470:17550 1:N:0:GAACCTAG+TCCGCATA
TTGCTGTCATTCACTCGCAGCACCCCACCATGGTACGTGCTATGCCTTAAATTACCATCGAAGTTGAA
TTCGGCTCCTGGTGCCTCGTGATTCGGGAAGAACCTCTTAACGAAACTATCCATTACTCTGAGTTTGC
+
FFFF,FFFFFFFFFFFFFFFFFFFFFFFFFFFFFFFFFFFFFFFFFFFFFFFFFFFFFFFFFFFFFFF
FFFFFFFFFFFFFFFFFFFFFFFFFFFFFFFFFFFFFFF:FFFFFFFFFFFFFFFFFFFFFFFFFFFF
@A00155:342:HHGFNDSXY:1:2108:15374:22075 1:N:0:GAACCTAG+TCCGCATA
TTGCTGTCATTCACTCGCAGCACCCCACCATGGTACGTGCTATGCCTTAAATTACCATCGAAGTTGAA
TTCGGCTCCTGGTGCCTCGTGATTCGGGAAGAACCTCTTAACGAAACTATCCATTACTCTGAGTTTGC
+
FFFFFFFFFFFFFFFFFFFFFFFFFFFFFFFFFFFFFFFFFFFFFFFFFFFFFFFFFFFFFFFFFFFF
FFFFFFFFFFFFFFFFFFFFFFFFFFFFFF:FFFFFFFFFFFFFFFFFFFFFFFF:FFFFFFFFFFFF
```

```
@A00155:342:HHGFNDSXY:1:1211:12997:5588 2:N:0:GAACCTAG+TCCGCATA
TTGCTGTCATTCACTCGCAGCACCCCACCATGGTACGTGCTATGCCTTAAATTACCATCGAAGTTGAA
TTCGGCTCCTGGTGCCTCGTGATTCGGGAAGAACCTCTTAACGAAACTATCCATTACTCTGAGTTTGC
+
:FF:FFFF,FF:F:FFF:FFFFFFFFFFF:FF:FFFFFF,F:FFF,FFFFFFFFFFFF::F:FFF,F:
FFFFF:FF:FFFFFF,FFFF,FFFFFF:F::FFFFF,FFFFF,FFFFFFFFF:FFFFFFFF:FFFFF:
@A00155:342:HHGFNDSXY:1:2305:24514:10567 1:N:0:GAACCTAG+TCCGCATA
TTGCTGTCATTCACTCGCAGCACCCCACCATGGTACGTGCTATGCCTTAAATTACCATCGAAGTTGAA
TTCGGCTCCTGGTGCCTCGTGATTCGGGAAGAACCTCTTAACGAAACTATCCATTACTCTGAGTTTGC
+
FFFFFFFFFFFFFFFFFFFFFFFFFFFFFFFFFFFFFFFFFFFFFFFFFFFFFFFFFFFFFFFFFFFF
FFFFFFFFFFFFFFFFFFFFFFFFFFFFFFFFFFFFFFFFFFFFFFFFFFF:FFFFFFFFFFFFFFFF
@A00155:342:HHGFNDSXY:1:1360:13178:29042 1:N:0:GAACCTAG+TCCGCATA
TTGCTGTCATTCACTCGCAGCACCCCACCATGGTACGTGCTATGCCTTAAATTACCATCGAAGTTGAA
TTCGGCTCCTGGTGCCTCGTGATTCGGGAAGAACCTCTTAACGAAACTATCCATTACTCTGAGTTTGC
+
FFFFFFFFFFFFFFFFFFFFFFFFFFFFFFFFFFFFFFFFF::FFFFFFFFFFFFFFFFFFFFFFFFF
FFFFFFFFFFFFFFFFFFFFFFFFFFFFFFFFFFFFFFFFFFFFFFFFFFFFF:FFFFFFFFF,FFFF
@A00155:342:HHGFNDSXY:1:1310:17192:34710 1:N:0:GAACCTAG+TCCGCATA
TTGCTGTCATTCACTCGCAGCACCCCACCATGGTACGTGCTATGCCTTAAATTACCATCGAAGTTGAA
TTCGGCTCCTGGTGCCTCGTGATTCGGGAAGAACCTCTTAACGAAACTATCCATTACTCTGAGTTTGC
+
FFFFFFFFFFFFFFFFFFFFFFFFFFFFFFFFFFFFFFFFFFFFFFF:FFFFFFFFFFFFFFFFFFFF
FFFFFFFFFFFFFFFFFFFFFFFFFFFFFFFFFFFFFFFFFFFFFFFFFFFFF:FFFFFFFFFFFFFF
@A00155:342:HHGFNDSXY:1:1267:3477:10113 1:N:0:GAACCTAG+TCCGCATA
TTGCTGTCATTCACTCGCAGCACCCCACCATGGTACGTGCTATGCCTTAAATTACCATCGAAGTTGAA
TTCGGCTCCTGGTGCCTCGTGATTCGGGAAGAACCTCTTAACGAAACTATCCATTACTCTGAGTTTGC
+
FFFFFFFFFFFFFFFFFFFFFFFFF:FFFFFF:FFFFFFFFFFFFFFFFFF,FFFFFFFFFFFFFFFF
FFFFFF:FFFFFFFFFFFFFFF:FFFFFFFFFFFFFF:FFFF:FFFFFFFFFFFF,FFFFFFFFFFFF
@A00155:342:HHGFNDSXY:1:2545:16938:4460 1:N:0:GAACCTAG+TCCGCATA
TTGCTGTCATTCACTCGCAGCACCCCACCATGGTACGTGCTATGCCTTAAATTACCATCGAAGTTGAA
TTCGGCTCCTGGTGCCTCGTGATTCGGGAAGAACCTCTTAACGAAACTATCCATTACTCTGAGTTTGC
+
FFFFFFFFFFFFFFFFFFFFFFFFFFFFFFFFFFFFFFFFFFFFFFFFFFFFFFFFFFFFFF:FFFFF
FFFFFFFFFFFFFFFFFFFFFFFFFFFFFFFFFFFFFFFFFFFFFFFFFFFFFFFFFFFFFFFFFFFF
@A00155:342:HHGFNDSXY:1:2545:16595:4617 1:N:0:GAACCTAG+TCCGCATA
TTGCTGTCATTCACTCGCAGCACCCCACCATGGTACGTGATATGCCTTAAATTACCATCGAAGTTGAA
TTCGGCTCCTGGTGCCTCGTGATTCGGGAAGAACCTCTTAACGAAACTATCCATTACTCTGAGTTTGC
+
FF:FFFFFFFF,FFF:FFF,FFFFF,F:,FF:F:F:FFF,FFFFFF,FFFFF:FFF:,FFFF:,F:F:
FFFFFFFFFFFFF,F,:,FFFF,FF:FFFFFFFFFFFFFFFF,FFFF,FFF:FFFF:,FFFFF,FFFF
@A00155:342:HHGFNDSXY:1:2555:20265:34773 1:N:0:GAACCTAG+TCCGCATA
TTGCTGTCATTCACTCGCAGCACCCCACCATGGTACGTGCTATGCCTTAAATTACCATCGAAGTTGAA
TTCGGCTCCTGGTGCCTCGTGATTCGGGAAGAACCTCTTAACGAAACTATCCATTACTCTGAGTTTGC
+
FFFFFFFFFFFFFFFFFFFFFFFFFFFFFFFFFF:FFFFFFFFFFFFFFFFFFFFFFFFFFFFFFFFF
FFFFFFFFFFFFFFFFFFFFFF:FFFFFFFFFFFFFFFFFFFFFFFFFFFFFFFFFFFFFFFFFFFFF
@A00155:342:HHGFNDSXY:1:2617:30409:15483 1:N:0:GAACCTAG+TCCGCATA
TTGCTGTCATTCACTCGCAGCACCCCACCATGGTACGTGCTATGCCTTAAATTACCATCGAAGTTGAA
TTCGGCTCCTGGTGCCTCGTGATTCGGGAAGAACCTCTTAACGAAACTATCCATTACTCTGAGTTTGC
+
FFFFFFFFFFF:FFFFFFFFFFFFFFFFFFFFFFFFFFFFFFFFFFFFFFFFFFFFFFFFFFFFFFFF
FFFFFFFFFFFFFFFFFFFFFFFFFFFFFFFFFFFFFFFFFFFFFFFFFFFF::FFFFFFFFFFFFFF
```

@A00155:342:HHGFNDSXY:1:1469:20790:7404 2:N:0:GAACCTAG+TCCGCATA
AGCTGTCATTCACTCGCAGCACCCCACCATGGTACGTGCTATGCCTTAAATTACCATCGAAGTTGAAT
TCGGCTCCTGGTGCCTCGTGATTCGGGAAGAACCTCTTAACGAAACTATCCATTACTCT
+
FFFFF:FFFFFFFFFFFFFFFFFFFFFFF:F:FFFFFFFFFFFFFFFFFFFFFFFFFFFFFFFFFFFF
FFFFFFFFFFFFFFFFFFFFFFFFFFFFFFFFFFFF,FFFFFFFFFFFFFFFFFFFFFF
@A00155:342:HHGFNDSXY:1:2239:31837:26757 1:N:0:GAACCTAG+TCCGCATA
TGCTGTCATTCACTCGCAGCACCCCACCATGGTACGTGCTATGCCTTAAATTCCCATCGAAGTTGAAT
TCGGCTCCTGGTGCCTCGTGATTCGGGAAGAACCTCTTAACGAAACTATCCATTACTCTGAGTTTG
+
F:::F:FF:FFFFFFFFFFFF:F:FFF:FFFFF:FFF:F,F:FF:::FFFF:,:F,,:FF,F:FF:FF
FFFF:FF,FFF,:FFFFF:F,,::FFFF,F:,:::,FFFFF:FF:FF,F:,:::,F,F:FFF:FFF
@A00155:342:HHGFNDSXY:1:1203:5204:3834 2:N:0:GAACCTAG+TCCGCATA
TGCTGTCATTCACTCGCAGCACCCCACCATGGTACGTGCTATGCCTTAAATTACCATCGAGGTTGAAT
TCGGCTCCTGGTGCCTCGTGATTCGGGAAGAACCTCTTAACGAAACTATCCATTACTCTGAGTTTGCT
+
FFFFFFFFFFFFFFFFFFFFFFFFFFFFFFFFFFFFFFFFFFFFFFFFF::FFFFFFFFF:FFFFFFF
FFFFFFFFFFFFFFF:FFFFFFFFFFFFFFFFFFFFFFFFFFFFFFFFFFFFFFFFFFFFFFFFFFF:
@A00155:342:HHGFNDSXY:1:2210:8079:24878 1:N:0:GAACCTAG+TCCGCATA
TGCTGTCATTCACTCGCAGCACCCCACCATGGTACGTGCTATGCCTTAAATTACCATCGAAGTTGAAT
TCGGCTCCTGGTGCCTCGTGATTCGGGAAGAACCTCTTAACGAAACTATCCATTACTCTGAGTTTGCT
+
FFFFFFFFFFFFFFFFFFFFFFFFFFFFFFFFFFFFFFFFFFFFFFFFFFFFFFFFFFFFFFFFFFFF
FFFFFFFFFFFFFFFFFFFFFFFFFFFF:FFFFFFFFFFFFFFFFFFFFFFFFFFFFFFFFFFFFFFF
@A00155:342:HHGFNDSXY:1:2462:23800:36417 2:N:0:GAACCTAG+TCCGCATA
TGCTGTCATTCACTCGCAGCACCCCACCATGGTACGTGCTATGCCTTAAATTACCATCGAAGTTGAAT
TCGGCTCCTGGTGCCTCGTGATTCGGGAAGAACCTCTTAACGAAACTATCCATTACTCTGAGTTTGCT
+
FFFFFFFFFFFFFFFFFFFFFFFFFFFFFFFFFFFFFFFFFFFFFFFFFFFFFFFFFFFFFFFFFFFF
FFFFFFFFFFFFFFFFFFFFFFFFFFFFFFFFFFFFFFFFFFFFFFFFFFFFFFFFFFFFFFFFFFFF
@A00155:342:HHGFNDSXY:1:1203:5023:3865 2:N:0:GAACCTAG+TCCGCATA
TGCTGTCATTCACTCGCAGCACCCCACCATGGTACGTGCTATGCCTTAAATTACCATCGAGGTTGAAT
TCGGCTCCTGGTGCCTCGTGATTCGGGAAGAACCTCTTAACGAAACTATCCATTACTCTGAGTTTGCT
+
FFFFFFFF:FFFFFFFFFFFFFFFFFFFFFFFFFFFFFFFFFFFFFFFFFFFFFFFFFFFFFFFFFFF
FFFFFFFFFFFFFFFFFFFFFFFFFFFFFFFFFFFFFFFFFFFFFFFFFFFFFFFFFFFFFFFFFFFF
@A00155:342:HHGFNDSXY:1:2503:31376:19100 1:N:0:GAACCTAG+TCCGCATA
TGCTGTCATTCACTCGCAGCACCCCACCATGGTACGTGCTATGCCTTAAATTACCATCGAAGTTGAAT
TCGGCTCCTGGTGCCTCGTGATTCGGGAAGAACCTCTTAACGAAACTATCCATTACTCTGAGTTTGCT
+
FFFFFFFFFFFFFFFFFFFFFFFFFFFFFFFFF:FFFFFFFFFFFFFFFFFFFFFFFFFFFFFFFF:,
FFFFFFFFFFFFFFFFFFFFFFFFFFFFFFFFFFFFFFFFFFFFFFF:FFFFFF,FFFFFFFFFFFFF
@A00155:342:HHGFNDSXY:1:1170:1723:12399 2:N:0:GAACCTAG+TCCGCATA
TGCTGTCATTCACTCGCAGCACCCCACCATGGTACGTGCTATGCCTTAAATTACCATCGAAGTTGAAT
TCGGCTCCTGGTGCCTCGTGATTCGGGAAGAACCTCTTAACGAAACTATCCATTACTCTGAGTTTGCT
+
FFFFFFFFFFFFFFFFFFFFFFFFFFFFFFFFFFFFFFFFFFFFFFFFFFFFFFFFFFFFFFFFFFFF
FFFFFFFFFFFFFFFFFFFFFFFFFFFFFFFFFFFFFFFFFFFFFFFFFFFFFFFF,FFFFFFFFFFF
@A00155:342:HHGFNDSXY:1:2223:6063:35697 2:N:0:GAACCTAG+TCCGCATA
TGCTGTCATTCACTCGCAGCACCCCACCATGGTACGTGCTATGCCTTAAATTACCATCGAAGTTGAAT
TCGGCTCCTGGTGCCTCGTGATTCGGGAAGAACCTCTTAACGAAACTATCCATTACTCTGAGTTTGCT
+
FFFFFFFFFFFFFFFFFFFFFFFFFFFFFFFFFFFFFFFFFFFFFFFFFFFFFFFFFFFFFFFFFFFF
FFFFFFFFFFFFFFFFFFFFFFFFFFFFFFFFFFFFFFF:FFFFFFFFFFFFFFFFFFFFFFFFFFFF

@A00155:342:HHGFNDSXY:1:1529:1027:11600 1:N:0:GAACCTAG+TCCGCATA
TGCTGTCATTCACTCGCAGCACCCCACCATGGTACGTGCTATGCCTTAAATTACCATCGAAGTTGAAT
TCGGCTCCTGGTGCCTCGTGATTCGGGAAGAACCTCTTAACGAAACTATCCATTACTCTGAGTTTGCT
+
FFFFFFFFFFFF:FFF:FFFFF:FFFF:FFFFF,FFFFFFFFFFFFF,,FFF:FFFFFFFFFFFFFFF
FFFFFFFF:FFFF:FFFFFF:FFFFFF:,FF:FFFFFF:F:FF,:FF:FFFFF:,F:FFF:F:FFFFF
@A00155:342:HHGFNDSXY:1:2274:8467:12493 1:N:0:GAACCTAG+TCCGCATA
TGCTGTCATTCACTCGCAGCACCCCACCATGGTACGTGCTATGCCTTAAATTACCATCGAAGTTGAAT
TCGGGTCCTGGTGCCTCGTGATTCGGGAAGAACCTCTTAACGAAACTATCCATTACTCTGAGTTTGCT
+
FFFFFFFFFFFFFFFFFFFFFFFFFFFFFFFFFFFFFFFFFFFFFFFFFFFFFFFFFFFFFFFFFFFF
FFFFFFFFFFFF:FFFFFFFFFFFFFFFFFFFFFFFFFFFFFFFFFFFFF:FFFFFFFFFFFFFFFFF
@A00155:342:HHGFNDSXY:1:2166:22887:30984 1:N:0:GAACCTAG+TCCGCATA
TGCTGTCATTCACTCGCAGCACCCCACCATGGTACGTGCTATGCCTTAAATTACCATCGAAGTTGAAT
TCGGCTCCTGGTGCCTCGTGATTCGGGAAGAACCTCTTAACGAAACTATCCATTACTCTGAGTTTGCT
+
FFFFFFFFFFFFFFFFFFFFFFFFFFFFFFFFFFFFFFFFFFFFFF,FFFFFFFFFFFFFFFFFFFFF
FFFFFFFFFFFFFFFFFFFFFFFFFFFFFFFFFFFFFFFFFFFFFFFFFFFFFFFFFFFFFFFFFFFF
@A00155:342:HHGFNDSXY:1:2210:5584:33583 1:N:0:GAACCTAG+TCCGCATA
TGCTGTCATTCACTCGCAGCACCCCACCATGGTACGTGCTATGCCTTAAATTACCATCGAAGTTGAAT
TCGGCTCCTGGTGCCTCGTGATTCGGGAAGAACCTCTTAACGAAACTATCCATTACTCTGAGTTTGCT
+
FFFFFFFFFFFFFFFFFFFFFFFFFFFFFFFFFFFFFFFFFFFFFFFFFFFF:FFFFFFFFFFFFFFF
FFFFFFFFFFFFFFFFFFFFFFFFFFFFFFFFFFFFFFFFFFFFFFFFFFFFFF:FFFFFFFFFFFFF
@A00155:342:HHGFNDSXY:1:1647:7961:14246 2:N:0:GAACCTAG+TCCGCATA
TGCTGTCATTCACTCGCAGCACCCCACCATGGTACGTGCTATGCCTTAAATTACCATCGAAGTTGAAT
TCGGCTCCTGGTGCCTCGTGATTCGGGAAGAACCTCTTAACGAAACTATCCATTACTCTGAGTTTGCT
+
FFFFFFFFFFFFFFFFFFFFFFFFFFFFFFFFFFFF:FFFFFFFFF:FFFFFFFF:F:FF:FFFFFFF
FFFFFFFFFFFFFFFFFFFFFFFFFFFFFFFFFFFFFFFFFFFFFF:FFFFFFFFFFFFFFFF,FFFF
@A00155:342:HHGFNDSXY:1:2143:22137:24862 2:N:0:GAACCTAG+TCCGCATA
TGCTGTCATTCACTCGCAGCACCCCACCATGGTACGTGCTATGCCTTAAATTACCATCGAAGTTGAAT
TCGGCTCCTGGTGCCTCGTGATTCGGGAAGAACCTCTTAACGAAACTATCCATTACTCTGAGTTTGCT
+
FFFFFFFFFFFFFFFFFFFFFFFFFFFFFFFF:FFFFFFFFFFFFFFF:FFFFFFFFFFFFFFFFFFF
FFFFFFFFFFFFFFFFFFFFFFFFFFFFFFFFFFFFFFFFFFFFFFFFFFFFFFFFFFFFFFFFFFFF
@A00155:342:HHGFNDSXY:1:2307:27887:34788 1:N:0:GAACCTAG+TCCGCATA
GCTGTCATTCACTCGCAGCACCCCACCATGGTACGTGCTATGCCTTAAATTACCATCGAAGTTGAATT
CGGCTCCTGGTGCCTCGTGATTCGGGAAGAACCTCTTAACGAAACTATCCATTACTCTGAGTTTGCTT
+
FFFFFFFFFFFFFFFFFFFFFFFFFFFFFFFFFFFFFFFFFFFFFFFFFFFFFFFFFFFFFFFFFFFF
FFFFFFFFFFFFFFFFFFFFFFFFFFFF:FFFFFFFFFFFFFFFFFFFFFFFFFFFFFFFFF,FFFFF
@A00155:342:HHGFNDSXY:1:2270:24921:3505 1:N:0:GAACCTAG+TCCGCATA
GCTGTCATTCACTCGCAGCACCCCACCATGGTACGTGCTATGCCTTAAATTACCATCGAAGTTGAATT
CGGCTCCTGGTGCCTCGTGATTCGGGAAGAACCTCTTAACGAAACTATCCATTACTCTGAGTTTGCTT
+
FFFFFFFFFFFFFFFFFFFFFFFFFFFFFFFFFFFFFFFFFFFFFFFFFFFFFFFFFFFFFFFFFFFF
FFFFFFFFFFFFFFFFFFFFFFFFFFFFFFFFFFFFFFFFFFFFFFFFFFFFF:FFFFFFFFFFFFFF
@A00155:342:HHGFNDSXY:1:2603:21721:29058 1:N:0:GAACCTAG+TCCGCATA
CTGTCATTCACTCGCAGCACCCCACCATGGTACGTGCTATGCCTTAAATTACCATCGAAGTTGAATTC
GGCTCCTGGTGCCTCGTGATTCGGGAAGAACCTCTTAACGAAACTATCCATTACTCTGAGTTTGCT
+
FFFFFFFFFFFFFFFFFFFFFFFFFFFFFFFFFFFFFFFFFFFFFFFF,FFFFFFFFFFFFFFFFFFF
:FFFFFFFFFFFFFFFFFFFFFFFFFFFFFFFFFFFFFFFFFFFFFF:FF:FFFFFF:FFF:FFFF

@A00155:342:HHGFNDSXY:1:1427:7554:36276 1:N:0:GAACCTAG+TCCGCATA
CTGTCATTCACTCGCAGCACCCCACCATGGTACGTGCTATGCCTTAAATTACCATCGAAGTTGAATTC
GGCTCCTGGTGCCTCGTGATTCGGGAAGAACCTCTTAACGAAACTATCCATTACTCTGAGTTTGCT
+
FFFFFFFFFFFFFFFFFFFFFFFFFFFFFFF:FFFFFF:FFFFFFFFFFFFFFFFFFFFFFFFFFFFF
FFFFFFFFFFFFFFFFFFFFFFFFFFFFFFFFFFFFFFFFFFFFFFFFFFFFFFFFFFFFFFFFFF
@A00155:342:HHGFNDSXY:1:2534:10294:8077 2:N:0:GAACCTAG+TCCGCATA
CTGTCATTCACTCGCAGCACCCCACCATGGTACGTGCTATGCCTTAAATTACCATCGAAGTTGAATTC
GGCTCCTGGTGCCTCGTGATTCGGGAAGAACCTCTTAACGAAACTATCCATTACTCTGAGTTTGCTTT
+
FFFFFFFFFFF:FFFFFFFFFFFFFFFFF:FFFFFFFFFFFFF::FFFF::FFFF,FFF:FF:FFFFF
:FFFFFFF:FF,FFFFF:FFFFFF,FFFFFFFF:FFFFFFFFFFFFFFFFFFFFFFFFFFFFFFFFFF
@A00155:342:HHGFNDSXY:1:1417:21838:20149 2:N:0:GAACCTAG+TCCGCATA
CTGTCAGTCACTCGCAGCACCCCACCATGGTACGTGCTATGCCTTAAATTACCATCGAAGTTGAATTC
GGCTCCTGGTGCCTCGTGATTCGGGAAGAACCTCTTAACGAAACTATCCATTACTCTGAGTTTGCTTT
+
F:FFFF,FFFFFFFFFFFFFFFF:FFFFFFFFFF:FFFFFFFFFFFFFFFFFF:FFFFFFFFFFFFFF
FFFFFFFFFFFFFFFFFFFFFFFFFFFFFFFFFFFFFFFFFFFFFFFFFFFFFFFFFFFFFFFFFFFF
@A00155:342:HHGFNDSXY:1:2642:2193:3787 1:N:0:GAACCTAG+TCCGCATA
CTGTCATTCACTCGCAGCACCCCACCATGGTACGTGCTATGCCTTAAATTACCATCGAAGTTGAATTC
GGCTCCTGGTGCCTCGTGATTCGGGAAGAACCTCTTAACGAAACTATCCATTACTCTGAGTTTGCTTT
+
FFFFFFFFFFFFF:FFFFFF:FFFFFFFFFFF,FFFFFFFFF,FF:FFFFFFFFFFFFFF:FFFFFF:
FFF:FFF:FFFF:FFFF:FFFFFFFFFFFFFFFFFFFFFFFFFFFFFF,FFFF:FFFFFFFFF:,FFF
@A00155:342:HHGFNDSXY:1:1548:28140:7701 1:N:0:GAACCTAG+TCCGCATA
CTGTCATTCACTCGCAGCACCCCACCATGGTACGTGCTATGCCTTAAATTACCATCGAAGTTGAATTC
GGCTCCTGGTGCCTCGTGATTCGGGAAGAACCTCTTAACGAAACTATCCATTACTCTGAGTTTGCTTT
+
FFFFFFFFFFFFFFFFFFFFFFFFFFFFFFFFFFFF:FFFFFFFFFFFFFFFFFFFFFFFFFFFFFFF
FFFFFFFFFFFFFFFFFFFFFFFFFFFFFFFFFFFFFFFFFFF:FFFFFFFFFFFFFFFFFFFFFFFF
@A00155:342:HHGFNDSXY:1:2356:6343:35086 1:N:0:GAACCTAG+TCCGCATA
CTGTCATTCACTCGCAGCACCCCACCATGGTACGTGCTATGCCTTAAATTACCATCGAAGTTGAATTC
GGCTCCTGGTGCCTCGTGATTCGGGAAGAACCTCTTAACGAAACTATCCATTACTCTGAGTTTGCTTT
+
FFFFFFFFFFFFFFFFFFFFFFFFFFFFFFFFFFFFFFFFFFFFFF:FFFFFFFF:FFFFFFFFFFFF
::FFFFFFFFFFFFFFFFFFFFFFFFFFFFFFFFFFFFFF:FFFFFFF:FFFFFFFFFFF,F:F:F:F
@A00155:342:HHGFNDSXY:1:2664:18991:6699 1:N:0:GAACCTAG+TCCGCATA
CTGTCATTCACTCGCAGCACCCCACCATGGTACGTGCTATGCCTTAAATTACCATCGAAGTTGAATTC
GGCTCCTGGTGCCTCGTGATTCGGGAAGAACCTCTTAACGAAACTATCCATTACTCTGAGTTTGCTTT
+
FFFFFFFFFFFFFFFFFFFFFFFFFFFFFFF:FFFFFFFFFFFFFFFFFFFFFFFFFFFFFFFFFFFF
FFFFFFFFFFFFFFFFFFFFFFFFFFFFFFFFFFFFFFF:FFFFFFFFFFFFFFFFFFFFFFFFFF,F
@A00155:342:HHGFNDSXY:1:1203:5023:3865 1:N:0:GAACCTAG+TCCGCATA
CTGTCATTCACTCGCAGCACCCCACCATGGTACGTGCTATGCCTTAAATTACCATCGAGGTTGAATTC
GGCTCCTGGTGCCTCGTGATTCGGGAAGAACCTCTTAACGAAACTATCCATTACTCTGAGTTTGCTTT
+
FFFFFFFFFFFFFFFFFFFF:FFFFFFFFFF:FFFFFFFFF:FFFFFFFFFFFFFFFFFFFFFFFFFF
FFFFFFFFFFFFFFFFFFFFFFFFFFFFFFFFFFFFFFFFFFFFFFFFFFFFFFFFFFFFFFFFFFFF
@A00155:342:HHGFNDSXY:1:1203:5204:3834 1:N:0:GAACCTAG+TCCGCATA
CTGTCATTCACTCGCAGCACCCCACCATGGTACGTGCTATGCCTTAAATTACCATCGAGGTTGAATTC
GGCTCCTGGTGCCTCGTGATTCGGGAAGAACCTCTTAACGAAACTATCCATTACTCTGAGTTTGCTTT
+
FFFFFFFFFFFFFFFFFFFFFFFFFFFFFFFFFFFFFFFFFFFFFFFFFFFFFFFFFFFFFFFFFFFF
FFFFFFFFFFFFFFFFFFFFFFFFFFF:F:FFFFFFFFFFFFFFFFFFFFFFFFFFFFFFFFFFFFFF

```
@A00155:342:HHGFNDSXY:1:1126:18466:3912 2:N:0:GAACCTAG+TCCGCATA
CTGTCATTCACTCGCAGCACCCCACCATGGTACGTGCTATGCCTTAAATTACCATCGAAGTTGAATTC
GGCTCCTGGTGCCTCGTGATTCGGGAAGAACCTCTTAACGAAACTATCCATTACTCTGAGTTTGCTTT
+
FFFFFFFFFFFFFFFFFFFFFFFFFF,FFFFFFFFFFFFFFFFFFFFFFFFFFFFFFFFFFFFFFFFF
FFFFFF:FFFFFFFFFFFFFFFFFFFFFFFFFFFFFFFFFFFFFFFFFFFFFFFFFFFFFFFFFFFFF
@A00155:342:HHGFNDSXY:1:1305:16360:35274 1:N:0:GAACCTAG+TCCGCATA
CTGTCATTCACTCGCAGCACCCCACCATGGTACGTGCTATGCCTTAAATTACCATCGAAGTTGAATTC
GGCTCCTGGTGCCTCGTGATTCGGGAAGAACCTCTTAACGAAACTATCCATTACTCTGAGTTTGCTTT
+
FFFFFFFFFFFFFFFFFFFFFFFFFFFFFFFFFFFFFFFFFFFFFFFFFFFFFFFFFFFFFFFFFFFF
FFFFFFFFFFF:FFFFFFFFFFFFFFFFFFFFFFFFFFFFFFFFFFFFFFFFFFFFFFFFFFFFFFFF
@A00155:342:HHGFNDSXY:1:1311:25699:15562 1:N:0:GAACCTAG+TCCGCATA
CTGTCATTCACTCGCAGCACCCCACCATGGTACGTGCTATGCCTTAAATTACCATCGAAGTTGAATTC
GGCTCCTGGTGCCTCGTGATTCGGGAAGAACCTCTTAACGAAACTATCCATTACTCTGAGTTTGCTTT
+
FFFFFFFFFFFFFFFFFFFFFFFFFFFFFFFFFFFFFFFFFFFFFFFFFFFFFFFFFFFFFFFFFFFF
FFFFFFFFFFFFFFFFFFFFFFFFFFFFFFFFFFFFFFFFFFFFFFFF,FFFFFFFFFFFF:FFFFFF
@A00155:342:HHGFNDSXY:1:2316:32597:34835 1:N:0:GAACCTAG+TCCGCATA
CTGTCATTCACTCGCAGCACCCCACCATGGTACGTGCTATGCCTTAAATTACCATCGAAGTTGAATTC
GGCTCCTGGTGCCTCGTGATTCGGGAAGAACCTCTTAACGAAACTATCCATTACTCTGAGTTTGCTTT
+
FFFFFFFFFFFFFFFFFFFFFF:FFFFFFFFFFFFFFFFFFFFFFFFF:FFFFFFFFFFFFFFFFFFF
FFFFFFFFFFFFFFFFFFF,FFFFFFFFFFFFFFFFFFFFFFFFF:FFFF:FFFFFFFFFFFFFFFFF
@A00155:342:HHGFNDSXY:1:1170:1723:12399 1:N:0:GAACCTAG+TCCGCATA
CTGTCATTCACTCGCAGCACCCCACCATGGTACGTGCTATGCCTTAAATTACCATCGAAGTTGAATTC
GGCTCCTGGTGCCTCGTGATTCGGGAAGAACCTCTTAACGAAACTATCCATTACTCTGAGTTTGCTTT
+
FFF:FFFFFFFFFFFFFFFFFF:FFFFFFFFFFFFFFFFFFFFFFFFFFFFFFFFFFFFFFFFFFFFF
FFFFFFFFFFFFFFFFFFFFFFFFFF:F:FFFFFFFFFFFFFFFFFFFFFFFFFFFFFFFFFFF:FFF
@A00155:342:HHGFNDSXY:1:2139:2157:35978 2:N:0:GAACCTAG+TCCGCATA
CTGTCATTCACTCGCAGCACCCCACCATGGTACGTGCTATGCCTTAAATTACCATCGAAGTTGAATTC
GGCTCCTGGTGCCTCGTGATTCGGGAAGAACCTCTTAACGAAACTATCCATTACTCTGAGTTTGCTTT
+
FFFFFFF:FFFFFFFFFFFF:FFFFFFFFFFFFFFF:FFFFF:FFFFFFFF:FFFFFFFFFFFFFFFF
FFFFFFFFFFFFFFFFFFFFFFFFFFFFFFFFFFFFFFFFFFFFFFFFFFFFFFFFFFFFFFFFFFFF
@A00155:342:HHGFNDSXY:1:2143:22137:24862 1:N:0:GAACCTAG+TCCGCATA
CTGTCATTCACTCGCAGCACCCCACCATGGTACGTGCTATGCCTTAAATTACCATCGAAGTTGAATTC
GGCTCCTGGTGCCTCGTGATTCGGGAAGAACCTCTTAACGAAACTATCCATTACTCTGAGTTTGCTTT
+
FFFFFFFFFFFFFFFFFFFFFFFFFFFFFFFFFFFFFFFFFFFFFFFFFFFFFFFFFFFFFFFFFFFF
FFFFFFFFFFFFFFFFFFFFFFFFFFFFFFFFFFFFFFFFFFFFFFFFFFFFFFFFFFFFFFFFFF,F
@A00155:342:HHGFNDSXY:1:2117:7726:17409 1:N:0:GAACCTAG+TCCGCATA
CTGTCATTCACTCGCAGCACCCCACCATGGTACGTGCTATGCCTTAAATTACCATCGAAGTTGAATTC
GGCTCCTGGTGCCTCGTGATTCGGGAAGAACCTCTTAACGAAACTATCCATTACTCTGAGTTTGCTTT
+
FFFFFFFFFFFFFFFFFFFFFFFFFFFFFFFFFFFFFFFFFFFFFFFFFFFFFFFFFFFFFFFFFFFF
FFFFFFFFFFF:FFFFFFFFFFFFFFFFFFFFFFFFFFFFFFFFFFFFFFFFFFFFFFFFFFFFFFFF
@A00155:342:HHGFNDSXY:1:2342:16857:29966 1:N:0:GAACCTAG+TCCGCATA
CTGTCATTCACTCGCAGCACCCCACCATGGTACGTGCTATGCCTTAAATTACCATCGAAGTTGAATTC
GGCTCCTGGTGCCTCGTGATTCGGGAAGAACCTCTTAACGAAACTATACATTACTCTGAGTTTGCTTT
+
FFFFFFFFFFFFFFFFFFFFFFFFFFFFFFFFFFFFFFFFFFFFFFFFFFFFFFFFFFFFFFFFFFFF
FFFFFFFFFFFFFFFFFFFFFFFFFFFFFFFFFFFFFFFFFFFFFFFFFFFFFFFFFFFFFFFFFFFF
```

```
@A00155:342:HHGFNDSXY:1:2175:12111:8155 1:N:0:GAACCTAG+TCCGCATA
CTGTCATTCACTCGCAGCACCCCACCATGGTACGTGCTATGCCTTAAATTACCATCGAAGTTGAATTC
GGCTCCTGGTGCCTCGTGATTCGGGAAGAACCTCTTAACGAAACTATCCATTACTCTGAGTTTGCTTT
+
FFFFFFFFFFFFFFFFFFFFFFFFFFFFFFFFFFFFFFFFFFFFFFFFFFFFFFFFFFFFFFFFFFFF
FFFFFFFFFFFFFFFFFFFFFFFFFFFFFFFFFFFFFFFFFFFFFFFFFFFFFFFFFFFFFFFFFFF:
@A00155:342:HHGFNDSXY:1:1359:19361:1110 2:N:0:GAACCTAG+TCCGCATA
TGTCATTCACTCGCAGCACCCCACCATGGTACGTGCTATGCCTTAAATTACCATCGAAGTTGAATTCG
GCTCCTGGTGCCTCGTGATTCGGGAAGAACCTCTTAACGAAACTATCCATTACTCTGAGTTTGCTTT
+
FFFFF,FFFFFFFFFFFFFFFFFFFFFFFFFFFFFFFFFFFFFFFFFFF:FFFFFFFFFFFFFFFFFF
FFFFF:FFFFFFFFFFFFF:FFFFFFFFFFFFFFFFFFFFFFFFFFFFFFFFFFFFFFFFFFFFFFF
@A00155:342:HHGFNDSXY:1:1215:7536:16047 2:N:0:GAACCTAG+TCCGCATA
GTCATTCACTCGCAGCACCCCACCATGGTACGTGCTATGCCTTAAATTACCATCGAAGTTGAATTCGG
CTCCTGGTGCCTCGTGATTCGGGAAGAACCTCTTAACGAAACTATCCATTACTCTGAGTTTGCTTTTA
+
FFFFFFFFFFFFFFFFFFFFFFFFFFF:FFFF:FFFFFFFFFFFFF:FFFFFFFFFFFFFFFFFFFFF
FFFFFFFFFF:FFFFFFFFFFFFFFFFFFFFFFFFFFFFFFFFFFFFFFFFFFFFFFFFF:FFFFFFF
@A00155:342:HHGFNDSXY:1:1420:29423:21512 2:N:0:GAACCTAG+TCCGCATA
GTCATTCACTCGCAGCACCCCACCATGGTACGTGCTATGCCTTAAATTACCATCGAAGTTGAATTCGG
CTCCTGGTGCCTCGTGATTCGGGAAGAACCTCTTAACGAAACTATCCATTACTCTGAGTTTGCTTTTA
+
FFFFFFFFFFFFFFFFFFFFFFFFFFFFFFFFFF,FFFFFFFFFFFF:FFFFFFFFFFFFFFFFFFFF
FFFFFFFFFFFFFFFFFFFFFFFFFFFFF:FFFFFFFFFFFFFFFFFFFFFFFFFFFFFFFFFFFFFF
@A00155:342:HHGFNDSXY:1:2114:22101:15029 2:N:0:GAACCTAG+TCCGCATA
GTCATTCACTCGCAGCACCCCACCATGGTACGTGCTATGCCTTAAATTACCATCGAAGTTGAATTCGG
CTCCTGGTGCCTCGTGATTCGGGAAGAACCTCTTAACGAAACTATCCATTACTCTGAGTTTGCTTTTA
+
:FFF:FF:FF::FFFFFFFFFFFFFFFFFFFFFFFFFFFFFFFFFFF:FFFFFFF:FFFFFFFFFFFF
FFFFFFFFFFFFFFFFFFFFFFFFFFFFFFFFFFFFFFFFFFFFFFFFFFFFFFFFFFFFFFFFFFFF
@A00155:342:HHGFNDSXY:1:1534:13449:10222 2:N:0:GAACCTAG+TCCGCATA
TCATTCACTCGCAGCACCCCACCATGGTACGTGCTATGCCTTAAATTACCATCGAAGTTGAATTCGGC
TCCTGGTGCCTCGTGATTCGGGAAGAACCTCTTAACGAAACTATCCATTACTCTGAGTTTGCTTTTA
+
FFFFFFFFFFFFFFFFFF:FFFFFFFF,F,,F:FF:FF,FFFFFF,FF:FFFF:FF,FFFFFFFFF,F
FF::FFFFFFF:F:F:FF:FFFFF:F:F,:FFFFFFFFFFFF:FFFFFFFFFFFFFFFFF,FFFFFF
@A00155:342:HHGFNDSXY:1:2604:2392:7138 1:N:0:GAACCTAG+TCCGCATA
TCATTCACTCGCAGCACCCCACCATGGTACGTGCTATGCCTTAAATTACCATCGAAGTTGAATTCGGC
TCCTGGTGCCTCGTGATTCGGGAAGAACCTCTTAACGAAACTATCCATTACTCTGAGTTTGCTTTTAG
+
FFFFFFFFFF:FFFFFFFFFFFFFFFFFFFFFFFFFFFFFFFFFFFFFF:FFFFFFFFFFFFFFFFFF
FFFFFFFFFFFFFFFF:FFFFFFFF:FFFFFFFFFFFFFFFFFFFFFFFFFFFFFFFFFF,FFFFFFF
@A00155:342:HHGFNDSXY:1:2517:14850:20415 1:N:0:GAACCTAG+TCCGCATA
TCATTCACTCGCAGCACCCCACCATGGTACGTGCTATGCCTTAAATTACCATCGAAGTTGAATTCGGC
TCCTGGTGCCTCGTGATTCGGGAAGAACCTCTTAACGAAACTATCCATTACTCTGAGTTTGCTTTTAG
+
FFFFFFFFFFFFFFFFFFFFFFFFFFFFFFFFFFFFFFFFFFFFFFFFFFFFFFFFFFFFFFFFFFFF
FFFFFFFFFFFFFFFFFFFFFFFFFFFFFFFFFFFFFFFFFFFFFFFFFFFFFFFFFFFFFFF:FFFF
@A00155:342:HHGFNDSXY:1:2458:20790:30953 1:N:0:GAACCTAG+TCCGCATA
TCATTCACTCGCAGCACCCCACCATGGTACGTGCTATGCCTTAAATTACCATCGAAGTTGAATTCGGC
TCCTGGTGCCTCGTGATTCGGGAAGAACCTCTTAACGAAACTATCCATTACTCTGAGTTTGCTTTTAG
+
FFFFFFFFF:FFFFFFFFFFFFFFFFFFFFFFFFF:FFFFFFFFFFFFFFFFFFFFFFFFFFFFFFFF
FFFFFFFFFFFFFFFFFFFFFFFFFFFFFFFFFF:FFFFFFFFFFFFFFFFFFFFFFFF:FF::FFFF
```

```
@A00155:342:HHGFNDSXY:1:2548:4634:20509 2:N:0:GAACCTAG+TCCGCATA
CATTCACTCGCAGCACCCCACCATGGTACGTGCTATGCCTTAAATTACCATCGAAGTTGAATTCGGCT
CCTGGTGCCTCGTGATTCGGGAAGAACCTCTTAACGAAACTATCCATTACTCTGAGTTTGCTTTTAGC
+
FF:FFFFFFFFFFFFFFFFFFFFFFFF::FFF:FFFF:FFFFFFF,FFF:FFFFFFFFFFFFFFFFFF
FFFFFFF,FFFFFFFFFFFFFFFFFFFFFFFFFFFFFFFFFFFFFFFFFFFFFFFFFFFFFFFFFFFF
@A00155:342:HHGFNDSXY:1:1146:9525:10755 1:N:0:GAACCTAG+TCCGCATA
CATTCACCCGCAGCACCCCACCATGGTACGTGCTATGCCTTAAATTACCATCGAAGTTGAATTCGGCT
CCTGGTGCCTCGTGATTCGGGAAGAACCTCTTAACGAAACTATCCATTACTCTGAGTTTGCTTTTAGC
+
FFFFFFFFFFFFFFFFFFFFFFFFFFFFFFFFFFFFFF:FFFFFFFFFFFFFFFFFFFFFFFFFFFFF
FFFFFFFF:FFFFFFFFFFFFFFFFFFFF:FFFFFFFFFFFFFF,FFFF:FFFFFFFFFFFF:FFF:F
@A00155:342:HHGFNDSXY:1:2534:25961:10066 2:N:0:GAACCTAG+TCCGCATA
CATTCACTCGCAGCACCCCACCATGGTACGTGCTATGCCTTAAATTACCATCGAAGTTGAATTCGGCT
CCTGGTGCCTCGTGATTCGGGAAGAACCTCTTAACGAAACTATCCATTACTCTGAGTTTGCTTTTAGC
+
FF,FFFFFFFFFFFFFFFFFFFFFFFFFFFFFFFFFFFFFFFFFFFFFFFFFFFFFFFFFFFFFFFFF
FFFFFFFFFFFFFFFFFFFFFFFFFFFFFFFFFFFFFFFFFFFFFFFFFFFFFFFFFFFFFFFFFFFF
@A00155:342:HHGFNDSXY:1:2673:24008:2988 2:N:0:GAACCTAG+TCCGCATA
ATTCACTCGCAGCACCCCACCATGGTACGTGCTATGCCTTAAATTACCATCGAAGTTGAATTCGGCTC
CTGGTGCCTCGTGATTCGGGAAGAACCTCTTAACGAAACTATCCATTACTCTGAGTTTGCTTTTAGCT
+
FFFFFFFFFFFFFFFFFFFFFFFFFFFFFFFFFFFFFFFFFFFFFFFFFFFFFFFFFFFFFFFFFFFF
FFFFFFFFFFFFFFFFFFFFFFFFFFFFFFFFFFFFFFFFFFFFFFFFFFFFFFFFFFFFFFFFFFFF
@A00155:342:HHGFNDSXY:1:2504:2672:27633 2:N:0:GAACCTAG+TCCGCATA
ATTCACTCGCAGCACCCCACCATGGTACGTGCTATGCCTTAAATTACCATCGAAGTTGAATTCGGCTC
CTGGTGCCTCGTGATTCGGGAAGAACCTCTTAACGAAACTATCCATTACTCTGAGTTTGCTTTTAGCT
+
FFFFFFFFFFFFFFFFFF:FFF,FFFF:FFFFFFFFFFFFFFFF,FFFFFFFFFFFFFF:FFFFFFFF
FFFFFFFFFFFFFFFFFFFFFFFFFFFFFF,:FFFFFFFFFFFFF,FFFFFFFFFFFFFFFFFFFFFF
@A00155:342:HHGFNDSXY:1:2240:18412:34507 1:N:0:GAACCTAG+TCCGCATA
ATTCACTCGCAGCACCCCACCATGGTACGTGCTATGCCTTAAATTACCATCGAAGTTGAATTCGGCTC
CTGGTGCCTCGTGATTCGGGAAGAACCTCTTAACGAAACTATCCATTACTCTGAGTTTGCTTTTAGCT
+
FFFFFFFFFFFFFFFF:FFFFFFFFFFFFFFFF:FFFFFFFFFF:F,,FFFFFFFFFFFFFF:FFFFF
FF:FFFFFFFFFFFFFFFFFFFFFFFFFFFFFFFFFFF:FF::,,FFFFFF:FFFF:FFF:FFFFFF:
@A00155:342:HHGFNDSXY:1:2604:2392:7138 2:N:0:GAACCTAG+TCCGCATA
ATTCACTCGCAGCACCCCACCATGGTACGTGCTATGCCTTAAATTACCATCGAAGTTGAATTCGGCTC
CTGGTGCCTCGTGATTCGGGAAGAACCTCTTAACGAAACTATCCATTACTCTGAGTTTGCTTTTAGCT
+
FFFFFFFFFFFFFFFFF:F:FFFFFFFFF:FF:FFFFF:FFFFFFFFFF:FFFFFFFFFF:FFFFFFF
FFFFFFFFF:FFFFFFFF:FFF:FFFF::FFFFFFFFFFFFFFFFFFFFFFFFFFFFFFFF:FFFFFF
@A00155:342:HHGFNDSXY:1:1211:12997:5588 1:N:0:GAACCTAG+TCCGCATA
ATTCACTCGCAGCACCCCACCATGGTACGTGCTATGCCTTAAATTACCATCGAAGTTGAATTCGGCTC
CTGGTGCCTCGTGATTCGGGAAGAACCTCTTAACGAAACTATCCATTACTCTGAGTTTGCTTTTAGCT
+
FFFFFFFFFFFFFFFFFFFFFFFFFFFFFFFFFFFFFFFFFFFFFFFFFFFFFFFFFFFFFFFFFFFF
FFFFFFFFFFFFFFFFFFFFFFFF:FFFFFFFFFFFFFFF:FFFFFFFFFFFFFFFFFFFFFFFFFFF
@A00155:342:HHGFNDSXY:1:1214:20247:8594 1:N:0:GAACCTAG+TCCGCATA
TTCACTCGCAGCACCCCACCATGGTACGTGCTATGCCTTAAATTACCATCGAAGTTGAATTCGGCTCC
TGGTGCCTCGTGATTCGGGAAGAACCTCTTAACGAAACTATCCATTACTCTGAGTTTGCT
+
FFFFFFFFFFFFFFFFFFFFFFFFFFFFFFFFFFFFFFFFFFFFFFFFFFFFFFFFFFFFFFFFFFFF
FFFFFFFFFFFFFFFFFFFFFFFFFFFFFFFFFFFFFFFFFFFF:FFFFFFFFFFFFFFF
```

```
@A00155:342:HHGFNDSXY:1:1534:30608:21371 1:N:0:GAACCTAG+TCCGCATA
TTCACTCGCAGCACCCCACCATGGTACGTGCTATGCCTTAAATTACCATCGAAGTTGAATTCGGCTCC
TGGTGCCTCGTGATTCGGGAAGAACCTCTTAACGAAACTATCCATTACTCTGAGTTTGCT
+
F,FFFFFFFFFFFFFFFFFFFFFFFFFFFFFFFFFFFFFFFFFFFFFFFFFFFFFFFF:FFFFFFFFF
FFFFFFFFFFFFFFFFFFFFFFFFFFFFFFFFFFFFFFFFFFFFFFFFFFFFFFF:FFFFF
@A00155:342:HHGFNDSXY:1:1509:5972:19382 1:N:0:GAACCTAG+TCCGCATA
TTCACTCGCAGCACCCCACCATGGTACGTGCTATGCCTTAAATTACCATCGAAGTTGAATTCGGCTCC
TGGTGCCTCGTGATTCGGGAAGAACCTCTTAACGAAACTATCCATTACTCTGAGTTTGCT
+
FFF:FFFFFFFFFFFFFFFF:F:FFFFFFFFFFFFF,FFF:F:F,FFFF,FFFFF:FFFFFF:FFFFF
FFFFFFFFFFFFF,FFFFFFFFFFFFFFFFFFFFFFFFFFFF:FFFFFFFFFFFFFFFFF
@A00155:342:HHGFNDSXY:1:2673:24008:2988 1:N:0:GAACCTAG+TCCGCATA
TTCACTCGCAGCACCCCACCATGGTACGTGCTATGCCTTAAATTACCATCGAAGTTGAATTCGGCTCC
TGGTGCCTCGTGATTCGGGAAGAACCTCTTAACGAAACTATCCATTACTCTGAGTTTGCTTTTAGCTT
+
FFFFFFFFFFFFFFFFFFFFFFFFFFFFFFFFFFFFFFFFFFFFFFFFFFFFFFFFFFF,FFFFFFFF
FFFFFFFFFFFFFFFFFFFFFFFFFFFFFFFFFFFFFFFFFFFFFFFFFF:FFFFFFFFFF:FFFFFF
@A00155:342:HHGFNDSXY:1:2651:4942:19163 2:N:0:GAACCTAG+TCCGCATA
TCACTCGCAGCACCCCACCATGGTACGTACTATGCCTTAAATTACCATCGAAGTTGAATTCGGCTCCT
GGTGCCTCGTGATTCGGGAAGAACCTCTTAACGAAACTATCCATTACTCTGAGTTTGCTTTTAGCTTG
+
FFFFFFFFFFFFFFFFFFFFFFFFFFF:FF,F:FFFFFFF,FF::FFFFFF,FFFFFFFFFF:FFFFF
FFFFFFFFFFFFFFFFFFFFFFFFFFFFFFFFFFFFFF:FFFFFFFFFFFFFFFFFFFFFFFFFFFFF
@A00155:342:HHGFNDSXY:1:1278:7600:8296 2:N:0:GAACCTAG+TCCGCATA
TCACTCGCAGCACCCCACCATGGTACGTGCTATGCCTTAAATTACCATCGAAGTTGAATTCGGCTCCT
GGTGCCTCGTGATTCGGGAAGAACCTCTTAACGAAACTATCCATTACTCTGAGTTTGCTTTTAGCTTG
+
FFFFFFFFFFFFFFFFFFFFFFFFFFFFF:FFFFFFFFFFFFFFFFFFFF:FFFFFFFFFFFFFFFFF
FFFFFFFFFFFFFFFFFFFFFFFFFF:FFFFFFFFFFFFFFFFFFFFFFF:FFFFFFFFFFFFF:FFF
@A00155:342:HHGFNDSXY:1:2210:5584:33583 2:N:0:GAACCTAG+TCCGCATA
CACTCGCAGCACCCCACCATGGTACGTGCTATGCCTTAAATTACCATCGAAGTTGAATTCGGCTCCTG
GTGCCTCGTGATTCGGGAAGAACCTCTTAACGAAACTATCCATTACTCTGAGTTTGCTTTTAGCTTGG
+
FFFFFFFFFFFFFFFFFFFFFFFFFFFFFFFFFFFFFFF::FFFFFFFFFFFFFFFF,FFFFFFFFFF
F:FFFFFFFFFFFFFFFFFFFFFFFFF:FFFFFFFFFFFFFFFFFFFFFFFFFFFFF:FFFFFFFFFF
@A00155:342:HHGFNDSXY:1:2210:8079:24878 2:N:0:GAACCTAG+TCCGCATA
CACTCGCAGCACCCCACCATGGTACGTGCTATGCCTTAAATTACCATCGAAGTTGAATTCGGCTCCTG
GTGCCTCGTGATTCGGGAAGAACCTCTTAACGAAACTATCCATTACTCTGAGTTTGCTTTTAGCTTGG
+
FFFFF:FFFFFFFFF,FF:,FF:FFFFFFFFFFF,FFFF:F,FFFFFFFF:FFFFFF:FFFFFFFFFF
FFFFFFFFFFFFFFFFFFFFF:FFFFFF,FFFFFFFFFFFFFFFFFFFFFFF:FFFFFFFFFFFFFFF
@A00155:342:HHGFNDSXY:1:2166:22887:30984 2:N:0:GAACCTAG+TCCGCATA
CACTCGCAGCACCCCACCATGGTACGTGCTATGCCTTAAATTACCATCGAAGTTGAATTCGGCTCCTG
GTGCCTCGTGATTCGGGAAGAACCTCTTAACGAAACTATCCATTACTCTGAGTTTGCTTTTAGCTTGG
+
FFFFFFFFFFFFFFFFFFFFFFFFFFFFFFFFFFFFFF:FFFFFFFFFFF,FFFFFFFFFFFFFFFFF
FFFFFFFFFFFFFFFFFFFFFFFFFFFFFFFFFFFFFFFFFFFFFFFFFF:FFFFFFFFFFFFFFFFF
@A00155:342:HHGFNDSXY:1:1467:28339:6449 2:N:0:GAACCTAG+TCCGCATA
ACTCGCAGCACCCCACCATGGTACGTGCTATGCCTTAAATTACCATCGAAGTTGAATTCGGCTCCTGG
TGCCTCGTGATTCGGGAAGAACCTCTTAACGAAACTATCCATTACTCTGAGTTTGCTTT
+
FFFFFFFFFFFFFFFFFFFFFFFFFFFFFFFFFFFFFFFF,FFF:FFFF:FFFFFFFFFFFFFFFFFF
FFFFFFFFFFFFFFFFFFFFFFFFFFFFFFFFFFFFFFFFFFFFFFFFFFFFFFFFFFF
```

```
@A00155:342:HHGFNDSXY:1:2303:12283:5008 2:N:0:GAACCTAG+TCCGCATA
ACTCGCAGCACCCCACCATGGTACGTGCTATGCCTTAAATTACCATCGAAGTTGAATTCGGCTCCTGG
TGCCTCGTGATTCGGGAAGAACCTCTTAACGAAACTATCCATTACTCTGAGTTTGCTTTTAGCTTGGG
+
FFFFFFFFFFFFFFFFFFFFFFFFFFFFFFFFFF,FFFFFFFFFFFFFFFFFFFFFFFFFFFFFFFFF
FFFFFFFFFFFFFFFFFFFFFFFFFFFFFFFFFFFFFFFFFFFFFFFFFFFF:FFFFFFFFFFFFFFF
@A00155:342:HHGFNDSXY:1:1606:8115:20807 2:N:0:GAACCTAG+TCCGCATA
ACTCGCAGCACCCCACCATGGTACGTGCTATGCCTTAAATGACCATCGAAGTTGAATTCGGCTCCTGG
TGCCTCGTGATTCGGGAAGAACCTCTTAACGAAACTATCCATTACTCTGAGTTTGCTTTTAGCTTGGG
+
FF:FFFF:FFF:FFFFFFFFFFF:F:,FFFFFFFFFFFFF,F:FF:FFFFFFFFFFFFFFFFFFFFFF
FFFFFFFFFFFFFFFFFFFFFFFFFFFFFFFFFFFFFF:FFFFFFFFFFFFFFFFFF:FFF:FFFF,F
@A00155:342:HHGFNDSXY:1:1535:16405:8703 2:N:0:GAACCTAG+TCCGCATA
ACTCGCAGCACCCCACCATGGTACGTGCTATGCCTTAAATTACCATCGAAGTTGAATTCGGCTCCTGG
TGCCTCGTGATTCGGGAAGAACCTCTTAACGAAACTATCCATTACTCTGAGTTTGCTTTTAGCTTGGG
+
FFFFFFF:F,FFF:FFF:FF,FFFFFF,F,FFFFFFFFFFFFFFF:FFFFFF,FFF:FFFFF:FFFFF
FFFFFFFFFFFFFFFFFFF:FFFF:FFFFFFFF,FFFFFFFFFFFFFFFFFFFFFFFFFFFFF,FFFF
@A00155:342:HHGFNDSXY:1:2120:3179:13542 2:N:0:GAACCTAG+TCCGCATA
CTCGCAGCACCCCACCATGGTACGTGCTATGCCTTAAATTACCATCGAAGTTGAATTCGGCTCCTGGT
GCCTCGTGATTCGGGAAGAACCTCTTAACGAAACTATCCATTACTCTGAGTTTGCTTTTAGCTTGGGT
+
FFFFFFFFFFFFFFFF::FFFFFFFFFFF,FFFFFFFFF:FFF,FFFFFFFFFFFFFFFFFFFFFFFF
FFFFFFFFF:FFFFFFFFFFFFFFFF,FFFFFFFFFFFFFFFFFFFFFFFFFFFFFFFF,FFFFFFF:
@A00155:342:HHGFNDSXY:1:1178:26304:4805 2:N:0:GAACCTAG+TCCGCATA
CTCGCAGCACCCCACCATGGTACGTGCTATGCCTTAAATTACCATCGAAGTTGAATTCGGCTCCTGGT
GCCTCGTGATTCGGGAAGAACCTCTTAACGAAACTATCCATTACTCTGAGTTTGCTTTTAGCTTGGGT
+
F,FFF:FFFFFFF::F,F:F:FFFFFFFFFFFFFFFF:F:FFF::FF:FFFFFFFFFFFFFFFFFFF:
FFFFFFFFFFFFFFFFFFFFFF,F:FF,FFFFFFFFFFFFFF:FFFFFFFFFFFFFFFFFFFFFFFFF
@A00155:342:HHGFNDSXY:1:1521:8106:28056 2:N:0:GAACCTAG+TCCGCATA
TCGCAGCACCCCACCATGGTACGTGCTATGCCTTAAATTACCATCGAAGTTGAATTCGGCTCCTGGTG
CCTCGTGATTCGGGAAGAACCTCTTAACGAAACTATCCATTACTCTGAGTTTGCTTTTAGCTTGGGTT
+
FFFFFFFFFFFFFFFFFF:FFFFFFFFFFF:FFFFFFF:FFFFFFFFFFFFFFFF:FFFFFFFFFFFF
FFFFFFFFFFFFFFFFFFFFFFFFFFFFFFFFFFFFFFFFFFFFFFFFFFFFFFF:FFFFFFFFFFF:
@A00155:342:HHGFNDSXY:1:1521:7916:28385 2:N:0:GAACCTAG+TCCGCATA
TCGCAGCACCCCACCATGGTACGTGCTATGCCTTAAATTACCATCGAAGTTGAATTCGGCTCCTGGTG
CCTCGTGATTCGGGAAGAACCTCTTAACGAAACTATCCATTACTCTGAGTTTGCTTTTAGCTTGGGTT
+
FFFFFFF,FFFFFFFFFFFFFFF,FFFFFFFFF:FFFFFFFFFFFFFFFFFFFFFFFFFFFFFFFFFF
FFFFFFFFFFFFFFFFFFFFFFFFFFFFFFFFF:FFFFFFFFFFFFFFFFFFFFFFFFFFFFFFFFFF
@A00155:342:HHGFNDSXY:1:2644:32099:31626 2:N:0:GAACCTAG+TCCGCATA
TCGCAGCACCCCACCATGGTACGTGCTATGCCTTAAATTACCATCGAAGTTGAATTCGGCTCCTGGTG
CCTCGTGATTCGGGAAGAACCTCTTAACGAAACTATCCATTACTCTGAGTTTGCTTTTAGCTTGGGTT
+
FFFFFFFFFFFFFFFFFFFFFFFFF:FF,FFFFF:FFF,FFF:FFF::FFFFFFFFFFFFFFFFFF:F
FFFFFFFFFFFFFFF:FFFFFFFFFFFFFFFFFFFFFFFFFFFFFFFFFFFFFFFFF:FFFFFFFFFF
@A00155:342:HHGFNDSXY:1:2503:31376:19100 2:N:0:GAACCTAG+TCCGCATA
TCGCAGCACCCCACCATGGTACGTGCTATGCCTTAAATTACCATCGAAGTTGAATTCGGCTCCTGGTG
CCTCGTGATTCGGGAAGAACCTCTTAACGAAACTATCCATTACTCTGAGTTTGCTTTTAGCTTGGGTT
+
FFFFFFFFFFFF,FFF:FFFFFFFFFFFFFFFFFF:::FFF::FFF:FFFFF,,FFFFFFFFFFFFFF
FFFFFFFFFFFFFFFFFFFFFFFFF,:FF::FF:FFFFF:FFFFFFFFFFFFFFFFFFFFFFFFFFFF
```

@A00155:342:HHGFNDSXY:1:1469:20790:7404 1:N:0:GAACCTAG+TCCGCATA
GCAGCACCCCACCATGGTACGTGCTATGCCTTAAATTACCATCGAAGTTGAATTCGGCTCCTGGTGCC
TCGTGATTCGGGAAGAACCTCTTAACGAAACTATCCATTACTCTGAGTTTGCTTTTAGC
+
FFFFFFFFFFFFFFFFFFFFFFFFFFFFFFF:FFFFFFFFFFFFFFFFFFFFFFFFFFFFFFFFFFFF
FFFFFFFFFFFFFFFFFFFFFFFFFFFFF:FF,FFFFFFFFFF:FFFFFFFFFFFFFFF
@A00155:342:HHGFNDSXY:1:1414:3332:31688 1:N:0:GAACCTAG+TCCGCATA
GCAGCACCCCACCATGGTACGTGCTATGCCTTAAATTACCATCGAAGTTGAATTCGGCTCCTGGTGCC
TCGTGATTCGGGAAGAACCTCTTAACGAAACTATCCATTACTCTGAGTTTGCTTTTAGCTTGGGTTCG
+
FFFFFFFFFFFFFFFFFFFFFFFFFFFFFFFFFFFFFFFFFFFFFFFFFFFFFFFFFFFFFFFFFFFF
FFFFFF::FFFFFFFF:FFFFFFFFFFFFFFFFFFFFFFFFFFFFFFFFFFFFF,F:F:FFFFFFFFF
@A00155:342:HHGFNDSXY:1:2437:2944:28729 1:N:0:GAACCTAG+TCCGCATA
GCAGCACCCCACCATGGTACGTGCTATGCCTTAAATTACCATCGAAGTTGAATTCGGCTCCTGGTGCC
TCGTGATTCGGGAAGAACCTCTTAACGAAACTATCCATTACTCTGAGTTTGCTTTTAGCTTGGGTTCG
+
FFFFFFFFFFFFFFFFFFFFFFFFFFFFF:F:FFFF:FFFFFFFFFFFFFFFFFFFFFFFFFF,FFFF
FFFFFFFFFFFFFFFFFF,FFFFFFFFFFFFFFFF,F:FF:FFFFFFFFFF:FF,FF:FFFFFFFF,F
@A00155:342:HHGFNDSXY:1:2161:8214:18380 1:N:0:GAACCTAG+TCCGCATA
CAGCACCCCACCATGGTACGTGCTATGCCTTAAATTACCATCGAAGTTGAATTCGGCTCCTGGTGCCT
CGTGATTCGGGAAGAACCTCTTAACGAAACTATCCATTACTCTGAGTTTGCTTTTAGCTTGGGTTCGT
+
FFFFFFFFFFFFFFFFFFFFFFFFFFFFFFFFFFFFFFFFFFFFFFFFFFFFFFFFFFFFFFFFFFFF
FFFFFFFFFFFFFFFFFFFFFFFFFFFFFFFFFFFFFFFFFFFFFFFFFFFFFFFFFFFF:FFFFFFF
@A00155:342:HHGFNDSXY:1:1308:1633:14340 1:N:0:GAACCTAG+TCCGCATA
CAGCACCCCACCATGGTACGTGCTATGCCTTAAATTACCATCGAAGTTGAATTCGGCTCCTGGTGCCT
CGTGATTCGGGAAGAACCTCTTAACGAAACTATCCATTACTCTGAGTTTGCTTTTAGCTTGGGTTCGT
+
FFFFFFFFFFFFFFFFFFFFFFFFFFFF,FFFF:FFFFFFF:FF:FFF:FFFFFFFFFFFFFFFFFFF
FFF,FFFFFFFFFFFFFFFFFFFFFFFFF:FFFFFFFFFFFFFFFFFFFFFFFFFFFFFFFFFFFFF:
@A00155:342:HHGFNDSXY:1:2303:12283:5008 1:N:0:GAACCTAG+TCCGCATA
CAGCACCCCACCATGGTACGTGCTATGCCTTAAATTACCATCGAAGTTGAATTCGGCTCCTGGTGCCT
CGTGATTCGGGAAGAACCTCTTAACGAAACTATCCATTACTCTGAGTTTGCTTTTAGCTTGGGTTCGT
+
FFFFFFF:FFFFFFFFFFFFFFFFFFFFFFFFFFFFFFFFFFFFFFFFFFF:FFFF:FF:FFFFFFFF
FFFFFFFFFF:FFFFFFFFFFFFFFFFFFFFFFF:FFF:FFFFFFF:FFFFF:FFFFFF:F:F,FFF:
@A00155:342:HHGFNDSXY:1:2363:11641:33833 1:N:0:GAACCTAG+TCCGCATA
CAGCACCCCACCATGGTACGTGCTATGCCTTAAATTACCATCGAAGTTGAATTCGGCTCCTGGTGCCT
CGTGATTCGGGAAGAACCTCTTAACGAAACTATCCATTACTCTGAGTTTGCTTTTAGCTTGGGTTCGT
+
FFFFFFFFFFFFFFFFFFFFFFFFFFFFFFFFFFFFFFFFFFFFFFFFFFFFFFFFFFFFFFFFFFFF
FFFFFFFFFFFFFFFFFFFFFFFFFFFFFFFFFFFFFFFFFFFFFFFFFFFFFFFFFFFFFFFFFFFF
@A00155:342:HHGFNDSXY:1:2161:8142:18443 1:N:0:GAACCTAG+TCCGCATA
CAGCACCCCACCATGGTACGTGCTATGCCTTAAATTACCATCGAAGTTGAATTCGGCTCCTGGTGCCT
CGTGATTCGGGAAGAACCTCTTAACGAAACTATCCATTACTCTGAGTTTGCTTTTAGCTTGGGTTCGT
+
FFFFFFFFFFFFFFFFFFFFFFFFFFFFFFFFFFFFFFFFFFFFFFF:FFFFFFFFFFFFFFFFFFFF
FFFFFFFFFFFFFFFFFFFFFFFF:FFFFFFFFF,FFFFFFFFFFFFFFFFFFFFFFFFFFFFFF:FF
@A00155:342:HHGFNDSXY:1:2458:8341:3787 1:N:0:GAACCTAG+TCCGCATA
CAGCACCCCACCATGGTACGTGCTATGCCTTAAATTACCATCGAAGTTGAATTCGGCTCCTGGTGCCT
CGTGATTCGGGAAGAACCTCTTAACGAAACTATCCATTACTCTGAGTTTGCTTTTAGCTTGGGTTCGT
+
FFFFFFFFFFFFFFFFFFFFFFFFFFFFFFFFF:FFFFF:FFFFFFFFFFFFFFFFFFFFFFFFFFFF
FFFFFFFFFFFFFFFFFFFFFFFFFFFFFFFFFFFFFFFFFFFFFFFFFFFFFFFFFFFFFFFFFFF:

```
@A00155:342:HHGFNDSXY:1:1230:13096:4633 1:N:0:GAACCTAG+TCCGCATA
CAGCACCCCACCATGGTACGTGCTATGCCTTAAATTACCATCGAAGTTGAATTCGGCTCCTGGTGCCT
CGTGATTCGGGAAGAACCTCTTAACGAAACTATCCATTACTCTGAGTTTGCTTTTAGCTTGGGTTCGT
+
FFFFFFFFFFFFFFFFFFFFFFFFFFFFFFFFFFFFFFFFFFFFFFFFFFFFFFFFFFFFFFFFFFFF
FFFFFFFFFFFFFFFFFFFFFFFFFFFFFFFFFFFFFF:FFF:FFFFFFFFFF,FFFFFFFFFFFFFF
@A00155:342:HHGFNDSXY:1:2115:5303:30561 2:N:0:GAACCTAG+TCCGCATA
CAGCACCCCACCATGGTACGTGCTATGCCTTAAATTACCATCGAAGTTGAATTCGGCTCCTGGTGCCT
CGTGATTCGGGAAGAACCTCTTAACGAAACTATCCATTACTCTGAGTTTGCTTTTAGCTTGGGTTCGT
+
FFFFFFFFFFFFFFFFFFFFFFFFF:FFF:FFFFFFFFF:FFFFFFFFFFFFFFFFFFFFFFFFFFFF
FFF:FFFFFFFFFFFFFFFFFFFFFFF,FFFFFFFF:FFF:FFFFFFFFFFFFFFFFFFFFFFFFFFF
@A00155:342:HHGFNDSXY:1:2418:6822:33411 2:N:0:GAACCTAG+TCCGCATA
AGCACCCCACCATGGTACGTGCTATGCCTTAAATTACCATCGAAGTTGAATTCGGCTCCTGGTGCCTC
GTGATTCGGGAAGAACCTCTTAACGATACTATCCATTACTCTGAGTTTGCTTTTAGCTTGGGTTCGTG
+
FFFFFFFFFFFFFFFFFFFFFFFF:FFFFFFFFFF,FFFFFFFFFFFFFFFFFFFFFFFF,FFF:FFF
:FFFFFFF:FFFFFFFFFFFFFFFFF,FFFFF:,F:FFFFFFFFFFFFFFFFFFFFFFF:FF:FFFFF
@A00155:342:HHGFNDSXY:1:2360:31684:22294 1:N:0:GAACCTAG+TCCGCATA
AGCACCCCACCATGGTACGTGCTATGCCTTAAATTACCATCGAAGTTGAATTCGGCTCCTGGTGCCTC
GTGATTCGGGAAGAACCTCTTAACGAAACTATCCATTACTCTGAGTTTGCTTTTAGCTTGGGTTCGTG
+
FFFFFFFFFFFFFFFFFFFFFFFFFFFFFFFFFFFFFFFFFFFFFFFFFFFFFFFFFFF:FFFFFFFF
FFFFFFFFFFFFFFFFFFFFFFFFFFF:FFFFFFFFFFFFFFFFF:FFFFFFFFFFFFFFFFF:FFFF
@A00155:342:HHGFNDSXY:1:1126:18466:3912 1:N:0:GAACCTAG+TCCGCATA
AGCACCCCACCATGGTACGTGCTATGCCTTAAATTACCATCGAAGTTGAATTCGGCTCCTGGTGCCTC
GTGATTCGGGAAGAACCTCTTAACGAAACTATCCATTACTCTGAGTTTGCTTTTAGCTTGGGTTCGTG
+
FFFFFFFFFFFFFFFFFFFFFFFFFFFFFFFF::FFFFFFFFFFFFFFFFFFFFFFFFFFFFFFFFFF
FFFFFFFFFFFFFFFFFFFFFFFFFFFFFFFFFFFFFFFFFFF:FFFFFFF:FFFFFFFFFFFFFFFF
@A00155:342:HHGFNDSXY:1:1414:3332:31688 2:N:0:GAACCTAG+TCCGCATA
AGCACCCCACCATGGTACGTGCTATGCCTTAAAGTACCATCGAAGTTGAATTCGGCTCCTGGTGCCTC
GTGATTCGGGAAGAACCTCTTAACGAAACTATCCATTACTCTGAGTTTGCTTTTAGCTTGGGTTCGTG
+
FFFFFFFFFFFFFFFFFFFF,FFF:FFF:FFFF,FFFFFFFFFFFFFFFFFFFFFFFFFFFFFFFFFF
FFFFFFFFFFFFFFFFFFFFF:FFFFFFFFFFFF,FFFFFFFFFFFFFFFFFFFFFFFFFFFFFFFFF
@A00155:342:HHGFNDSXY:1:1240:1832:9424 1:N:0:GAACCTAG+TCCGCATA
AGCACCCCACCATGGTACGTGCTATGCCTTAAATTACCATCGAAGTTGAATTCGGCTCCTGGTGCCTC
GTGATTCGGGAAGAACCTCTTAACGAAACTATCCATTACTCTGAGTTTGCTTTTAGCTTGGGTTCGTG
+
FFFFFFFFFFFFFFFFFFFFFFFFFFFFFFFF::FFFFFFFFFFFFFFFFFFFFFFFFFFFFFFFFFF
FFFFFFFFFFFFFFFFFFFFFFFFFFFFFFFFFFFFFFFFFFFFFFFFFFFFFFFFFFFFFFFF:FFF
@A00155:342:HHGFNDSXY:1:1461:5249:20541 2:N:0:GAACCTAG+TCCGCATA
AGCACCCCACCATGGTACGTGCTATGCCTTAAATTACCATCGAAGTTGAATTCGGCTCCTGGTGCCTC
GTGATTCGGGAAGAACCTCTTAACGAAACTATCCATTACTCTGAGTTTGCTTTTAGCTTGGGTTCGTG
+
FFFFF:FFFFFFF,FFFFFFFFFFFFFFFFFFFFFFFFFFFFFF:FFFFFF,FFFFFFFF:FFFFFFF
FFFFFFFFFFFFFFFFFFFFFFFFFFFFFFFFFFFFFFFFFFFFFFFFFFFFFFFFFFFFFFFFFFFF
@A00155:342:HHGFNDSXY:1:2225:2627:25426 1:N:0:GAACCTAG+TCCGCATA
AGCACCCCACCATGGTACGTGCTATGCCTTAAATTACCATCGAAGTTGAATTCGGCTCCTGGTGCCTC
GTGATTCGGGAAGAACCTCTTAACGAAACTATCCATTACTCTGAGTTTGCTTTTAGCTTGGGTTCGTG
+
FFFFFFFFFFFFFFFFFFFFFFFFFFFFFFFFFFFFFFFFFFFFFFFFFFFFFFFFFFFFFFFFFFFF
FFFF,FFFFFFFFFFFFFF:FFFFFFFFFFFFFFFFFFFFFFF:FFFFFFF,FFF,FFFFFFFFFFFF
```

```
@A00155:342:HHGFNDSXY:1:2403:23782:36573 1:N:0:GAACCTAG+TCCGCATA
AGCACCCCACCATGGTACGTGCTATGCCTTAAATTACCATCGAAGTTGAATTCGGCTCCTGGTGCCTC
GTGATTCGGGAAGAACCTCTTAACGAAACTATCCATTACTCTGAGTTTGCTTTTAGCTTGGGTTCGTG
+
FFFFFFFFFFFFFFFFFFFFFFFFFFFFF:FFFFFFFFFFFFFFFFFFFFFFFFFFFFFFFFFFFFFF
FFFF,FFFFFFFFFFFFFFFFFFFFFFFFFFFFFFFFFFFFFFFFFFFFFF:FFFFFFFFFFFFFFFF
@A00155:342:HHGFNDSXY:1:1263:31421:7404 2:N:0:GAACCTAG+TCCGCATA
AGCACCCCACCATGGTACGTGCTATGCCTTAAATTACCATCGAAGTTGAATTCGGCTCCTGGTGCCTC
GTGATTCGGGAAGAACCTCTTAACGAAACTATCCATTACTCTGAGTTTGCTTTTAGCTTGGGTTCGTG
+
FFFFFFFFFFFFFFFFFFFFFFFFFFFFFFFFFFF:FF:FFFFFFF:FFFFFFFFFFFFFFFFFFFFF
FFFFFFFFFFFFFFFFFFFFFF,FFFFFFFFFFFFFFFFFFFFFFFFFFFFFFFFFFFFFFFFFFFFF
@A00155:342:HHGFNDSXY:1:1165:30798:16845 1:N:0:GAACCTAG+TCCGCATA
AGCACCCCACCATGGTACGTGCTATGCCTTAAATTACCATCGAAGTTGAATTCGGCTCCTGGTGCCTC
GTGATTCGGGAAGAACCTCTTAACGAAACTATCCATTACTCTGAGTTTGCTTTTAGCTTGGGTTCGTG
+
FFFFFFFFFFFFFFFFFFFFFFFFFFFFFFFFFFFFFFFFFFFFFFFFFFFFFFFFFFFFFFFFFFFF
FFFFFFFFFFFFFFFFFFFFFFFFFFFFFFFFFF:F,FFFF:FFFFFFFFFF:FFFFFFFFFFFFF:F
@A00155:342:HHGFNDSXY:1:2360:28854:22216 1:N:0:GAACCTAG+TCCGCATA
AGCACCCCACCATGGTACGTGCTATGCCTTAAATTACCATCGAAGTTGAATTCGGCTCCTGGTGCCTC
GTGATTCGGGAAGAACCTCTTAACGAAACTATCCATTACTCTGAGTTTGCTTTTAGCTTGGGTTCGTG
+
FFFFFFFFFFFFFFFFFFFFFFF,FFFFFFFFFFFFFFFFFFFFFFFFFF,FFFFFFFFFFFFFFFFF
FFFFFFFFFFFFFFFFFFFFFFFFFFFFFFFFFFFFFFFFFFFFFFFFFFF:FFFFF:FFFFFFFFFF
@A00155:342:HHGFNDSXY:1:2607:17192:1767 1:N:0:GAACCTAG+TCCGCATA
AGCACCCCACCATGGTACGTGCTATGCCTTAAATTACCATCGAAGTTGAATTCGGCTCCTGGTGCCTC
GTGATTCGGGAAGAACCTCTTAACGAAACTATCCATTACTCTGAGTTTGCTTTTAGCTTGGGTTCGTG
+
FFFFFFFFFFFFFFFFFFFFFFFFFFFFFFFFFFFFFFFFFFFFFFFFFFFFFFFFFFFFFFFFFFFF
FFFFFFFFFFFFFFFFFFFFFFFFFFFFFF:FFFFFFFFFFFFFFFFFFFFFFFFFF:FFFFFFFFFF
@A00155:342:HHGFNDSXY:1:1417:21838:20149 1:N:0:GAACCTAG+TCCGCATA
AGCACCCCACCATGGTACGTGCTATGCCTTAAATTACCATCGAAGTTGAATTCGGCTCCTGGTGCCTC
GTGATTCGGGAAGAACCTCTTAACGAAACTATCCATTACTCTGAGTTTGCTTTTAGCTTGGGTTCGTG
+
FFFFFFFFFFFFFFFFFFFFFFFFFFFFFFFFFFFFFFFFFFFFFFFFFFFFFFFFFFFFFFFFFFFF
FFFFFFFFFFFFFFFFFFFFFFFFFFFFFFFFFFFFFFFFFFFFFFFFFFFFFFFFFFFFFFFFFFFF
@A00155:342:HHGFNDSXY:1:1369:23131:13651 1:N:0:GAACCTAG+TCCGCATA
AGCACCCCACCATGGTACGTGCTATGCCTTAAATTACCATCGAAGTTGAATTCGGCTCCTGGTGCCTC
GTGATTCGGGAAGAACCTCTTAACGAAACTATCCATTACTCTGAGTTAGCTTTTAGCTTGGGTTCGTG
+
FFFFFFFFFFFFFFFFFFFFFFFFFFFFF:FFFFFFFFFFFFFFFFFFFFFFFFFFFFFFFFFFFFFF
FFFFFFFFFFFFFFFFFFFFFFFFFFFFFFFFFFFFFFFFFFFFFF,FFF,:FF:FFFFFFFFFFFF
@A00155:342:HHGFNDSXY:1:1617:27561:32910 1:N:0:GAACCTAG+TCCGCATA
GCACCCCACCATGGTACGTGCTATGCCTTAAATTACCATCGAAGTTGAATTCGGCTCCTGGTGCCTCG
TGATTCGGGAAGAACCTCTTAACGAAACTATCCATTACTCTGAGTTTGCTTTTAGCTTGGGTTCGTG
+
FFFFFFFFFFFFFFFFFFFFFF,FFFFFFFFFFFFFFFF:FFFFFFFFFFFFFFFFFFFFFFFFFFFF
FFFFFFFFFFFFFFFFFFFFFFFFFF:FFFFFFFFFFFFFFFFF:FFFF:,F:FFFFFFFFF:FFFF
@A00155:342:HHGFNDSXY:1:1359:19361:1110 1:N:0:GAACCTAG+TCCGCATA
GCACCCCACCATGGTACGTGCTATGCCTTAAATTACCATCGAAGTTGAATTCGGCTCCTGGTGCCTCG
TGATTCGGGAAGAACCTCTTAACGAAACTATCCATTACTCTGAGTTTGCTTTTAGCTTGGGTTCGTG
+
FFFFFFFFFFFFFFFFFFFFFFFFFFFFFFFFFFFFFFFFFFFFFFFFFFFFFFFFFFFFFFFFFFFF
FFF:FFFFFFFFFFFFFFFFFFFFFFFFFFFFFFFFFFFF:FFFFFFFFFF,FFFFFFFFFFFFFFF
```

```
@A00155:342:HHGFNDSXY:1:1458:32253:11287 2:N:0:GAACCTAG+TCCGCATA
GCACCCCACCATGGTACGTGCTATGCCTTAAATTACCATCGAAGTTGAATTCGGCTCCTGGTGCCTCG
TGATTCGGGAAGAACCTCTTAACGAAACTATCCATTACTCTGAGTTTGCTTTTAGCTTGGGTTCGTGA
+
FFFFFFFFFFF:FF,FFFFFFFFFFFFFFFF:::FFF:FFFF,FFFFFFF:FFFFFFF:FFFFFFFFF
FFF:FFFFFFFFFFFFFFFF:,FFFFFFFFFFFFFFFF:FFFFFF,:FFFFFFFFFFFFFFFFFFFFF
@A00155:342:HHGFNDSXY:1:2211:15881:10582 1:N:0:GAACCTAG+TCCGCATA
GCACCCCACCATGGTACGTGCTATGCCTTAAATTACCATCGAAGTTGAATTCGGCTCCTGGTGCCTCG
TGATTCGGGAAGAACCTCTTAACGAAACTATCCATTACTCTGAGTTTGCTTTTAGCTTGGGTTCGTGA
+
FFFFFFFFFFFFFFFFFFFFFFFFFFFFFFFFFFFFFFFFFFFFFFFFFFFFFFFFFFFFFFFFFFFF
FFFFFFFFFF:FFFFFFFFFFFFFFFFFFFFFFFFFFFFF:FFFFFFF:FF:FFFFFFFFFFFFFFFF
@A00155:342:HHGFNDSXY:1:2342:16857:29966 2:N:0:GAACCTAG+TCCGCATA
GCACCCCACCATGGTACGTGCTATGCCTTAAATTACCATCGAAGTTGAATTCGGCTCCTGGTGCCTCG
TGATTCGGGAAGAACCTCTTAACGAAACTATACATTACTCTGAGTTTGCTTTTAGCTTGGGTTCGTGA
+
FFFFFFFFFFFFFFFFFFFFFFFFFFFFFFFFFFFFFFFFFFFFFFFFFFFFFFFFFFFFFFFFFFFF
FFFFFFFFFFFFFFFFFFFFFFFFFFFFFFFFFF:FFFFFFFFFFF,FFFFFFFFFFFFFFFFFFFFF
@A00155:342:HHGFNDSXY:1:1234:21169:6245 2:N:0:GAACCTAG+TCCGCATA
CACCCCACCATGGTACGTGCTATGCCTTAAATTACCATCGAAGTTGAATTCGGCTCCTGGTGCCTCGT
GATTCGGGAAGAACCTCTTAACGAAACTATCCATTACTCTGAGTTTGCTTTTAGCTTGGGTTCGTGAG
+
FFFFFFFFFFFF:FFFF:FFFFFFFFFFFFFFFFFF,FFFFFFFFFFFFFFFF:FFFFFFFFFFFFFF
FFFFFFFFFFFFFFFFFFFFFFFFFFFF:FFFFF:FFFFFFFFFFFFFFFFFFFFFFFFFFFFFFFFF
@A00155:342:HHGFNDSXY:1:1305:16360:35274 2:N:0:GAACCTAG+TCCGCATA
ACCCCACCATGGTACGTGCTATGCCTTAAATTACCATCGAAGTTGAATTCGGCTCCTGGTGCCTCGTG
ATTCGGGAAGAACCTCTTAACGAAACTATCCATTACTCTGAGTTTGCTTTTAGCTTGGGTTCGTGAGA
+
FFFFF,FFFFFFFFFFFFFFFFFFFFFFFFFFFFFFFFFFFFFFFFF:FFFFFF:FFFFFFFFFFF:F
FFFFFFFFFFFFFFFFFFFFFFFFFFFFFFFFFFFFFFFFFFF,FFF:FFFFFFFFFFFFFFFFFFFF
@A00155:342:HHGFNDSXY:1:1311:25699:15562 2:N:0:GAACCTAG+TCCGCATA
ACCCCACCATGGTACGTGCTATGCCTTAAATTACCATCGAAGTTGAATTCGGCTCCTGGTGCCTCGTG
ATTCGGGAAGAACCTCTTAACGAAACTATCCATTACTCTGAGTTTGCTTTTAGCTTGGGTTCGTGAGA
+
FFFFFFFFFF:F:FFF,FF:FFFFFFFFFF:,FFFFFFF:,FFFFFFFFFFFFFFFFFFFFFFFFFFF
FFFFFFFFF:FFFFFFFFFFFFFFFFFFFFFFFFFFFFFFFFF:FFFFFFFFFFFFFFFFFFFFFFFF
@A00155:342:HHGFNDSXY:1:2609:26286:2080 2:N:0:GAACCTAG+TCCGCATA
ACCCCACCATTGTACGTGCTATGCCTTAAATTACCATCGAAGTTGAATTCGGCTCCTGGTGCCTCGTG
ATTCGGGAAGAACCTCTTAACGAAACTATCCATTACTCTGAGTTTGCTTTTAGCTTGGGTTCGTGAGA
+
FFFFFFFFF:,FFFFFFFFFFFFFF:FFFF:F:FFFFFFF:F:FFFFFFFFFFFF:FFFFFFFFF:FF
FFFFF:FFFFFFFFFFF:FFF:FFF:FFFFFFFFFFFFFFFFFFFFFFFFFFFFFFFFFFFFFF:FFF
@A00155:342:HHGFNDSXY:1:2437:8106:33411 2:N:0:GAACCTAG+TCCGCATA
ACCCCACCATGGTACGTGCTATGCCTTAAATTACCATCGAAGTTGAATTCGGCTCCTGGTGCCTCGTG
ATTCGGGAAGAACCTCTTAACGAAACTATCCATTACTCTGAGTTTGCTTTTAGCTTGGGTTCGTGAGA
+
FFFFFFFFFFFFFFFFFFF,FFFFF,,FFFFF,FF:FFFFFFFFFFFFFFFFFFFFFFFFFFFFFFFF
FFFFFFFFFFFFFFFF:FFFFFFFFFFFFFFFFFFFFFFFFFFFFFFFFFFFFFFFFFFFFFFFFFF
@A00155:342:HHGFNDSXY:1:2555:20265:34773 2:N:0:GAACCTAG+TCCGCATA
ACCCCACCATGGTACGTGCTATGCCTTAAATTACCATCGAAGTTGAATTCGGCTCCTGGTGCCTCGTG
ATTCGGGAAGAACCTCTTAACGAAACTATCCATTACTCTGAGTTTGCTTTTAGCTTGGGTTCGTGAGA
+
FFFFFFFFFFFFFFFFFFF:FFFFFFFFF,FF:FF:FFFF,FFFFFFF,FFFFFFFFFFFFFFFFFFF
FFFFFFFFFFFFFFFFFFFFFFFFFFFFFFFFFFFFFFFFFFFF,FFFFFFFFFFFFFFFFFFFFFFF
```

```
@A00155:342:HHGFNDSXY:1:1267:3477:10113 2:N:0:GAACCTAG+TCCGCATA
CCCCACCATGGTACGTGCTATGCCTTAAATTACCATCGAAGTTGAATTCGGCTCCTGGTGCCTCGTGA
TTCGGGAAGAACCTCTTAACGAAACTATCCATTACTCTGAGTTTGCTTTTAGCTTGGGTTCGTGAGAA
+
FFFFFF:FFFF:FF,FF:FF:,FF,FF:FFFFFFFFFFFFFFFFFFFF,FFFFFFFFFFFFFF:FFFF
FFFFFFFFFFFFFFFFFFFFFFFFFFFFFFFFFFFFFFFFFFFFF,FFFFFFFFFFFFFFFFFFFFFF
@A00155:342:HHGFNDSXY:1:1571:22092:33019 1:N:0:GAACCTAG+TCCGCATA
CCCCACCATGGTACGTGCTATGCCTTAAATTACCATCGAAGTTGAATTCGGCTCCTGGTGCCTCGTGA
TTCGGGAAGAACCTCTTAACGAAACTATCCATTACTCTGAGTTTGCTTTTAGCTTGGGTTCGTGAGAA
+
FFFFFFFFFFFFFFFFFFFFFFFFFFFFFFFFFF:FFFFFFFFFFFFFFFFFFFFFFFFFFFFFFFFF
FFFFFFFFFFFFF:FFFFFFFFFFFF,FF:FFFFF:FFFFFFFFFFFFFFFFFFFFFFFFFFFFFFF:
@A00155:342:HHGFNDSXY:1:1250:21766:32362 1:N:0:GAACCTAG+TCCGCATA
CCCCACCATGGTACGTGCTATGCCTTAAATTACCATCGAAGTTGAATTCGGCTCCTGGTGCCTCGTGA
TTCGGGAAGAACCTCTTAACGAAACTATCCATTACTCTGAGTTTGCTTTTAGCTTGGGTTCGTGAGAA
+
FFFFFFFFFFFFFFFFFFFFFFFFFFFFFFFFFFFFFFFFFFFFFFFFFFFFFFFFFFFFFFFFFFFF
FFFFFFFFFFFFFFFFFFFFFFFFFFFFFF:FFF:FFFFFFFFFFFFFFFFFFFFFFFFFFFFFFFFF
@A00155:342:HHGFNDSXY:1:2566:13847:28855 2:N:0:GAACCTAG+TCCGCATA
CCCACCATGGTACGTGCTATGCCTTAAATTACCATCGAAGTTGAATTCGGCTCCTGGTGCCTCGTGAT
TCGGGAAGAACCTCTTAACGAAACTATCCATTACTCTGAGTTTGCTTTTAGCTTGGGTTCGTGAGAAT
+
FFFFFFFFFFFFFFF:FFFFFF:FFFFFFFFFFFFFFFFFFFFFFFFFFFFFFFFFFFFFFFFFFFFF
FFFFFFFFFFFFFFFFFFFFFFFFFFFFFFFFFF:FFFFFFF:FFFFFFFFFFFFFFFFFFFFFFFFF
@A00155:342:HHGFNDSXY:1:2458:20790:30953 2:N:0:GAACCTAG+TCCGCATA
CCCACCATGGTACGTGCTATGCCTTAAATTACCATCGAAGTTGAATTCGGCTCCTGGTGCCTCGTGAT
TCGGGAAGAACCTCTTAACGAAACTATCCATTACTCTGAGTTTGCTTTTAGCTTGGGTTCGTGAGAAT
+
FFFFFFFFFFFFFFFFFFFFFFFFFFF:FFFFFFFFFFFFFFFFFFFFFFFFFFFFFFFFFFFFFFFF
FFFFFFFFFFFFFFFFFFFFFFFFFFFFFFFFFFFFFFFFFFFFFFFFFFFFFFFFFFFFFFFFFFFF
@A00155:342:HHGFNDSXY:1:1529:1027:11600 2:N:0:GAACCTAG+TCCGCATA
CCCACCATGGTACGTGCTATGCCTTAAATTACCATCGAAGTTGAATTCGGCTCCTGGTGCCTCGTGAT
TCGGGAAGAACCTCTTAACGAAACTATCCATTACTCTGAGTTTGCTTTTAGCTTGGGTTCGTGAGAAT
+
FFF:F,,,FFFFFFFFFFFFFFFF:F:F,,FFF,FFFFFFF:FF:,FFFFFFFFFFF:FFF:FFFF:F
FFFFFFFFFFFFFFFFFFFFFFFF:F,:FFFFFFFFFFFFFF:FF,FFF:FF:FFFFFFFFFFFF:F,
@A00155:342:HHGFNDSXY:1:2175:12111:8155 2:N:0:GAACCTAG+TCCGCATA
CCCACCATGGTACGTGCTATGCCTTAAATTACCATCGAAGTTGAATTCGGCTCCTGGTGCCTCGTGAT
TCGGGAAGAACCTCTTAACGAAACTATCCATTACTCTGAGTTTGCTTTTAGCTTGGGTTCGTGAGAAT
+
FFFFFFFFFFFFFFFFFFFFFFFFFFFFFFFFFFFFFFFFFFFFFFFFFFFFFFFFFFFFFFFFFFFF
FFFFFFFFFFFFFFF:FFFFFFFFFFFFFFFFFFFFFFFFFFFFFFFFFFFFFFFFFFFFFFFFFFFF
@A00155:342:HHGFNDSXY:1:2252:10963:10958 2:N:0:GAACCTAG+TCCGCATA
CCCACCATGGTACGTGCTATGCCTTAAATTACCATCGAAGTTGAATTCGGCTCCTGGTGCCTCGTGAT
TCGGGAAGAACCTCTTAACGAAACTATCCATTACTCTGAGTTTGCTTTTAGCTTGGGTTCGTGAGAAT
+
FFF:FFFFFFFFFF:FFFFFFFFFFFFFFFFFFFFFFFFFFFFFFFFFFFFFFFFFFFFFFFFF:FFF
FFFFFFFFFFFFFFFFFFFFFFFFFFFFFFFFFFFFFFFFFFFFFFFFFFFFFFFFFFFFFFFFFFFF
@A00155:342:HHGFNDSXY:1:1122:19714:20917 2:N:0:GAACCTAG+TCCGCATA
CCCACCATGGTACGTGCTATGCCTTAAATTACCATCGAAGTTGAATTCGGCTCCTGGTGCCTCGTGAT
TCGGGAAGAACCTCTTAACGAAACTATCCATTACTCTGAGTTTGCTTTTAGCTTGGGTTCGTGAGAAT
+
FFFFFFFFFFFFFFFFFFFFFFFFFFFFFFFFFFFFFFFFFFFFFFFFFFFFFFFFFFFFFFFFFFFF
FFFFFFFFFFFFFFFFFFFFFFFFFFFFFFFFFFFFFFFFFFFFFFFFFFFFFFFFFFFFFFFFFFFF
```

```
@A00155:342:HHGFNDSXY:1:1310:17192:34710 2:N:0:GAACCTAG+TCCGCATA
CCCACCATGGTACGTGCTATGCCTTAAATTACCATCGAAGTTGAATTCGGCTCCTGGTGCCTCGTGAT
TCGGGAAGAACCTCTTAACGAAACTATCCATTACTCTGAGTTTGCTTTTAGCTTGGGTTCGTGAGAAT
+
FFFFFFFFFFFFFFFFFFFFFFFFFFFFFFFFFFFFFFFFF:FFFFFFFFFFFFFFFFFFFFFFFFFF
FFFFFF,FFFFFFFFFFFFFFFFFFFFFFFFFFFFFFFFFFFFFF:FFFFFFFFFFFFFFFFFFFFFF
@A00155:342:HHGFNDSXY:1:1308:1633:14340 2:N:0:GAACCTAG+TCCGCATA
CCCACCATGGTACGTGCTATGCCTTAAATTACCATCGAAGTTGAATTCGGCTCCTGGTGCCTCGTGAT
TCGGGAAGAACCTCTTAACGAAACTATCCATTACTCTGAGTTTGCTTTTAGCTTGGGTTCGTGAGAAT
+
FFFFFFFF:FFFF:,FFFF:FFF,FFF,F:,FF:FFFF,FFFFFFFFFFFFFFFFFFFFFF:FFFFFF
FFFFFFFFFFFF:FFFF,FFFFFFFFFFFFFFFFFFFFFFF:FFF,FFFFFFFFFFF:FFFFFFFFFF
@A00155:342:HHGFNDSXY:1:2429:12201:15107 2:N:0:GAACCTAG+TCCGCATA
CCCACCATGGTACGTGCTATGCCTTAAATTACCATCGAAGTTGAATTCGGCTCCTGGTGCCTCGTGAT
TCGGGAAGAACCTCTTAACGAAACTATCCATTACTCTGAGTTTGCTTTTAGCTTGGGTTCGTGAGAAT
+
FFFFFFFFFFFFFFFFF:FFFFF,FFFFFFFFFFFFFFFFFF:FFFFFFFFFFFFFFFFFFFFFFFFF
FFFFFFFFFFFFFFFF:FFFFFFFFFFFF:FFFFFFFFFFFFFFFFFFFFFFFFFFFFFFFFFFFFFF
@A00155:342:HHGFNDSXY:1:1240:1832:9424 2:N:0:GAACCTAG+TCCGCATA
CCCACCATGGTACGTGCTATGCCTTAAATTACCATCGAAGTTGAATTCGGCTCCTGGTGCCTCGTGAT
TCGGGAAGAACCTCTTAACGAAACTATCCATTACTCTGAGTTTGCTTTTAGCTTGGGTTCGTGAGAAT
+
FFFFF::,FFFFFFFFFFFFFFFFFFFFF,FFFFFFFFFFFFFF:FFFFFFFFFFFFFFFFFFFFFFF
FFFFFFFFFFFFFFFF,FFFFFFFFFFFFFFFFF:FFFFFFFFFFFFFFFFFFFFFFFFFFFFFFFFF
@A00155:342:HHGFNDSXY:1:2363:11641:33833 2:N:0:GAACCTAG+TCCGCATA
CCCACCATGGTACGTGCTATGCCTTAAATTACCATCGAAGTTGAATTCGGCTCCTGGTGCCTCGTGAT
TCGGGAAGAACCTCTTAACGAAACTATCCATTACTCTGAGTTTGCTTTTAGCTTGGGTTCGTGAGAAT
+
FFFFFFF:,,FFFFFF:FFFFFFFFF:F,,,F:FFFFF,FFFFFFFFFFFFFFFFFFFFFFFFFFFFF
FFFFFFFFFFFFFF:FFFFFFFFFFFFFFFFFFFFFFFFFFFFFFFF:FFFFFFFFF:FFFFFFFFFF
@A00155:342:HHGFNDSXY:1:2642:2193:3787 2:N:0:GAACCTAG+TCCGCATA
CCCACCATGGTACGAGCTATGCCTTAAATTACCATCGAAGTTGAATTCGGCTCCTGGTGCCTCGTGAT
TCGGGAAGAACCTCTTAACGAAACTATCCATTACTCTGAGTTTGCTTTTAGCTTGGGTTCGTGAGAAT
+
FFFFFFFFF,FF:F,,FFFFFFF,:FFF:FFFFFFF:FF:FFFFFF:FF:FFFF,F:F:,FFF::FF,
:F,:,FFFFFFFFF,:FFF:FFFF:FFF:FF:FF,FFFFF:FFFF,:F:F:F:FF::FF:F:FF:FFF
@A00155:342:HHGFNDSXY:1:2120:3179:13542 1:N:0:GAACCTAG+TCCGCATA
CCACCATGGTACGTGCTATGCCTTAAATTACCATCGAAGTTGAATTCGGCTCCTGGTGCCTCGTGATT
CGGGAAGAACCTCTTAACGAAACTATCCATTACTCTGAGTTTGCTTTTAGCTTGGGTTCGTGAG
+
FFFFFFFFFFFFFFFFFFFFFFFFFFFFFFFFFFFFFFFFFFFFFFFFFFFFFFFFFFFFFFFFFFFF
FFFFFFFFFFFFFFFFFFFF:FFFFFFFFFFFFFFFFFF:FFFFFF,FFFF,FFFFFFFFFFFF
@A00155:342:HHGFNDSXY:1:1178:26304:4805 1:N:0:GAACCTAG+TCCGCATA
CCACCATGGTACGTGCTATGCCTTAAATTACCATCGAAGTTGAATTCGGCTCCTGGTGCCTCGTGATT
CGGGAAGAACCTCTTAACGAAACTATCCATTACTCTGAGTTTGCTTTTAGCTTGGGTTCGTGAG
+
FFFFFFFFFFFFFFFFFFFFFFFFFFFFFFFFFFFFFFFFFFFFFFFFFFFFFFFFFFFFFFFFFFFF
FFFFFFFFFFFFFFFFFFFFFFFFFFFFFFFFFFFFFFFFFFFFFFFFFFFFFFFFFFFFFFFF
@A00155:342:HHGFNDSXY:1:1461:5249:20541 1:N:0:GAACCTAG+TCCGCATA
CCACCATGGTACGTGCTATGCCTTAAATTACCATCGAAGTTGAATTCGGCTCCTGGTGCCTCGTGATT
CGGGAAGAACCTCTTAACGAAACTATCCATTACTCTGAGTTTGCTTTTAGCTTGGGTTCGTGAG
+
FFFFFFFFFFFFFFFFFFFFFFFFFF:F:FFFF:FFFFFF:FFFF:FFFFFFFFFFFFFFFFFFFFFF
FFFFFFFFFFFFFFFFFFFFFFFFFFFFF:FFFFFFFFFFFFFFFF:FFFFFFFFFFFFFFFFF
```

@A00155:342:HHGFNDSXY:1:1278:7600:8296 1:N:0:GAACCTAG+TCCGCATA
CCACCATGGTACGTGCTATGCCTTAAATTACCATCGAAGTTGAATTCGGCTCCTGGTGCCTCGTGATT
CGGGAAGAACCTCTTAACGAAACTATCCATTACTCTGAGTTTGCTTTTAGCTTGGGTTCGTGAG
+
FFFFFFFFFFFFFFFFFFFFFFF:FFFFFFFFFFFFFFFFFFFFFFFFFFFFFFFFFFFFFFFFFFFF
FFFFFFFFFFFFFFFF:FFFFFFFFFFFFFF:FFFFFFFFFFFFFF,FFFF:FFFFFF:FFFFF
@A00155:342:HHGFNDSXY:1:1223:17173:19053 2:N:0:GAACCTAG+TCCGCATA
CCACCATGGTACGTGCTATGCCTTAAATTACCATCGAAGTTGAATTCGGCTCCTGGTGCCTCGTGATT
CGGGAAGAACCTCTTAACGAAACTATCCATTACTCTGAGTTTGCTTTTAGCTTGGGTTCGTGAGAATT
+
FFFFFFFFFFFFFFFFFFFFFFFFFFFFFFFFFFFFFFFFFFFFFFFFFFFFFFFFFFFFFFFFFFFF
FFFFFFFFFFFFFFFFFFFFFFFFFFFFFFFFFFFFFFFFFFFFFFFFFFFFFFFFFFFFFFFFFFFF
@A00155:342:HHGFNDSXY:1:2161:8142:18443 2:N:0:GAACCTAG+TCCGCATA
CCACCATGGTACGTGCTATGCCTTAAATTACCATCGAAGTTGAATTCGGCTCCTGGTGCCTCGTGATT
CGGGAAGAACCTCTTAACGAAACTATCCATTACTCTGAGTTTGCTTTTAGCTTGGGTTCGTGAGAATT
+
FFFFFFFFFFF,FFFFFFFF,FFFFFF,FFFF:FFFFFFFFFFFFFFFFFFFFFFFFFFFFFFFFFFF
FFFFFFFFFFFFFFFFFFFFFFFFFFFFFFFFFFFFFFFFFFFFFFFFFFFFFFFFFFFFFFFFFFFF
@A00155:342:HHGFNDSXY:1:2161:8214:18380 2:N:0:GAACCTAG+TCCGCATA
CCACCATGGTACGTGCTATGCCTTAAATTACCATCGAAGTTGAATTCGGCTCCTGGTGCCTCGTGATT
CGGGAAGAACCTCTTAACGAAACTATCCATTACTCTGAGTTTGCTTTTAGCTTGGGTTCGTGAGAATT
+
FFF:FFFFFFFFFFFFFFFFFF:FFFFFFFFFFFF:FFFFFFFFFFFFFFFFFFFFFFFFFFFFFFFF
FFFFFFFFFFFFFFFFFFFFFFFFFFFFFFFFFFFFFFFFFFFFFFFFFFFFFFFFFFFFFFFFFFFF
@A00155:342:HHGFNDSXY:1:1230:13096:4633 2:N:0:GAACCTAG+TCCGCATA
CCACCATGGTACGTGCTATGCCTTAAATTACCATCGAAGTTGAATTCGGCTCCTGGTGCCTCGTGATT
CGGGAAGAACCTCTTAACGAAACTATCCATTACTCTGAGTTTGCTTTTAGCTTGGGTTCGTGAGAATT
+
FFFFFF:FFFFFFFFFFFFF:FFFFFFFFFFFFFFFFFFFFFFFFFFFFFFFFFFFFFFFFFFFFFFF
FFFFFFFFFFFFFFFFFFFFFFFFFFFFFFF:F:FFFFFFFFFFFFFFFFFFFFFFFFFFFFFFFFFF
@A00155:342:HHGFNDSXY:1:2316:19831:33990 2:N:0:GAACCTAG+TCCGCATA
CCACCATGGTACGTGCTATGCCTTAAATTACCATCGAAGTTGAATTCGGCTCCTGGTGCCTCGTGATT
CGGGAAGAACCTCTTAACGAAACTATCCATTACTCTGAGTTTGCTTTTAGCTTGGGTTCGTGAGAATT
+
FFFFFFFFFFFFFFFFFFFFFFFFFFFFF:FFFFFFFFFFFFFFFFFFFFFFFFFFFFFFFFFFFF:F
FFFFFFFFFFFFF,FFFFFFFFFFFFFFFFFFFFFFFFFF:FFFFFFFFFFFFFFFFFFFFFFFFFFF
@A00155:342:HHGFNDSXY:1:2243:30617:26396 2:N:0:GAACCTAG+TCCGCATA
CACCATGGTACGTGCTATGCCTTAAATTACCATCGAAGTTGAATTCGGCTCCTGGTGCCTCGTGATTC
GGGAAGAACCTCTTAACGAAACTATCCATTACTCTGAGTTTGCTTTTAGCTTGGGTTCGTGAGA
+
FFFFFFFFFFFFFFFFFFFFFFF:FFF:FFFFFFFFFFFFFFFFFFFFFFFFFFFFFFFFFFFFFFFF
FFFFFFFFFFFFFF:FFFFFFFFFFFFFFFFFFFFFFFFFFFFFFFFFFFFFFFFFFFFFFFFF
@A00155:342:HHGFNDSXY:1:1253:27688:34287 2:N:0:GAACCTAG+TCCGCATA
CACCATGGTACGTGCTATGCCTTAAATTACCATCGAAGTTGAATTCGGCTCCTGGTGCCTCGTGATTC
GGGAAGAACCTCTTAACGAAACTATCCATTACTCTGAGTTTGCTTTTAGCTTGGGTTCGTGAGAATT
+
F:FFFFFFFFFFFFFFFFFFF,FFFFFF:FFFFFF:FFFFFFFFFFFFFFFFFFFFFFFFFFFFFFFF
FFFFFFFFFFFF:FFFFFFFFFFFFFFFFFFFFFFFFFFFFFF:FFFFFFFFFFFFFFFFFFFFFFF
@A00155:342:HHGFNDSXY:1:2166:25654:14951 2:N:0:GAACCTAG+TCCGCATA
CACCATGGTACGTGCTATGCCTTAAATTACCATCGAAGTTGAATTCGGCTCCTGGTGCCTCGTGATTC
GGGAAGAACCTCTTAACGAAACTATCCATTACTCTGAGTTTGCTTTTAGCTTGGGTTCGTGAGAATT
+
FFFFF:FF:FFFFFFFFFF:FFFFFFFFFFF,FFFFFFFFFFFFFFFFFFFFFFFFFFFFFFFFFFFF
FFFFFFFFFFFFFFFFFFFFFFFFFFF:FFFFFFFFFFFFFFFFFFFFFFFFFFFFFFFFFFFFFFF

```
@A00155:342:HHGFNDSXY:1:1253:27642:34147 2:N:0:GAACCTAG+TCCGCATA
CACCATGGTACGTGCTATGCCTTAAATTACCATCGAAGTTGAATTCGGCTCCTGGTGCCTCGTGATTC
GGGAAGAACCTCTTAACGAAACTATCCATTACTCTGAGTTTGCTTTTAGCTTGGGTTCGTGAGAATT
+
FFFF,FFF:FFFFFFF,FFF:FFFFFFFFFFFFFFF,FFFFFFFFFF:FFFFFFFFFFFFFFFFFFFF
FFFFFFFFFFFFFFFFFFFFFFFFFFFFFFFFFFFFFF:FFFFFFFFFF:FFFFFFFFFFFFFFFFF
@A00155:342:HHGFNDSXY:1:2114:11397:11146 2:N:0:GAACCTAG+TCCGCATA
CACCATGGTACGTGCTATGCCTTAAATTACCATCGAAGTTGAATTCGGCTCCTGGTGCCTCGTGATTC
GGGAAGAACCTCTTAACGAAACTATCCATTACTCTGAGTTTGCTTTTAGCTTGGGTTCGTGAGAATT
+
FF:F::FFF:FFFF:FFFFFFFFFFF:FFFFFFFFFFFFFFFFFFFFFFFFFFFFFFFFFFFFFFFFF
FFF:FFFF:FFFFFFFFFFFFFFFFFFFFFFFFFFFFFFFFFFFFFFFFFFFFFFFFFFFFFFFFFF
@A00155:342:HHGFNDSXY:1:2340:21332:34554 2:N:0:GAACCTAG+TCCGCATA
CACCATGGTACGTGCTATGCCTTAAATTACCATCGAAGTTGAATTCGGCTCCTGGTGCCTCGTGATTC
GGGAAGAACCTCTTAACGAAACTATCCATTACTCTGAGTTTGCTTTTAGCTTGGGTTCGTGAGAATTG
+
FF,F:FFFFF:FFFFFFFFFFFF:FFFFFF::F:FFFFFFFF:FFF:F,FF:FFFFFFFFFFF,:FFF
F:FF:FFFF:::F:FFFF,FF,FFFF:FFFFFFFFF,FFFFFFFFFFFFFFFFFFFFFFFFFFFFFFF
@A00155:342:HHGFNDSXY:1:1509:16993:25316 2:N:0:GAACCTAG+TCCGCATA
CACCATGGTACGTGCTATGCCTTAAATTACCATCGAAGTTGAATTCGGCTCCTGGTGCCTCGTGATTC
GGGAAGAACCTCTTAACGAAACTATCCATTACTCTGAGTTTGCTTTTAGCTTGGGTTCGTGAGAATTG
+
FFF:FFFF:FFFF:FFFF:,FFFF,:FF:FFFFFFFF,FFFFFFFFF,FFFFFFFFFFFFF:FFFFFF
FFFFFF:F,:FFFF,FFFFF:FFFF:FFF:FFFFFFFF:FFFFFFFF:FFFFFFFFFFFFFFFF:FFF
@A00155:342:HHGFNDSXY:1:1635:7437:23046 2:N:0:GAACCTAG+TCCGCATA
CACCATGGTACGTGCTATGCCTTAAATTACCATCGAAGTTGAATTCGGCTCCTGGTGCCTCGTGATTC
GGGAAGAACCTCTTAACGAAACTATCCATTACTCTGAGTTTGCTTTTAGCTTGGGTTCGTGAGAATTG
+
FFFFFFFFFFFFFFFFFFFFFFFFFFFFF:FFFFFFFFFFFFFFFFFFF,FFFFFFFFFFFFFFFFFF
FF:FFFFFFFFF:FFFFFFFFFFFFFFFFFFFFFFFFFFFFFFFFFFFFF:FFFFFFFFFFFFFFFFF
@A00155:342:HHGFNDSXY:1:1255:17833:12618 2:N:0:GAACCTAG+TCCGCATA
CACCATGGTACGTGCTATGCCTTAAATTACCATCGAAGTTGAATTCGGCTCCTGGTGCCTCGTGATTC
GGGAAGAACCTCTTAACGAAACTATCCATTACTCTGAGTTTGCTTTTAGCTTGGGTTCGTGAGAATTG
+
FFFFFFFFFFFFFF:F:FFFFFFFFFFFFF:FFFFFF:FFF,FFFFFFFFFFFFFFFFFF:FFF:FFF
FFFFFFFFFFFFFF:FFFFFFFFFFF:FFF:FFFFFFF:FFFFFFFFFFFFFFFFFFFFFFFF:FFFF
@A00155:342:HHGFNDSXY:1:1121:17888:22545 2:N:0:GAACCTAG+TCCGCATA
ACCATGGTACGTGCTATGCCTTAAATTACCATCGAAGTTGAATTCGGCTCCTGGTGCCTCGTGATTCG
GGAAGAACCTCTTAACGAAACTATCCATTACTCTGAGTTTGCTTTTAGCTTGGGTTCGTGAGAATT
+
FFF:FFF,FFF:FFFFFFFFFFFFFFFFFFFFFFFFFFFFFFFFFFFFFFFFFFFFFFFFFFFFFFFF
FFFFFFFFFFFFF:FFFFFFFFFFFFFFFFFFFFFFFF:FFFFFFFFFFFFFFFFFFFFFFFFFFF
@A00155:342:HHGFNDSXY:1:2270:24921:3505 2:N:0:GAACCTAG+TCCGCATA
ACCATGGTACGTGCTATGCCTTAAATTACCATCGAAGTTGAATTCGGCTCCTGGTGCCTCGTGATTCG
GGAAGAACCTCTTAACGAAACTATCCATTACTCTGAGTTTGCTTTTAGCTTGGGTTCGTGAGAATTGA
+
FFFFFFFFFFFFFFFFFFFFFFFFFFF:FFFFFFFFFFFFFF:FFFFFFFFFFF:FFFFF:FFFFFFF
FFFFFFFFFFFFFFFFFFFFFFFFFFFFFFFFFFFFFFFFFFFFFFFFFFFFFFFFFFFFFFFFFFFF
@A00155:342:HHGFNDSXY:1:1467:28339:6449 1:N:0:GAACCTAG+TCCGCATA
CCATGGTACGTGCTATGCCTTAAATTACCATCGAAGTTGAATTCGGCTCCTGGTGCCTCGTGATTCGG
GAAGAACCTCTTAACGAAACTATCCATTACTCTGAGTTTGCTTTTAGCTTGGGTTCGTG
+
FFFFFFFFFFFFFFFFFFFFFFFFFFFFFFFF:FFFFFFFFFFFFFFFFFFFFFFFFFFFFFFFFFFF
FFFFFFFFFFFFFFFFFFFFFFFFF:FF:FFF:FFFFFFFFF:FFFFF,FFFFFFFFFF
```

```
@A00155:342:HHGFNDSXY:1:1223:17173:19053 1:N:0:GAACCTAG+TCCGCATA
CCATGGTACGTGCTATGCCTTAAATTACCATCGAAGTTGAATTCGGCTCCTGGTGCCTCGTGATTCGG
GAAGAACCTCTTAACGAAACTATCCATTACTCTGAGTTTGCTTTTAGCTTGGGTTCGTGAGAATTGAA
+
FFFFFFFFFFFFFFFFFFFFFFFFFFFFFFFFFFFFFFFFFFFFFFFFFFFFFFFFFFFFFFFFFFFF
FFFFFFFFFFFFFFFFFFFFFFFFFFFF:FFF:FFFFFFFFFFFFFFFFFFFF:FFFFFFFFFFFFFF
@A00155:342:HHGFNDSXY:1:2247:21260:29637 1:N:0:GAACCTAG+TCCGCATA
CCATGGTACGTGCTATGCCTTAAATTACCATCGAAGTTGAATTCGGCTCCTGGTGCCTCGTGATTCGG
GAAGAACCTCTTAACGAAACTATCCATTACTCTGAGTTTGCTTTTAGCTTGGGTTCGTGAGAATTGAA
+
FFFFFFFFFFFFFFFFFF:FFFFFFFFFFFFFFFFFFFFFFFFFFFFFFFFFFFFFFFFFFFFFFFFF
FFFFFFFFFFFFFFFF:FFFFFFFFFFF:FFFFFFFFFFFFFFFFFFFFFFFFFFFFFF:FFFFFFFF
@A00155:342:HHGFNDSXY:1:1509:11505:27524 2:N:0:GAACCTAG+TCCGCATA
CATGGTACGTGCTATGCCTTAAATTACCATCGAAGTTGAATTCGGCTCCTGGTGCCTCGTGATTCGGG
AAGAACCTCTTAACGAAACTATCCATTACTCTGAGTTTGCTTTTAGCTTGGGTTCGTGAGAATTGAAA
+
FFFFFFFFF:::FFFFFFFFF:F::FFF,FFFFFFFFFFFFFFFFFFFFFFFFFFFFFFFFFFFFF:F
FFFFFFFFFFFFFFFFFFFFFF:FFFFFFFFFFFFFFFFFFFFFFFFFFFFFFFFFFFFFFFFFFFFF
@A00155:342:HHGFNDSXY:1:1423:31412:36605 2:N:0:GAACCTAG+TCCGCATA
CATGGTACGTGCTATGCCTTAAATTACCATCGAAGTTGAATTCGGCTCCTGGTGCCTCGTGATTCGGG
AAGAACCTCTTAACGAAACTATCCATTACTCTGAGTTTGCTTTTAGCTTGGGTTCGTGAGAATTGAAA
+
FF:FFFFFFFF:FFFFFFFFFFFFFFFF,FFFFFFFFFFFFFFFFFFFFFFF:FFFFFFFFFFFFFFF
FFFFFFFFFFFFFFFFFFFFFFFFFFFFFFFFFFFFFFFFFFFFFFFFFFFFFFFFFFFFFFFFFFFF
@A00155:342:HHGFNDSXY:1:2415:31928:36558 2:N:0:GAACCTAG+TCCGCATA
CATGGTACGTGCTATGCCTTAAATTACCATCGAAGTTGAATTCGGCTCCTGGTGCCTCGTGATTCGGG
AAGAACCTCTTAACGAAACTATCCATTACTCTGAGTTTGCTTTTAGCTTGGGTTCGTGAGAATTGAAA
+
FFFFF:FFFFFF:FFF:F:FF:FFFFFF:FFFFFFFFFFFFFFFFFFFFFFFFFFFFFFFFFFFFFFF
FFFFFFF:FF:FF:FFFFFFFFFFFFFFFFFFFFFF:FFFFFFFFFFFFFFFFFFFFFFFFFFFFFFF
@A00155:342:HHGFNDSXY:1:2437:2944:28729 2:N:0:GAACCTAG+TCCGCATA
CATGGTACGTGCTATGCCTTAAATTACCATCGAAGTTGAATTCGGCTCCTGGTGCCTCGTGATTCGGG
AAGAACCTCTTAACGAAACTATCCATTACTCTGAGTTTGCTTTTAGCTTGGGTTCGTGAGAATTGAAA
+
F:FF:FFF:F:FFFFFFFFFFF:FFFFFFF,FFFFFFFFFFFF,FFFF,FF,FFFFFFFFFFFFFFFF
FFFFFFFFF:FFFF:FFFFFFFF:FFFFFFF::FFFFFFFFFFFFFFFFFFFFFFFFFFFFFFF:FFF
@A00155:342:HHGFNDSXY:1:1366:12048:13557 2:N:0:GAACCTAG+TCCGCATA
CATGGTACGTGCTATGCCTTAAATTACCATCGAAGTTGAATTCGGCTCCTGGTGCCTCGTGATTCGGG
AAGAACCTCTTAACGAAACTATCCATTACTCTGAGTTTGCTTTTAGCTTGGGTTCGTGAGAATTGAAA
+
FFFFFFFFFFFFFFFFFFFFFFFFFF:FFFFFFFFFFFF:FFFFFFFFFFFFFFFFFFFFF:FFFFFF
:FFFFFFFFFFFFFFFFFFFFFF:FFFFFFFFFFFFFFFFFFFF:F:FFFFFF:FF:FFF:FFFFFFF
@A00155:342:HHGFNDSXY:1:1557:13711:5165 2:N:0:GAACCTAG+TCCGCATA
CATGGTACGTGCTATGCCTTAAATTACCATCGAAGTTGAATTCGGCTCCTGGTGCCTCGTGATTCGGG
AAGAACCTCTTAACGAAACTATCCATTACTCTGAGTTTGCTTTTAGCTTGGGTTCGTGAGAATTGAAA
+
FF:FF,FFFFFFFFFFFFF:FFFFFFFFFFFFFFFFFFFFFFFFFFFFFFFF:FFFFFFFFFFFFFFF
FFFFFFFFFFFFFFFFFFFFFFFFFFFFFFFFFFFFFFFFFFFFFFFFFFFFFFFFFFFFFFFFFFFF
@A00155:342:HHGFNDSXY:1:2644:18132:35587 2:N:0:GAACCTAG+TCCGCATA
CATGGTACGTGCTATGCCTTAAATTACCATCGAAGTTGAATTCGGCTCCTGGTGCCTCGTGATTCGGG
AAGAACCTCTTAACGAAACTATCCATTACTCTGAGTTTGCTTTTAGCTTGGGTTCGTGAGAATTGAAA
+
FFFFFFFFF:FFFFFFFFFFFF,FFFFF:FFFFFFFFFFFFFFFFFFFFFFFFFFFFFFFFFFFFFFF
FFFFFFFFFFFFFFFFFFFFFFFFFFFFFFFFFFFF:FFFFFFFFFFFFFFFFFFFFFFFFFFFFFFF
```

```
@A00155:342:HHGFNDSXY:1:2263:17996:8923 1:N:0:GAACCTAG+TCCGCATA
CATGGTACGTGCTATGCCTTAAATTACCATCGAAGTTGAATTCGGCTCCTGGTGCCTCGTGATTCGGG
AAGAACCTCTTAACGAAACTATCCATTACTCTGAGTTTGCTTTTAGCTTGGGTTCGTGAGAATTGAAA
+
FFFFFFFFFFFF:FFFFFFFFFF:FFFF,FFFFFFFFFFF:FFFFFFFFFFFFFFFFFFFFF:FFFFF
F:FFFFFFFFFFFFF:FFFFFFFFFFFFFFFFF:F:FFFFF:F:FFFFFFFFFFFFFFFFF:FFFFFF
@A00155:342:HHGFNDSXY:1:2502:27923:13933 2:N:0:GAACCTAG+TCCGCATA
CATGGTACGTGCTATGCCTTAAATTACCATCGAAGTTGAATTCGGCTCCTGGTGCCTCGTGATTCGGG
AAGAACCTCTTAACGAAACTATCCATTACTCTGAGTTTGCTTTTAGCTTGGGTTCGTGAGAATTGAAA
+
FFFFFFFFFFFFF:FFFFFFF:FFFFFFFF:F,FFFFFF:FFFFFFFFFFFFFFFFFFFFFFFF:FFF
FFFFFFFFFFFF:FFFFFF,FFFFFFFFFFFFFFFFFFFF:FF,FFFFFFFFFFFFFFFFFFFFFFF:
@A00155:342:HHGFNDSXY:1:1210:14977:29528 2:N:0:GAACCTAG+TCCGCATA
CATGGTACGTGCTATGCCTTAAATTACCATCGAAGTTGAATTCGGCTCCTGGTGCCTCGTGATTCGGG
AAGAACCTCTTAACGAAACTATCCATTACTCTGAGTTTGCTTTTAGCTTGGGTTCGTGAGAATTGAAA
+
F:FFFFFFFFFFFFFFFFFFFFFFFFFFFFFF,FFFFFFFFFFF:FFFFFFFFFFFFFFFFFFFFFFF
FFFFFFF:FF:F:FFFFFFFFFFFFFFFFFFFFFFFFFFFFFFFFFFFFFFFFFFFFFFFFFFFFFF
@A00155:342:HHGFNDSXY:1:1178:25446:1063 2:N:0:GAACCTAG+TCCGCATA
CATGGTACGTGCTATGCCTTAAATTACCATCGAAGTTGAATTCGGCTCCTGGTGCCTCGTGATTCGGG
AAGAACCTCTTAACGAAACTATCCATTACTCTGAGTTTGCTTTTAGCTTGGGTTCGTGAGAATTGAAA
+
F,FFFF:FFFFFFFF,FFFFFFFF:::FF,FFFFFFFFFFFFFFFFFFFFFFFFFFFFFFFFFF:FFF
FFFFF:FFFFF,FF:FFFFFFFFFFFF:FFFFFFFFFFFFFFFFFFFFFFFFFFFFFFFFFFFFFFFF
@A00155:342:HHGFNDSXY:1:1604:2799:4460 2:N:0:GAACCTAG+TCCGCATA
CATGGTACGTGCTATGCCTTAAATTACCATCGAAGTTGAATTCGGCTCCTGGTGCCTCGTGATTCGGG
AAGAACCTCTTAACGAAACTATCCATTACTCTGAGTTTGCTTTTAGCTTGGGTTCGTGAGAATTGAAA
+
FFF:FFFFFFFFFFFFFFFFFF:FF,:FFFFFFFFFFFFFFFFFF,FFFFFFFFFFFFFFFFFFFF:F
FFFFFF:FFF:FFFFFFFFFFFF,FFFFFFFFFFFFFFFFFFFFFF:FFFFFFFFFFFFFFFFFFFFF
@A00155:342:HHGFNDSXY:1:2263:17996:8923 2:N:0:GAACCTAG+TCCGCATA
CATGGTACGTGCTATGCCTTAAATTACCATCGAAGTTGAATTCGGCTCCTGGTGCCTCGTGATTCGGG
AAGAACCTCTTAACGAAACTATCCATTACTCTGAGTTTGCTTTTAGCTTGGGTTCGTGAGAATTGAAA
+
FFFFFFFFFFFFFFFFFFFFF:FFFFFFFFFFFFFFFFFFFFFFFFFFFFFFFFFFFFFFFFFFFFFF
FFFFFFFFFFFFFFFFFFFFFFFFFFFFFFFFFFFFFFFFFFFFFFFFFFFFFFFFFFFFFFFFFFFF
@A00155:342:HHGFNDSXY:1:1528:30915:10191 2:N:0:GAACCTAG+TCCGCATA
CATGGTACGTGCTATGCCTTAAATTACCATCGAAGTTGAATTCGGCTCCTGGTGCCTCGTGATTCGGG
AAGAACCTCTTAACGAAACTATCCATTACTCTGAGTTTGCTTTTAGCTTGGGTTCGTGAGAATTGAAA
+
FFFFFFFFFFFFFFF:,F,FFFFFFFFFFFFFFFFFF:FFFFFFFFF,FFFFFFFFFFFFFFFFFFFF
FFFFFFFFFFFFFF,FFFFFFFFFFFFFF:FFFFFFFFFF:FFFFFFFFFFFFFFFFFFFFFFFFFFF
@A00155:342:HHGFNDSXY:1:2403:23782:36573 2:N:0:GAACCTAG+TCCGCATA
CATGGTACGTGCTATGCCTTAAATTACCATCGAAGTTGAATTCGGCTCCTGGTGCCTCGTGATTCGGG
AAGAACCTCTTAACGAAACTATCCATTACTCTGAGTTTGCTTTTAGCTTGGGTTCGTGAGAATTGAAA
+
FF,FFFFFFFFFFFFFFFFFF:FF:FFFFFFFFFFFFFF:F,FFFFFFFFFFFFFFFFFFFFFFFFFF
FFFFFFFFFF,F:FFF:FFF:FFFFFFFFFFFFFFFFFFF:FFFFFFFFFFFFFFFFFFFFFFFFFFF
@A00155:342:HHGFNDSXY:1:2517:14850:20415 2:N:0:GAACCTAG+TCCGCATA
ATGGTACGTGCTATGCCTTAAATTACCATCGAAGTTGAATTCGGCTCCTGGTGCCTCGTGATTCGGGA
AGAACCTCTTAACGAAACTATCCATTACTCTGAGTTTGCTTTTAGCTTGGGTTCGTGAGAATTGAAAC
+
FFFFFFFFFFFFFFFFFFFFFFFFFFFFFFFFFFFFFFFFFFFF:FFFFFFFFFFFFFFFFFFFFFFF
FFFFFFFFFF:FFFFFFFFFFFFFFFFFFFFFFFFFFFFFFFFFFFFFFFFFFFFFFFFFFFFFFFFF
```

```
@A00155:342:HHGFNDSXY:1:2408:3278:5979 2:N:0:GAACCTAG+TCCGCATA
TGGTACGTGCTATGCCTTAAATTACCATCGAAGTTGAATTCGGCTCCTGGTGCCTCGTGATTCGGGAA
GAACCTCTTAACGAAACTATCCATTACTCTGAGTTTGCTTTTAGCTTGGGTTCGTGAGAATTGAAAC
+
:FFF,:F,FF:FFFFF,FFFFF::FFF:FFFFFFFFFFFFFFFFFF:FFFFFFF:FF:FFFFFFFFFF
FFFFFFFFF:F:FFFFFFFFFF:FFFFFF:FFFF:FFFFFFFFFFFFFFFFFFFFFFFFFFFFFFFF
@A00155:342:HHGFNDSXY:1:2330:13602:28119 2:N:0:GAACCTAG+TCCGCATA
TGGTACGTGCTATGCCTTAAATTACCATCGAAGTTGAATTCGGCTCCTGGTGCCTCGTGATTCGGGAA
GAACCTCTTAACGAAACTATCCATTACTCTGAGTTTGCTTTTAGCTTGGGTTCGTGAGAATTGAAACA
+
FFFFFFFFFFFFFFFFFF:FFF:FFFFFFFFFFFFFFF:FFFFFFFFFFFFFFFFFFFFFFFFFFFFF
FFFFFFFFFFFFFFFFFFFFFFFFFFFFFFFFFFFFFFFFFFFFFFFFFFFFFFFFFFFFFFFFFFFF
@A00155:342:HHGFNDSXY:1:2247:21260:29637 2:N:0:GAACCTAG+TCCGCATA
TGGTACGTGCTATGCCTTAAATTACCATCGAAGTTGAATTCGGCTCCTGGTGCCTCGTGATTCGGGAA
GAACCTCTTAACGAAACTATCCATTACTCTGAGTTTGCTTTTAGCTTGGGTTCGTGAGAATTGAAACA
+
F:FFFFF,FFFFFFFFFFFF:FFFFFFFFFFFFFFFFFFFFFFFFFFFFFFFFFFFFFFFFFFFFFFF
FFFFFFFF:FFFFFFFFFFFFFFFFFFFFFFFFFFFFFFFFFFFFFFFFFFFFFFFFFFFFFFFFFF
@A00155:342:HHGFNDSXY:1:2225:2627:25426 2:N:0:GAACCTAG+TCCGCATA
TGGTACGTGCTATGCCTTAAATTACCATCGAAGTTGAATTCGGCTCCTGGTGCCTCGTGATTCGGGAA
GAACCTCTTAACGAAACTATCCATTACTCTGAGTTTGCTTTTAGCTTGGGTTCGTGAGAATTGAAACA
+
FFFFFFFFFFFFFFFFFFFFFFFFFFF:FFFFFFFFFFFFFFFFFFFFFFFFFFFFFFFFFFFFFFFF
FFFFFFFFF:,FFFFFF:FFFFFFFFFFFFFFFF:FFFF:FFFFFFFFFFFFFFFFFFFFFFFFFFFF
@A00155:342:HHGFNDSXY:1:2211:15881:10582 2:N:0:GAACCTAG+TCCGCATA
TGGTACGTGCTATGCCTTAAATTACCATCGAAGTTGAATTCGGCTCCTGGTGCCTCGTGATTCGGGAA
GAACCTCTTAACGAAACTATCCATTACTCTGAGTTTGCTTTTAGCTTGGGTTCGTGAGAATTGAAACA
+
F:FFFFF:FFFFFFFFFFFFFFFFFFFFFFFFFFFFF,FFFFFFFFFFFFFFFFFFFFFFFFFFFFFF
FFFFFFFFFF:FFFFFFFFFFFFFFFFFFFFFFFFFFFFFFFFFFFFFFFFFFFFFFFFFFFFFFFFF
@A00155:342:HHGFNDSXY:1:2138:21386:8343 2:N:0:GAACCTAG+TCCGCATA
TGGTACGTGCTATGCCTTAAATTACCATCGAAGTTGAATTCGGCTCCTGGTGCCTCGTGATTCGGGAA
GAACCTCTTAACGAAACTATCCATTACTCTGAGTTTGCTTTTAGCTTGGGTTCGTGAGAATTGAAACA
+
FFFFFFFFFFFFFFFFFFFFFFFFFFFFFFFFFFFFFFFFFFFFFFFFFFFFFFFFFFFFFFFFFFFF
FFFFFFFFFFFFFFFFFFFFFFFF:FFFFFFFFFFFFFFFFFFFFFFFFFFFFFFFFFFFFFFFFFF
@A00155:342:HHGFNDSXY:1:2429:12201:15107 1:N:0:GAACCTAG+TCCGCATA
TGGTACGTGCTATGCCTTAAATTACCATCGAAGTTGAATTCGGCTCCTGGTGCCTCGTGATTCGGGAA
GAACCTCTTAACGAAACTATCCATTACTCTGAGTTTGCTTTTAGCTTGGGTTCGTGAGAATTGAAACA
+
FFFFFFFFFFFFFFFFFFFFFFFFFFFFFFFFFFFFFFFFFFFFFFFFFFFFFFFFFFFFFFFFFFFF
FFFFFFFFFFFFFFFFFFFFFFFFFFFFFFFFFFFFFFF:FFFFFFFFFFFFFFFFFFFFFFFFFFFF
@A00155:342:HHGFNDSXY:1:1574:18114:8907 2:N:0:GAACCTAG+TCCGCATA
TGGTACGTGCTATGCCTTAAATTACCATCGAAGTTGAATTCGGCTCCTGGTGCCTCGTGATTCGGGAA
GAACCTCTTAACGAAACTATCCATTACTCTGAGTTTGCTTTTAGCTTGGGTTCGTGAGAATTGAAACA
+
FFFFFFF:FFFFFFFFFFFFFFFF:FFFFFFFFFFFFFFFFFFFFFFFFFFFFFFFFFFFFFFFFFF
FFFFFF:FFFFFFFFFFFFFFFFFFFFFFFFFFFFFFFFFFFFFFFFFFFFFFFFFFFFFFFFFFFF
@A00155:342:HHGFNDSXY:1:1146:9525:10755 2:N:0:GAACCTAG+TCCGCATA
TGGTACGTGCTATGCCTTAAATTACCATCGAAGTTGAATTCGGCTCCTGGTGCCTCGTGATTCGGGAA
GAACCTCTTAACGAAACTATCCATTACTCTGAGTTTGCTTTTAGCTTGGGTTCGTGAGAATTGAAACA
+
FFFFFFFFFFFFFFFFFFFFFFFFFF:FFFFFFFFFFFFFFFFFFFFFFFFFF:FFFFFFFFFFFFFF
FFFFFFFFFFFFFFFFFFFFFFFFFFFFFFFFFF:F:FFFFFFFFFFFFFFFFFFFFFFFFFFFFFFF
```

@A00155:342:HHGFNDSXY:1:1219:32750:22889 2:N:0:GAACCTAG+TCCGCATA
TGGTACGTGCTATGCCTTAAATTACCATCGAAGTTGAATTCGGCTCCTGGTGCCTCGTGATTCGGGAA
GAACCTCTTAACGAAACTATCCATTACTCTGAGTTTGCTTTTAGCTTGGGTTCGTGAGAATTGAAACA
+
FFFFFFFFFFFFFFFFFFFFF:FFFFF:FFF,FFFFFFFFFFFFFFFFFFFFFFFFF:FFFFFFFFFF
FFFFFFF:F:FFFFFFFFFFFFFFFFFFFFFFFFFFFF,FFFFFFFFFFFFFFFFFFFFFFFFFFFFF
@A00155:342:HHGFNDSXY:1:1227:10917:21746 2:N:0:GAACCTAG+TCCGCATA
TGGTACGTGCTATGCCTTAAATTACCATCGAAGTTGAATTCGGCTCCTGGTGCCTCGTGATTCGGGAA
GAACCTCTTAACGAAACTATCCATTACTCTGAGTTTGCTTTTAGCTTGGGTTCGTGAGAATTGAAACA
+
FFFFF,F:FFFFFFF:FFFF::FFFFFFFFFFFFFFFFFFFFFFFFFFFFF:FFFFFFFFFFFFF:FF
FFFFFFFFFFFFFFFFFFFFF,FFFFFFFFFFFFFFFFFFF:FFFFFFFFFFFFFFFFFFFFFFFFFF
@A00155:342:HHGFNDSXY:1:1250:21766:32362 2:N:0:GAACCTAG+TCCGCATA
TGGTACGTGCTATGCCTTAAATTACCATCGAAGTTGAATTCGGCTCCTGGTGCCTCGTGATTCGGGAA
GAACCTCTTAACGAAACTATCCATTACTCTGAGTTTGCTTTTAGCTTGGGTTCGTGAGAATTGAAACA
+
:FFFFFFFFFFFFFFFFFFF:FFFFFFFFFFFFFFFFFFFFFFFFFFFFF:FFFFFFFFFFFFFFFFF
FFFFFFFFF:FFFFFFFFFFF:F:FFFFFFFFFFFFFF:FFFFFFFFFFFFFFFFFFFFFFFFFFFFF
@A00155:342:HHGFNDSXY:1:1437:25572:22388 2:N:0:GAACCTAG+TCCGCATA
TGGTACGTGCTATGCCTTAAATTACCATCGAAGTTGAATTCGGCTCCTGGTGCCTCGTGATTCGGGAA
GAACCTCTTAACGAAACTATCCATTACTCTGAGTTTGCTTTTAGCTTGGGTTCGTGAGAATTGAAACA
+
FFFFFFFFFFFFFF:FFFFFFF::F:FFFFFFFFFFFF,FFFFFFFFFFFFFFFFFFFFFFFFFFFFF
FFFFF,FFFFFFFFFFFFFFFF:F,FF,FFFFFFFFFFFFFFFFF:F:FFFFFFFFFFFFFFFFFFFF
@A00155:342:HHGFNDSXY:1:1122:19714:20917 1:N:0:GAACCTAG+TCCGCATA
TGGTACGTGCTATGCCTTAAATTACCATCGAAGTTGAATTCGGCTCCTGGTGCCTCGTGATTCGGGAA
GAACCTCTTAACGAAACTATCCATTACTCTGAGTTTGCTTTTAGCTTGGGTTCGTGAGAATTGAAACA
+
FFFFFFFFFFFFFFFFF:FFFFFFFFFFFFFFFFFFFFFFFFFFFFFFFFFFFFFFFFFF,FFFFFF,
FFFFFF:FFFFFFFF:FFFFFFF,FFFF:FFFFFFFFFFFFFFFFFFFFFFFFFFFFFFFFFFF:FFF
@A00155:342:HHGFNDSXY:1:2234:6732:31407 2:N:0:GAACCTAG+TCCGCATA
GGTACGTGCTATGCCTTAAATTACCATCGAAGTTGAATTCGGCTCCTGGTGCCTCGTGATTCGGGAAG
AACCTCTTAACGAAACTATCCATTACTCTGAGTTTGCTTTTAGCTTGGGTTCGTGAGAATTGAAACA
+
FFFFFFFFFFFFFFFFFFFFFFFFFFFFFFFFFFFFF,FFFFFFFFFFFFF:FFFFFFFFFFFFFFFF
FF:FFFFF:FF:FFFFFFFFFFFFFFFFFFFFFFFFFF:FFFFFFFFFFFFFFFFFFFFFFFFFFFF
@A00155:342:HHGFNDSXY:1:2607:17192:1767 2:N:0:GAACCTAG+TCCGCATA
GGTACGTGCTATGCCTTAAATTACCATCGAAGTTGAATTCGGCTCCTGGTGCCTCGTGATTCGGGAAG
AACCTCTTAACGAAACTATCCATTACTCTGAGTTTGCTTTTAGCTTGGGTTCGTGAGAATTGAAACA
+
:FFFFFF:FFFFFFFFFFFFFFFFFFFFFFFFFFFFFFFFFFFFFFFFFFFFFFFFF:FFFFFFFFFF
FFFFFFFFFFF:FFFFFFFFFFFFFFFFFFFFFFFFFFFFFFFFFFFFFFFFFFFFFFFFFFFFFFF
@A00155:342:HHGFNDSXY:1:1458:29875:34006 2:N:0:GAACCTAG+TCCGCATA
GGTACGTGCTATGCCTTAAATTACCATCGAAGTTGAATTCGGCTCCTGGTGCCTCGTGATTCGGGAAG
AACCTCTTAACGAAACTATCCATTACTCTGAGTTTGCTTTTAGCTTGGGTTCGTGAGAATTGAAACAA
+
FFFFFFFFFFFFFFFFFFFFF:FFFFFFF:FFFFFFFFFFFFFFFFFFFFF:FFFF:FFFFFFFFFFF
FFFFFFFFFFFFFFFFFFFFFF,FFFFFFFFFF:FFFFFFFFFFFFFFFFFFFFFFFFFFFFFFFFFF
@A00155:342:HHGFNDSXY:1:1165:15103:19132 2:N:0:GAACCTAG+TCCGCATA
GGTACGTGCTATGCCTTAAATTACCATCGAAGTTGAATTCGGCTCCTGGTGCCTCGTGATTCGGGAAG
AACCTCTTAACGAAACTATCCATTACTCTGAGTTTGCTTTTAGCTTGGGTTCGTGAGAATTGAAACAA
+
FFFFFFFFFFFFFFF:FFFFFFFFF:FFF:FFFFFFFFFFFFFFFFFFFFFFFFFFFFFFFFFFFFFF
FFF:FFFFFFFFFFFFFFFF:FFFFFFFFFFFFFFFFFFFFFFFFFFFFFFFFFFFFFFFFFFFFFF:FFFFFFF

```
@A00155:342:HHGFNDSXY:1:2545:16595:4617 2:N:0:GAACCTAG+TCCGCATA
GGTACGTGCTATGCCTTAAATTACCATCGAAGTTGAATTCGGCTCCTGGTGCCTCGTGATTCGGGAAG
AACCTCTTAACGAAACTATCCATTACTCTGAGTTTGCTTTTAGCTTGGGTTCGTGAGAATTGAAACAA
+
FFFFFFFFFFFFFFFFFFF:FF,FF:FFF:,FFFFFFFFFFFFFFFFFFFFFFFFFFFFFFFFF:F:F
F:FFFF::,:F,FFFFFFFFFFFFFFF:FFFFFF,FFFFFFFFFFFFFFFFFFFFFFFFFFF,F,FFF
@A00155:342:HHGFNDSXY:1:2458:8341:3787 2:N:0:GAACCTAG+TCCGCATA
GGTACGTGCTATGCCTTAAATTACCATCGAAGTTGAATTCGGCTCCTGGTGCCTCGTGATTCGGGAAG
AACCTCTTAACGAAACTATCCATTACTCTGAGTTTGCTTTTAGCTTGGGTTCGTGAGAATTGAAACAA
+
F:FFFF,FFFFFFFF:FFF,F:FF:FFFFFFFFFFFFFFFFFFFFFFFFFFFFFFFFFFFFFFFFFFF
FFFFF:FFFFFFFFFFFFFFFFFFFF:FFFFFFFFFFFFFFFFFFFFFFFFFFFFFFFFFFFFFFFFF
@A00155:342:HHGFNDSXY:1:2545:16938:4460 2:N:0:GAACCTAG+TCCGCATA
GGTACGTGCTATGCCTTAAATTACCATCGAAGTTGAATTCGGCTCCTGGTGCCTCGTGATTCGGGAAG
AACCTCTTAACGAAACTATCCATTACTCTGAGTTTGCTTTTAGCTTGGGTTCGTGAGAATTGAAACAA
+
FFFFFFFFFFFFFFFFFFFFF,FFFFFFFFFFFFFFFFFFFFFFFFFFFFFFFFFFFFFFFFFFFFFF
FFFFFFFFFFFFFFFFFFFFFFFFFFFFFFFFFFFFFFFFFFFFFF:FF:FFFFFFFFFFFFFFFFFF
@A00155:342:HHGFNDSXY:1:2305:24514:10567 2:N:0:GAACCTAG+TCCGCATA
GGTACGTGCTATGCCTTAAATTACCATCGAAGTTGAATTCGGCTCCTGGTGCCTCGTGATTCGGGAAG
AACCTCTTAACGAAACTATCCATTACTCTGAGTTTGCTTTTAGCTTGGGTTCGTGAGAATTGAAACAA
+
FF,FFFFFF:FFFFFFFFFFFFFFFFFFFFFFFFFFFFFFFFFFFFFFFFFFFFFFFFFFFFFFFFFF
FFFFFFFFFFFFFFFFFFFFFF:FFFFFFFFFFFFFFFFFFFFFFFFFFFFFFFFFFFFFFFFFFFFF
@A00155:342:HHGFNDSXY:1:2264:20473:30843 2:N:0:GAACCTAG+TCCGCATA
GGTACGTGCTATGCCTTAAATTACCATCGAAGTTGAATTCGGCTCCTGGTGCCTCGTGATTCGGGAAG
AACCTCTTAACGAAACTATCCATTACTCTGAGTTTGCTTTTAGCTTGGGTTCGTGAGAATTGAAACAA
+
FFFFFFFFFFFFFFFFFFFFFFFFFFFFFFFFFFFFFFFFFFFFFFFFFFFFFFFFFFFFFFFFFFFF
FFFFFFFFFFFFFFFFFFFFFFFFFFFFFFFFFFFFFFFFFFFFFFFFFFFFFFFFFFFFFFFFFFFF
@A00155:342:HHGFNDSXY:1:2360:31684:22294 2:N:0:GAACCTAG+TCCGCATA
GGTACGTGCTATGCCTTAAATTACCATCGAAGTTGAATTCGGCTCCTGGTGCCTCGTGATTCGGGAAG
AACCTCTTAACGAAACTATCCATTACTCTGAGTTTGCTTTTAGCTTGGGTTCGTGAGAATTGAAACAA
+
FFFFFF:FFFFFFFFFFF,FFFFFFFFFF:FFFFFF:FFFFFFFFFFFFFFFFFFFFFFFFFFFFFFF
FFFFFF:F:FFFFFFFFFFFFFF:FFFFFFFFFFFFFFFFF,FFFFFFFFFFFFFFFFFFFFFFFFFF
@A00155:342:HHGFNDSXY:1:2617:30409:15483 2:N:0:GAACCTAG+TCCGCATA
GGTACGTGCTATGCCTTAAATTACCATCGAAGTTGAATTCGGCTCCTGGTGCCTCGTGATTCGGGAAG
AACCTCTTAACGAAACTATCCATTACTCTGAGTTTGCTTTTAGCTTGGGTTCGTGAGAATTGAAACAA
+
FFFFFFFFFFFFFFFFFFFFFFFFFFFFFFFFFFFFFFFFFFFFFFFFFFFFFFFFFFFFFFFFFFFF
FF:F:FFFFFFFFFFFFFFFFFFFFFFFFFFFFFFFFFFFFFFF:FFFFFFFFFFFFFFFFFFFFFFF
@A00155:342:HHGFNDSXY:1:2360:28854:22216 2:N:0:GAACCTAG+TCCGCATA
GGTACGTGCTATGCCTTAAATTACCATCGAAGTTGAATTCGGCTCCTGGTGCCTCGTGATTCGGGAAG
AACCTCTTAACGAAACTATCCATTACTCTGAGTTTGCTTTTAGCTTGGGTTCGTGAGAATTGAAACAA
+
FFFFFFFF:FFFFFFFFFFFFFFFFFFF:FFFFFFFFFFFFFFFFFFFFFFFFFFFFFFFFFFFFFFF
FFFFFFFFFFFFFFFFFFFFFFFFFFFFFFFFFFFFFFFFFFFFFFFFFFFFFFFFFFFFFFFFFFFF
@A00155:342:HHGFNDSXY:1:1124:31467:28839 2:N:0:GAACCTAG+TCCGCATA
GTACGTGCTATGCCTTAAATTACCATCGAAGTTGAATTCGGCTCCTGGTGCCTCGTGATTCGGGAAGA
ACCTCTTAACGAAACTATCCATTACTCTGAGTTTGCTTTTAGCTTGGGTTCGTGAGAATTGAAACA
+
FFF:FFFFFFFFFFFFFF:::,FF,FFFFFFFFFFF:FFFFFFFFFFFFFFFFFFF:FFFFFFFFFFF
FFFFFF,F:FFFFFFFFFFFFFFFFFFFFFFF::FFFFFFFFFFF:FFFFFFFFFFFFFFFFFFFF
```

```
@A00155:342:HHGFNDSXY:1:2323:20509:12085 2:N:0:GAACCTAG+TCCGCATA
GTACGTGCTATGCCTTAAATTACCATCGAAGTTGAATTCGGCTCCTGGTGCCTCGTGATTCGGGAAGA
ACCTCTTAACGAAACTATCCATTACTCTGAGTTTGCTTTTAGCTTGGGTTCGTGAGAATTGAAACA
+
FF:FFFFFFFFFFFFF:FFFF,FF,FFF:FFFFFFFFFFFFF:FF:FF:FFFFFFFFFFFFFFFFFFF
FFFFF:FFFFFFFFFFFFFFFFFFFFFFFFFFFFFFFFF:FFFFFFFFFFFFFFFFFFFFFFFFFF
@A00155:342:HHGFNDSXY:1:2114:6976:18270 2:N:0:GAACCTAG+TCCGCATA
TTACGTGCTATGCCTTAAATTACCATCGAAGTTGAATTCGGCTCCTGGTGCCTCGTGATTCGGGAAGA
ACCTCTTAACGAAACTATCCATTACTCTGAGTTTGCTTTTAGCTTGGGTTCGTGAGAATTGAAACA
+
,FFF,F,FF:F,,,F::F,F:,F:FFF,,FFFF,:F,F,FF:FFF:FFFF,,F,:FFF:FFF::FF,F
F:F:FF:FF,FF,FFFFFF:,FF:F:,FFF:FFF,F,:FFF:FFFFFFFFFFFFFFFFFFFFFFFF
@A00155:342:HHGFNDSXY:1:1201:31919:35697 2:N:0:GAACCTAG+TCCGCATA
GTACGTGCTATGCCTTAAATTACCATCGAAGTTGAATTCGGCTCCTGGTGCCTCGTGATTCGGGAAGA
ACCTCTTAACGAAACTATCCATTACTCTGAGTTTGCTTTTAGCTTGGGTTCGTGAGAATTGAAACA
+
FFFFFFFFFFFFFF,FFFF:FFFF,FFFFFFFFFF:FFFFFFFFF:FFFF,FFFFFFFFFFFFFFFFF
FFFFFFFFFFFFFFFFFFFFFFFFFFFFFFFFFFFFFFFFFFFFFFFFFFFFFFFFFFFFFF:FFF
@A00155:342:HHGFNDSXY:1:2678:23104:10942 2:N:0:GAACCTAG+TCCGCATA
GTACGTGCTATGCCTTAAATTACCATCGAAGTTGAATTCGGCTCCTGGTGCCTCGTGATTCGGGAAGA
ACCTCTTAACGAAACTATCCATTACTCTGAGTTTGCTTTTAGCTTGGGTTCGTGAGAATTGAAACA
+
FFFFFFFFFFFF:FFFFFFFFF:FFFFFFFFFFFFFFFFFFFFFFFFFFFFFFFFFFFFFFFFFFFFF
FFFFFFFFFFFFFFFFFFFFFFFFFFFFFFFFFFFFFFFFFF,FFFFFFFFFFFFFFFFFFFFFFF
@A00155:342:HHGFNDSXY:1:2618:7636:6731 2:N:0:GAACCTAG+TCCGCATA
GTACGTGCTATGCCTTAAATTACCATCGAAGTTGAATTCGGCTCCTGGTGCCTCGTGATTCGGGAAGA
ACCTCTTAACGAAACTATCCATTACTCTGAGTTTGCTTTTAGCTTGGGTTCGTGAGAATTGAAACAA
+
FFFFFF,FFFF,FFFFFFFFF,:FFFFFFFFFFFFF:FFFFFFFFFFFFFFFFFFFFFFFFF,FFFFF
FFFFF:FFFFFFFFFFFFFFFFFFFFFFFFFFFFFFFFFFFFFFFFFFFFFFFFFFFFFFFFFFFFF
@A00155:342:HHGFNDSXY:1:1255:17833:12618 1:N:0:GAACCTAG+TCCGCATA
TACGTGCTATGCCTTAAATTACCATCGAAGTTGAATTCGGCTCCTGGTGCCTCGTGATTCGGGAAGAA
CCTCTTAACGAAACTATCCATTACTCTGAGTTTGCTTTTAGCTTGGGTTCGTGAGAATTGAAACAAAG
+
,FFFF:FFF,FFF::FFFFFFFFFFFFFFFFFFFFFFFFFFFFFFFF::FF:FFFFFFFFFFFFFFFF
FF:FFFFFFFFFFFFFFFFFFFFFFFFF:FF,,:FFF,FFFF,FFFFFFFFFFFFF,FFFFFFFF::F
@A00155:342:HHGFNDSXY:1:2521:1063:4304 2:N:0:GAACCTAG+TCCGCATA
ACGTGCTATGCCTTAAATTACCATCGAAGTTGAATTCGGCTCCTGGTGCCTCGTGATTCGGGAAGAAC
CTCTTAACGAAACTATCCATTACTCTGAGTTTGCTTTTAGCTTGGGTTCGTGAGAATTGAAACA
+
FFF,:,,F,FFF:FFFFF,FFFF:F:FF:FFFFF:,FF,F:FFF,:FFF::FFF:F,:,::FFF:FFF
F:FF:FF:,FFFFFF:FFFFFFF:F:::FFFFFF,:FFFFF:FFFF:FFF::FFFFF:FFFFFF
@A00155:342:HHGFNDSXY:1:1470:28085:24893 2:N:0:GAACCTAG+TCCGCATA
ACGTGCTATGCCTTAAATTACCATCGAAGTTGAATTCGGCTCCTGGTGCCTCGTGATTCGGGAAGAAC
CTCTTAACGAAACTATCCATTACTCTGAGTTTGCTTTTAGCTTGGGTTCGTGAGAATTGAAACA
+
FF:F:FFFFFFF,FFF,FFFFF,,FFF:::FFFFFFFFFFF:F:F:FFFFFFFFFF:FFFFFFFFFFF
FFFFFFFFFFFFFFFFFFFFFFF:FFFFFFFFFFFFFFF:FFFFFFFFFFFFFFFFFFFFFFFF
@A00155:342:HHGFNDSXY:1:2511:2401:30733 2:N:0:GAACCTAG+TCCGCATA
ACGTGCTATGCCTTAAATTACCATCGAAGTTGAATTCGGCTCCTGGTGCCTCGTGATTCGGGAAGAAC
CTCTTAACGAAACTATCCATTACTCTGAGTTTGCTTTTAGCTTGGGTTCGTGAGAATTGAAACAAAGC
+
:FFFFFFFF::FFFFFFFFFFFFFFFFFFFFFF,FFFFFFFFFFFFFFFFF:FFFFFFFFF,FFFFFF
F:F,FFFFFFFFFFFFFFFFFFFFFFFFFFFF,F:FFFF::FFFFFF:FFFFFFFFFFFFFFFFFFFF
```

```
@A00155:342:HHGFNDSXY:1:1658:32470:17550 2:N:0:GAACCTAG+TCCGCATA
ACGTGCTATGCCTTAAATTACCATCGAAGTTGAATTCGGCTCCTGGTGCCTCGTGATTCGGGAAGAAC
CTCTTAACGAAACTATCCATTACTCTGAGTTTGCTTTTAGCTTGGGTTCGTGAGAATTGAAACAAAGC
+
FFFFFFFFFFFFF,FF,FFFFFFFFFFFFFFFFFFFFFFFFFFFFFFFFFFFFFFFFFFFFFFFFF:FFFF
FFFFFF:FFFFFFFFFFFFF:FFFFFFFFFFFFFFFFFFFFFFFFFFFFFFFFFFFFFFFFFFFFF:FFF
@A00155:342:HHGFNDSXY:1:1617:27561:32910 2:N:0:GAACCTAG+TCCGCATA
ACGTGCTATGCCTTAAATTACCATCGAAGTTGAATTCGGCTCCTGGTGCCTCGTGATTCGGGAAGAAC
CTCTTAACGAAACTATCCATTACTCTGAGTTTGCTTTTAGCTTGGGTTCGTGAGAATTGAAACAAAGC
+
FFFFFFFFFFFFFFFFFFFFFF,FFFFFFFFFFFFFFFFFFFFFFFFFFFFFFFFFFFFFFFFFFFFF
FFF:FFFFFF:FFFFFFFFFFFFFFFFFFF,FFFFFFFFFFFFFFFFFFFFFFFFFFFFFFFFFFFFF
@A00155:342:HHGFNDSXY:1:1165:9046:27868 2:N:0:GAACCTAG+TCCGCATA
ACGTGCTATGCCTTAAATTACCATCGAAGTTGAATTCGGCTCCTGGTGCCTCGTGATTCGGGAAGAAC
CTCTTAACGAAACTATCCATTACTCTGAGTTTGCTTTTAGCTTGGGTTCGTGAGAATTGAAACAAAGC
+
FFFFFFFFFF:FFFFFFF:FFFFFFFFFFFFF:::FFFFFFFFFFFFFFFFFFFFFFFFFFFF:FFFF
FFF:FFFFFFFFFF:FFFFFFFFFFFFFFFFFFFFFFFFFFFFFFFFFFFFFFFFFFFFF:,FFFFFF
@A00155:342:HHGFNDSXY:1:1135:10963:5102 2:N:0:GAACCTAG+TCCGCATA
CGTGCTATGCCTTAAATTACCATCGAAGTTGAATTCGGCTCCTGGTGCCTCGTGATTCGGGAAGAACC
TCTTAACGAAACTATCCATTACTCTGAGTTTGCTTTTAGCTTGGGTTCGTGAGAATTGAAACAAAGCT
+
FFFFFFFFFFFFFF::FFFFFFFFFFFFFFFFFFFFFFFFFFFFFFFFFFFFFFFFFFFFFFFFFFFF
FF:FFFFFFFFFFFFFFFFFFFFFFFFFF,FFFFFFFFFFFFFFFFFFFFFFFFFFFFFF:FFFFFFF
@A00155:342:HHGFNDSXY:1:2205:32389:34131 2:N:0:GAACCTAG+TCCGCATA
CGTGCTATGCCTGAAATTACCATCGAAGTTGAATTCGGCTCCTGGTGCCTCGTGATTCGGGAAGAACC
TCTTAACGAAACTATCCATTACTCTGAGTTTGCTTTTAGCTTGGGTTCGTGAGAATTGAAACAAAGCT
+
FFFFF,FFFFFF,F,,:,:FFFFFF::FFFFFF::FFFFF:FFFF:FFF:FFFFFF:FFFFF:FFF:F
FFFF:FFFFF,FFFFFFFFFFF:FFFFFF,,FFFF:,:FF:FFFFF:FFFFFFFFFFF:,FFFFFFFF
@A00155:342:HHGFNDSXY:1:2459:22128:22874 2:N:0:GAACCTAG+TCCGCATA
CGTGCTATGCCTTAAATTACCATCGAAGTTGAATTCGGCTCCTGGTGCCTCGTGATTCGGGAAGAACC
TCTTAACGAAACTATCCATTACTCTGAGTTTGCTTTTAGCTTGGGTTCGTGAGAATTGAAACAAAGCT
+
FFFF:FFFF:FFFF:FFFFFFFFFFFFFFFFFFFFFFFFFFFFFFFFFFFFFF:FFFFFFFFFFFFFF
FFFFFFFFFFFFFFFFFFFFFFFFFFFFFFFFFFF:FFFFFFFFFFFFFFFFFFFFFFFFFFFFFFFF
@A00155:342:HHGFNDSXY:1:1526:10303:15389 2:N:0:GAACCTAG+TCCGCATA
CGTGCTATGCCTTAAATTACCATCGAAGTTGAATTCGGCTCCTGGTGCCTCGTGATTCGGGAAGAACC
TCTTAACGAAACTATCCATTACTCTGAGTTTGCTTTTAGCTTGGGTTCGTGAGAATTGAAACAAAGCT
+
FFFFFFFFFFFFFFFF:FFFF:FFFFFFFFFFFFFFFFFFFFFFFFFFFFFFFFFFFFFFFFFFFFFF
FF:FFFFFFFFFFFFFFFFFFFFFFFFFFF:FFFFFFFFFFFFFFFFFFFFFFFFFFFFFFFFFFFFF
@A00155:342:HHGFNDSXY:1:2459:26666:23657 2:N:0:GAACCTAG+TCCGCATA
CGTGCTATGCCTTAAATTACCATCGAAGTTGAATTCGGCTCCTGGTGCCTCGTGATTCGGGAAGAACC
TCTTAACGAAACTATCCATTACTCTGAGTTTGCTTTTAGCTTGGGTTCGTGAGAATTGAAACAAAGCT
+
FFFF:FFFFFFFFFFFFFFFFFFFFFFFFFFFFFFFFFFFFFFFFFFFFFFFFFFFFFFFFFFFFFFF
FFFFFFFFFFFFFFFFFFFFFFFFFFFFFFFFFFFFFFFFFFFFFFFFFFFFFFFFFFFFFFFFFFFF
@A00155:342:HHGFNDSXY:1:1644:27787:14732 2:N:0:GAACCTAG+TCCGCATA
CGTGCTATGCCTTAAATTACCATCGAAGTTGAATTCGGCTCCTGGTGCCTCGTGATTCGGGAAGAACC
TCTTAACGAAACTATCCATTACTCTGAGTTTGCTTTTAGCTTGGGTTCGTGAGAATTGAAACAAAGCT
+
FFFFFFFFFFFF:FF:FFFFFFFFFFFFFFFFFFFFFF:FFFFFFFFFFFFFFFFFFFFFFFFFFFFF
FF:F,FFFFFFFFFFFFFF:FFFFFF:FF,FFFFFFFFFF,FFFFFFFFFFFFFFFFFFF:FFFFFFF
```

@A00155:342:HHGFNDSXY:1:2333:15383:29857 2:N:0:GAACCTAG+TCCGCATA
CTTGCTATGCCTTAAATTACCATCGAAGTTGAATTCGGCTCCTGGTGCCTCGTGATTCGGGAAGAACC
TCTTAACGAAACTATCCATTACTCTGAGTTTGCTTTTAGCTTGGGTTCGTGAGAATTGAAACAAAGCT
+
F,FFFF:FFFFFFFF:F:FFFFFFFFFFFFFFFFFFFFFFFFFFFFFFFFFFFFFFFFFFFFFFFFFF
FFF:FFFFFFFFFFFFFFFFFFFFFFFFF:FFFFFFFFFFFFFFFFFFFFFFFFFFFFFFFFFFFFFF
@A00155:342:HHGFNDSXY:1:2117:1362:15123 2:N:0:GAACCTAG+TCCGCATA
CGTGCTATGCCTTAAATTACCATCGAAGTTGAATTCGGCTCCTGGTGCCTCGTGATTCGGGAAGAACC
TCTTAACGAAACTATCCATTACTCTGAGTTTGCTTTTAGCTTGGGTTCGTGAGAATTGAAACAAAGCT
+
:F:F:FFFF,:,FFFFFFFF:F,FFFFFF:FFFFFFFFF:FFFFFFFFFF:F::FFFFFF:FFFFFFF
:F::FFFFFFFFFFFFFFFFFF:F:FFF:FFFF:FFFFFFFFFFF:FFFFFFFFFFFFFFFFFFFFFF
@A00155:342:HHGFNDSXY:1:2117:7726:17409 2:N:0:GAACCTAG+TCCGCATA
CGTGCTATGCCTTAAATTACCATCGAAGTTGAATTCGGCTCCTGGTGCCTCGTGATTCGGGAAGAACC
TCTTAACGAAACTATCCATTACTCTGAGTTTGCTTTTAGCTTGGGTTCGTGAGAATTGAAACAAAGCT
+
FFFFFFFFF:FFFFFFFFFFFFFFFFFFFFFFFFFFFFFFFFFFFFFFFFFF:FFFFFFFFFFFFFFF
FFFFFFFFFFFFFFFFFFFFFFFFFF:FFFFFFFFFFFFFFFFFFFFFFFFFFFFFFFFFFFFFFFFF
@A00155:342:HHGFNDSXY:1:2132:29658:27117 2:N:0:GAACCTAG+TCCGCATA
CGTGCTATGCCTTAAATTACCATCGAAGTTGAATTCGGCTCCTGGTGCCTCGTGATTCGGGAAGAACC
TCTTAACGAAACTATCCATTACTCTGAGTTTGCTTTTAGCTTGGGTTCGTGAGAATTGAAACAAAGCT
+
FFFFFF:FFFFFFFFFFFFFF,FFFFFFFFFF,FFFFFFFFFFFFFFFFFFFFFFFFFFFFFFFFFFF
FFF:FFFFFFFFF:FFFFFFFFFFFFFFF:FFFFFFFFFFFFFFFFFFFFFFFFFFFFFFFFFFFFFF
@A00155:342:HHGFNDSXY:1:2133:28122:1752 2:N:0:GAACCTAG+TCCGCATA
CGTGCTATGCCTTAAATTACCATCGAAGTTGAATTCGGCTCCTGGTGCCTCGTGATTCGGGAAGAACC
TCTTAACGAAACTATCCATTACTCTGAGTTTGCTTTTAGCTTGGGTTCGTGAGAATTGAAACAAAGCT
+
FFFFFFFFFFFFF:FFFFFFF:FFFFFFFFFFFFFFFFFFFFFFFFFFFFFFFFFFFFFFFFFFFFFF
FFFFFFFFFFFFFFFFFFFFFFFFFFFFFFF:FFFFFFFFFFFFFFFFFFFFFFFFFFFFFF:FFFFF
@A00155:342:HHGFNDSXY:1:1361:5104:32064 2:N:0:GAACCTAG+TCCGCATA
CGTGCTATGCCTTAAATTACCATCGAAGTTGAATTCGGCTCCTGGTGCCTCGTGATTCGGGAAGAACC
TCTTAACGAAACTATCCATTACTCTGAGTTTGCTTTTAGCTTGGGTTCGTGAGAATTGAAACAAAGCT
+
FFFF:FFFFFFFFFFF:,FFFFFFFFFFFFFFFFFFFFFFFFFFFFFFFFFFFFFFFFFFFFFFFFFF
FFFF:FFFFFFFFFFFFFFFF:FFFFFFFFFFFFFFFFFFFFFFF:FFFFFFFFFFFFFFFFFFFF,F
@A00155:342:HHGFNDSXY:1:1107:24397:4946 2:N:0:GAACCTAG+TCCGCATA
GTGCTATGCCTTAAATTACCATCGAAGTTGAATTCGGCTCCTGGTGCCTCGTGATTCGGGAAGAACCT
CTTAACGAAACTATCCATTACTCTGAGTTTGCTTTTAGCTTGGGTTCGTGAGAATTGAAA
+
FFFFFFFFFF:FFFFFF:FFFFFFFFFFFFF:FFFFFFFFFFFFFFFFFFFFFFFFFFFFFFFFFFFF
FFFF:FFFFFFFFFFFFFFFFFFFFFFFFFFFFFFFFFFFFFFFFFFFFFFFFFFFFFF
@A00155:342:HHGFNDSXY:1:2537:26313:1344 2:N:0:GAACCTAG+TCCGCATA
GTGCTATGCCTTAAATTACCATCGAAGTTGAATTCGGCTCCTGGTGCCTCGTGATTCGGGAAGAACCT
CTTAACGAAACTATCCATTACTCTGAGTTTGCTTTTAGCTTGGGTTCGTGAGAATTGAAA
+
FFFFFF,FFFFFFFF,FFF:,:F:FFFFFFFF,FFFFFFFFFFFFFFFFFF:FFFFFFFFF:FF:FF,
FFFFFF:FFFFF:FFFFFFFFFFFFFFFFFFFF:FF:FFFFFFFFFFFFFFFFFFFFFFF
@A00155:342:HHGFNDSXY:1:2444:28782:3897 2:N:0:GAACCTAG+TCCGCATA
GTGCTATGCCTTAAATTACCATCGAAGTTGAATTCGGCTCCTGGTGCCTCGTGATTCGGGAAGAACCT
CTTAACGAAACTATCCATTACTCTGAGTTTGCTTTTAGCTTGGGTTCGTGAGAATTGAAACA
+
FFFFFFFFFFFFFFFFFFFF,FFFF,FFFFFFFFFFFFFFFFFFFFFFFFFFFFFFFFFFFFFFFFFF
,FFFFFFFFF:FFFFFFFFFFFFFFFFFFFFFFFFFFF:FFFFFFFFF:FFFFFFFFFFFFF

```
@A00155:342:HHGFNDSXY:1:2307:9290:34397 2:N:0:GAACCTAG+TCCGCATA
GTGCTATGCCTTAAATTACCATCGAAGTTGAATTCGGCTCCTGGTGCCTCGTGATTCGGGAAGAACCT
CTTAACGAAACTATCCATTACTCTGAGTTTGCTTTTAGCTTGGGTTCGTGAGAATTGAAACA
+
FFFFFFFFFFFFFFFFFFFF:FFFFFFFFFFF:FFFFFFFF:FFFFFFFFFFFFFFFFFFFFFFFFFF
FFFFFFFFFFFFFFFFFFFFFFFFFFFFFFF:FFFFFFFFFFFFFFFFFFFFFFFFFFFFFFF
@A00155:342:HHGFNDSXY:1:1571:22092:33019 2:N:0:GAACCTAG+TCCGCATA
GTGCTATGCCTTAAATTACCATCGAAGTTGAATTCGGCTCCTGGTGCCTCGTGATTCGGGAAGAACCT
CTTAACGAAACTATCCATTACTCTGAGTTTGCTTTTAGCTTGGGTTCGTGAGAATTGAAACAAAGCT
+
FF:FFFFFFFFFFFFFFFFF,FFFF,FFFFFFFFFFFFFFFFFFFFFFFFFFFFFFFFFFFFFF:FFF
F:FFFF:FFFFFF:FFFFFFFFFFFFFFFFFFF:FFFFFFFFFFFFFFFFFFFFFFFFFFFFF,FFFF
@A00155:342:HHGFNDSXY:1:1369:23131:13651 2:N:0:GAACCTAG+TCCGCATA
GTGCTATGCCTTAAATTACCATCGAAGTTGAATTCGGCTCCTGGTGCCTCGTGATTCGGGAAGAACCT
CTTAACGAAACTATCCATTACTCTGAGTTTGCTTTTAGCTTGGGTTCGTGAGAATTGAAACAAAGCTT
+
FFFFFF:FFF,FFFFFFFFFFFFFFFFFFFFFFFFFFFFFFFFFFF:FFFFFFFFFFFFFFFFFFFFF
F:F:FFFFFFF:FFFFFFFFFFFFFFFFF,FFFFFFFFFFFFFFFFFFFFFFFFFFFFFFFFFFFFFF
@A00155:342:HHGFNDSXY:1:1672:14018:34381 2:N:0:GAACCTAG+TCCGCATA
GTGCTATGCCTTAAATTACCATCGAAGTTGAATTCGGCTCCTGGTGCCTCGTGATTCGGGAAGAACCT
CTTAACGAAACTATCCATTACTCTGAGTTTGCTTTTAGCTTGGGTTCGTGAGAATTGAAACAAAGCTT
+
F,FFFFFFFFFFFFF:FFFFFFFFF:FFFFF:FFFFF:FFFFFFFFFFFFFFFFFFFFFFF,FFFFFFF
FFFFFFFFFFFFFFFFFFFFFFFFFFFF:FFFFF:FFFFFFFFFFFFFFFFFFFFFFFFFFFFFFFFFF
@A00155:342:HHGFNDSXY:1:2464:15393:35070 2:N:0:GAACCTAG+TCCGCATA
GTGCTATGCCTTAAATTACCATCGAAGTTGAATTCGGCTCCTGGTGCCTCGTGATTCGGGAAGAACCT
CTTAACGAAACTATCCATTACTCTGAGTTTGCTTTTAGCTTGGGTTCGTGAGAATTGAAACAAAGCTT
+
FFFFFFFFFFFFFFFFFFFFFFFFFFFFFFFFFFFFFFFFFFFF:FFFFFFFFFFFFFFFFFFFFFFFF
FFFFFFFFFFFFFFFFFFFFFFFFFFFF,FFFFFFFFFFFFFFFFFFFFFFFF,FFFFFFFFFFFFFFF
@A00155:342:HHGFNDSXY:1:1165:30798:16845 2:N:0:GAACCTAG+TCCGCATA
GTGCTATGCCTTAAATTACCATCGAAGTTGAATTCGGCTCCTGGTGCCTCGTGATTCGGGAAGAACCT
CTTAACGAAACTATCCATTACTCTGAGTTTGCTTTTAGCTTGGGTTCGTGAGAATTGAAACAAAGCTT
+
FFFFFFFFFFFFFFFFFFFF,FFFFFFFFFFFFFFFFFFFFFFFF:FFFFFFFFFFFFFFFFFFFFFFF
FFFF:FFFFFFFFFFFFFFFFFFFFFFFFFFFFFFFFFFFFFFFFFFFFFFFFFFFFFFFFFFFFFFFF
@A00155:342:HHGFNDSXY:1:2168:30572:29888 2:N:0:GAACCTAG+TCCGCATA
GTGCTATGCCTTAAATTACCATCGAAGTTGAATTCGGCTCCTGGTGCCTCGTGATTCGGGAAGAACCT
CTTAACGAAACTATCCATTACTCTGAGTTTGCTTTTAGCTTGGGTTCGTGAGAATTGAAACAAAGCTT
+
FF,:FFFFFFFFFFFF,FFFFFFFF,FFFFFF:FFFFFFFFFFFFF,FFFF:FFFFFF:FFFFFFFFF
FFFFFFFFFFFFFFFFF:FFFFFFFFFFFFFFFFFFFFFFFFFFFFFFFFFFFFFFFFFFFFFFFFFF
@A00155:342:HHGFNDSXY:1:1521:31602:4711 2:N:0:GAACCTAG+TCCGCATA
GTGCTATGCCTTAAAGTACCATCGAAGTTGAATTCGGCTCCTGGTGCCTCGTGATTCGGGAAGAACCT
CTTAACGAAACTATCCATTACTCTGAGTTTGCTTTTAGCTTGGGTTCGTGAGAATTGAAACAAAGCTT
+
FFFFFFFFFFFF:F,,FFFFFFFFFFFFFFF:FFFFFFFFFFFFFFFFFFFFFFFFFFFFFFFFFFFFF
FF:F:FFFFFFFFFFFFFFFFFFFFFFF:::FFFFFF,FFFFFFFFFFFFFFFFFFFFFFFFFFF:FFFF
@A00155:342:HHGFNDSXY:1:2240:18412:34507 2:N:0:GAACCTAG+TCCGCATA
GTGCTATGCCTTAAATTACCCTCGAAGTTGAATTCGGCTCCTGGTGCCTCGTGATTCGGGAAGAACCT
CTTAACGAAACTATCCATTACTCTGAGTTTGCTTTTAGCTTGGGTTCGTGAGAATTGAAACAAAGCTT
+
FFFFFFFFFF:F:F:FFFFF,FF:FF:F::FFFFFF:F,FFFFFFFFFFFFFFFFFFFFFFFFFF:FFF
FFFF,FFFFFFFFFFFFFFFFFFFFFFFFFFFFFFFFFF:FFFFF:FFFFFFFFFFFFFFFFFFFFFFFF
```

```
@A00155:342:HHGFNDSXY:1:2664:18991:6699 2:N:0:GAACCTAG+TCCGCATA
GTGCTATGCCTTAAATTACCATCGAAGTTGAATTCGGCTCCTGGTGCCTCGTGATTCGGGAAGAACCT
CTTAACGAAACTATCCATTACTCTGAGTTTGCTTTTAGCTTGGGTTCGTGAGAATTGAAACAAAGCTT
+
FFFFFFFFFF:FFFFFFFFF:FFFFFFFFFFFFFFFFFFFFFFFFFFFFFFFFFFFFFFFFFFFFFF
FFFFFFFFFFFFFFFFFFFFFFFFFFFFFFFFFFFFFFFFFFFFFFFFFFFFFFFFFFFFFFFFFFFF
@A00155:342:HHGFNDSXY:1:1360:13178:29042 2:N:0:GAACCTAG+TCCGCATA
GTGCTATGCCTTAAATTACCATCGAAGTTGAATTCGGCTCCTGGTGCCTCGTGATTCGGGAAGAACCT
CTTAACGAAACTATCCATTACTCTGAGTTTGCTTTTAGCTTGGGTTCGTGAGAATTGAAACAAAGCTT
+
FFFFFFFFFFFFFFFFFFFF::FFFF:FFFFF,FFFFFFFFFFFFFFFFFFFFFFFFFFFFFFFFFFF
FFFFFFFFF:FFFFFFF:FFFFFFFFF:FFFF:FFFFFFFFFFFFFFFFFFFFFFFFFFFFFFFFFFF
@A00155:342:HHGFNDSXY:1:2218:10122:35243 1:N:0:GAACCTAG+TCCGCATA
GTGCTATGCCTTAAATTACCATCGAAGTTGAATTCGGCTCCTGGTGCCTCGTGATTCGGGAAGAACCT
CTTAACGAAACTATCCATTACTCTGAGTTTGCTTTTAGCTTGGGTTCGTGAGAATTGAAACAAAGCTT
+
FFFFFFFFFFFFFFFFFFFFFFFFFFFFFFFFFFFFFFFFFFFFFFFFFFFFFFFFFFFFFFFFFFFF
FFFFFFFFFFFFFFFFFFFFFFFFF:FFFFFFFFFFFFFFFFFFFFFFFFFFFFFFFFFFFFFFFFFF
@A00155:342:HHGFNDSXY:1:1462:10131:16344 2:N:0:GAACCTAG+TCCGCATA
GTGCTATGCCTTAAATTACCATCGAAGTTGAATTCGGCTCCTGGTGCCTCGTGATTCGGGAAGAACCT
CTTAACGAAACTATCCATTACTCTGAGTTTGCTTTTAGCTTGGGTTCGTGAGAATTGAAACAAAGCTT
+
F,:FFF:FFFFFFFF,FFFF,FF:F:FFFFFFFF:FFFFFFFFFFFFFFFFFF:FFFFFFFFFFFFFF
FFFFFFF,F:FF:FFFFFFF:FFFF::F:FFFFFF:FF:FFFFF:F:FFFFFFFFF::FF,FFFFFFF
@A00155:342:HHGFNDSXY:1:1460:16197:24377 2:N:0:GAACCTAG+TCCGCATA
GTGCTATGCCTTAAATTACCATCGAAGTTGAATTCGGCTCCTTGTGCCTCGTGATTCGGGAAGAACCT
CTTAACGAAACTATCCATTACTCTGAGTTTGCTTTTAGCTTGGGTTCGTGAGAATTGAAACAAAGCTT
+
FFFFFFFFFFFFFFF:F:FFFFFFFFFFFFFFFFFFFFFFFFFFFFFFFFFFFFFFFFFFFFFFFFFF
FFFFFFFFFFFFFFFFFFFFFFFFFFFFFFFFFFF,:FFFFFFFFFFFFFFFFFFFFFFFFFFFFFFF
@A00155:342:HHGFNDSXY:1:1269:9561:29105 2:N:0:GAACCTAG+TCCGCATA
GTGCTATGCCTTAAATTACCATCGAAGTTGAATTCGGCTCCTGGTGCCTCGTGATTCGGGAAGAACCT
CTTAACGAAACTATCCATTACTCTGAGTTTGCTTTTAGCTTGGGTTCGTGAGAATTGAAACAAAGCTT
+
FFF:FFFF,F,FFFFFFFFFFFFFFFFFFFFFFFFFFFF:F:FFFFFFF,F:FFFF,F,,FFFFFFFF
FFFFFFFFFFFF:FFFFFFFF:F:FFF:FFFF::FFFFFFFFFF:FFFFFFFFFFFFFFFFFFFF:FF
@A00155:342:HHGFNDSXY:1:2359:6162:8218 2:N:0:GAACCTAG+TCCGCATA
GTGCTATGCCTTAAATTACCATCGAAGTTGAATTCGGCTCCTGGTGCCTCGTGATTCGGGAAGAACCT
CTTAACGAAACTATCCATTACTCTGAGTTTGCTTTTAGCTTGGGTTCGTGAGAATTGAAACAAAGCTT
+
FFFFFFFFFFFFF:F::FFFFFFFFFFFFFF:FFFFFFFFFF:FFFFFFFFF:FFFFFFFFFFFFFF:
F:F,:FFFFFFF:FFFFFFFFFFFFFFFFFFFFFFFFF,FFFFFF:FFFFFFFFFFFFFFFFFFFFFF
@A00155:342:HHGFNDSXY:1:2364:23475:8422 2:N:0:GAACCTAG+TCCGCATA
GTGCTATGCCTTAAATTACCATCGAAGTTGAATTCGGCTCCTGGTGCCTCGTGATTCGGGAAGAACCT
CTTAACGAAACTATCCATTACTCTGAGTTTGCTTTTAGCTTGGGTTCGTGAGAATTGAAACAAAGCTT
+
FFFFFFFFFFFFFF:FFFFF,FFFFFFFFFFFFFFFFFFFFFFFFFFFFFFFFFFFF:FFFFFFFFFF
FFFFFFFFFFFFFFFFF,FFFFFFFFFFFFFFFFFFFFFFFFFFFFFFFFFFFFFFFFFFFFFFFFFF
@A00155:342:HHGFNDSXY:1:2502:9417:5619 2:N:0:GAACCTAG+TCCGCATA
GTGCTATGCCTTAAATTACCATCGAAGTTGAATTCGGCTCCTGGTGCCTCGTGATTCGGGAAGAACCT
CTTAACGAAACTATCCATTACTCTGAGTTTGCTTTTAGCTTGGGTTCGTGAGAATTGAAACAAAGCTT
+
FFF:FFF:FFFFFFF:FFFFFFFFFFFFF:FFFFFFFFFFFFFFFFFFFFFF:FFFFFFFFFFFFFFF
FF:FF:FFFFFFFFFFFFFFFFFFFFFFFFFF,FFFFFFFFFFFFFFFFFFFFFFFFFFFFFFFFFFF
```

```
@A00155:342:HHGFNDSXY:1:2456:32786:7607 2:N:0:GAACCTAG+TCCGCATA
GTGCTATGCCTTAAATTACCATCGAAGTTGAATTCGGCTCCTGGTGCCTCGTGATTCGGGAAGAACCT
CTTAACGAAACTATCCATTACTCTGAGTTTGCTTTTAGCTTGGGTTCGTGAGAATTGAAACAAAGCTT
+
F:FFFFFFFFFFFFFFFFFFFFFFFF:F:FFFFFFFFFFFFFFFFFFFFFFFFFFFFFFFFFFFFFFF
FFFFFF:FFFFFFFFFFFFFF:FFFFFFFFFFFFFFFFFFFFFFFFFFFFFFFFFFFFFFFFFFFFFF
@A00155:342:HHGFNDSXY:1:1333:30427:32236 2:N:0:GAACCTAG+TCCGCATA
GTGCTATGCCTTAAATTACCATCGAAGTTGAATTCGGCTCCTGGTGCCTCGTGATTCGGGAAGAACCT
CTTAACGAAACTATCCATTACTCTGAGTTTGCTTTTAGCTTGGGTTCGTGAGAATTGAAACAAAGCTT
+
FFFFFFFFFFFFFFFFFFFFFFFFFFFFFFFFFFFFFFFFFFFFFFF:FFFFFFFFFFFFFFFFFFFF
FF:F:FFFFFFFFFFFFFFFFFFFFFF:FFFF:FFFFFFFFFFFFFFFFFFFFFFFFFFFFFFFFFFF
@A00155:342:HHGFNDSXY:1:1402:1759:6605 2:N:0:GAACCTAG+TCCGCATA
GTGCTATGCCTTAAATTACCATCGAAGTTGAATTCGGCTCCTGGTGCCTCGTGATTCGGGAAGAACCT
CTTAACGAAACTATCCATTACTCTGAGTTTGCTTTTAGCTTGGGTTCGTGAGAATTGAAACAAAGCTT
+
FFF:FFFF,FFFFFFF,FFF:FFFFFF,FFFFFFFF:FFFFFFFFFFF:F:FFFFFFFFFFFFFFFF:
,FFFFFFFFFFFF:FFF:FFFF:FFF:FFFF,:FFF,FFFFFFFFFFF::FFFFFFFFFFFFFF:FFF
@A00155:342:HHGFNDSXY:1:1377:12635:12602 2:N:0:GAACCTAG+TCCGCATA
GTGCTATGCCTTAAATTACCATCGAAGTTGAATTCGGCTCCTGGTGCCTCGTGATTCGGGAAGAACCT
CTTAACGAAACTATCCATTACTCTGAGTTTGCTTTTAGCTTGGGTTCGTGAGAATTGAAACAAAGCTT
+
FFFFFFFFFFFFFFFFFFFF:FFFF::FFFF:FFFFFFFFFFFFFFFFFFF:FFFFFFFFFFFFFFFF
FFFFFFFFFFFFFFFFFF::FFFFFFFFFFFFFFFFFFFFFFFFFFFFFFFFFFFFFFFFFFFFFFFF
@A00155:342:HHGFNDSXY:1:1377:12491:12101 2:N:0:GAACCTAG+TCCGCATA
GTGCTATGCCTTAAATTACCATCGAAGTTGAATTCGGCTCCTGGTGCCTCGTGATTCGGGAAGAACCT
CTTAACGAAACTATCCATTACTCTGAGTTTGCTTTTAGCTTGGGTTCGTGAGAATTGAAACAAAGCTT
+
FFFFF:F,F:FF,:,FFFFFFF,:FF:FFFFFFF:FFFFF:FF:FF:FFFFF:FFFF:FFFF,FF,F,
F:FFF,FFF:FF,:F,:FFFFFFFFFFFFFFFFF,FFFFFF:FFF:F,FFF:FFFF:FF:F,F:FFF
@A00155:342:HHGFNDSXY:1:1355:18692:3834 2:N:0:GAACCTAG+TCCGCATA
GTGCTATGCCTTAAATTACCATCGAAGTTGAATTCGGCTCCTGGTGCCTCGTGATTCGGGAAGAACCT
CTTAACGAAACTATCCATTACTCTGAGTTTGCTTTTAGCTTGGGTTCGTGAGAATTGAAACAAAGCTT
+
FFFFFFFFFFFFFFFFFFFFFFFFFFFFFFFFFFFFFFFFFFFFFFFFFFFFFFFFFFFFFFFFFFFF
FFFFFFFFFFFFFFFFFFFFFFFFFFFFFFFFFFFFFFFFFFFFFFFFFFFFFFFFFFFFFFFFFFFF
@A00155:342:HHGFNDSXY:1:1219:32750:22889 1:N:0:GAACCTAG+TCCGCATA
TGCTATGCCTTAAATTACCATCGAAGTTGAATTCGGCTCCTGGTGCCTCGTGATTCGGGAAGAACCTC
TTAACGAAACTATCCATTACTCTGAGTTTGCTTTTAGCTTGGGTTCGTGAGAATTGAAACAAAGCTTA
+
FFFFFFF:FFFFFFFFFFFFFFFFFFFFFFFFFFFFFFFF:FFFFFFFFFFFFFFFFFFFFFFFFFFF
FFFFFFFFFFF:FFFFFFFFFFFFFFFFFFF,F:FFFFFFFFFFFFF:FFFFFFFFFFFFFFFFFF:F
@A00155:342:HHGFNDSXY:1:2248:29658:22670 2:N:0:GAACCTAG+TCCGCATA
GCTATGCCTTAAATTACCATCGAAGTTGAATTCGGCTCCTGGTGCCTCGTGATTCGGGAAGAACCTCT
TAACGAAACTATCCATTACTCTGAGTTTGCTTTTAGCTTGGGTTCGTGAGAATTGAAACA
+
:FFFFF:FFFFFFFFF::FF:FFFFFFFFFFFFFFFFFFFFFF:FFF:FF:FFFFFF:FF:FFFFFFF
F:FFFFFFFFFF:FFFFFFFFFFF:FFFF:FFFFFFFFFFFFFFFFFF:FFFFFFFFFFF
@A00155:342:HHGFNDSXY:1:2558:1750:34867 2:N:0:GAACCTAG+TCCGCATA
GCTATGCCTTAAATTACCATCGAAGTTGAATTCGGCTCCTGGTGCCTCGTGATTCGGGAAGAACCTCT
TAACGAAACTATCCATTACTCTGAGTTTGCTTTTAGCTTGGGTTCGTGAGAATTGAAACAAAGC
+
FFFFFFFFFFF,F:,FFF,:FFFFFFFFF:,F,F:FFFFFFFF::FFFFF,FFF,FFFFFFFFFFFFF
:::FFFFFFFFFFFF,:FFFF:FFFF:F:FFFFF:FF:F:FFFFFFFFFFF::,FF:FFF:FFF
```

```
@A00155:342:HHGFNDSXY:1:1326:11469:33630 2:N:0:GAACCTAG+TCCGCATA
GCTATGCCTTAAATTACCATCGAAGTTGAATTCGGCTCCTGGTGCCTCGTGATTCGGGAAGAACCTCT
TAACGAAACTATCCATTACTCTGAGTTTGCTTTTAGCTTGGGTTCGTGAGAATTGAAACAAAGC
+
FFFFFFF:FFFFFFFFFFFFF:FFFFFFFFFFFFFFFFFFFFFF:FFFFFFFFF:FF,FFFFFFFFFF
FFFFFFFFFFFFF:FFFFFFFF:FFFFFFFFFFFFF,FFFFFFFFFFFFFFFFFFFFFFFFFFF
@A00155:342:HHGFNDSXY:1:1530:18313:8124 2:N:0:GAACCTAG+TCCGCATA
GCTATGCCTTAAATTACCATCGAAGTTGAATTCGGCTCCTGGTGCCTCGTGATTCGGGAAGAACCTCT
TAACGAAACTATCCATTACTCTGAGTTTGCTTTTAGCTTGGGTTCGTGAGAATTGAAACAAAGCT
+
FFFFFFFFFFFFFFFFFF:FFFFFFFFFFFFFFFFFFFFFFF,FFFFFFFFFFFFFFFFFFFFFFFFF
FFFFFFFFFFFFFFFFFFF:FFFFFFFFFFFFFFFFFFFFFFFFFFFFFFFFFFFFFFFFFFFFF
@A00155:342:HHGFNDSXY:1:2359:21251:2159 2:N:0:GAACCTAG+TCCGCATA
GCTATGCCTTAAATGACCATCGAAGTTGAATTCGGCTCCTGGTGCCTCGTGATTCGGGAAGAACCTCT
TAACGAAACTATCCATTACTCTGAGTTTGCTTTTAGCTTGGGTTCGTGAGAATTGAAACAAAGCTT
+
FFFFFFFFF:F,FF,FFFFFFFFFF,FFFFFFFFFFFFFFFFFFFFFFFFFFFFFFFFFFFFFFF,FF
FFFFFFFFFFFFFFFFFFFFFFFFFFFFFFFFFFFFFFFFFFFFFFFFFFFFFFFFFFFFFFFFFF
@A00155:342:HHGFNDSXY:1:1453:22516:2409 2:N:0:GAACCTAG+TCCGCATA
CTATGCCTTAAATTACCATCGAAGTTGAATTCGGCTCCTGGTGCCTCGTGATTCGGGAAGAACCTCTT
AACGAAACTATCCATTACTCTGAGTTTGCTTTTAGCTTGGGTTCGTGAGAATTGAAACAA
+
FFFFFF:FFFF,FFFFF,FFFF:FFFFFFFFFFFFFFFFFF,FFFFFF:FFFFFFFFFFFFFFFFFFF
FFFFFFFFF:FFFFFFFFFFFFFFF:FFFFFFFFFFFFFFFFFFFFFFFFFFFFFFFFFF
@A00155:342:HHGFNDSXY:1:2472:30770:2300 2:N:0:GAACCTAG+TCCGCATA
CTATGCCTTAAATTACCATCGAAGTTGAATTCGGCTCCTGGTGCCTCGTGATTCGGGAAGAACCTCTT
AACGAAACTATCCATTACTCTGAGTTTGCTTTTAGCTTGGGTTCGTGAGAATTGAAACAA
+
FF:FFFFFFF:FFFFFF:FFFF:FFFFFFFFFFFFFFFFFFFFFFFFFFFFFFFFFFFFFFFFFFF:F
FFFFFFFFFFFFFFFFFFFFFFFFFFFFFFFFFFFFFFFFFFFFFFFFFFFFFFFFFFFF
@A00155:342:HHGFNDSXY:1:1232:4209:10410 2:N:0:GAACCTAG+TCCGCATA
CTATGCCTTAAATTACCATCGAAGTTGAATTCGGCTCCTGGTGCCTCGTGATTCGGGAAGAACCTCTT
AACGAAACTATCCATTACTCTGAGTTTGCTTTTAGCTTGGGTTCGTGAGAATTGAAACAAAGCTT
+
FFFFFFFFFFFFFFFFF,FFFF,FFFFFFFFFFFFFFFFFFFFFFFFFFFFFF:FFFFFFFFFFFFF:
F:FFFFFF:FFFFFFFFFFFFFFFFFFFFFFFFFFFFFFFFFFFFFFFFFFFFFF:FFFFFFFFF
@A00155:342:HHGFNDSXY:1:2243:30617:26396 1:N:0:GAACCTAG+TCCGCATA
TATGCCTTAAATTACCATCGAAGTTGAATTCGGCTCCTGGTGCCTCGTGATTCGGGAAGAACCTCTTA
ACGAAACTATCCATTACTCTGAGTTTGCTTTTAGCTTGGGTTCGTGAGAATTGAAACAAAGCTT
+
FFFFFFFFFFFFFFFFFFFFFFFFFFFFFFFFFFFFFFFFFFFFFFFFFFFFFFFFFFFFFFFFFFFF
FFFFFFFFFFFFFFFFFFFFFFFFFFFFFFFFFFFFFFFFFFFFFFFFFFFFFFFFFFFFFFFF
@A00155:342:HHGFNDSXY:1:1253:27642:34147 1:N:0:GAACCTAG+TCCGCATA
TATGCCTTAAATTACCATCGAAGTTGAATTCGGCTCCTGGTGCCTCGTGATTCGGGAAGAACCTCTTA
ACGAAACTATCCATTACTCTGAGTTTGCTTTTAGCTTGGGTTCGTGAGAATTGAAACAAAGCTTAGG
+
FFFFFFFFFFFFFFFFFFFFFFFFF:F:F:FFFFFFFF:FFFFFFFFFFFFFFFF,FFFFFFFFFFFF
FFFFFFFFFFFFFFFFFFFFFFFFFFFF:FFFFFFFFFFFFFFFFFFFFFFFFFFFFFFFF:FFF:F
@A00155:342:HHGFNDSXY:1:2114:11397:11146 1:N:0:GAACCTAG+TCCGCATA
TATGCCTTAAATTACCATCGAAGTTGAATTCGGCTCCTGGTGCCTCGTGATTCGGGAAGAACCTCTTA
ACGAAACTATCCATTACTCTGAGTTTGCTTTTAGCTTGGGTTCGTGAGAATTGAAACAAAGCTTAGG
+
FFFFFFFFFFFFF:FFFFFFFFFFFFFFFFFFFFFFFFFFFFFFFFFFFFFFFFFFFFFFFFFFFFFF
FFFFFFFFFFFFFFFFFFFFFFFFFFFFF,FFFFFFFFFFF:FFFFFFFFF:FFFFFFFFFFFFFFF
```

@A00155:342:HHGFNDSXY:1:2166:25654:14951 1:N:0:GAACCTAG+TCCGCATA
TATGCCTTAAATTACCATCGAAGTTGAATTCGGCTCCTGGTGCCTCGTGATTCGGGAAGAACCTCTTA
ACGAAACTATCCATTACTCTGAGTTTGCTTTTAGCTTGGGTTCGTGAGAATTGAAACAAAGCTTAGG
+
FFFFF:FFFFFFFFFFFFFFFFFFFFFFFFFFFFFFFFFFFFFFFFFFFFFFFFFFFFFFFFFF
FFFFFFFFFFFFFFFFFFFFFFFFFFFFFFFFFFFFFFFFFFFFFFFFFFFFFFFFFFFFFFFFFFF
@A00155:342:HHGFNDSXY:1:1253:27688:34287 1:N:0:GAACCTAG+TCCGCATA
TATGCCTTAAATTACCATCGAAGTTGAATTCGGCTCCTGGTGCCTCGTGATTCGGGAAGAACCTCTTA
ACGAAACTATCCATTACTCTGAGTTTGCTTTTAGCTTGGGTTCGTGAGAATTGAAACAAAGCTTAGG
+
FFFFFFFFFFFFFFFFFFFFFFFFFFFFFFFFFFFFFFFFFFFFFFFFFFFFFFFFFFFFFFFFFFFF
FFFFFFFFFFFFFFFFFFFFFFFF:FFFFFFFFFFFFFFFFFFFFFFFFFFFFFFFFFF:FFFFFFF
@A00155:342:HHGFNDSXY:1:2359:6162:8218 1:N:0:GAACCTAG+TCCGCATA
TATGCCTTAAATTACCATCGAAGTTGAATTCGGCTCCTGGTGCCTCGTGATTCGGGAAGAACCTCTTA
ACGAAACTATCCATTACTCTGAGTTTGCTTTTAGCTTGGGTTCGTGAGAATTGAAACAAAGCTTAGGG
+
FFFFFFFFFFFFFFFFFFFFFFFFFFFFFFFFFFFFFFFFFFFFFFFFFFFFFFFFFFFFFFF:FFFF
FFFFFFFFFFFFFFFFFFF:FFFFFFFFF:FFFFFFFF:FFF:F,:FFFFF:FFFFFFFF:FFFFFFF
@A00155:342:HHGFNDSXY:1:1423:31412:36605 1:N:0:GAACCTAG+TCCGCATA
TATGCCTTAAATTACCATCGAAGTTGAATTCGGCTCCTGGTGCCTCGTGATTCGGGAAGAACCTCTTA
ACGAAACTATCCATTACTCTGAGTTTGCTTTTAGCTTGGGTTCGTGAGAATTGAAACAAAGCTTAGGG
+
FFFFFFFFFFFFFFFFFFFFFFFFFFFF:FFFFFFFFFFFFFFFFFFFFFFFFFFFFFFFFFFFFFFF
FFFFFFFFFFFF:FFFFFFFFFF,FFFFFFFFFFFFFFFFF:FF:FFFFFFFF::FFFFFFF,FFFFF
@A00155:342:HHGFNDSXY:1:1178:25446:1063 1:N:0:GAACCTAG+TCCGCATA
TATGCCTTAAATTACCATCGAAGTTGAATTCGGCTCCTGGTGCCTCGTGATTCGGGAAGAACCTCTTA
ACGAAACTATCCATTACTCTGAGTTTGCTTTTAGCTTGGGTTCGTGAGACTTGAAACAAAGCTTAGGG
+
FFFFFF:FFFF,FFFFFF:FFFF,F,:FFF,FF:FFFFFFF:FFFFF:FFF,F:FFFFFFFFFFFFF:
FF,FFFFFF:,FFFFFFFFFFFF:FFF,FF,FFFFFFFFFFFF:FFF:F,:FFFFFFFFFF,FFFFFF
@A00155:342:HHGFNDSXY:1:2138:21386:8343 1:N:0:GAACCTAG+TCCGCATA
TATGCCTTAAATTACCATCGAAGTTGAATTCGGCTCCTGGTGCCTCGTGATTCGGGAAGAACCTCTTA
ACGAAACTATCCATTACTCTGAGTTTGCTTTTAGCTTGGGTTCGTGAGAATTGAAACAAAGCTTAGGG
+
FFFFFFF:FFFFFFFFFFFFFFFFFFFFFFFFFFFFFFFFFFFFFFFFFFFFFFFFFFFFFFFFFFFF
FFFFFFFFFFFFFFFFFFFFFFFFFFFFFFFFFFFFFFFFFFFFFFFFFF:FFFFFFFFFFFFFFFFF
@A00155:342:HHGFNDSXY:1:2133:28122:1752 1:N:0:GAACCTAG+TCCGCATA
TATGCCTTAAATTACCATCGAAGTTGAATTCGGCTCCTGGTGCCTCGTGATTCGGGAAGAACCTCTTA
ACGAAACTATCCATTACTCTGAGTTTGCTTTTAGCTTGGGTTCGTGAGAATTGAAACAAAGCTTAGGG
+
FFFFFFFFFFFFFFFFFFFFFFFFFFFFFFFFFFFFFFFFFFFFFFFFFFFFFFFFFFFFFFFFFFFF
FFFFFFFFFFFFFFFFFFFFFFFFFFFFFFFFFFFFFFFFFFFFFFFFFFFFFFFFF:FFF:FFFFFF
@A00155:342:HHGFNDSXY:1:1210:14977:29528 1:N:0:GAACCTAG+TCCGCATA
TATGCCTTAAATTACCATCGAAGTTGAATTCGGCTCCTGGTGCCTCGTGATTCGGGAAGAACCTCTTA
ACGAAACTATCCATTACTCTGAGTTTGCTTTTAGCTTGGGTTCGTGAGAATTGAAACAAAGCTTAGGG
+
FFFFFFFFFFFFFFFFFFFFFFFFFFFFFFFFFFFFFFFFFFFFFFFFFFF:FFFFFFFFFFFFFFFF
FFFFFFFFFFFFFFFFFFFFFFFFFFFFFFFFFFFFFFFFFFFFFFFFF:FFFFFFF:FFFFFFFFFF
@A00155:342:HHGFNDSXY:1:2132:29658:27117 1:N:0:GAACCTAG+TCCGCATA
TATGCCTTAAATTACCATCGAAGTTGAATTCGGCTCCTGGTGCCTCGTGATTCGGGAAGAACCTCTTA
ACGAAACTATCCATTACTCTGAGTTTGCTTTTAGCTTGGGTTCGTGAGAATTGAAACAAAGCTTAGGG
+
FFFFFFFFFFFFFFFFFFFFFFFFFFFFFFFFFFFFFFFFFFFFFFFFFFFFFFFFFFFFFFFFFFFF
FFFFFFFFFFFFFFFFFFFFFFFFFFFFF:FFFFFFFFFFF:FFFFFFFFFFFFFFFF:FFFFFFFFF

```
@A00155:342:HHGFNDSXY:1:2502:27923:13933 1:N:0:GAACCTAG+TCCGCATA
TATGCCTTAAATTACCATCGAAGTTGAATTCGGCTCCTGGTGCCTCGTGATTCGGGAAGAACCTCTTA
ACGAAACTATCCATTACTCTGAGTTTGCTTTTAGCTTGGGTTCGTGAGAATTGAAACAAAGCTTAGGG
+
FFFFFFFFFFFFFFFFFFFFFFFFFFFFFFFFFFFFFFFFFFFFFFFFFFFFFFFFFFFFFFFFFFFF
FFFFFFFFFFFF:FFFFFFFFFFFFFFFF:FFFFFFFFFFFFFFFFFFF:FFFFF:FFFFFFF,FFFF
@A00155:342:HHGFNDSXY:1:2330:13602:28119 1:N:0:GAACCTAG+TCCGCATA
TATGCCTTAAATTACCATCGAAGTTGAATTCGGCTCCTGGTGCCTCGTGATTCGGGAAGAACCTCTTA
ACGAAACTATCCATTACTCTGAGTTTGCTTTTAGCTTGGGTTCGTGAGAATTGAAACAAAGCTTAGGG
+
FFFFFFFFFFFFFFFFFFFFF:FFFFFFFFFFFFFFFFFFFFFFFFFFFFFFFFFFFFFFFFFFFFFF
FFFFFFFFFFFFFFFFFF::FFFFFFFFFFFFFFF:FFFFF:FFFFFFFFFFFFFFFFFFF:F::FFF
@A00155:342:HHGFNDSXY:1:1121:17888:22545 1:N:0:GAACCTAG+TCCGCATA
ATGCCTTAAATTACCATCGAAGTTGAATTCGGCTCCTGGTGCCTCGTGATTCGGGAAGAACCTCTTAA
CGAAACTATCCATTACTCTGAGTTTGCTTTTAGCTTGGGTTCGTGAGAATTGAAACAAAGCTTAGG
+
FFFFFF:FFFFFFFFFFFFFFFFFFFFFFFFFFFFFFFFFFFFFFFFFFFFFFFFFFFFFFFFFFFFF
FFFFFFFFFFFFFFFFFFFFFFFFFFFFFFFFFFFFFFFFFFFFFFFFFFFFFFFFFFFFFFFFFF
@A00155:342:HHGFNDSXY:1:2408:3278:5979 1:N:0:GAACCTAG+TCCGCATA
ATGCCTTAAATTACCATCGAAGTTGAATTCGGCTCCTGGTGCCTCGTGATTCGGGAAGAACCTCTTAA
CGAAACTATCCATTACTCTGAGTTTGCTTTTAGCTTGGGTTCGTGAGAATTGAAACAAAGCTTAGGGT
+
:FFFFF,FFFFFFFFFFFFFFFFFFFFFFFFFFFFFFFFFFFFF,FFFFFFFFFFFFFFFFFFFFFFF
FFFFFFFFFFFFFFFFFFFFFFFFFFFFFFFF:FFFFFF:FFFFFFFF,FFFFFFF:FFFF:,FF,FF
@A00155:342:HHGFNDSXY:1:1613:25599:10473 2:N:0:GAACCTAG+TCCGCATA
GCCTTAAATTACCATCGAAGTTGAATTCGGCTCCTGGTGCCTCGTGATTCGGGAAGAACCTCTTAACG
AAACTATCCATTACTCTGAGTTTGCTTTTAGCTTGGGTTCGTGAGAATTGAAACAAAGC
+
FFF,:FFFFFFFFF,FFFFFFFFFFFFFFFFFFFFFFFF:FFFFFFF:FFF:F:FFFF:FFF:FF:F:
,FFFFFFFF,FFF:FF:FFFFF::FFF:F,FFFFFFFFFFFFFFFFFFFFFFFFFFFFF
@A00155:342:HHGFNDSXY:1:2315:14615:18662 2:N:0:GAACCTAG+TCCGCATA
GCCTTAAATTACCATCGAAGTTGAATTCGGCTCCTGGTGCCTCGTGATTCGGGAAGAACCTCTTAACG
AAACTATCCATTACTCTGAGTTTGCTTTTAGCTTGGGTTCGTGAGAATTGAAACAAAGCT
+
FFFFFFFF:FFFF,FFFF:FFFFFF:FFFFFFFFFFFFFFFFFFFFFFFFFFFF:FFFFFFFFF,,FF
FFFF,FFFFF::FFFFFFFFFFFFFFFF:FF:FFFFFFFFFFFFF:FFFF:F:FF:FFFF
@A00155:342:HHGFNDSXY:1:2305:7048:19774 2:N:0:GAACCTAG+TCCGCATA
GCCTTAAATTACCATCGAAGTTGAATTCGGCTCCTGGTGCCTCGTGATTCGGGAAGAACCTCTTAACG
AAACTATCCATTACTCTGAGTTTGCTTTTAGCTTGGGTTCGTGAGAATTGAAACAAAGCT
+
FFF:,FFFFFFFFF:FFFFFFFF:FFF:FFFFFFFFFFFFF:FFFFFFFFF,FFFFFFFFFFF,F:::
FFFFFFFFFFFFFFFFFF:FFFFFFFFFFFFFFFFFFFFFFFFFFFFFFFFFFFFFF:FF
@A00155:342:HHGFNDSXY:1:1638:11198:11052 2:N:0:GAACCTAG+TCCGCATA
GCCTTAAATTACCATCGAAGTTGAATTCGGCTCCTGGTGCCTCGTGATTCGGGAAGAACCTCTTAACG
AAACTATCCATTACTCTGAGTTTGCTTTTAGCTTGGGTTCGTGAGAATTGAAACAAAGCT
+
FFFFFFFFF,FFFF:FFFFFFFFF,FFFFFFFFFFFFFFFFFFFFFFFFFFFFFFFFFFFFFFFFFF
FFFFFFFFFFFFFFFFFFFFFFFFFFFFFFFFFFFFFFFFFFFFFFFFFFFFFFFFFFFF
@A00155:342:HHGFNDSXY:1:1557:13711:5165 1:N:0:GAACCTAG+TCCGCATA
GCCTTAAATTACCATCGAAGTTGAATTCGGCTCCTGGTGCCTCGTGATTCGGGAAGAACCTCTTAACG
AAACTATCCATTACTCTGAGTTTGCTTTTAGCTTGGGTTCGTGAGAATTGAAACAAAGCTTAGGGTTT
+
,FF,FFFFFFFFFF,FFFF:FF:FFFFFFFF,:F,,FFFFF,F:FFFFFFFFFFF:FF:F:FFFFFFF
FFF::FFFFFFFF:FFFFFFFFF,F,FFFF,FFF,FF,F,FFF:FF,,FFFFF:FFFFF:FF,FFFFF
```

```
@A00155:342:HHGFNDSXY:1:2574:9182:24283 2:N:0:GAACCTAG+TCCGCATA
CCTTAAATTACCATCGAAGTTGAATTCGGCTCCTGGTGCCTCGTGATTCGGGAAGAACCTCTTAACGA
AACTATCCATTACTCTGAGTTTGCTTTTAGCTTGGGTTCGTGAGAATTGAAACAAAGCT
+
FFFFF:FFFFFF:FFFFFFFFFFFFFFFFFFFFFFF:FFFFFFFFFFFFFFFFFFFFFFFF,FFFFFF
FFFFFFFFFFFFF:FFFFFFFFFFFFFFFFFFFFFFFFFFFFFFFFFFFFFFFFFFFFF
@A00155:342:HHGFNDSXY:1:1373:22345:6590 2:N:0:GAACCTAG+TCCGCATA
CCTTAAATTACCATCGAAGTTGAATTCGGCTCCTGGTGCCTCGTGATTCGGGAAGAACCTCTTAACGA
AACTATCCATTACTCTGAGTTTGCTTTTAGCTTGGGTTCGTGAGAATTGAAACAAAGCTT
+
FFFFFFFFFFFFFFFFFFFFFFFFFFFFFFFFFFFFFFFFFFF,FFFFFFFFFFFFFFFFFFFFFFFF
FFFFFFFFFFFFFFFFFFFFFFFF:FFFFF:FFFFFFFFFFFFFFFFFFFFFFFFFFFFF
@A00155:342:HHGFNDSXY:1:2607:25916:23234 2:N:0:GAACCTAG+TCCGCATA
CCTTAAATTACCATCGAAGTTGAATTCGGCTCCTGGTGCCTCGTGATTCGGGAAGAACCTCTTAACGA
AACTATCCATTACTCTGAGTTTGCTTTTAGCTTGGGTTCGTGAGAATTGAAACAAAGCTT
+
FFFFFFFFFFFFFFFFFFFFFFFFFFFFFFFFFFFFFFFFFFFFFFFFFFFFFFFFFFFFFFFF:FFF
FFFFFFFFFFFFFFFFFFFF,FFFFFFFFFFFFFFFFFFFFFFFFFFFFFFFFFFFFFFF
@A00155:342:HHGFNDSXY:1:2618:7636:6731 1:N:0:GAACCTAG+TCCGCATA
CCTTAAATTACCATCGAAGTTGAATTCGGCTCCTGGTGCCTCGTGATTCGGGAAGAACCTCTTAACGA
AACTATCCATTACTCTGAGTTTGCTTTTAGCTTGGGTTCGTGAGAATTGAAACAAAGCTTAGGGTTTA
+
FFFFFFFFFFFFFFFFFFFFFFFFFF:FFFFFFFFFFFFFFFFFFFFFFFFFFFFFFFFFFFFFFF:F
FFFFFFF:FFFFFFFFFFFFFFFFFFFFFFFFFFF:FFFFFFFFFFFFFFFF:FFFFFFFFFFF:FFFF
@A00155:342:HHGFNDSXY:1:1165:15103:19132 1:N:0:GAACCTAG+TCCGCATA
CCTTAAATTACCATCGAAGTTGAATTCGGCTCCTGGTGCCTCGTGATTCGGGAAGAACCTCTTAACGA
AACTATCCATTACTCTGAGTTTGCTTTTAGCTTGGGTTCGTGAGAATTGAAACAAAGCTTAGGGTTTA
+
FFFFFFFFFFFFFFFFFFFFFFFFFFFFFFFFFFFFFFFFFFFFFFFFFFFFFFFFFFFFFFFFFFFF
FFFFFFFFFFFFFFFFFFFFFFFFFFFFFFFFFFFFFFFFFFFFFFFFFFFFFFFFFF,FFFFFFFFF
@A00155:342:HHGFNDSXY:1:2521:1063:4304 1:N:0:GAACCTAG+TCCGCATA
TTAAATTACCATCGAAGTTGAATTCGGCTCCTGGTGCCTCGTGATTCGGGAAGAACCTCTTAACGAAA
CTATCCATTACTCTGAGTTTGCTTTTAGCTTGGGTTCGTGAGAATTGAAACAAAGCTTAGGGTTTAG
+
FFFFFFFFFFFFFFFFFFFFFFFFFFFFFFFFFFFFFFFFFFFFFFFFFFFFFFFF:FFFFF:FFFFF
FFFFFFFFFFFF:FFFFFFFF:FFFF::,FFFF:FF,:FFFF:FFF,F:F:FF,,:FF:FF,FFFFF
@A00155:342:HHGFNDSXY:1:2234:6732:31407 1:N:0:GAACCTAG+TCCGCATA
TTAAATTACCATCGAAGTTGAATTCGGCTCCTGGTGCCTCGTGATTCGGGAAGAACCTCTTAACGAAA
CTATCCATTACTCTGAGTTTGCTTTTAGCTTGGGTTCGTGAGAATTGAAACAAAGCTTAGGGTTTAG
+
FFFFFFFFFFFFFFFFFFFFFFFFFFFFFFFFFFFFFFFFFFFFFFFFFFF:FFFFFFFF:FFFFFFF
FF:FFFFFFFFFFFFFFFFFFFFFFFFFFFFFFFFFFFFFFFF:FFFFFFFFFFF:F,F:FFF:FFF
@A00155:342:HHGFNDSXY:1:2264:20473:30843 1:N:0:GAACCTAG+TCCGCATA
TTAAATTACCATCGAAGTTGAATTCGGCTCCTGGTGCCTCGTGATTCGGGAAGAACCTCTTAACGAAA
CTATCCATTACTCTGAGTTTGCTTTTAGCTTGGGTTCGTGAGAATTGAAACAAAGCTTAGGGTTTAGT
+
FFFFFFFFFFFFFFFFFFFFFFFFFFFFFFFFFFFFFFFFFFFFFFFFFFFFFFFFFFFFFFFFFFFF
FFFFFF:FFFFFFFFFFFFFFFFFFFFFFFFFFFFFFFFFFFFFFFFFFFFFFFFFFFFFFFFFFFFF
@A00155:342:HHGFNDSXY:1:2323:20509:12085 1:N:0:GAACCTAG+TCCGCATA
TAAATTACCATCGAAGTTGAATTCGGCTCCTGGTGCCTCGTGATTCGGGAAGAACCTCTTAACGAAAC
TATCCATTACTCTGAGTTTGCTTTTAGCTTGGGTTCGTGAGAATTGAAACAAAGCTTAGGGTTTAG
+
FFFFFFFFFFFFFFFFFFFFFFFFFFFFFFFFFFFFFFFFFFFFFFFFFFFFFFFFFFFFFFFFFFFF
FF:FFFFFFFFFFFFFFFFFFFFFFFFFFFFFFFFFFFFFFFFFFFFFFFFF:FF,FFFFFFF:FF
```

```
@A00155:342:HHGFNDSXY:1:1201:31919:35697 1:N:0:GAACCTAG+TCCGCATA
TAAATTACCATCGAAGTTGAATTCGGCTCCTGGTGCCTCGTGATTCGGGAAGAACCTCTTAACGAAAC
TATCCATTACTCTGAGTTTGCTTTTAGCTTGGGTTCGTGAGAATTGAAACAAAGCTTAGGGTTTAG
+
FFFFFFFFFFFFFFFFFFFFFFF:FFFFFFFFFFFFFFFFFFFFFFFFFFFFF:FFFFFFFFFF:FF:
FFFFFFFF,FFFFFFFFFFFF,::FFFFFFFF::FFFFFFFFFFFFFFFFFFFFFFFFFFFFFFFF
@A00155:342:HHGFNDSXY:1:2678:23104:10942 1:N:0:GAACCTAG+TCCGCATA
TAAATTACCATCGAAGTTGAATTCGGCTCCTGGTGCCTCGTGATTCGGGAAGAACCTCTTAACGAAAC
TATCCATTACTCTGAGTTTGCTTTTAGCTTGGGTTCGTGAGAATTGAAACAAAGCTTAGGGTTTAG
+
FFFFFFFFFFFFFFFFFFFFFFFFFFFFFFFFFFFFFFFFFFFFFFFFFFFFFFFFFFFFFFFFFFFF
FFFFFFFFFFFFFFFFFFFFFFFFFFFFFFFFFFFFFFFFFFFFFFFFFFFFFF:FFFFFFFFFFF
@A00155:342:HHGFNDSXY:1:1124:31467:28839 1:N:0:GAACCTAG+TCCGCATA
TAAATTACCATCGAAGTTGAATTCGGCTCCTGGTGCCTCGTGATTCGGGAAGAACCTCTTAACGAAAC
TATCCATTACTCTGAGTTTGCTTTTAGCTTGGGTTCGTGAGAATTGAAACAAAGCTTAGGGTTTAG
+
FFFFFFFFFFFFFFFFFFFFFFFFFFFFFFFFFFFFFFFFFFFFFFFFFF:FFFFFFFFFFFFFFFFF
FFFFFFFFFFFFFFFF:FFFFFFFFFFFFFFFFFFFFFFF:FFFFFFFFFFFFFF,FFFFFFFFFF
@A00155:342:HHGFNDSXY:1:2555:1479:36965 1:N:0:GAACCTAG+TCCGCATA
TAAATTACCATCGAAGTTGAATTCGGCTCCTGGTGCCTCGTGATTCGGGAAGAACCTCTTAACGAAAC
TATCCATTACTCTGAGTTTGCTTTTAGCTTGGGTTCGTGAGAATTGAAACAAAGCTTAGGGTTTAGTT
+
FFFFFFFFFFFFFFFFFF:FFFFF::FF,:FFFFFF,FF,FF:FFF,FFFFFFF:FF:FFFF:FFFFF
FFFFFFFFFFF:FFFFFFFF,FFFF:,FFFFFF:F,FFF:F,FFF:,::FF:FF:F,FFFFFFFFFFF
@A00155:342:HHGFNDSXY:1:2456:32786:7607 1:N:0:GAACCTAG+TCCGCATA
TAAATTACCATCGAAGTTGAATTCGGCTCCTGGTGCCTCGTGATTCGGGAAGAACCTCTTAACGAAAC
TATCCATTACTCTGAGTTTGCTTTTAGCTTGGGTTCGTGAGAATTGAAACAAAGCTTAGGGTTTAGTT
+
FFFFFFFFFFFFFFFFFFFFFFFFFFFFFFFFFFFFFFFFFFFFFFFFFFFFFFFFFFFFFFFFFFFF
FFFFFFFFFFFFFFFF:FFFFFFFFFFFFFFFFFFFFFFFFFFFFF:FFFFFFF,FFFFFFFFFFFFF
@A00155:342:HHGFNDSXY:1:2114:6985:18286 1:N:0:GAACCTAG+TCCGCATA
AAATTACCATCGAAGTTGAATTCGGCTCCTGGTGCCTCGTGATTCGGGAAGAACCTCTTAACGAAACT
ATCCATTACTCTGAGTTTGCTTTTAGCTTGGGTTCGTGAGAATTGAAACAAAGCTTAGGGTTTAG
+
FFFF:FFFFFFFFFFFFFFFFFFFFFFFFFFFFFFFFFFFFFFFFF:FF:,FFFFFFFFFFFFFFFFF
FFF:FFFFFFFFFFFFFFFFFFFFF,FFFFFFFFFF:FFFFFFFFFFFFFFF,FF:F:FF,FFFF
@A00155:342:HHGFNDSXY:1:1470:28085:24893 1:N:0:GAACCTAG+TCCGCATA
AATTACCATCGAAGTTGAATTCGGCTCCTGGTGCCTCGTGATTCGGGAAGAACCTCTTAACGAAACTA
TCCATTACTCTGAGTTTGCTTTTAGCTTGGGTTCGTGAGAATTGAAACAAAGCTTAGGGTTTAG
+
FFFFFFFFFFFFFFFF:FFFFF,FFF::FFFFFFFFFFFFFFFFFFFFFFFF:FFFFFFFFF:FFFFF
FFFFFFF:F:FFFFFFFF:FFFFFFFFFFFFFFFFFFFF::FFFFFFF,F,F,FF,FFFFFFFF
@A00155:342:HHGFNDSXY:1:2205:32389:34131 1:N:0:GAACCTAG+TCCGCATA
AATTACCATCGAAGTTGAATTCGGCTCCTGGTGCCTCGTGATTCGGGAAGAACCTCTTAACGAAACTA
TCCATTACTCTGAGTTTGCTTTTAGCTTGGGTTCGTGAGAATTGAAACAAAGCTTAGGGTTTAGTTAG
+
FFFFFFFFFFFFFFFFFFFFFFFFFFFFFFFFFFFFFF:FFF:FFFF:FFF:FFFFFF:FFFF:FF:F
:FF::F:FFF,FFFF:FFF:F,FFFF,FFFFFFFF,FFFF::FFF::FF,FFFF:FFFF:,FFFFF,F
@A00155:342:HHGFNDSXY:1:1521:31602:4711 1:N:0:GAACCTAG+TCCGCATA
ATTACCATCGAAGTTGAATTCGGCTCCTGGTGCCTCGTGATTCGGGAAGAACCTCTTAACGAAACTAT
CCATTACTCTGAGTTTGCTTTTAGCTTGGGTTCGTGAGAATTGAAACAAAGCTTAGGGTTTAGTTAGC
+
FFFFFFFFFFFFFFFFFFFFFFFFFFFFFFFFFFFFFFFFFFFFFFF:FFFFFFFFFFFFFF:FFF:F
FFFFFFFFFFFFFFFFFFFFFFFFFFFFFFFFFFFF:F:FFFFFFFFFFFFF:FFFFFFFFFFFFFFF
```

```
@A00155:342:HHGFNDSXY:1:1462:10131:16344 1:N:0:GAACCTAG+TCCGCATA
ATTACCATCGAAGTTGAATTCGGCTCCTGGTGCCTCGTGATTCGGGAAGAACCTCTTAACGAAACTAT
CCATTACTCTGAGTTTGCTTTTAGCTTGGGTTCGTGAGAATTGAAACAAAGCTTAGGGTTTAGTTAGC
+
FFFFFFFFFFFFFFFFFFFF:FFFFFFF:FFFFFFFFFF:FFF:FFF:F:FFFFFFFFFFFFFFFF
FFFFFFFFFFFFFFFFFFFFFFFFFFFFFF:FFF,FFFFFFFF:FFFFF:FFFFFFFFFFFFFFFFF
@A00155:342:HHGFNDSXY:1:2502:9417:5619 1:N:0:GAACCTAG+TCCGCATA
ATTACCATCGAAGTTGAATTCGGCTCCTGGTGCCTCGTGATTCGGGAAGAACCTCTTAACGAAACTAT
CCATTACTCTGAGTTTGCTTTTAGCTTGGGTTCGTGAGAATTGAAACAAAGCTTAGGGTTTAGTTAGC
+
FFFFFFFFFFFFFFFFFFFFFFFFFFFFFFFFFFFFFFFFFFFFFFFFFFFFFFFFFFFFFFFFFFFF
FFFFFFFFFFFFFFFFFFFFFFFFFFFFFFFFFF:FFFFFFFFFFFFFFFFFFFF:FFFF:FFFFFFFF
@A00155:342:HHGFNDSXY:1:2333:15383:29857 1:N:0:GAACCTAG+TCCGCATA
ATTACCATCGAAGTTGAATTCGGCTCCTGGTGCCTCGTGATTCGGGAAGAACCTCTTAACGAAACTAT
CCATTACTCTGAGTTTGCTTTTAGCTTGGGTTCGTGAGAATTGAAACAAAGCTTAGGGTTTAGTTAGC
+
FFFFFFFFFFFFFFFFFFFFFFFFFFFFFFFFFFFFFFFFFFFFFFFFFFFFFFFFFFFFFFFFFFFF
FFFFFFFFFFFFFFFFFFFFFFFFFFFFFFFFFFFFFFFFFFFFFFFFF:FFFFFFFFFF:FFFFFFFFF
@A00155:342:HHGFNDSXY:1:1107:24397:4946 1:N:0:GAACCTAG+TCCGCATA
TTACCATCGAAGTTGAATTCGGCTCCTGGTGCCTCGTGATTCGGGAAGAACCTCTTAACGAAACTATC
CATTACTCTGAGTTTGCTTTTAGCTTGGGTTCGTGAGAATTGAAACAAAGCTTAGGGTTT
+
FFFFFFFFFFFFFFFFFFFFFFFFFFFFFFFFFFFFFFFFFFFFFFFFFFFFFFFFFFFFFFFFFFFF
FFFFFFFFFFFFFFFFFFFF:FFFFFFFFFFFFFFFFFFFFFFFF:FFFFFFFF:FFFFFFF
@A00155:342:HHGFNDSXY:1:2537:26313:1344 1:N:0:GAACCTAG+TCCGCATA
TTACCATCGAAGTTGAATTCGGCTCCTGGTGCCTCGTGATTCGGGAAGAACCTCTTAACGAAACTATC
CATTACTCTGAGTTTGCTTTTAGCTTGGGTTCGTGAGAATTGAAACAAAGCTTAGGGTTT
+
FFFFFFFFFFFFFFFFFFFFFFFFFFFFFFFFFFFFFFFFFFFFFFFFFFFFFFFFFFFFFFFFFFFF
,FFFFFFFFFFFFF:,FFFFFFF:FF:FFFFFFFFFFFFFFFFFFFFFFFFF,FF:FFFFF
@A00155:342:HHGFNDSXY:1:2307:9290:34397 1:N:0:GAACCTAG+TCCGCATA
TTACCATCGAAGTTGAATTCGGCTCCTGGTGCCTCGTGATTCGGGAAGAACCTCTTAACGAAACTATC
CATTACTCTGAGTTTGCTTTTAGCTTGGGTTCGTGAGAATTGAAACAAAGCTTAGGGTTTAG
+
FFFFFFFFFFFFFFFFFFFFFFFFFFFFFFFFFFFFFFFFFFFFFFFFFFFFFFFFFFF:FFFFFFFF
FFFFFFFFFFFFFFFFFFFF:FF,FFFFFFFFFFFFFF:FFFFFFFFFF:FFFFFFFFF:FF
@A00155:342:HHGFNDSXY:1:2444:28782:3897 1:N:0:GAACCTAG+TCCGCATA
TTACCATCGAAGTTGAATTCGGCTCCTGGTGCCTCGTGATTCGGGAAGAACCTCTTAACGAAACTATC
CATTACTCTGAGTTTGCTTTTAGCTTGGGTTCGTGAGAATTGAAACAAAGCTTAGGGTTTAG
+
FFFFFFFFFFFFFFFFFFFFFFFFFFFFFFFFFFFFFFFFFFFFFFFFFFFFFFFFFFFFFFFFFFFF
FFFFFFFFFFFFFFFFFFFFFFFFFFFFFFFFFFFFFFFFFFFFFFFFFFFFFFFFFFFFFF
@A00155:342:HHGFNDSXY:1:2518:4218:28119 1:N:0:GAACCTAG+TCCGCATA
ACCATCGAAGTTGAATTCGGCTCCTGGTGCCTCGTGATTCGGGAAGAACCT
+
FFFFFFFFFFFFFFFFFFFFFFFFFFFFFFFFFFFFFFFFFFFFFFFFFFF
@A00155:342:HHGFNDSXY:1:2248:29658:22670 1:N:0:GAACCTAG+TCCGCATA
ACCATCGAAGTTGAATTCGGCTCCTGGTGCCTCGTGATTCGGGAAGAACCTCTTAACGAAACTATCCA
TTACTCTGAGTTTGCTTTTAGCTTGGGTTCGTGAGAATTGAAACAAAGCTTAGGGTTTAG
+
FFFFF:FFFFFFFFFFFFFFFFFFFF:FFFFFF::FFFF:FFFFFFFF:FFFFFFF:FFFFFFFFFFF
FFFFFFFFF:FFFF:,FFFFFFFFFFFFFFFFFF:FFFFFF,FFF::FFFFFFFF,FFFF
@A00155:342:HHGFNDSXY:1:1326:11469:33630 1:N:0:GAACCTAG+TCCGCATA
ACCATCGAAGTTGAATTCGGCTCCTGGTGCCTCGTGATTCGGGAAGAACCTCTTAACGAAACTATCCA
```

```
TTACTCTGAGTTTGCTTTTAGCTTGGGTTCGTGAGAATTGAAACAAAGCTTAGGGTTTAGTTAGC
+
FFFFFFFFFFFFFFFFFFFFFFFFFFFFFFFF:FFFFFFFFFFFFFFFFFFFFFFFFFFFFFFFFFFF
:FFFFFFFFFFFFFFFFFFFFFFFFFFFFFFFFFFFFFFFFFFF:FF:FFFFFFFFFFFFFFFFF
@A00155:342:HHGFNDSXY:1:1530:18313:8124 1:N:0:GAACCTAG+TCCGCATA
ACCATCGAAGTTGAATTCGGCTCCTGGTGCCTCGTGATTCGGGAAGAACCTCTTAACGAAACTATCCA
TTACTCTGAGTTTGCTTTTAGCTTGGGTTCGTGAGAATTGAAACAAAGCTTAGGGTTTAGTTAGC
+
FFFFFFFFFFFFFFFFFFFFFFFFFFFFFFFFFFFFFFFFFFFFFFFFFFFFFFFFF:FFFFFFFFFF
FFFFFFFFFFFFFFFFFFFFFF:FFFFFFFFFFFFFFFFFFFFFFFFFFFFFFFFFFFFFFFFFF
@A00155:342:HHGFNDSXY:1:2359:21251:2159 1:N:0:GAACCTAG+TCCGCATA
ACCATCGAAGTTGAATTCGGCTCCTGGTGCCTCGTGATTCGGGAAGAACCTCTTAACGAAACTATCCA
TTACTCTGAGTTTGCTTTTAGCTTGGGTTCGTGAGAATTGAAACAAAGCTTAGGGTTTAGTTAGCC
+
FFFFFFFFFFFFFFFFFFFFFFFFFFFFFFFFFFFFFFFFFFFFFFFFFFFFFFFFFFFFFFFFFFFF
FF:FFFFFFFFFFFFFFFFFFFFFFFFFFFF:FFFFFFFFFFFFFFF:FFFFFFFFFFFFFFFFFF
@A00155:342:HHGFNDSXY:1:2472:30770:2300 1:N:0:GAACCTAG+TCCGCATA
CCATCGAAGTTGAATTCGGCTCCTGGTGCCTCGTGATTCGGGAAGAACCTCTTAACGAAACTATCCAT
TACTCTGAGTTTGCTTTTAGCTTGGGTTCGTGAGAATTGAAACAAAGCTTAGGGTTTAGT
+
FFFFFFFFFFFFFFFFFFFFFFFFFFFFFFFFFFFFFFFFFFFFFFFFFFFFFFFFFFFFFFFFFFFF
FFFFFFFFFFFFFFFFFFFFFFFFFFFFFFFFFFFFFFFFFFFFF:FFFFFFFFFFFFFF
@A00155:342:HHGFNDSXY:1:1453:22516:2409 1:N:0:GAACCTAG+TCCGCATA
CCATCGAAGTTGAATTCGGCTCCTGGTGCCTCGTGATTCGGGAAGAACCTCTTAACGAAACTATCCAT
TACTCTGAGTTTGCTTTTAGCTTGGGTTCGTGAGAATTGAAACAAAGCTTAGGGTTTAGT
+
FFFFFFFFFFFFFFFFFFFFFFFFFFFFFFFFFFFFFFFFFFFFFFFFFFFFFFFFFFFFFFFFFFFF
FFFFFFFFFFFFFFFF:FFFFFFFFFFFFFFFFFFFFFFFFFFFFFF:FFFFFFFFFF:F
@A00155:342:HHGFNDSXY:1:1232:4209:10410 1:N:0:GAACCTAG+TCCGCATA
CCATCGAAGTTGAATTCGGCTCCTGGTGCCTCGTGATTCGGGAAGAACCTCTTAACGAAACTATCCAT
TACTCTGAGTTTGCTTTTAGCTTGGGTTCGTGAGAATTGAAACAAAGCTTAGGGTTTAGTTAGCC
+
FFFFFFFFFFFFFFFFFFFFFFFFFFFFFFFFFFFFFFFFFFFFFFFFFFFFFFFFFFFFFFFFFFFF
FFFFFFFFFFFFFFFFFFFFFFFFFFFFFFFFFFFFFFFFFFFFFFF:FFFFFFFFFFFFFFFFF
@A00155:342:HHGFNDSXY:1:1638:11198:11052 1:N:0:GAACCTAG+TCCGCATA
CGAAGTTGAATTCGGCTCCTGGTGCCTCGTGATTCGGGAAGAACCTCTTAACGAAACTATCCATTACT
CTGAGTTTGCTTTTAGCTTGGGTTCGTGAGAATTGAAACAAAGCTTAGGGTTTAGTTAGC
+
FFFFFFFFFFFFFFFFFFFFFFFFFFFFFFFFFFFFFFFFFFFFFFFFFFFFFFFFFFFFFFFFFFFF
:FFFF:FFFFF:FFFFFFFF:FFFFFFFFFF,FFFFFFFFFFFFF:FFFFFFFFFFFFFF
@A00155:342:HHGFNDSXY:1:2315:14615:18662 1:N:0:GAACCTAG+TCCGCATA
CGAAGTTGAATTCGGCTCCTGGTGCCTCGTGATTCGGGAAGAACCTCTTAACGAAACTATCCATTACT
CTGAGTTTGCTTTTAGCTTGGGTTCGTGAGAATTGAAACAAAGCTTAGGGTTTAGTTAGC
+
FFFFFFFFFFFFFFFFFFFFFFFFFFFFFFFF:FFFFFFFFFFFFFFFFFFFFF:FFF,FFFFFFFFF
FFF:FFFFFFF::FFFFFFFFFFFFFFFFFFFFFFFFFFFFFFF:F:FFFFFFFFFFFFF
@A00155:342:HHGFNDSXY:1:1613:25599:10473 1:N:0:GAACCTAG+TCCGCATA
CGAAGTTGAATTCGGCTCCTGGTGCCTCGTGATTCGGGAAGAACCTCTTAACGAAACTATCCATTACT
CTGAGTTTGCTTTTAGCTTGGGTTCGTGAGAATTGAAACAAAGCTGAGGGTTTAGTTAGC
+
FFFFFFFFFFFF:F,,:FFFFFFFFFFF:FFF,:FFF:FFFFF:FFFF:FF:FFFFFFFFFFF,:FFF
FFFF:,F:FF,F:FFFFFF:FFFFF::FF:FFFF,FFFFFF,FF,,FFF:FFFFFFFF,F
@A00155:342:HHGFNDSXY:1:2574:9182:24283 1:N:0:GAACCTAG+TCCGCATA
GAAGTTGAATTCGGCTCCTGGTGCCTCGTGATTCGGGAAGAACCTCTTAACGAAACTATCCATTACTC
```

```
TGAGTTTGCTTTTAGCTTGGGTTCGTGAGAATTGAAACAAAGCTTAGGGTTTAGTTAGC
+
FFFFFFFFFFFFFFFFFFFFFFFFFFFFFFFFFFFFFFFFFFFFFFFFFFFFFFFFF:FFFFFFFFFF
FFFFFFFFFFFFFFFFFFFFFFFFFFFFFFFFFFFFFFF:FFFFFFFFFF:FF,FFFFF
@A00155:342:HHGFNDSXY:1:2305:7048:19774 1:N:0:GAACCTAG+TCCGCATA
GAAGTTGAATTCGGCTCCTGGTGCCTCGTGATTCGGGAAGAACCTCTTAACGAAACTATCCATTACTC
TGAGTTTGCTTTTAGCTTGGGTTCGTGAGAATTGAAACAAAGCTTAGGGTTTAGTTAGC
+
FFFFFF:FFFFFF:F:FFFF:FFFFFFFFFFFFFFFFFFFFFFFFFF:FFFF:FFFFFFFFFFFFFFF
FFF:FFFF:FFFFFFFFFFFFFFFFFFFFFFFFFFFF:FFFF:,FF::FFFFFFFFF:,
@A00155:342:HHGFNDSXY:1:1373:22345:6590 1:N:0:GAACCTAG+TCCGCATA
GAAGTTGAATTCGGCTCCTGGTGCCTCGTGATTCGGGAAGAACCTCTTAACGAAACTATCCATTACTC
TGAGTTTGCTTTTAGCTTGGGTTCGTGAGAATTGAAACAAAGCTTAGGGTTTAGTTAGCC
+
FFFFFFFFFFFFFFFFFFFFFFFFFFFFFFFFFFFFFFFFFFFFFFFFFFFFFFFFFFFFFFFFFFFF
FFFFFFFFFFFFFFFFFFFFFFFFF::FFFFFFFFFFFFFFFFFFFFFFFFFFFFFF:FF
@A00155:342:HHGFNDSXY:1:2607:25916:23234 1:N:0:GAACCTAG+TCCGCATA
GAAGTTGAATTCGGCTCCTGGTGCCTCGTGATTCGGGAAGAACCTCTTAACGAAACTATCCATTACTC
TGAGTTTGCTTTTAGCTTGGGTTCGTGAGAATTGAAACAAAGCTTAGGGTTTAGTTAGCC
+
FFFFFFFFFFFFFFFFFFFFFFFFFFFFFFFFFFFFFFFFFFFFFFFFFFFFFFFFFFFFFFFFFFFF
FFFFFFFFFFFFFFFFFFFFFFFFFFFFFFFFFFFFFFFFFFFFFFFFFF:FFFFFFFFF
@A00155:342:HHGFNDSXY:1:1329:21142:13777 1:N:0:GAACCTAG+TCCGCATA
TGCCTCGTGATTCGGGAAGAACCTCTTAACGAAACTATCCATTACTCTGAGT
+
FFFFFFFFFFFFFFFFFFFFFFFFFFFFFFFFFFFFFFFFF,FFFFFFFFFF
```
