# Supplementary material for: First Detection and Molecular Characterization of Apple Stem Grooving Virus, Apple Chlorotic Leaf Spot Virus, and Apple Hammerhead Viroid in Loquat in Spain
Source: Plants (Basel). 2021 Oct 25;10(11):2293. doi: 10.3390/plants10112293 (PMC8624106; doi:10.3390/plants10112293)
Supplement: Supplementary file 1 [file plants-10-02293-s001.zip › Mapping_reads_AHVd.pdf]

@A00155:342:HHGFNDSXY:1:1101:16297:9236 1:N:0:GAACCTAG+TCCGCATA
TGTCCACTTAAGGACTCACCTCTGGCCTATGGTCATCTCAATGCCGAGAGGGATGTGGAGGGCGAGAG
AGAGCGACTTCTCTCGGGCCCACAGCCTTACAGCTGTGGAGCACGGTATCCTCTGCCGAAACAGAGGT
+
FFFFFFFFFFFFFFFFFFFFFFFFFFFFFFFFFFFFFFFFFFFFFFFFFFFFF,F,FFFFFFFFFFF
FFFFFFFFFFFFFFFFFFFFFFFFFFFFFFFFFFFFFFFFFFFFFFFFFFFFFFFFFFFFFFFFFFFF
@A00155:342:HHGFNDSXY:1:1101:25382:14262 1:N:0:GAACCTAG+TCCGCATA
TCACCTCTGGCCTATGGTCATCTCAATGCCGAGAGGGATGTGGAGGGCTAGAGAGAGCGACTTCTCTC
GGGCCCACAGCCTTACAGCTGTGGAGCACGGTATCCTCTGCCGAAACAGAGGTTGGACAAGACCGGAG
+
FFFFFFFFF:FFF,,FFFFFFFFFFF,,FFFF:FFFFFFFFFFFF:FF,F,FFFFFF,FFFFF:FFFF
:FFFFFFFFFFFFFFFFFF:FFFFF:FFFF,FFFFFFF,:F:FFFFFFFFFFFFF:FFFF,FF,:,FF
@A00155:342:HHGFNDSXY:1:1101:29740:22216 1:N:0:GAACCTAG+TCCGCATA
GAGAGGGATGTGGAGGGCGAGAGAGAGCGACTTCTCTCGGGCCCACAGCCTTACAGCTGTGGAGCACG
GTATCCTCTGCCGAAACAGAGGTTGGACAAGACCGGAGGGGTCTCCTGGTTCCAAAGGAGATGTACTC
+
FFFFFFFFFFFFFFFFFFFFFFFFFFFFFFFFFFFFFFFFFFFFFFFFFFFFFFFFFFFFFFFFFFFF
FFFFFFFFFFFFFFFFFFFFFFFFFFFFFFFFFFFFFFFFFFFFFFF:FFFFFFFFFFFFFFFFFFFF
@A00155:342:HHGFNDSXY:1:1101:27055:25895 1:N:0:GAACCTAG+TCCGCATA
TGTGGAGGGCGAGAGAGAGCGACTTCTCTCGGGCCCACAGCCTTACAGCTGTGGAGCACGGTATCCTC
TGCCGAAACAGAGGTTGGACAAGACCGGAGGGGTCTCCTAGTTCCAAAGGAGATGTACTCCGGG
+
FFFFFFFFFFFFFFFFFFFFFFFFFFFFFFFFFFFFFFFFFFFFFFFFFFFFFFFFFFFFFFFFFFFF
FFFFFFFFFFFFFFFFFFFFFFFFFFFFF:FFFFFFFFFFFFFFFFFFFFFFFFFFFFFFFFFFFF
@A00155:342:HHGFNDSXY:1:1101:27281:25974 1:N:0:GAACCTAG+TCCGCATA
TGTGGAGGGCGAGAGAGAGCGACTTCTCTCGGGCCCACAGCCTTACAGCTGTGGAGCACGGTATCCTC
TGCCGAAACAGAGGTTGGACAAGACCGGAGGGGTCTCCTAGTTCCAAAGGAGATGTACTCCGGG
+
FFFFFFFFFFFFFFFFFFFFFFFFFFFFFFFFFFFFFFFFFFFFFFFFFFFFFFFFFFFFFFFFFFFF
FF:FFFFFFFFFFFFFFFFFFFFFFFFFFFFFFFFFFFFF:FFFFFFFFFFFFFFFFFFFFFFFFF
@A00155:342:HHGFNDSXY:1:1102:6081:1376 1:N:0:GAACCTAG+TCCGCATA
GGATGTGGAGGGCGAGAGAGAGCGACTTCTCTCGGGCCCACAGCCTTACAGCTGTGGAGCACGGTATC
CTCTGCCGAAACAGAGGTTGGACAAGACCGGAGGGGTCTCCTAGTTCCAAAGGAGATGTACTCCGGGC
+
FF:FFFFFFFFFFFFFFFFF:F,FFFFFFFFFFF,FFFFFFF,FFFFFF,,:FFFFFFFFFFFFFFFF
FFFFFFFFFFFFFFFFFFFFFFFFFFFFF:FFFFF:FFFFFFFFFFFFFFFFFFFFFFFFFFFFFFFF
@A00155:342:HHGFNDSXY:1:1102:15284:16250 1:N:0:GAACCTAG+TCCGCATA
GATCAGCTATGTCCACTTAAGGACTCACCTCTGGCCTATGGTCATCTCAATGCCGAGAGGGATGTGGA
GGGCGAGAGAGAGCGACTTCTCTCGGGCCCACAGCCTTACAGCTGTGGAGCACGGTATCCTCTGCCGA
+
FFFFFFFFFF:FFFFFFFFFFFFFFFFFFFFFFFFFFF:FFFFFFFFFFFFFFFFFFFFFFFFF,FFFFF
FFFFFFFFFFFFFFFFFFFFFFFFFFFFFFFFFFFFFF:FFFFFFFFFFFFFFFFFFFFFFFFFFFFFF
@A00155:342:HHGFNDSXY:1:1102:10303:21840 1:N:0:GAACCTAG+TCCGCATA
TCTCAATGCCGAGAGGGATGTAGAGGGCGAGAGAGAGCGACTTCTCTCGGGCCCACAGCCTTACAGCT
GTGGAGCACGGTATCCTCTGCCGAAACAGAGGTTGGACAAGACCGGAGGGGTCTCCTAGTTCCAAAGG
+
FFFFFFFFFFFFF:FFFFFF:F:FFFFFFFFFFFFFFFFFF:FFFFFFFFFFFFFFFFFF:FFFFFFF
FFFFFFFFFFFFFFFFFFFFFFFFFFFFFFFFFFFFFFFFF:FFFFFFFFFFFFFFFFFFFFFFFFFF
@A00155:342:HHGFNDSXY:1:1103:31855:6621 1:N:0:GAACCTAG+TCCGCATA
CTTCTCTCGGGCCCACAGCCTTACAACTGTGGAGCACGGTATCCTCTGCCGAAACAGAGGTTGGACAA
GACCGGAGGGGTCTCCTAGTTCCAAAGGAGATGTACTCCGGGCTTGTTCACGACCTACCGTGTAAGTC
+
FFFF:FFFFFFFFFFFFFFFFFFFFFFFFFFFFFFFFFFFFFFFFFFFFFFFFFFFFFFFF:FFFFFF
FFFFFFFFFFFFFFFFF:FFFFFFFFFF:FFFFFFFFFFFFFFFFFFFFFFFFFFFFFFFFF,FFFFFFF

@A00155:342:HHGFNDSXY:1:1103:29595:12289 1:N:0:GAACCTAG+TCCGCATA
GTCGCTCTCTCTCGCCCTCCACATCCCTCTCGGCATTGAGATGACCATAGGCCAGAGGTGAGTCCTTA
AGTGGACACAGCTGATCTAAGGCGGTGTGGTGGGGCATGGGTTTGAACCCCCATGACGGTCGGAGTCT
+
FFFFFFFFFFFFFFFFFFFFFFFFFFFFFFFFFFFFFFFFFFFFFFFFFFFFFFFFFFFFFFFFFFFFFF
FFFFFFF:FFFFFFFFFFFFFFFFFFFFFFFFFFF:FFFFFFFFFFFFFFFFFFFFFFFFFFFFFFFFFF
@A00155:342:HHGFNDSXY:1:1103:21025:18865 1:N:0:GAACCTAG+TCCGCATA
ACCTCTGTTTCGGCAGAGGATACCGTGCTCCACAGCTGTAAGGCTGTGGGCCCGAGAGAAGTCGCTCT
CTCTCGCCCTCCACATCCCTCTCGGCATTGAGATGACCATAGGCCAGAGGAGAGTCCTTAAGTGGACA
+
FFFFFFFFFFFFFFFFFFFFFFFFFFFFFFFFFFFFFFFFFFFFFFFFFFFFFFFFFFFFFFFFFFFFFF
FFFFFFFFFFFFFFF:FFFFF:FFFF::FFFFF:FFFF,FFFFFFFFFFFFFFFFFFFFFFFFFFFF:FF
@A00155:342:HHGFNDSXY:1:1103:8929:19492 1:N:0:GAACCTAG+TCCGCATA
ATCTCAATGCCGAGAGGGATGTGGAGGGCGAGAGAGAGCGACTTCTCTCGGGCCCACAGCCTTACAGC
TGTGGAGCACGGTATCCTCTGCCGAAACAGAGGTTGGACAAGACCGGAGGGGTCTCCTAGTTCCAAAG
+
FFFFFFFFFFFFFFFFFFFFFFF:FFFFFFFFFFFFFFF:FFFFFFFFFFFFFFFFFFFFFFFFFFFFFF
FFFFFFFFFFFFFFFFFFFFFFFFFFFFF,FFFF:FFFFFFFFFFFFFFFFFFFFFFFFFFFFFFFFFFF
@A00155:342:HHGFNDSXY:1:1103:27715:28792 1:N:0:GAACCTAG+TCCGCATA
GAGGGCGAGAGAGAGCGACTTCTCTCGGGCCCACAGCCTTACAGCTGTGGAGCACGGTATCCTCTGCC
GAAACAGAGGTTGGACAAGACCGGAGGGGTCTCCTAGTTCCAAAGGAGATGTACTCCGGGCTTGTTCA
+
FFFFFFFFFFFFFFFFFFFFFFFFFFFFFFFFFFFFFFFFFFFFFFFFFFFFFFFFFFFFFFFFFFFFFF
FFFFFFFFFFFFFFFFFFFFFFFFFFFFFFFFFFFFFFFFFFFFFFFFFFFFFFFFFFFFFFF:FFFFFF
@A00155:342:HHGFNDSXY:1:1104:8458:9752 1:N:0:GAACCTAG+TCCGCATA
TCCACTTAAGGACTCTCCTCTGGCCTATGGTCATCTCAATGCCGAGAGGGATGTGGAGGGCGAGAGAG
AGCGACTTCTCTCGGGCCCACAGCCTTACAGCTGTGGAGCACGGTATCCTCTGCCGAAACAGAGGTTG
+
FFFFFFFFFFFFFFFFFFFFFF,FFFFFFFFFFFFFFFFFFF,FF:FFFFF:FFFFFFFFFFFFFFFFFF
FFFFFFFFFFFFFFFFFFFFFFFF:FFFFFFFFFFFFFFFFFFFFFFFFFFFFFFFFFF:FFFFFFFFFF
@A00155:342:HHGFNDSXY:1:1104:18647:20447 1:N:0:GAACCTAG+TCCGCATA
ATCTCAATGCCGAGAGGGATGTGGAGGGCGAGAGAGAGCGACTTCTCTCGGGCCCACAGCCTTACAGC
TGTGGAGCACGGTATCCTCTGCCGAAACAGAGGTTGGACAAGACCGGAGGGGTCTCCTGGTTCCAAAG
+
,FFFFF:F:FFFFFF:FFFFFFFFFF:FFFFFF,F,:FFFFFFFFFFFFFFFFFFFFFFFFFF:F:FFFF
FFF:F,FFFFFFFFFFFFF:F,FFFFFFFFF,F:FFFFFFFFFFFFFFFFF:FFFFFFFFF,FFFFFFFF
@A00155:342:HHGFNDSXY:1:1104:27769:31735 1:N:0:GAACCTAG+TCCGCATA
AGGGCGAGAGAGAGCGACTTCTCTCGGGCCCACAGCCTTACAGCTGTGGAGCACGGTATCCTCTGCCG
AAACAGAGGTTGGACAAGACCGGAGGGGTCTCCTAGTTCCAAAGGAGATGTACTCCGGGCTTGTTCAC
+
FFFFFFFFFFFFFFFFFFFFFFFFFFFFFFFF:FFFFFFFFFFFFFFF:FFFFFFFFFFFFFFFF:FFFF
FFFFFFFFFFFFFFFFFFFFFFFFFFFFFFFFFFF:FFFFFFFFF:FFFFFFFFFFFFFFFFFFFFF:FF
@A00155:342:HHGFNDSXY:1:1105:30517:6778 1:N:0:GAACCTAG+TCCGCATA
TTACAGCTGTGGAGCACGGTATCCTCTGCCGAAACAGAGGTTGGACAAGACCGGAGGGGTCTCCTAGT
TCCAAAGGAGATGTACTCCGGGCTTGTTCACGACCTACCGTGTAAGTCGTAGTCTAGTAGGCTACCTG
+
FFFFFFFFFFFFFFFFFFFFFFFFFFFFFFFFFFFFFFFFFFFFFFFFFFFFFFFFFFFFFFFFFFFFFF
FFFFFFFFFFFFFFFFFFFFFFFFFFFFFFFFFFFFFFFFFFFFF,FFFFFFFFFFFFFFFFFFFFFFFF
@A00155:342:HHGFNDSXY:1:1105:2257:19366 1:N:0:GAACCTAG+TCCGCATA
ACCTCTGTTTCGGCAGAGGATACCGTGCTCCACAGCTGTAAGGCTGTGGGCCCGAGAGAAGTCGCTCT
CTCTCGCCCTTCACATCCCTCTCGGCATTGAGATGACCATAGGCCAGAGGTGAGTCCTTAAGTGGACA
+
FF:F:FFFF:FFFFFFFFFFFF,FFFFFFFFFFFFFFF:FFF,FFFFF:F,FFFFFFFFFFFFFFFFFFF
:FFFFFFFFFFFFFFFFFFFFFFFFFFFFFFFFFFFFFF,FF:FFF:FFFFFFFFFFFFFFFFFFFFFFF

@A00155:342:HHGFNDSXY:1:1106:31919:20384 1:N:0:GAACCTAG+TCCGCATA
ATCTCAATGCCGAGAGGGATGTGGAGGGCGAGAGAGAGCGACTTCTCTCGGGCCCACAGCCTTACAGC
TGTGGAGCACGGTATCCTCTGCCGAAACAGAGGTTGGACAAGACCGGAGGGGTCTCCTA
+
FFFFFFFFFFFFFFFFFFFFF:FFFFFFFFFFFFFFFFFFFFFFFFFFFFFFFFFFFFFFFFFFFFFF
FFFFFFFFFFFFFFFFFFFFFFFFFFFFFFFFFFFFFFFFFFFFFFFFFFFFFFFFFFF
@A00155:342:HHGFNDSXY:1:1106:17951:21245 1:N:0:GAACCTAG+TCCGCATA
GGATGTGGAGGGCGAGAGAGAGCGACTTCTCTCGGGCCCACAGCCTTACAGCTGTGGAGCACGGTATC
CTCTGCCGAAACAGAGGTTGGACAAGACCGGAGGGGTCTCCTGGTTCCAAAGGAGATGTACTCC
+
FFFFFFFFFFFFFFFFFFFFFFFFFFFFFFFFFFFFFFFFFFFFFFFFFFFFFFFFFFFF:FFFFFFF
FFFFFFFFFFFFFFFFFFFFFFFFFFFFFFFFFFFFFFFFFFFFFFFFFFFFFFFFFFFFFFFF
@A00155:342:HHGFNDSXY:1:1106:32850:23062 1:N:0:GAACCTAG+TCCGCATA
AGGGATGTGGAGGGCGAGAGAGAGCGACTTCTCTCGGGCCCACAGCCTTACAGCTGTGGAGCACGGTA
TCCTCTGCCGAAACAGAGGTTGGACAAGACCGGAGGGGTCTCCTAGTTCCAAAGGAGATGTACTCCGG
+
FFFFFFFFFFFFFFFFFFFFFFFFFFFFFFFFFFFFFFFFFFFFFFFFFFFFFFFFFFFFFFFFFFFF
FFFFFFFFFFFFFFFFFFFFFFFFFFFFFFFFFFFFFFFFFFFFFFFFFFFFFFFFFFFFFFFFFFFF
@A00155:342:HHGFNDSXY:1:1106:7636:27148 1:N:0:GAACCTAG+TCCGCATA
GATGTGGAGGGCGAGAGAGAGCGACTTCTCTCGGGCCCACAGCCTTACAGCTGTGGAGCACGGTATCC
TCTGCCGAAACAGAGGTTGGACAAGACCGGAGGGGTCTCCTAGTTCCAAAGGAGATGTACTCCGGGCT
+
FFFFFFFFFFFFFFFFFFFFFFFFFFFFFFFFFFFFFFFFFFFFFF:FFFFFFFFFFFFFFFFFFFFF
FFFFFFFFFFFFFFFFFFFFFFFFFFFFFFFFFFFFFFFFFFFFFFFFFFFFFFFFFFFFFFFFFFFF
@A00155:342:HHGFNDSXY:1:1106:12554:28369 1:N:0:GAACCTAG+TCCGCATA
GATGTACTCCGGGCTTGTTCACGACCTACCGTGTAAGTCGTAGTCTAGTAGGCTACCTGACGAGTCCT
TTTTAGGACGAAACTTACACAACTCAAAGAGTAACGACGGTTTCGTCCTTGGAACGGACTCATCAGGG
+
FFFFFFFFFFFFFFFFFFFFFFFFFFFFFFFFFFFFFFFFFFFFFFFFFFF:FFFFFFFFFFFFFFFF
:FFFFFFFFFFFFFFFFFFFFFFFFFFFFFFFFFFFFFFFFFFFFFFFFFFFFFFFFFFFFFFFFFFF
@A00155:342:HHGFNDSXY:1:1106:25681:32784 1:N:0:GAACCTAG+TCCGCATA
TCCTCTGCCGAAACAGAGGTTGGACAAGACCGGAGGGGTCTCCTAGTTCCAAAGGAGATGTACTCCGG
GCTTGTTCACGACCTACCGTGTAAGTCGTAGTCTAGTAGGCTACCTGACGAGTCCTTTTTAGGACGAA
+
FFFFFFFFFFFFFFFFFFFFFFFFFFFFFFFFFFFFFFFFFFFFFFFFFFFFFFFFFFFFFFFFFFFF
FFFFF,FFFFFFFFFFFFFFFFFFFFFFFFFFFFFFFFFFFFFFFFFFFFFFFFFFFFFFFFFF
@A00155:342:HHGFNDSXY:1:1107:26775:8124 1:N:0:GAACCTAG+TCCGCATA
TGTTTCGGCAGAGGATACCGTGCTCCACAGCTGTAAGGCTGTGGGCCCGAGAGAAGTCGCTCTCTCTC
GCCCTCTACATCCCTCTCGGCATTGAGATGACCATAGGCCAGAGGTGAGTCCTTAAGTGGACACAGCT
+
FFFFFFFFFFFFFFFFFFFFFFFFFFFFFFFFF:FFFFFFFFFFFFFFF:FFFFFFFFFFFFFFFFFF
FFFFFFFFFFFFFFFFFFFFFFFFFFFFFFFF:FFFF:FFFFFFFFFFFFFFFFFFFFFFFFFFFFFF
@A00155:342:HHGFNDSXY:1:1107:19913:21762 1:N:0:GAACCTAG+TCCGCATA
AATGCCGAGAGGGATGTGGAGGGCGAGAGAGAGCGACTTCTCTCGGGCCCACAGCCTTACAGCTGTGG
AGCACGGTATCCTCTGCCGAAACAGAGGTTGGACAAGACCGGAGGGGTCTCCTAGTTCCAAAGGAGAT
+
FFFFFFFFFFFFFFFFFFFFFFFFFFFFFFFFFFFFFFFFFFFFFFFFFFFFFFFFFFFFFFFFFFFF
FFFFFFFFFFFFFFFFFFFFFFFFFFFFFFFFFFFFFFFFFFFFFFFFFFFFFFFFFFFFFFFFFFFF
@A00155:342:HHGFNDSXY:1:1108:22299:2691 1:N:0:GAACCTAG+TCCGCATA
GGATGTGGAGGGCGAGAGAGAGCGACTTCTCTCGGGCCCACAGCCTTACAGCTGTGGAGCACGGTATC
CTCTGCCGAAACAGAGGTTGGACAAGACCGGAGGGGTCTCCTAGTTCCAAAGGAGATGTACTCC
+
FFFFFFFFFFFFFFFFFFFFFFFFFF:FFFFFFFFFFFFFFFFFFFFFFFFFFFFFFFFFFFFFFFFF
FFFFFFFFFFFFFFFFF:FFFFFFFFFFFFFFFFFFFFFFFFFFFFFFFFFFFFFFFFFFFFFF

```
@A00155:342:HHGFNDSXY:1:1108:23068:12007 1:N:0:GAACCTAG+TCCGCATA
AATGCCGAGAGGGATGTGGAGGGCGAGAGAGAGCGACTTCTCTCGGGCCCACAGCCTTACAGCTGTGG
AGCACGGTATCCTCTGCCGAAACAGAGGTTGGACAAGACCGGAGGGGTCTCCTAGTTCCAAAGGAGAT
+
FFFFFFFFFFFFFF:F,FFFFFFFFFFFFFFFFFFFFFFFFFFFFFFFFFFFFFFFFFFFFFFFFFFF
FFFFFFFFFFFFFFFFFFFFFFFFFFFFFFFFFFFFFFFFFFFFFFFFFFFFFFFFFFFFFFFFFFFF
@A00155:342:HHGFNDSXY:1:1108:8721:14591 1:N:0:GAACCTAG+TCCGCATA
CGACTTCTCTCGGGCCCACAGCCTTACAGCTGTGGAGCACGGTATCCTCTGCCGAAACAGAGGTTGGA
CAAGACCGGAGGGGTCTCCTAGTTCCAAAGGAGATGTACTCCGGGCTTGTTCACGACCTACCGTGTAA
+
FFFFFFFFFFFFFF:FFFFFFFFFFFFFFFFFFFFFFFF,FFF:FFFFFFFFFFFFFFFFFFFFFFFF
FFFFFFFFFFFFFFFFFFFFFFFFFFFFFFFFFFFFFFFFFFF:FFFFFFFFFFFFFFFFFFFFFF::
@A00155:342:HHGFNDSXY:1:1108:8901:14904 1:N:0:GAACCTAG+TCCGCATA
CGACTTCTCTCGGGCCCACAGCCTTACAGCTGTGGAGCACGGTATCCTCTGCCGAAACAGAGGTTGGA
CAAGACCGGAGGGGTCTCCTAGTTCCAAAGGAGATGTACTCCGGGCTTGTTCACGACCTACCGTGTAA
+
FFFFFFFFFFFFFFFF,FFF,FF:FFFF,F,F:::,F:F,FFF:FFFFFFFFFFFFFFFFFFFFFFFF
FFFFFFFFFFFFFFFFFFFFFFFFFFFFFFF::FFFFFFFFFF:FF:FFFFF:FFFFFFFFFFFFF:F
@A00155:342:HHGFNDSXY:1:1108:26323:15358 1:N:0:GAACCTAG+TCCGCATA
GGATGTGGAGGGCGAGAGAGAGCGACTTCTCTCGGGCCCACAGCCTTACAGCTGTGGAGCACGGTATC
CTCTGCCGAAACAGAGGTTGGACAAGACCGGAGGGGTCTCCTAGTTCCAAAGGAGATGTACTCC
+
FFFFF:FFFFFFFFFFFFFFFFFFFF:FFFFFFFFFFFFFFFFFFFFFFFFFFFFFFFFFFFFFFFFF
FFFFFFFFFFFFFFFFFFFFFFFFFFFFFFFFFFFFFFFFFFFFFFFFFFFFFFFFFFFFFFFF
@A00155:342:HHGFNDSXY:1:1108:16703:16642 1:N:0:GAACCTAG+TCCGCATA
CTCTGCCGAAACAGAGGTTGGACAAGACCGGAGGGGTCTCCTAGTTCCAAAGGAGATGTACTCCGGGC
TTGTTCACGACCTACCGTGTAAGTCGTAGTCTAGTAGGCTACCTGACGAGTCCTTTTTAGGACGAAAC
+
FFFFFFFFFFFFFFFFFFFFFFFFFFFFFFFFFFFFFFFFFFFFFFFFFFFFFFFFFFFFF:FFFFFF
FFFFFFFFFFFFFFFFFFFFF,FFFFFFF,FFFFF:FFFFFFFFFFFFFFFFFFFFFFFFFFFFF:FF
@A00155:342:HHGFNDSXY:1:1109:19370:2628 1:N:0:GAACCTAG+TCCGCATA
CTTCTCTCGGGCCCACAGCCTTACAGCTGTGGAGCACGGTATCCTCTGCCGAAACATAGGTTGGACAA
GACCGGAGGGGTCTCCTAGTTCCAAAGGAGATGTACTCCGGGCTTGTTCACGACCTACCGTGTAAGTC
+
FFFFFFFFFFFFFFFFFFFFFFFFFFF,FFFFFFFFFFFFFFFFFFFFFFFFFFFFFFFFFFFFFFFF
FFFFFFFFFFFFFFFFFFFFFFFFFFFFFFFFFFFFFFFFFFF:FFFFFFFFFFFFFFFFFFFFFFFF
@A00155:342:HHGFNDSXY:1:1109:16161:24220 1:N:0:GAACCTAG+TCCGCATA
GAGCGACTTCTCTCGGGCCCACAGCCTTACAGCTGTGGAGCACGGTATCCTCTGCCGAAACAGAGGTT
GGACAAGACCGGAGGGGTCTCCTAGTTCCAAAGGAGATGTACTCCGGGCTTGTTCACGACCTACCGTG
+
FFFFFFFFFFFFFFFFFFFFFFFFFFFFFFFFFFFFFFFFFFFFFFFFFFFFFFFFFFFFFFFFFFFF
FFFFFFFFFFFFFFFFFFFFFFFFFFFFFFFFFFFFFFFFFFFFFFFFFFFFFFFFFFFFFFFFFFFF
@A00155:342:HHGFNDSXY:1:1109:16595:28729 1:N:0:GAACCTAG+TCCGCATA
ATCCTCTGCCGAAACAGAGGTTGGACAAGACCGGAGGGGTCTCCTAGTTCCAAAGGAGATGTACTCCG
GGCTTGTTCACGACCTACCGTGTAAGTCGTAGTCTAGTAGGCTACCTGACGAGTCCTTTTTAGGACGA
+
FFFFFFFFFFFFFFFFFFFFFFFFFFFFFFFFFFFFFFFFFFFFFFFFFFFFFFFFFFFFFFFFFFFF
FFFFFFFFFFFFFFFFFFFFFFFF:FFFFFFFFFFFFFFFFFFFFFFFFFFFFFFFFFF:FFFFFFFF
@A00155:342:HHGFNDSXY:1:1110:9751:20290 1:N:0:GAACCTAG+TCCGCATA
ATCCTCTGCCGAAACAGAGGTTGGACAAGACCGGAGGGGTCTCCTAGTTCCAAAGGAGATGTACTCCG
GGCTTGTTCACGACCTACCGTGTAAGTCGTAGTCTAGTAGGCTACCTGACGAGTCCTTTTTAGGACGA
+
FFFFFFFFFFFFFFFFFFFFFFFFFFFFFFFFF:F,,FFFFFFFFFFFFFFFFFFFFFFFFFFFFFFF
FFFFFFFFFFFFFF,FFFFFFFFFFFFFFFFFFFFFFFFF:FFFFFFFFFFFFFFFF:FFFFFFFFFF
```

@A00155:342:HHGFNDSXY:1:1110:12771:36699 1:N:0:GAACCTAG+TCCGCATA
CTTCTCTCGGGCCCACAGCCTTACAGCTGTGGAGCACGGTATCCTCTGCCGAAACAGAGGTTGGACAA
GACCGGAGGGGTCTCCTAGTTCCAAAGGAGATGTACTCCGGGCTTGTTCACGACCTACCGTGTAAGTC
+
FFFFFFFFFFFFFFFFFFFF:FFFFFFFFFFFFFFFFFFFFFFFFFFFFFFFF,FFFFFF:FFFFFF:
FFFFFFFFFFFFFFFFFFFFFFFFFFFFFFFFFFFFFFFFFFFFFFFFFFFFFFFFFFFFFFFFFFFF
@A00155:342:HHGFNDSXY:1:1111:18810:2785 1:N:0:GAACCTAG+TCCGCATA
CGACTTCTCTCGGGCCCACAGCCTTACAGCTGTGGAGCACGGTATCCTCTGCCGAAACAGAGGTTGGA
CAAGACCGGAGGGGTCTCCTAGTTCCAAAGGAGATGTACTCCGGGCTTGTTCACGACCTACC
+
FFFFFFFFFFFFFFFFFFFFFFFFFFFFFFFFFFFFFFFFFFFFFFFFFFFFFFFFFFFFFFFFFFFF
FFFFFFFFFFFFFFFFFFFFFFFFFFFFFFFFFFFFFFFFFFFFFFFFFFFFFFFFFFFFFF
@A00155:342:HHGFNDSXY:1:1111:11026:15796 1:N:0:GAACCTAG+TCCGCATA
AAGGACTCTCCTCTGGCCTATGGTCATCTCAATGCCGAGAGGGATGTGGAGGGCGAGAGAGAGCGACT
TCTCTCGGGCCCACAGCCTTACAGCTGTGGAGCACGGTATCCTCTGCCGAAACAGAGGTTGGACAAGA
+
FFFFFFFFFFFFFFFFFFFFFFFFFFFFFFFFFFFFFFFFFFFFFFFFFFFFFFFFFFFFFFFFFFFF
FFFFFFFFFFFFFFFFFFFFFFFFFFFFFFFFFFFFFFFFFFFFFFFFFFFFFFFFFFFFFFFFFFFF
@A00155:342:HHGFNDSXY:1:1111:26892:22294 1:N:0:GAACCTAG+TCCGCATA
AGATCAGCTGTGTCCACTTAAGGACTCACCTCTGGCCTATGGTCATCTCAATGCCGAGAGGGATGTGA
AGGGCGAGAGAGAGCGACTTCTCTCGGGCCCACAGCCTTACAGCTGTGGAGCACGGTATCCTCTGCCG
+
FFFFFFFFFFFFFFFFFFFFFFFFFFFFFFFFFFFFFFFFFFFFFFFFFFFFFFFFFFFFFFF:FFFF
FFFFFFFFFFFFFFFFFFFFFFFFFFFFFFFFFFFFFFFFFFFFFFFFFFFFFFFFFFFFFFFFFFFF
@A00155:342:HHGFNDSXY:1:1111:30553:24565 1:N:0:GAACCTAG+TCCGCATA
GCGACTTCTCTCGGGCCCACAGCCTTACAGCTGTGGAGCACGGTATCCTCTGCCGAAACAGAGGTTGG
ACAAGACCGGAGGGGTCTCCTAGTTCCAAAGGAGATGTACTCCGGGCTTGTTCACGACCTACCG
+
FFFFFFFFFFFFFFFFFFFFFFFFFFFFFFFFFFFFFFFFFFFF:FFFFFFFFFFFFFFFFFFFFFFF
FFFFFFFFFFFFFFFFFFFFFFFFFFFFFFFFFFFFFFFFFFFFFFFFFFF:FFFFFFFFFFFF
@A00155:342:HHGFNDSXY:1:1112:4670:5791 1:N:0:GAACCTAG+TCCGCATA
GGGCGAGAGAGAGCGACTTCTCTCGGGCCCACAGCCTTACAGCTGTGGAGCACGGTATCCTCTGCCGA
AACAGAGGTTGGACAAGACCGGAGGGGTCTCCTAGTTCCAAAGGAGATGTACTCCGGGCTTGTTCACG
+
FFFF:F,FFFFFFFFFFFFFFF,FFFFFFFFF,FFFFFFFF:FFFFFFFFFF,FFF:FFFFFFFF,FF
FF:FFFF:FFFFFFFF,FFFFFFFFFFFFFFFFFFFFFFFFFFFFFFFFFFFFFFFFFFFFFF:FFF
@A00155:342:HHGFNDSXY:1:1112:16595:8500 1:N:0:GAACCTAG+TCCGCATA
CTTCTCTCGGGCCCACAGCCTTACAGCTGTGGAGCACGGTATCCTCTGCCGAAACAGAGGTTGGACAA
GACCGGAGGGGTCTCCTAGTTCCAAAGGAGATGTACTCCGGGCTTGTTCACGACCTACCGTGTAA
+
FFFFFFFFFFFFFFFFFFFFFF:FFFFFFFFFFFFFFFFFFFFFFFFFFFFFFFFFFFFFFFFFFFFF
F:FFFFFFFFFFFFFFFFFFFFFFFFFFFFFFFFFFFFFFFFF:FFFFFFFFFFFFFFFFFFFFF
@A00155:342:HHGFNDSXY:1:1112:8757:21324 1:N:0:GAACCTAG+TCCGCATA
CTTCTCTCGGGCCCACAGCCTTACAGCTGTGGAGCACGGTATCCTCTGCCGAAACAGAGGTTGGACAA
GACCGGAGGGGTCTCCTGGTTCCAAAGGAGATGTACTCCGGGCTTGTTCACGACCTACCGTGTAAGTC
+
FFFFFFFFFFFFFFFFFFFFFFFFFFFFFFF::FFFFFFFFFFFFFFFFFFFFFFFFFFFFFFFFFFF
FFFF:FFFFFFFFFFFFFFFFFFFFFFFFFFFFFFFFFFFFFFFFFFFFFFFFFF:FFFFFFFFFFF
@A00155:342:HHGFNDSXY:1:1112:25220:21746 1:N:0:GAACCTAG+TCCGCATA
AGGGATGTGGAGGGCGAGAGAGAGCGACTTCTCTCGGGCCCACAGCCTTACAGCTGTGGAGCACGGTA
TCCTCTGCCGAAACAGAGGTTGGACAAGACCGGAGGGGTCTCCTAGTTCCAAAGGAGATGTACTCCGG
+
FFFFF:F:FFF:FFFFFFFFFFFFFFFFFFFFFFF:FFFF:FFFFFFFFFFFFFFFFFFFFFFFFFFF
FFFFFFFFFFFFFFFFFFFF:FFFFFFFFFFFFFFFFFFFFFFFFFFFFFFFFFFFFFFFFFFFFFF

```
@A00155:342:HHGFNDSXY:1:1112:16477:25238 1:N:0:GAACCTAG+TCCGCATA
AAGGACTCACCTCTGGCCTATGGTCATCTCAATGCCGAGAGGGATGTGAAGGGCGAGAGAGAGCGACT
TCTCTCGGGCCCACAGCCTTACAGCTGTGGAGCACGGTATCCTCTGCCGAAACAGAGGTTGGACAAGA
+
FFFFFFFFFFFFFFFFFFF:FFFFFFFFFFFFFFFFFFFFFFFFFFFFFFFFFFFFFFFFFFFFFFFF
FFFFFFFFFFFFFFFFFFFFFFFFFFFFFFFFFFFFFFFFFFFFFFFFFFFFFFFFFFFFFFFFF:FF
@A00155:342:HHGFNDSXY:1:1113:27877:13917 1:N:0:GAACCTAG+TCCGCATA
GCGACTTCTCTCGGGCCCACAGCCTTACAGCTGTGGAGCACGGTATCCTCTGCCGAAACAGAGGTTGG
ACAAGACCGGAGGGGTCTCCTAGTTCCAAAGGAGATGTACTCCGGGCTTGTTCACGACCTACCGTGTA
+
FFFFFFFFFFFFFFFFFFFFFFFFFFFFFFFFFFFFFFFFFFFFFFFFFFFFFFFFFFFFFFFF:FFF
FFFFFFFFFFFFFFFFFFFFFFFFFFFFFFFFFFFFFFFFFFFFFFFFFFFFFFFFFFFFFFFFFFFF
@A00155:342:HHGFNDSXY:1:1113:20609:25567 1:N:0:GAACCTAG+TCCGCATA
TGTTTCGGCAGAGGATACCGTGCTCCACAGCTGTAAGGCTGTGGGCCCGAGAGAAGTCGCTCTCTCTC
GCCCTCTACATCCCTCTCGGCATTGAGATGACCATAGGCCAGAGGTGAGTCCTTAAGTGGACACAGCT
+
FFFFFFFFFFFFFFFFFFFFFFFFFFFFFFFFFFFFFFFFFFFFFFFFFFFFFFFFFFFFFFFFFFFF
FFFFFFFFFFFFFFFFFFFFFFF:FFFFFFFFFFFFFFFFFFFFFFFFFFFFFFFFFFFFFF,FFFFF
@A00155:342:HHGFNDSXY:1:1113:17345:28150 1:N:0:GAACCTAG+TCCGCATA
ATCTCAATGCCGAGAGGGATGTGGAGGGCGAGAGAGAGCGACTTCTCTCGGGCCCACAGCCTTACAGC
TGTGGAGCACGGTATCCTCTGCCGAAACAGAGGTTGGACAAGACCGGAGGGGTCTCCTAGTTCCAAAG
+
FFFFFFFFFFFFFFFFFFFFFFFFFFFFFFFFFFFFFFFFFFFFFFFFFFFFFFFFFFFFFFFFFFFF
FFFFFFFFFFFFFFFFFFFFFFFFFFFFFFFFFFFFFFFFFFFFFFFFFFFFFFFFFFFFFFFFFFFF
@A00155:342:HHGFNDSXY:1:1114:15646:16000 1:N:0:GAACCTAG+TCCGCATA
GGGCGAGAGAGAGCGACTTCTCTCGGGCCCACAGCCTTACAGCTGTGGAGCACGGTATCCTCTGCCGA
AACAGAGGTTGGACAAGACCGGAGGGGTCTCCTGGTTCCAAAGGAGATGTACTCCGGGCTTGTTCACG
+
FFFFFFFFFFFFFFFFFFFFFFFFFFFFFFFFFFFFFFFFFFFFFFFFFFFFFFFFFFFFFFFFFFFF
FFFFFFFFFFFFFFFFFFFFFFFFFFFFFFFFFFFFFFFFFFFFFFFFFFFFFFFFFFFFFFFFFFFF
@A00155:342:HHGFNDSXY:1:1114:25021:21371 1:N:0:GAACCTAG+TCCGCATA
ACCTCTGTTTCGGCAGAGGATACCGTGCTCCACAGCTGTAAGGCTGTGGGCCCGAGAGAAGTCGCTCT
CTCTCGCCCTCTACATCCCTCTCGGCATTGAGATGACCATAGGCCAGAGGTGAGTCCTTAAGTGGACA
+
FFFFFFFFFFFFFFFFFFFFFFFFFFFFFFFFFFFFFFFFF,FFFFFFFFFFFFFFFFFFFFFFFFFF
FFFFFFFFFFFFFFFFF:FFFFFFFFFFFFFFFFFFFFFFFFFFFFFFFFFFFFFFFFFFF:FFFFF:
@A00155:342:HHGFNDSXY:1:1114:30391:32737 1:N:0:GAACCTAG+TCCGCATA
AATGCCGAGAGGGATGTAGAGGGCGAGAGAGAGCGACTTCTCTCGGGCCCACAGCCTTACAGCTGTGG
AGCACGGTATCCTCTGCCGAAACAGAGGTTGGACAAGACCGGAGGGGTCTCCTAGTTCCAAAGGAGAT
+
FFFFFFFFFFFFFFFF:FFFFFFFFFFFFFFFFFFFFFFFF:FFFFFFFFFFFFFFFFFFF:FFFFF,
FFFFFFFFFFFFFFF:FFFFFFFFFFFFFFFFFFFFFFFFFFFFFFFFFFFFFFFFFFFFFFFFFFF:
@A00155:342:HHGFNDSXY:1:1115:17463:34992 1:N:0:GAACCTAG+TCCGCATA
GTCATCTCAATGCCGAGAGGGATGTGGAGGGCGAGAGAGAGCGACTTCTCTCGGGCCCACAGCCTTAC
AGCTGTGGAGCACGGTATCCTCTGCCGAAACAGAGGTTGGACAAGACCGGAGGGGTCTCCTAG
+
FFFFFFFFFFFFFFFFFFFFFFFFFFFFFFFFFFFFFFFFFFFFFFFFFFFFFF:FFFFFFFFFFFFF
FFFFFFFFFFFFFFFFFFFFFFFFFFFFFFFFFFFFFFFFFFFF:FFFFFFFFFFFFFFFFFF
@A00155:342:HHGFNDSXY:1:1116:9091:9502 1:N:0:GAACCTAG+TCCGCATA
GAGGGCGAGAGAGAGCGACTTCTCTCGGGCCCACAGCCTTACAGCTGTGGAGCACGGTATCCTCTGCC
GAAACAGAGGTTGGACAAGACCGGAGGGGTCTCCTAGTTCCAAAGGAGATGTACTCCGGGCTTGTTCA
+
FFFFFFFFFFFFFFFFFFFFFFFFFFFFFFFFFFFFFF:FFFFFFFFFF:FFFFFFFFFFFFFFFFFF
FFFFFFFFFFFFFFFFFFFFFFFFFFFFFFFFFFF:FFFFFFFFFFFFFFFFFFF:FFFFFFFFFFFF
```

@A00155:342:HHGFNDSXY:1:1116:13630:12727 1:N:0:GAACCTAG+TCCGCATA
GTCATCTCAATGCCGAGAGGGATGTGGAGGGCGAGAGAGAGCGACTTCTCTCGGGCCCACAGCCTTAC
AGCTGTGGAGCACGGTATCCTCTGCCGAAACAGAGGTTGGACAAGACCGGAGGGGTCTCCTAG
+
FFFFFFFFFFFFFFFFFFFFFFFFFFFFFFFFFFFFFFFFFFFFFFFFFFFFFFFFFFFFFFFFFFFF
FFFFFFFFFFFFFFFFFFFFFFFFFFFFFFFFFFFFFFFFFFFFFFFFFFFFFFFFFFFFFFF
@A00155:342:HHGFNDSXY:1:1116:29225:21010 1:N:0:GAACCTAG+TCCGCATA
ACCGTGCTCCACAGCTGTAAGGCTGTGGGCCCGAGAGAAGTCGCTCTCTCTCGCCCTCCACATCCCTC
TCGGCATTGAGATGACCATAGGCCAGAGGTGAGTCCTTAAGTGGACACAGCTGATCTAAGGCGGTGTG
+
FFFFFFFFFFFFFFFFFFFFFFFFFFFFFFFFFFFFFFFFFFFF:FFFFF:FFFFFFFFFFFFFFFFF
FFFFFF:FFFFFFFFFFFFFFFFFFFFFFFFFFFFFFFFFFFFFFFFF,FFFF:FFFFFFFFFFF:FFF
@A00155:342:HHGFNDSXY:1:1116:30897:24659 1:N:0:GAACCTAG+TCCGCATA
GACTTCTCTCGGGCCCACAGCCTTACAGCTGTGGAGCACGGTATCCTCTGCCGAAACAGAGGTTGGAC
AAGACCGGAGGGGTCTCCTAGTTCCAAAGGAGATGTACTCCGGGCTTGTTCACGACCTACCGTGTAAG
+
FFFFFFFFFFFFFFFFFFFFFFF:FFFFFFFFFFFFFFFFFF:FFFFFFFFFFFFFFFFFFFFFFFFF
FFFFFFFFFFFFFFFFFFFFFFFFFFFFFFFFFFFFFFFFFFFFFFFFFFFFFFFFFFFFFFFFFFFF
@A00155:342:HHGFNDSXY:1:1116:30915:25066 1:N:0:GAACCTAG+TCCGCATA
GACTTCTCTCGGGCCCACAGCCTTACAGCTGTGGAGCACGGTATCCTCTGCCGAAACAGAGGTTGGAC
AAGACCGGAGGGGTCTCCTAGTTCCAAAGGAGATGTACTCCGGGCTTGTTCACGACCTACCGTGTAAG
+
FFFFFFFFFFFFFFFFFFFFFFFFFFFFFFFFFFFFFFFFFFFFFFFFFFFFFFFFFFFFFFFFFFFF
FF:FFFFFFFFFFFFFFFFFFFFFFFFFFFFFFFFFFFFFFFFFFFFFFFFFFFFFFFFFFFFFFFFF
@A00155:342:HHGFNDSXY:1:1118:28031:7232 1:N:0:GAACCTAG+TCCGCATA
CTCCACAGCTGTAAGGCTGTGGGCCCGAGAGAAGTCGCTCTCTCTCGCCCTCCACATCCCTCTCGGCA
TTGAGATGACCATAGGCCAGAGGTGAGTCCTTAAGTGGACACAGCTGATCTAAGGCGGTG
+
FFFFFFFFFFFFFFFFFFFFFFFFFFFFFFF:F:FFFFFFFFFFFFFFFFFFFFFFFFFFFFFFFFFF
FFFFFFFFFFFFFFFFFFFFFFFFFFFFF,FFFFFFFFFFFFFFFFFFFFFFFFFFFFFF
@A00155:342:HHGFNDSXY:1:1118:9932:11616 1:N:0:GAACCTAG+TCCGCATA
CTGCCGAAACAGAGGTTGGACAAGACCGGAGGGGTCTCCTAGTTCCAAAGGAGATGTACTCCGGGCTT
GTTCACGACCTACCGTGTAAGTCGTAGTCTAGTAGGCTACCTGACGAGTCCTTTTTAGGACGAAACTT
+
FFFFFFFF:,FFFFFF:FFFFF::,FF,:F::FFFFF::F,FFF:FFFFFFFFFFFFFFFFFFFF:FF
FFF::F:FFF,FF,,:FFFFF:FFFFFFFFFFFF:FFFFF:FFFFFFF:F,FFFFFFFF:F:FFFFFF
@A00155:342:HHGFNDSXY:1:1118:31792:18505 1:N:0:GAACCTAG+TCCGCATA
AGAGAGAGCGACTTCTCTCGGGCCCACAGCCTTACAGCTGTGGAGCACGGTATCCTCTGCCGAAACAG
AGGTTGGACAAGACCGGAGGGGTCTCCTAGTTCCAAAGGAGATGTACTCCGGGCTTGTTCACGACCTA
+
FFFFFFFFFFFFFFFFFFFFFFFFFFFFFFF::F:FFFFFFFFFFFFFFFFFFFFFFFFFFFFFFFFF
FFFFFFFFFFFFFFFFFFFFFFFFFFFFFFFFFFFFFFFFFFFFF,F:FFFFFFFFFFFFFFFFF:F:
@A00155:342:HHGFNDSXY:1:1119:22869:25410 1:N:0:GAACCTAG+TCCGCATA
CTTCTCTCGGGCCCACAGCCTTACAGCTGTGGAGCACGGTATCCTCTGCCGAAACAGAGGTTGGACAA
GACCGGAGGGGTCTCCTAGTTCCAAAGGAGATGTACTCCGGGCTTGTTCACGACCTACCGTGTAAGTC
+
FFFFFFFFFFFFFFFFFFFF,F:FFFFFFFFFFFFFFFFFFFFFFFFFFFFFFFFFFF:FFFFFFFFF
FFFFFFFFFFFFFFFFFFFFFFFFFFFFFFFFFFFFFFFFFFFFFFFFFFFFFFFFFFFFFFFFFFFF
@A00155:342:HHGFNDSXY:1:1119:20564:26710 1:N:0:GAACCTAG+TCCGCATA
TACTCCGGGCTTGTTCACGACCTACCGTGTAAGTCGTAGTCTAGTAGGCTACCTGACGAGTCCTTTTT
AGGACGAAACTTACACAACTCGAAGAGTAACGACGGTTTCGTCCTTGGAACGGACTCATCAGGGAGCC
+
FFFFFFFFFFFFFFFFFFFFFFFFFFFFFFFFFFFFFFFFFFFFFFFFFFFFFFFFFFFFFFFF:FFF
FFFFFFFFFFFFFFFFFFFFFFFFFFFF:FFFFFFFFFFFFFFFFFFFF:FFFFFFFFFFFFFFFFFF

```
@A00155:342:HHGFNDSXY:1:1120:1814:3818 1:N:0:GAACCTAG+TCCGCATA
AGAGAGAGCGACTTCTCTCGGGCCCACAGCCTTACAGCTGTGGAGCACGGTATCCTCTGCCGAAACAG
AGGTTGGACAAGACCGGAGGGGTCTCCTGGTTCCAAAGGAGATGTACTCCGGGCTTGTTCACGACCTA
+
FFFFFFF:FFFFFF,FFF:F,F,FF:FF,FFFFFFF:::FFFFFFFFF:FFFFFFF:FFFFFFFFFFF
FFFFFF,FFFFFFFFFFFFFFFF:FFFFFFFFFFFFFFFFFFFFFFFFFF,FFFFFFFFFFFFFFFFFF
@A00155:342:HHGFNDSXY:1:1120:5620:12978 1:N:0:GAACCTAG+TCCGCATA
GAGAGCGACTTCTCTCGGGCCCACAGCCTTACAGCTGTGGAGCACGGTATCCTCTGCCGAAACAGAGG
TTGGACAAGACCGGAGGGGTCTCCTAGTTCCAAAGGAGATGTACTCCGGGCTTGTTCACGACCTACCG
+
FFFFFFFFFFFFFFFFFFFFFFFFFFFFF,FFFFFFFFFFFFFFFFFFF,FFFFFFFFFFFFFFFFFF
FFFFFFFFFFFFFFFFFFFFFFFFFFFFFFFFFFFFFFFFFFFFFFFFFFFFFF:FFFFFFFFFFFFF
@A00155:342:HHGFNDSXY:1:1120:24144:28494 1:N:0:GAACCTAG+TCCGCATA
GAGGGCGAGAGAGAGCGACTTCTCTCGGGCCCACAGCCTTACAGCTGTGGAGCACGGTATCCTCTGCC
GAAACAGAGGTTGGACAAGACCGGAGGGGTCTCCTAGTTCCAAAGGAGATGTACTCCGGGCTTGTTCA
+
FFFFF:FFFFFFFFFFFFFFFFFFFFFFFFFFFFFFFFFFFFFFFFFFFFFFFFFFFFFFFFFFFFF:
FFFFFFFFFFFFFFFFFFFFFFFFFFFFFFFFFFFFFFFFFFFFFFFFFFFFFFFFFFFFFFFFFFFF
@A00155:342:HHGFNDSXY:1:1120:29197:32268 1:N:0:GAACCTAG+TCCGCATA
ACCTCTGTTTCGGCAGAGGATACCGTGCTCCACAGCTGTAAGGCTGTGGGCCCGAGAGAAGTCGCTCT
CTCTCGCCCTTCACATCCCTCTCGGCATTGAGATGACCATAGGCCAGAGGTGAGTCCTTAAGTGGACA
+
FFFFFFFFFFFFFFFFFFFFFFFFFFFFFFFFFFFFFFFFF,FFFFFFFFFFFFFFFFFFFFFFFFFF
FFFFFFFFFFFFFFFFFFFFFFFFFFFFF:FFFFFFFFFFFFFFFFFFFFFFFFFFFFFFFFFFFFFF:
@A00155:342:HHGFNDSXY:1:1121:11053:8015 1:N:0:GAACCTAG+TCCGCATA
CTCAATGCCGAGAGGGATGTGGAGGGCGAGAGAGAGCGACTTCTCTCGGGCCCACAGCCTTACAGCTG
TGGAGCACGGTATCCTCTGCCGAAACAGAGGTTGGACAAGACCGGAGGGGTCTCCTAGTTCCAAAGGA
+
FFFFFFFFFFFFFFFFFFFFFFFFFFFFFFFFFFFFFFFFFFFFFFFFFFFFFFFFFFFFFFFFFFFF
FFFFFF:FFFFFFFFFFFFFFFFFFFFFFFFFF:FFFFFFFFFFFFFFFFFFFFFFFFFFFFFFFFFF
@A00155:342:HHGFNDSXY:1:1121:11234:8202 1:N:0:GAACCTAG+TCCGCATA
CTCAATGCCGAGAGGGATGTGGAGGGCGAGAGAGAGCGACTTCTCTCGGGCCCACAGCCTTACAGCTG
TGGAGCACGGTATCCTCTGCCGAAACAGAGGTTGGACAAGACCGGAGGGGTCTCCTAGTTCCAAAGGA
+
FFFFFFFFFFFFFFFFFFFFFFFFFFFFFFFFFFFFFFFFFFFFFFFFFFFFFFF:FFFFFFFFFFFF
FFFFFFFFFFFFFFFFFFFFFFFFFFFFFFFFFFFFFFFFFFFFFFFFFFFFFFFFFFFFFFFFFFFF
@A00155:342:HHGFNDSXY:1:1121:11704:10394 1:N:0:GAACCTAG+TCCGCATA
CTCTGCCGAAACAGAGGTTGGACAAGACCGGAGGGGTCTCCTGGTTCCAAAGGAGATGTACTCCGGGC
TTGTTCACGACCTACCGTGTAAGTCGTAGTCTAGTAGGCTACCTGACGAGTCCTTTTTAGGACGAAAC
+
FFFFFFFFFFFF,FFFFFFFFFFFFFFFFFFFFFFFFFFFFFFF:FFFFFFFFFFFFFF:FFFFFFFF:FF
FFFFFFFFFFFFF:FFFFFFFFFFFFFFFFFFFFFFFFFFFFF:FFFFFFFFFFFFFFFFFFFFFFFFFFFF
@A00155:342:HHGFNDSXY:1:1121:28989:15217 1:N:0:GAACCTAG+TCCGCATA
CAGCCTTACAGCTGTGGAGCACGGTATCCTCTGCCGAAACAGAGGTTGGACAAGACCGGAGGGGTCTC
CTGGTTCCAAAGGAGATGTACTCCGGGCTTGTTCACGACCTACCGTGTAAGTCGTAGTCTAGTAGGCT
+
FFFFFFFFFFFFFFFFFFFFFFFFFFFFFFFFFFFFFFFFFFFFFFFFFFFFFFFFFFFFFFFFFFFF
FFFFFFFFFFFFFFFFFFFFFFFFFFFFFFFFFFFFFFFFFFFFFFFFF:FFFFFFFFFFFFFFFFFF
@A00155:342:HHGFNDSXY:1:1121:32922:15483 1:N:0:GAACCTAG+TCCGCATA
AGGCCAGAGGTGAGTCCTTAAGTGGACACAGCTGATCTAAGGCGGTGTGGTGGGGCATGGGTTTGAAC
CCCCATGACGGTCGGAGTCTAGTAGGCTCCCTGATGAGTCCGTTCCAAGGACGAAACCGTCGTTACTC
+
FFFFFFFFFFFFFFFFFFFFFFFFFFFFFFFFFFFFFFFFFFFFFF:FFFFFFFFFFFFFFFFFFFFFF
FFFFFFFFFFFFFFFFFF:FFFFFFFFFFFFFFFFFFFFFFFFFFFFFFFFFFFFFFFFFFFFFFFFFF
```

```
@A00155:342:HHGFNDSXY:1:1121:32967:15499 1:N:0:GAACCTAG+TCCGCATA
AGGCCAGAGGTGAGTCCTTAAGTGGACACAGCTGATCTAAGGCGGTGTGGTGGGGCATGGGTTTGAAC
CCCCATGACGGTCGGAGTCTAGTAGGCTCCCTGATGAGTCCGTTCCAAGGACGAAACCGTCGTTACTC
+
FFFFFFFFFFFFFFFFFFFFFFFFFFF:FFFFFF:FFFFFFFFFFFF,FFFFFFFFFFFFFFFFFFFF
FFFFFFFFFFFFFFFFF:F,FFFFFFFFFFFFFF:FFFFFFFFFFFFFFFFFFFFFFFF:FFFFFF:F
@A00155:342:HHGFNDSXY:1:1121:17219:25300 1:N:0:GAACCTAG+TCCGCATA
CGGTAGGTCGTGAACAAGCCCGGAGTACATCTCCTTTGGAACTAGGAGACCCCTCCGGTCTTGTTCAA
CCTCTGTTTCGGCAGAGGATACCGTGCTCCACAGCTGTAAGGCTGTGGGCCCGAGAGAAGTCGCTCTC
+
FFFFFFFFFFFFFFFFF,FFFFFFFFFFFF:FFF:FFFFFFFFFFFFFFFFFFFFFFFFFFFFFFFFF
FFFFFFFFFFFF:FFFFFF:FFFFFFFFFFFFFFFFF:FFFFFFFF:FFFFFFFFFFFFFFF:FFFFF
@A00155:342:HHGFNDSXY:1:1121:1217:27649 1:N:0:GAACCTAG+TCCGCATA
CGAGAGAGAGCGACTTCTCTCGGGCCCACAGCCTTACAGCTGTGGAGCACGGTATCCTCTGCCGAAAC
AGAGGTTGGACAAGACCGGAGGGGTCTCCTAGTTCCAAAGGAGATGTACTCCGGGCTTGTTCACGACC
+
:FFFFFFFFFFFFFFFFFFFFFFFFFF::,FFF,:,F:FFFF,FFFFF::FFFFFFFF:FFFFFF:FF
FFFFFFFFF,FF:FFF:FFF:FFFFFFFF:,FFFFF:FFFFFFFFFF:FFFFFFFFFFFFFFFFFFFF
@A00155:342:HHGFNDSXY:1:1122:19660:6950 1:N:0:GAACCTAG+TCCGCATA
ACTTCTCTCGGGCCCACAGCCTTACAGCTGTGGAGCACGGTATCCTCTGCCGAAACAGAGGTTGGACA
AGACCGGAGGGGTCTCCTGGTTCCAAAGGAGATGTACTCCGGGCTTGTTCACGACCTACCGTGTAAGT
+
FFFFFFFFFFFFFFFFFFFFFFF,FFFFFFFFFFFFFFFFF,FFFFFFFFFFFFFFFFFFFFFFFFFF
FFFFFFFFFFFFFFFFFFFFFFFFFFFFFFFFFFFFFFFFFFFF:FFFFFFFFFFFFFFFFFFFFFFF
@A00155:342:HHGFNDSXY:1:1122:20609:7341 1:N:0:GAACCTAG+TCCGCATA
ACCTCTGTTTCGGCAGAGGATACCGTGCTCCACAGCTGTAAGGCTGTGGGCCCGAGAGAAGTCGCTCT
CTCTCGCCCTTCACATCCCTCTCGGCATTGAGATGACCATAGGCCAGAGGTGAGTCCTTAAGTGGACA
+
FFFFFFFFFFFFFFFFFFFFFFFFFFFFFFFFFFFFFFFFFFFFFFFFFFFFFFFFFFFFFFFFFFFF
FFFFFFFFFFFFFFFFFFFFFFFFFFF:FF:FFFFFFFFFFFFFFFFFFFFFFFFFFFFFFFFFFFF:
@A00155:342:HHGFNDSXY:1:1122:10710:8046 1:N:0:GAACCTAG+TCCGCATA
CTCTGCCGAAACAGAGGTTGGACAAGACCGGAGGGGTCTCCTAGTTCCAAAGGAGATGTACTCCGGGC
TTGTTCACGACCTACCGTGTAAGTCGTAGTCTAGTAGGCTACCTGACGAGTCCTTTTTAGGACGAAAC
+
FFFFFFFFFFFFFFFFFFFFFFFFFFFFFFFFFFFFFFFFFFFFFFFFFFFFFFFFFFFFFFFFFFFF
F:FFFFFFFFFFFFFFFFFFFFFFFFFFFFFFFFFFFFFFFFFFFFFFFFFFFFFFFFFFFFFFFFFF
@A00155:342:HHGFNDSXY:1:1122:22553:34976 1:N:0:GAACCTAG+TCCGCATA
ACCTCTGTTTCGGCAGAGGATACCGTGCTCCACAGCTGTAAGGCTGTGGGCCCGAGAGAAGTCGCTCT
CTCTCGCCCTCCACATCCCTCTCGGCATTGAGATGACCATAGGCCAGAGGTGAGTCCTTAAGTGGACA
+
FFFFFFFFFFFFFFFFFFFFFFFFFFFFFFFFFFFFFFFFFFFFFFFFFFFFFFFFFFF:FFFFFFFF
FFFFFFFFFFFFFFFFFFFFFFFFFFFFFFFFFFFFFFFFFFFFFFFFFFFFFFFFFFFFFFFFFFFF
@A00155:342:HHGFNDSXY:1:1123:26015:7686 1:N:0:GAACCTAG+TCCGCATA
GTCATCTCAATGCCGAGAGGGATGTGGAGGGCGAGAGAGAGCGACTTCTCTCGGGCCCACAGCCTTAC
AGCTGTGGAGCACGGTATCCTCTGCCGAAACAGAGGTTGGACAAGACCGGAGGGGTCTCCTAGTTCCA
+
FFFFFFFFFFFFFFFFFFFFFFFFFFFFFFFFFFFFFFFFFFFFFFFFFFFFFFFFFFFFFFFFFFFF
FFFFFFFFFFFFFFFFFFFFFFFFFFFFFFFFF:F:FFF:FFFFFFFFFFFFFFFFFFFFFFFFFFFF
@A00155:342:HHGFNDSXY:1:1123:27444:17049 1:N:0:GAACCTAG+TCCGCATA
GTATCCTCTGCCGAAACAGAGGTTGGACAAGACCGGAGGGGTCTCCTAGTTCCAAAGGAGATGTACTC
CGGGCTTGTTCACGACCTACCGTGTAAGTCGTAGTCTAGTAGGCTACCTGACGAGTCCTTTTTAGGAC
+
FFFFFFFFFFFFFFFFFFFFFFFFFFFFFFFFFFFFFFFFFFFFFFFFFFFFFFFFFFFF:FFF:FFF
FFFFFFFFFFFFFFFFFFFFFFFFFFFFFFFFFFFFFFFFFFFFFFFFFFFFFFFFFFFFFFFFFFFF
```

@A00155:342:HHGFNDSXY:1:1123:12653:28228 1:N:0:GAACCTAG+TCCGCATA
AGCGACTTCTCTCGGGCCCACAGCCTTACAGCTGTGGAGCACGGTATCCTCTGCCGAAACAGAGGTTG
GACAAGACCGGAGGGGTCTCCTAGTTCCAAAGGAGATGTACTCCGGGCTTGTTCACGACCTACCGTGT
+
FFFFFFFFFFFFFFFFFFFFFFFFFFF,FFFFFFFFFFFFFFFFF:FFFFFFFFFFFFFFFFFFFFFF
FFFFFFFFFFFFFFFFFFFFFFFFFFFFFFFFFFFFFFF:FFFFFFFFFFFFFFFFFFFFFFFFFFFF
@A00155:342:HHGFNDSXY:1:1124:32090:10410 1:N:0:GAACCTAG+TCCGCATA
GTATCCTCTGCCGAAACAGAGGTTGGACAAGACCGGAGGGGTCTCCTAGTTCCAAAGGAGATGTACTC
CGGGCTTGTTCACGACCTACCGTGTAAGTCGTAGTCTAGTAGGCTACCTGACGAGTCCTTTTTAGGAC
+
FFFFFFFFFFFFFFFFFFFFFFF:FFFFFFFFFFFFFFFFFFFFFFFFFFFFFFFFFFFFFFFFFFFF
FFFFFFFF:FFFFFFFFFFFFFFFFFFFFFFFFFFF:FFFFFFFFFFFFFFFFFFFFFF:FFFFFFFF
@A00155:342:HHGFNDSXY:1:1124:19587:25019 1:N:0:GAACCTAG+TCCGCATA
GGATGTGGAGGGCGAGAGAGAGCGACTTCTCTCGGGCCCACAGCCTTACAGCTGTGGAGCACGGTATC
CTCTGCCGAAACAGAGGTTGGACAAGACCGGAGGGGTCTCCTAGTTCCAAAGGAGATGTAGTCC
+
FFFFFFFFFFFFFFFFFFFFFFFFFFFFFFFFFFFFFFFFFFFFFFFFFFFFFFFFFFFFFFFFFFFF
FFFFFFFFFFFFFFFFFFFFFFFFF:FFFFFFFFFFFFFFFFFFFFFFFFFFFFFFFFFF,FFF
@A00155:342:HHGFNDSXY:1:1124:11785:26381 1:N:0:GAACCTAG+TCCGCATA
TGTGGAGGGCGAGAGAGAGCGACTTCTCTCGGGCCCACAGCCTTACAGCTGTGGAGCACGGTATCCTC
TGCCGAAACAGAGGTTGGACAAGACCGGAGGGGTCTCCTGGTTCCAAAGGAGATGTACTCCGGGCTTG
+
FFFFFFFFFFFFFFFFFFFFFFFFFFFFFFF:FFFFFFFFFFFFFFFFFFFFFFFFFFFFFFFFFFFF
FFFFFFFFFFFFFFFFFFFFFFFFFFFFFFFFFFFFFFFFFFFFFFFFFFFFFFFFFFFFFFFFFFFF
@A00155:342:HHGFNDSXY:1:1124:19397:27226 1:N:0:GAACCTAG+TCCGCATA
TATCCTCTGCCGAAACAGAGGTTGGACAAGACCGGAGGGGTCTCCTAGTTCCAAAGGAGATGTACTCC
GGGCTTGTTCACGACCTACCGTGTAAGTCGTAGTCTAGTAGGCTACCTGACGAGTCCTTTTTAGGACG
+
FFFFFFFFFFFFFFFFFF:FFFFFFFFFFFFFFFFFFFFFFFFFFFFFFFFFFFFFFFFFFFFFFFFF
FFFFFFFFFFFFFFFFFFFFFFFFFFFFFFFFFFFFFFFFFFFFFFFFFFFFFFFFFFFFFFFFFFFF
@A00155:342:HHGFNDSXY:1:1124:2166:28385 1:N:0:GAACCTAG+TCCGCATA
CGAGAGAGAGCGACTTCTCTCGGGCCCACAGCCTTACAGCTGTGGAGCACGGTATCCTCTGCCGAAAC
AGAGGTTGGACAAGACCGGAGGGGTCTCCTAGTTCCAAAGGAGATGTACTCCGGGCTTGTTCACGACC
+
FFFFFFFFFFFFFFFFFFFFFFFFFFFFFFFFFFFFFFFFFFFFFFFFFFFFFFFFFFFFFFFFFFFF
FFFFFFFFFFFFFF:FFFFFFFFFFFFFFFFFFFFFFFFFFFFFFFFFFFFFFFFFFFFFFFFFFFFF
@A00155:342:HHGFNDSXY:1:1124:2492:31172 1:N:0:GAACCTAG+TCCGCATA
CTTCTCTCGGGCCCACAGCCTTACAGCTGTGGAGCACGGTATCCTCTGCCGAAACAGAGGTTGGACAA
GACCGGAGGGGTCTCCTAGTTCCAAAGGAGATGTACTCCGGGCTTGTTCACGACCTACCGTGTAAGTC
+
FFFFFFFFFFFFFFFFFFFFFFFFFFFFFFFFFFFFFFFFFFFFFFFFFFFFFFFFFFFFFFFFFFFF
FFFFFFFFFFFFFFFFFFFFFFFFFFFFFFFFFFFF:FFFFFFFFFFFFFFFFFFF:FFFFFFFFFFF
@A00155:342:HHGFNDSXY:1:1125:26648:4053 1:N:0:GAACCTAG+TCCGCATA
GTATCCTCTGCCGAAACAGAGGTTGGACAAGACCGGAGGGGTCTCCTGGTTCCAAAGGAGATGTACTC
CGGGCTTGTTCACGACCTACCGTGTAAGTCGTAGTCTAGTAGGCTACCTGACGAGTCCTTTTTAGGAC
+
FFFFFFFFFFFFFFFFFFFFFFFFFFFFFF,FFFFFFFFF:FFFFFFFFFFFFFFFFFFFFFFFFFFF
FFFFFFFFFFFFFFFFFFFFFFFFFFFFFFFFFFFFFFFFFFFFFFFFFFFFFFFFFFFFFFFFFFFF
@A00155:342:HHGFNDSXY:1:1125:25726:10222 1:N:0:GAACCTAG+TCCGCATA
CACCTCTGGCCTATGGTCATCTCAATGCCGAGAGGGATGTGGAGGGCGAGAGAGAGCGACTTCTCTCG
GGCCCACAGCCTTACAGCTGTGGAGCACGGTATCCTCTGCCGAAACAGAGGTTGGACAAGACCGGAG
+
FFFFFFFFFFFFFFFFFFFFFFFFFFFFFFFFFFFFFFF:FFFFFFFFFFFFFFFFFFFFFFFFFFFF
FFFFFFFFFFFFFFFFFFFFFFFFFFFFFFFFFFFFFFFFFFFFFFFFFFFFFFFFFFFFFFFFFFF

```
@A00155:342:HHGFNDSXY:1:1125:21829:10645 1:N:0:GAACCTAG+TCCGCATA
GTCCACTTAAGGACTCTCCTCTGGCCTATGGTCATCTCAATGCCGAGAGGGATGTGGAGGGCGAGAGA
GAGCGACTTCTCTCGGGCCCACAGCCTTACAGCTGTGGAGCACGGTATCCTCTGCCGAAACAGAGGTT
+
FFFFFFFFFFFFFFFFFFFFFFFFFFFFFFFFFFFFFFFFFFFFFFFFFFFFFFFFFFFFFFFFF:FF
FFFFFFFFFFFFFFFFFFFFFFFFFFFFFFFFFFFFFFF:FFFFFF:FFFFFFFFFFFFFF:FFFFFF
@A00155:342:HHGFNDSXY:1:1125:23818:15749 1:N:0:GAACCTAG+TCCGCATA
TCGGCAGAGGATACCGTGCTCCACAGCTGTAAGGCTGTGGGCCCGAGAGAAGTCGCTCTCTCTCGCCC
TCCACATCCCTCTCGGCATTGAGATGACCATAGGCCAGAGGTGAGTCCTTAAGTGGACAC
+
FFFFFFFFFFFFFFFFFFFF:FFFFFFFFFFFFFFFFFFFFFFFFFFFFFFFFFFFFFFFFFFFFFFF
FFFFFFFFFFFFFFFFFFFFFFFFFFFFFFFFFFFFFFFFFFFFFFFFFFFF:FFFFFFF
@A00155:342:HHGFNDSXY:1:1125:29378:22247 1:N:0:GAACCTAG+TCCGCATA
GGACTCACCTCTGGCCTATGGTCATCTCAATGCCGAGAGGGATGTGAAGGGCGAGAGAGAGCGACTTC
TCTCGGGCCCACAGCCTTACAGCTGTGGAGCACGGTATCCTCTGCC
+
FFFFFFFFFFFFFFFFFFFFFFFFFFFFFFFFF:FFFFFFFFFFFFFFFFFF:FFFFFFFFFFFFFFF
FFFFFFFFFFFFFFFFFFFFFFFFFFFFFFFFFFFFFFFFFFFFFF
@A00155:342:HHGFNDSXY:1:1125:17418:29246 1:N:0:GAACCTAG+TCCGCATA
CCATGCCCCGCCACACCGCCTTAGATCAGCTGTGTCCACTTAAGGACTCACCTCTGGCCTATGGTCAT
CTCAATGCCGAGAGGGATGTGGAGGGCGAGAGAGAGCGACTTCTCTCGGGCCCACAGCCTTACAGCTG
+
FFFFFFFFFFFFFFFFFFFF:FFFFFFFFFFFFFFFFFFFFFFFFFFFFFFFFFFFFFFFFFFFFFFF
FFFFFFFFFFFFFFFFFFFFFFFFFFFFFFFFFFFFFFFFFFFFFFFFFFFFFFFFFFFFFFFFFFFF
@A00155:342:HHGFNDSXY:1:1126:17553:13949 1:N:0:GAACCTAG+TCCGCATA
GAGAGAGCGACTTCTCTCGGGCCCACAGCCTTACAGCTGTGGAGCACGGTATCCTCTGCCGAAACAGA
GGTTGGACAAGACCGGAGGGGTCTCCTAGTTCCAAAGGAGATGTACTCCGGGCTTGTACACGACCTAC
+
FFFFFFFFFFFFFFFFFFFFFFFFFFFFFF:FFFFFFFFFFFFFFFFFFFFFFFFFFFFFFFFFFFFF
FFFFFFFFFFFFFFFFFFFFFFFFFFFFFFFFFFFFFFFFFFFFFFFFFFFFFFFFFFFFFFFFFFFF
@A00155:342:HHGFNDSXY:1:1126:13548:36417 1:N:0:GAACCTAG+TCCGCATA
GAGGGCGAGAGAGAGCGACTTCTCTCGGGCCCACAGCCTTTCAGCTGTGGAGCACGGTATCCTCTGCC
GAAACAGAGGTTGGACAAGACCGGAGGGGTCTCCTAGTTCCAAAGGAGATGTACTCCGGGCTTGTTCA
+
FFFFFFFFFFFFFFFFFFFFFFFFFFFFFFFFFFFFFFFFFFFFFFFFFFFFFFFFFFFFFFFFFFFF
FFFFFFFFFF,FFFFFFF:F,FFFFFFFFFFFFFFFFFFFFFFFF:FFFFF,FFFFFFF:FFFFFFFF
@A00155:342:HHGFNDSXY:1:1127:1958:6762 1:N:0:GAACCTAG+TCCGCATA
AGAGAGAGCGACTTCTCTCGGGCCCACAGCCTTACAGCTGTGGAGCACGGTATCCTCTGCCGAAACAG
AGGTTGGACAAGACCGGAGGGGTCTCCTGGTTCCAAAGGAGATGTACTCCGGGCTTGTTCACGACCTA
+
FFFFFFFFFFFFFFFFFFF,,F:,:FFFFFFFFFFFFFFFFFFFFFFFF:FFFFFFFFFFFFFFFFFF
FFFFFFFFFFFFFFFFFFFFFFFFFF:FFFFFFFFFFFFFFFFFFFFFFFFFFFFFFFFFFFFFFFFF
@A00155:342:HHGFNDSXY:1:1127:2862:11490 1:N:0:GAACCTAG+TCCGCATA
ATCTCAATGCCGAGAGGGATGTGGAGGGCGAGAGAGAGCGACTTCTCTCGGGCCCACAGCCTTACAGC
TGTGGAGCACGGTATCCTCTGCCGAAACAGAGGTTGGACAAGACCGGAGGGGTCTCCTAGTTCCAAAG
+
FFFFFFFF:FFFFFFFF,FFFFFFF:F:FFFFF:FF:FFFFFFFFFFFFF,FFFFFFFF,FFFFFFFF
FFFFFFFFFFFFFFFFFFFFFFFFFFFFFFFFFF:FFFFFFFFF:FFFFFFFFFFFFFFFFFFFFFFF
@A00155:342:HHGFNDSXY:1:1127:27968:14356 1:N:0:GAACCTAG+TCCGCATA
GTCATCTCAATGCCGAGAGGGATGTGGAGGGCGAGAGAGAGCGACTTCTCTCGGGCCCACAGCCTTAC
AGCTGTGGAGCACGGTATCCTCTGCCGAAACAGAGGTTGGACAAGACCGGAGGGGTCTCCTTGTTCCA
+
FFFFFFFFFFFFFFFFFFFFFFFFFFFFFFFFFFFFFFFFFFFFFFFFFFFFFFFFFFFFFFFFFFFF
FFFFFFFFFFFFFFFFFFFFFFFFFFFFFFFFFFFFFFFFFFFF,FFFFFFFFFFFFFFFFFFFFFFF
```

@A00155:342:HHGFNDSXY:1:1127:18349:19993 1:N:0:GAACCTAG+TCCGCATA
CAGCCTTACAGCTGTGGAGCACGGTATCCTCTGCCGAAACAGAGGTTGGACAAGACCGGAGGGGTCTC
CTAGTTCCAAAGGAGATGTACTCCGGGCTTGTTCACGACCTACCGTGTAAGTCGTAGTCTAGTAGGCT
+
FFFFFFF:FFFFFFFFFFFFFFFFF:FFFFFFFFFFFFFFFFFFFFFFFFFFFFFFFFFFFFFFFFFF
FFFFFFFFFFFFFFFFFFFFFFFFFFFFFFFFFFFFFFFFFFFFFFFFF:FFFFFFFFFFFFFFFFFF
@A00155:342:HHGFNDSXY:1:1127:28185:28354 1:N:0:GAACCTAG+TCCGCATA
ATCTCAATGCCGAGAGGGATGTGGAGGGCGAGAGAGAGCGACTTCTCTCGGGCCCACAGCCTTACAGC
TGTGGAGCACGGTATCCTCTGCCGAAACAGAGGTTGGACAAGACCGGAGGGGTCTCCTAGTTCCAAAG
+
FFFFFFFFFFFFFFFFFFFF:FFFFFFFFFFFFFFFFFFFFFFFFFFFFFFFFFFFFFFFFFFFFFFF
FFFFFFFFFFFFFFFFFFFFFFFFFFFFFFFFFFFFFFFFFFFFFFFFFFFFFFFFF:FFFFFFFFFF
@A00155:342:HHGFNDSXY:1:1127:27877:28510 1:N:0:GAACCTAG+TCCGCATA
ATCTCAATGCCGAGAGGGATGTGGAGGGCGAGAGAGAGCGACTTCTCTCGGGCCCACAGCCTTACAGC
TGTGGAGCACGGTATCCTCTGCCGAAACAGAGGTTGGACAAGACCGGAGGGGTCTCCTAGTTCCAAAG
+
FFFFFFFFFFFFFFFFFFF,FFFFFFFFFFFFFFFFFFFFFFFFFFFFFFFFFFFFFFFFFFFFFFFF
FFFFFFFFFFFFFFFF:FFFFFFFFFFFFFFFFFFFFFFFFFFFFFF:FFFFFFFFFF,FFFFFFFFF
@A00155:342:HHGFNDSXY:1:1127:28718:28682 1:N:0:GAACCTAG+TCCGCATA
TCTCAATGCCGAGAGGGATGTGGAGGGCGAGAGAGAGCGACTTCTCTCGGGCCCACAGCCTTACAGCT
GTGGAGCACGGTATCCTCTGCCGAAACAGAGGTTGGACAAGACCGGAGGGGTCTCCTAGTTCCAAAG
+
::::FFFFFFFF::FFFF:FFFFFF:FF:F:FFFFFFFFFFFFFF:,FFFFFFFFFFFF:FF,FFFFF
FFFFFF,FFF:FF:F,FFFFFFFFFFFFFFFFF:,FFFFFFFFFFFF,F,FFFFF,F:,FFF:FFF:
@A00155:342:HHGFNDSXY:1:1127:8856:31015 1:N:0:GAACCTAG+TCCGCATA
TCTCTCGGGCCCACAGCCTTACAGCTGTGGAGCACGGTATCCTCTGCCGAAGCAGAGGTTGGACAAGA
CCGGAGGGGTCTCCTAGTTCCAAAGGAGATGTACTCCGGGCTTGTTCACGACCTACCGTGTAAGTCGT
+
FFFFFFFFFFFFFFFFFFFFFFFFFFFFFFFFFFFFFFFFFFFFFFFFFFF,FFFFFFFFFFFFFFFF
FFFFFFFFFFFFFF:FFFFFFF:FFFFFFFFFFFFFFFFFFFFFFFFFFFFFFFFFFFFFFFFFFFFF
@A00155:342:HHGFNDSXY:1:1127:16875:31250 1:N:0:GAACCTAG+TCCGCATA
CCCACCACACCGCCTTAGATCAGCTGTGTCCACTTAAGGACTCACCTCTGGCCTATGGTCATCTCAAT
GCCGAGAGGGATGTGGAGGGCGAGAGAGAGCGACTTCTCTCGGGCCCACAGCCTTACAGCTGTGGAGC
+
FFFFFFFFFFFFFF:FFFFFFFFFFFFFFFFFFF:FFFFFFFFFFFFFFFFFFFFFFFFFFFFFFFFF
FFFFFFFFFFFFF:FFFFFFFFFFFFFFFFFFFFFFFFFFFFFFFFFFFFFFFFFFFFFFFFFFFFFF
@A00155:342:HHGFNDSXY:1:1128:21549:7091 1:N:0:GAACCTAG+TCCGCATA
GGATGTGGAGGGCGAGAGAGAGCGACTTCTCTCGGGCCCACAGCCTAACAGCTGTGGAGCACGGTATC
CTCTGCCGAAACAGAGGTTGGACAAGACCGGAGGGGTCTCCTAGTTCCAAAGGAGATGTACTCC
+
FFFFFFFFFFFFFFFFFFFFFFFFFFFFFFFFFFFFFFFFFFFFFFFFFFFFFFFFFFFF:FFFFFFF
FFFFFFFFFFFFFFFFFFFFFFFFFFFFFFFFFFFFFFFFFFFFFFFFFFFFFFFFFFFFFFFF
@A00155:342:HHGFNDSXY:1:1128:25753:15468 1:N:0:GAACCTAG+TCCGCATA
GAGAGGGATGTGGAGGGCGAGAGAGAGCGACTTCTCTCGGGCCCACAGCCTTACAGCTGTGGAGCACG
GTATCCTCTGCCGAAACAGAGGTTGGACAAGACCGGAGGGGTCTCCTAGTTCCAAAGGAGATGTACTC
+
FFFFFFFFFFFFFFFFFFFFFFFFFFFFFFFFFFFFFFFFFFFFFFFFFFFFFFFFFFFFFFFFFFFF
FFFFFFFFFFFFFFFFFFFFFFFFFFFFFFFFFFFFFFFFFFFFFFFFFFFFFFFFFFFFFFFFFFFF
@A00155:342:HHGFNDSXY:1:1129:31638:9377 1:N:0:GAACCTAG+TCCGCATA
AGAGCGACTTCTCTCGGGCCCACAGCCTTACAGCTGTGGAGCACGGTATCCTCTGCCGAAACAGAGGT
TGGACAAGACCGGAGGGGTCTCCTAGTTCCAAAGGAGATGTACTCCAGGCTTGTTCACGACCTACCGT
+
FFFFFFFFFFFFFFFFFFFFFFFFFFFFFFFFFFFFFFFF:FFF:FFFFFFFFFFFFFFFFFFFFF:F
FFFFFFFFFFFFFFFFFFFFFFFFFFFF:FFFFFFFFFFFFFFFFFFFFFFFFFFFFFFFFFFFFFFF

```
@A00155:342:HHGFNDSXY:1:1129:17282:15765 1:N:0:GAACCTAG+TCCGCATA
GTTCAAACCCATGCCCCGCCACACCGCCTTAGATCAGCTGTGTCCACTTAAGGACTCACCTCTGGCCT
ATGGTCATCTCAATGCCGAGAGGGATGTGGAGGGCGAGAGAGAGCGACTTCTCTCGGGCCCACAGCCT
+
F:FFFFFFF:FFFFFFFFFFFFF:FFF:FF:FFFFFFFFFFFFF:F:FFF,FFFFFFFF:,F:FF:FF
FFFFFFFFFFFFFFFFFFFFFFFFF,FFFFFFFFFFFFFFFFFFFFFFFFFFFFFFF:FFFFFFFFFF
@A00155:342:HHGFNDSXY:1:1129:17264:24314 1:N:0:GAACCTAG+TCCGCATA
CACCTCTGGCCTATGGTCATCTCAATGCCGAGAGGGATGTGGAGGGCGAGAGAGAGCGACTTCTCTCG
GGCCCACAGCCTTACAGCTGTGGAGCACGGTATCCTCTGCCGAAACAGAGGTTGGACAAGACCGGAGG
+
FFFFFFFFFFFF:FFFFFFFFFFFFFFFFFFFFFFFFFFFFFFFFFFFFFFFFFFFFFF,FFFFFFF
FFFFFFFFFFF:FFFFFFFFFFFFFFFFFFFFFFFFFFFFFFFFFFFFFFFFFFFFFFFFFFFFFFFF
@A00155:342:HHGFNDSXY:1:1129:23466:32925 1:N:0:GAACCTAG+TCCGCATA
AGAGAGAGCGACTTCTCTCGGGCCCACAGCCTTACAGCTGTGGAGCACGGTATCCTCTGCCGAAACAG
AGGTTGGACAAGACCGGAGGGGTCTCCTAGTTCCAAAGGAGATGTACTCCGGGCTTGTTCACGACCTA
+
FFFFFFFFFFFFFFFFFFFFFFFFFFFFFFFFFFFFFFFFFFFFFFFFFFF:FFFFFFFFFFFFFFFF
FFFFFFFFFFF:FF:FFFFFFFFFFFFFFFFFFFFFFFFFFFFFFFFFFFFFFFFFFFFFFFFFFFFF
@A00155:342:HHGFNDSXY:1:1130:11379:8453 1:N:0:GAACCTAG+TCCGCATA
CAGCCTTACAGCTGTGGAGCACGGTATCCTCTGCCGAAACAGAGGTTGGACAAGACCGGAGGGGTCTC
CTAGTTCCAAAGGAGATGTACTCCGGGCTTGTTCACGACCTACCGTGTAAGTCGTAGTCTAGTAGGCT
+
FFFFFFFFFFFFFFFFFFFFFFFFFFFFFFFFFFFFFFFFFFFFFFFFFFFFFFFFFFFFFFFFFFFF
FFFFFFFFFFFFFFFFFFFFFFFFFFFFFFFFFFFFFFFFFFFFFFFFFFFFFFFFFFFF:FFFFFFF
@A00155:342:HHGFNDSXY:1:1130:29324:9408 1:N:0:GAACCTAG+TCCGCATA
TTAAGGACTCTCCTCTGGCCTATGGTCATCTCAATGCCGAGAGGGATGTGGAGGGCGAGAGAGAGCGA
CTTCTCTCGGGCCCACAGCCTTACAGCTGTGGAGCACGGTATCCTCTGCCGAAACAGAGGTTGGACAA
+
FFFFFFFFFFFFFFFFF:FFF:FFFF:FFFFFFFFFFFFFFFFFFFFFFFFFFFFFFFFFFFFFFFFF
FFFFFFFFFFFFFFFFFF,FFFFFFFFFFFFFF::FFFFFFFFFFFFF:FFFFFFFFFFFFFFFFFFF
@A00155:342:HHGFNDSXY:1:1131:10809:12759 1:N:0:GAACCTAG+TCCGCATA
GAGAGGGATGTGGAGGGCGAGAGAGAGCGACTTCTCTCGGGCCCACAGCCTTACAGCTGTGGAGCACG
GTATCCTCTGCCGAAACAGAGGTTGGACAAGACCGGAGGGGTCTCCTAGTTCCAAAGGAGATGTACTC
+
FFFFFFFFFFFFFFFFFFFFFFFFFFFFFFFFFFFFFFFFFFFFFFFFFFFFFFFFFFFFFFFFFFFF
FFFFFFFFFFFFFFFFFFFFFFFFFFFFFFFFFFFFFFFFFFFFFFFFFFFFFFFFFFFFFFFFFFFF
@A00155:342:HHGFNDSXY:1:1131:23023:16689 1:N:0:GAACCTAG+TCCGCATA
GCGACTTCTCTCGGGCCCACAGCCTTACAGCTGTGGAGCACGGTATCCTCTGCCGAAACAGAGGTTGG
ACAAGACCGGAGGGGTCTCCTAGTTCCAAAGGAGATGTACTCCGGGCTTGTTCACGACCTACCGTGTA
+
FFFFFFFFFFFFFFFFFFFFFFFFFFFFFFFFFFFFFFFFFFFFFFFFFFFFFFFFFFFFFFFFFFFF
FFFFFFFFFFFFFFFFFFFFF,FFFFFFFFFFFFFFFFFFFFFFFFFFFFFFFFFFFFFFFFFFFFFF
@A00155:342:HHGFNDSXY:1:1132:11451:8171 1:N:0:GAACCTAG+TCCGCATA
GGTATCCTCTGCCGAAACAGAGGTTGGACAAGACCGGAGGGGTCTCCTAGTTCCAAAGGAGATGTACT
CCGGGCTTGTTCACGACCTACCGTGTAAGTCGTAGTCTAGTAGGCTACCTGACGAGTCCTTTTTAGGA
+
FFFFFFFFFFFFFFFFFFFFFFFFFFFFFFFFFFFFFFFFFFFFFFFFFFFFFFFFFFFFFFFFFFFF
FFFFFFFFFFFFFFFFFFFFFFFFFFFFFFFFFFFFFFFFFFFFFFFFFFFFFFFFFFFFFFFFFFFF
@A00155:342:HHGFNDSXY:1:1132:17897:27070 1:N:0:GAACCTAG+TCCGCATA
TGTGGAGGGCGAGAGAGAGCGACTTCTCTCGGGCCCACAGCCTTACAGCTGTGGAGCACGGTATCCTC
TGCCGAAACAGAGGTTGGACAAGACCGGAGGGGTCTCCTAGTTCCAAAGGAGATGTACTCCGGG
+
FFFFFFFFFFFFFFFFFFFFFFFFFFFFFFFFFFFFFFFFFFFFFFFFFFFFF:FFFFFFFFFFFFFF
FFFFFFFFFFFFFFFFFFFFFFFFFFFFFFFFFFFFFFFFFFFFFFFFFFFFFFFFFFFFFFFF
```

@A00155:342:HHGFNDSXY:1:1132:4589:34366 1:N:0:GAACCTAG+TCCGCATA
GGATGTGGAGGGCGAGAGAGAGCGACTTCTCTCGGGCCCACAGCCTTACAGCTGTGGAGCACGGTATC
CTCTGCCGTAACAGAGGTTGGACAAGACCGGAGGGGTCTCCTAGTTCCAAAGGAGATGTACTCC
+
FFFFFFFFFFFFFFFFFFFFFFFFFF,FFFFFFFFFFFFFFFFFFFF,FFFFFFFFFFFF:FFFFFFF
FFFFFFFFFFFFFFFFFFFFFFFFFFFFFFFFFFFFFFFFFFFFFFFFFFFFFFFFFFFFFFFF
@A00155:342:HHGFNDSXY:1:1132:8440:36965 1:N:0:GAACCTAG+TCCGCATA
TGTTTCGGCAGAGGATACCGTGCTCCACAGCTGTAAGGCTGTGGGCCCGAGAGAAGTCGCTCTCTCTC
GCCCTCCACATCCCTCTCGGCATTGAGATGACCATAGGCCAGAGGTGAGTCCTTAAGTGGACACAGCT
+
FFFFFFFFFFFFFFFFFFFFFFFFFFFFFFFFFFFFFFFFFFFFFFFFFFFFFFFFFFFFFFFFFFFF
FFFFFFFFFFFFFFFFFFFFFFFFFFFFFF:FFFFFFFFFFFFFFFFFFFF:FFFFFFF:FFFFFFFF
@A00155:342:HHGFNDSXY:1:1132:15221:37027 1:N:0:GAACCTAG+TCCGCATA
AGGGATGTGGAGGGCGAGAGAGAGCGACTTCTCTCGGGCCCACAGCCTTACAACTGTGGAGCACGGTA
TCCTCTGCCGAAACAGAGGTTGGACAAGACCGGAGGGGTCTCCTAGTTCCAAAGGAGATGTACTCCGG
+
FFFFFFFFFFFFFFFFFFFFFFFFFFFFFFFFFFFFFFFF:FFFFFFFFFFFFFFFF:FFFFFFFFFF
FFFFFFFFFFFFFFFFFFFFFFFFFFF:FF:FFF:FFFFFFFFFF:FFFFFF:FFFFFFFFFFFFFFF
@A00155:342:HHGFNDSXY:1:1133:17779:8923 1:N:0:GAACCTAG+TCCGCATA
CACCTCTGGCCTATGGTCATCTCAATGCCGAGAGGGATGTGAAGGGCGAGAGAGAGCGACTTCTCTCG
GGCCCACAGCCTTACAGCTGTGGAGCACGGTATCCTCTGCCGAAACAGAGGTTGGACAAGACCGGAGG
+
FFFFFFFFFFFFFFFFFFFFFFFFFFFFFFFFFFFFFFFFFFFFFFFFFFFFFFFFFFFFFFFFFFFF
FFFFFFFFFFFFFFFFFFFFFFFFFFFFFFFFFFFFFFFFFFFFFFFFFFFFFFFFFFFFFFFFFFFF
@A00155:342:HHGFNDSXY:1:1133:31620:10786 1:N:0:GAACCTAG+TCCGCATA
CTCAATGCCGAGAGGGATGTAGAGGGCGAGAGAGAGCGACTTCTCTCGGGCCCACAGCCTTACAGCTG
TGGAGCACGGTATCCTCTGCCGAAACAGAGGTTGGACAAGACCGGAGGGGTCTCCTAGTTCCAAAGGA
+
FFFFFFFFFFFFFFFFFFF:FFFFFFFFFFFFFFFFFFFFF:FFFFFFFFFFFFFFFFFFFFFFFFFF
FFFFFFFFFFFFFFFFFFFFFFFFFFFFFFFFFFFFFFFFFFFFFFFFFFFFFFFFFFFFFFFFFFFF
@A00155:342:HHGFNDSXY:1:1133:17580:12273 1:N:0:GAACCTAG+TCCGCATA
CACCTCTGGCCTATGGTCATCTCAATGCCGAGAGGGATGTGAAGGGCGAGAGAGAGCGACTTCTCTCG
GGCCCACAGCCTTACAGCTGTGGAGCACGGTATCCTCTGCCGAAACAGAGGTTGGACAAGACCGGAGG
+
FFFFFFFFFFFFFFFFFFFFFFFFFFFFFFFF:FFFFFFFFFFFFFFFFFFFFFFFFFFFFFFFFFFF
FFFFFFFFFFFFFFFFFFFFFFFFFFFFFFFFFFFFFFFFFFFFFF:FFFFFFFFFFFFFFFFFFFFF
@A00155:342:HHGFNDSXY:1:1133:28791:19163 1:N:0:GAACCTAG+TCCGCATA
AGAGAGAGCGACTTCTCTCGGGCCCACAGCCTTACAGCTGTGGAGCACGGTATCCTCTGCCGAAACAG
AGGTTGGACAAGACCGGAGGGGTCTACTAGTTCCAAAGGAGATGTACTCCGGGCTTGTTCACGACCTA
+
FFFFFFFF:FFFFFFFFFFF:FF:FFFF,FF:FFFFF:FFF:F:F,FF,FFFFF:FFFFF:FFFFFFF
FFFFF:FFFFF:FFFFFFFFFFFFFF:FFFFFFFFFFFFFFF:FFFFFFFFFF:F:FFFFFFFFFFFF
@A00155:342:HHGFNDSXY:1:1133:1994:33755 1:N:0:GAACCTAG+TCCGCATA
AAGGACTCACCTCTGGCCTATGGTCATCTCAATGCCGAGAGGGATGTGAAGGGCGAGAGAGAGCGACT
TCTCTCGGGCCCACAGCCTTACAGCTGTGGAGCACGGTATCCTCTGCCGAAACAGAGGTTGGACAAGA
+
FFFFFFFFFFFFFFFFFFFFFFFFFFFFFFFFFFFFFFFF:FFFFFFFFFFFFFFFFFFFFFFFFFFF
FFFFFFFFFFFFFFFFFFFFFFFFFFFFFFFFFFFFFFFFFFFFFFFFFFFFFFFFFFFFFFFFFFFF
@A00155:342:HHGFNDSXY:1:1134:25834:26005 1:N:0:GAACCTAG+TCCGCATA
TGTCCACTTAAGGACTCACCTCTGGCCTATGGTCATCTCAATGCCGAGAGGGATGTGGAGGGCGAGAG
AGAGCGACTTCTCTCGGGCCCACAGCCTTACAGCTGTGGAGCACGGTATCCTCTGCCGAAACAGAGGT
+
FFFFFFFFFFFFFFFFFFFFFFFFFFFFFFFFFFFFFFFFFFFFFFFFFF:FFFFFFFFFFFF:FFFF
FFF:FFFFFFFFFFFFFFFFFFFFFFFF:FFFFFFFFFFFFFFFFFF,F:FFFFFFFFFFFFFFFFFF

```
@A00155:342:HHGFNDSXY:1:1135:13693:19351 1:N:0:GAACCTAG+TCCGCATA
TTAAGGACTCACCTCTGGCCTATGGTCATCTCAATGCCGAGAGGGATGTGGAGGGCGAGAGAGAGCGA
CTTCTCTCGGGCCCACAGCCTTACAGCTGTGGAGCACGGTATCCTCTGCCGAAACAGAGGTTGGACAA
+
FFFFFFFFFFFFFFFFFFFFFFFFFFFFFFFFFFFFFFFFF:FFFFFF,FFFFFFFFFFFF:FFFFFF
FFFFFFFFFFFFFFFFFFFFFFFFFFFFFFFFFFFFFFFF,FFFFFFFFFFFFFFFFFFFFFFFFFFF
@A00155:342:HHGFNDSXY:1:1136:17842:6245 1:N:0:GAACCTAG+TCCGCATA
AATGCCGAGAGGGATGTGGAGGGCGAGAGAGAGCGACTTCTCTCGGGCCCACAGCCTTACAGCTGTGG
AGCACGGTATCCTCTGCCGAAACAGAGGTTGGACAAGACCGGAGGGGTCTCCTAGTTCCAAAGGAGAT
+
FFFFFFFFFFFFFFFFFFFFFFFFFFFFFFFFFFFFFFFFFFFFFFFFFFFFFFFFFFFFFFFFFFFF
FFFFFFFFFFFFFFFFFFFFFFFFFFFFFFFFFFFFFFFFFFFFFFFFFFFF:FFFFFFFFFFFFFFF
@A00155:342:HHGFNDSXY:1:1136:9462:16783 1:N:0:GAACCTAG+TCCGCATA
AGGGCGAGAGAGAGCGACTTCTCTCGGGCCCACAGCCTTACAGCTGTGGAGCACGGTATCCTCTGCCG
AAACAGAGGTTGGACAAGACCGGAGGGGTCTCCTAGTTCCAAAGGAGATGTACTCCGGGCTTGTTTAC
+
FFFFFFFFFF:FFFFFFFFFFFFFFF:FFFFFFFFFFFFFFFFFFFFFFFFFFFFFFFFFFFFFFFFF
FFFFFFFFFFFFFFFFFFFFFFFFFFFFFFFFFFFFFFFFFFFFFFFFFFFFFFFFFFFFFFFFF:F:FF:F
@A00155:342:HHGFNDSXY:1:1136:4128:35728 1:N:0:GAACCTAG+TCCGCATA
GGACTCTCCTCTGGCCTATGGTCATCTCAATGCCGAGAGGGATGTGGAGGGCGAGAGAGAGCGACTTC
TCTCGGGCCCACAGCCTTACAGCTGTGGAGCACGGTATCCTCTGCCGAAACAGAGGTTGGACAAGACC
+
FFFFFFFFFFFFFFFFFFFFFFFFFFFFFFFFFFFFFFFFFFFFFFFFFFFFFFFFFFFFFFFFFFFF
FFFFFFFFFFFFFFFFFFFFFFFFFFFFFFFFFFFFFFFFFFFFFFFFFFFFFFFFFFFFFFFFFFFF
@A00155:342:HHGFNDSXY:1:1136:3803:35916 1:N:0:GAACCTAG+TCCGCATA
GGACTCTCCTCTGGCCTATGGTCATCTCAATGCCGAGAGGGATGTGGAGGGCGAGAGAGAGCGACTTC
TCTCGGGCCCACAGCCTTACAGCTGTGGAGCACGGTATCCTCTGCCGAAACAGAGGTTGGACAAGACC
+
FFFFFFFFFFFFFFFFFFFFFFF:FFFFFFFFFFFFFFFFF,FFFFFFFFFFFFFFFFFFFFFFFFFF
FFFFFFFFFFFFFFFFFFFFFFFFFFFFFFFFFFFF:FFFFFFFFFFFFFFFFFFFFFFFFFFFF:FF
@A00155:342:HHGFNDSXY:1:1137:21947:5181 1:N:0:GAACCTAG+TCCGCATA
GTATCCTCTGCCGAAACAGAGGTTGGACAAGACCGGAGGGGTCTCCTAGTTCCAAAGGAGATGTACTC
CGGGCTTGTTCACGACCTACCGTGTAAGTCGTAGTCTAGTAGGCTACCTGACGAGTCCTTTTTAGGAC
+
FFFFFFFFFFFFFFFFFFFFFFF:FFFFFFFFFFFFFFFFFFFFFFFFFFFFFFFFFFFFFFFFFFFF
FFFFF:FFFFFFFFFFFFFFFFFFFFFFFFFFFFFFFFFFFFFFFFFFFFFFFFFFFFFFFFFFFFFF
@A00155:342:HHGFNDSXY:1:1137:31882:35070 1:N:0:GAACCTAG+TCCGCATA
GGCCAGAGGTGAGTCCTTAAGTGGACACAGCTGATCTAAGGCGGTGTGGCGGGGCATGGGTTTGAACC
CCCATGACGGTCGGAGTCTAGTAGGCTCCCTGATGAGTCCGTTCCAAGGACGAAACCGTCGTTACTCT
+
FFFFFFFFFFFFFFFFFFFFFFFFFF:FFFFFFFFFFFFFFFFFFFFFFFFFFFFFFFFFFFFFFFFF
FFFFFFFFFFFFFFFFFFFFFFFFFFFFFFFFFFFFFFFFFFFFFFFFFFFFF,FFFFFFFFFFFFFF
@A00155:342:HHGFNDSXY:1:1138:2094:6872 1:N:0:GAACCTAG+TCCGCATA
CGACTTCTCTCGGGCCCACAGCCTTACAGCTGTGGAGCACGGTATCCTCTGCCGAAACAGAGGTTGGA
CAAGACCGGAGGGGTCTCCTAGTTCCAAAGGAGATGTACTCCGGGCTTGTTCACGACCTACCGTGTAA
+
:FFFFFFFFFFF:FFFFFFF:FFF:F:FFFFFFFFFF,FFFF,::FF::FFFF:FFFFF,FF:F::FF
FFFF:FF,FFFFFFFFFF,FFF,FF,FFF:FFFFFFFFFF::F:FFFFF:FFFFFFFFFFFFFFFFFF
@A00155:342:HHGFNDSXY:1:1138:23402:11866 1:N:0:GAACCTAG+TCCGCATA
GCTGTGTCCACTTAAGGACTCACCTCTGGCCTATGGTCATCTCAATGCCGAGAGGGATGTGGAGGGCG
AGAGAGAGCGACTTCTCTCGGGCCCACAGCCTTACAGCTGTGGTGCACGGTATCCTCTGCCGAAACAG
+
FFFFFFFFFFFFFFFFFFFFFFFFFFFFFFFFFFFFFFFFFFFFFFFFFFFFFFFFFFFFFFFFFFFF
FFFFFFFFFFFFFFFFFFFFFFFFFFFFFFFFFFFFFFFFFFFFFFFFFFFFFFFFFFFFFFFFFFFF
```

@A00155:342:HHGFNDSXY:1:1138:2383:13103 1:N:0:GAACCTAG+TCCGCATA
ACCTCTGTTTCGGCAGAGGATACCGTGCTCCACAGCTGTAAGGCTGTGTGCCCGAGAGAAGTCGCTCT
CTCTCGCCCTCCACATCCCTCTCGGCATTGAGATGACCATAGGCCAGAGGTGAGTCCTTAAGTGGAC
+
FFFFFFFFFFFFFFFFFFFFFFF,FF:FFFFFFFFFFFFFFF,FFFFF,FFFFFFFFFFFFFFFFFFF
FFFFFFFFFFFFFFFFFFFFFFFFF::F,FFFFFFFFFFFFFF,FFFFFFFFFFFF::FFFFFFFFF
@A00155:342:HHGFNDSXY:1:1138:2645:27398 1:N:0:GAACCTAG+TCCGCATA
ATCTCAATGCCGAGAGGGATGTGGAGGGCGAGAGAGAGCGACTTCTCTCGGGCCCACAGCCTTACAGC
TGTGGAGCACGGTATCCTCTGCCGAAACAGAGGTTGGACAAGACCGGAGGGGTCTCCTGGTTCCAAAG
+
FFFFFFFFFFFFFFFFFFFFFFFFFFFFFFFFFFFFFFFFFFFFFFFFFFFFFFFFFFFFFFFFFFFF
FFFFFFF:FFFFFFFFFFFFFFFFFFFFFFFFFFFFFFFFFFFFFFFFFFFFFFFFFFFFFFFFFFFF
@A00155:342:HHGFNDSXY:1:1139:19081:9424 1:N:0:GAACCTAG+TCCGCATA
ATGCCGAGAGGGATGTGGAGGGCGAGAGAGAGCGACTTCTCTCGGGCCCACAGCCTTACAGCTGTGGA
GCACGGTATCCTCTGCCGAAACAGAGGTTGGACAAGACCGGAGGGGTCTCCTAGTTCCAAAGGAGA
+
FFFFFFFFFFFFF,:FF:FF:FFFFFFFF:FFFFFFFFFF:FFFFFFFFFFFFF:,FFFFFFFFF,FF
FFFFFFFFFFFF,FFF::FFFFFFFF:FFFFFFFF:FFF:FF:FFFFFFFFFFFFFF,:FFFFFFF
@A00155:342:HHGFNDSXY:1:1139:21766:12446 1:N:0:GAACCTAG+TCCGCATA
TCTCTCGGGCCCACAGCCTTACAGCTGTGGAGCACGGTATCCTCTGCCGAAACAGAGGTTGGACAAGA
CCGGAGGGGTCTCCTAGTTCCAAAGGAGATGTACTCCGGGCTTGTTCACGACCTACCGTGTAAGTCGT
+
FFFFFFFFFFFFFFFFFFFFFFFFFFFFF:FFFFFFFFFFFFFFFFFFFFFFFFFFFFFFFFFFFFFF
FFFFFFFFFFFFFFFFFFFFFFFFFFFFFFFF:FFFFFFFFFFFFFFFFFFFFFFFFFFFFFFFFFFF
@A00155:342:HHGFNDSXY:1:1140:4300:2738 1:N:0:GAACCTAG+TCCGCATA
GGATGTGGAGGGCGAGAGAGAGCGACTTCTCTCGGGCCCACAGCCTTACAGCTGTGGAGCACGGTATC
CTCTGCCGAAACAGAGGTTGGACAAGACCGGAGGGGTCTCCTAGTTCCAAAGGAGATGTACTCC
+
FFFFFFFFFFFFFFFFFFFFFFFFFFFFFFFFFFFFFFFFFFFFFFFFFFFFFFFFFFFFFFFFFFFF
FFFFFFFFFFFFFFFFFFFFFFFFFFFFFFFFFFFFFFFFFFFFFFFFFFFFFFFFFFFFFFFF
@A00155:342:HHGFNDSXY:1:1140:30834:5729 1:N:0:GAACCTAG+TCCGCATA
CTTCTCTCGGGCCCACAGCCTTACAGCTGTGGAGCACGGTATCCTCTGCCGAAACAGAGGTTGGACAA
GACCGGAGGGGTCTCCTAGTTCCAAAGGAGATGTACTCCGGGCTTGTTCACGACCTACCGTGTAAGTC
+
FFFFFFFFFFFFFFFFFFFFFFFFFFFFFFFFFFFFFFFFFFFFFFFFFFFFFFFFFFFFFFFFFFFF
FFFFFFFFFFFFFFFFFFFFFFFFFFFFFFFFFFFFFFFFFFFFFFFFFFFFFFFFFFFFFFFFFFFF
@A00155:342:HHGFNDSXY:1:1140:5050:10488 1:N:0:GAACCTAG+TCCGCATA
GGGCGAGAGAGAGCGACTTCTCTCGGGCCCACAGCCTTACAGCTGTGGAGCACGGTATCCTCTGCCGA
AACAGAGGTTGGACAAGACCGGAGGGGTCTCCTGGTTCCAAAGGAGATGTACTCCGGGCTTGTTCACG
+
FFFFFFFFFFFFFFFFFFFFFFFFFFFFFFF,F,FFFFFFFFFFFFFFFFFFFFFFFFFF:FFFFFFF
FFF:F,FFFFFFFFFFFFFFFFFFFFFFFFFFFFFFFFFFFFFFFFFFFFFFFFF:FFFFFFFFFFF:
@A00155:342:HHGFNDSXY:1:1140:31693:12759 1:N:0:GAACCTAG+TCCGCATA
CTTAAGGACTCACCTCTGGCCTATGGTCATCTCAATGCCGAGAGGGATGTGGAGGGCGAGAGAGAGCG
ACTTCTCTCGGGCCCACAGCCTTACAGCTGTGGAGCACGGTATCCTCTGCCGAAACAGAGGTTGGACA
+
FFFFFFFFFFFFFFFFFFFFFF,FFFFFFFFFFFFFFFFFFFFFFFF,F,FFFFFFFFFFFFFFFFFF
FFFFFFFFFFFFFFFFFFFFFFFFFFFFFFFFFFFFFFFFFFFFFFFFFFFFFFFFFFFFFFFFFFFF
@A00155:342:HHGFNDSXY:1:1140:24189:16203 1:N:0:GAACCTAG+TCCGCATA
ATCTCAATGCCGAGAGGGATGTAGAGGGCGAGAGAGAGCGACTTCTCTCGGGCCCACAGCCTTACAGC
TGTGGAGCACGGTATCCTCTGCCGAAACAGAGGTTGGACAAGACCGGAGGGGTCTCCTAGTTCCAAAG
+
FFFFFFFFFFFFFF:FFFFFF:FFFFFFFFFFFFFFFFFFFF:FFFFFFFFFFFFFF:FFFFFFFFFF
FFFFFFFFFFFFFFFFFFFFFFFFFFFFFFFFF::FFFFFFFFFFFFFFFFFFFFFFFFFFFFFFFFF

```
@A00155:342:HHGFNDSXY:1:1140:30129:16971 1:N:0:GAACCTAG+TCCGCATA
GTCATCTCAATGCCGAGAGGGATGTGGAGGGCGAGAGAGAGCGACTTCTCTCGGGCCCACAGCCTTAC
AGCTGTGGAGCACGGTATCCTCTGCCGAAACAGAGGTTGGACAAGACCGGAGGGGTCTCCTGGTTCCA
+
FFFFFFFFFFFFFFFFFFFFFFFFFFFFFFFFFFFFFFFFFFFFFFFFFFFFFFFFFFFFFFFFFFFF
FFFFFFFFFFFFFFFFFFFFFFFFFFFFFFFFFFFFFFFFFFFFFFFFFFFFFFFFFFFFFFFFFFFF
@A00155:342:HHGFNDSXY:1:1140:28510:20713 1:N:0:GAACCTAG+TCCGCATA
GCGACTTCTCTCGGGCCCACAGCCTTACAGCTGTGGAGCACGGTATCCTCTGCCGAAACAGAGGTTGG
ACAAGACCGGAGGGGTCTCCTAGTTCCAAAGGAGATGTACTCCGGGCTTGTTCACGACCTACCGTGTA
+
FFFFFFFFFFFFFFFFFFFFFFFFFFFFFFFFF:FFFFFFFFFFFFFFFFFFFFFFFFFFFFFFFFFF
FFFF:FFFFFFFFFFFFFFFFFFFFFFFFFFFFFFFFFFFFFFFFFFFFFFFFFFFFFFFFFFFFFFF
@A00155:342:HHGFNDSXY:1:1141:14832:3067 1:N:0:GAACCTAG+TCCGCATA
GTCCACTTAAGGACTCTCCTCTGGCCTATGGTCATCTCAATGCCGAGAGGGATGTGGAGGGCGAGAGA
GAGCGACTTCTCTCGGGCCCACAGCCTTACAGCTGTGGAGCACGGTATCCTCTGCCGAAACAGAGGTT
+
FFFFFFFFFFFFFFFFFFFFFFFFFFFFFFFFFFFFFFFFFFFFFFFFFFFFFFFFFFFFFFFFFFFF
FFFFFFFFFFFFFFFFFFFFFFFFFFFFFF,FF:FFFFFFFFFFFFFFFFFFFFFFFFFFFFFFFFFF
@A00155:342:HHGFNDSXY:1:1141:32624:10113 1:N:0:GAACCTAG+TCCGCATA
ATCTCAATGCCGAGAGGGATGTGGAGGGCGAGAGAGAGCGACTTCTCTCGGGCCCACAGCCTTACAGC
TGTGGAGCACGGTATCCTCTGCCGAAACAGAGGTTGGACAAGACCGGAGGGGTCTCCTGGTTCCAAAG
+
FFFFFFFFFFFFFFFFFFFFF:FFFFFFFFFFFFFFFFFF:FFFFFFFFFFFFFFFFFFFFF:F:FFFF
FFFFFFFF,FFFFF:FFFFFFFFFFFFFFFFFFFFFF:FF:FFFFFFFFFFFFFFFFFFFFFFFFFFF
@A00155:342:HHGFNDSXY:1:1141:23692:26897 1:N:0:GAACCTAG+TCCGCATA
GAGAGGGATGTGGAGGGCGAGAGAGAGCGACTTCTCTCGGGCCCACAGCCTTACAGCTGTGGAGCACG
GTATCCTCTGCCGAAACAGAGGTTGGACAAGACCGGAGGGGTCTCCTGGTTCCAAAGGAGATGTACT
+
FFFFFFFFFFFFFFFFFFFFFFFFFFFFFFFFFFFFFFFFFFFFFFFFFFFFFFFFFFFFFFFFFFFF
FFFFFFFFFFFFFFFFFFFFFFFFFFFFFFFFFFFFFFFFFFFFFFFFFFFFFFFFFFFFFFFFFFF
@A00155:342:HHGFNDSXY:1:1141:12147:29669 1:N:0:GAACCTAG+TCCGCATA
TATCCTCTGCCGAAACAGAGGTTGGACAAGACCGGAGGGGTCTCCTAGTTCCAAAGGAGATGTACTCC
GGGCTTGTTCACGACCTACCGTGTAAGTCGTAGTCTAGTAGGCTACCTGACGAGTCCTTTTAGGACG
+
FFFFFFFFFFFFFFFFFFFFFFFFFFFFFFFFFFFFFFFFFFFFFFFFFFFFFFFFFFFFFFFFFFF:
FFFFFFFFFFFFFFFFFFFFFFFFF:FFFFFFFFFFFFFFFFFFFFFFFFFFFFFFFFFFFFFFFFFF
@A00155:342:HHGFNDSXY:1:1141:30228:33019 1:N:0:GAACCTAG+TCCGCATA
AGGACTCACCTCTGGCCTATGGTCATCTCAATGCCGAGAGGGATGTGAAGGGCGAGAGAGAGCGACTT
CTCTCGGGCCCACAGCCTTACAGCTGTGGAGCACGGTATCCTCTGCCGAAACAGAGGTTGGACAAGAC
+
FFFFFFFFFFFFFFFFFFFFFFFFFFFFFFFFFFFFFFFFFFFFFFFFFFFFFFFFFFFFFFFFFFFF
FFFFFFFFFFFFFFFFFFFFFFFFFFFFFFFFFFFFFFFFFFFFFFFFFFFFFFFFFFFFFFFFFFFF
@A00155:342:HHGFNDSXY:1:1141:25174:33160 1:N:0:GAACCTAG+TCCGCATA
CCTTACAGCTGTGGAGCACGGTATCCTCTGCCGAAACAGAGGTTGGACAAGACCGGAGGGGTCTCCTA
GTTCCAAAGGAGATGTACTCCGGGCTTGTTCACGACCTACCGTGTAAGTCGTAGTCTAGTAGGCTACC
+
FF:FFFFFFFFFFFFFFFFFFFFFFFFFFFFFFFFFFFFFFFFFFFFFFFFFFFFFFFFFFFFFFFFF
FFFFFFFFFFFFFFFFFFFFFFFFFFFFFFFFFFFFFFFFFFFFFF:FFFFFFFFFFFFFFFFFFFFF
@A00155:342:HHGFNDSXY:1:1141:30047:33927 1:N:0:GAACCTAG+TCCGCATA
AGGACTCACCTCTGGCCTATGGTCATCTCAATGCCGAGAGGGATGTGAAGGGCGAGAGAGAGCGACTT
CTCTCGGGCCCACAGCCTTACAGCTGTGGAGCACGGTATCCTCTGCCGAAACAGAGGTTGGACAAGAC
+
FFFFFFFFFFFFFFFFFF:FFFFF:FFFFFFFFFFFFFFFFFF:FFFFFFFFFFFFFFFFFFFFFFFF
FFFFFFFFFFFFFFFFFFFFFFFFFFFFFFFFFFFFFFFFFFFFFFFFFFFFFFFFFFFFFFFFFFFF
```

@A00155:342:HHGFNDSXY:1:1142:30391:29168 1:N:0:GAACCTAG+TCCGCATA
TTCTCTCGGGCCCACAGCCTTACAGCTGTGGAGCACGGTATCCTCTGCCGAAACAGAGGTTGGACAAG
ACCGGAGGGGTCTCCTAGTTCCAAAGGAGATGTACTCCGGGCTTGTTCACGACCTACCGTGTAAGTCG
+
FFFFFFFFFFFFFFFFFFFFFFFFFFFFFFFFFFFFFFFFFFFFFFFFFFFFFFFFFFFFFFFFFFFF
FFFFFFFFFFFFFFFFFFFFFFFFFFFFFFFFFFFFFFFFFFFFFFFFFFFFFFFFFFFFFFF:FFFF
@A00155:342:HHGFNDSXY:1:1143:23113:22608 1:N:0:GAACCTAG+TCCGCATA
GAGAGCGACTTCTCTCGGGCCCACAGCCTTACAGCTGTGGAGCACGGTATCCTCTGCCGAAACAGAGG
TTGGACAAGACCGGAGGGGTCTCCTGGTTCCAAAGGAGATGTACTCCGGGCTTGTTCAC
+
FFFFFFFFFFFFFFFFFFFFFFFFFFFFFFFFFFFFFFFFFFFFFFFFFFFFFFFFFFFFFFFFFFFF
FFFFFFFF:FFFFFFFFFFFFFFFFFFFFFFFFFFFFFFFFFFFFFFFFFF
@A00155:342:HHGFNDSXY:1:1144:17734:4398 1:N:0:GAACCTAG+TCCGCATA
GTATCCTCTGCCGAAACAGAGGTTGGACAAGACCGGAGGGGTCTCCTAGTTCCAAAGGAGATGTACTC
CGGGCTTGTTCACGACCTACCGTGTAAGTCGTAGTCTAGTAGGCTACCTGACGAGTCCTTTTTAGGAC
+
FFFFFFFFFFFFFFFFFFFFFFFFFFFFFFFFFFFFFFFFFFFFFFFFFFFFFFFFFFFFFFFFFFFF
FFFFFFFFFFFFFFFFFFFFFFFFFFFFFFFFFFFFF:FFFFFFFFFFFFFFFFFFFFFFFFFFFFFF
@A00155:342:HHGFNDSXY:1:1144:27543:27023 1:N:0:GAACCTAG+TCCGCATA
ATCTCAATGCCGAGAGGGATGTGGAGGGCGAGAGAGAGCGACTTCTCTCGGGCCCACAGCCTTACAGC
TGTGGAGCACGGTATCCTCTGCCGAAACAGAGGTTGGACAAGACCGGAGGGGTCTCCTAGTTCCAAAG
+
FFFFFFFFFFFFFFFFFFFFF:FFFFFFFFFFFFFFFFFFFFFFFFFFFFFFFFFFFFFFFFFFFFFF
FFFFFFFFFFFFFFFFFFFFFFFFFFFFFFFFFFFFFFFFFFFFFFFFFFFFFFFFF:FFFFFFFFFF
@A00155:342:HHGFNDSXY:1:1144:27661:27070 1:N:0:GAACCTAG+TCCGCATA
ATCTCAATGCCGAGAGGGATGTGGAGGGCGAGAGAGAGCGACTTCTCTCGGGCCCACAGCCTTACAGC
TGTGGAGCACGGTATCCTCTGCCGAAACAGAGGTTGGACAAGACCGGAGGGGTCTCCTAGTTCCAAAG
+
FFFFFFFFFFFFFFFFFF:FFFFFFFFFFFFFFFFFFFFFFFFFFFFFFFFFFFFFFFFFFFFFFFFF
FFFFFFFFFFFFFFFFFFFFFFFFFFFFFFFFFFFFFFFFFFFFFFFFFFFFFFFFFFFFFFFFFFFF
@A00155:342:HHGFNDSXY:1:1144:28727:31767 1:N:0:GAACCTAG+TCCGCATA
TCCTCTGCCGAAACAGAGGTTGGACAAGACCGGAGGGGTCTCCTAGTTCCAAAGGAGATGTACTCCGG
GCTTGTTCACGACCTACCGTGTAAGTCGTAGTCTAGTAGGCTACCTGACGAGTCCTTTTTAGGACGAA
+
FFFFFFFFFFFFFFFFFFFFFFFFFFFFFFFFFFFFFFFFFFFFFFFFFFFFFFFFFFFFFFFFFFFF
FFFFFFFFFFFFFFFFFFFFFFFFFFFFFFFFFFFFFFFFFFFFFFFFFFFFFFFFFFFFFFFFFFFF
@A00155:342:HHGFNDSXY:1:1144:8187:33739 1:N:0:GAACCTAG+TCCGCATA
CAGCCTTACAGCTGTGGAGCACGGTATCCTCTGCCGAAACAGAGGTTGGACAAGACCGGAGGGGTCTC
CTAGTTCCAAAGGAGATGTACTCCGGGCTTGTTCACGACCTACCGTGTAAGTCGTAGTCTCGTAGGCT
+
FFFFFFFFFFFF,FF:FF:FFFFFFFFFF:F:FFF,FFF,FFF:FFFF:F:FFFFFFFFFFF::FFFF
,FFFFF:FFFFFFFFFFFFFF:FF::FF,F::FFFF:FFF:F:FF,F:FFF:FF,FFF:F,FFFFFFF
@A00155:342:HHGFNDSXY:1:1144:25979:35430 1:N:0:GAACCTAG+TCCGCATA
ACCTCTGTTTCGGCAGAGGATACCGTGCTCCACAGCTGTAAGGCTGTGGGCCCGAGAGAAGTCGCTCT
CTCTCGCCCTCCACATCCCTCTCGGCATTGAGATGACCATAGGCCAGAGGAGAGTCCTT
+
FFFFFFFFFFFFFFFFFFFFFFFFFFFFFFFFFFFF:FFFFFFFFFFFFFFFFFFFFFFFFFFFFFFF
FFFFFFFFFFFFFFFFFFFFFFFFFFFF:F:FFFFFFFFFFFFFFFFFFFFFFFFFFFF
@A00155:342:HHGFNDSXY:1:1145:11939:1564 1:N:0:GAACCTAG+TCCGCATA
AAGGACTCACCTCTGGCCTATGGTCATCTCAATGCCGAGAGGGATGTGAAGGGCGAGAGAGAGCGACT
TCTCTCGGGCCCACAGCCTTACAGCTGTGGAGCACGGTATCCTCTGCCGAAACAGAGGTT
+
FFFFFFFFFFFFFFFFFFFFFFFFFFFFFFFFFFFFFFFFFFFFFFFFFFFFFFFFFFFFFFFFFFFF
FFFFFFFFFFFFFFFFFFFFFFFFFFFFFFFFFFFFFFFFFFFFFFFFFFFFFFFFFFFF

```
@A00155:342:HHGFNDSXY:1:1145:3197:4742 1:N:0:GAACCTAG+TCCGCATA
GACAAGACCGGAGGGGTCTCCTAGTTCCAAAGGAGATGTACTCCGGGCTTGTTCACGACCTACCGTGT
AAGTCGTAGTCTAGTAGGCTACCTGACGAGTCCTTTTTAGGACGAAACTTACACAACTCGAAGAGTAA
+
FFFFFF:FFFFFFFFFFFFFFFFFFFFFFFFFFFFFFFFFFFFFFFFFFFFFFFFFFFFFFFFFFFFF
FFFFFFFFFFFFFFFFFFFFFFFFFFFFFFFFFFFFFFFFFFFFFFFFFFFFFFFFFFFFFFFFFFFF
@A00155:342:HHGFNDSXY:1:1145:3106:4773 1:N:0:GAACCTAG+TCCGCATA
GACAAGACCGGAGGGGTCTCCTAGTTCCAAAGGAGATGTACTCCGGGCTTGTTCACGACCTACCGTGT
AAGTCGTAGTCTAGTAGGCTACCTGACGAGTCCTTTTTAGGACGAAACTTACACAACTCGAAGAGTAA
+
FFFFFFFFFFFFFFFFFFFFFFFFFFFFFFFFFFFF:FFFFFFFFFFFFFFFFFFFFFFFFFFFFFFF
FFFFFFFFFFFFFFFFFFFFFFFFFFFFFFFFFFFFFFFFFFFFFFFFFFFFFFFFFFFFFFFFFFFF
@A00155:342:HHGFNDSXY:1:1145:14154:8813 1:N:0:GAACCTAG+TCCGCATA
TTCTCTCGGGCCCACAGCCTTACAGCTGTGGAGCACGGTATCCTCTGCCGAAACAGAGGTTGGACAAG
ACCGGAGGGGTCTCCTAGTTCCAAAGGAGATGTACTCCGGGCTTGTTCACGACCTACCGTGTAAGTCG
+
FFFFFFFFFFFFFFFFFFFFFFFFFFFFFFFFFFFFFFFFFFFFFFFFFFFFFFFFFFFFFFFFFFFF
FFFFFFFFFFFFFFFFFFFFFFFFFFFFFFFFFFFFFFFFFFFFFFFFFFFFFFFFFFFFFFFFFFFF
@A00155:342:HHGFNDSXY:1:1145:21775:19225 1:N:0:GAACCTAG+TCCGCATA
GAGGGCGAGAGAGAGCGACTTCTCTCGGGCCCACAGCCTTACAGCTGTGGAGCACGGTATCCTCTGCC
GAAACAGAGGTTGGACAAGACCGGAGGGGTCTCCTAGTTCCAAAGGAAATGTACTCCGGGC
+
FFFFFFFFFFFFFFFFFFFFFFFFFFFFFFFFFFFFFFFFFFF:FFFFFFFFFF:FFFFFFFFFFFFF
FFFFFFFFFFFFFFFFFFFFFFFFFFFFFFFFFFFFFFFFFFFFFFFF:FFFFFFFFFFFFF
@A00155:342:HHGFNDSXY:1:1146:16721:4022 1:N:0:GAACCTAG+TCCGCATA
GGATGTGGAGGGCGAGAGAGAGCGACTTCTCTCGGGCCCACAGCCTTACAGCTGTGGAGCACGGTATC
CTCTGCCGAAACAGAGGTTGGACAAGACCGGAGGGGTCTCCTAGTTCCAAAGGAGATGTACTCC
+
FFFFFFFFFFFFFFFFFFFFFFFFFFFFFFFFFFFFFFFFFFFFF:FFFFFFFFFFFFFFFFFFFFFF
FFFFFFFFFFFFFFFFFFFFFFFFFFFFFFFFFFFFFFFFFFFFFFFFFFFFFFFFFFFFFFFF
@A00155:342:HHGFNDSXY:1:1146:23610:6277 1:N:0:GAACCTAG+TCCGCATA
GGATGTGGAGGGCGAGAGAGAGCGACTTCTCTCGGGCCCACAGCCTTACAGCTGTGGAGCACGGTATC
CTCTGCCGAAACAGAGGTTGGACAAGACCGGAGGGGTCTCCTAGTTCCAAAGGAGATGTACTCC
+
FFFFFFFFFFFFFFFFFFFFFFFFFFFFFFFFFFFFFFFFFFFFFFFFFFFFFFFFFFFFFFFFFFFF
FFFFFFFFFFFFFFFFFFFFFFFFFFFFFFFFFFFFFFFFFFFFFFFFFFFFFFFFFFFFFFFF
@A00155:342:HHGFNDSXY:1:1146:25229:20478 1:N:0:GAACCTAG+TCCGCATA
AGAGAGAGCGACTTCTCTCGGGCCCACAGCCTTACAGCTGTGGAGCACGGTATCCTCTGCCGAAACAG
AGGTTGGACAAGACCGGAGGGGTCTCCTGGTTCCAAAGGAGATGTACTCCGGGCTTGTTCACGACCTA
+
FFFFFFFFFFFFFFFFFFFFFFFFFFFFFFFFFFFFFFFFFFFFFFFFFFFFFFFFFFFFF:FFFFFF
FFFFFFFFFFFFFFFFFFFFFFFFFFFFFFFFFFFFFFFFFFFFFFFFFFFFFF:FFFFFFFFFFFFF
@A00155:342:HHGFNDSXY:1:1146:29740:31078 1:N:0:GAACCTAG+TCCGCATA
TTACAGCTGTGGAGCACGGTATCCTCTGCCGAAACAGAGGTTGGACAAGACCGGAGGGGTCTCCTAGT
TCCAAAGGAGATGTACTCCGGGCTTGTTCACGACCTACCGTGTAAGTCGTAGTCTAGTAGGCTACCT
+
FFFFF:FF,FFFFFFFFFFFFF::FFFFFFFF,FFF:FFFFFFFFFFFFFF:FFFFFF:FFFFF,F
F,FFFFFFFFFFF:FFF:F,FFFFFF:F,FFFF:FFFFF:F:FFFFFF,FF,FFFFFFF:FFFFFFF
@A00155:342:HHGFNDSXY:1:1146:30508:33974 1:N:0:GAACCTAG+TCCGCATA
GGGCGAGAGAGAGCGACTTCTCTCGGGCCCACAGCCTTACAGCTGTGGAGCACGGTATCCTCTGCCGA
AACAGAGGTTGGACAAGACCGGAGGGGTCTCCTAGTTCCAAAGGAGATGTACTCCGGGCTTGTTCACG
+
FFFFFFFFFFFFFFFFFFFFFFFFFFFFFFFFFFFFFFFFFFFFFFFFFFFFFFFFFFFFFFFFFFFF
FFFFFFFFFFFFFFFFFFFFFFFFFFFFFFFFFFFFFFFFFFFFFFFFFFFFFFFFFFFFFFFFFFFF
```

@A00155:342:HHGFNDSXY:1:1147:30770:8437 1:N:0:GAACCTAG+TCCGCATA
CCCGCCACACCGCCTTAGATCAGCTGTGTCCACTTAAGGACTCACCTCTGGCCTATGGTCATCTCAAT
GCCGAGAGGGATGTGGAGGGCGAGAGAGAGCGACTTCTCTCGGGCCCACAGCCTTACAGCTGTGGAGC
+
FFFFFFFFFFFFFF:FFFFFFFFFFFFFFFFFFFFFFFFFFFFFFFFFFFFFFFFFFFFFFFFFFFFF
FFFFFFFFFFFFFFFFFFFFFFFFFFFFFFFFFFFFFFFFFFFFFFFFFFFFFFFFFFFF:FFFFFFF
@A00155:342:HHGFNDSXY:1:1147:30391:27007 1:N:0:GAACCTAG+TCCGCATA
ACCTCTGTTTCGGCAGAGGATACCGTGCTCCACAGCTGTAAGGCTGTGGGCCCGAGAGAAGTCGCTCT
CTCTCGCCCTCCACATCCCTCTCGGCATTGAGATGACCATAGGCCAGAGGAGAGTCCTTAAG
+
FFFFFFFFFFFFFFFFFFFFFFFFFFFFFFFFFFFFFFFFFFFFFFFFFFFFFFFFFFFFFFFFFFFF
FFFFFFFFFFFFFFFFFFFFFFFFFF,FFFFFFFFFFFFFFFFFFFFFFFFFFFFFFFFFFF
@A00155:342:HHGFNDSXY:1:1148:29098:3630 1:N:0:GAACCTAG+TCCGCATA
AGAGAGAGCGACTTCTCTCGGGCCCACAGCCTTACAGCTGTGGAGCACGGTATCCTCTGCCGAAACAG
AGGTTGGACAAGACCGGAGGGGTCTCCTGGTTCCAAAGGAGATGTACTCCGGGCTTGTTCACGACCTA
+
FFFFFFFFFFFFFFFFFFFFFFFFFFFFFFFFFFFFFFFFFFFFFFFFFFFFFFFFFFFFFFFFFFFF
FFFFFFFFFFFFFFFFFFFFFFFFFFFFFFFFFFFFFFFFFFFFFFFFFFFFFFFFFFFFFFFFFFFF
@A00155:342:HHGFNDSXY:1:1148:8947:27038 1:N:0:GAACCTAG+TCCGCATA
TCCTCTGCCGAAACAGAGGTTGGACAAGACCGGAGGGGTCTCCTGGTTCCAAAGGAGATGTACTCCGG
GCTTGTTCACGACCTACCGTGTAAGTCGTAGTCTAGTAGGCTACCTGACGAGTCCTTTTTAGGACGAA
+
FFFFFFFFFFFFFFFFFFFFFFFFFFFFFFFFFFFFFFFFFFFFFFFFFFFFFFFFFFFFFFFFFFFF
FF,FFFFFFFFF:FFFFFFFFFFFFFFFFFFFFFFFFFFFFFFFFFFFFFFFFFFFFFFFFFFFFFFF
@A00155:342:HHGFNDSXY:1:1148:6542:28260 1:N:0:GAACCTAG+TCCGCATA
GGGCGAGAGAGAGCGACTTCTCTCGGGCCCACAGCCTTACAGCTGTGGAGCACGGTATCCTCTGCCGA
AACAGAGGTTGGACAAAACCGGAGGGGTCTCCTAGTTCCAAAGGAGATGTACTCCGGGCTTGTTCACG
+
FFFFFFFFFFFFFFFFFFFFFFFFFFFFFFFFFFFFF::FFFFFFFFFFFF:FFFF,FFFFFFFFFFF
FFFFFFFFFFFFFFFFF:FFFFFFFFFFFFFFFFFFFFFFFFFFFFFFFFFFFFFFFFFFFFFFFFFF
@A00155:342:HHGFNDSXY:1:1149:27398:2347 1:N:0:GAACCTAG+TCCGCATA
ATGCCGAGAGGGATGTGGAGGGCGAGAGAGAGCGACTTCTCTCGGGCCCACAGCCTTACAGCTGTGGA
GCACGGTATCCTCTGCCGAAACAGAGGTTGGACAAGACCGGAGGGGTCTCCTGGTTCCAAAGGAGATG
+
FFFFFFFFFFFFF:FFFFFFFFFFFFFFFFFFFFFFFFFFFFFF:FFFFFFFFFFFFFFFFFFFFFFF
FFFFFFFFFFFFFFFFFFFFFFFFFFFFFFFFFFFFFFFFFFFFFFFFFFFFFFFFFFFFFFFFFFFF
@A00155:342:HHGFNDSXY:1:1149:24044:19617 1:N:0:GAACCTAG+TCCGCATA
CTTCTCTCGGGCCCACAGCCTTACAGCTGTGGAGCACGGTATCCTCTGCCGAAACAGAGGTTGGACAA
GACCGGAGGGGTCTCCTAGTTCCAAAGGAGATGTACTCCGGGCTTGTTCACGACCTACCGTGTAAGTC
+
FFFFFFFFFFFFFFFFFFFF::FFFFFFFFFFFFFFFFFFFFFFFFFFFFFFFFFFFFFFFFFFFFFF
F:FFFFFFFFFFFFFFFFFFFFFFFFFFFFFFFFFFFFFFFFFFFFFFFFFFFFFFFFFFFFFFFFFF
@A00155:342:HHGFNDSXY:1:1149:28980:26725 1:N:0:GAACCTAG+TCCGCATA
CAGCCTTACAGCTGTGGAGCACGGTATCCTCTGCCGAAACAGAGGTTGGACAAGACCGGAGGGGTCTC
CTAGTTCCAAAGGAGATGTACTCCGGGCTTGTTCACGACCTACCGTGTAAGTCGTAGTCTAGTAGGCT
+
FFFFFFFFFFFFFFFFFFFFFFFFFFFFFFFFFFFFFFFFFFFFFFFFFFFFFFFFFFFFFFFFFFFF
FFFFFFFFFFFFFFFF,FF,FFFFFFFFFFFFFFFFFFFFFFFFF:FF,FFFFFFFFFFFFFFFFFFF
@A00155:342:HHGFNDSXY:1:1150:23140:6934 1:N:0:GAACCTAG+TCCGCATA
GTCATCTCAATGCCGAGAGGGATGTGGAGGGCGAGAGAGAGCGACTTCTCTCGGGCCCACAGCCTTAC
AGCTGTGGAGCACGGTATCCTCTGCCGAAACAGAGGTTGGACAAGACCGGAGGGGTCTCCTAGTTCCA
+
FFFFFFFFFFFFFFFFFFFFFFFFFFFFFFFFFFFFFFFFFFFFFFFFFFFFFFFFFFFFFFFFFFFF
FFFFFFFFFFFFFFFFFFFFFFFFFFFFFFFFFFFFFFFFFFFFFFFFFFFFFFFFFFFF:FFFFFFF

```
@A00155:342:HHGFNDSXY:1:1150:27353:10535 1:N:0:GAACCTAG+TCCGCATA
ACTTCTCTCGGGCCCACAGCCTTACAGCTGTGGAGCACGGTATCCTCTGCCGAAACAGAGGTTGGACA
AGACCGGAGGGGTCTCCTAGTTCCAAAGGAGATGTACTCCGGGCTTGTTCACGACCTACCGTGTAAGT
+
FFFFFFFFFFFFFFFFFFFFFFFFFFFFFFFFFFFFFFFFFFFFFFFFFFFFFFFFFFFFFFFFFFFF
FFFFFFFFFFFFFFFFFFFFFFFFFFFFFFFFFFFFFFFFFFFFFFFFFFFFFFFFFFFFFFFFFFFF
@A00155:342:HHGFNDSXY:1:1150:26910:29747 1:N:0:GAACCTAG+TCCGCATA
GGATGTAGAGGGCGAGAGAGAGCGACTTCTCTCGGGCCCACAGCCTTACAGCTGTGGAGCACGGTATC
CTCTGCCGAAACAGAGGTTGGACAAGACCGGAGGGGTCTCCTAGTTCCAAAGGAGATGTACTCC
+
FFFFFF:FFFFFFFFFFFFFFF:FFFFFFFFFFFFFFFFFFFFFFFFFFFFFFFFFFFFFFFFFFFFF
FFFFF:FFFFFFFF:FFFFFFFFFFFFFFFFFFFFFFFFFFFFFFFFFFFFFFF:FFFFFFFFF
@A00155:342:HHGFNDSXY:1:1150:22607:35352 1:N:0:GAACCTAG+TCCGCATA
GGATGTAGAGGGCGAGAGAGAGCGACTTCTCTCGGGCCCACAGCCTTACAGCTGTGGAGCACGGTATC
CTCTGCCGAAACAGAGGTTGGACAAGACCGGAGGGGTCTCCTAGTTCCAAAGGAGATGTACTCC
+
FFFFF:FFFFFFFFFFFFFFFFFFFFFFFFFFFFFFFFFFFFFFFFFFFFFFFFFFFFFFFFFFFFFF
FFFFFFFFFFFFFFFFFFFFFFFFFFFFFFFFFFFFFFFFFFFFFFFFFFFFFFFFFFFFFFFF
@A00155:342:HHGFNDSXY:1:1150:29713:37043 1:N:0:GAACCTAG+TCCGCATA
TCCACTTAAGGACTCACCTCTGGCCTATGGTCATCTCAATGCCGAGAGGGATGTAGAGGGCGAGAGAG
AGCGACTTCTCTCGGGCCCACAGCCTTACAGCTGTGGAGCACGGTATCCTCTGCCGAAACAGAGGTTG
+
FFFFFFFFFFFFFFFFFFFFFFFFFFFFFFFFFFFFFFFFFFFFFFFFFFFFFFFFFFFFFFFFFFFF
FFFFFFFFFFFFFFFFFFFFFFFFFFFFFFFFFFFFFFFFFFFFFFFFFFFFFFFFFFFFFFFFFFFF
@A00155:342:HHGFNDSXY:1:1151:7274:13025 1:N:0:GAACCTAG+TCCGCATA
CTTCTCTCGGGCCCACAGCCTTACAGCTGTGGAGCACGGTATCCTCTGCCGAAACAGAGGTTGGACAA
GACCGGAGGGGTCTCCTAGTTCCAAAGGAGATGTACTCCGGGCTTGTTCACGACCTACCGTGTAAGTC
+
FFFFFFFFFFFFFFFFFFFFFFFFFFFFFFFFFFFFFFFFFFFFFFFFFFFFFFFFFFFFFFFFFFFF
FFFFFFFFFFF:FFFFFFFFFFFFFFFFFFFFFFFF:F:FFFFFFFFFFFFFFFFFFFFFFFFFFFFF
@A00155:342:HHGFNDSXY:1:1151:30092:15624 1:N:0:GAACCTAG+TCCGCATA
CCTTACAGCTGTGGAGCACGGTATCCTCTGCCGAAACAGAGGTTGGACAAGACCGGAGGGGTCTCCTA
GTTCCAAAGGAGATGTACTCCGGGCTTGTTCACGACCTACCGTGTAAGTCGTAGTCTAGTAGGCTACC
+
FFFFFFFFFFFFFFFFFFFFFFFFFFFFFFFFFFFFFFFFFFFFFFFFFFFFFFFFFFFFFFFFFFFF
FFFFFFFFFFFFFFFFFFFFFFFFFFFFFFFFFFFFFFFFFFFFFFFFFFFFFFFFFFFFFFFFFFFF
@A00155:342:HHGFNDSXY:1:1151:26404:19006 1:N:0:GAACCTAG+TCCGCATA
TCGGCAGAGGATACCGTGCTCCACAGCTGTAAGGCTGTGGGCCCGAGAGAAGTCGCTCTCTCTCGCCC
TCTACATCCCTCTCGGCATTGAGATGACCATAGGCCAGAGGTGAGTCCTTAAGTG
+
FFFFFFFFFFFFFFFFFFFFFFFFFFFFFFFFFFFFFFFFFFFFFFFFFFFFFFFFFFFFFFFFFFFF
FFFFFFFFFFFFFFFFFFFFFFFFFFFFF:FFFFFFFFFFFFFFFFFFFFFFFFF
@A00155:342:HHGFNDSXY:1:1151:5177:34413 1:N:0:GAACCTAG+TCCGCATA
AGCGACTTCTCTCGGGCCCACAGCCTTACAGCTGTGGAGCACGGTATCCTCTGCCGAAACAGAGGTTG
GACAAGACCGGAGGGGTCTCCTGGTTCCAAAGGAGATGTACTCCGGGCTTGTTCACGACCTACCGTGT
+
FFFFFFFFFFFFFFFFF,FFFFFFFFFFFFF:FFFF:FFFFFF:FFFFFFF,F,FFFFFFFFFFFFFF
FFFFFF:FFFFFFFFFFFFFFFFFFF:FFFFFFFFFFFFFFFFFFFFFFFFFFFFFFFFFFFFFFF:F
@A00155:342:HHGFNDSXY:1:1152:5104:2722 1:N:0:GAACCTAG+TCCGCATA
AGCGACTTCTCTCGGGCCCACAGCCTTACAGCTGTGGAGCACGGTATCCTCTGCCGAAACAGAGGTTG
GACAAGACCGGAGGGGTCTCCTGGTTCCAAAGGAGATGTACTCCGGGCTTGTTCACGACCTACCGTGT
+
FFFFFFFFFFFFFFFFFFFFFFFFFFFFFFFFFFF,FFFFFFFFFFFFFFFFFFFFFFFFFFF:FFFF
FFFFFFFFFFFFFFFFFFFFFFFFFFFFFFFFFFFFFFFFFFFFFFFFFFFF:F,FFFFFFFFFFFFF
```

@A00155:342:HHGFNDSXY:1:1152:2853:11569 1:N:0:GAACCTAG+TCCGCATA
AATGCCGAGAGGGATGTGGAGGGCGAGAGAGAGCGACTTCTCTCGGGCCCACAGCCTTACAGCTGTGG
AGCACGGTATCCTCTGCCGAAACAGAGGTTGGACAAGACCGGAGGGGTCTCCTGGTTCCAAAGGAGAT
+
FFFFFFFFFFFFFFFFFFF,F,FFFFFFFFFFFFFFFFF:FFFFFFFFFFFFFFFFFFFFFFFFFF
FFFFFFFFFFFFFFFF:FFFFFFFFFF:FFFFFFFFFFFFFFFFFFFFFFFFFFFFFFF:FFFFFFFFF
@A00155:342:HHGFNDSXY:1:1152:6171:15562 1:N:0:GAACCTAG+TCCGCATA
AATGCCGAGAGGGATGTGGAGGGCGAGAGAGAGCGACTTCTCTCGGGCCCACAGCCTTACAGCTGTGG
AGCACGGTATCCTCTGCCGAAACAGAGGTTGGACAAGACCGGAGGGGTCTCCTAGTTCCAAAGGAGAT
+
FFFFFFFFFFFFFFFFFFFFFFFFFFFFFFFFFFFFFFFFFF:FFFFFFFFFFFFFFFFFFFFFFFFF
FFFFFFFFFFFFFFFFFFFFFFFFFFFFFFFFFFFFFFFFFFFFFFFFFFFFFFFFFFFFFFFFFFFF
@A00155:342:HHGFNDSXY:1:1152:18331:21809 1:N:0:GAACCTAG+TCCGCATA
AATGCCGAGAGGGATGTGGAGGGCGAGAGAGAGCGACTTCTCTCGGGCCCACAGCCTTACAGCTGTGG
AGCACGGTATCCTCTGCCGAAACAGAGGTTGGACAAGACCGGAGGGGTCTCCTGGTTCCAAAGGAGAT
+
FFFFFFFFFFFFFFFFFFFFFFFFFF:FFFFFFFFFF:FFFFFFFFFFFFFFFFFFFFFFFFFFFFFF
FFFFFFFFFFFFFFFFFFFFFFFFFFFF:FFFFFFFFFFFFFFFFFFFFFFFFFFFFFFFFFFFFFFF:
@A00155:342:HHGFNDSXY:1:1152:18041:24032 1:N:0:GAACCTAG+TCCGCATA
GACTTCTCTCGGGCCCACAGCCTTACAGCTGTGGAGCACGGTATCCTCTGCCGAAACAGAGGTTGGAC
AAGACCGGAGGGGTCTCCTAGTTCCAAAGGAGATGTACTCCGGGCTTGTTCACGACCTACCGTGTAAG
+
FFFFFFFFFFFFFFFFFFFFFFFFFFFFFFFF:FFFFFFFFFFFFFFFFFFFFFFFFFFFFFFFFFFF
FFFFFFFFFFFFFFFFFFFFFFFFFFFFFFFFFFFFFFFFFFFFFFFFFFFFFFFFFFFFFFFFFFFF
@A00155:342:HHGFNDSXY:1:1152:18123:33630 1:N:0:GAACCTAG+TCCGCATA
GGCGAGAGAGAGCGACTTCTCTCGGGCCCACAGCCTTACAGCTGTGGAGCACGGTATCCTCTGCCGAA
ACAGAGGTTGGACAAGACCGGAGGGGTCTCCTAGTTCCAAAGGAGATGTACTCCGGGCTTGTTCACGA
+
FFFFFFFFFFFFFFFFFFFFFFFFFFFFFFFFFFFFFFFFFFFFFFFFFFF:FFFFFFFFFFFFFFFF
FFFFFFFFFFFFFFFFFFFFFFFFFFFFFFFFFFFFFFFFFFFFFFFFFFFFFFFFFFFFFFFFFFFF
@A00155:342:HHGFNDSXY:1:1152:9173:34632 1:N:0:GAACCTAG+TCCGCATA
TCTCTCGGGCCCACAGCCTTACAGCTGTGGAGCACGGTATCCTCTGCCGAAACAGAGGTTGGACAAGA
CCGGAGGGGTCTCCTAGTTCCAAAGGAGATGTACTCCGGGCTTGTTCACGACCTACCGTGTAAGTCGT
+
FFFFFFFFFFFFFFFFFFFFFFFFFFFFFFFFFFFFFFFF:FFFFFFFFFFFFFFFFFFF,FFFFFFFF:
FFFFFFFFFFFFFFFFFFFFFFFFFFFFFFFFFFFFFFFFFFFFFFFFFFFFF:FFFFFFFFFFFFFF
@A00155:342:HHGFNDSXY:1:1153:27977:2879 1:N:0:GAACCTAG+TCCGCATA
CCTTACAGCTGTGGAGCACGGTATCCTCTGCCGAAACAGAGGTTGGACAAGACCGGAGGGGTCTCCTA
GTTCCAAAGGAGATGTACTCCGGGCTTGTTCACGACCTACCGTGTAAGTCGTAGTCTAGTAGGCTACC
+
F,,F:FFF,FFFF,FFFFF:F:FF,FFF::FFFFFFF::F:FFF,FF:FFF:FFFFFF:FFFFFFFFF
FF:FFF:FF,FFFFFFF,FFFFFFFFFF,:FFFFFF,FFF,FFF:FFFFFFFF,FF,F:FFF,FFFFF
@A00155:342:HHGFNDSXY:1:1153:25599:16172 1:N:0:GAACCTAG+TCCGCATA
ACCTCTGTTTCGGCAGAGGATACCGTGCTCCACAGCTGTAAGGCTGTGGGCCCGAGAGAAGTCGCTCT
CTCTCGCCCTTCACATCCCTCTCGGCATTGAGATGACCATAGGCCAGAGGTGAGTCCTTAAGTGGAC
+
FF:FFFFF,FFFFFFFFF:FFFFFFFFFFFFFFFFFFFFFFFFFFFFFFFFFFFFFFFFFFFFFFFFF
FFFFFFFFFF:FFFF,FFFFFFFFFF:FFFFFFFFFFFFFFFFFFFFFFFFFFFFFFFFFFF:FFFFF
@A00155:342:HHGFNDSXY:1:1154:31665:3724 1:N:0:GAACCTAG+TCCGCATA
CTGGCCTATGGTCATCTCAATGCCGAGAGGGATGTGAAGGGCGAGAGAGAGCGACTTCTCTCGGGCCC
ACAGCCTTACAGCTGTGGAGCACGGTATCCTCTGCCGAAACAGAGGTTGGACAAGACCGGAG
+
FFFFFFFFFFFFFFFFFFFFFFFFFFFFFFFFFFFFFFFFFFFFFFFFFFFFFFFFFFFFFFFFFFFF
FFFFFFFFFFFFFFFFFFFFFFFFFFFFFFFFFFFFFFFFFFFFFF,FFFFFFFFFFFFFFF

```
@A00155:342:HHGFNDSXY:1:1154:7672:26835 1:N:0:GAACCTAG+TCCGCATA
CTTCTCTCGGGCCCACAGCCTTACAGCTGTGGAGCACGGTATCCTCTGCCGAAACAGAGGTTGGACAA
GACCGGAGGGGTCTCCTGGTTCCAAAGGAGATGTACTCCGGGCTTGTTCACGACCTACCGTGTAAGTC
+
FFFFFFFFFFFFFFFFFFFFFFFFFFFFFF:FFF:FFFFFFFFFFFFFFFFFFFFFFFFFFF:FFFFF
FFFFFFFFFFFFFFFFFFFFFFFFFFFFFFFFFFFFFFFFFFFFFFFFFFFFFFFFFFFFFFFF:FFF
@A00155:342:HHGFNDSXY:1:1155:25391:3724 1:N:0:GAACCTAG+TCCGCATA
GGATGTGGAGGGCGAGAGAGAGCGACTTCTCTCGGGCCCACAGCCTTACAGCTGTGGAGCACGGTATC
CTCTGCCGAAACAGAGGTTGGACAAGACCGGAGGGGTCTCCTATTTCCAAAGGAGATGTACTCC
+
FFFFFFFFFFFFFFFFFFFFFFFFFFFFFFFFFFFFFF:FFFFFFFFFFFFFFFFFFFFFFFFFFFFF
FFFFFFFFFFFFFFFFFFFFFFFFFFFFFFFFFFFFFFFFFFFFFFFFFFFFFFFFFFFFFFFF
@A00155:342:HHGFNDSXY:1:1155:22462:16282 1:N:0:GAACCTAG+TCCGCATA
CTTCTCTCGGGCCCACAGCCTTACAGCTGTGGAGCACGGTATCCTCTGCCGAAACAGAGGTTGGACAA
GACCGGAGGGGTCTCCTAGTTCCAAAGGAGATGTACTCCGGGCTTGTTCACGACCTACCGTGTAAGTC
+
FFFFFFFFFFFFFFFFFFFFFFFFFFFFFFFFFFFFFFFFFFFFFFFFFFFFFFFFFFFFFFFFFFFF
F:FFFFFFFFFFFFFFFFFFFFFFFFFFFFFFFFFFFFFFFFFFFFFFFFFFFFFFFFFF,FFFFF:F
@A00155:342:HHGFNDSXY:1:1155:16351:25488 1:N:0:GAACCTAG+TCCGCATA
ACCTCTGTTTCGGCAGAGGATACCGTGCTCCACAGCTGTAAGGCTGTGGGCCCGAGAGAAGTCGCTCT
CTCTCGCCCTTCACATCCCTCTCGGCATTGAGATGACCATAGGCCAGAGGTGAGTCCTTAAGTGGACA
+
FFFFFFFFFFFFFFFFFFFFFFFFFFFFFFFFFFFFFFFFFFFFFFFFFFFFFFFFFFF:FFFFFFFF
FFFFFFFFFFFFFFFFFFFFFFFFFFFFFFFFFFFFFF:FFFFFFFFFFFFFFFFFFFF,FFFFFFFF
@A00155:342:HHGFNDSXY:1:1155:16821:26146 1:N:0:GAACCTAG+TCCGCATA
ACCTCTGTTTCGGCAGAGGATACCGTGCTCCACAGCTGTAAGGCTGTGGGCCCGAGAGAAGTCGCTCT
CTCTCGCCCTTCACATCCCTCTCGGCATTGAGATGACCATAGGCCAGAGGTGAGTCCTTAAGTGGACA
+
FFFFFFFFFFFFFFFFFFFFFFFFFFFFFFFFFFFFFFFFFFFFFFFFFFFFFFFFFFFFFFFFFFFF
FFFFFFFFFFFFFFFFFFFFFFFFFFFFFFFFFFFFFF,FFFFFFFFFFFFFFFFFFFFFFFFFFFFF
@A00155:342:HHGFNDSXY:1:1155:10637:23296 1:N:0:GAACCTAG+TCCGCATA
AGATCAGCTGTGTCCACTTAAGGACTCACCTCTGGCCTATGGTCATCTCAATGCCGAGAGGGATGTGG
AGGGCGAGAGAGAGCGACTTCTCTCGGGCCCACAGCCTTACAGCTGTGGAGCACGGTATCCTCTGCCG
+
FFFFFFFFFFFFFFFFFFFFFFFFFFFFFFFFFFFFFFFFFFFFFFFFFFFFFFFF:FFFFFFFFFFF
FFFFFFFFFFFFFFFFFFFFFFFFFFFFFFFFFFFFFFFFFFFFFFFFFFFFFFFFF:FFFFFFFFFF
@A00155:342:HHGFNDSXY:1:1155:28013:35728 1:N:0:GAACCTAG+TCCGCATA
TGTGTCCACTTAAGGACTCACCTCTGGCCTATGGTCATCTCAATGCCGAGAGGGATGTGGAGGGCGAG
AGAGAGCGACTTCTCTCGGGCCCACAGCCTTACAGCTGTGGAGCACGGTATCCTCTGCCGAAACAGAG
+
FFFFFFFFFFFFFFFFFFFFFFFFFFFFFFFFFFFFFFFFFFFFFFFFFFFFFFFFFFFFFFFFFFFF
FFFFFFFFFFFFFFFFFFFFFFFFFFFFFFFFFFFFFFFF,F:FFFFFFFFFFFFFFFFFFFFFFFFF
@A00155:342:HHGFNDSXY:1:1156:1479:1830 1:N:0:GAACCTAG+TCCGCATA
CTCCTCTGGCCTATGGTCATCTCAATGCCGAGAGGGATGTGGAGGGCGAGAGAGAGCGACTTCTCTCG
GGCCCACAGCCTTACAGCTGTGGAGCACGGTATCCTCTGCCGAAACAGAGGTTGGACAAGACCGGAGG
+
FFFFFFFFFFFFFFFFFFFFFF:FFFFFFF,FFFFFF:F,FFFFFFFFF:FF:F:FFFFFFFFFFFFF
FFFFF:FFFF:F:FF:FF,FFFFFFFFFFF:FFFFFFFFFFFFF:FFFFFF,FFFFFFFFFFFFFFFF
@A00155:342:HHGFNDSXY:1:1156:31792:11992 1:N:0:GAACCTAG+TCCGCATA
TAGTCTAGTAGGCTACCTGACGAGTCCTTTTTAGGACGAAACTTACACAACTCGAAGAGTAACGACGG
TTTCGTTCTTGGAACGGACTCATCAGGGAGCCTACTAGACTCCGACCGTCATGGGGGTTCAAACCCAT
+
:FFF:FFFFFFFFFFFFFFFFFFFFFFFFFFFFFFFFF:FFFFFFFFFFFFFFFF,FFFFFFFFFFFF
:FFFF,FFFFFFFFFFFFFFFFFFFFFFFFFFFFFFFFFF,FFFFFFFFFFFFFFFFFFFFFFFFFFF
```

@A00155:342:HHGFNDSXY:1:1156:13774:12446 1:N:0:GAACCTAG+TCCGCATA
AATGCCGAGAGGGATGTGGAGGGCGAGAGAGAGCGACTTCTCTCGGGCCCACAGCCTTACAGCTGTGG
AGCACGGTATCCTCTGCCGAAACAGAGGTTGGACAAGACCGGAGGGGTCTCCTAGTTCCAAAGGAGAT
+
FFFFFFFFFFFFF:FFFFFFFFFFFFFFFFFFFFFFFFFFFFFFFF:FFFFFFFFFFFFFFFFFFFFF
FFFFFFFFFFFFFFFFFFFFFFFFFFFFFFFFFFFF:FFFFF,FFFFFFFFFFFFFFFFFFFFFFFFF
@A00155:342:HHGFNDSXY:1:1156:16866:20995 1:N:0:GAACCTAG+TCCGCATA
ATCTCAATGCCGAGAGGGATGTGGAGGGCGAGAGAGAGCGACTTCTCTCGGGCCCACAGCCTTACAGC
TGTGGAGCACGGTATCCTCTGCCGAAACAGAGGTTGGACAAGACCGGAGGGGTCTCCTAGTTCCAAAG
+
FFFFFFFFFFFFFFFFFFFFFFFFFFFFFFFFFFFFFFFFFFFFFFFFFFFFFFFFFFFFFFFFFFFF
FFFFFFFFFFFFFFFFFFFFFFFFFFFFFFFFFFFFFFFFFFFFFFFFFFFFFFFFFFFFFFFFFFFF
@A00155:342:HHGFNDSXY:1:1156:11098:32894 1:N:0:GAACCTAG+TCCGCATA
TGCCGAGAGGGATGTGGAGGGCGAGAGAGAGCGACTTCTCTCGGGCCCACAGCCTTACAGCTGTGGAG
CACGGTATCCTCTGCCGAAACAGAGGTTGGACAAGACCGGAGGGGTCTCCCAGTTCCAAAGGAGATGT
+
FFFFFFFFFFFF:FFFFFFFFFFFFFFFFFFFFFFFFFFFFFFFFFFFFFFFFFF,FFFFFFFFFFFF
FFFFFFFFFFFFFFFFFFFFFFFFFFFFFFFFFFFFFFFFFFFFFFFFFFFFFFFFFFFFFFFFFFFF
@A00155:342:HHGFNDSXY:1:1156:10538:34366 1:N:0:GAACCTAG+TCCGCATA
GCGAGAGAGAGCGACTTCTCTCGGGCCCACAGCCTTACAGCTGTGGAGCACGGTATCCTCTGCCGAAA
CAGAGGTTGGACAAGACCGGAGGGGTCTCCTAGTTCCAAAGGAGATGTACTCCGGGCTTGTTCACGAC
+
FFFFFFFFFFFFFFFFFFFFFFFFFFFFFFFFFFFFFFFFFFFFFFFFFFFFFF:FFFFFFFFFFFFF
FFFFFFFFFFFFFFFFFFFFFFFFFFFFFFF:FFFF:FFFFFFFFFFFFFFFFFFFFFFFFFFFFFFF
@A00155:342:HHGFNDSXY:1:1157:5077:3396 1:N:0:GAACCTAG+TCCGCATA
TCCACTTAAGGACTCACCTCTGGCCTATGGTCATCTCAATGCCGAGAGGGATGTGAAGGGCGAGAGAG
AGCGACTTCTCTCGGGCCCACAGCCTTACAGCTGTGGAGCACGGTATCCTCTGCCGAAACAGAGGTTG
+
FFFFFFFFFFFFFFFFFFF,FFFFFFFFFFFFFFFFFFFFF::FFFFFFFFFFFFFFFFFFFFFFFFF
FFFFFFFFFFFFFFFFFFFFFFFFFFFFFFF:FFFFFFFFFFFFFFFFFFFFFFFFFFFFFFFFFFFF
@A00155:342:HHGFNDSXY:1:1157:32054:7717 1:N:0:GAACCTAG+TCCGCATA
CTGGCCTATGGTCATCTCAATGCCGAGAGGGATGTGGAGGGCGAGAGAGAGCGACTTCTCTCGGGCCC
ACAGCCTTACAGCTGTGGAGCACGGTATCCTCTGCCGAAACAGAGGTTGGACAAGACCGGAGGGGTCT
+
FFFFFFFFFFFFF:FFFFFFFFFFFFFFFFFFFF:FFFFFFFFFFFFFFFFFFFFFFFFFFFFFFFFF
FFFFFFFF:FFFFFFFFFFFFFFFFFFFFFFFFFFFFFFFFFFFFFFFFFFFFFFFFFFFFFFFFFFF
@A00155:342:HHGFNDSXY:1:1157:21757:19914 1:N:0:GAACCTAG+TCCGCATA
ACTTCTCTCGGGCCCACAGCCTTACAGCTGTGGAGCACGGTATCCTCTGCCGAAACAGAGGTTGGACA
AGACCGGAGGGGTCTCCTAGTTCCAAAGGAGATGTACTCCGGGCTTGTTCACGACCTACCGTGTAAGT
+
FFFFFFFFFFFFFFFFFFFFFFFFFFFFFFFFFFFFFFFFFFFFFFFFFFFFFFFFFFFFFFFFFFFF
FFFFFFFFFFFFFFFFFFFFFFFFFFFFFFFFFFFFFFFFFFFFFFFFFFFFFFFFFFFFFFFFFFFF
@A00155:342:HHGFNDSXY:1:1158:24569:10254 1:N:0:GAACCTAG+TCCGCATA
TGTCCACTTAAGGACTCACCTCTGGCCTATGGTCATCTCAATGCCGAGAGGGATGTGGAGGGCGAGAG
AGAGCGACTTCTCTCGGGCCCACAGCCTTACAGCTGTGGAGCACGGTATCCTCTGCCGAAACAGAGGT
+
FFFFFFFFFFFFFFFFFFFFFFFFFFFF:FFFFFFFFFFFFFFFFFFFFFFFFFFFFFFFFFFFFFFF
FFFFFFFF:FFFFFFFFFFFFFFFFFFFFFFFFFFFFFFFFFFFFFFFFFFFFFFFFFFFFFFFFFFF
@A00155:342:HHGFNDSXY:1:1158:7410:14168 1:N:0:GAACCTAG+TCCGCATA
GGATGTAGAGGGCGAGAGAGAGCGACTTCTCTCGGGCCCACAGCCTTACAGCTGTGGAGCACGGTATC
CTCTGCCGAAACAGAGGTTGGACAAGACCGGAGGGGTCTCCTAGTTCCAAAGGAGATGTACTCC
+
FFFFFFFFFFFFFFFFFFFFFFFFFFFFFFFFF:FFFFFFFFFFFFFFFFFFFFFFFFFFFFFFFFFF
FFFFFFFFFFFFFFFFFFFFFFFFFFFF:FFFFFFFFFFFFFFFFFFFFFFFFFFFFFFFFFFF

```
@A00155:342:HHGFNDSXY:1:1158:2889:20181 1:N:0:GAACCTAG+TCCGCATA
GAGAGGGATGTGGAGGGCGAGAGAGAGCGACTTCTCTCGGGCCCACAGCCTTACAGCTGTGGAGCACG
GTATCCTCTGCCGAAACAGAGGTTGGACAAGACCGGAGGGGTCTCCTAGTTCCAAAGGAGATGTACTC
+
FFFFFFFFFFFFFF:,FFFFFFFFFFFF:FFFFFF:FFFFFFFFFFFFFFFFF:FFFFFFFFFFFFF:
FFFFFFFFFFFFFFFFFFFFFF:FFFFFFFF,FFFFFFFFFFFFFFFFFFFFFFFFFFFFFFFFFFFFF
@A00155:342:HHGFNDSXY:1:1158:2456:20275 1:N:0:GAACCTAG+TCCGCATA
GAGAGGGATGTGGAGGGCGAGAGAGAGCGACTTCTCTCGGGCCCACAGCCTTACAGCTGTGGAGCACG
GTATCCTCTGCCGAAACAGAGGTTGGACAAGACCGGAGGGGTCTCCTAGTTCCAAAGGAGATGTACTC
+
FFFFFFFFFFFFFFFFFFFFFFFFF,FFFFFFFFFF,,FFFFFFFFFFFFFFFFFFFFFFFFFFFFF:
FFFFFFFFFFFFFFFFFFFFFFFFFFFFFFFFF:F:FFFFFFFFF:FFFFFF:FF:FFFFFFFFFFFFF
@A00155:342:HHGFNDSXY:1:1159:14443:32362 1:N:0:GAACCTAG+TCCGCATA
GTGTCCACTTAAGGACTCACCTCTGGCCTATGGTCATCTCAATGCCGAGAGGGATGTAGAGGGCGAGA
GAGAGCGACTTCTCTCGGGCCCACAGCCTTACAGCTGTGGAGCACGGTATCCTCTGCCGAAACAGAGG
+
FFFFFFFFFFFFFFFFFFFFFFFFFFFFFFFFFFFFFFFFFFFFFFFFF:FFF,FFFFFFFFFFFFF
FFFFFFFFFFFFFFFFFFFFFFFFFFFFFFFFFFFFFFFFFFFFFFFFFFFFFFFFFFFFFFFFFFFFF
@A00155:342:HHGFNDSXY:1:1159:4716:35994 1:N:0:GAACCTAG+TCCGCATA
CCGAGAGGGATGTGGAGGGCGAGAGAGAGCGACTTCTCTCGGGCCCACAGCCTTACAGCTGTGGAGCA
CGGTATCCTCTGCCGAAACAGAGGTTGGACAAGACCGGAGGGGTCTCCTAGTTCCAAAGGAGATGTAC
+
FFFFFFFFFFFFFFFFFFFFFFFFFFFFFFFFFFFFFFFFFFFFFFFFFFFFFFFFFFFFFFFFFFFFF
FFFF:FFFFFFFFFFFFFFFFFFFFFFFFFFFFFFFFFFFFFFFFFFFFFFFFFFFFFFFFFFFFFFFF
@A00155:342:HHGFNDSXY:1:1160:15926:11412 1:N:0:GAACCTAG+TCCGCATA
ACCTCTGTTTCGGCAGAGGATACCGTGCTCCACAGCTGTAAGGCTGTGGGCCCGAGAGAAGTCGCTCT
CTCTCGCCCTCTACATCCCTCTCGGCATTGAGATGACCATAGGCCAGAGGTGAGTCCTTAAGTGGACA
+
FFFFFFFFFFFFFFFFFFFFFFFFFFFFFFFFFFFFFFFFFFFFFFFFFFFFFFFFFFFFFFFFFFFFF
FFFFFFFFFFFFFFFFFFFFFFFFFFFFFFFFFFFFFFFFFFFFFFFFFFFFFFFFFFFFFFFFFFFFF
@A00155:342:HHGFNDSXY:1:1160:4200:19883 1:N:0:GAACCTAG+TCCGCATA
AGAGAGAGCGACTTCTCTCGGGCCCACAGCCTTACAGCTGTGGAGCACGGTATCCTCTGCCGAAACAG
AGGTTGGACAAGACCGGAGGGGTCTCCTAGTTCCAAAGGAGATGTACTCCGGGCTTGTTCACGACCTA
+
FF:FF:FFFFFFFFFFFFFFF,,:FFFF::FFFFFFFFFFFFFFFFFFFFFFFFFFFFFFFFF:FFFF
FFFFFFFFFFF:FFFFFFFFFFFFFFFFFFFFFFFFFFFFF:FFFFFFFFFFFFFFFFFFFFFFFFFFF
@A00155:342:HHGFNDSXY:1:1161:8775:1814 1:N:0:GAACCTAG+TCCGCATA
CAGCCTTACAGCTGTGGAGCACGGTATCCTCTGCCGAAACAGAGGTTGGACAAGACCGGAGGGGTCTC
CTAGTTCCAAAGGAGATGTACTCCGGGCTTGTTCACGACCTACCGTGTAAGTCGTAGTCTAGTAGGC
+
FFFFF::FFFFFFFFFFFFFFFFFF,FFFFFFFFFFFFFF:F:FFFFFFFFFFFFFFFF:FFFFFFFF
FFFFFFFFFFFFFFFFFFFFFFFFFFFFFF:FFFFFFFFFFFFFFF:F:F,FFFFFFF,F:FFFFFFF
@A00155:342:HHGFNDSXY:1:1161:28709:26193 1:N:0:GAACCTAG+TCCGCATA
AGCGACTTCTCTCGGGCCCACAGCCTTACAGCTGTGGAGCACGGTATCCTCTGCCGAAACAGAGGTTG
GACAAGACCGGAGGGGTCTCCTAGTTCCAAAGGAGATGTACTCCGGGCTTGTTCACGACCTACCGTGT
+
FFF:FFFFFFFFFFFFFFFFFFFFFFFFFFFFFFFFFFFFFFFFFFFFFFFFFFFFFFFFFFFFFFFFF
FFFFFFFFFFFFFFFFFFFFFFFFFFFFFFF:FFFFFFFFFFFFFFFFFFFFFFFFFFFFFFFFFFFFF
@A00155:342:HHGFNDSXY:1:1161:21739:30342 1:N:0:GAACCTAG+TCCGCATA
CTTCTCTCGGGCCCACAGCCTTACAGCTGTGGAGCACGGTATCCTCTGCCGAAACAGAGGTTGGACAA
GACCGGAGGGGTCTCCTAGTTCCAAAGGAGATGTACTCCGGGCTTGTTCACGACCTACCGTGTAAGTC
+
FFFFFF:FFFFFFFFFFFFFFFFFFFFF:FFFFFFFFFFFFFFFFFFFFFFFFFFFFFFFFFFFFFFFF
FFFFFFFFFFFFFFFFFFFFFFFFFFFFFFFFFFFFFFFFFFFFFFFFFFFFFFFFFFFFFFFFFFFFF
```

```
@A00155:342:HHGFNDSXY:1:1161:23258:33254 1:N:0:GAACCTAG+TCCGCATA
TTACAGCTGTGGAGCACGGTATCCTCTGCCGAAACAGAGGTTGGACAAGACCGGAGGGGTCTCCTAGT
TCCAAAGGAGATGTACTCCGGGCTTGTTCACGACCTACCGTGTAAGTCGTAGTCTAGTAGGCTACCTG
+
FFFFFFFFFFFFFFFFFFFFFFFFFFFFFFFFFFFFFF:FFFFFFFF:FFFFFFFFFFFFFFFFFFFFFF
FFFFFFFFFFFFFFFFFFFFFFFFFFFFFFFFFFFFFFFFFFFFFFFFFFFFFFFFFFFFFFFFF:FFFF
@A00155:342:HHGFNDSXY:1:1161:3983:36636 1:N:0:GAACCTAG+TCCGCATA
AGAAGTCGCTCTCTCTCGCCCTCCACATCCCTCTCGGCATTGAGATGACCATAGGCCAGAGGTGAGTC
CTTAAGTGGACACAGCTGATCTAAGGCGGTGTGGCGGGGCATGGGTTTGAACCCCCATGACGGTCGGA
+
FFFFFFFFFFFFFFFFFFFFF:FFFFFFFFFFFFFFFFFFFFFFFFFFFFFFFFFFFFFFFFFFFFFFFF
FFFFFFFFFFFFFFFFFFFFFFFFFFFFFFFFFFFFFFFFFFF:FFFFFFFFFFFFFFFFFFFFFFFFFF
@A00155:342:HHGFNDSXY:1:1162:7798:10864 1:N:0:GAACCTAG+TCCGCATA
CTTCTCTCGGGCCCACAGCCTTACAGCTGTGGAGCACGGTATCCTCTGCCGAAACAGAGGTTGGACAA
GACCGGAGGGGTCTCCTAGTTCCAAAGGAGATGTACTCCGGGCTTGTTCACGACCTACCGTGTAAGTC
+
FFFFFFFFFFFFFFFFFFFF,FFFFFFF:FFFFFFFFFFFFFF,FFFFFFFFFFFFFFFFFFFFFFFFFF
FFFFFFFFFFFFFFFFFFFFFFFFFFFFFFFFFFFFFFFFFFFFFFFFFFFFFFFFFFFFFF:FFFFFFF
@A00155:342:HHGFNDSXY:1:1163:25943:3458 1:N:0:GAACCTAG+TCCGCATA
CTTCTCTCGGGCCCACAGCCTTACAGCTGTGGAGCACGGTATCCTCTGCCGAAACAGAGGTTGGACAA
GACCGGAGGGGTCTCCTAGTTCCAAAGGAGATGTACTCCGGGCTTGTTCACGACCTACCGTGTAAGTC
+
FFFFFFFFFFFFFFFFFFFFFFFFFFFFFFFFFFFFFFFFFFFFFFFFFFFFFFFFFFFFFFFFFFFFFF
FFFFFFFFFFFFFFFFFFFFFFFFFFFFFFFFFFFFFFFFFFFFFFFFFFFFFFFFFFFFFFFFFF:FFF
@A00155:342:HHGFNDSXY:1:1163:7952:29637 1:N:0:GAACCTAG+TCCGCATA
CTTCTCTCGGGCCCACAGCCTTACAGCTGTGGAGCACGGTATCCTCTGCCGAAACAGAGGTTGGACAA
GACCGGAGGGGTCTCCTGGTTCCAAAGGAGATGTACTCCGGGCTTGTTCACGACCTACCGTGTAAGTC
+
FFFFF:F:FFFFFFFFFFFFFFFFFFFFFFFFFFFFFFFFFFFFFFFFFFFFFFFFFFFFFFFFFFFFFF
F:FFFFFFFFFFFFFFFFFF,FFFFFFFFFFFFFFFFFFFFFF,FFFFFFFFFFFFFFFFFFFFFFFFFF
@A00155:342:HHGFNDSXY:1:1164:3956:17331 1:N:0:GAACCTAG+TCCGCATA
AGGGATGTGGAGGGCGAGAGAGAGCGACTTCTCTCGGGCCCACAGCCTTACAGCTGTGGAGCACGGTA
TCCTCTGCCGAAACAGAGGTTGGACAAGACCGGAGGGGTCTCCTGGTTCCAAAGGAGATGTACTCCGG
+
FFFFFFFFFFFFFFFFFFFFFFFFFFFFFFFFFFFFFFFFFFFFFFFFFFFFFFFFFFFFFFFFFFFFFF
FFFFFFFFFFFFFFFFFFFFFFFFFFFFFFFFFFFFFFFFFFFFFFFFFFFFFFFFFFFFFFFFFFFFFF
@A00155:342:HHGFNDSXY:1:1164:7355:29387 1:N:0:GAACCTAG+TCCGCATA
CAGCCTTACAGCTGTGGAGCACGGTATCCTCTGCCGAAACAGAGGTTGGACAAGACCGGAGGGGTCTC
CTGGTTCCAAAGGAGATGTACTCCGGGCTTGTTCACGACCTACCGTGTAAGTCGTAGTCTAGTAGGCT
+
FFFFFFFFFFFFFFFFFFFFFFFFFFFFFFFFFFFFFFFFFFFFFFFFFFFFFFFFFFFFFFFFFFFFFF
FFFFFFFFFFFFFFFFFFFFFFFFFFFFFFFFFFFFFFFFFFFFFFFFFFFFFFFFFFFFFFFFFFFFFF
@A00155:342:HHGFNDSXY:1:1165:16929:34788 1:N:0:GAACCTAG+TCCGCATA
CCTTACAGCTGTGGAGCACGGTATCCTCTGCCGAAACAGAGGTTGGACAAGACCGGAGGGGTCTCCCA
GTTCCAAAGGAGATGTACTCCGGGCTTGTTCACGACCTACCGTGTAAGTCGTAGTCTAGTAGGCTACC
+
FFFF,FFFFFF:FFFFF:FFFFFFFFFFF:FFFFFFFFFFFFFFFFFFFFFF:FFFF,FF::FFFFFFF,
FFFFFF:FFFFFF,FFFFFFFFFFF,FFFFFFFFFFFFF:FF:FFFFFFFFFFF:FFFF:FFFFFF,FFF
@A00155:342:HHGFNDSXY:1:1166:17047:2613 1:N:0:GAACCTAG+TCCGCATA
CCTTACAGCTGTGGAGCACGGTATCCTCTGCCGAAACAGAGGTTGGACAAGACCGGAGGGGTCTCCCA
GTTCCAAAGGAGATGTACTCCGGGCTTGTTCACGACCTACCGTGTAAGTCGTAGTCTAGTAGGCTACC
+
FFFFFFFFFFFFFFFFFFFFFFFFFFFFFFFFFFFFFFFFFFFFFFFFFFFFFFFFFFFFFFFFFFFFFF
FFFFFFFFFFFFFFFFFFFFFFFFFFFFFFFFFFFFFFFFFFFFFFFFFFFFFFFFFFFFFFFFFFFFFF
```

```
@A00155:342:HHGFNDSXY:1:1166:22571:19664 1:N:0:GAACCTAG+TCCGCATA
CAATGCCGAGAGGGATGTGGAGGGCGAGAGAGAGCGACTTCTCTCGGGCCCACAGCCTTACAGCTGTG
GAGCACGGTATCCTCTGCCGAAACAGAGGTTGGACAAGACCGGAGGGGTCTCCTAGTTCCAAAGGAGA
+
FFFFFFFFFFFFFFF:FFFFFFFFFFFFFFFFFFFFFFFFFFFFFFFFFFFFFFFFFFF:FFFFFFFF
FFFFFFFFFFFFFFFFFFFFFFFFFFFFFFFFFFFFFFFFFFFFFFFFFFFFFFFFFFFFFFFFFFFF
@A00155:342:HHGFNDSXY:1:1166:12852:30890 1:N:0:GAACCTAG+TCCGCATA
AGGGCGAGAGAGAGCGACTTCTCTCGGGCCCACAGCCTTACAGCTGTGGAGCACGGTATCCTCTGCCG
AAACAGAGGTTGGACAAGACCGGAGGGGTCTCCTGGTTCCAAAGGAGATGTACTCCGGGCTTGTTCAC
+
FF:F,FFFFFFFFFFFF:,FFFFFFF,F:FFF::,FFFFFFFFFF:F:FFFFFFF:FFFF:FFF:FFF
FFFFFFF:F,FFFFFFFFFFF:FFFFF,FFFFFF::FFFF,FFFFFFFFFFFF,::F:,FFFFFFFFF
@A00155:342:HHGFNDSXY:1:1166:13937:33207 1:N:0:GAACCTAG+TCCGCATA
AGGGCGAGAGAGAGCGACTTCTCTCGGGCCCACAGCCTTACAGCTGTGGAGCACGGTATCCTCTGCCG
AAACAGAGGTTGGACAAGACCGGAGGGGTCTCCTGGTTCCAAAGGAGATGTACTCCGGGCTTGTTCAC
+
FFFFFFFFFFFFFFFFFFFFFFFFFFFFFFFFFFFFFFFFFFFFFFFFFFFFFFFFFFFFFFFFFFFF
FFFFFFFFFFFFFFFFFFFFFFF:FFFFFFFFFFFFFFFFFFFFFFFFFFFFFFFFFFFFFFFFFFFF
@A00155:342:HHGFNDSXY:1:1167:3188:1282 1:N:0:GAACCTAG+TCCGCATA
CACAGCTTTACAGCTGTGGAGCACGGTATCCTCTGCCGAAACAGAGGTTGGACAAGACCGGAGGGGTC
TCCTAGTTCCAAAGGAGATGTACTCCGGGCTTGTTCACGACCTACCGTGTAAGTCGTAGTCTAGTAGG
+
FFFFFFFFFFFFFFFFFFFFF,FFF,FFFFFFFFFFFFFFFFFFFFFFFFFFFFFFFFFFFFFFFFFF
FFFFFFFFFFFFFFFFFFFFFFFFF:FFFFFFFFFFFFFFFFFFFFFFFFF:FFFFFFFFFFFFFFFF
@A00155:342:HHGFNDSXY:1:1167:17445:3803 1:N:0:GAACCTAG+TCCGCATA
AGGGCGAGAGAGAGCGACTTCTCTCGGGCCCACAGCCTTACAGCTGTGGAGCACGGTATCCTCTGCCG
AAACAGAGGTTGGACAAGACCGGAGGGGTCTCCTAGTTCCAAAGGAGATGTACTCCGGGCTTGTTCAC
+
FFFFFFFFF:FFFFFFFFFFFFFFFFFFFFFFFFFFFFFFFFFFFFFFFFFFFFFFF:FFFFFFFFFF
FFFFFFFFFFFFFFFFF:FFFFFFFFFFFFFFFFFFFFFFFFFFFFFFFFFFFFFFFFFFFFFFFFFF
@A00155:342:HHGFNDSXY:1:1167:15031:11835 1:N:0:GAACCTAG+TCCGCATA
ATCTCAATGCCGAGAGGGATGTAGAGGGCGAGAGAGAGCGACTTCTCTCGGGCCCACAGCCTTACAGC
TGTGGAGCACGGTATCCTCTGCCGAAACAGAGGTTGGACAAGACCGGAGGGGTCTCCTAGTTCCAAAG
+
FFFFFFFFFFFFFFFFFFFFF:FFFFFFFFFFFFFFFFFFFFFFFFFFFFFFFFFFFFFFFFFFFFFF
FFFFFFFFFFFFF:FFFFFFFFFFFFFFFFFFFFFFFFFFFFFFFFFFFFFFFFFFFFFFFFFFFFFF
@A00155:342:HHGFNDSXY:1:1167:20546:14998 1:N:0:GAACCTAG+TCCGCATA
GAGAGCGACTTCTCTCGGGCCCACAGCCTTACAGCTGTGGAGCACGGTATCCTCTGCCGAAACAGAGG
TTGGACAAGACCAGAGGGGTCTCCTAGTTCCAAAGGAGATGTACTCCGGGCTTGTTCACGACCTACCG
+
FFFFFFFFFFFFFFFFFFFFFFFFFFFFFFFFFFFFFFFFFFFFFFFF:FFFFFFFFFFFFFFFFFFF
FFFFFFFFFFFFFFFFFFFFFFFFFFFFFFFFFFFFFFFFFFFFFFFFFFFFFFFFFFFFFFFFFFFF
@A00155:342:HHGFNDSXY:1:1167:14687:18474 1:N:0:GAACCTAG+TCCGCATA
TTCTCTCGGGCCCACAGCCTTACAGCTGTGGAGCACGGTATCCTCTGCCGAAACAGAGGTTGGACAAG
ACCGGAGGGGTCTCCTAGTTCCAAAGGAGATGTACTCCGGGCTTGTTCACGACCTACCGTGTAAGTCG
+
FFFFFFFFFFFFFFF:FFFFFFFFFFFFFFFFFFFFFFFFFFFFFFFFFFFFFFFFFFFFFFFFFFFF
FFFFFFFFFFFFFFFFFFFFFFFFFFFFFFFFFFFFFFFFFF,FFFFFFFFFFFFFFFFFFFFFFFFF
@A00155:342:HHGFNDSXY:1:1167:21088:24017 1:N:0:GAACCTAG+TCCGCATA
AGAGAGAGCGACTTCTCTCGGGCCCACAGCCTTACAGCTGTGGAGCACGGTATCCTCTGCCGAAACAG
AGGTTGGACAAGACCGGAGGGGTCTCCTAGTTCCAAAGGAGATGTACTCCGGGCTTGTTCACGACCTA
+
FFFFFFFFFFFFFFFFFFFFFFFFFFFFFFFFFFFFFFFFFFFFFFFFFFFFFFFFFFFFFFFFFFFF
FFFFFFFFFFFFFFFFFFFFFFFFFFFFFFFFFFFFFFFFFFFFFFFFFFFFF,FFFFFFFFFFFFFF
```

```
@A00155:342:HHGFNDSXY:1:1167:8829:24111 1:N:0:GAACCTAG+TCCGCATA
GAGAGGGATGTGGAGGGCGAGAGAGAGCGACTTCTCTCGGGCCCACAGCCTTACAGCTGTGGAGCACG
GTATCCTCTGCCGAAACAGAGGTTGGACAAGACCGGAGGGGTCTCCTAGTTCCAAAGGAGATGTACTC
+
FFFFFFFFFFFFFFFFFFFFFFFFFFFFFFFFFFFFFFFFFFFFFFFFFFFFFFFFFFFFFFFFFFFF
FFFFFFFFFFFFFFFFFFFFFFFFFFFFFFFFFFFFFFFFFFFFFFFFFFFFFFFFFFFFFFFFFFFF
@A00155:342:HHGFNDSXY:1:1167:18105:26647 1:N:0:GAACCTAG+TCCGCATA
AGCTGTGTCCACTTAAGGACTCACCTCTGGCCTATGGTCATCTCAATGCCGAGAGGGATGTGAAGGGC
GAGAGAGAGCGACTTCTCTCGGGCCCACAGCCTTACAGCTGTGGAGCACGGTATCCTCTGCCGAAAC
+
FFF:FFFFFFFFFFF,FFFFFFFFFFFFFFFFF:FFF,FFFFFFFFFF:FFFF:FFFF:FFFFFFFFF
FFFFFFFFFFFFFFFFFFFFFFFFFFFFF:FFFFFFF:FFFFFFFFFFFFFFFFFFFFFFFFFFFFF
@A00155:342:HHGFNDSXY:1:1167:20094:32534 1:N:0:GAACCTAG+TCCGCATA
AGAGAGAGCGACTTCTCTCGGGCCCACAGCCTTACAGCTGTGGAGCACGGTATCCTCTGCCGAAACAG
AGGTTGGACAAGACCGGAGGGGTCTCCTAGTTCCAAAGGAGATGTACTCCGGGCTTGTTCACGACCTA
+
FFFFFFFFFFFFFFFFFFFFFFFFFFFFFFFFFFFFFFFFFFFFFFFFFFFFFFFFFFFFF:FFFFFF
FFFFFFFFFFF:FFFFFFFFFFFFFFFFFFFFFFFFFFFFFFFFFFFFFFFFFFFFFFFFFFFFFFFF
@A00155:342:HHGFNDSXY:1:1167:17824:35462 1:N:0:GAACCTAG+TCCGCATA
TTACAGCTGTGGAGCACGGTATCCTCTGCCGAAACAGAGGTTGGACAAGACCGGAGGGGTCTCCTAGT
TCCAAAGGAGATGTACTCCGGGCTTGTTCACGACCTACCGTGTAAGTCGTAGTCTAGTAGGCTACCTG
+
FFFFFFFFFFFFFFFFFFF:FFFFFFFFFFFFFFFFFFFFFFFFFFFFFFFFFFFFFFFFFFFFFFFF
FFFFFFFFFFFFFFFFFFFFFFF:FF:FFFFFFFFFFFFFFFFFFFFFFFFFFFFFFFFFFFF:FF:F
@A00155:342:HHGFNDSXY:1:1168:18539:8015 1:N:0:GAACCTAG+TCCGCATA
TATCCTCTGCCGAAACAGAGGTTGGACAAGACCGGAGGGGTCTCCTAGTTCCAAAGGAGATGTACTCC
GGGCTTGTTCACGACCTACCGTGTAAGTCGTAGTCTAGTAGGCTACCTGACGAGTCCTTTTTAGGACG
+
FFFFFFFFFFFFFFFFFFFFFFFFFFFFF:FFFFFFFFFFFFFFFFFFFFFFFFFFFFFFFFFFFFFF
FFFFFFFFFFFFFFFFFFFFFFFFF:FFFFFFFFFFFFFFFFFFFFFFFFFFFFFFFFFFFF:FFFFF
@A00155:342:HHGFNDSXY:1:1168:29912:12524 1:N:0:GAACCTAG+TCCGCATA
ACCTCTGTTTCGGCAGAGGATACCGTGCTCCACAGCTGTAAGGCTGTGGGCCCGAGAGAAGTCGCTCT
CTCTCGCCCTCCACATCCCTCTCGGCATTGAGATGACCATAGGCCAGAGGAGAGTCCTTAAGTGGACA
+
FFFFFFFFFFFFFFFFFFFFFFFFFFFFFFFFFFFFFFFFFFFFFF:FFFFFFFFFFFFFFFFFFFFF
FFFFFFFFFFFFFFFFFFFFFFFFFFFFFFFFFFFFFFFFFFFFFFFFFFFFFFFFFFFFFFFFFFFF
@A00155:342:HHGFNDSXY:1:1169:26657:1689 1:N:0:GAACCTAG+TCCGCATA
CTCAATGCCGAGAGGGATGTGGAGGGCGAGAGAGAGCGACTTCTCTCGGGCCCACAGCCTTACAGCTG
TGGAGCACGGTATCCTCTGCCGAAACAGAGGTTGGACAAGACCGGAGGGGTCTCCTGGTTCCAAAGGA
+
FFFFFFFFFFFFFFFFF:FFFFFFFFFFFFFFFFFFFFFFFFFFFFFFFFFFFFFFFFF,FFFFFF:F
:FFFFFFFFFFFFFFFFFFFFFFFFFFFFFFFFFFFFFF:FFFFFFFFFFFFFFFFFFFFFFFFFFFF
@A00155:342:HHGFNDSXY:1:1169:29794:9032 1:N:0:GAACCTAG+TCCGCATA
GGCAGAGGATACCGTGCTCCACAGCTGTAAGGCTGTGGGCCCGAGAGAAGTCGCTCTCTCTCGCCCTC
CACATCCCTCTCGGCATTGAGATGACCATAGGCCAGAGGTGAGTCCTTAAGTGGACA
+
FFFFFFFFFFFFF:FFFFFFFFFFF:FFFF:FFFFFFFFFFFFFFFFFFF:FFFFFFFFFFFFFFFFF
FFFF,FFFFF:FF:FF,:FFFF:FFFFFFFFFFFFFFFFFFFFFFF,FFF::FFFF:
@A00155:342:HHGFNDSXY:1:1169:3441:15655 1:N:0:GAACCTAG+TCCGCATA
TAAGTGGACACAGCTGATCTAAGGCGGTGTGGCGGGGCATGGGTTTGAACCCCCATGACGGTCGGAGT
CTAGTAGGCTCCCTGATGAGTCCGTTCCAAGGACGACACCGTCGTTACTCTTCGAGTTGTGTAAGTTT
+
FFFFFFFFFFFFFFFFFFFFFFFFFFFFFFFFFFFFFFFFFFFFFFFFFFFFFFFFFFFFFFFFFFFF
FFFFFFFFFFFFFFFFFFFFFFFFFFFFFFFFFFF,FFFFFFFFFFFFFFFFFFFFFFFFFFFFFFFF
```

```
@A00155:342:HHGFNDSXY:1:1170:15691:1611 1:N:0:GAACCTAG+TCCGCATA
CTTCTCTCGGGCCCACAGCCTTACAGCTGTGGAGCACGGTATCCTCTGCCGAAACAGAGGTTGGACAA
GACCGGAGGGGTCTCCTAGTTCCAAAGGAGATGTACTCCGGGCTTGTTCACGACCTACCGTGTAAG
+
FFFFFFFFFFFFFFFFFFFF:FFFFFFFFFFFFFFFFFFFFFFFFFFFFFFFFFFFFFFFFFFFFFFF
FFFFFFFFFFFFFFFFFFFFFFFFFFFFFFFFFFFFFFFFFFFFFFFFFFFFFFFFFFFF:FFFFF
@A00155:342:HHGFNDSXY:1:1170:19985:13088 1:N:0:GAACCTAG+TCCGCATA
AAAGACTCACCTCTGGCCTATGGTCATCTCAATGCCGAGAGGGATGTGGAGGGCGAGAGAGAGCGACT
TCTCTCGGGCCCACAGCCTTACAGCTGTGGAGCACGGTATCCTCTGCCGAAACAGAGGTTGGACAAGA
+
FFFFFFFFFFFFFFFFFFFFFFFFFFFFFFFFFFFFFFFFFFFFFFFFFFFFFFFFFFFFFFFFFFFF
FFFFFFFFFFFFFFFFFFFFFFFFFFFFFFFFFFFFFF:FFFFFFFFFFFFFFFFFFFFF:FFFFF:F
@A00155:342:HHGFNDSXY:1:1170:2474:13730 1:N:0:GAACCTAG+TCCGCATA
ACCTCTGTTTCGGCAGAGGATACCGTGCTCCACAGCTGTAAGGCTGTGGGCCCGAGAGAAGTCGCTCT
CTCTCGCCCTCCACATCCCTCTCGGCATTGAGATGACCATAGGCCAGAGGTGAGTCCTTAAGTGGACA
+
FFFFFFFFFFFFFFFFFFFFFFFFFFFFFFFF:FF,FFFFFFFFFFFFFFFFFFFFFFFFFFFFFFFF
FFFFFFFFFFFFFFFFFFFFFFFFFFFF,FFFFFFFFFFFFFFFFFFFFFFFFFFFFFFFFF:FFFFF
@A00155:342:HHGFNDSXY:1:1170:29478:22263 1:N:0:GAACCTAG+TCCGCATA
GAGCGACTTCTCTCGGGCCCACAGCCTTACAGCTGTGGAGCACGGTATCCTCTGCCGAAACAGAGGTT
GGACAAGACCGGAGGGGTCTCCTAGTTCCAAAGGAGATGTACTCCGGGCTTGTTCACGACCTACCGTG
+
FFFFFFFFFFFFFFFFFFFFFFFFFFFFFFFFFFFFFFFFFFFFFFFFFFFFFFFFFFFFFFFFFFFF
FFFFFF:FFFFFFFFFFFFFFFFFFFFFFFFFFFFFFFFFFFFFFFFFFFFFFFFFFFFFFFFFFFFF
@A00155:342:HHGFNDSXY:1:1171:29378:10191 1:N:0:GAACCTAG+TCCGCATA
AAGGACTCACCTCTGGCCTATGGTCATCTCAATGCCGAGAGGGATGTGAAGGGCGAGAGAGAGCGACT
TCTCTCGGGCCCACAGCCTTACAGCTGTGGAGCACGGTATCCTCTGCCGAAACAGAGGTTGGACAAGA
+
FFFFFFFFFFFFFFFFFFFFFFFFFFFFFFFFFFFFFFF:FFFFFFFFFFFFFFFFFFFF:FFFFFFF
FFFFFFFFFFFFFFFFFFFFFFFFFFFFFFFFFFFFFFFFFFFFFFFFFFFFFFFFFFFFFFFFFFFF
@A00155:342:HHGFNDSXY:1:1171:31738:16626 1:N:0:GAACCTAG+TCCGCATA
AAAGAGAGCGACTTCTCTCGGGCCCACAGCCTTACAGCTGTGGAGCACGGTATCCTCTGCCGAAACAG
AGGTTGGACAAGACCGGAGGGGTCTCCTAGTTCCAAAGGAGATGTACTCCGGGCTTGTTCACGACCTA
+
FFFFFFFFFFFFFFFFFFFFFFFFFFFFFFFFFFFFFFFFFFFFFF:FFFFFFFFFFFFFFFFFFFFF
FFFFFFFFFFFFFFFFFFFFFFFF:FFFFFFFFFFFFFFFFFFFFFFFFFFFF:FFFFFFFFFFFFFF
@A00155:342:HHGFNDSXY:1:1171:30761:21950 1:N:0:GAACCTAG+TCCGCATA
CTTCTCTCGGGCCCACAGCCTTACAGCTGTGGAGCACGGTATCCTCTGCCGAAACAGAGGTTGGACAA
GACCGGAGGGGTCTCCTAGTTCCAAAGGAGATGTACTCCGGGCTTGTTCACGACCTACCGTGTAAGTC
+
FFFFFFFFFFFFFFFFFFFFFFFFFFFFFFFFFFFFFFFFFFFFFFFFFFFFFFFFFFFFFFFFFFFF
FFFFFFFFFFFFFFFFF:FFFFFFFFFFFFFFFFFFFFFFFFFFFFFFFFFFFFFFFFFFFFFFFFFF
@A00155:342:HHGFNDSXY:1:1172:27850:6261 1:N:0:GAACCTAG+TCCGCATA
TATCCTCTGCCGAAACAGAGGTTGGACAAGACCGGAGGGGTCTCCTGGTTCCAAAGGAGATGTACTCC
GGGCTTGTTCACGACCTACCGTGTAAGTCGTAGTCTAGTAGGCTACCTGACGAGTCCTTTTTAGGACG
+
FFFFFFFFFFFFFFFFFFFFFFFFFFFFF,FFFFFFFFFFFFFFFFFFFFFFFFFFFFFFFFFFFFFF
FFFFFFFFFFFFFFFFFFFFFFFFFFFFFFFFFFFF,FFFFFFFFFFFFFFFFF:FFF:FFFFFFFFF
@A00155:342:HHGFNDSXY:1:1172:22164:23563 1:N:0:GAACCTAG+TCCGCATA
TATCCTCTGCCGAAACAGAGGTTGGACAAGACCGGAGGGGTCTCCTAGTTCCAAAGGAGATGTACTCC
GGGCTTGTTCACGACCTACCGTGTAAGTCGTAGTCTAGTAGGCTACCTGACGAGTCCTTTTTAGGACG
+
FFFFFFFFFFFFFFFFFFFFFFFFFFFFFFFFFFFFFFFFFFFFFFFFFFFFFFFFFFFF:FFFFFFF
FFFFFFFFFFFFFFFF:FFFF:FFFFFFFFFFFFFFFFFFFFFFFFFFFFFFFFFFFFFFFFFFFFFF
```

@A00155:342:HHGFNDSXY:1:1173:12066:3599 1:N:0:GAACCTAG+TCCGCATA
CCTTACAGCTGTGGAGCACGGTATCCTCTGCCGAAACAGAGGTTGGACAAGACCGGAGGGGTCTCCTG
GTTCCAAAGGAGATGTACTCCGGGCTTGTTCACGACCTACCGTGTAAGTCGTAGTCTAGTAGGCTACC
+
FFFFFFFFFFFFFFFFFFFFFFFFFFFFFFFFFFFFFFFFFFFFFFFFFFFFFFFFFFFFFFFFFFFF
FFFFFFFFFFFFFFFFFFFFFFFFFFFFFFFFFFFFFFFFFFFFFFFFFFFFFFFFFFFFFFFFFFFF
@A00155:342:HHGFNDSXY:1:1173:11207:6558 1:N:0:GAACCTAG+TCCGCATA
GTCCACTTAAGGACTCACCTCTGGCCTATGGTCATCTCAATGCCGAGAGGGATGTGGAGGGCGAGAGA
GAGCGACTTCTCTCGGGCCCACAGCCTTACAGCTGTGGAGCACGGTATCCTCTGCCGAAACAGAGGTT
+
FFFFFFFFFFFFFFFFFFFFFFFFFFFFFFFFFFFFFFFFFFFFFFFFFFFFFFFFFFFFFFFFFFFF
FFFFFFFFFFFFFFFFFFFFFFFFFFFFFFFFFFFFFFFFF:FFFFFFFFFFFFFFFFFFFFFFFFFF
@A00155:342:HHGFNDSXY:1:1173:29975:11945 1:N:0:GAACCTAG+TCCGCATA
GTATCCTCTGCCGAAACAGAGGTTGGACAAGACCGGAGGGGTCTCCTAGTTCCAAAGGAGATGTACTC
CGGGCTTGTTCACGACCTACCGTGTAAGTCGTAGTCTAGTAGGCTACCTGACGAGTCCTTTTTAGGAC
+
FFFFFFFFFFFFFFFFFFFFFFFFFFFFFFFFFFFFFFFFFFFFFFFFFFFFFFFFFFFFFFFFFFFF
FFFFFFFFFFFFFFFFFFFFFFFFFFFFFFFFFFFFFFFFFFFFFFFFFFFFFFFFFFFFFFFFFFFF
@A00155:342:HHGFNDSXY:1:1173:8314:15264 1:N:0:GAACCTAG+TCCGCATA
GGATGTGGAGGGCGAGAGAGAGCGACTTCTCTCGGGCCCACAGCCTTACAGCTGTGGAGCACGGTATC
CTCTGCCGAAACAGAGGTTGGACAAGACCGGAGGGGTCTCCTAGTTCCAAAGGAGATGTACTCC
+
FFFFFFFFFFFFFFFFFFFFFFFFFFFFFFFFFFFFFFFFFFFFFFFFFFFFFFFFFFFFFFFFFFFF
FFFFFFFFFF:FFFFFFFFFFFFFFFFFFFFFFFFFFFFFFFFFFFFFFFFFFFFF
@A00155:342:HHGFNDSXY:1:1173:16830:16892 1:N:0:GAACCTAG+TCCGCATA
CTTCTCTCGGGCCCACAGCCTTACAGCTGTGGAGCACGGTATCCTCTGCCGAAACAGAGGTTGGACAA
GACCGGAGGGGTCTCCTAGTTCCAAAGGAGATGTACTCCGGGCTTGTTCACGACCTACCGTGTAAGTC
+
FFFFFFFFFFFFFFFFFFFFFFFFFFFFFFFFFFFFFFFFFFFFFFFFFFFFFFFFFFFFFFFFFFFF
FFFFFFFFFFFFFFFF:FFFFFFFFFFFFFFFFFFFFFFFFFFFFFF:FFFFFFFFFFFFFFFFFFFF
@A00155:342:HHGFNDSXY:1:1174:26865:8437 1:N:0:GAACCTAG+TCCGCATA
ATCTCAATGCCGAGAGGGATGTGGAGGGCGAGAGAGAGCGACTTCTCTCGGGCCCACAGCCTTACAGC
TGTGGAGCACGGTATCCTCTGCCGAAACAGAGGTTGGACAAGACCGGAGGGGTCTCCTAGTTCCAAAG
+
FFFFFFFFFF:FFFFF:FFFFFFFF,:F,:F,FFF:F,FFFF:FFFFFFFFF:FFFFFF:FFFFFFFF
FFFFFFFFFFFF,:FFFFFF:FFFFFFFFFF:F,FFFFFFFFFF:FFFFFFFFFFFFFF:F:F:F:FF
@A00155:342:HHGFNDSXY:1:1174:23936:21652 1:N:0:GAACCTAG+TCCGCATA
ACTTACACGGTAGGTCGTGAACAAGCCCGGAGTACATCTCCTTTGGAACTCGGAGACCCCTCCGGTCT
TGTCCAACCTCTGTTTCGGCAGAGGATACCGTGCTCCACAGCTGTAAGGCTGTGGGCCCGAGAGAAG
+
FFFFFF,F:F:FFFFFFFF:F:F,FFFFFFFF::FFF:FF:F:FFFFFF:,F,FFFF:FFFFF,F:F:
,F,FFF:F:FFFFF:FFFFFFFFF:F:,,:FFFFFFF:FF:FF:F:FFFFFFFF:FFFFFFFFFFFF
@A00155:342:HHGFNDSXY:1:1174:28899:23891 1:N:0:GAACCTAG+TCCGCATA
ACCGTGCTCCACAGCTGTAAGGCTGTGGGCCCGAGAGAAGTCGCTCTCTCTCGCCCTCCACATCCCTC
TCGGCATTGAGATGACCATAGGCCAGAGGTGAGTCCTTAAGTGGACACAGCTGATCTAAGGCGGTGTG
+
FFFFFFFF:FFFFFFFFFFFFFFFFFFFFFFFFFFFFFFF:FFFFFFFFFFFF,FFFFFFFFFFFFFF
FFFFFFFFFFFFF:FFFFFFFFFFFFFFFFFFFFFFFFFFFFF:FFFFFFFFFFFFFFFFFFFFFFFF
@A00155:342:HHGFNDSXY:1:1174:7708:26490 1:N:0:GAACCTAG+TCCGCATA
ACCTCTGTTTCGGCAGAGGATACCGTGCTCCACAGCTGTAAGGCTGTGGGCCCGAGAGAAGTCGCTCT
CTCTCGCCCTTCACATCCCTCTCGGCATTGAGATGACCATAGGCCAGAGGTGAGTCCTTAAGTGGACA
+
FFFFFFFFFFFFFFFFFFFFFFFFFFFFFFFFFFFFFFFFFFFFFFFFFFFFFFFFFFFFFFFFFFFF
FFFFFFFFFFFFFFFFFFFFFFFFFFFFFFFFFFFFFFFFFFFFFFF:FFFFFFFFFFFFFFFFFFFF

```
@A00155:342:HHGFNDSXY:1:1176:21404:15389 1:N:0:GAACCTAG+TCCGCATA
TTACAGCTGTGGAGCACGGTATCCTCTGCCGAAACAGAGGTTGGACAAGACCGGAGGGGTCTCCTAGT
TCCAAAGGAGATGTACTCCGGGCTTGTTCACGACCTACCGTGTAAGTCGTAGTCTAGTAGGCTACCTG
+
FF:FFFFFF:FFFFFFFFFFFFFFFFFFFFFFFFFFF:FFFFFFFFFFF:FFFFFFFFFFFFFFFFFF
FFFFFFFFFFFFFFFFFFFFFFFFFFFFFFFFFFFFFFFFFFFFFFFFFFFFFFFFFFFFFFFFFFFF
@A00155:342:HHGFNDSXY:1:1176:4788:20807 1:N:0:GAACCTAG+TCCGCATA
AGCGACTTCTCTCGGGCCCACAGCCTTACAGCTGTGGAGCACGGTATCCTCTGCCGAAACAGAGGTTG
GACAAGACCGGAGGGGTCTCCTAGTTCCAAAGGAGATGTACTCCGGACTTGTTCACGACCTACCGTGT
+
FFFFFFFFFFFFFFFFFFFFFFFFFFFFFFFFFFFFFFFFFFFFFFFFFFFFFFFFFFFFFFFFFFFF
FFFFFFFFFFFFFFFFFFFFFFFFFFFFFFFFFFFFFFFFFFFFFFFFFFFFFFFFFFFFFFFFFFFF
@A00155:342:HHGFNDSXY:1:1176:1958:20791 1:N:0:GAACCTAG+TCCGCATA
GGGATGTGGAGGGCGAGAGAGAGCGACTTCTCTCGGGCCCACAGCCTTACAGCTGTGGAGCACGGTAT
CCTCTGCCGAAACAGAGGTTGGACAAGACCGGAGGGGTCTCCTAGTTCCAAAGGAGATGTACTCCGGG
+
FFFFFFFFFFFFFFFFFFFFFFF:FFFFFFFFFFFFFFFFFFFFFFFFFFFFFFFFFFFFFFFFFFFF
FFFFFFFFFFFFFFFFFFFFFFFFFFFFFFFFFFFFFFFFFFFFFFFFFFFFFFFFFFFFFFFFFFFF
@A00155:342:HHGFNDSXY:1:1176:19750:22169 1:N:0:GAACCTAG+TCCGCATA
TTCTCTCGGGCCCACAGCCTTACAGCTGTGGAGCACGGTATCCTCTGCCGAAACAGAGGTTGGACAAG
ACCGGAGGGGTCTCCTAGTTCCAAAGGAGATGTACTCCGGGCTTGTTCACGACCTACCGTGTAAGTCG
+
FFFFFFFFFFFFFFFFFFFFFFFFFFFFFFFFFFFFFFF:FFFFFFFFFFFFFFFFFFFFFFFFFFFF
FFFFFFFFFFFFFFFFFFFFFFFFFFFFFFFFFFFFFFFFFFFFFFFFFFFFFFFFFFFFFFFFFFFF
@A00155:342:HHGFNDSXY:1:1176:3522:28604 1:N:0:GAACCTAG+TCCGCATA
CCGCCACACCGCCTTAGATCAGCTGTGTCCACTTAAGGACTCACCTCTGGCCTATGGTCATCTCAATG
CCGAGAGGGATGTGGAGGGCGAGAGAGAGCGACTTCTCTCGGGCCCACAGCCTTACAGCTGTGGAGCA
+
FFFFFFFFFFFFFFFFFFFFFF,FFFFFFFFF,FFFFFFFFFF:FFFFF,FF:FFFFFFFFFFFFFFF
FFFFFFFFFF,FFFFFFFFFFFFFF:F:FFFFF:FFFFFFFFFFFFFFFFF:FFFFFFFFFFFFFF:
@A00155:342:HHGFNDSXY:1:1176:25174:28995 1:N:0:GAACCTAG+TCCGCATA
GCGACTTCTCTCGGGCCCACAGCCTTACAGCTGTGGAGCACGGTATCCTCTGCCGAAACAGAGGTTGG
ACAAGACCGGAGGGGTCTCCTAGTTCCAAAGGAGATGTACTCCGGGCTTGTTCACGACCTACCGTGTA
+
FFFFFFFFFFFFFFFFFFFF:FFFFFFFFFFFFFFFFFF:FFFFFFFFFFFFFFFFFFFFFFFFFFFF
FFFFFFFFFFFFFFFFFFFFFFFFFFFFFFFFFFFFFFFFFFFFFFFFFFFF:FFFFFFFFFF,FFF
@A00155:342:HHGFNDSXY:1:1177:22489:4648 1:N:0:GAACCTAG+TCCGCATA
GAGCGACTTCTCTCGGGCCCACAGCCTTACAGCTGTGGTGCACGGTATCCTCTGCCGAAACAGAGGTT
GGACAAGACCGGAGGGGTCTCCTAGTTCCAAAGGAGATGTACTCCGGGCTTGTTCACGACCTACCGTG
+
FFFFFFFFFFFFFFFFFFFFFFFFFFFFFFFFFFFFFFFFFFFFFF,FFFFFFFFFFFFFFFFFFFFF
FFFFFFFFFFFFFFFFFFFFFFFFFFFFFFFFFFFFFFFFFFFFFFFFFFFFFFFFFFFFFFFFFFFF
@A00155:342:HHGFNDSXY:1:1177:16288:12884 1:N:0:GAACCTAG+TCCGCATA
GCTCCACAGCTGTAAGGCTGTGGGCCCGAGAGAAGTCGCTCTCTCTCGCCCTCCACATCCCTCTCGGC
ATTGAGATGACCATAGGCCAGAGGTGAGTCCTTAAGTGGACACAGCTGATCTAAGGCGGTGTGGTGGG
+
FFFFFFFFFFFFFFFFFFFFFFFFFFFFFFFFFFFFFFFFFFFFFFFFFFFFFFFFFFFFF:FFFFFF
F:FFFFFFFFFFFFFFFFFFFFFFFFF:FFFFFF:FFFFFFFFFFFF:F:FFFFFFFFFF,FFFFFFFF
@A00155:342:HHGFNDSXY:1:1177:31702:13714 1:N:0:GAACCTAG+TCCGCATA
GTATCCTCTGCCGAAACAGAGGTTGGACAAGACCGGAGGGGTCTCCTAATTCCAAAGGAGATGTACTC
CGGGCTTGTTCACGACCTACCGTGTAAGTCGTAGTCTAGTAGGCTACCTGACGAGTCCTTTTTAGGAC
+
FFFFFFFFFFFFFFFFFFFFFFFFFFFFFFFFFFFFFFFFFFFFFFFFFFFFFFFFFFFFFFFFFFFF
FFFFFF,FFFFFFFFFFFFFFFFFFFFFFFFFFFFFF:FFFFFFFFFFFFFFFFFFFFFFFFFFFFFFF
```

```
@A00155:342:HHGFNDSXY:1:1178:2790:30499 1:N:0:GAACCTAG+TCCGCATA
TGGTCATCTCAATGCCGAGAGGGATGTGGAGGGCGAGAGAGAGCGACTTCTCTCGGGCCCACAGCCTT
ACAGCTGTGGAGCACGGTATCCTCTGCCGAAACAGAGGTTGGACAAGACCGGAGGGGTCTCCTAGTTC
+
FFFFFFFFFFFFFFFFFFFFFFFFFFFFFFFF:F:FFF:FFFFFFFFFFFFFFFFFFFFFFFFFFFFFF
FFFFFFFFFFFFFFFFFFFFFFFFFFFFFFFFFFFFFFFFFFF:FFFF:FFFFFFFFFFFFFFFFFFFFF
@A00155:342:HHGFNDSXY:1:1201:20546:3474 1:N:0:GAACCTAG+TCCGCATA
TTCTCTCGGGCCCACAGCCTTACAGCTGTGGAGCACGGTATCCTCTGCCGAAACAGAGGTTGGACAAG
ACCGGAGGGGTCTCCTAGTTCCAAAGGAGATGTACTCCGGGCTTGTTCACGACCTACCGTGTAAGTCG
+
FFFFFFFFFFFFFFFFFFFFFFFFFFFFFFFFFFFFFFFFFFFFFFFFFFFFFFFFFFFFFFFFFFFFF
FFFFFFFFFFFFFFFFFFFFFFFFF:FFFFFFFFFFFFFFFFFFFFFFFFFFFFFFFFFFFFFFFFFFF
@A00155:342:HHGFNDSXY:1:1201:32280:5447 1:N:0:GAACCTAG+TCCGCATA
TTACAGCTGTGGAGCACGGTATCCTCTGCCGAAACAGAGGTTGGACAAGACCGGAGGGGTCTCCTAGT
TCCAAAGGAGATGTACTCCGGGCTTGTTCACGACCTACCGTGTAAGTCGTAGTCTAGTAGGCTACCTG
+
FFFFFFFFFFFFFFFFFFFFFFFFFFFFFFFFFFFFFFFFFFFFFFFFFFFFFFFFFFFFFFFFFFFFF
FFFFFFFFFFFFFF:FFFFFFFFFFFFFFFFFFFFFFFFFFFFFFFFFFFFFFFFFFFFFFFFFFFFFF
@A00155:342:HHGFNDSXY:1:1201:3549:6417 1:N:0:GAACCTAG+TCCGCATA
GAGGGCGAGAGAGAGCGACTTCTCTCGGGCCCACAGCCTTACAGCTGTGGAGCACGGTATCCTCTGCC
GAAACAGAGGTTGGACAAGACCGGAGGGGTCTCCTGGTTCCAAAGGAGATGTACTCCGGGCTTGTTCA
+
FFFFFFFF,FFFFFFFFFFFFFFFF:FFFFFFFFFFFFFFFFFFFFFFFFFFFF,FFFFFFFFFFFFFF
FF:FFFFFFFFFFFFFFFFFFFFFFFFFFFFFFFFFFFFFFFFFFFFFFFFFFFFFFFFFFFFFFFFFF
@A00155:342:HHGFNDSXY:1:1201:10963:25269 1:N:0:GAACCTAG+TCCGCATA
ATGCCGAGAGGGATGTGGAGGGCGAGAGAGAGCGACTTCTCTCGGGCCCACAGCCTTACAGCTGTGGA
GCACGGTATCCTCTGCCGAAACAGAGGTTGGACAAGACCGGAGGGGTCTCCTAGTTCCAAAGGAGATG
+
FFFFFFFFFFFFFFFFFFFFFFFFFFFFFFFFFFFFF:FFFFFFFFFFFFFFFFFF:FFFFFFFFFFFF
FFFFFFFFFFFFFFFFFFFFFFFFFFFFFFFFFFFFFFFFFFFFFFFFFFFFFFFFFFFFFFFFFFFFF
@A00155:342:HHGFNDSXY:1:1201:27154:34272 1:N:0:GAACCTAG+TCCGCATA
AGCGACTTCTCTCGGGCCCACAGCCTTACAGCTGTGGAGCACGGTATCCTCTGCCGAAACAGAGGTTG
GACAAGACCGGAGGGGTCTCCTAGTTCCAAAGGAGATGTACTCCGGGCTTGTTCACGACCTACCGTGT
+
FFFFFFFFFFFFFFFFFFFFFFFFFFFFFFFFFFFFFFFFFFFFFFFFFFFFFFFFFFFFFFFFFFFFF
FFFFFFFFFFFFFFFFFFFFFFFFFFFFFFFFFF,FFFFFFFFFFFFFFFFFFFFFFFFFFFFFFFFFF
@A00155:342:HHGFNDSXY:1:1202:12147:25379 1:N:0:GAACCTAG+TCCGCATA
GGATGTGGAGGGCGAGAGAGAGCGACTTCTCTCGGGCCCACAGCCTTACAGCTGTGGAGCACGGTATC
CTCTGCCGAAACAGAGGTTGGACAAGACCGGAGGGGTCTCCTAGTTCCAAAGGAGATGTACTCC
+
FFFFFFFFFFFFFFFFFFFFFFFFFFFFFFFFFFFFFFFFFFFFFFFFFFFFFFFFFFFFFFFFFFFFF
FFFFFFFFFFFFFFFFFFFFFFFFFFFFFFFFFFFFFFFFFFFFFFFFFFFFFFFFFFFFFFFF
@A00155:342:HHGFNDSXY:1:1202:10474:27962 1:N:0:GAACCTAG+TCCGCATA
AGAGAGAGCGACTTCTCTCGGGCCCACAGCCTTACAGCTGTGGAGCACGGTATCCTCTGCCGAAACAG
AGGTTGGACAAGACCGGAGGGGTCTCCTGGTTCCAAAGGAGATGTACTCCGGGCTTGTTCACGACCTA
+
FFFFFFFFFFFFFFFFFFFFFFFFFFFFFFFFFF:FFFFFFFFFFFFFFFFFFFFFFFFFFFFFFFFFF
FFFFFFFFFFFFFFFFFFFFFFFFFFFFFFFFFFFFFFFFFFFFFFFFFFFFFFFFFFFFFFFFFFFFF
@A00155:342:HHGFNDSXY:1:1202:6207:34788 1:N:0:GAACCTAG+TCCGCATA
GAGCGACTTCTCTCGGGCCCACAGCCTTACAGCTGTGGAGCACGGTATCCTCTGCCGAAACAGAGGTT
GGACAAGACCGGAGGGGTCTCCTAGTTCCAAAGGAGATGTACTCCGGGCTTGTTCACGACCTACCGTG
+
FFFFFFFFFFFFFFFFFFFFFF:FFFFFFFFFFFFFFFFFFFFFFFFFFFFFFFFFFFFFFFFFFFFFF
:FFFFFFFFFFFFFFFFFFFFFFFFF:FFFFFFFFFFFFFFFFFFFFFFFFFF:FFFFF:FFFFFFFFFF
```

```
@A00155:342:HHGFNDSXY:1:1202:6804:35509 1:N:0:GAACCTAG+TCCGCATA
AGGGCGAGAGAGAGCGACTTCTCTCGGGCCCACAGCCTTACAGCTGTGGAGCACGGTATCCTCTGCCG
AAACAGAGGTTGGACAAGACCGGAGGGGTCTCCTAGTTCCAAAGGAGATGTACTCCGGGCTTGTTCAC
+
FFFFFFFFFFFFFFFFFFFFFFFFFFFFFFFFFFFFFFFF,FFFFFFFFFFFFFFFFFFFFFFFFFFFFF
FFFFFFFFFFFFFFFFFFFFFFFFFFFFFFFFFFFFFFFFFFFFFFFFFFFFFFFFFFFFFFFFFFFFFF
@A00155:342:HHGFNDSXY:1:1203:31629:9330 1:N:0:GAACCTAG+TCCGCATA
CTGTGTCCACTTAAGGACTCACCTCTGGCCTATGGTCATCTCAATGCCGAGAGGGATGTGGAGGGCGA
GAGAGAGCGACTTCTCTCGGGCCCACAGCCTTACAGCTGTGGAGCACGGTATCCTCTGCCGAAACAGA
+
FFFFFFFF:FFFFFFFFFFFFFFFFFFFFFFFFFFFFFFFFFFFFFFFFFFFFFFFFFFFFFFFFFFFF
FFFFFFFFFFF:FFFFFFFFFFFFFFF:FFFFFFFFFF:FFFFFFFFFFFF,FFFFFFFFFFFFFFFFFF
@A00155:342:HHGFNDSXY:1:1203:27208:15107 1:N:0:GAACCTAG+TCCGCATA
AGAGAGAGCGACTTCTCTCGGGCCCACAGCCTTACAGCTGTGGAGCACGGTATCCTCTGCCGAAACAG
AGGTTGGACAAGACCGGAGGGGTCACCTAGTTCCAAAGGAGATGTACTCCGGGCTTGTTCACGACCTA
+
FFFFFFFFFFFFFFFFFFFFFFFFFFFFFFFFFF:FFFFFFFFFFFFFFFFFFFFFFFFFFFFFFFFFFF
FFFFFFFFFFFFFFFFFFFFFFFFFFFFFFFFFFFFFFFFFFFFFF:FFFFFFFFFFFFFFFFFFFFFFF
@A00155:342:HHGFNDSXY:1:1203:26856:15186 1:N:0:GAACCTAG+TCCGCATA
AGAGAGAGCGACTTCTCTCGGGCCCACAGCCTTACAGCTGTGGAGCACGGTATCCTCTGCCGAAACAG
AGGTTGGACAAGACCGGAGGGGTCACCTAGTTCCAAAGGAGATGTACTCCGGGCTTGTTCACGACCTA
+
FFFFFFFFFFFFFFFFFFFFFFFFFFFFFFFFFFFFFFFFFFFFFFFFFFF:FFFFFFFFFFFFFFFFFF
FFFFFFFFFFF:FFFFFFFFFFFFFFFFFFFFFFFFFFFFFFFFFFFFFFFFFFFFFFFFFFFFFFFFF:
@A00155:342:HHGFNDSXY:1:1203:7735:19335 1:N:0:GAACCTAG+TCCGCATA
CGAGAGAGAGCGACTTCTCTCGGGCCCACAGCCTTACAGCTGTGGAGCACGGTATCCTCTGCCGAAAC
AGAGGTTGGACAAGACCGGAGGGGTCTCCTGGTTCCAAAGGAGATGTACTCCGGGCTTGTTCACGACC
+
FFFFFFFFFFFFFFFFFFFFFF,FFFFFFFFFFFFFF:F:FFFFFFFFFFFFFFFFFFFFFFF:FFFFFF
FFFFFFFFFFFFFFFFFFFFFFFFFFFFFFFFFFFFFFFFFFFFFFFFFFFFFFFFFFFFFFFFFFFFFF
@A00155:342:HHGFNDSXY:1:1203:27561:32158 1:N:0:GAACCTAG+TCCGCATA
ATCTCAATGCCGAGAGGGATGTGGAGGGCGAGAGAGAGCGACTTCTCTCGGGCCCACAGCCTTACAGC
TGTGGAGCACGGTATCCTCTGCCGAAACAGAGGTTGGACAAGACCGGAGGGGTCTCCTAGTTCCAAAG
+
FFFFFFFFFFFFFFFFFFFFFFFFFFFFFFFFFFFFFFFFFFFFFFFFFFFFFFFFFFFFFFFFFFFFFF
FFFFFFFFFFFFFFFFFFFFFFFFFFFFFFFFFFFFFFFFFFFFFFFFFFFFFFFFFFFFFFFFFFFFFF
@A00155:342:HHGFNDSXY:1:1204:5819:2926 1:N:0:GAACCTAG+TCCGCATA
CCTCTGGCCTATGGTCATCTCAATGCCGAGAGGGATGTGAAGGGCGAGAGAGAGCGACTTCTCTCGGG
CCCACAGCCTTACAGCTGTGGAGCACGGTATCCTCTGCCGAAACAGAGGTTGGACAAGAC
+
FFFFFFFFFF:FFFFFFFFFFFFFFFF:FFFFFFFFFF:FF,FFFFFF:FFFFFFFFFFFFFFFFFFFFF
FFFFFFFFFFF:FFFFFFFFFFFFFF:FFF:FFFFFFFFFFFFFFFFFFFFFFFFFFFFF
@A00155:342:HHGFNDSXY:1:1204:17996:8234 1:N:0:GAACCTAG+TCCGCATA
AGAGAGAGCGACTTCTCTCGGGCCCACAGCCTTACAGCTGTGGAGCACGGTATCCTCTGCCGAAACAG
AGGTTGGACAAGACCGGAGGGGTCTCCTAGTTCCAAAGGAGATGTACTCCGGGCTTGTTCACG
+
FFFFFFFFFFFFFFFFFFFFFFFFFFFFFFFFFFFFFFFFFFFFFFFFFFFFFFFFFFFFFFFFFFFFFF
FFFFFFFFFFFFFFFF:FFFFFFFFFFFFFFFFFFFFFFFFFFFFFFFFFFFFFFFFFFFFFF
@A00155:342:HHGFNDSXY:1:1204:4309:11271 1:N:0:GAACCTAG+TCCGCATA
GCTGTGTCCACTTAAGGACTCACATCTGGCCTATGGTCATCTCAATGCCGAGAGGGATGTGGAGGGCG
AGAGAGAGCGACTTCTCTCGGGCCCACAGCCTTACAGCTGTGGAGCACGGTATCCTCTGCCGAAACAG
+
FFFFFFFFFFFFFFFFFFFFFFFF,F:F:FFF,,FFF:FFFFFFFFFFF,:FFFFFFFFFFFFFFFFFFF
FFFFFFFFFFFFFFFFFFFFFFFFF:FFFFFFFFFFFF,FFFFFFFFFFFFFFFFFFFFFFFFFFFFFFF
```

```
@A00155:342:HHGFNDSXY:1:1204:19361:12038 1:N:0:GAACCTAG+TCCGCATA
ACCTCTGTTTCGGCAGAGGATACCGTGCTCCACAGCTGTAAGGCTGTGGGCCCGAGAGAAGTCGCTCT
CTCTCGCCCTCTACATCCCTCTCGGCATTGAGATGACCATAGGCCAGAGGTGAGTCCTTAAGTGGACA
+
FFFFFFFFFFFFFFFFFFFFFFFFFFFFFFFFFFFFFFFFFFFFFFFFFFFFFFFFFFFFFFFFFFFF
FFFFFFFFFFFFFFFFFFFFFFFFFFFFFFFFFFFFFFFFFFFFFFFFFFFFFFFFFFFFFFFFFFFF
@A00155:342:HHGFNDSXY:1:1204:24831:34444 1:N:0:GAACCTAG+TCCGCATA
GGTCATCTCAATGCCGAGAGGGATGTGGAGGGCGAGAGAGAGCGACTTCTCTCGGGCCCACAGCCTTA
CAGCTGTGGAGCACGGTATCCTCTGCCGAAACAGAGGTTGGACAAGACCGGAGGGGTCTCCTAGTTCC
+
FFFFFFFFFFFFFFFFFFFFFFFFFFFFFFFFFFFFFFFFFFFFFFFFFFFFFFFFFFFFFFFFFFFF
FFFFFFFFFFFFFFFFF:FFFFFFFFFFFF:FFFFFFFFFFFFFFFFFFFFFFFFFFFFFFFFFFFFF
@A00155:342:HHGFNDSXY:1:1205:21070:6167 1:N:0:GAACCTAG+TCCGCATA
CACCTCTGGCCTATGGTCATCTCAATGCCGAGAGGGATGTGGAGGGCGAGAGAGAGCGACTTCTCTCG
GGCCCACAGCCTTACAGCTGTGGAGCACGGTATCCTCTGCCGAAACAGAGGTTGGACAAGACCGGAGG
+
FFFFFFFFFFFFFFFFFFFFFFFFFFFFFFFFFFFFFFFFFFFFFFFFFF:FFFFFFFFFFFFFFFFF
FFFFFFFFFFFFFFFFFFFFFFFFFFFFFFFFFFFFFFFFFFFFFFFFFFFFFFFFFF:FFFFFFFFF
@A00155:342:HHGFNDSXY:1:1205:12156:14810 1:N:0:GAACCTAG+TCCGCATA
CCAACCTCTGTTTCGGCAGAGGATACCGTGCTCCACAGCTGTAAGGCTGTGGGCCCGAGAGAAGTCGC
TCTCTCTCGCCCTCCACATCCCTCTCGGCATTGAGATGACCATAGGCCAGAGGTGAGTCCTTAAGTGG
+
FFFFFFFFFFFFFFFFFFFFFFFFFFFFFFFFFFFFFFFFFFFFFFFFFFFFFFFFFFFFFFFFFFFF
FFFFFFFFFFFFFFFFFFFFFFFFFFFFFFFFFFFFFFFFFFFFFFFFFFFFFFFFFFFFFFFFFFFF
@A00155:342:HHGFNDSXY:1:1205:23502:15264 1:N:0:GAACCTAG+TCCGCATA
AGTCTAGTAGGCTACCTGACGAGTCCTTTTTAGGACGAAACTTACACAACTCGAAGAGTAACGACGGT
TTCGTCCTTGGAACGGACTCATCAGGGAGCCTACTAGACTCCGACCGTCATGGGGGTTCAAACCCATG
+
FFFFFFFFFFFFFFFFFFFFFFFFFFFFFFFFFFFFF,FFF,FFFFFFFFFFFFFFFFFFFFFFFFFF
FFFFFFFFFFFFFFFFFFFFFFFFFFFFFFFFFFFFFF:FFFFFFFFFFFFFFFFFFFFFF:FFFFFF
@A00155:342:HHGFNDSXY:1:1205:27272:20948 1:N:0:GAACCTAG+TCCGCATA
AGGGATGTGGAGGGCGAGAGAGAGCGACTTCTCTCGGGCCCACAGCCTTACAGCTGTGGAGCACGGTA
TCCTCTGTCGAAACAGAGGTTGGACAAGACCGGAGGGGTCTCCTAGTTCCAAAGGAGATGTACTCCGG
+
FFFFF:FFFFFFFFFFFFFFFFFFFFFFFFFFFFFFFFFFFFFFFFFFFFFFFFFFFFFFFFFFFFFF
FFFFFFFFFFFFFFFFFFFFFFFFFFFFFFFFFFFFF:FFFFFFFFFFFFFFFFFFFFFFFFFFFFFF
@A00155:342:HHGFNDSXY:1:1205:18123:23015 1:N:0:GAACCTAG+TCCGCATA
CTCTGGCCTATGGTCATCTCAATGCCGAGAGGGATGTGAAGGGCGAGAGAGAGCGACTTCTCTCGGGC
CCACAGCCTTACAGCTGTGGAGCACGGTATCCTCTGCCGAAACAGAGGTTGGACAAGACCGGAGGGGT
+
FFFFFFFFFFFF:FFFFFFFFFFFFFFFFFFFFFFFF:FFFFFFFFFFFFFFFFFFF:F,FFFFFF,F
FFFFFFFF:FFFFFFFFFFFFF:FFFFFFFFFFFFFF:FFFF:FFFFFFFFFFFFFFFFFF::F,FFF
@A00155:342:HHGFNDSXY:1:1205:3938:34585 1:N:0:GAACCTAG+TCCGCATA
TGCCGAGAGGGATGTAGAGGGCGAGAGAGAGCGACTTCTCTCGGGCCCACAGCCTTACAGCTGTGGAG
CACGGTATCCTCTGCCGAAACAGAGGTTGGACAAGACCGGAGGGGTCTCCTAGTTCCAAAGGAGATG
+
FFFFFFFFFFFFFF,,FFFFFFFFFFFFFFFFFFF,FFFFFF:FFFFFFF:FFF:F,FFFFFFFFFFF
FFFFFFFFFFFFFFFFFFFFFFFFFFFFFFFFFFFFFFFFFFFFFFFFFFFFFFFFFFFFFFFFFFF
@A00155:342:HHGFNDSXY:1:1206:26323:2018 1:N:0:GAACCTAG+TCCGCATA
GTAAGTCGTAGTCTAGTAGGCTACCTGACGAGTCCTTTTTAGGACGAAACTTACACAACTCGAAGAGT
AACGACGGTTTCGTCCTTGGAACGGACTCATCAGGGAGCCTACTAGACTCCGACCGTCATGGGG
+
FFFFFFFFFFFFFFFFFFFFFFFFFFFFFFFFFFFFFFFFFFFFFFFFFFFFFFFFFFFFFFFFFFFF
F:F:FFF:FFFFFFFFFFF:FFFFFFFFFFFFFFFFFFFFFFFFFFFFFFFFFFFFF:FFFFFF
```

```
@A00155:342:HHGFNDSXY:1:1206:20980:9392 1:N:0:GAACCTAG+TCCGCATA
GAGCGACTTCTCTCGGGCCCACAGCCTTACAGCTGTGGAGCACGGTATCCTCTGCCGAAACAGAGGTT
GGACAAGACCGGAGGGGTCTCCTGGTTCCAAAGGAGATGTACTCCGGGCTTGTTCACGACCTACCGTG
+
FFFFFFFFFFFFFFFFFFFFFFFFFFFFFFFFFFFFFFFFFFFFFFFFFFFFFFFFFFFFFFFFFFFF
FFFFFFFFFFFFFFFFFFFFFFFFFFFFFFFFFFFFFFFFFFFFFFFFFFFFFFFFFFFFFFFFFFFF
@A00155:342:HHGFNDSXY:1:1206:5665:14278 1:N:0:GAACCTAG+TCCGCATA
CTTCTCTCGGGCCCACAGCCTTACAGCTGTGGAGCACGGTATCCTCTGCCGAAACAGAGGTTGGACAA
GACCGGAGGGGTCTCCTAGTTCCAAAGGAGATGTACTCCGGGCTTGTTCACGACCTACCG
+
FFFFFFFFFFFFFFFFFFFFF:FFFFFFFFF,FFFFFFF:FFFFFFFFFFFFFFFFFFFFFFFFFFFF
FFFFFFFFFFFFFFFFFFFFFFFFFFFFFFF,FFF,FFFFF:FFFFFFFFFFFFFFFFFF
@A00155:342:HHGFNDSXY:1:1206:1940:21950 1:N:0:GAACCTAG+TCCGCATA
CCACAGCCTTACAGCTGTGGAGCACGGTATCCTCTGCCGAAACAGAGGTTGGACAAGACCGGAGGGGT
CTCCTAGTTCCAAAGGAGATGTACTCCGGGCTTGTTCACGACCTACCGTGTAAGTCGTAGTCTAGTAG
+
FFFFFFFFFFFFFFFFFFFFFFFFFFFFFFFFFFFFFFFFFFF:FFFFFFFFFFFFFFFFFFFFFFFF
FFFFFFFFFFFFFFFFFFFFFFFFFFFFFFFFFFFFFFFFFFFFFFFFFFFFFFFFFFFFFFFFFFFF
@A00155:342:HHGFNDSXY:1:1206:26250:30796 1:N:0:GAACCTAG+TCCGCATA
TAGATCAGCTGTGTCCACTTAAGGACTCACCTCTGGCCTATGGTCATCTCAATGCCGAGAGGGATGTG
GAGGGCGAGAGAGAGCGACTTCTCTCGGGCCCACAGCCTTACAGCTGTGGAGCACGGTATCCT
+
FFFFFFFFFFFFFFFFFFFFFFF:FFFFFFFFFFFFFFFFFFFFFFFFFFFFFFFFFFFFFFFFFFFF
FFFFFFFFFFFFFFFFFFFFFFFFFFFFFFFFFFFFFFFFFFFFFF:FFFFFFFFFFFFFFFF
@A00155:342:HHGFNDSXY:1:1207:21016:17253 1:N:0:GAACCTAG+TCCGCATA
GGGCGAGAGAGAGCGACTTCTCTCGGGCCCACAGCCTTACAGCTGTGGAGCACGGTATCCTCTGCCGA
AACAGAGGTTGGACAAGACCGGAGGGGTCTCCTAGTTCCAAAGGAGATGTACTCCGGGCTTGTTCACG
+
FFFFFFFFFFFFFFFFFFFFFFFFFFFFFFFFFFFFFFFFFFFFFFFFFFFFFFFFFFFFFFFFFFFF
FFFFFFFFFFFFFFFFFFFFFFFFFFFFFFFFFFFFFFFFFFFFFFFFFF:FFFFFFFFFFFFFFFFF
@A00155:342:HHGFNDSXY:1:1207:10366:18944 1:N:0:GAACCTAG+TCCGCATA
ACTTCTCTCGGGCCCACAGCCTTACTGCTGTGGAGCACGGTATCCTCTGCCGAAACAGAGGTTGGACA
AGACCGGAGGGGTCTCCTAGTTCCAAAGGAGATGTACTCCGGGCTTGTTCACGACCTACCGTGTAAGT
+
FF:FFFFFFFFFFFFFFFFFFFFFFFFFFFFFFFFFFFFFFFFFF:FFFFFFFFFFFFFFFFFFFFFF
FFFFFFFFFFFFFFFFFFFFFFFFFFFFFFFFFFFFFFFFFFFFFFFFFFFFFFFFFFFFFFFFFFFF
@A00155:342:HHGFNDSXY:1:1208:32072:2550 1:N:0:GAACCTAG+TCCGCATA
GAGAGGGATGTGGAGGGCGAGAGAGAGCGACTTCTCTCGGGCCCACAGCCTTACAGCTGTGGAGCACG
GTATCCTCTGCCGAAACAGAGGTTGGACAAGACCGGAGGGGTCTCCTAGTTCCAAAGGAGATGTACTC
+
F:FFFFFFFFFFFFFFFFFFFFFFFFFFFFFFFFFFFFFFFFFFFFFFFFFFFFFFFFFFFFFFFFFF
FF:FFFFFFFFFFFFFFFF:FFFFFFFFFFFFFFFFFFFFFFFFFFFFFFFFFFFFFFFFFFFFFFFF
@A00155:342:HHGFNDSXY:1:1208:4806:9784 1:N:0:GAACCTAG+TCCGCATA
AGGGATGTAGAGGGCGAGAGAGAGCGACTTCTCTCGGGCCCACAGCCTTACAGCTGTGGAGCACGGTA
TCCTCTGCCGAAACAGAGGTTGGACAAGACCGGAGGGGTCTCCTAGTTCCAAAGGAGATGTACTCCGG
+
FFFFFFFFFFFFFFFF:FFFFFFFFFFFFFFFFFF:FFFFFF::FFF,F,FFFFFFFFFFFFFFFFFF
FFFFFFFFFFFFFFFFFFFFFFFFFFFFFFFFFFFFFFFFFFFFFF:FFFFFFFFFFFF:F:FFFFFF
@A00155:342:HHGFNDSXY:1:1208:19434:11162 1:N:0:GAACCTAG+TCCGCATA
AGGGATGTGGAGGGCGAGAGAGAGCGACTTCTCTCGGGCCCACAGCCTTACAGCTGTGGAGCACGGTA
TCCTCTGCCGAAACAGAGGTTGGACAAGACCGGAGGGGTCTCCTAGTTCCAAAGGAGATG
+
FFFFFFFFFFFFFFFFFFFFFFFFFFFFFFFFFFFFFFFFFFFFFFFFFFFFFFFFFFFFFFFFFFFF
FFFFFFFFFFFFFFFFFFFFFFFFFFFFFFFFFFFFFFFFFFFFFFFFFFFFFFFFFFFF
```

```
@A00155:342:HHGFNDSXY:1:1209:2293:5932 1:N:0:GAACCTAG+TCCGCATA
TGTGGAGGGCGAGAGAGAGCGACTTCTCTCGGGCCCACAGCCTTACAGCTGTGGAGCACGGTATCCTC
TGCCGAAACAGAGGTTGGACAAGACCGGAGGGGTCTCCTAGTTCCAAAGGAGATGTACTCCGGG
+
FFFFFFFFFFFF:FFFFFFFFFFFFFFFFFFFFF,FFFFFFFFFFFFFFFFFF:FFFFFFFFFFFFFF
FFFFFFFFFFF:FFFFFFFFFFFFFFFFFFFFFFFFFFFFFFFFFFFFFFFFFFFFFFFFFFFF
@A00155:342:HHGFNDSXY:1:1209:4101:5963 1:N:0:GAACCTAG+TCCGCATA
AGAGAGAGCGACTTCTCTCGGGCCCACAGCCTTACAGCTGTGGAGCACGGTATCCTCTGCCGAAACAG
AGGTTGGACAAGACCGGAGGGGTCTCCTGGTTCCAAAGGAGATGTACTCCGGGCTTGTTCACG
+
F:FFF:F:FFF,FFF:FF:F,FF,FFFF:FFFFFFFFFF,F:FFFFFFF,F,FFFF:FFFFFF,FFFF
:FFFFF,FFF::FFFFFFFFFFFFFFFFFF:FFFFFFF:FFFF:FFFFFFFFF,FFFF:FFFF:
@A00155:342:HHGFNDSXY:1:1209:32723:16673 1:N:0:GAACCTAG+TCCGCATA
GCGAGAGAGAGCGACTTCTCTCGGGCCCACAGCCTTACAGCTGTGGAGCACGGTATCCTCTGCCGAAA
CAGAGGTTGGACAAGACCGGAGGGGTCTCCTAGTTCCAAAGGAGATGTACTCCGGGCTTGTTCACGAC
+
FFFFFFFFFFFFFFFFFFFFFFFFFFFFFFFF:FFFFFFFFFFFFFFFFFFFFFFFFFFFFFFFFFFF
FFFFFFFFFFFFFF:FFFFFFFFFFFFFFFFFFFFFFFFFFFFFFFFFFFFFFFFFFFFFFFFFFFFF
@A00155:342:HHGFNDSXY:1:1209:4264:17331 1:N:0:GAACCTAG+TCCGCATA
CTATGGTCATCTCAATGCCGAGAGGGATGTGGAGGGCGAGAGAGAGCGACTTCTCTCGGGCCCACAGC
CTTACAGCTGTGGAGCACGGTATCCTCTGCCGAAACAGAGGTTGGACAAGACCGGAGGGGTCTCCTAG
+
FFFFFFFFFFFFFFFFFFFFFFFFFFFFFFFFFFFFFFFFFFFFFFFFFFFFFFFFFFFFFFFFFFFF
FFF,FFFFFFFFFFFFFFFF:FFFFFFFFFFFFFFFFFFFFFFFFFFFFFFFFFFFFFFFFFFFFFFF
@A00155:342:HHGFNDSXY:1:1209:24903:31501 1:N:0:GAACCTAG+TCCGCATA
CAATGCCGAGAGGGATGTGGAGGGCGAGAGAGAGCGACTTCTCTCGGGCCCACAGCCTTACAGCTGTG
GAGCACGGTATCCTCTGCCGAAACAGAGGTTGGACAAGACCGGAGGGGTCTCCTAGTTCCAAAG
+
FFFFFFFFFFFFFFFFFFFFFFFFFFFFFFFFFFFFFF:FFFFFFFFFFFFFFFFFFFFFFFFFFFFF
FFFFFFFFFFFFFFFFFFFFFFFFFFFFFFFFFFFFFFFFFFFFFFFFFFFFFFFFFFFFFFFF
@A00155:342:HHGFNDSXY:1:1209:32922:31735 1:N:0:GAACCTAG+TCCGCATA
GGATGTAGAGGGCGAGAGAGAGCGACTTCTCTCGGGCCCACAGCCTTACAGCTGTGGAGCACGGTATC
CTCTGCCGAAACAGAGGTTGGACAAGACCGGAGGGGTCTCCTAGTTCCAAAGGAGATGTACTCC
+
FFFFFFFFFFFFFFFFFFFFFFFFFFFFFFFF:FFFFFFFFFFFFFFFFFFFF,FFFFFFFFFFFFFF
FFFFFFFF:FFFFFFFFFFFFFFF:FFFFFFFFFFFFF:FFFFFFFFFFFFFFFFFFFFFFFFFF
@A00155:342:HHGFNDSXY:1:1210:3369:16720 1:N:0:GAACCTAG+TCCGCATA
AGATCAGCTGTGTCCACTTAAGGACTCACCTCTGGCCTATGGTCATCTCAATGCCGAGAGGGATGTAG
AGGGCGAGAGAGAGCGACTTCTCTCGGGCCCACAGCCTTACAGCTGTGGAGCACGGTATCCTCTGCCG
+
FFFFFFFFFFFFFFF:FF,FFFFFFFFFFFFFFFFFFFFFFF:FFFFFFFFFFFFFFFFFFFFFFFFF
FFFFFFFFFFFFFFFFFFFFFFFFFFFFFFFFFFFFFFFFFFFFFFFFFFFFFFFFFFFFFFFFFFFF
@A00155:342:HHGFNDSXY:1:1210:15537:20854 1:N:0:GAACCTAG+TCCGCATA
CTTCTCTCGGGCCCACAGCCTTACAGCTGTGGAGCACGGTATCCTCTGCCGAAACAGAGGTTGGACAA
GACCGGAGGGGTCTCCTAGTTCCAAAGGAGATGTACTCCGGGCTTGTTCACGACCTACCGTGTAAGTC
+
FFFFFFFFFFFFFFFFFFFFFFFFFFFFFFFFFFFFFFFFFFFFFFFFFFFFFFFFFFFFFFFFFFFF
FFFFFFFFFFFFFFFFFFFFFFFFFFFFFFFFFFFFFFFFFFFFFFFFFFFFFFFFFFFFFFFF:FFF
@A00155:342:HHGFNDSXY:1:1210:11487:23359 1:N:0:GAACCTAG+TCCGCATA
CTCAATGCCGAGAGGGATGTGGAGGGCGAGAGAGAGCGACTTCTCTCGGGCCCACAGCCTTACAGCTG
TGGAGCACGGTATCCTCTGCCGAAACAGAGGTTGGACAAGACCGGAGGGGTCTCCTAGTTCCAAAGGA
+
FFFFFFFFFFFFFFFFFFFFFFFFFFFFFFFFFFFFFFFFFFFFFFFFFFFFFFFFFFFFFFFFFFFF
FFFFFFFFFFFFFFFFFFFFFFFFFFFFFFFFFFFFFFFFFFFFFFFFFFFFFFFFFFFFFFFFFFFF
```

@A00155:342:HHGFNDSXY:1:1211:29324:3333 1:N:0:GAACCTAG+TCCGCATA
GAGCGACTTCTCTCGGGCCCACAGCCTTACAGCTGTGGTGCACGGTATCCTCTGCCGAAACAGAGGTT
GGACAAGACCGGAGGGGTCTCCTAGTTCCAAAGGAGATGTACTCCGGGCTTGTTCACGACCTACCGTG
+
FFFFFFFFFFFFFFFFFFFFFFFFFFFFFFFFFFFFFFFFFFFFFFFFFFFFFFFFFFFFFFFFFFF:
FFFFFFFFFFFFFFFFFFFFFFFF:FFFFFFF:FFFFFFFFFFFFFFFFFFFFFFFFFFFFFFFFFFF
@A00155:342:HHGFNDSXY:1:1211:3631:35900 1:N:0:GAACCTAG+TCCGCATA
ACCTCTGTTTCGGCAGAGGATACCGTGCTCCACAGCTGTAAGGCTGTGGGCCCGAGAGAAGTCGCTCT
CTCTCGCCCTTCACATCCCTCTCGGCATTGAGATGACCATAGGCCAGAGGTGAGTCCTTAAGTGGACA
+
FFFFFFFFFFFFFFFF:FFFFFFFFFFFFFFFFFFFFFFFFFFFFFFFFFFFFFFFFFFFFFFFFFFF
FFFFFFFFFFFFFFFFFFFFFFFFFFF:FFFFFFFFFFFFFFFFFFFFFFFFFFFFFFFFFFFFFFFF
@A00155:342:HHGFNDSXY:1:1212:19199:6778 1:N:0:GAACCTAG+TCCGCATA
TGTTTCGGCAGAGGATACCGTGCTCCACAGCTGTAAGGCTGTGGGCCCGAGAGAAGTCGCTCTCTCTC
GCCCTCCACATCCCTCTCGGCATTGAGATGACCATAGGCCAGAGGTGAGTCCTTAAGTGGACACAGCT
+
FFFFFFFFFFFFFFFFFFFFFFFFFFFFFFFFFFFFFFFFFFFFFFFFFFFFFFFFFFFFFFFFFFFF
FFFFFFFFFFFFFFFFFFFFFFFFFFFFFFFFFFFFFFFFFFFFFFFFFFFFFFFFFFFFFFFFFFFF
@A00155:342:HHGFNDSXY:1:1212:5493:15170 1:N:0:GAACCTAG+TCCGCATA
GCTCCACAGCTGTAAGGCTGTGGGCCCGAGAGAAGTCGCTCTCTCTCGCCCTCCACATCCCTCTCGGC
ATTGAGATGACCATAGGCCAGAGGTGAGTCCTTAAGTGGACACAGCTGATCTAAGGCGGTGTGGTGGG
+
FFFFFFFFFFFFFFFFFFFFFFFFFFFFFFFFFFFFF,FFFFFFFFFFFFFFFFFFFFFFFFFFFFFF
:FFFFFFFFFFFFFFFFFFFFFFFFFFFFFFFF:FFFFFFFFFFFFFFFFFFFFFFFFFFFFFFFFFF
@A00155:342:HHGFNDSXY:1:1212:7925:17534 1:N:0:GAACCTAG+TCCGCATA
TGTGGAGGGCGAGAGAGAGCGACTTCTCTCGGGCCCACAGCCTTACAGCTGTGGAGCACGGTATCCTC
TGCCGAAACAGAGGTTGGACAAGACCGGAGGGGTCTCCTAGTTCCAAAGGAGATGTACTCCGGG
+
FFFFFFFFFFFFFFFFFFFFFFFFFFFFFFFFFFFFFFFFFFFFFFFFFFFF:FFFFFFFFFFFFFFF
FFFFFFFFFFFFFFFFFFFFFFF:FFFFFFFFFFFFF:FFFFFFFFF:FFFFFFFFFFFFFFFF
@A00155:342:HHGFNDSXY:1:1212:16242:23641 1:N:0:GAACCTAG+TCCGCATA
CTTCTCTCGGGCCCACAGCCTTACAGCTGTGGAGCACGGTATCCTCTGCCGAAACAGAGGTTGGACAA
GACCGGAGGGGTCTCCTAGTTCCAAAGGAGATGTACTCCGGGCTTGTTCACGACCTACCGTGTAAGTC
+
FFFFFFFFFFFFFFFFFFFF:FFFFFFFFFFFFFFFFFFFFFFFFFFFFFFFFFFFFFFFFFFFFFFF
FFFFFFFFFFFFFFFFFFFFFFFFFFFFFFFFFFFFFFFFFFFFFFFFFFFFFFFFFFFFFFFFFFFF
@A00155:342:HHGFNDSXY:1:1212:8187:28354 1:N:0:GAACCTAG+TCCGCATA
GCGACTTCTCTCGGGCCCACAGCCTTACAGCTGTGGAGCACGGTATCCTCTGCCGAAACAGAGGTTGG
ACAAGACCGGAGGGGTCTCCTGGTTCCAAAGGAGATGTACTCCGGGCTTGTTCACGACCTACCGTGTA
+
:FFFF:FFFFFFFFFFFFFFFFFFFFFFFFFF:FFFFFFFFFFFF:FFFFFFFFFFFFFFFFFFFF:F
FFFF:FFFFFFFF:FFFFFFFFFFFFFFFF:FFFFFFFFFFFFFFF:,FFFFFFFFFFFFFFF:FFFF
@A00155:342:HHGFNDSXY:1:1212:24053:35446 1:N:0:GAACCTAG+TCCGCATA
AGGGCGAGAGAGAGCGACTTCTCTCGGGCCCACAGCCTTACAGCTGTGGAGCACGGTATCCTCTGCCG
AAACAGAGGTTGGACAAGACCGGAGGGGTCTCCTAGTTCCAAAGGAGATGTACTCCGGGCTTGTTCAC
+
FFFFFFFFFFFFFFFFFFFFFFFFFFFFFFFFFFFFFFFFFFFFFFFFFFFFFFFFFFFFFFFFFFFF
FFFFFFFFFFFFFFFFFFFF:FFFFFFFFFFFFFFFFFFFFFFFFFFFFFFF:FFFFFFFFFFFFFFF
@A00155:342:HHGFNDSXY:1:1213:32805:3474 1:N:0:GAACCTAG+TCCGCATA
GTATCCTCTGCCGAAACAGAGGTTGGACAAGACCGGAGGGGTCTCCTAGTTCCAAAGGAGATGTACTC
CGGGCTTGTTCACGACCTACCGTGTAAGTCGTAGTCTAGTAGGCTACCTGACGAGTCCTTTTTAGGAC
+
FFFFFFFFFFFFFFFFFFFFFFFFFFFFFFFFFFFFFFFFFFF,FF:FFFFFFFFFFFFFFFFFFFFF
FFFFFFFF:FFFFFFFFFFFFFFFFFFFFFFFFFFFF:FFFFFFFFFFFFFFFFFFFFFFFFFFFFFF

```
@A00155:342:HHGFNDSXY:1:1213:30083:11631 1:N:0:GAACCTAG+TCCGCATA
TTCTCTCGGGCCCACAGCCTTACAGCTGTGGAGCACGGTATCCTCTGCCGAAACAGAGGTTGGACAAG
ACCGGAGGGGTCTCCTGGTTCCAAAGGAGATGTACTCCGGGCTTGTTCACGACCTACCGTGTAAG
+
FFFFFFFFFFFFFFFFFFF:FFFFFFFFFFFFFFFFFFFFFFFFFFFFFFFFFFFFFFFFFFFFFFFF
FFFFF:FFFFFFFFFFFFFFFFFFFFFFFFFFFFFFFFFFFFFFFFFFFFFFFFFFFFF:FF,:F
@A00155:342:HHGFNDSXY:1:1213:22110:18865 1:N:0:GAACCTAG+TCCGCATA
AGGGCGAGAGAGAGCGACTTCTCTCGGGCCCACAGCCTTACAGCTGTGGAGCACGGTATCCTCTGCCG
AAACAGAGGTTGGACAAGACCGGAGGGGTCTCCTAGTTCCAAAGGAGATGTACTCCGGGCTTGTTCAC
+
FFFF:FFFFFFFFFFFFFFFFFFFFFFFFFFFFFFFFFF,FFFFFFFFFFFFFFFFFFFFFFFFFFFF
F:FFFFFFFFFFFFF:FFFFFFFFFFFFFFFFFFFFFFFFFFFFFFFFFFFFFFFFFFFFFFFFFFFF
@A00155:342:HHGFNDSXY:1:1213:10285:20306 1:N:0:GAACCTAG+TCCGCATA
TTCTCTCGGGCCCACAGCCTTACAGCTGTGGAGCACGGTATCCTCTGCCGAAACAGAGGTTGGACAAG
ACCGGAGGGGTCTCCTAGTTCCAAAGGAGATGTACTCCGGGCTTGTTCACGACCTACCGTGTAAGTCG
+
FFFFFFFFFFFFFFFFFFFFFFFFFFFF,FFFFFFFFFFFFFFFFFFFFFFFFFFFFFFFFFFFFFFF
FFFFFFFFFFFFFFFFFFFFFFFFFFFFFFFFFFF:FFFFFFFFFFFFFFFFFFFFFFFFFFFFFFFF
@A00155:342:HHGFNDSXY:1:1213:19307:36808 1:N:0:GAACCTAG+TCCGCATA
ACTTCTCTCGGGCCCACAGCCTTACAGCTGTGGAGCACGGTATCCTCTGCCGAAACAGAGGTTGGACA
AGACCGGAGGGGTCTCCTAGTTCCAAAGGAGATGTACTCCGGGCTTGTTCACGACCTACCGTGTAAGT
+
FF:FFFFFFFFFFFFFFFFFFFFFFFFFFFFFFFFF:FFFFFFFFFFFFFFFFFFFFFFFFFFFFFFF
FFFFFFFFFFFFFFFFFFFFFFFFFFFFFFFFFFFFFFFFFFFFFFF,FFFFFFFFFF:FFFFFFFFF
@A00155:342:HHGFNDSXY:1:1214:8784:5776 1:N:0:GAACCTAG+TCCGCATA
ATCCTCTGCCGAAACAGAGGTTGGACAAGACCGGAGGGGTCTCCTAGTTCCAAAGGAGATGTACTCCG
GGCTTGTTCACGACCTACCGTGTAAGTCGTAGTCTAGTAGGCTACCTGATGAGTCCTTTTTAGGACGA
+
FFFFFFFFFFFFFFFFFFFFFFFFFFFFFFFFFFFFFFFFFFFFFFFFFFFFFFFFFFFFFFFFFFFF
FFFFFFFFFFFFFFFFFFFFFFFFFFFFFFFFFFF:F:FFFFFFFFFFFFFFFFFFFFFFFFFFFFFF
@A00155:342:HHGFNDSXY:1:1214:23936:11725 1:N:0:GAACCTAG+TCCGCATA
GACTTCTCTCGGGCCCACAGCCTTACAGCTGTGGAGCACGGTATCCTCTGCCGAAACAGAGGTTGGAC
AAGACCGGAGGGGTCTCCTAGTTCCAAAGGAGATGTACTCCGGGCTTGTTCACGACCTACCGTGTAAG
+
FFFFFFFFFFFFFFFFFFFFFF:FFFFFFFFFFFFFFFFFFF:FFFFFFFFFFFFFFFFFFFFFFFFF
FF:FFFFFFFFFFFFFFFFFFFFFFFFFFFFFFFFFFFFFFFFFFFFFFFFFFFFFFFFFFFFFFF:F
@A00155:342:HHGFNDSXY:1:1214:28601:18114 1:N:0:GAACCTAG+TCCGCATA
CTTCTCTCGGGCCCACAGCCTTACAGCTGTGGAGCACGGTATCCTCTGCCGAAACAGAGGTTGGACAA
GACCGGAGGGGTCTCCTAGTTCCAAAGGAGATGTACTCCGGGCTTGTTCACGACCTACCGTGTAAGTC
+
FFFFFFFFFFFFFFFFF:FFFFFFFFFFFFFFFFFFFFFFFFFFFFFFFFFFFFFFFFFFFFFFFFFF
FFF:FFFF,FFFFFFFFFFFFFFFFFFFFFFFFF,FFFFFF:FFFFFFFFFFFFFFFFFFFFF:FFFF
@A00155:342:HHGFNDSXY:1:1214:5150:21746 1:N:0:GAACCTAG+TCCGCATA
GACTTCTCTCGGGCCCACAGCCTTACAGCTGTGGAGCACGGTATCCTCTGCCGAAACAGAGGTTGGAC
AAGACCGGAGGGGTCTCCTAGTTCCAAAGGAGATGTACTCCGGGCTTGTTCACGACCTACCGTGTAAG
+
FFFFFFFFFFFFFFFFFFFFFFFFFFFFFFFFF,FFFFFFFFFFFFFFFFFFFFFFFFFFFFFFFFFF
FFFFFFFFFFFFFFFFFFFFFFFFFFFFFFFFFFFFFFFFFFFFFFFFFFFFFFFFFFFFFFFFFFFF
@A00155:342:HHGFNDSXY:1:1214:25066:23766 1:N:0:GAACCTAG+TCCGCATA
CTCAATGCCGAGAGGGATGTGGAGGGCGAGAGAGAGCGACTTCTCTCGGGCCCACAGCCTTACAGCTG
TGGAGCACGGTATCCTCTGCCGAAACAGAGGTTGGACAAGACCGGAGGGGTCTCCTAGTTCCAAAGGA
+
FFFFFFFFFFFFFFFF:FFFFFFFFFFFFFFFFFFFFFFFFFFFFFFFFFFFFFFFFFFF,,FFFFFF
FFFFFFFFFFFFFFFFFFFFFFFFFFF:FFFFFFFFFFFFFFFFFFFFFFFFFFFFFFFFFFFFFFFF
```

```
@A00155:342:HHGFNDSXY:1:1214:28519:36229 1:N:0:GAACCTAG+TCCGCATA
CTTCTCTCGGGCCCACAGCCTTACAGCTGTGGAGCACGGTATCCTCTGCCGAAACAGAGGTTGGACAA
GACCGGAGGGGTCTCCTAGTTCCAAAGGAGATGTACTCCGGGCTTGTTCACGACCTACCGTGTAAGTC
+
FFFFFFFFFFFFFFFFFFFFFFFFFFFFFFFFFFFFFFFF:FFFFFFFFFFFFFF:FFFFFFFFFFFF
FFFFFFFFFFFFFFFFFFFFFFFFFFFFFFFFFFFF:FFFFFFFFFFFFFFFFFFFFFFFFFFFFFFF
@A00155:342:HHGFNDSXY:1:1215:14543:11459 1:N:0:GAACCTAG+TCCGCATA
CTCTGGCCTATGGTCATCTCAATGCCGAGAGGGATGTAGAGGGCGAGAGAGAGCGACTTCTCTCGGGC
CCACAGCCTTACAGCTGTGGAGCACGGTATCCTCTGCCGAAACAGAGGTTGGACAAGACCGGAGGGGT
+
FFFFFFFFFFFFFFFFFFFFFFFFFFFFFFFFFFFFF:FFFFFFFFFFFFFFFFFFFFFFFFFFFFFF
FFFFFFFFFFFFFFFFFFFFFFFFFFFFFFFFFFFFFFFF:FF:FFFFFFFFFFFFFFFFFFFFFFFF
@A00155:342:HHGFNDSXY:1:1215:14000:30279 1:N:0:GAACCTAG+TCCGCATA
AGAGAGAGCGACTTCTCTCGGGCCCACAGCCTTACAGCTGTGGAGCACGGTATCCTCTGCCGAAACAG
AGGTTGGACAAGACCGGAGGGGTCTCCTAGTTCCAAAGGAGATGTACTCCGGGCTTGTTCACGACCTA
+
FFFFFFFFFFFF,FFFFFFFFFFFFFFFFFFFFFFFFFFFFFFFFFFFFFF:FFFFFFFFFFFFFFFF
FFFFFFFFFFFFFFFFFFFFFFFFFFFFFFFFFFFFFFFFFFFFFFFFFFFFFFFFFFFFFFFFFFF:
@A00155:342:HHGFNDSXY:1:1216:31195:4789 1:N:0:GAACCTAG+TCCGCATA
GGATGTGGAGGGCGAGAGAGAGCGACTTCTCTCGGGCCCACAGCCTTACAGCTGTGGAGCACGGTATC
CTCTGCCGAAACAGAGGTTGGACAAGACCGGAGGGGTCTCCTAGTTCCAAAGGAGATGTACTCC
+
FFFFFFFFFFFFFFFFFFFFFFFFFFFFFFFFFFFFFFFFFFFFFFFFFFFFFFFFFFFFFFFFF,FF
FFFFFFFFFFFFFFFFFFFFFFFFFFFFFFFFFFFFFFFFFFFFFFFFFFFFFFFFFFFFFFFF
@A00155:342:HHGFNDSXY:1:1216:15338:6918 1:N:0:GAACCTAG+TCCGCATA
AGAGAGAGCGACTTCTCTCGGGCCCACAGCCTTACAGCTGTGGAGCACGGTATCCTCTGCCGAAACAG
AGGTTGGACAAGACCGGAGGGGTCTCCTAGTTCCAAAGGAGATGTACTCCGGGCTTGTTCACGACCTA
+
FFFFFFFFFFFFFFFFFFFFFFFFFFFFFFFFFFFFFFFFFFFFFFFFFFFFFFFFFFFFFFFFFFFF
FFFFFFFFFFFFFFFFFFFFFFFFFFFFFFFFFFFFFFFFFFFFFFFFFFFFFFFF:FFFFFFFFFFF
@A00155:342:HHGFNDSXY:1:1216:12409:12837 1:N:0:GAACCTAG+TCCGCATA
ATAGGCCAGAGGTGAGTCCTTAAGTGGACACAGCTGATCTAAGGCGGTGTGGCGGGGCATGGGTTTGA
ACCCCCATGACGGTCGGAGTCTAGTAGGCTCCCTGATGAGTCCGTTCCAAGGACGACACCGTCGTTAC
+
FFFFFFFFFFFFFFFFFFFFFFFFFFFFFFFFFFFF:FFFFFFFFFFFFFFFFFFFFFFFFFFFFFFF
FFFFFFFFFFFFFFFFFFFFFFFFFFFFFFFFFFFFFFFFFFFFFFFFFFFFFFF,FFFFFFFFFFFF
@A00155:342:HHGFNDSXY:1:1216:8061:30608 1:N:0:GAACCTAG+TCCGCATA
AGCGACTTCTCTCGGGCCCACAGCCTTACAGCTGTGGAGCACGGTATCCTCTGCCGAAACAGAGGTTG
GACAAGACCGGAGGGGTCTCCTAGTTCCAAAGGAGATGTACTCCGGGCTTGTTCACGACCTACCGTGT
+
FFFFFFFFFF,FFFFFFFFFFF,FFFFFFF:FFFFF:FFFFFFFFFFFFFFF,FFFFFFFFFFFFFFF
:FFFF,FFFFFFFFFFFFFFFFF::FFFFFFFFFFFFFFFF:,FFFFFFFFFFFFFFFFFFFFFFFFF
@A00155:342:HHGFNDSXY:1:1217:31647:13870 1:N:0:GAACCTAG+TCCGCATA
TTCTCTCGGGCCCACAGCCTTACAGCTGTGGAGCACGGTATCCTCTGCCGAAACAGAGGTTGGACAAG
ACCGGAGGGGTCTCCTAGTTCCAAAGGAGATGTACTCCGGGCTTGTTCACGACCTACCGTGTAAGTCG
+
FFFFFFFFFFFFFFFFFFFFFFFFFFFFFFFFFFFFFFFFFFFFFFFFFFFFFFFFFFFFFFFFFFFF
FFFFFFFFFFFFFFFFFFFFFFFFFFFFFFFFFFFFFFFFFF,FFFFFFFFFFFFFFFFFFFFFFFFF
@A00155:342:HHGFNDSXY:1:1217:7835:15781 1:N:0:GAACCTAG+TCCGCATA
TCCACTTAAGGACTCACCTCTGGCCTATGGTCATCTCTATGCCGAGAGGGATGTGGAGGGCGAGAGAG
AGCGACTTCTCTCGGGCCCACAGCCTTACAGCTGTGGAGCACGGTATCCTCTGCCGAAACAGAGGTTG
+
FFFFFFFFFFFFFFFFFFFFFFFFFFFFFFFFFFFFFFFFFFFFFFFFFF:FFFFFFFFFFFFFFFFF
FFFFFFFFFFFFFFFFFFFFFFF:FFFFFFFFFFFFFFFFFFFFFFFFFFFFFFFFFFFFFFFFFFFF
```

```
@A00155:342:HHGFNDSXY:1:1217:24496:19742 1:N:0:GAACCTAG+TCCGCATA
GAGCGACTTCTCTCGGGCCCACAGCCTTACAACTGTGGAGCACGGTATCCTCTGCCGAAACAGAGGTT
GGACAAGACCGGAGGGGTCTCCTAGTTCCAAAGGAGATGTACTCCGGGCTTGTTCACGACCTACCGTG
+
FFFFFFFFFFFFFFFFFFFFFFFFFFFFFFFFFFFFFFFFFFFFFFFFFFFFFFFFFFFFFFFFFFFF
FFFFFFFFFFFFFFFFFFFFFFFFFFFFFFFFFFFFFFFFFFFFFFFFFFFFFFFFFFFFFFFF:FFF
@A00155:342:HHGFNDSXY:1:1217:1651:30436 1:N:0:GAACCTAG+TCCGCATA
GAGCGACTTCTCTCGGGCCCACAGCCTTACAACTGTGGAGCACGGTATCCTCTGCCGAAACAGAGGTT
GGACAAGACCGGAGGGGTCTCCTAGTTCCAAAGGAGATGTACTCCGGGCTTGTTCACGACCTACCGTG
+
FFFFFFFFFFFFFFF:FFFF:FFFFFFFFFFFFFFFFFFFFFFFFFFFFFFFFFFFF,FFFFFFFFFF
FFFFFFFFFFFFFFFFFFFFFFFFFFFFFF:FFFFFFFFFFFFFFFFFFFFFFFF,FFFFFFFFFFFF
@A00155:342:HHGFNDSXY:1:1217:3061:31908 1:N:0:GAACCTAG+TCCGCATA
ATCTCAATGCCGAGAGGGATGTAGAGGGCGAGAGAGAGCGACTTCTCTCGGGCCCACAGCCTTACAGC
TGTGGAGCACGGTATCCTCTGCCGAAACAGAGGTTGGACAAGACCGGAGGGGTCTCCTAGTTCCAAAG
+
FFFFFFFFFFFFFFFFFFFFFFFFF:FF:FFFFFFFFFF:FFF:FFFFF:FFFFFFF:FFFFFFFFFF
FFFFFFFFFFFFFFFFFFFFFFFFFFFFFFFFFFFFFFFFFFFFFFFFFFFFFFFFFFFFFFFFFF:F
@A00155:342:HHGFNDSXY:1:1218:3504:21183 1:N:0:GAACCTAG+TCCGCATA
CTCTGGCCTAAGGTCATCTCAATGCCGAGAGGGATGTGGAGGGCGAGAGAGAGCGACTTCTCTCGGGC
CCACAGCCTTACAGCTGTGGAGCACGGTATCCTCTGCCGAAACAGAGGTTGGACAAGACCGGAGGGGT
+
FFFFFFFFFFFFFFFFFFFFFFF:FFFFFFFFFFFF:FFFFF,FFFFFFFFFFFFFFFFFFFFFFFFF
FFFFFFFFFFFFFFFFFFFFFFFFFFFFFFFFFFFFFFFFFFFFFFFFFFFFFFFFFFFFFFFFFFFF
@A00155:342:HHGFNDSXY:1:1218:17499:27633 1:N:0:GAACCTAG+TCCGCATA
GGCAGAGGATACCGTGCTCCACAGCTGTAAGGCTGTGGGCCCGAGAGAAGTCGCTCTCTCTCGCCCTC
CACATCCCTCTCGGCATTGAGATGACCATAGGCCAGAGGAGAGTCCTTAAGTGGACA
+
FFFFFFFFFFFFFFFFFFFFFFFFFFFFFFFFFFFFFFFFFFFFFFFFFFFFFFFFFFFFFFFFFFFF
FFFFFFFFFFFFFFFF:FFFFFFFFFFFFFFFFFFFFFFFFFFFFFFFFFFFFFFFF
@A00155:342:HHGFNDSXY:1:1218:9742:29637 1:N:0:GAACCTAG+TCCGCATA
CTCTCGGGCCCACAGCCTTACAGCTGTGGAGCACGGTATCCTCTGCCGAAACAGAGGTTGGACAAGAC
CGGAGGGGTCTCCTAGTTCCAAAGGAGATGTACTCCGGGCTTGTTCACGACCTACCGTGTAAGTCGTA
+
FFFFFFFFFFFFFFFFFF:FFFFFFFFFFFFFFFFFFFFFFFFFFFFFFFFFFFFFFFFFFFFFF,FF
FFFFFFFFFFFFFFFFFFFFFFFFFFFFFFFFFFFFFFFFFFFFFFFFFFFFFFFFFFFFFFFFFFFF
@A00155:342:HHGFNDSXY:1:1218:15917:33567 1:N:0:GAACCTAG+TCCGCATA
GGGCGAGAGAGAGCGACTTCTCTCGGGCCCACAGCCTTACAGCTGTGGAGCACGGTATCCTCTGCCGA
AACAGAGGTTGGACAAGACCGGAGGGGTCTCCTAGTTCCAAAGGAGATGTACTCCGGGCTTGTTCACG
+
FFFFFFFFFFFFFFFFFFFFFFFFFFFFFFFFFFFFFFFFFFFFFFFFFFFFFFFFFFFFFFFFFFFF
FFFFFFFFFFFFFFFFFFFFFFFFFFFFFFFFFFFFFFFFFFFFFFFFFFFFF:FF:FFFFFFFFFFF
@A00155:342:HHGFNDSXY:1:1218:25229:36855 1:N:0:GAACCTAG+TCCGCATA
TTACAGCTGTGGAGCACGGTATCCTCTGCCGAAACAGAGGTTGGACAAGACCGGAGGGGTCTCCTAGT
TCCAAAGGAGATGTACTCCGGGCTTGTTCACGACCTACCGTGTAAGTCGTAGTCTAGTAGGCTACCTG
+
FF,FFFFFFFFFFFFFFFFFFFFFFFFFFFFFFFFFFFFFFFFFFFFFFFFFFFFFFFFFFFFFFFFF
FFFFFFFFFFFFFFFFFFFFFFFFFFFFFFFFFFFFFFFFFFFFFFFFFFFFFFF:FFFFFFFFFFFF
@A00155:342:HHGFNDSXY:1:1219:14850:16313 1:N:0:GAACCTAG+TCCGCATA
CGACTTCTCTCGGGCCCACAGCCTTACAGCTGTGGAGCACGGTATCCTCTGCCGAAACAGAGGTTGGA
CAAGACCGGAGGGGTCTCCTGGTTCCAAAGGAGATGTACTCCGGGCTTGTTCACGACCTACCGTGTAA
+
FFFFFFFFFFFFFFFFFFFFFFFFF:FFFFFFFFFFFFFFFFFFFFFFFFFFFFFFFFFFFFFFFFFF
FFFFFFFFFFFFFFFFFFFFFFFFFFFFFFFFFFFFFFFFFFFFFFFFFFFFFFFFFFFFFFFFFFFF
```

@A00155:342:HHGFNDSXY:1:1219:15917:16313 1:N:0:GAACCTAG+TCCGCATA
CGACTTCTCTCGGGCCCACAGCCTTACAGCTGTGGAGCACGGTATCCTCTGCCGAAACAGAGGTTGGA
CAAGACCGGAGGGGTCTCCTGGTTCCAAAGGAGATGTACTCCGGGCTTGTTCACGACCTACCGTGTAA
+
FFFFFFFFFFFFFFFFFFFFFFFFF:FFFFFFFFFFFFFFFFFFFFFFFFFFFFFFFFFFFFFFFFFF
FFFFFFFFFFFFFFFFFFFFFFFFFFFFFFFFFFFFFFFFFFFFFFFFFFFFFFFFFFFFFFF:FF,F
@A00155:342:HHGFNDSXY:1:1219:9200:27352 1:N:0:GAACCTAG+TCCGCATA
CCTCTGGCCTATGGTCATCTCAATGCCGAGAGGGATGTGAAGGGCGAGAGAGAGCGACTTCTCTCGGG
CCCACAGCCTTACAGCTGTGGAGCACGGTATCCTCTGCCGAAACAGAGGTTGGAC
+
FFFFFFFFFFFFFFFFFFFFFFFFFFFFFFFFFFFFFFFFFFFFFFFFFFFFFFFFFFFFFFFFFFFF
FFFFFFFFFFFFFFFFFFFFFFFFFFFFFFFFFFFFFFFFFFFFFFFFFFFFFFF
@A00155:342:HHGFNDSXY:1:1219:10845:29575 1:N:0:GAACCTAG+TCCGCATA
CTCTGGCCTATGGTCATCTCAATGCCGAGAGGGATGTGAAGGGCGAGAGAGAGCGACTTCTCTCGGGC
CCACAGCCTTACAGCTGTGGAGCACGGTATCCTCTGCCGAAACAGAGGTTGGACAAGACCGGAG
+
FFFFFFFFFFFFFFFFFFFFFFFFFFFFFFFFFFFFFFFFFFFFFFFFFFFFFFFFFFFFFFFFFFFF
FFFFFFFFF:FFFFFFFFFFFFFFFFFFFFFFFFFFFFFFFFFFFFFF,FFFFFFFFFFFFFFF
@A00155:342:HHGFNDSXY:1:1220:13666:16861 1:N:0:GAACCTAG+TCCGCATA
CCACCACACCGCCTTAGATCAGCTGTGTCCACTTAAGGACTCACCTCTGGCCTATGGTCATCTCAATG
CCGAGAGGGATGTGGAGGGCGAGAGAGAGCGACTTCTCTCGGGCCCACAGCCTTACAGCTGTGGAGCA
+
FFFFFFFFFFFFFFFFFFFFFFFFFFFFFFFFFFFFFFFFFFFFFFFFFFFFFFFFFFFFFFFFFFFF
FFFFFFFFFF,FFFFFFFFFFFFFFFFFFFFFF:FFFF:FFFFFFFFFFFFF:FFFFFFFFFFFFFFF
@A00155:342:HHGFNDSXY:1:1220:26901:21684 1:N:0:GAACCTAG+TCCGCATA
CTTCTCTCGGGCCCACAGCCTTACAGCTGTGGAGCACGGTATCCTCTGCCGAAACAGAGGTTGGACAA
GACCGGAGGGGTCTCCTAGTTCCAAAGGAGATGTACTCCGGGCTTGTTCACGACCTACCGTGTAAGT
+
FFFF:FFFFF::FFFFFF:F,FF:F:,FFF,,F,FF:FF,:FFFFF,FF,,FFF:F,FFFFF,:FFFF
FFF,F,F,,FFFFFFF,FFFFF:FFFFFFFFF,FF:,:,FFFFF,::F,F,FF,FFFFFFFFFFF:F
@A00155:342:HHGFNDSXY:1:1220:5828:36730 1:N:0:GAACCTAG+TCCGCATA
GAGCGACTTCTCTCGGGCCCACAGCCTTACAGCTGTGGAGCACGGTATCCTCTGCCGAAACAGAGGTT
GGACAAGACCGGAGGGGTCTCCTAGTTCCAAAGGAGATGTACTCCGGGCTTGTTCACGACCTACCGTG
+
FFFFFFFFFFFFFFFFFFFFFFFFFF:FFFFF,FFFFFFFFFFFFF:FFFFFFFFFFFFFFFFFFFFF
FFFFFFFFFFFFFFFFFFFFFFFFFFFFFFFFFFFFFFFFFFFFFFFFFFFFFFFFFFFFFFFFFFFF
@A00155:342:HHGFNDSXY:1:1221:9661:2315 1:N:0:GAACCTAG+TCCGCATA
CACGGTAGGTCGTGAACAAGCCCGGAGAACATCTCCTTTGGAACTAGGAGACCCCTCCGGTCTTGTCC
AACCTCTGTTTCGGCAGAGGATACCGTGCTCCACAGCTGTAAGGCTGTGGGCCCGAGAGAAGTCGCTC
+
FFFFFFFFFFFFFFFFFFFFFFFFFFF,FFFFFFFFFFFFFFFFFFFFFFFFFFFFFFFFFFFFFFFF
FFFFFFFFFFFFFFFFFFFFFFFFFFFFFFFFFFFFFFFFFFFFFFFFFFFFFFFFFFFFFFFFFFFF
@A00155:342:HHGFNDSXY:1:1221:6207:19476 1:N:0:GAACCTAG+TCCGCATA
CTCTCGCCCTCCACATCCCTCTCGGCATTGAGATGACCATAGGCCAGAGGTGAGTCCTTAAGTGGACA
CAGCTGATCTAAGGCGGTGTGGCGGGGCATGGGTTTGAACCCCCATGACGGTCGGAGTCTAGTAGGCT
+
FFFFFFFFFFFFFFFFFFFFFFFFFF,F,FFFFFFFF,FFFFFFFFFFFFFFFFFFFFFFFFFFFFF,
FFFFFF:FFFF:FFFFFFFFFFFFF,F:FFFFFFFFFFFFFFFFFFFFFFFFFFFFF:FFF:FFFFFF
@A00155:342:HHGFNDSXY:1:1221:9950:19789 1:N:0:GAACCTAG+TCCGCATA
TTAAGGACTCTCCTCTGGCCTATGGTCATCTCAATGCCGAGAGGGATGTGGAGGGCGAGAGAGAGCGA
CTTCTCTCGGGCCCACAGCCTTACAGCTGTGGAGCACGGTATCCTCTGCCGAAACAGAGGTTGGACAA
+
FFFFFFFFFFFFFF:FFFF:F::FFFFFFFFFFFF:F:F:F:FFFF,:FFFFFFFF:FFFFFFFF:FF
,FFFFF:F:FFFFF:F,FFF:FFFF,:FF::FFF:FFF:FFFFFFFFFFFFFF:FFFFFFFFFFFFFF

```
@A00155:342:HHGFNDSXY:1:1221:20726:27618 1:N:0:GAACCTAG+TCCGCATA
AGAGAGAGCGACTTCTCTCGGGCCCACAGCCTTACAGCTGTGGAGCACGGTATCCTCTGCCGAAACAG
AGGTTGGACAAGACCGGAGGGGTCTCCTAGTTCCAAAGGAGATGTACTCCGGGCTTGTTCACGACCTA
+
FFFFFFFFFFFFFFFFFFFFFFFFFFFFFFFFFFFFFFFFFFFFFFFFFFFFFF:FFFFFFFFFFFFF
FFFFFFFFFFFFFFFFFFFFFFFFFFFFFFFFFFFFFFFFFFFFFFFFFFFFFFFFFFF:FFFFFFFF
@A00155:342:HHGFNDSXY:1:1221:32190:34945 1:N:0:GAACCTAG+TCCGCATA
GAGCGACTTCTCTCGGGCCCACAGCCTTACAGCTGTGGAGCACGGTATCCTCTGCCGAAACAGAGGTT
GGACAAGACCGGAGGGGTCTCCTAGTTCCAAAGGAGATGTACTCCGGGCTTGTTCACGACCTACCGT
+
FFFFFFFFFFFFFFFFFFFFFFFFFFFFFFFFFFF,:FFFFFFFFFFFFFFFFFFFFFFFFFFFFFFF
FFFFFF,FFFFFFFFFFFFFFFFF,FFFFFFFFFFFFFFF:FFFFFFFFFFFFFF:FFFFFF:FFF:
@A00155:342:HHGFNDSXY:1:1222:6696:3662 1:N:0:GAACCTAG+TCCGCATA
AGAGAGAGCGACTTCTCTCGGGCCCACAGCCTTACAGCTGTGGAGCACGGTATCCTCTGCCGAAACAG
AGGTTGGACAAGACCGGAGGGGTCTCCTTGTTCCAAAGGAGATGTACTCCGGGCTTGTTCACGAC
+
FFFFFFFFFFFFFFFFFFFFFFFFFFFFFFFFFFFFFFFFFFFFFFFFFFFFFFFFFFFFFFFFFFFF
FFFFFFFFFFFFFFFFFFFFFFFFFFFFFFFFFFFF,FFFFFFFFFFFFFFFFFFFFFFFFFFFF
@A00155:342:HHGFNDSXY:1:1223:32407:16031 1:N:0:GAACCTAG+TCCGCATA
GGACTCACCTCTGGCCTATGGTCATCTCAATGCCGAGAGGGATGTGAAGGGCGAGAGAGAGCGACTTC
TCTCGGGCCCACAGCCTTACAGCTGTGGAGCACGGTATCCTCTGCCGAAACAGAGGTTGGACAA
+
FFFFFFFFFFFFFFFFF:FFFFFFFFFFFFFFFFFFFFFFFFFFFFFFFFFFFFFFFFFFFFFFFFFF
FFFFFFFFFFFFFFFFFF:FFFFFFFFFFFFFFFFFFFFFFFFFFFFFFFFFFFFFFFFFFFFFFFFFFFFFFFFF:F
@A00155:342:HHGFNDSXY:1:1223:14172:30076 1:N:0:GAACCTAG+TCCGCATA
GAGGGCGAGAGAGAGCGACTTCTCTCGGGCCCACAGCCTTACAGCTGTGGAGCACGGTATCCTCTGCC
GAAACAGAGGTTGGACAAGACCGGAGGGGTCTCCTAGTTCCAAAGGAGATGTACTCCGGGCTTGTTCA
+
FFFFFFFFFFFFFFFFFFFFFFFFFFFFFFFFFFFFFFFFFFFFFFFFFFFFFFFFF:FFFFFFFFFF
FFFFFFFFFFFFFFFFFFFFFFFFFFFFFFFFFFFFFFFFFFFFFFFFFFF:FFFF:FFFFFFFFFFF
@A00155:342:HHGFNDSXY:1:1223:17824:31704 1:N:0:GAACCTAG+TCCGCATA
TGTGTCCACTTAAGGACTCACCTCTGGCCTATGGTCATCTCAATGCCGAGAGGGATGTAGAGGGCGAG
AGAGAGCGACTTCTCTCGGGCCCACAGCCTTACAGCTGTGGAGCACGGTATCCTCTGCCGAAACAGAG
+
FFFFF:FF,:FFFFFFFFFFFFFFFFFFFFF:FF:FFFFFFFFFF::FFFFFFF:FF:FFFFFFFFFF
FFFFFFFFF:FFFFFFFFFFFFFFFFF:F:FFFFFFF:FFF:FFFFF:FFFFFFFFF:FFFF,FFF:F
@A00155:342:HHGFNDSXY:1:1223:11198:35415 1:N:0:GAACCTAG+TCCGCATA
TATCCTCTGCCGAAACAGAGGTTGGACAAGACCGGAGGGGTCTCCTAGTTCCAAAGGAGATGTACTCC
GGGCTTGTTCACGACCTACCGTGTAAGTCGTAGTCTAGTAGGCTACCTGACGAGTCCTTTTTAGGACG
+
FFFFFFFFFFFFFFFFFFFFFFFFFFFFF:FFFFFFFFFFFFFFFFFFFFFFFFFFFFFFFFFFFFFF
FFFFFFFFFFFFFFFFFFFFFFFFFFFFFFFFFFFFFFFFFFFFFFFFFFFFFFFFFFFFFFFFFFFF
@A00155:342:HHGFNDSXY:1:1224:2537:11835 1:N:0:GAACCTAG+TCCGCATA
GTCCACTTAAGGACTCACCTCTGGCCTATGGTCATCTCAATGCCGAGAGGGATGTGGAGGGCGAGAGA
GAGCGACTTCTCTCGGGCCCACAGCCTTACAGCTGTGGAGCACGGTATCCTCTGCCGAAACAGAGGTT
+
FFFFFFFFFFFFFFFFFFFFFFFFFFFFFFFFFFFFFFFFFFFFFFFFFFFFFFFFFF:FFFFFFFFF
FFFFFFFFFFFFFFFFFFFFFFFFFFFFFFFFFFFFFFFFFFFFFFFFF:FFFFFFFFFFFFFFFFFF
@A00155:342:HHGFNDSXY:1:1224:2889:19523 1:N:0:GAACCTAG+TCCGCATA
TGGTCATCTCAATGCCGAGAGGGATGTGGAGGGCGAGAGAGAGCGACTTCTCTCGGGCCCACAGCCTT
ACAGCTGTGGAGCACGGTATCCTCTGCCGAAACAGAGGTTGGACAAGACCGGAGGGGTCTCCTAGTTC
+
FFFFFFFFFFFFFFFFFFFFFFFFFFFFFFFFFFFFFFFFFFFFFFFFFFFFFFFFFFFFFFFFFFFF
FFFFFFFFFFFFFFFFFFFFFFFFFFFFFFFFFFFFFFFFFFFFFFFFFFFFFFFFFFFFFFFFFFFF
```

@A00155:342:HHGFNDSXY:1:1224:20356:32487 1:N:0:GAACCTAG+TCCGCATA
GAGAGGGATGTAGAGGGCGAGAGAGAGCGACTTCTCTCGGGCCCACAGCCTTACAGCTGTGGAGCACG
GTATCCTCTGCCGAAACAGAGGTTGGACAAGACCGGAGGGGTCTCCTAGTTCCAAAGGAGATG
+
FFFFFFFFFF:FFFFFFFFFFFFFFFFFFFFFFFFFFFFFFFFFFFFFFFFFFFFFFFFFFFFFF
FFFFFFFFFFFFFFFFFFFFFFFFFFFFFFFFFFFFFFFFFFFFFFFFFFFFF:FFFFFFFFF
@A00155:342:HHGFNDSXY:1:1224:27145:32690 1:N:0:GAACCTAG+TCCGCATA
ACTTCTCTCGGGCCCACAGCCTTACAGCTGTGGAGCACGGTATCCTCTGCCGAAACAGAGGTTGGACA
AGACCGGAGGGGTCTCCTAGTTCCAAAGGAGATGTACTCCGGGCTTGTTCACGACCTACC
+
FFFFFFFFFFFFFFFF,FFFFF:FFF::FFFFFFF:FFFFFFFFFF,FFFFFFFFFFFFFFFFF:FFF
FFFFFF:FFFFFFFFFFFFFFFFFFFFFFFFF:::FFFF:FFFFF,FFFFFFFFFF:FFF
@A00155:342:HHGFNDSXY:1:1224:1325:33254 1:N:0:GAACCTAG+TCCGCATA
ACAGAGGTTGGACAAGACCGGAGGGGTCTCCTGGTTCCAAAGGAGATGTACTCCGGGCTTGTTCACGA
CCTACCGTGTAAGTCGTAGTCTAGTAGGCTACCTGACGAGTCCTTTTTAGGACGAAACTTAC
+
FFFFFFFFFFFFFFFFFFFFFFFFFFFFFFFFFFFFFFFFFFFFFFFFFFFFFFFFFFFFFFFFFFFF
FFFFFFFFFFFFFFFFFFFFF::FFFFFFFFFFFFFFFFFFFFFFFFFFFFFFFF,:FFFFF
@A00155:342:HHGFNDSXY:1:1224:6479:36448 1:N:0:GAACCTAG+TCCGCATA
GAGAGGGATGTGGAGGGCGAGAGAGAGCGACTTCTCTCGGGCCCACAGCCTTACAGCTGTGGAGCACG
GTATCCTCTGCCGAAACAGAGGTTGGACAAGACCGGAGGGGTCTCCTAGTTCCAAAGGAGATGTAATC
+
:FFF:FFFFFF:FFFFFFFFFFFFFF:FFFF:F,F:F:FFFFFF:F:F,F,FFFFF:FFF:,FFFFFF
F:FFF,F,FF::,FFFFFFF:F,,:FFFFFFF,,FFFF:::,FF:FFF:,FFFFFFF,FFF:F:F,F:
@A00155:342:HHGFNDSXY:1:1225:29279:2816 1:N:0:GAACCTAG+TCCGCATA
GATGTGGAGGGCGAGAGAGAGCGACTTCTCTCGGGCCCACAGCCTTACAGCTGTGGAGCACGGTATCC
TCTGCCGAAACAGAGGTTGGACAAGACCGGAGGGGTCTCCTAGTTCCAAAGGAGATGTACTCCGGGCT
+
FFFFFFFFFFFFFFFFFFFFFFFFFFFFFFFF,FFFFFF:FFFFFFFFFFFFFFFFFFFFFFFFFF:F
FFFFFFFFFFFFFFFFFFFFFFFFFFFFFFFFFFFFFFFFFFFFFFFFFFFFFFFFFFFFFFFFFFFF
@A00155:342:HHGFNDSXY:1:1225:15944:13698 1:N:0:GAACCTAG+TCCGCATA
CTATGGTCATCTCAATGCCGAGAGGGATGTGGAGGGCGAGAGAGAGCGACTTCTCTCGGGCCCACAGC
CTTACAGCTGTGGAGCACGGTATCCTCTGCCGAAACAGAGGTTGGACAAGACCGGAGGGGTCTCCTAG
+
FFFFFFFFFFFFFFFFFFFFFFFFFFFFFFFFFFFFFFFFFFFFFFFFFFFFFFFFFFFFFFFFFFFF
FFFFFFFFFFFFFFFFFFFFFFFFFFFFFFFFFFFFFFFFFFFFFFFFFFFFFFFFFFFFFFFFFFFF
@A00155:342:HHGFNDSXY:1:1225:30743:18850 1:N:0:GAACCTAG+TCCGCATA
AGAGAGAGCGACTTCTCTCGGGCCCACAGCCTTACAGCTGTGGAGCACGGTATCCTCTGCCGAAACAG
AGGTTGGACAAGACCGGAGGGGTCTCCTAGTTCCAAAGGAGATGTACTCCGGGCTTGTTCACGACCTA
+
FFFFFFFFFFFFFFFFFFFFFFFFFFFFFFFFFFFFFFFFFFFFFFFFFFFFFFFFFFFFFFFFFFFF
FFFFFFFFFFFFFFFFFFFFFFFFFFFFFFFFFFFFFFFFFFFFFFFFFFFFFFFFFFFFFFFFFFFF
@A00155:342:HHGFNDSXY:1:1225:23402:31062 1:N:0:GAACCTAG+TCCGCATA
AGAGAGCGACTTCTCTCGGGCCCACAGCCTTACAGCTGTGGAGCACGGTATCCTCTGCCGAAACAGAG
GTTGGACAAGACCGGAGGGGTCTCCTGGTTCCAAAGGAGATGTACTCCGGGCTTGTTCACGACCTACC
+
FFFFFFFFFFFFFFFFFFFFFFFFFFFFFFFFFFFFFFFFFFFFFFFFFFFFFFFFFFFFFFFFFFFF
FFFFFFFFFFFFFFFFFFFFFFFFFFFFFFFFFFFFFFFFFFFFFFFFFFFFFFFFFFFFFFFF:FFF
@A00155:342:HHGFNDSXY:1:1225:22480:36730 1:N:0:GAACCTAG+TCCGCATA
GAGAGGGATGTGGAGGGCGAGAGAGAGCGACTTCTCTCGGGCCCACAGCCTTACAGCTGTGGAGCACG
GTATCCTCTGCCGAAACAGAGGTTGGACAAGACCGGAGGGGTCTCCTAGTTCCAAAGGAGATGTACTC
+
FFFFFFFF,FFFFFFFFFFFFFFFFFFFFFFFFFFFFFFFFF:FFF:FFFFFFFFFFFFF
FFFFFFFFFFFFFFFFFFFFFFFFFFFFFFFFFFFFFFFFFFFFFFF:FFFFFFFFFFFFFFFFFFFF

@A00155:342:HHGFNDSXY:1:1226:6813:1611 1:N:0:GAACCTAG+TCCGCATA
GAGCGACTTCTCTCGGGCCCACAGCCTTACAGCTGTGGAGCACGGTATCCTCTGCCGAAACAGAGGTT
GGACAAGACCGGAGGGGTCTCCTAGTACCAAAGGAGATGTACTCCGGGCTTGTTCACGACCTACCGTG
+
FFFFFFFFFFFFFFFFFFFFFFFFFFFF::FFFFFFFFFFFFFFFFFFFFFFFFFFFFFFFFFFFFFF
FFFFFFFFFFFFFFFFFFFFFFFFFFFFFFFFFFFFFFFFFFFFFFF:FFFFFFFFFFFFFFFFFFFF
@A00155:342:HHGFNDSXY:1:1226:19786:30467 1:N:0:GAACCTAG+TCCGCATA
ACCTCTGTTTCGGCAGAGGATACCGTGCTCCACAGCTGTAAGGCTGTGGGCCCGAGAGAAGTCGCTCT
CTCTCGCCCTCCACATCCCTCTCGGCATTGAGATGACCATAGGCCAGAGGTGAGTCCTTAAGTGGACA
+
FFFFFFFFFFFFFFFFF:FF,FFFFFFFFFFFFFFFFFFFFFFFFFFFFFFFFFFFFFFFFFFFFFFF
FFFFFFFFFFFFFFFFFFFFFFFFFFFFFFFFFFFFFFFFFFFFFFFFFFFFFFFFFFFFFFFFFFFF
@A00155:342:HHGFNDSXY:1:1227:14841:3333 1:N:0:GAACCTAG+TCCGCATA
GGCGAGAGAGAGCGACTTCTCTCGGGCCCACAGCCTTACAGCTGTGGAGCACGGTATCCTCTGCCGAA
ACAGAGGTTGGACAAGACCGGAGGGGTCTCCTAGTTCCAAAGGAGATGTACTCCGGGCTTGTTCACGA
+
FFFFFFFFFFFFFFFFFFFFFFFFFFFFFFFFFFFFFFFFFFFFFFFFFFFFFFF,FFFFFFFFFFFF
FFFFFFFFFFFFFFFFFFFFFFFFFFFFFFFFFFFFFFFFFFFFFFFFFFFFFFFFFFFFFFF:FFFF
@A00155:342:HHGFNDSXY:1:1227:27208:17738 1:N:0:GAACCTAG+TCCGCATA
CATCTCAATGCCGAGAGGGATGTGGAGGGCGAGAGAGAGCGACTTCTCTCGGGCCCACAGCCTTACAG
CTGTGGAGCACGGTATCCTCTGCCGAAACAGAGGTTGGACAAGACCGGAGGGGTCTCCTAGTTCCAAA
+
FFFFFFFFFFFFFFFFFFFFFFFFFFFFFFFFFFFFFFFFFFFFFFFFFFFFFFFFFFFFFFFFFFFF
FFFFFFFFFFFFFFFFFFFFFFFFFFFFFFFFFFFFFFFFFFFFFFFFFFFFFFFFFFFFFFFFFFF:
@A00155:342:HHGFNDSXY:1:1227:18258:27289 1:N:0:GAACCTAG+TCCGCATA
CCACATTCCTCTCGGCATTGAGATGACCATAGGCCAGAGGTGAGTCCTTAAGTGGACACAGCTGATCT
AAGGCGGTGTGGCGGGGCATGGGTTTGAACCCCCATGACGGTCGGAGTCTAGTAGGCTCCCTGATGAG
+
FFFFFFFFFFFFFFFFFFFFFFFFFFFFFFFFFFFFFFFFFFFFFFFFFFFFFFFFFFFFFFFFFFFF
FFFFFFFFFFFFFFFFFFFFFFFFFFFFFFFFFFFFFFFFFFFFFFFFFFFFFFFFFFFFFFFFFFFF
@A00155:342:HHGFNDSXY:1:1227:4327:27586 1:N:0:GAACCTAG+TCCGCATA
TTAAAGACTCACCTCTGGCCTATGGTCATCTCAATGCCGAGAGGGATGTGGAGGGCGAGAGAGAGCGA
CTTCTCTCGGGCCCACAGCCTTACAGCTGTGGAGCACGGTATCCTCTGCCGAAACAGAGGTTGGACAA
+
FFFFF:FFFFFFFFFFF,:FFFFF:F:FFF:F:FFFFFFFFFFFFFFFFFFFFFFFFFFFFFFFFFFF
:F:FFFFFFFFFFFFFFFFFFFFFFFFFFFFFFFFFFFFF:FFFFFFFFFFFFFF:FFFFFFFFFFFF
@A00155:342:HHGFNDSXY:1:1227:21233:27680 1:N:0:GAACCTAG+TCCGCATA
CTTCTCTCGGGCCCACAGCCTTACAGCTGTGGAGCACGGTATCCTCTGCCGAAACAGAGGTTGGACAA
GACCGGAGGGGTCTCCTAGTTCCAAAGGAGATGTACTCCGGGCTTGTTCACGACCTACCGTGTAA
+
::FFFFFFFF:FFFFFFFFFFFFFFFF:F,FFFFFFFFF,FFFFFFFFFFFFF,FFFF::FFFFF:FF
:FFF:FFFF:FF:FF,FFF:F,FFFFF:FFFFFFFFFFFFFFF:FFFFFFFFF:FFFF:FFFFF:
@A00155:342:HHGFNDSXY:1:1227:18620:28322 1:N:0:GAACCTAG+TCCGCATA
CCACATTCCTCTCGGCATTGAGATGACCATAGGCCAGAGGTGAGTCCTTAAGTGGACACAGCTGATCT
AAGGCGGTGTGGCGGGGCATGGGTTTGAACCCCCATGACGGTCGGAGTCTAGTAGGCTCCCTGATGAG
+
FFFFFFFFFFFFFFFFFFFFFFFFFFFFFFFFFFFFFFFFFFFFFFFFFFFFFFFFFFFFFFFFFFFF
FFFFFFFFFFFFFFFFFFFFFFFFFFFFFFFFFFFFFFFFFFFFFFFFFFFFFFFFFFFFFFFFFFFF
@A00155:342:HHGFNDSXY:1:1228:17544:16814 1:N:0:GAACCTAG+TCCGCATA
ATCTCAATGCCGAGAGGGATGTGGAGGGCGAGAGAGAGCGACTTCTCTCGGGCCCACAGCCTTACAGC
TGTGGAGCACGGTATCCTCTGCCGAAACAGAGGTTGGACAAGACCGGAGGGGTCTCCTAGTTCCAAAG
+
FFFFFFFFFFFFFFFFFFFFFFFFFFFFFFFFFF:FFFFFFFFFFFFFFFFFFFFFFFFFFFFFFFFF
FFFFFFFFFFFFFFFFFFFFFFFFFFFFFFFFFFFFFFFF:FFFFFFFFFFFFFFFFFFFFFFFFFFF

```
@A00155:342:HHGFNDSXY:1:1228:6108:17487 1:N:0:GAACCTAG+TCCGCATA
TGTTTCGGCAGAGGATACCGTGCTCCACAGCTGTAAGGCTGTGGGCCCGAGAGAAGTCGCTCTCTCTC
GCCCTTCACATCCCTCTCGGCATTGAGATGACCATAGGCCAGAGGTGAGTCCTTAAGTGGACACAGCT
+
,FFFFFFFFFFFFFFFFFFFFF:FFFF:FFFFFFFFFF:FFFFFFFFFFFFFFFFFFFFFFFFFFFFF
FFFFFFFFFFFFFFFFFFF:FF::::FFF:F:FFFFF:FFFFFFFFFFFFFFFFFFFFFFFFFFFFFF
@A00155:342:HHGFNDSXY:1:1228:10511:18223 1:N:0:GAACCTAG+TCCGCATA
TCTCTCGGGCCCACAGCCTTACAGCTGTGGAGCACGGTATCCTCTGCCGAAACAGAGGTTGGACAAGA
CCGGAGGGGTCTCCTAGTTCCAAAGGAGATGTACTCCGGGCTTGTTCACGACCTACCGTGTAAG
+
FFFFFFFFFFFFFFFFFFFF,FFFFFFFFFFFFFFFFFFFFFFFFFFFFFFFFFFFFFFFFFFFFFFF
FFFFFFFFFFFFFFFFFFFFFFFFFFFFFFFFFFFFFFFFFFFFFFFFFFFFFFFFFFF,FF::F
@A00155:342:HHGFNDSXY:1:1228:22851:20181 1:N:0:GAACCTAG+TCCGCATA
GGTATCCTCTGCCGAAACAGAGGTTGGACAAGACCGGAGGGGTCTCCTAGTTCCAAAGGAGATGTACT
CCGGGCTTGTTCACGACCTACCGTGTAAGTCGTAGTCTAATAGGCTACCTGACGAGTCCTTTTTAGGA
+
FFFFFFFFFFFFFFFFFFFFFFFFFFFFFFFFFFFFFFFFFFFFFFFFFFFFFFFFFFFFFFFFFFFF
FFFFFFFFFFFFFFFFFFFFFFFFFFF:FFFFFFFFFFFFFFFFFFFFFFFFFFFFFFFFFFFFFFFF
@A00155:342:HHGFNDSXY:1:1228:21983:21840 1:N:0:GAACCTAG+TCCGCATA
GGTATCCTCTGCCGAAACAGAGGTTGGACAAGACCGGAGGGGTCTCCTAGTTCCAAAGGAGATGTACT
CCGGGCTTGTTCACGACCTACCGTGTAAGTCGTAGTCTAATAGGCTACCTGACGAGTCCTTTTTAGGA
+
FFFFFFFFFFFFFFFFFFFFFFFFFFFFFFFFFFFFFFFFFFFFFFFF:FFFFFFFFFFFFFFFFFFF
FFFFFFFFFFFFFFFFFFF:FFFFFFF,FFFFFFFFFFFFFFFFFFFFFFFFFFFFFFFFFFFFFFFF
@A00155:342:HHGFNDSXY:1:1228:3794:23813 1:N:0:GAACCTAG+TCCGCATA
GAGCGACTTCTCTCGGGCCCACAGCCTTACAGCTGTGGAGCACGGTATCCTCTGCCGAAACAGAGGTT
GGACAAGACCGGAGGGGTCTCCTAGTTCCAAAGGAGATGTACTCCGGGCTTGTTCACGACCTACCGTG
+
FFFFFFFFFFFFFFFFFFFFFFFFFFFFFFFFFFFFFFFFFFFFFFFFFFFFFFFFFFFFFFFFFFFF
FFFFFF:FFFFFFFFFFFFFFFFFFFFFFFFFFFFFFFFFFFFFFFFFFFFFFFFFFFFFFFFF:FFF
@A00155:342:HHGFNDSXY:1:1228:27977:27054 1:N:0:GAACCTAG+TCCGCATA
TAGTCTAGTAGGCTACCTGACGAGTCCTTTTTAGGACGAAACTTACACAACTCGAAGAGTAACGACGG
TTTCGTCCTTGGAACGGACTCATCAGGGAGCCTACTAGACTCCGACCGTCATGGGGGTTCAAACCCAT
+
FFFFFFFFFFFFFFFFFFFFFFFFFFFF:FFFFFFFFFFFFFFFFFFFFFFFFFFFFFFFFFFFFFFF
FFFFFFF:FFFFFFFFFFFFFFFFFFFFFFFFFFFFFFFFFFFFFFFFFFFFFFF:FFFF,FFFFF
@A00155:342:HHGFNDSXY:1:1228:4390:33270 1:N:0:GAACCTAG+TCCGCATA
AGGACTCACCTCTGGCCTATGGTCATCTCAATGCCGAGAGGGATGTAGAGGGCGAGAGAGAGCGACTT
CTCTCGGGCCCACAGCCTTACAGCTGTGGAGCACGGTATCCTCTGCCGAAACAGAGGTTGGACAAGAC
+
FFFFFFFFFFFFFFFFFFFFFFFFFFFFFFFFFF:FFF,FFFFF:F,FFFFFFFFFFFFFFFFFFFFF
FFFFFFFFFFFFFFFFFFFFFFFFFFFFFFFFFFFFFFFF:FFFFFFFFFFFF:FFFFFFFFFFFFFF
@A00155:342:HHGFNDSXY:1:1229:24252:10300 1:N:0:GAACCTAG+TCCGCATA
AAGGACTCTCCTCTGGCCTATGGTCATCTCAATGCCGAGAGGGATGTGGAGGGCGAGAGAGAGCGACT
TCTCTCGGGCCCACAGCCTTACAGCTGTGGAGCACGGTATCCTCTGCCGAAACAGAGGTTGGACAAGA
+
FFFFFFFFFFFFFFFFFFFFFFFFFFFFFFFFFFFFFFFFFFFFFFF:FFFFFFFFFFFFFFFFFFFF
FFFFFFFFFFFFFFFFFFFFFFFFFFFFFFFFFFFFFFFFFFFFFFFFFFFF,FFFFF:,FFFFFFFFF
@A00155:342:HHGFNDSXY:1:1229:1868:16438 1:N:0:GAACCTAG+TCCGCATA
TGCCCCACCACACCGCCTTAGATCAGCTGTGTCCACTTAAGGACTCACCTCTGGCCTATGGTCATCTC
AATGCCGAGAGGGATGTGGAGGGCGAGAGAGAGCGACTTCTCTCGGGCCCACAGCCTTACAGCTGTGG
+
FFFFFFFFFFFFFFFFF:FFFFFFFFFFFFFFFF:FFFFFFFFFFFFFFFFFFFFFFFFFFFFFFFFFF
FFFFFFF:FFFFFF:FFFFFFFFFFFFFFFFFFFFFFFFFFFFFFFFFFFFFFFFFFFFFFFFFFFF
```

```
@A00155:342:HHGFNDSXY:1:1229:26684:23218 1:N:0:GAACCTAG+TCCGCATA
TCCTCTGCCGAAACAGAGGTTGGACAAGACCGGAGGGGTCTCCTGGTTCCAAAGGAGATGTACTCCGG
GCTTGTTCACGACCTACCGTGTAAGTCGTAGTCTAGTAGGCTACCTGACGAGTCCTTTTTAGGACGAA
+
FFFFFFFFFFFFFFFFFFFFFFFFFFFFFFFFFFFFFFFFFFFFFFFFFFFFFFFFFFFFFFFFFFFF
FFFFFFFFFFFFFFFFFFFFFFFFFFFFFFFFFFFFF:FFFFFFFFFFFFFFFFFFFF:FFFFFFFFFF
@A00155:342:HHGFNDSXY:1:1230:13955:3176 1:N:0:GAACCTAG+TCCGCATA
TGGTCATCTCAATGCCGAGAGGGATGTGGAGGGCGAGAGAGAGCGACTTCTCTCGGGCCCACAGCCTT
ACAGCTGTGGAGCACGGTATCCTCTGCCGAAACAGAGGTTGGACAAGACCGGAGGGGTCTCCTAGTTC
+
FFFFFFFFFFFFFFFFFFFFFFFFFFFFFFFFFFFFFFFFFFFFFFFFFFFFFFFFFFFFFFFFFFFF
FFFFFFFFFFFFFFFF:FFFFFFFFFFFFF,FFFFFFFFFFFFFFF:FFFFFFFFFFFFFFFFFFFFFF
@A00155:342:HHGFNDSXY:1:1230:26187:6417 1:N:0:GAACCTAG+TCCGCATA
ACTTCTCTCGGGCCCACAGCCTTACAGCTGTGGAGCACGGTATCCTCTGCCGAAACAGAGGTTGGACA
AGACCGGAGGGGTCTCCTAGTTCCAAAGGAGATGTACTCCGGGCTTGTTCACGACCTACCGTGTAAGT
+
FFFFFFFFFFFFFFFFFFFFFFFF:FFFFFFFFFFFFFFFFFFFF:FFFFFFFFFFFFFFFFFFFFFFF
FFFFFFFFFFFFFFFFFFFFFFFFFFFFFFFFFFFFFFFFFFFFFFFFFFFFFFFFFFFFFFFFFFFF
@A00155:342:HHGFNDSXY:1:1230:1533:11412 1:N:0:GAACCTAG+TCCGCATA
GGGCGAGAGAGAGCGACTTCTCTCGGGCCCACAGCCTTACAGCTGTGGAGCACGGTATCCTCTGCCGA
AACAGAGGTTGGACAAGACCGGAGGGGTCTCCTAGTTCCAAAGGAGATGTACTCCGGGCTTGTTCACG
+
FFFFFFFFFFFFFFFFFFFFFFFFFFFFFFFFFFFFFFFFFFF,FFFFFFFFFFFFFFFFFFFFFFFF:
FFFFFFFFFFFFFFFFFFFFFFFFFFFFFFFFFFF,FFFFFFFF:FFFFF:F:FFFFFFFFFFFFF:FF
@A00155:342:HHGFNDSXY:1:1230:11749:12571 1:N:0:GAACCTAG+TCCGCATA
TCTGTTTCGGCAGAGGATACCGTGCTCCACAGCTGTAAGGCTGTGGGCCCGAGAGAAGTCGCTCTCTC
TCGCCCTTCACATCCCTCTCGGCATTGAGATGACCATAGGCCAGAGGTGAGTCCTTAAGTGGACACAG
+
FFFFFFFFFFFFFFFFFFFFFFFFFFFFFFFFFFFFFFFFFFFFFFFFFFFFFFFFFFFFFFFFFFFF
FFFFFFFFFFFFFFFFFFFFFFFFFFFFFFFFFFFFFFFFFFFFFFFFFFFFFFFFFFFFFFFFFFFF
@A00155:342:HHGFNDSXY:1:1230:19325:13636 1:N:0:GAACCTAG+TCCGCATA
CCCACAGCCTTACAGCTGTGGAGCACGGTATCCTCTGCCGAAACAGAGGTTGGACAAGAACGGAGGGG
TCTCCTAGTTCCAAAGGAGATGTACTCCGGGCTTGTTCACGACCTACCGTGTAAGTCGTAGTCTAGTA
+
FFFFFFFFFFFFFFFFFFFFFFFFFFFFFFFFFFFFFFFFFFFFFFFFFFFFFFFFFFFFFFFFFFFF
FFFFFFFFFFFFFFFFFFFFFFFFFFFFFFFFFFFFFFFFFFFFFFFFFFFFFFFFFFFFFFFFFFFF
@A00155:342:HHGFNDSXY:1:1230:29017:34773 1:N:0:GAACCTAG+TCCGCATA
CTTCTCTCGGGCCCACAGCCTTACAGCTGTGGAGCACGGTATCCTCTGCCGAAACAGAGGTTGGACAA
GACCGGAGGGGTCTCCTAGTTCCAAAGGAGATGTACTCCGGGCTTGTTCACGACCTACCGTGTAAGTC
+
FFFFFFFFFFFFFFFFFFFFFFFFFFFFFFFFFFFFFFFFFFFFFFFFFFFFFFFFFFFFFFFFFFFF
FFFFFFFFFFFFFFFFFFFFFFFFFFFFFFFFFFFFFFFFFFFFFFFFFFFFFFFFFF:FFFFFFFFF
@A00155:342:HHGFNDSXY:1:1231:18475:12790 1:N:0:GAACCTAG+TCCGCATA
GGATGTGGAGGGCGAGAGAGAGCGACTTCTCTCGGGCCCACAGCCTTACAGCTGTGGAGCACGGTATC
CTCTGCCGAAACAGAGGTTGGACAAGACCGGAGGGGTCTCCTAGTTCCAAAGGAGATGTACTCC
+
FFFFFFFFFFFFFFFFFFFFFFFFFFFFFFFFFFFFFFFFFFFFFFFFFFF:FFFFFFFFFFFFFFFFF
FFFFFFFFFFFFFFFFFFFFFFFFFFFFFFFFFFFFFFFFFFFFFFFFFFFFFFFFFFFFFFFF
@A00155:342:HHGFNDSXY:1:1231:2618:20369 1:N:0:GAACCTAG+TCCGCATA
TCCTCTGCCGAAACAGAGGTTGGACAAGACCGGAGGGGTCTCCTAGTTCCAAAGGAGATGTACTCCGG
GCTTGTTCACGACCTACCGTGTAAGTCGTAGTCTAGTAGGCTACCTGACGAGTCCTTTTTAGGACGAA
+
FFFFFFFFFFFFFFFFFFFFFFFFFFFFFFFFF,FFFFFFFFFFFFFFFFFFFFFFFFFFFFFFFFFFF
FFFFFFFFFFFFFFFFFFFFFFFFFFFFFFFFFFFFFFFFFFFFFFFFFFFFFFFFFFFFFFFFFFFF
```

```
@A00155:342:HHGFNDSXY:1:1231:26404:34162 1:N:0:GAACCTAG+TCCGCATA
AAGGACTCACCTCTGGCCTATGGTCATCTCAATGCCGAGAGGGATGTGAAGGGCGAGAGAGAGCGACT
TCTCTCGGGCCCACAGCCTTACAGCTGTGGAGCACGGTATCCTCTGCCGAAACAGAGGTTGGAC
+
FFFFFFFFFFFFFFFFFFF,FFFFFFFFFFFFFFFFF:F,FFFF::FF,FFFFFFFFFFFFFFFFFFF
FFFFFFFFFFFFFFFFFFFFFFFFFFFFFFFFFFFF:FFFFFFFFFFFFFFFFFFFFFFFFFFFFFFF
@A00155:342:HHGFNDSXY:1:1232:18014:1313 1:N:0:GAACCTAG+TCCGCATA
GAGGGCGAGAGAGAGCGACTTCTCTCGGGCCCACAGCCTTACAGCTGTGGAGCACGGTATCCTCTGCC
GAAACAGAGGTTGGACAAGACCGGAGGGGTCTCCTGGTTCCAAAGGAGATGTACTCCGGGCTTGTTCA
+
FFFFFFFFFFFFFFFFFFFF:FFFFFFFFFFFFFFFFFFFFFFFFFFFFFFFFFFFFFFFFFFFFFFF
FFFFFFFFFFF,FFFFFFFFFFFF:FFFFFF:FFFFFFFFFFFFFFFFFFFFFFFFFFFFFFF:FF:FFF
@A00155:342:HHGFNDSXY:1:1232:12409:19476 1:N:0:GAACCTAG+TCCGCATA
ACCTCTGTTTCGGCAGAGGATACCGTGCTCCACAGCTGTAAGGCTGTGGGCCCGAGAGAAGTCGCTCT
CTCTCGCCCTCCACATCCCTCTCGGCATTGAGATGACCATAGGCCAGAGGAGAGTCCTTAAG
+
FFFFFFFFFFFFFFFFFFFFFFFFFFFFFFFFFFFFFFFFFFFFFFFFFFFFFFFFFFFFFFFFFFFF
FFFFFFFFFFFFFFFFFFFFFFFFFFFFFFFFFFFFFFFFFFFFFFFFFFF,FFFFFFFFFFF
@A00155:342:HHGFNDSXY:1:1232:31105:20353 1:N:0:GAACCTAG+TCCGCATA
CTTCTCTCGGGCCCACAGCCTTACAGCTGTGGAGCACGGTATCCTCTGCCGAAACAGAGGTTGGACAA
GACCGGAGGGGTCTCCTAGTTCCAAAGGAGATGTACTCCGGGCTTGTTCACGACCTACCGTGTAAGTC
+
FFFFFFFFFFFFFFFFFFFFFFFFFFFFFFFFFFFFFFFFFFFFFFFFFFFFFFFFFFFFF:FFFFFFF
FFFFFFFFFFFFFFFFFFFFFFFFFFFFFFFFFFFFFFFFFFFFF:FFFFFFFFFFFFFFFFFFFFFFFFF
@A00155:342:HHGFNDSXY:1:1232:3839:22576 1:N:0:GAACCTAG+TCCGCATA
GTCATCTCAATGCCGAGAGGGATGTGGAGGGCGAGAGAGAGCGACTTCTCTCGGGCCCACAGCCTTAC
AGCTGTGGAGCACGGTATCCTCTGCCGAAACAGAGGTTGGACAAGACCGGAGGGGTCTCCTAGTTCCA
+
FFFFFFFFFFFFFFFFFF:FFFFFFFFFFFFFFFFF,FFFFFFFFFFFFFFFFFFFFFFFFFFFFFF:FF
FFFFFFFFFFFFFFFF:FFFFFFFFF,FFFFFFFFFFFFFFFF:FFF:FFFFFFFFFFFFFFFFFFFF
@A00155:342:HHGFNDSXY:1:1232:30391:31767 1:N:0:GAACCTAG+TCCGCATA
AGCGACTTCTCTCGGGCCCACAGCCTTACAGCTGTGGAGCACGGTATCCTCTGCCGAAACAGAGGTTG
GACAAGACCGGAGGGGTCTCCTGGTTCCAAAGGAGATGTACTCCGGGCTTGTTCACGACCTACCGTGT
+
FFFFFFFFFFFFFFFFFFFFFFFFFFFFFFFFFFFFFFFFFFFFFFFFFFFFFFFFFFFFFFFFFFFF
FFF:FFFFFFFFFFFFFFFFFFFFFFFFFFFFFFFFFFFFFFFFFFFFFFFFFFFFFFFFFFFFFF:FF
@A00155:342:HHGFNDSXY:1:1233:12726:9909 1:N:0:GAACCTAG+TCCGCATA
CAATGCCGAGAGGGATGTGGAGGGCGAGAGAGAGCGACTTCTCTCGGGCCCACAGCCTTACAGCTGTG
GAGCACGGTATCCTCTGCCGAAACAGAGGTTGGACAAGACCGGAGGGGTCTCCTAGTTCCAAAGGAGA
+
FFF,FFFF::FFFFF,FFFFFFFFFFFFFFFFFF,F:F::F:FFF::FFFFFFF:FF,FFFFFF:F,:
FF:FFF,FFFFF,:FF,FFFFFF:FFF:,FFFFFFFFF:FF::FFFF:FFFFFFFFFFFFF:FF:FFF
@A00155:342:HHGFNDSXY:1:1233:4417:26647 1:N:0:GAACCTAG+TCCGCATA
GAGCGACTTCTCTCGGGCCCACAGCCTTACAGCTGTGGAGCACGGTATCCTCTGCCGAAACAGAGGTT
GGACAAGACCGGAGGGGTCTCCTAGTTCCAAAAGAGATGTACTCCGGGCTTGTTCACGACCTACCGTG
+
FFFFFFFFFFFFFFFFFFFFF:FFFF:FFFFFFFFF:FFFFFFFFFFFFFFFFFFFFFFFFFFFFFFFF
FFFFFFFFFFFFFFFFFFFFFFFFFFFFFFFFFFFFFFFFFFFF:FFFFFFFFFFFFFFFFFFFFFFFF
@A00155:342:HHGFNDSXY:1:1234:18656:28416 1:N:0:GAACCTAG+TCCGCATA
ATCTCAATGCCGAGAGGGATGTGGAGGGCGAGAGAGAGCGACTTCTCTCGGGCCCACAGCCTTACAGC
TGTGGAGCACGGTATCCTCTGCCGAAACAGAGGTTGGACAAGACCGGAGGGGTCTCCTGGTTCCAAAG
+
FFFFFFFFFFFFFFFFFFFFFFFFFFFFFFFFFFFFFFFFFFFFFFFF:FFFFFFFFFFFFFFFFF:F:
FFFFFFFFFFFFFFFFFFFFFFFFFFFFFFFFFFFFFFFFFF::FFFFFFFFFFFFFFFFFFFFFFFFFF
```

```
@A00155:342:HHGFNDSXY:1:1235:2211:1501 1:N:0:GAACCTAG+TCCGCATA
GAGAGGGATGTGGAGGGCGAGAGAGAGCGACTTCTCTCGGGCCCACAGCCTTACAGCTGTGGAGCACG
GTATCCTCTGCCGAAACAGAGGTTGGACAAGACCGGAGGGGTCTCCTAGTTCCAAAGGAGATGTACTC
+
FFFFFFFFFFFFFFFFFFFFFFFFFFFFFFFFFFFFFFFFFFFFFFFFFFFFFFFFFFFFFFFFFFFF
FFFFFFFFFFFFFFFFF:FFFFFFFFFFFFFFFFFFFFFFFFFFFFFFFFFFFFFFFFFFFFFFFFFF
@A00155:342:HHGFNDSXY:1:1235:1696:4272 1:N:0:GAACCTAG+TCCGCATA
TTAAGGACTCACCTCTGGCCTATGGTCATCTCAATGCCGAGAGGGATGTGAAGGGCGAGAGAGAGCGA
CTTCTCTCGGGCCCACAGCCTTACAGCTGTGGAGCACGGTATCCTCTGCCGAAACAGAGGTTGGACAA
+
FFFFFFFFFFFFFF,FFFFFFFFFFFFFFFFFFFFFF,F:FF,,FFF:FFFFF:FFFFFFFFFFFFFF
FFFFFFFFF:FFFFFFFFFFFFFF:FFFFF,FFFFFFFFFFF:F,FFFFFFF:FFFFFFF:FF,F,FF
@A00155:342:HHGFNDSXY:1:1235:20491:13808 1:N:0:GAACCTAG+TCCGCATA
TCCTCTGCCGAAACAGAGGTTGGACAAGACCGGAGGGGTCTCCTGGTTCCAAAGGAGATGTACTCCGG
GCTTGTTCACGACCTACCGTGTAAGTCGTAGTCTAGTAGGCTACCTGACGAGTCCTTTTTAGGACGAA
+
FFFFFFFFFFFFFFFFFFFFFFFFFFFFFFFFFFFFFFFFFFFFFFFFFFFFFFFFFFFFFFFFFFFF
FFFFFFFFFFFFFFFFFFFFFFF:FFFFFFFFFFFFFFFFFFFFFFFFFFFFFFFFFFFFFFFFFFFF
@A00155:342:HHGFNDSXY:1:1235:12952:28275 1:N:0:GAACCTAG+TCCGCATA
CACCTCTGGCCTATGGTCATCTCAATGCCGAGAGGGATGTGAAGGGCGAGAGAGAGCGACTTCTCTCG
GGCCCACAGCCTTACAGCTGTGGAGCACGGTATCCTCTGCCGAAACAGAGGTTGGACAAGACCGGAGG
+
FFFFFFF:FFFFFFFFFFFFFFFFFFFF:FFFFFFFFFFFFFFFFFFFFFFFFFFFFFFF:FFFFFFF
FFFFFFF:FFFFFFFFFFFFFFFF:FFFFFFFFFFFFFFFFFFFF,:FFFFFFFFFFFF:FFFFFFFF
@A00155:342:HHGFNDSXY:1:1236:16866:4836 1:N:0:GAACCTAG+TCCGCATA
GCCCACAGCCTTACAGCTGTGGAGCACGGTATCCTCTGCCGAAACAGAGGTTGGACAAGACCGGAGGG
GTCTCCTAGTTCCAAAGGAGATGTACTCCGGGCTTGTTCACGACCTACCGTGTAAGTCGTAGTCTAGT
+
FFFFFFFFFFFF:FFFFFFFFFFFFFFFFFFFFFFFFFFFFFFFFFFFFFFFFFFFFFFFFFFFFFF
FFFFFFFFFFFFFFFFFFFFFFFFFFFFFFFFFFFFFFFFFFFFFFFFFFFFFFFFFFFFFFFFFFFF
@A00155:342:HHGFNDSXY:1:1236:28040:25567 1:N:0:GAACCTAG+TCCGCATA
ACTTCTCTCGGGCCCACAGCCTTACAGCTGTGGAGCACGGTATCCTCTGCCGAAACAGAGGTTGGACA
AGACCGGAGGGGTCTCCTAGTACCAAAGGAGATGTACTCCGGGCTTGTTCACGACCTACCGTGTAAGT
+
FFFFFFFFFFFFFFFFFFFF:FFFFFFFFFFFFFFFFFFFF:FFFFFFFFFFFFFFFFFFFFFFFFFF
FFFFFFFFFFFFFFFFFFFFFFFFFFFF:FFFFFFFFF:FF:FFFFFFFFFFFFFFFFFFFFFFFFFF
@A00155:342:HHGFNDSXY:1:1237:15655:16329 1:N:0:GAACCTAG+TCCGCATA
CTGTGTCCACTTAAGGACTCACCTCTGGCCTATGGTCATCTCAATGCCGAGAGGGATGTAGAGGGCGA
GAGAGAGCGACTTCTCTCGGGCCCACAGCCTTACAGCTGTGGAGCACGGTATCCTCTGCCGAAACAGA
+
FFFFFFFFFFFFFFFFFFFFFFFFFFFFFFFFFFFFFFFFFFFFFFFFFFFFFFFFFFFFFFFFFFFF
FFFFFFFFFFFFFFFFFFFFFFFFFFFFFFFFFFFFFFFFFFFFFFFFFFFFFFFFFFFFFFFFFFFF
@A00155:342:HHGFNDSXY:1:1237:3088:27665 1:N:0:GAACCTAG+TCCGCATA
TGCCGAGAGGGATGTGGAGGGCGAGAGAGAGCGACTTCTCTCGGGCCCACAGCCTTACAGCTGTGGAG
CACGGTATCCTCTGCCGAAACAGAGGTTGGACAAGACCGGAGGGGTCTCCTAGTTCCAAAGGAGATGT
+
FFFFFFFFFFFFFFFFFFFFFFFFFFFFFFFFFFFFFFFFFFFFFFFFFFFFFFFFFFFFFFFFFFFF
FFFFFFFFFFFFFFFFFFFFFFFFFFFFFFFFFFFFFFFFFFFFFFFFFFFFFFFFFFFFFFFFFFFF
@A00155:342:HHGFNDSXY:1:1237:17626:31548 1:N:0:GAACCTAG+TCCGCATA
GAGGGCGAGAGAGAGCGACTTCTCTCGGGCCCACAGCCTTACAGCTGTGGAGCACGGTATCCTCTGCC
GAAACAGAGGTTGGACAAGACCGGAGGGGTCTCCTAGTTCCAAAGGAGATGTACTCCGGGCTTGTTCA
+
FFFFFFFFFFFFFFFFFFFFFFFFFFFFFFFFFFFFFFFFFFFFFFFFFFFFFFFFFFFFFFFFFFFF
FFFFFFFFFFFFFFFFFFFFFFFFFFFFFFFFFFFFFFFFFFFFFF,FFFFF:FFFFFFFFFFFFFFF
```

```
@A00155:342:HHGFNDSXY:1:1238:27434:9674 1:N:0:GAACCTAG+TCCGCATA
CTCAATGCCGAGAGGGATGTGGAGGGCGAGAGAGAGCGACTTCTCTCGGGCCCACAGCCTTACAGCTG
TGGAGCACGGTATCCTCTGCCGAAACAGAGGTTGGACAAGACCGGAGGGGTCTCCTGGTTCCAAAGGA
+
FFFFFFFFFFFFFFFFFFFFFFFFFFFFFFFFFFFFFFFFFFFFFFFFFFFFFFFFFFFFFFFFFFFF
FFFFFFFFFFFFFFFFFFFFFFFFFFFFFFFFFFFFFFFFFFFFFFFFFFFF:FFFFFFFFFFFFFFF
@A00155:342:HHGFNDSXY:1:1240:28230:4758 1:N:0:GAACCTAG+TCCGCATA
ATGCCGAGAGGGATGTAGAGGGCGAGAGAGAGCGACTTCTCTCGGGCCCACAGCCTTACAGCTGTGGA
GCACGGTATCCTCTGCCGAAACAGAGGTTGGACAAGACCGGAGGGGTCTCCTAGTTCCAAAGGAGATG
+
FFFFFFFFFFFFFFF:FFFFFFFFFFFFFFFFF:FFFFFFFFFFFFFFFFFFFFFFFFFFFFFFFFFF
FFFFFFFFFFF:FFFFFFFFFFFFFFFFFFFFFFFFFFFFFFFFFFFFFFFFFFFFFFFFFFFFFFFF
@A00155:342:HHGFNDSXY:1:1240:16233:6934 1:N:0:GAACCTAG+TCCGCATA
TTACAGCTGTGGAGCACGGTATCCTCTGCCGAAACAGAGGTTGGACAAGACCGGAGGGGTCTCCTAGT
TCCAAAGGAGATGTACTCCGGGCTTGTTCACGACCTACCGTGTAAGTCGTAGTCTAGTAGGCTACCTG
+
F:FFFFFFFFFFFFFFFFFFFFFFFFFFFFFFFFFFFFFFFFFFFFFFFFFFFFFFFFFFFFFFFFFF
FFFFFFFFFFFFFFFFFFFFFFFFFFFFFFFFFFFFFFFFFFFFFFFFF:FFFFFFFFFFFFFFFFFF
@A00155:342:HHGFNDSXY:1:1240:4137:9408 1:N:0:GAACCTAG+TCCGCATA
GTGTCCACTTAAGGACTCACCTCTGGCCTATGGTCATCTCAATGCCGAGAGGGATGTAGAGGGCGAGA
GAGAGCGACTTCTCTCGGGCCCACAGCCTTACAGCTGTGGAGCACGGTATCCTCTGCCGAAACAGAGG
+
FFFFFFFFFFFFFFFFFFFFFFFFFFFFFFFFF,FFFF:FFFFFFF:FFFFF:FFFFFFFFFFFFFFF
FFFFFFFFFFFFFFFFFFFFFFFFFFFFFFFFFFFFFFFFFF:F:FFFFFFFFFFFFFFFFFFFFFFF
@A00155:342:HHGFNDSXY:1:1240:22083:10708 1:N:0:GAACCTAG+TCCGCATA
CAGCCTTACAGCTGTGGAGCACGGTATCCTCTGCCGAAACAGAGGTTGGACAAGACCGGAGGGGTCTC
CTAGTTCCAAAGGAGATGTACTCCGGGCTTGTTCACGACCTACCGTGTAAGTCGTAGTCTAGTAGGCT
+
FFFFF:FFFFFFFFFFFFFFFFFFF:FFFFFFFFFFFFFFFFFFFFFFFFFFFFFFFFFFFFFFFFFF
FFFFFFFFFFFFFFFFFFFFFFFFFFFF:FFFFFFFFFFFFFFF:FFFFFFFFFFFFFFFFFFFFFFF
@A00155:342:HHGFNDSXY:1:1240:6370:10864 1:N:0:GAACCTAG+TCCGCATA
CAGCCTTACAGCTGTGGAGCACGGTATCCTCTGCTGAAACAGAGGTTGGACAAGACCGGAGGGGTCTC
CTAGTTCCAAAGGAGATGTACTCCGGGCTTGTTCACGACCTACCGTGTAAGTCGTAGTCTAGTAGGCT
+
FFFFF:FFFFFFFFFFFFFFFFFFFFFFFFFFFFFFFFFFFFFFFFFFFFF,FFFFFFFFFFFFFFFF
FFFFFFFFFFFFFFFFFFFFFFFFFFFF:FFFFFFFFFFFFFFFFFFFFFFFFFFFFFFFFFFFFFFF
@A00155:342:HHGFNDSXY:1:1240:4580:18881 1:N:0:GAACCTAG+TCCGCATA
CTTCTCTCGGGCCCACAGCCTTACAGCTGTGGAGCACGGTATCCTCTGCCGAAACAGAGGTTGTACAA
GACCGGAGGGGTCTCCTAGTTCCAAAGGAGATGTACTCCGGGCTTGTTCACGACCTACCGTGTAAGTC
+
FFFFF:FF,FFFFFFFF:F::,:FFFFF:,,FF,F:F:F::,FF,F,FF,FFFFFFFFF:FFF,FFFF
:FF:F:F,F:,FFFFFFFFFFFFFF::,FFFF,FFFFFF:F:F,,::FFFF:F,FFFFFFFF,FF:FF
@A00155:342:HHGFNDSXY:1:1240:21025:27414 1:N:0:GAACCTAG+TCCGCATA
TTAGATCAGCTGTGTCCACTTAAGGACTCACCTCTGGCCTATGGTCATCTCAATGCCGAGAGGGATGT
GAAGGGCGAGAGAGAGCGACTTCTCTCGGGCCCACAGCCTTACAGCTGTGGAGCACGGTATCCTCTGC
+
FFFFFFFFFFFFFFFFFF:FFFFFFFFFFFFFFFFFFFFFFFFFFFFFFFFFF,FFFFFFFFFFF:FF
FFFFFFFFFFFF:FFFFFFFFFFFFFFFFFFFFFFFFFFFF,FFFFFFFFFFFFFFFFFF,FFFFFFFF
@A00155:342:HHGFNDSXY:1:1240:20754:28604 1:N:0:GAACCTAG+TCCGCATA
TTAGATCAGCTGTGTCCACTTAAGGACTCACCTCTGGCCTATGGTCATCTCAATGCCGAGAGGGATGT
GAAGGGCGAGAGAGAGCGACTTCTCTCGGGCCCACAGCCTTACAGCTGTGGAGCACGGTATCCTCTGC
+
FFFFFFFFFFFFFFFFFFFF,FFFFFFFFFFFFFFFFFFFFFFFFFFFFFFFFFFFFFFFFFFFFFFF
FFFFFFFFFFFFFFFFFFFFFFFFFFFFFFFFFFFFFFFFFFFFFFFFFFFFFFFFFFFF:FFFFFFF
```

```
@A00155:342:HHGFNDSXY:1:1240:4255:31344 1:N:0:GAACCTAG+TCCGCATA
CTGGCCTATGGTCATCTCAATGCCGAGAGGGATGTGAAGGGCGAGAGAGAGCGACTTCTCTCGGGCCC
ACAGCCTTACAGCTGTGGAGCACGGTATCCTCTGCCGAAACAGAGGTTGGACAAGACCGGAG
+
FFFFFFFFFFFFFFFFFFFFFFFFFFFFFFF,FF:FFFFFFFFFFFFFFFFFFFFFFFFFFFFFFFFF
FFFFFFFF,FFFFFFFFFFFFFFFFF:FFFFFFFFFFFFFF:FFFF:FFFFFFFFFFFFFFF
@A00155:342:HHGFNDSXY:1:1240:17427:34021 1:N:0:GAACCTAG+TCCGCATA
GACTTCTCTCGGGCCCACAGCCTTACAGCTGTGGAGCACGGTATCCTCTGCCGAAACAGAGGTTGGAC
AAGACCGGAGGGGTCTCCTAGTTCCAAAGGAGATGTACTCCGGGCTTGTTCACGACCTACCGTGTAAG
+
FFFFFFFFFFFFFFFFFFFFFFFF,FFFFF:FFFFFFFFFFFFFFF:FFFFFFFFFFFFFFFFFFFFF
FF:FFFFFFFFFFFFFFFFFF,FFFFFFFFFFFFFF:FFFFFFFF,FFFFFFFFFFFFFFFFFFFFF:
@A00155:342:HHGFNDSXY:1:1241:6361:3114 1:N:0:GAACCTAG+TCCGCATA
GAGGGCGAGAGAGAGCGACTTCTCTCGGGCCCACAGCCTTACAGCTGTGGAGCACGGTATCCTCTGCC
GAAACAGAGGTTGGACAAGACCGGAGGGGTCTCCTAGTTCCAAAGGAGATGTACTCCGGGCTTGTTCA
+
FFFFFFFFFFFFFFFFFFFFFFFFF:FFF,FFFFFFFFFFFFFF:FFF:FFFFFFFFFFFFFFFFFFF
FFFFFFFFFFFFFFFFFFFFFFFFFFFFFFFFFFFFFFFFFFFFFFFFFFFFFFFFFFFFFFFFFFFF
@A00155:342:HHGFNDSXY:1:1241:27416:11365 1:N:0:GAACCTAG+TCCGCATA
GGATGTGGAGGGCGAGAGAGAGCGACTTCTCTCGGGCCCACAGCCTTACAGCTGTGGAGCACGGTATC
CTCTGCCGAAACAGAGGTTGGACAAGACCGGAGGGGTCTCCTAGTTCCAAAGGAGATGTACTCC
+
FFFFFFFFFFFFFFFFFFFFFFFFF:FFFFFFFFFFFFFFFFFFFFFFFFFFFFFFFFFFFFFFFFFF
FFFFFFFFFFFFFFFFFFFFFFFFFFFFFFFFFFFFFFFFFFFFFFFFFFFFFFFFFFFFFFFF
@A00155:342:HHGFNDSXY:1:1241:9661:33379 1:N:0:GAACCTAG+TCCGCATA
GACTTCTCTCGGGCCCACAGCCTTACAGCTGTGGAGCACGGTATCCTCTGCCGAAACAGAGGTTGGAC
AAGACCGGAGGGGTCTCCTAGTTCCAAAGGAGATGTACTCCGGGCTTGTTCACGACCTACCGTGTAAG
+
FFFFFFFFFFFFFFFFFFFFFFFFFFFFFFFFFFFFFFFFFFFFFFFFFFFFFFFFFFFFFFFFFFFF
FFFFFFFFFFFFFFFFFFFFFFFFFFFFFFFFFFFFFFFFFFFFFFFFFFFFFFFFFFFFFFFFFF:F
@A00155:342:HHGFNDSXY:1:1242:6271:1141 1:N:0:GAACCTAG+TCCGCATA
CCATGCCCCGCCACACCGCCTTAGATCAGCTGTGTCCACTTAAGGACTCACCTCTGGCCTATGGTCAT
CTCAATGCCGAGAGGGATGTGGAGGGCGAGAGAGAGCGACTTCTCTCGGGCCCACAGCCTTACAGCTG
+
FFFFFFFFFFFFFFFFFFF:F,FFFFFFFFFFFFFFFFF:FFFFFFFFFFFFFFFFFFFFFFFFFFFF
FFFFFFFFFFFFFFFFFFFFFFFFFFFFFFFFFFFFFFFFFFFFFFFFFFFFFFFFFFFFFFFFFFFF
@A00155:342:HHGFNDSXY:1:1242:6207:28025 1:N:0:GAACCTAG+TCCGCATA
AGAGAGCGACTTCTCTCGGGCCCACAGCCTTACAGCTGTGGAGCACGGTATCCTCTGCCGAAACAGAG
GTTGGACAAGACCGGAGGGGTCTCCTAGTTCCAAAGGAGATGTACTCCGGGCTTGTTCACGACCTACC
+
FFFFFFFFFFFFFFFFFFFFFFFFFFFFFFFFFFFFFFFFFFFFFFFFF:FFFFFFFFFFFFFFFFFF
FFFFFFFFFFFFFFFFFFFFFFFFFFFFFFFFFFFFFFFF:FFFFFFFFFFFFFFFFFFFFFFFFFFF
@A00155:342:HHGFNDSXY:1:1243:31855:10347 1:N:0:GAACCTAG+TCCGCATA
CCATGCCCCGCCACACCGCCTTAGATCAGCTGTGTCCACTTAAGGACTCACCTCTGGCCTATGGTCAT
CTCAATGCCGAGAGGGATGTGGAGGGCGAGAGAGAGCGACTTCTCTCGGGCCCACAGCCTTACAGCTG
+
FFFFFFFFFFFFFFFFFFFF:FFFF:FFFFFFFFFFFFFF,F,FFFFFFFFFFFFFFFFFFFFFFFFF
FFFFFFFFFFFFFFFFF,FFFFFFFFFFFFFFFFFFFFFFFFFFFFFFFFFFFFFFFFF:F:FFFFFF
@A00155:342:HHGFNDSXY:1:1243:8748:19272 1:N:0:GAACCTAG+TCCGCATA
AGAGAGAGCGACTTCTCTCGGGCCCACAGCCTTACAGCTGTGGAGCACGGTATCCTCTGCCGAAACAG
AGGTTGGACAAGACCGGAGGGGTCTCCTAGTTCCAAAGGAGATGTACTCCGGGCTTGTTCACGACC
+
FFFFFFFFFFFFFFFFFFFFFFFFFFFFF:FFFFFFFFFFFF:FFFFFFFFFFF:FFFFFFFFFFFFF
FFFFFFFFFFFFFFFFFFFFFFFFF:FFFF:FFFFFFFFFFFFFFFFFFFFFFFFF:FFFFFFFF:
```

```
@A00155:342:HHGFNDSXY:1:1243:18611:20384 1:N:0:GAACCTAG+TCCGCATA
AGACTCCGACCGTCATGGGGGTTCAAACCCATGCCCCACCACACCGCCTTAGATCAGCTGTGTCCACT
TAAGGACTCACCTCTGGCCTATGGTCATCTCAATGCCGAGAGGGATGTGGAGGGCGAGAGAGAGCGAC
+
FFFFFFFFFFF:FFFFFFFFFFFFFFFFFFFFFFFFFFFFFFFFFFFFFFFFFFFFFFFFFFFFFFFF
FFFFF:FFFFFFFFFFFFFFFFFF:FFFFFFFFFFFFFFFFFFF:FFFFFFFFFFFFFFFFFFFFFFFF
@A00155:342:HHGFNDSXY:1:1243:29451:30514 1:N:0:GAACCTAG+TCCGCATA
AGAGAGAGCGACTTCTCTCGGGCCCACAGCCTTACAGCTGTGGAGCACGGTATCCTCTGCCGAAACAG
AGGTTGGACAAGACCGGAGGGGTCTCCTAGTTCCAAAGGAGATGTACTCCGGGCTTGTTCACGACCTA
+
FFFFFFFFFFFFFFFFFFFFFFFFFFFFFFFFFFFFFFFFFFFFFFFFFFFFFFF:FFFFFFFFFFFF
FFFFFFFFFFFFFFFFFFFFFFFFFFFFFFFFFFFFFFFFFFFFFFFFFFFFFFFFFFFFFFFFFFFF
@A00155:342:HHGFNDSXY:1:1244:26856:6167 1:N:0:GAACCTAG+TCCGCATA
CTTCTCTCGGGCCCACAGCCTTACAGCTGTGGAGCACGGTATCCTCTGCCGAAACAGAGGTTGGACAA
GACCGGAGGGGTCTCCTGGTTCCAAAGGAGATGTACTCCGGGCTTGTTCACGACCTACCGTGTAAGTC
+
FFFFFFFFFFFFFFFFFFFFFFFFFFFFFFFFFFFFFFFFFFFFFFFFFFFFFFFFFFFFFFFFFFFF
FFFFFFFFFFFFFFFFFFFFFFFFFFFFFFFFFFFFFFFFFFFFFFFFFFFFFFFFF:FFFFFFFFF
@A00155:342:HHGFNDSXY:1:1244:20998:8422 1:N:0:GAACCTAG+TCCGCATA
GAGGGCGAGAGAGAGCGACTTCTCTCGGGCCCACAGCCTTACAGCTGTGGAGCACGGTATCCTCTGCC
GAAACAGAGGTTGGACAAGACCGGAGGGGTCTCCTGGTTCCAAAGGAGATGTACTCCGGGCTTGTTCA
+
FFFF:FFFFFFFFF::FFFFFFFFFFF:FFFFFFFFFF,FFF:FFFFFFFFFFFF:F::F::FFF:FF
FFFFFFFFFFFFFFFFF:FFFFFFFFFFFFFFFFFFFFFFFFFFFFFFFFFFF:FFFFFFFFFFFFFF:FF
@A00155:342:HHGFNDSXY:1:1244:32931:10300 1:N:0:GAACCTAG+TCCGCATA
CTTCTCTCGGGCCCACAGCCTTACAGCTGTGGAGCACGGTATCCTCTGCCGAAACAGAGGTTGGACAA
GACCGGAGGGGTCTCCTAGTTCCAAAGGAGATGTACTCCGGGCTTGTTCACGACCTACCGTGTAAGTC
+
FFFFFFFFFFFFFFFFFFFFFFFFFFFFFFFFFFFFFFFF:FFFFFFFFFFFFFFFFFFFFFFFFFFF
FFF:FFFFFFFFFFFFFFFFFFFFFFFFFF:FFFFFFFFFFFFF:FFFFFFFFFFFFFFFFFFFFFFFF
@A00155:342:HHGFNDSXY:1:1244:5846:33536 1:N:0:GAACCTAG+TCCGCATA
GAGCGACTTCTCTCGGGCCCACAGCCTTACAGCTGTGGAGCACGGTATCCTCTGCCGAAACAGAGGTT
GGACAAGACCGGAGGGGTCTCCTGGTTCCAAAGGAGATGTACTCCGGGCTTGTTCACGACCTACCGTG
+
FFFFFFFFFFFFFFFFFFFFFFFFFFFFFFFFFFFFFFFFFFFFFFFFFFFFFFFFFFFFFFFFFFFF
FFFFFFFFFFFFFFFFFFFFFFFFFFFFFFFFFFFFFFFFFFFFFFFFFFFFFFFFFFFFFFFFFFFF
@A00155:342:HHGFNDSXY:1:1245:22788:4319 1:N:0:GAACCTAG+TCCGCATA
ATCTCAATGCCGAGAGGGATGTGGAGGGCGAGAGAGAGCGACTTCTCTCGGGCCCACAGCCTTACAGC
TGTGGAGCACGGTATCCTCTGCCGAAACAGAGGTTGGACAAGACCGGAGGGGTCTCCTAGTTCCAAAG
+
FFFFFFFFFFFFFFFFFFFFFFFFFFFFFFFFFFFFFFFFFFFFFFFFFFFFFFFFFFFFFFFFFFFF
FFFFFFFFFFFFFFFFFFFFFFFFFFFFFFFFFFFFFFFFFFFFFFFFFFFFFFFFFFFFFFFFFF:F
@A00155:342:HHGFNDSXY:1:1245:14018:8015 1:N:0:GAACCTAG+TCCGCATA
CTCAATGCCGAGAGGGATGTGGAGGGCGAGAGAGAGCGACTTCTCTCGGGCCCACAGCCTTACAGCTG
TGGAGCACGGTATCCTCTGCCGAAACAGAGGTTGGACAAGACCGGAGGGGTCTCCTGGTTCCAAAGGA
+
FFFFFFFFFFFFFFFFFFF,FFFFFFFFFFFFFFFFFFFFFFFFFFFFFFFFFFFFFFFFFFFFFFFF
FFFFFFFFFFFFFFFFFFFFFFFFFFFFFFFFFFFFFFFFFFFFFFFFFFFFFFFFFFFFFFFFFFFF
@A00155:342:HHGFNDSXY:1:1245:21558:27054 1:N:0:GAACCTAG+TCCGCATA
AGAGAGAGCGACTTCTCTCGGGCCCACAGCCTTACAGCTGTGGAGCACGGTATCCTCTGCCGAAACAG
AGGTTGGACAAGACCGGAGGGGTCTCCTAGTTCCAAAGGAGATGTACTCCGGGCTTGTTCACGACCTA
+
FFFFFFFFFFFFFFFFFFFFFFFFFFFFFFFFFFFFFFFFFFFFFFFFFFFFFFFFFFFFFFFFFFFF
FFFFFFFFFFFFFFFFFFFFFFFFFFFFFFFFFFFFFFFFFFFFFFFFFFF:FFFFFFFFFFFFFFFF
```

```
@A00155:342:HHGFNDSXY:1:1245:14986:30953 1:N:0:GAACCTAG+TCCGCATA
GAGAGGGATGTGGAGGGCGAGAGAGAGCGACTTCTCTCGGGCCCACAGCCTTACAGCTGTGGAGCACG
GTATCCTCTGCCGAAACAGAGGTTGGACAAGACCGGAGGGGTCTCCTAGTTCCAAAGGAGATGTACTC
+
F:FFFFFFFFFFFFFFFFFFFFFFFFFFFFFFFFFFFFFFFFFFFFFFFFFFFFFFFFFFFFFFFFFF
FFFFFFFFFFFFFF,FFFFFFFFFFFFFFFFFFFFFFFFFFFFFFFFFFFFFFFFFFFFFFFFFFFFF
@A00155:342:HHGFNDSXY:1:1246:11921:10457 1:N:0:GAACCTAG+TCCGCATA
AGAGAGAGCGACTTCTCTCGGGCCCACAGCCTTACAGCTGTGGAGCACGGTATCCTCTGCCGAAACAG
AGGTTGGACAAGACCGGAGGGGTCTCCTAGTTCCAAAGGAGATGTACTCCGGGCTTGTTCACGACCT
+
FFFFFFFFFFFFFFFFFFFFFFFFFFFFFFFFFFFFFFFFFF:FFFFFFFFFFFFFFFFFFFFFFFFF
FFFFFFFFFFFFFFFFFFFFFFFFFFFFFFFFFFFFFFFFFFFFFFFFFFFFFFFFFFFFFFFFFFF
@A00155:342:HHGFNDSXY:1:1246:17363:14215 1:N:0:GAACCTAG+TCCGCATA
ATGCCGAGAGGGATGTAGAGGGCGAGAGAGAGCGACTTCTCTCGGGCCCACAGCCTTACAGCTGTGGA
GCACGGTATCCTCTGCCGAAACAGAGGTTGGACAAGACCGGAGGGGTCTCCTAGTTCCAAAGGAGATG
+
FFFFFFFFFFFFFFFFFFFFFFFFFFFFFFFFFFFFFFFFFFFFFFFFFFFFFFFFFFFFFFFFFFFF
FFFFFFFFFFFFFFFFFFFFFFFFFFFFFFFFFFFFFFFFFFFFFFFFFFFFFFFFFFFFFFFFFFFF
@A00155:342:HHGFNDSXY:1:1246:23999:14747 1:N:0:GAACCTAG+TCCGCATA
CGAGAGAGAGCGACTTCTCTCGGGCCCACAGCCTTACAGCTGTGGAGCACGGTATCCTCTGCCGAAAC
AGAGGTTGGACAAGACCGGAGGGGTCTCCTAGTTCCAAAGGAGATGTACTCCGGGCTTGTTCACGACC
+
:FFFFFFFFFFFFFFFFFFFFFFFFFFFFFFF,FFFFFFFFFFFFFFFFFFFFFFFFFFFFFFFFFFF
FFFFFFFFFFFFFFFFFFFFFFFFFFFFFF:FFFFF:FFF::FFFFFFFFFFFFFFFFFFFFFFFFFF
@A00155:342:HHGFNDSXY:1:1247:30779:13463 1:N:0:GAACCTAG+TCCGCATA
GTCTAGTAGGCTACCTGACGAGTCCTTTTTAGGACGAAACTTACACAACTCGAAGAGTAACGACGGTT
TCGTCCTTGGAACGGACTCATCAGGGAGCCTACTAGACTCCGACCGTCATGGGGGTTCAAACCCATGC
+
FFFFFFFFFFFFFFFFFFFFFFFFFFFFFFFFFFFFFFFFFFFFFFFFFFFFFFFFFFFFFFFFFFFF
FFFFFF:FFFFFFFFFFFFFFFFFFFFFFFFFFFFFFFFFFFFFFFFFFFFFFFFFFFFFFFFFFFFF
@A00155:342:HHGFNDSXY:1:1247:6225:20384 1:N:0:GAACCTAG+TCCGCATA
GGATGTGGAGGGCGAGAGAGAGCGACTTCTCTCGGGCCCACAGCCTTACAGCTGTGGAGCACGGTATC
CTCTGCCGAAACAGAGGTTGGACAAGACCGGAGGGGTCTCCTAGTTCCAAAGGAGATGTACTCC
+
FFFFF::FFF,FFFFFFFFFFFFFFF:F:FFFFF:FFFFFF,FFF,,,FFFF:FFF:FFFFFFFFFFF
FFFFFFFFFFFFFFFFFFFFF,FF::FFF:FFFFF:FFFFFF:FFFF:FFFFFFFFFF:FFFFF
@A00155:342:HHGFNDSXY:1:1247:23854:34194 1:N:0:GAACCTAG+TCCGCATA
AGAGAGAGCGACTTCTCTCGGGCCCACAGCCTTACAGCTGTGGAGCACGGTATCCTCTGCCGAAACAG
AGGTTGGACAAGACCGGAGGGGTCTCCTAGTTCCAAAGGAGATGTACTCCGGGCTTGTTCACGACCTA
+
FFFFFFFFFFFFFFFFFFFFFFFFFFFFFFFFFFFFFFFFFFFFFFFFFFFFFFFFFFFFFFFFFFFF
FFFFFFFFFFFFFFFFFFFFFFFFFFFFFFFFFFFFFFFFFFFFFFFFFFFFFFFFFFFFFFFFFFFF
@A00155:342:HHGFNDSXY:1:1247:4969:34366 1:N:0:GAACCTAG+TCCGCATA
TTCTCTCGGGCCCACAGCCTTACAGCTGTGGAGCACGGTATCCTCTGCCGAAACAGAGGTTGGACAAG
ACCGGAGGGGTCTCCTAGTTCCAAAGGAGATGTACTCCGGGCTTGTTCACGACCTACCGTGTAAGTCG
+
FFFFFFFFFFFFFFFF,FFFF::FFF:FFFFFFFFFFFFFFFF:FFFF:FFFFFFFFFFFFFFFFFF,
FFFFFFFFFFFFFFFFFFFFFFFFFFFF:FFFFFFFFFFFF,,FFFFFFFFFFFFFFFFFFFFFFFFF
@A00155:342:HHGFNDSXY:1:1248:14371:23093 1:N:0:GAACCTAG+TCCGCATA
CTCTCTCTCGCCCTCCACATCCCTCTCGGCATTGAGATGACCATAGGCCAGAGGTGAGTCCTTAAGTG
GACACAGCTGATCTAAGGCGGTGTGGCGGGGCATGGGTTTGAACCCCCATGACGGTCGGAGTCTAGTA
+
FFFFFFFFFFFFFFFFFFFFFFFFFFFFFFFFFFFFFFFFFF,FFFFFFFFFFFFFFFFFFFFFFFFF
FFFFFFFFFFFFFFFFFFFFFFFFFFFFFFFFFFFFF:FFFFFFFFFFFFFFFFFFFFFFFFFFFFFF
```

@A00155:342:HHGFNDSXY:1:1248:19497:35321 1:N:0:GAACCTAG+TCCGCATA
ATCTCAATGCCGAGAGGGATGTGGAGGGCGAGAGAGAGCGACTTCTCTCGGGCCCACAGCCTTACAGC
TGTGGAGCACGGTATCCTCTGCCGAAACAGAGGTTGGACAAGACCGGAGGGGTCTCCTAGTTCCAAAG
+
FFFFFFFFFF,FFFFFFFFFFFFFFFFFFFFFFFF:FFFFF:FFFFFFFFFFFFFFFFFF,FFFFF,F
FFFFFF:FFF,FFFFFFF:FFFFFFFF::FFFF:::FF:FF:FFFFFFFF,FFFFFFFFFF:FFFFFF
@A00155:342:HHGFNDSXY:1:1249:27046:15922 1:N:0:GAACCTAG+TCCGCATA
CGAGAGAGAGCGACTTCTCTCGGGCCCACAGCCTTACAGCTGTGGAGCACGGTATCCTCTGCCGAAAC
AGAGGTTGGACAAGACCGGAGGGGTCTCCTAGTTCCAAAGGAGATGTACTCCGGGCTTGTTCACGACC
+
FFFFFFFFFFFFFFFFFFFFFFFFFFFFFFFFFFFFFFFFFFFFFFFFFFFFFFFFFFFFFFFFFFFF
FFFFFFFFFFFFFFFFFFFFFFFFFFFFFFFFFFFFFFFFFFFFFFFFFFFFFFFFFFFFFFFFFFFF
@A00155:342:HHGFNDSXY:1:1249:22299:20415 1:N:0:GAACCTAG+TCCGCATA
TATGGTCATCTCAATGCCGAGAGGGATGTGGAGGGCGAGAGAGAGCGACTTCTCTCGGGCCCACAGCC
TTACAGCTGTGGAGCACGGTATCCTCTGCCGAAACAGAGGTTGGACAAGACCGGAGGGGTCTCCTAGT
+
FFFFFFFFFFFFFFFFFFFFF:FFFFFFFFFFFFFFFFFFFFFFFFFFFFFFFFFFFFFFFFFFFFFF
,FFFFFFFFFFFFFFFFFFFFFFFFFFFFFFFFFFFFFFFFFFFFFFFFFFFFFFFFFFFFFFFF:FF
@A00155:342:HHGFNDSXY:1:1249:8006:26036 1:N:0:GAACCTAG+TCCGCATA
GATGTGGAGGGCGAGAGAGAGCGACTTCTCTCGGGCCCACAGCCTTACAGCTGTGGAGCACGGTATCC
TCTGCCGAAACAGAGGTTGGACAAGACCGGAGGGGTCTCCTAGTTCCAAAGGAGATGTACTCCGGGCT
+
FFFFFFFFFFFFFFFFFFFFFFFFFFFFFFFFFFFF,FFFFFFF:FFFFFFFFFFFFFFFFFFFFFFF
FFFFFFFFFFFFFFFFF,FFFFFF,FFFFFFFFFFFFFFFFFFFFFF:FFFFFFFFFFFFFFFFFFFF
@A00155:342:HHGFNDSXY:1:1249:28330:33458 1:N:0:GAACCTAG+TCCGCATA
ATAGGCCAGAGGTGAGTCCTTAAGTGGACACAGCTGATCTAAGGCGGTGTGGCGGGGCATGGGTTTGA
ACCCCCATGACGGTCGGAGTCTAGTAGGCTCCCTGATGAGTCCGTTCCAAGGACGAAACCGTCGTTAC
+
FFFFFFF:FFF:FFFFFFFFFFFFFFFFF:FFFFFFFFFFFFF:,FFFFFFFFFFFF:FFFFFFFF,F
FFFFF:FFFFFFFFF,,FFFFFFFFFFF:FFFFFFFFFFF,F,FFFFFFF::FFFF,:FFFFFF:F::
@A00155:342:HHGFNDSXY:1:1249:26964:35571 1:N:0:GAACCTAG+TCCGCATA
AGCGACTTCTCTCGGGCCCACAGCCTTACAGCTGTGGAGCACGGTATCCTCTGCCGAAACAGAGGTTG
GACAAGACCGGAGGGGTCTCCTGGTTCCAAAGGAGATGTACTCCGGGCTTGTTCACGACCTACCGTGT
+
FFFFFFFFFFFFFFFFFFFFFFFFFFFFFFFFFFFF:FFFFFFFFFFF:FFFFFFFFFFFFFFFFFFF
FFFFFFFFFFFFFFFFFFFFFFF:FFFFFFFFFFFFFF:FFFFFFFFFFFFFFFFFFFF:FFFFFFFF
@A00155:342:HHGFNDSXY:1:1250:32985:2973 1:N:0:GAACCTAG+TCCGCATA
CTATGGTCATCTCAATGCCGAGAGGGAGGTGGAGGGCGAGAGAGAGCGACTTCTCTCGGGCCCACAGC
CTTACAGCTGTGGAGCACGGTATCCTCTGCCGAAACAGAGGTTGGACAAGACCGGAGGGGTCTCCTAG
+
FF:FFFFFFFFFFFFFFFFFFFFFFFF,F:FFFFFFFFFFFFFFFFFFFF,:FFFFFFFFFFFFFFFF
FFF:F,FFFFFFFFFFFFFFFFFFFFFFFFFFFFFFFFFFF,FFF,F:FFFFFFFFFFFFFFFFFF,F
@A00155:342:HHGFNDSXY:1:1250:29640:8108 1:N:0:GAACCTAG+TCCGCATA
TGCCGAGAGGGATGTGGAGGGCGAGAGAGAGCGACTTCTCTCGGGCCCACAGCCTTACAGCTGTGGAG
CACGGTATCCTCTGCCGAAACAGAGGTTGGACAAGACCGGAGGGGTCTCCTAGTTCCAAAGGAGATGT
+
FFFFFFFFFFFF:FFFFFFFF:FFFFFFFFFFFFFFFFFFFFFFFFFFFFFFFFFFFFFFFFFFFFFF
FFFFFFFFFFFFFFFFFFFFFFFFFFFFFFFFFFFFFFFFFFFFFFFFFFF:FFFFFFFFFFFFFFFF
@A00155:342:HHGFNDSXY:1:1250:30553:27915 1:N:0:GAACCTAG+TCCGCATA
GGGCGAGAGAGAGCGACTTCTCTCGGGCCCACAGCCTTACAGCTGTGGAGCACGGTATCCTCTGCCGA
AACAGAGGTTGGACAAGACCGGAGGGGTCTCCTAGTTCCAAAGGAGATGTACTCCGGGCTTGTTCACG
+
FFFFFFFFFFFFFFFFFFFFFFFFFFFFFFFFFFFF:FFFFFFFFFFFFFFFFFFFFFFFFFFFFFFF
FFFFFFFFFFFFFFFFFFFFFFFFFFFFFFFFFFFFFFFFFFFFFFFFFFFFFFFFFFFFFFFFFFFF

```
@A00155:342:HHGFNDSXY:1:1250:3893:34256 1:N:0:GAACCTAG+TCCGCATA
ACCGTGCTCCACAGCTGTAAGGCTGTGGGCCCGAGAGAAGTCGCTCTCTCTCGCCCTTCACATCCCTC
TCGGCATTGAGATGACCATAGGCCAGAGGTGAGTCCTTAAGTGGACACAGCTGATCTAAGGCGGTGTG
+
FFFFFFFFFFFFFFFFFFFF:FFFFFF:FFFFF:FFFFFFFFFFFFFFFF:FFFFFFFFFFFFFF
FFFFFFFFFFFFFFFFFFFFFFFFFFFFFFFFFFFFFFFFFFFFFFFF:FFFFFFFFFFF::FFF
@A00155:342:HHGFNDSXY:1:1251:30192:6433 1:N:0:GAACCTAG+TCCGCATA
AGACTCACCTCTGGCCTATGGTCATCTCAATGCCGAGAGGGATGTGGAGGGCGAGAGAGAGCGACTTC
TCTCGGGCCCACAGCCTTACAGCTGTGGAGCACGGTATCCTCTGCCGAAACAGAGGTTGGACAAGACC
+
FFFFFFFFFFFFFFFFFFFFFFFFFFFFFFFFFFFFFFFFF:FFFFFFFFFFFFFFFFFFFFFFFFF
FFFFFFFFFFFFFFFFFFFFFFFFFFFFFFFFFFFFFFFFF:FFFFFFFFFFFFFFFFFFFFFFFFFF
@A00155:342:HHGFNDSXY:1:1251:32316:15843 1:N:0:GAACCTAG+TCCGCATA
GGATGTAGAGGGCGAGAGAGAGCGACTTCTCTCGGGCCCACAGCCTTACAGCTGTGGAGCACGGTATC
CTCTGCCGAAACAGAGGTTGGACAAGACCGGAGGGGTCTCCTAGTTCCAAAGGAGATGTACTCC
+
FFFFF,FFFFFFFFFFFFFFF:FFF:FF:FFFFFFFFFFFFFF:FFFFFFFFFFFFFFFFFFFFF:
FFFFFFFFFFFFFFFFFFFFF:FF,FFFFFFFFFFFFFFFF:FFFFFFFFFFFFFFFFFFFFF
@A00155:342:HHGFNDSXY:1:1252:16550:4225 1:N:0:GAACCTAG+TCCGCATA
CTTCTCTCGGGCCCACAGCCTTACAGCTGTGGAGCACGGTATCCTCTGCCGAAACAGAGGTTGGACAA
GACCGGAGGGGTCTCCTAGTTCCAAAGGAGATGTACTCCGGGCTTGTTCACGACCTACCGTGTAAGTC
+
FFFFFFFFFFFFFFFFFFFFFFFFFFFFFFFFFFFFFFFFFFFFFFFFFFFFFFFFFFFFFFFFFFF
FFFFFFFFFFFFFFFFFFFFFFFFFFFFFFFFFFFFFFFFFFFFFFFFFFFFFFFFFFFFFFFFFFF
@A00155:342:HHGFNDSXY:1:1252:14805:12164 1:N:0:GAACCTAG+TCCGCATA
TGTTTCGGCAGAGGATACCGTGCTCCACAGCTGTAAGGCTGTGGGCCCGAGAGAAGTCGCTCTCTCTC
GCCCTCCACATCCCTCTCGGCATTGAGATGACCATAGGCCAGAGGTGAGTCCTTAAGTGGACACAGCT
+
FFFFFFFFF:FFFFFFFFFFFFFFFFFFFFFFFFFFFFFFFFFFFFFFFFFFFFFFFFFFFFFFFFF
FFFFFFFFFFFFFFFFFFFFFF,FFFFFFFFFFFFFFFFFFFFFFFFFFFFFFFFFFFFFFFFFFFF
@A00155:342:HHGFNDSXY:1:1252:32145:24815 1:N:0:GAACCTAG+TCCGCATA
CAATGCCGAGAGGGATGTAGAGGGCGAGAGAGAGCGACTTCTCTCGGGCCCACAGCCTTACAGCTGTG
GAGCACGGTATCCTCTGCCGAAACAGAGGTTGGACAAGACCGGAGGGGTCTCCTAGTTCCAAAGGAGA
+
FFFFFFFFFFFFFF:FF:FFFFFFFFFFFFFFFFFFFFFFFFFFFFFFFFFFF,FFFFFFFFFFFFF
FFFFFFFFFFFFFFFFFFFFFFFFFFFFFFFFFFF:FFFFFFFFFFFFFFFFFFFFFFFFFFFFFFF
@A00155:342:HHGFNDSXY:1:1252:7265:30264 1:N:0:GAACCTAG+TCCGCATA
GTCATCTCAATGCCGAGAGGGATGTAGAGGGCGAGAGAGAGCGACTTCTCTCGGGCCCACAGCCTTAC
AGCTGTGGAGCACGGTATCCTCTGCCGAAACAGAGGTTGGACAAGACCGGAGGGGTCTCCTAGTTCCA
+
FFFFFFFFFFFFFFFFFFFFFF:F,FFFF:FFFFF:FFFFFFFFFFFFFFFFFFF,FFFFFFFFF:FF
FFFFFFFFFFFFFFFFFF:FFFF:FF:FFFFFF::FFFFFFFFFFFFFFFFFFFFFFFFFFFFFFFF
@A00155:342:HHGFNDSXY:1:1253:2817:4993 1:N:0:GAACCTAG+TCCGCATA
GTCATCTCAATGCCGAGAGGGATGTGGAGGGCGAGAGAGAGCGACTTCTCTCGGGCCCACAGCCTTAC
AGCTGTGGAGCACGGTATCCTCTGCCGAAACAGAGGTTGGACAAGACCGGAGGGGTCTCCTAGTTCCA
+
FFFFFFFFFFFFFFFFFFFFFFF:FFFFF,FFFFFFFFFFFFF:FFFFFFFFFFFFFFFFFFFFFFFF
FFFFFFFFFFFFFFFFFFFFFFFFFFFFFFFFFFFFFFFFFFFFFFFF:FFFFFFFFFFFFFFFFFFF
@A00155:342:HHGFNDSXY:1:1253:26648:5306 1:N:0:GAACCTAG+TCCGCATA
ATGCCGAGAGGGATGTGGAGGGCGAGAGAGAGTGACTTCTCTCGGGCCCACAGCCTTACAGCTGTGGA
GCACGGTATCCTCTGCCGAAACAGAGGTTGGACAAGACCGGAGGGGTCTCCTAGTTCCAAAGGAGATG
+
FFFFFFFFFFFF:FF,FFFFFFFFFFFFFFFFFFFFFFFFFFFFF:FFFFFFFFFFFFFFFFFFFFFF
FFFFFFFFFFFFFFFFFFFFFFFFFFFFFFFFFFFFFFFFFFFFFFFFFFFF:FFFFFFFFFFFFFFF
```

```
@A00155:342:HHGFNDSXY:1:1253:9317:12117 1:N:0:GAACCTAG+TCCGCATA
GGACTCACCTCTGGCCTATGGTCATCTCAATGCCGAGAGGGATGTGAAGGGCGAGAGAGAGCGACTTC
TCTCGGGCCCACAGCCTTACAGCTGTGGAGCACGGTATCCTCTGCC
+
FFFFFFFFFFFFFFFFFFFFFFFFFFFFFFFFFFFFFFFFFFFFFFFFFF:FFFFFFFFFFFFFFFFF
FFFFFFFFFFFFFFFFFFFFFFFFFFFFFFFFFFFFFFFFFFFFFF
@A00155:342:HHGFNDSXY:1:1253:4589:27571 1:N:0:GAACCTAG+TCCGCATA
CTCTCGGGCCCACAGCCTTACAGCTGTGGAGCACGGTATCCTCTGCCGAAACAGAGGTTGGACAAGAC
CGGAGGGGTCTCCTAGTTCCAAAGGAGATGTACTCCGGGCTTGTTCACGACCTACCGTGTAAGTCGTA
+
FFFFFFFFFFFFFFFFFFFFFFFFFFF:FFFFFFFFFFFFFFFFFFFFFFFFFFFFFFFFFFFFFFFF
FFFFFFFFFFFFFFFFFFFFFFFFFFFFFFFFFFFFFFFF:FFFFFFFFFFFFFFFFFFFFFFFFFFF
@A00155:342:HHGFNDSXY:1:1254:5873:20776 1:N:0:GAACCTAG+TCCGCATA
GCGACTTCTCTCGGGCCCACAGCCTTACTGCTGTGGAGCACGGTATCCTCTGCCGAAACAGAGGTTGG
ACAAGACCGGAGGGGTCTCCTAGTTCCAAAGGAGATGTACTCCGGGCTTGTTCACGACCTACCGTGTA
+
FFFFFFFFFFFFFFFFFFFFFFFFFFFFFFFFFFFFF:FFFFFFFFFFFFFFFFFFFFFFFFFFFFFF
FFFFFFFFFFFFFFFFFFFFFFFFFFFFFFFFFFFFF:FFFFFFFF:FFFFFFFFFFFFFFFFFFFFF
@A00155:342:HHGFNDSXY:1:1255:1922:3724 1:N:0:GAACCTAG+TCCGCATA
GAGCGACTTCTCTCGGGCCCACAGCCTTACAGCTGTGGAGCACGTTATCCTCTGCCGAAACAGAGGTT
GGACAAGACCGGAGGGGTCTCCTAGTTCCAAAGGAGATGTACTCCGGGCTTGTTCACGACCTACCGTG
+
FFFFFFFFFFFFFFFFFFFFFFFFFFFFFFFFFFFFFFFFFFFFFFFFFFFFFFFFFFFFFFFFFFFF
FFFFFFFFFFFFFFFFFFFFFFFFFFFFFFFFFFFFFFFFFFFFFFFFFFFFFFFFFFFFFFFFFFFF
@A00155:342:HHGFNDSXY:1:1255:2220:6840 1:N:0:GAACCTAG+TCCGCATA
GGATGTGGAGGGCGAGAGAGAGCGACTTCTCTCGGGCCCACAGCCTTACAGCTGTGGAGCACGGTATC
CTCTGCCGTAACAGAGGTTGGACAAGACCGGAGGGGTCTCCTAGTTCCAAAGGAGATGTACTCC
+
FFFFFFFFFFFFFFFFFFFFFFFFFFFFFFFFFFFFFFFFFFFFFFFFFFFFFFFFFFFFFFFFFFFF
FFFFFFFFFFFFFFFFFFFFFFFFFFFFFFFFFFFFFFFFFFFFFFFFFFFFFFFFFFFFFFFF
@A00155:342:HHGFNDSXY:1:1255:4544:15687 1:N:0:GAACCTAG+TCCGCATA
CAATGCCGAGAGGGATGTAGAGGGCGAGAGAGAGCGACTTCTCTCGGGCCCACAGCCTTACAGCTGTG
GAGCACGGTATCCTCTGCCGAAACAGAGGTTGGACAAGACCGGAGGGGTCTCCTAGTTCCAAAGGAGA
+
FFFFFFFFFFFFFFFFFFFFFFFFFFFFFFFFFFFFFFF:FFFFFFFFF:FFFFFFFFFFFFFFFFFF
FFFFFFFFFFFFFFFFFFFFFFFFFFF:FFFFFFFFFFFFFFFFFFFFFFFFFFFF:FFFFF,FFFFFFF
@A00155:342:HHGFNDSXY:1:1255:2871:16892 1:N:0:GAACCTAG+TCCGCATA
GAGCGACTTCTCTCGGGCCCACAGCCTTACAGCTGTGGAGCACGGTATCCTCTGCCGAAACAGAGGTT
GGACAAGACCGGAGGGGTCTCCTAGATCCAAAGGAGATGTACTCCGGGCTTGTTCACGACCTACCGTG
+
FFFFFFFFFFFFF:FFF:F:FFF,F:FF::FFFFFFFFFFFFF:,:FF,:FFFFFFFFFFFFFFFFFF
FFFFFFFFFFFFF:FFFF:FFFFF,FFFFFFFFFFFFFFFFFFFFFFFFFFFFFFFFFFFFFFFFFFF
@A00155:342:HHGFNDSXY:1:1255:2193:33818 1:N:0:GAACCTAG+TCCGCATA
TTCTCTCGGGCCCACAGCCTTACAGCTGTGGAGCACGGTATCCTCTGCCGAAACAGAGGTTGGACAAG
ACCGGAGGGGTCTCCTAGTTCCAAAGGAGATGTACTCCGGGCTTGTTCACGACCTACCGTGTAAGTCG
+
FFFFFFFFFFFFFFFFF:FFFF:FFF:FFFFFF:FFFFFFFFFFFF:FF:FFFFFFFFFFFFFFFFFF
FFFFFFFFFFFFFFFFFFFFFFFFFFFFF,FFFFFFFF,:FF:FFFFFF:FFFFFFFFFF:FFFFFFFF
@A00155:342:HHGFNDSXY:1:1258:23421:11992 1:N:0:GAACCTAG+TCCGCATA
GGATGTGGAGGGCGAGAGAGAGCGACTTCTCTCGGGCCCACAGCCTTACAGCTGTGGAGCACGGTATC
CTCTGCCGAAACAGAGGTTGGACAAGACCGGAGGGGTCTCCTGGTTCCAAAGGAGATGTACTCC
+
FFFFF:FFFFFFFFFFFFFFFFFFFFFFFFFFFFFF:FFFFFFFFFF:F:FFFFFFFFFFFFFFFFFFF
FFFFFFFFFFFFFFFFFFFFFFFFFFFFFFFFFFFFFFFFFFFFFFFFFFFFFFFFFFFFFFFF
```

```
@A00155:342:HHGFNDSXY:1:1258:1958:29403 1:N:0:GAACCTAG+TCCGCATA
TTCTCTCGGGCCCACAGCCTTACAGCTGTGGAGCACGGTATCCTCTGCCGAAACAGAGGTTGGACAAG
ACCGGAGGGGTCTCCTGGTTCCAAAGGAGATGTACTCCGGGCTTGTTCACGACCTACCGTGTAAGTCG
+
FFFFFFFFFFFFFFFFFFFFFFFFFFFFFFFFFFFFF:FFFFFF:FFFFFFFFFFFFFFFFFFFFFFF
FFFFFFFFFFFFFFFFFFFFFFFFFFFFFFFFFFFFF,FFFFF:FFFFFFFFFFFFFFFFFFFFFFFF
@A00155:342:HHGFNDSXY:1:1259:32678:15499 1:N:0:GAACCTAG+TCCGCATA
GGCGAGAGAGAGCGACTTCTCTCGGGCCCACAGCCTTACAGCTGTGGAGCACGGTATCCTCTGCCGAA
ACAGAGGTTGGACAAGACCGGAGGGGTCTCCTAGTTCCAAAGGAGATGTACTCCGGGCTTGTTCACGA
+
FF:FFFFFFFFFFFFFFFFFFFFFFFFFFFFFFFFFFFFFFFFFFFFFF:FFFFF:FFFFFFFFFFFF
FFFFFFFFFFFFFFFFFFFFFFFFFFFFFFFF,FFFF:FFFFFFFFFF:FFFFFFFFFFFFFFFFF:F
@A00155:342:HHGFNDSXY:1:1259:15420:18208 1:N:0:GAACCTAG+TCCGCATA
AGAGGGATGTGGAGGGCGAGAGAGAGCGACTTCTCTCGGGCCCACAGCCTTACAGCTGTGGAGCACGG
TATCCTCTGCCGAAACAGAGGTTGGACAAGACCGGAGGGGTCTCCTAGTTCCAAAGGAGATGTACTCC
+
:FFFFFFFFFFFFFFFFFFFFFFFFFFFFFFFFFFFFFFFFFFFFFFFFFFFFFFFFFFFFFFFFFFF
FFFFFFFFFFFFFFFFFFFFFFFFFFFFFFFFFFFFFFFFFFFFFFFFFFFFFFFFFFFFFFF:FFFF
@A00155:342:HHGFNDSXY:1:1259:2636:21057 1:N:0:GAACCTAG+TCCGCATA
CAATGCCGAGAGGGATGTAGAGGGCGAGAGAGAGCGACTTCTCTCGGGCCCACAGCCTTACAGCTGTG
GAGCACGGTATCCTCTGCCGAAACAGAGGTTGGACAAGACCGGAGGGGTCTCCTAGTTCCAAAGGAGA
+
FFFFFFFFFFFFFFF,FFFFFF:,FFFFF,FFFFFFFFF:FFFFFFFFFFFFFFFFFFFFFFFFFFFF
FFFFFFFFFFFFFFFFFFFFFFFFFFFF:FFFFFFFFFFFFFFFFFF:FFFFFFFFFFFFFFFFFFFF
@A00155:342:HHGFNDSXY:1:1260:17137:14325 1:N:0:GAACCTAG+TCCGCATA
CAGCCTTACAGCTGTGGAGCACGGTATCCTCTGCCGAAACAGAGGTTGGACAAGACCGGAGGGGTCTC
CTAGTTCCAAAGGAGATGTACTCCGGGCTTGTTCACGACCTACCGTGTAAGTCGTAGTCTAGTAGGCT
+
FFFFFFFFFFFFFFFFFFFFFFFFFFFFFFFFFFFFFFFFFFFFFFFFFFFFFFFFFFFFFFFFFFFF
FFFFFFFFFFFFFFFFFFFFFFFFFFFFF:FFFFFFFFFFFFFFFFFFFFFFFFFFFFFFFFFFFFFF
@A00155:342:HHGFNDSXY:1:1260:23032:30044 1:N:0:GAACCTAG+TCCGCATA
ATCTCAATGCCGAGAGGGATGTGGAGGGCGAGAGAGAGCGACTTCTCTCGGGCCCACAGCCTTACAGC
TGTGGAGCACGGTATCCTCTGCCGAAACAGAGGTTGGACAAGACCGGAGGGGTCTCCTAGTTCCAAAG
+
FFFFFFFFFFFFFFFFFFFFFFFFFFFFFFFFFFFFFFFFFFFFFFFFFFFFFFFFFFFFF:FFFFFF
FFFFFFFFFFFFFFFFFFFFFFFFFFFFFFFFFFFFFFFFFFFFFFFFFFFFFFFFFFFFFFFFFFFF
@A00155:342:HHGFNDSXY:1:1260:9118:30311 1:N:0:GAACCTAG+TCCGCATA
GGATGTGGAGGGCGAGAGAGAGTGACTTCTCTCGGGCCCACAGCCTTACAGCTGTGGAGCACGGTATC
CTCTGCCGAAACAGAGGTTGGACAAGACCGGAGGGGTCTCCTAGTTCCAAAGGAGATGTACT
+
FFFFFFFFFFFFFFFFFFFFFFFFFFFFFFFFFFFFFFFFFFFFFFFFFFFFFFFFFFFFFFFFFFFF
FFFFFFFFFFFFFFFFFFFFFFFFFFFFFFF:FFFFFFFFFFFFFFFFFFFFFFFFFFFFFF
@A00155:342:HHGFNDSXY:1:1260:7672:32471 1:N:0:GAACCTAG+TCCGCATA
TCCACTTAAGGACTCACCTCTGGCCTATGGTCATCTCAATGCCGAGAGGGATGTGAAGGGCGAGAGAG
AGCGACTTCTCTCGGGCCCACAGCCTTACAGCTGTGGAGCACGGTATCCTCTGCCGAAACAGAGGTTG
+
FFFFFFFFFFFFFFFFFF:FFFFFFFFFFFFFFFFFFFF:FFFFFFFFFFF,FFFFFFFFFFFFFFFF
FFFFFFFFFFFFFFFFFFFFFFFFFFFFFFFFFFFFFFFFFFFFFFFFFFFFFFFFFFFFFFFFFFFF
@A00155:342:HHGFNDSXY:1:1261:30924:7764 1:N:0:GAACCTAG+TCCGCATA
GGATGTAGAGGGCGAGAGAGAGCGACTTCTCTCGGGCCCACAGCCTTACAGCTGTGGAGCACGGTATC
CTCTGCCGAAACAGAGGTTGGACAAGACCGGAGGGGTCTCCTAGTTCCAAAGGAGATGTACTCC
+
FFFFFFFFFFFFFFFFFFFFFF:FFFFFFFFFFFFFFFFFFFFFFF:FFFFFFFFFFFFFFFFFFFFF
FFFFFFFFFFFFFFFFFFFFFFFFFFFFFFFFFFFFFFFFFFFFFFFFFFFFFFFFFFFF:FFF
```

```
@A00155:342:HHGFNDSXY:1:1261:15664:16000 1:N:0:GAACCTAG+TCCGCATA
TATGCCGAGAGGGATGTGGAGGGCGAGAGAGAGCGACTTCTCTCGGGCCCACAGCCTTACAGCTGTGG
AGCACGGTATCCTCTGCCGAAACAGAGGTTGGACAAGACCGGAGGGGTCTCCTAGTTCCAAAGGAGAT
+
FFFFFFFFFFFFFFFFFFFFFFFFFFFFFFFFFFFFFFFFFFFFFFFFFFFFFFFFFFFFFFFFFFFF
FFFFFFFFFFFFFFFFFFFFFFFFFFFFFFFFFFFFFFFFFFFFFFFFFFFFFFFFFFFFFFFFFFFF
@A00155:342:HHGFNDSXY:1:1261:6388:27430 1:N:0:GAACCTAG+TCCGCATA
GATGTGGAGGGCGAGAGAGAGCGACTTCTCTCGGGCCCACAGCCTTACAGCTGTGGAGCACGGTATCC
TCTGCCGAAACAGAGGTTGGACAAGACCGGAGGGGTCTCCTGGTTCCAAAGGAGATGTACTCCGGGCT
+
FFFFFFFFFFFFFFFFFFFFFFFFFFFFFFFF:FFFFFFFFFFFFFFFFFFFFFFFFFFFFFFFFFFF
FFFFFFFFFFFFFFFFFFFFFFFFFFFFFFFFFFFFFFFFFFFFFFFFFFFFFFFFFFFFFFFFFFFF
@A00155:342:HHGFNDSXY:1:1261:32669:32863 1:N:0:GAACCTAG+TCCGCATA
TCCTCTGCCGAAACAGAGGTTGGACAAGACCGGAGGGGTCTCCTAGTTCCAAAGGAGATGTACTCCGG
GCTTGTTCACGACCTACCGTGTAAGTCGTAGTCTAGTAGGCTACCTGACGAGTCCTTTTTAGGAC
+
FFFFFFFFFFFFFFFFFFFFFFFFFFFFFFFFFFFFFFFFFFFFFFFFFFFFFFFFFFFFFFFFFFFF
FF,FFFFFFFFFFFFFFFFFFFFFF:FFFFF:FFFFF:FFFF:FFFFFFFFFFFFFFFFFFFFFF
@A00155:342:HHGFNDSXY:1:1261:8594:34601 1:N:0:GAACCTAG+TCCGCATA
GAGCGACTTCTCTCGGGCCCACAGCCTTACAGCTGTGGAGCACGGTATCCTCTGCCGAAACAGAGGTT
GGACAAGACCGGAGGGGTCTCCTAGTTCCAAAGGAGATGTACTCCGGGCTTGTTCACGACCTACCGTG
+
FFFFFFFFFFFFFFFFFFFFFFFFFFFFFFFFFFFFFFFFFFFFFFFFFFFFFFFFFFFFFFFFFFFF
FFFFFFFFFFFFFFFFFFFFFFFFFFFFFFFFFFFFFFFFFFFFFFFFF:FFFFFFFFFFFFFFFFFF
@A00155:342:HHGFNDSXY:1:1262:23999:12117 1:N:0:GAACCTAG+TCCGCATA
CGAGAGGGATGTGGAGGGCGAGAGAGAGCGACTTCTCTCGGGCCCACAGCCTTACAGCTGTGGAGCAC
GGTATCCTCTGCCGAAACAGAGGTTGGACAAGACCGGAGGGGTCTCCTAGTTCCAAAGGAGATGTAC
+
FFFFFFFFF:FFFFFFFFFFFFFFFFFFFFFFFFFFFFFFFFFFFFFFFFFFFFFFFFFFFFFFFFFF
FFFFFFFFFFFFFFFFFFFFFFFFFFFFFFFFFFFFFFFFFFFFFFFFFFFFFFFFFFFFFFFFFFF
@A00155:342:HHGFNDSXY:1:1262:28465:16814 1:N:0:GAACCTAG+TCCGCATA
TGGTCATCTCAATGCCGAGAGGGATGTGGAGGGCGAGAGAGAGCGACTTCTCTCGGGCCCACAGCCTT
ACAGCTGTGGAGCACGGTATCCTCTGCCGAAACAGAGGTTGGACAAGACCGGAGGGGTCTCCTAGTTC
+
FFFFFFFFFFFFFFFFFFFFFFFFFFFFFFFFFFFFFFFFFFFFFFFFFFFFFFFFFFFFFFFFFFFF
FFFFFFF:FFFFFFFFFFFFFFFFFFFFFFFFFFFFFFFFFFFFFFF:FFFFFFFFFFFFFFFFFFFF
@A00155:342:HHGFNDSXY:1:1262:30671:32346 1:N:0:GAACCTAG+TCCGCATA
CTTAAGGACTCTCCTCTGGCCTATGGTCATCTCAATGCCGAGAGGGATGTGGAGGGCGAGAGAGAGCG
ACTTCTCTCGGGCCCACAGCCTTACAGCTGTGGAGCACGGTATCCTCTGCCGAAACAGAGGTTGGACA
+
FFFFFFFFFFFFFFFFFFFFFFFFFFFFFFFFFFFFFFFFFFFFFFFFFFFFFFFFFFFFFFFFFFFF
FFFFFFFFFFFFFFFFFFFFFFFFFFFFFFFFFFFFFFFFFFFFFFFFFFFFFFFFFFFFFFFFFFFF
@A00155:342:HHGFNDSXY:1:1263:32515:4977 1:N:0:GAACCTAG+TCCGCATA
GTCCACTTAAGGACTCTCCTCTGGCCTATGGTCATCTCAATGCCGAGAGGGATGTGGAGGGCGAGAGA
GAGCGACTTCTCTCGGGCCCACAGCCTTACAGCTGTGGAGCACGGTATCCTCTGCCGAAACAGAGGTT
+
FFFFFFF,FFFFFFFFFFFFFFFFFFFFFFFFF::F:FFFFFFFFFFFFFFF:FFFFFFFFFFFFFFF
FFFFFFFFFFFFFFFFFFFFFFFFFF,F:FFFF:FFFFFFFFF:F:FFFFFFFFFFFFFFFFFFFFFF
@A00155:342:HHGFNDSXY:1:1264:15872:7153 1:N:0:GAACCTAG+TCCGCATA
GAGCGACTTCTCTCGGGCCCACAGCCTTACAGCTGTGGAGCACGGTATCCTCTGCCGAAACAGAGGTT
GGACAAGACCGGAGGGGTCTCCTAGTTCCAAAGGAGATGTACTCCGGGCTTGTTCACGA
+
FFFFFFFFFFFFFFFFFFFFFFFFFFFFFFFFFFFFFFFFFFFFFFFFFFFFFFFFFFFFFFFFFFFF
FF:FFFFFFFFFFFFFFFFFFFFFFFFFFFFFFFFFFFFFFFFFFFFF:FFFFFFFFFF
```

@A00155:342:HHGFNDSXY:1:1264:23249:22341 1:N:0:GAACCTAG+TCCGCATA
TCCTCTGCCGAAACAGAGGTTGGACAAGACCGGAGGGGTCTCCTGGTTCCAAAGGAGATGTACTCCGG
GCTTGTTCACGACCTACCGTGTAAGTCGTAGTCTAGTAGGCTACCTGACGAGTCCTTTTTAGGACGAA
+
FFFFFFFFFFFFFFFFFFFFFFFFFFFFFFFFFFFFFFFFFFFFFFFFFFFFFFFFFFFFFFFFFFFF
FFFFFFFFFFFFFFFFFFFFFFFFFFFFFFFFFFFFFFFFFFFFFFFFFFFFFFFFFFFFFFFFFFFF
@A00155:342:HHGFNDSXY:1:1264:9995:22811 1:N:0:GAACCTAG+TCCGCATA
GAGCGACTTCTCTCGGGCCCACAGCCTTACAGCTGTGGAGCACGGTATCCTCTGCCGAAACAGAGGTT
GGACAAGACCGGAGGGGTCTCCTAGTTCCAAAGGAGATGTACTCCGGGCTTGTTCACGACCTACCGT
+
:F:FFF,,F,FFFFFFF,,FFFF:FF,:,:FFF,:F,FFF:FFFF:F,FF:FF,FF,F,F,F:FFF,,
:,FFFF,FF::,F,F:FF::FFFF,:FFF:FFFF:F:F:,,F,FFFF,FFFFF:FF:,FF,FFF,:F
@A00155:342:HHGFNDSXY:1:1264:5367:36401 1:N:0:GAACCTAG+TCCGCATA
ATGCCGAGAGGGATGTAGAGGGCGAGAGAGAGCGACTTCTCTCGGGCCCACAGCCTTACAGCTGTGGA
GCACGGTATCCTCTGCCGAAACAGAGGTTGGACAAGACCGGAGGGGTCTCCTAGTTCCAAAGGAGATG
+
FFFFFFFFFFFFFFFF:FFFFFF:FFFFFFFFFFFFFFFFFFFFFFFFFFFFFFFFFFFFFFFFFFFF
FFFFFFFFFFFFFFFFFFFFFFFFFFFFFFFFFFFFFFFF:FFFFFFFFFFFFFFFFFFFF:FFFFFF
@A00155:342:HHGFNDSXY:1:1264:6198:36965 1:N:0:GAACCTAG+TCCGCATA
GCGACTTCTCTCGGGCCCACAGCCTTACAGCTGTGGAGCACGGTATCCTCTGCCGAAACAGAGGTTGG
ACAAGACCGGAGGGGTCTCCTAGTTCCAAAGGAGATGTACTCCGGGCTTGTTCACGACCTACCGTGTA
+
FFFFFFFFFFFFFFFFFFFFF:FFFFFFFFFFFFFFFFFFFFFFFFFFF:FFFFFFFFFFFFFFFF,F
FFFFFFF:FFFFFFFFFFFFFFFFFFFFFFFFFFFFFFFFFFFFFFFFFFFFF:FFF:FFFFFFFFFF
@A00155:342:HHGFNDSXY:1:1265:17047:6402 1:N:0:GAACCTAG+TCCGCATA
GAGCGACTTCTCTCGGGCCCACAGCCTTACAGCTGTGGAGCACGGTATCCTCTGCCGAAACAGAGGTT
GGACAAGACCGGAGGGGTCTCCTGGTTCCAAAGGAGATGTACTCCGGGCTTGTTCACGACCTACCGTG
+
FFFFFFFFFFFFFFFFFFFFFFFFFFFFFFFFFFFFFFFFFFFFFFFFFFFFFFFFFFFFFFFFFFFF
FFFFFF,FFFFFFFFFFFFFFFFFFFFFFFFFFFFFFFFFFFFFFFFFFFFFFFFFFFFFFFFFFFFF
@A00155:342:HHGFNDSXY:1:1265:16532:8234 1:N:0:GAACCTAG+TCCGCATA
GGTCTTGTCCAACCTCTGTTTCGGCAGAGGATACCGTGCTCCACAGCTGTAAGGCTGTGGGCCCGAGA
GAAGTCGCTCTCTCTCGCCCTCTACATCCCTCTCGGCATTGAGATGACCATAGGCCAGAGGTGAGTCC
+
FFFFFFFFFFFFFFFF:FFFFFFFFFFFFFFFFFFFFFFFFFFFFFFFFFFFFFFFFFFFFFFFFFFF
FFFFFFFFFFFFFFFFFFFFFFFFFFFFFFFFFFFFFFFFFFFFFFFFFFFFFFFF:FFFFFFFFFF:
@A00155:342:HHGFNDSXY:1:1265:25418:13385 1:N:0:GAACCTAG+TCCGCATA
GGATGTGGAGGGCGAGAGAGAGCGACTTCTCTCGGGCCCACAGCCTTACAGCTGTGGAGCACGGTATC
CTCTGCCGAAACAGAGGTTGGACAAGACCGGAGGGGTCTCCTAGTTCCAAAGGAGATGTACTCC
+
FFFFF,FFFFFFFFFFFFFFFFFFFFFFFFFFFFFFFFFFFF,FFFF,FFFFFFFFFFFFFFFFFFFF
FFFFFFFFF,FFFFFFFFFFFFFFFFFFFFFFFFFFFFFFFFFFFFFFFFFFFFFFFFFFFFFF
@A00155:342:HHGFNDSXY:1:1265:30201:24142 1:N:0:GAACCTAG+TCCGCATA
ATCTCAATGCCGAGAGGGATGTGGAGGGCGAGAGAGAGCGACTTCTCTCGGGCCCACAGCCTTACAGC
TGTGGAGCACGGTATCCTCTGCCGAAACAGAGGTTGGACAAGACCGGAGGGGTCTCCTAGTTCCAAAG
+
FFFFFFFFFFFFFFFFFFF:FFFFFFFFFFFFF:FFFFFFF:FFFFFFFFFFFFFFFFFFFFFFFFFF
FFFFFF:FFFFFFFFFFFFFFFFFFFFFFFFFFFFFFFFFFFFFFFFFFFFFFFFFFFFFFFFFFFFF
@A00155:342:HHGFNDSXY:1:1265:3305:30827 1:N:0:GAACCTAG+TCCGCATA
GCCCCGCCACACCGCCTTAGATCAGCTGTGTCCACTTAAGGACTCACCTCTGGCCTATGGTCATCTCA
ATGCCGAGAGGGATGTGGAGGGCGAGAGAGAGCGACTTCTCTCGGGCCCACAGCCTTACAGCTGTGGA
+
FFFFFFFFFFFFFFFFFFFFF:FFFFFFFFFFFFFFFFFFFFFFFFFFFFFFFFFFFFFFFFFFFFFF
FFFFFFFFFFFFFFFFFFFFFFFFFFFFFFFFFFFFFFFFFFFFFFFFFFFFFFFFFFFFFFFFFFFF

```
@A00155:342:HHGFNDSXY:1:1266:16260:2597 1:N:0:GAACCTAG+TCCGCATA
AGAGAGAGCGACTTCTCTCGGGCCCACAGCCTTACAGCTGTGGAGCACGGTATCCTCTGCCGAAACAG
AGGTTGGACAAGACCGGAGGGGTCTCCTGGTTCCAAAGGAGATGTACTCCGGGCTTGTTCACGACCTA
+
FFFFFFFFFFFFFF:FFFFFFF:FFFFFFFFFFFFFFFFFFFFFFF:FFFFFFFFFFFFFFFFFFFFF
FFFFFFFFFFF:FFFFFFFFFFFFFFFFFFFFFFFFFFFFFFFFFFFFFFFFFFFFFFFFFFFFFFFF
@A00155:342:HHGFNDSXY:1:1266:16423:2973 1:N:0:GAACCTAG+TCCGCATA
AGAGAGAGCGACTTCTCTCGGGCCCACAGCCTTACAGCTGTGGAGCACGGTATCCTCTGCCGAAACAG
AGGTTGGACAAGACCGGAGGGGTCTCCTGGTTCCAAAGGAGATGTACTCCGGGCTTGTTCACGACCT
+
FFFFFFFFFFFFFFFFFFFFFFFFFFFFFFFFFFFFFFFFFFFFFFFFFFF:FFFFFFFFFFFF:FFF
FFFFFFFFFFFFFFFFFFFFFFFFFFFFF:FFFFFFFFFFFFFFFFFFFFFFFFFFFFFFFFFFFFF
@A00155:342:HHGFNDSXY:1:1266:7798:22827 1:N:0:GAACCTAG+TCCGCATA
GTCATCTCAATGCCGAGAGGGATGTGGAGGGCGAGAGAGAGCGACTTCTCTCGGGCCCACAGCCTTAC
AGCTGTGGAGCACGGTATCCTCTGCCGAAACAGAGGTTGGACAAGACCGGAGGGGTCTCCTAGTTCCA
+
FFFFFFFFFFFFFFFFFFFFFFFF,FFFFFFFFFFFFF:FFFFFFF:FFFFFFFFFFFFFFFFFFFFF
FFFFFFFFFFFFFFFF:FFFFFFFFFFFFFFFFFFFFFFFFFFFFFFFFFFFFFFFFFFFFFFFFFF
@A00155:342:HHGFNDSXY:1:1267:10465:9502 1:N:0:GAACCTAG+TCCGCATA
TTACAGCTGTGGAGCACGGTATCCTCTGCCGAAACAGAGGTTGGACAAGACCGGAGGGGTCTCCTAGT
TCCAAAGGAGATGTACTCCGGGCTTGTTCACGACCTACCGTGTAAGTCGTAGTCTAGTAGGCTACCTG
+
:FFFFFFFF,FFFFFFFFF:FFFFFFF:FFFFFFFFFFFF:,FFFFFF:FFFFFFFFFFFFFFFFFFF
FFF:FFFFFFFFFFFF,FFF::FFFFFFFFFFFFFFFFFFFFFFFF:FFF:FFFFFFFFFF:FFFFFF
@A00155:342:HHGFNDSXY:1:1267:16197:35837 1:N:0:GAACCTAG+TCCGCATA
GCGAGAGAGAGCGACTTCTCTCGGGCCCACAGCCTTACAGCTGTGGAGCACGGTATCCTCTGCCGAAA
CAGAGGTTGGACAAGACCGGAGGGGTCTCCTAGTTCCAAAGGAGATGTACTCCGGGCTTGTTCACGAC
+
F:FFFFFFFFFFFFFFFFFFFFFFFFFFFFFFFFFFFFFFF,FFFFFFFFFFFFFFFFFFFFFFFFFF
FF:FFFF:FFFFFFFFFFFFFFFFFFFFFFFFFFFFFFFFFFFFFFFFFFFFFFFFFFFF,FFFFFFF
@A00155:342:HHGFNDSXY:1:1268:11993:4413 1:N:0:GAACCTAG+TCCGCATA
GAGGGCGAGAGAGAGCGACTTCTCTCGGGCCCACAGCCTTACAGCTGTGGAGCACGGTATCCTCTGCC
GAAACAGAGGTTGGACAAGACCGGAGGGGTCTCCTGGTTCCAAAGGAGATGTACTCCGGGCTTGTTCA
+
FFFFFFFFFFFFFFFFFFFFFFFFFFFFFFF:FFFFFFFFFFF:FFFFFFFFFFFFFFFFFFFFFFFF
FFFFFFFFFFFFFFFFFFF:FFFFFFFFFFFFFFFFFFFFFFFFFFFFFFFFFFFFFFFFFFFFFFF:
@A00155:342:HHGFNDSXY:1:1268:12735:11678 1:N:0:GAACCTAG+TCCGCATA
GGATGTGGAGGGCGAGAGAGAGCGACTTCTCTCGGGCCCACAGCCTTACAGCTGTGGAGCACGGTATC
CTCTGCCGAAACAGAGGTTGGACAAGACCGGAGGGGTCTCCTGGTTCCAAAGGAGATGTACTCC
+
FFFFFFFFFFFFFFFFFFFFFFFFFFFFFFFFFFFFFFFFFFFFFFFFFFFFFFFFFFFFFFFFFFFF
FFFFFFFFFFFFFFFFFFFFFFFFFFFFFFFFFFFFFFFFFFFFFFFFFFFFFFFFFFFFFFFF
@A00155:342:HHGFNDSXY:1:1268:13024:18161 1:N:0:GAACCTAG+TCCGCATA
CTCAATGCCGAGAGGGATGTGGAGGGCGAGAGAGAGCGACTTCTCTCGGGCCCACAGCCTTACAGCTG
TGGAGCACGGTATCCTCTGCCGAAACAGAGGTTGGACAAGACCGGAGGGGTCTCCTAGTTCCAAAGGA
+
FFFFFFFFFFFFFFFFF:FFFFFFFFFFFFFFFFFFFFFF:FFFFFFFFFFFFFFFFFFFFFFFFFFF
FFFFFFFFFFFFFFFFFFFFFFFFFFFFFFFFFFFFFFFF:FFFFFFFFFFFFFFFFFFFFFFFFFFF
@A00155:342:HHGFNDSXY:1:1268:13367:19539 1:N:0:GAACCTAG+TCCGCATA
TCCTCTGCCGAAACAGAGGTTGGACAAGACCGGAGGGGTCTCCTAGTTCCAAAGGAGATGTACTCCGG
GCTTGTTCACGACCTACCGTGTAAGTCGTAGTCTAGTAGGCTACCTGACGAGTCCTTTTTAGGACGAA
+
,FFFFFFFFFFFFFFFFFFFFFFFFF:,FFFFFFFFFFFFFF:FFFFFFFFFFF:FFFFFFFFFFFFF
FFFFFFFFFFFFFFFFFFFFF:FFFFFFFF:FFFFFFFFFFFFFFFFFFFFFFFFFFFFFFFFFFFFF
```

@A00155:342:HHGFNDSXY:1:1268:3260:21543 1:N:0:GAACCTAG+TCCGCATA
TTAAGGACTCTCCTCTGGCCTATGGTCATCTCAATGCCGAGAGGGATGTGGAGGGCGAGAGAGAGCGA
CTTCTCTCGGGCCCACAGCCTTACAGCTGTGGAGCACGGTATCCTCTGCCGAAACAGAGGTTGGACAA
+
FFFFFFFFFFFFFFFFFFFFFFFFFFFFFFFFFFFFFFF:FFFFFFF,FFFFFFFFFFFFFFFFFFFF
FFFFFFFFFFFFFFFFFFFFFFFFFFFFFFFFFFF:FFFFFFFFFFFFFFFFFFFFFFFFFFFFFF,F
@A00155:342:HHGFNDSXY:1:1268:24813:22764 1:N:0:GAACCTAG+TCCGCATA
CCTTACAGCTGTGGAGCACGGTATCCTCTGCCGAAACAGAGGTTGGACAAGACCGGAGGGGTCTCCCA
GTTCCAAAGGAGATGTACTCCGGGCTTGTTCACGACCTACCGTGTAAGTCGTAGTCTAGTAGGCTACC
+
FFFFFFFFFFFFFFFFFFFFFFFFFFFFFFFFFFFFFFFFFFFFFFFFFF:FFFFFFFFFFFFFFFFF
FFFFF:FFFFFFFFFFFFFFFFFFFFFFFFFFFFFFFFFFFFFFFFFFFFFFFFFFFFFFFFFFFFFF
@A00155:342:HHGFNDSXY:1:1268:5846:25989 1:N:0:GAACCTAG+TCCGCATA
GGATGTGGAGGGCGAGAGAGAGCGACTTCTCTCGGGCCCACAGCCTTACAGCTGTGGAGCACGGTATC
CTCTGCCGAAACAGAGGTTGGACAAGACCGGAGGGGTCTCCTAGTTCCAAAGGAGATGTACTCC
+
FFFFFFFFFFFFFFFF:FFFFFFFFFFFFFFFFFFFFFFFFFFFFFFFFFFFFF:FFFFFFFFFFFFF
FFFFFFFFFFFFFFFFFFFFFFFFFFFFFFFFFFFFFFFFF:FFFFFFFFFFFFFFFFFFFFFF
@A00155:342:HHGFNDSXY:1:1269:8223:10629 1:N:0:GAACCTAG+TCCGCATA
TGCCGAGAGGGATGTAGAGGGCGAGAGAGAGCGACTTCTCTCGGGCCCACAGCCTTACAGCTGTGGAG
CACGGTATCCTCTGCCGAAACAGAGGTTGGACAAGACCGGAGGGGTCTCCTAGTTCCAAAGGAG
+
FFF:FFFFFFFFFF,FFFFFFFFFFFFFFFFFFFFFFFFFFFFFFFFFFFFFFFFFFFFFFFFFFFFF
FFFFFFFFFFFFFFFFFFFFFFFFFFFFFFFFFFFFFFFFFFFFFFFFFFFFFFFFFFFFFFFF
@A00155:342:HHGFNDSXY:1:1269:9607:19727 1:N:0:GAACCTAG+TCCGCATA
AGAGCGACTTCTCTCGGGCCCACAGCCTTACAGCTGTGGAGCACGGTATCCTCTGCCGAAACAGAGGT
TGGACAAGACCGGAGGGGTCTCCTAGTTCCAAAGGAGATGTACTCCGGGCTTGTTCACGACCTACCGT
+
FFFFFFFFFFFFFFFFFFFFFFFFFFFFFFFFFFFFFFFFFFFFFFFFFFFFFFFFFFFFFFFFFFFF
FFFFFFFFFFFFFFFFFFFFFFFFFFFFFFFFFF:FFFFFFFFFFFFFFFFFFFFFFFFFFFFFFFFF
@A00155:342:HHGFNDSXY:1:1269:8594:22138 1:N:0:GAACCTAG+TCCGCATA
AGAGCGACTTCTCTCGGGCCCACAGCCTTACAGCTGTGGAGCACGGTATCCTCTGCCGAAACAGAGGT
TGGACAAGACCGGAGGGGTCTCCTAGTTCCAAAGGAGATGTACTCCGGGCTTGTTCACGACCTACCGT
+
FFFFFFFFFFFFFFFFFFFFFFFFFFFFFFFFFF,:FFFFFFFFFFFFFFFFFFFFFFFFFFFFFFFF
FFFFFFFFFFFFFFFFFFFFFFFFFFFFFFFFFFFFFFFFFFFFFFFFFFFFFFFFFFFFFFFFFFFF
@A00155:342:HHGFNDSXY:1:1269:31123:25175 1:N:0:GAACCTAG+TCCGCATA
CGAGAGGGATGTGGAGGGCGAGAGAGAGCGACTTCTCTCGGGCCCACAGCCTTACAGCTGTGGAGCAC
GGTATCCTCTGCCGAAACAGAGGTTGGACAAGACCGGAGGGGTCTCCTAGTTCCAAAGGAGATGTACT
+
FFFFFFFFFFFFFFFFFFFFFFFFFFFFFFFFFFFFFFFFFFFFFFFFFFFFFFFFFFFFFFFFFFFF
FFFFFFFFFFFFFFFFFFFFFFFFFFFFFFFFFFFFFFFFFFFFFFFFFFFFFFFFFFFFF:FFFFFF
@A00155:342:HHGFNDSXY:1:1269:28004:32174 1:N:0:GAACCTAG+TCCGCATA
CTATGGTCATCTCAATGCCGAGAGGGATGTGGAGGGCGAGAGAGAGCGACTTCTCTCGGGCCCACAGC
CTTACAGCTGTGGAGCACGGTATCCTCTGCCGAAACAGAGGTTGGACAAGACCGGAGGGGTCTCCTGG
+
FFFFFFFFFFFFFFFFFFFFFF:FFFFFFFFFFFFFFFFFFFFFFFFFFFFFFFFFFFFFFFFFFFFF
FFFFFFFFFFFFFFFFFFFFFFFFFFFFFFFFFFFFFFFFFFFFFFFFFFFFFFFFFFFFFFFFFFFF
@A00155:342:HHGFNDSXY:1:1270:23167:1689 1:N:0:GAACCTAG+TCCGCATA
GAGGGCGAGAGAGAGCGACTTCTCTCGGGCCCACAGCCTTACAGCTGTGGAGCACGGTATCCTCTGCC
GAAACAGAGGTTGGACAAGACCGGAGGGGTCTCCTAGTTCCAAAGGAGATGTACTCCGGGCTTGTTCA
+
FFFFFFFFFFFFFFFFFFFFFFFFFFFFFFFFFFFFFFFFFFFFFFFFFFFFFF:FFFFFFFFFFFFF
FFFFFFFFF:FFFFFFF:FFFFFFFFFFFFFFFFFFFFFFFFFFFFFFFFFFFFFFFFFFFFFFFFFF

```
@A00155:342:HHGFNDSXY:1:1270:20735:4742 1:N:0:GAACCTAG+TCCGCATA
ACTTCTCTCGGGCCCACAGCCTTACAGCTGTGGAGCACGGTATCCTCTGCCGAAACAGAGGTTGGACA
AGACCGGAGGGGTCTCCTGGTTCCAAAGGAGATGTACTCCGGGCTTGTTCACGACCTACCGTGTAAGT
+
FF:FFFFFFFFFFFFFFFFFFFF:FFFFFFFFFFFFFFFFFFFFFFFFFFFFFFFFFFFFFFFFFFFF
F:FFFFFFF:FFFFFFFFFFFFFFFFFFFFFFFFFFFFFFFFFF:FFFFFFFFFFFFFFFFFFFFFFF
@A00155:342:HHGFNDSXY:1:1270:15555:10833 1:N:0:GAACCTAG+TCCGCATA
TCGGCAGAGGATACCGTGCTCCACAGCTGTAAGGCTGTGGGCCCGAGAGAAGTCGCTCTCTCTCGCCC
TCCACATCCCTCTCGGCATTGAGATGACCATAGGCCAGAGGTGAGTCCTTAAGTGGACACA
+
FFFFFFFFFFFFFFFFFFFFFFFFFFFFFFFFFFFFFFFFFFFFFFFFFFFFFFFFFFFFFFFFFFFF
FFFFFFFFFFFFFFFFFFFFFFFFFFFFFFFFFFFFFFFFFFFFFFF:FFFFF:FFFFFFF
@A00155:342:HHGFNDSXY:1:1270:9625:26459 1:N:0:GAACCTAG+TCCGCATA
GGATGTGGAGGGCGAGAGAGAGCGACTTCTCTCGGGCCCACAGCCTTACAGCTGTGGAGCACGGTATC
CTCTGCCGAAACAGAGGTTGGACAAGACCGGAGGGGTCTCCTAGTTCCAAAGGAGATGTACTCC
+
FFFFFFFFFFFFFFFFFFFFFF,FFFFFFFFFFFFFFFFFFFFFFFF:FFFFFFFFFFFFFFFFFFFF
FFFFFFFFFFFFFFFFFFFFFFFFFFFFFFFFFFFFFFFFFFFFFFFFFFFFFFFF:FFFFFFF
@A00155:342:HHGFNDSXY:1:1271:18430:2284 1:N:0:GAACCTAG+TCCGCATA
TACGACTTACACGGTAGGTCGTGAACAAGCCCGGAGTACATCTCCTTTGGAACTAGGAGACCCCTCCG
GTCTTGTCCAACCTCTGTTTCGGCAGAGGATACCGTGCTCCACAGCTGTAAGGCTG
+
FFFFFFFFFFFFFFFFFFFFFFFFFFFFFFFFFFFFFFFFFFFFF:FFFFFFFFFFFFFFFFFFFFFF
FFFFFFFFFFFFFFFFFFFFFFFFFFFFFFFFFFFFFFFFFFFFFFFFFFFFFFFF
@A00155:342:HHGFNDSXY:1:1271:20374:6339 1:N:0:GAACCTAG+TCCGCATA
CTTCTCTCGGGCCCACAGCCTTACAGCTGTGGAGCACGGTATCCTCTGCCGAAACAGAGGTTGGACAA
GACCGGAGGGGTCTCCTAGTTCCAAAGGAGATGTACTCCGGGCTTGTTCACGACCTACCGTGTAAGTC
+
F:FFFFFFFFFFFFFFFFFFFFFFFFFFFFFFFFFFFFFFFFFFFFFFFFFFFFFFFFFFFFFFFFFF
:FFFFFFFFFFFFFFFFFFFFFFFFFFFFFFFFFFFFFFFFFFFFFFFFFFFFFFFFFFFFFFF:FFF
@A00155:342:HHGFNDSXY:1:1271:5918:16783 1:N:0:GAACCTAG+TCCGCATA
TTCTCTCGGGCCCACAGCCTTACAGCTGTGGAGCACGGTATCCTCTGCCGAAACAGAGGTTGGACAAG
ACCGGAGGGGTCTCCTAGTTCCAAAGGAGATGTACTCCGGGCTTGTTCACGACCTACCGTGTAAGTCG
+
FFFFFFFFFFFFFFFFFFFFFFFFFFFFFFFFFFFFFFFFFFFFFFFFFFFFFFFFFFFFFFFFFFFF
FFF:FFFFFFFFFFFFFFFFFFFFFFFFFFFFFFF:FFFFFFF:FFFF:FFFFFFFFFFFFFFFFFFF
@A00155:342:HHGFNDSXY:1:1271:3830:18051 1:N:0:GAACCTAG+TCCGCATA
TGACGGTCGGAGTCTAGTAGGCTCCCTGATGAGTCCGTTCCAAGGACGAAACCGTCGTTACTCTTTGA
GTTGTGTAAGTTTCGTCCTAAAAAGGACTCGTCAGGTAGCCTACTAGACTACGACTTACACGGTAGGT
+
FFFFFFFFFFFFFFFFFFFFFFF:FFFFFFFFF,FFF,FFFFFFFFFFFFFFF:FFF:FFFFFF,FFF
FFFFFFFFFFFFFFFFFFFFFFFFFFF:F:FFFFFFFFFFF,FFFFFFFFFFFFFFFFFF:FFF:FF:
@A00155:342:HHGFNDSXY:1:1271:25889:28040 1:N:0:GAACCTAG+TCCGCATA
CGACTTCTCTCGGGCCCACAGCCTTACAGCTGTGGAGCACGGTATCCTCTGCCGAAACAGAGGTTGGA
CAAGACCGGAGGGGTCTCCTAGTTCCAAAGGAGATGTACTCCGGGCTTGTTCACGACCTACCGTGTAA
+
FFFF:FFFFFFFFFFFFFFFFFFFFFFFFFFFFFFF:FFFFFFFFFFFFFFFFFFFFFFFFFFFFFFF
FFFFFFFFFFFFFFFFFFFFFFFFFFFFFFFFFFFFFFFFFFFFFFFFFFFFFFFFFFFFFFFFFFFF
@A00155:342:HHGFNDSXY:1:1271:21052:30499 1:N:0:GAACCTAG+TCCGCATA
ACCTCTGTTTCGGCAGAGGATACCGTGCTCCACAGCTGTAAGGCTGTGGGCCCGAGAGAAGTCGCTCT
CTCTCGCCCTTCACATCCCTCTCGGCATTGAGATGACCATAGGCCAGAGGTGAGTCCTTAAGTGGACA
+
FFFFFFFFFFFFFFFFFFFFFFFFFFFFFFFFFFFFFFFFFFFFFFFFFFFFFFFFFFFFFFFFFFFF
FFFFFFFFFFFFFFFFFFFFFFFFFFF:FFFFFFFFFFFFFFFFFFFFFFFFFFFFFFFFFFFFFFFF
```

```
@A00155:342:HHGFNDSXY:1:1272:8974:2440 1:N:0:GAACCTAG+TCCGCATA
TTCTCTCGGGCCCACAGCCTTACAGCTGTGGAGCACGGTATCCTCTGCCGAAACAGAGGTTGGACAAG
ACCGGAGGGGTCTCCTAGTTCCAAAGGAGATGTACTCCGGGCTTGTTCACGACCTACCGTGTAAGTCG
+
FFF:FFFF:FFFF:FF:FFFFFFF:FFF:FFFFFFFFFFFFFF:FFFFFFFFFF:FFFFFFFFFFFF:
FFFFFFFFFFFFFFFFFFFFFFFFFFFFFFFFFF:FFFFFFF:FFFFFFFFFFFFFFFFFFFF,FFFF
@A00155:342:HHGFNDSXY:1:1272:8305:11397 1:N:0:GAACCTAG+TCCGCATA
GAGCGACTTCTCTCGGGCCCACAGCCTTACAGCTGTGGAGCACGGTATCCTCTGCCGAAACAGAGGTT
GGACAAGACCGGAGGGGTCTCCTAGTTCCAAAGGAGATGTACTCCGGGCTTGTTCACGACCTACCGTG
+
FFFFFFFFFFFFFFFFFFFFFFFFFFFFFFFFFFFFFFFFFFFFFFFFFFFFFFFFFFFFFFFFFFFF
FFFFFFFFFFFFFFFFFFFFFFFFFFFFFFFFFFFFFFFFFFFFFFFFFFFFFFFFFFFFFFFFFFFF
@A00155:342:HHGFNDSXY:1:1272:20690:11553 1:N:0:GAACCTAG+TCCGCATA
GGATGTGGAGGGCGAGAGAGAGCGACTTCTCTCGGGCCCACAGCCTTACAGCTGTGGAGCACGGTATC
CTCTGCCGAAACAGAGGTTGGACAAGACCGGAGGGGTCTCCTAGTTCCAAAGGAGATGTACTCC
+
FFF,FFFFFFFFFFFFFFFFFFFFFF:FFFFFFFFFFFFFFFFFFFFFFFFFFFFFFFFFFFFFFFFF
FFFFFFFFFFFFFFFFFFFFFFFFFFFFFFFFFFFFFFFFFF,F:FFFFFFFFFFFFFFFFFFF
@A00155:342:HHGFNDSXY:1:1272:17381:18161 1:N:0:GAACCTAG+TCCGCATA
TGGTCATCTCAATGCCGAGAGGGATGTGGAGGGCGAGATAGAGCGACTTCTCTCGGGCCCACAGCCTT
ACAGCTGTGGAGCACGGTATCCTCTGCCGAAACAGAGGTTGGACAAGACCGGAGGGGTCTCCTAGTTC
+
FFFFFFFFFFFFFFFFFFFFFFFFFFFFFFFFFFFFFFFFFFFFFFFFFFFFFFFFFFFFFFFFFFFF
FFFFFFFFFFFFFFFFFFFFFFFFFFFFFFFFFFFFFFFFFFFFFFFFFFFFFFFFFFFFFFFFFFFF
@A00155:342:HHGFNDSXY:1:1272:16441:20729 1:N:0:GAACCTAG+TCCGCATA
CTGTGTCCACTTAAGGACTCACCTCTGGCCTATGGTCATCTCAATGCCGAGAGGGATGTGGAGGGCGA
GAGAGAGCGACTTCTCTCGGGCCCACAGCCTTACAGCTGTGGAGCACGGTATCCTCTGCCGAAACAGA
+
FFFF:FFFFFFFFFFFFFFFFFFFFFFFFFFFFFFFFFFFFFFFFFFFFFFFFFFFFFF:FFFFFFF
FFFFFFFFFFFFFFFFFFFFFFF:FFFFFFFFFFFF:FFFFFFFFFFFFFFFFFFFFFFFFFFFFF:F
@A00155:342:HHGFNDSXY:1:1272:12138:30405 1:N:0:GAACCTAG+TCCGCATA
AGGGATGTAGAGGGCGAGAGAGAGCGACTTCTCTCGGGCCCACAGCCTTACAGCTGTGGAGCACGGTA
TCCTCTGCCGAAACAGAGGTTGGACAAGACCGGAGGGGTCTCCTAGTTCCAAAGGAGATGTACTCCGG
+
FFFFFFFFFFFFFFFFFFFFFFFFFFFFF:FFFFFFFFFFFFFFFFFFFFFFFFFFFFFFFFFFFFFF
FFFFFFFFFFFFFFFFFFFFFFFFFFFFFFFFFFFFFFFFFFFFFFFFFFFFFFFFFFFFFFFFFFFF
@A00155:342:HHGFNDSXY:1:1272:10095:34820 1:N:0:GAACCTAG+TCCGCATA
GACTTCTCTCGGGCCCACAGCCTTACAGCTGTGGAGCACGGTATCCTCTGCCGAAACAGAGGTTGGAC
AAGACCGGAGGGGTCTCCTGGTTCCAAAGGAGATGTACTCCGGGCTTGTTCACGACCTACCGTGTAAG
+
FFF:FFFFFFFFFFFFFFFFFFF:FFFFFFFFFFFFFFFFFFFFFFFFFFFFFFFFFFFF:FFFFFFF
FF,FFFFFFFFFFFFFFFFFFFFFFFFFFFFFFFFFFFFFFF,FF:FFFFFFFFFFFFFFFFFFFF,F
@A00155:342:HHGFNDSXY:1:1272:3369:34851 1:N:0:GAACCTAG+TCCGCATA
GAGAGGGATGTGGAGGGCGAGAGAGAGCGACTTCTCTCGGGCCCACAGCCTTACAGCTGTGGAGCACG
GTATCCTCTGCCGAAACAGAGGTTGGACAAGACCGGAGGGGTCTCCTAGTTCCAAAGGAGATGTACTC
+
F:FFFFFFFFFFFF:FFFFFFFFF:FFFFFFFFFFFFFFFFFFFF:FFFFFFFFFFFFFFFFFFFFFF
:FFFFFFFFFFF,FFFFFFFFFFFFFFFFFFFFF,:FFFF:FFFF:FF,FF,FFFFFFFFFFFFFFFF
@A00155:342:HHGFNDSXY:1:1272:29604:35164 1:N:0:GAACCTAG+TCCGCATA
AGGGCGAGAGAGAGCGACTTCTCTCGGGCCCACAGCCTTACAGCTGTGGAGCACGGTATCCTCTGCCG
AAACAGAGGTTGGACAAGACCGGAGGGGTCTCCTAGTTCCAAAGGAGATGTACTCCGGGCTTGTTCAC
+
FFFFFFFFFFFFFFFFFFFFFFFFFFFFFFFFFFFFFFFFFFFFFFFFFFFFFFFFF,FFFFFFFFFF
FFFFFFFFFFFFFFFFFFFFFFFFFFFFFFFFFFFFFFFFFFFFFFFFFFFFFFFFFFFFFFFFFFFF
```

```
@A00155:342:HHGFNDSXY:1:1273:20329:2065 1:N:0:GAACCTAG+TCCGCATA
CAGCCTTACAGCTGTGGAGCACGGTATCCTCTGCCGAAACAGAGGTTGGACAAGACCGGAGGGGTCTC
CTAGTTCCAAAGGAGATGTACTCCGGGCTTGTTCACGACCTACCGTGTAAGTCGTAGTCTAGTAGGCT
+
FFFFFFFFFFFFFFFFFFFFFFFFFFFFFFFFFFFFFFFF:FFFFFFFFFF:FFFFFFFFFFFFFF
FFFFFFFFFFFFFFFFFFFFFFFFFFFFFFFFFFFFFFFFFFFFFFFFFFFFFFFFFFFFFFFFFFFF
@A00155:342:HHGFNDSXY:1:1273:18620:2832 1:N:0:GAACCTAG+TCCGCATA
CAGCCTTACAGCTGTGGAGCACGGTATCCTCTGCCGAAACAGAGGTTGGACAAGACCGGAGGGGTCTC
CTAGTTCCAAAGGAGATGTACTCCGGGCTTGTTCACGACCTACCGTGTAAGTCGTAGTCTAGTAGGCT
+
FFFFFFFFFFFFFFFFFFFFFFFFFFFFFFFFFFFFFFFFFFFFFFFFFF:FFFFFFFFFFFFFFFFF
FFFFFFFFF:FFFFFFFFFFFFFFFFFFFFFFFFFFFFFFFFFFFFFFFF::FFFFFFFFFF:FFFFFFF
@A00155:342:HHGFNDSXY:1:1273:10276:8547 1:N:0:GAACCTAG+TCCGCATA
ACTTCTCTCGGGCCCACAGCCTTACAGCTGTGGAGCACGGTATCCTCTGCCGAAACAGAGGTTGGACA
AGACCGGAGGGGTCTCCTAGTTCCAAAGGAGATGTACTCCGGGCTTGTTCACGACCTACCGTGTAAGT
+
:FFFFFFFFFFFFFFFFFFFFF:FFFFFFFFFFFFFFFFFFFFFFFFFFFFFFFFFFFFFFFFFFFFF
FFFFFFFFFFFFFFFFFFFFFFFFFFFFFFFFFFFFFFFFFFFFFFFFFF:FFFFFFFFFFFFFF:FF
@A00155:342:HHGFNDSXY:1:1273:10438:11804 1:N:0:GAACCTAG+TCCGCATA
CTTCTCTCGGGCCCACAGCCTTACAGCTGTGGAGCACGGTATCCTCTGCCGAAACAGAGGTTGGACAA
GACCGGAGGGGTCTCCTAGTTCCAAAGGAGATGTACTCCGGGCTTGTTCACGACCTACCGTGTAAGTC
+
FFFFFFFFFFFFFFFFFFFFFFFFFFFFFFFFFFFFFFFFFFFFFFFFFFFFFFFFFFFFFFFFFFFF
FFFFFFFFFFFFFFFFFFFFFFFFFFFFFFFFFFFFFFFFFFFFFFFFFFFFFFFFFFFFFFFFFFFF
@A00155:342:HHGFNDSXY:1:1273:31349:16579 1:N:0:GAACCTAG+TCCGCATA
CTTCTCTCGGGCCCACAGCCTTACAGCTGTGGAGCACGGTATCCTCTGCCGAAACAGAGGTTGGACAA
GACCGGAGGGGTCTCCTAGTTCCAAAGGAGATGTACTCCGGGCTTGTTCACGACCTACCGTGTAAGTC
+
FFFFFFFFFFFFFFFFFFFF:FFFFFFFFFFFFFFFFFFFFFFFFFFFFFFFFFFFFFFFFFFFFFFF
FFFFFFFFFFFFFFFFFFFFFFFFFFFFFFFFFFFFFFFFFFFFFFFFFFFFFFFFFFFFFFFFFFFF
@A00155:342:HHGFNDSXY:1:1273:18548:23688 1:N:0:GAACCTAG+TCCGCATA
CTCAATGCCGAGAGGGATGTGGAGGGCGAGAGAGAGCGACTTCTCTCGGGCCCACAGCCTTACAGCTG
TGGAGCACGGTATCCTCTGCCGAAACAGAGGTTGGACAAGACCGGAGGGGTCTCCTAGTTCCAAAGGA
+
FFFFFFFFFFFFFFFFFFFFFFFFFFFFFFFFFFFFFFFFFFFFFFFFFFFFFFFFFFFFFFFFFFFF
FFFFFFFFFFFFFFFFFFFFFFFFFFFFFFFFFFFFFFFFFFFFFFFFFFFFFFFFFFFFFFFFFFFF
@A00155:342:HHGFNDSXY:1:1273:19407:25394 1:N:0:GAACCTAG+TCCGCATA
CTTCTCTCGGGCCCACAGCCTTACAGCTGTGGAGCACGGTATCCTCTGCCGAAACAGAGGTTGGACAA
GACCGGAGGGGTCTCCTGGTTCCAAAGGAGATGTACTCCGGGCTTGTTCACGACCTACCGTGTAAGTC
+
FFFFFFFFFFFFFFFFFFFFFFFFFFFFFFFFFFFFFFFFFFFFFFFFFFFFFFFFFFFFFFFFFFFF
FFFFFFFFFFFFFFFFFFFFFFFFFFFFFFFFFFFFFFFFFFFFFFFFFFFFFFFFFFFFFFFFFFFF
@A00155:342:HHGFNDSXY:1:1273:9182:26349 1:N:0:GAACCTAG+TCCGCATA
GTCATCTCAATGCCGAGAGGGATGTGGAGGGCGAGAGAGAGCGACTTCTCTCGGGCCCACAGCCTTAC
AGCTGTGGAGCACGGTATCCTCTGCCGAAACAGAGGTTGGACAAGACCGGAGGGGTCTCCTAGTTCCA
+
FFFFFFFFFFFFFFFFF:F:FF::FFFF:FFFFFFF:FFFFF,FFFFFFFFFFFFFFFFFFFFFFFFF
FFFFFFFFFFFFFFFFFF:FFFFFFFFFFFF::F:FFFFFFFFFFFFFFFFFFFFFFFFFFF,FFFFFF
@A00155:342:HHGFNDSXY:1:1274:6008:23672 1:N:0:GAACCTAG+TCCGCATA
GCGACTTCTCTCGGGCCCACAGCCTTACAGCTGTGGAGCACGGTATCCTCTGCCGAAACAGAGGTTGG
ACAAGACCGGAGGGGTCTCCTAGTTCCAAAGGAGATGTACTCCGGGCTTGTTCACGACCTACCGTGTA
+
FFFFFFFFFFFFFFFFFFFFFF:F:FFFFFFFFFFFFFFFF:FF:FFFFFFFFFFFFFFFFFFFFFFF
FFFFFFFFFFFFFFFFFFFFFFFFFFFFFFFFFFFFFFFFFFFFFFFFFFFFFFFFFFFFFFFFFFFF
```

```
@A00155:342:HHGFNDSXY:1:1274:23357:33771 1:N:0:GAACCTAG+TCCGCATA
TTCTCTCGGGCCCACAGCCTTACAGCTGTGGAGCACGGTATCCTCTGCCGAAACAGAGGTTGGACAAG
ACCGGAGGGGTCTCCTAGTTCCAAAGGAGATGTACTCCGGGCTTGTTCACGACCTACCGTGTAAGTCG
+
:FFFFFFFFFFFFFFFFFFFFFFFFFFFFFFFFFFFFFFFFFFFFFFFFFFFFFFFFFFFFFFFFFFF
FFFFFFFFFFFFFFFFFFFFFFFFFFFFFFFFF:FFFFFFFFFFFFFFFFFFFFFFFFFFFFFFFFFF
@A00155:342:HHGFNDSXY:1:1274:22525:36464 1:N:0:GAACCTAG+TCCGCATA
TCCTCTGCCGAAACAGAGGTTGGACAAGACCGGAGGGGTCTCCTAGTTCCAAAGGAGATGTACTCCGG
GCTTGTTCACGACCTACCGTGTAAGTCGTAGTCTAGTAGGCTACCTGACGAGTCCTTTTTAGGACGAA
+
FFFFFFFFFFFFFFFFFFFFFFFFFF:FFFFFFFFFFFFFFFFFFFFFFFFFFFFFFFFFFFFFFFFF
FFFFFFFFFFFFFFFFFFFFFFFF:FFFFFFFFFFFFFFFFFFFFFFFFFFFFFFFFFF,FFFFFFFFF
@A00155:342:HHGFNDSXY:1:1275:26901:2957 1:N:0:GAACCTAG+TCCGCATA
GAGCGACTTCTCTCGGGCCCACAGCCTTACAGCTGTGGAGCACGGTATCCTCTGCCGAAACAGAGGTT
GGACAAGACCGGAGGGGTCTCCTAGTTCCAAAGTAGATGTACTCCGGGCTTGTTCACGACCTACCGTG
+
F:FF:F:FFFF:FFFFF,FFFFFF:FFF:FFFFF:FFF:FF::FFF,FFF,FFFFFF:::FFF:FFFF
FFFFFF,FFFFF::FFFF:FFF:FF:F:FFFFF:,F,,:F:,:FFFFFF,FFFFF,FF,FFF,FF::F
@A00155:342:HHGFNDSXY:1:1275:10809:7279 1:N:0:GAACCTAG+TCCGCATA
TATCCTCTGCCGAAACAGAGGTTGGACAAGACCGGAGGGGTCTCCTAGTTCCAAAGGAGATGTACTCC
GGGCTTGTTCACGACCTACCGTGTAAGTCGTAGTCTAGTAGGCTACCTGACGAGTCCTTTTTAGG
+
FFFFFFFFFFFFFFFFFFFFFFFFFFFFFFFFFFFFFFFFFFFFFFFFFFFFFFFFFFFFFFFFFFFF
FFFF:FFFFFFFFFFFFFFFFFFFFFFFFFFFFFFFFFFFFFFFFFFFFFFFFFFFFFF:FFFF
@A00155:342:HHGFNDSXY:1:1275:30617:22952 1:N:0:GAACCTAG+TCCGCATA
CTCCTCTGGCCTATGGTCATCTCAATGCCGAGAGGGATGTGGAGGGCGAGAGAGAGCGACTTCTCTCG
GGCCCACAGCCTTACAGCTGTGGAGCACGGTATCCTCTGCCGAAACAGAGGTTGGACAAGACCGGAGG
+
FFFFFFFFFFFFFFFFFFFFFFFFFFFFFFFFFFFFFFF:FFFFFFFFFFFFFFFFFFFFFFFFFFFF
FFFFFFFFFFFFFFFFFFFFFFFFFFFFFFFFFFFFFFFFFFFFFFFFFFFFFFFFFFFFFFFFFFFF
@A00155:342:HHGFNDSXY:1:1275:32190:27712 1:N:0:GAACCTAG+TCCGCATA
CACATCCCTCTCGGCATTGAGATGACCATAGGCCAGAGGTGAGTCCTTAAGTGGACACAGCTGATCTA
AGGCGGTGTGGCGGGGCATGGGTTTGAACCCCCATGACGGTCGGAGTCTAGTAGGCTCCCTGATGAGT
+
FFFFFFFFFF:FFFFFFFFFFFFFFFFFFFFFFFFFF:F:FFFFFFFFFFFFFFFFFFFFFFFFFFFF
FFFFFFFFFFFFFFFFFFFFFFFF:FFFFFFFFFFFFFFFFFFFFFFFFFFFFFFFFFFFFFFFFFFF
@A00155:342:HHGFNDSXY:1:1276:25247:2566 1:N:0:GAACCTAG+TCCGCATA
CCACATCCCTCTCGGCATTGAGATGACCATAGGCCAGAGGTGAGTCCTTAAGTGGACACAGCTGATCT
AAGGCGGTGTGGCGGGGCATGGGTTTGAACCCCCATGACGGTCGGAGTCTAGTAGGCTCCCTGATGAG
+
FFFFFFFFFFFFFFFFFFFFFFFFFFFFFFFFFFFFFFFFFFFFFFFFFFFFFFFFFFFFFFFF:FFF
FFFFFFFFFFFFFFFFFFFFFFFFFFFFFFFFFFFFFFFFFFFFFFFFFFFFFFFFFFFFFFFFFFFF
@A00155:342:HHGFNDSXY:1:1276:8594:14184 1:N:0:GAACCTAG+TCCGCATA
TGTGTCCACTTAAGGACTCACCTCTGGCCTATGGTCATCTCAATGCCGAGAGGGATGTAGAGGGCGAG
AGAGAGCGACTTCTCTCGGGCCCACAGCCTTACAGCTGTGGAGCACGGTATCCTCTGCCGAAACAGAG
+
FFFFFFFFFFFFFFFFFF:FFFFFF:FF,::FFFFF:FFFFFFFFFFFFFFFFFF:FFFFFFF:FFF,
FFFF:FFFFFFFFFFFFFFFFFFFFFFFFFFFFFFFFFFFFFFFFFFFFFFFFFFFFFFFFFFFFFFF
@A00155:342:HHGFNDSXY:1:1276:4860:14512 1:N:0:GAACCTAG+TCCGCATA
TCTCAATGCCGAGAGGGATGTGGAGGGCGAGAGAGAGCGACTTCTCTCGGGCCCACAGCCTTACAGCT
GTGGAGCACGGTATCCTCTGCCGAAACAGAGGTTGGACAAGACCGGAGGGGTCTCCTGGTTCCAAAGG
+
FFFFFFFFFFFFFFFFFFFFFFFF:FFFFFFFFFFFFFFFFF:FFFFFFFFFFFFFFFFFFFFFFFFFF
FFFFFFFFFFFFFFFFFFFFFFFFFFFFFFFFFFFFFFFFFFFFFF:FFFFFFFFFFFFFFFFFFFFF
```

```
@A00155:342:HHGFNDSXY:1:1276:9435:14763 1:N:0:GAACCTAG+TCCGCATA
TGTGTCCACTTAAGGACTCACCTCTGGCCTATGGTCATCTCAATGCCGAGAGGGATGTAGAGGGCGAG
AGAGAGCGACTTCTCTCGGGCCCACAGCCTTACAGCTGTGGAGCACGGTATCCTCTGCCGAAACAGAG
+
FFFFFFFFFFFFFFFFFFFFFFFFFFFFFFFFFFFFFFF:FFFFFFFFFFFFFFFFFF:FFFFFFFFF
FFFFFFFFFFFFFFFFFFFFFFFFFFFFFFFFFFFFFFFFFFFFFFFFFFFFFFFFFFFFFFFFFFFF
@A00155:342:HHGFNDSXY:1:1276:12301:21793 1:N:0:GAACCTAG+TCCGCATA
TCTCAATGCCGAGAGGGATGTGGAGGGCGAGAGAGAGCGACTTCTCTCGGGCCCACAGCCTTACAGCT
GTGGAGCACGGTATCCTCTGCCGAAACAGAGGTTGGACAAGACCGGAGGGGTCTCCTGGTTCCAAAGG
+
FFFFFFFFFFFFFFFFFF:FFFFFFFFFFFFFFFFFFFFFFFFFFFFFFFFFFFFFFFFF:FFFFFFF
FFFFFFFFFFFFFFFFFFFFFFFFFFFFFFFFFFFFFFFF:FFFFFFFFFFFFFFFFFFFFFFFFFFF
@A00155:342:HHGFNDSXY:1:1276:15438:27602 1:N:0:GAACCTAG+TCCGCATA
AGATCAGCTGTGTCCACTTAAGGACTCACCTCTGGCCTATGGTCATCTCAATGCCGAGAGGGATGTGA
AGGGCGAGAGAGAGCGACTTCTCTCGGGCCCACAGCCTTACAGCTGTGGAGCACGGTATCCTCTGCCG
+
FFFFFFFFFFFFFFFFFFFFFFFFFFFFFFFFFFFFFF:FFFFFFFFFFFFFFFFFFFFFFFFFFFFF
FFFFFFFFFFFFFFFFFF:FFFFFFFFFFFFFFFFFFFFFFFFFFFFFFFFF:FFFFFFFFFFFFFFF
@A00155:342:HHGFNDSXY:1:1277:12138:2973 1:N:0:GAACCTAG+TCCGCATA
GTCCACTTAAGGACTCACCTCTGGCCTATGGTCATCTCAATGCCGAGAGGGATGTAGAGGGCGAGAGA
GAGCGACTTCTCTCGGGCCCACAGCCTTACAGCTGTGGAGCACGGTATCCTCTGCCGAAACAGAGGTT
+
FFFFFFFFFFFFFFFFFFFFFFFFFFFFFFFFFFFFFFFFFFFFFFFFFFFFFFFFFFFFFFFFFFFF
FFFFFFFFFFFFFFFFFFFFFFFFFF,FFFFFFFFFFFFFFFFFFFFFFFFFFFFFFFFFFFFFFFFF
@A00155:342:HHGFNDSXY:1:1277:8974:4006 1:N:0:GAACCTAG+TCCGCATA
CTCTCTCGCCCTCCACATCCCTCTCGGCATTGAGATGACCATAGGCCAGAGGTGAGTCCTTAAGTGGA
CACAGCTGATCTAAGGCGGTGTGGCGGGGCATGGGTTTGAACCCCCATGACGGTCGGAGTCTAGTAGG
+
FFFFFFFFFFFFFFFFFFFFFFFFFFFFFFFFFFFFFFFFFFFFFFFFFFFFFFFFFFFFFFFFFFFF
FFFFFFFFFFFFFFFFFFFFFFFFFFFFFFFFFFFFFFFFFFFFFFFFFFFFFFFFFFFFFFFFFFFF
@A00155:342:HHGFNDSXY:1:1277:14344:16626 1:N:0:GAACCTAG+TCCGCATA
TTACAGCTGTGGAGCACGGTATCCTCTGCCGAAACAGAGGTTGGACAAGACCGGAGGGGTCTCCTAGT
TCCAAAGGAGATGTACTCCGGGCTAGTTCACGACCTACCGTGTAAGTCGTAGTCTAGTAGGCTACCTG
+
FFFFFFFFFFFFFFFFFFFFFFFFFFFFFFFFFFFFFFFFFFFFFFFFFFFFFFFFFFFFFFFFFFFF
FFFFFFFFFFFFFFFFFFFFFFFFFFFFFFFFFFFFFFFFFFFFFFFF,FFFFFFFFFFFFFFFFFFF
@A00155:342:HHGFNDSXY:1:1277:18855:35462 1:N:0:GAACCTAG+TCCGCATA
CAGCTGTGGAGCACGGTATCCTCTGCCGAAACAGAGGTTGGACAAGACCGGAGGGGTCTCCTAGTTCC
AAAGGAGATGTACTCCGGGCTTGTTCACGACCTACCGTGTAAGTCGTAGTCTAGTAGGCTACCTGACG
+
FFFFFFFFFFFFFFFFFFFFFFFFFFFFFFFFFFFFFFFFFFFFF:FFFFFFFFFFFFFFFFF:FFFF
FFFFFFFFFFFFFFFFFFFFFFFFFFFFFFFFFFFFFFFFFFFFFFFFFFFFFFFFFFFFFFFFFFFF
@A00155:342:HHGFNDSXY:1:1278:6696:5822 1:N:0:GAACCTAG+TCCGCATA
ACCTCTGTTTCGGCAGAGGATACCGTGCTCCACAGCTGTAAGGCTGTGGGCCCGAGAGAAGTCGCTCT
CTCTCGCCCTCCACATCCCTCTCGGCATTGAGATGACCATAGGCCAGAGGAGAGTCCTTAAGTGGACA
+
FFFFFFF:FFFFFFFFFFFFFFFFFFFFFFFFFFFFFFFFF:F:FFFF,FFFFFFFFFFFFFFFFFFF
FFFFFFFFFFF:FFFFFFFFFFFFFFF:FFFFFFFFFFF:FF:FFFFFFFFFFFFFF:F:FFFFFFF:
@A00155:342:HHGFNDSXY:1:1278:3649:6558 1:N:0:GAACCTAG+TCCGCATA
ACCTCTTTTTCGGCAGAGGATACCGTGCTCCACAGCTGTAAGGCTGTGGGCCCGAGAGAAGTCGCTCT
CTCTCGCCCTCCACATCCCTCTCGGCATTGAGATGACCATAGGCCAGAGGTGAGTCCTTAAGTGGACA
+
FFFFFFFFFFFFFFFFFFFFFFFFFFFFFFFFFFFFFFFFFFFFFFFFFFFFFFFFFFF,FFFFFFFF
FFFFFFFFFFFFFFFFFFFFFFFFFF,FFFFFFFFFFFFFFFFFFFFFFFFFFFFFFFFFFFFFFFFF
```

@A00155:342:HHGFNDSXY:1:1278:12780:16579 1:N:0:GAACCTAG+TCCGCATA
CCGAGAGGGATGTGGAGGGCGAGAGAGAGCGACTTCTCTCGGGCCCACAGCCTTACAGCTGTGGAGCA
CGGTATCCTCTGCCGAAACAGAGGTTGGACAAGACCGGAGGGGTCTCCTAGTTCCAAAGGAGATGTAC
+
F,,FF:,FFFFF:FFFFFFFFFFF:FFFF:FFFF,,FFFFF:,F,FFFF,FFFFF:F:FFF,::F,FF
F::FFFFFFFFFFF:FFF:F,F,FFFF,FFF::FFF,FFF:FFFFFFF,F:F,F:FFFF,F:FF:FFF
@A00155:342:HHGFNDSXY:1:1278:9182:16799 1:N:0:GAACCTAG+TCCGCATA
TGCCGAGAGGGATGTGGAGGGCGAGAGAGAGCGACTTCTCTCGGGCCCACAGCCTTACAGCTGTGGAG
CACGGTATCCTCTGCCGAAACAGAGGTTGGACAAGACCGGAGGGGTCTCCCAGTTCCAAAGGAGATGT
+
FFFFFFFFFFFFFFFFFFFFFF:FFFFFFFFFFFFFFFFFFFFFFFFFFFFFFFFFFFFFFFFFFFFF
FFFFFFFFFFFFFFFFFFF:FFFFFFFFFFFFFFFFFFFFFFFFFFFFFFFFFFFFFFFFFFFFFFFF
@A00155:342:HHGFNDSXY:1:1278:19397:36526 1:N:0:GAACCTAG+TCCGCATA
CTTCTCTCGGGCCCACAGCCTAACAGCTGTGGAGCACGGTATCCTCTGCCGAAACAGAGGTTGGACAA
GACCGGAGGGGTCTCCTAGTTCCAAAGGAGATGTACTCCGGGCTTGTTCACGACCTACCGTGTAAGTC
+
FFFFFFFFFFFFFFFFFFFFFFFFFFFFFFFFFFFFFFFFFFFFFFFFFFFFFFFFFFFFFFFFFFFF
:FFFFFFFFFFFFFFFFFFFFFFFFFFFFFFFFFFFFFFFFFFFFFFFFFFFFFFFFFFFFFFFFFFF
@A00155:342:HHGFNDSXY:1:1301:13973:1078 1:N:0:GAACCTAG+TCCGCATA
GTCATCTCAATGCCGAGAGGAATGTGGAGGGCGAGAGAGAGCGACTTCTCTCGGGCCCACAGCCTTAC
AGCTGTGGAGCACGGTATCCTCTGCCGAAACAGAGGTTGGACAAGACCGGAGGGGTCTCCTAGTTCCA
+
FFFFFFFFFFFFFFFFFFFFF,FFFFFFFFFFFFFFFFFFFFFFFFFFFFFFFFFFFFFFFFFFFFFF
FFFFFFFFFFFFFFFFFFFFFFFFFFFFFFFFFFFFFFFFFFFFFFFFFFFFFFFFFFFFF:FFFFFF
@A00155:342:HHGFNDSXY:1:1301:5330:12038 1:N:0:GAACCTAG+TCCGCATA
GGACAAGACCGGAGGGGTCTCCTAGTTCCAAAGGAGATGTACTCCGGGCTTGTTCACGACCTACCGTG
TAAGTCGTAGTCTAGTAGGCTACCTGACGAGTCCTTTTTAGGACGAAACTTACACAACTCAAAG
+
FFFFFF:FFFFFFFFFFFFFFFFFFFFFFFFFFFFFFF,FFFFFFFF,,FFFFFFFFFFFFFFFFFFF
FFFFF:FFF:FF:FFFFFFFFFFFFFFF:FFFFF:FFFFFFFFFFFFFFFFFFFFFFFFFFFFF
@A00155:342:HHGFNDSXY:1:1301:11758:25300 1:N:0:GAACCTAG+TCCGCATA
TCAATGCCGAGAGGGATGTGGAGGGCGAGAGAGAGCGACTTCTCTCGGGCCCACAGCCTTACAGCTGT
GGAGCACGGTATCCTCTGCCGAAACAGAGGTTGGACAAGACCGGAGGGGTCTCCTAGTTCCAAAGGAG
+
FFFFFFFFFFFFFFFFFF,FFFFFFFFFFFFFFFFFF,FFFFFFFFFFFFFFFFFFFFFFFFFFFFFF
FF:FFFFFFFFFFFFFFFFFFFFFFFFFFFFFFFFFF:FFF,FF:FFFFFFFFFFFFF,FFFFFFFFF
@A00155:342:HHGFNDSXY:1:1301:11632:25676 1:N:0:GAACCTAG+TCCGCATA
TCAATGCCGAGAGGGATGTGGAGGGCGAGAGAGAGCGACTTCTCTCGGGCCCACAGCCTTACAGCTGT
GGAGCACGGTATCCTCTGCCGAAACAGAGGTTGGACAAGACCGGAGGGGTCTCCTAGTTCCAAAGGAG
+
FFFFFFFFFFFFFFFF:FFFFFFFFFFFFFFFFFFFFFFFFFFFFFFFFFFFFFFFFFFFFFFFFFFF
FFFFFFFFFF:FF:FFFFFFFFFFFFFFFFFFFFFFFFFFFFFFFFFFFFFFFFFFFFFFFFFFFFFF
@A00155:342:HHGFNDSXY:1:1301:25572:26835 1:N:0:GAACCTAG+TCCGCATA
GATGTGGAGGGCGAGAGAGAGCGACTTCTCTCGGGCCCACAGCCTTACAGCTGTGGAGCACGGTATCC
TCTGCCGAAACAGAGGTTGGACAAGACCGGAGGGGTCTCCTAGTTCCAAAGGAGATGTACTCCGGGCT
+
FFFFFFFFFFFFFFFFFFFFFFFFFFFFFFFFFFFFFFFF:FFFF:FFFFFFFFFFFFFFFFFFFFFF
FFFFFFFFFFFFFFFFFFFFFFFFFFFFFFFFFFFFFFFFFFFFFFFFFFFFFFFFFFFFFFFFFFFF
@A00155:342:HHGFNDSXY:1:1301:14181:35321 1:N:0:GAACCTAG+TCCGCATA
GGGCCCACAGCCTTACAGCTGTGGAGCACGGTATCCTCTGCCGAAACAGAGGTTGGACAAGACCGGAG
GGGTCTCCTAGTTCCAAAGGAGATGTACTCCGGGCTTGTTCACGACCTACCGTGTAAGTCG
+
FFFFFFFFFFFF:FFFFFFFFFFFFFFFFFFFFFFFFFFFFFFFFFFFFFFFFFFFFFFFFFFFFFFF
FFFFFFFFFFFFFFFFFFFFFFFFFFFFFFFFFFFFFFFFFFFFFFFFFFFFFFFFFFFFF

```
@A00155:342:HHGFNDSXY:1:1303:8585:8437 1:N:0:GAACCTAG+TCCGCATA
GGCGAGAGAGAGCGACTTCTCTCGGGCCCACAGCCTTACAGCTGTGGAGCACGGTATCCTCTGCCGAA
ACAGAGGTTGGACAAGACCGGAGGGGTCTCCTAGTTCCAAAGGAGATGTACTCCGGGCTTGTTCACGA
+
FFFFFFFFFFFFFFFFFFFFFF:FFFFF:FFFFFFFFFFF:FFFFFFFFFF:FFFFFFFFFFFFFFFF
FFFFFFFFFFFFFFFF:FFFFFFFFFFFFFFFFFFFFFFFFFFFFFFFFFFFFFFFFFFFFFFFFFFF
@A00155:342:HHGFNDSXY:1:1303:3341:9752 1:N:0:GAACCTAG+TCCGCATA
CAATGCCGAGAGGGATGTAGAGGGCGAGAGAGAGCGACTTCTCTCGGGCCCACAGCCTTACAGCTGTG
GAGCACGGTATCCTCTGCCGAAACAGAGGTTGGACAAGACCGGAGGGGTCTCCTAGTTCCAAAGGAGA
+
FFFFFFFFFFFFFFFFFFFFFFFFFF:FFFFFF:,FFFFFFFFFFF,FFFFF:F:FFFFFFFFFFFFF
FFFFFFFFFFFFFFFFFFFFFFFFFFFF,FFFFFFFF,FFFFFFFFFFFFF:FF:FFFFFF,FFFFFF
@A00155:342:HHGFNDSXY:1:1303:20012:18364 1:N:0:GAACCTAG+TCCGCATA
AGAGAGAGCGACTTCTCTCGGGCCCACAGCCTTACAGCTGTGGAGCACGGTATCCTCTGCCGAAACAG
AGGTTGGACAAGACCGGAGGGGTCTACTAGTTCCAAAGGAGATGTACTCCGGGCTTGTTCACGACCTA
+
FFFFFFFFFFFFFFFFFFFFFFFFFFFFFFFF:FFFFFFFFFFFFFFFFFFFFFFFFFFFFFFFFFFF
FFFFFFFFFFFFFFFFFFFFFFFFFFFFFF:FFFFFFFFFFFFFFFFFFFFFFFFFFFFFFFFFFFFF
@A00155:342:HHGFNDSXY:1:1304:12554:2033 1:N:0:GAACCTAG+TCCGCATA
ACCTCTGTTTCGGCAGAGGATACCGTGCTCCACAGCTGTAAGGCTGTGGGCCCGAGAGAAGTCGCTCT
CTCTCGCCCTTCACATCCCTCTCGGCATTGAGATGACCATAGGCCAGAGGTGAGTCCTTAAGTGGACA
+
FFFFFF:FFFFFFFFFFFFFFFFFFFFFFFFFFFFFFFFFFFFFFFFFFFFFFFFFFFFFFFFFFFFF
FFFFFFFFFFFFFFFFFFFFFFFFFFFFFFFFFFFFFFFFFFFFFFFFFFFFFFFFFFFFFFFFFFFF
@A00155:342:HHGFNDSXY:1:1304:18973:27430 1:N:0:GAACCTAG+TCCGCATA
CACCTCTGGCCTATGGTCATCTCAATGCCGAGAGGGATGTGGAGGGCGAGAGAGAGCGACTTCTCTCG
GGCCCACAGCCTTACAGCTGTGGAGCACGGTATCCTCTGCCGAAACAGAGGTTGGACAAGACCGGAGG
+
FFFFFFFFFFFFFFFFFFFFFFFFFFFFFFFFFFFFFFFFFFFFFFFFFFFFFFFFFFFFFFFFFFFF
FFFFFFFFFFFFFFFFFFFFFFFFFFFFFF:FFFFFFFFFFFFFFFFFFFFFFFFFFFFFFFFFFFFF
@A00155:342:HHGFNDSXY:1:1304:19452:36464 1:N:0:GAACCTAG+TCCGCATA
CACCTCTGGCCTATGGTCATCTCAATGCCGAGAGGGATGTGAAGGGCGAGAGAGAGCGACTTCTCTCG
GGCCCACAGCCTTACAGCTGTGGAGCACGGTATCCTCTGCCGAAACAGAGGTTGGACAAGACCGGAG
+
FFFFFFFFFFFFFFFFFFFF:FFFFFFFFF:FFFFFFFFFFFFFFFFFFFFF:FFFFFFFFFFFFFFF
FFFFFFFFFFF:FFFFFFFFFF:FFFFFFFFFFFFFFFFFFFFFFFFFFFFFFFFFFFFFFFFFFFF
@A00155:342:HHGFNDSXY:1:1305:8793:15342 1:N:0:GAACCTAG+TCCGCATA
AGAGAGAGCGACTTCTCTCGGGCCCACAGCCTTACAGCTGTGGAGCACGGTATCCTCTGCCGAAACAG
AGGTTGGACAAGACCGGAGGGGTCTCCTAGTTCCAAAGGAGATGTACTCCGGGCTTGTTCACGACCTA
+
FFFFFF,FFFFFFFFFFFFFFFFFFFFFF:FFFFFFFFF::F,FFFFFFFFFFFFFFFFFFFFFFFFF
FFFFFFFFF,FFFFFFFFFFFFFFFFFFF,FFFFFFFFFF:FFFFFFFFFFFFFFFFFFFFFFFFFFF
@A00155:342:HHGFNDSXY:1:1305:16893:16783 1:N:0:GAACCTAG+TCCGCATA
CTTCTCTCGGGCCCACAGCCTTACAGCTGTGGAGCACGGTATCCTCTGCCGAAACAGAGGTTGGACAA
GACCGGAGGGGTCTCCTAGTTCCAAAGGAGATGTACTCCGGGCTTGTTCACGACCTACCGTGTAAGTC
+
FFFFFFFFFFFFFFFFFFFFFFFFFFFFFFFFFFF:FFFFFFFFFFFFFFFFFFFFFFFFFFFFFFFF
F,FFFFFFFFFFFFFFFFFFFFFFFFFFFFFFFFFFFFFFFFFFFFFFFFFFFFFFFFFFFFFFFFFF
@A00155:342:HHGFNDSXY:1:1306:11379:4131 1:N:0:GAACCTAG+TCCGCATA
ATGCCGAGAGGGATGTGGAGGGCGAGAGAGAGCGACTTCTCTCGGGCCCACAGCCTTACAGCTGTGGA
GCACGGTATCCTCTGCCGAAACAGAGGTTGGACAAGACCGGAGGGGTCTCCTGGTTCCAAAGGAGATG
+
F:FF:FFFFFFFF,FFFFFFFFFFFFFFFFF,,FFF::FFFF:FFFFFFFFFF,FFFFFFFFFFFF,F
FFFF:FFFFFFFFFF:FFFFFFFFFFFFFFFFFF:FF:F:FFFFFFFFFFFFFFFFFFFFFFFFFFFF
```

@A00155:342:HHGFNDSXY:1:1306:9001:4899 1:N:0:GAACCTAG+TCCGCATA
ATGCCGAGAGGGATGTGGAGGGCGAGAGAGAGCGACTTCTCTCGGGCCCACAGCCTTACAGCTGTGGA
GCACGGTATCCTCTGCCGAAACAGAGGTTGGACAAGACCGGAGGGGTCTCCTGGTTCCAAAGGAGATG
+
FFFFFFFFFFFFFFFFFFFFFFFFFFFFFFFFFFFF:FFFFFFFFFFFFFFFFFFFFFFFFFFFFFFF
FFFFFFFFFFFFFFFFFFFFFFFFFFFFFFFFFF:F:FFFFFFFFFFFFFFFFFFFFFFFFFFFFFFF
@A00155:342:HHGFNDSXY:1:1306:32244:6010 1:N:0:GAACCTAG+TCCGCATA
ACCTCTGTTTCGGCAGAGGATACCGTGCTCCACAGCTGTAAGGCTGTGGGCCCGAGAGAAGTCGCTCT
CTCTCGCCCTCCACATCCCTCTCGGCATTGAGATGACCATAGGCCAGAGGAGAGTCCTTAAGTGGACA
+
FFFFFFFFFFFFFFFFFFFFFFFFFFFFFFFFFFFFFF:FFFFFFFFFFFFFFFFFFFFFFFFFFFFF
FFFFFFFFFFFFFFFFFFFFFFFFFFFF:FFFFFFFFFFFFFFFFFFFFFFFFFFFFFFFFFFFFFFF
@A00155:342:HHGFNDSXY:1:1306:16938:6590 1:N:0:GAACCTAG+TCCGCATA
GAGAGGGATGTGGAGGGCGAGAGAGAGCGACTTCTCTCGGGCCCACAGCCTTACAGCTGTGGAGCACG
GTATCCTCTGCCGAAACAGAGGTTGGACAAGACCGGAGGGGTCTCCTAGTTCCAAAGGAGATGTACTC
+
FFFFFFFFFFFFFFFFFFFFFFFFFFFFFFFFFFFFFFFFFFFFFFFFFFFFFFFFFFFFFFFFFFFF
FFFFFFFFFFFFFFFFFFFFFFFFFFFFFFFFFFFFFFFFFFFFFFFFFFFFFFFFFFFFFFFFFFFF
@A00155:342:HHGFNDSXY:1:1306:2239:14575 1:N:0:GAACCTAG+TCCGCATA
CTTCTCTCGGGCCCACAGCCTTACAGCTGTGGAGCACGGTATCCTCTGCCGAAACAGAGGTTGGACAA
GACCGGAGGGGTCTCCTGGTTCCAAAGGAGATGTACTCCGGGCTTGTTCACGACCTACCGTGTAAGTC
+
FFFFFFFFFFFFFFFFFFFFFFFFFFFFFFFFFFFFFFFFFFFFFF:FFFFFFFFFFFF:FFFFFFFF
FFFFFFFFFFFFFFFFFFFFFFFFFFFFFFFFFFFFFFFFFFFFFFFFF:FFFFFFFFFFFFFFFFFF
@A00155:342:HHGFNDSXY:1:1306:3206:28025 1:N:0:GAACCTAG+TCCGCATA
CCTTACAGCTGTGGAGCACGGTATCCTCTGCCGAAACAGAGGTTGGACAAGACCGGAGGGGTCTCCTA
GTTCCAAAGGAGATGTACTCCGGGCTTGTTCACGACCTACCGTGTAAGTCGTAGTCTAGTAGGCTACC
+
FFFFFFFFFFFFFFFFFFFF:FFFFFFFFFFFFFFFFFF:FFFFFFFFF,F:FFFFFFFFFFFFFFFF
FFFFFFFFFFFFFFFFFFFFFFFFFFFFFFFFF:FFFFFFFFFFFFFFFFFFFFFFFFFFFFFFFFFF
@A00155:342:HHGFNDSXY:1:1307:23927:3223 1:N:0:GAACCTAG+TCCGCATA
CCTCTGGCCTATGGTCATCTCAATGCCGAGAGGGATGTAGAGGGCGAGAGAGAGCGACTTCTCTCGGG
CCCACAGCCTTACAGCTGTGGAGCACGGTATCCTCTGCCGAAACAGAGGTTGGACAAGAC
+
FFFFFFFFFFFFFFFFFFFFFFFFFFFF:FFFFFF,FF:FFFFFFFFFFFFFFFFFFFFFFFFFFFFF
FFFFFFFFFFFFFFFFFF:FFFFFFFFFFFFFFFFFFFFFFFFFFFFFFFFFFFFFFFFF
@A00155:342:HHGFNDSXY:1:1307:32922:12853 1:N:0:GAACCTAG+TCCGCATA
AGATCAGCTGTGTCCACTTAAGGACTCACCTCTGGCCTATGGTCATCTCAATGCCGAGAGGGATGTGA
AGGGCGAGAGAGAGCGACTTCTCTCGGGCCCACAGCCTTACAGCTGTGGAGCACGGTATCCTCTGCCG
+
FFFFFFFFFFFFFFFFFFFFFFFFFFFFFFFFFFFFFF,FFF:FFFFFFFFFFFFFFF:FFFFFFFFF
FFFFFFFFFFFFFFFFFF,FFFFFFFFFFFFFFFFFFFFFF:FFFFFFFFFFFFFFFFFFFFFFFFFF
@A00155:342:HHGFNDSXY:1:1307:9308:29042 1:N:0:GAACCTAG+TCCGCATA
AGAGAGAGCGACTTCTCTCGGGCCCACAGCCTTACAGCTGTGGAGCACGGTATCCTCTGCCGAAACAG
AGGTTGGACAAGACCGGAGGGGTCTCCTAGTTCCAAAGGAGATGTACTCCGGGCTTGTTCACGACCTA
+
FFFFFFFFFFFFFFFFFF:FFFFFFF:FFFFFFFFFFFFF:F:FFFFFFFFFFFFFFFF:FFFFFFFF
FFFFFFFFFFFFFFFFFFFFFFFFFFFFFFFFFFFFFFFFFFFFFFFFF:FFFFFFFFFFFFFFFFF
@A00155:342:HHGFNDSXY:1:1307:6325:34741 1:N:0:GAACCTAG+TCCGCATA
GAGCGACTTCTCTCGGGCCCACAGCCTTACAGCTGTGGAGCACGGTATCCTCTGCCGAAACAGAGGTT
GGACAAGACCGGAGGGGTCTCCTAGTTCCAAAGGAGATGTACTCCGGGCTTGTTCACGACCTACCGTG
+
FF,FFFFF:FFFFFFFFFFFFFFFFFF,FFFFFFFF:FFFFFFFFFFFFFFFFF,FFFFFF:FF,FFF
F:FFF:FFFFFFFFFFFFFFFFFFFFFFFFFFFFFFFFFFFFFFFFFFF:FFFFFFFFFFFFFFFFFF

```
@A00155:342:HHGFNDSXY:1:1307:9155:35790 1:N:0:GAACCTAG+TCCGCATA
AGAGAGAGCGACTTCTCTCGGGCCCACAGCCTTACAGCTGTGGAGCACGGTATCCTCTGCCGAAACAG
AGGTTGGACAAGACCGGAGGGGTCTCCTAGTTCCAAAGGAGATGTACTCCGGGCTTGTTCACGACCTA
+
FFFFFFFFFFF:FFFFFFF:FFFFFFFFFFF:FFFFFFFFFFFFFFFFFFFFFFFFFFFFFFFFFFFF
FFFFFFFFFFFFFFFFFFFFFFFFFFFFFFFFFFFFFFFFFFFFFFFFFFFF:FFFFFFFFFFFFFFF
@A00155:342:HHGFNDSXY:1:1308:30770:7216 1:N:0:GAACCTAG+TCCGCATA
CTTCTCTCGGGCCCACAGCCTTACAGCTGTGGAGCACGGTATCCTCTGCCGAAACAGAGGTTGGACAA
GACCGGAGGGGTCTCCTAGTTCCAAAGAAGATGTACTCCGGGCTTGTTCACGACCTACCGTGTAAGTC
+
FFFFFFFFFFFFFFFFFFFFFFFFFFFFFFFFFFFFFFFFFFFFFFFFFFFFFFFFFFFFFFFFFFFF
FFFFFFFFFFFFFFFFFFFFFFFFFFFFFFFFFFFFFFFFFFFFFFFFFFFFFFFFFFFFFFFFFFFF
@A00155:342:HHGFNDSXY:1:1308:17824:25880 1:N:0:GAACCTAG+TCCGCATA
TTCTCTCGGGCCCACAGCCTTACAGCTGTGGAGCACGGTATCCTCTGCCGAAACAGAGGTTGGACAAG
ACCGGAGGGGTCTCCTAGTTCCAAAGGAGATGTACTCCGGGCTTGTTCACGACCTACCGTGTAAGTCG
+
FFFFFFFFFFFFFFFFFFFFFFFFFFFFFFFFFFFFFFFFFFFFFFFFFFFFFFFFFFFFFFFFFFFF
FFFFFFFFFFFFFFFFFFFFFFFFFFFFFFFFFFFFFFFFFFFFFFFFFFFFFFFFFFFFFFFFFFFF
@A00155:342:HHGFNDSXY:1:1309:19000:15702 1:N:0:GAACCTAG+TCCGCATA
CAATGCCGAGAGGGATGTGGAGGGCGAGAGAGAGCGACTTCTCTCGGGCCCACAGCCTTACAGCTGTG
GAGCACGGTATCCTCTGCCGAAACAGAGGTTGGACAAGACCGGAGGGGTCTCCTAGTTCCAAAGGAGA
+
FFFFFFFFFFFFFFFFFFFFFFFFFFFFFFFFFFFFFFFFFFFFFFFFFFFFFFFFFFFFFFFFFFFF
FFFFFFFFFFFFFFFFFFFFF:FFFFFFF:FFFFFFFFFFFFFFFFFFFFFFFFFFFFFFFFFFFFFF
@A00155:342:HHGFNDSXY:1:1309:14317:21621 1:N:0:GAACCTAG+TCCGCATA
CCTTACAGCTGTGGAGCACGGTATCCTCTGCCGAAACAGAGGTTGGACAAGACCGGAGGGGTCTCCTG
GTTCCAAAGGAGATGTACTCCGGGCTTGTTCACGACCTACCGTGTAAGTCGTAGTCTAGTAGGCTACC
+
FFFFFFFFFFFFFFFFFFFFFFFFFFFFFFFFFFFFFFFFFFFFFFFFFFFFFFFFFFFFFFFFFFFF
FFFFFFFFFFFFFFFFFFFFFFFFFFFFFFFFFFFFFFFFFFFFFFFFFFFFFFFFFFFFFFFFFFFF
@A00155:342:HHGFNDSXY:1:1309:17011:23281 1:N:0:GAACCTAG+TCCGCATA
GAGGGCGAGAGAGAGCGACTTCTCTCGGGCCCACAGCCTTACAGCTGTGGAGCACGGTATCCTCTGCC
GAAACAGAGGTTGGACAAGACCGGAGGGGTCTCCTGGTTCCAAAGGAGATGTACTCCGGGCTTGTTCA
+
FFFFFFFFFFFFFFFFFFFFFFFFFFFFFFFFFFF:FFFFFFFFFFFFF:FFFFFFFFFFFFFFFFFF
FFFFFFFFFFFFFFFFFFFFFFFFFFFFFFFFFFFFFFFFFFFFFFFFFFFFFFFFFFFFFFFFFFFF
@A00155:342:HHGFNDSXY:1:1310:12463:1438 1:N:0:GAACCTAG+TCCGCATA
CACCTCTGGCCTATGGTCATCTCAATGCCGAGAGGGATGTGAAGGGCGAGAGAGAGCGACTTCTCTCG
GGCCCACAGCCTTACAGCTGTGGAGCACGGTATCCTCTGCCGAAACAGAGGTTGGACAAGACCGGAGG
+
FFFFFFFFFFFFFFFFFFFFFFFFFFFFFFFFFFFFFFFFFFFFFFFFFFFFFFFFFFFFFFFFFFF:
FFFFFFFFFFFFFFFFFFFFFFFFFFFFFFFFFF:FFFFFFFFFFFFFFFFFFFFFFFF:FFFFFFFFF
@A00155:342:HHGFNDSXY:1:1310:32000:13416 1:N:0:GAACCTAG+TCCGCATA
GGATGTAGAGGGCGAGAGAGAGCGACTTCTCTCGGGCCCACAGCCTTACAGCTGTGGAGCACGGTATC
CTCTGCCGAAACAGAGGTTGGACAAGACCGGAGGGGTCTCCTAGTTCCAAAGGAGATGTACTCC
+
FFFFFFFFFFFFFFFFFFFFFFFFFFF:FFFFFFFFFFFFFFFFFFFFFFFFFF:FFFFFFFFFFFFFF
FFFFFFFFFFFFFFFFFFFFFFFFFFFFFFFFFFFFFFFFFFFFFFFFFFFFFFFFFFFFFFFF
@A00155:342:HHGFNDSXY:1:1310:24930:13667 1:N:0:GAACCTAG+TCCGCATA
GTATCCTCTGCCGAAACAGAGGTTGGACAAGACCGGAGGGGTCTCCTGGTTCCAAAGGAGATGTACTC
CGGGCTTGTTCACGACCTACCGTGTAAGTCGTAGTCTAGTAGGCTACCTGACGAGTCCTTTTTAGGAC
+
FFFFFFFFFFFFFFFFFFFFFFFFFFFFFFFFFFFFFF:FFFFFFFFFFFFFFFFFFFFFFFFFFFFF
FFFFF:FFFFFFFFFFFFFFFFFFFFFFFFFFFFFFFF:FFFFFFFFFFFFFFFFFFFFFFFFFFFFFF
```

```
@A00155:342:HHGFNDSXY:1:1310:20383:16658 1:N:0:GAACCTAG+TCCGCATA
CCTACTAGACTCCGACCGTCATGGGGGTTCAAACCCATGCCCCACCACACCGCCTTAGATCAGCTGTG
TCCACTTAAGGACTCACCTCTGGCCTATGGTCATCTCAATGCCGAGAGGGATGTGGAGGGCGAGAGAG
+
FFFFFFFFFFFFFFFFFFFFFFFFFFFF:FFFFFFFFFFFFFFFFFFFFFFFFFFFFFFFFFFFFFFF
FFFFFFFFFFFFFFFFFFFFFFFFFFFFFFFFFFFFFFFFFFFFFFFFFFF:FF,FFFFFFFFFFFFFF
@A00155:342:HHGFNDSXY:1:1310:9489:28385 1:N:0:GAACCTAG+TCCGCATA
TTCTCTCGGGCCCACAGCCTTACAGCTGTGGAGCACGGTATCCTCTGCCGAAACAGAGGTTGGACAAG
ACCGGAGGGGTCTCCTAGTTCCAAAGGAGATGTACTCCGGGCTTGTTCACGACCTACCGTGTAAGTCG
+
FFFFFFFFFFFFFFFFFFFFFFFFFFFFFFFFFFFFFFFFFFFFFFFFFFFFFFFFFFFFFFFFFFF:
FFFFFFFFFFFFFFFFFFFFFFFFFFFFFFFFFFFFFFFFFFFFFFFFFFFFFFFFFFFFFFFFFFFF
@A00155:342:HHGFNDSXY:1:1310:30671:28995 1:N:0:GAACCTAG+TCCGCATA
CCTTACAGCTGTGGTGCACGGTATCCTCTGCCGAAACAGAGGTTGGACAAGACCGGAGGGGTCTCCTA
GTTCCAAAGGAGATGTACTCCGGGCTTGTTCACGACCTACCGTGTAAGTCGTAGTCTAGTAGGCTACC
+
FFFFFFFFFFFFF:FFFFFFFFFFFFFFFFFFFFFFFFFFFFFFFFFFFFFFFFFFFFFFFFFFFFFF
FFFFFFFFFFFFFFFFFFFFFFFFFFFFFFFFFFFFFFFFFFFFFFFFFFFFFFFFFFFFFFFFFFFF
@A00155:342:HHGFNDSXY:1:1311:18005:1517 1:N:0:GAACCTAG+TCCGCATA
GAGGGCGAGAGAGAGCGACTTCTCTCGGGCCCACAGCCTTACAGCTGTGGAGCACGGTATCCTCTGCC
GAAACAGAGGTTGGACAAGACCGGAGGGGTCTCCTAGTTCCAAAGGAGATGTACTCCGGGCTTGTTCA
+
FFFFF:FFFFFFFFFFFFFFFFFFFFFFFFFFFFFFFFFFF:FFFFFFFFFFFFFFFFFFFFFFFFFFF
,FFFFFFFFFFF,FFFFF:FF::FFFFFFFFFFFF:FF:FFFFF:,FFFFFFFFFF:F,FFFFFF::F
@A00155:342:HHGFNDSXY:1:1311:7328:9142 1:N:0:GAACCTAG+TCCGCATA
GGATGTGGAGGGCGAGAGAGAGCGACTTCTCTCGGGCCCACAGCCTTACAGCTGTGGAGCACGGTATC
CTCTGCCGAAACAGAGGTTGGACAAGACCGGAGGGGTCTCCTAGTTCCAAAGGAGATGTACTCC
+
FFFFFFFFFFFFFFFFFFFFFFFFFFFFFFFFFFFFFFFFFFFFFFF:FFFFFFFFFFFFFFFFFFFF
FFFFFFFFFFFFFFFFFFFFFFFFFFFFFFFFFFFFFFFFFFFFFFFFFFFFFFFFFFFFFFFF
@A00155:342:HHGFNDSXY:1:1311:26784:10426 1:N:0:GAACCTAG+TCCGCATA
CTTCTCTCGGGCCCACAGCCTTACAGCTGTGGAGCACGGTATCCTCTGCCGAAACAGAGGTTGGACAA
GACCGGAGGGGTCTCCTAGTTCCAAAGGAGATGTACTCCGGGCTTGTTCACGACCTACCGTGTAAGTC
+
FFFFFFFFFFFFFFFFFFFFFFFFFFFFFFFFFFFFFFFFFFFFFFFFFFFFFFFFFFFFFFFFFFFF
FFFFFFFFFFFFFFFFFFFFFFFFFFFFFFFFFFFFFFFFFFFFFFFFFFFFFFFFFFFFFFFFFFFF
@A00155:342:HHGFNDSXY:1:1311:12174:19883 1:N:0:GAACCTAG+TCCGCATA
GTCCACTTAAGGACTCTCCTCTGGCCTATGGTCATCTCAATGCCGAGAGGGATGTGGAGGGCGAGAGA
GAGCGACTTCTCTCGGGCCCACAGCCTTACAGCTGTGGAGCACGGTATCCTCTGCCGAAACAGAGGTT
+
FFFFFFFFFFFFFFFFFFFFFFFFFFFFFFFFFFFFFFFFFFFFFFFFFFFFFFFFFFFFFFFFFFFF
FFFFFFFFFFFFFFFFFFFFFFFFFFFFFFFFFFFFFFFFFFFFFFFFFFFFFFFFFFFFFFFFFFFF
@A00155:342:HHGFNDSXY:1:1311:27380:27054 1:N:0:GAACCTAG+TCCGCATA
TTCTCTCGGGCCCACAGCCTTACAGCTGTGGAGCACGGTATCCTCTGCCGAAACAGAGGTTGGACAAG
ACCGGAGGGGTCTCCTAGTTCCAAAGGAGATGTACTCCGGGCTTGTTCACGACCTACCGTGTAAGTCG
+
FFFFFFFFFFFFFFFFFFFFFFFFFFFFFFFFFFFFFFFF:FFFFFFFFFFFFFFFFFFFFFFFFFFF
FFFFFFFFFFFFFFFFFFFFFFFFFFFF:FFFFFFFFFFFFFFFFFFFFFFFFFFFFFFFFFFF:FFFF
@A00155:342:HHGFNDSXY:1:1311:18412:29528 1:N:0:GAACCTAG+TCCGCATA
TGCTCCACAGCTGTAAGGCTGTGGGCCCGAGAGAAGTCGCTCTCTCTCGCCCTCTACATCCCTCTCGG
CATTGAGATGACCATAGGCCAGAGGTGAGTCCTTAAGTGGACACAGCTGATCTAAGGCGGT
+
FFFFFFFFFFFFFFFFFFFFFFFFFFFFFFFFFFFFFFFFFFFFFFFFFFFFFFFFFFFFFFFFFFFF
FFFFFFFFFFFFFFFFFFFFFFFFFFFFFFFFFFFFFFFFFFFFFFFFFFFFFFFFFFFFF
```

```
@A00155:342:HHGFNDSXY:1:1311:22959:31579 1:N:0:GAACCTAG+TCCGCATA
ACTTCTCTCGGGCCCACAGCCTTACAGCTGTGGAGCACGGTATCCTCTGCCGAAACAGAGGTTGGACA
AGACCGGAGGGGTCTCCTGGTTCCAAAGGAGATGTACTCCGGGCTTGTTCACGACCTACCGTGTAAGT
+
FFFFFFFFFFFFFFFFFFFFFFFFFFFFFFFFFFFFFFFFFFFFFFFFFFFFFFFFFFFFFFFFFFFF
FFFFFFFFFFFFFFFFFFFFFFFFFFFFFFFFFFFFFFFFFFFFFFFFFFFFFFFFFFFFFFFFFFF:
@A00155:342:HHGFNDSXY:1:1311:9570:32064 1:N:0:GAACCTAG+TCCGCATA
TTCTCTCGGGCCCACAGCCTTACAGCTGTGGAGCACGGTATCCTCTGCCGAAACAGAGGTTGGACAAG
ACCGGAGGGGTCTCCTAGTTCCAAAGGAGATGTACTCCGGGCTTGTTCACGACCTACCGTGTAAGTCG
+
FFFFFFFFFFFFFFFFFFFFFFFFFFFFFFFFFFFFFFFFFFFFFFFFFFFFFFFFFFFFFFFFFFFF
FFFFFFFFFFFFFFFFFFFFFFFFFFFFFFFFFFFFFF::FFFFFFFFFFFFFFFFFFFFFFFFFFF:
@A00155:342:HHGFNDSXY:1:1311:25418:36182 1:N:0:GAACCTAG+TCCGCATA
CTATGCCGAGAGGGATGTGGAGGGCGAGAGAGAGCGACTTCTCTCGGGCCCACAGCCTTACAGCTGTG
GAGCACGGTATCCTCTGCCGAAACAGAGGTTGGACAAGACCGGAGGGGTCTCCTAGTTCCAAAGGAGA
+
FFFFFFFFFFFFFFFFFFFFFFFFF,FFFFFFFFFFFFFFFFFFFFFFFFFFFFFFFF::FFFFFFFF
:FFFFFFFFFFFFFFFFFFFFFFFFFFFFFFFFFFFF:FFFFFFFF,FFFFFFFFFFFFFFFFFFFFF
@A00155:342:HHGFNDSXY:1:1312:14705:1720 1:N:0:GAACCTAG+TCCGCATA
AGGGATGTGGAGGGCGAGAGAGAGCGACTTCTCTCGGGCCCACAGCCTTACAGCTGTGGAGCACGGTA
TCCTCTGCCGAAACAGAGGTTGGACAAGACCGGAGGGGTCTCCTAGTTCCAAAGGAGATGTACTCCGG
+
FFFFFFFFFFFFFFFFFFFFFFFFFFFFFFFFFFFFFFFFFFFFFFFFFFFFFFFFFFFFFFFFFFFF
FFFFFFFFFFFFFFFFFFFFFFFFFFFFFFFFFFFFFFFFFFFFFFFFFFFFFFFFFFFFFFFFFFFF
@A00155:342:HHGFNDSXY:1:1312:23755:4053 1:N:0:GAACCTAG+TCCGCATA
ATCTCAATGCCGAGAGGGATGTGGAGGGCGAGAGAGAGCGACTTCTCTCGGGCCCACAGCCTTACAGC
TGTGGAGCACGGTATCCTCTGCCGAAACAGAGGTTGGACAAGACCGGAGGGGTCTCCTAGTTCCAAAG
+
FFFFFFFFFF,FFFFFFFFFF:FFFF:FFFFFFFFFFF:FFFFFFFFFFFFFFFFFFFFFF:FFFFFF
FFFFFFFFFFFFFFFFFFFFFFFFFFFFFFFFFFFFFFFFFFFFFFFFFFFFFFFFF:FFFFFFFFF
@A00155:342:HHGFNDSXY:1:1312:3233:10629 1:N:0:GAACCTAG+TCCGCATA
TGCCGAGAGGGATGTGGAGGGCGAGAGAGAGCGACTTCTCTCGGGCCCACAGCCTTACAGCTGTGGAG
CACGGTATCCTCTGCCGAAACAGAGGTTGGACAAGACCGGAGGGGTCTCCTAGTTCCAAAGGAGATGT
+
FFFFFFFFFFFFFF:FF,FFFF:FFFFFFFF,FFFFFFFFFFFFFFFFFFFFFFFFFFFFFFFFFFFF
FFFFFFFFFFFFFFFFFFFFFFFFFFFFFFFFFFFFFFFFFFFFFFFFFFFFFFFFFFFFFFFFFFFF
@A00155:342:HHGFNDSXY:1:1312:14769:30452 1:N:0:GAACCTAG+TCCGCATA
CTTCTCTCGGGCCCACAGCCTTACAGCTGTGGAGCACGGTATCCTCTGCCGAAACAGAGGTTGGACAA
GACCGGAGGGGTCTCCTAGTTCCAAAGGAGATGTACTCCGGGCTTGTTCACGACCTACCGTGTAAGTC
+
FFFF,FFFFFFFFFFFFFFFFFFFFFFFFFFFFFFFFFFFFFFFFFFFFFFFFFFFFFFFFFFFFFFF
FFFFFFFFFFFFFFFFFFFFFFFFFFFFFFFFFFFFFFFFFFFFFFFFFFFFFFFFFFFFFFFFFFFF
@A00155:342:HHGFNDSXY:1:1312:9579:36401 1:N:0:GAACCTAG+TCCGCATA
GCGACTTCTCTCGGGCCCACAGCCTTACAGCTGTGGAGCACGGTATCCTCTGCCGAAACAGAGGTTGG
ACAAGACCGGAGGGGTCTCCTGGTTCCAAAGGAGATGTACTCCGGGCTTGTTCACGACCTACCG
+
,FFF:F,FF,F::,,FFF:FF,FF,FFFF,FFFFFFF,FFF,,,FFFFF::F:FFFFF:F:FF,:F,F
:FFFF::FFFFF,FFFFF,FF,F:FFF:FF:FF:F,F:F:F,F,FFFFF::FFF:F:FF:FF:F
@A00155:342:HHGFNDSXY:1:1312:29414:36746 1:N:0:GAACCTAG+TCCGCATA
GGATGTGGAGGGCGAGAGAGAGCGACTTCTCTCGGGCCCACAGCCTTACAGCTGTGGAGCACGGTATC
CTCTGCCGAAACAGAGGTTGGACAAGACCGGAGGGGTCTCCTAGTTCCAAAGGAGATGTACTCC
+
FFFFFFFFFFFFFFFFFFFFFFFFFFFFFFFFFFFFFFFFFFFFFFFFFFFFFFFFFFFFFFFFFFFF
FFFFFFFFFFFFFFFFFFFFFFFFFFFFFFFFF:FFFFFFF:FFFFFFFFFFFFFFFFFFFFFF
```

```
@A00155:342:HHGFNDSXY:1:1313:19759:13009 1:N:0:GAACCTAG+TCCGCATA
AGAGAGAGCGACTTCTCTCGGGCCCACAGCCTTACAGCTGTGGAGCACGGTATCCTCTGCCGAAACAG
AGGTTGGACAAGACCGGAGGGGTCTCCTAGTTCCAAAGGAGATGTACTCCGGGCTTGTTCACGACCTA
+
FFFFFFFFFFFF:FFFFFFF:FFFFFFF:FF:F:FFFFF,FFFFFF:F,F,:F:,FFF,FFFFFFFFF
FF:F::FF,FF:F:FFFF:F:,FFFFF,FFF:F:FFF:FFFFF,FFF,,FFFF:FFFF,FFF:FFFF:
@A00155:342:HHGFNDSXY:1:1313:13675:16626 1:N:0:GAACCTAG+TCCGCATA
TGGTCATCTCAATGCCGAGAGGGATGTGGAGGGCGAGAGAGAGCGACTTCTCTCGGGCCCACAGCCTT
ACAGCTGTGGAGCACGGTATCCTCTGCCGAAACAGAGGTTGGACAAGACCGGAGGGGTCTCCTAGTTC
+
FFFFFFFFFFFFFFFFFFFFFFFFFFFFFFFFFFFFFFFFFFFFFFFFFFFFFFFFFFFFFFFFFFFF
FFFFFFFFFFFFFFFFFFFFFFFFFFFFFFFFFFFFFFFFFFFFFFFF:FFFFFFFFFFFFFFFFFFF
@A00155:342:HHGFNDSXY:1:1313:19018:19492 1:N:0:GAACCTAG+TCCGCATA
ATGCCGAGAGGGATGTGGAGGGCGAGAGAGAGCGACTTCTCTCGGGCCCACAGCCTTACAGCTGTGGA
GCACGGTATCCTCTGCCGAAACAGAGGTTGGACAAGACCGGAGGGGTCTCCTGGTTCCAAAGGAGATG
+
FFFFFF:FFFFFFFFFFFFFFFFFFFFFFFFFFFFFFFFFFFF:FFFFFFFFFFFFFFFFFFFFFFFF
FFFFFFFFFFFFFFFFFFFFFFFFFFFFFFFFFFFFFFFFFFFFFFFFFFFFFFFFFFFFFFFFFFFF
@A00155:342:HHGFNDSXY:1:1313:6596:34585 1:N:0:GAACCTAG+TCCGCATA
GGATGTGGAGGGCGAGAGAGAGCGACTTCTCTCGGGCCCACAGCCTTACAGCTGTGGAGCACGGTATC
CTCTGCCGAAACAGAGGTTGGACAAGACCGGAGGGGTCTCCTAGTTCCAAAGGAGATGTACTCC
+
FFFFF:FFFFFFFFFFFFFFFFFFFFFFFFFFFFFFFFFFFFFFF:F:FFFF:F:FFFFFFFFFFFFF
FFFFFFFFFFFFFFFFF:FFFFFFFFFFFFFFFFFFFFFFFFFFFFFFFFFFFFFFFFFFFFFF
@A00155:342:HHGFNDSXY:1:1314:9308:4617 1:N:0:GAACCTAG+TCCGCATA
ACTTCTCTCGGGCCCACAGCCTTACAGCTGTGGAGCACGTTATCCTCTGCCGAAACAGAGGTTGGACA
AGACCGGAGGGGTCTCCTAGTTCCAAAGGAGATGTACTCCGGGCTTGTTCACGACCTACCGTGTAAGT
+
FF:FFFFFFFFFFFFFFFFF::,FFFFFFFF:FFFFFF:,:FFFFFFF:FFFFFFFFFFFFFFFFFFF
FFFFFFFFFFFFFFFFFFFFFFFFFFFF,FFFFFFFFFFF::FFFFFFFFFFFFFFFFFFFFFFFFFF
@A00155:342:HHGFNDSXY:1:1314:4246:14638 1:N:0:GAACCTAG+TCCGCATA
TCTCTCGGGCCCACAGCCTTACAGCTGTGGAGCACGGTATCCTCTGCCGAAACAGAGGTTGGACAAGA
CCGGAGGGGTCTCCTAGTTCCAAAGGAGATGTACTCCGGGCTTGTTCACGACCTACCGTGTAAGTCGT
+
FFFFFFFFFFFFFFFFFF:FFFFFFFFFFFFFFF,FFFFFFFFFFFFFFFFFFFFFFFFFFFFFFFFF
FFFFFFFFFFFFFFFFFFFFFFFFFFFFFFFFFFFFFFFFFFFFFFFFFFFFFFFFFFFFFF:FFFF:
@A00155:342:HHGFNDSXY:1:1315:2871:6089 1:N:0:GAACCTAG+TCCGCATA
AGGGCGAGAGAGAGCGACTTCTCTCGGGCCCACAGCCTTACAGCTGTGGAGCACGGTATCCTCTGCCG
AAACAGAGGTTGGACAAGACCGGAGGGGTCTCCTAGTTCCAAAGGAGATGTACTCCGGGCTTGTTCAC
+
FFF::FFFFFFFFFFFFFFF,FFFFFFF:,FFFFFFFF:FFF::FFFF:FFFFFFFFFFFFFFFFFFF
FFFFFFFFFFFF:FFFFFFFFF:FFFFFFFFFFFFFFF,FFFF:FFFFFFFF:FFFFFFFFFFFFFFF
@A00155:342:HHGFNDSXY:1:1315:11143:12211 1:N:0:GAACCTAG+TCCGCATA
ACTTCTCTCGGGCCCACAGCCTTACAGCTGTGGAGCACGGTATCCTCTGCCGAAACAGAGGTTGGACA
AGACCGGAGGGGTCTCCTAGTTCCAAAGGAGATGTACTCCGGGCTTGTTCACGACCTACCGTGTAAGT
+
FF,FFFFFFFFFFFFFFFFFFFFFFFFFFFFFFFFFFFFFFFFFFFFFFFFFFFFFFFFFFFFFFFFF
F:FFFFFFFFFFFFFFFFFFFFFFFFFFFFFFFFFFFFFFFFFFFFFFFFFFFFFFFFFFF,FFFFFF
@A00155:342:HHGFNDSXY:1:1315:19524:15045 1:N:0:GAACCTAG+TCCGCATA
AGAGAGAGCGACTTCTCTCGGGCCCACAGCCTTACAGCTGTGGAGCACGGTATCCTCTGCCGAAACAG
AGGTTGGACAAGACCGGAGGGGTCTCCTGGTTCCAAAGGAGATGTACTCCGGGCTTGTTCACGACCTA
+
FFFFFFFFFFFFFFFFFFFFFFFFFFFFFFFFFFFFFFFFFFFFFFFFFFFFFFFFFFFFFFFFFFFF
FFFFFFFFFFFFFFFFFFFFFFFFF:FFFFFFFFFFFFFFFFFFFFFFFFFFFFFFFFFFF:FFFFFF
```

```
@A00155:342:HHGFNDSXY:1:1315:18973:23046 1:N:0:GAACCTAG+TCCGCATA
GGATGTGGAGGGCGAGAGAGAGCGACTTCTCTCGGGCCCACAGCCTTACAGCTGTGGAGCACGGTATC
CTCTGCCGAAACAGAGGTTGGACAAGACCGGAGGGGTCTCCTGGTTCCAAAGGAGATGTACTCC
+
FFFFFFFFFFFFFFFFFFFFFFFFFFFFFFFFFFFFFFFFFFFFFFFFFFFFFFFFFFFFFFFFFFFF
FFFFFFFFFFFFFFFFFFFFFFFFFFFFFFFFFFFFFFFFFFFFFFFFFFFFFFFFFFFFFFFF
@A00155:342:HHGFNDSXY:1:1315:18285:26334 1:N:0:GAACCTAG+TCCGCATA
ACTTCTCTCGGGCCCACAGCCTTACAGCTGTGGAGCACGGTATCCTCTGCCGAAACAGAGGTTGGACA
AGACCGGAGGGGTCTCCTAGTTCCAAAGGAGATGTACTCCGGGCTTGTTCACGACCTACCGTGTAAGT
+
FFFFFFFFFFFFFFFFFFFFFFFFFFFFFFFFFFFFFFFFFFFFFFFFFFFFFFFFFFFFFFFFFFFF
FFFFFFFFFFFFFFFFFFFFFFFFFFFFFFFFFFFFFFFFFFFFFFFFFFFFFFFFFFFFFFFFFFFF
@A00155:342:HHGFNDSXY:1:1315:23900:27790 1:N:0:GAACCTAG+TCCGCATA
GAGCGACTTCTCTCGGGCCCACAGCCTTACAGCTGTGGAGCACGGTATCCTCTGCCGAAACAGAGGTT
GGACAAGACCGGAGGGGTCTCCTAGTTCCAAAGGAGATGTACTCCGGGCTTGTTCACGACCTACCGTG
+
FFFFFFFFFFFFFFFFFFFFFFFFFFFFFFFFFFFFFFFFFFFFFFFFFFFFFFFFFFFF:FFFFFFF
FFFFFFFFFFFFFFFFFFFFFFFFFFFFFFFFFFFFFFFFFFFFFFFFF:FFFFFFFFFFFFFFFFFF
@A00155:342:HHGFNDSXY:1:1315:25409:32158 1:N:0:GAACCTAG+TCCGCATA
CTCTGCCGAAACAGAGGTTGGACAAGACCGGAGGGGTCTCCTAGTTCCAAAGGAGATGTACTCCGGGC
TTGTTCACGACCTACCGTGTAAGTCGTAGTCTAGTAGGCTACCTGACGAGTCCTTTTTAGGACGAAAC
+
FFFFFFFFFFFFFFFFFFFFFFFFFFFFFFFFFFFFFFFFFFFFFFFFFFFFFFFFFFFFFFFFFFFF
FFFFFFFFFFFFFFFFFFFFFFFFFFFFFFFFFFFFFFFF:FFFFFFFFFFFFFFFFFFF:FFFFFFF
@A00155:342:HHGFNDSXY:1:1315:1615:36605 1:N:0:GAACCTAG+TCCGCATA
AAGGCGAGAGAGAGCGACTTCTCTCGGGCCCACAGCCTTACAGCTGTGGAGCACGGTATCCTCTGCCG
AAACAGAGGTTGGACAAGACCGGAGGGGTCTCCTAGTTCCAAAGGAGATGTACTCCGGGCTTGTTCAC
+
FFFFFFFFFFFFFFFFFFFFF:FFFFFFFFFF,F,,,FFFFFF,FFFF::F:FFFFFFFFFFF:FFFF
,FFF:FFFFFFFFFFFFFFFFFFFFFFFFFFFFFFFFFFFFF,:FFFFFFFFFFFFFFFFFFFFFFFF
@A00155:342:HHGFNDSXY:1:1316:31656:2926 1:N:0:GAACCTAG+TCCGCATA
AGAGAGAGCGACTTCTCTCGGGCCCACAGCCTTACAGCTGTGGAGCACGGTATCCTCTGCCGAAACAG
AGGTTGGACAAGACCGGAGGGGTCTCCTAGTTCCAAAGGAGATGTACTCCGGACTTGTTCACGACCTA
+
FFFFFFFFFFFFFFFFFFFFFFFFFFFFFFFFFFFFFFFFFFFFFFFFFFFFFFFFFFFFFFFFFFFF
FFFFFFFFFFFFFFFFFFFFFFFFFFFFFFFFFFFFFFFFFFFFFFFFFFFFFFFFFFFFFFFFFFF:
@A00155:342:HHGFNDSXY:1:1316:30418:2942 1:N:0:GAACCTAG+TCCGCATA
CCCACCACACCGCCTTAGATCAGCTGTGTCCACTTAAGGACTCACCTCTGGCCTATGGTCATCTCAAT
GCCGAGAGGGATGTGGAGGGCGAGAGAGAGCGACTTCTCTCGGGCCCACAGCCTTACAGCTGTGGAGC
+
FFFFFFFFFFFFFFFFFFFFF:FFFFFFFFFFFFFFFFFFFFFFFFFFFFFFFFFFF:FFFFFFFFFF
F:FFFFFFFFF:FFFFFFFFFFFFFFFFFF:FFFFFFFFFFFFFFFFFFFFFFFF:FFFFFFFF:FFF
@A00155:342:HHGFNDSXY:1:1316:6759:35336 1:N:0:GAACCTAG+TCCGCATA
CCCACAGCCTTACAGCTGTGGAGCACGGTATCCTCTGCCGAAACAGAGGTTGGACAAGACCGGAGGGG
TCTCCTAGTTCCAAAGGAGATGTACTCCGGGCTTGTTCACGACCTACCGTGTAAGTCGTAGTCTAGTA
+
FFFFFFFFFFFFFFFFFFFFFFFFFFFFFFFFFFFFFFFFFFFFFFFFFFFFFFFFFFFFFFFFFFFF
FFFFFFFFFFFFFFFFFFFFFFFFFFFF:FFFFFFFFFFFFFFFFFFFFFFFFFFFFFFFFFFFFFFF
@A00155:342:HHGFNDSXY:1:1317:17101:2550 1:N:0:GAACCTAG+TCCGCATA
AGCGACTTCTCTCGGGCCCACAGCCTTACAGCTGTGGAGCACGGTATCCTCTGCCGAAACAGAGGTTG
GACAAGACCGGAGGGGTCTCCTAGTTCCAAAGGAGATGTACTCCGGGCTTGTTCACGACCTACCGTGT
+
FFFFFFFFFFFFFFFFFFFFFFFFFFFFFFFFFFFFFFFFFFFFFFFFFFFFFFFFFFFFFFFFFFFF
FFFFFFFFFFFFFFFFFFFFFFFFFF:FFFFFFFFFFFFFFFFFFFFFFFFFFFFFFFFFFFFFFFFF
```

```
@A00155:342:HHGFNDSXY:1:1317:19036:6402 1:N:0:GAACCTAG+TCCGCATA
GAGCGACTTCTCTCGGGCCCACAGCCTTACAGCTGTGGAGCACGGTATCCTCTGCCGAAACAGAGGTT
GGACAAGACCGGAGGGGTCTCCTGGTTCCAAAGGAGATGTACTCCGGGCTTGTTCACGACCTACCGTG
+
FFFFFFFFFFFFFFFFFFFFFFFFFFF:FFFFFFFFFFFFFFFFFFFFFFFFFFFFFFFFFFFFFFFF
FFFFFFFFFFFFFFFFFFFFFFFFFFFFFFFFFFFFFFFFFFFFFFFFFFFFFFFFFFFFFFFFFFFF
@A00155:342:HHGFNDSXY:1:1317:20229:6527 1:N:0:GAACCTAG+TCCGCATA
GAGCGACTTCTCTCGGGCCCACAGCCTTACAGCTGTGGAGCACGGTATCCTCTGCCGAAACAGAGGTT
GGACAAGACCGGAGGGGTCTCCTGGTTCCAAAGGAGATGTACTCCGGGCTTGTTCACGACCTACCGTG
+
FFFFFFFFFFFFFFFFFFFFFFFFFFFFFFFFFFFFFFFFFFFFFFFFFFFFFFFFFFFFFFFFFFFF
FFFFFFFFFFFFFFFFFFFFFFFFFFFFFFFFFFFFFFFFFFFFFFFFFFFFFFFFFFFFFFFFF:F:F
@A00155:342:HHGFNDSXY:1:1317:14651:8641 1:N:0:GAACCTAG+TCCGCATA
GAGCGACTTCTCTCGGGCCCACAGCCTTACAGCTGTGGAGCACGGTATCCTCTGCCGAAACAGAGGTT
GGACAAGACCGGAGGGGTCTCCTAGTTCCAAAGGAGATGTACTCCGGGCTTGTTCACGACCTACCGTG
+
FFFFFFFFFFFFFFFFFFFFFFFFFFFF:FFFFFFFF:FFFFFFFFFFFFFFFFFFFFFFFFFFF:FF
FFFFFFFFFFFFFFFFFFFFFFFF:FFFFFFF,FFFFFFFFFFFFFFFFFFFFFFFFFFFFFF,FFF:F
@A00155:342:HHGFNDSXY:1:1317:29369:10645 1:N:0:GAACCTAG+TCCGCATA
TGCCGAGAGGGATGTGGAGGGCGAGAGAGAGCGACTTCTCTCGGGCCCACAGCCTTACAGCTGTGGAG
CACGGTATCCTCTGCCGAAACAGAGGTTGGACAAGACCGGAGGGGTCTCCTGGTTCCAAAGGAGATGT
+
FFFFFFFFFFFFFFFFFFFFFFFFFFFFFFFFFFFFFFFFFFFFFFFFFFFFFFFFFFFFFFFFFFFF
FFFFFFFFFFFFFFFFFFFFFFFFFFFFFFFFFFFFFFFFFFFFFFFFFFFFFFFFFFFFFFFFFFFF
@A00155:342:HHGFNDSXY:1:1317:23628:14418 1:N:0:GAACCTAG+TCCGCATA
CGACTTCTCTCGGGCCCACAGCCTTACAGCTGTGGAGCACGGTATCCTCTGCCGAAACAGAGGTTGGA
CAAGACCGGAGGGGTCTCCTAGTTCCAAAGGAGATGTACTCCGGGCTTGTTCACGACCTACCGTGTAA
+
FFFFFFFFFFFFFFFFFFFFFFFFFFFFFFFFFFFFFFFFFFFFFFFFFFFFFFFFFFFFFFFFFFFF
FFFFFFFFFFFFFFFFFFFFFFFFFFFFFFFFFFFFFFFFFFFFFFFFFFFFFFFFFFFFFFFFFFFF
@A00155:342:HHGFNDSXY:1:1317:21052:14465 1:N:0:GAACCTAG+TCCGCATA
ATGCCGAGAGGGATGTAGAGGGCGAGAGAGAGCGACTTCTCTCGGGCCCACAGCCTTACAGCTGTGGA
GCACGGTATCCTCTGCCGAAACAGAGGTTGGACAAGACCGGAGGGGTCTCCTAGTTCCAAAGGAGATG
+
FFFFFFFFFFFFFFFF:FFFFFFFFFFFFFFFFFFFFFFFFFFFFFFFFFFFFFFFFF,FFFFFFFFF
FFFFFFFFFFFFFFFFFFFFFFFFFFFFFFFFFFFFFFFFFFFFFFFFFFFFFF:FFFFFFFFFFFFF
@A00155:342:HHGFNDSXY:1:1317:8983:14606 1:N:0:GAACCTAG+TCCGCATA
CTTCTCTCGGGCCCACAGCCTTACAGCTGTGGAGCACGGTATCCTCTGCCGAAACAGAGGTTGGACAA
GACCGGAGGGGTCTCCTAGTTCCAAAGGAGATGTACTTCGGGCTTGTTCACGACCTACCGTGTAAGTC
+
FFFFFFFFFFFFFFFFFFFFFFFFFFFFFFFFFFFFFFFFFFFFFFFFFFFFFFFFFFFFFFFFF:F:F
FFFFFFFFFFFFFFFFFFFFFFFFFF:FFFFFFF:FFFFFFFFFFFFFFFFFFFFFFFFFFFFFFFFFF
@A00155:342:HHGFNDSXY:1:1318:22968:4789 1:N:0:GAACCTAG+TCCGCATA
CTCTGGCCTATGGTCATCTCAATGCCGAGAGGGATGTGAAGGGCGAGAGAGAGCGACTTCTCTCGGGC
CCACAGCCTTACAGCTGTGGAGCACGGTATCCTCTGCCGAAACAGAGGTTGGACAAGACCGGAGGGGT
+
FFFFFFFFFFFFFFFFFFFFFFFFFFFFFFFFFFFFFFFFFFFFFFFFFFFFFFFFFFFFFFFFFFFF
FFFFFFFFFFFFFFFFFFFFFFFFFFFFF,FFFFFFFFFFFFFFFFFFFFFFFFFFFFFFFFFFFFFF
@A00155:342:HHGFNDSXY:1:1318:32796:21872 1:N:0:GAACCTAG+TCCGCATA
AGAGAGCGACTTCTCTCGGGCCCACAGCCTTACAGCTGTGGAGCACGGTATCCTCTGCCGAAACAGAG
GTTGGACAAGACCGGAGGGGTCTCCTGGTTCCAAAGGAGATGTACTCCGGGCTTGTTCACGACCTACC
+
FFFFFFFFFFFFFFFFFFFFFFFFFFFFFF:FFFFFFFFFFFFFFFFFFFFFFFFFFFFFFFFFFFFF
FFFFFFFFFFFFFFFFFFFFFFFFFFFFFFFFFFFFFFFFFFFFFFFF:FF:FFFFFFFFFFFFFFF
```

@A00155:342:HHGFNDSXY:1:1319:21197:3349 1:N:0:GAACCTAG+TCCGCATA
TGGTCATCTCAATGCCGAGAGGGATGTGGAGGGCGAGAGAGAGCGACTTCTCTCGGGCCCACAGCCTT
ACAGCTGTGGAGCACGGTATCCTCTGCCGAAACAGAGGTTGGACAAGACCGGAGGGGTCTCCTAGTTC
+
FFFFFFFFFFFFFFFFFFF:FFFFFFFFFFFFFFFFFFFFFFFFFFFFFFFFFFFFFFFFFFFFFFFF
FFFFFFFFFFFFFFFFFFFFFFFFFFFFFFFFFFFFFFFFFFFFFFFFFFFFFFFFFFFFFFFFFFFF
@A00155:342:HHGFNDSXY:1:1319:12807:8140 1:N:0:GAACCTAG+TCCGCATA
AGCGACTTCTCTCGGGCCCACAGCCTTACAGCTGTGGAGCACGGTATCCTCTGCCGAAACAGAGGTTG
GACAAGACCGGAGGGGTCTCCTAGTTCCAAAGGAGATGTACTCCGGGCTTGTTCACGACCTACCGTGT
+
FFFFFFFFFFFFFFFFFFFFFFFFFFFFFFFFFFFFFFFFFFFFFFFFFFFFFFFFFFFFFFFFFFFF
FFFFFFFFFFFFFFFFFFFFF:FFFFFFFFFFFFFFFFFFFFFFFFFFFFFFFFFFFFFFFFFFFFFF
@A00155:342:HHGFNDSXY:1:1320:12029:13088 1:N:0:GAACCTAG+TCCGCATA
GAGCGACTTCTCTCGGGCCCACAGCCTTACAGCTGTGGAGCACGGTATCCTCTGCCGAAACAGAGGTT
GGACAAGACCGGAGGGGTCTCCTAGTTCCAAAGGAGATGTACTCCGGGCTTGTTCACGACCTACCGTG
+
FFFFFFFFFFFFFFFFFFFFFFFFFFFFFFFFFFFFFFFFFFFFFFFFFFFFFFFFFFFFFFFFFFFF
FFFFFFFFFFFFFFFFFFFFFFFFFFFFFFFFFFFFFFFFFFFFFFFFFFFFFFFFFFFFFFFFFFFF
@A00155:342:HHGFNDSXY:1:1320:17734:17769 1:N:0:GAACCTAG+TCCGCATA
CTTCTCTCGGGCCCACAGCCTTACAGCTGTGGAGCACGGTATCCTCTGCCGAAACAGAGGTTGGACAA
GACCGGAGGGGTCTCCTAGTTCCAAAGGAGATGTACTCCGGGCTTGTTCACGACCTACCGTGTAAG
+
FFFFFFFFFFFFFFFFFFFFFFFFFFFFFFFFFFFFFFFFFFFFFFFFFFFFFFFFFFFFFFFFFFFF
FFFFFFFFFFFFFFFFFFFFFFFFFFFFFFFFFFFFFFFFFFFFFFFFFFFFFFFFFFFFFFFFFF
@A00155:342:HHGFNDSXY:1:1320:5023:18208 1:N:0:GAACCTAG+TCCGCATA
CTTCTCTCGGGCCCACAGCCTTACAGCTGTGGAGCACGGTATCCTCTGCCGAAACAGAGGTTGGACAA
GACCGGAGGGGTCTCCTAGTTCCAAAGGAGATGTACTCCGGGCTTGTTCACGACCTACCGTGTAAGTC
+
FFFFFFFFFFFFFFFFFFFFFFF:FFFFFFFFF:FFFFFFFFFFFFF,FFFFFFFFFFFFFFFFFFFF
FFFFFFFFFFFFFFFFFFFFFFFFFFFFFF:FFFFFFFFFFFFFFFFFFFFFFFFFFFFFFFFFFFFF
@A00155:342:HHGFNDSXY:1:1320:31259:20525 1:N:0:GAACCTAG+TCCGCATA
ACTTACACGGTAGGTCGTGAACAAGCCCGGAGTACATCTCCTTTGGAACTAGGAGACCCCTCCGGTCT
TGTCCAACCTCTGTTTCGGCAGAGGATACCGTGCTCCACAGCTGTACGGCTGTGGGCCCGAGAGAAGT
+
FF:FFFFFFF::FFFFF:FFFFFFFFFFFFFFFFF:FFF:F,F,FFFFF:FF:FFFFFFFFFFFFFF:
FFFFFFFFFFFFFFFFF:,FF:FFF,FFFFFF,:FFFFFFFFFF::FF,:FFFFFFF,FF:FF:F:FF::
@A00155:342:HHGFNDSXY:1:1320:17282:21778 1:N:0:GAACCTAG+TCCGCATA
AGCGACTTCTCTCGGGCCCACAGCCTTACAGCTGTGGAGCACGGTATCCTCTGCCGAAACAGAGGTTG
GACAAGACCGGAGGGGTCTCCTGGTTCCAAAGGAGATGTACTCCGGGCTTGTTCACGACCTACCGTGT
+
FFFFFFFFF:FFFFFFFFFFFFFFFFFFFFFFFFFFFFFFFFFFFFFFFFFFFFFFFFFFFFFFFFFF
FFFFFFFFFFFFFFFFFFFFFFFFFFFFFFFFFFFFFF:FFFFF:FFFFFFFFFFFF:FFFFFFFFFF
@A00155:342:HHGFNDSXY:1:1320:23032:23437 1:N:0:GAACCTAG+TCCGCATA
TTCTCTCGGGCCCACAGCCTTACAGCTGTGGAGCACGGTATCCTCTGCCGAAACAGAGGTTGGACAAG
ACCGGAGGGGTCTCCTAGTTCCTAAGGAGATGTACTCCGGGCTTGTTCACGACCTACCGTGTAAGTCG
+
FFFFFFFFFFFFFFFFFFF,FFFFFFFFFFFFFFFFFFFFFFFFFFFFFFFFFF:FFFFFFFFFFFFF
FFFFFFFFFFFFFFFFFFFFFFFFFFFFFFFFFFFFFFFFFFF:FFFFFFFFFFFFFFFF:FFFFFFF
@A00155:342:HHGFNDSXY:1:1320:23782:24205 1:N:0:GAACCTAG+TCCGCATA
TTCTCTCGGGCCCACAGCCTTACAGCTGTGGAGCACGGTATCCTCTGCCGAAACAGAGGTTGGACAAG
ACCGGAGGGGTCTCCTAGTTCCTAAGGAGATGTACTCCGGGCTTGTTCACGACCTACCGTGTAAGTCG
+
FFFFFFFFFFFFFFFFFFFFFFFFFFFFFFFFFFFFFFF:FFFFFFFFFFFFFFFFFFFFFFFFFFFF
FFFFFFFFFFFFFFFFFFFFFFFFFFFFFFFFFFFFFFFFFFFFFFFFFFFFFFFFFFFFFFFFFFFF

```
@A00155:342:HHGFNDSXY:1:1321:11333:7717 1:N:0:GAACCTAG+TCCGCATA
CCTTACAACTGTGGAGCACGGTATCCTCTGCCGAAACAGAGGTTGGACAAGACCGGAGGGGTCTCCTA
GTTCCAAAGGAGATGTACTCCGGGCTTGTTCACGACCTACCGTGTAAGTCGTAGTCTAGTAGGCTACC
+
FFFFFFFFFFFFFFFFFFFFFFFFFFFFFFFFFFFFFFFFFFFFFFFFFFFFFFFFFFFFFFFFFFFF
FFFFFFFFFFFFFFFFFFFFFFFFFFFFFFFFFFFFFFFFFFFFFFFFFFFFFFFFFFFFFFFFFF:F
@A00155:342:HHGFNDSXY:1:1321:25373:16282 1:N:0:GAACCTAG+TCCGCATA
AGGGCGAGAGAGAGCGACTTCTCTCGGGCCCACAGCCTTACAGCTGTGGAGCACGGTATCCTCTGCCG
AAACAGAGGTTGGACAAGACCGGAGGGGTCTCCTAGTTCCAAAGGAGATGTACTCCGGGCTTGTTCAC
+
FFFF:FFFFFFFFFFFFFFFFFFFFFFFFFFF:FFFFFF,FFFFFFFFFFFFFFFFFFFFFFFFFFFF
FFFFFFFFFFFFFFFFFFFFFFFFFFFFFF:FFFFFFFFFFFFF:FFFFFF,:FFFFFFFFFFFFFFF
@A00155:342:HHGFNDSXY:1:1321:15031:16845 1:N:0:GAACCTAG+TCCGCATA
TGTCCACTTAAGGACTCACCTCTGGCCTATGGTCATCTCAATGCCGAGAGGGATGTGGAGGGCGAGAG
AGAGCGACTTCTCTCGGGCCCACAGCCTTACAGCTGTGGAGCACGGTATCCTCTGCCGAAACAGAGGT
+
FFFFFFFFFFFFFFFFFFFFFFFFFFFFFFFFFFFFFFFFFFFFFFFFFFFFFFFFFFFFFFFFFFFF
FFFFFFFFFFFFFFFFFFFFFFFFFFFFFFFFFFFFFFFFFFFFFFFFFFFFFFFFFFFFFFFFFFFF
@A00155:342:HHGFNDSXY:1:1321:15311:18364 1:N:0:GAACCTAG+TCCGCATA
CCTTACAGCTGTGGAGCACGGTATCCTCTGCCGAAACAGAGGTTGGACAAGACCGGAGGGGTCTCCTA
GTTCCAAAGGAGATGTACTCCGGGCTTGTTCACGACCTACCGTGTAAGTCGTAGTCTAGTAGGCTACC
+
FF,F,FFFFFFFFFFFFFFFFFFFFFFFFFFFFFFFFFFFFFFFFFFFFFFFFFFFFFFFFFFFFFFF
FFFFFFFFF:FFFFFFFFFF:FFFFFFFFFFFFFFFFFFFFFFFFFF,FFFFFFFFFFFFFFFFFFFF:
@A00155:342:HHGFNDSXY:1:1321:19623:31688 1:N:0:GAACCTAG+TCCGCATA
TGGTCATCTCAATGCCAAGAGGGATGTGGAGGGCGAGAGAGAGCGACTTCTCTCGGGCCCACAGCCTT
ACAGCTGTGGAGCACGGTATCCTCTGCCGAAACAGAGGTTGGACAAGACCGGAGGGGTCTCCTAGTTC
+
FFFFF:FFFFFFFFFFFFFFFFFF:FFFFFFFFFFFFFFFFFFFFFFFFFFFFFFFFFFFFFFFFFFFF
FFFFFFFFFFFFFFFFFFFFFFFFFFFFFFFFFFFFFFFFFFFFFFFFFFF:FFFFFFFFFFFFFFFF
@A00155:342:HHGFNDSXY:1:1322:11279:5462 1:N:0:GAACCTAG+TCCGCATA
CTCAATGCCGAGAGGGATGTGGAGGGCGAGAGAGAGCGACTTCTCTCGGGCCCACAGCCTTACAGCTG
TGGAGCACGGTATCCTCTGCCGAAACAGAGGTTGGACAAGACCGGAGGGGTCTCCTAGTTCCAAAGGA
+
FFFFFFFFFFFFFFFFFFFFFFFFFFFFFFFFFFFFFFFFFFFFFFFFFFFFFFFFFFFFFFFFFFFF
FFFFFFFFFFFFFFFFFFFFFFFFFFFFFFFFFFFFFFFFFFFFFFFFFFFFFFFFFFFFFFFFFFFF
@A00155:342:HHGFNDSXY:1:1322:15167:9784 1:N:0:GAACCTAG+TCCGCATA
CTTCTCTCGGGCCCACAGCCTTACAGCTGTGGAGCACGGTATCCTCTGCCGAAACAGAGGTTGGACAA
GACCGGAGGGGTCTCCTAGTTCCAAAGGAGATGTACTCCGGGCTTGTTCACGACCTACCGTGTAAGTC
+
FFFFFFFFFFFFFFFFFFFFFFFFFFFFFFFFFFFFFFFFFFFFFFFFFFFFFFFFFFFFFFFFFFFF
FFFFFFFFFFFFFFFFFFFFFFFFFFFFFFFFFFFFFFFFFFFFFFFFFFFFFFFFFFFFFFFFFFFF
@A00155:342:HHGFNDSXY:1:1322:25211:21825 1:N:0:GAACCTAG+TCCGCATA
TTCTCTCGGGCCCACAGCCTTACAGCTGTGGAGCACGGTATCCTCTGCCGAAACAGAGGTTGGACAAG
ACCGGAGGGGTCTCCTAGTTCCAAAGGAGATGTACTCCGGGCTTGTTCACGACCTACCGTGTAAGTCG
+
,FFFFFFFFFFFFFFFFFFFF:FFFFFFFFFFFFFFFFF,FFFFFFFFFFFFFFFFFFFFFFFFFFFF
FFFFFFFFFFFFFFFFFFFFFFFFFFFF:FFFFFFFFFFFFFFFFFFFFF:FFFFFFFFFFFFF,FFFF
@A00155:342:HHGFNDSXY:1:1322:27010:36370 1:N:0:GAACCTAG+TCCGCATA
AAGGACTCACCTCTGGCCTATGGTCATCTCAATGCCGAGAGGGATGTGGAGGGCGAGAGAGAGCGACT
TCTCTCGGGCCCACAGCCTTACAGCTGTGGAGCACGGTATCCTCTGCCGAAACAGAGGTTGGAC
+
FFFFFFFFFFFFFFFFFFFFFFFFFFFFFFFFFFFFF:FFFFFFFFFFFFFFFFFFFFFFFFFFFFF:
FFFFFFFFFFFFFFFFFFFFFFFFFFFFFFFFFFFFFFFFFFFFFFFFFFFFFFFFFFFFFFF
```

```
@A00155:342:HHGFNDSXY:1:1323:9516:1720 1:N:0:GAACCTAG+TCCGCATA
TCCTCTGCCGAAACAGAGGTTGGACAAGACCGGAGGGGTCTCCTAGTTCCAAAGGAGATGTACTCCGG
GCTTGTTCACGACCTACCGTGTAAGTCGTAGTCTAGTAGGCTACCTGACGAGTCCTTTTTAGGACGAA
+
FFFFFFFFFFFFFFFFFFFFFFFFFFFFFFFFFFFFFFFFFFFFFFFFFFFFFFFFFFFFFFFFFFFF
FFFFFFFFFFFFFFFFFFFFFFFFFFFFFFFFFFFFFFFFFFFFFFFFFFFFFFFFF:FFFFFFFFFF
@A00155:342:HHGFNDSXY:1:1323:23891:3411 1:N:0:GAACCTAG+TCCGCATA
GGATATGGAGGGCGAGAGAGAGCGACTTCTCTCGGGCCCACAGCCTTACAGCTGTGGAGCACGGTATC
CTCTGCCGAAACAGAGGTTGGACAAGACCGGAGGGGTCTCCTAGTTCCAAAGGAGATGTACTCCGGGC
+
FFF:FFFFFFFFFFFFFFFFFFFFFFFFFFFFFFFFFFFFFFFFFFFFFFFFFFFFFFFFFFFFFFFF
FFFFFFFFFFFFFFFFFFFFFFFFF:FFFFFFFFFFFFFFFFFFFFFFFFFFFFFFFF:FFFFFFFFF
@A00155:342:HHGFNDSXY:1:1324:17047:4711 1:N:0:GAACCTAG+TCCGCATA
GGATGTGGAGGGCGAGAGAGAGCGACTTCTCTCGGGCCCACAGCCTTACAGCTGTGGAGCACGGTATC
CTCTGCCGAAACAGAGGTTGGACAAGACCGGAGGGGTCTCCTAGTTCCAAAGGAGATGTACTCC
+
FFFFFFFFFFFFFFFFFFFFFFFFFFFFFFFFFFFFFFFFFFFFFFFFFFFFFFFFFFFFFFFFFFFF
FFFFFFFFFFFFF:FFFFFFFFFFFFFFFFFFF:FFFFFFFFFFFFFFFFFFFFFFFFFFFFFF
@A00155:342:HHGFNDSXY:1:1324:2953:13933 1:N:0:GAACCTAG+TCCGCATA
GGATGTGGAGGGCGAGAGAGAGCGACTTCTCTCGGGCCCACAGCCTTACAGCTGTGGAGCACGGTATC
CTCTGCCGAAACAGAGGTTGGACAAGACCGGAGGGGTCTCCTAGTTCCAAAGGAGATGTACTCC
+
F::FF,FFFFFFFFFFF,FFF::FF:FFFFF:F::,F:FFFF,F,:,FFFF:FFF,FF,FFFF:FFF,
F:FFFFFFFFFFFFF::FFFFFFFF:FFFF:FFFF:FFF,FFFFFFFF,FFFFFFFFFFFFFFF
@A00155:342:HHGFNDSXY:1:1324:30924:15311 1:N:0:GAACCTAG+TCCGCATA
ACTTCTCTCGGGCCCACAGCCTTACAGCTGTGGAGCACGGTATCCTCTGCCGAAACAGAGGTTGGACA
AGACCGGAGGGGTCTCCTAGTTCCAAAGGAGATGTACTCCGGGCTTGTTCACGACCTACCGTGTAAGT
+
FFFFFFFFFFFFFFFFFFFFFF,:FFFFFFFFFFFFFFFFFFFFFFFFFFFFFFFFFFFFFFFFFFFF
FFFFFFFFFFFFFFFFFFFFFFFFFFFFFFFFFFFFFFFFFFFFFFFFFFFFFFFFFFFFFFFFFFFF
@A00155:342:HHGFNDSXY:1:1324:31521:19633 1:N:0:GAACCTAG+TCCGCATA
GGATGTGGAGGGCGAGAGAGAGCGACTTCTCTCGGGCCCACAGCCTTACAGCTGTGGAGCACGGTATC
CTCTGCCGAAACAGAGGTTGGACAAGACCGGAGGGGTCTCCTAGTTCCAAAGGAGATGTACTCC
+
FFFFFFFFFFFFFFFFFFFFFFFFFFFFFFFFFFFFFFFFFFF:FFFFFFFFFFFFFFFFFFFFFFFF
FFFFFFFFFFFFFFFFF:FFFFFFFFFFFFFFFFFFFFFFFF,FFFFFFFFFFFFFFF:FFFFF
@A00155:342:HHGFNDSXY:1:1324:4300:22592 1:N:0:GAACCTAG+TCCGCATA
GTCATCTCAATGCCGAGAGGGATGTAGAGGGCGAGAGAGAGCGACTTCTCTCGGGCCCACAGCCTTAC
AGCTGTGGAGCACGGTATCCTCTGCCGAAACAGAGGTTGGACAAGACCGGAGGGGTCTCCTAGTTCCA
+
FFFFFFFFFFFFFFFFFFFFF:FFFFFFFFFF:,FFFFFFFFFFFFFFFFFF,F:FFFFFFFFFFFFF
FFFFFFFFFFFFFFFFFFFFFFFFFFFFFFFFF:FFFFFFFFFFFFFFFFFFF:FFFFFFFFFFFFFF
@A00155:342:HHGFNDSXY:1:1324:16767:27117 1:N:0:GAACCTAG+TCCGCATA
GCGACTTCTCTCGGGCCCACAGCCTTACAGCTGTGGAGCACGGTATCCTCTGCCGAAACAGAGGTTGG
ACAAGACCGGAGGGGTCTCCTGGTTCCAAAGGAGATGTACTCCGGGCTTGTTCACGACCTACCG
+
FFFFFFFFFFFFFFFFFFFFFFFFFFFFFFFFFFFFFFFFFFFFFFFFFFFFFFFFFFFFFFFFFFFF
FFFFFFFFFFFFFFFFFFFFFFFFFFFFFFFFFFFFFFFFFFFFFFFFFFFFFFFFFFF:FFF
@A00155:342:HHGFNDSXY:1:1325:16541:20995 1:N:0:GAACCTAG+TCCGCATA
GAGCGACTTCTCTCGGGCCCACAGCCTTACAGCTGTGGAGCACGGTATCCTCTGCCGAAACAGAGGTT
GGACAAGACCGGAGGGGTCTCCTGGTTCCAAAGGAGATGTACTCCGGGCTTGTTCACGACCTACCGTG
+
FFFFFFFFFFFFFFFFFFFFFFFFFFFFFFFFFFFFFFFFFFFFFFFFFFFFFFFFFFFFFFFFFFFF
FFFFFFFFFFFFFFFFFFFFFFFFFFFFFFFFFFFFFFFFFFFFFFFFFFFFFFFFFFFFFFFFFFFF
```

```
@A00155:342:HHGFNDSXY:1:1325:25418:32800 1:N:0:GAACCTAG+TCCGCATA
ACCTCTGTTTCGGCAGAGGATACCGTGCTCCACAGCTGTAAGGCTGTGGGCCCGAGAGAAGTCGCTCT
CTCTCGCCCTTCACATCCCTCTCGGCATTGAGATGACCATAGGCCAGAGGTGAGTCCTTAAGTGGACA
+
FFFFFFFFFFFFFFFFFFFFFFFFFFFFFFFFFFFF:FFFFFFFFFFFFFFFFFFFFFFFFFFFFFFF
FFFFFFFFFFFFFFFFFFFFFFFFFFFFFF:FFFFFFFFFFFFFFFFFFFFFFFFFFF,FFFFFFFFF
@A00155:342:HHGFNDSXY:1:1325:2121:34663 1:N:0:GAACCTAG+TCCGCATA
TACCGTGTAAGTCGTAGTCTAGTAGGCTACCTGACGAGTCCTTTTTAGGACGAAACTTACACAACTCA
AAGAGTAACGACGGTTTCGTCCTTGGAACGGACTCATCAGGGAGCCTACTAGACTCCGACCGTCATGG
+
FFFFFFFFFFFFFFFFFFFFFFFFFFFFF:FFFF:FFFFFFFFFFFFFFFFFFFFFFFFFFFFFFFFF
FFFFFFFFFFFFFFFFFFFFFFFFFFFFFFFFFFFFFFFFFFFFFFFFFFFFFFFFFFFFFFFFFF:F
@A00155:342:HHGFNDSXY:1:1326:13792:3834 1:N:0:GAACCTAG+TCCGCATA
ATCTCAATGCCGAGAGGGATGTGGAGGGCGAGAGAGAGCGACTTCTCTCGGGCCCACAGCCTTACAGC
TGTGGAGCACGGTATCCTCTGCCGAAACAGAGGTTGGACAAGACCGGAGGGGTCTCCTAGTTCCAAAG
+
FFFFFFFFFFFFFFFFFFFFFFFFFFFFFFFFFFFF:FFFFFFFFFFFFFFFFFFFFFFFFFFFFFFF
FFFFFFFFFFFFFFFFFFFFFFFFFFF,:FFFFFFFFFFFFFFFFFFFFFFFFFFFFFFFFFFFFFFF
@A00155:342:HHGFNDSXY:1:1326:12192:18129 1:N:0:GAACCTAG+TCCGCATA
ATCCTCTGCCGAAACAGAGGTTGGACAAGACCGGAGGGGTCTCCTAGTTCCAAAGGAGATGTACTCCG
GGCTTGTTCACGACCTACCGTGTAAGTCGTAGTCTAGTAGGCTACCTGACGAGTCCTTTTTAGGACGA
+
FFFFFFFFFFFFFFFFFFFFFFFFFFFFFFFFFFFFFFFFFFFFFFFFFFFFFFFFFFFFFFFFFFFF
FFFFFFFFFFFFFFFFFFFFFFFF:FFFFFFFFFFFFFFFFFFFFFFFFFFFFFFFFFFFFFFFFFFF
@A00155:342:HHGFNDSXY:1:1326:13973:21778 1:N:0:GAACCTAG+TCCGCATA
GGGCGAGAGAGAGCGACTTCTCTCGGGCCCACAGCCTTACAGCTGTGGAGCACGGTATCCTCTGCCGA
AACAGAGGTTGGACAAGACCGGAGGGGTCTCCTAGTTCCAAAGGAGATGTACTCCGGGCTTGTTCACG
+
FFFFFFFFFFFFFFFFFFFFFFFFFFFFFFFFFFFFFFFFFFFFFFFFFFFFFFFFFFFFFFFFFFFF
FFFFFFFFFFFFFFFFFFF:FFFFFFFFFFFFFFFFFFFFFFFFFFFFFFFFFFFFFFFFFFFFFFF:
@A00155:342:HHGFNDSXY:1:1326:29405:28588 1:N:0:GAACCTAG+TCCGCATA
TCCCTCTCGGCATTGAGATGACCATAGGCCAGAGGTGAGTCCTTAAGTGGACACAGCTGATCTAAGGC
GGTGTAGCGGGGCATGGGTTTGAACCCCCATGACGGTCGGAGTCTAGTAGGCTCCCTGATGAGTCCGT
+
FFFFFFFFFFFF:FFFFFFFFFFFFFFFFFFFFFFFFFFFFFFFFFFFFFFFFFFFFFFFFFFFFFF
FFFFFFFFFFFFFFFFFFFFFFFFFFFFFFFFFFFFFFFFFFFFFFFFFFFFFFFFFFFFFFFFFFFF
@A00155:342:HHGFNDSXY:1:1326:10095:30436 1:N:0:GAACCTAG+TCCGCATA
CACCTCTGGCCTATGGTCATCTCAATGCCGAGAGGGATGTGGAGGGCGAGAGAGAGCGACTTCTCTCG
GGCCCACAGCCTTACAGCTGTGGAGCACGGTATCCTCTGCCGAAACAGAGGTTGGACAAGACCGGAGG
+
FFFFFFFFFFFFFFFFFFFFFFFFFFFFF::FFFFFFFFFFFFFFFFFFFFFF:FFFFF:FFFFFFFF
FFFFFFFFFFFFFFFFFFFFFFFFFF:FFFFFFFFFFFFFFFFFFFFFFFFFFFFFFFFFFFFFFFFF
@A00155:342:HHGFNDSXY:1:1326:4752:33896 1:N:0:GAACCTAG+TCCGCATA
CAATGCCGAGAGGGATGTAGAGGGCGAGAGAGAGCGACTTCTCTCGGGCCCACAGCCTTACAGCTGTG
GAGCACGGTATCCTCTGCCGAAACAGAGGTTGGACAAGACCGGAGGGGTCTCCTAGTTCCAAAGGAGA
+
FFFFFFFFFFFFFFF:FFFFFFFFFFFFFFFFFFFFFFFFFFFFFFFFFFFFFFFFFFFFFFFFFFFF
:FFFFFFFFFFF:FFFFFFFFFFFFFFFF:FFFFFFF:FFFFFFFFFFFFFFFFFFFFFFFFFFFFFF
@A00155:342:HHGFNDSXY:1:1327:11623:4429 1:N:0:GAACCTAG+TCCGCATA
GATGTGGAGGGCGAGAGAGAGCGACTTCTCTCGGGCCCACAGCCTTACAGCTGTGGAGCACGGTATCC
TCTGCCGAAACAGAGGTTGGACAAGACCGGAGGGGTCTCCTAGTTCCAAAGGAGATGTACTCCGGGCT
+
FFFFFFFFFFFFFFFFFFFFFFFFFFFFFFFFFFFFFFFFFFFFFFFFFFFFFFFFFFFFFFFFFFFF
FFFFFFFFFFFFFFFFFFFFFFFFFFFFFFFFFFFFFFFFFFFFFFFFFFFFFFFFFFFFFFFFFFFF
```

```
@A00155:342:HHGFNDSXY:1:1327:26964:8672 1:N:0:GAACCTAG+TCCGCATA
CTCTGCCGAAACAGAGGTTGGACAAGACCGGAGGGGTCTCCTAGTTCCAAAGGAGATGTACTCCGGGC
TTGTTCACGACCTACCGTGTAAGTCGTAGTCTAGTAGGCTACCTGACGAGTCCTTTTTAGGACGAAAC
+
FFFFFFFFFFFFFFFFFFFFFFFFFFFFFFFFFFFFFFFFFFFFFFFFFFFF,FFFFFFFFFFFFFFF
FFFFFFFFFFFFFFFFFFFFFFFFFFFFFFFFFFFFFFFFFFFFFFFFFFFFFFFFFFFFFFFFFFFF
@A00155:342:HHGFNDSXY:1:1327:5014:17848 1:N:0:GAACCTAG+TCCGCATA
ACTTCTCTCGGGCCCACAGCCTTACAGCTGTGGAGCACGGTATCCTCTGCCGAAACAGAGGTTGGACA
AGACCGGAGGGGTCTCCTAGTTCCAAAGGAGATGTACTCCGGGCTTGTTCACGACCTACCGTGTAAGT
+
FFFFFFFFFFFFFFFFFFFFFFFFFFFFFFFFFF:FFFFF::FFFFFFFFFFFFFFFFFFFFFFFFFF
FFFFFFFFFFFFFFFFFFFFFFFFFFFFFFFFFFFFFFFFFFF:FFFFFFFFFFFFFFFFFFFFFF:FF
@A00155:342:HHGFNDSXY:1:1327:30572:25504 1:N:0:GAACCTAG+TCCGCATA
CTGGCCTATGGTCATCTCAATGCCGAGAGGGATGTGGAGGGCGAGAGAGAGCGACTTCTCTCGGGCCC
ACAGCCTTACAGCTGTGGAGCACGGTATCCTCTGCCGAAACAGAGGTTGGACAAGACCGGAGGGGTCT
+
FFFFFFFFFFFFFFFFFFFFFFFFFFF,FFF,FFFFFFFFFFFFFFFFFFFFFF:FFFFF:FFFFFFF
FFFFFFFF:F:FFFF:FFFFFFFFFFFFFFFFFFFFFFFF:F:FFFFFFFFFFFFFFFFFFFFFFFFFF
@A00155:342:HHGFNDSXY:1:1327:24813:32722 1:N:0:GAACCTAG+TCCGCATA
CTCTGGCCTATGGTCATCTCAATGCCGAGAGGGATGTAGAGGGCGAGAGAGAGCGACTTCTCTCGGGC
CCACAGCCTTACAGCTGTGGAGCACGGTATCCTCTGCCGAAACAGAGGTTGGACAAGACCGGAGGGGT
+
FFFFFFFFFFFFFFFFFFFFFFFFFFFFFFFFFF:FFF:FFFFFFFFFFFFFFFFFFFFFFFFFFFFF
FFFFFFFFFFFFFFFFFFFFFFFFFFFFFFFFFFFFFFFFFFFFFFFFFFFFFFFFFFFFFFFFFFFF
@A00155:342:HHGFNDSXY:1:1327:25446:32753 1:N:0:GAACCTAG+TCCGCATA
CTCTGGCCTATGGTCATCTCAATGCCGAGAGGGATGTAGAGGGCGAGAGAGAGCGACTTCTCTCGGGC
CCACAGCCTTACAGCTGTGGAGCACGGTATCCTCTGCCGAAACAGAGGTTGGACAAGACCGGAGGGGT
+
FFFFFFFFFFFFFFFFFFFFFFFFFFFFFFFFFFFFF,FFFFFFFFFFFFFFFFFFFFFFFFFFFFFF
FFFFFFFFFFFFFFFFFFFFFFFFFFFFFFFFFFFFFFFFFFFFFFFFFFFFFFFFFFFFFFFFFFFF
@A00155:342:HHGFNDSXY:1:1327:26096:33348 1:N:0:GAACCTAG+TCCGCATA
GAGCGACTTCTCTCGGGCCCACAGCCTTACAGCTGTGGAGCACGGTATCCTCTGCCGAAACAGAGGTT
GGACAAGACCGGAGGGGTCTCCTAGTTCCAAAGGAGATGTACTCCGGGCTTGTTCACGACCTACCGTG
+
,FFF:FFFFFFFFFFF:FFFFFFFFFFFFFFFF::,F,F,FFFFFFFFFFFFFF:F:FFFFFFFF:FF
:FFFFF,FFFFFFFF::FFF,FFF:FF:FFFFFFFFFFFFFFFFFFF:FFFFFFFFFFFFF:F,F,::
@A00155:342:HHGFNDSXY:1:1328:22263:8077 1:N:0:GAACCTAG+TCCGCATA
GTCCACTTAAGGACTCACCTCTGGCCTATGGTCATCTCAATGCCGAGAGGGATGTAGAGGGCGAGAGA
GAGCGACTTCTCTCGGGCCCACAGCCTTACAGCTGTGGAGCACGGTATCCTCTGCCGAAACAGAG
+
FFF,:,F,FFFFFFFFFFFFFF,FFFFFFFF::FFFFF:FFFFFF:FFFFFFFFF:FFFF:FFFFFFF
FFFFFFFFFFFF:F:FFFFFFFF:FFFFFFFFFFFFF:FFFFFFF:FFF:FFFFFFFFF:FFFF:
@A00155:342:HHGFNDSXY:1:1328:19723:18991 1:N:0:GAACCTAG+TCCGCATA
CTTCTCTCGGGCCCACAGCCTTACAGCTGTGGAGCACGGTATCCTCTGCCGAAACAGAGGTTGGACAA
GACCGGAGGGGTCTCCTAGTTCCAAAGGAGATGTACTCCGGGCTTGTTCACGACCTACCGTGTAAGTC
+
FFFFFFF:F:FFFFFFFFFFFFFFFFFFFFFFFFFFFFFFFFFFFFFFFFFFF:FFFFFF:FFFFF
FFFFFFFFFFFFFFFFFFFFFFFFFFFF:FFFFFFFFFFFF:FFFFFFFFFF:F:FFFFF:FFFF:FFF
@A00155:342:HHGFNDSXY:1:1328:5909:21652 1:N:0:GAACCTAG+TCCGCATA
GGATGTGGAGGGCGAGAGAGAGCGACTTCTCTCGGGCCCACAGCCTTACAGCTGTGGAGCACGGTATC
CTCTGCCGAAACAGAGGTTGGACAAGACCGGAGGGGTCTCCTAGTTCCAAAGGAGATGTACTCC
+
FFFFF:FFFFFFFFFFFFFFFFFFFFFFFFFFFFFFFFFFFFFFFFFFFFFFFFFFFFFFFFFFFFFF
FFFFFFFFFFFFFFFFFFFFFFFFFFFFFFFFFFFFFFFFFFFFFFFFFFFFFFFFFFFFFFFF
```

```
@A00155:342:HHGFNDSXY:1:1328:5656:21590 1:N:0:GAACCTAG+TCCGCATA
GGATGTGGAGGGCGAGAGAGAGCGACTTCTCTCGGGCCCACAGCCTTACAGCTGTGGAGCACGGTATC
CTCTGCCGAAACAGAGGTTGGACAAGACCGGAGGGGTCTCCTAGTTCCAAAGGAGATGTACTCC
+
FFFFFFFFFFFFFFFFFFFFFFF:FFFFFFFFFFFFFFFFFFFFF:FFFFFFFFFFFFFFFFFFFFFF
FFFFFFFFFFFFFFFFFF:FFFFFFFFF:FFFFFFFFFFFFFFFFFFFFFFFFFF,FFFFFFFF
@A00155:342:HHGFNDSXY:1:1328:17300:33270 1:N:0:GAACCTAG+TCCGCATA
GTCGCTCTCTCTCGCCCTCCACATCCCTCTCGGCATTGAGATGACCATAGGCCAGAGGTGAGTCCTTA
AGTGGACACAGCTGATCTAAGGCGGTGTGGCGGGGCATGGGTTTGAACCCCCATGACGGTCGGAGTCT
+
FFFFFFFFFFFFFFFFFFFFFFFFFFFFFFFFFFFFFFFFFFFFFFFFFFFFFFFFFFFFFFFFFFFF
FFFFFFFFFFFFFFFFFFF:FFFFFFFFFFFFFFFFFFFFFFFFFFFFFFFFFFFFFFFFFFFFFF::FFFF
@A00155:342:HHGFNDSXY:1:1329:1759:7420 1:N:0:GAACCTAG+TCCGCATA
GAGAGCGACTTCTCTCGGGCCCACAGCCTTACAGCTGTGGAGCACGGTATCCTCTGCCGAAACAGAGG
TTGGACAAGACCGGAGGGGTCTCCTAGTTCCAAAGGAGATGTACTCCGGGCTTGTTCACGACCTACCG
+
FFFFFFFFFFFFFFFFFFFFFFFFFFFFFFFFF:FFFFFFFFFFFFFFFFFFFFFFFFFFFFFFFFFF
FFFFFFFFF:FFFFFFFFFFFFFFFFFFFFFFFFFFFFFFFFFFFFF:FFFFFFFFFFFFFFFFFFFF
@A00155:342:HHGFNDSXY:1:1329:24858:21371 1:N:0:GAACCTAG+TCCGCATA
GATGTGGAGGGCGAGAGAGAGCGACTTCTCTCGGGCCCACAGCCTTACAGCTGTGGAGCACGGTATCC
TCTGCCGAAACAGAGGTTGGACAAGACCGGAGGGGTCTCCTAGTTCCAAAGGAGATGTACT
+
FFFFFF,FFFFFFFFF:FFF::FFFF:FFFFF,FFFFFFFFFFFFFFFFFFF:FFFFFFFFFFF,FF:
FFFFFFFFFFFFFFF:FF,FFFFFFFF::,FF,:FFFFFFFF:FFFFFFF,FFFFFFFFFF
@A00155:342:HHGFNDSXY:1:1329:18358:21731 1:N:0:GAACCTAG+TCCGCATA
CTCTGGCCTATGGTCATCTCAATGCCGAGAGGGATGTGAAGGGCGAGAGAGAGCGACTTCTCTCGGGC
CCACAGCCTTACAGCTGTGGAGCACGGTATCCTCTGCCGAAACAGAGGTTGGACAAGACCGGAGGGGT
+
FFFFFFFFFFFFFFFFFFFFFFFFFFFFFFFFFFFFFFFFFFFFFFFFFFFFFFFFFFFFFFFFFFFF
FFFFFFFFFFFFFFFFFFFFFFFFFFFFFFFFFFFFFFFFFFFFFFFFFFFFFFFFFFFFFFFFFFFF
@A00155:342:HHGFNDSXY:1:1329:9399:22091 1:N:0:GAACCTAG+TCCGCATA
CGACTTCTCTCGGGCCCACAGCCTTACAGCTGTGGAGCACGGTATCCTCTGCCGAAACAGAGGTTGGA
CAAGACCGGAGGGGTCTCCTAGTTCCAAAGGAGATGTACTCCGGGCTTGTTCACGACCTACC
+
FFFFFFFFFFFFFFFFFFFFFFFFFFFFFFFFFFFFFFFFFFFFFFFFFFFFFFFFFFFFFF,FFFFF
FFFFFFFFFFFFFFFFFFFFFFFFFFFFFFFFFFFFFFFFFFFFFFFFFFFFFFFFFFFFFF
@A00155:342:HHGFNDSXY:1:1329:9688:23312 1:N:0:GAACCTAG+TCCGCATA
TTACAGCTGTGGAGCACGGTATCCTCTGCCGAAACAGAGGTTGGACAAGACCGGAGGGGTCTCCTAGT
TCCAAAGGAGATGTACTCCGGGCTTGTTCACGACCTACCGTGTAAGTCGTAGTCTAGTAGGCTACCTG
+
FFFFFFFFFFFFFFFFFFFFFFFFFFFFFFFFFFFFFFFFFFFFFFFFFFFFFFFFFFFFFFFFFFFF
FFFFFFFFFFFFFFFFFFFFFFF:FFFFFFFFFFFFFFFFFFFFFFFFFFFFFFFFFFFFFFFFFFFF
@A00155:342:HHGFNDSXY:1:1329:17065:33708 1:N:0:GAACCTAG+TCCGCATA
ATCTCAATGCCGAGAGGGATGTAGAGGGCGAGAGAGAGCGACTTCTCTCGGGCCCACAGCCTTACAGC
TGTGGAGCACGGTATCCTCTGCCGAAACAGAGGTTGGACAAGACCGGAGGGGTCTCCTAGTTCCAAAG
+
FFFFFFFFFFFFFFFFFFF:FFFFFFFFFFFFFFFFFFFFFFFFFFFFF:FFFFFFFFFFFFFFFFFF
FFFFFFFFFFFFF:FFFFFFFFFFFFFFFFFFFFFFFFFFFFFFFFFFFFFFFFF:F:FFFFFFF,:F
@A00155:342:HHGFNDSXY:1:1329:17291:36292 1:N:0:GAACCTAG+TCCGCATA
ATCTCAATGCCGAGAGGGATGTAGAGGGCGAGAGAGAGCGACTTCTCTCGGGCCCACAGCCTTACAGC
TGTGGAGCACGGTATCCTCTGCCGAAACAGAGGTTGGACAAGACCGGAGGGGTCTCCTAGTTCCAAAG
+
FFFFFFFFFFFFFFFFFFFFFFFFFFFFFFFFFFFFFFFFFFFFFFFFFFFFFFFFFFFFFFFFFFFF
FFFF:FFFFFFFFFFFFFFFFFFFFFFF,FFFFF,FF:FFFFFFFFFFFFFF:FFFFFFFFFFFFFFF
```

```
@A00155:342:HHGFNDSXY:1:1330:21043:2362 1:N:0:GAACCTAG+TCCGCATA
CTCAATGCCGAGAGGGATGTGGAGGGCGAGAGAGAGCGACTTCTCTCGGGCCCACAGCCTTACAGCTG
TGGAGCACGGTATCCTCTGCCGAAACAGAGGTTGGACAAGACCGGAGGGGTCTCCTAGTTCCAAAGGA
+
FFFFFFFFFFFFFFFFFFF,FFFFFFFFFFFFFFF:FFFFFFFFFFFFFFFFFFFFFFFFFFFFFFFF
FFFFFFFFFFFFFFFFFFFFFFFFFFFFFFFFFFFFFFFFFFFFFFFFFFFFFFFFFFFFFFF:FFFFF
@A00155:342:HHGFNDSXY:1:1330:24397:24173 1:N:0:GAACCTAG+TCCGCATA
GGATGTAGAGGGCGAGAGAGAGCGACTTCTCTCGGGCCCACAGCCTTACAGCTGTGGAGCACGGTATC
CTCTGCCGAAACAGAGGTTGGACAAGACCGGAGGGGTCTCCTAGTTCCAAAGGAGATGTACTCC
+
FFFFFFFFFFFFFFFFFFFFFFFFFFFFFFFFFFF,FFFFFFFFFFFFFFFF:FFFFFFFFFFFFFFF
F:FFFFFFFFFFFFFFFFFFFFFFF:FFFFFFFFFFFFFFFFFFFFFFFFFFFFFFFFFFFFFFFF
@A00155:342:HHGFNDSXY:1:1330:5023:36495 1:N:0:GAACCTAG+TCCGCATA
GAGAGGGATGTAGAGGGCGAGAGAGAGCGACTTCTCTCGGGCCCACAGCCTTACAGCTGTGGAGCACG
GTATCCTCTGCCGAAACAGAGGTTGGACAAGACCGGAGGGGTCTCCTAGTTCCAAAGGAGATGTACTC
+
FFF,FFFFFFFFFFFFFFFFFFFFFFFFFFFFFFFFFFFFFFFFFFFFFFFFFFFFF:FFFFFFFFFF
FFFFFFFFFFFFFFFFFFFFFFFFFFFFFFFFFFFFFFFFFFFFFFFFFFFFFFFFFFFFFFFFFFFF
@A00155:342:HHGFNDSXY:1:1331:11822:9408 1:N:0:GAACCTAG+TCCGCATA
CTCTGGCCTATGGTCATCTCAATGCCGAGAGGGATGTGGAGGGCGAGAGAGAGCGACTTCTCTCGGGC
CCACAGCCTTACAGCTGTGGAGCACGGTATCCTCTGCCGAAACAGAGGTTGGACAAGACCGGAGGGGT
+
FFFFFFFFFFFFFFFFFFFFFFFFFFFFFFFFFFFFFFFFFFFFFFFFFFFFFFFFFFFFFFFFFFFF
FFFF:FFFFFFFFFFFFFFFFFFFFFFFFFFFFFFFFFFFFFFFFFFFFFFFFFF:FFFFFFFFFFFF
@A00155:342:HHGFNDSXY:1:1331:31783:10473 1:N:0:GAACCTAG+TCCGCATA
GATCAGCTGTGTCCACTTAAGGACTCACCTCTGGCCTATGGTCATCTCAATGCCGAGAGGGATGTGGA
GGGCGAGAGAGAGCGACTTCTCTCGGGCCCACAGCCTTACAGCTGTGGAGCACGGTATCCTCTGCCGA
+
FFFFFFFFFFFFFFFFFFFFFFFFFFFFFFFFFFFFFFFFFFFFFFFFFFFFFFFFFFFFFFFFFFFF
FFFFFFFFFFFFFFFFFFFFFFFFFFFFFFFFFFFF:FFFFFFFFFFFFFF:FFFFFFFFFFFFFFFF
@A00155:342:HHGFNDSXY:1:1331:31919:10614 1:N:0:GAACCTAG+TCCGCATA
GATCAGCTGTGTCCACTTAAGGACTCACCTCTGGCCTATGGTCATCTCAATGCCGAGAGGGATGTGGA
GGGCGAGAGAGAGCGACTTCTCTCGGGCCCACAGCCTTACAGCTGTGGAGCACGGTATCCTCTGCCGA
+
FFFFFFFFFFFFFFFFFFFFFFFFFFFFFFFFFFFFFF,FFFFFFFFFFFFFFFFFFFFFF:FFFFFF
FFFFFFFFFFFFFFFFFFFFFFFFFFFFFFFFFFFFFF:FFFFFFFFFFFFFFFFFFFFFFFFFFFFF
@A00155:342:HHGFNDSXY:1:1332:23656:9079 1:N:0:GAACCTAG+TCCGCATA
ATGCCCCGCCACACCGCCTTAGATCAGCTGTGTCCACTTAAGGACTCACCTCTGGCCTATGGTCATCT
CAATGCCGAGAGGGATGTGGAGGGCGAGAGAGAGCGACTTCTCTCGGGCCCACAGCCTTACAGCTGTG
+
FFFFFFFFFFFFFFFFFFFFFFFFFFFFFFFFFFFFF,FFFFFFFFFFFFFFFFFFFFFFFFFFFFFF
FFFFFFFFFFFFFFF,FFFFFFFFFFFFFFFFFFFFFFFFFFFFFFFFFFFFFFFFFFFFFFFFFFFF
@A00155:342:HHGFNDSXY:1:1332:25337:11647 1:N:0:GAACCTAG+TCCGCATA
AGGCCAGAGGTGAGTCCTTAAGTGGACACAGCTGATCTAAGGCGGTGTGGCGGGGCATGGGTTTGAAC
CCCCATGACGGTCGGAGTCTAGTAGGCTCCCTGATGAGTCCGTTCCAAGGACGAAACCGTCGTTACTC
+
FFFFFFFFFFFFFFFFFFFFFFFFFFFFFFFFFFFFFFFFFFFFFFFFFFFFFFFFFFFFFFFFFFFF
FFFFFFFFFFFFFFFFFFFFFFFFFFFFFFFFFFFFFFFFFFFFFFFFFFFFFF,FFFFFFFFFFFFF
@A00155:342:HHGFNDSXY:1:1332:30725:19382 1:N:0:GAACCTAG+TCCGCATA
CGAGAGAGAGCGACTTCTCTCGGGCCCACAGCCTTACAGCTGTGGAGCACGGTATCCTCTGCCGAAAC
AGAGGTTGGACAAGACCGGAGGGGTCTCCTAGTTCCAAAGGAGATGTACTCCGGGCTTGTTCACGACC
+
FFFFFFF:FFFFFFFFFFFFFF::FFFFFFFF:FF:FFFFF:FFFFFFFFFFFFFFFFF:FFFFFFFF
FFFFFFFFFFFFFFFF:FFFFFFFFFFFFFFFF:FFFFFF:FFFFFF:FFFFFFFFFFFFFF,FFFFF
```

```
@A00155:342:HHGFNDSXY:1:1332:24632:35540 1:N:0:GAACCTAG+TCCGCATA
TGTGGCGGGGCATGGGTTTGAACCCCCATGACGGTCGGAGTCTAGTAGGCTCCCTGATGAGTCCGTTC
CAAGGACGACACCGTCGTTACTCTTCGAGTTGTGTAAGTTTCGTCCTAAAAAGGACTCGTCAGGTAGC
+
FFFFFFFFFFFFFFFFFFFFFFFFFFFFFFFFFFFFFFFFFFFFFFFFFFFFFFFFFFFFFFFFFFFF
FFFFFFFF,FFFFFFFFFFFFFFFFF:F,FFFFFFFFFF:FFFFFFFFFFFFFFFFFFFFFFFFFFFF
@A00155:342:HHGFNDSXY:1:1333:19849:2394 1:N:0:GAACCTAG+TCCGCATA
CTTCTCTCGGGCCCACAGCCTTACAGCTGTGGAGCACGGTATCCTCTGCCGAAACAGAGGTTGGACAA
GACCGGAGGGGTCTCCTAGTTCCAAAGGAGATGTACTCCGGGCTTGTTCACGACCTACCGTGTAAGTC
+
FFFFFFFFFFFFFFFFFFFFFFFFFFFFFFFFFFFFFFFFFFFFFFFFFFFFFFFFFFFFFFFFFFFF
FFFFFFFFFFFFFFFF:FFFFFFFFFFFFFFFFFFFFFFFFFFFFFFFFFFFFFFFFFFFFFFFFFF
@A00155:342:HHGFNDSXY:1:1333:23312:9111 1:N:0:GAACCTAG+TCCGCATA
AGAGAGAGCGACTTCTCTCGGGCCCACAGCCTTACAGCTGTGGAGCACGGTATCCTCTGCCGAAACAG
AGGTTGGACAAGACCGGAGGGGTCTCCTAGTTCCAAAGGAGATGTACTCCGGGCTTGTTCACGACCTA
+
FFFFFFFFFFFFFFFFFFFFFFFFFFFFFFFFF,FFFFFFFFFFFFFFFFFFFFFFFFFFFFFFFFFF
FFFFFFFFFFF:FFFFFFFFFFFFFFFFFFFFFF:FFFFFFFFFFFFFFFF:FFFFFFFFF:FFFFFF
@A00155:342:HHGFNDSXY:1:1333:18783:14544 1:N:0:GAACCTAG+TCCGCATA
TCCACTTAAGGACTCTCCTCTGGCCTATGGTCATCTCAATGCCGAGAGGGATGTGGAGGGCGAGAGAG
AGCGACTTCTCTCGGGCCCACAGCCTTACAGCTGTGGAGCACGGTATCCTCTGCCGAAACAGAGGTTG
+
FFFFFF::FFFFFFFFFFFFFFFFFFF:FFFFFFFFFFFFFFFFFF:FFFF,FFFFFFFFFFFFFFFF
FFFFFF:FFFFFFFFFFFFFFF:F:FFF:F:FFFFFFFFFFF,FFFFFFFFF:FFFFF:FFFFFFFFF
@A00155:342:HHGFNDSXY:1:1333:23285:17487 1:N:0:GAACCTAG+TCCGCATA
CTTCTCTCGGGCCCACAGCCTTACAGCTGTGGAGCACGGTATCCTCTGCCGAAACAGAGGTTGGACAA
GACCGGAGGGGTCTCCTAGTTCCAAAGGAGATGTACTCCGGGCTTGTTCACGACCTACCGTGTAAGTC
+
FFFFFFFFFFFFFFFFFFFFFFFFFFFFFFFFFFFFFFFFFFFFFFFFFFFFFFFFFFFFFFFFFFFF
F:FFFFFFFFF:FFFFFFFFFFFFFFFFFFFFFFFFFFFFFFFFFFFFFFFFFFFFFFFFFFFF:FFF
@A00155:342:HHGFNDSXY:1:1333:5150:20838 1:N:0:GAACCTAG+TCCGCATA
AGAGAGAGCGACTTCTCTCGGGCCCACAGCCTTACAGCTGTGGAGCACGGTATCCTCTGCCGAAACAG
AGGTTGGACAAGACCGGAGGGGTCTCCTAGTTCCAAAGGAGATGTACTCCGGACTTGTTCACGACCTA
+
FFFFFFFFFFFFFFFFFFFFFFFFFFFFFFFFF,F:FFFFFFFFFFFFFFFFFFFFFFFFFFFFFFFF
FFFFFFFFFFFFFFFFFFFFFFFFFFFF:FFFFFFFFFFFFFFFFFFFFFFFFFFFFFFFFFFFFFFF
@A00155:342:HHGFNDSXY:1:1334:31937:1282 1:N:0:GAACCTAG+TCCGCATA
AGAGAGAGCGACTTCTCTCGGGCCCACAGCCTTACAGCTGTGGAGCACGGTATCCTCTGCCGAAACAG
AGGTTGGACAAGACCGGAGGGGTCTCCTAGTTCCAAAGGAGATGTACTCCGGGCTTGTTCACGACCTA
+
FFFFFFFFFFFFFFFFFFFFFFFFFFFFFFFFFFFFFFFFFFF:FFF:FFF:FFFFFFFFFFFFFFFF
FFFFFFF:FF:FFFFFFFFFFFFFFFFFFFFFFFFFFFFFFFFFF:FFFFFFFF:FFFFFFFFFFFFF
@A00155:342:HHGFNDSXY:1:1334:14181:13182 1:N:0:GAACCTAG+TCCGCATA
TCCTCTGCCGAAACAGAGGTTGGACAAGACCGGAGGGGTCTCCTAGTTCCAAAGGAGATGTACTCCGG
GCTTGTTCACGACCTACCGTGTAAGTCGTAGTCTAGTAGGCTACCTGACGAGTCCTTTTTAGGACGAA
+
FFFFFFFFFFFFFFFFFFFFFFFFFFFFFFFFFFFFFFFFFFFFFFFFFFFFFFFFFFFFFFFFFFFF
FFFFFFFFFFFFFFFFFFFFFFFFFFFFFFFFFFFFFFFFFFFFFFFFFFFFFFFFFFFFFFFFFFFF
@A00155:342:HHGFNDSXY:1:1335:30825:6652 1:N:0:GAACCTAG+TCCGCATA
GAGGGCGAGAGAGAGCGACTTCTCTCGGGCCCACAGCCTTACAGCTGTGGAGCACGGTATCCTCTGCC
GAAACAGAGGTTGGACAAGACCGGAGGGGTCTCCTGGTTCCAAAGGAGATGTACTCCGGGCTTGTTCA
+
FFFFFFFFFFFFFFFFFFFFFFFFFFFFFFFFFFFFFFFFFFFFFFFFFFFFFFFFFFFFFFFFFFFF
FFFFFFFFFFFFFFFFFFFFFFFFFFFFFFFFFFFFFFFFFFFFFFFFFFFFFFFFFFFFFFFFFFFF
```

@A00155:342:HHGFNDSXY:1:1335:25102:7983 1:N:0:GAACCTAG+TCCGCATA
GAGCGACTTCTCTCGGGCCCACAGCCTTACAGCTGTGGAGCACGGTATCCTCTGCCGAAACAGAGGTT
GGACAAGACCGGAGGGGTCTCCTAGTTCCAAAGGAGATGTACTCCGGGCTTGTTCACGACCTACCGTG
+
FFFFFFFFFFFFFFFFFFFFFFFFFF::FFFFFFFFF:FFFFFFF:FFFFFFFFFFFFFFFFFFFF
FFFFFFFFFFFFFFFFFFFFF::FF:FFF,FFFFFFFFFFFFFFFFFFFFFFFFFFFFFFFFFFFF
@A00155:342:HHGFNDSXY:1:1335:10321:14325 1:N:0:GAACCTAG+TCCGCATA
GATGTGGAGGGCGAGAGAGAGCGACTTCTCTCGGGCCCACAGCCTTACAGCTGTGGAGCACGGTATCC
TCTGCCGATACAGAGGTTGGACAAGACCGGAGGGGTCTCCTAGTTCCAAAGGAGATGTACTCCGGGCT
+
FFFFFFFFFFFFFFFFFFFFFFFFFFFFFFFFFFFFFFFFFFFFFFFFFFFFFFFFFFFFFFFFFF
FFFFFFFFFFFFFFFFFFFFFFFFFFFFFFFFFFFFFFFFFFFFFFFFFFFFFFFFFFFFFFFFFF
@A00155:342:HHGFNDSXY:1:1336:25825:4069 1:N:0:GAACCTAG+TCCGCATA
AGCGACTTCTCTCGGGCCCACAGCCTTACAGCTGTGGAGCACGGTATCCTCTGCCGAAACAGAGGTTG
GACAAGACCGGAGGGGTCTCCTAGTTCCAAAGGAGATGTACTCCGGGCTTGTTCACGACCTACCGTGT
+
FFFFFFFFFFFFFFFFFFFFFFFFFFFFFFFFFFFFFF:FFFFFFFFFFFFFFFFFFFFFFFFFFF
FFFFFFFFFFFFFFFFFFFFFFFFFFFFFFFFFFFFFFFFFFFFFFFFFFFFFFFFFFFFFFFFFF
@A00155:342:HHGFNDSXY:1:1336:14850:4946 1:N:0:GAACCTAG+TCCGCATA
GCGACTTCTCTCGGGCCCACAGCCTTACAGCTGTGGAGCACGGTATCCTCTGCCGAAACAGAGGTTGG
ACAAGACCGGAGGGGTCTCCTAGTTCCAAAGGAGATGTACTCCGGGCTTGTTCACGACCTACCGTGTA
+
FFFFFFFFFFFFFFFFFFFFFFFFFFFFFFFFFFFFFFFFFFFFFFFFFF:FFFFFFFF:FFFFFF
FFFFFFFFFFFFFFFFFFFFFFFFFFFFFFFFFFFFFFFFFFFFFFFFFFFFFFFFFFFFFF:F:F
@A00155:342:HHGFNDSXY:1:1336:24062:9095 1:N:0:GAACCTAG+TCCGCATA
TTAGATCAGCTGTGTCCACTTAAGGACTCACCTCTGGCCTATGGTCATCTCAATGCCGAGAGGGATGT
AGAGGGCGAGAGAGAGCGACTTCTCTCGGGCCCACAGCCTTACAGCTGTGGAGCACGGTATCCTCTGC
+
:FFFFFFFFFFFFFFFFFFFFF:FFFFFFFFFFFFFFFFFFFFFFFFFFFFFFFFFFFFFFFFFFF
FFFFFFFFFFFFFFFFFFFFFFFFFFF:FFFFFFFFFF,FFFFFFFFFFFFFFFFFFFFFFFFFFF
@A00155:342:HHGFNDSXY:1:1336:17481:17957 1:N:0:GAACCTAG+TCCGCATA
GAGGGCGAGAGAGAGCGACTTCTCTCGGGCCCACAGCCTTACAGCTGTGGAGCACGGTATCCTCTGCC
GAAACAGAGGTTGGACAAGACCGGAGGGGTCTCCTAGTTCCAAAGGAGATGTACTCCGGGCTTGTTCA
+
FFFFFFFFFFFFFFFFFFFFFFFFFFFFFFFFFFFFFFFFFFFFFFFFFFFFFFFFFFFFFFFFFF
FFFFFFFFFFFFFFFFFFFFFFFFFFFFFF:FFFFFFFFFFFFFFFFFFFFFFFFFFFFFFFFFFF
@A00155:342:HHGFNDSXY:1:1336:16875:24925 1:N:0:GAACCTAG+TCCGCATA
TTCTCTCGGGCCCACAGCCTTACAGCTGTGGAGCACGGTATCCTCTGCCGAAACAGAGGTTGGACAAG
ACCGGAGGGGTCTCCTAGTTCCAAAGGAGATGTACTCCGGGCTTGTTCACGACCTACCGTGTAAGTCG
+
F:FFFFF:F:FFFFFFFFFFFFFFFFFFFF:FFFFFFF:FFFFFFFFFFFFFFFFFFFFFFFFFFF
FFFFFFFFFFFFFFFFFFFFFFF:FFFFFFFFFFFFFFFFF::FFFFFFFFFFFFFFFFFF::FFF
@A00155:342:HHGFNDSXY:1:1337:14814:3630 1:N:0:GAACCTAG+TCCGCATA
CTCTGGCCTATGGTCATCTCAATGCCGAGAGGGATGTGAAGGGCGAGAGAGAGCGACTTCTCTCGGGC
CCACAGCCTTACAGCTGTGGAGCACGGTATCCTCTGCCGAAACAGAGGTTGGACAAGACCGGAGGGGT
+
FFFFFFFFFFFFFFFFFFFFF:FFFFFFFFFFFF:F,FFFFFFFFFFFFFFFFFFFFFFFFFFFFF
FFFFFFFFFF:FFFFFFFFFFFFFFFFFFFFFFFFFFFFFFFFFF:FFFFFFFFFFFFFFFFFFFF
@A00155:342:HHGFNDSXY:1:1337:10945:5854 1:N:0:GAACCTAG+TCCGCATA
CTTCTCTCGGGCCCACAGCCTTACAGCTGTGGAGCACGGTATCCTCTGCCGAAACAGAGGTTGGACAA
GACCGGAGGGGTCTCCTAGTTCCAAAGGAGATGTACTCCGGGCTTGTTCACGACCTACCGTGTAAGTC
+
FFFFFFFFFFFFFFFFFFFF:FFFFFFFFFFFFFFFFFFFFFFFFFFFFFFFFFFFFFFFFFFF:F
F:FFFFFFFFFFFFFFFFFFFFFF,FFFFF:FFFFFFFFFFFFFFFFFFFFFFFFFFFFFFF,FFF

@A00155:342:HHGFNDSXY:1:1337:22598:13322 1:N:0:GAACCTAG+TCCGCATA
ACCTCTGTTTCGGCAGAGGATACCGTGCTCCACAGCTGTAAGGCTGTGGGCCCGAGAGAAGTCGCTCT
CTCTCGCCCTTCACATCCCTCTCGGCATTGAGATGACCATAGGCCAGAGGTGAGTCCTTAAGTGGACA
+
FFFFFFFFFFFFFFFFFFFFFFFFFFFFFFFFFFFFFFFFFFFFFFFFFFFFFFFFFFFFFFFFFFFF
FFFFFFFFFFFFFFFFFFFFFFFFFFFF:FFFFFFFFFFFFFFFFFFFFFFFFFFFFF:FFFFFFFFF
@A00155:342:HHGFNDSXY:1:1337:19361:25504 1:N:0:GAACCTAG+TCCGCATA
AGAGAGAGCGACTTCTCTCGGGCCCACAGCCTTACAGCTGTGGAGCACGGTATCCTCTGCCGAAACAG
AGGTTGGACAAGACCGGAGGGGTCTCCTGGTTCCAAAGGAGATGTACTCCGGGCTTGTTCACGACCTA
+
FFFFFFFFFFFFFFFFFFFFFFFFFFFFFFFFFFFFFFFFFFFFFFFFFFFFFFFFFFFFFFFFFFFF
FFFFFFFFFFFFFFFFFFFFFFFFFFFFFFFFFFFFFFFFFFFFFFFFFFFFFFFFFFFFFFFFFFFF
@A00155:342:HHGFNDSXY:1:1337:1859:30107 1:N:0:GAACCTAG+TCCGCATA
CTTCTCTCGGGCCCACAGCCTTACAGCTGTGGAGCACGGTATCCTCTGCCGAAACAGAGGTTGGACAA
GACCGGAGGGGTCTCCTAGTTCCAAAGGAGATGTACTCCGGGCTTGTTCACGACCTACCGTGTAAGTC
+
FFFFFFFFFFFFFFFFFFFFFFFFFFFFFFFFF,FFFFFFFFFFFFFF:FFFFFFFFFFFFFFFFFFF
FFFFFFFFFFFFFFFFFFFFFFFF,FFFFFFFFFFFFFFFFFFFFFFFFFFFFFFFFFFFFFFFFFFF
@A00155:342:HHGFNDSXY:1:1337:24713:34460 1:N:0:GAACCTAG+TCCGCATA
GTCATCTCAATGCCGAGAGGGATGTGGAGGGCGAGAGAGAGCGACTTCTCTCGGGCCCACAGCCTTAC
AGCTGTGGAGCACGGTATCCTCTGCCGAAACAGAGGTTGGACAAGACCGGAGGGGTCTCCTAGTTCCA
+
FFFFFFFFFFFFFFFFFFFFFFFFFFFFFFFFFFFFFFFF:FFFFFFFFFFFFFFFFFFFFFFFFFFF
FFFFFFFFFFFFFFFFFFFFFFFFFFFFFFFFFFFFFFFFFFFFFFFFFFFFFFFFFFFFFFFFFFFF
@A00155:342:HHGFNDSXY:1:1337:11143:35916 1:N:0:GAACCTAG+TCCGCATA
CATCTCAATGCCGAGAGGGATGTGGAGGGCGAGAGAGAGCGACTTCTCTCGGGCCCACAGCCTTACAG
CTGTGGAGCACGGTATCCTCTGCCGAAACAGAGGTTGGACAAGACCGGAGGGGTCTCCTGGTTCCAAA
+
FFFFFFFFFFFFFFFFFFFFFF:FFFFFFFFFFFFFFFFFFFFFFFFFFFFFFFFFFFFFFFFFFFFF
FFFFFFFFFFFFFF:FFFFFFFFFFFFFFFFFFFFFFFFFFFFFFFFFFFFFFFFFFFFFFFFFFFFF
@A00155:342:HHGFNDSXY:1:1338:11388:30765 1:N:0:GAACCTAG+TCCGCATA
ATCTCAATGCCGAGAGGGATGTGGAGGGCGAGAGAGAGCGACTTCTCTCGGGCCCACAGCCTTACAGC
TGTGGAGCACGGTATCCTCTGCCGAAACAGAGGTTGGACAAGACCGGAGGGGTCTCCTAGTTCCAAAG
+
FFFFFFFFFF:FFFFFFFF:F:FFFFFFFFFFFFFFFFFFFFFFFFFFFFFFFFFFFFFFFFFFFFFF
FFFFFFFFFFFFFFFFFFFFFFFFFFFFFFFFFFFFFFFFFFFFFFFFFFFFFFFFFFFFFFFFFFFF
@A00155:342:HHGFNDSXY:1:1339:29432:5932 1:N:0:GAACCTAG+TCCGCATA
ATCTCAATGCCGAGAGGGATGTAGAGGGCGAGAGAGAGCGACTTCTCTCGGGCCCACAGCCTTACAGC
TGTGGAGCACGGTATCCTCTGCCGAAACAGAGGTTGGACAAGACCGGAGGGGTCTCCTAGTTCCAAAG
+
FFFFFFFFFFFFFFFFFFFFFFFFFFFFFFFFFFFFFFFFFF:FFFFFFFFFFFFFFFFFFFFFFFFF
FFFFFFFFFFFFFFFFFFFFFFFFFFFFFFFFFFFFFFFFFFFFFFFFFFFFFFFFFFFFFFFFFFFF
@A00155:342:HHGFNDSXY:1:1339:19976:7560 1:N:0:GAACCTAG+TCCGCATA
AGCGACTTCTCTCGGGCCCACAGCCTTACAGCTGTGGAGCACGGTATCCTCTGCCGAAACAGAGGTTG
GACAAGACCGGAGGGGTCTCCTAGTTCCAAAGGAGATGTACTCCGGGCTTGTTCACGACCTACCGTGT
+
FFFFFFFFFFFFFFFFFFFFFFFFFFFFFFFFFFFFFFFFFFFFFFFFFFFFFFFFFFFFFFFFFFFF
FFFFFFFFFFFFFFFFFFFFFFFFFFFFFFFFFFFFFFFFFFFFFFFFFFFFFFFFFFFFFFFFFFFF
@A00155:342:HHGFNDSXY:1:1339:23656:8703 1:N:0:GAACCTAG+TCCGCATA
CTCAATGCCGAGAGGGATGTGGAGGGCGAGAGAGAGCGACTTCTCTCGGGCCCACAGCCTTACAGCTG
TGGAGCACGGTATCCTCTGCCGAAACAGAGGTTGGACAAGACCGGAGGGGTCTCCTAGTTCCAAAGGA
+
FFFFFFFFFFFFFFFFFFFFFFFFFFFFFFFFFFFFFFFFFFFFFFFFFFFFFFFFFFFFFFFFFFFF
FFFFFFFFFFFFFFFFFFFFFFFFFFFFFFFFFFFFFFFFFFFFFFFFFFFFFFFFFFFFFFFFFFFF

```
@A00155:342:HHGFNDSXY:1:1339:28980:8750 1:N:0:GAACCTAG+TCCGCATA
TGTGGAGGGCGAGAGAGAGCGACTTCTCTCGGGCCCACAGCCTTTCAGCTGTGGAGCACGGTATCCTC
TGCCGAAACAGAGGTTGGACAAGACCGGAGGGGTCTCCTAGTTCCAAAGGAGATGTACTCCGGG
+
FFF:FF:FFFFFFFFFFFFFFFFFFFFFFFF:FF:FFFFFFFFFFFFFFFF,FFFFFF,:FFFF:FF
FFFFFFFFFFFFFFFFFFFFFFF:FFFFFFFFF,FFFFFFF::FFFFFFFFFFFFFFFFFFF:FF
@A00155:342:HHGFNDSXY:1:1339:30563:9142 1:N:0:GAACCTAG+TCCGCATA
TGTGGAGGGCGAGAGAGAGCGACTTCTCTCGGGCCCACAGCCTTTCAGCTGTGGAGCACGGTATCCTC
TGCCGAAACAGAGGTTGGACAAGACCGGAGGGGTCTCCTAGTTCCAAAGGAGATGTACTCCGGG
+
,F,FFFF,FFFFFFFF:FFFFFFFFFFFF,FFFF,FFFFFFFFFFFFF,FFF:FFFFFFFFFFFF:FF
F:,FFFFFFF:FF:FFFFFFFF:FF,FFFFFFFFFF,FFF:FFFFFFFFFFFFFFFFFFFF:FFF
@A00155:342:HHGFNDSXY:1:1339:4390:11600 1:N:0:GAACCTAG+TCCGCATA
TTAAGGACTCTCCTCTGGCCTATGGTCATCTCAATGCCGAGAGGGATGTGGAGGGCGAGAGAGAGCGA
CTTCTCTCGGGCCCACAGCCTTACAGCTGTGGAGCACGGTATCCTCTGCCGAAACAGAGGTTGGACAA
+
FFFFFFFFFFFFFFFFFFFFFFFFFFFFFFFFFFFFFFFFFFFFF::FFFFFFFFFFFFFFFFFFFF
FFFFFFFFFFFFFFFFFFFFFFF:FFFFFFFFFFFFFFFFF:FFFFFFFFFFFFFFFFFFFFFFFFFFF
@A00155:342:HHGFNDSXY:1:1339:13557:12727 1:N:0:GAACCTAG+TCCGCATA
CTCTGTTTCGGCAGAGGATACCGTGCTCCACAGCTGTAAGGCTGTGGGCCCGAGAGAAGTCGCTCTCT
CTCGCCCTCCACATCCCTCTCGGCATTGAGATGACCATAGGCCAGAGGTGAGTCCTTAAGTGGACACA
+
FFFFFFFFFFFFFFFFFFFFFFFFFFFFFFFFFFFFFFFFFFFFFFFFFFFFFFFFFFFFFFFFFFFF
FFFFFFFFFFFFFFFFFFFFFFFFFFFFFFFFFFFFFFFFFFFFFFFFFFFFFFFFFFFFFFFFFFFF
@A00155:342:HHGFNDSXY:1:1339:26901:24972 1:N:0:GAACCTAG+TCCGCATA
TGTCCACTTAAGGACTCACCTCTGGCCTATGGTCATCTCAATGCCGAGAGGGATGTGAAGGGCGAGAG
AGAGCGACTTCTCTCGGGCCCACAGCCTTACAGCTGTGGAGCACGGTATCCTCTGCCGAAACAGAGGT
+
FFFFFFFFFFFFFFFFFFFFFFFFFFFFFFFFFFFFFFFFFFFFFF:FFFFFFFFFFFFFFFFFFFFF
FFFFFFFFFFFFFFFFFFFFFFFFFFFFFFFFFFFFFFFFF:FFFFFFFFFFFFFFFFFFFFFFFFFF
@A00155:342:HHGFNDSXY:1:1340:23900:9627 1:N:0:GAACCTAG+TCCGCATA
CTTCTCTCGGGCCCACAGCCTTACAGCTGTGGAGCACGGTATCCTCTGCCGAAACAGAGGTTGGACAA
GACCGGAGGGGTCTCCTAGTTCCAAAGGAGATGTACTCCGGGCTTGTTCACGACCTACCGTGTAAGTC
+
FFFFFFFFFFFFFFFFFFFFFFFFFFFFFFFFFFFFFFFFFFFFFFFFFFFFFFFFFFFFFFFFFFFF
FFFFFFFFFFFFFFFFFFFFFFFFFFFFFFFFFFFFFFFFFFFFFFFFFFFFFFFFFFFFFFFFFFFF
@A00155:342:HHGFNDSXY:1:1340:10194:13761 1:N:0:GAACCTAG+TCCGCATA
GTAACCTCTGCCGAAACAGAGGTTGGACAAGACCGGAGGGGTCTCCTAGTTCCAAAGGAGATGTACTC
CGGGCTTGTTCACGACCTACCGTGTAAGTCGTAGTCTAGTAGGCTACCTGACGAGTCCTTTTTAGGAC
+
FFF,FFFFFFFFFFFFFFFFFFFFFFFFFFFFFFFFFFFFFFFFFFFFFFFFFFFFFFFFFFFFFFFF
FFFFFFFFFFFFFFFFFFFFFFFFFFFFFFFFFFFFFFFFFFFFFFFFFFFFFFFFFFFFFFFFFFFF
@A00155:342:HHGFNDSXY:1:1340:11758:31469 1:N:0:GAACCTAG+TCCGCATA
CTTCTCTCGGGCCCACAGCCTTACAGCTGTGGAGCACGGTATCCTCTGCCGAAACAGAGGTTGGACAA
GACCGGAGGGGTCTCCTAGTTCCAAAGGAGATGTACTCCGGGCTTGTTCACGACCTACCGTGTAAGTC
+
FFFFFFFFFF:FFFFFFFF:FFFF:FFFFFFFFFFFF:F:FFFFFFFFFFFFF:FF:FFFFFFFFFF
FFF,FFFFFFFFFFFFFFFFFFFFFFFFFFFFFFFFFFFFFFFF:FFFFFFFFF,FFFFFFFFFFFFF
@A00155:342:HHGFNDSXY:1:1341:17427:7216 1:N:0:GAACCTAG+TCCGCATA
TTAGATCAGCTGTGTCCACTTAAGGACTCACCTCTGGCCTATGGTCATCTCAATGCCGAGAGGGATGT
AGAGGGCGAGAGAGAGCGACTTCTCTCGGGCCCACAGCCTTACAGCTGTGGAGCACGGTATCCTCTGC
+
:FFFFFFFFFFFFFFFFFFFF:F:FFFFFFFFFFFFFFFFFFFFFFFFFFFFFFFF:FFFFFFFFF:FF
,FFFFFFFFFFFFFFFFFFFFFFFFFFFFFFFFFFFFFFFFFFFFFFFFFFFFFFFFFFF:FFFFFFFF
```

```
@A00155:342:HHGFNDSXY:1:1341:9001:7686 1:N:0:GAACCTAG+TCCGCATA
GGATGTGGAGGGCGAGAGAGAGCGACTTCTCTCGGGCCCACAGCCTTACAGCTGTGGAGCACGGTATC
CTCTGCCGAAACAGAGGTTGGACAAGACCGGAGGGGTCTCCTGGTTCCAAAGGAGATGTACTCC
+
FFFFFFFFFFFFFFFFFFFFFFFFFFFFFFFFFFFFFFFFFFFFFFFFFFFFFFFFFFFFFFFFFFFF
FFFFFFFFFFFFFFFFFFFFFFFFFF:FFFFFFFFFFFFFFFFFFFFFFFFFFFFFFFFFFFFF
@A00155:342:HHGFNDSXY:1:1341:3134:22388 1:N:0:GAACCTAG+TCCGCATA
TGCCGAGAGGGATGTAGAGGGCGAGAGAGAGCGACTTCTCTCGGGCCCACAGCCTTACAGCTGTGGAG
CACGGTATCCTCTGCCGAAACAGAGGTTGGACAAGACCGGAGGGGTCTCCTAGTTCCAAAGGAG
+
FFFFFFFFFFFFFFFF:FFFFFFFFFFFFFFFFFFFFFFFFFFFFFFFFFFFFFFFFFFFFFFFFFFF
FFFFFFFFFFFFFFFFFFFFFFF:FFFFFFFFFFFFFFFFFFFFFFFFFFFFFFFFFF:FFFFF
@A00155:342:HHGFNDSXY:1:1341:29279:23547 1:N:0:GAACCTAG+TCCGCATA
TAGATCAGCTGTGTCAACTTAAGGACTCACCTCTGGCCTATGGTCATCTCAATGCCGAGAGGGATGTG
GAGGGCGAGAGAGAGCGACTTCTCTCGGGCCCACAGCCTTACAGCTGTGGAGCACGGTATCCTCTGCC
+
FFFFFFFFFFFFFFFFFFFFFFFFFFFFFFFFFFFFFFFFFFFFFFFFFFFFFFFFFFFFFFFFFFFF
FFFFFFFFFFFFFFFFFFFFFFFFFFFFFFFFFFFFFFFFFFFFFFFFFFFFFFFFFFFFFFFFFFFF
@A00155:342:HHGFNDSXY:1:1341:17662:26036 1:N:0:GAACCTAG+TCCGCATA
CGCTCTCTCTCGCCCTCCACATCCCTCTCGGCATTGAGATGACCATAGGCCAGAGGTGAGTCCTTAAG
TGGACACAGCTGATCTAAGGCGGTGTGGTGGGGCATGGGTTTGAACCCCCATGACGGTCGGAGTCTAG
+
FFFFFFFFFFFFFFFFFFFFFFFFFFFFFFFFFFFFFFFFFFFFFFFFFFFFFFFFFFFFFFFFFF:F
FFFFFFFFFFFFFFFFFFFFFFF:FFFFFFFFFFFFFFFFFFFFFFFFFFFFFFFFFFFFFFF,FFFF
@A00155:342:HHGFNDSXY:1:1341:18602:29575 1:N:0:GAACCTAG+TCCGCATA
CGCTCTCTCTCGCCCTCCACATCCCTCTCGGCATTGAGATGACCATAGGCCAGAGGTGAGTCCTTAAG
TGGACACAGCTGATCTAAGGCGGTGTGGTGGGGCATGGGTTTGAACCCCCATGACGGTCGGAGTCTAG
+
FFFFFFFFFFFFFFFFFFFFFFFFFFF,FFFFFFFFFFFFFFFFFFFFFFF:FFFFFFFFFFFFFFFF
FFFFFFFFFFFFFFFFFFFFFFF:F:FF,FFFFFFFFFFFF:F::FFFFFFFFFFFFFFFF:FFF,FF
@A00155:342:HHGFNDSXY:1:1342:16342:10817 1:N:0:GAACCTAG+TCCGCATA
GGATGTGGAGGGCGAGAGAGAGCGACTTCTCTCGGGCCCACAGCCTTACAGCTGTGGAGCACGGTATC
CTCTGCCGAAACAGAGGTTGGACAAGACCGGAGGGGTCTCCTAGTTCCAAAGGAGATGTACT
+
FF:FF::FFFFFFFFFFFFFFFFFFFF:FFFFFFFFFFFFFFFFFFFFFFFFFFFFFFFFFFFF,FFF
FFFFFFFFFFFFFFFFFFFFFFFFF,FFFFFFFFFFFFFFFFFFF:FFFF:FFFFF:FFFFF
@A00155:342:HHGFNDSXY:1:1342:22299:12868 1:N:0:GAACCTAG+TCCGCATA
CTTCTCTCGGGCCCACAGCCTTACAGCTGTGGAGCACGGTATCCTCTGCCGAAACAGAGGTTGGACAA
GACCGGAGGGGTCTCCTAGTTCCAAAGGAGATGTACTCCGGGCTTGTTCACGACCTACCGTGTAAGTC
+
FFFFFFFFFFFFFFFFFFFFFFFFFFFFFFFFFFFFFFFFFFFFFFFF:FFFFFFFFFFFFFFFFFFF
FFFFFFFFFFFFFFFFFFFFFFFFFF:FFFFFFFFFFF:FFFFFFFFFFFFFFFFFFFFFFFFFFFFF
@A00155:342:HHGFNDSXY:1:1342:28149:13604 1:N:0:GAACCTAG+TCCGCATA
CCACATCCCTCTCGGCATTGAGATGACCATAGGCCAGAGGTGAGTCCTTAAGTGGACACAGCTGATCT
AAGGCGGTGTGGCGGGGCATGGGTTTGAACCCCCATGACGGTCGGAGTCTAGTAGGCTCCCTGATGAG
+
FFFFFFFFFFFF:FFFFFFFF,FFFFFFFFFF,FFFFFFFFFFFFFFFFFFFFFFFFFFFFFFFFFFF
FFFFFFF:FFFFFFFFFFFFFFFF:FFFFFFFFFFFFFFFFFFFFFFFFFFFFFFFFFFFFFFFFFF
@A00155:342:HHGFNDSXY:1:1342:2293:18114 1:N:0:GAACCTAG+TCCGCATA
TTCTCTCGGGCCCACAGCCTTACAGCTGTGGAGCACGGTATCCTCTGCCGAAACAGAGGTTGGACAAG
ACCGGAGGGGTCTCCTGGTTCCAAAGGAGATGTACTCCGGGCTTGTTCACGACCTACCGTGTAAGTCG
+
FFFFFFFFFFFFFFFFFFFFFFFFFFFFFFFFFFFFFFFFFFFFFFFFFFFFFFFFFFFFFFFFFFFF
FFFFFFFFFFFFFFFFFFFFFFFFFFFFFFFFFFFFFFFFFFFFFFFFFFFFFFFFFFFFFFFFFFFF
```

@A00155:342:HHGFNDSXY:1:1342:13286:24220 1:N:0:GAACCTAG+TCCGCATA
ACCTCTGTTTCGGCAGAGGATACCGTGCTCCACAGCTGTAAGGCTGTGGGCCCGAGAGAAGTCGCTCT
CTCTCGCCCTCCACATCCCTCTCGGCATTGAGATGACCATAGGCCAGAGGAGAGTCCTTAAGTGGACA
+
FFFFFFFFFFFFFFFFFFFFFFFFFFFFFFFFFFFFFFFFFFFFFFFFFFFFFFFFFFFFFFFFFFFF
FFFFFFFFFFFFFFFFFFFFFFFFFFFFFFFFFFFF:FFFFFFFFFFFFFFFFFFFFFFFFFFFFFF:
@A00155:342:HHGFNDSXY:1:1343:12481:17973 1:N:0:GAACCTAG+TCCGCATA
GAGAGGGATGTGGAGGGCGAGAGAGAGCGACTTCTCTCGGGCCCACAGCCTTACAGCTGTGGAGCACG
GTATCCTCTGCCGAAACAGAGGTTGGACAAGACCGGAGGGGTCTCCTAGTTCCAAAGGAGATGTACTC
+
FFFFFFFFFFFFFFFFFFFFFFFFFFFFFFFFFFFFFFFFFFFFFFFFFFFFFFFFFFFFFFFFFFFF
FFFFFFFFFFFFFFFFFFFFFFFFFFFFFFFFFFFFFFFFFFFFFFFFFFFFFFFFFFFFFFFFFFFF
@A00155:342:HHGFNDSXY:1:1343:11831:18912 1:N:0:GAACCTAG+TCCGCATA
GAGAGGGATGTGGAGGGCGAGAGAGAGCGACTTCTCTCGGGCCCACAGCCTTACAGCTGTGGAGCACG
GTATCCTCTGCCGAAACAGAGGTTGGACAAGACCGGAGGGGTCTCCTAGTTCCAAAGGAGATGTACTC
+
FFFFFFFFFFFFFFFFFFFFFFFFFFFFFFF:FFFF:FFFFFFFFFFFFFFFFFFFFFFFFFFFFFFF
FFFFFFFFFFFFFFFFFFFFFFFFFFFFFFFFFFFFFFFFFFFFFFFFFFFFFFFFFFFFFFFFFFFF
@A00155:342:HHGFNDSXY:1:1344:19253:7686 1:N:0:GAACCTAG+TCCGCATA
TTAGATCAGCTGTGTCCACTTAAGGACTCACCTCTGGCCTATGGTCATCTCAATGCCGAGAGGGATGT
GGAAGGCGAGAGAGAGCGACTTCTCTCGGGCCCACAGCCTTACAGCTGTGGAGCACGGTATCCTCTGC
+
FFFFFFFFFFFFFFFFFFFFFFFFFFFFFFFFFFFFFFFFFFFFFFFFFFFFFFFFFFFFFFFFFFFF
FFFFFFFFFFFFFFFFFFFFFFFFFFFFFFFFFFFFFFFFFFFFFFFFFFFFFFFFFFFFFFFFFFFF
@A00155:342:HHGFNDSXY:1:1344:30020:12743 1:N:0:GAACCTAG+TCCGCATA
CTTCTCTCGGGCCCACAGCCTTACAGCTGTGGAGCACGGTATCCTCTGCCGAAACAGAGGTTGGACAA
GACCGGAGGGGTCTCCTAGTTCCAAAGGAGATGTACTCCGGGCTTGTTCACGACCTACCGTGTAAGTC
+
FFFFFFFFFFFFFFFFFFFFFFFFFFFFFFFFFFFFFFFFFFFFFFFFFFFFFFFFFFFFFFFFFFFF
:FFFFFFFFFFFFFFFFFFFFFFFFFFFFFFFFFFFFFFFFFFFFFFFFFFFFFFFFFFFFFFF:FFF
@A00155:342:HHGFNDSXY:1:1344:11623:34272 1:N:0:GAACCTAG+TCCGCATA
CGAGAGGGATGTGGAGGGCGAGAGAGAGCGACTTCTCTCGGGCCCACAGCCTTACAGCTGTGGAGCAC
GGTATCCTCTGCCGAAACAGAGGTTGGACAAGACCGGAGGGGTCTCCTAGTTCCAAAGGAGATGTACT
+
FF:F::FFFFFFFFFFFFFFFFFFFFFFFFFFFFFFFFFFF:FFFFFF,FF::FFF:FFFFFFFFFFF
FFFFFFF:FFFFFFFFFFFF,FF:FFFFFFF:FFFFFFFFFFFFFFFF:FFFFFFFFFFFFFFFFFFF
@A00155:342:HHGFNDSXY:1:1345:18548:3490 1:N:0:GAACCTAG+TCCGCATA
TCGGCAGAGGATACCGTGCTCCACAGCTGTAAGGCTGTGGGCCCGAGAGAAGTCGCTCTCTCTCGCCC
TCCACATCCCTCTCGGCATTGAGATGACCATAGGCCAGAGGTGAGTCCTTAAGTGGACACA
+
FFFFFFFFFFFFFFFFFFFFFFFFFFFFFFFFFFFFFFFFFFFFFFFFFFFFFFFFFFFFFFFFFFFF
FFFFFFFFFFFFFFFFFF:FFFFFFFFFFFFFFFFFFFFFFFFFFFFFFFFFFFFFFFFFF
@A00155:342:HHGFNDSXY:1:1345:30825:21652 1:N:0:GAACCTAG+TCCGCATA
TTCTCTCGGGCCCACAGCCTTACAGCTGTGGAGCACGGTATCCTCTGCCGAAACAGAGGTTGGACAAG
ACCGGAGGGGTCTCCTAGTTCCAAAGGAGATGTACTCCGGGCTTGTTCACGACCTACCGTGTAAGTCG
+
FFFFFFFFFFFFFFFFFFFFFFFFFFFFFFFFFFFFFFF:F,FFFFFFFFFFFFFFFFFFFFFFFFF:
FFFFFFFFFFFFFF:FFFF:FFFFFFFFFFFFFFFFFFFFFFFFFF:FFFFFFFFFFFFFFFFFFFFF
@A00155:342:HHGFNDSXY:1:1345:23529:27618 1:N:0:GAACCTAG+TCCGCATA
CTATGGTCATCTCAATGCCGAGAGGGATGTGGAGGGCGAGAGAGAGCGACTTCTCTCGGGCCCACAGC
CTTACAGCTGTGGAGCACGGTATCCTCTGCCGAAACAGAGGTTGGACAAGACCGGAGGGGTCTCCTGG
+
FFFFFFFFFFFFFFFFFFFFFFFFFFFFFFFFFFFFFFFFFFFFFFFFFFF:FFFFFFFFFFFFFFFF
FFFFFFFFFFFFFFFFFFFFFFFFFFFFFFFFFFFF:FFFFFFFFFFFFFFFFFFFFFFFFFFFFFF:

```
@A00155:342:HHGFNDSXY:1:1345:24288:28307 1:N:0:GAACCTAG+TCCGCATA
CTCAATGCCGAGAGGGATGTAGAGGGCGAGAGAGAGCGACTTCTCTCGGGCCCACAGCCTTACAGCTG
TGGAGCACGGTATCCTCTGCCGAAACAGAGGTTGGACAAGACCGGAGGGGTCTCCTAGTTCCAAAGGA
+
FFFFFFFFFF:FFFFFFFFFFFFFFFFFFFFFFFFFFFFFFFFFFFFFFFFFFFFFFFFFFFFFFFFFFFFF
FFFFFFFFFFFFFFFFFFFFFFFFFFFFFFFFFFFFFFFFF:FFFFFFFFFFFFFFFFFFFFFFFFFF
@A00155:342:HHGFNDSXY:1:1345:22028:32440 1:N:0:GAACCTAG+TCCGCATA
CTCAATGCCGAGAGGGATGTAGAGGGCGAGAGAGAGCGACTTCTCTCGGGCCCACAGCCTTACAGCTG
TGGAGCACGGTATCCTCTGCCGAAACAGAGGTTGGACAAGACCGGAGGGGTCTCCTAGTTCCAAAGGA
+
FFFFFFFFFFFFFFFFFFFFFFFFFFFFFFFFFFFFFFFFFFF:FFFFFFFFFFFFFFFFFFFFFF:F
FFFFFFFFFFFFFFFFFFFFFFFFFFFFFFFFFFFFFFFFFFFFFFFFFFFFFFFFFFFFFFFFFFFF
@A00155:342:HHGFNDSXY:1:1345:12156:36354 1:N:0:GAACCTAG+TCCGCATA
AGGGCGAGAGAGAGCGACTTCTCTCGGGCCCACAGCCTTACAGCTGTGGAGCACGGTATCCTCTGCCG
AAACAGAGGTTGGACAAGACCGGAGGGGTCTCCTAGTTCCAAAGGAGATGTACTCCGGGCTTGTTCAC
+
FFFFFFFFFFFFFFFFFFFFFFFFFFFFFFFFFFFFFFFFFFFFFFFFFFFFFFFFFFFFFFFFFFFF
FFFFFFFFFFFFFFFFF:FFFFFFFFFFFFFFFFFFFFFFFFFFFFFFFFFFFFFFFFFFFFFFFFFF
@A00155:342:HHGFNDSXY:1:1346:19190:5290 1:N:0:GAACCTAG+TCCGCATA
CTCCTCTGGCCTATGGTCATCTCAATGCCGAGAGGGATGTGGAGGGCGAGAGAGAGCGACTTCTCTCG
GGCCCACAGCCTTACAGCTGTGGAGCACGGTATCCTCTGCCGAAACAGAGGTTGGACAAGACCGGAGG
+
FFFFFFFFFFFFFFFFFFFFFFFFFFFFFFFFFFFFFFFFFFFFFFFFFFFFFFFFFFFFFFFFFFFF
FFFFFFFFFFFFFFFFFFFFFFFFFFFFFFFFFFFFFFFFFFFFFFFFFFFFFFFFFFFFFFFFFFFF
@A00155:342:HHGFNDSXY:1:1346:12735:18004 1:N:0:GAACCTAG+TCCGCATA
AGGACTCACCTCTGGCCTATGGTCATCTCAATGCCGAGAGGGATGTGAAGGGCGAGAGAGAGCGACTT
CTCTCGGGCCCACAGCCTTACAGCTGTGGAGCACGGTATCCTCTGCCGAAACAGAGGTTGGACAAGAC
+
FFFFFFFFFFFFFFFFFFFFFFFFFFFFFFFFFFFFFFFFFFFFFFFFFFFFFFFFFFFFFFFFFFFF
FFFFFFFFFFFFFFFFFFFFFFFFFFFFFFFFFFFFFFFFFFFFFFFFFFFFFFFFFFFFFFFFFFFF
@A00155:342:HHGFNDSXY:1:1346:28094:20744 1:N:0:GAACCTAG+TCCGCATA
CTGCCGAAACAGAGGTTGGACAAGACCGGAGGGGTCTCCTAGTTCCAAAGGAGATGTACTCCGGGCTT
GTTCACGACCTACCGTGTAAGTCGTAGTCTAGTAGGCTACCTGACGAGTCCTTTTTAGGACGAAACTT
+
FFFFFFFFFFFFFFFFFFFFFFFFFFFFFFFFFFFFFFFFFFFFFFFFFFFFFFFFFFFFFFFFFFFF
FFFFFFFFFFFFFFFFFFFFFFFFFFFFFFFFF:FFFFFFFFFFFFFFFFFFFFFFFFFFFFFFFFFF
@A00155:342:HHGFNDSXY:1:1346:14552:22216 1:N:0:GAACCTAG+TCCGCATA
TCTCTCGGGCCCACAGCCTTACAGCTGTGGAGCACGGTATCCTCTGCCGAAACAGAGGTTGGACAAGA
CCGGAGGGGTCTCCTAGTTCCAAAGGAGATGTACTCCGGGCTTGTTCACGACCTACCGTGTAAGTCGT
+
FFFFFFFFFFFFFFFFFFFFFFFFFFFFFFFFFFFFFFFFFFFFFFFFFFFFFFFFFFFFFFFFFF:F
FFFFFFFFFFFFFFFFFFFFFFFFFFFFF:FFFFFFFFFFFFFFFFFFFFFFFFFFFFFFFFFFFFFF
@A00155:342:HHGFNDSXY:1:1346:9977:30671 1:N:0:GAACCTAG+TCCGCATA
GGATGTGGAGGGCGAGAGAGAGCGACTTCTCTCGGGCCCACAGCCTTACAGCTGTGGAGCACGGTATC
CTCTGCCGAAACAGAGGTTGGACAAGACCGGAGGGGTCTCCTGGTTCCAAAGGAGATGTACTCC
+
FFFFFFFFFFFFFFFF:FFFFFFFFFFFFFFFFFFFFFFFFFFFFFFFFFFFFFFFFFFFFFFFFF:F
FFFFFFFFFFFFFFFFFFFFFFFFFFFFFFFFFFFFFFFFFFFFFFFFFFFFFFFFFFFFFFFF
@A00155:342:HHGFNDSXY:1:1347:12147:1110 1:N:0:GAACCTAG+TCCGCATA
AGGTCATCTCAATGCCGAGAGGGATGTGGAGGGCGAGAGAGAGCGACTTCTCTCGGGCCCACAGCCTT
ACAGCTGTGGAGCACGGTATCCTCTGCCGAAACAGAGGTTGGACAAGACCGGAGGGGTCTCC
+
FFFFFFFFFFFFFFFFFFFFFFFFFFFFFFFFFFFFFFFFFFFFFFFFFFFFFFFFFFFFFFFFFFFF
FFFFFFFFFFFFFFFFFFFFFFFFFFFFFFFFFFFFFFFFFFFFFFFFFFFF,FFFFFFFFF
```

```
@A00155:342:HHGFNDSXY:1:1347:22055:24345 1:N:0:GAACCTAG+TCCGCATA
ACTTCTCTCGGGCCCACAGCCTTACAGCTGTGGAGCACGGTATCCTCTGCCGAAACAGAGGTTGGACA
AGACCGGAGGGGTCTCCTAGTTCCAAAGGAGATGTACTCCGGGCTTGTTCACGACCTACCGTGTAAGT
+
FFFFFFFFFFFFFFFFFFFFFFFFFFFFFFFFFFFFFFFF:FFFFFFFFFFFFF:FFFFFFFFFFFFF
FFFFFFFFFFFFFFFFFFFFFF:FFFFFFFFFFFFFFFFFFFFFFFFFFFFFFFFFFFFFFFFFFFFF
@A00155:342:HHGFNDSXY:1:1347:28004:28573 1:N:0:GAACCTAG+TCCGCATA
ACTTCTCTCGGGCCCACAGCCTTACAGCTGTGGAGCACGGTATCCTCTGCCGAAACAGAGGTTGGACA
AGACCGGAGGGGTCTCCTAGTTCCAAAGGAGATGTACTCCGGGCTTGTTCACGACCTACCGTGTAAGT
+
FFFFFFFFFFFFFFFFFFFFFFFFFFFFFFFFFFFFFFFFFFFFFFFFFFFFFFFFFFFFFFFFFFFF
FFFFFFFFFFFFFFFFFFFFFFFFFFFFFFFFFFFFFFFFFFFFFFFFFFFFFFFFFFFFFFFFFFFF
@A00155:342:HHGFNDSXY:1:1348:17716:8531 1:N:0:GAACCTAG+TCCGCATA
GTCATCTCAATGCCGAGAGGGATGTGGAGGGCGAGAGAGAGCGACTTCTCTCGGGCCCACAGCCTTAC
AGCTGTGGAGCACGGTATCCTCTGCCGAAACAGAGGTTGGACAAGACCGGAGGGGTCTCCTAGTTCCA
+
FFFFFFFFFFFFFFFFFFFFFFFFFFFFFFFFFFFFFFFFFFFFFFFFFFFFFFFFFFFFFFFFFFFF
FFFFFFFFFFFFFFF,FFFFFFFFFFFFFFFFFFFFFFFFFFFFFFFFFFFFFFFFFFFFFFFFFFFF
@A00155:342:HHGFNDSXY:1:1348:12228:18035 1:N:0:GAACCTAG+TCCGCATA
GTGTGGCGGGGCATGGGTTTGAACCCCCATGACGGTCGGAGTCTAGTAGGCTCCCTGATGAGTCCGTT
CCAAGGACGAAACCGTCGTTACTCTTTGAGTTGTGTAAGTTTCGTCCTAAAAAGGACTCGTCAGGTAG
+
FFFFFFFFFFFFFFFFFFFFFFFFFFFFFFFFFFFFFFFFFFFFFFFFFFFFFFFFFFFFFFFFFFFF
FFFFFFFFFFFFFFFFFFFFFFFFFFFFFFFFFFFFFFFFFFFFFFFFFFFFFFFFFFFFFFFFFFFF
@A00155:342:HHGFNDSXY:1:1348:2519:22138 1:N:0:GAACCTAG+TCCGCATA
ACGGCTGTGGAGCACGGTATCCTCTGCCGAAACAGAGGTTGGACAAGACCGGAGGGGTCTCCTAGTTC
CAAAGGAGATGTACTCCGGGCTTGTTCACGACCTACCGTGTAAGTCGTAGTCTAGTAGGCTACCT
+
FF,FFFFFFFFFFFFFFFFFF:FFFFFFFFFFFFFFFFFFFFFFFFFFFFFF,FFFFFFFFFFFFFFFFFF
FFFFFFFFFFFF:FFF:FF:FFFFFFFFFFFFFFFFFFFFFFFFFFF:FF:FFFFFFFF:FFFFFF
@A00155:342:HHGFNDSXY:1:1348:14705:27305 1:N:0:GAACCTAG+TCCGCATA
TGTCCACTTAAGGACTCTCCTCTGGCCTATGGTCATCTCAATGCCGAGAGGGATGTGGAGGGCGAGAG
AGAGCGACTTCTCTCGGGCCCACAGCCTTACAGCTGTGGAGCACGGTATCCTCTGCCGAAACAGAGGT
+
FFFFFFFFFFFFFFFFFFFFFFFFFFFFFFFFFFFFFFFFFFFFFFFFFFFFFFFFFFFFFFFFFFFF
FFFFFFFFFFFFFFFFFFFFFFFFFFFFFFFFFFFFFFFFFFFFFFFFFFFFFFFFFFFFFFFFFFFF
@A00155:342:HHGFNDSXY:1:1349:20329:2221 1:N:0:GAACCTAG+TCCGCATA
TTACAGCTGTGGAGCACGGTATCCTCTGCCGAAACAGAGGTTGGACAAGACCGGAGGGGTCTCCTAGT
TCCAAAGGAGATGTACTCCGGGCTTGTTCACGACCTACCGTGTAAGTCGTAGTCTAGTAGGCTACCTG
+
FFFFFFFFFFFFFFFFFFFFFFFFFFFFFFFFFFFFFFFFFFFFFFFFFFFFFFFFFFFFFFFFFFFF
FFFFFFFFFFFFFFFFFFFFFFFFFFFFFFFFFFFFFFFFFFFFFFFFFFFFFFFFFFFFFFFFFFFF
@A00155:342:HHGFNDSXY:1:1349:27579:6950 1:N:0:GAACCTAG+TCCGCATA
GAGCGACTTCTCTCGGGCCCACAGCCTTACAGCTGTGGAGCACGGTATCCTCTGCCGAAACAGAGGTT
GGACAAGACCGGAGGGGTCTCCTAGTTCCAAAGGAGATGTACTCCGGACTTGTTCACGACCTACCGTG
+
FFFFFFFFFFFFFFFFFFFFFFFFFFFFFFFFFFFFFFFFFFFFFF,FFFFFFFFFFFFFFFFFFFFF
FFFFFFFFFFFFFFFFFFFFFFFFFFFFFFFFFFFFFFFFFFFFFFFFFFFFFFFFFFFFFFFFFFFF
@A00155:342:HHGFNDSXY:1:1349:12436:13510 1:N:0:GAACCTAG+TCCGCATA
CCGAGAGGGATGTGGAGGGCGAGAGAGAGCGACTTCTCTCGGGCCCACAGCCTTACAGCTGTGGAGCA
CGGTATCCTCTGCCGAAACAGAGGTTGGACAAGACCGGAGGGGTCTCCTGGTTCCAAAGGAGATGTAC
+
FFFFFFFFFFFFFFFFFFFFFFFFFFFFFFFFFF:FFFFFFFFFFFFFFFFFFFFFFFFFF:FFFFFFFF
FFFFFFFFFFFFFFFFFFFFFFFFFFFFFFFFFFFFFFFFFFFFFFFFFFFFFFFFFFFFFFFFFFFF
```

```
@A00155:342:HHGFNDSXY:1:1350:5529:8625 1:N:0:GAACCTAG+TCCGCATA
TATCCTCTGCCGAAACAGAGGTTGGACAAGACCGGAGGGGTCTCCTAGTTCCAAAGGAGATGTACTCC
GGGCTTGTTCACGACCTACCGTGTAAGTCGTAGTCTAGTAGGCTACCTGACGAGTCCTTTTTAGGACG
+
FFFFFFFFFFFFFFFFFFFFFFFFFFFFFFFFFFFFFFFFFFFFFFFFFFFFFFFFFFFFFFFFFFFF
FFFFFFFFFFFFFFFFFFFFFFFFFFFFFFFFFFFF:FFFFFFFFFFFFFFFFFFFFFFFFFFFFFFF
@A00155:342:HHGFNDSXY:1:1350:24975:10958 1:N:0:GAACCTAG+TCCGCATA
GCGAGAGAGAGCGACTTCTCTCGGGCCCACAGCCTTACAGCTGTGGAGCACGGTATCCTCTGCCGAAA
CAGAGGTTGGACAAGACCGGAGGGGTCTCCTAGTTCCAAAGGAGATGTACTCCGGGCTTGTTCACGAC
+
FFFF:FFFFFFFFFFFFFFFFFFFFFFFFFFFFFFFFFFFFFFFF,FFFF:FFFFFFFFF:FFF,FFF
FFFFFFFFFFFFFFFFFFFFFFFFFF:FFFFFFFFF:FFFF:FFFFFFFFFFFFFFFFFFFFFFFFFF
@A00155:342:HHGFNDSXY:1:1350:28293:14137 1:N:0:GAACCTAG+TCCGCATA
GCTCCACAGCTGTAAGGCTGTGGGCCCGAGAGAAGTCGCTCTCTCTCGCCCTCCACATCCCTCTCGGC
ATTGAGATGACCATAGGCCAGAGGTGAGTCCTTAAGTGGACACAGCTGATCTAAGGCGGTGTGGTGGG
+
FFFFFFFFFFFFFFFFFFFFFFFFFFFFFFFFFFFFFFFFFFFFFFFFFFFFFFFFFFFFFFFFFFFF
F:FFFFFFFFFFFFFFFFFFFFFFFFFFFFFFFFFFFFFFFFFFFFFFFFFFFFFFFFFFFFFFFFFF
@A00155:342:HHGFNDSXY:1:1350:11406:20149 1:N:0:GAACCTAG+TCCGCATA
TCGGCAGAGGATACCGTGCTCCACAGCTGTAAGGCTGTGGGCCCGAGAGAAGTCGCTCTCTCTCGCCC
TCCACATCCCTCTCGGCATTGAGATGACCATAGGCCAGAGGTGAGTCCTTAAGTGGACACA
+
FFFFFFFFFFFFFFFFFFFFFFFFFFFFFFFFF:FFFFFFFFFFFFFFFFFFFFFFFFFFFFFFFFFF
FFFFFFFFFFFFFFFF:FFFFFFFFFFFFFFF:FFFFFFFFFFFFFFFFFFF:FFFFFFFF
@A00155:342:HHGFNDSXY:1:1350:25916:28432 1:N:0:GAACCTAG+TCCGCATA
ATCTCAATGCCGAGAGGGATGTGGAGGGCGAGAGAGAGCGACTTCTCTCGGGCCCACAGCCTTACAGC
TGTGGAGCACGGTATCCTCTGCCGAAACAGAGGTTGGACAAGACCGGAGGGGTCTCCTAGTTCCAAAG
+
FFFFFFFFFFFF:F:FFFFFFFF:FFFFFFFFFFFFFFFFFFFFFFFFFFFFFFFFFFFFFFFFFFFF
FFFFFFF:FFFFFFFFFFFFFFFFFFFFFFFFFFFFFFFFFFFFFFFFFFFFFFFFFFFFFFFFFFFF
@A00155:342:HHGFNDSXY:1:1350:17987:36432 1:N:0:GAACCTAG+TCCGCATA
GATGTGGAGGGCGAGAGAGAGCGACTTCTCTCGGGCCCACAGCCTTACAGCTGTGGAGCACGGTATCC
TCTGCCGAAACAGAGGTTGGACAAGACCGGAGGGGTCTCCTAGTTCCAAAGGAGATGTACTCCGGGCT
+
FFFFFFFFFFFFFFFFFFFFFFFFFFFFFFFFFFFFFFFFFFFFFFFFFFFFFFFFFFFFFFFFFFFF
FFFFFFFFFFFFFFFFFF:FFFFFFFFFFFFFFFFFF:FFFFFFFFFFFFFFFFFFFFFFFFFFFFFF
@A00155:342:HHGFNDSXY:1:1351:8712:2769 1:N:0:GAACCTAG+TCCGCATA
GGATGTAGAGGGCGAGAGAGAGCGACTTCTCTCGGGCCCACAGCCTTACAGCTGTGGAGCACGGTATC
CTCTGCCGAAACAGAGGTTGGACAAGACCGGAGGGGTCTCCTAGTTCCAAAGGAGATGTACTCC
+
FFFFFFFFFFFFFFFFFFFFFFFFFFFFFFFFFFFFFFFFFFFFFFFFFFFFFFFFFFFFFFFFFFFF
FFFFFFFFFFFFFFFFFFFFFFFFFFFFFFFFFFFFFFFFFFFFFFFFFFFFFFFFFFFFFFFF
@A00155:342:HHGFNDSXY:1:1351:10854:9048 1:N:0:GAACCTAG+TCCGCATA
CGACTTCTCTCGGGCCCACAGCCTTACAGCTGTGGAGCACGGTATCCTCTGCCGAAACAGAGGTTGGA
CAAGACCGGAGGGGTCTCCTAGTTCCAAAGGAGATGTACTCCGGGCTTGTTCACGACCTACCGTGTAA
+
FFFFFFFFFFFFFFFFFFFFFFFFFFFFFFFFFFFFFFFFFFFFFFFFFFFFFFFFFFFFFFFFFFFF
F:FFFFFFFFFFFFFFFFFFFFFFFFFFFFFFFFFFFFFFFFFFFFFFFFFFFFFFFFFFFFFFFFFF
@A00155:342:HHGFNDSXY:1:1351:9751:15468 1:N:0:GAACCTAG+TCCGCATA
ATCCTCTGCCGAAACAGAGGTTGGACAAGACCGGAGGGGTCTCCTAGTTCCAAAGGAGATGTACTCCG
GGCTTGTTCACGACCTACCGTGTAAGTCGTAGTCTAGTAGGCTACCTGACGAGTCCTTTTTA
+
FFFF:FFFFFFFFFFF,F:F:F:FFFFF:F,FFFFFFFFF:FFFFFF:FFFFFF,FFFFFF,F,FFF:
:FFFFF:FFFFFFFFFFF:FFFFF,F:FF:FFFFF,:F:FFF:FFF::FFFFFFFFFFF:FF
```

```
@A00155:342:HHGFNDSXY:1:1351:17852:26428 1:N:0:GAACCTAG+TCCGCATA
TGGTCATCTCAATGCCGAGAGGGATGTGGAGGGCGAGAGAGAGCGACTTCTCTCGGGCCCACAGCCTT
ACAGCTGTGGAGCACGGTATCCTCTGCCGAAACAGAGGTTGGACAAGACCGGAGGGGTCTCCTAGTTC
+
FFFFFFFFFFFFFFFFFFFFFFFFFFFFFFFFFFFFFFFFFFFFFFFFFFFFFFFFFFFFFFFFFFFF
:FFFFFFFFFFFFFFFFFFFFFFFFFFFFFFFFFFFFFFFFFFFFFFFFFFFFFFFFFFFFFF:FFFFF
@A00155:342:HHGFNDSXY:1:1351:22761:28635 1:N:0:GAACCTAG+TCCGCATA
ACTTCTCTCGGGCCCACAGCCTTACAGCTGTGGAGCACGGTATCCTCTGCCGAAACAGAGGTTGGACA
AGACCGGAGGGGTCTCCTAGTTCCAAAGGAGATGTACTCCGGGCTTGTTCACGACCTACCGTGTAAGT
+
FFFFFFFFFFFFFFFFFFFFFFFFFFFFFFFFFFFFFFFF:FFFFFFFFFFFFFFFFFFFFFFFFFFF
F:FFFFFFFFFFFFFFFFFFFFFFFFFFFFFFFFFFFFFF::FFFFFFFF:FFFFFFFFFFFFFFFFF
@A00155:342:HHGFNDSXY:1:1351:26096:33035 1:N:0:GAACCTAG+TCCGCATA
GAGCGACTTCTCTCGGGCCCACAGCCTTACAGCTGTGGAGCACGGTATCCTCTGCCGAAACAGAGGTT
GGACAAGACCGGAGGGGTCTCCTAGTTCCAAAGGAGATGTACTCCGGGCTTGTTCACGACCTACCGTG
+
:FFFFFFFFFFFFFFFFFFFFFFFFFFFFFFF,FFFFFFFFFFFFFFFFFFFF:FFFFF:FFFF:FF
FF:FFF:FF:F:F:F,FFFFFFFFFFFFFFFFF:FFFFFFFFFFFFF:FF,FFFFFFFFFFFFFFFFF
@A00155:342:HHGFNDSXY:1:1352:24569:4586 1:N:0:GAACCTAG+TCCGCATA
GGGCGAGAGAGAGCGACTTCTCTCGGGCCCACAGCCTTACAGCTGTGGAGCACGGTATCCTCTGCCGA
AACAGAGGTTGGACAAGACCGGAGGGGTCTCCTAGTTCCAAAGGAGATGTACTCCGGGCTTGTTCACG
+
FFFFFF:FFFFFF:FFFFFFFFFFFFFFFFFFFFFFFFFFFFFFFF:FFFFFFFFFFFFFFF:FFFFF:
FFFFFFFFFFF:FFFFFFFFFFFFFFFFFF:FFFFFFFFFFFFFFFFFFFFFFFFFFFFFFFFF:FFFF
@A00155:342:HHGFNDSXY:1:1352:24505:4726 1:N:0:GAACCTAG+TCCGCATA
GGGCGAGAGAGAGCGACTTCTCTCGGGCCCACAGCCTTACAGCTGTGGAGCACGGTATCCTCTGCCGA
AACAGAGGTTGGACAAGACCGGAGGGGTCTCCTAGTTCCAAAGGAGATGTACTCCGGGCTTGTTCACG
+
FFFFFFFFFFFFFFFFFFFFFFFFFFFFFFFFFFFFFFFFFFFFFFFFFFFFFFFFFFFFFFFFFFFF
FFFFFFFFFFFFFFFFFFFFFFFFFFFFFFFFFFFFFFFFFFFFFFFFFF:FFFFFFFFFFFFFFFFF
@A00155:342:HHGFNDSXY:1:1352:5068:9204 1:N:0:GAACCTAG+TCCGCATA
GAGGGCGAGAGAGAGCGACTTCTCTCGGGCCCACAGCCTTACAGCTGTGGAGCACGGTATCCTCTGCC
GAAACAGAGGTTGGACAAGACCGGAGGGGTCTCCTAGTTCCAAAGGAGATGTACTCCGGGCTTGTTCA
+
FFF:FFFFFFFFFFFFFFFFFF,FFFFF,FFFFFFFF,FFFFFFFFFFFFFFFFFFFFF:FFFFFFFF
FFFFFFFFFFFF:FFFFF:FFFFFFFFFFFFFFFFF:FFFFFFF:FF:FFFFFFFFF:FFFFFFFFFF
@A00155:342:HHGFNDSXY:1:1352:28619:14418 1:N:0:GAACCTAG+TCCGCATA
GAGCGACTTCTCTCGGGCCCACAGCCTTACAGCTGTGGTGCACGGTATCCTCTGCCGAAACAGAGGTT
GGACAAGACCGGAGGGGTCTCCTAGTTCCAAAGGAGATGTACTCCGGGCTTGTTCACGACCTACCGTG
+
FFFFFFFFFFFFFFFFFFFFFFFFFFFFFFFFFFFF:FFFFFFFF:FFFFFFFFFFFFFFFFFFFFFF
FFFFFFFFFFFFFFFFFFFFFFFFFFFFFFFFFF:FFFFFFFFFFFFFFFFFFFFFFFFFFFFFFFFF
@A00155:342:HHGFNDSXY:1:1352:8386:18897 1:N:0:GAACCTAG+TCCGCATA
GAGCGACTTCTCTCGGGCCCACAGCCTTACAGCTGTGGAGCACGGTATCCTCTGCCGAAACAGAGGTT
GGACAAGACCGGAGGGGTCTCCTAGTTCCAAAGGAGATGTACTCCGGGCTTGTTCACGACCTACCGTG
+
FFFFFFFFFFFFFFFFFFFFFFFFFFFFFF:FFFFFFFFFFFFFFFFFFFFFFFFFFFFFFFFFFFFF
FFFFFFFFFFFFFFFFFFFFFFFFFFFFFFFFFFFFFFFFFFFFFFFFFFFFFFFFFFFFFFFFFFFF
@A00155:342:HHGFNDSXY:1:1352:8232:18944 1:N:0:GAACCTAG+TCCGCATA
GAGCGACTTCTCTCGGGCCCACAGCCTTACAGCTGTGGAGCACGGTATCCTCTGCCGAAACAGAGGTT
GGACAAGACCGGAGGGGTCTCCTAGTTCCAAAGGAGATGTACTCCGGGCTTGTTCACGACCTACCGTG
+
FFFFFFFFFFFFFFFFFFFFFFFFFFFFFFFFFFFFFFFFFFFFFFFFFFFFFFFFFFFFFFFFFFFF
FFFFFFFFFFFFFFFFFFFFFFFFFFFFFFFFFFFFFFFFFFFFFFFFFFFFFFFFFFFFFFFFFFFF
```

```
@A00155:342:HHGFNDSXY:1:1352:19795:19022 1:N:0:GAACCTAG+TCCGCATA
CTTCTCTCGGGCCCACAGCCTTACAGCTGTGGAGCACGGTATCCTCTGCCGAAACAGAGGTTGGACAA
GACCGGAGGGGTCTCCTGGTTCCAAAGGAGATGTACTCCGGGCTTGTTCACGACCTACCGTGTAAGTC
+
FFFFFFFFFFFFFFFFFFF:FF:FFFFFFFFFFFFFFFFFFFFFFFFFFFFFFFFFFFFFFFFFFFFF
,FFFFFFFFFFFFFFFFFFFFFFFFFFFFFFFFFFFFFFFFF:FFFFFFFFFFFFFFFFFFFFFFFFFF
@A00155:342:HHGFNDSXY:1:1352:19895:27649 1:N:0:GAACCTAG+TCCGCATA
GATCAGCTGTGTCCACTTAAGGACTCACCTCTGGCCTATGGTCATCTCAATGCCGAGAGGGATGTGAA
GGGCGAGAGAGAGCGACTTCTCTCGGGCCCACAGCCTTACAGCTGTGGAGCACGGTATCCTCTGCCGA
+
FFFFFFFFFFFFFFFFFFFFFFFFFFFFFFFFFFFFFFFFFFFFFFFFFFFFFFFFFFFFFFFFFFFF
FFFFFFFFFFF:FFFFFFFFFFFFFFFFFFFFFFFFFFFFFFFFFFFFFFFFFFFFFFFFFFFFFFFF
@A00155:342:HHGFNDSXY:1:1352:26323:34272 1:N:0:GAACCTAG+TCCGCATA
CCATGCCCCGCCACACCGCCTTAGATCAGCTGTGTCCACTTAAGGACTCACCTCTGGCCTATGGTCAT
CTCAATGCCGAGAGGGATGTGGAGGGCGAGAGAGAGCGACTTCTCTCGGGCCCACAGCCTTACAGCTG
+
FFFFFFFFFFFFFFFFFFFFFFFFFFFFFFFFFFFFFFFFFFFFFFFFFFFFFFFFFFFF:FFFFFFF
FFFFFFFFFFFFFFFFFFFFFFFFFFFFFFFFFFFFFFFFFFFFFFFFFFFFFFFFFFFFFFFFFFFF
@A00155:342:HHGFNDSXY:1:1353:16396:12947 1:N:0:GAACCTAG+TCCGCATA
AGCGACTTCTCTCGGGCCCACAGCCTTACAGCTGTGGAGCACGGTATCCTCTGCCGAAACAGAGGTTG
GACAAGACCGGAGGGGTCTCCTAGTTCCAAAGGAGATGTACTCCGGGCTTGTTCACGACCTACCGTGT
+
FFFFFFFFFFFFFFFFFFFFFFFFFFFFFFFFFFFFFFFFFFFFFF:FFFFFFFFFFFFFFFFFFFFF
FFFFF:FFFFFFFFFFFFFFFFFFFFFFFFFFFFFFFFFFFFFFFF::FFFFFFF:FFFFFFFFFFFFF
@A00155:342:HHGFNDSXY:1:1353:31304:32095 1:N:0:GAACCTAG+TCCGCATA
CTTCTCTCGGGCCCACAGCCTTACAGCTGTGGAGCACGGTATCCTCTGCCGAAACAGAGGTTGGACAA
GACCGGAGGGGTCTCCTAGTTCCAAAGGAGATGTACTCCGGGCTTGTTCACGACCTACCG
+
FFFFFFFFFFFFFFFFFFFFFFFFFFFFFFFFFFFFFFFFFFFFFFFFFFFFFFFFFFFFFFFFFFFF
FFFFFFFFFFFFFFFFFFFFFFFFFFFFFF:FFFFFFFFFFFF:FFFFFFFFFFFFFF:F
@A00155:342:HHGFNDSXY:1:1354:7437:8077 1:N:0:GAACCTAG+TCCGCATA
CATCCCTCTCGGCATTGAGATGACCATAGGCCAGAGGTGAGTCCTTAAGTGGACACAGCTGATCTAAG
GCGGTGTGGCGGGGCATGGGTTTGAACCCCCATGACGGTCGGAGTCTAGTAGGCTCCCTGATGAGTCC
+
FFFFFFFFFFFFFFFFF:FFFFFFFFFFFF:FFF,FF:FFFFFFFFFFFFFFFFFFFFFFFFFFFFFF
FFFFFFFFFFFFFFF:FFFFFFFFFF:FFFFFFFFFFFFFFFFFFFF:FFFFFFFFFFFFFFFFFFFF
@A00155:342:HHGFNDSXY:1:1354:8947:11036 1:N:0:GAACCTAG+TCCGCATA
GGATGTGGAGGGCGAGAGAGAGCGACTTCTCTCGGGCCCACAGCCTTACAGCTGTGGAGCACGGTATC
CTCTGCCGAAACAGAGGTTGGACAAGACCGGAGGGGTCTCCTGGTTCCAAAGGAGATGTACTCC
+
FF:FF:FFFFFFFFFFFFFFFFFFFFFFFFFFFFFFFFFFFFFFFFFFFFFFFFFFFFFFFFFFFFFF
FFFFFFFFFFFFFFFFFFFFFFFFFFFFFFFFFFFFFFFFFFFFFFFFFFFFFFFFFFFFFFFF
@A00155:342:HHGFNDSXY:1:1355:14271:10457 1:N:0:GAACCTAG+TCCGCATA
TCTCGGGCCCACAGCCTTACAGCTGTGGAGCACGGTATCCTCTGCCGAAACAGAGGTTGGACAAGACC
GGAGGGGTCTCCTAGTTCCAAAGGAGATGTACTCCGGGCTTGTTCACGACCTACCGTGTAAGTCGTAG
+
FFFFFFFFFFFFFFFFFFFFFFFFFFFFFFFFFFFFFFFFFFFFFFFFFFFFFFFFFFFFFFFFFFFF
FFFFFFFFFFFFFFFFFFFF:FFFFFFFFFFFFFFFFFFFFFFFFFFFF:FFFFFFFFFFFFFFFFFFF
@A00155:342:HHGFNDSXY:1:1355:21359:16094 1:N:0:GAACCTAG+TCCGCATA
AAGACTCACCTCTGGCCTATGGTCATCTCAATGCCGAGAGGGATGTGGAGGGCGAGAGAGAGCGACTT
CTCTCGGGCCCACAGCCTTACAGCTGTGGAGCACGGTATCCTCTGCCGAAACAGAGGTTGGACAAGAC
+
FFFFFFFFFFFFFFFFFFFFFFFFFFFFFFFFFFFFFFFFFFFFF:FFFFFFFFFFFFFFFFFFFFFFF
FFFFFFFFFFFFFFFFFFFFFFFFFFFFFFFFFFFFFFFFFFFFFFFFFFFFFFFFFFFFFFFFFF:FF
```

@A00155:342:HHGFNDSXY:1:1355:2998:28698 1:N:0:GAACCTAG+TCCGCATA
GGATGTGGAGGGCGAGAGAGAGCGACTTCTCTCGGGCCCACAGCCTTACAGCTGTGGAGCACGGTATC
CTCTGCCGAAACAGAGGTTGGACAAGACCGGAGGGGTCTCCTAGTTCCAAAGGAGATGTACTCC
+
FFFFFFFFFFFFFFFFFFFFFFFFFFFFFFFFFFFFFFFFFF:FFFFFFFFFFF:FFFFFFFFFF,FF
FFFFFFFFFFFFFFFFFFFFFFFFFFFFFFFFFFFFFFFFFFFFFFFFFFFFFFFFFFFFFFFF
@A00155:342:HHGFNDSXY:1:1355:31774:29997 1:N:0:GAACCTAG+TCCGCATA
AGAGAGAGCGACTTCTCTCGGGCCCACAGCCTTACAGCTGTGGAGCACGGTATCCTCTGCCGAAACAG
AGGTTGGACAAGACCGGAGGGGTCTCCTGGTTCCAAAGGAGATGTACTCCGGGCTTGTTCACGACCTA
+
F:FFFFFFFFFFF:FFFFF:FFFF:FFFFFFF:FFF:FFFFF:FFFFF,:F:FFFFF:FFF::FFFFF
FF:FFFF:FFFFFFFFF::FFFFF:FFFFFFFFFFFFFFFFFFFFFFFFFF:FFF:F,FFFF:FFFFF
@A00155:342:HHGFNDSXY:1:1355:26765:31344 1:N:0:GAACCTAG+TCCGCATA
CCACCACACCGCCTTAGATCAGCTGTGTCCACTTAAGGACTCACCTCTGGCCTATGGTCATCTCAATG
CCGAGAGGGATGTGGAGGGCGAGAGAGAGCGACTTCTCTCGGGCCCACAGCCTTACAGCTGTGGAGCA
+
FFFFFFFFFFFFFFFFFF:FFFFFFFFFFFFFF:FFFFFFFFFFFFFFFFFFFFFFFFFFFFFFFFFF
FFFFFFFFFF:FFFFFFFFFFFFFFFFFFFFFFFFFFFFFFFFFFFFFFFFFFFFFFFFFFFFFFFFF
@A00155:342:HHGFNDSXY:1:1356:9507:4679 1:N:0:GAACCTAG+TCCGCATA
AAGGACTCACCTCTGGCCTATGGTCATCTCAATGCCGAGAGGGATGTGAAGGGCGAGAGAGAGCGACT
TCTCTCGGGCCCACAGCCTTACAGCTGTGGAGCACGGTATCCTCTGCCGAAACAGAGGTTGGAC
+
FFFFFFFFFFFFFFFFFFFFFFFFFFFFFFFFFFFFFFFFFFFFFFFFFFFFFFFFFFFFFFFFFFFF
FFFFFFFFFFFFFFFFFFFFFFFFFFFFFFFFFFFFFFFFFFFFFFFFFFFFFFFFFFFFFFFF
@A00155:342:HHGFNDSXY:1:1356:19533:11992 1:N:0:GAACCTAG+TCCGCATA
TTCTCTCGGGCCCACAGCCTTACAGCTGTGGAGCACGGTATCCTCTGCCGAAACAGAGGTTGGACAAG
ACCGGAGGGGTCTCCTAGTTCCAAAGGAGATGTACTCCGGGCTTGTTCACGACCTACCGTGTAAGTC
+
FFFFFFFFFFFFFFFFFFFFFFFF:FFFFFFFFFFFFFFFFFFF:FFFFFF:F:FFFFFFFF:FFFFF
FFF,FFFFFFFFFFFFF:FFFFFFFFFF:FFFFFFFFFFFFFFFFFFFFFFFFFFFFFFFFFFFFFF
@A00155:342:HHGFNDSXY:1:1356:24370:13197 1:N:0:GAACCTAG+TCCGCATA
GAGCGACTTCTCTCGGGCCCACAGCCTTACAGCTGTGGAGCACGGTATCCTCTGCCGAAACAGAGGTT
GGACAAGACCGGAGGGGTCTCCTAGTTCCAAAGGAGATGTACTCCGGGCTTGTTCACGACCTACCGTG
+
FFFFFFFFFFFFFFFFFFFFFFFFFFFFFFFFFFFFFFFFFFFFFFFFFFFFFFFFFFFFFFFFFFFF
FFF:FFFFFFFFFFFFFFFFFFFFFFFFFFFFFFFFFFFFFFFFFFF:FFFFFFFFFFFFFFFFFFFF
@A00155:342:HHGFNDSXY:1:1356:24695:18865 1:N:0:GAACCTAG+TCCGCATA
TGCCGAGAGGGATGTGGAGGGCGAGAGAGAGCGACTTCTCTCGGGCCCACAGCCTTACAGCTGTGGAG
CACGGTATCCTCTGCCGAAACAGAGGTTGGACAAGACCGGAGGGGTCTCCTAGTTCCAAAGGAGATGT
+
FFFFFFFFFFFFFFFFFFFFFFFFFFFFFFFFFFFFFFFFFFFFFFFFFFFFFFFFFFFFFFFFFFFF
FFFFFFFFFFFFFFFFFFFFFFFFFFFFFFFFFFFFFFFFFFFFFFFFFFFFFFFFFFFFFFFFFFFF
@A00155:342:HHGFNDSXY:1:1356:21296:23907 1:N:0:GAACCTAG+TCCGCATA
CTTCTCTCGGGCCCACAGCCTTACAGCTGTGGAGCACGGTATCCTCTGCCGAAACAGAGGTTGGACAA
GACCGGAGGGGTCTCCTAGTTCCAAAGGAGATGTACTCCGGGCTTGTTCACGACCTACCGTGTAAGTC
+
FFFFFFFFFFFFFFFFFFFFFFFFFFFFFFFFFFFFFFFFFFFFFFFFFFFFFFFFFFFFFFFFFFFF
,FFFFFFFFFFFFFFFFFFFFFFFFFFFFFFFFFFFFFFFFFFFFFFFFFFFFFFFFFFFFFFFFFFF
@A00155:342:HHGFNDSXY:1:1357:11216:7764 1:N:0:GAACCTAG+TCCGCATA
TGGTCATCTCAATGCCGAGAGGGATGTGGAGGGCGAGAGAGAGCGACTTCTCTCGGGCCCACAGCCTT
ACAGCTGTGGAGCACGGTATCCTCTGCCGAAACAGAGGTTGGACAAGACCGGAGGGGTCTCCTAGTTC
+
FFFFFFFFFFFFFFFFFFFFFFFFFFFFFFFFFFFFFFFFFFFFFFFFFFFFFFFFFFFFFFFFFFFF
FFFFFFFFFFFFFFFFFFFFFFFFFFFFFFFFFFFFFFFFFFFFFFFFFFFFFFFFFFFFFFFFFFFF

```
@A00155:342:HHGFNDSXY:1:1357:29460:14904 1:N:0:GAACCTAG+TCCGCATA
CCTCTGGCCTATGGTCATCTCAATGCCGAGAGGGATGTGAAGGGCGAGAGAGAGCGACTTCTCTCGGG
CCCACAGCCTTACAGCTGTGGAGCACGGTATCCTCTGCCGAAACAGAGGTTGGACAAGACCGGA
+
FFFFFFFFFFFFFFFFFFFFFFFFFFFFFFFFFFFFFFFFFFFFFFFFFFFFFFFFFFFFFFFFFFFF
FFFFFFFFFFFFFFFFFFFFFFFFFFFFFFFFFFFFFFFFFFFFFFFFFFFFFFFFFFFFFFFF
@A00155:342:HHGFNDSXY:1:1357:14633:29841 1:N:0:GAACCTAG+TCCGCATA
CTCAATGCCGAGAGGGATGTGGAGGGCGAGAGAGAGCGACTTCTCTCGGGCCCACAGCCTTACAGCTG
TGGAGCACGGTATCCTCTGCCGAAACAGAGGTTGGACAAGACCGGAGGGGTCTCCTAGTTCCAAAGGA
+
FFFFFFFFFFFFFFFFFFFFFFFFFFFFFFFFFFFFFFFFFFFFFFFFFFFFFFFFFFFFFFFFFFFF
FFFFFFFFFFFFFFFFFFFFFFFFFFFFFFFFFFFF:FFFFFFFFFFFFFFFFFFFFFFFFFFFFFFF
@A00155:342:HHGFNDSXY:1:1357:21088:32002 1:N:0:GAACCTAG+TCCGCATA
GATGTAGAGGGCGAGAGAGAGCGACTTCTCTCGGGCCCACAGCCTTACAGCTGTGGAGCACGGTATCC
TCTGCCGAAACAGAGGTTGGACAAGACCGGAGGGGTCTCCTAGTTCCAAAGGAGATGTACTCCGGGCT
+
FF,F:FFFFFFFFFFFFFFFFFFFFFFFFFFFFFFFFFFFFFFFFFFFFFF:FFFFFFFFFFFFF
FFFFFFFFFFFFFFFFFFFFFFFF:FFFFFFFFFFFFFFFFFFFFFFFFFFFFFFFFFF:FFFFFFFF
@A00155:342:HHGFNDSXY:1:1357:30644:35650 1:N:0:GAACCTAG+TCCGCATA
TGCCGAGAGGGATGTGGAGGGCGAGAGAGAGCGACTTCTCTCGGGCCCACAGCCTTACAGCTGTGGAG
CACAGTATCCTCTGCCGAAACAGAGGTTGGACAAGACCGGAGGGGTCTCCTAGTTCCAAAGGAGATGT
+
FFFFFFF:FFFFFF:FFFFFF:FFFFFFFFFFFFFFFFFFFFFFFFFFFFFFFFFFFFFFFFFFFFFF
F:FFFFFFFFFFFFFFFFFFFFFFFFFFFFFFFFFFFFFF,FFFFFFFFFFFFFFFFFFFFFFFFFFF
@A00155:342:HHGFNDSXY:1:1358:14190:9502 1:N:0:GAACCTAG+TCCGCATA
AGGACTCACCTCTGGCCTATGGTCATCTCAATGCCGAGAGGGATGTAGAGGGCGAGAGAGAGCGACTT
CTCTCGGGCCCACAGCCTTACAGCTGTGGAGCACGGTATCCTCTGCCGAAACAGAGGTTGGACAAGAC
+
FFFFFFFFFFFFFFFFFFFFFFFFFFFFFFFFFFFFFFFFF:FFFFFFFFFFFFFFFFFFFFFFFFFF
FFFFFFFFFFFFFFFFFFFFFFFFFFFFFFFFF,FFFFFFFFFFFFFFF:FFFFFFFFFFFFFFFFFF
@A00155:342:HHGFNDSXY:1:1358:26449:12258 1:N:0:GAACCTAG+TCCGCATA
GGGCGAGAGAGAGCGACTTCTCTCGGGCCCACAGCCTTACAGCTGTGGAGCACGGTATCCTCTGCCGA
AACAGAGGTTGGACAAGACCGGAGGGGTCTCCTAGTTCCAAAGGAGATGTACTCCGGGCTTGTTCACG
+
FFFFFFFFFFFFFFFFFFFFFFFFFFFFF:FFFFFFFFFFFFFFFFFFFFFFFFFFFFFFFFFFFFFF
FFFFFFFFFFFFFFFFFFFFFFFFFFFFFFFFFFFFFFFFFFFFFFFFFFFFFFFFFFFFFFFFFFFF
@A00155:342:HHGFNDSXY:1:1358:20844:18646 1:N:0:GAACCTAG+TCCGCATA
ACTTCTCTCGGGCCCACAGCCTTACAGCTGTGGAGCACGGTATCCTCTGCCGAAACAGAGGTTGGACA
AGACCGGAGGGGTCTCCTGGTTCCAAAGGAGATGTACTCCGGGCTTGTTCACGACCTACCGTGTAAGT
+
FFFFFFFFFFFFFFFFFFFFFFFFFFFFFFFFFFFFFFFFFFFFFFFFFFFFFFFFFFFFFFFFFFFF
FFFFFFFFFFFFFFFFFFFFFFFFFF:FFFFF:FFFFFFFFFFFFFFFFFFFFFFFFFFFFFFFFFFF
@A00155:342:HHGFNDSXY:1:1358:15646:26678 1:N:0:GAACCTAG+TCCGCATA
TGTCCACTTAAGGACTCACCTCTGGCCTATGGTCATCTCAATGCCGAGAGGGATGTGGAGGGCGAGAG
AGAGCGACTTCTCTCGGGCCCACAGCCTTACAGCTGTGGAGCACGGTATCCTCTGCCGAAACAGAGGT
+
FFFFFFFFFFFFFFFFFFFFFFFFFFFFFFFFFFFFFFFFFFFFFFFFFFFFFFFFFFF:FFFFFFFF
FFFFFFFFFFFFFFFFFFFFFFFFFFFFFFFFFFFFFFFFFFFFFFFFFFFFFFFFFFFFFFFFFFFF
@A00155:342:HHGFNDSXY:1:1358:29758:32643 1:N:0:GAACCTAG+TCCGCATA
GAGAGGGATGTAGAGGGCGAGAGAGAGCGACTTCTCTCGGGCCCACAGCCTTACAGCTGTGGAGCACG
GTATCCTCTGCCGAAACAGAGGTTGGACAAGACCGGAGGGGTCTCCTAGTTCCAAAGGAGATGTACTC
+
FFFFFFFFFFFFFFFFFFFFFFFFFFFFFFFFFFFFFFFFFFFFFFFFFFFFFFFF,FFFFFFFFFFF
FFFFFFFFFFFFFFFFFFFFFFFFFFFFFFFFFFFFFFFFFFFFFFFFFFFFFFFFFFFFF:FFFFFF
```

@A00155:342:HHGFNDSXY:1:1359:8594:5321 1:N:0:GAACCTAG+TCCGCATA
TGTCCACTTAAGGACTCACCTCTGGCCTATGGTCATCTCAATGCCGAGAGGGATGTGGAGGGCGAGAG
AGAGCGACTTCTCTCGGGCCCACAGCCTTACAGCTGTGGAGCACGGTATCCTCTGCCGAAACAGAGGT
+
FFFFFFFFFFFFFFFFFFFFFFFFFFFF,FFFFFFFFFFFFFFFFFFFFFFFFFFFFFFFFFFFFFFF
FFFFFFFFFFFFFFFFFFFFFFFFFFFFFFFFFFFFFFFFFFFFFFFFFFFFFFFFFFFFFFFFFFFF
@A00155:342:HHGFNDSXY:1:1359:16595:15953 1:N:0:GAACCTAG+TCCGCATA
CTATGGTCATCTCAATGCCGAGAGGGATGTGGAGGGCGAGAGAGAGCGACTTCTCTCGGGCCCACAGC
CTTACAGCTGTGGAGCACGGTATCCTCTGCCGAAACAGAGGTTGGACAAGACCGGAGGGGTCTCCTAG
+
FFFFFFFFFFFFFFFFFFFFFFFFFFFFFFFFFFFFFFFFFFFFFFFFFFFFFFFFFFFFFFFFFFFF
FFFFFFFFFFFFFFFF:FFFFFFFFFFFFFFFFFFFFFFFFFFFFFFFFFFFFFFFFFFFFFFFFFFF
@A00155:342:HHGFNDSXY:1:1359:21603:34209 1:N:0:GAACCTAG+TCCGCATA
ATCTCAATGCCGAGAGGGATGTGGAGGGCGAGAGAGAGCGACTTCTCTCGGGCCCACAGCCTTACAGC
TGTGGAGCACGGTATCCTCTGCCGAAACAGAGGTTGGACAAGACCGGAGGGGTCTCCTGGTTCCAAAG
+
FFFFFFFFFFFFFFFFFFFFFFFFFFFFFFFFFFFFFFFFFFFFFFFFFFFFFFFFFFF:FF:FFFFF
FFFFFFFFFFFFFFFFFFFFFFFFFFFFFFFFFFFFFFFFFFFFFFFFFFFFFFFFFFFFFF:FFFF:
@A00155:342:HHGFNDSXY:1:1359:14036:36605 1:N:0:GAACCTAG+TCCGCATA
ACCTCTGTTTCGGCAGAGGATACCGTGCTCCACAGCTGTAAGGCTGTGGGCCCGAGAGAAGTCGCTCT
CTCTCGCCCTCCACATCCCTCTCGGCATTGAGATGACCATAGGCCAGAGGAGAGTCCTTAAGTGGACA
+
FFFFFFFFFFFFFFFFFFFFFFFFFFFFFFFFFFFFFFFFFFFFFFFFFFFFFFFFFFFFFFFFFFFF
FFFFFFFFFFFFFFFFFFFFFFFFFFFFFFFFFFFFFFFFFFFFFFFFFFFFFFFFFFFFFFFFFFF:
@A00155:342:HHGFNDSXY:1:1360:25220:8406 1:N:0:GAACCTAG+TCCGCATA
GGATGTGGAGGGCGAGAGAGAGCGACTTCTCTCGGGCCCACAGCCTTACAGCTGTGGAGCACGGTATC
CTCTGCCGAAACAGAGGTTGGACAAGACCGGAGGGGTCTCCTAGTTCCAAAGGAGATGTACTCC
+
FFFFFFFFFFFFFFFFFFFFFFFFFFFFFFFFFFFFFFFFFFFFFFFFFFFFFFFFFFFFFFFFFFFF
FFFFFFFF:FFFFFFFFFFFFFFFF:FFFFFFFFFFFFFFFFFFFFFFFFFFFFFFFFFFFFFF
@A00155:342:HHGFNDSXY:1:1360:18394:16752 1:N:0:GAACCTAG+TCCGCATA
TTACAGCTGTGGAGCACGGTATCCTCTGCCGAAACAGAGGTTGGACAAGACCGGAGGGGTCTCCTAGT
TCCAAAGGAGATGTACTCCGGGCTTGTTCACGACCTACCGTGTAAGTCGTAGTCTAGTAGGCTACCTG
+
FF:FFFFFFFFFFFFFFFFFFFFFFFFFFFFFFFFF,FFFFFFFFFFFFFFFFFFFFFFFFFFFFFFF
FFFFFFFFFFFFFFFFFFFFFFFFFFFFFFFFFFFFFFFFFFFFFFFFFFFFFFFFFFFFFFFFFFFF
@A00155:342:HHGFNDSXY:1:1360:13431:36245 1:N:0:GAACCTAG+TCCGCATA
CACATCCCTCTCGGCATTGAGATGACCATAGGCCAGAGGTGAGTCCTTAAGTGGACACAGCTGATCTA
AGGCGGTGTGGCGGGGCATGGGTTTGAACCCCCATGACGGTCGGAGTCTAGTAGGCTCCCTGATGAGT
+
FFFFFFFFFFFFFFFFFFFFFFFFFFFFFFFFFFFFFFFFFFFFFFFFFFFFFFFFFFFFFFFF:FFF
FFFFFF:FFFFFFFFFFFFFFFFFFFFFFFFFFFFFFFFFFFFFFFFFFFFFFFFFFFFFFFFFFFFF
@A00155:342:HHGFNDSXY:1:1361:22137:1720 1:N:0:GAACCTAG+TCCGCATA
AGGGATGTAGAGGGCGAGAGAGAGCGACTTCTCTCGGGCCCACAGCCTTACAGCTGTGGAGCACGGTA
TCCTCTGCCGAAACAGAGGTTGGACAAGACCGGAGGGGTCTCCTAGTTCCAAAGGAGATGTACTCCGG
+
FFFFFFF:FFFFFFFFFFFFFFFFFFFF:FFFFFFFFFFFFFFFFFFFFFFFFFFFFFFFFFFFFFFF
FFFFFFF:FFFFFFFFFFFFFFFFFFFFFFFFFFFFFFFFFFFFFFFFFFFFFFFFFFFFFFFFFFFF
@A00155:342:HHGFNDSXY:1:1361:17788:12383 1:N:0:GAACCTAG+TCCGCATA
GAGCGACTTCTCTCGGGCCCACAGCCTTACAGCTGTGGAGCACGGTATCCTCTGCCGAAACAGAGGTT
GGACAAGACCGGAGGGGTCTCCTAGTTCCAAAGGAGATGTACTCCGGGCTTGTTCACGACCTACCGTG
+
FFFFFFFFFFFFFFFFFFFFFFFFFFFFFFFFFFFFFFFFFFFFFFFFFFFFFFFFFFFFFFFFFFFF
FFFFFFFFFFFFFFFFFFFFFFFFFFFFFFFFFFFFFFFFFFFFFFFFFFFFFFFFFFFFFFFFFFFF

```
@A00155:342:HHGFNDSXY:1:1361:29152:13056 1:N:0:GAACCTAG+TCCGCATA
GAGAGGGATGTAGAGGGCGAGAGAGAGCGACTTCTCTCGGGCCCACAGCCTTACAGCTGTGGAGCACG
GTATCCTCTGCCGAAACAGAGGTTGGACAAGACCGGAGGGGTCTCCTAGTTCCAAAGGAGATGTACTC
+
FFFFFFFFFFFFFFFFFFFFFFFFFFFFFFFFFFFFFFFFFFFFFFFFFFFFFFFFFFFFFFFFFFFF
FFFFFFFFFFFFFFFFFFFFFFFFFFFFFFFFFFFFFFFFFFFFFFFFFFFFFFFFFFFFFFFFFFFF
@A00155:342:HHGFNDSXY:1:1361:29469:13228 1:N:0:GAACCTAG+TCCGCATA
GAGAGGGATGTAGAGGGCGAGAGAGAGCGACTTCTCTCGGGCCCACAGCCTTACAGCTGTGGAGCACG
GTATCCTCTGCCGAAACAGAGGTTGGACAAGACCGGAGGGGTCTCCTAGTTCCAAAGGAGATGTACTC
+
FFFFFFFF,FFFFFFFFFFFFFFFFFFFFFFFFFFFFFFFFFFFFFFFFFFFFFFFFFFFFFFFFFFF
FFFFFFFFFFFFFFFFFFFFFFFFFFFFFFFFFFFFFFFFFFFFFFFFFFFFFFFFFFFFFFFFFFFF
@A00155:342:HHGFNDSXY:1:1361:27959:19163 1:N:0:GAACCTAG+TCCGCATA
TGCCGAGAGGGATGTGGAGGGCGAGAGAGAGCGACTTCTCTCGGGCCCACAGCCTTACAGCTGTGGAG
CACGGTATCCTCTGCCGAAACAGAGGTTGGACAAGACCGGAGGGGTCCCCTAGTTCCAAAGGAGATGT
+
FFFFFFFFFFFFFFFFFFFFFFFFFFFFFFFF:FFFFFFFFFFF:FFFFFFFFFFFFFFFFFFFFFFF
FFFFFFFFFFFFFFFFFFFFFFFFFFFFFFFFFFFFFFFFFFFFFFFFFFFFFFFFFFFFFFFFFFFF
@A00155:342:HHGFNDSXY:1:1361:27742:19695 1:N:0:GAACCTAG+TCCGCATA
TGCCGAGAGGGATGTGGAGGGCGAGAGAGAGCGACTTCTCTCGGGCCCACAGCCTTACAGCTGTGGAG
CACGGTATCCTCTGCCGAAACAGAGGTTGGACAAGACCGGAGGGGTCTCCTAGTTCCAAAGGAGATGT
+
FFFFFFFFFFFFFFFFFFFFFFFFFFFFFFFFFFFFFFFFFFFFFFFFFFFFFFFFFFFFFFFFFFFF
FF:FFFFFFFFFFFFFFFFFFFFFFFFFFFFFFFFFFF:FFFFFFFFFFFFFFFFFFFFFFFFFFFFF
@A00155:342:HHGFNDSXY:1:1361:9426:22138 1:N:0:GAACCTAG+TCCGCATA
GCTGTGTCCACTTAAGGACTCACCTCTGGCCTATGGTCATCTCAATGCCGAGAGGGATGTGAAGGGCG
AGAGAGAGCGACTTCTCTCGGGCCCACAGCCTTACAGCTGTGGAGCACGGTATCCTCTGCCGAAACAG
+
FFFFFFFFFFFFFFFFFFFFFFFFFFFFFFFFFF,FFFFF:FFFFFFFFF:FFFFFFFFFFFF,FFFF
FFFFFFFFFFF:FF:FFFF::FFFFFFFFF,FFFFFFF:FFFFFFFFFF:FFFF,FF:FF:FFFFFF:
@A00155:342:HHGFNDSXY:1:1362:4101:2801 1:N:0:GAACCTAG+TCCGCATA
GTCCAACCTCTGTTTCGGCAGAGGATACCGTGCTCCACAGCTGTAAGGCTGTGGGCCCGAGAGAAGTC
GCTCTCTCTCGCCCTCCACATCCCTCTCGGCATTGAGATGACCATAGGCCAGAGGAGAGTCCTTAAGT
+
FFFFFFFFFFFFFFFFFFFFFFFFFFFFFFFFFFFFF:FFFFFFFFFFFFFFFFFFFFFFFFFFFFFF
FFFFFFFFFFFFFFFFFFFFFFFFFFFFFFFFFF:F:FFFFFFFFFFFFFFFFFFFFFFFFFFFFF:F
@A00155:342:HHGFNDSXY:1:1362:24487:23985 1:N:0:GAACCTAG+TCCGCATA
AAGGCGAGAGAGAGCGACTTCTCTCGGGCCCACAGCCTTACAGCTGTGGAGCACGGTATCCTCTGCCG
AAACAGAGGTTGGACAAGACCGGAGGGGTCTCCTAGTTCCAAAGGAGATGTACTCCGGGCTTGTTCAC
+
FFFFFFFFFFFFFFFFFFFFFFFFFFFFFFFFFFFFFFFFFFFFFFFFFFFFFFF:FFFFFFFFFFFF
FFFFFFFFFFFFFFFFFFFFFFFFFFFFFFFFFFFFFFFFFFFFFFFFFFF:FFFFFFFFFFFFFFFF
@A00155:342:HHGFNDSXY:1:1362:12364:24251 1:N:0:GAACCTAG+TCCGCATA
GAGAGGGATGTGGAGGGCGAGAGAGAGCGACTTCTCTCGGGCCCACAGCCTTACAGCTGTGGAGCACG
GTATCCTCTGCCGAAACAGAGGTTGGACAAGACCGGAGGGGTCTCCTAGTTCCAAAGGAGATGTACTC
+
FFFFFFFFFFFFFFFFFFFFFFFFFFFFFFFFFFFFFFFFFFFFFFFFFF:FFFFFFFFFFFFFFFFF
FFFFFFFFFFFFFFFFFFFFFFFFFFFFFFFFFFFFFFFFFFFFFFFFFFFFFFFFFFFFFFFFFFFF
@A00155:342:HHGFNDSXY:1:1362:1714:34303 1:N:0:GAACCTAG+TCCGCATA
AAAGACTCACCTCTGGCCTATGGTCATCTCAATGCCGAGAGGGATGTGGAGGGCGAGAGAGAGCGACT
TCTCTCGGGCCCACAGCCTTACAGCTGTGGAGCACGGTATCCTCTGCCGAAACAGAGGTTGGACAAGA
+
FF:FFFFFFFFFFFF:FFFFF,FF:FFFFFFFFF,FFFFFFFF::FFF,:F:FFFFFFFFFF:FFFFF
FFFFFFFFFFFFFFFFFFFFFFFFFFFFFFFFFF::FFFFFFFFFFFFF,FFFFFFFFFFFFF:FFFF
```

```
@A00155:342:HHGFNDSXY:1:1362:1488:36730 1:N:0:GAACCTAG+TCCGCATA
TCCACTTAAGGACTCACCTCTGGCCTATGGTCATCTCAATGCCGAGAGGGATGTGAAGGGCGAGAGAG
AGCGACTTCTCTCGGGCCCACAGCCTTACAGCTGTGGAGCACGGTATCCTCTGCCGAAACAGAGGTTG
+
FFFFFFFFFFFFFFFFFFFFFF,FFFFF:FFFFFFFFFFFFFFFFFFFFFFFFFFFFFFFFFFFFFFF
FFFFF:FFFF:FFFF:FF:FFFFFFFFFFFFFFFFFFFFFFFFFFFFFFFFFFFFFFFFFFFFFFFFF
@A00155:342:HHGFNDSXY:1:1363:20347:12054 1:N:0:GAACCTAG+TCCGCATA
GCCCCGCCACACCGCCTTAGATCAGCTGTGTCCACTTAAGGACTCACCTCTGGCCTATGGTCATCTCA
ATGCCGAGAGGGATGTGGAGGGCGAGAGAGAGCGACTTCTCTCGGGCCCACAGCCTTACAGCTGTGGA
+
FFFFFFFFFFFFFFFFFFFFFF:FFFFFFFFFFFFFF:FFFFFFFFFFFFFFFFFFFFFFFFFFFFFF
FFFFFFFFFF:FFFFFFFFFFFFFF:FFFFFFFFFFFFFFFFFFFFFFFFFFFFFFFFFFFFFFFFFF
@A00155:342:HHGFNDSXY:1:1363:26955:18176 1:N:0:GAACCTAG+TCCGCATA
AGAGAGCGACTTCTCTCGGGCCCACAGCCTTACAGCTGTGGAGCACGGTATCCTCTGCCGAAACAGAG
GTTGGACAAGACCGGAGGGGTCTCCTAGTTCCAAAGGAGATGTACTCCGGGCTTGTTCACGACCTACC
+
FFFFFFFFFFFFFFFFFFFFFFFFFFFFFFFFFFFFFFFFFFFFFFFFFFFFFFFFFFFFFFFFFFFF
FFFFFFFFFFFFFFFFFFFFFFFFFFFFFFFFFFFFFFFF:FFFFFFFFFFFFFFFFF:FFFFFFFFF
@A00155:342:HHGFNDSXY:1:1363:24560:24643 1:N:0:GAACCTAG+TCCGCATA
CTCCTCTGGCCTATGGTCATCTCAATGCCGAGAGGGATGTGGAGGGCGAGAGAGAGCGACTTCTCTCG
GGCCCACAGCCTTACAGCTGTGGAGCACGGTATCCTCTGCCGAAACAGAGGTTGGACAAGACCGGAGG
+
FFFFFFFFFFFFFFFFFFFFFFFFFFFFFFFFFFFFFFFFFFFFFFFFFFFFFFFFFFFFFFFFFFFF
FFFFFFFFFFFFFFFFFFFFFFFFFFFFFFFFFFFFFFFFFFFFFFFFFFFFFFFFFFFFFFFFFFFF
@A00155:342:HHGFNDSXY:1:1363:6144:29262 1:N:0:GAACCTAG+TCCGCATA
AGAGAGAGCGACTTCTCTCGGGCCCACAGCCTTACAGCTGTGGAGCACGGTATCCTCTGCCGAAACAG
AGGTTGGACAAGACCGGAGGGGTCTCCTAGTTCCAAAGGAGATGTACTCCGGGCTTGTTCACGACCTA
+
FFFFFFFFFFFFFFFFFFFFFFFFFFFF,FFFFFFFFFFFF:FFFFFFFFFFFFFFFFFFFFFFFFFF
FFFFFFFFFFFFFFFFFFFFFFFFFFFFFFFFFFFFFFFFFFFFFFFFFFFFF:FFFFFF:FFFFFFF
@A00155:342:HHGFNDSXY:1:1363:2880:33661 1:N:0:GAACCTAG+TCCGCATA
GAGAGGGATGTAGAGGGCGAGAGAGAGCGACTTCTCTCGGGCCCACAGCCTTACAGCTGTGGAGCACG
GTATCCTCTGCCGAAACAGAGGTTGGACAAGACCGGAGGGGTCTCCTAGTTCCAAAGGAGATGTACTC
+
FFFFFFFFFFFFFFFFFFFFFFFFFFFFFFFFFFFFFFF:FF:FFFFFFFFFFFFFFFFFFFFFFFFF
FFFFFFFFFFFFFFFFFFFFFFFFFFFFFFFFFFFFFFFFFFFF:FFFFFFFFFFFFFFFFFFFF:FF
@A00155:342:HHGFNDSXY:1:1364:25699:14622 1:N:0:GAACCTAG+TCCGCATA
GAGAGGGATGTGGAGGGCGAGAGAGAGCGACTTCTCTCGGGCCCACAGCCTTACAGCTGTGGAGCACG
GTATCCTCTGCCGAAACAGAGGTTGGACAAGACCGGAGGGGTCTCCTAGTTCCAAAGGAGATGTACTC
+
FFFFFFFFFFFFFFFFFFFFFFFFFFFFFFFFFFFFFFFFFFFFFFFFFFFFFFFFFFFFFFFFFFFF
FFFFFFFFFFFFFFFFFFFFFFFFFFFFFFFFFFFFFFFFFFFFFFFFFFFFFFFFFFFFFFFFFFFF
@A00155:342:HHGFNDSXY:1:1364:28782:18239 1:N:0:GAACCTAG+TCCGCATA
GTCATCTCAATGCCGAGAGGGATGTGGAGGGCGAGAGAGAGCGACTTCTCTCGGGCCCACAGCCTTAC
AGCTGTGGAGCACGGTATCCTCTGCCGAAACAGAGGTTGGACAAGACCGGAGGGGTCTCCTGGTTCCA
+
FFFFFFFFFFFFFFFFFFFFFFF:FFFFFFFFFFFFFFFFFFFFFFFFFFFFFFFFFFFFFFFFFFFF
FFFFFFFFFFFFFFFFFFFFFFFFFFFFFFFFFFFFFFFFFFFFFFFFFFFFFFFFFFFFFFFFF:FF
@A00155:342:HHGFNDSXY:1:1364:30355:19617 1:N:0:GAACCTAG+TCCGCATA
TCCTCTGCCGAAACAGAGGTTGGACAAGACCGGAGGGGTCTCCTAGTTCCAAAGGAGATGTACTCCGG
GCTTGTTCACGACCTACCGTGTAAGTCGTAGTCTAGTAGGCTACCTGACGAGTCCTTTTTAGGACGAA
+
FFFFFFFFFFFFFFFFFFFFFFFFFFFFFFFFFFFFFFFFFFFFFFFFFFFFFFFFFFFFFFFFFFFF
FFFFFFFFFFFFFFFFFFFFFFFFFFFFFFFFFFFFFFFFFFFFFFFFFFFFFFFFFF:FFFFFFFFF
```

@A00155:342:HHGFNDSXY:1:1364:21233:27336 1:N:0:GAACCTAG+TCCGCATA
CTTCTCTCGGGCCCACAGCCTTACAGCTGTGGAGCACGGTATCCTCTGCCGAAACAGAGGTTGGACAA
GACCGGAGGGGTCTCCTAGTTCCAAAGGAGATGTACTCCGGGCTTGTTCACGACCTACCGTGTAAGTC
+
FFFFFFFFFFFFFFFFFFFFFFFFFFFFFFFFFFFFFFFFFFFFFFFFFFFFFFFFFFFFFFFFFFFF
FFFFFFFFFFFFFFFFFFFFFFFFFFFFFFFFFFFFFFFFFFFFFFFFFFFFFFFFFFFFFFFFFFFF
@A00155:342:HHGFNDSXY:1:1364:25328:35399 1:N:0:GAACCTAG+TCCGCATA
GGGCGAGAGAGAGCGACTTCTCTCGGGCCCACAGCCTTACAGCTGTGGAGCACGGTATCCTCTGCCGA
AACAGAGGTTGGACAAGACCGGAGGGGTCTCCTGGTTCCAAAGGAGATGTACTCCGGGCTTGTTCACG
+
FFFFFFFFFFFFFFFFFFFFFFFFFFFFFFFFFFFFFFFFFFFFFFFFFFFFFFFFFFFFFFFFFFFF
FFFFFFFFFFFFFFFFFFFFFFFFFFFFFFFFF:FFFFFFFFFFFFFFFFFFFFFFFFFFFFFFFFFF
@A00155:342:HHGFNDSXY:1:1365:4255:7983 1:N:0:GAACCTAG+TCCGCATA
CGAGAGGGATGTAGAGGGCGAGAGAGAGCGACTTCTCTCGGGCCCACAGCCTTACAGCTGTGGAGCAC
GGTATCCTCTGCCGAAACAGAGGTTGGACAAGACCGGAGGGGTCTCCTAGTTCCAAAGGAGATGTACT
+
FFFFFFFFFFF:FFFFFFFFFFFFFFFFFFFFFFFFFFFFFFFFFFFF:FF,F:FFFFFFFFFFFFFF
FFF:FFFFFFFFF:FFFFFFFFFFFFFFFFF:FFFFFFFFFFFFFFFFFFFFFFFFFFFFFFFFFFFF
@A00155:342:HHGFNDSXY:1:1365:32723:15201 1:N:0:GAACCTAG+TCCGCATA
AGGGCGAGAGAGAGCGACTTCTCTCGGGCCCACAGCCTTACAGCTGTGGAGCACGGTATCCTCTGCCG
AAACAGAGGTTGGACAAGACCGGAGGGGTCTCCTAGTTCCAAAGGAGATGTACTCCGGGCTTGTTCAC
+
FFFFFFFFFFFFFFFFFFFFFFFFFFFFFFFFFFFFFFFFFFFFFFFFFFFFFFFFFFFFFFFFFFFF
FFFFFFFFFFFFFFFFFFFFFFFFFFFFFFFFFFFFFFFFFFFFFFFFFFFFFFFFFFFFFFFFFFFF
@A00155:342:HHGFNDSXY:1:1365:24252:18474 1:N:0:GAACCTAG+TCCGCATA
GAGAGGGATGTGGAGGGCGAGAGAGAGCGACTTCTCTCGGGCCCACAGCCTTACAGCTGTGGAGCACG
GTATCCTCTGCCGAAACAGAGGTTGGACAAGACCGGAGGGGTCTCCTGGTTCCAAAGGAGATGTACTC
+
FFFFFFFFFFFFFFF:FFFFFFFF:FFFFFFFFFFFFFFFFFFFFFFFFFFFFFFFFFFFFFFFFFFF
FFFFFFFFFFFFFFFFFFFFFFFFFFFFFFFFFFFFFFFFFFFFFFFFFFFFFFFFFFFFFFFFFFFF
@A00155:342:HHGFNDSXY:1:1365:18566:23876 1:N:0:GAACCTAG+TCCGCATA
CTTCTCTCGGGCCCACAGCCTTACAGCTGTGGAGCACGGTATCCTCTGCCGAAACAGAGGTTGGACAA
GACCGGAGGGGTCTCCTAGTTCCAAAGGAGATGTACTCCGGGCTTGTTCACGACCTACCGTGTAAGTC
+
FFFFFFFFFFFFFFFFFFFFFFFFFFFFFFFFFFFFFFFFFFFFFFFFFFFFFFFFFFFFFFFFFFFF
FFFFFFFFFFFFFFFFFFFFFFFFFFFFFFFFFFFFFFFFFFFFFFFFFFFFFFFFFFFFFFFFFFFF
@A00155:342:HHGFNDSXY:1:1366:31602:8844 1:N:0:GAACCTAG+TCCGCATA
GGGCGAGAGAGAGCGACTTCTCTCGGGCCCACAGCCTTACAGCTGTGGAGCACGGTATCCTCTGCCGA
AACAGAGGTTGGACAAGACCGGAGGGGTCTCCTAGTTCCAAAGGAGATGTACTCCGGGCTTGTTCACG
+
FFFFFFFFFFFFFFFFFFFFFFFFFFFFFFFFFFFFF::FFFFFFFFFFFFFFFFF:FFFFFFFFFFF
FFFFFFFFFFFFFFF:FFFFFFFFFFFFFFFFFFFFFFFFFFFFFFFFFF:FFFFFFFFFFFFFFFFF
@A00155:342:HHGFNDSXY:1:1366:6488:8907 1:N:0:GAACCTAG+TCCGCATA
CTCAATGCCGAGAGGGATGTGGAGGGCGAGAGAGAGCGACTTCTCTCGGGCCCACAGCCTTACAGCTG
TGGAGCACGGTATCCTCTGCCGAAACAGAGGTTGGACAAGACCGGAGGGGTCTCCTAGTTCCAAAGGA
+
FFFFFFFFFFFFFFFFFFFFFFFFFFFFFFFFFFFFFFFFFFFFFFFFFFFFFFFFFFFFFFFFFFFF
FFFFFFFFFFFFFFFFFFFFFFFFFFFFFFF:FFFFFFFFFFFFFFFFFFFFFFFFFFFFFFFFFFFF
@A00155:342:HHGFNDSXY:1:1366:16848:9095 1:N:0:GAACCTAG+TCCGCATA
TGTGGAGGGCGAGAGAGAGCGACTTCTCTCGGGCCCACAGCCTTACAGCTGTGGAGCACGGTATCCTC
TGCCGAAACAGAGGTTGGACAAGACCGGAGGGGTCTCCTGGTTCCAAAGGAGATGTACTCCGGG
+
:FFFFFFFFFFFFFFFFFFFFFFFFFFFFFFFFFFFFFFFFFFFFFFFFFFFF:FFFFFFFFFFFFFF
FFFFFFFFFFFFFFFFFFFFFFFFFFFFFFFFFFFFFFFFFFFFFFFFFFFFFFFFFFFFFFFFFFFF

```
@A00155:342:HHGFNDSXY:1:1366:12382:12947 1:N:0:GAACCTAG+TCCGCATA
GGATGTGGAGGGCGAGAGAGAGCGACTTCTCTCGGGCCCACAGCCTTACAGCTGTGGAGCACGGTATC
CTCTGCCGAAACAGAGGTTGGACAAGACCGGAGGGGTCTCCTAGTTCCAAAGGAGATGTACTCC
+
FFFFFFFFFFFFFFFFFFFFFFFFFFFFFFFFFFFFFFFFFFFFFFFFFFFFFFFFFFFFFFFFFFFF
FFFFFFFFFFFFFFFFFFFFFFFFFFFFFFFFFFFFFFFFFFFFFFFFFFFFFFFFFFFFFFFF
@A00155:342:HHGFNDSXY:1:1366:10827:13416 1:N:0:GAACCTAG+TCCGCATA
TCTCAATGCCGAGAGGGATGTGGAGGGCGAGAGAGAGCGACTTCTCTCGGGCCCACAGCCTTACAGCT
GTGGAGCACGGTATCCTCTGCCGAAACAGAGGTTGGACAAGACCGGAGGGGTCTCCTGGTTCCAAAGG
+
FFFFFFFFFFFFFFFFFFFF,FFFFFFFFFFFFFFFFFFFF:FFFFFFFFFFFFFFFFFF:F:FFFFF
FFFFFFFFFFFFFFFFFFFFFFFFFFFFFFFFFFFFFFFFFFFFFFFFFFFFFFFFFFFFFFFFFFFF
@A00155:342:HHGFNDSXY:1:1366:10655:20979 1:N:0:GAACCTAG+TCCGCATA
ACAAGCCCGGAGTACATCTCCTTTGGAACTAGGAGACCCCTCCGGTCTTGTCCAACCTCTGTTTCGGC
AGAGGATACCGTGCTCCACAGCTGTAAGGCTGTGGGCCCGAGAGAAGTCGCTCTCTCTCGCCCTCTAC
+
FFFFFFFFFFFFFFFFFFFFFFFFFFFFFFFFFFFFFFFFFFFFFFFFFFFFFFFFFFFFFFFFFFFF
FFFFFFFFFFFFFFFFFFFFFFFFFFFFFFFFFFFFFFFFFFFFF,FFFFFFFFFFFFFFFFFFFFFF
@A00155:342:HHGFNDSXY:1:1366:7419:28651 1:N:0:GAACCTAG+TCCGCATA
GTCATCTCAATGCCGAGAGGGATGTGGAGGGCGAGAGAGAGCGACTTCTCTCGGGCCCACAGCCTTAC
AGCTGTGGAGCACGGTATCCTCTGCCGAAACAGAGGTTGGACAAGACCGGAGGGGTCTCCTAGTTCCA
+
FFFFFFFFFFFFFFFFFFFFFFFFFFFFFFFFFFFFFFFFFFFFFFFFFFFFFFFFFFFFFFFFFFFF
FFFFFFFFFFFFFFFFFFFFFFFFFFFFFFFFFFFFFFFFFFFF,FFFFFFFFFFFFFFFFFFFFFFF
@A00155:342:HHGFNDSXY:1:1367:32714:3724 1:N:0:GAACCTAG+TCCGCATA
AGCGACTTCTCTCGGGCCCACAGCCTTACAGCTGTGGAGCACGGTATCCTCTGCCGAAACAGAGGTTG
GACAAGACCGGAGGGGTCTCCTGGTTCCAAAGGAGATGTACTCCGGGCTTGTTCACGACCTACCGTGT
+
FFFFFFFFFFFFFFFFFFFFFFFFFFFFFFFFFFFFFFFFFFFFF:FFFFFFFFFFF:FFFFFFFFFF
FFFFFFFFFFFFFFFFFFFFFFFFFFFFFFFFFFFFFFFFFFFFFFFFFFF:FFFFFFFFF:FFFFFF
@A00155:342:HHGFNDSXY:1:1367:10881:6934 1:N:0:GAACCTAG+TCCGCATA
GCGACTTCTCTCGGGCCCACAGCCTTACAGCTGTGGAGCACGGTATCCTCTGCCGAAACAGAGGTTGG
ACAAGACCGGAGGGGTCTCCTAGTTCCAAAGGAGATGTACTCCGGGCTTGTTCACGACCTACCGTGTA
+
FFFFFFFFFFFFFFFFFFFFFFFFFFFFFFFFFFFFFFFFFFFFFFFFFFFFFFFFFFFFFFFFFFFF
FFFF:FFFFFFFFFFFFFFFFFFFFFFFFFFFFFFFFFFFFFFFFFFFFFFFFFFFFFFFFFFFFFFF
@A00155:342:HHGFNDSXY:1:1367:23375:18991 1:N:0:GAACCTAG+TCCGCATA
TCGGCAGAGGATACCGTGCTCCACAGCTGTAAGGCTGTGGGCCCGAGAGAAGTCGCTCTCTCTCGCCC
TCCACATCCCTCTCGGCATTGAGATGACCATAGGCCAGAGGTGAGTCCTTAAGTGGACACA
+
FFFFFF:FFFFFFFFFFFFFFFFFFFFFFFFFFFFFFFF:FFFFFFFFFFFF:FFFFFFFFFFFFFFF
FFFFFFFFFFFFFFFFFFFFFFFFFFFFFFFFFFFFFFFFFFFFFFFFFFFFFFF:FFFFF
@A00155:342:HHGFNDSXY:1:1367:20907:19977 1:N:0:GAACCTAG+TCCGCATA
ATCTCAATGCCGAGAGGGATGTGGAAGGCGAGAGAGAGCGACTTCTCTCGGGCCCACAGCCTTACAGC
TGTGGAGCACGGTATCCTCTGCCGAAACAGAGGTTGGACAAGACCGGAGGGGTCTCCTAGTTCCAAAG
+
FFFFFFFFFFFFFFFFFFFFFFFFFFFFFFFFFFFFFFFFFFFFFFFFFFFFFFFFFFFFFFFFFFFF
FFFFFFFFFFFFFFFFFFFFFFFFFFF:F:FFFFFFFFFFFFFFFFFFFFFFFFFFFFFFFFFFFFFF
@A00155:342:HHGFNDSXY:1:1367:26024:22075 1:N:0:GAACCTAG+TCCGCATA
TCGGCAGAGGATACCGTGCTCCACAGCTGTAAGGCTGTGGGCCCGAGAGAAGTCGCTCTCTCTCGCCC
TCCACATCCCTCTCGGCATTGAGATGACCATAGGCCAGAGGTGAGTCCTTAAGTGGACACA
+
FFFFFFFFFFFFFFFFFFFFFFFFFFFFFFFFFFFFFFFFFFFFFFFFFF:FFFFFFFFFFFFFFFFF
FFFFFFFFFFFFFFFFFF:FFFFFFFFFFFFFFFFFFF:FFFFFFFFFFFFFFFFFFFFFF
```

```
@A00155:342:HHGFNDSXY:1:1367:27498:35493 1:N:0:GAACCTAG+TCCGCATA
AGGGCGAGAGAGAGCGACTTCTCTCGGGCCCACAGCCTTACAGCTGTGGAGCACGGTATCCTCTGCCG
AAACAGAGGTTGGACAAGACCGGAGGGGTCTCCTAGTTCCAAAGGAGATGTACTCCGGGCTTGTTCAC
+
FFFFFFFFFFFFFFFFFFFFFFFFFFFFFFFFFFFFFFFFFFFFFFFFFFFFFFFFFFFFFFFFFFFF
FFFFFFFFFFFFFFFFFFFF:FFFFFFFFFFFFFFF:FFFFFFFFFFFFFF:FFFFFFFFFFFFFFFF
@A00155:342:HHGFNDSXY:1:1368:3794:4335 1:N:0:GAACCTAG+TCCGCATA
CTTCTCTCGGGCCCACAGCCTTACAGCTGTGGAGCACGGTATCCTCTGCCGAAACAGAGGTTGGACAA
GACCGGAGGGGTCTCCTAGTTCCAAAGGAGATGTACTCCGGGCTTGTTCACGACCTACCGTGTAAGTC
+
FFFFFFFFFFFFFFFFFFFFFFFFFFFFFFFFFFFFFFFFFFFFFFFFFFFFFFFFFFFFFFFFFFFF
FFFFFFFFFFFFFFFFFFFFFFFFFFFFFFFFFFFFFFFFFFFFFFFFFFFFFFFFFFFFFFFFFFFF
@A00155:342:HHGFNDSXY:1:1368:2239:7028 1:N:0:GAACCTAG+TCCGCATA
AGAGAGAGCGACTTCTCTCGGGCCCACAGCCTTACAGCTGTGGAGCACGGTATCCTCTGCCGAAACAG
AGGTTGGACAAGACCGGAGGGGTCTCCTAGTTCCAAAGGAGATGTACTCCGGGCTTGTTCACGACCTA
+
FF:FFFFFFFFFFFFFFFFFFFFFFFFFFFFFFFFFFFFFFFFFFFF:FFFFFFFFFFFFFFFFFFFF
FFFFFFFFFFFFFFFFFFFFFFFFFFFFFFFFFFFFFFFFFFFFFFFFFF:F:FFFFFFFFFFFFFFF
@A00155:342:HHGFNDSXY:1:1368:11822:7905 1:N:0:GAACCTAG+TCCGCATA
CTATGGTCATCTCAATGCCGAGAGGGATGTGGAGGGCGAGAGAGAGCGACTTCTCTCGGGCCCACAGC
CTTACAGCTGTGGAGCACGGTATCCTCTGCCGAAACAGAGGTTGGACAAGACCGGAGGGGTCTCCTAG
+
FFFFFFFFFFFFFFFFFFFFFFFFFFFFFFFFFFFFFFFFFFFFFFFFFFFFFFFFFFFFFFFFFFFF
FFFFFFFFFFFFFFFFFFFFFFFFFFFFFFFFFFFFFFFFFFFFFFFFFFFFFFFFFFFFFFFFFFFF
@A00155:342:HHGFNDSXY:1:1368:11785:7936 1:N:0:GAACCTAG+TCCGCATA
CTATGGTCATCTCAATGCCGAGAGGGATGTGGAGGGCGAGAGAGAGCGACTTCTCTCGGGCCCACAGC
CTTACAGCTGTGGAGCACGGTATCCTCTGCCGAAACAGAGGTTGGACAAGACCGGAGGGGTCTCCTAG
+
FFFFFFFFFFFFFFFFFFFFFFFFFFFFFFFFFFFFFFFFFFFFFFFFFFF:FFFFFFFFFFFFFFFF
FFFFFFFFFFFFFFFFFFFFFFFFFFFFFFFFFFFFFFFFFFFFFFFFFFFFFFFFFFFFFFFFFFFF
@A00155:342:HHGFNDSXY:1:1368:7771:20118 1:N:0:GAACCTAG+TCCGCATA
AGAGAGAGCGACTTCTCTCGGGCCCACAGCCTTACAGCTGTGGAGCACGGTATCCTCTGCCGAAACAG
AGGTTGGACAAGACCGGAGGGGTCTCCTAGTTCCAAAGGAGATGTACTCCGGGCTTGTTCACGACCTA
+
FFFFFFFFFFFFFFFFFFFFFFFFFFFFFFFFFFFFFFFF:F,FFFFFFFFFFFFFFFFFFFFFFFFF
FFFFFFFFFFFF:FFFFFFFFFFFFFFFFFFFFFFFFFFFFFFFFFFFFFF:FFFFFFFFFFFFFFFF
@A00155:342:HHGFNDSXY:1:1368:10456:22326 1:N:0:GAACCTAG+TCCGCATA
AGAGAGAGCGACTTCTCTCGGGCCCACAGCCTTACAGCTGTGGAGCACGGTATCCTCTGCCGAAACAG
AGGTTGGACAAGACCGGAGGGGTCTCCTAGTTCCAAAGGAGATGTACTCCGGGCTTGTTCACGACCTA
+
FFFFFFFFFFFFFFFFFFFFFFFFFFFFFFFF:FFFFFFFFFFFFFFFFFFFFFFFFFFFFFFFFFFF
FFFFFFFFFFFFFFFFFFFFFFFFFFFFFFFFFFFFFFFFFFFFFFFFFFFFFFFFFFFFFFFFFFF:
@A00155:342:HHGFNDSXY:1:1368:29658:33348 1:N:0:GAACCTAG+TCCGCATA
GGATGTGGAGGGCGAGAGAGAGCGACTTCTCTCGGGCCCACAGCCTTACAGCTGTGGAGCACGGTATC
CTCTGCCGAAACAGAGGTTGGACAAGACCGGAGGGGTCTCCTGGTTCCAAAGGAGATGTACTCC
+
FFFFFFFFFFFFFFFFFFFFFFFF,FF,FFFFFFFFFF,FFFFFFFFFFFFFFFFFFFFFFFFFFFFF
FFFFFFFFFFFFFFFFFFFFFFFFFF:FFFFFFFFFFFFFFFFFFFFFFFFFFFFFFFFFFFFFFF
@A00155:342:HHGFNDSXY:1:1369:31448:1595 1:N:0:GAACCTAG+TCCGCATA
GGATGTAGAGGGCGAGAGAGAGCGACTTCTCTCGGGCCCACAGCCTTACAGCTGTGGAGCACGGTATC
CTCTGCCGAAACAGAGGTTGGACAAGACCGGAGGGGTCTCCTAGTTCCAAAGGAGATGTA
+
FF:FF::FFFFFFFFFFFFFFFFFFFFFFFFFFFFFFFFFFFFFFFFFFFFFFFFFFFFFFFFFFFFF
FFFFFFFFFFFFFFFFFFFFFFFF::FFFFFFFFFFFFFFFF:FFFFFFFFFFFFFFFFF
```

```
@A00155:342:HHGFNDSXY:1:1369:4128:8390 1:N:0:GAACCTAG+TCCGCATA
GAGCGACTTCTCTCGGGCCCACAGCCTTACAGCTGTGGAGCACGGTATCCTCTGCCGAAACAGAGGTT
GGACAAGACCGGAGGGGTCTCCTAGTTCCAAAGGAGATGTACTCCGGGCTTGTTCACGACCTACCGTG
+
FFFFFFFFFFFFFFFFFFFFFFFFFFFFFFFFFFFFFFFFFFFFFF,FFFFFFFFFFFFFFFFFFFFF
FFFFFFFFFFFFFFFFFFFFFFFFFFFFFFFFFFFFFFFFFFFFFFFFFFFFFFFFF:FFF:FFFFFF
@A00155:342:HHGFNDSXY:1:1369:25536:8578 1:N:0:GAACCTAG+TCCGCATA
AGCGACTTCTCTCGGGCCCACAGCCTTACAGCTGTGGAGCACGGTATCCTCTGCCGAAACAGAGGTTG
GACAAGACCGGAGGGGTCTCCTAGTTCCAAAGGAGATGTACTCCGGGCTTGTTCACGACCTACCGTGT
+
FFFFFFFFFFFFFFFFFFFFFFFFFFFFFFFFFFFFFFF:FFFFF:FFFFFFFFFFFFFFFFFFFFFF
FFFFFFFFFFFFFFFFFFFFFFFFFFF:FFFFFFFF:FFFFFFFFFFF:FFFFFFFFFFFFFFFFFFF
@A00155:342:HHGFNDSXY:1:1369:14199:17127 1:N:0:GAACCTAG+TCCGCATA
GAGCGACTTCTCTCGGGCCCACAGCCTTACAGCTGTGGAGCACGGTATCCTCTGCCGAAACAGAGGTT
GGACAAGACCGGAGGGGTCTCCTAGTTCCAAAGGAGATGTACTCCGGGCTTGTTCACGACCTACCGTG
+
FFFF,FFFFFFFFFFFFFFFFFFFFFFFFFFFF:FFFFFFFFFFFFFF:FFFFF,FFFFF:FFFFF:F
FFFFFFFFF,F:FF:,FF:FFFFFFF:FFFFF:::F:,F,FFFF:FF,FFFFFFFFF,FF,FFFFF,F
@A00155:342:HHGFNDSXY:1:1369:24198:19601 1:N:0:GAACCTAG+TCCGCATA
TGTCCACTTAAGGACTCACCTCTGGCCTATGGTCATCTCAATGCCGAGAGGGATGTGGAGGGCGAGAG
AGAGCGACTTCTCTCGGGCCCACAGCCTTACAGCTGTGGAGCACGGTATCCTCTGCCGAAACAGAGGT
+
FFFFFFFFFFFFFFFFFFFFFFFFFFFFFFFFFFFFFFFFFFFFFFFFFFFFF:FFFFFFFFFFFFFF
FFFFFFFFFFFFFFFFFFFFFFFFFFF:FFFFFFFFFFFFFFFFFFFFFFFFFFFFFFFFFFFFFFFF
@A00155:342:HHGFNDSXY:1:1369:26992:22467 1:N:0:GAACCTAG+TCCGCATA
CTCTCGGGCCCACAGCCTTACAGCTGTGGAGCACGATATCCTCTGCCGAAACAGAGGTTGGACAAGAC
CGGAGGGGTCTCCTAGTTCCAAAGGAGATGTACTCCGGGCTTGTTCACGACCTACCGTGTAAGTCGTA
+
FFFFFFFFFFFFFFFFF:FFFFFFFFFFFFFFFFFFF:FFFFFFFFFFFFFFFFFFFFFFFFFFF:FF
FFFFFFFFFFFFF,FFFFFFF:FFFFFFFFFFFFFFFFFFFFFFFF:FFFFFFFFFFFFFFFFFFFFF
@A00155:342:HHGFNDSXY:1:1370:14913:6872 1:N:0:GAACCTAG+TCCGCATA
GCGACTTCTCTCGGGCCCACAGCCTTACAGCTGTGGAGCACGGTATCCTCTGCCGAAACAGAGGTTGG
ACAAGACCGGAGGGGTCTCCTAGTTCCAAAGGAGATGTACTCCGGGCTTGTTCACGACCTACCGTGTA
+
FFFFFFF:FFFFFFFFFFFFFFFFFFFFFFFFFFFFFFFFFFFFFFFFF:FFF:FFFFFF
FFFFF:FFFF:FFFFFFFFFFFFFFFFFFFFFFFFFFFFFFFFFFFFFFFFFFFFFFFFFFFFF,FFF
@A00155:342:HHGFNDSXY:1:1370:24180:7451 1:N:0:GAACCTAG+TCCGCATA
TGTGGAGGGCGAGAGAGAGCGACTTCTCTCGGGCCCACAGCCTTACAGCTGTGGAGCACGGTATCCTC
TGCCGAAACAGAGGTTGGACAAGACCGGAGGGGTCTCCTAGTTCCATAGGAGATGTACTCCGGG
+
FFFFFFFFFFFFFFFFFFFFFFFFFFFFFFFFFFFFFFFFFF:FFFFFFFFFFFFFFFFFFFFFFFFF
FFFFFFFFFFFFFFFFFFFFFFFFFFFFFFFFFFFFFFFFFFFFFFFFFFFFFFFFFFFFFFFF
@A00155:342:HHGFNDSXY:1:1370:13476:11115 1:N:0:GAACCTAG+TCCGCATA
CCTTACAGCTGTGGAGCACGGTATCCTCTGCCGAAACAGAGGTTGGACAAGACCGGAGGGGTCTCCTA
GTTCCAAAGGAGATGTACTCCGGGCTTGTTCACGACCTACCGTGTAAGTCGTAGTCTAGTAGGCTACC
+
FF:FFFFFFFFFFFFFFFFFFFFFFFFFFFFFFFFFFFFFFFFFFFFFFFFFFFFFFFFFFFFFFFF
FFFFFFFFFFFFFFFFFFFFFFFFF,FF,FFFFFFFFFFFFFFFFFFFFFFFFFFFFFFF,FFFFFFF
@A00155:342:HHGFNDSXY:1:1370:18828:20635 1:N:0:GAACCTAG+TCCGCATA
ATCTCAATGCCGAGAGGGATGTAGAGGGCGAGAGAGAGCGACTTCTCTCGGGCCCACAGCCTTACAGC
TGTGGAGCACGGTATCCTCTGCCGAAACAGAGGTTGGACAAGACCGGAGGGGTCTCCTAGTTCCAAAG
+
FFFFFFFFFFFFFFFFFFF:FFFFFFFFFFFFFFFFFFFFFFFFFFFFFFFFFFFFFFFFFFFFFFFF
FFFFFFFFFFFFFFFFFFFFFFFFFFFFF:FFFFFFFFFFF,:FFFFFFFFFFFFFFFFFFFFFFFFF
```

```
@A00155:342:HHGFNDSXY:1:1371:20699:16391 1:N:0:GAACCTAG+TCCGCATA
AGCTGTGGAGCACGGTATCCTCTGCCGAAACAGAGGTTGGACAAGACCGGAGGGGTCTCCTAGTTCCA
AAGGAGATGTACTCCGGGCTTGTTCACGACCTACCGTGTAAGTCGTAGTCTAGTAGGCTACCTGACGA
+
FFFFFFFFF:FFFFFFFFFFFFFFFFFFFFFFFFFFFFFFFFFFFFFFFFFFFFFFF:FFFFFFFFFF
FFFFFFFFFFFFFFFFFFFFFFFFF:FFFFF:FFFFFFFFF:FFFFFFFFF:FFFFFFFFFFFFFFFF
@A00155:342:HHGFNDSXY:1:1371:12698:21668 1:N:0:GAACCTAG+TCCGCATA
AGCGACTTCTCTCGGGCCCACAGCCTTACAGCTGTGGAGCACGGTATCCTCTGCCGAAACAGAGGTTG
GACAAGACCGGAGGGGTCTCCTAGTTCCAAAGGAGATGTACTCCGGGCTTGTTCACGACCTACCG
+
FFFFFFFFFFFFFFFFFFFFFFFFFFFFFFFFFFFFFFFFFFFFFFFFFFFFFFFFFFFFFFFFFFFF
FFFFFFFFFFFFFFFFFFFFFFFFFFFFFFFFFFFFFFFFFFFFFFFFFFFFFFFFFFFFFFFFF
@A00155:342:HHGFNDSXY:1:1371:29496:23109 1:N:0:GAACCTAG+TCCGCATA
ATGCCGAGAGGGATGTAGAGGGCGAGAGAGAGCGACTTCTCTCGGGCCCACAGCCTTACAGCTGTGGA
GCACGGTATCCTCTGCCGAAACAGAGGTTGGACAAGACCGGAGGGGTCTCCTAGTTCCAAAGGAGATG
+
FFFFFFFFFFFFFFFFFFFFFFFFFFFFFFFFFFFFFFFFFFFFFFFFFFFFFFFFFFFFFFFFFFFF
FFFFFFFFFFFFFFFFFFFFFFFFFFFFFFFFFFFFFFFFFFFFFFFFFFFFFFFFFFFFFFFFFFFF
@A00155:342:HHGFNDSXY:1:1372:24044:10285 1:N:0:GAACCTAG+TCCGCATA
AAGGACTCTCCTCTGGCCTATGGTCATCTCAATGCCGAGAGGGATGTGGAGGGCGAGAGAGAGCGACT
TCTCTCGGGCCCACAGCCTTACAGCTGTGGAGCACGGTATCCTCTGCCGAAACAGAGGTTGGACAAGA
+
FFFFFFFFFFFFFFFFFFFFFFFFFFFFFFFFFFFFFFFFFFFFFF:FFFFFFFFFFFFFFFFFF:FF
FFFFFFFFFFFFFFFFFFFFFFFF:FFFFFFFFFFFFFFFFFFFFFFFFFFFFFFFFFFFFFFFFFF
@A00155:342:HHGFNDSXY:1:1372:5755:12367 1:N:0:GAACCTAG+TCCGCATA
TTCTCTCGGGCCCACAGCCTTACAGCTGTGGAGCACGGTATCCTCTGCCGAAACAGAGGTTGGACAAG
ACCGGAGGGGTCTCCTAGTTCCAAAGGAGATGTACTCCGGGCTTGTTCACGACCTACCGTGTAAGTCG
+
FFFFFFFFFFFFFFFFFFFFF:FFFFFFFF:FFFFFFFFFFFFFFFFFFFFFFFFFFFFFFFFFFFFF
FFFFFFFFFFFFFFFFFFFFFFFFFFFFFFFFFFFF:FFFFFFFFFFFFFFFFFFFFFFFF:FFFFFF
@A00155:342:HHGFNDSXY:1:1372:28004:15890 1:N:0:GAACCTAG+TCCGCATA
CCACAGCTGTAAGGCTGTGGGCCCGAGAGAAGTCGCTCTCTCTCGCCCTCCACATCCCTCTCGGCATT
GTGATGACCATAGGCCAGAGGTGAGTCCTTAAGTGGACACAGCTGATCTAAGGCGGTGTGGCGGGGCA
+
FFFFFFFFFFFFFFF:FFFFFFFFFFFFFFFFFFFFFFFFFFFFFFFFFFFFFFFFFFFFFFFFFF,:
FFF:FFFFFFFFFFFFFFFFFFFFFFF:FFFFFFFFFFFFFFFFFFFFFFFFFFFFFFFFFFFFFFFF
@A00155:342:HHGFNDSXY:1:1372:9191:28119 1:N:0:GAACCTAG+TCCGCATA
CCGAGAGGGATGTGGAGGGCGAGAGAGAGCGACTTCTCTCGGGCCCACAGCCTTACAGCTGTGGAGCA
CGGTATCCTCTGCCGAAACAGAGGTTGGACAAGACCGGAGGGGTCTCCTAGTTCCAAAGGAGATGTAC
+
FFFFFFFFFFFFFFFFFFFFFFFFFFFFFFFFFFFFFFFFFFFFFFFFFFFFFFFFFFF:FFFFFFFF
FFFFFFFFFFFFFFFF:FFFFFFFFFFFFFFFFFFFFFFFFFFFFFFFFFFFFFFFFFFFFFFFFFF:
@A00155:342:HHGFNDSXY:1:1372:22372:29559 1:N:0:GAACCTAG+TCCGCATA
TTAGATCAGCTGTGTCCACTTAAGGACTCACCTCTGGCCTATGGTCATCTCAATGCCGAGAGGGATGT
GGAGGGCGAGAGAGAGCGACTTCTCTCGGGCCCACAGCCTTACAGCTGTGGAGCACGGTATCC
+
FFFFFFFFFFFFFFFFFFFF:FFFFFFFFFFFFFFFFFFFFFFFFFFFFFFFFFFFFFFFFFFFF:FF
FFFFFFFFFFFFFFFFFFFFFFFFFFFFFFFFFFFFFFFFFFFFFFFFFFFFFFFFFFFFFFF
@A00155:342:HHGFNDSXY:1:1372:16007:32972 1:N:0:GAACCTAG+TCCGCATA
ACTTCTCTCGGGCCCACAGCCTTACAGCTGTGGAGCACGGTATCCTCTGCCGAAACAGAGGTTGGACA
AGACCGGAGGGGTCTCCTAGTTCCAAAGGAGATGTACTCCGGGCTTGTTCACGACCTACCGTGTAAGT
+
FFFFFFFFFFFFFFFFFFFFFFFFFFFFFFFFFFFFFFFFFFFFFFFFFFFFFFFFFFFFFFFFFFFF
FFFFFFFFFFFFFFFFFFFFFFFFFFFFFFFFFFFFFFFFFFFFFFFF:FFFFFFFFFFFFFFFFFFF
```

```
@A00155:342:HHGFNDSXY:1:1373:12219:12070 1:N:0:GAACCTAG+TCCGCATA
GAGAGAGCGACTTCTCTCGGGCCCACAGCCTTACAGCTGTGGAGCACGGTATCCTCTGCCGAAACAGA
GGTTGGACAAGACCGGAGGGGTCTCCTAGTTCCAAAGGAGATGTACTCCGGGCTTGTTCACG
+
FFFFFFFFFFFFFFFFFFFFFFFFFFFFFFFFFFFFFFFFFFFFFFFFFFFFFFFFFFFFFFFFFFFF
FFFFFFFFFFFFFFFFFFFFFFFFFFFFFFFFFFFFFFFFFFFFFFFFFFFFFFFFFFFFFF
@A00155:342:HHGFNDSXY:1:1373:12219:25723 1:N:0:GAACCTAG+TCCGCATA
CTCTGGCCTATGGTCATCTCAATGCCGAGAGGGATGTGAAGGGCGAGAGAGAGCGACTTCTCTCGGGC
CCACAGCCTTACAGCTGTGGAGCACGGTATCCTCTGCCGAAACAGAGGTTGGACAAGACCGGAGGGGT
+
FFFFFFFFFFFFFFFFFFFFFF:FFFFFF,FFFFFFFFFFFFFFFFFFFFFFFFFFFFFFFFFFFFFF
FFFFFFFFFFFFFFFFFFFFFFF:FFFFFFFFFFFFFFFFFFFFFFFFFFFFFFFFFFFFFFFFFFFF
@A00155:342:HHGFNDSXY:1:1373:9055:27759 1:N:0:GAACCTAG+TCCGCATA
AGCGACTTCTCTCGGGCCCACAGCCTTACAGCTGTGGAGCACGGTATCCTCTGCCGAAACAGAGGTTG
GACAAGACCGGAGGGGTCTCCTGGTTCCAAAGGAGATGTACTCCGGGCTTGTTCACGACCTACCGTGT
+
FFFFFF,FFFFFFFFFFFFFFFFFFFFFFFFFFFFFFFFFFFFFFFFFFFFFFFFFFF,FFFFFFFFF
FFFFFFFFFFFFFFFFFFFFFFFFFFFFFFFFFFFFFFFFFFFFFFFFFFFFFFFFFFFFFFFFFFFF
@A00155:342:HHGFNDSXY:1:1373:9435:28103 1:N:0:GAACCTAG+TCCGCATA
AGCGACTTCTCTCGGGCCCACAGCCTTACAGCTGTGGAGCACGGTATCCTCTGCCGAAACAGAGGTTG
GACAAGACCGGAGGGGTCTCCTGGTTCCAAAGGAGATGTACTCCGGGCTTGTTCACGACCTACCGTGT
+
FFFFFFFFFFFFFFFFFFFFFFFFFFFFFFFFF:FFFFFFFFFFFFFFFFFFFFFFFFFFFF:FFFFF
FFFFFFFFFFFFFFFFFFFFFFFFFFFFFFFFFFFFFFFFFFFFFFFFFFFFFFFFFFFFFFFFF:FF
@A00155:342:HHGFNDSXY:1:1373:28004:31861 1:N:0:GAACCTAG+TCCGCATA
CTCAATGCCGAGAGGGATGTGGAGGGCGAGAGAGAGCGACTTCTCTCGGGCCCACAGCCTTACAGCTG
TGGAGCACGGTATCCTCTGCCGAAACAGAGGTTGGACAAGACCGGAGGGGTCTCCTAGTTCCAAAGGA
+
FFFFFFFFFFFFFFFFFFF:FFFFFFFFFFFFFFFFFFFFFFFFFFFFFFFFFFFFFFFFFFFFFFFF
FFFFFFFFFFFFFFFFFFFFFFFFFFFFFFFFFFFFFFFFFFFFFFFFFFFFFFFFFFFFFFFFFFFF
@A00155:342:HHGFNDSXY:1:1374:27308:15280 1:N:0:GAACCTAG+TCCGCATA
CTCAATGCCGAGAGGGATGTAGAGGGCGAGAGAGAGCGACTTCTCTCGGGCCCACAGCCTTACAGCTG
TGGAGCACGGTATCCTCTGCCGAAACAGAGGTTGGACAAGACCGGAGGGGTCTCCTAGTTCCAAAGGA
+
FFFFFFFFFFFFFFFFFFFFFFFFFFFFFFFFFF:FFFFFFFFFFFFFFFFFFFFFFFFFFFFFFFFF
FFFFFFFFFFFFFF:FFFFFFFFFFFFFFFFFFFFFFFFFFFFFFFFFFFFFFFFFFFFFFFFFFFFF
@A00155:342:HHGFNDSXY:1:1374:5032:19476 1:N:0:GAACCTAG+TCCGCATA
CAATGCCGAGAGGGATGTGGAGGGCGAGAGAGAGCGACTTCTCTCGGGCCCACAGCCTTACAGCTGTG
GAGCACGGTATCCTCTGCCGAAACAGAGGTTGGACAAGACCGGAGGGGTCTCCTAGTTCCAAAGGAGA
+
FFFFFFFFFFFFFFF:FFFFFFF,,FFFFFFFFFFFFFFFFFFFFFFFFFFFFFFFF:FFFFFFFFFF
FFFFFFFFF:FFFFFFFFFFFFFFFFFFFFFFFFFFFFFFFFFFFFFFFFFFFFFFFFFFFFFFFFFF
@A00155:342:HHGFNDSXY:1:1374:20193:21966 1:N:0:GAACCTAG+TCCGCATA
GAGCGACTTCTCTCGGGCCCACAGCCTTACAACTGTGGAGCACGGTATCCTCTGCCGAAACAGAGGTT
GGACAAGACCGGAGGGGTCTCCTAGTTCCAAAGGAGATGTACTCCGGGCTTGTTCACGACCTACCGTG
+
FFFFFFFFFFFFFFFFFFFFFFFFFFFFFFFFFFFFFFFFFFFFFFFFFFFFFFFFFFFFFFFFFFFF
FFFFFFFFFFFFFFFFFFFFFFFFFFFFFFFFFFFFFFFFFFFFFFFFFFFFFFFFFFFFFFFFFFFF
@A00155:342:HHGFNDSXY:1:1375:29559:10034 1:N:0:GAACCTAG+TCCGCATA
CGAGAGGGATGTGGAGGGCGAGAGAGAGCGACTTCTCTCGGGCCCACAGCCTTACAGCTGTGGAGCAC
GGTATCCTCTGCCGAAACAGAGGTTGGACAAGACCGGAGGGGTCTCCTAGTTCCAAAGGAGATGTA
+
:FF:FF,:FFF,:FFFFFFFF:FFFF:FFFFFFFFFFFF:F:FF:FF::F,F,FFFFFFFF:FF:FFF
:FFF,FFFFFFF,FFFFFFFFF:F:FFFFFF:FFFFFFFFFF:FF:FF::F,:FFFFF,FF:,::F
```

@A00155:342:HHGFNDSXY:1:1375:6108:16548 1:N:0:GAACCTAG+TCCGCATA
GATCAGCTGTGTCCACTTAAGGACTCACCTCTGGCCTATGGTCATCTCAATGCCGAGAGGGATGTGAA
GGGCGAGAGAGAGCGACTTCTCTCGGGCCCACAGCCTTACAGCTGTGGAGCACGGTATCCTCTGCCGA
+
FFFFFFFFFFFFFFFFF,FFFFFFFFFFFFFFFFFFF:FFFFFFFFFFFFFFFFFFFFFFFFFFFFFF
FFFFFFFFFFFFFFFFFFFFFFFFFFFFFFFFFFFFFFFFF:FFFFFFFFFFFFFFFFFFFFFFFFFF
@A00155:342:HHGFNDSXY:1:1375:20916:26193 1:N:0:GAACCTAG+TCCGCATA
ACCTCTGTTTCGGCAGAGGATACCGTGCTCCACAGCTGTAAGGCTGTGGGCCCGAGAGAAGTCGCTCT
CTCTCGCCCTCCACATCCCTCTCGGCATTGAGATGACCATAGGCCAGAGGAGAGTCCTTAAGTGGACA
+
FFFFFFFFFFFFFFFFFFFFFFFFFFFFFFFFFFFFFFFFFFFFFFFFFFFFFFFFFFFFFFFFFFFF
FFFFFFFFFFFFFFFFFFFFFFFFFFFFFFFFFFFFFFFFFFFFFFFFFFFFFFFFFFFFFFFFFFFF
@A00155:342:HHGFNDSXY:1:1375:21233:26490 1:N:0:GAACCTAG+TCCGCATA
ACCTCTGTTTCGGCAGAGGATACCGTGCTCCACAGCTGTAAGGCTGTGGGCCCGAGAGAAGTCGCTCT
CTCTCGCCCTCCACATCCCTCTCGGCATTGAGATGACCATAGGCCAGAGGAGAGTCCTTAAGTGGACA
+
FFFFFFFFFFFFFFFFFFFFFFFFFFFFFFFFFFFFFFFFFFFFFFFFFFFFFF:FFFFFFFFFFFFF
FFFFFFFFFFFFFFFFFFFFFFFFFFFFFFFFFFFFFFFFFFFFFFFFFFFFFFFFFFFFFF:FFFFF
@A00155:342:HHGFNDSXY:1:1375:1036:36980 1:N:0:GAACCTAG+TCCGCATA
TTAAGGACTCACCTCTGGCCTATGGTCATCTCAATGCCGAGAGGGATGTAGAGGGCGAGAGAGAGCGA
CTTCTCTCGGGCCCACAGCCTTACAGCTGTGGAGCACGGTATCCTCTGCCGAAACAGAGGTTGGACAA
+
F,:FFFFFFFFFFFFFFFFF::,:FFF:FFFF,FFFFFF:F:FFFF,F,,FFFFFFFFFFFFFFFFFF
F::FFF:FFFFFFFFFFFFF:FFFFFF:F,FFFFF:FFFF,FFFFFFFFFFF:FFFFFFF,FFF:FFF
@A00155:342:HHGFNDSXY:1:1376:28420:5963 1:N:0:GAACCTAG+TCCGCATA
ACTTCTCTCGGGCCCACAGCCTTACAGCTGTGGAGCACGGTATCCTCTGCCGAAACAGAGGTTGGACA
AGACCGGAGGGGTCTCCTAGTTCCAAAGGAGATGTACTCCGGGCTTGTTCACGACCTACCGTGTAAGT
+
FFFFFFFFFFFFFFFFFFFFFFFFFFFFFFFFFFFFFFFFFFFFFFFFFFFFFFFFFFFFFFFFFFFF
FFFFFFFFFFFFFFFFFFFFFFFFFFFFFFFFFFFFFFFFFFFFFFFFFFFFFFFFFFFFFFFF,FFF
@A00155:342:HHGFNDSXY:1:1376:8603:6402 1:N:0:GAACCTAG+TCCGCATA
CCCACAGCCTTACAGCTGTGGAGCACGGTATCCTCTGCCGAAACAGAGGTTGGACAAGACCGGAGGGG
TCTCCTGGTTCCAAAGGAGATGTACTCCGGGCTTGTTCACGACCTACCGTGTAAGTCGTAGTCTAGTA
+
FFFFFFFFF:FFFFFFFFFFFFFFFFFFFFFFFFFFFFFFFFFFFFFFFFFFFFF:F:FFFFFFFFFF
FFFFFFFFFFFFFFFFFFFFFFFFFFFFFFFFFFFFFFFFFFFFFFFFFFFFFFFFFFFFFFFFFFFF
@A00155:342:HHGFNDSXY:1:1376:8829:6574 1:N:0:GAACCTAG+TCCGCATA
CCCACAGCCTTACAGCTGTGGAGCACGGTATCCTCTGCCGAAACAGAGGTTGGACAAGACCGGAGGGG
TCTCCTGGTTCCAAAGGAGATGTACTCCGGGCTTGTTCACGACCTACCGTGTAAGTCGTAGTCTAGTA
+
FFFFFFFFFFFFFFFFFFFFFFFFFFFFFFFFFFFFFFFFFFFFFFFFFFFFFFFFFFFFFFFFFFFF
FFFFFFFFFFFFFFFFFFFFFFFFFFFFFFFFF:FFFFFFFFFFFFFFFFFFFFFFFFFFFFFFFFFF
@A00155:342:HHGFNDSXY:1:1376:17020:19570 1:N:0:GAACCTAG+TCCGCATA
AAGGACTCTCCTCTGGCCTATGGTCATCTCAATGCCGAGAGGGATGTGGAGGGCGAGAGAGAGCGACT
TCTCTCGGGCCCACAGCCTTACAGCTGTGGAGCACGGTATCCTCTGCCGAAACAGAGGTTGGAC
+
FFFFFFFFFFFFFFFFFFFFFFFFFFFFFFFFFFFFFFFFFFFFFFFFFFFFFFFFFFFFFFFFFFFF
FFFFFFFFFFFFFFFFFFFFFFFFFFFFFFFFFFFFFFFFFFFFFFFFFFFFFFFFFF:FFFFF
@A00155:342:HHGFNDSXY:1:1376:26666:34178 1:N:0:GAACCTAG+TCCGCATA
GACTTCTCTCGGGCCCACAGCCTTACAGCTGTGGAGCACGGTATCCTCTGCCGAAACAGAGGTTGGAC
AAGACCGGAGGGGTCTCCTGGTTCCAAAGGAGATGTACTCCGGGCTTGTTCACGACCTACCGTGTAAG
+
FFFFFFFFFFFFFFFFFFFFFFFFFFFFFFFFFFFFFFFFFFFFFFFFFFFFFFFFFFFFFFFFFFFF
FFFFFFFFFFFFFFFFFFFFFFFFFFFFFFFFFFFFFFFFFFFFFFFFFFFFFFFFFFFFFFFFFFFF

```
@A00155:342:HHGFNDSXY:1:1377:3125:9283 1:N:0:GAACCTAG+TCCGCATA
ACCTCTGTTTCGGCAGAGGATACCGTGCTCCACAGCTGTAAGGCTGTGGGCCCGAGAGAAGTCGCTCT
CTCTCGCCCTCCACATCCCTCTCGGCATTGAGATGACCATAGGCCAGAGGTGAGTCCTTAAGTGGACA
+
FFFFFFFFFFFFFFFFFFFFFFFFFFFFFFFFFFFFFFFFFFFFFFFFFFFFFFFFFFFFFFFFFFFF
FFFFFFFFFFFFFFFFFFFFFFFFFF:FFFFFFFFFFFFFFFFFFFFFFFFFFFFFFFFFFFFFFFFF
@A00155:342:HHGFNDSXY:1:1377:4661:18521 1:N:0:GAACCTAG+TCCGCATA
GACTCACCTCTGGCCTATGGTCATCTCAATGCCGAGAGGGATGTGGAGGGCGAGAGAGAGCGACTTCT
CTCGGGCCCACAGCCTTACAGCTGTGGAGCACGGTATCCTTTGCCGAAACAGAGGTTGGAC
+
FFFFFF,FFFFFFFFFFFFFFFFF:FFFFFFFFFFF,FF:FFFFFFFFFFFFFFFFFFFFFFFFFFFF
FFFFFFFFFFFFFFFFFFFFFFFFFFFFFFFFFFFFFFF:F:FFFFFF:FFFFFFFFFF
@A00155:342:HHGFNDSXY:1:1377:6813:27633 1:N:0:GAACCTAG+TCCGCATA
CTTCTCTCGGGCCCACAGCCTTACAGCTGTGGAGCACGGTATCCTCTGCCGAAACAGAGGTTGGACAA
GACCGGAGGGGTCTCCTGGTTCCAAAGGAGATGTACTCCGGGCTTGTTCACGACCTACCGTGTAAGTC
+
FFFFFFFFFFFFFFFFFFFFFFFFFFFFFFFFFFFFFFFFFFFFFFFFFFFFFFFFFFFFFFFFFFFF
FFFFFFFFFFFFFFFFFFFFFFFFFFFFFFFFFFF:FFFFFFFFFFFFFFFFFFFFFFFFFFFFF:FFF
@A00155:342:HHGFNDSXY:1:1378:15609:14058 1:N:0:GAACCTAG+TCCGCATA
ATCTCAATGCCGAGAGGGATGTGGAGGGCGAGAGAGAGCGACTTCTCTCGGGCCCACAGCCTTACAGC
TGTGGAGCACGGTATCCTCTGCCGAAACAGAGGTTGGACAAGACCGGAGGGGTCTCCTAGTTCCAAAG
+
FFFFFFFFFFFFFFFFFFFFFFFFFFFFFFFFFFFFFFFFFFFFFFFFFFFFFFFFFFFFFFFFFFFF
FFFFFFFFFFFFF:FFFFFFFFFFFFFFFFFFFFFFFFFFFFFFFFFFFFFFFFFFFFFFFFFFFFFF
@A00155:342:HHGFNDSXY:1:1378:25870:22592 1:N:0:GAACCTAG+TCCGCATA
GGCCAGAGGTGAGTCCTTAAGTGGACACAGCTGATCTAAGGCGGTGTGGTGGGGCATGGGTTTGAACC
CCCATGACGGTCGGAGTCTAGTAGGCTCCCTGATGAGTCCGTTCCAAGGACGAAACCGTCGTTACTC
+
:FFFFFFFFFFFFFFFFFFF:FFFFFFFF,FFFFFFF:FFF:FFFFFFFFFFFFF:FFFFFFFFFFFF
FFFF:FFFFFFFFFFF:FFF,FFFFFFFFF:FFFFFFFF:F:F:FF,,FFFFFFFFFFFFFFFFFFF
@A00155:342:HHGFNDSXY:1:1378:21694:32925 1:N:0:GAACCTAG+TCCGCATA
TCTCAATGCCGAGAGGGATGTGGAGGGCGAGAGAGAGCGACTTCTCTCGGGCCCACAGCCTTACAGCT
GTGGAGCACGGTATCCTCTGCCGAAACAGAGGTTGGACAAGACCGGAGGGGTCTCCTAGTTCCAAAGG
+
FFFFFFFFFFFFFFFFFFFFFFFFFFFFFFFFFFFFFFFFFFFFFFFFFFFFFFFFFFFFFFFFFFFF
FFFF:FFFFFFFFFFFFFFFFFFFFFFFFFFFFFFFFFFFFFFFFFFFFFFFFFFFFFFFFFFFFFFF
@A00155:342:HHGFNDSXY:1:1401:28574:3317 1:N:0:GAACCTAG+TCCGCATA
CTCTGGCCTATGGTCATCTCAATGCCGAGAGGGATGTAGAGGGCGAGAGAGAGCGACTTCTCTCGGGC
CCACAGCCTTACAGCTGTGGAGCACGGTATCCTCTGCCGAAACAGAGGTTGGACAAGACCGGAGGGGT
+
FFFFFFFFFFFFFFFFFFFFFFF:FFFFFFFFFF::F,FFFF:FFFFFFFFFFFFFFFFFFFFFFFFF
FFFFFFFFFFFFFFFFFFFFFFFFFFFFFFFFFFFFFFFFFFFFFFFFFFFFFFFFFFFFFFFFFFFF
@A00155:342:HHGFNDSXY:1:1401:26205:17597 1:N:0:GAACCTAG+TCCGCATA
CCACATCCCTCTCGGCATTGAGATGACCATAGGCCAGAGGTGAGTCCTTAAGTGGACACAGCTGATCT
AAGGCGGTGTGGCGGGGCATGGGTTTGAACCCCCATGACGGTCGGAGTCTAGTAGGCTCCCTGATGAG
+
FFFFFFFFFFFFFFFFFFFFFFFFFFFFFFFF:FFF,FFFFFFFFFFFFFFFFFFFFFFFFFFFFFFF
FFFFFFFFFFFFFFFFFFFFFFFFFFFFFFFFFFFFFFFFFFFFFFFFFFFFFFFFFFFFFFFFFFFF
@A00155:342:HHGFNDSXY:1:1401:22254:23750 1:N:0:GAACCTAG+TCCGCATA
ACTTCTCTCGGGCCCACAGCCTTACAGCTGTGGAGCACGGTATCCTCTGCCGAAACAGAGGTTGGACA
AGACCGGAGGGGTCTCCTAGTTCCAAAGGAGATGTACTCCGGGCTTGTTCACGACCTACCGTGTAAGT
+
FFFFFFFFFFFFFFFFFFFFFFFFFF:FFFFFFFFFFFFFFFFF,FFFFFF:FFFF,FFFFFFFFFFF
FFFFFFFFFFFFFFFF,FFFFFFFFFFFFFFFFFFFFFFFFFFFF:FFFFFFFFFFFF,FFFFFFFFFF
```

@A00155:342:HHGFNDSXY:1:1401:12933:26616 1:N:0:GAACCTAG+TCCGCATA
TTAAGGACTCTCCTCTGGCCTATGGTCATCTCAATGCCGAGAGGGATGTGGAGGGCGAGAGAGAGCGA
CTTCTCTCGGGCCCACAGCCTTACAGCTGTGGAGCACGGTATCCTCTGCCGAAACAGAGGTTGGACAA
+
FFFFFFFFFFFFFFFFFFFF:FFFFFFFFFFFFFFF:FFFFFFFFF:FFFFFFFFFFFF:FFFFFFFF
FFFFFFFFFFFFFFFFFFFFFFFFFFFFFFFFFFFFFFFFFFFFFFFFFFFFFFFFFFFFFFFFFFFF
@A00155:342:HHGFNDSXY:1:1402:25464:5791 1:N:0:GAACCTAG+TCCGCATA
GGGCGAGAGAGAGCGACTTCTCTCGGGCCCACAGCCTTACAGCTGTGGAGCACGGTATCCTCTGCCGA
AACAGAGGTTGGACAAGACCGGAGGGGTCTCCTAGTTCCAAAGGAGATGTACTCCGGGCTTGTTCACG
+
FFF:FFFFFFFFFFFFFFFFFFFFFFFFFFFFFFFFFFFFFFFFFFFFFFFFFFFFFFFFFFFFFFFF
FFFFFFFFFFFFFFFFFFFFFFFFFFFFFFFFFFFFFF:FFFFFFFFFFFFF:FFFFFFFFFFFFFFF
@A00155:342:HHGFNDSXY:1:1402:22110:30859 1:N:0:GAACCTAG+TCCGCATA
TCCTCTGCCGAAACAGAGGTTGGACAAGACCGGAGGGGTCTCCTAGTTCCAAAGGAGATGTACTCCGG
GCTTGTTCACGACCTACCGTGTAAGTCGTAGTCTAGTAGGCTACCTGACGAGTCCTTTTTAGGACGAA
+
FFFFFFFFFFFFFFFFFFFFFFFFFFFFFFFFFFFFFFFFFFFFFFFFFFFFFFFFFFFFFFFFFFFF
FF:FFFFFFFFFFFFFFFFFFFFFFFFFFFFFFFFFFFFFFFFFFFFFFFFFFFFF,FFFFFFFFFFF
@A00155:342:HHGFNDSXY:1:1403:21630:6261 1:N:0:GAACCTAG+TCCGCATA
TTACAGCTGTGGAGCACGGTATCCTCTGCCGAAACAGAGGTTGGACAAGACCGGAGGGGTCTCCTAGT
TCCAAAGGAGATGTACTCCGGGCTTGTTCACGACCTACCGTGTAAGTCGTAGTCTAGTAGGCTACCTG
+
FFFFFFFFFFFFFFFFFFFFFFFFF:FFFFFFFFFFFFFFFFFFFF:FFFFFFFFFFFFFFFFFFFFF
FFFFFFFFFFF:FFFFFFFFFFF:FFFFFFFFFFFFFFFFFFFF:FFFFFFFFFFFFFFFFFFFFF:F
@A00155:342:HHGFNDSXY:1:1403:8829:8359 1:N:0:GAACCTAG+TCCGCATA
GAGAGAGCGACTTCTCTCGGGCCCACAGCCTTACAGCTGTGGAGCACGGTATCCTCTGCCGAAACAGA
GGTTGGACAAGACCGGAGGGGTCTCCTGGTTCCAAAGGAGATGTACTCCGGGCTTGTTCACGACCTAC
+
FFFFFFFFFFFFFFFFFFFFFFFFFFFFFFFFFFFF:FFFFFFFFFFF:F:FFFFFFFFFFFFFFFFF
FFFFFFFFFFFFFFFFFFFFFFFFFFFFFFFFFFFFF::FFFFFFFFFFFFFFFFFFFFFF:FFFFFF
@A00155:342:HHGFNDSXY:1:1403:3577:8844 1:N:0:GAACCTAG+TCCGCATA
AGAGAGAGCGACTTCTCTCGGGCCCACAGCCTTACAGCTGTGGAGCACGGTATCCTCTGCCGAAACAG
AGGTTGGACAAGACCGGAGGGGTCTCCTAGTTCCAAAGGAGATGTACTCCGGGCTTGTTCACGACCTA
+
FFFFFFFFFFFFFFFFFFFFFFFFFFFF:FFFFFFF:FFFFFFFFFFFFFFFF:FFFFFFFFFFFFFF
FFFFF:FFFFFFFFFFFFFFFFFFFFFFFFFFFFFFFFFFFFFFFFFFFFFFFFFFFFFFFFFFFFFF
@A00155:342:HHGFNDSXY:1:1403:26982:15060 1:N:0:GAACCTAG+TCCGCATA
CTTCTCTCGGGCCCACAGCCTTACAGCTGTGGAGCACGGTATCCTCTGCCGAAACAGAGGTTGGACAA
GACCGGAGGGGTCTCCTAGTTCCAAAGGAGATGTACTCCGGGCTTGTTCACGACCTACCGTGTAAGTC
+
FFFFFFFFFFFFFFFFFFFF:FFFFF:FFFFFF:FFFFFFFFFFFFFFFFFFFFFFFFFFFFFFFFFF
FFFF:,,FFFFFFFFFFFFFFFFFFFFFFFFFFFFFFFFF:FFFFFFFFFFFFFFFFFFFFFFFFFFF
@A00155:342:HHGFNDSXY:1:1403:27597:15092 1:N:0:GAACCTAG+TCCGCATA
GCTGTGTCCACTTAAGGACTCACCTCTGGCCTATGGTCATCTCAATGCCGAGAGGGATGTAGAGGGCG
AGAGAGAGCGACTTCTCTCGGGCCCACAGCCTTACAGCTGTGGAGCACGGTATCCTCTGCCGAAACAG
+
FFFFFFFFFFFFFFFFFFFFFFFFFFFFFFFFFFFFFFFFFFFFFFFFFFFFFFFFFFFFFFFFFFFF
FFFFFFFFFFFFFFFFFFFFFFFFFFFFFFFF:FFFFFFFFFFFFFFFFFFFFFF:FFFFFFFFFFFF
@A00155:342:HHGFNDSXY:1:1403:27272:15154 1:N:0:GAACCTAG+TCCGCATA
CTTCTCTCGGGCCCACAGCCTTACAGCTGTGGAGCACGGTATCCTCTGCCGAAACAGAGGTTGGACAA
GACCGGAGGGGTCTCCTAGTTCCAAAGGAGATGTACTCCGGGCTTGTTCACGACCTACCGTGTAAGTC
+
FFFFFFFF:FFFFFFFFFFFFF:FFFFFF,FFFFFFFFFFFF:FFFFFFFFFFF::FFFF:FFFFFFF
FFFFFFFFF:FFFFFFFFFFFFFFFFFFFFFF:FFF,FFFFFFFFFFFFFFFFFFF:FFFFFFF:FFF

```
@A00155:342:HHGFNDSXY:1:1403:18358:35227 1:N:0:GAACCTAG+TCCGCATA
CTCCACAGCTGTAAGGCTGTGGGCCCGAGAGAAGTCGCTCTCTCTCGCCCTCCACATCCCTCTCGGCA
TTGAGATGACCATAGGCCAGAGGTGAGTCCTTAAGTGGACACAGCTGATCTAAGGCGGTGTGGCGGGG
+
FFFFFFFFFFFFFFFFFFFFFFFFFFFFFFFFF:FFFFFFFFFFFFFFFFFFFFFF,FFFFFFFFFF
FFF:FFFFFFFFFFFFFFFFFFF,FFFFFFFFFFFFFFFFFFFFFFFFFFFFFFFFFF:FFFFFFFFF
@A00155:342:HHGFNDSXY:1:1403:18204:35274 1:N:0:GAACCTAG+TCCGCATA
CTCCACAGCTGTAAGGCTGTGGGCCCGAGAGAAGTCGCTCTCTCTCGCCCTCCACATCCCTCTCGGCA
TTGAGATGACCATAGGCCAGAGGTGAGTCCTTAAGTGGACACAGCTGATCTAAGGCGGTGTGGCGGGG
+
FFFFFFFFFFFFFFFFFFFFFFFFFFFFFFFFFFFFFFFFFFFFFFFFFFFFFFFFFFFFFFFFFFF:
FFFFFFFFFFFFFFFFFFFFFFFFFFFFFFFFFFFFFFFFFFFFFFFFFFFFFFFFFFFFFFFFFFFF
@A00155:342:HHGFNDSXY:1:1404:6479:1501 1:N:0:GAACCTAG+TCCGCATA
ATCTCAATGCCGAGAGGGATGTGGAGGGCGAGAGAGAGCGACTTCTCTCGGGCCCACAGCCTTACAGC
TGTGGAGCACGGTATCCTCTGCCGAAACAGAGGTTGGACAAGACCGGAGGGGTCTCCTAGTTCCAAAG
+
FFFFFFFFFFFFFFFFFFFFFFFFFFFFFFFFFFFFFFFFFFFFFFFFFFFFFFFFFFFFFFFFFFFF
FFFFFFFFFFFFFFFFFFFFFFFFFFFFFFFFFFFFFFFFFFFFFFFFFFFFFFFFFFFFFFFFFFFF
@A00155:342:HHGFNDSXY:1:1404:16613:2331 1:N:0:GAACCTAG+TCCGCATA
CCGCCACACCGCCTTAGATCAGCTGTGTCCACTTAAGGACTCACCTCTGGCCTATGGTCATCTCAATG
CCGAGAGGGATGTGGAGGGCGAGAGAGAGCGACTTCTCTCGGGCCCACAGCCTTACAGCTGTGGAGCA
+
FFFFFFFFFFFFF:FFFFFFFFFFFFFFFFF:FFFFFFFFFFFFFFFFFFFFFFFFFFFFFFFFF,:FF
FFFFFFFFFF,FFFFFFFFFFFFFFFFFFFFFF:FFFFFFFFFFFFFFFFFFFF:FFFFFFFFFFFFF
@A00155:342:HHGFNDSXY:1:1404:6903:16423 1:N:0:GAACCTAG+TCCGCATA
GTCCACTTAAGGACTCACCTCTGGCCTATGGTCATCTCAATGCCGAGAGGGATGTGAAGGGCGAGAGA
GAGCGACTTCTCTCGGGCCCACAGCCTTACAGCTGTGGAGCACGGTATCCTCTGCCGAAACAGAGGTT
+
FFFFFFF:FFFFFFFFFFFFFFFFFFFFFFFFFF:FFFFFFFFF:FFFFFFFFFFFFFFFFFFFFFFFF
FFFFFFFFFFFFFFFFFFFFFFFFFFF:FFFFFFFFFFFFFFFFFFFFFFFFFFFFFFFFFFFFFFFF
@A00155:342:HHGFNDSXY:1:1404:7491:17096 1:N:0:GAACCTAG+TCCGCATA
AGTTTCGTCCTAAAAAGGACTCGTCAGGTAGCCTACTAGACTACGACTTAC
+
::FFFFFFFFF:FF:FFFFFFFFFFFFFFFFFFFFFFFFFFFFFFFFFFFF
@A00155:342:HHGFNDSXY:1:1404:3667:19774 1:N:0:GAACCTAG+TCCGCATA
CTTCTCTCGGGCCCACAGCCTTACAGCTGTGGAGCACGGTATCCTCTGCCGAAACAGAGGTTGGACAA
GACCGGAGGGGTCTCCTAGTTCCAAAGGAGATGTACTCCGGGCTTGTTCACGACCTACCGTGTAAGTC
+
FFFFFFFFFFFFFFFFFFFFFFFFFFFFFFFFFFFFFFFFFFFFFFFFFFFFFFFFF:FFFFFFFFFF
FFFFFFFFFFFFFFFFFFFFFFFFFFFFFFFFFFFFFFFFFFFFFFFFFFFFFFFFFFFFFFFFFFFF
@A00155:342:HHGFNDSXY:1:1405:10149:21261 1:N:0:GAACCTAG+TCCGCATA
CTCAATGCCGAGAGGGATGTAGAGGGCGAGAGAGAGCGACTTCTCTCGGGCCCACAGCCTTACAGCTG
TGGAGCACGGTATCCTCTGCCGAAACAGAGGTTGGACAAGACCGGAGGGGTCTCCTAGTTCCAAAGGA
+
FFFFFFFFFFFFFFFFFFFFFFFFFFFFFFFFFFFFFFFFFFFFFFFFFFFFFFFFFFFFFF:FFFFFF
FFFFFFFFFFFFFFFFFFFF:FFFFFFFFFFFFFFFFFFFFFFFFFFFFFFFFFFFFFFFFFFFFFFF
@A00155:342:HHGFNDSXY:1:1406:4065:2237 1:N:0:GAACCTAG+TCCGCATA
ACTTCTCTCGGGCCCACAGCCTTACAGCTGTGGAGCACGGTATCCTCTGCCGAAACAGAGGTTGGACA
AGACCGGAGGGGTCTCCTAGTTCCAAAGGAGATGTACTCCGGACTTGTTCACGACCTACCGTGTAAGT
+
FFFFFFFFFFFFFFFFFFFFFFFFFFFFFFFFFFFFFFFFFFFFFFFFFFFFFFFFF:FFFFFFFFF
FFFFFFFFFFFFFFFFFFFFFFFFFFFFFFFFFFFFFFFFFFFFFFFFFFFFFFFFFFFFFFFFFFFF
@A00155:342:HHGFNDSXY:1:1406:30156:30608 1:N:0:GAACCTAG+TCCGCATA
CACGGTATCCTCTGCCGAAACAGAGGTTGGACAAGACCGGAGGGGTCTCCTAGTTCCAAAGGAGATGT
```

```
ACTCCGGGCTTGTTCACGACCTACCGTGTAAGTCGTAGTCTAGTAGGCTACCTGACGAGTCCTTTTTA
+
FFFFFFFFF:FFFFF:FFFFFFFFFFFFFFFFFFFFFFFFFFFFFFFFFFFFFFFFFFFFFFFFFFFF
FFFFFFFFFFFFFFFFFFFFFFFFFFFFFFFFFFFFFFFFFFFFFFFFFFF:FFFFFFFFFFFFFFFF
@A00155:342:HHGFNDSXY:1:1406:28158:33818 1:N:0:GAACCTAG+TCCGCATA
GGCGAGAGAGAGCGACTTCTCTCGGGCCCACAGCCTTACAGCTGTGGAGCACGGTATCCTCTGCCGAA
ACAGAGGTTGGACAAGACCGGAGGGGTCTCCTAGTTCCAAAGGAGATGTACTCCGGGCTTGTTCACGA
+
FFFFFFFFFFFFFFFFFFFFFFFFFFFFFFFFFFFFFFFFFFFFFFFFFFFFFFFFFFFFFFFF:FFF
FFFFFFFFFFFFFFFFFFFFFFFFFFFFFFFFFFFFFFFFFFFFFFFFFFFFFFFFFFFFFFFFFFFF
@A00155:342:HHGFNDSXY:1:1407:9661:7889 1:N:0:GAACCTAG+TCCGCATA
AGGGCGAGAGAGAGCGACTTCTCTCGGGCCCACAGCCTTACAGCTGTGGAGCACGGTATCCTCTGCCG
AAACAGAGGTTGGACAAGACCGGAGGGGTCTCCTAGTTCCAAAGGAGATGTACTCCGGGCTTGTTCAC
+
FFFFFFFFFFFFFFFFFFFFFFFFFFFFFFFFFFFFFFFFFFFFFFFFFFFFFFFFFFFFFFFFFFFF
FFFFFFFFFFFFFFFFFFFFFFFFFFFFFFFFFFFFFFFF:FFFFFFFFFFFFFFFFFFFFFFFFF,FF
@A00155:342:HHGFNDSXY:1:1407:3432:25191 1:N:0:GAACCTAG+TCCGCATA
GTCATCTCAATGCCGAGAGGGATGTAGAGGGCGAGAGAGAGCGACTTCTCTCGGGCCCACAGCCTTAC
AGCTGTGGAGCACGGTATCCTCTGCCGAAACAGAGGTTGGACAAGACCGGAGGGGTCTCCTAGTTCCA
+
FFFFFFFFFFFFFFFFFFFFFFFF:FFFFFFFF,FFFFFF:FFFFFFFFFFFFFFFFFFFFFFFFFFF
FFFFFFFFFFFFFFFFFFFFFFFFFFFFFFFFFFFFFFFFFFFFFFFFFFFFFFFFFFFFFFFFFFFF
@A00155:342:HHGFNDSXY:1:1408:30029:12602 1:N:0:GAACCTAG+TCCGCATA
GAGGGCGAGAGAGAGCGACTTCTCTCGGGCCCACAGCCTTACAGCTGTGGAGCACGGTATCCTCTGCC
GAAACAGAGGTTGGACAAGACCGGAGGGGTCTCCTAGTTCCAAAGGAGATGTACTCCGGGCTTGTTCA
+
FFFFF:FFFFFFFFFFFFFFFFFFFFFFFFFFFFFFFFFFFFFFFFFFFFFFFFFFFFFFFFFFFFFF
FFFFFFFFFFFFFFFFFFFFFFFFFFFFFFFFFFFF:FFFFFFFFFFFFFFFFFFFFFFFFFFFFFFF
@A00155:342:HHGFNDSXY:1:1408:17508:12680 1:N:0:GAACCTAG+TCCGCATA
AAAGACTCACCTCTGGCCTATGGTCATCTCAATGCCGAGAGGGATGTGGAGGGCGAGAGAGAGCGACT
TCTCTCGGGCCCACAGCCTTACAGCTGTGGAGCACGGTATCCTCTGCCGAAACAGAGGTTGGACAAGA
+
FFF:FFF,FFF:F,FFFFFFFFFFFFFFFFFFFF,F:FFFF::FFFFF:FFF:FFFFFFFF:F:,FFF
FFFFFFFF:F:FFFFFFF:FFFF,F,:,::FFFFF::FFFF,:FFFF:FFFFFFFF,F:FFFFFFFFF
@A00155:342:HHGFNDSXY:1:1408:29523:14732 1:N:0:GAACCTAG+TCCGCATA
GAGCGACTTCTCTCGGGCCCACAGCCTTACAGCTGTGGAGCACGGTATCCTCTGCCGAAACAGAGGTT
GGACAAGACCGGAGGGGTCTCCTGGTTCCAAAGGAGATGTACTCCGGGCTTGTTCACGACCTACCGTG
+
FFFFFFFFFFFFFF:FFFFFFFFFFFFFFFFFFFFFFFFFFFFFFFFFFFFFFFFFFFFFFFFFFFFF
FFFFFFFFFFFFFFFFFFFFFFFFFFFFFFFFFFFFFFFFFFFFFFFFFFFFFFFFFFFFFFFFFFFF
@A00155:342:HHGFNDSXY:1:1408:17571:18897 1:N:0:GAACCTAG+TCCGCATA
AGCGACTTCTCTCGGGCCCACAGCCTTACAGCTGTGGAGCACGGTATCCTCTGCCGAAACAGAGGTTG
GACAAGACCGGAGGGGTCTCCTAGTTCCAAAGGAGATGTACTCCGGGCTTGTTCACGACCTACCGTGT
+
FFFFFFF:FFFFFFFFFFFFFFFFFFFFFFFFFFFFF:FFFFFFFFFFFFFFFFFFFFFFFFFFFFFF
FFFFFFFFFFFFFFFFFFFFFFFF:FFFFFFFFFFFFFFFFFFFFFFFFFFFFFFFFFFFFFFFFFFF
@A00155:342:HHGFNDSXY:1:1408:18891:29387 1:N:0:GAACCTAG+TCCGCATA
ACTTCTCTCGGGCCCACAGCCTTACAGCTGTGGAGCACGGTATCCTCTGCCGAAACAGAGGTTGGACA
AGACCGGAGGGGTCTCCTAGTTCCAAAGGAGATGTACTCCGGGCTTGTTCACGACCTACCGTGTAAG
+
FFFFFFFFFFFFFFFFFFFFFFFFFFFFFFFFFFFFFFFF:FFFFFFFFFFF:FFFFFFFFFFFF:FFF
FFFFFFFFFFFFFFFFFFFFFFFFFFFF:FFFFFFFFFFF,FFFFFFFFFFFFFFFFFFFFFFFFFFF
@A00155:342:HHGFNDSXY:1:1408:19759:31923 1:N:0:GAACCTAG+TCCGCATA
AGAGAGAGCGACTTCTCTCGGGCCCACAGCCTTACAGCTGTGGAGCACGGTATCCTCTGCCGAAACAG
```

AGGTTGGACAAGACCGGAGGGGTCTCCTAGTTCCAAAGGAGATGTACTCCGGGCTTGTTCACGACCTA
+
FFFFFFFFFFFFFFFFFFFFFFFFFFFFFFFFFFFFFFFFFFFFFFFFFFFFFFFFFFFFFFFFFFFF
FFFFFFFFFFFF:FFFFFFFFFFFFFFFFFFFFFFFFFFFFFFFFFFFFFFFFFFFFFFFFFFFFFFF
@A00155:342:HHGFNDSXY:1:1409:20745:5259 1:N:0:GAACCTAG+TCCGCATA
TCCTCTGCCGAAACAGAGGTTGGACAAGACCGGAGGGGTCTCCTAGTTCCAAAGGAGATGTACTCCGG
GCTTGTTCACGACCTACCGTGTAAGTCGTAGTCTAGTAGGCTACCTGACGAGTCCTTTTTAGGACGAA
+
FFFFFFFFFFFFFFFFFFFFFFFFFFFFFFFFFFFFFFFFFFFFFFFFFFFFFFFFFFFFFFFFFFFF
FFFFFFFFFFFFFFFFFFFFFFFFFFFFFFFFFFFFFFFFFFFFFFFFFFFFFFFFFFFFFFFFFFFF
@A00155:342:HHGFNDSXY:1:1409:17381:7889 1:N:0:GAACCTAG+TCCGCATA
CTTCTCTCGGGCCCACAGCCTTACAGCTGTGGAGCACGGTATCCTCTGCCGAAACAGAGGTTGGACAA
GACCGGAGGGGTCTCCTGGTTCCAAAGGAGATGTACTCCGGGCTTGTTCACGACCTACCGTGTAAGTC
+
FFFFFFFFFFFFFFFFFFFFFFFFFFFFFFFFFFFFFFFFFFFFFFFFFFFFFFFFFFFFFFFFFFFF
FFFFFFFFFFFFFFFFFFFFFFFFFFFFFFFFFFFFFFFFFFFFFFFFFFFFFFF:FFFFFFFFFFFF
@A00155:342:HHGFNDSXY:1:1409:13512:14935 1:N:0:GAACCTAG+TCCGCATA
GAGGGCGAGAGAGAGCGACTTCTCTCGGGCCCACAGCCTTACAGCTGTGGAGCACGGTATCCTCTGCC
GAAACAGAGGTTGGACAAGACCGGAGGGGTCTCCTAGTTCCAAAGGAGATGTACTCCGGGCTTGTTCA
+
FFFFFFFFFFFFFFFFFFFFFFFFFFFFFFFFFFFFFFFFFFFFFFFFFFFFFFFFFFFFFFFFFFFF
FFFFFFFFFFFFFFFFF:FFFFFFFFFFFFFFFFFFFFFFFFFFFFFFFFFFFFFFFFFFFFFFFFFF
@A00155:342:HHGFNDSXY:1:1409:6641:17942 1:N:0:GAACCTAG+TCCGCATA
GCGACTTCTCTCGGGCCCACAGCCTTACAGCTGTGGAGCACGGTATCCTCTGCCGAAACAGAGGTTGG
ACAAGACCGGAGGGGTCTCCTAGTTCCAAAGGAGATGTACTCCGGGCTTGTTCACGACCTACCGTGTA
+
FFFFFFFFFFFFFFFFFFFFFFFFFFFFFFFFFFFF:FFFFF:FFFFF:FFFFFFFFFFFFFFFFFFF
FFFFFFFFFFFFFFFFFFFFFFFFFFFFFFFFFFFFFFFFFFFFFFFFFFFFFFFFFFFFFFFFFFFF
@A00155:342:HHGFNDSXY:1:1409:19081:22200 1:N:0:GAACCTAG+TCCGCATA
AGAGAGAGCGACTTCTCTCGGGCCCACAGCCTTACAGCTGTGGAGCACGGTATCCTCTGCCGAAACAG
AGGTTGGACAAGACCGGAGGGGTCTCCTAGTTCCAAAGGAGATGTACTCCGGGCTTGTTCACGACCTA
+
FFFFFFFFFFFFFFFFFFFFFFFFFFFFFFFFFFFFFFFFFFFFFFFFFFFFFFFFFFFFFFFFFFFF
FFFFFFFFFFFFFFFFFFFFFFFFFFFFFFFFFFFFFFFFFFFFFFFFFFFFFFFFFFFFFFFFFFFF
@A00155:342:HHGFNDSXY:1:1409:28664:25144 1:N:0:GAACCTAG+TCCGCATA
GTATCCTCTGCCGAAACAGAGGTTGGACAAGACCGGAGGGGTCTCCTAGTTCCAAAGGAGATGTACTC
CGGGCTTGTTCACGACCTACCGTGTAAGTCGTAGTCTAGTAGGCTACCTGACGAGTCCTTTTTAGGAC
+
FFFFFFFFFFFFFFFFFFFFFFFFFFFFFFFFFFFFFFFFFFFFFFFFFFFFFFFFFFFFFFFFFFFF
FFFFFFFFFFFFFFFFFFFFFFFFFF:FFFFFFFFFFFFFFFFFFFFFFFFFFFFFFFFFFFFFFFFF
@A00155:342:HHGFNDSXY:1:1409:29116:25426 1:N:0:GAACCTAG+TCCGCATA
GTATCCTCTGCCGAAACAGAGGTTGGACAAGACCGGAGGGGTCTCCTAGTTCCAAAGGAGATGTACTC
CGGGCTTGTTCACGACCTACCGTGTAAGTCGTAGTCTAGTAGGCTACCTGACGAGTCCTTTTTAGGAC
+
FFFFFFFFFFFFFFFFFFFFFFFFFFFFFFFFFFFFFFFFFFFFFFFFFFFFFFFFFFFFFFFFFFFF
FFFFFFFFFFFFFFFFFFFFFFFFFFFFFFFFFFFFFFFFFFFFFFFFFFFFFFFFFFFFFFFF:FFF
@A00155:342:HHGFNDSXY:1:1409:11614:29716 1:N:0:GAACCTAG+TCCGCATA
ACTTCTCTCGGGCCCACAGCCTTACAGCTGTGGAGCACGGTATCCTCTGCCGAAACAGAGGTTGGACA
AGACCGGAGGGGTCTCCTAGTTCCAAAGGAGATGTACTCCGGGCTTGTTCACGACCTACCGTGTAAGT
+
FFFFFFFFFFFFFFFFFFFFFFFFFFFFFFFFFFFFFFFFFFFFFFFFFFFFFFFFFFFFFFFFFFFF
FFFFFFFFFFFFFFFFFFFFFFFFFFFFFFFFFFFFFFFFFFFFFFFFFFFFFFFFFFFFFFFFFFFF
@A00155:342:HHGFNDSXY:1:1410:9805:8547 1:N:0:GAACCTAG+TCCGCATA
GAGAGGGATGTGGAGGGCGAGAGAGAGCGACTTCTCTCGGGCCCACAGCCTTACAGCTGTGGAGCACG

```
GTATCCTCTGCCGAAACAGAGGTTGGACAAGACCGGAGGGGTCTCCTAGTTCCAAAGGAGATGTACTC
+
FFFFFFFFFFFFFFFFFFFFFFFFFFFFFFFFFFFFFFFFFFFFFFFFFFFFFFFFFFFFFFFFFFFF
FFFFFFFFFFFFFFFFFFFFFFFFFFFFFFFFFFFFFFFFFFFFFFFFFFFFFFFFFFFFFFFFFFFF
@A00155:342:HHGFNDSXY:1:1410:6696:22044 1:N:0:GAACCTAG+TCCGCATA
GAGCGACTTCTCTCGGGCCCACAGCCTTACAGCTGTGGAGCACGGTATCCTCTGCCGAAACAGAGGTT
GGACAAGACCGGAGGGGTCTCCTAGTTCCAAAGGAGATGTACTCCGGGCTTGTTCACGACCTACCGTG
+
FFFFFFFFFFFFFFFFFFFFFFFFFFFFFFFFFFFFFFFFFFFFFFFFFFFFFFFFFFFFFFFFFFFF
FFFFFFFFFFFFFFFFFFFFFFFFFFFFFFFFFFFFFFFFFFFFFFFFFFFFFFFFFFFFFFFFFFFF
@A00155:342:HHGFNDSXY:1:1410:1597:26459 1:N:0:GAACCTAG+TCCGCATA
CCTTACAGCTGTGGTGCACGGTATCCTCTGCCGAAACAGAGGTTGGACAAGACCGGAGGGGTCTCCTA
GTTCCAAAGGAGATGTACTCCGGGCTTGTTCACGACCTACCGTGTAAGTCGTAGTCTAGTAGGCTACC
+
FFFFFFFFFFF:FFFFFF:FFFFFFFFFFFFFFFFFFFFFFFFFFFFFFFF::FFFFFFFFFFFFFFF
FFFFF:FFFFFFFFFFFFFFFFFFFFFFFFFFF:FFFFFFF:FFFFFFFFFFFFFFFFFFFFFFFFFF
@A00155:342:HHGFNDSXY:1:1410:2121:27649 1:N:0:GAACCTAG+TCCGCATA
ACTTCTCTCGGGCCCACAGCCTTACAGCTGTGGAGCACGGTATCCTCTGCCGAAACAGAGGTTGGACA
AGACCGGAGGGGTCTCCTAGTTCCAAAGGAGATGTACTCCGGGCTTGTTCACGACCTACCGTGTAAGT
+
FFFFFFFFFFFFFFFFFFFF:FFFFFFFFFFFFFFFFFF:FFFFFFFFFFFFFFFFFFFFFFFFFFFF
FF:FFFFFFFFFFFFFFFFFFFFFFFFFFFFFFFFFFFFFF,FFFFFFFFFFFFFFFFFFFFFFFFFF
@A00155:342:HHGFNDSXY:1:1410:25319:35697 1:N:0:GAACCTAG+TCCGCATA
GAGCGACTTCTCTCGGGCCCACAGCCTTACAGCTGTGGAGCACGGTATCCTCTGCCGAAACAGAGGTT
GGACAAGACCGGAGGGGTCTCCTAGTTCCAAAGGAGATGTACTCCGGGCTTGTTTACGACCTACCGTG
+
FFFFFFFFFFFFFFFFFFFFFFFFFFFFFFFFFFFFFFFFFFFFFFFFFFFFFFFFFFFFFFFFFFFF
FFFFFFFFFFFFFFFFFFFFFFFFFFFFFFFFFFFFFFFFFFFFFFFFFFFFFFFFFFFFFFFFFFFF
@A00155:342:HHGFNDSXY:1:1411:26756:11193 1:N:0:GAACCTAG+TCCGCATA
CCACATCCCTCTCGGTATTGAGATGACCATAGGCCAGAGGTGAGTCCTTAAGTGGACACAGCTGATCT
AAGGCGGTGTGGCGGGGCATGGGTTTGAACCCCCATGACGGTCGGAGTCTAGTAGGCTCCCTGATGAG
+
FFFFFFFFFFFFFFFF:FFFFFFFFFFFFFFFFFFFFFFFFFFFFFFFFFFFFFFFFFFFFFFFFFFF
FFFFFFFFFFFFFFFFFFFFFFFFFFFFFFFFFFFFFFFFFFFFFFFFFFFFFFFFFFFFFFFFFFFF
@A00155:342:HHGFNDSXY:1:1411:9136:23202 1:N:0:GAACCTAG+TCCGCATA
TGGTCATCTCAATGCCGAGAGGGATGTGGAGGGCGAGAGAGAGCGACTTCTCTCGGGCCCACAGCCTT
ACAGCTGTGGAGCACGGTATCCTCTGCCGAAACAGAGGTTGGACAAGACCGGAGGGGTCTCCTAGTTC
+
FFFFFFFFFFFFFFFF:FFFFFFFFFFFFFFFFFFFFFFFFFFFFFFFFFFFFFFFFFFFFFFFFF,F
FFFFFFFFFFFFFFFFFFFFFFFFFFFFFFFFFFFFFFFFFFFFFFFFFFFFFFFFFFFFFFFFFFFF
@A00155:342:HHGFNDSXY:1:1412:10945:17033 1:N:0:GAACCTAG+TCCGCATA
GGATGTGGAGGGCGAGAGAGAGCGACTTCTCTCGGGCCCACAGCCTTACAGCTGTGGAGCACGGTATC
CTCTGCCGAAACAGAGGTTGGACAAGACCGGAGGGGTCTCCTAGTTCCAAAGGAGATGTACTCC
+
FFF:FFFFFFFFFFFFFFFFFFFFFFFFFFFFFFFFFFFFFFFFF:F:FFFFFFFFFFFFFFFFFFFF
FFFFFFFFFF:FFFFFFFFFFFFFFFFFFFFFFFFFFFFFFFFFFFFFFFFFFFFFFFFFFFF
@A00155:342:HHGFNDSXY:1:1412:21649:17315 1:N:0:GAACCTAG+TCCGCATA
AGCGACTTCTCTCGGGCCCACAGCCTTACAGCTGTGGAGCACGGTATCCTCTGCCGAAACAGAGGTTG
GACAAGACCGGAGGGGTCTCCTAGTTCCAAAGGAGATGTACTCCGGGCTTGTTCACGACCTACCGTGT
+
FFFFFFFFFFFFFFFFFFFFFFFFFFFFFFFFFFFFFFFFFFFFFFFFFFFFFFFFFFFFFFFFFFFF
FFFFFFFFFFFFFFFFFFFFFFFFFFFFFFFFFFFFFFFFFFFFFFFFFFFFFFFFFFFFF:FFFFFF
@A00155:342:HHGFNDSXY:1:1413:7943:1532 1:N:0:GAACCTAG+TCCGCATA
AAGGACTCACCTCTGGCCTATGGTCATCTCAATGCCGAGAGGGATGTGAAGGGCGAGAGAGAGCGACT
```

TCTCTCGGGCCCACAGCCTTACAGCTGTGGAGCACGGTATCCTCTGCCGAAACAGAGGTTGGACAAGA
+
FFFFFFFFFFFFFFFFFFFFFFFFFFFFFFFFFFFFFFFFFFF:FFFFFFFFFFFFFFFFFFFFFFFF
FFFFFFFFFFFFFFFFFFFFFFFFFFFFFFFFFFFFFFFFFFFFFFFFFFFFFFFFFFFFFFFFFFFF
@A00155:342:HHGFNDSXY:1:1413:31512:2206 1:N:0:GAACCTAG+TCCGCATA
ATCTCAATGCCGAGAGGGATGTGGAGGGCGAGAGAGAGCGACTTCTCTCGGGCCCACAGCCTTACAGC
TGTGGAGCACGGTATCCTCTGCCGAAACAGAGGTTGGACAAGACCGGAGGGGTCTCCTAGTTCCAAAG
+
FFFFFFFFFFFFFFFFFFFFFFFFFFFFFFFFFFFFFFFFFFFFFFFFFFFFFFFFFFFFFFFFFFFF
FFFFFFFFFFFFFFFFFFFFFFFFFFFFFFFFFFFFFFFFFFFFFFFFFFFFFFFFFF:FFFFFFFFF
@A00155:342:HHGFNDSXY:1:1413:3739:21496 1:N:0:GAACCTAG+TCCGCATA
GGATGTGGAGGGCGAGAGAGAGCGACTTCTCTCGGGCCCACAGCCTTACAGCTGTGGAGCACGGTATC
CTCTGCCGAAACAGAGGTTGGACAAGACCGGAGGGGTCTCCTAGTTCCAAAGGAGATGTACTCC
+
FFFFFFFFFFFFFFFFFFFFFFFFFFFFFFFFFFFFFFFFFFFFFFFFFFFFFFFFFFFFFFFFFFFF
FFFFFFFFFFFFFFFFFFFFFFFFFFFFF:FFFFFFFFFFFFFFFFFFFFFFFF,FFFFFFFFFFF
@A00155:342:HHGFNDSXY:1:1413:30183:34444 1:N:0:GAACCTAG+TCCGCATA
CTTCTCTCGGGCCCACAGCCTTACAGCTGTGGAGCACGGTATCCTCTGCCGAAACAGAGGTTGGACAA
GACCGGAGGGGTCTCCTAGTTCCAAAGGAGATGTACTCCGGGCTTGTTCACGACCTACCGTGTAA
+
FFFFFFFFFFFFFFFFFFFF:F,FFFFFFFFFFFFFFFFFFFFFFFFF:FFFFFFFFFFFFFFFFF:F
FFFFFFFFFFFFFFFFFFFFFFFFFFFFFFFFFFF:FFFFFFFFFFFFFFFFFFFFFFFFFFFFFF
@A00155:342:HHGFNDSXY:1:1414:11017:2002 1:N:0:GAACCTAG+TCCGCATA
TGTTTCGGCAGAGGATACCGTGCTCCACAGCTGTAAGGCTGTGGGCCCGAGAGAAGTCGCTCTCTCTC
GCCCTCTACATCCCTCTCGGCATTGAGATGACCATAGGCCAGAGGTGAGTCCTTAAGTGGACACAGCT
+
FFFFFFFFFFFFFFFFFFFFFFFFFFFFFFFFFFFFFFF:FFFFFFFFFFFFFFFFFFFFFFFFFFFF
FFFFFFFFFFFFFFFFFFFFFFFFFF:FFFFFFFFFFFFFFFFFFFFFFFFFFFFFFFFFFFFFFFFF
@A00155:342:HHGFNDSXY:1:1414:30002:4100 1:N:0:GAACCTAG+TCCGCATA
CGAGAGAGAGCGACTTCTCTCGGGCCCACAGCCTTACAGCTGTGGAGCACGGTATCCTCTGCCGAAAC
AGAGGTTGGACAAGACCGGAGGGGTCTCCTTGTTCCAAAGGAGATGTACTCCGGGCTTGTTCACGACC
+
FFFFFFFFFFFFFFFFFFFFFFFFFFFFFFFFFFFFFF:FFFFFFFFFFFFFFFFFFFFFFFFFFFFF
FFFFFFFFFFFFFFFFFFFFFF:FFFFFFFFFFFFFFFFFFFFFFFFFFFFFFFFFFFFFFFFFFFFF
@A00155:342:HHGFNDSXY:1:1414:7853:27242 1:N:0:GAACCTAG+TCCGCATA
CCACATCCCTCTCGGCATTGAGATGACCATAGGCCAGAGGTGAGTCCTTAAGTGGACACAGCTGATCT
AAGGCGGTGTGGCGGGGCATGGGTTTGAACCCCCATGACGGTCGGAGTCTAGTAGGCTCCCTGATGAG
+
FFFFFFFFFFFFFFFFF::FFFFFFFFF,FFFFFFFFFFFFFFFFFFFFFFFFFFFFFFFFFFFFFFF
FFFFFFFFFFFFFFFFFFFFFFFFFF:FFFFFFFFFFFFFFFFFFFFFFFFFFFFFFFFFFFFFFFFF
@A00155:342:HHGFNDSXY:1:1415:6867:1047 1:N:0:GAACCTAG+TCCGCATA
GACTCACCTCTGGCCTATGGTCATCTCAATGCCGAGAGGGATGTGGAGGGCGAGAGAGAGCGACTTCT
CTCGGGCCCACAGCCTTACAGCTGTGGAGCACGGTATCCTTTGCCGAAACAGAGGTTGGAC
+
FFFFFFFFFFFFFFFFFFFFFFFFFFFFFFFFFF:FFFFFF,FFFF:FFFFFFFFFFFFFFFFFFFFF
FFFFFFFFFFFFFFFFFFFFFFFFF:FFFFFFFFFFFFFFFFFFFFFFFFFF:FFFFFFFFFF
@A00155:342:HHGFNDSXY:1:1415:28384:5149 1:N:0:GAACCTAG+TCCGCATA
TCGGCAGAGGATACCGTGCTCCACAGCTGTAAGGCTGTGGGCCCGAGAGAAGTCGCTCTCTCTCGCCC
TCTACATCCCTCTCGGCATTGAGATGACCATAGGCCAGAGGTGAGTCCTTAAGTGGACACA
+
FFFFFFFFFFFFFFFFFFFFFFFFFFFFFFFFFFFFFFFFFFFFFFFFFFFFFFFFFFFFFFFFFFFF
FFFFFFFFFFFFFFFFFFFFFFFFFFFFFFFFFFFFFFFFFFFFFFFFFFFFFFFFFFFFFF
@A00155:342:HHGFNDSXY:1:1415:10791:17174 1:N:0:GAACCTAG+TCCGCATA
AGGGATGTGGAGGGCGAGAGAGAGCGACTTCTCTCGGGCCCACAGCCTTACAGCTGTGGAGCACGGTA

```
TCCTCTGCCGAAACAGAGGTTGGACAAGACCGGAGGGGTCTCCTAGTTCCAAAGGAGATGTACT
+
FFFFFFFFFFFFFFFFFFFFFFFFFFFFFFFFFFFFFFFFFFFFFFFFFFFFFFFFFFFFFFFFFFFF
FFFFFFFFFFFFFFFFFFFFFFFFFFFFFFFFFFFFFFFFFFFFFFFFFFFFFFFFFFFFFFFF
@A00155:342:HHGFNDSXY:1:1415:20311:19351 1:N:0:GAACCTAG+TCCGCATA
CTCCTCTGGCCTATGGTCATCTCAATGCCGAGAGGGATGTGGAGGGCGAGAGAGAGCGACTTCTCTCG
GGCCCACAGCCTTACAGCTGTGGAGCACGGTATCCTCTGCCGAAACAGAGGTTGGACAAGACCGGAGG
+
FFFFFFFFFFFFFFFFFFFFFFFFFFFFFFFFFFFFFFFFFFFFFFFFFFFFFFFFFFFFF:FFFFFF
FFFFFFFFFFFFFFFFFFFFFFFFFFFFFFFFFFFFFFFFFFFFFFFFFFFFFFFFFFFFFFFFFFFF
@A00155:342:HHGFNDSXY:1:1416:1877:1297 1:N:0:GAACCTAG+TCCGCATA
CTTCTCTCGGGCCCACAGCCTTACAGCTGTGGAGCACGGTATCCTCTGCCGAAACAGAGGTTGGACAA
GACCGGAGGGGTCTCCTAGTTCCAAAGGAGATGTACTCCGGGCTTGTTCACGACCTACCGTGTAAGTC
+
FFFFF:FFFFFFFFFFFFFFFFFFFFFFFFFFFFFFFFFFFFFFFFFFFFFFFFFFFFFFFFFFFFFF
FFFFFFFFFFFFFFFFFFFFFFFFFFFFFFFFFFFFFFF:FFFFFF::FFFFFFFFFFFFFFFFFFFF
@A00155:342:HHGFNDSXY:1:1416:15176:6699 1:N:0:GAACCTAG+TCCGCATA
ACCCCTCCGGTCTTGTCCAACCTCTGTTTCGGCAGAGGATACCGTGCTCCACAGCTGTAAGGCTGTGG
GCCCGAGAGAAGTCGCTCTCTCTCGCCCTCTACATCCCTCTCGGCATTGAGATGACCATAGGCCAGAG
+
FFFFFFFFFFFFF:FFFFFFFFFFFFFFFFFFFF,FFFFFFFFFFFFFFFFFF:FFFFFFFFFFFF:FF
FFFFFFFFFFFFFFFFFFFFFFFFFFFFFFFFFFF:FFFFFFFFFFFFFFFFFFFFFFFFFFFFFFFF
@A00155:342:HHGFNDSXY:1:1417:24243:8406 1:N:0:GAACCTAG+TCCGCATA
GATGTGGAGGGCGAGAGAGAGCGACTTCTCTCGGGCCCACAGCCTTACAGCTGTGGAGCACGGTATCC
TCTGCCGAAACAGAGGTTGGACAAGACCGGAGGGGTCTCCTAGTTCCAAAGGAGATGTACTCCGGGCT
+
FFFFFFFFFFFFFFFFFFFFFFFFFFFF:FFFFFFFFFFFFFFFFFFFFFFFFFFFFFFFFFFFFFFF
FFFFFFFFFFF:FFFFFFFFFFFFFFFFFFFFFFFFFFFFFFFFFFFFFFFFFFFFFFFFFFFFFFFF
@A00155:342:HHGFNDSXY:1:1417:20347:19132 1:N:0:GAACCTAG+TCCGCATA
CTTCTCTCGGGCCCACAGCCTTACAGCTGTGGAGCACGGTATCCTCTGCCGAAACAGAGGTTGGACAA
GACCGGAGGGGTCTCCTGGTTCCAAAGGAGATGTACTCCGGGCTTGTTCACGACCTACCGTGTAAGTC
+
FFFFFFFFFFFFFFFFFFFF:FFFFFFFFFFFFFFFFFFFFFFFFFFFFFFFFFFFFF:FFFFFFFFF
FFFFFFFFFFFFFFFFFFFFFFFFFFFFFFFFFFFFFFFFFFFFFFFFFFFFFFFF:FFFFFFFFFFF
@A00155:342:HHGFNDSXY:1:1417:20184:23077 1:N:0:GAACCTAG+TCCGCATA
GAGAGGGATGTAGAGGGCGAGAGAGAGCGACTTCTCTCGGGCCCACAGCCTTACAGCTGTGGAGCACG
GTATCCTCTGCCGAAACAGAGGTTGGACAAGACCGGAGGGGTCTCCTAGTTCCAAAGGAGATGTACTC
+
FFFFFFFFFFFFFFFFFFFFFFFFFFFFFFFFFFFFFFFFFFFFFFFFFFFFFFFFFFFFFFFFFFFF
FFFFFFFFFFFFFFFFFFFFFFFFFFFFFFFFFFFFFFFFFFFFFFFFFFFFFFFFFFFFFFFFFFFF
@A00155:342:HHGFNDSXY:1:1418:10402:2096 1:N:0:GAACCTAG+TCCGCATA
TGCCCCGCCACACCGCCTTAGATCAGCTGTGTCCACTTAAGGACTCACCTCTGGCCTATGGTCATCTC
AATGCCGAGAGGGATGTGGAGGGCGAGAGAGAGCGACTTCTCTCGGGCCCACAGCCTTACAGCTGTGG
+
::FFFFF:FFFFFF:FF:FFFFFFFFFFFFFFFFFFF,FFFFFFFFFFFFFFFFFFFFFFFFFFFFFF
FFFFFFF:FFFFFFFF:FFFFFFFFFFFFFFFFFFFFFFFFFFFFFFFFFFFFFFFFFFFFFFFFFFF
@A00155:342:HHGFNDSXY:1:1418:5810:8641 1:N:0:GAACCTAG+TCCGCATA
GTAAGGCTGTGGGCCCGAGAGAAGTCGCTCTCTCTCGCCCTCCACATCCCTCTCGGCATTGAGATGAC
CATAGGCCAGAGGTGAGTCCTTAAGTGGACACAGCTGATCTAAGGCGGTGTGGCGGGGCATGGGTTTG
+
FFFFFFFFFFFFFFFFFFFFFFFFFFFFFFFFFFFFFFFFFFFFFFFFFFFFFFFFF,FFFFFFFFFF
FFFFFFFFFFFFFFFFFFFFFFFFFFFFFFFFFFFFFFFFFFFFFFFFFFFFFFFFFFFFFFFF:FFF
@A00155:342:HHGFNDSXY:1:1418:8558:15029 1:N:0:GAACCTAG+TCCGCATA
CTCAATGCCGAGAGGGATGTGGAGGGCGAGAGAGAGCGACTTCTCTCGGGCCCACAGCCTTACAGCTG
```

TGGAGCACGGTATCCTCTGCCGAAACAGAGGTTGGACAAGACCGGAGGGGTCTCCTAGTTCCAAAGGA
+
FFFFFFFFFFFFFFFFFFFFFFFFFFFFFFFFFFFFFFFFFFFFFFFFFFFFFFFFFFFFFFFFFFFF
FFFFFFFFFFFFFFFFFFFFFFFFFFFFFFFFFFFFFFFFFFFFFFFFFFFFFFFFFFFFFFFFFFFF
@A00155:342:HHGFNDSXY:1:1418:5927:19179 1:N:0:GAACCTAG+TCCGCATA
ATCTCAATGCCGAGAGGGATGTGGAGGGCGAGAGAGAGCGACTTCTCTCGGGCCCACAGCCTTACAGC
TGTGGAGCACGGTATCCTCTGCCGAAACAGAGGTTGGACAAGACCGGAGGGGTCTCCTAGTTCCAAAG
+
FFFFFFFFFFFF:FFFFF:FFFFFFFFFFFFFFFFFFFFFFFFFFFFFFFFFFFFFFFFFFFFFFFFF
FFFFFFFFFFFFFFFFFFFFFFFFFFFFFFFFFFFFFFFF:FFFFFFFFFFFFFFFFFFFFFFFFFFF
@A00155:342:HHGFNDSXY:1:1418:24704:23641 1:N:0:GAACCTAG+TCCGCATA
ACTTCTCTCGGGCCCACAGCCTTACAGCTGTGGAGCACGGTATCCTCTGCCGAAACAGAGGTTGGACA
AGACCGGAGGGGTCTCCTAGTTCCAAAGGAGATGTACTCCGGGCTTGTTCACGACCTACCGTGTAAGT
+
FFFFFFFFFFFFFFFFFFFFFFFFFFFFFFFFFFFFFFFFF:FFFFFFFFFFFFFFFFFFFFFFFFFF
FFFF:FFFFFFFFFFFFFFFFFFFFFFFFFFFFFFFFFFFFFFFFFFFFFFFFFFFFFFFFF:FFFFFF
@A00155:342:HHGFNDSXY:1:1418:16179:27790 1:N:0:GAACCTAG+TCCGCATA
CTCAATGCCGAGAGGGATGTGGAGGGCGAGAGAGAGCGACTTCTCTCGGGCCCACAGCCTTACAGCTG
TGGAGCACGGTATCCTCTGCCGAAACAGAGGTTGGACAAGACCGGAGGGGTCTCCTAGTTCCAAAGGA
+
FFFFFFFFFFFFFFFFFFFF:FFFFFFFFFFFFFFFFFFFFFFFFFFFFFFFFFFFFFFFFF:FFFFFF
FFFFFFFFFFFFFFFFFFFFFFFFFFFFFFFFFFFFFFFFFFFFFFFFFFFFFFFFFFFFFFF:FFFFF
@A00155:342:HHGFNDSXY:1:1418:20952:28291 1:N:0:GAACCTAG+TCCGCATA
CTTCTCTCGGGCCCACAGCCTTACAGCTGTGGAGCACGGTATCCTCTGCCGAAACAGAGGTTGGACAA
GACCGGAGGGGTCTCCTAGTTCCAAAGGAGATGTACTCCGGGCTTGTTCACGACCTACCGTGTAAGTC
+
FFFFFFFFFFFFFFFFFFFFFFF,FFFFFFFFFFFFFFFFFFFFFFFFFFFFFFFFFFFFFFFFFFFF
:FFFFFFFFFFFFFFFFFFFFFFFFFFFFFFFFFFFFFFFFFFF:FFFF:FFFFFFFFFFFFFFFFFF
@A00155:342:HHGFNDSXY:1:1419:14199:2628 1:N:0:GAACCTAG+TCCGCATA
AGCGACTTCTCTCGGGCCCACAGCCTTACAGCTGTGGAGCACGGTATCCTCTGCCGAAACAGAGGTTG
GACAAGACCGGAGGGGTCTCCTAGTTCCAAAGGAGATGTACTCCGGGCTTGTTCACGACCTACCGTGT
+
FFFFFFFFFFFFFFFFFFFFFFFFFFFFFFFFFFFFFFFFFFFFFFFFFFFFFFFFFFFFFFFFFFFF
FFFFF:FFFFFFFFFFFFFFFFFFFFFFFFFFFFFFFFFFFFFFFFFFFFFFFFFFFFFFFFFFFFFF
@A00155:342:HHGFNDSXY:1:1419:7310:14622 1:N:0:GAACCTAG+TCCGCATA
CTTCTCTCGGGCCCACAGCCTTACAGCTGTGGAGCACGGTATCCTCTGCCGAAACAGAGGTTGGACAA
GACCGGAGGGGTCTCCTAGTTCCAAAGGAGATGTACTCCGGGCTTGTTCACGACCTACCGTGTAAGTC
+
FFFFFFFFFFFFFFFFFFFFFFFFFFFFF:FFFFFFFFFFFFFFFFFFFFFFFFFFFFFFFFFFFFFF
FFFFFFFFFFFFFFFFFFFFFFFFFFFFFFFFFFFFFFFFFFFFFFFFFFFFFFFFFFFFFFFF,FFF
@A00155:342:HHGFNDSXY:1:1419:24189:20494 1:N:0:GAACCTAG+TCCGCATA
GTAAGGCTGTGGGCCCGAGAGAAGTCGCTCTCTCTCGCCCTCCACATCCCTCTCGGCATTGAGATGAC
CATAGGCCAGAGGTGAGTCCTTAAGTGGACACAGCTGATCTAAGGCGGTGTGGCGGGGCATGGGTTTG
+
FFFFFFFFFFFFFFFFFFFFFFFFFFFFFFFFFFFFFFFFFFFFFFFFFFFFFFFFF:FFFFFFFFFF
FFF:FFFFFFFFFFFFFFFFFFFFFFFFFFF::FFFFFFFFFFFFFFFFFFFFFFFFFFFFFFFFFFF
@A00155:342:HHGFNDSXY:1:1420:12011:12211 1:N:0:GAACCTAG+TCCGCATA
AGAGCGACTTCTCTCGGGCCCACAGCCTTACAGCTGTGGAGCACGGTATCCTCTGCCGAAACAGAGGT
TGGACAAGACCGGAGGGGTCTCCTAGTTCCAAAGGAGATGTACTCCAGGCTTGTTCACGACCTACCGT
+
FFFFFFFFFFFFFFFFFF:FFFFFFFFFFFFFFFFFFFFFFFFFFFF:FFFFF:FFFFFFFFFFFFFF,F
FFFFFFFFFFFFFFFF:FFFFFFFFFFFFFFFFFFFFFFFFFFFFFF:FFFFFFFFFFF:FFFFFFFFFFF
@A00155:342:HHGFNDSXY:1:1420:31946:21621 1:N:0:GAACCTAG+TCCGCATA
GAGAGGGATGTAGAGGGCGAGAGAGAGCGACTTCTCTCGGGCCCACAGCCTTACAGCTGTGGAGCACG

```
GTATCCTCTGCCGAAACAGAGGTTGGACAAGACCGGAGGGGTCTCCTAGTTCCAAAGGAGATGTACTC
+
FFFFFFFF:FFFFF:FFFFFFFFFFFFFFFF,FFFFFFFFFFFFFFFFFFFFFFFFF:FFFFFFFFFF
FFFFFFFFFFFFFF:FFFFFFFFFFFFFFFFFFFFFFFFFFFFFFFFFFFFFFFFFFFFFFFFFFFFF
@A00155:342:HHGFNDSXY:1:1420:10773:28040 1:N:0:GAACCTAG+TCCGCATA
TGTTTCGGCAGAGGATACCGTGCTCCACAGCTGTAAGGCTGTGGGCCCGAGAGAAGTCGCTCTCTCTC
GCCCTTCACATCCCTCTCGGCATTGAGATGACCATAGGCCAGAGGTGAGTCCTTAAGTGGACACAGCT
+
FFFFFFFFFFFFFFFFFFFFFFFFFFFFFFFFFFFFFFFFFFFFFFFFFFFFFF:FFFFFFFFFFFFF
FFFFFFFFFFFFFFFFFFFFFFFFFFFFFFFFFFFFFFFFFFFFFFFFFFFFFFFFFFFFFFFFFFFF
@A00155:342:HHGFNDSXY:1:1420:32190:28307 1:N:0:GAACCTAG+TCCGCATA
CAATGCCGAGAGGGATGTGGAGGGCGAGAGAGAGCGACTTCTCTCGGGCCCACAGCCTTACAGCTGTG
GAGCACGGTATCCTCTGCCGAAACAGAGGTTGGACAAGACCGGAGGGGTCTCCTAGTTCCAAAGGAGA
+
FFFFFFFFFFFFFFFFFFFFFFFFFFFFFFFFFFFFFFFFFFFFFFFFFFFFFFFFFFFFFFFFFFFF
FFFFFFFF:FFFFFFFFFFFFFFFFFFFFFFFFFFFFFFF:FFFFFFFFFFFFF:FFFFFFFFFFFFF
@A00155:342:HHGFNDSXY:1:1420:20094:33004 1:N:0:GAACCTAG+TCCGCATA
GCGACTTCTCTCGGGCCCACAGCCTTACAGCTGTGGAGCACGGTATCCTCTGCCGAAACAGAGGTTGG
ACAAGACCGGAGGGGTCTCCTAGTTCCAAAGGAGATGTACTCCGGGCTTGTTCACGACCTACCGTGTA
+
FFFF:,FFFFFFFFFFFFFFFFFFFFFFFFFFFFFFFFFFFFFFFFFFFFFFFFFFFFFFFFFFFFFF
FFFF:FFFFFFFFFFFFFFFFFFFFFFFFFFFFFFFFFFFFFF:FFF:FFFFFFFFFFFFFFFFFFFF
@A00155:342:HHGFNDSXY:1:1421:27606:5431 1:N:0:GAACCTAG+TCCGCATA
GGATGTGGAGGGCGAGAGAGAGCGACTTCTCTCGGGCCCACAGCCTTACAGCTGTGGAGCACGGTATC
CTCTGCCGAAACAGAGGTTGGACAAGACCGGAGGGGTCTCCTAGTTCCAAAGGAGATGTACTCC
+
FFF:FFFFFFFFFFFFFF:FFFFFFFFFFFFFFFFFFFFFFFFFF,F:FFFFFFFF:FFFFFFFFFFF
FFFFFFFFFFFFFFFFFFFFFFFFFFFF,FFF:FF:FFFFFFFFFFFFF:FFFFFF:FFFF:FF
@A00155:342:HHGFNDSXY:1:1421:4164:7044 1:N:0:GAACCTAG+TCCGCATA
GATGTGGAGGGCGAGAGAGAGCGACTTCTCTCGGGCCCACAGCCTTACAGCTGTGGAGCACGGTATCC
TCTGCCGAAACAGAGGTTGGACAAGACCGGAGGGGTCTCCTAGTTCCAAAGGAGATGTACTCCGGGCT
+
FFFFFFFFFFFFFFFFFFFFFFFFFFFFFFFFFFFFFFFFFFFFFFFFFFFFFFFFFFFFFFFFFFFF
FFFFFFFFFFFFFFFFFFFFFFFFFFFFFFFFFFFFFFFFFFFFFFFFFFFFFFFFFFFFFFFFFFFF
@A00155:342:HHGFNDSXY:1:1422:5050:14090 1:N:0:GAACCTAG+TCCGCATA
ATCTCAATGCCGAGAGGGATGTGGAGGGCGAGAGAGAGCGACTTCTCTCGGGCCCACAGCCTTACAGC
TGTGGAGCACGGTATCCTCTGCCGAAACAGAGGTTGGACAAGACCGGAGGGGTCTCCTGGTTCCAAAG
+
FFFFFFFFFFFF:FFFFFFFFFFFFFFFFFFFFFFFFFFFFFFFFFFFFFFFFFFFFFFFFFF:FFFF
FF:FFFFFFFFFF:FFFFFFFFFFFFFFFFFFFFFFFFFFF:FFFFFFFFFFFFFFFFFFFFFFFFFF
@A00155:342:HHGFNDSXY:1:1422:15826:34788 1:N:0:GAACCTAG+TCCGCATA
GATGTGGAGGGCGAGAGAGAGCGACTTCTCTCGGGCCCACAGCCTTACAGCTGTGGAGCACGGTATCC
TCTGCCGAAACAGAGGTTGGACAAGACCGGAGGGGTCTCCTAGTTCCAAAGGAGATGTACTCCGGGCT
+
FF:FFFFFFFFFFFFFFFFFFFFFFFFFFFFFFFFFFFFFFFFFFFFFFFFFFFFFFFFFFFFFFFFF
FFFFFFFFFFFFFFFFFFFFFFFFFFFFFFFFFFFFFFFFF:FFFFFFFFFFFFFFFFFFFFFFFFFF
@A00155:342:HHGFNDSXY:1:1422:23800:35603 1:N:0:GAACCTAG+TCCGCATA
GAGGGCGAGAGAGAGCGACTTCTCTCGGGCCCACAGCCTTACAGCTGTGGAGCACGGTATCCTCTGCC
GAAACAGAGGTTGGACAAGACCGGAGGGGTCTCCTAGTTCCAAAGGAGATGTACTCCGGGCTTGTTCA
+
FFFFFFFFFFFFFFFFFFFFFFFFFF:FFFFFFFFFFFFFFFFFFFFFFFFFFFFFFFFFFFFFFFFF
:FFFFFFFFFFFFFFFFFFFFFFFFFFFFFFFFFF:FFFFFFFFFFFFFFFFFFFFFFFFFFFFFFFF
@A00155:342:HHGFNDSXY:1:1422:11749:35837 1:N:0:GAACCTAG+TCCGCATA
ATCTCAATGCCGAGAGGGATGTGGAGGGCGAGAGAGAGCGACTTCTCTCGGGCCCACAGCCTTACAGC
```

```
TGTGGAGCACGGTATCCTCTGCCGAAACAGAGGTTGGAAAAGACCGGAGGGGTCTCCTAGTTCCA
+
FF:::FFF:F,,FFF,,:FF,:F,F::::FF,F:FF:,,,FF:F,F,,F,:,:F:FFFFF:FFFFFFF
:,,,:FFFF:,FFFFFF,FFF:F:FFF,::FF:,FFFF,FF,::,,FFFFFFFFF,F:F:,FF,F
@A00155:342:HHGFNDSXY:1:1423:3974:17581 1:N:0:GAACCTAG+TCCGCATA
TTAAGGACTCTCCTCTGGCCTATGGTCATCTCAATGCCGAGAGGGATGTGGAGGGCGAGAGAGAGCGA
CTTCTCTCGGGCCCACAGCCTTACAGCTGTGGAGCACGGTATCCTCTGCCGAAACAGAGGTTGGACAA
+
FFFFFFFFFFFFFFFFFFFFFFFFFFFFFFFFFFFFFFFFFFFFFFFFFF:FFFFFFFFFFFFFFFFF
FFFFFFFFFFFFFFFFFFFFFFFFFFFFFFFFFFFFFFFF:FFFFFFFFFF:FFFFFFFFFFFFFFFF
@A00155:342:HHGFNDSXY:1:1423:8341:27993 1:N:0:GAACCTAG+TCCGCATA
CTTCTCTCGGGCCCACAGCCTTACAGCTGTGGAGCACGGTATCCTCTGCCGAAACAGAGGTTGGACAA
GACCGGAGGGGTCTCCTGGTTCCAAAGGAGATGTACTCCGGGCTTGTTCACGACCTACCGTGTAAGTC
+
FFFFFFFFFFFFFFFFFFFFFFFFFFFFFFFFFFFFFFFFFFFFFFFFFFFFFFFFFFFFFFFFFFFF
FFFFFFFFFFFFFFFFFFFFFFFFFFFFFFFFFFFFFFFFFFFFFFFFFFFFFFFFFFFFFFFFFFFF
@A00155:342:HHGFNDSXY:1:1423:16658:35978 1:N:0:GAACCTAG+TCCGCATA
TCCACTTAAGGACTCACCTCTGGCCTATGGTCATCTCAATGCCGAGAGGGATGTGAAGGGCGAGAGAG
AGCGACTTCTCTCGGGCCCACAGCCTTACAGCTGTGGAGCACGGTATCCTCTGCCGAAACAGAGGTTG
+
FFFFFFFFFFFFFFFFFFFFFFFFFFFFFFFFFFFFFFFFFFFFFFFFFFFFFFFFFFFFFFFFFFFF
FFFFFFFFFFFFFFFFFFFFFFFFFFFFFFFFFFFFFFFFFFFFFFFFFFFFFFFFFFFFFFFFFFFF
@A00155:342:HHGFNDSXY:1:1425:5954:5384 1:N:0:GAACCTAG+TCCGCATA
ATCTCTATGCCGAGAGGGATGTGGAGGGCGAGAGAGAGCGACTTCTCTCGGGCCCACAGCCTTACAGC
TGTGGAGCACGGTATCCTCTGCCGAAACAGAGGTTGGACAAGACCGGAGGGGTCTCCTAGTTCCAAAG
+
FFFFFFFFFFFF:FFFFFFFFFFFFFFFFFFFFFFFFFFFFFFFFFFFFFFFFFFFFFFFFFFFFFFF
FFFFFFFFFFFFFFFFFFFFFFFFFFFFFFFFFFFFFFFFF:FFFFFFFFFFFFFFFFFFFFFF:FFF
@A00155:342:HHGFNDSXY:1:1425:5348:12477 1:N:0:GAACCTAG+TCCGCATA
ATGCCGAGAGGGATGTGGAGGGCGAGAGAGAGCGACTTCTCTCGGGCCCACAGCCTTACAGCTGTGGA
GCACGGTATCCTCTGCCGAAACAGAGGTTGGACAAGACCGGAGGGGTCTCCTAGTTCCAAAGGAGAT
+
FFFFFFFFFFFFFFFFFFFFFFFFFFFFFFFFFFFFFFFFFFFFFFFFFFFFFFFFFFFFFFFFFFFF
FFFFFFFFFFFFFFFFFFFFFFFFFFFFFFFFFFFFFFFFFFFFFFFFFFFFFFFFFFFFFFFFFFF
@A00155:342:HHGFNDSXY:1:1425:28393:29653 1:N:0:GAACCTAG+TCCGCATA
AGATCAGCTGTGTCCACTTAAGGACTCACCTCTGGCCTATGGTCATCTCAATGCCGAGAGGGATGTGA
AGGGCGAGAGAGAGCGACTTCTCTCGGGCCCACAGCCTTACAGCTGTGGAGCACGGTATCCTCTGCCG
+
FFFFFFFFFFFFFFFFFFFFFFFFFFFFFFFFFFFFFFFFF,FFFFFFFF:FFFFFFFFFFFFFFFFF
FFFFFFFFFFFFFFFFFFFFFFFFFFFFFFFFFFFFFFFFFFFFFFFFFFFF:FFFFFFFFFFFFFFF
@A00155:342:HHGFNDSXY:1:1425:24930:33552 1:N:0:GAACCTAG+TCCGCATA
GGATGTAGAGGGCGAGAGAGAGCGACTTCTCTCGGGCCCACAGCCTTACAGCTGTGGAGCACGGTATC
CTCTGCCGAAACAGAGGTTGGACAAGACCGGAGGGGTCTCCTAGTTCCAAAGGAGATGTACTCC
+
FFFFF,FFFFFFFFFFFFFFFFFFF:FFFFFFFFFFFFFFFFFFFFF:FFFFFFFFFFFFFFFFFFFF
FFFFFFFFFFFFFFFFFFFFFFFFFFFFFFFFFFFFFFFFFFFFFFFFFFFFFFFFFFFFFFFF
@A00155:342:HHGFNDSXY:1:1426:16920:21057 1:N:0:GAACCTAG+TCCGCATA
ACCTCTGTTTCGGCAGAGGATACCGTGCTCCACAGCTGTAAGGCTGTGGGCCCGAGAGAAGTCGCTCT
CTCTCGCCCTCCACATCCCTCTCGGCATTGAGATGACCATAGGCCAGAGGAGAGTCCTTAAGTGGACA
+
FFFFFFFFFFFFFFFFFFFFFFFFFFFFFFFFFFFFFFFFFFFFFFFFFFFFFFFFF:FFFFFFFFFF
FFFFFF:FFFFFFFFFFFFFFFFFFFF:FFFFFFFFFFFFFFFFFFFFFFFFFFFFFFFFFFFFFFF:
@A00155:342:HHGFNDSXY:1:1426:7771:29951 1:N:0:GAACCTAG+TCCGCATA
GAGCGACTTCTCTCGGGCCCACAGCCTTACAGCTGTGGAGCACGGTATCCTCTGCCGAAACAGAGGTT
```

GGACAAGACCGGAGGGGTCTCCTAGTTCCAAAGGAGATGTACTCCGGGCTTGTTCACGACCTACCGTG
+
F:FFFFFFFFFFFFFFFFF:FFF:FFFFFFFFFFFFFFFFFFFFFFFFFFFFFFFFFFFFFFFFFFFF
FFFFFFFFFFFFFFFFFFFFFFFFFFFFFFFFFFFFFFFFFFFFFFFFFFFFFFFFFFFFFF:FFFFFF
@A00155:342:HHGFNDSXY:1:1426:8133:30483 1:N:0:GAACCTAG+TCCGCATA
TGCCCCGCCACACCGCCTTAGATCAGCTGTGTCCACTTAAGGACTCACCTCTGGCCTATGGTCATCTC
AATGCCGAGAGGGATGTGGAGGGCGAGAGAGAGCGACTTCTCTCGGGCCCACAGCCTTACAGCTGTGG
+
FFFFFFFFFFFFFFFFFFFFFFFFFFFFFFFFFFFFFFFFFFFFFFFFFFFFFFFFFFFFFFFFFFFF
FFFFFFFFFFFFFFFFFFFFFFFFFFFFFFFFFFFFFFFFFFFFFFFFFFFFFFFFFFFFFFFFFFFF
@A00155:342:HHGFNDSXY:1:1426:28438:31203 1:N:0:GAACCTAG+TCCGCATA
AAGGACTCACCTCTGGCCTATGGTCATCTCAATGCCGAGAGGGATGTGGAGGGCGAGAGAGAGCGACT
TCTCTCGGGCCCACAGCCTTACAGCTGTGGAGCACGGTATCCTCTGCCGAAACAGAGGTTGGAC
+
FFFFFFFFFFFFFFFFFFFFFFFFFFFFFFFFFFFFFFFF:FFFFFFFFFFFFFFFFFFFFFFFFFFF
FFFFFFFFFFFFFFFFFFFFFFFFFFFFFFFFFFFFFFFFFFFFFFFFFFFFFFFFFFFFFFFF
@A00155:342:HHGFNDSXY:1:1428:11324:8672 1:N:0:GAACCTAG+TCCGCATA
CCACCACACCGCCTTAGATCAGCTGTGTCCACTTAAGGACTCACCTCTGGCCTATGGTCATCTCAATG
CCGAGAGGGATGTGGAGGGCGAGAGAGAGCGACTTCTCTCGGGCCCACAGCCTTACAGCTGTGGAGCA
+
FFFFFFFFFFFFFFFFF:FFFFFFFFFFFFFFFFFFFFFFFFFFFFFFFFFFFFFFFFFFFFFFFFFF
FFFFFFFFFF:FFFFFFFFFFFFFFFF:FF:FFFFFFFFFFFFFFFFFFFFFFFFFFFFFFFFFFFFF
@A00155:342:HHGFNDSXY:1:1428:30156:13823 1:N:0:GAACCTAG+TCCGCATA
TCCTCTGCCGAAACAGAGGTTGGACAAGACCGGAGGGGTCTCCTGGTTCCAAAGGAGATGTACTCCGG
GCTTGTTCACGACCTACCGTGTAAGTCGTAGTCTAGTAGGCTACCTGACGAGTCCTTTTTAGGACGAA
+
FFFFFFFFFFFFFFFFFFFFFFFFFFFFFFFFFFFFFFFFFFF:FFFFFFFFFFFFFFFFFFFFFFFF
FFFFFFFFFFFFFFFFFFFFFFFFFFFFFFFFFFFFFFFFFFFFFFFFFFFFFFFF,F:FFFFFFFFF
@A00155:342:HHGFNDSXY:1:1429:9227:16094 1:N:0:GAACCTAG+TCCGCATA
TTACAGCTGTGGAGCACGGTATCCTCTGCCGAAACAGAGGTTGGACAAGACCGGAGGGGTCTCCTAGT
TCCAAAGGAGATGTACTCCGGGCTTGTTCACGACCTACCGTGTAAGTCGTAGTCTAGTAGGCTAC
+
FFFFFFFFFFFFFFFFFFFFFFFFFFFFFFFFFFFFFFFFFFFFFFFFFFFFFFFFFFFFFFFFFFFF
FFFFFFFFFFFFFFFFFF:FFFFFFFFFF:FFFFFFFF:FFFFFFFFFFFFFFFFFFFFFFFFF:F
@A00155:342:HHGFNDSXY:1:1429:3938:23907 1:N:0:GAACCTAG+TCCGCATA
CGACTTCTCTCGGGCCCACAGCCTTACAGCTGTGGAGCACGGTATCCTCTGCCGAAACAGAGGTTGGA
CAAGACCGGAGGGGTCTCCTAGTTCCAAAGGAGATGTACTCCGGGCTTGTTCACGACCTACCGTGTAA
+
FFFFFFFFFFFFFFFFFFFFFFFFFFFFFFFFFFFFFFFFFFFFFFFFFFFFFFFFFFFFFFFFFFFF
FFFFFFFFFFFFFFFFFFFFFFFFFFFFFFFFFFFFFFFFFFFFFFFFFFFFFFFFFFFFFFFFFFFF
@A00155:342:HHGFNDSXY:1:1429:31078:26256 1:N:0:GAACCTAG+TCCGCATA
ACTTAAGGACTCACCTCTGGCCTATGGTCATCTCAATGCCGAGAGGGATGTGGAGGGCGAGAGAGAGC
GACTTCTCTCGGGCCCACAGCCTTACAGCTGTGGAGCACGGTATCCTCTGCCGAAACAGAGGTTGGAC
+
FFFFFFFFFFFFFFFFFFFFFFFFFFFFFFFFFFFFFFFFFFFFFFFFFFFFFFFFFFFFFFFFFFFF
FFFFFFFFFFFFFFFFFFFFFFFFFFFFFFFFFFFFFFFFFFFFF:FFFFFFFFFFFFFFFFFFFFFF
@A00155:342:HHGFNDSXY:1:1429:16233:28855 1:N:0:GAACCTAG+TCCGCATA
TGCCCCGCCACACCGCCTTAGATCAGCTGTGTCCACTTAAGGACTCACCTCTGGCCTATGGTCATCTC
AATGCCGAGAGGGATGTGGAGGGCGAGAGAGAGCGACTTCTCTCGGGCCCACAGCCTTACAGCTGTGG
+
:FFFFFFF:FFFFFFFFFFFFF:FFFFFFFFF:F,:FFFFFFF:FFFFF,FFFFF:FFFFFFFFFFF
:FFF,:F,FFFFFF,:,FFFFFFFFFF,FFF:FFFFF,FFFFFFFFFFFFFFFFFFFFFFFFFFFFFF
@A00155:342:HHGFNDSXY:1:1430:13901:21026 1:N:0:GAACCTAG+TCCGCATA
AGCGACTTCTCTCGGGCCCACAGCCTTACAGCTGTGGAGCACGGTATCCTCTGCCGAAACAGAGGTTG

GACAAGACCGGAGGGGTCTCCTAGTTCCAAAGGAGATGTACTCCGGGCTTGTTCACGACCTACCGTGT
+
FFFFFFFFFFF:FFFF,FFFFFFF::FFFFFFF,FF::FFFFF:FFFFF:F,,FF:FFFFF:FFFFF,
,FFFF:F:F:F,F,:FFFFFFFFFF:FF,FFF:FFFF:FFFFF:F::,:,:,,,FFFFFFF:F:FF,:
@A00155:342:HHGFNDSXY:1:1430:21694:28510 1:N:0:GAACCTAG+TCCGCATA
TTAAGGACTCTCCTCTGGCCTATGGTCATCTCAATGCCGAGAGGGATGTGGAGGGCGAGAGAGAGCGA
CTTCTCTCGGGCCCACAGCCTTACAGCTGTGGAGCACGGTATCCTCTGCCGAAACAGAGGTTGGACAA
+
FFFFFFFFFFFFFFFFFFFFFFFFFFFFFFFFFFFFFFFFFFFFFFF:FFFFFFFFFFFFFFFFFFFF
FFFFFFFFFFFFFFFFFFFFFF:FFFFFFFFFFFFFFFF:FFFFFFFFFFFFFFFFFFFFFFFFFFFF
@A00155:342:HHGFNDSXY:1:1430:2609:29716 1:N:0:GAACCTAG+TCCGCATA
GGTATCCTCTGCCGAAACAGAGGTTGGACAAGACCGGAGGGGTCTCCTAGTTCCAAAGGAGATGTACT
CCGGGCTTGTTCACGACCTACCGTGTAAGTCGTAGTCTAGTAGGCTACCTGACGAGTCCTTTTTAGGA
+
FFFFFFFFFFFFFFFFFFFFFFFFFFFFFFFFFFFFFFFFFFFFFFFFFFFFFFFFFFFFFFFFFFFF
FFFFFFFFFFFFFFFFFFFFFFFFFFFFFFFFFFFFFFFFFFFFFFFFFFFFFFFFFFFFFFFFFFFF
@A00155:342:HHGFNDSXY:1:1430:20907:35728 1:N:0:GAACCTAG+TCCGCATA
GAGAGAGCGACTTCTCTCGGGCCCACAGCCTTACAGCTGTGGAGCACGGTATCCTCTGCCGAAACAGA
GGTTGGACAAGACCGGAGGGGTCTCCTAGTTCCAAAGGAGATGTACTCCGGGCTTGTTCACGACCTAC
+
FFFFFFF,FFFF:FFF:FFF:FFFFFF::F:FF:F:FFFFFFF::FF::FFFFFFFFFFFFFFFFFFF
FFFFF:FFFFFFFF,FFF:F:FFFF:FFFFF:FFFFF,FFFF:FFFF:FFFF:FFFFFFFF:FFFFFF
@A00155:342:HHGFNDSXY:1:1431:29821:10927 1:N:0:GAACCTAG+TCCGCATA
TGTGGAGGGCGAGAGAGAGCGACTTCTCTCGGGCCCACAGCCTTACAGCTGTGGAGCACGGTATCCTC
TGCCGAAACAGAGGTTGGACAAGACCGGAGGGGTCTCCTAGTTCCAAAGGAGATGTACTCCGGG
+
:F:FFFFFFFFFFFFFFFFFFFFFFFFFFFFFFFF:FFFFFFFFFFFFFFFFFFFFFFFFFF:FFFFF
FFFFFFFFFFFFFFFFFFFFFFFFFFFFFFFFFFFFFFFFFFFFFFFFFFFFFFFFFFFFFFFF
@A00155:342:HHGFNDSXY:1:1431:32994:18270 1:N:0:GAACCTAG+TCCGCATA
AGCGACTTCTCTCGGGCCCACAGCCTTACAGCTGTGGAGCACGGTATCCTCTGCCGAAACAGAGGTTG
GACAAGACCGGAGGGGTCTCCTAGTTCCAAAGGAGATGTACTCCGGGCTTGTTCACGACCTACCGTGT
+
FFFF:FFFFFFFFFFFFFFFF,FFFFFFFFFFFFFFFFFF:FFFFFFFFFFFFFFFFFFFFFFFFFFF
FFFFFFFFFFFFFFFFFFFFF,FFFFFFFFFFFFFF:FFFFFFFFFFF::FFFFFFFFFFFFFFFFFF
@A00155:342:HHGFNDSXY:1:1431:29767:20196 1:N:0:GAACCTAG+TCCGCATA
CCTTACAGCTGTGGAGCACGGTATCCTCTGCCGAAACAGAGGTTGGACAAGACCGGAGGGGTCTCCTG
GTTCCAAAGGAGATGTACTCCGGGCTTGTTCACGACCTACCGTGTAAGTCGTAGTCTAGTAGGCTACC
+
FFFFFFFFFFFFFFFFFFFFFFFFFFFFFFFFFFFFFFFFFFFFFFFFFFFFFFFFFFFFFFFFFFFF
FFFFFFFFFFFFFFFFFFFFFFFFFFFFFFFFFFFFFFFFFFFFFF:FFFFFFFFFFFFFFFFFFFFF
@A00155:342:HHGFNDSXY:1:1431:17716:20369 1:N:0:GAACCTAG+TCCGCATA
CTTCTCTCGGGCCCACAGCCTTACAGCTGTGGAGCACGGTATCCTCTGCCGAAACAGAGGTTGGACAA
GACCGGAGGGGTCTCCTAGTTCCAAAGGAGATGTACTCCGGGCTTGTTCACGACCTACCGTGTAAGTC
+
FFFFFFFFFFFFFFFFFFFFFF:FFFFFFFFFFFFFFFFFFFFFFFFFFFFFFFFFFFFFFFFFFFFF
FFFFFFFFFFFFFFFFFFFFFFFFFFFFFFFFFFFFFFFFFFFFFFFFFFFFFFFFFFFFFFFFFFFF
@A00155:342:HHGFNDSXY:1:1431:18213:24956 1:N:0:GAACCTAG+TCCGCATA
CTTCTCTCGGGCCCACAGCCTTACAGCTGTGGAGCACGGTATCCTCTGCCGAAACAGAGGTTGGACAA
GACCGGAGGGGTCTCCTAGTTCCAAAGGAGATGTACTCCGGGCTTGTTCACGACCTACCGTGTAAGTC
+
FFFFFFFFFFFFFFFFFFFFFF:FFFFFFFFFFFFFFFFFFFFFFFFFFFFFFFFFFFFFFFFFFFFF
FFFFFFFFFFFFFFFFFFFFFFFFFFFFFFFFFFFFFFFFFFFFFFFFFFFFFFFFFFFFFFF::FFF
@A00155:342:HHGFNDSXY:1:1431:17029:28009 1:N:0:GAACCTAG+TCCGCATA
TTAAGGACTCACCTCTGGCCTATGGTCATCTCAATGCCGAGAGGGATGTGAAGGGCGAGAGAGAGCGA

```
CTTCTCTCGGGCCCACAGCCTTACAGCTGTGGAGCACGGTATCCTCTGCCGAAACAGAGGTTGGACAA
+
FFFFFFFFFFFFFFFFFFFFFFFFFFFFFFFFFFFFFFFFFFFFFFFFF,FFFFFFFFFFFFFFFFFFFF
FFFFFFFFFFFFFFFFFFFFFF:FFFFFFFFFFFFFFFFFFFFFFFFFFFFFFFFFFFFFFFFFFFFFFF
@A00155:342:HHGFNDSXY:1:1432:30219:6793 1:N:0:GAACCTAG+TCCGCATA
CAATGCCGAGAGGGATGTGGAGGGCGAGAGAGAGCGACTTCTCTCGGGCCCACAGCCTTACAGCTGTG
GAGCACGGTATCCTCTGCCGAAACAGAGGTTGGACAAGACCGGAGGGGTCTCCTAGTTCCAAAGGAGA
+
FFFFFFFFFFFFFFFFFFFFFFFFFFFFFFFFFFFFFFFFF:FFFFFFFFFFFFFFFFFFFFFFFFFFFFF
FFFFFFFFFFFFFFFFFFFFFFFFFFFFFFFFFFFFFFFFFFFFFFFFFFFFFFFFFFFFFFFFFFFFFFF
@A00155:342:HHGFNDSXY:1:1432:19904:22467 1:N:0:GAACCTAG+TCCGCATA
TTCTCTCGGGCCCACAGCCTTACAGCTGTGGAGCACGGTATCCTCTGCCGAAACAGAGGTTGGACAAG
ACCGGAGGGGTCTCCTAGTTCCAAAGGAGATGTACTCCGGGCTTGTTCACGACCTACCGTGTAAGTCG
+
FFFFFFFFFFFFFFFFFFFFFFFFFFFFFFFFFFFFFFFFFFFFFFFFFFFFFFFFFF:FFFFFFFFFFF
FFFFFFFFFFFFFFFFFFFFFFFFFFFFFFFFFFFFFFFFFFFFF:FFFFFFFFFFFFFFFFFFFFFFFFFF
@A00155:342:HHGFNDSXY:1:1433:9028:8234 1:N:0:GAACCTAG+TCCGCATA
GGCCTATGGTCATCTCAATGCCGAGAGGGATGTGGAGGGCGAGAGAGGGCGACTTCTCTCGGGCCCAC
AGCCTTACAGCTGTGGAGCACGGTATCCTCTGCCGAAACAGAGGTTGGACAAGACCGGAGGGGTCTCC
+
FFFFFFFFFFFFFFFFFFFFFFFFFFFFFFFFFFFFFFFFFFFFFFFFFFFFFFFFFFFFFFFFFFFFFFF
FFFFFFFFFFFFFFF:FFFFFFFFFFFFFFFFFFFFFFFFFFFFFFFFFFFFFFFFFFFFFFFFFFFFFFF
@A00155:342:HHGFNDSXY:1:1433:27670:14121 1:N:0:GAACCTAG+TCCGCATA
GTATCCTCTGCCGAAACAGAGGTTGGACAAGACCGGAGGGGTCTCCTGGTTCCAAAGGAGATGTACTC
CGGGCTTGTTCACGACCTACCGTGTAAGTCGTAGTCTAGTAGGCTACCTGACGAGTCCTTTTTAGGAC
+
FFFFFFFFFFFFFFFFFFFFFFFFFFFFFFFFFFFFFFFFFFFFFFFFFFFFFFFFFFFFFFFFFFFFFFF
FFFFFFFFFFFFFFFFFFFFFFFFFFFFFFFFFFFFFFFFFFFFFFFFFFFFFFFFFFFFFFFFFFF:FFF
@A00155:342:HHGFNDSXY:1:1433:29161:26443 1:N:0:GAACCTAG+TCCGCATA
CTCTGGCCTATGGTCATCTCAATGCCGAGAGGGATGTGAAGGGCGAGAGAGAGCGACTTCTCTCGGGC
CCACAGCCTTACAGCTGTGGAGCACGGTATCCTCTGCCGAAACAGAGGTTGGACAAGACCGGAGGGGT
+
FFFF:FFFFFF:FF,FFFFFFFF:F,FF:FFFF,:F::FFF:,FFFFFFFFF:FF,:FFFFFFFFFFF
FFFFFFFF,FFFFFFFF:FFFFF::FFFFFF,,FFFFFFFFF:FFFFF:,FFFFFFFFFF::FFFFFF
@A00155:342:HHGFNDSXY:1:1433:5638:27696 1:N:0:GAACCTAG+TCCGCATA
GTCCAACCTCTGTTTCGGCAGAGGATACCGTGCTCCACAGCTGTAAGGCTGTGGGCCCGAGAGAAGTC
GCTCTCTCTCGCCCTTCACATCCCTCTCGGCATTGAGATGACCATAGGCCAGAGGTGAGTCCTTAAGT
+
FFFFFFFFFFFFFFFFFFFFFFFFFFFFFFFFFFFFFFFFFFF:FFFFFFFFFFFFFFFFFFFFFFFFFFF
FFFFFFFFFFFFFFFFFFFFFFFFFFFFFFFFFF,FFFFFFFFFFFFFFFFFFFFFFFFFFFFFFFFFFFF
@A00155:342:HHGFNDSXY:1:1433:7554:32017 1:N:0:GAACCTAG+TCCGCATA
CGACTTCTCTCGGGCCCACAGCCTTACAGCTGTGGAGCACGGTATCCTCTGCCGAAACAGAGGTTGGA
CAAGACCGGAGGGGTCTCCTAGTTCCAAAGGAGATGTACTCCGGGCTTGTTCACGACCTACCGTGTAA
+
FFFFF::FFFFFFFFFFFFFF:FFFFFFFF:FF,FFFFF::FFF,FF::FFFF,FFFFF:FFF:F:FF
FFFFFFFF:F:FFFFFFFF,FFFFFFFFFF:FFF:FFF,F,FFFFF,FFFFFFFFF:FFFFF::F:FF
@A00155:342:HHGFNDSXY:1:1433:22336:35477 1:N:0:GAACCTAG+TCCGCATA
GGCATTGAGATGACCATAGGCCAGAGGTGAGTCCTTAAGTGGACACAGCTGATCTAAGGCGGTGTGGC
GGGGCATGGGTTTGAACCCCCATGACGGTCGGAGTCTAGTAGGCTCCCTGATGAGTCCGTTCCAAGGA
+
FFFFFFFFFFFFFFFFFFFFFFFFFFFFFFFFFFFFFFFFFFFFFFFFFFFFFFFFFFFFFFFFFFFFFFF
FFFFFFFFFFFFFFFFFFFFFFFFFFFFFFFFFFFFFFFFFFFFFFFFFFFFFFFFFFFFFFFFFFFFFFF
@A00155:342:HHGFNDSXY:1:1433:22625:35603 1:N:0:GAACCTAG+TCCGCATA
GGCATTGAGATGACCATAGGCCAGAGGTGAGTCCTTAAGTGGACACAGCTGATCTAAGGCGGTGTGGC
```

GGGGCATGGGTTTGAACCCCCATGACGGTCGGAGTCTAGTAGGCTCCCTGATGAGTCCGTTCCAAGGA
+
FFFF,FFFFFFFFFFFFFFFFFFFFFFFFFFFFFFFFFFFFFFF:FFFFFFFFFFFFFFFFFFFFFFF
FFFFFFFFFFFFFFFFFFFFFFFFFFFFFFFFFFFFFFFFFFFFFFFFFFFFFFFFFFFFFFFFFFFF
@A00155:342:HHGFNDSXY:1:1434:16089:13573 1:N:0:GAACCTAG+TCCGCATA
GTCATCTCAATGCCGAGAGGGATGTGGAGGGCGAGAGAGAGCGACTTCTCTCGGGCCCACAGCCTTAC
AGCTGTGGAGCACGGTATCCTCTGCCGAAACAGAGGTTGGACAAGACCGGAGGGGTCTCCTAGTTCCA
+
FFFFFFFFFFFFFFF:FFFFFF:FFFFFFFFFFFFFFFFFFFFFFFFFFFFFFFFFFFFFFFFFFFFF
FFFFFFFFFFFFFFFFFFFFFFFFFFFFFFFFF:FFFFFFFFFFFFF,FFFFFFFFFFFFFFFFFFFF
@A00155:342:HHGFNDSXY:1:1434:13449:31454 1:N:0:GAACCTAG+TCCGCATA
GAGCGACTTCTCTCGGGCCCACAGCCTTACAGCTGTGGAGCACGGTATCCTCTGCCGAAACAGAGGTT
GGACAAGACCGGAGGGGTCTCCTAGTTCCAAAGGAGATGTACTCCGGGCTTGTTCACGACCTACCGTG
+
FFFFFFFFFFFFFFFFFFFFFFFFFFFFFFFFFFFFFFFFFFFFFFFFFFFFFFFFFFFFFFFFFFFF
FFFFFFFFFFFFFFFFFFFFFFFFFFFFFFFFFFFFFFFFFFFFFFFFFFFFFFFFFFFFFFFFFFFF
@A00155:342:HHGFNDSXY:1:1434:22607:31751 1:N:0:GAACCTAG+TCCGCATA
TGTTTCGGCAGAGGATACCGTGCTCCACAGCTGTAAGGCTGTGGGCCCGAGAGAAGTCGCTCTCTCTC
GCCCTCCACATCCCTCTCGGCATTGAGATGACCATAGGCCAGAGGTGAGTCCTTAAGTGGACACAGCT
+
FFFFFFFFFFFFFFFFFFFFFFFFFFFFFFFFFFFFFFFFFFFFFFFFFFFFFFFFFFFFFFFFFFFF
FFFFFFFFFFFFFFFFFFFFFFFFFFFFFFFFFFFFFFFFFFFFFFFFFFFFFFFFFFFFFFFFFFFF
@A00155:342:HHGFNDSXY:1:1435:3396:3521 1:N:0:GAACCTAG+TCCGCATA
GTATCCTCTGCCGAAACAGAGGTTGGACAAGACCGGAGGGGTCTCCTAGTTCCAAAGGAGATGTACTC
CGGGCTTGTTCACGACCTACCGTGTAAGTCGTAGTCTAGTAGGCTACCTGACGAGTCCATTTTAG
+
FFFFFFFFFFFFFFFFFFFFFFFFFFFFFFFFFFFFFFFFFFFFFFFFFFFFFFFFFFFFFFFFFFFF
FFFFFFFFFFFFFFFFFFFFFFFFFFFFFFFFFFFFFFFFFFFFFFFFFFFFFFFFFFFFFFFFF
@A00155:342:HHGFNDSXY:1:1436:29767:2033 1:N:0:GAACCTAG+TCCGCATA
ACTTCTCTCGGGCCCACAGCCTTACAGCTGTGGAGCACGGTATCCTCTGCCGAAACAGAGGTTGGACA
AGACCGGAGGGGTCTCCTAGTTCCAAAGGAGATGTACTCCGGGCTTGTTCACGACCTACCGTGTAAGT
+
FFFFFFFFFFFFFFFFFFFFFFFFFFFFFFFFFFFFFFFFFFFFFFFFFFFFFFFFFFFFFFFFFFFF
FFFFFFFFFFFFFFFFFFFFFFFFFFFFFFFFFFFFFFFFFFFFFFFFFFFFFFFFFFFFFFFFFFFF
@A00155:342:HHGFNDSXY:1:1436:6876:15687 1:N:0:GAACCTAG+TCCGCATA
GAGCGACTTCTCTCGGGCCCACAGCCTTACAGCTGTGGAGCACGGTATCCTCTGCCGAAACAGAGGTT
GGACAAGACCGGAGGGGTCTCCTGGTTCCAAAGGAGATGTACTCCGGGCTTGTTCACGACCTACCGTG
+
FFFFFFFFFFFFFFFFFFF:FFFFFFFF,FFFFFFFFFFFFFFFFF,FFFFFFFFFFFFFFFFFFFFF
FFFFFFFFFFFFFFFFFFFFFFFFFFFFFFFFFFFFFFFFFFFFFFFFFFFFFFFFFFFFFFFFFFFF
@A00155:342:HHGFNDSXY:1:1436:7392:17863 1:N:0:GAACCTAG+TCCGCATA
ATCTCAATGCCGAGAGGGATGTGGAGGGCGAGAGAGAGCGACTTCTCTCGGGCCCACAGCCTTACAGC
TGTGGAGCACGGTATCCTCTGCCGAAACAGAGGTTGGACAAGACCGGAGGGGTCTCCTGGTTCCAAAG
+
FFFFFFFFFFFFFFFFFFFFFFFFFFFFFFFFFFFFFFFFFFFFFFFFFF:FFFFFFFFFFFFFFFFF
FFFFFFFFFFFFF:FFFFFFFFFFFFFFFFFFFFFFFFFFF,FFFFFFFFFFFFFFFFFFFFFFFFFF
@A00155:342:HHGFNDSXY:1:1436:17273:18787 1:N:0:GAACCTAG+TCCGCATA
CTCAATGCCGAGAGGGATGTGGAGGGCGAGAGAGAGCGACTTCTCTTGGGCCCACAGCCTTACAGCTG
TGGAGCACGGTATCCTCTGCCGAAACAGAGGTTGGACAAGACCGGAGGGGTCTCCTAGTTCCAAAGGA
+
FFFFFFFFFFFFFFFFFFFFFFFFFFFFFFFFF:FFFFFFFFFFFFFFFFFFFFFFFFF,FFFFFFFF
:FFFFFFFFFFFFFFFFFFFFFFFFFFFFFFFFFFFFFFFFFFFFFFFFFFFFFFFFFFFFFFFFFFF
@A00155:342:HHGFNDSXY:1:1436:10167:25614 1:N:0:GAACCTAG+TCCGCATA
GAGCGACTTCTCTCGGGCCCACAGCCTTACAGCTGTGGAGCACGGTATCCTCTGCCGAAACAGAGGTT

GGACAAGACCGGAGGGGTCTCCTAGTTCCAAAGGAGATGTACTCCGGGCTTGTTCACGACCTACCGTG
+
FFFFFFFFFFFFFFFFFFFFFFFFFFFFFFFFFFFFFFFFFFFFFFFFFFFFFFFFFFFFFFFFF:FF
FFFFFFFFFFFFFFFFFFFFFFFFFFFFF:FFFFFFFFFFFFFFFFFFFFFFFFFFFFFFFFFFFFFF
@A00155:342:HHGFNDSXY:1:1437:25979:12571 1:N:0:GAACCTAG+TCCGCATA
GGACTCTCCTCTGGCCTATGGTCATCTCAATGCCGAGAGGGATGTGGAGGGCGAGAGAGAGCGACTTC
TCTCGGGCCCACAGCCTTACAGCTGTGGAGCACGGTATCCTCTGCCGAAACAGAGGTTGGACAAGACC
+
FFFFFFFFFFFFFFFFFFFFFFFFFFFFFFFFFFFFFFFFFF:FFFFFFFFFFFFFFFFFFFFFFFFF
FFFFFFFFFFFFFFFFFFFFFFFFFFFFFFFFFFFF:FFFFFFFFFFFFFFFFFFFFFFFFFFFFFFF
@A00155:342:HHGFNDSXY:1:1437:30174:22842 1:N:0:GAACCTAG+TCCGCATA
TGTAGAGGGCGAGAGAGAGCGACTTCTCTCGGGCCCACAGCCTTACAGCTGTGGAGCACGGTATCCTC
TGCCGAAACAGAGGTTGGACAAGACCGGAGGGGTCTCCTAGTTCCAAAGGAGATGTACTCCGGGCTTG
+
FFFFFFFFFFFFFFFFFF:FFFFFFFFFFFF:FFFFFFFF:FFF:FFFFFFFFFFFFFFFFFFFFFFF
FFFFFFFFFFFFFFFFFFFFFFFFFFFFFFFFFFFFFFFFFFFFFFFFF,FFFFFFFFFFF:FFFFFF
@A00155:342:HHGFNDSXY:1:1438:31159:13432 1:N:0:GAACCTAG+TCCGCATA
CCTTACAGCTGTGGAGCACGGTATCCTCTGCCGAAACAGAGGTTGGACAAGACCGGAGGGGTCTCCTG
GTTCCAAAGGAGATGTACTCCGGGCTTGTTCACGACCTACCGTGTAAGTCGTAGTCTAGTAGGCTACC
+
FFFF:FFFFFFFFFFFFFFFFFFFFFFFFFFFFFFFFFFFFFFFFF:FF:FFFFFFFFFFFFFFFFFF
FFFFFFFFFFFFFFFFFFFFFFFFFFFFFFFFFFFFFFFFFFFFFFFFFFFFFFFFFFFFFFFFFFFF
@A00155:342:HHGFNDSXY:1:1438:9598:14199 1:N:0:GAACCTAG+TCCGCATA
TCCACTTAAGGACTCACCTCTGGCCTATGGTCATCTCAATGCCGAGAGGGATGTAGAGGGCGAGAGAG
AGCGACTTCTCTCGGGCCCACAGCCTTACAGCTGTGGAGCACGGTATCCTCTGCCGAAACAGAGGTTG
+
FFFFFFFFFFFFFFFFFFFFFFFFFFFFFFFFFFFFFFFFFFFFFFFFFFFFFFFFFFFFFFFFFFFF
FFFFFFFFFFFFFFFFFFFFFFFFFFFFFFFFFFFFFFFFFFFFFFFFFFFFFFFFFFFFFFFFFFFF
@A00155:342:HHGFNDSXY:1:1438:29559:15671 1:N:0:GAACCTAG+TCCGCATA
ATGCCGAGAGGGATGTGGAGGGCGAGAGAGAGCGACTTCTCTCGGGCCCACAGCCTTACAGCTGTGGA
GCACGGTATCCTCTGCCGAAACAGAGGTTGGACAAGACCGGAGGGGTCTCCCAGTTCCAAAGGAGATG
+
FF:FFFFFFFFFFFFFFFFFFFFFFFFFFFFFFFFFFFFFFFFFFFFFFFFFFFFFFFFFFFFFFFFF
FFFFFFF:FFFFFFFFFFFFFFFFFFFFFFFFFFFFFFFFFFFFFFFFFFFFFFFFFFFFFFFFFFFF
@A00155:342:HHGFNDSXY:1:1438:17264:20275 1:N:0:GAACCTAG+TCCGCATA
GTATCCTCTGCCGAAACAGAGGTTGGACAAGACCGGAGGGGTCTCCTAGTTCCAAAGGAGATGTACTC
CGGGCTTGTTCACGACCTACCGTGTAAGTCGTAGTCTAGTAGGCTACCTGACGAGTCCT
+
FFFFFFFFFFFFFFFFFFFFFFFFFFFFFFFFFFFFFFFFFFFFFFFFFFFFFFFFFFFFFFFF:FFF
FFFF:FFFFFFFFFFFFFFFFFFFFFFFFFFFFFFFFFFFFFFFFFFFFFFFFFFFFF
@A00155:342:HHGFNDSXY:1:1438:8495:24345 1:N:0:GAACCTAG+TCCGCATA
ACTTCTCTCGGGCCCACAGCCTTACAGCTGTGGAGCACGGTATCCTCTGCCGAAACAGAGGTTGGACA
AGACCGGAGGGGTCTCCTAGTTCCAAAGGAGATGTACTCCGGGCTTGTTCACGACCTACCGTGTAAGT
+
FFF:FFFFFFFFFFFFFFFFFF:FFFFFFFFFFFFFFFFFF:FFFFFFFFFFFFFFFFFFFFFFFFFF
FFFFFFFFFFFFFFFFFFFFFFFFFFFFFFFFFFFFFFFFFFFFFFF:FF:FFFFFFFFFFFFF:FFF
@A00155:342:HHGFNDSXY:1:1438:29957:36933 1:N:0:GAACCTAG+TCCGCATA
AAGGACTCACCTCTGGCCTATGGTCATCTCAATGCCGAGAGGGATGTAGAGGGCGAGAGAGAGCGACT
TCTCTCGGGCCCACAGCCTTACAGCTGTGGAGCACGGTATCCTCTGCCGAAACAGAGGTTGGACAAGA
+
FFFFFFFFFFFFFFFFFFFFFF:FFFFFFFFFFFFFFFFFFFFFFFFFFFFFFFFFFFFFFFFFFFFF
FFFFFFFFFFFFFFFFFFFFFFFFFFFFFFFFFFFFFFFFFFFFFFFFFFFFFFFFFFFFFFFFFFFF
@A00155:342:HHGFNDSXY:1:1439:13639:2472 1:N:0:GAACCTAG+TCCGCATA
TGCCGAGAGGGATGTGGAGGGCGAGAGAGAGCGACTTCTCTCGGGCCCACAGCCTTACAGCTGTGGAG

```
CACGGTATCCTCTGCCGAAACAGAGGTTGGACAAGACCGGAGGGGTCTCCTAGTTCCAAAGGAGATGT
+
FFFFFFFFFFFFFFFFFFFFFFFFFFFFFFFF:FFFFFFFFFFFFFFFFFFFFFFFFFFFFFFFFFFF
FFFFFFFFFFFFFFFFFFFFFFFFFFFFFFFFFFFFFFFFFFFFFFFFFFFFFFFFFFF,FFFFFFFF
@A00155:342:HHGFNDSXY:1:1439:19443:23766 1:N:0:GAACCTAG+TCCGCATA
GGATGTGGAGGGCGAGAGAGAGCGACTTCTCTCGGGCCCACAGCCTTACAGCTGTGGAGCACGGTATC
CTCTGCCGAAACAGAGGTTGGACAAGACCGGAGGGGTCTCCTAGTTCCAAAGGAGATGTACTCC
+
FFFFFFFFFFFFFFFFFFFFFFFFFFFFFFFFFFFFFFFFFFFFFFFFFFFFFFFFFFFFFFFFFFFF
FFFFFFFFFFFFFFFFFFFFFFFFFFFFFFFFFFFFFFFFFFFFFFFFFFFFFFFFFFFFFFFF
@A00155:342:HHGFNDSXY:1:1439:20735:24408 1:N:0:GAACCTAG+TCCGCATA
TCTCTCGGGCCCACAGCCTTACAGCTGTGGAGCACGGTATCCTCTGCCGAAACAGAGGTTGGACAAGA
CCGGAGGGGTCTCCTAGTTCCAAAGGAGATGTACTCCGGGCTTGTTCACGACCTACCGTGTAAGTCGT
+
FFFFFFFFFFFFFFFFFFFFFFFFFFFFFFFFFFFFFFFFFFFFFFFFFFFFFFFFFFFFFFFFFFFF
FFFFFFFFFFFFFFFFFFFFFFFFFFFFFFFFFFFFFFFFFFFFFFFFFFFFFFFFFFFFFFFFFFFF
@A00155:342:HHGFNDSXY:1:1439:19768:24768 1:N:0:GAACCTAG+TCCGCATA
GGATGTGGAGGGCGAGAGAGAGCGACTTCTCTCGGGCCCACAGCCTTACAGCTGTGGAGCACGGTATC
CTCTGCCGAAACAGAGGTTGGACAAGACCGGAGGGGTCTCCTAGTTCCAAAGGAGATGTACTCC
+
FFFFFFFFFFFFFFFFFFFFFFFFFFFFFFFFFFFFFFFFFFFFFFFFFFFFFFFFFFFFFFFFFFFF
FFFFFFFFFFFFFFF:FFFFFFF:FFFFFFFFFFFFFFFFFF:FFFFFFFFFFFFFFFF,FFFF
@A00155:342:HHGFNDSXY:1:1439:9444:27273 1:N:0:GAACCTAG+TCCGCATA
TCGGCAGAGGATACCGTGCTCCACAGCTGTAAGGCTGTGGGCCCGAGAGAAGTCGCTCTCTCTCGCCC
TCTACATCCCTCTCGGCATTGAGATGACCATAGGCCAGAGGTGAGTCCTTAAGTG
+
FFFFFFFFFFFFFFFFFFFFFFFFFFFFFFFFFFFFFFFFFFFFFFFFFFF:FFFFFFFFFFFFFFFF
FFFFFFFFFFFFFFFFFFFFFFFFFFFFFFFFFFFFFFFFFFFFFFFFFFFFFFF
@A00155:342:HHGFNDSXY:1:1440:29857:17065 1:N:0:GAACCTAG+TCCGCATA
ATCTCAATGCCGAGAGGGATGTAGAGGGCGAGAGAGAGCGACTTCTCTCGGGCCCACAGCCTTACAGC
TGTGGAGCACGGTATCCTCTGCCGAAACAGAGGTTGGACAAGACCGGAGGGGTCTCCTAGTTCCAAAG
+
FFFFFFFFFFFFFFFF,FF:F:FFF,FFFFFFFFFFFFFFFF,FFF:FFFFFFFFFFFFFFFFFFFFF
FFFFFFFFFFFFFFFFFFFFFFFFFFFFFFFFFFF,FFFFF:FFFFFFFFFFFFFFFFFFFFFFFFFF:
@A00155:342:HHGFNDSXY:1:1440:21866:22388 1:N:0:GAACCTAG+TCCGCATA
CCCACAGCCTTACAGCTGTGGAGCACGGTATCCTCTGCCGAAACAGAGGTTGGACAAGACCGGAGGGG
TCTCCTAGTTCCAAAGGAGATGTACTCCGGGCTTGTTCACGACCTACCGTGTAAGTCGTAGTCTAGTA
+
FFFFFF:FFFFFFFFFFFFFFFFFFFFFFFFFFFFFFFFFFFFFFFF,FFFFFFFF,FFFFFFF:FF
FFFFFFFFFFFFFFFFFFFFFFFFFFFFFFFFFFFFFFFFFFFFFFF,FFFFFFFFFF:FF,F::FFFFF
@A00155:342:HHGFNDSXY:1:1440:5665:35133 1:N:0:GAACCTAG+TCCGCATA
ACCTCTGTTTCGGCAGAGGATACCGTGCTCCACAGCTGTAAGGCTGTGGGCCCGAGAGAAGTCGCTCT
CTCTCGCCCTCCACATCCCTCTCGGCATTGAGATGACCATAGGCCAGAGGAGAGTCCTTAAGTGGACA
+
FFFFFFFFFFFFFFFFFFFFFFFFFFFFFFFFFFFFFFFFFFFFFFFFFFFFFFFFFFFFFFFFFFFF
FFFFFFFFFFFFFFFFFFFFFFFFFFFFFFFFFFFFFFFFFFFFFFFF:FFF,FFFFFFFFFFFFFFFF:
@A00155:342:HHGFNDSXY:1:1440:24460:36088 1:N:0:GAACCTAG+TCCGCATA
AGAGGGATGTGGAGGGCGAGAGAGAGCGACTTCTCTCGGGCCCACAGCCTTACAGCTGTGGAGCACGG
TATCCTCTGCCGAAACAGAGGTTGGACAAGACCGGAGGGGTCTCCTAGTTCCAAAGGAGATGTACTCC
+
FFFFFFFFFFFFFFFFFFFFFFFFFFFFFFFFFFFFFFFFFFFFFFFFFFFF:FFFFFFFFFFFFFFF
FFFFFFFFFFFFFFFFFFFFFFFFFFFFFFFFFFFFFFFFFFFFFFFFFFFFFFFFFFFFFFFFFFFF
@A00155:342:HHGFNDSXY:1:1441:21811:5697 1:N:0:GAACCTAG+TCCGCATA
GCGAGAGAGAGCGACTTCTCTCGGGCCCACAGCCTTACAGCTGTGGAGCACGGTATCCTCTGCCGAAA
```

```
CAGAGGTTGGACAAGACCGGAGGGGTCTCCTAGTTCCAAAGGAGATGTACTCCGGGCTTGTTCACGAC
+
FFFFFFFFFFFFFFFFFFFFFFFFFFFFFFFFFFFFFFFFFFFFFFFFFFFFFF:FFFFFFFFFFFFF
FFFFFFFFFFFFFFFFFFFFFFFFFFFFFFFFFFFFFFFFFFFFFFFFFFFFFFFFFF:FFFFFFFFF
@A00155:342:HHGFNDSXY:1:1441:28004:11256 1:N:0:GAACCTAG+TCCGCATA
CATCTCAATGCCGAGAGGGATGTGGAGGGCGAGAGAGAGCGACTTCTCTCGGGCCCACAGCCTTACAG
CTGTGGAGCACGGTATCCTCTGCCGAAACAGAGGTTGGACAAGACCGGAGGGGTCTCCTAGTTCCAAA
+
FFFFFFFFFFFFFFFFFFF::F,FFFFFFFFFFFFFFFFFFFFFFFFFFFFFFFFFFFFFFF:F:FFF
FFFFFFFFFFFFFFFFFFFFFFFFFFFFFFFFFFFFFFFFFFFFFFFFFFFFFFFFFFFFFFFFFFFF
@A00155:342:HHGFNDSXY:1:1441:9362:14262 1:N:0:GAACCTAG+TCCGCATA
GAGAGGGATGTGGAGGGCGAGAGAGAGCGACTTCTCTCGGGCCCACAGCCTTACAGCTGTGGAGCACG
GTATCCTCTGCCGAAACAGAGGTTGGACAAGACCGGAGGGGTCTCCTAGTTCCAAAGGAGATGTACTC
+
FFFFFFFFFFFFFFFFFFFFFFFFFFFFFFFFFFFFFFFFFFFFFFFFFFFFFFFFFFFFFFFFFFFF
FFFFFFFFFFFFFFFFFFFFFFFFFFFFFFFFFFFFFFFFFFFFFFFFFFFFFFFFFFFFFFFFFFFF
@A00155:342:HHGFNDSXY:1:1441:8269:19914 1:N:0:GAACCTAG+TCCGCATA
CTATGGTCATCTCAATGCCGAGAGGGATGTGGAGGGCGAGAGAGAGCGACTTCTCTCGGGCCCACAGC
CTTACAGCTGTGGAGCACGGTATCCTCTGCCGAAACAGAGGTTGGACAAGACCGGAGGGGTCTCCTAG
+
FFFFFFFFFFFFFFFFFFFFFFFFFFFFFFFFFFFFFFFFFFFFFFFFFF,FFFF:FFFFFFFFFFFF
FFF:FFFFFFFFFFFFFFFFFFFFFFFFFFFFFFFFFFFFF:FFFFFFFFFFFFFFFFFFFFFFFFFF
@A00155:342:HHGFNDSXY:1:1441:31024:24596 1:N:0:GAACCTAG+TCCGCATA
CTTCTCTCGGGCCCACAGCCTTACAGCTGTGGAGCACGGTATCCTCTGCCGAAACAGAGGTTGGACAA
GACCGGAGGGGTCTCCTGGTTCCAAAGGAGATGTACTCCGGGCTTGTTCACGACCTACCGTGTAAGTC
+
FFFFFFFFFFFFFFFFFFFF,F,FFFFFFFFFF:FFFFFFFFFFFFFFFFFFFFFFFFFFFFFFFFFF
FFFFFFFFFFFFFFFFFFFFFFFFFFFFFFFFFFFFFFFFFFFFFFFFFFFFFFFFFFFFFFFFFFFF
@A00155:342:HHGFNDSXY:1:1441:1515:28729 1:N:0:GAACCTAG+TCCGCATA
CGACTTCTCTCGGGCCCACAGCCTTACAGCTGTGGAGCACGGTATCCTCTGCCGAAACAGAGGTTGGA
CAAGACCGGAGGGGTCTCCTAGTTCCAAAGGAGATGTACTCCGGGCTTGTTCACGACCTACCGTGTAA
+
FFFFFFFFFFFFFF:FFFFFFFFFFFFFFFFFFFFFFFF:FFFFFFFFFFFFFFFFF:FFFFFFFFFF
FFFFFFFFFFFFFFFFFFFFFFFFFFFF:FFFFFFFFFFFFFFFFFFFFFF:FF:FFFFFFFFFFFFF
@A00155:342:HHGFNDSXY:1:1442:30391:2143 1:N:0:GAACCTAG+TCCGCATA
TATGGAGGGCGAGAGAGAGCGACTTCTCTCGGGCCCACAGCCTTACAGCTGTGGAGCACGGTATCCTC
TGCCGAAACAGAGGTTGGACAAGACCGGAGGGGTCTCCTAGTTCCAAAGGAGATGTACTCCGGG
+
:FFFFFFFFFFFFFFFFFFFFFFFFFFFFFFFFFFFFFFFFFFFFFFFFFFFFFFFFFFFFF:FFFFF
FF:FFFFFFFFFFFFFFFFFF:FFFFFFFFFFFFFFFFFFFFFFFFFFFFFFFFFFFFFFFFFF
@A00155:342:HHGFNDSXY:1:1442:1976:3286 1:N:0:GAACCTAG+TCCGCATA
CGAGAGGGATGTGGAGGGCGAGAGAGAGCGACTTCTCTCGGGCCCACAGCCTTACAGCTGTGGAGCAC
GGTATCCTCTGCCGAAACAGAGGTTGGACAAGACCGGAGGGGTCTCCTAGTTCCAAAGGAGATGTAC
+
FFFFFFFFFFFFFFFFFFFFFFFFFFFFFFFFFFFFFFFFFFFFFFFFFFFFFFFFFFFFFFFFFFFF
FFFFFFFFFFFFFFFFFFFFFFFFFFFFFFFFFFFFFFFFFFFFFFFFFFFFFFFFFFFFFFFFFFF
@A00155:342:HHGFNDSXY:1:1442:17318:14137 1:N:0:GAACCTAG+TCCGCATA
TGTAGAGGGCGAGAGAGAGCGACTTCTCTCGGGCCCACAGCCTTACAGCTGTGGAGCACGGTATCCTC
TGCCGAAACAGAGGTTGGACAAGACCGGAGGGGTCTCCTAGTTCCAAAGGAGATGTACTCCGGG
+
FFFFFFFFFFFFFFFFFFFFFFFFFFFFFFFFFFFFFFFFFFFFFFFFFFFFFFFFFFFFFFFFFFFF
FFFFFFFFFFFFFFFFFFFFFFFFFFFFFFFFFFFFFFFFFFFFFFFFFFFFFFFFFFFFFFFF
@A00155:342:HHGFNDSXY:1:1442:17481:14418 1:N:0:GAACCTAG+TCCGCATA
TGTAGAGGGCGAGAGAGAGCGACTTCTCTCGGGCCCACAGCCTTACAGCTGTGGAGCACGGTATCCTC
```

TGCCGAAACAGAGGTTGGACAAGACCGGAGGGGTCTCCTAGTTCCAAAGGAGATGTACTCCGGG
+
FFFFFFFFFFFFFFFFFFFFFFFFFFFFFFFFFFFFFFFFFF:FFFFFFFFFFFFFFFFFFFFFFFFF
FFFFFFFFFFFFFFFFFFFFFFFFFFFFFFFFFFFFFFFFFFFFFFFFFFFFFF::FFFFFFF
@A00155:342:HHGFNDSXY:1:1443:5918:14779 1:N:0:GAACCTAG+TCCGCATA
CAGCCTTACAGCTGTGGAGCACGGTATCCTCTGCCGAAACAGAGGTTGGACAAGACCGGAGGGGTCTC
CTAGTTCCAAAGGAGATGTACTCCGGGCTTGTTCACGACCTACCGTGTAAGTCGTAGTCTAGTAGGCT
+
FFFFFFF:FFFF:FFFFFFFFFFFFFFFFFFFFFFFFFFFFFFFFFFFFFFFFFFFFFFFFFFFFFFF
FFFFFFFFFFFFFFFFFFFFFFFFF:FFFFFFFFFFFFFFFFF,FFFFFF:FF:FFFFFFFFFFFFFFF
@A00155:342:HHGFNDSXY:1:1443:19479:20635 1:N:0:GAACCTAG+TCCGCATA
TCCTCTGCCGAAACAGAGGTTGGACAAGACCGGAGGGGTCTCCTAGTTCCAAAGGAGATGTACTCCGG
GCTTGTTCACGACCTACCGTGTAAGTCGTAGTCTAGTAGGCTACCTGACGAGTCCTTTTTAGGACGAA
+
FFFFFFFFFFFFFFFFFFFFFFFFFFFFFFFFFFFFFFFFFFFFFFFFFFFFFFFFFFFFFFFFFFFF
FFFFFFFFFFFFFFFFFFFFFFFFFFFFFFFFFFFFFFFFFFFFFFFFFFFFFFFFFFFFFFFFFFFF
@A00155:342:HHGFNDSXY:1:1444:22218:1110 1:N:0:GAACCTAG+TCCGCATA
AGGGATGTAGAGGGCGAGAGAGAGCGACTTCTCTCGGGCCCACAGCCTTACAGCTGTGGAGCACGGTA
TCCTCTGCCGAAACAGAGGTTGGACAAGACCGGAGGGGTCTCCTAGTTCCAAAGGAGATGTACTCCGG
+
FFFFFFFFFFFFFFFFFFFFFFFFFFFFFFFFFFFFFFFF:FFFFFFF:FFFFFFFFFFFFFF:FFFF:
FFFFFFFFFFFFFFFFFFFFFFFFFF:FFFFFFFF:FFFFFFFFFFFFFFF:FFFFFFFFFFFFFFFFF
@A00155:342:HHGFNDSXY:1:1444:29206:5666 1:N:0:GAACCTAG+TCCGCATA
GGATGTGGAGGGCGAGAGAGAGCGACTTCTCTCGGGCCCACAGCCTTACAGCTGTGGAGCACGGTATC
CTCTGCCGAAACAGAGGTTGGACAAGACCGGAGGGGTCTCCTAGTTCCAAAGGAGATGTACTC
+
F:FFF,FFFF:F::FFFFFFFFFFF,FFFFFFFF:F:FFFFFFFFFFF,FFFFFFFFFF,FFF:FFFF
FFFFFFF,FFF,FFFFFFFFFF:F:FFFFFFF:FF:FFFFFFFFFFF:FFF::FFFFFFFFFF
@A00155:342:HHGFNDSXY:1:1444:14805:6120 1:N:0:GAACCTAG+TCCGCATA
ATCTCAATGCCGAGAGGGATGTGGAGGGCGAGAGAGAGCGACTTCTCTCGGGCCCACAGCCTTACAGC
TGTGGAGCACGGTATCCTCTGCCGAAACAGAGGTTGGACAAGACCGGAGGGGTCTCCTAGTTCCAAAG
+
FFFFFFFFFFFFFFFFFF:FFFFFFFFFFFFFFFFFFFFFFFFFFFFFFFFFFFFFFFFFFFFFFFFF
FFFFFFFFFFFFFFFFFFFFFFFFFFFFFFFFFFFFFFFFFFFFFFFFFFFFFF:FFFFFFFFFFFFF
@A00155:342:HHGFNDSXY:1:1444:31458:12007 1:N:0:GAACCTAG+TCCGCATA
AGCGACTTCTCTCGGGCCCACAGCCTTACAGCTGTGGAGCACGGTATCCTCTGCCGAAACAGAGGTTG
GACAAGACCGGAGGGGTCTCCTGGTTCCAAAGGAGATGTACTCCGGGCTTGTTCACGACCTACCGTGT
+
FFFFFFFFFFFFFFFFFFFFFFFFFFFFFFFFFFFFFFFFFFFFFFFF:FFFFFFFFFFFFFFFFFFFF
FFFFFFFFFFFFFFFFFFFFFFFFFFFFFFFFFFFFFFFFFFFFFFFFFFFFFFFFFFFFFFFFFFFF
@A00155:342:HHGFNDSXY:1:1444:8983:23970 1:N:0:GAACCTAG+TCCGCATA
GTATCCTCTGCCGAAACAGAGGTTGGACAAGACCGGAGGGGTCTCCTGGTTCCAAAGGAGATGTACTC
CGGGCTTGTTCACGACCTACCGTGTAAGTCGTAGTCTAGTAGGCTACCTGACGAGTCCTTTTTAGGAC
+
FFFFFFFFFFFFFFFFFFFFFFFFFFFFFFFFFFFFFFFFFFFFFFFFFFFFFFFFFFFFFFFFFFFF
FFFFFFFFFFFFFFFFFFFFFFFFFFFFFFFFFFFFFFFFFFFFFFFFFFFFFFFFFFFFFFFFFFFF
@A00155:342:HHGFNDSXY:1:1444:8965:24032 1:N:0:GAACCTAG+TCCGCATA
GTATCCTCTGCCGAAACAGAGGTTGGACAAGACCGGAGGGGTCTCCTGGTTCCAAAGGAGATGTACTC
CGGGCTTGTTCACGACCTACCGTGTAAGTCGTAGTCTAGTAGGCTACCTGACGAGTCCTTTTTAGGAC
+
FFFFFFFFFFFFFFFFFFFFFFFFFFFFFFFFFFFFFFFFFFFFFFFFFFFFFFFFFFFFFFFFFFFF
FFFFFFFFFFFFFFFFFFFFFFFFFFFFFFFFFFFFFFFFFFFFFFFFFFFFFFFFFFFFFFFFFFFF
@A00155:342:HHGFNDSXY:1:1444:15664:24142 1:N:0:GAACCTAG+TCCGCATA
TGTCCACTTAAGGACTCACCTCTGGCCTATGGTCATCTCAATGCCGAGAGGGATGTAGAGGGCGAGAG

```
AGAGCGACTTCTCTCGGGCCCACAGCCTTACAGCTGTGGAGCACGGTATCCTCTGCCGAAACAGAGGT
+
FFFFFFFFFFFFFFFFFFFFFFFFFFFFFFFFFFFFFFFFFFFFFFFFFFFFFFFFFFFFFFFFFFFF
FFFFFFFFF:FFFFFFFFFFFFFFFFFFFFFFFFFFFFFFFFFFFFFFFFFFFFFFFFFFFFFFFFFF
@A00155:342:HHGFNDSXY:1:1444:8223:30671 1:N:0:GAACCTAG+TCCGCATA
AGAGAGCGACTTCTCTCGGGCCCACAGCCTTACAGCTGTGGAGCACGGTATCCTCTGCCGAAACAGAG
GTTGGACAAGACCGGAGGGGTCTCCTAGTTCCAAAGGAGATGTACTCCGGGCTTGTTCACG
+
FFFFFFFFFFFFFFFFFFFFFFFFFFFFFFFFFFFFFFFFFFFFFFFFFFFFFFFFFFFFFFFFFFFF
FFFFFFFFFFFFFFFFFFFFFFFFFFFFFFFFFFFFFFFFFFFFFFFF:FFFFFFFFFFFF
@A00155:342:HHGFNDSXY:1:1445:2636:21214 1:N:0:GAACCTAG+TCCGCATA
AATGCCGAGAGGGATGTGGAGGGCGAGAGAGAGCGACTTCTCTCGGGCCCACAGCCTTACAGCTGTGG
AGCACGGTATCCTCTGCCGAAACAGAGGTTGGACAAGACCGGAGGGGTCTCCTAGTTCCAAAGGAGAT
+
FFFFFFFFFFFFFFFFFFFFFFF:FFF:FFFFFF:FFFFFFFFFFFFFFFF:FFFFFFFFFFFFFFFF
FFFFFFFFFFFFFFFFFFFFFFFFFFFFFFFFFFFF,FFFFFFFF:FFFFFFFFFFFFFFFFF:F:FF
@A00155:342:HHGFNDSXY:1:1445:23701:28291 1:N:0:GAACCTAG+TCCGCATA
CTTCTCTCGGGCCCACAGCCTTACAGCTGTGGAGCACGGTATCCTCTGCCGAAACAGAGGTTGGACAA
GACCGGAGGGGTCTCCTAGTTCCAAAGGAGATGTACTCCGGGCTTGTTCACGACCTACCGTGTAA
+
FFFFFFFFFFFFFFFFFFFFFF:FFFFFFFFFFFFFFFFFFFFFFFFFFFFFFFFFFFFF:FFFFFFF
FFFFFFFFFFFFFFFFFFFFFFFFFFFFFFFFFFFFFFFFFFFFFFFFFFFFFFFFFFFFFFFFF
@A00155:342:HHGFNDSXY:1:1446:8603:12132 1:N:0:GAACCTAG+TCCGCATA
GGATGTGGAGGGCGAGAGAGAGCGACTTCTCTCGGGCCCACAGCCTTACAGCTGTGGAGCACGGTATC
CTCTGCCGAAACAGAGGTTGGACAAGACCGGAGGGGTCTCCTAGTTCCAAAGGAGATGTA
+
FFFFFFFFFFFFFFFFFFFFFF:FFFFFFFFFFFFFFFFFFFFF,FFFFFFFFFFFFFFFFFFFFFFF
FFFFFFFFFFFFFFFFFFFFFFFF:FFFFFFFFFFFFFFFFFFFFFFFFFFFFFFFFFF
@A00155:342:HHGFNDSXY:1:1446:12915:22044 1:N:0:GAACCTAG+TCCGCATA
TTAGATCAGCTGTGTCCACTTAAGGACTCACCTCTGGCCTATGGTCATCTCAATGCCGAGAGGGATGT
GGAGGGCGAGAGAGAGCGACTTCTCTCGGGCCCACAGCCTTACAGCTGTGGAGCACGGTATCCTCTGC
+
FFFFFFFFFFFFFFFFFFFFFFFFFFFFFFFFFFFFFFFF:FFFFFFFFFFFFFFFFFFFFFFFFFFF
FFFFFFFFFFFFFFFFFFFFFFFFFFFFFFFFFFFFFFFFFFFFFFFFFFFFFFFFFFFFFFFFFFFF
@A00155:342:HHGFNDSXY:1:1446:11379:36166 1:N:0:GAACCTAG+TCCGCATA
CTCTGGCCTATGGTCATCTCAATGCCGAGAGGGATGTGAAGGGCGAGAGAGAGCGACTTCTCTCGGGC
CCACAGCCTTACAGCTGTGGAGCACGGTATCCTCTGCCGAAACAGAGGTTGGACAAGACCGGAGGGGT
+
FFFFFFFFFFFFFFFFFFFFFFFFFFFFFFFFFFFFFFFFFFFFFFFFFFFFFFFFFFFFFFFFFFFF
FFFFFFFFFFFFFFFFFFFFFFFFFFFFFFFFFFFFFFFFFFFFFFFFFFFFFFFFFFFFFFFFFFFF
@A00155:342:HHGFNDSXY:1:1447:8250:11522 1:N:0:GAACCTAG+TCCGCATA
GGATGTAGAGGGCGAGAGAGAGCGACTTCTCTCGGGCCCACAGCCTTACAGCTGTGGAGCACGGTATC
CTCTGCCGAAACAGAGGTTGGACAAGACCGGAGGGGTCTCCTAGTTCCAAAGGAGATGTACTCC
+
FFFFFFFFFFFFFFFFFFF:FFFFFFFFFFFFFFFFF:FFFFFFFFFFFFFFFFFFFFFFFFFFFFFF
FFFFFFFFFFFFFFFFFFFFFFFFFFFFFFFFFFFFFFFFFFF,FFFFFFFFFFFFFFFFFFFF
@A00155:342:HHGFNDSXY:1:1447:3332:15718 1:N:0:GAACCTAG+TCCGCATA
AGAGAGAGCGACTTCTCTCGGGCCCACAGCCTTACAGCTGTGGAGCACGGTATCCTCTGCCGAAACAG
AGGTTGGACAAGACCGGAGGGGTCTCCTAGTTCCAAAGGAGATGTACTCCGGGCTTGTTCACGACCTA
+
FFFFFFFFFFFFFFFFFFFFFFFFFFFFFFF:FFFFFFFFFFFFFFFFF:F:FFF:FFFFFFFFFFFF
FFFFFFFFFFFFFFFFFFFFFFFFFFFFFFFFFFFFFFFFFFFFFFFFFFFFFFFFFFFFFFFFFFFF
@A00155:342:HHGFNDSXY:1:1447:6849:17206 1:N:0:GAACCTAG+TCCGCATA
TGTCCACTTAAGGACTCACCTCTGGCCTATGGTCATCTCAATGCCGAGAGGGATGTGGAGGGCGAGAG
```

```
AGAGCGACTTCTCTCGGGCCCACAGCCTTACAGCTGTGGAGCACGGTATCCTCTGCCGAAACAGAGGT
+
FFFFFFFFFFFFFFFFFFFFFFFFFFFFFFFFFFFFFFFFFFFFFFF:FF:FFFF:FFFFFFFFFFF
FFFFFFFFFFFFFFFFFFFFFFFFFFFFF,FFFFFFFFFFFFFFFF:FFFFFFFFFFFFFFFFF:FFF
@A00155:342:HHGFNDSXY:1:1447:25500:26240 1:N:0:GAACCTAG+TCCGCATA
TTAAGGACTCACCTCTGGCCTATGGTCATCTCAATGCCGAGAGGGATGTGGAGGGCGAGAGAGAGCGA
CTTCTCTCGGGCCCACAGCCTTACAGCTGTGGAGCACGGTATCCTCTGCCGAAACAGAGGTTGGACAA
+
FFFFFFFFFFFFFFFFFFFF:FFFFFFFFFFFFFFFFFFFFFFFFFFFFFFFFFFFFFFFFFFFFFFF
FFFFFFFFFFFFFFFFFFFFFFFFFFFFFFFFFFFFFFFFFFFFFFFFFFFFFFFFFFFFFFFFFFFF
@A00155:342:HHGFNDSXY:1:1448:17698:12790 1:N:0:GAACCTAG+TCCGCATA
AAGGACTCACCTCTGGCCTATGGTCATCTCAATGCCGAGAGGGATGTAGAGGGCGAGAGAGAGCGACT
TCTCTCGGGCCCACAGCCTTACAGCTGTGGAGCACGGTATCCTCTGCCGAAACAGAGGTTGGACAAGA
+
FFFFFFFFFFFFFFFFFFFFFFFFFFFFFFFFFFFFFFFFFFFFF:FFFFFFFFFFFFFFFFFFFFFFF
FFFFFFFFFFFFFFFFFFFFFFFFFFFFFFFFFF:FFFFFFFFFFFFFFFFFFFFFFFFFFFFFFFFF
@A00155:342:HHGFNDSXY:1:1448:18005:13166 1:N:0:GAACCTAG+TCCGCATA
AAGGACTCACCTCTGGCCTATGGTCATCTCAATGCCGAGAGGGATGTAGAGGGCGAGAGAGAGCGACT
TCTCTCGGGCCCACAGCCTTACAGCTGTGGAGCACGGTATCCTCTGCCGAAACAGAGGTTGGACAAGA
+
FFFFFFFFFFFFFFFFFFFFFFFFFFFFFFFFFFFFFFFFFFFFF:FFFFFFFFFFFFFFFFFFFFFFF
FFFFFFFFFFFFFFFFFFFFFFFFFFFFFFFFFFFFFFFFFFFFFFFFFFFFFFFFFFFFFFFFFFFF
@A00155:342:HHGFNDSXY:1:1448:29098:18161 1:N:0:GAACCTAG+TCCGCATA
CTATGGTCATCTCAATGCCGAGAGGGATGTGGAGGGCGAGAGAGAGCGACTTCTCTCGGGCCCACAGC
CTTACAGCTGTGGAGCACGGTATCCTCTGCCGAAACAGAGGTTGGACAAGACCGGAGGGGTCTCCTAG
+
FF:FFFFFFFFFFFFFFFFFFFFF:FF:F:FFFFFFFFFFFFFFFFFFFFFFFFFFFFFFFFFFFFFF
FFFFFFFFFFFFFFFFFFFFFFFFFFFFFFFFFFFFFFFFFFFFFFFFFFFFFFFFFFFFFFFFFF:F
@A00155:342:HHGFNDSXY:1:1449:8250:10081 1:N:0:GAACCTAG+TCCGCATA
CTTCTCTCGGGCCCACAGCCTTACAGCTGTGGAGCACGGTATCCTCTGCCGAAACAGAGGTTGGACAA
GACCGGAGGGGTCTCCTAGTTCCAAAGGAGATGTACTCCGTGCTTGTTCACGACCTACCGTGTAAGTC
+
FFFFFFFFFFFFFFFFFFFFFFFFFFFFFFFFFFFFFFFFFFFFFFFFFFFFFFFFFFFFFFFFFFFF
F:FFFFFFFFFFFFFFFFFFFFFFFFFFFFFFFFFFFFFFFFFFFFFFFFFFFFF:FFFFFFFFFFF
@A00155:342:HHGFNDSXY:1:1449:13304:36213 1:N:0:GAACCTAG+TCCGCATA
CCTTACAGCTGTGGAGCACGGTATCCTCTGCCGAAACAGAGGTTGGACAAGACCGGAGGGGTCTCCTG
GTTCCAAAGGAGATGTACTCCGGGCTTGTTCACGACCTACCGTGTAAGTCGTAGTCTAGTAGGCTACC
+
FFFFFFFFFFFFFFFFFFFFFFFFFFFFFFFFFFFFFFFFFFFFFFFFFFFFFFFFFFFFFFFFFFFF
FFFFFFF:FFFFF:FFFFFFFFFFFFFFFFFFFFFFFFFFFFFFFFFFFFFFFFFFFFFFFFFFFFFF
@A00155:342:HHGFNDSXY:1:1450:27425:2613 1:N:0:GAACCTAG+TCCGCATA
GTCATCTCAATGCCGAGAGGGATGTAGAGGGCGAGAGAGAGCGACTTCTCTCGGGCCCACAGCCTTAC
AGCTGTGGAGCACGGTATCCTCTGCCGAAACAGAGGTTGGACAAGACCGGAGGGGTCTCC
+
FFFFFFFFFFFFFFFFFFFFFFFFFFFFFFFFFFFFFFFFFFFFFFFFFFFFFFFFFFFFFFFFFFFF
FFFFFFFFFFFFFFFFFFFFFFFFFFFFFFFFFFFFFFFFFFFFF:FFFFFFFFFFFFFFF
@A00155:342:HHGFNDSXY:1:1450:5240:2863 1:N:0:GAACCTAG+TCCGCATA
TGTGTCCACTTAAGGACTCACCTCTGGCCTATGGTCATCTCTATGCCGAGAGGGATGTGGAGGGCGAG
AGAGAGCGACTTCTCTCGGGCCCACAGCCTTACAGCTGTGGAGCACGGTATCCTCTGCCGAAACAGAG
+
FFFFFFFFFFFFFFFFFFFFFFFFFFFF:FFF,FFFFFFFFFFFFF::FFFFFFFFFFFFFFFFFFFF
:FFFFFFFFFFFFFFFF:F,FFFFFFFFFFFFFFFFFFFFFFFFFFFFFFFFFFFFFFFFFFFFFFFF
@A00155:342:HHGFNDSXY:1:1450:31521:9486 1:N:0:GAACCTAG+TCCGCATA
TCTCTCGGGCCCACAGCCTTACAGCTGTGGAGCACGGTATCCTCTGCCGAAACAGAGGTTGGACAAGA
```

CCGGAGGGGTCTCCTAGTTCCAAAGGAGATGTACTCCGGGCTTGTTCACGACCTACCGTGTAAGTCGT
+
FFFFFFFFFFFFFFFFFFFFFFFFFFFFFFFFFFFFF:FFFFFFFFFFFFFFFFFFFFFFFFFFFFFF
FFFFFFFFFFFFFFFFFFFFFFFFFFFFFFFFFFFFFFFFFFFFFFFFFFFFFFFFFFFFFFF:FFFFF
@A00155:342:HHGFNDSXY:1:1450:31485:9518 1:N:0:GAACCTAG+TCCGCATA
TCTCTCGGGCCCACAGCCTTACAGCTGTGGAGCACGGTATCCTCTGCCGAAACAGAGGTTGGACAAGA
CCGGAGGGGTCTCCTAGTTCCAAAGGAGATGTACTCCGGGCTTGTTCACGACCTACCGTGTAAGTCGT
+
FFFFFFFFFFFFFFFFFFFFFFFFFFFFFFFFFFFFF:FFFFFFFFFFFFFFFFFFFFFFFFFF:F:F
FFFFFFFFFFFFFF:FFFFFFFFFFFFFFFFFFFFF:FFFFFFFFFFFFFFFFFFFFFFFFFFFFFFFF
@A00155:342:HHGFNDSXY:1:1450:12409:16376 1:N:0:GAACCTAG+TCCGCATA
AATGCCGAGAGGGATGTGGAGGGCGAGAGAGAGCGACTTCTCTCGGGCCCACAGCCTTACAGCTGTGG
AGCACGGTATCCTCTGCCGAAACAGAGGTTGGACAAGACCGGAGGGGTCTCCTAGTTCCAAAGGAGAT
+
FFFFFFFFFFFFFFFFFFFFFFFFFFFFFFFFFFFFFFFFFFFFFFFFFFFFFFFFFFFFFFFFFFFF
FFF:FFFFFFFFFFFFFFFFFFFFFFFFFFFFFFFFFFFFFFFFFFFFFFFFFFFFFFFFFFFFFFFF
@A00155:342:HHGFNDSXY:1:1450:9100:16783 1:N:0:GAACCTAG+TCCGCATA
ACTTCTCTCGGGCCCACAGCCTTACAGCTGTGGAGCACGGTATCCTCTGCCGAAACAGAGGTTGGACA
AGACCGGAGGGGTCTCCTAGTTCCAAAGGAGATGTACTCCGGGCTTGTTCACGACCTACCGTGTAAGT
+
FFFFFFFFFFFFFFFFFFFFFFFFFFFFFFFFFFFFFFFFFFFFFFFFFFFFF:FFFFFFFFFFFFFF
FFFFFFFFFFFFFFFFFFFFFFFFFFFFFFFFFFFFFFFFFFFFFFFFFFFFFFFFFFFFFFFFF,FF
@A00155:342:HHGFNDSXY:1:1450:5312:17362 1:N:0:GAACCTAG+TCCGCATA
AGATCAGCTGTGTCCACTTAAGGACTCACCTCTGGCCTATGGTCATCTCAATGCCGAGAGGGATGTAG
AGGGCGAGAGAGAGCGACTTCTCTCGGGCCCACAGCCTTACAGCTGTGGAGCACGGTATCCTCTGCCG
+
FFFFFFFFFFFFFFFFFFFFFFFFFFFF:FFFFFFFFFFFFFFFFFFFFFFFFFFFFFFFFFFF,F,FF
FFFFFFFFFFFFFFFFFFFFFFFFFFFFFFFFFFFFFFFFFFFFFFFFFFFFFFFFFFFFFFFFFFFF
@A00155:342:HHGFNDSXY:1:1451:10999:4100 1:N:0:GAACCTAG+TCCGCATA
GAGCGACTTCTCTCGGGCCCACAGCCTTACAGCTGTGGAGCACGGTATCCTCTGCCGAAACAGAGGTT
GGACAAGACCGGAGGGGTCTCCTAGTTCCAAAGGAGATGTACTCCGGGCTTGTTCACGACCTACCGTG
+
FFFFFFFFFFFFFFFFFFFFFF:FFFFFFFFFFFFFFFFFFFFFFFFFFFFFFFFFFFFFFFFFFFFF
FFFFFFFFFFFFFFFFFFFFFFFFFFFF:FFFFFFFFFFFFFFFFFFFFFFFFFFFFFFFFFF:FFFFF
@A00155:342:HHGFNDSXY:1:1451:11812:5040 1:N:0:GAACCTAG+TCCGCATA
GGCCAGAGGTGAGTCCTTAAGTGGACACAGCTGATCTAAGGCGGTGTGGCGGGGCATGGGTTTTAACC
CCCATGACGGTCGGAGTCTAGTAGGCTCCCTGATGAGTCCGTTCCAAGGACGAAACCGTCGTTACTCT
+
FFFFFFFFFFFFFFFFFFFFFFFFFFFFFFFFFFFFFFFFFFFFFFFFFFFFFFFFFFFFFFFFFFFF
FFFFFFFFFFFFFFFFFFFFFFFFFFFFFFFFFFFF:FFFFFFFFFFFFFFFF,FFFFFFFFFFFFFF
@A00155:342:HHGFNDSXY:1:1451:4390:11318 1:N:0:GAACCTAG+TCCGCATA
CAATGCCGAGAGGGATGTGGAGGGCGAGAGAGAGCGACTTCTCTCGGGCCCACAGCCTTACAGCTGTG
GAGCACGGTATCCTCTGCCGAAACAGAGGTTGGACAAGACCGGAGGGGTCTCCTAGTTCCAAAGGAGA
+
FFFFFFFFFFFFFFFFFFFFFFFF,FFFFFFFFFFFFFFFFFFFFFFFFFFFFFFFFF,FFFFFFFFFFF
FFFFFFFFFFFFFFFFFFFFFFFFFFFFFFFFFFFFF,FFFFFFFFFFFFFFFFFFFFFFFFFFFFFFF
@A00155:342:HHGFNDSXY:1:1451:16179:17331 1:N:0:GAACCTAG+TCCGCATA
GCGAGAGAGAGCGACTTCTCTCGGGCCCACAGCCTTACAGCTGTGGAGCACGGTATCCTCTGCCGAAA
CAGAGGTTGGACAAGACCGGAGGGGTCTCCTAGTTCCAAAGGAGATGTACTCCGGGCTTGTTCACGAC
+
FFFFFFFFFFFFFFFFFFFFFFFFFFFFF:FFFFFFFF,FFFFFFFFFFFFFFFFFFFFFFFFFFFFFF
FFFFFFFFFFFF:FFFFFFFFFFF:FFFFFFFFFFFFFFFFFFFFFFFFFFFFFFFFFFFFFFFFFFFF
@A00155:342:HHGFNDSXY:1:1451:27715:27978 1:N:0:GAACCTAG+TCCGCATA
AAGGACTCACCTCTGGCCTATGGTCATCTCAATGCCGAGAGGGATGTGAAGGGCGAGAGAGAGCGACT

TCTCTCGGGCCCACAGCCTTACAGCTGTGGAGCACGGTATCCTCTGCCGAAACAGAGGTTGGACAAGA
+
FFFFFFFFFFFFFFFFFFFFFFFFFFFFFFFFFFFFFFFFFFF:FFFFFFFFFFFFFFFFFFFFFFFFFFFF
FFFF:FFFFFFFFFFFFFFFFFFFFFFFFFFFFFFFF:FFFF:FFFFFFFFFFFFFFFFFFFFFFFFFFFFFF
@A00155:342:HHGFNDSXY:1:1451:11840:29418 1:N:0:GAACCTAG+TCCGCATA
CTTCTCTCGGGCCCACAGCCTTACAGCTGTGGAGCACGGTATCCTCTGCCGAAACAGAGGTTGGACAA
GACCGGAGGGGTCTCCTAGTTCCAAAGGAGATGTACTCCGGGCTTGTTCACGACCTACCGTGTAAGTC
+
FFFFFFFFFFFFFFFFFFFF,FFFF:FFF,FFFFF:FF:FFFFFFFFFFFFFFFFFFF:FFFFFFFFF
:FFFFFFFF,FFFFF:F:FFFFFFFFFFFFFF:FFFFFF,FFFF:FFFFFFFFFFFFFFFFFFFFFFFF
@A00155:342:HHGFNDSXY:1:1451:18213:31657 1:N:0:GAACCTAG+TCCGCATA
GAGCGACTTCTCTCGGGCCCACAGCCTTACAGCTGTGGAGCACGGTATCCTCTGCCGAAACAGAGGTT
GGACAAGACCGGAGGGGTCTCCTAGTTCCAAAGGAGATGTACTCCGGGCTTGTTCACGACCT
+
FFFFFFFFF:,FFFFFFFF,FFFFFFFFFFFFFFF,FFFFFFFFFFFFFFFFFFFFFFFFFFF:FFFFF
FFFFFF:FFFFFFFFFFFFF:FFFFFFFFFFFFFF:FFFFFFF:F::FFFFFFFFF:FFFFFF
@A00155:342:HHGFNDSXY:1:1451:23647:35149 1:N:0:GAACCTAG+TCCGCATA
CTCTGGCCTATGGTCATCTCAATGCCGAGAGGGATGTGGAGGGCGAGAGAGAGCGACTTCTCTCGGGC
CCACAGCCTTACAGCTGTGGAGCACGGTATCCTCTGCCGAAACAGAGGTTGGACAAGACCGGAGGGGT
+
FFFFFFFFFFFFFFFFFFFFFFFFFFFF:FFFFFFFFFFFFFFFFFFFFFFFFFFFFFFFFFFFFFFFFF
FFFFFFFFFFFFFFFFFFFFFFFFFFFFFFFFFFFFFFFFFFFFFFFFFFFFFFFFFFFFFFFFFFFF
@A00155:342:HHGFNDSXY:1:1452:30418:1031 1:N:0:GAACCTAG+TCCGCATA
AGGGATGTGGAGGGCGAGAGAGAGCGACTTCTCTCGGGCCCACAGCCTTACAGCTGTGGAGCACGGTA
TCCTCTGCCGAAACAGAGGTTGGACAAGACCGGAGGGGTCTCCTAGTTCCAAAGGAGATGTACTCCGG
+
FFFFFFFFFFFFFFFFFFFFFFFFFFFFFFFFFFFFFFFFFFFFFFFFFFFFFFFFFFFFFFFFFFFF
FFFFFFFFFFFFFFFFFFFFFFFFFFFFFFFFFFFFFFFFFFFFFFFFFFFFFFFFFFFFFFFFFFFF
@A00155:342:HHGFNDSXY:1:1452:7609:31172 1:N:0:GAACCTAG+TCCGCATA
CAAGCCCGGAGTACATCTCCTTTGGAACTAGGAGACCCCTCCGGTCTTGTCCAACCTCTGTTTCGGCA
GAGGATACCGTGCTCCACAGCTGTAAGGCTGTGGGCCCGAGAGAAGTCGCTCTCTCTCGCCCTCCACA
+
FFFFFFFFFFFFFFFFFFFFFFFFFFFFFFFFFFFFFFFFFFFFFFFFFFFFFFFFFFFFFFFFFFFF
FFFFFFFFFFFFFFFFFFFFFFFFFFFFFFFFFFFFFFFFFFFFF:FFFFFFFFFFFFFFFFFFFFFFF
@A00155:342:HHGFNDSXY:1:1452:7581:31187 1:N:0:GAACCTAG+TCCGCATA
CAAGCCCGGAGTACATCTCCTTTGGAACTAGGAGACCCCTCCGGTCTTGTCCAACCTCTGTTTCGGCA
GAGGATACCGTGCTCCACAGCTGTAAGGCTGTGGGCCCGAGAGAAGTCGCTCTCTCTCGCCCTCCACA
+
FFFFFFFFFFFFFFFFFFFFFFFFFFFFFFFFFFFFFFFFFFFFFFFFFFFFFFFFFFFFFFFFFFFF
F:FFFFFFFFFFFFFFFFFFFFFFFFFFFFFFFFFFFFFFFFFFFFFFFFFFFFFFFFFFFFFFFFFF
@A00155:342:HHGFNDSXY:1:1453:30029:1047 1:N:0:GAACCTAG+TCCGCATA
TGGTCATCTCAATGCCGAGAGGGATGTGGAGGGCGAGAGAGAGCGACTTCTCTCGGGCCCACAGCCTT
ACAGCTGTGGAGCACGGTATCCTCTGCCGAAACAGAGGTTGGACAAGACCGGAGGGGTCTCCTAGTTC
+
FFFFFFFFFFFFFFFFFFFFFFFFF,FFFFFFFFFFFFFFFFFFFFFFFFFFFFFFFFFFFFFFFFFF:
FFFFFFFFFFFFFFFFFF,FFFFFFFFFFFFFF:FFFFFFFFFFF::FFFFFFFFFFFFFFFF:FFFF
@A00155:342:HHGFNDSXY:1:1453:15176:11271 1:N:0:GAACCTAG+TCCGCATA
AGAGAGAGCGACTTCTCTCGGGCCCACAGCCTTACAGCTGTGGAGCACGGTATCCTCTGCCGAAACAG
AGGTTGGACAAGACCGGAGGGGTCTCCTAGTTCCAAAGGAGATGTACTCCGGGCTTGTTCACGAC
+
FFFFFFFFFFFFFFFFFFFFFFFFFFFF:FFFFFFFFFFFFFFFFFFFFFFFFFFFFFFFFFFFFFFFF
FFFFFFFFFFFFFFFFFFFFFFFFFFFFFF:FFFFF:FFFFFFFFFFFFFFFFFFFFFFFFFFFFF
@A00155:342:HHGFNDSXY:1:1453:17571:12164 1:N:0:GAACCTAG+TCCGCATA
GGATGTGGAGGGCGAGAGAGAGCGACTTCTCTCGGGCCCACAGCCTTACAGCTGTGGAGCACGGTATC

```
CTCTGCCGAAACAGAGGTTGGACAAGACCGGAGGGGTCTCCTAGTTCCAAAGGAGATGTACTCC
+
FFFFFFFFFFFFFFFFFFFFFFFFFFFFFFFFFFFFFFFFFFFFFFFFFFFFFFFFFFFFFFFFFFFF
FFFFFFFFFFFF:FFFFFFFFFFFFFFFFFFFFFFFFFFFFFFFFFFFFF:FFFFFFFFFFFFFF
@A00155:342:HHGFNDSXY:1:1453:24135:21966 1:N:0:GAACCTAG+TCCGCATA
TCCTCTGCCGAAACAGAGGTTGGACAAGACCGGAGGGGTCTCCTAGTTCCAAAGGAGATGTACTCCGG
GCTTGTTCACGACCTACCGTGTAAGTCGTAGTCTAGTAGGCTACCTGACGAGTCCTTTTTAGGACGAA
+
FFFFFFFFFFFFFFFFFFFFFFFFFFFFFFFFFFFFFFFFFFFFFFFFFFFFFFFFFFFFFFFFFFFF
FFFFFFFFFFFFFFFFFFFFFFFFFFFFFFFFFFFFFFFFFFFFFFFFFFFFFFFFFFFFFFFFFFFF
@A00155:342:HHGFNDSXY:1:1454:5086:22044 1:N:0:GAACCTAG+TCCGCATA
CTCCTCTGGCCTATGGTCATCTCAATGCCGAGAGGGATGTGGAGGGCGAGAGAGAGCGACTTCTCTCG
GGCCCACAGCCTTACAGCTGTGGAGCACGGTATCCTCTGCCGAAACAGAGGTTGGACAAGACCGGAGG
+
FFFFFFFFFFFFFFFFFFFFFFFFFFFFFFFFFFFF:FF:FFFFFFFFFFFFFFFFFFFFFFFFFFFF
FFFFFFFFFFFFFFFFFFFFFFFFFFFFFFFFFFFFFFFFFFFFFFFFFFFFFFFFFFFF:FFFFFFF
@A00155:342:HHGFNDSXY:1:1454:10926:34256 1:N:0:GAACCTAG+TCCGCATA
GAGAAGGATGTGGAGGGCGAGAGAGAGCGACTTCTCTCGGGCCCACAGCCTTACAGCTGTGGAGCACG
GTATCCTCTGCCGAAACAGAGGTTGGACAAGACCGGAGGGGTCTCCTAGTTCCAAAGGAGATGTACTC
+
FFFFFFFFFFFFFFFFFFFFFFFFFFFFFFFFFFFFFFFFFFFFFFFFFFFFFFFFFFFFFFFFFFFF
FFFFFFFFFFFFFFFFFFFFFFFFFFFFFFFFFFFFFFFFFFFFFFFFFFFFFFFFFFFFFFFFFFFF
@A00155:342:HHGFNDSXY:1:1454:28122:36104 1:N:0:GAACCTAG+TCCGCATA
TTCTCTCGGGCCCACAGCCTTACAGCTGTGGAGCACGGTATCCTCTGCCGAAACAGAGGTTGGACAAG
ACCGGAGGGGTCTCCTAGTTCCAAAGGAGATGTACTCCGGGCTTGTTCACGACCTACCGTGTAAGTCG
+
FFFFFFFFFFFFFFFFFFFFFFFFFFFFFFFFFFFFFFFFFFFFFFFFFFFFFFFFFFFFFFFFFFFF
FFFFFFFFFFFFFFFFFFFFFFFFFFFFFFFFFFFFFFFFFFFFFFFFFFFFFFFFFFFFFFFFFFFF
@A00155:342:HHGFNDSXY:1:1455:28592:7858 1:N:0:GAACCTAG+TCCGCATA
CAGCTGTGGAGCACGGTATCCTCTGCCGAAACAGAGGTTGGACAAGACCGGAGGGGTCTCCTGGTTCC
AAAGGAGATGTACTCCGGGCTTGTTCACGACCTACCGTGTAAGTCGTAGTCTAGTAGGCTACCTGACG
+
FFFFFFFFFFFFFFFFFFFFFFFFFFFFFFFFFFFFFFFFFFFFFFFFFFFFFFFFFFFFF:FFFFFF
FFFFFFFFFFFFFFFFFFFFFFFFFFFFFFFFFFFFF:FFFFFFFFFFF:FFFFFFFFFF,FFFFFFF
@A00155:342:HHGFNDSXY:1:1455:6424:18411 1:N:0:GAACCTAG+TCCGCATA
GTCATCTCAATGCCGAGAGGGATGTAGAGGGCGAGAGAGAGCGACTTCTCTCGGGCCCACAGCCTTAC
AGCTGTGGAGCACGGTATCCTCTGCCGAAACAGAGGTTGGACAAGACCGGAGGGGTCTCCTAGTTCCA
+
FFFFFFFFFFFFFFFFFFFFF,,F,F:FF:FFFFFFFFFF,FFFF:FFFFFFFF:FFFFFFFFFF:F
:FFFFFFFFFFFFFFFFFFFFFFFFFFFF:FF:FFFFF,FFFFFFFFFFFF:FFFFFFFFFFFFFFFF
@A00155:342:HHGFNDSXY:1:1455:14705:25144 1:N:0:GAACCTAG+TCCGCATA
AGAGAGAGCGACTTCTCTCGGGCCCACAGCCTTACAGCTGTGGAGCACGGTATCCTCTGCCGAAACAG
AGGTTGGACAAGACCGGAGGGGTCTCCTAGTTCCAAAGGAGATGTACTCCGGGCTTGTTCACGACCTA
+
FFFFFFFFFFFFFFFFFFFFFFFFFFFFFFFFFFFFFFFFFFFFFFFFFFFFFFFFFFFFFFFFFFFF
FFFFFFFFFFFFFFFFFFFFFFFFFFFFFFFFFFFFFFFFFFFF:FFFFFFFFFFFFFFFFFFFFFFF
@A00155:342:HHGFNDSXY:1:1455:10339:26819 1:N:0:GAACCTAG+TCCGCATA
TTAGATCAGCTGTGTCCACTTAAGGACTCACCTCTGGCCTATGGTCATCTCAATGCCGAGAGGGATGT
GGAGGGCGAGAGAGAGCGACTTCTCTCGGGCCCACAGCCTTACAGCTGTGGAGCACGGTATCCTCTGC
+
FFFFFFFFFFFFFFFFFFFFFFFFFFFFFFFFFFFFFFFFFFFFFFFFFFFFFFFFFF:F:FFFFFFF
FFFFFFFFFFFFFFFFFFFFFFFFFFFFFFFFF:FFFFFFFFFFFFFFFFFFFFFFFFFFFFFFFFFF
@A00155:342:HHGFNDSXY:1:1455:23267:33927 1:N:0:GAACCTAG+TCCGCATA
TTACAGCTGTGGAGCACGGTATCCTCTGCCGAAACAGAGGTTGGACAAGACCGGAGGGGTCTCCTAGT
```

```
TCCAAAGGAGATGTACTCCGGGCTTGTTCACGACCTACCGTGTAAGTCGTAGTCTAGTAGGCTACCTG
+
:FFFFFFFFFFFFFFFFFFFFFFFFFFFFFFFFFFFFFFFFFFFFFFFFFFFFFFFFFFFFFFFFFFF
FFFFFFFFFFFFFFFFFFFFFFFFFFFFFFFFFFFFFFFFFFFFFFFFFFFFFFFFFFFFFFFFFFFF
@A00155:342:HHGFNDSXY:1:1456:10601:14465 1:N:0:GAACCTAG+TCCGCATA
ACTTCTCTCGGGCCCACAGCCTTACTGCTGTGGAGCACGGTATCCTCTGCCGAAACAGAGGTTGGACA
AGACCGGAGGGGTCTCCTAGTTCCAAAGGAGATGTACTCCGGGCTTGTTCACGACCTACCGTGTAAGT
+
FFFFFFFFFFFFFFFFFFFFFFFFFFFFFFFFFFFFFFFFFFFFFFFFFFFFFFFFFFFFFFFFFFFF
FFFFFFFFFFFFFFFFFFFFFFFFFFFFFFFFFFFFFFFFFFFFFFFFFFFFFFFFFFFFFFFFFFFF
@A00155:342:HHGFNDSXY:1:1457:19922:8155 1:N:0:GAACCTAG+TCCGCATA
ATCTCAATGCCGAGAGGGATGTAGAGGGCGAGAGAGAGCGACTTCTCTCGGGCCCACAGCCTTACAGC
TGTGGAGCACGGTATCCTCTGCCGAAACAGAGGTTGGACAAGACCGGAGGGGTCTCCTAGTTCCAAAG
+
FFFFFFFFFFFFFFFFFFF,FFFFFFFFFFFFFFFFFFFFFFFFFFFFFFFFFFFFFFFFFFFFFFFF
FFFFFFFFFFFFFFFFFFFFFFFFFFFFFFFFFFFFFFFFFFFFFFFFFFFFFFFFFFFFFFFFFF:F
@A00155:342:HHGFNDSXY:1:1457:17481:13228 1:N:0:GAACCTAG+TCCGCATA
ACCTCTGTTTCGGCAGAGGATACCGTGCTCCACAGCTGTAAGGCTGTGGGCCCGAGAGAAGTCGCTCT
CTCTCGCCCTCCACATCCCTCTCGGCATTGAGATGACCATAGGCCAGAGGAGAGTCCTTAAGTGGACA
+
FFFFFFFFFFFFFFFFFFFFFFFFFFFFFFFFFFFF:FFFFFFFFFFFFFFFFFFFFFFFFFFFFFFF
FFFFFFFFFFFFFFFFFFFFFFFFFFF,FFFFFFFFFFFFFFFFFFFFFFFFFFFFFFFF:FFFFFFFF
@A00155:342:HHGFNDSXY:1:1457:27796:25927 1:N:0:GAACCTAG+TCCGCATA
CACCTCTGGCCTATGGTCATCTCAATGCCGAGAGGGATGTGAAGGGCGAGAGAGAGCGACTTCTCTCG
GGCCCACAGCCTTACAGCTGTGGAGCACGGTATCCTCTGCCGAAACAGAGGTTGGACAAGACCGGAG
+
FFFFFFFFFFFFFF:FFFFFFFFFFFFFFFFFFFFFFFF:FFFFFFFFFFFFFFFFFFFFFFFFFFFF
FFFFFFFFFFFFFFFFFFFFFFFFFFFFFFFFFFFFFFFFFFFFFFFFFF:FFFFFFF:FFFFF:F
@A00155:342:HHGFNDSXY:1:1458:1289:14215 1:N:0:GAACCTAG+TCCGCATA
TTAAGGACTCTCCTCTGGCCTATGGTCATCTCAATGCCGAGAGGGATGTGGAGGGCGAGAGAGAGCGA
CTTCTCTCGGGCCCACAGCCTTACAGCTGTGGAGCACGGTATCCTCTGCCGAAACAGAGGTTGGACAA
+
FFFFFF:FFFFFFFFFFF:FFFFFFFFFFFFFFFFFFFFFFFFFFFFFF:FFFFFFFFFFFFFFFFFF
FFFFFFFFFFFFFFFFFFFF:FFFFFFFFFFFFFFFFFFF,FFF:FFFFFFFFFFFFFFFF:FF:FFF
@A00155:342:HHGFNDSXY:1:1458:10637:28087 1:N:0:GAACCTAG+TCCGCATA
TTCTCTCGGGCCCACAGCCTTACAGCTGTGGAGCACGGTATCCTCTGCCGAAACAGAGGTTGGACAAG
ACCGGAGGGGTCTCCTAGTTCCAAAGGAGATGTACTCCGGGCTTGTTCACGACCTACCGTGTAAGTCG
+
FFFFFFFFFFFFFFFFFFFFFFFFFFFFFFFFFFFFFFFFFFFFFFFFFFFFFFFFFFFFFFFFFFFF
FFFFFFFFFFFFFFFFFFFFFFFFFFFFFFFFFFFFFFFFFF:FFFFFFFFFFFFFFFFFFFFFFFFF
@A00155:342:HHGFNDSXY:1:1458:25852:33614 1:N:0:GAACCTAG+TCCGCATA
TTACAGCTGTGGAGCACGGTATCCTCTGCCGAAACAGATGTTGGACAAGACCGGAGGGGTCTCCTAGT
TCCAAAGGAGATGTACTCCGGGCTTGTTCACGACCTACCGTGTAAGTCGTAGTCTAGTAGGCTACCT
+
F,FFFFFFFF::FFFFFFFFF:FFFFFFFFF:FFFFFF,,:FFFF:FF,FFFF:FFF:F:FFFFFF,:
FFFF:FFFFFFF,FF,,FF,F:FF,,,FFFF:FFF:FFFF:F:FFFF:FFFFFFFFFFF::FF,:FF
@A00155:342:HHGFNDSXY:1:1459:3613:13823 1:N:0:GAACCTAG+TCCGCATA
ATCCTCTGCCGAAACAGAGGTTGGACAAGACCGGAGGGGTCTCCTGGTTCCAAAGGAGATGTACTCCG
GGCTTGTTCACGACCTACCGTGTAAGTCGTAGTCTAGTAGGCTACCTGACGAGTCCTTTTTAGGACGA
+
FFFFFFFFFFFFFFFFFFFFFFFFFF,FFFFFFFFFFFFFFFFFFFFFFFFFF:FFFFFFFFFFFFFFF
FFFFFFFFFFFFFFFFFFFFFFFFFFFFFFFFFFFFFFFF,:FFFFFFFFFFFFFFFFFFFFFFFFFFF
@A00155:342:HHGFNDSXY:1:1459:6813:15327 1:N:0:GAACCTAG+TCCGCATA
TGTCCACTTAAGGACTCACCTCTGGCCTATGGTCATCTCAATGCCGAGAGGGATGTAGAGGGCGAGAG
```

AGAGCGACTTCTCTCGGGCCCACAGCCTTACAGCTGTGGAGCACGGTATCCTCTGCCGAAACAGAGGT
+
FFFFFFFF:FFFFFFFFFFFFFFFFFFFFFFFFFFFFFFFFFFFFFFFFFFF:FFF:FFFFFFFFFFF
FFFFFFFFFFFFFFFFFFFFFFFFFFFFFFFFFFFFFFFFFFFFFFFFFFFFFFFFFFFFFF:FFFFF
@A00155:342:HHGFNDSXY:1:1459:16857:26616 1:N:0:GAACCTAG+TCCGCATA
TATGGAGGGCGAGAGAGAGCGACTTCTCTCGGGCCCACAGCCTTACAGCTGTGGAGCACGGTATCCTC
TGCCGAAACAGAGGTTGGACAAGACCGGAGGGGTCTCCTAGTTCCAAAGGAGATGTACTCCGGG
+
:FFFFFFFFFFFFFFFFFFFFFFFFFFFFFFFFFFFFFFFFFFFFFFFFFFFFFFFF:FFFF,FFFFF
FFFFFFFFFFFFFFFFFFFFFFFFFFFFFFFFFFFFFFFFFFFFFFFFFFFFFFFFFFFFFFFF
@A00155:342:HHGFNDSXY:1:1459:12391:28902 1:N:0:GAACCTAG+TCCGCATA
ATCTCAATGCCGAGAGGGATGTGGAGGGCGAGAGAGAGCGACTTCTCTCGGGCCCACAGCCTTACAGC
TGTGGAGCACGGTATCCTCTGCCGAAACAGAGGTTGGACAAGACCGGAGGGGTCTCCTAGTTCCAAAG
+
FFFFFFFFFFFFFFFFFFFFFFFFFFFFFFFFFFFFFFFFFFFFFFFFFFFFFFFFFFFFFFFFFFFF
FFFFFFFFFFFFFFFFFFFFFFFFFFFFFFFFFFFFFFFFFFFFFFFFFFFFFFFFFFFFFFFFFFFF
@A00155:342:HHGFNDSXY:1:1459:5918:31626 1:N:0:GAACCTAG+TCCGCATA
CTGTGTCCACTTAAGGACTCACCTCTGGCCTATGGTCATCTCAATGCCGAGAGGGATGTGGAGGGCGA
GAGAGAGCGACTTCTCTCGGGCCCACAGCCTTACAGCTGTGGAGCACGGTATCCTCTGCCGAAACAGA
+
F:,FF:FFFF::FF:FFF:FFFFFFFFFFFFFFFFFFFFFFFF:FF:FF:FFFFFFF,::FFFFFFFFF
FF:FFFFFFFFFFFFFFFFFFFFFFFFFFFFFFFFFFF::FFFFFFFFF::FF::F:FFFFFFFFFFF
@A00155:342:HHGFNDSXY:1:1460:21603:2832 1:N:0:GAACCTAG+TCCGCATA
ACCTCTGGCCTATGGTCATCTCAATGCCGAGAGGGATGTGGAGGGCGAGAGAGAGCGACTTCTCTCGG
GCCCACAGCCTTACAGCTGTGGAGCACGGTATCCTCTGCCGAAACAGAGGTTGGACAAGAC
+
FFFFFFFFFFFFFFFFFFFFFFFFFFFFFFFFFFFFFFFFFFFFFFFFFFFFFFFFFFFFFFFFFFFF
FFFFFFFFFFFFFFFFFFFFFFFFFFFFFFFFFFFFFFFFFFFFFFFFFFFFFFFFFFFFF
@A00155:342:HHGFNDSXY:1:1460:20645:32800 1:N:0:GAACCTAG+TCCGCATA
GGCGAGAGAGAGCGACTTCTCTCGGGCCCACAGCCTTACAGCTGTGGAGCACGGTATCCTCTGCCGAA
ACAGAGGTTGGACAAGACCGGAGGGGTCTCCTAGTTCCAAAGGAGATGTACTCCGGGCTTGTTCACGA
+
FFFFFFFFFFFFFFFFFFFFFFFFFFFFFFFFFFFF:FFFFFFFFFFFFFFFFFFFFFFFFFFFFFFF
FFFFFFFFFFFFFFF,FFFFFFFFFFFFFFFFFFFFFFFFFFFFFFFFFFFFFFFFFFFFFFFFFFFF
@A00155:342:HHGFNDSXY:1:1461:1371:11443 1:N:0:GAACCTAG+TCCGCATA
GGCAGAGGATACCGTGCTCCACAGCTGTAAGGCTGTGGGCCCGAGAGAAGTCGCTCTCTCTCGCCCTT
CACATCCCTCTCGGCATTGAGATGACCATAGGCCAGAGGTGAGTCCTTAAGTGGACACAGCTGA
+
FFFFFFFFFF,FFFFFFFFFFFFFFFFFFFFFFFFFFFF:FFFFFFFFFFFFFFFFFFFFFFFFFFFF
FFFFFFFFFFFFFFFFFFFFFFFFF:FFFFFFFFFFFFFFFFFFFFFFFFFFFFFFFFFFFFFF
@A00155:342:HHGFNDSXY:1:1461:7997:19382 1:N:0:GAACCTAG+TCCGCATA
GGGCGAGAGAGAGCGACTTCTCTCGGGCCCACAGCCTTACAGCTGTGGAGCACGGTATCCTCTGCCGA
AACAGAGGTTGGACAAGACCGGAGGGGTCTCCTAGTTCCAAAGGAGATGTACTCCGGGCTTGTTCACG
+
FFFFFFFFFFFFFFFFFFFFFFFFFFFFF:FFFFFFFFF:FFFFFFFFFFFFFFFFFFFFFFFFFFFF:
FFFFFFFFFFFFFFFFFFFFFFFFFFFFFFFFFFFF:FFFFFFFFFFFFFFFFFFFFFFFFFFFFFFF:
@A00155:342:HHGFNDSXY:1:1461:19732:22514 1:N:0:GAACCTAG+TCCGCATA
CCTCTGGCCTATGGTCATCTCAATGCCGAGAGGGATGTGAAGGGCGAGAGAGAGCGACTTCTCTCGGG
CCCACAGCCTTACAGCTGTGGAGCACGGTATCCTCTGCCGAAACAGAGGTTGGACAAGAC
+
FFFFFFFFFFFFFFFFFFFFFFFFFFFFFFFFFFFFFFFFFFFFFFFFFFFFFFFFFFFFFFFFFFFF
FFFFFFFFFFFFFFFFFFFFFFFFFFFFFFFFFFFFFFFFFFFFFFFFFFFFFFFFFFFF
@A00155:342:HHGFNDSXY:1:1461:10511:24079 1:N:0:GAACCTAG+TCCGCATA
GGGCGAGAGAGAGCGACTTCTCTCGGGCCCACAGCCTTACAGCTGTGGAGCACGGTATCCTCTGCCGA

AACAGAGGTTGGACAAGACCGGAGGGGTCTCCTAGTTCCAAAGGAGATGTACTCCGGGCTTGTTCACG
+
FFFFFFFFFFFFFFFFFFFFFFFFFFFFFFFFFFFFFFFFFFFFFFFFFFF:FFFFFFFFFFFFFFFF
FFFFFFFFFFFFFFFFFFFFFFFFFFFFFFFFFFFFFFFFFFFFFFFFFFFFFFFFFFFFFFFFFFFF
@A00155:342:HHGFNDSXY:1:1461:31060:31767 1:N:0:GAACCTAG+TCCGCATA
GCGACTTCTCTCGGGCCCACAGCCTTACAGCTGTGGAGCACGGTATCCTCTGCCGAAACAGAGGTTGG
ACAAGACCGGAGGGGTCTCCTAGTTCCAAAGGAGATGTACTCCGGGCTTGTTCACGACCTACCGAGT
+
F,:FF,FF:FF,FF,FFFFFFFFF,FFFF,FF::F,FFFFFF:FF:::FFFF,,FFFFFFFF,FF,F,
FFFFFF,F,FFFFFFFFFFFFFF,:FFFFFF,F,FFFFFFFF,FFFF:::FFFF:FFFFFFF:F,FF
@A00155:342:HHGFNDSXY:1:1462:4679:3458 1:N:0:GAACCTAG+TCCGCATA
TATCCTCTGCCGAAACAGAGGTTGGACAAGACCGGAGGGGTCTCCTAGTTCCAAAGGAGATGTACTCC
GGGCTTGTTCACGACCTACCGTGTAAGTCGTAGTCTAGTAGGCTACCTGACGAGTCCTTTTTAGGAC
+
FFFFFFFFFFFFFFFFFFFFFFFFFFFFFFF:FFFFFFF:FFFFFFFFFFFFFFFFFFFFFFFFFFFF
FFFFFFFFFFFFFFFFFFFFFFFFFFFFFFFFFFFFFFFFFFFF,FFFFFFFFFFFFFFFFFFF:FF
@A00155:342:HHGFNDSXY:1:1463:5593:1877 1:N:0:GAACCTAG+TCCGCATA
ATCCTCTGCCGAAACAGAGGTTGGACAAGGCCGGAGGGGTCTCCTAGTTCCAAAGGAGATGTACTCCG
GGCTTGTTCACGACCTACCGTGTAAGTCGTAGTCTAGTAGGCTACCTGACGAGTCCTTTTTAGGACGA
+
FFFFFFFFFFFFFFFFFFFFFFFFFFFFF,FFFFFFFFFFFFFFFFFFFFFFFFFFFFFFFFFFFFFF
FFFFFFFFFFFFFFFFFFFFFFFF:FFFFFFFFFFFFFFFFFF:FFFFFFFFFFFFFFFFFFFFFFFF
@A00155:342:HHGFNDSXY:1:1463:2058:5462 1:N:0:GAACCTAG+TCCGCATA
GAGAGGGATATGGAGGGCGAGAGAGAGCGACTTCTCTCGGGCCCACAGCCTTACAGCTGTGGAGCACG
GTATCCTCTGCCGAAACAGAGGTTGGACAAGACCGGAGGGGTCTCCTAGTTCCAAAGGAGATGTACTC
+
FFFFFFFFFFFFFFFFF,FFFFFFFFFFFFFFFFFFFFF:FFFFF:FF,FFFFFFFFFFFFFFFFFF:
FFFFFFFFFFFFF:F:FFFFFFFFFFFFFFFFFFFFFFF,FFFFFFFFFFFFFFFFFFFFFFFFFFFF
@A00155:342:HHGFNDSXY:1:1464:29107:4272 1:N:0:GAACCTAG+TCCGCATA
GAGAGAGCGACTTCTCTCGGGCCCACAGCCTTACAGCTGTGGAGCACGGTATCCTCTGCCGAAACAGA
GGTTGGACAAGACCGGAGGGGTCTCCTGGTTCCAAAGGAGATGTACTCCGGGCTTGTTCACGACCTAC
+
FFFFFFFFFFFFFFFFFFFFFFFFFFFFFFFFFFFFFFFFFFFFFFFFFFFFFFFFFFFFFFFFFFFF
FFFFFFFFFFFFFFFFFFFFFFFFFFFFFFFFFFFFFFFFFFFFFFFFFFFFFFFFFFFFFFFFF:FF
@A00155:342:HHGFNDSXY:1:1464:7346:33943 1:N:0:GAACCTAG+TCCGCATA
GCGACTTCTCTCGGGCCCACAGCCTTACATCTGTGGAGCACGGTATCCTCTGCCGAAACAGAGGTTGG
ACAAGACCGGAGGGGTCTCCTAGTTCCAAAGGAGATGTACTCCGGGCTTGTTCACGACCTACCGTGTA
+
FFFFFFFFFFFFFFFFF:FFFFFFFFFFFFFFFFFFFFF::FFFFFFFFFFFFFFFFFFFFFFFFFFF
FFFF,FFFFFFFFFFFFFFFFF:FFFFFFFFFFFFFFFFFFFFFFFFFFFFFFFFFFFFFFFFFFFFF
@A00155:342:HHGFNDSXY:1:1464:28103:36198 1:N:0:GAACCTAG+TCCGCATA
TGGTCATCTCTATGCCGAGAGGGATGTGGAGGGCGAGAGAGAGCGACTTCTCTCGGGCCCACAGCCTT
ACAGCTGTGGAGCACGGTATCCTCTGCCGAAACAGAGGTTGGACAAGACCGGAGGGGTCTCCTAGTTC
+
FFFFFFFFFFFFFFFFFFFFFFFFFFFFFFFFFFFFFF:FFFFFFFFFFFFFFFFFFFFFFFFFFFFF
FFFFFFFFFFFFFFFFFFFFFFFFFFFFFFFFFFFFFFFFFFFFFFFFFFFFFFFFFFFFFFFFFFFF
@A00155:342:HHGFNDSXY:1:1465:25825:11334 1:N:0:GAACCTAG+TCCGCATA
CTTCTCTCGGGCCCACAGCCTTACAGCTGTGGAGCACGGTATCCTCTGCCGAAACAGAGGTTGGACAA
GACCGGAGGGGTCTCCTAGTTCCAAAGGAGATGTACTCCGGGCTTGTTCACGACCTACCGTGTAAGTC
+
FFFFFFFFFFFFFFFFFFFFFFFFFFFFFFFFFFFFFFFFFFFFFFFFFFFFFFFFFFFFFFFFFFFF
FFFFFFFFFFFFFFFFFFFFFFFFFFFFFFFFFF:FFFFFFFFFFFFFFFFFFFFFFFFFFFFFFFFF
@A00155:342:HHGFNDSXY:1:1465:14904:12900 1:N:0:GAACCTAG+TCCGCATA
CTTCTCTCGGGCCCACAGCCTTACAGCTGTGGAGCACGGTATCCTCTGCCGAAACAGAGGTTGGACAA

```
GACCGGAGGGGTCTCCTAGTTCCAAAGGAGATGTACTCCGGGCTTGTTCACGACCTACCGTGTAAGTC
+
FF:FFFFFFFFFFFFFFFFFFFFFF:FFFFFFF:FFFFFFFFFFFFFFFFFFFFFFFFFFFFF,FFFF
FFFF:FFFFFFFFFFFFFFFFFFFFFFFF:FFFFFFFFFFFFFFFFFFFFFFFFFFFFFF,FFFFFFF
@A00155:342:HHGFNDSXY:1:1465:29713:15906 1:N:0:GAACCTAG+TCCGCATA
CTTCTCTCGGGCCCACAGCCTTACAGCTGTGGAGCACGGTATCCTCTGCCGAAACAGAGGTTGGACAA
GACCGGAGGGGTCTCCTAGTTCCAAAGGAGATGTACTCCGGGCTTGTTCACGACCTACCGTGTAAGTC
+
FFF:FFFFFFFFFF:FFFFF:FFFFFFFFFFFFFFFF::FFF,FFF:FFF,FFFFFFFFFFFFFFFFF
FFFFFFFFFFFFFFFFFFFFFFFFFFFFFFFFFFFFFFFF:FFFFF:FFF:FFFFFFFFF:F:F,:FF
@A00155:342:HHGFNDSXY:1:1465:30364:21731 1:N:0:GAACCTAG+TCCGCATA
CTCAATGCCGAGAGGGATGTGGAGGGCGAGAGAGAGCGACTTCTCTCGGGCCCACAGCCTTACAGCTG
TGGAGCACGGTATCCTCTGCCGAAACAGAGGTTGGACAAGACCGGAGGGGTCTCCTAGTTCCAAAGGA
+
FFFFFFFFFFFFFFFFFFF:FFFFFFFFFFFFFFFFFFFFFFFFFFFFFFFFFFFFFFFFFFFFFFFF
FFFFFFFFFFFFFFFFFFFFFFFFFFFFFFFFFFFFFFFF:FFF:FFFFFFFFFFFFFFFFFFFFFFF
@A00155:342:HHGFNDSXY:1:1465:16242:27868 1:N:0:GAACCTAG+TCCGCATA
CCTCTGGCCTATGGTCATCTCAATGCCGAGAGGGATGTGGAGGGCGAGAGAGAGCGACTTCTCTCGGG
CCCACAGCCTTACAGCTGTGGAGCACGGTATCCTCTGCCGAAACAGAGGTTGGACAAGACCGGAGGGG
+
FFFFFFFFFFFFFFFFFFFFFFFFFFFFFF:FFFF:FFFFFFFFFFFFFFFFFFF:F:FFFFF:FFFF
FFFFFFFFFFFFFFFFFFFFFFFFFFFFFFFFFFFFFFFFFFFFFFFFFFFFFFFFFFFFFFFFFFFF
@A00155:342:HHGFNDSXY:1:1465:22128:31485 1:N:0:GAACCTAG+TCCGCATA
TTCTCTCGGGCCCACAGCCTTACAGCTGTGGAGCACGGTATCCTCTGCCGAAACAGAGGTTGGACAAG
ACCGGAGGGGTCTCCTAGTTCCAAAGGAGATGTACTCCGGGCTAGTTCACGACCTACCGTGTAAGTCG
+
FFFFFFFFFFFFFFFFFFFFFFFFFFFFFFFFFFFFFFFFFFFFFFFFFFFFFFFFFFFFFFFFFFFF
FFFFFFFFFFFFFFFFFFFFFFFFFFFFFFFFFFFFFFFFFFFFFFFFFF:FFFFFFFFFF:F:FFFF
@A00155:342:HHGFNDSXY:1:1465:3721:32769 1:N:0:GAACCTAG+TCCGCATA
CTTCTCTCGGGCCCACAGCCTTACAGCTGTTGAGCACGGTATCCTCTGCCGAAACAGAGGTTGGACAA
GACCGGAGGGGTCTCCTAGTTCCAAAGGAGATGTACTCCGGGCTTGTTCACGACCTACCGTGTAAGTC
+
:FFFFFFFFFFFFFFFF,FFFFFFFFFFFF,,FFFFFFFFFFFFFFFFFFFFFF:FFFFFFFF:FFFF
FFFFFFFFFFFFFFFFFFFFFFFFFFFFFFFFFFFFFFFFFFFFFFFFFFFFFFFFFFFF:FFFFFFF
@A00155:342:HHGFNDSXY:1:1466:3043:3286 1:N:0:GAACCTAG+TCCGCATA
TGTGGAGGGCGAGAGAGAGCGACTTCTCTCGGGCCCACAGCCTTACAGCTGTGGAGCACGGTATCCTC
TGCCGAAACAGAGGTTGGACAAGACCGGAGGGGTCTCCTAGTTCCAAAAGAGATGTACTCCGGG
+
FFFFFFFFFFFFFFFFFFFFFFFFFFFFFFFFFFFFFFFFFFFF:FFFFFFFFFFFFFFFFF:FFFFF
FFFFFFFFF:FFFFFFFFFFFFF:FFFFFFFFFFFFFFFFFFFFFFFFFFFFFFFFFFFFFFFF
@A00155:342:HHGFNDSXY:1:1466:28854:6214 1:N:0:GAACCTAG+TCCGCATA
TGCTCCACAGCTGTAAGGCTGTGGGCCCGAGAGAAGTCGCTCTCTCTCGCCCTCCACATCCCTCTCGG
CATTGAGATGACCATAGGCCAGAGGTGAGTCCTTAAGTGGACACAGCTGATCTAAGGCGGTGTGGTGG
+
FFFFFFFFFFFFFFFFFFFFFFFFFFFFFFFFFFFFFFFFFFFFFFFFFFFFFFFFFFFFFFFFFFFF
FF:FFFFFFFFFFFFFFFFFFFFFFFFFFFFFFFFFFFFFFFFFFFFFFFFFFFFFFFFF:FFFFFFF
@A00155:342:HHGFNDSXY:1:1466:17897:31422 1:N:0:GAACCTAG+TCCGCATA
CTTCTCTCGGGCCCACAGCCTTACAGCTGTGGAGCACGGTATCCTCTGCCGAAACAGAGGTTGGACAA
GACCGGAGGGGTCTCCTAGTTCCAAAGGAGATGTACTCCGGGCTTGTTCACGACCTACCGTGTAAGTC
+
FFFFFFFFFFFFFFFFFFFFFFFFFFFFFFFFFFFFFFFFFFFFFFFFFFFFFFFFFFFFFFFFFFFF
F:FFFFFFFFFFFFFFF:FFFFFFFFFFFFFFFFFFFFFFFFFFFFFFFFFFFFFFFFFFFFFFFFFF
@A00155:342:HHGFNDSXY:1:1468:31566:5776 1:N:0:GAACCTAG+TCCGCATA
GGATGTGGAGGGCGAGAGAGAGCGACTTCTCTCGGGCCCACAGCCTTACAGCTGTGGAGCACGGTATC
```

```
CTCTGCCGAAACAGAGGTTGGACAAGACCGGAGGGGTCTCCTGGTTCCAAAGGAGATGTACTCC
+
FFFFFFFFFFFFFFFFFFFFFFFFFF:FFFFFFFF:FFFFFFFFF:FFF:FFFFFFFFFFFFFFFFFF
FFFFFFFFFFFFFFFFFFFFFFFFFFFFFFFFFFFFFF:FFFFFFFFFFFFFFFFFFFFFFFFFF
@A00155:342:HHGFNDSXY:1:1468:30888:15405 1:N:0:GAACCTAG+TCCGCATA
TGGTCATCTCAATGCCGAGAGGGATGTGGGGGGCGAGAGAGAGCGACTTCTCTCGGGCCCACAGCCTT
ACAGCTGTGGAGCACGGTATCCTCTGCCGAAACAGAGGTTGGACAAGACCGGAGGGGTCTCCTAGTTC
+
FFFFFFFFFFFFFFFFFFFFFFFFFF:FFFFFFFFFFFFFFFFFFFFFFFFFFFFFFFFFFFFFFFFF
FFFFFF:FFFFFFFFFFFFFFF:FFFFFFFFFFFFFFF,FFFFFFFFFFFFFFFFFFFFFFFFFFFF:F
@A00155:342:HHGFNDSXY:1:1469:15881:5760 1:N:0:GAACCTAG+TCCGCATA
CAGCCTTACAGCTGTGGAGCACGGTATCCTCTGCCGAAACAGAGGTTGGACAAGACCGGAGGGGTCTC
CTAGTTCCAAAGGAGATGTACTCCGGGCTTGTTCACGACCTACCGTGTAAGTCGTAGTCTAGTAGGCT
+
FFFFFFFFFFFFFFFFFFFFFFFFFFFFFFFFFFFFFFFFFFFFFFFFFFFFFFFFFFFFFFFFFFFF
FFFFFFFFFFFFFFFFFFFFFFFFFFFFFFFFFFFFFFFFFFFFFFFFFFFFFFFFFFFFFFFFFFFF
@A00155:342:HHGFNDSXY:1:1469:17544:8891 1:N:0:GAACCTAG+TCCGCATA
GAGCGACTTCTCTCGGGCCCACAGCCTTACAGCTGTGGAGCACGGTATCCTCTGCCGAAACAGAGGTT
GGACAAGACCGGAGGGGTCTCCTAGTTCCAAAGGAGATGTACTCCGGGCTTGTTCACGACCTACCGTG
+
FFFFFFFFFFFFFFFFFFFFFFFFFFFFFFFFFFFFFFFFFFFFFF:FFFFFFFFFFFFFFFFFFFFF
FFFFFFFFFFFFFFFFFFFFFFFFFFFFFFFFFFFF:FFFFFFFFFFFFFFFFFFFFFFFFFF,FFFFF
@A00155:342:HHGFNDSXY:1:1469:32678:12430 1:N:0:GAACCTAG+TCCGCATA
GACTTCTCTCGGGCCCACAGCCTTACAGCTGTGGAGCACGGTATCCTCTGCCGAAACAGAGGTTGGAC
AAGACCGGAGGGGTCTCCTAGTTCCAAAGGAGATGTACTCCGGGCTTGTTCACGACCTACCGTGTAAG
+
FFFFFFFFFFFFFFFFFFFFFFF,,FFF:FFFFFFFFFFFFFFFFFFFFFFFFFFFFFFFFFFFFFFF
FFFFFFFFFFFFFFFFFFFFFFFFFFFFFFFFFFFFFFFFFFFFFFFFFFFFFFFFFFFFFFFF:FFF
@A00155:342:HHGFNDSXY:1:1469:30056:16157 1:N:0:GAACCTAG+TCCGCATA
ATCTCAATGCCGAGAGGGATGTGGAGGGCGAGAGAGAGCGACTTCTCTCGGGCCCACAGCCTTACAGC
TGTGGAGCACGGTATCCTCTGCCGAAACAGAGGTTGGACAAGACCGGAGGGGTCTCCTAGTTCCATAG
+
FFFFFFFFFFFFFFFFFFFFFFFFFFFFFFFFFFFFFFFFFF:FF,FFF:FFFFFFFF:FFFFFFFF
FFF:FFFFFFFFFFFFFFFFFFFFFFFFFF:FFFFFFFFFFFFFFFFFF:FFFFFFFFFFFF:FFFFF
@A00155:342:HHGFNDSXY:1:1470:7618:14434 1:N:0:GAACCTAG+TCCGCATA
CTCTGCCGAAACAGAGGTTGGACAAGACCGGAGGGGTCTCCTAGTTCCAAAGGAGATGTACTCCGGGC
TTGTTCACGACCTACCGTGTAAGTCGTAGTCTAGTAGGCTACCTGACGAGTCCTTTTTAGGACGAAAC
+
FFFFFFFFFFFFFFFFFFFFFFFF,FFFFFFFFFFFFFFFFFFFFFFFFFFFFFFFFFFFFFFFFFFF
FFFFFF:FFFFFFFFFFFFFFFFFFFFFFFFFFFFFFFFFFFFFFFFFFFFFFFFFFFFFFFFF:FFF
@A00155:342:HHGFNDSXY:1:1470:20934:21652 1:N:0:GAACCTAG+TCCGCATA
ACCTCTGTTTCGGCAGAGGATACCGTGCTCCACAGCTGTAAGGCTGTGGGCCCGAGAGAAGTCGCTCT
CTCTCGCCCTTCACATCCCTCTCGGCATTGAGATGACCATAGGCCAGAGGTGAGTCCTTAAG
+
FFFFFFFFFFFFFFFFFF:FFFFFFFFFFFFFFFFFFFFFFFFFFFFFFFFFFFFFFFFFFFFFFFFF
FFFFFFFFFFFFFFFFFFFFFFFFFFFF,FFFFFFFFFFFFFFFFFFFFFFFFFFFFFFFFFFF:
@A00155:342:HHGFNDSXY:1:1470:19741:26631 1:N:0:GAACCTAG+TCCGCATA
GGGCGAGAGAGAGCGACTTCTCTCGGGCCCACAGCCTTACAGCTGTGGAGCACGGTATCCTCTGCCGA
AACAGAGGTTGGACAAGACCGGAGGGGTCTCCTAGTTCCAAAGGAGATGTACTCCGGGCTAGTTCACG
+
F:,FFFFFFFFFFFFFFFFFFFFFF:,:FFFFFFFFFFFFFFFFFFFFFFFFFF,FFFFFFFFFFFFF
FFFF,FFFF,,FFFFFFFFFFFFFFFFFFFFFFFFFFF:FFFF,FFFFFFFFFF:F:FFF,FFFFFFF
@A00155:342:HHGFNDSXY:1:1471:13982:6887 1:N:0:GAACCTAG+TCCGCATA
TCAATGCCGAGAGGGATGTGGAGGGCGAGAGAGAGCGACTTCTCTCGGGCCCACAGCCTTACAGCTGT
```

```
GGAGCACGGTATCCTCTGCCGAAACAGAGGTTGGACAAGACCGGAGGGGTCTCCTAGTTCCAAAGGAG
+
FFFFFFFFFFF:FFFF:FFFFFFFFFFFFFFFFFFFFFFFFFFFFFFFFFFFFFFFFFFF:FFFFFFF
FFFFFFFFFFFFFFFFFFFFFFFFFFFFFFFFFFFFFFFFFFFFFFFFFFFFFFFFFFFFFFFFFFFF
@A00155:342:HHGFNDSXY:1:1471:5701:9768 1:N:0:GAACCTAG+TCCGCATA
GAGCGACTTCTCTCGGGCCCACAGCCTTACAGCTGTGGAGCACGGTATCCTCTGCCGAAACAGAGGTT
GGACAAGACCGGAGGGGTCTCCTGGTTCCAAAGGAGATGTACTCCGGGCTTGTTCACGACCTACCGTG
+
:FFFFFFFFFFFFFFFFFFFFFFFFFFF:F,FFFF:FFFF:FFFFFF,FFFFFF:FFFFFFFF:FFF
FFFFF:FFFFFFFFFFFFFFFFFFFFFFFFFFFFFFFFF:FFFFFFFFFF:FFFFFFFFFFFFFFFFFF
@A00155:342:HHGFNDSXY:1:1471:28682:15530 1:N:0:GAACCTAG+TCCGCATA
CTTCTCTCGGGCCCACAGCCTTACAGCTGTGGAGCACGGTATCCTCTGCCGAAACAGAGGTTGGACAA
GACCGGAGGGGTCTCCTAGTTCCAAAGGAGATGTACTCCGGGCTTGTTCACGACCTACCGTGTAAGTC
+
FFFFFFFFFFFFFFFFFFFFFFFFFFFFFFFFFFF:FFFFFFFFFFFFFFFFFFFFFFFFFFFFFFFF
FFFFFFFFFFFFFFFFFFFFFFFFFFFFF:FFFFFFFFFFFFFFFFFFFFFFFFFFFFFFFFFFFFFFF
@A00155:342:HHGFNDSXY:1:1471:22525:22341 1:N:0:GAACCTAG+TCCGCATA
GAGAGAGCGACTTCTCTCGGGCCCACAGCCTTACAGCTGTGGAGCACGGTATCCTCTGCCGAAACAGA
GGTTGGACAAGACCGGAGGGGTCTCCTAGTTCCAAAGGAGATGTACTCCGGGCTTGTTCACGACCTAC
+
FFFFFFFFFFFFFFFFFFFFFFFFFFFFFFFFFFFFFFFFFFFFFFFFFFFFFFFFFFFFFFFFFFFF
FFFFFFFFFFFFFFFFFFFFFFFFFFFFFFFFFFFFFFFFFFFFFFFFFFFFFFFFFFFFFFFFFFFF
@A00155:342:HHGFNDSXY:1:1471:23954:23750 1:N:0:GAACCTAG+TCCGCATA
GAGAGAGCGACTTCTCTCGGGCCCACAGCCTTACAGCTGTGGAGCACGGTATCCTCTGCCGAAACAGA
GGTTGGACAAGACCGGAGGGGTCTCCTAGTTCCAAAGGAGATGTACTCCGGGCTTGTTCACGACCTAC
+
FFFFFFFFFFFFFFFFFFFFFFFFFFFFFFFFFFFFFFFFFFFFFFFFFFFFFFFFFFFFFFFFFFFF
FFFFFFFFFF:FFFFFFFFFFFFFFFFFFFFFFFFFFFFFFFFFFFFFFFFFFFFFFFFFFFFFFF:F
@A00155:342:HHGFNDSXY:1:1471:13557:24377 1:N:0:GAACCTAG+TCCGCATA
CTCAATGCCGAGAGGGATGTGGAGGGCGAGAGAGAGCGACTTCTCTCGGGCCCACAGCCTTACAGCTG
TGGAGCACGGTATCCTCTGCCGAAACAGAGGTTGGACAAGACCGGAGGGGTCTCCTGGTTCCAAAGGA
+
FFFFFFFFFFFFFFFFFFFFFFFFFFFFFFFFFFFFFFFFFFFFFFFFFFFFFFFFFFFFFFFFFFFF
FFFFFFFFFFFFFFFFFFFFFFFFFFFFFFFFFFFFFFFFFFFFFFFFFFFFFFFFFFFFFFFFFFFF
@A00155:342:HHGFNDSXY:1:1471:12228:24925 1:N:0:GAACCTAG+TCCGCATA
CTCAATGCCGAGAGGGATGTGGAGGGCGAGAGAGAGCGACTTCTCTCGGGCCCACAGCCTTACAGCTG
TGGAGCACGGTATCCTCTGCCGAAACAGAGGTTGGACAAGACCGGAGGGGTCTCCTGGTTCCAAAGGA
+
FFFFFFFFFFFFFFFFFFFFFFFFFFFFFFFFFFFFFFFFFFFFFFFFFFFFFFFFFFFFFFFFFFFF
FFFFFFFFFFFFFFFFFFFFFFFFFFFFFFFFFFFFFFFFFFFFFFFFFFFFFFFFFFFFFFFFFFFF
@A00155:342:HHGFNDSXY:1:1471:25861:25457 1:N:0:GAACCTAG+TCCGCATA
GAGAGCGACTTCTCTCGGGCCCACAGCCTTACAGCTGTGGAGCACGGTATCCTCTGCCGAAACAGAGG
TTGGACAAGACCAGAGGGGTCTCCTAGTTCCAAAGGAGATGTACTCCGGGCTTGTTCACGACCTACCG
+
FFFFFFFFFFFFFFFFFFFFFFFFFFFFFF:FFFFFFFFFFFFFFFFFFFFFFFFFFFFFFFFFFFFF
FFFFFFFFFFFFFFFFFFFFFFFFFFF:FFFFFFFFFFFFFF:FFFFFFFFFFFFFFFFFFFFFFFFFF
@A00155:342:HHGFNDSXY:1:1471:9688:34272 1:N:0:GAACCTAG+TCCGCATA
TCCTCTGCCGAAACAGAGGTTGGACAAGACCGGAGGGGTCTCCTGGTTCCAAAGGAGATGTACTCCGG
GCTTGTTCACGACCTACCGTGTAAGTCGTAGTCTAGTAGGCTACCTGACGAGTCCTTTTTAGGACGAA
+
FFFFFFFFFFFFFFFFFFFFFFFFFFF:FFFFFFFF,FFFFFFFFFFFFFFFFFFFFFFFFFFFFFFF
FFFFFFFFFFFFFFFFFFFFFFFFFFFFFFFFFFFFFFFFFFFFFFFFFFFFFFFFFFFFFFFFFFFF
@A00155:342:HHGFNDSXY:1:1472:2076:4679 1:N:0:GAACCTAG+TCCGCATA
CTTCTCTCGGGCCCACAGCCTTACAGCTGTGGAGCACGGTATCCTCTGCCGAAACAGAGGTTGGACAA
```

```
GACCGGAGGGGTCTCCTAGTTCCAAAGGAGATGTACTCCGGGCTTGTTCACGACCTACCG
+
FFFFFFFFFFFFFFFFFFFFFFFFFFFFFFFFFFFFFFFFFFFFFFFFFFFFFFFFFFFFFFFFFFFF
FFFFFFFFFFFFFFFFFFFFFFFFFFFFFFFFFFFF,FF:FFFFFFFFFFFFFFFFFFF
@A00155:342:HHGFNDSXY:1:1472:8567:23876 1:N:0:GAACCTAG+TCCGCATA
GGATGTGGAGGGCGAGAGAGAGCGACTTCTCTCGGGCCCACAGCCTTACAGCTGTGGAGCACGGTATC
CTCTGCCGAAACAGAGGTTGGACAAGACCGGAGGGGTCTCCTGGTTCCAAAGGAGATGTACTCC
+
FFFFFFFFFFFFFFFFFFFFFFFFFFFFFFFFFFFFFFFFFFFFFFFFFFFFFFFFFFFFFFFFFFFF
FFFFFFFFFFFFFFFFFFFFFFFFFFFFFFFFFFFFFFFFFFFFFFFFFFFFFFFFFFFFFFFF
@A00155:342:HHGFNDSXY:1:1472:3016:25128 1:N:0:GAACCTAG+TCCGCATA
AGGGCGAGAGAGAGCGACTTCTCTCGGGCCCACAGCCTTACAGCTGTGGAGCACGGTATCCTCTGCCG
AAACAGAGGTTGGACAAGACCGGAGGGGTCTCCTAGTTCCAAAGGAGATGTACTCCGGGCTTGTTCAC
+
FFFFFFFFFFFFFFFFFFFFFFFFFFFF::FFFFFFFFF:FFFFFFFFFFFFFFFFFFFFFFFFFFFF
FFFFFFFFFFFFFFFFFFFFFFFFFFFFFFFFFFFFFFFFFFFFFFFFFFFFFFFF:FFFFFFFFFFF
@A00155:342:HHGFNDSXY:1:1472:19388:26490 1:N:0:GAACCTAG+TCCGCATA
CTCTCGCCCTCCACATCCCTCTCGGCATTGAGATGACCATAGGCCAGAGGTGAGTCCTTAAGTGGACA
CAGCTGATCTAAGGCGGTGTGGCGGGGCATGGGTTTGAACCCCCATGACGGTCGGAGTCTAGTAGGCT
+
FFFFFFFFFFFFFFFFFFFFFFFFFFFFFFFFFFFFFFFFFFFFFFFFFFFFFFFFFFFFFFFFFFFF
FFFFFFFFFFFFFFFFFFFFFFFFFFFFFFFFFFFFFFFFFFFFFFFFFFFFFFFFFFFFFFFFFFFF
@A00155:342:HHGFNDSXY:1:1472:28971:32064 1:N:0:GAACCTAG+TCCGCATA
CCACATCCCTCTCGGCATTGAGATGACCATAGGCCAGAGGTGAGTCCTTAAGTGGACACAGCTGATCT
AAGGCGGTGTGGCGGGGCATGGGTTTGAACCCCCATGACGGTCGGAGTCTAGTAGGCTCCCTGATGAG
+
FFFFFFFFFFFFFFFFFF:FFFFFFFFFFFFFFFFFFFFFFFFFFFFFFFFFFFFFFFF:FFFFFFFFF
FFFFFFFFFFFFFFFFFFFFFFFFFFFFFFFFFFFFFFFFFFFFFFFFFFFFFFFFFFFFFFFFFFFF
@A00155:342:HHGFNDSXY:1:1473:13521:12226 1:N:0:GAACCTAG+TCCGCATA
CCTTACAGCTGTGGAGCACGGTATCCTCTGCCGAAACAGAGGTTGGACAAGACCGGAGGGGTCTCCTA
GTTCCAAAGGAGATGTACTCCGGGCTTGTTCACGACCTACCGTGTAAGTCGTAGTCTAGTAGGCTACC
+
FFFFFFFFFFFFFFFFFFFFFFFFFFFFFFFFFFFFFFFFFFF:FFFFFFFFFFFFFFFFFFFFFFFFF
FFFFFFFFFFFFFFFFFFFFFFFFFFFFF:FFFFFFFFFFFFFFFFFFFFFFFFFFFFFFFFFFFFFFF
@A00155:342:HHGFNDSXY:1:1473:14344:34757 1:N:0:GAACCTAG+TCCGCATA
TGTCCACTTAAGGACTCACCTCTGGCCTATGGTCATCTCAATGCCGAGAGGGATGTGGAGGGCGAGAG
AGAGCGACTTCTCTCGGGCCCACAGCCTTACAGCTGTGGAGCACGGTATCCTCTGCCGAAACAGAGGT
+
FFFFFFFFFFFFFFFFFFFFFFFFFFFFFFFFFFFFFFFFFFFFFFFFFFFFF:F:FFFFFFFFFFFF
FFFFFFFFFFFFFFFFFFFFFFFFFFFFFFFFFFFFFFFFFFFFFFFFFFFFFFFFFFFFFFFFFFFF
@A00155:342:HHGFNDSXY:1:1474:10945:10457 1:N:0:GAACCTAG+TCCGCATA
AGGGCGAGAGAGAGCGACTTCTCTCGGGCCCACAGCCTTACAGCTGTGGAGCACGGTATCCTCTGCCG
AAACAGAGGTTGGACAAGACCGGAGGGGTCTCCTAGTTCCAAAGGAGATGTACTCCGGGCTTGTTCAC
+
FFFFFFFFFFFFFFFFFFFFFFFFFFFFFFFFFFFFFFFFFFFFFFFFFFFFFFFFFFFFFFFFFFFF
FFFFFFFFFFFFFFFFFFFFFFFFFFFFFFFFFFFFFFFFFFFFFFFFFFFFFFFFFFFFFFFFFFFF
@A00155:342:HHGFNDSXY:1:1474:14271:12461 1:N:0:GAACCTAG+TCCGCATA
GAGCGACTTCTCTCGGGCCCACAGCCTTACAGCTGTGGAGCACGGTATCCTCTGCCGAAACAGAGGTT
GGACAAGACCGGAGGGGTCTCCTAGTTCCAAAGGAGATGTACTCCGGGCTTGTTCACGACCTACCGTG
+
FFFFFFFFFFFFFFFFFFFFFFFFFFFFFFFFFFFFFFFFFFFFFFFFFFFFFFFFFFFFFFFFFFFF
FFFFFF:FFFFFFFFFFFFFFFFFFFFFFFFFFF:FFFFFFFFFFFFFFFFFFFFFFFF:FFFFFFFFF
@A00155:342:HHGFNDSXY:1:1474:19533:13808 1:N:0:GAACCTAG+TCCGCATA
AGGGATGTGGAGGGCGAGAGAGAGCGACTTCTCTCGGGCCCACAGCCTTACAGCTGTGGAGCACGGTA
```

```
TCCTCTGCCGAAACAGAGGTTGGACAAGACCGGAGGGGTCTCCTAGTTCCAAAGGAGATGTACTCCGG
+
FFFFFFF:FFFFFFFFFFFFFFFFFFFFF:FFFFFFFFFFFFFFFFFFFFFFFFFFFFFFFFFFFFFFFF
FFFFFFFFFFFFFFFFFFFFFFFFFFFFFFFFFFFFFFFFFFFFFFFFFFFFFFFFFFFFFFFFFFFF
@A00155:342:HHGFNDSXY:1:1474:28239:18458 1:N:0:GAACCTAG+TCCGCATA
CCATGCCCCGCCACACCGCCTTAGATCAGCTGTGTCCACTTAAGGACTCACCTCTGGCCTATGGTCAT
CTCAATGCCGAGAGGGATGTGGAGGGCGAGAGAGAGCGACTTCTCTCGGGCCCACAGCCTTACAGCTG
+
FFFFFFFFFFFFFFFFFFFF:FFFFFFFFFFFFFFFFFFF,FFFFFFFFFFFFFFFFF:FFFFFFFFF
FFFFFFFFFFFFFFFFF::FFFFFFFFFFFFFFFFFFFFFFFFFFFFFFFFFFFFFFFFF:FFFFFF,F
@A00155:342:HHGFNDSXY:1:1474:16260:18474 1:N:0:GAACCTAG+TCCGCATA
ATGTGGAGGGCGAGAGAGAGCGACTTCTCTCGGGCCCACAGCCTTACAGCTGTGGAGCACGGTATCCT
CTGCCGAAACAGAGGTTGGACAAGACCGGAGGGGTCTCCTAGTTCCAAAGGAGATGTACTCCGGGCTT
+
FFFFFFFFF:FFFFFFFFFFFFFFFFFFFFFFFFFFFFFFFFFFFFFFFFFFFFFFFFFFFFFFFFFF
FFFFFFFFFFFFFFFFFFFFFFFF:FFFFFFFFFFFFFFFF:FFFFFFFFFFFFFFFFFFFFFFFFFFF
@A00155:342:HHGFNDSXY:1:1475:3206:1063 1:N:0:GAACCTAG+TCCGCATA
ACTTCTCTCGGGCCCACAGCCTTACAGCTGTGGAGCACGGTATCCTCTGCCGAAACAGAGGTTGGACA
AGACCGGAGGGGTCTCCTGGTTCCAAAGGAGATGTACTCCGGGCTTGTTCACGACCTACCGTGTAAGT
+
FFFFFFFFFFFFFFFFFFFFFFFFFFFFFFFF,FFFFFFFFFFFFFFFFFFFFFFFFF:FFFFFFFFF
F,FFFFFFFFFFFFFFFFFFFFFFFFFFFFFFFFFFFFFFFFFFFFFFFFFFFFFFFFFFFFFFFFFF
@A00155:342:HHGFNDSXY:1:1475:13096:11021 1:N:0:GAACCTAG+TCCGCATA
ACTAGACTACGACTTACACGGTAGGTCGTGAACAAGCCCGGAGTACATCTCCTTTGGAACTAGGAGAC
CCCTCCGGTCTTGTCCAACCTCTGTTTCGGCAGAGGATACCGTGCTCCACAGCTGTAAGGCTGTGGGC
+
FFFFFFFFFF:FFF,FFFF,FFFFFFFFFFFFFFFFFFFF:FFFFFFFFFFFFF:FFF:FFF:FFFFF
FFFFFFFFFFFFFFFFFFFFFFFFFFFFFFFFFFFFFFFFFFFFFFFFFFFFFFFFFFFFFFFFFFFF
@A00155:342:HHGFNDSXY:1:1475:23366:11522 1:N:0:GAACCTAG+TCCGCATA
GCGACTTCTCTCGGGCCCACAGCCTTACAGCTGTGGAGCACGGTATCCTCTGCCGAAACAGAGGTTGG
ACAAGACCGGAGGGGTCTCCTGGTTCCAAAGGAGATGTACTCCGGGCTTGTTCACGACCTACCGTGTA
+
FFFFFFFFFFFFFFFFFFFFFFFFFFFFFFFFFFFFFF:FFFFFFFFFFFFFFFF:FFFFFFFFFFF
FFFFFFFFFFFFFFFFFFFFFFFFFFFFFFFFFFFFFFFFFFFFFFF:FFFFFFFFFFFFFFFFFFFF
@A00155:342:HHGFNDSXY:1:1476:22092:28823 1:N:0:GAACCTAG+TCCGCATA
CTTCTCTCGGGCCCACAGCCTTACAGCTGTGGAGCACGGTATCCTCTGCCGAAACAGAGGTTGGACAA
GACCGGAGGGGTCTCCTAGTTCCAAAGGAGATGTACTCCGGGCTTGTTCACGACCTACCGTGTAAGTC
+
FFFFFFFFFFFFFFFFFFFFFFFFFFFFFFFFFFFFFFFFFFFFFFFFFFFFFFFFFFFFFFFFFFFF
FFFFFFFFFFFFFFFFFFFFFFFFFFFFFFFFFFFFFFFFFFFFFFFFFFFF:FFFFFFFFFFF:FFF
@A00155:342:HHGFNDSXY:1:1476:4218:29465 1:N:0:GAACCTAG+TCCGCATA
AGCGACTTCTCTCGGGCCCACAGCCTTACAGCTGTGGAGCACGGTATCCTCTGCCGAAACAGAGGTTG
GACAAGACCGGAGGGGTCTCCTAGTTCCAAAGGAGATGTACTCCGGGCTTGTTCACGACCTACC
+
:FFFFFFFFFFFFFFFFFFF:FFFFFFFFF::FFFFFFFFFFFFFF,FFFFFFFFFFFFFFFFFFFFF
FFFFF,FFF:FFFFFFFFFFFFFFFFFFFFFFFFFFFF:FFFFF:FFFFFFFFFFFFFFFFFFFF
@A00155:342:HHGFNDSXY:1:1476:7428:33552 1:N:0:GAACCTAG+TCCGCATA
ATCCTCTGCCGAAACAGAGGTTGGACAAGACCGGAGGGGTCTCCTAGTTCCAAAGGAGATGTACTCCG
GGCTTGTTCACGACCTACCGTGTAAGTCGTAGTCTAGTAGGCTACCTGACGAGTCCTTTT
+
FFFFFFFFFF:FFFFFFFFFFFFFFFFFFFF:FFFFFFFFFFFFFFFFFFFFFFFFFFFFFFFFFFFFF
FFFFFFFFFFFFFFFFFFFFFFFF,FFFFFFFFFFFFFFFFFFFFFFFFFFFFFFFFFFFF
@A00155:342:HHGFNDSXY:1:1477:24451:5196 1:N:0:GAACCTAG+TCCGCATA
TTCTCTCGGGCCCACAGCCTTACAGCTGTGGAGCACGGTATCCTCTGCCGAAACAGAGGTTGGACAAG
```

```
ACCGGAGGGGTCTCCTAGTTCCAAAGGAGATGTACTCCGGGCTTGTTCACGACCTACCGTGTAAGTCG
+
:FFFF:FFF:FFFFFFFFF,:F:FFFF:F::FFFFF,FF,:FFFFFFFFFF:FFFFF,F,FFFFFFF,
FFFFFF:F:FFFFFFFFFFFF,FFF:FF,FFFFFFFFFFFFFFF:FFFFFFFFFFFFF:FFFFFFFF:
@A00155:342:HHGFNDSXY:1:1477:4417:11240 1:N:0:GAACCTAG+TCCGCATA
GGATGTAGAGGGCGAGAGAGAGCGACTTCTCTCGGGCCCACAGCCTTACAGCTGTGGAGCACGGTATC
CTCTGCCGAAACAGAGGTTGGACAAGACCGGAGGGGTCTCCTAGTTCCAAAGGAGATGTACTCC
+
FFFFFFFFFFFFFFF:FFFFFFFFFFFFFFFFFFFFFFFFFFFFFF:FFFFFFFFFFFFFFFFFFFFF
FFFFFFFFFFFFFFFFFFF:FFFFFFFFFFFF:FFFFFFFFFFFFFFFFFFFFFFFFFF:FFFFF
@A00155:342:HHGFNDSXY:1:1477:32967:14027 1:N:0:GAACCTAG+TCCGCATA
GACTTCTCTCGGGCCCACAGCCTTACAGCTGTGGAGCACGGTATCCTCTGCCGAAACAGAGGTTGGAC
AAGACCGGAGGGGTCTCCTAGTTCCAAAGGAGATGTACTCCGGGCTTGTTCACGACCTACCGTGTAAG
+
FFFFFFFFFFFFFFFFFFFFFF,:FFFFFFFFFFFFFFFFFF:FFFFFFFFFFFFFFFFFFFFFFFFF
FFFFFFFFFFFFFFFFFFFFFFFFFFFFFFFFFFFFFFFFFFFFF:FFFFFFFFFFFFFFFFFFFFFF
@A00155:342:HHGFNDSXY:1:1477:16251:32831 1:N:0:GAACCTAG+TCCGCATA
ACTTCTCTCGGGCCCACAGCCTTACAGCTGTGGAGCACGGTATCCTCTGCCGAAACAGAGGTTGGACA
AGACCGGAGGGGTCTCCTAGTTCCAAAGGAGATGTACTCCGGGCTTGTTCACGACCTACCGTGTAAGT
+
FFFFFFFFFFFFFFFFFFFFFFFFFFFFFFFFFFFFFFFFFFFFFFFFFFFFFFFFFFFFFFFFFFFF
FFFFFFFFFFFFFFFFFFFFFFFFFFFFFFFFFFFFFFFFFFFFFFFFFFFFFFFFFFFFFFFFFFFF
@A00155:342:HHGFNDSXY:1:1478:2239:9690 1:N:0:GAACCTAG+TCCGCATA
GCGACTTCTCTCGGGCCCACAGCCTTACAGCTGTGGAGCACGGTATCCTCTGCCGAAACAGAGGTTGG
ACAAGACCGGAGGGGTCTCCTGGTTCCAAAGGAGATGTACTCCGGGCTTGTTCACGACCTACC
+
FF:FFFFFF:FFFFFFF,:FFFFFFFF,FFFFFFF,FFFFFFFFFF,:FFFFFFFFFFFFFFFFFFF:
FFFF,FFFFFFFFFFFFFFFFFFFFFFFFF,FF,FF,FFFFFFFFFFFFFFFFFFFFFFFFFF
@A00155:342:HHGFNDSXY:1:1478:27190:13103 1:N:0:GAACCTAG+TCCGCATA
ACTTCTCTCGGGCCCACAGCCTTACAGCTGTGGAGCACGGTATCCTCTGCCGAAACAGAGGTTGGACA
AGACCGGAGGGGTCTCCTAGTTCCAAAGGAGATGTACTCCGGGCTTGTTCACGACCTACCGTGTAAGT
+
FFFFFFF:FFFFFFFFFFFFFFFFFFFFFFFFFFFFFFFFFFFFFFFFFFFFFFFFFF:FFFFFFFFF
F:,FFFFFFFFFFFFFFFFFFFFFFFFFFFFFFFFFFFFFFFFFF:FFFFFFFFFFFFFFFFFFFFFF
@A00155:342:HHGFNDSXY:1:1478:29550:24205 1:N:0:GAACCTAG+TCCGCATA
AAGGACTCACCTCTGGCCTATGGTCATCTCAATGCCGAGAGGGATGTGAAGGGCGAGAGAGAGCGACT
TCTCTCGGGCCCACAGCCTTACAGCTGTGGAGCACGGTATCCTCTGCCGAAACAGAGGTTGGACAAGA
+
FFFFFFFFFFFFFFFFFFFFFFFFFFFFFFFFFFFFFFFFFFFFFFFFFFFFFFFFFFFFFFFFFFFF
FFFFFFFFFFFFFFFFFFFFFFFFFFFFFFFFFFFFFFFFFFFFFFFFFFFFFFFFFFFFFFFFFFFF
@A00155:342:HHGFNDSXY:1:1501:32389:5447 1:N:0:GAACCTAG+TCCGCATA
ATCTCAATGCCGAGAGGGATGTGGAGGGCGAGAGAGAGCGACTTCTCTCGGGCCCACAGCCTTACAGC
TGTGGAGCACGGTATCCTCTGCCGAAACAGAGGTTGGACAAGACCGGAGGGGTCTCCTAGTTCCAAAG
+
FFFFFFFFFFFFFF,FFFFFF,FFFFFFFFFFFFFFFFFFFFFFFFFFFFFFFFFFFFFFFFFFFFFF
FFFFFFFFFFFFFFFFFFFFFFFFFFFFFFF:FFFFFFFFF:FFFFFFFFFFFFFFFFFFFFFFFFFFF
@A00155:342:HHGFNDSXY:1:1501:10682:16673 1:N:0:GAACCTAG+TCCGCATA
TTCTCTCGGGCCCACAGCCTTACAGCTGTGGAGCACGGTATCCTCTGCCGAAACAGAGGTTGGACAAG
ACCGGAGGGGTCTCCTAGTTCCAAAGGAGATGTACTCCGGGCTTGTTCACGACCTACCGTGTAAGTCG
+
FFFFFFFFFFFFFFFFFFFFF:FFFFFFFFFFFFFFFFFFFFFFFFFFFFFFFFFFFFFFFFFFFFFF
FFFFFFFFFFFFFFFFFFFFFFFFFFFFFFFFFFFFFFFFFFF:FFFFFFFFFFFFFFFFFFFF:FFFF
@A00155:342:HHGFNDSXY:1:1501:15546:17519 1:N:0:GAACCTAG+TCCGCATA
TGTGTCCACTTAAGGACTCACCTCTGGCCTATGGTCATCTCAATGCCGAGAGGGATGTGGAGGGCGAG
```

```
AGAGAGCGACTTCTCTCGGGCCCACAGCCTTACAGCTGTGGAGCACGGTATCCTCTGCCGAAACAGAG
+
FFFFFFFFFFFFFFFFFFFFFFFFFFFFFFFFFFFFFFFFFFFFFFFFFFFFFFFFFFFFFFFFFFFF
FFFFFFFFFFFFFFFFFFFFFFFFFFFFFFFFFFFFFFFFFFFFFFFFFFFFFFFFFFFFFFFFFFFF
@A00155:342:HHGFNDSXY:1:1501:20030:34428 1:N:0:GAACCTAG+TCCGCATA
GGATGTGGAGGGCGAGAGAGAGCGACTTCTCTCGGGCCCACAGCCTTACAGCTGTGGAGCACGGTATC
CTCTGCCGAAACAGAGGTTGGACAAGACCGGAGGGGTCTCCTAGTTCCAAAGGAGATGTACTCC
+
FFFFFFFFFFFFFFFFFFFFFFFFFFFFFFFFFFFFFFFFFFFFFFFFFFFFFFFFFFFFFFFFFFFF
FFFFFFFFFFFFFFFFFFFFFFFFFFFFFFFFFFFFFFFFFFFFFFFFFFFFFFFFFFF:FFFF
@A00155:342:HHGFNDSXY:1:1502:27796:2910 1:N:0:GAACCTAG+TCCGCATA
CTCTGCCGAAACAGAGGTTGGACAAGACCGGAGGGGTCTCCTAGTTCCAAAGGAGATGTACTCCGGGC
TTGTTCACGACCTACCGTGTAAGTCGTAGTCTAGTAGGCTACCTGACGAGTCCTTTTTAGGACGAAAC
+
FFFFFFFFFFFFFFFFFFFFFFFFFFFFFFFFFFFFFFFFFFFFFFFFFFF:FFFFFFFFFFFFFFFF
FFFFFFFFFFFFFFFFFFFFFFFFFFFFF:FFFFFFFFFFFFFFFFFFFFFFFFFFFFFFFFFFFFFF
@A00155:342:HHGFNDSXY:1:1502:14262:22874 1:N:0:GAACCTAG+TCCGCATA
ACCTCTGTTTCGGCAGAGGATACCGTGCTCCACAGCTGTAAGGCTGTGGGCCCGAGAGAAGTCGCTCT
CTCTCGCCCTCCACATCCCTCTCGGCATTGAGATGACCATAGGCCAGAGGAGAGTCCTTAAGTGGACA
+
FFFFFFFFFFFFFFFFFFFFFFFFFFFFFFFFFFFFFFFFFFFFFFFFFFFFFFFFFFFF,FFFFFFF
FFFFFFFFFFFFFFF:FFFFFFFFFFFFFFFFFFFFFFFFFFFFFFFFFFFFFFFFFFFFFF:FFFFF
@A00155:342:HHGFNDSXY:1:1502:11605:35399 1:N:0:GAACCTAG+TCCGCATA
GGATGTGGAGGGCGAGAGAGAGCGACTTCTCTCGGGCCCACAGCCTTACAGCTGTGGAGCACGGTATC
CTCTGCCGAAACAGAGGTTGGACAAGACCGGAGGGGTCTCCTAGTTCCAAAGGAGATGTACTCC
+
FFF:FFFFFFFFFFFFFFFFFFFFFFFFFFFFFF,FFFFFFFFFFFFFFFFFFFFFFFFFFFFFFFFF
FFFFFFFFFFFFFFFFFFFFFFFFFFFFFFFFFFFFFFFFFFFFFFFFFFFFFFFFFFFFFFFF
@A00155:342:HHGFNDSXY:1:1502:10203:36918 1:N:0:GAACCTAG+TCCGCATA
GGATGTGGAGGGCGAGAGAGAGCGACTTCTCTCGGGCCCACAGCCTTACAGCTGTGGAGCACGGTATC
CTCTGCCGAAACAGAGGTTGGACAAGACCGGAGGGGTCTCCTAGTTCCAAAGGAGATGTACTCC
+
FFFFFFFFFFFFFFFFFFFFFFFFFFFFFFFFFFFFFFFFFFFFFF,F:FFFF:FFFFFFFFFFFFFF
FFFFFFFFFFFFFFFFFFFFFFFF:FFFFFFFFFFFFFFFFFFFFFFFFFFFFFFFFFFFFFFF
@A00155:342:HHGFNDSXY:1:1503:20057:3850 1:N:0:GAACCTAG+TCCGCATA
CTTCTCTCGGGCCCACAGCCTTACAGCTGTGGAGCACGGTATCCTCTGCCGAAACAGAGGTTGGACAA
GACCGGAGGGGTCTCCTAGTTCCAAAGGAGATGTACTCCGGGCTTGTTCACGACCTACCGTGTAAGTC
+
FFFFFFFFFFFFFFFFFFFFFFFFFFFFFFFFFFFFFFFFFFFFFFFFFFFFFFFFFFFFFFFFFFFF
FFFFFFFFFFFFFFFFFF:FFFFFFFFFFFFFFFFFFFFFFFFFFFFFFFFFFFFFFFFFFFFFFFFF
@A00155:342:HHGFNDSXY:1:1503:26558:4492 1:N:0:GAACCTAG+TCCGCATA
ATCTCAATGCCGAGAGGGAGGTGGAGGGCGAGAGAGAGCGACTTCTCTCGGGCCCACAGCCTTACAGC
TGTGGAGCACGGTATCCTCTGCCGAAACAGAGGTTGGACAAGACCGGAGGGGTCTCCTAGTTCCAAAG
+
FF:F:FFFFFFFFFFFFFF,:FFFFFFFFFFFFFF:FFFFFF:FFFFFFFFFFFFFFFFFFFFFFFFF
FFFF:FF:FFFFF:FF:FFFFFF:FFFFFFFFFFFFFFFFF,FFF:FFFFFFFFFFFFFFFFFFFFFF
@A00155:342:HHGFNDSXY:1:1503:23204:7075 1:N:0:GAACCTAG+TCCGCATA
ACCTCTGTTTCGGCAGAGGATACCGTGCTCCACAGCTGTAAGGCTGTGGGCCCGAGAGAAGTCGCTCT
CTCTCGCCCTTCACATCCCTCTCGGCATTGAGATGACCATAGGCCAGAGGTGAGTCCTTAAGTGGACA
+
FFFFFFFFFFFFFFFFFFFFFFFFFFFFFFFFFFFFFFFFFFFFFFFFFFFFFFFFFFFFFFFFFFFF
FFFFFFFFFFFFFFFFFFFFFFFFFFFFFFFFFFFFFFFFF,FFFFFFFFFFFFFFFFFFFFFFFFF:
@A00155:342:HHGFNDSXY:1:1503:11080:7467 1:N:0:GAACCTAG+TCCGCATA
CGAGAGGGATGTGGAGGGCGAGAGAGAGCGACTTCTCTCGGGCCCACAGCCTTACAACTGTGGAGCAC
```

```
GGTATCCTCTGCCGAAACAGAGGTTGGACAAGACCGGAGGGGTCTCCTAGTTCCAAAGGAGATGTACT
+
FFFFFFFFFFFFFFFFFFFFFFFFFFFFFFFFFFFFFFFFFFFFFFFFFFFFFFFFFFFFFFFFFFFF
FFFFFFFFFFFFFFFFFFFFFFFFFFFFFFFF:FFFFFFFFFFFFFFFFFFFFFFFFFFFFFFFFFFF
@A00155:342:HHGFNDSXY:1:1503:14082:20713 1:N:0:GAACCTAG+TCCGCATA
TGCCGAGAGGGATGTGGAGGGCGAGAGAGAGCGACTTCTCTCGGGCCCACAGCCTTACAGCTGTGGAG
CACGGTATCCTCTGCCGAAACAGAGGTTGGACAAGACCGGAGGGGTCTCCTAGTTCCAAAGGAGATGT
+
FFFFFFFFFFFFFFFFFFFFFFFFFFFFFFFFFFFFFFFFFFFFFFFFFFFFFFFFFFF:FFFFFFFF
FFFFFFFFFFFFFFFFFFFFFFFFFFFFFFFFFFFFFFFFFFFFFFFFFFFFFFFFFFF:FFFFFFFF
@A00155:342:HHGFNDSXY:1:1503:13476:29183 1:N:0:GAACCTAG+TCCGCATA
GAGGGCGAGAGAGAGCGACTTCTCTCGGGCCCACAGCCTTACAGCTGTGGAGCACGGTATCCTCTGCC
GAAACAGAGGTTGGACAAGACCGGAGGGGTCTCCTAGTTCCAAAGGAGATGTACTCCGGGCTTGTTCA
+
FFFFFFFFFFFFFFFFFFFFFFFFFFFFFFFFFFFFFFFFFFFFFFFFFFFFFFFFFFFFFFFFFFFF
FFFFFFFFFFFFFFFFFFF:FFFFFFFFFFFFFFFFFFFFFFFFFFFFFFFFFFFFFFFFFFFFFFFF
@A00155:342:HHGFNDSXY:1:1503:5529:32142 1:N:0:GAACCTAG+TCCGCATA
AGCGACTTCTCTCGGGCCCACAGCCTTACAGCTGTGGAGCACGGTATCCTCTGCCGAAACAGAGGTTG
GACAAGACCGGAGGGGTCTCCTAGTTCCAAAGGAGATGTACTCCGGGCTTGTTCACGACCTACCGTGT
+
FFFFFFFFFFFFFFFFFFFFFFFFFF:FFFFFFFFFFFFFFFFFFFFFFFF:FFFF:FFFFFFFFFFFF
FFFFFFFFFFFFFFFFFFFFFFFFFFFFFFFFFFFFFFFFFFFFFF:FFFFFFFFFFFFFFFFFFFFF
@A00155:342:HHGFNDSXY:1:1503:22137:35258 1:N:0:GAACCTAG+TCCGCATA
TGTAGAGGGCGAGAGAGAGCGACTTCTCTCGGGCCCACAGCCTTACAGCTGTGGAGCACGGTATCCTC
TGCCGAAACAGAGGTTGGACAAGACCGGAGGGGTCTCCTAGTTCCAAAGGAGATGTACTCCGGG
+
FFFFFFFFFFFFFFFFFFFFFFFFFFFFFFFFFFFFFFFFFFFFFFFFFFFFFFFFFFFFFFFFFFFF
FFFFFFFFFFFFFFFFFFFFFFFFFFFFFFFFFFFFFFFFFFFFFFFFFF:FFFFFFFFFFFFFF
@A00155:342:HHGFNDSXY:1:1503:22318:35258 1:N:0:GAACCTAG+TCCGCATA
TGTAGAGGGCGAGAGAGAGCGACTTCTCTCGGGCCCACAGCCTTACAGCTGTGGAGCACGGTATCCTC
TGCCGAAACAGAGGTTGGACAAGACCGGAGGGGTCTCCTAGTTCCAAAGGAGATGTACTCCGGG
+
FFFFFFFFFFFFFFFFFFFFFFFFFFFFFFFFFFFFFFFFFFFFFFFFFFFFFFFFFFFFFFFFFFFF
FFFFFFFFFFFFFFFFFFFFFFFFFFFFFFFFFFFFFFFFFFFFFFFFFFFFFFFFFFFFFFFF
@A00155:342:HHGFNDSXY:1:1504:6786:3035 1:N:0:GAACCTAG+TCCGCATA
TCCACTTAAGGACTCACCTCTGGCCTATGGTCATCTCAATGCCGAGAGTGATGTGGAGGGCGAGAGAG
AGCGACTTCTCTCGGGCCCACAGCCTTACAGCTGTGGAGCACGGTATCCTCTGCCGAAACAGAGGTTG
+
:FFF:FFFFFFFFFFFFFFFFF:FFFFFFFFFFFFFFFFF:FF:FFF:,F:FF:FFFF:FFFFFFFFF
FFFFFFFFFFFFF:FFF:FFFFFF,FFFF,FFFFFFFFFF:FFFF:FFFFFFFFFF:FFFFFFF:F:F
@A00155:342:HHGFNDSXY:1:1505:17852:11177 1:N:0:GAACCTAG+TCCGCATA
TTAAGGACTCTCCTCTGGCCTATGGTCATCTCAATGCCGAGAGGGATGTGGAGGGCGAGAGAGAGCGA
CTTCTCTCGGGCCCACAGCCTTACAGCTGTGGAGCACGGTATCCTCTGCCGAAACAGAGGTTGGACAA
+
FFFFFFFFFFFFFFFFFFFFFFFF:FFFFFFFFFFFFFFFFFFFFFFFFFFFFFF:FFFFFFFFFFFFF
FFFFFFFFFFFFFFFFFFFFFFFFFFFFFFFFFFFFFFFFFFFFFFFFFFFFFFFFFFFFFFFFFFFF
@A00155:342:HHGFNDSXY:1:1505:16857:24236 1:N:0:GAACCTAG+TCCGCATA
GCGACTTCTCTCGGGCCCACAGCCTTACAGCTGTGGAGCACGGTATCCTCTGCCGAAACAGAGGTTGG
ACAAGACCGGAGGGGTCTCCTAGTTCCAAAGGAGATGTACTCCGGGCTTGTTCACGACCTACCGTGT
+
:FFFFF:::,,:F::FFFFFF,FF:,FF:,FFFFF:FFFFF:,,F:FF,F:FF,,FFFFFF:FFFFFF
:FF::FF:,:FFFFFFFF,,FFF,FF:FFFF:FFFFFF:F:FFFF:FF,:FF:F:,FF,,FF,,,FF
@A00155:342:HHGFNDSXY:1:1505:6479:26115 1:N:0:GAACCTAG+TCCGCATA
ACCTCTGTTTCGGCAGAGGATACCGTGCTCCACAGCTGTAAGGCTGTGGGCCCGAGAGAAGTCGCTCT
```

CTCTCGCCCTCCACATCCCTCTCGGCATTGAGATGACCATAGGCCAGAGGAGAGTCCTTAAGTGGACA
+
FFFFFFFFFFFFFFFFF,FFFFFF:FFFFFFFF:FFF:FFFFFFFFFF:FFFFFFF:FF:FFFFFFFF
FFFFFFFFFFFFFFFFFFFFFFFFFFF,F,FFFFFFFFFFFFFFFFFFFFFFFFF:FFFFFFFFFFFF
@A00155:342:HHGFNDSXY:1:1505:5005:31955 1:N:0:GAACCTAG+TCCGCATA
ACCTCTGTTTCGGCAGAGGATACCGTGCTCCACAGCTGTAAGGCTGTGGGCCCGAGAGAAGTCGCTCT
CTCTCGCCCTCCACATCCCTCTCGGCATTGAGATGACCATAGGCCAGAGGAGAGTCCTTAAGTGGACA
+
FFFFFFFFFFFFFFFFFFFFFF:FFFFFFFFFFFFFFFF,FFFFFFFFFFFFFFFFFFFFFFFFFFFF
FFFFFFFFFFFFFFFFFFFFFFFFFFF:FFFFFFFFFFFFFFFFFFFFFFFFFFFFFFFFFFFFFFFF
@A00155:342:HHGFNDSXY:1:1506:23095:21668 1:N:0:GAACCTAG+TCCGCATA
TAGATCAGCTGTGTCCACTTAAGGACTCACCTCTGGCCTATGGTCATCTCTATGCCGAGAGGGATGTG
GAGGGCGAGAGAGAGCGACTTCTCTCGGGCCCACAGCCTTACAGCTGTGGAGCACGGTATCCTCTGCC
+
FFFFFFFFFFFFFFFFFFFFFFFFFFFFFFFFFFFFFFFFFFFFFFFFFFFFFFFF:FFFFFF:FFF
F:FFFFFFFFFFFFFFFFFFFFFFFFFFFFFFFFFFFFFFFFFFFFFFFFFFFFFFF:FFFFFFFFF
@A00155:342:HHGFNDSXY:1:1506:32018:35399 1:N:0:GAACCTAG+TCCGCATA
TGTCCACTTAAGGACTCTCCTCTGGCCTATGGTCATCTCAATGCCGAGAGGGATGTGGAGGGCGAGAG
AGAGCGACTTCTCTCGGGCCCACAGCCTTACAGCTGTGGAGCACGGTATCCTCTGCCGAAACAGAGGT
+
FFFFFFFFFFFFFFFFFFFFFFFFFFFFFFFFFFFFFFFFFFFFFFFFFFFFF:FFFFFFFFFFFFFF
FFFFFFFFFFFFFFFFFFFFFFFFFFFFFFFFFFFFFFFFFFFFFFFFF:FFFFFFFFFFFFFFF:FFFF
@A00155:342:HHGFNDSXY:1:1507:22733:12242 1:N:0:GAACCTAG+TCCGCATA
TCTCTCGGGCCCACAGCCTTACAGCTGTGGAGCACGGTATCCTCTGCCGAAACAGAGGTTGGACAAGA
CCGGAGGGGTCTCCTAGTTCCAAAGGAGATGTACTCCGGGCTTGTTCACGACCTACCGTGTAAGTCGT
+
FFFFFFFFFFFFFFFFFFFFFFFFFFFFFFFFFFFFFFFFFFFFFFFFFFFFFFFFFFFFFFFFF:FF
FFFFFFFFFFFFFFFFFFFFFFFFFFFFFFFFFFFFFFFFFFFFFFFFFFFFFFFFFFFFFFFFFFFF
@A00155:342:HHGFNDSXY:1:1507:18593:30718 1:N:0:GAACCTAG+TCCGCATA
CCTTACAGCTGTGGAGCACGGTATCCTCTGCCGAAACAGAGGTTGGACAAGACCGGAGGGGTCTCCTA
GTTCCAAAGGAGATGTACTCCGGGCTTGTTCACGACCTACCGTGTAAGTCGTAGTCTAGTAGGCTACC
+
FF,FFFFFFFFFFFFFFFFFFFFFFFFFFFFFFFFFFFFFFFFFFFFFFFFFFFFFFFFFFFFFFFFF
FFFFFFFFFFFFFFFFFFFFFFFFFFF,FFFFFFFFFFFFFFFFFFF:FFFFFFFFFFFFFFFFFFFFF
@A00155:342:HHGFNDSXY:1:1508:3360:3959 1:N:0:GAACCTAG+TCCGCATA
GGATGTGGAGGGCGAGAGAGAGCGACTTCTCTCGGGCCCACAGCCTTACAGCTGTGGAGCACGGTATC
CTCTGCCGAAACAGAGGTTGGACAAGACCGGAGGGGTCTCCTAGTTCCAAAGGAGATGTACT
+
FFFFFFFFFFFFFFFFFFFFFFFFFFFFFFFFFFFFFFFFFFFFFFFFFFFFFFFFFFFFFFFFFFFF
FFFFFFFFFFFFFFFFFFF:FFFFFFFFFFFFFFFFFFFFFFFFFFFFFFF,FFFFFFFFFFF
@A00155:342:HHGFNDSXY:1:1508:31530:23312 1:N:0:GAACCTAG+TCCGCATA
CAATGCCGAGAGGGATGTAGAGGGCGAGAGAGAGCGACTTCTCTCGGGCCCACAGCCTTACAGCTGTG
GAGCACGGTATCCTCTGCCGAAACAGAGGTTGGACAAGACCGGAGGGGTCTCCTAGTTCCAAAGGAGA
+
FFFFFFFFFFFFFFFFFFFFFFFFFFFFFFFFFFFFFFFF:FFFFFFFFFFFFFFFFFFFFFFFFFFF
FFFFFFFFFFFFFFFFFFFFFFFFFFFFFFFFFFFFFFFFFFFFFFFFFFFFFFFFFFFFFFFFFFFF
@A00155:342:HHGFNDSXY:1:1508:2826:32002 1:N:0:GAACCTAG+TCCGCATA
CTTCTCTCGGGCCCACAGCCTTACAGCTGTGGAGCACGGTATCCTCTGCCGAAACAGAGGTTGGACAA
GACCGGAGGGGTCTCCTAGTTCCAAAGGAGATGTACTCCGGACTTGTTCACGACCTACCGTGTA
+
FFFFFFFFFFFFFFFFFFFFFF:FFFFFFF:FFFFFFFFFFFFFFFFF,FFFFFFFFFFFFFFFFFFF
FFFFFFFFFFFFFFFFFFFFFFFFFF,FFFFFFFFFFFFFFFF:FFFFFFFFFFFFFFFFFFFFF,
@A00155:342:HHGFNDSXY:1:1508:5005:35900 1:N:0:GAACCTAG+TCCGCATA
CTTCTCTCGGGCCCACAGCCTTACAGCTGTGGAGCACGGTATCCTCTGCCGAAACAGAGGTTGGACAA

GACCGGAGGGGTCTCCTAGTTCCAAAGGAGATGTACTCCGGACTTGTTCACGACCTACCGTGTA
+
FFFFFFFFFFFFFFFFFFFFFF,FFF:FFFFF:,FFFF,FFFFFFFFFFFFFFFFFFFFFFFFFFFFF
F:FFFFFFFFFFFFFFFFFFFFFF:F:FFFFFFFFFFFFFFFFFFFFF:FFFFFFFFFFFFFFF
@A00155:342:HHGFNDSXY:1:1509:3956:25097 1:N:0:GAACCTAG+TCCGCATA
TTACAGCTGTGGAGCACGGTATCCTCTGCCGAAAAAGAGGTTGGACAAGACCGGAGGGGTCTCCTAGT
TCCAAAGGAGATGTACTCCGGGCTTGTTCACGACCTACCGTGTAAGTCGTAGTCTAGTAGGCTACCTG
+
FFFFFFFFFFFFFFFFFFFFFFFFFF:FFFFF:,F:F:FFFFFFF:FFFFFFFFFFFFFFFFFFFFF
FFF:FFFF:FFFFFFFFF:FFFFFFFFFFFFFFF:FFFFFF,FFFFFFFFF:FF:FFFFFFFFFFFF
@A00155:342:HHGFNDSXY:1:1510:9570:12242 1:N:0:GAACCTAG+TCCGCATA
AGGGATGTGGAGGGCGAGAGAGAGCGACTTCTCTCGGGCCCACAGCCTTACAGCTGTGGAGCACGGTA
TCCTCTGCCGAAACAGAGGTTGGACAAGACCGGAGGGGTCTCCTAGTTCCAAAGGAAATGTACTCCGG
+
FFFFFFFFFFFFFFFFFFFFFFFFFFFFFFFFFFFFFFFFFFFFFFFFF:FFFFFFFFFFFFFFFFFF
FFFFFFFFFFFFFFFFFFFFFFFFFFFFFFFFFFFFFFFFFFFFFFFFFFFFFFFFFFFFFFFFFFFF
@A00155:342:HHGFNDSXY:1:1510:2248:31657 1:N:0:GAACCTAG+TCCGCATA
GCCGAAACAGAGGTTGGACAAGACCGGAGGGGTCTCCTAGTTCCAAAGGAGATGTACTCCGGGCTTGT
TCACGACCTACCGTGTAAGTCGTAGTCTAGTAGGCTACCTGACGAGTCCTTTTTAGGACGAAACTTAC
+
FFFFFFFFFFFFFFFFFFFFFFFFFFFFFFFFFFFFFFFFFFFFF,FFFFFFFFFFFFFFFFFFFFFF
FFFFFFFFFFFFFFFFFFFFFFFFFFFFFFFFFFFFFFFFFFFFFFFFFFFFFFFFFFFFFFFFFFFF
@A00155:342:HHGFNDSXY:1:1510:2926:31861 1:N:0:GAACCTAG+TCCGCATA
GCCGAAACAGAGGTTGGACAAGACCGGAGGGGTCTCCTAGTTCCAAAGGAGATGTACTCCGGGCTTGT
TCACGACCTACCGTGTAAGTCGTAGTCTAGTAGGCTACCTGACGAGTCCTTTTTAGGACGAAACTTAC
+
FFFFFFFFFFFFFFFFFFFFF::FFFFFFFFFFFFFFFFFFFFFFFFFFFFFFFFFFFFFFFFFFFFF
FFFFFFFFFFFFF,FFFFFFFFFFFFFFFFFFFFFFFFFFFFFFFFFFFFFFFFFFFFFFFFFFFFFF
@A00155:342:HHGFNDSXY:1:1511:3477:11960 1:N:0:GAACCTAG+TCCGCATA
ATGCCGAGAGGGATGTGGAGGGCGAGAGAGAGCGACTTCTCTCGGGCCCACAGCCTTACAGCTGTGGA
GCACGGTATCCTCTGCCGAAACAGAGGTTGGACAAGACCGGAGGGGTCTCCTAGTTCCAAAGGAGATG
+
FFFFFFFFFFFFF:FFFFF:F:F,FFFFFFFFFFFFFFFFFFF:FFFFFFF:,FFFFFFFFFFFFFFF
FFFFFFFFFFFFFFFFFFFFF,:FFF,FFFFFFFF:FFFFFFFFFFF,FFFFFFFFFFFFFFFFFFFF
@A00155:342:HHGFNDSXY:1:1511:32768:14810 1:N:0:GAACCTAG+TCCGCATA
TGTGGAGGGCGAGAGAGAGCGACTTCTCTCGGGCCCACAGCCTTACAGCTGTGGAGCACGGTATCCTC
TGCCGAAACAGAGGTTGGACAAGACCGGAGGGGTCTCCTAGTTCCAAAGGAGATGTACTCCGGG
+
FFFFFFFFFFFFFFFFFFFFFFFFFFFFFFFFFFFFFFFFFFFFFFFFFFFFFFFFFFF:FFFFFFFF
:FFFFFFFFFFFFFFFFFFFFFFFFFFFFFFFFFFFFFFFFFFFFFFFFFFFFFFFFFFFFFFF
@A00155:342:HHGFNDSXY:1:1511:2645:16438 1:N:0:GAACCTAG+TCCGCATA
ATGCCGAGAGGGATGTAGAGGGCGAGAGAGAGCGACTTCTCTCGGGCCCACAGCCTTACAGCTGTGGA
GCACGGTATCCTCTGCCGAAACAGAGGTTGGACAAGACCGGAGGGGTCTCCTAGTTCCAAAGGAGAT
+
FFFFF:FFFFFFFFFFF:FFFFFFFFFFFFFF::FFFFFF,FFFFFFFFFFFFFFFFFFFFFFFFFFF
FF:FFFFFFFFFFFFFFFFFFFFFFFFFFFFFFFFFFFFFFFFFFFFFFFFFFFFFFFFFFFFFFFFF
@A00155:342:HHGFNDSXY:1:1511:27218:32910 1:N:0:GAACCTAG+TCCGCATA
TTACAGCTGTGGAGCACGGTATCCTCTGCCGAAACAGAGGTTGGACAAGACCGGAGGGGTCTCCTAGT
TCCAAAGGAGATGTACTCCGGGCTTGTTCACGACCTACCGTGTAAGTCGTAGTCTAATAGGCTACCTG
+
,FFFFFFFFFFFFFFF:FFFFFFFFFFFFFFFFFFFFFFFFFFFFFFFFFFFF:FFFFFFFFFFFFF
FFFFFFFFFFFFFFFFFFFFFFFFFFFFFFFFFFFF:,,FFFFFFFFF:FFFFFFFFFFF,FFFFFF
@A00155:342:HHGFNDSXY:1:1513:8630:19257 1:N:0:GAACCTAG+TCCGCATA
GGATGTGGAGGGCGAGAGAGAGCGACTTCTCTCGGGCCCACAGCCTTACAGCTGTGGAGCACGGTATC

CTCTGCCGAAACAGAGGTTGGACAAGACCGGAGGGGTCTCCTGGTTCCAAAGGAGATGTACTCC
+
FFFFF:FFFFFFFFFFFF:,FFFFFFFFFFF:FFF:FFFFFFFFFFFFFFFFFFFFFFFFFFFFF:FF
FFFFFFFFFFFFFFFFFFFFFFFFFFFFFFFFFFFFFFFFFFFFFFFFFFFFFFFFFFFFFFFF
@A00155:342:HHGFNDSXY:1:1513:17978:19789 1:N:0:GAACCTAG+TCCGCATA
GGATGTGGAGGGCGAGAGAGAGCGACTTCTCTCGGGCCCACAGCCTTACAGCTGTGGAGCACGGTATC
CTCTGCCGAAACAGAGGTTGGACAAGACCGGAGGGGTCTCCTGGTTCCAAAGGAGATGTACTCC
+
FFFFFFFFFFFFFFFFFFFFFFFFFFFFFFFFFFFFFFFFFFFFFFFFFFFFFFFFFFFFFFFF:FFF
FFFFFFFFFFFFFFFFFFFFFFFFFFFFFFFFFFFFFFFFFFFFFFFFFFFFFFFFFFFFFFFF
@A00155:342:HHGFNDSXY:1:1513:17472:22983 1:N:0:GAACCTAG+TCCGCATA
CTTCTCTCGGGCCCACAGCCTTACAGCTGTGGTGCACGGTATCCTCTGCCGAAACAGAGGTTGGACAA
GACCGGAGGGGTCTCCTAGTTCCAAAGGAGATGTACTCCGGGCTTGTTCACGACCTACCGTGTAAGTC
+
FFFFFFFFFFFFFFFFFFFFFFFFFFFFFFFFFFFFFFFFFFFFFFFFFFFFFFFFFFFFFFFFFFFF
FFFFFFFFFFFFFFFFFFFFFFFFFFFFFFFFFFFFFFFFFFFFFFFFFFFFFFFFFFFFFFFFFFFF
@A00155:342:HHGFNDSXY:1:1513:11397:31469 1:N:0:GAACCTAG+TCCGCATA
CACCTCTGGCCTATGGTCATCTCAATGCCGAGAGGGATGTGAAGGGCGAGAGAGAGCGACTTCTCTCG
GGCCCACAGCCTTACAGCTGTGGAGCACGGTATCCTCTGCCGAAACAGAGGTTGGACAAGACCGGAG
+
FFFFFFFFFFFFFFFFFFFFFFFFFFFFFFFFFFFFFFF:FFFFFFFFFFFFFFFFFFFFFFFFFFFF
FFFFFF:FFFFFFFFFFFFFFFFFFFFFFFFFFFFFFFFFFFF:FFFFFFF,FFFFFFF:FFFFFFF
@A00155:342:HHGFNDSXY:1:1514:28863:20134 1:N:0:GAACCTAG+TCCGCATA
TGTTTCGGCAGAGGATACCGTGCTCCACAGCTGTAAGGCTGTGGGCCCGAGAGAAGTCGCTCTCTCTC
GCCCTCCACATCCCTCTCGGCATTGAGATGACCATAGGCCAGAGGTGAGTCCTTAAGTGGACACAGCT
+
FFF:FFFFFFFFFFFFFFFFFFFFFFFFFFF:FFFFFFFFFFFFFFFFFFFFFFFFFFFFFFFFFFFF
FFFFFFFFF:,FFFFFFFFFFF,FFFFFFFFFFF::FFFFFFFFFFFFFFFFFFFFFFFFFFFFFFFF
@A00155:342:HHGFNDSXY:1:1514:7102:23124 1:N:0:GAACCTAG+TCCGCATA
GAGAGAGCGACTTCTCTCGGGCCCACAGCCTTACAGCTGTGGAGCACGGTATCCTCTGCCGAAACAGA
GGTTGGACAAGACCGGAGGGGTCTCCTAGTTCCAAAGGAGATGTACTCCGGGCTTGTTCACGACCTAC
+
FFFFFFFFFFFFFFFFFFFFFFFFFFFFFFFF:FFFFFFFFFFFFFFFFFFFFFFFFFFFFFFFFFFF
FFFFFFFFFFFFFFFFFFFFFF:FFFFFFFFFFFFFF,FFFFFF:FFFFFFFFFFFFFFFFFFFFFFF
@A00155:342:HHGFNDSXY:1:1515:22092:2957 1:N:0:GAACCTAG+TCCGCATA
GGGCGAGAGAGAGCGACTTCTCTCGGGCCCACAGCCTTACAGCTGTGGAGCACGGTATCCTCTGCCGA
AACAGAGGTTGGACAAGACCGGAGGGGTCTCCTGGTTCCAAAGGAGATGTACTCCGGGC
+
FFFFFFFFFFFFFFFFFF:FFFFFFFFFFFFFFFFFFFFFFFFFFFFFFFFFFFFFFFFFFFFFFFFF
FFFFFFFFFFFFFFFFFFFFFFFFFFFFFFFF:FFFFFFFFFFFFFFFFFFFFFFFFFF
@A00155:342:HHGFNDSXY:1:1515:18747:14951 1:N:0:GAACCTAG+TCCGCATA
GCGACTTCTCTCGGGCCCACAGCCTTACAGCTGTGGAGCACGGTATCCTCTGCCGAAACAGAGGTTGG
ACAAGACCGGAGGGGTCTCCTAGTTCCAAAGGAGATGTACTCCGGGCTTGTTCACGACCTACCGTGTA
+
FFFFFFFFFFFFFFFFFFFFFFFFFFFFFFFFFFFFFFFFFFFFFFFFFFFFFFFFFFFFF:FFFFFF
FFFFFFFFFFFFFFFFFFFFFFFFFFFFFFFFFFFFFFFFFFFFFFFFFFFFFFFFFFFFFFFFFFFF
@A00155:342:HHGFNDSXY:1:1515:11659:18709 1:N:0:GAACCTAG+TCCGCATA
AGAGAGAGCGACTTCTCTCGGGCCCACAGCCTTACAGCTGTGGAGCACGGTATCCTCTGCCGAAACAG
AGGTTGGACAAGACCGGAGGGGTCTCCTGGTTCCAAAGGAGATGTACTCCGGGCTTGTTCACGACCTA
+
FFFFFFFFFFFFFFFFFFFFFFFFFFFFFFFFFFFFFFFFFFFFFFFFFFFFFFFFFFFFFFFFFFFF
FFFFFFFFFFFFFFFFFFFFFFFFFFFFFFFFFFFFFFFFFFFFFFFFFFFFFFFFFFFFFFFFFFFF
@A00155:342:HHGFNDSXY:1:1515:19759:20588 1:N:0:GAACCTAG+TCCGCATA
CTTCTCTCGGGCCCACAGCCTTACAGCTGTGGAGCACGGTATCCTCTGCCGAAACAGAGGTTGGACAA

GACCGGAGGGGTCTCCTAGTTCCAAAGGAGATGTACTCCGGGCTTGTTCACGACCTACCGTGTAAGTC
+
FFFFFFFFFFFFFFFFFFFFFFFFFFFFFFFFFFFFFFFFFF:FFFFFFFFFFFFFFFFFFFFFFFFFFF
:FF:FFFFFFFFFFFFFFFFFFFFFFFFFFFFFFFFFFFFFFFFFFFFFFFFFFFFFFFFFFFFFFFFFF
@A00155:342:HHGFNDSXY:1:1515:13919:28260 1:N:0:GAACCTAG+TCCGCATA
ATCTCAATGCCGAGAGGGATGTGGAGGGCGAGAGAGAGCGACTTCTCTCGGGCCCACAGCCTTACAGC
TGTGGAGCACGGTATCCTCTGCCGAAACAGAGGTTGGACAAGACCGGAGGGGTCTCCTAGTTCCAAAG
+
FFFFFFFFFFFFFFFFFFFFFFFFFFFFFFFFFFFFFFFFFFFFFF,FFFFFFFFFFFFFFFFFFFFFF
FFFFFFFFFFFFFFFFFFFFFFFFFFFFFFFFFFFFFFFFFFFFFFFFFFFFFFFFFFFFFFFFFFFFFF
@A00155:342:HHGFNDSXY:1:1516:31774:3881 1:N:0:GAACCTAG+TCCGCATA
GGATGTGGAGGGCGAGAGAGAGCGACTTCTCTCGGGCCCACAGCCTTACAGCTGTGGAGCACGGTATC
CTCTGCCGAAACAGAGGTTGGACAAGACCGGAGGGGTCTCCTAGTTCCAAAGGAGATGTACTCC
+
FFFFFFFFFFFFFFFFFFFFFFFFFFFFFFFFFFFFFFFFFFFFFFFFFFFFFFFFFFFFFFFFFFFFFF
FFFFFFFFFFFFFFFFFFFFFFFFFFFFFFFFFFFFFFFFFFF,FFFFFFFFFFFFFFFFFFFFF
@A00155:342:HHGFNDSXY:1:1516:13847:25692 1:N:0:GAACCTAG+TCCGCATA
AGGACTCACCTCTGGCCTATGGTCATCTCAATGCCGAGAGGGATGTGAAGGGCGAGAGAGAGCGACTT
CTCTCGGGCCCACAGCCTTACAGCTGTGGAGCACGGTATCCTCTGCCGAAACAGAGGTTGGACAAGAC
+
FFFFFFFFFFFFFFFFFFFFFFFFFFFFFFFFFFFFFFFFFFF:FF:FFFFFFFFFFFFFFFFFFFFFFF
FFFFFFFFFFFFFFFFFFFFFFFFFFFFFFFFFFFFFFFFFFFFFFFFFFFFFFFFFFFFFFFFFFFFFF
@A00155:342:HHGFNDSXY:1:1516:22498:25833 1:N:0:GAACCTAG+TCCGCATA
ACCATAGGCCAGAGGTGAGTCCTTAAGTGGACACAGCTGATCTAAGGCGGTGTAGCGGGGCATGGGTT
TGAACCCCCATGACGGTCGGAGTCTAGTAGGCTCCCTGATGAGTCCGTTCCAAGGACGAAACCGTCGT
+
FFFFFFFFFFFFFFFFFFFFFFFFFFFFFFFFFFFFFFFFFFF:FFFFFFFFFFFFFFFFFFFFFFFFFF
FFFFFFFFFFFFFFFFFFFFFFFFFFFFFFFFFFFFFFFFFFFFFFFFFFFFFFFFFFFFFFFFFFFFFF
@A00155:342:HHGFNDSXY:1:1516:20672:25895 1:N:0:GAACCTAG+TCCGCATA
GAGGGCGAGAGAGAGCGACTTCTCTCGGGCCCACAGCCTTACAGCTGTGGAGCACGGTATCCTCTGCC
GAAACAGAGGTTGGACAAGACCGGAGGGGTCTCCTAGTTCCAAAGGAGATGTACTCCGGGCTTGTTCA
+
FFFFFFFFFFFF:FFFFFFFFFFFFFFFFFF:FF:FFFFFFFFFFFFFFF:FFF:FFFFFFFFFFFFFF
FF:FFF:FF:FFFFFFFFF:F:FFFFFFFFFFFFFFFFFFFFFFFFFFFFFFFFFFFFFFFFFFFFFFFF
@A00155:342:HHGFNDSXY:1:1517:8811:11209 1:N:0:GAACCTAG+TCCGCATA
TCCACTTAAGGACTCACCTCTGGCCTATGGTCATCTCAATGCCGAGAGGGATGTGAAGGGCGAGAGAG
AGCGACTTCTCTCGGGCCCACAGCCTTACAGCTGTGGAGCACGGTATCCTCTGCCGAAACAGAGGTTG
+
FF:FFFFFFFFFFFFFFFFFFFFFFFFFFFFFFFFFFFFFFFFF:FFFFFFFFFFFFF:FFFFFFFFFF
FFFFFFFFFFFFFFFFF,FFFF,FFFFFFFFFFFFFFFFFFFFF:FFFFFFFFF:FFFFFFFFFFFFFF
@A00155:342:HHGFNDSXY:1:1517:26937:13542 1:N:0:GAACCTAG+TCCGCATA
ACTTCTCTCGGGCCCACAGCCTTACAGCTGTGGAGCACGGTATCCTCTGCCGAAACAGAGGTTGGACA
AGACCGGAGGGGTCTCCTAGTTCCAAAGGAGATGTACTCCGGGCTTGTTCACGACCTACCGTGTAAGT
+
FFFFFFFFFFFFFFFFFFFFFFFFFFFFFF,FFFFFF:FFFFFFFFFFFFFFFFFFFFFFFFFFFFFFF
FFFFFFFFFFFFFFFFFFFFFFF:FFFFFFFFFFFFFFFF:FFFF:FFFFFFFFFFFF:FFFFFFFFFF
@A00155:342:HHGFNDSXY:1:1517:12554:13745 1:N:0:GAACCTAG+TCCGCATA
CAATGCCGAGAGGGATGTAGAGGGCGAGAGAGAGCGACTTCTCTCGGGCCCACAGCCTTACAGCTGTG
GAGCACGGTATCCTCTGCCGAAACAGAGGTTGGACAAGACCGGAGGGGTCTCCTAGTTCCAAAGGAGA
+
FFFFFFFFFFFFFFFFFFFFFFFFFFFFFFFFFFFFFFFFFFFF:FFFFFFFFFFFFF:FFFFFF:FFF
FFFFFFFF:FFFFFFFFFFFF:FFFFFFFFFFFFFFFFFFFFFFFFFFFFFFFFF::FFFFFFFFFFFFF
@A00155:342:HHGFNDSXY:1:1517:24867:18537 1:N:0:GAACCTAG+TCCGCATA
GACTTCTCTCGGGCCCACAGCCTTACAGCTGTGGAGCACGGTATCCTCTGCCGAAACAGAGGTTGGAC

AAGACCGGAGGGGTCTCCTAGTTCCAAAGGAGATGTACTCCGGGCTTGTTCACGACCTACCGTGTAAG
+
FFFFFFFFFFFFFFFFFFFFFFFFFFFFFFFF:FFFFFFFFF:FFFFFFFFFFFFFFFFFFFFFFFFF
FFFFF:FFFFFFFFFFFFF:FFFFFFFFFFFFFFFFFFFFFFFFFFFFFFFFFFFFFFFFFFFFFFFF
@A00155:342:HHGFNDSXY:1:1518:6686:7435 1:N:0:GAACCTAG+TCCGCATA
AGGGCGAGAGAGAGCGACTTCTCTCGGGCCCACAGCCTTACAGCTGTGGAGCACGGTATCCTCTGCCG
AAACAGAGGTTGGACAAGACCGGAGGGGTCTCCTAGTTCCAAAGGAGATGTACTCCGAGCTTGTTCAC
+
FFFFFFFFFFFFFFFFFFFFFFFFFFFFFFFFFFFFFFFFFFFFFFFFFFFF:FFFFFFFFFF:FFFF
FFFFFF:FFFFFFFFFFFFFF:FFFFFFFFFFFFFFFFFFFFFFFFFF:F::FFFFFFFFFFFFFFFF
@A00155:342:HHGFNDSXY:1:1518:23330:9142 1:N:0:GAACCTAG+TCCGCATA
TGCCGAGAGGGATGTGGAGGGCGAGAGAGAGCGACTTCTCTCGGGCCCACAGCCTTACAGCTGTGGAG
CACGGTATCCTCTGCCGAAACAGAGGTTGGACAAGACCGGAGGGGTCTCCTAGTTCCAAAGGAGATGT
+
FFFFFFF:FFFF,FFFFFFFFFFFFFFFFFFFFFFFFFFFFFFFFFFFFFFFFFFFFFFFFFFFFFFF
FFFFFFFFFFFFFFFFFFFFFFFFFFFFFFFFFFFFFFFFFFFFFFFFFFFFFFFFFFFFFFFFFFFF
@A00155:342:HHGFNDSXY:1:1518:25825:11052 1:N:0:GAACCTAG+TCCGCATA
AGCTGTGTCCACTTAAGGACTCACCTCTGGCCTATGGTCATCTCAATGCCGAGAGGGATGTGAAGGGC
GAGAGAGAGCGACTTCTCTCGGGCCCACAGCCTTACAGCTGTGGAGCACGGTATCCTCTGCCGAAACA
+
FFFFFFFFFFFFFFFFFFFFFFFF,FFFFFFFFFFFFFFFFFFFFFFFFFFFFFFFFFFFFFFFFFFF
FFFFFFFFFFFFFFF:FFFFFFFF,FFFFFFFF,FFFFFFFFFFFFFFFFFFFFFFFFFFFFFF:F,:
@A00155:342:HHGFNDSXY:1:1518:1425:27132 1:N:0:GAACCTAG+TCCGCATA
ACCGTCATGGGGGTTCAAACCCATGCCCCGCCACACCGCCTTAGATCAGCTGTGTCCACTTAAGGACT
CACCTCTGGCCTATGGTCATCTCAATGCCGAGAGGGATGTGGAGGGCGAGAGAGAGCGACTTCTCTCG
+
F,FF::FFF,:F:F::FFFFFFFF:FF:F,::FFF:::F:F:FF,FF:FFFFF:FF:F::F:F:FFFF
,F,,FFFFF,,FFF,:FF:,:FFF:F,,F,F,,,FFF:FFFFFF:,FF:FFFFF,FFF,F:,:FFFFF
@A00155:342:HHGFNDSXY:1:1519:30400:2253 1:N:0:GAACCTAG+TCCGCATA
CTCCACATCCCTCTCGGCATTGAGATGACCATAGGCCAGAGGTGAGTCCTTAAGTGGACACAGCTGAT
CTAAGGCGGTGTGGCGGGGCATGGGTTTGAACCCCCATGACGGTCGGAGTCTAGTAGGCTCCCTGATG
+
FFFFFF:FFFFFFFF:FF,FFF:FFFFFFFFFFFFFFF:FFFFFFFFF:FFFFFFFFFFFFFFFFFFF
FFF,FFFFFFFFFFFFFFFFFF:FF:FFFFFFFFFFFFFFFFFFFFFFF:FFFFFFFFFFFFFFF:FF
@A00155:342:HHGFNDSXY:1:1519:2880:20541 1:N:0:GAACCTAG+TCCGCATA
CGAGAGAGAGCGACTTCTCTCGGGCCCACAGCCTTACAGCTGTGGAGCACGGTATCCTCTGCCGAAAC
AGAGGTTGGACAAGACCGGAGGGGTCTCCTAGTTCCAAAGGAGATGTACTCCGGGCTTGTTCACGACC
+
FFFFFFFFFFF:FFFFFFFFFFFFFFFFFFFFFFFFFF,FF:FFFFFFFFFFFFFFFFFFFF,FFFFF
FFFF,FF:FFFFFFFF:FFFFF:FFFFFFFFFFFFFFFFFFFFFFFFFFFF:FFFFFFFFFFFFFFFF
@A00155:342:HHGFNDSXY:1:1519:3305:27258 1:N:0:GAACCTAG+TCCGCATA
TAAGTGGACACAGCTGATCTAAGGCGGTGTGGCGGGGCATGGGTTTGAACCCCCATGACGGTCGGAGT
CTAGTAGGCTCCCTGATGAGTCCGTTCCAAGGACGACACCGTCGTTACTCTTCGAGTTGTGTAAGTTT
+
FFFFFFFFFFFFFFFFFFFFFFFFFFFFFFFFFFFFFFFFFFFFFFFFFFFFFFFFFFFFFFFFFFFF
FFFFFFFFFFFFFFFFFFFFFFFFFFFFFFFFFFF:,:FFFFFFFFF:FFFFFFFFFFFFFFFFFFFF
@A00155:342:HHGFNDSXY:1:1519:12029:31093 1:N:0:GAACCTAG+TCCGCATA
ACCTCTGTTTCGGCAGAGGATACCGTGCTCCACAGCTGTAAGGCTGTGGGCCCGAGAGAAGTCGCTCT
CTCTCGCCCTTCACATCCCTCTCGGCATTGAGATGACCATAGGCCAGAGGTGAGTCCTTAAGTGGACA
+
:FFFFFFFFFFFFFFFFFFF,FFFFFFF:FFF:FFFFFFF:,FFFFFF:FFF:FFFFFFFFFFFF,F:
:FFF,FFFF,:FFFFFFFF,FFF:FFF,FFF:,FFFFF:,FFF,FF:FFFFFFFFFFFFFF:,,:::F
@A00155:342:HHGFNDSXY:1:1520:25120:2190 1:N:0:GAACCTAG+TCCGCATA
CCACATCCCTCTCGGCATTGAGATGACCATAGGCCAGAGGTGAGTCCTTAAGTGGACACAGCTGATCT

```
AAGGCGGTGTGGCGGGGCATGGGTTTGAACCCCCATGACGGTCGGAGTCTAGTAGGCTCCCTGATGAG
+
FFFFFFFFFFFFFFFFFFFFFFFFFFFFFFFFFFFFFFFFFFFFFFFFFFFFFFFFFFFFFFFFFFFF
FFFFFFFFFFFFFFFFFFFFFFFFFFFFFFFFFFFFFFFFFFFFFFFFFFFFFFFFFFFFFFFFFFF:
@A00155:342:HHGFNDSXY:1:1520:17879:6089 1:N:0:GAACCTAG+TCCGCATA
TCCACTTAAGGACTCACCTCTGGCCTATGGTCATCTCAATGCCGAGAGGGATGTGAAGGGCGAGAGAG
AGCGACTTCTCTCGGGCCCACAGCCTTACAGCTGTGGAGCACGGTATCCTCTGCCGAAACAGAGGTTG
+
FFFFFFFFFFFFFFFFFFFFFFFFFFFFFFFFFFFFFFFFFFFFFFFFFFFFFFFFFFFFFFFFFFFF
FFFFFFFFFFFFFFFFFFFFFFFFFFFFFFFFFFFFFFFFFFFFF:FFFFFFFFFFFFFFFFFFFFFF
@A00155:342:HHGFNDSXY:1:1520:15456:8187 1:N:0:GAACCTAG+TCCGCATA
TCCACTTAAGGACTCACCTCTGGCCTATGGTCATCTCAATGCCGAGAGGGATGTGAAGGGCGAGAGAG
AGCGACTTCTCTCGGGCCCACAGCCTTACAGCTGTGGAGCACGGTATCCTCTGCCGAAACAGAGGTTG
+
:FFFFFFFFFFFFFFFFFFFFFFFFFFFFFFFFFFFFFFFFFFFFFF:FFFFFFFFFFF:FFFFFFFF
FFF:FFFFFFFFFFFFFFFFFFFFFFFFF:FFFFFFFFFF:FFF:FFFFFFFFFFFF:F:FFFFFFFF
@A00155:342:HHGFNDSXY:1:1520:3495:10958 1:N:0:GAACCTAG+TCCGCATA
ATCTCAATGCCGAGAGGGATGTGGAGGGCGAGAGAGAGCGACTTCTCTCGGGCCCACAGCCTTACAGC
TGTGGAGCACGGTATCCTCTGCCGAAACAGAGGTTGGACAAGACCGGAGGGGTCTCCTGGTTCCAAAG
+
FFFFFFFFFF:FF:FF:FFFF,,FFFFFFFFFFFFFFF:FF:FFFFFFFFFFFFFFFFFFFFF:FFFF
FFFFFFFFFFFFF,FFFFFFFFFFFFFFFFFFFFFFFFFFFFFFFFFFFFFF:FFFFFFFFFFFFFFF
@A00155:342:HHGFNDSXY:1:1520:21721:23516 1:N:0:GAACCTAG+TCCGCATA
TGGTCATCTCAATGCCGAGAGGGATGTGGAGGGCGAGAGAGAGCGACTTCTCTCGGGCCCACAGCCTT
ACAGCTGTGGAGCACGGTATCCTCTGCCGAAACAGAGGTTGGACAAGACCGGAGGGGTCTCCTAGTTC
+
FFFFFFFFFFFFFFFFFFFFFFFFFFFFFFFFFFFFFFFFFFFFFFFFFFFFFFFFFFFFFFFFFF::
:FFFFFFFFFFFFFFFFFFFFFFFFFFFFFF,FFFFFFFFFFFFFFFFFFFFFFFFFFFFFFFFFFFF
@A00155:342:HHGFNDSXY:1:1520:4842:26506 1:N:0:GAACCTAG+TCCGCATA
CCTTACAGCTGTGGAGCACGGTATCCTCTGCCGAAACAGAGGTTGGACAAGACCGGAGGGGTCTCCTG
GTTCCAAAGGAGATGTACTCCGGGCTTGTTCACGACCTACCGTGTAAGTCGTAGTCTAGTAGGCTACC
+
FFFFFFFFFFFFFFFFFFFFFFFFFFFFFFFFFFFFFFFFFFFFFFFFFFFFFFFFFFFFFFFFFFFF
FFFFFFFFFFFFFFFFFFFFFFFFFFFFFFFFFFFFFFFFFFFFFFFFFFFFFFFFFFFFFFFFFFFF
@A00155:342:HHGFNDSXY:1:1520:7563:34507 1:N:0:GAACCTAG+TCCGCATA
TATCCTCTGCCGAAACAGAGGTTGGACAAGACCGGAGGGGTCTCCTAGTTCCAAAGGAGATGTACTCC
GGGCTTGTTCACGACCTACCGTGTAAGTCGTAGTCTAGTAGGCTACCTGACGAGTCCTT
+
FFFFF:FFFF::FFFFFFF:FFFFFFFFF,F,FF,FFFFFFFFFFFFFFFFFFFF,,FFFFFFFFFF,
,FFFFFFFFFFF,FFFFF:FFFFFF,:FFFFFFFFFFF,FFFFFFFF:FFFFFFFF,FF
@A00155:342:HHGFNDSXY:1:1521:23005:21324 1:N:0:GAACCTAG+TCCGCATA
TTAGATCAGCTGTGTCCACTTAAGGACCCACCTCTGGCCTATGGTCATCTCAATGCCGAGAGGGATGT
GGAGGGCGAGAGAGAGCGACTTCTCTCGGGCCCACAGCCTTACAGCTGTGGAGCACGGTATCCTCTGC
+
:FFFFFFFFFFFFFFFFFFFFFFFFFF:FFFFFFFFFFFFFFFFFFFFFFFFFFFFFFFFFFFFFFFF
FFFFFFFFFFFFFFFFFFFFFFFFFFFFFFFFFFFFFFFFFFFFFFFFFFFFFFFFFFFFFFFFFFFF
@A00155:342:HHGFNDSXY:1:1522:23737:20087 1:N:0:GAACCTAG+TCCGCATA
ACCTCTGTTTCGGCAGAGGATACCGTGCTCCACAGCTGTAAGGCTGTGGGCCCGAGAGAAGTCGCTCT
CTCTCGCCCTCCACATCCCTCTCGGCATTGAGATGACCATAGGCCAGAGGAGAGTCCTTAAGTGGACA
+
FFFFFFFFFFFFFFFFFFFFFFFFFFFFFFFFFFFFFFFFF:FFFFFFFFFFFFFFFFFFFFFFFFFF
FFFFFFFFFFFFFFFFFFFFFFFFFFFFFF,F:FFFFFFFFFFFFFFFFFFFFFFFFFFFFFFFFFFF
@A00155:342:HHGFNDSXY:1:1522:18249:23265 1:N:0:GAACCTAG+TCCGCATA
TGTGGAGGGCGAGAGAGAGCGACTTCTCTCGGGCCCACAGCCTTACAGCTGTGGAGCACGGTATCCTC
```

```
TGCCGAAACAGAGGTTGGACAAGACCGGAGGGGTCTCCTAGTTCCAAAGGAGATGTACTCCGGG
+
FFFFFFFFFFFFFFFFFFFFFFFFFFFFFFFFFFFFFFFFFFFFFFFFFFFFFFFFFFFFFFFFFFFFFFFF
FFFFFFFFFFFFFFFFFFFFFFFFFFFFFFFFFFFFFFFFFFFFFFFFFFFFFFFFFFFFFFFF
@A00155:342:HHGFNDSXY:1:1522:16143:26725 1:N:0:GAACCTAG+TCCGCATA
ATCTCAATGCCGAGAGGGATGTAGAGGGCGAGAGAGAGCGACTTCTCTCGGGCCCACAGCCTTACAGC
TGTGGAGCACGGTATCCTCTGCCGAAACAGAGGTTGGACAAGACCGGAGGGGTCTCCTAGTTCCAAAG
+
FFFFFFFFFFFFFFFFFFFFFF:FFFFFFFFFFFFFFFFFFFFFFFFFFFFFFFFFFFFFFFFFFFFFFFFF
FFFFFFFFFFFFFFFFFFFFFFFFFFFFFFFFFFFFFFFFFFFFFFFF:FFFFFFFFFFFFFFFFFFFFF
@A00155:342:HHGFNDSXY:1:1522:5656:32424 1:N:0:GAACCTAG+TCCGCATA
CCTTACAGCTGTGGAGCACGGTATCCTCTGCCGAAACAGAGGTTGGACAAGACCGGAGGGGTCTCCTA
GTTCCAAAGGAGATGTACTCCGGGCTTGTTCACGACCTACCGTGTAAGTCGTAGTCTAG
+
FFFFFFFFFFFFFFFFFFFFFFFFF:FFFFFF:FFFFFFFF,FFFFFFFFFFFFFFFFFFFFFFFFFFFF
FFFFFFFFFFFFFFFFFFFFFFFFFFFFFFFFFFF:FFFFF:FFFFFFFFFFFFFFFFFFF
@A00155:342:HHGFNDSXY:1:1523:18991:8766 1:N:0:GAACCTAG+TCCGCATA
ACATCCCTCTCGGCATTGAGATGACCATAGGCCAGAGGTGAGTCCTTAAGTGGACACAGCTGATCTAA
GGCGGTGTGGCGGGGCATGGGTTTGAACCCCCATGACGGTCGGAGTCTAGTAGGCTCCCTGATGAGTC
+
F:FF:,F:FFF,F,:F:FFFFF:FFFFFFFFF:FFFFFFFFFFFFFFF:F,F:FFF,FF::FF,FFFF
FF:,FFFFFFFF,F:FFFFF,F:FFFFFFFFFFF:FFFFFF:F,FFFFFFFFFF::F,FFFFF:FFFF
@A00155:342:HHGFNDSXY:1:1523:26503:20995 1:N:0:GAACCTAG+TCCGCATA
TTCTCTCGGGCCCACAGCCTTACAGCTGTGGAGCACGGTATCCTCTGCCGAAACAGAGGTTGGACAAG
ACCGGAGGGGTCTCCTAGTTCCAAAGGAGATGTACTCCGGGCTTGTTCACGACCTACCGTGTAAGTC
+
FFFFFFFFFFFFFFFFFFFFFFFFFFFFFFFFFFFFFFFFFFFFFFFFFFFFFFFFFFFFFFFFFFFFFFFF
FFFFFFFFFFFFFFFFFFFFFFFFFFFFFFFFFFFFFFFFFFFFFFFFFFFFFFFFFFFFFFFFFFF
@A00155:342:HHGFNDSXY:1:1524:6126:24471 1:N:0:GAACCTAG+TCCGCATA
TGTAGAGGGCGAGAGAGAGCGACTTCTCTCGGGCCCACAGCCTTACAGCTGTGGAGCACGGTATCCTC
TGCCGAAACAGAGGTTGGACAAGACCGGAGGGGTCTCCTAGTTCCAAAGGAGATGTACTCCGGG
+
FF:FFFFFFFFFFFFFFFFFFFFFFFFFFFFFFFFFFFFFFFFFF,FFF:FFFFFFFFFFFF:FFFFF
FFFFFFFFFFFFFFFFFFFFFFFFFFFFFFFFFFFFFFFFFFFFFFFFFFFFFFFFFFFFFFFF
@A00155:342:HHGFNDSXY:1:1524:1597:26647 1:N:0:GAACCTAG+TCCGCATA
GTCATCTCAATGCCGAGAGGGATGTAGAGGGCGAGAGAGAGCGACTTCTCTCGGGCCCACAGCCTTAC
AGCTGTGGAGCACGGTATCCTCTGCCGAAACAGAGGTTGGACAAGACCGGAGGGGTCTCCTAGTTCCA
+
FFFFFFFFF:FFFFF:F:FFFF,FFFF:FFFFFFFFFFFFFFFFFFFFFFFFFFFFFFF:FFFF:FFFF
FFFFFF:FFFF:FFFF:FFFFFF:FFFFF::FF,FFFFFFF:FFF,:FFF,FFFFF:FF:FFFFFFFF
@A00155:342:HHGFNDSXY:1:1525:23059:6668 1:N:0:GAACCTAG+TCCGCATA
AGCGACTTCTCTCGGGCCCACAGCCTTACAGCTGTGGAGCACGGTATCCTCTGCCGAAACAGAGGTTG
GACAAGACCGGAGGGGTCTCCCAGTTCCAAAGGAGATGTACTCCGGGCTTGTTCACGACCTACCGTGT
+
FFFFFFFFFFFFFFFFFFFFFFFFFFFFFFFFFFFFFFFFFFFFFFFFFFFFFFFFFFFFFFFFFFFFFFFF
FFFFFFFFFFFFFFFFFFFFFFFFFFFFFFFFFFFFFFFFFFFFFFFFFFFFF,F,FFFFFFFFFFF
@A00155:342:HHGFNDSXY:1:1525:23068:6872 1:N:0:GAACCTAG+TCCGCATA
AGCGACTTCTCTCGGGCCCACAGCCTTACAGCTGTGGAGCACGGTATCCTCTGCCGAAACAGAGGTTG
GACAAGACCGGAGGGGTCTCCCAGTTCCAAAGGAGATGTACTCCGGGCTTGTTCACGACCTACCGTGT
+
FFFFFFFFFFFFF:FFFFFFFFFFFFFFFFFFFFFFFFFFFFFFFFFFFFFFFFF:FFFFFFFFFFFFFF
FFFFFFFFFFFFFFFFFFFFFFFFFFFFFFFFFFFFFFFFFF:FFFFFFFFFFFFFFFFFFFFFFFFFFF
@A00155:342:HHGFNDSXY:1:1525:23077:6887 1:N:0:GAACCTAG+TCCGCATA
AGCGACTTCTCTCGGGCCCACAGCCTTACAGCTGTGGAGCACGGTATCCTCTGCCGAAACAGAGGTTG
```

```
GACAAGACCGGAGGGGTCTCCCAGTTCCAAAGGAGATGTACTCCGGGCTTGTTCACGACCTACCGTGT
+
FFFFFFFFFFFFFFFFFFFFFFFFF:FFFFFFFFFFFFFFFFFFFFFFFFFFFFFFFFFFFFFFFFFF
FFFFFFFFFFFFFFFFFFFFFFFFFFFFFFFFFFFFFFFFFFFFFFFFFFFFFFFFFFFFFFFFFFFF
@A00155:342:HHGFNDSXY:1:1525:9733:12179 1:N:0:GAACCTAG+TCCGCATA
ACTTCTCTCGGGCCCACAGCCTTACAGCTGTGGAGCACGGTATCCTCTGCCGAAACAGAGGTTGGACA
AGACCGGAGGGGTCTCCTGGTTCCAAAGGAGATGTACTCCGGGCTTGTTCACGACCTACCGTGTAAGT
+
FF:FFFFFFFFFFFFFFFFFFFFFFFFFFFFFFFFFFFFFFFFFFFFFFFFFFFFFFFFFFFFFFFFF
FFFFFFFFFFFFFFFFFFFFFFFFFFFFFFFFFFFFFFFFFFFFFFFFFFFFFFFFFFF:FFFFFFFF
@A00155:342:HHGFNDSXY:1:1525:29839:13526 1:N:0:GAACCTAG+TCCGCATA
CTCAATGCCGAGAGGGATGTGGAGGGCGAGAGAGAGCGACTTCTCTCGGGCCCACAGCCTTACAGCTG
TGGAGCACGGTATCCTCTGCCGAAACAGAGGTTGGACAAGACCGGAGGGGTCTCCTAGTTCCAAAGGA
+
FF:FFFFFFFFFFFFFFFFFF:FFFFFFFFFFFFFFFFFFFFFFFFFFFFFFFFFFFFFFFFFFFFFF
FFFFFFFFFFFFFFFFFFFFFFFFFFFFFFFFFFFFFFFFFFFFFFFFFFFFFFFFFFFFFFFFFFFF
@A00155:342:HHGFNDSXY:1:1525:24569:22185 1:N:0:GAACCTAG+TCCGCATA
ACTTCTCTCGGGCCCACAGCCTTACAGCTGTGGAGCACGGTATCCTCTGCCGAAACAGAGGTTGGACA
AGACCGGAGGGGTCTCCTAGTTCCAAAGGAGATGTACTCCGGGCTTGTTCACGACCTACCGTGTAAGT
+
FFFFFFFFFFFFFFFFFFFFFFFFFFFFFFFFFFFFFFFFF,FFFFFFFFFFFFFFFFFFFFFFFFFF
FFFFFFFFFFFFFFFFFFFFFFFFFFFFFFFFFFFFFFFFFFFFFFFFFFFFFFFFFFFFFFFFFFFF
@A00155:342:HHGFNDSXY:1:1525:24388:22279 1:N:0:GAACCTAG+TCCGCATA
ACTTCTCTCGGGCCCACAGCCTTACAGCTGTGGAGCACGGTATCCTCTGCCGAAACAGAGGTTGGACA
AGACCGGAGGGGTCTCCTAGTTCCAAAGGAGATGTACTCCGGGCTTGTTCACGACCTACCGTGTAAGT
+
FFFFFFFFFFFFFFFFFFFFF,FFFFFFFFFFFFFFFFFFFFFFFFFF:FFFFFFFFFFFFFFFFF:F
FFFFFFFFFFFFFFFFF:FFFFFFFFFFFFFFFFFF,FFFFFFFFF:FFFFFFFFFFFFFFFFFFFF:
@A00155:342:HHGFNDSXY:1:1526:26078:30499 1:N:0:GAACCTAG+TCCGCATA
GGATGTAGAGGGCGAGAGAGAGCGACTTCTCTCGGGCCCACAGCCTTACAGCTGTGGAGCACGGTATC
CTCTGCCGAAACAGAGGTTGGACAAGACCGGAGGGGTCTCCTAGTTCCAAAGGAGATGTACTCC
+
FFF:FFFFFFFFFFFFFFF:FF:FFFFF:FFFFFFFFFFFFFFFFFFFFFFFFFFFFFFFFFFFFFFF
FFFFFFFFFFFFFFFFFFFFFFFFFFFFFFFFFFFFFFFFFFFFFFFFF:FFFFFFFFFFFFFF
@A00155:342:HHGFNDSXY:1:1527:27760:5259 1:N:0:GAACCTAG+TCCGCATA
GGATGTGGAGGGCGAGAGAGAGCGACTTCTCTCGGGCCCACAGCCTTACAGCTGTGGAGCACGGTATC
CTCTGCCGAAACAGAGGTTGGACAAGACCGGAGGGGTCTCCTAGTTCCAAAGGAG
+
FFFFFFFFFFFFFFFFFFFFFFFFFFFFFFFFFF,FFFFFFFFFFFFFFFFFFFFFFFFFFFFFFFFF
FFFFFFFFFF:FFFFFFFFFFFFFFFFFFFFFFFFFFFFFFFFFFFFFFFFFFFF
@A00155:342:HHGFNDSXY:1:1527:3794:36652 1:N:0:GAACCTAG+TCCGCATA
TGTCCACTTAAGGACTCACCTCTGGCCTATGGTCATCTCAATGCCGAGAGGGATGTGGAGGGCGAGAG
AGAGCGACTTCTCTCGGGCCCACAGCCTTACAGCTGTGGAGCACGGTATCCTCTGCCGAAACAGAGGT
+
FFFFFFFFFFFFFFFFFFFFFFFFFFFFFFFFFFFFFFFFFFFFFFFFFFFFFFFFFFFFFFFFFFFF
FFFFFFFFFFFFFFFFFFFFFFFFFFFFFFFFFFFFFFFFFFFFFFF:FFFFFFFFFFFFFFFFFFFF
@A00155:342:HHGFNDSXY:1:1527:3812:36714 1:N:0:GAACCTAG+TCCGCATA
TGTCCACTTAAGGACTCACCTCTGGCCTATGGTCATCTCAATGCCGAGAGGGATGTGGAGGGCGAGAG
AGAGCGACTTCTCTCGGGCCCACAGCCTTACAGCTGTGGAGCACGGTATCCTCTGCCGAAACAGAGGT
+
FFFFFFFFFFFFFFFFFFFFFFFFFFFFFFFFFFFFFFFFFFF::FFFFF,F:FF,FFFFFFFFFFFF
FFFFFFFFFFFFFFFFFFFFFFFFFFFFFFFFFFFFFFFFFFFFFFFFFFFFFFFFFFFFFFFFFFFF
@A00155:342:HHGFNDSXY:1:1527:3269:36746 1:N:0:GAACCTAG+TCCGCATA
TGTCCACTTAAGGACTCACCTCTGGCCTATGGTCATCTCAATGCCGAGAGGGATGTGGAGGGCGAGAG
```

```
AGAGCGACTTCTCTCGGGCCCACAGCCTTACAGCTGTGGAGCACGGTATCCTCTGCCGAAACAGAGGT
+
FFFFFFFFFFFFFFFFFFFFFFFFFFFFFFFFFFFFFFFFFFFFFFFFFF:FFFFFFFFFFFFFFFFF
FFFFFFFFFFFFFFF,FFFFFFFFFFFFFFFFFFFF,FFFFFFFFFF:FFFFFFFFFFFFFFFFFFFF
@A00155:342:HHGFNDSXY:1:1528:3757:3302 1:N:0:GAACCTAG+TCCGCATA
TGTCCACTTAAGGACTCACCTCTGGCCTATGGTCATCTCAATGCCGAGAGGGATGTGGAGGGCGAGAG
AGAGCGACTTCTCTCGGGCCCACAGCCTTACAGCTGTGGAGCACGGTATCCTCTGCCGAAACAGAGGT
+
FFFFFFFFFFFFFFFFFFFFFFFFFF:FFFFFFFFFFFFFFFFFFFFFFF,FFF:F:FFFFFFFFFFF
FFFFFFFFFFFFFFFFFFFFFFFFFFFFF:FFFFFFFFFFFFFFFFFFFFFFFFFFFFFFFFFFFFFF
@A00155:342:HHGFNDSXY:1:1528:5575:13557 1:N:0:GAACCTAG+TCCGCATA
GTGTCCACTTAAGGACTCACCTCTGGCCTATGGTCATCTCAATGCCGAGAGGGATGTAGAGGGCGAGA
GAGAGCGACTTCTCTCGGGCCCACAGCCTTACAGCTGTGGAGCACGGTATCCTCTGCCGAAACAGAGG
+
F:FFFFFFFFFFFFFF:FFFFFFFFFFFFFFF,FFFFFFFFFFFFFFFFFFFFFFFFFFFFFFFFFFF
FFFFFFFFFFFFFFFFFFF:FFFFFFFFF:F:FFFFFFFFFFFFFFFFF,FFFFFFFFFFFFFFFFFF
@A00155:342:HHGFNDSXY:1:1528:27715:32017 1:N:0:GAACCTAG+TCCGCATA
GAGAGGGATGTAGAGGGCGAGAGAGAGCGACTTCTCTCGGGCCCACAGCCTTACAGCTGTGGAGCACG
GTATCCTCTGCCGAAACAGAGGTTGGACAAGACCGGAGGGGTCTCCTAGTTCCAAAGGAGATGTACTC
+
FFFFFFFFFFFFFFFFFFF:FFFFFFF:FFFFFFF:FFFFFFFFFFFFFFFFFFFFFFFFFFFFFFFF
FFFFFFFFFFFFFFFFFFFFFFFFFFFFFFFFFFFFFFFFFFFFFFFFFFFFFFFFFFFFFFFFFFFF
@A00155:342:HHGFNDSXY:1:1529:29297:1344 1:N:0:GAACCTAG+TCCGCATA
TGTCCACTTAAGGACTCACCTCTGGCCTATGGTCATCTCAATGCCGAGAGGGATGTGGAGGGCGAGAG
AGAGCGACTTCTCTCGGGCCCACAGCCTTACAGCTGTGGAGCACGGTATCCTCTGCCGAAACAGAGGT
+
FFFFFFFFFFFFFFFFFFFFFFFFF:FFFFFFFFFFFFFFFFF:FFFFFF:FFF:FFFFFFFFF:FFFF
FFFFFFFFFFFFFFFF:FFFFFFFFFFFFFFFF:FFFFFFFFFF:FFFFFFFFFFFFFFFFFFFFFFF
@A00155:342:HHGFNDSXY:1:1529:8006:5995 1:N:0:GAACCTAG+TCCGCATA
GTATCCTCTGCCGAAACAGAGGTTGGACAAGACCGGAGGGGTCTCCTAGTTCCAAAGGAGATGTACTC
CGGGCTTGTTCACGACCTACCGTGTAAGTCGTAGTCTAGTAGGCTACCTGACGAGTCCTTTTTAGG
+
FFFFFFFFFFFFFFFFFFFFFFFFFFFFFF:FFFFFFFFFFFFFFFFFFFFFFFFFFFFFFFFFFFFF
FFFFFFFFFFFFFFFFFFFFFFFFFFFFFFFFFFFFFFFFF,FFFFFFFFFFFFFFFFFF:FFFFF
@A00155:342:HHGFNDSXY:1:1529:22996:7717 1:N:0:GAACCTAG+TCCGCATA
TCCGGTCTTGTCCAACCTCTGTTTCGGCAGAGGATACCGTGCTCCACAGCTGTAAGGCTGTGGGCCCG
AGAGAAGTCGCTCTCTCTCGCCCTTCACATCCCTCTCGGCATTGAGATGACCATAGGCCAGAGGTGAG
+
FFFFFFFFFFFFFFFFFFFFFFFFFFFF:FFFFFFFFFFFFFFFFFFFFFFFFFFFFFFFFFFFFFFF
FFFFF:FFFFF:FFFFFFFFFFFFFFFFFFFFFFFFFFFFFF,F:FFFFFFFFFFFFFFFFFFFFFFF
@A00155:342:HHGFNDSXY:1:1529:3251:9095 1:N:0:GAACCTAG+TCCGCATA
ACCATAGGCCAGAGGTGAGTCCTTAAGTGGACACAGCTGATCTAAGGCGGTGTAGCGGGGCATGGGTT
TGAACCCCCATGACGGTCGGAGTCTAGTAGGCTCCCTGATGAGTCCGTTCCAAGGACGAAACCGTCGT
+
FFFFFFFFFFFFFFFFFFFFFFFFFFFFFFFFFFFFFFFFFFFFFFFFFFFFFFFFFFFFFFFFFFFF
FFFFFFFFFFFFFFFFFFFFFFFFFFFFFFFFFFFFFFFFFFFFFFFFFFFFFFFFFFFFFFFF,FFF
@A00155:342:HHGFNDSXY:1:1529:29460:10895 1:N:0:GAACCTAG+TCCGCATA
CCTTACAGCTGTGGAGCACGGTATCCTCTGCCGAAACAGAGGTTGGACAAGACCGGAGGGGTCTCCTA
GTTCCAAAGGAGATGTACTCCGGGCTTGTTCACGACCTACCGTGTAAGTCGTAGTCTAGTAGGCTACC
+
FF:F:FFFFFFFFFFFFFF:FFFFFFFFFFFFFFFFFFFFFFFFFFFFFFFF:FFFFFFFFFFFFFFFF
FFFFFFFFFFFFFFFFFFFFFFFFFFFFFFFFFFFFFFFFFF:FFFFFFFFFFFFF:FF:FFFFFFFF
@A00155:342:HHGFNDSXY:1:1529:31665:15781 1:N:0:GAACCTAG+TCCGCATA
GAGCGACTTCTCTCGGGCCCACAGCCTTACAGCTGTGGAGCACGGTATCCTCTGCCGAAACAGAGGTT
```

GGACAAGACCGGAGGGGTCTCCTAGTTCCAAAGGAGATGTACTCCGGGCTTGTTCACGACCTACCGTG
+
FFFFFFFFFFFFFFFFFFFFFFFFFFF:FFFFFFFFFFFFFFFFFF:FFFFFFFFFFFFFFFFFFFFF
FFFFFFFFFFFFFFFFFFFFFFFFFFFFFFFFFFFFFFFFFFFFFFFFFFFFFFFFFFFFFFFFFFFF
@A00155:342:HHGFNDSXY:1:1529:9227:35227 1:N:0:GAACCTAG+TCCGCATA
GAGGGCGAGAGAGAGCGACTTCTCTCGGGCCCACAGCCTTACAGCTGTGGAGCACGGTATCCTCTGCC
GAAACAGAGGTTGGACAAGACCGGAGGGGTCTCCTAGTTCCAAAGGAGATGTACTCCGGGCTTGTTCA
+
FFFFFFFFFFFFFFFFFFFFFFFFFFFFFFFFFFFFFFFFFFFFFFFF:FFFFFFFFFFFFFFFFFFF
FFFFFFFFFFFFFFFFFFFFFFFFFFFFFFFFFFFFFFFFFFFFFFFFFFFFFFFFFFFFFFFFFFFF
@A00155:342:HHGFNDSXY:1:1530:9543:22279 1:N:0:GAACCTAG+TCCGCATA
CACTTAAGGACTCACCTCTGGCCTATGGTCATCTCAATGCCGAGAGGGATGTGAAGGGCGAGAGAGAG
CGACTTCTCTCGGGCCCACAGCCTTACAGCTGTGGAGCACGGTATCCTCTGCCGAAACAGAGGT
+
FFFFFFFFFFFFFFFFFFFFFFFFFFFFFFFFFFFFFFFFFFFFFFFFFF:FFFFFFFFFFFFFFFFF
FFFFFFFFFFFFFFFFFFFFFFFFFFFFFFFFFFFFFFFFFFFFFFFFFFFFFFFFFFFFFFFF
@A00155:342:HHGFNDSXY:1:1530:1841:22811 1:N:0:GAACCTAG+TCCGCATA
AAGGACTCTCCTCTGGCCTATGGTCATCTCAATGCCGAGAGGGATGTGGAGGGCGAGAGAGAGCGACT
TCTCTCGGGCCCACAGCCTTACAGCTGTGGAGCACGGTATCCTCTGCCGAAACAGAGGTTGGACAAGA
+
FFFFFFFFFFFFFFFFFFFFFFFF:FFFFFFFFFF:FFFFFFFFFFFFFFFFFFFFFFFFFFFFFFFF
FFFFFFFFFFFFFFFFFFFF,FFFF:FFFFFFFFFFFFFFFFFFFF,FFFFFF,FFFFFFFF:FFFFF
@A00155:342:HHGFNDSXY:1:1530:11559:26960 1:N:0:GAACCTAG+TCCGCATA
TATCCTCTGCCGAAACAGAGGTTGGACAAGACCGGAGGGGTCTCCTAGTTCCAAGGGAGATGTACTCC
GGGCTTGTTCACGACCTACCGTGTAAGTCGTAGTCTAGTAGGCTACCTGACGAGTCCTTTTTAGGACG
+
FFFFFFFFFFF:FFFFFFFFFFFFFFFFFFFFFFFFFFFF:FFFFFFFFFFFFFF,FFFFFFFFFFFFF
FFFFFFFFFFFFFFFFFFFFFFFFFFFFFFFFFFFFFFFF:FFFFFFFFFFFFFFFFF:FFFFFFFF:
@A00155:342:HHGFNDSXY:1:1530:10673:27117 1:N:0:GAACCTAG+TCCGCATA
AGGGCGAGAGAGAGCGACTTCTCTCGGGCCCACAGCCTTACAGCTGTGGAGCACGGTATCCTCTGCCG
AAACAGAGGTTGGACAAGACCGGAGGGGTCTCCTGGTTCCAAAGGAGATGTACTCCGGGCTTGTTCAC
+
FFFFFFFFFFFFFFFFFF,FFFFFFF:FFFFFFFFFFFF,FFFF:FFFFF:F,FFFFFFFFFFFFFFF
FFFFFFFF,FFFFFFFF,FFFFFFFFF:FFFFFFFF:FFF,FFFFFFFFFFFFFFFF,FFFFFFF:FF
@A00155:342:HHGFNDSXY:1:1531:27127:21605 1:N:0:GAACCTAG+TCCGCATA
CTCAATGCCGAGAGGGATGTGGAGGGCGAGAGAGAGCGACTTCTCTCGGGCCCACAGCCTTTCAGCTG
TGGAGCACGGTATCCTCTGCCGAAACAGAGGTTGGACAAGACCGGAGGGGTCTCCTAGTTCCAAAGGA
+
FFFFFFFFFFFFFFFFFFFFFFFF,FF:FFFFFFFFFFFFFFFFFFFFFFFFFFFFF:FFFFFFFFFF
FFFFFFFFFFFFFFFFFFFFFFFFFFFFFFFFFFFFFFFFFFFFFFFFFFFFFFFFFFFFFFFFFFFF
@A00155:342:HHGFNDSXY:1:1531:20139:23688 1:N:0:GAACCTAG+TCCGCATA
GAGAGCGACTTCTCTCGGGCCCACAGCCTTACAGCTGTGGAGCACGGTATCCTCTGCCGAAACAGAGG
TTGGACAAGACCGGAGGGGTCTCCTAGTTCCAAAGGAGATGTACTCCGGGCATGTTCACGACCTACCG
+
FFFFFFFFFFFFFFFFFFFFFFFFFFFFFFFFFFFFFFFFFFFFFFFFFFFFFFFFFFFFFFFFFFFF
FFFFFFFFFFFFFFFFFFFFFFFFFFFFFFFFFFFFFFFFFFFFFFFFFFFFFFFFFFFFFFFFFFFF
@A00155:342:HHGFNDSXY:1:1531:29749:34788 1:N:0:GAACCTAG+TCCGCATA
GGCGAGAGAGAGCGACTTCTCTCGGGCCCACAGCCTTACAGCTGTGGAGCACGGTATCCTCTGCCGAA
ACAGAGGTTGGACAAGACCGGAGGGGTCTCCTAGTTCCAAAGGAGATGTACTCCGGGCTTGTTCACGA
+
FFFFFFFFFFFFFFFFFFFFFF:FFFFFFFFFFFFFFFFFFF:FF,FFFFF:FFFFFFFFFF:FFFF
FFFFFFFFFFFFFFF:FFFF:FFF:FFFFFFFFFFFFFFFFFFFFFFFFFFFFFF:FFFFFFFFFFF
@A00155:342:HHGFNDSXY:1:1532:9064:1595 1:N:0:GAACCTAG+TCCGCATA
TCAATGCCGAGAGGGATGTAGAGGGCGAGAGAGAGCGACTTCTCTCGGGCCCACAGCCTTACAGCTGT

GGAGCACGGTATCCTCTGCCGAAACAGAGGTTGGACAAGACCGGAGGGGTCTCCTAGTTCCAAAGGAG
+
FFFFFFFFFFFFFF:FFFFFFFFFF:FFFFFFFFFFF:F,FFF::F,F:FFFFFFF:F:FFFFFFF:F
FFFFF:FFFFFFFFFFFFFFFF:FFFFFFF,:FFFFFF:FF:F:FFFF,FFFFFFFFFFFFFFFFF:F
@A00155:342:HHGFNDSXY:1:1532:31186:11318 1:N:0:GAACCTAG+TCCGCATA
CACCTCTGGCCTATGGTCATCTCAATGCCGAGAGGGATGTGAAGGGCGAGAGAGAGCGACTTCTCTCG
GGCCCACAGCCTTACAGCTGTGGAGCACGGTATCCTCTGCCGAAACAGAGGTTGGACAAGACCGGAG
+
FFFFFFFFFFFF:FFFFFFFFFFF:FFFFFFF:FFFFFFFFFFFFFFFFFFFFFFFFFFFFFFFFFFF
FFFFFFFFFFFFFFFFFFFFFFFFFFFFFFFFFFFFFFFFFFFFFFFFFFFFFFFFFFFFFFFFFFF
@A00155:342:HHGFNDSXY:1:1532:22525:24283 1:N:0:GAACCTAG+TCCGCATA
CGAGAGGGATGTAGAGGGCGAGAGAGAGCGACTTCTCTCGGGCCCACAGCCTTACAGCTGTGGAGCAC
GGTATCCTCTGCCGAAACAGAGGTTGGACAAGACCGGAGGGGTCTCCTAGTTCCAAAGGAGATGTACT
+
FF::FFFFFFFFFFFFFFFFFFFFFFFFFFFFFFFFFFFFFFFFFFFFFFFFFFFFFFFFFFFFFFFF
FFFFFFFFFFF:FFFFFFFFFFFFFFFFF:FFFFFFFFFFFFFFFFFFFFFFFFFFFF:FFFFFFFFF
@A00155:342:HHGFNDSXY:1:1532:31774:25614 1:N:0:GAACCTAG+TCCGCATA
CCGAGAGGGATGTGGAGGGCGAGAGAGAGCGACTTCTCTCGGGCCCACAGCCTTACAGCTGTGGAGCA
CGGTATCCTCTGCCGAAACAGAGGTTGGACAAGACCGGAGGGGTCTCCTAGTTCCAAAGGAGATGTAC
+
FFFFFFFFFFFFFFFFFFFFFFFFFFFFFFFFFFFFFFFFFFFFFFFFFFFFF:FFFFFFFFFFFFFF
FFFFFFFFFFFFFFFFFFFFFFFFF:FFF,FF:FFFFFFFFFFFFFFFFFFFFFFFFFFFFFFFFFFF
@A00155:342:HHGFNDSXY:1:1532:26449:30608 1:N:0:GAACCTAG+TCCGCATA
GAGCGACTTCTCTCGGGCCCACAGCCTTACAGCTGTGGAGCACGGTATCCTCTGCCGAAACAGAGGTT
GGACAAGACCGGAGGGGTCTCCTAGTTCCAAAGGAGATGTACTCCGGGCTTGTTCACGACCTACCGTG
+
FFFFFFFFFFFFFFFFFFFFFFFFFFFFFFFFFFFFFFFFFFFFFFFFFFFFFFFFFFFFFFFFFFFF
FFFFFFFFFFFFFFFFFFFFFFFFFFFFFFFFFFFFFFFFFFFFFFFFFFFFFFFFFFFFFFFFFFFF
@A00155:342:HHGFNDSXY:1:1532:8712:33113 1:N:0:GAACCTAG+TCCGCATA
GGATGTGGAGGGCGAGAGAGAGCGACTTCTCTCGGGCCCACAGCCTTACAGCTGTGGAGCACGGTATC
CTCTGCCGAAACAGGGGTTGGACAAGACCGGAGGGGTCTCCTAGTTCCAAAGGAGATGTACTCC
+
FFFFF:FF:FFFFF:FFFFFFFFFFFFFFFFFFFFF:FFFFFFFFFFF:F:FFFFFFFFFFFFFFF:FF
FFFFFFFFFFFFFFFFFFFFFFFFFFFFFFFF:FFFFFFFFFFFFFFFFFFFFFFFFFFFFFFFFF
@A00155:342:HHGFNDSXY:1:1534:14787:11068 1:N:0:GAACCTAG+TCCGCATA
AAGGACTCTCCTCTGGCCTATGGTCATCTCAATGCCGAGAGGGATGTGGAGGGCGAGAGAGAGCGACT
TCTCTCGGGCCCACAGCCTTACAGCTGTGGAGCACGGTATCCTCTGCCGAAACAGAGGTTGGACAAGA
+
FFFFFFFFFFFFFFFFFF:FFFFFFFFFFFFFFFFFFFFFFFFFFFFFFFFFFFFFFFFFFFFFFFFF
FFFFFFFFFFFFFFFFFFFF,FFFFFFFFFFFFFFFFFFFFFFFFFFFFFFFFF:FFFFFFFFFFFFFF
@A00155:342:HHGFNDSXY:1:1534:13810:18584 1:N:0:GAACCTAG+TCCGCATA
TATCCTCTGCCGAAACAGAGGTTGGACAAGACCGGAGGGGTCTCCTGGTTCCAAAGGAGATGTACTCC
GGGCTTGTTCACGACCTACCGTGTAAGTCGTAGTCTAGTAGGCTACCTGACGAGTCCTTTTTAGGACG
+
FFFFFFFFFFFFFFFFFFFFFFFFFFFFFFFFFFFFFFFFFFFFFFFFFFFFFFFFFFFFFFFFFFFF
FFFFFFFFFFFFFFFFFFFFFFFFFFFFFFFFFFFFFFFFFFFFFFFFFFFFFFFFFFFFFFFFFFFF
@A00155:342:HHGFNDSXY:1:1535:8079:9251 1:N:0:GAACCTAG+TCCGCATA
GGATGTGGAGGGCGAGAGAGAGCGACTTCTCTCGGGCCCACAGCCTTACAGCTGTGGAGCACGGTATC
CTCTGCCGAAACAGAGGTTGGACAAGACCGGAGGGGTCTCCTAGTTCCAAAGGAGATGTACTCC
+
F:F:FF:FFFFFFFFFFFF:FFFFFFF,FFFFF,F,FFFFFFFFFF,FFF::::F:FF:FFF:FFFFF
:,FFFFFFFF:FF:FFFF:F:FFFF,FFF:FFFF:FFFFF:,FFFFF,FFFFFFFFF::FFFFF
@A00155:342:HHGFNDSXY:1:1535:25382:16485 1:N:0:GAACCTAG+TCCGCATA
CTTCTCTCGGGCCCACAGCCTTACAGCTGTGGAGCACGGTATCCTCTGCCGAAACAGAGGTTGGACAA

```
GACCGGAGGGGTCTCCTAGTTCCAAAGGAGATGTACTCCGGGCTTGTTCACGACCTACCGTGTAAGTC
+
FFFFFFFFFF:FFFFFFFFFF::FFFF,FFFFFFFFFFFFFFFFFFFFFFFFFFFFFFFFFFFF:FFF
FFFFFFFFFFFFFFFFFFFFFFFFFFFFFFFFFFFFFFFFFFFFFFFFFFFFFFFFFFFFFFFF:FFF
@A00155:342:HHGFNDSXY:1:1535:15194:23578 1:N:0:GAACCTAG+TCCGCATA
CTCAATGCCGAGAGGGATGTGGAGGGCGAGAGAGAGCGACTTCTCTCGGGCCCACAGCCTTACAGCTG
TGGAGCACGGTATCCTCTGCCGAAACAGAGGTTGGACAAGACCGGAGGGGTCTCCTAGTTCCAAAGGA
+
FFFFFFFFFFFF:FFFF:F:FFFFFFFFFFFFFFFFFFFFFFF:F:FFFFFFFFFFFFFFFFFFFFFF
FFFFFFFFFFFFFFFFFFFFFFFFFFFFFFFFFFFFFFFFFFFFFFFFFFFFFFFFFFFFFFF,FFFF
@A00155:342:HHGFNDSXY:1:1535:15727:24314 1:N:0:GAACCTAG+TCCGCATA
TGCCGAGAGGGATGTAGAGGGCGAGAGAGAGCGACTTCTCTCGGGCCCACAGCCTTACAGCTGTGGAG
CACGGTATCCTCTGCCGAAACAGAGGTTGGACAAGACCGGAGGGGTCTCCTAGTTCCAAAGGAGATGT
+
FFFFFFFFFFFFFFFFFFFFFFFFFFFFFFFFFFFFFFFFFFFFFFFFFFFFFFFFFF:FFFFFFFFF
FFFFFFFFFFFFFFFFFFFFFFFFFFFFFFFFFFFFFFFFFFFFFFFFFFFFFFFFFFFFFFFFFFFF
@A00155:342:HHGFNDSXY:1:1535:6099:27993 1:N:0:GAACCTAG+TCCGCATA
GTCATCTCAATGCCGAGAGGGATGTGGAGGGCGAGAGAGAGCGACTTCTCTCGGGCCCACAGCCTTAC
AGCTGTGGAGCACGGTATCCTCTGCCGAAACAGAGGTTGGACAAGACCGGAGGGGTCTCCTAGTTCCA
+
FFFFFFFFFFFFFFFFF:FFFF:FFFFFFFFF,FFFFFFFFFFFFFFFFFFFFFFFFFFFFFFFFFFF
FFFFFFFFFFFFFFFFFFFFFFFFFFFFFFFFFFFFFFFFFFFFFFFFFFFFFFFFFFFFFFFFFFFF
@A00155:342:HHGFNDSXY:1:1535:23213:28917 1:N:0:GAACCTAG+TCCGCATA
ATCTCAATGCCGAGAGGGATGTAGAGGGCGAGAGAGAGCGACTTCTCTCGGGCCCACAGCCTTACAGC
TGTGGAGCACGGTATCCTCTGCCGAAACAGAGGTTGGACAAGACCGGAGGGGTCTCCTAGTTCCAAAG
+
FFFFFFFFFFFFFFFFFFFFFF:F:FFFFFFFFFFFFFFFFFFFFFFFFFFFFFFFFFFFFFFFFFFF
FFFFFFFFFFFFFFFFFFFFFFFFFFFFFFFFFFFFFFFFFFFFFFFFFFFFFFFFFFFFFFFFFFFF
@A00155:342:HHGFNDSXY:1:1535:6632:29700 1:N:0:GAACCTAG+TCCGCATA
GTCATCTCAATGCCGAGAGGGATGTGGAGGGCGAGAGAGAGCGACTTCTCTCGGGCCCACAGCCTTAC
AGCTGTGGAGCACGGTATCCTCTGCCGAAACAGAGGTTGGACAAGACCGGAGGGGTCTCCTAGTTCCA
+
FFFFFFFFFFFFFFFFFFFFFFFFFFFFFFFFFFFFFFFFFFFFFFFFFFFFFFFFFFFFFFFFFFFF
FFFFFFFFFFFFFFFFFFFFFFFFFFFFFFFFFFFFFFFFFFFFFFFFFFFFFFFFFFFFFFFFFFFF
@A00155:342:HHGFNDSXY:1:1535:17083:33552 1:N:0:GAACCTAG+TCCGCATA
GGATGTGGAGGGCGAGAGAGAGCGACTTCTCTCGGGCCCACAGCCTTACAGCTGTGGAGCACGGTATC
CTCTGCCGAAACAGAGGTTGGACAAGACCGGAGGGGTCTCCTAGTTCCAAAGGAGATGTACTCC
+
FFFFFFFFFFFFFFFFFFFFFFFFFFFFFFFFFFFFFFFFFFFFFFFFFFFFFFFFFFFFFFFFFFFF
FFFFFFFFFFFFFFFFFFFFFFFFFFFFFFFFFFFFFFFFFFFFFFFFFFFFFFFFFFFFFFFF
@A00155:342:HHGFNDSXY:1:1536:7817:2065 1:N:0:GAACCTAG+TCCGCATA
CAGCCTTACAGCTGTGGAGCACGGTATCCTCTGCCGAAACAGAGGTTGGACAAGACCGGAGGGGTCTC
CTGGTTCCAAAGGAGATGTACTCCGGGCTTGTTCACGACCTACCGTGTAAGTCGTAGTCTAGTAGGCT
+
FFFFFFFFFFFFFFFFFFFFFFFFFFFFFFFFFFFFFFFFFFFFFFFFFFFFFFFFFFFFFFFFFFFF
FFFFFFFFFF:FFFFFFFFFFFFFFFFFF:FFFFFFFFFFFFFFFFFFFFFFFFFFFF:FFFF:FFFFF
@A00155:342:HHGFNDSXY:1:1536:20564:9392 1:N:0:GAACCTAG+TCCGCATA
CGAGAGAGAGCGACTTCTCTCGGGCCCACAGCCTTACAGCTGTGGAGCACGGTATCCTCTGCCGAAAC
AGAGGTTGGACAAGACCGGAGGGGTCTCCTAGTTCCAAAGGAGATGTACTCCGGGCTTGTTCACGACC
+
FFFFFFFFFFF:F:FFFFFFFF::F:FFFFFFFFFFFFFFF:FFFFFFF:FFFFFFFFFFFFFFFFFF
FFFFFFFFFFFFFFFFFFFFFFFFFFFFFFFF:FFFFFFFFFFFFFFFFFFFFFFFFFFFFFFFFFFF
@A00155:342:HHGFNDSXY:1:1536:14841:29700 1:N:0:GAACCTAG+TCCGCATA
GGATGTGGAGGGCGAGAGAGAGCGACTTCTCTCGGGCCCACAGCCTTACAGCTGTGGAGCACGGTATC
```

```
CTCTGCCGAAACAGAGGTTGGACAAGACCGGAGGGGTCTCCTGGTTCCAAAGGAGATGTACTCC
+
FFFFFFFFFFFFFFFFFFFFFFFFFFFFFFFFFFFFFFFFFFFFFFFFFFFFFFFFFFFFFFFFFFFF
FFFFFFFFFFFFFFFFFFFFFFFFFFFFFFFFFFFFFFFFFFFFFFFFFFFFFFFFFFFFFFFF
@A00155:342:HHGFNDSXY:1:1537:6099:3787 1:N:0:GAACCTAG+TCCGCATA
CTTCTCTCGGGCCCACAGCCTTACAGCTGTGGAGCACGGTATCCTCTGCCGAAACAGAGGTTGGACAA
GACCGGAGGGGTCTCCTAGTTCCAAAGGAGATGTACTCCGGGCTTGTTCACGACCTACCGTGTAAGTC
+
FFFFFFFFFFFFFFFFFFFFFFF:FFFFFFFFFFFFFFFFFFFFFFFFFFFFFFFFFFFFFFFFFFFF
FFFFFFFFFFFFFFFFFFFFFFFFFFFFFFFFFFFFFFFFFFFFFFFFFFFFFFFFFFFFFFFF,FFF
@A00155:342:HHGFNDSXY:1:1537:7075:7294 1:N:0:GAACCTAG+TCCGCATA
CTCAATGCCGAGAGGGATGTGGAGGGCGAGAGAGAGCGACTTCTCTCGGGCCCACAGCCTTACAGCTG
TGGAGCACGGTATCCTCTGCCGAAACAGAGGTTGGACAAGACCGGAGGGGTCTCCTAGTTCCAAAGGA
+
FFFFFFFFFFFFFFFF:FFFFFFFFFFFFFFFFFFFFFFFFFFFFFFFFFFFFFFFFFFFFFFFFFFF
FFFFFFFFFFFFFFFFFFFFFFFFFFFFFFFFFFFFFFFFFFFFFFFFFFFFFFFFFFFFFFF:FFFF
@A00155:342:HHGFNDSXY:1:1537:7527:13338 1:N:0:GAACCTAG+TCCGCATA
CTTCTCTCGGGCCCACAGCCTTACAGCTGTGGAGCACGGTATCCTCTGCCGAAACAGAGGTTGGACAA
GACCGGAGGGGTCTCCTAGTTCCAAAGGAGATGTACTCCGGGCTTGTTCACGACCTACCGTGTAAGTC
+
FFFFFFFFFFFFFFFFFFFFFFFFFFFFFFFFFFFFFFFFFFFFFFFFFFFFFFFFFFFFFFFFFFFF
FFFFFFFFFFFFFFFFFFFFFFFFFFFFFFFFFFF:FF:FFFFFFFFFFFFFF:FFFFFFFFFFFFFF
@A00155:342:HHGFNDSXY:1:1537:14950:17394 1:N:0:GAACCTAG+TCCGCATA
CTTCTCTCGGGCCCACAGCCTTACAGCTGTGGAGCACGGTATCCTCTGCCGAAACAGAGGTTGGACAA
GACCGGAGGGGTCTCCTGGTTCCAAAGGAGATGTACTCCGGGCTTGTTCACGACCTACCGTGTAAGTC
+
FFFFFFFFFFFFFFFFFFFFFFFFFFFFFFFFFFFFFFFFFFFFFFFFFFFFFFFFFFFFFFFFFFFF
:FFFFFFFFFFFFFFFFFFFFFFFFF:FFFFFFFFFFFFFFFFFFFFFFFFFFFFFFFFFFFFFFFFF
@A00155:342:HHGFNDSXY:1:1537:12355:25958 1:N:0:GAACCTAG+TCCGCATA
CTTCTCTCGGGCCCACAGCCTTACAGCTGTGGAGCACGGTATCCTCTGCCGAAACAGAGGTTGGACAA
GACCGGAGGGGTCTCCTGGTTCCAAAGGAGATGTACTCCGGGCTTGTTCACGACCTACCGTGTAAGTC
+
FFFFFFFFFFFFFFFFFFFFFFFFFFFFFFFFFFFFFFFFFFFFFFF:F:FFFFFFFFFFFFFFFFFF
FFFFFFFFFFFFF:FFFFFFFFFFF:FFFFFFFFFFFFFFFFFFFFFFFFFFFFFFFFFFFFFFFFFF
@A00155:342:HHGFNDSXY:1:1538:22426:3724 1:N:0:GAACCTAG+TCCGCATA
CAAGCCCGGAGTACATCTCCTTTGGAACTAGGAGACCCCTCCGGTCTTGTCCAACCTCTGTTTCGGCA
GAGGATACCGTGCTCCACAGCTGTAAGGCTGTGGGCCCGAGAGAAGTCGCTCTCTCTCGCCCTCCACA
+
FFFFFFFFFFFFFFFFFFFFFFFFFFFFFFFFFFFFFFFFFFFFFFFFFFFFFFFFFFFFFFFF:FFF
FFFFFFFFFFF:FFFFFFFFFFFF:FFFFFFFFFFFFFFFFFFFFFFFFFFFFFFFFFFFFFFFFFF:
@A00155:342:HHGFNDSXY:1:1538:2112:14638 1:N:0:GAACCTAG+TCCGCATA
GAGCGACTTCTCTCGGGCCCACAGCCTTACAGCTGTGGAGCACGGTATCCTCTGCCGAAACAGAGGTT
GGACAAGACCGGAGGGGTCTCCTAGTTCCAAAGGAGATGTACTCCGGGCTTGTTCACGAC
+
FFFFFFF:FFFF:FFFFFFFFFFFFFFFFFFFFFFFFFFFFFFFFFFFFFFFFFFFFFFFFFFFFFFF
FFFFFFFFFFFFFFFFFFFFFFFF:FFFFFFFFFFFFFFFFFFFFFFFFFFFFFFFFFFF
@A00155:342:HHGFNDSXY:1:1538:5186:35243 1:N:0:GAACCTAG+TCCGCATA
CTCTCGCCCTCCACATCCCTCTCGGCATTGAGATGACCATAGGCCAGAGGTGAGTCCTTAAGTGGACA
CAGCTGATCTAAGGCGGTGTGGCGGGGCATGGGTTTGAACCCCCATGACGGTCGGAGTCTAGTAGGCT
+
FFFFFFFFFFFFFFFFFFFFFFFFFFFFFFFFFFFFFFFFFFFFFFFFFFFFFFFFFFFFFFFFFFFF
FFFFFFFFFFFFFFFFFFFFFFFFFFFFFFFFFFFFFFFFFF:FFFFFFFFFFFFFFFFFFFFFF:FFF
@A00155:342:HHGFNDSXY:1:1539:4987:1611 1:N:0:GAACCTAG+TCCGCATA
CTCTGGCCTATGGTCATCTCAATGCCGAGAGGGATGTGAAGGGCGAGAGAGAGCGACTTCTCTCGGGC
```

CCACAGCCTTACAGCTGTGGAGCACGGTATCCTCTGCCGAAACAGAGGTTGGACAAGACCGGAGGGGT
+
FFFFFFFFFFFFFFFFFFFFFF:FFFFFF,FFF,,FFFF:FFFFFFFFFFFFFFFFFFFFFFFFFFFF
FFFF,FFFFF,F:FFFFFFFFFFFFFF:FFFFFF:FFFFF:FF:FFFFFFFFFFFFFFFFFFFFFFFF
@A00155:342:HHGFNDSXY:1:1539:27245:6433 1:N:0:GAACCTAG+TCCGCATA
GAGAGGGATGTAGAGGGCGAGAGAGAGCGACTTCTCTCGGGCCCACAGCCTTACAGCTGTGGAGCACG
GTATCCTCTGCCGAAACAGAGGTTGGACAAGACCGGAGGGGTCTCCTAGTTCCAAAGGAGATGTACTC
+
FFFFFFFFFFFFFFFFF:FFFFFFFFFFFFFFFFFFFFFFFFFFFFFFFFFFF:FF,FFFFFFFFFF
FFF:FFFFFFFFFFFFFFFFFFFFFFFFFFFFFFFFFFF:FFFFF:FFFFFFFFFFFFFFFFFFFFFF
@A00155:342:HHGFNDSXY:1:1539:2202:11819 1:N:0:GAACCTAG+TCCGCATA
TGTGGAGGGCGAGAGAGAGCGACTTCTCTCGGGCCCACAGCCTTACAGCTGTGGAGCACGGTATCCTC
TGCCGAAACAGAGGTTGGACAAGACCGGAGGGGTCTCCTAGTTCCAAAGGAGATGTACTCCGGG
+
FFFFFFFFFFFFFFFFFFFFFFFFFFFFFFFFFFFFFFFFFFFFF:FFFFFFFFFFFFFFFFFFFFFF
FFFFFFFFFFFFFFFFFFFFFFFFFFFFFFFFFFFFFFFFFFFFFFF:FFFFFFFFFFFFFFFF
@A00155:342:HHGFNDSXY:1:1539:15664:20228 1:N:0:GAACCTAG+TCCGCATA
GAGAGGGATGTGGAGGGCGAGAGAGAGCGACTTCTCTCGGGCCCACAGCCTTACAGCTGTGGAGCACG
GTATCCTCTGCCGAAACAGAGGTTGGACAAGACCGGAGGGGTCTCCTAGTTCCAAAGGAGATGTACTC
+
FFFFFFFFFFFFFFFFFFFFFFFFFFFFFFFFFFFFFFFFFFFFFFFFFFFFFFFFFFFFFFFFFFFF
FFFFFFFFFFFFFFFFFFF,FFFFFFFFFFFFFFFFFFFFFFFFFFFFFFFFFFFFFFFFF:FFFFFF
@A00155:342:HHGFNDSXY:1:1539:28357:21292 1:N:0:GAACCTAG+TCCGCATA
CGACTTCTCTCGGGCCCACAGCCTTACAGCTGTGGAGCACGGTATCCTCTGCCGAAACAGAGGTTGGA
CAAGACCGGAGGGGTCTCCTGGTTCCAAAGGAGATGTACTCCGGGCTTGTTCACGACCTACCGTG
+
FFFFFFFFFFFFFFFFFFFFFFFFFF:FFFFFFFFFFFFF:FFFFFFFFFFFFFFFFFFFFFF:FFFFF
FFFFFFFFFFFFFFFFFFFFFFFF,FFFFFF:FFFFFFFFFFFFFFFFFFFFFFFFFFFFFFFF:FFF
@A00155:342:HHGFNDSXY:1:1540:30680:2268 1:N:0:GAACCTAG+TCCGCATA
TGCTCCACAGCTGTAAGGCTGTGGGCCCGAGAGAAGTCGCTCTCTCTCGCCCTCCACATCCCTCTCGG
CATTGAGATGACCATAGGCCAGAGGTGAGTCCTTAAGTGGACACAGCTGATCTAAGGCGGTGTGGC
+
FFFFFFFFFFFFFFFFFFFFFFFFFFFFFFFFFFFFFFFFFFFFFFFFFFFFFFFFFFFFFFFFFFFF
FF:FFFFFFFFFFFFFFFFFFFFFFFFFFFFFFFFFFFFFFFFFFFFFFFFFFFFFFFFFFFFFFF
@A00155:342:HHGFNDSXY:1:1540:18728:6151 1:N:0:GAACCTAG+TCCGCATA
CTTCTCTCGGGCCCACAGCCTTACAGCTGTGGAGCACGGTATCCTCTGCCGAAACAGAGGTTGGACAA
GACCGGAGGGGTCTCCTAGTTCCAAAGGAGATGTACTCCGGGCTTGTTCACGACCTACCGTGTAAGTC
+
FFFFFFFFFFFFFFFFFFFFFFFFFFFFFFFFFFFFFFFFFFFFFFFFFFFFFFFFFFFFFFFFFFFF
FFFFFFFFFFFFFFFFFFFFFFFFFFFFFFFFFFFFFFFFFFFFFFFFFFFFFFFFFFFFFFFFFFFF
@A00155:342:HHGFNDSXY:1:1540:18647:7952 1:N:0:GAACCTAG+TCCGCATA
CTTCTCTCGGGCCCACAGCCTTACAGCTGTGGAGCACGGTATCCTCTGCCGAAACAGAGGTTGGACAA
GACCGGAGGGGTCTCCTAGTTCCAAAGGAGATGTACTCCGGGCTTGTTCACGACCTACCGTGTAAGTC
+
FFFFFFFFFFFFFFFFFFFFFFFFFFFFFFFFFFFFFFFF:FFFFFFFFFFFFFFFFFFFFFFFFFFF
FFFFFFFFFFFFFFFFFFFF:FFFFFFFFFFFFFFF:FFFFFFFFFFFFFFFFFFFFFFFFFFFFFFF
@A00155:342:HHGFNDSXY:1:1540:16685:12790 1:N:0:GAACCTAG+TCCGCATA
GAGCGACTTCTCTCGGGCCCACAGCCTTACAACTGTGGAGCACGGTATCCTCTGCCGAAACAGAGGTT
GGACAAGACCGGAGGGGTCTCCTAGTTCCAAAGGAGATGTACTCCGGGCTTGTTCACGACCTACCGTG
+
FFFFFFFFFFFFFFFFFFFFFFFFFFFFFFFFFFFFFFFFFFFFFFFFFFFFFFFFFFFFFFFFFFFF
FFFFFFFFFFFFFFFFFFFFFFFFFFFFFFFFFFFFFFFFFFFFFFFFFFFFFFFFFFFFFFFFFFF:
@A00155:342:HHGFNDSXY:1:1540:5963:30201 1:N:0:GAACCTAG+TCCGCATA
TCTCTCGGGCCCACAGCCTTACAGCTGTGGAGCACGGTATCCTCTGCCGAAACAGAGGTTGGACAAGA

```
CCGGAGGGGTCTCCTGGTTCCAAAGGAGATGTACTCCGGGCTTGTTCACGACCTACCGTGTAAGTCGT
+
FFFFFFFFFFFFFFFFFFFFFFFFFFFFFFFFFFFFFFFFFFFFFFFFFFFFFFFFFFFFFFFFFFFF
FFFFFFFFFFFFFFFF:FFFFFFFF,FFFFFFFFFFFFFFFFFFFFFFFFFFFFFFFFFFFFFFFFFF
@A00155:342:HHGFNDSXY:1:1540:6596:33082 1:N:0:GAACCTAG+TCCGCATA
AGCGACTTCTCTCGGGCCCACAGCCTTACAGCTGTGGAGCACGGTATCCTCTGCCGAAACAGAGGTTG
GACAAGACCGGAGGGGTCTCCTAGTTCCAAAGGAGATGTACTCCGGGCTTGTTCACGACCTACCGTGT
+
FFFFFFFFFFFFFFFFFFFFFFFFFFFFFFFFFFFFFFFFFFFFFFFFFFFFFFFFFFFFFFFFFFFF
FFFFFFFFFFFFFFFFFFFFFFFFFFFFFFFFF:FFFFFF:FFFFFFFFFFFFFFFFFFFFFFFFFFF
@A00155:342:HHGFNDSXY:1:1541:17716:15452 1:N:0:GAACCTAG+TCCGCATA
CTCTGGCCTATGGTCATCTCAATGCCGAGAGGGATGTGGAGGGCGAGAGAGAGCGACTTCTCTCGGGC
CCACAGCCTTACAGCTGTGGAGCACGGTATCCTCTGCCGAAACAGAGGTTGGACAAGACCGGAGGGGT
+
FFFFFFFF:FFFFFFFFFFFFFFFFFF:FFFFFF:FFFFFFFFFFFFFFFFFFFFFFFFFFFFFFFFF
FFFFFFFFFFFFFFFFFFFFFFFFFFFFFFFFFFFFFFFFFFFFFFFFFFFFFFFFFFFFFFFFFFFF
@A00155:342:HHGFNDSXY:1:1541:25012:18850 1:N:0:GAACCTAG+TCCGCATA
CACCTCTGGCCTATGGTCATCTCAATGCCGAGAGGGATGTAGAGGGCGAGAGAGAGCGACTTCTCTCG
GGCCCACAGCCTTACAGCTGTGGAGCACGGTATCCTCTGCCGAAACAGAGGTTGGACAAGACCGGAGG
+
FFFFFFFFFFFFFFFFFFFFFFFFFFFFFFFFFFFFFFFFFFFFFFFFFFFFFFFFFFFFFFFFFFFF
FFFFFFFFFFFFFFFFFFFFFFFFFFFFFFFFFFFFFFFFFFFFFFFFFFFFFFFFFFFFFFFFFFFF
@A00155:342:HHGFNDSXY:1:1541:12355:22013 1:N:0:GAACCTAG+TCCGCATA
ACTACGACTTACACGGTAGGTCGTGAACAAGCCCGGAGTACATCTCCTTTGGAACTAGGAGACCCCTC
CGGTCTTGTCCAACCTCTGTTTCGGCAGAGGATACCGTGCTCCACAGCTGTAAGGCTGTGGGCCCGAG
+
FFFFFFFFF,FFFFFFFFFFFFF:FFFFF:FFFFFFF:FFFFFFFFFFFFFFFFFFFFFFFFFFFFFF
FFFFFFFFFFF::F:::FFFFFFFFFFF:FFFFFFFFFFFFFF:FFF::FFFFFFFFFFFFFFFFFFF
@A00155:342:HHGFNDSXY:1:1541:27172:28541 1:N:0:GAACCTAG+TCCGCATA
TCCCTCTCGGCATTGAGATGACCATAGGCCAGAGGTGAGTCCTTAAGTGGACACAGCTGATCTAAGGC
GGTGTGGCGGGGCATGGGTTTAAACCCCCATGACGGTCGGAGTCTAGTAGGCTCCCTGATG
+
FFFFFFFFFFFFFFF:FFFFFFFFFFFFFFFFFFFFFFFFFFFFFFFFFFFFFFFFFFFFFFFFFFFF
FFFFFFFFFFFFFFFFFFFFFFFFFFFFFFFFFFFF:FFFFFFFFFFFFFFFFFFFFFFFF
@A00155:342:HHGFNDSXY:1:1541:19569:30686 1:N:0:GAACCTAG+TCCGCATA
TGTTTCGGCAGAGGATACCGTGCTCCACAGCTGTAAGGCTGTGGGCCCGAGAGAAGTCGCTCTCTCTC
GCCCTCCACATCCCTCTCGGCATTGAGATGACCATAGGCCAGAGGTGAGTCCTTAAGTGGACACAGCT
+
FFFFFFFFFFFFFFFFFFFFFFFFFFFFFFFFFFFFFFFFFFFFFFFFFFFFFFFFFFFF:FFFFFFF
FFFFFFFFFFFF:FFFFFFFFFF:FFFFFFFFFFFFFFFFFFFFFFFFFFFFFFFFFFFFFFF,FFFFF
@A00155:342:HHGFNDSXY:1:1542:15121:14184 1:N:0:GAACCTAG+TCCGCATA
GGGATGTGGAGGGCGAGAGAGAGCGACTTCTCTCGGGCCCACAGCCTTACAGCTGTGGAGCACGGTAT
CCTCTGCCGAAACAGAGGTTGGACAAGACCGGAGGGGTCTCCTGGTTCCAAAGGAGATGTACTCCGGG
+
FFFFFFFFFFFFFFFFFFFFFFFFFFFFFFFFFFFFFFFFFFFFFFFFFFFFFFFFFFFFFFFFFFFF
FFFFFFFFFFFFFFFFFFFFFFFFFFFFFFFFFFFFFFFFFFFFFFFFFFFFFFFFFFFFFFFFFFFF
@A00155:342:HHGFNDSXY:1:1542:28420:22623 1:N:0:GAACCTAG+TCCGCATA
ACCTCTGTTTCGGCAGAGGATACCGTGCTCCACAGCTGTAAGGCTGTGGGCCCGAGAGAAGTCGCTCT
CTCTCGCCCTCCACATCCCTCTCGGCATTGAGATGACCATAGGCCAGAGGAGAGTCCTTAAGTGGACA
+
FFFFFFFFFFFFFFFFFFFFFFFFFFFFFFFFFFFFFFFFFFFFFFFFFFFFFFFFFFFFFFFFFFFF
FFFFFFFFFFFFFFFFFFFFFFFFFFFFFFFFFFFFFFFFFFFFFFFFFFFFFFFFFFFFFFFFFFFF
@A00155:342:HHGFNDSXY:1:1542:12472:24878 1:N:0:GAACCTAG+TCCGCATA
AGAGAGAGCGACTTCTCTCGGGCCCACAGCCTTACAGCTGTGGAGCACGGTATCCTCTGCCGAAACAG
```

AGGTTGGACAAGACCGGAGGGGTCTCCTAGTTCCAAAGGAGATGTACTCCGGGCTTGTTCACGACCT
+
FFFFFFFFFFFFFFFFFFFFFFFFFFFFFFFFFFFFFFFFFFFFFFFFFFFFFFFFFFFFFFFFFFFF
FFFFFFFFFFFFFFFFFFFFFFFFFFFFFFFFFFFFFFFFFFFFFFFFFFFFFFFFFFFFFFFFFFF
@A00155:342:HHGFNDSXY:1:1542:26069:24972 1:N:0:GAACCTAG+TCCGCATA
AGGGCGAGAGAGAGCGACTTCTCTCGGGCCCACAGCCTTACAGCTGTGGAGCACGGTATCCTCTGCCG
AAACAGAGGTTGGACAAGACCGGAGGGGTCTCCTAGTTCCAAAGGAGATGTACTCCGGGCTTGTTCAC
+
FFFFFFFFFFFFFFFFFFFFFFFFFFFFFFFFFFFFFFFFFFFFFFFFFFFFFFFFFFFFFFFFFFFF
FFFFFFFFFFFFFFFF:FFFFFFFFFFFFFFFFFFFFFF:FFFFFFFFFFF:FFFFFF:FFFFFFFFF
@A00155:342:HHGFNDSXY:1:1542:6497:27712 1:N:0:GAACCTAG+TCCGCATA
GATGTGGAGGGCGAGAGAGAGCGACTTCTCTCGGGCCCACAGCCTTACAGCTGTGGAGCACGGTATCC
TCTGCCGAAACAGAGGTTGGACAAGACCGGAGGGGTCTCCTAGTTCCAAAGGAGATGTACTCCGGGCT
+
FFFFFFFFFFFFFFFFFFFFFFFFFFFFFFFFFFFFFFFFFFFFFFFFFFFFFFFFFFFFFFFFFFFF
FFFFFFFFFFFFFFFFFFFFFFFFFFFFFFFFFFFFFFFFFFFFFFFFFFFF:FFFFFFFFFFFF:FF
@A00155:342:HHGFNDSXY:1:1542:2266:27868 1:N:0:GAACCTAG+TCCGCATA
CTTCTCTCGGGCCCACAGCCTTACAGCTGTGGAGCACGGTATCCTCTGCCGAAACAGAGGTTGGACAA
GACCGGAGGGGTCTCCTAGTTCCAAAGGAGATGTACTCCGGGCTTGTTCACGACCTACCGTGTAAG
+
FFFFFFFFFFFFFFFFFFFFFF,FFFF:FFFFFFFFFFFFFFFFFFFFFFFFFFFFFFFFFFFFFFFF
FFFFFFFFFFFFFFFFFFFFFFFFFFFFFFFFFFFFFFFFFFFFFFFFFFFFFFFFFFFFFFFFFF
@A00155:342:HHGFNDSXY:1:1542:23240:32471 1:N:0:GAACCTAG+TCCGCATA
TCCTCTGCCGAAACAGAGGTTGGACAAGACCGGAGGGGTCTCCTGGTTCCAAAGGAGATGTACTCCGG
GCTTGTTCACGACCTACCGTGTAAGTCGTAGTCTAGTAGGCTACCTGACGAGTCCTTTTAGGACGAA
+
FFF,F:FFFFF,FFFFFFF:FFFFFFFFFFFFFFFFFFFFFFFFFFFFFFFF:FFFF:FFFFFFFFFF
FF:,F,FFFFFFFFFF,FF,FFFFF,FFFFFFFFFFF,FFFFF:FFFFFFFFFF:F,,FFFFFFFF:F
@A00155:342:HHGFNDSXY:1:1543:3197:15734 1:N:0:GAACCTAG+TCCGCATA
GGACTCTCCTCTGGCCTATGGTCATCTCAATGCCGAGAGGGATGTGGAGGGCGAGAGAGAGCGACTTC
TCTCGGGCCCACAGCCTTACAGCTGTGGAGCACGGTATCCTCTGCCGAAACAGAGGTTGGACAAGACC
+
FFFFFFFFFFFFFFFFFFFFFFFFFFFFFFFFF:F:F:FFF:FFFFFFFFFFFFFFFFFFFFFFFFFF
FFFFFFFFFFFFFFFFFFFFFFFFFFFFFFFFFFFFFFFFFFFFFFFFFFFFFFFFFFFFFFFFFFFF
@A00155:342:HHGFNDSXY:1:1543:13991:23156 1:N:0:GAACCTAG+TCCGCATA
TTCTCTCGGGCCCACAGCCTTACAGCTGTGGAGCACGGTATCCTCTGCCGAAACAGAGGTTGGACAAG
ACCGGGGGGGTCTCCTAGTTCCAAAGGAGATGTACTCCGGGCTTGTTCACGACCTACCG
+
FFFFFFFFFFFFFFFFFFFFFFFFFFFFFFFFFFFFFFF:FFFFFFFFFFFFFFFFFFFFFFFFFFFF
FFFFFFFFFFFFFFFFFFFFFFFFFFFFFFFFFFFFFFFFFFFFFFFF:FFFFFFFFFF
@A00155:342:HHGFNDSXY:1:1543:7844:29324 1:N:0:GAACCTAG+TCCGCATA
CTGGCCTATGGTCATCTCAATGCCGAGAGGGATGTGGAGGGCGAGAGAGAGCGACTTCTCTCGGGCCC
ACAGCCTTACAGCTGTGGAGCACGGTATCCTCTGCCGAAACAGAGGTTGGACAAGACCGGAGGGGTCT
+
FFFFFFFFFFFFFFFFFFFFFFFFFFF:FFFFFFFFFFFF:FFFFFFFFFFFFFFFFFFFFFFFFFFF
FFFFFFF:FFFFFFFFFFFFFFFFFFFFFFFFFFF:FFFFFFFFFFFFFFFFFFFFFFFFFFFFFFFF
@A00155:342:HHGFNDSXY:1:1544:26603:3975 1:N:0:GAACCTAG+TCCGCATA
ACCTCTGTTTCGGCAGAGGATACCGTGCTCCACAGCTGTAAGGCTGTGGGCCCGAGAGAAGTCGCTCT
CTCTCGCCCTCCACATCCCTCTCGGCATTGAGATGACCATAGGCCAGAGGAGAGTCCTTAAGTGGACA
+
FFFFFFFFFFFFFFFFFFFFFFFFFFFFFFFFFFFFFFFFFFFFFFFFFFFFFFFFFFFFFFFFFFFF
FFFFFFFFFFFFFFFFFFFFFFFFFFFFFFFFFFFFFFFFFFFFFFFFFFFFFFFFFFFFFFFFFFFF
@A00155:342:HHGFNDSXY:1:1544:8034:16313 1:N:0:GAACCTAG+TCCGCATA
GTATCCTCTGCCGAAACAGAGGTTGGACAAGACCGGAGGGGTCTCCTAGTTCCAAAGGAGATGTACTC

```
CGGGCTTGTTCACGACCTACCGTGTAAGTCGTAGTCTAGTAGGCTACCTGACGAGTCCTTTTTAGGAC
+
FFFFFFFFFFFFFFFFFFFFFFFFFFFFFFFFFFFFFFFFFFFFFFFFFFFFFFFFFFFFFFFFFFFF
FFFFFFFF:FFFFFFFFFFFFFFFFFFFFFFFFFFFFFFFFFFFFFFFFFFFFFFFFFFFFFFFFFF
@A00155:342:HHGFNDSXY:1:1544:28411:18505 1:N:0:GAACCTAG+TCCGCATA
TTCTCTCGGGCCCACAGCCTTACAGCTGTGGAGCACGGTATCCTCTGCCGAAACAGAGGTTGGACAAG
ACCGGAGGGGTCTCCTAGTTCCAAAGGAGATGTACTCCGGGCTTGTTCACGACCTACCGTGTAAGTCG
+
FFFFFFFFFFFFFFFFFFF:,FFFFFFFFFFFFFFFFFF,FFFFFFFFFFFFFFFFFFFFFFFFFFFFF
FFFFF:FFFFFFFFFFFFFFFFFFFFFFFFFFFFFFFFFFFFFFFFFFFFFFFFFFFFFFFFF,FFFF
@A00155:342:HHGFNDSXY:1:1544:28420:18521 1:N:0:GAACCTAG+TCCGCATA
TTCTCTCGGGCCCACAGCCTTACAGCTGTGGAGCACGGTATCCTCTGCCGAAACAGAGGTTGGACAAG
ACCGGAGGGGTCTCCTAGTTCCAAAGGAGATGTACTCCGGGCTTGTTCACGACCTACCGTGTAAGTCG
+
FFFFFFFFFFFFFFFFFF:FFFFFFFF:FF:FFFFFFFFFFFFFFFFFFFFFFFFFFFFFFFFFFFFF
FFFFFF,FFFFFFFFFFFFFFFFFFFFF,FFFFFFF,FFFFFFFFFFFFFFFFFFFFFFFFFFFFFFF
@A00155:342:HHGFNDSXY:1:1544:27760:20290 1:N:0:GAACCTAG+TCCGCATA
TCCACTTAAGGACTCACCTCTGGCCTATGGTCATCTCAATGCCGAGAGGGATGTGGAGGGCGAGAGAG
AGCGACTTCTCTCGGGCCCACAGCCTTACAGCTGTGGAGCACGGTATCCTCTGCCGAAACAGAGGTTG
+
FFFFFFFFFFFFFFFFFFFFFFFFFFFFFFFFFFFFFFFFFFFF:FFFF:F,FFFFFFFFFFFFFF
FFFFFFFFFFFFFFFFFFFFFFFFF:FFFFFFFFFFFFF:FF:FFFFFFFFFFFFFFFFFFFFFFFFFF
@A00155:342:HHGFNDSXY:1:1544:13883:26882 1:N:0:GAACCTAG+TCCGCATA
AGGGCGAGAGAGAGCGACTTCTCTCGGGCCCACAGCCTTACAGCTGTGGAGCACGGTATCCTCTGCCG
AAACAGAGGTTGGACAAGACCGGAGGGGTCTCCTAGTTCCAAAGGAGATGTACTCCGGGCTTGTTCAC
+
FFFFFFFFFFFFFFFFFFFFFFFFFFFFFFFFFFFFFFFFFFFFFFFFFFFFFFFFFF:FFFFFFFFF
FFFFFFFFFFFFFFFFFFFFFFFFFFFFFFFFFFFFFFFFFFFFFFFFFFFFFFFFFFFFFFFFFFFF
@A00155:342:HHGFNDSXY:1:1544:27868:28933 1:N:0:GAACCTAG+TCCGCATA
TCCACTTAAGGACTCACCTCTGGCCTATGGTCATCTCAATGCCGAGAGGGATGTGGAGGGCGAGAGAG
AGCGACTTCTCTCGGGCCCACAGCCTTACAGCTGTGGAGCACGGTATCCTCTGCCGAAACAGAGGTTG
+
FFFFFFFF::FF:FFFF,FFFFF::FFFF:FFFFFF:FFF:F:FFFFF:FFFFFFFFFFFFFFFFFFF
FFFF:FFFFFFFFFFF:F,FFF:FF:,FFF:FFF:FFFFFFFFF:FFF:FFFFFFFFF:FFFF,FFFF
@A00155:342:HHGFNDSXY:1:1545:29496:8891 1:N:0:GAACCTAG+TCCGCATA
CAATGCCGAGAGGGATGTAGAGGGCGAGAGAGAGCGACTTCTCTCGGGCCCACAGCCTTACAGCTGTG
GAGCACGGTATCCTCTGCCGAAACAGAGGTTGGACAAGACCGGAGGGGTCTCCTAGTTCCAAAGGAGA
+
FFFFFFFFFFFFFFFFFFFFFFFFFFFFFFFFFFFFFFFFFFFFFFFFFFFFFFFFFFFFFFFFFFFF
FFFFFFFFFFFFFFFFFFFFFFFFFFFFFFFFFFFFFFFFFFFFFFFFFFFFFFFFFFFFFFFFFFFF
@A00155:342:HHGFNDSXY:1:1545:5520:10645 1:N:0:GAACCTAG+TCCGCATA
GGACTCACCTCTGGCCTATGGTCATCTCAATGCCGAGAGGGATGTGGAGGGCGAGAGAGAGCGACTTC
TCTCGGGCCCACAGCCTTACAGCTGTGGAGCACGGTATCCTCTGCCGAAACAGAGGTTGGACAAGACC
+
FFF:FFFFFFFFFFFFF,FFFF:FFFFFFFF,FFFFFFF,FFFFFF:F:FFFFFFFFFFFFFFFFFF
FF:FFF:FFFFFFFFFFFFFFFFFFFFFFFFFFFFFFFFFFFFFFFF:FFFFFFFFF:FFFF,F:FFF
@A00155:342:HHGFNDSXY:1:1545:16450:12884 1:N:0:GAACCTAG+TCCGCATA
CGACTTCTCTCGGGCCCACAGCCTTACAGCTGTGGAGCACGGTATCCTCTGCCGAAACAGAGGTTGGA
CAAGACCGGAGGGGTCTCCTGGTTCCAAAGGAGATGTACTCCGGGCTTGTTCACGACCTACCGTGTAA
+
FFFF,FFFFFFFFFFFFFFFFFFFFFFFFFFFFFFFFFFFFFFFFFFFFFFFFFFFFFFFFFFFFFFF
FFFFFF:FFFFFFFFFFFFFFFFFFFFFFFFF,FFFFFFFFFFFFFF:FFFFFFFFFFFF,FFF:FFFF
@A00155:342:HHGFNDSXY:1:1546:9995:3051 1:N:0:GAACCTAG+TCCGCATA
CTTCTCTCGGGCCCACAGCCTTACAGCTGTGGAGCACGGTATCCTCTGCCGAAACAGAGGTTGGACAA
```

```
GACCGGAGGGGTCTCCTGGTTCCAAAGGAGATGTACTCCGGGCTTGTTCACGACCTACCGTGTAAGTC
+
FFFFFFFFFFFFFFFFFFFFFFFFFFFFF:FFFFFFFFFFFFFFFFFFFFFFFFFFF,FFFFFFFFFF
FFFFFFFFFFFFFFFFFFFFFFFFF:FFFFFFFFFF:FFFFFF,FFFFFFFFFFFFFFFFFFFFFFFF
@A00155:342:HHGFNDSXY:1:1546:32696:10207 1:N:0:GAACCTAG+TCCGCATA
GAGCGACTTCTCTCGGGCCCACAGCCTTACAGCTGTGGAGCACGGTATCCTCTGCCGAAACAGAGGTT
GGACAAGACCGGAGGGGTCTCCTAGTTCCAAAGGAGATGTACTCCGGACTTGTTCACGACCTACCGTG
+
FFFFFFFFFFFFFFFFFFFFFF,FFF:FFFFFFFFFFFFFFFFFFFFFFFFFFFFFFFFFFFFFFFFF
FFFFFFFFFFFFFFFFFFFFFFFFFFFFFFFFFFFFFFFFFFFFFFFFF:FFFFFFFFFFFFFFFFFF
@A00155:342:HHGFNDSXY:1:1547:12789:14935 1:N:0:GAACCTAG+TCCGCATA
TGCCGAGAGGGATGTGGAGGGCGAGAGAGAGCGACTTCTCTCGGGCCCACAGCCTTACAGCTGTGGAG
CACGGTATCCTCTGCCGAAACAGAGGTTGGACAAGACCGGAGGGGTCTCCTAGTTCCAAAGGAGATGT
+
FFFFFFFFFFFFFFFFFFFFFFFFFFFFFFFFFFFFFFFFFFFFFFFFFFFFFFFFFFFFFFFFFFFF
FFFFFFFFFFFFFFFFFFFFFFFFFFFFFFFFF:FFFFFF:FFFFFFFFFFFFFFFFFFFFFFF,FFF
@A00155:342:HHGFNDSXY:1:1547:20627:26850 1:N:0:GAACCTAG+TCCGCATA
ACTTCTCTCGGGCCCACAGCCTTACAGCTGTGGAGCACGGTATCCTCTGCCGAAACAGAGGTTGGACA
AGACCGGAGGGGTCTCCTAGTTCCAAAGGAGATGTACTCCGGGCTTGTTCACGACCTACCGTGTAAGT
+
FFFFFFFFFFFFFFFFFFFFFFF:FFFFFFFFFFFFFFFFFFFFFFFFFFFFFFFF:FFFFFFFFFFF
FFFFFFFFFFFFFFFFFFFFFFFFFFFFFFFFFFFFFFFFFFFFFFFFFFFFFFFFF:FFF,FFF:FF
@A00155:342:HHGFNDSXY:1:1548:22318:16470 1:N:0:GAACCTAG+TCCGCATA
ACCTCTGTTTCGGCAGAGGATACCGTGCTCCACAGCTGTAAGGCTGTGGGCCCGAGAGAAGTCGCTCT
CTCTCGCCCTCCACATCCCTCTCGGCATTGAGATGACCATAGGCCAGAGGAGAGTCCTTAAGTGGACA
+
FFFFFFFFFFFFFFFFFFFFFFFFFFFFFFFFFFFFFFFFFFFFFFFFFFFFFFFFFFFFFFF:FFFF
FFFFFFFFFFFFFFFFFFFFFFFFFFFFFFFFFFFFFFFFFFFFFFFFFFFFFFFFFFFFFFFFFFFF
@A00155:342:HHGFNDSXY:1:1548:20980:30060 1:N:0:GAACCTAG+TCCGCATA
ATCCTCTGCCGAAACAGAGGTTGGACAAGACCGGAGGGGTCTCCTAGTTCCAAAGGAGATGTACTCCG
GGCTTGTTCACGACCTACCGTGTAAGTCGTAGTCTAGTAGGCTACCTGACGAGTCCTTTTTAGGACGA
+
FFFFFFFFFFFFFFFFFFFFFFFFFFFFFFFFFFFFFFFFFFFFFFFFFFFFFFFFFFFFFFFFFFFF
FFFFFFFFFFFFFFFFFFFFFFFFFFFFFFFFFFFFFFFFFFFFFFFFFFFFFFFFFFFFFFFFFFFF
@A00155:342:HHGFNDSXY:1:1549:25482:8766 1:N:0:GAACCTAG+TCCGCATA
GAGCGACTTCTCTCGGGCCCACAGCCTTACAGCTGTGGAGCACGGTATCCTCTGCCGAAACAGAGGTT
GGACAAGACCGGAGGGGTCTCCTGGTTCCAAAGGAGATGTACTCCGGGCTTGTTCACGAC
+
FFFFFFFFFFFFFFFFFFFFFFFFFFFFFFFFF::FFFFFFFFFFFFFFFFFFFFFFFFFFFFFFFFF
FFFFFFFFFFFFFFFFFFFFFFFFFFFFFFFFFFFFFFFFFFFFFFFFFFFFFFFFFFFF
@A00155:342:HHGFNDSXY:1:1550:22191:2848 1:N:0:GAACCTAG+TCCGCATA
AGAGAGAGCGACTTCTCTCGGGCCCACAGCCTTACAGCTGTGGAGCACGGTATCCTCTGCCGAAACAG
AGGTTGGACAAGACCGGAGGGGTCTCCTAGTTCCAAAGGAGATGTACTCCGGGCTTGTTCACGACCTA
+
FFFFFFFFFFFFFFFFFFFFFFFFFFFFFFFFFFFFFFFFFFFFFFFFFFFFFFFFFFFFFFFFFFFF
FFFFFFFFFFFFFFFFFFFFFFFFFFFFFFFFFFFFFFFFFFFFFFFFFFFFFFFFFFFFFFFFFFFF
@A00155:342:HHGFNDSXY:1:1550:13476:12680 1:N:0:GAACCTAG+TCCGCATA
GGATGTGGAGGGCGAGAGAGAGCGACTTCTCTCGGGCCCACAGCCTTACAGCTGTGGAGCACGGTATC
CTCTGCCGAAACAGAGGTTGGACAAGACCGGAGGGGTCTCCTAGTTCCAAAGGAGATGTAC
+
FFFFFFFFFFFFFFFFFFFFFFFFFFFFFFFFFFFFFFFFFFFFFFFFFFFFFFFFFFFFFFFF:FFF
FFFFFFFFFFFFFFFF:FFFFFFFFFFFFFFFFFFFFFFFFFFFFFFFFFFFFFFFFFFF
@A00155:342:HHGFNDSXY:1:1550:25337:17221 1:N:0:GAACCTAG+TCCGCATA
GAGAGGGATGTGGCGGGCGAGAGAGAGCGACTTCTCTCGGGCCCACAGCCTTACAGCTGTGGAGCACG
```

```
GTATCCTCTGCCGAAACAGAGGTTGGACAAGACCGGAGGGGTCTCCTAGTTCCAAAGGAGATGTACTC
+
:FFF::FF,,,FF,FFFF:,FFFF:FF,F:F,F,FF::F,:FF:,:FFFF,FF:F:F:FF:FF:FFFF
FFFFFFFFFFF:FFFFFFFFFFFFF:FFFF,FFFF:F,FFFFFFFFFFFFFFFF,:F,F,FFFF:FFF
@A00155:342:HHGFNDSXY:1:1550:16387:21825 1:N:0:GAACCTAG+TCCGCATA
CAATGCCGAGAGGGATGTGGAGGGCGAGAGAGAGCGACTTCTCTCGGGCCCACAGCCTTACAGCTGTG
GAGCACGGTATCCTCTGCCGAAACAGAGGTTGGACAAGACCGGAGGGGTCTCCTAGTTCCAAAGGAGA
+
FFFFFFFFFFFFFF:FF:FFFFFFFFFFFFFFFFFFFFFFFFFFFFFFFFFFFFFFFFFFFFFFFFFF
FFFFFFFFFFFFFFFFFFFFFFFFFFFFFFFFFFFFFFFFFFFFFFFFFFFFFFFFFFFFFFFFFFFF
@A00155:342:HHGFNDSXY:1:1550:11903:25300 1:N:0:GAACCTAG+TCCGCATA
GGATGTGGAGGGCGAGAGAGAGCGACTTCTCTCGGGCCCACAGCCTTACAGCTGTGGAGCACGGTATC
CTCTGCCGAAACAGAGGTTGGACAAGACCGGAGGGGTCTCCTAGTTCCAAAGGAGATGTACTCC
+
FFF,FFFFFFFFFFFFFFFFFFFFFFFFFFFFF::FFF:FFFFFFFFFFFFFFFFFFFFFFFFFFFFF
:FFFFFFFFFFFF:FF,FFFFFFFF,FF:FFFFFFFFFFFFFFFFF:FFFFF:FFFFFFFFFFF
@A00155:342:HHGFNDSXY:1:1550:11767:26318 1:N:0:GAACCTAG+TCCGCATA
GGATGTGGAGGGCGAGAGAGAGCGACTTCTCTCGGGCCCACAGCCTTACAGCTGTGGAGCACGGTATC
CTCTGCCGAAACAGAGGTTGGACAAGACCGGAGGGGTCTCCTAGTTCCAAAGGAGATGTACTCC
+
FFFFFFFFFFFFFFFFFFFFFFFFFFFFFFFFFFFFFFFFFFFFFFFFFFFFFFFFFFFFFFFFFFFF
FFFFFFFFFFFFFFFFFFFFFFFFFFFFFFFFFFFFFFFFFFFFFFFFFFFFFFFFFFFFFFFF
@A00155:342:HHGFNDSXY:1:1551:23122:2707 1:N:0:GAACCTAG+TCCGCATA
AGGGATGTGGAGGGCGAGAGAGAGCGACTTCTCTCGGGCCCACAGCCTTACAGCTGTGGAGCACGGTA
TCCTCTGCCGAAACAGAGGTTGGACAAGACCGGAGGGGTCTCCTAGTTCCAAAGGAGATGTACTCCGG
+
FFFFFFFFFFFFFFFFFFFFFFFFFFFFFFFFFFFFFFFFFFFFFFFFFFFFFFFFFFFFFFFFFFFF
FFFFFFFFFFFFFFFFFF:FFFFFFFF,FFFFFFFFFFFFFFFFFFFFFFFFFFFFFFFFFFFFF:FF
@A00155:342:HHGFNDSXY:1:1551:20057:4163 1:N:0:GAACCTAG+TCCGCATA
TCGGCAGAGGATACCGTGCTCCACAGCTGTAAGGCTGTGGGCCCGAGAGAAGTCGCTCTCTCTCGCCC
TTCACATCCCTCTCGGCATTGAGATGACCATAGGCCAGAGGTGAGTCCTTAAGTGGAC
+
FFFFFFFFFFFFFFFFFFFFFFFFFFFFFFFFFFFFFFFFFFFFFFFFFFFFFFFFFFFFFFFFFFFF
FFFFFFFFFFFFFFFFFFFFFFFFFFFFFFFFFFFFFFFFFFFFFFFFFF:FFFFFFF
@A00155:342:HHGFNDSXY:1:1551:31485:20290 1:N:0:GAACCTAG+TCCGCATA
GGGCGAGAGAGAGCGACTTCTCTCGGGCCCACAGCCTTACAGCTGTGGAGCACGGTATCCTCTGCCGA
AACAGAGGTTGGACAAGACCGGAGGGGTCTCCTAGTTCCAAAGGAGATGTACTCCGGGCTTGTTCAC
+
FFFFFFFFFFFFFFFFFFFFFFFFFFFFFFFFFFFFFFFFFFFFFFFFFFFFFFF:F:FFFFFFFFF
FFFFFFFFFFFFFFF:FFFFFFFFFFFFFFFFF:FFFFFFFFFFFFFFFFFFFFFFFFFFFFFFFFFF
@A00155:342:HHGFNDSXY:1:1552:16260:3881 1:N:0:GAACCTAG+TCCGCATA
GAGCGACTTCTCTCGGGCCCACAGCCTTACAGCTGTGGAGCACGGTATCCTCTGCCGAAACAGAGGTT
GGACAAGACCGGAGGGGTCTCCTAGTTCCAAAGGAGATGTACTCCGGGCTTGTTCACGACCTACCGTG
+
F:FFF,:FFFFFFFFFFFFFFFFFFFF:FFFFFFFFFFFFF:FFFF,F:FFFFFFFFFF:FFFFFFFF
FFFFFFFFFFFFFFFFFFFFFFFFF:FFFFF:F:FFFFFFFFFFFFFFFF:FFFFFFFFFFFF:FFF:F
@A00155:342:HHGFNDSXY:1:1552:4824:6120 1:N:0:GAACCTAG+TCCGCATA
CTCCTCTGGCCTATGGTCATCTCAATGCCGAGAGGGATGTGGAGGGCGAGAGAGAGCGACTTCTCTCG
GGCCCACAGCCTTACAGCTGTGGAGCACGGTATCCTCTGCCGAAACAGAGGTTGGACAAGACCGGAGG
+
FFFFFFFFFFFFFFFFFFFFFFFFFFFFFF:FF:FFFFFFFFF,FFFFFF::FFFFFFFFFFFFFFFF
FFFFFFFFFFF:FFFFFFFFF:FFFFFFFFFFFFFFFFFFFFFFFFFFFFFFFFFFFFFFFFFFFFFF
@A00155:342:HHGFNDSXY:1:1552:22688:10723 1:N:0:GAACCTAG+TCCGCATA
CACAGCTGATCTAAGGCGGTGTGGCGGGGCATGGGTTTGAACCCCCATGACGGTCGGAGTCTAGTAGG
```

CTCCCTGATGAGTCCGTTCCAAGGACGAAACCGTCGTTACTCTTCGAGTTGTGTAAGTTTCGTCCTAA
+
FFFFFFFFFFFFFFFFFFFFFF:FFFFFFFFFFFFFFFFFFFFFFFFFFFFFFFFFFFFFFFFFFFFF
FFFFFFFFFFFFFFFFFFFFFFFFFFF:FFFFFFFFFFFF:FFFFFFFFFFFFF:FFFFFFFFFF:FF
@A00155:342:HHGFNDSXY:1:1552:27055:13338 1:N:0:GAACCTAG+TCCGCATA
CTCCTCTGGCCTATGGTCATCTCAATGCCGAGAGGGATGTGGAGGGCGAGAGAGAGCGACTTCTCTCG
GGCCCACAGCCTTACAGCTGTGGAGCACGGTATCCTCTGCCGAAACAGAGGTTGGACAAGACCGGAGG
+
FFFFFFFFFFFFFFF:FFFFFFFFFFFF:F:FFFFFFFF:FFFFFFFFFFFFFFFFFFFFFFFFFFFF
F:F:FFFFFF:FFFFFF:FFFFFFFFFFFFFFFFFFFFFF:FFFFFFFFFFFFFFFFFFFFFFFFFFF
@A00155:342:HHGFNDSXY:1:1552:19180:20932 1:N:0:GAACCTAG+TCCGCATA
CTTCTCTCGGGCCCACAGCCTTACAGCTGTGGAGCACGGTATCCTCTGCCGAAACAGAGGTTGGACAA
GACCGGAGGGGTCTCCTAGTTCCAAAGGAGATGTACTCCGGGCTTGTTCACGACCTACCGTGTAAGTC
+
FFFFFF:FFF,F::FFFFFFFFFFFF,FFF:FFFFF,,FFFFF,,FFFFFFFFFFFFFFFFFFFFFFF
FFF:FFF:F,FFF:FF:FFF:FFFFFFFF:F,FFFFFF,F:FFF:FFFF,F::FFFFFFF:FFF:F:F
@A00155:342:HHGFNDSXY:1:1552:32090:23249 1:N:0:GAACCTAG+TCCGCATA
GAGGGCGAGAGAGAGCGACTTCTCTCGGGCCCACAGCCTTACAGCTGTGGAGCACGGTATCCTCTGCC
GAAACAGAGGTTGGACAAGACCGGAGGGGTCTCCTAGTTCCAAAGGAGATGTACTCCGGGCTTGTTCA
+
FFFFFFFFFFFFFFFFFFFFFFFFFFFFFFFFFFFFFFFFFFFFFFFFFFFFFFFFFFFFFFFFFFFF
FFFFFFFFFFFFFFFFFFFFFFFFFFFFFFFFFFF:FFFFFFFFFFFFFFFFFFFFFFFFFFFFFFFF
@A00155:342:HHGFNDSXY:1:1553:13810:9314 1:N:0:GAACCTAG+TCCGCATA
ACTTCTCTCGGGCCCACAGCCTTACAGCTGTGGAGCACGGTATCCTCTGCCGAAACAGAGGTTGGACA
AGACCGGAGGGGTCTCCTAGTTCCAAAGGAGATGTACTCCGGGCTTGTTCACGACCTAC
+
FFFFFFFFFFFFFFFFFFFFFFFFFFFFFFFFFFFFFFFFFFFFFFFFFFFFFFFFFFFFFFFFFFFF
FFFFFFFFFFFFFFFFFFFFFFFF:FFFFFFFFFFFFFFFFFFFFFFFFFFFFFFFFFF
@A00155:342:HHGFNDSXY:1:1553:25807:14904 1:N:0:GAACCTAG+TCCGCATA
GAGCGACTTCTCTCGGGCCCACAGCCTTACAGCTGTGGAGCACGGTATCCTCTGCCGAAACAGAGGTT
GGACAAGACCGGTGGGGTCTCCTAGTTCCAAAGGAGATGTACTCCGGGCTTGTTCACGACCTACCGTG
+
FFFFFFFFFFFFFFFFFFFFFFFFFFFFFFFFFFFFFFFFFFFFFFFFFFFFFFFFFFFFFFFFFF:F
FFFFFFFFFFFFFFFFFFFFFFFFFFFFFFFFFFFFFFFFFFFFFFFFFFFFFFFFFFFFFFFFFFFF
@A00155:342:HHGFNDSXY:1:1553:23113:19163 1:N:0:GAACCTAG+TCCGCATA
ACCTCTGTTTCGGCAGAGGATACCGTGCTCCACAGCTGTAAGGCTGTGGGCCCGAGAGAAGTCGCTCT
CTCTCGCCCTCTACATCCCTCTCGGCATTGAGATGACCATAGGCCAGAGGTGAGTCCTTAAGTGGACA
+
FFFFFFFFFFFFFFFFFFFFFFFFFFFFFFFFFFFFFFFFFFFFFFFFFFFFFFFFFFFFFFFFFFFF
FFFFFFFFFFFFFFFFFFFFFFFFFFF:FFFFFFFFFFFFFFFFFFFFFFFFFFFFFFFFFFFFFFFF
@A00155:342:HHGFNDSXY:1:1553:7554:23563 1:N:0:GAACCTAG+TCCGCATA
TGTGGAGGGCGAGAGAGAGCGACTTCTCTCGGGCCCACAGCCTTACAGCTGTGGAGCACGGTATCCTC
TGCCGAAACAGAGGTTGGACAAGACCGGAGGGGTCTCCTAGTTCCAAAGGAGATGTACTCCGGGCTTG
+
FFFFFFFFFFFFFFFFFFFFFFFFFFFFFFFFFFFFFFFFFFFFFFFFFFFFFFFFFFFFFFFFFFFF
FFFFFFFFFFFFFFFFFFFFFF:FFFFFFFFFFFFFFFFFFFFFFFFFFFFFFFFFFFFFFFFFFFFF
@A00155:342:HHGFNDSXY:1:1553:24334:28604 1:N:0:GAACCTAG+TCCGCATA
CTTCTCTCGGGCCCACAGCCTTACAGCTGTGGAGCACGGTATCCTCTGCCGAAACAGAGGTTGGACAA
GACCGGAGGGGTCTACTAGTTCCAAAGGAGATGTACTCCGGGCTTGTTCACGACCTACCGTGTAAGTC
+
FFFFFFFFFFFFFFFFFFFFFF:FFFFFFFFFFFFFFFFFFFFFFFFFF:FFFFFFF:F,FFFFFF:F
:FFFFFFFFFFFFF,FFF:FFFFFFFFFFFFFFFFFFFFF:FFFFFFFFFFFFFFFFFFFFFFFFFFF
@A00155:342:HHGFNDSXY:1:1553:9073:34585 1:N:0:GAACCTAG+TCCGCATA
ATCTCAATGCCGAGAGGGATGTGGAGGGCGAGAGAGAGCGACTTCTCTCGGGCCCACAGCCTTACAGC

TGTGGAGCACGGTATCCTCTGCCGAAACAGAGGTTGGACAAGACCGGAGGGGTCTCCTAGTTCCAAAG
+
FFFFFFFFFFFFFFFFFFF:FFFFFFFFFFFFFFFFFFFFFFFFFFFFFFFFFFFFFFFFFFFFFFFF
FFFFFFFFFFFFFFFFFFFFFFFFFFFFFFFFFFFFFFFFFFFFFFFFFFFFFFFFFFFFFFFFFFFF
@A00155:342:HHGFNDSXY:1:1554:29008:7200 1:N:0:GAACCTAG+TCCGCATA
ACATCCCTCTCGGCATTGAGATGACCATAGGCCAGAGGTGAGTCCTTAAGTGGACACAGCTGATCTAA
GGCGGTGTGGCGGGGCATGGGTTTGAACCCCCATGACGGTCGGAGTCTAGTAGGCTCCCTGATGAGTC
+
FF:FFFFFFFFFFFF:FFFFFFFFFFFFFFFFFFFFFFFFFFFFFFFFFFFFFFFFF:FFFFFFFFFF
FFFFFFFFFFFFFFFFFFFFFFFFFFFFFFFFFFFFFFFFFFFFFFFFFFFFFFF:FFFFFFFFFFFF
@A00155:342:HHGFNDSXY:1:1554:17644:9314 1:N:0:GAACCTAG+TCCGCATA
GAGCGACTTCTCTCGGGCCCACAGCCTTACAGCTGTGGAGCACGGTATCCTCTGCCGAAACAGAGGTT
GGACAAGACCGGAGGGGTCTCCTAGTTCCAAAGGAGATGTACTCCGGGCTTGTTCACGACCTACCGTG
+
FFFFFFFFFFFFFFFFFFFFFFFFFFFFFFFFFFFFFFFFFFFFFFFFFFFFFFFFFFFFFFFFFFFF
FFFFFFFFFFFFFFFFFFFFFFFFFFFFFFFFFFFFFFFFFFFFFFFFFFFFFFFFFFFFFFFFFFFF
@A00155:342:HHGFNDSXY:1:1554:6008:13745 1:N:0:GAACCTAG+TCCGCATA
AGAGAGAGCGACTTCTCTCGGGCCCACAGCCTTACAGCTGTGGAGCACGGTATCCTCTGCCGAAACAG
AGGTTGGACAAGACCGGAGGGGTCTCCTAGTTCCAAAGGAGATGTACTCCGGGCTTGTTC
+
FFFFFFFFFFFFFFFFFFFFFFFFFFFFFFFFFFFFFFFFF,FFFFFFFFFFFFFFFFFFFFFFFFFF
FFFFFFFFFFFFFF:FFFFFFFFFFFFFFFFFFF,FFFFFFFFFF::FFF:FFFFFFFFF
@A00155:342:HHGFNDSXY:1:1554:13666:14606 1:N:0:GAACCTAG+TCCGCATA
TTCTCTCGGGCCCACAGCCTTACAGCTGTGGAGCACGGTATCCTCTGCCGAAACAGAGGTTGGACAAG
ACCGGAGGGGTCTCCTAGTTCCAAAGGAGATGTACTCCGGGCTTGTTCACGACCTACCGTGTAAGTCG
+
FFFFFFFFFFFFFFFFFFFFF:FFFFFFFFFFFFFFFFFFFFFFFFFFFFFFFFFFFFFFFFFFFFFF
FFFFFFFFFFFFFFFFFFFFFFFFFFFFFFFFFFFFFFFFFFFFFFFFFFFFFFFFFFFFFFFFFFFF
@A00155:342:HHGFNDSXY:1:1554:17219:24079 1:N:0:GAACCTAG+TCCGCATA
ACTTCTCTCGGGCCCACAGCCTTACAGCTGTGGAGCACGGTATCCTCTGCCGAAACAGAGGTTGGAGA
AGACCGGAGGGGTCTCCTAGTTCCAAAGGAGATGTACTCCGGGCTTGTTCACGACCTACCGTGTAAGT
+
FFFFFFFFFFFFFFFFFFFFFFFFFFFFFFFFFFFFFFFFFFFFFFFFFFFFFFFFFFFFFF:FFFFF
FF:FFFFFFFFFFF,FFFFF:FFFFF:FFFFFFFFFFFFFFFFFF,FFFFFFFFFF:FFFFFFFF:FF
@A00155:342:HHGFNDSXY:1:1554:17173:24659 1:N:0:GAACCTAG+TCCGCATA
ACTTCTCTCGGGCCCACAGCCTTACAGCTGTGGAGCACGGTATCCTCTGCCGAAACAGAGGTTGGAGA
AGACCGGAGGGGTCTCCTAGTTCCAAAGGAGATGTACTCCGGGCTTGTTCACGACCTACCGTGTAAGT
+
FF:FFFFFFFFFFFFFFFFFFFFFFFFFFFFFFFFFFFFFFFFFFFFFFFFFFFFFFFFFFFFFFFFF
FFFFFFFFFFFFFFFFFFFFFFFFFFFFFFFFFFFFFFFFFFFFFFFFFFFFFFFFFFFFFFFFFFFF
@A00155:342:HHGFNDSXY:1:1554:12093:34648 1:N:0:GAACCTAG+TCCGCATA
GTGGAGGGCGAGAGAGAGCGACTTCTCTCGGGCCCACAGCCTTACAGCTGTGGAGCACGGTATCCTCT
GCCGAAACAGAGGTTGGACAAGACCGGAGGGGTCTCCTAGTTCCAAAGGAGATGTACTCCGGGCTTGT
+
FFFFFFFFFFFFFFFFFFFFFFFFFFFFFFFFFFFFFFFFFFFFFFFFFFFFFFFFFFFFFFFFFFFF
FFFFFFFFFFFFFFFFFFFFFFFFFFFFFFFFFFFFFFFFFFFFFFFFFFFFFFFFFFFFFFFFFFFF
@A00155:342:HHGFNDSXY:1:1554:11677:35305 1:N:0:GAACCTAG+TCCGCATA
GTGGAGGGCGAGAGAGAGCGACTTCTCTCGGGCCCACAGCCTTACAGCTGTGGAGCACGGTATCCTCT
GCCGAAACAGAGGTTGGACAAGACCGGAGGGGTCTCCTAGTTCCAAAGGAGATGTACTCCGGGCTTGT
+
FFFFFFFFFFFFFFFFFFFFFFFFFFFFFFFFFF:FFFFFFFFFFFFFFFFFFFFFFFFFFFFFFFFF
:FFFFFFFFFFFF:FFFFFFF:FFFFFFFFFFFFFFFFFFFFFFFFFFFFFFFFFFFFFFFFFFFFFF
@A00155:342:HHGFNDSXY:1:1554:13738:35336 1:N:0:GAACCTAG+TCCGCATA
GTGGAGGGCGAGAGAGAGCGACTTCTCTCGGGCCCACAGCCTTACAGCTGTGGAGCACGGTATCCTCT

```
GCCGAAACAGAGGTTGGACAAGACCGGAGGGGTCTCCTAGTTCCAAAGGAGATGTACTCCGGGCTTGT
+
FFFFFFFFFFFFFFFFFFFFFFFFFFFFFFFFFFFFFFFFFFFFFFFFFFFFFFFFFFFFFFFFFFFF
FFFFFFFFFFFFFFFFFFFFFFFFFFFFFFFFFFFFFFFFFFFFFFFFFFFFFFFFFFFFFFFFFFFF
@A00155:342:HHGFNDSXY:1:1555:26476:4570 1:N:0:GAACCTAG+TCCGCATA
GTCATCTCAATGCCGAGAGGGATGTGGAGGGCGAGAGAGAGCGACTTCTCTCGGGCCCACAGCCTTAC
AGCTGTGGAGCACGGTATCCTCTGCCGAAACAGAGGTTGGACAAGACCGGAGGGGTCTCCTAGTTCCA
+
FFFFFFFFFFFFFFFFFFFFFFFFFFFFFFFFFFFFFFFFFFFFFFFFFFFFFFFFFFFFFFFFFFFF
FFFFF:FFFFFFFFFFFFFFFFFFFFFFF:F:FFFFFFFFFFFFFFFFFFFFFFFFFFFFFFFFFFFF
@A00155:342:HHGFNDSXY:1:1555:15004:8625 1:N:0:GAACCTAG+TCCGCATA
CAATGCCGAGAGGGATGTGGAGGGCGAGAGAGAGCGACTTCTCTCGGGCCCACAGCCTTACAGCTGTG
GAGCACGGTATCCTCTGCCGAAACAGAGGTTGGACAAGACCGGAGGGGTCTCCTGGTTCCAAAGGAGA
+
FFFFFFFFFFFFFFFFF:FFFFFFFFFFFFFFFFFFFFFFFFFFFFFFFFFFFFFFFFFFFFFFFFFF
FFFFFFFFFFFFFFFFFFFFFFFFFFFFFFFFFFFFF:FFFF:FFFFFFFFFFFFFFFFFFF:FFFFF
@A00155:342:HHGFNDSXY:1:1555:2772:34914 1:N:0:GAACCTAG+TCCGCATA
TGTGGAGGGCGAGAGAGAGCGACTTCTCTCGGGCCCACAGCCTTACAGCTGTGGAGCACGGTATCCTC
TGCCGAAACAGAGGTTGGACAAGACCGGAGGGGTCTCCTAGTTCCAAAGGAGATGTACTCCGGG
+
FFFFFFFFFFFFFFFFF:FFFFFFFFFFF:,F:FFFFFFFFFFFFFF:FFFFFFFFFFFFFF:FFFFF
FF:FFFFFFFFFFFFFFFFFFF:FFFFFFF:F,FFFFFFFFFFFFFFFFFFFFFFFFFFFFFFF
@A00155:342:HHGFNDSXY:1:1556:7563:12336 1:N:0:GAACCTAG+TCCGCATA
CTCCTCTGGCCTATGGTCATCTCAATGCCGAGAGGGATGTGGAGGGCGAGAGAGAGCGACTTCTCTCG
GGCCCACAGCCTTACAGCTGTGGAGCACGGTATCCTCTGCCGAAACAGAGGTTGGACAAGACCGGAGG
+
FFFFFFFFFFFFFFFFFFFFFFFFFFFFFF:FFFFFFFFFFFFFFFFFFFFFFFFFFFFFFFFFFFFF
FFFFFFFFFFFFFFFFFFFFFFFFFFFFFFFFFFFFFFFFFFFFFFFFFFFFFFFFFFFFFFFFFFFF
@A00155:342:HHGFNDSXY:1:1556:7500:18270 1:N:0:GAACCTAG+TCCGCATA
GTCATCTCAATGCCGAGAGGGATGTGGAGGGCGAGAGAGAGCGACTTCTCTCGGGCCCACAGCCTTAC
AGCTGTGGAGCACGGTATCCTCTGCCGAAACAGAGGTTGGACAAGACCGGAGGGGTCTCCTAGTTCCA
+
FFFFFFFFFFFF,FFFFFFFF::FFFFFFFFFFFFFFFFFFFFFFFFFFFFFFFFFFF:FFFFFFFFF
FFFFFFFFFFFFFFFFFFFFFFFFFFFFFFFFFFF,FFFFFFFFFFFFFFFFFFFFFFFFFFFFFFFF
@A00155:342:HHGFNDSXY:1:1556:9326:25911 1:N:0:GAACCTAG+TCCGCATA
AAGGACTCTCCTCTGGCCTATGGTCATCTCAATGCCGAGAGGGATGTGGAGGGCGAGAGAGAGCGACT
TCTCTCGGGCCCACAGCCTTACAGCTGTGGAGCACGGTATCCTCTGCCGAAACAGAGGTTGGACAAGA
+
FFFFFFFFFFFFFFFFFFFFFFFFFFFFFFFFFFF,FFFFFFFF:F:FFFFFFFFFFFFFFFFFFFFF
FFFFFFFFFFFFFFFFFFFF,FFFFFFFFFFFFFFFFFFFFFFFFFFFFFFFFFFFFFFFFFFFFFFF
@A00155:342:HHGFNDSXY:1:1557:27914:3396 1:N:0:GAACCTAG+TCCGCATA
TGGTCATCTCAATGCCGAGAGGGATGTAGAGGGCGAGAGAGAGCGACTTCTCTCGGGCCCACAGCCTT
ACAGCTGTGGAGCACGGTATCCTCTGCCGAAACAGAGGTTGGACAAGACCGGAGGGGTCTCCTAGTTC
+
FFFFFFFFFFFFFFFFFFFFFFFFFFFFFFFFFFFFFFFFFFFFFFFFFFFFFFFFFFFFFFFFFFFF
FFFFFFFFFFFFFFFFFFFFFFFFFFFFFFFFFFFFFFFFFFFFFFFFFFFFFFFFFFFFFFFFFFFF
@A00155:342:HHGFNDSXY:1:1557:15881:32503 1:N:0:GAACCTAG+TCCGCATA
GGATGTGGAGGGCGAGAGAGAGCGACTTCTCTCGGGCCCACAGCCTTACAGCTGTGGAGCACGGTATC
CTCTGCCGAAACAGAGGTTGGACAAGACCGGAGGGGTCTCCTAGTTCCAAAGGAGATGTACT
+
FFFFFFFFFFFFFFFFFFFFFFFFFFFFFFFFFFFFFFFFFFFFFFFFFFFFFFFFFFFFFFFFF:FF
FFFFFFFFFFFFFFFFFFFFFFFFFFFFFFFFFFFFFFFFFFFFFFF:FFFFFFFFFFFFFF
@A00155:342:HHGFNDSXY:1:1558:10077:17722 1:N:0:GAACCTAG+TCCGCATA
GTATCCTCTGCCGAAACAGAGGTTGGACAAGACCGGAGGGGTCTCCTAGTTCCAAAGGAGATGTACTC
```

```
CGGGCTTGTTCACGACCTACCGTGTAAGTCGTAGTCTAGTAGGCTACCTGACGAGTCCTTTTTAGGAC
+
FFFFFF:FFFFFFFFFFFFFFFFFFF:FFFFFFFFFFFFFFFFFFFFFFFFFFFFFFFFFFFFFFFFFFF
FFFFFFFFFFFFFFFFFFFFFFFFFFFFFF:FFFFFFFFFFF:FFFFFFFFFFFFFFFFFFFFFFFFFFF
@A00155:342:HHGFNDSXY:1:1558:13006:28401 1:N:0:GAACCTAG+TCCGCATA
GGATGTGGAGGGCGAGAGAGAGCGACTTCTCTCGGGCCCACAGCCTTACAGCTGTGGAGCACGGTATC
CTCTGCCGAAACAGAGGTTGGACAAGACCGGAGGGGTCTCCTAGTTCCAAAGGAGATGTACTCC
+
FFFFFFFFFFFFFFFFFFFFFFFFFFFFFFFFFFFFFFFFFFFFFFFFFFFFFFFFFFFFFFFFFFFFF
FFFFFF:FFFFFFFFFFFFFFFF:F,FFFFFFFFFFFFFFFFFFFFFFFFFFFFFFFFFFFFFFF
@A00155:342:HHGFNDSXY:1:1558:4444:31516 1:N:0:GAACCTAG+TCCGCATA
TGGAGGGCGAGAGAGAGCGACTTCTCTCGGGCCCACAGCCTTACAGCTGTGGAGCACGGTATCCTCTG
CCGAAACAGAGGTTGGACAAGACCGGAGGGGTCTCCTAGTTCCAAAGGAGATGTACTCCGGGCTTGTT
+
FFFFFFFFFFFFFFFFFFFFFFFF:FFFFFFFFFFFFFFFFFFFFFFFFFFFFFFFFFFFFFFFFFFFF
FFFFFFFFFFFFFFFFFFFFFFFFFFFFFFFFFFFFFFFFFF:FFFFFFFFFFFFFFFFFFFFFFFFFFF
@A00155:342:HHGFNDSXY:1:1558:23285:36996 1:N:0:GAACCTAG+TCCGCATA
AGAGAGAGCGACTTCTCTCGGGCCCACAGCCTTACAGCTGTGGAGCACGGTATCCTCTGCCGAAACAG
AGGTTGGACAAGACCGGAGGGGTCTCCTAGTTCCAAAGGAGATGTACTCCGGGCTTGTTCACGAC
+
FFFFFFFFFFFFFFFFFFFFFFFFFFFFFFFFFFFFFFFFFFFFFFFFFFFFFFFFFFFFFFFFFFFFF
FFFFFFFFFF:FFFFFFFFFFFFFFFFFFFFFFFFFFFFFFFFFFFFFFFFFFFFFFFFFFFFFFF
@A00155:342:HHGFNDSXY:1:1559:20220:3662 1:N:0:GAACCTAG+TCCGCATA
TGTGTCCACTTAAGGACTCACCTCTGGCCTATGGTCATCTCAATGCCGAGAGGGATGTGAAGGGCGAG
AGAGAGCGACTTCTCTCGGGCCCACAGCCTTACAGCTGTGGAGCACGGTATCCTCTGCCGAAACAGAG
+
FFFFFFFFFFFFFFFFFFFFFFFFFFFFFFFFFFFFFFFFFFFFFFFFFFFFFFFFFFFFFF:FFFFFF
FFFFFFFFFFFFFFFFFFFFFFFFFFFFFFFFFFFFFFFFFFFFFFFFFFFFFFFFFFFFFFFFFFFFF
@A00155:342:HHGFNDSXY:1:1559:30246:6120 1:N:0:GAACCTAG+TCCGCATA
GTCTTGTCCAACCTCTGTTTCGGCAGAGGATACCGTGCTCCACAGCTGTAAGGCTGTGGGCCCGAGAG
AAGTCGCTCTCTCTCGCCCTCCACATCCCTCTCGGCATTGAGATGACCATAGGCCAGAGGTG
+
FFFFFFFFFFFFFFFFFFFFFFFFFFFFFFFFFFFFFFFFFFFFFFFFFFFFFFFFFFFFFFFFFFFFF
FFFFFFFFFFFFFFFFFFFFFFFFFFFFFFFFFFFFFFFFFFFFFFFFFFFFFFFFFFFFFFF
@A00155:342:HHGFNDSXY:1:1559:22236:12070 1:N:0:GAACCTAG+TCCGCATA
GATGTGGAGGGCGAGAGAGAGCGACTTCTCTCGGGCCCACAGCCTTACAGCTGTGGAGCACGGTATCC
TCTGCCGAAACAGAGGTTGGACAAGACCGGAGGGGTCTCCTAGTTCCAAAGGAGATGTACTCCGGGCT
+
FFFFFFFFFFFFFFFFFFFFFFFFFFFFFFFFFFFFFFFFFFFFFFFFFFFFFFFFFFFFFFFFFFFFF
FFFFFFFFFFFFFFFFFFFFFFFFFFFFFFFFFFFFFFFFFFFFFFFFFFFFFFFFFFFFFFFFFFFFF
@A00155:342:HHGFNDSXY:1:1559:24894:17769 1:N:0:GAACCTAG+TCCGCATA
ACCCCTCCGGTCTTGTCCAACCTCTGTTTCGGCAGAGGATACCGTGCTCCACAGCTGTAAGGCTGTGG
GCCCGAGAGAAGTCGCTCTCTCTCGCCCTCTACATCCCTCTCGGCATTGAGATGACCATAGGCCAGAG
+
FFFFFFFFFFFF:,FFFFFFFFFFFFFFFFFFFFFFFFFFFFFFFFFFFFFFFFFFF:FFFFFFFFF
FFFFFFFFFFFFFFFFFFFFFFFFFFFFFFFFFFFFFFFFFFFFFFFFFFFFFFFF:F:FFF:FFFF
@A00155:342:HHGFNDSXY:1:1559:23574:33520 1:N:0:GAACCTAG+TCCGCATA
TGTCCACTTAAGGACTCACCTCTGGCCTATGGTCATCTCAATGCCGAGAGGGATGTGAAGGGCGAGAG
AGAGCGACTTCTCTCGGGCCCACAGCCTTACAGCTGTGGAGCACGGTATCCTCTGCCGAAACAGAGGT
+
FFFFFFFFFFFFFFFFFFFFFFFFFFFFFFFFFFFFFFFFFFFFFFFFFFFFFFFFFFFFFFFFFFFFF
:FFFFFFFFFFFFFFFFFFFFFFFFFFFFFFFFFFFFFFFFFFFFFFFFFFFFFFFFFFFFFFFFFFFF
@A00155:342:HHGFNDSXY:1:1560:20274:6887 1:N:0:GAACCTAG+TCCGCATA
GGGCGAGAGAGAGCGACTTCTCTCGGGCCCACAGCCTTACAGCTGTGGAGCACGGTATCCTCTGCCGA
```

```
AACAGAGGTTGGACAAGACCGGAGGGGTCTCCTGGTTCCAAAGGAGATGTACTCCGGGCTTGTTCACG
+
::FFFFFF:FFFFFFFFF,FFFFFF:FFFFFFFFFF,FFFFFFFFFFFFFFFFF:FFFFFFFF:FFFF
FFF::FFFFFFFFFFFFFFFFFFFFFFFFF,FFFFFFFFFFFFF,FFFFFFFFFFFFFF:FFFFFFFF:
@A00155:342:HHGFNDSXY:1:1561:10619:2065 1:N:0:GAACCTAG+TCCGCATA
CTTCTCTCGGGCCCACAGCCTTACAGCTGTGGAGCACGGTATCCTCTGCCGAAACAGAGGTTGGACAA
GACCGGAGGGGTCTCCTGGTTCCAAAGGAGATGTACTCCGGGCTTGTTCACGACCTACCGTGT
+
FFFFFFFFFFFFFFFFFFFFFFFFFFFFFFFFFFFFFFFFFFFFFFFFFFFFFFFFFFFFFFFFFFFF
FFFFFFFFFFFFFFFFFFFFFFFFFFFFFFFFFFFFFFFFFFFFFFFFFFFFFFFFFFFFFFF
@A00155:342:HHGFNDSXY:1:1561:32461:18912 1:N:0:GAACCTAG+TCCGCATA
TTAAGGACTCTCCTCTGGCCTATGGTCATCTCAATGCCGAGAGGGATGTGGAGGGCGAGAGAGAGCGA
CTTCTCTCGGGCCCACAGCCTTACAGCTGTGGAGCACGGTATCCTCTGCCGAAACAGAGGTTGGACAA
+
FFFFFFFFFFFFFFFFFFFFFFFFFFFFF:FFFFFFFFFFFFFFFFFFFFFFFFFFFFFFFFFFFFFF
FFFFFFFFFFFFFFFFFFFFFFFFFFFFFFFFFFFFFFFFFFFFFFFFFFFFFFFFF:FFFFFFFFFF
@A00155:342:HHGFNDSXY:1:1561:28357:24424 1:N:0:GAACCTAG+TCCGCATA
CTCTGGCCTATGGTCATCTCAATGCCGAGAGGGATGTAGAGGGCGAGAGAGAGCGACTTCTCTCGGGC
CCACAGCCTTACAGCTGTGGAGCACGGTATCCTCTGCCGAAACAGAGGTTGGACAAGACCGGAGGGGT
+
FFFFFFFFFFFFF:FF:FFFFFFFFFFFFFFF:F:FFFFF,FF:FFFFFFFFFFFFFFFFFFFF:FFF
FFFFFFF::FFFFFFFFFFFFFFFFFFFFFFFFFFFF:FFFFF:F:F:FFF:FFFFF:FF:FFFFFFFF
@A00155:342:HHGFNDSXY:1:1561:14968:25034 1:N:0:GAACCTAG+TCCGCATA
GGACTCACCTCTGGCCTATGGTCATCTCAATGCCGAGAGGGATGTGAAGGGCGAGAGAGAGCGACTTC
TCTCGGGCCCACAGCCTTACAGCTGTGGAGCACGGTATCCTCTGCCGAAACAGAGGTTGGAC
+
FFFFFFFFFFFFFFFFFFFFFFFFFFFFFFFFFFFFFFFFFFFFFFFFFFFFFFFFFFFFFFFFFFFF
FFFFFFFFFFFFFFFFFFFFFFFFFFFFFFFFFFFFFFFFFFFFFFFFFFFFFFFF,FFFFFFFF
@A00155:342:HHGFNDSXY:1:1561:3640:25363 1:N:0:GAACCTAG+TCCGCATA
TGTCCACTTAAGGACTCTCCTCTGGCCTATGGTCATCTCAATGCCGAGAGGGATGTGGAGGGCGAGAG
AGAGCGACTTCTCTCGGGCCCACAGCCTTACAGCTGTGGAGCACGGTATCCTCTGCCGAAACAGAGGT
+
FFFFFFFFFFFFFFFFFFFFFFFFFFFFFFFFFFFFFFFFFFFFFFFFFFFFFFFFFFFFFFFFFFFF
FFFFFF,FFFFFFFFFFFFFFFFFFFF:F,FFFFFFFFFFFF:FFFFFFFFFFFFFFFFFFFFFFFFF
@A00155:342:HHGFNDSXY:1:1562:30770:2738 1:N:0:GAACCTAG+TCCGCATA
AGAGAGAGCGACTTCTCTCGGGCCCACAGCCTTACAGCTGTGGAGCACGGTATCCTCTGCCGAAACAG
AGGTTGGACAAGACCGGAGGGGTCTCCTGGTTCCAAAGGAGATGTACTCCGGGCTTGTTCAC
+
FFFFFFFFFFFF:FFFFFFFFFFFFFFFFFFFFFFFFFFFFFFFFFFFFFFFFFFFFFFFFFFFFFFF
FFFFFFFFFFFFFFFFFFFFFFFFFFFFFFFFFFFFFFFFFF:FFFFFFFFFFFFFFFFFFF
@A00155:342:HHGFNDSXY:1:1562:4869:7921 1:N:0:GAACCTAG+TCCGCATA
GCTGTGTCCACTTAAGGACTCACCTCTGGCCTATGGTCATCTCAATGCCGAGAGGGATGTGAAGGGCG
AGAGAGAGCGACTTCTCTCGGGCCCACAGCCTTACAGCTGTGGAGCACGGTATCCTCTGCCGAAACAG
+
FFFFFFFFFFFFFFFFFFFFFFFFFFFFFFFFF,FFFFFFFFFFFFFFFFFFF:FFFFFFFFF:FFFFF
FFFFFFFFFFFFFFFFFFFFFFFFFFFFFFFFFFFFFFFFFFFFFFFFFFFFFFFFFFFFFFFFFFFF
@A00155:342:HHGFNDSXY:1:1562:13422:26490 1:N:0:GAACCTAG+TCCGCATA
CGAGAGGGATGTGGAGGGCGAGAGAGAGCGACTTCTCTCGGGCCCACAGCCTTACAGCTGTGGAGCAC
GGTATCCTCTGCCGAAACAGAGGTTGGACAAGACCGGAGGGGTCTCCTAGTTCCAAAGGAGATGTACT
+
,FFFF,F:FFF:F:F,F,F,FFFF,FFFF:F:FFFF:F:,,,F,FF:F:F,FFFFF,:FFF,,FFFFF
FFFF:F:FFFFFFFFFF,FFFFFF:F,F:FFFFFF,FFF:F,F:FFFFF:FF:FFFFFFF:FFF:FFF
@A00155:342:HHGFNDSXY:1:1563:15826:7044 1:N:0:GAACCTAG+TCCGCATA
AGCGACTTCTCTCGGGCCCACAGCCTTACAGCTGTGGAGCACGGTATCCTCTGCCGAAACAGAGGTTG
```

```
GACAAGACCGGAGGGGTCTCCTAGTTCCAAAGGAGATGTACTCCGGGCTTGTTCACGACCTACCGTGT
+
FFFFFFFFF:FFFFFFFFFFFFFFF:FFFFFFFFFFFFFFFFFFFFFFFFFFFF:FFFFFFFFFFFFF
FFFFFFFFFFFFFFFFFFFFFFFFFFFFFFFFFFFFFFFFFFFFFFFFFFFFF:FFFFFFFFF:F:FF
@A00155:342:HHGFNDSXY:1:1563:9037:14826 1:N:0:GAACCTAG+TCCGCATA
TCTCTCGGGCCCACAGCCTTACAGCTGTGGAGCACGGTATCCTCTGCCGAAACAGAGGTTGGACAAGA
CCGGAGGGGTCTCCTAGTTCCAAAGGAGATGTACTCCGGGCTTGTTCACGACCTACCGTGTAAGTCGT
+
FFFFFFFFFFFFFFFFFF:FFF::FFFFFFFFFFFFFFFFFFFFFFFFFFFFFFFFFFFFFFFFFFFF
FFFFFFFFFFFFFFFFFFFFFFFFFFFFFFFFFFFFFFFFF,FFFFFFFFFFFFFFFFFFFFFFFFFF
@A00155:342:HHGFNDSXY:1:1563:15953:15843 1:N:0:GAACCTAG+TCCGCATA
GAGCGACTTCTCTCGGGCCCACAGCCTTACAGCTGTGGAGCACGGTATCCTCTGCCGAAACAGAGGTT
GGACAAGACCGGAGGGGTCTCCTAGTTCCAAAGGAGATGTACTCCGGGCTTGTTCACGACCTACCGTG
+
FFFFFFFFFFFFFFFFFFFFFFFFFFFFFFFFFFFFFFFFFFFFFFFFFFFFFFFFFFFFFFFFFFFF
FFFFFFFFFFFFFFFFFFFFFFFFFFFFFFFFFFFFFFFFFFFFFFFFFFFFFFFFFFFFFFFFFFFF
@A00155:342:HHGFNDSXY:1:1563:1515:31673 1:N:0:GAACCTAG+TCCGCATA
AGCACGGTATCCTCTGCCGAAACAGAGGTTGGACAAGACCGGAGGGGTCTCCTGGTTCCAAAGGAGAT
GTACTCCGGGCTTGTTCACGACCTACCGTGTAAGTCGTAGTCTAGTAGGCTACCTGACGAGTCCTTTT
+
FFFFFFFFFFFFFFFFFFFFFFFF:FFFFFFFFFFFFFFFFFFFFFFFFFFFFFFFFFFFFFFFFFFF
FFFFFFFFFFFFFFFFF,FFFFFFFFFFFFFFFFFFFFFFFFFFFFFFFFFFFFFFFFFFFFFFFFFF
@A00155:342:HHGFNDSXY:1:1563:2772:35008 1:N:0:GAACCTAG+TCCGCATA
GGATGTGGAGGGCGAGAGAGAGCGACTTCTCTCGGGCCCACAGCCTTACAGCTGTGGTGCACGGTATC
CTCTGCCGAAACAGAGGTTGGACAAGACCGGAGGGGTCTCCTAGTTCCAAAGGAGATGTACTCC
+
FFFFFFFFFFFFFFFFFFFFFFFFFFFFFFFFFFFFFFFFFFFFFFFFFFFFFFFFFFFFFFFFFFFF
FFFFFFFFFFFFFFFFFFFFFFFFF,FFFFFFFFFFFFFFFFFFFFFFF:FFFFFFFFFFFFFF
@A00155:342:HHGFNDSXY:1:1564:8486:34225 1:N:0:GAACCTAG+TCCGCATA
CTTCTCTCGGGCCCACAGCCTTACAGCTGTGGAGCACGGTATCCTCTGCCGAAACAGAGGTTGGACAA
GACCGGAGGGGTCTCCTAGTTCCAAAGGAGATGTACTCCGGGCTTGTTCACGACCTACCGTGTAAGTC
+
FFFFFFFFFFFFFFFFFFFFFFFFFFFFFFFFFFFFFFFFFFFFFFFFFFFFFFFFFFFFFFFFFFFF
FFFFFFFFFFFFFFFFFFFFFFFFFFFFFFFFFFFFFFFFFFFFFFFFFFFFFFFFFFFFFFFFFFFF
@A00155:342:HHGFNDSXY:1:1565:30373:6277 1:N:0:GAACCTAG+TCCGCATA
TTCTCTCGGGCCCACAGCCTTACAGCTGTGGAGCACGGTATCCTCTGCCGAAACAGAGGTTGGACAAG
ACCGGAGGGGTCTCCTAGTTCCAAAGGAGATGTACTCCGGGCTTGTTCACGACCTACCGTGTAAGTCG
+
F,F:F,FF::FFFFFFFFFFFF,FFFF:FFFFFFFF,FFFFF:FFFFFFFFFFFFFFFF:FFFFFFFF
F:FFFFF,::FFFFF:FFF:F,FFFFFFFFFFFFFFFFFFFFFFFFF,FFFFFFFFFFFFFFFFFFFF
@A00155:342:HHGFNDSXY:1:1565:23014:19366 1:N:0:GAACCTAG+TCCGCATA
GAGAGAGCGACTTCTCTCGGGCCCACAGCCTTACAGCTGTGGAGCACGGTATCCTCTGCCGAAACAGA
GGTTGGACAAGACCGGAGGGGTCTCCTGGTTCCAAAGGAGATGTACTCCGGGCTTGTTCACGACCTAC
+
FFFFFFFFFFFFFFFFFFFFFFFFFFFFFFFFFFFFFFFFFFFFFFFFFFFFFFFFFFFFFFFFFFFF
FFFFFFFFFFFFFFFFFFFFFFFFFFFFFFFFFFFFFFFFFFFFFFFFFFFFFFFFFFFFFFFFFFFF
@A00155:342:HHGFNDSXY:1:1565:32307:26694 1:N:0:GAACCTAG+TCCGCATA
TTCTCTCGGGCCCACAGCCTTACAGCTGTGGAGCACGGTATCCTCTGCCGAAACAGAGGTTGGACAAG
ACCGGAGGGGTCTCCTAGTTCCAAAGGAGATGTACTCCGGGCTTGTTCACGACCTACCGTGTAAGTCG
+
FFFFFFFFFFFFFFFFFFFFFFFFFFFFFFFFFFFFFFFFFFFFFFFFFFFFFFFFFFFFFFFFFFFF
FFFFFFFFFFFFFFF:FFFFFFFFFFFFFFFFFFFFFFFFFF::FFFFFFFFFFFFFFFFFFFFFFFF
@A00155:342:HHGFNDSXY:1:1565:29134:33473 1:N:0:GAACCTAG+TCCGCATA
AGAGAGAGCGACTTCTCTCGGGCCCACAGCCTTACAGCTGTGGAGCACGGTATCCTCTGCCGAAACAG
```

AGGTTGGACAAGACCGGAGGGGTCTCCTAGTTCCAAAGGAGATGTACTCCGGGCTTGTTCACGACCTA
+
FFFFFFFFFFFFFFFFFFFFFFFFFFFF:FFFFFFFFFFFFFFFFFFFFFFFFFFFFFFFFFFFFFFFFF
FFFFFFFFFFFFFFFFFFFFF:FFFFFFF:FFFFFFFFFFFFFFFFFFFFFFFFFFFFFFFFFFFFFFFFF
@A00155:342:HHGFNDSXY:1:1566:30445:1391 1:N:0:GAACCTAG+TCCGCATA
GAGCGACTTCTCTCGGGCCCACAGCCTTACAGCTGTGGAGCACGGTATCCTCTGCCGAAACAGAGGTT
GGACAAGACCGGAGGGGTCTCCTAGTTCCAAAGGAGATGTACTCCGGGCTTGTTCACGACCTACCGTG
+
FFFFFFFFFFFFFFFFFFFFFFFFFFFFFFFFFFFFFFFFFFFFFFFFFFFFFFFFFFFFFFFFFFFFF
FFFFFFFFFFFFFFFFFFFFFFFFFFFFFFFFFFFFFFFFFFFFFFFFFFFFFFFFFFFFFFFFFFFFF
@A00155:342:HHGFNDSXY:1:1566:31991:2942 1:N:0:GAACCTAG+TCCGCATA
GAGCGACTTCTCTCGGGCCCACAGCCTTACAGCTGTGGAGCACGGTATCCTCTGCCGAAACAGAGGTT
GGACAAGACCGGAGGGGTCTCCTAGTTCCAAAGGAGATGTACTCCGGGCTTGTTCACGACCTACCGTG
+
FFFFF:FFFFFFFFFFFFFFFFFFFFFFFFFFFFFFFFFFFFFFFFFFFFFFFFFFFFFFFFFFFFFF
FFFFFFFFFFFFFFFFFFFFFFFFF:FFFFFFFFFFFFFFFFFFFFFFFFFFFFFFFFFFFFFFF:FFFFF
@A00155:342:HHGFNDSXY:1:1566:11288:8829 1:N:0:GAACCTAG+TCCGCATA
CCTCTGGCCTATGGTCATCTCAATGCCGAGAGGGATGTGGAGGGCGAGAGAGAGCGACTTCTCTCGGG
CCCACAGCCTTACAGCTGTGGAGCACGGTATCCTCTGCCGAAACAGAGGTTGGACAAGACCGGA
+
FFFFFFFFFFFFFFFFFFFFFFFFFFFFFFFFF:FF:F:FFFFFFFFFFFFFFFFFFFFFFFFFFFFFFF
FFFFFFFFFFFFFFFFFFFFFFFFFFFFFFFFFFFFFFFFFFFFFFFFFFFFFFFFFFFFFFFFF
@A00155:342:HHGFNDSXY:1:1566:18258:13823 1:N:0:GAACCTAG+TCCGCATA
TTACAGCTGTGGAGCACGGTATCCTCTGCCGAAACAGAGGTTGGACAAGACCGGAGGGGTCTCCTAGT
TCCAAAGGAGATGTACTCCGGGCTTGTTCACGACCTACCGTGTAAGTCGTAGTCTAGTAGGCTACCTG
+
FFFFFFFFFFFFFFFFFFFFFFFFFFFFFFFFFFFFFFFFFFFFFFFFFFFFFFFFFFFFFFFFFFFFF
FFFFFFFFFFFFFFFFFFFFFFFFFFFFFFFFFFFFFFFFFFFFFFFFFFFFFFFFFFFFFFFFFFFFF
@A00155:342:HHGFNDSXY:1:1566:19289:24158 1:N:0:GAACCTAG+TCCGCATA
TTCTCTCGGGCCCACAGCCTTACAGCTGTGGAGCACGGTATCCTCTGCCGAAACAGAGGTTGGACAAG
ACCGGAGGGGTCTCCTGGTTCCAAAGGAGATGTACTCCGGGCTTGTTCACGACCTACCGTGTAAGTCG
+
FFFFFFFFFFFFFFFFFFFFFFFFFFFFFFFFFFFFFFFFFFFFFFFFFFFFFFF:FFFFFFFFFFFFF
FFFFFFFFFFFFFFFFFFFFFFFFFFFFFFFFFFFFFFFFFFFFFFFFFFFFFFFFFFFFFFFFFFFFF
@A00155:342:HHGFNDSXY:1:1566:31367:26663 1:N:0:GAACCTAG+TCCGCATA
ATCTCAATGCCGAGAGGGATGTGGAGGGCGAGAGAGAGCGACTTCTCTCGGGCCCACAGCCTTACAGC
TGTGGAGCACGGTATCCTCTGCCGAAACAGAGGTTGGACAAGATCGGAGGGGTCTCCTAGTTCCAAAG
+
FFFFFFFFFFFFFFFFFF:FFFFFFFFFFFFFFFFFFFFFFFFFFFFFFFFFFFFFFFFFFFFFFFFFF
FFFFFFFFFFFFFFFFFFFFFFFFFFFFFFFFFFFFFFFFFFFFFFFFFFFFFFF:FFFFFFFFFFFFF
@A00155:342:HHGFNDSXY:1:1566:28113:34961 1:N:0:GAACCTAG+TCCGCATA
CCGAAACAGAGGTTGGACAAGACCGGAGGGGTCTCCTAGTTCCAAAGGAGATGTACTCCGGGCTTGTT
CACGACCTACCGTGTAAGTCGTAGTCTAGTAGGCTACCTGATGAGTCCTTTTTAGGACGAAAC
+
FFFFFFFFFFFFFFFFFFFFFFFFFFFFFFFFFFFFFFFFFFFFFFFFFFFFFFFFFFFFFFFF:FFFF
FFFFFFFFFFFFFF:FFFFFFFFFFFFFFFFFFFFFFFFFFFFFFFFFFFFFFFFFFFFFF:FF
@A00155:342:HHGFNDSXY:1:1566:6903:35556 1:N:0:GAACCTAG+TCCGCATA
ACTTCTCTCGGGCCCACAGCCTTACAGCTGTGGAGCACGGTATCCTCTGCCGAAACAGAGGTTGGACA
AGACCGGAGGGGTCTCCTAGTTCCAAAGGAGATGTACTCCGGGCTTGTTCACGACCTACCGTGTAAGT
+
FFFFFFFFFFFFFFFFFFFFFFFFFFFFFFFFFFFFFFFFFFFFFFFFFFFFFFF,FFFFFFFFFFFFF
FFFFFFFFFFFFFFFFFFFFFFFFFFFFFFFFFFFFFFFFFFFF:FFFFFFFFFFFFFFFF:FFFFFF
@A00155:342:HHGFNDSXY:1:1567:4661:2394 1:N:0:GAACCTAG+TCCGCATA
AGCGACTTCTCTCGGGCCCACAGCCTTACAGCTGTGGAGCACGGTATCCTCTGCCGAAACAGAGGTTG

```
GACAAGACCGGAGGGGTCTCCTAGTTCCAAAGGAGATGTACTCCGGGCTTGTTCACGACCTACCGTGT
+
FFFFFFFFFFFFFFFFFFFFFFFFFFFFFFFFFFFFFFFFFFFFFF,FFFFFFFFFFFFFFFFFFFFFFF
FFFFFFFFFFFFFFFFFFFFFFFFFFFFFFFFFFFFFFFFFFFFFFFFFFFFFFFFFFFFFFFFFFFFFF
@A00155:342:HHGFNDSXY:1:1567:8467:10269 1:N:0:GAACCTAG+TCCGCATA
TTACAGCTGTGGAGCACGGTATCCTCTGCCGAAACAGAGGTTGGACAAGACCGGAGGGGTCTCCTAGT
TCCAAAGGAGATGTACTCCGGGCTTGTTCACGACCTACCGTGTAAGTCGTAGTCTAGTAGGCTACCTG
+
FFFFFFFFFFFFFFFFFFFFFFFFFFFFFFFFFFFFFFFFFFFFFFFFFFFFFFFFFFFFFFFFFFFFFF
FFFFFFFFFFFFFFFFFFFFFFF:FFFFFFFF:FFFFFFFFFFFFFFFFFFFFFFFFFFFFFFFFFFFFF
@A00155:342:HHGFNDSXY:1:1567:23990:10911 1:N:0:GAACCTAG+TCCGCATA
CCTTACAGCTGTGGAGCACGGTATCCTCTGCCGAAACAGAGGTTGGACAAGACCGGAGGGGTCTCCTA
GTTCCAAAGGAGATGTACTCCGGGCTTGTTCACGACCTACCGTGTAAGTCGTAGTCTAGTAGGCTACC
+
FFFFFFFFFFFFFFFFFFFFFFFFFFFFFFFFFFFFFFFFFFFFFFFFFFFFFFFFFFFFFFFFFFFFFF
FFFFFFFFFFFFFFFFFFFFFFFFFF:FFFFFFFFFFFFFFFFFFFFFFFFFF,FFFFFFFFFFFFFFFF
@A00155:342:HHGFNDSXY:1:1567:9778:13792 1:N:0:GAACCTAG+TCCGCATA
GAGAGCGACTTCTCTCGGGCCCACAGCCTTACAGCTGTGGAGCACGGTATCCTCTGCCGAAACAGAGG
TTGGACAAGACCGGAGGGGTCTCCTAGTTCCAAAGGAGATGTACTCCGGGCTTGTTCACGACCTACCG
+
FFFFFFFFF:FFFFFFFFFFFFFFFFFFFFFFFFFFFFFFFFFFFFFFFFFFFFFFFFFFFFFFFFFFFF
FFFFFFFF:FFFFFFFFFFFFFFFFFFFFFFFFFFFFFFFFFFFFFFFFFFFFFF:FFFFFFFFFFFFFF
@A00155:342:HHGFNDSXY:1:1567:7545:16626 1:N:0:GAACCTAG+TCCGCATA
TTACAGCTGTGGAGCACGGTATCCTCTGCCGAAACAGAGGTTGGACAAGACCGGAGGGGTCTCCTAGT
TCCAAAGGAGATGTACTCCGGGCTTGTTCACGACCTACCGTGTAAGTCGTAGTCTAGTAGGCTACCTG
+
FFFFFFFFFFFFFFFFFFFFFFFFFFFFFFFFFFFFFFFFFFFFFFFFFF:FFFFFFFFFFFFFFFFFFF
FFFFFFFFFFFFFFFFFFFFFFFFFFFFFFFFFFFFFFFFFFFFFFFFFFFFFFFFFFFFFFFFFFFFFF
@A00155:342:HHGFNDSXY:1:1567:29514:23046 1:N:0:GAACCTAG+TCCGCATA
AAGGACTCACCTCTGGCCTATGGTCATCTCTATGCCGAGAGGGATGTGGAGGGCGAGAGAGAGCGACT
TCTCTCGGGCCCACAGCCTTACAGCTGTGGAGCACGGTATCCTCTGCCGAAACAGAGGTTGGACAAGA
+
FFFFFFFFFFFFFFFFFFFFFFFFFFFFFFFFFFFFFFFFFFFFF:FFFFFFFFFFFFFFFFFFFFFFFF
FFFFFFFFFFFFFFFFFFFFF:FFFFFFFFFFFFFFFFFFFFFFFFFFFFFFFFFFFFFFFFFFFFFFFF
@A00155:342:HHGFNDSXY:1:1567:27805:30358 1:N:0:GAACCTAG+TCCGCATA
GTATCCTCTGCCGAAACAGAGGTTGGACAAGACCGGAGGGGTCTCCTAGTTCCAAAGGAGATGTACTC
CGGGCTTGTTCACGACCTACCGTGTAAGTCGTAGTCTAGTAGGCTACCTGACGAGTCCTTTTTAGGAC
+
FFFFFFFFFFFFFFFFFFFFFFFFFFFFFFFFFFFFFFFFFFFFFFFFFFFFFFFFFFFFFFFFFFFFFF
FFFFFFFFFFFFFFFFFFFFFFFFFFFFFFFFFFFFFFFFFFFFFFFFFFFFFFFFFFFFFFFFFFFFFF
@A00155:342:HHGFNDSXY:1:1568:31575:29183 1:N:0:GAACCTAG+TCCGCATA
TTAAGGACTCACCTCTGGCCTATGGTCATCTCAATGCCGAGAGGGATGTGGAGGGCGAGAGAGAGCGA
CTTCTCTCGGGCCCACAGCCTTACAGCTGTGGAGCACGGTATCCTCTGCCGAAACAGAGGTTGGACAA
+
F:FFFFFFFFFFFFFFFFFFFFFFFFFFFFFFFFFFFFFFFFFFFFFFFF:FFFFFFFFFFFFFFFFFFF
FFFFFFFFFFFFFFFFFFFFFFFFFFFFFFFFFFFF:FFFF,FFFFFFFFFFFFFFFFFFFFFFFFFF,F
@A00155:342:HHGFNDSXY:1:1569:9046:7263 1:N:0:GAACCTAG+TCCGCATA
CTTCTCTCGGGCCCACAGCCTTACAGCTGTGGAGCACGGTATCCTCTGCCGAAACAGAGGTTGGACAA
GACCGGAGGGGTCTCCTAGTTCCAAAGGAGATGTACTCCGGGCTTGTTCACGACCTACCGTGTAAGTC
+
FFFFFFFFFFFFFFFFFFFFFFFFFFFFFFFFFFFFFFFFFFFFFFFFFFFFFFFFFFFFFFFFFFFFFF
FFFFFFFFFFFFFFFFFFFFFFFFFFFFFFFFFFFFFFFFFFFFFFFFFFFFFFFFFFFFFFFFFFFFFF
@A00155:342:HHGFNDSXY:1:1569:1542:17534 1:N:0:GAACCTAG+TCCGCATA
GATCAGCTGTGTCCACTTAAGGACTCACCTCTGGCCAATGGTCATCTCAATGCCGAGAGGGATGTGGA
```

GGGCGAGAGAGAGCGACTTCTCTCGGGCCCACAGCCTTACAGCTGTGGAGCACGGTATCCTCTGC
+
FFFFFFFFFFFFFFFFFFFFFFFFFFFFFFFFFFFFFFFFFFFFFFFFFFFFFFFFFFFFFFFFFFFF
FFFFFFF:F:F:FFF:FFFFFFFFFFFFFFFFFFFFFFF:FFFFFFFFFFFFFFFFFFFFFFFFF
@A00155:342:HHGFNDSXY:1:1569:22553:20509 1:N:0:GAACCTAG+TCCGCATA
AGAGAGAGCGACTTCTCTCGGGCCCACAGCCTTACAGCTGTGGAGCACGGTATCCTCTGCCGAAACAG
AGGTTGGACAAGACCGGAGGGGTCTCCTGGTTCCAAAGGAGATGTACTCCGGGCTTGTTCACGACCTA
+
FFFFFFFFFFFFFFFFFFFFFFFFFFFFFFFFFFFFFFFFFFFFFFFFFFFFFFFFFFFFFFFFFFFF
FFFFFFFFFFF:FFFFFF:FFFFFFFFFFFFFFFFFFFFFFFFFFFFFFFFFFFFFFFFFFFFFFFFF
@A00155:342:HHGFNDSXY:1:1569:21522:21073 1:N:0:GAACCTAG+TCCGCATA
TTCTCTCGGGCCCACAGCCTTACAGCTGTGGAGCACGGTATCCTCTGCCGAAACAGAGGTTGGACAAG
ACCGGAGGGGTCTCCTAGTTCCAAAGGAGATGTACTCCGGGCTTGTTCACGACCTACCGTGTAAGTCG
+
FFFFFFFFFFFFFFFFFFFFFFFFFFFFFFFFFFFFFFFFFFFFFFFFFFFFFFFFFFFFFFFFFFFF
FFFFFFFFFFFFFFFF:FFFFF:FFFFFFFFFFFFFFFFFFFFFFFFFFFF:FFFFFFFFFFFFFFFF
@A00155:342:HHGFNDSXY:1:1569:29668:26882 1:N:0:GAACCTAG+TCCGCATA
CTCCTCTGGCCTATGGTCATCTCAATGCCGAGAGGGATGTGGAGGGCGAGAGAGAGCGACTTCTCTCG
GGCCCACAGCCTTACAGCTGTGGAGCACGGTATCCTCTGCCGAAACAGAGGTTGGACAAGACCGGAGG
+
FFF,F:F:F:F,FFF,:FFFFFF:FFFF::FF:FFFFFFFFFFF,FFFFF::F:F:FFF:F,FFFF:F
FF:F,FFF:FFFFFFFFFFFFFFFFFFFF,FFF,FFFFFFFFFF:FF,FF,,FFFFFFFFFFFF:F::
@A00155:342:HHGFNDSXY:1:1569:12780:30076 1:N:0:GAACCTAG+TCCGCATA
CTATGGTCATCTCAATGCCGAGAGGGATGTGGAGGGCGAGAGAGAGCGACTTCTCTCGGGCCCACAGC
CTTACAGCTGTGGAGCACGGTATCCTCTGCCGAAACAGAGGTTGGACAAGACCGGAGGGGTCTCCTAG
+
:F:FFFFFFFFFFFFFFFFF:FFFFFFFFFFFFFFFFFFFFFFFFFFFF:FFFFFFFFFFFFFFFFFF
FFFFFFFF,FFFF:FFFFFFFFFFFFFFFFFFFFFFFFFFFFFFFFFFF:FFFFFFFFFFFFF:FFFF
@A00155:342:HHGFNDSXY:1:1570:10592:5525 1:N:0:GAACCTAG+TCCGCATA
ATCTCAATGCCGAGAGGGATGTGGAGGGCGAGAGAGAGCGACTTCTCTCGGGCCCACAGCCTTACAGC
TGTGGAGCACGGTATCCTCTGCCGAAACAGAGGTTGGACAAGACCGGAGGGGTCTCCTGGTTCCAAAG
+
FFFFFFFFFFFFFFFFFFFFFFFFFFFFFFFFFFFFFFF:FFFFFFFFFFFFFFFFFFFFFFFFFFFF
FFFFFFFFFFFFFFFFFFFFFFFFFFFFFFFFFFFFFFFFFFFFFFFFFFFFFFFFFFFFFFFFFFFF
@A00155:342:HHGFNDSXY:1:1570:14253:8672 1:N:0:GAACCTAG+TCCGCATA
CTCGGGCCCACAGCCTTACAGCTGTGGAGCACGGTATCCTCTGCCGAAACAGAGGTTGGACAAGACCG
GAGGGGTCTCCTAGTTCCAAAGGAGATGTACTCCGGGCTTGTTCACGACCTACCGTGTAAGTCGTAGT
+
FFFFFFFFFFFFFFFFFFFFFFFFFFFFFFFFFFF:FFFFFFFFFFFFFFFFFFFFFFFFFFFFFFFF
FFFFFFFFFFFFFFFFFFFFFFFFFFFFFFFFFFFFFFFFFFFFFFFFFFFFFFFFFFFFFFFFFFFF
@A00155:342:HHGFNDSXY:1:1570:29252:8719 1:N:0:GAACCTAG+TCCGCATA
TCCTCTGCCGAAACAGAGGTTGGACAAGACCGGAGGGGTCTCCTAGTTCCAAAGGAGATGTACTCCGG
GCTTGTTCACGACCTACCGTGTAAGTCGTAGTCTAGTAGGCTACCTGACGAGTCCTTTTTAGGACGAA
+
FFFFFFFFFFFFFFFFFFFFFFFFFFF:FFFFFFFFFFFFFFFFFFFFFFFFFFFFFFFFFFFFFFFF
FFFFFFFFFFFFFFFFFFFFFFFFFFFFFFFFFFFFFFFFFFFFFFFFFFFFFFFFFFFFFFFFFFFF
@A00155:342:HHGFNDSXY:1:1571:14145:2942 1:N:0:GAACCTAG+TCCGCATA
CTTCTCTCGGGCCCACAGCCTAACAGCTGTGGAGCACGGTATCCTCTGCCGAAACAGAGGTTGGACAA
GACCGGAGGGGTCTCCTAGTTCCAAAGGAGATGTACTCCGGGCTTGTTCACGACCTACCGTGTAAGTC
+
:FFFFFFFFFFFFFFFFFFFFFFFFFF:F,:FFFFFF,FFFFFFFFFFFFFFFFFFFFFFFFFFFFFF
FFFFFFFFFFFFFFFFFFFFFFFFFFFFFF:FFFFFFFFFFFFFFFFFFFFFFFFFFFFF:FFFFFFF
@A00155:342:HHGFNDSXY:1:1571:22281:8453 1:N:0:GAACCTAG+TCCGCATA
GGCGAGAGAGAGCGACTTCTCTCGGGCCCACAGCCTTACAGCTGTGGAGCACGGTATCCTCTGCCGAA

```
ACAGAGGTTGGACAAGACCGGAGGGGTCTCCTAGTTCCAAAGGAGATGTACTCCGGGCTTGTTCACGA
+
FFFFFFFFFFFF:FFFFFFFFFFFFFFFFFFFFFFFFFFFFFFFFFFFFFFFFFFFFFFFFFFFFFFF
FFFFFFFFFFFFFFFFFFFFFFFFFFFFFFFFFFFFFFFFFF:FFFFFFFFFFFFFFFF,FFFFFFFFF
@A00155:342:HHGFNDSXY:1:1571:14443:11600 1:N:0:GAACCTAG+TCCGCATA
GAGCGACTTCTCTCGGGCCCACAGCCTTACAGCTGTGGAGCACGGTATCCTCTGCCGAAACAGAGGTT
GGACAAGACCGGAGGGGTCTCCTAGTTCCAAAGGAGATGTACTCCGGGCTTGTTCACGACCTACCGTG
+
FFFFFFF:FFFFFFFFFFFFFFFFFFFFFFFFFFFFFFFFFFFF:FFFFFFF:FFFFFFFFFFFF
FFFFFFFFFFFFFFFFFFFFFFFFFFFFFFFFFFFFFFFFFFFFFFFFFFFFFFFFFFFFFFFFFFFF
@A00155:342:HHGFNDSXY:1:1571:18665:29215 1:N:0:GAACCTAG+TCCGCATA
ACCTCTGTTTCGGCAGAGGATACCGTGCTCCACAGCTGTAAGGCTGTGGGCCCGAGAGAAGTCGCTCT
CTCTCGCCCTTCACATCCCTCTCGGCATTGAGATGACCATAGGCCAGAGGTGAGTCCTTAAGTGGACA
+
FFFFFFFFFFFFFFFFFFFFFFFFFFFFFFFFFFFFFFFFFFFFFFFFFFFFFFFFFFFFFFFFFFFF
FFFFFFFFFFFFFFFFFFFFFF,FFFFF:FFFFFFFFFF:FFFFFFFFFFFFFFFFFFFFF:FFFF::F
@A00155:342:HHGFNDSXY:1:1571:18855:36057 1:N:0:GAACCTAG+TCCGCATA
ACCTCTGTTTCGGCAGAGGATACCGTGCTCCACAGCTGTAAGGCTGTGGGCCCGAGAGAAGTCGCTCT
CTCTCGCCCTTCACATCCCTCTCGGCATTGAGATGACCATAGGCCAGAGGTGAGTCCTTAAGTGGAC
+
FFFFFFFFFFFFFFFFFFFFFFFFFFFFFFFFFFFF:FFFFFFF:FFFFFFFFFFFFFFFFFFFFFFF
FFFFFFFFFF:FFFFFFFFFFFFFFFFF,FFFFFFFFF:FFFFFFFFFFFFFFFFFFFFFFFFFFFF
@A00155:342:HHGFNDSXY:1:1571:18783:36683 1:N:0:GAACCTAG+TCCGCATA
ACCTCTGTTTCGGCAGAGGATACCGTGCTCCACAGCTGTAAGGCTGTGGGCCCGAGAGAAGTCGCTCT
CTCTCGCCCTTCACATCCCTCTCGGCATTGAGATGACCATAGGCCAGAGGTGAGTCCTTAAGTGGACA
+
FFFFFFFFFFFFFFFFFFFFFFFFFFFFFFFFFFFFFFFFFFFFFFFF:FFFFFFFFFFFFFFFFFFF
FFFFFFFFFFFFFFFFFFFFFFFFFFFFFFFFFFFFFFFFFFFFFFFF:FFFFFFFFFFFFFFFFFFF:
@A00155:342:HHGFNDSXY:1:1572:30581:4726 1:N:0:GAACCTAG+TCCGCATA
CTCTGCCGAAACAGAGGTTGGACAAGACCGGAGGGGTCTCCTAGTTCCAAAGGAGATGTACTCCGGGC
TTGTTCACGACCTACCGTGTAAGTCGTAGTCTAGTAGGCTACCTGACGAGTCCTTTTTAGGACGAAAC
+
FFFFFFFFFFFFFFFFFFFFFFFFFFFFFFFFFFFFFFFFFFFFFFFFFFFFFFFFFFFFFFFFFFFF
FFFFFFFFFFFFFFFFFFFFFFFFFFFFFFFFFFF:FFFFFFFFFFFFFFFFFFFFFFFFFFFFFFFF
@A00155:342:HHGFNDSXY:1:1572:21332:7905 1:N:0:GAACCTAG+TCCGCATA
CAATGCCGAGAGGGATGTGGAGGGCGAGAGAGAGCGACTTCTCTCGGGCCCACAGCCTTACAGCTGTG
GAGCACGGTATCCTCTGCCGAAACAGAGGTTGGACAAGACCGGAGGGGTCTCCTGGTTCCAAAGGAG
+
FFFFFFFFFFFFFFFFF:FFFFFFFFFFFFFFFFFFFFFFFFFFFFFFFFFFFFFFFFFFFFFFFFFF
FFFFFFFFFFFFFFFFFFFFFFFFFFFFFFFFFFFFFFFFFFFFFFFFFFFFFFFFFFFFFFFFFFF
@A00155:342:HHGFNDSXY:1:1572:26937:19492 1:N:0:GAACCTAG+TCCGCATA
GAGGGCGAGAGAGAGCGACTTCTCTCGGGCCCACAGCCTTACAGCTGTGGAGCACGGTATCCTCTGCC
GAAACAGAGGTTGGACAAGACCGGAGGGGTCTCCTAGTTCCAAAGGAGATGTACTCCGGGCTTGTTCA
+
FFFFFFFFFFFFFFFFFFFFFFFFFFFFFFFFFFFFFFFFFFFFFFFFFFFFFFFFFFFFFFFFFFFF
FFFFFFFFFFFFFFFFFFFFFFFFFFFFFFFFFFFFFFFFFFFFFFFFFFFFFFFFFFFFFFFFFFFF
@A00155:342:HHGFNDSXY:1:1572:31376:24361 1:N:0:GAACCTAG+TCCGCATA
CCTTACAGCTGTGGAGCACGGTATCCTCTGCCGAAACAGAGGTTGGACAAGACCGGAGGGGTCTCCTG
GTTCCAAAGGAGATGTACTCCGGGCTTGTTCACGACCTACCGTGTAAGTCGTAGTCTAGTAGGCTACC
+
FFFFFFFFFFFFFFFFFFFFFFFFFFFFFFFFFFFFFFFFFFFFFFFFFFFFFFFFFFFFFFFFFFFF
FFFFFFFFFFFFFFFFFFFFFFFFFFFFFFFFFFFF:FFFFFFFFFFFFFFFFFFFFFFFFFFFFFFF
@A00155:342:HHGFNDSXY:1:1572:12753:25676 1:N:0:GAACCTAG+TCCGCATA
CTTCTCTCGGGCCCACAGCCTTACAGCTGTGGAGCACGGTATCCTCTGCCGAAACAGAGGTTGGACAA
```

GACCGGAGGGGTCTCCTAGTTCCAAAGGAGATGTACTCCGGGCTTGTTCACGACCTACCGTGTAAGTC
+
FFFFFFFFFFFFFFFFFFFF:FFFFFFFFFFFFFFFFFF:FFFFFFFFFFFFFFFFFFFFFFFFFFFF
FFFFFFFFFFFFFFFFFFFFFFFFFFFFFFFFFFFFFFFFFFFFFFFFFFFFFFFFFFFF:FFFFFFF
@A00155:342:HHGFNDSXY:1:1572:22055:26725 1:N:0:GAACCTAG+TCCGCATA
ACCTCTGTTTCGGCAGAGGATACCGTGCTCCACAGCTGTAAGGCTGTGGGCCCGAGAGAAGTCGCTCT
CTCTCGCCCTCCACATCCCTCTCGGCATTGAGATGACCATAGGCCAGAGGTGAGTCCTTAAGTGGAC
+
FFFFFFFFFFFFFFFFFFFFFFFFFFFFFFFFFFFFFFFFFFFFFFFFFFFFFFFFFFFFFFFFFFFF
FFFFFFFFFFFFFFFFFFFFFFFFFFFFFFFFFFFFFFFFFFFFFFFF:FFFFFFFFFFFFFFFFFF
@A00155:342:HHGFNDSXY:1:1572:25708:29763 1:N:0:GAACCTAG+TCCGCATA
GGATGTGGAGGGCGAGAGAGAGCGACTTCTCTCGGGCCCACAGCCTTACAGCTGTGGAGCACGGTATC
CTCTGCCGAAACAGAGGTTGGACAAGACCGGAGGGGTCTCCTAGTTCCAAAGGAGATGTACTCC
+
FFFFF:FFFFFFFFFFFFFFFFFFFFFFFFFFFFFFFFFFFFFFFFF:FFFFFFFFFFFFFFFFFFFF
FFFFFFFFFFFFFFFFFFFFFFFFFFFFFFFFFFFFFFFFFFFFFFFFFFFFFFFFFFFFFFFF
@A00155:342:HHGFNDSXY:1:1573:10357:6809 1:N:0:GAACCTAG+TCCGCATA
TCCACTTAAGGACTCTCCTCTGGCCTATGGTCATCTCAATGCCGAGAGGGATGTGGAGGGCGAGAGAG
AGCGACTTCTCTCGGGCCCACAGCCTTACAGCTGTGGAGCACGGTATCCTCTGCCGAAACAGAGGTTG
+
FFFFFF:FFFFFFFFFFFFFFFFFFFFFFFFFFFFFFFFFFFFFFF,FFFF,FFFFFFFFFFFFFFFF
FFFFFFFFFFFFFFFFFFFFFFFFF:F:FFFFFFFFFFFFFFFFFFFFFFFFFFFFFFFFFFFFFFFF
@A00155:342:HHGFNDSXY:1:1573:9579:19930 1:N:0:GAACCTAG+TCCGCATA
CTTCTCTCGGGCCCACAGCCTTACAGCTGTGGAGCACGGTATCCTCTGCCGAAACAGAGGTTGGACAA
GACCGGAGGGGTCTCCTAGTTCCAAAGGAGATGTACTCCGGGCTTGTTCACGACCTACCGTGTAAGTC
+
FFFFFFFFFFFFFFFFFFFFFFFFFFFFFFFFFFFFFFFFFFFFFFFFFFFFFFFFFFFFFFFFFFFF
FFFFFFFFFFFFFFFFFFFFFFFFFFF:FFFFFFFFFFFFFFFFFFFFFFFFFFFFFFFFFFFFFFFF
@A00155:342:HHGFNDSXY:1:1573:5746:21496 1:N:0:GAACCTAG+TCCGCATA
CTTCTCTCGGGCCCACAGCCTTACAGCTGTGGAGCACGGTATCCTCTGCCGAAACAGAGGTTGGACAA
GACCGGAGGGGTCTCCTAGTTCCAAAGGAGATGTACTCCGGGCTTGTTCACGACCTACCGTGTAAGTC
+
FFFFFFFFFFFFFFFFFFFFFFFFF,FFFFFFFFFF:FFFFFFFFFFF:FFFFFF:FFFFFFFFFFFF
FFFFFFFFFFFFFFFFFFFFFFFFFFFFFFFFFFFFFFFFFFFFFFFFFFF:FFFFFFFFFFFFFFFF
@A00155:342:HHGFNDSXY:1:1573:21305:28839 1:N:0:GAACCTAG+TCCGCATA
ACTTCTCTCGGGCCCACAGCCTTACAGCTGTGGAGCACGGTATCCTCTGCCGAAACAGAGGTTGGACA
AGACCGGAGGGGTCTCCTAGTTCCAAAGGAGATGTACTCCGGGCTTGTTCACGACCTACCGTGTAAGT
+
FFFFFFFFFFFFFFFFFFFFFFFFFFFFFFFFFFFFFFFFFFFFFFFFFFFFFFFFFFFFFFFFFFFF
FFFFFFFFFFFFFFFFFFFFFFFFFFFFFFFFFFFFFFFFFFFFFFFFFFFFFFFFFFFFFFFFFFFF
@A00155:342:HHGFNDSXY:1:1573:25889:30201 1:N:0:GAACCTAG+TCCGCATA
CTTCTCTCGGGCCCACAGCCTTACAGCTGTGGAGCACGGTATCCTCTGCCGAAACAGAGGTTGGACAA
GACCGGAGGGGTCTCCTGGTTCCAAAGGAGATGTACTCCGGGCTTGTTCACGACCTACCGTGTAAGTC
+
FFFFFFFFFFFFFFFFFFFFFFFFFFFFFFFFFFFFFFFFFFFFFFFFFFFFFFFFFFFFFFFFFFFF
FFFFFFFFFFFFFFFFFFFFFFFFFFF:FF:FFFFFFFFFFFFFFFFFFF:FFFFFFFFFFFFFFFFFF
@A00155:342:HHGFNDSXY:1:1573:8504:33098 1:N:0:GAACCTAG+TCCGCATA
AGAGAGCGACTTCTCTCGGGCCCACAGCCTTACAGCTGTGGAGCACGGTATCCTCTGCCGAAACAGAG
GTTGGACAAGACCGGAGGGGTCTCCTAGTTCCAAAGGAGATGTACTCCGGGCTTGTTCACGACCTACC
+
FFFFFFFFFFFFFFFFFFFFFFFFFFFFFFFFFFFFFFFFFFFFFFFFFFFFFFFFFFFFFFFFFFFF
FFFFFFFFFF,FFFFFFFFFFFFF:FFFFFFFFFFFFFFFFFFFFFFFFFFFFFFFFFFFFFFFFFFF
@A00155:342:HHGFNDSXY:1:1573:8250:33599 1:N:0:GAACCTAG+TCCGCATA
AGAGAGCGACTTCTCTCGGGCCCACAGCCTTACAGCTGTGGAGCACGGTATCCTCTGCCGAAACAGAG

```
GTTGGACAAGACCGGAGGGGTCTCCTAGTTCCAAAGGAGATGTACTCCGGGCTTGTTCACGACCTACC
+
FFFFFFFFFFFFFFFFFFFFFFFFFFFFFFFFFFFFFFFFFFFFFFFFFFFFFFFFFFF:FFFFFFFF
FFFFFFFFFFFFFFFFFFFFFFFFFFFFFFFFFFFFFFFFFFFFFFFFFFFFF:FFFFFFFFFFFFFF
@A00155:342:HHGFNDSXY:1:1574:8684:29904 1:N:0:GAACCTAG+TCCGCATA
TCCTCTGCCGAAACAGAGGTTGGACAAGACCGGAGGGGTCTCCTAGTTCCAAAGGAGATGTACTCCGG
GCTTGTTCACGACCTACCGTGTAAGTCGTAGTCTAGTAGGCTACCTGACGAGTCCTTTTTAGGACGAA
+
,FFFFFFFFFFFFF:FFFFFFFFFF:F:FFFFFFF:FFFFFFF:FFFFFFF:FFFF,F,F,FFFFFFF
FF:::FFFFF,F:FFFFFF,FFFF,FFFFF:FF,FFFFFFFFFFF:FF,FFF,FFF,,:,F,FFFFFF
@A00155:342:HHGFNDSXY:1:1574:7319:32581 1:N:0:GAACCTAG+TCCGCATA
CTTCTCTCGGGCCCACAGCCTTACAGCTGTGGAGCACGGTATCCTCTGCCGAAACAGAGGTTGGACAA
GACCGGAGGGGTCTCCTAGTTCCAAAGGAGATGTACTCCGGGCTTGTTCACGACCTACCGTGTAAGTC
+
FFFFFFFFFFFFFFFFFFFFFFFFFFFFFFFFFFFFFFFFFFFFFFFFFFFFFFFFFFFFFFFFFFFF
FFFFFFFFFFFFFFFFFFFFFFFFFFFFFFFFFFFFFFFFFFFFFFFFFFFFFFFFFFFFFFFFFFFF
@A00155:342:HHGFNDSXY:1:1575:1551:1485 1:N:0:GAACCTAG+TCCGCATA
GCGACTTCTCTCGGGCCCACAGCCTTACAGCTGTGGAGCACGGTATCCTCTGCCGAAACAGAGGTTGG
ACAAGACCGGAGGGGTCTCCTAGTTCCAAAGGAGATGTACTCCGGGCTTGTTCACGACCTACCGTGTA
+
FFFFFFFFFFFFFFFFFFFFFFFFFFF:FFFFFFF:,,FFFFFFFFFFFFFFFFFFFFF:F:FFFFFF
FFFFFFFFFFFFFFFFFFFFFFFFFFFFFFFF:F:FFFFFF:FFFFF:FFFFFFFF::FFFFFFFFFF
@A00155:342:HHGFNDSXY:1:1575:3658:23359 1:N:0:GAACCTAG+TCCGCATA
CCTCTGCCGAAACAGAGGTTGGACAAGACCGGAGGGGTCTCCTAGCTCCAAAGGAGATGTACTCCGGG
CTTGTTCACGACCTACCGTGTAAGTCGTAGTCTAGTAGGCTACCTGACGAGTCCTTTTTAGGACGAA
+
FFFF:FFFFFFFFFFFFFFF:,FFFFFFFFFF:F:FFF,FFFFFF:FFFFFFFFFFFFFFFFFFF:FF
FF::FFF:FFFFFFFFFF,FFFF:FFFFFFFFFFFFF:F:FFF:F,FF:FFFFFF,FF:FFFFFFFF
@A00155:342:HHGFNDSXY:1:1575:13928:26083 1:N:0:GAACCTAG+TCCGCATA
GAGATGTACTCCGGGCTTGTTCACGACCTACCGTGTAAGTCGTAGTCTAGTAGGCTACCTGACGAGTC
CTTTTTAGGACGAAACTTACACAACTCGAAGAGTAACGACGGTTTCGTCCTTGGAACGGACTCATCA
+
FFFFFFFFFFFFFFFFFFFFFFFFFFFFFFFFFFFFFFFFFFFFFFFFFF,FFFFFFFFFFFFFFFFF
FFFFFFF,FFFFFFFF:FFFFFFFFFFFFFFFFFFFFFFFFFFFFFFFFFFFF:::FFFFFFFFFFF
@A00155:342:HHGFNDSXY:1:1576:6180:29951 1:N:0:GAACCTAG+TCCGCATA
ATCTCAATGCCGAGAGGGATGTGGAGGGCGAGAGAGAGCGACTTCTCTCGGGCCCACAGCCTTACAGC
TGTGGAGCACGGTATCCTCTGCCGAAACAGAGGTTGGACAAGACCGGAGGGGTCTCCTGGTTCCAAAG
+
FFFFFFFFFFFFFFFFFF:,FFFFFFFFFFFFFFFFFFFFFFFFFFFFFFFFFFFFFFFFFFFFFFFF
FFFFFFFFFFFFF:FFFFFFFFFFFFFFFFFFF:FFFFFFFFFFFFFFFFFFFFFFFFFFFFFFFFFF
@A00155:342:HHGFNDSXY:1:1576:1316:30138 1:N:0:GAACCTAG+TCCGCATA
GGCCAGAGGTGAGTCCTTAAGTGGACACAGCTGATCTAAGGCGGTGTAGCGGGGCATGGGTTTGAACC
CCCATGACGGTCGGAGTCTAGTAGGCTCCCTGATGAGTCCGTTCCAAGGACGAAACCGTCGTTACTCT
+
FFFFFFFFFFFFFFFFFFFFFFFFFF,FFFFFFFFFFFFFFFFFFFFFFFFFFFF,FFFFFFFFFFFF
FFFFFFFFFFFFFFFFFF:FFFFFFFFFFFFFFFFFFFFFFFFFFFFFFFFFF,FFFFFFFFFFFFFF
@A00155:342:HHGFNDSXY:1:1576:14488:32002 1:N:0:GAACCTAG+TCCGCATA
TATCCTCTGCCGAAACAGAGGTTGGACAAGACCGGAGGGGTCTCCTAGTTCCAAAGGAGATGTACTCC
GGGCTTGTTCACGACCTACCGTGTAAGTCGTAGTCTAGTAGGCTACCTGACGAGTCCTTTTTAGGACG
+
:FFFFFFFFFFFFFFFFFFFFFFFFFFFFFFFFFFFFFFFFFFFFFFFFFFFFFFFFFFFFFFFFFFF
FFFFF:FFFFFFFFFFFFFFFFFFFFFFFFFFFFFFFFFFFFFFFFFFFFFFFFFFFFF:FFFF
@A00155:342:HHGFNDSXY:1:1577:16721:11945 1:N:0:GAACCTAG+TCCGCATA
GACTTCTCTCGGGCCCACAGCCTTACAGCTGTGGAGCACGGTATCCTCTGCCGAAACAGAGGTTGGAC
```

```
AAGACCGGAGGGGTCTCCTAGTTCCAAAGGAGATGTACTCCGGGCTTGTTCACGACCTACCGTGT
+
FFFFFFFFFFFFFFFFFFFFFFFF,FFFFFFFFFFFFFFFFFFFFFFFFFFFFFFFFFFFFFFFFFF
FFFFFFFFFFFFFFFFFFFFFFFFFFFFFFFFFFFFFFFFFFFFFFFFFFFFFFFFFFFFFFF,FF
@A00155:342:HHGFNDSXY:1:1577:25446:20791 1:N:0:GAACCTAG+TCCGCATA
CTTCTCTCGGGCCCACAGCCTTACAGCTGTGGAGCACGGTATCCTCTGCCGAAACAGAGGTTGGACAA
GACCGGAGGGGTCTCCTAGTTCCAAAGGAGATGTACTCCGGGCTTGTTCACGACCTACCGTGTAAGTC
+
FFFFFFFFFFFFFFFFFFFFFF:FFFFFFFFF,FFFFFFFFFFFFFFFFFFFFFFFFFFFFFFFFFFF
FFFFFFFFFFFFFFFFFFFFFFFFFFFFFFFFFFFFFFFFFFFFFFFFFFFFFFFFFFFFFF:FFFFF
@A00155:342:HHGFNDSXY:1:1577:12952:26584 1:N:0:GAACCTAG+TCCGCATA
CTCTATGCCGAGAGGGATGTGGAGGGCGAGAGAGAGCGACTTCTCTCGGGCCCACAGCCTTACAGCTG
TGGAGCACGGTATCCTCTGCCGAAACAGAGGTTGGACAAGACCGGAGGGGTCTCCTAGTTCCAAAGGA
+
FFFFFFFFFFFFFFFFFFFFFFFFFFFFFFFFFF:FFFFFFFFFFFFFFFFFFFFFFFFFFFFFFFFF
FFFFFFFFFFFFFFFFF:FFFFFFFFFFFFFFFFFFFFFFFFFFFFFFFFFFFFFFFFFFFFFF,FFF
@A00155:342:HHGFNDSXY:1:1577:7835:30686 1:N:0:GAACCTAG+TCCGCATA
TTAAGGACTCACCTCTGGCCTATGGTCATCTCAATGCCGAGAGGGATGTGGAGGGCGAGAGAGAGCGA
CTTCTCTCGGGCCCACAGCCTTACAGCTGTGGAGCACGGTATCCTCTGCCGAAACAGAGGTTGGACAA
+
FFFFFFFFFFFFFFFFFFFFFFFFFFFFFF:FFFFFFFF:F:FFFFFFFFFFFFFFFFFFF:FFFFFF
FFFFFFFFFFFFFFFFFFFFFFFFFFFFFFFFFFFFFFFFFFFFFFFFFFFFFFFFFFFFFFFFFFFF
@A00155:342:HHGFNDSXY:1:1578:23845:10535 1:N:0:GAACCTAG+TCCGCATA
CTTCTCTCGGGCCCACAGCCTTACAGCTGTGGAGCACGGTATCCTCTGCCGAAACAGAGGTTGGACAA
GACCGGAGGGGTCTCCTAGTTCCAAAGGAGATGTACTCCGGGCTTGTTCACGACCTACCGTGTAAGTC
+
FFFFFFFFFFFFFFFFFFFFFFFFFFFFFFFFFFFFFFFFFFFFFFFFFFFFFFFFFFFFFFFFFFFF
:FFFFFFFFFFFFFFFFFFFFFFFFFFFFFFFFFFFFFFFFFFFFFFFFFFFFFFFFFFFFFFFFFFF
@A00155:342:HHGFNDSXY:1:1578:29405:11960 1:N:0:GAACCTAG+TCCGCATA
CTTCTCTCGGGCCCACAGCCTTACAGCTGTGGAGCACGGTATCCTCTGCCGAAACAGAGGTTGGACAA
GACCGGAGGGGTCTCCTAGTTCCAAAGGAGATGTACTCCGGGCTTGTTCACGACCTACCGTGTAAGTC
+
FFFFFFFFFFFFFFFFFFFFFFFFFFFFFFFFFFFFFFFFFFFFFFFFFFFFFFFFFFFFFFFFFFFF
FFFFFFFFFFFFFFFFFFFFFFFFFFFFFFFFFFFFFFFFFFF:FFFFFFFFFFFFFFFFFFFFFFFF
@A00155:342:HHGFNDSXY:1:1578:28167:13166 1:N:0:GAACCTAG+TCCGCATA
ATCCTCTGCCGAAACAGAGGTTGGACAAGACCGGAGGGGTCTCCTGGTTCCAAAGGAGATGTACTCCG
GGCTTGTTCACGACCTACCGTGTAAGTCGTAGTCTAGTAGGCTACCTGACGAGTCCTTTTTAGGACGA
+
FFFFFFFFFFFFFFFFFFFFFFFFFFFFFFFFFFFFFFFFFFFFFFFFFFFFFFFFFFFFFFFFFFFF
FFFFFFFFFFFFFFFFFFFFFFFFF:FFFFFFFFFFFFFFFFFFFFFFFFFFFFFFFFFFFFFFFFFF
@A00155:342:HHGFNDSXY:1:1578:22426:27242 1:N:0:GAACCTAG+TCCGCATA
GAGCGACTTCTCTCGGGCCCACAGCCTTACAGCTGTGGAGCACGGTATCCTCTGCCGAAACAGAGGTT
GGACAAGACCGGAGGGGTCTCCTAGTTCCAAAGGAGATGTACTCCGGGCTTGTTCACGACCTACCGTG
+
FFFFFFFFFFFFFFFFFFFFFFFFFFFFFFFFFFFFFFFFFFFFFFFFFFFFFFFFFFFFFFFFFFFF
FFFFFF:FFFFFFFFFFFFFFFFFFFFFFFFFFFFFFFFFFFFFFFFFFFFFFFFFFFFFFFFFFFFF
@A00155:342:HHGFNDSXY:1:1601:13449:14293 1:N:0:GAACCTAG+TCCGCATA
GGATGTGGAGGGCGAGAGAGAGCGACTTCTCTCGGGCCCACAGCCTTACAGCTGTGGAGCACGGTATC
CTCTGCCGAAACAGAGGTTGGACAAGACCGGAGGGGTCTCCTAGTTCCAAAGGAGATGTACTCC
+
FFFFFFFFFF:FFFFFFFFFFFFFF:FFFFFFFFFFFFFFFFFFFFF:FFFFFFFFFFFFFFFFFFFF
FFFFFFFFFFFFFFFFFFFFFFFFFFFFFFFFFFFFFFFFFFFFFFFFFFFFFFFFFFFFFFFF
@A00155:342:HHGFNDSXY:1:1601:9362:19492 1:N:0:GAACCTAG+TCCGCATA
ATCTCAATGCCGAGAGGGATGTGGAGGGCGAGAGAGAGCGACTTCTCTCGGGCCCACAGCCTTACAGC
```

```
TGTGGAGCACGGTATCCTCTGCCGAAACAGAGGTTGGACAAGACCGGAGGGGTCTCCTAGTTCCAAAG
+
FFFFFFFFFFFFFFFFFFFFFFFFFFFFFFFFFFFFFFFFFFFFFFFFFFFFFFFFFFFFFFFFFFFF
FFFFFFFFFFFFFFFFFFFFFFFFFFFFFFFFFFFFFFFFFFFFFFFFFFFFFFFFFF,FFFFFFFFF
@A00155:342:HHGFNDSXY:1:1601:11216:23766 1:N:0:GAACCTAG+TCCGCATA
GGGCGAGAGAGAGCGACTTCTCTCGGGCCCACAGCCTTACAGCTGTGGAGCACGGTATCCTCTGCCGA
AACAGAGGTTGGACAAGACCGGAGGGGTCTCCTGGTTCCAAAGGAGATGTACTCCGGGCTTGTTCACG
+
FFFFFFFFFFFFFFFFFFFFFFFFFFFFFFFFFFFFFFFFFFFFFFFFFFFFFFFFFFFFFFFFFFFF
FF:FFFFFFFFFFFFFFFF:FFFFFFFFFFFFFFFF:FFFFFFFFFFFFFFFFFFFFFF:FFFFFFFF
@A00155:342:HHGFNDSXY:1:1601:12373:25895 1:N:0:GAACCTAG+TCCGCATA
TGCCGAGAGGGATGTGGAGGGCGAGAGAGAGCGACTTCTCTCGGGCCCACAGCCTTACAGCTGTGGAG
CACGGTATCCTCTGCCGAAACAGAGGTTGGACAAGACCGGAGGGGTCTCCTAGTTCCAAAGGAGATGT
+
FFFFF:FFFFFFFFFFF:FFFFFFFFFFFFFFFFFFFF:FFFFFFFFFFFFFFFFFFFFFFFFFF:F:
FFFFF::FFFFFFFFFFFFFFFFFFFFFFFFFFF:FFFFFFFFFFFFFFFFFFFFFFFFF:FFFFFFF
@A00155:342:HHGFNDSXY:1:1601:16080:30655 1:N:0:GAACCTAG+TCCGCATA
GAGGGCGAGAGAGAGCGACTTCTCTCGGGCCCACAGCCTTACAGCTGTGGAGCACGGTATCCTCTGCC
GAAACAGAGGTTGGACAAGACCGGAGGGGTCTCCTGGTTCCAAAGGAGATGTACTCCGGGCTTGTTCA
+
FFFFFFFFFFFFFFFFFFFFFFFFFFFFFFFFFFFFFFFFFFFFFFFFFFFFFFFFFFFFFFFFFFFF
F:FFFFFFFFFFFFFFFFFFFFFFFFFFFFFFFFFFFFFFFFFFFFFFFFFFFFFFFFFFFFFFFFFF
@A00155:342:HHGFNDSXY:1:1601:14570:31704 1:N:0:GAACCTAG+TCCGCATA
GAGGGCGAGAGAGAGCGACTTCTCTCGGGCCCACAGCCTTACAGCTGTGGAGCACGGTATCCTCTGCC
GAAACAGAGGTTGGACAAGACCGGAGGGGTCTCCTGGTTCCAAAGGAGATGTACTCCGGGCTTGTTCA
+
,FFFFF,FFFFFFFFF,F:FFF:FFFF:FFFFFFF:::FFFFFFFFF,FFFF:F:F:F:FF,FFFF,:
FFFF:FFF,FFFF,FFFF,FFFF:FF,:FFFFFFFFFFFFFFFFFFFFFFFFFF:FF,FFF:FF:FFF
@A00155:342:HHGFNDSXY:1:1602:6153:15060 1:N:0:GAACCTAG+TCCGCATA
CTTCTCTCGGGCCCACAGCCTTACAGCTGTGGAGCACGGTATCCTCTGCCGAAACAGAGGTTGGACAA
GACCGGAGGGGTCTCCTAGTTCCAAAGGAGATGTACTCCGGGCTTGTTCACGACCTACCGTGTAAGTC
+
FFFFFFFFFFFFFFFF::FFFFFFFFFFFFFFFFFFFFFFFFFFFFFF,FFFFFFFFFFFFFFFFFFF
FFF:FFFFFFFFFFFFFFFFFFFFFFFFFFFFFFFFFFFFFFFFFFFFFFFFFFFFFFFFFFFF,FFF
@A00155:342:HHGFNDSXY:1:1603:10999:3881 1:N:0:GAACCTAG+TCCGCATA
AGGGCGAGAGAGAGCGACTTCTCTCGGGCCCACAGCCTTACAGCTGTGGAGCACGGTATCCTCTGCCG
AAACAGAGGTTGGACAAGACCGGAGGGGTCTCCTAGTTCCAAAGGAGATGTACTCCGGGCTTGTTCAC
+
FFFF:FFFFFFFFFFFFFFFFF:FFFFFFFFFFFFFFFFFFFFFFFFFFFFFFFFFFFFFFFFFFFFF
FFFFFFFFFFFFFFFFFFFF:FFFFFFFFFFFFFFFFFF:FFFFFFFFFF:FFFFFFFF:FFFFFFFF
@A00155:342:HHGFNDSXY:1:1603:1253:24987 1:N:0:GAACCTAG+TCCGCATA
ATCTCAATGCCGAGAGGGATGTGGAGGGCGAGAGAGAGCGACTTCTCTCGGGCCCACAGCCTTACAGC
TGTGGAGCACGGTATCCTCTGCCGAAACAGAGGTTGGACAAGACCGGAGGGGTCTCCTAGTTCCAAAG
+
FFFFFFFFFFFFFFFFF:FFFF:FFFFFFFFFFFFFFFFFFFFFFFFFFFFFFFFFFFFFFFFFFFFF
FF:FFFFFFFFFFFFFFFFFFFFFF:FFFFFFFFFFFFFF,FFFFFFFFFFFFFFFF:FF,FFFF:FF
@A00155:342:HHGFNDSXY:1:1604:25843:6668 1:N:0:GAACCTAG+TCCGCATA
GACTTCTCTCGGGCCCACAGCCTTACAGCTGTGGAGCACGGTATCCTCTGCCGAAACAGAGGTTGGAC
AAGACCGGAGGGGTCTCCTAGTTCCAAAGGAGATGTACTCCGGGCTTGTTCACGACCTACCGTGTAAG
+
FFFFF:FFFFFFFFFFFFFFFFFFFFFFFFFFFFFFFFFFFFFFFFFFFFFFFFFFFFFFFFFFFFFF
FFFFFFFFFFF:FFFFFFFFFFFFFFFFFFF:FFFFFFF:FFFFFFFFFFFFFFFFFFFFFFFFFFFF
@A00155:342:HHGFNDSXY:1:1604:26241:18129 1:N:0:GAACCTAG+TCCGCATA
CGACTTCTCTCGGGCCCACAGCCTTACAGCTGTGGAGCACGGTATCCTCTGCCGAAACAGAGGTTGGA
```

CAAGACCGGAGGGGTCTCCTAGTTCCAAAGGAGATGTACTCCGGGCTTGTTCACGACCTACCGTGTAA
+
FFFFFFFFFFFFFFFFFFFFFFFFFFFFFFFFFFFFFFFFFFFFFFFFFFFFFFFFFFFFFFFFFFFF
FFF::FF,FFFFFFFFFFFFFFFFF:FFFFFFFFFFFFFFFFFFFFFFFFFFFFFFFFFFFFFFFFFF
@A00155:342:HHGFNDSXY:1:1605:15944:18239 1:N:0:GAACCTAG+TCCGCATA
TGCCGAGAGGGATGTGGAGGGCGAGAGAGAGCGACTTCTCTCGGGCCCACAGCCTTACAGCTGTGGAG
CACGGTATCCTCTGCCGAAACAGAGGTTGGACAAGACCGGAGGGGTCTCCTGGTTCCAAAGGAGATGT
+
FFFFFFFFFFFFFFFFFFFFFFFFFFFFFFFFFFF:FFFFFFFFFFFFFFFFFF:FFFFFFFFFFFFF
FFFFFFFFFFFFFFFFFFFFFFF:FFFFFFFFFFFFFFFFFFFFFFFFFFFFFFFFFFFFFFFFFFFF
@A00155:342:HHGFNDSXY:1:1606:25319:2691 1:N:0:GAACCTAG+TCCGCATA
CTTCTCTCGGGCCCACAGCCTTACAGCTGTGGAGCACGGTATCCTCTGCCGAAACAGAGGTTGGACAA
GACCGGAGGGGTCTCCTAGTTCCAAAGGAGATGTACTCCGGGCTTGTTCACGACCTACCGTGTAAGTC
+
FFFFFFFFFFFFFFFFFFFFFFFFFFFFFFFFFFFFFFFFFFFFFFFFFFFFFFFFFFFFFFFFFFFF
FFFFFFFFFFFFFFFFF,FFFFFFFFFFFFFFFFFFFFFFFFFFFFFFFFFFFFFFFFFFFFFFFFFF
@A00155:342:HHGFNDSXY:1:1606:24596:8046 1:N:0:GAACCTAG+TCCGCATA
CTTCTCTCGGGCCCACAGCCTTACAGCTGTGGAGCACGGTATCCTCTGCCGAAACAGAGGTTGGACAA
GACCGGAGGGGTCTCCTAGTTCCAAAGGAGATGTACTCCGGGCTTGTTCACGACCTACCGTGTAAGTC
+
FFFFFFFFFFFFFFFFFFFFFF:FFFFFFFFFFFFFFFFFFFFFFFFFFFFFFFFFFFFFFFFFFFFF
FFFFFFFFFFFFFFFFFFFFFFFFFFFFFFFFFFFFFFFFFFFFFFFFFFFFFFFFFFFFFFFFFFFF
@A00155:342:HHGFNDSXY:1:1606:30418:11772 1:N:0:GAACCTAG+TCCGCATA
CAATGCCGAGAGGGATGTAGAGGGCGAGAGAGAGCGACTTCTCTCGGGCCCACAGCCTTACAGCTGTG
GAGCACGGTATCCTCTGCCGAAACAGAGGTTGGACAAGACCGGAGGGGTCTCCTAGTTCCAAAGGAGA
+
FFFFFFFFFFFFFF:FFFFFFFFFFFFFFFFFFFFFFFFFFFFFFFFFFFFFFFFFFFFFF:FFFFFF
FFFFFFFFFFFFFFFFFFFFFFFFFFFFFFFFFFFFFFFFFFFFFFFFFFFFFFFFFFFFFFFFFFFF
@A00155:342:HHGFNDSXY:1:1606:15591:24486 1:N:0:GAACCTAG+TCCGCATA
TGGTCATCTCAATGCCGAGAGGGATGTGGAGGGCGAGAGAGAGCGACTTCTCTCGGGCCCACAGCCTT
ACAGCTGTGGAGCACGGTATCCTCTGCCGAAACAGAGGTTGGACAAGACCGGAGGGGTCTCCTAGTTC
+
FFFFFFFFFFFFFFF:FFF,FFFFFFFFFFFFFFFFFFFFFFFFFFFFFFFFFFFFFFFFFFFFFFFF
FFFFFFFFFFFFFFFFFFFFFFFFFF:FFFFFFFFFFFF:FFFFFFFFFFFFFFFFFFFFF:FFFFFF
@A00155:342:HHGFNDSXY:1:1607:20175:13510 1:N:0:GAACCTAG+TCCGCATA
GTATCCTCTGCCGAAACAGAGGTTGGACAAGACCGGAGGGGTCTCCTAGTTCCAAAGGAGATGTACTC
CGGGCTTGTTCACGACCTACCGTGTAAGTCGTAGTCTAGTAGGCTACCTGACGAGTCCTTTTTAGGAC
+
FFFFFFFFFFFFFFFFFFFFFFFFFFFFFFFFFFFFFFFFFFFFFFFFFFFFFFFFFFFFFFFFFFFF
FFFFFFFFFFFFFFFFFFFFFFFFFFFFFFFFFFFF::FFFFFFFFFFFFFFFFFFFFF:FFFFFFFF
@A00155:342:HHGFNDSXY:1:1607:13123:23124 1:N:0:GAACCTAG+TCCGCATA
AGCGACTTCTCTCGGGCCCACAGCCTTACAGCTGTGGAGCACGGTATCCTCTGCCGAAACAGAGGTTG
GACAAGACCGGAGGGGTCTCCTAGTTCCAAAGGAGATGTACTCCGGACTTGTTCACGACCTACCGTGT
+
FFFFFFFFFFFFFFFFFFFFFFFFFFF:FFFFFFFFFFFFFFFFFFFFFFFFFFFFFFFFFFFFFFFF
FFFF:FFFFFFFFFFFFFFFFF:FFFFFFFFFFFFFFFFFFFFFFFFFFFFFFFFFFFFFF:F:FFFF
@A00155:342:HHGFNDSXY:1:1607:23411:27508 1:N:0:GAACCTAG+TCCGCATA
TAGTCTAGTAGGCTACCTGACGAGTCCTTTTTAGGACGAAACTTACACAACTCGAAGAGTAACGACGG
TTTCGTCCTTGGAACGGACTCATCAGGGAGCCTACTAGACTCCAACCGTCATGGGGGTTCAAACCCAT
+
FFFFFFFFFFFFFFFFFFFFFFFFFFFF:FFFFFFFFFFFFFFFFFFFF:FFFFFFFFFFFFFFFFFF
FFFF,FFFFFFFFFFFFFFFFFFFFFFFFFFFFFFFFFFFFFFFFFFFFFFFFFFFFFFFFFFFFFFF
@A00155:342:HHGFNDSXY:1:1607:21124:36793 1:N:0:GAACCTAG+TCCGCATA
AGACTCACCTCTGGCCTATGGTCATCTCAATGCCGAGAGGGATGTGGAGGGCGAGAGAGAGCGACTTC

```
TCTCGGGCCCACAGCCTTACAGCTGTGGAGCACGGTATCCTCTGCCGAAACAGAGGTTGGACAAGACC
+
FFFFFFFFFFFFFFFFFFFFFFFFFFFFFFFFFFFFFFFFFFFFFFFFFFFFFFFFFFFFFFFFFFFF
FFFFFFFFFFFFFFFFFFFFFFFFFFFFFFFFFFFFFFFFFFFFFFFFFFFFFFFFFFFFFFFF::FF
@A00155:342:HHGFNDSXY:1:1608:17716:5400 1:N:0:GAACCTAG+TCCGCATA
GGATGTGGAGGGCGAGAGAGAGCGACTTCTCTCGGGCCCACAGCCTTACAGCTGTGGAGCACGGTATC
CTCTGCCGAAACAGAGGTTGGACAAGACCGGAGGGGTCTCCTAGTTCCAAAGGAGATGTACTCC
+
FFFFFFFFFFFFFFFFFFFFFFFFFFFFFFFFFFFFFFFFFFFFFFFFFFFFF:FFFFFFFFFFFFFF
FFFFFFFFFFFFFFFFFFFFFFFFFFFFFFFFFFFFFFFFFFFFFFFFFFFFFFFFFFFF:FFFF
@A00155:342:HHGFNDSXY:1:1608:14326:7858 1:N:0:GAACCTAG+TCCGCATA
ACCTCTGTTTCGGCAGAGGATACCGTGCTCCACAGCTGTAAGGCTGTGGGCCCGAGAGAAGTCGCTCT
CTCTCGCCCTCCACATCCCTCTCGGCATTGAGATGACCATAGGCCAGAGGAGAGTCCTTAAGTGGACA
+
FFFFFFFFFFFFFFFFFFFFFFFFFFFFFFFFFFFFFFFFFFFFFFFFFFFFFFFFFFFFFFFFFFFF
FFFFFFFFFFFFFFFFFFFFFFFFFFFF,:FFFFFFFFFFFFFFFFFFFFFFFFFFFFFFFFFFFFFF
@A00155:342:HHGFNDSXY:1:1608:25364:11537 1:N:0:GAACCTAG+TCCGCATA
ACTTCTCTCGGGCCCACAGCCTTACAGCTGTGGAGCACGGTATCCTCTGCCGAAACAGAGGTTGGACA
AGACCGGAGGGGTCTCCTAGTTCCAAAGGAGATGTACTCCGGGCTTGTTCACGACCTACCGTGTAAG
+
FFFFFFFFF,FFFFFFFFFFFFF,FFFFFF,:FFFFFFFFFFFFFFFFF,F:FFFFFFFFF:FFFF,F
F,FFFFFFFFFFFFFF:FFFFFFFFFFFFFFFFFFF:FFFF,FFFFFFFFFF:FFFF,FFF:FFFFF
@A00155:342:HHGFNDSXY:1:1608:21169:15953 1:N:0:GAACCTAG+TCCGCATA
CTTCTCTCGGGCCCACAGCCTTACAGCTGTGGAGCACGGTATCCTCTGCCGAAACAGAGGTTGGACAA
GACCGGAGGGGTCTCCTGGTTCCAAAGGAGATGTACTCCGGGCTTGTTCACGACCTACCGTGTAAGTC
+
FFFFFFFFFFFFFFFFFFFFFFFFFFFFFFFFFFFFFFFFFFFFFFFFFFFFFFFFFFFFFFFFFFFF
FFFFFFFFFFFFFFFFFFFFFFFFFFFFFFFFFFFFFFFFFFFFFFFFFFFFFFFFF:FFFFFFFFFF
@A00155:342:HHGFNDSXY:1:1608:32217:27383 1:N:0:GAACCTAG+TCCGCATA
TGCTCCACAGCTGTAAGGCTGTGGGCCCGAGAGAAGTCGCTCTCTCTCGCCCTCCACATCCCTCTCGG
CATTGAGATGACCATAGGCCAGAGGTGAGTCCTTAAGTGGACACAGCTGATCTAAGGCGGTGTGGTGG
+
FFFFFFFFFFFFFFFFFFFFFFFFFFFFFFFFFFFFFFFFFFFFFFFFFFFFFFFFFFFFFFFFFFFF
FFFFF:FFFFFFFFFFFFFFFF:FFFFFFFFFFFFFFFFFFF:FFFFFFFFFFFFFFFFF:F:FFFFF
@A00155:342:HHGFNDSXY:1:1608:3540:28166 1:N:0:GAACCTAG+TCCGCATA
GGATGTGGAGGGCGAGAGAGAGCGACTTCTCTCGGGCCCACAGCCTTACAGCTGTGGAGCACGGTATC
CTCTGCCGAAACAGAGGTTGGACAAGACCGGAGGGGTCTCCTAGTTCCAAAGGAGATGTACTCC
+
FFFFF:FFFFFFFFFFFFFFF:FFFFFFFFFFFFFFF:FFFFFFFF,FFFFFFFFFFFFFF:FFFFF
FFFFFFFFFFFFFFFFFFFFFFFFFFFFFFFFFFFFFFFFFFFFFFFFFFFFFFFFFFFFFFFF
@A00155:342:HHGFNDSXY:1:1608:27154:32518 1:N:0:GAACCTAG+TCCGCATA
GGCGAGAGAGAGCGACTTCTCTCGGGCCCACAGCCTTACAGCTGTGGAGCACGGTATCCTCTGCCGAA
ACAGAGGTTGGACAAGACCGGAGGGGTCTCCTAGTTCCAAAGGAGATGTACTCCGGGCTTGTTCACGA
+
FFFFFFFFFFFFFFFFFFFFFFF,FFFFFFFFFFFFFFFFFFFFFFFFFF:FFFFFFFFFFFFFFFFFF
FFFFFFFFFFFFFFFFFFFFFFFFFFFFFFFFFFFFFFFFFFFFFFFFFFFFFFFFFFFFFFFFFFFF
@A00155:342:HHGFNDSXY:1:1608:23231:35994 1:N:0:GAACCTAG+TCCGCATA
GACTTCTCTCGGGCCCACAGCCTTACAGCTGTGGAGCACGGTATCCTCTGCCGAAACAGAGGTTGGAC
AAGACCGGAGGGGTCTCCTGGTTCCAAAGGAGATGTACTCCGGGCTTGTTCACGACCTACCGTGTAAG
+
F:FFFFFFFFFFFFFFFFFFFFF:FFFFFFFFFFFFFFFFFFFFFFFFFFFFFFFFFFFFFFFFFFFF
FFFFFFFFFFFFFFFFFFFFFFFFFFFFFFF:FFFFFFFFFFFFFFFFF:FFFFFFFFFFFFFFFFFF
@A00155:342:HHGFNDSXY:1:1609:19443:2973 1:N:0:GAACCTAG+TCCGCATA
TCAATGCCGAGAGGGATGTAGAGGGCGAGAGAGAGCGACTTCTCTCGGGCCCACAGCCTTACAGCTGT
```

```
GGAGCACGGTATCCTCTGCCGAAACAGAGGTTGGACAAGACCGGAGGGGTCTCCTAGTTCCAAAGGAG
+
FFFFFFFFFFFFFFFF:FFFFFFFFFFFFFFFFFFFFFFFFFFFFFFFFFFFFFFFFFFFFFFFFFFF
F:FFFFFFFFFFFFFFFFFFFFFFFFFFFFFFFFFFFF,FFFFFFFFFFFFFFFFFFFFFFFFFF:FFF
@A00155:342:HHGFNDSXY:1:1609:13105:23249 1:N:0:GAACCTAG+TCCGCATA
CTTCTCTCGGGCCCACAGCCTTACAGCTGTGGAGCACGGTATCCTCTGCCGAAACAGAGGTTGGACAA
GACCGGAGGGGTCTCCTAGTTCCAAAGGAGATGTACTCCGGGCTTGTTCACGACCTACCGTGTAAGTC
+
FFFFFFFFFFFFFFFFFFFFF:FFFFFFFFFFFFFFFFFFFFFFFFFFFFFFFFFFFFFFFFFFFFFF
FFFFFFFFFFFFFFFFFFFFFFFFFFFFFFFFFFFFFFFFFFFFFFFFFFFFFFFFFFFFFFFFFFFF
@A00155:342:HHGFNDSXY:1:1609:26910:29121 1:N:0:GAACCTAG+TCCGCATA
GTCATCTCAATGCCGAGAGGGATGTGGAGGGCGAGAGAGAGCGACTTCTCTCGGGCCCACAGCCTTAC
AGCTGTGGAGCACGGTATCCTCTGCCGAAACAGAGGTTGGACAAGACCGGAGGGGTCTCCTAGTTCCA
+
FFFFFFFFFFFFFFFFFFFFFFFFFFFFFFFFFFFFFFFFFFFFFFFFFFFFFFFFFFFFFFFFFFFF
FFFFFFFFFFFFFFFFFFFFFFFFFFFFFFFFFFFFFFFFFFFFFFFFFFFFFFFFFFFFF:FFFFFF
@A00155:342:HHGFNDSXY:1:1609:26313:33411 1:N:0:GAACCTAG+TCCGCATA
ACTTCTCTCGGGCCCACAGCCTTACAGCTGTGGAGCACGGTATCCTCTGCCGAAACAGAGGTTGGACA
AGACCGGAGGGGTCTCCTGGTTCCAAAGGAGATGTACTCCGGGCTTGTTCACGACCTACCGTGTAAGT
+
FFFFFFFFFFFFFFFFFFFFFFFFFFFFF:FFFFFFFFFFFFFFFFFFFFFFFFFFFFFFFFFFFFFF
FFFFFFFFFFFFFFFFFFFFFFFFFFFFFFFFFFFFFFFFFFFFFFFFFFFFFFFFFFFFFFFFFFFF
@A00155:342:HHGFNDSXY:1:1609:27326:34162 1:N:0:GAACCTAG+TCCGCATA
GTCATCTCAATGCCGAGAGGGATGTGGAGGGCGAGAGAGAGCGACTTCTCTCGGGCCCACAGCCTTAC
AGCTGTGGAGCACGGTATCCTCTGCCGAAACAGAGGTTGGACAAGACCGGAGGGGTCTCCTAGTTCCA
+
FFFFFFFFFFFFFFFFFFFFFFFFFFFFFFFFFFFFFFFFFFFFFFFFFFFFFFFFFFFFFFFFFFFF
FFFFFFFFFFFFFFFFFFFFFFFFFFFFFFFFFFFFFFFFFFFFFFFFFFFFFFFFFFFFFFFFFFFF
@A00155:342:HHGFNDSXY:1:1609:27263:34240 1:N:0:GAACCTAG+TCCGCATA
GTCATCTCAATGCCGAGAGGGATGTGGAGGGCGAGAGAGAGCGACTTCTCTCGGGCCCACAGCCTTAC
AGCTGTGGAGCACGGTATCCTCTGCCGAAACAGAGGTTGGACAAGACCGGAGGGGTCTCCTAGTTCCA
+
FFFFFFFFFFFFFFFFFFFFFFF:FFFFFFFFFFFFFFFFFFFFFFFFFFFFFFFFFFFFFFFFFFFF
FFFFFFFFFFFFFFFFFFFFFFFFFFFFFFFFFFFFFFFFFFFFFFFFFFFFFFFFFFFFFFFFFFFF
@A00155:342:HHGFNDSXY:1:1609:12897:36260 1:N:0:GAACCTAG+TCCGCATA
TGTCCACTTAAGGACTCACCTCTGGCCTATGGTCATCTCAATGCCGAGAGGGATGTGGAGGGCGAGAG
AGAGCGACTTCTCTCGGGCCCACAGCCTTACAGCTGTGGAGCACGGTATCCTCTGCCGAAACAGAGGT
+
FFFFFFFFFFFFFFFFFFFFFFFFFFFFFFFFFFFFFFFFFFFFFFFFFFFFFFFFFFFFFFFFFFFF
FFFFFFFFFFFFFFFFFFFFFFFFFFFFFFFFFFFFFFFFFFFFFFFFFFFFFFFFFFFFFFFFFFFF
@A00155:342:HHGFNDSXY:1:1609:12807:36385 1:N:0:GAACCTAG+TCCGCATA
TGTCCACTTAAGGACTCACCTCTGGCCTATGGTCATCTCAATGCCGAGAGGGATGTGGAGGGCGAGAG
AGAGCGACTTCTCTCGGGCCCACAGCCTTACAGCTGTGGAGCACGGTATCCTCTGCCGAAACAGAGGT
+
FFFFFFFFFFFF:FFFFFFFFFFFFFFFFFFFFFFFFFF:FFFFFFFFFFFFFFFFFFFFFFFFFFFF
FFFFFFFFF:FFFFFFFFFFFFFFFFFFF:FFFFFFFFFFFFFFFFFFFFFFFFFFFFFFFFFFFFFF
@A00155:342:HHGFNDSXY:1:1610:26657:6261 1:N:0:GAACCTAG+TCCGCATA
GAGAGGGATGTAGAGGGCGAGAGAGAGCGACTTCTCTCGGGCCCACAGCCTTACAGCTGTGGAGCACG
GTATCCTCTGCCGAAACAGAGGTTGGACAAGACCGGAGGGGTCTCCTAGTTCCAAAGGAGATGTACTC
+
FFFFFFFFFFFFFFFFFFFFFFFFFFFFFFFF,FFFFFFFFFFFFFFFFFFFFFFFFFFFFFFFFFFF
FFFFFFFFFFFFFFFFFFFFFFFFFFFFFFFFFFFFFFFFFFFFFFFFFFFFFFFFFFFFFFFFFFFF
@A00155:342:HHGFNDSXY:1:1610:14380:35070 1:N:0:GAACCTAG+TCCGCATA
ATCTCAATGCCGAGAGGGATGTAGAGGGCGAGAGAGAGCGACTTCTCTCGGGCCCACAGCCTTACAGC
```

TGTGGAGCACGGTATCCTCTGCCGAAACAGAGGTTGGACAAGACCGGAGGGGTCTCCTAGTTCCAAAG
+
FFFFFFFFFFFFFFFFFFFFFFFFFFFFFFFFFFFFFFFFFFFFFFFFFFFFFFFFFFFFFFF,:FFFFF
FFFFFFFFFFFFFFFFFFFFFFFFFFFFFFFFFFFFFFFFFFFFFFFFFFFFFFFFFFFFFFFFFFFFF
@A00155:342:HHGFNDSXY:1:1610:32461:35947 1:N:0:GAACCTAG+TCCGCATA
GTCATCTCAATGCCGAGAGGGATGTGGAGGGCGAGAGAGAGCGACTTCTCTCGGGCCCACAGCCTTAC
AGCTGTGGAGCACGGTATCCTCTGCCGAAACAGAGGTTGGACAAGACCGGAGGGGTCTCCTAGTTCCA
+
FFFFFFFFFFFFFFFFFFFFFFF:FFFFFFFFFFFFFFFFFFFFFFFFFFFFFFFFFFFFFFFFFFFFF
FFFFFFFFFFFFFFFFFFFFFFFFFFFFFFFFFFFFFFFFFFFFFFFFFFFFFFFFFFFFFFFFFFFFF
@A00155:342:HHGFNDSXY:1:1611:15727:10504 1:N:0:GAACCTAG+TCCGCATA
CTCAATGCCGAGAGGGATGTGGAGGGCGAGAGAGAGCGACTTCTCTCGGGCCCACAGCCTTACAGCTG
TGGAGCACGGTATCCTCTGCCGAAACAGAGGTTGGACAAGACCGGAGGGGTCTCCTAGTTCCAAAGGA
+
FFFFFFFFFFFFFFFFFFFFFFFFFFFFFFFFFFFFFFFFFFFFFFFFFFFFFFFFFFFFFFFFFFF:F
FFFFFFFFFFFFFFFFFFFFFFFFFFFFFFFFFFFFFFFFFFFFFFFFFFFFFFFFFFFFFFFF:FFFF
@A00155:342:HHGFNDSXY:1:1611:12418:12383 1:N:0:GAACCTAG+TCCGCATA
ACTTCTCTCGGGCCCACAGCCTTACAGCTGTGGAGCACGGTATCCTCTGCCGAAACAGAGGTTGGACA
AGACCGGAGGGGTCTCCTGGTTCCAAAGGAGATGTACTCCGGGCTTGTTCACGACCTACCGTGTAAGT
+
FFFFFFFFFFFFFFFFFFFFFFF::FFFFFFFFFFFFFFFFFFFFFFFFFFFFFFFFFFFFFFFFFFFF
FFFFFFFFFFFFFFFFFFFFFFFFFFFFFFFFFFFFFFFFFFFFF:FFFFFFFFFFFFFFFFFFFFFFF
@A00155:342:HHGFNDSXY:1:1611:7401:31125 1:N:0:GAACCTAG+TCCGCATA
GAGGGCGAGAGAGAGCGACTTCTCTCGGGCCCACAGCCTTACAGCTGTGGAGCACGGTATCCTCTGCC
GAAACAGAGGTTGGACAAGACCGGAGGGGTCTCCTAGTTCCAAAGGAGATGTACTCCGGGCTTGTTCA
+
:FFFFFFFFFFFFFFFFFFFFFF:FFFF:F,F,FFFF:FFFFFFFFFFF,FFFFFFFFF,FFFFFFFFF
FFFFFFFFFFFFFFFFFFFFFFFFFFFFFFFFFFFFFFFFFFFFF::FFFFFFFFFFFFFFFFFFFF:F
@A00155:342:HHGFNDSXY:1:1611:14742:34225 1:N:0:GAACCTAG+TCCGCATA
ATGCCGAGAGGGATGTAGAGGGCGAGAGAGAGCGACTTCTCTCGGGCCCACAGCCTTACAGCTGTGGA
GCACGGTATCCTCTGCCGAAACAGAGGTTGGACAAGACCGGAGGGGTCTCCTAGTTCCAAAGGAGATG
+
FFFFFFFFFFFFFFFFFFFFFFFFFFFFFFFFFFFFFFFFFFFFFFFFFFFFFFFF,FFFFFFFFFFFF
FFFFFFFFFFFFFFFFFFFFFFFFFFFFFFFFFFFFFFFFFFFFFFFFFFFFFFFFFFFFFFFFFFFFF
@A00155:342:HHGFNDSXY:1:1612:22290:6872 1:N:0:GAACCTAG+TCCGCATA
AGAGAGCGACTTCTCTCGGGCCCACAGCCTTACAGCTGTGGAGCACGGTATCCTCTGCCGAAACAGAG
GTTGGACAAGACCGGAGGGGTCTCCTAGTTCCAAAGGAGATGTACTCCGGGCTTGTTCACGACCTACC
+
FFFFFFFFFFFFFFFFFFFFFFFFFFFFFFFFFFFFFFFFFFFFFFFFFFFFFFFFFFFFFFFFFFFFF
FFFFFFFFFFFFFFFFFFFFFFFFFFFFFFFFFFFFF:FFFFFFFFFFFFFFFFFFFFFFF:FFFFFFF
@A00155:342:HHGFNDSXY:1:1612:29387:34663 1:N:0:GAACCTAG+TCCGCATA
AGAGGGATGTGGAGGGCGAGAGAGAGCGACTTCTCTCGGGCCCACAGCCTTACAGCTGTGGAGCACGG
TATCCTCTGCCGAAACAGAGGTTGGACAAGACCGGAGGGGTCTCCTAGTTCCAAAGGAGATGTACTCC
+
FFFFFFFFFFFFFFFFFFFFFFFFFFFFFFFFFFFFFFFFFFFFFFFFFFFFFFFFFFFFFFFFFFFFF
FFFFFFFFF:FFFFFFFFFFFFFFFFFFF:FF,FFFFFFFFFFFFFFFFFFFFFFFFFFFFFFFFFFFF
@A00155:342:HHGFNDSXY:1:1612:22064:35227 1:N:0:GAACCTAG+TCCGCATA
ACTTCTCTCGGGCCCACAGCCTTACAGCTGTGGAGCACGGTATCCTCTGCCGAAACAGAGGTTGGACA
AGACCGGAGGGGTCTCCTAGTTCCAAAGGAGATGTACTCCGGGCTTGTTCACGACCTACCGTGTAAG
+
FFFFFFFFFFFFFFFFFFFFFFFFFFFFFFFFFFFFFFFFFFFFFFFFFFFFFFFFFFFFFFF:FFFFFF
FFFFFFFFFFFFFFFFFFFFFFFFFFFFFFFFFFFFFFFFFFFFFFFFFFFFFFFFFFFFFFF,FFF:F
@A00155:342:HHGFNDSXY:1:1612:15022:36652 1:N:0:GAACCTAG+TCCGCATA
ATCTCAATGCCGAGAGGGATGTGGAGGGCGAGAGAGAGCGACTTCTCTCGGGCCCACAGCCTTACAGC

TGTGGAGCACGGTATCCTCTGCCGAAACAGAGGTTGGACAAGACCGGAGGGGTCTCCTAGTTCCAAAG
+
FFFFFFFFFFFFFFFFFFFFFFFFFFFFFFFFFFFFFFFFFFFFFFFFFFFFFFFFFFFF,FFFFFF
FFFFFFFFFFFFFFFFFFFFFFFFFFFFFFFFFFFFFFFFFFFFFFFFFFFFFFFFFFFFFFFFFFF
@A00155:342:HHGFNDSXY:1:1612:17888:36918 1:N:0:GAACCTAG+TCCGCATA
ACTTCTCTCGGGCCCACAGCCTTACAGCTGTGGAGCACGGTATCCTCTGCCGAAACAGAGGTTGGACA
AGACCGGAGGGGTCTCCTAGTTCCAAAGGAGATGTACTCCGGGCTTGTTCACGACCTACCGTGTAAG
+
FFFFFFFFFFFFFFFFFFFFF,F:FFFF:FFFFFFFFFFFFFFFFFFFFFFFFFFFFFFFFFFFFFF
FFFFFFFFFFFFFFFFFF:FFFFFFFFFFFFFFFFFFFFFFFFFFFFFFFFFFFFFFFFFFFFFFF
@A00155:342:HHGFNDSXY:1:1613:24334:21277 1:N:0:GAACCTAG+TCCGCATA
GAGGGCGAGAGAGAGCGACTTCTCTCGGGCCCACAGCCTTACAGCTGTGGAGCACGGTATCCTCTGCC
GAAACAGAGGTTGGACAAGACCGGAGGGGTCTCCTAGTTCCAAAGGAGATGTACTCCGGGCTTGTTCA
+
FFFFFFFFFFFFFFFFFFFFFFFFFFFFFFFFFFFFFFFFFFFFFFFFFFFFFFFFFFFFFFFFFFF
FFFFFFFFFFFFFFFFFFFFFFFFFFFFFFFFFFFFFFFFFFFFFFFFFFFFFFFFFFFFFFFFFFF
@A00155:342:HHGFNDSXY:1:1613:5963:23750 1:N:0:GAACCTAG+TCCGCATA
GTATCCTCTGCCGAAACAGAGGTTGGACAAGACCGGAGGGGTCTCCTAGTTCCAAAGGAGATGTACTC
CGGGCTTGTTCACGACCTACCGTGTAAGTCGTAGTCTAGTAGGCTACCTGAAGAGTCCTTTTTAGGAC
+
FFFFFFFFFFFFFFFFFFFFFFFFFFFFFFFFFFFFFFFFFFFFFFFFFFFFFFFFFFFFFFFFFFF
FFFFFFFFFFFFFFFFFFFFFFFFFFF:FFFFFFFFFFFFFFFFF:FFFFFFFFFFFFFFFFF:FFF
@A00155:342:HHGFNDSXY:1:1613:10013:24251 1:N:0:GAACCTAG+TCCGCATA
ATCTCAATGCCGAGAGGGATGTAGAGGGCGAGAGAGAGCGACTTCTCTCGGGCCCACAGCCTTACAGC
TGTGGAGCACGGTATCCTCTGCCGAAACAGAGGTTGGACAAGACCGGAGGGGTCTCCTAGTTCCAAAG
+
FFFFFFFFFFFFFFFFFFFFFFFFFFFFFFFFFFFFFFFFFFFFFFFFFFFFFFFFFFFFFFFFFFF
FFFFFFFFFFF:FFFFFFFFFFFFFFFFFFFFFFFFFFFFFFFFFFFFFFFFFFFFFFF,FFFFFFF
@A00155:342:HHGFNDSXY:1:1613:12988:29246 1:N:0:GAACCTAG+TCCGCATA
GCGACTTCTCTCGGGCCCACAGCCTTACAGCTGTGGAGCACGGTATCCTCTGCCGAAACAGAGGTTGG
ACAAGACCGGAGGGGTCTCCTAGTTCCAAAGGAGATGTACTCCGGGCTTGTTCACGACCTACCGTGTA
+
FFFFFFFFFFFFFFFFFFFFFFFFFFFFFFFFFFFFFFFFFFFFFFFFFFFFFFFFFFFFFFFFFFF
FFFFFFFFFFFFFFFFFFFFFFFFFFFFFFFFFFFFFFFFFFFFFFFFFFFFFFFFFFFFFFFFFFF
@A00155:342:HHGFNDSXY:1:1613:3170:31782 1:N:0:GAACCTAG+TCCGCATA
GGATGTAGAGGGCGAGAGAGAGCGACTTCTCTCGGGCCCACAGCCTTACAGCTGTGGAGCACGGTATC
CTCTGCCGAAACAGAGGTTGGACAAGACCGGAGGGGTCTCCTAGTTCCAAAGGAGATGTACTCC
+
:FFFFFFFFFFFF,FFFFFFFFFFFFFF:FFFFFFFFFFFFFF,F:FFFFFFFFFFFFFFFFFFFFF
FFFFFFFFFFFFFFFFFFFFFFF:FFFFFFFFFFFFFFFFFFFFFFFFFFFFFFFFFFFFFFF
@A00155:342:HHGFNDSXY:1:1614:4788:14418 1:N:0:GAACCTAG+TCCGCATA
AAAAAGGACTCGTCAGGTAGCCTACTAGACTACGACTTACACGGTAGGTCG
+
FFFFFFFFFFFFFFFFFFFFFFFFFFFFFFFFFFFFFFFFFFFFFFFFFFF
@A00155:342:HHGFNDSXY:1:1614:26160:20838 1:N:0:GAACCTAG+TCCGCATA
ATGCCGAGAGGGATGTGGAGGGCGAGAGAGAGCGACTTCTCTCGGGCCCACAGCCTTACAGCTGTGGA
GCACGGTATCCTCTGCCGAAACAGAGGTTGGACAAGACCGGAGGGGTCTCCTAGTTCCAAAGGAGATG
+
FFFFFFFFFFFF:FFFFFFFFFFFFFFFFF:FFFFFFFFFFFFFFFFFFFFFFFFFFFFFFFFFFFF
FFFFFFFFFFFFFFFFFFFFFFFFFFFFF:FFFFFFFFFFFFFFFFFFFFFFFFFFFFFFFFFFFFF
@A00155:342:HHGFNDSXY:1:1614:14796:30968 1:N:0:GAACCTAG+TCCGCATA
GTCATCTCAATGCCGAGAGGGATGTGGAGGGCGAGAGAGAGCGACTTCTCTCGGGCCCACAGCCTTAC
AGCTGTGGAGCACGGTATCCTCTGCCGAAACAGAGGTTGGACAAGACCGGAGGGGTCTCCTAGTTCCA
+

FFFFFFFFFFFFFFFFFFFFFFFFFFFFFFFFFFFFFFFFFFFFFFFFFFFFFFFFFFFFFFFFFFFF
FFFFFFFFFFFFFFFFFFFFFFFFFFFFF:FFFFFFFFFFFFFFFFFFFFFFFFFFFFFFFFFFFFFF
@A00155:342:HHGFNDSXY:1:1614:21043:32174 1:N:0:GAACCTAG+TCCGCATA
GAGCGACTTCTCTCGGGCCCACAGCCTTACAGCTGTGGAGCACGGTATCCTCTGCCGAAACAGAGGTT
GGACAAGACCGGAGGGGTCTCCTAGTTCCAAAGGAGATGTACTCCGGGCTTGTTCACGACCTACCGTG
+
FFFFFFFFFFFFF:FFFFF:FFFFFFFFFFFFFFF:FFFF,FFFFFFFFFFFFFFFFFFFFFFFFFFF
FFFFFFFFFFFFFFFFFFFFFFFFFFFFFFFFFFFFFFFFFFFFFFFFFFFFFFFFFFFFFFFFFFFF
@A00155:342:HHGFNDSXY:1:1615:18918:30060 1:N:0:GAACCTAG+TCCGCATA
CTTCTCTCGGGCCCACAGCCTTACAGCTGTGGAGCACGGTATCCTCTGCCGAAACAGAGGTTGGACAA
GACCGGAGGGGTCTCCTGGTTCCAAAGGAGATGTACTCCGGGCTTGTTCACGACCTACCGTGTAAGTC
+
F:FFFFFFFFFFFFFFFFFFFFFFFFF:FFFFFFFF,FFFFFFFFFFFFFFF:FFF:FFF,:F:FFFF
:FFF:,FF:FFFF:FFFFFFFFFFFFFFF:F:FFFF,FF,,FF:F:F::FFFFFF,FFFF,:FFFF,F
@A00155:342:HHGFNDSXY:1:1615:10999:32941 1:N:0:GAACCTAG+TCCGCATA
GTGTCCACTTAAGGACTCACCTCTGGCCTATGGTCATCTCAATGCCGAGAGGGATGTGAAGGGCGAGA
GAGAGCGACTTCTCTCGGGCCCACAGCCTTACAGCTGTGGAGCACGGTATCCTCTGCCGAAACAGAGG
+
FFFFFFFFFFFFFFFFFFFFFFFFFFFFFFFFFFFFFFFFFFFFFFFF:FFFF:F:FFFFFFFFFFFF
FFFFFFFFFF:FFF:FFFFFFFFFFFFFFFFFFFFFFFFFFFFFFFFF,FFFFFFFFFFFFFFFFFFF
@A00155:342:HHGFNDSXY:1:1616:18566:1266 1:N:0:GAACCTAG+TCCGCATA
GTGTCCACTTAAGGACTCACCTCTGGCCTATGGTCATCTCAATGCCGAGAGGGATGTGGAGGGCGAGA
GAGAGCGACTTCTCTCGGGCCCACAGCCTTACAGCTGTGGAGCACGGTATCCTCTGCCGAAACAGAGG
+
FFFFFFF:FFFFFFFFFFFFFFFFFFFFFFFFFFFFFFFFFFFFFFFFFFFF:FFFFFFFFFFFFFFF
FF:FFFFFFFFFFFFFFFFFFFFFFFFF:FFFFFFFFFFFFFFFFFFFFFFFFFFFFFFFFFFFFFFF
@A00155:342:HHGFNDSXY:1:1616:9299:25488 1:N:0:GAACCTAG+TCCGCATA
GAGAGGGATGTAGAGGGCGAGAGAGAGCGACTTCTCTCGGGCCCACAGCCTTACAGCTGTGGAGCACG
GTATCCTCTGCCGAAACAGAGGTTGGACAAGACCGGAGGGGTCTCCTAGTTCCAAAGGAGATGTACTC
+
FFFFFFFF,FFFFFFFFFFFFFFFFFFFFFFFFFFFFFFFF:FFFFFFFFFFFFFFFFFFFFFFFFFF
FFFFFFFFFFFFFFFFFFFFFFFFFFFFFFFFFFFFFFFFFFFFFFFFFFF:FFFFFFFFFFFFFFFF
@A00155:342:HHGFNDSXY:1:1616:3278:25770 1:N:0:GAACCTAG+TCCGCATA
TTACAGCTGTGGAGCACGGTATCCTCTGCCGAAACAGAGGTTGGACAAGACCGGAGGGGTCTCCTAGT
TCCAAAGGAGATGTACTCCGGGCTTGTTCACGACCTACCGTGTAAGTCGTAGTCTAGTAGGCTACCTG
+
FFFFFFFFFFFFFFFFFFFFFFF:FFFFFFFFFFF:FFFFFFFFFFFFFFFFFFFFFFFFFFFFFFFF
FFFFFFFFFFFFFFFFFFFFFFF:FFFFFFFFFFFF:FFFFFFF,FFFFFFFFFFFFFFFFFFFFFFF
@A00155:342:HHGFNDSXY:1:1617:30156:12164 1:N:0:GAACCTAG+TCCGCATA
TGTAGAGGGCGAGAGAGAGCGACTTCTCTCGGGCCCACAGCCTTACAGCTGTGGAGCACGGTATCCTC
TGCCGAAACAGAGGTTGGACAAGACCGGAGGGGTCTCCTAGTTCCAAAGGAGATGTACTCCGGG
+
FFFFFFFFFFFFFFFFFFFFFFFFFFFFFFFFFFFFFFFFFFFFFFFFFFFFFFFFFFFFFFFFFFFF
FFFFFFFFFFFFFFFFFFFFFFFFFFFFFFFFFFFFFFFFF,FFFFFFFFFFFFFFFFFFFFFF
@A00155:342:HHGFNDSXY:1:1617:19235:25817 1:N:0:GAACCTAG+TCCGCATA
CAATGCCGAGAGGGATGTAGAGGGCGAGAGAGAGCGACTTCTCTCGGGCCCACAGCCTTACAGCTGTG
GAGCACGGTATCCTCTGCCGAAACAGAGGTTGGACAAGACCGGAGGGGTCTCCTAGTTCCAAAGGAGA
+
FFFFFFFFFFFFFFFFF:FFFFFFFFFFFFFFFFFFFFFFFFFFFFFFFFFFFFFFFFFFFFFFFFFF
FFFFFFFFFFFFFFFFFFFFFFFFFFFFFFFFFFFFFFFFFFFFFFFFFFFFF:FFFFFFFFFFFFFF
@A00155:342:HHGFNDSXY:1:1619:10366:7232 1:N:0:GAACCTAG+TCCGCATA
TGCCGAGAGGGATGTGGAGGGCGAGAGAGAGCGACTTCTCTCGGGCCCACAGCCTTACAGCTGTGGAG
CACGGTATCCTCTGCCGAAACAGAGGTTGGACAAGACCGGAGGGGTCTCCTGGTTCCAAAGGAGAT
+

FFFFFFFFFFF,FFFFFFFFFFFFFFFFFFFFFFFFFFFFFFFFFFFFFFFFFFFFFFFFFFFFFFFF
FFFFFFFFFFFFFFFFFFFFFFFFFFFFFFFFFFFFFFFFFFFFFFFFFFFFFFFFFFFFFFFFFF
@A00155:342:HHGFNDSXY:1:1619:10239:7326 1:N:0:GAACCTAG+TCCGCATA
TGCCGAGAGGGATGTGGAGGGCGAGAGAGAGCGACTTCTCTCGGGCCCACAGCCTTACAGCTGTGGAG
CACGGTATCCTCTGCCGAAACAGAGGTTGGACAAGACCGGAGGGGTCTCCTGGTTCCAAAGGAGAT
+
FFFFFFFFFFFF:FFFFFFFFFFFFFFFFFFFFFFFFFFFFFFFFFFFFFFFFFFFFFFFFFFFFFF
FFFFFFFFFFFFFFFFFFFFFFFFFFFFFFFFFFFFFFFFFFFFFFFFFFFFFFFFFFFFFFFFFF
@A00155:342:HHGFNDSXY:1:1619:12735:12680 1:N:0:GAACCTAG+TCCGCATA
GCGACTTCTCTCGGGCCCACAGCCTTACAGCTGTGGAGCACGGTATCCTCTGCCGAAACAGAGGTTGG
ACAAGACCGGAGGGGTCTCCTAGTTCCAAAGGAGATGTACTCCGGGCTTGTTCACGACCTACC
+
FFFFFFFFFFFFFFFFFFFFFFFFFFFFFFFFFFFFFFFFFFFFFFFFFFFFFFFFFFFFFFFFFFFF
FFFFFFFFFFFFFFFFFFFFFFFFFFFFFFFFFFFFFFFFFFFFFFFFFFFFFFFFFFFFFFF
@A00155:342:HHGFNDSXY:1:1619:9064:28401 1:N:0:GAACCTAG+TCCGCATA
GAGGGATGTAGAGGGCGAGAGAGAGCGACTTCTCTCGGGCCCACAGCCTTACAGCTGTGGAGCACGGT
ATCCTCTGCCGAAACAGAGGTTGGACAAGACCGGAGGGGTCTCCTAGTTCCAAAGGAGATGTACTCCG
+
FFFFFFFFFFF:FFFFFFFFFFFFFFFFFFFFFFFFFFFFFFFFFFFFFFFFFFFFFFFFFFFFFFFF
FFFFFFFFFFFFFFFFFFFFFFFFFFFFFFFFFFFFFFFFFFFFFFFFFFFFFFFFFFFFFF,FFFFF
@A00155:342:HHGFNDSXY:1:1619:30716:32831 1:N:0:GAACCTAG+TCCGCATA
CTTCTCTCGGGCCCACAGCCTTACAGCTGTGGAGCACGGTATCCTCTGCCGAAACAGAGGTTGGACAA
GACCGGAGGGGTCTCCTAGTTCCAAAGGAGATGTACTCCGGGCTTGTTCACGACCTACCGTGTAAGTC
+
FFFFFFFFFFFFFFFFFFFFFFFFFFFFFFFFFFFFFFFF:FFFFFFFFFFFFFFFFFFFFFFFFFFF
FFFFFFFFFFFFFFFFFFFFFFFFFFFFFFFFFFFFFFFFFFFFFFFFFFFFFFFF:FFFFFFFFFFF
@A00155:342:HHGFNDSXY:1:1619:31331:33238 1:N:0:GAACCTAG+TCCGCATA
CTTCTCTCGGGCCCACAGCCTTACAGCTGTGGAGCACGGTATCCTCTGCCGAAACAGAGGTTGGACAA
GACCGGAGGGGTCTCCTAGTTCCAAAGGAGATGTACTCCGGGCTTGTTCACGACCTACCGTGTAAGTC
+
FFFFFFF,,FFFFFFFFFFF,F:FFFF:::FFFFFFFFFFFFFFFFF:FFFFFFFFFFF:FFFFFFFF
:F:F,FF::FFFFF:FFFFF,FFFFF:,F:::FFFFFF:FFFF:FFFFFFF:FFFFFF,FFFFF:,F:
@A00155:342:HHGFNDSXY:1:1619:9453:36495 1:N:0:GAACCTAG+TCCGCATA
TCTGCCGAAACAGAGGTTGGACAAGACCGGAGGGGTCTCCTAGTTCCAAAGGAGATGTACTCCGGGCT
TGTTCACGACCTACCGTGTAAGTCGTAGTCTAGTAGGCTACCTGACGAGTCCTTTTTAGGACGAAACT
+
FFFFFFFFFFFFFFFFFFFFFFFFFFFFFFFF:FFFFFFFFFFFFFFFFFFFFFFFFFFFFFFFFFFF
FFFFFFFFFFFFFFFFFFF:FFFFFFFFFFFFFFFFFFFFFFFFFF:FFFFFFFFFFFFFFF:FFFFF
@A00155:342:HHGFNDSXY:1:1620:8196:1689 1:N:0:GAACCTAG+TCCGCATA
TCTGCCGAAACAGAGGTTGGACAAGACCGGAGGGGTCTCCTAGTTCCAAAGGAGATGTACTCCGGGCT
TGTTCACGACCTACCGTGTAAGTCGTAGTCTAGTAGGCTACCTGACGAGTCCTTTTTAGGACGAAACT
+
FFFFFFFFFFFFFFFFFFFFFFFFFFFFFFFFFFFFFFFFFFFFFFFFFFFFFFFFFFFFFFFFFFFF
FFFFFFFFFFFFFFFFFFFFFFFFFFFFFFF:FFFFFFFFFFFFFFFFFFFFF:FFFFFFFFFFFFFF
@A00155:342:HHGFNDSXY:1:1620:8929:3458 1:N:0:GAACCTAG+TCCGCATA
GAGAGGGATGTGGAGGGCGAGAGAGAGCGACTTCTCTCGGGCCCACAGCCTTACAGCTGTGGAGCACG
GTATCCTCTGCCGAAACAGAGGTTGGACAAGACCGGAGGGGTCTCCTAGTTCCAAAGGAGATGTACTC
+
FFFFFFFFFFFFFFFFFFFFFFFFFFFFFFFFFFFFFFFFFFFFFFFFFFFFFFFFFFFFFFFFFFFF
FFFFFFFFFFFFFFFFFFFFFFFFFFFFFFFFFFFFFFFFFFFFFFFFFFFFFFFFFFFFFFFFFFFF
@A00155:342:HHGFNDSXY:1:1620:21151:10911 1:N:0:GAACCTAG+TCCGCATA
CACATCCCTCTCGGCATTGAGATGACCATAGGCCAGAGGTGAGTCCTTAAGTGGACACAGCTGATCTA
AGGCGGTGTGGTGGGGCATGGGTTTGAACCCCCATGACGGTCGGAGTCTAGTAGGCTCCCTGATGAGT
+

```
FFFFFFFFFFFFFFFFFFFFFFFFFFFFFFFFFFFFFFFFFFFFFFFFFFFFFFFFFF:FFFFFFFFF
FFFFFFFFFFFFFFFFFFFFFFFFFFFFFFFFFFFFFFFFFFFFFFFFFFF:FFFFFFFFFF:FFFFF
@A00155:342:HHGFNDSXY:1:1620:31177:21574 1:N:0:GAACCTAG+TCCGCATA
GGATGTGGAGGGCGAGAGAGAGCGACTTCTCTCGGGCCCACAGCCTTACAGCTGTGGAGCACGGTATC
CTCTGCCGAAACAGAGGTTGGACAAGACCGGAGGGGTCTCCTAGTTCCAAAGGAGATGTACTCC
+
FFFFFFFFFFFF:FFFFFFFFFF:FFFFFFFFFFFFFFFFFFFFFFFFFFFFFFFF:FFFFFFFFFFF
FFFFFFFFFFFFFFFFFFFFFFFFFFFFFFFFFFFFFFFFFFFFFFFFFFFFFFFFFFFFFFFF
@A00155:342:HHGFNDSXY:1:1620:15157:22451 1:N:0:GAACCTAG+TCCGCATA
ACTTCTCTCGGGCCCACAGCCTTACAGCTGTGGAGCACGGTATCCTCTGCCGAAACAGAGGTTGGACA
AGACCGGAGGGGTCTCCTAGTTCCAAAGGAGATGTACTCCGGGCTTGTTCACGACCTACCGTGTAAGT
+
FFFFFFFFFFFFFFFFFFFFFFFFFFFFFFFFFFFFFFFFFFFFFFFFFFFFFFFFFFFFFFFFFFFF
FFFFFFFFFFFFFFFFFFFFFFFFFFFFFFFFFFFFFFFFFFFFFFFFFFFFFFFFFFFFFFFFFFFF
@A00155:342:HHGFNDSXY:1:1620:28583:31203 1:N:0:GAACCTAG+TCCGCATA
GAGCGACTTCTCTCGGGCCCACAGCCTTACAGCTGTGGAGCACGGTATCCTCTGCCGAAACAGAGGTT
GGACAAGACCGGAGGGGTCTCCTGGTTCCAAAGGAGATGTACTCCGGGCTTGTTCACGACCTACCGTG
+
FFFFFFFFFFFFFFFFFFFFFFFFFFFFFFFFFFFFFFF:FFFFFF:FFFFF:FFFFFFFFFFFFFFF
FFFFFFFFFFFFFFFFFFFFFFFFFFFFFFFFFFFFFFFF:FFFFFFFF:FFFFFFFFFFFFFFFF:F
@A00155:342:HHGFNDSXY:1:1621:27769:3208 1:N:0:GAACCTAG+TCCGCATA
ATCTCAATGCCGAGAGGGATGTGGAGGGCGAGAGAGAGCGACTTCTCTCGGGCCCACAGCCTTACAGC
TGTGGAGCACGGTATCCTCTGCCGAAACAGAGGTTGGACAAGACCGGAGGGGTCTCCTAGTTCCAAAG
+
FF::FFFFF:FFFFF::FFFFFF,FFFFF,FFFFFF::,FFFFFFFFFFFF:FFFFFF:FFFFFFFFF
::FFFFF:FFFFFFFFFFFFFF:FFFFFFFF:FF:FFF:F:FF,FFFFFFF:FF:F:FFFFFF:FFFF
@A00155:342:HHGFNDSXY:1:1622:25156:7670 1:N:0:GAACCTAG+TCCGCATA
TGTCCACTTAAGGACTCTCCTCTGGCCTATGGTCATCTCAATGCCGAGAGGGATGTGGAGGGCGAGAG
AGAGCGACTTCTCTCGGGCCCACAGCCTTACAGCTGTGGAGCACGGTATCCTCTGCCGAAACAGAGGT
+
FFFFFFFFFFFFFFFFFFFFFFFFFFFFFFFFFFFFFFFFFFFFFFFFFFFFFFFFFFFFFFFFFFFF
FFFFFFFFFFFFFFFFFFFFFFFFFFF:FFFFFFFFFFFFFFFFFFFFFFFFFFFFFFFFFFFFFFFF
@A00155:342:HHGFNDSXY:1:1622:21404:15515 1:N:0:GAACCTAG+TCCGCATA
CCACATCCCTCTCGGCATTGAGATGACCATAGGCCAGAGGTGAGTCCTTAAGTGGACACAGCTGATCT
AAGGCGGTGTGGCGGGGCATGTGTTTGAACCCCCATGACGGTCGGAGTCTAGTAGGCTCCCTGATG
+
F:FF::F:,F:FF:,,F,FFF,:F,F:,F:F,FF,FFF::FFF,,,FFFF:F,:FFFF:FFFF,F:,:
,FFFF,,FF,:,FF,FF,FFF,:F,FF::F,FF,FFFFF,FFF:F:F,F,,FF,,F:FFF,F,:FF
@A00155:342:HHGFNDSXY:1:1622:18783:30577 1:N:0:GAACCTAG+TCCGCATA
GGATGTGGAGGGCGAGAGAGAGCGACTTCTCTCGGGCCCACAGCCTTACAGCTGTGGAGCACGGTATC
CTCTGCCGAAACAGAGGTTGGACAAGACCGGAGGGGTCTCCTAGTTCCAAAGGAGATGTACTCC
+
FFFFFFFFFFFFFFFFFFFFFFFFFFFFFFFFFFFFFFFFFFFFFFFFFFFFFFFFFFFFFFFFFFFF
FFFFFFFFFFFFFFFFFFFFFFFFFFFFFFFFFFFFFFFFFFFFFFFF:FFFFFFFFFFFFFFF
@A00155:342:HHGFNDSXY:1:1622:18747:30859 1:N:0:GAACCTAG+TCCGCATA
GGATGTGGAGGGCGAGAGAGAGCGACTTCTCTCGGGCCCACAGCCTTACAGCTGTGGAGCACGGTATC
CTCTGCCGAAACAGAGGTTGGACAAGACCGGAGGGGTCTCCTAGTTCCAAAGGAGATGTACTCC
+
FFFFFFFFFFFFFFFFFFFFFFFFFFFFFFFFFFFFFFFFFFF:FFFFFFFFFFF:FFFFFFFFFFFF
FFFFFFFFFFFFFFFFFFFFFFFFFFFFFFFFFFFFFFFFFFFFFFFFFFFFFFFFFFFFFFFF
@A00155:342:HHGFNDSXY:1:1623:4915:2957 1:N:0:GAACCTAG+TCCGCATA
CACCTCTGGCCTATGGTCATCTCAATGCCGAGAGGGATGTGGAGGGCGAGAGAGAGCGACTTCTCTCG
GGCCCACAGCCTTACAGCTGTGGAGCACGGTATCCTCTGCCGAAACAGAGGTTGGACAAGACCGGAGG
+
```

FFFFFFFFFFFFFFFFFFFFFFFFFFFFFFFFFFFFFFFFFFFFFFFFFFFFFFFFFFFFFFFFFFFF
FFFFFFFFFFFFFFFFFFFFFFFFFFFFFFF:FFFFFFFFFFFFFFFFFFFFFFFFFFFFFFFFFFFF
@A00155:342:HHGFNDSXY:1:1623:21504:5290 1:N:0:GAACCTAG+TCCGCATA
TATCCTCTGCCGAAACAGAGGTTGGACAAGACCGGAGGGGTCTCCTAGTTCCAAAGGAGATGTACTCC
GGGCTTGTTCACGACCTACCGTGTAAGTCGTAGTCTAGTAGGCTACCTGACGAGTCCTTTTTAGGACG
+
FFFFFFFFFFFFFFFFFFFFFFFFFFFFFFFFFFFFFFFFFFFFFFFFFFFFFFFFFFFFFFFFFFFF
FFFFFFFFFFFFFFFFFFFFFFFFFFFFFFFFFFFFFFFFFFFFFFFFFFFFFFFFFFFFFFFFFFFF
@A00155:342:HHGFNDSXY:1:1623:20961:7232 1:N:0:GAACCTAG+TCCGCATA
TTAAGGACTCACCTCTGGCCTATGGTCATCTCAATGCCGAGAGGGATGTGAAGGGCGAGAGAGAGCGA
CTTCTCTCGGGCCCACAGCCTTACAGCTGTGGAGCACGGTATCCTCTGCCGAAACAGAGGTTGGACAA
+
FFFFFFFFFFFFFFFFFFFFFFFFFFFFFFFFFFFFFFFFFFFFFFFFFFFFFFFFFFFFFFFFFFFF
FFFFFFFFFFFFFFFFFFFF:FFFFFFFFFFFFFFFFFFFFFFFFFFFFFFFFFFFFFFFFFFFFFFF
@A00155:342:HHGFNDSXY:1:1623:27281:14888 1:N:0:GAACCTAG+TCCGCATA
TGCCCCGCCACACCGCCTTAGATCAGCTGTGTCCACTTAAGGACTCACCTCTGGCCTATGGTCATCTC
AATGCCGAGAGGGATGTGGAGGGCGAGAGAGAGCGACTTCTCTCGGGCCCACAGCCTTACAGCTGTGG
+
FFFFFFFFFFFFFFFFF:FFFFFFFFFFFFFFFFFFF:FFFFFFFFFFFFFFFFFFFFFFFFFFFFFF
FFFFFFF:FFFFFFFFFFFFFFFFFFFFFFFFFFFFF:FFFFFFFFFFFFFFFFFFFFFFFFFFFFFF
@A00155:342:HHGFNDSXY:1:1623:3188:33317 1:N:0:GAACCTAG+TCCGCATA
GATGTGGAGGGCGAGAGAGAGCGACTTCTCTCGGGCCCACAGCCTTACAGCTGTGGAGCACGGTATGC
TCTGCCGAAACAGAGGTTGGACAAGACCGGAGGGGTCTCCTAGTTCCAAAGGAGATGTACTCCGGGCT
+
FFFFFFFFFFFFFFFFFFFFFFFFFFFFFFFFFFFFFFFFFFF:FFFFFFFFFFFFFFFFFFFFFF:F
FFFFFFFFFFFFFFFFFFFFFFFF:FFFFFFFFFFFFFFFFFFFFFFFFFFFFFFFFFFFFFFFFFF
@A00155:342:HHGFNDSXY:1:1623:5159:33974 1:N:0:GAACCTAG+TCCGCATA
AGCGACTTCTCTCGGGCCCACAGCCTTACAGCTGTGGAGCACGGTATCCTCTGCCGAAACAGAGGTTG
GACAAGACCGGAGGGGTCTCCTAGTTCCAAAGGAGATGTACTCCGGGCTTGTTCACGACCTACCGTGT
+
FFFFFFFFFFFFFFFFFFFFFFFFFFFFFFFFFFFFFFFFFFFFFFFFFFFFFFFFFFFFFFFFFFFF
FFFFFFFFFFFFFFFFFFFFFFFFFFFFFFFFFFFFFFFFFFFFFFFFFFFFFFFFFFFFFFFFFFFF
@A00155:342:HHGFNDSXY:1:1623:2555:35383 1:N:0:GAACCTAG+TCCGCATA
GGATGTGGAGGGCGAGAGAGAGCGACTTCTCTCGGGCCCACAGCCTTACAGCTGTGGAGCACGGTATC
CTCTGCCGAAACAGAGGTTGGACAAGACCGGAGGGGTCTCCTAGTTCCAAAGGAGATGTACTCC
+
FFFFFFFFFFFFFFFFFFFFFFFFFFFFFFFFFFFFFFFFFFFFFFFFFFFFFFFF:FFFFFFFFFFF
FFFFFFFFFFFFFF:FFFFFFFFFFFFFFFFFFFFFFFFFFFFFFFFFFFFFFFFFFFFFFFFF
@A00155:342:HHGFNDSXY:1:1624:12472:27633 1:N:0:GAACCTAG+TCCGCATA
GAGAGGGATGTGGAGGGCGAGAGAGAGCGACTTCTCTCGGGCCCACAGCCTTACAGCTGTGGAGCACG
GTATCCTCTGCCGAAACAGAGGTTGGACAAGACCGGAGGGGTCTCCTAGTTCCAAAGGAGATGTACTC
+
FFFFFFFFFFFFFFFFFFFFFFFFFFFFFFFF:FFFFF:FFFFFFFFFFFFFFFFFFFFFFFFFFFFF
FFFFFFFFFFFFFFFFFFFFFFFFFFFF:F,FFFFFFF:FFFFFFFFFF:FFFFFFFFFFFFFFFFFF
@A00155:342:HHGFNDSXY:1:1624:20573:34240 1:N:0:GAACCTAG+TCCGCATA
ATGCCCCGCCACACCGCCTTAGATCAGCTGTGTCCACTTAAGAACTCACCTCTGGCCTATGGTCATCT
CAATGCCGAGAGGGATGTGGAGGGCGAGAGAGAGCGACTTCTCTCGGGCCCACAGCCTTACAGC
+
FFFFFFFFFFFFFFFFFFFFFFFFFFFFFFFFFFFFFFFFFFFFFFFFFFFFFFFFFFFFFFFFFFFF
FFFFFFFFFFFFFFF,F:FFFFFFFFFFFFFFFFFFFFF:FFFFFFFFFFFFFFFFFFFFFFFF
@A00155:342:HHGFNDSXY:1:1624:21269:37043 1:N:0:GAACCTAG+TCCGCATA
ATGCCCCGCCACACCGCCTTAGATCAGCTGTGTCCACTTAAGAACTCACCTCTGGCCTATGGTCATCT
CAATGCCGAGAGGGATGTGGAGGGCGAGAGAGAGCGACTTCTCTCGGGCCCACAGCCTTACAGC
+

FFFFFFFFFFFFFFFFFF,FFFFFFFFFFFFFFFFFFFFFFFFFFFFFFFFFFFFFFFFFFFFFFFFF
FFFFFFFFFFFFFFF:FFFFFFFFFFFFFFFFFFFFFFFFFFFFFFFFFFFFFFFFFFFFFFFF
@A00155:342:HHGFNDSXY:1:1625:22110:1454 1:N:0:GAACCTAG+TCCGCATA
CACATCCCTCTCGGCATTGAGATGACCATAGGCCAGAGGTGAGTCCTTAAGTGGACACAGCTGATCTA
AGGCGGTGTGGCGGGGCATGGGTTTGAACCCCCATGACGGTCGGAGTCTAGTAGGCTCCCTGATGAG
+
FFF:FFFFFF:FFFFF,:FFFF:FFFFFFFFFFFFFFFFFFFFFFFF:FFFFFFFFFF:FFFFF:FFF
FFFFFF,FFFFFF:,FF,FF:F::FFFFFFFFFFFFFFFFFFFFFFFFFFF:FFFFFFFFFFFFFFF
@A00155:342:HHGFNDSXY:1:1625:30752:4022 1:N:0:GAACCTAG+TCCGCATA
ACCTCTGTTTCGGCAGAGGATACCGTGCTCCACAGCTGTAAGGCTGTGGGCCCGAGAGAAGTCGCTCT
CTCTCGCCCTCCACATCCCTCTCGGCATTGAGATGACCATAGGCCAGAGGAGAGTCCTTAAGTGGACA
+
FFFFFFFFFFFFFFFFFFFFFFFFFFFFFFFFFFFFFFFFFFFFFFFFFFFFFFFFFFF:FFFFFFFF
FFFFFFFFFFFFFFFFFFFFF:FFFFF:FFFFFFFFFFFFFFFFFFFFFFFFFFFFFF:FFFFFFFFF
@A00155:342:HHGFNDSXY:1:1625:11134:5181 1:N:0:GAACCTAG+TCCGCATA
TCTCTCGGGCCCACAGCCTTACAGCTGTGGAGCACGGTATCCTCTGCCGAAACAGAGGTTGGACAAGA
CCGGAGGGGTCTCCTAGTTCCAAAGGAGATGTACTCCGGGCTTGTTCACGACCTACCGTGTAAGTCGT
+
FFFFFFFFFFFFFFFFFFFFFFFFFFFFFFFFFFFFFFFFFFFFFFFFFFFFFFFF:FFFFFFFFFFF
FFFFFFFFFFFFFFFFFFFFFFFFFFFFFFFFFFFF:FFFFFFFFFFFFFFFFFFFFFFFFFFFFFFF
@A00155:342:HHGFNDSXY:1:1625:8684:5885 1:N:0:GAACCTAG+TCCGCATA
CAGCCTTACAGCTGTGGAGCACGGTATCCTCTGCCGAAACAGAGGTTGGACAAGACCGGAGGGGTCTC
CTAGTTCCAAAGGAGATGTACTCCGGGCTTGTTCACGACCTACCGTGTAAGTCGTAGTCTAGTAGGCT
+
FFFFFFFFFFFFFFFFFFFFFFFFFFFFFFFFFFFFFFFFFFFFFFFFFFFFFFFFFFFFFFFFFFFF
FFFFFFFFFFFFFFFFFFFFFFFFFFFF:FFFFFFFFFFFFFFFFFFFF:FFFFFFFFFFFFFFFFFF
@A00155:342:HHGFNDSXY:1:1625:16911:24643 1:N:0:GAACCTAG+TCCGCATA
AATGCCGAGAGGGATGTGGAGGGCGAGAGAGAGCGACTTCTCTCGGGCCCACAGCCTTACAGCTGTGG
AGCACGGTATCCTCTGCCGAAACAGAGGTTGGACAAGACCGGAGGGGTCTCCTAGATCCAAAGGAGAT
+
FFFFFFF:FFFFFFFFFFFFFFFFFFFFFFFFFFFFFFFF:FFFFFFFFFFFFFFFFFFFFFFFFFFF
FFFFFFFFFFFFFFFFFFFFFFFFFFFFFFFFFFFFFFFFFFFFFFFFFFFFFFFFFFFFF:FFFFFF
@A00155:342:HHGFNDSXY:1:1625:15239:26976 1:N:0:GAACCTAG+TCCGCATA
GAGAGGGATGTAGAGGGCGAGAGAGAGCGACTTCTCTCGGGCCCACAGCCTTACAGCTGTGGAGCACG
GTATCCTCTGCCGAAACAGAGGTTGGACAAGACCGGAGGGGTCTCCTAGTTCCAAAGGAGATGTACTC
+
FFFFFFFFFFFFFFFFFFFFFFFFFFFFFFFFFFFFFFFFFFFFFFFFFFFFFFFFFFFFFFFFFFFF
FFFFFFFFFFFFFFFFFFFFFFFFFFFFFFFFFFFFFFFFFFFFFFFFFFFFFFFFFFFFFFFFFFFF
@A00155:342:HHGFNDSXY:1:1626:14389:16391 1:N:0:GAACCTAG+TCCGCATA
TCTCAATGCCGAGAGGGATGTGGAGGGCGAGAGAGAGCGACTTCTCTCGGGCCCACAGCCTTACAGCT
GTGGAGCACGGTATCCTCTGCCGAAACAGAGGTTGGACAAGACCGGAGGGGTCTCCTAGTTCCAAAGG
+
FFFFFFFFFFFFFFFFFFFFFFFFFFFFFFFFFFFFFFFFFFFFFFFFFFFFFFFFFFFFFFFFFFFF
FFFFFFFFFFFFFFFFFFFFFFFFFFFFFFF:FFFFFFFFFFFFFFFFFFFFFFFFFFFFFFFFFFFF
@A00155:342:HHGFNDSXY:1:1626:3983:26428 1:N:0:GAACCTAG+TCCGCATA
CTCTGGCCTATGGTCATCTCAATGCCGAGAGGGATGTAGAGGGCGAGAGAGAGCGACTTCTCTCGGGC
CCACAGCCTTACAGCTGTGGAGCACGGTATCCTCTGCCGAAACAGAGGTTGGACAAGACCGGAGGGGT
+
FFFFFFFFF:FFFFFFFFFFFF:FFFF:FFFFFF:F:FFFFFFFFFFFFFF:FFFFFFFFFFFFFFFF
FFFFFFFFFF:FFFFFFFFFFFFFFFFFFFFFFFFFFFFFFFFFFFFFFFFFFFFFFFFFFFFFFFFF
@A00155:342:HHGFNDSXY:1:1626:15582:30232 1:N:0:GAACCTAG+TCCGCATA
CTTCTCTCGGGCCCACAGCCTTACAGCTGTGGAGCACGGTATCCTCTGCCGAAACAGAGGTTGGACAA
GACCGGAGGGGTCTCCTAGTTCCAAAGGAGATGTACTCCGGGCTTGTTCACGACCTACCGTGTAAGTC
+

```
F:FFFFFFFFFFFFFFFFFFFFFFFFFFFFFFFFFFFFFFFFFFFFFFFFFFFFFFF:FFFFFFFFFF
FFFFFFFFFFFFFFFFFFFFFFFFFFFFFF:FFFFFF:FFFFFFFFFFFFFFFFFFFFFFFFFFFFFF
@A00155:342:HHGFNDSXY:1:1626:1551:35963 1:N:0:GAACCTAG+TCCGCATA
CTATGGTCATCTCAATGCCGAGAGGGATGTGGAGGGCGAGAGAGAGCGACTTCTCTCGGGCCCACAGC
CTTACAGCTGTGGAGCACGGTATCCTCTGCCGAAACAGAGGTTGGACAAGACCGGAGGGGTCTCCTAG
+
FFFFFFFFFFFFFFFFF:FFFF:FFFFF,:FFFFFFFF:FFFFFFFF,F,FFFF:FFFFFF,FFFFF:
FFFFFFFFFFFFFFFF:FFFFFFFFFFFF:FFF:F:FFFFFFFFFF:FF:FFFFFFFFFFFFF,F:F:
@A00155:342:HHGFNDSXY:1:1627:23809:20556 1:N:0:GAACCTAG+TCCGCATA
ACCCCTCCGGTCTTGTCCAACCTCTGTTTCGGCAGAGGATACCGTGCTCCACAGCTGTAAGGCTGTGG
GCCCGAGAGAAGTCGCTCTCTCTCGCCCTCCACATCCCTCTCGGCATTGAGATGACCATAGGCCAGAG
+
FFFFFFFFFFFF:FFFFFFFFFFFFFFFFFFFFFFFF:FFFFFFFFFFFFFFFFFFFFFFFFFFFFFF
FFFFFFFFFFFFFFFFFFFFFFFFFFFF:FFFFFFFFFFFFFFFF::FFFFFFFFFFFFFFFFFFFFF
@A00155:342:HHGFNDSXY:1:1628:24343:5822 1:N:0:GAACCTAG+TCCGCATA
CGACTTCTCTCGGGCCCACAGCCTTACAGCTGTGGAGCACGGTATCCTCTGCCGAAACAGAGGTTGGA
CAAGACCGGAGGGGTCTCCTAGTTCCAAAGGAGATGTACTCCGGGCTTGTTCACGACCTACCGTGTAA
+
FFFFFFFFFFFFFFFFFFFFFFFFFFFFFFFFFFFFFFFFFFFFFFFFFFFFFFFFFFFFFFFFFFFF
FFFFFFFFFFFFFFFFFFFFFFFFFFFFFFFFFFFFFFFFFFFFFFFFFFF:FFFFFFFFFFFFFFFF
@A00155:342:HHGFNDSXY:1:1628:25265:34851 1:N:0:GAACCTAG+TCCGCATA
TTCTCTCGGGCCCACAGCCTTACAGCTGTGGAGCACGGTATCCTCTGCCGAAACAGAGGTTGGACAAG
ACCGGAGGGGTCTCCTAGTTCCAAAGGAGATGTACTCCGGGCTTGTTCACGACCTACCGTGTAAGTCG
+
FFFFFFFFFFFFFFFFFFFFFFFFFFFFFFFFFFFFFFFFFFFFFFFFFFFFFFFFFFFFFFFFFFFF
FFFFFFFFFFFFFFFFFFFFFFFFFFFFFFFFFFFFFFFFFFFFFFFFFFFFFFFFFFFFFFFFFFFF
@A00155:342:HHGFNDSXY:1:1629:25880:4288 1:N:0:GAACCTAG+TCCGCATA
AGAGAGAGCGACTTCTCTCGGGCCCACAGCCTTACAGCTGTGGAGCACGGTATCCTCTGCCGAAACAG
AGGTTGGACAAGACCGGAGGGGTCTCCTGGTTCCAAAGGAGATGTACTCCGGGCTTGTTCACGACCTA
+
FFFFFFFFFFFFFFFFFF:F:FFFFFFFFFFFF:FFFFFFFFFFFFFFFFFF:FFF:FFFFFFFFFFF
FFFFFFFFFFF:FFFFFFFFFFFFFFF:FFFFFFFFFFFFFFF:FFFFFFFFFFFFFFF:FFFFFFFF
@A00155:342:HHGFNDSXY:1:1629:13711:12962 1:N:0:GAACCTAG+TCCGCATA
GACTTCTCTCGGGCCCACAGCCTTACAGCTGTGGAGCACGGTATCCTCTGCCGAAACAGAGGTTGGAC
AAGACCGGAGGGGTCTCCTAGTTCCAAAGGAGATGTACTCCGGGCTTGTTCACGACCTACCGTGTAAG
+
FFFFFFFFFFFFFFFFFFFFFFFFFFFFFFFFFFFFFFFFFFFFFFFFFFFFFFFFFFFFFFFFFFFF
FFFFFFFFFFFFFFFFFFFFFFFFFFFFFFFFFFFFFFFFFFFFFFFFFFFFFFFFFFFFFFFFFFFF
@A00155:342:HHGFNDSXY:1:1629:23773:13385 1:N:0:GAACCTAG+TCCGCATA
CTCAATGCCGAGAGGGATGTGGAGGGCGAGAGAGAGCGACTTCTCTCGGGCCCACAGCCTTACAGCTG
TGGAGCACGGTATCCTCTGCCGAAACAGAGGTTGGACAAGACCGGAGGGGTCTCCTAGTTCCAAAGGA
+
FFFFFFFFFFFFFFFF:FFFFFFFFFFFFFFFFFFFFFFFFFFFFFFFFFFFFFFFFFFFFFFFFFFF
FFFFFFFFFFFFFFFFFFFFFFFFFFFFFFFFFFFFFFFFFFFFFFFFFFFFFFFFFFFFFFFFFFFF
@A00155:342:HHGFNDSXY:1:1629:25789:14465 1:N:0:GAACCTAG+TCCGCATA
AGCGACTTCTCTCGGGCCCACAGCCTTACAGCTGTGGAGCACGGTATCCTCTGCCGAAACAGAGGTTG
GACAAGACCGGAGGGGTCTCCTAGTTCCAAAGGAGATGTACTCCGGGCTTGTTCACGACCTACCGTGT
+
FFFFFFFFFFFFFFFFFFFF:FFFFFFFFFFFFFFFFF:FFFFFF:FFFFFFFFFFFFFFFFFFFFFF
FFFFFFFFFFFFFFFFFFFFFFFFFFFFFFFFFFFFFFFFFFFFFFFFFFFFFFFFFFFFFFFFFFFF
@A00155:342:HHGFNDSXY:1:1629:7762:21198 1:N:0:GAACCTAG+TCCGCATA
GAGCGACTTCTCTCGGGCCCACAGCCTTACAGCTGTGGAGCACGGTATCCTCTGCCGAAACAGAGGTT
GGACAAGACCGGAGGGGTCTCCTAGTTCCAAAGGAGATGTACTCCGGGCTTGTTCACGACCTACCGTG
+
```

FFFFFFFFFFFFFFFFFFFFFFFFFFFFFFFFFFFFFFFFFFFFFFFFFFFFFFFFFFFFFFFFFFFFFFFFFF
FFFFFFFFFFFFFFFFFFFFFFFFFFFFFFFFFFFFFFFFFFFFFFFFFFFFFFFFFFFFFFFFFFFFFFFFFF
@A00155:342:HHGFNDSXY:1:1629:26106:32111 1:N:0:GAACCTAG+TCCGCATA
TTCTCTCGGGCCCACAGCCTTGCAGCTGTGGAGCACGGTATCCTCTGCCGAAACAGAGGTTGGACAAG
ACCGGAGGGGTCTCCTAGTTCCAAAGGAGATGTACTCCGGGCTTGTTCACGACCTACCGTGTAAGTCG
+
FFFFFFFFFFFFFFFFFFFFFFFFFFFFFFFFFFFFFFFFFFFFFFFFFFFFFFFFFFFFFFFFFFFFFFFFFF
FFFFFFFFFFFFFFFFFFFFFFFFFFFFFFFFFFFFFFFFFFFFFFFFFFFFFFFFFFFFFFFFFFFFFFFFFF
@A00155:342:HHGFNDSXY:1:1629:6262:36104 1:N:0:GAACCTAG+TCCGCATA
TCCGGTCTTGTCCAACCTCTGTTTCGGCAGAGGATACCGTGCTCCACAGCTGTAAGGCTGTGGGCCCG
AGAGAAGTCGCTCTCTCTCGCCCTTCACATCCCTCTCGGCATTGAGATGACCATAGGCCAGAGGTGAG
+
FFFFFFFFFFFFFFFFFF:FFF:F,FFFFFF::FFF:FFFFFFFFFFFFFFFFFFFFFFFFFFFFFFFF
FFFFFFFFFFFFFFFFFFFFFFFFFFFFFFFFFFFFFFFFFFFFFFFFFFFFFFFFFFFFFFFFFFFFFFFFFF
@A00155:342:HHGFNDSXY:1:1630:8666:17910 1:N:0:GAACCTAG+TCCGCATA
GAGAGGGATGTGGAGGGCGAGAGAGAGCGACTTCTCTCGGGCCCACAGCCTTACAGCTGTGGAGCACG
GTATCCTCTGCCGAAACAGAGGTTGGACAAGACCGGAGGGGTCTCCTAGTTCCAAAGGAGATGTACTC
+
FFFFFFFFFFFFFFFFFFFFFFFFFFFFFFFFFFFFFFFFFFFFFFFFFFFFFFFFFFFFFFFFFFFFFFFFFF
FFFFFFFFFFFFFFFFFFFFFFFFFFFFFFFFFFFFFFFFFFFFFFFFFFFFFFFFFFFFFFFFFFFFFFFFFF
@A00155:342:HHGFNDSXY:1:1630:18927:35274 1:N:0:GAACCTAG+TCCGCATA
AATGCCGAGAGGGATGTAGAGGGCGAGAGAGAGCGACTTCTCTCGGGCCCACAGCCTTACAGCTGTGG
AGCACGGTATCCTCTGCCGAAACAGAGGTTGGACAAGACCGGAGGGGTCTCCTAGTTCCAAAGGAGAT
+
FFFFFFFFFFFFFFFFFFFFFFFFFFFFFFFFFFFF:FFFFFFFFFFFFFFFFFFFFFFFFFFFFFFF
FFFFFFFFFFFFFFFFFFFFFFFFFF:FFFFFFFFFFFFFFFFFFFFFFFFFFFFFFFFFFFFFFFFFFF
@A00155:342:HHGFNDSXY:1:1631:15845:16094 1:N:0:GAACCTAG+TCCGCATA
TGTTTCGGCAGAGGATACCGTGCTCCACAGCTGTAAGGCTGTGGGCCCGAGAGAAGTCGCTCTCTCTC
GCCCTCTACATCCCTCTCGGCATTGAGATGACCATAGGCCAGAGGTGAGTCCTTAAGTGGACACAGCT
+
FFFFFFFFFFFFFFFFFFFFFFFFFFFFF:FFFFF:FFFFFFFFFFFFFFFFFFFFFFFFFFFFFFFF
FFFFFFFFFFFFFFFFFFFFFFFFFFFFFFFFFFFFFFFFFFFFFFFFFFFFFFFFFFFFFFFFFFFF:
@A00155:342:HHGFNDSXY:1:1631:2908:16799 1:N:0:GAACCTAG+TCCGCATA
GGGCGAGAGAGAGCGACTTCTCTCGGGCCCACAGCCTTACAGCTGTGGAGCACGGTATCCTCTGCCGA
AACAGAGGTTGGACAAGACCGGAGGGGTCTCCTGGTTCCAAAGGAGATGTACTCCGGGCTTGTTCACG
+
FFFFFFFFFFFFFFFFFFFFFFFFFFFFFFFFF:FFFFFFFFFFFFFFFF,F:FFFFFF:FFFFFFFF
FFFF:FFFFFFFFFFF:FFFFFFFFFFFFFFFF::FFF:FFF:FFFFFFFFFF:FFFFFFFFFFFFFF
@A00155:342:HHGFNDSXY:1:1631:23484:19711 1:N:0:GAACCTAG+TCCGCATA
GAGAGGGATGTGGAGGGCGAGAGAGAGCGACTTCTCTCGGGCCCACAGCCTTACAGCTGTGGAGCACG
GTATCCTCTGCCGAAACAGAGGTTGGACAAGACCGGAGGGGTCTCCTAGTTCCAAAGGAGATGTACTC
+
FFFFFFFFFF:FFFFFFFFFFFFFFFFFFFFFFFFFFFFFFFFFFFFFFFFFFFFFFFFFFFFFFFFF
FFFFFFFFFFFFFFFFFFFFFFFFFFFFFFFFFFFFFFFFFFF:FFFFFFFFFFFFFFFFFFFFFFFF
@A00155:342:HHGFNDSXY:1:1631:1452:33035 1:N:0:GAACCTAG+TCCGCATA
CCTCTGGCCTATGGTCATCTCAATGCCGAGAGGGATGTGGAGGGCGAGAGAGAGCGACTTCTCTCGGG
CCCACAGCCTTACAGCTGTGGAGCACGGTATCCTCTGCCGAAACAGAGGTTGGACAAGAC
+
FFFFFFFF:FFFFFFFFFFFFFFFFFFFFF:FFFFFFFFFFFFFFF:FFFFF:FFFFFFFFFF:FFFF
FFFFFFFFFFFFFFFFFFFFFFFFFFFFFFFFFFFFFFFFFFF:FFFFFFFF:FFFF:F
@A00155:342:HHGFNDSXY:1:1632:27317:9471 1:N:0:GAACCTAG+TCCGCATA
CACAGCCTTACAGCTGTGGAGCACGGTATCCTCTGCCGAAACAGAGGTTGGACAAGACCGGAGGGGTC
TCCTAGTTCCAAAGGAGATGTACTCCGGGCTTGTTCACGACCTACCGTGTAAGTCGTAGTCTAGTAGG
+

FFFFFFFFFFFFFFFFFFFFFFFFFFFFFFFFFFFFFFFFFFFFFFFFFFFFFFFFFFFFFFFFFFFF
FFFFFFFFFFFFFFFFFFFFFFFFFFFFFFFFFFFFFFFFFFFFFFFFFFFFFFFFFFFFFFFFFFFF
@A00155:342:HHGFNDSXY:1:1632:20735:26506 1:N:0:GAACCTAG+TCCGCATA
AGAGAGAGCGACTTCTCTCGGGCCCACAGCCTTACAGCTGTGGAGCACGGTATCCTCTGCCGAAACAG
AGGTTGGACAAGACCGGAGGGGTCTTCTAGTTCCAAAGGAGATGTACTCCGGGCTTGTTCACGACCTA
+
FFFFFFFFFFFFFFFFFFFFFFFFFFFFFFFFFFFFFFFFFFFFFFFFFFFFFFFFFFFFFFFFFFFF
FFFFFFFFFFFFFFFFFFFFFFFFFFFFFFFFFFFFFFFFFFFFFFFFFFFFFFFFFFFFFFFFFFF:
@A00155:342:HHGFNDSXY:1:1633:29351:18349 1:N:0:GAACCTAG+TCCGCATA
GGGCGAGAGAGAGCGACTTCTCTCGGGCCCACAGCCTTACAGCTGTGGAGCACGGTATCCTCTGCCGA
AACAGAGGTTGGACAAGACCGGAGGGGTCTCCTAGTTCCAAAGGAGATGTACTCCGGGCTTGTTCACG
+
FFFFFFFFFFFFFFFF:FFFFFFFFFF:FFFFFFFFFFFFFFFFFFFFFFFFFFFFFFFF:FFFFFFF
FFFFFFFFFFFFFFFFFFFFFFFFFFFFFFFFF:FFFFFFFFFFFFFFFFFFFFFF:FFFFFFFFFFF
@A00155:342:HHGFNDSXY:1:1633:22842:22169 1:N:0:GAACCTAG+TCCGCATA
CGAGAGAGAGCGACTTCTCTCGGGCCCACAGCCTTACAGCTGTGGAGCACGGTATCCTCTGCCGAAAC
AGAGGTTGGACAAGACCGGAGGGGTCTCCTAGTTCCAAAGGAGATGTACTCCGGGCTTGTTCACGACC
+
FFFFFFFFFFFFFFFFFFFFFFFFFFFFFFFFFFFFFFFFFFFFFFFFFFFFFFFFFFFFFFFFFFFF
FFFFFFFFFFFFF:FFFFFFFFFFFFFFFFFFFFFFFFFFFFFFFFFFFFFFFFFFFFFFFFFFFFFF
@A00155:342:HHGFNDSXY:1:1633:30942:24925 1:N:0:GAACCTAG+TCCGCATA
CTCAATGCCGAGAGGGATGTGGAGGGCGAGAGAGAGCGACTTCTCTCGGGCCCACAGCCTTACAGCTG
TGGAGCACGGTATCCTCTGCCGAAACAGAGGTTGGACAAGACCGGAGGGGTCTCCTGGTTCCAAAGGA
+
FFFFFFFFFFFF:FFFFFFFFFFFFFFFFFFFFFFFFFFFFFFFFFFFFFFFFFFFFFFFFFFFFFFF
FFFFFFFFFFFFFFFFFFFFFFFFFFFFFFFFFFFFFFFFFFFFFFFFFFFFFFFFFFFFFFFFFFFF
@A00155:342:HHGFNDSXY:1:1634:20835:1094 1:N:0:GAACCTAG+TCCGCATA
GACTTCTCTCGGGCCCACAGCCTTACAGCTGTGGAGCACGGTATCCTCTGCCGAAACAGAGGTTGGAC
AAGACCGGAGGGGTCTCCTGGTTCCAAAGGAGATGTACTCCGGGCTTGTTCACGACCTACCGTGTAAG
+
FFFFFFFFFFFFFFFFFFFFFFFFFFFFFFFFFFFFFFFFFFFFFFFFFFFF:FFFFFFFFFFFFFFF
FFFFFFFFFFFFFFFFFFFF:FFFFFFFFFFFFFFFFFFFFFFFFFFFFFFFFFFFFFFFFFFFFFF:
@A00155:342:HHGFNDSXY:1:1634:29451:10786 1:N:0:GAACCTAG+TCCGCATA
GAGCGACTTCTCTCGGGCCCACAGCCTTACAGCTGTGGAGCACGGTATCCTCTGCCGAAACAGAGGTT
GGACAAGACCGGAGGGGTCTCCTAGTTCCAAAGGAGATGTACTCCGGGCTTGTTCACGACCTACCGTG
+
FFFFFFFFFFFFFFFFFFFFFFFFFFFFFFFFFFFFFFFFFFFFFFFFFFFFFFFFFFFFFFFFF:FF
FFFFFFFFFFFFFFFFFFFFFFFFFFFFFFFFFFFFFFFFFFFFFFFFFFFFFFFFFFFFF:FFFFFF
@A00155:342:HHGFNDSXY:1:1634:3947:33348 1:N:0:GAACCTAG+TCCGCATA
GTCCACTTAAGGACTCACCTCTGGCCTATGGTCATCTCAATGCCGAGAGGGATGTGGAGGGCGAGAGA
GAGCGACTTCTCTCGGGCCCACAGCCTTACAGCTGTGGAGCACGGTATCCTCTGCCGAAACAGAGGTT
+
FFFFFFFFFFFFFFFFFFFFFFFFFFFFFFFFFFFFFFFFFF:FFFFFFFFFFFFF:FFFFFFFFFFF
FFFFFFFFFFFFFFFFFFFFFFFFFFFFFFFFFFFFFFFFFFFFFFFFFFFFFFFFFFFFFFFFFFFF
@A00155:342:HHGFNDSXY:1:1635:17788:13009 1:N:0:GAACCTAG+TCCGCATA
AAGGACTCACCTCTGGCCTATGGTCATCTCAATGCCGAGAGGGATGTAGAGGGCGAGAGAGAGCGACT
TCTCTCGGGCCCACAGCCTTACAGCTGTGGAGCACGGTATCCTCTGCCGAAACAGAGGTTGGACAAGA
+
FFFFFFFFFFFFFFFFFFFFFFFFFFFFFFFFFFFFFFFFFFF:FFFFFFFFFFFFFFFFFFFFFFFF
FFFFFFFFFFFFFFFFFFFFFFFFFFFFFFFFFFFFFFFFFFFFFFFF:FFFFFFFFFFFFFFFFFFF
@A00155:342:HHGFNDSXY:1:1635:11062:21997 1:N:0:GAACCTAG+TCCGCATA
GTCATCTCAATGCCGAGAGGGATGTGGAGGGCGAGAGAGAGCGACTTCTCTCGGGCCCACAGCCTTAC
AGCTGTGGAGCACGGTATCCTCTGCCGAAACAGAGGTTGGACAAGACCGGAGGGGTCTCCTAGTTCCA
+

FFFFFFFFFF:F:FFF:F::FF,FFFFFFFFFFFFFFFFFFFFFFF::FFFFFFFFFFFFFFFFFFFF
FFFFFFFFFFFFFFFF:FFFFFF:FFF,FFFFF:FF:FFFFFFFFFFFFFFFFFFFFFFFFFFFFFFF
@A00155:342:HHGFNDSXY:1:1635:31439:28980 1:N:0:GAACCTAG+TCCGCATA
AGAGCGACTTCTCTCGGGCCCACAGCCTTACAGCTGTGGAGCACGGTATCCTCTGCCGAAACAGAGGT
TGGACAAGACCGGAGGGGTCTCCTGGTTCCAAAGGAGATGTACTCCGGGCTTGTTCACGAC
+
FFFFFFFFFFFFFFFFFFFFFFFFFFFFFFFFFF:FFFFFFFFFFFFFFFFFFFFFFFFFFFFFFFFF
FFFFFFF:FFFFFFFFFFFFFFFFFFFFFFFFFFFFFFFFF:FFFFFFFFFFFFFFFFFFF
@A00155:342:HHGFNDSXY:1:1635:15691:36057 1:N:0:GAACCTAG+TCCGCATA
TGTGTCCACTTAAGGACTCACCTCTGGCCTATGGTCATCTCAATGCCGAGAGGGATGTGAAGGGCGAG
AGAGAGCGACTTCTCTCGGGCCCACAGCCTTACAGCTGTGGAGCACGGTATCCTCTGCCGAAACAGAG
+
FFFFFFFFFFFFFFFFFFFFFFFFFFFFFFFF:FFFFFFF,FF::F,FFFFFFF,FFFFFFFFFFFFF
FFFFFFFFFFFFFFFFFFFFFFFFFFF,F:FFFF,FFFFF:FFFFFFFF:F,F:F:FFFFFFFF::F:
@A00155:342:HHGFNDSXY:1:1636:20980:16031 1:N:0:GAACCTAG+TCCGCATA
AGACTCACCTCTGGCCTATGGTCATCTCAATGCCGAGAGGGATGTGGAGGGCGAGAGAGAGCGACTTC
TCTCGGGCCCACAGCCTTACAGCTGTGGAGCACGGTATCCTCTGCCGAAACAGAGGTTGGACAAGACC
+
FFFFFFFFFFFFFFFFFFFFFFFFFFFFFFFFFFFFFFFFFFFFFFFFFFFFFFFFFFFFFFFFF:FF
FFFFFFFFFFFFFFFFFFFFFFFFFFFFFFFFFFFFFFFFFFF:FFFFFFF:FFFFFFFFFFFFFFFF
@A00155:342:HHGFNDSXY:1:1636:2889:22310 1:N:0:GAACCTAG+TCCGCATA
TCCTCTGCCGAAACAGAGGTTGGACAAGACCGGAGGGGTCTCCTAGTTCCAAAGGAGATGTACTCCGG
GCTTGTTCACGACCTACCGTGTAAGTCGTAGTCTAGTAGGCTACCTGACGAGTCCTTTTAGGACGAA
+
FFFFFFFFFFFFFFFFFFFFFFFFFFFF:FFFFFFFFFFFFFFFFFFFFFFFFFFFFFFFFFFFFFFF
FFFFFFFFFFFFFFFFFFFFFFFFFFFFFFFFFFFFFFFFFFFFFFFFFFFFFFFFFFFFFFFFFFFF
@A00155:342:HHGFNDSXY:1:1636:24532:22748 1:N:0:GAACCTAG+TCCGCATA
GAGGGCGAGAGAGAGCGACTTCTCTCGGGCCCACAGCCTTACAGCTGTGGAGCACGGTATCCTCTGCC
GAAACAGAGGTTGGACAAGACCGGAGGGGTCTCCTAGTTCCAAAGGAGATGTACTCCGGGCTTGTTCA
+
FFFFFFFFFFFFFFFFFFFFFFFFFFFFFFFFFFFFFFFFFFFFFFFFFFFFFFFFFF:FFFFFFFFF
FFFFFFFFFFFFFFFFFFFFFFFFFFFFFFFFFFFFFFFFFFFFFFFFFFFFFFFFFFFFFFFFFFFF
@A00155:342:HHGFNDSXY:1:1636:22797:28260 1:N:0:GAACCTAG+TCCGCATA
ACCTCTGTTTCGGCAGAGGATACCGTGCTCCACAGCTGTAAGGCTGTGGGCCCGAGAGAAGTCGCTCT
CTCTCGCCCTCCACATCCCTCTCGGTATTGAGATGACCATAGGCCAGAGGTGAGTCCTTAAGTGGACA
+
FFFFFFFFFFFFFFFFFFFFFFFFFFFFFFFFFFFFFFFFFFFFFFFFFFFFFFFFFFFF:FFFFFFF
FFFFFFFFFFFFFFFFFFFFFFFFFFFFFFFFFFFFFFFFFFFFFFFFFFFFFFFFFFFFFFFFFFF:
@A00155:342:HHGFNDSXY:1:1637:22761:1861 1:N:0:GAACCTAG+TCCGCATA
TGTTTCGGCAGAGGATACCGTGCTCCACAGCTGTAAGGCTGTGGGCCCGAGAGAAGTCGCTCTCTCTC
GCCCTTCACATCCCTCTCGGCATTGAGATGACCATAGGCCAGAGGTGAGTCCTTAAGTGGACACAGCT
+
FFFFFFFFFFFFFFFFFFFFFFFFFFFFFFFFFFFFFFFFFFFFFFFFFFFFFFFFFFFFFFFFFFFF
FFFFFFFFFFFFFFFFFFFFFFFFFFFFFFFFFFFFFFFFFFFFFFFFFFFFFFFFFFFFFFFFFFFF
@A00155:342:HHGFNDSXY:1:1637:27606:4492 1:N:0:GAACCTAG+TCCGCATA
CCCACAGCCTTACAGCTGTGGAGCACGGTATCCTCTGCCGAAACAGAGGTTGGACAAGACCGGAGGGG
TCTCCTAGTTCCAAAGGAGATGTACTCCGGGCTTGTTCACGACCTACCGTGTAAGTCGTAGTCTAGTA
+
FFFFFFFFFFFFFFFFFFFFFFFFFFFFFFFFFFFFFFF:FFFFFFFFFFFFFFFFFFFFFFFFFFFF
FFFFFFFFFFFFFFFFFFFFFFFFFFFFFFFFFFFFFFFFFFFFF:FFF:FFFFFFFFFFFFFFFFFF
@A00155:342:HHGFNDSXY:1:1637:29731:13244 1:N:0:GAACCTAG+TCCGCATA
CTCAATGCCGAGAGGGATGTGGAGGGCGAGAGAGAGCGACTTCTCTCGGGCCCACAGCCTTACAGCTG
TGGAGCACGGTATCCTCTGCCGAAACAGAGGTTGGACAAGACCGGAGGGGTCTCCTAGTTCCAAAGGA
+

FFFFFFFFFFFFFFFFFFF:F:FFFFFFFFFFFFFFFFFFFFFFFFFFFFFFFFFFFFFFF,FF:FFF
FFFF:FFFFFF:FFFFFFFFFFFFFFFFFFFFFFF:FFFFFFFFFFFFFFFFFFFF:FFFFFFFFFFF
@A00155:342:HHGFNDSXY:1:1637:24641:14199 1:N:0:GAACCTAG+TCCGCATA
TGGCCTATGGTCATCTCAATGCCGAGAGGGATGTAGAGGGCGAGAGAGAGCGACTTCTCTCGGGCCCA
CAGCCTTACAGCTGTGGAGCACGGTATCCTCTGCCGAAACAGAGGTTGGACAAGACCGGAGGGGTCTC
+
FFFFFFFFFFFFFFFFFFFFFFFFFFFFFFFFFFFFFFFFFFFFFFFFFFFFFFFFFFFFFFFFFFFF
FFFFFFFFFFFFFFFFFFFFFFFFFFFFFFFFFFFFFFFFFFFFFFFFFFFFFFFFFFFFFFFFFFFF
@A00155:342:HHGFNDSXY:1:1637:8160:16877 1:N:0:GAACCTAG+TCCGCATA
GGATGTGGAGGGCGAGAGAGAGCGACTTCTCTCGGGCCCACAGCCTTACAGCTGTGGAGCACGGTATC
CTCTGCCGAAACAGAGGTTGGACAAGACCGGAGGGGTCTCCTAGTTCCAAAGGAGATGTACTCC
+
FFFFFFFFFFFFFFFFFFFFFFFFFFFFFFFFFFFFFFFFFFFFFFFFFFF:FFFFFFFFFF,FF
FFFFFFFFFFFFFFFFFFFFFFFFFFFFFFFFFFFFFFFFFFFFFFFFFFFF:FFFFFFFFF
@A00155:342:HHGFNDSXY:1:1637:8467:17033 1:N:0:GAACCTAG+TCCGCATA
GGATGTGGAGGGCGAGAGAGAGCGACTTCTCTCGGGCCCACAGCCTTACAGCTGTGGAGCACGGTATC
CTCTGCCGAAACAGAGGTTGGACAAGACCGGAGGGGTCTCCTAGTTCCAAAGGAGATGTACTCC
+
FF:FFFFFFFFFFFFFFFFFFFFFFFFFFFFFFFFFFFFFFFFF:FFFFFFFFFFFFFFFFFFFFFFF
FFFFFFFFFFFFFFFFFFFFFFFFFF,FFFFFFFFFFFFFFFFFFFFFFFFFFFFFFFFFFFFFFF
@A00155:342:HHGFNDSXY:1:1637:32750:18380 1:N:0:GAACCTAG+TCCGCATA
TGCCGAGAAGGATGTGGAGGGCGAGAGAGAGCGACTTCTCTCGGGCCCACAGCCTTACAGCTGTGGAG
CACGGTATCCTCTGCCGAAACAGAGGTTGGACAAGACCGGAGGGGTCTCCTAGTTCCAAAGGAGATGT
+
FFFFFFFFFFFFFFFFFFFFFFFFFFFFFFFFFFFFFFFFFFFFFFFFFFFFFFFFFFFFFFFFFFFF
FFFFFFFFFFFFFFFFFFFFFFFFFFFFFFFFFFFFFFFFFFFFFFFFFFFFFFFFFFFFFFFFFFFF
@A00155:342:HHGFNDSXY:1:1638:18656:18270 1:N:0:GAACCTAG+TCCGCATA
AGAGAGAGCGACTTCTCTCGGGCCCACAGCCTTACAGCTGTGGAGCACGGTATCCTCTGCCGAAACAG
AGGTTGGACAAGACCGGAGGGGTCTCCTAGTTCCAAAGGAGATGTACTCCGGGCTTGTTCACGACCTA
+
FFFFFFFFFFFFFFFFFFFFFFFFFFFFFFFFFFFFFFFFFFFFFFFFFFFFFFFFFFFFFFFFFFFF
FFFFFFFFFFF:FFFFFFFFFFFFFFFFFFFFFFFFFFFFFFFFFFFFFFFFFFFFFFFFFF:FFFFFF
@A00155:342:HHGFNDSXY:1:1638:17662:18584 1:N:0:GAACCTAG+TCCGCATA
AGAGAGAGCGACTTCTCTCGGGCCCACAGCCTTACAGCTGTGGAGCACGGTATCCTCTGCCGAAACAG
AGGTTGGACAAGACCGGAGGGGTCTCCTAGTTCCAAAGGAGATGTACTCCGGGCTTGTTCACGACCTA
+
FFFFFFFFFFFF:FFFFFFFFFFFFFFFFFFFFFFFFFFFFFFFFFFFFFF:FFFFFFFFFFFFFFFF
FFFFFFFFFFFFFFFFFFFFFFFFF:FFFFFFFFFFFFFF,FFFFFFFFFFFFF:FFFFFF:FFFFFFF
@A00155:342:HHGFNDSXY:1:1638:12020:19335 1:N:0:GAACCTAG+TCCGCATA
GGCGAGAGAGAGCGACTTCTCTCGGGCCCACAGCCTTACAGCTGTGGAGCACGGTATCCTCTGCCGAA
ACAGAGGTTGGACAAGACCGGAGGGGTCTCCTAGCTCCAAAGGAGATGTACTCCGGGCTTGTTCACGA
+
FFFFFFFFFFFFFFFFFFFFFFFFFFFFFFFFFFFFFFFFFFFFFFFFFFFFFFFFFFFFFFFFFFFF
FFFFFFFFFFFFFFFFFFFFFFFFFFFFFFFFFFFFFFFFFFF:FFFFFFFFFFFFFFFFFFFFFFFFF
@A00155:342:HHGFNDSXY:1:1639:3513:6010 1:N:0:GAACCTAG+TCCGCATA
TGGTCATCTTAATGCCGAGAGGGATGTGGAGGGCGAGAGAGAGCGACTTCTCTCGGGCCCACAGCCTT
ACAGCTGTGGAGCACGGTATCCTCTGCCGAAACAGAGGTTGGACAAGACCGGAGGGGTCTCCTAGTTC
+
FFFFFFFFFFFFFFFFFFFFFFFFFFF:,FFFFFFFFFFFFFFFFFF:FFFFFFFFFFFFFFFFFFFFF
FFFFFFFFFFFFFFFFFFFFFFFFFFFF,FFFFFFFFFFFFFF:FFFFFFFFFFFFFFFFFFFFFFFFFF
@A00155:342:HHGFNDSXY:1:1639:4616:16282 1:N:0:GAACCTAG+TCCGCATA
TTAAGGACTCACCTCTGGCCTATGGTCATCTCAATGCCGAGAGGGATGTGGAGGGCGAGAGAGAGCGA
CTTCTCTCGGGCCCACAGCCTTACAGCTGTGGAGCACGGTATCCTCTGCCGAAACAGAGG
+

FFFFFFFFFFF:FFFFFFFFFFFFFF:FFFFFFFFFFFFFFFFFFF,FFFFFFFFFFFFFFFFFFFFF
F:FFFFFFFFFFFFFFFFFF,FFFFFFFFFFFFFFFFFFFFFFFFFFFFFFFFFF:FFFFF
@A00155:342:HHGFNDSXY:1:1639:4851:16407 1:N:0:GAACCTAG+TCCGCATA
TTAAGGACTCACCTCTGGCCTATGGTCATCTCAATGCCGAGAGGGATGTGGGGGGCGAGAGAGAGCGA
CTTCTCTCGGGCCCACAGCCTTACAGCTGTGGAGCACGGTATCCTCTGCCGAAACAGAGG
+
FFFFFFFFFFFFFFFFFFFFFFFFFFFFFFFFFFFFFFFFFFFFFFFFFFFFFFFFFFFFFFF:FFFF
FFFFFFFFFFFFFFFFFFFFFFF,FFFFFFFFFFFFFFFFF:FFFFFFFFFFFFFFFFFFFF
@A00155:342:HHGFNDSXY:1:1639:28673:16517 1:N:0:GAACCTAG+TCCGCATA
GCGACTTCTCTCGGGCCCACAGCCTTACAGCTGTGGAGCACGGTATCCTCTGCCGAAACAGAGGTTGG
ACAAGACCGGAGGGGTCTCCTAGTTCCAAAGGAGATGTACTCCGGGCTTGTTCACGACCTACCGTGTA
+
FFFFFFFFFFFFFFFFFFFFFFFFFFFFFFFFFFFFFFFFFFFFFFFFFFFFFFFFFFFFFFFFFFFF
FFFFFFFFFFFFFFFFFFFFFFFFFFFFFFFFFFFFFFFFFFFFFFFFFFFFFFFFFFFFFFFFFFFF
@A00155:342:HHGFNDSXY:1:1639:17607:21339 1:N:0:GAACCTAG+TCCGCATA
TCTGTTTCGGCAGAGGATACCGTGCTCCACAGCTGTAAGGCTGTGGGCCCGAGAGAAGTCGCTCTCTC
TCGCCCTTCACATCCCTCTCGGCATTGAGATGACCATAGGCCAGAGGTGAGTCCTTAAGTGGACACAG
+
FFFFFFFFFFFFFFFFFFFFFFFFFFFFFFFFFFFFFFFFFFFFFFFFFFFFFFFFFFFFFFFFFFFF
FFFFFFFFFFFFFFFFFFFFFFFFF:FFFFFFFFFFFFFFFFF,FFFFFFFFFFFFFFFFFFFFFFFFF
@A00155:342:HHGFNDSXY:1:1639:8350:25692 1:N:0:GAACCTAG+TCCGCATA
AAGGACTCACCTCTGGCCTATGGTCATCTCAATGCCGAGAGGGATGTAGAGGGCGAGAGAGAGCGACT
TCTCTCGGGCCCACAGCCTTACAGCTGTGGAGCACGGTATCCTCTGCCGAAACAGAGGTTGGACAAGA
+
FFFFFFFFFFFFFFFFFFFFFFFFFFFFFFFFFFFFFFFFFFFFFFFFFFFFFFFFFFFFFFFFFFFF
FFFFFFFFFFFFFFFFFFFFFFFFFFFFFFFFFFFFFFFFFFFFFFFFFFFFFFFFFFFFFFFFFFFF
@A00155:342:HHGFNDSXY:1:1639:29523:28635 1:N:0:GAACCTAG+TCCGCATA
TCTCTCGGGCCCACAGCCTTACAGCTGTGGAGCACGGTATCCTCTGCCGAAACAGAGGTTGGACAAGA
CCGGAGGGGTCTCCTAGTTCCAAAGGAGATGTACTCCGGGCTTGTTCACGACCTACCGTGTAAGTCGT
+
FFF,FFFFFFFFFFFFFFFFFFFFFFFFF,F:,,FFFFFFFF:FFFFFFFFFFFFFFFFFFFFFFFFFF
FFFFFFFFFFFFFFFFFFFFFFFFFFFFFFFFFFFFFFFF:FFF,FFFFFFFFFFFFFFFFFFFFFFFF
@A00155:342:HHGFNDSXY:1:1639:6135:29778 1:N:0:GAACCTAG+TCCGCATA
TTAAGGACTCACCTCTGGCCTATGGTCATCTCAATGCCGAGAGGGATGTGGAGGGCGAGAGAGAGCGA
CTTCTCTCGGGCCCACAGCCTTACAGCTGTGGAGCACGGTATCCTCTGCCGAAACAGAGG
+
FFFFFFFFFFFFFFFFFF:FFFFFFFFFFFFFFFFFFFFFFFFFFFFFF:FFFFFFFFFFFFFFFFFFF
FFFFFFFFFFFFFFFFFFFFFFFFFFFFFFFFFFFFFFFF,FFFFFFFFFFFFFFFFFFF
@A00155:342:HHGFNDSXY:1:1640:10583:3286 1:N:0:GAACCTAG+TCCGCATA
ATCTCAATGCCGAGAGGGATGTAGAGGGCGAGAGAGAGCGACTTCTCTCGGGCCCACAGCCTTACAGC
TGTGGAGCACGGTATCCTCTGCCGAAACAGAGGTTGGACAAGACCGGAGGGGTCTCCTAGTTCCAAAG
+
FFFFFFFFFFFFFFFFFFFFFFFFFFFFFFFFFFFFFFFFFFFFFFFFFFFFFFFFFFFFFFFFFFFF
FFFFFFFFFFFFFFFFFFFFFFFFFFFFFFFFFFFFFFFFFFFFFFFFFFFFFFFFFFFFFFFFFFFF
@A00155:342:HHGFNDSXY:1:1640:19623:10426 1:N:0:GAACCTAG+TCCGCATA
AGAGAGAGCGACTTCTCTCGGGCCCACAGCCTTACAGCTGTGGAGCACGGTATCCTCTGCCGAAACAG
AGGTTGGACAAGACCGGAGGGGTCTCCTAGTTCCAAAGGAGATGTACTCCGGGCTTGTTCACGACCTA
+
FFFFFFFFFFFFFFFFFFFFFFFFFFFFFFFFFFFFFFFFFFF:FFFFFFFFFFFFFFF::FFFFFFFF
FFFFF:FFFFFF:F:FFFF:FFFFFFFF:FFF::FFFFF:FFFFF:FFFF:F:FF,FFFFFFFFFFFF
@A00155:342:HHGFNDSXY:1:1640:25889:15107 1:N:0:GAACCTAG+TCCGCATA
GCTGTGTCCACTTAAGGACTCACCTCTGGCCTATGGTCATCTCAATGCCGAGAGGGATGTAGAGGGCG
AGAGAGAGCGACTTCTCTCGGGCCCACAGCCTTACAGCTGTGGAGCACGGTATCCTCTGCCGAAACAG
+

FFFFFFFFFFFFFFFF:FFFFFFFFFFFFFFFFFFFFFFFFFFFFFFFFFFFFFFFFFFFFFFFFFFF
FFFFFFFFFFFFFFFFFFFFFFFFFFFFFFFFFFFFFFFFFFFFFFFFFFFFFFFFFFFFFFFFFFFF
@A00155:342:HHGFNDSXY:1:1640:3721:28510 1:N:0:GAACCTAG+TCCGCATA
TGGTCATCTCAATGCCGAGAGGGATGTGGAGGGCGAGAGAGAGCGACTTCTCTCGGGCCCACAGCCTT
ACAGCTGTGGAGCACGGTATCCTCTGCCGAAACAGAGGTTGGACAAGACCGGAGGGGTC
+
FFFFFFFFFFFFFFFFFFF:FFF:FFFFFFFFFFFFFFFFFFFFFFF:FFFFFFFFFFFFFFFFFFFF
FFFFFFFFFFFFFFFFFF,FFFFFFFFFFFFFFFFFFFFFFFFFFFFFFFFFFFFFFFF
@A00155:342:HHGFNDSXY:1:1640:6903:32174 1:N:0:GAACCTAG+TCCGCATA
ACTTCTCTCGGGCCCACAGCCTTACAGCTGTGGAGCACGGTATCCTCTGCCGAAACAGAGGTTGGACA
AGACCGGAGGGGTCTCCTGGTTCCAAAGGAGATGTACTCCGGGCTTGTTCACGACCTACCGTGTAAG
+
FFFFFFFFFFFFFFFFFFFFFFFFFFFFFFFFFFFFFFFFF,FFFFFFFFFFFFFFFFFFFFFFFFFF
FFFFFFFFFFFFFFFFFFFFFFFFFFFFFFFFFFFFFFFFFFFFFFFFFFFFFFFFFFFFF:FF:FF
@A00155:342:HHGFNDSXY:1:1641:7392:4335 1:N:0:GAACCTAG+TCCGCATA
CTCTGCCGAAACAGAGGTTGGACAAGACCGGAGGGGTCTCCTAGTTCCAAAGGAGATGTACTCCGGGC
TTGTTCACGACCTACCGTGTAAGTCGTAGTCTAGTAGGCTACCTGACGAGTCCTTTTTAGGACGAAAC
+
FFFFFFFFFFFFFFFFFFFFFFFFFFFF:FFFFFFFFFFFFFFFFFFFFFFFFFFFFFFFFFFFFFFF
FFFFFFFFFFFFFFFFFFFFFFFFFFFFFFFFFFFFFFFF:FFFFFFFFFFFFFFFFFFFFFFFFFFF
@A00155:342:HHGFNDSXY:1:1641:3676:33379 1:N:0:GAACCTAG+TCCGCATA
CTTCTCTCGGGCCCACAGCCTTACAGCTGTGGAGCACGGTATCCTCTGCCGAAACAGAGGTTGGACAA
GACCGGAGGGGTCTCCTAGTTCCAAAGGAGATGTACTCCGGGCTTGTTCACGACCTACCGTGTAAGTC
+
FFFFFFFFFFFFFFFFFFFFFFFFFFFFFFFFFFFFFFFF:FFFFFFFFFFFFFFFFFFFFFFFFFFF
FFFFFFFFFFFFFFFFFFFFFFFFFFFFFFFFFFFFFFFFFFFFFFFFFFFFFFFFFFFFFFFF,FFF
@A00155:342:HHGFNDSXY:1:1642:7292:9956 1:N:0:GAACCTAG+TCCGCATA
TTACAGCTGTGGAGCACGGTATCCTCTGCCGAAACAGAGGTTGGACAAGACCGGAGGGGTCTCCTAGT
TCCAAAGGAGATGTACTCCGGGCTTGTTCACGACCTACCGTGTAAGTCGTAGTCTAGTAGGCTACCTG
+
FFFFFFFF:FFFFFFFFFFFFFFFFFFF,FFFFFFFFFF:FFFFFFFFFFFFFFFFFFFFFFFFFFFF
FFFFFFFFFFFFFFFFFFFFFF:F:FFFFFFFFFFFFFFFFFFFFFFFFFFFFFFFFFF:FFFFFFFF
@A00155:342:HHGFNDSXY:1:1642:10393:11663 1:N:0:GAACCTAG+TCCGCATA
TCTCTCGGGCCCACAGCCTTACAGCTGTGGAGCACGGTATCCTCTGCCGAAACAGAGGTTGGACAAGA
CCGGAGGGGTCTCCTAGTTCCAAAGGAGATGTACTCCGGGCTTGTTCACGACCTACCGTGTAAGTCGT
+
FFFFFFFFFFFFFFFFFFFFFFFFFFFFFFFFFFFFFFFFFFFFFFFFFFFFFFFFFFFFFFFFFFF:
FFFFFFFFFFFFFFFFFFFFFFFFFFFFFFFFFFFFFFFFFFFFFFFFFFFFFFFFFFFFFFFFFFFF
@A00155:342:HHGFNDSXY:1:1642:16604:12524 1:N:0:GAACCTAG+TCCGCATA
CTTACAGCTGTGGAGCACGGTATCCTCTGCCGAAACAGAGGTTGGACAAGACCGGAGGGGTCTCCTAG
TTCCAAAGGAGATGTACTCCGGGCTTGTTCACGACCTACCGTGTAAGTCGTAGTCTAGTAGGCTACCT
+
FFFFFFFFFFFFFFFFFFFFFFFFFFFFFFFFFFFFFFFFFFFFFFFFFFFFFFFFFFFFFFFFF,FF
FFFFFFFFFFFF:FFFFFFFFFFFFFFFFFFFFFFFFFFFFFFFFFFFFFFFFFFFFFFFFFFFFFF
@A00155:342:HHGFNDSXY:1:1642:13105:14043 1:N:0:GAACCTAG+TCCGCATA
GAGAGGGATGTGGAGGGCGAGAGAGAGCGACTTCTCTCGGGCCCACAGCCTTACAGCTGTGGAGCACG
GTATCCTCTGCCGAAACAGAGGTTGGACAAGACCGGAGGGGTCTCCTAGTTCCAAAGGAGATGTACTC
+
FFFFFFF:FFFFFFFFFFFFFFFFFFFFFFFFFFFFFFFFFFFFFFFFFFF:FFFFFFFF
FFFFFFFFF::FF:FFFFFF:FFFFFFFF:FFFFFFFFFFFFFFFFFFFFFFFFFFFFFFFFFFFFF
@A00155:342:HHGFNDSXY:1:1642:4426:23062 1:N:0:GAACCTAG+TCCGCATA
AGAAGTCGCTCTCTCTCGCCCTCCACATCCCTCTCGGCATTGAGATGACCATAGGCCAGAGGTGAGTC
CTTAAGTGGACACAGCTGATCTAAGGCGGTGTGGCGGGGCATGGGTTTGAACCCCCATGACGGTCGGA
+

FFFFFFFFFFFFFFFFFFFFFFFFFFFFFFFFFFFFFFFFFFFFFFFFFFFFFFFFFFFFFFFFFFFF
FFFFFFFFFFFFFFFFFF:FFFFFFFFFFFFFFFFFFFFFFFFFFFFFFFFFFFFFFFFFFFFFFFFF
@A00155:342:HHGFNDSXY:1:1642:22923:34178 1:N:0:GAACCTAG+TCCGCATA
GGATGTAGAGGGCGAGAGAGAGCGACTTCTCTCGGGCCCACAGCCTTACAGCTGTGGAGCACGGTATC
CTCTGCCGAAACAGAGGTTGGACAAGACCGGAGGGGTCTCCTAGTTCCAAAGGAGATGTACTCCGGGC
+
FFFFFFFFFFFFFFFFFFFFFFFFFF:FFFFF:FFFFFFFFFFFFFFFFFFFFFFFFFFFFFFFFFFF
FFFFFFFFFFFFFFFFFFFFFFFFFFFFFFFFFFFFFFFFFFFFFFF:FFFFFFFFFFFFFFFFFFF:
@A00155:342:HHGFNDSXY:1:1643:5801:3834 1:N:0:GAACCTAG+TCCGCATA
GAGCGACTTCTCTCGGGCCCACAGCCTTACAGCTGTGGAGCACGGTATCCTCTGCCGAAACAGAGGTT
GGACAAGACCGGAGGGGTCTCCTAGTTCCAAAGGAGATGTACTCCGGGCTTGTTCACGACCTACCGTG
+
FFFFFFFFFFFFFFFFFFFFFFFFFFFFFFFFFFFFFFFFFFFFFFFFFFFFFFFFFFFFFFFFFFFF
FFFFFFFFFFFFFFFFFFFFFFFFFFFFFFFFFFFFFFFFFFFFFFFFFFFFFFFFFFFFFFFFFFF:
@A00155:342:HHGFNDSXY:1:1643:32154:12680 1:N:0:GAACCTAG+TCCGCATA
AGGGATGTGGAGGGCGAGAGAGAGCGACTTCTCTCGGGCCCACAGCCTTACAGCTGTGGAGCACGGTA
TCCTCTGCCGAAACAGAGGTTGGACAAGACCGGAGGGGTCTCCTGGTTCCAAAGGAGATGTACTCCGG
+
FFFFFFFFFFFFFFFFFFFFFFFFFFFFFFFFFFFFFFFFFFFFFFF,F,FFFFFFFFFFFF:FFFFF
FFFFFFFFFFFFFFFFFFF:FFFFFFFFFFFFFFFFFFFFFFFFFFFFFFFFFFFFFFFFF:FFFFFF
@A00155:342:HHGFNDSXY:1:1643:14669:32002 1:N:0:GAACCTAG+TCCGCATA
GAGCGACTTCTCTCGGGCCCACAGCCTTACAGCTGTGGAGCACGGTATCCTCTGCCGAAACAGAGGTT
GGACAAGACCGGAGGGGTCTCCTAGTTCCAAAGGAGATGTACTCCGGGCTTGTTCACGACCTACCGTG
+
FFFFFFFFFFFFFFFFFFFFFFFFFFFFFFFFFFFFFFFFFFFFFFFFFFFFFFFFFFFFFFFFFFFF
FFFFFFFFFFFFFFFFFFFFFFFFFFFFFFFFFFFFFFFFFFFFFFFFFFFFFFFFFFFFFFFFFFFF
@A00155:342:HHGFNDSXY:1:1644:8278:7623 1:N:0:GAACCTAG+TCCGCATA
TGTAGAGGGCGAGAGAGAGCGACTTCTCTCGGGCCCACAGCCTTACAGCTGTGGAGCACGGTATCCTC
TGCCGAAACAGAGGTTGGACAAGACCGGAGGGGTCTCCTAGTTCCAAAGGAGATGTACTCCGGG
+
FFFFFFFFFFFFFFFFFFFFFFFFFFFFFFFF:FFFFFF:FFFFFFFFFFFFFFFFFFFFFFFFFFFF
FFFFFFFFFFFFFFFFFF:FFFFFFFFFFFFFFFFFFFFFFFFFFFFFFFFFFFFFFFFFFFFF
@A00155:342:HHGFNDSXY:1:1644:18439:12759 1:N:0:GAACCTAG+TCCGCATA
AAGGACTCTCCTCTGGCCTATGGTCATCTCAATGCCGAGAGGGATGTGGAGGGCGAGAGAGAGCGACT
TCTCTCGGGCCCACAGCCTTACAGCTGTGGAGCACGGTATCCTCTGCCGAAACAGAGGTTGGACAAGA
+
FFFFFFFFFFFFFFFFFFFFFFFFFFFFFFFFFFFFFFFFFFFFFFFFFFFFFFFFFFFFFFFFFFFF
FFFFFFFFFFFFFFFFFFFFFFFFFFFFFFFFFFFFF:FFFFFFFFFFFF:FFFFFFFFFFFFFFFFF
@A00155:342:HHGFNDSXY:1:1644:27832:20509 1:N:0:GAACCTAG+TCCGCATA
GAGCGACTTCTCTCGGGCCCACAGCCTTACAGCTGTGGAGCACGGTATCCTCTGCCGAAACAGAGGTT
GGACAAGACCGGAGGGGTCTCCTAGTTCCAAAAGAGATGTACTCCGGGCTTGTTCACGACCTACCGTG
+
FFFFFFFFFFFFFFFFFFFFFFFFFFFFFFFFFFFFFFFFFF:FFFFFFFFFFFFFFFFFFFFFFFFF
FFFFFFFFFFFFFFFFFFFFFFFFFFFFFFFFFFFFFFFFFFFFFFFFFFFFFFFFFFFFFFFFFFFF
@A00155:342:HHGFNDSXY:1:1644:3712:33379 1:N:0:GAACCTAG+TCCGCATA
GAGCGACATCTCTCGGGCCCACAGCCTTACAGCTGTGGAGCACGGTATCCTCTGCCGAAACAGAGGTT
GGACAAGACCGGAGGGGTCTCCTAGTTCCAAAGGAGATGTACTCCGGGCTTGTTCACGACCTACCGTG
+
FFFFFFFFFFFFFFFFFFFFFFF,FFFFFFFF:FFFFFFFFFFFFF:FFFFFFFFFFFFFFFFFFF:F
FFFFFFFFFFFFFFFFFFFFFFFFFFFFFFFFFFFFFFFFFFFFFFFFFFFFFFFFFFFFFFFFFFFF
@A00155:342:HHGFNDSXY:1:1644:10854:36417 1:N:0:GAACCTAG+TCCGCATA
GTCATCTCAATGCCGAGAGGGATGTGGAGGGCGAGAGAGAGCGACTTCTCTCGGGCCCACAGCCTTAC
AGCTGTGGAGCACGGTATCCTCTGCCGAAACAGAGGTTGGACAAGACCGGAGGGGTCTCCTAGTTCCA
+

```
FFFFFFFFFFFFFFFFFFFFFFFFFFFFFFFFFFFFFFFFFFFFFFFFFFFFFFFFFFFFFFFFFFFF
FFFFFFFFFFFFFFFFFFFFFFFFFFFFFFFFFFFFFFFFFFFFFFF:FFFFFFFFFFF:FFFFFFFF
@A00155:342:HHGFNDSXY:1:1645:6271:5619 1:N:0:GAACCTAG+TCCGCATA
TAAGTGGACACAGCTGATCTAAGGCGGTGTGGCGGGGCATGGGTTTGAACCCCCATGACGGTCGGAGT
CTAGTAGGCTCCCTGATGAGTCCGTTCCAAGGACGAAACCGTCGTTACTCTTTGAGTTGTGTAAGTTT
+
FFFFFFFFFFFFFFFFFFFFFF,FFFFF,FFFFFFFFFFFFFFFFFFFFF:FFFFFFFFFFFFFFFFF
FFFFFFFFFFFFFFFFFFFFFFFFFFFFFFFFFFFFFFFFFFFFFFFFFFF,FFFFFFFFFFFFFFFF
@A00155:342:HHGFNDSXY:1:1645:12048:16971 1:N:0:GAACCTAG+TCCGCATA
GGGCGAGAGAGAGCGACTTCTCTCGGGCCCACAGCCTTACAGCTGTGGAGCACGGTATCCTCTGCCGA
AACAGAGGTTGGACAAGACCGGAGGGGTCTCCTAGTTCCAAAGGAGATGTACTCCGGGCTTGTTCACG
+
F:FF,F,FFFFFFFFFFFFFFFFF,FFFFFFFFFFF,FF:F::FFF:FFFFFFFFFFFFFF:FFFFFF
FFFFFFFFFF:FFFFFFFFFFFFFF:FFFFFFF,FFFF:FFFFFFFFFFFFFFFF:F:FFF:,FFFFF
@A00155:342:HHGFNDSXY:1:1645:18078:24471 1:N:0:GAACCTAG+TCCGCATA
GTCATCTCAATGCCGAGAGGGATGTGGAGGGCGAGAGAGAGCGACTTCTCTCGGGCCCACAGCCTTAC
AGCTGTGGAGCACGGTATCCTCTGCCGAAACAGAGGTTGGACAAGACCGGAGGGGTCTCCTGGTTCCA
+
FFFFFFFFFFFFFFFFFFFFFFFFFFFFFFFFFFFFFFFFFFFFFFFFFFFFFFFFFFFFFFFFFFFF
FFFFFFFFFFFFFFFFFFFFFFFFFFFFFFFFFFFFFFFFFFFFFFFFFFF:FFFFFFFFFFFFFF:F
@A00155:342:HHGFNDSXY:1:1645:10140:26663 1:N:0:GAACCTAG+TCCGCATA
TCTTGTCCAACCTCTGTTTCGGCAGAGGATACCGTGCTCCACAGCTGTAAGGCTGTGGGCCCGAGAGA
AGTCGCTCTCTCTCGCCCTTCACATCCCTCTCGGCATTGAGATGACCATAGGCCAGAGGTGAGTCCTT
+
FFFFFFFFFFFFFFFFFFFFFFFFFFFFFFFFFFFFFFFFFFFFFFFFFFFFFFFFFFFFFFFFFFFF
FFFFFFFFFFFFFFFFFFFFFFFFFFFFFFFFFFFFFFFFFFFFFFFFFFFFFFFFFFFFFFFFFFFF
@A00155:342:HHGFNDSXY:1:1646:11605:2895 1:N:0:GAACCTAG+TCCGCATA
GATGTAGAGGGCGAGAGAGAGCGACTTCTCTCGGGCCCACAGCCTTACAGCTGTGGAGCACGGTATCC
TCTGCCGAAACAGAGGTTGGACAAGACCGGAGGGGTCTCCTAGTTCCAAAGGAGATGTACTCCGGGCT
+
FFFFFFFFFFFFFFFFFFFFFFFFFFFFFFFFFFFFFFFFFFFFFFFFFFFFFFFFFFFFFFFFFFFF
FFFFFFFFFFFFFFFFFFFFFFFFFFFFFFFFFFFFFFFFFFFFFFFFFFFFFFF:FFFFFFFFFFFF
@A00155:342:HHGFNDSXY:1:1646:24957:9142 1:N:0:GAACCTAG+TCCGCATA
CTCGGGCCCACAGCCTTACAGCTGTGGAGCACGGTATCCTCTGCCGAAACAGAGGTTGGACAAGACCG
GAGGGGTCTCCTAGTTCCAAAGGAGATGTACTCCGGGCTTGTTCACGACCTACCGTGTAAGTCGTAGT
+
FFFFFFFFFFFFFFFFFFFFFFFFFFFFFFFFFFFFFFFFFFFFFFFFFFFFFFFFFFFFFFFFFFFF
FFFFFFFFFFFFFFFFFFFFFFFFFFFFFFFFFFFFFFFFFFFFFFFFFFF:FFFFF:FFFFFFFFFF
@A00155:342:HHGFNDSXY:1:1646:19135:19946 1:N:0:GAACCTAG+TCCGCATA
GGATGTAGAGGGCGAGAGAGAGCGACTTCTCTCGGGCCCACAGCCTTACAGCTGTGGAGCACGGTATC
CTCTGCCGAAACAGAGGTTGGACAAGACCGGAGGGGTCTCCTAGTTCCAAAGGAGATGTACTCC
+
FFFFFFFFFFFFFFFFFFFFFFFFFFFFFFFFFFFFFFFFFFFFFFFFFFFFFFFFFFFFFFFFFFFF
FFFFFFFFFFFFFFFFFFFFFFFFFFFFFFFFFFFFFFFFFFFFFFFFFFFFFFFFFFFFFFFF
@A00155:342:HHGFNDSXY:1:1646:12635:23625 1:N:0:GAACCTAG+TCCGCATA
GGATGTGGAGGGCGAGAGAGAGCGACTTCTCTCGGGCCCACAGCCTTACAGCTGTGGAGCACGGTATC
CTCTGCCGAAACAGAGGTTGGACAAGACCGGAGGGGTCTCCTAGTTCCAAAGGAGATGTACTCC
+
FFFFFFFFFFFFFFFFFFFFFFFFFFFFFFFFFFFFFFFFFFFFFFFFFFFFFFFFFFFFFFFFFFFF
FFFFFFFFFFFFFF:FFFFFFFFFFFFFFFFFFFFFFFFFFFFFFFFFFFFFFFFFFFFFFFFF
@A00155:342:HHGFNDSXY:1:1646:8404:33959 1:N:0:GAACCTAG+TCCGCATA
CATCTCAATGCCGAGAGGGATGTGGAGGGCGAGAGAGAGCGACTTCTCTCGGGCCCACAGCCTTACAG
CTGTGGAGCACGGTATCCTCTGCCGAAACAGAGGTTGGACAAGACCGGAGGGGTCTCCTAGTTCCAAA
+
```

```
FFFFFFFFFFFFFFFFFFFFFFFFFFFFFFFF:FFFFFFFFFFFFFFFFFFFFFFFFFFFFFFFFFF
FFFFFFFFFFFFFFFFFFFFFFFFFFFFFFFFFFFF:FFFFFFFFFFFFFFFFFFFFFFFFFFFFFF
@A00155:342:HHGFNDSXY:1:1647:32886:1924 1:N:0:GAACCTAG+TCCGCATA
GGATGTGGAGGGCGAGAGAGAGCGACTTCTCTCGGGCCCACAGCCTTACAGCTGTGGAGCACGGCATC
CTCTGCCGAAACAGAGGTTGGACAAGACCGGAGGGGTCTCCTAGTTCCAAAGGAGATGTACTCC
+
FFF,FFFFFFFFFFFFFFFFFFFFFFFFFFFFFFFFFFFFFFFFFFFFFFF:FFFFFFFFFFFFFFF
FFFFFFFFFFFFFFFFFFFFFFFFFFFFFFFFFFFFFFFFFFFFFFFFFFFFFFFF:FFFFFFF
@A00155:342:HHGFNDSXY:1:1647:20130:9267 1:N:0:GAACCTAG+TCCGCATA
CAGCCTTACAGCTGTGGAGCACGGTATCCTCTGCCGAAACAGAGGTTGGACAAGACCGGAGGGGTCTC
CTAGTTCCAAAGGAGATGTACTCCGGGCTTGTTCACGACCTACCGTGTAAGTCGTAGTCTAGTAGGCT
+
FFFFFFFFFFFFFFFFFFFFFFFFFFFFFFFFFFFFFFFFFFFFFFFFFFFFFFFFFFFFFFFFFFF
FFFFFFFFFFFFFFFFFFFFFFFFFFFFFFFFFFFFFFFFFFFFFFFFFFFFFFFFFFFFFFFFFFF
@A00155:342:HHGFNDSXY:1:1647:3224:33724 1:N:0:GAACCTAG+TCCGCATA
TGTTTCGGCAGAGGATACCGTGCTCCACAGCTGTAAGGCTGTGGGCCCGAGAGAAGTCGCTCTCTCTC
GCCCTTCACATCCCTCTCGGCATTGAGATGACCATAGGCCAGAGGTGAGTCCTTAAGTGGACACAGCT
+
FFFFFFFFFFFFFFFFFFFFFFFFFFFFFFFFFFF:FF:FFFFF:FF,FFFFFFFFFFFFFFFFFFF
F:FFFFFFFFFFFFFF:FFFFFFFFFFFFFFFFFFFFFFFFFFFFF,FFFFFFFFFFFFFFFFFFFF
@A00155:342:HHGFNDSXY:1:1647:9462:35133 1:N:0:GAACCTAG+TCCGCATA
AGAGAGAGCGACTTCTCTCGGGCCCACAGCCTTACAGCTGTGGAGCACGGTATCCTCTGCCGAAACAG
AGGTTGGACAAGACCGGAGGGGTCTCCTAGTTCCAAAGGAGATGTACTCCGGGCTTGTTCACGACCTA
+
FFFFFFFFFFFFFFFFFFFFFFFFFFFFFFFFFFFFFFFFFFFFFFFFFFFFFFFFFFFFFFFFFFF
FFFFFFFFFFFFFFFFFFFFFFFFFFFFFFFFFFFFFFFFFFFFFFFFFFFFFFFFFFFFFFFFFF:
@A00155:342:HHGFNDSXY:1:1648:29206:31563 1:N:0:GAACCTAG+TCCGCATA
CTTCTCTCGGGCCCACAGCCTTACAGCTGTGGAGCACGGTATCCTCTGCCGAAACAGAGGTTGGACAA
GACCGGAGGGGTCTCCTAGTTCCAAAGGAGATGTACTCCGGGCTTGTTCACGACCTACCGTGTAAGTC
+
FFFFFFFFFFFFFFFFFFFFFFFFFFFFFFFFFFFFFFFFFFFFFFFFFFFFFFFFFFFFFFFFFFF
FFFFFFFFFFFFFFFFFFFFFFFFFFFFFFFFFFFFFFFFFFFFFFFFFFFFFFFF:FFFFFFFFFF
@A00155:342:HHGFNDSXY:1:1648:24695:32299 1:N:0:GAACCTAG+TCCGCATA
AGGGCGAGAGAGAGCGACTTCTCTCGGGCCCACAGCCTTACAGCTGTGGAGCACGGTATCCTCTGCCG
AAACAGAGGTTGGACAAGACCGGAGGGGTCTCCTAGTTCCAAAGGAGATGTACTCCGGGCTTGTT
+
FFFFFFFFFFFFFFFFFFFFFFF:FFFFFFFFFFFFFFFFFFFFFFFFFFFFFFFFFFFFFFFFFFF
FFFFFFFFFFFFFFFFFFFFFFFFFFFFFFFFFFFFFFFFFFFFFFFFFFFFFFFFFFFFFFFFF
@A00155:342:HHGFNDSXY:1:1648:7627:34366 1:N:0:GAACCTAG+TCCGCATA
TTCTCTCGGGCCCACAGCCTTACAGCTGTGGAGCACGGTATCCTCTGCCGAAACAGAGGTTGGACAAG
ACCGGAGGGGTCTCCTAGTTCCAAAGGAGATGTACTCCGGGCTTGTTCACGACCTACCGTGTAAGTCG
+
FFFFFFFFFFFFFFFFFFFFFFFFFFFFFFFFFFFFFFFFFFFFFFFFFFFFFFFFFFFFFFFFFFF
FFFFFFFFFFFFFFFFFFFFFFFFFFFFFFFFFFFFF:FFFFFFFFFFFFFFFFFFFFFFFFFFFFF
@A00155:342:HHGFNDSXY:1:1648:17653:34632 1:N:0:GAACCTAG+TCCGCATA
TTCTCTCGGGCCCACAGCCTTACAGCTGTGGAGCACGGTATCCTCTGCCGAAACAGAGGTTGGACAAG
ACCGGAGGGGTCTCCTAGTTCCAAAGGAGATGTACTCCGGGCTTGTTCACGACCTACCGTGTAAGTCG
+
:FFFFFFFFFFFFFFFFFFFFFFFFFFFFFFFFFFFFFFFFFFFFFFFFFFFFFFFFFFFFFFFFFF
FFFFFFFFFFFFFFFF:FFFFFFFFFFFFFFFFFFFFFFFFFFFFFFFFFFFFFFFFFFFFFFFFFF
@A00155:342:HHGFNDSXY:1:1649:28528:19993 1:N:0:GAACCTAG+TCCGCATA
GCGACTTCTCTCGGGCCCACAGCCTTACAGCTGTGGAGCACGGTATCCTCTGCCGAAACAGAGGTTGG
ACAAGACCGGAGGGGTCTCCTGGTTCCAAAGGAGATGTACTCCGGGCTTGTTCACGACCTACCGTGTA
+
```

FFFFFFFFFFFFFFFFFFFFFFFFFFFFFFFFFFFFFFFFFFFFFFFFFFFFFFFFFFFFFFFFFFFF
FFFFFFFFFFFFFFFFFFFFFFFFFFFFFFFFFFFFFFFFFFFFFFFFFFFFFFFFFFFFFFF:FFFF
@A00155:342:HHGFNDSXY:1:1649:8703:21261 1:N:0:GAACCTAG+TCCGCATA
GGGCGAGAGAGAGCGACTTCTCTCGGGCCCACAGCCTTACAGCTGTGGAGCACGGTATCCTCTGCCGA
AACAGAGGTTGGACAAGACCGGAGGGGTCTCCTAGTTCCAAAGGAGATGTACTCCGGGCTTGTTCACG
+
FFFFFFFFFFFFFFFFFFFFFFFFFFFFFFFFFFFFFFFFFFFFFFFFFFFFFFFFFFFFFFFFFFFF
FFFFFFFFFFFFFFFF:FFFFFFFFFFFFFFFFFFFFFFFFFFFFFFFFFFFFFFFFFFFFFFFFFFF
@A00155:342:HHGFNDSXY:1:1649:29939:24471 1:N:0:GAACCTAG+TCCGCATA
GTCCACTTAAGGACTCACCTCTGGCTTATGGTCATCTCAATGCCGAGAGGGATGTGGAGGGCGAGAGA
GAGCGACTTCTCTCGGGCCCACAGCCTTACAGCTGTGGAGCACGGTATCCTCTGCCGAAACAGAGGTT
+
FFFFFFFFFFFFFFFFFFFFFFFFFFFFFFFFFFFFFFFFFFFFFFF:FFFFFFFFFFFFFFF:FFFFF
FFFFFFFFFFFFFFFFFFFFFFFFFFF:FFFFFFFFFFFFFFFFFFFFFFFFFFFFFFFFFFFFFFFF
@A00155:342:HHGFNDSXY:1:1649:3613:36746 1:N:0:GAACCTAG+TCCGCATA
AAGGACTCACCTCTGGCCTATGGTCATCTCAATGCCGAGAGGGATGTGGAGGGCGAGAGAGAGCGACT
TCTCTCGGGCCCACAGCCTTACAGCTGTGGAGCACGGTATCCTCTGCCGAAACAGAGGTTGGACAAGA
+
FFFFFFFFFFFFFFFFFFFFFFFFFFFFFFFFFFFFF:F:FFFFFF:FFFFFFFFFFFFFFFFFFFF:
FFFFFFFFFFFFFFFFFFFFFFFFFFFFFFFFFFFFFFFFFFFFFFFFFFFFFFFFFFFFFFFFFFFF
@A00155:342:HHGFNDSXY:1:1650:18078:2581 1:N:0:GAACCTAG+TCCGCATA
CTGTGTCCACTTAAGGACTCACCTCTGGCCTATGGTCATCTCAATGCCGAGAGGGATGTAGAGGGCGA
GAGAGAGCGACTTCTCTCGGGCCCACAGCCTTACAGCTGTGGAGCACGGTATCCTCTGCCGAAACAGA
+
FFFFFFFFFFFFFFFFFFFFFFFFFFFFFFFFFFFFFFFFFFFFFFFFFFFFFFFFFFF,,FFFFFFFF
FFFFFFFFFFFFFFFFFFFFFFFFFFFFFFFFFFFFF:FFFFFFFFFFFFFFFFFFFF:FFFFFFFF
@A00155:342:HHGFNDSXY:1:1650:8558:21699 1:N:0:GAACCTAG+TCCGCATA
GTCATCTCAATGCCGAGAGGGATGTGGAGGGCGAGAGAGAGCGACTTCTCTCGGGCCCACAGCCTTAC
AGCTGTGGAGCACGGTATCCTCTGCCGAAACAGAGGTTGGACAAGACCGGAGGGGTCTCCTAGTTCCA
+
FFFFFFFFFFFFFFFFFFFFFFFF,FFFFFFFFFFFFF,FFFF:FFFFFFFFFFFFFFFFFFFFFFFFF
FFFFFFFFFFFFFFFF,FFFFFFFFFFFFFFFFFFFFFFFFFFFFFFFFFFFFFFFFFFFFFFFFFFF
@A00155:342:HHGFNDSXY:1:1650:31702:26616 1:N:0:GAACCTAG+TCCGCATA
CTTCTCTCGGGCCCACAGCCTTACAGCTGTGGAGCACGGTATCCTCTGCCGAAACAGAGGTTGGACAA
GACCGGAGGGGTCTCCTAGTTCCAAAGGAGATGTACTCCGGGCTTGTTCACGACCTACCGTGTA
+
FFFFFFFFFFFFFFFFFFFFFFFFFFF:FFFFFFFFFFFFFFFFFFFFFFFFF:FFFFFFFFFFFFFF
FFFFFFFFFFFFFFFFFFFFFFFFFFFFFFFFFFFFFFFFFFFFFFFFFFFFFFFFFFFFFFFF
@A00155:342:HHGFNDSXY:1:1650:14253:31751 1:N:0:GAACCTAG+TCCGCATA
GAGCGACTTCTCTCGGGGCCACAGCCTTACAGCTGTGGAGCACGGTATCCTCTGCCGAAACAGAGGTT
GGACAAGACCGGAGGGGTCTCCTAGTTCCAAAGGAGATGTACTCCGGGCTTGTTCACGACCTACCGTG
+
FFFFFFFFFFFFFFFFFFFFFFFFFFFFFFFFFFFFFFFFFFFFFFFFFFFFFFFFFFFFFFFFFFFF
FFFFFFFFFFFFFFFFFFFFFFFFFFFFFFFFFFFFFFFFFFFFFFFFFFFFFFFFFFFFFFFFFFFF
@A00155:342:HHGFNDSXY:1:1651:1353:6057 1:N:0:GAACCTAG+TCCGCATA
TTAAGGACTCACCTCTGGCCTATGGTCATCTCAATGCCGAGAGGGATGTGGAGGGCGAGAGAGAGCGA
CTTCTCTCGGGCCCACAGCCTTACAGCTGTGGAGCACGGTATCCTCTGCCGAAACAGAGGTTGGACAA
+
FFFFFFFFFFFFFFFFFFFFFFFF,FFFFFFFFFFFF,FFFF:FF:FFF:FFFFFFFFFFFFFFFFFF
F:FFFFFFFFFFFFFFFFFFFFFFFFFFFFFFFFFFFFFF:FFFFFFFFFFF:FFFFFFFFFFFFFFFF
@A00155:342:HHGFNDSXY:1:1651:18774:23109 1:N:0:GAACCTAG+TCCGCATA
TTAAGGACTCTCCTCTGGCCTATGGTCATCTCAATGCCGAGAGGGATGTGGAGGGCGAGAGAGAGCGA
CTTCTCTCGGGCCCACAGCCTTACAGCTGTGGAGCACGGTATCCTCTGCCGAAACAGAGGTTGGACAA
+

FFFFF:FFFFFFFFFFFFFFFFFFFFFFFFFFFFFFFFFFFFFFFFFFFFFFFFFFFFFFFFFF
FFFFFFFFFFFFFFFFFFFFFFFFFFFFFFFFFFFFFFFFFFFFFFFFFFFFFFFFFFFFFFFFFFFF
@A00155:342:HHGFNDSXY:1:1651:21468:26804 1:N:0:GAACCTAG+TCCGCATA
TCCTCTGCCGAAACAGAGGTTTGACAAGACCGGAGGGGTCTCCTAGTTCCAAAGGAGATGTACTCCGG
GCTTGTTCACGACCTACCGTGTAAGTCGTAGTCTAGTAGGCTACCTGACGAGTCCTTTTTAGGACGAA
+
FFFFFFFFFFFFFFFFFFFFFFFFFFFFFFFFFFFFFFFFFFFFFFFFFFFFFFFFFFFFFFFFFFFF
FFFFFFFFFFFFFFFFFFFFFFF:FFFFFFFFFFFFFFFFFFFFFFFFFFF:FFFFFF:FFFFFFFFF
@A00155:342:HHGFNDSXY:1:1651:2329:30013 1:N:0:GAACCTAG+TCCGCATA
TGTCCACTTAAGGACTCACCTCTGGCCTATGGTCATCTCAATGCCGAGAGGGATGTGAAGGGCGAGAG
AGAGCGACTTCTCTCGGGCCCACAGCCTTACAGCTGTGGAGCACGGTATCCTCTGCCGAAACAGAGGT
+
FFFFFF:FFFFFFFFFFFFFFFFFFF:FFFFFFFFFFFFFFFFFFF:FFFFFF:FFFFFFFF:FFFFF
FFFFFFFFFFFFFFFFF,FFFFFFFFFFFFFF:FFFFFFFF,FFFFFFFF:FFFFFFFFFFFFFFFFF
@A00155:342:HHGFNDSXY:1:1651:32027:34914 1:N:0:GAACCTAG+TCCGCATA
GAGCGACTTCTCTCGGGCCCACAGCCTTACAGCTGTGGAGCACGGTATCCTCTGCCGAAACAGAGGTT
GGACAAGACCGGAGGGGTCTCCTAGTTCCAAAGGAGATGTACTCCGGGCTTGTTCACGACCTACCGTG
+
FFFFFFFFFFFFFFFFFFFFFFFFFFFFFFFFFFFFFFFFFFFFFFFFF:FFFFFFFFFFFFFFFFFF
FFFFFFFFFFFFFFFFFFFFFFFFFFFFFFFFFFFFFFFFFFFFFFFFFFFFFFFFFFFFFFFFFFFF
@A00155:342:HHGFNDSXY:1:1652:25735:5259 1:N:0:GAACCTAG+TCCGCATA
AGGGATGTGGAGGGCGAGAGAGAGCGACTTCTCTCGGGCCCACAGCCTTACAGCTGTGGAGCACGGTA
TCCTCTGCCGAAACAGAGGTTGGACAAGACCGGAGGGGTCTCCTAGTTCCAAAGGAGATGTACTCCGG
+
FFFFFFFFFFFFFFFFFFFFFFFFFFFFFFFFFFFFFFFFFFFFFFFFFFFFFFFFFFFFFFFFFFFF
FFFFFFFFFFFFFFFFFFFFFFFFFFFFFFFFFFFFFFFFFFFFFFFFFFFFFFFFFFFFFFFFFFFF
@A00155:342:HHGFNDSXY:1:1652:16080:9674 1:N:0:GAACCTAG+TCCGCATA
TTCTCTCGGGCCCACAGCCTTACAGCTGTGGAGCACGGTATCCTCTGCCGAAACAGAGGTTGGACAAG
ACCGGAGGGGTCTCCTAGTTCCAAAGGAGATGTACTCCGGGCTTGTTCACGACCTACCGTGTAAGTCG
+
FFFFFFFFFFFFFFFFFFFFFFFFFFFFFFFFFFFFFFFFFFFFFFFFFFFFFFFFFFFFFFFFFFFF
FFFFFFFFFFFFFFFFFFFFFFFFFFFFFFFFFFFFFFFFFFFFFFFFFFFFFFFFFFFFFFFFFFFF
@A00155:342:HHGFNDSXY:1:1652:8748:16297 1:N:0:GAACCTAG+TCCGCATA
CACCTCTGGCCTATGGTCATCTCAATGCCGAGAGGGATGTGGAGGGCGAGAGAGAGCGACTTCTCTCG
GGCCCACAGCCTTACAGCTGTGGAGCACGGTATCCTCTGCCGAAACAGAGGTTGGACAAGACCGGAGG
+
FFFFFFFFFFFFFFFFFFFFFFFFFFFFFFFFFFFFF,FFFFFFFFFFFFFFFFFFFFFFFFFFFFFF
FFFFFFFFFFFFFFFFFFFFFFFFFFFFFFFFFFFFFFFFFFFFFFFFFFFFFFFFFFFFFFFFFFFF
@A00155:342:HHGFNDSXY:1:1652:18078:19335 1:N:0:GAACCTAG+TCCGCATA
CCCACAGCCTTACAGCTGTGGAGCACGGTATCCTCTGCCGAAACAGAGGTTGGACAAGACCGGAGGGG
TCTCCTAGTTCCAAAGGAGATGTACTCCGGGCTTGTTCACGACCTACCGTGTAAGTCGTAGTCTAGTA
+
FFFFFFFFFFFFFFFFFFFFFFFFFFFFFFFFFFFFFFFFFFFFFFFFFFFFFFFFFFFFFFFFFFFF
FFFFFFFFFFFFFFFFFFFFFFFFFFFFFFFFFFFFFFFFFFFFFFFFFFFFFFFFFFFFFFFFFFFF
@A00155:342:HHGFNDSXY:1:1652:19569:19601 1:N:0:GAACCTAG+TCCGCATA
ATCTCAATGCCGAGAGGGATGTGGAGGGCGAGAGAGAGCGACTTCTCTCGGGCCCACAGCCTTACAGC
TGTGGAGCACGGTATCCTCTGCCGAAACAGAGGTTGGACAAGACCGGAGGGGTCTCCTAGTTCCAAAG
+
:FFFFFFFFF:FFFFFFFFFFFFFFFFFFFFFFFFFFFFFFFFFFFFFFFFFFFFFFFFFFFFFFFF
FFFFFFFFFFFFFFFFFFFFFFFFFFFFF:FFFFFFFFFFFFFFFFFFFF:FFFFFFFFFFFFFFFFF
@A00155:342:HHGFNDSXY:1:1652:20446:24064 1:N:0:GAACCTAG+TCCGCATA
AGAGAGAGCGACTTCTCTCGGGCCCACAGCCTTACAGCTGTGGAGCACGGTATCCTCTGCCGAAACAG
AGGTTGGACAAGACCGGAGGGGTCTCCTGGTTCCAAAGGAGATGTACTCCGGGCTTGTTCACGACCTA
+

FFFFFFFFFFFFFFFFFFFFFFFFFFFFFFF:FFFFFFFFFFFFFFFFFFFFFFFFFFFFFFFF:FFF
FFFFFFFFFFFFFFFFFFFFFFFFFFFFFFFFFFFFFFFFFFFFFFFFFFFFFFFFFFFFFFFFFFF:
@A00155:342:HHGFNDSXY:1:1652:18539:32722 1:N:0:GAACCTAG+TCCGCATA
AAAGACTCACCTCTGGCCTATGGTCATCTCAATGCCGAGAGGGATGTGGAGGGCGAGAGAGAGCGACT
TCTCTCGGGCCCACAGCCTTACAGCTGTGGAGCACGGTATCCTCTGCCGAAACAGAGGTTGGACAAGA
+
FFFFFFFFFFFFFFFFFFFFFFFFFFFFFFFFFFFFFFFFFFFFFFFFFFFFFFFFFFFFFFFFFFFF
FFFFFFFFFFFFFFFFFFFFFFFFFFFFFFFFFFFFFFFFFFFFFFFFFFF:FFFFFFFFFFFFFFFF
@A00155:342:HHGFNDSXY:1:1653:15139:7451 1:N:0:GAACCTAG+TCCGCATA
CTTCTCTCGGGCCCACAGCCTTACAGCTGTGGAGCACGGTATCCTCTGCCGAAACAGAGGTTGGACAA
GACCGGAGGGGTCTCCTAGTTCCAAAGGAGATGTACTCCGGGCTTGTTCACGACCTACCGTGTA
+
FFFFFFFFFFFFFFFFFFFFFFFFFFF:FFFFFFFFFFFFFFFFFF:FFFFFFFFFFF:FFFFFFFFF
FFFFFFFFFFFFFFFFFFFFFFFFFFFF:FFFFFFFFFFF:FFFFFFFFFFFFFFFFFFF,F,F
@A00155:342:HHGFNDSXY:1:1653:6813:15734 1:N:0:GAACCTAG+TCCGCATA
CAATGCCGAGAGGGATGTAGAGGGCGAGAGAGAGCGACTTCTCTCGGGCCCACAGCCTTACAGCTGTG
GAGCACGGTATCCTCTGCCGAAACAGAGGTTGGACAAGACCGGAGGGGTCTCCTAGTTCCAAAGGAGA
+
FFFFFFFFFFFFFFFFFFFFFFFFFFFFFFFFFFFFFFFFFFFFFFFFFFFFFFFFFFFFFFFFFFFF
FFFFFFFFFFFFFFFFFFFFFFFFFFFFFFFFF:FFFFFFFFFFFFFFFFFFFFFFFFFFFFFFFFFF
@A00155:342:HHGFNDSXY:1:1653:31060:24189 1:N:0:GAACCTAG+TCCGCATA
AGAGAGAGCGACTTCTCTCGGGCCCACAGCCTTACAGCTGTGGAGCACGGTATCCTCTGCCGAAACAG
AGGTTGGACAAGACCGGAGGGGTCTCCTGGTTCCAAAGGAGATGTACTCCGGGCTTGTTCACGACCT
+
FFFFFFFFFFFFFFFFFFFFFFFFFF:FFFFFFFFFFFFFFFFFFFFFFFFFFFFFFFFFFFFFFFFF
FFFFFFFFFFFFFFFFFFFFFFFFFFFFFFFFFFFFFFFFFFFFFFFFFFFFFFFFFFFFFFFFFFF
@A00155:342:HHGFNDSXY:1:1653:21676:26944 1:N:0:GAACCTAG+TCCGCATA
GTCATCTCAATGCCGAGAGGGATGTGGAGGGCGAGAGAGAGCGACTTCTCTCGGGCCCACAGCCTTAC
AGCTGTGGAGCACGGTATCCTCTGCCGAAACAGAGGTTGGACAAGACCGGAGGGGTCTCCTAGTTCCA
+
FFFFFFFFFFFFFFFFFFFFFFFF,FFFFFFFFFFFFFFFFFFFFFFFFFFFFFFFFFFFFFFFFF,F
FFFFFFFFFFFFFFFFFFFFFFFFFFFFFFFFFFFFFFFFFFFFFFFFFFFFFFFFFFFFFFFFFFFF
@A00155:342:HHGFNDSXY:1:1653:20645:27821 1:N:0:GAACCTAG+TCCGCATA
CTCAATGCCGAGAGGGATGTGGAGGGCGAGAGAGAGCAACTTCTCTCGGGCCCACAGCCTTACAGCTG
TGGAGCACGGTATCCTCTGCCGAAACAGAGGTTGGACAAGACCGGAGGGGTCTCCTAGTTCCAAAGGA
+
FFFFFFFFFFFFFFFFFFFFFFFFFFFFFFFFFFF:F:FFFFFFFFFFFFFFFFFFFFF:FFFFFFFF
FFFFFFFFFFFFFFFFFFFFFFFFFFFFFFFFFFFFFFFFFFFFFFFFFFFFFFFFFFFFFFF,FFFF
@A00155:342:HHGFNDSXY:1:1653:2763:28667 1:N:0:GAACCTAG+TCCGCATA
AGAGAGCGACTTCTCTCGGGCCCACAGCCTTACAGCTGTGGAGCACGGTATCCTCTGCCGAAACAGAG
GTTGGACAAGACCGGAGGGGTCTCCTAGTTCCAAAGGAGATGTACTCCGGACTTGTTCACGACCTACC
+
FFFFFFFFFFFFFFFFFFFFF:FFFFFFFFFFFFFFFFFFFFFFFFFFFFFFFFFFFFFFFFFFFFFF
FFFFFFFFFFFFFFFFFFFFFFFF:FFFFFFFFFF:FF:FF:FFFFFFFFFFFFFFFFF,FFFFFFFF
@A00155:342:HHGFNDSXY:1:1653:24415:30749 1:N:0:GAACCTAG+TCCGCATA
GAGAGGGATGTGGAGGGCGAGAGAGAGCGACTTCTCTCGGGCCCACAGCCTTACAGCTGTGGAGCACG
GTATCCTCTGCCGAAACAGAGGTTGGACAAGACCGGAGGGGTCTCCCAGTTCCAAAGGAGATGTACTC
+
FFFFFFFFFF:FFFFFFFFFFFFFFFFFFFFFFFFFFFFFFFFFFFFFFFFFFFFFFFFFFFFFFFFF
FFFFFFFFFFFFFFFFFFF:FFFFFFFFFFFFFFFFFFFFFFFFFFFFFFFFFFFFFFFFFFFFFFFF
@A00155:342:HHGFNDSXY:1:1653:21377:32377 1:N:0:GAACCTAG+TCCGCATA
TCCACTTAAGGACTCACCTCTGGCCTATGGTCATCTCAATGCCGAGAGGGATGTGGAGGGCGAGAGAG
AGCGACTTCTCTCGGGCCCACAGCCTTACAGCTGTGGAGCACGGTATCCTCTGCCGAAACAGAGGTTG
+

FFFFFFFFFF:FFFFFF,FFFFFFFFFFFFFFFFFF,FFFFFFFFFFFFFF:FFFFFFFFFFFFFFF
FFFFFFFFFFFFFFFFFFFFFFFF:FFFF:F:FF,FFFFFF,FFFF:FFFFFFF:FFF:FFFFFF:FFF
@A00155:342:HHGFNDSXY:1:1653:26666:33677 1:N:0:GAACCTAG+TCCGCATA
GAGAGGGATGTGGAGGGCGAGAGAGAGCGACTTCTCTCGGGCCCACAGCCTTACAGCTGTGGAGCACG
GTATCCTCTGCCGAAACAGAGGTTGGACAAGACCGGAGGGGTCTCCCAGTTCCAAAGGAGATGTACTC
+
FFFFFFFFFFFFFFFFFFFFFFFFFFFFFFFFFFFFFFFFFFFFFFFFFFFFFFFFFFFFFFFFFFFF
FFFFFFFFFFFFFFFFFFFFFFFFFFFFFFFFFFFFFFFFFFFFFFFFFFFFFFFFFFFFFFFFFFFF
@A00155:342:HHGFNDSXY:1:1654:4065:2769 1:N:0:GAACCTAG+TCCGCATA
ATCTCAATGCCGAGAGGGATGTGGAGGGCGAGAGAGAGCGACTTCTCTCGGGCCCACAGCCTTACAGC
TGTGGAGCACGGTATCCTCTGCCGAAACAGAGGTTGGACAAGACCGGAGGGGTCTCCTGGTTCCAAAG
+
FF:F:F:FFFFFFFFFFFF:FFFF,FFFFFFFFFFFFFFF:FFFFFFFFF:FFFFFFFFFFFFFF:F
FFFFFFFFFFFFFFFFFFFFFFFFF:FFFFFFFFFFFFFFF,FFFFF,FFFFFFFFFFFFFFFFFFFF:
@A00155:342:HHGFNDSXY:1:1654:14733:6527 1:N:0:GAACCTAG+TCCGCATA
CAATGCCGAGAGGGATGTGGAGGGCGAGAGAGAGCGACTTCTCTCGGGCCCACAGCCTTACAGCTGTG
GAGCACGGTATCCTCTGCCGAAACAGAGGTTGGACAAGACCGGAGGGGTCTCCTAGTTCCAAAGGAGA
+
FFFFFFFF:FFFFFFFFFFFFFFFFFFFFFFFFFFFFFFFFFFFFFFFFFFFFFFFFFFFFFFFFFFF
FFFFFFFFFFFFFFFFFFFFFFFFFFFFFFFFFFFFFFFFFFFFFFFFFFFFFFFFFFFFFFF:FFFF
@A00155:342:HHGFNDSXY:1:1654:28492:10160 1:N:0:GAACCTAG+TCCGCATA
CCTTACAGCTGTGGAGCACGGTATCCTCTGCCGAAACAGAGGTTGGACAAGACCGGAGGGGTCTCCTG
GTTCCAAAGGAGATGTACTCCGGGCTTGTTCACGACCTACCGTGTAAGTCGTAGTCTAGTAGGCTACC
+
FFFFFFFFFFFFFFFFFFFFFFFFFFFFFFFFFFFFFFFFFFFFFFFFFFF:FFFFFFFFFFFFFFFF
FFFFFFFFFFFFFFFFFFFFFFFFFFFFFFFFFFFFFFFFFFFFFFFFFFFFFFFFFFFFFFFFFFFF
@A00155:342:HHGFNDSXY:1:1654:21721:11209 1:N:0:GAACCTAG+TCCGCATA
GATGTGGAGGGCGAGAGAGAGCGACTTCTCTCGGGCCCACAGCCTTACAGCTGTGGAGCACGGTATCC
TCTGCCGAAACAGAGGTTGGACAAGACCGGAGGGGTCTCCTAGTTCCAAAGGAGATGTACTCCGGGCT
+
FFFFFFFFFFFFFFFFFFFFFFFFFFFFFFFFFFFFFFFFFFFFFFFFFFFFFFFFFFFFFFFFFFFF
:FFFF:FFFFFFFFFFFFFFFFFF,FFFFFFFFFFFFFFFFFFFFFFFFFFFFFFFFFFFFFFFFFFF
@A00155:342:HHGFNDSXY:1:1654:29930:11616 1:N:0:GAACCTAG+TCCGCATA
ATCTCAATGCCGAGAGGGATGTGGAGGGCGAGAGAGAGCGACTTCTCTCGGGCCCACAGCCTTACAGC
TGTGGAGCACGGTATCCTCTGCCGAAACAGAGGTTGGACAAGACCGGAGGGGTCTCCTAGTTCCAAAG
+
FFFFFFFFFFFFFFFFFFFFFFFFFFFFFFFFFFFFFFFFFFFFFFFFFFFFFFFFFFFFFFFFFFFF
FFFFFFFFFFFFFFFFFFFFFFFFFFFFFFFFF:FFFFFFF,FFFFFFFFFFFFFFFFFFFFFFFFFF
@A00155:342:HHGFNDSXY:1:1654:21685:12430 1:N:0:GAACCTAG+TCCGCATA
GATGTGGAGGGCGAGAGAGAGCGACTTCTCTCGGGCCCACAGCCTTACAGCTGTGGAGCACGGTATCC
TCTGCCGAAACAGAGGTTGGACAAGACCGGAGGGGTCTCCTAGTTCCAAAGGAGATGTACTCCGGGCT
+
FFFF,FFFFFFFFFFFFFFFFFFFFFFFFFFFFFFFFFFFFFFFFFFFFFFFFFFFFFFFFFFFFFFF
FFFFFFFFFFFFFFFFFFFFFFFFFFFFFFFFFFFFFFFFFFFFFFFFFFFFFFFFFFF:FFFFFFFFF
@A00155:342:HHGFNDSXY:1:1654:5737:21324 1:N:0:GAACCTAG+TCCGCATA
CTCAATGCCGAGAGGGATGTGGAGGGCGAGAGAGAGCGACTTCTCTCGGGCCCACAGCCTTACAGCTG
TGGAGCACGGTATCCTCTGCCGAAACAGAGGTTGGACAAGACCGGAGGGGTCTCCTAGTTCCAAAGGA
+
FFFFFFFFFFFFFFFF,FFFFFFFFFFFFFFFFFFFFFFFF,FFFFFFFFFFFFFFFFFFFFFFFFFFF
FFFFFFFFFFFFFFFFFFFFFFFFFFFFFFFFFFFFFFFFFFFFFFFFFFFFFFFFFFFFFFFFFFFF
@A00155:342:HHGFNDSXY:1:1654:4580:22388 1:N:0:GAACCTAG+TCCGCATA
ATCTCAATGCCGAGAGGGATGTAGAGGGCGAGAGAGAGCGACTTCTCTCGGGCCCACAGCCTTACAGC
TGTGGAGCACGGTATCCTCTGCCGAAACAGAGGTTGGACAAGACCGGAGGGGTCTCCTAGTTCCAAAG
+

FFFFFFFFFFFFFF:FFFFFFFFFFFFFFFFFFFFFF:FFFFFFFFFFFFFFFFFFFFFFFFFFFFFFF
FFFFFFFFF:FFFFFFFFFFFFFFFFFFFFFFFFFFFFFFFFFFFFFFFFFFFFFFFFFFFFFFFFFF
@A00155:342:HHGFNDSXY:1:1655:14570:6872 1:N:0:GAACCTAG+TCCGCATA
CTCTGGCCTATGGTCATCTCAATGCCGAGAGGGATGTAGAGGGCGAGAGAGAGCGACTTCTCTCGGGC
CCACAGCCTTACAGCTGTGGAGCACGGTATCCTCTGCCGAAACAGAGGTTGGACAAGACCGGAGGGGT
+
FFFFFFFFFFFFFFFFFFFFFFFFFFFFFFFFF:FFFFFFFFFFFFFFFFFFFFFFFFFFFFFFFFFF
FFFFFFFF:FFFFFFFFFFFFFFFFFFFFFFFFFFFFFFFFFFFFFFFFFFFFFFFFFFFFFFFFFFF
@A00155:342:HHGFNDSXY:1:1655:8495:19116 1:N:0:GAACCTAG+TCCGCATA
TGCCGAAACAGAGGTTGGACAAGACCGGAGGGGTCTCCTAGTTCCAAAGGAGATGTACTCCGGGCTTG
TTCACGACCTACCGTGTAAGTCGTAGTCTAGTAGGCTACCTGACGAGTCCTTTTTAGGAC
+
FFFFFFFFFFFFFFFFFFFFFFFFFFFFFFFFFFFFFFFFFFFFFFFFFFFFFFFFFFFFFFFFFFFF
FFFFFFFFFFFFFFFFFFFFFF:F:FFFFFFFFFFFFFFFFFFFFFFFFFFFFFFFFFFFF
@A00155:342:HHGFNDSXY:1:1655:10294:20760 1:N:0:GAACCTAG+TCCGCATA
TTAAGGACTCTCCTCTGGCCTATGGTCATCTCAATGCCGAGAGGGATGTGGAGGGCGAGAGAGAGCGA
CTTCTCTCGGGCCCACAGCCTTACAGCTGTGGAGCACGGTATCCTCTGCCGAAACAGAGGTTGGAC
+
FFFFFFFFFFFFFFFFFFFFFFFFFFFFFFFFFFFFFFFFFFFFFFFFFFFFFFFFFFFFFFFFFFFF
FFFFFFFFFFFFFFFFFFFFFFFFFFFFFFFFFFFFFF,FFFFFFFFFFFFFFFFFFFFFFFFFFFF
@A00155:342:HHGFNDSXY:1:1655:31123:24518 1:N:0:GAACCTAG+TCCGCATA
ATGCCGAGAGGGATGTGGAGGGCGAGAGAGAGCGACTTCTCTCGGGCCCACAGCCTTACAGCTGTGGA
GCACGGTATCCTCTGCCGAAACAGAGGTTGGACAAGACCGGAGGGGTCTCCTAGTTCCAAAGGAGATG
+
FFFFFFFFFFFFFFFFFFFFFFFFFFFFFFFFFFFFFFFFF:FFFFFFFFFFFFFFFFFFFFFFFFFF
FFFFFFFFFFFFFFFFFFFFFFFFFFFFFFFFFFFFFFFFFFFFFFFFFFFFFFFFFFFF:FFFFFFF
@A00155:342:HHGFNDSXY:1:1656:9091:20807 1:N:0:GAACCTAG+TCCGCATA
ATCTCAATGCCGAGAGGGATGTGGAGGGCGAGAGAGAGCGACTTCTCTCGGGCCCACAGCCTTACAGC
TGTGGAGCACGGTATCCTCTGCCGAAACAGAGGTTGGACAAGACCGGAGGGGTCTCCTAGTTCCAAAG
+
FFFFFFFFFFFFFFFFFFFFFFFFFFFFFFFFFFFFFFFFFFF:FFFFFFFFFFFF:FFFFFFF:FFFF
FFFFFFFFFFFFFFFFFFFFFFFFFFFFFFFFFFFFFFFFFFFFFFFFFFFFFFFFFFFFFFFFFFFF
@A00155:342:HHGFNDSXY:1:1656:15980:22091 1:N:0:GAACCTAG+TCCGCATA
CAATGCCGAGAGGGATGTGGAGGGCGAGAGAGAGCGACTTCTCTCGGGCCCACAGCCTTACAGCTGTG
GAGCACGGTATCCTCTGCCGAAACAGAGGTTGGACAAGACCGGAGGGGTCTCCTAGTTCCAAAGGAGA
+
FFFFFFFFFFFFFFFFF,FFFFFFFFFFFFFFFFFFFFFFFFFFFFFFFFFFFFFFFFFFFFFFFFFF
FFFFFFFFFFFFFFFFFFFFFFF:FFFFF:FFFFFFFFFFFFFFFFFF:FFFFFFFFFFF:FFFFFFF
@A00155:342:HHGFNDSXY:1:1656:21178:24142 1:N:0:GAACCTAG+TCCGCATA
CGAGAGAGAGCGACTTCTCTCGGGCCCACAGCCTTACAGCTGTGGAGCACGGTATCCTCTGCCGAAAC
AGAGGTTGGACAAGACCGGAGGGGTCTCCTGGTTCCAAAGGAGATGTACTCCGGGCTTGTTCACGACC
+
FFFFFFFFFFFFFFFFFFFF:FFFFFFFFFFFFFFFFFFFFFFFFFFFFFFFFFFFFFFFFFFFFFFF
FFFFFFFFFFFFFFFFFFFFFFFFFFFFFFFFFFFFFFFFFFFFFFFFFFFFFFFFFFFFFFFFFFFF
@A00155:342:HHGFNDSXY:1:1656:19913:28369 1:N:0:GAACCTAG+TCCGCATA
CAATGCCGAGAGGGATGTAGAGGGCGAGAGAGAGCGACTTCTCTCGGGCCCACAGCCTTACAGCTGTG
GAGCACGGTATCCTCTGCCGAAACAGAGGTTGGACAAGACCGGAGGGGTCTCCTAGTTCCAAAGGAGA
+
FFFFFFFFFFFFFFFFFFFFFFFF:FFFFFFFFFFFFFFFFFFFFFFFFFFFFFFFFFF:FFFFFFFFF
FFFFFFFFFFFFFFFF:FFFFFFFFFFFFFFFFFFFFFFFFFFFFFFFFFFFFFFF,FFFFFFFFFF:FFF
@A00155:342:HHGFNDSXY:1:1656:27299:30326 1:N:0:GAACCTAG+TCCGCATA
CTTCTCTCGGGCCCACAGCCTTACAGCTGTGGAGCACGGTATCCTCTGCCGAAACAGAGGTTGGACAA
GACCGGAGGGGTCTCCTAGTTCCAAAGGAGATGTACTCCGGGCTTGTTCACGACCTACCGTGTAAGTC
+

```
:FF:FFFF,F:FFFFFF,FF:FFFFFFFF:FFFFFFFFFF:FFFFFFFF:F:FFFF:,FFF:FFF:FF
:FFF::F:,FFFFFFFFF,FFFFFFF::F,FF:FFFF,FF,FFFFFFFFFFFF:FFFFFFFFFFFF:F
@A00155:342:HHGFNDSXY:1:1657:10782:7607 1:N:0:GAACCTAG+TCCGCATA
CAATGCCGAGAGGGATGTGGAGGGCGAGAGAGAGCGACTTCTCTCGGGCCCACAGCCTTACAGCTGTG
GAGCACGGTATCCTCTGCCGAAACAGAGGTTGGACAAGACCGGAGGGGTCACCTAGTTCCAAAGGAGA
+
FFFFFFFFFFFFFFFFFFFFFFFFFFFFFFFFFFFFFFFFFFFFFFFFFFFFFFFFFFFFFFFFFFFF
FFFFFFFFF:FFFFFFFFFFFFFFFFFFFFFFFFF::,,F:,,F,,,:,F,FF,F:,:FFFFFF:F:F
@A00155:342:HHGFNDSXY:1:1657:3387:19977 1:N:0:GAACCTAG+TCCGCATA
CTGTGTCCACTTAAGGACTCACCTCTGGCCTATGGTCATCTCAATGCCGAGAGGGATGTGGAGGGCGA
GAGAGAGCGACTTCTCTCGGGCCCACAGCCTTACAGCTGTGGAGCACGGTATCCTCTGCCGAAACAGA
+
FFFFFFFFFFFFFFFFFFFFFFFFFFFFF,FFFFFFFFFFFFFFFFFFFFFFFFFFFFFFFFFFFFFF
FFFFFFFFFFFFFFFFFFFFFFFF:FFFFFFFFF:FFFFFFFFFFFFFFFFFFFFFFFFFFFFFFFFF
@A00155:342:HHGFNDSXY:1:1657:16251:32111 1:N:0:GAACCTAG+TCCGCATA
TCGGCAGAGGATACCGTGCTCCACAGCTGTAAGGCTGTGGGCCCGAGAGAAGTCGCTCTCTCTCGCCC
TCTACATCCCTCTCGGCATTGAGATGACCATAGGCCAGAGGTGAGTCCTTAAGTGGACACA
+
FFFFFFFFFFFFFFFFFFFFFFFFFFFFFFFFFFF:FFFFFFFFFFFFFFFFFFFFFFFFFFFFFFF:
FFFFFFFFFFFFFFFFFF:FFFFFFFFFFFFFFFFFFFFFFFFFFFFFFFFFFFFFFFFFFFFFFFFFF
@A00155:342:HHGFNDSXY:1:1657:9715:32471 1:N:0:GAACCTAG+TCCGCATA
CTTCTCTCGGGCCCACAGCCTTACAGCTGTGGAGCACGGTATCCTCTGCCGAAACAGAGGTTGGACAA
GACCGGAGGGGTCTCCTAGTTCCAAAGGAGATGTACTCCGGGCTTGTTCACGACCTACCGTGTAAGTC
+
FFFFFFFFFFFFFFFFFFFFFFF:FFFFFFFFFFFFFFFFFFFFFFFFF,FFFFFFFFFFFFFFFFFF
FFFFFFFFFFFFFFFFFFFFFFFFFFFFF,FFFFFFFFFF:FFFFFFFFFFFFFFFFFFFFFFFFFFF
@A00155:342:HHGFNDSXY:1:1658:29731:17848 1:N:0:GAACCTAG+TCCGCATA
GATCAGCTGTGTCCACTTAAGGACTCACCTCTGGCCTATGGTCATCTCAATGCCGAGAGGGATGTGAA
GGGCGAGAGAGAGCGACTTCTCTCGGGCCCACAGCCTTACAGCTGTGGAGCACGGTATCCTCTGCCGA
+
FFFFFFFFFFFFFFFFFFFFFFFFFFFFFFFFFFFFFFFFFFFFFFFFFFFFFFFFFFFFFFFFFFFF
FFFFFFFFFFFFFFFFFFFFFFFFFFFFFFFFFFFFFFFFFFFFFFFFFFFFFFFFFFFFFFFFFFFF
@A00155:342:HHGFNDSXY:1:1659:7383:7232 1:N:0:GAACCTAG+TCCGCATA
GTCATCTCAATGCCGAGAGGGATGTGGAGGGCGAGAGAGAGCGACTTCTCTCGGGCCCACAGCCTTAC
AGCTGTGGAGCACGGTATCCTCTGCCGAAACAGAGGTTGGACAAGACCGGAGGGGTCTCCTAGTTCCA
+
FFFFFFFFFFFFFFFFFFFFF:FF,FFFFFFFF:FFFFFFFFFFF,FFFFFFFFFFFFFFFFFFFFFF
:FFFFFFF:FFFFFFFFFFFFFFFFFFFFFFFFFFFFFFFFFFFFFFFFFFFFFFFFFFFFFFFFFFF
@A00155:342:HHGFNDSXY:1:1659:13458:18537 1:N:0:GAACCTAG+TCCGCATA
GAGCGACTTCTCTCGGGCCCACAGCCTTACAGCTGTGGAGCACGGTATCCTCTGCCGAAACAGAGGTT
GGACAAGACCGGAGGGGTCTCCTAGTTCCAAAGGAGATGTACTCCGGGCTTGTTCACGACCTACCGTG
+
FFFFFFFFFFFFFFFFFFFFFFFFFFFFFFFFFFFFFFFFFFFFFFFFFFFFFFFFFFFFFFFFFFFF
FFFFFFFFFFFFFFFFFFFFFFFFFFFFFFFFFFFFFFFFFFFFFFFFFFFFFFFFFFFFFFFFFFFF
@A00155:342:HHGFNDSXY:1:1660:5141:2691 1:N:0:GAACCTAG+TCCGCATA
ACCTCTGTTTCGGCAGAGGATACCGTGCTCCACAGCTGTAAGGCTGTGGGCCCGAGAGAAGTCGCTCT
CTCTCGCCCTTCACATCCCTCTCGGCATTGAGATGACCATAGGCCAGAGGTGAGTCCTTAAGTGGACA
+
FFFFFFFFFFFF:FFFFFFFFFF:FF,FFFFF:FFFFFFFFFFFFF:F:F::FFFFFFFFFFFFFFFF
FFFFFFF:FFFFFFFFFFFFFFFFFFFFFFFFFFFFFFF:FF,FFFFFFFFFFFF:FFFF:FFFFFF:
@A00155:342:HHGFNDSXY:1:1660:2293:19366 1:N:0:GAACCTAG+TCCGCATA
GGCAGAGGATACCGTGCTCCACAGCTGTAAGGCTGTGGGCCCGAGAGAAGTCGCTCTCTCTCGCCCTT
CACATCCCTCTCGGCATTGAGATGACCATAGGCCAGAGGTGAGTCCTTAAGTGGAC
+
```

FF:FF:FFFFFFFFF:FF:FFFFFFFFFFFF:FFFFF:FFFFFFFFFFFFFFFFFFFFFFFFFFFFFFF
FFFFFFFFFFFF:FFFFF,:FFFF:FFFFFFFFFFFFFFFFFFFFFFFFFF:FFFFF
@A00155:342:HHGFNDSXY:1:1660:21287:19914 1:N:0:GAACCTAG+TCCGCATA
TGTCCACTTAAGGACTCACCTCTGGCCTATGGTCATCTCAATGCCGAGAGGGATGTGAAGGGCGAGAG
AGAGCGACTTCTCTCGGGCCCACAGCCTTACAGCTGTGGAGCACGGTATCCTCTGCCGAAACAGAGGT
+
FFFFFFFFFFFFFFFFFFFFFFFFFFFFFFFFFFFFFFFFFFFFFFFFFFFFFFFFFFFFFFFFFFFFF
FFFFFFFFFFFFFFFFFFFFFFFFFFFFFFFFFFFFFFFFFFFFFFFF,FFFFFFFFFFFFFFFFFFFF
@A00155:342:HHGFNDSXY:1:1660:10999:20102 1:N:0:GAACCTAG+TCCGCATA
TTAAGGACTCACCTCTGGCCTATGGTCATCTCAATGCCGAGAGGGATGTAGAGGGCGAGAGAGAGCGA
CTTCTCTCGGGCCCACAGCCTTACAGCTGTGGAGCACGGTATCCTCTGCCGAAACAGAGGTTGGACAA
+
FFFFFFFFFFFFFFFFFFFF:FFFFFFF::FFFFFFFFF:FFFFFFFFFFFFFF:FFFFFFF:FFFFFF
FFFFFFFFF,FFFF:FF:FFFFFFFF:FFFFFFF:FFFFFFFFFFFFFFFFFFFF,FF:FF:FFFFFFF
@A00155:342:HHGFNDSXY:1:1661:24849:8296 1:N:0:GAACCTAG+TCCGCATA
AGAGAGAGCGACTTCTCTCGGGCCCACAGCCTTACAGCTGTGGAGCACGGTATCCTCTGCCGAAACAG
AGGTTGGACAAGACCGGAGGGGTCTCCTAGTTCCAAAGGAGATGTACTCCGGGCTTGTTCACGACCTA
+
FFFFFFFFFFFFFFFFFFFFFFFFFFFFFFFFFFFFFFFFFFFFFFFFFFFFFFFFFFFFFFFFFFFFF
FFFFFFFFFFFFFFFFFFFFFFFFFFFFFFFFFFFFFFFFFFFFFFFFF:FFFFFFFFFFFFFFFFFFF
@A00155:342:HHGFNDSXY:1:1661:18376:17065 1:N:0:GAACCTAG+TCCGCATA
GTCATCTCAATGCCGAGAGGGATGTGGAGGGCGAGAGAGAGCGACTTCTCTCGGGCCCACAGCCTTAC
AGCTGTGGAGCACGGTATCCTCTGCCGAAACAGAGGTTGGACAAGACCGGAGGGGTCTCCTAGTTCCA
+
FFFFFFFFFFFFFFFFFFFFFFFF:FFFFFFFFFFFFFFFFFFFFFFFFFFFFFFFFFFFFFFFFFFFF
FFFFFFFFFFFFFFFFFFFFFFFFFFFFFFFFFFFFFFFFFFFFFFFFFFFFFFFFFFFFFFFFFFFFF
@A00155:342:HHGFNDSXY:1:1661:26078:18881 1:N:0:GAACCTAG+TCCGCATA
CCAACCTCTGTTTCGGCAGAGGATACCGTGCTCCACAGCTGTAAGGCTGTGGGCCCGAGAGAAGTCGC
TCTCTCTCGCCCTTCACATCCCTCTCGGCATTGAGATGACCATAGGCCAGAGGTGAGTCCTTAAGTGG
+
FFFFFFFFFFFFFFFFFFFFFFFFFFFFFFFFFFFFFFFFFFFFFFFFFFFFFFFFFFFFFFFFFFFFF
FFFFFFFFFFFFFFFFFFFFFFFFFFFFFFFFFFFFFFFFFF:FFFFFF,FFFFFFFFFFFFFFFFFFF
@A00155:342:HHGFNDSXY:1:1661:27064:19116 1:N:0:GAACCTAG+TCCGCATA
CTTCTCTCGGGCCCACAGCCTTACAGCTGTGGAGCACGGTATCCTCTGCCGAAACAGAGGTTGGACAA
GACCGGAGGGGTCTCCTAGTTCCAAAGGAGATGTACTCCGGGCTTGTTCACGACCTACCGTGTAAGTC
+
FFFFFFFFFFFFFFFFFFFFFFFFFFFFFFFFFFFFFFFFFFFFFFFFFFFFFFFFFFFFFFFFFFFFF
:FFFFFFFFFFFFFFFFFFFFFFFFFFFFFFFFFFFFFFFFFF:FFFFFFFFFFFFFFFFFFFFFFFFF
@A00155:342:HHGFNDSXY:1:1661:22453:20807 1:N:0:GAACCTAG+TCCGCATA
GACTTCTCTCGGGCCCACAGCCTTACAGCTGTGGAGCACGGTATCCTCTGCCGAAACAGAGGTTGGAC
AAGACCGGAGGGGTCTCCTAGTTCCAAAGGAGATGTACTCCGGGCTTGTTCACGACCTACCGTGTAAG
+
FFF:FFFFFFFFFFFFFFFFFFFFFFFFFFFFFFFFFFFFFFFFFFFFFFFFFFFFFFFFFFFFFFFFF
FFFFFFFFFFFFFFFFFFFFFFFFFFFFFFFFFFFFFF,FFFFFFFFFF,FFFFFFFFFFFFFFFFFF:
@A00155:342:HHGFNDSXY:1:1661:13304:28322 1:N:0:GAACCTAG+TCCGCATA
TGGTCATCTCAATGCCGAGAGGGATGTGGAGGGCGAGAGAGAGCGACTTCTCTCGGGCCCACAGCCTT
ACAGCTGTGGAGCACGGTATCCTCTGCCGAAACAGAGGTTGGACAAGACCGGAGGGGTCTCCTGGTTC
+
FFFFFFFFFFFFFFFFFFFFFFFFFFFFFFFFFFFFFFFFFFFFFFFFF:FFFFFFFFFFFFFFFFFFF
FFFFFFF:FFFFFFFFFFFFFFFFFFFFFFFFFFFFFFFFFFFFFFFFFFFFFF:FFFFFFFFFFFFFF
@A00155:342:HHGFNDSXY:1:1662:26901:17957 1:N:0:GAACCTAG+TCCGCATA
CCTCTGGCCTATGGTCATCTCAATGCCGAGAGGGATGTGAAGGGCGAGAGAGAGCGACTTCTCTCGGG
CCCACAGCCTTACAGCTGTGGAGCACGGTATCCTCTGCCGAAACAGAGGTTGGACAAGACCGGAGGGG
+

```
FFFFFFFFFFFFFFFFFFFFFFFFFFFFFFFFFFFFFFFFFFFFFFFFFFFFFFFFFF:FFFFFFFFF
FFFFFFFFFFFFFFFFFFFFFFFFFFFFFFFFFFFFFFFFFFFFFFFFFF:FFFFFFFFFFFFFFFFF
@A00155:342:HHGFNDSXY:1:1662:14262:36933 1:N:0:GAACCTAG+TCCGCATA
CACAGCCTTACAGCTGTGGAGCACGGTATCCTCTGCCGAAACAGAGGTTGGACAAGACCGGAGGGGTA
TCCTAGTTCCAAAGGAGATGTACTCCGGGCTTGTTCACGACCTACCGTGTAAGTCGTAGTCTAGTAGG
+
FFFFFFFFFFFFFFFFFFFFFFFFFFFFFFFFFFFFFFFFFFFFF:FFFFFFFFFFFFFFFFFFFFF:F
FFFFFFFFFFFFFFFFFFFFFFFFFFFFFFFFFFFFFFFFFFFFFFFFFFFFFFFFFFFFFFFFFFFF
@A00155:342:HHGFNDSXY:1:1663:6180:4867 1:N:0:GAACCTAG+TCCGCATA
CCACATCCCTCTCGGCATTGAGATGACCATAGGCCAGAGGTGAGTCCTTCAGTGGACACAGCTGATCT
AAGGCGGTGTGGCGGGGCATGGGTTTGAACCCCCATGACGGTCGGAGTCTAGTAGGCTCCCTGATGAG
+
FFFFFFFFFFFFFFFFFFFFFF,FFFFFF:FF,F:FFFFFFFFFFFFFF,FFFFFFFFF:FFF,FFFF
FFFFFFF:FFFFFFFFFFFFFFFFFFFFFFFFFFFFFFFFFFF:FFFFF:FF:FFFFFFFFFFFFFFFF
@A00155:342:HHGFNDSXY:1:1663:11831:13150 1:N:0:GAACCTAG+TCCGCATA
ATGCCGAGAGGGATGTGGAGGGCGAGAGAGAGCGACTTCTCTCGGGCCCACAGCCTTACAGCTGTGGA
GCACGGTATCCTCTGCCGAAACAGAGGTTGGACAAGACCGGAGGGGTCTCCTAGTTCCAAAGGAGATG
+
FFFFFFFFFFFFFFFFFFFFFFFFFFFFFFFFFFFFFFFF:FFFFFFFFFFFFFFFFFFFFFFFFFF:F
FFFF:FFFFFFFFFFFFFFFFFFFFFFFFFFFFFFFFF,FFFFF:FFFFFF:FFFFFFFFFFFFFFFFFF
@A00155:342:HHGFNDSXY:1:1663:24035:25927 1:N:0:GAACCTAG+TCCGCATA
ACTTCTCTCGGGCCCACAGCCTTACAGCTGTGGAGCACGGTATCCTCTGCCGAAACAGAGGTTGGACA
AGACCGGAGGGGTCTCCTGGTTCCAAAGGAGATGTACTCCGGGCTTGTTCACGACCTACCGTGTAAGT
+
FFFFFFFFFFFFFFFFFFFFFFFFFFFFFFFFFFFFFFFFFFFFFFFFFFFFFFFFFFFFFFFFFFFF
FFFFFFFFFFFFFFFFFFFFFFFFFFFFFFFFFFFFFFFFF:FFFFFFFFFFFFFFFFFFFFFFFFFFF
@A00155:342:HHGFNDSXY:1:1663:12310:31485 1:N:0:GAACCTAG+TCCGCATA
CAGCCTTACAGCTGTGGAGCACGGTATCCTCTGCCGAAACAGAGGTTGGACAAGACCGGAGGGGTCTC
CTAGTTCCAAAGGAGATGTACTCCGGGCTTGTTCACGACCTACCGTGTAAGTCGTAGTCTAGTAGGCT
+
FFFFFFFFFFFFFFFFFFFFFFFFF:FFFFFFFFFFFFFFFFFFFFFFFFFFFFFFFFFFFFFFFFFF
FFFFFFFFFFFFFFFFFFFFFFFFFFFFFFFFFFFFFFFFFFFFFFFFFFFFFF:FFFFFFFFFFFF
@A00155:342:HHGFNDSXY:1:1664:3640:4601 1:N:0:GAACCTAG+TCCGCATA
TAGGCCAGAGGTGAGTCCTTAAGTGGACACAGCTGATCTAAGGCGGTGTAGCGGGGCATGGGTTTGAA
CCCCCATGACGGTCGGAGTCTAGTAGGCTCCCTGATGAGTCCGTTCCAAGGACGAAACCGTCGTTACT
+
FFFFFFFFFFFFFF:FF:FFFF,,:FFFFFFFF,FFFFFFFFFFFFFFFFFFFF,:FFF:FFFFFFFF
FFFFFFFFF:FFFFFFFFFFFFFFFFFFFFFF:FFFFFFFFFFFFFFFFFFFFF,,FFFFFFFFFFF
@A00155:342:HHGFNDSXY:1:1665:18521:1313 1:N:0:GAACCTAG+TCCGCATA
CTCAATGCCGAGAGGGATGTGGAGGGCGAGAGAGAGCGACTTCTCTCGGGCCCACAGCCTTACAGCTG
TGGAGCACGGTATCCTCTGCCGAAACAGAGGTTGGACAAGACCGGAGGGGTCTCCTAGT
+
FFFFF:FFFFFFFFFFFFFFFFFFFFFFFFFFFFFFFFFF,FFFFFFFFFFFFFFFFFFFFFFFFFFF
FFFFFFFFFFFFFFFFFFFFFFFFFFFFFFFFFFFFFFFFFFFFFFFFFFFFFFFFFFFF
@A00155:342:HHGFNDSXY:1:1665:30101:23563 1:N:0:GAACCTAG+TCCGCATA
TATCCTCTGCCGAAACAGAGGTTGGACAAGACCGGAGGGGTCTCCTGGTTCCAAAGGAGATGTACTCC
GGGCTTGTTCACGACCTACCGTGTAAGTCGTAGTCTAGTAGGCTACCTGACGAGTCCTTTTTAGGACG
+
FFFFFFFFFFFFFFFFFFFF:::FFFFFFFFFFFFFFFFF:FFFFFF:FFFFFFF:FFFFF,FFFFFF
FFFF:FFF:FFFFFFFFFFFFFFFFFFFFFFFFFFFFFF:FFFFFFFFFFFF,FFFFFFFFFFFFFFFF
@A00155:342:HHGFNDSXY:1:1665:30291:23766 1:N:0:GAACCTAG+TCCGCATA
GATCAGCTGTGTCCACTTAAGGACTCACCTCTGGCCTATGGTCATCTCAATGCCGAGAGGGATGTGAA
GGGCGAGAGAGAGCGACTTCTCTCGGGCCCACAGCCTTACAGCTGTGGAGCACGGTATC
+
```

FFFFFFFFFFFFFFFFFFFFFFFFFFFFFFFFFFFFFFFFFFFFFFFFFFFFFFFFFFFFFFFFFFFFFF
FFFFFFFFFFFFFFFFFFFFFFFFFFFFFFFFFFFFFFFFFFFFFFFFFFFFFFFFFFFFF
@A00155:342:HHGFNDSXY:1:1665:5547:34804 1:N:0:GAACCTAG+TCCGCATA
CTTCTCTCGGGCCCACAGCCTTACAGCTGTGGAGCACGGTATCCTCTGCCGAAACAGAGGTTGGACAA
GACCGGAGGGGTCTCCTGGTTCCAAAGGAGATGTACTCCGGGCTTGTTCACGACCTACCGTGTAAGTC
+
FFFFFFFFFFFFFFFFFFFFFFFFFFFFFFFFFFF:FFFFFFFFFFFFFFFFFFFFFFFFFFFFFFFFFF
FFFFFFFFFFFFFFFFFFFFFFFFFFFFFFFFFFFFFFFF,F:FFFFFFFFFFFFFFFFFFFFFFFFFFF
@A00155:342:HHGFNDSXY:1:1666:27914:4022 1:N:0:GAACCTAG+TCCGCATA
TGCCGAGAGGGATGTGGAGGGCGAGAGAGAGCGACTTCTCTCGGGCCCACAGCCTTACAGCTGTGGAG
CACGATATCCTCTGCCGAAACAGAGGTTGGACAAGACCGGAGGGGTCTCCTAGTTCCAAAGGAGATGT
+
FFFFFFFFFFFF:FFFFFFFFFFFFFFFFFFFFFFFFFFFFFF:FFFFFFFFFFFFFFFFFFFFFFFFFF
FFFFFFFFFFFFFFFFFFFFFFFF:FFFFFFFFFFFFFFFFFFFFFFFFFFFFFFFFFFFF:FFFFFFFF
@A00155:342:HHGFNDSXY:1:1666:6253:10441 1:N:0:GAACCTAG+TCCGCATA
CTCAATGCCGAGAGGGATGTGGAGGGCGAGAGAGAGCGACTTCTCTCGGGCCCACAGCCTTACAGCTG
TGGAGCACGGTATCCTCTGCCGAAACAGAGGTTGGACAAGACCGGAGGGGTCTCCTAGTTCCAAAGGA
+
FFFFFFFFFFFF:F,FF:FFFFFFFF,F:FFF:FFFFFFFFFFFFFFFFFF:FFFFFFFFFFF,FFFFFF
:FFFFFFFFFFFFFFFFFFFF,FF:FFFF,FFF,FFFFF:FFFFFFFFFFFFFFFFFFFFFFFFFFFFFF
@A00155:342:HHGFNDSXY:1:1666:29279:18161 1:N:0:GAACCTAG+TCCGCATA
ATCTCAATGCCGAGAGGGATGTGGAGGGCGAGAGAGAGCGACTTCTCTCGGGCCCACAGCCTTACAGC
TGTGGAGCACGGTATCCTCTGCCGAAACAGAGGTTGGACAAGACCGGAGGGGTCTCCTGGTTCCAAAG
+
FFF:FFFFFFFFFFFFFF:FFFFFFFF:FFFFFFFFFFFFFFFFFF:FFF:::F:FFFFFFFFFFFFFFF
FFFFFFF::FFFFF:FFFFFFFFFFFFFFFFFFFFFFFFFFF,FFFFFFFFFFFFFFFFFFFFFFFFFFF
@A00155:342:HHGFNDSXY:1:1666:17363:19038 1:N:0:GAACCTAG+TCCGCATA
GCGACTTCTCTCGGGCCCACAGCCTTACAGCTGTGGAGCACGGTATCCTCTGCCGAAACAGAGGTTGG
ACAAGACCGGAGGGGTCTCCTAGTTCCAAAGGAGATGTACTCCGGGCTTGTTCACGACCTACCGTGTA
+
FFFFFFFFFFFFFFFFFFFFFFFFFFFFFFFFFFFFFFFFFFFFFFFFFFFFFFFFFFFFFFFFFFFFFF
FFFFFFFFFFFFFFFFFFFFFFFFFFFFFFFFFFFFFFFFFFFFFFFFFFFFFFFFFFFFFFFFFFFFFF
@A00155:342:HHGFNDSXY:1:1667:6126:1799 1:N:0:GAACCTAG+TCCGCATA
CTTCTCTCGGGCCCACAGCCTTACAGCTGTGGAGCACGGTATCCTCTGCCGAAACAGAGGTTGGACAA
GACCGGAGGGGTCTCCTGGTTCCAAAGGAGATGTACTCCGGGCTTGTTCACGACCTACCGTGTAAGTC
+
FFFFFFFFFF:FFFFFFFFFFFF:FFFFFFFFFFFFFFFFFFFFFFFFFFFFFFFFFFFFFFFFFFFFFF
FFFFFFFFFFFFFFFFFFFFFFFFF:FFFFFFFF:FFFF:FFFFFFF:FFFFFFFFFFFFFF,FFFFFFF
@A00155:342:HHGFNDSXY:1:1667:3477:30467 1:N:0:GAACCTAG+TCCGCATA
GGGCGAGAGAGAGCGACTTCTCTCGGGCCCACAGCCTTACAGCTGTGGAGCACGGTATCCTCTGCCGA
AACATAGGTTGGACAAGACCGGAGGGGTCTCCTAGTTCCAAAGGAGATGTACTCCGGGCTTGTTCACG
+
FFFFFFFFFFFFFFFFFFFFFFFFFFFFFFFFFFFFFFFFFF:FFFFFFFFFFFFFFFFFFFFFFFFFFF
FFFFFF:F:FFFFFFFFFFFFFFFFFFFFFF,FFFFFFFFFFFFFFFFFFFFFFFFFFFFFFFFFFFFFF
@A00155:342:HHGFNDSXY:1:1668:26530:3004 1:N:0:GAACCTAG+TCCGCATA
GAGCGACTTCTCTCGGGCCCACAGCCTTACAGCTGTGGAGCACGGTATCCTCTGCCGAAACAGAGGTT
GGACAAGACCGGAGGGGTCTCCTAGTTCCAAAGGAGATGTACTCCGGGCTTGTTCACGACCTACCGTG
+
FFFFFFFFFFFFFFFFFFFFFFFFFFFFFFFFFFFFFFFFFFFFFFFFFFFFFFFFFFFFFFFFFFFFFF
FFFFFFFFFFFFFFFFFFFFFFFFFFFFFFFFFFFFFFFFFFFFFFFFFFFFFFFFFFFFFFFFFF:FFF
@A00155:342:HHGFNDSXY:1:1668:24632:16250 1:N:0:GAACCTAG+TCCGCATA
GGGCGAGAGAGAGCGACTTCTCTCGGGCCCACAGCCTTACAGCTGTGGAGCACGGTATCCTCTGCCGA
AACAGAGGTTGGACAAGACCGGAGGGGTCTCCTAGTTCCAAAGGAGATGTACTCCGGGCTTGTTCACG
+

FFFFFFFFFFFFFFFFFFFFFFFFFFFFFFFFFFFFFFFFFFF:FFFFFFFFFFFFFFFFFFFFFFFF
FFFFFFFFFFFFFFFF,FFFFFFFFFFFFFFFFFFFFFFFFFFFFFFFFFFFFFFFFFFFFFFFFFF
@A00155:342:HHGFNDSXY:1:1668:18466:16752 1:N:0:GAACCTAG+TCCGCATA
CTCTATGCCGAGAGGGATGTGGAGGGCGAGAGAGAGCGACTTCTCTCGGGCCCACAGCCTTACAGCTG
TGGAGCACGGTATCCTCTGCCGAAACAGAGGTTGGACAAGACCGGAGGGGTCTCCTAGTTCCAAAGGA
+
FFFFFFFFFFFFFFFFFFFFFFFFFFFFFFFFFF:FFFFFFFFFFFFFFFFFFFFF,FFFFFFFFFFF
FFFFFFFFFFFFFFFFFFFFF:FFFFFFFFFFFF:FFFFFFFFFFFFFFFFFFFFFFFFFFFFFFFFF
@A00155:342:HHGFNDSXY:1:1668:13584:18067 1:N:0:GAACCTAG+TCCGCATA
TGTGGAGGGCGAGAGAGAGCGACTTCTCTCGGGCCCACAGCCTTACAGCTGTGGAGCACGGTATCCTC
TGCCGAAACAGAGGTTGGACAAGACCGGAGGGGTCTCCTAGTTCCAAAGGAGATGTACT
+
FFFFFFFFF:FFFFFFFFFFFFFFFFFFFFFFFFFFFFFFFFFFFFFFFFFFFFFFFFFFFFFFFFFF
FFFFFFFFFFFFFFFFFFFFFFFFFFFFFFFFFFFFFFFFFFFFFFFFFFFFFFFFFFF
@A00155:342:HHGFNDSXY:1:1668:13648:18082 1:N:0:GAACCTAG+TCCGCATA
TGTGGAGGGCGAGAGAGAGCGACTTCTCTCGGGCCCACAGCCTTACAGCTGTGGAGCACGGTATCCTC
TGCCGAAACAGAGGTTGGACAAGACCGGAGGGGTCTCCTAGTTCCAAAGGAGATGTACT
+
FF,FFFFFFFFFFFFFFFFFFFFFFFFFFFFFFFFFFFFFFFFFFFFFFFFFFFFFFFFFFFFFFFFF
FFFFFFFFFFFFFFFFFFFFFFFFFFFFFFFFFFFFFFFFFFFFFFFFFFFFFFF:FF
@A00155:342:HHGFNDSXY:1:1668:11758:19664 1:N:0:GAACCTAG+TCCGCATA
TGTGGAGGGCGAGAGAGAGCGACTTCTCTCGGGCCCACAGCCTTACAGCTGTGGAGCACGGTATCCTC
TGCCGAAACAGAGGTTGGACAAGACCGGAGGGGTCTCCTAGTTCCAAAGGAGATGTACT
+
FFFFFFFFFFFFFFFFFFFFFFFFFFFFFFFFFFFFFFFFFFFFFFF:FFFFFFFFFFFFFFFFFFFF
FFFFFFFFFFFFFFFFFFFFFFFFFFFFFFFFFFFFFFFFFFFFFFFFFFFFFFFFFFF
@A00155:342:HHGFNDSXY:1:1668:15311:22623 1:N:0:GAACCTAG+TCCGCATA
GGGCGAGAGAGAGCGACTTCTCTCGGGCCCACAGCCTTACAGCTGTGGAGCACGGTATCCTCTGCCGA
AACAGAGGTTGGACAAGACCGGAGGGGTCTCCTAGTTCCAAAGGAGATGTACTCCGGGCTTGTTCACG
+
FFFFFFFFFFFFFFFFFFFFFFFFFFFFFFFFFFFFFFFFFFFFFFFFFFFFFFFFFF:FFFFFFFFF
FFFFFFFFFFFFF:FF:FFFFFFFFFFFFFFFFFFFFFFFFFFFFFFFFFFFFFF:FFFFFFFF:F:
@A00155:342:HHGFNDSXY:1:1669:29776:10723 1:N:0:GAACCTAG+TCCGCATA
AGCGACTTCTCTCGGGCCCACAGCCTTACAGCTGTGGAGCACGGTATCCTCTGCCGAAACAGAGGTTG
GACAAGACCGGAGGGGTCTCCTGGTTCCAAAGGAGATGTACTCCGGGCTTGTTCACGACCTACCGTGT
+
FFFFFFFFFFFFFFFFFFFFFFFFFFFFFFFFFFFFFFFFFFFFFFFFFFFFFFFFFFFFFFFFFFFF
FFFFFFFFFFFFFFFFFFFFFFF:FFFFFFFFFFFFFFFFFFFFFFFFFFFFFFFFFFFFFFFFFFFF
@A00155:342:HHGFNDSXY:1:1669:30436:11835 1:N:0:GAACCTAG+TCCGCATA
AGAGAGAGCGACTTCTCTCGGGCCCACAGCCTTACAGCTGTGGAGCACGGTATCCTCTGCCGAAACAG
AGGTTGGACAAGACCGGAGGGGTCTCCTAGTTCCAAAGGAGATGTACTCCGGGCTTGTTCACGACCTA
+
FFFFFFFFFFFFFFFFFFFFF:FFFFFFFFFFFFFFFFF:FFFFFFFFFFFFFFFFFFFFFFFFFFFF
FFFFFFFFFFF:FFFFFFFFFFFFFFFFFFFFFFFFF:FFFFFFFFFFFFFFFF:FFFFFFFFFFFFF
@A00155:342:HHGFNDSXY:1:1669:30960:21825 1:N:0:GAACCTAG+TCCGCATA
GAGCGACTTCTCTCGGGCCCACAGCCTTACAGCTGTGGAGCACGGTATCCTCTGCCGAAACAGAGGTT
GGACAAGACCGGAGGGGTCTCCTAGTTCCAAAGGAGATGTACTCCGGGCTTGTTCACGACCTACCGTG
+
FFFFFFFFFFFFFFFFFFFFFFFFFFFFFFFFFFFFFFFFFFFFFFFFFFFFFFFFFFFFFFFFFFFF
FF:FFFFFFFFFFFFFFFFFFFFFFFFFFFFFFFFFFFF:FFFFFFFFFFFFFFFFFFFFFFFFFFFF
@A00155:342:HHGFNDSXY:1:1669:19714:22106 1:N:0:GAACCTAG+TCCGCATA
GGATGTGGAGGGCGAGAGAGAGCGACTTCTCTCGGGCCCACAGCCTTACAGCTGTGGAGCACGGTATC
CTCTGCCGAAACAGAGGTTGGACAAGACCGGAGGGGTCTCCTAGTTCCAAAGGAGATGTACTCC
+

FFFFFFFFFFFFFFFFFFFFFFFFFFFFFFFFFFFFFFF:FFFFFFFFFFFFFFFFFFFFFFFFFFFF
FFFFFFFFFFFFFFFFF:FFFFFFFFFFFFFFFFFFFFFFFFFFFFFF:FFFFFFFFFF,:FFF
@A00155:342:HHGFNDSXY:1:1669:30165:24361 1:N:0:GAACCTAG+TCCGCATA
GAGCGACTTCTCTCGGGCCCACAGCCTTACAGCTGTGGAGCACGGTATCCTCTGCCGAAACAGAGGTT
GGACAAGACCGGAGGGGTCTCCTAGTTCCAAAGGAGATGTACTCCGGGCTTGTTCACGACCTACCGTG
+
FFFFFFFFFFFFFFFFFFFFFFFFFFFFFFFFFFFFFFFFFFFFFFFFFFFFFFFFFFFFFFFFFFFF
FFFFFFFFFFFFFFFFFFFFFFFFFFFFFFFFFFFFFFFFFFFFFFFFFFFFFFFFFFFFFFFFFFFF
@A00155:342:HHGFNDSXY:1:1669:27733:29888 1:N:0:GAACCTAG+TCCGCATA
AGAGAGAGCGACTTCTCTCGGGCCCACAGCCTTACAGCTGTGGAGCACGGTATCCTCTGCCGAAACAG
AGGTTGGACAAGACCGGAGGGGTCTCCTAGTACCAAAGGAGATGTACTCCGGGCTTGTTCACGACCTA
+
F,F:FFFFFFFFFFFFFF,:FFFFFF:FFF,F::FFF:F:FFFF:FFFF,:,FFF:FF,F,,FFFFF,
FF:,F:FFFFF:F,F:FFF,,,FFFF:FF,,,,FFFF:,FFFFFFFFFFFF,:FFF:F,:F:F::FFF
@A00155:342:HHGFNDSXY:1:1670:6795:9659 1:N:0:GAACCTAG+TCCGCATA
CTCTGGCCTATGGTCATCTCAATGCCGAGAGGGATGTGAAGGGCGAGAGAGAGCGACTTCTCTCGGGC
CCACAGCCTTACAGCTGTGGAGCACGGTATCCTCTGCCGAAACAGAGGTTGGACAAGACCGGAGGGGT
+
FFFFFFFFFFFFFFFFFFFFFFFFFFF:FFFF:FFFFFFF:FFFFFFFF,FFFFFFFFFFFFFFFFF:
FFFFFFFFFFFFFFFFFFFFFFF:FFFFFFFFFFFFFFFFFFFF:FFFFFFFFFFFFFFFFFFFFFFF
@A00155:342:HHGFNDSXY:1:1670:28546:23249 1:N:0:GAACCTAG+TCCGCATA
GAGAGAGCGACTTCTCTCGGGCCCACAGCCTTACAGCTGTGAAGCACGGTATCCTCTGCCGAAACAGA
GGTTGGACAAGACCGGAGGGGTCTCCTAGTTCCAAAGGAGATGTACTCCGGGCTTGTTCACGACCTAC
+
FFFFFFFFFFFFFFFFFFFFFFFFFFFFFFFFFFFFFFFFFFFFFFFF:FFFFFFFFFFFFFFFFFFF
FFFFF:FFFFFFFFFFFFFFFFFFFFFFFFFFFF:FFFFFFFFFFFFFFFFFFFFFFFFFFFFFFFFF
@A00155:342:HHGFNDSXY:1:1670:12373:24893 1:N:0:GAACCTAG+TCCGCATA
CTCAATGCCGAGAGGGATGTGGAGGGCGAGAGAGAGCGACTTCTCTCGGGCCCACAGCCTTACAGCTG
TGGAGCACGGTATCCTCTGCCGAAACAGAGGTTGGACAAGACCGGAGGGGTCTCCTAGTTCCAAAGGA
+
FFFFFFFFFFFFFFFFFFFFFFFFFFFFFFFFFFFFFFFFFFFFFFFFFFFFFFFFFFFFFFFFFFFF
FFFFFFFFFFFFFFFFFFFFFFFFFFFFFFFFFFFFFFFFFFFFFFFFFFFFFFFFFFFFF:FFFFFF
@A00155:342:HHGFNDSXY:1:1671:9878:2973 1:N:0:GAACCTAG+TCCGCATA
GGATGTAGAGGGCGAGAGAGAGCGACTTCTCTCGGGCCCACAGCCTTACAGCTGTGGAGCACGGTATC
CTCTGCCGAAACAGAGGTTGGACAAGACCGGAGGGGTCTCCTAGTTCCAAAGGAGATGTACTCC
+
FFFFFFFFFFFFFFFFFFFFFFFFFFFFFFFFFFFFFFFFFFFFFFFFFFFFFFFFFFFFF:FFFFFF
FFFFFFFFFFFFFFFFFFFFFFFFFFFFFFFFFFFFFFFFFFFFFFFFFFFFFFFFFFFFFFFF
@A00155:342:HHGFNDSXY:1:1671:3549:5165 1:N:0:GAACCTAG+TCCGCATA
CTGCTCTCGGGCCCACAGCCTTACAGCTGTGGAGCACGGTATCCTCTGCCGAAACAGAGGTTGGACAA
GACCGGAGGGGTCTCCTAGTTCCAAAGGAGATGTACTCCGGGCTTGTTCACGACCTACCGTGTAAGTC
+
FF,FFFFFFFFFFFFFFF:F,FFFFFFFFFFFFFFF,FFFFFFFFFF:FFFFF::FFFFFF:FFFFFF
FFFF,F,,FF:FFFFFFFFFFFFFFFFF:FFFFFFFFFFFF:FF,F,FFFF:F:FFFFFFFFFFFFFF
@A00155:342:HHGFNDSXY:1:1671:3712:5916 1:N:0:GAACCTAG+TCCGCATA
CTTCTCTCGGGCCCACAGCCTTACAGCTGTGGAGCACGGTATCCTCTGCCGAAACAGAGGTTGGACAA
GACCGGAGGGGTCTCCTAGTTCCAAAGGAGATGTACTCCGGGCTTGTTCACGACCTACCGTGTAAGTC
+
FFFFFFFFFFFFFFFFFFFFFFFFFFFF:FFFFFFFFFFFFFFFFFFFFFFFFFFFFFFFFFFFFFFF
FFFFFFFF:FFFFFFFFFFFFFFFFFFFFFFFFFFFFFFF,FFFFFFFFFFFFFFFFFFFFFFFFFFF
@A00155:342:HHGFNDSXY:1:1671:7482:13448 1:N:0:GAACCTAG+TCCGCATA
GGCGAGAGAGAGCGACTTCTCTCGGGCCCACAGCCTTACAGCTGTGGAGCACGGTATCCTCTGCCGAA
ACAGAGGTTGGACAAGACCGGAGGGGTCTCCTAGTTCCAAAGGAGATGTACTCCGGGCTTGTTCACGA
+

:FFFFFFFFFFFFFFFFFFFFFFFFFFFFFFFFFF:F:FFFFFFFFFFFF:FFFFFFFFFFFFFFFFF
FFFF:FFFFFFFFFFFFFFFFFFFFFFFFFFFFFFFFFFFFFFFFFFFFFFFFFFFFFFFFFFFFFFF
@A00155:342:HHGFNDSXY:1:1671:22489:17707 1:N:0:GAACCTAG+TCCGCATA
ATCTCAATGCCGAGAGGGATGTGGAGGGCGAGAGAGAGCGACTTCTCTCGGGCCCACAGCCTTACAGC
TGTGGAGCACGGTATCCTCTGCCGAAACAGAGGTTGGACAAGACCGGAGGGGTCTCCTGGTT
+
FFFFFFFFFFFFFFFFFFF:FFFF:FFFFFFFFFFFFFFFFFFFFFFFFFFFFFFFFFFFFFFFFFF
FFFFFFFFFFFFFFFFFFFFFFFFFFFFFFFFFFFFFFFFFFFFFFFFFFFFFFFFFFFFFF
@A00155:342:HHGFNDSXY:1:1671:15383:20932 1:N:0:GAACCTAG+TCCGCATA
ATGCCGAGAGGGATGTGGAGGGCGAGAGAGAGCGACTTCTCTCGGGCCCACAGCCTTACAGCTGTGGA
GCACGGTATCCTCTGCCGAAACAGAGGTTGGACAAGACCGGAGGGGTCTCCTAGTTCCAAAGGAGATG
+
FFFFFFFFFFFFFFF:FFFFFFFFFFFFFFFFFFFFFFFFFFFFFFFFFFFFFFFF:FFFFFFFFFFF
FFFFFFFFFFFFFFFFFFFFFFFFFFFFFFFFFFFFFFFFFFFFFFFFFFFF:F:FFFFFFFFFFFFF
@A00155:342:HHGFNDSXY:1:1671:14850:30874 1:N:0:GAACCTAG+TCCGCATA
CTGTGTCCACTTAAGGACTCACCTCTGGCCTATGGTCATCTCAATGCCGAGAGGGATGTGGAGGGCGA
GAGAGAGCGACTTCTCTCGGGCCCACAGCCTTACAGCTGTGGAGCACGGTATCCTCTGCCGAAACA
+
FFFFFFFFFFFFFFFFFFFFFFFFFFFFFFF:FFFFFFFFFFFFFFFFFFFFFFFFFFFFFFFFFFFF
FFFFFFFFFFFFFFFFFFFFFFFFFFFFFFFFFFFFFFFFFFFFFFFFFFFFFFFFFFFFFFFFFF
@A00155:342:HHGFNDSXY:1:1671:17002:33724 1:N:0:GAACCTAG+TCCGCATA
CATCTCAATGCCGAGAGGGATGTGGAGGGCGAGAGAGAGCGACTTCTCTCGGGCCCACAGCCTTACAG
CTGTGGAGCACGGTATCCTCTGCCGTAACAGAGGTTGGACAAGACCGGAGGGGTCTCCTAGTTCCAAA
+
FFFFFFFFFFFFFFFFFFFFFFFFFFFFFFFFFFFFFFFFFFFFFFFFFFFFFFFFFFFFFFFFFFFF
FFFFFFFFFFFFFFFFFFFFFFFFFFFFFFFFFFFFFFFFFFFFFFFFFFFFFFFFFFFFFFFFFFFF
@A00155:342:HHGFNDSXY:1:1671:3115:35352 1:N:0:GAACCTAG+TCCGCATA
ACTTCTCTCGGGCCCACAGCCTTACAGCTGTGGAGCACGGTATCCTCTGCCGAAACAGAGGTTGGACA
AGACCGGAGGGGTCTCCTAGTTCCAAAGGAGATGTACTCCGGGCTTGTTCACGACCTACC
+
FFFFFFFFFF,FFFFFF:FFFF:::,FFFFFFF:FF,F,FF:FF:F,,:FFFF,FF:FFFF:FFFFFF
FF,FFF:FFFFFFFFFFFFFFFFFFFFFFFFFFFFF,F:FFFFFF:FFFF,FF:FFFFFF
@A00155:342:HHGFNDSXY:1:1672:20021:3161 1:N:0:GAACCTAG+TCCGCATA
GACTTCTCTCGGGCCCACAGCCTTACAGCTGTGGAGCACGGTATCCTCTGCCGAAACAGAGGTTGGAC
AAGACCGGAGGGGTCTCCTAGTTCCAAAGGAGATGTACTCCGGGCTTGTTCACGACCTACCGTGTAAG
+
:FFFFF,FFFFFFFFFFFFFFFF:FFF,F:F:FFFF:,FFFFF::FFF:FFF:FFFFF:FFF:FFFFF
FF:FFFFFFFFFFFFFFFFFFFFFFFFF:FFFF:FF:FFFFFFFFFFF:FFFFFFFFFFFFFFF,F:F
@A00155:342:HHGFNDSXY:1:1672:24207:3302 1:N:0:GAACCTAG+TCCGCATA
CTTCTCTCGGGCCCACAGCCTTACAGCTGTGGAGCACGGTATCCTCTGCCGAAACAGAGGTTGGACAA
GACCGGAGGGGTCTCCTGGTTCCAAAGGAGATGTACTCCGGGCTTGTTCACGACCTACCGTGTAAGTC
+
FFFFFFFFFFFFFFFFFFFFFFFFFFFFFFFFFFFFFFFF:FFFFFFFFFFFFFFFFFFFFFFFFFFF
F:FFFFFFFFFFFFFFFFFFFFFFFFFFFFFFFFFFFFFFFFFFFFFFFFFFFFFFFFFFFFFFFFFF
@A00155:342:HHGFNDSXY:1:1672:14498:13322 1:N:0:GAACCTAG+TCCGCATA
CTCAATGCCGAGAGGGATGTGGAGGGCGAGAGAGAGCGACTTCTCTCGGGCCCACAGCCTTACAGCTG
TGGAGCACGGTATCCTCTGCCGAAACAGAGGTTGGACAAGACCGGAGGGGTCTCCTAGTTCCAAAGGA
+
FFFFFFFFFFFFFFFFFFF:FFFFFFFFFFFFFFFFFFFFFFFFFFFFFFFFFFFFFFFFFFFFFFFF
FFFFFFFFFFFFFFFFFFFFFFFFFFFFFFFFFFFFFFFFFFFFF:FFFFFFFFFFFFFFFFFFFFFF
@A00155:342:HHGFNDSXY:1:1672:20148:14465 1:N:0:GAACCTAG+TCCGCATA
GCTGTGTCCACTTAAGGACTCACCTCTGGCCTATGGTCATCTCAATGCCGAGAGGGATGTAGAGGGCG
AGAGAGAGCGACTTCTCTCGGGCCCACAGCCTTACAGCTGTGGAGCACGGTATCCTCTGCCGAAACAG
+

```
FFFFFFFFFFFF:FFFFFFFFFFFFFFFFFFFFFFFFFFFFFFFFFFFFFFFFFFFFFFFFFFFFFFF
FFFFFFFFFFFFFFFFFFFFFFFFFFFFFFFFFFFFFFFFFFFFFFFFFFFFFFFFFFFFFFFFFFFF
@A00155:342:HHGFNDSXY:1:1672:29704:20275 1:N:0:GAACCTAG+TCCGCATA
ATCTCAATGCCGAGAGGGATGTGGAGGGCGAGAGAGAGCGACTTCTCTCGGGCCCACAGCCTTACAGC
TGTGGAGCACGGTATCCTCTGCCGAAACAGAGGTTGGACAAGACCGGAGGGGTCTCCTAGTTCCAAAG
+
FFFFFFFFFFFFFF,FFFFFF:FFFFFFFFF:FFFFFFFFFFFFFFFFFFFFFFFFF:FFFF:FFFFF
FFFFFFFFFFFFFFFFFFFFFFFFFFF:FFFFFFFFFFFFF,FFFFFFFFFFFFFFFFFFFFFFF:FF
@A00155:342:HHGFNDSXY:1:1672:11044:28322 1:N:0:GAACCTAG+TCCGCATA
CTTCTCTCGGGCCCACAGCCTTACAGCTGTGGAGCACGGTATCCTCTGCCGAAACAGAGGTTGGACAA
GACCGGAGGGGTCTCCTAGTTCCAAAGGAGATGTACTCCGGGCTTGTTCACGACCTACCGTGTAAGTC
+
FFFFFFFFFFFFFFFFFFFFFFFFFFFFFFFFFFFFFFFFFFFFFFFFFFFF:FFFFFFFFF:FFFFF
:FFFFFFFFFFF:FFFFFFFFFFFFFFFFFFFFFFFFFFFFFFFFFFFFFF:FFFFFF,FFFFFFFFF
@A00155:342:HHGFNDSXY:1:1672:25229:32878 1:N:0:GAACCTAG+TCCGCATA
ATGACCATAGGCCAGAGGTGAGTCCTTAAGTGGACACAGCTGATCTAAGGCGGTGTGGCGGGGCATGG
GTTTGAACCCCCATGACGGTCGGAGTCTAGTAGGCTTCCTGATGAGTCCGTTCCAAGGACGAAACCGT
+
FFFFFFFFFFFFFFFFFFFFFFFFFFFFFFFFFFFFFFFFFFFFFFFFFFFFFFFFFFFFFFFFFFFF
FFFFFFFFFFFFFFFFFFFFFFFFFFFFFFFFFFF:FFFFFF,FFFFFFFFFFFFFFFFFFFF::FFFF
@A00155:342:HHGFNDSXY:1:1672:9390:35790 1:N:0:GAACCTAG+TCCGCATA
GACTTCTCTCGGGCCCACAGCCTTACAGCTGTGGAGCACGGTATCCTCTGCCGAAACAGAGGTTGGAC
AAGACCGGAGGGGTCTCCTAGTTCCAAAGGAGATGTACTCCGGGCTTGTTCACGACCTACCGTGTAAG
+
FFFFFFFFFFFFFFFFFFFFFFFFFFFFFFFFFFFFFFFFFFF:FFFFFFFFFFFFFFFFFFFFFFFF
FFFFFFFFFFFFFFFFFFFFFFFFFFFFFFFFFFFFFFFFFFFFFFFFFFFFF:FFFFFFFFFFFFFF
@A00155:342:HHGFNDSXY:1:1673:9326:4460 1:N:0:GAACCTAG+TCCGCATA
ATCTCAATGCCGAGAGGGATGTAGAGGGCGAGAGAGAGCGACTTCTCTCGGGCCCACAGCCTTACAGC
TGTGGAGCACGGTATCCTCTGCCGAAACAGAGGTTGGACAAGACCGGAGGGGTCTCCTAGTTCCAAAG
+
FFFFFFFFFFFFFFFFFFFFFFFFF:FFFFFFFFFFFFFFFFFFFFFFFFFFFFFFFFFFFFFFFFFF
FFFFFFFFFFFFFFFFFFFFFFFFFFFFFFFFFFFFFFFFFFFFFFFFFFFFFFFFFFFFFFFFFFFF
@A00155:342:HHGFNDSXY:1:1673:27118:4648 1:N:0:GAACCTAG+TCCGCATA
GCTGTGTCCACTTAAGGACTCACCTCTGGCCTATGGTCATCTCAATGCCGAGAGGGATGTGAAGGGCG
AGAGAGAGCGACTTCTCTCGGGCCCACAGCCTTACAGCTGTGGAGCACGGTATCCTCTGCCGAAACAG
+
FF:FFFFFFFFFFFFFFFFFFFFFFFFFFF:FFFFFFFFFFFFFFFFFFF:FFFFFFFF,FFFFFFFF
FFFFFFFFFFFFFFFFFFFFFFFFFFFFFFFFFFFFFFFFFFFFFFFFFFFFFFFFFFFFFFFFFFFF
@A00155:342:HHGFNDSXY:1:1673:18710:4930 1:N:0:GAACCTAG+TCCGCATA
GGATGTGGAGGGCGAGAGAGAGCGACTTCTCTCGGGCCCACAGCCTTACAGCTGTGGAGCACGGTATC
CTCTGCCGAAACAGAGGTTGGACAAGACCGGAGGGGTCTCCTAGTTCCAAAGGAGATGTACTCC
+
FFF:FFFFFFFFFFFFFFFFFFFFFFFFFFFFFFFFFFFFFFFFFFFFFFFFFFFFFFFFFFFFFFFF
FFFFFFFFFFFFFFFFFFFFFFFFFFFFFFFFFFFFFFFFFFFFFFFFFFFFFFFFFFFFFFFF
@A00155:342:HHGFNDSXY:1:1673:32524:4993 1:N:0:GAACCTAG+TCCGCATA
AGAGAGAGCGACTTCTCTCGGGCCCACAGCCTTACAGCTGTGGAGCACGGTATCCTCTGCCGAAACAG
AGGTTGGACAAGACCGGAGGGGTCTCCTAGTTCCAAAGGAGATGTACTCCGGGCTTGTTCACGACCT
+
FFFFFFFFF:FFFFFFFFFFFFFFFFFFFFF,:FFFFFFFFFFFFFFFF:FFFFFFFFFFFFFFFFFF
FFF,FFFFFFF:FFF:FFFFFFFFFFFFFFF:FFFFFFFFFF:FFFFFFFFF:F:FFFFFF:FFFFF
@A00155:342:HHGFNDSXY:1:1673:25238:6370 1:N:0:GAACCTAG+TCCGCATA
CTTCTCTCGGGCCCACAGCCTTACAGCTGTGGAGCACGGTATCCTCTGCCGAAACAGAGGTTGGACAA
GACCGGAGGGGTCTCCTAGTTCCAAAGGAGATGTACTCCGGGCTTGTTCACGACCTACCGTGTAAGTC
+
```

```
FFFFFFFFFFFFFFFFFFFFF:FFFFFFFFFFFFFFFFFFFFFFFFFFFFFFFFFFF:FFFFFFFFF
FFFFFFFFFFFFFFFFFFFFFF:FFFFFFFFFFFFFFFFFFFF:FFFFFFFFFFFF,FFFFFFFF
@A00155:342:HHGFNDSXY:1:1673:7048:31391 1:N:0:GAACCTAG+TCCGCATA
GAGGGCGAGAGAGAGCGACTTCTCTCGGGCCCACAGCCTTACAGCTGTGGAGCACGGTATCCTCTGCC
GAAACAGAGGTTGGACAAGACCGGAGGGGTCTCCTAGTTCCAAAGGAGATGTACTCCGGGCTTGTTCA
+
FFFFFFFFFFFFFF:FFF:FFFFFFFFFF,,F,F,FFFFFFFFFFFF,,FFFFFF:FFFFFFFFFF
FFFF:FFFFF:FF:FFFFFFFFFFFFFFFFFFFFFF,FFFFFFFFFFFFFFFFFFFFFF:FFFFFFF
@A00155:342:HHGFNDSXY:1:1674:7455:5635 1:N:0:GAACCTAG+TCCGCATA
AGCGACTTCTCTCGGGCCCACAGCCTTACAGCTGTGGAGCACGGTATCCTCTGCCGAAACAGAGGTTG
GACAAGACCGGAGGGGTCTCCTAGATCCAAAGGAGATGTACTCCGGGCTTGTTCACGACCTACCGTGT
+
FFFFFFFFFFFFFF:FFFFFFFF:FFFFFFFFFFFFF,FFFFF,FFFFFFFFFFFFFFF:FFFFF
FFFFF:FFFFFFFFFFFFFFFFFF,FFFFF:FFFFFFFFFFFFFF,:FFF,FF:F:FFFFFFFF,:F
@A00155:342:HHGFNDSXY:1:1674:25545:8375 1:N:0:GAACCTAG+TCCGCATA
GTCATCTCAATGCCGAGAGGGATGTGGAGGGCGAGAGAGAGCGACTTCTCTCGGGCCCACAGCCTTAC
AGCTGTGGAGCACGGTATCCTCTGCCGAAACAGAGGTTGGACAAGACCGGAGGGGTCTCCTAGTTCCA
+
FFFFFFFFFFFFFFFFFFFFFFFFFFFF:FFFFFFFFFFFFFFFFFFFFFFFFFFFFFFFFFFFFFF:F
FFFFFFFFFFFFFFFFFFFFFFFFFFFFFFFFFFFFFFFFFFFFFFFFFFFFFFFFFFFFFFFFFFFF
@A00155:342:HHGFNDSXY:1:1674:3803:16908 1:N:0:GAACCTAG+TCCGCATA
CTTCTCTCGGGCCCACAGCCTTACAGCTGTGGAGCACGGTATCCTCTGCCGAAACAGAGGTTGGACAA
GACCGGAGGGGTCTCCTAGTTCCAAAGGAGATGTACTCCGGGCTTGTTCACGACCTACCGTGTAAGTC
+
FFFFFFFFFFFFFFFFFFFFFFFFFFFFFFF::FFFFFFFFFFFFFFFFFFFFFFFFFFFFFFFFFFF
FFFFFFFFFFFFFFFFFFFFFFFFFFFFFFFFFFFFFFFFFFF:FFFFFFFFFFFFFFFFFFFFFFFF
@A00155:342:HHGFNDSXY:1:1674:23990:26099 1:N:0:GAACCTAG+TCCGCATA
CCGAGAGGGATGTAGAGGGCGAGAGAGAGCGACTTCTCTCGGGCCCACAGCCTTACAGCTGTGGAGCA
CGGTATCCTCTGCCGAAACAGAGGTTGGACAAGACCGGAGGGGTCTCCTAGTTCCAAAG
+
FFFFF:FFFFFFFFFFFFFFFFFFFFFFFFFFFFFFFFFFFFFFFFFFFFFFFFFFFFFFFFFFFFFF
,FFFFFF:FFFFFFFFFFFFFFFFFFF:FFFFFFFFFFFFFF:FFFFFFFF:FFFFFF
@A00155:342:HHGFNDSXY:1:1674:25084:26490 1:N:0:GAACCTAG+TCCGCATA
AGGGCGAGAGAGAGCGACTTCTCTCGGGCCCACAGCCTTACAGCTGTGGAGCACGGTATCCTCTGCCG
AAACAGAGGTTGGACAAGACCGGAGGGGTCTCCTAGTTCCAAAGGAGATGTACTCCGGGCTTGTTCAC
+
FFFFFFFFFFFFFFFFFF:FFFFFFFFFFFFFFFFFFFFFFFFFFFFFFFFFF:F:FFFFFFFFFFFF
FFFFFFFFFFFFFFFFFFFFFFFFFFFFFFFFFFFFFFFFFFFFFFFFFFFFFFFFFFFFFFFFFFFF
@A00155:342:HHGFNDSXY:1:1674:15691:26663 1:N:0:GAACCTAG+TCCGCATA
TTACAGCTGTGGAGCACGGTATCCTCTGCCGAAACAGAGGTTGGACAAGACCGGAGGGGTCTCCTAGT
TCCAAAGGAGATGTACTCCGGGCTTGTTCACGACCTACCGTGTAAGTCGTAGTCTAGTAGGCTACCTG
+
FFFFFFFFFFFFFFFFFFFFFFFFFFFFFFFFFFFFFFFFFFFFFFFFFFFFFFFFFFFFFFFFFFFF
FFFFFFFFFFFFFFFFFFFFFFFFFFFFFFFFFFFFFFFFFFFFFFFFFFFFFFFFFFFFFFFFFFFF
@A00155:342:HHGFNDSXY:1:1674:7600:31219 1:N:0:GAACCTAG+TCCGCATA
CTCAATGCCGAGAGGGATGTGGAGGGCGAGAGAGAGCGACTTCTCTCGGGCCCACAGCCTTACAGCTG
TGGAGCACGGTATCCTCTGCCGAAACAGAGGTTGGACAAGACCGGAGGGGTCTCCTAGTTCCAAAGGA
+
FFFFFFFFFFFFFFFFF:FFFFFFFFFFFFFFFFFFFFFFFFFFFFFFFFFFFFFFFFFFFFFFFFFF
FFFFFFFFFFFFFFFFFFFFFFFFF:FFFFFFFFFFFFFFFFFFFFFFFFFFFFFFFFFFFFFFFFFF
@A00155:342:HHGFNDSXY:1:1675:28863:17942 1:N:0:GAACCTAG+TCCGCATA
GGGCGAGAGAGAGCGACTTCTCTCGGGCCCACAGCCTTACAGCTGTGGAGCACGGTATCCTCTGCCGA
AACAGAGGTTGGACAAGACCGGAGGGGTCTCCTAGTTCCAAAGGAGATGTACTCCGGGCTTGTTCACG
+
```

FFFFFFFFFFFFFFFFFFFFFFFFFFFFFFFFFFFFFFFFFFFFFFFFFFFFFFFFFFFFFFFFFFFF
FFFF:FFFFFFFFF:FFFFFFFFFFFFFFFFFFFFFFFFFFFFFFFFFFFFFFFFFFFFFFFF:FFFFF
@A00155:342:HHGFNDSXY:1:1675:12608:29716 1:N:0:GAACCTAG+TCCGCATA
AGGGCGAGAGAGAGCGACTTCTCTCGGGCCCACAGCCTTACAGCTGTGGAGCACGGTATCCTCTGCCG
AAACAGAGGTTGGACAAGACCGGAGGGGTCTCCTAGTTCCAAAGGAGATGTACTCCGGGCTTGTTCAC
+
FFFFFFFFFFFFFFFFFFFFFFFFFFFFFFFFFFFFFF:FFFFFFFFFFFFFFFFFFFFFFFFFFFFF
FFFFFFFFFFFFFFFFFFFFFFFFFFFFFFFFFFFF:FFFFFFFFFFFFFFFFFFFFFFFFFFFFFFF
@A00155:342:HHGFNDSXY:1:1675:3558:35274 1:N:0:GAACCTAG+TCCGCATA
CTTCTCTCGGGCCCACAGCCTTACAGCTGTGGAGCACGGTATCCTCTGCCGAAACAGAGGTTGGACAA
GACCGGAGGGGTCTCCTAGTTCCAAAGGAGATGTACTCCGGGCTTGTTCACGACCTACCGTGTAAGTC
+
FFFFFFFFFFFFFFFFFFFF:FFFF,FFFFFFFFFF:F,FFFFFFF:FFF:FFFFFF:FFFFFFFFF
FFFFFFFFFFFFFFFFFFFFFFFFFFFFFFFFFFFF:FFFFFFFFFFFFFFFFFFFFFFF:FFF,FFF
@A00155:342:HHGFNDSXY:1:1675:8865:36605 1:N:0:GAACCTAG+TCCGCATA
CTATGGTCATCTCAATGCCGAGAGGGATGTGGAGGGCGAGAGAGAGCGACTTCTCTCGGGCCCACAGC
CTTACAGCTGTGGAGCACGGTATCCTCTGCCGAAACAGAGGTTGGACAAGACCGGAGGGGTCTCCTAG
+
FFFFFFFFFFFFFFFFFF:FFF:FFFFF:FFFFFFFFF:FFFFFFF:FFFFFFFFFFFFFFFFFFFF
FFFFFFFFFFFFFFFFFFFFFFFFFFFFFFFFFFFFFFFFFFFFFFFFFF,FFFF:FFFFFFFFFFFF:
@A00155:342:HHGFNDSXY:1:1676:26549:6386 1:N:0:GAACCTAG+TCCGCATA
GAGAGGGATGTGGAGGGCGAGAGAGAGCGACTTCTCTCGGGCCCACAGCCTTACAGCTGTGGAGCACG
GTATCCTCTGCCGAAACAGAGGTTGGACAAGACCGGAGGGGTCTCCTGGTTCCAAAGGAGATGTACTC
+
FFFFFFFFFFFFFFFFFFFFFFFFFFFFFFFFFFFFFFFFFFFFFFFFFFFFFFFFFFFFFFFFFFFF
FFFFFFFFFFFFFFFFFFFFFFFFFFFFFFFFFFFFFFFFFFFFFFFFFFFFFFFFFFFFFFFF:FFF
@A00155:342:HHGFNDSXY:1:1676:7075:23484 1:N:0:GAACCTAG+TCCGCATA
GATGTGGAGGGCGAGAGAGAGCGACTTCTCTCGGGCCCACAGCCTTACAGCTGTGGAGCACGGTATCC
TCTGCCGAAACAGAGGTTGGACAAGACCGGAGGGGTCTCCTAGTTCCAAAGGAGATGTAC
+
F::FFFFFFFFFFFFFFFFFFFFFFFFFFFFFFFFFFFFFFFFFFFFFFFFFFFFFFFFFFFFFFFFF
FFFFFFFF:FFFFFFFFFFFFFFFFFFFFFFFFFFFFFFFFFFFFFFFFFFFFFFFFFFF
@A00155:342:HHGFNDSXY:1:1676:27471:24706 1:N:0:GAACCTAG+TCCGCATA
CTCGGGCCCACAGCCTTACAGCTGTGGAGCACGGTATCCTCTGCCGAAACAGAGGTTGGACAAGACCG
GAGGGGTCTCCTAGTTCCAAAGGAGATGTACTCCGGGCTTGTTCACGACCTACCGTGTAAGTCGTAGT
+
FFFFFFFFFFFFFFFFFFFFFFFFFFFFFFFFFFFFFFFFFFFFFFFFFFFFFFFFFFFFFFF:FFFF
FFFFFFFFFFFFFFFFFFFFFFFFFFFFFFFFFFFFFFFFFFFFFFFFFFFFFFFFFFFFFFFFFFF:
@A00155:342:HHGFNDSXY:1:1677:2781:4899 1:N:0:GAACCTAG+TCCGCATA
CAGCCTTACAGCTGTGGAGCACGGTATCCTCTGCCGAAACAGAGGTTGGACAAGACCGGAGGGGTCTC
CTAGTTCCAAAGGAGATGTACTCCGGGCTTGTTCACGACCTACCGTGTAAGTCGTAGTCTAGTAGGCT
+
FFFFFFFFFFFFFFFFFFFFF,F,FFFFFFF::,FFFFFFFFF:FFFFFF:FFFF:FFFFFFFFFFF
FFFFFFFFFFFFFFFFFFFFFFFFF,FFFFFFFFFF,FFF:FFFFFFFFFFFFFFFFFFFFFFFFFFF
@A00155:342:HHGFNDSXY:1:1677:27841:31798 1:N:0:GAACCTAG+TCCGCATA
GAGCGACTTCTCTCGGGCCCACAGCCTTACAGCTGTGGAGCACGGTATCCTCTGCCGAAACAGAGGTT
GGACAAGACCGGAGGGGTCTCCTAGTTCCAAAGGAGATGTACTCCGGGCTTGTTCACGACCTACCGTG
+
FFFFFFFFFFFFFFFFFFFFFFFFFFFFFFFFFFFFFFFFFFFFFFFFFFFFFFFFFFFFFFFFFFFF
FFFFFF:FFFFFFFFFFFFFFFFFFFFFFFFFFFFFFFFFFFFFFFFFFFFFFFFFFFFFFFFFFF:F
@A00155:342:HHGFNDSXY:1:1678:16206:13088 1:N:0:GAACCTAG+TCCGCATA
GGATGTGGAGGGCGAGAGAGAGCGACTTCTCTCGGGCCCACAGCCTTACAGCTGTGGAGCACGGTATC
CTCTGCCGAAACAGAGGTTGGACAAGACCGGAGGGGTCTCCTGGTTCCAAAGGAGATGTACTCC
+

FFFFFFFFFFFFFFFFFFFFFFFFFFFFFFFFFFFFFFFFFFFFFFFFFFFFFFFFFFFFFFFFFFFF
FFFFFFFFFFFFFFFFFFFFFFFFFFFFFFFFFFFFFFFFFFFFFFFFFFFFFFFFFFFFFFFF
@A00155:342:HHGFNDSXY:1:1678:25464:14904 1:N:0:GAACCTAG+TCCGCATA
ACTTCTCTCGGGCCCACAGCCTTACAGCTGTGGAGCACGGTATCCTCTGCCGAAACAGAGGTTGGACA
AGACCGGAGGGGTCTCCTAGTTCCAAAGGAGATGTACTCCGGGCTTGTTCACGACCTACC
+
FFFFFFFFFFFFFFFFFFFFFFFFFFFFFFFFFF:FFFFFFFFFFFFFFFFFFFFFFFFFFFFFFFFF
FFFFFFFFFFFFFFFFFFFFFFFFFFFFFFFFFFFFFFFFFFFFFFFFFFFFFFFFFFFF
@A00155:342:HHGFNDSXY:1:1678:16604:19758 1:N:0:GAACCTAG+TCCGCATA
TAGAGGGCGAGAGAGAGCGACTTCTCTCGGGCCCACAGCCTTACAGCTGTGGAGCACGGTATCCTCTG
CCGAAACAGAGGTTGGACAAGACCGGAGGGGTCTCCTAGTTCCAAAGGAGATGTACTCCGGGCTTGTT
+
FFFFFFFFFFFFFFFFFFFFFFFFFFFFFFFFFFFFFFFFFFFFFFFFFFFFFFFFFFFFFFFFFFFF
FFFFFFFFFFFFFFFFFFFFFFFFFFFFFFFFFFFFFFFFFFFFFFFFFFFFFFFFFFFFFFFFFFFF
@A00155:342:HHGFNDSXY:1:1678:27281:24565 1:N:0:GAACCTAG+TCCGCATA
CAATGCCGAGAGGGATGTGGAGGGCGAGAGAGAGCGACTTCTCTCGGGCCCACAGCCTTACAGCTGTG
GAGCACGGTATCCTCTGCCGAAACAGAGGTTGGACAAGACCGGAGGGGTCTCCTAGTTCCAAAGGAGA
+
FFFFFFFFFFFFFFFFFFFFFFFFFFFFFFFFFFF:FFFFFFFFFFFFFFFFFFFFFFFFFFFFFFFF
FFFFFFFFFFFFFFFFFFFFFFFFFFFFFFFFFFFFFFFFFFFFFFFFFFFFFFFFFFFFFFFFFFFF
@A00155:342:HHGFNDSXY:1:2101:28465:29152 1:N:0:GAACCTAG+TCCGCATA
ATCTCAATGCCGAGAGGGATGTGGAGGGCGAGAGAGAGCGACTTCTCTCGGGCCCACAGCCTTACAGC
TGTGGAGCACGGTATCCTCTGCCGAAACAGAGGTTGGACAAGACCGGAGGGGTCTCCAAGTTCCAAAG
+
FFFFFFFFFFFFFFFFFFFFFFFFFFFFFFFFFFFFFFFFFFFFFFFFFFFFFFFFFFFFFFFFFFFF
FFFFFFFFFFFFFFFFFFFFFFFFFFFFFFFFFFFFFFFFFFFFFFFFFFFFFFFFFFFFFFFFFFFF
@A00155:342:HHGFNDSXY:1:2101:7256:29684 1:N:0:GAACCTAG+TCCGCATA
CCCACAGCCTTACAGCTGTGGAGCACGGTATCCTCTGCCGAAACAGAGGTTGGACAAGACCGGAGGGG
TCTCCTAGTTCCAAAGGAGATGTACTCCGGGCTTGTTCACGACCTACCGTGTAAGTCGTAGTCTAGTA
+
FF:FFFFFFFFFF:FFFFFF,FFFFFFFFFFFFFFF:FFFFFFFFFF:FFFFFFFFFFFFFFFF,:FF
FFFFFFFF::FFFFFFFFFFF:FFFFFF:FFFFFFFFFFFFFFFFFFFFFFFFFFFFFFFFFFFFFFF
@A00155:342:HHGFNDSXY:1:2101:32172:36448 1:N:0:GAACCTAG+TCCGCATA
GAAACCGTCGTTACTCTTCGAGTTGTGTAAGTTTCGTCCTAAAAAGGACTCGTCAGGTAGCCTACTAG
ACTACGACTTACACGGTAGGTCGTGAACAAGCCCGGAGTACATCTCCTTTGGAACTAGGAGACCCCTC
+
FFFFFFFFFFFFFFFF:FFFFFFFFFFFFFFFFFFFF:FFFFFFFFFFFFF:FFFFFFF:FFFFFFF,
FFFFFFFFFFF:FFFFFFFFFFFFFFF:FF,FFFFFFFFFFF,,F:FFFFFFFF:FFFFFFF:FFF:F
@A00155:342:HHGFNDSXY:1:2102:29658:4789 1:N:0:GAACCTAG+TCCGCATA
ATCTCAATGCCGAGAGGGATGTGGAGGGCGAGAGAGAGCGACTTCTCTCGGGCCCACAGCCTTACAGC
TGTGGAGCACGGTATCCTCTGCCGAAACAGAGGTTGGACAAGACCGGAGGGGTCTCCAAGTTCCAAAG
+
FFFFFFFFFFFFFFFFFFFFFFFFFFFFFFFFFFFFFFFFFFFFFFFFFFFFFFFFFFFFFFFFFFFF
FFFFFFFFFFFFFFFFFFFFFFFFFFFFFFFFFFFFFFFFFFFFFFFFFFFFFFFFFFFFFFFFFFFF
@A00155:342:HHGFNDSXY:1:2102:4616:8609 1:N:0:GAACCTAG+TCCGCATA
TGCCCCGCCACACCGCCTTAGATCAGCTGTGTCCACTTAAGGACTCACCTCTGGCCTATGGTCATCTC
AATGCCGAGAGGGATGTGGAGGGCGAGAGAGAGCGACTTCTCTCGGGCCCACAGCCTTACAGCTGTGG
+
FFFFFFFFFFFFFFFFF,FFFFFFFFFFFFFFFFFFFFFFFFFFFFFFFFFFFFFFFFFFFFFFFFFF
FFFFFFFFFFFFFFFFFFFFFFF:FFFFFFFFFFFFFFFFFFFFFFFFFFFFFFFFF:FFFFFFFFFF
@A00155:342:HHGFNDSXY:1:2102:13467:10535 1:N:0:GAACCTAG+TCCGCATA
GCTGTGTCCACTTAAGGACTCACCTCTGGCCTATGGTCATCTCAATGCCGAGAGGGATGTGGAGGGCG
AGAGAGAGCGACTTCTCTCGGGCCCACAGCCTTACAGCTGTGGTGCACGGTATCCTCTGCCGAAACAG
+

FFFFFFFFFFFFFFFFFFFFFFFFFFFFFFFFFFFFFFFFFFFFFFFFFFFFFFFFFFFFFFFFFFFF
FFFFFF:FFFFF:FFFFFFFFFFFFFFFFFFFF,FFFFFF,FFFFFFFFFFFFFFFFFFFFFFFFFFF
@A00155:342:HHGFNDSXY:1:2102:4300:11005 1:N:0:GAACCTAG+TCCGCATA
TGCCCCGCCACACCGCCTTAGATCAGCTGTGTCCACTTAAGGACTCACCTCTGGCCTATGGTCATCTC
AATGCCGAGAGGGATGTGGAGGGCGAGAGAGAGCGACTTCTCTCGGGCCCACAGCCTTACAGCTGTGG
+
FFFFFFFF:FFFFFFFF:F:FF:FFFFFFFFFFFFFF:FFFFFFFFFFFFFFFFFFFFFFFFFFFFFF
FFFFFFF:FFF:FFFFFFFFFFFFFFFFFFFF:FFFFFFFFFFFFFFFFFFFFFFFFFFFFFFFFFFF
@A00155:342:HHGFNDSXY:1:2102:13087:23218 1:N:0:GAACCTAG+TCCGCATA
GTGTCCACTTAAGGACTCACCTCTGGCCTATGGTCATCTCTATGCCGAGAGGGATGTGGAGGGCGAGA
GAGAGCGACTTCTCTCGGGCCCACAGCCTTACAGCTGTGGAGCACGGTATCCTCTGCCGAAACAGAGG
+
FFFFFFFFFFFFFFFFFFFFFFFFFFFFFFFFFFFFFFFFFFFFFFF:FFFFFFFFFFFFFFFFFFFF
FFF:FFFFFFFFFFFFFFFFFFFFFFFFFFFFFFFFFFFFFFFFFFFFFFFFFFFFFFFFFFFFFFFF
@A00155:342:HHGFNDSXY:1:2102:13096:23328 1:N:0:GAACCTAG+TCCGCATA
GTGTCCACTTAAGGACTCACCTCTGGCCTATGGTCATCTCTATGCCGAGAGGGATGTGGAGGGCGAGA
GAGAGCGACTTCTCTCGGGCCCACAGCCTTACAGCTGTGGAGCACGGTATCCTCTGCCGAAACAGAGG
+
FFFFFFFFFFFFFFFFFFFFFFFFFFFFFFFFFFFFFFFFFFFFFFFFFFFFFFFFFFFFFFFFFFFF
FFFFFFFFFFFFFFFFFFFFFFFFFFFF:FFFFFFFFFFFF:FFFFFFFFFFFFFFFFFFFFFFFFFF
@A00155:342:HHGFNDSXY:1:2102:11026:33708 1:N:0:GAACCTAG+TCCGCATA
TGCCGAGAGGGATGTAGAGGGCGAGAGAGAGCGACTTCTCTCGGGCCCACAGCCTTACAGCTGTGGAG
CACGGTATCCTCTGCCGAAACAGAGGTTGGACAAGACCGGAGGGGTCTCCTAGTTCCAAAGGAGATGT
+
FFFFFFFFFFFFFFFFFFFFFFFFFFFFFFFFFFFFFFFFFFFFFFFFFFFFFFFFFFFFFFFFFFFF
FFFFFFFFFFFFFFFFFFFFFFFFFFFFFFFFFFFFFFFFFFFFFFFFFFFFFFFFFFFFFFFFFFFF
@A00155:342:HHGFNDSXY:1:2103:28357:10457 1:N:0:GAACCTAG+TCCGCATA
ACTTCTCTCGGGCCCACAGCCTTACAGCTGTGGAGCACGGTATCCTCTGCCGAAACAGAGGTTGGACA
AGACCGGAGGGGTCTCCTGGTTCCAAAGGAGATGTACTCCGGGCTTGTTCACGACCTACCGTGTAAGT
+
FFFFFFFFFFFFFFFFFFFFFFFFFFFFFFFFFFFFFFFFFFFFFFFFFFFFFFFFFFFFFFFFFFFF
FFFFFFFFFFFFFFFFFFFFFFFFFFFFFFFFFFFFFFFFFFFFFFFFFFFFFFFFFFFFFFFFFFFF
@A00155:342:HHGFNDSXY:1:2103:9525:14606 1:N:0:GAACCTAG+TCCGCATA
GAGAGGGATGTGGAGGGCGAGAGAGAGCGACTTCTCTCGGGCCCACAGCCTTACAGCTGTGGAGCACG
GTATCCTCTGCCGAAACAGAGGTTGGACAAGACCGGAGGGGTCTCCTAGTTCCAAAGGAGATGTACTC
+
FFFFFFFFFFFFFFFFFFFFFFFFFFFFFFFFFFFFFFFFFFFFFFFFFFFFFFFFFFFFFFFFFFFF
FFFFFFFFFFFFFFF:FFFFFFFFFFFFFFFFFFFFFFFFFFFFFFFFFFFFFFFFFFFFFFFFFFFF
@A00155:342:HHGFNDSXY:1:2103:22498:19977 1:N:0:GAACCTAG+TCCGCATA
CACCTCTGGCCTATGGTCATCTCAATGCCGAGAGGGATGTGGAGGGCGAGAGAGAGCGACTTCTCTCG
GGCCCACAGCCTTACAGCTGTGGAGCACGGTATCCTCTGCCGAAACAGAGGTTGGACAAGACCGGAGG
+
FFFFFFFFFFFF:FFFFFFFFFFFFFFFFFFFFFFFFFFFFFFFFFFFFFFFFFFFFFFFFFFFFFF
FFFFFFFFFFFFFFFFFFFFFFFFFFFFFFFFFFFFFFFFFFFFFFFFFFFFFFFFFFF:FFFFFFFF
@A00155:342:HHGFNDSXY:1:2103:22263:24361 1:N:0:GAACCTAG+TCCGCATA
CAATGCCGAGAGGGATGTAGAGGGCGAGAGAGAGCGACTTCTCTCGGGCCCACAGCCTTACAGCTGTG
GAGCACGGTATCCTCTGCCGAAACAGAGGTTGGACAAGACCGGAGGGGTCTCCTAGTTCCAAAGGAGA
+
FFFFFFFFFFFFFFFFFFFF:FFFFFFFFFFF:FFFFFFFFFFFFFFFFFFFFFFFF::FFFFFFFFF
FFFFFFF:FFFFFFFFFFFFFFFFFFFFFFFFF,FFFFFFF:FFFFFFFFFFF,FFFFFFFFFFFFFF
@A00155:342:HHGFNDSXY:1:2103:27389:28166 1:N:0:GAACCTAG+TCCGCATA
TCCTCTGCCGAAACAGAGGTTGGACAAGACCGGAGGGGTCTCCTAGTTCCAAAGGAGATGTACTCCGG
GCTTGTTCACGACCTACCGTGTAAGTCGTAGTCTAGTAGGCTACCTGACGAGTCCTTTTTAGGACGAA
+

```
FFFFFFFFFFFFFFFFFFFFFFFFFFFFFFFFFFFFFFFFFFFFFFFFFFFFFFFFFFFFFFFFFFFF
FFFFFFFFFFFFFFFFFFFFFFFFFFFFFFFFFFFFFFFFFFFFFFFFFFFFFFFFFFFFFFFFFFFF
@A00155:342:HHGFNDSXY:1:2104:17390:6214 1:N:0:GAACCTAG+TCCGCATA
TGTAGAGGGCGAGAGAGAGCGACTTCTCTCGGGCCCACAGCCTTACAGCTGTGGAGCACGGTATCCTC
TGCCGAAACAGAGGTTGGACAAGACCGGAGGGGTCTCCTAGTTCCAAAGGAGATGTACTCCGGG
+
FFFFFFFFFFFFFFFFFFFFFFFFFFFFFFFFFFFFFFFFFFFFFFFFFFFFFFFFFFFFFFFFFFFF
FFFFFFFFFFFFFFFFFFFFFFFFFFFFFFFFFFFFFFFFFFFFFFFFFFFFFFFFFFFFFFFF
@A00155:342:HHGFNDSXY:1:2104:25183:11851 1:N:0:GAACCTAG+TCCGCATA
AGCGACTTCTCTCGGGCCCACAGCCTTACAGCTGTGGAGCACGGTATCCTCTGCCGAAACAGAGGTTG
GACAAGACCGGAGGGGTCTCCTAGTTCCAAAGGAGATGTACTCCGGGCTTGTTCACGACCTACCGTGT
+
FFFFFFFFFFFFFFFFFFFFFFFFFFFFFFFFFFFFFFFFFFFFFFFFFFFFFFFFFFFFFFFFFFFF
FFFFFFFFFFFFFFFFFFFFFFFFFFFFFFFFFFFFFFFFFFFFFFFFFFFFFFFFFFFFFFFFFFFF
@A00155:342:HHGFNDSXY:1:2104:8133:17080 1:N:0:GAACCTAG+TCCGCATA
AGGGCGAGAGAGAGCGACTTCTCTCGGGCCCACAGCCTTACAGCTGTGGAGCACGGTATCCTCTGCCG
AAACAGAGGTTGGACAAGACCGGAGGGGTCTCCTAGTTCCAAAGGAGATGTACTCCGAGCTTGTTCAC
+
FFFFFFFFFFFFFFFFFFFFFFFFFFFFFFFFFFFFFF:FFF:FFFFFFFFFFFFFFFFFFFFFFFFF
FFFFFFFFFFFFFFFFFFFFFFFFFFFFFFFFF::FFFFFFFFFFF:FFFFFFFFFFFFFFFFF:FFF
@A00155:342:HHGFNDSXY:1:2104:31060:24158 1:N:0:GAACCTAG+TCCGCATA
CTTCTCTCGGGCCCACAGCCTTACAGCTGTGGAGCACGGTATCCTCTGCCGAAACAGAGGTTGGACAA
GACCGGAGGGGTCTCCTAGTTCCAAAGGAGATGTACTCCGGGCTTGTTCACGACCTACCGTGTAAGTC
+
FFFFFFFFFFFFFFFFFFFFFFFFFFFFFFFFFFFFF:FFFFFFFFFFFFFFFFFFFFFFFFFFFFFF
FFFFFFFFFFFFFFFFFFFFFFFFFFFFFFFFFFFFFFFFFFFFFFFFFFFFFFFFFFFFFFFFFFFF
@A00155:342:HHGFNDSXY:1:2104:8630:25269 1:N:0:GAACCTAG+TCCGCATA
TGTTTCGGCAGAGGATACCGTGCTCCACAGCTGTAAGGCTGTGGGCCCGAGAGAAGTCGCTCTCTCTC
GCCCTTCACATCCCTCTCGGCATTGAGATGACCATAGGCCAGAGGTGAGTCCTTAAGTGGACACAGCT
+
FFFFFFFFFFFFFFFFFFFFFFFFFFFFFFFFFFFFFFFFFFFFFFFFFFFFFFFFFFFFFFFFFFFF
FFFFFFFFFFFFFFFFFFFFFFFFFFFFFFFFFFFFFFFFFFFFFFFFFFFFFFFFFFFFFFFFFFFF
@A00155:342:HHGFNDSXY:1:2104:18945:26381 1:N:0:GAACCTAG+TCCGCATA
ACCTCTGTTTCGGCAGAGGATACCGTGCTCCACAGCTGTAAGGCTGTGGGCCCGAGAGAAGTCGCTCT
CTCTCGCCCTCCACATCCCTCTCGGCATTGAGATGACCATAGGCCAGAGGAGAGTCCTTAAGTGGACA
+
FFFFFFFFFFFFFFFFFFFFFFFFFFFFFFFFFFFFFFFFFFFFFFFFFFFFFFFFFFFFFFFFFFFF
FFFFFFFFFFFFFFFFFFFFFFFFFFFFFFFFFFFFFFFFFFFFFFFFFFFFFFFFFFFFFFFFFFFF
@A00155:342:HHGFNDSXY:1:2104:21169:30577 1:N:0:GAACCTAG+TCCGCATA
ACCTCTGTTTCGGCAGAGGATACCGTGCTCCACAGCTGTAAGGCTGTGGGCCCGAGAGAAGTCGCTCT
CTCTCGCCCTCCACATCCCTCTCGGCATTGAGATGACCATAGGCCAGAGGAGAGTCCTTAAGTGGACA
+
FFFFFFFFFFFFFFFFFFFFFFFFFFFFFFFFFFFFFFFFFFFFFFFFFFFFFFFFFFF:FFFFFFFF
FFFFFFFFFFFFFFF:FFFFFFFFFFFFFFFFFFFFFFFFFFFFFFFFFF:FFFFFFFFFFFFFFFFF
@A00155:342:HHGFNDSXY:1:2104:6840:30812 1:N:0:GAACCTAG+TCCGCATA
CAGCCTTACAGCTGTGGAGCACGGTATCCTCTGCCGAAACAGAGGTTGGACAAGACCGGAGGGGTCTC
CTAGTTCCAAAGGAGATGTACTCCGGGCTTGTTCACGACCTACCGTGTAAGTCGTAGTCTAGTAGGCT
+
FFFFFFF:FFFFFFFFFFFFFFFFFFFFFFFFFFFFFFFF:FFFFFFFFFFFFFFFFFFFFFFFFFFF
FFFFFFFFFFFFFFFFFFFFFFFFFFFFFFFFFFFFFFFFFFFFFFFFFFFFFFFFFFFFFFFFFFFF
@A00155:342:HHGFNDSXY:1:2104:26142:31203 1:N:0:GAACCTAG+TCCGCATA
TGCCGAGAGGGATGTAGAGGGCGAGAGAGAGCGACTTCTCTCGGGCCCACAGCCTTACAGCTGTGGAG
CACGGTATCCTCTGCCGAAACAGAGGTTGGACAAGACCGGAGGGGTCTCCTAGTTCCAAAGGAGATGT
+
```

FFFFFFFFFFFFFFFFFFFFFFFFFFFFFFFFFFFFFFFFFFFFFFFFFFFFFFFFFFFFFFFFFFFF
FFFFFFFFFFFFFFFFFFFFFFFFFFFFFFFFFFFFFFFFFFFFFFFFFFFFFFFFFFFFF:FFFFFF
@A00155:342:HHGFNDSXY:1:2104:7780:32628 1:N:0:GAACCTAG+TCCGCATA
ATCCTCTGCCGAAACAGAGGTTGGACAAGACCGGAGGGGTCTCCTAGTTCCAAAGGAGATGTACTCCG
GGCTTGTTCACGACCTACCGTGTAAGTCGTAGTCTAGTAGGCTACCTGACGAGTCCTTTTTAGGACGA
+
FFFFFFFFFFFFFFFFFFFFFFFFFFFFFFFFFFFFFFFFFFFFFFFFFFFFFFFFFFFFFFFFFFFF
FFFFFFFFFFFFFFFFFFFFFFFFFFFFFFFFFFFFFFFFFFF:FFFFFFFFFFFFFF:FFFFFFFFF
@A00155:342:HHGFNDSXY:1:2105:4743:1908 1:N:0:GAACCTAG+TCCGCATA
GACTTCTCTCGGGCCCACAGCCTTACAGCTGTGGAGCACGGTATCCTCTGCCGAAACAGAGGTTGGAC
AAGACCGGAGGGGTCTCCTAGTTCCAAAGGAGATGTACTCCGGGCTTGTTCACGACCTACCGTGTAAG
+
FFFFFFFFFF:FFFF:FFFFFFFFFFFFFFFFFFFFFFFFFFFFFFFFFF:FFFFF:FFFFFFFFFFF
FFFFFFFFFF:FFFFFF:FFFFFFFF:FFF:FFFFF:FFFFFFFFFFFFFFFFFFFFFFFFF,FFF,F
@A00155:342:HHGFNDSXY:1:2105:25798:4272 1:N:0:GAACCTAG+TCCGCATA
AGGGATGTGGAGGGCGAGAGAGAGCGACTTCTCTCGGGCCCACAGCCTTACAGCTGTGGAGCACGGTA
TCCTCTGCCGAAACAGAGGTTGGACAAGACCGGAGGGGTCTCCTAGTTCCAAAGGAGATGTACTCCGG
+
FFFFFFFFFFFFFFFFFFFFFFFFFFFFFFFFFFFFFFFFFFFFFFFFFFFFFFFFFFFFFFFFFFFF
FFFFFFFFFFFFFFFFFFFFFFFFFFFFFFFFFFFFFFFFFFFFFFFFFFFFFFFFFFFFFFFFFFFF
@A00155:342:HHGFNDSXY:1:2106:22634:1423 1:N:0:GAACCTAG+TCCGCATA
CTTCTCTCGGGCCCACAGCCTTACAGCTGTGGAGCACGGTATCCTCTGCCGAAACAGAGGTTGGACAA
GACCGGAGGGGTCTCCTAGTTCCAAAGGAGATGTACTCCGGGCTTGTTCACGACCTACCGTGTAAGT
+
FFFFFFFFFFFFFFFFFFFFFFFFFFFFFFFFFFFFFFFFFFFFFFFFFFFFFFFFF:FFFFFFFFFF
FFFFFFFFFFFFFFFFFFFFFFFFFFFFFFFFFFFFFFFFFFFFFFFFFFFFFFFFF:FFFFFFFFF
@A00155:342:HHGFNDSXY:1:2106:9959:5650 1:N:0:GAACCTAG+TCCGCATA
GGACTCACCTCTGGCCTATGGTCATCTCAATGCCGAGAGGGATGTAGAGGGCGAGAGAGAGCGACTTC
TCTCGGGCCCACAGCCTTACAGCTGTGGAGCACGGTATCCTCTGCCGAAACAGAGGTTGGACAAGACC
+
FFFFFFFFFFFFFFFFFFFFFFFFFFFFFFFFFFFFFFFFFFFFFFFFFFFFFFFFFFFFFFFFFFFF
FFFFFFFFFFFFFFFFFFFFFFFFFFFFFFFFFFFFFFFFFFFFFFFFFFFFFFFFFFFFFFFFFFFF
@A00155:342:HHGFNDSXY:1:2106:22435:15013 1:N:0:GAACCTAG+TCCGCATA
GGATGTGGAGGGCGAGAGAGAGCGACTTCTCTCGGGCCCACAGCCTTACAGCTGTGGAGCACGGTATC
CTCTGCCGAAACAGAGGTTGGACAAGACCGGAGGGGTCTCCTAGTTCCAAAGGAGATGTACTCC
+
FFFFFFFFFFFFFFFFFFFFFFFFFFFFFFFFFFFFFFFFFFFFFFFFFFFFFFFFFFFFFFFFFFFF
FFFFFFFFFFFFFFFFFFFFFFFFFFFFFFFFFFFFFFFFFFFFFFFFFFFFFFFFFFFFF:FF
@A00155:342:HHGFNDSXY:1:2107:25509:9878 1:N:0:GAACCTAG+TCCGCATA
TGCCGAGAGGGATGTGGAGGGCGAGAGAGAGCGACTTCTCTCGGGCCCACAGCCTTACAGCTGTGGAG
CACGGTATCCTCTGCCGAAACAGAGGTTGGACAAGACCGGAGGGGTCTCCTAGTTCCAAAGGAGATGT
+
FFFFFFFFFFFFFFFFFFFFFFFFFFFFFFFFFFFFFFFFFFFFFFFFFFFFFFFFFFFFFFFFFFFF
FFFFFFFFFFFFFFFFFFFFFFFFFFFFFFFFFFFFFFFFFFFFFFFFFFFFFFFFFFFFFFFFFFFF
@A00155:342:HHGFNDSXY:1:2107:10592:35712 1:N:0:GAACCTAG+TCCGCATA
ACTTCTCTCGGGCCCACAGCCTTACAGCTGTGGAGCACGGTATCCTCTGCCGAAACAGAGGTTGGACA
AGACCGGAGGGGTCTCCTAGTTCCAAAGGAGATGTACTCCGGGCTTGTTCACGACCTACCGTGTAAGT
+
FFFFFFFFFFFFFFFFFFFFFFFFFFFFFFFFFFFFFFFFFFFFFFFFFFFFFFFFFFFFFFFFFFFF
FFFFFFFFFFFFFFFFFFFFFFFFFFFFFFFFFFFFFFFFFFFFFFFFFFFFFFFFFFFFFFFFF,F:
@A00155:342:HHGFNDSXY:1:2107:2085:36198 1:N:0:GAACCTAG+TCCGCATA
TGTGGCGGGGCATGGGTTTGAACCCCCATGACGGTCGGAGTCTAGTAGGCTCCCTGATGAGTCCGTTC
CAAGGACGAAACCGTCGTTACTCTTTGAGTTGTGTAAGTTTCGTCCTAAAAAGGACTCGTCAGG
+

FFFFFFFFFFFFFFFFFFFFFFFFFFFFFFFFFFFFFFFFFFFFFFFFFFFFFFFFFFFF:FFFFFFFFF
FFFFFFFFF:FFFFFFFFFFFFFFFFFFFFFFFFFFFFFFFFFF:FF:FFFFFF:FFFFFFFFF:F
@A00155:342:HHGFNDSXY:1:2108:24144:30561 1:N:0:GAACCTAG+TCCGCATA
GCGAGAGAGAGCGACTTCTCTCGGGCCCACAGCCTTACAGCTGTGGAGCACGGTATCCTCTGCCGAAA
CAGAGGTTGGACAAGACCGGAGGGGTCTCCTAGTTCCAAAGGAGATGTACTCCGGGCTTGTTCACGAC
+
FFFFFFFFFFFFFFFFFFFFFFFFFFFFFFFFFFFFFFFFFFFFFFFFFFFFFFFFFFFFFFFFFFFF
FFFFFFFFFFFF:FFFFFFFFFFFFFFFFFFFFFFFFFFFFFFFFFFFFFFFFFFFFFFFFFFFFFFF
@A00155:342:HHGFNDSXY:1:2109:18882:6699 1:N:0:GAACCTAG+TCCGCATA
ACCTCTGTTTCGGCAGAGGATACCGTGCTCCACAGCTGTAAGGCTGTGGGCCCGAGAGAAGTCGCTCT
CTCTCGCCCTCCACATCCCTCTCGGCATTGAGATGACCATAGGCCAGAGGTGAGTCCTTAAGTGGACA
+
FFFFFFFFFFFFFFFFFF:F:FFFFFFFFFF:FFFF,FFFFFFFFFFFFFFFFFFFFFFFFFFFFFFF
F:FFFFFFFFFF,FFFF:FFF:FFFFF:FFFFFFFFFFFFFFFFFFFFF:FFFFFFFFFF:FF,FFFF
@A00155:342:HHGFNDSXY:1:2109:10890:9987 1:N:0:GAACCTAG+TCCGCATA
GGACTCACCTCTGGCCTATGGTCATCTCAATGCCGAGAGGGATGTGGAGGGCGAGAGAGAGCGACTTC
TCTCGGGCCCACAGCCTTACAACTGTGGAGCACGGTATCCTCTGCCGAAACAGAGGTTGGACAAGACC
+
FFFFFFFFFFFFFFFFFFFFFFFFFFFFFFFFFFFFFFFFFFFFFFFFFFFFFFFFFFFFFFFFFFFF
FFFFFFFFFFFFFFFFFFFFFFFFFFFFFFFFFFFFFFFFFFFFFFFFFFFFFFFFFFFFFFFF,FFF
@A00155:342:HHGFNDSXY:1:2109:15863:13463 1:N:0:GAACCTAG+TCCGCATA
ATCTCAATGCCGAGAGGGATGTGGAGGGCGAGAGAGAGCGACTTCTCTCGGGCCCACAGCCTTACAGC
TGTGGAGCACGGTATCCTCTGCCGAAACAGAGGTTGGACAAGACCGGAGGGGTCTCCTAGTTCCAAAG
+
FFFFFFFFFFFFFFFFFFFFFFFFFFFFFFFFFFFFFFFFFFFFFFFFFFFFFFFFFFFFFFFFFFFF
FFFFFFFFFFFFFFFFFFFFFFFFFFFFFFFFFFFFFFFFFFFFFFFFFFFFFFFFFFFFFFFFFFFF
@A00155:342:HHGFNDSXY:1:2109:8431:23171 1:N:0:GAACCTAG+TCCGCATA
CTCAATGCCGAGAGGGATGTGGAGGGCGAGAGAGAGCGACTTCTCTCGGGCCCACAGCCTTACAGCTG
TGGAGCACGGTATCCTCTGCCGAAACAGAGGTTGGACAAGACCGGAGGGGTCTCCTAGTTCCAAAGGA
+
FFFFFFFFFFFFFFFFFFFFFFFFFFFFFFFFFFFFFFFFFFFFFFFFFFFFFFFFFFFF:FFFFFFFF
FFFFFFFFFFFFFFFFFFFFFFFFFFFFFFFFFFFFFFFFFFFFFFFFFFFFFFFFFFFFFFFFFFFF
@A00155:342:HHGFNDSXY:1:2110:25708:4335 1:N:0:GAACCTAG+TCCGCATA
AGCGACTTCTCTCGGGCCCACAGCCTTACAGCTGTGGAGCACGGTATCCTCTGCCGAAACAGAGGTTG
GACAAGACCGGAGGGGTCTCCTGGTTCCAAAGGAGATGTACTCCGGGCTTGTTCACGACCTACCGTGT
+
FFFFFFFFFFFFFFFFFFFFFFFFFFFFFFFFFFFFFFFFFFFFFFFFFFFFFFFFFFFFFFFFFFFF
FFFFFFFFFFFFFFFFFFFFFFFFFFFFFFFFFFFFFFFFFFFFFFFFFFFFFFFFFFFFFFFFFFFF
@A00155:342:HHGFNDSXY:1:2111:17065:6778 1:N:0:GAACCTAG+TCCGCATA
ATGCCGAGAGGGATGTGGAGGGCGAGAGAGAGCGACTTCTCTCGGGCCCACAGCCTTACAGCTGTGGA
GCACGGTATCCTCTGCCGAAACAGAGGTTGGACAAGACCGGAGGGGTCTCCTAGTTCCAAAGGAGATG
+
FFFFFFFFFFFFF:FFFFFFFFFFFFFFFFFFFFFFFFFFFFFFFFFFFFFFFFFFFFFFFFFFFFFF
FFFFFFFFFFFFFFFFFFFFFFFFFFF,FFFFFFFFFFFFFFFFFFFFFFFFFF:FFFFFFFFFFFFFF
@A00155:342:HHGFNDSXY:1:2111:7907:13463 1:N:0:GAACCTAG+TCCGCATA
ACCTCTGTTTCGGCAGAGGATACCGTGCTCCACAGCTGTAAGGCTGTGGGCCCGAGAGAAGTCGCTCT
CTCTCGCCCTTCACATCCCTCTCGGCATTGAGATGACCATAGGCCAGAGGTGAGTCCTTAAGTGGACA
+
FFFFFFFFFFFFFFFFFFFFFFFFFFFFFFFFFFFFFFFFFFFFFFFFFFFFFFFFFFFFFFFFFFFF
FFFFFFFFFFFFFFFFFFFFFFFFFFFFFFFFFFFFFFFFFFFFFFFFFFFFFF:F:FFFFFFFFFFF
@A00155:342:HHGFNDSXY:1:2111:27136:31140 1:N:0:GAACCTAG+TCCGCATA
CTTCTCTCGGGCCCACAGCCTTACAGCTGTGGAGCACGGTATCCTCTGCCGAAACAGAGGTTGGACAA
GACCGGAGGGGTCTCCTAGTTCCAAAGGAGATGTACTCCGGGCTTGTTCACGACCTACCGTGTAAGTC
+

FFFFFFFFFFFFFFFFFFFFFFFFFFFFFFFFFFFFFFFFFFFFFFFFFFFFFFFFFFFFFFFFFFFFFF
FFFFFFFFFFFFFFFFFFFFFFFFFFFFFFFFFFFFFFFFFFFFFFFFFFFFFFFFFFFFFFFFFFFFFF
@A00155:342:HHGFNDSXY:1:2112:14597:3380 1:N:0:GAACCTAG+TCCGCATA
AAGGACTCACCTCTGGCCTATGGTCATCTCAATGCCGAGAGGGATGTGAAGGGCGAGAGAGAGCGACT
TCTCTCGGGCCCACAGCCTTACAGCTGTGGAGCACGGTATCCTCTGCCGAAACAGAGGTTGGACAAGA
+
FFFFFFFFFFFFFFFFFFFFFFFFFFFFFFFFFFFFFFFFFFFFFFFFFFFFFFFFFFFFFFFFFFFFFF
FFFFFFFFFFFFFFFFFFFFFFFFFFFFFFFFFFFFFFFFFFFFFFFFFFFFFFFFFFFFFFFFFFFFFF
@A00155:342:HHGFNDSXY:1:2112:3188:26146 1:N:0:GAACCTAG+TCCGCATA
ACTTCTCTCGGGCCCACAGCCTTACAGCTGTGGAGCACGGTATCCTCTGCCGAAACAGAGGTTGGACA
AGACCGGAGGGGTCTCCTAGTTCCAAAGGAGATGTACTCCGGGCTTGTTCACGACCTACCGTGTAAGT
+
FFFFFFFFFFFFFFFFFFFFFFFFFFFFFFFFFFFFFFFFFFFFFFFFFFFFFFFFFFFFFFFFFFFFFF
F:FFFFFFFFFFFFFFFFFFFFFFFFFFFFFFFFFFFFFFFFFFFFFFFFFFFFFFFFFFF:FFFF:FFF
@A00155:342:HHGFNDSXY:1:2112:13566:31939 1:N:0:GAACCTAG+TCCGCATA
GGATGTAGAGGGCGAGAGAGAGCGACTTCTCTCGGGCCCACAGCCTTACAGCTGTGGAGCACGGTATC
CTCTGCCGAAACAGAGGTTGGACAAGACCGGAGGGGTCTCCTAGTTCCAAAGGAGATGTACTCC
+
FFFFFFFFFFFFFFFFFFFFFFFFFFFFFFFFFFFFFFFFFFFFFFFFFFFFFFFFFFFFFFFFFFFFFF
FFFFFFFFFFFFFFFFFFFFFFFFFFFFFFFFFFFFFFFFFFFFFFFFFFFFFFFFFFFFFFFF
@A00155:342:HHGFNDSXY:1:2112:3378:33144 1:N:0:GAACCTAG+TCCGCATA
AAAGACTCACCTCTGGCCTATGGTCATCTCAATGCCGAGAGGGATGTGGAGGGCGAGAGAGAGCGACT
TCTCTCGGGCCCACAGCCTTACAGCTGTGGAGCACGGTATCCTCTGCCGAAACAGAGGTTGGACAAGA
+
FFFFFFFFFFFFFFFFFFFFFFFFFFFFFFFFFFFFFFFFFFFFFFFFFFFFFFFFFFFFFFFFFFFFF:
FFFFFFFFFFFFFFFFFFFFFFFFFFFFF:FFF,FFFFF:FFFFFFFFFFF,FFFFFFFFFFFF:FFF
@A00155:342:HHGFNDSXY:1:2112:10022:35603 1:N:0:GAACCTAG+TCCGCATA
GAGCGACTTCTCTCGGGCCCACAGCCTTACAGCTGTGGAGCACGGTATCCTCTGCCGAAACAGAGGTT
GGACAAGACCGGAGGGGTCTCCTAGTTCCAAAGGAGATGTACTCCGGGCTTGTTCACGACCTACCGTG
+
FFFFFFFFFFFFFFFFFFFFFFFFFFFFFFFFFFFFFFFFFFFFFFFFFFFFFFFFFFFFFFFFFFFFFF
FFFFFFFFFFFFFFFFFFFFFFFFFFFFFFFFFFFFFFFFFFFFFFFFFFFFFFF:FFFFFFFFFFFF
@A00155:342:HHGFNDSXY:1:2113:29306:6277 1:N:0:GAACCTAG+TCCGCATA
GGATGTGGAGGGCGAGAGAGAGCGACTTCTCTCGGGCCCACAGCCTTACAGCTGTGGAGCACGGTATC
CTCTGCCGAAACAGAGGTTGGACAAGACCGGAGGGGTCTCCTAGTTCCAAAGGAGATGTACTCC
+
FFF,FFFFFFFFFFFFFFFFFFFFFFFFFFFFFFFFFFFFFFFFFFFFFFFFFFFFFFFFFFFFFFFFFF
FFFFFFFFFFFFFFFFFFFFFFFFFF:FFFFFFFFFFFFFFFFFFFFFFFFFFFFFFFFFFFFF
@A00155:342:HHGFNDSXY:1:2113:13223:7513 1:N:0:GAACCTAG+TCCGCATA
ACTTCTCTCGGGCCCACAGCCTTACAGCTGTGGAGCACGGTATCCTCTGCCGAAACAGAGGTTGGACA
AGACCGGAGGGGTCTCCTAGTTCCAAAGGAGATGTACTCCGGGCTTGTTCACGACCTACCGTGTAAGT
+
FFFFFFFFFFFFFFFFFFFFFFFFFFFFFFFFFFFFFFFFFFFFFFFFFFFFFFFFFFFFFFFFFFFFFF
FFFFFFFFFFFFFFFFFFFFFFFFFFFFFFFFFFFFFFFFFFFFFFFFFFFFFFFFFFFFFFFFFFFFFF
@A00155:342:HHGFNDSXY:1:2113:23574:13479 1:N:0:GAACCTAG+TCCGCATA
GAGAGCGACTTCTCTCGGGCCCACAGCCTTACAGCTGTGGAGCACGGTATCCTCTGCCGAAACAGAGG
TTGGACAAGACCGGAGGGGTCTCCTAGTTCCAAAGGAGATGTACTCCGGGCTTGTTCACGACCTACCG
+
FFFFFFFFFFFFFFFFFFFFFFFFFFFFFFFFFFFFFFFFFFFFFFFFFFFFFFFFFFFFFFFFFFFFFF
FFFFFFFFFFF:FFFFFFFFFFFFFFFFFF:FFFFFFFFFFFFFFFFFFFFFFFFFFFFFFFFFFFFF:F
@A00155:342:HHGFNDSXY:1:2113:20844:23062 1:N:0:GAACCTAG+TCCGCATA
CTCAATGCCGAGAGGGATGTGGAGGGCGAGAGAGAGCGACTTCTCTCGGGCCCACAGCCTTACAGCTG
TGGTGCACGGTATCCTCTGCCGAAACAGAGGTTGGACAAGACCGGAGGGGTCTCCTAGTTCCAAAGGA
+

FFFFFFFFFF:FFFFFFFFFFFFFFFFFFFFFFFFFFFFFFFFFFFFFFFFFFFFFFFFFFFF:FFFF
FFFFFFFFFFFFFFFFFFFFFFFFFFFFFFFFFFFFFFFFFFFFFFFFFFFFFFFFFFFFFFFFFFFF
@A00155:342:HHGFNDSXY:1:2113:8883:31501 1:N:0:GAACCTAG+TCCGCATA
TCCACTTAAGGACTCTCCTCTGGCCTATGGTCATCTCAATGCCGAGAGGGATGTGGAGGGCGAGAGAG
AGCGACTTCTCTCGGGCCCACAGCCTTACAGCTGTGGAGCACGGTATCCTCTGCCGAAACAGAGGTTG
+
FFF:FF,FF:FFFF,FFFFFFFF,FF,FFFFFFFFFFFFFFFFFFF:FFFF:F,FFF:FFFFFFFFFF
FFFFFFFF:FFFFFFFF:FFFFFFF,FF,FFFFF:FF:FF:FFFFF:FFF:FFFFFFFF:FFF:FFFF
@A00155:342:HHGFNDSXY:1:2113:10050:32111 1:N:0:GAACCTAG+TCCGCATA
TCCACTTAAGGACTCTCCTCTGGCCTATGGTCATCTCAATGCCGAGAGGGATGTGGAGGGCGAGAGAG
AGCGACTTCTCTCGGGCCCACAGCCTTACAGCTGTGGAGCACGGTATCCTCTGCCGAAACAGAGGTTG
+
FFFFFFFFFFFFFFFFFFFFFFFFFFFFFFFFFFFFFFFFFFFFFFFFFFFFFFFFFFFFFFFFFFFF
FFFFFFFFFFFFFFFFFFFFFFFFFFFFFFFFFFFFFFFFFFFFFFFFFFFFFFFFFFFFFFFFFFFF
@A00155:342:HHGFNDSXY:1:2113:26901:32831 1:N:0:GAACCTAG+TCCGCATA
GCGAGAGAGAGCGACTTCTCTCGGGCCCACAGCCTTACAGCTGTGGAGCACGGTATCCTCTGCCGAAA
CAGAGGTTGGACAAGACCGGAGGGGTCTCCTAGTTCCAAAGGAGATGTACTCCGGGCTTGTTCACGAC
+
FFFFFFFFFFFFFFFFFFFFFFFFFFFFFFFFFFFFFFFFFFFFFFFFFFFFFFFFFFFFFFFFFFFF
FFFFFFFFFFFFFFFFFFFFFFFFFFFFFFFFFFFF:FFFFFFFFFFFFFFFFFFFFFFFFFFFF:FF
@A00155:342:HHGFNDSXY:1:2113:27724:35822 1:N:0:GAACCTAG+TCCGCATA
AGGGATGTGGAGGGCGAGAGAGAGCGACTTCTCTCGGGCCCACAGCCTTACAGCTGTAGAGCACGGTA
TCCTCTGCCGAAACAGAGGTTGGACAAGACCGGAGGGGTCTCCTAGTTCCAAAGGAGATGTACTCCGG
+
FFFFFFFFFFFFFFFFFFFFFFFFFFFFFFFFFFFFFFFFFFFFFFFFFFFFFFFFFFFFFFFFFFFF
FFFFFFFFFFFFFFFFFFFFFFFFFFFFFFFFFFFFFFFFFFFFFFFFFF:FFFFFFFFFFFFFFFFF
@A00155:342:HHGFNDSXY:1:2114:26341:8061 1:N:0:GAACCTAG+TCCGCATA
CTTCTCTCGGGCCCACAGCCTTACAGCTGTGGAGCACGGTATCCTCTGCCGAAACAGAGGTTGGACAA
GACCGGAGGGGTCTCCTAGTTCCAAAGGAGATGTACTCCGGGCTTGTTCACGACCTACCA
+
FFFFFFFFFFFFFFFFFFFFFFFFFFFFFFFFFFFFFFFFFFFFFFFFFFFFFFFFFFFFFFFFFFFF
FFFFFFFFFFFFFFFFFFFFFFFFFFFFFFFFFFFFFFFFFFFFFFFFFFFFFFFFFFFF
@A00155:342:HHGFNDSXY:1:2114:2284:20447 1:N:0:GAACCTAG+TCCGCATA
AGGGCGAGAGAGAGCGACTTCTCTCGGGCCCACAGCCTTACAGCTGTGGAGCACGGTATCCTCTGCCG
AAACAGAGGTTGGACAAGACCGGAGGGGTCTCCTAGTTCCAAAGGAGATGTACTCCGGGCTTGTTCAC
+
FF,FFFFFFF:FF,FFFFFFFFFFFFFFFFFFFF:FF:FFF:,FFFFF:F:FFFFF:FF,F:,FFFF:
FF::FFF,:FFFFF,FFFFFF:FFFFFFFF:FF:FFFFF,FF:,FFFFF,,FF:FFF:FFFFFFFFFF
@A00155:342:HHGFNDSXY:1:2114:21441:32017 1:N:0:GAACCTAG+TCCGCATA
CTATGGTCATCTCAATGCCGAGAGGGATGTGGAGGGCGAGAGAGAGCGACTTCTCTCGGGCCCACAGC
CTTACAGCTGTGGAGCACGGTATCCTCTGCCGAAACAGAGGTTGGACAAGACCGGAGGGGTCTCCTAG
+
FFFFFFFFFFFFFFFFFFFFFFFFFFFFFFFFFFFFFFFFFFFFFFFFFFFFFFFFFFFFFFFFFFFF
FFFFFFFFFFFFFFFFFFFFFFFFFFFFFFFFFFFFFFFFFFFFFFFFFFFFFFFFFFFFFFFFFFFF
@A00155:342:HHGFNDSXY:1:2115:5258:35744 1:N:0:GAACCTAG+TCCGCATA
GGATGTGGAGGGCGAGAGAGAGCGACTTCTCTCGGGCCCACAGCCTTACAGCTGTGGAGCACGGTATC
CTCTGCCGAAACAGAGGTTGGACAAGACCGGAGGGGTCTCCTGGTTCCAAAGGAGATGTACTCC
+
FFFFFFFFFFFFFFFFFFFFFFFFFFFFFFFFFF:FFFF:FFFFFFFFFFFFFFFFFFFFFFFFFFFF
FFFFFFFFFFFFFFFFFFFFFFFFF:FFFFFFFFFFFFFFFFFFFFFFFFFFFFFFFFFFFFFF
@A00155:342:HHGFNDSXY:1:2116:18150:33019 1:N:0:GAACCTAG+TCCGCATA
CTTCTCTCGGGCCCACAGCCTTACAGCTGTGGAGCACGGTATCCTCTGCCGAAACAGAGGTTGGACAA
GACCGGAGGGGTCTCCTAGTTCCAAAGGAGATGTACTCCGGGCTTGTTCACGACCTACC
+

FFFFFFFFFFFFFFFFFFFFFFFFFFFFFFFFFFFFFFFFFFFFFFFFFFFFFFFFFFFFFFFFFFFFFFF
FFFFFFFFFFFFFFFFFFFFFFFFFFFFFFFFFFFFFFFFFFFFFFFFFFFFFFFFFFFFF
@A00155:342:HHGFNDSXY:1:2117:3549:19132 1:N:0:GAACCTAG+TCCGCATA
TCGGCAGAGGATACCGTGCTCCACAGCTGTAAGGCTGTGGGCCCGAGAGAAGTCGCTCTCTCTCGCCC
TCCACATCCCTCTCGGCATTGAGATGACCATAGGCCAGAGGAGAGTCCTTAAGTG
+
FFFFFFFFFFFFFFFFFFFFFFFFFFFFFFFFFFFFFFFFFFFFFFFFFFFFFFFFFFFFFFFFFFFFFFF
FFFFFFFFFFFFFFFFFFFFFFFFFFFFFFFFFFFFFFFFFFFFFFFFFFFFFFFFF
@A00155:342:HHGFNDSXY:1:2118:27778:6104 1:N:0:GAACCTAG+TCCGCATA
GAGCGACTTCTCTCGGGCCCACAGCCTTACAGCTGTGGAGCACGGTATCCTCTGCCGAAACAGAGGTT
GGACAAGACCGGAGGGGTCTCCTAGTTCCAAAGGAGATGTACTCCGGGCTTGTTCACGACCTACCGTG
+
FF:FFFFFFFFFFFFFFFFFFFFFFFFFFFFFFFFFFFFFFFFFFFFFFFFFFFFFFFFFFFFFFFFFFFF
FFFFFFFFFFFFFFFFFFFFFFFFFFFFFFFFF:FFFFFFFFFFFFFFFFFFFFFFFFFFFFFFFFFFFF
@A00155:342:HHGFNDSXY:1:2118:1533:6558 1:N:0:GAACCTAG+TCCGCATA
GGCAGAGGATACCGTGCTCCACAGCTGTAAGGCTGTGGGCCCGAGAGAAGTCGCTCTCTCTCGCCCTC
TACATCCCTCTCGGCATTGAGATGACCATAGGCCAGAGGTGAGTCCTTAAGTGGACACAGCTGATCT
+
FFFFFFFFFFFFFF:FFFFFFFFFFFFF:FFFF:FFF:FFFFFFF:FFFF,F,FFF:FFFFF:FFFFF
FFFFFFFFFFF,FFFFF,FF:F,:FFFF,:FFFF:FFFFFFFF,FF:,FFF,FFF:FFFFFF:FFFF
@A00155:342:HHGFNDSXY:1:2118:16929:7733 1:N:0:GAACCTAG+TCCGCATA
AGAGAGAGCGACTTCTCTCGGGCCCACAGCCTTACAGCTGTGGAGCACGGTATCCTCTGCCGAAACAG
AGGTTGGACAAGACCGGAGGGGTCTCCTGGTTCCAAAGGAGATGTACTCCGGGCTTGTTCACGACCTA
+
FFFFFFFFFFFFFFFFFFFFFFFFFFFFFFFFFFFFFFFFFFFFFFFFFFF:FFFFFFFFFFFFFFFF
FFFFFFFFFFFFFFFFFFFFFFFFFFFFFFFFFFFFFFFFFFFFFFFFFFFFFFFFFFFFFFFFFFFF
@A00155:342:HHGFNDSXY:1:2118:32072:10473 1:N:0:GAACCTAG+TCCGCATA
AGCGACTTCTCTCGGGCCCACAGCCTTACAGCTGTGGAGCACGGTATCCTCTGCCGAAACAGAGGTTG
GACAAGACCGGAGGGGTCTCCTAGTTCCAAAGGAGATGTACTCCGGGCTTGTTCACGACCTACCGTGT
+
FFFFFFFFFFFFFFFFFFF:FFFFFFFFFFFFFFFFFFFFFFFFFFFFFFFFFFFFFFFFFFFFFFFF
FFFFFFFFFFFFFFFFFFFFFFFFFFFFFFFFFFFFFFFFFFFFFFFFFF:FFFFFFFFFFFFFFFFF
@A00155:342:HHGFNDSXY:1:2118:24523:36166 1:N:0:GAACCTAG+TCCGCATA
CTTCTCTCGGGCCCACAGCCTTACAGCTGTGGAGCACGGTATCCTCTGCCGAAACAGAGGTTGGACAA
GACCGGAGGGGTCTCCTAGTTCCAAAGGAGATGTACTCCGGGCTTGTTCACGACCTACCGTGTAAGTC
+
FFFFFFFFFFFFFFFFFFFFFFFFFFFFFFFFFFFFFFFFFFFFFFFFFFFFFFFFFFFFFFFFFFFF
FFFFFFFFFFFFFFFFFFFFFFFFFFFFFFFFFFFFFFFFFFFFFFFFFFFFFFFFFFFFFFFFFFFF
@A00155:342:HHGFNDSXY:1:2119:2130:5337 1:N:0:GAACCTAG+TCCGCATA
CTTCTCTCGGGCCCACAGCCTTACAGCTGTGGAGCACGGTATCCTCTGCCGAAACAGAGGTTGGACAA
GACCGGAGGGGTCTCCTAGTTCCAAAGGAGATGTACTCCGGGCTTGTTCACGACCTACC
+
FFFFFFFFFFFFFFFFFFFFFFFFFFFFFFFFFFFFFFFFFFFFFFFFFFFFFFFFFFFFFFFFFFFF
:FFFFFFFFFFFFFFFFFFFFFFFFFFFFFFFFFFFFFFFFFFFFFFFFF:FFFFFFFFF
@A00155:342:HHGFNDSXY:1:2119:23918:12571 1:N:0:GAACCTAG+TCCGCATA
ACCCCTCCGGTCTTGTCCAACCTCTGTTTCGGCAGAGGATACCGTGCTCCACAGCTGTAAGGCTGTGG
GCCCGAGAGAAGTCGCTCTCTCTCGCCCTCCACATCCCTCTCGGCATTGAGATGACCATAGGCCAGAG
+
FFFFFFFFFFFFFFFFFFFFFFFFFFFFFFFFFFFFFFFFFFFFFFFFFFFFFFFFFFFFFFFFFFFF
FFFFFFFFFFFFFFFFFFFFFFFFFFFFFFFFFFFFFFFFFFFFFFFFFFFFFFFFFFFFFFFFFFFF
@A00155:342:HHGFNDSXY:1:2120:6605:6355 1:N:0:GAACCTAG+TCCGCATA
TGACCATAGGCCAGAGGTGAGTCCTTAAGTGGACACAGCTGATCTAAGGCGGTGTGGCGGGGCATGGG
TTTGAACCCCCATGACGGTCGGAGTCTAGTAGGCTCCCTGATGAGTCCGTTCCAAGGACGACACCGTC
+

```
FFFFFFFFFFFFFFFFFFFFFFFFFFFFFFFFFFFFFFFFFFFFFFFFFFFFFFFFFFFFFFFFFFFF
FFFFFFFFFFFFFFFFFFFFFFFFFFFFFFFFFFFFFFFFFFFFFFFFFFFFFFFFFFFF,F,FFFFF
@A00155:342:HHGFNDSXY:1:2120:27787:9596 1:N:0:GAACCTAG+TCCGCATA
GAGAGGGATGTGGAGGGCGAGAGAGAGCGACTTCTCTCGGGCCCACAGCCTTACAGCTGTGGAGCACG
GTATCCTCTGCCGAAACAGAGGTTGGACAAGACCGGAGGGGTCTCCTAGTTCCAAAGGAGATGTACTC
+
F:FFFFFFFFFFFFFFFFFFFFFFFFFFFFFFFFFFFFFFFFFFFFFFFFFFFFFFFFFFFFFFFFFF
FFFFFFFFFFFFFFFFFFFFFFFFFFFFFFFFFFFFFFFFFFFFFFFFFFFFFFFFFFFFFFFFFFFF
@A00155:342:HHGFNDSXY:1:2120:29279:12680 1:N:0:GAACCTAG+TCCGCATA
GAGAGCGACTTCTCTCGGGCCCACAGCCTTACAGCTGTGGAGCACGGTATCCTCTGCCGAAACAGAGG
TTGGACAAGACCGGAGGGGTCTCCTAGTTCCAAAGGAGATGTACTCCGGGCTTGTTCACGACCTACCG
+
FFFFFFFFFFFFFFFFFFFFFFFFFFFFFFFFFFFFFFFFFFFFFFFFFFFFFFFFFFFFFFFFFFFF
FFFFFFFFFFFFFFFFFFFFFFFFFFFFFFFFFFFFFFFFFFFFFFFFFFF,FFFFFFFFFFFFFFFF
@A00155:342:HHGFNDSXY:1:2120:25446:21324 1:N:0:GAACCTAG+TCCGCATA
AGCGACTTCTCTCGGGCCCACAGCCTTACAGCTGTGGAGCACGGTATCCTCTGCCGAAACAGAGGTTG
GACAAGACGGGAGGGGTCTCCTAGTTCCAAAGGAGATGTACTCCGGGCTTGTTCACGACCTACCGTGT
+
F:FFFFFFFFFFFFFFFFFFFF:FFFFFFFFFFFFFFFFFFFFFFFFFFFFFFFFFFFFFFFFFF:FF
FFFFFFFFFFFFFFFFFFFFFFFFFFFFFFFFFFFFFFFFFFFFFFFFFFF:FFFFFFFFFFFFFFFF
@A00155:342:HHGFNDSXY:1:2120:28212:23422 1:N:0:GAACCTAG+TCCGCATA
GAGAGGGATGTGGAGGGCGAGAGAGAGCGACTTCTCTCGGGCCCACAGCCTTACAGCTGTGGAGCACG
GTATCCTCTGCCGAAACAGAGGTTGGACAAGACCGGAGGGGTCTCCTAGTTCCAAAGGAGATGTACTC
+
FFFFFFFFFFFFFFFFFFFFFFFFFFFFFFFFFFFFFFFFFFFFFFFFFFFFFFFFFFFFFFFFFFFF
FFFFFFFFFFFFFFFFFFFFFFFFFFFFFFFFFFFFFFFFFFFFFFFFFFFFFFFFFFFFFFFFFFFF
@A00155:342:HHGFNDSXY:1:2120:15935:23860 1:N:0:GAACCTAG+TCCGCATA
TCCTCTGCCGAAACAGAGGTTGGACAAGACCGGAGGGGTCTCCTAGTTCCAAAGGAGATGTACTCCGG
GCTTGTTCACGACCTACCGTGTAAGTCGTAGTCTAGTAGGCTACCTGACGAGTCCTTTTTAGGACGAA
+
FFFFFFFFFFFFFFFFFFFFFFFFFFFFFFFFFFFFFFFFFFFFFFFFFFFFFFFFFFFFFFFFFFFF
FFFFFFFFFFFFFFFFFFFFFFFFFFFFFFFFFFFFFFFFFFFFFFFFFFFFFFFFFFFFFFFFFFFF
@A00155:342:HHGFNDSXY:1:2120:31910:34522 1:N:0:GAACCTAG+TCCGCATA
ATCCTCTGCCGAAACAGAGGTTGGACAAGACCGGAGGGGTCTCCTAGTTCCAAAGGAGATGTACTCCG
GGCTTGTTCACGACCTACCGTGTAAGTCGTAGTCTAGTAGGCTACCTGACGAGTCCTTTTTAGGACGA
+
FFFFFFFFFFFFFFFFFFFFFFFFFFFFFFFFFFFFFFFFFFFFFFFFFFFFFFFFFFFFFFFFFFFF
FFFFFF:FFFFFFF:FFFFFFFFFFFFFFFFFFFFFFFFFFFFFFFFFFFFFFFFFFFFFFFFF:FF:
@A00155:342:HHGFNDSXY:1:2121:5159:18349 1:N:0:GAACCTAG+TCCGCATA
CTTCTCTCGGGCCCACAGCCTTACAGCTGTGGAGCACGGTATCCTCTGCCGAAACAGAGGTTGGACAA
GACCGGAGGGGTCTCCTAGTTCCAAAGGAGATGTACTCCGGGCTTGTTCACGACCTACCGTGTAAGTC
+
FFFFFFFFFFFFFFFFFFFFFFFFFFFFFFFFFFFFFFFFFFFFFFFFFF:FFFFFFF:FFFFFFFFF
:FFFFFFFFFFFFFFFFFFFFF,FFFFFFFFFFFFFFFFFFFFFFFFFFFF,FFFFFFFFFFFFFFFF
@A00155:342:HHGFNDSXY:1:2121:3522:34021 1:N:0:GAACCTAG+TCCGCATA
ATCTCAATGCCGAGAGGGATGTGGAGGGCGAGAGAGAGCGACTTCTCTCGGGCCCACAGCCTTACAGC
TGTGGAGCACGGTATCCTCTGCCGAAACAGAGGTTGGACAAGACCGGAGGGGTCTCCTAGTTCCAAAG
+
FFFFFFFFFFFFFFFFFFF:FFFFFFFFFFFFFFFFFFFFFFFFFFFFFFFFFFFFFFFFFFFFFFFF
FFFFFFFFFFFFFFFFFFFFFFFFFFFFFFFFFFFFFFFFFFFFFFFFFFFFFFFFFFFFFFFFFFFF
@A00155:342:HHGFNDSXY:1:2122:18069:15562 1:N:0:GAACCTAG+TCCGCATA
CTTCTCTCGGGCCCACAGCCTTACAGCTGTGGAGCACGGTATCCTCTGCCGAAACAGAGGTTGGACAA
GACCGGTGGGGTCTCCTAGTTCCAAAGGAGATGTACTCCGGGCTTGTTCACGACCTACCGTGTAAGTC
+
```

F:FFFFFFFFFFFFFFFFFFF:FFFFF:FFFFFFFFFFFFFFFFFFFFFFFFFFFFFFFFFFFFFFFF
FFFFFFFFFFFFFFFFF:FFFFFFFFFFFFFFFFFFFFFFFFFFFFFFFFFFFFFFFFFFF:FFFFF
@A00155:342:HHGFNDSXY:1:2122:29857:17691 1:N:0:GAACCTAG+TCCGCATA
CTTCTCTCGGGCCCACAGCCTTACAGCTGTGGAGCACGGTATCCTCTGCCGAAACAGAGGTTGGACAA
GACCGGAGGGGTCTCCTAGTTCCAAAGGAGATGTACTCCGGGCTTGTTCACGACCTACCGTGTAAGTC
+
FFFFFFFFFFFFFFFFFFFFFF:FFFFFFFFFFFFFFFFFFFFFFFFFFFFFFFFFFFFFFFFFFFFF
FFFFFFFFFFFFFFFFFFFFFFFFFFFFFFFFFFFFFFFFFFFFFFFFFFFFFFFFFFFFFFFFFFFF
@A00155:342:HHGFNDSXY:1:2122:29342:23782 1:N:0:GAACCTAG+TCCGCATA
GACTTCTCTCGGGCCCACAGCCTTACAGCTGTGGAGCACGGTATCCTCTGCCGAAACAGAGGTTGGAC
AAGACCGGAGGGGTCTCCTGGTTCCAAAGGAGATGTACTCCGGGCTTGTTCACGACCTACCGTGTAAG
+
FFFFFFFFFFFFFFFFFFFFFFFFFFFFFFFFFFFFFFFFFFFFFFFFFFFFFFFFFFFFFFFFFFFF
FFFFFFFFFFFFFFFFFFFFFFFFFFFFFFFFFFFFFFFFFFFFFFFFFFFFFFFFFFFF:FFFFFFF
@A00155:342:HHGFNDSXY:1:2122:19199:33238 1:N:0:GAACCTAG+TCCGCATA
CTTCTCTCGGGCCCACAGCCTTACAGCTGTGGAGCACGGTATCCTCTGCCGAAACAGAGGTTGGACAA
GACCGGAGGGGTCTCCTAGTTCCAAAGGAGATGTACTCCGGGCTTGTTCACGACCTACCGTGTAAGTC
+
FFFFFFFFFFFFFFFFFFFFFFFFFFFFFFFFFFFFFFFFFFFFFFFFFFFFFFFFFFFFFFFFFFFF
FFFFFFFFFFFFFFFFFFFFFFFFFFFFFFFFFFFFFFFFFFFFFFFFFFFFFFFFFFFFFFFF:FFF
@A00155:342:HHGFNDSXY:1:2122:1362:36511 1:N:0:GAACCTAG+TCCGCATA
GAGCGACTTCTCTCGGGCCCACAGCCTTACAGCTGTGGAGCACGGTATCCTCTGCCGAAACAGAGGTT
GGACAAGACCGGAGGGGTCTCCTAGTTCCAAAGGAGATGTACTCCGGGCTTGTTCACGACCTACCGTG
+
F:FFFFFFFFFFFFFFFFFFFFFFFFFFFFFFFFFFFFFFFFFFFFFFFFFFFFFFFFFFF:F:FFFF
FFFFFF:FFFFFFFFFFFFFFFFF,FFFFFFFFFFF:FFFFFFFFFFFFFFFFFF:FFFFFFF:FF:F
@A00155:342:HHGFNDSXY:1:2123:12210:6574 1:N:0:GAACCTAG+TCCGCATA
ACTTCTCTCGGGCCCACAGCCTTACAGCTGTGGAGCACGGTATCCTCTGCCGAAACAGAGGTTGGACA
AGACCGGAGGGGTCTCCTAGTTCCAAAGGAGATGTACTCCGGGCTTGTTCACGACCTACCGTGTAAGT
+
FFFFFFFFFFFFFFFFFFFFFFFFFFFFFFFFFFFFFFFFFFFFFFFFFFFFFFFFFFFFFFFFFFFF
FFFFFFFFFFFFFFFFFFFFFFFFFFFFFFFFFFFFFFFFFFFFFFFFFFFFFFFFFFFFFFFFFFFF
@A00155:342:HHGFNDSXY:1:2123:18954:9111 1:N:0:GAACCTAG+TCCGCATA
CTTACAGCTGTGGAGCACGGTATCCTCTGCCGAAACAGAGGTTGGACAAGACCGGAGGGGTCTCCTAG
TTCCAAAGGAGATGTACTCCGGGCTTGTTCACGACCTACCGTGTAAGTCGTAGTCTAGTAAGCTACCT
+
FFF:FFFFFFFFFFFFFFFFFFFFFFFFFFFFFFFFFFFFFFFFFFFFFFFFFFFFFFFFFFFFFFFF
FFFFFFFFFFFFFFFFFFFFFFFFFFFFFFFFFFFFFFFFFFFFFFFFFFFFFFFFFFFFFFFFFFFF
@A00155:342:HHGFNDSXY:1:2123:9968:14935 1:N:0:GAACCTAG+TCCGCATA
GCGACTTCTCTCGGGCCCACAGCCTTACAGCTGTGGAGCACGGTATCCTCTGCCGAAACAGAGGTTGG
ACAAGACCGGAGGGGTCTCCTAGTTCCAAAGGAGATGTACTCCGGGCTTGTTCACGACCTACCG
+
FFFFFFFFFFFFFFFFFFFFFFFFFFFFFFFFFFFFFFFFFFFFFFFFFFFFFFFFFFFFFFFFFFFF
FFFF:FFFFFFFFFFFFFFFFFFFFFFFFFFFFFFFFFFFFFFFFFFFFFFFFFFFFFFFFFFF
@A00155:342:HHGFNDSXY:1:2123:4544:20415 1:N:0:GAACCTAG+TCCGCATA
ACTTCTCTCGGGCCCACAGCCTTACAGCTGTGGAGCACGGTATCCTCTGCCGAAACAGAGGTTGGACA
AGACCGGAGGGGTCTCCTAGTTCCAAAGGAGATGTACTCCGGGCTTGTTCACGACCTACC
+
FFFFFFFFFFFFFFFFFFFFFFFFFFFFFFFFFFFFFFFFFFFFFFFFFFFFFFFFFFFFFFFFFFFF
FF:FFFFFFFFFFFFFF:FFFFFFFFFFFFFFFFFFFFFFFFFFFFFFFFFFFFFFFFFFFF
@A00155:342:HHGFNDSXY:1:2124:3450:28667 1:N:0:GAACCTAG+TCCGCATA
TTACAGCTGTGGAGCACGGTATCCTCTGCCGAAACAGAGGTTGGACAAGACCGGAGGGGTCTCCTAGT
TCCAAAGGAGATGTACTCCGGGCTTGTTCACGACCTACCGTGTAAGTCGTAGTCTAGTAGGCTACCTG
+

FFFFFFFFFFFFFFFFFF,FFFFFFFFFFFFFFFFFFFFFFFFFFFFFFFFFFFFFFFFFFFFFFFFF
FFFFFFFFFFFFFFFFFFFFFFFFFFFFFFF:FFFFFFFFFFFFFFFFFFFFFFFFFFFFFFFFFFFF
@A00155:342:HHGFNDSXY:1:2124:31882:31156 1:N:0:GAACCTAG+TCCGCATA
GAGGGCGAGAGAGAGCGACTTCTCTCGGGCCCACAGCCTTACAGCTGTGGAGCACGGTATCCTCTGCC
GAAACAGAGGTTGGACAAGACCGGAGGGGTCTCCTAGTTCCAAAGGAGATGTACTCCGGGCTTGTTCA
+
:FFFFFFFFFFFFFFFFFF,FFFFFFFFFFF:FF:,FF:FFFF:FFFFFFFFFFFF:FFFF:FFFFFF
FFFFFFFFFFFFFF:FFF:FF:::FFF,F,FFFFFFFFF:FFFFFFFFFFFFFFFFF:,F:::F:FFF
@A00155:342:HHGFNDSXY:1:2125:23231:10755 1:N:0:GAACCTAG+TCCGCATA
AGCGACTTCTCTCGGGCCCACAGCCTTACAGCTGTGGAGCACGGTATCCTCTGCCGAAACAGAGGTTG
GACAAGACCGGAGGGGTCTCCTAGTTCCAAAGGAGATGTACTCCGGGCTTGTTCACGACCTACCGTGT
+
FFFFFFFFFFFFFFFFFFFFFFFFFF:FFFFFFFFFFFFFFFFFFFFFFFFFFFFFFFFFFFFFFFFF
FFFFFFFFFFFFFFFFFFFFFFFFFFFFFFFFFFFFFFFFFFFFFFFFFFFFFFFFFFFFFFFFFFFF
@A00155:342:HHGFNDSXY:1:2125:2917:12806 1:N:0:GAACCTAG+TCCGCATA
AGGGCGAGAGAGAGCGACTTCTCTCGGGCCCACAGCCTTACAGCTGTAGAGCACGGTATCCTCTGCCG
AAACAGAGGTTGGACAAGACCGGAGGGGTCTCCTAGTTCCAAAGGAGATGTACTCCGGGCTTGTTC
+
FFFFFFFFFFF:FFFFFFFFFFFFFFFFFFFFFFFFFFFFFFFFFFFFFFFFFFFFFFFFFFFFFFFF
FFFFFFFFFFFFFFFFFFFFFFFFFFFFFFFFFFFFFFFFFFFFFFFFFFFFFFFFFFFFFFFFFF
@A00155:342:HHGFNDSXY:1:2125:1940:18004 1:N:0:GAACCTAG+TCCGCATA
CCACATCCCTCTCGGCATTGAGATGACCATAGGCCAGAGGTGAGTCCTTAAGTGGACACAGCTGATCT
AAGGCGGTGTGGCGGGGCATGGGTTTGAACCCCCATGACGGTCGGAGTCTAGTAGGCTCCCTGATGAG
+
FFFFFFFFFFFFFFFFFF:F:FFFFFFFFFFFFFFFFFFFFFFFFFFFFFFFFFFFF:FFFFFFFFFF
FFFFFFF,FFFFFFFFFF,FFFFFF:FFFFFFFFFFFFFFF:FFF:F:F:FFF:FFF,FFFFFF:FFF
@A00155:342:HHGFNDSXY:1:2125:7500:22247 1:N:0:GAACCTAG+TCCGCATA
GAGCGACTTCTCTCGGGCCCACAGCCTTACAGCTGTGGAGCACGGTATCCTCTGCCGAAACAGAGGTT
GGACAAGACCGGAGGGGTCTCCTAGTTCCAAAGGAGATGTACTCCGGGCTTGTTCACGACCTACCGTG
+
FFFFFFFFFFFFFFFFFFFFFFFFFFFFFFFFFFFFF:FFFFFFFFFFFFFFFFFFFFFFFFFFFFFF
FFFFFFFFF:FFFFFFFFFFFFFFFFFFFFFFFFFFFFFFFFFFFFFFFFFFFFFFFFFFFFFFFF:F
@A00155:342:HHGFNDSXY:1:2126:1994:5541 1:N:0:GAACCTAG+TCCGCATA
GGATGTGGAGGGCGAGAGAGAGCGACTTCTCTCGGGCCCACAGCCTTACAGCTGTGGAGCACGGTATC
CTCTGCCGAAACAGAGGTTGGACAAGACCGGAGGGGTCTCCTGGTTCCAAAGGAGATGTACTCC
+
FFF:FFFFFFFFFFFFFFFFFFFFFFFFFFFFFFFFFFFFFFFFF:FFFFFFFFFFFFFFFFFFFFFF
FFFF:FFFF:FFFFFFFFFFFFFFFFFFFFFFFFFFFFFFFFFFFFFFFFF:FFFFFFFFFFFF
@A00155:342:HHGFNDSXY:1:2126:30743:25394 1:N:0:GAACCTAG+TCCGCATA
GCATTGAGATGACCATAGGCCAGAGGTGAGTCCTTAAGTGGACACAGCTGATCTAAGGCGGTGTAGCG
GGGCATGGGTTTGAACCCCCATGACGGTCGGAGTCTAGTAGGCTCCCTGATGAGTCCGTTCCAAGGAC
+
FFFFFFFFFFFFFFFFFFFFFFFFFFFFFFFFFFFFFFFFFFF,FFFFFFFFFFFFFFFFFFFFFFFF
FFFFFFFFFFFFFFFFFFFFFFFFFFFFFFFFFFFFFFFFFFFFFFFFFFFFFFFFFFFFFFFFFFFF
@A00155:342:HHGFNDSXY:1:2127:22580:3082 1:N:0:GAACCTAG+TCCGCATA
GGATGTGGAGGGCGAGAGAGAGCGACTTCTCTCGGGCCCACAGCCTTACAGCTGTGGAGCACGGTATC
CTCTGCCGAAACAGAGGTTGGACAAGACCGGAGGGGTCTCCTAGTTCCAAAGGAGATGTACT
+
FFFFFFFFFFFFFFFFFFFFFFFFFFFFFFFFFFFFFFFFFFFFFFFFFFFFFFFFFFFFFFFFFFFF
FFFFFFFFFFFFFFFFFFFFFFFFFFFFFFFFFFFFFFFFFFFFFFFFFFFFFFFFFFF:FF
@A00155:342:HHGFNDSXY:1:2127:30798:9549 1:N:0:GAACCTAG+TCCGCATA
GGATGTGGAGGGCGAGAGAGAGCGACTTCTCTCGGGCCCACAGCCTTACAGCTGTGGAGCACGGTATC
CTCTGCCGAAACAGAGGTTGGACAAGACCGGAGGGGTCTCCTAGTTCCAAAGGAGATGTACTCC
+

FFF:FFFFFFFFFFFFFFFFFFFFFFFFFFFFFFFFFFFFFFFFFFFFFFFFFFFF:FFFFFFFFFFF
FFFFFFFFFFFFFFFFFFFFFFFFFFFFFFFFFFFFFFFFFFFFFFFFFFFFFFFFFFFFFFFF
@A00155:342:HHGFNDSXY:1:2127:2817:33113 1:N:0:GAACCTAG+TCCGCATA
GGATGTGGAGGGCGAGAGAGAGCGACTTCTCTCGGGCCCACAGCCTTACAGCTGTGGAGCACGGTATC
CTCTGCCGAAACAGAGGTTGGACAAGACCGGAGGGGTCTCCTAGTTCCAAAGGAGATGTACTCC
+
FFFFFFFFFFFFFFFFFFFFFFFFFFFFFFFFFFFFFFFFFFFFFFFFFFFFFFFFFFFFFFFFFFFF
FFFFFFFFFFFFFFFFFFFFFFFFFF:FFFFFFFFFFFFFFFFFFFFFFFFFFFFFFFFFFFFF
@A00155:342:HHGFNDSXY:1:2129:6497:2347 1:N:0:GAACCTAG+TCCGCATA
TCCTCTGCCGAAACAGAGGTTGGACAAGACCGGAGGGGTCTCCTGGTTCCAAAGGAGATGTACTCCGG
GCTTGTTCACGACCTACCGTGTAAGTCGTAGTCTAGTAGGCTACCTGACGAGTCCTTTTTAGGACGAA
+
,FFFFFF,FFFFFFF,FF:F,FFFFFFFFFF:FF::F,:FFFFFFFFFFFF:FF:FFFFF:FFF,FFF
FF,:FFFFFFFFFFFFF,FFFFFFFFFFFFFFFFFFFFFFFFFF,FFFFFFFF:FF:F,FF:,F,FFF
@A00155:342:HHGFNDSXY:1:2129:15944:10504 1:N:0:GAACCTAG+TCCGCATA
CGAGAGGGATGTAGAGGGCGAGAGAGAGCGACTTCTCTCGGGCCCACAGCCTTACAGCTGTGGAGCAC
GGTATCCTCTGCCGAAACAGAGGTTGGACAAGACCGGAGGGGTCTCCTAGTTCCAAAGGAGATGTACT
+
FFFFFFFFFFFFFFFFFFFFFFFFFFFFFFFFFFFFFFFFFFFFFFFFFFFFFFFFFFFFFFFFFFFF
FFFFFFFFFFFFFFFFFFFFFFFFFFFFFFFFFFFFFFFFFFFFFFFFFFFFFFFFFFFFFFFFFFFF
@A00155:342:HHGFNDSXY:1:2129:4878:10911 1:N:0:GAACCTAG+TCCGCATA
CCTTACAGCTGTGGAGCACGGTATCCTCTGCCGAAACAGAGGTTGGACAAGACCGGAGGGGTCTCCTA
GTTCCAAAGGAGATGTACTCCGGGCTTGTTCACGACCTACCGTGTAAGTCGTAGTCTAGTAGGCTACC
+
FFFFFFFFFFFFFFFFFFFFFFFFFFFFFFFFFFFFFFFFFFFFFFFFFFFFF:FFFFFFFFFFFFFF
FFFFFFF:FFFFFFFFFFFFFFFFFFFFFFFFFFFFFFFFFFFFFFFFFFFFFFFFFFFFFFFFFFFF
@A00155:342:HHGFNDSXY:1:2129:21423:13604 1:N:0:GAACCTAG+TCCGCATA
ATGCCGAGAGGGATGTGGAGGGCGAGAGAGAGCGACTTCTCTCGGGCCCACAGCCTTACAGCTGTGGA
GCACGGTATCCTCTGCCGAAACAGAGGTTGGACAAGACCGGAGGGGTCTCCTGGTTCCAAAGGAGATG
+
FFFFFFFFFFFFFFFFFFFFFFFFFFFFFFFFFFFFFFFFFFFFFFFFFFFFFFFFFFFFFFFFFFFF
FFFFFFFFFFFFFFFFFFFFFFFFFFFFFFFFFFFFFFFFFFFFFFFFFFFFFFFFFFFFFFFFFFFF
@A00155:342:HHGFNDSXY:1:2129:9489:18051 1:N:0:GAACCTAG+TCCGCATA
ACCTCTGTTTCGGCAGAGGATACCGTGCTCCACAGCTGTAAGGCTGTGGGCCCGAGAGAAGTCGCTCT
CTCTCGCCCTCCACATCCCTCTCGGCATTGAGATGACCATAGGCCAGAGGAGAGTCCTTAAGTGGAC
+
FFFFFFF,F,F:,F,FF:FFFFFFFF:FFFFF:FF,FFFFFF,FF:FFF:F:FFFF:FFF,:,::F:F
F:FFFF:FFFF:F,F,,:FFF::,FFF,F,FFFFFFFFF:F::F,::FFFF:FFFFF,,FFF:F:FF
@A00155:342:HHGFNDSXY:1:2129:30879:20619 1:N:0:GAACCTAG+TCCGCATA
CTTCTCTCGGGCCCACAGCCTTACAGCTGTGGAGCACGGTATCCTCTGCCGAAACAGAGGTTGGACAA
GACCGGAGGGGTCTCCTGGTTCCAAAGGAGATGTACTCCGGGCTTGTTCACGACCTACCGTGTAAGTC
+
F:FFFFFFFFFFFFFFF:FFFFF:F:FFF,FFFFFFF,FFFFF::FFFF:FFFF:FFFF:FFFFFFFF
FFFFF:F,FF:F,FF:FF::FFFFFFF,FFFFFFFFF:F:F:FFF:FFFFFFFFFFFF,FFFFFF::F
@A00155:342:HHGFNDSXY:1:2129:19217:23187 1:N:0:GAACCTAG+TCCGCATA
CTTCTCTCGGGCCCACAGCCTTACAGCTGTGGAGCACGGTATCCTCTGCCGAAACAGAGGTTGGACAA
GACCGGAGGGGTCTCCTAGTTCCAAAGGAGATGTACTCCGGGCTTGTTCACGACCTACCGTGTAAGTC
+
FFFFFFFFFFFFFFFFFFFFFFFFFFFFFFFFFFFFFFFFFFFFFFFFFFFFFFFFFFFFFFFFFFFF
FFFFFFFFFFFFFFFFFFFFFFFFFFFFFFF:FFFFFFFFFFFFFFFFFFFFFFFFFFFFFFFFFFFF
@A00155:342:HHGFNDSXY:1:2129:20772:24940 1:N:0:GAACCTAG+TCCGCATA
GTCATCTCAATGCCGAGAGGGATGTGGAGGGCGAGAGAGAGCGACTTCTCTCGGGCCCACAGCCTTAC
AGCTGTGGAGCACGGTATCCTCTGCCGAAACAGAGGTTGGACAAGACCGGAGGGGTCTCCTGGTTCCA
+

FFFFFFFFFFFFF:FFF:FFFF:FFFFFFFFFFFFFFFFFFFF:FFFF:FFFFFFFFFFFFFFF,FFF
FFFFFFFFFFFFFFFFFFFFFFFFFFFFFFFFFFFFFFFFFFFFFFFFFFFFFFFFFFFFFFFFFFFF
@A00155:342:HHGFNDSXY:1:2129:12527:26349 1:N:0:GAACCTAG+TCCGCATA
GGATGTAGAGGGCGAGAGAGAGCGACTTCTCTCGGGCCCACAGCCTTACAGCTGTGGAGCACGGTATC
CTCTGCCGAAACAGAGGTTGGACAAGACCGGAGGGGTCTCCTAGTTCCAAAGGAGATGTACTCC
+
FFFFFFFFFFFFFFFFFFFFFFFFFFFFFFFFFFFFFFFFFFFFFFFFFFFFFFFFFFFFFFFFFFFF
FFFFFFFFFFFFFFFFFFFFFFFFFFFFFFFFFFFFFFFFFFFFFFFFFFFFFFFFFFFFFFFF
@A00155:342:HHGFNDSXY:1:2129:7581:29653 1:N:0:GAACCTAG+TCCGCATA
CTTCTCTCGGGCCCACAGCCTTACAGCTGTGGAGCACGGTATCCTCTGCCGAAACAGAGGTTGGACAA
GACCGGAGGGGTCTCCTAGTTCCAAAGGAGGTGTACTCCGGGCTTGTTCACGACCTACCGTGTAAGTC
+
FFFFFFFFFFFFFFFFFFF:F:FFFFF,FFFFFFFFFFFFFFFFFFFFFFFFFFFFFFFFFFFFFFFF
FFF:FFFF:,FFFFFFFFFF:FFFF:FFFFF,:FFFFFFFFFFF,F:FFFFFFFFFFFF:F:FFFFFF
@A00155:342:HHGFNDSXY:1:2130:18819:1767 1:N:0:GAACCTAG+TCCGCATA
AAAGACTCACCTCTGGCCTATGGTCATCTCAATGCCGAGAGGGATGTGGAGGGCGAGAGAGAGCGACT
TCTCTCGGGCCCACAGCCTTACAGCTGTGGAGCACGGTATCCTCTGCCGAAACAGAGGTTGGACAAGA
+
FFFFFFFFFFFFFFFFFFFFFFFFFFFFFFFFFFFFFFF:FFFFFFFFFFFFFFFFFFFFFFFFFFFF
FFFFFFFFFFFFFFFFFFFFFFFFFFFFFFFFFFFFFFFFFFFFFFFFFFFFFFFFFFFFFFFFFFFF
@A00155:342:HHGFNDSXY:1:2130:28302:14152 1:N:0:GAACCTAG+TCCGCATA
ACCTCTGTTTCGGCAGAGGATACCGTGCTCCACAGCTGTAAGGCTGTGGGCCCGAGAGAAGTCGCTCT
CTCTCGCCCTCTACATCCCTCTCGGCATTGAGATGACCATAGGCCAGAGGTGAGTCCTTAAGTGGACA
+
FFFFFFFFFF:FFFF:F:FFFFFFFFFFFFFF:FFFFFFFFFFFFFFFFFFFFFF:FFFF:::FFFFF
FFFFFFFF:FFFFFFFFFFFFFFFFFFFFFFFFFFFFFFFFFFFFFFFF:FFFFFFFF:FFFFFFFFF
@A00155:342:HHGFNDSXY:1:2130:5520:22420 1:N:0:GAACCTAG+TCCGCATA
AAGGACTCACCTCTGGCCTATGGTCATCTCAATGCCGAGAGGGATGTGAAGGGCGAGAGAGAGCGACT
TCTCTCGGGCCCACAGCCTTACAGCTGTGGAGCACGGTATCCTCTGCCGAAACAGAGGTTGGACAAGA
+
FFFFFFFFFFFFFFFFFFFFFFFFFFFFFFFFFFFFFFFFFFFFFFFFFFFFFFFFFFFFFFFFFFFF
FFFFFFFFFFFFFFFFFFFFFFFFFFFFFFFFFFFFFFFFFFFFFFFFFFFFFFF:FFFFFFFFFFFF
@A00155:342:HHGFNDSXY:1:2130:25635:25128 1:N:0:GAACCTAG+TCCGCATA
CGACTTCTCTCGGGCCCACAGCCTTACAGCTGTGGAGCACGGTATCCTCTGCCGAAACAGAGGTTGGA
CAAGACCGGAGGGGTCTCCTAGTTCCAAAGGAGATGTACTCCGGGCTTGTTCACGACCTACCGTGTAA
+
FFFFF:FFFFFFFFFFFFFFFFFFFFFFFFF,FFFFFFFFF:F:FFF,FF:F,F:FFFFFFFFFF,FFF
FFF,FFF::F::FFFFFFF,FF,FFFFFFF:F,FFFFFFFF:::FFFFF,FFFFFFFFFFF:,:F,FF
@A00155:342:HHGFNDSXY:1:2130:26612:25160 1:N:0:GAACCTAG+TCCGCATA
GGATGTGGAGGGCGAGAGAGAGCGACTTCTCTCGGGCCCACAGCCTTACAGCTGTGGAGCACGGTATC
CTCTGCCGAAACAGAGGTTGGACAAGACCGGAGGGGTCTCCTAGTTCCAAAGGAGATGTACTCC
+
FFFFFFFFFFFFFFFFFFFFFFFFFFFFFFFFFFFFFFFFFFFFFFFFFFFFFFFFFFFFFFFFFFFF
FFFFFFFFFFFFFFFFFFFFFFFFFFFFFFFFFFFFFFFFFFFFFFFFFFFFFFFFFFFFFFFF
@A00155:342:HHGFNDSXY:1:2130:19768:27461 1:N:0:GAACCTAG+TCCGCATA
AGCGACTTCTCTCGGGCCCACAGCCTTACAGCTGTGGAGCACGGTATCCTCTGCCGAAACAGAGGTTG
GACAAGACCGGAGGGGTCTCCTGGTTCCAAAGGAGATGTACTCCGGGCTTGTTCACGACCTACCGTGT
+
FFFFFFFFFFFFFFFFFFFFFFFFF:FFFFFFFFFFFFFFFFFFFFFFFFFFFFFFFFFFFFFFFFFF
FFFFFFFFFFFFFFFFFFFFFFFFFFFFFFFFFFFFFF:FFFFFFFFFFFFFFFFFFFFFF,FFFFFF
@A00155:342:HHGFNDSXY:1:2130:20103:27665 1:N:0:GAACCTAG+TCCGCATA
AGCGACTTCTCTCGGGCCCACAGCCTTACAGCTGTGGAGCACGGTATCCTCTGCCGAAACAGAGGTTG
GACAAGACCGGAGGGGTCTCCTGGTTCCAAAGGAGATGTACTCCGGGCTTGTTCACGACCTACCGTGT
+

FFFFFFFFFFFFFFFFFFFFFFFFFFFFFFFFFFFFFFFFFFFFFFFFFFFFFFFFFFFFFFFFFFFFFF
FFFFFFFFFFFFFFFFFFFFFFFFFFFFFFFFFFFFFFFFFFFFFFFFFFFFFFFFFFFFFFFFFFFFFF
@A00155:342:HHGFNDSXY:1:2130:25301:32252 1:N:0:GAACCTAG+TCCGCATA
TTAGATCAGCTGTGTCCACTTAAGGACTCACCTCTGGCCTATGGTCATCTCAATGCCGAGAGGGATGT
GGAGGGCGAGAGAGAGCGACTTCTCTCGGGCCCACAGCCTTACAGCTGTGGAGCACGGTATCCTCTGC
+
FFFFFFFFFFFFFFFFFFFFFFFFFFFFFFFFFFFFFFFFFFFFFFFFFFFFFFFFFFFFFFFFFFFFFF
FFFFFFFFFFFFFFFFFFFFFFFFFFFFFFFFFFFFFFFFFFFFFFFFFFFFFFFFFFFFFF,FFFFFFFF
@A00155:342:HHGFNDSXY:1:2132:24858:23249 1:N:0:GAACCTAG+TCCGCATA
CGACTTCTCTCGGGCCCACAGCCTTACAGCTGTGGAGCACGGTATCCTCTGCCGAAACAGAGGTTGGA
CAAGACCGGAGGGGTCTCCTAGTTCCAAAGGAGATGTACTCCGGGCTTGTTCACGACCTACC
+
FFFFFFFFFFFFFFFFFFFFFFFFFFFFFFFFFFFFFFFFFFFFFFFFFFFFFFFFFFFFFFFFFFFFFF
FFFFFFFFFFFFFFFFFFFFFFFFFFFFFFFFFFFFFFFFFFFFFFFFFFFFFFFFFFF:FF
@A00155:342:HHGFNDSXY:1:2133:32506:4429 1:N:0:GAACCTAG+TCCGCATA
CTCAATGCCGAGAGGGATGTAGAGGGCGAGAGAGAGCGACTTCTCTCGGGCCCACAGCCTTACAGCTG
TGGAGCACGGTATCCTCTGCCGAAACAGAGGTTGGACAAGACCGGAGGGGTCTCCTAGTTCCAAAGGA
+
FFF::FFFFFFFFFFF:,FFFFFFFFFFFFFFFFFFFFFFFFFFF:FFFFFFFFFFFFFFF,FFFFFF
FFFFFFFFFFF:FFFFFFFFFFFFFFFFFFF:FFF,FFFFFFFF:FFFFFFFFFFF,FFFFFFFFFFF
@A00155:342:HHGFNDSXY:1:2133:20302:13510 1:N:0:GAACCTAG+TCCGCATA
CTTCTCTCGGGCCCACAGCCTTACAGCTGTGGAGCACGGTATCCTCTGCCGAAACAGAGGTTGGACAA
GACCGGAGGGGTCTCCTGGTTCCAAAGGAGATGTACTCCGGGCTTGTTCACGACCTACCGTGTAAGTC
+
FFFFFFFFFFFFFFFFFFFFFFFFFFFFFFFFFFFFFFFFFFFFFFFFFFFFFFFFFFFFFFFFFFFF
FFFFFFFFFFFFFFFFFFFFFFFFFFFFFFFFFFFFFFFFFFFFFFF:FFFFFFFFFFFFFFFFFFFF
@A00155:342:HHGFNDSXY:1:2133:21612:15280 1:N:0:GAACCTAG+TCCGCATA
GATCAGCTGTGTCCACTTAAGGACTCACCTCTGGCCTATGGTCATCTCAATGCCGAGAGGGATGTGAA
GGGCGAGAGAGAGCGACTTCTCTCGGGCCCACAGCCTTACAGCTGTGGAGCACGGTATCCTCTGCCGA
+
FFFFFFFFFFFFFFFFFFFFFFFFFFFFFFFFFFFFFFFFFFFFFFFFFFFFFFFFFFFFFF:FFFFFF
FFFFFFFFFFFFFFFFFFFFFFFFFFFFFFFFFFFFFFFFFFFFFFFFFFFFFFFFFFFFFFFFFFFFF
@A00155:342:HHGFNDSXY:1:2133:20356:22279 1:N:0:GAACCTAG+TCCGCATA
CTTCTCTCGGGCCCACAGCCTTACAGCTGTGGAGCACGGTATCCTCTGCCGAAACAGAGGTTGGACAA
GACCGGAGGGGTCTCCTGGTTCCAAAGGAGATGTACTCCGGGCTTGTTCACGACCTACCGTGTAAGTC
+
FFFFFFFFFFFFFFFFFFFFFFFFFFFFFFFFFFFFFFFFFFFFFFFFFFFFFFFFFFFFFFFFFFFF
FFFFFFFFFFFFFFFFFFFFFFFFFFFFFFFFFFFFFFFFFFF:FFFFFFFFFFFFFFFFFFFFFFFF
@A00155:342:HHGFNDSXY:1:2134:15365:4930 1:N:0:GAACCTAG+TCCGCATA
TTAAGGACTCACCTCTGGCCTATGGTCATCTCAATGCCGAGAGGGATGTAGAGGGCGAGAGAGAGCGA
CTTCTCTCGGGCCCACAGCCTTACAGCTGTGGAGCACGGTATCCTCTGCCGAAACAGAGGTTGGACAA
+
FFFFFFFFFFFFFFFFFFFFFFFFFFFFFF::FFFFFFFFFFFFFFFFFFFFFFFFFFFFFFFFFFFF
F:FFFFFFFFFFFFFFFFFFF:FFFFFFFFFFFFFFFFF:FFFFFFFFFFFF:FFFFFFFFFFFFF:F
@A00155:342:HHGFNDSXY:1:2134:30798:8641 1:N:0:GAACCTAG+TCCGCATA
CTTCTCTCGGGCCCACAGCCTTACAGCTGTGGAGCACGGTATCCTCTGCCGAAACAGAGGTTGGACAA
GACCGGAGGGGTCTCCTAGTTCCAAAGGAGATGTACTCCGGGCTTGTTCACGACCTACCGTGTAAGTC
+
FFFFFFFFFFFFFFFFFFFFFFFFFFFFFFFFFFFFFFFFFFFFFFFFFFFFFFFFFFFFFFFFFFFF
FFF:FFFFFFFFFFFFFFFFFFFFFFFFFFFFFFFFFFFFFFFFFFFFFFFFFFFFFFFF:FFFFFFF
@A00155:342:HHGFNDSXY:1:2134:15781:12258 1:N:0:GAACCTAG+TCCGCATA
GATGTGGAGGGCGAGAGAGAGCGACTTCTCTCGGGCCCACAGCCTTACAGCTGTGGAGCACGGTATCC
TCTGCCGAAACAGAGGTTGGACAAGACCGGAGGGGTCTCCTAGTTCCAAAGGAGATGTACTCCGGGCT
+

FFFFFFFFFFFFFFFFFFFFFFFFFFFFFFFFFFFFFFFFFFFFFFFFFFFFFFFFFFFFFFFFFFFF
FFFFFFFFFFFFFFFFFFFFFFFFFFFFFFFFFFFFFFFFFFFFFFFFFFFFFFFFFFFFFFFFFFFF
@A00155:342:HHGFNDSXY:1:2134:8784:30452 1:N:0:GAACCTAG+TCCGCATA
CTTCTCTCGGGCCCACAGCCTTACAGCTGTGGAGCACGGTATCCTCTGCCGAAACAGAGGTTGGACAA
GACCGGAGGGGTCTCCTAGTTCCAAAGGAGATGTACTCCGGGCTTGTTCACGACCTACCGTGTAAGTC
+
FFFFFFFFFFFFFFFFFFFF:FFFFFFFFFFFFFFFFFFFFFFFFFFFFFFFFFFFFFFFFFFFFFFF
FFFFFFFFFFFFFFFFFFFFFFFFFFFFFFFFFFFFFFFFFFFFFFFFFFFFFFFFFFFFFFFFFFFF
@A00155:342:HHGFNDSXY:1:2135:25599:1141 1:N:0:GAACCTAG+TCCGCATA
AGGGATGTAGAGGGCGAGAGAGAGCGACTTCTCTCGGGCCCACAGCCTTACAGCTGTGGAGCACGGTA
TCCTCTGCCGAAACAGAGGTTGGACAAGACCGGAGGGGTCTCCTAGTTCCAAAGGAGATGTACTCCGG
+
FFFFFF::F:FF:FFFFFFFFFFFFFFFFFFFFFFFFFFFFFFFFFFFFFFF:FFFFFFFFFFFFFFF
FFFFFFFFFFFFF:FFFFFFFFFFFFFFFF:FFFFFFF:FFFFFFF:FFFFFFFFF:FFFFFFFF:FF
@A00155:342:HHGFNDSXY:1:2135:26603:12900 1:N:0:GAACCTAG+TCCGCATA
GTATCCTCTGCCGAAACAGAGGTTGGACAAGACCGGAGGGGTCTCCTGGTTCCAAAGGAGATGTACTC
CGGGCTTGTTCACGACCTACCGTGTAAGTCGTAGTCTAGTAGGCTACCTGACGAGTCCTTTTTAGGAC
+
FFFFFFFFFFFFFFFFFFFFFFFFFFFFFFFFFFFFFFFFFFFFFFFFFFFFFFFFFFFFFFFFFFFF
FFFFFFFFFFFFFFFFFFFFFFFFFFFFFFFFFFFFFFFFFFFFFFFFFFFFFFFFFFFFFFFFF:FF
@A00155:342:HHGFNDSXY:1:2135:30662:14669 1:N:0:GAACCTAG+TCCGCATA
TTAAGGACTCTCCTCTGGCCTATGGTCATCTCAATGCCGAGAGGGATGTGGAGGGCGAGAGAGAGCGA
CTTCTCTCGGGCCCACAGCCTTACAGCTGTGGAGCACGGTATCCTCTGCCGAAACAGAGGTTGGACAA
+
FFFFFFFFFFFFFFFFFFFFFFFFFFFFFFFFFFFFFFFFFFFFFFFFFFFFFFFFFFFFFFFFFFFF
FFFFFFFFFFFFFFFFFFFFFFFFFFFFFFFFFFFFFFFFFFFFFFFFFFFFFFFFFFFFFFFFFFFF
@A00155:342:HHGFNDSXY:1:2135:4517:21465 1:N:0:GAACCTAG+TCCGCATA
CTTCTCTCGGGCCCACAGCCTTACAGCTGTGGAGCACGGTATCCTCTGCCGAAACAGAGGTTGGACAA
GACCGGAGGGGTCTCCTAGTTCCAAAGGAGATGTACTCCGGGCTTGTTCACGACCTACCGTGTAAGTC
+
FFFFFFFFFFFFFFFFFFFFFFFFFFFFFFFFFFFFFFFFFFFFFFFFFFFFFFFFFFFFFFFFFFFF
FFFFFFFFFFFFFFFFFFFFFFFFFFFFF:FFFFFFFFFFFFFFFFFFFFFFFFFFFFFFFFFFFFFF
@A00155:342:HHGFNDSXY:1:2135:31521:30718 1:N:0:GAACCTAG+TCCGCATA
AAGGCTGTGGGCCCGAGAGAAGTCGCTCTCTCTCGCCCTCCACATCCCTCTCGGCATTGAGATGACCA
TAGGCCAGAGGTGAGTCCTTAAGTGGACACAGCTGATCTAAGGCGGTGTGGCGGGGCATGGGTTTGAA
+
FFFFFFFFFFFFFFFFFFFFFFFFFFFFFFFFFFFFFFFFFFFFFFFFFFFFFFFFFFFFFFFFFFFF
FFFFFFFFFFFFFFFFFFFFFFFFFFFFFFFFFFFFFFFFFFFFFFFFFFFFFFFFF:FFFFFFFFFF
@A00155:342:HHGFNDSXY:1:2135:21667:32033 1:N:0:GAACCTAG+TCCGCATA
AGAGAGAGCGACTTCTCTCGGGCCCACAGCCTTACAGCTGTGGAGCACGGTATCCTCTGCCGAAACAG
AGGTTGGACAAGACCGGAGGGGTCTCCTAGTTCCAAAGGAGATGTACTCCGGGCTTGTTCACGACCTA
+
FFFFFFFFFFFFFFFFFFFFFFFFFFFFFFFFFFFFFFFFFFFFFFFFFFFFFFFFFFFFFFFFFFFF
FFFFFFFFFFFFFFFFFFFFFFFFFFFFFFFFFFFFFFFFFFFFFFFFFFFFFFFFFFFFFFFFFFFF
@A00155:342:HHGFNDSXY:1:2135:21314:33395 1:N:0:GAACCTAG+TCCGCATA
AGAGAGAGCGACTTCTCTCGGGCCCACAGCCTTACAGCTGTGGAGCACGGTATCCTCTGCCGAAACAG
AGGTTGGACAAGACCGGAGGGGTCTCCTAGTTCCAAAGGAGATGTACTCCGGGCTTGTTCACGACCTA
+
FFFFFFFFFFFFFFFFFFFFFFFFFFFFFFFFFFFFFFFFFFFFFFFFFFFFFFF:FFFFFFFFFFFF
FFFFFFFFFFFFFFFFFFFFFFFFFFFFFFFFFFFFFFFFFFFFFFFFFFFFFFFFFFFFFFFFFFFF
@A00155:342:HHGFNDSXY:1:2136:23737:3239 1:N:0:GAACCTAG+TCCGCATA
AGAGAGAACGACTTCTCTCGGGCCCACAGCCTTACAGCTGTGGAGCACGGTATCCTCTGCCGAAACAG
AGGTTGGACAAGACCGGAGGGGTCTCCTAGTTCCAAAGGAGATGTACTCCGGGCTTGTTCACGACCTA
+

FFFFFFFFFFFFFFFFFFFFFFFFFFFFFFFFFFFFFFFFFFFFFFFFFFFFFFFFFFFFFFFFFFFFFFFF
FFFFFFFFFFFFFFFFFFFFFFFFFFFFFFFFFFFFFFFFFFFFFFFFFFFFFFFFFFFF:FFFFFFFFFFFF
@A00155:342:HHGFNDSXY:1:2136:26567:22138 1:N:0:GAACCTAG+TCCGCATA
TTCTCTCGGGCCCACAGCCTTACAGCTGTGGAGCACGGTATCCTCTGCCGAAACAGAGGTTGGACAAG
ACCGGAGGGGTCTCCCAGTTCCAAAGGAGATGTACTCCGGGCTTGTTCACGACCTACCGTGTAAGTCG
+
FFFFFFFFFFFFFFFFFF:FFFFFFFFFFFFFFFFFFFFFFFFFFFFFFFFFFFFFFFFFFFFFFFFFFFFF
FFFFFFFFFFFFFFFFFFFFFFFFFFFFFFFFFFFFFFFFFFFFFFFFFFFFFFFFFFFFFFFFFFFFF:FF
@A00155:342:HHGFNDSXY:1:2137:6153:3537 1:N:0:GAACCTAG+TCCGCATA
GTCATCTCAATGCCGAGAGGGATGTGGAGGGCGAGAGAGAGCGACTTCTCTCGGGCCCACAGCCTTAC
AGCTGTGGAGCACGGTATCCTCTGCCGAAACAGAGGTTGGACAAGACCGGAGGGGTCTCCTAGTTCCA
+
FFFFFFFFFFFFFFFFFFFFFFFFFFFFFFFFFFFFFFFFFFFFFFFFFFFFFFFFFFFFFFFFFFFFFFFF
FFFFFFFFFFFFFFFFFFFFFFFFFFFFFFFFFFFFFFFFFFFFFFFFFFFFFFFFFFFFFFFFFFFFFFFF
@A00155:342:HHGFNDSXY:1:2137:23493:8046 1:N:0:GAACCTAG+TCCGCATA
TGTTTCGGCAGAGGATACCGTGCTCCACAGCTGTAAGGCTGTGGGCCCGAGAGAAGTCGCTCTCTCTC
GCCCTCCACATCCCTCTCGGCATAGAGATGACCATAGGCCAGAGGTGAGTCCTTAAGTGGACACAGCT
+
FFFFFFFFFFFFFFFFFFFFFFFFFFFFFFFFFFFFFFFFFFFFFFFFFFFFFFFFFFFFFFFFFFFFFFFF
FFFFFFFFFFFFFFFFFFFFFFFFFFFFFFFFFFFFFFFFFFFFFFFFFFFFFFFFFFFFFFFFFFFFFFFF
@A00155:342:HHGFNDSXY:1:2137:16387:9361 1:N:0:GAACCTAG+TCCGCATA
GAGCGACTTCTCTCGGGCCCACAGCCTTACAGCTGTGGAGCACGGTATCCTCTGCCGAAACAGAGGTT
GGACAAGACCGGAGGGGTCTCCTAGTTCCAAAGGAGATGTACTCCGGGCTTGTTCACGACCTACCGTG
+
FFFFFFFFFFFFFFFFFFFFFFFFFFFFFFFFFFFFFFFFFFFFFFFFFFFFFFFFFFFFFFFFFFFFFFFF
FFFFFFFFFFFFFFFFFFFFFFFFFFFFFFFFFFFFFFFFFFFFFFFFFFFFFFFFFFFFFFFFFFFFFFFF
@A00155:342:HHGFNDSXY:1:2137:10872:13463 1:N:0:GAACCTAG+TCCGCATA
GAGCGACTTCTCTCGGGCCCACAGCCTTACAGCTGTGGAGCACGGTATCCTCTGCCGAAACAGAGGTT
GGACAAGACCGGAGGGGTCTCCTAGTTCCAAAGGAGATGTACTCCGGGCTTGTTCACGACCTACCGTG
+
FFFFFFFFFFFFFFFFFFFFFFFFFFFFFFFFFFFFFFFFFFFFFFFFFFFFFFFFFFFFFFFFFFFFFFFF
FFFFFFFFFFFFFFFFFFFFFFFFFFFFFFFFFFFFFFFFFFFF,FFFFFFFFFFFFFFFFFFFFFFFFFFF
@A00155:342:HHGFNDSXY:1:2137:28836:22811 1:N:0:GAACCTAG+TCCGCATA
GAGCGACTTCTCTCGGGCCCACAGCCTTACAGCTGTGGAGCACGGTATCCTCTGCCGAAACAGAGGTT
GGACAAGACCGGAGGGGTCTCCTAGTTCCAAAGGAGATGTACTCCGGGCTTGTTCACGA
+
FFFFFFFFFFFFFFFFFFFFFFFFFFFFFFFFFFFFFFFFFFFFFFFFFFFFFFFFFFFFFFFFFFFFFFFF
FFFFFF:FFFFFFFFFFFFFFFFFFFFFFFFFFFFFFFFFFFFFFFFFFFFFFFFFFFF
@A00155:342:HHGFNDSXY:1:2138:11288:4946 1:N:0:GAACCTAG+TCCGCATA
AGAGAGAGCGACTTCTCTCGGGCCCACAGCCTTACAGCTGTGGAGCACGGTATCCTCTGCCGAAACAG
AGGTTGGACAAGACCGGAGGGGTCTCCTGGTTCCAAAGGAGATGTACTCCGGGCTTGTTCACGACCTA
+
FFFFF:FFFFFFFFFFF:F:FFFFFFFFFFFFFFFF:FFFFFFFFFFFFFFFF:FFFFFF,:FFFFFF
F,F::F,FFFFFFFFFFFFFFFFFFFF:FFFFFFFFFF:FFFFFFFFFF:FF:FF,FFF:FFFFFFFF
@A00155:342:HHGFNDSXY:1:2138:15076:10097 1:N:0:GAACCTAG+TCCGCATA
TATGCCGAGAGGGATGTGGAGGGCGAGAGAGAGCGACTTCTCTCGGGCCCACAGCCTTACAGCTGTGG
AGCACGGTATCCTCTGCCGAAACAGAGGTTGGACAAGACCGGAGGGGTCTCCTAGTTCCAAAGGAGAT
+
FFFFFFFFFFFFFFFFFFFFFFFFFFFFFFFFFFFFFFFFFFFFFFFFFFFFFFFFFFFFFFFFFFFFFFFF
FFFFFFFFFFFFFFFFFFFFFFFFFFFFFFFFFFFFFFFFFFFFFFFFFFFFFFFFFFFFFFFFFFFFFFFF
@A00155:342:HHGFNDSXY:1:2138:23294:16031 1:N:0:GAACCTAG+TCCGCATA
CCCTCCACATCCCTCTCGGCATTGAGATGACCATAGGCCAGAGGTGAGTCCTTAAGTGGACACAGCTG
ATCTAAGGCGGTGTGGCGGGGCATGGGTTTGAACCCCCATGACGGTCGGAGTCTAGTAGGCTTCCTGA
+

FFFFFFFFFFFFFFFFFFFF::FFFFFFFFFFF:FFFFFFFFFFFFFFFFFFFFFFF:FFFFFFFFFF
FFFFFFFFFFF:FFFF:FF,FFFFFF:FFFFFFFFF:FFFFFFFFFF:FFFFFFFFFFFFFFFFFFFF
@A00155:342:HHGFNDSXY:1:2138:2302:19257 1:N:0:GAACCTAG+TCCGCATA
CTTACAGCTGTGGAGCACGGTATCCTCTGCCGAAACAGAGGTTGGACAAGACCGGAGGGGTCTCCTAG
TTCCAAAGGAGATGTACTCCGGGCTTGTTCACGACCTACCGTGTAAGTCGTAGTCTAGTAGGCTACCT
+
FFFFFFFFFFFFFFFFFFFFFFFFFFFFFFFFFFFFFFFFFFFFFFFFFFFFFFFFFFFFFFFFFFFF
FFFFFFFFFFFFFFFFFFFFFFFFFFFFFFFFFFFFFFFFFFFFFFFFFFFFFFFFFFFFFFFF:FFF
@A00155:342:HHGFNDSXY:1:2138:11559:31250 1:N:0:GAACCTAG+TCCGCATA
GGATGTAGAGGGCGAGAGAGAGCGACTTCTCTCGGGCCCACAGCCTTACAGCTGTGGAGCACGGTATC
CTCTGCCGAAACAGAGGTTGGACAAGACCGGAGGGGTCTCCTAGTTCCAAAGGAGATGTACTCC
+
FFFFFFFFFFFFFFFFFFFFFFFFFFFFFFFFFFFFFFFFFFFFFFFFFFFFFFFFFFFFFFFFFFFF
FFFFFFFFFFFFFFFFFFFFFFFFFFFFFFFFFFFFFFFFFFFFFFFFFFFFFFFFFFFFFFFF
@A00155:342:HHGFNDSXY:1:2138:1063:33301 1:N:0:GAACCTAG+TCCGCATA
AGAGAGCGACTTCTCTCGGGCCCACAGCCTTACAGCTGTGGAGCACGGTATCCTCTGCCGAAACAGAG
GTTGGACAAGACCGGAGGGGTCTCCTAGTTCCAAAGGAGATGTACTCCGGGCTTGTTCACGAC
+
FF:F,FFF:FFF,FFFF:FFFFFFFFFFFFFFFFFFFFFF:F,F:FFFFFFFFFFFFFF:F::FFF:F
FFFFFFFFFFFF:FFFFFFFFF:F:FFF:FFFFFFFFFF,FFFFFF:FF:F:FFFFF,FFFFF
@A00155:342:HHGFNDSXY:1:2139:7021:12900 1:N:0:GAACCTAG+TCCGCATA
GAGCGACTTCTCTCGGGCCCACAGCCTTACAGCTGTGGAGCACGGTATCCTCTGCCGAAACAGAGGTT
GGACAAGACCGGAGGGGTCTCCTAGTTCCAAAGGAGATGTACTCCGGGCTTGTTCACGACCTACCGTG
+
FFFFFFFFFFFFFFFFFFFFFFFFFFFFFFFFFFFFF:FFFFFFFF:FF:FFFFFFFFFFFFFFFFFF
FFFFFFFFFFFFFFFFFFFFFFFFF:FF:FFFFFFFFFFFFFFFFFFFFF:FFFFFFFFFFFFFFFFF
@A00155:342:HHGFNDSXY:1:2139:27471:31563 1:N:0:GAACCTAG+TCCGCATA
TCCTCTGCCGAAACAGAGGTTGGACAAGACCGGAGGGGTCTCCTAGTTCCAAAGGAGATGTACTCCGG
GCTTGTTCACGACCTACCGTGTAAGTCGTAGTCTAGTAGGCTACCTGACGAGTCCTTTTTAGGAC
+
FFFFFFFFFFFFFFFFFFFFFFFFFFFFFFFFFFFFFFFFFFFFFFFFFFFFFFFFFFFFFFFFFFFF
FFFFFFFFFFFFFFFFFFFFFFFFFFFFFFFFFFFFFFFFFFFFFFFFFFFFFFFFFFFFFFFFF
@A00155:342:HHGFNDSXY:1:2140:19768:14121 1:N:0:GAACCTAG+TCCGCATA
TGCCGAGAGGGATGTGGAGGGCGAGAGAGAGCGACTTCTCTCGGGCCCACAGCCTTACAGCTGTGGAG
CACGGTATCCTCTGCCGAAACAGAGGTTGGACAAGACCGGAGGGGTCTCCTGGTTCCAAAGGAGATGT
+
FFFFFFFFFFFFFFFFFFFFFFFFFFFFFFFFFFFFFFFFFFFFFFFFFFFFFFFFFFFFFFFFFFFF
FFFFFFFFFFFFFFFFFFFFFFFFFFFFFFFFFFFFFFFFFFFFFFFFFFFFFFFFFFFFFFFFFFFF
@A00155:342:HHGFNDSXY:1:2140:17734:16078 1:N:0:GAACCTAG+TCCGCATA
CTCAATGCCGAGAGGGATGTGGAGGGCGAGAGAGAGCGACTTCTCTCGGGCCCACAGCCTTACAGCTG
TGGAGCACGGTATCCTCTGCCGAAACAGAGGTTGGACAAGACCGGAGGGGTCTCCTAGTTCCAAAGGA
+
FFFFFFFFFFFFFFFFFFFFFFFFFFFFFFFFFFFFFFFFFFFFFFFFFFFFFFFFFFFFFFFFFFFF
FFFFFFFFFFFFFFFFFFFFFFFFFFFFFFFFFFFFFFFFFFFFFFFFFFFFFFFFFFFFFFFFFFFF
@A00155:342:HHGFNDSXY:1:2140:16351:16532 1:N:0:GAACCTAG+TCCGCATA
GAGGGCGAGAGAGAGCGACTTCTCTCGGGCCCACAGCCTTACAGCTGTGGAGCACGGTATCCTCTGCC
GAAACAGAGGTTGGACAAGACCGGAGGGGTCTCCTAGTTCCAAAGGAGATGTACTCCGGGCTTGTTCA
+
FFFFFFFFFFFFFFFFFFFFFFFFFFFFFFFFFFF:FFFFFFFFFFFFFFFFFFFFFF:FFFFFFFFF
FFFFFFFFFFFFFFFF:FFFFFFFFFFFFFFFFFFFFFFFFFFFFFFFFFFFFFFFFFFFFFFFFFF
@A00155:342:HHGFNDSXY:1:2140:9896:22827 1:N:0:GAACCTAG+TCCGCATA
CTCAATGCCGAGAGGGATGTGGAGGGCGAGAGAGAGCGACTTCTCTCGGGCCCACAGCCTTACAGCTG
TGGAGCACGGTATCCTCTGCCGAAACAGAGGTTGGACAAGACCGGAGGGGTCTCCTGGTTCCAAAGGA
+

FFFFFFFFFFFFFFFFFFFFFFFFFFFFFFFFFFFFFFFFFFFFFFFFFFFFFFFFFFFFFFFFFFFF
FFFFFFFFFFFFFFFFFFFFFFFFFFFFFFFFFFFFFFFFFFFFFFFFFFFFFFFFFFFFFFFFFFFF
@A00155:342:HHGFNDSXY:1:2140:19669:29324 1:N:0:GAACCTAG+TCCGCATA
GAGAGAGAGCGACTTCTCTCGGGCCCACAGCCTTACAGCTGTGGAGCACGGTATCCTCTGCCGAAACA
GAGGTTGGACAAGACCGGAGGGGTCTCCTAGTTCCAAAGGAGATGTACTCCGAGCTTGTTCACGACCT
+
FFFFFFFFFFFFFF:FFFFFFFFFFFFFFFFFFFFFFFFFFFFFFFFFFFFFFFFFFFFFFFFFFFFF
FFFFFFFFFFFFFFFFFFFFFFFFFFFFFF:FFFFFFFFFFFFFFFFFFFFFFFFFFFFFFFFFFFFF
@A00155:342:HHGFNDSXY:1:2140:23665:30483 1:N:0:GAACCTAG+TCCGCATA
CTATGGTCATCTCAATGCCGAGAGGGATGTGGAGGGCGAGAGAGAGCGACTTCTCTCGGGCCCACAGC
CTTACAGCTGTGGAGCACGGTATCCTCTGCCGAAACAGAGGTTGGACAAGACCGGAGGGGTCTCCTAG
+
FFFFFFFFFFFFFFFFFFFF:FFFFFFFFFFFFFFFFFFFFFFFFFFFFFFFFFFFFFFFFFFFFFFF
FFFFFFFFFFFFFFFFFFFFFFFFFFFFFFFFFFFFFFFFFFFFFFFFFFFFFFFFFFFFFFFFFFFF
@A00155:342:HHGFNDSXY:1:2140:23918:31548 1:N:0:GAACCTAG+TCCGCATA
CTCTGGCCTATGGTCATCTCAATGCCGAGAGGGATGTGGAGGGCGAGAGAGAGCGACTTCTCTCGGGC
CCACAGCCTTACAGCTGTGGAGCACGGTATCCTCTGCCGAAACAGAGGTTGGACAAGACCGGAGGGGT
+
FFFFFFFFFFFFFFFFFFFFFFFFFFFFFFFFFFFF:FFFFFFFFFFFFFFFFFFF:FFFFFFFFFF
FFFFFFFFFFFFFFFFFFFFFFFFFFFFFFFFFFFFFFFFFFFFFFFFFFFFFFFFFFFFFFFFFFFF
@A00155:342:HHGFNDSXY:1:2140:2935:35289 1:N:0:GAACCTAG+TCCGCATA
ATGCCGAGAGGGATGTGGAGGGCGAGAGAGAGCGACTTCTCTCGGGCCCACAGCCTTACAGCTGTGGA
GCACGGTATCCTCTGCCGAAACAGAGGTTGGACAAGACCGGAGGGGTCTCCTAGTTCCAAAGGAGATG
+
FFFFFFFFFFFFFFFFFFFFFFFFFFFFFFFFFFFFFFFFFFFFFFFFFFFFFFFFFFFFFFFFFFFF
FFFFFFFFFFFFFFFFFFFFFFFFFFFFFFFFFFFFFFFFFFFFFFFFFFFFFFFFFFFFFFFFFFFF
@A00155:342:HHGFNDSXY:1:2141:10872:2127 1:N:0:GAACCTAG+TCCGCATA
GTCATCTCAATGCCGAGAGGGATGTGGAGGGCGAGAGAGAGCGACTTCTCTCGGGCCCACAGCCTTAC
AGCTGTGGAGCACGGTATCCTCTGCCGAAACAGAGGTTGGACAAGACCGGAGGGGTCTCCTAGTTCCA
+
FFFFFFFFFFFFFFFFFFFFFFFFFFFFFFFFFFFFFFFFFFFFFFFFFFFFFFFFFFFFFFFFFFFF
FFFFFFFFFFFFFFFFFFFFFFFFFFFFFFFFFFFFFFFFFFFFFFFFFFFFFFFFFFFFFFFFFFFF
@A00155:342:HHGFNDSXY:1:2141:30011:3333 1:N:0:GAACCTAG+TCCGCATA
ATCTCAATGCCGAGAGGGATGTGGAGGGCGAGAGAGAGCGACTTCTCTCGGGCCCACAGCCTTACAGC
TGTGGAGCACGGTATCCTCTGCCGAAACAGAGGTTGGACAAGACCGGAGGGGTCTCCTGGTTCCAAAG
+
FFFFFFFFFFFFFFFFFFFFFF,FFFFFFFFFFFFFFFFFFFFFFFFFFFFFFFFFFFFFFFFFFFFF
FF:FFFFFFFFFFFFFFFFFFFFFF:FFFFFFFFFFFFFFFFFFFFFFFFFFFFFFFFFFFFFFFFFF
@A00155:342:HHGFNDSXY:1:2141:11397:7513 1:N:0:GAACCTAG+TCCGCATA
GCCCCACCACACCGCCTTAGATCAGCTGTGTCCACTTAAGGACTCACCTCTGGCCTATGGTCATCTCA
ATGCCGAGAGGGATGTGGAGGGCGAGAGAGAGCGACTTCTCTCGGGCCCACAGCCTTACA
+
FFFFFFFFFFFFFFFFFFFFFFFFFFFFFFFFFFFFFFFFFFFFFFFFFFFFFFFFFFFFFFFFFFFF
FFFFFFFFFFFFFFFFFFFFFFFFFFFFFFFFFFFFFFFFFFFFFFFFFFFFFFFFFFFF
@A00155:342:HHGFNDSXY:1:2141:32805:9017 1:N:0:GAACCTAG+TCCGCATA
CTGTGTCCACTTAAGGACTCACCTCTGGCCTATGGTCATCTCAATGCCGAGAGGGATGTGGAGGGCGA
GAGAGAGCGACTTCTCTCGGGCCCACAGCCTTACAGCTGTGGAGCACGGTATCCTCTGCCGAAACAGA
+
FFFFFFFFF:FFFFFFFFFFFFFFFFFFFFFFFFFFFFFFFFFFFFFFFFFFFFFFFFFFFFFFFFFF
FFFFFFFFFFFFFFFFFFFFFFFFFFFFFFFFFFFFFFFFFFFFFFFFFFFFFFFFFFFFFFFFFFF:
@A00155:342:HHGFNDSXY:1:2141:6858:9799 1:N:0:GAACCTAG+TCCGCATA
CAATGCCGAGAGGGATGTGGAGGGCGAGAGAGAGCGACTTCTCTCGGGCCCACAGCCTTACAGCTGTG
GAGCACGGTATCCTCTGCCGAAACAGAGGTTGGACAAGACCGGAGGGGTCTCCTAGTTCCA
+

FFFFFFFFFFFFFFF:FFFFFFFFFFFFFFFFFFFFFFFFFFFFFFFFFFFFFFFFFFFFFFFFFFFF
FFFFFFF:F:FFFFFFFFFFFFFFFFFFF:FFFFFFFFFFFFFFFFFFFFFFFFFFFFFFF
@A00155:342:HHGFNDSXY:1:2141:19660:10238 1:N:0:GAACCTAG+TCCGCATA
ACCTCTGGCCTATGGTCATCTCAATGCCGAGAGGGATGTAGAGGGCGAGAGAGAGCGACTTCTCTCGG
GCCCACAGCCTTACAGCTGTGGAGCACGGTATCCTCTGCCGAAACAGAGGTTGGACAAGACCGGAGGG
+
FFFFFFFFFFFFFFFFFFFFFFFFFFFFFFFFFFF:FFFFFFFFFFFFFFFFFFFFFFFFFFFFFFFF
FFFFFFFFFFFFFFFFFFFFFFFFFFFFFFFFFFFFFFFFFFFFFFFFFFFFFFFFFFFFFFFFFFFF
@A00155:342:HHGFNDSXY:1:2141:31295:24095 1:N:0:GAACCTAG+TCCGCATA
AGAGAGAGCGACTTCTCTCGGGCCCACAGCCTTACAGCTGTGGAGCACGGTATCCTCTGCCGAAACAG
AGGTTGGACAAGACCGGAGGGGTCTCCTAGTTCCAAAGGAGATGTACTCCGGGCTTGTTCACGACCT
+
FFFFFFFFFFFFFFFFFFFFFFFFFFFFFFFFFFFFFFFFFFFFFFFFFFFFFFFFFFFFFFFFFFFF
FFF:FFFFFFFFFFFFFFFFFFFFFFFF:FFFFF:FFFFFFFFFF:FFF:FFFFFFFFFFFFFFFFF
@A00155:342:HHGFNDSXY:1:2141:22571:25426 1:N:0:GAACCTAG+TCCGCATA
GCTCCACAGCTGTAAGGCTGTGGGCCCGAGAGAAGTCGCTCTCTCTCGCCCTCCACATCCCTCTCGGC
ATTGAGATGACCATAGGCCAGAGGTGAGTCCTTAAGTGGACACAGCTGATCTAAGGCGGTGTGGTGGG
+
FFFFFFFFFFFFFFFFFFFFFFFFFFFFFFFFFFFFFFFFFFFFFFFFFFFFFFFFFFFFFFFFFFFF
FFFFFFFFFFFFF:FFFFFFFFFFFFFFFFFFFFFFFFFFFFFFFFFFFFFFFFFFFFFFFFFFFFFFFF
@A00155:342:HHGFNDSXY:1:2141:17852:29559 1:N:0:GAACCTAG+TCCGCATA
GGGCGAGAGAGAGCGACTTCTCTCGGGCCCACAGCCTTACAGCTGTGGAGCACGGTATCCTCTGCCGA
AACAGAGGTTGGACAAGACCGGAGGGGTCTCCTAGTTCCAAAGGAGATGTACTCCGGGCTTGTTCACG
+
FFFFFFFFFFFFFFFFFFFFFFFFFFFFFFFFFFFFFFFFFFFFFFFFFFFFFFFFFF:FFFFFFFFF
FFFFFFFFFFFFFFFFFFFFFFFFFFFFFFFFFFFFFFFFFFFFFFFFFFFFFFFFFFFFFFFFFFFF
@A00155:342:HHGFNDSXY:1:2142:16966:12524 1:N:0:GAACCTAG+TCCGCATA
GAGAGGGATGTGGAGGGCGAGAGAGAGCGACTTCTCTCGGGCCCACAGCCTTACAGCTGTGGAGCACG
GTATCCTCTGCCGAAACAGAGGTTGGACAAGACCGGAGGGGTCTCCTAGTTCCAAAGGAGATGTACTC
+
FFFFFFFFFFFFFFFFFFFFFFFFFFFFFFFFFFFFFFFFFFFFFFFFFFFFFFFFFFFFFFFFFFFF
FFFFFFFFFFFFFFFFFFFFFFFFFFFFFFFFFFFFFFFFFFFFFFFFFFFFFFFFFFFFFFFFFFFF
@A00155:342:HHGFNDSXY:1:2143:29966:1407 1:N:0:GAACCTAG+TCCGCATA
ACTTCTCTCGGGCCCACAGCCTTACAGCTGTGGAGCACGGTATCCTCTGCCGAAACAGAGGTTGGACA
AGACCGGAGGGGTCTCCTAGTTCCAAAGGAGATGTACTCCGGGCTTTTTCACGACCTACCGTGTAAG
+
FFFFFFFFFFFFFFFFFFFFFFFFFFFFFFFFFFFFFFFFF,FFFFFFFFFFFFFFFFFFFFFFFFFF
FFFFFFFFFFFFFFFFFFFFFFFFFFFFFFFFFFFFFFFFFFFFFFFFFFFFFFFFFFFFFFFFFFF
@A00155:342:HHGFNDSXY:1:2143:10682:6965 1:N:0:GAACCTAG+TCCGCATA
GGATGTGGAGGGCGAGAGAGAGCGACTTCTCTCGGGCCCACAGCCTTACAGCTGTGGAGCACGGTATC
CTCTGCCGAAACAGAGGTTGGACAAGACCGGAGGGGTCTCCTAGTTCCAAAGGAGATGTACTCC
+
FFFFFFFFFFFFFFFFFFFFFFFFFFFFFFFFFFFFFFFFFFFFFFFFFFFFFFFFFFFFFFFFFFFF
FFFFFFFFFFFFFFFFFFFFFFFFFFFFFFFFFFFFFFFFFFFFFFFFFFFFFFFFFFFFFFFF
@A00155:342:HHGFNDSXY:1:2143:11812:9111 1:N:0:GAACCTAG+TCCGCATA
GCTGTGTCCACTTAAGGACTCACCTCTGGCCTATGGTCATCTCAATGCCGAGAGGGATGTAGAGGGCG
AGAGAGAGCGACTTCTCTCGGGCCCACAGCCTTACAGCTGTGGAGCACGGTATCCTCTGCCGAAACAG
+
F:FFFFFFFFFFFFFFFFFFFFFFFFFFFFFFFFFF:FFFFFF:FFFFFFFFFFFFFFFFFFFFF:FF
FFFFFFFFF:FFFFFFF:FFFFFFFFFF:FF,FFF::FFFFF,FFFFFFFFFFFFFFFFFFFFF,FFF
@A00155:342:HHGFNDSXY:1:2143:3992:9659 1:N:0:GAACCTAG+TCCGCATA
CTCCTCTGGCCTATGGTCATCTCAATGCCGAGAGGGATGTGGAGGGCGAGAGAGAGCGACTTCTCTCG
GGCCCACAGCCTTACAGCTGTGGAGCACGGTATCCTCTGCCGAAACAGAGGTTGGACAAGACCGGAGG
+

```
FFFFFFFFFFFFFFFFFFFFFFFFFFFFFFFFFFFFFFFFFFFFFFFFFFFFFFFFFFFFFFFFFFFF
FFFFFFFFFFFFFFFFFFFFFFFFFFFFFFF:FFFFFFFFFFFFFFFFFFFFFFFFFFFFFFFFFFFF
@A00155:342:HHGFNDSXY:1:2143:7898:13886 1:N:0:GAACCTAG+TCCGCATA
CTCAATGCCGAGAGGGATGTGGAGGGCGAGAGAGAGCGACTTCTCTCGGGCCCACAGCCTTACAGCTG
TGGAGCACGGTATCCTCTGCCGAAACAGAGGTTGGACAAGACCGGAGGGGTCTCCTAGTTCCAAAGGA
+
FFFFFFFFFFFFFFFFFFFFFFFFFFFFFFFFFFFFFFFFFFFFFFFFFFFFFFFFFFFFFFFFFFFF
FFFFFFFFFFF,FFFFFFFFFFFFFFFFFFFFFFFFFFFFFFFFFFFFFFFFFFFFFFFFFFFFFFFF
@A00155:342:HHGFNDSXY:1:2143:4553:25786 1:N:0:GAACCTAG+TCCGCATA
GGATGTGGAGGGCGAGAGAGAGCGACTTCTCTCGGGCCCACAGCCTTACAGCTGTGGAGCACGGTATC
CTCTGCCGAAACAGAGGTTGGACAAGACCGGAGGGGTCTCCTAGTTCCAAAGGAGATG
+
FFFFFFFFFFFFFFFFFFFFFFFFFFFFFFFFFFFFFFFFFFFFFFFFFFFFFFFFFFFFFFFFFFFF
FFFFFFFFFFFFFFFFFFFFFFFFFFFFFFFFFFFFFFFFFFFFFFFFFFFFFFFFFF
@A00155:342:HHGFNDSXY:1:2144:20039:9298 1:N:0:GAACCTAG+TCCGCATA
AGAGAGAGCGACTTCTCTCGGGCCCACAGCCTTACAGCTGTGGAGCACGGTATCCTCTGCCGAAACAG
AGGTTGGACAAGACCGGAGGGGTCTCCTGGTTCCAAAGGAGATGTACTCCGGGCTTGTTCACGACCTA
+
FFFFFFFFFFFFFFFFFFFFFFFFFFFFFFFFFFFFFFFFFFFFFFFFFFFFFFFFFFFFFFFFFFFF
FFFFFFFFFFFFFFFFFFFFFFFFFFFFFFFFFFFFFFFFFFFFFFFFFFFFFFFFFFFFFFFFFFFF
@A00155:342:HHGFNDSXY:1:2144:27579:9799 1:N:0:GAACCTAG+TCCGCATA
TTAAGGACTCACCTCTGGCCTATGGTCATCTCAATGCCGAGAGGGATGTGGAGGGCGAGAGAGAGCGA
CTTCTCTCGGGCCCACAGCCTTACAGCTGTGGAGCACGGTATCCTCTGCCGAAACAGAGG
+
FFFFFFFFFFFFFFFFFFFFFFFFFFFFFFFFFFFFFFFFFFFFFFFF:FFFFFFFFFFFFFFFFFFF
FFFFFFFFFFFFFFFFFFFFFFFFFFFFFFFFFFFFFFFF,FFFFFFFFFFFFFFFFFFF
@A00155:342:HHGFNDSXY:1:2144:11577:33567 1:N:0:GAACCTAG+TCCGCATA
AAGGACTCACCTCTGGCCTATGGTCATCTCAATGCCGAGAGGGATGTGGAGGGCGAGAGAGAGCGACT
TCTCTCGGGCCCACAGCCTTACAGCTGTGGAGCACGGTATCCTCTGCCGAAACAGAGGTTGGACAAGA
+
FFFFFFFFFFFFFFFFFFFFFFFFFFFFFFFFFFFFFFFFFFFFFFFFFFFFFFFFFFFFFFFFFFF:
FFFFFFFFFFFFFFFFFFFFFFFFFFFFFFFFFFFFFFFFFFFFFFFFFFFFFFFFFFFFFFFFFFFF
@A00155:342:HHGFNDSXY:1:2145:12355:4319 1:N:0:GAACCTAG+TCCGCATA
ACCTCTGTTTCGGCAGAGGATACCGTGCTCCACAGCTGTAAGGCTGTGGGCCCGAGAGAAGTCGCTCT
CTCTCGCCCTCCACATCCCTCTCGGCATTGAGATGACCATAGGCCAGAGGTGAGTCCTTAAGTGGACA
+
FFFFFFFFFFFFFFFFFFFFFFFFFFFFFFFFFFFFFFFFFFFFFFFFFFFFFFFFFFFFFFFFFFFF
FFFFFFFFFFFFFFFFFFFFFFFFFFF,FFFFFFFFFFFFFFFFFFFFFFFFFFFFFFFFFFFFFFFF
@A00155:342:HHGFNDSXY:1:2145:16568:6731 1:N:0:GAACCTAG+TCCGCATA
CTGCCGAAACAGAGGTTGGACAAGACCGGAGGGGTCTCCTAGTTCCAAAGGAGATGTACTCCGGGCTT
GTTCACGACCTACCGTGTAAGTCGTAGTCTAGTAGGCTACCTGACGAGTCCTTTTTAGGACGAAACTT
+
FFFFFFFFF:F:FFFFFFFFFFFFFFFFFFFFFFFFFFFFFFFFFFFFFFFFFFFFFFFFFFFFFFFF
FFFFFFFFFFFFFFFFFFFFFFFFFFFFFFFFFFFFFFFFFFFFFFFFFFFFFFFFFFFFFFFFFFFF
@A00155:342:HHGFNDSXY:1:2145:2718:25363 1:N:0:GAACCTAG+TCCGCATA
GGACTCTCCTCTGGCCTATGGTCATCTCAATGCCGAGAGGGATGTGGAGGGCGAGAGAGAGCGACTTC
TCTCGGGCCCACAGCCTTACAGCTGTGGAGCACGGTATCCTCTGCCGAAACAGAGGTTGGACAA
+
FFFFFFFFFFFFFFFFFFFFFFFFFFFFFFFFFFFFFFFFFFFFFFFFFFFFFFFFFFFFFFFFFFFF
FFFFFFFFFFFFFFFF:FFFFFFFFFFFFFFFFFFFFFFFFFFFFFFFFFFFFFFFFFFFFFF
@A00155:342:HHGFNDSXY:1:2146:24207:1955 1:N:0:GAACCTAG+TCCGCATA
CACCTCTGGCCTATGGTCATCTCAATGCCGAGAGGGATGTGGAGGGCGAGAGAGAGCGACTTCTCTCG
GGCCCACAGCCTTACAGCTGTGGAGCACGGTATCCTCTGCCGAAACAGAGGTTGGACAAGACCGGAGG
+
```

```
FFFFFFFFFFFFFFFFFFFFFFFFFFFFFFFFFFFF::FFFFFFFFFFFFFFFFFFFFFFFFFFFFFF
FFFFFFFFFFFFF:FFFFFFFFFFFFFFFFFFFFFFFFFFFFFFFFFFFFFFFFFFFFFFFFFFFFFF
@A00155:342:HHGFNDSXY:1:2146:22896:5196 1:N:0:GAACCTAG+TCCGCATA
AGCGACTTCTCTCGGGCCCACAGCCTTACAGCTGTGGAGCACGGTATCCTCTGCCGAAACAGAGGATG
GACAAGACCGGAGGGGTCTCCTAGTTCCAAAGGAGATGTACTCCGGGCTTGTTCACGACCTACCGTGT
+
FFFFFF:FF:FFFFF:F:FFFFFFF,FFFFFFF:FFFFFFFFFFFFFFFFF,FFFFFFFFFFF,,F,
FFFFF:FFFFFFFFFFFFFFFFFFFFFFFFFF:FFFFFFFFFFFF,FFFFF,FF:FFFFFFFFF,FFF:
@A00155:342:HHGFNDSXY:1:2146:30544:8641 1:N:0:GAACCTAG+TCCGCATA
GCGAGAGAGAGCGACTTCTCTCGGGCCCACAGCCTTACAGCTGTGGAGCACGGTATCCTCTGCCGAAA
CAGAGGTTGGACAAGACCGGAGGGGTCTCCTAGTTCCAAAGGAGATGTACTCCGGGCTTGTTCACGAC
+
FFFFFFFFFFFFFFFFFFFFFFFFFFFFFFFFFFFFFFFFFFFFFFFFFFFFFFFFFFFFFFFFFFFF
FFFFFF:FFFFFFFFFFFFFFFFFFFFFFFFFFFFFFFFFFFFFFFFFFFFFFFFFFFFFFFFF:FFF
@A00155:342:HHGFNDSXY:1:2146:12084:12493 1:N:0:GAACCTAG+TCCGCATA
AGCGACTTCTCTCGGGCCCACAGCCTTACAGCTGTGGAGCACGGTATCCTCTGCCGAAACAGAGGTTG
GACAAGACCGGAGGGGTCTCCTGGTTCCAAAGGAGATGTACTCCGGGCTTGTTCACGACCTACCGTGT
+
FFFFFFFFFFFFFFFFFFFFFFFFFFFFFFFFFFFFFFFFFFFFFFFFFFFFFFFFFFFFFFFFFFFF
FFFFFFFFFFFFFFFFFFFFFFFFFFFFFFFFFFFFFFFFFFFFFFFFFFFFFFFFFFFFFFFFFFFF
@A00155:342:HHGFNDSXY:1:2146:3974:18427 1:N:0:GAACCTAG+TCCGCATA
GAGCGACTTCTCTCGGGCCCACAGCCTTACAGCTGTGGAGCACGGTATCCTCTGCCGAAACAGAGGTT
GGACAAGACCGGAGGGGTCTCCTAGTTCCAAAGGAGATGTACTCCGGGCTTGTTCACGACCTACCGTG
+
FFFFFFFFFFFFFFFFFFFFFFFFFFFFFFFFFFFFFFFFFFFFFFF:FFFFFFFFFFFFFFFFFFFF
FFFFFFFFFFFFFFFFFFFFFFFFFFFFFFFFFFFFFFFFFFFFFFFFFFFFFFFFFFFFFF,FFF,F
@A00155:342:HHGFNDSXY:1:2146:14181:21230 1:N:0:GAACCTAG+TCCGCATA
GGATGTAGAGGGCGAGAGAGAGCGACTTCTCTCGGGCCCACAGCCTTACAGCTGTGGAGCACGGTATC
CTCTGCCGAAACAGAGGTTGGACAAGACCGGAGGGGTCTCCTAGTTCCAAAGGAGATGTACTCCGGGC
+
FFFFFFFFFFFFFFFFFFFFFFFFFFFFFFFFFFFFFFFFFFFFFFFFFFFFFFFFFFFFFFFFFFFF
FFFFFFFFFFFFFFFFFFFFFFFFFFFFFFFFFFFFFFFFFFFFFFFFFFFFFFFFFFFFFFFFFFFF
@A00155:342:HHGFNDSXY:1:2146:8422:29293 1:N:0:GAACCTAG+TCCGCATA
GTATCCTCTGCCGAAACAGAGGTTGGACAAGACCGGAGGGGTCTCCTAGTTCCAAAGGAGATGTACTC
CGGGCTTGTTCACGACCTACCGTGTAAGTCGTAGTCTAGTAGGCTACCTGACGAGTCCTTTTTAGGAC
+
FFFFFFFFFFFFFFFFFFFFFFFFFFFFFFFFFFFFFFFFFFFFFFFFFFFFFFFFFFFFFFFFFFFF
FFFFFFFFFFFFFFFFFFFFFFFFFFFFFFFFFFFFFFFF:FFFFFFFFFFFFFFFFFFFFFFFFFFF
@A00155:342:HHGFNDSXY:1:2146:4942:36166 1:N:0:GAACCTAG+TCCGCATA
GTATCCTCTGCCGAAACAGAGGTTGGACAAGACCGGAGGGGTCTCCTAGTTCCAAAGGAGATGTACTC
CGGGCTTGTTCACGACCTACCGTGTAAGTCGTAGTCTAGTAGGCTACCTGACGAGTCCTTTTTAGGAC
+
FF:F,FFFFFFFFFFFFFFFFFFFFFFFFFFFFFFFFFFFFFFFFFFF:FFFFFFFFFFFFF:FFFF
FF:,FFFF:FFFFFFFFFFFFFFFFFF:FFFFFFFFFFF,FFFFFFFFFFFFFFFFFFFFFFFF,FFF
@A00155:342:HHGFNDSXY:1:2147:14823:7717 1:N:0:GAACCTAG+TCCGCATA
TGTGGAGGGCGAGAGAGAGCGACTTCTCTCGGGCCCACAGCCTTACAGCTGTGGAGCACGGTATCCTC
TGCCGAAACAGAGGTTGGACAAGACCGGAGGGGTCTCCTGGTTCCAAAGGAGATG
+
FFFFFFFFFFFFFFFFFFFFFFFFFFFFFFFFFFFFFFFFFFFFFFFFFFFFFFFFFFFFFFFFFFFF
FFFFFF,FF:FFFFFFFFFFFFFFFFFFFFFFFFFFFFFFFFFFFFFFFFFFFF
@A00155:342:HHGFNDSXY:1:2147:10465:8500 1:N:0:GAACCTAG+TCCGCATA
ATGCCGAGAGGGATGTGGAGGGCGAGAGAGAGCGACTTCTCTCGGGCCCACAGCCTTACAGCTGTGGA
GCACGGTATCCTCTGCCGAAACAGAGGTTGGACAAGACCGGAGGGGTCTCCTGGTTCCAAAGGAGATG
+
```

```
FFFFFFFFFFFFF,,FFFFF:FF,FFFFFFFFFFFFF:FFF:FFFFFFFFFFFFFF,,FFFFFF:FFF
FFFFFFFFFFFFFFFFFFFFFFFFFFFFF::FFFFFFFFFFFFFFFFFFFFFFFFFFFFF::FF:FFF
@A00155:342:HHGFNDSXY:1:2147:28944:8844 1:N:0:GAACCTAG+TCCGCATA
ACTTCTCTCGGGCCCACAGCCTTACAGCTGTGGAGCACGGTATCCTCTGCCGAAACAGAGGTTGGACA
AGACCGGAGGGGTCTCCTAGTTCCAAAGGAGATGTACTCCGGGCTTGTTCACGACCTACCGTGTAAGT
+
FFFFFFFFFFFFFFFFFFFFFFFFFFFFFFFFFFFFFFFFFFFFFFFFFFFFFFFFFFFFFFFFFFFF
F,FFFFFFFFFFFFFFFFF:FFFFFFFFFFFFFFFFFFFFFFFFFFFFFFFFFFFFFFFFFFFFFFFF
@A00155:342:HHGFNDSXY:1:2147:10285:18458 1:N:0:GAACCTAG+TCCGCATA
GCGACTTCTCTCGGGCCCACAGCCTTACAGCTGTGGAGCACGGTATCCTCTGCCGAAACAGAGGTTGG
ACAAGACCGGAGGGGTCTCCTAGTTCCAAAGGAGATGTACTCCGGGCTTGTTCACGACCTACCGTGTA
+
FFFFFFFFFFFFFFFFFFFFFFFFFFFFFFFFFFFFFFFFFFFFFFFFFFFFFFFFFFFFFFFFFFFF
FFFFF:FFFFFFFFFFFFFFFFFFFFFFFFFFFFFFFFFFFFFFFFFFFFFFFFFFFFFFFFFFFFFF
@A00155:342:HHGFNDSXY:1:2148:23303:32362 1:N:0:GAACCTAG+TCCGCATA
CGACTTCTCTCGGGCCCACAGCCTTACAGCTGTGGAGCACGGTATCCTCTGCCGAAACAGAGGTTGGA
CAAGACCGGAGGGGTCTCCTAGTTCCAAAGGAGATGTACTCCGGGCTTGTTCACGACCTACCGTGTAA
+
FFFFFFFFFFFFFFFFFFFFFFFFFFFFFFFFFFFFFFFFFFFFFFFFFFFFFFFFFFFFFFFFFFFF
FFFFFFFFFFFFFFFFFFFFFFFFFFFFFFFFFFFFFFFFFFFFFFFFFFFFFFFFFFFFFFFFFFFF
@A00155:342:HHGFNDSXY:1:2149:17770:14199 1:N:0:GAACCTAG+TCCGCATA
TACTCCGGGCTTGTTCACGACCTACCGTGTAAGTCGTAGTCTAGTAGGCTACCTGACGAGTCCTTTTT
AGGACGAAACTTACACAACTCAAAGAGTAACGACGGTTTCGTCCTTGGAACGGACTCATCA
+
FFFFFFFFFFFFFFFFFFFFFFFFFFFFFFFFFFFFFFFFFFFFFFFFFFFFFFFFFFFFFFFFFFFF
FFFFFFFFFFFFFFFFFFFFFFFFFFFFFFFFFFFFFFFFFFFFFFFFFFFFFFFFFFFFF
@A00155:342:HHGFNDSXY:1:2149:30318:18740 1:N:0:GAACCTAG+TCCGCATA
ACCTCTGTTTCGGCAGAGGATACCGTGCTCCACAGCTGTAAGGCTGTGGGCCCGAGAGAAGTCGCTCT
CTCTCGCCCTCCACATCCCTCTCGGCATTGAGATGACCATAGGCCAGAGGTGAGTCCTTAAGTGGACA
+
FFFFFFFFFFFFFFFFFFFFFFFFFFFFFFFFFFFFFFFFFFFFFFFFFFFFFFFFFFF:FFFFFFFF
FFFFFFFFFFFFFFFFFFFFFFFFFFF,FFFFFFFFFFFFFFFFFFFFFFFFFFFFFFFFFFFFFFFF
@A00155:342:HHGFNDSXY:1:2149:23240:35509 1:N:0:GAACCTAG+TCCGCATA
GAGAGGGATGTAGAGGGCGAGAGAGAGCGACTTCTCTCGGGCCCACAGCCTTACAGCTGTGGAGCACG
GTATCCTCTGCCGAAACAGAGGTTGGACAAGACCGGAGGGGTCTCCTAGTTCCAAAGGAGATGTACTC
+
FFFFFFFFFFFFFFFFFFFFFFFFFFFFFFFFFFFFFFFFFFFFFF:FFF:FFFFFFFFFFFFFFFFF
FFFFFFFFFFFFFFFFFFFFFF:FFFFFFFFFFFFFFFFFFFFFFFF:FFFFFFFFFFFFFFFFFFFF
@A00155:342:HHGFNDSXY:1:2151:10185:20290 1:N:0:GAACCTAG+TCCGCATA
GGATGTGGAGGGCGAGAGAGAGCGACTTCTCTCGGGCCCACAGCCTTACAGCTGTGGAGCACGGTATC
CTCTGCCGAAACAGAGGTTGGACAAGACCGGAGGGGTCTCCTGGTTCCAAAGGAGATGTACTCC
+
FFFFFFFFFFFFFFFFFFFFFFFFFFFFFFFFFFFFFFFFFFFFFFFFFFFFFFFFFFFFFFFFFFFF
FFFFFFFFFFFFFFFFFFFFFFFFFFFFFFFFFFFFFFFFFFFFFFFFFFFFFFFFFFFFFFFF
@A00155:342:HHGFNDSXY:1:2151:4734:32111 1:N:0:GAACCTAG+TCCGCATA
GGGCGAGAGAGAGCGACTTCTCTCGGGCCCACAGCCTTACAGCTGTGGAGCACGGTATCCTCTGCCGA
AACAGAGGTTGGACAAGACCGGAGGGGTCTCCTGGTTCCAAAGGAGATGTACTCCGGGCTTGTTCACG
+
FFF:FFFFFFFFFFFFFFFF:FFFFFFFFFFFFFFFFFFFFFFFFFFFFFFFFFF:FFFFFFFFFFFF
FFFFFFFFFFFFFFF:FFF:FFFFF:F,FFFF,FFFFFFFFFFFFFFFFFFFFFFFFFFFFF:FFFFF
@A00155:342:HHGFNDSXY:1:2151:4128:34162 1:N:0:GAACCTAG+TCCGCATA
GGGCGAGAGAGAGCGACTTCTCTCGGGCCCACAGCCTTACAGCTGTGGAGCACGGTATCCTCTGCCGA
AACAGAGGTTGGACAAGACCGGAGGGGTCTCCTGGTTCCAAAGGAGATGTACTCCGGGCTTGTTCACG
+
```

```
FFFFFFFFFFFFFFFFFFFFFFFFFFFFFFFFFFFFFFFFFFFFFFFFFFFFFFFF:FFFFFFFFFFF
:FFFFFFF,FFFFFFFFFFFFFFFFFFFFFFFFFFFFFFFFFFFFFFFFFFFFFFFFFFFFFFFFFFF
@A00155:342:HHGFNDSXY:1:2152:6569:15405 1:N:0:GAACCTAG+TCCGCATA
ATCTCAATGCCGAGAGGGATGTGGAGGGCGAGAGAGAGCGACTTCTCTCGGGCCCACAGCCTTACAGC
TGTGGAGCACGGTATCCTCTGCCGAAACAGAGGTTGGACAAGACCGGAGGGGTCTCCAAGTTCCAAAG
+
FFFFFFFFFFFFFFFFFFFFFFFFFFFF:FFFFFFFFF:FFFFFFFFFFFFFFFFFFFFFFF,FFFFF
FFFFFFFFF,FFF:FFFFFFFFFFFFFFFFFFFFFFFFFFFFFFFFFFF:FFFFFFFFF:FFFFFFFF:
@A00155:342:HHGFNDSXY:1:2152:3857:17096 1:N:0:GAACCTAG+TCCGCATA
GTCATCTCAATGCCGAGAGGGATGTGGAGGGCGAGAGAGAGCGACTTCTCTCGGGCCCACAGCCTTAC
AGCTGTGGAGCACGGTATCCTCTGCCGAAACAGAGGTTGGACAAGACCGGAGGGGTCTCCTAGTTCCA
+
FFFFFFFFFFFFFFFFFFFFFFFF:FFFFFFFFFFFFFFFFFFFFFFFFFFFFFFFFFFFFFFFFFFF
FFFFFFFFFFFFFFFFFFFFFFFFFFFFFFFFFFFFFFFFFFFFFF:FFFFFFFFFFFFFFFFFFFFF
@A00155:342:HHGFNDSXY:1:2152:32235:17801 1:N:0:GAACCTAG+TCCGCATA
GGCGAGAGAGAGCGACTTCTCTCGGGCCCACAGCCTTACAGCTGTGGAGCACGGTATCCTCTGCCGAA
ACAGAGGTTGGACAAGACCGGAGGGGTCTCCTAGTTCCAAAGGAGATGTACTCCGGGCTTGTTCACG
+
FFFFFFFFFFFFFFFFFFFFFFFFFFFFFFFFFFFFFFFFFFFFFFF:FFFFFFFFFFFFFFFFFFFF
FFFFFFFFFFFFFFF,FFFFFFFFFFFFFFFFFFFFFFFFFFFFFFFFFFFFFF::FFFFFFFFFFF
@A00155:342:HHGFNDSXY:1:2152:4463:20369 1:N:0:GAACCTAG+TCCGCATA
ATGCCGAGAGGGATGTAGAGGGCGAGAGAGAGCGACTTCTCTCGGGCCCACAGCCTTACAGCTGTGGA
GCACGGTATCCTCTGCCGAAACAGAGGTTGGACAAGACCGGAGGGGTCTCCTAGTTCCAAAGGAGAT
+
FFFFF:FFF,::FFFFFFFFFFFFFFFFFFFFFFFFFFFFFFFFFFFF:FFFFFFFFFFFFFFFFFFF
FFFF:FFFFFFFFFF,FFFFFFFFFFFFFFFFFFF,FFFFFFFFFFFFF:FFFFFFFFFFF,F:FF
@A00155:342:HHGFNDSXY:1:2152:11153:25692 1:N:0:GAACCTAG+TCCGCATA
GAGCGACTTCTCTCGGGCCCACAGCCTTACAGCTGTGGAGCACGGTATCCTCTGCCGAAACAGAGGTT
GGACAAGACCGGAGGGGTCTCCTGGTTCCAAAGGAGATGTACTCCGGGCTTGTTCACGACCTACCGTG
+
FFFFFFFF:FFFFFF:FFFFFFFFFFFFFFFFFFFFFF:FFFFFFFFFFFFFFF:FFFFFFFFFFFFF:
FFFFFFFFFFF:FFFFFFF:FFFFF:FFFFFFF,FFFFF,FFFF,F,FF:FFFFFFFFFFFF:F:FFF
@A00155:342:HHGFNDSXY:1:2153:10818:15342 1:N:0:GAACCTAG+TCCGCATA
GCGACTTCTCTCGGGCCCACAGCCTTACAGCTGTGGAGCACGGTATCCTCTGCCGAAACAGAGGTTGG
ACAAGACCGGAGGGGTCTCCTAGTTCCAAAGGAGATGTACTCCGGGCTTGTTCACGACCTACCGTGTA
+
FFFFFFFFFFFFFFFFFFFFFFFFFFFFFFFFFFFFFFFFFFFFFFFFFFFFFFFFFFFFFFFFFFFF
FFFFFFFFFFFFFFFFFFFFFFFFFFFFFFFFFFFFFFFFFFFFFFFFFFFFFFFFFFFFFFFFFF:F
@A00155:342:HHGFNDSXY:1:2153:7907:18129 1:N:0:GAACCTAG+TCCGCATA
TTCTCTCGGGCCCACAGCCTTACAGCTGTGGAGCACGGTATCCTCTGCCGAAACAGAGGTTGGACAAG
ACCGGAGGGGTCTCCTAGTTCCAAAGGAGATGTACTCCGGGCTTGTTCACGACCTACCGTGTAAGTC
+
FFFFFFFFFFFFFFFFFFFFFFFFFFFFFFFFFFFFFFFFFFFFFFFFFFFF:FFFFFFFFFFFFFFF
FFFFFFFFFFFFFFFFFFFFFFFFFFFFFFFFFFFFFFFFFFFFFFFFFFFFFFFFFFFFFFFFFFF
@A00155:342:HHGFNDSXY:1:2153:17454:30217 1:N:0:GAACCTAG+TCCGCATA
AGGGCGAGAGAGAGCGACTTCTCTCGGGCCCACAGCCTTACAGCTGTGGAGCACGGTATCCTCTGCCG
AAACAGAGGTTGGACAAGACCGGAGGGGTCTCCTAGTTCCAAAGGAGATGTACTCCGGGCTTGTTCAC
+
FFFFFFFFFFFFFFFFFFFFFFFFFFFFFFFFFFFFFFFFFFFFFFFFFFFFFFFFFFFFFFFFFFFF
FFFFFFFFFFFFFFFFFFFFFFFFFFFFFFFFFFFFFFFFFFFFFFFFFFFFFFFFFFFFFFFFFFFF
@A00155:342:HHGFNDSXY:1:2153:24885:33818 1:N:0:GAACCTAG+TCCGCATA
GGATGTAGAGGGCGAGAGAGAGCGACTTCTCTCGGGCCCACAGCCTTACAGCTGTGGAGCACGGTATC
CTCTGCCGAAACAGAGGTTGGACAAGACCGGAGGGGTCTCCTAGTTCCAAAGGAGATGTACTCC
+
```

```
FFFFFF:FFFFFFFFFFFFFFFFFFFFFFFFFFFFFFFFFFFFFFFFFFFFFFFFFFFFFF:FF
FFFFFFFFFFFFFFFFFFFFFFFFFFFFFFFFFFFFFFFFFF:FFFFFFFFFFFFFFFFFFFFF
@A00155:342:HHGFNDSXY:1:2153:10854:36135 1:N:0:GAACCTAG+TCCGCATA
GAGGGCGAGAGAGAGCGACTTCTCTCGGGCCCACAGCCTTACAGCTGTGGAGCACGGTATCCTCTGCC
GAAACAGAGGTTGGACAAGACCGGAGGGGTCTCCTAGTTCCAAAGGAGATGTACTCCGGGCTTGTTCA
+
FFFFFFFFFFFFFFFFFFFFFFFFFFFFFFFFFFFFFFFFFFFFFFFFFFFFFFFFFFFFFFFFFFFF
FFFFFFFFFFFFFFFFFFFFFFFFFFFFFFFFFFFFFFFFFFFFFFFFFFFFFFFFFFFFFFFFFFFF
@A00155:342:HHGFNDSXY:1:2154:29062:16438 1:N:0:GAACCTAG+TCCGCATA
CTCAATGCCGAGAGGGATGTGGAGGGCGAGAGAGAGCGACTTCTCTCGGGCCCACAGCCTTACAGCTG
TGGAGCACGGTATCCTCTGCCGAAACAGAGGTTGGACAAGACCGGAGGGGTCTCCTGGTTCCAAAGGA
+
FFFFFFFFFFFFFFFFFFFFFFFFFFFFFFFFFFFFFFFFFFFFFFFFFFFFFFFFFFFFFFFFFFFF
FFFFFFFFFFFFFFFFFFFFFFFFFFFFFFFFFFFFFFFFFFFFFFFFFFFFFFFFFFFFFFFFFFFF
@A00155:342:HHGFNDSXY:1:2154:28899:21950 1:N:0:GAACCTAG+TCCGCATA
GAGAGGGATGTGGAGGGCGAGAGAGAGCGACTTCTCTCGGGCCCACAGCCTTACAGCTGTGGAGCACG
GTATCCTCTGCCGAAACAGAGGTTGGACAAGACCGGAGGGGTCTCCTAGTTCCAAAGGAGATGTACTC
+
FFFFFFFFFFFFFFFFFFFFFFFFFFFFFFFFFFFFFFFFFFFFFFFFFFFFFFFFFFFFFFFFFFFF
FFFFFFFFFFFFFFFFFFFFFFFFFFFFFFFFFFFFFFFFFFFFFFFFFFFFFFFFFFFFFFFFFFFF
@A00155:342:HHGFNDSXY:1:2155:1452:4069 1:N:0:GAACCTAG+TCCGCATA
GGATGTGGAGGGCGAGAGAGAGCGACTTCTCTCGGGCCCACAGCCTTACAGCTGTGGAGCACGGTATC
CTCTGCCGAAACAGAGGTTGGACAAGACCGGAGGGGTCTCCTAGTTCCAAAGGAGATGTACT
+
FFFFFFFFFFFFFFFFFFFFFFFFFFFFF:FFFFFFFFFFFFFFFFFFFFFFFFFFFFFFFFFFFFFF
FFF:FFFFFFFFF:FFFFFFFFF:FF:FFFF:FFFFFFFFFFFFFFFFFFF,FFFFFFF:FF
@A00155:342:HHGFNDSXY:1:2155:17933:25848 1:N:0:GAACCTAG+TCCGCATA
CCTTACAGCTGTGGAGCACGGTATCCTCTGCCGAAACAGAGGTTGGACAAGACCGGAGGGGTCTCCTA
GTTCCAAAGGAGATGTACTCCGGACTTGTTCACGACCTACCGTGTAAGTCGTAGTCTAGTAGGCTACC
+
FF:FFFFFFFFFFFFFFFFFFFFFFFFFFFFFFFFFFFFFFFFFFFFFFFFFFFFFFFFFFFFFFFFF
FFFFFFFFFFFFFFFFFFFFFFFFFFFFFFFFFFFFFF,FFFFFFFFFFFFFFFFFFFFFFFFFFFFF
@A00155:342:HHGFNDSXY:1:2155:18376:27148 1:N:0:GAACCTAG+TCCGCATA
GGCCAGAGGTGAGTCCTTAAGTGGACACAGCTGATCTAAGGCGGTGTGGCGGGGCATGGGTTTGAACC
CCCATGACGGTCGGAGTCTAGTAGGCTCCCTGATGAGTCCGTTCCAAGGACGACACCGTCGTTACTCT
+
FFFFFFFFFFFFFFFFFFFFFFFFFFFFFFFFFFFFFFFFFFFFFFFFFFFFFFFFFFFFFFFF:FFF
FFFFFFFFFFFFFFFFFFFFFFFFFFFFFFFFFFFFFFFFFFFFFFFFFFFF,,FFFFFFFFFFFFFF
@A00155:342:HHGFNDSXY:1:2156:8504:3035 1:N:0:GAACCTAG+TCCGCATA
GGATGTAGAGGGCGAGAGAGAGCGACTTCTCTCGGGCCCACAGCCTTACAGCTGTGGAGCACGGTATC
CTCTGCCGAAACAGAGGTTGGACAAGACCGGAGGGGTCTCCTAGTTCCAAAGGAGATGTACTCC
+
FFFFFFFFFFFFFFFFFFFFFFFFFFFFFFFFFFFFFFFFFFFFFFFFFFFFFFFFFFFFFFFFFFFF
FFFFFFFFFFFFFFFFFFFFFFFFF:FF,FFFFFFFFFFFFFFFFFFFFF,FFFFFFFFFFF:F
@A00155:342:HHGFNDSXY:1:2156:32145:3677 1:N:0:GAACCTAG+TCCGCATA
CAAGCCCGGAGTACATCTCCTTTGGAACTAGGAGACCCCTCCGGTCTTGTCCAACCTCTGTTTCGGCA
GAGGATACCGTGCTCCACAGCTGTAAGGCTGTGGGCCCGAGAGAAGTCGCTCTCTCTCGCCCTCTACA
+
FFFFFFFFFFFFFFFFFFFFFFFFFFFFFFFFFFFFFFFFFFFFFFFFFFFFFFFFFFFFFFFFFFFF
FFFFFFFFFFFFFFFFFFFFFFFFFFFFFFFFFFFFFFFFFFFFFFFFFFFFFFFFFFFFFFFFFFFF
@A00155:342:HHGFNDSXY:1:2156:17580:4100 1:N:0:GAACCTAG+TCCGCATA
CTCAATGCCGAGAGGGATGTAGAGGGCGAGAGAGAGCGACTTCTCTCGGGCCCACAGCCTTACAGCTG
TGGAGCACGGTATCCTCTGCCGAAACAGAGGTTGGACAAGACCGGAGGGGTCTCCTAGTTCCAAAGGA
+
```

FFFFFFFFFFFFFFFFFFFFFFFFFFFFFFFFFFFFFFFFFFFFFFFFFFFFFFFFFFFFFFFFFFFFFFFF
FFFFFFFFFFFFFFFFFFFFFFFFFFFFFFFFFFFFFF:FFFFFFFFFFFFFFFFFFFFFFFFFFFFFFFFF
@A00155:342:HHGFNDSXY:1:2156:7726:20290 1:N:0:GAACCTAG+TCCGCATA
TGCCCCGCCACACCGCCTTAGATCAGCTGTGTCCACTTAAGGACTCACCTCTGGCCTATGGTCATCTC
AATGCCGAGAGGGATGTGGAGGGCGAGAGAGAGCGACTTCTCTCGGGCCCACAGCCTTACAGCTGTGG
+
FFFFFFFFFFFFFFFFFFFFFFFFFFFFFFFFFFFFFFFFFFFFFFFFFFFFFFFFFFFFFFFFFFFFFFFF
FFFFFFFFFFFFFFFFFFFFFFFFFFFFFFFFFFFFFFFFFFFFFFFFFFFFFFFFFFFFFFFFFFFFFFFF
@A00155:342:HHGFNDSXY:1:2156:7120:21308 1:N:0:GAACCTAG+TCCGCATA
TGCCCCGCCACACCGCCTTAGATCAGCTGTGTCCACTTAAGGACTCACCTCTGGCCTATGGTCATCTC
AATGCCGAGAGGGATGTGGAGGGCGAGAGAGAGCGACTTCTCTCGGGCCCACAGCCTTACAGCTGTGG
+
FFFFFFFFFFFFFFFFFFFFFFFF:,FFFFFFFFF,:F:FFFFF:FFFFFFFFFFF:FFFFFFFFFF
F:FFFFF,::FF,FFF,FFFFFFFFFFF:FF:FFF:FFFFFFFFFFFFFFFFFFFFFF:FFFFFF:FF
@A00155:342:HHGFNDSXY:1:2156:5638:21903 1:N:0:GAACCTAG+TCCGCATA
AGAGAGAGCGACTTCTCTCGGGCCCACAGCCTTACAGCTGTGGAGCACGGTATCCTCTGCCGAAACAG
AGGTTGGACAAGACCGGAGGGGTCTCCTAGTTCCAAAGGAGATGTACTCCGGGCTTGTTCACGACCTA
+
FFFFFFFFFFFFFFFFFFFFFFFFFFFFFFFFFFFFFFFFFFFFFFFFFFFFFFFFFFFF:FFFFFFFF
FFFFFFFFFFFFFFFFFFFFFFFFFFFFFFFFFFFFFFFFFFFFFFFFFFFFFFFFFFFFFFFFFFFFFFFF
@A00155:342:HHGFNDSXY:1:2156:7446:22874 1:N:0:GAACCTAG+TCCGCATA
CCTTACAGCTGTGGAGCACGGTATCCTCTGCCGAAACAGAGGTTGGACAAGACCGGAGGGGTCTCCTA
GTTCCAAAGGAGATGTACTCCGGGCTTGTTCACGACCTACCGTGTAAGTCGTAGTCTAGTAGGCTACC
+
FFFFFFF:FFFFFF:FFFFFFFFFFFFFFFFFFFFFFFFFFFFFFFFFFFFFFFFFFFFFFFFFFFFFFF
FFFFFFFFFFFFFFFFFFFFFFFFFFFFFFFFFFFFFFFFFFFFF:FFFF:FFFFFFFFFFFFFFFFF
@A00155:342:HHGFNDSXY:1:2157:24279:14074 1:N:0:GAACCTAG+TCCGCATA
AGAGAGAGCGACTTCTCTCGGGCCCACAGCCTTACAGCTGTGGAGCACGGTATCCTCTGCCGAAACAG
AGGTTGGACAAGACCGGAGGGGTCTCCTAGTTCCAAAGGAGATGTACTCCGGGCTTGTTCACGACCTA
+
FFFFFFFFFFFFFFFFFFFFFFFFFFFFFFFF:FFFFFFFFFFFFFFFFFFFFFFFFFFFFFFFFFFFF
FFFFFFFFFFFFFFFFFFFFFFFFFFFFFFFFFFFFFFFFFFFFFFFFFFFFFFFFFFFFFFFFFFFFF:
@A00155:342:HHGFNDSXY:1:2157:26232:15013 1:N:0:GAACCTAG+TCCGCATA
GATGTAGAGGGCGAGAGAGAGCGACTTCTCTCGGGCCCACAGCCTTACAGCTGTGGAGCACGGTATCC
TCTGCCGAAACAGAGGTTGGACAAGACCGGAGGGGTCTCCTAGTTCCAAAGGAGATGTACTCCGGGCT
+
FFFFFFFFFFFFFFFFFFFFFFFFFFFFFFFFFFFFFFFFFFFFFFFFFFFFFFFFFFFFFFFFFFFFFFFF
FFFFFFFFFFFFFFFFFFFF:FFFFFFFFFFFFFFFFFFFFFFFFFFFFFFFFFFFFFFFFFFFFFFFF
@A00155:342:HHGFNDSXY:1:2157:9010:15875 1:N:0:GAACCTAG+TCCGCATA
CTTCTCTCGGGCCCACAGCCTTACAGCTGTGGAGCACGGTATCCTCTGCCGAAACAGAGGTTGGACAA
GACCGGAGGGGTCTCCTGGTTCCAAAGGAGATGTACTCCGGGCTTGTTCACGACCTACCGTGTAAGTC
+
FFFFFFFFFFFFFFFFFFFFFFFFFFFFFFFFFFFFFFFFFFFFFFFFFFFFFFFFFFFFFFFFFFFFFFFF
F:FFFFFFFFFFFFFFFFFFFFFFFFFFFFFFFFFFFFFFFFFFFFFFFFFFFFFFFFFFFFFFFFFFFF
@A00155:342:HHGFNDSXY:1:2157:16034:22060 1:N:0:GAACCTAG+TCCGCATA
ATCTCAATGCCGAGAGGGATGTAGAGGGCGAGAGAGAGCGACTTCTCTCGGGCCCACAGCCTTACAGC
TGTGGAGCACGGTATCCTCTGCCGAAACAGAGGTTGGACAAGACCGGAGGGGTCTCCTAGTTCCAAAG
+
FFF:FFFFFFFFFFFFFFFFFFFFFFFFFFFFFFFFFFFFFFFFFFFFFFFFFFFFFFFFFFFFFFFFFF
FFFFFFFFFFFFFFFFFFFFFFFFFFFFFFFFFFFFFFFFFFFFFFFFFFFFFFFFFFFF:FFFFFFFFF
@A00155:342:HHGFNDSXY:1:2158:11397:14152 1:N:0:GAACCTAG+TCCGCATA
CAATGCCGAGAGGGATGTAGAGGGCGAGAGAGAGCGACTTCTCTCGGGCCCACAGCCTTACAGCTGTG
GAGCACGGTATCCTCTGCCGAAACAGAGGTTGGACAAGACCGGAGGGGTCTCCTAGTTCCAAAGGAGA
+

```
FFFFFFFFFFFFFFFFFFFFFFFFFFFFFFFFFFFFFFFFFFFFFFFFFFFFFFFFFFFFFFFFFFFF
FFFFFFFFFFFFFFFFFFFFFFFFFFFFFFFFFFFFFFFFFFFFFFFFFFFFFFFFFFFFFFFFFFFF
@A00155:342:HHGFNDSXY:1:2158:10185:16407 1:N:0:GAACCTAG+TCCGCATA
GAGCGACTTCTCTCGGGCCCACAGCCTTACAGCTGTGGAGCACGGTATCCTCTGCCGAAACAGAGGTT
GGACAAGACCGGAGGGGTCTCCTAGTTCCAAAGGAGATGTACTCCGGGCTTGTTCACGAC
+
FFFFFFFFFFFFFFFFFFFFFFFFFFFFFFFFFFFFFFFFFFFFFFFFFFFFFFFFFFFFFFFFFFFF
FFFFFFFFFFFFFFFFFFFFFFFFFFFFFFFFFFFFFFFFFFFFFFFFFFFFFFFFFFFF
@A00155:342:HHGFNDSXY:1:2158:12111:26663 1:N:0:GAACCTAG+TCCGCATA
AGCGACTTCTCTCGGGCCCACAGCCTTACAGCTGTGGAGCACGGTATCCTCTGCCGAAACAGAGGTTG
GACAAGACCGGAGGGGTCTCCTAGTTCCAAAGGAGATGTACTCCGGGCTTGTTCACGACCTACCGTGT
+
FFFFFFFFFFFFFFFFFFFFFFFFFFFFFFFFFFFFFFFFFFFFFFFFFFFFFFFFFFFFFFFFFFFF
FFFFFFFFFFFFFFFFFFFFF:FFFFFFFFFFFFFFFFFFFFFFFFFFFFF:FFFF:FFFFFFFFFFFFF
@A00155:342:HHGFNDSXY:1:2158:8703:30091 1:N:0:GAACCTAG+TCCGCATA
TCCTCTGCCGAAACAGAGGTTGGACAAGACCGGAGGGGTCTCCTAGTTCCATAGGAGATGTACTCCGG
GCTTGTTCACGACCTACCGTGTAAGTCGTAGTCTAGTAGGCTACCTGACGAGTCCTTTTTAGGACGAA
+
FFFFFFFFFFFFFFFFFFFFFFFFFFFFFFFFFFFFFFFFFFFFFFFFFFFFFFFFFFFFFFFFFFFF
FFF:FFFFFFFFFFF:FFFFFF:F:FFFFFFFFFFFFFFFFFFFFFFFFFFFFFFFFFFFFFFFFFFFFFF
@A00155:342:HHGFNDSXY:1:2159:13187:27367 1:N:0:GAACCTAG+TCCGCATA
CTCTGGCCTATGGTCATCTCAATGCCGAGAGGGATGTGAAGGGCGAGAGAGAGCGACTTCTCTCGGGC
CCACAGCCTTACAGCTGTGGAGCACGGTATCCTCTGCCGAAACAGAGGTTGGACAAGAC
+
FFFFFFFFFFFFFFFFFFFFFFFFFFFFFFFFFFFFFFFFFFFFFFFFFFFFFFFFFFFFFFFFFFFF
FFFFFFFFFFFFFFFFFFFFFFFFFFFFFFFFFFFFFFFFFFFFFFFFFFFFFFFFFFF
@A00155:342:HHGFNDSXY:1:2159:27950:29168 1:N:0:GAACCTAG+TCCGCATA
CTTCTCTCGGGCCCACAGCCTTACAGCTGTGGAGCACGGTATCCTCTGCCGAAACAGAGGTTGGACAA
GACCGGAGGGGTCTCCTAGTTCCAAAGGAGATGTACTCCGGGCTTGTTCACGACCTACCGTGTAAGTC
+
FFFFFFFFFFFFFFFFFFFFFFFFFFFFFFFFFFFFFFFFFFFFFFFFFFFFFFFFFFFFFFFFFFFF
FFFFFFFFFFFFFFFFFFFFFFFFFFFFFFFFFFFFFFFFFFFF:FFFFFFFFFFF::FFFFFFFFFF
@A00155:342:HHGFNDSXY:1:2159:26982:30812 1:N:0:GAACCTAG+TCCGCATA
CTTCTCTCGGGCCCACAGCCTTACAGCTGTGGAGCACGGTATCCTCTGCCGAAACAGAGGTTGGACAA
GACCGGAGGGGTCTCCTAGTTCCAAAGGAGATGTACTCCGGGCTTGTTCACGACCTACCGTGTAAGTC
+
FFFFFFFFFFFFFFFFFFFFFFFFFFFFFFFFFFFFFFFFFFFFFFFFFFFFFFFFFFFFFFFFFFFF
:FFFFFFFFFFFFFFFFFFFFFFFFFFFFFFFFFFFFFFFFFFFFFFFFFFFFFFFF:FFFFFFFFFF
@A00155:342:HHGFNDSXY:1:2159:21169:33520 1:N:0:GAACCTAG+TCCGCATA
CAATGCCGAGAGGGATGTGGAGGGCGAGAGAGAGCGACTTCTCTCGGGCCCACAGCCTTACAGCTGTG
GAGCACGGTATCCTCTGCCGAAACAGAGGTTGGACAAGACCGGAGGGGTCTCCTAGTTCCAAAGGAGA
+
FFFFFFFFFFFFFFF:FFFFFFFFFFFFFFFFFFFFFFFFFFFFFFFFFFFFFFFFFFFFFFFFFFFF
FFFFFFFFFFFFFFFFFFFFFFFFFFFFFFFFFFF,FFFFFFFFFFFFFFFFFFFFFFFFFFFFFFFF
@A00155:342:HHGFNDSXY:1:2159:20907:36385 1:N:0:GAACCTAG+TCCGCATA
TCAATGCCGAGAGGGATGTGGAGGGCGAGAGAGAGCGACTTCTCTCGGGCCCACAGCCTTACAGCTGT
GGAGCACGGTATCCTCTGCCGAAACAGAGGTTGGACAAGACCGGAGGGGTCTCCTAGTTCCAAAG
+
F:FFF::::::::::F,:,,,F:FF,:FF::F,:F:F::,:,:,,,:,,,F:FFF,:,F,F,F:,F,F
:,FF::,F::F,,F:FF,FF:F:::FFF:F,FF:,:FFFF,,F:F::FF,,:F,,F:FFF,F::F
@A00155:342:HHGFNDSXY:1:2160:23918:12289 1:N:0:GAACCTAG+TCCGCATA
GTCATCTCAATGCCGAGAGGGATGTGGAGGGCGAGAGAGAGCGACTTCTCTCGGGCCCACAGCCTTAC
AGCTGTGGAGCACGGTATCCTCTGCCGAAACAGAGGTTGGACAAGACCGGAGGGGTCTCCTAGTTCCA
+
```

FFFFFFFFFFFFFFF:FFFFFFFFFFFFFFFFFFFFFFFFFFFFFFFFFFFFFFFFFFFFFFFFFFFFF
FFFFFFFFFFFFFFFFFFFFFFFFFFFFFF:FFFFFFFFFFFFFFFFFFFFFFFFFFFFFFFFFFFFFF
@A00155:342:HHGFNDSXY:1:2160:23927:12367 1:N:0:GAACCTAG+TCCGCATA
GTCATCTCAATGCCGAGAGGGATGTGGAGGGCGAGAGAGAGCGACTTCTCTCGGGCCCACAGCCTTAC
AGCTGTGGAGCACGGTATCCTCTGCCGAAACAGAGGTTGGACAAGACCGGAGGGGTCTCCTAGTTCCA
+
FFFFFFFFFFFFFFF,FFFFFFFFFFFFFFFFFFFFFFFFFFFFFFFFFFFFFFFFFFFFFFFFFFFFF
FFFFFFFFFFFFFFFFFFFFFFFF::FFFFFFFFFFFFFFFFFFFFFFFFFFFFFFFFFFFFFFFFF,F
@A00155:342:HHGFNDSXY:1:2160:26666:18490 1:N:0:GAACCTAG+TCCGCATA
CATCTCAATGCCGAGAGGGATGTGGAGGGCGAGAGAGAGCGACTTCTCTCGGGCCCACAGCCTTACAG
CTGTGGAGCACGGTATCCTCTGCCGAAACAGAGGTTGGACAAGACCGGAGGGGTCTCCTAGTTCCAAA
+
FFFFFFFFFFFFFFFFFFFFFFFFFFFFFFFFFFFFFFFFFFFFFFFFFFFFFFFFFFFFFFFFFFFF
FFFFFFFFFFFFFFFFFFFFFFFFFFFFFFFFFFFFFFFFFFFFFFFFFFFFFFFFFFFFFFFFFFFF
@A00155:342:HHGFNDSXY:1:2160:23149:20854 1:N:0:GAACCTAG+TCCGCATA
TTAGATCAGCTGTGTCCACTTAAGGACTCACCTCTGGCCTATGGTCATCTCAATGCCGAGAGGGATGT
GGAGGGCGAGAGAGAGCGACTTCTCTCGGGCCCACAGCCTTACAGCTGTGGAGCACGGTATCCTCTGC
+
FFFFFFFFFFFFFFFFFFFFFFFFFFFFFFFFFFF:FFFFFFFFFFFFFFFFFFFFFFFFFFFFF,FFF
FFFFFFFFFFFFFFFFFFFFFFFFFFFFFFFFFFFFFFFFFFFFFFFFFFFFFFFFFFFFFFFFFFFF
@A00155:342:HHGFNDSXY:1:2161:31051:3881 1:N:0:GAACCTAG+TCCGCATA
TCCTCTGCCGAAACAGAGGTTGGACAAGACCGGAGGGGTCTCCTGGTTCCAAAGGAGATGTACTCCGG
GCTTGTTCACGACCTACCGTGTAAGTCGTAGTCTAGTAGGCTACCTGACGAGTCCTTTTTAGGACGAA
+
FFFFFFFFFFFFFFFFFFFFFFFFFFFFFFFFFFFFFFFFFFFFFFFFFFFFFFFFFFFFFFFFFFFF
FFFFFFFFFFFFFFFFFFFFFFFFFFFFFFFFFFFFFFFFFFFFFFFFFFFFFFFFFFFFFFFFFFFF
@A00155:342:HHGFNDSXY:1:2161:22516:10488 1:N:0:GAACCTAG+TCCGCATA
CTTCTCTCGGGCCCACAGCCTTACAGCTGTGGAGCACGGTATCCTCTGCCGAAACAGAGGTTGGACAA
GACCGGAGGGGTCTCCTGGTTCCAAAGGAGATGTACTCCGGGCTTGTTCACGACCTACCGTGTAAGTC
+
FFFFFFFFFFFFFFFFFFFFFFFFFFFFFFFFFFFFFFFFFFFFFFFFFFFFFFFFFFFFFFFFFFFF
FFFFFFFFFFFFFFFFFFFFFFFFFFFFFFFFFFFFFFFFFFFFFFFFFFFFFFFFFFFFFFFFFFFF
@A00155:342:HHGFNDSXY:1:2161:24677:26099 1:N:0:GAACCTAG+TCCGCATA
GGATGTGGAGGGCGAGAGAGAGCGACTTCTCTCGGGCCCACAGCCTTACAACTGTGGAGCACGGTATC
CTCTGCCGAAACAGAGGTTGGACAAGACCGGAGGGGTCTCCTAGTTCCAAAGGAGATGTACTCC
+
FFFFFFFFFFFFFFFFFFFFFFFFFFFFFFFFFFFFFFFFFFFFFFFFFFFFFFFFFFFFFFFFFFFF
FFFFFFFFFFFFFFFFFFFFFFFFFFFFFFFFFFFFFFFFFFFFFFFFFFFFFFFFFFFFFFFF
@A00155:342:HHGFNDSXY:1:2161:14913:28260 1:N:0:GAACCTAG+TCCGCATA
CCTCTGTTTCGGCAGAGGATACCGTGCTCCACAGCTGTAAGGCTGTGGGCCCGAGAGAAGTCGCTCTC
TCTCGCCCTCTACATCCCTCTCGGCATTGAGATGACCATAGGCCAGAGGTGAGTCCTTAAGTGGACAC
+
FFFFFFFFFFFFFFFFFFFFFFFFFFFFFFFFFFFFFFFFFFFFFFFFFFFFFFFFFFFFFFFFFFFF
FFFFFFFFFFFFFFFFFFFFFFFFFFFFFFFFFFFFFFFFFFFFFFFFFFFFFFFFFFFFFFFFFFFF
@A00155:342:HHGFNDSXY:1:2161:20193:30044 1:N:0:GAACCTAG+TCCGCATA
CGAGAGAGAGCGACTTCTCTCGGGCCCACAGCCTTACAGCTGTGGAGCACGGTATCCTCTGCCGAAAC
AGAGGTTGGACAAGACCGGAGGGGTCTCCTAGTTCCAAAGGAGATGTACTCCGGGCTTGTTCACGACC
+
FFFFFFFFFFFFFFFFFFFFFFFFFFFFFFFFFFFFFFFFFFFFFFFFFFFFFFFFFFFFFFFFFFFF
FFFFFFFFFFFFFFFFFFFFFFFFFFFFFFFFFFFF:FFFFFFFFFFFFFFFFFFFFFFFFFFFF:FFFF
@A00155:342:HHGFNDSXY:1:2162:4924:9893 1:N:0:GAACCTAG+TCCGCATA
TATCCTCTGCCGAAACAGAGGTTGGACAAGACCGGAGGGGTCTCCTAGTTCCAAAGGAGATGTACTCC
GGGCTTGTTCACGACCTACCGTGTAAGTCGTAGTCTAGTAGGCTACCTGACGAGTCCTTTTTAGGACG
+

```
FFFFFFFFFFFFFFFFFF:F:FFFFFFFFFFFFFFFFFFFFFFFFFFFFFFFFFFFFFFFFFFF:FFF
FFFFFFFFFFFFFFFFFFFFFFFFF:FFFF:FFFFFFFFF:FFFFFFFFFFFFFFFFF:,FFF,FFFF
@A00155:342:HHGFNDSXY:1:2162:2121:17472 1:N:0:GAACCTAG+TCCGCATA
GAGCGACTTCTCTCGGGCCCACAGCCTTACAGCTGTGGAGCACGGTATCCTCTGCCGAAACAGAGGTT
GGACAAGACCGGAGGGGTCTCCTAGTTCCAAAGGAGATGTACTCCGGGCTTGTTCACGACCTACCGTG
+
FFFFFFFFFFFFFFFFFFFFFFFFFFF:FFFFFFFFFFFFFFFFFFFFFFFFFFFFFFFFFFFFFFFF
FFFFFFFFFFFFFFFF:FFFFFFFFFFFFFFFFFFFFFFFFFFFFFFFFF:FFFFFFFFFFFFFFFFF
@A00155:342:HHGFNDSXY:1:2163:30147:6073 1:N:0:GAACCTAG+TCCGCATA
AGAGAGAGCGACTTCTCTCGGGCCCACAGCCTTACAGCTGTGGAGCACGGTATCCTCTGCCGAAACAG
AGGTTGGACAAGACCGGAGGGGTCTCCTAGTTCCAAAGGAGATGTACTCCGGGCTTGTTCACGACCTA
+
FFFFFFFFFFFFFFFFFFFFFFFFFFFFFFFFFFFFFFFFFFFFFFFFFFFFFFFFFFFFFFFFFFFF
FFFFFFFFFFFFFFFFFFFFFFFFFFFFFFFFFFFFFFFFFFFFFFFFFFFFFFFFFFFFFFFFFFFF
@A00155:342:HHGFNDSXY:1:2163:15230:33567 1:N:0:GAACCTAG+TCCGCATA
GGATGTGGAGGGCGAGAGAGAGCGACTTCTCTCGGGCCCACAGCCTTACAGCTGTGGAGCACGGTATC
CTCTGCCGAAACAGAGGTTGGACAAGACCGGAGGGGTCTCCTGGTTCCAAAGGAGATGTACTCC
+
FFFFFFFFFFFFFFFFFFFFFFFFFFFFFFFFFFFFFFFFFFFFFFFFFFFFFFFFFFFFFFFFFFFF
FFFFFFFFFFFFFFFFFFFFFFFFFFFFFFFFFFFFFFFFFFFFFFFFFFFFFFFFFFFFFFFF
@A00155:342:HHGFNDSXY:1:2164:13765:32158 1:N:0:GAACCTAG+TCCGCATA
GGATGTGGAGGGCGAGAGAGAGCGACTTCTCTCGGGCCCACAGCCTTACAGCTGTGGAGCACGGTATC
CTCTGCCGAAACAGAGGTTGGACAAGACCGGAGGGGTCTCCTAGTTCCAAAGGAGATGTACTC
+
FFFFFFFFFFFFFFFFFFFFFFFFFFFFFFFFFFFFFFFFFFFFFFFFFFFFFFFFFFFFFFFFFFFF
FFFFFFFFFFFFFFFFFFFFFFFFFFFFFFFFFFFFFFFFFFFFFFFFFFFFFFFFFFFFFFF
@A00155:342:HHGFNDSXY:1:2165:24994:3035 1:N:0:GAACCTAG+TCCGCATA
AATGCCGAGAGGGATGTGGAGGGCGAGAGAGAGCGACTTCTCTCGGGCCCACAGCCTTACAGCTGTGG
AGCACGGTATCCTCTGCCGAAACAGAGGTTGGACAAGACCGGAGGGGTCTCCTAGTTCCAAAGGAGAT
+
FFFFFFFFFFFFFFFFFFFFFFFFFFFFFFFFFFFFFFFFFFFFFFFFFFFFFFFFFFFFFFFFFFFF
FFF:FFFFFFFFFFFFFFFFFFFFFFFFFFFFFFFFFFFFFFFFFFFFFFFFFFFFFFFFFFFFFFFF
@A00155:342:HHGFNDSXY:1:2165:21079:17018 1:N:0:GAACCTAG+TCCGCATA
GTCATCTCAATGCCGAGAGGGATGTGGAGGGCGAGAGAGAGCGACTTCTCTCGGGCCCACAGCCTTAC
AGCTGTGGAGCACGGTATCCTCTGCCGAAACAGAGGTTGGACAAGACCGGAGGGGTCTCCTAGTTCCA
+
FFFFFFFFFFFFFFFFFFFFFFFFFFFFFFFFFFFFFFFFFFFFFFFFFFFFFFFFFFFFFFFFFFFF
FFFFFFFFFFFFFFFFFFFFFFFFFFFFFFFFFFFFFFFFFFFFFFFFFFFFFFFFFFFFFFFFFFFF
@A00155:342:HHGFNDSXY:1:2165:18195:29152 1:N:0:GAACCTAG+TCCGCATA
ACTTCTCTCGGGCCCACAGCCTTACAGCTGTGGAGCACGGTATCCTCTGCCGAAACAGAGGTTGGACA
AGACCGGAGGGGTCTCCTAGTACCAAAGGAGATGTACTCCGGGCTTGTTCACGACCTACCGTGTAAGT
+
FFFFFFFFFFFFFFFFFFFFFFFFFFFFFFFFFFFFFFFFFFFFFFFFFFFFFFFFFFFFFFFFFFFF
FFFFFFFFFFFFFFFFFFFFFFFFFFFFFFFFFFFFFFFFFFFFFFFFFFFFFFFFFFFFFFFFFFFF
@A00155:342:HHGFNDSXY:1:2166:16125:21840 1:N:0:GAACCTAG+TCCGCATA
GCGACTTCTCTCGGGCCCACAGCCTTACAGCTGTGGAGCACGGTATCCTCTGCCGAAACAGAGGTTGG
ACAAGACCGGAGGGGTCTCCTAGTTCCAAAGGAGATGTACTCCGGGCTTGTTCACGACCTACCGTGTA
+
FFFFFFFFFFFFFFFFFFFFFFFFFFFFFFFFFFFFFFFFFFFFFFFFFFFFFFFFFFFFFFFFFFFF
FFFFFFFFFFFFFFFFFFFFFFFFFFFFFFFFFFFFFFFFFFFFFFFFFFFFFFFFFFFFFFFFFF:F
@A00155:342:HHGFNDSXY:1:2166:30228:32174 1:N:0:GAACCTAG+TCCGCATA
GAGCGACTTCTCTCGGGCCCACAGCCTTACAGCTGTGGAGCACGGTATCCTCTGCCGAAACAGAGGTT
GGACAAGACCGGAGGGGTCTCCTAGTTCCAAAGGAGATGTACTCCGGGCTTGTTCACGACCTACCGTG
+
```

FFFFFFFFFFFFFFFFFFFFFFFFFFFFFFFFFFFFFFFFF:FFFFFFFFFFFFFFFFFFFFFFFFFF
FFFFFFFFFFFFFFFFFFFFFFFFFFFFFFFFFFFFFFFFFFFFFFFFFFFFFFFFFFFFFFFFFFFF
@A00155:342:HHGFNDSXY:1:2168:8486:1251 1:N:0:GAACCTAG+TCCGCATA
ATCCTCTGCCGAAACAGAGGTTGGACAAGACCGGAGGGGTCTCCTATTTCCAAAGGAGATGTACTCCG
GGCTTGTTCACGACCTACCGTGTAAGTCGTAGTCTAGTAGGCTACCTGACGAGTCCTTTTTAGGACGA
+
F:F::FFFFFFFFFFFF:FFFFFFFFFFFFFF,:F:FF,F,FFF,F,FFF:FFFF,F,F,:,FF,F:,
,,,F:F:FFFF:FFFFFF,:FFFFF:,,F:F:FFFFFFFFFFFF,,FF:F:FFFFF,FFF,F,FFFFF
@A00155:342:HHGFNDSXY:1:2168:19732:35540 1:N:0:GAACCTAG+TCCGCATA
AGAGAGAGCGACTTCTCTCGGGCCCACAGCCTTACAGCTGTGGAGCACGGTATCCTCTGCCGAAACAG
AGGTTGGACAAGACCGGAGGGGTCTCCTAGTTCCAAAGGAGATGTACTCCGGGCTTGTTC
+
FFFFFFFFFFFFFFFFFFFFFFFFFFFFFFFFFFFFFFFFFF:FFFFFFFFFFFFFFFFFFFFFFFFF
FFFFFFFFFFF:FFFFFFFFFFFFFFFF:FFFFFFFFFFFFFFFFFFFFFFFFFF,FFFF
@A00155:342:HHGFNDSXY:1:2169:21314:11600 1:N:0:GAACCTAG+TCCGCATA
CACATCCCTCTCGGCATTGAGATGACCATAGGCCAGAGGTGAGTCCTTAAGTGGACACAGCTGATCTA
AGGCGGTGTGGCGGGGCATGGGTTTGAACCCCCATGACGGTCGGAGTCTAGTAGGCTCCCTGATGAGT
+
FFFFFFFFFF:FFFFFFFFFFFFFFFFFFFFFFFFFFFFFFFFFFFFFFFFFFFFFFFFFFFFFFFFF
FFFFFFFF:FFFFFFFFFFFFFFFFF:FFFFFFFFFFFFFFFFFFFFF,FFFFF:FFFFFFFFFFFFFF
@A00155:342:HHGFNDSXY:1:2169:4716:24001 1:N:0:GAACCTAG+TCCGCATA
AGCGACTTCTCTCGGGCCCACAGCCTTACAGCTGTGGAGCACGGTATCCTCTGCCGAAACAGAGGTTG
GACAAGACCGGAGGGGTCTCCTAGTTCCAAAGGAGATGTACTCCGGGCTTGTTCACGACCTACCGTGT
+
FFFFFFFFFFFFFFFFFFFFFFFFF:FFFFFFFFFFFFFFFFFFFFFFFFFFFFFFFFFFFFFFFFFF
FFFFFFFFFFFFFFFFFFFFFF:FFFFFFF:FFFFFFFFFFFFFFFFFFFFFFFFFFFFFF,FFFFFF
@A00155:342:HHGFNDSXY:1:2169:1145:28557 1:N:0:GAACCTAG+TCCGCATA
ACTTCTCTCGGGCCCACAGCCTTACAGCTGTGGAGCACGGTATCCTCTGCCGAAACAGAGGTTGGACA
AGACCGGAGGGGTCTCCTGGTTCCAAAGGAGATGTACTCCGGGCTTGTTCACGACCTACCGTGTAAGT
+
:FFFFFFFFFFFFFFFFFFFFFFFFFFFFFFFFFFFFFFFFFF,FFFFFFFFF:FFFFFFFFFFFFFF
FF,FFFFFFFFFFFFFFFFFFFFFFF,::FFFFFFFFF:FF:FFF:FFFF:FFF:FFFFFFFFFFFFF
@A00155:342:HHGFNDSXY:1:2169:3224:31908 1:N:0:GAACCTAG+TCCGCATA
ATGCCGAGAGGGATGTGGAGGGCGAGAGAGAGCGACTTCTCTCGGGCCCACAGCCTTACAGCTGTGGA
GCACGGTATCCTCTGCCGAAACAGAGGTTGGACAAGACCGGAGGGGTCTCCTAGTTCCAAAGGAG
+
FFFFFFFFFFF:F:FFFFFFFFF:FFFFFFFFFFFFFFFFFFFFFFFFFFFFFFFFFFFFFFFFFFFF
FFFFFFFFFFFFFFFFFFFFFFFFFFFF,FFFFFFFFFFFFFFFFFFFFFFFFFFFFFFFFFFFF
@A00155:342:HHGFNDSXY:1:2169:26946:34914 1:N:0:GAACCTAG+TCCGCATA
ATCTCAATGCCGAGAGGGATGTAGAGGGCGAGAGAGAGCGACTTCTCTCGGGCCCACAGCCTTACAGC
TGTGGAGCACGGTATCCTCTGCCGAAACAGAGGTTGGACAAGACCGGAGGGGTCTCCTAGTTCCAAAG
+
FFFFFFFFFFFFFFFFFFFFFFFFFFFFFFFFFFFFFFFFFFFFFFFFFFFFFFFFFFFFFFFFFFFF
FFFFFFFFFFFFFFFFFFFFFFFFFFFFFFFFFFFFFFFFFFFFFFFFFFFFFFFFFFFFFFFFFFFF
@A00155:342:HHGFNDSXY:1:2169:25907:36996 1:N:0:GAACCTAG+TCCGCATA
TTCTCTCGGGCCCACAGCCTTACAGCTGTGGAGCACGGTATCCTCTGCCGAAACAGAGGTTGGACAAG
ACCGGAGGGGTCTCCTAGTTCCAAAGGAGATGTACTCCGGGCTTGTTCACGACCTACCGTGTAAGTCG
+
FFFFFFFFFF:FFFFFFFF:FFFFFFFFFFFFFFFFFFFFFFFFF:FFFFFFFFFFFFFFFFFFFFFF
FFFFFFFFFFFFFFFFFFFFFFF:FFFFFFFFFFFFFFFFFFFFFFFFFFFFFFFFFFFFFFFFFFFF
@A00155:342:HHGFNDSXY:1:2170:25717:1282 1:N:0:GAACCTAG+TCCGCATA
TTCTCTCGGGCCCACAGCCTTACAGCTGTGGAGCACGGTATCCTCTGCCGAAACAGAGGTTGGACAAG
ACCGGAGGGGTCTCCTAGTTCCAAAGGAGATGTACTCCGGGCTTGTTCACGACCTACCGTGTAAGTCG
+

FFFFFFFFFFFFFFFFFFFFFFFFFFFFFFFFFFFFFFFFFFFFFFFFFFFFFFFFFFFFFFFFFFFF
FFFFFFFFFFFFFFFFFFFFFFFFFFFFFFFFFFFFFFFFFFFFFFFF:FFFFFFFFFFFFFFFFFFF
@A00155:342:HHGFNDSXY:1:2170:28402:6715 1:N:0:GAACCTAG+TCCGCATA
TGCCGAGAGGGATGTAGAGGGCGAGAGAGAGCGACTTCTCTCGGGCCCACAGCCTTACAGCTGTGGAG
CACGGTATCCTCTGCCGAAACAGAGGTTGGACAAGACCGGAGGGGTCTCCTAGTTCCAAAGGAG
+
FFFFF:FFFFFF:FFFFFFFFFFFFFFFFFFFFFFFFFFFFFFFFFFFFFFFFFFFFFFFFFFFFFFF
FFFFFFFFFFFFFFFFFFFFFFFFFFFFFFFFFFFFFFFFFFFFFFFFFFFFFFFFFFFF:FFFFF
@A00155:342:HHGFNDSXY:1:2170:2998:8750 1:N:0:GAACCTAG+TCCGCATA
TTACAGCTGTGGAGCACGGTATCCTCTGCCGAAACAGAGGTTGGACAAGACCGGAGGGGTCTCCTGGT
TCCAAAGGAGATGTACTCCGGGCTTGTTCACGACCTACCGTGTAAGTCGTAGTCTAGTAGGCTACCTG
+
FFFFFFFFFFFFFFFFFFFFFFFFFFFFFFFFFFFFFFFFFFFFFFFFFFFFFFFFFFFFFFFFFFFF
FFFFFFFFFFFFFFFFFFFFFFFFFFFFFFFFFFFFFFFFFFFFFFFFFFFFFFFFFFFFFFFFFFFF
@A00155:342:HHGFNDSXY:1:2170:3242:9361 1:N:0:GAACCTAG+TCCGCATA
TTACAGCTGTGGAGCACGGTATCCTCTGCCGAAACAGAGGTTGGACAAGACCGGAGGGGTCTCCTGGT
TCCAAAGGAGATGTACTCCGGGCTTGTTCACGACCTACCGTGTAAGTCGTAGTCTAGTAGGCTACCTG
+
FFFFFFFFFF:FFFFFFFFFFFFFFFFFFFFFFFFFFFFFFFFFFFFFFFFFFFFFFFFFFFFFFFFF
FFFFFFFFFFFFFFFFFFF:FFFFFFFFFFFFFFFFFFFFFFFFFFFFFFFFFFFFFFFF:FFFF:FF
@A00155:342:HHGFNDSXY:1:2170:32696:11710 1:N:0:GAACCTAG+TCCGCATA
ATGGGGGTTCAAACCCATGCCCCGCCACACCGCCTTAGATCAGCTGTGTCCACTTAAGGACTCACCTC
TGGCCTATGGTCATCTCAATGCCGAGAGGGATGTGGAGGGCGAGAGAGAGCGACTTCTCT
+
FFFFFFFFFFFFFFFFFFFFFFFFFFFFFFFFFFFFFFF:FFFFFFFFFFFFFFFFFFFFFFFFFFFF
FFFFFFFFFFFFFFFFFFFFFFFFFFFFFFF,FFFFFFFFFFFFFFFFFFFFFFFFFFFFFF
@A00155:342:HHGFNDSXY:1:2170:6705:24377 1:N:0:GAACCTAG+TCCGCATA
ATGCCGAGAGGGATGTGGAGGGCGAGAGAGAGCGACTTCTCTCGGGCCCACAGCCTTACAGCTGTGGA
GCACGGTATCCTCTGCCGAAACAGAGGTTGGACAAGACCGGAGGGGTCTCCTAGTTCCAAAGGAGATG
+
FFFFFFFFFFFFF:FFFFFFFFFFFFFFFFFFFFFFFFFFFFFFFFFFFFFFFFFFFFFFFFFFFFFF
FFFFFFFFFFF:FFFFFFFFF:FFFFFFFFFFFFFFFFFFFFFFFFFFFFFFFFFFFFFFFFFFFFFF
@A00155:342:HHGFNDSXY:1:2170:16378:28134 1:N:0:GAACCTAG+TCCGCATA
TTCTCTCGGGCCCACAGCCTTACAGCTGTGGAGCACGGTATCCTCTGCCGAAACAGAGGTTGGACAAG
ACCGGAGGGGTCTCCTAGTTCCAAAGGAGATGTACTCCGGGCTTGTTCACGACCTACCGTGTAAGTCG
+
FFFFFFFFFFFFFFFFFFFFFFFFFFFFFFFFFFFFFFFFFFFFFFFFFFFFFFFFFFFFFFFFFFFF
FFFFFFFFFFFFFFFFFFFFFFFFFFFFFFFFFFFFFFFFFFFFFFFFFFFFFFFFFFFFFFFFFFFF
@A00155:342:HHGFNDSXY:1:2170:14064:29857 1:N:0:GAACCTAG+TCCGCATA
GAGAGGGATGTGGAGGGCGAGAGAGAGCGACTTCTCTCGGGCCCACAGCCTTACAGCTGTGGAGCACG
GTATCCTCTGCCGAAACAGAGGTTGGACAAGACCGGAGGGGTCTCCTAGTTCCAAAGGAGATGTACT
+
FFF:FFFFFFFFFFFFFFFFFFFFFFFFFFFFFFFFFFFFFFFFFFFFFFFFFFFFFFFFFFFFFFFF
FFFFFFFFFFFFFFFFFFFFFFFFFFFFFFFFFFFFFFFFFFFFFFFFFFFFFFFFFFFFFFFFFFF
@A00155:342:HHGFNDSXY:1:2171:10728:7044 1:N:0:GAACCTAG+TCCGCATA
AATGCCGAGAGGGATGTGGAGGGCGAGAGAGAGCGACTTCTCTCGGGCCCACAGCCTTACAGCTGTGG
AGCACGGTATCCTCTGCCGAAACAGAGGTTGGACAAGACCGGAGGGGTCTCCTAGTTCCAAAGGAGAT
+
FFFFFFFFFFFFFFFFFFFFFFFFFFFFFFFFFFFFFFFFFFFFFFFFFFFFFFFFFFFFFFFFFFFF
FFFFFFFFFFFFFFFFFFFFFFFFFFFFFFFFFFFFFFFFFFFFFFFFFFFFFFFFFFFFFFFFFFFF
@A00155:342:HHGFNDSXY:1:2171:15826:18474 1:N:0:GAACCTAG+TCCGCATA
AAGGACTCACCTCTGGCCTATGGTCATCTCAATGCCGAGAGGGATGTAGAGGGCGAGAGAGAGCGACT
TCTCTCGGGCCCACAGCCTTACAGCTGTGGAGCACGGTATCCTCTGCCGAAACAGAGGTTGGACAAGA
+

FFFFFFFFFFFFFFFFFFFFFFFFFFFFFFFFFFFFFFFFFFFFFFFFFFFFFFFFFFFFFFFFFFFF
FFFFFFFFFFFFFFFFFFFFFFFFFFFFFFFFFFFFFFFFFFFFFFFFFFFFFFFFFFFFFFFFFFFF
@A00155:342:HHGFNDSXY:1:2171:27190:21684 1:N:0:GAACCTAG+TCCGCATA
TGTCCACTTAAGGACTCTCCTCTGGCCTATGGTCATCTCAATGCCGAGAGGGATGTGGAGGGCGAGAG
AGAGCGACTTCTCTCGGGCCCACAGCCTTACAGCTGTGGAGCACGGTATCCTCTGCCGAAACAGAGGT
+
FFFFFFFFFFF:FFFFF:FFFFFFFFFFFFFFFFFFFFFFFFFFFFFFFFFFF,FFFFFFFFFFFFFF
FFFFFFFFFFFFF:F:FFFFF:FFFFFFFFFFFFFFFFFFFFFFFFFFFFFFFFFFFF:FFFFFFFFF
@A00155:342:HHGFNDSXY:1:2172:30897:11882 1:N:0:GAACCTAG+TCCGCATA
GAGAGGGATGTGGAGGGCGAGAGAGAGCGACTTCTCTCGGGCCCACAGCCTTACAGCTGTGGAGCACG
GTATCCTCTGCCGAAACAGAGGTTGGACAAGACCGGAGGGGTCTCCTGGTTCCAAAGGAGATGTACTC
+
FFFFFFFFFFFFFFFFFFFFFFFFFFFFFFFFFFFFFFFFFFFFFFFFFFFFFFFFFFFFFFFFFFFF
FFFFFFFFFFFFFFFFFFFFFFFFFFFFFFFFFFFFFFFFFFFFFF:FFFFFFFFFFFFFFFFFFFFF
@A00155:342:HHGFNDSXY:1:2172:29143:27383 1:N:0:GAACCTAG+TCCGCATA
GGGCGAGAGAGAGCGACTTCTCTCGGGCCCACAGCCTTACAGCTGTGGAGCACGGTATCCTCTGCCGA
AACAGAGGTTGGACAAGACCGGAGGGGTCTCCTAGTTCCAAAGGAGATGTACTCCGGGC
+
FFFFFFFF,FFFFFFFF,FFFFF,F,FF,FFFFFF:FFFFF,FFFFF,FF,FFFFFFF,F:F:F:,FF
FF:FFFF,,,FFF:FF,FFFFFFFF:FFFFFFFFFFFF:FFFFFF:F,FFFFF,:F:::
@A00155:342:HHGFNDSXY:1:2172:13910:33223 1:N:0:GAACCTAG+TCCGCATA
CTTCTCTCGGGCCCACAGCCTTACAGCTGTGGAGCACGGTATCCTCTGCCGAAACAGAGGTTGGACAA
GACCGGAGGGGTCTCCTAGTTCCAAAGGAGATGTACTCCGGGCTTGTTCACGACCTACC
+
FFFFFFFFFFFFFFFFFFFFFFFFFFFFFFFFFFFFFFFFFFFFFFFFFFFFFFFFFFFFFFFFFFFF
FFFFFFFFFFFFFFFFFFFFFFFFFFFFFFFFFFFFFFFFFFFFFFFFFFFFFFFFFFF
@A00155:342:HHGFNDSXY:1:2172:13566:33285 1:N:0:GAACCTAG+TCCGCATA
CTTCTCTCGGGCCCACAGCCTTACAGCTGTGGAGCACGGTATCCTCTGCCGAAACAGAGGTTGGACAA
GACCGGAGGGGTCTCCTAGTTCCAAAGGAGATGTACTCCGGGCTTGTTCACGACCTACC
+
FFFFFFFFFFFFFFFFFFFFFFFFFFFFFFFFFFFFFFFFFFFFFFFFFFFFFFFFFFFFFFFFFFFF
FFFFFFFFFFFFFFFFFFFFFFFFFFFFFFFFFFFFFFFFFFFFFFFFFFFFFFFFFFF
@A00155:342:HHGFNDSXY:1:2173:15600:1579 1:N:0:GAACCTAG+TCCGCATA
CTTCTCTCGGGCCCACAGCCTTACAACTGTGGAGCACGGTATCCTCTGCCGAAACAGAGGTTGGACAA
GACCGGAGGGGTCTCCTAGTTCCAAAGGAGATGTACTCCGGGCTTGTTCACGACCTACCGTGTAAGTC
+
FFFFFFFFFFFFFFFFFFFFFFFFFFFFFFFFFFFFFFFFFFFFFFFFFFFFFFFFFFFFFFFFFFFF
FFFFFFFFFFFFFFFFFFFFFFFF:FFFFFFFFFFFFFFFFFFFFFFFFFFFFFFFFFFFFFFF:FF
@A00155:342:HHGFNDSXY:1:2173:12608:9549 1:N:0:GAACCTAG+TCCGCATA
GGATACCGTGCTCCACAGCTGTAAGGCTGTGGGCCCGAGAGAAGTCGCTCTCTCTCGCCCTCCACATC
CCTCTCGGCATTGAGATGACCATAGGCCAGAGGTGAGTCCTTAAGTGGACACAGCTGATCTAAGGCGG
+
FFFFFFFFFFFFFFFFFFFFFFFFFFFFFFFFFFFFFFFFFFF:FFFFFFFFFFFFFFFFFFFFFFFF
FFFFFFFFFFFFFFFFFFFFFFFFFFFFFFFFFFFFFFFFFFFFFFFFFFFFFFFFFFFFFFFFFFFF
@A00155:342:HHGFNDSXY:1:2173:25473:12508 1:N:0:GAACCTAG+TCCGCATA
ACCTCTGTTTCGGCAGAGGATACCGTGCTCCACAGCTGTAAGGCTGTGGGCCCGAGAGAAGTCGCTCT
CTCTCGCCCTCCACATCCCTCTCGGCATTGAGATGACCATAGGCCAGAGGAGAGTCCTTAAGTGGACA
+
FFFFFFFFFFFFFFFFFFFFFFFFFFFFFFFFFFFFFFFFFFFFFFFFFFFFFFFFFFFFFFFFF:FF
FFFFFFFFFFFFFFFFFFFFFFFFFFFFFFFFFFFFFFFFFFFFFFFFFFFFFFFFFFFFFFFFFFFF
@A00155:342:HHGFNDSXY:1:2173:8305:17315 1:N:0:GAACCTAG+TCCGCATA
GGGCGAGAGAGAGCGACTTCTCTCGGGCCCACAGCCTTACAGCTGTGGAGCACGGTATCCTCTGCCGA
AACAGAGGTTGGACAAGACCGGAGGGGTCTCCTAGTTCCAAAGGAGATGTACTCCGGGCTTGTTCACG
+

```
FFFFFFFFFFFFFFFFFFFFF:FFFFFFFFFFFFFFFFFFFFFFFFFFFFFFFFFFFF:FFFFFFFF:
FFFFFFFFFFFFFFFFFFFFFFFFFFFFFFFFFFFFFFFFFFFFFFFFFFFFFFFFFFFFFFFFF:FF
@A00155:342:HHGFNDSXY:1:2173:5791:26866 1:N:0:GAACCTAG+TCCGCATA
GGATGTGGAGGGCGAGAGAGAGCGACTTCTCTCGGGCCCACAGCCTTACAGCTGTGGAGCACGGTATC
CTCTGCCGAAACAGAGGTTGGACAAGACCGGAGGGGTCTCCTAGTTCCAAAGGAGATGTACTCC
+
FFFFFFFFFFFFFFFFFFFFFFFFFFFFFFFFFFFFFFFFFFFFFFFFFFFFFFFFFFFFFFFFFFFF
FFFFFFFFFFFF:F,FFFFFFFF:FFFFFFFFFFFFFFFFFFFFFFFFFFFFFFFFFFFFFFFF
@A00155:342:HHGFNDSXY:1:2173:22435:28416 1:N:0:GAACCTAG+TCCGCATA
GTATCCTCTGCCGAAACAGAGGTTGGACAAGACCGGAGGGGTCTCCTAGTTCCAAAGGAGATGTACTC
CGGGCTTGTTCACGACCTACCGTGTAAGTCGTAGTCTAGTAGGCTACCTGACGAGTCCTT
+
FFFFFFFFFFFFFFFFFFFFFFFFFFFFFFFFFFFFFFFFFFFFFFFFFFFFFFFFFFFFFFFFFF:F
FFFFFFFFFFFFFFFFFFFFFFFFFFFFFF:FFFFFFFFFFFFFFFFFFFFFFFFFFFFF
@A00155:342:HHGFNDSXY:1:2174:5575:1188 1:N:0:GAACCTAG+TCCGCATA
TTACAGCTGTGGAGCACGGTATCCTCTGCCGAAACAGAGGTTGGACAAGACCGGAGGGGTCTCCTAGT
TCCAAAGGAGATGTACTCCGGGCTTGTTCACGACCTACCGTGTAAGTCGTAGTCTAGTAGGCTACCTG
+
FFFFFFFFF:FFFFFFFFFFFFFFFFFFFFFFFFFFFFFFFFFFFFFF,FFFFFFFFFFFFFFFFFFF
FFFFFFFFFFFFFFFFFFFFFFFFFFFFFFFFFFFFFFFF,FFF:FFFFFFFFFFFFFFFFFFFFFFF
@A00155:342:HHGFNDSXY:1:2175:6244:9831 1:N:0:GAACCTAG+TCCGCATA
ATCTCTATGCCGAGAGGGATGTGGAGGGCGAGAGAGAGCGACTTCTCTCGGGCCCACAGCCTTACAGC
TGTGGAGCACGGTATCCTCTGCCGAAACAGAGGTTGGACAAGACCGGAGGGGTCTCCTAGTTCCAAAG
+
FFFFFFFFFFFF:FFFFFFFF:FFFFFFFFFFFFFFFFFFFF:FFFFFFFFFFF:FFFFFFFFFFFFF
FFFFFFFFFFFFFFFFFFFFFFFFFF:FFFFFFFFFFFFFFFFFFFFFFFFFFFFFFFFFFFFFFFFF
@A00155:342:HHGFNDSXY:1:2175:10800:11428 1:N:0:GAACCTAG+TCCGCATA
AAGGACTCACCTCTGGCCTATGGTCATCTCAATGCCGAGAGGGATGTGGAGGGCGAGAGAGAGCGACT
TCTCTCGGGCCCACAGCCTTACAGCTGTGGAGCACGGTATCCTCTGCCGAAACAGAGGTTGGACAAGA
+
FFFFFFFFFFFFFFFFFFFFFFFFFFFFFFFFFFFFFFFFFFFFFFFFFFFFFFFFFFFFFFFFFFFF
FFFFFFFFFFFFFFFFFFFFFFFFFFFFFFFFFFFFFFFFFFFFFFFFFFFFFFFFFFFFFFFFFFFF
@A00155:342:HHGFNDSXY:1:2175:9372:20071 1:N:0:GAACCTAG+TCCGCATA
GTATCCTCTGCCGAAACAGAGGTTGGACAAGACCGGAGGGGTCTCCTAGTTCCAAAGGAGATGTACTC
CGGGCTTGTTCACGACCTACCGTGTAAGTCGTAGTCTAGTAGGCTACCTGACGAGTCCTTTTTAGGAC
+
FFFFFFFFFFFFFFFFFFFFFFFFFFFFFF:FFFFFFFFFFFFFFFFFFFFFFFFFFFFFFFFFFFFF
FFFFFFFFFFFFFFFFFFFFFFFFFFFFFFFFFFFFFFFFFFFFFFFFFFFFFFFFFFFFFFFFFFFF
@A00155:342:HHGFNDSXY:1:2175:19090:21214 1:N:0:GAACCTAG+TCCGCATA
TGTGGAGGGCGAGAGAGAGCGACTTCTCTCGGGCCCACAGCCTTACAGCTGTGGAGCACGGTATCCTC
TGCCGAAACAGAGGTTGGACAAGACCGGAGGGGTCTCCTAGTTCCAAAGGAGATGTACTCCGGG
+
F:FFFFFFFFFFF:FFFFFFFFFFFFFFFFF,F:FFFFFF:FFFFFFFFF:F,FF:FFFFFFFFFFFF
F:FFFFFFFFFF::FFFFFFFF,FFF:FFFFFFF:FFFFFF:FFFFFFFFFFFFFFFFF:FFFF
@A00155:342:HHGFNDSXY:1:2175:22182:25097 1:N:0:GAACCTAG+TCCGCATA
GAGAGGGATGTGGAGGGCGAGAGAGAGCGACTTCTCTCGGGCCCACAGCCTTACAGCTGTGGAGCACG
GTATCCTCTGCCGAAACAGAGGTTGGACAAGACCGGAGGGGTCTCCTAGTTCCAAAGGAGATGTACTC
+
FFFFFFFFFFFFFFFFFFFFFFFFFFFFFFFFFFFFFFFFFFFFFFFFFFFFFFFFFFFFFFFFFFFF
FFFFFFFFFFFFFFFFFFFFFFFFFFFFFF:FFFFFFFFFFFFFFF:FFFFFFFFFFFFFFFFFFFFF
@A00155:342:HHGFNDSXY:1:2175:30581:29966 1:N:0:GAACCTAG+TCCGCATA
CTCTGGCCTATGGTCATCTCAATGCCGAGAGGGATGTGGAGGGCGAGAGAGAGCGACTTCTCTCGGGC
CCACAGCCTTACAGCTGTGGAGCACGGTATCCTCTGCCGAAACAGAGGTTGGACAAGACCGGAGGGGT
+
```

FFFFFFFFFFFFFFFFFFFFFFFFFFFFFFFFFFFFFFFFFFFFFFF:FFF:FFFFFFFFFFFFFFFF
FFFFFFFFFFFFFFFFFFFFFFFFFFFFFFFFFFFFFFFFFFFFFFFFFFFFFFFFFFFFFFFFFFFFFF
@A00155:342:HHGFNDSXY:1:2175:25482:33473 1:N:0:GAACCTAG+TCCGCATA
CCTCTGCCGAAACAGAGGTTGGACAAGACCGGAGGGGTCTCCTAGTTCCAAAGGAGATGTACTCCGGG
CTTGTTCACGACCTACCGTGTAAGTCGTAGTCTAGTAGGCTACCTGACGAGTCCTTTTTAGGACGAAA
+
FFFFFFFFFFFFFFFFFFFFFFFFFF::FFFFFFFFFFFF:FFFFFFFFFFFFFFFFFFFFFFFFFFFF
FFFFFFFFFFFFFFFFFFFFFFFFFFFF::FFF:FFFFFFFFFFFFFFFFFFFFFFFFFFF:FFFFFFFFFF
@A00155:342:HHGFNDSXY:1:2176:16803:16501 1:N:0:GAACCTAG+TCCGCATA
AATGCCGAGAGGGATGTGGAGGGCGAGAGAGAGCGACTTCTCTCGGGCCCACAGCCTTACAGCTGTGG
AGCACGGTATCCTCTGCCGAAACAGAGGTTGGACAAGACCGGAGGGGTCTCCTAGTTCCAAAGGAGAT
+
FFFFFFFFFFFFFFFFFFFFFFFFFFFFFFFFFFFFFFFFFFFFFFFFFFFFFFFFFFFFFFFFFFFFFF
FFFFFFFFFFFFFFFFFFFFFFFFFFFFFFFFFFFFFFFFFFFFFFFFFFFFFFFFFFFFFFFFFFFFFF
@A00155:342:HHGFNDSXY:1:2176:16957:16517 1:N:0:GAACCTAG+TCCGCATA
AATGCCGAGAGGGATGTGGAGGGCGAGAGAGAGCGACTTCTCTCGGGCCCACAGCCTTACAGCTGTGG
AGCACGGTATCCTCTGCCGAAACAGAGGTTGGACAAGACCGGAGGGGTCTCCTAGTTCCAAAGGAGAT
+
FFFFFFF:FFFFFFFFFFFFFFFFFFFFFFFFFFFFFFFFFFFFFFFFFFFFFFFFFFFFFFFFFFFFF
FFFFFFFFFFFFFFFFFFFFFFFFFFFFF:FFF:FFFF:FFFFFFFFFFFFFFFFFFF:FF:FFFFFFFFF
@A00155:342:HHGFNDSXY:1:2176:20193:28761 1:N:0:GAACCTAG+TCCGCATA
CAAGCCCGGAGTACATCTCCTTTGGAACTAGGAGACCCCTCCGGTCTTGTCCAACCTCTGTTTCGGCA
GAGGATACCGTGCTCCACAGCTGTAAGGCTGTGGGCCCGAGAGAAGTCGCTCTCTCTCGCCCTCCACA
+
FFFFFFFFFFFFFFFFFFFFFFFFFFFFFFFFFFFFFFFFFFFFFFFFFFFFFFFFFFFFFFFFFFFFFF
FFFFFFFFFFFFFFFFFFFFFFFFFFFFFFFFFFFFFFFFFFFFFFFFFFFFFFFFFFFFFFFFFFFFFF
@A00155:342:HHGFNDSXY:1:2177:25536:13150 1:N:0:GAACCTAG+TCCGCATA
GCGACTTCTCTCGGGCCCACAGCCTTACAGCTGTGGAGCACGGTATCCTCTGCCGAAACAGAGGTTGG
ACAAGACCGGAGGGGTCTCCTGGTTCCAAAGGAGATGTACTCCGGGCTTGTTCACGACCTACCG
+
FFFFFFFFFFFFFFFFFFFFFFFF:FFFFFFFFFFFFFFFFFFFFFFFFFFFFFFFFFFFFFFFFFFFFF
FFFFFFFFFFFFFFFFFFFFFFFFFFFFFFFFFFFFFFFFFFFFF:FFFFFFFFFFFFFFFF:F
@A00155:342:HHGFNDSXY:1:2177:5801:25661 1:N:0:GAACCTAG+TCCGCATA
GAGGGCGAGAGAGAGCGACTTCTCTCGGGCCCACAGCCTTACAGCTGTGGAGCACGGTATCCTCTGCC
GAAACAGAGGTTGGACAAGACCGGAGGGGTCTCCTAGTTCCAAAGGAGATGTACTCCGGGCTTGTTCA
+
FFF,FFFFFFFFFFFF,FFFFFFFFFFFFFFFFFFFFFFFFFFFFFFFFFFFFFFFF:,FFF::FFFFF
F:FFFFFF,F::FFFFFFFFF:FFFFFFF:F:FFFFFF:F,FFFFFFFF:FFFFFFF:FFFFFFFFFF
@A00155:342:HHGFNDSXY:1:2177:29704:27383 1:N:0:GAACCTAG+TCCGCATA
TGTGTCCACTTAAGGACTCACCTCTGGCCTATGGTCATCTCAATGCCGAGAGGGATGTAGAGGGCGAG
AGAGAGCGACTTCTCTCGGGCCCACAGCCTTACAGCTGTGGAGCACGGTATCCTCTGCCGAAACAGAG
+
FFFFFFFFFFFFFFFFFFFFFFFFFFFFFFFFFFFFFFFFFFFFFFFFFFFFFFFFFFFFFFFFFFFFFF
FFFFFFFFFFFFFFFFFFFFFFFFFFFFFFFFFFFFFFFFFFFFFFFFFFFFFFFFFFFFFFFFFFFFFF
@A00155:342:HHGFNDSXY:1:2177:29514:27461 1:N:0:GAACCTAG+TCCGCATA
TGTGTCCACTTAAGGACTCACCTCTGGCCTATGGTCATCTCAATGCCGAGAGGGATGTAGAGGGCGAG
AGAGAGCGACTTCTCTCGGGCCCACAGCCTTACAGCTGTGGAGCACGGTATCCTCTGCCGAAACAGAG
+
FFFFFFFFFFFFFFFFFFFFFFFFFFFFFFF:FFFFFFFFFFFFFFFFFFFFFFFFFFFFFFFFFFFFFF
FFFFFFFFFFFFFFFFFFFFFFFFFFFFFFFFFFFFFFFFFFFFFFFFFFFFFFFFFFFFFFFFFFFFFF
@A00155:342:HHGFNDSXY:1:2177:29668:27602 1:N:0:GAACCTAG+TCCGCATA
TGTGTCCACTTAAGGACTCACCTCTGGCCTATGGTCATCTCAATGCCGAGAGGGATGTAGAGGGCGAG
AGAGAGCGACTTCTCTCGGGCCCACAGCCTTACAGCTGTGGAGCACGGTATCCTCTGCCGAAACAGAG
+

FFFFFFFFFFFFFFFFFFFFFFFFFFFFFFFFFFFFFFFFFFFFFFFFFF:FFFF:FFFFFFFFFFFF
FFFFFFFFFFFFFFFFFFFFFFFFFFFFFFFFFFFFFF:FFFFFFFFFFFFFFFFFFFFFFFFFFFFF
@A00155:342:HHGFNDSXY:1:2178:7853:4100 1:N:0:GAACCTAG+TCCGCATA
GGGCGAGAGAGAGCGACTTCTCTCGGGCCCACAGCCTTACAGCTGTGGAGCACGGTATCCTCTGCCGA
AACAGAGGTTGGACAAGACCGGAGGGGTCTCCTGGTTCCAAAGGAGATGTACTCCGGGCTTGTTCACG
+
FFFFFFFFFFFFFFFFFFFFFFFFFFFFFFFFFFFFFFFFFFFFFFFFFFFFFFFFFFFFFFFFFFFF
FFFFFFFFFFFFFFFFFFFF:FFFFFFFFFFFFFFFFFFFFFFFFFFFF:FFFFFFFFFFFFFFFFFF
@A00155:342:HHGFNDSXY:1:2178:28203:6809 1:N:0:GAACCTAG+TCCGCATA
GGATGTGGAGGGCGAGAGAGAGCGACTTCTCTCGGGCCCACAGCCTTACAGCTGTGGAGCACGGTATC
CTCTGCCGAAACAGAGGTTGGACAAGACCGGAGGGGTCTCCTAGTTCCAAAGGAGATGTACTCC
+
FFFFF:FFFFFFFFFFFFFFFFFFFFFFFFFFFFFFFFFFFFFFF:FFFFFFFFFFFFFFFFFFFFFF
FFFFFFFFFFFFFF:FFFFFFFFFFFFFFFFFFFFFFFFFFFFFFFFFFFFFFFFFFFFFFFFF
@A00155:342:HHGFNDSXY:1:2178:24532:8750 1:N:0:GAACCTAG+TCCGCATA
GAGGGCGAGAGAGAGCGACTTCTCTCGGGCCCACAGCCTTACAGCTGTGGAGCACGGTATCCTCTGCC
GAAACAGAGGTTGGACAAGACCGGAGGGGTCTCCTAGTTCCAAAGGAGATGTACTCCGGGCTTGTTCA
+
FFFFFFFFFFFFFFFFFFFFFFFFFFFFFFFFFFFFFFFFFFFFFFFFFFFFFFFFFFFFFFFFFFFF
FFFFFFFFFFFFFFFFFFFFFFFFFFFFFFFFFFFFFFFFFFFFFFFFFFFFFFFFFFFFFFFFFFFF
@A00155:342:HHGFNDSXY:1:2201:15212:9862 1:N:0:GAACCTAG+TCCGCATA
CCGCCACACCGCCTTAGATCAGCTGTGTCCACTTAAGGACTCACCTCTGGCCTATGGTCATCTCAATG
CCGAGAGGGATGTGGAGGGCGAGAGAGAGCGACTTCTCTCGGGCCCACAGCCTTACAGCTGTGGAGC
+
FF,FFFFF:FFF:FFFFFFFFFFFFFFFF:FFF::FF:FFFFFFFFFF::FF:FFFFFFF,FFFFFFF
:FFF:FFFFF:FFFFF,FFFFFFFFFFFFFFFFFFFFFFF,FFFFFFFFFFFFFFFFFFFFFFF:FF
@A00155:342:HHGFNDSXY:1:2201:8088:27586 1:N:0:GAACCTAG+TCCGCATA
GAGGGCGAGAGAGAGCGACTTCTCTCGGGCCCACAGCCTTACAGCTGTGGAGCACGGTATCCTCTGCC
GAAACAGAGGTTGGACAAGACCGGAGGGGTCTCCTAGTTCCAAAGGAGATGTACTCCGGGCTTGTTCA
+
FFFFFFFF,FFFFFFFFFFFFFFFFFFFFFFFF:FFFFFF,FFFFFFFFFFFFFFFFFFFFFFFFFFF
FFFFFF,FFFFFFFFFFF,FF:FFFFFFFFFFFFFF:FFFFFFFFFFFFFFFFFFFFFFFFFFFFFFF
@A00155:342:HHGFNDSXY:1:2201:8015:27649 1:N:0:GAACCTAG+TCCGCATA
GAGGGCGAGAGAGAGCGACTTCTCTCGGGCCCACAGCCTTACAGCTGTGGAGCACGGTATCCTCTGCC
GAAACAGAGGTTGGACAAGACCGGAGGGGTCTCCTAGTTCCAAAGGAGATGTACTCCGGGCTTGTTCA
+
FFFFFFFFFFFFFFFFFFFFFFFFFFFFFFFFFFFFFFFFFFFFFFFFFFFFFFF:FFFFFFFFFFFF
FFFFFFFFFFFFFFF,FFF:FFFFFFFFFFFFF,FFFFFFFFFFF:FFFF,FFFFFFFFFFFFFFFFF
@A00155:342:HHGFNDSXY:1:2201:23656:29371 1:N:0:GAACCTAG+TCCGCATA
CTCTGGCCTATGGTCATCTCAATGCCGAGAGGGATGTGGAGGGCGAGAGAGAGCGACTTCTCTCGGGC
CCACAGCCTTACAGCTGTGGAGCACGGTATCCTCTGCCGAAACAGAGGTTGGACAAGACCGGAGGGGT
+
FFFFFFFFFFFFFFFFFFFFFFFFFFFFFFFFF::FFFFFFFFFFFFFFFFFFFFFFFFFFFFFFFFF
FFFFFFFFFFFFFFFFFFFFFFFFFFFFFFFFFFFFFFFFFFFFF:FFFFFFFFFFFFFFFFFFFFFF
@A00155:342:HHGFNDSXY:1:2201:18674:36088 1:N:0:GAACCTAG+TCCGCATA
ATGCCGAGAGGGATGTGGAGGGCGAGAGAGAGCGACTTCTCTCGGGCCCACAGCCTTACAGCTGTGGA
GCACGGTATCCTCTGCCGAAACAGAGGTTGGACAAGACCGGAGGGGTCTCCTGGTTCCAAAGGAGATG
+
FFFFFFFFFF:FFFFFFFFFFFFFFFFFFFFFFFFFFFFFFFFFFFFFFFFFFFFFFFFFFFFFFFFF
FFFFFFFFFFFFFFFFFFFFFFFFFFFFFFFFFFFFFFFFFFFFFFFFFFFFFFFFFFFFFFFFFFFF
@A00155:342:HHGFNDSXY:1:2201:25581:36495 1:N:0:GAACCTAG+TCCGCATA
GTCATCTCAATGCCGAGAGGGATGTGGAGGGCGAGAGAGAGCGACTTCTCTCGGGCCCACAGCCTTAC
AGCTGTGGAGCACGGTATCCTCTGCCGAAACAGAGGTTGGACAAGACCGGAGGGGTCTCCTAGTTCCA
+

```
FFFFFFFFFFFFFFF,F:FFFFFFFFFFFFFFFFF:FFFFFFFFFFFFFFFFFFFFFFFFFFF:FFFF
:FFFFFFF:FF:FFFFFFFFFFFFF:FFFFFFFFFFFFFFFFFF:FFFFFFFFFFFFF:F,FF:FFFF
@A00155:342:HHGFNDSXY:1:2202:26892:4820 1:N:0:GAACCTAG+TCCGCATA
AGCGACTTCTCTCGGGCCCACAGCCTTACAGCTGTGGAGCACGGTATCCTCTGCCGAAACAGAGGTTG
GACAAGACCGGAGGGGTCTCCTAGTTCCAAAGGGGATGTACTCCGGGCTTGTTCACGACCTACCGTGT
+
FFFFFFFFFFFFFFFFFFFFFFFFFFF:FFFFFFFFFFFFFFFFFFFFFFFFFFFFFFFFFFFFFFFF
FFFFFFFFFFFFFFFFFFFFFFFFFFFFFFFF:FFFFFFFFFFFFFFFFFFFFFFF:FFFFFFFFFFF
@A00155:342:HHGFNDSXY:1:2202:24144:7921 1:N:0:GAACCTAG+TCCGCATA
CTCTGGCCTATGGTCATCTCAATGCCGAGAGGGATGTGGAGGGCGAGAGAGAGCGACTTCTCTCGGGC
CCACAGCCTTACAGCTGTGGAGCACGGTATCCTCTGCCGAAACAGAGGTTGGACAAGACCGGAGGGGT
+
FFFFFFFFFFFFFFFFFFFFFFFFFFFFFFFFFFFFFFFFFFFFFFFFFFFFFFFFFFFFFFFFFFFF
FFFFFFFFFF:FFFFFF:FFFFFFFFFFFFFFFFFFFFFFFFFFFFFFFFFFFFFFFFFFFFFFF:F:
@A00155:342:HHGFNDSXY:1:2202:24903:9048 1:N:0:GAACCTAG+TCCGCATA
TGCCGAGAGGGATGTGGAGGGCGAGAGAGAGCGACTTCTCTCGGGCCCACAGCCTTACAGCTGTGGAG
CACGGTATCCTCTGCCGAAACAGAGGTTGGACAAGACCGGAGGGGTCTCCTAGTTCCAAAGGAGATGT
+
FFFFFFFFFFFF:F:FFFFFFFFFFFFFFFFFFFFFFFFFFFFFFFFFFFFFFFFFFFFFFFFFFFFF
FFFFFFFFFFFFFFFFFFFFFFFFFFFFFFFFFFFFFFFFFFFFFFFFFFFFFFFFFFFFFFFFFFFF
@A00155:342:HHGFNDSXY:1:2202:27010:12258 1:N:0:GAACCTAG+TCCGCATA
CCTTACAGCTGTGGAGCACGGTATCCTCTGCCGAAACAGAGGTTGGACAAGACCGGAGGGGTCTCCTG
GTTCCAAAGGAGATGTACTCCGGGCTTGTTCACGACCTACCGTGTAAGTCGTAGTCTAGTAGGCTACC
+
FFFFFFFFFFFFFFFFFFFFFFFFFFFFFFFFFFFFFFFFFFFFFFFFFFFFFFFFFFFFFFFFFFFF
FFFFFFFFFFFFFFFFFF:FFFFFFFFFFFFFFFFFFFFFFFFFFFFFFFFFFFFFFFFFFFFFFFFF
@A00155:342:HHGFNDSXY:1:2202:26621:17628 1:N:0:GAACCTAG+TCCGCATA
ACCTCTGTTTCGGCAGAGGATACCGTGCTCCACAGCTGTAAGGCTGTGGGCCCGAGAGAAGTCGCTCT
CTCTCGCCCTCCACATCCCTCTCGGCATTGAGATGACCATAGGCCAGAGGTGAGTCCTTAAGTGGACA
+
FFFFFFFFFFFFFFFFFFFFFFFFFFFFFFFFFFFF:FFFFFFFFFFFFFFFFFFFFFFFFFFFFFFF
FFFFFFFFFFFFFFFFFFFFFFFFFFF:FFFFFFFFFFFFFFFFFFFFFFFFFFFFFFFFFFFFFFFF
@A00155:342:HHGFNDSXY:1:2202:25464:20979 1:N:0:GAACCTAG+TCCGCATA
CAATGCCGAGAGGGATGTAGAGGGCGAGAGAGAGCGACTTCTCTCGGGCCCACAGCCTTACAGCTGTG
GAGCACGGTATCCTCTGCCGAAACAGAGGTTGGACAAGACCGGAGGGGTCTCCTAGTTCCAAAGGAGA
+
FFFFFFFFFFFFFFFFFFFFFFFFFFFFFFFFFFFFFFFFFFFFFFFFFFFFFFFFFFFFFFFFFFFF
FFFFFFFFFFFFFFFFFFFFFFFFFFFF:FFFFFFF,FFFFFFFFFFFFFFFFFFFFFFFF,:FFFFF
@A00155:342:HHGFNDSXY:1:2202:32036:31109 1:N:0:GAACCTAG+TCCGCATA
CCTCTGGCCTATGGTCATCTCAATGCCGAGAGGGATGTGAAGGGCGAGAGAGAGCGACTTCTCTCGGG
CCCACAGCCTTACAGCTGTGGAGCACGGTATCCTCTGCCGAAACAGAGGTTGGACAAGACCGGA
+
FFFFFFFFFFFFFFFFFFFFFFFFFFFFFFFFFFFFFFFFFFFFFFFFFFFFFFFFFFFFFFFFFFFF
FFFFFFFFFFFFFFFFFFFFFFFFFFFFFFFFFFFFFFFFFFFFFFFFFFFFFFFFFFFFFFFF
@A00155:342:HHGFNDSXY:1:2204:2003:1391 1:N:0:GAACCTAG+TCCGCATA
TTACAGCTGTGGAGCACGGTATCCTCTGCCGAAACAGAGGTTGGACAAGACCGGAGGGGTCTCCTAGT
TCCAAAGGAGATGTACTCCGGGCTTGTTCACGACCTACCGTGTAAGTCGTAGTCTAGTAGGCTACCTG
+
:FFFFFFFFFFFFFFFFFFFFFFFFFFFFFFFFFFFFFFFFFFFFFFFFFFFFFFFFFFFFFFFFFFF
FFFFFFFFFFFFFFFFFFFFFFFFFFFFFFFFFFFFFFFFFFFF:FFFFF:FFFFFFFF:FFF:FFFF
@A00155:342:HHGFNDSXY:1:2204:12228:28025 1:N:0:GAACCTAG+TCCGCATA
AAGGACTCACCTCTGGCCTATGGTCATCTCAATGCCGAGAGGGATGTGGAGGGCGAGAGAGAGCGACT
TCTCTCGGGCCCACAGCCTTACAGCTGTGGAGCACGGTATCCTCTGCCGAAACAGAGGTTGGACAAGA
+
```

FFFFFFFFFFFFFFFFFFFFFFFFFFFFFFFFFFFFFFFFFFFFFFFFFFFFFFFFFFFFFFFFFFFF
FFFFFFFFFFFFFFFFFFFFFFFFFFFFFFFFFFFFFFFFFFFFFFFFFFFFFFFFFFFFFFFFFFFF
@A00155:342:HHGFNDSXY:1:2205:27624:4867 1:N:0:GAACCTAG+TCCGCATA
GAGAGCGACTTCTCTCGGGCCCACAGCCTTACAGCTGTGGAGCACGGTATCCTCTGCCGAAACAGAGG
TTGGACAAGACCGGAGGGGTCTCCTAGTTCCAAAGGAGATGTACTCCGGGCTTGTTCACGACCTACCG
+
FFFFFFFFFFFFFFFFFFFFFFFFFFFFFFFFFFFFFFFFFFFFFFFFFFFFFFFFFFFFFFFFFFFF
FFFFFFFFFFFFFFFFFFFFFFFFFFFFFFFFFF:FFFFFFFFFF:FFFFFFFFFFFFFFFFFFFFFF
@A00155:342:HHGFNDSXY:1:2205:17770:9157 1:N:0:GAACCTAG+TCCGCATA
CACCTCTGGCCTATGGTCATCTCAATGCCGAGAGGGATGTGGAGGGCGAGAGAGAGCGACTTCTCTCG
GGCCCACAGCCTTACAGCTGTGGAGCACGGTATCCTCTGCCGAAACAGAGGTTGGACAAGACCGGAGG
+
FFFFFFFFFFFFFFFFFFFFFFFFFFFFFFFFFFFFFFFFFFFFFFFFFFFFFFFFFFFFFFFFFFFF
FFFFFFFFFFFFFFFFFFFFFFFFFFFFFFFFFFFFFFFFFFFFFFFFFFFFFFFFFFFFFFFFFFFF
@A00155:342:HHGFNDSXY:1:2205:6334:13182 1:N:0:GAACCTAG+TCCGCATA
CTTCTCTCGGGCCCACAGCCTTACAGCTGTGGAGCACGGTATCCTCTGCCGAAACAGAGGTTGGACAA
GACCGGAGGGGTCTCCTAGTTCCAAAGGAGATGTACTCCGGGCTTGTTCACGACCTACCGTGTAAGTC
+
FFFFFFFFFFFFFFFFFFFFF,FFFFFFFFFFFFFFF,FF:FF:FFFFFFFFFFFFFFFFFFFFFFFF
,FFFFFFFFFFFFFFFFFFFFFFFFFFFFFFFFFFFFFFFFFFFFFFFF:FFFFFFFFFFFFFF:FFF
@A00155:342:HHGFNDSXY:1:2205:14696:15233 1:N:0:GAACCTAG+TCCGCATA
TTAAGGACTCTCCTCTGGCCTATGGTCATCTCAATGCCGAGAGGGATGTGGAGGGCGAGAGAGAGCGA
CTTCTCTCGGGCCCACAGCCTTACAGCTGTGGAGCACGGTATCCTCTGCCGAAACAGAGGTTGGACAA
+
FFFFFFFFFFFFFFFFFFFFFFFFFFFFFFFFFFFFFFFFFFFFFFFFFFFFFFFFFFFFFFFFFFFF
FFFFFFFFFFFFFFFFFFF,FFFFFFFFFFFFFF:FFFFFFFFFFFFFFFFFFFFFFFFFFFFFFFFF
@A00155:342:HHGFNDSXY:1:2205:3929:15687 1:N:0:GAACCTAG+TCCGCATA
GATGTGGAGGGCGAGAGAGAGCGACTTCTCTCGGGCCCACAGCCTTACAGCTGTGGAGCACGGTATCC
TCTGCCGAAACAGAGGTTGGACAAGACCGGAGGGGTCTCCTAGTTCCAAAGGAGATGTACTCCGGGCT
+
FFFFF:FFFFFFFFFFFFFFFFFFFFFFFFFFFFFFFFFFFFFFFFFFFFFFFFFFFFFFFFFFFFFF
FFFFFFFFFFFFFFFFFFFFFFFFFFFFFFFFFFFFFFFFFFFFF:FF,FFFFFFFFFFFFFFFFFFF
@A00155:342:HHGFNDSXY:1:2205:29134:18161 1:N:0:GAACCTAG+TCCGCATA
GGTCTTGTCCAACCTCTGTTTCGGCAGAGGATACCGTGCTCCACAGCTGTAAGGCTGTGGGCCCGAGA
GAAGTCGCTCTCTCTCGCCCTTCACATCCCTCTCGGCATTGAGATGACCATAGGCCAGAG
+
FFFFFFFFFFFFFFFFFFFFFFFFFFFFFFFFFFFFFFFFFFFFFFFFFFFFFFFFFFFFFFFFFFFF
FFFFFFFFFFFFFFFFFFFFFFFFFFFFFFFFFFFFFFFFFFFFFFFFFFFFFFFFFFFF
@A00155:342:HHGFNDSXY:1:2205:13774:21277 1:N:0:GAACCTAG+TCCGCATA
GAGGGCGAGAGAGAGCGACTTCTCTCGGGCCCACAGCCTTACAGCTGTGGAGCACGGTATCCTCTGCC
GAAACAGAGGTTGGACAAGACCGGAGGGGTCTCCTAGTTCCAAAGGAGATGTACTCCGGGCTTGTTCA
+
FFFFFFFFFFFFFF:FFFFF:FF:FFF:FFFFFFFFFFFFFFFFFFFFFFFFFFF::F:F:,FFF,FF
FFFFFF:FF,FFFFFFFFFFFF:FF:FFFFFFFFFFFFF::FFFFFFFFFFFFFFF:,FFFFFFF:FF
@A00155:342:HHGFNDSXY:1:2205:6216:22467 1:N:0:GAACCTAG+TCCGCATA
GACTTCTCTCGGGCCCACAGCCTTACAGCTGTGGAGCACGGTATCCTCTGCCGAAACAGAGGTTGGAC
AAGACCGGAGGGGTCTCCTAGTTCCAAAGGAGATGTACTCCGGGCTTGTTCACGACCTACCGTGTAAG
+
FFFFFFFFFFFFFFFFFFFFFFFF:FFFF:FFFFFFFFFFFFFFFFFFFFFFFFFFFFFFFFFFFFFF
:FFFFFFFFFFFFFFFFFF,FFFFFFFFFFFFFFFFFFFFFFFFF:FFFFFFFFFFFFFFFF:F:FFF
@A00155:342:HHGFNDSXY:1:2205:22544:22498 1:N:0:GAACCTAG+TCCGCATA
TTCTCTCGGGCCCACAGCCTTACAGCTGTGGAGCACGGTATCCTCTGCCGAAACAGAGGTTGGACAAG
ACCGGAGGGGTCTCCTAGTTCCAAAGGAGATGTACTCCGGGCTTGTTCACGACCTACCGTGTAAGTCG
+

FFFFFFFFFFFFFFFFFFFFFFFFFFFFFFF:FFFFFFFFFFFFFFFFFFFFFFFFFFFFFFFFFFF
FFFFFFFFFFFFFFFFFFFFFFFFFFFFFFFFFFFFFFFFFFFFFFFFFFFFFFFFFFFFFFFFFFF
@A00155:342:HHGFNDSXY:1:2206:7030:8688 1:N:0:GAACCTAG+TCCGCATA
AGAGAGAGCGACTACTCTCGGGCCCACAGCCTTACAGCTGTGGAGCACGGTATCCTCTGCCGAAACAG
AGGTTGGACAAGACCGGAGGGGTCTCCTAGTTCCAAAGGAGATGTACTCCGGGCTTGTTCACGACCTA
+
FFFFFFFFFFFFFFFFFFFFFFFFFFFFFFFFFFFFFFFFFFFFFFFFFFFFFFFFFFFFFFFFFFF
FFFFFFFFFFFF:FFFFFFFFFFFFFFFFFFFFFFFFFFFFFFFFFFFFFFFFF:FFFFFFFFFFFF:
@A00155:342:HHGFNDSXY:1:2206:7274:15812 1:N:0:GAACCTAG+TCCGCATA
GAGGGCGAGAGAGAGCGACTTCTCTCGGGCCCACAGCCTTACAGCTGTGGAGCACGGTATCCTCTGCC
GAAACAGAGGTTGGACAAGACCGGAGGGGTCTCCTAGTTCCAAAGGAGATGTACTCCGGGCTTGTTCA
+
FFFFFFFFFFFFFFFFFFFFFFFFFFFF:FFFFFFFFFFFFFFFFFFFFFFFFFFFFFFFFFFFFFFF
FFFFFFFFFFFFFFFFFFFFFFFFFFFFFFFFFFFFFFF:FFFFFFFFFFFFFFFFFFFFFFFFFFFFFF
@A00155:342:HHGFNDSXY:1:2206:9245:26052 1:N:0:GAACCTAG+TCCGCATA
AAGGACTCTCCTCTGGCCTATGGTCATCTCAATGCCGAGAGGGATGTGGAGGGCGAGAGAGAGCGACT
TCTCTCGGGCCCACAGCCTTACAGCTGTGGAGCACGGTATCCTCTGCCGAAACAGAGGTTGGACAAGA
+
FFFFFFFFFFFFFFF:FFFFFFFFFFFFFFFFFFFFFFFFFFFFF:FFFFFFFFFFFFFFFFFFFFFFF
FFFFFFFFFFFFFFFFFFFF,F:FFFFFFFFFFFFFFFFFFFFFFFFFFFFFFF:FFFFFFFFFFFFFF
@A00155:342:HHGFNDSXY:1:2207:27670:4163 1:N:0:GAACCTAG+TCCGCATA
TTCTCTCGGGCCCACAGCCTTACAGCTGTGGAGCACGGTATCCTCTGCCGAAACAGAGGTTGGACAAG
ACCGGAGGGGTCTCCTGGTTCCAAAGGAGATGTACTCCGGGCTTGTTCACGACCTACCGTGTAAGTCG
+
FFFFFFFFFFFFFFFFFFFFFFFFFFFFFFFFFFFFFFFFFFFFFFFFFFFFFFFFFFFFFFFFFFF
FFFFFFFFFFFFFFFFFFFFFFFFFFFFFFFFFFFFFFFFFFF:FFFFFFFFFFFF,FFFFFFFFFFF
@A00155:342:HHGFNDSXY:1:2207:15700:5290 1:N:0:GAACCTAG+TCCGCATA
AGGGCGAGAGAGAGCGACTTCTCTCGGGCCCACAGCCTTACAGCTGTGGAGCACGGTATCCTCTGCCG
AAACAGAGGTTGGACAAGACCGGAGGGGTCTCCTAGTTCCAAAGGAGATGTACTCCGGGCTTGTTCAC
+
FFFFFFFFFFFFFFFFFFFFFFFFFFFFFFFFFFFFFFFF,FFFFFFFFFFFFFFFFFFFFFFFFFFFFF
FFFFFFFFFFFFFFFFFFFFFFFFFFFFFFFFFFFFFFFFFFFFFFFFF:FFFFFFFFFFFFFFFFFF
@A00155:342:HHGFNDSXY:1:2207:20555:6308 1:N:0:GAACCTAG+TCCGCATA
TCCACATCCCTCTCGGCATTGAGATGACCATAGGCCAGAGGTGAGTCCTTAAGTGGACACAGCTGATC
TAAGGCGGTGTGGCGGGGCATGGGTTTGAACCCCCATGACGGTCGGAGTCTAGTAGGCTCCCTGATGA
+
FFFFFFFFFFFFFFFFFFFFFFFFFFFFFFFFFFFFFFFFFFFFFFFFFFFFFFFFFFFFFFFFFFF
FFFFFFFFFFFFFFFFFFFFFFFFFFFFFFFFFFFFFFFFFFFFFFFFFFFFFFFFFFFFFFFFFFF
@A00155:342:HHGFNDSXY:1:2207:30183:17534 1:N:0:GAACCTAG+TCCGCATA
TTACAGCTGTGGAGCACGGTATCCTCTGCCGAAACAGAGGTTGGACAAGACCGGAGGGGTCTCCTAGT
TCCAAAGGAGATGTACTCCGGGCTTGTTCACGACCTACCGTGTAAGTCGTAGTCTAGTAGGCTACCTG
+
FF:FFFFFFFFFFFFFFFFFFFFFFFFFFFFFFFFFFFFFFFFFFFFFFFFFFFFFFFFFFFFFFFF
FFFFFFFFFFFFFF:FFFFFFFFFFFFFFFFFFFFFFFFFFFFFFFFFFFFFFFFFFFFFFFFFFFF
@A00155:342:HHGFNDSXY:1:2207:10122:18082 1:N:0:GAACCTAG+TCCGCATA
CCGCCACACCGCCTTAGATCAGCTGTGTCCACTTAAGGACTCACCTCTGGCCTATGGTCATCTCAATG
CCGAGAGGGATGTGGTGGGCGAGAGAGAGCGACTTCTCTCGGGCCCACAGCCTTACAGCTGTGGAGCA
+
FFFFFFFFFFFFFFFFFFFFFFFFFFFFFFFFFFFFFFFFFFFFFFFFFF:FFFFFFFFFFFFFFFF
FFFFFFFFFFFFFFFFFFFFFFFFFFFFFFFFFFFFFFFFFFFFFFFFFFFFFFFFFFFFFFFFFFF
@A00155:342:HHGFNDSXY:1:2207:10547:18724 1:N:0:GAACCTAG+TCCGCATA
CCGCCACACCGCCTTAGATCAGCTGTGTCCACTTAAGGACTCACCTCTGGCCTATGGTCATCTCAATG
CCGAGAGGGATGTGGTGGGCGAGAGAGAGCGACTTCTCTCGGGCCCACAGCCTTACAGCTGTGGAGCA
+

FFFFFFFFFFFFFFFFFFFFFFFFFFFFFFFFFFFFFFFFFFFFFFFFFFFFFFFFFFFFFFFFFFFF
FFFFFFFFFFFFFFFFFFFFFFFFFFFFFFFFFFFFFFFFFFFFFFFFFFFFFFFFFFFFFFFFFFFF
@A00155:342:HHGFNDSXY:1:2207:10393:19241 1:N:0:GAACCTAG+TCCGCATA
CCGCCACACCGCCTTAGATCAGCTGTGTCCACTTAAGGACTCACCTCTGGCCTATGGTCATCTCAATG
CCGAGAGGGATGTGGTGGGCGAGAGAGAGCGACTTCTCTCGGGCCCACAGCCTTACAGCTGTGGAGCA
+
FF:FFFFFFFFFFFFFFFFFFFFFFFF:FFFFFFFFFFFFFFFFFFFFFFFFFFFFFFFFFFFFFFFF
FFFFFFFFFFFFFFFFFFFFFFFFFFFFF:FFFFFFFFFFFFFFFFFFFFFFFFFFFF:FFFFFFFFF
@A00155:342:HHGFNDSXY:1:2207:32190:27774 1:N:0:GAACCTAG+TCCGCATA
GAGAGGGATGTGGAGGGCGAGAGAGAGCGACTTCTCTCGGGCCCACAGCCTTACAGCTGTGGAGCACG
GTATCCTCTGCCGAAACAGAGGTTGGACAAGACCGGAGGGGTCTCCTAGTTCCAAAGGAGATGTACTC
+
FFFFFFFFFFFFFFFFFFFFFFFFFFFFFFF,FFFFFFFFFFFFFFFFFFF:FFFFFFFFFFF:FFFF
FFFFFFFFFFFFFF:FFFFFFFFFFFFFF:FFFFFFFFFFFFFFFFFFFFFFFFFFFFFFFFFFFFFF
@A00155:342:HHGFNDSXY:1:2207:2437:36902 1:N:0:GAACCTAG+TCCGCATA
GCGAGAGAGAGCGACTTCTCTCGGGCCCACAGCCTTACAGCTGTGGAGCACGGTATCCTCTGCCGAAA
CAGAGGTTGGACAAGACCGGAGGGGTCTCCTAGTTCCAAAGGAGATGTACTCCGGGCTTGTTCACGAC
+
FFFFFFFFFFFFFFFFFFF:FFFFFFFFFFFFFFFFFFFFFFFFFFFFFFFFFF:FFFFFFFFFFFFF
FFFFFFFFFFFFFFFFFFFFFFFFFFFFFFFFFFFFFFFFFFFFFFFFFFFFFFFFFFFFFFFFFFFF
@A00155:342:HHGFNDSXY:1:2208:2808:3912 1:N:0:GAACCTAG+TCCGCATA
GCTCCACAGCTGTAAGGCTGTGGGCCCGAGAGAAGTCGCTCTCTCTCGCCCTCCACATCCCTCTCGGC
ATTGAGATGACCATAGGCCAGAGGTGAGTCCTTAAGTGGACACAGCTGATCTAAGGCGGTGTGGCGGG
+
FFFFFFFFFFFFFFFFFFFFFFFFFFFFFFFFFFFFFFFFFFFFFFFFFFFFFFFFFFFFFFFFFFFF
FFFFFFFFFFFFFFFFFFFFFFFFFFFFFFFFFFFFFFFFFFFFFFFFFFFFFFFFFFFFFFFFFFFF
@A00155:342:HHGFNDSXY:1:2208:2790:4131 1:N:0:GAACCTAG+TCCGCATA
GCTCCACAGCTGTAAGGCTGTGGGCCCGAGAGAAGTCGCTCTCTCTCGCCCTCCACATCCCTCTCGGC
ATTGAGATGACCATAGGCCAGAGGTGAGTCCTTAAGTGGACACAGCTGATCTAAGGCGGTGTGGCGGG
+
FFFFFFFFFFFFFFFFFFFFFFFFFFFFFFFFFFFFFFFFFFFFFFFFFFFFFFFFFFFFFFFFFFFF
FFFFFF:FFFFFFFF:FFFFFFFFFFFFFFFFFFFFFFFFFFFFFFFFFFFFFFFFFFFFFFFFFFFF
@A00155:342:HHGFNDSXY:1:2208:19994:12132 1:N:0:GAACCTAG+TCCGCATA
AGAGAGAGCGACTTCTCTCGGGCCCACAGCCTTACAGCTGTGGAGCACGGTATCCTCTGCCGAAACAG
AGGTTGGACAAGACCGGAGGGGTCTCCTAGTTCCAAAGGAGATGTACTCCGGGCTTGTTCACGACCT
+
FFFFFF:FFFFFFFFFFFFFFFFFFFFFFFFFFFFFFFFFFFFFFFFFFFFFFFFFFFFFFFFFFFFF
FFFFFFFFFFFFFFFFFFFFFFFFFFFFFFFFFFFF:FFF:FFFFFFFFFFFFFFFFF:FFFFFFFF
@A00155:342:HHGFNDSXY:1:2208:18059:18333 1:N:0:GAACCTAG+TCCGCATA
ATGCCCCGCCACACCGCCTTAGATCAGCTGTGTCCACTTAAGGACTCACCTCTGGCCTATGGTCATCT
CAATGCCGAGAGGGATGTGGAGGGCGAGAGAGAGCGACTTCTCTCGGGCCCACAGCCTTACAGCTGTG
+
FFFFFFFFFFFFFFFFFF:FFFFFFFFFFFFFFFFFFF:FFFFFFFFFFFFFFFFFFFFFFFFFFFFF
FFFFFFFFFFFFFFFFF:FFFFFFFFFFFFFFFFFFFFF:FFFFFFFFFFFFFFFFFFFFFFFFFFFF
@A00155:342:HHGFNDSXY:1:2209:16134:4257 1:N:0:GAACCTAG+TCCGCATA
TCAATGCCGAGAGGGATGTGGAGGGCGAGAGAGAGCGACTTCTCTCGGGCCCACAGCCTTACAGCTGT
GGAGCACGGTATCCTCTGCCGAAACAGAGGTTGGACAAGACCGGAGGGGTCTCCTAGTTCCAAAGGAG
+
FFFFFFFFFFFFFFFFFFFFFFFFFFFFFFFFFFFFFFFFFFFFFFFFFFFFFFFFFFF:FFFFFFFF
FFFFFFFFF:FFFFFFFFFFFF:FFF:FF:FFFFFF:FFFFFFFFFF:FFFFFFF,:FFFFFFFFFFF
@A00155:342:HHGFNDSXY:1:2209:26151:6230 1:N:0:GAACCTAG+TCCGCATA
AGAGAGAGCGACTTCTCTCGGGCCCACAGCCTTACAGCTGTGGAGCACGGTATCCTCTGCCGAAACAG
AGGTTGGACAAGACCGGAGGGGTCTCCTGGTTCCAAAGGAGATGTACTCCGGGCTTGTTCACGACCTA
+

FFFFFFFFFFFFFFFFFFFFFFFFFFFFFFFFFFFFFFFFFFFFFFFFFFFFFFFFFFFFFFFFFFFF
FFFFFFFFFFFFFFFFFFFFFFFFFFFFFFFFFFFFFFFFFFFFFFFFFFFFFF:FFFFFFFFFFFFF
@A00155:342:HHGFNDSXY:1:2209:4996:7044 1:N:0:GAACCTAG+TCCGCATA
AGCGACTTCTCTCGGGCCCACAGCCTTACAGCTGTGGAGCACGGTATCCTCTGCCGAAACAGAGGTTG
GACAAGACCGGAGGGGTCTCCTAGTTCCAAAGGAGATGTACTCCGGGCTTGTTCACGACCTACC
+
FFFFFFFFFFFFFFFFFFFFFFFFFFFFFFFFFFFFFFFFFFFFFFFFFFFFFFFFFFFFFFFFFFFF
FFFFFFFFFFFFFFFFFFFFF:FFFFFFFFFFFFFFFFFFFFFFFFFFFFFFFFFFFFFFFFFF
@A00155:342:HHGFNDSXY:1:2209:28628:10989 1:N:0:GAACCTAG+TCCGCATA
CAATGCCGAGAGGGATGTGGAGGGCGAGAGAGAGCGACTTCTCTCGGGCCCACAGCCTTACAGCTGTG
GAGCACGGTATCCTCTGCCGAAACAGAGGTTGGACAAGACCGGAGGGGTCTCCTAGTTCCAAAG
+
FFFFFFFFFFFFFFFFFFFFFFFFFFFFFFFFFFFFFFFFFFFFFFFFFFFFFFFFFF:,FFFFFFFF
FFFFFFFFFFFFFFFFFFFFFFFFFFFFFFFFFFFFFFFFFFFFFFFFFFFFFFFFFFFFFFFF
@A00155:342:HHGFNDSXY:1:2209:14452:19069 1:N:0:GAACCTAG+TCCGCATA
CGCTCTCTCTCGCCCTCCACATCCCTCTCGGCATTGAGATGACCATAGGCCAGAGGTGAGTCCTTAAG
TGGACACAGCTGATCTAAGGCGGTGTGGTGGGGCATGGGTTTGAACCCCCATGACGGTCGGAGTCTAG
+
FFFFFFFFFFFFFFFFFFFFFFFFFFFFFFFFFFFFFFFFFFFFFFFFFFFFFFFFFFFFFFFFFFFF
FFFFFFFFFFFFFFFFFFFFFFFFFFFFFFFFFFFFFFFFFFFFFFFFFFFFFFFFFFFFFFFFFFFF
@A00155:342:HHGFNDSXY:1:2209:19443:19319 1:N:0:GAACCTAG+TCCGCATA
GGATGTGGAGGGCGAGAGAGAGCGACTTCTCTCGGGCCCACAGCCTTACAGCTGTGGAGCACGGTATC
CTCTGCCGAAACAGAGGTTGGACAAGACCGGAGGGGTCTCCTAGTTCCAAAGGAGATGTACTCC
+
FFF:FFFFFFFFFFFFFFFFFFFFFFFFFFFFFFFFFFFFFFFFFFFFFFFFFFFFFFFFFFFFFFFF
FFFFFFFFFFFFFFFFFFFFFFFFFFFFFFFFFFFFFFFFFFFFFFFFFFFFFFFFFFFFFFFF
@A00155:342:HHGFNDSXY:1:2209:32154:20635 1:N:0:GAACCTAG+TCCGCATA
GGATGTGGAGGGCGAGAGAGAGCGACTTCTCTCGGGCCCACAGCCTTACAGCTGTGGAGCACGGTATC
CTCTGCCGAAACAGAGGTTGGACAAGACCGGAGGGGTCTCCTAGTTCCAAAGGAGATGTACTCC
+
FF,FF:FFFFFFFFFFFFFFFFFFFFFFFFFFFFFFFFFFFFFFFFFFFFFFFFFFFFFFFFFFF:FF
FFFFFFFFFFFFFFFFFFFFFFFFFFFFFFFFFFFFFFFFFFFFFFFFFFFFF:FFFFFFFFFF
@A00155:342:HHGFNDSXY:1:2209:12020:23312 1:N:0:GAACCTAG+TCCGCATA
TGTCCACTTAAGGACTCACCTCTGGCCTATGGTCATCTCAATGCCGAGAGGGATGTGAAGGGCGAGAG
AGAGCGACTTCTCTCGGGCCCACAGCCTTACAGCTGTGGAGCACGGTATCCTCTGCCGAAACAGAGGT
+
FFFFFFFFFFFFFFFFFFFFFFFFFFFFFFFFFFFFFFFFFFFFFFFFFFFFFFFFFFFFFFFFFFFF
FFFFFFFFFFFFFFFFFFFFFFFFFFFFFFFFFFFFFFFFFFFFFFFFFFFFFFFFFFF:FFFFFFFF
@A00155:342:HHGFNDSXY:1:2210:23204:1564 1:N:0:GAACCTAG+TCCGCATA
ACCTCTGTTTCGGCAGAGGATACCGTGCTCCACAGCTGTAAGGCTGTGGGCCCGAGAGAAGTCGCTCT
CTCTCGCCCTCCACATCCCTCTCGGCATTGAGATGACCATAGGCCAGAGGAGAGTCCTTAAG
+
FFFFFFFFFFFFFFFFFFFFFFFFFFFFFFFFFFFFFFFFFFFFFFFFFFFFFFFFFFFFFFFFFFFF
FFFFFFFFFFFFFFFFFFFFFFFFFFF:FFFFFFFFFFFFFFFFFFFFFFFFFFFFFFFFFF
@A00155:342:HHGFNDSXY:1:2210:23466:7999 1:N:0:GAACCTAG+TCCGCATA
CTTCTCTCGGGCCCACAGCCTTACAGCTGTGGAGCACGGTATCCTCTGCCGAAACAGAGGTTGGACAA
GACCGGAGGGGTTTCCTAGTTCCAAAGGAGATGTACTCCGGGCTTGTTCACGACCTACCGTGTAAGTC
+
FFFFFFFFFFFFFFFFFFFFFFFFFFFFFFFFFFFFFFFFFFFFFFFFFFFFFFFFFFFFFFFFFFFF
FFFFFFFFFFFFFFFFFFFFFFFFFFFFFFFFFFFFFFFFFFFFF:FFFFFFFFFFFFFFFFFFFFFF
@A00155:342:HHGFNDSXY:1:2210:15582:23281 1:N:0:GAACCTAG+TCCGCATA
ACCTCTGTTTCGGCAGAGGATACCGTGCTCCACAGCTGTAAGGCTGTGGGCCCGAGAGAAGTCGCTCT
CTCTCGCCCTTCACATCCCTCTCGGCATTGAGATGACCATAGGCCAGAGGTGAGTCCTTAAGTGGACA
+

```
FFFFFFFFFFFFFFFFFFFFFFFFFFFFFFFFFFFFFFFFFFFFFFFFFFFFFFFFFFFFFFFFFFFF
FFFFFFFFFFFF:FFFFFFFFFFFFFFFFFFFFFFFFFFFFFFFFFFFFFFFFFFFFFFFFFFFFFFF
@A00155:342:HHGFNDSXY:1:2210:32027:28401 1:N:0:GAACCTAG+TCCGCATA
CGACTTCTCTCGGGCCCACAGCCTTACAGCTGTGGAGCACGGTATCCTCTGCCGAAACAGAGGTTGGA
CAAGACCGGAGGGGTCTCCTAGTTCCAAAGGAGATGTACTCCGGGCTTGTTCACGACCTACCGTGTAA
+
FFFFFFFFFFFFFFFFFFFFFFFFFFFFFFFFFFFFFFFFFFFFFFFFFFFFFFFFFFFFFFFFFFFF
FFFFFFFFFFFFFFFFFFFF,FFFFFFFFFFFFFFFFFFFFFFFFFFFFFFFFFFFFFFFFFFFFFFF
@A00155:342:HHGFNDSXY:1:2211:30165:2628 1:N:0:GAACCTAG+TCCGCATA
GTCCACTTAAGGACTCACCTCTGGCCTATGGTCATCTCAATGCCGAGAGGGATGTGGAGGGCGAGAGA
GAGCGACTTCTCTCGGGCCCACAGCCTTACAGCTGTGGAGCACGGTATCCTCTGCCGAAACAGAGGTT
+
FFFFFFFFFFFFFFFFFFFFFFFFFFFFFFFFFFFFFFFFFFFFFFF:FFFFF:F:FFFFFFFFFFFF
FFFFFFFFFFFFFFFFFFFFFFFFFFFFFFFFFFFFFFFFFFFFFFFFFFFFFFFFFFFFFFFFFFFF
@A00155:342:HHGFNDSXY:1:2211:16568:28432 1:N:0:GAACCTAG+TCCGCATA
CTTCTCTCGGGCCCACAGCCTTACAGCTGTGGAGCACGGTATCCTCTGCCGAAACAGAGGTTGGACAA
GACCGGAGGGGTCTCCTAGTTCCAAAGGAGATGTACTCCGGACTTGTTCACGACCTACCGTGTAAGTC
+
FFFFFFFFFFFFFFFFFFFFFF:FFFFFFFFFFFFFFFFFFFFFFFFFFFFFFFFFFFFFFFFFFFFF
FFFFFFFFFFFFFFFFFFFFFFFFFFFFFFFFFFFFFFFFFFFFFFFFFFFFFFFFFFF,FFFFF,F
@A00155:342:HHGFNDSXY:1:2211:16975:32017 1:N:0:GAACCTAG+TCCGCATA
GAGAGGGATGTGGAGGGCGAGAGAGAGCGACTTCTCTCGGGCCCACAGCCTTACAGCTGTGGAGCACG
GTATCCTCTGCCGAAACAGAGGTTGGACAAGACCGGAAGGGTCTCCTAGTTCCAAAGGAGATGTACTC
+
FFFFFFFFFFFFFFFFFFFFFFFFFFFFFFFFFFFFFFFFFFFFFFFFFFFFF,FFFFFFFFFFFFFF
FFFFFFFFFFFFFFFFFFFFFFFFFFFFFFFFFFFFFFFFFFFFFF,FFFFFFFFFFFFFFFFFFFFFF
@A00155:342:HHGFNDSXY:1:2212:19687:22153 1:N:0:GAACCTAG+TCCGCATA
CCTTACAGCTGTGGAGCACGGTATCCTCTGCCGAAACAGAGGTTGGACAAGACCGGAGGGGTCTCCTG
GTTCCAAAGGAGATGTACTCCGGGCTTGTTCACGACCTACCGTGTAAGTCGTAGTCTAGTAGGCTACC
+
FFFFFFFFFFFFFFFFFFFFFFFFFFFFFFFFFFFFFFFFFFFFFFFFFFFFFFFFFFFFFFFFFFFF
FFFFFFFFFFFFFFFFFFFFFFFFFFFFFFFFFFFFFFFFFFFFFFFFFFFFFFFFFFFFFFFFFFFF
@A00155:342:HHGFNDSXY:1:2212:18231:30499 1:N:0:GAACCTAG+TCCGCATA
TTACACGGTAGGTCGTGAACAAGCCCGGAGTACATCTCCTTTGGAACTAGGAGACCCCTCCGGTCTTG
TCCAACCTCTGTTTCGGCAGAGGATACCGTGCTCCACAGCTGTAAGGCTGTGGGCCCGAGAGAAGTCG
+
FFFFFFFFFFFFFFFFFFFFFFFFFFFFFFFFFFFFFFFFFFFFFFFFFFFFFFFFFFFFFFFFFFFF
FFFFFFFFF:FFFFFFFFFFFFFFFFFFFFFFFFFFFFFFFFFFFFFFFFFFFFFFFFFFFFFFFFFF
@A00155:342:HHGFNDSXY:1:2212:16107:34679 1:N:0:GAACCTAG+TCCGCATA
GAGCGACTTCTCTCGGGCCCACAGCCTTACAGCTGTGGAGCACGGTATCCTCTGCCGAAACAGAGGTT
GGACAAGACCGGAGGGGTCTCCTAGTTCCAAAGGAGATGTACTCCGGGCTTGTTCACGACCTACCG
+
FFFFFFFFFFFFFFFFFFFFFFFFFFFFFFFFFFFFFFFFFFFFFFFFFFFFFFFFFFFFFFF:FFFF
FFFFFFFFFFFFFFFFFFF:F:FFFFFFFFFFFFFFFFFFFFFFFFFFFF:FFFFFFFFFFF::FF
@A00155:342:HHGFNDSXY:1:2213:20066:12414 1:N:0:GAACCTAG+TCCGCATA
GACTTCTCTCGGGCCCACAGCCTTACAGCTGTGGAGCACGGTATCCTCTGCCGAAACAGAGGTTGGAC
AAGACCGGAGGGGTCTCCTAGTTCCAAAGGAGATGTACTCCGGGCTTGTTCACGACCTACCGTGTAAG
+
FFFFFFFFFFFFFFFFFFFFFFFFFFFFFFFFFFFFFFFFFFFFFFFFFFFFFFFFFFFFFFFFFFFF
FF:FFFFFFFFFF:FFFFFFFFFFFFFFFFFFFFFFFFFFFFFFFFFFFFFFFFFFFFFFFFFFFFFF
@A00155:342:HHGFNDSXY:1:2214:11116:25441 1:N:0:GAACCTAG+TCCGCATA
ATCTCAATGCCGAGAGGGATGTAGAGGGCGAGAGAGAGCGACTTCTCTCGGGCCCACAGCCTTACAGC
TGTGGAGCACGGTATCCTCTGCCGAAACAGAGGTTGGACAAGACCGGAGGGGTCTCCTAGTTCCAAAG
+
```

```
FFFFFFFFFFFFFFFFFFFFFFFFFFF:FFFFFFFFFFFFFFFFFFFFFFFFFFFFFFFFFFFFFFFF
FFFFFFFFFFFFFFFF,FFFFFFFFF:FFFFFFFFFFFFFFFFFFFFFFFFFFFFFFFFFFFFFFFFF
@A00155:342:HHGFNDSXY:1:2214:15157:25457 1:N:0:GAACCTAG+TCCGCATA
AGATCAGCTGTGTCCACTTAAGGACTCACCTCTGGCCTATGGTCATCTCAATGCCGAGAGGGATGTGA
AGGGCGAGAGAGAGCGACTTCTCTCGGGCCCACAGCCTTACAGCTGTGGAGCACGGTATCCTCTGCCG
+
FFFFFFFFFFFFFFFFFFFFFFFFFFFFFFFFFFFFFFFFFFFFFFFFFFFFFFFFFFFFFFF,FFFF
FFFFFFFFFFFFFFFFFFFFFFFFFFFFFFFFFFFFFFFFFFFFFFFFFFFFFFFFF,FFFFFFFFFF
@A00155:342:HHGFNDSXY:1:2214:27236:25676 1:N:0:GAACCTAG+TCCGCATA
CACAGCCTTACAGCTGTGGAGCACGGTATCCTCTGCCGAAACAGAGGTTGGACAAGACCGGAGGGGTC
TCCTAGTTCCAAAGGAGATGTACTCCGGGCTTGTTCACGACCTACCGTGTAAGTCGTAGTCTAGTAGG
+
FFFFFFF:FFFFFFFFFFFFFFFFFFFFFFFFFFFFFFFFFFFFFFFFFFFFFFFFFFFFFFFFFFFF
FFFFFFFFFFFFFF:FFFFFFF:FFFFFFFFFFFFFFFFFFFFFFFFFFFFFFFFFFFFFFF:FFFFF
@A00155:342:HHGFNDSXY:1:2214:2067:30499 1:N:0:GAACCTAG+TCCGCATA
GGACTCACCTCTGGCCTATGGTCATCTCAATGCCGAGAGGGATGTGGAGGGCGAGAGAGAGCGACTTC
TCTCGGGCCCACAGCCTTACAGCTGTGGAGCACGGTATCCTCTGCCGAAACAGAGGTTGGACAAGACC
+
FFFFFFFFFFFFFFFFFFFFFFFFFFFFFFFFFFFFFFFFFFFFFFFFFFFFFFFFFFFFFFFFFFFF
FFFFFFFFFFFFFFFFFFFFFFFFFFFFFFFFFFFFFFFFFFFFFFFFFFFFFFFFFFFFFFFFFFFF
@A00155:342:HHGFNDSXY:1:2215:25807:3724 1:N:0:GAACCTAG+TCCGCATA
ACCGTGCTCCACAGCTGTAAGGCTGTGGGCCCGAGAGAAGTCGCTCTCTCTCGCCCTCCACATCCCTC
TCGGCATTGAGATGACCATAGGCCAGAGGTGAGTCCTTAAGTGGACACAGCTGATCTAAGGCGGTGTG
+
FFFFFFFFFFFFFFFFFFFFF:FF,FFFFF,FFFFFFFFFFFFFFFFFFFF:FF:FF:F:FF:FFFFF
FF:FFF:FFFFFFFFFFF:FF,FFFFFFFFFFFFFFFFFFF,F,FFFFFFFFFFFFFFFFF:FFFF,F
@A00155:342:HHGFNDSXY:1:2215:5656:10974 1:N:0:GAACCTAG+TCCGCATA
ACCTCTGTTTCGGCAGAGGATACCGTGCTCCACAGCTGTAAGGCTGTGGGCCCGAGAGAAGTCGCTCT
CTCTCGCCCTTCACATCCCTCTCGGCATTGAGATGACCATAGGCCAGAGGTGAGTCCTTAAGTGGACA
+
FFFFFFFFFFFFFFFFFFFFFFFFFFFFFFFFFFFFFFFFF,FFFFFFFFFFFFFFFFFFFFFFFFFF
FFFFFFFFFFFFFFFFFFFFFFFFFFFFFFFFFFFFFFFFFFFFFFFF,FFFFFFFF:FFF:FFF:,F
@A00155:342:HHGFNDSXY:1:2215:5710:21308 1:N:0:GAACCTAG+TCCGCATA
GGATGTGGAGGGCGAGAGAGAGCGACTTCTCTCGGGCCCACAGCCTTACAGCTGTGGAGCACGGTATC
CTCTGCCGAAACAGAGGTTGGACAAGACCGGAGGGGTCTCCTAGTTCCAAAGGAGATGTACTCC
+
FFFFFFFFFFFFFFFFFFFFFFFFFFFFFFFFFFFFFFFFFFFFFFFFFFFFFFFFFFFFFFFFFFFF
FFFFFFFFFFFFFFFFFFFFFFFFFFFFFFFF:FFFFFFFFFF:FFF,FFFFFFFFFFFFFFFF
@A00155:342:HHGFNDSXY:1:2215:5186:27696 1:N:0:GAACCTAG+TCCGCATA
TCGGCAGAGGATACCGTGCTCCACAGCTGTAAGGCTGTGGGCCCGAGAGAAGTCGCTCTCTCTCGCCC
TTCACATCCCTCTCGGCATTGAGATGACCATAGGCCAGAGGTGAGTCCTTAAGTGGACACA
+
FFFFFFF,:FFFFF:FFFFFFF:FFF,FFFFFFFFFF:FFFFFFF,FFFF:FFFFFFFFFFFFFFFF,
FFFFFFFFFFFFFFFFF,FFFFFFFFFFFFFFF:FFFFFFFFFFFF:FFFFFFFFFFFFFF
@A00155:342:HHGFNDSXY:1:2215:16315:34851 1:N:0:GAACCTAG+TCCGCATA
GGATGTGGAGGGCGAGAGAGAGCGACTTCTCTCGGGCCCACAGCCTTACAGCTGTGGAGCACGGTATC
CTCTGCCGAAACAGAGGTTGGACAAGACCGGAGGGGTCTCCTAGTTCCAAAGGAGATGTACTCC
+
FFFFFFFFFFFFFFFFFFFFFFFFFFFFFFFFFFFFFFFFFFFFFFFFFFFF:FFFFFFFFFFFFFFF
FFFFFFFFFFFFFFFFF:FFFFFFFFFFFFFFFFFFFFFFFFFFFFFFFFFFFFFFFFFFFFFF
@A00155:342:HHGFNDSXY:1:2216:20066:3082 1:N:0:GAACCTAG+TCCGCATA
CTTCTCTCGGGCCCACAGCCTTACAGCTGTGGAGCACGGTATCCTCTGCCGAAACAGAGGTTGGACAA
GACCGGAGGGGTCTCCTAGTTCCAAAGGAGATGTACTCCGGGCTTGTTCACGACCTACCGTGTAAGTC
+
```

```
FFFFFFFFFFFFFFFFFFFFFFFFFFFFFFFFFFFFFFFFFFFFFFFFFFFFFFFFFFFFFFFFFFFF
FFFFFFFFFFFFFFFFFFFFFFFFFFFFFFFFFFFFFFFFFFFFFFFFFFFFFFFFFFFFFFFF:FFF
@A00155:342:HHGFNDSXY:1:2216:9263:7795 1:N:0:GAACCTAG+TCCGCATA
CTTCTCTCGGGCCCACAGCCTTACAGCTGTGGAGCACGGTATCCTCTGCCGAAACAGAGGTTGGACAA
GACCGGTGGGGTCTCCTAGTTCCAAAGGAGATGTACTCCGGGCTTGTTCACGACCTACCGTGTAAGTC
+
FFFFFFFFFFFFFFFFFFFFFFFFFFFFFFFFFFFFFFFFFFFFFFFFFFFFFFFFFFFFFFFFFFFF
FFFFFFFFFFFFFFFFFFFFFFFFFFFFFFFFFFFFFFFFFFFFFFFFFFFFFFFFFFFFFFFFFFFF
@A00155:342:HHGFNDSXY:1:2216:32072:28541 1:N:0:GAACCTAG+TCCGCATA
ACCTCTGTTTCGGCAGAGGATACCGTGCTCCACAGCTGTAAGGCTGTGGGCCCGAGAGAAGTCGCTCT
CTCTCGCCCTCCACATCCCTCTCGGCATTGAGATGACCATAGGCCAGAGGAGAGTCCTTAAG
+
FFFFFFFFFFFFFFFFFFFFFFFFFFFFFFFFFFFFFFFFFFFFFFFFFFFFFFFFFFFFFFFFFFFF
FFFFFFFFFFFFFFFFFFFFFFFFFFF:FFFFFFFFFFFFFFFFFFFFFFFFFFFFFFFFFF
@A00155:342:HHGFNDSXY:1:2217:1542:6042 1:N:0:GAACCTAG+TCCGCATA
ACCTCTGTTTCGGCAGAGGATACCGTGCTCCACAGCTGTAAGGCTGTGGGCCCGAGAGAAGTCGCTCT
CTCTCGCCCTCCACATCCCTCTCGGCATTGAGATGACCATAGGCCAGAGGTGAGTCCTTAAGTGGACA
+
F:FFFFFFFFFFFFFFFFFFFFFFFFFFFFFF,FFFFFFFFFF,FF,FFFFFFFFFFFF,FFF:FFFF
FFFFF:FFF:FFF:FFF,F,FFFFFF:,FFFFFF:FF:FFFFF:FFF:FFFFFFFF:FFFF,,,F,FF
@A00155:342:HHGFNDSXY:1:2217:7220:13870 1:N:0:GAACCTAG+TCCGCATA
CTCTGGCCTATGGTCATCTCAATGCCGAGAGGGATGTGGAGGGCGAGAGAGAGCGACTTCTCTCGGGC
CCACAGCCTTACAGCTGTGGAGCACGGTATCCTCTGCCGAAACAGAGGTTGGACAAGACCGGAGGGGT
+
FFF:FFFFF,FFFFFFFFFFFFFFFFFFFFFFFF:FFFFFFFFFFFFFFFFFFFFFFFFFFFFFFFFF
FFFFFF:FFFFFFFFFFFFFFFFFFFFFFFFFFFFFFFFFFFFFFFFFFFFFFFFFFFFFFFFFF:FF
@A00155:342:HHGFNDSXY:1:2217:12988:13996 1:N:0:GAACCTAG+TCCGCATA
GGATGTGGAGGGCGAGAGAGAGCGACTTCTCTCGGGCCCACAGCCTTACAGCTGTGGAGCACGGTATC
CTCTGCCGAAACAGAGGTTGGACAAGACCGGAGGGGTCTCCTAGGTCCAAAGGAGATGTACTCC
+
FFFFFFFFFFFFFFFFFFFFFFFFFFFFFFFFFFFFFFFFFFFFFFFFFFFFFFFFFFFFFFFFFFFF
FFFFFFFFFFFFFFFFFFFFFFFFF:FFFFFFFFFFFFFFFFFFFFFFFFFFFFFFFFFF:FFFF
@A00155:342:HHGFNDSXY:1:2217:7401:15436 1:N:0:GAACCTAG+TCCGCATA
GTATCCTCTGCCGAAACAGAGGTTGGACAAGACCGGAGGGGTCTCCTAGTTCCAAAGGAGATGTACTC
CGGGCTTGTTCACGACCTACCGTGTAAGTCGTAGTCTAGTAGGCTACCTGACGAGTCCTTTTTAGGAC
+
FF:FFFFFFFFFFFFFFFFFFFFFFFFFFF:FFFFFFFFFFFFFFFFFFFFFFFFFFFFFFFFFFFFF
FFFFFFFFFFFFFFFFFFFFFFFFFFFFFFFFFFFFFFFFFFFFFFFF,FFFFFFFFFFFFFFFFF:FF
@A00155:342:HHGFNDSXY:1:2217:30481:20619 1:N:0:GAACCTAG+TCCGCATA
TCCTCTGCCGAAACAGAGGTTGGACAAGACCGGAGGGGTCTCCTAGTTCCAAAGGAGATGTACTCCGG
GCTTGTTCACGACCTACCGTGTAAGTCGTAGTCTAGTAGGCTACCTGACGAGTCCTTTTTAGGACGAA
+
FFFFFFFFFFFFFFFFFFFFFFFFFFFFFFFFFFFFFFFFFFFFFFFFFFFFFFFFFFFFFFFFFFFF
F:FFFFFFFFFFFFFFFFFFFFFFFFFFFFFFFFFFFFFFF:FFFFFFFFFFFFFFFFF:FFFFFFFFFF
@A00155:342:HHGFNDSXY:1:2217:27651:21261 1:N:0:GAACCTAG+TCCGCATA
GAGAGAGCGACTTCTCTCGGGCCCACAGCCTTACAGCTGTGGAGCACGGTATCCTCTGCCGAAACAGA
GGTTGGACAAGACCGGAGGGGTCTCCTAGTTCCAAAGGAGATGTACTCCGGGCTTGTTC
+
FFFFFFFFFFFFFFFFFFFFFFFF:FFFFFFFFFFFFFFFFFFFFFFFFFFFFFFFFFFFFFFFFFFF
FFFFFFFFFFFFFFFFFFFFFFFFFFFFFFFFFFFFFFFFFFFFFFFFFFFFFFFFFFFFF
@A00155:342:HHGFNDSXY:1:2217:10285:28040 1:N:0:GAACCTAG+TCCGCATA
TTCTCTCGGGCCCACAGCCTTACAGCTGTGGAGCACGGTATCCTCTGCCGAAACAGAGGTTGGACAAG
ACCGGAGGGGTCTCCTAGTTCCAAAGGAGATGTACTCCGGGCTTGTTCACGACCTACCGTGTAAGTCG
+
```

```
FFFFFFFFFFFFFFFFFFFFFFFFFFFFFFFFFFFFFFFFFFFFFFFFFFFFFFFFFFFFFFFFFFFF
FFFFFFFFFFFFFFF:FFFFFFFFFFFFFFFFFFFFFFFFFFFFFFFFFFFFFFFFFFFFFFFFFFFF
@A00155:342:HHGFNDSXY:1:2217:20383:33912 1:N:0:GAACCTAG+TCCGCATA
GAGAGAGCGACTTCTCTCGGGCCCACAGCCTTACAGCTGTGGAGCACGGTATCCTCTGCCGAAACAGA
GGTTGGACAAGACCGGAGGGGTCTCCTAGTTCCAAAGGAGATGTACTCCGGGCTTGTTCACGACCTAC
+
FFFFFFFFFFFFFFFFFFFFFFFFFFFFFFFFFFFFFFFFFFFFFFFFFFFFFFFFFFFFFFFFFFFF
FFFFFFFFFFFFFFFFFFFFFFFFFFFFFFFFFFFFFFFFFFFFFFFFFFFFFFFFFFFFFFFFFFFF
@A00155:342:HHGFNDSXY:1:2217:26729:35196 1:N:0:GAACCTAG+TCCGCATA
TCAATGCCGAGAGGGATGTAGAGGGCGAGAGAGAGCGACTTCTCTCGGGCCCACAGCCTTACAGCTGT
GGAGCACGGTATCCTCTGCCGAAACAGAGGTTGGACAAGACCGGAGGGGTCTCCTAGTTCCAAAGGAG
+
FFFFFFFFFFFFFFFFFFFFFFFFFFFFFFFFFFFFFFFFFFFFFFFFFFFFFFFFFF:FFFFFFFFF
FFFFFFFFFFFFFFFFFFFFFFFFFFFFFFFFFFFFFFFFFFFFFFFFFFFFFFFFFFFFFFFFFFFF
@A00155:342:HHGFNDSXY:1:2218:24252:9737 1:N:0:GAACCTAG+TCCGCATA
ATCTCAATGCCGAGAGGGATGTAGAGGGCGAGAGAGAGCGACTTCTCTCGGGCCCACAGCCTTACAGC
TGTGGAGCACGGTATCCTCTGCCGAAACAGAGGTTGGACAAGACCGGAGGGGTCTCCTAGTTCCAAAG
+
FFFFFFFFFFFFFFFFFFFFFFFFFFFFFFFFFFFFFFFFFFFFFFFFFFFFFFFFFFFFFFFFFFFF
FFFFFFFFFFFFF:FFFFFFFFFFFFFFFFFFFFFFFFFFFFFFFFFFFFFFFFFFFFFFFFFFFFFF
@A00155:342:HHGFNDSXY:1:2218:4209:13291 1:N:0:GAACCTAG+TCCGCATA
CTCAATGCCGAGAGGGATGTGGAGGGCGAGAGAGAGCGACTTCTCTCGGGCCCACAGCCTTACAGCTG
TGGAGCACGGTATCCTCTGCCGAAACAGAGGTTGGACAAGACCGGAGGGGTCTCCTAGTTCCAAAGGA
+
FFFFFFFFFFF,:FF::,F,FFFFF:FFFFFFFFFFFFFFFFF,FFFFFFFF,FFFFFF::FFF:FFF
,FFFFFF:FFFF,FFFFFF:F:F:F:FFFFFFFFFFFFFFFFFFFFFFFFFF:FFF:FFFFFFFFF,F
@A00155:342:HHGFNDSXY:1:2218:8260:16391 1:N:0:GAACCTAG+TCCGCATA
AAGGACTCACCTCTGGCCCATGGTCATCTCAATGCCGAGAGGGATGTGGAGGGCGAGAGAGAGCGACT
TCTCTCGGGCCCACAGCCTTACAGCTGTGGAGCACGGTATCCTCTGCCGAAACAGAGGTTGGACAAGA
+
FFFFFFFFFFFFF,FF:FFFFFFFFFFFFFFFFFFFFFFFFFFFFF:FFFFFFFFFF:FFFFFFFFFF
:FFFFFFFFFFFFFFFFF:F:FFFFFFFFFFFF,FFF:FFFFFFFFFFFFF:FFFFFFFF:FFFF:FF
@A00155:342:HHGFNDSXY:1:2219:17481:3239 1:N:0:GAACCTAG+TCCGCATA
GGGCGAGAGAGAGCGACTTCTCTCGGGCCCACAGCCTTACAGCTGTGGAGCACGGTATCCTCTGCCGA
AACAGAGGTTGGACAAGACCGGAGGGGTCTCCTAGTTCCAAAGGAGATGTACTCCGGGCTTGTTCACG
+
FFFFFF:F:FFFFF:FFF,F,,FFFF,FFFFFFFF:FFF:FFFFFFFFFFFF:FFFFFFFFFF::FFF
FFFFFF:,FFFFFFFFFF:FFFFFF:F,FFFFFFFFFFFFFFFFFFFFFFFFFFF,:FFFFFFFFFF:
@A00155:342:HHGFNDSXY:1:2219:23972:23844 1:N:0:GAACCTAG+TCCGCATA
GGATGTGGAGGGCGAGAGAGAGCGACTTCTCTCGGGCCCACAGCCTTACAGCTGTGGAGCACGGTATC
CTCTGCCGAAACAGAGGTTGGACAAGACCGGAGGGGTCTCCTAGTTCCAAAGGAGATGTACTCC
+
FFFFFFFFFFFFFFFFFFFFFFFFFFFFFFFFFFFFFFFFFFFFFFFFFFFFFFFFFFFFFFFFFFFF
FFFFFFFFFFFFFFFFFFFFFFFFFFFFFFFFFF:FFFFFFFFFFFFFFFFFFFFFFFFFFFFFFF
@A00155:342:HHGFNDSXY:1:2219:4309:26960 1:N:0:GAACCTAG+TCCGCATA
ACCTACCGTGTAAGTCGTAGTCTAGTAGGCTACCTGACGAGTCCTTTTTAGGACGAAACTTACACAAC
TCGAAGAGTAACGACGGTTTCGTCCTTGGAACGGACTCATCAGGGAGCCTACTAGACTCCGACCGTCA
+
FFFFFFFFFFFFFFFFFFF:F:FFFFFFFFFFFFFFFFFF:FFFFFFFFFF:F,FFFF:FFFFFFFFF
:FFFFFF:FFFFFFFFFFF:F:FFFFFFFF:,FFF,FFFFFFFFFF:F,FFFFFFFFFF:FFFF:FFF
@A00155:342:HHGFNDSXY:1:2219:4689:33943 1:N:0:GAACCTAG+TCCGCATA
GATGTGGAGGGCGAGAGAGAGCGACTTCTCTCGGGCCCACAGCCTTACAACTGTGGAGCACGGTATCC
TCTGCCGAAACAGAGGTTGGACAAGACCGGAGGGGTCTCCTAGTTCCAAAGGAGATGTACTCCGGGCT
+
```

```
FFFFFFFFFFFFFFFFFFFFFFFFFFFFFFFFFFFFFFFFFFFFFFFFFFFFFFFFFFFFFFFFFFFF
FFFFFFFFFFFFFFFFFFFFFFFFFFFFFFFFFFFFFFFFFFFFFFFFFFFFFFFFFFFFFFFFFFFF
@A00155:342:HHGFNDSXY:1:2220:21441:4085 1:N:0:GAACCTAG+TCCGCATA
AAAGACTCACCTCTGGCCTATGGTCATCTCAATGCCGAGAGGGATGTGGAGGGCGAGAGAGAGCGACT
TCTCTCGGGCCCACAGCCTTACAGCTGTGGAGCACGGTATCCTCTGCCGAAACAGAGGTTGGAC
+
FFFFFFFFFFFFFFFFFFFFFFF:FFFFFFFFFFFFFFFFFFFFFFFFFFFFFFFFFFFFFFFFFFFF
FFFFFFFFFFFFFFFFFFFFFFFFFFFFFFFFFFFFFFFFFFFFFFFFFFFFFFFFFFFFFFFF
@A00155:342:HHGFNDSXY:1:2220:1542:26271 1:N:0:GAACCTAG+TCCGCATA
ATGCCGAGAGGGATGTGGAGGGCGAGAGAGAGCGACTTCTCTCGGGCCCACAGCCTTACAGCTGTGGA
GCACGGTATCCTCTGCCGAAACAGAGGTTGGACAAGACCGGAGGGGTCTCCTAGTTCCAAAGGAGATG
+
FFFFFFFFFFFFFFFFFFFFFFFFFFFFFFFFFFFFFFFFFFFFFFFFFFFFFFFFFFFFFFFFFFFF
FFFFFFFFFFFFFFFFFFFFFFFFFFFFFFFFFFFFFFFFFFFFFFFFFFFFFFFFFFFFFFFFFFFF
@A00155:342:HHGFNDSXY:1:2220:20482:29982 1:N:0:GAACCTAG+TCCGCATA
GAGCGACTTCTCTCGGGCCCACAGCCTTACAGCTGTGGAGCACGGTATCCTCTGCCGAAACAGAGGTT
GGACAAGACCGGAGGGGTCTCCTGGTTCCAAAGGAGATGTACTCCGGGCTTGTTCACGACCTACCGTG
+
FFFFFFFFFFFFFFFFFFFFFFFFFFFFFFFFFFFFFFFFFFFFFFFFFFFFFFFFFFFFFFFFFFFF
FFFFFFFFFFFFFFFFFFFFFFFFFFFFFFFFFFFFFFFFFFFFFFFFFFFFFFFFFFFFFFFFFFFF
@A00155:342:HHGFNDSXY:1:2220:16532:36636 1:N:0:GAACCTAG+TCCGCATA
ATGCCGAGAGGGATGTGGAGGGCGAGAGAGAGCGACTTCTCTCGGGCCCACAGCCTTACAGCTGTGGA
GCACGGTATCCTCTGCCGAAACAGAGGTTGGACAAGACCGGAGGGGTCTCCTGGTTCCAAAGGAGATG
+
FFFFFFFFFFFFFFFF:FFFFFFFFFFFFFFFFFFFFFFFFFFFFFFFFFFFFFFFFFFFFFFFFFFF
FFFFFFFFFFFFFFFFFFFFFFFFFFFFFFFFFFFFFFFFFFFFFFFFFFFFFFFFFFFFFFFFFFFF
@A00155:342:HHGFNDSXY:1:2221:12852:3082 1:N:0:GAACCTAG+TCCGCATA
CTTCTCTCGGGCCCACAGCCTTACAGCTGTGGAGCACGGTATCCTCTGCCGAAACAGAGGTTGGACAA
GACCGGAGGGGTCTCCTGGTTCCAAAGGAGATGTACTCCGGGCTTGTTCACGACCTACCGTGTAAGTC
+
FFFFFFFFF:FFFFFFFF:FF:FFFFFFFFFFFFFF:FFFFFFFFFF:FFFFFFFFFFFFFFFFFFFF
FFF:,FFF,:FFFFFFF:F:FFFFFF:FFFFFF,FFFF::F:FFFFFFFF:F:FFF:,FF,FFF,FFF
@A00155:342:HHGFNDSXY:1:2221:18602:7999 1:N:0:GAACCTAG+TCCGCATA
ATGCCGAGAGGGATGTAGAGGGCGAGAGAGAGCGACTTCTCTCGGGCCCACAGCCTTACAGCTGTGGA
GCACGGTATCCTCTGCCGAAACAGAGGTTGGACAAGACCGGAGGGGTCTCCTAGTTCCAAAGGAGATG
+
FFFFFFFFFFFFFFFFFFFFFFFFFFFFFFFFFFFFFFFFFFFFFFFFFFFFFFFFFFFFFFFFFFFF
FFFFFFFFFFFFFFFFFFFFFFFFFFFFFFFFFFFFFFFFFFFFFFFFFFFFFFFFFFFFFFFFFFFF
@A00155:342:HHGFNDSXY:1:2221:4435:9518 1:N:0:GAACCTAG+TCCGCATA
GAGAGAGCGACTTCTCTCGGGCCCACAGCCTTACAGCTGTGGAGCACGGTATCCTCTGCCGAAACAGA
GGTTGGACAAGACCGGAGGGGTCTCCTGGTTCCAAAGGAGATGTACTCCGGGCTTGTTCA
+
FFFFFFFFFFFFFFFFFFFFFFFFFFFFFFFFFFFFFFFFFFFFFFFFFFFFFFFFFFFFFFFFFFFF
FFFFFFFFFFF:FFFFFFFFFFFFFFFFFFFF:FFFFFFFFFFFFFFFFFFFFFFFFFFF
@A00155:342:HHGFNDSXY:1:2221:30011:15045 1:N:0:GAACCTAG+TCCGCATA
GAGGGCGAGAGAGAGCGACTTCTCTCGGGCCCACAGCCTTACAGCTGTGGAGCACGGTATCCTCTGCC
GAAACAGAGGTTGGACAAGACCGGAGGGGTCTCCTAGTTCCAAAGGAGATGTACTCCGGGCTTGTTCA
+
FFFFFFFFFFFFFFFFFFFFFFFFFFFFFFFFFFFFFFFFFFFFFFFFFFFFFFFFFFFFFFFFFFFF
FFFF:F:FFFFFFFFFFFFFFFFFFFFFFFFFFFFFFFFFFFFFF:FFFFFFFFFFFFFFFFFFFFFF
@A00155:342:HHGFNDSXY:1:2221:28962:16016 1:N:0:GAACCTAG+TCCGCATA
GAGGGCGAGAGAGAGCGACTTCTCTCGGGCCCACAGCCTTACAGCTGTGGAGCACGGTATCCTCTGCC
GAAACAGAGGTTGGACAAGACCGGAGGGGTCTCCTAGTTCCAAAGGAGATGTACTCCGGGCTTGTTCA
+
```

FFFFFFFFFFFFFFFFFFFFFFFFFFFFFFFFFFFFFFFFFFFFFFFFFFFFFFFFFFFFFFFFFFFF
FFFFFFFFFFFFFFFFFFF:FFFFFFFFFFFFFFFFFFFFFFFFFFFFFFFFFFFFFFFFFFFFFFFF
@A00155:342:HHGFNDSXY:1:2222:17635:15186 1:N:0:GAACCTAG+TCCGCATA
AAGGACTCTCCTCTGGCCTATGGTCATCTCAATGCCGAGAGGGATGTGGAGGGCGAGAGAGAGCGACT
TCTCTCGGGCCCACAGCCTTACAGCTGTGGAGCACGGTATCCTCTGCCGAAACAGAGGTTGGACAAGA
+
FFFFFFFFFFFFFFFFFFFFFFFFFFFFFFFFFFFFFFFFFFFFFFFFFFFFFFFFFFFFFFFFFFFF
FFFFFFFFFFFFFFFFFFFFFFFFFFFFFFFFFFFFFFFFFFFFFFFFFFFFFFFFFFFFFFFFF:FF
@A00155:342:HHGFNDSXY:1:2222:20980:21668 1:N:0:GAACCTAG+TCCGCATA
GCGAGAGAGAGCGACTTCTCTCGGGCCCACAGCCTTACAGCTGTGGAGCACGGTATCCTCTGCCGAAA
CAGAGGTTGGACAAGACCGGAGGGGTCTCCTGGTTCCAAAGGAGATGTACTCCGGGCTTGTTCACGAC
+
FFFFFFFFFFFFFFFFFFFFFFFFFFFFFFFFFFFFFFFFFFFFFFFFFFFFFFFFFFFFFFFFFFFF
FFFFFFFFFFFFFFFFFFFFFFFFFFFFFFFFFFFFFFFFFFFFFFFFFFFFFFFFFFFFFFFFF:FF
@A00155:342:HHGFNDSXY:1:2222:7600:28119 1:N:0:GAACCTAG+TCCGCATA
GGATGTGGAGGGCGAGAGAGAGCGACTTCTCTCGGGCCCACAGCCTTACAGCTGTGGAGCACGGTATC
CTCTGCCGAAACAGAGGTTGGACAAGACCGGAGGGGTCTCCTGGTTCCAAAGGAGATGTACTCC
+
FFFFFFFFFFFFF:FFFFFFFFFFFFFF:FFFF:FFFFFFFFFFFFFFFFFFFFFFFFFFFFFFFFFF
FFFFFFFFFFF:FFFFFFFFFFFFFFFFF:FFFFFFFFFFFFFFFFFFFFFFFFFFFFFFFFFF
@A00155:342:HHGFNDSXY:1:2222:19180:30608 1:N:0:GAACCTAG+TCCGCATA
AAGGACTCTCCTCTGGCCTATGGTCATCTCAATGCCGAGAGGGATGTGGAGGGCGAGAGAGAGCGACT
TCTCTCGGGCCCACAGCCTTACAGCTGTGGAGCACGGTATCCTCTGCCGAAACAGAGGTTGGACAAGA
+
FFFFFFFFFFFFFFFFFFFFFFFFFFFFFFFFFFFFFFFFFFFFF:FFFFFFFFFFFFFFFFFFFFFFFF
FFFFFFFFFFFFFFFFFFFFFFFFFFFFFFFFFFFFFFFFFFFFFFFFFFFFFFFFFFFFFFFFFFFF
@A00155:342:HHGFNDSXY:1:2223:31711:11256 1:N:0:GAACCTAG+TCCGCATA
GAGCGACTTCTCTCGGGCCCACAGCCTTACAGCTGTGGAGCACGGTATCCTCTGCCGAAACAGAGGTT
GGACAAGACCGGAGGGGTCTCCTAGTTCCAAAGGAGATGTACTCCGGGCTTGTTCACGACCTACCGTG
+
FFFFF:FFFFFFFFFFFFFFFFFFFFFFFFFFFFFFFFFFFFFFFFFFFFFFFFFF:FFFFFFFFF
FFFFFFFFFFFFFFFFFFFFFFFFFFFFFFFFFFFFFFFFFFFFFFFFFFFFFFFFFFFFFFFFFFFF
@A00155:342:HHGFNDSXY:1:2223:32081:11835 1:N:0:GAACCTAG+TCCGCATA
GAGCGACTTCTCTCGGGCCCACAGCCTTACAGCTGTGGAGCACGGTATCCTCTGCCGAAACAGAGGTT
GGACAAGACCGGAGGGGTCTCCTAGTTCCAAAGGAGATGTACTCCGGGCTTGTTCACGACCTACCGTG
+
FFFFFFFFFFFFFFFFFFFFFFFFFFFFFFFFFFFFFFFFFFFF:FFF:FFFFFFFFFFFFFFFFF
FFFFF:FFFFFFFFFFFFFFFFFFFFFFFF:FFFFFFFFFFFFFFFFFFFFFFFFFFFF:FFFF:FFFFF
@A00155:342:HHGFNDSXY:1:2224:7301:3051 1:N:0:GAACCTAG+TCCGCATA
GTGGAGCACGGTATCCTCTGCCGAAACAGAGGTTGGACAAGACCGGAGGGGTCTCCTAGTTCCAAAGG
AGATGTACTCCGGGCTTGTTCACGACCTACCGTGTAAGTCGTAGTCTAGTAGGCTACCTGACGAGTCC
+
FFFFFFFFFFFFFFFFFFFFFFFFFFFFFFFFFFFFFFFFFFFFFFFFFFFFFFFFFFFFFFFFFFFF
FFFFFFFFFFFFFFF:FFFFFFFFFFFFFFFFFFFFFFFFF,FFFFFFFFFF:FF:FFFFFFFFFFFF
@A00155:342:HHGFNDSXY:1:2224:20772:11099 1:N:0:GAACCTAG+TCCGCATA
GGGCGAGAGAGAGCGACTTCTCTCGGGCCCACAGCCTTACAGCTGTGGAGCACGGTATCCTCTGCCGA
AACAGAGGTTGGACAAGACCGGAGGGGTCTCCTAGTTCCAAAGGAGATGTACTCCGGGCTTGTTCACG
+
FFFFFFFFFF:FFFFFFFFFFFFFFFFFFFFFFFFFFFFFFFFFFFFFFFFFFFFFFFFFFFFFFFFF
FFFFFFFFFFFFFFFFFFFFFFFFFFFFFFFFFF:FFFFFFFFFFFFFFFFFFFFFFFFFFFFFFFFF
@A00155:342:HHGFNDSXY:1:2224:21775:11428 1:N:0:GAACCTAG+TCCGCATA
GGGCGAGAGAGAGCGACTTCTCTCGGGCCCACAGCCTTACAGCTGTGGAGCACGGTATCCTCTGCCGA
AACAGAGGTTGGACAAGACCGGAGGGGTCTCCTAGTTCCAAAGGAGATGTACTCCGGGCTTGTTCACG
+

FFF:FFFFFFFFFFFFFFFFFFFFFFFFFFFFFFFFFFFFFFFFFFFFFFFFFFFFFFFFFFFFFFFF
FFFFFFFFFFFFFFFF,FFFF:FFFFFFFFFFFFFFFFFFFFF:FFFFFFFFFFF:FFFFFFFFFFF
@A00155:342:HHGFNDSXY:1:2224:12301:17848 1:N:0:GAACCTAG+TCCGCATA
AAGGACTCTCCTCTGGCCTATGGTCATCTCAATGCCGAGAGGGATGTGGAGGGCGAGAGAGAGCGACT
TCTCTCGGGCCCACAGCCTTACAGCTGTGGAGCACGGTATCCTCTGCCGAAACAGAGGTTGGACAAGA
+
FFFFFFFFFFFFFFFFFFFFFFFFFFFFFFFFFFFFFFFFFFFFFFFFFFFFFFFFFFFFFFFFFFFF
FFFFFFFFFFFFFFFFFFFFFFFFFFFFFFFFFFFFFFFFFFFFFFFFFFFFFFFFFFFFFFFFFFFF
@A00155:342:HHGFNDSXY:1:2224:19452:30420 1:N:0:GAACCTAG+TCCGCATA
GGATGTGGAGGGCGAGAGAGAGCGACTTCTCTCGGGCCCACAGCCTTACAGCTGTGGAGCACGGTATC
CTCTGCCGAAACAGAGGTTGGACAAGACCGGAGGGGTCTCCTAGTTCCAAAGGAGATGTACTCC
+
FFFFFFFFFFFFFFFFFFFFFFFFFFFFFFFFFFFFFFFFFFFFFFFFFFFFFFFFFFFFFFFFFFFF
FFFFFFFFFFFFFFFFFFFFFFFFFFFFFFFFFFFFFFFFFFFFFF:FFFF:FFFFFFFFFFFFF:
@A00155:342:HHGFNDSXY:1:2225:27588:26506 1:N:0:GAACCTAG+TCCGCATA
CTTCTCTCGGGCCCACAGCCTTACAGCTGTGGAGCACGGTATCCTCTGCCGAAACAGAGGTTGGACAA
GACCGGAGGGGTCTCCTAGTTCCAAAGGAGATGTACTCCGGGCTTGTTCACGACCTACCGTGTAAGTC
+
FFFFFFFFFFFFFFFFFFFFFFFFFFFFFFF,FFFFFFFFFFFFFFFFFFFFFFFFFFFFFFFFFFFF
FFFFFFFFFFFFFFFFFFFFFFFFFFFFFFFFFFFFFFFFFFFFFFFFFFFFFFFFFFFFFFFFFFFF
@A00155:342:HHGFNDSXY:1:2226:10936:16360 1:N:0:GAACCTAG+TCCGCATA
AATGCCGAGAGGGATGTGGAGGGCGAGAGAGAGCGACTTCTCTCGGGCCCACAGCCTTACAGCTGTGG
AGCACGGTATCCTCTGCCGAAACAGAGGTTGGACAAGACCGGAGGGGTCTCCTAGTTCCAAAGGAGAT
+
FFFFFFFFFFFFFFFFFFFFFFFFFFFFFFFFFFFFFFFFFFFFFFFFFFFFFFFFFFFFFFFFFFFF
FFFFFFFFFFFFFFFFFFFFF:FFFFFFFFFFFFFFFFFFFFFFFFFFFFFFF:FFFFFFFFFFFFFF
@A00155:342:HHGFNDSXY:1:2226:30689:17033 1:N:0:GAACCTAG+TCCGCATA
TCGGCAGAGGATACCGTGCTCCACAGCTGTAAGGCTGTGGGCCCGAGAGAAGTCGCTCTCTCTCGCCC
TCCACATCCCTCTCGGCATTGAGATGACCATAGGCCAGAGGTGAGTCCTTAAGTGGACACA
+
FFFFFFFFFFFFFFFFFFFFFFFFFFFFFFFFFFFFFFFFFFFFFFFFFFFFFFFFFFFFFFFFFFFF
FFFFFFFFFFFFFFFFFF:FFFFFFFFFFFFFFFFFFFFFFFFFFFFFFFFFFFFFFFFFF
@A00155:342:HHGFNDSXY:1:2226:1181:21324 1:N:0:GAACCTAG+TCCGCATA
GGATGTGGAGGGCGAGAGAGAGCGACTTCTCTCGGGCCCACAGCCTTACAGCTGTGGAGCACGGTATC
CTCTGCCGAAACAGAGGTTGGACAAGACCGGAGGGGTCTCCTAGTTCCAAAGGAGATGTAC
+
FFFFFFFFFFFFFF:FFFFFFFFFFFFFFFFFFFFFF:FFFFFFFFFF::FFFFFFFFFFF:FFFFFFF
FFFFFFFFFFFFFFF:,FFFFFFFF:FFFFFFFFFFF:FFFFFFFF:FFF:,F:FFFFFF:
@A00155:342:HHGFNDSXY:1:2226:19967:23453 1:N:0:GAACCTAG+TCCGCATA
CAATGCCGAGAGGGATGTGGAGGGCGAGAGAGAGCGACTTCTCTCGGGCCCACAGCCTTACAGCTGTG
GAGCACGGTATCCTCTGCCGAAACGGAGGTTGGACAAGACCGGAGGGGTCTCCTAGTTCCAAAGGAGA
+
FFFFFFFFFFFFFFFFFFFFFFFFFFFFFFFFFFFFF:FFFFFFFFFFFFFFFFFFFFFFFFFFFFFF
FFFFFFFFFFFFFFFFFFFFFFFF,FFFFFFFFFFF:FFFFFFFF:FFFFFFFFF:FFFFFFFFFF
@A00155:342:HHGFNDSXY:1:2226:12255:25160 1:N:0:GAACCTAG+TCCGCATA
TTCTCTCGGGCCCACAGCCTTACAGCTGTGGAGCACGGTATCCTCTGCCGAAACAGAGGTTGGACAAG
ACCGGAGGGGTCTCCTAGTTCCAAAGGAGATGTACTCCGGGCTTGTTCACGACCTACCGTGTAAGTCG
+
FFFFFFFFFFFFFFFFFFFFFFFFFFFFFFFFFFFFFFFFFFFFFFFFFFFFFFFFFFFFFFFFFFFF
FFFFFFFFFFFFFFFFFFFFFFFFFFFFFFFFFFFFFFFFFFFFFFFFFFFFFFFFFFFFFFFFFFFF
@A00155:342:HHGFNDSXY:1:2226:13657:28620 1:N:0:GAACCTAG+TCCGCATA
AGAGAGAGCGACTTCTCTCGGGCCCACAGCCTTACAGCTGTGGAGCACGGTATCCTCTGCCGAAACAG
AGGTTGGACAAGACCGGAGGGGTCTCCTAGTTCCAAAGGAGATGTACTCCGGACTTGTTCACGACCTA
+

```
FFFFFFFFFFFFFFFFFFFFFFFFFFFFFFFFFFFFFFFFFFFFFFFFFFFFFFFFFFFFFFFFFFFF
FFFFFFFFFFFFFFFFFFFFFFFFFFFFFFFFFFFFFFFFFFFFFFFFFFFFFFFFFFFFFFFFFFF:
@A00155:342:HHGFNDSXY:1:2226:9182:29042 1:N:0:GAACCTAG+TCCGCATA
TGTCCAACCTCTGTTTCGGCAGAGGATACCGTGCTCCACAGCTGTAAGGCTGTGGGCCCGAGAGAAGT
CGCTCTCTCTCGCCCTTCACATCCCTCTCGGCATTGAGATGACCATAGGCCAGAGGTGAGTCCTTAAG
+
FFFFFFFFFFFFFFFFFFFFFFFFFFFFFFFFFFFFFFFFFFFFFFFFFFFFFFFFFFFFFFFFFFFF
FFFFFFFFFFFFFFFFFFFFFFFFFFFFFFFFFFFFFFFFFFFFFFFFFF:FFFFFFFFFFFFFFFFF
@A00155:342:HHGFNDSXY:1:2226:20283:30326 1:N:0:GAACCTAG+TCCGCATA
GGATGTGGAGGGCGAGAGAGAGCGACTTCTCTCGGGCCCACAGCCTTACAGCTGTGGAGCACGGTATC
CTCTGCCGAAACAGAGGTTGGACAAGACCGGAGGGGTCTCCTAGTTCCAAAGGAGATGTACTCC
+
FFFFFFFFFFFFFFFFFFFFFFFFFFFFFFFFFFFFFFFFFFFFFFFFFFFFFFFFFFFFFFFFFFFF
FFFFFFFFFFFFFFFFFFFFFFFFFFFFFFFFFFFFFFFFFF:FFFFFFFFFFFFFFFFFFFFF
@A00155:342:HHGFNDSXY:1:2227:3323:6746 1:N:0:GAACCTAG+TCCGCATA
TTACAGCTGTGGAGCACGGTATCCTCTGCCGAAACAGAGGTTGGACAAGACCGGAGGGGTCTCCTAGT
TCCAAAGGAGATGTACTCCGGGCTTGTTCACGACCTACCGTGTAAGTCGTAGTCTAGTAGGCTACCTG
+
FFFFFFFFFFFFFFFFFFFFFFFFFFFFFFFFFFFFFFFFFFFFFFFFFFFFFFFFFFFFFFFFFFFF
FFFFFFFFFFFFFFFFFFFFFFFFFFFFFFFF:FFFFFFFFFFF:FFFFFFFFFFFFFFFFFFFFFFF
@A00155:342:HHGFNDSXY:1:2227:9019:13291 1:N:0:GAACCTAG+TCCGCATA
CTTCTCTCGGGCCCACAGCCTTACAGCTGTGGAGCACGGTATCCTCTGCCGAAACAGAGGTTGGACAA
GACCGGAGGGGTCTCCTAGTTCCAAAGGAGATGTACTCCGGGCTTGTTCACGACCTACCGTGTAAGTC
+
FFFFFFFFFFFFFFFFFFFFFF,FFFFFFFFFFFFFFFFFFFFFFFFFFFFFFFFFFFFFFFFFFFFF
FFFFFFFFFFFFFFFFFFFFFFFFFFFFFFFFFFFFFFFFFFFFFFFFFFFFFFFFFFFFFFFFFFFF
@A00155:342:HHGFNDSXY:1:2227:22580:15295 1:N:0:GAACCTAG+TCCGCATA
GAGAGCGACTTCTCTCGGGCCCACAGCCTTACAGCTGTGGAGCACGGTATCCTCTGCCGAAACAGAGG
TTGGACAAGACCGGAGGGGTCTCCTAGTTCCAAAGGAGATGTACTCCGGGCTTGTTCACGACCTACCG
+
FFFFFFFFFFFFFFFFFFFFFFFFFFFFFFFFFFFFFFFFFFFFFFFFFFFFFFFFFFFFFFFFFFFF
FFFFFFFFFFFFFFFFFFFFFFFFFFFFFFFFFFFFFFFFFFFFFF:FFFFFFFFFFFFFFFFFFF:F
@A00155:342:HHGFNDSXY:1:2227:30662:20212 1:N:0:GAACCTAG+TCCGCATA
AGAGAGAGCGACTTCTCTCGGGCCCACAGCCTTACAGCTGTGGAGCACGGTATCCTCTGCCGAAACAG
AGGTTGGACAAGACCGGAGGGGTCTCCTAGTTCCAAAGGAGATGTACTCCGGGCTTGTTCACGACCTA
+
FFFFFFFFFFFFFFFFFFFFFFFFFFFFFFFFFFFFFFFFFFFFFFFFFFFFFFFFFFFFFFFFFFFF
FFFFFFF:FFFFFFF:FFFFFFFFFFFFFFFFFFFFFFFFFFFFFFFFFFFFFFFFFFFF:F:FFFFF
@A00155:342:HHGFNDSXY:1:2227:1624:36276 1:N:0:GAACCTAG+TCCGCATA
AGAGAGAGCGACTTCTCTCGGGCCCACAGCCTTACAGCTGTGGAGCACGGTATCCTCTGCCGAAACAG
AGGTTGGACAAGACCGGAGGGGTCTCCTAGTTCCAAAGGAGATGTACTCCGGACTTGTTCACGACCTA
+
FFFFFFFFFFFFFFFFFFF:FFFFFFFFFFFFFFFFFFFFFFFFFFFFFFFFFFFFFFFFFFFFFFFF
FF:FFFFFFFFFFF:FFFFFFFFFFFFFFFFFFFFFFFFFFFFFFFFFFFFFFFFFFFFFFFFFFFFF
@A00155:342:HHGFNDSXY:1:2228:12210:2566 1:N:0:GAACCTAG+TCCGCATA
GAGAGCGACTTCTCTCGGGCCCACAGCCTTACAGCTGTGGAGCACGGTATCCTCTGCCGAAACAGAGG
TTGGACAAGACCGGAGGGGTCTCCTGGTTCCAAAGGAGATGTACTCCGGGCTTGTTCACGACCTACCG
+
FFFFFFFFFFFFFFFFFFFFFFFFFFFFFFFFFFFFFFFFFFFFFFFFFFFFFFFFFFFFFFFFFFFF
FFFFFFFFFFFFFFFFFFFFFFFFFFFFFFFFFFFFFFFFFFFFFFFFFFFFFFFFFFFFFFFFFFF:
@A00155:342:HHGFNDSXY:1:2228:8938:4257 1:N:0:GAACCTAG+TCCGCATA
GGCGAGAGAGAGCGACTTCTCTCGGGCCCACAGCCTTACAGCTGTGGAGCACGGTATCCTCTGCCGAA
ACAGAGGTTGGACAAGACCGGAGGGGTCTCCTAGTTCCAAAGGAGATGTACTCCGGGCTTGTTCACGA
+
```

:F:FFFFFFFFF:FFF:FFFFFFFFFFFFFFFFFFFFFFF:FFFFF:FF:FFFFFFFFFFFFFFFFFF
FFFFFFF,FFFFFFF:FFFFFFF:FF:FFFFFF:FFFFFFFFFF:FFFFF,FF,F::FFFFFFFFF,F
@A00155:342:HHGFNDSXY:1:2228:22498:10551 1:N:0:GAACCTAG+TCCGCATA
ATCCTCTGCCGAAACAGAGGTTGGACAAGACCGGAGGGGTCTCCTGGTTCCAAAGGAGATGTACTCCG
GGCTTGTTCACGACCTACCGTGTAAGTCGTAGTCTAGTAGGCTACCTGACGAGTCCTTTTTAGGACGA
+
FFFFFFFFFFFFFFFFFFFFFFFFFFFFFFFFFFFFFFFFFFFFFFFFFFFFFFFFFFFFFFFFFFFF
FFFFFFFFFFFFFFFFFFFFFFFFF:FFFFFFFFFFFFFFFFFF:FFFFFFFFFFFFFFFFFFFFFFFFF
@A00155:342:HHGFNDSXY:1:2228:3287:15013 1:N:0:GAACCTAG+TCCGCATA
ATCCTCTGCCGAAACAGAGGTTGGACAAGACCGGAGGGGTCTCCTAGTTCCAAAGGAGATGTACTCCG
GGCTTGTTCACGACCTACCGTGTAAGTCGTAGTCTAGTAGGCTACCTGACGAGTCCTTTTTAGGACGA
+
FFFFFFFFFFFFFFFFFFFFFFFFFFFFFF:FF:FFFFFFFFFFFFFFFFFFFFFFFFFFFFFFFFFFF
FFFFFFFFFFFFFFFFFFFFFFFFF,FFFFFFFFFFFFFFFFFFFFFFFFFFFFFFF:FFFFFFFFFF:F
@A00155:342:HHGFNDSXY:1:2228:8015:28181 1:N:0:GAACCTAG+TCCGCATA
AGAGAGAGCGACTTCTCTCGGGCCCACAGCCTTACAGCTGTGGAGCACGGTATCCTCTGCCGAAACAG
AGGTTGGACAAGACCGGAGGGGTCTCCTAGTTCCAAAGGAGATGTACTCCGGGCTTGTTCACGACCTA
+
FFFFFFFFFFFFFFFFFFFFFFFFFFFFFFFFF:FFFFFFFFFFFFFFFF:FFFFFFFFFFFFFFFFFF
FFFFFFFFFFFFFF:FFFFFFFFFFFFFFFFFFFFFFFFFFFFFFF:FFF:FFFFFFFFFF:FFFFFFFF:
@A00155:342:HHGFNDSXY:1:2228:21784:29262 1:N:0:GAACCTAG+TCCGCATA
GTCGCTCTCTCTCGCCCTCCACATCCCTCTCGGCATTGAGATGACCATAGGCCAGAGGTGAGTCCTTA
AGTGGACACAGCTGATCTAAGGCGGTGTGGCGGGGCATGGGTTTGAACCCCCATGACGGTCGGAGTC
+
:FFFFFF:,:FFFFFFFFFFFFFFFF:FF:F:FFFFFFF:FFFF::FFF,FF,::,F:FFFFFFFF::
FF,,:FF:FF::FFFFFFFF,FF,F:FFFFFFF:FFF,::FFF:FFFFFFFFF,FFFFFFF,:FF:F
@A00155:342:HHGFNDSXY:1:2228:29903:31892 1:N:0:GAACCTAG+TCCGCATA
TCTCTCGGGCCCACAGCCTTACAGCTGTGGAGCACGGTATCCTCTGCCGAAACAGAGGTTGGACAAGA
CCGGAGGGGTCTCCTAGTTCCAAAGGAGATGTACTCCGGGCTTGTTCACGACCTACCGTGTAAGTC
+
FFFF::F::FFFF:FFFFFF:FF,:FFFFFF:FFFFFFFFFFFFFFFFFFFF:FFFF:F:F:FFFF:F
F::FF:F,FFFFFFF:FF,F,F:FFFFFFFFFFFFF:FFF:F:FFFFFF,FF,FFFFFFFFFFFFF
@A00155:342:HHGFNDSXY:1:2228:10402:36761 1:N:0:GAACCTAG+TCCGCATA
AGAGAGAGCGACTTCTCTCGGGCCCACAGCCTTACAGCTGTGGAGCACGGTATCCTCTGCCGAAACAG
AGGTTGGACAAGACCGGAGGGGTCTCCTGGTTCCAAAGGAGATGTACTCCGGGCTTGTTCACGACCTA
+
FFFFFF:FFFFFFFFFFFFFFFFFFFFFFFFFFFFFFFFFFFFFFFFFFFFFFFFFFFFFFFFFFFFF
FFFFFF::FFF,FFFFFFFFFFFFFFFFFFFFFFFFFFFFFFFFFFFFFFFFFFF:FFFFFFFFFFFF
@A00155:342:HHGFNDSXY:1:2229:5511:1235 1:N:0:GAACCTAG+TCCGCATA
TGTTTCGGCAGAGGATACCGTGCTCCACAGCTGTAAGGCTGTGGGCCCGAGAGAAGTCGCTCTCTCTC
GCCCTTCACATCCCTCTCGGCATTGAGATGACCATAGGCCAGAGGTGAGTCCTTAAGTGGACACAGCT
+
FFFFFFFFFFFFFFFFFFFFFFFFFFFFFFFFFFFFFFFFFFFFFFFFFFFFFF:FFFFFFFFFFFFF
FFFFFFFFFFFFFFFFFFFFFFFFFFFFFFF:FFFFFFFFFFFFFFFFFFFFFFFFFFFFFFFFFFFFF
@A00155:342:HHGFNDSXY:1:2229:1515:3270 1:N:0:GAACCTAG+TCCGCATA
GGATGTGGAGGGCGAGAGAGAGCGACTTCTCTCGGGCCCACAGCCTTACAGCTGTGGAGCACGGTATC
CTCTGCCGAAACAGAGGTTGGACAAGACCGGAGGGGTCTCCTGGTTCCAAAGGAGATGTACTCC
+
FFFFFFFFFFFFFFFFFFFFFFFFFFFFFFFFFFFFFFFFFFFFFF:FFFFFFFFFFFFF,FFFFFFFF
FFFFFFFFFFFF,FFFFFFF:FFFFFFF::FFFFFFFFFFFFFFFFFFFFFFFFFFFF,FFFFF:
@A00155:342:HHGFNDSXY:1:2229:14615:8547 1:N:0:GAACCTAG+TCCGCATA
ACTTCTCTCGGGCCCACAGCCTTACAGCTGTGGAGCACGGTATCCTCTGCCGAAACAGAGGTTGGACA
AGACCGGAGGGGTCTCCTAGTTCCAAAGGAGATGTACTCCGGGCTTGTTCACGACCTACCGTGTAAGT
+

FFFFFFFFFFFFFFFFFFFFFFFFFFFFFFFFFFFFFFFFFFFFFFFFFFFFFFFFFFFFFFFFFFFFFF
FFFFFFFFFFFFFFFFFFFFFFFFFFFFFFFFFFFFFFFFFFFFFFFFFFFFFFFFFFFFFFFFFFFFFF
@A00155:342:HHGFNDSXY:1:2229:25889:8750 1:N:0:GAACCTAG+TCCGCATA
TGTCCACTTAAGGACTCTCCTCTGGCCTATGGTCATCTCAATGCCGAGAGGGATGTGGAGGGCGAGAG
AGAGCGACTTCTCTCGGGCCCACAGCCTTACAGCTGTGGAGCACGGTATCCTCTGCCGAAACAGAGGT
+
FFFFFFFFFFFFFFFFFFFFFFFFFFFFFFFFFFFFFFFFFFFFFFFFFFFFFFFFFFFFFFFFFFFFFF
FFFFFFFFFFFFFFFFFFFFFFFFFFFF,F,FFFFFFFFFFFFFFFFFFFFFFFFFFFFFFFFFFFFFFF
@A00155:342:HHGFNDSXY:1:2229:1515:16110 1:N:0:GAACCTAG+TCCGCATA
TCTGGCCTATGGTCATCTCAATGCCGAGAGGGATGTGGAGGGCGAGAGAGAGCGACTTCTCTCGGGCC
CACAGCCTTACAGCTGTGGAGCACGGTATCCTCTGCCGAAACAGAGGTTGGACAAGACCGGAGGGGTC
+
FFFFFFFFFFFFFFFFFFFFFFFFFFFFFFFFFFFFFFFFFFFFFFFFFFFF:FFFFFFFFFFFFFFFFF
FFFFFF:FFFFFFFFFFFFFFFFFFFFFFFFFFFFFFF:FFF,FFF:FFFFFF,FFFF::F:FFFFFFFF
@A00155:342:HHGFNDSXY:1:2229:21088:18505 1:N:0:GAACCTAG+TCCGCATA
GCTGTGTCCACTTAAGGACTCACCTCTGGCCTATGGTCATCTCAATGCCGAGAGGGATGTGGAGGGCG
AGAGAGAGCGACTTCTCTCGGGCCCACAGCCTTACAGCTGTGGAGCACGGTATCCTCTGCCGAAACAG
+
FFFFFFFFFFFFFFFFFFFFFFFFFFFFFFFFFFFFFFFFFFFFFFFFFFFFFFFFFFFFFFFFFFFFFF
FFFFFF:FFFFFFFFFFFFFFFFFFFFFFFFFFFFFFFFFFFFFFFFFFFFFF,FFFFFFFFFFFFFFFF
@A00155:342:HHGFNDSXY:1:2229:11198:21355 1:N:0:GAACCTAG+TCCGCATA
CTTCTCTCGGGCCCACAGCCTTACAGCTGTGGAGCACGGTATCCTCTGCCGAAACAGAGGTTGGACAA
GACCGGAGGGGTCTCCTAGTTCCAAAGGAAATGTACTCCGGGCTTGTTCACGACCTACCGTGTAAGTC
+
FFFFFFFFFFFFFFFFFFFF:FFFFFFFFFFFFFFFFFFFFFFFFFFFFFFFFFFFFFFFFFFFFFFFFF
FFFFFFFFFFFFFFFFFFFFFFFFFFFFFFF:FFFFFFFFFFFFFFFFFFFFFFFFFFFFFFFFFFFFFF
@A00155:342:HHGFNDSXY:1:2229:7636:34507 1:N:0:GAACCTAG+TCCGCATA
CTTCTCTCGGGCCCACAGCCTTACAGCTGTGGAGCACGGTATCCTCTGCCGAAACAGAGGTTGGACAA
GACCGGAGGGGTCTCCTAGTTCCAAAGGAGATGTACTCCGGGCTTGTTCACGACCTACCGTGTAAG
+
FFFFFFFFFFFFFFFFFFFFFFFFFFFFFFFFFFFFFFFFFFFFFFFFFFFFFFFFFFFFFFFFFFFFFF
FFFFFFFFFFFFFFFFFFFFFFFFFFFFFFFFFFFFFFFFFFFFFFFFFFFFFFFFFFFFFF:FFFFF
@A00155:342:HHGFNDSXY:1:2229:5394:37043 1:N:0:GAACCTAG+TCCGCATA
CTTCTCTCGGGCCCACAGCCTTACAGCTGTGGAGCACGGTATCCTCTGCCGAAACAGAGGTTGGACAA
GACCGGAGGGGTCTCCTAGTTCCAAAGGAGATGTACTCCGGGCTTGTTCACGACCTACCGTGTAAG
+
F,FFFFFFFFFFFFFFFFFFFFFFFFFFFFFFFFFFFFFFFFFFFFFFFFFFFFFFFFFFFFFFFFFFF:
FFFFFFFFFFFFFFFFFFFFFFFFFFFFFFFFFFFFFFFFFFFFFFFFFFFFFFFFFFFFFF,FFFFF
@A00155:342:HHGFNDSXY:1:2230:27823:23406 1:N:0:GAACCTAG+TCCGCATA
AGAGAGAGCGACTTCTCTCGGGCCCACAGCCTTACAGCTGTGGAGCACGGTATCCTCTGCCGAAACAG
AGGTTGGACAAGACCGGAGGGGTCTCCTAGTTCCAAAGGAGATGTACTCCGGGCTTGTTCACGACCTA
+
FFFFFFFFFFFFFFFFFFFFFFFFFFFFFFFFFFFFFFFFFFFFFFFFFFF:FFFFFFFFFF:FFFFFFF
FFFFFFFFFFFFFFFF,FFFFFFFFFFFFFFFFFFFFFFFFFFFFFFFFFF:FFFFF:FFF:FFFFFFFF
@A00155:342:HHGFNDSXY:1:2230:28339:29684 1:N:0:GAACCTAG+TCCGCATA
AGGGATGTAGAGGGCGAGAGAGAGCGACTTCTCTCGGGCCCACAGCCTTACAGCTGTGGAGCACGGTA
TCCTCTGCCGAAACAGAGGTTGGACAAGACCGGAGGGGTCTCCTAGTTCCAAAGGAGATGTACTCCGG
+
FFFFFFFFFFFFFF:FFFFFFFFFFFFFFFFFFFFFFFFFFFFFFFFFFFFFFFF:FFFFFFFFFFFFFF
FFFFFFFFFFFFFFFFFFFFFFFFFFFF:FFFFFFFFFFFFFFFFFF:FFF:FFFFFFFFFFFFFFFFFF
@A00155:342:HHGFNDSXY:1:2230:29107:31015 1:N:0:GAACCTAG+TCCGCATA
AGGGATGTAGAGGGCGAGAGAGAGCGACTTCTCTCGGGCCCACAGCCTTACAGCTGTGGAGCACGGTA
TCCTCTGCCGAAACAGAGGTTGGACAAGACCGGAGGGGTCTCCTAGTTCCAAAGGAGATGTACTCCGG
+

FFFFFFFFFFFFFFFFFFFFFFFFFFFFFFFFFFFFFFFFFFFFFFFFFFFFFFFFFFFFFFFFFFFF
FFFFFFFFFFFFFFFFFFFFFFFFFFFFFFFFFFFFFFFFFFFF:FFFFFFFFFFFFFFFFFFFFFFF
@A00155:342:HHGFNDSXY:1:2230:14733:33959 1:N:0:GAACCTAG+TCCGCATA
ATCTCAATGCCGAGAGGGATGTGGAGGGCGAGAGAGAGCGACTTCTCTCGGGCCCACAGCCTTACAGC
TGTGGAGCACGGTATCCTCTGCCGAAACAGAGGTTGGACAAGACCGGAGGGGTCTCCTAGTTCCAAAG
+
FFFFFFFFF:FFFFFFFFFFFFFFFFF:FFFFFFFFFFFFFFFFFFFFFFFFFFFFFFFFFFFFFFFF
FFFFFFFFFFFFFFFFFFFFFFFFFFFFFFFFFFFFFFFFFFFFFFFFFFFFFFFFFFFFFFFFFFFF
@A00155:342:HHGFNDSXY:1:2231:8892:2550 1:N:0:GAACCTAG+TCCGCATA
GAGCGACTTCTCTCGGGCCCACAGCCTTACAGCTGTGGAGCACGGTATCCTCTGCCGAAACAGAGGTT
GGACAAGACCGGAGGGGTCTCCTAGTTCCAAAGGAGATGTACTCCGGGCTTGTTCACGACCTACCGTG
+
FFFFFFFFFFFFFFFFFFFFFFFFFFFFFFFFFFFFFFFFFFFFFFF:FFFFFFFFFFFFFFFFFFFF
FFFFFFFFFFFFFFFFFFFFFFFFFFFFFFFFFFFFFFFF:FFFFFFFFFF,FFFFFFFFFFFFFFFF
@A00155:342:HHGFNDSXY:1:2231:9218:7529 1:N:0:GAACCTAG+TCCGCATA
GAGCGACTTCTCTCGGGCCCACAGCCTTACAGCTGTGGAGCACGGTATCCTCTGCCGAAACAGAGGTT
GGACAAGACCGGAGGGGTCTCCTAGTTCCAAAGGAGATGTACTCCGGGCTTGTTCACGACCTACCGTG
+
FFFFFFFFFFFFFFFFFFFFF:FFFFFFFFFFFFFFFFFFFFFFFFFFFF:FFF:FFFFFFFFFFFFFFF
FFFFFFFFFF:FFFFFFFFFFFFFFFFF:FFFFFFFFFFFFFFFFFFFFFFFFFFFF:FFFFFFFFFFFF:
@A00155:342:HHGFNDSXY:1:2231:20220:16720 1:N:0:GAACCTAG+TCCGCATA
GAGCGACTTCTCTCGGGCCCACAGCCTTACAGCTGTGGAGCACGGTATCCTCTGCCGAAACAGAGGTT
GGACAAGACCGGAGGGGTCTCCTGGTTCCAAAGGAGATGTACTCCGGGCTTGTTCACGACCTACCGTG
+
FFFFFFFFFFFFFFFFFFFFFFFFFFFFFFFFFFFFFFFFFFFFFFFFFFFFFFFFFFFFFFFFFFFF
FFFFFFFFFFFFFFFFFFFFFFFFFFFFFFFFFFFFFFFFFFFFFFFFFFFFFFFFFFFFFFFFFFFF
@A00155:342:HHGFNDSXY:1:2231:16034:24126 1:N:0:GAACCTAG+TCCGCATA
GGATGTGGAGGGCGAGAGAGAGCGACTTCTCTCGGGCCCACAGCCTTACAGCTGTGGAGCACGGTATC
CTCTGCCGAAACAGAGGTTGGACAAGACCGGAGGGGTCTCCTAGTTCCAAAGGAGATGTACTCC
+
FFFFFFFFFFFFFFFFFFFFFFFFFFFFFFFFFFFFFFFFFFFFF:FFFFFFFFFFFFFFFFFFFFFF
FFFFFFFFFF:FFFFFFFFFFFFFFF:FFFFFFFFFFFFFFFFFFFFFFFFFFFFFFFFF,FFFF
@A00155:342:HHGFNDSXY:1:2231:16324:25254 1:N:0:GAACCTAG+TCCGCATA
CTTCTCTCGGGCCCACAGCCTTACAGCTGTGGAGCACGGTATCCTCTGCCGAAACAGAGGTTGGACAA
GACCGGAGGGGTCTCCTGGTTCCAAAGGAGATGTACTCCGGGCTTGTTCACGACCTACCGTGTAAGTC
+
FFFFFFFFFFFFFFFFFFFFFFFFFFFFFFFFFFFFFFFFFFFFFFFFFFFFFFFFFFFFFFF:FFFF
FFFFFFFFFFFFFFFFFFFFFFFFFFFFFFFFFFFFFFFFFFF:FFFFFFFFFFFFFFFFFFFFFFFF
@A00155:342:HHGFNDSXY:1:2231:28004:31673 1:N:0:GAACCTAG+TCCGCATA
CGAGAGAGAGCGACTTCTCTCGGGCCCACAGCCTTACAGCTGTGGAGCACGGTATCCTCTGCCGAAAC
AGAGGTTGGACAAGACCGGAGGGGTCTCCTAGTTCCAAAGGAGATGTACTCCGGGCTTGTTCACGACC
+
FFFFFFFFFFFFFFFFFFFFFF:FFFFFFF:FFFFFFFFFFFFFFF:FFFFFFFFFFFFFFFFFFFFF
FFFFFFFFFFFFFFFFFFFFFFFFFFFFFFFFFFFFFFFFFFFFFFFFFFFFFFFFFFFFFFFFFFFF
@A00155:342:HHGFNDSXY:1:2232:18891:11162 1:N:0:GAACCTAG+TCCGCATA
CAGCCTTACAGCTGTGGAGCACGGTATCCTCTGCCGAAACAGAGGTTGGACAAGACCGGAGGGGTCTC
CTGGTTCCAAAGGAGATGTACTCCGGGCTTGTTCACGACCTACCGTGTAAGTCGTAGTCTAGTAGGCT
+
FFFFFFFFFFFFFFFFFFFFFFFFFFFFFFFFFFFFFFFFFFFFFFFFFFFFFFFFFFFFFFFFFFFF
FFFFFFFFFFFFFFFFFFFFFFFFFFFFFFFFFFFFFFFFFFFFFFFFFFFFFFFFFFFFFFFFFFFF
@A00155:342:HHGFNDSXY:1:2232:32353:18944 1:N:0:GAACCTAG+TCCGCATA
GGATGTGGAGGGCGAGAGAGAGCGACTTCTCTCGGGCCCACAGCCTTACAGCTGTGGAGCACGGTATC
CTCTGCCGAAACAGAGGTTGGACAAGACCGGAGGGGTCTCCTAGTTCCAAAGGAGATGTACTCC
+

```
FFFFFFFF:FFFFFFFFFFFFFFFFFFFFFFFFFFFFFFFFFFFFFFFFFFFFFFFFFFFFFFFFF
FFFFFFFFFFFFFFFFFFFFFFFFFFFFFFFFFFFFFFFFFFFF:FFFFFFFFFFFFFFFFFFFFF
@A00155:342:HHGFNDSXY:1:2232:13928:21292 1:N:0:GAACCTAG+TCCGCATA
GGGCGAGAGAGAGCGACTTCTCTCGGGCCCACAGCCTTACAGCTGTGGAGCACGGTATCCTCTGCCGA
AACAGAGGTTGGACAAGACCGGAGGGGTCACCTAGTTCCAAAGGAGATGTACTCCGGGCTTGTTCACG
+
FFFFFFFFFFFFFFFFFFFFFFFFFFFFFFFFFF:FFFFFFFFFFFFFFFFFFFFFFFFFFFFFFFFF
FFFFFFFFFFFFFFFFFFFFFFFFFFFFFFFFFFFFFFFFFFFFFFFFFFFFFF::FFFFFFFFFFFF
@A00155:342:HHGFNDSXY:1:2232:18511:22654 1:N:0:GAACCTAG+TCCGCATA
TTACAGCTGTGGAGCACGGTATCCTCTGCCGAAACAGAGGTTGGACAAGACCGGAGGGGTCTCCTAGT
TCCAAAGGAGATGTACTCCGGGCTTGTTCACGACCTACCGTGTAAGTCGTAGTCTAGTAGGCTACCTG
+
FFFFFFFFFFFFFFFFFFFFFFFFFFFFFFFFFFFFFFFFFFFFFFFFFFFFFFFFFFFFFFFFFFFF
FFFFFFFFFFFFFFFFFFFFFFFFFFFFFFFFFFFFFFFFFFFFFFFFFFFFFFF:FFFFFFFFFFFF
@A00155:342:HHGFNDSXY:1:2232:24288:31782 1:N:0:GAACCTAG+TCCGCATA
AGCGACTTCTCTCGGGCCCACAGCCTTACAGCTGTGGAGCACGGTATCCTCTGCCGAAACAGAGGTTG
GACAAGACCGGAGGGGTCTCCTAGTTCCAAAGGAGATGTACTCCGGGCTTGTTCACGACCTACCGTGT
+
FFFFFFFFFFFFFFFFFFFFFFFFFFFFFFFFFFFFFFFFFFFFFFFFFFFFFFFFFFFFFFFFFFFF
FFFF:FFFFFFFFFFFFFFFFFFFFFFFFFFFFFFFFFFFFFFFFFFFFFFFFFFFFFFFFFFFFFFF
@A00155:342:HHGFNDSXY:1:2232:19461:33786 1:N:0:GAACCTAG+TCCGCATA
GGGCGAGAGAGAGCGACTTCTCTCGGGCCCACAGCCTTACAGCTGTGGAGCACGGTATCCTCTGCCGA
AACAGAGGTTGGACAAGACCGGAGGGGTCTCCTAGTTCCAAAGGAGATGTACTCCGGGCTTGTTCACG
+
FFFFFFFFFFFFFFFFFFFFFFFFFFFFFFFFFFFFFFFFFFFFFFFFFFFFFFFFFFFFFFFFFFFF
FFFFFFFFFFFFFFFFFFFFFFFFFFFFFFFFFFFFFFFFFFFFFFFFFFFFFFFFFFFFFFFFFFFF
@A00155:342:HHGFNDSXY:1:2232:31313:36808 1:N:0:GAACCTAG+TCCGCATA
AGCGACTTCTCTCGGGCCCACAGCCTTACAGCTGTGGAGCACGGTATCCTCTGCCGAAACAGAGGTTG
GACAAGACCGGAGGGGTCTCCTGGTTCCAAAGGAGATGTACTCCGGGCTTGTTCACGACCTACCGTGT
+
FFFFFFFFFFFFFFFFFFFFFFFFFFFFFFFFFFFFFFFFFFFFFFFFFFFFFFFFFFFFFFFFFFFF
FFFFFFFF,FFFFFFFFFFFFFFFFFFFFFFFFFFFFFFFFFFFFFFFFFFFFFFFFFFFFFFFFFF:
@A00155:342:HHGFNDSXY:1:2233:6027:23578 1:N:0:GAACCTAG+TCCGCATA
ATCTCAATGCCGAGAGGGATGTAGAGGGCGAGAGAGAGCGACTTCTCTCGGGCCCACAGCCTTACAGC
TGTGGAGCACGGTATCCTCTGCCGAAACAGAGGTTGGACAAGACCGGAGGGGTCTCCTAGTTCCAAAG
+
FFFFFFFFFF:FFFFFFFFFFFFFFFFFFFFFFFFFFFFFFFFFFFFFFFFFFFFF::FF:FFFFFF
FFFFFF:FFFFFFFFFFFFFFFFFFFFFFFFFFFFFFFFFFFFFFFFFFFFFFFFFFFFFFFFFFFFF
@A00155:342:HHGFNDSXY:1:2233:5990:24612 1:N:0:GAACCTAG+TCCGCATA
ATCTCAATGCCGAGAGGGATGTAGAGGGCGAGAGAGAGCGACTTCTCTCGGGCCCACAGCCTTACAGC
TGTGGAGCACGGTATCCTCTGCCGAAACAGAGGTTGGACAAGACCGGAGGGGTCTCCTAGTTCCAAAG
+
FFFFFFFFFFFFFFFFFFFFF,FFFFFFFFFFFFFFFFFFFFFFFFFFFFFFFFFFFFFFFF:FFFF
FFFFFFFFFFFFFFFFFFFFFFFFFFFFFFFFFFFFFFFFFF:F,FFFFFFFFFFFF:FFFFFFFFFF
@A00155:342:HHGFNDSXY:1:2233:22824:25081 1:N:0:GAACCTAG+TCCGCATA
TTAAAGACTCACCTCTGGCCTATGGTCATCTCAATGCCGAGAGGGATGTGGAGGGCGAGAGAGAGCGA
CTTCTCTCGGGCCCACAGCCTTACAGCTGTGGAGCACGGTATCCTCTGCCGAAACAGAGGTTGGACAA
+
FFFFFFFFFFFFFFFFFFFFFFFFFFFFFFFFFFFFF:FFFFFFFFF:FFFFFF:FFFFF:FFFFF:F
FFFFFFFFFF:FFFFFFFFFFFFFFFFF:FFFFFFFF::FFFFFFFFFFFFFFFFFFFFFFFFFFFFF
@A00155:342:HHGFNDSXY:1:2233:19578:32362 1:N:0:GAACCTAG+TCCGCATA
GGATGTAGAGGGCGAGAGAGAGCGACTTCTCTCGGGCCCACAGCCTTACAGCTGTGGAGCACGGTATC
CTCTGCCGAAACAGAGGTTGGACAAGACCGGAGGGGTCTCCTAGTTCCAAAGGAGATGTACTCC
+
```

FFFFFFFFFFFFFFFFFFFFFFFFFFFFFFFFFFFFFFFFFFFFFFFFFF:FFFFFFFFFFFFFFFFF
FFFFFFFFFFFFFFFFFFFFFFFFFFFFFFFFFFFFFFFFFFFFFFFFFFFFFFFFFFFFFFFF
@A00155:342:HHGFNDSXY:1:2234:23683:1767 1:N:0:GAACCTAG+TCCGCATA
AACCCATGCCCCACCACACCGCCTTAGATCAGCTGTGTCCACTTAAGGACTCACCTCTGGCCTATGGT
CATCTCAATGCCGAGAGGGATGTGGAGGGCGAGAGAGAGCGACTTCTCTCGGGCCCACAGCCTTACAG
+
F:FFFFFFFFFFFFFFFFFFFFFFFFFFFF,FFFFFFFFFFFFFFFFFFFFFFFFFFFFFFFFFFFFF
FFFFFFFFFFFFFFFFFFF:FFFFFFFFFF:FFFFFFFFFFFFFFFFFFFFFFFFFFFFFFFFFFFFF
@A00155:342:HHGFNDSXY:1:2234:22869:15546 1:N:0:GAACCTAG+TCCGCATA
CAATGCCGAGAGGGATGTGGAGGGCGAGAGAGAGCGACTTCTCTCGGGCCCACAGCCTTACAGCTGTG
GAGCACGGTATCCTCTGCCGAAACAGAGGTTGGACAAGACCGGAGGGGTCTCCTAGTTCCAAAGGAGA
+
FFFFFFFFFFFFFFFFFFFFFFFFFFFFFFFFFFFFFFFFFFFFFFFFFFFFFFFFFFFFFFFFFFFF
FFFFFFFFFFFFFFFFFFFFFFFFFFFFFFFFFFFFFFFFFFFFFFFFFFFFFFFFFFFFFFFFFFFF
@A00155:342:HHGFNDSXY:1:2234:16993:32988 1:N:0:GAACCTAG+TCCGCATA
CTTCTCTCGGGCCCACAGCCTTACAGCTGTGGAGCACGGTATCCTCTGCCGAAACAGAGGTTGGACAA
GACCGGAGGGGTCTCCTAGTTCCAAAGGAGATGTACTCCGGGCTTGTTCACGACCTACCGTGTAAGTC
+
FFFFFFFFFFFFFFFFFFFFFFFFFFFFFFFFFFFFFFFFFFFFFFFFFFFFFFFFFFFFFFFFFFFF
FFFFFFFFFFFFFFFFFFFFFFFFFFFFFFFFFFFFFFFFFFFFFFFFFFFFFFFFFFFFFFFFFFFF
@A00155:342:HHGFNDSXY:1:2234:4218:33599 1:N:0:GAACCTAG+TCCGCATA
TCTCTCGGGCCCACAGCCTTACAGCTGTGGAGCACGGTATCCTCTGCCGAAACAGAGGTTGGACAAGA
CCGGAGGGGTCTCCTAGTTCCAAAGGAGATGTACTCCGGGCTTGTTCACGACCTACCGTGTAAGTCGT
+
FFFFFFFFFFFFFFFFFFFF,FFFFFFFFFFFFFFFFFFFFFFFFFFFFFFFFFFFFFFFFFFFFFFF
FFFFFFFFFFFFFFFFFFFFFFFFFFFFFFFFFFFFFFFFFFFFFFFFFFFFFFFFFFFFFFFFFFFF
@A00155:342:HHGFNDSXY:1:2234:20455:36292 1:N:0:GAACCTAG+TCCGCATA
TTAGATCAGCTGTGTCCACTTAAGGACTCACCTCTGGCCTATGGTCATCTCAATGCCGAGAGGGATGT
AGAGGGCGAGAGAGAGCGACTTCTCTCGGGCCCACAGCCTTACAGCTGTGGAGCACGGTATCCTCTGC
+
FFFFFFFFFFFFFFFFFFFFFFFFFFFFFFFFFFFFFFFFFFFFFFFFFFFFFFFFFF:F,FFFFFFF
FFFFFFFFFFFFFFFFFFFFFFFFFFFFFFFFFFFFFFFFFF:FFFFFFFFFFFFFFFFFFFFFFFFF
@A00155:342:HHGFNDSXY:1:2235:4074:14278 1:N:0:GAACCTAG+TCCGCATA
GGATGTGGAGGGCGAGAGAGAGCGACTTCTCTCGGGCCCACAGCCTTACAGCTGTGGAGCACGGTATC
CTCTGCCGAAACAGAGGTTGGACAAGACCGGAGGGGTCTCCTAGTTCCAAAGGAGATGTACTCC
+
FFFFFFFFFFFFFFFFFFFFFFFFFFFFFFFFFFFFFFFFFFFFFFF:FFFFFFFFFFFFFFFFFFFF
FFFFFFFFFFFFFFFFF:FFF:FFFFFFFFFFFFFFFFFFFFFFFFFFFFFFFFFF:FFFFFFF
@A00155:342:HHGFNDSXY:1:2235:21251:20509 1:N:0:GAACCTAG+TCCGCATA
CACAGCCTTACAGCTGTGGAGCACGGTATCCTCTGCCGAAACAGAGGTTGGACAAGACCGGAGGGGTC
TCCTAGTTCCAAAGGAGATGTACTCCGGGCTTGTTCACGACCTACCGTGTAAGTCGTAGTCTAGTAGG
+
FFFFFFFFFFFFFFFFFFFFFFFFFFFFFFFFFFFFFFFFFFFFFFFFFFFFFFFFFFFFFFFFFFFF
FFFFFFFFFFFFFFFFFFFFFF:FFFFFFF,FFFFFFFFFFFFFFFF:F:F,F:FFFFFFFFFFFFFF
@A00155:342:HHGFNDSXY:1:2236:25635:5901 1:N:0:GAACCTAG+TCCGCATA
GGATGTGGAGGGCGAGAGAGAGCGACTTCTCTCGGGCCCACAGCCTTACAGCTGTGGAGCACGGTATC
CTCTGCCGAAACAGAGGTTGGACAAGACCGGAGGGGTCTCCTGGTTCCAAAGGAG
+
FFFFFFFFFFFFFFFFFFFFFFFFFFFFFFFFFFFFFFFFFFFFFFFFFFFFFFFFFFFFFFFFFFFF
FFFFFFFFFFFFFFFFFFFFFFFFFFFFFFFFFFFFFFFFFFFFFFFFFF,FFFF
@A00155:342:HHGFNDSXY:1:2236:11930:9377 1:N:0:GAACCTAG+TCCGCATA
CTATGGTCATCTCAATGCCGAGAGGGATGTGGAGGGCGAGAGAGAGCGACTTCTCTCGGGCCCACAGC
CTTACAGCTGTGGAGCACGGTATCCTCTGCCGAAACAGAGGTTGGACAAGACCGGAGGGGTCTCCTA
+

```
FFFFF::FFFFFFFFFFFF,F:FFFFFFFFFFFFF,FFF:FFFFFFFFFFF,FFFFF,F:FFFF:FFF
FF:FFFF,:FFFFFFFF:FFFF:FFFFFFFFFFFF:,:FFFFF,:FF:FFFF,F:F:FF::,FFFFF
@A00155:342:HHGFNDSXY:1:2236:2989:33254 1:N:0:GAACCTAG+TCCGCATA
AGAGAGAGCGACTTCTCTCGGGCCCACAGCCTTACAGCTGTGGAGCACGGTATCCTCTGCCGAAACAG
AGGTTGGACAAGACCGGAGGGGTCTCCTGGTTCCAAAGGAGATGTACTCCGGGCTTGTTCACGACCTA
+
FFFFFFFFFFFFFFFFFFFFFFFFFFFFFFFFFFFFFFFFFFFFF:FFFFFFFFFFFFFFFFFFFFFF
FFFFFFFFFFFFFFFFFFFFFFFFFFFFFFFFFFFFFFFFFFFFFFFFFFFFFFFFFFFFFFFFFFFF
@A00155:342:HHGFNDSXY:1:2237:7509:16877 1:N:0:GAACCTAG+TCCGCATA
CAATGCCGAGAGGGATGTGGAGGGCGAGAGAGAGCGACTTCTCTCGGGCCCACAGCCTTACAGCTGTG
GAGCACGGTATCCTCTGCCGAAACAGAGGTTGGACAAGACCGGAGGGGTCTCCTAGTTCCAAAGGAGA
+
FFFFFFFFFF:FFFF,FFFFFFF:FFFFFFFFFFFFFF:FFFFFFFFFFFFFFFFFFFF,FFFFFFFF
FFFFFFFFFFFFFFFFFFFFFFFFFFFFFFFFFFFFFFFFFFFFFFFF:FFFFFFFFFFFFFFFFFFF
@A00155:342:HHGFNDSXY:1:2237:17146:18662 1:N:0:GAACCTAG+TCCGCATA
GGATGTGGAGGGCGAGAGAGAGCGACTTCTCTCGGGCCCACAGCCTTACAGCTGTGGAGCACGGTATC
CTCTGCCGAAACAGAGGTTGGACAAGACCGGAGGGGTCTCCTAGTTCCAAAGGAGATGTACTCC
+
FFFFFFFFFFFFFFFFFFFFFFFFFFFFFFFFFFFFFFFFFFFFFFFFFFFFFFFFFFFFFFFFFFFF
FFFFFFFFFFFFFF:FFFFFFFFFFFFFFFFFFFFFFFFFFFFFFFFFFFFFFFFFFFFFFFFF
@A00155:342:HHGFNDSXY:1:2238:21766:1423 1:N:0:GAACCTAG+TCCGCATA
AGAGAGAGCGACTTCTCTCGGGCCCACAGCCTTACAGCTGTGGAGCACGGTATCCTCTGCCGAAACAG
AGGTTGGACAAGACCGGAGGGGTCTCCTGGTTCCAAAGGAGATGTACTCCGGGCTTGTTCACGACCTA
+
FFFFFFFFFFFFFFFFFFFFFFFFFFFFFFFFFFFFFFFFFFFFFFFFFFFFFFFFFFFFFFFFFFFF
FFFFFFFFFFFFFFFFFFFFFFFFFFFFFFFFFFFFFFFFFFFFFFFFFFFFFFFFFFFFFFFFFFFF
@A00155:342:HHGFNDSXY:1:2238:3278:15624 1:N:0:GAACCTAG+TCCGCATA
GGATGTGGAGGGCGAGAGAGAGCGACTTCTCTCGGGCCCACAGCCTTACAGCTGTGGAGCACGGTATC
CTCTGCCGAAACAGAGGTTGGACAAGACCGGAGGGGTCTCCTAGTTCCAAAGGAGATGTACTC
+
FFF:FFFFFFFFFFFFFFFFFFFFFFFFFF:FFFFFFFFFFFF:FFFFFFFFFFFFFFFFFFFFFFFF
FFFFFFFFFFFFFFFFFFFFFFFFFFFFFFFFFFFFFFF,FFFFFFFF:FFFFFFFFFFFFFF
@A00155:342:HHGFNDSXY:1:2238:23583:31250 1:N:0:GAACCTAG+TCCGCATA
CAATGCCGAGAGGGATGTGGAGGGCGAGAGAGAGCGACTTCTCTCGGGCCCACAGCCTTACAGCTGTG
GAGCACGGTATCCTCTGCCGAAACAGAGGTTGGACAAGACCGGAGGGGTCTCCTAGTTCCAAAGGAGA
+
FFFFFFFFFFFFFFFFFFFFFFFFFFFFFFFFFFFFFFFFFFFFFFFFFFFFFFFFFFFFFFFFFFFF
FFFFFFFFFFFFFFFFFFFFFFFFFFFFFFFFFFFFFFFFFFFFFFFFFFFFFFFFFFFFFFFFFFFF
@A00155:342:HHGFNDSXY:1:2239:27778:9987 1:N:0:GAACCTAG+TCCGCATA
GAGAGAGCGACTTCTCTCGGGCCCACAGCCTTACAGCTGTGGAGCACGGTATCCTCTGCCGAAACAGA
GGTTGGACAAGACCGGAGGGGTCTCCTAGTTCCAAAGGAGATGTACTCCGGGCTTGTTCACGACCTAC
+
FFFFFFFFFFFFFFFFFFFFFFFFF:FFFFFFFFFFFFFFFFFFFFFFFFFFFFFFFFF:FFFFFFFF
FFFF:FFFFF,FFF:FFFFFFFFFFFFFFF:FFFFFF:FFFFFFFFF,FFFFFFFFFFFFFFFFFFFF
@A00155:342:HHGFNDSXY:1:2239:30047:12696 1:N:0:GAACCTAG+TCCGCATA
GAGAGAGCGACTTCTCTCGGGCCCACAGCCTTACAGCTGTGGAGCACGGTATCCTCTGCCGAAACAGA
GGTTGGACAAGACCGGAGGGGTCTCCTAGTTCCAAAGGAGATGTACTCCGGGCTTGTTCACGACCTAC
+
FFFFFFFFFFFFFFFFFFFFFFFFFFFFFFFFFFFFFFFFFFFFFFFFFFFFFFFFFFFFFFFFFFFF
FFFFFFFFFFFFF:FFFFFFFF:FFFFFFFFFFFFFFFFFFFFFFFFFFFFFFFFFFFFF:FFFFFFF
@A00155:342:HHGFNDSXY:1:2239:10773:20807 1:N:0:GAACCTAG+TCCGCATA
CCTTACAGCTGTGGAGCACGGTATCCTCTGCCGAAACAGAGGTTGGACAAGACCGGAGGGGTCTCCTA
GTTCCAAAGGAGATGTACTCCGGGCTTGTTCACGACCTACCGTGTAAGTCGTAGTCTAGTAGGCTACC
+
```

FFFFFFFFFFFFFFFFFFFFFFFFFFFFFFFFFFFFFFFFFFFFFFFFFFFFFFFFFFFFFFFFFFFFFFFF
FFFFFFFFFFFFFFFFFFFFFFFFFFFFFFFFFFFFFFFFFFFFFFFFFFFFFF::FFFFFF:FFFFFFF
@A00155:342:HHGFNDSXY:1:2239:29984:35665 1:N:0:GAACCTAG+TCCGCATA
CTTCTCTCGGGCCCACAGCCTTACAGCTGTGGAGCACGGTATCCTCTGCCGAAACAGAGGTTGGACAA
GACCGGAGGGGTCTCCTAGTTCCAAAGGAGATGTACTCCGGGCTTGTTCACGACCTACCGTGTAAGTC
+
FFFFFFFFFFFFFFFFF:FFFFFFFFFFFFFFFFFFFFFFFFFFFFFFFFFFFFFFFFFFFFFFFFFFFF
FFFFFFFFFFFFFFFFFFFFFFFFFFFFFFFFFFFFFFFFFFFFFFFFFFFFFFFFFFF:FFFFF:FFF
@A00155:342:HHGFNDSXY:1:2240:29460:11678 1:N:0:GAACCTAG+TCCGCATA
ATGCCCCGCCACACCGCCTTAGATCAGCTGTGTCCACTTAAGGACTCACCTCTGGCCTATGGTCATCT
CAATGCCGAGAGGGATGTGGAGGGCGAGAGAGAGCGACTTCTCTCGGGCCCACAGCCTTACAGCTGTG
+
FFFFFFFFFFFFFFFFFFFFFFFFFFFFFFFFFFFFFFFFFFFFFFFFFFFFFFFFFFFFFFFFFFFFFFFF
FFFFFFFFFFFFFFFFFFFFFFFFFFFFFFFFFFFFFFFFFFFFFFFFFFFFFFFFFFFFFFFFFFFFFFFF
@A00155:342:HHGFNDSXY:1:2240:15311:12038 1:N:0:GAACCTAG+TCCGCATA
GCGACTTCTCTCGGGCCCACAGCCTTACAGCTGTGGAGCACGGTATCCTCTGCCGAAACAGAGGTTGG
ACAAGACCGGAGGGGTCTCCTAGTTCCAAAGGAGATGTACTCCGGGCTTGTTCACGACCTACCGTGTA
+
FFFFFFFFFFFFFFFFFFFFFFFFFFFFFFFFFFFFFFFFFFFFFFFFFFFFFFFFFFFFFFFFFFFFFFFF
FFFFFFFFFFFFFFFFFFFFFFFFFFFFFFFFFFFFFFFFFFFFFFFFFFFFFFFFFFFFFFFFFFFFFFFF
@A00155:342:HHGFNDSXY:1:2240:6216:25848 1:N:0:GAACCTAG+TCCGCATA
AGCGACTTCTCTCGGGCCCACAGCCTTACAGCTGTGGAGCACGGTATCCTCTGCCGAAACAGAGGTTG
GACAAGACCGGAGGGGTCTCCTAGTTCCAAAGGAGATGTACTCCGGGCTTGTTCACGACCTACCGTGT
+
FFFFFFFFFFFFFFFFFFFFFFFFFFFFFFFFFFFFFFFFFFFFFFFFFFFFFFFFFFFFFFFFFFFFFFFF
FFFFFFFFFFFFFFFFFFFFFFFFFFFFFFFFFFFFFFFF:FFFFFFFFFFFFFFFFFFFFFFFFFFFFFFF
@A00155:342:HHGFNDSXY:1:2240:23900:27539 1:N:0:GAACCTAG+TCCGCATA
CTTCTCTCGGGCCCACAGCCTTACAGCTGTGGAGCACGGTATCCTCTGCCGAAACAGAGGTTGGACAA
GACCGGAGGGGTCTCCTAGTTCCAAAGGAGATGTACTCCGGGCTTGTTCACGACCTACCGTGTAAGTC
+
FFFFFFFFFFFFFFFFFFFFFFFFFFFFFFFFFFFFFFFFFFFFFFFFFFFFFFFFFFFFFFFFFFFFFFFF
FFFFFFFFFFFFFFFFFFFFFFFFFFFFFFFFFFFFFFFFFFFFFF,FFFFFFFFFFFFFFFFFFFFF:FFF
@A00155:342:HHGFNDSXY:1:2240:16116:31626 1:N:0:GAACCTAG+TCCGCATA
AGAGAGAGCGACTTCTCTCGGGCCCACAGCCTTACAGCTGTGGAGCACGGTATCCTCTGCCGAAACAG
AGGTTGGACAAGACCGGAGGGGTCTCCTGGTTCCAAAGGAGATGTACTCCGGGCTTGTTCACGACCTA
+
FFFFFFFFFFFFFFFFFFFFFFFFFFFFFFFFFF,FFFFFFFFFFFFFFFFFFFFFFFFFFFFFFFFFFFFF
FFFFFFFFFFFFFFFFFFFFFFFFFFFFFFFFFFFFFFFFFFFFFFFFFFFFFFFFFFFFFFFFFFFFFFF:
@A00155:342:HHGFNDSXY:1:2240:20437:32503 1:N:0:GAACCTAG+TCCGCATA
GAGGGCGAGAGAGAGCGACTTCTCTCGGGCCCACAGCCTTACAGCTGTGGAGCACGGTATCCTCTGCC
GAAACAGAGGTTGGACAAGACCGGAGGGGTCTCCTAGTTCCAAAGGAGATGTACTCCGGGCTTGTTCA
+
:FFFFF,FFF,FFFFF,FFF:::F:::::FFFFFFFFF:FFF:FFF:FF,F,FFFF,FFFFFFFFFFF
FFF,FFFF,FF,FFFF,:,FFF,FFFFFFF::FF:F,:FFFFFFFFFFFFF,FFFFFFFF,FF::FFF
@A00155:342:HHGFNDSXY:1:2240:12617:36839 1:N:0:GAACCTAG+TCCGCATA
CCACCACACCGCCTTAGATCAGCTGTGTCCACTTAAGGACTCACCTCTGGCCTATGGTCATCTCAATG
CCGAGAGGGATGTGGAGGGCGAGAGAGAGCGACTTCTCTCGGGCCCACAGCCTTACAGCTGTGGAGCA
+
FFFFFFFFFFFFFFFFFFFFFFFFFFFFFFFFFFFFFFFFFFFFFFFFFFFFFFFFFFFFFFFFFFFFFFFF
FFFFF:FFF:FFFFFFFFFFFFFFFFFFFFFFFFFFFFFFFFFFFFFFFFFFFFFFFFFFFFFFFFFFFFFF
@A00155:342:HHGFNDSXY:1:2241:26377:1078 1:N:0:GAACCTAG+TCCGCATA
TGGTCATCTCAATGCCGAGAGGGATGTGGAGGGCGAGAGAGAGCGACTTCTCTCGGGCCCACAGCCTT
ACAGCTGTGGAGCACGGTATCCTCTGCCGAAACAGAGGTTGGACAAGACCGGAGGGGTC
+

FFFFFFFFFFFFFFFFFFFFFFFFF:F,FFFFFFFFFFFFFFFFFFFFF:FFFFFFFFFFFFFFFFFFF
FFFFF:FFFFFFFFFFFFFFFFFFFFFFFFFFFFFFFFFFFFFFFFFFFFFFFFFFFFFFF
@A00155:342:HHGFNDSXY:1:2241:24939:16908 1:N:0:GAACCTAG+TCCGCATA
GGGCGAGAGAGAGCGACTTCTCTCGGGCCCACAGCCTTACAGCTGTGGAGCACGGTATCCTCTGCCGA
AACAGAGGTTGGACAAGACCGGAGGGGTCTCCTAGTTCCAAAGGAGATGTACTCCGGGCTTGTTCACG
+
FFFFFFFFFFFFF:FFFFFFFFFFFF,FFFFFFFFFFFFFFFFFF:FFFF:FFFFFFFFFFFF:F:FF
FFFFFFFFFFFFFFFFF:FFFFFFFFFFFFFFF:FFFFFFFFFFF:FFF:FFFFFFFFF:FFFFFFFFFFF:
@A00155:342:HHGFNDSXY:1:2241:31738:25269 1:N:0:GAACCTAG+TCCGCATA
TGCCGAGAGGGATGTGGAGGGCGAGAGAGAGCGACTTCTCTCGGGCCCACAGCCTTACAGCTGTGGAG
CACGGTATCCTCTGCCGAAACAGAGGTTGGACAAGACCGGAGGGGTCTCCTAGTTCCAAAGGAGATG
+
FFFFFFFFFFFF,FFFFFFFFFFFFFFF:FFFFFFFFFFFFFFFFFFFFFFF,FF:FFFFFFFFFFFFF
FFFFFFF,FFFFFFFFF:FFFFFFFF:FFFF:FFFFFFFFFFFFFFFFFFF,FFFFFFFFFFF,FF:F
@A00155:342:HHGFNDSXY:1:2241:21432:26991 1:N:0:GAACCTAG+TCCGCATA
TTAAGGACTCTCCTCTGGCCTATGGTCATCTCAATGCCGAGAGGGATGTGGAGGGCGAGAGAGAGCGA
CTTCTCTCGGGCCCACAGCCTTACAGCTGTGGAGCACGGTATCCTCTGCCGAAACAGAGGTTGGACAA
+
FFFFFFFFFFFFFFFFFFFFFFFFFFFFFFFFFFFFFFFFFFFFFF:FFFFFFFFFFFFFFFFFFFFFF
F:FFFFFFFFFFFFFFFFFFFFFFFFFFFFFFFFFFFFFFFFFFFFFFFFFFFFFFFF,FFFFFFFFFF
@A00155:342:HHGFNDSXY:1:2241:29649:32800 1:N:0:GAACCTAG+TCCGCATA
CGACTTCTCTCGGGCCCACAGCCTTACAGCTGTGGAGCACGGTATCCTCTGCCGAAACAGAGGTTGGA
CAAGACCGGAGGGGTCTCCTAGTTCCAAAGGAGATGTACTCCGGGCTTGTTCACGACCTACCGTGTA
+
FFFF,FFFF:FFFFFFFFFFFFFFFFFFFFFFFFFFFFFFFFFFFFFFFFFFFFFFFFFFFFFFFFFFF
FFFFFFFFFFFFFFFFFFFFFFFFFFFFFFFFFFFFFFFFFFFFFFFFFFFFFFFFFFFFFFFFFFFF
@A00155:342:HHGFNDSXY:1:2242:6470:1078 1:N:0:GAACCTAG+TCCGCATA
CCTCTGCCGAAACAGAGGTTGGACAAGACCGGAGGGGTCTCCTAGTTCCAAAGGAGATGTACTCCAGG
CTTGTTCACGACCTACCGTGTAAGTCGTAGTCTAGTAGGCTACCTGACGAGTCCTTTTTAGGACGAAA
+
FFFFFFFFFFFFFFFFFFFFFFFFFFFFFFFFFFFFFFFFFFFFFFFFFFFFFFFFF:FFFFFFFFFFF
FFFFFFFFFFFFFFFFFFFFFFFFFFFFFFFFFFFFFFFFFFFFFFFFFFFFFFFFF,FFFFFFFFFFFF
@A00155:342:HHGFNDSXY:1:2242:6298:22576 1:N:0:GAACCTAG+TCCGCATA
GAGCGACTTCTCTCGGGCCCACAGCCTTACAGCTGTGGAGCACGGTATCCTCTGCCGAAACAGAGGTT
GGACAAGACCGGAGGGGTCTCCTAGTTCCAAAGGAGATGTACTCCGGGCTTGTTCACGACCTACCGTG
+
FFFFFFFFFFFFFFFFFFFFFFFFFFF:FFF:FFFFFFFFFFFFFFFF:FFFF:FFFFFFFFFFFFFFFF
FFFFFFFFFFFFFFFFFFFF:FFFFFFFFFFFFFFFFFFFFFFFFFFFFFFFFFFFFFFFFFFFFFFFFFF
@A00155:342:HHGFNDSXY:1:2243:15501:4977 1:N:0:GAACCTAG+TCCGCATA
CTTCTCTCGGGCCCACAGCCTTACAGCTGTGGAGCACGGTATCCTCTGCCGAAACAGAGGTTGGACAA
GACCGGAGGGGTCTCCTAGTTCCAAAGGAGATGTACTCCGGGCTTGTTCACGACCTACCGTGTAAGTC
+
FFFFFFFFFFFFFFFFFFFFFFFFFFFFFFFFFFFFFFFFFFFFFFFFFFFFFFFFFFFFFFFFFFFFFF
FFFFFFFFFFFFFFFFFFFFFFFFFFFFFFFFFFFFFFFFFFFFFFFFFFFFFFFFFFFFFFFFFFFFFF
@A00155:342:HHGFNDSXY:1:2243:30653:17973 1:N:0:GAACCTAG+TCCGCATA
AGAGCGACTTCTCTCGGGCCCACAGCCTTACAGCTGTGGAGCACGGTATCCTCTGCCGAAACAGAGGT
TGGACAAGACCGGAGGGGTCTCCTGGTTCCAAAGGAGATGTACTCCGGGCTTGTTCACGACCTACCGT
+
FFFFFFFFFFFFFFFFFFFFFFFFFFFFFFFFFFFFFFFFFFFFFFFFFFFFFFFFFFFFFFFFFFFFFF
FFFFFFFFFFFFFFFFFFFFFFFFFFFFFFFFFFFFFFFFFFFFFFFFFFFF:FFFFFFFFFFFFFFFFF
@A00155:342:HHGFNDSXY:1:2243:12048:23202 1:N:0:GAACCTAG+TCCGCATA
GATGTGGAGGGCGAGAGAGAGCGACTTCTCTCGGGCCCACAGCCTTACAGCTGTGGAGCACGGTATCC
TCTGCCGAAACAGAGGTTGGACAAGACCGGAGGGGTCTCCTGGTTCCAAAGGAGATGTACTCCGGGCT
+

FFFFFFFFFFFFFFFFFFFFFFFFFFFFFFFFFFFFFFFFFFFFFFFFFFFFFFFFFFFFFFFFFFFF
FFFFFFFFFFFFFFFFFFFFFFFFFFFFFFFFFFFFFFFFFFFFFFFFFFFFFFFFFFFFFFFFFFFF
@A00155:342:HHGFNDSXY:1:2243:4237:23610 1:N:0:GAACCTAG+TCCGCATA
GATGTAGAGGGCGAGAGAGAGCGACTTCTCTCGGGCCCACAGCCTTACAGCTGTGGAGCACGGTATCC
TCTGCCGAAACAGAGGTTGGACAAGACCGGAGGGGTCTCCTAGTTCCAAAGGAGATGTACTCCGGGCT
+
FFFFFFFFFFFFFFFFFFFFFFFFFFFFFFFFFFFFFFFFFFFFFFFFFFFFFFFFFFFFFFFF:FFF
FFFFFFFFFFFFFFFFFFFFFFFFFFFFFFF:FFFFFFFFFFFFFFFFFF,FFFFFF:FFFFFFFFFF
@A00155:342:HHGFNDSXY:1:2244:20166:11397 1:N:0:GAACCTAG+TCCGCATA
CACCTCTGGCCTATGGTCATCTCAATGCCGAGAGGGATGTGGAGGGCGAGAGAGAGCGACTTCTCTCG
GGCCCACAGCCTTACAGCTGTGGAGCACGGTATCCTCTGCCGAAACAGAGGTTGGACAAGACCGGAGG
+
FFFFFFFFFFFFFFFFFFFFFFFFFFFFFFFFFFFFFFFFFFFFFFFFFFFFFFFFFFFFFFFFFFFF
FFFFFFFFFFFFFFFFFFFFFFFFFFFFFFFFFFFFFFFFFFFFFFFFFFFFFFFFFFFFFFFFFFFF
@A00155:342:HHGFNDSXY:1:2244:18638:29011 1:N:0:GAACCTAG+TCCGCATA
GGGCGAGAGAGAGCGACTTCTCTCGGGCCCACAGCCTTACAGCTGTGGAGCACGGTATCCTCTGCCGA
AACAGAGGTTGGACAAGACCGGAGGGGTCTCCTGGTTCCAAAGGAGATGTACTCCGGGCTTGTTCACG
+
FFF:FFFFFFFFFFFFFFFFFF:FF,FFF:FFFFF:FF:FFFFFFFFFFFFFFFFFFFFFFFFFFFFF
FFFFFFFFFFFFFFFFFFFFFFFFFFF:FFFFFFFFFFFFFFFFFFFFFFFFFFFFFFFFFFFFFFFF
@A00155:342:HHGFNDSXY:1:2244:19108:29450 1:N:0:GAACCTAG+TCCGCATA
GGGCGAGAGAGAGCGACTTCTCTCGGGCCCACAGCCTTACAGCTGTGGAGCACGGTATCCTCTGCCGA
AACAGAGGTTGGACAAGACCGGAGGGGTCTCCTGGTTCCAAAGGAGATGTACTCCGGGCTTGTTCACG
+
FFFFFFFFFFFFFFFFFFFFFFFFFFFFFFFFFFFFFFFFFFFFFFFFFFFFFFFFFFFFFFFFFFFF
FFFFFFFFFFFFFFFFF:FFFFFFFFFFFFFFFFFFFFFFFFFFFFFFFFFFFFFFFFFFFFFFF:FF
@A00155:342:HHGFNDSXY:1:2244:3956:36307 1:N:0:GAACCTAG+TCCGCATA
GGACTCTCCTCTGGCCTATGGTCATCTCAATGCCGAGAGGGATGTGGAGGGCGAGAGAGAGCGACTTC
TCTCGGGCCCACAGCCTTACAGCTGTGGAGCACGGTATCCTCTGCCGAAACAGAGGTTGGACAAGACC
+
FFFFFFFFFFFFFFFFFFFFFFFFFFFFFFFFFFFFFFFFFFFFFFFFFFFFFFFFFFFFFFFFFFFF
FFFFFFFFFFFFFFFFFFFFFFFFFFFFFFFFFFFFFFFFFFFFFFFFFFFFFFFFFFFFFFFFFFFF
@A00155:342:HHGFNDSXY:1:2245:9146:17895 1:N:0:GAACCTAG+TCCGCATA
CGACTTCTCTCGGGCCCACAGCCTTACAGCTGTGGAGCACGGTATCCTCTGCCGAAACAGAGGTTGGA
CAAGACCGGAGGGGTCTCCTGGTTCCAAAGGAGATGTACTCCGGGCTTGTTCACGACCTACCGTGTAA
+
FFFFFFFFFFFFFFFFFFFFFFFFFFFFFFFF:FFFFFFFFFFFFFFFFFFFFFFFFFFFFFFFFFFF
FFFFFFFFFFFFFFFFFFFFFFFFFFFFFFFFFFFFFFFFFFFFFFFFFFFFFFFFFFFFFFFFFFF:
@A00155:342:HHGFNDSXY:1:2245:19117:23265 1:N:0:GAACCTAG+TCCGCATA
ATCTCAATGCCGAGAGGGATGTGGAGGGCGAGAGAGAGCGACTTCTCTCGGGCCCACAGCCTTACAGC
TGTGGAGCACGGTATCCTCTGCCGAAACAGAGGTTGGTCAAGACCGGAGGGGTCTCCTA
+
FFFFFFFFFFFF,FFFFFFFFFFFFFFFFFFFFFFFFFF:FFFFFFFFFFFFFFFFFFFFFFFFFFFF
FFFFFFFFFFFFFFFFFFFFFFFFFFFFFFFFFFFFFFFFFF:FFFFFFFFFFFFFFFF
@A00155:342:HHGFNDSXY:1:2245:5276:32894 1:N:0:GAACCTAG+TCCGCATA
TGCCGAGAGGGATGTGGAGGGCGAGAGAGAGCGACTTCTCTCGGGCCCACAGCCTTACAGCTGTGGAG
CACGGTATCCTCTGCCGAAACAGAGGTTGGACAAGACCGGAGGGGTCTCCTAGTTCCAAAGGAGATGT
+
FFFFFFFFFFF,FFFFFFFFFFFFFFFFFFFFFFFFFFFFFFFFFFFFFFFFFFF:,FFFFFFFF:FF
F:FFF:FFFFFFFFFFFFFF:FFFFFFFFFFFFFF:FFFFFFFFFFFFFF:FFFFFFFFFFFFFFFFF
@A00155:342:HHGFNDSXY:1:2245:5828:35665 1:N:0:GAACCTAG+TCCGCATA
TCCACTTAAGGACTCACCTCTGGCCTATGGTCATCTCAATGCCGAGAGGGATGTGGAGGGCGAGAGAG
AGTGACTTCTCTCGGGCCCACAGCCTTACAGCTGTGGAGCACGGTATCCTCTGCCGAAACAGAGGTTG
+

FFFFFFFFFFFFFFFFFFFFFFFFFFFFFFFFFFFFFFFFFFFFFFFFFFFFFFFFFFFFFFFFFFFF
FFFFFFFFFFFFFFFFFFFFFFFFFFFFFFFFFFFFFFFFFFFFFFFFFFFFFFFFFFFFFFFFFFFF
@A00155:342:HHGFNDSXY:1:2245:20509:36542 1:N:0:GAACCTAG+TCCGCATA
GGATGTGGAGGGCGAGAGAGAGCGACTTCTCTCGGGCCCACAGCCTTACAGCTGTGGAGCACGGTATC
CTCTGCCGAAACAGAGGTTGGACAAGACCGGAGGGGTCTCCTAGTTCCAAAGGAGATGTACTCC
+
FFFFFFFFFFFFFFFFFFFFFFFFFFFFFFFFFFFFFFFFFFFFFFFFFFFFFFFFFFFFFFFFFFFF
FFFFFFFFFFFFFFFFFFFFFFFFFFFFFFFFFFFFFFFFFFFFFFFFFFFFFFFFFFFFFFFF
@A00155:342:HHGFNDSXY:1:2246:6741:12602 1:N:0:GAACCTAG+TCCGCATA
GGGCGAGAGAGAGCGACTTCTCTCGGGCCCACAGCCTTACAGCTGTGGAGCACGGTATCCTCTGCCGA
AACAGAGGTTGGACAAGACCGGAGGGGTCTCCTAGTTCCAAAGGAGATGTACTCCGGGCTTGTTCACG
+
FFFFFFFFFF:FFFFFFFFFFFFFFFFFFFFFFFFFFFFFFFFFFFF:FFFFFFFFF:FFFFFFFFFF
FFFFFFFFFF:FFFFFFFFFFFF:FFFFFFFFF:FFFFFFFF,FFFFFFFFFFF:F:FFFFFFFFFFF
@A00155:342:HHGFNDSXY:1:2246:8585:24502 1:N:0:GAACCTAG+TCCGCATA
CTCTGCCGAAACAGAGGTTGGACAAGACCGGAGGGGTCTCCTAGTTCCAAAGGAGATGTACTCCGGGC
TTGTTCACGACCTACCGTGTAAGTCGTAGTCTAGTAGGCTACCTGACGAGTCCTTTTTAGGACGAAAC
+
F:FFFFFFFFFFF:FFFFF:FFFFFFFFF:FFFFFFFFFFFFF:FFFFFFF,FF:FF:FFFFFFFFFF
FFFF:FFFFF,FFFFFFFFFFFFFFFFFFFFFF:FFFFFFFFFFFFFFFFFFFFFFFFF:F:FFFF:F
@A00155:342:HHGFNDSXY:1:2246:18521:28714 1:N:0:GAACCTAG+TCCGCATA
GACTTCTCTCGGGCCCACAGCCTTACAGCTGTGGAGCACGGTATCCTCTGCCGAAACAGAGGTTGGAC
AAGACCGGAGGGGTCTCCTAGTTCCAAAGGAGATGTACTCCGGGCTTGTTCACGACCTACCGTGTAAG
+
FFFFFFFFFFFFFFFFFFFFFF,FFFFFFFFFFFFFFFFFFFFFFFFFFFFFFFFFFFFFFFFFFFFF
FFFFFFFFFFFFFFFFFFFFFFFFFFFFFFFFFFFFFFFFFFFFFFFFFFFFFFFFFFFFFFFFFFFF
@A00155:342:HHGFNDSXY:1:2246:13105:30295 1:N:0:GAACCTAG+TCCGCATA
TCGGCAGAGGATACCGTGCTCCACAGCTGTAAGGCTGTGGGCCCGAGAGAAGTCGCTCTCTCTCGCCC
TCCACATCCCTCTCGGCATTGAGATGACCATAGGCCAGAGGTGAGTCCTTAAGTGGACACA
+
FFFFFFFFFFFFFFFFFFFFFFFFFFFFFFFFFFFFFFFFFFFFFFFFFFFFFFFFFFFFFFFFFFFF
FFFFFFFFFFFFFFFFFFFFFFFFFFFFFFFFFFFFFFFFFFFFFFFFFFFFFFFFFF:FF
@A00155:342:HHGFNDSXY:1:2247:15257:10504 1:N:0:GAACCTAG+TCCGCATA
GAGCGACTTCTCTCGGGCCCACAGCCTTACAGCTGTGGAGCACGGTATCCTCTGCCGAAACAGAGGTT
GGACAAGACCGGAGGGGTCTCCTAGTTCCAAAGGAGATGTACTCCGGGCTTGTTCACGACCTACCGTG
+
FFFFFFFFFFFFFFFFFFFFFFFFFFFFFFFFFFFFFFFFFFFFFFFFFFFFFFFFFFFFFFFFFFFF
FFFFFFFFFFFFFFFFFFFFFFFFFFFFFFF:FFFFFFFFFFFFFFFFFFFFFFFFFFFFFFFFFFFF
@A00155:342:HHGFNDSXY:1:2247:10538:23218 1:N:0:GAACCTAG+TCCGCATA
AGCGACTTCTCTCGGGCCCACAGCCTTACAGCTGTGGAGCACGGTATCCTCTGCCGAAACAGAGGTTG
GACAAGACCGGAGGGGTCTCCTGGTTCCAAAGGAGATGTACTCCGGGCTTGTTCACGACCTACCGTGT
+
FFFFFFFFFFFFFFFFFFFFFFFFFFFFFFFFFFFFFFFFFFFFFFFFFFFFFFFFFFFFFFFFFFFF
FFFFFFFFFFFFFFFFFFFFFFFFFFFFFFFFFFFFFF:FFFFFFFFFFFFFFFFFFFFFFFFFFFFF
@A00155:342:HHGFNDSXY:1:2248:32759:24627 1:N:0:GAACCTAG+TCCGCATA
TAGACTACGACTTACACGGTAGGTCGTGAACAAGCCCGGAGTACATCTCCTTTGGAACCAGGAGACCC
CTCCGGTCTTGTCCAACCTCTGTTTCGGCAGAGGATACCGTGCTCCACAGCTGTAAGGCTGTGGGCCC
+
FFFFFFFFFFFFFFFFFFFFFFFFFFFFFFFFFFFFFFFFFFFFFFFFFFFFFFFFFFFFFFFF:FFF
FFFFFFFFFFFFFFFFFFFFFFFFFFFFFFFFFFFFFFFFFFFFFFFFFFFFFFFFFFFFFFFFFFFF
@A00155:342:HHGFNDSXY:1:2248:21287:30311 1:N:0:GAACCTAG+TCCGCATA
TCCTCTGCCGAAACAGAGGTTGGACAAGACCGGAGGGGTCTCCTAGTTCCAAAGGAGATGTACTCCGG
GCTTGTTCACGACCTACCGTGTAAGTCGTAGTCTAGTAGGCTACCTGACGAGTCCTTTTTAGGACGAA
+

FFFFFFFFFFFFFFFFFFFFFFFFFFFFFFFFFFFFFFFFFFFFFFFFFFFFFFFFFFFFFFFFFFF
FFFFFFFFFFFFFFFFFFFFFFFFFFFFFFFFFFFFFFFFFF:FFFFFFFFFFFFFFFFFFFFFFFF
@A00155:342:HHGFNDSXY:1:2248:23303:36746 1:N:0:GAACCTAG+TCCGCATA
ATCTCAATGCCGAGAGGGATGTGGAGGGCGAGAGAGAGCGACTTCTCTCGGGCCCACAGCCTTACAGC
TGTGGAGCACGGTATCCTCTGCCGAAACAGAGGTTGGACAAGACCGGAGGGGTCTCCTAGTTCCAAAG
+
FFFFFFFFFFFFFFFFFFFFFFFFFFFFFFFFFFFFFFFFFFFFFFFFFFFFFFFFFFFFFFFFFFF
FFFFFFFFFFFFFFFFFFFFFFFFFFFFFFFFFFFFFFFFFFFFFFFFFFFFFFFFFFFFFFFFFFF
@A00155:342:HHGFNDSXY:1:2249:11451:17628 1:N:0:GAACCTAG+TCCGCATA
ACCTCTGTTTCGGCAGAGGATACCGTGCTCCACAGCTGTAAGGCTGTGGGCCCGAGAGAAGTCGCTCT
CTCTCGCCCTCCACATCCCTCTCGGCATTGAGATGACCATAGGCCAGAGGTGAGTCCTTAAGTGGACA
+
FFFFFFFFFFFFFFFFFFFFFFFFFFFFFFFFFFFFFFFFFFFFFFFFFFFFFFFFFF,FFFFFFFF
FFFFFFFFFFFFFFFFFFFFFFFFFFFFFFFFFFFFFFFFFFFFFFFFFFFFFFFFFFFFFFFFFFF
@A00155:342:HHGFNDSXY:1:2249:11487:18411 1:N:0:GAACCTAG+TCCGCATA
ACCTCTGTTTCGGCAGAGGATACCGTGCTCCACAGCTGTAAGGCTGTGGGCCCGAGAGAAGTCGCTCT
CTCTCGCCCTCCACATCCCTCTCGGCATTGAGATGACCATAGGCCAGAGGTGAGTCCTTAAGTGGACA
+
FFFFFFFFFFFFFFFFFFFFFFFFFFFFFFFFFFFFFFFFFFFFFFFFFFFFFFFFFFFFFFFFFFF
FFFFFFFFFFFFFFFFFFFFFFFFFFFFFFFFFFFFFFFFFFFFFFFFFFFFFFFFFFFFF:FFFFF
@A00155:342:HHGFNDSXY:1:2250:15356:32565 1:N:0:GAACCTAG+TCCGCATA
ATCTCAATGCCGAGAGGGATGTAGAGGGCGAGAGAGAGCGACTTCTCTCGGGCCCACAGCCTTACAGC
TGTGGAGCACGGTATCCTCTGCCGAAACAGAGGTTGGACAAGACCGGAGGGGTCTCCTAGTTCCAAAG
+
FFFFFFFFFFFFFFFFFF:FFFFFFFFFFFFFFFFFFFFFFFFFFFFFFFFFFFFFFFFFFFFFFFF
FFFFFFFFFFFFFFFFFFFFFFFFFFFFFFFFFFFFFFFFFFFFFFFFFFFFFFFFFFFFFFFFFFF
@A00155:342:HHGFNDSXY:1:2251:24505:20541 1:N:0:GAACCTAG+TCCGCATA
CTGTGTCCACTTAAGGACTCACCTCTGGCCTATGGTCATCTCAATGCCGAGAGGGATGTGGTGGGCGA
GAGAGAGCGACTTCTCTCGGGCCCACAGCCTTACAGCTGTGGAGCACGGTATCCTCTGCCGAAACAGA
+
FFFFFFFFFFFFFFFFFFFFFFFFFFFFFFFFFFFFFFFFFFFFFFFFFFFFFFFFFFFFFFFFFFF
FFFFFFFFFFFFFFFFFFFFFFFFFFFFFFFFFFFFFFFFFFFFFFFFFFFFFFFFFFFFFFFFFFF
@A00155:342:HHGFNDSXY:1:2251:8874:34929 1:N:0:GAACCTAG+TCCGCATA
GGTATCCTCTGCCGAAACAGAGGTTGGACAAGACCGGAGGGGTCTCCTAGTTCCAAAGGAGATGTACT
CCGGGCTTGTTCACGACCTACCGTGTAAGTCGTAGTCTAGTAGGCTACCTGACGAGTCCTTTTTAGGA
+
FFFFFFFFFFFFFFFFFFFFFFFFFFFFFFFFFFFFFFFFFFFFFFFFFFFFFFFFFFFFFFFFFFF
FFFFFFFFFFFFFFFFFFFFFFFFFFFFFFFFFFFFFFFFFFFFFFFFFFFFFFFFFFFFFFFFFFF
@A00155:342:HHGFNDSXY:1:2252:9950:18724 1:N:0:GAACCTAG+TCCGCATA
GGACTCTCCTCTGGCCTATGGTCATCTCAATGCCGAGAGGGATGTGGAGGGCGAGAGAGAGCGACTTC
TCTCGGGCCCACAGCCTTACAGCTGTGGAGCACGGTATCCTCTGCCGAAACAGAGGTTGGACAAGACC
+
FFFFFFFFFFFFFFFFFFFFFFFFFFFFFFFFFFFFFFFFFFFFFFFFFFFFFFFFFFFFFFFFFFF
FFFFFFFFFFFFFFFFFFFFFFFFFFFFFFFFFFFFFFFFFFFFFFFFFFFFFFFFFFFFFFFFFFF
@A00155:342:HHGFNDSXY:1:2252:12662:32628 1:N:0:GAACCTAG+TCCGCATA
TGTAGAGGGCGAGAGAGAGCGACTTCTCTCGGGCCCACAGCCTTACAGCTGTGGAGCACGGTATCCTC
TGCCGAAACAGAGGTTGGACAAGACCGGAGGGGTCTCCTAGTTCCAAAGGAGATGTACTCCGGG
+
FFFFFFFFFFFFFFFFFFFFFFFFFFFFFFFFFFFFFFFFFFFFFFFFFFFFFFFFFFFFFFFFFFF
FFFFFFFFFFFFFFFFFFFFFFFFFFFFFFFFFFFFFFFFFFFFFFFFFFFFFFFFFFFFFFFF
@A00155:342:HHGFNDSXY:1:2252:8241:33833 1:N:0:GAACCTAG+TCCGCATA
TCCACTTAAGGACTCACCTCTGGCCTATGGTCATCTCAATGCCGAGAGGGATGTGAAGGGCGAGAGAG
AGCGACTTCTCTCGGGCCCACAGCCTTACAGCTGTGGAGCACGGTATCCTCTGCCGAAACAGAGGTTG
+

```
FFFFFFFFFFFFFFFFFFFFFFFFFFFFFFFFFFFFFFFFFFFFFFFFFFFFFFFFFFFFFFFFFFFF
FFFFFFFFFFFFFFFFFFFFFFFFFFFFFFFFFFFFFFFFFFFFFF:FFFFFFFFFFFFFFFFFFFFF
@A00155:342:HHGFNDSXY:1:2253:26630:13636 1:N:0:GAACCTAG+TCCGCATA
CAATGCCGAGAGGGATGTGGAGGGCGAGAGAGAGCGACTTCTCTCGGGCCCACAGCCTTACAGCTGTG
GAGCACGGTATCCTCTGCCGAAACAGAGGTTGGACAAGACCGGAGGGGTCTCCTGGTTCCAAAGGAGA
+
FFFFFFFFFFFFFFFFFFFFFFFFFFFFFFFFFFFFFFFFFFFFFFFFFFFFFFFFFFFFFFFFFFFF
FFFFFFFFFFFFFFFFFFFFFFFFFFFFFFFFFFFFFFFFFFFFFFFFFFFFFFFFFFFFFFFFFFFF
@A00155:342:HHGFNDSXY:1:2253:4689:17879 1:N:0:GAACCTAG+TCCGCATA
CTCGGGCCCACAGCCTTACAGCTGTGGAGCACGGTATCCTCTGCCGAAACAGAGGTTGGACAAGACCG
GAGGGGTCTCCTAGTTCCAAAGGAGATGTACTCCGGGCTTGTTCACGACCTACCGTGTAAGTCGTAGT
+
FFFFFFFFFFFFFFFFFFFFFFFFFFFFFFFFFFFFFFFFFFFFFFFFFFFFFFFFFFFFFFFFFFFF
FFFFFFFFFFFFFFFFFFFFFFFFFFFFFFFFFFFFFFFFFFFFFFFFFFFFFFFFFFFFFFFFFFFF
@A00155:342:HHGFNDSXY:1:2254:28293:4993 1:N:0:GAACCTAG+TCCGCATA
AGAGAGAGCGACTTCTCTCGGGCCCACAGCCTTACAGCTGTGGAGCACGGTATCCTCTGCCGAAACAG
AGGTTGGACAAGACCGGAGGGGTCTCCTAGTTCCAAAGGAGATGTACTCCGGGCTTGTTC
+
FFFFFFFFFFFFFFFFFFFFFFFFFFFFFFFFFFFFFFFFFFFFFFFFFFFFFFFFFFFFFFFFFFFF
FFFFFFFFFFF:FFFFFFFFFFFFFFFFFFFFFFFFFFFFFFFFFFFFFFF:FFFFFFFFF
@A00155:342:HHGFNDSXY:1:2254:3206:7513 1:N:0:GAACCTAG+TCCGCATA
GGGCGAGAGAGAGCGACTTCTCTCGGGCCCACAGCCTTACAGCTGTGGAGCACGGTATCCTCTGCCGA
AACAGAGGTTGGACAAGACCGGAGGGGTCTCCTAGTTCCATAGGAGATGTACTCCGGGCTTGTTCACG
+
FFFFFFFFFF:F,F:FFFFFFF,FFFFF:FF:FFFFFFFFF:FFFFFFFFFFFF:FFFFFFFFFFFFF
FFFF:FFFFFFFFFFFFFF::FF:FFF,FFF:FFFFFFFFF,FFFFFFFFFFFFFF:FFFFFFFFFFF
@A00155:342:HHGFNDSXY:1:2254:28185:11099 1:N:0:GAACCTAG+TCCGCATA
GTCATCTCAATGCCGAGAGGGATGTAGAGGGCGAGAGAGAGCGACTTCTCTCGGGCCCACAGCCTTAC
AGCTGTGGAGCACGGTATCCTCTGCCGAAACAGAGGTTGGACAAGACCGGAGGGGTCTC
+
FFFFFFFFFFFFFFFFFFFFFFFFFFFFFFFFFFFFFFFFFFFFFFFFFFFFFFFFFFFFFFFFFFFF
FFFFFFFFFFFFFFFFFFFFFFFFFFFFFFFFFFFFFFFFFFFFFFFFFFFFFFFFFFF
@A00155:342:HHGFNDSXY:1:2254:2691:12884 1:N:0:GAACCTAG+TCCGCATA
TCCTCTGCCGAAACAGAGGTTGGACAAGACCGGAGGGGTCTCCTAGTTCCAAAGGAGATGTACTCCGG
GCTTGTTCACGACCTACCGTGTAAGTCGTAGTCTAGTAGGCTACCTGACGAGTCCTTTTTAGGACGAA
+
FFFFFFFFFFFFFFFFFFFFFFFFFFFFFFFFFFFFFFFFFFFFFFFFFFFFFFFFFFFFFFFFFFFF
FFFFFFFFFFFFFFFFFFFFFFFFFFFFFFFFFFFFFFFFFFFFFFFFFFFFFFFFFFFFFFFFFFFF
@A00155:342:HHGFNDSXY:1:2254:25843:13432 1:N:0:GAACCTAG+TCCGCATA
CCACATCCCTCTCGGCATTGAGATGACCATAGGCCAGAGGTGAGTCCTTAAGTGGACACAGCTGATCT
AAGGCGGTGTGGCGGGGCATGGGTTTGAACCCCCATGACGGTCGGAGTCTAGTAGGCTCCCTGATGAG
+
FFFFFFFFFFFFFFFFFFFFFFFFFFFFFFFFFFFFFFFFFFFFFFFFFFFFFFFFFFFFFFFFFFFF
FFFFFFFFFFFFFFFFFFFFFFFFFFFFFFFFFFFFFFFFFFFFFFFFFFFFFFFFFFFFFFFFFFFF
@A00155:342:HHGFNDSXY:1:2254:28863:22764 1:N:0:GAACCTAG+TCCGCATA
GGATGTGGAGGGCGAGAGAGAGCGACTTCTCTCGGGCCCACAGCCTTACAGCTGTGGAGCACGGTATC
CTCTGCCGAAACAGAGGTTGGACAAGACCGGAGGGGTCTCCTAGTTCCAAAGGAGATGTACTCC
+
FFFFFFFFF:FFFFFFF:FFFFFFFFFFFFFFFFFFFFFFFFFFF:FFFFFFFFF:F,FFFFFFFFFF
FFFFFFFFFFFFFFFFFFFF:FFFFFFFFFFF:FFFFFFFFFFF,FFFF:FF,FFFFFFFFF,F
@A00155:342:HHGFNDSXY:1:2254:8241:27633 1:N:0:GAACCTAG+TCCGCATA
GAGCGACTTCTCTCGGGCCCACAGCCTTACAGCTGTGGAGCACGGTATCCTCTGCCGAAACAGAGGTT
GGACAAGACCGGAGGGGTCTCCTAGTTCCAAAGGAGATGTACTCCGGGCTTGTTCACGACCTACCGTG
+
```

FFFFFFFFFFFFFFFFFFFFFFFFFFFFFFFFFFFFFFFFFFFFFFFFFFFFFFFFFFFFFFFFFFFF
FFFFFFFFFFFFFFFFFFFFFFFFFFFFFFFFFFFFFFFFFFFFFFFFFFFFF,FFFFF:FFFFFFF
@A00155:342:HHGFNDSXY:1:2254:9805:27743 1:N:0:GAACCTAG+TCCGCATA
CTCAATGCCGAGAGGGATGTGGAGGGCGAGAGAGAGCGACTTCTCTCGGGCCCACAGCCTTACAGCTG
TGGAGCACGGTATCCTCTGCCGAAACAGAGGTTGGACAAGACCGGAGGGGTCTCCTAGTTCCAAAGGA
+
FFFFFFF:FFFFFFFFFFF:FFFFFFFFFFFFFFFFFFFFFFFFFFFFFFFFFFFFFFFFFFFFFFFF
FFFFFFFFFFF,FFFFFFFFFFFFFFFFFFFFFFFFF,FFFFFFFFFFFFFFFFFFFFFFFFFFFFFF
@A00155:342:HHGFNDSXY:1:2255:10456:18662 1:N:0:GAACCTAG+TCCGCATA
ACCTCTGTTTCGGCAGAGGATACCGTGCTCCACAGCTGTAAGGCTGTGGGCCCGAGAGAAGTCGCTCT
CTCTCGCCCTCTACATCCCTCTCGGCATTGAGATGACCATAGGCCAGAGGTGAGTCCTTAAGTGGAC
+
FFFFFFFFFFFFFFFFFFFFFFFFFFFFFFFFFFFFFFFFFFFFFFFFFFFFFFFFFFFFFFFFFFFF
FFFFFFFFFFFFFFFFFFFFFFFFFFFFFFFFFFFFFFFFFFFFFFFFFFFFFFFFFFFFFFFFFFF
@A00155:342:HHGFNDSXY:1:2255:24542:29935 1:N:0:GAACCTAG+TCCGCATA
GAGCGACTTCTCTCGGGCCCACAGCCTTACAGCTGTGGAGCACGGTATCCTCTGCCGAAACAGAGGTT
GGACAAGACCGGAGGGGTCTCCTGGTTCCAAAGGAGATGTACTCCGGGCTTGTTCACGACCTAC
+
FFFFFFFFFFFFFFFFFFFFFFFFFFFFFFFFFFFFFFFFFFFFFFFFFFFFFFFFFFFFFFFFFFFF
FFFFFFFFFFFFFFFFFFFFFFFFFFFFFFFFFFFFFFFFFFFFFFFF::FFFFFFFFFFFFFF
@A00155:342:HHGFNDSXY:1:2255:22896:31782 1:N:0:GAACCTAG+TCCGCATA
CTTCTCTCGGGCCCACAGCCTTACAGCTGTGGAGCACGGTATCCTCTGCCGAAACAGAGGTTGGACAA
GACCGGAGGGGTCTCCTGGTTCCAAAGGAGATGTACTCCGGGCTTGTTCACGACCTACCGTGTAAGTC
+
FFFFFFFFFFFFFFFFFFFFFFFFFFFFFFFFFFFFFFFFFFFFFFFFFFFFFFFFFFFFFFFFFFFF
FFFFFFFFFFFFFFFFFFFFFFFFFFFFFFFFFFFFFFFFFFFFFFFFFFFFFFFFFFFFFFFFFFFF
@A00155:342:HHGFNDSXY:1:2256:15998:1078 1:N:0:GAACCTAG+TCCGCATA
TTCTCTCGGGCCCACAGCCTTACAGCTGTGGAGCACGGTATCCTCTGCCGAAACAGAGGTTGGACAAG
ACCGGAGGGGTCTCCTAGTTCCAAAGGAGATGTACTCCGGGCTTGTTCACGACCTACCGTGTAAGTCG
+
FFFFFFFFFFFFFFFFFFFFFFFFFFFFFFFFFFFFFFFFFFFFFFFFFFFFFFFFFFFFFFFFFFFF
FFFFFFFFFFFFFFFFFFFFFFFFFFFFFFFFFFFFFFFFFFFFFFFFFFFFFFFFFFFFFFFFFFFF
@A00155:342:HHGFNDSXY:1:2256:10050:1391 1:N:0:GAACCTAG+TCCGCATA
GAGCGACTTCTCTCGGGCCCACAGCCTTACAGCTGTGGAGCACGGTATCCTCTGCCGAAACAGAGGTT
GGACAAGACCGGAGGGGTCTCCTAGTTCCAAAGGAGATGTACTCCGGGCTTGTTCACGACCTACCGTG
+
FFFFFFFFFFFFFFFFFFFFFFFFFFFF:FFFFFFFFFFFFFFFFFFFFFFFFFFFFFFFFFFFFFFF
FFFFFFFFFFFFFFFFFFFFFFFFFFFFFFFFFFFFFFFFFFFFFFFFFFFFFFFFFFFFFFFFFFFF
@A00155:342:HHGFNDSXY:1:2256:16333:3004 1:N:0:GAACCTAG+TCCGCATA
CACCTCTGGCCTATGGTCATCTCAATGCCGAGAGGGATGTGAAGGGCGAGAGAGAGCGACTTCTCTCG
GGCCCACAGCCTTACAGCTGTGGAGCACGGTATCCTCTGCCGAAACAGAGGTTGGACAAGACCGGAGG
+
FFFFFFFFFFFFFFFFFFFFFFFFFFFFFFFFFFF:,FFFFFFFFFFFFFFFFFFFFFFFFFFFFFFF
FFFFFFFFFFFF:FFFFFFFFFFFFFFFFFFFFFFFFFFFFFF,FFFFFFF:FFFFFFFFFFFFFFFF
@A00155:342:HHGFNDSXY:1:2256:3034:7310 1:N:0:GAACCTAG+TCCGCATA
TAGATCAGCTGTGTCCACTTAAGGACTCACCTCTGGCCTATGGTCATCTCAATGCCGAGAGGGATGTG
GAGGGCGAGAGAGAGCGACTTCTCTCGGGCCCACAGCCTTACAGCTGTGGAGCACGGTATCCTCTGCC
+
FFFFFFFFFFFFFFFFFFFFFFFFFFFFFFFFFFFFFFFFFFFFFFFFFFFFFFFFFFFFFFFFFFFF
FFFFFFFFFFFFFFFFFFFFFFFFFFFFFFFFFFFFFFFFFFFFFFFFFFFFFFFFFFFFFFFFFFFF
@A00155:342:HHGFNDSXY:1:2256:9995:16203 1:N:0:GAACCTAG+TCCGCATA
CTCGGGCCCACAGCCTTACAGCTGTGGAGCACGGTATCCTCTGCCGAAACAGAGGTTGGACAAGACCG
GAGGGGTCTCCTAGTTCCAAAGGAGATGTACTCCGGGCTTGTTCACGACCTACCGTGTAAGTCGTAGT
+

FFFFFFFFFFFFFFFFFFFFFFFFFFFFFFFFFFFFFFFF:FFFFFFFFFFF,FFFFFFFFFFFFFFF
FFFFFFFFFFFFFFFFFFF:FFFFFFFFFFFFFF,FFFFF:FFFFF:FFFFFFFFFFFFFFFFFFFFFF:
@A00155:342:HHGFNDSXY:1:2256:32714:23766 1:N:0:GAACCTAG+TCCGCATA
GGATGTGGAGGGCGAGAGAGAGCGACTTCTCTCGGGCCCACAGCCTTACAGCTGTGGAGCACGGTATC
CTCTGCCGAAACAGAGGTTGGACAAGACCGGAGGGGTCTCCTAGTTCCAAAGGAGATGTACTCC
+
FFFFFFFFFFFFFFFFFFFFFFFFFFFFFFFFFFFFFFFFFFFFFFFFFFFFFFFFFFFFFFFFFFFF
FFFFFFFFFFFFFFFFFFFFFFFFFF:FFFFFFFFFFFFFFFFFFFFF:FFFFFFFFFFFFFFF
@A00155:342:HHGFNDSXY:1:2256:4010:29669 1:N:0:GAACCTAG+TCCGCATA
CTTAAGGACTCTCCTCTGGCCTATGGTCATCTCAATGCCGAGAGGGATGTGGAGGGCGAGAGAGAGCG
ACTTCTCTCGGGCCCACAGCCTTACAGCTGTGGAGCACGGTATCCTCTGCCGAAACAGAGGTTGG
+
FFFFFFFFFFFFFFFFFF:FFFFFFFFFFFFFFFFFFFFFFFFFFFFFFFFFFFFFFFFFFFFFFFFF
FFFFFFFFFFFFFFFFFFFFFFFFFFFFFFFFFFFFFFFFFFFFFFFFFFFFFFFFF:FFFF:FFF
@A00155:342:HHGFNDSXY:1:2256:13521:31735 1:N:0:GAACCTAG+TCCGCATA
ACTTCTCTCGGGCCCACAGCCTTACAGCTGTGGAGCACGGTATCCTCTGCCGAAACAGAGGTTGGACA
AGACCGGAGGGGTCTCCTGGTTCCAAAGGAGATGTACTCCGGGCTTGTTCACGACCTACCGTGTAAGT
+
FFFFFFFFFFF:FFFFFFF:FF:FFFFFFFF,:FFFF:FFFF:FFFFFF:FFFF:FFFF:FFFFFF:F
FF:F,FFFFFFFFFFF:FFFFFFFFFFF:FFFFFFFFFF:F:F:FFFFFFFFFFFFFF:,FF:FFFF:
@A00155:342:HHGFNDSXY:1:2257:12102:1219 1:N:0:GAACCTAG+TCCGCATA
ACTTCTCTCGGGCCCACAGCCTTACAGCTGTGGAGCACGGTATCCTCTGCCGAAACAGAGGTTGGACA
AGACCGGAGGGGTCTCCTGGTTCCAAAGGAGATGTACTCCGGGCTTGTTCACGACCTACCGTGTAAGT
+
FFFFFFFFFFFFFFFFFFFFFFFFFFFFFFFFFFFFFFFFFFFFFFFFFFFFFFFFFFFFFFFFFFFF
FFFFFFFFFFFFFFFFFFFFFFFFFFFFFFFFFFFFFFFFFFFFFFFFFFFFFFFFFFFFFFFFFFFF
@A00155:342:HHGFNDSXY:1:2257:18900:16282 1:N:0:GAACCTAG+TCCGCATA
ATCTCTATGCCGAGAGGGATGTGGAGGGCGAGAGAGAGCGACTTCTCTCGGGCCCACAGCCTTACAGC
TGTGGAGCACGGTATCCTCTGCCGAAACAGAGGTTGGACAAGACCGGAGGGGTCTCCTAGTTCCAAAG
+
FFFFFFFFFFFFFFFFFFFFFFFFFFFFFFFFFFFFFFFFFFFFFFFFFFFFFFFFFFFFFFFFFFFF
FFFFFFFFFFFFFFFFFFFFFFFFFFFFFFFFFFFFFFFFFFFFFFFFFFFFFFFFFF:FFFFFFFFF
@A00155:342:HHGFNDSXY:1:2257:6686:16517 1:N:0:GAACCTAG+TCCGCATA
AAGGACTCACCTCTGGCCTATGGTCATCTCAATGCCGAGAGGGATGTGAAGGGCGAGAGAGAGCGACT
TCTCTCGGGCCCACAGCCTTACAGCTGTGGAGCACGGTATCCTCTGCCGAAACAGAGGTTGGACAAGA
+
FFFFFFFFFFFFFFFFFFFFFFFFFFFFFFFFFFFFFFFFFFFFFFFFFFFFFFFFFFFFFFFFFFFF
FFFFFFFFFFFFFFFFFFFFFFFFFFF,FFFFFFFFFFFFFFFFFFFFFFFFFFFFFFFFFFFFFFFF
@A00155:342:HHGFNDSXY:1:2257:18032:17033 1:N:0:GAACCTAG+TCCGCATA
ATCTCTATGCCGAGAGGGATGTGGAGGGCGAGAGAGAGCGACTTCTCTCGGGCCCACAGCCTTACAGC
TGTGGAGCACGGTATCCTCTGCCGAAACAGAGGTTGGACAAGACCGGAGGGGTCTCCTAGTTCCAAAG
+
FFFFFFFFFFFFFFFFFFFFFFFFFFFFFFFFFFFFFFFFFFF:FFFFFFFFFFFFFFFF:FFFF
FFFFFFFFFFFFFFFFFFFFFFFFFFFFFFFFFFFFFFFFFF,FFFFFFFFFFFFFFFFFFFFFFFFFF
@A00155:342:HHGFNDSXY:1:2258:7889:1564 1:N:0:GAACCTAG+TCCGCATA
TTCTCTCGGGCCCACAGCCTTACAGCTGTGGAGCACGGTATCCTCTGCCGAAACAGAGGTTGGACAAG
ACCGGAGGGGTCTCCTAGTTCCAAAGGAGATGTACTCCGGGCTTGTTCACGACCTACCGTGTAAGTCG
+
FFFFFFFFFFFFFFFFFFFFFFFFFFFFFFFFFFFFFFFFFFFFFFFFFFFFFFFFFFFFFFFFFFFF
FFFFFFFFFFFFFFFFFFFFFFFFFFFFFFFFFFFFFFFFFFFFFFFFFFFF:FFFFFFFFFFFFFFF:
@A00155:342:HHGFNDSXY:1:2258:8721:20102 1:N:0:GAACCTAG+TCCGCATA
GGAGGGCGAGAGAGAGCGACTTCTCTCGGGCCCACAGCCTTACAGCTGTGGAGCACGGTATCCTCTGC
CGAAACAGAGGTTGGACAAGACCGGAGGGGTCTCCTGGTTCCAAAGGAGATGTACTCCGGGCTTGTTC
+

FFFFFFFFFFFFFFFFFFFFFFFFFFFFFFFFFFFFFFFFFFFFFFFFFFFFFFFFFFFFFFFFFFFFFFFF
FF:FFFFFFFFFFFFFFFFFFFFFFFFFFFFFFFFFFFFFFFFFFFFFFFFFFFFFFFFFFFFFFFFFFFFFF
@A00155:342:HHGFNDSXY:1:2258:21603:20964 1:N:0:GAACCTAG+TCCGCATA
AAGGACTCACCTCTGGCCTATGGTCATCTCAATGCCGAGAGGGATGTGGAGGGCGAGAGAGAGCGACT
TCTCTCGGGCCCACAGCCTTACAGCTGTGGAGCACGGTATCCTCTGCCGAAACAGAGGTTGGACAAGA
+
FFFFFFFFFFFFFFFFFFFFFFFFFFFFFFFFFFFFFFFFFF:FFFFFFFFFFFFFFFFFFFFFFFFFFFFF
FFFFFFFFFFFFFFFFFFFFFFFFFFFFFFFFFFFFFFFFFFFFFFFFFFFFFFFFFFFFFF,FFFFFFFFF
@A00155:342:HHGFNDSXY:1:2258:10963:24674 1:N:0:GAACCTAG+TCCGCATA
TGCCCCACCACACCGCCTTAGATCAGCTGTGTCCACTTAAGGACTCACCTCTGGCCTATGGTCATCTC
AATGCCGAGAGGGATGTGGAGGGCGAGAGAGAGCGACTTCTCTCGGGCCCACAGCCTTACAGCTGTGG
+
FFFFFFFFFFFFFFFFFFFFFFFFFFF:FFFFFFFFFFFFFFFFFFFFFFFFFFFFFF,FFFFFFFFFF
F:FFFFFFFFFFFF:FFFFFFFFFFFFFFFF:FFFF:FFFFFFFFFFFFF:FFFFFFFFFFFFFFFFF
@A00155:342:HHGFNDSXY:1:2258:5556:33786 1:N:0:GAACCTAG+TCCGCATA
CCAACCTCTGTTTCGGCAGAGGATACCGTGCTCCACAGCTGTAAGGCTGTGGGCCCGAGAGAAGTCGC
TCTCTCTCGCCCTCCACATCCCTCTCGGCATTGAGATGACCATAGGCCAGAGGAGAGTCCTTAAGTGG
+
FFFF,FFFFFFF:FFFFFFFFFFFFFFFFFFFFFFFFFFF:FFF,FFF:FF:FFFFFFFFFFF,FFFFF
FF:FFFFFFFFFFFFFFF,FFFFFFFFFFF,FFFFFFFFFF:FFFFFFFFFFFFFFF:FFFF:FFFFF
@A00155:342:HHGFNDSXY:1:2259:22788:5321 1:N:0:GAACCTAG+TCCGCATA
TATGGTCATCTCAATGCCGAGAGGGATGTGGAGGGCGAGAGAGAGCGACTTCTCTCGGGCCCACAGCC
TTACAGCTGTGGAGCACGGTATCCTCTGCCGAAACAGAGGTTGGACAAGACCGGAGGGGTCTCCTAGT
+
FFFFFFFFFFFFFFFFFFFFFFFFFFFFFFFFFFFFFFFFFFFFFFFFFFFFFFFFFFFFFFFFFFFF
:FFFFFFFFFFFFFFFFFFFFFFFFFFFFFFFFFFFFFFFFFFFFFFFFFFFFFFFFFFFFFFFFFFF
@A00155:342:HHGFNDSXY:1:2259:15953:29904 1:N:0:GAACCTAG+TCCGCATA
ACCTCTGTTTCGGCAGAGGATACCGTGCTCCACAGCTGTAAGGCTGTGGGCCCGAGAGAAGTCGCTCT
CTCTCGCCCTTCACATCCCTCTCGGCATTGAGATGACCATAGGCCAGAGGTGAGTCCTTAAGTGGACA
+
FFFFFFFFFFFFFFFFFFFFFFFFFFFFFFFFFFFFFFFFFFFFFFFFFFFFFFFFFFFFFFFFFFFF
FFFFFFFFFFFFFFFFFFFFFFFFFFFFFFFFFFFFFFFFFFFFFFFFFFFFFFFFFFFF,FFFFFFFF
@A00155:342:HHGFNDSXY:1:2260:16694:15154 1:N:0:GAACCTAG+TCCGCATA
GACTTCTCTCGGGCCCACAGCCTTACAGCTGTGGAGCACGGTATCCTCTGCCGAAACAGAGGTTGGAC
AAGACCGGAGGGGTCTCCTAGTTCCAAAGGAGATGTACTCCGGGCTTGTTCACGACCTACCGTGTAAG
+
FFFFFFFFFFFFFFFFFF:FFFFFFFFFFFFFFFFFFFFFFFFFFFFFFFFFFFFFFFFFFFFF:FFFF
FF:FFFFFFFFFFFFFFFFFFFFFFFFFFFFFFFFFFFFFFFFF,FFFFFFFFFFFFFFFFFFFFFF
@A00155:342:HHGFNDSXY:1:2260:2808:19695 1:N:0:GAACCTAG+TCCGCATA
CCTTACAGCTGTGGAGCACGGTATCCTCTGCCGAAACAGAGGTTGGACAAGACCGGAGGGGTCTCCTA
GTTCCAAAGGAGATGTACTCCGGGCTTGTTCACGACCTACCGTGTAAGTCGTAGTCTAGTAGGCTACC
+
FFFFFFFFFFFFFFFFFFFFFFFFFFFFFFFFFFFFFF:FFFFFFFFFFFF:FFFFFFFFFFFFFFFF
FFFFFFFFF:FFFFFFFFF:FFFFFFFFFFFFFFFF:FF:FFFFFFFFFF:FFFFFFFFFFFFFFFFF
@A00155:342:HHGFNDSXY:1:2261:18656:5243 1:N:0:GAACCTAG+TCCGCATA
GGATGTGGAGGGCGAGAGAGAGCGACTTCTCTCGGGCCCACAGCCTTACAGCTGAGGAGCACGGTATC
CTCTGCCGAAACAGAGGTTGGACAAGACCGGAGGGGTCTCCTAGTTCCAAAGGAGATGTACTCC
+
FFFFFFFFFFFFFFFFFFFFFFFFFFFFFFFFFFFFFFFFFFFFFFFFFFFFFFF,FFFFFFFFFFFFF
FFFFFFFFFFFFFFFFFFFFFFFFFFFFFFFFFFFFFFFFFFFFFFFFFFFFFFFFFFFFFFFFF
@A00155:342:HHGFNDSXY:1:2261:30092:9142 1:N:0:GAACCTAG+TCCGCATA
CAGCCTTACAGCTGTGGAGCACGGTTTCCTCTGCCGAAACAGAGGTTGGACAAGACCGGAGGGGTCTC
CTAGTTCCAAAGGAGATGTACTCCGGGCTTGTTCACGACCTACCGTGTAAGTCGTAGTCTAGTAGGCT
+

FFFFFFFFFFFFFFFFFFFFFFFFFFFFFFFFFFFFFFFFFFFFFFFFFFFFFFFFFFFFFFFFFFFF
FFF:FFFFFFFFFFFFFFFFFFFFFFFFFFFFFFFFFFFFFFFFFFFFFFFFFFFFFFFFFFFFFFFF
@A00155:342:HHGFNDSXY:1:2261:28094:9471 1:N:0:GAACCTAG+TCCGCATA
ATCTCAATGCCGAGAGGGATGTGGAGGGCGAGAGAGAGCGACTTCTCTCGGGCCCACAGCCTTACAGC
TGTGGAGCACGGTATCCTCTGCCGAAACAGAGGTTGGACAAGACCGGAGGGGTCTCCTGGTTCCAAAG
+
FFFFFFFFFFFFFFFFFF:FFFFFFFFFFFFFFFFFFFFFFF:FFFFFFFFFFFFFFFFFFFFFFFFF
FFFFFFFFFFFFFFFFFFFFFFFFFFFFFFFFFFFFFFFFFFFFFFFFFFFFFFFFFFFFFFFFFFFF
@A00155:342:HHGFNDSXY:1:2261:7482:15295 1:N:0:GAACCTAG+TCCGCATA
GTATCCTCTGCCGAAACAGAGGTTGGACAAGACCGGAGGGGTCTCCTAGTTCCAAAGGAGATGTACTC
CGGGCTTGTTCACGACCTACCGTGTAAGTCGTAGTCTAGTAGGCTACCTGACGAGTCCTTTTTAGGAC
+
FFFFFFFFFFFFFFFFFFFFFFFFFFFFFFFFFFFFFFFFFFFFFFFFFFFFFFFFFFFFFFFFFFFF
FFFFFFFFFFFFFFFFFFFFFFFFFFFFFFFFFFFFF,FFFFFFFFFFFFFFFFFFFFFFFFFFFFFF
@A00155:342:HHGFNDSXY:1:2261:9588:27712 1:N:0:GAACCTAG+TCCGCATA
CAATGCCGAGAGGGATGTGGAGGGCGAGAGAGAGCGACTTCTCTCGGGCCCACAGCCTTACAGCTGTG
GAGCACGGTATCCTCTGCCGAAACAGAGGTTGGACAAGACCGGAGGGGTCTCCTAGTTCCAAAGGAGA
+
FFFFFFFFFFFFFFFFFFFFFFFFFFFFFFFFFFFFFFFFFFFFFFFFFFFFFFFFFFFFFFFFFFFF
FFFFFFFFFFFFFFFFFFFFFFFFF:FFFFFFFFFFFFFFFFFFFFFFFFFFFFFFFFFFFFFFFFFF
@A00155:342:HHGFNDSXY:1:2262:22715:6104 1:N:0:GAACCTAG+TCCGCATA
AGCGACTTCTCTCGGGCCCACAGCCTTACAGCTGTGGAGCACGGTATCCTCTGCCGAAACAGAGGTTG
GACAAGACCGGAGGGGTCTCCTAGTTCCAAAGGAGATGTACTCCGGGCTTGTTCACGACCTACCGTGT
+
FFFFFFFFFFFFFFFFFFFFFFFFFFFFFFFFFFFFFFFFFFFFF,FFFFFFFFFFFFFFFFFFFFFF
FFFFFFFFFFFFFFFFFFFFFFFFFFFFFFFFFFFFFFFFFFFFFFFFFFFFFFFFFFFFFFFFFFFF
@A00155:342:HHGFNDSXY:1:2262:15962:6872 1:N:0:GAACCTAG+TCCGCATA
CTTACAGCTGTGGAGCACGGTATCCTCTGCCGAAACAGAGGTTGGACAAGACCGGAGGGGTCTCCTAG
TTCCAAAGGAGATGTACTCCGGGCTTGTTCACGACCTACCGTGTAAGTCGTAGTCTAGTAGGCTACCT
+
FFFFFFFFFFFFFFFFFFFFFFFFFFFFFFFFFFFFFFFFFFFFFFFFFFFFFFFFFFFFFFFFFFFF
FFFFFFFFFFFFFFFFFFFFFFFFFFFFFFFFFFFFFFFFFFFFFFFFFFFFFFFFFFFFFFFFFFFF
@A00155:342:HHGFNDSXY:1:2262:9372:31000 1:N:0:GAACCTAG+TCCGCATA
AGAGAGAGCGACTTCTCTCGGGCCCACAGCCTTACAGCTGTGGAGCACGGTATCCTCTGCCGAAACAG
AGGTTGGACAAGACCGGAGGGGTCTCCTGGTTCCAAAGGAGATGTACTCCGGGCTTGTTCACGACCTA
+
FFFFFFFFFFFFFFFFFFFFFFFFFFFFFFFFFFFFFFFFFFFFFFFFFFFFFFFFFFFFFFFFFFFF
FFFFFFFFFFFFFFFFFFFFFFFFFFFFFFFFFFFFFFFFFFFFFFFFFFFFFFFFFFFFFFFFFFFF
@A00155:342:HHGFNDSXY:1:2263:14326:16908 1:N:0:GAACCTAG+TCCGCATA
GGATGTGGAGGGCGAGAGAGAGCGACTTCTCTCGGGCCCACAGCCTTACAGCTGTGGAGCACGGTATC
CTCTGCCGAAACAGAGGTTGGACAAGACCGGAGGGGTCTCCTAGTTCCAAAGGAGATGTACTCC
+
FFF:FFFFFFFFFFFFFFFFFFFFFFFFFFFFFFFFFFFFFFFFFFFFFFFFFFFFFFFFFFFFFFFF
FFFFFFFFFFFFFFFFFFFFFFFFFFFFFFFFFFFFFFFFFFFFFFFFFFFFFFFFFFFFFFFF
@A00155:342:HHGFNDSXY:1:2263:9815:18270 1:N:0:GAACCTAG+TCCGCATA
GTCATCTCAATGCCGAGAGGGATGTGGAGGGCGAGAGAGAGCGACTTCTCTCGGGCCCACAGCCTTAC
AGCTGTGGAGCACGGTATCCTCTGCCGAAACAGAGGTTGGACAAGACCGGAGGGGTCTCCTGGTTCCA
+
FFFFFFFFFFFFFFFFFFFFFFFFFFFFFFFFFFFFFFFFFFFFFFFFFFFFFFFFFFFFFFFFFFFF
FFFFFFFFFFFFFFFFFFFFFFFFFFFFFFFFFFFFFFFFFFFFFFFFFFFFFFFFFFFFFFFFFFFF
@A00155:342:HHGFNDSXY:1:2264:22535:18474 1:N:0:GAACCTAG+TCCGCATA
CAGCCTTACAGCTGTGGAGCACGGTATCCTCTGCCGAAACAGAGGTTGGACAAGACCGGAGGGGTCTC
CTAGTTCCAAAGGAGATGTACTCCGGGCTTGTTCACGACCTACCGTGTAAGTCGTAGTCTAGTAGGCT
+

FFFFFFFFFFFFFFFFFFFFFFFFFFFFFFFFFFFFFFFFFFFFFFFFFFFFFFFFFFFFFFFFFFFFF
FF:FFFFFFFFFFFFFFFFFFFFFFFFFFFFFFFFFFFFFFF:FFFFF:FFFFFFFFFFFFFFFFFFFF
@A00155:342:HHGFNDSXY:1:2264:22824:20791 1:N:0:GAACCTAG+TCCGCATA
GTATCCTCTGCCGAAACAGAGGTTGGACAAGACCGGAGGGGTCTCCTAGTTCCAAAGGAGATGTACTC
CGGGCTTTTTCACGACCTACCGTGTAAGTCGTAGTCTAGTAGGCTACCTGACGAGTCCTTTTTAGGAC
+
FFFFFFFFFFFFFFFFFFFFFFFFFFFFFFFFFFFFFFFFFFFFFFFFFFFFFFFFFFF:FFFF:F:FF
FFFFF:FFFFFFFFFFFFFFFFFFFFFFFFFFFFFFFFFFFFFFFFFFFFFFFFFFFFFFFF:FFFFFF
@A00155:342:HHGFNDSXY:1:2264:20482:25880 1:N:0:GAACCTAG+TCCGCATA
CCTCTGTTTCGGCAGAGGATACCGTGCTCCACAGCTGTAAGGCTGTGGGCCCGAGAGAAGTCGCTCTC
TCTCGCCCTCCACATCCCTCTCGGCATTGAGATGACCATAGGCCAGAGGTGAGTCCTTAAGTGGACAC
+
FFFFFFFFFFFFFFFFFFFFFFFFFFFFFFFFFFFFFFFF:FFFFFFFFFFFFFFFFFFFFFFFFFFFF
FFFFFFFFFFFFFF:FFF:FFFFFFFFFFFFF:FFFFF:FFFFFFFFFFFFFFFFFFFFFFF:FFFFFF
@A00155:342:HHGFNDSXY:1:2265:10565:12806 1:N:0:GAACCTAG+TCCGCATA
CTTAAGGACTCACCTCTGGCCTATGGTCATCTCAATGCCGAGAGGGATGTGAAGGGCGAGAGAGAGCG
ACTTCTCTCGGGCCCACAGCCTTACAGCTGTGGAGCACGGTATCCTCTGCCGAAACAGAGGTTGGACA
+
FFFFFFFFFFFFFFFFFFFFFFFFFFFFFFFFFFFFFFFFFFFFFFFFFFFF:FFFFFFFFFFFFFFFF
FF:FFFFFFFFFFFFFFFFFFFFFFFFFFF::FFFFFFFFFFFFFFF:FFF,FFFFFFFFFFFFFFFFF
@A00155:342:HHGFNDSXY:1:2265:6967:15029 1:N:0:GAACCTAG+TCCGCATA
ATGCCGAGAGGGCTGTGGAGGGCGAGAGAGAGCGACTTCTCTCGGGCCCACAGCCTTACAGCTGTGGA
GCACGGTATCCTCTGCCGAAACAGAGGTTGGACAAGACCGGAGGGGTCTCCTAGTTCCAAAGGAGATG
+
FFF:F:FF:,FF,FFFFFFFFF:FFFFFFFFFFFF:FFFFFFFFFFFF:FFFF:,,FFFFFF:FFFF:F
FFFFF,F:FF:FFFFFFFFFFF:F:FFFFFFFFFFFFFF,FFFFFFFFFFFFFFFFFFFF::F:FFFF:
@A00155:342:HHGFNDSXY:1:2265:4689:16438 1:N:0:GAACCTAG+TCCGCATA
GCGACTTCTCTCGGGCCCACAGCCTTACAGCTGTGGAGCACGGTATCCTCTGCCGAAACAGAGGTTGG
ACAAGACCGGAGGGGTCTCCTGGTTCCAAAGGAGATGTACTCCGGGCTTGTTCACGACCTACCGTGTA
+
FFFFFFFFFFFFFFFFFFFFFFFFFFFFFFFFFFFFFFFFFFFFFFFFFFFFFFFFFFFFFFFFFFFFF
FFFF:FFFFFFFFFFFFFFF:FFF:FFFFFFFFFFFFFFFFF:FFFFFFFFFFFF:FFFFFFFFFFFFF
@A00155:342:HHGFNDSXY:1:2265:19633:30671 1:N:0:GAACCTAG+TCCGCATA
CATGCCCCGCCACACCGCCTTAGATCAGCTGTGTCCACTTAAGGACTCACCTCTGGCCTATGGTCATC
TCAATGCCGAGAGGGATGTGGAGGGCGAGAGAGAGCGACTTCTCTCGGGCCCACAGCCTTACAGCTGT
+
FFFFFFFFFFFFFFFFFFFFFFFFFFFFFFFFFFFFFFFF:FFFFFFFFFFFFFFFFFF,FFFFFFFFF
FFFFFFFFFFFFFFFFFFFFFFFFFFFFFFFFFFFFFFFFFFFFFFFFFFFFFFFFFFFFFFFFFFFFF
@A00155:342:HHGFNDSXY:1:2265:7681:32550 1:N:0:GAACCTAG+TCCGCATA
CCACATCCCTCTCGGCATTGAGATGACCATAGGCCAGAGGTGAGTCCTTAAGTGGACACAGCTGATCT
AAGGCGGTGTGGCGGGGCATGGGTTTGAACCCCCATGACGGTCGGAGTCTAGTAGGCTCCCTGATGAG
+
FFFFFFFFFFFFFFFFFFFFFFFFFFFFFFFFFFFF:FFFFFFFFFFFFFFFFFFFFFFF,FFFFFFFF
FFFFFFFFFFFFFFFFFFFFFFFFFFFFFFFFF:FFFFFFFFFFFFFF:FFFFFFFFFFFFFFFFFFFF
@A00155:342:HHGFNDSXY:1:2265:25437:35399 1:N:0:GAACCTAG+TCCGCATA
ATCTCAATGCCGAGAGGGATGTGGAGGGCGAGAGAGAGCGACTTCTCTCGGGCCCACAGCCTTACAGC
TGTGGAGCACGGTATCCTCTGCCGAAACAGAGGTTGGACAAGACCGGAGGGGTCTCCTAGTTCCAAAG
+
FFFFFFFFFFFF:FFFFFFFFFFFFFFFFFFFFFFFFFFFFFFFFFFFFFFFFFFFFFFFFFFFFFFF
FFFFFFFFFFFFFFFFFFFFFFFFFFFFFFFFFFFFFFFFFFFFFFFFFFFFFFFFFFFFFFFFFFFFF
@A00155:342:HHGFNDSXY:1:2266:18168:29763 1:N:0:GAACCTAG+TCCGCATA
AATGCCGAGAGGGATGTGGAGGGCGAGAGAGAGCGACTTCTCTCGGGCCCACAGCCTTACAGCTGTGG
AGCACGGTATCCTCTGCCGAAACAGAGGTTGGACAAGACCGGAGGGGTCTCCTAGTTCCAAAGGAGA
+

FFFFFFFFFFFFFFFFFFFFFFFFFFFFFFFFFFFFFFFFFFFFFFFFFFFFFFFFFFFFFFFFFFFF
FFFFFFFFFFFFFFFFFFFFFFFFFFFFFFFFFFFFFFFFFFFFFFFFFFFFFFFFFFFFFFFFFFF
@A00155:342:HHGFNDSXY:1:2267:3188:1846 1:N:0:GAACCTAG+TCCGCATA
ATGCCGAGAGGGATGTAGAGGGCGAGAGAGAGCGACTTCTCTCGGGCCCACAGCCTTACAGCTGTGGA
GCACGGTATCCTCTGCCGAAACAGAGGTTGGACAAGACCGGAGGGGTCTCCTAGTTCCAAAGGAGATG
+
FFFFFFFFFFFFFFFF:FFFFFFFFFFFFFFFFFFFFFFFFFFFFFFFFFFF:FFFFFFFFFFFFFFF
FFFFFFF:FFFFFFFFFFFFFF:FFFF:FFFFFFFF:FF,FFFFFFFFFFFFFFFFFFFFFF,FFFFF
@A00155:342:HHGFNDSXY:1:2267:25111:3427 1:N:0:GAACCTAG+TCCGCATA
CTCTATGCCGAGAGGGATGTGGAGGGCGAGAGAGAGCGACTTCTCTCGGGCCCACAGCCTTACAGCTG
TGGAGCACGGTATCCTCTGCCGAAACAGAGGTTGGACAAGACCGGAGGGGTCTCCTAGTTCCAAAGGA
+
FFFFFFFFFFFFFFFFFFFFFFFFFFFFFFFFFFFFFFFFFFFFFFFFFFFFFFFFFFFFFFFFFFFF
FFFFFFFFFFFFFFFFFFFFFFFFFFFFFFFFFFFFFFFFFFFFFFFFFFFFFFFFFFFFFFFFFFFF
@A00155:342:HHGFNDSXY:1:2267:10285:9064 1:N:0:GAACCTAG+TCCGCATA
CTCTGCCGAAACAGAGGTTGGACAAGACCGGAGGGGTCTCCTAGTTCCAAAGGAGATGTACTCCGGGC
TTGTTCACGACCTACCGTGTAAGTCGTAGTCTAGTAGGCTACCTGACGAGTCCTTTTTAGGACGAAAC
+
FFFFFFFFFFF:FFFFFFFF:FFFFFFFFFFFFFFFFFFFFFFFFFF:FF:FFFFFFF::FFFFFFFF
FFFFFFFFFFFFFFFFFFFFFF:FFFFFFFFFFFFFFFFFFFFFFFFFFFFFF:FFFFFFFFFFFFFF
@A00155:342:HHGFNDSXY:1:2267:4065:9502 1:N:0:GAACCTAG+TCCGCATA
AGGGATGTGGAGGGCGAGAGAGAGCGACTTCTCTCGGGCCCACAGCCTTACAGCTGTGGAGCACGGTA
TCCTCTGCCGAAACAGAGGTTGGACAAGACCGGAGGGGTCTCCTAGTTCCAAAGGAAATGTACTCCGG
+
FFFFF,FFFFFFFFFFFFFFFFFFF:FFFFFFFFFFFFFFFFFFFFFFFFFFFFFFFFFFFFFFFFFF
FFFFFFFFFFFFFFFFFF:FFFFFFFFFFFFFFFFFF:FFFFFFFFFFFFFFFFFFFFFFFFFFFFFF
@A00155:342:HHGFNDSXY:1:2267:27877:10191 1:N:0:GAACCTAG+TCCGCATA
GGATGTGGAGGGCGAGAGAGAGCGACTTCTCTCGGGCCCACAGCCTTACAGCTGTGGAGCACGGTATC
CTCTGCCGAAACAGAGGTTGGACAAGACCGGAGGGGTCTCCTAGTTCCAAAGGAGATGTACTCC
+
FFFFF,FFFF:::FFFFFFFFFF:FFFFF:FFFFF:FFFFFFFFF:F:FFFFFFFFFFF::FFFFF:F
:FFF:F,FFFFFFFFFFF:F,FFFFFFFF,FF:FF:FFFFFFFF:FFFFFFFFFFFF:FF:FFF
@A00155:342:HHGFNDSXY:1:2267:3830:30264 1:N:0:GAACCTAG+TCCGCATA
CACCTCTGGCCTATGGTCATCTCAATGCCGAGAGGGATGTGGAGGGCGAGAGAGAGCGACTTCTCTCG
GGCCCACAGCCTTACAGCTGTGGAGCACGGTATCCTCTGCCGAAACAGAGGTTGGACAAGACCGGAG
+
FFFFFFFFFFFFFFFFFFFFFFFFFFFFFFFFFFFFFFFFFFFFFFFFFFFFFFFFFFFFFFFFFFFF
FFFFFFFFFFFFFFFFFFFFFFFFFFFFFFFFFFFFFFFFFFFFFFFFFFFFFFFFFFFFFFFFFFF
@A00155:342:HHGFNDSXY:1:2268:28275:9345 1:N:0:GAACCTAG+TCCGCATA
GAGAGGGATGTGGAGGGCGAGAGAGAGCGACTTCTCTCGGGCCCACAGCCTTACAGCTGTGGAGCACG
GTATCCTCTGCCGAAACAGAGGTTGGACAAGACCGGAGGGGTCTCCTAGTTCCAAAGGAGATGTACTC
+
FFFFFFFFFFFFFFFFFFFFFFFFFFFFFFFFFFFFFFFFFFFFFFFFFFFFFFFFFFFFFFFFFFFF
FFFFFFFFFFFFFFFFFFFFFFFFFFFFFFFFFFFFFFFFFFFFFFFFFFFFFFFFFFFFFFFF:FFF
@A00155:342:HHGFNDSXY:1:2269:5683:4883 1:N:0:GAACCTAG+TCCGCATA
TTCTCTCGGGCCCACAGCCTTACAGCTGTGGAGCACGGTATCCTCTGCCGAAACAGAGGTTGGACAAG
ACCGGAGGGGTCTCCTAGTTCCAAAGGAGATGTACTCCGGGCTTGTTCACGACCTACCGTGTAAGTC
+
FFFFFFFFFFFFFFFFFFFFFFFFFFFFFFFFFFFFFFFFFFFFFFFFFFFFFFFFFFFFFFFFFFFF
FFFFFFFFFFFFFFFFFFFFFFFFFFFFFFFFFFFFFFFFFFFFFFFFFFFFFFFFFFFFFFFFFFF
@A00155:342:HHGFNDSXY:1:2269:17300:14575 1:N:0:GAACCTAG+TCCGCATA
GGACTCTCCTCTGGCCTATGGTCATCTCAATGCCGAGAGGGATGTGGAGGGCGAGAGAGAGCGACTTC
TCTCGGGCCCACAGCCTTACAGCTGTGGAGCACGGTATCCTCTGCCGAAACAGAGGTTGGACAAGACC
+

```
FFFFFFFFFFFFFFFFFFFFFFFFFFFFFFFFFFFFFFFFFFF:FFFFFFFFFFFFFFFFFFFFFFFFF
FFFFFFFFFFFFFFFFFFFFFFFFFFFFFFFFFFFFFFFFFFFFFFFFFFFFFFFFFFFFFFFFFFFFF
@A00155:342:HHGFNDSXY:1:2269:8359:16063 1:N:0:GAACCTAG+TCCGCATA
TGCCGAGAGGGATGTAGAGGGCGAGAGAGAGCGACTTCTCTCGGGCCCACAGCCTTACAGCTGTGGAG
CACGGTATCCTCTGCCGAAACAGAGGTTGGACAAGACCGGAGGGGTCTCCTAGTTCCAAAGGAGATGT
+
FFF:FFFFFFFFFFF:FFF:FFFFFFFFFFFFFF:FFFFFFFFFFFFFFFFFFFFFFFFFFFFFFFFFF
FFFFFFFFFFFFFFFFFFFFFFFFFF:FFFFFFFFFFFFFFFFFFFFFFF,FFFFFFFFF:FFFFFFFF
@A00155:342:HHGFNDSXY:1:2269:5367:20682 1:N:0:GAACCTAG+TCCGCATA
ACCTCTGTTTCGGCAGAGGATACCGTGCTCCACAGCTGTAAGGCTGTGGGCCCGAGAGAAGTCGCTCT
CTCTCGCCCTCCACATCCCTCTCGGCATTGAGATGACCATAGGCCAGAGGAGAGTCCTTAAGTGGACA
+
FFFFFFFFFFFFFFFFFFFFFFFFFFFFFFFFFFFFFFFFFFFFFFFFFFFFFFFFFFFFFFFFFFFFF
FFFFFFFFFFFFFFFFFFFFFFFFFFFFFFFFFFFFFFFFFFFFFFFFFFFFFFFFFFFFFFFFFFFFF
@A00155:342:HHGFNDSXY:1:2269:16622:32784 1:N:0:GAACCTAG+TCCGCATA
CTGGCCTATGGTCATCTCAATGCCGAGAGGGATGTGGAGGGCGAGAGAGAGCGACTTCTCTCGGGCCC
ACAGCCTTACAGCTGTGGAGCACGGTATCCTCTGCCGAAACAGAGGTTGGACAAGACCGGAGGGGTCT
+
FFFFFFFFFFFFFFFFFFFFFFFFFFFFFFFFFFFFFFFFFFFFFFFFFF:FFFFFFFFFFFFFFFFFF
FFFFFFFFFFFFFFFFFFFFFFFFFFFFFFFFFFFFFFFFFFFFFFFFFFFFFFFFFFFFFFFFFFFFF
@A00155:342:HHGFNDSXY:1:2269:17526:37012 1:N:0:GAACCTAG+TCCGCATA
CGACTTCTCTCGGGCCCACAGCCTTACAGCTGTGGAGCACGGTATCCTCTGCCGAAACAGAGGTTGGA
CAAGACCGGAGGGGTCTCCTAGTTCCAAAGGAGATGTACTCCGGGCTTGTTCACGACCTACCGTGTAA
+
FFFFFFFFFFFFFFFFFFFFFFFFFFFF:FFFFFFFFFFFFFFFFFFFFFFFFFFFFFFFFFFFFFFFF
FFF:FFFFFFFFFF:FFFFFFFFFFFFFFFFFFFFFFFFFFF:FFFFFFFFFFFFFFFFFFFFFFFFFF
@A00155:342:HHGFNDSXY:1:2270:25418:14669 1:N:0:GAACCTAG+TCCGCATA
GGATGTAGAGGGCGAGAGAGAGCGACTTCTCTCGGGCCCACAGCCTTACAGCTGTGGAGCACGGTATC
CTCTGCCGAAACAGAGGTTGGACAAGACCGGAGGGGTCTCCTAGTTCCAAAGGAGATGTACTCC
+
FFFFFFFFFFFFFFFFFFFFFFFFFFFFFFFFFFFFFFFFFFFFFFFFFFFFFFFFFFFFFFFFFFFFF
FFFFFFFFFFFFFFFFFFFFFFFFFFFFFFFFFFFFFFFFFFFFFFFFFFF:FFFFFFFFFFFF
@A00155:342:HHGFNDSXY:1:2270:30400:15154 1:N:0:GAACCTAG+TCCGCATA
AAGGACTCACCTCTGGCCTATGGTCATCTCAATGCCGAGAGGGATGTGAAGGGCGAGAGAGAGCGACT
TCTCTCGGGCCCACAGCCTTACAGCTGTGGAGCACGGTATCCTCTGCCGAAACAGAGGTTGGACAAGA
+
FFFFFFFFFFFFFFFFFFFFFFFFFFFFFFFFFFFFFFFFFFFFFFFFFFFFFFFFFFFFFFFFFFFFF
FFFFFFFFFFFFFFFFFFFFFFFFFFFFFFFFFFFFFFFFFFFFFFFFFFFFFFF:FFFFFFFFFFF:F
@A00155:342:HHGFNDSXY:1:2270:30418:15374 1:N:0:GAACCTAG+TCCGCATA
AAGGACTCACCTCTGGCCTATGGTCATCTCAATGCCGAGAGGGATGTGAAGGGCGAGAGAGAGCGACT
TCTCTCGGGCCCACAGCCTTACAGCTGTGGAGCACGGTATCCTCTGCCGAAACAGAGGTTGGACAAGA
+
FFFFFFFFFFFFFFFFFFFFFFFFFFFFFFFFFFFFFFFFFFFFFFFFFFFFFFFFFFFFFFFFFFFFF
FFFFFFFFFFFFFFFFFFFFFFFFFFFFFFFFFFFFFFFFFFFFFFFFFFFFFFFFFFF:FFFFFFFFF
@A00155:342:HHGFNDSXY:1:2270:23954:26412 1:N:0:GAACCTAG+TCCGCATA
TTACAGCTGTGGAGCACGGTATCCTCTGCCGAAACAGAGGTTGGACAAGACCGGAGGGGTCTCCTAGT
TCCAAAGGAGATGTACTCCGGGCTTGTTCACGACCTACCGTGTAAGTCGTAGTCTAGTAGGCTACCTG
+
FFFFFFFFFFFFFFFFFFFFFFFFFFFFFFFFFFFFFF,FFFFFFFFFFFFFFFFFFFFFFFFFFFFFF
FFFFFFFFFF:FFFFFFFFFFFFFFFFFFFFFFFFFFFFFFFFFFFFFFFFFFFFFFFFFFFFFFFFFF
@A00155:342:HHGFNDSXY:1:2270:23240:35728 1:N:0:GAACCTAG+TCCGCATA
TCAATGCCGAGAGGGATGTGGAGGGCGAGAGAGAGCGACTTCTCTCGGGCCCACAGCCTTACAGCTGT
GGAGCACGGTATCCTCTGCCGAAACAGAGGTTGGACAAGACCGGAGGGGTCTCCTAGTTCCAAAGGA
+
```

```
FFFFFF::FFFFF::FFFFFFFF:F,FF,FFF::F,FF,FF:F:FFF,F:F:F:FFFFFFF,FF::FF
FFFFFFFFFFFFFFFFFFF:FFF::F:FFFFFFFFFFFFF:FFFFFFFFF:FFFF:FFFF::FFFF:
@A00155:342:HHGFNDSXY:1:2271:10520:9721 1:N:0:GAACCTAG+TCCGCATA
TTACAGCTGTGGAGCACGGTATCCTCTGCCGAAACAGAGGTTGGACAAGACCGGAGGGGTCTCCTAGT
TCCAAAGGAGATGTACTCCGGGCTTGTTCACGACCTACCGTGTAAGTCGTAGTCTAGTAGGCTACCTG
+
FFFFFFF:FFFFFFFFFFFFFFFFFFFFFFFFFFFFFFFFFFFFFFFFFFFFFFFFFFFFFFFFFFF:
FFFFFFFFFFFFF:FFFFFFFFFFFFFFFFFFFFFFFFFFFFFFFFFFFFFFFF:FFFFF:FFFFF
@A00155:342:HHGFNDSXY:1:2271:27109:11898 1:N:0:GAACCTAG+TCCGCATA
ACTTCTCTCGGGCCCACAGCCTTACAGCTGTGGAGCACGGTATCCTCTGCCGAAACAGAGGTTGGACA
AGACCGGAGGGGTCTCCTAGTTCCAAAGGAGATGTACTCCGGGCTTGTTCACGACCTACCGTGTAAGT
+
FFFFFFFFFFFFFFFFFFFFFFFFFFFFFFFFFFFFFFFFFFFFFFFFFFFFFFFFFFFFFFFFFFFF
FFFF:FFFFFFFFFFFFFFFFFFFFFFFFFFFFFFFFFFFFFFFFFFFFFFFFFFFFFFFFFFFFFFF
@A00155:342:HHGFNDSXY:1:2271:27163:13338 1:N:0:GAACCTAG+TCCGCATA
ACTTCTCTCGGGCCCACAGCCTTACAGCTGTGGAGCACGGTATCCTCTGCCGAAACAGAGGTTGGACA
AGACCGGAGGGGTCTCCTAGTTCCAAAGGAGATGTACTCCGGGCTTGTTCACGACCTACCGTGTAAGT
+
FFFFFFFFFFFFFFFFFFFFFFFFFFFFFFFFFFFFFFFFFFFFFFFFFFFFFFFFFFFFFFFFFFFF
FF:FFFFFFFFFFFFFFFFFFFFFFFFFFFFFFFFFFFFFFFFF,FFFFFFF:FFFFFFFFFFFFFFF
@A00155:342:HHGFNDSXY:1:2271:31720:15374 1:N:0:GAACCTAG+TCCGCATA
GGATGTGGAGGGCGAGAGAGAGCGACTTCTCTCGGGCCCACAGCCTTACAGCTGTGGAGCACGGTATC
CTCTGCCGAAACAGAGGTTGGACAAGACCGGAGGGGTCTCCTAGTTCCAAAGGAGATGTACTCC
+
FFFFFFFFFFFFFFFFFFFFFFFFFF,FFFFFFFFFFFFFFFFFFFFFFFFFFFFFFFFFFFFFFFFF
FFFFFFFFFFFFFFFFFFFFFFFFFFFFFFFFFFFFFFFFFFFFFFFFFFFFFFFFFFFFFFFF
@A00155:342:HHGFNDSXY:1:2271:5213:32221 1:N:0:GAACCTAG+TCCGCATA
GAGGGCGAGAGAGAGCGACTTCTCTCGGGCCCACAGCCTTACAGCTGTGGAGCACGGTATCCTCTGCC
GAAACAGAGGTTGGACAAGACCGGAGGGGTCTCCTAGTTCCAAAGGAGATGTACTCCGGGCTTGTTCA
+
FFFFFFF:FFFFFFFFFFFFFF:FFFFFFFFFFFFFFFFFFFFFFFFFFFFFFFFFFFFFFFFFFFFF
FFFFFFFFFFFFFFFFFFFFFFFFFFFFFFFFFFFFFFFFFFFFFFFFFFFFFFFFFFFFFFFFFFFF
@A00155:342:HHGFNDSXY:1:2272:18041:3959 1:N:0:GAACCTAG+TCCGCATA
ATGCCGAGAGGGATGTGGAGGGCGAGAGAGAGCGACTTCTCTCGGGCCCACAGCCTTACAGCTGTGGA
GCACGGTATCCTCTGCCGAAACAGAGGTTGGACAAGACCGGAGGGGTCTCCTAGTTCCAAAGGAGATG
+
FFFFFFFFFFFFFFFFFFFFFFFFFFFFFFFFFFFFFFFFFFFFFFFFFFFFFFFFFFFFFFFFFFFF
FFFFFFFFFFFFFFFFFFFFFFFFFFFFFFFFFFFFFFFFFFFFFFFFFFFFFFFFFFFFFFFFFFFF
@A00155:342:HHGFNDSXY:1:2272:29767:15499 1:N:0:GAACCTAG+TCCGCATA
CTTCTCTCGGGCCCACAGCCTTACAGCTGTGGAGCACGGTATCCTCTGCCGAAACAGAGGTTGGACAA
GACCGGAGGGGTCTCCTAGTTCCAAAGGAGATGTACTCCGGGCTTGTTCACGACCTACCGTGTAAGTC
+
FFFF,F,FFFFF::FFF:FFF,FFF:FFFFFFFFFFFFF:F,FF,FFF:FFFFFFFFFFF,FFFFFFF
FFFF:,FFFFF:FFFFF,FFFFFFFF::FFFFFFFFF,:F:FFFFFFFFF:FFFFFFF,FF::FFF,:
@A00155:342:HHGFNDSXY:1:2272:8865:26647 1:N:0:GAACCTAG+TCCGCATA
ACCTCTGTTTCGGCAGAGGATACCGTGCTCCACAGCTGTAAGGCTGTGGGCCCGAGAGAAGTCGCTCT
CTCTCGCCCTCCACATCCCTCTCGGCATTGAGATGACCATAGGCCAGAGGAGAGTCCTTAAGTGGACA
+
FFFFFFFFFFFFFFFFFFFFFFFFFFFFFFFFFFFFFFFFFFFFFFFFFFFFFFFFFFFFFFFFFFFF
FFFFFFFFFFFFFFFFFFFFFFFFFFFFFFFFFFFFFFFFFFFFFFFFFFFFFFFFFFFFFFFFFFFF
@A00155:342:HHGFNDSXY:1:2272:14145:28745 1:N:0:GAACCTAG+TCCGCATA
GGATGTGGAGGGCGAGAGAGAGCGACTTCTCTCGGGCCCACAGCCTTACAGCTGTGGAGCACGGTATC
CTCTGCCGAAACAGAGGTTGGACAAGACCGGAGGGGTCTCCTAGTTCCAAAGGAG
+
```

```
FFFFFFFFFFFFFFFFFFFFFFFFFFFFFFFFFFFFFFFFFFFFFFFFFFFFFFFFFFFFFFFFFFFF
FFFFFFFFFFFFFFFFFFFFFFFFFFFFFFFFFFFFFFFFFFFFFFFFFFFFFFF
@A00155:342:HHGFNDSXY:1:2272:9769:32941 1:N:0:GAACCTAG+TCCGCATA
ACCTCTGTTTCGGCAGAGGATACCGTGCTCCACAGCTGTAAGGCTGTGGGCCCGAGAGAAGTCGCTCT
CTCTCGCCCTCCACATCCCTCTCGGCATTGAGATGACCATAGGCCAGAGGAGAGTCCTTAAGTGGACA
+
FFFFFFFFFFFFFFFFFFFFFFFFFFFFFFFFFFFFFFFFFFFFFFFF:FFFFFFFFFFFFFFFFFFF
FFFFFFFFFFFFFFFFFFFFFFFFFFFFFFFFFFFFFFFFFFFFFFFFFFFFFFFFFFFFFFFFFFF:
@A00155:342:HHGFNDSXY:1:2272:4942:35133 1:N:0:GAACCTAG+TCCGCATA
ACCTCTGTTTCGGCAGAGGATACCGTGCTCCACAGCTGTAAGGCTGTGGGCCCGAGAGAAGTCGCTCT
CTCTCGCCCTCCACATCCCTCTCGGCATTGAGATGACCATAGGCCAGAGGTGAGTCCTTAAGTGGACA
+
FFFFFFFFFFFFFFFFFFFFFFFFFFFFFFFFFFFFFFFFFFFFFFFFFFFFFFFFFFFFFFFFFFFF
FFFFFFFFFFFFFFFFFFFFFFFFFFF,FFFFFFFFFFFFFFFFFFFFFFFFFFFFFFFFFFFFFFFF
@A00155:342:HHGFNDSXY:1:2272:18484:36824 1:N:0:GAACCTAG+TCCGCATA
AATGCCGAGAGGGATGTGGAGGGCGAGAGAGAGCGACTTCTCTCGGGCCCACAGCCTTACAGCTGTGG
AGCACGGTATCCTCTGCCGAAACAGAGGTTGGACAAGACCGGAGGGGTCTCCTGGTTCCAAAGGAGAT
+
FFFFFFFFFFFFFFFFFFFFFFFFFFFFFFFFFFFFFFFFFFFFFFFFFFFFFFFFFFFFFFFFFFFF
FFFFFFFFFFFFFFFFFFFFFFFFFFFFFFFFFFFFFFFFFFFFFFFFFFFFFFFFFFFFFFFFFFFF
@A00155:342:HHGFNDSXY:1:2273:19298:8829 1:N:0:GAACCTAG+TCCGCATA
GGATGTGGAGGGCGAGAGAGAGCGACTTCTCTCGGGCCCACAGCCTTACAGCTGTGGAGCACGGTATC
CTCTGCCGAAACAGAGGTTGGACAAGACCGGAGGGGTCTCCTAGTTCCAAAGGAGATGTACTCC
+
FFFFFFFFFFFFFFFFFFFFFFFFFFFFFFFFFFFFFFFFFFFFFFFFFFFFFFFFFFFFFFFFFFFF
FFFFFFFFFFFFFFFFFFFFFFFFFFFFFFFFFFFFFFFFFFFFFFFFFFFFFFFFFFFFFFFF
@A00155:342:HHGFNDSXY:1:2273:19407:11083 1:N:0:GAACCTAG+TCCGCATA
GAGGGCGAGAGAGAGCGACTTCTCTCGGGCCCACAGCCTTACAGCTGTGGAGCACGGTATCCTCTGCC
GAAACAGAGGTTGGACAAGACCGGAGGGGTCTCCTAGTTCCAAAGGAGATGTACTCCGGGCTTGTTCA
+
FFFFFFFFFFFFFFFFFFFFFFFFFFFFFFFFFFFFFFFFFFFFFFFFFFFFFFFFFFFFFFFFFFFF
FFFFFFFFFFFFFFFFFFFFFFFFFFFFFFFFFFFFFFFFFFFFFFFFFFFFFFFFFFFFFFFFFFFF
@A00155:342:HHGFNDSXY:1:2273:2239:29262 1:N:0:GAACCTAG+TCCGCATA
TTAAGGACTCTCCTCTGGCCTATGGTCATCTCAATGCCGAGAGGGATGTGGAGGGCGAGAGAGAGCGA
CTTCTCTCGGGCCCACAGCCTTACAGCTGTGGAGCACGGTATCCTCTGCCGAAACAGAGGTTGGACAA
+
FFFFFFFF:FFFFFFFFFFFFFFFFFFFFFFFFFFFF,FFFFFFFFFFFFFF:FFFF:FFFFF:FFF,
:FFFFFFFFFFFFFFFF:FFFFFFFFFFFFFFFFFFFFFFFFF:FFFFF:FFFF,FFF:FFFFFFFFF
@A00155:342:HHGFNDSXY:1:2273:29432:31391 1:N:0:GAACCTAG+TCCGCATA
CTTCTCTCGGGCCCACAGCCTTACAGCTGTGGAGCACGGTATCCTCTGCCGAAACAGAGGTTGGACAA
GACCGGAGGGGTCTCCTAGTTCCAAAGGAGATGTACTCCGGGCTTGTTCACGACCTACCGTGTAAGTC
+
FFFFFFFFFFFFFFFFFFFFF:FFFFFFFFFFFFFFFFFFFFFFFF:FFFFFFFFFFFFFFFFFFFFF
FFFFFFFFFFFFFFFFFFF:FFFFFFFFFFFFF:FFFFFFFFF,FFFFFFFFFFFFFF:F:FFFFF,F
@A00155:342:HHGFNDSXY:1:2273:27416:36417 1:N:0:GAACCTAG+TCCGCATA
TTCTCTCGGGCCCACAGCCTTACAGCTGTGGAGCACGGTATCCTCTGCCGAAACAGAGGTTGGACAAG
ACCGGAGGGGTCTCCTAGTTCCAAAGGAGATGTACTCCGGGCTTGTTCACGACCTACCGTGTAAGTCG
+
FFFFFFFFFFFFFFFFFFFFFFFFFFFFFFFFFFFFFFFFFFFFFFFFFFFFFFFFFFFFFFFFFFF:
FFFFFFFFFFFFFFFFFFFFFFFFFFFFFFFFFFFFFFFFFFFFFFFFFFFFFFFFFFFFFFFFFFFF
@A00155:342:HHGFNDSXY:1:2274:19605:17691 1:N:0:GAACCTAG+TCCGCATA
ACCTCTGTTTCGGCAGAGGATACCGTGCTCCACAGCTGTAAGGCTGTGGGCCCGAGAGAAGTCGCTCT
CTCTCGCCCTCCACATCCCTCTCGGCATTGAGATGACCATAGGCCAGAGGAGAGTCCTTAAGT
+
```

FFFFFFFFFFFFFFFFFFFFFFFFFFFFFFFFFFFFFFFFFFFFFFFFFFFFFFFFFFFFFFFFFFFF
FFFFFFFFFFFFFFFFFFFFFFFFFFFFFFFFFFFFFFFFFFFFFFFFFFFFFFFFFFFFFFF
@A00155:342:HHGFNDSXY:1:2275:12472:15421 1:N:0:GAACCTAG+TCCGCATA
TTGTCCAACCTCTGTTTCGGCAGAGGATACCGTGCTCCACAGCTGTAAGGCTGTGGGCCCGAGAGAAG
TCGCTCTCTCTCGCCCTTCACATCCCTCTCGGCATTGAGATGACCATAGGCCAGAGGTGAGTCCTTAA
+
FFFFFFFFFFFFFFFFFFFFFFFFFFFFFFFFFFFFFFFFFFFFFFFFFFFFFFFFFFFFFFFFFFFF
FFFFFFFFFFFFFFFFFFFFFFFFFFFFFFFFFFFFFFFFFFFFFFFFFFFFFFFFFFFFFFFFFFFF
@A00155:342:HHGFNDSXY:1:2275:16414:30639 1:N:0:GAACCTAG+TCCGCATA
CTTCTCTCGGGCCCACAGCCTTACAGCTGTGGAGCACGGTATCCTCTGCCGAAACAGAGGTTGGACAA
GACCGGAGGGGTCTCCTAGTTCCAAAGGAGATGTACTCCGGACTTGTTCACGACCTACCGTGTAAGTC
+
FFFFFFFFFFFFFFFFFFFF:FFFFFFFFFFFFFFFFFFFFFFFFFFFFFFFFFFFFFFFFFFFFFFF
FFFFFFFFFFFFFFFFFFFFFFFFFFFFFFFFFFFFFFFFFFFFFFFFFFFFFFFFFFFFFFFFFFFF
@A00155:342:HHGFNDSXY:1:2275:18502:33098 1:N:0:GAACCTAG+TCCGCATA
GGGCGAGAGAGAGCGACTTCTCTCGGGCCCACAGCCTTACAGCTGTGGAGCACGGTATCCTCTGCCGA
AACAGAGGTTGGACAAGACCGGAGGGGTCTCCTAGTTCCAAAGGAGATGTACTCCGGGCTTGTTCACG
+
FFFFFFFFFFFFFFFFF:FFFFFFFFFFFFFFFFFFFFFFFFFFFFFFFFFFFFFFFFFFFFFFFFFF
FFFFFFFFFFFFFFFFFFFFFFFFFFFFFFFFFFFFFFFFFFFFFFFFFFFFFFFFFFFFFFFFF,FF
@A00155:342:HHGFNDSXY:1:2276:16839:3317 1:N:0:GAACCTAG+TCCGCATA
ATCCTCTGCCGAAACAGAGGTTGGACAAGACCGGAGGGGTCTCCTAGTTCCAAAGGAGATGTACTCCG
GGCTTGTTCACGACCTACCGTGTAAGTCGTAGTCTAGTAGGCTACCTGACGAGTCCTTTTTAGGACGA
+
FFFFFFFFFFFFFFFFFFFFFFFFFFFFFFFFFFFFFFFFFFFFFFFFFFFFFFFFFFFFFFFFFFFF
FFFFFFFFFFFFFFFFFFFFFFFF:FFFFFFFFFFFFFFFFFFFFFFFFFFFFFFFFFFFFFFFFFF
@A00155:342:HHGFNDSXY:1:2276:28438:9721 1:N:0:GAACCTAG+TCCGCATA
CTCAATGCCGAGAGGGATGTGGAGGGCGAGAGAGAGCGACTTCTCTCGGGCCCACAGCCTTACAGCTG
TGGAGCACGGTATCCTCTGCCGAAACAGAGGTTGGACAAGACCGGAGGGGTCTCCTAGTTCCAAAGGA
+
FFFFFFFFFFFFFFFFFFFFFFFFFFFFFFFFFFFFFFFFFFFFFFFFFFFFFFFFFFFFFFFFFFFF
FFFFFFFFFFFFFFFFFFFFFFFFFFFFFFFFFFFFFFFFFFFFFFFFFFFFFFFFFFFFFFFFF:FF
@A00155:342:HHGFNDSXY:1:2276:8151:17581 1:N:0:GAACCTAG+TCCGCATA
CCTTACAGCTGTGGAGCACGGTATCCTCTGCCGAAACAGAGGTTGGACAAGACCGGAGGGGTCTCCTA
GTTCCAAAGGAAATGTACTCCGGGCTTGTTCACGACCTACCGTGTAAGTCGTAGTCTAGTAGGCTACC
+
FFFFFFFFFFFFFFFFFFFFFFFFFFFFFFFFFFFFFFFFFFFFFFFFFFFFFFFFFFFFFFFFFFFF
FFFFFFFFFFFFFFFFFFFFFFFFFFFFFFFFFFFFFFFFF:FFF:FFFFFFFFFFFFFFFFFFFFFF
@A00155:342:HHGFNDSXY:1:2276:18249:27680 1:N:0:GAACCTAG+TCCGCATA
TCCTCTGCCGAAACAGAGGTTGGACAAGACCGGAGGGGTCTCCTGGTTCCAAAGGAGATGTACTCCGG
GCTTGTTCACGACCTACCGTGTAAGTCGTAGTCTAGTAGGCTACCTGACGAGTCCTTTTTAGGACGAA
+
FFFFFFFFFFFFFFFFFFFFFFFFFFFFFFFFFFFFFFFFFFFFF:FFFFFFFFFFFFFFFFFFFFFF
FFFFFFFFFFFFFFFFFFFFFFFFFFFFFFFFFF:FFFFFFFFFFFFFFFFFFFFF:F:FFFFFF:FF
@A00155:342:HHGFNDSXY:1:2276:20148:33724 1:N:0:GAACCTAG+TCCGCATA
CTCAATGCCGAGAGGGATGTGGAGGGCGAGAGAGAGCGACTTCTCTCGGGCCCACAGCCTTACAGCTG
TGGAGCACGGTATCCTCTGCCGAAACAGAGGTTGGACAAGACCGGAGGGGTCTCCTAGTTCCAAAGGA
+
FFFFFFFFFFFFFFFFFFFFFFFFFFFFFFFFFFFFFFFFFFFFFFFFFFFFFFFFFFFFFFFFFFFF
FFFFFFFFFFFFFFFFFFFFFFFFFFFFFFFFFFFFFFFFFFFFFFFFFFFFFFFFFFFFFFFFFFFF
@A00155:342:HHGFNDSXY:1:2277:25590:4038 1:N:0:GAACCTAG+TCCGCATA
AGCGACTTCTCTCGGGCCCACAGCCTTACAGCTGTGGAGCACGGTATCCTCTGCCGAAACAGAGGTTG
GACAAGACCGGAGGGGTCTCCTAGTTCCAAAGGAGATGTACTCCGGGCTTGTTCACGACCTACCGTGT
+

FFFFFFFFFFFFFFFFFFFFFFFFFFFFFFFFFFFFFFFFFFFFFFFFFFFFFFFFFFFFFFFFFFFF
FFFFFFFFFFFFFFFFFFFFFFFFFFFFFFFFFFFFFFFFFFFFFFFFFFFFFFFFFFFFFFFFF:FF
@A00155:342:HHGFNDSXY:1:2277:16938:4554 1:N:0:GAACCTAG+TCCGCATA
TGGTCATCTCAATGCCGAGAGGGATGTGGAGGGCGAGAGAGAGCGACTTCTCTCGGGCCCACAGCCTT
ACAGCTGTGGAGCACGGTATCCTCTGCCGAAACAGAGGTTGGACAAGACCGGAGGGGTCTCCTAGTTC
+
FFFFFFFFFFFFFFFFFFFFFFFFFFFFFFFFFFFFFFFFFFFFFFFFFFFFFFFFFFFFFFFFFFFF
FFFFFFFFFFFFFFFFFFFFFFFFFFFFFFFFFFFFFFFFFFFFFFFFFFFFFFFFFFFFFFFFFFFF
@A00155:342:HHGFNDSXY:1:2277:2889:14450 1:N:0:GAACCTAG+TCCGCATA
AATGCCGAGAGGGATGTAGAGGGCGAGAGAGAGCGACTTCTCTCGGGCCCACAGCCTTACAGCTGTGG
AGCACGGTATCCTCTGCCGAAACAGAGGTTGGACAAGACCGGAGGGGTCTCCTAGTTCCAAAGGAGAT
+
FFFFFFFFFFFFFFFFFFFFFFFFFFFFFFFFFFFFFFFFFFFFFFFFFFFFFFFFFFFFFFFFFFFF
F:FFFFFFFFFFFFFF:FFFFFFFFFFFFFFFFFFFFFFFFFFFFFFFFFFFFFFFFFFFFFFFFFFF
@A00155:342:HHGFNDSXY:1:2277:1678:33395 1:N:0:GAACCTAG+TCCGCATA
ACTTCTCTCGGGCCCACAGCCTTACAGCTGTGGAGCACGGTATCCTCTGCCGAAACAGAGGTTGGACA
AGACCGGAGGGGTCTCCTAGTTCCAAAGGAGATGTACTCCGGGCTTGTTCACGACCTACCGTGTAAGT
+
FFFFFFFFFFFFFFFFFFFFFF,FFFFFFF:FFFFFFFFF::FFFFFFFFFFF,FFFFFFFF:FFFF:
F:FFFFFFFFFFFFFFFFFF:FFFFF:FFFFFFFFFFFFFFFFF:FFFFFFFFFFF,:FFFFFFF,FF
@A00155:342:HHGFNDSXY:1:2278:23448:12602 1:N:0:GAACCTAG+TCCGCATA
GTCATCTCAATGCCGAGAGGGATGTGGAGGGCGAGAGAGAGCGACTTCTCTCGGGCCCACAGCCTTAC
AGCTGTGGAGCACGGTATCCTCTGCCGAAACAGAGGTTGGACAAGACCGGAGGGGTCTCCTAGTTCCA
+
FFFFFFFFFFFFFFFFF:FFFF:FFFFFFFFFFFFFFFFFFFFFFFFFFFFFFFFFFFFFFFFFF:F:F
FFFFFFFFFFFFFFFFFFFFFFFFFFFFFFFFFFFFFFFFFFFF:FFFFFFFFFFFFFFFFFFFFFFF
@A00155:342:HHGFNDSXY:1:2278:23393:12665 1:N:0:GAACCTAG+TCCGCATA
GTCATCTCAATGCCGAGAGGGATGTGGAGGGCGAGAGAGAGCGACTTCTCTCGGGCCCACAGCCTTAC
AGCTGTGGAGCACGGTATCCTCTGCCGAAACAGAGGTTGGACAAGACCGGAGGGGTCTCCTAGTTCCA
+
FFFFFFFFFFFFFFF:FFFFFFFFFFFFFFFFF:FFFFFFFFFFF:FFFFFFFFFFFFFFFFFFFFFF
FFFFFFFFFFFFFFFFFFFFFFFFFFFFFFFFFFFFFFFFFFFFFFFFFFFFFFFFFFFFFFFFFFFF
@A00155:342:HHGFNDSXY:1:2278:15673:17613 1:N:0:GAACCTAG+TCCGCATA
CTTCTCTCGGGCCCACAGCCTTACAGCTGTGGAGCACGGTATCCTCTGCCGAAACAGAGGTTGGACAA
GACCGGAGGGGTCTCCTAGTTCCAAAGGAGATGTACTCCGGGCTTGTTTACGACCTACCGTGTAAGTC
+
FFFFFFFFFFFFFFFFFFFFFFFFFFFFFFFFFFFFFFFFFFFFFFFFFFFFFFFFFFFFFFFFFFFF
FFFFFFFFFFFFFFFFFFFFFFFFFFFFFFFFFFFFFFFFFFFFFFFFFFFFFFFFFFFFFFFFFFFF
@A00155:342:HHGFNDSXY:1:2278:20500:22404 1:N:0:GAACCTAG+TCCGCATA
TGTGGAGGGCGAGAGAGAGCGACTTCTCTCGGGCCCACAGCCTTACAGCTGTGGAGCACGGTATCCTC
TGCCGAAACAGAGGTTGGACAAGACCGGAGGGGTCTCCTAGTTCCAAAGGAGATGTACTCCGGG
+
FFFF:FFFFFFFFFFFFFFFFFFFFFFFFFFFFFFFFFFFFFFFFFFFFFFFFFF:FFFFFFFFFF
FFFFFFFFFFFFFFFFFFFFFFFFFFFFFFFFFFFFFFFFFFFFFFFFFFFFFFFFFFFFFFFF
@A00155:342:HHGFNDSXY:1:2301:28085:1689 1:N:0:GAACCTAG+TCCGCATA
TGTTTCGGCAGAGGATACCGTGCTCCACAGCTGTAAGGCTGTGGGCCCGAGAGAAGTCGCTCTCTCTC
GCCCTCTACATCCCTCTCGGCATTGAGATGACCATAGGCCAGAGGTGAGTCCTTAAGTGGACACAGCT
+
FFFFFFFFFF:FFFFFFFF:FFFF:FFFFFF:FFFFF,FFFFF:FFF:FFFFFFFFFF:F:FFFFFFF
,FFFFFFFFFFFF,FFFF:FFF:FFFFFFFFFFF,F:FFFFFF:FFFFFFFFFFFFFF:FFFFFFF:F
@A00155:342:HHGFNDSXY:1:2301:26910:15749 1:N:0:GAACCTAG+TCCGCATA
CTTCTCTCGGGCCCACAGCCTTACAGCTGTGGAGCACGGTATCCTCTGCCGAAACAGAGGTTGGACAA
GACCGGAGGGGTCTCCTGGTTCCAAAGGAGATGTACTCCGGGCTTGTTCACGACCTACCGTGTAAGTC
+

FFFFFFFFFFFFFFFFFFFFFFFFFFFFFFFFFFFFFFFFFFFFFFFFFFFFFFFFFFFFFFFFFFFF
FFFFFFFFFFFFFFFFFFFFFFFFFFFFFFFFFFFFFFFFFFFFFFFFFFFFFFFFFFFFFFFFFFFF
@A00155:342:HHGFNDSXY:1:2301:20374:25285 1:N:0:GAACCTAG+TCCGCATA
TCTCTCGCCCTCCACATCCCTCTCGGCATTGAGATGACCATAGGCCAGAGGTGAGTCCTTAAGTGGAC
ACAGCTGATCTAAGGCGGTGTGGTGGGGCATGGGTTTGAACCCCCATGACGGTCGGAGTC
+
FFFFFFFFFFFF:FFFFF,FF:FFFFFFFFFF:FFFFFFFFF::,FFFF::,FFFFF,FFFFFFFFF,
:FF:FFFF:FFFF,FF:FFFFFFFF,:FFF:,F:,FFFFFFFF::FFFFFFFFFFFFFFF
@A00155:342:HHGFNDSXY:1:2302:31159:29121 1:N:0:GAACCTAG+TCCGCATA
AGAGAGAGCGACTTCTCTCGGGCCCACAGCCTTACAGCTGTGGAGCACGGTATCCTCTGCCGAAACAG
AGGTTGGACAAGACCGGAGGGGTCTCCTAGTTCCAAAGGAGATGTACTCCGGGCTTGTTCACG
+
FFFFFFFFFFFFFFFFFFFFFFFFFFFFFFFFFFFFFFFFFFFFFFFFFFFFFFFFFFFFFFFFFFFF
FFFFFFFFFF:FFFFFFFF:FFFFFFFFFFFFFFFFFFFFFFFF:FFFFFFFFFFFFFFFFFF
@A00155:342:HHGFNDSXY:1:2303:23719:1235 1:N:0:GAACCTAG+TCCGCATA
AAGGCTGTGGGCCCGAGAGAAGTCGCTCTCTCTCGCCCTCCACATCCCTCTCGGCATTGAGATGACCA
TAGGCCAGAGGTGAGTCCTTAAGTGGACACAGCTGATCTAAGGCGGTGTGGCGGGGCATGGGTTTGAA
+
FFFFFFFFFFFFFFFFFFFFFFFFFFFFFFFFFFFFFFFFFFFFFFFFFFFFFFFFFFFFFFFFFFFF
FFFFFFFFFFFFFFFFFFFFFFFFFFFFFFFFFFFFFFFFFFFFFFFFFFFFFFFFFFFFFFF:FFFF
@A00155:342:HHGFNDSXY:1:2303:23502:1423 1:N:0:GAACCTAG+TCCGCATA
AAGGCTGTGGGCCCGAGAGAAGTCGCTCTCTCTCGCCCTCCACATCCCTCTCGGCATTGAGATGACCA
TAGGCCAGAGGTGAGTCCTTAAGTGGACACAGCTGATCTAAGGCGGTGTGGCGGGGCATGGGTTTGAA
+
FFFFFFFFFFFFFFFFFFFFFFFFFFFFFFFFFFFFFFFFFFFFFFFFFFFFFFFFFFFFFFFFFFFF
FFFFFFFFFFFFFFFFFFFFFFFFFFFFFFFFFFFFFFFFFFFFFFFFFFFFFFFFFFFFFFFFFFFF
@A00155:342:HHGFNDSXY:1:2303:18448:6010 1:N:0:GAACCTAG+TCCGCATA
GAGCGACTTCTCTCGGGCCCACAGCCTTACAGCTGTGGAGCACGGTATCCTCTGCCGAAACAGAGGTT
GGACAAGACCGGAGGGGTCTCCTAGTTCCAAAGGAGATGTACTCCGGGCTTGTTCACGACCTACCGTG
+
FFFFFFFFFFFFFFFFFFFFFFFFFFFFFFFFFFFFF:FF:FFFFFFFFFFFFFFFFFFFFFFF:FFF
FFFFFFFFFFFFFFFFFFFFFFFFFFFFFFFFFFFFFFFFFFFFFFFFFFFFFFFFF:FFFFFFFF:F
@A00155:342:HHGFNDSXY:1:2303:13602:10488 1:N:0:GAACCTAG+TCCGCATA
CCCTCCACATCCCTCTCGGCATTGAGATGACCATAGGCCAGAGGTGAGTCCTTAAGTGGACACAGCTG
ATCTAAGGCGGTGTGGCGGGGCATGGGTTTGAACCCCCATGACGGTCGGAGTCTAGTAGGCTCCCTGA
+
FFFFFFFFFFFFFFFFFFFFFFFFFFFFFFFFFFFFFFFFFFFFFFFFFFFFFFFFFFFFFFFFFFFF
FFFFFFFFFFFFFFFFFFFFFFFFFFFFFFFFFFFFFFFFFFFFFFFFFFFFFFFFFFFFFFFFFFFF
@A00155:342:HHGFNDSXY:1:2303:28601:26976 1:N:0:GAACCTAG+TCCGCATA
GAGAGAGCGACTTCTCTCGGGCCCACAGCCTTACAGCTGTGGAGCACGGTATCCTCTGCCGAAACAGA
GGTTGGACAAGACCGGAGGGGTCTCCTGGTTCCAAAGGAGATGTACTCCGGGCTTGTTCACGACCTAC
+
FFFFFFFFFFFFFFFFFFFFFFFFFFFFFFFF,FFFFFFFFFFFFFFFFF:FFFFFFFFFFFFFFFFF
FFFFFFFFFFFFFFFFFFFFFFFFFFFFFFFFFFFFFFFFFFFFFFFFFFFFFFFFFFFFFFFFFFFF
@A00155:342:HHGFNDSXY:1:2303:9570:33692 1:N:0:GAACCTAG+TCCGCATA
TGTCCACTTAAGGACTCACCTCTGGCCTATGGTCATCTCAATGCCGAGAGGGATGTGAAGGGCGAGAG
AGAGCGACTTCTCTCGGGCCCACAGCCTTACAGCTGTGGAGCACGGTATCCTCTGCCGAAACAGAGGT
+
FFFFFFFFFFFFFFFFFFFFFFFFFFFFFFFFFFFFFFFFFFFFFFFFFFFFFFFFFFFFFFFFFFFF
FFFFFFFFFFFFFFFFFFFFFFFFFFFFFFFFFFFFFFFFFFFFFFFFFFFFFFFFFFFFFF:FFFFF
@A00155:342:HHGFNDSXY:1:2304:14172:5431 1:N:0:GAACCTAG+TCCGCATA
TCGGCAGAGGATACCGTGCTCCACAGCTGTAAGGCTGTGGGCCCGAGAGAAGTCGCTCTCTCTCGCCC
TTCACATCCCTCTCGGCATTGAGATGACCATAGGCCAGAGGTGAGTCCTTAAGTGGACACA
+

FFFFFFFFFFFFFFFFFFFFFFFFFFFFFFFFFFFFFFFFFFFFFFFFFFFFFFFFFFFFFFFFFFFF
FFFFFFFFFFFFFFFFFFFFFFFFFFFFFFFFFFFFFFFFFFFFFFFFFFFFFFFFFFFFF
@A00155:342:HHGFNDSXY:1:2304:14136:6433 1:N:0:GAACCTAG+TCCGCATA
TCGGCAGAGGATACCGTGCTCCACAGCTGTAAGGCTGTGGGCCCGAGAGAAGTCGCTCTCTCTCGCCC
TTCACATCCCTCTCGGCATTGAGATGACCATAGGCCAGAGGTGAGTCCTTAAGTGGACACA
+
FFFFFFFFFFFFFFFFFFFFFFFFFFFFFFFFFFFFFFFFFFFFFFFFFFFFFFFFFFFFFFFFFFFF
FFFFFFFFFFFFFFFFFFFFFFFFFFFFFFFFFFFFFFFFFFFFFFFFFFFFFFFFFFFFF
@A00155:342:HHGFNDSXY:1:2304:21332:11193 1:N:0:GAACCTAG+TCCGCATA
GAGCGACTTCTCTCGGGCCCACAGCCTTACAGCTGTGGAGCACGGTATCCTCTGCCGAAACAGAGGTT
GGACAAGACCGGAGGGGTCTCCTAGTTCCAAAGGAGATGTACTCCGGGCTTGTTCACGACCTACCGTG
+
FFFFFFFFFFFFFFFFFFFFFFFFFFFFFFFFFFFFFFFFFFFFFFFFFFFFFFFFFFFFFFFFFFFF
FFFFFFFFFFFFFFFFFFFFFFFFFFFFFFFFFFFFF:FFFFFFFFFFFFFFFFFFFFFFFFFFFFFF
@A00155:342:HHGFNDSXY:1:2304:16848:28917 1:N:0:GAACCTAG+TCCGCATA
CGGCAGAGGATACCGTGCTCCACAGCTGTAAGGCTGTGGGCCCGAGAGAAGTCGCTCTCTCTCGCCCT
CTACATCCCTCTCGGCATTGAGATGACCATAGGCCAGAGGTGAGTCCTTAAGTGGACACAGCTGATCT
+
FFFFFFFFFFFFFFFFFFFFFFFFFFFFFFFFFFFFFFFFFFFFFFFFFFFFFFFFFFFFFFFFFFFF
FFFFFFFFFFFFFFFFF:FFFFFFFFFFFFFFFFFFFFFFFFFFFFFFFFFFFFFFFFFFFFFFFFFF
@A00155:342:HHGFNDSXY:1:2305:11686:2127 1:N:0:GAACCTAG+TCCGCATA
AGAGAGAGCGACTTCTCTCGGGCCCACAGCCTTACAGCTGTGGAGCACGGTATCCTCTGCCGAAACAG
AGGTTGGACAAGACCGGAGGGGTCTCCTAGTTCCAAAGGAGATGTACTCCGGGCTTGTTCACGACCTA
+
FFFFFFFFFFFFFFFFFFFFFFFFFFFFFFFFFFFFFFFFFFFFFFFFFFFFFFFFFFFFFFFFFFFF
FFFFFFFFFFFFFFFFFFFFFFFFFFFFFFFFFFFFFFFFFFFFFFFFFFFFFFFFFFFFFFFFFFFF
@A00155:342:HHGFNDSXY:1:2305:19895:24486 1:N:0:GAACCTAG+TCCGCATA
TAGTCTAGTAGGCTACCTGACGAGTCCTTTTTAGGACGAAACTTACACAACTCGAAGAGTAACGACGG
TTTCGTTCTTGGAACGGACTCATCAGGGAGCCTACTAGACTCCGACCGTCATGGGGGTTCAAACCCAT
+
FFFFFFFFFFFFFFFFFFFFFFFFFFFFFFFFFFFFFFFFFFFFFFFFFFFFFFFFFFFFFFFFFFFF
FFFFFFFFFFFFFFFFFFFFFFFFFFFFFFFFFFFFFFFFFFFFFFFFFFFFFFFFFFFFFFFFFFF:
@A00155:342:HHGFNDSXY:1:2305:4951:31735 1:N:0:GAACCTAG+TCCGCATA
CCTCTGGCCTATGGTCATCTCAATGCCGAGAGGGATGTGAAGGGCGAGAGAGAGCGACTTCTCTCGGG
CCCACAGCCTTACAGCTGTGGAGCACGGTATCCTCTGCCGAAACAGAGGTTGGACAAGAC
+
FFFFFFFFFF,FFFFFFFFFFFFFFFFFFFFFFF:FF,FFFFFFFFFFFFFFFFFFFFFFFFFFFFFF
FFFFFFFFF:FFFFFFFFFFFFFFFFFFFFFFFFF:FFFFF:FFFFFFFFFFFFFFFFFF
@A00155:342:HHGFNDSXY:1:2306:32217:2300 1:N:0:GAACCTAG+TCCGCATA
TTCTCTCGGGCCCACAGCCTTACAGCTGTGGAGCACGGTATCCTCTGCCGAAACAGAGGTTGGACAAG
ACCGGAGGGGTCTCCTGGTTCCAAAGGAGATGTACTCCGGGCTTGTTCACGACCTACCGTGTAAGTCG
+
FFFFFFFFFFFFFFFFFFFFFFFFFFFFFFFFFFFFFFFFFFFFFFFFFFFFFFFFFFFFFFFFFFFF
FFFFFFFFFFFFFFFFFFFFFFFFFFFFFFFFFFFFFFFFFFFFFFFFFFFFFFFFFFFFFFFFFFFF
@A00155:342:HHGFNDSXY:1:2306:25590:13213 1:N:0:GAACCTAG+TCCGCATA
CAATGCCGAGAGGGATGTGGAGGGCGAGAGAGAGCGACTTCTCTCGGGCCCACAGCCTTACAGCTGTG
GAGCACGGTATCCTCTGCCGAAACAGAGGTTGGACAAGACCGGAGGGGTCTCCTAGTTCCAAAGGAGA
+
FFFFFFFFFFFFFFFFFFFFFFFFFFFFFFFFFFFFFFFFFFFFFFFFFFFFFFFFFFFFFFFFFFFF
FFFFFFFFFFFFFFFFFFFFFFFFFFFFFFFFFFFFF:FFFFFFFFFFFFFFFFFFFFFFFFFFFFFF
@A00155:342:HHGFNDSXY:1:2306:11324:29559 1:N:0:GAACCTAG+TCCGCATA
AGAGAGAGCGACTTCTCTCGGGCCCACAGCCTTACAGCTGTGGAGCACGGTATCCTCTGCCGAAACAG
AGGTTGGACAAGACCGGAGGGGTCTCCTAGTTCCAAAGGAGATGTACTCCGGGCTTGTTCACGACCTA
+

```
FFFFFFFFFFFFFFFFFFFFFFFF:FFF:FFFFFFFFFFFFFFFFFFFFFFFFFFFFFFFFFFFFFFF
:FFFFFFFFFFFFFFFFFFFFFFFFFFFFFFFFFFFFFFFFFFFFFFFFFFFFFFFFFF:FFFFFFF
@A00155:342:HHGFNDSXY:1:2306:24297:32769 1:N:0:GAACCTAG+TCCGCATA
GATGTAGAGGGCGAGAGAGAGCGACTTCTCTCGGGCCCACAGCCTTACAGCTGTGGAGCACGGTATCC
TCTGCCGAAACAGAGGTTGGACAAGACCGGAGGGGTCTCCTAGTTCCAAAGGAGATGTACTCCGGGCT
+
FFFFFFFFFFFFFFFFFFFFFFFFFFFFFFFFFFFFFFFFFFFFFFFFFFFFFFFFFFFFFFFFFFFF
FFFFFFFFFFFFFFFFFFFFFFFFFFFFFFFFFFFFFFFFFF:FFFFFFFFFFFFFFFFFFFFFFFFF
@A00155:342:HHGFNDSXY:1:2306:30129:33849 1:N:0:GAACCTAG+TCCGCATA
GGATGTAGAGGGCGAGAGAGAGCGACTTCTCTCGGGCCCACAGCCTTACAGCTGTGGAGCACGGTATC
CTCTGCCGAAACAGAGGTTGGACAAGACCGGAGGGGTCTCCTAGTTCCAAAGGAGATGTACTCC
+
FFFFFFFFFFFFFFFFFFFFFFFFFFFFFFFFFFFFFFFFFFFFFFFFFFFFFFFFFFFFFFFFFFFF
FFFFFFFFFFFFFFFFFFFFFFFFFFFFFFFFFFFFFFFFFFFFFFFFFFFFFFFFFFFFFFFF
@A00155:342:HHGFNDSXY:1:2307:12337:1125 1:N:0:GAACCTAG+TCCGCATA
TCTCTCGGGCCCACAGCCTTACAGCTGTGGAGCACGGTATCCTCTGCCGAAACAGAGGTTGGACAAGA
CCGGAGGGGTCTCCTAGTTCCAAAGGAGATGTACTCCGGGCTTGTTCACGACCTACCGTGTAAGTCGT
+
FFFFFFFFFFFFFFFFFFFFFFFFFFFFFFFFFFFFFFFFFFFFFFFFFFFFFFFFFFFFFFFFFFFF
FFFFFFFFFFFFFFFFFFFFFFFFFFFFFFFFFFFFFFFFFF:FFFFFFFFFFFFFFFFFFFFFFFFF
@A00155:342:HHGFNDSXY:1:2307:16667:5619 1:N:0:GAACCTAG+TCCGCATA
GGATGTGGAGGGCGAGAGAGAGCGACTTCTCTCGGGCCCACAGCCTTACAGCTGTGGAGCACGGTATC
CTCTGCCGAAACAGAGGTTGGACAAGACCGGAGGGGTCTCCTAGTTCCAAAGGAGATGTACTCC
+
FFFFF:FFFFFFFFFFFFFFFFFFFFFFFFFFFFFFFFFFFFFFFFFFFFFF:FFFFFFFFFFFFFFF
FFFFFFFFFFFFFFFFFFFFFFFFFF:FFFFFFFFFFFFFFF,FFFFFFFFFFFFF,FFFFFFF
@A00155:342:HHGFNDSXY:1:2308:9308:6214 1:N:0:GAACCTAG+TCCGCATA
GGATGTGGAGGGCGAGAGAGAGCGACTTCTCTCGGGCCCACAGCCTTACAGCTGTGGAGCACGGTATC
CTCTGCCGAAACAGAGGTTGGACAAGACCGGAGGGGTCTCCTAGTTCCAAAGGAGATGTACTCC
+
FFFFFFFFFFFFFFFFFFFFFFFFFFFFFFFFFFFFFFFFFFFFFFFFFFFFFFFFFFFFFFFFFFFF
FFFFFFFFFFFFFFFFFFFFFFFFFFFFFFFFFFFFFFFFFFFFFFFFFFFFFFFFFFFFFFFF
@A00155:342:HHGFNDSXY:1:2308:3839:7795 1:N:0:GAACCTAG+TCCGCATA
ATCTCAATGCCGAGAGGGATGTGGAGGGCGAGAGAGAGCGACTTCTCTCGGGCCCACAGCCTTACAGC
TGTGGAGCACGGTATCCTCTGCCGAAACAGAGGTTGGACAAGACCGGAGGGGTCTCCTAGTTCCAAAG
+
FFFFFFFFF,FFF:FFFFF:FF,FFFFFF,FFFFF:FFFFFFFFFF,FF,FFFFFFFFFFFFF:F:F,
FFFFFFFFFFFFF:FFFFFFFFFFFFFFFFF:F,FF:FFFFFFFFFFFFFFFFFFFFFFFFFFFFFFF
@A00155:342:HHGFNDSXY:1:2308:10511:30373 1:N:0:GAACCTAG+TCCGCATA
CTTCTCTCGGGCCCACAGCCTTACAGCTGTGGAGCACGGTATCCTCTGCCGAAACAGAGGTTGGACAA
GACCGGAGGGGTCTCCTGGTTCCAAAGGAGATGTACTCCGGGCTTGTTCACGACCTACCGTGTAAGTC
+
FFFFFFFFFFFFFFFFFFFFFFFFFFFFFFFFFFFFFFFFFFFFFFFFFFFFFFFFFFFFFFFFFFFF
FFFFFFFFFFFFFFFFFFFFFFF,FFFFFFFFFFFFFFFFFF:FFFFFFFFFFFFFFFFFFFFFFFFF
@A00155:342:HHGFNDSXY:1:2308:10926:30843 1:N:0:GAACCTAG+TCCGCATA
CTTCTCTCGGGCCCACAGCCTTACAGCTGTGGAGCACGGTATCCTCTGCCGAAACAGAGGTTGGACAA
GACCGGAGGGGTCTCCTGGTTCCAAAGGAGATGTACTCCGGGCTTGTTCACGACCTACCGTGTAAGTC
+
FFFFFFFFFFFFFFFFFFFFFFFFFFFFFFFFFFFFFFFFFFFFFFFFFFFFFFF:FFFFFFFFFFFF
FFFFFFFFFFFFFFFFFFFFFFFFFFFFFFFFFF:FFFFFFFFFFFFFFFFFFFFFFFFFFFFFFFFF
@A00155:342:HHGFNDSXY:1:2308:17065:34428 1:N:0:GAACCTAG+TCCGCATA
CCACAGCCTTACAGCTGTGGAGCACGGTATCCTCTGCCGAAACAGAGGTTGGACAAGACCGGAGGGGT
CTCCTAGTTCCAAAGGAGATGTACTCCGGGCTTGTTCACGACCTACCGTGTAAGTCGTAGTCTAGTAG
+
```

FFFFFFFFFFFFFFFFFFFFFFFFFFFFFFFFFFFFFFFFFFFFFFFFFFFFFFFFFFFFFFFFFFFF
FFFFFFFFFFFFFFFFFFFFFFFFFFFFFFF:FFFFFFFFFFFFFFFFFFFFFFFFFFFFFFFFF:FF
@A00155:342:HHGFNDSXY:1:2308:30201:35258 1:N:0:GAACCTAG+TCCGCATA
GGATGTGGAGGGCGAGAGAGAGCGACTTCTCTCGGGCCCACAGCCTTACAGCTGTGGAGCACGGTATC
CTCTGCCGAAACAGAGGTTGGACAAGACCGGAGGGGTCTCCTAGTTCCAAAGGAGATG
+
FFFFFFFFFFFFFFFFFFFFFFFFFFFFFFFFFFFFFFFFFFFFFFFFFFFFFFFFFFFFFFFFFFFF
FFFFFFFFFFFFFFFFFFFFFFFFFFFFFFF:FFFFFFFFFFFFFFFFFFFFFFFFFF
@A00155:342:HHGFNDSXY:1:2309:3115:4319 1:N:0:GAACCTAG+TCCGCATA
CGACTTCTCTCGGGCCCACAGCCTTACAGCTGTGGAGCACGGTATCCTCTGCCGAAACAGAGGTTGGA
CAAGACCGGAGGGGTCTCCTAGTTCCAAAGGAGATGTACTCCGGGCTTGTTCACGACCTACCGTGTAA
+
FFFFFFFFFFFFFFFFFFFFFFFFFFFFFFFFFFFFFFFFFFFFFFFFFFFFFFFFFFFFFFFFF:FF
FFFFFFFFFFFFF:FFFFFFFFFFFFFFFF:FFFFFFFFFF:FFFFFFFFFFFFFFFFFFFFFF,,FF
@A00155:342:HHGFNDSXY:1:2309:11388:4617 1:N:0:GAACCTAG+TCCGCATA
GAGCGACTTCTCTCGGGCCCACAGCCTTACAGCTGTGGAGCACGGTATCCTCTGCCGAAACAGAGGTT
GGACAAGACCGGAGGGGTCTCCTGGTTCCAAAGGAGATGTACTCCGGGCTTGTTCACGACCTACCGTG
+
FF:F:FFF,FFFFFFFFFF:FFF:FFFFFFFFFFF,FFFFFFFF:FFFFFF::FFF:FFFFF:FF:FF
FFFFFFFFF:FFFF:FFFFFFFFF:FFFFFFFFFFFFFF,FFFFF:,FF::F:FFF,FFF,,FFFFFF
@A00155:342:HHGFNDSXY:1:2309:11216:8891 1:N:0:GAACCTAG+TCCGCATA
GGATGTAGAGGGCGAGAGAGAGCGACTTCTCTCGGGCCCACAGCCTTACAGCTGTGGAGCACGGTATC
CTCTGCCGAAACAGAGGTTGGACAAGACCGGAGGGGTCTCCTAGTTCCAAAGGAGATGTACTCC
+
FFFFFFFFFFFFFFFFFFFFFFFFFFFFFFFFFFFFFFFFFFFFFFFFFFFFFFFFFFFFFFFFFFFF
FFFFFFFFFFFFFFFFFFFFFFFFFFFFFFFFFFFFFFFFFFFFFFFFFFFFFFFFFFFFFFFF
@A00155:342:HHGFNDSXY:1:2309:11243:24533 1:N:0:GAACCTAG+TCCGCATA
TGTTTCGGCAGAGGATACCGTGCTCCACAGCTGTAAGGCTGTGGGCCCGAGAGAAGTCGCTCTCTCTC
GCCCTCCACATCCCTCTCGGCATTGAGATGACCATAGGCCAGAGGTGAGTCCTTAAGTGGACACAGCT
+
FFFFFFFFFFFFFFFFFFFFFFFFFFFFFFFFFFFFFFFFFFFFFFFFFFFFFFFFFFFFFFFFFFFF
FFFFFFFFFFFFFFFFFFFFFFFFFFFFFFFFFFFFFFFFFFFFFFFFFFFFFFFFFFFFFFFF:FFF
@A00155:342:HHGFNDSXY:1:2309:10827:33708 1:N:0:GAACCTAG+TCCGCATA
TGTGGAGGGCGAGAGAGAGCGACTTCTCTCGGGCCCACAGCCTTACAGCTGTGGAGCACGGTATCCTC
TGCCGAAACAGAGGTTGGACAAGACCGGAGGGGTCTCCTAGTTCCAAAGGAGATGTACTCCGGG
+
FFFFFFFFFFFFFFFFFFFFFFFFFFFFFFFFFFFFFFFFFFFFFFFFFFFFFFFFFFFFFF:FFFFF
FFFFFFFFFFFFFFFFFFFFFFFFFFFFFFFFFFFFFFFFFFFFFFFFFFFFFFFFFFFFFFFF
@A00155:342:HHGFNDSXY:1:2310:29478:3630 1:N:0:GAACCTAG+TCCGCATA
CTCTGCCGAAACAGAGGTTGGACAAGACCGGAGGGGTCTCCTAGTTCCAAAGGAGATGTACTCCGGGC
TTGTTCACGACCTACCGTGTAAGTCGTAGTCTAGTAGGCTACCTGACGAGTCCTTTTTAGGACGAAAC
+
FFFFFFFFFFFFFFFFFFFFFFFFFFFFFFFFFFFFFFFFFFFFFFFFFFFFFFFFFFFFFFFFFFFF
FFFFFFFFFFFFFFFFFFFFFFFFFFFFFFFFFFFFFFFFFFFFFFFFFFFFFFFFFFFFFFFFFFFF
@A00155:342:HHGFNDSXY:1:2310:20039:15092 1:N:0:GAACCTAG+TCCGCATA
AGATCAGCTGTGTCCACTTAAGGACTCACCTCTGGCCTATGGTCTTCTCAATGCCGAGAGGGATGTGG
AGGGCGAGAGAGAGCGACTTCTCTCGGGCCCACAGCCTTACAGCTGTGGAGCACGGTATCCTCTGCCG
+
FFFFFFFFFFFFFFFFFFFFFFFFFFFFFFFFFFFFFFFFFFFFFFFFFFFFFFFFFFFFFFF,FFFF
FFFFFFFFFFFFFFFFFFFFFFFFFFFFFFFFFFFFFFFFFFFFFFFFFFFFFFFFFFFFFFFFFFFF
@A00155:342:HHGFNDSXY:1:2310:21630:27305 1:N:0:GAACCTAG+TCCGCATA
GTGTGGTGGGGCATGGGTTTGAACCCCCATGACGGTCGGAGTCTAGTAGGCTCCCTGATGAGTCCGTT
CCAAGGACGAAACCGTCGTTACTCTTCGAGTTGTGTAAGTTTCGTCCTAAAAAGGACTCGTCAGGTAG
+

```
FFFFFFFFFFFFFFFFFFFFFFFFFFFFFFFFFFFFFFFFFFF:FFFFFFFFFFFFFFFFFFFFFFFF
FFFFFFFFFFFFFFFFFFFFFFFFFFFFFFFFF:FFFFFFFFFFFFF:FFFF:FFFF:FFFFFFFFFF
@A00155:342:HHGFNDSXY:1:2311:1389:10661 1:N:0:GAACCTAG+TCCGCATA
GGATGTGGAGGGCGAGAGAGAGCGACTTCTCTCGGGCCCACAGACTTACAGCTGTGGAGCACGGTATC
CTCTGCCGAAACAGAGGTTGGACAAGACCGGAGGGGTCTCCTAGTTCCAAAGGAGATGTACT
+
:FFFFF,F::F:FFFFFF,F:FF::F:FFFF,FFFFF,F:FFF,,FFF:,F,FF,FF:F,F,F:FFFF
:FFFF:,FF,F,:F,:F:FFF:F:,:,:FF,,FF:FFF:,F:,FFF,:F,:F,FF,,,F:,F
@A00155:342:HHGFNDSXY:1:2311:11740:12430 1:N:0:GAACCTAG+TCCGCATA
AGAGAGAGCGACTTCTCTCGGGCCCACAGCCTTACAGCTGTGGAGCACGGTATCCTCTGCCGAAACAG
AGGTTGGACAAGACCGGAGGGGTCTCCTAGTTCCAAAGGAGATGTACTCCGGGCTTGTTCA
+
FFFFFFFFFFFFFFFFFFFFFFFFFFFF:FFFFFFFFFFFFFFFFFFFFFFFFFFFFFFFFFFFFFFF
FFFFFFFFFFFFFFFFFFFFFFFFFFFFFFFFFFFFFFFFFFFFFFFFFFFFFFFFFFFFF
@A00155:342:HHGFNDSXY:1:2311:15709:15984 1:N:0:GAACCTAG+TCCGCATA
GGACTCACCTCTGGCCTATGGTCATCTCAATGCCGAGAGGGATGTGGAGGGCGAGAGAGAGCGACTTC
TCTCGGGCCCACAGCCTTACAGCTGTGGAGCACGGTATCCTCTGCCGAAACAGAGGTTGGACAAGACC
+
FFFFFFFFF:FFFFFFFFFFFFFFFFFFFFFFFFF:FFFFFFFF,FFFFFFFFFFFFFFFFFFFFFFF
FFFFFFFFFFFFFFFFF:FFFFFFFFFFFFFFFFFFFFFFFFFFFFFFFFFFFFFFFFFFFFFFFFFF
@A00155:342:HHGFNDSXY:1:2311:25771:25770 1:N:0:GAACCTAG+TCCGCATA
TTCTCTCGGGCCCACAGCCTTACAGCTGTGGAGCACGGTATCCTCTGCCGAAACAGAGGTTGGACAAG
ACCGGAGGGGTCTCCTAGTTCCAAAGGAGATGTACTCCGGGCTTGTTCACGACCTACCGTGTAAGTCG
+
FFFFFFFFFFFFFFFFFFFFFFFFFFFFFFFFFFFFFFFFFFFFFFFFFFFFFF:FFFFFFFFFFFFF
FFFFFFFFFFFFFFFFFFFFFFFFFFFFFFFFFFFFFFFFFFFFFFFFFFFFFFFFF:FFFFFFFFFF
@A00155:342:HHGFNDSXY:1:2311:12093:31986 1:N:0:GAACCTAG+TCCGCATA
TGGTCATCTCAATGCCGAGAGGGATGTGGAGGGCGAGAGAGAGCGACTTCTCTCGGGCCCACAGCCTT
ACAGCTGTGGAGCACGGTATCCTCTGCCGAAACAGAGGTTGGACAAGACCGGAGGGGTCTCCTAGTTC
+
FFFFFFFFFFFFFFFFFFFFFFFFFFFFFFFFFFFFFFFFFFFFFFFFFFFFFFFFFFFFFFFFFFFF
FFFFFFFFFFFFFFFFFFFFFFFFFFFFFFFFFFFFFF:FFFFFFFFFFFFFFFFFFFFFFFFFFFFF
@A00155:342:HHGFNDSXY:1:2311:8938:32753 1:N:0:GAACCTAG+TCCGCATA
GTGTGGCGGGGCATGGGTTTGAACCCCCATGACGGTCGGAGTCTAGTAGGCTCCCTGATGAGTCCGTT
CCAAGGACGAAACCGTCGTTACTCTTTGAGTTGTGTAAGTTTCGTCCTAAAAAGGACTCGTCAGGTAG
+
FFFFFFFFFFFFFFFFFFFFFFFFFFFFFFFFFFFFFFFFFFFFFFFFFFFFFFFFFFFFFFFFFFFF
FFFFFFFFFFFFFFFFFFFFFFFFFFFFFFFFFFFFFFFFFFFFFFF:FFFFFFFFFFFFFFFFFFFF
@A00155:342:HHGFNDSXY:1:2313:4074:4069 1:N:0:GAACCTAG+TCCGCATA
TTCTCTCGGGCCCACAGCCTTACAGCTGTGGAGCACGGTATCCTCTGCCGAAACAGAGGTTGGACAAG
ACCGGAGGGGTCTCCTAGTTCCAAAGGAGATGTACTCCGGGCTAGTTCACGACCTACCGTGTAAGTCG
+
FFFFFFFFFFFF:FFFFFFFFFFFFFFFFFFFFF:FFFFFFFFFFFFFFFFFFFFFFFFFFFFFFF:F
FFFFFFFFFFFFFFFFFFFFFFFFFFFFFFFFFFFFFFFFFFFFFFFFFFFFFFFFFFFFFFFFFFFF
@A00155:342:HHGFNDSXY:1:2313:28366:15452 1:N:0:GAACCTAG+TCCGCATA
AGCGACTTCTCTCGGGCCCACAGCCTTACAGCTGTGGAGCACGGTATCCTCTGCCGAAACAGAGGTTG
GACAAGACCGGAGGGGTCTCCTAGTTCCAAAGGAGATGTACTCCGGGCTTGTTCACGACCTACCGTGT
+
FFFFFFFFFFFFFFFFFFFFFFFFFFFFFFFFFFFFFFFFFFFFFFFFFFFFFFFFFFFF:FFFFFFF
FFF:FFFFFFFFFFFFFFFFFFFFFFFFFFFFFFFFFFFFFFFFFFFF:FFFFFFFFFFFFFFFFFFF
@A00155:342:HHGFNDSXY:1:2314:5141:17096 1:N:0:GAACCTAG+TCCGCATA
GATGTAGAGGGCGAGAGAGAGCGACTTCTCTCGGGCCCACAGCCTTACAGCTGTGGAGCACGGTATCC
TCTGCCGAAACAGAGGTTGGACAAGACCGGAGGGGTCTCCTAGTTCCAAAGGAGATGTACTCCGGGCT
+
```

```
FF:FFFFFFFFFFFFFFFFFFFFFFFFFFFFFFFFFFFFFFFFFFFFFFFFFFFFFFFFF,FFFFFFF
FFFFFFFFFFFFFF:FFFFFFFFFFFFFFFFFFFFFFFFFFFFFFFFFFFFFFFFFFFFFFFFFFFFF
@A00155:342:HHGFNDSXY:1:2314:17788:19460 1:N:0:GAACCTAG+TCCGCATA
GTCATCTCAATGCCGAGAGGGATGTGGAGGGCGAGAGAGAGCGACTTCTCTCGGGCCCACAGCCTTAC
AGCTGTGGAGCACGGTATCCTCTGCCGAAACAGAGGTTGGACAAGACCGGAGGGGTCTCCTAGCTCCA
+
FFFFFFFFFFFFFFFFFFFFFFFFFFFFFFFFFFFFFFFFFFFFFFFFFFFFFFFFFFFFFFFFFFFF
FFF:FFFFFFFFFFFFFFFFFFFFFFFFFFFFFFFFFFFFFFFFFFFFFFFFFFFFFFFFFFFFFFFF
@A00155:342:HHGFNDSXY:1:2314:6117:30217 1:N:0:GAACCTAG+TCCGCATA
TGTAGAGGGCGAGAGAGAGCGACTTCTCTCGGGCCCACAGCCTTACAGCTGTGGAGCACGGTATCCTC
TGCCGAAACAGAGGTTGGACAAGACCGGAGGGGTCTCCTAGTTCCAAAGGAGATGTACTCCGGG
+
FFFFFFFF:FFFFFFFFFFFFFFFFFFFFFFFFFFFFFFFFFFFFFFFFFF:FFFFFFFFFF:FFFFF
FFFFFFFFFFFFFFFFFFFFFF:FFFFFFFFFFFFFFFFFFFFFFFFFFFFFFFFFFFFFFFFF
@A00155:342:HHGFNDSXY:1:2314:15772:32315 1:N:0:GAACCTAG+TCCGCATA
CTCAATGCCGAGAGGGATGTGGAGGGCGAGAGAGAGCGACTTCTCTCGGGCCCACAGCCTTACAGCTG
TGGAGCACGGTATCCTCTGCCGAAACAGAGGTTGGACAAGACCGGAGGGGTCTCCTAGTTCCAAAGGA
+
FFFFFFFFFFFFFFFFFFFFFFFFFFFFFFFFFFFFFFFFFFFFFFFFFFFFFFFFFFFFF,FFFFFF
FFFFFFFFFFFFFFFFFFFFFFFFFFFFFFFFFFFFFFFFFFFFFFFFFFFFFFFFFFFFFFFFFFFF
@A00155:342:HHGFNDSXY:1:2315:15926:14418 1:N:0:GAACCTAG+TCCGCATA
TATCCTCTGCCGAAACAGAGGTTGGACAAGACCGGAGGGGTCTCCTGGTTCCAAAGGAGATGTACTCC
GGGCTTGTTCACGACCTACCGTGTAAGTCGTAGTCTAGTAGGCTACCTGACGAGTCCTTTTTAGGACG
+
FFFFFFFFFFFFFFFFFFFFFFFFFFFFFFFFFFFFFFFFFFFFFFFFFFFFFFFFFFFFFFFFFFFF
FFFFFFFFFFFFFFFFFFFFFFFFFFFFFFFFFFFFF:FFFFFFFFFFFFFFF:FFFFFFFFFFFFFF
@A00155:342:HHGFNDSXY:1:2315:28999:21465 1:N:0:GAACCTAG+TCCGCATA
TGTGGAGGGCGAGAGAGAGCGACTTCTCTCGGGCCCACAGCCTTACAGCTGTGGAGCACGGTATCCTC
TGCCGAAACAGAGGTTGGACAAGACCGGAGGGGTCTCCTAGTTCCAAAGGAGATGTACTCCGGG
+
FFFFFFFFFFFFFFFFFFFFFFFFFFFFFFFFFFF:FFFFFFFFFFFFFFFFFFFFFFFFFFFFFFFF
FFFFFFFFFFFFFFFFFFFFFFFFF:FFFFFFFFFFFFF:FFFFFFFFFFFFFFFFFFFFFFFF
@A00155:342:HHGFNDSXY:1:2315:16685:28761 1:N:0:GAACCTAG+TCCGCATA
CTCAATGCCGAGAGGGATGTGGAGGGCGAGAGAGAGCGACTTCTCTCGGGCCCACAGCCTTACAGCTG
TGGAGCACGGTATCCTCTGCCGAAACAGAGGTTGGACAAGACCGGAGGGGTCTCCTAGTTCCAAAGGA
+
FFFFFFFFFFFFFFFFFFFFFFF:FFFFFFFFFFFFFFFF:FFFFFFFFFFFFFFFFFFFFFFFFFFF
FFFFFFFFFFFFFFFFFFFFFFF:FFFFFFFFFFFFFFFFFFFFFFFFFFFFFFFFFFFFFFFFFFFF
@A00155:342:HHGFNDSXY:1:2315:25753:31532 1:N:0:GAACCTAG+TCCGCATA
CACCTCTGGCCTATGGTCATCTCAATGCCGAGAGGGATGTAGAGGGCGAGAGAGAGCGACTTCTCTCG
GGCCCACAGCCTTACAGCTGTGGAGCACGGTATCCTCTGCCGAAACAGAGGTTGGACAAGACCGGAGG
+
:F:FFFF::FFFFF::FFF:FF:FFFFFFFFFF:,FFFF:F:FFF:FFFFF,FFF::FFFFFFFFFF,
:FFFFFFF::FFFFFFFFFFFFFFFFFF,FFFF:,FFFFFFFFFFFFFFF:FFFFFFFFFFFFF:FF:
@A00155:342:HHGFNDSXY:1:2316:2293:4648 1:N:0:GAACCTAG+TCCGCATA
GGGCGAGAGAGAGCGACTTCTCTCGGGCCCACAGCCTTACAGCTGTGGAGCACGGTATCCTCTGCCGA
AACATAGGTTGGACAAGACCGGAGGGGTCTCCTAGTTCCAAAGGAGATGTACTCCGGGCTTGTTCACG
+
FFFFF:FFFFF:FFFFFFFFFFFFFFFF:FFFFFFFFFFFFFFFFFFFFFFFFFFFFFFF:FFFFFFF
FFFFFFFFFFFFFFFFF:FFFFFFFFFFFFFFFFFFFFFFFFFFFFFFFFFFFFFFFFFFFFFFFFFF
@A00155:342:HHGFNDSXY:1:2316:28528:21151 1:N:0:GAACCTAG+TCCGCATA
ATCTCAATGCCGAGAGGGATGTGGAGGGCGAGAGAGAGCGACTTCTCTCGGGCCCACAGCCTTACAGC
TGTGGAGCACGGTATCCTCTGCCGAAACAGAGGTTGGACAAGACCGAAGGGGTCTCCTAGTTCCAAAG
+
```

FFFFFFFFFFFFFFFFFFFFFFFFFFFFFFFFFFFFFFFFFFFFFFFFFFFFFFFFFFFFFFFFFFFF
FFFFFFFFFFFFFFFFFFFFFFFFFFFF:FFFFFFFFFFFFFFFFFFFFFFFFFFFFFFFFFF:FFFF
@A00155:342:HHGFNDSXY:1:2316:9263:22576 1:N:0:GAACCTAG+TCCGCATA
CTTCTCTCGGGCCCACAGCCTTACAGCTGTGGAGCACGGTATCCTCTGCCGAAACAGAGGTTGGACAA
GACCGGAGGGGTCTCCTAGTTCCAAAGGAGATGTACTCCGGGCTTGTTCACGACCTACCGTGTAAGTC
+
FFFFFFFFFFFFFFFFFFFFFFFFFFFFFFFFFFFFFFFFFFFFFFFFFFFFFFFFFFFFFFFFFFFF
FFFFFFFFFFFFFFFFFF:FFFFFFFFFFFFFFFFFFFFFFFFFFFFFFFFFFFFFFFFFFFFFFF:F
@A00155:342:HHGFNDSXY:1:2317:14931:4429 1:N:0:GAACCTAG+TCCGCATA
ACAGCTGTGGAGCACGGTATCCTCTGCCGAAACAGAGGTTGGACAAGACCGGAGGGGTCTCCTAGTTC
CAAAGGAGATGTACTCCGGGCTTGTTCACGACCTACCGTGTAAGTCGTAGTCTAGTAGGCTACCTGAC
+
FFFFFFFFFFFFFFFFFFFFFFFFFFFFFFFFFFFFFFFFFFFFFFFFFFFFFFFFFFFFFFFFFFFF
FFFFFFFFFFFFFFFFFFFFFFFFFFFFFFFFFFFFFFFFFFFFFFFFFFFFFFFFFFFFFFFFFFFF
@A00155:342:HHGFNDSXY:1:2317:15203:4586 1:N:0:GAACCTAG+TCCGCATA
CTGTGGAGCACGGTATCCTCTGCCGAAACAGAGGTTGGACAAGACCGGAGGGGTCTCCTAGTTCCAAA
GGAGATGTACTCCGGGCTTGTTCACGACCTACCGTGTAAGTCGTAGTCTAGTAGGCTACCTGAC
+
:F:F::F:FFF::FFFFFFF:FF:FFFFFF,F:,F,,FF,FFFFFF,:FF,FFF:FF:FF:FF:,FFF
:FF:FF:,FFF,F,FFFFFF:FFFF,F,::F,F,FFFFF:FFFFF:,FFFF:FF,F,F:FFFFF
@A00155:342:HHGFNDSXY:1:2317:2645:29716 1:N:0:GAACCTAG+TCCGCATA
AGGGATGTGGAGGGCGAGAGAGAGCGACTTCTCTCGGGCCCACAGCCTTACAGCTGTGGAGCACGGTA
TCCTCTGCCGAAACAGAGGTTGGACAAGACCGGAGGGGTCTCCTAGTTCCAAAGGAGATGTACTCCGG
+
FFFFF,FFFFFFFFFFFFFFFFFFFFFFFFFFFFFFFFFFFFFFFFFFFFFFFFFFFFFFFFFFFFFF
FFFFFFFFFFFFFFFFFFFFFFFFFFFFFFFFFFFFFFFFFFFFFFFFFFFFFFFFFFFFFFFFFFFF
@A00155:342:HHGFNDSXY:1:2317:30906:33160 1:N:0:GAACCTAG+TCCGCATA
GGCGAGAGAGAGCGACTTCTCTCGGGCCCACAGCCTTACAGCTGTGGAGCACGGTATCCTCTGCCGAA
ACAGAGGTTGGACAAGACCGGAGGGGTCTCCTAGTTCCAAAGGAGATGTACTCCGGGCTTGTTCACGA
+
FFFFFFFFFFFFFFFFFFFFFFFFFFFFFFFFFFFFFFFFFFFFFFFFFFFFFFF:FFFFFFFFFFFF
FFFFFFFFFFFFFFFFFFFFFFFFFFFFFFFFFFFFFFFFFFFFFFFFFFFFFFFFFFFFFFFFFFFF
@A00155:342:HHGFNDSXY:1:2319:12274:4679 1:N:0:GAACCTAG+TCCGCATA
CGACTTCTCTCGGGCCCACAGCCTTACAGCTGTGGAGCACGGTATCCTCTGCCGAAACAGAGGTTGGA
CAAGACCGGAGGGGTCTCCTAGTTCCAAAGGAGATGTACTCCGGGCTTGTTCACGACCTACCGTGTAA
+
FFFFFFFFFFFFFFFFFFFFFFFFFFFFFFFFFFFFFFFFFFFFFFFFFFFFFFFFFFFFFFFFFFFF
FF:FFFFFFFFFFFFFFFFFFFFFFFFFFFFFFFFFFFFFFFFFFFFFFFFFFFFFFFFFFFF:FFFF
@A00155:342:HHGFNDSXY:1:2319:9616:19179 1:N:0:GAACCTAG+TCCGCATA
GAGCGACTTCTCTCGGGCCCACAGCCTTACAGCTATGGAGCACGGTATCCTCTGCCGAAACAGAGGTT
GGACAAGACCGGAGGGGTCTCCTAGTTCCAAAGGAGATGTACTCCGGGCTTGTTCACGACCTACCGTG
+
FFFFFFFFFFFFFFFFFFFFFFFFFFFFFFFFFFFFFFFFFFFFFFFFFFFFFFFFFFFFFFFFFFFF
FFFFFFFFFFFFFFFFFFFFFFFFFFFFFFFFFFFFFFFFFFFFFFFFFFFFFFFFFFFFFFFFFFFF
@A00155:342:HHGFNDSXY:1:2319:31647:36010 1:N:0:GAACCTAG+TCCGCATA
CTTCTCTCGGGCCCACAGCCTTACAGCTGTGGAGCACGGTATCCTCTGCCGAAACAGAGGTTGGACAA
GACCGGAGGGGTCTCCTAGTTCCAAAGGAGATGTACTCCGGGCTTGTTCACGACCTACCGTGTAAGTC
+
FFFFFFFFFFFFFFFFFFFFFFFFFFFFFFFFFFFFFFFFFFFFFFFFFFFFFFFFFFFFFFFFFFFF
FFFFFFFFFFFFFFFFFFFFFFFFFFFFFFFFFFFFFFFFFFFFFFFFFFFFFFFFFFFFFFFFFFFF
@A00155:342:HHGFNDSXY:1:2320:20591:18646 1:N:0:GAACCTAG+TCCGCATA
TTAGATCAGCTGTGTCCACTTAAGGACTCACCTCTGGCCTATGGTCATCTCAATGCCGAGAGGGATGT
AGAGGGCGAGAGAGAGCGACTTCTCTCGGGCCCACAGCCTTACAGCTGTGGAGCACGGTATCCTCTGC
+

FFFFFFFFFFFFFFFFFFFFFFFFFFFFFFFFFFFFFFFFFFFFFFFFFFFFFFFFFFFFFFFFFFFF
FFFFFFFFFFFFFFFFFFFFFFFFFFFFFFFFFFFFFFFFFFFFFFFFFFFFFFFFFFFFFFFFFFFF
@A00155:342:HHGFNDSXY:1:2320:32750:26334 1:N:0:GAACCTAG+TCCGCATA
ATGCCGAGAGGGATGTGGAGGGCGAGAGAGAGCGACTTCTCTCGGGCCCACAGCTTTACAGCTGTGGA
GCACGGTATCCTCTGCCGAAACAGAGGTTGGACAAGACCGGAGGGGTCTCCTAGTTCCAAAG
+
FFFFFFFFFFFFFFFFFFFFFFFFFFFF:FFFFFFFFFFFFFFFFFFFFFFFFFFFFFFFFFFFFFFF
FFFFFFFFFFFFFFFFFFFFFFFFFFFF:FFFFFFFFFFFFFFFFFFFFFFFFFFFFFFFFF
@A00155:342:HHGFNDSXY:1:2320:31828:27806 1:N:0:GAACCTAG+TCCGCATA
ATGCCGAGAGGGATGTGGAGGGCGAGAGAGAGCGACTTCTCTCGGGCCCACAGCTTTACAGCTGTGGA
GCACGGTATCCTCTGCCGAAACAGAGGTTGGACAAGACCGGAGGGGTCTCCTAGTTCCAAAG
+
FFFFFFFFFFFFFFFFFFFFFFFFFFFFFFFFFFFFFFFFFFFFFFFFFFFFFFFFFFFFFFFFFFFF
FFFFFFF:FFFFFFFFFFFFFFFFFFFFFFFFFFFFFFFFFFFFFFFFFFFF:FFFFFFFF:
@A00155:342:HHGFNDSXY:1:2321:15121:5541 1:N:0:GAACCTAG+TCCGCATA
ACTTCTCTCGGGCCCACAGCCTTACAGCTGTGGAGCACGGTATCCTCTGCCGAAACAGAGGTTGGACA
AGACCGGAGGGGTCTCCTAGTTCCAAAGGAGATGTACTCCGGGCTTGTTCACGACCTACCGTGTAAGT
+
FFFFFFFFFFFFFFFFFFFFFFFFFFFFFFFFFFFFFFFFFFFFFFFFFFFFFFFFFFFFFFFFFFFF
FFFFFFFFFFFFFFFFFFFFFFFFFFFFFFFFFFFFFFFFFFFFFFFFFFFFFFFFFFFFFFFFFFFF
@A00155:342:HHGFNDSXY:1:2321:4933:25723 1:N:0:GAACCTAG+TCCGCATA
ATCTCAATGCCGAGAGGGATGTGGAGGGCGAGAGAGAGCGACTTCTCTCGGGCCCACAGCCTTACAGC
TGTGGAGCACGGTATCCTCTGCCGAAACAGAGGTTGGACAAGACCGGAGGGGTCTCCTAGTTCCAAAG
+
FFFFFFFFFFFF:FFFFFFFFFFFFFFFFFFFFFFFFFFFFFFFFFFFFFFFFFFFFFFFFFF:FFFF
FFFFFFFFFFFFFFFFFFFFFFFFFFFFFFFFFFFFFFFFFFFFFFFFFFFFFFFFFF:FFFFFFFFF
@A00155:342:HHGFNDSXY:1:2322:10004:8954 1:N:0:GAACCTAG+TCCGCATA
AGAGAGAGCGACTTCTCTCGGGCCCACAGCCTTACAGCTGTGGAGCACGGTATCCTCTGCCGAAACAG
AGGTTGGACAAGACCGGAGGGGTCTCCTGGTTCCAAAGGAGATGTACTCCGGGCTTGTTCACGACCT
+
FFFFFFFFFFFFFFFFFFFFFFFFFFFFFFFFFFFFFFFFFFFFFFFFFFFF,FFFFFFF:FFFFFF
FFFFFFFFFFFFFFFFFFFFFFFFFFFFFFFFFFFFFFFFFFFFFFFFFFFF:FFFFFFFFFFFFFFF
@A00155:342:HHGFNDSXY:1:2322:11595:13463 1:N:0:GAACCTAG+TCCGCATA
CCTACTAGACTCCGACCGTCATGGGGGTTCAAACCCATGCCCCACCACACCGCCTTAGATCAGCTGTG
TCCACTTAAGGACTCACCTCTGGCCTATGGTCATCTCAATGCCGAGAGGGATGTGGAGGGCGAGAGAG
+
FFFFFFFFFFFFFFFFFFFFFFFFFFFFFFFFFFFFFFFFFFFFFFFFFFFFFFF:FFFFFFFFFFFF
FFFFFFFFFFFFFFFFFFFFFFFFFFFFFFFFFFFFFFFFFFFFFFFFFFFFFFFFFFFFFFFFFFFF
@A00155:342:HHGFNDSXY:1:2322:21106:18787 1:N:0:GAACCTAG+TCCGCATA
CTCTGCCGAAACAGAGGTTGGACAAGACCGGAGGGGTCTCCTAGTTCCAAAGGAGATGTACTCCGGGC
TTGTTCACGACCTACCGTGTAAGTCGTAGTCTAGTAGGCTACCTGACGAGTCCTTTTTAGGACGAAAC
+
FFFFFFFFFFFF:FFFFFFFFFFFFFFFFFFFFFFFFFFFFFFFFFFFFFFFFFFFFFFFFFFFFFFF
FFFFFFFFFFFFFFFFFFFFFFFFFFFFFFFFFFFFFF:FFFFFFFFFFFFFFFFFFFFFFFFFFFFF:
@A00155:342:HHGFNDSXY:1:2322:12364:20556 1:N:0:GAACCTAG+TCCGCATA
AGAGAGAGCGACTTCTCTCGGGCCCACAGCCTTACAGCTGTGGAGCACGGTATCCTCTGCCGAAACAG
AGGTTGGACAAGACCGGAGGGGTCTCCTAGTTCCAAAGGAGATGTACTCCGGGCTTGTTCACGACCTA
+
FFFFFFFFFFFFFFFFFFFFFF:FFFFFFFFFFFFFFFFFFFFFFFFFFFFFFFFFFFFFFFFFFFFF
FFFFFFFFFFFFFFFFFFFFFFFFFFFFFFFFFFFFFFFFFFFFFFFFFFFFFFFFFFFFFFFFFFF:
@A00155:342:HHGFNDSXY:1:2322:12373:20572 1:N:0:GAACCTAG+TCCGCATA
AGAGAGAGCGACTTCTCTCGGGCCCACAGCCTTACAGCTGTGGAGCACGGTATCCTCTGCCGAAACAG
AGGTTGGACAAGACCGGAGGGGTCTCCTAGTTCCAAAGGAGATGTACTCCGGGCTTGTTCACGACCTA
+

FFFFFFFFFFFF:FFFFFFFFFFFFFFFFFF,FFFF:F:FFFFF:FFFF::FFFF,FFFFFFFFFF::
FFFFFFFFFFF:FFFFFF:FFFFFFFFFFFFFFFF:FF:FFFFFFFFF:FF:F:,F:F:FFFFFFF:F
@A00155:342:HHGFNDSXY:1:2322:25608:24643 1:N:0:GAACCTAG+TCCGCATA
CTTCTCTCGGGCCCACAGCCTTACAGCTGTGGAGCACGGTATCCTCTGCCGAAACAGAGGTTGGACAA
GACCGGAGGGGTCTCCTGGTTCCAAAGGAGATGTACTCCGGGCTTGTTCACGACCTACCGTGTAAGTC
+
FFFFFFFFFFFFFFFFFFFFFFFFFFFFFFFFFFFFFFFFFFFFFFFFFFFFFFFFFFFFFFFFFFFF
FFFFFFFFFFFFFFFFFFFFFFFFFFFFFFFFFFFFFFFFFFFFFFFFFFFFFFFFFFFFFFFFFFFF
@A00155:342:HHGFNDSXY:1:2322:5593:28651 1:N:0:GAACCTAG+TCCGCATA
GAGAGGGATGTGGAGGGCGAGAGAGAGCGACTTCTCTCGGGCCCACAGCCTTACAGCTGTGGAGCACG
GTATCCTCTGCCGAAACAGAGGTTGGACAAGACCGGAGGGGTCTCCTGGTTCCAAAGGAGATGTACT
+
FFFFFFFFFFFFFFFFFFFFFFFFFFFFFFFFFFFFFFFFFFFFFFFFFFFFFFFFFFFFFFFFFFFF
FFFFFFFFFFFFFFFFFFFFFFFFFFFFFFFFFFFFFFFFFFFFFFFFFFFFFFF:FFFFFFFFFFF
@A00155:342:HHGFNDSXY:1:2322:3821:31501 1:N:0:GAACCTAG+TCCGCATA
ATCTCAATGCCGAGAGGGATGTAGAGGGCGAGAGAGAGCGACTTCTCTCGGGCCCACAGCCTTACAGC
TGTGGAGCACGGTATCCTCTGCCGAAACAGAGGTTGGACAAGACCGGAGGGGTCTCCTAGTTCCAAAG
+
FFFFFFFFFFFFFFFFFFFFFFFFFFFFFFFFFFFFFFFFFFFFFFFFFFFFFFFFFFFFFFF,FFFF
FFFFFFFFFFFFFFFFFFFFFFFFFFFFFFFFFFFFFFFFFFFFFFFFFFFFFFFFFFFFFFFFFFFF
@A00155:342:HHGFNDSXY:1:2323:4191:2487 1:N:0:GAACCTAG+TCCGCATA
CTTCTCTCGGGCCCACAGCCTTACAGCTGTGGAGCACGGTATCCTCTGCCGAAACAGAGGTTGGACAA
GACCGGAGGGGTCTCCTGGTTCCAAAGGAGATGTACTCCGGGCTTGTTCACGACCTACCGTGTAAGTC
+
FFFFFFFFFFFFFFFFFFFFFFFFFFFFFFFFFFFFFFFFFFFFFFFFFFFFFFFFFFFFFFFFFFFF
FFFFFFFFFFFFFFFFFFFFFFFFFFFFFFFFFFFFFFFFFFFFFFFFFFFFFFFFFFFFFFFF:FFF
@A00155:342:HHGFNDSXY:1:2323:2862:5666 1:N:0:GAACCTAG+TCCGCATA
GGATGTAGAGGGCGAGAGAGAGCGACTTCTCTCGGGCCCACAGCCTTACAGCTGTGGAGCACGGTATC
CTCTGCCGAAACAGAGGTTGGACAAGACCGGAGGGGTCTCCTAGTTCCAAAGGAGATGTACT
+
FFFFFFFFFFFFFFFFFFFFFFFFFFFFFFFFFFFFFFFFFFFFFFFFFFFFFFFFFFFFFFFFFFFF
FFFFFFFFFFFFFFFFFFFFFFFFFFFFFFFFFFFFFFFFFFFFFFFFFFFFFFFFFFFFFF
@A00155:342:HHGFNDSXY:1:2323:11333:13636 1:N:0:GAACCTAG+TCCGCATA
TCGGCAGAGGATACCGTGCTCCACAGCTGTAAGGCTGTGGGCCCGAGAGAAGTCGCTCTCTCTCGCCC
TTCACATCCCTCTCGGCATTGAGATGACCATAGGCCAGAGGTGAGTCCTTAAGTGGACACA
+
FFFFFFFFFFFFFFFFFFFFFFFFFFFFFFFFFFFFFFFFFFFFFFFFFFFFFFFFFFFFFFFFFFFF
FFFFFFFFFFFFFFFFFFFFFFFFFFFFFFFFFFFFFFFFFFFFFFFFFFFFFFFFFFFFF
@A00155:342:HHGFNDSXY:1:2323:20509:22357 1:N:0:GAACCTAG+TCCGCATA
AGAGCGACTTCTCTCGGGCCCACAGCCTTACAGCTGTGGAGCACGGTATCCTCTGCCGAAACAGAGGT
TGGACAAGACCGGAGGGGTCTCCTAGTTCCAAAGGAGATGTACTCCAGGCTTGTTCACGACCTACCGT
+
FFFFFFFFFFFFFFFFFFFFFFFFFFF:FFFFFFFFFFFFFFFFFFFFFFFFFFFFFFFFFFFFFFFF
FFFFFFFFFFFFFFFFFFFFFFFFFFFFFFFFFFFFFFFFFFFFFFFFFFFFFFFFFFFFFFFFFFFF
@A00155:342:HHGFNDSXY:1:2323:22336:22576 1:N:0:GAACCTAG+TCCGCATA
TGGTCATCTCAATGCCGAGAGGGATGTGGAGGGCGAGAGAGAGCGACTTCTCTCGGGCCCACAGCCTT
ACAGCTGTGGAGCACGGTATCCTCTGCCGAAACAGAGGTTGGACAAGACCGGAGGGGTCTCCTAGTTC
+
FFFFFFFFFFFFFFFFFFFFFFFFFFFFFFFFFFFFFFFFFFFFFFFFFFFFFFFFFFFFFFFFFFFF
FFFFFFFFFFFFFFFFFFFFFFFFFFFFFFFFFFFFFFFFFFFFFFFFFFFFFFFFFFFFF:FFFFFF
@A00155:342:HHGFNDSXY:1:2323:12807:23171 1:N:0:GAACCTAG+TCCGCATA
GGGCGAGAGAGAGCGACTTCTCTCGGGCCCACAGCCTTACAGCTGTGGAGCACGGTATCCTCTGCCGA
AACAGAGGTTGGACAAGACCGGAGGGGTCTCCTGGTTCCAAAGGAGATGTACTCCGGGCTTGTTCAC
+

FFFFFFFFFFFFFFFFFFFFFFFFFFFFFFFFFFFFFFFFFFFF:FF,FFFFF:FFFFFFFF:FF:FF
FFFFFFFFFFFFFFFFFF:FFFFF:FFFFFFFFFFFFF,FFF:FFFFFFFF:FF,FFFFFFFFFF:F
@A00155:342:HHGFNDSXY:1:2323:7753:30170 1:N:0:GAACCTAG+TCCGCATA
TGTCCACTTAAGGACTCACCTCTGGCCTATGGTCATCTCAATGCCGAGAGGGATGTGGAGGGTGAGAG
AGAGCGACTTCTCTCGGGCCCACAGCCTTACAGCTGTGGAGCACGGTATCCTCTGCCGAAACAGAGGT
+
FFFFFFFFFF:FFFFFFFFFFFFFFFFFFFFFFFFF:FFFFFFFFFFFFFFFFFFFFFFFFFFFFFFF
FFFFFFFFFFFFFFFFFFFFFFFFFFFFFFFFFFFFFFFFFFFFFFFFFF:FFFF:FFFFFFFFFFFF
@A00155:342:HHGFNDSXY:1:2323:5385:31454 1:N:0:GAACCTAG+TCCGCATA
GGATGTGGAGGGCGAGAGAGAGCGACTTCTCTCGGGCCCACAGCCTTACAGCTGTGGAGCACGGTATC
CTCTGCCGAAACAGAGGTTGGACAAGACCGGAGGGGTCTCCTGGTTCCAAAGGAGATGTACTCC
+
FFFFFFFFFFFFFFFFFFFFFFFFFFFFFFFFFFFFFFFFFFFFFFFFFFFFFFFFFFFFFFFFFFFF
FFFFFFFFFFFFFFFFFFFFFFFFFFFFFFFFFFFFFFFFFFFFFFF:FF:FFFFFFFFFFFFF
@A00155:342:HHGFNDSXY:1:2324:5656:5494 1:N:0:GAACCTAG+TCCGCATA
TGTTTCGGCAGAGGATACCGTGCTCCACAGCTGTAAGGCTGTGGGCCCGAGAGAAGTCGCTCTCTCTC
GCCCTCCACATCCCTCTCGGCATAGAGATGACCATAGGCCAGAGGTGAGTCCTTAAGTGGACACAGCT
+
FFFFFFFFFFFFFFFFFFFFFFFFFFFFFFFFFFFFFFFFFFFFFFFFF:FFFFFFFFFFFFFFFFFF
FFFFFFFFFFFFFFFFFFFFFFFFFFFFFFFFFFFFFFFFFFFFFFFFFFFFFFFFFFFFFFFFFFFF
@A00155:342:HHGFNDSXY:1:2324:29984:22263 1:N:0:GAACCTAG+TCCGCATA
AGAGAGAGCGACTTCTCTCGGGCCCACAGCCTTACAGCTGTGGAGCACGGTATCCTCTGCCGAAACAG
AGGTTGGACAAGACCGGAGGGGTCTCCTGGTTCCAAAGGAGATGTACTCCGGGCTTGTTCACGACCTA
+
FFFFFFFFFFFF:FFFFFFFFFFFFFFFFFFFFFFFFFFFFFFFFF:FFFFFFFFFFFFFFFFFFF:F
FFFFFFFFF:FFFFFFFFFFFFFFFFFFFFF:FFFFFFFFFFFFFFFFFFFFFF:FFFFFFFFFFFFF
@A00155:342:HHGFNDSXY:1:2325:20130:5290 1:N:0:GAACCTAG+TCCGCATA
AAAGACTCACCTCTGGCCTATGGTCATCTCAATGCCGAGAGGGATGTGGAGGGCGAGAGAGAGCGACT
TCTCTCGGGCCCACAGCCTTACAGCTGTGGAGCACGGTATCCTCTGCCGAAACAGAGGTTGGACAAGA
+
FFFFFFFFFFFFFFFFFFFFFFFFF,FFFFFFFFFFFFFFFFFFFFFFFFFFFFFFFFFFF:FFFFFF
FFFFFFFFFFFFFFFFFFFFFFFFFFFFFFFFFFFFFFFFFFFFFFFFFFFFFFFFFFFFFFFFFFFF
@A00155:342:HHGFNDSXY:1:2325:10176:6277 1:N:0:GAACCTAG+TCCGCATA
TTACAGCTGTGGAGCACGGTATCCTCTGCCGAAACAGAGGTTGGACAAGACCGGAGGGGTCTCCTGGT
TCCAAAGGAGATGTACTCCGGGCTTGTTCACGACCTACCGTGTAAGTCGTAGTCTAGTAGGCTACCTG
+
:F:FFFFFFFFFFFFFFFFFFFFFFFFFFFFFFFFFFFFFFFFFFFFFFFFFFFFFFFFFFFFFFFF
FFFFFFFFFFFFFFFFFFFFFFFFFFFFFFFFFFFFFF:F,FFFFFFFF,FFFFF,:FFFFFFFFFFF
@A00155:342:HHGFNDSXY:1:2325:19388:7733 1:N:0:GAACCTAG+TCCGCATA
CGACTTCTCTCGGGCCCACAGCCTTACAGCTGTGGAGCACGGTATCCTCTGCCGAAACAGAGGTTGGA
CAAGACCGGAGGGGTCTCCTAGTTCCAAAGGAGATGTACTCCGGGCTTGTTCACGACCTACC
+
FFFFFFFFFFFFFFFFFFFFFFFFFFFFFFFFFFFFFFFFFFFFFFFFFFFFFFFFFFFFFFFFFFFF
FF:FFFFFFFFFFFFFFFFFFFFFFFFFFFFFFFFFFFFFFFFFFFFFFFFFFFFFFFFFFF
@A00155:342:HHGFNDSXY:1:2325:11514:11068 1:N:0:GAACCTAG+TCCGCATA
AGGGATGTGGAGGGCGAGAGAGAGCGACTTCTCTCGGGCCCACAGCCTTACAGCTGTGGAGCACGGTA
TCCTCTGCCGAAACAGAGGTTGGACAAGACCGGAGGGGTCTCCTAGTTCCAAAGGAGATGTACTCCGG
+
FFFFFFFFFFFFFFFFFFFFFFFFFFFFFFFFFFFFFFFFFFFFFFFFFFFFFFFFFFFFFFFFFFFF
FFFFFFFFFFFFFF:FFFFFFFFFFFFFFFFFFFFFFFFFFFFFFFFFFFFFFFFFFFFFFFFFFFFF
@A00155:342:HHGFNDSXY:1:2325:24053:15374 1:N:0:GAACCTAG+TCCGCATA
CAGCCTTACAGCTGTGGAGCACGGTATCCTCTGCCGAAACAGAGGTTGGACAAGACCGGAGGGGTCTC
CTAGTTCCAAAGGAGATGTACTCCGGGCTTGTTCACGACCTACCGTGTAAGTCGTAGTCTAGTAGGCT
+

```
FFFFFFFFFFFFFFFFFFFFFFFFFFFFFFFFFFFFFFFFFFFFF:FFFFFFFFFFFFFFFFFFFFFF
FFFFFFFFFFFFFFFFFFFFFFFFFFFFFFFFFFFFFFFFFFFFFFFFFFFFFFFFFFFFFFFFFFFF
@A00155:342:HHGFNDSXY:1:2325:29333:15843 1:N:0:GAACCTAG+TCCGCATA
CTTCTCTCGGGCCCACAGCCTTACAGCTGTGGAGCACGGTATCCTCTGCCGAAACAGAGGTTGGACAA
GACCGGAGGGGTCTCCTAGTTCCAAAGGAGATGTACTCCGGGCTTGTTCACGACCTACCGTGTAAGTC
+
FFFFFFFFFFFFFFFFFFFFFFFFFFFFFFFFFFFFFFFFFFFFFFFFFFFFFFFFFFFFF:FFFFFF
FFFFFFFFFFFFFFFF:FFFFFFFFFFFFFFFFFFFFFFFFFFFFFFFFFFFFFFFFFFFFFFFFFF
@A00155:342:HHGFNDSXY:1:2325:29062:19132 1:N:0:GAACCTAG+TCCGCATA
CGAGAGAGAGCGACTTCTCTCGGGCCCACAGCCTTACAGCTGTGGAGCACGGTATCCTCTGCCGAAAC
AGAGGTTGGACAAGACCGGAGGGGTCTCCTAGTTCCAAAGGAGATGTACTCCGGGCTTGTTCACGACC
+
FFFFFFFFFFFFFFFFFFFFFFFFFFFFFFFFFFFFFFF:FFFFFFFFFFFFFFFFFFFFFFFFFFFF
FFFFFFFFFFFFFFFFFFFFFFFFFFFFFF:FFFFFFFFFFFFFFFFFFFFFFFFFFFFFFFFFFFFF
@A00155:342:HHGFNDSXY:1:2326:22182:10285 1:N:0:GAACCTAG+TCCGCATA
AGAGAGAGCGACTTCTCTCGGGCCCACAGCCTTACAGCTGTGGAGCACGGTATCCTCTGCCGAAACAG
AGGTTGGACAAGACCGGAGGGGTCTCCTAGTTCCAAAGGAGATGTACTCCGGGCTTGTTCACGAC
+
FFFFFFFFF:FFFFFFFFFFFFFFFFFFFFFFFFFFFFFFFFFFFFFFFFFFFFFFFFFFFFFFFFFF
FFFFFFFFFFFFFFFFFFFFFFFFFFFFFFFFFFFFFFFFFFFFFFFFFFFFFFFFFFFFFFFFF
@A00155:342:HHGFNDSXY:1:2326:21025:16203 1:N:0:GAACCTAG+TCCGCATA
GAGCGACTTCTCTCGGGCCCACAGCCTTACAGCTATGGAGCACGGTATCCTCTGCCGAAACAGAGGTT
GGACAAGACCGGAGGGGTCTCCTAGTTCCAAAGGAGATGTACTCCGGGCTTGTTCACGACCTACCGTG
+
FFFFFFFFFFFFFFFFFFFFFFFFFFFFFFFFFF:FFFFFFFFFFFFFFFFFFFFFFFFFFFFFFFFF
FFFFFFFFFFFFFFF:FFFFFFFFFFFFFFFFFFFFFFFFFFFFFFFFFFFFFFFFFFFFFFFFFFFF
@A00155:342:HHGFNDSXY:1:2326:32018:18490 1:N:0:GAACCTAG+TCCGCATA
CTTCTCTCGGGCCCACAGCCTTACAGCTGTGGAGCACGGTATCCTCTGCCGAAACAGAGGTTGGACAA
GACCGGAGGGGTCTCCTGGTTCCAAAGGAGATGTACTCCGGGCTTGTTCACGACCTACCGTGTAAGTC
+
FFFFFFFFFFFFFFFFFFFFFFFFFFFFFFFFFFFFFFFFFFFFFFFFFFFFFFFFFFFFFFFFFFF:
FFFFFFFFFFFFFFFFFFFFFFFFFFFFFFFFFFFFFFFFFFFFFFFFFFFFFFFF:FFFFFF:FFFF
@A00155:342:HHGFNDSXY:1:2326:32832:32049 1:N:0:GAACCTAG+TCCGCATA
TTAAGGACTCACCTCTGGCCTATGGTCATCTCAATGCCGAGAGGGATGTAGAGGGCGAGAGAGAGCGA
CTTCTCTCGGGCCCACAGCCTTACAGCTGTGGAGCACGGTATCCTCTGCCGAAACAGAGGTTGGACAA
+
FFFF,FFFFFFFFFFFFFFFFFFFFFFFFFFFFFFFFFFFFFFFFFFFFFFFFFFFF:FFFFFFFFFF
:FFFFFFFFFFFFFFFFFFFFFFFFFFFFFFFFFFFFFFFFFFFFFFF:FFFF:FFFFFFFFFFFFFF
@A00155:342:HHGFNDSXY:1:2327:9561:2362 1:N:0:GAACCTAG+TCCGCATA
CGGCAGAGGATACCGTGCTCCACAGCTGTAAGGCTGTGGGCCCGAGAGAAGTCGCTCTCTCTCGCCCT
CTACATCCCTCTCGGCATTGAGATGACCATAGGCCAGAGGTGAGTCCTTAAGTGGACACAGCTGATCT
+
FFFFFFFFFFFFFFFFFFFFFFFFFFFFFFFFFFFFFFFFFFFFFFFFFFFFFFFFFFFFFFFFFFFF
FFFFFFFFFFFFFFFFFFFF,FFFFFFFFFFFFFFFFFFFFFFFFFFFFFFFFFFFFFFFFFFFFFF
@A00155:342:HHGFNDSXY:1:2327:31828:21292 1:N:0:GAACCTAG+TCCGCATA
GTCATCTCAATGCCGAGAGGGATGTGGAGGGCGAGAGAGAGCGACTTCTCTCGGGCCCACAGCCTTAC
AGCTGTGGAGCACGGTATCCTCTGCCGAAACAGAGGTTGGACAAGACCGGAGGGGTCTCCTAGTTCCA
+
FFFFFFFFFFFFFFFFFFFFFFFFFFFFFFFFFFFFFFFFFFFFF:FFFFFFFFFFFFFFFFFFFFFF
FFFFFFFFFFFFFFFFFFFFFFFFFFFFFFFFFFFFFFFFFFFFFFFFFFFFFFFFFFFFFFFFF:FF
@A00155:342:HHGFNDSXY:1:2327:12255:23093 1:N:0:GAACCTAG+TCCGCATA
AGAGAGAGCGACTTCTCTCGGGCCCACAGCCTTACAGCTGTGGAGCACGGTATCCTCTGCCGAAACAG
AGGTTGGACAAGACCGGAGGGGTCTCCTAGTTCCAAAGGAGATGTACTCCGGGCTTGTTCACGACCTA
+
```

FFFFFFFFFFFFFFFFFFFFFFFFFFFFFFFFFFFFFFFFFFFFFFFFFFFF:FFFFFFFFFFFF
FFFFFFFFFFFFFFFFFFFFFFFFFFFFFFFFFFFFFFFFFFFFFFFFFFFFFFFFFFFFFFFFF:F:
@A00155:342:HHGFNDSXY:1:2327:24017:23891 1:N:0:GAACCTAG+TCCGCATA
ATCTCAATGCCGAGAGGGATGTGGAGGGCGAGAGAGAGCGACTTCTCTCGGGCCCACAGCCTTACAGC
TGTGGAGCACGGTATCCTCTGCCGAAACAGAGGTTGGACAAGACCGGAGGGGTCTCCTAGTTCCAAAG
+
FFFFFFFFFFFFFFFFFFFFFFFFFFFFFFFFFFFFFFFFFFFFFFFFFFFFFFFFFFFFFFFFFFFF
FFFFFFFFFFFFFFFFFFFFFFFFFFFFFFFFFF:FFFFFFFFFFFFFFFFFFFFFFFFFFFFFFFFF
@A00155:342:HHGFNDSXY:1:2327:31195:24580 1:N:0:GAACCTAG+TCCGCATA
CTCAATGCCGAGAGGGATGTAGAGGGCGAGAGAGAGCGACTTCTCTCGGGCCCACAGCCTTACAGCTG
TGGAGCACGGTATCCTCTGCCGAAACAGAGGTTGGACAAGACCGGAGGGGTCTCCTAGTTCCAAAGGA
+
FFFFFFFFFFFFFFFFFFFFFFFFFFFFFFFFFFFFFFFFFFFFFFFFFFFFFFF,FFFFFFFFFFFF
FFFFFFFFFFFFFFFFFFFFFFFFF:FFFFFFFFFFFFFFFFFFF:FFFFFFFFFFFFFFFFFFFFFFF
@A00155:342:HHGFNDSXY:1:2327:6922:30577 1:N:0:GAACCTAG+TCCGCATA
TTAAGGACTCACCTCTGGCCTATGGTCATCTCAATGCCGAGAGGGATGTGGAGGGCGAGAGAGAGCGA
CTTCTCTCGGGCCCACAGCCTTACAGCTGTGGAGCACGGTATCCTCTGCCGAAACAGAGGTTGGACAA
+
FFFFFFFFFFFFFFFFFFFFFFFFFFFFFFFFFFFFFFFFFFFFFFFFFFFFFFFFFFFFFFFFFF:F
FFFFFFFFFFFFFFFFFFFFFFFFFFFFFFFFFFFFFFFFFFFFFFFFFFFFFFFFFFFFFFFFFFFF
@A00155:342:HHGFNDSXY:1:2327:30563:32252 1:N:0:GAACCTAG+TCCGCATA
ACCTCTGTTTCGGCAGAGGATACCGTGCTCCACAGCTGTAAGGCTGTGGGCCCGAGAGAAGTCGCTCT
CTCTCGCCCTCCACATCCCTCTCGGCATTGAGATGACCATAGGCCAGAGGAGAGTCCTT
+
FFFFFFFFFFFFFFFFFFFFFFFFFFFFFFFFFFFFFFFFFFFFFFFFFFFFFFFFFFFFFFFFFFFF
FFFFFFFFFFFFFFFFFFFFFFFFFFF:FFFFFFFFFFFFFFFFFFFFFFFFFFFFFFF
@A00155:342:HHGFNDSXY:1:2327:31033:32690 1:N:0:GAACCTAG+TCCGCATA
CAGCCTTACAGCTGTGGAGCACGGTATCCTCTGCCGAAACAGAGGTTGGACAAGACCGGAGGGGTCTC
CTAGTTCCAAAGGAGATGTACTCCGGGCTTGTTCACGACCTACCGTGTAAGTCGTAGTCTAGTAGGCT
+
FFFFFF:FFFFFFFFFFFFFFFFFFFFFFFFFFFFFFFFFFFFFFFFFFFFFFFFFFFFFFFFFFFFF
FFFFFFFFFFFFFFFFFFFFFFFFFFFFFFFFFFFFFFFFFFFFFFFFFFFFFFFFFF:FFFFFFFF
@A00155:342:HHGFNDSXY:1:2327:30770:32831 1:N:0:GAACCTAG+TCCGCATA
CAGCCTTACAGCTGTGGAGCACGGTATCCTCTGCCGAAACAGAGGTTGGACAAGACCGGAGGGGTCTC
CTAGTTCCAAAGGAGATGTACTCCGGGCTTGTTCACGACCTACCGTGTAAGTCGTAGTCTAGTAGGCT
+
FFFFFFFFFFFFFF:FFFFFFFFFFFFFFFFFFFFFFFFFFFFFFFFFFFFFFFFFFFFFFFFFFFFF
FFFFFFFFFFFFFFFFFFFFFFFFFFFFFFFFFFFFFFFFFFFF:FFFFFFFFFFFFFFFFFFFFFF
@A00155:342:HHGFNDSXY:1:2327:1542:36605 1:N:0:GAACCTAG+TCCGCATA
CGAGAGAGAGCGACTTCTCTCGGGCCCACAGCCTTACAGCTGTGGAGCACGGTATCCTCTGCCGAAAC
AGAGGTTGGACAAGACCGGAGGGGTCTCCTAGTTCCAAAGGAGATGTACTCCGGGCTTGTTCACGACC
+
FFFFFFFFFFFFFFFFFFFFFFFFFFFFFFFFFFFFFFFFFFFFFFFFFFFFFFFFFFFFFFFFFFF:
FFFFFFFFFFFFFFFFFFFFFFFFFFFFFFFFFFFFFFFFFFFFFFFFF:FFFFFF,,FFFFFFFFFF:
@A00155:342:HHGFNDSXY:1:2328:29930:10895 1:N:0:GAACCTAG+TCCGCATA
TCCTCTGCCGAAACAGAGGTTGGACAAGACCGGAGGGGTCTCCTAGTTCCAAAGGAGATGTACTCCGG
GCTTGTTCACGACCTACCGTGTAAGTCGTAGTCTAGTAGGCTACCTGACGAGTCCTTTTTAGGACGAA
+
FFFFFFFFFFFFFFFFFFFFFFFFFFFFFFFFFFFFFFFFFFFFFFFFFFFFFFFFFFFFFFFFFFFF
FF:FFFFFFFFFFFFFFFFFFFFFFFFFFFFFFFFFFFFFFFFFFFFFFFFFFFFFFF::FFFFFFFFF
@A00155:342:HHGFNDSXY:1:2328:24560:34632 1:N:0:GAACCTAG+TCCGCATA
GGCCCACAGCCTTACAGCTGTGGTGCACGGTATCCTCTGCCGAAACAGAGGTTGGACAAGACCGGAGG
GGTCTCCTAGTTCCAAAGGAGATGTACTCCGGGCTTGTTCACGACCTACCGTGTAAGTCGTAGTCTAG
+

FFFFFFFFFFFFFFFFFFFFFFFFFFFFFFFFFFFFFFFFFFFFFFFFFFFFFFFFFFFFFFFFFFFF
FFFFFFFFFFFFFFFFFFFFFFFFFFFFFFFFFFFFFFFFFFFFFFFFFFFFFFFFFFFFFFFFFFFF
@A00155:342:HHGFNDSXY:1:2329:13675:6887 1:N:0:GAACCTAG+TCCGCATA
ACCTCTGTTTCGGCAGAGGATACCGTGCTCCACAGCTGTAAGGCTGTGGGCCCGAGAGAAGTCGCTCT
CTCTCGCCCTTCACATCCCTCTCGGCATTGAGATGACCATAGGCCAGAGGTGAGTCCTTAAGTGGACA
+
FFFFFFFFFFFFFFFFFFFFFFFFFFFFFFFFFFFFFFFFFFFFFFFFFFFFFFFFFFFFFFFFFFFF
FFFFFFFFFFFFFFFFFFFFFFFFFFFFFFFFFFFFFFFFFFFFFFFFFFFFFFFFFFFFFFFFFFF:
@A00155:342:HHGFNDSXY:1:2329:5972:18443 1:N:0:GAACCTAG+TCCGCATA
ATCTCAATGCCGAGAGGGATGTGGAGGGCGAGAGAGAGCGACTTCTCTCGGGCCCACAGCCTTACAGC
TGTGGAGCACGGTATCCTCTGCCGAAACAGAGGTTGGACAAGACCGGAGGGGTCTCCTAGTTCCAAAG
+
FFFFFFFFFFFFFFFFFFFFFFFFFFFFFFFFFFFFFFFFFFFFFFFFFFFFFFFFFFFFFFFFFFFF
FFFFFFFFFFFFFFFFFFFFFFFFFFFFFFFFFFFFFFFFFF:FFFFFFFFFFFFFFFFFFFFFFFFF
@A00155:342:HHGFNDSXY:1:2329:25825:32816 1:N:0:GAACCTAG+TCCGCATA
ATCTCAATGCCGAGAGGGATGTGGAGGGCGAGAGAGAGCGACTTCTCTCGGGCCCACAGCCTTACAGC
TGTGGAGCACGGTATCCTCTGCCGAAACAGAGGTTGGACAAGACCGGAGGGGTCTCCTAGTTCCAAAG
+
FFFFFFFF:F::F:FFFF::FFFFFF:FFFFFFFFFFFF:FFFFFFFFFF,FF:FF:FFFFFFFFF::
FF:FFFFFFFF::FFFFFFFFFFFFFFFFFFF:FFFFFFFF,FF:::FFFFFFFFF:FFFFFFFFFFF
@A00155:342:HHGFNDSXY:1:2330:25690:12414 1:N:0:GAACCTAG+TCCGCATA
CCCACAGCCTTACAGCTGTGGAGCACGGTATCCTCTGCCGAAACAGAGGTTGGACAAGACCGGAGGGG
TCTCCTAGTTCCAAAGGAGATGTACTCCGGGCTTGTTCACGACCTACCGTGTAAGTCGTAGTCTAGTA
+
FFFFFFFFFFFFFFFFFFFFFFFFFFFFFFFFFFFFFFFFFFFFFFFFFFFFFFFFFFFFFFFFFFFF
FFFFFFFFFFFFFFFFFFFFFFFFFFFFFFFFFFFFFFFFFFFFFFFFFFFFFFFFFFFFFFFFFFFF
@A00155:342:HHGFNDSXY:1:2330:25753:21981 1:N:0:GAACCTAG+TCCGCATA
GAGAGCGACTTCTCTCGGGCCCACAGCCTTACAGCTGTGGAGCACGGTATCCTCTGCCGAAACAGAGG
TTGGACAAGACCGGAGGGGTCTCCTAGTTCCAAAGGAGATGTACTCCGGGCTTGTTCACGAC
+
FFFFFFFFFFFFFFFF:FFFFFFFFFFFFFFFFFFFFFFFFFFFFFFFFFFFFFFFFFFFFFFFFFFF
FFFFFFFFFFFFFFFFFFFFFFFFFFFFFF:FFFFFFFFFFFFFFFFFFFFFFFFFFFFFFF
@A00155:342:HHGFNDSXY:1:2330:27434:25520 1:N:0:GAACCTAG+TCCGCATA
GAGAGCGACTTCTCTCGGGCCCACAGCCTTACAGCTGTGGAGCACGGTATCCTCTGCCGAAACAGAGG
TTGGACAAGACCGGAGGGGTCTCCTAGTTCCAAAGGAGATGTACTCCGGGCTTGTTCACGAC
+
FFFFFFFFFFFFFFFFFFFFFFFFFFFFFFFFFFFFFFFFFFFFFFFFFFFFFFFFFFFFFFFFFFFF
FFFFFFFFFFFFFFFFFFFFFFFFFFFFFFFFFFFFFFFFFFFFFFFFFFFFFFFFFFFFFF
@A00155:342:HHGFNDSXY:1:2330:11171:26882 1:N:0:GAACCTAG+TCCGCATA
AGGGATGTGGAGGGCGAGAGAGAGCGACTTCTCTCGGGCCCACAGCCTTACAGCTGTGGAGCACGGTA
TCCTCTGCCGAAACAGAGGTTGGACAAGACCGGAGGGGTCTCCTAGTTCCAAAGGAGATGTACT
+
FFF:F:FFFFFFFFFFFFFFFFF:FFFFFFFFFFFFFFFFFFF:FFFFFFFFFFFFFFFFFFFFFFFF
FFFFFFFFFFFFFFFFFFFFFFFFFFF:FFFFFFFFFFFFFFFFFFFFFFFFFFFFFFFFFFFF
@A00155:342:HHGFNDSXY:1:2330:20121:27101 1:N:0:GAACCTAG+TCCGCATA
CTCTATGCCGAGAGGGATGTGGAGGGCGAGAGAGAGCGACTTCTCTCGGGCCCACAGCCTTACAGCTG
TGGAGCACGGTATCCTCTGCCGAAACAGAGGTTGGACAAGACCGGAGGGGTCTCCTAGTTCCAAAGGA
+
FFFFFFFFFFFFFFFFF:FFFFFFFFFFFFFFFFFFFFFFFFFFFFFFFFFFFFFFFFFFFFFFFFFF
FFFFFFFFFFFFFFFFFFFFFFFFFFFFFFFFFFFFFFFFFFFFFFFFFFFFFFFFFFFFFFFFFFFF
@A00155:342:HHGFNDSXY:1:2330:20374:27195 1:N:0:GAACCTAG+TCCGCATA
CTCTATGCCGAGAGGGATGTGGAGGGCGAGAGAGAGCGACTTCTCTCGGGCCCACAGCCTTACAGCTG
TGGAGCACGGTATCCTCTGCCGAAACAGAGGTTGGACAAGACCGGAGGGGTCTCCTAGTTCCAAAGGA
+

FFFFFF:FFF:,F:FFF:FFFFFFFFFFFFFFF:F:FFFF:FFFFFFFFFFFFFFFFFFFFFFFFF:FF
FFFFFFFFFFF:FFFFFFFFFFFFF:FFFFFFFFFFFF:FFFFFFFFFF:FFFFFFFFFFFFFFFFFF
@A00155:342:HHGFNDSXY:1:2330:30635:27618 1:N:0:GAACCTAG+TCCGCATA
GAGCGACTTCTCTCGGGCCCACAGCCTTACAGCTGTGGAGCACGGTATCCTCTGCCGAAACAGAGGTT
GGACAAGACCGGAGGGGTCTCCTAGTTCCAAAGGAGATGTACTCCGGGCTTGTTCACGACCTACCGTG
+
:FFF:F::F:FFFFFF,FFFFFFFFFFFFFFFFFFFF:FFFFF:FFFFF:FFF,FF,F:FFFFF::F:
:FFFFF,FF,,FFF:FFFFFFFFFFFFF:FFFF:FFFF,FFFF,F::F:,FFFFFFF,FFFFFFF:,F
@A00155:342:HHGFNDSXY:1:2330:23647:27665 1:N:0:GAACCTAG+TCCGCATA
GAGCGACTTCTCTCGGGCCCACAGCCTTACAGCTGTGGAGCACGGTATCCTCTGCCGAAACAGAGGTT
GGACAAGACCGGAGGGGTCTCCTAGTTCCAAAGGAGATGTACTCCGGGCTTGTTCACGACCTACCGTG
+
FFFFFFFFFFFFFFFFFFFFFFFFFFFFFFFFFFFFFFFFFFFFFFF:FFFFFFFFFFFFFFFFFFFF
FFFFFFFFFFFFFFFFFFFFFFFFFFFFFFFFFFFFFFFFFF:FFF:FFFFFFFFFFFFFFFFFFFFF
@A00155:342:HHGFNDSXY:1:2330:26268:30921 1:N:0:GAACCTAG+TCCGCATA
GAGAGGGATGTGGAGGGCGAGAGAGAGCGACTTCTCTCGGGCCCACAGCCTTACAGCTGTGGAGCACG
GTATCCTCTGCCGAAACAGAGGTTGGACAAGACCGGAGGGGTCTCCTAGTTCCAAAGGAG
+
FFFFFFFFFFFFFFFFFFFFFFFFFFFFFFFFFFFFFFFFFFFFFFFFFFFFFFFFFFFFFFFFFFFF
FFFFFFFFFFFFFFFFFFFFFFFFFFFFFFFFFFFFFFFFFFFFFFFFFFFFFFFFFFFF
@A00155:342:HHGFNDSXY:1:2330:23574:31454 1:N:0:GAACCTAG+TCCGCATA
GAGAGGGATGTGGAGGGCGAGAGAGAGCGACTTCTCTCGGGCCCACAGCCTTACAGCTGTGGAGCACG
GTATCCTCTGCCGAAACAGAGGTTGGACAAGACCGGAGGGGTCTCCTAGTTCCAAAGGAG
+
FFFFFFFFFFFFFFFFFFFFFFFFFFFFFFFFFFFFFFFFFFFFFFFFFFFFFFFFFFFFFFFFFFFF
FFFFFFFFFFFFFFFFFFFFFFFFFFFFFFFFFFFFFFFFFF:FFFFFFFFFFFFF,FFF:F
@A00155:342:HHGFNDSXY:1:2331:6542:27445 1:N:0:GAACCTAG+TCCGCATA
CCCACCACACCGCCTTAGATCAGCTGTGTCCACTTAAGGACTCACCTCTGGCCTATGGTCATCTCAAT
GCCGAGAGGGATGTGGAGGGCGAGAGAGAGCGACTTCTCTCGGGCCCACAGCCTTACAGCTGTGGAGC
+
FFFFFFFFFFFFFFFFFFFFFF:FFFFFFFFFFFFFFFFFFFFFFFFFFFFFFFFFFFFFFFFFFFFF
FFFFFFFFFFFFFFFFFFFFFFFFFFFFFFFFFFFFFFFFFFFFFFFFFFFFFFFFFFFFFFFFFFFF
@A00155:342:HHGFNDSXY:1:2332:7708:12618 1:N:0:GAACCTAG+TCCGCATA
AGAGAGAGCGACTTCTCTCGGGCCCACAGCCTTACAGCTGTGGAGCACGGTATCCTCTGCCGAAACAG
AGGTTGGACAAGACCGGAGGGGTCTCCTGGTTCCAAAGGAGATGTACTCCGGGCTTGTTCACGACCTA
+
FFFFFFFFFFFFFFFFFFFFFFFFFFFFFFFFFFFFFFFFFFFFFFFFFFFFFFFFFFFFFFFFFFFF
FFFFFFFFFFFFFFFFFFFFFFFFFFFFFFFFFFFFFFFFFFFFFFFFFFFFFFFFFFFFFFF:FFF:
@A00155:342:HHGFNDSXY:1:2332:28429:19038 1:N:0:GAACCTAG+TCCGCATA
CCTCTGGCCTATGGTCATCTCAATGCCGAGAGGGATGTGGAGGGCGAGAGAGAGCGACTTCTCTCGGG
CCCACAGCCTTACAGCTGTGGAGCACGGTATCCTCTGCCGAAACAGAGGTTGGACAAGAC
+
FFFFFFFFFFFFFFFFFFFF,FFFF:F:FFFFFFF:F,FFFF:FFFFFFFFFFFFFFFFFFFFFFFFF
FFFFFFFFFFF:FFFFFFFFFFFFFFFFFFFFF:FFFFFFFFFFFFFFFFFF:FFFFFFFF
@A00155:342:HHGFNDSXY:1:2332:15637:34021 1:N:0:GAACCTAG+TCCGCATA
ACTTCTCTCGGGCCCACAGCCTTACAGCTGTGGAGCACGGTATCCTCTGCCGAAACAGAGGTTGGACA
AGACCGGAGGGGTCTCCTAGTTCCAAAGGAGATGTACTCCGGGCTTGTTCACGACCTACCGTGTAAGT
+
FFFFFFFFFFFFFFFFFFFFFFFFFFFFFFFFFFFFFFFFFFFFFFFFFFFFFFFFFFFFFFFFFFFF
FFFFFFFFFFFFFFFFFFFFFFFFFFFFFFFFFFFFFFFFFFFFFFFFFFFFFFFFFFFFFFFFFFFF
@A00155:342:HHGFNDSXY:1:2332:15456:34053 1:N:0:GAACCTAG+TCCGCATA
ACTTCTCTCGGGCCCACAGCCTTACAGCTGTGGAGCACGGTATCCTCTGCCGAAACAGAGGTTGGACA
AGACCGGAGGGGTCTCCTAGTTCCAAAGGAGATGTACTCCGGGCTTGTTCACGACCTACCGTGTAAGT
+

```
FFFFFFFFFFFFFFFFFFFFFFFFFFFFFFFFFFFFF:FFFFFFFFFFFFFFFFFFFFFFFFFFFFFF
FFFFFFFFFFFFFFFFFFFFFFFFFFFFFFFFFFFFFFFFFFFFFFFFFFFFFFFFFFFF:FFFFFFF
@A00155:342:HHGFNDSXY:1:2333:1136:10817 1:N:0:GAACCTAG+TCCGCATA
CAATGCCGAGAGGGATGTAGAGGGCGAGAGAGAGCGACTTCTCTCGGGCCCACAGCCTTACAGCTGTG
GAGCACGGTATCCTCTGCCGAAACAGAGGTTGGACAAGACCGGAGGGGTCTCCTAGTTCCAAAGGAG
+
FF:FFFFF:F:FFFFFFFF::FFFF,FF,FFF,FFF:FFFFFFF:FFF:F,:FFF:FFFF,FFFFFFF
F,F,FFF:FFFFFFFFF:FF:,F:FF:FFFFFFFF:FF:FFFFFFF,FFFFF:FFFFFFF,:,FF:F
@A00155:342:HHGFNDSXY:1:2333:6008:11804 1:N:0:GAACCTAG+TCCGCATA
GGATGTGGAGGGCGAGAGAGAGCGACTTCTCTCGGGCCCACAGCCTTACAGCTGTGGAGCACGGTATC
CTCTGCCGAAACAGAGGTTGGACAAGACCGGAGGGGTCTCCTAGTTCCAAAGGAGATGTACTCC
+
FFFFF:FFFFFFFFFFFFFFFFFFFFFFFFFFFFFFFFFF:FFFFFFFFFFFFFFFFFFFFFFFFFFF
FFFFFFFFFFFFFFFFFFFFFFFFFFFFFFFFFFFFFFFFFFFFFFFFFFFFFFFFFFFFFFF:
@A00155:342:HHGFNDSXY:1:2333:12961:12571 1:N:0:GAACCTAG+TCCGCATA
GGATGTAGAGGGCGAGAGAGAGCGACTTCTCTCGGGCCCACAGCCTTACAGCTGTGGAGCACGGTATC
CTCTGCCGAAACAGAGGTTGGACAAGACCGGAGGGGTCTCCTAGTTCCAAAGGAGATGTACTCCGGGC
+
FFFFFFFFFFFFFFFFFFFFFFFFFFFFFFFFFFFFFFFFFFFFFFFFFFFFFFFFFFFFFFFFFFFF
FFFFFFFFFFFFFFFFFFFFFFFFFFFFFFFFFFFFFFFFFFFFFFFFFFFFFFFFFFFFFFFF:FFF
@A00155:342:HHGFNDSXY:1:2333:21097:35712 1:N:0:GAACCTAG+TCCGCATA
GGATGTGGAGGGCGAGAGAGAGCGACTTCTCTCGGGCCCACAGCCTTACAGCTGTGGAGCACGGTATC
CTCTGCCGAAACAGAGGTTGGACAAGACCGGAGGGGTCTCCTAGTTCCAAAGGAGATGTACTCC
+
FFFFFFFFFFFFFFFFFFFFFFFFFFFFFFFFFFFFFFFFFFFFFFFFFFFFFFFFFFFFFFFFFFFF
FFFFFFFFFFFFFFFFFFFFFFFFFFFFFFFFFFFFFFFFFFFFFFFFFFFFFFFFFFFFFFFF
@A00155:342:HHGFNDSXY:1:2334:25129:13072 1:N:0:GAACCTAG+TCCGCATA
TGTCCACTTAAGGACTCACCTCTGGCCTATGGTCATCTCAATGCCGAGAGGGATGTGGAGGGCGAGAG
AGAGCGACTTCTCTCGGGCCCACAGCCTTACAGCTGTGGAGCACGGTATCCTCTGCCGAAACAGAGGT
+
FFFFFFFFFFFFFFFFFFFFFFFFFFFFFFFFFFFFFFFFFFFFFFFFFFFFFFFFFFFFFFFFFFFF
FFFFFFFFFFFFFFFFFFFFFFFFFFFFFFFFFFFFFFFFFFFFFFFFFFFFFFFFFFFFFFFFFFFF
@A00155:342:HHGFNDSXY:1:2334:11180:15530 1:N:0:GAACCTAG+TCCGCATA
AGAGAGAGCGACTTCTCTCGGGCCCACAGCCTTACAGCTGTGGAGCACGGTATCCTCTGCCGAAACAG
AGGTTGGACAAGACCGGAGGGGTCTCCTAGTTCCAAAGGAGATGTACTCCGGGCTTGTTCACGACCTA
+
FFFFFFFFFFFFFFFFFFFFFFFFFFFFFFFFFFFFFFFFFFFFFFFFFFFFFFFFFFFFFFFFFFFF
FFFFFFFFFFFFFFFFFFFFFFFFFFFFFFFFFFFFFFFFFFFFFFFFFFFFFFFFFFFFFFFFFF::
@A00155:342:HHGFNDSXY:1:2334:24406:18677 1:N:0:GAACCTAG+TCCGCATA
CTTCTCTCGGGCCCACAGCCTTACAGCTGTGGAGCACGGTATCCTCTGCCGAAACAGAGGTTGGACAA
GACCGGAGGGGTCTCCTAGTTCCAAAGGAGATGTACTCCGGGCTTGTTCACGACCTACCGTGTAAGTC
+
FFFFFFFFFFFFFFFFFFFFFFFFFFFFFFFFFFFFFFFFFFFFFFFFFFFFFFFFFFFFFFFFFFFF
FFFFFFFFFFFFFFFFFFFFFFFFFFFFFFFFFFFFFFFFFFFFFFFFFFFFFFFFFFFFFFFFFFFF
@A00155:342:HHGFNDSXY:1:2334:21269:28369 1:N:0:GAACCTAG+TCCGCATA
CTTCTCTCGGGCCCACAGCCTTACAGCTGTGGAGCACGGTATCCTCTGCCGAAACAGAGGTTGGACAA
GACCGGAGGGGTCTCCTGGTTCCAAAGGAGATGTACTCCGGGCTTGTTCACGACCTACCGTGTAAGTC
+
FFFFFFFFFFFFFFFFFFFFFFFFFFFFFFFFFFFFFFFFFFFFFFFFFFFFFFFFFFFFFFFFFFFF
FFFFFFFFFFFFFFFFFFFFFFFFFFFFFFFFFF:FFFFFFFF:FFFFFFFFFFFFFFFFFFFFFFFF
@A00155:342:HHGFNDSXY:1:2334:8630:29778 1:N:0:GAACCTAG+TCCGCATA
GATCAGCTGTGTCCACTTAAGGACTCACCTCTGGCCTATGGTCATCTCAATGCCGAGAGGGATGTGGA
GGGCGAGAGAGAGCGACTTCTCTCGGGCCCACAGCCTTACAGCTGTGGAGCACGGTATCCTCTGCCGA
+
```

```
FFFFFFFFFFFFFFFFFFFFFFFFFFFFFFFFFFFFFFFFFFFFFFFFFFFFFFFFFFFFFFFFFFFF
FFFFFFFFFFFFFFFFFFFFFFFFFFFFFFFFFFFFFFFFFFFFFFFFFFFFFFFFFFFFFFFFFFFF
@A00155:342:HHGFNDSXY:1:2334:20907:36448 1:N:0:GAACCTAG+TCCGCATA
GGATGTGGAGGGCGAGAGAGAGCGACTTCTCTCGGGCCCACAGCCTTACAGCTGTGGAGCACGGTATC
CTCTGCCGAAACAGAGGTTGGACAAGACCGGAGGGGTCTCCTAGTTCCAAAGGAGATG
+
FFFFFFFFFFFFFFFFFFFFFFFFFFFFFFFFFFFFFFFFFFFFFFFFFFFFF::FFFFFFFFFF:FF
FFFFFFFFFFFFFFFFFFFFFFFFFFFFFFFFFFFFFFFFFFFFFFFFFFFFFFF:FF
@A00155:342:HHGFNDSXY:1:2335:9498:19695 1:N:0:GAACCTAG+TCCGCATA
GAGGGCGAGAGAGAGCGACTTCTCTCGGGCCCACAGCCTTACAGCTGTGGAGCACGGTATCCTCTGCC
GAAACAGAGGTTGGACAAGACCGGAGGGGTCTCCTAGTTCCAAAGGAGATGTACTCCGGGCTTGTTCA
+
FFFFFFFFFFFFFFFFFFFF:FFFFFFFFFFFFFFFFFFFFFFFFFFFFFFFFFFFFF:FFFFFFFFF
FFFFFFFFFFFFFFFFFFFFFFFFFFFFFFFFFFFFFF:FFFFFFFFFFFF:FFFFFFFFFFFFFFFF
@A00155:342:HHGFNDSXY:1:2335:31801:29919 1:N:0:GAACCTAG+TCCGCATA
CTTCTCTCGGGCCCACAGCCTTACAGCTGTGGAGCACGGTATCCTCTGCCGAAACAGAGGTTGGACAA
GACCGGAGGGGTCTCCTAGTTCCAAAGGAGATGTACTCCGGGCTTGTTCACGACCTACCGTGTAAGTC
+
FF,FFFFFFFFFFFFFFFFFFFFFFFFFFFFFFFFFFFFFFFFFFFFFFFFFFFFFFFFFFFFFFFFF
FFFFFFFFFFFFFFFFFFFFFFFFFFFFFFFFFFFFFFFFFFFFFFFFFFFFFFFFFFFFFFFFFFFF
@A00155:342:HHGFNDSXY:1:2335:8142:34820 1:N:0:GAACCTAG+TCCGCATA
CTCTGCCGAAACAGAGGTTGGACAAGACCGGAGGGGTCTCCTAGTTCCAAAGGAGATGTACTCCGGGC
TTGTTCACGACCTACCGTGTAAGTCGTAGTCTAGTAGGCTACCTGACGAGTCCTTTTTAGGACGAAAC
+
FFFFFFFFFFFFFFFFFFFFFFFFFFFFFFFFFFFFFFFFFFFFFFFFFFFFFFFFFFFFFFFFFFFF
FFFFFFFFFFFFFFFFFFFFFFFFFFFFFFFFFFFFFFFFFFFFFFFFFFFFFFFFFFFFFFFFFFFF
@A00155:342:HHGFNDSXY:1:2335:6343:35900 1:N:0:GAACCTAG+TCCGCATA
GAGAGCGACTTCTCTCGGGCCCACAGCCTTACAGCTGTGGAGCACGGTATCCTCTGCCGAAACAGAGG
TTGGACAAGACCGGAGGGGTCTCCTAGTTCCAAAGGAGATGTACTCCGGGCTTGTTCACG
+
FFFFFFFFFFFFFF:FFFFFFFFFFFFF::FFFFFFFFFFFFFFF:FFFFFFFF:FFFFFFFFFFFFF
,:FFFFFFFFFFFFFFFFFFFFFFFFFFFFFFFFFFFFFFFF:FFFFFFFFFF:FFFFFF
@A00155:342:HHGFNDSXY:1:2336:26865:12477 1:N:0:GAACCTAG+TCCGCATA
ACTTCTCTCGGGCCCACAGCCTTACAGCTGTGGAGCACGGTATCCTCTGCCGAAACAGAGGTTGGACA
AGACCGGAGGGGTCTCCTAGTTCCAAAGGAGATGTACTCCGGGCTTGTTCACGACCTACCGTGTAAGT
+
FFFFFFFFFFFFFFFFFFFFFFFFFFFFFFFFFFFF:FFFFFFFFFFFFFFFFFFFFFFFFFFFFFFF
FFFFFFFFFFFFFFFFFFFFFFFFFFFFFFFFFFFFFFFFFFFFFFFFFFFFFFF:FFFFFFFFFFFF
@A00155:342:HHGFNDSXY:1:2336:19063:16814 1:N:0:GAACCTAG+TCCGCATA
GTCATCTCAATGCCGAGAGGGATGTGGAGGGCGAGAGAGAGCGACTTCTCTCGGGCCCACAGCCTTAC
AGCTGTGGAGCACGGTATCCTCTGCCGAAACAGAGGTTGGACAAGACCGGAGGGGTCTCCTAGTTCCA
+
FFFFFFFFFFFFFFFFFFFFFFFFFFFFFFFFFFFFFFFFFFFFFFFFFFFFFFFFFFFFFFFFFFFF
FFFFFFFFFFFFFFFFFFFFFFFFFFFFFFFFFFFFFFFFFFFFFFFFFFFFFFFFFFFFFFFFFFFF
@A00155:342:HHGFNDSXY:1:2336:12563:24784 1:N:0:GAACCTAG+TCCGCATA
TTCTCTCGGGCCCACAGCCTTACAGCTGTGGAGCACGGTATCCTCTGCCGAAACAGAGGTTGGACAAG
ACCGGAGGGGTCTCCTAGTTCCAAAGGAGATGTACTCCGGGCTTGTTCACGACCTACCGTGTAAGTCG
+
FFFFFFFFFFFFFFFFFFFFF::FFFFF:FFFFF:FFFFFFFFFFF:FFFFFFFFFFFFFFFFFFFF:
FFFFFFFFFFFFFFFFFFFFFFFFFFFFFFFFFFFFF:FFFFFFFFFFFFFFFFFFF,FFFFFFFFFF
@A00155:342:HHGFNDSXY:1:2337:14181:14434 1:N:0:GAACCTAG+TCCGCATA
AGACTCACCTCTGGCCTATGGTCATCTCAATGCCGAGAGGGATGTGGAGGGCGAGAGAGAGCGACTTC
TCTCGGGCCCACAGCCTTACAGCTGTGGAGCACGGTATCCTCTGCCGAAACAGAGGTTGGACAA
+
```

FFFFFFFFFFFFFFFFFFFFFFFFFFFFFFFFFFFFFFFFFFFFFFFFFFFFFFFFFFFFFFFFFFFFFFFFFFFFF
FFFFFFFFFFFFFFFFFFFFFFFFFFFFFFFFFFFFF:FFFFFFFFFFFFFFFFFFFFFFFFFFFF
@A00155:342:HHGFNDSXY:1:2337:27245:18490 1:N:0:GAACCTAG+TCCGCATA
TATCCTCTGCCGAAACAGAGGTTGGACAAGACCGGAGGGGTCTCCTAGTTCCAAAGGAGATGTACTCC
GGGCTTGTTCACGACCTACCGTGTAAGTCGTAGTCTAGTAGGCTACCTGACGAGTCCTTTTTAGGACG
+
FFFFFFFFFFFFFFFFFFFFFFFFFFFFFFFFFFFFFFFFFFFFFFFFFFFFFFFFFFFFFFFFFFFFFFFFFFFFF
FFFFFFFFFFFFFFFFFFFFFFFFFFFFFFFFFFFFFFFFFFFFFFFFFFFFFFFF,:FFFFFF:
@A00155:342:HHGFNDSXY:1:2337:10619:29246 1:N:0:GAACCTAG+TCCGCATA
TCCTCTGCCGAAACAGAGGTTGGACAAGACCGGAGGGGTCTCCTAGTTCCAAAGGAGATGTACTCCGG
GCTTGTTCACGACCTACCGTGTAAGTCGTAGTCTAATAGGCTACCTGACGAGTCCTTTTTAGGACGAA
+
FFFFFFFFFFFFFFFFFF:FFFFFFFFFFFFFFFFFFFFFFFFFFFFFFFFFFFFFFFFFFFFFFFFFFF
FF:FFFFFFFFFFFFFFFFFFFFFFFFFFFFFFFFFFFFFFFFFFFFFFFFFFFFFF,FFFFFFFFF
@A00155:342:HHGFNDSXY:1:2337:12717:29246 1:N:0:GAACCTAG+TCCGCATA
CATCTCAATGCCGAGAGGGATGTGGAGGGCGAGAGAGAGCGACTTCTCTCGGGCCCACAGCCTTACAG
CTGTGGAGCACGGTATCCTCTGCCGAAACAGAGGTTGGACAAGACCGGAGGGGTCTCCTGGTTCCAAA
+
FFFFFFFFFFFFFFFFFFFFFFFFFFFFFFFFFFFFFFFFFFFFFFFFFFFFFFFFFFFFF:FFFFF
FFFFFFFFFFFFFFFFFFFFFFFFFFFFFFFFFFFFFFFFFFFFFFFFFFFFFFFFFFFFFFFFFFFFFFFFFFFFF
@A00155:342:HHGFNDSXY:1:2337:6045:30248 1:N:0:GAACCTAG+TCCGCATA
TGCCCCGCCACACCGCCTTAGATCAGCTGTGTCCACTTAAGGACTCACCTCTGGCCTATGGTCATCTC
AATGCCGAGAGGGATGTGGAGGGCGAGAGAGAGCGACTTCTCTCGGGCCCACAGCCTTACAGCTGTGG
+
FFFFFFFFFFFFFFFFFFFFFFFFFFFFFFFFFFFFFFFFFFFFFFFFFFFFFFFFFFFFFFFFFFFFFFFFFFFFF
FFFFFFFFFFFFFFFFFFFFFFFFFFFFFFFFFFFFFFFFFFFFFFFFF:FFFFFFFFFFFFFFFFFF
@A00155:342:HHGFNDSXY:1:2337:23556:36526 1:N:0:GAACCTAG+TCCGCATA
GCGAGAGAGAGCGACTTCTCTCGGGCCCACAGCCTTACAGCTGTGGAGCACGGTATCCTCTGCCGAAA
CAGAGGTTGGACAAGACCGGAGGGGTCTCCTAGTTCCAAAGGAGATGTACTCCGGGCTTGTTCACGAC
+
FFFFFFFFFFFFFFFFFFFFFFFFFFFFFFFFFFFFFFFFFFFFFFFFFFFFFFFFFFFFFFFFFFFFFFFFFFFFF
FFFFFFFFFFFFFFFFFFFFFFFFFFFFFFFFFFFFFFFFFFFFFFFFFFFFFFFFFFFFFFFFFFFFFFFFFFFFF
@A00155:342:HHGFNDSXY:1:2338:21133:3771 1:N:0:GAACCTAG+TCCGCATA
AGGGATGTGGAGGGCGAGAGAGAGCGACTTCTCTCGGGCCCACAGCCTTACAGCTGTGGAGCACGGTA
TCCTCTGCCGAAACAGAGGTTGGACAAGACCGGAGGGGTCTCCTAGTTCCAAAGGAGATGTACTCCGG
+
FFFFFFFFFFFFFFFFFFFFFFFFFFFFFFFFFFFFFFFFFFFFFFFFFFFFF:FFFFFFFFFFFFF
FFFFFFFFFFFFFFFFFFFFFFFFFFFFFFFFFF:FFFFFFFFFFFFFFFFFFFFFFFFFFFFFFFFFF
@A00155:342:HHGFNDSXY:1:2338:27236:5478 1:N:0:GAACCTAG+TCCGCATA
AGAGAGAGCGACTTCTCTCGGGCCCACAGCCTTACAGCTGTGGAGCACGGTATCCTCTGCCGAAACAG
AGGTTGGACAAGACCGGAGGGGTCTCCTAGTTCCAAAGGAGATGTACTCCGGGCTTGTTCACGACCTA
+
FFFFFFFFFFFFFFFFFFFFFFFFFFFFFFFFFFFFFFFFFFFFFFFFFFFFFFFFFFFFFFFFFFFFFFFFFFFFF
FFFFFFFFFFFFFFFFFFFFFFFFFFFFFFFFFFFFFFFFFFFFFFFFFFFFFFFFFFFFFFFFFFFFFFFFFFFFF
@A00155:342:HHGFNDSXY:1:2338:30553:11882 1:N:0:GAACCTAG+TCCGCATA
AGCGACTTCTCTCGGGCCCACAGCCTTACAGCTGTGGAGCACGGTATCCTCTGCCGAAACAGAGGTTG
GACAAGACCGGAGGGGTCTCCTAGTTCCAAAGGAGATGTACTCCGGGCTTGTTCACGACCTACCGTGT
+
FFFFFFFFFFFFFFFFFFFFFFFFF:FFFFFFFFFFFFFFFFFFFFFFFFFFFFFFFFFFFFFFFFFFFF
FFFFF,FFFFFFFFFFFFFFFFF:FFFFFFFFFFFFFFFFFFFFFFFFFFFFFFFFF:FFFFFFFFFFF
@A00155:342:HHGFNDSXY:1:2338:22896:12587 1:N:0:GAACCTAG+TCCGCATA
TGCCGAGAGGGATGTGGAGGGCGAGAGAGAGCGACTTCTCTCGGGCCCACAGCCTTACAGCTGTGGAG
CACGGTATCCTCTGCCGAAACAGAGGTTGGACAAGACCGGAGGGGTCTCCTAGTTCCAAAGGAGATGT
+

FFFFF:FFFFFFFFFFFFFFFFFFFFFFFFFFFFFFFFFFFFFFFFFFFFFFFFFFFF:FFFFFFFFFFF
FFFFFFFFFFFFFFFFFFFFFFFFFFFFFFFFFFFFFFFFFFFFFFFFFFFFFFFFFFFFFFFFFFFFFF
@A00155:342:HHGFNDSXY:1:2338:21097:13416 1:N:0:GAACCTAG+TCCGCATA
AGAGAGAGCGACTTCTCTCGGGCCCACAGCCTTACAGCTGTGGAGCACGGTATCCTCTGCCGAAACAG
AGGTTGGACAAGACCGGAGGGGTCTCCTGGTTCCAAAGGAGATGTACTCCGGGCTTGTTCACGACCTA
+
FFFFFFFFFFFFFFFFFFFFFFFFFFFFFFFF,FFFFFFFFFFFFFFFFF:,FFFFFFFFFFFFFFFF:F
:F:FFFF,FFFFFF:FF:FFFF:FFFF,:FFFFF,,,FFFFFFFFF:F:FFFF,:FFF,::F:,F:FF
@A00155:342:HHGFNDSXY:1:2338:22562:16016 1:N:0:GAACCTAG+TCCGCATA
CAGCCTTACAGCTGTGGAGCACGGTATCCTCTGCCGAAACAGAGGTTGGACAAGACCGGAGGGGTCTC
CTAGTTCCAAAGGAGATGTACTCCGGGCTTGTTCACGACCTACCGTGTAAGTCGTAGTCTAGTAGGCT
+
FFFFFFFFFFFFFFFFFFFFFFFFFF,FFFFFFFFFFFFFFFFFFFFFFFFFFFFFFFFFFFFFFFFFFF
FFFFFFFFFFFFFFFFFFFFFFFFFFFFF,FFFFFFFFFFFFFFFFF:FF:FFFFFFFFFFFFFFFFFFF
@A00155:342:HHGFNDSXY:1:2338:20211:17675 1:N:0:GAACCTAG+TCCGCATA
CACCTCTGGCCTATGGTCATCTCAATGCCGAGAGGGATGTGAAGGGCGAGAGAGAGCGACTTCTCTCG
GGCCCACAGCCTTACAGCTGTGGAGCACGGTATCCTCTGCCGAAACAGAGGTTGGACAAGACCGGAGG
+
FFFFFFFFFFFFFFFFFFFFFFFFFFFFFFFFFFFFFF:FFFFFFFFFFFFFFFFFFFFFFFFFFFFFFF
FFFFFFFFFFFFFFFFFFFFFFFFFFFFFFFFFFFFFFFFFFFFFFFFFFFFFFFFFFFFFFFFFFFFFF
@A00155:342:HHGFNDSXY:1:2338:30798:23171 1:N:0:GAACCTAG+TCCGCATA
AGAGAGAGCGACTTCTCTCGGGCCCACAGCCTTACAGCTGTGGAGCACGGTATCCTCTGCCGAAACAG
AGGTTGGACAAGACCGGAGGGGTCTCCTAGTTCCAAAGGAGATGTACTCCGGGCTTGTTCACGACCTA
+
FFFFFFFFF:FFFFFFFFFFFFFFFFFFFFFFFFFFFFFFFFFFFFFFFFFFFFFFFFFFFFFFFFF:FF
FFFFFFFFFFFFFFFFFFFFFFFFFFF,FFFF:FFFFFFFFFFFFFFFFFFFF:FFFFFFFFFFFFFFF:
@A00155:342:HHGFNDSXY:1:2339:23321:4961 1:N:0:GAACCTAG+TCCGCATA
TTACAGCTGTGGAGCACGGTATCCTCTGCCGAAACAGAGGTTGGACAAGACCGGAGGGGTCTCCTAGT
TCCAAAGGAGATGTACTCCGGGCTAGTTCACGACCTACCGTGTAAGTCGTAGTCTAGTAGGCTACCTG
+
:FFFFFFFFFFFFFFFFFFFFFFFFFFFFFFFFFFFFFFFFFFFFFFFFFFFFFFFFFFFFFFFFFFFFF
FFFFFFFFFFFFFFFFFFFFFFFFFFFFFFFFFFFFFFFFFFFFFFFFFFFFFFFFFFFFFFFFFFFFFF
@A00155:342:HHGFNDSXY:1:2339:22869:19648 1:N:0:GAACCTAG+TCCGCATA
GAGCGACTTCTCTCGGGCCCACAGCCTTACAGCTGTGGAGCACGGTATCCTCTGCCGAAACAGAGGTT
GGACAAGACCGGAGGGGTCTCCTAGTTCCAAAGGAGATGTACTCCGGGCTTGTTCACGACCTACCGTG
+
FFFFFFFFFFFFFFFFFFFFFFFFFFFFFFFFFFFFFFFFFFFFFFFFFFFFFFFFFFFFFFFFFFFFFF
FFFFFFFFFFFFFFFFFFFFFFFFFFFFFFFFFFFFFFFFFFFFFFFFFFFFFFFFFFFFFFFFFFFFFF
@A00155:342:HHGFNDSXY:1:2339:24614:22357 1:N:0:GAACCTAG+TCCGCATA
CGAGAGAGAGCGACTTCTCTCGGGCCCACAGCCTTACAGCTGTGGAGCACGGTATCCTCTGCCGAAAC
AGAGGTTGGACAAGACCGGAGGGGTCTCCTAGTTCCAAAGGAGATGTACTCCGGGCTTGTTCACGACC
+
FFFFFFFFFFFFFFFFFFFFFFFFFF:FFFFFFFFFFFFFFFFFFFFF:FFFFFFFFFFFFFFFFFFFFF
FFFFFFFFFFFFFFFFFFFFFFFFFFFFFF:FFFFFFFFFFFFFFFFFFFFFFFFFFFFFFFFFFFFFFF
@A00155:342:HHGFNDSXY:1:2339:9986:22733 1:N:0:GAACCTAG+TCCGCATA
AGAGAGAGCGACTTCTCTCGGGCCCACAGCCTTACAGCTGTGGAGCACGGTATCCTCTGCCGAAACAG
AGGTTGGACAAGACCGGAGGGGTCTCCTAGTTCCAAAGGAGATGTACTCCGGGCTTGTTCACGACCTA
+
FFFFFFFFFFFFFFFFFFFFFFFFFFFFFFFFFFFFFFFFFFFFFFFFFFFFFFFFFFFFFFFFFFFFFF
FFFFFFFFFFFFFFFFFFFFFFFFFFFFFFFFFFFFFFFFFFFFFFFFFFFFFFFFF:FFFFFFFFFFFF
@A00155:342:HHGFNDSXY:1:2339:18249:30342 1:N:0:GAACCTAG+TCCGCATA
TGTGGAGGGCGAGAGAGAGCGACTTCTCTCGGGCCCACAGCCTTACAGCTGTGGAGCACGGTATCCTC
TGCCGAAACAGAGGTTGGACAAGACCGGAGGGGTCTCCTGGTTCCAAAGGAGATGTACTCCGGG
+

```
FFFFFFFFFFFFFFFFFFFFFFFFFFFFFFFFFFFFFFFFFFFFFFFFFFFFFFFFFFFFFFFFFFFF
FFFFFFFFFFFFFFFFFFFFFF:FFFFFFFFFFFFFFFFFFFFFFFFFFFFFFFFFFFFFFFFF
@A00155:342:HHGFNDSXY:1:2339:16007:33880 1:N:0:GAACCTAG+TCCGCATA
AGAGAGAGCGACTTCTCTCGGGCCCACAGCCTTACAGCTGTGGAGCACGGTATCCTCTGCCGAAACAG
AGGTTGGACAAGACCGGAGGGGTCTCCTAGTTCCAAAGGAGATGTACTCCGGGCTTGTTCACGACCTA
+
FFFFFFFFFFFFFFFFFFFFFFFFFFFFFFFFFFFFFFFFFFFFFFFFFFFFFFFFFFFFFFFFFFFF
FFFFFFFFFFFFFFFFFFFFFFFFFFFFFFFF:FFFFFFFFFFFFFFFFFFFFFFFFFFFFFFFFFFF
@A00155:342:HHGFNDSXY:1:2339:7292:35884 1:N:0:GAACCTAG+TCCGCATA
GGACTCACCTCTGGCCTATGGTCATCTCAATGCCGAGAGGGATGTAGAGGGCGAGAGAGAGCGACTTC
TCTCGGGCCCACAGCCTTACAGCTGTGGAGCACGGTATCCTCTGCCGAAACAGAGGTTGGACAAGACC
+
FFFFFFFFFFFFFFFFFFFFFFFFFFFFFFFFFFFFFFFFFFFFF:FFFFFFFFFFFFFFFFFFFFFF
FFFFFFFFFFFFFFFFF:FFFFFFFFFFFFFFFFFFFFFFFFFFFFFFFFFFFFFFFFFFFFFFFFFF
@A00155:342:HHGFNDSXY:1:2340:12147:19366 1:N:0:GAACCTAG+TCCGCATA
GACTTCTCTCGGGCCCACAGCCTTACAGCTGTGGAGCACGGTATCCTCTGCCGAAACAGAGGTTGGAC
AAGACCGGAGGGGTCTCCTAGTTCCAAAGGAGATGTACTCCGGGCTTGTTCACGACCTACCGTGTAAG
+
FFFFFFFFFFFFFFFFFFFFFFFFFFFFFFFFFFFFFFFFFFFFFFFFFFFFFFFFFFFFFFFFFFFF
FFFFFFFFFFFFFFFFFFFFFFFFFFFFFFFFFFFFFFFFFFFFFFFFFFFFFFFFFFFFFFFFFFFF
@A00155:342:HHGFNDSXY:1:2341:32416:1172 1:N:0:GAACCTAG+TCCGCATA
TTACAGCTGTGGAGCACGGTATCCTCTGCCGAAACAGAGGTTGGACAAGACCGGAGGGGTCTCCTAGT
TCCAAAGGAGATGTACTCCGGGCTTGTTCACGACCTACCGTGTAAGTCGTAGTCTAGTAGGCTACCTG
+
,F:FFFF:FFFFFFFFFFFFF:FFFFFFFFFF:FFFFFFFFFFF:F,FFFFFFFFFFFFFFFFFFFFF
FFFFFFFFFFFFFFFFFFFFFFFFFF:FFFFFFFFFFFFFFFFFFFFFFFFFFFFFFFFFFFFFFFFF
@A00155:342:HHGFNDSXY:1:2341:32262:1313 1:N:0:GAACCTAG+TCCGCATA
TTACAGCTGTGGAGCACGGTATCCTCTGCCGAAACAGAGGTTGGACAAGACCGGAGGGGTCTCCTAGT
TCCAAAGGAGATGTACTCCGGGCTTGTTCACGACCTACCGTGTAAGTCGTAGTCTAGTAGGCTACCTG
+
FFFFFFF,::F::FF:F:FFFFFFFFFFFF,FFFF,FFF:FFFFFFFFFFF:FFFFFFFFFFFFFFF:
FFFFFFFF:FF:FFFFFFFF:FF,,,FFF:F::FFFFFFFFFF,FFFFF:FFFFF,:FFFFFFFFFFF
@A00155:342:HHGFNDSXY:1:2341:22327:2707 1:N:0:GAACCTAG+TCCGCATA
CTCAATGCCGAGAGGGATGTGGAGGGCGAGAGAGAGCGACTTCTCTCGGGCCCACAGCCTTACAGCTG
TGGAGCACGGTATCCTCTGCCGAAACAGAGGTTGGACAAGACCGGAGGGGTCTCCTAGTTCCAAAGGA
+
FFFFFFFFFFFFFFFFFFFFFFFFFFFFFFFFFFFFFFFFFFFFFFFFFFFFFFFFFFFFF:FFFFFF
FFFFFFFFFFFFFFFFFFFFFFFFFFFFFFFFFFFFFFFFFFFFFFFFFFFFFFFFFFFFF:FFFFFF
@A00155:342:HHGFNDSXY:1:2341:17870:5134 1:N:0:GAACCTAG+TCCGCATA
GGGCGAGAGAGAGCGACTTCTCTCGGGCCCACAGCCTTACAGCTGTGGAGCACGGTATCCTCTGCCGA
AACAGAGGTTGGACAAGACCGGAGGGGTCTCCTAGTTCCAAAGGAGATGTACTCCGGGCTTGTTCACG
+
FFFFFFFFFFFFFFFFFFFFFFFFFFFFFFFFFFFF:FFFFFFFFFFFFFFFFFFFFFFFFFFFFFFF
FFFFFFFFFFFFFFFFFFFFFFFFFFFFFFFFFFFFFFFFFFFFFFFFFFFFFFFFFFFF:FFFFFFF
@A00155:342:HHGFNDSXY:1:2341:19153:7701 1:N:0:GAACCTAG+TCCGCATA
GGGCGAGAGAGAGCGACTTCTCTCGGGCCCACAGCCTTACAGCTGTGGAGCACGGTATCCTCTGCCGA
AACAGAGGTTGGACAAGACCGGAGGGGTCTCCTAGTTCCAAAGGAGATGTACTCCGGGCTTGTTCACG
+
FFFFFFFFFFFFFFFFFFFF:FFFFFFFFFFFFFFFFFFFFFFFFFFFFFFFFFFFFFFFFFFFFFFF
FFFFFFFFFFFFFFFFFFFFFFFFFFFFFFFFFFFFFFFFFFFFFFFFFFFFFFFFFFFFFFFFFFFF
@A00155:342:HHGFNDSXY:1:2341:30409:8907 1:N:0:GAACCTAG+TCCGCATA
GGGCGAGAGAGAGCGACTTCTCTCGGGCCCACAGCCTTACAGCTGTGGAGCACGGTATCCTCTGCCGA
AACAGAGGTTGGACAAGACCGGAGGGGTCTCCTAGTTCCAAAGGAGATGTACTCCGGGCTTGTTCACG
+
```

FFFFFFFFFFFFFFFFFFFFFFFFFFFFFFFFFFFFFFFFFFFFFFFFF,FFFFFFFFFFFF,FFF,F
FFFFFFFFFF:FFFFFFFFF:FFFFFFFFFFFFFF,FFFFFFFFFFF:FFFFFFFFFFFFFFFFFFFF
@A00155:342:HHGFNDSXY:1:2341:14669:12680 1:N:0:GAACCTAG+TCCGCATA
CTCTGGCCTATGGTCATCTCAATGCCGAGAGGGATGTGGAGGGCGAGAGAGAGCGACTTCTCTCGGGC
CCACAGCCTTACAGCTGTGGAGCACGGTATCCTCTGCCGAAACAGAGGTTGGACAAGACCGGAGGGGT
+
FFFFFFFFFFFFFFF:FFFFFFFFFFFFFFFFFFFFFFFFFFFFFFFFFFFFFFFFFFFFFF:FFFFF
FFFFFFFFFFFFFFFFFFFFFFFFFFFFFFFFFFFFFFFFFFFFFFFFFFFFFFF::FFFFFFFFFFF
@A00155:342:HHGFNDSXY:1:2341:29143:14105 1:N:0:GAACCTAG+TCCGCATA
AGAGCGACTTCTCTCGGGCCCACAGCCTTACAGCTGTGGAGCACGGTATCCTCTGCCGAAACAGAGGT
TGGACAAGACCGGAGGGGTCTCCTAGTTCCAAAGGAGATGTACTCCGGGCTTGTTCACGACCTACCGT
+
FFFFFFFFFFFFFFFFFFFFFFFFFFFFFFFFFFFFFFFFFFFFFFFFFFFFFFFFFFFFFFFFFFFF
FFFFFFFFFFFFFFFFFF:FFFF:FFFFFFFFFF:FFFFFFFFFFFFFFFFF:FF,FFFFFFFFFFFF
@A00155:342:HHGFNDSXY:1:2341:13503:26506 1:N:0:GAACCTAG+TCCGCATA
GAGCGACTTCTCTCGGGCCCACAGCCTTACAGCTGTGGAGCACGTTATCCTCTGCCGAAACAGAGGTT
GGACAAGACCGGAGGGGTCTCCTAGTTCCAAAGGAGATGTACTCCGGGCTTGTTCACGACCTACCGTG
+
FFFFFFF:FFFFFFFFFFFFFFFFFFFFFFFFFFFFFFFFFFF:FFFFFFFFFFFFFFFFFFFFFFFF
FFFFFFFFFFFFFFFFFFFFFFFFFFFFFFFFFFFFFFFFFFFFFFFFFFFFFFFFFFFFFFFFFF:F
@A00155:342:HHGFNDSXY:1:2341:12237:31078 1:N:0:GAACCTAG+TCCGCATA
AGAGAGAGCGACTTCTCTCGGGCCCACAGCCTTACAGCTGTGGAGCACGGTATCCTCTGCCGAAACAG
AGGTTGGACAAGACCGGAGGGGTCTCCTAGTTCCAAAGGAGATGTACTCCGGGCTTGTTCACGACCTA
+
FFFFFFFFFFFFFFFFFFFFFFFFFFFFFFFFFFFFFFFFFFFFFFFFFFFFFFFFFFFFFFFFFFFF
FFFFFFFFFFFFFFFFFFFFFFFFFFFFFFFFFFFFFFFFFFFFFFFFFFFFFFFFFFFFFFFFFFFF
@A00155:342:HHGFNDSXY:1:2341:22191:32784 1:N:0:GAACCTAG+TCCGCATA
TTACAGCTGTGGAGCACGGTATCCTCTGCCGAAACAGAGGTTGGACAAGACCGGAGGGGTCTCCTGGT
TCCAAAGGAGATGTACTCCGGGCTTGTTCACGACCTACCGTGTAAGTCGTAGTCTAGTAGGCTACCTG
+
FFFFFFFFFFFFFFFFFFFFFFFFFFFFFFFFFFFFFFFFFFFFFFFF:FFFFFFFFFFFFFFFFFFF
FFFFFFFFFFFFFFFFFFFFFFFFFFFFFFFFFFFFFFFFFFFFFFFFFFFFFFFFFFFFFFFFFFFF
@A00155:342:HHGFNDSXY:1:2341:13205:33724 1:N:0:GAACCTAG+TCCGCATA
AGAGAGAGCGACTTCTCTCGGGCCCACAGCCTTACAGCTGTGGAGCACGGTATCCTCTGCCGAAACAG
AGGTTGGACAAGACCGGAGGGGTCTCCTAGTTCCAAAGGAGATGTACTCCGGGCTTGTTCACGACCTA
+
FFFFFFFFFFFFFFFFFFFFFFFFFFFFFFFFFFFFFFFFFFFFFFFFFFFFFFFFFFFFFFFFFFFF
FFFFFFFFFFFFFFFFFFFFFFFFFFFFFFFFFFFFFFFFFFFFFFFFFFFFFFFFFFFFFFFFFFFF
@A00155:342:HHGFNDSXY:1:2341:15989:35133 1:N:0:GAACCTAG+TCCGCATA
ACCCCTCCGGTCTTGTCCAACCTCTGTTTCGGCAGAGGATACCGTGCTCCACAGCTGTAAGGCTGTGG
GCCCGAGAGAAGTCGCTCTCTCTCGCCCTCCACATCCCTCTCGGCATTGAGATGACCATAGGCCAGAG
+
FFFFFFFFFFFFFFFFFFFFFFFFFFF:FFFFFFFFFFFFFFFFFFFFFFFFFFFFFFFFFFFFFFFF
FFFFFFFFFFFFFFFFFFFFFFFFFFFFFFFFFFFFFFFFFFFFFFFFFFFFFFFFFFFFFFFFFFFF
@A00155:342:HHGFNDSXY:1:2342:30969:22748 1:N:0:GAACCTAG+TCCGCATA
GAGCGACTTCTCTCGGGCCCACAGCCTTACAGCTGTGGAGCACGGTATCCTCTGCCGAAACAGAGGTT
GGACAAGACCGGAGGGGTCTCCTAGTTCCAAAGGAGATGTACTCCGGGCTTGTTCACGACCTACCGTG
+
FFFFFFFFFFFFFFFFFFFFFFFFFFFFFFFFFFFFFFFFFFFFFFFFFFFFFFFFFFFFFFFFFFFF
FFFFFFFFFFFFFFFFFFFFFFFFFFFFFFFFFFFFFFFFFFFFFFFFFFFFFFFFFFFFFFFFFFFF
@A00155:342:HHGFNDSXY:1:2342:24189:34240 1:N:0:GAACCTAG+TCCGCATA
GAGAGCGACTTCTCTCGGGCCCACAGCCTTACAGCTGTGGAGCACGGTATCCTCTGCCGAAACAGAGG
TTGGACAAGACCGGAGGGGTCTCCTGGTTCCAAAGGAGATGTACTCCGGGCTTGTTCACGACCTACCG
+

FFFFFFFFFFFFFFFFFFFFFFFFFFFFFFFFFFFFFFFFFFFFFFFFFFFFFFFFFFFFFFFFFFFF
FFFFFFFFFFFFFFFFFFFFFFFFFFFFFFFFFFFFFFFFFFFFFFFFFFFFFFFFFFFFFFFFFFFF
@A00155:342:HHGFNDSXY:1:2344:10131:33348 1:N:0:GAACCTAG+TCCGCATA
CTCAATGCCGAGAGGGATGTGGAGGGCGAGAGAGAGCGACTTCTCTCGGGCCCACAGCCTTACAGCTG
TGGAGCACGGTATCCTCTGCCGAAACAGAGGTTGGACAAGACCGGAGGGGTCTCCTGGTTCCAAAGGA
+
FFFFFFFFF,F:FFFFFF,F:FFFFF,FF:F,FFFFFFF:FFFFFFF:FF,FFFFF,FFFFFFFF,FF
:FFFFFFF:FFFFFFFF,F,FFFFFF,:F:FFF:FFFFFFFFFF:FF:FFFFFFFF,::FFFFFF:,F
@A00155:342:HHGFNDSXY:1:2345:1090:3912 1:N:0:GAACCTAG+TCCGCATA
GGATGTGGAGGGCGAGAGAGAGCGACTTCTCTCGGGCCCACAGCCTTACAGCTGTGGAGCACGGTATC
CTCTGCCGAAACAGAGGTTGGACAAGACCGGAGGGGTCTCCTAGTTCCAAAGGAGATGTACTC
+
FFFFFFF:FFFFFF,FFFFF:FFFF,FFF:FFFFFFF,FFFFFFFFFFFFFFFFFFFF:FFFFFF:F
FFFFFF,FF:F::F,:FFFFFFFF:F:FFFF:FFFFFFFFFFFFFFFFF:FFFFFFFFF,FFF
@A00155:342:HHGFNDSXY:1:2345:7247:9095 1:N:0:GAACCTAG+TCCGCATA
CTTCTCTCGGGCCCACAGCCTTACAGCTGTGGAGCACGGTATCCTCTGCCGAAACAGAGGTTGGACAA
GACCGGAGGGGTCTCCTAGTTCCAAAGGAGATGTACTCCGGGCTTGTTCACGACCTACCGTGTAAGTC
+
FFFFFFFFFFFFF:FFFFFFFFFFFFFFFFFFFFFFFFFFFFFFFFFFFFFFFFFFFFFFFFFFFFFF
FFFFFFFFFFFFFFFFFFFFFFFFFFFFFFFFFFFFF:FFFFFFFFFFFFFFFFFFFFFFFFFFFFFF
@A00155:342:HHGFNDSXY:1:2346:16613:10692 1:N:0:GAACCTAG+TCCGCATA
AGGGATGTGGAGGGCGAGAGAGAGCGACTTCTCTCGGGCCCACAGCCTTACAGCTGTGGAGCACGGTA
TCCTCTGCCGAAACAGAGGTTGGACAAGACCGGAGGGGTCTCCTAGTTCCAAAGGAGATGTACTCCGG
+
FFFFFFFFFFFFFFFFFFFFFFFFFFFFFFFFFFFFFFFFFFFFFFFFFFFFFFFFFFFFFFFFFFFF
FFFFFFFFFFFFFFFFFFFF,FFFFFFFFFFFFFFFFFFFFFFFFFFFFFFFFFFFFFFFFFFFFFF:
@A00155:342:HHGFNDSXY:1:2346:20175:22341 1:N:0:GAACCTAG+TCCGCATA
TGTCCACTTAAGGACTCACCTCTGGCCTATGGTCATCTCTATGCCGAGAGGGATGTGGAGGGCGAGAG
AGAGCGACTTCTCTCGGGCCCACAGCCTTACAGCTGTGGAGCACGGTATCCTCTGCCGAAACAGAGGT
+
FFFF,F,FFFF:FFFFFFF:FFFFF,FFFFFFF:FFFFFFFFFFF,FFF,FF:F:FFF:FFF:FFFF:
:FFFF:FF:F:FFFFFFFF:FFFFF:FFFFFFFFFFFFFF::,F,FFFFFFFFFFFFFFFFFFFFFFF
@A00155:342:HHGFNDSXY:1:2346:10411:25316 1:N:0:GAACCTAG+TCCGCATA
TCAATGCCGAGAGGGATGTGGAGGGCGAGAGAGAGCGACTTCTCTCGGGCCCACAGCCTTACAGCTGT
GGAGCACGGTATCCTCTGCCGAAACAGAGGTTGGACAAGACCGGAGGGGTCTCCTAGTTCCAAAGGAG
+
FFFFFF::FFF:FFFF,:FF,F:FFFFFFFF:FFFF:::FFFFF:FFFF:FFFFF:FFFFF:F:F:::
FFF:FFF,FF:F:FFFFF:FFFFFFFFFF,FF:FFFFFFFF:,FFFFFFFFFFFFF:,FFFFFFFFF:
@A00155:342:HHGFNDSXY:1:2347:3911:2127 1:N:0:GAACCTAG+TCCGCATA
CTCAATGCCGAGAGGGATGTAGAGGGCGAGAGAGAGCGACTTCTCTCGGGCCCACAGCCTTACAGCTG
TGGAGCACGGTATCCTCTGCCGAAACAGAGGTTGGACAAGACCGGAGGGGTCTCCTAGTTCCAAAGGA
+
FFFFFFFFFFFFFFFFFFFFFFFFFFFFFFFFFFFFFFFFFFFFF:FFFFFFFFFFFFFFFFFFFFFF
FFFFFFFFFFFFFFFFFFFFFFFFFFFFFFFFFFFFFFFFFFFFFFFFFFFFFFFFFFFFFFFFFFFF
@A00155:342:HHGFNDSXY:1:2347:10529:4726 1:N:0:GAACCTAG+TCCGCATA
GGATGTGGAGGGCGAGAGAGAGCGACTTCTCTCGGGCCCACAGCCTTACAGCTGTGGAGCACGGTATC
CTCTGCCGAAACAGAGGTTGGACAAGACCGGAGGGGTCTCCTAGTTCCAAAGGAGATGTACTCC
+
FFFFFFFFFFFFFFFFFFFFFFFFFFFFFFFFFFFFFFFFFFFFFFFFFFFFFFFFFFFFFFFFFFFF
FFFFFFFFFFFFFFFFFFFFFFFFFFFFFFFFFFFFFFFFFFFFFFFFFFFFFFFFFFFFFF:FFFF
@A00155:342:HHGFNDSXY:1:2347:10682:5306 1:N:0:GAACCTAG+TCCGCATA
GGATGTGGAGGGCGAGAGAGAGCGACTTCTCTCGGGCCCACAGCCTTACAGCTGTGGAGCACGGTATC
CTCTGCCGAAACAGAGGTTGGACAAGACCGGAGGGGTCTCCTAGTTCCAAAGGAGATGTACTCC
+

FFFFFFFFFFFFFFFFFFFFFFFFFFFFFFFFFFFFFFFFFFFFFFFFFFFFFFFFFFFFFFFFFFFFFFFFFFFFF
FFFFFFFFFFFFFFFFFFFFFFFFFFFFFFFFFFFFFFFFFFFFFFFFFFFFFFFFFFFFFFFFFFFFFFFFFF
@A00155:342:HHGFNDSXY:1:2348:27805:7905 1:N:0:GAACCTAG+TCCGCATA
CTCAATGCCGAGAGGGATGTGGAGGGCGAGAGAGAGCGACTTCTCTCGGGCCCACAGCCTTACAGCTG
TGGAGCACGGTATCCTCTGCCGAAACAGAGGTTGGACAAGACCGGAGGGGTCTCCTAGTTCCAAAGGA
+
FFFFFFFFFFFFFFFFFFFFFFFFFFFFFFFFFFFFFFFFFFFFFFFFFFFFFFFFFFFFFFFFFFFFFFFFFFFFF
FFFFFFFFFFFFFFFFFFFFFFFFFFFFFFFFFFFFFFFFFFFFFFFFFFFFFFFFFFFFFFFFFFFFFFFFFFFFF
@A00155:342:HHGFNDSXY:1:2348:21585:8187 1:N:0:GAACCTAG+TCCGCATA
AGAGAGAGCGACTTCTCTCGGGCCCACAGCCTTACAGCTGTGGAGCACGGTATCCTCTGCCGAAACAG
AGGTTGGACAAGACCGGAGGGGTCTCCTAGTTCCAAAGGAGATGTACTCCGGGCTTGTTCACGACCTA
+
FFFFFFFFFFFFFFFFFFFFFFFFFFFFFFFFFFFFFFFFFFFFFFFFFFFFFFFFFFFFFFFFFFFFFFFFFFFFF
FFFFFFFFFFFFFFFFFFFFFFFFFFFFFFFFFFFFFFFFFFFFFFFFFFFFFFFFFFFFFFFFFFFFFFFFFFFFF
@A00155:342:HHGFNDSXY:1:2348:7301:21277 1:N:0:GAACCTAG+TCCGCATA
ATCTCAATGCCGAGAGGGATGTGGAGGGCGAGAGAGAGCGACTTCTCTCGGGCCCACAGCCTTACAGC
TGTGGAGCACGGTATCCTCTGCCGAAACAGAGGTTGGACAAGACCGGAGGGGTCTCCTTGTTCCAAAG
+
FF,F,FFFFFFFFFF:FF:FFFFFFFFFFFFFFFFFFFFFF:FF:FFFFFFFFF,FFF:FFFFFFFF,
FFFFFF,FF:F:FFFFFFFFFFFFFFFFF,F::FFFFFFFFF:FFFFFF:FFFFFFFF:FFFFFFFFF
@A00155:342:HHGFNDSXY:1:2348:24388:21527 1:N:0:GAACCTAG+TCCGCATA
GGGCGAGAGAGAGCGACTTCTCTCGGGCCCACAGCCTTACAGCTGTGGAGCACGGTATCCTCTGCCGA
AACAGAGGTTGGACAAGACCGGAGGGGTCTCCTAGTTCCAAAGGAGATGTACTCCGGGCTTGTTCACG
+
FFFFFFFFFFFFFFFFFFFFFFFFFFFFFFFFFFFFFFFFFFFFFFFFFFFFFFFFFFFFFFFFFFF:FF
FFFFFFFFFFFFFFFFFFFFFFFFFFFFFFFFFFFFFFFFFFFFFFFFFFFFFFFFFFFFFFFFFFFFFFFFFFFFF
@A00155:342:HHGFNDSXY:1:2348:2691:21621 1:N:0:GAACCTAG+TCCGCATA
GAGAGCGACTTCTCTCGGGCCCACAGCCTTACAGCTGTGGAGCACGGTATCCTCTGCCGAAACAGAGG
TTGGACAAGACCGGAGGGGTCTCCTAGTTCCAAAGGAGATGTACTCCGGGCTTGTTCACGACCTACCG
+
FFFFFFFFFFFFFFFFFFFFFFFFFFFFFFFFFFFFFFFFFFFFFFFFFFFFFFFFFFFFFFFFFFFFFFFFFFFFF
FFFFFFF:FFFFFFFFFFFFFFFFFFFFFFFFFFFFFFFFFFFFFFFFFFFFFFFFFFFFFFFFFFFFFF
@A00155:342:HHGFNDSXY:1:2348:15338:22357 1:N:0:GAACCTAG+TCCGCATA
AGAGAGAGCGACTTCTCTCGGGCCCACAGCCTTACAGCTGTGGAGCACGGTATCCTCTGCCGAAACAG
AGGTTGGACAAGACCGGAGGGGTCTCCTAGTTCCAAAGGAGATGTACTCCGGGCTTGTTCACGACCTA
+
FFFFFFFFFFFFFFFFFFFFFFFFFFFFFFFFFFFFFFFFFFFFFFFFFFFFFFFFFFFFFFFFFFFFFFFFFFFFF
FFFFFFFFFFF:FFFFFFFFFFFFFFFFFFFFFFFFFFFFFFFFFFFFFFFFFFFFFFFFFFFFFFFF
@A00155:342:HHGFNDSXY:1:2348:2284:22545 1:N:0:GAACCTAG+TCCGCATA
CACAGCCTTACAGCTGTGGAGCACGGTATCCTCTGCCGAAACAGAGGTTGGACAAGACCGGAGGGGTC
TCCTAGTTCCAAAGGAGATGTACTCCGGGCTTGTTCACGACCTACCGTGTAAGTCGTAGTCTAGTAGG
+
FFFFFFFFFF:FFFFFFFFFFFFFFFFFFFFFFFFFFFFFFFFFF:FFFFFFFFFFFFFFFFFFFFFFF
FFFFFFFFFFFFFFFFFFFFFFF:FFF:FFFFFFFFFFFFFFFFF,FFFFFFFFFFFFFFF:FF:FFFF
@A00155:342:HHGFNDSXY:1:2348:31033:27774 1:N:0:GAACCTAG+TCCGCATA
GAGCGACTTCTCTCGGGCCCACAGCCTTACAGCTGTGGAGCACGGTATCCTCTGCCGAAACAGAGGTT
GGACAAGACCGGAGGGGTCTCCTAGTTCCAAAGGAGATGTACTCCGGGCTTGTTCACGACCTACCGTG
+
FFFFFFFFFFFFFFFFFFFFFFFFFFFFFFFFFFFFFFFFFFFFFFFFFFFFFFFFFFFFFFFFFFFFFFFFFFFFF
FFFFFFFFFFFFFFFFFFFFFFFFF:FFFFFFFFFF:FFFFFFFFFFFFFF:FFF:FF:FFFFFFF:FF
@A00155:342:HHGFNDSXY:1:2348:7066:29074 1:N:0:GAACCTAG+TCCGCATA
GTATCCTCTGCCGAAACAGAGGTTGGACAAGACCGGAGGGGTCTCCTAGTTCCAAAGGAGATGTACTC
CGGGCTTGTTCACGACCTACCGTGTAAGTCGTAGTCTAGTAGGCTACCTGACGAGTCCTTTTTAGGAC
+

```
FFFFFFFFFFFFFFFFFFFFFFFFFFFFFFFFFFFFFFFFFFFFFFFFFFFFFFFFFFFFFFFFFFFF
FFFFFFFFFFFFFFFFFFFFFFFFFFFFFFFFFFFFFFFFFFFFFFFFFFFFFFFFFFF:FFFFFFFF
@A00155:342:HHGFNDSXY:1:2349:22363:3270 1:N:0:GAACCTAG+TCCGCATA
GAGAGAGCGACTTCTCTCGGGCCCACAGCCTTACAGCTGTGGTGCACGGTATCCTCTGCCGAAACAGA
GGTTGGACAAGACCGGAGGGGTCTCCTAGTTCCAAAGGAGATGTACTCCGGGCTTGTTCACGACCTAC
+
FFFFFFFFFFFFFFFFFFFFFFFFFFFFFFFFFFFFFFFFFFFFFFFFFFFFFFFFFFFFFFFFFFFF
FFFFFFFFFFFFFFFFFFFFFFFFFFFFFFFFFFFFFFFFFFFFFFFFFFFFFFFFFFFFFFFFFFFF
@A00155:342:HHGFNDSXY:1:2349:27407:20369 1:N:0:GAACCTAG+TCCGCATA
AGCGACTTCTCTCGGGCCCACAGCCTTACAGCTGTGGAGCACGGTATCCTCTGCCGAAACAGAGGTTG
GACAAGACCGGAGGGGTCTCCTAGTTCCAAAGGAGATGTACTCCGGGCTTGTTCACGACCTACCGTGT
+
FFFFFFFFFFFFFFFFFFFFFFFFFFFFFFFFFFFFFFFFFFFFFFFFFFFFFFFFFFFFFFFFFFFF
FFFFFFFFFFFFFFFFFFFFFFFFFFFFFFFFFFFFFFFFFFFFFFFFFFFFFFFFFFFFFFFFFFFF
@A00155:342:HHGFNDSXY:1:2350:19542:3865 1:N:0:GAACCTAG+TCCGCATA
CACCTCTGGCCTATGGTCATCTCAATGCCGAGAGGGATGTGAAGGGCGAGAGAGAGCGACTTCTCTCG
GGCCCACAGCCTTACAGCTGTGGAGCACGGTATCCTCTGCCGAAACAGAGGTTGGACAAGACCGGAGG
+
FFFFFFFFFFFFFFFFFFFFFFFFFFFFFFFFFFFFFFFFFFFFFFFFFFFFFFFFFFFFFFFFFFFF
FFFFFFFFFFFFFFFFFFFFFFFFFFFFFFFFFFFFFFFFFFFFFFFFFFFFFFFFFFFFFFFFFFFF
@A00155:342:HHGFNDSXY:1:2350:13304:11663 1:N:0:GAACCTAG+TCCGCATA
ATGCCGAGAGGGATGTGGAGGGCGAGAGAGAGCGACTTCTCTCGGGCCCACAGCCTTACAGCTGTGGA
GCACGGTATCCTCTGCCGAAACAGAGGTTGGACAAGACCGGAGGGGTCTCCTAGTTCCAAAGGAGATG
+
FFFFFFFFFFFFFFFFFFFFFFFFFFFFFFFFFFFFFFFFFFFFFFFFFFFFFF:FFFFFFFFFFFFF
FFFFFFFFFFFFFFFFFFFFFFFFFFFFFFFFFFFFFFFFFFFFFFFFFFFFFFFFFFFFFFFFFFFF
@A00155:342:HHGFNDSXY:1:2350:4716:17895 1:N:0:GAACCTAG+TCCGCATA
TCTCTCGGGCCCACAGCCTTACAGCTGTGGAGCACGGTATCCTCTGCCGAAACAGAGGTTGGACAAGA
CCGGAGGGGTCTCCTAGTTCCAAAGGAGATGTACTCCGGGCTTGTTCACGACCTACCGTGTAAGTCGT
+
FFFFFFFFFFFFFFFFFFFF:FFFFFFFFFFFFFFFFFFFFFFFFF:FFFFFFFFFFFFFFFFFFFFF
FFFFFFFFFFFFFFFFFFFFFFFFFFFFFFFFFFFFFFFFFFFFFFFFFFFFFFFFFFFFFFFFFFFF
@A00155:342:HHGFNDSXY:1:2350:18150:26349 1:N:0:GAACCTAG+TCCGCATA
CTCTGCCGAAACAGAGGTTGGACAAGACCGGAGGGGTCTCCTAGTTCCAAAGGAGATGTACTCCGGGC
TTGTTCACGACCTACCGTGTAAGTCGTAGTCTAGTAGGCTACCTGACGAGTCCTTTTTAGGACGAAAC
+
FFFFFFFFFFFFFFFFF:FFFFFFFFFFFFFFFFFFFFFFF:FFFFFFFFFFFFFFFFFFFFFFFFFF
FFFFFFFFFFFFFFFFFFFFFFFFFFFFFFFFFFFFFFFF:FFFFFFFFFFFFFFFFFFFFFFF::FF
@A00155:342:HHGFNDSXY:1:2350:27905:29183 1:N:0:GAACCTAG+TCCGCATA
GGCCAGAGGTGAGTCCTTAAGTGGACACAGCTGATCTAAGGCGGTGTGGCGGGGCATGGGTTTGAACC
CCCATGACGGTCGGAGTCTAGTAGGCTCCCTGATGAGTCCGTTCCAAGGACGAAACCGTCGTTACTCT
+
FFFFFFFFFFFFFFFFFFFFFFFFFFFFFFFFFFFFFFFFFFFFFFFFFFFFFFFFFFFFFFFFFFFF
FFFFFFFFFFFFFFFFFFFFFFFFFFFFFFFFFFFFFFFFFFFFFFFFFFFFF,FFFFFFFFFFFFFF
@A00155:342:HHGFNDSXY:1:2350:26350:32534 1:N:0:GAACCTAG+TCCGCATA
TATCCTCTGCCGAAACAGAGGTTGGACAAGACCGGAGGGGTCTCCTAGTTCCAAAGGAGATGTACTCC
GGGCTTGTTCACGACCTACCGTGTAAGTCGTAGTCTAGTAGGCTACCTGACGAGTCCTTTTTAGGACG
+
FFFFFFFFFFFFFFFFFFFFFFFFFFFFFFFFFFFFFFFFFFFFFFFFFFFFFFFFFFFFFFFFFFFF
FFFFFFFFFFFFFFFFFFFFFFFFFFFFFFFFFFFFFFFFFFFFFFFFFFFFFFFFFFFFFFFFFFFF
@A00155:342:HHGFNDSXY:1:2350:27308:34287 1:N:0:GAACCTAG+TCCGCATA
TGTCCACTTAAGGACTCACCTCTGGCCTATGGTCATCTCAATGCCGAGAGGGATGTGGAGGGCGAGAG
AGAGCGACTTCTCTCGGGCCCACAGCCTTACAGCTGTGGAGCACGGTATCCTCTGCCGAAACAGAGGT
+
```

```
FFFFFFFFFFFFFFFFFFFFFFFFFFFFFFFFFFFFFFFFFF:FFFFFFFFFFFF:FFF:FFFFFFFF
FFFFFFFFFFFFFFFFFFFFFFFFFFFFFFFFFFFFFFFFFFFFFFFFFFFFFFFFFFFFFFFFFFFF
@A00155:342:HHGFNDSXY:1:2351:15076:10880 1:N:0:GAACCTAG+TCCGCATA
TCCTCTGCCGAAACAGAGGTTGGACAAGACCGGAGGGGTCTCCTAGTTCCAAAGGAGATGTACTCCGG
GCTTGTTCACGACCTACCGTGTAAGTCGTAGTCTAGTAGGCTACCTGACGAGTCCTTTTTAGGACGAA
+
FFFFFFFFFFFFFFFFFFFFFFFFFFFFFFFFFFFFFFFFFFFFFFFFFFFFFFFFFFFFFFFFFFFF
FF:FFFFFFFFFFFFFFFFFFFFFFFFFFFFFFFFFFFFFFFFFFFFFFFFFFFFFFFFFFFFFFFFF
@A00155:342:HHGFNDSXY:1:2351:28556:17409 1:N:0:GAACCTAG+TCCGCATA
CTTCTCTCGGGCCCACAGCCTTACAGCTGTGGAGCACGGTATCCTCTGCCGAAACAGAGGTTGGACAA
GACCGGAGGGGTCTCCTAGTTCCAAAGGAGATGTACTCCGGGCTTGTTCACGACCTACCGTGTAAGTC
+
FFFFFFFFFFFFFFFFFFFFFFFFFFFFFFFFFFFFFFFFFFFFFFFFFFFFFFFFFFFFFFFFFFFF
FFFFFFFFFFFFFFFFFFFFFFFFFFFFFFFFFFFFFFFFFFFFFFFFFFFFFFFFFFFFFFFFFFFF
@A00155:342:HHGFNDSXY:1:2351:24966:30639 1:N:0:GAACCTAG+TCCGCATA
TCTGTTTCGGCAGAGGATACCGTGCTCCACAGCTGTAAGGCTGTGGGCCCGAGAGAAGTCGCTCTCTC
TCGCCCTTCACATCCCTCTCGGCATTGAGATGACCATAGGCCAGAGGTGAGTCCTTAAGTGGACACAG
+
FFFFFFFFFF:FFFFFFFFFFFFFFFFFFFFFFFFFFFFFFFFFFFFFFFFFFFFFFFFFFFFFFFFF
FFFFFFFF:F:FFFFFFFFFFFFFF:FFFFFFFFFFFFFFFFFFFFFFFFFFFFFFFFFFFFFFFFFF
@A00155:342:HHGFNDSXY:1:2352:29143:8343 1:N:0:GAACCTAG+TCCGCATA
CTTCTCTCGGGCCCACAGCCTTACAGCTGTGGAGCACGGTATCCTCTGCCGAAACAGAGGTTGGACAA
GACCGGAGGGGTCTCCTAGTTCCAAAGGAGATGTACTCCGGGCTTGTTCACGACCTACCGTGTAAGTC
+
FFFFFFFFFFFFFFFFFFFFFFFFFFFFFFFFFFFFFFFFFFFFFFFFFFFFFFFFFFFFFFFFFFFF
FFFF:FFFFFFFFFFFFFFFFFFFFFFFFFFFFFFFFFFFFFFFFFFFFFFFFFFFFFF:FFFFFFF
@A00155:342:HHGFNDSXY:1:2352:13160:16423 1:N:0:GAACCTAG+TCCGCATA
GAGGGCGAGAGAGAGCGACTTCTCTCGGGCCCACAGCCTTACAGCTGTGGAGCACGGTATCCTCTGCC
GAAACAGAGGTTGGACAAGACCGGAGGGGTCTCCTAGTTCCAAAGGAGATGTACTCCGGGCTTGTTCA
+
FFFFFFFFFFFFFFFFFFFFFFFFFFFFFFFFFFFFFFFFFFFFFFFFFFFFFFFFFFFFFFFFFFFF
FFFFFFFFFFFFFFFFFFFFFFFFFFFFFFFFFFFFFFFFFFFFFFFFFFFFFFFFFFFFFFFFFFFF
@A00155:342:HHGFNDSXY:1:2352:18774:22420 1:N:0:GAACCTAG+TCCGCATA
GAGAGCGACTTCTCTCGGGCCCACAGCCTTACAGCTGTGGAGCACGGTATCCTCTGCCGAAACAGAGG
TTGGACAAGACCGGAGGGGTCTCCTAGTTCCAAAGGAGATGTACTCCGGGCTTGTTCACGACCTACCG
+
FFFFFFFFFFFF,:FFFF:FF,,FFFFFFFFFFFFFFFFFFF,:FFFFFFF,FFFF:FFFFFFF:F:F
FFFFFFFFFFFFFFFF,FFFF:,F:F,:FF,FFF:,FFFFFFF,FFF,FFFF,:FFFFF,F,F,FF,F
@A00155:342:HHGFNDSXY:1:2353:12572:5760 1:N:0:GAACCTAG+TCCGCATA
CCACATCCCTCTCGGCATTGAGATGACCATAGGCCAGAGGTGAGTCCTTAAGTGGACACAGCTGATCT
AAGGCGGTGTGGCGGGGCATGGGTTTGAACCCCCATGACGGTCGGAGTCTAGTAGGCTCCCTGATGAG
+
FFFFFFFFFFFFFFFFFFFFFFFFFFFF:FFFFFFFFFFFFFFFFFFFFFFFFFFFFFFFFFFFFFFF
,FFFFFFFFFFFFFFFFFFFFFFFFFFFFFFFFFFFFFFFFFFFFFFFFFFFFFFFFFFFFFFFFFFF
@A00155:342:HHGFNDSXY:1:2353:5394:6104 1:N:0:GAACCTAG+TCCGCATA
GGCCTATGGTCATCTCAATGCCGAGAGGGATGTGGAGGGCGAGAGAGAGCGACTTCTCTCGGGCCCAC
AGCCTTACAGCTGTGGAGCACGGTATCCTCTGCCGAAACAGAGGTTGGACAAGACCGGAGGGGTCTCC
+
FFFFFFFFFFFFFFFFFFFFFFFFFFFFFFFFFFFFFFFFFFFFFFFFFFFFFFFFFFFF:FFFFFFF
FFFFFF::FFFFFFFFFFFFFFFFFFFFFFFFFFFFFFFFFFFFFFFFFFFFFFFFFFFF:FFFFFFF
@A00155:342:HHGFNDSXY:1:2353:29315:13119 1:N:0:GAACCTAG+TCCGCATA
ACCCCTCCGGTCTTGTCCAACCTCTGTTTCGGCAGAGGATACCGTGCTCCACAGCTGTAAGGCTGTGG
GCCCGAGAGAAGTCGCTCTCTCTCGCCCTCCACATCCCTCTCGGCATTGAGATGACCATAGGCCAGAG
+
```

FFFFFFFFFFFFFFFFFFFFFFFFFFFFFFFFFFFFFFFFFFFFFFFFFFFFFFFFFFFFFFFFFF:
FFFFFFFFFFFFFFFFFFFFFFFFFFFFFFFFFFFFFFFFFFFFF:FFFFFFFFFFFFFFFFFFFFF
@A00155:342:HHGFNDSXY:1:2353:14018:14434 1:N:0:GAACCTAG+TCCGCATA
ACTTCTCTCGGGCCCACAGCCTTACAGCTGTGGAGCACGGTATCCTCTGCCGAAACAGAGGTTGGACA
AGACCGGAGGGGTCTCCTGGTTCCAAAGGAGATGTACTCCGGGCTTGTTCACGACCTACCGTGTAAGT
+
FFFFFFFFFFFFFFFFFFFFFFFFFFFFFFFFFFFFFFFFFFFFFFFFFFFFFFFFFFFFFFFFFFF
FFFFFFFFFFFFFFFFFFFFFFFFFFFFFFFFFFFFFFFFFFFFFFFFFFFFFFFFFFFFFFFFFFF
@A00155:342:HHGFNDSXY:1:2353:15302:18192 1:N:0:GAACCTAG+TCCGCATA
GTCCACTTAAGGACTCACCTCTGGCCTATGGTCATCTCAATGCCGAGAGGGATGTGGAGGGCGAGAGA
GAGCGACTTCTCTCGGGCCCACAGCCTTACAGCTGTGGAGCACGGTATCCTCTGCCGAAACAGAGGTT
+
FFFFFFFFFF:FFFFFFFFFFFFFFFF:FFFFFFFFFFFFFFFFFFFFFFFFFFFFFFFFFFFFFFF
F:FFFFFFFFFFFFFFFFFFFFFFFFFFFFFFFFFFFFFFFFFFFFFFFFFFFFFFFFFFFFFFFFF
@A00155:342:HHGFNDSXY:1:2353:20699:21308 1:N:0:GAACCTAG+TCCGCATA
GAGCGACTTCTCTCGGGCCCACAGCCTTACAGCTGTGGAGCACGTTATCCTCTGCCGAAACAGAGGTT
GGACAAGACCGGAGGGGTCTCCTAGTTCCAAAGGAGATGTACTCCGGGCTTGTTCACGACCTACCGTG
+
FFFFFFFFFFFFFFFFFFFFFFFFFFFFFFFFFFFFFFFFFFFFFFFFFFFFFFFFFFFFFFFFFFF
FFFFFFFFFFFFFFFFFFFFFFFFFFFFFFFFFFFFFFFFFFFFFFFFFFFFFFFFFFFFFFFFFFF
@A00155:342:HHGFNDSXY:1:2353:5837:34585 1:N:0:GAACCTAG+TCCGCATA
AGCGACTTCTCTCGGGCCCACAGCCTTACAGCTGTGGAGCACGGTATCCTCTGCCGAAACAGAGGTTG
GACAAGACCGGAGGGGTCTCCTGGTTCCAAAGGAGATGTACTCCGGGCTTGTTCACGACCTACCGTGT
+
FFFFFFFFFFFFFFFFFFFFFFFFFFFFFFFFFFFFFFFFFFFFFFFFFFFF:FFFFFFFFFFFFF,
FFFFFFFF:FFFFFF,:FFFFFFFFFFFFFFFFFFFFFFFFFFF:FFFFFF:FFFFFFF:::,F,FF
@A00155:342:HHGFNDSXY:1:2354:12066:2065 1:N:0:GAACCTAG+TCCGCATA
CAATGCCGAGAGGGATGTAGAGGGCGAGAGAGAGCGACTTCTCTCGGGCCCACAGCCTTACAGCTGTG
GAGCACGGTATCCTCTGCCGAAACAGAGGTTGGACAAGACCGGAGGGGTCTCCTAGTTCCAAAGGAGA
+
FFFFFFFFFFFFFFFFFFFFFFFFFFFFFFFFFFFFFFFFFFFFFFFFFFFFFFFFFFFFFFFFFFF
FFFF:FFFFFFFFFFFFFFFFFFFFFFFFFFFFFFFFFFFFFFFFFFFFFFFFFFFFFFF:FFFFFF
@A00155:342:HHGFNDSXY:1:2354:21658:3959 1:N:0:GAACCTAG+TCCGCATA
GCTGTGTCCACTTAAGGACTCACCTCTGGCCTATGGTCATCTCAATGCCGAGAGGGATGTAGAGGGCG
AGAGAGAGCGACTTCTCTCGGGCCCACAGCCTTACAGCTGTGGAGCACGGTATCCTCTGCCGAAACA
+
FFFFFF,:FFFF,FFF:FFFFFFFFFF:,:F,F,FFFFF:FFFFF,::F:FFFFF,F:F:FFFF:F,F
::F:FFFFF,FF,,F:FFFFF:,F,FFFF:,,FFFF,FF:F::FF,FFF,,:FFFFFF,:::FFFFF
@A00155:342:HHGFNDSXY:1:2354:32099:17754 1:N:0:GAACCTAG+TCCGCATA
GCGACTTCTCTCGGGCCCACAGCCTTACAGCTGTGGAGCACGGTATCCTCTGCCGAAACAGAGGTTGG
ACAAGACCGGAGGGGTCTCCTAGTTCCAAAGGAGATGTACTCCGGGCTTGTTCACGACCTACCGTGTA
+
FFFFFFFFFFFFFFFFFFFFFFFFF:FFFFFFFFFFFFFFFFFFFFFFFFFFFFFFFFFFFFFFFFF
FFFFFFFFFFFFFFFFFFFFFFFFFFFFFFFFFFFFFFFFFFFFFFFFFFFFFFFFFFFFFFF:FFF
@A00155:342:HHGFNDSXY:1:2354:6234:19617 1:N:0:GAACCTAG+TCCGCATA
GTCATCTCAATGCCGAGAGGGATGTGGAGGGCGAGAGAGAGCGACTTCTCTCGGGCCCACAGCCTTAC
AGCTGTGGAGCACGGTATCCTCTGCCGAAACAGAGGTTGGACAAGACCGGAGGGGTCTCCTAGTTCCA
+
FFFFFFFFFFFFFFFFF:FFFFFFFFFFFFFFFFFFFFFFFFFFFFFFFFFFFFFFFFFFFFFFFFF
FFFFFFFFFFFFFFFFFFFFFFFFFFFFFFFFFF:FFFFFFFFFFFFFFFFFFFFFFFFFFFFFFFF
@A00155:342:HHGFNDSXY:1:2354:23827:26788 1:N:0:GAACCTAG+TCCGCATA
GAGAGAGCGACTTCTCTCGGGCCCACAGCCTTACAGCTGTGGAGCACGGTATCCTCTGCCGAAACAGA
GGTTGGACAAGACCGGAGGGGTCTCCTAGTTCCAAAGGAGATGTACTCCGGGCTTGTTCACGACCTAC
+

FFFFFFFFFFFFFFFFFFFFFFFFFFFFFFFFFFFFFFFFFFFFFFFFFFFFFFFFFFFFFFFFFFFF
FFFFFFFFFFFFFFFFFFFFFFFFFFFFFFFFFFFFFFFFFFFFFFFFFFFFFFFFFFFFFFFFFFFF
@A00155:342:HHGFNDSXY:1:2356:27760:20290 1:N:0:GAACCTAG+TCCGCATA
ACTTCTCTCGGGCCCACAGCCTTACAGCTGTGGAGCACGGTATCCTCTGCCGAAACAGAGGTTGGACA
AGACCGGAGGGGTCTCCTAGTTCCAAAGGAGATGTACTCCGGGCTTGTTCACGACCTACCGTGTAAGT
+
FFFFFFFFFFFFFFFFFFFFFFFFFFFFFFFFFFFFFFFFFFFFFFFFFFFFFFFFFFFFFFFFFFFF
FFFFFFFFFFFFFFFFFFFFFFFFFFFFFFFFFFFFFFFFFFFFFFFFFFFFFFFFFFFFFFFFFFF:
@A00155:342:HHGFNDSXY:1:2357:12156:1031 1:N:0:GAACCTAG+TCCGCATA
TGGTCATCTCAATGCCGAGAGGGATGTGGAGGGCGAGAGAGAGCGACTTCTCTCGGGCCCACAGCCTT
ACAGCTGTGGAGCACGGTATCCTCTGCCGAAACAGAGGTTGGACAAGACCGGAGGGGTCTCCTAGTTC
+
FFFFFFFFFFFFFFFFFFFFFFFFFFFFFFFFFFFFFFFFFFFFFFFFFFFFFFFFFFFFFFFFFFF:
FFFFFFFFFFFFFFFFFFFFF,FFFFFFFFFFFFFFFFFFFFFFFFFFFFFFFFFFFFFFFFFFFFFF
@A00155:342:HHGFNDSXY:1:2357:6822:2691 1:N:0:GAACCTAG+TCCGCATA
TGCCGAGAGGGATGTGGAGGGCGAGAGAGAGCGACTTCTCTCGGGCCCACAGCCTTACAGCTGTGGAG
CACGGTATCCTCTGCCGAAACAGAGGTTGGACAAGACCGGAGGGGTCTCCTAGTTCCAAAGGAGATGT
+
FFFFFFFFFFFF:FFFFFFFFFFFFFFFFFFFFFFFFFFFFFFFFFFFFFFFFFFFFFFFFFFFFFFF
FFFFFFFFFFFFFFFFFFFFFFFFFFFFFFFFFFFFFFFFFFFFFFFFFFFFFFFFFFFFFFFFFFFF
@A00155:342:HHGFNDSXY:1:2357:29487:5462 1:N:0:GAACCTAG+TCCGCATA
CCTCTGGCCTATGGTCATCTCAATGCCGAGAGGGATGTGAAGGGCGAGAGAGAGCGACTTCTCTCGGG
CCCACAGCCTTACAGCTGTGGAGCACGGTATCCTCTGCCGAAACAGAGGTTGGACAAGACCGG
+
FFFFFFFFFFFFFFFFFFFFFFFFFFFF,FFFFFFFFFFFFFFFFFFFFFFFFFFFF::FFFFFFFFF
FF::FFFFFFFFFFFFFFFFFFFF:FF,F:FFFF,FFFFFFF,FFF:F,,:FF:FFFFFFFFF
@A00155:342:HHGFNDSXY:1:2357:15221:13698 1:N:0:GAACCTAG+TCCGCATA
AGCGACTTCTCTCGGGCCCACAGCCTTACAGCTGTGGAGCACGGTATCCTCTGCCGAAACAGAGGTTG
GACAAGACCGGAGGGGTCTCCTAGTTCCAAAGGAGATGTACTCCGGGCTTGTTCACGACCTACCGTGT
+
FFFFFFFFFFFFFFFFFFFFFFFFFFF:FFFFFFFFFFFFFFFFFFFFFFFFFFFFFFFFFFFFFFFF
FFFFFFFFFFFFFFFFFFFFFF:FFFFFFFFFFFFFFFFFFFFFFFFFFFFFFFFFFFFFFFFFFFFF
@A00155:342:HHGFNDSXY:1:2357:2284:15217 1:N:0:GAACCTAG+TCCGCATA
GCGACTTCTCTCGGGCCCACAGCCTTACAGCTGTGGAGCACGGTATCCTCTGCCGAAACAGAGGTTGG
ACAAGACCGGAGGGGTCTCCTAGTTCCAAAGGAGATGTACTCCGGGCTTGTTCACGACCTACCGTGTA
+
FFFFFFFFFFFFFFFFFFFFFFFFFFFFFF:FFFFFFFFFFFFFFFFFFF:FFFFFFFFFFFF:FFFF
FFFF,FFFFF:FFFFFFFFFFFFFFFFF::FFFFFFFF::FFFFFF:FFFFF:FFF::FFFFF:F:FF
@A00155:342:HHGFNDSXY:1:2357:9652:27821 1:N:0:GAACCTAG+TCCGCATA
CTCAATGCCGAGAGGGATGTAGAGGGCGAGAGAGAGCGACTTCTCTCGGGCCCACAGCCTTACAGCTG
TGGAGCACGGTATCCTCTGCCGAAACAGAGGTTGGACAAGACCGGAGGGGTCTCCTAGTTCCAAAGGA
+
FFFFFFFFFFFF:FFFFFFFFFFFFFFF:FFFFFFFFFFFFFFFFFFFFFFFFFFFFFFFFFFFFFFF
FFFFFFFFFFFFFFFFFFFFFFFFFFFFFFFFFFFFFFFFFFFFFFFFFFFFFFFFFFFFFFFFFFFF
@A00155:342:HHGFNDSXY:1:2357:28058:34522 1:N:0:GAACCTAG+TCCGCATA
GTCATCTCAATGCCGAGAGGGATGTAGAGGGCGAGAGAGAGCGACTTCTCTCGGGCCCACAGCCTTAC
AGCTGTGGAGCACGGTATCCTCTGCCGAAACAGAGGTTGGACAAGACCGGAGGGGTCTCC
+
FFFFFFFFFFFFFFFFFFFFFFFFFFFFFFFFFFFFFFFFFFFFFFFFFFFFFFFFFFFFFFFFFFFF
FFFFFFFFFFFFFFFFFFFFFFFFFFFFFFFFFFFFFFFFFFFFFFFFFFFFFFFFFFFF
@A00155:342:HHGFNDSXY:1:2358:31168:6277 1:N:0:GAACCTAG+TCCGCATA
GTGTGGCGGGGCATGGGTTTGAACCCCCATGACGGTCGGAGTCTAGTAGGCTCCCTGATGAGTCCGTT
CCAAGGACGAAACCGTCGTTACTCTTTGAGTTGTGTAAGTTTCGTCCTAAAAAGGACTCGTCAGGTAG
+

FFFF:FFFFFFFFFFFFFFFFFFFFFFFFFFFFFFFFFFFFFFFFFFFFFFFFFFFFFFFFFFFFFFF
FFFFFFFFFFFFFFFFFFFF:FFFFF:FFFFFF:FFFFFFFFFFFFF::FFFFF:FFFFFFFFFFF:F
@A00155:342:HHGFNDSXY:1:2358:15745:6903 1:N:0:GAACCTAG+TCCGCATA
GGATGTGGAGGGCGAGAGAGAGCGACTTCTCTCGGGCCCACAGCCTTACAGCTGTGGAGCACGGTATC
CTCTGCCGAAACAGAGGTTGGACAAGACCGGAGGGGTCTCCTGGTTCCAAAGGAGATGTACTCC
+
FFFFFFFFFFFFFFFFFFFFFFFFFFFFFFFFFFFFFFFFFFFFFFF:FFFFFFFFFFFFFFFFFFFF
FFFFFFFFFFF:FFFFFFFFFFFFFFFFFFFFFFFFFFFFF:FF:FFFFFFFFFFFFFFFFFFF
@A00155:342:HHGFNDSXY:1:2358:24786:7560 1:N:0:GAACCTAG+TCCGCATA
GGGCGAGAGAGAGCGACTTCTCTCGGGCCCACAGCCTTACAGCTGTGGAGCACGGTATCCTCTGCCGA
AACAGAGGTTGGACAAGACCGGAGGGGTCTCCTAGTTCCAAAGGAGATGTACTCCGGGCTTGTTCACG
+
FFFFFFFFFFFFFFFFFFFFFFFFFFFFFFFFFFFFFFFFFFFFFFFFFFFFFFFFFFFFFFFFFFFF
FFFFFFFFFFFFFFFFFFFFFFFFFFFFFFFFFFFFFFFFFFFFFFFFFFFFFFFFFFFFFFFFFFFF
@A00155:342:HHGFNDSXY:1:2358:16486:26287 1:N:0:GAACCTAG+TCCGCATA
GAGAGGGATGTGGAGGGCGAGAGAGAGCGACTTCTCTCGGGCCCACAGCCTTACAGCTGTGGAGCACG
GTATCCTCTGCCGAAACAGAGGTTGGACAAGACCGGAGGGGTCTCCTAGTTCCAAAGGAGATGTACTC
+
FFFFFFFFFF:FFFFFFFFFFFFFFFFFFFFFFFFFFFFFFFFFFFFFFFFFFFFFFFFFFFFFFFFF
FFFFFFFFFFFFFFFFFFFFFFFFFFFFF:FFFFFFFFFFFFFFFFFFFFFFFFFFFFFFF:FFFFFF
@A00155:342:HHGFNDSXY:1:2358:30300:29700 1:N:0:GAACCTAG+TCCGCATA
CTCTGGCCTATGGTCATCTCAATGCCGAGAGGGATGTGAAGGGCGAGAGAGAGCGACTTCTCTCGGGC
CCACAGCCTTACAGCTGTGGAGCACGGTATCCTCTGCCGAAACAGAGGTTGGACAAGACCGG
+
FFFFFFFFFFFFFFFFFFFFFF::FFFFFFFFFFFFFFFF:FFFFFFFFFFFFFFFFFFFFFFFFFFF
FFFFFFFFFFFFFFFFFFFFFFFFFFFFFFFFFFFFFFFFFFFFFFFFFFFFFFFFFFFFFF
@A00155:342:HHGFNDSXY:1:2359:10737:6903 1:N:0:GAACCTAG+TCCGCATA
GTGTCCACTTAAGGACTCACCTCTGGCCTATGGTCATCTCAATGCCGAGAGGGATGTAGAGGGCGAGA
GAGAGCGACTTCTCTCGGGCCCACAGCCTTACAGCTGTGGAGCACGGTATCCTCTGCCGAAACAGAG
+
F:FFFFFFF,FFFFFF,FFFFFFFFFFFFFFFFFFFFFFFFFFFF:FFFFFFF:FFFFFFFF,FFFFF
FFFF:FFFFFFFFFFFFFFFFFFFFF:FFF:FF:F:F,F:FFFFFFFFFFFFF,FFF:FFFFFFFFF
@A00155:342:HHGFNDSXY:1:2359:2663:11992 1:N:0:GAACCTAG+TCCGCATA
ATCTCAATGCCGAGAGGGATGTAGAGGGCGAGAGAGAGCGACTTCTCTCGGGCCCACAGCCTTACAGC
TGTGGAGCACGGTATCCTCTGCCGAAACAGAGGTTGGACAAGACCGGAGGGGTCTCCTAGTTCCAAAG
+
FFFFFFFFFFFFFF:FFFF,FF:FFFFFFFFFFFFFFFFFFFFFFFFFFFFFFFFFFFFFFFFFFFFF
FFFFFFFFFFFFFFFFFFFFFFFFFFFFFFFFFFFFFFFF,FFFFFFFFFFFFFFFFFFFFFFFFFFF
@A00155:342:HHGFNDSXY:1:2359:2492:24032 1:N:0:GAACCTAG+TCCGCATA
AGAGAGAGCGACTTCTCTCGGGCCCACAGCCTTACAGCTGTGGAGCACGGTATCCTCTGCCGAAACAG
AGGTTGGACAAGACCGGAGGGGTCTCCTAGTTCCAAAGGAGATGTACTCCGGGCTTGTTCACGACCT
+
FFFFFFFFFFFFFFFFFFFFFFFFFFFFFFFFFFFFF:FFFFFFFFFFFFFFFFFFFF:FFFFFFFFF
FFFFFFFFFFF:FFFFFFFFFFFFFFFFFFFFFFFFFFFFFFFFFFFFF:F:FFFFFFFFFF:FFFF
@A00155:342:HHGFNDSXY:1:2359:22761:32800 1:N:0:GAACCTAG+TCCGCATA
CTTCTCTCGGGCCCACAGCCTTACAGCTGTGGAGCACGGTATCCTCTGCCGAAACAGAGGTTGGACAA
GACCGGAGGGGTCTCTTAGTTCCAAAGGAGATGTACTCCGGGCTTGTTCACGACCTACCGTGTAAGTC
+
FFFFFFFFFFFFFFFFFFFFFFFFFFFFFFFFFFFFFFFFFFFFFFFFFFFFFFFFFFFFFFFFFFFF
:FFFFFFFFFFFFFFFFFFFFFFFFFFFFFFFFFFFFFFFFFFFFFFFFFFFFFFFFFFFFFFFFFFF
@A00155:342:HHGFNDSXY:1:2360:29966:3349 1:N:0:GAACCTAG+TCCGCATA
GAGCGACTTCTCTCGGGCCCACAGCCTTACAGCTGTGGAGCACGGTATCCTCTGCCGAAACAGAGGTT
GGACAAGACCGGAGGGTTCTCCTAGTTCCAAAGGAGATGTACTCCGGGCTTGTTCACGACCTACCGTG
+

FFFFFFFFFFFFFFFFFFFFFFFFFFFFFFFFFFFFFFFFFFFFFFFFFFFFFFFFFFFFFFFFFFFFFF
FFFFFFFFFFFFFFFFFFFFFFFFFFFFFFFFFFFFFFFFFFFFFFFFFFFFFFFFFFFFFFFFFFFF:F
@A00155:342:HHGFNDSXY:1:2360:26729:11240 1:N:0:GAACCTAG+TCCGCATA
GAGCGACTTCTCTCGGGCCCACAGCCTTACAGCTGTGGAGCACGGTATCCTCTGCCGAAACAGAGGTT
GGACAAGACCGGAGGGGTCTCCTGGTTCCAAAGGAGATGTACTCCGGGCTTGTTCACGACCTACCGTG
+
FFFFFFFFFFFFFFFFFFFFFFFFFFFFFFFFFFFFFFFFFFFFFFFFFFFFFFFFFFFFFFFFF:FFFF
FFFFFFFFFFFFFFFFFFFFFFFFFFFFFFFFFFFFFFFFFFFFFFFFFFFFFFFFFFFFFFFFFFFFFF
@A00155:342:HHGFNDSXY:1:2360:21658:24064 1:N:0:GAACCTAG+TCCGCATA
ATGCCGAGAGGGATGTGGAGGGCGAGAGAGAGCGACTTCTCTCGGGCCCACAGCCTTACAGCTGTGGA
GCACGGTATCCTCTGCCGAAACAGAGGTTGGACAAGACCGGAGGGGTCTCCTAGTTCCAAAGGAGATG
+
FFFFFFFFFFFFFFFFFFFFFFFFFFFFFFFFFFFFFFFFFFFFFFFFFFFFFFFFFFFFFFFFFFFFFF
FFFFFFFFFFFFFFFFFFFFFFFFFFFFFFFFFFFFFFFFFFFFFFFFFFFFFFFFFFFFFFFFFFFFFF
@A00155:342:HHGFNDSXY:1:2360:29704:26506 1:N:0:GAACCTAG+TCCGCATA
TGGTCATCTCAATGCCGAGAGGGATGTGGAGGGCGAGAGAGAGCGACTTCTCTCGGGCCCACAGCCTT
ACAGCTGTGGAGCACGGTATCCTCTGCCGAAACAGAGGTTGGACAAGACCGGAGGGGTCTCCTAGTTC
+
FFFFFFFFFFFFFF:FFFFFFFFF:FFFFFFFFFFFFFFFFFFFFFFFF:FFFFFFFFFFFFFFFFFFFF
FFFFFFFFFFFFFFFFFFFFFFFFFFFFFFFFFFFFFFFFFFFFFFFFFFFFFFFFFFFFFFFFFFFFFF
@A00155:342:HHGFNDSXY:1:2361:13711:11240 1:N:0:GAACCTAG+TCCGCATA
ACCTCTGTTTCGGCAGAGGATACCGTGCTCCACAGCTGTAAGGCTGTGGGCCCGAGAGAAGTCGCTCT
CTCTCGCCCTTCACATCCCTCTCGGCATTGAGATGACCATAGGCCAGAGGTGAGTCCTTAAGTGGACA
+
FFFFFFFFFFFFFFFFFFFFF:FFFFFFFFFFFFFFFFFFFFFFFFFFFFFFFFFFFFFFFFFFFFFFFF
FFFFFFFFFFFFFFFFFFFFFFFFFFFFFFFFFFFFFFFFFFFFFFFFFFFFFFFFFFFFFFFFFFFFFF
@A00155:342:HHGFNDSXY:1:2361:19361:30170 1:N:0:GAACCTAG+TCCGCATA
TGTGGAGGGCGAGAGAGAGCGACTTCTCTCGGGCCCACAGCCTTACAGCTGTGGAGCACGGTATCCTC
TGCCGAAACAGAGGTTGGACAAGACCGGAGGGGTCTCCTAGTTCCAAAGGAGATGTACTCCGGGCTTG
+
:FFFFFFFFFFFFFFFFFFFFFFFFFFFFFFFFFFFFFFFFFFFFFFFFFFFFFFFFFFFFFFFFFFFFF
FFFFFFFFFFFFFFFFFFFFFFFFF:FFFFFFFFFFFFFFFFFFFFFFFFFFFFFFFFFFFFFFFFFFFF
@A00155:342:HHGFNDSXY:1:2361:13621:31469 1:N:0:GAACCTAG+TCCGCATA
AGCCTTACAGCTGTGGAGCACGGTATCCTCTGCCGAAACAGAGGTTGGACAAGACCGGAGGGGTCTCC
TAGTTCCAAAGGAGATGTACTCCGGGCTTGTTCACGACCTACCGTGTAAGTCGTAGTCTAGTAGGCTA
+
FFFFFFFFFFFFFFFFFFFFFFFFFFFFFFFFFFFFFFFFFFFFFFFFFFFFFFFFFFFFFFFFFFFFFF
FFFFFFFFFFFFFFFFFFFFFFFFFFFFFFFFFFFFFFFFFFFFFFFFFFFFFFFFFFFFFFFFFFFFFF
@A00155:342:HHGFNDSXY:1:2361:31801:37027 1:N:0:GAACCTAG+TCCGCATA
TCTCTCGCCCTCCACATCCCTCTCGGCATTGAGATGACCATAGGCCAGAGGTGAGTCCTTAAGTGGAC
ACAGCTGATCTAAGGCGGTGTGGCGGGGCATGGGTTTGAACCCCCATGACGGTCGGAGTCTAGTAGGC
+
FFFF:FFFFFFFFFFFF:FFFFFFF:FFFF:FFFFFF:FFFFFFFFFF:FFFFFFFFFF:FFFFFFF:FF
F:FF:FFFFFFFFFFFFFF:FFFF:FFFFFFFFFFFFFFF:FFFFFFFFFFFFFFFFFFFFFFFFFFFFF
@A00155:342:HHGFNDSXY:1:2362:20518:1767 1:N:0:GAACCTAG+TCCGCATA
CGTCCTAAAAAGGACTCGTCAGGTAGCCTACTAGACTACGACTTACACGGTAGGTCGTGAACAAGCCC
GGAGTACATCTCCTTTGGAACTAGGAGACCCCTCCGGTCTTGTCCAACCTCTGTTTCGGCAGAGGATA
+
FFFFFFFFFFFFFFFFFFFFFFFFFFFFFFFFFFFFFFFFFFFFFFFFFFFFFFFFFFFFFFFFFFFFFF
FFFFFFFFFFFFFFFFFFF:FFFFFFFFFFFFFFFFFFFFFFFFFFFFFFFFFFFFFFFFFFFFFFFFFF
@A00155:342:HHGFNDSXY:1:2362:32253:25254 1:N:0:GAACCTAG+TCCGCATA
CGAGAGGGATGTGGAGGGCGAGAGAGAGCGACTTCTCTCGGGCCCACAGCCTTACAGCTGTGGAGCAC
GGTATCCTCTGCCGAAACAGAGGTTGGACAAGACCGGAGGGGTCTCCTAGTTCCAAAGGAGATGTACT
+

FFFF,FFFFFFFFFFFFF:FFFFF:FF:FFFF:FFFFFFFFFFFFFFFFFFFFFFFFFFFFFFFFFFF
FFF:FFFF:FFFFFFFFFFFFFFFFFFFFFF,FFFFFFFFFFFFFFFFFFFFFFF:FFFFFFFFFFFF
@A00155:342:HHGFNDSXY:1:2362:6515:34162 1:N:0:GAACCTAG+TCCGCATA
GGATGTAGAGGGCGAGAGAGAGCGACTTCTCTCGGGCCCACAGCCTTACAGCTGTGGAGCACGGTATC
CTCTGCCGAAACAGAGGTTGGACAAGACCGGAGGGGTCTCCTAGTTCCAAAGGAGATGTACTCC
+
FFFFFFFFFFFFFFFFFFFFFFF:FFFFFFFFFFFFFFFFFFFFFFFFFFFFFFFFFFFFF,F:FFFF
FFF:F:FFFFFFFFFF:FFFFFFFFFFFFF:FFF:FFFFFFFFFFFFFFF:FFFFFFFFFFFFF
@A00155:342:HHGFNDSXY:1:2362:6506:34178 1:N:0:GAACCTAG+TCCGCATA
GGATGTAGAGGGCGAGAGAGAGCGACTTCTCTCGGGCCCACAGCCTTACAGCTGTGGAGCACGGTATC
CTCTGCCGAAACAGAGGTTGGACAAGACCGGAGGGGTCTCCTAGTTCCAAAGGAGATGTACTCC
+
FFFFFFFFFFFFFFFFFFFFFFFFFFFFFFFFFFFFFFFFFFFFFFFFFF:FFF:FFFFFFFFFFFFF
FFFFFFFFFFFFFFFFFFFFFFFFFFFFFFFF:FFFFFFF,FFFFFFFFFFFFFFFFFFFFFFF
@A00155:342:HHGFNDSXY:1:2362:31946:36871 1:N:0:GAACCTAG+TCCGCATA
TTCTCTCGGGCCCACAGCCTTACAGCTGTGGAGCACGGTATCCTCTGCCGAAACAGAGGTTGGACAAG
ACCGGAGGGGTCTCCTAGTTCCAAAGGAGATGTACTCCGGGCTTGTTCACGACCTACCGTGTAAGTCG
+
FFFFFFFFF:FFF:FF:FFF:FFFFFF:FF,:FF:FFFFFFFFFFFF:FFFFFFF:FFF:FFFFFFFF
FFFF:::FF:FFFFFFFFFFFF:FFF:FF:FFFFFFFFF::F:FF,FF:F:FFFFFFFFFF:F,:FFF
@A00155:342:HHGFNDSXY:1:2363:16848:34178 1:N:0:GAACCTAG+TCCGCATA
TACCGTGTAAGTCGTAGTCTAGTAGGCTACCTGACGAGTCCTTTTTAGGACGAAACTTACACAACTCG
AAGAGTAACGACGGTTTCGTCCTTGGAACGGACTCATCAGGGAGCCTACTAGACTCCGACCGTCATGG
+
FFFFF,FFFFFFFFFFFFFFFFF:FFFFFFFFFFFFFFFFF:FFFFFFFFFF:FFF:FFFFFFFF:FF
FFFFFFFFFFFFFFFFFFFFFFFFFFFFFFFFFFFFFFFFFFFFFF:FFF,FFFFFFFFFFFFFF:FF
@A00155:342:HHGFNDSXY:1:2364:3712:8484 1:N:0:GAACCTAG+TCCGCATA
GTCATCTCAATGCCGAGAGGGATGTGGTGGGCGAGAGAGAGCGACTTCTCTCGGGCCCACAGCCTTAC
AGCTGTGGAGCACGGTATCCTCTGCCGAAACAGAGGTTGGACAAGACCGGAGGGGTCTCCTAGTTCCA
+
FFFFFFFFFFFFFFFFF:FFFFFFFFFFFFFFFFFFFFFFFFFF::FFFFFFFFFFFFFFFFFFFFFF
FFFFFFFFFFFFFFFFFFFFFFFFFFFFFFFFFFFFFFFFFFFFFFFFFFFFFFFFFFFF:FFFFFF:
@A00155:342:HHGFNDSXY:1:2364:32560:36025 1:N:0:GAACCTAG+TCCGCATA
GGGCGAGAGAGAGCGACTTCTCTCGGGCCCACAGCCTTACAGCTGTGGAGCACGGTATCCTCTGCCGA
AACAGAGGTTGGACAAGACCGGAGGGGTCTCCTGGTTCCAAAGGAGATGTACTCCGGGCTTGTTCACG
+
FFFFFFFFFFFFFFFFF:FFFFFFFFFFFFFFFFFFFFFFFFFFFFFFFFFFFFFFFFFFFFFFFFFF
FFFFFFFFFFFF,FFFFFFFFFFFFFFFFFFF,FFFFFFFFFFFFFFFFFFFFFFFFFFFFFFFFFFF
@A00155:342:HHGFNDSXY:1:2365:15501:1564 1:N:0:GAACCTAG+TCCGCATA
GAGAGGGATGTAGAGGGCGAGAGAGAGCGACTTCTCTCGGGCCCACAGCCTTACAGCTGTGGAGCACG
GTATCCTCTGCCGAAACAGAGGTTGGACAAGACCGGAGGGGTCTCCTAGTTCCAAAGGAGATGTACTC
+
FFFFFFFFFFFFFFFFFFFFFFFFFFFFFFFFFFFFFFFFFFFFFFFFFFFFFFFFFFFFFFFFFFFF
FFFFFFFFFFFFFFFFFFFFFFFFFFFFFF:FFFFFFFFFFFFFFFFFFFFFFFFFFFFFFFFFFFFF
@A00155:342:HHGFNDSXY:1:2365:18186:13009 1:N:0:GAACCTAG+TCCGCATA
TTAGATCAGCTGTGTCCACTTAAGGACTCACCTCTGGCCTATGGTCATCTCAATGCCGAGAGGGATGT
AGAGGGCGAGAGAGAGCGACTTCTCTCGGGCCCACAGCCTTACAGCTGTGGAGCACGGTATCCTCTGC
+
FFFFFFF:FFFFFFFFFFFFFFFFFFFFFFFFFFFFFFFFFFFFFFFFFFFFFFFFFFFFFFFFFFFF
FFFFFFFFFFFFFFFFFFFFFFFFFFFFFFFFFFFFFFFFFFFFFFFFFFFFFFFFFFFFFFFFFFFF
@A00155:342:HHGFNDSXY:1:2365:17526:16094 1:N:0:GAACCTAG+TCCGCATA
TCTCTCGGGCCCACAGCCTTACAGCTGTGGAGCACGGTATCCTCTGCCGAAACAGAGGTTGGACAAGA
CCGGAGGGGTCTCCTGGTTCCAAAGGAGATGTACTCCGGGCTTGTTCACGACCTACCGTGTAAGTCGT
+

FFFFFFFFFFFFFFFFFFFF,FFFFFFFFFFFFFFFFFFFFFFFFFFFFFFFFFFFFFFFFFFFFFFF
FFFFFFFFFFFFFFFFFFFFFFFFFFFFFFFFFFFFFFFFFFFFFFF:FFFFFFFFFFFF:FFFFFFF
@A00155:342:HHGFNDSXY:1:2365:7175:22842 1:N:0:GAACCTAG+TCCGCATA
CTATGGTCATCTCAATGCCGAGAGGGATGTGGAGGGCGAGAGAGAGCGACTTCTCTCGGGCCCACAGC
CTTACAGCTGTGGAGCACGGTATCCTCTGCCGAAACAGAGGTTGGACAAGACCGGAGGGGTCTCCTAG
+
FF:FFFFFFFFFFFFFFFFFFFFFFFFFFFFFFFFFFFFFFFFFFFFFFFFFFFFFFFFFFFFFFFFF
FFFFFFFFFFFFFFFFFFFFFFFFFFFFFFFFFFFFFFFFFFFFFFFFFFFFFFFFFFFFFFFFFFFF
@A00155:342:HHGFNDSXY:1:2366:23827:4805 1:N:0:GAACCTAG+TCCGCATA
GAGAGGGATGTGGAGGGCGAGAGAGAGCGACTTCTCTCGGGCCCACAGCCTTACAGCTGTGGAGCACG
GTATCCTCTGCCGAAACAGAGGTTGGACAAGACCGGAGGGGTCTCCTAGTTCCAAAGGAGATGTACTC
+
FFFFFFFFFFFFFFFFFFFFFFFFFFFFFFFFFFFFFFFFFFFFFFFFFFF:FFFFFFFFFFFFFFFF
FFFFFFFFFFFFFFFFFFFFFFFFFFFFFFFFFFFFFFFFFFFFFFFFFFFFFFFFFFFFFFFFFFFF
@A00155:342:HHGFNDSXY:1:2366:23267:12383 1:N:0:GAACCTAG+TCCGCATA
ACTTCTCTCGGGCCCACAGCCTTACAGCTGTGGAGCACGGTATCCTCTGCCGAAACAGAGGTTGGACA
AGACCGGAGGGGTCTCCTAGTTCCAAAGGAGATGTACTCCGGGCTTGTTCACGACCTACCGTGTAAGT
+
FFFFFFFFFFFFFFFFFFFFFFFFFFFFFFFFFFFFFFFFFFFFFFFFFFFFFFFFFFFFFFFFFFFF
FFFFFFFFFFFFFFFFFFFFFFFFFFFFFFFFFFFFFFFFFFFFFFFFFFFFFFFFFFFFFFFFFFFF
@A00155:342:HHGFNDSXY:1:2366:11180:17096 1:N:0:GAACCTAG+TCCGCATA
AGAGAGAGTGACTTCTCTCGGGCCCACAGCCTTACAGCTGTGGAGCACGGTATCCTCTGCCGAAACAG
AGGTTGGACAAGACCGGAGGGGTCTCCTAGTTCCAAAGGAGATGTACTCCGGGCTTGTTCACGACCTA
+
FFF:FFFFFFFFFFFFFFFFFFFFFFFFFFFFFFFFFFFFFFFFFFFFFFFFFFFFFFFFFFFFFFFF
FFFFFFFFFFF:FFFFFFFFFFFFFFFFFFFFFFFFFFFFFFFFFFFFFFFFFFFFFFFF
@A00155:342:HHGFNDSXY:1:2366:20636:23672 1:N:0:GAACCTAG+TCCGCATA
GAGAGCGACTTCTCTCGGGCCCACAGCCTTACAGCTGTGGAGCACGGTATCCTCTGCCGAAACAGAGG
TTGGACAAGACCGGAGGGGTCTCCTAGTTCCAAAGGAGATGTACTCCGGGCATGTTCACGACCTACCG
+
FFFFFFFFFFFFFFFFFFFFFFFFFFFFFFFFFFFFFFFFFFFFFFFFFFFFFFFFFFFFFFFFFFFF
FFFFFFFFFFFFFFFFFFFFFFFFFFFFFFFFFFFFFFFFFFFFFFFFFFFFFFFFFFFFFFF:FFFF
@A00155:342:HHGFNDSXY:1:2366:25889:26757 1:N:0:GAACCTAG+TCCGCATA
AGAGAGAGCGACTTCTCTCGGGCCCACAGCCTTACAGCTGTGGAGCACGGTATCCTCTGCCGAAACAG
AGGTTGGACAAGACCGGAGGGGTCTCCTGGTTCCAAAGGAGATGTACTCCGGGCTTGTTCACG
+
FFFFFFFFFFFFFFFFFFFFFFFFFFFFFFFFFFFFFFFFFFFFFFFFFFFFFFFFFFFFFFFFFFFF
FFFFFFFFFFFFFFFFFFFFFFFFFFFFFFFFFFFFFFFFFFFFFFFFFFFFFFFFFFFFFFF
@A00155:342:HHGFNDSXY:1:2366:26124:27320 1:N:0:GAACCTAG+TCCGCATA
AGAGAGAGCGACTTCTCTCGGGCCCACAGCCTTACAGCTGTGGAGCACGGTATCCTCTGCCGAAACAG
AGGTTGGACAAGACCGGAGGGGTCTCCTGGTTCCAAAGGAGATGTACTCCGGGCTTGTTCACG
+
FFFFFFFFFFFFFFFFFFFFFFFFFFFFFFFFFFFFFFFFFFFFFFFFFFFFFFFFFFFFFFFFFFFF
FFFFFFFFFFF,FFFFFFFFFFFFFFFFFFFFFFFFFFFFFFFF:FFFFFFFFFFFFFFFFFF
@A00155:342:HHGFNDSXY:1:2366:1542:28526 1:N:0:GAACCTAG+TCCGCATA
TTAGATCAGCTGTGTCCACTTAAGGACTCACCTCTGGCCTATGGTCATCTCAATGCCGAGAGGGATGT
AGAGGGCGAGAGAGAGCGACTTCTCTCGGGCCCACAGCCTTACAGCTGTGGAGCACGGTATCCTCT
+
:FFF:FFFFFFFFFF:FFFF:FFF:FFFFFFF:FFFFFFFFFFFFFFF:FFFFFFFFFFFFFFFFFFF
FF:,FFFF,FFFFFFFFFFFFFFFFFFFFFFFFFFFFFFFFFFFFFFFFFFFFFFFFFFFFFFFF
@A00155:342:HHGFNDSXY:1:2366:2835:29700 1:N:0:GAACCTAG+TCCGCATA
TTAGATCAGCTGTGTCCACTTAAGGACTCACCTCTGGCCTATGGTCATCTCAATGCCGAGAGGGATGT
AGAGGGCGAGAGAGAGCGACTTCTCTCGGGCCCACAGCCTTACAGCTGTGGAGCACGGTATCCTCT
+

```
FFFFFFFFFFFFFFF::FFF:FFFFFFFFFFFFFFFFFFFFFFFFFFFFFFFFFFFFFFFFFFFFFFF
FFFFFFFFFFFFFFFFFFFFFFFFFFFFFFFFFFFFFFFFFFFFFFFFFFFFFFFFFFFFFFFFFFFFF
@A00155:342:HHGFNDSXY:1:2366:24406:32268 1:N:0:GAACCTAG+TCCGCATA
GAGAGGGATGTAGAGGGCGAGAGAGAGCGACTTCTCTCGGGCCCACAGCCTTACAGCTGTGGAGCACG
GTATCCTCTGCCGAAACAGAGGTTGGACAAGACCGGAGGGGTCTCCTAGTTCCAAAGGAGATGTACTC
+
FFFFFFFFFFFFFFFFFFFFFFFFFFFFFFFFFFFFFFFFFFFFFFFFFFFFFFFFFFFFFFFFFFFFF
FFFFFFFFFFFFFFFFFFFFFFFFFFFFFFFF:FFFFFFFFFFFFFFFFFFFFFFFFFFFFFFFFFFFF
@A00155:342:HHGFNDSXY:1:2366:26928:34538 1:N:0:GAACCTAG+TCCGCATA
AGCGACTTCTCTCGGGCCCACAGCCTTACAGCTGTGGAGCACGGTATCCTCTGCCGAAACAGAGGTTG
GACAAGACCGGAGGGGTCTCCTAGTTCCAAAGGAGATGTACTCCGGGCTTGTTCACGACCTACCGTGT
+
FFFFFFFFFFFFFFFFFFFFFFFFFFFFFFFFFFFFFFFFFFFFFFFFFFFFFFFFFFFFFFFFFFFFF
FFFFFFFFFFFFFFFFFFFFFFFFFFFFFFFFFFFFFFFFFFFFFFFFFFFFFFFFFFFFFFFFFFFFF
@A00155:342:HHGFNDSXY:1:2366:25292:35556 1:N:0:GAACCTAG+TCCGCATA
GACTTCTCTCGGGCCCACAGCCTTACAGCTGTGGAGCACGGTATCCTCTGCCGAAACAGAGGTTGGAC
AAGACCGGAGGGGTCTCCTAGTTCCAAAGGAGATGTACTCCGGGCTTGTTCACGACCTACCGTGT
+
FFFFFFFFFFFFFFFFFFFFFFFFFFFFFFFFFFFFFFFFFFFFFFFFFFFFFFFFFFFFFFFFFFFFF
FFFFFFFFFFFFFFFFFFFFFFFFFFFFFFFFFFFFFFFFFFFFFFFFFFFFFFFFFFFFFFFFFF
@A00155:342:HHGFNDSXY:1:2368:23122:1892 1:N:0:GAACCTAG+TCCGCATA
TCCTCTGCCGAAACAGAGGTTGGACAAGACCGGAGGGGTCTCCTGGTTCCAAAGGAGATGTACTCCGG
GCTTGTTCACGACCTACCGTGTAAGTCGTAGTCTAGTAGGCTACCTGACGAGTCCTTTTTAGGACGAA
+
FFFFFFFFFFFFFFFFFFFFFFFFFFFFFFFFFFFFFFFFFFFFFFFFFFFFFFFFFFFFFFFFFFFFF
FFF:FFFFFFFFFFFFFFFFFFFFFFFFFFFFFFFFFFFFFFFFFFFFFFFFFFFFFFFFFFFFFFFFF
@A00155:342:HHGFNDSXY:1:2368:29740:4554 1:N:0:GAACCTAG+TCCGCATA
AGGTCGTGAACAAGCCCGGAGTACATCTCCTTTGGAACCAGGAGACCCCTCCGGTCTTGTCCAACCTC
TGTTTCGGCAGAGGATACCGTGCTCCACAGCTGTAAGGCTGTGGGCCCGAGAGAAGTCGCTCTCTCTC
+
FFFFFFFFFFFFFFFFFFFFFFFFFFFFFFFFFFFFFFFFFFFFFFFFFFFFFFFFFFFFFFFFFFFFF
FFFFFFFFFFFFFFFFFFFFFFFFFFFFFFFFFFFFFFFFFFFFFFFFFFFFFFFFFFFFFFFFFFFFF
@A00155:342:HHGFNDSXY:1:2368:19551:14747 1:N:0:GAACCTAG+TCCGCATA
TTACAGCTGTGGAGCACGGTATCCTCTGCCGAAACAGAGGTTGGACAAGACCGGAGGGGTCTCCTAGT
TCCAAAGGAGATGTACTCCGGGCTTGTTCACGACCTACCGTGTAAGTCGTAGTCTAGTAGGCTACCTG
+
FFFFFFFFFFFFFFFFFFFFFFFFFFFFFFFFFFFFFFFFFFFFFFFFFFFFFFFFFFFFFFFFFFFFF
FFFFFFFFFFFFFFFFFFFFFFFFFFFFFFFFFFFFFFFFFFFFFFFFFFFFFFFFFFFFFFFFFFFFF
@A00155:342:HHGFNDSXY:1:2368:18502:16344 1:N:0:GAACCTAG+TCCGCATA
TTACAGCTGTGGAGCACGGTATCCTCTGCCGAAACAGAGGTTGGACAAGACCGGAGGGGTCTCCTAGT
TCCAAAGGAGATGTACTCCGGGCTTGTTCACGACCTACCGTGTAAGTCGTAGTCTAGTAGGCTACCTG
+
FFFFFFFFFFFFFFFFFFFFFFFFFFFFFFFFFFFFFFFFFFFFFFFFFFFFFFFFFFFFFFFFFFFFF
FFFFFFFFFFFFFFFFFFFFFFFFFFFFFFFFFFFFFFFFF:FFFFFFFFFFFFFFFFFFFFFFFFFFF
@A00155:342:HHGFNDSXY:1:2368:21323:16783 1:N:0:GAACCTAG+TCCGCATA
GAGAGGGATGTGGAGGGCGAGAGAGAGCGACTTCTCTCGGGCCCACAGCCTTACAGCTGTGGAGCACG
GTATCCTCTGCCGAAACAGAGGTTGGACAAGACCGGAGGGGTCTCCTAGTTCCAAAGGAGATGTACTC
+
FFFFFFFFFFFFFFFFFFFFFFFFFFFFFFFFFFFFFFFFFFFFFFFFFFFFFFFFFFFFFFFFFFFFF
FFFFFFFFFFFFFFFFFFFFFFFFFFFFFFFFFFFFFFFFFFFFFFFFFFFFFFFFFFFFFFFFFFFFF
@A00155:342:HHGFNDSXY:1:2368:19551:23766 1:N:0:GAACCTAG+TCCGCATA
TTACAGCTGTGGAGCACGGTATCCTCTGCCGAAACAGAGGTTGGACAAGACCGGAGGGGTCTCCTAGT
TCCAAAGGAGATGTACTCCGGGCTTGTTCACGACCTACCGTGTAAGTCGTAGTCTAGTAGGCTACCTG
+
```

```
:FFFFFFFFFFFFFFFFFFF:FFFFFFFFFFFFFFFFFFFFFFFFFFFFFFFFFFFFFFFFFFFFFFF
FFFFFFFFFFFFFFFFFFFFFFFFFFFFFFFFFFFFFFFFFFFFFFFFFFFFFFFFFFFF:FFFFFFF
@A00155:342:HHGFNDSXY:1:2369:31693:7122 1:N:0:GAACCTAG+TCCGCATA
CCTTACAGCTGTGGAGCACGGTATCCTCTGCCGAAACAGAGGTTGGACAAGACCGGAGGGGTCTCCTA
GTTCCAAAGGAGATGTACTCCGGGCTTGTTCACGACCTACCGTGTAAGTCGTAGTCTAGTAGGCTACC
+
FF:FFFFFFFFFFFFFFFFFFFFFFFFFFFFFFFFFFFFFFF:FFFFFFFFFFFFFFFFFFFFFFF:F
FFFFFFFFFFFFFFFF:FFFFFFFFFFFFFFFFFFFFFFFFFFFFF,FFFFFFFFFFFFFFFFFFFFF
@A00155:342:HHGFNDSXY:1:2369:4933:8938 1:N:0:GAACCTAG+TCCGCATA
TGTCCACTTAAGGACTCTCCTCTGGCCTATGGTCATCTCAATGCCGAGAGGGATGTGGAGGGCGAGAG
AGAGCGACTTCTCTCGGGCCCACAGCCTTACAGCTGTGGAGCACGGTATCCTCTGCCGAAACAGAGGT
+
FFFFFFFFFFFFFFFFFFFFFFFFFFFFFFFFFFFFFFFFFFFFFFFFFF:FFFFFFFFFFFFFFFFF
FFFFFFFFFFFFFFFFFFFFFFFFFFF:FFFFFF:FFFFFF::FFFFFFFFFFF:FFFFFFFFFFFFF
@A00155:342:HHGFNDSXY:1:2369:28185:14794 1:N:0:GAACCTAG+TCCGCATA
CTCTCGGGCCCACAGCCTTACAGCTGTGGAGCACGATATCCTCTGCCGAAACAGAGGTTGGACAAGAC
CGGAGGGGTCTCCTAGTTCCAAAGGAGATGTACTCCGGGCTTGTTCACGACCTACCGTGTAAGTCGTA
+
FF:FFFFFFFFFFFFFFFF:FFFFFFFFFF:FFFFFFFFFFFFFFFFFFFFFFFFFFFFFFFFFFFFF
FFFFFFFFFFFFFFFFFFF:FFFFFFFFFFFFFFFFFFFFFFFFFFFF:FFFFFFFFFFFF:FFFFFF
@A00155:342:HHGFNDSXY:1:2369:25997:23406 1:N:0:GAACCTAG+TCCGCATA
CACCTCTGGCCTATGGTCATCTCAATGCCGAGAGGGATGTGGAGGGCGAGAGAGAGCGACTTCTCTCG
GGCCCACAGCCTTACAGCTGTGGAGCACGGTATCCTCTGCCGAAACAGAGGTTGGACAAGACCGGAGG
+
FFFFFFFFFFFFFFFFFFFFFFFFFFFFFFFF:FFFFFFFFFFFFFFFFFFFFFFFFFFFFFFFFFFF
FFFFFFFFFFFFFFFFFFFFFFFFFFFFFFFFFFFFFFFFFFFFFFFFFFFFFFFFFFFFFFFFFFFF
@A00155:342:HHGFNDSXY:1:2370:31738:15718 1:N:0:GAACCTAG+TCCGCATA
AAGGACTCACCTCTGGCCTATGGTCATCTCAATGCCGAGAGGGATGTGAAGGGCGAGAGAGAGCGACT
TCTCTCGGGCCCACAGCCTTACAGCTGTGGAGCACGGTATCCTCTGCCGAAACAGAGGTTGGACAAGA
+
:FFFFFFFFFFFFFFFFFFFFFFFFFFFFFFFFFFFFFFFFFFFFFFFFFFFFFFFFFFFFFFFFFFF
FFFFFFFFFFFFFFFFFFFFFFFFFFFFFFFFFFFFFFFFFFFFFFFFFF:FFFFFFFFFFFFFFFFF
@A00155:342:HHGFNDSXY:1:2370:32759:20838 1:N:0:GAACCTAG+TCCGCATA
CGACTTCTCTCGGGCCCACAGCCTTACAGCTGTGGAGGACGGTATCCTCTGCCGAAACAGAGGTTGGA
CAAGACCGGAGGGGTCTCCTAGTTCCAAAGGAGATGTACTCCGGGCTTGTTCACGACCTACCGTGTAA
+
FFFFFFFFFFFFFFFFFFFFFFFFFFFFFFFFFFFFFFFFFFFFFFFFFFFFFFFFFFFFFFFFFFFF
FFFFFFFFFFFFFFFFFFFFFFFFFFFFFFFFFFFFFFFFFFFFFFFFFFFFFFFFFFFFFFF:FFFF
@A00155:342:HHGFNDSXY:1:2370:1154:21465 1:N:0:GAACCTAG+TCCGCATA
GTGTCCACTTAAGGACTCACCTCTGGCCTATGGTCATCTCTATGCCGAGAGGGATGTGGAGGGCGAGA
GAGAGCGACTTCTCTCGGGCCCACAGCCTTACAGCTGTGGAGCACGGTATCCTCTGCCGAAACAGAGG
+
FFFFF:F:FF:FFFF:FF::FFFFFF,,FFFFFF:FFFFFFFFF:FF:FFFF:FFFFFFFFFFFFFF:
FFF:F::FFFFFFFFFFFFF:FFFF:FFFFFFFFFFFFF,FFFFFFFFFFFFFFFFF:FF::F,F:FF
@A00155:342:HHGFNDSXY:1:2370:14036:33630 1:N:0:GAACCTAG+TCCGCATA
GCTGTGTCCACTTAAGGACTCACCTCTGGCCTATGGTCATCTCAATGCCGAGAGGGATGTAGAGGGCG
AGAGAGAGCGACTTCTCTCGGGCCCACAGCCTTACAGCTGTGGAGCACGGTATCCTCTGCCGAAACAG
+
FFFFFFFFFFFFFFFFFFFFFFFFFFFFFFFFFFFFFFFFFFFFFFFFFF:FFFFFFFFFFFFFFFFF
FFFFFFFFFFFFFFFFFFFFFFFFFFFFFFFFFFFFFFFFFFFFFFFFFFFFFFFFFFFFFFFFFFFF
@A00155:342:HHGFNDSXY:1:2371:13946:1501 1:N:0:GAACCTAG+TCCGCATA
GCTGTGTCCACTTAAGGACTCACCTCTGGCCTATGGTCATCTCAATGCCGAGAGGGATGTAGAGGGCG
AGAGAGAGCGACTTCTCTCGGGCCCACAGCCTTACAGCTGTGGAGCACGGTATCCTCTGCCGAAACAG
+
```

FFFFFFFFFFFFFFFFFFFFFFFFFFFFFFFFFFFFFFFFFFFFFFFFFFFFFFFFFFFFF:FFFFFFF
FFFFFFFFFFFFFFFFFFFFFFFFFFFFFFFFFFFFFFFFFFFFFFFFFFFFFFFFFFFFFFFFFFFFF
@A00155:342:HHGFNDSXY:1:2371:5665:19539 1:N:0:GAACCTAG+TCCGCATA
GTCATCTCAATGCCGAGAGGGATGTGGAGGGCGAGAGAGAGCGACTTCTCTCGGGCCCACAGCCTTAC
AGCTGTGGAGCACGGTATCCTCTGCCGAAACAGAGGTTGGACAAGACCGGAGGGGTCTCCTGGTTCCA
+
FFFFFFFFFFFFFFFFFFFFFFFFFFFFFFFFFFFFFFFFFFFFFFFFFFFFFFFFFFFFFFFFFFFFF
FFFFFFFFFFFF:FFFFFFFFFFFFFFFFFFFFFFFFFFFFFFFFFFFFFFFFFFFFFFFFFF::FFFF
@A00155:342:HHGFNDSXY:1:2371:12500:20760 1:N:0:GAACCTAG+TCCGCATA
AGCGACTTCTCTCGGGCCCACAGCCTTACAGCTGTGGAGCACGGTATCCTCTGCCGAAACAGAGGTTG
GACAAGACCGGAGGGGTCTCCTAGTTCCAAAGGAGATGTACTCCGGGCTTGTTCACGACCTACCGTGT
+
FFFFFFFFFFFFFFFFFFFFFFFFFFFFFFFFFFFFFFFFFFFFF:FFFFFFFFFFFFFFFFFFFFFFF
FFFFFFFFFFFFFFFFFFFFFFFFFFFFFFFFFFFFFFFFFFFFFFFFFFFFFFFFFFFFFFFFFFFFF
@A00155:342:HHGFNDSXY:1:2371:12518:20791 1:N:0:GAACCTAG+TCCGCATA
AGCGACTTCTCTCGGGCCCACAGCCTTACAGCTGTGGAGCACGGTATCCTCTGCCGAAACAGAGGTTG
GACAAGACCGGAGGGGTCTCCTAGTTCCAAAGGAGATGTACTCCGGGCTTGTTCACGACCTACCGTGT
+
FFFFFFFFFFFFFFFFFFFFFFFFFFFFFFFFFFFFFFFFFFFFFFFFFFFFFFFFFFFFFFFFFFFFF
FFFFFFFFFFFFFFFFFFFFFFFFFFFFFFFFFFFFFFFFFFFFFFFFFFFFFFFFFFFFFFFFFFFFF
@A00155:342:HHGFNDSXY:1:2371:23213:23124 1:N:0:GAACCTAG+TCCGCATA
TCTCAATGCCGAGAGGGATGTAGAGGGCGAGAGAGAGCGACTTCTCTCGGGCCCACAGCCTTACAGCT
GTGGAGCACGGTATCCTCTGCCGAAACAGAGGTTGGACAAGACCGGAGGGGTCTCCTAGTTCCA
+
FFFFFFFFFFFFFFFFFFFFFFFFFFFFFFFFFFFFFFFFFFFFFF:FFFFFFFFFFFFFFF:FFFFFF
FFFFFFFFFFFF,FFFFFFFFFFFFFFFFFFFFFF,FFFFFFFFFFFFFFFFFFFF:FFFFFFFFFF
@A00155:342:HHGFNDSXY:1:2371:28736:28964 1:N:0:GAACCTAG+TCCGCATA
CGAGAGAGAGCGACTTCTCTCGGGCCCACAGCCTTACAGCTGTGGAGCACGGTATCCTCTGCCGAAAC
AGAGGTTGGACAAGACCGGAGGGGTCTCCTAGTTCCAAAGGAGATGTACTCCGGGCTTGTTCACGACC
+
FFFFFFFFFFFFFFFFFFFFFFFFFFFFFFFFFFFFFFFFFFFFFFFFFFFFFFFFFFFFFFFFFFFFF
FFFFFF:FFFFFFFFFFFFFFFFFFFFFFFFFFFFFFFFFFFFFFFF:FFFFFFFFFFFFFFFFFFFFF
@A00155:342:HHGFNDSXY:1:2372:10619:5760 1:N:0:GAACCTAG+TCCGCATA
ACCTCTGTTTCGGCAGAGGATACCGTGCTCCACAGCTGTAAGGCTGTGGGCCCGAGAGAAGTCGCTCT
CTCTCGCCCTTCACATCCCTCTCGGCATTGAGATGACCATAGGCCAGAGGTGAGTCCTTAAGTGGACA
+
FFFFFFFFFFFFFFFFFFFFFFFFFFFFFFFFFFFFFFFFFFFFFFFFFFFFFFFFFFFFFFFFFFFFF
FFFFFFFFFFFFFFFFFFFFFFFFFFFFFFFFFFFFFFFFFFFFFFFFFFFFFFFFFFFFFFFFF:FFF
@A00155:342:HHGFNDSXY:1:2372:3911:7169 1:N:0:GAACCTAG+TCCGCATA
TGTAGAGGGCGAGAGAGAGCGACTTCTCTCGGGCCCACAGCCTTACAGCTGTGGAGCACGGTATCCTC
TGCCGAAACAGAGGTTGGACAAGACCGGAGGGGTCTCCTAGTTCCAAAGGAGATGTACTCCGGG
+
FF:FFFFFFFFFFFFFFF:FFFFFFFF:FFF:F:F,FFF:FFFFFFFF,FFFFFFFFFFFFFFFFFFF
F:,FFFFFFF:FFF:FF,FFFFFFFF::FFF:FFFFFFF:FFFFFFFFF:FFFFFFFFFFFFFF
@A00155:342:HHGFNDSXY:1:2372:15148:8469 1:N:0:GAACCTAG+TCCGCATA
TTAGATCAGCTGTGTCCACTTAAGGACTCACCTCTGGCCTATGGTCATCTCAATGCCGAGAGGGATGT
GGAGGGCGAGAGAGAGCGACTTCTCTCGGGCCCACAGCCTTACAGCTGTGGAGCACGGTATCCTCTGC
+
:FFFFFFFFFFFFFFFFFFFFFFFFFFF:FFFFFFFFFFFF:FFFFFFFFFFFFFFFFFF:FF:FFFFF
F:FFFFFFFFFFFFFFFFFFFFFFFFFFFFFFFFFFFFFF,FFFFFFFFFFFFFFFFFFFFFFFFFFFF
@A00155:342:HHGFNDSXY:1:2372:6542:16235 1:N:0:GAACCTAG+TCCGCATA
AGGGCGAGAGAGAGCGACTTCTCTCGGGCCCACAGCCTTACAGCTGTGGAGCACGGTATCCTCTGCCG
AAACAGAGGTTGGACAAGACCGGAGGGGTCTCCTAGTTCCAAAGGAGATGTACTCCGGGCTTGTTCAC
+

FFFFFFFFFFFFFFFFFFFFF:FFFFFFFFFFFFFFFFFFFFFFFFFFFFFFFFFFFFFFF:FFFFFF
FFFFFFFFFFFFFFFFFFFFFFFFFFFFFFFFFFFFFFFFFFFFFFFFFFFFFFFFFFFFFFFFFFFF
@A00155:342:HHGFNDSXY:1:2372:8621:21089 1:N:0:GAACCTAG+TCCGCATA
GAGAGGGATGTGGAGGGCGAGAGAGAGCGACTTCTCTCGGGCCCACAGCCTTACAGCTGTGGAGCACG
GTATCCTCTGCCGAAACAGAGGTTGGACAAGACCGGAGGGGTCTCCTAGTTCCAAAGGAGATGTACTC
+
FFFFFFFFFF:FFFFFFFFFFFFFFFFFFFFFFFFFFFFFFFFFFFFFFFFFFFFFFFFFFFFFFFFF
FFFFFFFFFFFFFFFFFFFFFFFFFFFFFFFFFFFFFFFFFFFFFFFFFFFFFFF:FFFFFFFFFFFF
@A00155:342:HHGFNDSXY:1:2372:5773:26428 1:N:0:GAACCTAG+TCCGCATA
GGACTCACCTCTGGCCTATGGTCATCTCAATGCCGAGAGGGATGTGGAGGGCGAGAGAGAGCGACTTC
TCTCGGGCCCACAGCCTTACAGCTGTGGAGCACGGTATCCTCTGCCGAAACAGAGGTTG
+
FFFFFFFFFFFFF:FFFFFFFFFFFFFFFFFFFFFFFFFFFFFFFFFFFFFFFFFFFFFFFFFFF:FF
FFFFFFFFFFFFFFFFFFFFFFFFFFFFFFFFFFFFFFFFFFFFFFFFFFFFFFFFFFF
@A00155:342:HHGFNDSXY:1:2372:5728:30013 1:N:0:GAACCTAG+TCCGCATA
GGACTCACCTCTGGCCTATGGTCATCTCAATGCCGAGAGGGATGTGGAGGGCGAGAGAGAGCGACTTC
TCTCGGGCCCACAGCCTTACAGCTGTGGAGCACGGTATCCTCTGCCGAAACAGAGGGTG
+
FFFFFFFFFFFFFFFFFFFFFFFFFFFFFFFFFFFFFFFFFFFFFFFFFFF,FFFFFFF:F:FF::FF
F:FFFFFF:FFF:F:FF,:FFFFFF:FF,F:,:FF:,F::FFFF,FF:F,,,F,FF,,F
@A00155:342:HHGFNDSXY:1:2372:7075:36542 1:N:0:GAACCTAG+TCCGCATA
AGAGAGAGCGACTTCTCTCGGGCCCACAGCCTTACAGCTGTGGAGCACGGTATCCTCTGCCGAAACAG
AGGTTGGACAAGACCGGAGGGGTCTCCTAGTTCCAAAGGAGATGTACTCCGGGCTTGTTCACGAC
+
FFFFFFFFFFFFFFFFFF:FF:FFFFFFF,FFFFFFF,FFF::FFFFFFFFFFFFFFFF:FFFFFFFF
FF,FFF:FFFFF:F:F:FFFFFFFF:FFFF::FFFFFF:FFF:F,FFFFFF,FF,FFFFFFFFFF
@A00155:342:HHGFNDSXY:1:2373:28881:12242 1:N:0:GAACCTAG+TCCGCATA
GGATGTAGAGGGCGAGAGAGAGCGACTTCTCTCGGGCCCACAGCCTTACAGCTGTGGAGCACGGTATC
CTCTGCCGAAACAGAGGTTGGACAAGACCGGAGGGGTCTCCTAGTTCCAAAGGAGATGTACTCC
+
FFF:FFFFFFFFFFFFFFFFFFF:FFFFFFFFFFFFFFFFFFFFFFFFFFFFFFFFFFFFFFFFFFFF
FFFFFFFFFFFFFFFFFFFFFFFFFFFFFFFFFFFFFFFFFFFFFF:FFFFFFFFFFFFFFFFF
@A00155:342:HHGFNDSXY:1:2373:28031:23484 1:N:0:GAACCTAG+TCCGCATA
TTACAGCTGTGGAGCACGGTATCCTCTGCCGAAACAGAGGTTGGACAAGACCGGAGGGGTCTCCTAGT
TCCAAAGGAGATGTACTCCGGGCTTGTTCACGACCTACCGTGTAAGTCGTAGTCTAGTAGGCTACCTG
+
FFFFFFFFFFFFFFFFFFFFFFFFFFFFFFFFFFFFF:FFFFFFFFFFFFFFF:FFFFFFFFFFFFFF
FFFFFFFFFFFFFFFFFF,FF:FFFFFFFFFFFFFFFFFFFFFFFFFFFFFFFFFFFFF:F:FFFF:F
@A00155:342:HHGFNDSXY:1:2373:3025:23923 1:N:0:GAACCTAG+TCCGCATA
GAGCGACTTCTCTCGGGCCCACAGCCTTACAGCTGTGGAGCACGGTATCCTCTGCCGAAACAGAGGTT
GGACAAGACCGGAGGGGTCTCCTAGTTCCAAAGGAGATGTACTCCGGGCTTGTTCACGACCTACCGTG
+
FFFFFFFFFFFFFFFFFFFFFFFFFF:FFFFFFFFFFFFFFFFFFFFFFFFFFFFFFFFFFFFFFFFF
FFFFFFFFFFFFFFFFFFFFFFFFFFFFFFFFFFFFFFFFFFFFFFFFFFFFFFFFFFFFFFFFFF:F
@A00155:342:HHGFNDSXY:1:2373:21820:28980 1:N:0:GAACCTAG+TCCGCATA
CTTCTCTCGGGCCCACAGCCTTACAGCTGTGGAGCACGGTATCCTCTGCCGAAACAGAGGTTGGACAA
GACCGGAGGGGTCTCCTGGTTCCAAAGGAGATGTACTCCGGGCTTGTTCACGACCTACCGTGTAAGTC
+
FFFFFFFFFFFFFFFF:FFF:FFFFFFFFFFFFFFFFFFFFFFFFFFFFFFFFFFFFFFFFFF:FFFF
FFFFFFFF:FFFFFFF:FFFFFFFFFFFFFF:FFFFF:FFFFFFFFF:FFFFFFFFFFFFFFFFFFFF
@A00155:342:HHGFNDSXY:1:2374:12952:1188 1:N:0:GAACCTAG+TCCGCATA
GTCATCTCAATGCCGAGAGGGATGTGGAGGGCGAGAGAGAGCGACTTCTCTCGGGCCCACAGCCTTAC
AGCTGTGGAGCACGGTATCCTCTGCCGAAACAGAGGTTGGACAAGACCGGAGGGGTCTCCTAGTTCCA
+

```
FFFFFFFFFFFFFFFFFFFFFFFFFFFFFFFFFFFFFFFFFFFFFFFFFFFFFFFFFFFFFFFFFFFFFF
FFFFFFFFFFF:FFFFFFFFFFFFFFFFFFF:FFFFFFFFFFFF:FFFFFFFFFFFFFFFFFFFFFFFF
@A00155:342:HHGFNDSXY:1:2374:19886:2613 1:N:0:GAACCTAG+TCCGCATA
ACTTCTCTCGGGCCCACAGCCTTACAGCTGTGGAGCACGGTATCCTCTGCCGAAACAGAGGTTGGACA
AGACCGGAGGGGTCTCCTAGTTCCAAAGGAGATGTACTCCGGGCTTGTTCACGACCTACCGTGTAAGT
+
FFFFFFFFFFFFFFFFFFFFFFFFFFFF:FFFFFFFFFFFFFFFFFFFFFFFFFFFFFFFFFFFFFFFF
F,FFFFFFFFFFFFFFFFFFFFFFFFFFFFFFFFFFFFFFFFFFFFFFFFFFFFFFFFFFFFFFFFF:FF
@A00155:342:HHGFNDSXY:1:2374:20121:6778 1:N:0:GAACCTAG+TCCGCATA
GAGCGACTTCTCTCGGGCCCACAGCCTTACAACTGTGGAGCACGGTATCCTCTGCCGAAACAGAGGTT
GGACAAGACCGGAGGGGTCTCCTAGTTCCAAAGGAGATGTACTCCGGGCTTGTTCACGACCTACCGTG
+
:FFFFFFFFFFF,:FFFFFFFFFFFF:FFFFFFFFFF,FF:FFF::FFF:FFF:FFFFFFFFFFFF:F
F:FFFF,FFFFFFFFFFFF:F:FFF,F:FFFFF:FFFFFFFFFFFF:FFFFFFF:FFFF,F,FFFF:F
@A00155:342:HHGFNDSXY:1:2374:15628:13964 1:N:0:GAACCTAG+TCCGCATA
CTTCTCTCGGGCCCACAGCCTTACAGCTGTGGAGCACGGTATCCTCTGCCGAAACAGAGGTTGGACAA
GACCGGAGGGGTCTCCTAGTTCCAAAGGAGATGTACTCCGGGCTTGTTCACGACCTACCGTGTAAGTC
+
FFFFFFFFFFFFFFFFFFFFFFFFFFFFFFFFFFFFFFFFFFFFFFFFFFFFFFFFFFFFFFFFFFFFFF
FFFFFFFFFFFFFFFFFFFFFFFFFFFFFFFFFFFFFFFFFFFFFFFFFFFFFFFFFFFFFFFFFFFFFF
@A00155:342:HHGFNDSXY:1:2374:1732:15953 1:N:0:GAACCTAG+TCCGCATA
GTATCCTCTGCCGAAACAGAGGTTGGACAAGACCGGAGGGGTCTCCTAGTTCCAAAGGAGATGTACTC
CGGGCTTGTTCACGACCTACCGTGTAAGTCGTAGTCTAGTAGGCTACCTGACGAGTCCTTTTTAGGAC
+
FF,FFFFFFFFFFFFFFFFFFFFFFFFFFFFFFFFFF:FFFFFFFFFF:FFFFFFFFFFFFF:FFFFFF
FFFFFFFFF:FFFFFFFFFFFFFF,F,F::FF:FFFF,F:FFFF::FF::FFFFFFFFF:,FFFFFFF
@A00155:342:HHGFNDSXY:1:2374:9245:20008 1:N:0:GAACCTAG+TCCGCATA
TATCCTCTGCCGAAACAGAGGTTGGACAAGACCGGAGGGGTCTCCTAGTTCCAAAGGAGATGTACTCC
GGGCTTGTTCACGACCTACCGTGTAAGTCGTAGTCTAGTAGGCTACCTGACGAGTCCTT
+
FFFFFFFFFFFFFFFFFFFFFFFFFFFFFFFFFFFFFFFFFFFFFFFFFFFFFFFFFFFFFF:FFFFF
FFFFFFFFFFFFFFFFFFFFFFFFFFFFFFFFFFFFFFFFFFFFFFFFFFFFFFFFFFF
@A00155:342:HHGFNDSXY:1:2374:6506:24471 1:N:0:GAACCTAG+TCCGCATA
GTCCACTTAAGGACTCTCCTCTGGCCTATGGTCATCTCAATGCCGAGAGGGATGTGGAGGGCGAGAGA
GAGCGACTTCTCTCGGGCCCACAGCCTTACAGCTGTGGAGCACGGTATCCTCTGCCGAAACAGAGGTT
+
FFFFFFFFFFFFFFFFFFFFFFFF,FFFFFF:FFFFFFFFFFFFFFFFFFFFFFFFFFFFFFFFFFFFF
FFFFFFFFFFFFFFFFFFFFFFFFFFFFFFFFFFFFFFFFFFFFFFFFFFFFFFFFFFFFFFFFFFFFFF
@A00155:342:HHGFNDSXY:1:2375:24316:2362 1:N:0:GAACCTAG+TCCGCATA
GAGGGCGAGAGAGAGAGACTTCTCTCGGGCCCACAGCCTTACAGCTGTGGAGCACGGTATCCTCTGCC
GAAACAGAGGTTGGACAAGACCGGAGGGGTCTCCTAGTTCCAAAGGAGATGTACTCCGGGCTTGTTCA
+
FFFFFFFFFFFFFFFFFFFFFFFFFFFFFFFFFFFFFFFFF:FFFFFFFFFFFFFFFFFFFFFFFFFFFF
FFFFFFFFFFFFFFFFFFFFFFFFFFFFFFFFFFFFFFFFFFFFFFFFFFFFFFFFFFFFFFFFFFFFFF
@A00155:342:HHGFNDSXY:1:2375:10257:31156 1:N:0:GAACCTAG+TCCGCATA
CCCACAGCCTTACAGCTGTGGAGCACGGTATCCTCTGCCGAAACAGAGGTTGGACAAGACCGGAGGGG
TCTCCTGGTTCCAAAGGAGATGTACTCCGGGCTTGTTCACGACCTACCGTGTAAGTCGTAGTCTAGTA
+
FFFFFFFFFFF:FFFFFFFFFFFFFFFFFFFFFFFFFFFFFFFFFFFFFFFFFFFFFFFFFFFFFFFFFF
FFFFFFFFFFFFFFFFFFFF:FFFFFFFFFFFFFFFFFFFFFFFFFFFFFFFFFFFFFFFFFFFFFF:FF
@A00155:342:HHGFNDSXY:1:2375:12545:32894 1:N:0:GAACCTAG+TCCGCATA
AGGGATGTGGAGGGCGAGAGAGAGCGACTTCTCTCGGGCCCACAGCCTTACAGCTGTGGAGCACGGTA
TCCTCTGCCGAAACAGAGGTTGGACAAGACCGGAGGGGTCTCCTAGTTCCAAAGGAGATGTAC
+
```

,FFFFFFFFFFFFFFFFFFFFFFFFF:FFF:FFFFFFFFFFFFFFFFFFFFFFFFFFFFFFFFFFF
FFFFFFFFFFFFFFFFFFFFFFFFFFFFFFFFFFFFFFFFFFFFFFFFFFFFFFFFFFFFF
@A00155:342:HHGFNDSXY:1:2376:26955:11318 1:N:0:GAACCTAG+TCCGCATA
CTTCTCTCGGGCCCACAGCCTTACAGCTGTGGAGCACGGTATCCTCTGCCGAAACAGAGGTTGGACAA
GACCGGAGGGGTCTCCTAGTTCCAAAGGAGATGTACTCCGGGCTTGTTCACGACCTACCGTGTAAGTC
+
FFFFFFFFFFFFFFFFFFFFFFFFFFFFFFFFFFFFFFFFFFFFFFFFFFFFFFFFFFFFFFFFFF
FFFFFFFFFFFFFFFFFFFFFFFFFFFFFFFFFFFFFFFFFFFFFFFFFFFFFFFFFFFFFF:FFF
@A00155:342:HHGFNDSXY:1:2376:7030:30389 1:N:0:GAACCTAG+TCCGCATA
TGTCCACTTAAGGACTCACCTCTGGCCTATGGTCATCTCAATGCCGAGAGGGATGTGAAGGGCGAGAG
AGAGCGACTTCTCTCGGGCCCACAGCCTTACAGCTGTGGAGCACGGTATCCTCTGCCGAAACAGAGGT
+
FFFFFFFFFFFFFFFFFFFFFFFFFFFFFFFFFFFFFFFFFFFFFFFFFFFFFFFFFFFFFFFFFF
FFFFFFFFFFFFFFFFFFFFFFFFFFFFFFFFFFFFFFFFFFFFFFFFFFFFFFFFFFFFFFFFFF
@A00155:342:HHGFNDSXY:1:2376:10863:30671 1:N:0:GAACCTAG+TCCGCATA
GAGGGCGAGAGAGAGCGACTTCTCTCGGGCCCACAGCCTTACAGCTGTGGAGCACGGTATCCTCTGCC
GAAACAGAGGTTGGACAAGACCGGAGGGGTCTCCTAGTTCCATAGGAGATGTACTCCGGGCTTGTTCA
+
FFFFFFFFFFFFFFFFFFFFFFFFFFFFFFFFFFFFFFFFFFFFFFFFFFFFFFFFFFFFFFFFFF
FFFFFFFFFFFFFFFFFFFFFFFFFFFFFF:FFFFFFFFFFFFF,FFFF:FFFFFFFFFFFFFFFF
@A00155:342:HHGFNDSXY:1:2376:11107:30686 1:N:0:GAACCTAG+TCCGCATA
GAGGGCGAGAGAGAGCGACTTCTCTCGGGCCCACAGCCTTACAGCTGTGGAGCACGGTATCCTCTGCC
GAAACAGAGGTTGGACAAGACCGGAGGGGTCTCCTAGTTCCATAGGAGATGTACTCCGGGCTTGTTCA
+
FFFFFFFFFFFFFFFFFFFFFFFFFFFFFFFFFFFFFFFFFFFFFFFFFFFFFFFFFFFFFFFFFF
FFFFFFFFFFFFFFFFF:FFFFFFFFFFFFFFFFFFFFFFFFFFFFF:FFFFFFFFFFFFFFFFFF
@A00155:342:HHGFNDSXY:1:2376:15013:35790 1:N:0:GAACCTAG+TCCGCATA
CTCAATGCCGAGAGGGATGTGGAGGGCGAGAGAGAGCGACTTCTCTCGGGCCCACAGCCTTACAGCTG
TGGAGCACGGTATCCTCTGCCGAAACAGAGGTTGGACAAGACCGGAGGGGTCTCCTAGTTCCAAAGGA
+
FFFFFFFFFFFFFFFFFFFFFFFFFFFFFFFFFFFFFFFFFFFFFFFFFFFFFFFFFFFFFFFFFF
FFFFFFFFFFFFFFFFFFFFFFFFFFFFFFFFFFFFFFFFFFFFFFFFFFFFFFFFFFFFFFFFFF
@A00155:342:HHGFNDSXY:1:2377:27335:7686 1:N:0:GAACCTAG+TCCGCATA
AGAGAGAGTGACTTCTCTCGGGCCCACAGCCTTACAGCTGTGGAGCACGGTATCCTCTGCCGAAACAG
AGGTTGGACAAGACCGGAGGGGTCTCCTAGTTCCAAAGGAGATGTACTCCGGGCTTGTTCACGACCTA
+
FFFFFFFFFFFFFF:FFFFFFFFFFFFFFFFFFFFFFFFFFFFFFFFFFFFFFFFFFFFFFFFFFF
FFFFFFFFFFFFFFFFFFFFFFFFFFFFFFFFFFFFFFFFFFFFFFFFFFFFFFFFFFFFFFFFFF
@A00155:342:HHGFNDSXY:1:2377:28131:17018 1:N:0:GAACCTAG+TCCGCATA
CTCAATGCCGAGAGGGATGTGGAGGGCGAGAGAGAGCGACTTCTCTCGGGCCCACAGCCTTACAGCTG
TGGTGCACGGTATCCTCTGCCGAAACAGAGGTTGGACAAGACCGGAGGGGTCTCCTAGTTCCAAAGGA
+
FFFFFFFFFFFFFFFF:FFFFFFFFFFFFFFFFFFFFFFFFFFFFFFFFFFFFFFFFFFFFFFFFF
FFFFFFFFFFFFFFFFFFFFFFFFFFFFFFFFFFFFFFFFFFFFFFFFFFFFFFFFFFFFFFFFFF
@A00155:342:HHGFNDSXY:1:2377:9236:21527 1:N:0:GAACCTAG+TCCGCATA
CTGTGTCCACTTAAGGACTCACCTCTGGCCTATGGTCATCTCAATGCCGAGAGGGATGTGGAGGGCGA
GAGAGAGCGACTTCTCTCGGGCCCACAGCCTTACAGCTGTGGAGCACGGTATCCTCTGCCGAAACAGA
+
FFFFFFFFFFFFFFFFFFFFFFFFFFFFFFFFFFFFFFFFFFFFFFFFFFFFFFFFFFFFFFFFFF
FFFFFFFFFFFFFFFFFFFFFFFFFFFFFFFFFFFFFFFFFFFFFFFFFFFFFFFFFFFFFFFFFF
@A00155:342:HHGFNDSXY:1:2377:27326:32659 1:N:0:GAACCTAG+TCCGCATA
TTACAGCTGTGGAGCACGGTATCCTCTGCCGAAACAGAGGTTGGACAAGACCGGAGGGGTCTCCTAGT
TCCAAAGGAGATGTACTCCGGGCTTGTTCACGACCTACCGTGTAAGTCGTAGTCTAGTAGGCTACCTG
+

```
FFFFFFFFFFFFFFFFFFFFFFFFFFFFFFFFFFFFFFFFFFFFFFFFF:FFFFFFFFFFFFFFFFFF
FFFFFFFFFFFFFFFFFFFFFFFFFFFFFFFFFFFFFFFFFFFFFFFFFFFFFFFFFFFFFFFFFFFF
@A00155:342:HHGFNDSXY:1:2378:29622:4225 1:N:0:GAACCTAG+TCCGCATA
AGCGACTTCTCTCGGGCCCACAGCCTTACAGCTGTGGAGCACGGTATCCTCTGCCGAAACAGAGGTTG
GACAAGACCGGAGGGGTCTCCTAGTTCCAAAGGAGATGTACTCCGGGCTTGTTCACGACCTACCGTGT
+
FF,FFFFFFFFFFF,FF:FFFFFFFFFFFFFF,FFFFFFFFFFFFFFFFFFFFFF:FFFFFFFFFFFF
:FFFF,FFFFFFFFFFF:FFFFFF:FFFFFFFFFFFFFFFFFFFF:FFFFFFFF:FFFFFFFF,FFFF
@A00155:342:HHGFNDSXY:1:2378:7401:6699 1:N:0:GAACCTAG+TCCGCATA
GGTCTTGTCCAACCTCTGTTTCGGCAGAGGATACCGTGCTCCACAGCTGTAAGGCTGTGGGCCCGAGA
GAAGTCGCTCTCTCTCGCCCTCCACATCCCTCTCGGCATTGAGATGACCATAGGCCAGAGGTGAGTCC
+
FF:FFFFFFFFFFFFFFFFFFFFFFFFFFFF:FFFF:,F:FFFFFFFFFFFF,,F::FFFF:FFFFF:
F:FFFFFFFFF:FFFF:FFFFFFF:FFFF,FFFF:FFFFFFFFFFF:FFFFF:,FFFFF,FFFFFFFF
@A00155:342:HHGFNDSXY:1:2378:2989:10770 1:N:0:GAACCTAG+TCCGCATA
CTTCTCTCGGGCCCACAGCCTTACAGCTGTGGAGCACGGTATCCTCTGCCGAAACAGAGGTTGGACAA
GACCGGAGGGGTCTCCTAGTTCCAAAGGAGATGTACTCCGGGCTTGTTCACGACCTACCGTGTAAGTC
+
FFFFFFFFFFFFFF:FFFFFFFFFFFFFFFFFFFFFFFFFFFFFFFFFFFFFFFFFFFFFFFFFFFFF
:FFFFFF:FFFFFFFFFFFFFFFFFFFFFFFFFFFFFFFFFFF,FFFFFFFFFFFFFFFFFFFF:FFF
@A00155:342:HHGFNDSXY:1:2378:5981:24158 1:N:0:GAACCTAG+TCCGCATA
GAGGGCGAGAGAGAGCGACTTCTCTCGGGCCCACAGCCTTACAGCTGTGGAGCACGGTATCCTCTGCC
GAAACAGAGGTTGGACAAGACCGGAGGGGTCTCCTAGTTCCAAAGGAGATGTACTCCGGGCTTGTTCA
+
FFFFFFFFFFFFFFFFFFFFFFFFFFFFFFFFFFFFFFFFFFFFFFFFFFFFF:FFFFFFFFFFFFFF
F::FFFFFFFFFFFFFFFFFFFFFFFFFFFFFFFFFFFFFFFFFFFFFFFFFFFFFFFFFFFFFFFFF
@A00155:342:HHGFNDSXY:1:2378:12961:32769 1:N:0:GAACCTAG+TCCGCATA
TATCCTCTGCCGAAACAGAGGTTGGACAAGACCGGAGGGGTCTCCTAGTTCCAAAGGAGATGTACTCC
GGGCTTGTTCACGACCTACCGTGTAAGTCGTAGTCTAGTAGGCTACCTGACGAGTCCTTTTTAGGACG
+
FFFFFFFFFFFFFFFFFFFFFFFFFFFFFFFFFFFFFFFFFFFFFFFFFFFFFFFFFFFFFFFFFFFF
FFFFFFFFFFFFFFFFFFFFFFFFFFFFFFFFFFFFFFFFFFFFFFF:FFFFFFFFFFFFFFFFFFFF
@A00155:342:HHGFNDSXY:1:2378:32958:34209 1:N:0:GAACCTAG+TCCGCATA
CTCTGGCCTATGGTCATCTCAATGCCGAGAGGGATGTGAAGGGCGAGAGAGAGCGACTTCTCTCGGGC
CCACAGCCTTACAGCTGTGGAGCACGGTATCCTCTGCCGAAACAGAGGTTGGACAAGACCGGAGGGGT
+
F:FFFFFFF:FFFFFFFFFFFFFFFFFF:,FFFFFFFFF,FFFFFFFFFFF,F:FFFFFFFFFFFFF:
FFFFFFF:,FFFFFFFF:FFFF:FFFF:FFFFFFFFFFFFFFFFFFFFFFFFFFFFFFFFFFFFFFFF
@A00155:342:HHGFNDSXY:1:2401:16215:5744 1:N:0:GAACCTAG+TCCGCATA
GGATGTGGAGGGCGAGAGAGAGCGACTTCTCTCGGGCCCACAGCCTTACAGCTGTGGAGCACGGTATC
CTCTGCCGAAACAGAGGTTGGACAAGACCGGAGGGGTCTCCTAGTTCCAAAGGAGATGTACTCC
+
FFFFFFFFFFFFFFFFFFFFFFFFFFFFFFFFFFFFFFFFFFFFFFFFFFFFFFFFFFFFFFFFFFFF
:FFFFFFFFFFFFFFFFFFFFFFFFFFFFFFFFFFFFFFFFFFFFFFFFFFFFFFFFFFFFFFF
@A00155:342:HHGFNDSXY:1:2401:16315:5791 1:N:0:GAACCTAG+TCCGCATA
GGATGTGGAGGGCGAGAGAGAGCGACTTCTCTCGGGCCCACAGCCTTACAGCTGTGGAGCACGGTATC
CTCTGCCGAAACAGAGGTTGGACAAGACCGGAGGGGTCTCCTAGTTCCAAAGGAGATGTACTCC
+
FFFFFFFFFFFFFFFFFFFFFFFFFFFFFFFFFFFFFFFFFFFFFFFFFFFFFF:F:FFFFFFFFFFF
FFFFFFFFFFFFFFFFFFFFFFFFFFFF:FFFFFFFFFFFFFFFFF:FFFFFFFFFFFFFFFFF
@A00155:342:HHGFNDSXY:1:2401:10004:9518 1:N:0:GAACCTAG+TCCGCATA
TCTCTCGGGCCCACAGCCTTACAGCTGTGGAGCACGGTATCCTCTGCCGAAACAGAGGTTGGACAAGA
CCGGAGGGGTCTCCTAGTTCCAAAGGAGATGTACTCCGGGCTTGTTCACGACCTACCGTGTAAGTCGT
+
```

```
FFFFFFFFFFFFFFFFFFFFFFFFFFFFFFFFFF,FFFFFF,FFFFFF:FFFFFFFFFFFFFFFFFFF
FFFFFFFF:FFFFFFFFFFFFFFFF,FFFFFFFFFFFFFFFFFFFFFFFFFFFFFFFFFFFFFFFFFF
@A00155:342:HHGFNDSXY:1:2401:8287:32002 1:N:0:GAACCTAG+TCCGCATA
CTATGGTCATCTCAATGCCGAGAGGGATGTGGAGGGCGAGAGAGAGCGACTTCTCTCGGGCCCACAGC
CTTACAGCTGTGGAGCACGGTATCCTCTGCCGAAACAGAGGTTGGACAAGACCGGAGGGGTCTCCTA
+
FFFFFFFFFFFFFFFFFFFFFFFFFFFFFFFFFFFFFFFFFF:F:FFFFFFFFFFFFFFFFFFFFFFF
FFFFFFFFFFFFFFFFFFFFFFFFFFFFFFFFFFFFFFFFFFFFFFFFFFFFFFFFFFFFFFFFFFF
@A00155:342:HHGFNDSXY:1:2402:28944:6496 1:N:0:GAACCTAG+TCCGCATA
GCGACTTCTCTCGGGCCCACAGCCTTACAGCTGTGGAGCACGGTATCCTCTGCCGAAACAGAGGTTGG
ACAAGACCGGAGGGGTCTCCTGGTTCCAAAGGAGATGTACTCCGGGCTTGTTCACGACCTACCGTGTA
+
FFFFFFFFFFFFFFFFFFFFFFFFFFFFFFFFFFFFFFFFFFFFFFFFFFFFFFFFFFFFFFFFFFFF
FFFF:FFFFFFFFFFFFFF:FFFFFFFFFFFFFFFFFFFFFFFFFFFFFFFFFFF:FFFFFF:FFFFF
@A00155:342:HHGFNDSXY:1:2402:29116:9298 1:N:0:GAACCTAG+TCCGCATA
CTTCTCTCGGGCCCACAGCCTTACAGCTGTGGAGCACGGTATCCTCTGCCGAAACAGAGGTTGGACAA
GACCGGAGGGGTCTCCTAGTTCCAAAGGAGATGTACTCCGGGCTTGTTCACGACCTACCGTGTAAGTC
+
FFFFFFFFFFFFFFFFFFFFFFFFFFFFFFFFFFFFFFFFFFFFFFFFFFFFFFFFFFFFFFFFFFFF
FFFFFFFFFFFFFFFFFFFFFFFFFFFFFFFF:FFFFFFFFFFFFFFFFFFFFFFFFFFFFFFFFFFF
@A00155:342:HHGFNDSXY:1:2402:25870:11256 1:N:0:GAACCTAG+TCCGCATA
GGATGTGGAGGGCGAGAGAGAGCGACTTCTCTCGGGCCCACAGCCTTACAGCTGTGGAGCACGGTATC
CTCTGCCGAAACAGAGGTTGGACAAGACCGGAGGGGTCTCCTAGTTCCAAAGGAGATGTACTCC
+
FFFFFFFFFFFFFFFFFFFFFFFFFFFFFFFFFFFFFFFFFFFFFFFFFFFFFFFFFFFFFFFFFFFF
FFFFFFFFFFFFFFFFFFFFFFFFFFFFFFFFFFFFFFFFFFFFFFFFFFFFFFFFFFFFFFFF
@A00155:342:HHGFNDSXY:1:2402:2871:16642 1:N:0:GAACCTAG+TCCGCATA
TAAGGACTCTCCTCTGGCCTATGGTCATCTCAATGCCGAGAGGGATGTGGAGGGCGAGAGAGAGCGAC
TTCTCTCGGGCCCACAGCCTTACAGCTGTGGAGCACGGTATCCTCTGCCGAAACAGAGGTTGGACAAG
+
:FFFFFFFFFFFFFFF:FFFFFFFFFFFFFFFFFFFFFFFFFFFF::FFFFFFFFFFF:FFFFFFFFF
FFFFFFFFFFFFFFFFFFFFFF:FFFFFFFFFFF:F,FFFFF,FFFF,F:FF::,:FFFFFFFFFFF:
@A00155:342:HHGFNDSXY:1:2402:30563:21042 1:N:0:GAACCTAG+TCCGCATA
CTCAATGCCGAGAGGGATGTGGAGGGCGAGAGAGAGCGACTTCTCTCGGGCCCACAGCCTTACAGCTG
TGGAGCACGGTATCCTCTGCCGAAACAGAGGTTGGACAAGACCGGAGGGGTCTCCTAGTTCCAAAGG
+
FFFFFFFFFFFFFFFFF:FFFFFFFFFFFFFFFFFFFFFFFFFFFFF:FFFFFFFFFFFFFFFFFFFF
FFFFFFFFFFFFFFFFFFFFFFFFFFFFFFFFF:FFFFFFFFFFFFFF:FFFFFFFFFFFFFFFFFF
@A00155:342:HHGFNDSXY:1:2403:12572:6668 1:N:0:GAACCTAG+TCCGCATA
CTTCTCTCGGGCCCACAGCCTTACAGCTGTGGAGCACGGTATCCTCTGCCGAAACAGAGGTTGGACAA
GACCGGAGGGGTCTCCTAGTTCCAAAGGAGATGTACTCCGGGCTTGTTCACGACCTACCGTGTAAGTC
+
FFFFFFFFFFFFFFFFFFFFFFFFFFFFFFFFFFFFFFFFFFFFFFFFFFFFFFFFF:FFFFFFFFFF
F:FFFFFFFFFFFFFFFFFFFFFFFFFFFFF:FFFFFFFFFFFFFFFFF:FFFFFFFFFFFFFFFFFF
@A00155:342:HHGFNDSXY:1:2403:22218:11569 1:N:0:GAACCTAG+TCCGCATA
CCATGCCCCGCCACACCGCCTTAGATCAGCTGTGTCCACTTAAGGACTCACCTCTGGCCTATGGTCAT
CTCAATGCCGAGAGGGATGTGGAGGGCGAGAGAGAGCGACTTCTCTCGGGCCCACAGCCTTACAGCTG
+
FFFFFFFFFFFFFFFFFFFFFFFF::FFFFFFFFFFFFFFFFFFFFFFFFFFFFFFFFFFFFFFFFFF
FFFFFFFFFFFFFFFFFFFFFFFFFFFFFFFFFFFFFFFFFFFFFFFFFFFFFFFFFFFFFFFFFFFF
@A00155:342:HHGFNDSXY:1:2403:30680:14325 1:N:0:GAACCTAG+TCCGCATA
ATGCCCCGCCACACCGCCTTAGATCAGCTGTGTCCACTTAAGGACTCACCTCTGGCCTATGGTCATCT
CAATGCCGAGAGGGATGTGGAGGGCGAGAGAGAGCGACTTCTCTCGGGCCCACAGCCTTACAGCTGTG
+
```

FFFFFFFFFFFFFFFFFF:FFFFFFFFFFFFFFFFFFFFFFFFFFFFFFFFFFFFFFFFFFFFFFFFF
:FFFFFFFFFFFFFF,FFFFFFFFFFFFFFFFFFFFFFFFFFFFFFFFFFFFFFFFFFFFFFFFFFFF
@A00155:342:HHGFNDSXY:1:2403:2564:15452 1:N:0:GAACCTAG+TCCGCATA
ACCTCTGTTTCGGCAGAGGATACCGTGCTCCACAGCTGTAAGGCTGTGGGCCCGAGAGAAGTCGCTCT
CTCTCGCCCTCCACATCCCTCTCGGCATTGAGATGACCATAGGCCAGAGGAGAGTCCTTAAGTGGACA
+
FFFFFFFFFFFFFFFFFFFFFFFFFFFFFFFFFFFFFFFFFFFFFFFFFFFFFFFFFFFFFFFFFFFF
FFFFFFFFFFFFFF:FFFFFFFFFFFFFFFFFFFFFF:FFFFFFFFFFFFFFFFFFFFFFFFFFF:FF
@A00155:342:HHGFNDSXY:1:2403:27959:17284 1:N:0:GAACCTAG+TCCGCATA
GAGAGAGCGACTTCTCTCGGGCCCACAGCCTTACAGCTGTGGAGCACGGTATCCTCTGCCGAAACAGA
GGTTGGACAAGACCGGAGGGGTCTCCTAGTTCCAAAGGAGATGTACTCCGGGCTTGTTCACGACCTAC
+
FFFFFFFFFFFFFFFFFFFFFFFFFFFFFFFFFFFFFFFFFFFFFFFFF:FFFFFFFFFFFFFFFFFF
FFFFFFFFFFFFFFFFFFFFFFFFFFFFFFFFFFFFFFFFFFFFFFFFFFFFFFFFFFFFFFFFFFFF
@A00155:342:HHGFNDSXY:1:2403:27480:17707 1:N:0:GAACCTAG+TCCGCATA
GAGAGAGCGACTTCTCTCGGGCCCACAGCCTTACAGCTGTGGAGCACGGTATCCTCTGCCGAAACAGA
GGTTGGACAAGACCGGAGGGGTCTCCTAGTTCCAAAGGAGATGTACTCCGGGCTTGTTCACGACCTAC
+
FFFFFFFFFFFFFFFFFFFFFFF:FFFFFFFFFFFFFFFFFFFFFFFFFFFFFFFFFFFFFFFFFFFF
FFFFFFFFFFFFFFFFFFFFFFFFFFFFFFFFFFFFFFFFFFFFFFFFFFFFFFFFFFFFFFFFFFFF
@A00155:342:HHGFNDSXY:1:2403:28583:18208 1:N:0:GAACCTAG+TCCGCATA
GAGAGAGCGACTTCTCTCGGGCCCACAGCCTTACAGCTGTGGAGCACGGTATCCTCTGCCGAAACAGA
GGTTGGACAAGACCGGAGGGGTCTCCTAGTTCCAAAGGAGATGTACTCCGGGCTTGTTCACGACCTAC
+
FFFFFFFFFFFFFFFF:FFFFFFFFFFFFFF:FFFFFFFFFFFFFFFFFFFFFFFFFFFFFFFFFFFF
F:FFFFFFFFFFFFFFFFFF:FFFFFFFFFFFFFF:F:FFFFFF::FFFFFFFFFFFFFFFFFFFFFF
@A00155:342:HHGFNDSXY:1:2403:1588:32925 1:N:0:GAACCTAG+TCCGCATA
GTCATCTCAATGCCGAGAGGGATGTGGAGGGCGAGAGAGAGCGACTTCTCTCGGGCCCACAGCCTTAC
AGCTGTGGAGCACGGTATCCTCTGCCGAAACAGAGGTTGGACAAGACCGGAGGGGTCTCCTAGTTCCA
+
FFFFFFFFFFFFFFFFFFFFFF,FFF:FFFFFFFFFFFFFFFFFFFFF:F,FFFFFFFFFFFFFFFFF
FFFFFFFFFFF:FFF::FFFFFFFFFF:FFFFFFFFFFFFFFFFFFFFFFFFF:FFFFFF:FF:FFFF
@A00155:342:HHGFNDSXY:1:2404:19696:1000 1:N:0:GAACCTAG+TCCGCATA
TGGCCTATGGTCATCTCAATGCCGAGAGGGATGTGGAGGGCGAGAGAGAGCGACTTCTCTCGGGCCCA
CAGCCTTACAGCTGTGGAGCACGGTATCCTCTGCCGAAACAGAGGTTGGACAAGACCGGAGG
+
FFFFFFFFFFFFFFFFFFFFFFFFFFFF:FF:F:FFFFFFFFFFFFFFF:FFFFFFFFFFFFFFFFFF
FFFFF,F,FFFFFFFFFFFFFFFFFFFFFFFFFFFFFFFFFFFFF,:FFFFFFFFFFFFFFF
@A00155:342:HHGFNDSXY:1:2404:7554:5932 1:N:0:GAACCTAG+TCCGCATA
TCCTCTGGCCTATGGTCATCTCAATGCCGAGAGGGATGTGGAGGGCGAGAGAGAGCGACTTCTCTCGG
GCCCACAGCCTTACAGCTGTGGAGCACGGTATCCTCTGCCGAAACAGAGGTTGGACAAGACCGGAGG
+
FFF:FFFFFFFFFFFFFFFFFFFFFFFFFFF:FFFFFF,FFFF:F:FFFFFFF:FFFFFFFFFFFFFF
FFFFFFFFFF:F:FFFFFFFFFFFFFFFFFF:FFFF:F:FFFFFFFFFFF:FFFFFFFFFFFFFFFF
@A00155:342:HHGFNDSXY:1:2404:16161:8030 1:N:0:GAACCTAG+TCCGCATA
CCGAGAGGGATGTGGAGGGCGAGAGAGAGCGACTTCTCTCGGGCCCACAGCCTTACAGCTGTGGAGCA
CGGTATCCTCTGCCGAAACAGAGGTTGGACAAGACCGGAGGGGTCTCCTGGTTCCAAAGGAGATGTAC
+
FFFFFFFFFFFFFFFFFFFFFFFFFFFFFFFFFFFFFFFFFFFFFFFFFFFFFFFFFFFFFFFFFFFF
FFFF,FFFFFFFFFFFFFFFFFFFFFFFFFFFFFFFFFFFFFFFFFFFFFFFFFFFFFFFFFFFFFFF
@A00155:342:HHGFNDSXY:1:2404:22914:9079 1:N:0:GAACCTAG+TCCGCATA
GTATCCTCTGCCGAAACAGAGGTTGGACAAGACCGGAGGGGTCTCCTAGTTCCAAAGGAGATGTACTC
CGGGCTTGTTCACGACCTACCGTGTAAGTCGTAGTCTAGTAGGCTACCTGACGAGTCCTTTTTAGGAC
+

FFFFFFFFFFFFFFFFFFFFFFFFFFFFFFFFFFFFFFFFFFFFFFFFFFFFFFFFFFFFFFFFFFFFFF
FFFFFFFFFFFFFFFFFFFFFFFFFFFFFFFFFFFFFFFFFFFFFFF:FFFFFFFFFFFFF:FFFFFFFF
@A00155:342:HHGFNDSXY:1:2404:3568:11146 1:N:0:GAACCTAG+TCCGCATA
CTCTGGCCTATGGTCATCTCAATGCCGAGAGGGATGTGAAGGGCGAGAGAGAGCGACTTCTCTCGGGC
CCACAGCCTTACAGCTGTGGAGCACGGTATCCTCTGCCGAAACAGAGGTTGGACAAGACCGGAGGGGT
+
FFFFFFFFFFFFFFFFFFFFFFFFFFFFFFFFFFFFFF,FFFFFFFFFFFFFFFFFFFFFFFFFFFFFFF
FFFFFFFFFFFFFFFFFFFFFFFFF,FFFFFFFFFFFFFFFFFFFFFFFFFFFFFFFFFFFFFFFFFFFF
@A00155:342:HHGFNDSXY:1:2404:3070:11350 1:N:0:GAACCTAG+TCCGCATA
CTCTGGCCTATGGTCATCTCAATGCCGAGAGGGATGTGAAGGGCGAGAGAGAGCGACTTCTCTCGGGC
CCACAGCCTTACAGCTGTGGAGCACGGTATCCTCTGCCGAAACAGAGGTTGGACAAGACCGGAGGGGT
+
FFFFFFFF:FFFFF:FF:FFFFFFFFFFF:FFFFFFFFFFFF,FFFFFF,:FFFFF,FFFFFFFFFFF
:FFFFFFFFF:,F:FFFFF:FFFFFFFFFFF,FFF:FFFF:FF:FFFF:FFFFFFFF,FFFFFFFFFF
@A00155:342:HHGFNDSXY:1:2404:26892:13745 1:N:0:GAACCTAG+TCCGCATA
AATGCCGAGAGGGATGTGGAGGGCGAGAGAGAGCGACTTCTCTCGGGCCCACAGCCTTACAGCTGTGG
AGCACGGTATCCTCTGCCGAAACAGAGGTTGGACAAGACCGGAGGGGTCTCCTGGTTCCAAAGGAGA
+
FF:FFF:FFFF:FFFF:FFFF:FFFFFFFF:FFFFF::FFFFFFF,FF:FFFFFFFFFFFFFFFF,FF
FFFFFF::F:FFFFFFFFFF:FFFFF:FFFFFFFF:FFFFFFFFFFFFFFFFFFFFFFFFFFFF:FFF
@A00155:342:HHGFNDSXY:1:2404:30472:13870 1:N:0:GAACCTAG+TCCGCATA
CGAGAGGGATGTGGAGGGCGAGAGAGAGCGACTTCTCTCGGGCCCACAGCCTTACAGCTGTGGAGCAC
GGTATCCTCTGCCGAAACAGAGGTTGGACAAGACCGGAGGGGTCTCCTGGTTCCAAAGGAGATGTACT
+
F:FFFFFFF,:FF:FFF:FFFFFFF:FFFFFFFFFFFF:F:FF:FFFFFFF,:F::FFFF,FFFF:FF
::FFFFF:FFFF,FFFFFFFF,FFF:FFFFFFFFFFFFFFFFFFFFFFFF:FFFFFFFF:FFFFF:FFF
@A00155:342:HHGFNDSXY:1:2404:24813:23046 1:N:0:GAACCTAG+TCCGCATA
CTTCTCTCGGGCCCACAGCCTTACAGCTGTGGAGCACGGTATCCTCTGCCGAAACAGAGGTTGGACAA
GACCGGAGGGGTCTCCTAGTTCCAAAGGAGATGTACTCCGGGCTTGTTCACGACCTACCGTGTAAGTC
+
FFFFFFFFFFFFFFFFFFFFFFFFFFFFFFFFFFFFFFFFFFFFFFFFFFFFFFFFFFFFFFFFFFFFFF
FFFFFFFFFFFFFFFFFFFFFFFFFFFFFFFFFFFFFFFFFFFFFFFFFFFFFFFFFFFFFFFFFFFFFF
@A00155:342:HHGFNDSXY:1:2404:14082:34741 1:N:0:GAACCTAG+TCCGCATA
TTACAGCTGTGGAGCACGGTATCCTCTGCCGAAACAGAGGTTGGACAAGACCGGAGGGGTCTCCTAGT
TCCAAAGGAGATGTACTCCGGGCTTGTTCACGACCTACCGTGTAAGTCGTAGTCTAGTAGGCTACCTG
+
FFFFFFFFFFFFFFFFFFFF:FFFFFFFFFFFFFFFFFFFFFFFFFFFFFFFFFFFFFFFFFFFFFFFFF
FFFFFFFFFFFFFFFFFFFFFFFFFFFFFFFFFFFFFFFFFFFFFFFFFFFFFFFFFFFFFFFFFFFFFF
@A00155:342:HHGFNDSXY:1:2405:26775:4053 1:N:0:GAACCTAG+TCCGCATA
TGGTCATCTCAATGCCGAGAGGGATGTGGAGGGCGAGAGAGAGCGACATCTCTCGGGCCCACAGCCTT
ACAGCTGTGGAGCACGGTATCCTCTGCCGAAACAGAGGTTGGACAAGACCGGAGGGGTCTCCTAGTTC
+
FFFFFFFFFFFFFFFFFFF:FFFF,FFFFFFFFFFFFFFFFFFFFFFFFFFFFFFFFFFFFFFFFFFFFF
FFFFFFFFFFFFFFFFFF:FFFFFFFFFFFFFFFFFFFFFFFFFFFFFFFFFFFFFFFFFFFFFFFFFFF
@A00155:342:HHGFNDSXY:1:2405:25021:6402 1:N:0:GAACCTAG+TCCGCATA
GAAGTCGCTCTCTCTCGCCCTCCACATCCCTCTCGGCATTGAGATGACCATAGGCCAGAGGTGAGTCC
TTAAGTGGACACAGCTGATCTAAGGCGGTGTGGTGGGGCATGGGTTTGAACCCCCATGACGGTCGGAG
+
:FFFFFFFFFFFFFFFFFFFFFFFFF:FFFFFFFFFFFFF:FFFFFFFFFFFFFFFFFFFFFFFFFFFF
FFFFFFFFFFFFFFFFFFFFFF,FFFFFFFF:FFFFFFFFFFFFFFFFFFFFFFFFFFFFFFFFFFFFFF
@A00155:342:HHGFNDSXY:1:2405:13187:25520 1:N:0:GAACCTAG+TCCGCATA
CAAGCCCGGAGTACATCTCCTTTGGAACTAGGAGACCCCTCCGGTCTTGTCCAACCTCTGTTTCGGCA
GAGGATACCGTGCTCCACAGCTGTAAGGCTGTGGGCCCGAGAGAAGTCGCTCTCTCTCGCCCTCCACA
+

FFF:FF:FFFFFFFFFFFFFFF:F:FFFFFFFFFFFFFFFFFFF:F,FFFFFFFFF,F:FF,FFFFFF
FFFFFFFFFFFFF,:FFFFF:FFFFFFFFFF:F:FFFF:FFFFF:F:FFFFFFFFFFFFFFF,FFFFF
@A00155:342:HHGFNDSXY:1:2405:28827:29904 1:N:0:GAACCTAG+TCCGCATA
AAGGACTCACCTCTGGCCTATGGTCATCTCAATGCCGAGAGGGATGTAGAGGGCGAGAGAGAGCGACT
TCTCTCGGGCCCACAGCCTTACAGCTGTGGAGCACGGTATCCTCTGCCGAAACAGAGGTTGGACAAGA
+
FFFFFFFFFFFFFFFFFFFFFFFF,FFFFFFFFFFFFFF:FFFFFFFFFF:F:FFFFFFFFFFFFFFF
FFFFFFFFFFFFFFFFFFFFFFFFFFFFFFFFFFFFFFFFFFFFFFFFFFFFFFFFFFFFFFFFFFFF
@A00155:342:HHGFNDSXY:1:2406:6768:6042 1:N:0:GAACCTAG+TCCGCATA
AATGCCGAGAGGGATGTAGAGGGCGAGAGAGAGCGACTTCTCTCGGGCCCACAGCCTTACAGCTGTGG
AGCACGGTATCCTCTGCCGAAACAGAGGTTGGACAAGACCGGAGGGGTCTCCTAGTTCCAAAGGAGAT
+
FFFFFFFFFFFFFFFFFFFFFFF,FFFFFFFFF,FFFFFFFFF:FFFFFFFFFF:FFFFFFFFF,FF:
FFFFFFFFFFFFFFFFFFFFFFFF::FF:FFFF,:F,FFF::FFFFFFFFFFFFFF:FFFFFFF:FFF
@A00155:342:HHGFNDSXY:1:2406:4770:9095 1:N:0:GAACCTAG+TCCGCATA
ACCTCTGTTTCGGCAGAGGATACCGTGCTCCACAGCTGTAAGGCTGTGGGCCCGAGAGAAGTCGCTCT
CTCTCGCCCTTCACATCCCTCTCGGCATTGAGATGACCATAGGCCAGAGGTGAGTCCTTAAGTGGACA
+
FFFFFFFFFFF,FFFFFFFFFFFFFFFFFFFFFFFFFFFFFFFFFFFFFFFFFFFFFFFFFFFFFFFF
FFFF:FFFFFFFFF:FFFFFFFFFFFFFFFFFFFFFFF,FFFFFFF,FFFF,FF:FFFFFFFF:FFF:
@A00155:342:HHGFNDSXY:1:2406:11369:15170 1:N:0:GAACCTAG+TCCGCATA
GAGCGACTTCTCTCGGGCCCACAGCCTTACAGCTGTGGAGCACGGTATCCTCTGCCGAAACAGAGGTT
GGACAAGACCGGAGGGGTCTCCTAGTTCCAAAGGAGATGTACTCCGGGCTTGTTCACGA
+
FFFFFFFFFFFFFFFFFFFFFFFFFFFFFFFFFFFFFFFFFFFFFFFFFFFFFFFFFFFFFFFFFFFF
FFFFFFFFFFFFFFFFFFFF:FFFFFFFFFFFFFFFFFFFFFFFFFFFFFFFFFFFFF
@A00155:342:HHGFNDSXY:1:2406:28248:17879 1:N:0:GAACCTAG+TCCGCATA
TTCTCTCGGGCCCACAGCCTTACAGCTGTGGAGCACGGTATCCTCTGCCGAAACAGAGGTTGGACAAG
ACCGGAGGGGTCTCCTGGTTCCAAAGGAGATGTACTCCGGGCTTGTTCACGACCTACCGTGTAAGTCG
+
FFFFFFFFFFFFFFFFFFFFFFFFFFFFFFFFFFFFFFFFFFFFFFFFFFFFFFFFFFFFFFFFFFFF
:FFFFFFFFFFFFFFFFFFFFFFFFFFFFFFFFFFFFFFFFFFFFFFFFFFFFFFFFFFFFFF:FFFF
@A00155:342:HHGFNDSXY:1:2406:3992:18333 1:N:0:GAACCTAG+TCCGCATA
AGCGACTTCTCTCGGGCCCACAGCCTTACAGCTGTGGAGCACGGTATCCTCTGCCGAAACAGAGGTTG
GACAAGACCGGAGGGGTCTCCTAGTTCCAAAGGAGATGTACTCCGGGCTTGTTCACGACCTACCGTGT
+
FFFFFFFFFFFFFFFFFFFFFFFF:FF,FFFFFFFFFFFFFFFFFFFFFF,:,:FFFFFFFFFFFF:F
FFFFFFFFFFFFFFFFFFFFFFFFFFFFF:F:FFF:FFFFFFFFFFFFFFFFF:FF,FFFFFFFFF::
@A00155:342:HHGFNDSXY:1:2406:6479:21668 1:N:0:GAACCTAG+TCCGCATA
ATCTCAATGCCGAGAGGGATGTGGAGGGCGAGAGAGAGCGACTTCTCTCGGGCCCACAGCCTTACAGC
TGTGGAGCACGGTATCCTCTGCCGAAACAGAGGTTGGACAAGACCGGAGGGGTCTCCTGGTTCCAAAG
+
FFFFFFFFFFFF:F:FFFFFFFFFFFFFFFFFFFFFFFFFF,FFFFFFFFFFFFFFFFFFFFFFFFFF
FFFFFFFFFFFF:FFFFFF:FFFFFFFF:FFFFFFFFFFFFFFFFFFFFFFFFFFFFFF:FFFFFFFF
@A00155:342:HHGFNDSXY:1:2406:11098:21840 1:N:0:GAACCTAG+TCCGCATA
GAGCGACTTCTCTCGGGCCCACAGCCTTACAGCTGTGGAGCACGGTATCCTCTGCCGAAACAGAGGTT
GGACAAGACCGGAGGGGTCTCCTAGTTCCAAAGGAGATGTACTCCGGGCTTGTTCACGACCTACC
+
FFFFFFFFFFFFFFFFFFFFFFFFFFFF:FFFFFFFFFFFFFFFFFFFFFFFFFFFFFFFFFFFFFFF
FFFFFFFFFFFFFFFFFFFFFFFFFFFFFFFFFFFFFFFFFFFFFFFFFFFFFFFFFFFFFFFFF
@A00155:342:HHGFNDSXY:1:2406:27037:23547 1:N:0:GAACCTAG+TCCGCATA
CTTCTCTCGGGCCCACAGCCTTACAGCTGTGGAGCACGGTATCCTCTGCCGAAACAGAGGTTGGACAA
GACCGGAGGGGTCTCCTAGTTCCAAAGGAGATGTACTCCGGGCTTGTTCACGACCTACCGTGTAAGTC
+

```
FFFFFFFFFFFFFFFFFFFFFFFFFFFFFFFFFFFFFFFFFFFFFFFFFFFFFFFFFFFFFFFFFFFF
FFFFFFFFFFFFFFFFFFFFFFFFFFFFFFFFFFFFFFFFFFFFFFFFFFFFFFFFFFFFFFFFFFFF
@A00155:342:HHGFNDSXY:1:2407:28619:35869 1:N:0:GAACCTAG+TCCGCATA
GAGGGCGAGAGAGAGCGACTTCTCTCGGGCCCACAGCCTTACAGCTGTGGAGCACGGTATCCTCTGCC
GAAACAGAGGTTGGACAAGACCGGAGGGGTCTCCTAGTTCCAAAGGAGATGTACTCCGGGCTTGTTCA
+
:F:FF,FFFFFFFFFFFF:FFFFFF:FFFF:FF:FF,F:FFFFF,F:FFFF,FFFFFFFFF,FF:,FF
FF:FFFFFFF:FFFFFFF,FFFFFF:F:F:FF:FF:FFFFFFFF,FFFFF:FFFFFFFFFFF,:FFFF
@A00155:342:HHGFNDSXY:1:2407:9778:36182 1:N:0:GAACCTAG+TCCGCATA
AGAGAGAGCGACTTCTCTCGGGCCCACAGCCTTACAACTGTGGAGCACGGTATCCTCTGCCGAAACAG
AGGTTGGACAAGACCGGAGGGGTCTCCTAGTTCCAAAGGAGATGTACTCCGGGCTTGTTCACGACCTA
+
FFFFFFFFFFFFFFFFFFFFFFFFFFFFFFFFFFFFFFFFFFFFFFFFFFF,FFFFFFFFFFFFFFFF
FFFFFFFFFFF,FFFFFFFFFFFFFFFFFFFFFFFFFFFFFFFFFFFFFFFFFFF:FFFFFFFF
@A00155:342:HHGFNDSXY:1:2408:1570:11068 1:N:0:GAACCTAG+TCCGCATA
TCCTCTGCCGAAACAGAGGTTGGACAAGACCGGAGGGGTCTCCTAGTTCCAAAGGAGATGTACTCCGG
GCTTGTTCACGACCTACCGTGTAAGTCGTAGTCTAGTAGGCTACCTGATGAGTCCTTTTTAGGACGAA
+
FFFFFFFFFFFFFFFFFFFFFFFFFFFFFFFFFFF:FFFFFFFF::FFFFFFFFFFFFFFFFFFFFFF
FFFFFFFFFFFFFFFFFFFFFFFFFFFFFFFFFFFF:FFFFF:FFFFFFFFFF:FFFFFFFFFFFFFF
@A00155:342:HHGFNDSXY:1:2408:25138:17691 1:N:0:GAACCTAG+TCCGCATA
ACCTCTGTTTCGGCAGAGGATACCGTGCTCCACAGCTGTAAGGCTGTGGGCCCGAGAGAAGTCGCTCT
CTCTCGCCCTCCACATCCCTCTCGGCATTGAGATGACCATAGGCCAGAGGAGAGTCCTTAAGTGGACA
+
FFFFFFFFFFFFF:FFFFFFFFFFFFFFFFFFFFFFFFFFFFFFFFFFFFFFFF:FFFFFFFFFFFFF
FF:FFFFFFFFFFF:FFFFFFFFFFF,FFFFFFFFFFF,F:FFFFFFFFFFFFFFFFFFFFFFFFFFF
@A00155:342:HHGFNDSXY:1:2408:30969:27289 1:N:0:GAACCTAG+TCCGCATA
CTTCTCTCGGGCCCACAGCCTTACAGCTGTGGAGCACGGTATCCTCTGCCGAAACAGAGGTTGGACAA
GACCGGAGGGGTCTCCTAGTTCCAAAGGAGATGTACTCCGGGCTTGTTCACGACCTACC
+
F,FFFFFFFFFFFFFFFFFFFFFFFFFFFFFFFFFFFFFFFFFFFFFFFFFFFFFFFFFFFFFFFFFF
FFF::FFFFFFFFFFFFFFFFFFFFFFFFFFFF:FFFFFFFFFF,FFFFFFFFFFF:FF
@A00155:342:HHGFNDSXY:1:2408:3622:30780 1:N:0:GAACCTAG+TCCGCATA
GAGAGAGCGACTTCTCTCGGGCCCACAGCCTTACAGCTGTGGAGCACGGTATCCTCTGCCGAAACAGA
GGTTGGACAAGACCGGAGGGGTCTCCTAGTTCCAAAGGAGATGTACTCCGGGCTTGTTCACGACCTAC
+
FFFFFFFFFFFFFFF:FFF,FFFFFFF,F:FFFFFFFFFFF:FFFF:F,FFFFFFFFFFFFFFFFFFF
FFFF::FFFFFFF,FFF:FFFFFFFFFFFFFFFFFFFFFFFFFF,FF,FFFFFFFFFFF:FFFFFFFF
@A00155:342:HHGFNDSXY:1:2408:7970:31078 1:N:0:GAACCTAG+TCCGCATA
TGTGGAGGGCGAGAGAGAGCGACTTCTCTCGGGCCCACAGCCTTACAGCTGTGGAGCACGGTATCCTC
TGCCGAAACAGAGGTTGGACAAGACCGGAGGGGTCTCCTAGTTCCAAAGGAGATGTACTCCGGG
+
FFFFFFFFFFFFFFFFFF:FFFFFFFFFFFF,FFFFFFFFFFFF:,F:FFF:FFFF,FFFFFFFF,FF
F,FFFFFFFFFF:FFFFFFFFF,:F:FFFFF,FFFFFFFFFF:FFFFFFFFFFFFFFFFFFFFF
@A00155:342:HHGFNDSXY:1:2408:17454:35321 1:N:0:GAACCTAG+TCCGCATA
CTTCTCTCGGGCCCACAGCCTTACAGCTGTGGAGCACGGTATCCTCTGCCGAAACAGAGGTTGGACAA
GACCGGAGGGGTCTCCTAGTTCCAAAGGAGATGTACTCCGGGCTTGTTCACGACCTACCGTGTAAGTC
+
FFFFFFFFFFFFFFFFFFFFFFFFFFFFFFFFFFFFFFFFFFFFFFFFFFFFFFFFFFFFFFFFFFFF
FFFFFFFFFFFFFFFFFFFFFFFFFFFFFFFFFFFFFFFFFFFFFFFFFFFFFFFFFFFFFFFFFFFF
@A00155:342:HHGFNDSXY:1:2408:21667:35822 1:N:0:GAACCTAG+TCCGCATA
AGAGAGAGCGACTTCTCTCGGGCCCACAGCCTTACAGCTGTGGAGCACGGTATCCTCTGCCGAAACAG
AGGTTGGACAAGACCGGAGGGGTCTCCTAGTTCCAAAGGAGATGTACTCCGGGCTTGTTCACGACCTA
+
```

FFFFFFFFF:FFFFFFF:FF,FFFFFFFFFFF:FFFFFFF:FFFFFFFF::FFFFFFF:FFFFFF,FF
F,FFFF:FFFF:FFFF:FF:FFFFFF:FFFFFFFFFFFFF:FFF,F:FFF,FFFF,FFFFFF:FFFFF
@A00155:342:HHGFNDSXY:1:2408:17924:35947 1:N:0:GAACCTAG+TCCGCATA
ACTTCTCTCGGGCCCACAGCCTTACAGCTGTGGAGCACGGTATCCTCTGCCGAAACAGAGGTTGGACA
AGACCGGAGGGGTCTCCTAGTTCCAAAGGAGATGTACTCCGGGCTTGTTCACGACCTACCGTGTAAG
+
FFFFFFFFFFFFFFFFFFFFFFFFFFFFFFFFFFFFFFFFFFFFFFFFFFF:FFFFFFFFFFFFFFFF
FFFFFFFFFFFFFFFFFFFFFFFFFFFFFFFFFFFFFFFFFFFFFFFFFFFFFFFFF:FFFFFFFFF
@A00155:342:HHGFNDSXY:1:2409:28447:15843 1:N:0:GAACCTAG+TCCGCATA
TTACAGCTGTGGAGCACGGTATCCTCTGCCGAAACAGAGGTTGGACAAGACCGGAGGGGTCTCCTAGT
TCCAAAGGAGATGTACTCCGGGCTTGTTCACGACCTACCGTGTAAGTCGTAGTCTAGTAGGCTACCTG
+
FFFFFFFFFFFFFFFFFFFFFFFFFFFFFFFFFFFFFFFFFFFFFFFFFFFFFFFFFFFFFFFFFFFF
FFFFF:FFFFFFFFFFFFFFFFFFFFFFFFFFFFFFFFFFFFFFFFFFFFFFFFFFFFFFFFFFFFFF
@A00155:342:HHGFNDSXY:1:2409:20618:26710 1:N:0:GAACCTAG+TCCGCATA
AGAGAGAGCGACTTCTCTCGGGCCCACAGCCTTACAGCTGTGGAGCACGGTATCCTCTGCCGAAACAG
AGGTTGGACAAGACCGGAGGGGTCTCCTAGTTCCAAAGGAGATGTACTCCGGGCTTGTTCACGACCTA
+
FFFFFFFFFFFFFFFFFFFFFFFFFFFFFFFFFFFFFFFFFFFFFFFFFFFFFFFFFFFFFFFFFFFF
FFFFFFFFFFFFFFF:FFFFFFFFFFFFFFFFFFFFFFFFFFFFFFFFFFFFFFFFFFFFFFFFFFFF
@A00155:342:HHGFNDSXY:1:2410:21630:16720 1:N:0:GAACCTAG+TCCGCATA
GGATGTGGAGGGCGAGAGAGAGCGACTTCTCTCGGGCCCACAGCCTTACAGCTGTGGAGCACGGTATC
CTCTGCCGAAACAGAGGTTGGACAAGACCGGAGGGGTCTCCTAGTTCCAAAGGAGATGTACT
+
FFFFFFFFFFFFFFFFFFFFFFFFFFFFFFFFFFFFFFFFFFFFFFFFFFFFFFFFFFFFFFFFFFFF
FFFFFFFFFFFFFFFFFFFFFFFFFFFFFFFFFFFFFFFFFFFFFFFFFFFFFFFFFFFFFF
@A00155:342:HHGFNDSXY:1:2410:12264:19820 1:N:0:GAACCTAG+TCCGCATA
GAGAGGGATGTGGAGGGCGAGAGAGAGCGACTTCTCTCGGGCCCACAGCCTTACAGCTGTGGAGCACG
GTATCCTCTGCCGAAACAGAGGTTGGACAAGACCGGAGGGGTCTCCTAGTTCCAAAGGAGATGTACTC
+
FFFFFFFFFFFFFFFFFFFFFFFFFFFFFFFFFFFFFFFFFFFFFFFFFFFFFFFFFFFFFFFFFFFF
FFFFFFFFFFFFFFFFFFFFFFFFFFFFFFFFFFFFFFFFFFFFFFFFFFFFFFFFFFFFFFFFFFFF
@A00155:342:HHGFNDSXY:1:2411:13566:1094 1:N:0:GAACCTAG+TCCGCATA
CTCAATGCCGAGAGGGATGTAGAGGGCGAGAGAGAGCGACTTCTCTCGGGCCCACAGCCTTACAGCTG
TGGAGCACGGTATCCTCTGCCGAAACAGAGGTTGGACAAGACCGGAGGGGTCTCCTAGTTCCAAAGGA
+
FFFFFFFFFFFFFFFFFFFFFFFFFFFFFFFFFFFFFFFFFFFFFFFFFFFFFFFFFFFFFFFFFFFF
FFFFFFFFFFFFFFFFFFFFFFFF:FFFFFFFFFFFFFF:FFFFFFFFFFFFFFFFFFFFFFFFFFFF
@A00155:342:HHGFNDSXY:1:2411:27218:29058 1:N:0:GAACCTAG+TCCGCATA
GGATGTGGAGGGCGAGAGAGAGCGACTTCTCTCGGGCCCACAGCCTTACAGCTGTGGAGCACGGTATC
CTCTGCCGAAACAGAGGTTGGACAAGACCGGAGGGGTCTCCTAGTTCCAAAGGAGATGTACTCC
+
FFFFFFFFFFFFFFFFFFFFFFFFFFFFFFFFFFF:FFFFFFFFFFFFFFFFFFFFFFFFFFFFFFFF
FFFFFFFFFFFFFFFFFFFFFFFFFFFFFFFFFFFFFFFF,FFFFFFFFF:FFFFFF:FFFFFF
@A00155:342:HHGFNDSXY:1:2411:29071:30264 1:N:0:GAACCTAG+TCCGCATA
ATCTCAATGCCGAGAGGGATGTAGAGGGCGAGAGAGAGCGACTTCTCTCGGGCCCACAGCCTTACAGC
TGTGGAGCACGGTATCCTCTGCCGAAACAGAGGTTGGACAAGACCGGAGGGGTCTCCTAGTTCCAAAG
+
FFFFFFFFFFFFFFFFFFFFFF,FFFFFFFFFFFFFFFFFFF:FFFFFFFFFFFFFFFFFFFFFFFFF
FFFFFFFFFFFFFFFFFFFFFFFFFFFFFFFFFF:FFFFFFFFFFFFFFFFFFFFFFFFFFFFFFFFF
@A00155:342:HHGFNDSXY:1:2412:20826:4836 1:N:0:GAACCTAG+TCCGCATA
GGCATTGAGATGACCATAGGCCAGAGGTGAGTCCTTAAGTGGACACAGCTGATCTAAGGCGGTGTGGC
GGGGCATGGGTTTGAACCCCCATGACGGTCGGAGTCTAGTAGGCTCCCTGATGAGTCCGTTCCAAGGA
+

FFFFFFFFFFFFFFFFFFFFFFFFFFFFFFFFFFFFFFFFFFFFFFFFFFFFFFFFFFFFFFFFFFFF
FFFFFFFFFFFFFFFFFFFFFFFFFFFFFFFFFFFFFFFFFFFFFFFFFFFFFFFFFFFFFFFFFFFF
@A00155:342:HHGFNDSXY:1:2412:22896:11177 1:N:0:GAACCTAG+TCCGCATA
GGCATTGAGATGACCATAGGCCAGAGGTGAGTCCTTAAGTGGACACAGCTGATCTAAGGCGGTGTGGC
GGGGCATGGGTTTGAACCCCCATGACGGTCGGAGTCTAGTAGGCTCCCTGATGAGTCCGTTCCAAGGA
+
FFFFFFFFFFFFFFFFFFFFFFFFFFFFFFFFFFFFFFFFFFFFFFFFFFFFFFFFFFFFFFFFFFFF
FFFFFFFFFFFFFFFFFFFFFFFFFFFFFFFFFFFFFFFFF:FFFFFFFFFFFFFFFFFFFFFFFFFF
@A00155:342:HHGFNDSXY:1:2412:10167:20823 1:N:0:GAACCTAG+TCCGCATA
GGATGTGGAGGGCGAGAGAGAGCGACTTCTCTCGGGCCCACAGCCTTACAGCTGTGGAGCACGGTATC
CTCTGCCGAAACAGAGGTTGGACAAGACCGGAGGGGTCTCCTGGTTCCAAAGGAGATGTACTCC
+
FFFFFFFFFFFFFFFFFFFFFFFFFFFFFFFFFFFFFFFFFFFFFFFFFFFFFFFFFFFFFFFFFFFF
FFFFFFFFFFFFFFFFFFFFFFFFFFFFFFFFFFFFFFFFFFFFFFFFFFFF:FFFFFFFFFFF
@A00155:342:HHGFNDSXY:1:2412:26268:23156 1:N:0:GAACCTAG+TCCGCATA
CAATGCCGAGAGGGATGTGGAGGGCGAGAGAGAGCGACTTCTCTCGGGCCCACAGCCTTACAGCTGTG
GAGCACGGTATCCTCTGCCGAAACAGAGGTTGGACAAGACCGGAGGGGTCTCCTAGTTCCAAAGGAGA
+
FFFFFFFFFFFFFFFFFFFFFFFFFFFFFFFFFFFFFFFFFFFFFFFFFFFFFFFFFFFFFFFFFFFF
FFFFFFFFFFFFFFFFFFFFFFFFFFFFFFFFFFFFFFFFFFFFFFFFFFFFFFFFFFFFFFFFFFFF
@A00155:342:HHGFNDSXY:1:2412:25310:27978 1:N:0:GAACCTAG+TCCGCATA
AGCCTTACAGCTGTGGAGCACGGTATCCTCTGCCGAAACAGAGGTTGGACAAGACCGGAGGGGTCTCC
TAGTTCCAAAGGAGATGTACTCCGGGCTTGTTCACGACCTACCGTGTAAGTCGTAGTCTAGTAGGCTA
+
FFFFFFFFFFFFFFFFFFFFFFFFFFFFFFFFFFFFFFFFFFFFFFFFFFFFFFFFFFFFFFFFFFFF
FFFFFFFFFFFFFFFFFFFFFFFFFFFF:FFFFFFFFFFFFFFFFFFFFFFFFFFFFFFFFFF:FFFF
@A00155:342:HHGFNDSXY:1:2412:24668:29590 1:N:0:GAACCTAG+TCCGCATA
AGCCTTACAGCTGTGGAGCACGGTATCCTCTGCCGAAACAGAGGTTGGACAAGACCGGAGGGGTCTCC
TAGTTCCAAAGGAGATGTACTCCGGGCTTGTTCACGACCTACCGTGTAAGTCGTAGTCTAGTAGGCTA
+
FFFFFFFFFFFFFFFFFFFFFFFFFFFFFFFFFFFFFFFFFFFFFFFFFFFFFFFFFFFFFFFFFFFF
FFFFFFFFFFFFFFFFFFFFFFFFFFFFFFFFFFFFFFFFFFFFFFFFFFFFFFFFFFFFFFFFFFFF
@A00155:342:HHGFNDSXY:1:2413:10474:13463 1:N:0:GAACCTAG+TCCGCATA
CTTCTCTCGGGCCCACAGCCTTACAGCTGTGGAGCACGGTATCCTCTGCCGAAACAGAGGTTGGACAA
GACCGGAGGGGTCTCCTAGTTCCAAAGGAGATGTACTCCGGGCTTGTTCACGACCTACCGTGTAAGTC
+
FFFFFFFFFFFFFFFFFFFFFFFFFFFFFFFFFFFFFFFFFFFFFFFFFFFFF::FFFFFFFFFFFFF
FFFFFFFFFFFFFFFFFFFFFFFFF:FFFFFFFFFFFFFFFFFFFFFFFFFFFFFFFFFFFFFFFFFF
@A00155:342:HHGFNDSXY:1:2413:19199:19742 1:N:0:GAACCTAG+TCCGCATA
CTTCTCTCGGGCCCACAGCCTTACAGCTGTGGAGCACGGTATCCTCTGCCGAAACAGAGGTTGGACAA
GACCGGAGGGGTCTCCTAGTTCCAAAGGAGATGTACTCCGGGCTTGTTCACGACCTACCGTGTAAGTC
+
FFFFFFFFFFFFFFFFFFFFFFFFFFFFFFFFFFFFFFFFFFFFFFFFFFFFFFFFFFFFFFFFFFFF
FFFFFFFFFFFFFFFFFFFFFFFFFFFFFFFFFFFFFFFFFFFFFFFFFFFFF:FFFFFFFFFFFFFF
@A00155:342:HHGFNDSXY:1:2413:28239:21371 1:N:0:GAACCTAG+TCCGCATA
GGATGTAGAGGGCGAGAGAGAGCGACTTCTCTCGGGCCCACAGCCTTACAGCTGTGGAGCACGGTATC
CTCTGCCGAAACAGAGGTTGGACAAGACCGGAGGGGTCTCCTAGTTCCAAAGGAGATGTACTCC
+
FFFFFFFFFFFFFFFFFFFFFFFFFF:FFFFFFFFFFFFFFFFFFFFFFFFFFFFFFFFFFFFFFFFF
FFFFFFFFFFFFFF:FFFFFFFFFFFFFFFFFFFFFFFFFFFFFFFFFFFFFFFFFFFFFFFFF
@A00155:342:HHGFNDSXY:1:2413:3441:26209 1:N:0:GAACCTAG+TCCGCATA
CTTCTCTCGGGCCCACAGCCTTACAGCTGTGGAGCACGGTATCCTCTGCCGAAACAGAGGTTGGACAA
GACCGGAGGGGTCTCCTGGTTCCAAAGGAGATGTACTCCGGGCTTGTTCACGACCTACCGTGTAAGTC
+

FFFFFFFFFFFFFFFFFFFFFFFFFFFFF:FFFFFFFFFFFFFFFFFFFFFFFF:FFFFFFFFF:FFFF
FFFFFFFFFFFFFFFFFFFFFFFFFFFFFFFFFFFFFFFFFFFFFFFFFFFF:FFF:FFFFFFFFFFF
@A00155:342:HHGFNDSXY:1:2413:4517:27884 1:N:0:GAACCTAG+TCCGCATA
AGAGAGAGCGACTTCTCTCGGGCCCACAGCCTTACAGCTGTGGAGCACGGTATCCTCTGCCGAAACAG
AGGTTGGACAAGACCGGAGGGGTCTCCTGGTTCCAAAGGAGATGTACTCCGGGCTTGTTCACGACCTA
+
FFFFFFFFFFFFFF,F:FFF:FFFFFFFFFFFFFFFFFFFFF:F,FFFFF,FFFFFFFFF,FFFFFFF
FFFFF:FFFFF,FFFFFFFFF:FFFFFFFFFFFFFFFFFFFFFFFFFFFF,FF:FF:FFFF:FFFFFFF
@A00155:342:HHGFNDSXY:1:2414:3314:16344 1:N:0:GAACCTAG+TCCGCATA
CTTCTCTCGGGCCCACAGCCTTACAGCTGTGGAGCACGGTATCCTCTGCCGAAACAGAGGTTGGACAA
GACCGGAGGGGTCTCCTGGTTCCAAAGGAGATGTACTCCGGGCTTGTTCACGACCTACCGTGTAAGTC
+
FFFFFFFFFFFFFFFFFFFFFFFFFFFFFFFFFFFFFFFFFFFFFFFFFFFFFFFFFFFFFFFFFFFF
FFF:FFFFFFFFFFFFFFFFFFFFFFFFFFFFFFFFFF:FFFFFFFFFFFFFFFF:FFFFFFFFFFFFF
@A00155:342:HHGFNDSXY:1:2414:4218:17503 1:N:0:GAACCTAG+TCCGCATA
GCCCACAGCCTTACAGCTGTGGAGCACGGTATCCTCTGCCGAAACAGAGGTTGGACAAGACCGGAGGG
GTCTCCTAGTTCCAAAGGAGATGTACTCCGGGCTTGTTCACGACCTACCGTGTAAGTCGTAGTCTAGT
+
FFFFFFFFFFFFFFFFFFFFFFFFF:FFFFFFFFFFFFFFFFFFFFFFFFFFFFFFFFFFFFFFFFFF
FFFFFFFFFFFFFFFFFFFFFFFFFFFFFFFFFFFFFFFFFFFF:FFFFFFFFFFFFFFFFFFFFFFFF
@A00155:342:HHGFNDSXY:1:2414:32045:20290 1:N:0:GAACCTAG+TCCGCATA
ACTTCTCTCGGGCCCACAGCCTTACAGCTGTGGAGCACGGTATCCTCTGCCGAAACAGAGGTTGGACA
AGACCGGAGGGGTCTCCTAGTTCCAAAGGAGATGTACTCCGGACTTGTTCACGACCTACCGTGTAAGT
+
FFFFFFFFFFFFFFFFFFFFFFFFFFFFFFFFFFFFFFFFFFFFFFFFFFFFFFFFFFFFFFFFFFFF
FFFFFFFFFFFFFFFFFFFFFFFFFFFFFFFFFFFFFFFFFFFFFFFFFFFFFFF:FFFFFFFFFFFF
@A00155:342:HHGFNDSXY:1:2414:16631:28322 1:N:0:GAACCTAG+TCCGCATA
TCCTCTGCCGAAACAGAGGTTGGACAAGACCGGAGGGGTCTCCTAGTTCCAAAGGAGATGTACTCCGG
GCTTGTTCACGACCTACCGTGTAAGTCGTAGTCTAGTAGGCTACCTGACGAGTCCTTTTTAGGACGAA
+
FFFFF,FFFFFFFFFFFFF:FFFFFFFFFFFF:FFFFFFFFFFFFF:FFFFFFFFFFFFFFFFFFFFFF
FFFFF:FF:FFFFFFFFFFFFFFFFFFFFFFFFFFFFFFFFFFFFFFFFFFFFFFFFFFFFFFFFFFF
@A00155:342:HHGFNDSXY:1:2414:4119:28354 1:N:0:GAACCTAG+TCCGCATA
CCCACAGCCTTACAGCTGTGGAGCACGGTATCCTCTGCCGAAACAGAGGTTGGACAAGACCGGAGGGG
TCTCCTAGTTCCAAAGGAGATGTACTCCGGGCTTGTTCACGACCTACCGTGTAAGTCGTAGTCTAGTA
+
FFFFFFFFFFFFFFFFFFFFFFFFF,FFFF,FFFFFFFFFFFF:FFF:FFFF:FFFFFFFF:FFFFFFF
FFFFFFFFFFFFFFFFFFFFFFFFFFFF,FFFF,FFFFFFFFFFFFFFF,FFFF,FFFFF,F,FFFFFF
@A00155:342:HHGFNDSXY:1:2414:23068:31829 1:N:0:GAACCTAG+TCCGCATA
TTCTCTCGGGCCCACAGCCTTACAGCTGTGGAGCACGGTATCCTCTGCCGAAACAGAGGTTGGACAAG
ACCGGAGGGGTCTCCTAGTTCCAAAGGAGATGTACTCCGGGCTTGTTCACGACCTACCGTGTAAGTCG
+
FFFFFFFFFFFFFFFFFFFFFFFFFFFFFFFFFFFFFFFF:FFFFFFFFFFFFFFFFFFFFFFFFFFFF
FFFFFFFFFFFFFFFFFFFFFFFFFFFFFFFFFFFFFFFFFFF::FFFFFFFFFFFFFFFFFFFFFFFF
@A00155:342:HHGFNDSXY:1:2415:10185:8516 1:N:0:GAACCTAG+TCCGCATA
AGAGAGAGCGACTTCTCTCGGGCCCACAGCCTTACAGCTGTGGAGCACGGTATCCTCTGCCGAAACAG
AGGTTGGACAAGACCGGAGGGGTCTCCTAGTTCCAAAGGAGATGTACTCCGGGCTTGTTCACGACCT
+
F:FFFFFFFFFFFFFFFFFFFFFFFFFFF:FFFFFFFFFFFFFFFFFFFFFFFFFFFFFFFFFFFFFFF
FFFFFFFFFFFFFFFFFFFFFFFFFFFFFFFFFFFFFFFFFFFFFFFFFFFFFFFFFFFFFFFFFFF
@A00155:342:HHGFNDSXY:1:2415:28628:14278 1:N:0:GAACCTAG+TCCGCATA
CTTCTCTCGGGCCCACAGCCTTACAGCTGTGGAGCACGGTATCCTCTGCCGAAACAGAGGTTGGACAA
GACCGGAGGGGTCTCCTGGTTCCAAAGGAGATGTACTCCGGGCTTGTTCACGACCTACCGTGTAAGTC
+

```
FFFFFFFFFFFFFFFFFFFFFFFFFFFFFFFFFFFFFFFFFFFFFFFFFFFFFFFFFFFFFFFFFFFF
FFFFFFFFFFFFFFFFFFFFFFFFFFFFFFFFFFFFFFFFFFFFFFFFFFFFFFFFFFFFFFFFFFFF
@A00155:342:HHGFNDSXY:1:2415:19379:20870 1:N:0:GAACCTAG+TCCGCATA
TGTTTCGGCAGAGGATACCGTGCTCCACAGCTGTAAGGCTGTGGGCCCGAGAGAAGTCGCTCTCTCTC
GCCCTCCACATCCCTCTCGGCATTGAGATGACCATAGGCCAGAGGTGAGTCCTTAAGTGGACACAGCT
+
FFFFFFFFFFFFFFFFFFFFFFFFFF:FFFFFFFFFFFFFFFFFFFFFFFFFFFFFFFFFFFFFFFFF
FFFFFFFFFFFFFFFFFFFFFFFFFFFFFFFFFFFFFFFFFFFFFFFFFFF:FFFFFFFFFFFFFFFF
@A00155:342:HHGFNDSXY:1:2416:32868:4429 1:N:0:GAACCTAG+TCCGCATA
GATGTGGAGGGCGAGAGAGAGCGACTTCTCTCGGGCCCACAGCCTTACAGCTGTGGAGCACGGTATCC
TCTGCCGAAACAGAGGTTGGACAAGACCGGAGGGGTCTCCTAGTTCCAAAGGAGATGTACTCCGGGCT
+
FFFFFFFFFFFFFFFFFFFFFFFFFFFFFFFFFFFFFFFFFFFFFFFFFFFFFFFFFFFFFFFFFFFF
FFFFFFFFFFFFF:FFFFFFFFFFFFFFFFFFFFFFFFFFFFFFFFFFFFFFFFFFFFFFFF:FFFFFF
@A00155:342:HHGFNDSXY:1:2416:27046:9471 1:N:0:GAACCTAG+TCCGCATA
TATCCTCTGCCGAAACAGAGGTTGGACAAGACCGGAGGGGTCTCCTGGTTCCAAAGGAGATGTACTCC
GGGCTTGTTCACGACCTACCGTGTAAGTCGTAGTCTAGTAGGCTACCTGACGAGTCCTTTTTAGGACG
+
FFFFFFFFFFFFFFFFFFFFFFFFFFFFFFFFFFFFFFFFFFFFFFFFFFFFFFFFFFFFFFFFFFFF
FFFFFFFFFFFFFFFFFFFFFFFFFFFFFFFFFFFFFFFFFFFFFFFFFFFFFF:FFFFFF,FFFF
@A00155:342:HHGFNDSXY:1:2416:6054:22717 1:N:0:GAACCTAG+TCCGCATA
CCAACCTCTGTTTCGGCAGAGGATACCGTGCTCCACAGCTGTAAGGCTGTGGGCCCGAGAGAAGTCGC
TCTCTCTCGCCCTCCACATCCCTCTCGGCATTGAGATGACCATAGGCCAGAGGCGAGTCCTTAA
+
:FFFFFFFFFFFFFFFFFFFFFFF:FFFF:::FFFFFFFF:FF,FFFFFFFFFF:FFFFFF,FFFFFFF
:FFFFF:FFFFFFFFFFFF:FF:,FFF:FFF::FFFFF:F,,FF:FFFF:F:F,:FFFFF,::F
@A00155:342:HHGFNDSXY:1:2417:28537:3505 1:N:0:GAACCTAG+TCCGCATA
ACAGCTGTGGAGCACGGTATCCTCTGCCGAAACAGAGGTTGGACAAGACCGGAGGGGTCTCCTAGTTC
CAAAGGAGATGTACTCCGGGCTTGTTCACGACCTACCGTGTAAGTCGTAGTCTAGTAGGCTACCTGAC
+
FFFFFFFFFFFFFFFFFFFFFFFFFFFFFFFFFFFFFFFFFFFFFFFFFFFFFFFFFFFFFFFFFFFF
FFFFFFFFFFFFFFFFFFFFFFFFFFFFFFFFFFFFFFFFF:FFFFFFFFFFFFFFFFFFFFFFFFFF
@A00155:342:HHGFNDSXY:1:2417:17228:5650 1:N:0:GAACCTAG+TCCGCATA
GAGCGACTTCTCTCGGGCCCACAGCCTTACAGCTGTGGAGCACGGTATCCTCTGCCGAAACAGAGGTT
GGACAAGACCGGAGGGGTCTCCTAGTTCCAAAGGAGATGTACTCCGGGCTTGTTCACGACCTACCGTG
+
FFFFFFFFFFFFFFFFFFFFFFFFFFFFFFFFFFFFFFFFFFFFFFFFFFFFFFFFFFFFFFFFFFFF
FFFFFF:FFFFFFFFFFFFFFFFFFFFFFFFFFFFFFFFFFFFFFFFFFFFFFFFFFFFFFFFFFFFF
@A00155:342:HHGFNDSXY:1:2417:18439:6183 1:N:0:GAACCTAG+TCCGCATA
ACCTCTGTTTCGGCAGAGGATACCGTGCTCCACAGCTGTAAGGCTGTGGGCCCGAGAGAAGTCGCTCT
CTCTCGCCCTCCACATCCCTCTCGGCATTGAGATGACCATAGGCCAGAGGTGAGTCCTTAAGTGGACA
+
FFFFFFFFFFFFFFFFFFFFFFFFFFFFFFFFFFFFFFFFFFFFFFFFFFFFFFFFFFFFFFFFFFFF
FFFF:FFFFFFFFFFFFFFFFFFFFFFFFFFFFFFFFFF,F:F:FFFFFFFFFFFFFFFFFFFFFFFFF
@A00155:342:HHGFNDSXY:1:2417:19009:6825 1:N:0:GAACCTAG+TCCGCATA
ACCTCTGTTTCGGCAGAGGATACCGTGCTCCACAGCTGTAAGGCTGTGGGCCCGAGAGAAGTCGCTCT
CTCTCGCCCTCCACATCCCTCTCGGCATTGAGATGACCATAGGCCAGAGGTGAGTCCTTAAGTGGACA
+
FFFFFFFFFFFFFFFFFFFFFFFFFFF:FFFFFFFFFFFFFFFFFFFFFFFFFFFFFFFFF:FFFFFFFF
FFFFF:FFFFFFFFFFFFFFFFFFFFFFFFFFFFFFFFFFFFFFFFFFFFFFFFFFFFFFFFFFFFFF
@A00155:342:HHGFNDSXY:1:2417:30201:20353 1:N:0:GAACCTAG+TCCGCATA
GAGAGGGATGTGGAGGGCGAGAGAGAGCGACTTCTCTCGGGCCCACAGCCTTACAGCTGTGGAGCACG
GTATCCTCTGCCGAAACAGAGGTTGGACAAGACCGGAGGGGTCTCCTAGTTCCAAAGGAGATGTACTC
+
```

FFFFFFFFFFFFFFFFFFFFFFFFFFFFFFFFFFFFFFFFFFFFFFFFFFFFF:FFFFFFFFFFFFFF
FF:FFFFFFFFFFFFFFFFFFFFFFFFFFFFFFFFFFFFFFFFFFFFFFFFFFFFFFFFFFFFFFFFF
@A00155:342:HHGFNDSXY:1:2417:10348:25488 1:N:0:GAACCTAG+TCCGCATA
TTAAGGACTCTCCTCTGGCCTATGGTCATCTCAATGCCGAGAGGGATGTGGAGGGCGAGAGAGAGCGA
CTTCTCTCGGGCCCACAGCCTTACAGCTGTGGAGCACGGTATCCTCTGCCGAAACAGAGGTTGGACAA
+
:,FF:FF,:FFFF:F:F,FFF:F:,FFFFFFFFF,FFFFFF,:FFF:::FFFFFFFFFFFFFFFFFFF
FFF:FFFFFFFFFFFFFFFFFFFFFF:FF,F:FFFFFFFFFFFFFFFFF:FFFFFFFFFFFFFFFFFFF
@A00155:342:HHGFNDSXY:1:2419:7211:3552 1:N:0:GAACCTAG+TCCGCATA
ACCTCTGTTTCGGCAGAGGATACCGTGCTCCACAGCTGTAAGGCTGTGGGCCCGAGAGAAGTCGCTCT
CTCTCGCCCTTCACATCCCTCTCGGCATTGAGATGACCATAGGCCAGAGGTGAGTCCTTAAGTGGACA
+
FFFFFFFFFFFFFFFF:FFFFFFFFFFFFFFFFFFFFFFFFFFFFFFFFFFFFFFFFFFFFFFFFFFF
FFFFFFFFFFFFFFFFFFFFFFFFFFFF:FFFFFFFFFFFFFFFFFFFFFFFFFFFFFFFFFFFFFFF
@A00155:342:HHGFNDSXY:1:2419:20943:25332 1:N:0:GAACCTAG+TCCGCATA
CCCACAGCCTTACAGCTGTGGAGCACGGTATCCTCTGCCGAAACAGAGGTTGGACAAGACCGGAGGGG
TCTCCTAGTTCCAAAGGAGATGTACTCCGGGCTTGTTCACGACCTACCGTGTAAGTCGTAGTCTAGTA
+
:FFFFFFFFFFFFF:F:FFFFFFFFFFFFFF::FF,FF:FFFFFF,FF:FF,,F:FF,FFF:,FFFFF
:FFF:,F,FF:FFFFF:FFFFFFFFFFFFFF:FFFFFFFFFFFF,,FFFF:FFFF::FFF:F:FFF:F
@A00155:342:HHGFNDSXY:1:2420:2862:8547 1:N:0:GAACCTAG+TCCGCATA
AGCGACTTCTCTCGGGCCCACAGCCTTACAGCTGTGGAGCACGGTATCCTCTGCCGAAACAGAGGTTG
GACAAGACCGGAGGGGTCTCCTAGTTCCAAAGGAGATGTACTCCGGGCTTGTTCACGACCTACCGTGT
+
FFFFFFFFFFFFFFFFFFFFFFFFFFFFFFFFFFFFFFFFFFFFFFFFFFFFFFFFFFFFFFF:F,:FF
FFFFFFFFFFFFFFFFFFFFFFF:FFFFFFFFFFFF,FFFFF:FFFFFFFFFFFFFFFFFFFFFFFF:FF
@A00155:342:HHGFNDSXY:1:2420:28700:21731 1:N:0:GAACCTAG+TCCGCATA
CTCAATGCCGAGAGGGATGTAGAGGGCGAGAGAGAGCGACTTCTCTCGGGCCCACAGCCTTACAGCTG
TGGAGCACGGTATCCTCTGCCGAAACAGAGGTTGGACAAGACCGGAGGGGTCTCCTAGTTCCAAAGGA
+
FFFFFFFFFFFF:FFFFFFFFFFFFFFFFFFFFFFFFFFFFFFFFFFFFFFFFFFFFFFFFFFFFFFF
FFFFFFFFFFFFFFFFFFFFF:FFFFFFFFFFFFFFFFFFFFFFFFFFFFFFFFFFFFFFFFFFF:FFF
@A00155:342:HHGFNDSXY:1:2420:23176:22905 1:N:0:GAACCTAG+TCCGCATA
CTTCTCTCGGGCCCACAGCTTTACAGCTGTGGAGCACGGTATCCTCTGCCGAAACAGAGGTTGGACAA
GACCGGAGGGGTCTCCTAGTTCCAAAGGAGATGTACTCCGGGCTTGTTCACGACCTACC
+
FFFFFFFFFFFFFFFFFFFFFFFFFFFFFFFFFFFFFFFFFFFFFFFFFFFFF:FFFFFFFFFFFFFF
FFFFFFFFFFFFFFFFFFFFFFFFFFFFFFFFFFFFFFFFFFFFFFFFFFFFFFFFFFF
@A00155:342:HHGFNDSXY:1:2421:25292:2487 1:N:0:GAACCTAG+TCCGCATA
GAGCGACTTCTCTCGGGCCCACAGCCTTACAGCTGTGGAGCACGGTATCCTCTGCCGAAACAGAGGTT
GGACAAGACCGGAGGGGTCTCCTAGTTCCAAAGGAGATGTACTCCGGGCTTGTTCACGACCTACCGTG
+
FFFFFFFFFFFFFFFFFFFFFFFFFFFFFFFFFFFFFFFFFFFFFFFFFFFFFFFFFFFFFFFFFFFF
FFFFFFFFFFFFFFFFFFFFFFFFFFFFFFFFFFFFFFFFFFFFFFFFFFFFFFFFFFFFFFFFFFFF
@A00155:342:HHGFNDSXY:1:2421:21983:15389 1:N:0:GAACCTAG+TCCGCATA
CCTCTGGCCTATGGTCATCTCAATGCCGAGAGGGATGTGAAGGGCGAGAGAGAGCGACTTCTCTCGGG
CCCACAGCCTTACAGCTGTGGAGCACGGTATCCTCTGCCGAAACAGAGGTTGGACAAGACCGGAGGGG
+
FFFFFFFFFFFFFFFFFFFFFFFFFFFFFFFFFFFFFFFFFFFFFFFFFFFFFFFFFFFFFFFFFFFF
FFFFFFFFFFFFFFFFFFFFFFFFFFFFFFFFFFFFFFFFFFFFFFFFFFFFFFF:FFFFFFFFFFFF
@A00155:342:HHGFNDSXY:1:2421:6858:15530 1:N:0:GAACCTAG+TCCGCATA
TGGTCATCTCAATGCCGAGAGGGATGTGGAGGGCGAGAGAGAGCGACTTCTCTCGGGCCCACAGCCTT
ACAGCTGTGGAGCACGGTATCCTCTGCCGAAACAGAGGTTGGACAAGACCGGAGGGGTCTCCTAGTTC
+

```
FFFFFFFFFFFFFFFFFFFFFFFFFFFFFFFFFFFFFFFFFFFF:FFFFFFFFFFFFFFFFFFFFF:FF
FFFFFFFFFFFFFFFFFFFFFFFFFFFFFFF:FFFFFFFFFFFFFFFFFFFFFFFFFFFFFFFFFFFFF
@A00155:342:HHGFNDSXY:1:2421:19669:27539 1:N:0:GAACCTAG+TCCGCATA
TGTCCACTTAAGGACTCACCTCTGGCCTATGGTCATCTCAATGCCGAGAGGGATGTGAAGGGCGAGAG
AGAGCGACTTCTCTCGGGCCCACAGCCTTACAGCTGTGGAGCACGGTATCCTCTGCCGAAACAGAGGT
+
FFFFFFFFFFFFFFFFFFFFFFFFFFFFFFFFFFFFFFFFFFFFFFFFFFFFFFFFFFFFFFFFFFFFF
FFFFFFFFFFFFFFFFFFFFFFFFFFFFFFFFFFFFFFFFFFFFFFFFFFFFFFFFFFFFFFFFFFFFF
@A00155:342:HHGFNDSXY:1:2422:32235:1141 1:N:0:GAACCTAG+TCCGCATA
TGTAGAGGGCGAGAGAGAGCGACTTCTCTCGGGCCCACAGCCTTACAGCTGTGGAGCACGGTATCCTC
TGCCGAAACAGAGGTTGGACAAGACCGGAGGGGTCTCCTAGTTCCAAAGGAGATGTACTCCGGG
+
:FFFFFFFFFFFFFFFFFFFFFF,FFFFFFFFFFFFFFFFFFFFFFFFFFFFFFFFFFFFFFFFFFFFF
FFFFFFFFFFFFFFFFFFFFFFFFFFFFFFFFFFFFFFFFFFFFFFFFFFFFFFFFFFFFFFFFF
@A00155:342:HHGFNDSXY:1:2422:9073:2174 1:N:0:GAACCTAG+TCCGCATA
TGGTCATCTCAATGCCGAGAGGGATGTGGAGGGCGAAAGAGAGCGACTTCTCTCGGGCCCACAGCCTT
ACAGCTGTGGAGCACGGTATCCTCTGCCGAAACAGAGGTTGGACAAGACCGGAGGGGTCTCC
+
FFFFFFFFFFFFFFFFFFFFFFFFFFFFFFFFFFFFFFFFFFFFFFFFFFFFFFFFFFFFFFFFFFFFF
FFFFFFFFFFFFFFFFFFFFFFFFFFFFFFFFFFFFFFFFFFFFFFFFFFFFFFFFFFFFFFF
@A00155:342:HHGFNDSXY:1:2422:4580:3662 1:N:0:GAACCTAG+TCCGCATA
CTCTGGCCTATGGTCATCTCAATGCCGAGAGGGATGTGAAGGGCGAGAGAGAGCGACTTCTCTCGGGC
CCACAGCCTTACAGCTGTGGAGCACGGTATCCTCTGCCGAAACAGAGGTTGGACAAGACCGGAGGGGT
+
FFFFFF:FFFFFFFFFFFFFFFFFFFFFFFFFFFFFF:FFFFFFFFFFFFFFFFFFFFFFFFFFFFFFF
FFFF:FFFFFFFF,FFFFFFFFFFFFFFFFFFFFFFFFFFFFFFFFFFFFFFFFFFFFFFFFFF:FFFF
@A00155:342:HHGFNDSXY:1:2422:28275:10692 1:N:0:GAACCTAG+TCCGCATA
AGCGACTTCTCTCGGGCCCACAGCCTTACAGCTGTGGAGCACGGTATCCTCTGCCGAAACAGAGGTTG
GACAAGACCGGAGGGGTCTCCTAGTTCCAAAGGAGATGTACTCCGGGCTTGTTCACGACCTACCGTGT
+
FFFFFFFFFFFFFFFFFF:FFFFFFFFFFFFFFFFFFFFFFFFFFFFFFFFFFFFFFFFFF:FFFFFF
FFFFFFFFFFFFFFFFFFFFFFFFFFFFFFFFFFFFFFFFFFFF,FFFFFFFFFFFFFFFFFFFFFFFF
@A00155:342:HHGFNDSXY:1:2422:27082:15076 1:N:0:GAACCTAG+TCCGCATA
TCTCTCGGGCCCACAGCCTTACAGCTGTGGAGCACGGTATCCTCTGCCGAAACAGAGGTTGGACAAGA
CCGGAGGGGTCTCCTGGTTCCAAAGGAGATGTACTCCGGGCTTGTTCACGACCTACCGTGTAAGTCGT
+
FFFFFFFFFFFFFFFFFFFFFFFFFFFFFFFFF:FFFFFFFFFFFFFFFFFFFFFFFFFFFFFFFFFFF
FFFFFFFFFFFFFFFFFFFFFFFFFFFFFFFFFFFFFFFFFFFFFFFFFFFFFFFFFFFFFFFFFFFFF
@A00155:342:HHGFNDSXY:1:2422:28537:17942 1:N:0:GAACCTAG+TCCGCATA
TAGATCAGCTGTGTCCACTTAAGGACTCACCTCTGGCCTATGGTCATCTCAATGCCGAGAGGGATGTG
AAGGGCGAGAGAGAGCGACTTCTCTCGGGCCCACAGCCTTACAGCTGTGGAGCACGGTATCCTCT
+
,FFF:FFFF:FF,FF:FF,FFFF,FFF:F:FFF:FFF:F:F,,,FFFF:FF:F,,,,F:F:FFFFFFF
::FF:FFFFFFFF:FFFF:,FF:F:F:FFF::F:F,,F:F:F:,FF,,FFF:,:FFF,FF,FFFF
@A00155:342:HHGFNDSXY:1:2422:26304:29857 1:N:0:GAACCTAG+TCCGCATA
TCAATGCCGAGAGGGATGTAGAGGGCGAGAGAGAGCGACTTCTCTCGGGCCCACAGCCTTACAGCTGT
GGAGCACGGTATCCTCTGCCGAAACAGAGGTTGGACAAGACCGGAGGGGTCTCCTAGTTCCAAAGGAG
+
FFFFFFFFFFFFFFFFF:FFFFFFFFFFFFFFFFFFFFFFFFFFFFFFFFFFFFFFFFFFFFFFFFFFF
FFFFFFFFFFFFFFFFFFFFFFFFFFFFF:FFFFFFFFFFFFFFFFFFFFFFFFFFFFFFFFFFFFFFF
@A00155:342:HHGFNDSXY:1:2423:31250:34006 1:N:0:GAACCTAG+TCCGCATA
GAGCGACTTCTCTCGGGCCCACAGCCTTACAGCTGTGGAGCACGGTATCCTCTGCCGAAACAGAGGTT
GGACAAGACCGGAGGGGTCTCCTAGTTCCAAAGGAGATGTACTCCGGGCTTGTTCACGACCTACCGTG
+
```

FFFFFFFFFFFFFFFFFFFFFFFFFFFFFFFFFFFFFFFFFFFFFF:FFFFFFFFFFFFFFFFFFFFF
FFFFF:FFFFFFFFFFFFFFFFF:FFFFFFFFFF,FFFFF:FFFFFFFFFFFFFFFFFFFFF:FFF:F
@A00155:342:HHGFNDSXY:1:2424:12373:12931 1:N:0:GAACCTAG+TCCGCATA
AAGGACTCACCTCTGGCCTATGGTCATCTCAATGCCGAGAGGGATGTAGAGGGCGAGAGAGAGCGACT
TCTCTCGGGCCCACAGCCTTACAGCTGTGGAGCACGGTATCCTCTGCCGAAACAGAGGTTGGACAAGA
+
FFFFFFFFFFFFFFFFFFFFFFFFFFFFFF:FFFFFFFFFFFFFFFFFFFFFFFFFFFFFFFFFFFFF
FFFFFFFFFFFFFFFFFFFFFFFFFFFFFFFFFFFFFFFFFFFFFFFFFFFFFFFFFFFFFFFFFFFF
@A00155:342:HHGFNDSXY:1:2424:12445:14434 1:N:0:GAACCTAG+TCCGCATA
AAGGACTCACCTCTGGCCTATGGTCATCTCAATGCCGAGAGGGATGTAGAGGGCGAGAGAGAGCGACT
TCTCTCGGGCCCACAGCCTTACAGCTGTGGAGCACGGTATCCTCTGCCGAAACAGAGGTTGGACAAGA
+
FFFFFFFFFFFFFFFFFFFFFFFFFFFFFFFFFFFFFFFFFFFFFFFFFFFFFFFFFFFFFFFFFFFF
FFFFFFFFFFFFFFFFFFFFFFFFFFFFFFFFFFFFFFFFFFFFFFFFFFFFFFFFFFFFFFFF:FFF
@A00155:342:HHGFNDSXY:1:2425:19144:2769 1:N:0:GAACCTAG+TCCGCATA
CGGCAGAGGATACCGTGCTCCACAGCTGTAAGGCTGTGGGCCCGAGAGAAGTCGCTCTCTCTCGCCCT
CTACATCCCTCTCGGCATTGAGATGACCATAGGCCAGAGGTGAGTCCTTAAGTGGACACAGCTGATCT
+
FFFFFFFFFFFFFFFFFFFFFFFFFFFFFFFFFFFFFFFFF:FFFFFFFFFFFFFFFFFFFFFFF:FF
FFFF:FFFFFFFFFFFFFFFFFFFFFFFFFFF,FFFFFFFFFFFFF:FFFFFFFFFFFFFFFFFFFFF
@A00155:342:HHGFNDSXY:1:2425:27389:21245 1:N:0:GAACCTAG+TCCGCATA
ACCGCCTTAGATCAGCTGTGTCCACTTAAGGACTCACCTCTGGCCTATGGTCATCTCAATGCCGAGAG
GGATGTGAAGGGCGAGAGAGAGCGACTTCTCTCGGGCCCACAGCCTTACAGCTGTGGAGCACGGTATC
+
FFFFFFFFFFFFFFFFFFFFFFFFFFFFFFFFFFFFFFFFFFFFFFFFFFFFFFFFFFFFFFFFFFFF
FFFFFFFFFFFFFFFFFFFFFFFFFFFFFFFFFFFFFFFFFFFFFFFFFFFFFFFFFFFFFFFFFFFF
@A00155:342:HHGFNDSXY:1:2425:14253:27461 1:N:0:GAACCTAG+TCCGCATA
GGACTCACCTCTGGCCTATGGTCATCTCAATGCCGAGAGGGATGTGAAGGGCGAGAGAGAGCGACTTC
TCTCGGGCCCACAGCCTTACAGCTGTGGAGCACGGTATCCTCTGCCGAAACAGAGGTTGGACAA
+
FFFFFFFFFFFFFFFFFFFFFFFFFFFFFFFFFFFFFFFFFFFFFFFFFFFFFFFFFFFFFFFFFFFF
FFFFFFFFFFFFFFFFFFFFFFFFFFFFFFFFFFFFFFFFFFFFFFFFFFFFFFFFFFFFFFFF
@A00155:342:HHGFNDSXY:1:2426:16107:3270 1:N:0:GAACCTAG+TCCGCATA
CTTCTCTCGGGCCCACAGCCTTACAGCTGTGGAGCACGGTATCCTCTGCCGAAACAGAGGTTGGACAA
GACCGGAGGGGTCTCCTAGTTCCAAAGGAGATGTACTCCGGGCTTGTTCACGACCTACCGTGTAAGTC
+
FFFFFFFFFFFFFFFFFFFFFFFFFFFFFFFFFFFFFFFF:FFFFFFFFFFFFFFFFFFFFFFFFFFF
FFFFFFFFFFFFFFFFFFFFFFFFFFFFFF:FFFFFFFFFFFFFFFFFFFFFFFFFFFFFFFFF,FFF
@A00155:342:HHGFNDSXY:1:2426:20491:19163 1:N:0:GAACCTAG+TCCGCATA
CGAGAGAGAGCGACTTCTCTCGGGCCCACAGCCTTACAGCTGTGGAGCACGGTATCCTCTGCCGAAAC
AGAGGTTGGACAAGACCGGAGGGGTCTCCTAGTTCCAAAGGAGATGTACTCCGGGCTTGTTCACGACC
+
FFFFFFFFFFFFFFFFFFFFFFFFFFFFFFFFFFFFFFFFFFFFFFFFFFFFFFFFFFFFFFFFFFFF
FFFFFFFFFFFFFFFFFFFFFFFFFFFFFFFFFFFFFFFFFFFFFFFFFFFFFFFFFFFFFFFFFFFF
@A00155:342:HHGFNDSXY:1:2426:14163:26522 1:N:0:GAACCTAG+TCCGCATA
GGATGTGGAGGGCGAGAGAGAGCGACTTCTCTCGGGCCCACAGCCTTACAGCTGTGGAGCACGGTATC
CTCTGCCGAAACAGAGGTTGGACAAGACCGGAGGGGTCTCCTAGTTCCAAAGGAGATGTACTCC
+
FFF,FFFFFFFFFFFFFFFFFFFFFF:FFFFFFFFFFFFFFFFFFFFFFFFFFFFFFFFFFFFFFFFF
FFFFFFFFFFFFFFFFFFFFFFFFFFFFFFFFFFFFFFFFFFFFFFFFFFFFFFFFFFFFFFFF
@A00155:342:HHGFNDSXY:1:2426:14208:26537 1:N:0:GAACCTAG+TCCGCATA
GGATGTGGAGGGCGAGAGAGAGCGACTTCTCTCGGGCCCACAGCCTTACAGCTGTGGAGCACGGTATC
CTCTGCCGAAACAGAGGTTGGACAAGACCGGAGGGGTCTCCTAGTTCCAAAGGAGATGTACTCC
+

FFFFFFFFFFFFFFFFFFFFFFFFFFFFFFFFFFFFFFFFFFFFFFFFFFFFFFFFFFFFFFFFF:FF
FFFFFFFFFFFFFFFFFFFFFFFFFFFFFFFFFFFFFFFFFFFFFFFFFFFFFFFFFFFFFFFF
@A00155:342:HHGFNDSXY:1:2426:19623:30248 1:N:0:GAACCTAG+TCCGCATA
ACTTCTCTCGGGCCCACAGCCTTACAGCTGTGGAGCACGGTATCCTCTGCCGAAACAGAGGTTGGACA
AGACCGGAGGGGTCTCCTAGTTCCAAAGGAGATGTACTCCGGGCTTGTTCACGACCTACCGTGTAAGT
+
FFFFFFFFFFFFFFFFFFFF:FFFFFFFFFFFFFFFFFFFFFFFFFFFFFFFFFFFFFFFFFFFFFFF
FFFFFFFF,FFFFFFFFFFFFFFFFFFFFFFFFFFFFFFFFFFFFFFFFFFFFFFFFFFFFFFFFFFF
@A00155:342:HHGFNDSXY:1:2427:9308:1548 1:N:0:GAACCTAG+TCCGCATA
GGATGTAGAGGGCGAGAGAGAGCGACTTCTCTCGGGCCCACAGCCTTACAGCTGTGGAGCACGGTATC
CTCTGCCGAAACAGAGGTTGGACAAGACCGGAGGGGTCTCCTAGTTCCAAAGGAGATGTACTCC
+
FFFFFF:FFFFFFFFFFFFFFFFFFFFFFFFFFFFFFFFFFF:FFF:FFFFFFFFFFFFFFFFFFFFF
FFFFFFFFFFFFFFFFFFFFFFFF,FFFFFFFFFFFFFFFFF:FFFFFFFFFFFFFFFFFFFFF
@A00155:342:HHGFNDSXY:1:2427:27190:2926 1:N:0:GAACCTAG+TCCGCATA
AGGGCGAGAGAGAGCGACTTCTCTCGGGCCCACAGCCTTACAGCTGTGGAGCACGGTATCCTCTGCCG
AAACAGAGGTTGGACAAGACCGGAGGGGTCTCCTAGTTCCAAAGGAGATGTACTCCGGGCT
+
FFFFFFFFFFFFFFFFFFFFFF:FFFFFFFFFFFF:FFFFFFFF:FFF,FFFFFFFFFFFFFFF:FFF
FFFF:FFF:FFFFFFFFFFFFFFFFFFFFFFFFFFFFFFFFFFFFFFFFFFF:FFFFFFFF
@A00155:342:HHGFNDSXY:1:2427:5168:9565 1:N:0:GAACCTAG+TCCGCATA
CAATGCCGAGAGGGATGTGGAGGGCGAGAGAGAGCGACTTCTCTCGGGCCCACAGCCTTACAGCTGTG
GAGCACGGTATCCTCTGCCGAAACAGAGGTTGGACAAGACCGGAGGGGTCTCCTAGTTCCAAAGGAGA
+
FFFFFFFFFF:FFFFFFFFFFFFFFFFFFFFFFFFFFFFFFFFFFFFFFFFFFFFFFFFFFFFFFFFF
FFFFFFFFFFFFFFFFFFFFFFFFFFFFF:FFFFFFFF:FFFFFFFFFFFFFFFFFFFF:FFFFFFFF
@A00155:342:HHGFNDSXY:1:2427:29930:10144 1:N:0:GAACCTAG+TCCGCATA
TATCCTCTGCCGAAACAGAGGTTGGACAAGACCGGAGGGGTCTCCTGGTTCCAAAGGAGATGTACTCC
GGGCTTGTTCACGACCTACCGTGTAAGTCGTAGTCTAGTAGGCTACCTGACGAGTCCTTTTTAGGACG
+
FFFFFFFFFFFFFFFFFFFFFFFFFFFFFFFFFFFFFFFFFFFFFFFFFFFF:FFFFFFFFFFFFFFF
FFFF:FFFFFFFFFFFFFFFFFFFFFFFFFFFFFFFFFFFFFF:FFFFFFFFFFFFFFF:FFFFFFFF
@A00155:342:HHGFNDSXY:1:2427:5077:11725 1:N:0:GAACCTAG+TCCGCATA
CAATGCCGAGAGGGATGTGGAGGGCGAGAGAGAGCGACTTCTCTCGGGCCCACAGCCTTACAGCTGTG
GAGCACGGTATCCTCTGCCGAAACAGAGGTTGGACAAGACCGGAGGGGTCTCCTAGTTCCAAAGGAGA
+
FFFFFFF,FFFFFFFFFFFFFF:FFFFFFFFFFFFFFFFFFFFFFFFFFFFFF,FFFFFFFFFFFFFF
FFFFFFFFFFFFFFFFFFFFFFFFFFFFFFF:FFFFFFFFFFFFFFFFFFFFFFFFFFFFFFFFFFFF
@A00155:342:HHGFNDSXY:1:2427:20085:17425 1:N:0:GAACCTAG+TCCGCATA
CGGTAGGTCGTGAACAAGCCCGGAGTACATCTCCTTTGGAACTAGGAGACCCCTCCGGTCTTGTTCAA
CCTCTGTTTCGGCAGAGGATACCGTGCTCCACAGCTGTAAGGCTGTGGGCCCGAGAGAAGTCGCTCTC
+
FFFFFFFFFFFFFFFFFFFFFFFFFFFFFFFFFFFFFFFFFFFFFFFFFFFFFFFFFFFFFFFFFFFF
FFFFFFFFFFFFFFFFFFFFFFFFFFFFFFFFFFFFFFFFFFFFFFFFFFFFFFFFFFFFFFFFFFFF
@A00155:342:HHGFNDSXY:1:2427:6813:23625 1:N:0:GAACCTAG+TCCGCATA
CTCAATGCCGAGAGGGATGTGGAGGGCGAGAGAGAGCGACTTCTCTCGGGCCCACAGCCTTACAGCTG
TGGAGCACGGTATCCTCTGCCGAAACAGAGGTTGGACAAGACCGGAGGGGTCTCCTAGTTCCAAAGGA
+
FFFFFFFFFF:FFF:FFFF:F:FFF:FFFFFFFFFFFFFFF:FFFFFFFFFFFFFFFFFFF,:FFFFF
FFFFFFFFFFFFFFFF:FFFF:FFFF,FFFFFFFFF,FFFFFF:FFFFFFFFF,FFFF,FFFF:FF:F
@A00155:342:HHGFNDSXY:1:2427:26494:23672 1:N:0:GAACCTAG+TCCGCATA
TATGGTCATCTCAATGCCGAGAGGGATGTGGAGGGCGAGAGAGAGCGACTTCTCTCGGGCCCACAGCC
TTACAGCTGTGGAGCACGGTATCCTCTGCCGAAACAGAGGTTGGACAAGACCGGAGGGGTCTCCTAG
+

FFF:FFFFFF:FFFFFFFFFFFFFFFFF,:FF,FFFFFFFFFFFFFFFFFFFFFFFFF:FFFFF:FFF
:FFFFF:FFFFFFFF:FFFFFFF:FFFFFFFFFFFFFFF,FF:FFFFFFFF:F:FFFFFFFFFFFF
@A00155:342:HHGFNDSXY:1:2427:3387:35383 1:N:0:GAACCTAG+TCCGCATA
ATCTCAATGCCGAGAGGGATGTGGAGGGCGAGAGAGAGCGACTTCTCTCGGGCCCACAGCCTTACAGC
TGTGGAGCACGGTATCCTCTGCCGAAACAGAGGTTGGACAAGACCGGAGGGGTCTCCTA
+
FFFFFFFFFFFFFFFFFFFFFFFFFFFFFFFFFFFFFFFFFFFFFFFFFFFFFFFFFFFFFFFFFFFF
FFFFFFFFFFFFF:FFFFFFF:FFFFFFFFFFFFFFFFFFF:FF:FFFFFFFFFFFFFF
@A00155:342:HHGFNDSXY:1:2428:11487:15092 1:N:0:GAACCTAG+TCCGCATA
GGATGTAGAGGGCGAGAGAGAGCGACTTCTCTCGGGCCCACAGCCTTACAGCTGTGGAGCACGGTATC
CTCTGCCGAAACAGAGGTTGGACAAGACCGGAGGGGTCTCCTAGTTCCAAAGGAGATGTACTCC
+
FFFFFFFFFFFFFFFFFFFFFFFFFFFFFFFFFFFFFFFFFFFFFFFFFFFFFFFFFFFFFFFFF:FF
FFFFFFFFFFFFFFFFFFFFFFFFFFFFFFFFFFFFFFFFFFFFFFFFFFFFFFFFFFFFFFFF
@A00155:342:HHGFNDSXY:1:2428:20464:20713 1:N:0:GAACCTAG+TCCGCATA
GCGACTTCTCTCGGGCCCACAGCCTTACAGCTGTGGAGCACGGTATCCTCTGCCGAAACAGAGGTTGG
ACAAGACCGGAGGGGTCTCCTAGTTCCAAAGGAGATGTACTCCGGGCTTGTTCACGACCTACCGTGTA
+
FFFFFFFFFFFFFFFFFFFFFFFFFFFFFFFFFFFFFFFFFFFFFFFFFFFFFFFFFFFFFFFFFFFF
FFFF:FFFFFFFFFFFFFFFF:FFFFFFFFFFFFFFFFFFFFFFFFFF:FFFFFFFFFFFFFFFFFFF
@A00155:342:HHGFNDSXY:1:2429:14281:7748 1:N:0:GAACCTAG+TCCGCATA
TCTCTCGGGCCCACAGCCTTACAGCTGTGGAGCACGGTATCCTCTGCCGAAAAAGAGGTTGGACAAGA
CCGGAGGGGTCTCCTAGTTCCAAAGGAGATGTACTCCGGGCTTGTTCACGACCTACCGTGTAAGTC
+
FFFFF::FFFFF:FFF:F,:FFF:F:FF:FFF,FFFF::F:FFF:,FF,FFFFFFF:FFF,FF:FF:F
F,FFFF:FF:FFFFFF::FFFFFF,:F:FFF:FFFF:,F:,FF::FFF:FFF:FFFFF,FFFFFFF
@A00155:342:HHGFNDSXY:1:2429:22851:10441 1:N:0:GAACCTAG+TCCGCATA
GGATGTGGAGGGCGAGAGAGAGCGACTTCTCTCGGGCCCACAGCCTTACAGCTGTGGAGCACGGTATC
CTCTGCCGAAACAGAGGTTGGACAAGACCGGAGGGGTCTCCTAGTTCCAAAGGAGATGTACTCC
+
FFF:FFFFFFFFFFFFFFFFFFFFFFFFFFFFFFFFFFFFFFFFFF,FFFFFFFFFFFFFFFFFFFFF
FFFFFFFFFFFFFFFFFFFFFFFFFFFFFFFFFFFFFFFFFFFFFFFFFFFFFFFFFFFFFFFF
@A00155:342:HHGFNDSXY:1:2429:24957:13432 1:N:0:GAACCTAG+TCCGCATA
GGATGTGGAGGGCGAGAGAGAGCGACTTCTCTCGGGCCCACAGCCTTACAGCTGTGGAGCACGGTATC
CTCTGCCGAAACAGAGGTTGGACAAGACCGGAGGGGTCTCCTAGTTCCAAAGGAGATGTACTCC
+
FFFFFFFFFFFFFFFFFFFFFFFFFFFFFFFFF:FFF:FFFFFFFFF:FFFFFFFFFFFFFF:FF:FF
FFFFFF:FF:FFFFFF:FFFFFFFF:FFFFFFFFFFFFFFFFFFFFF:FFFF:FFFFF:FFFFF
@A00155:342:HHGFNDSXY:1:2429:19849:23156 1:N:0:GAACCTAG+TCCGCATA
GGATGTGGAGGGCGAGAGAGAGCGACTTCTCTCGGGCCCACAGCCTTACAGCTGTGGAGCACGGTATC
CTCTGCCGAAACAGAGGTTGGACAAGACCGGAGGGGTCTCCTAGTTCCAAAGGAGATGTACTCC
+
FFFFFFFFFFFFFFFFFFFFFFFFFFFFFFFFFFFFFFF:FFFFFFFFFFFFFFFFFFFFFFFFFFFF
FFFFFFFFFFFFFFFFFFFFFFFFFFFFFFFFFFFFFFFFFFFFFFFFFFFFFFFFFFFFFFFF
@A00155:342:HHGFNDSXY:1:2430:24017:12900 1:N:0:GAACCTAG+TCCGCATA
GTCATCTCAATGCCGAGAGGGATGTGGAGGGCGAGAGAGAGCGACTTCTCTCGGGCCCACAGCCTTAC
AGCTGTGGAGCACGGTATCCTCTGCCGAAACAGAGGTTGGACAAGACCGGAGGGGTCTCCTAGTTCCA
+
FFFFFFFFFFFFFFFFFFFFFFFFFFFFFFFFFFFFFFFFFFFFFFFFFFFFFFFFFFFFFFFFFFFF
FFFFFFFFFFFFFFFFFFFFFFFFFFFFFFFFFFFFFFFFFFFFFFFFFFFFFFFFFFFFFFFFFFFF
@A00155:342:HHGFNDSXY:1:2430:19009:13088 1:N:0:GAACCTAG+TCCGCATA
GTGTGGTGGGGCATGGGTTTGAACCCCCATGACGGTCGGAGTCTAGTAGGCTCCCTGATGAGTCCGTT
CCAAGGACGAAACCGTCGTTACTCTTCGAGTTGTGTAAGTTTCGTCCTAAAAAGGACTCGTCAGGTAG
+

FFFFFFFFFFFFFFFFFFFFFFFFFFFFFFFFFFFFFFFFFFFFFFFFFFFFFFFFFFFFFFFFFFFF
FFFFFFFFFFFFFFFFFFFFFFFFFFFFFFF,:FFF:FFFFFFFFFFFFFFFFFFFFFFFFFFFFFFFF
@A00155:342:HHGFNDSXY:1:2430:4164:21292 1:N:0:GAACCTAG+TCCGCATA
TTAAGGACTCACCTCTGGCCTATGGTCATCTCAATGCCGAGAGGGATGTGGAGGGCGAGAGAGAGCGA
CTTCTCTCGGGCCCACAGCCTTACAGCTGTGGAGCACGGTATCCTCTGCCGAAACAGAGGTTGGACAA
+
FFFF:FFFFFFFFFFFFFFFF:FFFFFFFFFFFFFFFFFFFFFFFFF:FFFFFFFFFFFFFFFFFFFFF
FFFFFFFFFFFFFFFFFFFFFFFFFFFFFFFFFFFFFFFFFFFFFFFFFFFFFFFFFFFF:FFFFFFF
@A00155:342:HHGFNDSXY:1:2430:2591:30937 1:N:0:GAACCTAG+TCCGCATA
TGTTTCGGCAGAGGATACCGTGCTCCACAGCTGTAAGGCTGTGGGCCCGAGAGAAGTCGCTCTCTCTC
GCCCTTCACATCCCTCTCGGCATTGAGATGACCATAGGCCAGAGGTGAGTCCTTAAGTGGACACAGCT
+
FFFFFFFFFFFFFFFFFFFFFFFFFFFFFFFFFFFFFFFFFFFFFFFFFFFFFFFFFFFFFFFFFFFF
FFFFFFFFFFFFFFFFFFFFFFFFFFFFFFFFFFFFFF:FFFFFFFFFFFFFFF:FFFFFFFFFFFFFF
@A00155:342:HHGFNDSXY:1:2431:30156:10160 1:N:0:GAACCTAG+TCCGCATA
AATGCCGAGAGGGATGTGGAGGGCGAGAGAGAGCGACTTCTCTCGGGCCCACAGCCTTACAGCTGTGG
AGCACGGTATCCTCTGCCGAAACAGAGGTTGGACAAGACCGGAGGGGTCTCCTAGTTCCAAAGGAGAT
+
FFFFFFFFFFFFFF:F:FFFFF:FFFFFFFFFFFFFFFFFF:FFFFFFFFFFFFFFFFFFF:FFFFFF
FFFFFFFFFFFFFFFFFFFFFFFFFFFFFFFFFFFFFFFFFFFFFFFFFFFF:FFFFFFFFFFFFFF
@A00155:342:HHGFNDSXY:1:2431:30065:15984 1:N:0:GAACCTAG+TCCGCATA
CTTCTCTCGGGCCCACAGCCTTACAGCTGTGGAGCACGGTATCCTCTGCCGAAACAGAGGTTGGACAA
GACCGGAGGGGTCTCCTAGTTCCAAAGGAGATGTACTCCGGGCTTGTTCACGACCTACCGTGTAAGTC
+
FFFFFFFFFFFFFFFFFFFFFFFFFFFFFFFFFFFFFFFFFFFFFFFFFFFFFFFFFFFFFFFFFFFF
FFFFFFFFFFFFFFFFFFFFFFFFFFFFFFFFFFFFFFFFFFFFFFFFFFFFFFFFFFFFFFFF:FFF
@A00155:342:HHGFNDSXY:1:2431:6352:17816 1:N:0:GAACCTAG+TCCGCATA
CTTCTCTCGGGCCCACAGCCTTACAACTGTGGAGCACGGTATCCTCTGCCGAAACAGAGGTTGGACAA
GACCGGAGGGGTCTCCTAGTTCCAAAGGAGATGTACTCCGGGCTTGTTCACGACCTACCGTGTAAGTC
+
FFFFFFFFFFFFFFFFFFFFFFFFFFFFFFFFFFFFFFFF:FFFFFFFFFFFFFFFFFFFFFFFFFFF
FFFFF:FFFFFFFFFFFFFFFFFF:FFFFFFFFF:FFFFF,FFFFFFFFFFFFF:F:,FF:FF,FFFF
@A00155:342:HHGFNDSXY:1:2431:26684:20087 1:N:0:GAACCTAG+TCCGCATA
GAGAGGGATGTGGAGGGCGAGAGAGAGCGACTTCTCTCGGGCCCACAGCCTTACAGCTGTGGAGCACG
GTATCCTCTGCCGAAACAGAGGTTGGACAAGACCGGAGGGGTCTCCTAGTTCCAAAGGAGATGTACTC
+
FFF:FFFFFFFFFFFFFFFFFFFFFFFFFFFFFFFFFFFFFFFFFFFFFFFFFFFFFFFFFFFFFFFF
FFFFFFFFFFFFFFFFFFFFFFFFFFFFFFFFFFFFFFFFFFFFFFFFFFFFFFFFFFFFFFFFFFFF
@A00155:342:HHGFNDSXY:1:2431:13232:25535 1:N:0:GAACCTAG+TCCGCATA
GCTGTGTCCACTTAAGGACTCACCTCTGGCCTATGGTCATCTCAATGCCGAGAGGGATGTGGAGGGCG
AGAGAGAGCGACTTCTCTCGGGCCCACAGCCTTACAGCTGTGGAGCACGGTATCCTCTGCCGAAACAG
+
FFFFFFFFFFFF:FFFFFFFFFFFFFFFFFFFFFFFFFFFFFFFFFFFFFFFFFFFFFFFFFFFFFF
FFFFFFFFFFFFFFFFFFFFFFFFFFFFFFFFFFFFFFFFFFFFFFF:FFFFFFFFFFFFFFFFFFFF
@A00155:342:HHGFNDSXY:1:2431:20292:30373 1:N:0:GAACCTAG+TCCGCATA
GACCAGCTGTGTCCACTTAAGGACTCACCTCTGGCCTATGGTCATCTCAATGCCGAGAGGGATGTGGA
GGGCGAGAGAGAGCGACTTCTCTCGGGCCCACAGCCTTACAGCTGTGGAGCACGGTATCCTCTGCCGA
+
FFFFFFFFFFFFFFFFFFFFFFFFFFFFFFFFFFFFFFFFFFFFFFFFFFFFFFFFFFFFFFFFFFFF
:FFFFFFFFFFFFFFFFF:FFFFFFFFFFFFFFFFFFFFFFFF:FFFFFFFFFFFFFFFFFFFFFFFFFFF
@A00155:342:HHGFNDSXY:1:2431:14344:30436 1:N:0:GAACCTAG+TCCGCATA
AGATCAGCTGTGTCCACTTAAGGACTCACCTCTGGCCTATGGTCATCTCAATGCCGAGAGGGATGTAG
AGGGCGAGAGAGAGCGACTTCTCTCGGGCCCACAGCCTTACAGCTGTGGAGCACGGTATCCTCTGCCG
+

FFFFFFFFFFFFFFFFFFFF:FFFFFFFFFFFFFFFFFFFFFFFFFFFFFFFFFF:FFFFFFFFFFFF
FFFFFFFFFFFFFFF::FFFFFFFFFFFFFFFFFFFFFFFFFFFFFFFFFFFFFFFFFFFFFFFFFFF
@A00155:342:HHGFNDSXY:1:2431:26124:35931 1:N:0:GAACCTAG+TCCGCATA
CTTCTCTCGGGCCCACAGCCTTACAGCTGTGGAGCACGGTATCCTCTGCCGAAACAGAGGTTGGACAA
GACCGGAGGGGTCTCCTGGTTCCAAAGGAGATGTACTCCGGGCTTGTTCACGACCTACCGTGTAAGTC
+
FFFFFFFFFFFFFFFFFFFFFFFFFFFFFFFFFFFFFFFFFFFFFFFFFFFFFFFFFFFFFFFFFFFF
FFFFFFF:FFFFFFFFFFFFFFFFFFFFFFFFFFFFFF:FFFFFFFFFFFFFFFFFFFFFFFFFFFFF
@A00155:342:HHGFNDSXY:1:2432:16369:4163 1:N:0:GAACCTAG+TCCGCATA
GATCAGCTGTGTCCACTTAAGGACTCACCTCTGGCCTATGGTCATCTCAATGCCGAGAGGGATGTGGA
GGGCGAGAGAGAGCGACTTCTCTCGGGCCCACAGCCTTACAGCTGTGGAGCACGGTATCCTCTGCCGA
+
FFFFFFFFFFFFFFFFFFFFFFFFFFFFFFFFFFFFFFFFFFFFFFFFFFFFFFFFFFFFFFFFFFFF
FFFFFFFFFFFFFFFFFFFFFF:FFFFFFFFFFFFFFFFFFFFFFFFFFFFFFFFFFFFFFFFFFFFF
@A00155:342:HHGFNDSXY:1:2432:11749:20807 1:N:0:GAACCTAG+TCCGCATA
CGGTAGGTCGTGAACAAGCCCGGAGTACATCTCCTTTGGAACTAGGAGACCCCTCCGGTCTTGTCCAA
CCTCTGTTTCGGCAGAGGATACCGTGCTCCACAGCTGTAAGGCTGTGGGCCCGAGAGAAGTCGCTCTC
+
FFFFFFFFFFFFFFFFFFFFFFFFFFFFFFFFFFFFFFFFFFFFFFFFFFFFFFF:FFFFF:FFFFFF
FFFFFFFFFF:FFFFFFFFFFFF:FFFFFFFFFFFFFFFFFFFFFFFFFFFFFFFFFFFFFFFFFFFF
@A00155:342:HHGFNDSXY:1:2432:26802:29496 1:N:0:GAACCTAG+TCCGCATA
GATGTGGAGGGCGAGAGAGAGCGACTTCTCTCGGGCCCACAGCCTTACAGCTGTGGAGCACGGTATCC
TCTGCCGAAACAGAGGTTGGACAAGACCGGAGGGGTCTCCTAGTTCCAAAGGAGATGTACTCCGGGCT
+
FFFFFFFFFFFFFFFFFFFFFFFFFFFFF,FF:FF:FFFFFFFFFFFFFFFFFFFFFFFFFFFFFFFF
FFFFFFFFFFFFFFFF:FFFFFFFFFF:FFFFFFFFFFFF:FFFFFF:FFFFFFFFFF:FFFFFF:FF
@A00155:342:HHGFNDSXY:1:2432:3866:33677 1:N:0:GAACCTAG+TCCGCATA
GTCATCTCAATGCCGAGAGGGATGTGGAGGGCGAGAGAGAGCGACTTCTCTCGGGCCCACAGCCTTAC
AGCTGTGGAGCACGGTATCCTCTGCCGAAACAGAGGTTGGACAAGACCGGAGGGGTCTCCTAGTTCCA
+
FFFFFFFFFFFFFFF:FFF:FFFFFFFFFFFFFFFFFFFFFFFFFFFFFFFFFFFFFFFFFFFFFFFF
FFFFFFFFFFFFFFFFFFFFFFFFFFFFFFFFFFFFFFFF:FFFFFFFFFFFFFFFF:FFF:FFFFFF
@A00155:342:HHGFNDSXY:1:2432:15266:33943 1:N:0:GAACCTAG+TCCGCATA
GAGCGACTTCTCTCGGGCCCACAGCCTTACAGCTGTGGAGCACGGTATCCTCTGCCGAAACAGAGGTT
GGACAAGACCGGAGGGGTCTCCTGGTTCCAAAGGAGATGTACTCCGGGCTTGTTCACGACCTACCGTG
+
FFFFFFFFFFFFFFFFFFFFFFFFFFFFFFFFFFFFFFFFFFFFFFFFFFFFFFFFFFFFFFFFFFFF
FFFFFFFFFFFFFFFFFFFFFFFFFFFFFFFFFFFFFFFFFFFFFFFFFFFFFFFFFFFFFFFFFFFF
@A00155:342:HHGFNDSXY:1:2433:13819:7764 1:N:0:GAACCTAG+TCCGCATA
GGATGTAGAGGGCGAGAGAGAGCGACTTCTCTCGGGCCCACAGCCTTACAGCTGTGGAGCACGGTATC
CTCTGCCGAAACAGAGGTTGGACAAGACCGGAGGGGTCTCCTAGTTCCAAAGGAGATGTACTCC
+
FFFFFFFFFFFFFFFFFFFFFFFFFFFFFFFFFFFFFFFFFFFFFFFFFFFFFFFFFFFFFFFFFFFF
FFFFFFFFFFFFFFFFFFFFFFFFFFFFFFFFFFFFFFFFFFFFFFFFFFFFFFFFFFFFFFFF
@A00155:342:HHGFNDSXY:1:2433:17300:13823 1:N:0:GAACCTAG+TCCGCATA
GAGCGACTTCTCTCGGGCCCACAGCCTTACAGCTGTGGAGCACGGTATCCTCTGCCGAAACAGAGGTT
GGACAAGACCGGAGGGGTCTCCTAGTTCCAAAGGAGATGTACTCCGGGCTTGTTCACGACCTACCGTG
+
FFFFFFFFFFFFFFFFFFFFFFFFFFFFFFFFFFFFFFFF:FFFFFFFFFFFFFFFFFFFFFFFFFFF
FFFFFFFFFFFFFFFFFFFFFFFFFFFFFFFFFFFFFFFFFFFFFFFFFFFFFFFFFFFFFFFFFFFF
@A00155:342:HHGFNDSXY:1:2434:9914:4820 1:N:0:GAACCTAG+TCCGCATA
GGATGTGGAGGGCGAGAGAGAGCGACTTCTCTCGGGCCCACAGCCTTACAGCTGTGGAGCACGGTATC
CTCTGCCGAAACAGAGGTTGGACAAGACCGGAGGGGTCTCCTAGTTCCAAAGGAGATGTACTCC
+

```
FFFFFFFFFFFFFFFFFFFFFFFFFFFFFFFFFFFFFFFFFFFFFFFFFFFFFFFFFFFFFFFFFFFF
FFFFFFFFFFFFFFFFFFFFFFFFFFFFFFFFFFFFFFFFFFFFFFFFFFFFFFFFFFFFFFFF
@A00155:342:HHGFNDSXY:1:2434:8232:10614 1:N:0:GAACCTAG+TCCGCATA
AGCGACTTCTCTCGGGCCCACAGCCTTACAGCTGTGGAGCACGGTATCCTCTGCCGAAACAGAGGTTG
GACAAGACCGGAGGGGTCTCCTAGTTCCAAAGGAGATGTACTCCGGGCTTGTTCACGACCTACCGTGT
+
FFFFFFFFFFFFFFFFFFFFFFFFFFFFFFFFFFFFFFFFFFFFF:FFFFFFFFFFFFFFFFFFFFFF
FFFFFFFFFFFFFFFFFFFFFFFFFFFFFFFFFFFFFFFFFFFFFFFFFFFFFFFFFFFFFFFFFFFF
@A00155:342:HHGFNDSXY:1:2434:16251:17143 1:N:0:GAACCTAG+TCCGCATA
TTCTCTCGGGCCCACAGCCTTACAGCTGTGGAGCACGGTATCCTCTGCCGAAACAGAGGTTGGACAAG
ACCGGAGGGGTCTCCTAGTTCCAAAGGAGATGTACTCCGGGCTTGTTCACGACCTACCGTGTAAGTCG
+
FFFFFFFFFFFFFFFFFFFFFFFFFFFFFFFFFFFFFFFFFFFFFFFFFFFFFFFFFFFFFFFFFFFF
FFFFFFFFFFFFFFFFFFFFFFFFFFFFFFFFFFFFFFFFFFFFFFFFFFFFFFFFFFFFFFFFFFFF
@A00155:342:HHGFNDSXY:1:2434:18638:25128 1:N:0:GAACCTAG+TCCGCATA
AGAGAGAGCGACTTCTCTCGGGCCCACAGCCTTACAGCTGTGGAGCACGGTATCCTCTGCCGAAACAG
AGGTTGGACAAGACCGGAGGGGTCTCCTAGTTCCAAAGGAGATGTACTCCGGGCTTGTTCACGACCTA
+
FFFFFFFFFFFFFFFFFFFFFFFFFFFFFFFFFFFFFFFFFFFFFFFFFFFFFFFFFFFFFFFFFFFF
FFFFFFFFFFFFFFFFFFFFFFFFFFFFFFFFFFFFFFFFFFFFFFFFFFFFFFFFFFFFFF:FFFFF
@A00155:342:HHGFNDSXY:1:2434:30355:25128 1:N:0:GAACCTAG+TCCGCATA
GAGCGACTTCTCTCGGGCCCACAGCCTTACAGCTGTGGAGCACGGTATCCTCTGCCGAAACAGAGGTT
GGACAAGACCGGAGGGGTCTCCTAGTTCCAAAGGAGATGTACTCCGGGCTTGTTCACGACCTACCGTG
+
FFFFFFFFFFFFFFFFFFFFFFFF:F:FFFFFFFFFFFFFFFFFFFFFFFFFFFFFFFFFFFFFFFFF
FFFFFFFFFFFFFFFFFFFFFFFF:FFFFFFFFFFFFFFFFFFFFFF:FFFFFFFFFFFFFFF:FFFF
@A00155:342:HHGFNDSXY:1:2434:20139:27696 1:N:0:GAACCTAG+TCCGCATA
AGAGAGAGCGACTTCTCTCGGGCCCACAGCCTTACAGCTGTGGAGCACGGTATCCTCTGCCGAAACAG
AGGTTGGACAAGACCGGAGGGGTCTCCTAGTTCCAAAGGAGATGTACTCCGGACTTGTTCACGACCTA
+
FFFFFFFFFFFFFFFFFFFFFFFFFFFFFFFFFFFFFFFFFFFFFFFFFFFFFFFFFFFFFFFFFFFF
FFFFFFFFFFFFFFFFFFFFFFFFFFFFFFFFFFFFFFFFFFFFFFFFFFFFFFFFFFFFFFFFFFFF
@A00155:342:HHGFNDSXY:1:2434:10465:31923 1:N:0:GAACCTAG+TCCGCATA
TCTCTCTCGCCCTCCACATCCCTCTCGGCATTGAGATGACCATAGGCCAGAGGTGAGTCCTTAAGTGG
ACACAGCTGATCTAAGGCGGTGTGGTGGGGCATGGGTTTGAACCCCCATGACGGTCGGAGTCTAGTAG
+
FFFFFFFFFFFFFFFFFFFFFFFFFFFFFFFFFFFFFFFFFFFFFFFFFFFFFFFFFFFFFFFFFFFF
FF:FFFF:FFFFFFFFFFFF,FFFFFFFFFFFFFFFFFFF:FFFFFFFFFFFFFFFFFFFFFFFFFFF
@A00155:342:HHGFNDSXY:1:2434:1597:34037 1:N:0:GAACCTAG+TCCGCATA
ACTTCTCTCGGGCCCACAGCCTTACAGCTGTGGAGCACGGTATCCTCTGCCGAAACAGAGGTTGGACA
AGACCGGAGGGGTCTCCTAGTTCCAAAGGAGATGTACTCCGGGCTTGTTCACGACCTACCGTGTAAGT
+
FF:FFFFFFFFFFFFFFFFFFFFFFFFF:FFFFFFFFFFFFFFFFFFFFFFFFFFFFFFFFFFFFFFF
FFFFFFFFFFFFFFFFFFFFFFFFFFFF:FFFFFFFFFFFFFFFFFFFFFFFFFFFFFFFFFFFFFFF
@A00155:342:HHGFNDSXY:1:2434:22083:36949 1:N:0:GAACCTAG+TCCGCATA
GAGCGACTTCTCTCGGGCCCACAGCCTTACAGCTGTGGAGCACGGTATCCTCTGCCGAAACAGAGGTT
GGACAAGACCGGAGGGGTCTCCTAGTTCCAAAGGAGATGTACTCCGGGCTTGTTCACGACCTACCGTG
+
FFFFFFFFFFFFFFFFFFFFFFFFFFFFFFFFFFFFFFFFFFFFFFFFFFFFFFFFFFFFFFFFFFFF
FFFFFFFFFFFFFFFFFFFFFFFFFFFFFFFFFFFFFFFFFFFFFFFFFFFFFFFFFFFFFFFFFFFF
@A00155:342:HHGFNDSXY:1:2435:31521:4288 1:N:0:GAACCTAG+TCCGCATA
AGGTCGTGAACAAGCCCGGAGTACATCTCCTTTGGAACTAGGAGACCCCTCCGGTCTTGTCCAACCTC
TGTTTCGGCAGAGGATACCGTGCTCCACAGCTGTAAGGCTGTGGGCCCGAGAGAAGTCGCTCTCTCTC
+
```

FFF,FFFFFFFFF:FFFFFFFFFFFFFFFFFFFFFFFF:F:FFFFFFFF:F::FFF:FF:FF:FFF:F
FFFFFF:FFFFFFFFFFFFFFFFF:FFFFFF,FFFFFFFF,FFFFFFFFFFFFFF:F,FFFFFFFFFF
@A00155:342:HHGFNDSXY:1:2435:11044:18584 1:N:0:GAACCTAG+TCCGCATA
GCGACTTCTCTCGGGCCCACAGCCTTACAGCTGTGGAGCACGGTATCCTCTGCCGAAACAGAGGTTGG
ACAAGACCGGAGGGGTCTCCTAGTTCCAAAGGAGATGTACTCCGGGCTTGTTCACGACCTACCGTGTA
+
F::F:FFFFF:FFFFFFFF:FFFF,FFFFFFF,:FF:,FFFFF:FFFFFFFFFFFFFFFFFFFFF,,F
FFFFFFF:F,F,FF,FFF,F:FFFF,FFFF::FFFF:FFFFFFFFFFFFFFFFF:FFFFFF:FFF:,F
@A00155:342:HHGFNDSXY:1:2435:6705:19335 1:N:0:GAACCTAG+TCCGCATA
CTTCTCTCGGGCCCACAGCCTTACAGCTGTGGAGCACGGTATCCTCTGCCGAAACAGAGGTTGGACAA
GACCGGAGGGGTCTCCTAGTTCCAAAGGAGATGTACTCCGGGCTTGTTCACGACCTACCGTGTAAGTC
+
FFFFFFFFFFFFFFFFFFFFFFFFFFFFFFFFFFFFFFFFFFFFFFFFFFFFFFFFFFFFFFFFFFFF
FFFFFFFFFFFFFFFFFFFFFFFFFFFFFFFFFFFFFFFFF:FFFFFFFFFFFFFFFFFFFFFFFFFF
@A00155:342:HHGFNDSXY:1:2435:2727:23876 1:N:0:GAACCTAG+TCCGCATA
AGCGACTTCTCTCGGGCCCACAGCCTTACAGCTGTGGAGCACGGTATCCTCTGCCGAAACAGAGGTTG
GACAAGACCGGAGGGGTCTCCTAGTTCCAAAGGAGATGTACTCCGGACTTGTTCACGACCTACCGTGT
+
FFFFFFFFFFFFFFFFFFFFFFFFFFFFFFFFFFFFFFFFFFFFF:FFFFFFFFFFFFFFFFFFFFFF
FFFFFFFFFFFFFFFFFFFFFFFFFFFFFFFFFFFFFFFFFFFFFFFFFFFFFFFF:FF:FFFFFFFF
@A00155:342:HHGFNDSXY:1:2435:5547:29136 1:N:0:GAACCTAG+TCCGCATA
TCTCTCGGGCCCACAGCCTTACAGCTGTGGAGCACGGTATCCTCTGCCGAAACAGAGGTTGGACAAGA
CCGGAGGGGTCTCCTAGTTCCAAAGGAGATGTACTCCGGGCTTGTTCACGACCTACCGTGTAAGTCGT
+
FFFFFFFFFFFFFFFFFFFFFFFFFFFFFFFFFFFFFF:FFFFFFFFFFFFFFFFFFFFFFFFFFFFF
FFFFFFFFFFFFFFFFFFFFFFFFFFFFFFFFFFFFFFFFF:FFFFFFFFFFFFFFFFFF:FFFFFFF
@A00155:342:HHGFNDSXY:1:2435:23854:31407 1:N:0:GAACCTAG+TCCGCATA
GAGAGCGACTTCTCTCGGGCCCACAGCCTTACAGCTGTGGAGCACGGTATCCTCTGCCGAAACAGAGG
TTGGACAAGACCGGAGGGGTCTCCTAGTTCCAAAGGAGATGTACTCCGGGCTTGTTCACGACCTACCG
+
FFFFFFFFFFFFFFFFFFFFFFFFFFFFFFFFFFFFFFFFFFFFFFFFFFFFFFFFFFFFFFFFFFFF
FFFFFFFFFFFFFFFFFFFFFFFFFFFFFFFFFFFFFFFFFFFFFFFFFFFFFFFFFFFFFFFFFFFF
@A00155:342:HHGFNDSXY:1:2435:14805:33677 1:N:0:GAACCTAG+TCCGCATA
GAGAGAGCGACTTCTCTCGGGCCCACAGCCTTACAGCTGTGGAGCACGGTATCCTCTGCCGAAACAGA
GGTTGGACAAGACCGGAGGGGTCTCCTAGTTCCAAAGGAGATGTACTCCGGGCTTGTTCACGACCTAC
+
FFFFFFFFFFFFFFFFFFFFFFFFFFFFFFFFFFFFFFFFFFFFFFFFFFFFFFFFFFFFFFFFFFFF
FFFFFFFFFFFFFFFFFFFFFFFFFFFFFFFFFFFFFFFFFFFFFFFFFFFFFFFFFFFFFFFFFFFF
@A00155:342:HHGFNDSXY:1:2436:23258:18630 1:N:0:GAACCTAG+TCCGCATA
GTATCCTCTGCCGAAACAGAGGTTGGACAAGACCGGAGGGGTCTCCTAGTTCCAAAGGAGATGTACTC
CGGGCTTGTTCACGACCTACCGTGTAAGTCGTAGTCTAGTAGGCTACCTGACGAGTCCTTTTTAGGAC
+
FFFFFFFFFFFFFFFFFFFFFFFFFFFFFFFFFFFFFFFFFFFFFFFFFFFFFFFFFFFFFFFFFFFF
FFFFFFFFFFFFFFFFFFFFFFFFFFFFFFFFFFFFFFFFFFFFFFFFFFFFFFFFFFFFFFFFFFFF
@A00155:342:HHGFNDSXY:1:2436:9381:36558 1:N:0:GAACCTAG+TCCGCATA
GGATGTGGAGGGCGAGAGAGAGCGACTTCTCTCGGGCCCACAGCCTTACAGCTGTGGAGCACGGTATC
CTCTGCCGAAACAGAGGTTGGACAAGACCGGAGGGGTCTCCTAGTTCCAAAGGAGATGTACTCCGGGC
+
FFFFFFFFFFFFFFFFFFFFFFFFFFFFFFFFFFFFFFFFFFFFFFFFFFFFFFFFFFFFFFFFFFFF
FFFFFFFFFFFFFFFFFFFFFFFFFFFFFFFFFFFFFFFFFFFFFFFFFFFFFFFFFFFFFFFFFFFF
@A00155:342:HHGFNDSXY:1:2437:29812:1642 1:N:0:GAACCTAG+TCCGCATA
GTAAGTCGTAGTCTAGTAGGCTACCTGACGAGTCCTTTTTAGGACGAAACTTACACAACTCGAAGAGT
TACGACGGTTTCGTCCTTGGAACGGACTCATCAGGGAGCCTACTAGACTCCGACCGTCATGGG
+

```
FFFFFFFFFFFFFFFFFFFFFFFFFFFFFFFFFFFF:FFFFFFFFFFFFFFFFFFFFFFFFFFFFFFF
FFFFFFFFFFF:FFFFFFFFFFFFFFFFFFFFFFFFF:FFFFFFFFFFFFFFFFFFFFFFF:F
@A00155:342:HHGFNDSXY:1:2437:15962:23249 1:N:0:GAACCTAG+TCCGCATA
AGAGAGAGCGACTTCTCTCGGGCCCACAGCCTTACAGCTGTGGAGCACGGTATCCTCTGCCGAAACAG
AGGTTGGACAAGACCGGAGGGGTCTCCTAGTTCCAAAGGAGATGTACTCCGGGCTTGTTCACGACCTA
+
FFFFFFFFFFFFFFFFFFFFFFFFFFFFFFFFFFFFFFFF:FFFFFFFFFFFFFFFFFFFFFFFFFFF
FFFFFFFFFFFFFFFFFFFFFFFFFFFFFFFFFFFFFFFFFFFFFFFFFFFFFFFFFFFFFFFFFFFF
@A00155:342:HHGFNDSXY:1:2437:13602:24580 1:N:0:GAACCTAG+TCCGCATA
TCCTCTGCCGAAACAGAGGTTGGACAAGACCGGAGGGGTCTCCTAGTTCCAAAGGAGATGTACTCCGG
GCTTGTTCACGACCTACCGTGTAAGTCGTAGTCTAATAGGCTACCTGACGAGTCCTTTTTAGGACGAA
+
FFFFFFFFFFFFFFFFFFFFF:FFFFFFFFFFFFFFFFFFFFFFFFFFFFFF,FFFFFFFFFFFFFFF
FFFFFFFF:FFFFFFFFFFFFFFFFFFFFFFFFFF:FFFFFFFFFFFFFFF:FFFFFFFFFFFFF:FF
@A00155:342:HHGFNDSXY:1:2437:14859:28385 1:N:0:GAACCTAG+TCCGCATA
CTTCTCTCGGGCCCACAGCCTTACAGCTGTGGAGCACGGTATCCTCTGCCGAAACAGAGGTTGGACAA
GACCGGAGGGGTCTCCTGGTTCCAAAGGAGATGTACTCCGGGCTTGTTCACGACCTACCGTGTAAGTC
+
FFFFFFFFFFFFFFFFFFFFFFFFFFFFFFFFFFFFFFFFFFFFFFFFFFFFFFFFFFFFFFFFFFFF
FFFFFFFFFFFFFFFFFFFFFFFFFFFFFFFFFFFFFFFFFFFFFFFFFFFFFFFFFFFFFFFFFFFF
@A00155:342:HHGFNDSXY:1:2437:13178:30514 1:N:0:GAACCTAG+TCCGCATA
GAGGGCGAGAGAGAGCGACTTCTCTCGGGCCCACAGCCTTACAGCTGTGGAGCACGGTATCCTCTGCC
GAAACAGAGGTTGGACAAGACCGGAGGGGTCTCCTGGTTCCAAAGGAGATGTACTCCGGGCTTGTTCA
+
FFFFFFFFFFFFFFFFFFFFFFFFFFFFFFFFFFFFFFFFFFFFFFFFFFFFFFFFFFFFFFFFFFFF
FFFFFFFFFFFFFFFFFFFFFFFFFFFFFFFFFFFFFFFFFFFFFFFFFFFFFFFFFFFFFF:FFFFF
@A00155:342:HHGFNDSXY:1:2437:26865:34428 1:N:0:GAACCTAG+TCCGCATA
AATGCCGAGAGGGATGTGGAGGGCGAGAGAGAGCGACTTCTCTCGGGCCCACAGCCTTACAGCTGTGG
AGCACGGTATCCTCTGCCGAAACAGAGGTTGGACAAGACCGGAGGGGTCTCCTAGTTCCAAAGGAGAT
+
FFFFFFF:FFFFFFFFFFFFFFFFFFFFFFFFFFFFFFFFFFFFFFFFFFFFF:FFFFFFFFFFFFFF
FFFFFFFFFFFFFFFFFFFFFFFFFFFFFFFFFFFF:FFFFFFFFFFFFFFFFFFFFFFFFFFFFFFF
@A00155:342:HHGFNDSXY:1:2437:3558:35430 1:N:0:GAACCTAG+TCCGCATA
TGTTTCGGCAGAGGATACCGTGCTCCACAGCTGTAAGGCTGTGGGCCCGAGAGAAGTCGCTCTCTCTC
GCCCTCCACATCCCTCTCGGCATTGAGATGACCATAGGCCAGAGGTGAGTCCTTAAGTGGACACAGCT
+
FFFFFFFFFFFFFFFFFFFFFFFFFFFFFFFFFFFFFFFFFFFFFFFFFFFFFFFFFFFFFFFFFFFF
FFFFFFFFFFFFFFFFFFFFFFFFFFFFFFFFFFFFFFFFFFFFFFFFFFFFFFFFFFFFFFFFFFFF
@A00155:342:HHGFNDSXY:1:2439:10149:22451 1:N:0:GAACCTAG+TCCGCATA
CACGGTATCCTCTGCCGAAACAGAGGTTGGACAAGACCGGAGGGGTCTCCTAGTTCCAAAGGAGATGT
ACTCCGGGCTTGTTCACGACCTACCGTGTAAGTCGTAGTCTAGTAGGCTACCTGACGAGTCCTTTTTA
+
FFFFFFFFFFFFFFFFFFFFFFFFFFFFFFFFFFFFFFFFFFFFFFFFFFFFFFFFFFFFFFFFFFFF
FFFFFFFFFFFFFFFFFF:FFFFFFFFFFFFFFFFFFFFFFFFFFFFFFFF,FFFFFFFFF:F:FFFF
@A00155:342:HHGFNDSXY:1:2440:13910:12900 1:N:0:GAACCTAG+TCCGCATA
GAGAGGGATGTAGAGGGCGAGAGAGAGCGACTTCTCTCGGGCCCACAGCCTTACAGCTGTGGAGCACG
GTATCCTCTGCCGAAACAGAGGTTGGACAAGACCGGAGGGGTCTCCTAGTTCCAAAGGAGATGTACTC
+
FFFFFFFFFFFFFFFFFFFFFFFFFFFFFFFFFFFFFFFFFFFFFFFFFFFFFFFFFFFFFFFFFFFF
FFFFFFFFFFFFFFFFFFFFFFFFFF:FFFFFFFFFFFFFFFFFFFFFFFFFFFFFFFFFFFFFFFFF
@A00155:342:HHGFNDSXY:1:2440:25337:31156 1:N:0:GAACCTAG+TCCGCATA
AGAGAGAGCGACTTCTCTCGGGCCCACAGCCTTACAGCTGTGGAGCACGGTATCCTCTGCCGAAACAG
AGGTTGGACAAGACCGGAGGGGTCTCCTAGTTCCAAAGGAGATGTACTCCGGGCTTGTTCACGACCTA
+
```

```
FFFFFFFFFFFFFFFFFFFFFFFFFFFFFFFFFFFFFFFFFFFFFFFFFFFFFFFFFFFFFFFFFFFF
FFFFFFFFFFFFFFFFFFFFFFFFFFFFFFFFFFFFFFFFFFFFFFFFFFFFFFFFFFFFFFFFFFFF
@A00155:342:HHGFNDSXY:1:2440:25201:31641 1:N:0:GAACCTAG+TCCGCATA
AGAGAGAGCGACTTCTCTCGGGCCCACAGCCTTACAGCTGTGGAGCACGGTATCCTCTGCCGAAACAG
AGGTTGGACAAGACCGGAGGGGTCTCCTAGTTCCAAAGGAGATGTACTCCGGGCTTGTTCACGACCTA
+
FFFFFFFFFFFFFFFFFFFFFFFFFFFFFFFFFFFFFFFFFFFFFFFFFFFFFFFFFFFFFFFFFFFF
FFFFFFFFFFFFFFFFFFFFFFFFFFFFFFFFFFFFFFFFFFFFFFFFFFFFFFFFFFFFFFFFFFFF
@A00155:342:HHGFNDSXY:1:2440:15320:32816 1:N:0:GAACCTAG+TCCGCATA
CCACCACACCGCCTTAGATCAGCTGTGTCCACTTAAGGACTCACCTCTGGCCTATGGTCATCTCAATG
CCGAGAGGGATGTGGAGGGCGAGAGAGAGCGACTTCTCTCGGGCCCACAGCCTTACAGCTGTGGAGCA
+
FFFFF,FF:FFFF:F:FFFFFFFFFFFF,F:::FFF,:FFFFFFFFFF,FFF:F:FFFFFFFFFFFFF
FFFFFFFFFF,FFFFFFFFFFFFF:FFFFFFFFFFF:FFFFFFFFFFFFFFFFFFFF:FF,:FFFFF:
@A00155:342:HHGFNDSXY:1:2440:26648:35149 1:N:0:GAACCTAG+TCCGCATA
GCTGTGTCCACTTAAGGACTCACCTCTGGCCTATGGTCATCTCAATGCCGAGAGGGATGTAGAGGGCG
AGAGAGAGCGACTTCTCTCGGGCCCACAGCCTTACAGCTGTGGAGCACGGTATCCTCTGCCGAAACAG
+
FFFFFFFFFFFFFFFFFFFFFFFFFFFFFFFFFFFFFFFFFFFFFFFFFFFFFFFFF:FF:FFFFFFF
FFFFFFFFFFFFFFFFFFFFFFFFFFFFFFFFFFFFFFFFFFFFFFFFFFFFFFFFFFFFFFFFFFFF
@A00155:342:HHGFNDSXY:1:2441:14507:15468 1:N:0:GAACCTAG+TCCGCATA
CCTCTGGCCTATGGTCATCTCAATGCCGAGAGGGATGTGGAGGGCGAGAGAGAGCGACTTCTCTCGGG
CCCACAGCCTTACAGCTGTGGAGCACGGTATCCTCTGCCGAAACAGAGGTTGGACAAGAC
+
FFFFFFFFFFFFF:FFFFFFFFFFFFFFFFFF:FF,FF:FFFFFFFFFFFFFFFFFFFFFFFFF:FFF
FFFFFFFFFFFF:FFFFF:FFFF:FFFFFFFFFFFFFFFFFFF:FFF:FFFFFFFFFFFF
@A00155:342:HHGFNDSXY:1:2441:5502:15749 1:N:0:GAACCTAG+TCCGCATA
ACCGCCTTAGATCAGCTGTGTCCACTTAAGGACTCACCTCTGGCCTATGGTCATCTCAATGCCGAGAG
GGATGTGAAGGGCGAGAGAGAGCGACTTCTCTCGGGCCCACAGCCTTACAGCTGTGGAGCACGGTATC
+
FFFFFFFFFFFFFFFFFFFFFFFFFFFFFFFFFFFFFFFFFFFFFFFFFFFFFFFFFFFFFFFFFFFF
FFFFFFFF:FFFFFFFFFFFFFFFFFFFFFFFFFFFFFFFFFFFFFFFFFFFFFFFFFFFFFFF:FF
@A00155:342:HHGFNDSXY:1:2442:2573:2879 1:N:0:GAACCTAG+TCCGCATA
GTCATCTCAATGCCGAGAGGGATGTGGAGGGCGAGAGAGAGCGACTTCTCTCGGGCCCACAGCCTTAC
AGCTGTGGAGCACGGTATCCTCTGCCGAAACAGAGGTTGGACAAGACCGGAGGGGTCTCCTAGTTCCA
+
FFFFFFFFFFFFFFFFF:FFFFFFFFFFFFFFFFFFFFFFFFFFFFFFFFFFFFFFFFFFFFFFFFFF
FFFFFFFFFFFFFFF:FFFFFFFFFFFFFFFFFFFFFFFFFFFFFFFFFFFFFFFFFFFFFFF:FFFF
@A00155:342:HHGFNDSXY:1:2442:15094:14231 1:N:0:GAACCTAG+TCCGCATA
GGATGTGGAGGGCGAGAGAGAGCGACTTCTCTCGGGCCCACAGCCTTACAGCTGTGGAGCACGGTATC
CTCTGCCGAAACAGAGGTTGGACAAGACCGGAGGGGTCTCCTAGTTCCAAAGGAGATGTACTCC
+
FFF,FFFFFFFFFFFFFFFFFFFFFFFFFFFFFFFFF:FFFFFFFF,FFFFF:FFFFFFFFFFFF,FF
FFFFFFFFFFFFFFFFF,FFFFFFFFFFFF:FFFFFFFFFFFFFFFFFFFFFFFFFFFFFFFFF
@A00155:342:HHGFNDSXY:1:2442:5909:17675 1:N:0:GAACCTAG+TCCGCATA
TGTCCACTTAAGGACTCTCCTCTGGCCTATGGTCATCTCAATGCCGAGAGGGATGTGGAGGGCGAGAG
AGAGCGACTTCTCTCGGGCCCACAGCCTTACAGCTGTGGAGCACGGTATCCTCTGCCGAAACAGAGGT
+
FFFFFFFFFFFFFFFFFFFFFFFFFFFFFFFFFFFFFFFFFFFFFFFFFFFFFFFFFFFFFFFFFFFF
FFFFFFFFFFFFFFFFFFFFFFFFFFFFF:FFFFFFFFFFFFFFFFFFFFFFFFFFFFFFFFFFFFFF
@A00155:342:HHGFNDSXY:1:2442:5936:17816 1:N:0:GAACCTAG+TCCGCATA
TGTCCACTTAAGGACTCTCCTCTGGCCTATGGTCATCTCAATGCCGAGAGGGATGTGGAGGGCGAGAG
AGAGCGACTTCTCTCGGGCCCACAGCCTTACAGCTGTGGAGCACGGTATCCTCTGCCGAAACAGAGGT
+
```

FFFFF,FFF:FFFFFFFF:FFFFF:FFFFFFFFFFFFFFFFFFFFFFFFF:FF,F:FFFFFFFFFFFF
FFFFFFFFFFFFF,FF:FFFFFFFFFFFF:FFFF:FFF,FFFF:FF:F:FFFFFFFFFFFFFFFF:F:
@A00155:342:HHGFNDSXY:1:2442:29640:21762 1:N:0:GAACCTAG+TCCGCATA
GTCATCTCAATGCCGAGAGGGATGTGGAGGGCGAGAGAGAGCGACTTCTCTCGGGCCCACAGCCTTAC
AGCTGTGGAGCACGGTATCCTCTGCCGAAACAGAGGTTGGACAAGACCGGAGGGGTCTCCTAGTTCCA
+
FFFFFFFFFFFFFFFFFFFFFF:FFFFFFFFFFFFFFFFFFFFFFFFFFFFFFFFFFFFFFFFFFFFF
FFFFFFFFFFFFFFFFFFFFFFFFFFFFFFFFFFFFFFFFFFFF:FFFFFFFFFFFFFFFFFFFFFFF
@A00155:342:HHGFNDSXY:1:2442:11514:31015 1:N:0:GAACCTAG+TCCGCATA
GGATGTGGAGGGCGAGAGAGAGCGACTTCTCTCGGGCCCACAGCCTTACAGCTGTGGAGCACGGTATC
CTCTGCCGAAACAGAGGTTGGACAAGACCGGAGGGGTCTCCTGGTTCCAAAGGAGATGTACTCC
+
FFFFFFFFFFFFFFFFFFFFFFFFFFFFFFFFFFFFFFFFFFFFFFFFFFFFFFFFFFFFFFFFFFFF
FFFFFFFFFFFFFFFFFFFFFFFFFFFFFFFFFFFFFFFFFFFFFFFFFFFFFFFFFFFFFFFF
@A00155:342:HHGFNDSXY:1:2442:32850:33489 1:N:0:GAACCTAG+TCCGCATA
CTTCTCTCGGGCCCACAGCCTTACAGCTGTGGAGCACGGTATCCTCTGCCGAAACAGAGGTTGGACAA
GACCGGAGGGGTCTCCTCGTTCCAAAGGAGATGTACTCCGGGCTTGTTCACGACCTACCGTGTAAGTC
+
FFFFFFFFFFFFFFFFFFFFFFFFFFFFFFFFFFFFFFFFFFFFFFFFFFFFFFFFFFFFFFFFFFFF
FFFFFFFFFFFFFFFFFFFFFFFFFFFFFFFFFFFFFFFFFFFFFFFFF:FFFFFFFFFFFFFFFFFF
@A00155:342:HHGFNDSXY:1:2442:4779:34413 1:N:0:GAACCTAG+TCCGCATA
ATCTCAATGCCGAGAGGGATGTGGAGGGCGAGAGAGAGCGACTTCTCTCGGGCCCACAGCCTTACAGC
TGTGGAGCACGGTATCCTCTGCCGAAACAGAGGTTGGACAAGACCGGAGGGGTCTCCTAGTTCCTAAG
+
FFFFFFFFFFFFFFFFFFF:FFF:FFFFFFFFFFFFFFFFFFFFFFFFFFFFFFFFFFF:FFFFFFFF
FFFFFFFFFFFFFFFFFFFFFFFFFFFFFFFFF:FFFFFFFFFF:FFFFF,FFFFFFF:FFFFFF:FF
@A00155:342:HHGFNDSXY:1:2443:21847:8641 1:N:0:GAACCTAG+TCCGCATA
AGAGAGAGCGACTTCTCTCGGGCCCACAACCTTACAGCTGTGGAGCACGGTATCCTCTGCCGAAACAG
AGGTTGGACAAGACCGGAGGGGTCTCCTAGTTCCAAAGGAGATGTACTCCGGGCTTGTTCACGACCTA
+
FFFFFFFFFFFFFFFFFFFFFFFFFFFFFFFFFFFFFFFFFFFFFFFFFFFFFFFFFFFFFFFFFFFF
FFFFFFFFFFFFFFFFFFFFFFFFFFFFFFFFFFFFFFFFFFFFFF:FFFFFFFFFFFFFFFFFF:FF
@A00155:342:HHGFNDSXY:1:2443:11876:27226 1:N:0:GAACCTAG+TCCGCATA
ATCTCAATGCCGAGAGGGATGTGGAGGGCGAGAGAGAGCGACTTCTCTCGGGCCCACAGCCTTACAGC
TGTGGAGCACGGTATCCTCTGCCGAAACAGAGGTTGGACAAGACCGGAGGGGTCTCCTAGTTCCAAAG
+
FFFFFFFFFFFFFFFFFFF:FFFFFFFFFFFFFFFFFFFFFFFFFFFFFFFFFFFFFFFFFFFFFFFF
FFFFFFFFFFFFFFFFFFFFFFFFFFFFFFFFFFFFFFFFFFFFFFFFFFFFFFFFFFFFFFFFFFFF
@A00155:342:HHGFNDSXY:1:2444:28664:28244 1:N:0:GAACCTAG+TCCGCATA
CTTCTCTCGGGCCCACAGCCTTACAGCTGTGGAGCACGGTATCCTCTGCCGAAACAGAGGTTGGACAA
GACCGGAGGGGTCTCCTAGTTCCAAAGGAGATGTACTCCGGGCTTGTTCACGACCTACCGTGTAAGTC
+
FFFFFFFFFFFFFFFFFFFFFFFFFFFFFFFFFFFFFFFFFFFFFFFFFFFFFFFFFFFFFFFFFFFF
FFFFFFFFFFFFFFFFFFFFFFFFFFFFFFFFFFFFFFFFFFFFFFFFFFFFFFFFFFFFFFFFF:FFF
@A00155:342:HHGFNDSXY:1:2444:32117:30217 1:N:0:GAACCTAG+TCCGCATA
TTCTCTCGGGCCCACAGCCTTACAGCTGTGGAGCACGGTATCCTCTGCCGAAACAGAGGTTGGACAAG
ACCGGAGGGGTCTCCTAGTTCCAAAGGAGATGTACTCCGGGCTTGTTCACGACCTACCGTGTAAGTCG
+
FFFFFFFFFFFFFFFFFFFFF,FFFFFFFFFFFFFFFFFFFFFFFFFFFFFFFFFFFFFFFFFFFFFF
FFFFFFFFFFFFFFFFFFFFFFFFFFFFFFFFFFFFFFFFFFFFFFFFFFFFFFFFFFFFFFF:F,FF
@A00155:342:HHGFNDSXY:1:2444:29008:37043 1:N:0:GAACCTAG+TCCGCATA
AAGGACTCTCCTCTGGCCTATGGTCATCTCAATGCCGAGAGGGATGTGGAGGGCGAGAGAGAGCGACT
TCTCTCGGGCCCACAGCCTTACAGCTGTGGAGCACGGTATCCTCTGCCGAAACAGAGGTTGGACAAGA
+

```
FFFFFFFFFFFFFFFFFFFFFFFFFFFFFFFFFFFFFFFFFFFFFFFFFFFFFFFFFFFFFFFFFFFFFF
FFFFFFFFFFFFFFFFFFFFFFFFFFFFFFFFFFFFFFFFFFFFFFFFFFFFFFFFFFFFFFFFFFFFFF
@A00155:342:HHGFNDSXY:1:2445:26567:3255 1:N:0:GAACCTAG+TCCGCATA
CCTCCACATCCCTCTCGGCATTGAGATGACCATAGGCCAGAGGTGAGTCCTTAAGTGGACACAGCTGA
TCTAAGGCGGTGTGGCGGGGCATGGGTTTGAACCCCCATGACGGTCGGAGTCTAGTAGGCTCCCTGAT
+
FFFFFFFFFFFFFFFFFFFFF:FFFFFFFFFFFFFFFFFFFFFFFFFFFFFFFFFFFFFFFFFFFFFFFF
FFFFFFFFFFFFFFFFFFFFFFFFFFFFFFFFFFFFFFFFFFFFFFFFFFFFFFFFFFFFFFFFFFFFFF
@A00155:342:HHGFNDSXY:1:2445:14498:8218 1:N:0:GAACCTAG+TCCGCATA
GTCATCTCAATGCCGAGAGGGATGTGGAGGGCGAGAGAGAGCGACTTCTCTCGGGCCCACAGCCTTAC
AGCTGTGGAGCACGGTATCCTCTGCCGAAACAGAGGTTGGACAAGACCGGAGGGGTCTCCTAGTTCCA
+
FFFFFFFFFFFFFFFFFFFFFFFFFFFFFFFFFFFFFFFFFFFFFFFFFFFFFFFFFFFFFFFFFFFFFF
FFFFFFFFFFFFFFFFFFFFFFFFFFFFFFFFFFFFFFFFFFFFFFFFFFFFFFFFFFFFFFFFFFFFFF
@A00155:342:HHGFNDSXY:1:2445:31340:22044 1:N:0:GAACCTAG+TCCGCATA
CTTAAGGACTCTCCTCTGGCCTATGGTCATCTCAATGCCGAGAGGGATGTGGAGGGCGAGAGAGAGCG
ACTTCTCTCGGGCCCACAGCCTTACAGCTGTGGAGCACGGTATCCTCTGCCGAAACAGAGGTTGGACA
+
FFFFFFFFFFFFFFFFFFFFFFFFFFFFFFFFFFFFFFFFFFFFFFFFFFFFFFFFFFFFFFFFFFFFFF
:FFFFFFFFFFFFFFFFFFFF:FFFFFFFFFFFFFFFFFFFFFFFFFFFFFFFFFFFFFFFFFFFFF::F
@A00155:342:HHGFNDSXY:1:2445:7030:31892 1:N:0:GAACCTAG+TCCGCATA
GTCATCTCAATGCCGAGAGGGATGTGGAGGGCGAGAGAGAGCGACTTCTCTCGGGCCCACAGCCTTAC
AGCTGTGGAGCACGGTATCCTCTGCCGAAACAGAGGTTGGACAAGACCGGAGGGGTCTCCTAGTTCCA
+
FFFFFFFFFFFFFFFFFFFFFFFFFFFFFFFFFFFFFFFFFFFFFFFFFFFFFFFFFFFFFFFFFFFFFF
FFFFFFFFFFFFFFFFFFFFFFFFFFFFFFFFFFFFFFFFFFFFFFFFFFFFFFFFFFFFFF,FFFFFF
@A00155:342:HHGFNDSXY:1:2446:18710:20964 1:N:0:GAACCTAG+TCCGCATA
AGCGACTTCTCTCGGGCCCACAGCCTTACAGCTGTGGAGCACGGTATCCTCTGCCGAAACAGAGGTTG
GACAAGACCGGAGGGGTCTCCTAGTTCCAAAGGAGATGTACTCCGGGCTTGTTCACGACCTACCGTGT
+
FFFFFFFFFFFFFFFFFFFFFFFFFFFFFFFFFFFFFFFFFFFFFFFFFFFFFFFFFFFFFFFFFFFFFF
FFFFFFFFFFFFFFFFFFFFFFFFFFFFFFFFFFFFFFFFFFFFFFFFF:FFFFFFFFFFFFFFFFFFF
@A00155:342:HHGFNDSXY:1:2447:9968:8422 1:N:0:GAACCTAG+TCCGCATA
GGGCGAGAGAGAGCGACTTCTCTCGGGCCCACAGCCTTACAGCTGTGGAGCACGGTATCCTCTGCCGA
AACAGAGGTTGGACAAGACCGGAGGGGTCTCCTAGTTCCAAAGGAGATGTACTCCGGGCTTGTTCACG
+
FFFFFFFFFFFFFFFFFFFFFFFFFFFFFFFFFFFFFFFFFFFFFFFFFFFFFFFFFFFFFFFFFFFFFF
FFFFFFFFFFFFFFFFFFFFFFFFFFFFFFFFFFFFFFFFFFFFFFFFFFFFFFFFFFFFFFFFFFFFFF
@A00155:342:HHGFNDSXY:1:2447:8187:24909 1:N:0:GAACCTAG+TCCGCATA
TGTCCACTTAAGGACTCACCTCTGGCCTATGGTCATCTCAATGCCGAGAGGGATGTGGAGGGCGAGAG
AGAGCGACTTCTCTCGGGCCCACAGCCTTACAGCTGTGGAGCACGGTATCCTCTGCCGAAACAGAGGT
+
FFFFFFFFFFFFFFFFFFFFFFFFFFFFFFFF:FFFFFFFFFFFFFFFFFFFFFFFFFFFFFFFFFFFFF
FFFFFFFFFFFFFFFFFFFFFFFFFFFFFFFFFFFFFFFFFFFFFFFFFFFFFFFFFFFFFFFFFFFFFF
@A00155:342:HHGFNDSXY:1:2448:8910:1986 1:N:0:GAACCTAG+TCCGCATA
ACTTCTCTCGGGCCCACAGCCTTACAGCTGTGGAGCACGGTATCCTCTGCCGAAACAGAGGTTGGACA
AGACCGGAGGGGTCTCCTAGTTCCAAAGGAGATGTACTCCGGGCTTGTTCACGACCTACCGTGTAAGT
+
FFFFFFFFFFFFFFFFFFFFFFFFFFFFFFFFFFFFFFFFFFFFFFFFFFFFFFFFFFFFFFFFFFFFFF
FFFFFFFFFFFFFFFFFFFFFFFFFFF:FFFFFFFFFFFFFFFFFFFFFFFFFFFFFFFFFFFFFFFFFF
@A00155:342:HHGFNDSXY:1:2448:12662:23109 1:N:0:GAACCTAG+TCCGCATA
CACCTCTGGCCTATGGTCATCTCAATGCCGAGAGGGATGTGGAGGGCGAGAGAGAGCGACTTCTCTCG
GGCCCACAGCCTTACAGCTGTGGAGCACGGTATCCTCTGCCGAAACAGAGGTTGGACAAGACCGGAGG
+
```

FFFFFFFFFFFFFFFFFFFFFFFFFFFFFFFFFFFFFFFFFFFFFFFFFFFFFFFFFFFFFFFFFFFFFF
FFFFFFFFFFFFFFFFFFFFFFFFFFFFFFFFFFFFFFFFFFFFFFFFFFFFFFFFFFFFF:FFFFFFFF
@A00155:342:HHGFNDSXY:1:2448:24885:24956 1:N:0:GAACCTAG+TCCGCATA
AGATCAGCTGTGTCCACTTAAGGACTCACCTCTGGCCTATGGTCATCTCAATGCCGAGAGGGATGTGA
AGGGCGAGAGAGAGCGACTTCTCTCGGGCCCACAGCCTTACAGCTGTGGAGCACGGTATCCTCTGCCG
+
FFFFFFFFFFFFFFFFFFFFFFFFFFFFFFFFFFFFFFFFFFFFFFFFFFFFFFFFFFFFFFFFFFFFFF
FFFFFFFFFFFFFFFFFFFFFFFFFFFFFFFFFFFF:FFFFFF:FFFFFFFFFFFFFFFFFFFFFFFFF
@A00155:342:HHGFNDSXY:1:2448:31458:25066 1:N:0:GAACCTAG+TCCGCATA
CTCTGGCCTATGGTCATCTCAATGCCGAGAGGGATGTGGAGGGCGAGAGAGAGCGACTTCTCTCGGGC
CCACAGCCTTACAGCTGTGGAGCACGGTATCCTCTGCCGAAACAGAGGTTGGACAAGACCGGAGGGGT
+
FFFFFFFFFFFFFFFFFFF:F:FFFFFF:FFF:FF:FFFFFFFFFFFFFFFFFFFFFFFFFFFFFFFF
FFFFFFFF:FFFFFFFFFFF:FFFFFFFFFFFFFFFFFFFFFFFFFFFFF,FFFFFFFFFFFFFFFFFF
@A00155:342:HHGFNDSXY:1:2449:13910:21543 1:N:0:GAACCTAG+TCCGCATA
GAGCGACTTCTCTCGGGCCCACAGCCTTACAGCTGTGGAGCACGGTATCCTCTGCCGAAACAGAGGTT
GGACAAGACCGGAGGGGTCTCCTAGTTCCAAAGGAGATGTACTCCGGGCTTGTTCACGACCTACCGTG
+
FFFFFFFFFFFFFFFFFFFFFFFFFFFFFFFFFFFFFFFFFFFFFFFFFFFFFFFFFFFFFFFFFFFFFF
FFFFFFF:FFFFFFFFFFFFFFFFFFFFFFFFFFFFFFFFFFFFFFFFFFFFFFFFFFFFFFFFFF:F
@A00155:342:HHGFNDSXY:1:2449:26332:27962 1:N:0:GAACCTAG+TCCGCATA
AGAGAGCGACTTCTCTCGGGCCCACAGCCTTACAGCTGTGGAGCACGGTATCCTCTGCCGAAACAGAG
GTTGGACAAGACCGGAGGGGTCTCCTAGTTCCAAAGGAGATGTACTCCGGGCTTGTTCACGACCTACC
+
F,FFFFFFFFFFFFFFFFF:FFFFFFFF:FFFFFFFFFFFFFFFFFFFFFFFF:FFFFFFFFFFFFFFF
FFFF:FFFFFF:FFF:FFFFF:FFFFFF:FFFFFFFFFFFFFFFFFFFFFFFF:FFFFFFFFFFFFFFF
@A00155:342:HHGFNDSXY:1:2450:19560:2707 1:N:0:GAACCTAG+TCCGCATA
TTAAGGACTCACCTCTGGCCTATGGTCATCTCAATGCCGAGAGGGATGTGGAGGGCGAGAGAGAGCGA
CTTCTCTCGGGCCCACAGCCTTACAGCTGTGGAGCACGGTATCCTCTGCCGAAACAGAGGTTGGACAA
+
FFFFFFFFFFFFFFFFFFFFFFFFFFFFFFFFFFFFFFFFFFFFFFF:FFFFFFFFFFFFFFFFFFFFFF
FFFFFFFFFFFFFFFFFFFFFFFFFFFFFFFFFFFFFFFFFFFFFFFFFFFFFFFFFF:FFFFFFFFFFF
@A00155:342:HHGFNDSXY:1:2450:15203:9377 1:N:0:GAACCTAG+TCCGCATA
GTCATCTCAATGCCGAGAGGGATGTGGAGGGCGAGAGAGAGCGACTTCTCTCGGGCCCACAGCCTTAC
AGCTGTGGAGCACGGTATCCTCTGCCGAAACAGAGGTTGGACAAGACCGGAGGGGTCTCCTAGTTCCA
+
FFFFFFFFFFFFFFF:FFFFFFFFFFFFFFFFFFFFFFFFFFFFFFFFFFFFFFFFFFFFFFFFFFFFFF
FFFFFFFFFFFFFFFFFFFFFFFFFFFFFFFFFFFFFFFFFFFFFFFFFFFFFFFFFFFFFFFFFFFFFF
@A00155:342:HHGFNDSXY:1:2450:6225:10081 1:N:0:GAACCTAG+TCCGCATA
ACTTCTCTCGGGCCCACAGCCTTACAGCTGTGGAGCACGGTATCCTCTGCCGAAACAGAGGTTGGACA
AGACCGGAGGGGTCTCCTAGTTCCAAAGGAGATGTACTCCGGGCTTGTTCACGACCTACCGTGTAAGT
+
FFFFFFFFFFFFFFFFFFFFFFFFFFFFFFFFFFFFFFFFFFFFFFFFFFFFFFFFFFFFFFFFFFFFFF
FFFFFFFFFFFFFFFFFFFFFFFFFFFFFFFFFFFFFFFFFFFFF:FFFFFFFFFFFFFFFFFFFFFFFF
@A00155:342:HHGFNDSXY:1:2450:2645:23328 1:N:0:GAACCTAG+TCCGCATA
TGCCCCACCACACCGCCTTAGATCAGCTGTGTCCACTTAAGGACTCACCTCTGGCCTATGGTCATCTC
AATGCCGAGAGGGATGTGGAGGGCGAGAGAGAGCGACTTCTCTCGGGCCCACAGCCTTACAGCTGTGG
+
FFFFFFFFFFFFFFFFFFFFFFFFFFFFFFFFFFFFFFFFFFFFFFFFFFFFFFFFFFFFFFFFFFFFFF
FFFFFFFFFFFFFFFFFFFFFFFFFFFFFFFFFFFFFFFFFFFFFFFFFFFFFFFF:FFFFFFFFFFFFF
@A00155:342:HHGFNDSXY:1:2450:7563:29496 1:N:0:GAACCTAG+TCCGCATA
AAGGACTCACCTCTGGCCTATGGTCATCTCAATGCCGAGAGGGATGTGGAGGGCGAGAGAGAGCGACT
TCTCTCGGGCCCACAGCCTTACAGCTGTGGAGCACGGTATCCTCTGCCGAAACAGAGGTTGGACAAGA
+

FFFFFFFFFFFFFFFFFFFFFFFFFFFFFFFFFFFFFFFFFFFFFFFFFFFFFFFFFFFFFFFFFFFFFFFFFFF
FFFFFFFFFFFFFFFFFFFFFFFFFFFFFFFFFFFFFFFFFFFFFFFFFFFFFFFFFFFF:FFFFFFFFFFFFFF
@A00155:342:HHGFNDSXY:1:2450:6867:29763 1:N:0:GAACCTAG+TCCGCATA
GACTTCTCTCGGGCCCACAGCCTTACAGCTGTGGAGCACGGTATCCTCTGCCGAAACAGAGGTTGGAC
AAGACCGGAGGGGTCTCCTAGTTCCAAAGGAGATGTACTCCGGGCTTGTTCACGACCTACCGTGTAAG
+
FFFFFFFFFFFFFFFFFFFFFF,FFFFFFFFFFFFFFFFFFFFFFFFFFFFFFFFFFFFFFFFFFF:FFF
FFFFFFFFFFFFFFFFFFFFFFFFFFFFFFFFFFFFFFFFFFFFF:FFFFFFFFFFFFFFFFFFFFFFF
@A00155:342:HHGFNDSXY:1:2450:31738:30686 1:N:0:GAACCTAG+TCCGCATA
TTACAGCTGTGGAGCACGGTATCCTCTGCCGAAACAGAGGTTGGACAAGACCGGAGGGGTCTCCTAGT
TCCAAAGGAGATGTACTCCGGGCTTGTTCACGACCTACCGTGTAAGTCGTAGTCTAGTAGGCTACCTG
+
FFFFFFFFFFFFFFFFFFFF:FFFFFFFFFFFFFFFFFFFFFFFFFFFFFFFFFFFFFFFFFFFFFFF
FFFFFFFFFFFFFFFFFFFFFFFFFFFFFFFFFFFFFFFFFFFFFFFFFFFFFFFFFFFFFFFFFFFF
@A00155:342:HHGFNDSXY:1:2450:32298:34194 1:N:0:GAACCTAG+TCCGCATA
GGATGTAGAGGGCGAGAGAGAGCGACTTCTCTCGGGCCCACAGCCTTACAGCTGTGGAGCACGGTATC
CTCTGCCGAAACAGAGGTTGGACAAGACCGGAGGGGTCTCCTAGTTCCAAAGGAGATGTACTCC
+
FFF:FFFFFFFFFFFFFFFFFFFFFFFFFFFFFFFFFFFF:FFFFFFFFFFFFFFFFFF:FFFFFFF
FFFFFFFFFFFFFFFFFFFFFFFFFFFFFFFFFFFFFFFFFFFFFFFFFFFFFFFFFFFF:FFFFFF
@A00155:342:HHGFNDSXY:1:2452:25355:1846 1:N:0:GAACCTAG+TCCGCATA
CCGAGAGGGATGTGGAGGGCGAGAGAGAGCGACTTCTCTCGGGCCCACAGCCTTACAGCTGTGGAGCA
CGGTATCCTCTGCCGAAACAGAGGTTGGACAAGACCGGAGGGGTCTCCTGGTTCCAAAGGAGATGTAC
+
FFFFFFFFFFFFFFFFFFFFFFFFFFFFFFFFFFFFFFFFFFFFFFFFFFFFFFFFFFFFFFFFFFFF
FFFFFFFFFFFFFFFFFFFFFFFFFFFFFFFFFFFFFFFFFFFFFFFFFFFFFFFFFFFFFFFFFFFF
@A00155:342:HHGFNDSXY:1:2452:26729:4726 1:N:0:GAACCTAG+TCCGCATA
GCTCCACAGCTGTAAGGCTGTGGGCCCGAGAGAAGTCGCTCTCTCTCGCCCTCCACATCCCTCTCGGC
ATTGAGATGACCATAGGCCAGAGGTGAGTCCTTAAGTGGACACAGCTGATCTAAGGCGGTGTGGTGGG
+
FFFFFFFFFFFFFFFFFFFFFFFFFFFFFFFFFFFFFFFFFFFFFFFFFFFFFFFFFFFFFFFFFFFF
FFFFFFFFFFFFFFFFF:FFFFFFFFFFFFFFFFFFFFFFFFFFFFFFFFFFFFFFFFFFFFFFFFFF
@A00155:342:HHGFNDSXY:1:2452:11053:7232 1:N:0:GAACCTAG+TCCGCATA
GGACTCACCTCTGGCCTATGGTCATCTCAATGCCGAGAGGGATGTAGAGGGCGAGAGAGAGCGACTTC
TCTCGGGCCCACAGCCTTACAGCTGTGGAGCACGGTATCCTCTGCCGAAACAGAGGTTGGACAAGACC
+
FFFFFFFFFFFFFFFFFFFFFFFFFFFFFFFFFFFFFFFFFFFFFFFFFFFFFFFFFFFFFFFFFFFF
FFFFFFFFFFFFFFFFFFFFFFFFFFFFFFFFFFFFFFFFFFFFFFFFFFFFFFFFFFFFFFFFFFFF
@A00155:342:HHGFNDSXY:1:2452:22779:14638 1:N:0:GAACCTAG+TCCGCATA
CCCGGAGTACATTTCCTTTGGAACTAGGAGACCCCTCCGGTCTTGTCCAACCTCTGTTTCGGCAGAGG
ATACCGTGCTCCACAGCTGTAAGGCTGTGGGCCCGAGAGAAGTCGCTCTCTCTCGCCCTCCACA
+
FFFFFFFFFFFFFFFFFFFFFFFFFFFFFFFFFFFFFFFFFFFFFFFFFFFFFFFFFFFFFFFFFFFF
FFFFFFFFFFFFFFFFFFFFFFFFFFFFFFFFFFFFFFFFFF:FFFFFFFFFFFFFFFFFFFF,FF
@A00155:342:HHGFNDSXY:1:2452:3079:23578 1:N:0:GAACCTAG+TCCGCATA
TGCCGAGAGGGATGTGGAGGGCGAGAGAGAGCGACTTCTCTCGGGCCCACAGCCTTACAGCTGTGGAG
CACGGTATCCTCTGCCGAAACAGAGGTTGGACAAGACCGGAGGGGTCTCCTAGTTCCAAAGGAGATGT
+
FFFFFFFFFFFFFFFFFFFFFFFFFFFFFFFFFFFFFFFFFFFFFFFFFFFFFFFFFFFFFFFFFFFF
FFFFFFFFFFFFFFFFFFFFFFFFF:FFFFFFFFFFFFFFFFFFFFFFFFFFFFFFFFFFFF:FFFFFFFF
@A00155:342:HHGFNDSXY:1:2452:11388:26788 1:N:0:GAACCTAG+TCCGCATA
AGGGATGTGGAGGGCGAGAGAGAGCGACTTCTCTCGGGCCCACAGCCTTACAGCTGTGGAGCACGGTA
TCCTCTGCCGAAACAGAGGTTGGACAAGACCGGAGGGGTCTCCTAGTTCCAAAGGAGATGTACTCCGG
+

```
FFFFFFFFFFFFFFFFFFFFFFFFFFFFFFFFFFFFFFFFFFFFFFFFFFFFFFFFFFFFFFFFFFFF
FFFFFFFFFFFFFFFFFFFF:FFFFFFFFFFFFFFFFFFFFFFFFFFFFFFFFFFFFFFFFFFFFFFF
@A00155:342:HHGFNDSXY:1:2452:26594:34554 1:N:0:GAACCTAG+TCCGCATA
ACTTCTCTCGGGCCCACAGCCTTACAGCTGTGGAGCACGGTATCCTCTGCCGAAACAGAGGTTGGACA
AGACCGGAGGGGTCTCCTGGTTCCAAAGGAGATGTACTCCGGGCTTGTTCACGACCTACCGTGTAAGT
+
FFFFFFFFFFFFFFFFFFFFFFFFFFFFFFFFFFFFFFFFFFFFFFFFFFFFFFFFFFFFFFFFFFFF
FFFFFFFFFFFFFFFFFFFFFFFFFFFFFFFFFFFFFFFFFFFFFF,FFFFFFFFFFFF:FFFFFFFFF
@A00155:342:HHGFNDSXY:1:2452:17544:34601 1:N:0:GAACCTAG+TCCGCATA
TGTTTCGGCAGAGGATACCGTGCTCCACAGCTGTAAGGCTGTGGGCCCGAGAGAAGTCGCTCTCTCTC
GCCCTTCACATCCCTCTCGGCATTGAGATGACCATAGGCCAGAGGTGAGTCCTTAAGTGGACACAGCT
+
FFFFFFFFFFFFFFFFFFFFFFFFFFFFFFFFFFFFFFFFFFFFFFFFFFFFFFFFFFFFFFFFFFFF
FFFFFFFFFFFFFFFFFFFFFFFFFF:FFFFFFFFFFFFFFFFFFFFFFFFFFFFFFFFFFFFFFFFF
@A00155:342:HHGFNDSXY:1:2453:29830:3208 1:N:0:GAACCTAG+TCCGCATA
CTCCTCTGGCCTATGGTCATCTCAATGCCGAGAGGGATGTGGAGGGCGAGAGAGAGCGACTTCTCTCG
GGCCCACAGCCTTACAGCTGTGGAGCACGGTATCCTCTGCCGAAACAGAGGTTGGACAAGACCGGAGG
+
FFFFFFFFFFFFFFFFFFFFFFFFFFFFFFFFFFFFFFFF:FFFFFFFFFFFFFFFFFFFFFFFFFFF
FFFFFFFFFFFFFFFFFFFFFFFFFFFFFFFFFFFFFFFFFFFFFFFFFFFFFFFFFFFFFFFFFFFF
@A00155:342:HHGFNDSXY:1:2453:21404:21308 1:N:0:GAACCTAG+TCCGCATA
GAGAGAGCGACTTCTCTCGGGCCCACAGCCTTACAGCTGTGGAGCACGGTATCCTCTGCCGAAACAGA
GGTTGGACAAGACCGGAGGGGTCTCCTAGTTCCAAAGGAGATGTACTCCGGGCTTGTTCACGACCTAC
+
FFFFFFFFFFFFFFFFFFFFFFFFFFFFFFFFFFFFFFFFFFFFFFFFFFFFFFFFFFFFFFFFFFFF
FFFFFFFFFFFFF:FFFFFFFFFFFFFFFFFFFFFFFFFFFFFFFFFFFFFFFFFFFFFFFFFFFFFF
@A00155:342:HHGFNDSXY:1:2453:3097:24737 1:N:0:GAACCTAG+TCCGCATA
ATGCCGAGAGGGATGTGGGGGGCGAGAGAGAGCGACTTCTCTCGGGCCCACAGCCTTACAGCTGTGGA
GCACGGTATCCTCTGCCGAAACAGAGGTTGGACAAGACCGGAGGGGTCTCCTAGTTCCAAAGGAGATG
+
FFFFFFFFFFFFFFF,FFFFFFFFFFFFFFFFFFFFFFFFFFFFFFFFFFFFFFFFFFFFFFFFFFFF
FFFFFFFFFFFFFFFFFFFFFFFFFFFFFFFFFFF:FFFFFFFFFFFFFFFFFFFFFFFFFFFFFFFF
@A00155:342:HHGFNDSXY:1:2455:32895:11584 1:N:0:GAACCTAG+TCCGCATA
AAGACTCACCTCTGGCCTATGGTCATCTCAATGCCGAGAGGGATGTGGAGGGCGAGAGAGAGCGACTT
CTCTCGGGCCCACAGCCTTACAGCTGTGGAGCACGGTATCCTCTGCCGAAACAGAGGTTGGACAAGAC
+
FFF:FFFFFFFFFFFFFFFFFFFFFFFFFFFFFFFFFFFFFFFFFFFFFFFFFFFFFFFFFFFFFFFF
FFFFFFFFFFFFFFFFFFFFFFFFFFFFFFFFF:FFFFFFFFFFFFFFFFFFFFFFFFFFFFFFFF,F
@A00155:342:HHGFNDSXY:1:2455:8205:12414 1:N:0:GAACCTAG+TCCGCATA
AGTACATCTCCTTTGGAACTAGGAGACCCCTCCGGTCTTGTCCAACCTCTGTTTCGGCAGAGGATACC
GTGCTCCACAGCTGTAAGGCTGTGGGCCCGAGAGAAGTCGCTCTCTCTCGCCCTCCACATCCCTCTCG
+
FFFFFF:F:FF:FFFFFFF::FFF,FFFFFFFFFFFFFFFFFFFFF:FF:FFFFFFFF:FFFFFFFFF
FFFFFFFFFFFFFF,FFFFFFFFFFFFFFFFFFFFFFFFFFFFFFFFFFF:FFFFFFFFFFFFFFFFFF
@A00155:342:HHGFNDSXY:1:2455:25708:16391 1:N:0:GAACCTAG+TCCGCATA
TGGTCATCTCTATGCCGAGAGGGATGTGGAGGGCGAGAGAGAGCGACTTCTCTCGGGCCCACAGCCTT
ACAGCTGTGGAGCACGGTATCCTCTGCCGAAACAGAGGTTGGACAAGACCGGAGGGGTCTCCTAGTTC
+
FFFFFFFFFFFFFF,FFFF,FFFFFFFFFFFF:FFFFFFFFFFFFFFFFFFFFFFFFFFFFFFFFFFF
FFFFFFFFFFFFFFFFFFFFFFFFFFFFFFFFFFFFFFFF:FFFFFF:FFF:FFF:FFFFFFFFFFFF
@A00155:342:HHGFNDSXY:1:2456:24623:17644 1:N:0:GAACCTAG+TCCGCATA
GTCCAACCTCTGTTTCGGCAGAGGATACCGTGCTCCACAGCTGTAAGGCTGTGGGCCCGAGAGAAGTC
GCTCTCTCTCGCCCTCCACATCCCTCTCGGCATTGAGATGACCATAGGCCAGAGGTGAGTCTTTAAGT
+
```

FFFFFFFFFFFFFFFFFFFFFFFFFFFFFFFFFFFFFFFFFFFFFFFFFFFFFFFFFFFFFFFFFFFFFF
FFFFFFFFFFFFFFFFFFFFFFFFFFFFFFFFFFFFFFFFFFFFFFFFFFFF:FFFFFFFFFFFFFFFFF
@A00155:342:HHGFNDSXY:1:2456:3947:30906 1:N:0:GAACCTAG+TCCGCATA
GAGCGACTTCTCTCGGGCCCACAGCCTTACAGCTGTGGAGCACGGTATCCTCTGCCGAAACAGAGGTT
GGACAAGACCGGAGGGGTCTCCTAGTTCCAAAGGAGATGTACTCCGGGCTTGTTCACGACCTACCGTG
+
FFFFFFFFFFFFFF:FFFFFFFF,FFFF:FFFFFFFFFFFFFFFFFFFFFFFFFFFFFFFFFFFFFFFFF
FFFFFFFFFFFFFFFFFFFFFFFFF:FFFFFFFFFFFFFFFFFFFFFFFFFFFFFFFFFFFFFFFFF:FF
@A00155:342:HHGFNDSXY:1:2457:16884:20870 1:N:0:GAACCTAG+TCCGCATA
GTCCAACCTCTGTTTCGGCAGAGGATACCGTGCTCCACAGCTGTAAGGCTGTGGGCCCGAGAGAAGTC
GCTCTCTCTCGCCCTTCACATCCCTCTCGGCATTGAGATGACCATAGGCCAGAGGTGAGTCCTTAAGT
+
FFFFFFFFFFFFFFFFFFFFFFFFFFFFFFFFFFFFFFFFFF,FFFFFFFFFFFFFFFFFFFFFFFFFFF
FFFFFFFFFFFFFFFFFFFFFFFFFFFFFFFFFF,FFFFFFFFFFFFFFFFFFFFFFFFFFFFFFFFFFF
@A00155:342:HHGFNDSXY:1:2457:14904:26490 1:N:0:GAACCTAG+TCCGCATA
ATGCCGAGAGGGATGTGGAGGGCGAGAGAGAGCGACTTCTCTCGGGCCCACAGCCTTACAGCTGTGGA
GCACGGTATCCTCTGCCGAAACAGAGGTTGGACAAGACCGGAGGGGTCTCCTAGTTCCAAAGGAGATG
+
FFFFFFFF:FFF::FFFFFFFFFFFFFF,FFFFFFFFFFFFFFFFFFFFFFFFFFFFF:FFFFFFFFFF
FFFFFFFFFFFFFFFFFFFFFFFFFFFFFFFFFFFFFFFFFFFFFFFFFFFFFFFFFFFFFFFFFFFFFF
@A00155:342:HHGFNDSXY:1:2458:4309:3662 1:N:0:GAACCTAG+TCCGCATA
AGCGACTTCTCTCGGGCCCACAGCCTTACAGCTGTGGAGCACGGTATCCTCTGCCGAAACAGAGGTTG
GACAAGACCGGAGGGGTCTCCTAGTTCCAAAGGAGATGTACTCCGGGCTTGTTCACGACCTACCGTGT
+
FFFFFFFFFFFFFFFFFFFFFFFFFFFFFFFFFFFFFFFFFF:FFFFF::FFFFFFFFFFFFFFFFFFFF
FFFFFFFFFFFFFFFFF:FFFFFFFFFFFFF,:FFFFFFFFFFFFFFFFFFFFFF:FFFFFFFFF:FF
@A00155:342:HHGFNDSXY:1:2458:23981:23766 1:N:0:GAACCTAG+TCCGCATA
AGAGAGAGCGACTTCTCTCGGGCCCACAGCCTTACAGCTGTGGAGCACGGTATCCTCTGCCGAAACAG
AGGTTGGACAAGACCGGAGGGGTCTCCTAGTTCCAAAGGAGATATACTCCGGGCTTGTTC
+
FFFFFFFFFFFFFFFFFFFFFFFFFFFFFFFFFFFFFFFFFFFFFFFFFFFFFFFFFFFFFFFFFFFF,F
FFFFFFFFFFFFFFFFFFFFFFFFFFFFFFFFFFFFFFFFFFFFFFFFFFFFFFFFFFFF
@A00155:342:HHGFNDSXY:1:2458:20365:33223 1:N:0:GAACCTAG+TCCGCATA
TGTCCACTTAAGGACTCACCTCTGGCCTATGGTCATCTCAATGCCGAGAGGGATGTGGAGGGCGAGAG
AGAGCGACTTCTCTCGGGCCCACAGCCTTACAGCTGTGGAGCACGGTATCCTCTGCCGAAACAGAGGT
+
FFFFFFFFFFFFFFFFFFFFFFFFFFFFFFFFF:FFFFFFFFFFFFF:F:FFFFFFFFFFFFFFFFFFF
FFFFFFFF:FFFFFFFFFFFFFFFFFFFFFFFFFFFFFFFFFFFFFF,FFFFFFFFFFFFFFFFFFFF
@A00155:342:HHGFNDSXY:1:2459:2682:26365 1:N:0:GAACCTAG+TCCGCATA
TCTCGGGCCCACAGCCTTACAGCTGTGGAGCACGGTATCCTCTGCCGAAACAGAGGTTGGACAAGACC
GGAGGGGTCTCCTAGTTCCAAAGGAGATGTACTCCGGGCTTGTTCACGACCTACCGTGTAAGTCGTAG
+
FFFFFFFFFFFFFFFFF:FFFFFFFFFFFFFFFFFFFFFFFFFFFFFFFFFFFFFFFFFFFFFFFFFFF
FFFFFFFFFFFFFF,FFFFFFFFFFFFFFFFFFFFFFFFFFFFFFFFFFFFFFFF,FFFFF:FFFFFFFFF
@A00155:342:HHGFNDSXY:1:2459:15302:26428 1:N:0:GAACCTAG+TCCGCATA
CAGCCTTACAGCTGTGGAGCACGGTATCCTCTGCCGAAACAGAGGTTGGACAAGACCGGAGGGGTCTC
CTAGTTCCAAAGGAGATGTACTCCGGGCTTGTTCACGACCTACCGTGTAAGTCGTAGTCTAGTAGGCT
+
FFFFF,FFFFFFFFFFFFFFFFFFFFFFFFFFFFFFFFFFFFFFFFFFFFFFFFFFFFFFFFFFFFFFF
FFFFFFFFFFFFFFFFFFFFFFFFFFFFFFFFFFFFFFFFFFFFFFFFFFFFFFFFFFFFFFFFFFFFFF
@A00155:342:HHGFNDSXY:1:2460:30698:3677 1:N:0:GAACCTAG+TCCGCATA
TTCTCTCGGGCCCACAGCCTTACAGCTGTGGAGCACGGTATCCTCTGCCGAAACAGAGGTTGGACAAG
ATCGGAGGGGTCTCCTAGTTCCAAAGGAGATGTACTCCGGGCTTGTTCACGACCTACCGTGTAAGTCG
+

FFFFFFFFFFFFFFFFFFFFFFFFFFFFFFFFFFFFFFFFFFFFFFFFFFFFFFFFFFFFFFFFFFFFFF
FFFFFFFFFFFFFFFFFFFFFFFFFFFFFFFFFFFFFFFFFFFFFFFFFFFFFFFFFFFFFFFFFFFFFF
@A00155:342:HHGFNDSXY:1:2460:18620:4429 1:N:0:GAACCTAG+TCCGCATA
GAGCGACTTCTCTCGGGCCCACAGCCTTACAGCTGTGGAGCACGGTATCCTCTGCCGAAACAGAGGTT
GGACAAGACCGGAGGGGTCTCCTAGTTCCAAAGGAGATGTACTCCGGGCTTGTTCACGACCTACCGTG
+
FFFFFFFFFFFFFFFFFFFFFFFFFFFFFFFFFFFFFFFFFFFFFFFFFFFFFFFFFFFFFFFFFFFFFF
FFFFFFFFFFFFFFFFFFFFFFFFFFF:FFFFFFFFFFFFFFFFFFFFFFFFFFFFFFFFFFFFFFFFFF
@A00155:342:HHGFNDSXY:1:2460:8205:9878 1:N:0:GAACCTAG+TCCGCATA
GCGACTTCTCTCGGGCCCACAGCCTTACAGCTGTGGAGCACGGTATCCTCTGCCGAAACAGAGGTTGG
ACAAGACCGGAGGGGTCTCCTAGTTCCAAAGGAGATGTACTCCGGGCTTGTTCACGACCTACCGTGTA
+
FFFFFFFFFFFFFFFFFFFFFFFFFFFFFFFFFFFFFFFFFFFFFFFFFFFFFFFFFFFFFFFF:FFFFF
FFFF,FFFFFFFFFFFFFFFFFFFFFFFF:FFFFFFFFFFFFFFFFFFFFFFFFFFFFFFFFFFFFFFFF
@A00155:342:HHGFNDSXY:1:2460:30364:13463 1:N:0:GAACCTAG+TCCGCATA
GTAAGGCTGTGGGCCCGAGAGAAGTCGCTCTCTCTCGCCCTCCACATCCCTCTCGGCATTGAGATGAC
CATAGGCCAGAGGTGAGTCCTTAAGTGGACACAGCTGATCTAAGGCGGTGTGGCGGGGCATGGGTTTG
+
FFFFFFFFFFFFFFFFFFFFFFFFFFFFFFFFFFFFFFFFFFFFFFFFFFFFFFFFFFFFFFFFFFFFFF
FFFFFFFFFFFFFFFFFFFFFFFFFFFFFFF:FFFFFFFFFFFFFFFFFFFFFFFFFFFFFFFFFFFFFF
@A00155:342:HHGFNDSXY:1:2460:6605:23390 1:N:0:GAACCTAG+TCCGCATA
GTCGCTCTCTCTCGCCCTCCACATCCCTCTCGGCATTGAGATGACCATAGGCCAGAGGTGAGTCCTTA
AGTGGACACAGCTGATCTAAGGCGGTGTGGCGGGGCATGGGTTTGAACCCCCATGACGGTCGGAGTCT
+
F:FFFFFFFFFFFFF:FFFFFFFFFFFFFFFFFFFFFFFFFFFFFFFFFFFFF:FFFFFFFFFFFFFF
FFFFFFFFFFFFFFFFFFFFFFFFFFFFFFFFFFFFFFFFFF:FFFFFFFFFFFFFFFFFFFFFFFFFFF
@A00155:342:HHGFNDSXY:1:2460:5412:23860 1:N:0:GAACCTAG+TCCGCATA
CTTCTCTCGGGCCCACAGCCTTACAGCTGTGGAGCACGGTATCCTCTGCCGAAACAGAGGTTGGACAA
GACCGGAGGGGTCTCCTGGTTCCAAAGGAGATGTACTCCGGGCTTGTTCACGACCTACCGTGTAAGTC
+
FFFFFFFFFFFFFFFFFFFFFFFFFFFFFFFFFFFFFFFFFFFFFFFFFFFFFFFFFFFFFF:FFFFFFF
FFFFFFF:FFFFFFFFFFFFFFFFFFFFFFFFFFFFFFFFFFFFFF,FFFFFFFFFFF:FFFFFF:FFF:
@A00155:342:HHGFNDSXY:1:2461:5764:18082 1:N:0:GAACCTAG+TCCGCATA
ATCTCAATGCCGAGAGGGATGTGGAGGGCGAGAGAGAGCGACTTCTCTCGGGCCCACAGCCTTACAGC
TGTGGAGCACGGTATCCTCTGCCGAAACAGAGGTTGGACAAGACCGGAGGGGTCTCCTAGTTCCAAAG
+
FFFFFFFFFFFFFFFFFFFFFFFFFFFFFFFFFFFFFFFFFFFFFFFFFFFFFFFFFFFFFFFFFFFFFF
FFFFFFFFFFFFFFFFFFFFFFFFFFFFF:FFFF::FFFFFFFFFFFFFFFFFFFFFFFFFFFF:FFFFF
@A00155:342:HHGFNDSXY:1:2461:24270:21230 1:N:0:GAACCTAG+TCCGCATA
TGTAGAGGGCGAGAGAGAGCGACTTCTCTCGGGCCCACAGCCTTACAGCTGTGGAGCACGGTATCCTC
TGCCGAAACAGAGGTTGGACAAGACCGGAGGGGTCTCCTAGTTCCAAAGGAGATGTACTCCGGG
+
FFFFFFFFFFFFFFFFFFFFFFFFFFFFFFFFFFFFFFFFFFFFFFFFFFFFFFFFFFFFFFFFFFFFFF
FFFFFFFFFFFFFFFFFFFFFFFFFFFFFFFFFFFFFFFFFFFFFFFFFFFFFFFFFFFFFFFFFF
@A00155:342:HHGFNDSXY:1:2462:30996:26115 1:N:0:GAACCTAG+TCCGCATA
CTTCTCTCGGGCCCACAGCCTTACAGCTGTGGAGCACGGTATCCTCTGCCGAAACAGAGGTTGGACAA
GACCGGAGGGGTCTCCTAGTTCCAAAGGAGATGTACTCCGGGCTTGTTCACGACCTACCGTGTAAGTC
+
FFFFFFFFFFFFFFFFFFFFFFFFFFFFFFFFFFFFFFFFFF:FFFFFFFFFFFFFFFFFFFFFFFFFF:
FFFFFFFFFFFFFFFFFFFFFFFFFFFFFFFFFFFFFFFFFFFFFFFFFFFFFFFFFFFFFFFFFFFFFF
@A00155:342:HHGFNDSXY:1:2463:16251:12821 1:N:0:GAACCTAG+TCCGCATA
TTCTCTCGGGCCCACAGCCTTACAGCTGTGGAGCACGGTATCCTCTGCCGAAACAGAGGTTGGACAAG
ACCGGAGGGGTCTCCTAGTTCCAAAGGAGATGTACTCCGGGCTTGTTCACGACCTACCGTGTAAGTCG
+

FFFFFFFFF:FFFFFFF,F,F:FFFFFF,:FFFFF:FF:FFFFFF:FFF:FFFFFFFFFFFFFFFFF
FFFFFFFFFF:FFF,FFF:FF:FFF,FFFF:FFFFF,FFF:,FFF:FFFF:F:FFFF:F:FFFFFFFF
@A00155:342:HHGFNDSXY:1:2464:22453:2018 1:N:0:GAACCTAG+TCCGCATA
GAGAGGGATGTGGAGGGCGAGAGAGAGCGACTTCTCTCGGGCCCACAGCCTTACAGCTGTGGAGCACG
GTATCCTCTGCCGAAACAGAGGTTGGACAAGACCGGAGGGGTCTCCTAGTTCCAAAGGAGATGTACTC
+
FFFFFFFFFFFFFFFFFFFFFFFFFFFFFFFFFFFFFFFFFFFFFFFFFFFFFFFFFFF:FFFFFFFF
FF:FF:FFFFFFFFFFFFFFFFFFFFFFFFFFFFFFFFFFFFFFFFF:FFFFFFFFFFFFFFFFFFFF
@A00155:342:HHGFNDSXY:1:2464:22634:2926 1:N:0:GAACCTAG+TCCGCATA
GAGAGGGATGTGGAGGGCGAGAGAGAGCGACTTCTCTCGGGCCCACAGCCTTACAGCTGTGGAGCACG
GTATCCTCTGCCGAAACAGAGGTTGGACAAGACCGGAGGGGTCTCCTAGTTCCAAAGGAGATGTACTC
+
FFFFFFFFFFFFFFFFFFFFFFFFFFFFFFFFFFFFFFFFFFFFFFFFFFFFFFFFFFFFFFFFFFFF
FFFFFFFFFFFFFFFFFFFFFFFFFFFFFFFF:FFFFFFFFFFFFFFFFFFFFFFFFFFFFFFFFFFF
@A00155:342:HHGFNDSXY:1:2464:11722:13526 1:N:0:GAACCTAG+TCCGCATA
CTTCTCTCGGGCCCACAGCCTTACAGCTGTGGAGCACGGTATCCTCTGCCGAAACAGAGGTTGGACAA
GACCGGAGGGGTCTCCTAGTTCCAAAGGAGATGTACTCCGGGCTTGTTCACGACCTACC
+
FFFFFFFFFFFFFFFFFFFFFFFFFFFFFFFFFFFFFFF:FFFFFFFFFFFFFFFFFFFFFFFFFFFF
:FFFFFFFFFFFFFFFFFFFFFFFFFF:FFFFFFFFFFFFFFFFFFFFFFFFFFFFFFFFFF
@A00155:342:HHGFNDSXY:1:2464:28149:15828 1:N:0:GAACCTAG+TCCGCATA
GCCCACAGCCTTACAGCTGTGGAGCACGGTATCCTCTGCCGAAACAGAGGTTGGACAAGACCGGAGGG
GTCTCCTAGTTCCAAAGGAGATGTACTCCGGGCTTGTTCACGACCTACCGTGTAAGTCGTCGTCTAGT
+
,FFFFFF:FF,:FFFFF,FFF:FFFFFF:::,:FFFFFFFFFFFFF::FFF:FFFFFF,F:FFFFFF:
FFFFFFFFFFF:F,::FF:FFFFF,F:F:FFFF,FFFFF:FFFF:F:FFF,FFF,:FFFF,F:F:FFF
@A00155:342:HHGFNDSXY:1:2464:20943:36980 1:N:0:GAACCTAG+TCCGCATA
AGAGAGAGCGACTTCTCTCGGGCCCACAGCCTTACAGCTGTGGAGCACGGTATCCTCTGCCGAAACAG
AGGTTGGACAAGACCGGAGGGGTCTCCTAGTTCCAAAGGAGATGTACTCCGGGCTTGTTCACGACCTA
+
FFFFFFFFFFFFFFFFFFFFFFFFFFFFFFFFFFFFFFFFFFFFFFFFFFFFFFFFFFFFFFFFFFFF
FFFFFFFFFFFF:FFFFFFFFFFFFFFFFFFFFFFFFFFFFFFFFFFFFFFFFFFFFFFFFFFFFFF
@A00155:342:HHGFNDSXY:1:2465:32353:1626 1:N:0:GAACCTAG+TCCGCATA
GGATGTGGAGGGCGAGAGAGAGCGACTTCTCTCGGGCCCACAGCCTTACAGCTGTGGAGCACGGTATC
CTCTGCCGAAACAGAGGTTGGACAAGACCGGAGGGGTCTCCTAGTTCCAAAGGAGATGTACTCC
+
FFFFFFFFFFFFFFFFFFFFFFFFFFFFFFFFFFFFFFFFFFFFFFFFFFFFFFFFFFFFFFFFFFFF
FFFFFFFFFF,FFFFFFFFFFFFF:FFFFFFFFFFFFFFFFF,FFFFF:F:FFFFFFFFFFFFF
@A00155:342:HHGFNDSXY:1:2465:3061:8609 1:N:0:GAACCTAG+TCCGCATA
TCTCAATGCCGAGAGGGATGTGGAGGGCGAGAGAGAGCGACTTCTCTCGGGCCCACAGCCTTACAGCT
GTGGAGCACGGTATCCTCTGCCGAAACAGAGGTTGGACAAGACCGGAGGGGTCTCCTAGTTCCAAAGG
+
FFFFFFFFFFFFFFFFFFFFFFFFFFFFFFFFFFFFFFFFFFFFFFFFFFFFFFFFFFFFFFFFFFFF
FFFFFFFFFFFFFFFFFFFFFFFFFFFFFFFFFFFFFFFFFFFF:FFFFFFFFFFFFFFFFFFFFFFF
@A00155:342:HHGFNDSXY:1:2465:12020:11506 1:N:0:GAACCTAG+TCCGCATA
GGACTCACCTCTGGCCTATGGTCATCTCAATGCCGAGAGGGATGTAGAGGGCGAGAGAGAGCGACTTC
TCTCGGGCCCACAGCCTTACAGCTGTGGAGCACGGTATCCTCTGCCGAAACAGAGGTTGGACAAGACC
+
FFFFFFFFF:FFFFFFFFFFFFFFFFFFFFFFFFFFFFFFFFFFFFFFFFFFFFFFFFFFFFFFFFFF
FFFF:FFFFFFFFFFFFFFFFFFFFFFFFFFF:FFFF:F:FFFFFFFFFFFFFFFFFFFFFFFFFF:FFFFF
@A00155:342:HHGFNDSXY:1:2465:24596:14873 1:N:0:GAACCTAG+TCCGCATA
GACTTCTCTCGGGCCCACAGCCTTACAGCTGTGGAGCACGGTATCCTCTGCCGAAACAGAGGTTGGAC
AAGACCGGAGGGGTCTCCTAGTTCCAAAGGAGATGTACTCCGGGCTTGTTCACGACCTACCGTGTAAG
+

FFFFFFFFFFFFFFFFFFFFFFFFFFFFFFFFFFFFFFFFFFFFFFFFFFFFFFFFFFFFFFFFFFFF
FFFFFFFFFFFFFFFFFFFFFFFFFFFFFFFFFFFFFFFFFFFFFFFFFFFFFFFFFFFFFF:FFF,F
@A00155:342:HHGFNDSXY:1:2465:21486:17096 1:N:0:GAACCTAG+TCCGCATA
GGATGTGGAGGGCGAGAGAGAGCGACTTCTCTCGGGCCCACAGCCTTACAGCTGTGGAGCACGGTATC
CTCTGCCGAAACAGAGGTTGGACAAGACCGGAGGGGTCTCCTAGTTCCAAAGGAGATGTACTCC
+
FFF:FFFFFFFFFFFFFFFFFFFFFFFFFFFFFFFFFFFFFFFFFFFFFFFFFFFFFFFFFFFFFFFF
FFF:FFFFFFFFFFFFFFFFFFFFFFFFFFFFFFFFFFFFFFFFFFFFFFFFFFFFFFFFFFFF
@A00155:342:HHGFNDSXY:1:2465:12255:20901 1:N:0:GAACCTAG+TCCGCATA
GGACTCACCTCTGGCCTATGGTCATCTCAATGCCGAGAGGGATGTGAAGGGCGAGAGAGAGCGACTTC
TCTCGGGCCCACAGCCTTACAGCTGTGGAGCACGGTATCCTCTGCCGAAACAGAGGTTGGACAAGACC
+
FFFFFFFFFFFFFFFFFFFFFFFF:FFFFFFFFFFFFFFFFFFFFFFFFFFFFFFFFFF:FFFFFFFF
FFFFFFFFFFFFFFFFFFFFFFFFFFFFFFFFFFFFFFFFFFFFFFFF,FFFFFFFFFFFFFFFF:FF
@A00155:342:HHGFNDSXY:1:2465:31213:21793 1:N:0:GAACCTAG+TCCGCATA
CTTCTCTCGGGCCCACAGCCTTACAGCTGTGGAGCACGGTATCCTCTGCCGAAACAGAGGTTGGACAA
GACCGGAGGGGTCTCTTAGTTCCAAAGGAGATGTACTCCGGGCTTGTTCACGACCTACC
+
FFFFFFFFFFFFFFFFFFFFFFFFFFFFFFFFFFFFFFFFFFFFFFFFFFFFFFFFFFFFFFFFFFFF
FFFFFFFFFFFFFFFFFFFFFFF:FFFFFFFFFFFFFFFFFFFFFFFFFFFFFFFFFFF
@A00155:342:HHGFNDSXY:1:2465:5086:30749 1:N:0:GAACCTAG+TCCGCATA
GGATGTAGAGGGCGAGAGAGAGCGACTTCTCTCGGGCCCACAGCCTTACAGCTGTGGAGCACGGTATC
CTCTGCCGAAACAGAGGTTGGACAAGACCGGAGGGGTCTCCTAGTTCCAAAGGAGATGTACTCC
+
FF,FFFFFFFFFFFFFFFFFFFFFFFFFFFFFFFFFFFFFFFFFFFFFFFFFFFFFFFFFFFFFFFF
FFFFFFFFFFFFFFFFFFFFFFFFFFFFFFFFFFFFFFFFFFFFFFFFFFFFFFFFFFFFFFFF
@A00155:342:HHGFNDSXY:1:2466:30960:35415 1:N:0:GAACCTAG+TCCGCATA
TAGTCTAGTAGGCTACCTGACGAGTCCTTTTTAGGACGAAACTTACACAACTCGAAGAGTAACGACGG
TTTCGTCCTTGGAACGGACTCATCAGGGAGCCTACTAGACTCCGACCGTCATGGGGGTTCAAACCCA
+
FFFFFFFFFFFFFFFFFFFFFFFFFF,::FFFFFFFFFFFFFFFFFFFFFFFFFFFFFFFFFFFFFFF
FFFFFFFF:FFFFFFFFFF:FFFFFFFFFFFFFFFFFFFFFFFFFFFFFFFFFFFFFFFFFFFFFFF
@A00155:342:HHGFNDSXY:1:2467:16857:14434 1:N:0:GAACCTAG+TCCGCATA
TGTCCACTTAAGGACTCACCTCTGGCCTATGGTCATCTCAATGCCGAGAGGGATGTGAAGGGCGAGAG
AGAGCGACTTCTCTCGGGCCCACAGCCTTACAGCTGTGGAGCACGGTATCCTCTGCCGAAACAGAGGT
+
FFFFFFFFFFFF:FFFFFFFFFFFFFFFFFFFFFFFFFFFFFFFFF,FFFFFFFFFFFFFFFFFFFFF
FFFFFFFFFF,:FFFF:FFFFFFFFFFFFFFFFFFFFFFFFFFFFFF:FFFFFFFFFFFFFFFFFFF
@A00155:342:HHGFNDSXY:1:2467:6587:23735 1:N:0:GAACCTAG+TCCGCATA
AATGCCGAGAGGGATGTAGAGGGCGAGAGAGAGCGACTTCTCTCGGGCCCACAGCCTTACAGCTGTGG
AGCACGGTATCCTCTGCCGAAACAGAGGTTGGACAAGACCGGAGGGGTCTCCTAGTTCCAAAGGAGAT
+
FFFFFFFFFFFFFFFFFF:FFFFFFFFFFFFFFFFFFFF:FFFFFFFFFFFFFFFFFFFFFFF::FFF
FFFFFFFFFFFFFFFFFFFFFFFFFFFFF:F:FFFFFFFFFFFFFFFFFFFFFFFFFFFFFFFFFFFF
@A00155:342:HHGFNDSXY:1:2467:6542:23750 1:N:0:GAACCTAG+TCCGCATA
AATGCCGAGAGGGATGTAGAGGGCGAGAGAGAGCGACTTCTCTCGGGCCCACAGCCTTACAGCTGTGG
AGCACGGTATCCTCTGCCGAAACAGAGGTTGGACAAGACCGGAGGGGTCTCCTAGTTCCAAAGGAGAT
+
FFFFFF:FFFFFFFFFFFFFFFFFFFFFFFFFFFFFFFFFFFFFFFFFFFFFFFFFFFFFFFFFFFFF
FFFFFFFFFFFFFFFFFFFFFFFFFFFFFFFFFFFFFFFF:FFFFFFFFFFFFFFFFFFFFFFFFFFF
@A00155:342:HHGFNDSXY:1:2467:7337:25003 1:N:0:GAACCTAG+TCCGCATA
AATGCCGAGAGGGATGTAGAGGGCGAGAGAGAGCGACTTCTCTCGGGCCCACAGCCTTACAGCTGTGG
AGCACGGTATCCTCTGCCGAAACAGAGGTTGGACAAGACCGGAGGGGTCTCCTAGTTCCAAAGGAGAT
+

```
FFFFFFFFFFFFFF:F,FFFFFFFFFFF,FFFFFFFFFFFFFFFFFFFFFFFF:FF,:FFFFF:FFFF
FFFFFFFFFFFFFFFFFFFFFFFFFFFFFFFFFFFFFFFFFFFFFFF:FFFFFFFFFFFFFFFFFFFF
@A00155:342:HHGFNDSXY:1:2468:20862:2174 1:N:0:GAACCTAG+TCCGCATA
CTTCTCTCGGGCCCACAGCCTTACAGCTGTGGAGCACGGTATCCTCTGCCGAAACAGAGGTTGGACAA
GACCGGAGGGGTCTCCTGGTTCCAAAGGAGATGTACTCCGGGCTTGTTCACGACCTACCGTGTAAGTC
+
FFFFFFFFFFFFFFFFFFFFFFFFFFFFFFFFFFFFFFFFFFFFFFFFFFFFFFFFFFFFFFFFFFFF
FFFFFFFFFFFFFFFFFFFFFFFFFFFFFFFFFFFFFFFFFFFFFFFFFFFFFFFFFFFFFFFFFFFF
@A00155:342:HHGFNDSXY:1:2468:10004:6073 1:N:0:GAACCTAG+TCCGCATA
ACTTCTCTCGGGCCCACAGCCTTACAGCTGTGGAGCACGGTATCCTCTGCCGAAACAGAGGTTGGACA
AGACCGGAGGGGTCTCCTAGTTCCAAAGGAGATGTACTCCGGGCTTGTTCACGACCTACCGTGTAAGT
+
FFFFFFFFFFFFFFFFFFFFFFFFFFFFFFFFFFFFFFFFFFFFFFFFFFFFFFFFFFFFFFFF:FFF
FFFFFFFFFFFFFFFFFFFFFFFFFFFFFFFFFFFFFFFFFFFFFFFFFFFFFFFFFFFFFFFFF:,F
@A00155:342:HHGFNDSXY:1:2468:1678:13228 1:N:0:GAACCTAG+TCCGCATA
AGACTCACCTCTGGCCTATGGTCATCTCAATGCCGAGAGGGATGTGGAGGGCGAGAGAGAGCGACTTC
TCTCGGGCCCACAGCCTTACAGCTGTGGAGCACGGTATCCTCTGCCGAAACAGAGGTTGGACAAGACC
+
FFFFFFFFFFFFFFFFF,FFFFFFFFFFFFFFFFFFFFFFF:FFFFFFFFF:FFFFFFFFFFFFFFFF
FFFFFFFFFFFFFFFFFFFFFFFFFFFFFFFFFFFFFF:FFFFFFFFFF:FFFFFFFFFFFFFFFFFF
@A00155:342:HHGFNDSXY:1:2468:18285:16595 1:N:0:GAACCTAG+TCCGCATA
ATCTCAATGCCGAGAGGGATGTAGAGGGCGAGAGAGAGCGACTTCTCTCGGGCCCACAGCCTTACAGC
TGTGGAGCACGGTATCCTCTGCCGAAACAGAGGTTGGACAAGACCGGAGGGGTCTCCTAGTTCCAAAG
+
FFFFFFFFFFF:FFFFFFF:FFF:FFFFF:FFFF:F:FFFFFFFFFFFFFFFFFFFFFFFFFFFFFFF
FFFFFFFFFFFF:FFFFF:FFFFFFFFFF:FFFFF:FFF:FFFF,FFFFFFFFFFFFFFFFFFFFFFF
@A00155:342:HHGFNDSXY:1:2468:16043:19883 1:N:0:GAACCTAG+TCCGCATA
TACGACTTACACGGTAGGTCGTGAACAAGCCCGGAGTACATCTCCTTTGGAACTAGGAGACCCCTCCG
GTCTTGTCCAACCTCTGTTTCGGCAGAGGATACCGTGCTCCACAGCTGTAAGGCTGTG
+
FFFFFFFFFFFFFFFFFFFFFFFFFFFFFFFFFFFFFFFFFFFFFFFFFFFFFFFFFFFFFFFFFFFF
FFFFFFFFFFFFFFFFFFFFFFFFFFFFFFFFF:FFFFFFFFFFFFFFFFFFFFFFFF
@A00155:342:HHGFNDSXY:1:2469:20428:8719 1:N:0:GAACCTAG+TCCGCATA
GAGCGACTTCTCTCGGGCCCACAGCCTTACAGCTGTGGAGCACGGTATCCTCTGCCGAAACAGAGGTT
GGACAAGACCGGAGGGGTCTCCTAGTTCCAAAGGAGATGTACTCCGGGCTTGTTCACGACCTACCGTG
+
FFFFFFFFFFFFFFFFFFFFFFFFFF:FFFFFFFFFFFFFFFFFFFFFFFFFFFFFFFFFFFFFFFFF
FFFFFFFFFFFFFFFFFFFFFFFFFFFFFFFFFFFFFFFFFFFFFFFFFFFFFFFFFFFFFFFFFFFF
@A00155:342:HHGFNDSXY:1:2469:31114:14544 1:N:0:GAACCTAG+TCCGCATA
CAATGCCGAGAGGGATGTAGAGGGCGAGAGAGAGCGACTTCTCTCGGGCCCACAGCCTTACAGCTGTG
GAGCACGGTATCCTCTGCCGAAACAGAGGTTGGACAAGACCGGAGGGGTCTCCTAGTTCCAAAGGAGA
+
FFFFFFFFFFFFFFF,FFFFFFFFFFFFFFFFFFFFFFFFFFFFFFFFFFFFFFFFFFFFFFFFFFFF
FFFFFFFFFFFFFFFFFFFFFFFFFFFFFFFFFFFFFFFFFFFFFFFFFFFFFFFFFFFFFFFFFFFF
@A00155:342:HHGFNDSXY:1:2469:14760:26303 1:N:0:GAACCTAG+TCCGCATA
CCTCTGGCCTATGGTCATCTCAATGCCGAGAGGGATGTGGAGGGCGAGAGAGAGCGACTTCTCTCGGG
CCCACAGCCTTACAGCTGTGGAGCACGGTATCCTCTGCCGAAACAGAGGTTGGACAAGAC
+
:FFFFFFFFF:FF::FFF,,FFFFF:,FFFF,FFF,,FFFF:FF:FFFFFFFFF:FFFFFFF:FFFF:
::FFFFFFFFFFFFFFFFFF:FFFFFFFFFFFFFFFFF:FFFFFF:F,F,FFFFFFFFF:
@A00155:342:HHGFNDSXY:1:2470:23204:1689 1:N:0:GAACCTAG+TCCGCATA
GCGAGAGAGAGCGACTTCTCTCGGGCCCACAGCCTTACAGCTGTGGAGCACGGTATCCTCTGCCGAAA
CAGAGGTTGGACAAGACCGGAGGGGTCTCCTAGTTCCAAAGGAGATGTACTCCGGGCTTGTTCACGAC
+
```

FFFFFFFFFFFFFFFFFFFFFFFFFFFFFFFFFFFFFFFFFFFFFFFFFFFFFFFFFFFFFFFFFFFFFF
FFFFFFFFFFFFFFFFFFFFFFFFFFFFFFFFFFFFFFFFFFFFFFFFFFFFFFFFFFFFFFFFFFFFFF
@A00155:342:HHGFNDSXY:1:2470:2799:2206 1:N:0:GAACCTAG+TCCGCATA
GGACAAGACCGGAGGGGTCTCCTAGTTCCAAAGGAGATGTACTCCGGGCTTGTTCACGACCTACCGTG
TAAGTCGTAGTCTAGTAGGCTACCTGACGAGTCCTTTTTAGGACGAAACTTACACAACTCAAAGAGTA
+
FFFFFFFFFFFFFFFFFFFFFFFFFFFFFFFFFFFFFFFFFFFFFFFF:FFFFFFFFFFFFFFFFFFFF
FFFFFFFFFFFF:FFFFFFFFFFFFFFFFFFFFFFFFFFFFFFFFFFFFFFFFFFFFFFFFFFFFFFFF:
@A00155:342:HHGFNDSXY:1:2470:2582:3709 1:N:0:GAACCTAG+TCCGCATA
AGGGATGTGGAGGGCGAGAGAGAGCGACTTCTCTCGGGCCCACAGCCTTACAGCTGTGGAGCACGGTA
TCCTCTGCCGAAACAGAGGTTGGACAAGACCGGAGGGGTCTCCTGGTTCCAAAGGAGATGTACTCCGG
+
FFFFFFFFFFFFFFFFFFFFFFFFFFFFFFFFFFFFFFFFFFFFFFFFFFF:FFFFFFFFFFFFFFFFFF
FFFFFFFFFFFFFFFFFFFFFFFFFFFFFFFFFFFFFFFFFFFFFFFFFFFF,FFFFFFFFFFFFFFFFF
@A00155:342:HHGFNDSXY:1:2470:28980:35744 1:N:0:GAACCTAG+TCCGCATA
GGCATTGAGATGACCATAGGCCAGAGGTGAGTCCTTAAGTGGACACAGCTGATCTAAGGCGGTGTAGC
GGGGCATGGGTTTGAACCCCCATGACGGTCGGAGTCTAGTAGGCTCCCTGATGAGTCCGTTCCAAGGA
+
FFFFFFFFFFFFFFFFFFFFFF,FFFFFFFFFFFFFFFFFFFFFFFFFFFFFFFFFFFFFFFFFFFFFFF
FFFFFFFFFFFFFFFFFFFFFFFFFFFFFFFFFFFFFFFFFFFFFFFFFFFFFFFFFFFFFFFFFFFFFF
@A00155:342:HHGFNDSXY:1:2471:24415:8703 1:N:0:GAACCTAG+TCCGCATA
TGCCCCGCCACACCGCCTTAGATCAGCTGTGTCCACTTAAGGACTCACCTCTGGCCTATGGTCATCTC
AATGCCGAGAGGGATGTGGAGGGCGAGAGAGAGCGACTTCTCTCGGGCCCACAGCCTTACAGCTGTGG
+
FFFFFFFFFFFFFFFFF:FFFFFFFFFFFFFFFFFFFFFFFFFFFFF:FFFFFFFFFFFFFFFFFFFFFF
FFFF:FFFFFFFFFFFFFFFFFFFFFFFFF:FFFFFFFFFFFFFFFFFFFFFFFFFFFFFFFFFFFFFFF
@A00155:342:HHGFNDSXY:1:2471:14109:9267 1:N:0:GAACCTAG+TCCGCATA
AGCGACTTCTCTCGGGCCCACAGCCTTACAGCTGTGGAGCACGGTATCCTCTGCCGAAACAGAGGTTG
GACAAGACCGGAGGGGTCTCCTAGTTCCAAAGGAGATGTACTCCGGACTTGTTCACGACCTACCGTGT
+
FFFFFFFFFFFFFFFFFFFFFFFFFFFFFFFFFFFFFFFFFFFFFFFFFFFFFFFFFFFFFFFFFFFFFF
FFFFFFFFFFFFFFFFFFFFFFFFFFFFFFFFFFFFFFFFFFFFFFFFFFFFFFFFFFFFFFFFFFFFFF
@A00155:342:HHGFNDSXY:1:2471:7265:20431 1:N:0:GAACCTAG+TCCGCATA
CCTTACAGCTGTGGAGCACGGTATCCTCTGCCGAAACAGAGGTTGGACAAGACCGGAGGGGTCTCCTG
GTTCCAAAGGAGATGTACTCCGGGCTTGTTCACGACCTACCGTGTAAGTCGTAGTCTAGTAGGCTACC
+
FFFFFFFFFFFFFFFFFFFFFFFFFFFFFFFFFFFFFFFFFFFFFFFFFFF:FFFFFFFFFFFF:FFFF
FFFFFFFFFFFFFFFFFFFFFFFFFFFFFFFFFFFFFFFFFFFFFFFFFFFFFFFFFFFFFFFFFFFFFF
@A00155:342:HHGFNDSXY:1:2471:18566:28447 1:N:0:GAACCTAG+TCCGCATA
TGGAGGGCGAGAGAGAGCGACTTCTCTCGGGCCCACAGCCTTACAGCTGTGGAGCACGGTATCCTCTG
CCGAAACAGAGGTTGGACAAGACCGGAGGGGTCTCCTAGTTCCAAAGGAGATGTACTCCGGGCTTGTT
+
FFFFFFFFFFFFFFFFFFFFFFFFFFFFFFFFFFFFFFFFFFFFFFFFFFFFFFFFFFFFFFFFFFFFFF
FFFFFFFFFFFFFFFFFFFFFFFFFFFFFFFFFFFFFFFFFFFFFFFFFFFFFF:FFFFFFFFFFFFFF
@A00155:342:HHGFNDSXY:1:2471:2682:29653 1:N:0:GAACCTAG+TCCGCATA
CTCAATGCCGAGAGGGATGTGGAGGGCGAGAGAGAGCGACTTCTCTCGGGCCCACAGCCTTACAGCTG
TGGAGCACGGTATCCTCTGCCGAAACAGAGGTTGGACAAGACCGGAGGGGTCTCCTAGTTCCAAAGGA
+
FFFFFFFFFFFFFFFFF:FFFFFFF,FFFFFFFFFFFFFFFFFFFFFF,,FFFFFFFFFFFFFFFFFF
:FFFFFFFFFFFFFFFFFFFFFFFFFFFFFFFFFFFFFFFFFFFFFF::FFFFFFFFF:FFFFFFFF:FF
@A00155:342:HHGFNDSXY:1:2471:1832:30749 1:N:0:GAACCTAG+TCCGCATA
CTCAATGCCGAGAGGGATGTGGAGGGCGAGAGAGAGCGACTTCTCTCGGGCCCACAGCCTTACAGCTG
TGGAGCACGGTATCCTCTGCCGAAACAGAGGTTGGACAAGACCGGAGGGGTCTCCTAGTTCCAAAGGA
+

```
FFFFFFFFFFFFFFFFF:F,FFFFFFFFFFFF:FFFFFFFFFFFFFFFFFF:FFFFFFF,FFFFFF,F
FFFFFF:FFFFFFFFFFFFFFF:FFFFFFFFFFFFFFF:FFFFFFFFFFFFFFFFF,F,FFF,FFFFF
@A00155:342:HHGFNDSXY:1:2471:19859:32033 1:N:0:GAACCTAG+TCCGCATA
CTATGGTCATCTCAATGCCGAGAGGGATGTGGAGGGCGAGAGAGAGCGACTTCTCTCGGGCCCACAGC
CTTACAGCTGTGGAGCACGGTATCCTCTGCCGAAACAGAGGTTGGACAAGACCGGAGGGGTCTCCTAG
+
FFFFFFFFFFFFFFFFFFFFFFFFFFFFFFFFFFFFFFFFFFFFFFFFFFFFFFFFFFFFFFFFFFFF
FFFFFFFFFFFFFFFFFFFFFFFFFFFFFFFFFFFFFFFFFFFFFFFFFFFFFFFFFFFFFFFFFFFF
@A00155:342:HHGFNDSXY:1:2471:25925:32299 1:N:0:GAACCTAG+TCCGCATA
TGCCGAGAGGGATGTGGAGGGCGAGAGAGAGCGACTTCTCTCGGGCCCACAGCCTTACAGCTGTGGAG
CACGGTATCCTCTGCCGAAACAGAGGTTGGACAAGACCGGAGGGGTCTCCTAGTTCCAAAGGAGATGT
+
FFFFFFFFFFFFFFFFFFFFFFFFFFFFFFFFFFFFFFFFFFFFFFFFFFFFFFFFFFFFFFFFFFFF
FFFFFFFFFFFFFFFFFFFFFFFFFFFFFFFFFFFFFFFFFFFFFFFFFFFFFFFFFFFFFFFFFFFF
@A00155:342:HHGFNDSXY:1:2472:1841:13228 1:N:0:GAACCTAG+TCCGCATA
GAGAGGGATGTGGAGGGCGAGAGAGAGCGACTTCTCTCGGGCCCACAGCCTTACAGCTGTGGAGCACG
GTATCCTCTGCCGAAACAGAGGTTGGACAAGACCGGAGGGGTCTCCTAGTTCCAAAGGAG
+
FFFFFFFF,F:FF:FFF:FFFFFFFFF:FFF,FFFFFFFFFFFF:FFFFFFFFFFFFFFFFF,FFFFF
FF:FFFFFFFFFFFFFF:FFFFFFFFFF:,FFFFFF,FFFFFFFFFFFFFFFFFFFFF,F
@A00155:342:HHGFNDSXY:1:2472:15338:14904 1:N:0:GAACCTAG+TCCGCATA
GAGCGACTTCTCTCGGGCCCACAGCCTTACAGCTGTGGAGCACGGTATCCTCTGCCGAAACAGAGGTT
GGACAAGACCGGAGGGGTCTCCTAGTTCCAAAGAAGATGTACTCCGGGCTTGTTCACGACCTACCGTG
+
FFFFFFFFFFFFFFFFFFFFFFFFFFFFFFFFFFFFFFFFFFFFFFFFFFFFFFFFFFFFFFFFFFFF
FFFFFFFFFFFFFFFFFFFFFFFFFFFFFFFFFFFFFFFFFFFFFFFFFFFFFFFFFFFFFFFFFFFF
@A00155:342:HHGFNDSXY:1:2472:15492:15358 1:N:0:GAACCTAG+TCCGCATA
CTTCTCTCGGGCCCACAGCCTTACAGCTGTGGAGCACGGTATCCTCTGCCGAAACAGAGGTTGGACAA
GACCGGAGGGGTCTCCTAGTTCCAAAGGAGATGTACTCCGGGCTTGTTCACGACCTACCGTGTAAGTC
+
FFFFFFFFFFFFFFFFFFFFFFFFFFFFFFFFFFFFFFFFFFFFFFFFFFFFFFFFFFFFF:FFFFFF
FFFFFFFFFFFFFFFFFFFFFFFFFFFFFFFFFFFFFFFFFFFFFFFFFFFFFFFFFFFFFFFFFFFF
@A00155:342:HHGFNDSXY:1:2473:4309:10238 1:N:0:GAACCTAG+TCCGCATA
CTTCTCTCGGGCCCACAGCCTTACAGCTGTGGAGCACGGTATCCTCTGCCGAAACAGAGGTTGGACAA
GACCGGAGGGGTCTCCTAGTTCCAAAGGAGATGTACTCCGGGCTTGTTCACGACCTACCGTGTAAGTC
+
FFFFFFFFFFFFFFFFFFFF:FFFFFFFFF:,FFF:FFFF:FFFFFFF:FFF:FFFFFFFFFFFFFF:
FFFFFFFFF,F,:FFFFFF:FFFFFFFFFFFFFFFF:FFFFFFFFFFFF,FFFFFFFFFFFFFF:FFF
@A00155:342:HHGFNDSXY:1:2473:28827:28401 1:N:0:GAACCTAG+TCCGCATA
ACTTCTCTCGGGCCCACAGCCTTACAGCTGTGGAGCACGGTATCCTCTGCCGAAACAGAGGTTGGACA
AGACCGGAGGGGTCTCCTAGTTCCAAAGGAGATGTACTCCGGGCTTGTTCACGACCTACCGTGTAAGT
+
FFFFFFFFFFFFFFFFFFFFFFFFFFFFFFFFFFFFFFFFFFFFFFFFFFFFFFFFFFFFFFFFFFFF
FFFFFFFFFFFFFFFFFFFFFFFFFFFFFFFFFFFFFFFFFFFFFFFFFFFFFFFFFFFFFFFFFFFF
@A00155:342:HHGFNDSXY:1:2474:31620:10692 1:N:0:GAACCTAG+TCCGCATA
GCGACTTCTCTCGGGCCCACAGCCTTACAGCTGTGGAGCACGGTATCCTCTGCCGAAACAGAGGTTGG
ACAAGACCGGAGGGGTCTCCTAGTTCCAAAGGAGATGTACTCCGGGCTTGTTCACGACCTACCGTGTA
+
FFFFFFFFFFFFFFFFFFFFFFFFFFFFFFFFFFFFFFFFFFFFFFFFFFFFFFFFFFFFFFFFFFFF
FFFFFFFFFFFFFFFFFFFFFFFFFFFFFFFFFFFFFFFFFFFFFFFFFFFFFFFFFFFFFFFFFFFF
@A00155:342:HHGFNDSXY:1:2474:8296:16203 1:N:0:GAACCTAG+TCCGCATA
GGGCGAGAGAGAGCGACTTCTCTCGGGCCCACAGCCTTACAGCTGTGGAGCACGGTATCCTCTGCCGA
AACAGAGGTTGGACAAGACCGGAGGGGTCTCCTAGTTCCAAAGGAGATGTACTCCGGGCTTGTTCACG
+
```

FFFFFFFFFFFFFFFFFFFFFFFFFFFFFFF:FFFFFFFFFFFFFFFFFFFFFFFFFFFFFFFFFFFF
FFFFFFFFFFFFFFFFFFFFFFFFFFF:FFFFFFFFFFFFFFFFFFFFFFFFFFFFFFFFFFFFFFFF
@A00155:342:HHGFNDSXY:1:2474:27832:33912 1:N:0:GAACCTAG+TCCGCATA
CCATGCCCCGCCACACCGCCTTAGATCAGCTGTGTCCACTTAAGGACTCACCTCTGGCCTATGGTCAT
CTCAATGCCGAGAGGGATGTGGAGGGCGAGAGAGAGCGACTTCTCTCGGGCCCACAGCCTTACAGCTG
+
FFFFFFFFFFFFFFFFFFFFFFFFFFFFFFFFFFFFFFFFFFFFFFFFFFFFFFFFFFFFFFFFFFFF
FFFFFFFFFFFFFFFFFFFFFFFFFFFFFFFFFFFFFFFFFFFFFFFFFFFFFFFFFFFF:FFFFFFF
@A00155:342:HHGFNDSXY:1:2475:14118:7028 1:N:0:GAACCTAG+TCCGCATA
GATGTGGAGGGCGAGAGAGAGCGACTTCTCTCGGGCCCACAGCCTTACAGCTGTGGAGCACGGTATCC
TCTGCCGAAACAGAGGTTGGACAAGACCGGAGGGGTCTCCTGGTTCCAAAGGAGATGTACTCCGGGCT
+
FFFFFFFFFFFFFFFFFFFFFFFFFFFFFFFFFFFFFFFFFFFFFFFFFFFFFFFFFFFFFFFFFFFF
FFFFFFFFFFFFFFFFFFFFFFFFFFFFFFFFFFFFFFFFFFFFFFFFFFFFFFFFFFFFFFFFFFFF
@A00155:342:HHGFNDSXY:1:2475:23149:11083 1:N:0:GAACCTAG+TCCGCATA
GTATCCTCTGCCGAAACAGAGGTTGGACAAGACCGGAGGGGTCTCCTAATTCCAAAGGAGATGTACTC
CGGGCTTGTTCACGACCTACCGTGTAAGTCGTAGTCTAGTAGGCTACCTGACGAGTCCTTTTTAGGAC
+
FFFFFFFFFFFFFFFFFFFFFFFFFFFFFFFFFFFFFFFFFFFFFFFFFFFFF:FFFFFFFFFFFFFF
FFFFFFFFFFFFFFFFFFFFFFFFFFFFFFFFFFFFFFFFFFFFFFFFFFFFFFFFFFFFFFFFFFFF
@A00155:342:HHGFNDSXY:1:2475:5873:15013 1:N:0:GAACCTAG+TCCGCATA
GACTTCTCTCGGGCCCACAGCCTTACAGCTGTGGAGCACGGTATCCTCTGCCGAAACAGAGGTTGGAC
AAGACCGGAGGGGTCTCCTAGTTCCAAAGGAGATGTACTCCGGGCTTGTTCACGACCTACCGTGTAAG
+
FFFFFFFFFFFFFFFFFFFFFF:F:FF:FFFFFFFF:FFFFF:FFFFFFFFFFFFFFFFFFFFFF:FF
FFFFFFFFFFFFFFFFFFFFF:FFFFFFFFFFFFFFFFFF,FFFF:FFFFFFFFFFF:FF,F,F:FFF
@A00155:342:HHGFNDSXY:1:2475:23213:15922 1:N:0:GAACCTAG+TCCGCATA
CTTCTCTCGGGCCCACAGCCTTACAGCTGTGGAGCACGGTATCCTCTGCCGAAACAGAGGTTGGACAA
GACCGGAGGGGTCTCCTAGTTCCAAAGGAGATGTACTCCGGGCTTGTTCACGACCTACCGTGTAAGTC
+
FFFFFFFFFFFFFFFFFFFFFFFFFFFFFFFFFFFFFFFFFFFFFFFFFFFFFFFFFFFFFFFFFFFF
FFFFFFFFFFFFFFFFFFFFFFFFFFFFFFFFFFFFFFFFFFFFFFFFFFFFFFFFFFFFFFFFFFFF
@A00155:342:HHGFNDSXY:1:2475:28601:15953 1:N:0:GAACCTAG+TCCGCATA
AGATCAGCTGTGTCCACTTAAGGACTCACCTCTGGCCTATGGTCATCTCAATGCCGAGAGGGATGTGA
AGGGCGAGAGAGAGCGACTTCTCTCGGGCCCACAGCCTTACAGCTGTGGAGCACGGTATCCTCTGCCG
+
FFFFFFFFFFFFFFFFFFFFFFFFFFFFFFFFFFFFFFFFFFFFFFFFFFFFFFFFFFFFFFFFFFFF
FFFFFFFFFFFFFFFFFFFFFFFFFFFFFFFFFFFFFFFFFFFFFFFFFFFFFFFFFFFFFFFFFFFF
@A00155:342:HHGFNDSXY:1:2475:12500:24518 1:N:0:GAACCTAG+TCCGCATA
GACTTCTCTCGGGCCCACAGCCTTACAGCTGTGGAGCACGGTATCCTCTGCCGAAACAGAGGTTGGAC
AAGACCGGAGGGGTCTCCTAGTTCCAAAGGAGATGTACTCCGGGCTTGTTCACGACCTACC
+
FFFFFFFFFFFFFFFFFFFFFFFFFFFFFFFFFFFFFFFFFFFFFFFFFFFFFFFFFFFFFFFFFFFF
FFFFFFFFFFFFFFFFFFFFFFFFFFFFFFFFFFFFFFFFFFFFFF:FFFFFFFFFFFFFF
@A00155:342:HHGFNDSXY:1:2475:7789:26349 1:N:0:GAACCTAG+TCCGCATA
TTCTCTCGGGCCCACAGCCTTACAGCTGTGGAGCACGGTATCCTCTGCCGAAACAGAGGTTGGACAAG
ACCGGAGGGGTCTCCTAGTTCCAAAGGAGATGTACTCCGGGCTTGTTCACGACCTACCGTGTAAGTCG
+
FFFFFFFFFFFFFFFFFFFFFFFFFFFFFFFFFFFFFFFFFFFFFFFFFFFFFFFFFFFFFFFFFFFF
FFFFFFFFFFFFFFFFFFFFFFFFFFFFFFFFFFFFFFFFFFFFFFFF:FFFFFFFFFFFFFFFFFFF
@A00155:342:HHGFNDSXY:1:2475:12717:30436 1:N:0:GAACCTAG+TCCGCATA
CTCGGGCCCACAGCCTTACAGCTGTGGAGCACGGTATCCTCTGCCGAAACAGAGGTTGGACAAGACCG
GAGGGGTCTCCTAGTTCCAAAGGAGATGTACTCCGGGCTTGTTCACGACCTACCGTGTAAGTCGTAGT
+

```
FFFFFFFFFFFFFFFFFFFFFFFFFFFFFFFFFFFFFFFFFFFFFFFFFFFFFFFFFFFFFFFFFFFF
FFFFFFFFFFFFFFFFFFFF:FFFFFFFFFFFFFFFFFFFFFFFFFFFFFFFF:FFFFFFFFFFFFFFFF
@A00155:342:HHGFNDSXY:1:2476:9082:3505 1:N:0:GAACCTAG+TCCGCATA
CTCAATGCCGAGAGGGATGTGGAGGGCGAGAGAGAGCGACTTCTCTCGGGCCCACAGCCTTACAGCTG
TGGAGCACGGTATCCTCTGCCGAAACAGAGGTTGGACAAGACCGGAGGGGTCTCCTGGTTCCAAAGGA
+
FFFFFFFFFFFF:FFFFFFFFFFFFFFFFFFFFFFFFFFFFFFFFFFFFFFFFFFFFFFFFFFFFFFF
FFFFFFFFFFFFFFFFFFFFFFFFF:FFFFFFFFFFFFFFFFFFFFFFFFFFFFFFFFFF:FFFFFFFFF
@A00155:342:HHGFNDSXY:1:2476:23484:7373 1:N:0:GAACCTAG+TCCGCATA
CATCCCTCTCGGCATTGAGATGACCATAGGCCAGAGGTGAGTCCTTAAGTGGACACAGCTGATCTAAG
GCGGTGTGGCGGGGCATGGGTTTGAACCCCCATGACGGTCGGAGTCTAGTAGGCTCCCTGATGAGTCC
+
FFFFFFFFFFFFFF:FFFFFFFFFFFFFFFFFFFFFFF:FFFFFF:FFFFFFFFFFFFFFFFFFFFFFF
FFFFFFFFFFFFFFFFFFFFFFFFFFFFFFFFFFFFFFFFFFFFFFFFFFFFFFFFFFFFFFFFFFFF
@A00155:342:HHGFNDSXY:1:2476:26196:7373 1:N:0:GAACCTAG+TCCGCATA
GGGCGAGAGAGAGCGACTTCTCTCGGGCCCACAGCCTTACAGCTGTGGAGCACGGTATCCTCTGCCGA
AACAGAGGTTGGACAAGACCGGAGGGGTCTCCTAGTTCCAAAGGAGATGTACTCCGGGCTTGTTCACG
+
FFFFFFFFFFFFFFFFFFFFFFFFFFFFFFFFFFFFFFFFFFFFFFFFFFFFFFFFFFFFFFFFFFFF
FFFFFFFFFFFFFFFFFFFFFFFFFFFFFFFFFFFFFFFFFFFFFFFFFFFFFFFFFFFFFFFFFFFF
@A00155:342:HHGFNDSXY:1:2476:26096:7670 1:N:0:GAACCTAG+TCCGCATA
GGGCGAGAGAGAGCGACTTCTCTCGGGCCCACAGCCTTACAGCTGTGGAGCACGGTATCCTCTGCCGA
AACAGAGGTTGGACAAGACCGGAGGGGTCTCCTAGTTCCAAAGGAGATGTACTCCGGGCTTGTTCACG
+
FFFFFFFFFFFFFFFFFFFFFFFFFFFFFFFFFFFFFFFFFFFFFFFFFFFFFFFFFFFFFFFFFFFF
FFFFFFFFFFFFFFFF:FFFFFFFFFFFFFFFFFFFFFFFFFFFFFFFFFFFFFFFFFFFFFFFFFF
@A00155:342:HHGFNDSXY:1:2476:22978:7811 1:N:0:GAACCTAG+TCCGCATA
CATCCCTCTCGGCATTGAGATGACCATAGGCCAGAGGTGAGTCCTTAAGTGGACACAGCTGATCTAAG
GCGGTGTGGCGGGGCATGGGTTTGAACCCCCATGACGGTCGGAGTCTAGTAGGCTCCCTGATGAGTCC
+
FFFFFFFFFFFFFFFFFFFFFFFFFFFFFFFFFFFFFFFFFFFFFFFFFFFFFFFFFFFFFFFFFFFF
FFFFFFFFFFFFFFFFFFFFFFFFFFFFFFFFFFFFFFFFFFFFFFFFFFFFFFFFFFFFFFFFFFFF
@A00155:342:HHGFNDSXY:1:2476:21414:11428 1:N:0:GAACCTAG+TCCGCATA
CATCCCTCTCGGCATTGAGATGACCATAGGCCAGAGGTGAGTCCTTAAGTGGACACAGCTGATCTAAG
GCGGTGTGGCGGGGCATGGGTTTGAACCCCCATGACGGTCGGAGTCTAGTAGGCTCCCTGATGAGTCC
+
F:FFFFFFFFFFFF:FFFFFFFFFFFFFFFFFFFFFFFFFFFFFFFFFFFFFFFFFFFFFFF:FFFFF
FFFFFFFFFFFFFFFFFFFFFF:FFFFFFFFFFFFFFFFFFFFFFFFFFFFFFFFFFFFFFF:FFFFFFF
@A00155:342:HHGFNDSXY:1:2476:14696:16454 1:N:0:GAACCTAG+TCCGCATA
ATCTCAATGCCGAGAGGGATGTGGAGGGCGAGAGAGAGCGACTTCTCTCGGGCCCACAGCCTTACAGC
TGTGGAGCACGGTATCCTCTGCCGAAACAGAGGTTGGACAAGACCGGAGGGGTCTCCTAGTTCCAAAG
+
FFFFFFFFFFFFFFFFFFFFFFFFFFFFFFFFFFFFFFFFFFFFFFFFFFFFFFFFFFFFFFFFFFFF
FFFFFFFFFFFFFFFFFFFFFFFFFFFFFFFFFFFFFFFFFF:FFFFFFFFFFFFFFFFFFFFFFFFFF
@A00155:342:HHGFNDSXY:1:2476:1045:31923 1:N:0:GAACCTAG+TCCGCATA
TTCTCTCGGGCCCACAGCCTTACAGCTGTGGAGCACGGTATCCTCTGCCGAAACAGAGGTTGGACAAG
ACCGGAGGGGTCTCCTAGTTCCAAAGGAGATGTACTCCGGGCTTGTTCACGACCTACCGTGTAAGTCG
+
FFFFF:FFFFFFF:FFFF:FF:FFFFFFFFFFFFFFFFFFFFFFFFFFFFFFFFFFFF:FFFFFFFFFF
:FFFFFFFFFFFFFFFF:FFF:FFFFFFF:FFFFFFFFFFFFFFFFFFFFFF:FFFF,FFFF:FF:FFF
@A00155:342:HHGFNDSXY:1:2476:14705:35822 1:N:0:GAACCTAG+TCCGCATA
GAGCGACTTCTCTCGGGCCCACAGCCTTACAGCTGTGGAGCACGGTATCCTCTGCCGAAACAGAGGTT
GGACAAGACCGGAGGGGTCTCCTAGTTCCAAAGGAGATGTACTCCTGGCTTGTTCACGACCTACCGTG
+
```

```
FFFFFFFFFFFFFFFFFFFFFFFFFFFFFFFFFFFFFFFFFFFFFFFFFFFFFFFFFFFFFFFFFFFF
FFFFFF:FFFFFFFFFFFFFF:FFFFFFFFFFFFFFFFFFFFFFFFFFFFFFFFFFFFFFFFFFFFFF
@A00155:342:HHGFNDSXY:1:2477:10438:6449 1:N:0:GAACCTAG+TCCGCATA
GAGAGAAGTCGCTCTCTCTCGCCCTCCACATCCCTCTCGGCATTGAGATGACCATAGGCCAGAGGTGA
GTCCTTAAGTGGACACAGCTGATCTAAGGCGGTGTGGTGGGGCATGGGTTTGAACCCCCATGACGGTC
+
FFFFFFFFFFFFFFFFFFFFFFFFFFFFFFFFFFFFFFFFFFFFFFFFFFFFFFFFFFFFFFFFFFFF
FFFFFFFFFFFFFFFFFFFFFFFFFF:FFFFFFFFFFFFFFFF:FFFFFFFFFFFFFFFFFFFFFFFF
@A00155:342:HHGFNDSXY:1:2477:25780:18865 1:N:0:GAACCTAG+TCCGCATA
GGGCGAGAGAGAGCGACTTCTCTCGGGCCCACAGCCTTACAGCTGTGGAGCACGGTATCCTCTGCCGA
AACAGAGGTTGGACAAGACCGGAGGGGTCTCCTAGTTCCAAAGGAGATGTACTCCGGGCTTGTTCACG
+
FFFFFFFFFFFFFFFFFFFFFFFFFFFFFFFFFFFFFFFFFFFFFFFFFFFFFFFFFFFFFFFFFFFF
FFFFFFFFFFFFFFFF:FFFFFFFFFFFFFFFFFFFFFFFFFFFFFFFFFFFFFFFFFFFFFFFFFF
@A00155:342:HHGFNDSXY:1:2478:29740:9815 1:N:0:GAACCTAG+TCCGCATA
GGATGTGGAGGGCGAGAGAGAGCGACTTCTCTCGGGCCCACAGCCTTACAGCTGTGGAGCACGGTATC
CTCTGCCGAAACAGAGGTTGGACAAGACCGGAGGGGTCTCCTAGTTCCAAAGGAGATGTACTCC
+
F:FFFFFFFFFFFFFFFFFFFFFFFFFFFFFFFFFFFF:FFFFFFFF:FFFFFF:FFFFFFFFFFFFF
FFFFFFFFFFFFFFFF:,FFFFFFFFFFFFFFFFFFFFFFFFFFFFFFFFFFFFFFFFFFFFFF
@A00155:342:HHGFNDSXY:1:2478:13114:10394 1:N:0:GAACCTAG+TCCGCATA
CTTCTCTCGGGCCCACAGCCTTACAGCTGTGGAGCACGGTATCCTCTGCCGAAACAGAGGTTGGACAA
GACCGGAGGGGTCTCCTGGTTCCAAAGGAGATGTACTCCGGGCTTGTTCACGACCTACCGTGTAAGTC
+
FFFFFFFFFFFFFFFFFFFFFFFFFFFFFFFFFFFFFFFFFFFFFFFFFFFFFFFFFFFFFFFFFFFF
:FFFFFFFFFFFFFFFFFFFFFFFFFFFFFFFFFFFFFFFFFFFFFFFF:FFFFFFFFFFFFFFFFFF
@A00155:342:HHGFNDSXY:1:2478:14199:17440 1:N:0:GAACCTAG+TCCGCATA
AGACTCACCTCTGGCCTATGGTCATCTCAATGCCGAGAGGGATGTGGAGGGCGAGAGAGAGCGACTTC
TCTCGGGCCCACAGCCTTACAGCTGTGGAGCACGGTATCCTCTGCCGAAACAGAGGTTGGACAAGACC
+
FFFFFFFFFFFFFFFFFFFFFFFFFFFFFFFFFFFFFFFFFFFFFFFFFFFFFFFFFFFFFFFFFFFF
FFFFFFFFFFFFFFFFFFFFFFFFFFFFFFFFFFFFFFFFFFFFFFFF:FFFFFFFFFFFFFFFFFFF
@A00155:342:HHGFNDSXY:1:2478:28637:19899 1:N:0:GAACCTAG+TCCGCATA
GGATGTGGAGGGCGAGAGAGAGCGACTTCTCTCGGGCCCACAGCCTTACAGCTGTGGAGCACGGTATC
CTCTGCCGAAACAGAGGTTGGACAAGACCGGAGGGGTCTCCTAGTTCCAAAGGAGATGTACTCC
+
FFFFFFFFFFFFFFFFFFFFFFFFFF:FFFFFFFFFFFFFFFFFFFFFFFFFFFFFFFFFFFFFFFFF
FFFFFFFFFFFFF:FFF:FFFFFFFFFF:FFFFFFFFFFFFFFFF:FFFFFFFFFFFFFFFFFF
@A00155:342:HHGFNDSXY:1:2478:20292:23891 1:N:0:GAACCTAG+TCCGCATA
CCTTACAGCTGTGGAGCACGGTATCCTCTGCCGAAACAGAGGTTGGACAAGACCGGAGGGGTCTCCCA
GTTCCAAAGGAGATGTACTCCGGGCTTGTTCACGACCTACCGTGTAAGTCGTAGTCTAGTAGGCTACC
+
FFFFFFFFFFFFFFFFFFFFFFFFFFFFFFFFFFFFFFFFFFFFFFFFFFFFFFFFFFFFFFFFFFFF
FFFFFFFFFFFFFFFFFFFFFFFFFFFFFFFFFFFFFFFFFFFFFFFFFFFFFFFF:F,FFF:FFFFF
@A00155:342:HHGFNDSXY:1:2501:29170:13964 1:N:0:GAACCTAG+TCCGCATA
TGTGGAGGGCGAGAGAGAGCGACTTCTCTCGGGCCCACAGCCTTACAGCTGTGGAGCACGGTATCCTC
TGCCGAAACAGAGGTTGGACAAGACCGGAGGGGTCTCCTAGTTCCAAAGGAGATGTACTCCGGG
+
FFFFFFF:F:::FF:F:FF:FFFFFFFFFFFF:F:FFFF:F,F:F:FF:F,FF,FFFFF:FFFFF,F:
FFFF:FFFFF,FFF,FFFFFFFFFFFFFFF:FFFFFF:FF,FFF,FF:F:FFFFFFFFFFFFF:
@A00155:342:HHGFNDSXY:1:2502:17752:5525 1:N:0:GAACCTAG+TCCGCATA
ATCTCTATGCCGAGAGGGATGTGGAGGGCGAGAGAGAGCGACTTCTCTCGGGCCCACAGCCTTACAGC
TGTGGAGCACGGTATCCTCTGCCGAAACAGAGGTTGGACAAGACCGGAGGGGTCTCCTAG
+
```

```
FFFFFFFFFFFFFFFFFFFFFFFFFFFFFFFFFFFFFFFFFFFFFFFFFFFFFFFFFFFFFFFFFFFF
FFFFFFFFFFFFF,FFFFFFFFFFFFFFFFFFFFFFFFFFFFFFFFFFFFFFFFFFFFFFFF
@A00155:342:HHGFNDSXY:1:2502:10890:9111 1:N:0:GAACCTAG+TCCGCATA
ACTTCTCTCGGGCCCACAGCCTTACAGCTGTGGAGCACGGTATCCTCTGCCGAAACAGAGGTTGGACA
AGACCGGAGGGGTCTCCTAGTTCCAAAGGAGATGTACTCCGGGCTTGTTCACGACCTACCGTGTAAGT
+
FFFFFFFFFFFFFFFFFFFFFFFFFFFFFFFFFFFFFFFFFFFFFFFFFFFFFFFFFFFFFFFFFFFF
FFFFFFFFFFFFFFFFFFFFFFFFFFFFFFFFFFFFFFFFFFFFF,FFFFFFFFFFFFFFFFFFFFFF
@A00155:342:HHGFNDSXY:1:2502:8241:10285 1:N:0:GAACCTAG+TCCGCATA
AGGGCGAGAGAGAGCGACTTCTCTCGGGCCCACAGCCTTACAGCTGTGGAGCACGGTATCCTCTGCCG
AAACAGAGGTTGGACAAGACCGGAGGGGTCTCCTAGTTCCAAAGGAGATGTACTCCGGGCTTGTTCAC
+
FFFFFFFFFFFFFFFFFFFFFFFFFFFFFFFFFFFFFFFFFFFFFFFFFFFFFFFFFFFFFFFFFFFF
FFFFFFFFFFFFFFFFFFFFFFFFFFFFFFFFFFFFFFFFFFFFFFFFFFFFFFFFFFFFFFFFFFFF
@A00155:342:HHGFNDSXY:1:2502:25870:14700 1:N:0:GAACCTAG+TCCGCATA
GTCATCTCAATGCCGAGAGGAATGTGGAGGGCGAGAGAGAGCGACTTCTCTCGGGCCCACAGCCTTAC
AGCTGTGGAGCACGGTATCCTCTGCCGAAACAGAGGTTGGACAAGACCGGAGGGGTCTCCTAGTTCCA
+
FFFFFFFFFFFFFFFFFFFFF,:FFFFFFFFFFFFFFFFFFFFFFFFFFFFFFFFFFFFFFFFFF:FFF
FFFFFFFFFFFFFFFFFFFFFFFFFFFFFFFFFFFFFFFFFFFFFFFFFFFFFFFFFFFF:FFFFFFF
@A00155:342:HHGFNDSXY:1:2503:18846:8609 1:N:0:GAACCTAG+TCCGCATA
GCTGTGTCCACTTAAGGACTCACCTCTGGCCTATGGTCATCTCAATGCCGAGAGGGATGTGAAGGGCG
AGAGAGAGCGACTTCTCTCGGGCCCACAGCCTTACAGCTGTGGAGCACGGTATCCTCTGCCGAAACAG
+
FFFFFFFFFFFFFFFFFFFFFFFFFFFFFFFFFFFFFFFFFFFFFFFFFFFFFFFF:FFFFFFFFFFF
FFFFFFFFFFFFFFFFFFFFFFFFFFFFFFFFFFFFFFFFFFFFFFFFFFFFFFFFFFFFFFFFFFFF
@A00155:342:HHGFNDSXY:1:2503:28836:12289 1:N:0:GAACCTAG+TCCGCATA
ATCTCAATGCCGAGAGGGATGTGGAGGGCGAGAGAGAGCGACTTCTCTCGGGCCCACAGCCTTACAGC
TGTGGAGCACGGTATCCTCTGCCGAAACAGAGGTTGGACAAGACCGGAGGGGTCTCCTAGTTCCAAAG
+
FFFFFFFFFFFFFFFFFF:FFFFFFFFFFFFFFFFFFFFFFFFF:FFFFFFFFFFFFFFFFFFFFFFFF
FFFFFFFFFFFFFFFFFFFFFFFFFFFFFFFFFFFFFFFFFFFFFFFFFFFFFFFFFFFFFFFFFFFF
@A00155:342:HHGFNDSXY:1:2503:30635:18850 1:N:0:GAACCTAG+TCCGCATA
GTGTCCACTTAAGGACTCTCCTCTGGCCTATGGTCATCTCAATGCCGAGAGGGATGTGGAGGGCGAGA
GAGAGCGACTTCTCTCGGGCCCACAGCCTTACAGCTGTGGAGCACGGTATCCTCTGCCGAAACAGAGG
+
FFFFFFFFFFFFFFFFFFFFFFFFFFFFFFFFFFFFFFFFFFFFFFFFFFFFFFFFFFFFFFFFFFFF
FFFFFFFFFFFFFFFFFFFFFFFFFFFFFFFFFFFFFFFFFFFFFFFFFFFFFFFFFFFFFFFFFFFF
@A00155:342:HHGFNDSXY:1:2503:20437:36730 1:N:0:GAACCTAG+TCCGCATA
CTCAATGCCGAGAGGGATGTGGAGGGCGAGAGAGAGCGACTTCTCTCGGGCCCACAGCCTTACAGCTG
TGGAGCACGGTATCCTCTGCCGAAACAGAGGTTGGACAAGACCGGAGGGGTCTCCTGGTTCCAAAGGA
+
FFFFFFFFFFFFFFFFFFF:FFFFFFFFFFFFFFFFFFFFF:FFFFFFFFFFFFFFFFFFFFFFFFFF
FFFFFFFFFFFFFFFFFFFFFFFFFFFFFF:FFFFFFFFFFFFFFFFFFFFFFFFFFFFFFFFFF:FFF
@A00155:342:HHGFNDSXY:1:2504:20030:13166 1:N:0:GAACCTAG+TCCGCATA
ATCTCAATGCCGAGAGGGATGTAGAGGGCGAGAGAGAGCGACTTCTCTCGGGCCCACAGCCTTACAGC
TGTGGAGCACGGTATCCTCTGCCGAAACAGAGGTTGGACAAGACCGGAGGGGTCTCCTAGTTCCAAAG
+
FFFFFFFFFFFFFFFFFFFFFFFFF:FFFFFFFFFFFFFFFFFFFFFFFFFFFFFFFFFFFFFFF:FFFF
FFFFFFFFFFFFFFFFFFFFFFFFFFFFFFFFFFFFFFFFFFFFFFFFFFFFFFFFFF,FFFFFFFFF
@A00155:342:HHGFNDSXY:1:2504:18059:16517 1:N:0:GAACCTAG+TCCGCATA
AGCTGTGGAGCACGGTATCCTCTGCCGAAACAGAGGTTGGACAAGACCGGAGGGGTCTCCTAGTTCCA
AAGGAGATGTACTCCGGGCTTGTTCACGACCTACCGTGTAAGTCGTAGTCTAGTAGGCTACCTGACGA
+
```

FFFFFFFFFFFFFFFFFFFFFFFFFFFFFFFFFFFFFFFFFFFFFFFFFFFFFFFFFFFFFFFFFFFF
FFFFFFFFFFFFFFFFFFFFFFFFFFFFFFFFFFFFFFFFFFFFFFFFFFFFFFFFFFFFFFFFFFFF
@A00155:342:HHGFNDSXY:1:2504:23439:19758 1:N:0:GAACCTAG+TCCGCATA
GAGAGAGCGACTTCTCTCGGGCCCACAGCCTTACAGCTGTGGAGCACGGTATCCTCTGCCGAAACAGA
GGTTGGACAAGACCGGAGGGGTCTCCTAGTTCCAAAGGAGATGTACTCCGGGCTTGTTCACGACCTA
+
FFFFFFFF:F:FFFFFFFFFFFFFFFFFF:FFFFFFFFFFFFFFFFFFFF:FFFFFFFFFFFFFFFF
FFFFFFFFFFFFFFF:F:FF:FFFFFF:FF,FFFFF:FFFFFF:FFFFF::F:F:FFFFFFFFF,:F
@A00155:342:HHGFNDSXY:1:2504:17942:35070 1:N:0:GAACCTAG+TCCGCATA
GCGACTTCTCTCGGGCCCACAGCCTTACAGCTGTGGAGCACGGTATCCTCTGCCGAAACAGAGGTTGG
ACAAGACCGGAGGGGTCTCCTAGTTCCAAAGGAGATGTACTCCGGGCTTGTTCACGACCTACCGTGTA
+
FFFFFFFFFFFFFFFFFFFFFFFFFFFFFFFFFFFFFFFFFFFFFFFFFFFFFFFFFFFFFFFFFFFF
FFFFFFFFFFFFFFFFFFFFFFFFFFFFFFFFFFFFFFFFFFFFFFFFFFFF:FFFFFFFFFFFFFF
@A00155:342:HHGFNDSXY:1:2505:21676:6590 1:N:0:GAACCTAG+TCCGCATA
TGTTTCGGCAGAGGATACCGTGCTCCACAGCTGTAAGGCTGTGGGCCCGAGAGAAGTCGCTCTCTCTC
GCCCTCCACATCCCTCTCGGCATTGAGATGACCATAGGCCAGAGGTGAGTCCTTAAGTGGACACAGCT
+
FFFFFFFFFFFFFFFFFFFFFFFFFFFFFFFFFFFFFFFFFFFFFFFFFFFFFFFFFFFFFFFFFFFF
FFFFFFFFFFFFFFFFFFFFFFFFFFFFFFFFFFFFFFFFFFFFFFFFFFF:FFFFFFFF,FFFFFFFF
@A00155:342:HHGFNDSXY:1:2505:5113:16642 1:N:0:GAACCTAG+TCCGCATA
GGATGTGGAGGGCGAGAGAGAGCGACTTCTCTCGGGCCCACAGCCTTACAGCTGTGGAGCACGGTATC
CTCTGCCGAAACAGAGGTTGGACAAGACCGGAGGGGTCTCCTAGTTCCAAAGGAGATGTACTCC
+
FFFFF:FFFFFFFFFFFFFFFFFFFFFFFFFFFFFFFFFFFFFFF:FF:FFFFFFFFFFFFFFFFFFFF:FF
FFFFFFFFFFFFFFFFFFFFFFFFFFFFFFFFFFFFFFFFFFFFF:FFFFFF:FFFFFFFFFFFF
@A00155:342:HHGFNDSXY:1:2505:31096:35556 1:N:0:GAACCTAG+TCCGCATA
CTCTGCCGAAACAGAGGTTGGACAAGACCGGAGGGGTCTCCTAGTTCCAAAGGAGATGTACTCCGGGC
TTGTTCACGACCTACCGTGTAAGTCGTAGTCTAGTAGGCTACCTGACGAGTCCTTTTTAGGACGAAAC
+
FFFFFFFFFFFFFFFFFFFFFFFFFFFFFFFFFFFFFFFFFFFFFFFFFFFFFFFFFFFFFFFFFFFF
FFFFFFFFFFFFFFFFFFFFFFFFFFFFFFFFFFFFFFFFFFFFFFFFFFFFFFFFFFFFFFFFFFFF
@A00155:342:HHGFNDSXY:1:2506:24198:13056 1:N:0:GAACCTAG+TCCGCATA
GTAAGGCTGTGGGCCCGAGAGAAGTCGCTCTCTCTCGCCCTCCACATCCCTCTCGGCATTGAGATGAC
CATAGGCCAGAGGTGAGTCCTTAAGTGGACACAGCTGATCTAAGGCGGTGTGGCGGGGCATGGGTTTG
+
:FF::FFFFFFF,:FF:FF::FF:FFFFFF:F:FFFFFF:FFFFFFFFFFFFFF:F,F:,FFFF,FFF
:FFFF:FFFFFFFF:FFFFF::F,,FFFFFFFFFF:FF,FFFFFFFFF:FF:FFF::FFF,,FF,:FF
@A00155:342:HHGFNDSXY:1:2506:13458:15436 1:N:0:GAACCTAG+TCCGCATA
GAGAGGGATGTGGAGGGCGAGAGAGAGCGACTTCTCTCGGGCCCACAGCCTTACAGCTGTGGAGCACG
GTATCCTCTGCCGAAACAGAGGTTGGACAAGACCGGAGGGGTCTCCTGGTTCCAAAGGAGATGTACTC
+
FFFFFFFFFFFFFFFFFFFFFFFFFFFFFFFFFFFFFFFFFFFFFFFFFFFFFFFFFFFFFFFFFFFF
FFFFFFFFFFFFFFFFFFFFFFFFFFFFF:FFFFFFFFFFFFFFFFFFFFFFFFFFFFFFFFFFFFFFF
@A00155:342:HHGFNDSXY:1:2506:17372:16391 1:N:0:GAACCTAG+TCCGCATA
CTTCTCTCGGGCCCACAGCCTTACAACTGTGGAGCACGGTATCCTCTGCCGAAACAGAGGTTGGACAA
GACCGGAGGGGTCTCCTAGTTCCAAAGGAGATGTACTCCGGGCTTGTTCACGACCTACCGTGTAAG
+
FFFFFFFFFFFFFFFFFFFFFFFFFFFFFFFFFFFFFFFFFFFFFFFFFFFFFFFFFFFFFFFFFFFF
FFFFFFFFFFFFFFFFFFFFFFFFFFFFFFFFFFFFFFFFFFFFFFFFFFF:FFFFFFFFFFFFFF:F
@A00155:342:HHGFNDSXY:1:2506:10348:25739 1:N:0:GAACCTAG+TCCGCATA
ATCCTCTGCCGAAACAGAGGTTGGACAAGACCGGAGGGGTCTCCTAGTTCCAAAGGAGATGTATTCCG
GGCTTGTTCACGACCTACCGTGTAAGTCGTAGTCTAGTAGGCTACCTGACGAGTCCTTTTTAGGACGA
+

```
FFFFFFFFFFFFFFFFFFFFFFFFFFFFFFFFFFFFFFFFFFFFFFFFFFFFFFFFFFFFFFFFFFFFFF
FFFFFFFFFFFFFFFFFFFFFFFFFFFFFFFFFFFFFFFFFFFFFFFFFFFFFFFFFFFFFFFFFFFFFF
@A00155:342:HHGFNDSXY:1:2506:11749:27602 1:N:0:GAACCTAG+TCCGCATA
CTCAATGCCGAGAGGGATGTGGAGGGCGAGAGAGAGCGACTTCTCTCGGGCCCACAGCCTTACAGCTG
TGGAGCACGGTATCCTCTGCCGAAACAGAGGTTGGACAAGACCGGAGGGGTCTCCTAGTTCCAAAGGA
+
FFFFFFFFFFFFFFFFFFFFFFFFFFFFFFFFFFFFFFFFFFFFFFFFFFFFFFFFFFFFFFFFFFFFFF
FFFFFFFFFFFFFFFFFFFFFFFFF:FFFFFFFFFFFFFFFFFFFFFFFFFFFFFFFFFFFFFFFFFFFF
@A00155:342:HHGFNDSXY:1:2507:4435:2440 1:N:0:GAACCTAG+TCCGCATA
GGGCGAGAGAGAGCGACTTCTCTCGGGCCCACAGCCTTACAGCTGTGGAGCACGGTATCCTCTGCCGA
AACAGAGGTTGGACAAGACCGGAGGGGTCTCCTAGTTCCAAAGGAGATGTACTCCGGGCTTGTTCACG
+
F::FFFFF:FFFFFFFFFFFFFFFFFFFFFFFFFFFFFFFFFFF:FFFFFFFFFFFFFFFFFFFFFFFFF
FFFFFFFFFFFFFFFFFF:FF:FFFFFFFFFFFFFFFFFF:FFFFFFFFFFFFFFFF,FF,FFFFFFF
@A00155:342:HHGFNDSXY:1:2507:16812:14888 1:N:0:GAACCTAG+TCCGCATA
GGATGTAGAGGGCGAGAGAGAGCGACTTCTCTCGGGCCCACAGCCTTACAGCTGTGGAGCACGGTATC
CTCTGCCGAAACAGAGGTTGGACAAGACCGGAGGGGTCTCCTAGTTCCAAAGGAGATGTACTCC
+
FFF:FFFFFF:F:F:FFFFFFFFFFFFFFFFFFFFFFFFFFFFFFFFFFF,FFFF:FFFFFFFFFFFFFF
FFFFFFFFFFFFFFFFFFFFFFFFFFFFFFFFFFFFFFFFFFFFFFFFFFFFFFFFFFFFFFFFFF
@A00155:342:HHGFNDSXY:1:2507:9236:30420 1:N:0:GAACCTAG+TCCGCATA
GGATGTGGAGGGCGAGAGAGAGCGACTTCTCTCGGGCCCACAGCCTTACAGCTGTGGAGCACGGTATC
CTCTGCCGAAACAGAGGTTGGACAAGACCGGAGGGGTCTCCTAGTTCCAAAGGAGATGTACTCC
+
FFFFFFFFFFFFFFFFFFFFFFFFFFFFFFFFFFFFFF:FFFFFFFFFFFFFFFFFFFFFFFFFFFFFFF
FFFFFFFFFFFFFFFFFFFFFFFFFFFFFFFFFFFFFFFFFF,FFFFFFFFFFFFFFFFFFFFF
@A00155:342:HHGFNDSXY:1:2508:26648:14043 1:N:0:GAACCTAG+TCCGCATA
TAGGTCGTGAACAAGCCCGGAGTACATCTCCTTTGGAACCAGGAGACCCCTCCGGTCTTGTCCAACCT
CTGTTTCGGCAGAGGATACCGTGCTCCACAGCTGTAAGGCTGTGGGCCCGAGAGAAGTCGCTCTCTCT
+
FFFFFFF:F:F:FFFFFFFFFFFFFFFFFFFFFFFFFFFFFFFFFFFFFFFFFFFFFFFFFFFFFFFFFF
FFFFFFFFFFFFFFFFFFFFFFFFFFFFFFFFFFFFFFFFFFFFFFFFFF:FFFFFFFFFFFFFFFFFFF
@A00155:342:HHGFNDSXY:1:2509:25337:31688 1:N:0:GAACCTAG+TCCGCATA
CCATGCCCCGCCACACCGCCTTAGATCAGCTGTGTCCACTTAAGGACTCACCTCTGGCCTATGGTCAT
CTCAATGCCGAGAGGGATGTGGAGGGCGAGAGAGAGCGACTTCTCTCGGGCCCACAGCCTTACAGCTG
+
FFFFFFFFFFFFFFFFFFFFFFFFFFFFFFFFFFFFFFFFFFFFFFFFFFFFFFFFFFFFFFFFFFFFFF
FFFFFFFFFFFFFFFFFFFF:FFFFFFFFFFFFFFFFFFFFFFFFFFFFFFFFFFFFFFFFFFFFFFFFF
@A00155:342:HHGFNDSXY:1:2510:24099:8938 1:N:0:GAACCTAG+TCCGCATA
TGTCCACTTAAGGACTCACCTCTGGCCTATGGTCATCTCAATGCCGAGAGGGATGTGAAGGGCGAGAG
AGAGCGACTTCTCTCGGGCCCACAGCCTTACAGCTGTGGAGCACGGTATCCTCTGCCGAAACAGAGGT
+
FFFFFFFFFFFFFFFFFFFFFFFFFFFFFFFFFFFFFFFFFFFFFFFFFFFFFFFFFFFFFFFFFFFFFF
FFFFFFFFFFFFFFFFF:FFFFFFFFFFFFFFFFFFFFFFFFFFFFFFFFFFFFFFFFFFFFFFFFFFFF
@A00155:342:HHGFNDSXY:1:2510:2844:12023 1:N:0:GAACCTAG+TCCGCATA
GGCGAGAGAGAGCGACTTCTCTCGGGCCCACAGCCTTACAGCTGTGGAGCACGGTATCCTCTGCCGAA
ACAGAGGTTGGACAAGACCGGAGGGGTCTCCTAGTTCCAAAGGAGATGTACTCCGGGCTTGTTCACGA
+
FFFFFFFFFFFFFFFFFFFFFFFFFFFFFFFFFFFFF:FFFFFFFFFFFFFFFFFFFFFFFFFFFFF:FF
FFFFFFFFFFFFFFFFFFF:FFFFFFFFFFFFFFFFFFFFFFFFFFFFFFFFFF:FFF,FFFFFFFFFFFFF
@A00155:342:HHGFNDSXY:1:2510:26413:13072 1:N:0:GAACCTAG+TCCGCATA
GGGCGAGAGAGAGCGACTTCTCTCGGGCCCACAGCCTTACAGCTGTGGAGCACGGTATCCTCTGCCGA
AACAGAGGTTGGACAAGACCGGAGGGGTCTCCTGGTTCCAAAGGAGATGTACTCCGGGCTTGTTCACG
+
```

FFFFFFFFFFFFFFFFFFFFFFFFFFFFFFFFFFFFFFFFFFFFFFFFFFFFFFFFFFFFFFFFFFFFFF
FFFFFFFFFFFFFFFFFFFFFFFFFFFFFFFFFFFFFFFF:FFFFFFFFFF:FFFFFFFFFFFFFFFFFF
@A00155:342:HHGFNDSXY:1:2510:14425:17300 1:N:0:GAACCTAG+TCCGCATA
AGAGAGAGCGACTTCTCTCGGGCCCACAGCCTTACAGCTGTGGAGCACGGTATCCTCTGCCGAAACAG
AGGTTGGACAAGACCGGAGGGGTCTCCTAGTTCCAAAGGAGATGTACTCCGGGCTTGTTCACGACCTA
+
FFFFFFFFFFFFFFFFFFFFFFFFFFFFFFFFFFFFFFFFFFFF:FFFFFFFFFFFFFFFFFFFFFFFFF
FFFFFFFFFFFFFFFFFFFFFFFFFFFFFFFFFFFFFFFFFFFFFFFFFFFFFFFFFFFFFFFFFFFFFF
@A00155:342:HHGFNDSXY:1:2510:29017:35994 1:N:0:GAACCTAG+TCCGCATA
GAGGGCGAGAGAGAGCGACTTCTCTCGGGCCCACAGCCTTACAGCTGTGGAGCACGGTATCCTCTGCC
GAAACAGAGGTTGGACAAGACCGGAGGGGTCTCCTAGTTCCAAAGGAGATGTACTCCGGGCTTGTTCA
+
FFFFFFFFFFFFFFFFFFFFFFFFFFFFFFFFFFFFFFFFFFFFFFFFFFFFFFFFFFFFFFFFFFFFFF
FFFFFFFFFFFFFFFFFFFFFFFFFFFFFFFFFFFFFFFFFFFFFFFFFFFFFFFFFFFFFFFFFFFFFF
@A00155:342:HHGFNDSXY:1:2511:29071:5243 1:N:0:GAACCTAG+TCCGCATA
AGCGACTTCTCTCGGGCCCACAGCCTTACAGCTGTGGAGCACGGTATCCTCTGCCGAAACAGAGGTTG
GACAAGACCGGAGGGGTCTCCTAGTTCCAAAGGAGATGTACTCCGGGCTTGTTCACGACCTACCGTGT
+
FFFFFFFFFFFFFFFFFFFFFFFFFFFFFFFFFFFFFFFFFFFFFFFFFFFFFFFFFF:FFFFFFFFFFF
FFFFFFFFFFFFFFFFFFFFFFFFFFFFFFFFFFFFFFFFFFFFFFFFFFFFFFFFFFFFFFFFFFFFFF
@A00155:342:HHGFNDSXY:1:2511:29062:24424 1:N:0:GAACCTAG+TCCGCATA
CTTCTCTCGGGCCCACAGCCTTACAGCTGTGGAGCACGGTATCCTCTGCCGAAACAGAGGTTGGACAA
GACCGGAGGGGTCTCCTAGTTCCAAAGGAGATGTACTCCGGGCTTGTTCACGACCTACCGTGTAAGTC
+
FFFFFFFFFFFFFFFFFFFF:FFFFFFFFFFFFFFFFFFFFFFFFFFFFFFFFFFFFFFFFFFFFFFFFF
FFFFFFFFFFFFFFFFFFFFFFFFFFFFFFFFFFFFFFFFFFFFFFFFFFFFFFFFFFFFFFFFFFFFFF
@A00155:342:HHGFNDSXY:1:2511:4481:29230 1:N:0:GAACCTAG+TCCGCATA
CTTCTCTCGGGCCCACAGCCTTACAGCTGTGGAGCACGGTATCCTCTGCCGAAACAGAGGTTGGACAA
GACCGGAGGGGTCTCCTGGTTCCAAAGGAGATGTACTCCGGGCTTGTTCACGACCTACCGTGTAAGTC
+
FFFFFFFFFFFFFFFFFFFFFFFFFFFFFFFFFFFFFFFFFFFFFFFFFFFFFFFFFFFFFFFFFFFFFF
FFFFFFFFF:FFFFFFFFFFFFFFFFFFFFFFFFFFFFFFFFFFFFFFFFFFFFFFFFFFFFFFFFFFFF
@A00155:342:HHGFNDSXY:1:2511:31186:30859 1:N:0:GAACCTAG+TCCGCATA
ATCTCAATGCCGAGAGGGATGTAGAGGGCGAGAGAGAGCGACTTCTCTCGGGCCCACAGCCTTACAGC
TGTGGAGCACGGTATCCTCTGCCGAAACAGAGGTTGGACAAGACCGGAGGGGTCTCCTAGTTCCAAAG
+
FFFFFFFFFFFFFF:FFFFFFFFFFFFFFFFFFFFFFFFFFFFFFFFFFFFFFFFFFFFFFFFFFFFFFFF
FFFFFFFFFFFFF:FFFFFFFFFFFFFFFFFFFFFFFFFFFFFFFFFFFFFFFFFFFFFFFFFFFFFFFFFF
@A00155:342:HHGFNDSXY:1:2511:19840:33411 1:N:0:GAACCTAG+TCCGCATA
TGGTCATCTCAATGCCGAGAGGGATGTAGAGGGCGAGAGAGAGCGACTTCTCTCGGGCCCACAGCCTT
ACAGCTGTGGAGCACGGTATCCTCTGCCGAAACAGAGGTTGGACAAGACCGGAGGGGTCTCCTAGTTC
+
FFFFFFFFFFFFFFFFFFFFFFFFFFFFFFFFFFFFFFFFFFFFFFFFFFFFFFFFFFFFFFFFFFFFFF
FFFFFFFFFFFFFFFFFFFFFFFFFFFFFFFFFFFFFFFFFFFFFFFFFFFFFFFFFFFFFFFFFFFFFF
@A00155:342:HHGFNDSXY:1:2512:30807:11005 1:N:0:GAACCTAG+TCCGCATA
CTTCTCTCGGGCCCACAGCCTTACAGCTGTGGAGCACGGTATCCTCTGCCGAAACAGAGGTTGGACAA
GACCGGAGGGGTCTCCTAGTTCCAAAGGAGATGTACTCCGGGCTTGTTCACGACCTACCGTGTAAGTC
+
FFFFFFFFFFFFFFFFFFFFFFFFFFFFFFFFFFFFFFFFFFFFFFFFFFFFFFFFFFFFFFFFFFFFFF
FFFFFFFFFFFFFFFFFFFFFFFFFFFFFFFFFFFFFFFFFFFFFFFFFFFFFFFFFFFFFFFFFFFFFF
@A00155:342:HHGFNDSXY:1:2512:17580:25269 1:N:0:GAACCTAG+TCCGCATA
AGAGAGAGCGACTTCTCTCGGGCCCACAGCCTTACAGCTGTGGAGCACGGTATCCTCTGCCGAAACAG
AGGTTGGACAAGACCGGAGGGGTCTCCTGGTTCCAAAGGAGATGTACTCCGGGCTTGTTCACGACCTA
+

```
FFFFFFFFFFFFFFFFFFFFFFFFFFFFFFFFFFFFFFFFFFFFFFFFFFFFFFFFFFFFFFFFFFFF
FFFFFFFFFFFFFFFFFFFFFFFFFFFFFFFFFFFFFFFFFFFFFFFFFFFFFFFFFFFF:FFFFFFF
@A00155:342:HHGFNDSXY:1:2512:18376:25645 1:N:0:GAACCTAG+TCCGCATA
AGAGAGAGCGACTTCTCTCGGGCCCACAGCCTTACAGCTGTGGAGCACGGTATCCTCTGCCGAAACAG
AGGTTGGACAAGACCGGAGGGGTCTCCTGGTTCCAAAGGAGATGTACTCCGGGCTTGTTCACGACCTA
+
FFFFFFFFFFFFFFFFFF:FFFFFFFFFFFF:FFFFFFFFFFFFFFFFFFF:FF:FFFFFFFFFFFFF
FFFFFFFFFFFFFF:FFFFFFFFFFFFFFFFFFFFFFFFFFFFFFFFFFFFFF:FFFFFFFFFFFFF:
@A00155:342:HHGFNDSXY:1:2512:11749:30639 1:N:0:GAACCTAG+TCCGCATA
TTCTCTCGGGCCCACAGCCTTACAGCTGTGGAGCACGGTATCCTCTGCCGAAACAGAGGTTGGACAAG
ACCGGAGGGGTCTCCTAGTTCCAAAGGAGATGTACTCCGGGCTTGTTCACGACCTACCGTGTAAGT
+
F:FFFFF:FFFFFFFFFFF:FF,F:FF,FFFF,FFF,FFFFF,F,F,FFFFFF:FFFFFFFF::FFFF
FFF,FFF,FF:F:FFFFFF,FFFFFF:F:FF,:FFFFFFFFFF,:,FFF:,FFFFFF,,FFF,F:F
@A00155:342:HHGFNDSXY:1:2513:14281:20713 1:N:0:GAACCTAG+TCCGCATA
TTCTCTCGGGCCCACAGCCTTACAGCTGTGGAGCACGGTATCCTCTGCCGAAACAGAGGTTGGACAAG
ACCGGAGGGGTCTCCTGGTTCCAAAGGAGATGTACTCCGGGCTTGTTCACGACCTACCGTGTAAGTCG
+
FFFFFFFFFFFFFFFFFFFFFFFFFFFFFFFFFFFFFFFFFF:FFFFFFFFFFFFFFFFFFFFFFFF,
FFFFFFFFFFFFFFFFFFFFFFFFFFFFFFFFFFFFFFFFFF:FFFFFFFFFFFFFFFFFFFFFFFFF
@A00155:342:HHGFNDSXY:1:2513:17951:24878 1:N:0:GAACCTAG+TCCGCATA
GTCATCTCAATGCCGAGAGGGATGTGGAGGGCGAGAGAGAGCGACTTCTCTCGGGCCCACAGCCTTAC
AGCTGTGGAGCACGGTATCCTCTGCCGAAACAGAGGTTGGACAAGACCGGAGGGGTCTCCTAGTTCCA
+
FFFFFFFFFFFFFFFFFFFFFFFFFFFFFFFFFFFFFFFFFFFFF:FFFFFFFFFFFFFFFFFFFFFF
FFFFFFFFFFFFFFFFFFFFFFFFFFFFFFFFFFFFFF:FFFFFFFFFFFFFFFFFFFFFFFFFFFFF
@A00155:342:HHGFNDSXY:1:2513:1832:26021 1:N:0:GAACCTAG+TCCGCATA
TGCCGAGAGGGATGTGGAGGGCGAGAGAGAGCGACTTCTCTCGGGCCCACAGCCTTACAGCTGTGGAG
CACGGTATCCTCTGCCGAAACAGAGGTTGGACAAGACCGGAGGGGTCTCCTAGTTCCAAAGGAGATGT
+
FFFFF:FFFFFF,F:FFFFFFFFFFFFFFFFFFFFFFFFFFFFFFFFFFFFFFFFFFFFFFFFFFFFF
FFFFFF:FFFFFFFFFFFF:FFFFFF:FFFFF::F:FFFF:FFFFFFFFF,,FFFFF::FFF:FF:FF
@A00155:342:HHGFNDSXY:1:2514:14850:1376 1:N:0:GAACCTAG+TCCGCATA
ATCTCAATGCCGAGAGGGATGTGGAGGGCGAGAGAGAGCGACTTCTCTCGGGCCCACAGCCTTACAGC
TGTGGAGCACGGTATCCTCTGCCGAAACAGAGGTTGGACAAGACCGGAGGGGTCTCCTAGTTCCAAAG
+
FFFFFFFFF:,FFF,FFFFFFFF:FFFFFFFF:FFFFFFFFFFFFFFFFFFFFFFF:FFF,FFFFFFF
FF,FFFFFFFFFF,FFFFFFFFFFFFFF:FFFFFFFFF:FFFFFFFFFFFF::FFFFFFF,,::FFFF
@A00155:342:HHGFNDSXY:1:2514:19407:6762 1:N:0:GAACCTAG+TCCGCATA
TGTCCACTTAAGGACTCTCCTCTGGCCTATGGTCATCTCAATGCCGAGAGGGATGTGGAGGGCGAGAG
AGAGCGACTTCTCTCGGGCCCACAGCCTTACAGCTGTGGAGCACGGTATCCTCTGCCGAAACAGAGGT
+
FFFFFFFFFFFFFFFFFFFFFFFFFFFFFFFFFFFFFFFFFFFFFFFFFFFFFFFFFFFFFFFFFFFF
FFFFFFFFFFFFFFFFFFFFFFFFFFFFFFFFFFFFFFFFFFFFFFFFFFFFFFFFFFFFFFFFFFFF
@A00155:342:HHGFNDSXY:1:2514:27272:20196 1:N:0:GAACCTAG+TCCGCATA
AGCGACTTCTCTCGGGCCCACAGCCTTACAGCTGTGGAGCACGGTATCCTCTGCCGAAACAGAGGTTG
GACAAGACCGGAGGGGTCTCCTAGTTCCAAAGGAGATGTACTCCGGACTTGTTCACGACCTACCGTGT
+
FFFFFF:FFF:FF,FFF::FFFFFF:FFFFFFFFFFFFFFFFFFFFFF::FFFF:FFFF:F:FFFF,:
FFFFFFFF:,FFFFFFFFFFFFFFFFFFFFFFFFFFFFFFFFFFFFFFF:FFF,FFFFFFFFF,F,FF
@A00155:342:HHGFNDSXY:1:2514:28103:23015 1:N:0:GAACCTAG+TCCGCATA
CTTCTCTCGGGCCCACAGCCTTACAGCTGTGGAGCACGGTATCCTCTGCCGAAACAGAGGTTGGACAA
GACCGGAGGGGTCTCCTAGTTCCAAAGGAGATGTACTCCGGGCTTGTTCACGACCTACCGTGTAAGTC
+
```

```
FFFFFFFFFFFFFFFFFFFFFFFFFFFFFFFFFFFFFFFFFFFFFFFFFFFFFFFFFFFFFFFFFFFF
FFFFFFFFFFFFFFFFFFFFFFFFFFFFFFFFFFFFFFFFFFFFFFFFFFFFFFFFF:FFFFFFFFFF
@A00155:342:HHGFNDSXY:1:2514:19931:26647 1:N:0:GAACCTAG+TCCGCATA
GCGACTTCTCTCGGGCCCACAGCCTTACAGCTGTGGAGCACGGTATCCTCTGCCGAAACAGAGGTTGG
ACAAGACCGGAGGGGTCTCCTAGTTCCAAAGGAGATGTACTCCGGGCTTGTTCACGACCTACCGTGTA
+
FFFFFFFFFFFFFFFFFFFFFFFFFFFFFFFFFFFFFFFFFFFFF:FFFFFFFFFFFFFFFFFFFFFF
FFFFFFFFFFFFFFFFFFFFFFFFFFFFFFFFFFFFFFFFFFFFFFFFFF:FFFFFFFFFFFFFFFFF
@A00155:342:HHGFNDSXY:1:2514:16043:34538 1:N:0:GAACCTAG+TCCGCATA
GTAGGTCGTGAACAAGCCCGGAGTACATCTCCTTTGGAACCAGGAGACCCCTCCGGTCTTGTCCAACC
TCTGTTTCGGCAGAGGATACCGTGCTCCACAGCTGTAAGGCTGTGGGCCCGAGAGAAGTCGCTCTCTC
+
FFFFFFFFFFFFFFF,FFFFFFFFFFFFFFFFFFFFFFFFFFFFFFFFFFFFFFFF:FFFFFFFFFF
FFFFFFFFFFFFF:FFFFFFFFFFFFFFFFFFF:FFFFFFFFFFFFFFFFFFFFFFFFFFFFFFFFFF
@A00155:342:HHGFNDSXY:1:2515:17933:18082 1:N:0:GAACCTAG+TCCGCATA
CTCAATGCCGAGAGGGATGTAGAGGGCGAGAGAGAGCGACTTCTCTCGGGCCCACAGCCTTACAGCTG
TGGAGCACGGTATCCTCTGCCGAAACAGAGGTTGGACAAGACCGGAGGGGTCTCCTAGTTCCAAAGGA
+
F,FF:,FF:::FFF:FFF:FFFFFFFF:FFF,F:FFF,FFFFFFF,F:F,,F:FFFFFFFFFFFFFF
FFFFFFFFFF:FFF:F,FFFF,FF:FFFF:F:::FFFFF:FFF::FFF:FFFFF,F::FFF,FFF:FF
@A00155:342:HHGFNDSXY:1:2515:17390:22905 1:N:0:GAACCTAG+TCCGCATA
GGAGGGCGAGAGAGAGCGACTTCTCTCGGGCCCACAGCCTTACAGCTGTGGAGCACGGTATCCTCTGC
CGAAACAGAGGTTGGACAAGACCGGAGGGGTCTCCTGGTTCCAAAGGAGATGTACTCCGGGCTTGTTC
+
FFFFFFFFFFFFFFFFFFFFFFFFFFFFFFFFFFFFFFFFFFFFFFFFFFFFFFFFFFFFFFFFFFFF
FFFFFFFFFFFFFFFFFFFFFFFFFFFFFFFFFFFFFFFFFFFFFFFFFFFFFFFFFFFFFFFFFFFF
@A00155:342:HHGFNDSXY:1:2515:17436:24518 1:N:0:GAACCTAG+TCCGCATA
GTCCACTTAAGGACTCACCTCTGGCCTATGGTCATCTCAATGCCGAGAGGGATGTGGAGGGCGAGAGA
GAGCGACTTCTCTCGGGCCCACAGCCTTACAGCTGTGGAGCACGGTATCCTCTGCCGAAAC
+
FFFFFFFFFFFFFFFFFFFFFFFFFFFFFFFFFFFFF:FFFFFFFF:FFFFFFFFF,FFFFFFFFFF
FFFFFFFFFFFFFFFFFF:FFFFFFFFFFFFFFFF:FFFFFFF:FFFFFFFFFF:,FFFFF
@A00155:342:HHGFNDSXY:1:2515:30029:31360 1:N:0:GAACCTAG+TCCGCATA
GATCAGCTGTGTCCACTTAAGGACTCACCTCTGGCCTATGGTCATCTCAATGCCGAGAGGGATGTGGA
GGGCGAGAGAGAGCGACTTCTCTCGGGCCCACAGCCTTACAGCTGTGGAGCACGGTATCCTCTGC
+
FFFFFFFFFFFFFFFFFFFFFFFFFFFFFFFFFFFFFFFFFFFFFFFFFFFFFFFFFFFFFFFFFFFF
FFFFFFFFFFFFFFFFFFFFFFFFFFFFFFFFFFFFFFFFFFFFFFFFFFFFFFFFFFFFFFFFF
@A00155:342:HHGFNDSXY:1:2515:22218:36871 1:N:0:GAACCTAG+TCCGCATA
ATCTCAATGCCGAGAGGGATGTGGAGGGCGAGAGAGAGCGACTTCTCTCGGGCCCACAGCCTTACAGC
TGTGGAGCACGGTATCCTCTGCCGAAACAGAGGTTGGACAAGACCGGAGGGGTCTCCTAGTTCCAAAG
+
FFFFFFFFFFFFFFFFFFFFFFFFFFFFFFFFFFFFFFFFF:FFFFFFFFFFFFFFFFFFFFFFFFFF
FFFFFFFFFFFFFFFFFFFFFFFFFFFFFFFFFFFFFFFFFFFFFFFFFFFFFFFFFFFFFFFFFFFF
@A00155:342:HHGFNDSXY:1:2516:16025:6950 1:N:0:GAACCTAG+TCCGCATA
TGTGTCCACTTAAGGACTCACCTCTGGCCTATGGTCATCTCAATGCCGAGAGGGATGTGGAGGGCGAG
AGAGAGCGACTTCTCTCGGGCCCACAGCCTTACAGCTGTGGAGCACGGTATCCTCTGCCGAAACAGAG
+
:FFFFFFFFFFFFFFFFFFFFFFFFFFFFFFFFFFFFFFFFFFFFFFFFFFFFFFFFFFFFFFFFFFF
FFFFFFFFFFFFFFFFFFFFFFFFFFFFFFFFFFFFFFFFFFFFFFFFFFFFFFFFFFFFFFFFFFFF
@A00155:342:HHGFNDSXY:1:2516:21206:14450 1:N:0:GAACCTAG+TCCGCATA
CTTCTCTCGGGCCCACAGCCTTACATCTGTGGAGCACGGTATCCTCTGCCGAAACAGAGGTTGGACAA
GACCGGAGGGGTCTCCTAGTTCCAAAGGAGATGTACTCCGGGCTTGTTCACGACCTACCGTGTAAGTC
+
```

```
FFFFFFFFFFFFFFFFFFFFFFFFFFFFFFFFFFFFFFF:FFFFFFFFFFFFFFFFFFFFFFFFFFFF
FFFFFFFFFFFFFFFFFFFFFFFFFFFFFFFFFFFFFFFFFFFFFFFFFFFFFFFFFFFFFFFFFFFF
@A00155:342:HHGFNDSXY:1:2516:3405:20509 1:N:0:GAACCTAG+TCCGCATA
GAGAGAGAGCGACTTCTCTCGGGCCCACAGCCTTACAGCTGTGGAGCACGGTATCCTCTGCCGAAACA
GAGGTTGGACAAGACCGGAGGGGTCTCCTAGTTCCAAAGGAGATGTACTCCGGGCTTGTTCACGACCT
+
FFFFFFFFFFFFFFFFFFFFFFFFFFFFFFFFFFFFFFFFFFFFF:FFFFFFFFFFFFFFFFFFFFFF
FFFFFFFFFFFFFFFFFFFFFFFFFFFFFFFFFFFFFFFFF,FFFFFFFF,FFF:FFFFFFFFF:FFF
@A00155:342:HHGFNDSXY:1:2516:28546:22842 1:N:0:GAACCTAG+TCCGCATA
AGAGAGAGCGACTTCTCTCGGGCCCACAGCCTTACAGCTGTGGAGCACGGTATCCTCTGCCGAAACAG
AGGTTGGACAAGACCGGAGGGGTCTCCTAGTTCCAAAGGAGATGTACTCCGGGCTTGTTCACGACCTA
+
FFFFFFFFFFFFFFFFFFFFFFFFFF:FFFFFFFFFFFFFFFFFFFFFFFFFFF:FFFFFFFFFFFFF
FFFFFFFFFFFFFF:FFFFFFFFFFFFFFFFFFFFFFFFFFFFFFFFFFFFFFF:FFFFFFFFFFFFF
@A00155:342:HHGFNDSXY:1:2517:23529:1407 1:N:0:GAACCTAG+TCCGCATA
TGTGGAGGGCGAGAGAGAGCGACTTCTCTCGGGCCCACAGCCTTACAGCTGTGGAGCACGGTATCCTC
TGCCGAAACAGAGGTTGGACAAGACCGGAGGGGTCTCCTGGTTCCAAAGGAGATGTACTCCGGG
+
FF:FFFFFFFFFFFFFFFFFFFFFFFFFFFFFFFFFFFFFFF:FFFFFFFFFFFFFFFFFFFFFFFFF
FFFFFFFFFFFFFFFFFFFF:FFFFFFFFFFFFFFFFFFFFFFFFFFFFFFFF,FFFFFFFFFF
@A00155:342:HHGFNDSXY:1:2517:2582:11443 1:N:0:GAACCTAG+TCCGCATA
CATCTCAATGCCGAGAGGGATGTGGAGGACGAGAGAGAGCGACTTCTCTCGGGCCCACAGCCTTACAG
CTGTGGAGCACGGTATCCTCTGCCGAAACAGAGGTTGGACAAGACCGGAGGGGTCTCCTAGTTCCAAA
+
FFFFFFFFFFFFFFFFFFFFFFFFFFFFFFFFFFFFFFFFFFFFFFFFFFFFFFFFFFFFFFFFFFFF
FFFFFFFFFFFFFFFFFFFFFFFFFFFFFFFFFFFFFFFFFFFFFFFFFFFFFFFFFFFFFFFFFFFF
@A00155:342:HHGFNDSXY:1:2517:3007:17440 1:N:0:GAACCTAG+TCCGCATA
GGATGTGGAGGGCGAGAGAGAGCGACTTCTCTCGGGCCCACAGCCTTACAGCTGTGGAGCACGGTATC
CTCTGCCGAAACAGAGGTTGGACAAGACCGGAGGGGTCTCCTAGTTCCAAAGGAGATG
+
FFFFFFFFFFFFFFFFFFFFFFFFFFFFFFFFFFFFFFFFFFFFFFFFFFFFFFFFFFFFFFFFF:FF
FFFFFFFFFFFFFF:FFFFFFFFFFFFFFFFFFFFFFFFFFFFFFF:FFFFFFFFF,:
@A00155:342:HHGFNDSXY:1:2517:7726:18662 1:N:0:GAACCTAG+TCCGCATA
CAGCCTTACAGCTGTGGAGCACGGTATCCTCTGCCGAAACAGAGGTTGGACAAGACCGGAGGGGTCTC
CTAGTTCCAAAGGAGATGTACTCCGGGCTTGTTCACGACCTACCGTGTAAGTCGTAGTCTAGTAGGCT
+
FFFFFFFFFFFFFFFFFFFFFFFFFFFFFFFFFFFFFFFFFFFFFFFFFFFFFFFF:FFFFFFFFFFF
FFFFFFFFFFFFFF:FFFFFFFFFFFFFFFFFFFFFFFFFFFFFFFFFFF:FFFFFFFFFFFFFFFFF
@A00155:342:HHGFNDSXY:1:2517:2112:20838 1:N:0:GAACCTAG+TCCGCATA
GGACTCACCTCTGGCCTATGGTCATCTCAATGCCGAGAGGGATGTGGAGGGCGAGAGAGAGCGACTTC
TCTCGGGCCCACAGCCTTACAGCTGTGGAGCACGGTATCCTCTGCCGAAACAGAGGTTGGACAAGACC
+
FFFFFFFFFFFFFFFFFFFFFFFFFFFFFFFFFFFFFFFFF:FFFFFFFFFFFFFFFFFFFFFF,FFF
FFFFFFFFFFFFFFFFFFFFFFFFFFFFFFFFFFFFFFFFF:FFFFFFF:FFFFFFFFFFFFF::FFF
@A00155:342:HHGFNDSXY:1:2517:11939:29997 1:N:0:GAACCTAG+TCCGCATA
CCCTCCACATCCCTCTCGGCATTGAGATGACCATAGGCCAGAGGTGAGTCCTTAAGTGGACACAGCTG
ATCTAAGGCGGTGTGGTGGGGCATGGGTTTGAACCCCCATGACGGTCGGAGTCTAGTAGGCTCCCTGA
+
FFFFFFFFFFFFFFFFFFFFFFFFFFFFFFFFFFFFFFFFFFFFFFFFFFFFFFFFFFFFFFFFFFFF
FFFFFFFFFFFFFFFFFFFFFFFFFFFFFFFFFFFFFFFFFFFFFFFFFFFFFFFFFFFFFFFFFFFF
@A00155:342:HHGFNDSXY:1:2518:14696:3176 1:N:0:GAACCTAG+TCCGCATA
GACTTCTCTCGGGCCCACAGCCTTACAGCTGTGGAGCACGGTATCCTCTGCCGAAACAGAGGTTGGAC
AAGACCGGAGGGGTCTCCTAGTTCCAAAGGAGATGTACTCCGGGCTTGTTCACGACCTACCGTGTAAG
+
```

FFFFFFFFFFFFFFFFFFFFFFFFFFFFFFFFFFFFFFFFFFFFFFFFFFFFFFFFFFFFFFFFFFFF
FFFFFFFFFFFFFFFFFFFFFFFFFFFFFFFFFFFFFFFFFFFFFFFFFFFFFFFFFFFFFFFFFFFF
@A00155:342:HHGFNDSXY:1:2518:6985:17284 1:N:0:GAACCTAG+TCCGCATA
GGACTCACCTCTGGCCTATGGTCATCTCAATGCCGAGAGGGATGTGAAGGGCGAGAGAGAGCGACTTC
TCTCGGGCCCACAGCCTTACAGCTGTGGAGCACGGTATCCTCTGCCGAAACAGAGGTTGGACAAGACC
+
FFFFFFFFFFFFFFFFFFFFFFFFFFFFFFFFFFFFFFFFFFFFFFFFFFFFFFFFFFFFFFFFFFFF
FFFFFFFFFFFFFFFFFFFFFFFFFFFFFFFFFFFFFFFFFFFFFFFFFFFFFFFFFFFFFFFFFFFF
@A00155:342:HHGFNDSXY:1:2518:11948:28291 1:N:0:GAACCTAG+TCCGCATA
TTAAGGACTCACCTCTGGCCTATGGTCATCTCAATGCCGAGAGGGATGTGAAGGGCGAGAGAGAGCGA
CTTCTCTCGGGCCCACAGCCTTACAGCTGTGGAGCACGGTATCCTCTGCCGAAACAGAGGTTGGACAA
+
FFFFFFFFFFFFFFFFFFFFFFFFFFFFFFFFFFFFFFFFFFFFFFFFFFFFFFFFFFFFFFFFFFFF
FFFFFFFFFFFFFFFFFFFF,FFFFFF:FFFFFFFFFFFFFFFFFFFFFFFFFFFFFFFFFFFFFFFF
@A00155:342:HHGFNDSXY:1:2518:24478:31610 1:N:0:GAACCTAG+TCCGCATA
CTGTGTCCACTTAAGGACTCACCTCTGGCCTATGGTCATCTCAATGCCGAGAGGGATGTGAAGGGCGA
GAGAGAGCGACTTCTCTCGGGCCCACAGCCTTACAGCTGTGGAGCACGGTATCCTCTGCCGAAACAGA
+
FFFFFFFFFFFFFFFFFFFFFFFFFFFFFFFFFFFFFFFFFFFFFFFFFFFFFFFFFFFFFFFFFFFF
FFFFFFFFFFFFFFFFFFFFFFFFFFFFFFFFFFFFFFFFFFFFFFFFFFFFFFFFFFFFFFFFFFFF
@A00155:342:HHGFNDSXY:1:2518:6162:34428 1:N:0:GAACCTAG+TCCGCATA
CAATGCCGAGAGGGATGTGGAGGGCGAGAGAGAGCGACTTCTCTCGGGCCCACAGCCTTACAGCTGTG
GAGCACGGTATCCTCTGCCGAAACAGAGGTTGGACAAGACCGGAGGGGTCTCCTGGTTCCAAAGGAGA
+
FFFFFFFFFFFFFFFFFFFFFFFFFFFFFFF:FFFFF:FFFFFFFFFFFFFFFFFFF,:FFF,FFFFF
FFFFFFFFFFFFFFFFFFFFFFFFFFFFFFFF:FFFFFFFFFFFFFFFFFFF:FFFFFFFFFFFF:FF
@A00155:342:HHGFNDSXY:1:2519:13015:8249 1:N:0:GAACCTAG+TCCGCATA
GAGCGACTTCTCTCGGGCCCACAGCCTTACAGCTGTGGAGCACGGTATCCTCTGCCGAAACAGAGGTT
GGACAAGACCGGAGGGGTCTCCTAGTTCCAAAGGAGATGTACTCCGGGCTTGTTCACGACCTACCGTG
+
FFFFFFFFFFFFFFFFFFFFFFFFFFFFFFFFFFFFFFFFFFFFFFFFFFFFFFFFFFFFFFFFFFFF
FFFFFFFFFFFFFFFFFFFFFFFFFFFFFFFFFFFFFFFFFFFFFFFFFFFFFFFFFFFFFFFFFFFF
@A00155:342:HHGFNDSXY:1:2519:2013:16595 1:N:0:GAACCTAG+TCCGCATA
AAAGACTCACCTCTGGCCTATGGTCATCTCAATGCCGAGAGGGATGTGGAGGGCGAGAGAGAGCGACT
TCTCTCGGGCCCACAGCCTTACAGCTGTGGAGCACGGTATCCTCTGCCGAAACAGAGGTTGGACAAGA
+
FF:FFFFFFF:FFFFFFFFFFF:FFFFFF:FFFFF,FFF:FFFFFFFF:FFFFFFFFFFFFFFFFFFF
FFFFFFFFFFFFFFFFFFFFFFF,FFFFFFFFFFF:FFF:FFFFFF:FFFFFFFF::FFFFFFFFF:F
@A00155:342:HHGFNDSXY:1:2519:26928:18881 1:N:0:GAACCTAG+TCCGCATA
CCTTACAGCTGTGGAGCACGGTATCCTCTGCCGAAACAGAGGTTGGACAAGACCGGAGGGGTCTCCTA
GTTCCAAAGGAGATGTACTCCGGGCTTGTTCACGACCTACCGTGTAAGTCGTAGTCTAGTAGGCTACC
+
FFFFFFFFFFFFFFFFFFFFFFFFFFFFFFFFFFFFFFFFFFFFFFFFFFFFFFFFFFFFFFFFFFFF
FFFFFFFFFFFFFFFFFFFFFFFFFFFFFFFFFFFFFFFFFFFFFFFFFFFFFFFFFFFFFFFFFFF:
@A00155:342:HHGFNDSXY:1:2519:9679:22701 1:N:0:GAACCTAG+TCCGCATA
ATCTCAATGCCGAGAGGGATGTGGAGGGCGAGAGAGAGCGACTTCTCTCGGGCCCACAGCCTTACAGC
TGTGGAGCACGGTATCCTCTGCCGAAACAGAGGTTGGACAAGACCGGAGGGGTCTCCTAGTTCCAAAG
+
FFFFFFFFFFFFFFFFFFFFFFFFFFFFFFFFFFFFFFFFFFFFFFFFFFFFFFFFFFFFFFFFFFFF
FFFFFFFFFFFFFFFFFFFFFFFFFFFFFFFFFFFFFFFFFFFFFFFFFFFFFFFFFFFFFFFFFFFF
@A00155:342:HHGFNDSXY:1:2519:12228:22983 1:N:0:GAACCTAG+TCCGCATA
CTGGCCTATGGTCATCTCAATGCCGAGAGGGATGTGGAGGGCGAGAGAGAGCGACTTCTCTCGGGCCC
ACAGCCTTACAGCTGTGGAGCACGGTATCCTCTGCCGAAACAGAGGTTGGACAAGACCGGAGGGGTCT
+

FFFFFFFFFFFFFFFFFF:FFFFFFFFFFFFFF:FFFFFFFFFFFFFFFFFFFFFFFFFFFFFFFFFFFF
FFFFFFFFFFF:FFFFFFFFFFFFFFFFFFFFFFFFFFFFFFFFFFFFFFFFFFFF:FFFF:FFFFFFFF
@A00155:342:HHGFNDSXY:1:2519:7491:35978 1:N:0:GAACCTAG+TCCGCATA
CGACTTCTCTCGGGCCCACAGCCTTACAGCTGTGGAGCACGGTATCCTCTGCCGAAACAGAGGTTGGA
CAAGACCGGAGGGGTCTCCTAGTTCCAAAGGAGATGTACTCCGGGCTTGTTCACGACCTACCGTGTAA
+
FFFFFFFFFFFFFFFFFFFFFFFFFFFFFFFFFFFFFFFFFFFF,FFFFFFFFFFFFFFFFFFFFFFFFF
FFFFFFFFFFFFFFFFFFFFFFFFFFFFFFFFFFFFFFFFFF:FFFFFFFFFFFF:FFFFFFFFF:FFFF
@A00155:342:HHGFNDSXY:1:2520:7536:33520 1:N:0:GAACCTAG+TCCGCATA
TCCACATCCCTCTCGGCATTGAGATGACCATAGGCCAGAGGTGAGTCCTTAAGTGGACACAGCTGATC
TAAGGCGGTGTGGCGGGGCATGGGTTTGAACCCCCATGACGGTCGGAGTCTAGTAGGCTCCCTGATGA
+
FFFFFFFFFFFFFFFFFF:FFFFFFFFFFFFFFFFFFFFFFFFFFFFFFFFFFFFFFFFFFFFFFFFFFF
FFFFFFFFFFFFFFFFFFFFFFFFFFFFFFFFFFFFFFFFFFFFFFFFFFFFFFFFFFFFFFFFFFFFFF
@A00155:342:HHGFNDSXY:1:2521:28230:1658 1:N:0:GAACCTAG+TCCGCATA
ATGCCGAGAGGGATGTGGAGGGCGAGAGAGAGCGACTTCTCTCGGGCCCACAGCCTTACAGCTGTGGA
GCACGGTATCCTCTGCCGAAACAGAGGTTGGACAAGACCGGAGGGGTCTCCTGGTTCCAAAGGAGATG
+
FF:FFFFFFFFFFFFFF:FFFF:FFFFF:FFF:FFFFFFFFFFFFFFFFFFFFFFFFF,FFFFFFFFF:F
:FFFFFFF:F:FFFFFF,FFFFFFFFF:FFFFFFFFFFFFFFFFFFFFFFFFFFFFFFFFFFF,FFFFFF
@A00155:342:HHGFNDSXY:1:2521:7265:30483 1:N:0:GAACCTAG+TCCGCATA
AGGGCGAGAGAGAGCGACTTCTCTCGGGCCCACAGCCTTACAGCTGTGGAGCACGGTATCCTCTGCCG
AAACAGAGGTTGGACAAGACCGGAGGGGTCTCCTAGTTCCAAAGGAGATGTACTCCGGGCTTGTTCAC
+
FFFFFFFFFFFFFFFFFFFFFFFFFFFFFFFFFFF,FFFFFFFFFFFFFFFFFFFFFFFFFFFFFFFFFF
FFFFFFFFFFFFFFFFFFFFFFFFFFFFF:FFFFFFFFFFFFFFFFFFFFFFFFFFFFFFFFFFFFFFFF
@A00155:342:HHGFNDSXY:1:2522:9408:14904 1:N:0:GAACCTAG+TCCGCATA
AGGGCGAGAGAGAGCGACTTCTCTCGGGCCCACAGCCTTACAGCTGTGGAGCACGGTATCCTCTGCCG
AAACAGAGGTTGGACAAGACCGGAGGGGTCTCCTAGTTCCAAAGGAGATGTACTCCGGGCTTGTTC
+
FFFFFFFFFFFFFFFFFFFFFFFFFFFFFFFFFFFFFFFFFFFFFFFFFFFFFFFFFFFFFFFFFFFFFF
FFFFFFFFFFFFFFFFFFFFFFFFFFFFFFFFFFFFFFFFFFFFFFFFFFFFFFFFFFFFFFFFFFFF
@A00155:342:HHGFNDSXY:1:2522:7636:23171 1:N:0:GAACCTAG+TCCGCATA
TGTGGAGGGCGAGAGAGAGCGACTTCTCTCGGGCCCACAGCCTTACAGCTGTGGAGCACGGTATCCTC
TGCCGAAACAGAGGTTGGACAAGACCGGAGGGGTCTCCTAGTTCCAAAGGAGATGTACTCCGGG
+
FFFFFFFFFFFFFFFFFFFFFFFFFFFFFFFFFFFFFFFFFFFFFFFFFFFFFFFFFFFFFFFFFFFFFF
FF:FFFFFFFFFFFFFFFFFFFFFFFFFFFFFFFFFFFFFFFFFFFFFFFFFFFFFFFFFFFFFFF
@A00155:342:HHGFNDSXY:1:2522:23710:24142 1:N:0:GAACCTAG+TCCGCATA
GGATGTGGAGGGCGAGAGAGAGCGACTTCTCTCGGGCCCACAGCCTTACAGCTGTGGAGCACGGTATC
CTCTGCCGAAACAGAGGTTGGACAAGACCGGAGGGGTCTCCTAGTTCCAAAGGAGATGTACTCC
+
FFFFFFFFFFFFFFFFFFFFFFFFFFFFFFFFFFFFFFFFFFFFFFFFFFFFFFFFFFFFFFFFFFFFFF
FFFFFFFFFFFFFFFFFFFFFFFFFFFFFFFFFFFFFFFFFFFFFFFFFFFFFFFFFFFFFFFFFF
@A00155:342:HHGFNDSXY:1:2522:32741:24314 1:N:0:GAACCTAG+TCCGCATA
AGGGATGTGGAGGGCGAGAGAGAGCGACTTCTCTCGGGCCCACAGCCTTACAGCTGTGGAGCACGGTA
TCCTCTGCCGAAACAGAGGTTGGACAAGACCGGAGGGGTCTCCTAGTTCCAAAGGAGATGTACTCCGG
+
FFFFFFFFFFFF:FFFFFFFFFFFFFFFFF:FFFFFFF:FFFFFFFFFFFFFFFFFFFFFFFFFFFFFFF
FFFFFFFFFFFFFFFFFFFFFFFFFFFF:F,FFFFFFF:FFFFFFFFFFFFFFFFFFFFFFFF:FFFFFF
@A00155:342:HHGFNDSXY:1:2522:16613:33771 1:N:0:GAACCTAG+TCCGCATA
GGATGTGGAGGGCGAGAGAGAGCGACTTCTCTCGGGCCCACAGCCTTACAGCTGTGGAGCACGGTATC
CTCTGCCGAAACAGAGGTTGGACAAGACCGGAGGGGTCTCCTAGTTCCAAAGGAGATGTACTCC
+

FFFFF:FFFFFFFFFFFFFFFFFFFFFFFFFFFFFFFFFFFFFFFFFFFFFFFFFFFFFFFFFFFFFF
FFFFFFFFFFFFFFFFFFFFFFFFFFFFFFFFFFFFFFFFFFFFFFFFFFFFFFFFFFFFFFFFF
@A00155:342:HHGFNDSXY:1:2522:18973:35321 1:N:0:GAACCTAG+TCCGCATA
ATGCCCCGCCACACCGCCTTAGATCAGCTGTGTCCACTTAAGAACTCACCTCTGGCCTATGGTCATCT
CAATGCCGAGAGGGATGTGGAGGGCGAGAGAGAGCGACTTCTCTCGGGCCCACAGCCTTACAGC
+
FFFFFFFFFFFFFFFFFFFFFFFFFFFFFFFF:FFFFFFFFFFFFFFFFFFFFFFFFFFFFFFFFFFF
FFFFFFFFFFFFFFFFFFFFFFFFFFFFFFFFFFF:FFFFFFFFFFFFFFFFFFFFFFFFFFFFF
@A00155:342:HHGFNDSXY:1:2523:19117:4726 1:N:0:GAACCTAG+TCCGCATA
AGCGACTTCTCTCGGGCCCACAGCCTTACAGCTGTGGAGCACGGTATCCTCTGCCGAAACAGAGGTTG
GACAAGACCGGAGGGGTCTCCTGGTTCCAAAGGAGATGTACTCCGGGCTTGTTCACGACCTACCGTGT
+
FFFFFFFFFFFFFFFFFFFFFFFFFFFFFFFFFFFFFFFFFFFFFFF:FFFFFFFFFFFFFFFFFFFF
FFFFFFFFFFFFFFFFFFFFFFFFFFFF:FFFFFFFFFFFFFFFFFFFFFFFFF:FFFFFFFFFFFFFF
@A00155:342:HHGFNDSXY:1:2523:30553:19304 1:N:0:GAACCTAG+TCCGCATA
GACTCACCTCTGGCCTATGGTCATCTCAATGCCGAGAGGGATGTAGAGGGCGAGAGAGAGCGACTTCT
CTCGGGCCCACAGCCTTACAGCTGTGGAGCACGGTATCCTCTGCCGAAACAGAGGTTGGACAAGACCG
+
FFFFFFFFFFFFFFFFFFFFFFFFFFFFFFFFFFFFFFFFFFFFFFF:FFFFFFFFFFFFFFFFFFFF
FFFFFFFFFFFFFFFFFFFFFFFFFFFFFFFFFFFFFFFFFFFFFFFFFFFFFFFFFFFFFFFFFFFF
@A00155:342:HHGFNDSXY:1:2523:31186:19648 1:N:0:GAACCTAG+TCCGCATA
GACTCACCTCTGGCCTATGGTCATCTCAATGCCGAGAGGGATGTAGAGGGCGAGAGAGAGCGACTTCT
CTCGGGCCCACAGCCTTACAGCTGTGGAGCACGGTATCCTCTGCCGAAACAGAGGTTGGACAAGACCG
+
FFFFFFFFFFFFFFFFFFFFFFFFFFFFFFFFFFFFFFFFF:FFFFFFFFFFFFFFFFFFFFFFFFFF
FFFFFFFFFFFFFFFFFFFFFFFFFFFFFFFFFFFFFFFFFFFFFFFFFFFFFFF:FFFFFFF:FFFF
@A00155:342:HHGFNDSXY:1:2523:18105:23891 1:N:0:GAACCTAG+TCCGCATA
CTCTGGCCTATGGTCATCTCAATGCCGAGAGGGATGTGAAGGGCGAGAGAGAGCGACTTCTCTCGGGC
CCACAGCCTTACAGCTGTGGAGCACGGTATCCTCTGCCGAAACAGAGGTTGGACAAGACCGGAG
+
FFFFFFFFFFFFFFFFFFFFFFFFFFFFFFFFFFF,FFFFFFFFFFFFFFFFFFFFFFFFFFFFFFFF
FFFFFFFFFFFFFFFFFFFFFFFFFFFFFFFFFFFFFFFFFFFFFFFFFFFFFFFFFFFFFFFFF
@A00155:342:HHGFNDSXY:1:2523:15139:24267 1:N:0:GAACCTAG+TCCGCATA
ACTTCTCTCGGGCCCACAGCCTTACAGCTGTGGAGCACGGTATCCTCTGCCGAAACAGAGGTTGGACA
AGACCGGAGGGGTCTCCTAGTTCCAAAGGAGATGTACTCCGGGCTTGTTCACGACCTACCGTGTAAGT
+
FFFFFFFFFFFFFFFFFFFFFFFFFFFFFFFFFFFFFFFFFFFFFFFFFFFFFFFFFFFFFFFFFFFF
FFFFFFFFFFFFFFFFFFFFFFFFFFFFFFFFFFFFFFFFFFFFFFFFFFFFFFFFFFFFFFFFFFFF
@A00155:342:HHGFNDSXY:1:2523:28031:31093 1:N:0:GAACCTAG+TCCGCATA
GACTCACCTCTGGCCTATGGTCATCTCAATGCCGAGAGGGATGTAGAGGGCGAGAGAGAGCGACTTCT
CTCGGGCCCACAGCCTTACAGCTGTGGAGCACGGTATCCTCTGCCGAAACAGAGGTTGGACAAGACCG
+
FFFFFFFFFFFFFFFFFFFFFFFFFFFFFFFFFFFFFF:FFFFFFFFFFFFFFFFFFFFFFFFFFFFF
FFFFFFFFFFFFFFFFFFFFFFFFF:FFFFFFFFFFFFFFFFFFFFFFFFFFFFFF:FFFFFFFFFFFF
@A00155:342:HHGFNDSXY:1:2523:23493:36417 1:N:0:GAACCTAG+TCCGCATA
TCTCTCGGGCCCACAGCCTTACAGCTGTGGAGCACGGTATCCTCTGCCGAAACAGAGGTTGGACAAGA
CCGGAGGGGTCTCCTAGTTCCAAAGGAGATGTACTCCGGGCTTGTTCACGACCTACCGTGTAAGTCGT
+
FFFFFFFFFFFFFFFFFFFFFFFFFFFFFFFFFFFFFFFFFFFFFFFFFFFFFFFFFFFFFFFFFFFF
FFFFFFFFFFFFFFFFFFFFFFFFFFFFFFFFFFFFFFFFFFFFFFFFFFFFFFFFFFFFFF:FFFFF
@A00155:342:HHGFNDSXY:1:2524:5249:19225 1:N:0:GAACCTAG+TCCGCATA
AGAGAGAGCGACTTCTCTCGGGCCCACAGCCTTACAGCTGTGGAGCACGGTATCCTCTGCCGAAACAG
AGGTTGGACAAGACCGGAGGGGTCTCCTAGTTCCAAAGGAGATGTACTCCGGGCTTGTTCACGACCTA
+

FFFFFFFFFFFFFFFFFF::FFFFFFFFFFFFFFF:FFFFFFFFFFFFFFFFFFFFFFFFFFFF:FF:
F:FFFFFFFFFFFFFFFFFFFFFFFFF:FFFFFFFFFFFFFFFFFFFFF:FFFFFFFFFFFFFFFFFF
@A00155:342:HHGFNDSXY:1:2524:8024:20212 1:N:0:GAACCTAG+TCCGCATA
AGAGAGAGCGACTTCTCTCGGGCCCACAGCCTTACAGCTGTGGAGCACGGTATCCTCTGCCGAAACAG
AGGTTGGACAAGACCGGAGGGGTCTCCTAGTTCCAAAGGAGATGTACTCCGGGCTTGTTCACGACCT
+
FFFFFFFFFFFFFFFFFFFFFFFFFFFFFFFFFFFFFFFFFFFFFFFFFFFFFFFFFFFFFFFFFFFF
FFFFFFFFFFFFFFFF:FFFFFFFFFF:FFFFFFFFFFFFFFFFFFFFFFFFFFFFFFFFFFFFFFF
@A00155:342:HHGFNDSXY:1:2524:5556:27398 1:N:0:GAACCTAG+TCCGCATA
ACCTCTGTTTCGGCAGAGGATACCGTGCTCCACAGCTGTAAGGCTGTGGGCCCGAGAGAAGTCGCTCT
CTCTCGCCCTCCACATCCCTCTCGGCATTGAGATGACCATAGGCCAGAGGAGAGTCCTTAAGTGGACA
+
FFFFFFFFFFFFFFFFFFFFFFFFFFFFFFFFFFFFFFFFFFFFFF::FFFFF:FFFFFFFFFF:FFF
FFFFFFFF:FFFFF,:F,FFFFFFF:FFFFFFFFFFFFFFF,FFFFFFFFFFF:FFFFFF:F:FFFFF
@A00155:342:HHGFNDSXY:1:2524:1344:32784 1:N:0:GAACCTAG+TCCGCATA
TCCACTTAAGGACTCTCCTCTGGCCTATGGTCATCTCAATGCCGAGAGGGATGTGGAGGGCGAGAGAG
AGCGACTTCTCTCGGGCCCACAGCCTTACAGCTGTGGAGCACGGTATCCTCTGCCGAAACAGAGGTTG
+
F:FF:FFFFFFFFF:FFFFFFFF:FFFFFFFFFFFFFFFFFFFFFF:FFFFFFFFFFFFFFFFF:FFF
:FFFFFFFFFFFFFFFFFFFFFFFFFFFF:F:FFFFF:FF:FFFFFFFFFFFFFFF:F:,FF,FFFFF
@A00155:342:HHGFNDSXY:1:2525:18719:11303 1:N:0:GAACCTAG+TCCGCATA
GATCAGCTGTGTCCACTTAAGGACTCACCTCTGGCCTATGGTCATCTCAATGCCGAGAGGGATGTAGA
GGGCGAGAGAGAGCGACTTCTCTCGGGCCCACAGCCTTACAGCTGTGGAGCACGGTATCCTCTGCCGA
+
FFFFFFFFFFFFFFFFFFFFFFFFFFF:FFFFFFFFFFFFFFFFFFFFFFFFFFFFFFFFFFFFF:::
FFFFFFFFFFFFFFFFFFFFFFFFFF:FFFFFFFFFFFFFFF:FF,FFFFFFFFFFFFFFFFFFFFFF
@A00155:342:HHGFNDSXY:1:2525:30282:28260 1:N:0:GAACCTAG+TCCGCATA
ACCTCTGTTTCGGCAGAGGATACCGTGCTCCACAGCTGTAAGGCTGTGGGCCCGAGAGAAGTCGCTCT
CTCTCGCCCTCCACATCCCTCTCGGCATTGAGATGACCATAGGCCAGAGGAGAGTCCTTAAGTGGACA
+
FFFFFFFFFFFFFFFFFFFFFFFFFFFFFFFFFFFFFFFFFFFFFFFFFFFFFFFFFFFFFFFFFFFF
FFFFFFFFFFFFFFFFFFFFFFFFFFFFFFFFFFFFFFFFFFFFFFFFFFFFFFFFFFFFFFFFFFFF
@A00155:342:HHGFNDSXY:1:2526:25030:10582 1:N:0:GAACCTAG+TCCGCATA
TGTGTCCACTTAAGGACTCACCTCTGGCCTATGGTCATCTCAATGCCGAGAGGGATGTGGAGGGCGAG
AGAGAGCGACTTCTCTCGGGCCCACAGCCTTACAGCTGTGGAGCACGGTATCCTCTGCCGAAACAGAG
+
FFFFFFFF:FFFFFFFFFFFFFFFFFFFFFFFFFFFFFFFFFFFFFFFFFFFFFF:FFFFFFFFFFFF
FFFFFFFFFFFFFFFFFFFFFFFFFFFFFFFFFFFFFFFFFFFFFFFFFFFFFFFF:FFFFFFFFFFF
@A00155:342:HHGFNDSXY:1:2526:27163:19507 1:N:0:GAACCTAG+TCCGCATA
AGAGAGCGACTTCTCTCGGGCCCACAGCCTTACAGCTGTGGAGCACGGTATCCTCTGCCGAAACAGAG
GTTGGACAAGACCGGAGGGGTCTCCTAGTTCCAAAGGAGATGTACTCCGGGCTTGTTCACGACCTACC
+
FFFFFFFFFFFFFFFFFFFFFFFFFFFFFFFFFFFF:FFFFFFFFFFFFFFFFFFFF:FFFFFFFFFF
FFFFFFFFFFFFFFFFFF:FFFFFFFFFFFFFFFFFFFFFF,FFFFFFFFFFFFFFFFFFFFFFFFFF
@A00155:342:HHGFNDSXY:1:2526:27407:19711 1:N:0:GAACCTAG+TCCGCATA
AGAGAGCGACTTCTCTCGGGCCCACAGCCTTACAGCTGTGGAGCACGGTATCCTCTGCCGAAACAGAG
GTTGGACAAGACCGGAGGGGTCTCCTAGTTCCAAAGGAGATGTACTCCGGGCTTGTTCACGACCTACC
+
FFFFFFFFFFFFFFFFFFFFFFFFFFFFFFFFFFFFFFFFFFFFFFFFFFFFFFFFFFFFFFFFFFFF
FFFFFFFFFFFFFFFFFFFFFFFFFFFFFFFFFFFFFFFFFFFFFFFFFFFFFFFFFFFFFFFFFFFF
@A00155:342:HHGFNDSXY:1:2526:3459:28087 1:N:0:GAACCTAG+TCCGCATA
CTCAATGCCGAGAGGGATGTGGAGGGCGAGAGAGAGCGACTTCTCTCGGGCCCACAGCCTTACAGCTG
TGGAGCACGGTATCCTCTGCCGAAACAGAGGTTGGACAAGACCGGAGGGGTCTCCTGGTTCCAAAGGA
+

FFFFFFFFFFFFFFFFFFFFFFFFFFFFFFFFFFFFFFFFFFFFFFFFFFFFFFFFFFFFFFFFFFFF
FFFFFFFFFFFFFFFFFFFFFFFFFFFFFFFFFFFFFFFFFFFFFFFFFFFFFFFFFFFFFF:FFFFFF
@A00155:342:HHGFNDSXY:1:2526:19497:36479 1:N:0:GAACCTAG+TCCGCATA
GAGGGCGAGAGAGAGCGACTTCTCTCGGGCCCACAGCCTTACAGCTGTGGAGCACGGTATCCTCTGCC
GAAACAGAGGTTGGACAAGACCGGAGGGGTCTCCTGGTTCCAAAGGAGATGTACTCCGGGCTTGTTCA
+
FFFFF:FFFFFFFFFFFFFFFFFFFFFFFFFFFFFFFFFFFFFFFFFFFF:FFFFFFFFFFFFFF
FFFFFFFFFFFFFFFFFFFF:FFFFFFFFFFFFFFFFFFFFFFFFFFFFFFFFFFFFFFFFFFFFFFF
@A00155:342:HHGFNDSXY:1:2527:28456:5368 1:N:0:GAACCTAG+TCCGCATA
ACCTCTGTTTCGGCAGAGGATACCGTGCTCCACAGCTGTAAGGCTGTGGGCCCGAGAGAAGTCGCTCT
CTCTCGCCCTCCACATCCCTCTCGGCATTGAGATGACCATAGGCCAGAGGAGAGTCCTTAAGTGGACA
+
FFF:F:FFFFFFFFFFFFFFFFFFFFFFFFFFFFFFFFFFFFFFFFFFFFFFFFFFFFFFFF:FFFF
FFFFFFFFFFFFFFFFFFFFFFFFFFFFFFFFFFFFFFFFFFFFFFFFFFFFFFFFFFFF,FFFFFFF
@A00155:342:HHGFNDSXY:1:2527:22824:15060 1:N:0:GAACCTAG+TCCGCATA
GAGAGGGATGTGGAGGGCGAGAGAGAGCGACTTCTCTCGGGCCCACAGCCTTACAGCTGTGGAGCACG
GTATCCTCTGCCGAAACAGAGGTTGGACAAGACCGGAGGGGTCTCCTAGTTCCAAAGGAGATGTACTC
+
FFFFFFFFFFFFFFFFFFFFFFFFFFFFFFFFFFFFFFFFFFFFFFFFFFF:FFFFFFFFFFFFFF
FFFFFFFFFFFFFFFFFFFFFFFFFFFFFFFFFFFFFFFFFFFFFFFFFFFFFFFFFFFFFFFFFFFF
@A00155:342:HHGFNDSXY:1:2527:9281:15718 1:N:0:GAACCTAG+TCCGCATA
GCGACTTCTCTCGGGCCCACAGCCTTACAGCTGTGGAGCACGGTATCCTCTGCCGAAACAGAGGTTGG
ACAAGACCGGAGGGGTCTCCTGGTTCCAAAGGAGATGTACTCCGGGCTTGTTCACGACCTACCGTGTA
+
FFFFF::FFFFF,FFFFFFFFF:FFFFFF:FFFFFFFFFFFFFFFFFFFFFFF:FFFFFFFFFF:FF,,
FFFF:FF:F:FF:FFFF:F,:FFFF:FFFFF:FFFFFFFFFF:F,FFFFFFF:FF,FFFFFFFF,FFF
@A00155:342:HHGFNDSXY:1:2527:10755:17331 1:N:0:GAACCTAG+TCCGCATA
GTATCTTCTGCCGAAACAGAGGTTGGACAAGACCGGAGGGGTCTCCTAGTTCCAAAGGAGATGTACTC
CGGGCTTGTTCACGACCTACCGTGTAAGTCGTAGTCTAGTAGGCTACCTGACGAGTCCTTTTTAGGAC
+
FFFFFFFFFFFFFFFFFFFFFFFFFFFFFFFFFFFFFFFFFFFFFFFFFFFFFFFFFFFFFFFFFFFF
FFFFFFFFFFFFFFFFFFFFFFFFFFFFFFFFFFFFF,FFFFFFFFFFFFFFFFFFFFF:FFFFFFFF
@A00155:342:HHGFNDSXY:1:2527:15248:18818 1:N:0:GAACCTAG+TCCGCATA
TAGATCAGCTGTGTCCACTTAAGGACTCACCTCTGGCCTATGGTCATCTCAATGCCGAGAGGGATGTA
GAGGGCGAGAGAGAGCGACTTCTCTCGGGCCCACAGCCTTACAGCTGTGGAGCACGGTATCCTCTGCC
+
FFFFFFFFFFFFFFFFFFFFFFFFFFFFFFFFFFFFFFFFFFFFFFFFFFFFFFFFFFFFFFFFFFFF
FFFFFFFFFFFFFFFFFFFFFFFFFFFFFFFFFFFFFFFFFFFFFFFFFFFFFFFFFFFFFFFFFFFF
@A00155:342:HHGFNDSXY:1:2527:8043:27571 1:N:0:GAACCTAG+TCCGCATA
GAGAGGGATGTGGAGGGCGAGAGAGAGCGACTTCTCTCGGGCCCACAGCCTTACAGCTGTGGAGCACG
GTATCCTCTGCCGAAACAGAGGTTGGACAAGACCGGAGGGGTCTCCTAGTTCCAAAGGAGATGTACTC
+
FFFFFFFFFFFFFFFFFFFFFFFFFFFFFFFFFFFFFFFFFFFFFFFFFFFFFFFFFFFFFFFFFFFF
FFFFFFFFFFFFFFFFFFFFFFFFFFFFFFFFFFFFFFFFFFFFFFFFFFFFFFFFFFFFFFFF:FFF
@A00155:342:HHGFNDSXY:1:2527:3441:30530 1:N:0:GAACCTAG+TCCGCATA
ATCTCAATGCCGAGAGGGATGTGGAGGGCTAGAGAGAGCGACTTCTCTCGGGCCCACAGCCTTACAGC
TGTGGAGCACGGTATCCTCTGCCGAAACAGAGGTTGGACAAGACCGGAGGGGTCTCCTAGTTCCAAAG
+
FFFFFFFFFFFFFFFFFFFFFFFFFFFFFFFFFFFFFFFFFFFFFFFFFFFFFFFFFFFFFFFFFFFF
FFFFFFFFFFFFFFFFFFFFFFFFFFFFFFFFFFFFFFFFFFFFFFFFFFFFFFFFFFFFFFFFFFFF
@A00155:342:HHGFNDSXY:1:2527:2211:30655 1:N:0:GAACCTAG+TCCGCATA
ATCTCAATGCCGAGAGGGATGTGGAGGGCTAGAGAGAGCGACTTCTCTCGGGCCCACAGCCTTACAGC
TGTGGAGCACGGTATCCTCTGCCGAAACAGAGGTTGGACAAGACCGGAGGGGTCTCCTAGTTCCAAAG
+

```
FFFFFFFFFFFFFF:FFFFFFFFFFF:FFFFFFFFF,FFFFFFFFFFFFFFFFFFFFF:FFFFFFFFF
FFFFFFF:FF:FFFFFFFFFFFFFFFFFFFFFFFFFFFFFF,FFFFFFFFFFFFF:FFFFFFFFFFFF
@A00155:342:HHGFNDSXY:1:2527:10908:31156 1:N:0:GAACCTAG+TCCGCATA
CATAGGCCAGAGGTGAGTCCTTAAGTGGACACAGCTGATCTAAGGCGGTGTGGTGGGGCATGGGTTTG
AACCCCCATGACGGTCGGAGTCTAGTAGGCTCCCTGATGAGTCCGTTCCAAGGACGAAACCGTCGTTA
+
FFFFFFFFFFFFFFFFFFFFFFFFFFFFFFFFFFFFFFFFFFFFFFFFFFFFFFFFFFFFFFFFFFFF
FFFFFFFFFFFFFFFFFFFFFFFFFFFFFFFFFFFFFFFFFFFFFFFFFFFFFFFFFFFFFFFFFFFF
@A00155:342:HHGFNDSXY:1:2528:12789:9862 1:N:0:GAACCTAG+TCCGCATA
ACCTCTGTTTCGGCAGAGGATACCGTGCTCCACAGCTGTAAGGCTGTGGGCCCGAGAGAAGTCGCTCT
CTCTCGCCCTTCACATCCCTCTCGGCATTGAGATGACCATAGGCCAGAGGTGAGTCCTTAAGTGGAC
+
FFFFFFFFFFFFFFFFFFFFFFFFFFFFFFFFFFFFFFFFFFFFFFFFFFFFFFFFFFFFFFFFFFFF
FFFFFFFFFFFFFFFFFFFFFFFFFFFFFFFF:FFFFFFFFFFFFFFFFFFFFFFFFFFFFFFFFFF
@A00155:342:HHGFNDSXY:1:2528:19849:10316 1:N:0:GAACCTAG+TCCGCATA
AGAGAGAGCGACTTCTCTCGGGCCCACAGCCTTACAGCTGTGGAGCACGGTATCCTCTGCCGAAACAG
AGGTTGGACAAGACCGGAGGGGTCTCCTAGTTCCAAAGGAGATGTACTCCGGGCTTGTTCACG
+
FFFFFFFFFFFFFFFFFFFFFFFFFFFFFFFFFFFFFFFFFFFFFFFFFFFFFFFFFFFFFFFFFFFF
FFFFFFFFFFFFFF:FFFFFFFFFFFFFFFFFFFFFFFFFFFFFFFFFFFFFFFFFFFFFFFF
@A00155:342:HHGFNDSXY:1:2528:19325:20619 1:N:0:GAACCTAG+TCCGCATA
GTCCACTTAAGGACTCACCTCTGGCCTATGGTCATCTCAATGCCGAGAGGGATGTGAAGGGCGAGAGA
GAGCGACTTCTCTCGGGCCCACAGCCTTACAGCTGTGGAGCACGGTATCCTCTGCCGAAACAGAGGTT
+
FFFFFFFFFFFFFFFFFFFFFFFFFFFFFFFFFFFFFFFFFFFFFFFFFFFFFFFFFFFFFFFFFFFF
FFFFFFFFFFFFFFFFFFFFFFFFFFFFFFFFFFFFFFFFFFFFFFFFFFFFFFFFFFFFFFFFFFFF
@A00155:342:HHGFNDSXY:1:2528:19596:23657 1:N:0:GAACCTAG+TCCGCATA
GTCCACTTAAGGACTCACCTCTGGCCTATGGTCATCTCAATGCCGAGAGGGATGTGAAGGGCGAGAGA
GAGCGACTTCTCTCGGGCCCACAGCCTTACAGCTGTGGAGCACGGTATCCTCTGCCGAAACAGAGGTT
+
FFFFFFFFFFFFFFFFFFFFFFFFFFFFFFFFFFFFFFFFFFFFFFFFFFFFFFFFFFFFFFFFFFFF
FFFFFFFFFFFFFFFFFFFFFFFFFF:FFFFFFFFFFFFFF:FFFFFFFFFFFFFFFFFFFFFFFFFF
@A00155:342:HHGFNDSXY:1:2529:16333:14685 1:N:0:GAACCTAG+TCCGCATA
ACCTCTGTTTCGGCAGAGGATACCGTGCTCCACAGCTGTAAGGCTGTGGGCCCGAGAGAAGTCGCTCT
CTCTCGCCCTCCACATCCCTCTCGGCATTGAGATGACCATAGGCCAGAGGAGAGTCCTTAAGTGGACA
+
FFFFFFFFFFFFFFFFFFFFFFFFFFFFFFFFFFFFFFFFFFFFFFFFFFFFFFFFFFFFFFFFFFFF
FFFFFFFFFFFFFFFFFFFFFFFFFFFFFFFFFFFFFFFFFFFFFFFFFFFFFFFFFFFFFFFFFFFF
@A00155:342:HHGFNDSXY:1:2529:18349:20243 1:N:0:GAACCTAG+TCCGCATA
GGATGTGGAGGGCGAGAGAGAGCGACTTCTCTCGGGCCCACAGCCTTACAGCTGTGGAGCACGGTATC
CTCTGCCGAAACAGAGGTTGGACAAGACCGGAGGGGTCTCCTGGTTCCAAAGGAGATGTACTCC
+
FFFFFFFFFFFFFFFFFFFFFFFFFFFFFFFFFFFFFFFFFFFFFFFFFFFFFFFFFFFFFFFFFFFF
FFFFFFFFFFFFFFFFFFFFFFFFFFFFFFFFFFFFFFFFFFFFFFFFFFFFFFFFFFFFFFFF
@A00155:342:HHGFNDSXY:1:2529:8024:29763 1:N:0:GAACCTAG+TCCGCATA
CTCTGGCCTATGGTCATCTCAATGCCGAGAGGGATGTGAAGGGCGAGAGAGAGCGACTTCTCTCGGGC
CCACAGCCTTACAGCTGTGGAGCACGGTATCCTCTGCCGAAACAGAGGTTGGACAAGAC
+
FFFFFFFFFFFFFFFFFFFFFFFFFFFFFFFFFFFFFFFFFFFFFFFFFFFFFFFFFFF:FFFFFFFF
FFFFFFFFFFFFFFFFFFFFFFFFFFFFFFFFFFFFFFFFFFF:FFFFFFFFFFFFFFF
@A00155:342:HHGFNDSXY:1:2530:23185:5916 1:N:0:GAACCTAG+TCCGCATA
AGAGAGAGCGACTTCTCTCGGGCCCACAGCCTTACAGCTGTGGAGCACGGTATCCTCTGCCGAAACAG
AGGTTGGACAAGACCGGAGGGGTCTCCTAGTTCCAAAGGAGATGTACTCCGGGCTTGTTCACGACCTA
+
```

FFFFFFFFFFFFFFFFFFFFFFFFFFF:F,F,:FFFFFFFFFFFFFFFFFFFFFFFFFFFFFF:FFFF
FFFFFFFFFFFFFFFFFFFFFFFFFFFFFFFFFFFFFFFFFFFFF:FFFFFFFF:FF,FFFFFF:FFF
@A00155:342:HHGFNDSXY:1:2530:26476:10019 1:N:0:GAACCTAG+TCCGCATA
ATGCCGAGAGGGATGTAGAGGGCGAGAGAGAGCGACTTCTCTCGGGCCCACAGCCTTACAGCTGTGGA
GCACGGTATCCTCTGCCGAAACAGAGGTTGGACAAGACCGGAGGGGTCTCCTAGTTCCAAAGGAGATG
+
FFFFFFFFFFFFFFFFFFFFFFFFFFFFFFFFFFFFFFFFFFFFFFFF:FFFFFFFFFFFFFFFFFFF
FFFFFFF:FFFFFFFFFFFFFFFFFFFFFFFFFFFFFFFFFFFFFFFFFFFFFFFFFFFFFFFFFFFF
@A00155:342:HHGFNDSXY:1:2530:14769:26600 1:N:0:GAACCTAG+TCCGCATA
TGTCCACTTAAGGACTCACCTCTGGCCTATGGTCATCTCAATGCCGAGAGGGATGTAGAGGGCGAGAG
AGAGCGACTTCTCTCGGGCCCACAGCCTTACAGCTGTGGAGCACGGTATCCTCTGCCGAAACAGAGGT
+
FFFFFFFFFFFFFFFFFFFFFFFFFFFFFFFFFFFFFFFFFFFFFFFFFFFFFFFFFFFFFFFFFFFF
FFFFFFFFFFFFFFFFFFFFFFFFFFFFFFFFFFFFFFFFFFFFFFFFFFFFFFFFFFFFFFFFFFFF
@A00155:342:HHGFNDSXY:1:2530:2410:32033 1:N:0:GAACCTAG+TCCGCATA
CTCAATGCCGAGAGGGATGTGGAGGGCGAGAGAGAGCGACTTCTCTCGGGCCCACAGCCTTACAGCTG
TGGAGCACGGTATCCTCTGCCGAAACAGAGGTTGGACAAGACCGGAGGGGTCTCCTAGTTCCAAAGGA
+
FFFFFFFFFFFFFFFFF:FFFFFFFFFFFFFFFFFFFFF:FFFFFFFFFFFFFFFFFFFFFFFFFFFF
FFFFFFFFFFFFFFFFFFFFFFFFFFFFFFFFFF:FFFFFFFFFFFFFFFFFFFFFFFFFFFFFFFFF
@A00155:342:HHGFNDSXY:1:2531:27010:4711 1:N:0:GAACCTAG+TCCGCATA
AGAGGGATGTGGAGGGCGAGAGAGAGCGACTTCTCTCGGGCCCACAGCCTTACAGCTGTGGAGCACGG
TATCCTCTGCCGAAACAGAGGTTGGACAAGACCGGAGGGGTCTCCTAGTTCCAAAGGAGAT
+
FFFFFFFF:::FFFFF:FF:FFF:FFF:FF,F,FFFF:F,:FFFFFF:FFFFFFFFFFF:FFFFFFFF
FFFFFFF:F:F::F:FF:F:FF:FFFFFFFFFF:FFFF:FFFFFFFFFFF:FFFFFFFFFF
@A00155:342:HHGFNDSXY:1:2532:8657:1297 1:N:0:GAACCTAG+TCCGCATA
GGATGTGGAGGGCGAGAGAGAGCGACTTCTCTCGGGCCCACAGCCTTACAGCTGTGGAGCACGGTATC
CTCTGCCGAAACAGAGGTTGGACAAGACCGGAGGGGTCTCCTAGTTCCAAAGGAGATGTACTCC
+
FFFFFFFFFFFFFFFFFFFFFFFFFFFFFFFFFFFFFFFFFFFFFFFFFFFFFFFFFFFFFFFFFFFF
FFFFFFFFFFFFFFFFFFFFFFFFFFFFFFFFF:FFFFFFFFFFFFFFFFFFFFFFFFFFFFFF
@A00155:342:HHGFNDSXY:1:2532:19651:4272 1:N:0:GAACCTAG+TCCGCATA
CGAGAGGGATGTAGAGGGCGAGAGAGAGCGACTTCTCTCGGGCCCACAGCCTTACAGCTGTGGAGCAC
GGTATCCTCTGCCGAAACAGAGGTTGGACAAGACCGGAGGGGTCTCCTAGTTCCAAAGGAGATGTACT
+
FFFFFFFFF:FFFFFFFFFFFFFFFFFFFFFFFFFFFFFFFFFFFFFFFFFFFFFFFFFFFFFFFFFF
FF:FFFFFFFFFFFFFFFFF,FFFFFFFFF:FFFFFFFFFFFFFFFFFFFFFFFFFFFFFFFFFFFFF
@A00155:342:HHGFNDSXY:1:2532:3079:15468 1:N:0:GAACCTAG+TCCGCATA
TGCCGAGAGGGATGTGTAGGGCGAGAGAGAGCGACTTCTCTCGGGCCCACAGCCTTACAGCTGTGGAG
CACGGTATCCTCTGCCGAAACAGAGGTTGGACAAGACCGGAGGGGTCTCCTAGTTCCAAAGGAGATGT
+
FFFFFFFFFFFFFFFFFFFFFFFFFFFFFFFFFFFFFFFFFFFFFFFFFFFFFFFFFFFFFFFFFFFF
FFFFFFFFFFFFFFFFFFFFFFFFFFFFFFFFFFFFFFFFFFFFFFFFFF:FFFFFFFFFFFFFFFFF
@A00155:342:HHGFNDSXY:1:2532:30508:19664 1:N:0:GAACCTAG+TCCGCATA
TTCTCTCGGGCCCACAGCCTTACAGCTGTGGAGCACGGTATCCTCTGCCGAAACAGAGGTTGGACAAG
ACCGGAGGGGTCTCCTAGTTCCAAAGGAGATGTACTCCGGGCTTGTTCACGACCTACCGTGTAAGTCG
+
FFFFFFFFFFFFFFFFFFFFFFFFFFFFFFFFFFFFFFFFFFFFFFFFFFFFFFFFFFFFFFFFFFFF
FFFFFFFFFFFFFFFFF:FFFFFFFFFFFFFFFFFFFFFFFFFFFFFFFF,FFFFFFFFFFFFFFFF:
@A00155:342:HHGFNDSXY:1:2532:7591:22748 1:N:0:GAACCTAG+TCCGCATA
TAGTCTAGTAGGCTACCTGACGAGTCCTTTTTAGGACGAAACTTACACAACTCGAAGAGTAACGACGG
TTTCGTCCTTGGAACGGACTCATCAGGGAGCCTACTAGACTCCGACCGTCATGGGGGTTCAAACCCAT
+

```
FFFFFFFFFFFFFFFFFFFFFFFFFFFFFFFF:FFFFFFFFFFFFFFFFFFFFFFFFFFFFFFFFFF
FFFFFFFFFFFFFFFFFFFFFFFFFFFFFF:FFFFFFFFFFFFFFFF:FF:FFFFFFFFFFFFFFFF
@A00155:342:HHGFNDSXY:1:2532:6696:23422 1:N:0:GAACCTAG+TCCGCATA
TAGTCTAGTAGGCTACCTGACGAGTCCTTTTAGGACGAAACTTACACAACTCGAAGAGTAACGACGG
TTTCGTCCTTGGAACGGACTCATCAGGGAGCCTACTAGACTCCGACCGTCATGGGGGTTCAAACCCAT
+
FFFFFFFFFFFFFFFFFFFFFFFFFFFFFFFFFFFFFFFFFFFFFFF:FFFFFFFFFFFFFFFFFFF
FFFFFFFFFFFFFFFFFFFFFFFFFFFFFFFF:FFFFFFFFFFFFFFFFFFFFFFFFFFFFFFFF:F
@A00155:342:HHGFNDSXY:1:2532:10285:24439 1:N:0:GAACCTAG+TCCGCATA
GAGCGACTTCTCTCGGGCCCACAGCCTTACAGCTGTGGAGCACGGTATCCTCTGCCGAAACAGAGGTT
GGACAAGACCGGAGGGGTCTCCTAGTTCCAAAGGAGATGTACTCCGGGCTTGTTCACGACCTACCGTG
+
FFFFFFFFFFFFFFFFFFFFFFFFFFFFFFFFFFFFFFFFFFFFFFFFFFFFFFFFFFFFFFFFFFF
FFFFFFFFFFFFFFFFFFFFFFFFFFFFFFF,FFFFFFFFFFFFFFFFFFFFFFFFFFFFFFFFFFF
@A00155:342:HHGFNDSXY:1:2533:24840:8563 1:N:0:GAACCTAG+TCCGCATA
GAGACCCCTCCGGTCTTGTCCAACCTCTGTTTCGGCAGAGGATACCGTGCTCCACAGCTGTAAGGCTG
TGGGCCCGAGAGAAGTCTCTCTCTCTCGCCCTCCACATCCCTCTCGGCATTGAGATGAC
+
FFFFFFFFFFFFFFFFFFFFFFFFFFFFFFFFFFFFFFFFFFFFFFFFFFFFFFFFFFFFFFFFFFF
FFFFFFFFFFFFFFFFFFFFFFFFFFFFFFFFFFFFFFFFFFFFFFFFFFFFFFFFFFF
@A00155:342:HHGFNDSXY:1:2533:14579:21042 1:N:0:GAACCTAG+TCCGCATA
GTCGCTCTCTCTCGCCCTCCACATCCCTCTCGGCATTGAGATGACCATAGGCCAGAGGTGAGTCCTTA
AGTGGACACAGCTGATCTAAGGCGGTGTGGCGGGGCATGGGTTTGAACCCCCATGACGGTCGGAGTCT
+
FFFFFFFFFFFFFFFFFFFFFFF:FFFFFFFFFF,FFFFFFFFFFFFFFFFF:FFFFFFFFFFFFFF
FFFFFFFFFFFFFFFFFFF:FFFFFFFFFFFFFFFFFFFFFFFFFFFFFFFFFFFFFFFFFFFFF:F
@A00155:342:HHGFNDSXY:1:2534:15917:10113 1:N:0:GAACCTAG+TCCGCATA
TCTCTCTCGCCCTCCACATCCCTCTCGGCATTGAGATGACCATAGGCCAGAGGTGAGTCCTTAAGTGG
ACACAGCTGATCTAAGGCGGTGTGGCGGGGCATGGGTTTGAACCCCCATGACGGTCGGAGTCTAG
+
FFFFF::FFFFFFFFFFFFFFFFFFFFFFF:FFFFFFFFFF:FFFFFFF:FF,FFFFFFFFFFFFF:
FF:FF:FFFFFFFFFFFFFFFFFFFFFF:FFFFF:FFFFFFFFFFFFFFFFF:FFF,FFFFF:FF
@A00155:342:HHGFNDSXY:1:2534:6153:12054 1:N:0:GAACCTAG+TCCGCATA
TGGTCATCTCAATGCCGAGAGGGATGTGGAGGGCGAAAGAGAGCGACTTCTCTCGGGCCCACAGCCTT
ACAGCTGTGGAGCACGGTATCCTCTGCCGAAACAGAGGTTGGACAAGACCGGAGGGGTCTCC
+
FFFFFFFFFFFFFFFFFFFFFFFFFFFFFFFFFFFFFFFFFFFFFFFFFFFFFFFFFFFFFFFFFFF
FFFF:FFFFFFFFFFFFFFFFFFFFFFFFFFFFFFF:FFFFFFFFFFFFFFFFFFFFFFFFF
@A00155:342:HHGFNDSXY:1:2534:5746:12446 1:N:0:GAACCTAG+TCCGCATA
TGGTCATCTCAATGCCGAGAGGGATGTGGAGGGCGAAAGAGAGCGACTTCTCTCGGGCCCACAGCCTT
ACAGCTGTGGAGCACGGTATCCTCTGCCGAAACAGAGGTTGGACAAGACCGGAGGGGTCTCC
+
FFFFFFFFFFFFFFFFFFFFFFFFFFFFFFFFFFFFFFFFFFFFFFFFFFFFFFFFFFFFFFFFFFF
FFFFFFFFFFFFFFFFFFFFFFFFFFFFFFFFFFFFFFFFFFFFFFFFFFFFFFFFFFFFFF
@A00155:342:HHGFNDSXY:1:2535:28004:1235 1:N:0:GAACCTAG+TCCGCATA
CCTTACAGCTGTGGAGCACGGTATCCTCTGCCGAAACAGAGGTTGGACAAGACCGGAGGGGTCTCCTA
GTTCCAAAGGAGATGTACTCCGGGCTTGTTCACGACCTACCGTGTAAGTCGAAGTCTAGTAGGCT
+
FFFFFFFFFF,:FFFFFFFFFFFFF,FFFFFFFFFFF,FF,:FFFFFFF:FFFFFFFFFFFF,:F,:
,,,FFF,FFFFFFFFFFF:FFFFFFFFFFFF:FFF:FFFFFFFFFFFFFFF,FFFFF:FFFFFFF
@A00155:342:HHGFNDSXY:1:2535:20003:7795 1:N:0:GAACCTAG+TCCGCATA
AGGGATGTGGAGGGCGAGAGAGAGCGACTTCTCTCGGGCCCACAGCCTTACAGCTGTGGAGCACGGTA
TCCTCTGCCGAAACAGAGGTTGGACAAGACCGGAGGGGTCTCCTAGTTCCAAAGGAGATGTACTCCGG
+
```

```
FFFFFFFFFFFFFFFFFFFFFFFFFFFFFFFFFFFFFFFFFFFFFFFF:FFFFFFFFFFFFFFFFFF
FFFFFFFFFFFFFFFFFFFFFFFFFFFFFFFFFFFFFFFFFFFFFFFFFFFFFFFFFFFFFFFFFFF
@A00155:342:HHGFNDSXY:1:2535:12915:17910 1:N:0:GAACCTAG+TCCGCATA
CTGTGTCCACTTAAGGACTCACCTCTGGCCTATGGTCATCTCAATGCCGAGAGGGATGTGGAGGGCGA
GAGAGAGCGACTTCTCTCGGGCCCACAGCCTTACAGCTGTGGAGCACGGTATCCTCTGCCGAAACAGA
+
FFFFFFFFFFFFFFFFFFFFFFFFFFFFFFFFFFF,FFFFFFFFFFFFFFFFFFF:FFFFFFFFFFFF
FFFFFFFFFFFFFFFFFFFFFFFFFFFFFFFFFFFFFFFFFFFFFFFFFFFFFFFFFFFFFFFFFFFF
@A00155:342:HHGFNDSXY:1:2535:31051:18411 1:N:0:GAACCTAG+TCCGCATA
CGACTTCTCTCGGGCCCACAGCCTTACAGCTGTGGAGCACGGTATCCTCTGCCGAAACAGAGGTTGGA
CAAGACCGGAGGGGTCTCCTGGTTCCAAAGGAGATGTACTCCGGGCTTGTTCACGACCTACCGTGTAA
+
FFFFFFFFFFFFFFFFFFFFFFFFFF:FFFFFFFFFFFFFFFFFF:FFFFFFFFFF:FFFFFFFFFFFF
FFFFFFFFFFFFFFFFFFFFFFFFFFFFFFFFFFFFFFFFFFFFFFFFFFFFFFFFFFFFFFFFFFFF
@A00155:342:HHGFNDSXY:1:2535:30327:18599 1:N:0:GAACCTAG+TCCGCATA
CGACTTCTCTCGGGCCCACAGCCTTACAGCTGTGGAGCACGGTATCCTCTGCCGAAACAGAGGTTGGA
CAAGACCGGAGGGGTCTCCTGGTTCCAAAGGAGATGTACTCCGGGCTTGTTCACGACCTACCGTGTAA
+
FFFFFFFFFFFFFFFFFFFFFFFFFF,FFFFFFFFFFFFFFFFFFFFFFFFFFFFFFFFFFFFFFFFFF
FFFFFFFFFFFFFFFFFFFFFFFFFFFFFFFFFFF:FFFFFFFFFFFFFFFFFFFFFFFFFFFFFFFF
@A00155:342:HHGFNDSXY:1:2535:18484:19445 1:N:0:GAACCTAG+TCCGCATA
CAGCCTTACAGCTGTGGAGCACGGTATCCTCTGCCGAAACAGAGGTTGGACAAGACCGGAGGGGTCTC
CTAGTTCCAAAGGAGATGTACTCCGGGCTTGTTCACGACCTACCGTGTAAGTCGTAGTCTAGTAGGCT
+
FFFFFFFFFFFFFFFFFFFFFFFFFFFFFFFFFFFFFFFFFFFFFFFFFFFFFFFFFFFFFFFFFFFF
FFFFFFFFFFFFFFFFFFFFFFFFFFFFFFFFFFFFFFFFFFFFFFFFFFFFFFFFFFFFFFFFFFFF
@A00155:342:HHGFNDSXY:1:2535:11080:20087 1:N:0:GAACCTAG+TCCGCATA
CTGTGTCCACTTAAGGACTCACCTCTGGCCTATGGTCATCTCAATGCCGAGAGGGATGTGGAGGGCGA
GAGAGAGCGACTTCTCTCGGGCCCACAGCCTTACAGCTGTGGAGCACGGTATCCTCTGCCGAAACAGA
+
FFFFFFFFFFFFFFFFFFFFFFFFFFFFFFFFFFFFFFFFFFFFFFFFFFFFFFF:FFFFFFFFFFFF
FFFFFFFFFFFFFFFFFFFFFFFFFFFFFFFFFFFFFFFFFFFFFFFFFFFFFFFFFFFFFFFFFFFF
@A00155:342:HHGFNDSXY:1:2535:14805:23813 1:N:0:GAACCTAG+TCCGCATA
CACCTCTGGCCTATGGTCATCTCAATGCCGAGAGGGATGTGAAGGGCGAGAGAGAGCGACTTCTCTCG
GGCCCACAGCCTTACAGCTGTGGAGCACGGTATCCTCTGCCGAAACAGAGGTTGGACAAGACCGGAG
+
F:FFFFFFFFFFFFFFFFFFFFFFFFFFFFFFFFFFFFFFFFFFFFFFFFFFFFFFFFFFFFFFFFFF
FFFFFFFFFFFFFFFFFFFFFFFFFFFFFFFFFFFFFFFFFFFFFFFFFFFFFFFFFFFFFFFFFFF
@A00155:342:HHGFNDSXY:1:2535:22209:28213 1:N:0:GAACCTAG+TCCGCATA
CTCTCGCCCTCCACATCCCTCTCGGCATTGAGATGACCATAGGCCAGAGGTGAGTCCTTAAGTGGACA
CAGCTGATCTAAGGCGGTGTGGCGGGGCATGGGTTTGAACCCCCATGACGGTCGGAGTCTAGTAGGCT
+
FFFFFFFFFFFFFFFFFFFFFFFFFFFFFFFFFFFFFFFFFFFFFFFFFFFFFFFFFFFFFFFFFFFF
FFFFFFFFFFFFFFFFFFFFFFFFFFFFFFFFFFFFFFFFFFFFFFFFFFFFFFFFFFFFFFFFFFFF
@A00155:342:HHGFNDSXY:1:2535:6560:32362 1:N:0:GAACCTAG+TCCGCATA
GAGGGCGAGAGAGAGCGACTTCTCTCGGGCCCACAGCCTTACAGCTGTGGAGCACGGTATCCTCTGCC
GAAACAGAGGTTGGACAAGACCGGAGGGGTCTCCTAGTTCCAAAGGAGATGTACTCCGGGCTTGTTCA
+
FFFFFFFFFFFFFFFFFF:FFFFFFFFFFFFFFFFFFFFFFFFFFFFFFFFFFFFFFFFFFFFFFFFF
FFFFFFFFFFFFFFFFFFFFFFFFFFFFFFFFFFFF:FFFFFFFFFFFFFFFFFFFFFFFFFFFF:FFFF
@A00155:342:HHGFNDSXY:1:2536:16468:7686 1:N:0:GAACCTAG+TCCGCATA
TATCCTCTGCCGAAACAGAGGTTGGACAAGACCGGAGGGGTCTCCTAGTTCCAAAGGAGATGTACTCC
GGGCTTGTTCACGACCTACCGTGTAAGTCGTAGTCTAGTAGGCTACCTGACGAGTCCTTTTTAGGAC
+
```

FFFFFFFFFFFFFFFFFFFFFFFFFFFFFFFFFFFFFFF:FFFFFFFFFFFFFFFFFFFFFFFFFFFF
FFFFFFFFFFFFFFFFFFFFFFFFFFFF:FFFFFFFFFFFFFFFFFFFFFFFFFFFFFFFF,FFFFFFFF
@A00155:342:HHGFNDSXY:1:2536:24062:10692 1:N:0:GAACCTAG+TCCGCATA
GTCATCTCAATGCCGAGAGGGATGTGGAGGGCGAGAGAGAGCGACTTCTCTCGGGCCCACAGCCTTAC
AGCTGTGGAGCACGGTATCCTCTGCCGAAACAGAGGTTGGACAAGACCGGAGGGGTCTCCTAGTTCCA
+
FFFFFFFFFFFFFFFFFFFFFF,FFFFFFFFFFFFFFFFFFFFFFFFFFFFFFFFFFFFFFFFFFFF,F
FFFFFFFFFFFFFFFFFFFFFFFFFFFFFFFFFFFFFFFFFFFFFFFFFFFFFFFFFFFFFFFFFFFFF
@A00155:342:HHGFNDSXY:1:2536:1271:14497 1:N:0:GAACCTAG+TCCGCATA
GCGAGAGAGAGCGACTTCTCTCGGGCCCACAGCCTTACAGCTGTGGAGCACGGTATCCTCTGCCGAAA
CAGAGGTTGGACAAGACCGGAGGGGTCTCCTAGTTCCAAAGGAGATGTACTCCGGGCTTGTTCACGA
+
FFF,F:F:FFFFF,,FFFFFFFFFFFFFFFFFFFFFFFF:FFFFFFFFFFFFFFFFFFFFF:FFFF,:F
F:FFFFFFFF:FFF,,FFFFFFFFF:FFFFFFFFFFF:,,F:FF:FF:F:FFFFFFFFFFFF::FF:
@A00155:342:HHGFNDSXY:1:2536:22932:25113 1:N:0:GAACCTAG+TCCGCATA
AAGGACTCACCTCTGGCCTATGGTCATCTCAATGCCGAGAGGGATGTAGAGGGCGAGAGAGAGCGACT
TCTCTCGGGCCCACAGCCTTACAGCTGTGGAGCACGGTATCCTCTGCCGAAACAGAGGTTGGACAAGA
+
FFFFFFFFFFFFFFFFFFFFFFFFFFFFFFFFFFFFFFFFFFFFFFFFFFFFFFFFFFFFFFFFFFFFF
FFFFFFFFFFFFFFFFFFFFFFFFFFFFFFFFFFFFFFFFFFFFFFFFFFFFFFFFFFFFFFFFFFFFF
@A00155:342:HHGFNDSXY:1:2536:14181:28526 1:N:0:GAACCTAG+TCCGCATA
GCGACTTCTCTCGGGCCCACAGCCTTACAGCTGTGGAGCACGGTATCCTCTGCCGAAACAGAGGTTGG
ACAAGACCGGAGGGGTCTCCTAGTTCCAAAGGAGATGTACTCCGGGCTTGTTCACGACCTACCGTGTA
+
FFFFFFFFFFFFFFFFFFFFFFFFFFFFFFFFFF,FFFFFFFFFFFFFFFFFFFFFFFFFFFFFFFFFF
FFFFFFFF:FFFFFFFFFFFFFFFFFFFFFFFFFFFFFFFFFFFFFFFFFFFFFFFFFFFFFFFFFF:F
@A00155:342:HHGFNDSXY:1:2537:20528:4507 1:N:0:GAACCTAG+TCCGCATA
AGCGACTTCTCTCGGGCCCACAGCCTTACAGCTGTGGAGCACGGTATCCTCTGCCGAAACAGAGGTTG
GACAAGACCGGAGGGGTCTCCTAGTTCCAAAGGAGATGTACTCCGGGCTTGTTCACGACCTACCGTGT
+
F:FFFFFFFFFFFFFFFFFFFFFFFFFFFFFFFFFFFFFFFFFFFFFFFFFFFFFFFFFFFFFFFFFFF
FFFFFFFFFFFFFFFFFFFFFF:FFFFFFFFFFFFFFFFFFFFFFFFFFFFFFFFFFFFFFFFFFFFF:
@A00155:342:HHGFNDSXY:1:2537:28610:7670 1:N:0:GAACCTAG+TCCGCATA
GCGACTTCTCTCGGGCCCACAGCCTTACAGCTGTGGAGCACGGTATCCTCTGCCGAAACAGAGGTTGG
ACAAGACCGGAGGGGTCTCCTAGTTCCAAAGGAGATGTACTCCGGGCTTGTTCACGACCTACCGTGTA
+
FFFFFFFFFFFFFFFFFFFFFFFF:FFFFFFFFFFFFFFFFFFFFFFF:FFFFF:FFFFFFFFFFFFFF
FFFFFFF:FFFFFFFFFFFFFFFFFFFFFFFFFFFFFFFFFFFFFF:FFFFFFFFFFFFFFFFFFFFFF
@A00155:342:HHGFNDSXY:1:2537:16514:35634 1:N:0:GAACCTAG+TCCGCATA
CTTCTCTCGGGCCCACAGCCTTACAGCTGTGGAGCACGGTATCCTCTGCCGAAACAGAGGTTGGACAA
GACCGGAGGGGTCTCCTAGTTCCAAAGGAGATGTACTCCGGGCTTGTTCACGACCTACCGTGTAAGTC
+
FFFFFFFFFFFFFFFFFFFFFFFFFFFFFFFFFFFFFFFFFFFFFFFFFFFFFFFFFFFFFFFFFFFFF
FFFFFFFFFFFFFFFFFFFFFFFFFFFFFFFF:FFFFFFFFFFFFFFF:FFFFFFFFFFFFFFFFFFFF
@A00155:342:HHGFNDSXY:1:2538:23701:11036 1:N:0:GAACCTAG+TCCGCATA
AGAGAGAGCGACTTCTCTCGGGCCCACAGCCTTACAGCTGTGGAGCACGGTATCCTCTGCCGAAACAG
AGGTTGGACAAGACCGGAGGGGTCTCCTAGTTCCAAAGGAGATGTACTCCGGGCTTGTTCACGACCTA
+
FFFFFFFFFFFFFFFFFFFFFFFFFFFFFFFFFFFFFFFFFFFFFFFFFFFFFFF:FFFFFFFFFFFFF
FFFFFFFFFFFFFFFFFFFFFFFFFFFFFFFFFFFFFFFFFFFFFFFFFFFFFFFFFFFFFF:FFFFFF
@A00155:342:HHGFNDSXY:1:2538:28465:29058 1:N:0:GAACCTAG+TCCGCATA
AAGGACTCTCCTCTGGCCTATGGTCATCTCAATGCCGAGAGGGATGTGGAGGGCGAGAGAGAGCGACT
TCTCTCGGGCCCACAGCCTTACAGCTGTGGAGCACGGTATCCTCTGCCGAAACAGAGGTTGGACAAGA
+

```
FFF,FFFFFFFFF:FFFF,FFFFFFFFFFFFFFFFFFFFFFFF:FFFF:FFFFFFFFFFFF:FFFF,F
FFFFFFFFFFFFFFFFFFFFFFFFFFFFFF,FFF:FFF:FF:FFFFFFFFFFFFF,FFFFFFFFFFFF
@A00155:342:HHGFNDSXY:1:2539:7229:12070 1:N:0:GAACCTAG+TCCGCATA
AAGGACTCTCCTCTGGCCTATGGTCATCTCAATGCCGAGAGGGATGTGGAGGGCGAGAGAGAGCGACT
TCTCTCGGGCCCACAGCCTTACAGCTGTGGAGCACGGTATCCTCTGCCGAAACAGAGGTTGGACAAGA
+
FFFFFFFFFFFFFFFFFFFFFFFFFFFFFFFFFFFFFFFFFFFF:FFFFFFFFFFFFFFFFFFFF:FF
FFFFFFFFFFFFFFFFFFFFFFFFFFFFFFFFFFFFFF:FFFFFFFFFFFF:FFF:FFFFFFFFFFFF
@A00155:342:HHGFNDSXY:1:2539:2211:16752 1:N:0:GAACCTAG+TCCGCATA
CAGCCTTACAGCTGTGGAGCACGGTATCCTCTGCCGAAACAGAGGTTGGACAAGACCGGAGGGGTCTC
CTAGTTCCAAAGGAGATGTACTCCGGGCTTGTTCACGACCTACCGTGTAAGTCGTAGTCTAGTAGGCT
+
FFFFFFFFFFFFFFFFFFFFFFFFFFFFFFFFFFFFFFFFFFFFFFFFFFFFFFFFFFFFFFFFFFFF
FFFFFFFFFFFFFFFFFFFFFFFFFFFFFFFFFFFFFFFFFFFFFFFFFFFFFFFFFFFFFFFFF:FF
@A00155:342:HHGFNDSXY:1:2539:11586:35243 1:N:0:GAACCTAG+TCCGCATA
GAGCGACTTCTCTCGGGCCCACAGCCTTACAGCTGTGGAGCACGGTATCCTCTGCCGAAACAGAGGTT
GGACAAGACCGGAGGGGTCTCCTAGTTCCAAAGGAGATGTACTCCGGGCTTGTTCACGACCTACCGTG
+
FFFFFFFFFFFFFFFFFFFFFFFFFFFFFFFFFFFFFFFFFFFFFFFFFFFFFFFFFFFFFFFFFFFF
FFFFFFFFFFFFFFFFFFFFFFFFFFFFFFFFF:FFFFFFFFFFFFFFFFFFFFFFFFFFFFFFFFFF
@A00155:342:HHGFNDSXY:1:2540:25572:16814 1:N:0:GAACCTAG+TCCGCATA
GGATGTGGAGGGCGAGAGAGAGCGACTTCTCTCGGGCCCACAGCCTTACAGCTGTGGAGCACGGTATC
CTCTGCCGAAACAGAGGTTGGACAAGACCGGAGGGGTCTCCTAGTTCCAAAGGAGATGTACTCC
+
FFFFFFFFFFFFFFFFFFFFFFFFFFFFFFFFFFFFFFFFFFFFFFFFFFFFFFFFFFFFFFFFFFFF
FFFFFFFFFFFFFFFFFFFFFFFFFFFFFFFFFFFFFFFFFFFFFFFFFFFFFFFFFFFFFFFF
@A00155:342:HHGFNDSXY:1:2540:20880:18427 1:N:0:GAACCTAG+TCCGCATA
GCGACTTCTCTCGGGCCCACAGCCTTACAGCTGTGGAGCACGGTATCCTCTGCCGAAACAGAGGTTGG
ACAAGACCGGAGGGGTCTCCTAGTTCCAAAGGAGATGTACTCCGGGCTTGTTCACGACCTACCGTGTA
+
FFFFFFFFFFFFFFFFFFFFFFFF:FFFFFFFFFFFFFFFFFFFFFFFFFFFFFFFFFFFFFFFFFFF
FFFFFFFFFFFFFFFFFFFFFFFFFFFFFFFFFFFFFFFFFFFFFFFFFFFFFFFFFFFFFFFFFFFF
@A00155:342:HHGFNDSXY:1:2540:20835:18630 1:N:0:GAACCTAG+TCCGCATA
GCGACTTCTCTCGGGCCCACAGCCTTACAGCTGTGGAGCACGGTATCCTCTGCCGAAACAGAGGTTGG
ACAAGACCGGAGGGGTCTCCTAGTTCCAAAGGAGATGTACTCCGGGCTTGTTCACGACCTACCGTGTA
+
FFFFFFFFFFFFFFFFFFFFFFFFFFFFFFFFFFFFFFFFFFFFFFFFFFFFFFFFFFFFFFFF:FF
FFFFFFFFFFFFFFFFFFFFFFFFFFFFFFFFFFFFFFFFFFFFFFF:FFFFFFFFFFFFFFFF:FFF
@A00155:342:HHGFNDSXY:1:2541:23791:3020 1:N:0:GAACCTAG+TCCGCATA
ATCTCAATGCCGAGAGGGATGTGGAGGGCGAGAGAGAGCGACTTCTCTCGGGCCCACAGCCTTACAGC
TGTGGAGCACGGTATCCTCTGCCGAAACAGAGGTTGGACAAGACCGGAGGGGTCTCCTAGTTCCAAAG
+
FFFFFFFFFFFFFFFFFFFFFFFFFFFFFFFFFFFFFFFFFFFFFFFFFFFFFFFFFFFFFFF,FFFF
FFFFFFFFFFFFFFFFFFFFFFFFFFFFFFFFFFFFFFFFFFFFFFFFFFFFFFFFFFFFFFFFFFFF
@A00155:342:HHGFNDSXY:1:2541:16785:5165 1:N:0:GAACCTAG+TCCGCATA
GAGCGACTTCTCTCGGGCCCACAGCCTTACAGCTGTGGAGCACGGTATCCTCTGCCGAAACAGAGGTT
GGACAAGACCGGAGGGGTCTCCTAGTTCCAAAGGAGATGTACTCCGGGCTTGTTCACGACCTACCGTG
+
FFFFFFFFFFFFFFFFFFFFFFFFFFFFFFFFFFFFFFFFFFFFFFFFFFFFFFFFFFFFFFFFFFFF
FFFFFFFFFFFFFFFFFFFFFFFFFFFFFFFFFFFFFFFFFFFFFFFFFFFFFFFFFFFFFF:FFFFF
@A00155:342:HHGFNDSXY:1:2541:20573:17738 1:N:0:GAACCTAG+TCCGCATA
GAGAGAGCGACTTCTCTCGGGCCCACAGCCTTACAGCTGTGGAGCACGGTATCCTCTGCCGAAACAGA
GGTTGGACAAGACCGGAGGGGTCTCCTAGTTCCAAAGGAGATGTACTCCGGGCTTGTTCACGACCTA
+
```

F::F,:FFFFFF:FFFF::FFFF,FFF:FF:,F:F,FF:FFF:FFFFFFFF:FF:FF,F:FFFFFF:F
:,FFFFFF,FFFFFF:F,,:,:FF,,,:FFFFFFFFF:F:FF:FF:FF,FF:F,,::FF:F:FFFF:
@A00155:342:HHGFNDSXY:1:2541:28140:20948 1:N:0:GAACCTAG+TCCGCATA
CCTTACAGCTGTGGAGCACGGTATCCTCTGCCGAAACAGAGGTTGGACAAGACCGGAGGGGTCTCCTA
GTTCCAAAGGAGATGTACTCCGGGCTTGTTCACGACCTACCGTGTAAGTCGTAGTCTAGTAGGCTACC
+
FFFFFFFFFFFFFFFFFFFFFFFFFFFFFFFFFFFFFFFFFFFFFFFFFFFFFFFFFFFFFFFFFFFF
FFFFFFFFFFFFFFFFFFFFFFFFFFFFFFFFFFFFFFFFFFFFFFFFFFFFFFFFFFFFFFFFFFFF
@A00155:342:HHGFNDSXY:1:2541:4209:22623 1:N:0:GAACCTAG+TCCGCATA
ACAGCTGTGGAGCACGGTATCCTCTGCCGAAACAGAGGTTGGACAAGACCGGAGGGGTCTCCTGGTTC
CAAAGGAGATGTACTCCGGGCTTGTTCACGACCTACCGTGTAAGTCGTAGTCTAGTAGGCTACCTGAC
+
FFFFFFFFFFFFFFFFFFFFFFFFFFFFFFFFFFFFFFFFFFFFFF:F:FFFFFFFFFFFFFFF:FFF
FFF:FFFFFFFFFFFFFFFFFFFFFFFFFFFFFFFFFFFFFFFFFFFFFFFFFFFFFFFFFFFFFFFF
@A00155:342:HHGFNDSXY:1:2542:7301:6684 1:N:0:GAACCTAG+TCCGCATA
GAGAGGGATGTAGAGGGCGAGAGAGAGCGACTTCTCTCGGGCCCACAGCCTTACAGCTGTGGAGCACG
GTATCCTCTGCCGAAACAGAGGTTGGACAAGACCGGAGGGGTCTCCTAGTTCCAAAGGAGATGTACTC
+
FFFFFFFFFFFFFFFFFFFFFFFFFFFFFFFFFFFFFFFFFFFFFFFFFFFFFFFFFFFFFFFFFFFF
FF:FFFFFFFFFFFFFFFFFFFFFFFFFFFFFFFFFFFFFFFFFFFFFFFFFFFFFFFFFFFFFFFFF
@A00155:342:HHGFNDSXY:1:2542:32832:16078 1:N:0:GAACCTAG+TCCGCATA
TGCCGAGAGGGATGTGGAGGGCGAGAGAGAGCGACTTCTCTCGGGCCCACAGCCTTACAGCTGTGGAG
CACGGTATCCTCTGCCGAAACAGAGGTTGGACAAGACCGGAGGGGTCTCCTGGTTCCAAAGGAGAT
+
FFFFFFFFFFFFFFFFFFFFFFFFFFFFFFFFFFFFF:FFFFFFFFFFFFFFFFF,FFFFFFF:FFFF
FFFFFFFFFFFFFFFFFFFFFFF:FF,:FFFFFFF:F:FFFFFFFFFFFFFFFFFFFFFFFFFFF:
@A00155:342:HHGFNDSXY:1:2542:11360:22889 1:N:0:GAACCTAG+TCCGCATA
GGACTCACCTCTGGCCTATGGTCATCTCAATGCCGAGAGGGATGTGGAAGGCGAGAGAGAGCGACTTC
TCTCGGGCCCACAGCCTTACAGCTGTGGAGCACGGTATCCTCTGCCGAAACAGAGGTTGGACAAGACC
+
FFFFFFFFFFFFFFFFF:FFFFFFFFFFFFFFFFFFFFFFFFFFFFFFFFFFFFFFFFFFFFFFFFFF
FFFFFFFFFFFFFFFFFFFFFFFFFFFFFFFFFFFFFFFFFFFFFFFFFFFFFFFFFFFFFFFFFFFF
@A00155:342:HHGFNDSXY:1:2542:2220:28635 1:N:0:GAACCTAG+TCCGCATA
ACCTCTGTTTCGGCAGAGGATACCGTGCTCCACAGCTGTAAGGCTGTGGGCCCGAGAGAAGTCGCTCT
CTCTCGCCCTCCACATCCCTCTCGGCATTGAGATGACCATAGGCCAGAGGTGAGTCCTTAAGTGGACA
+
FFFFFFFFFFFFFFFFFFFFFFFFFFFFFFFFFFFFFFFFFFFFFFFFFFFFFF:FFFFFFFFFFFFF
FFFFFFFFFFFFFFFFFFFFFFFFFFFFFFFFFFFFFFFFFFFFFFFFFFFFFFFFFFFFFFFFFFFF
@A00155:342:HHGFNDSXY:1:2542:31096:29951 1:N:0:GAACCTAG+TCCGCATA
TATCCTCTGCCGAAACAGAGGTTGGACAAGACCGGAGGGGTCTCCTAGTTCCAAAGGAGATGTACTCC
GGGCTTGTTCACGACCTACCGTGTAAGTCGTAGTCTAGTAGGCTACCTGACGAGTCCTTTTTAGGACG
+
FFFFFFFFFFFFFFFFFFFFFFFFFFFFFFFFFFFFFFFFFFFFFFFFFFFFFFFFFFFFFFFFFFFF
FFFFFFFFFFFFFFFFFFFFFFFF::FFFFFFFFFFF:FFFFFF:FFFFFFF:FFFFF,FFFFFFFFF
@A00155:342:HHGFNDSXY:1:2542:13584:32878 1:N:0:GAACCTAG+TCCGCATA
ACTTCTCTCGGGCCCACAGCCTTACAGCTGTGGAGCACGGTATCCTCTGCCGAAACAGAGGTTGGACA
AGACCGGAGGGGTCTCCTAGTTCCAAAGGAGATGTACTACGGGCTTGTTCACGACCTACCGTGTAAGT
+
FFFFFFFF:F:FFFFF,FFFFFFFFFFF:FFFFF:FFFFF:FFFFFFFFFF:FFFFFFFFFFFFFF:F
F:FFF:FFFFF,FFFFF:FFFFFFFFFFFFFF,F,FFF,FFFFFFFFFF:FFFFFFFFFFFF:FFFFF
@A00155:342:HHGFNDSXY:1:2542:22236:33238 1:N:0:GAACCTAG+TCCGCATA
TTACAGCTGTGGAGCACGGTATCCTCTGCCGAAACAGAGGTTGGACAAGACCGGAGGGGTCTCCTAGT
TCCAAAGGAGATGTACTCCGGGCTTGTTCACGACCTACCGTGTAAGTCGTAGTCTAGTAGGCTACCTG
+

```
FFFFFFFFFFFFFFFFFFFFFFFFFFFFFFFFFFFFFFFFFFFFFFFFFFFFFFFFFFFFFFFFFFFF
FFFFFFFFFFFFFFFFFFFFFFFFFFFFFFFFFFFFFFFFFFFFFFFFFFFFFFFFFFFFFFFFFFFF
@A00155:342:HHGFNDSXY:1:2543:27959:4726 1:N:0:GAACCTAG+TCCGCATA
GCGACTTCTCTCGGGCCCACAGCCTTACAGCTGTGGAGCACGGTATCCTCTGCCGAAACAGAGGTTGG
ACAAGACCGGAGGGGTCTCCTAGTTCCAAAGGAGATGTACTCCGGGCTTGTTCACGACCTACCGTGTA
+
FFFFFFFFFFFFFFFFFFFFFFFFFFFFFFFFFFFFFFFFFFFFFFFFFFFFFFFFFFFFFFFFFFFF
FFFFFFFFFFFFFFFFFFFFFF:FFFFFFFFFFF:FFFFFFFFFFFFFFFFFFFFFFFFFFFFF:FFF
@A00155:342:HHGFNDSXY:1:2543:14000:6386 1:N:0:GAACCTAG+TCCGCATA
CTGTGTCCACTTAAGGACTCACCTCTGGCCTATGGTCATCTCAATGCCGAGAGGGATGTGGAGGGCGA
GAGAGAGCGACTTCTCTCGGGCCCACAGCCTTACAGCTGTGGAGCACGGTATCCTCTGCCGAAACAGA
+
FFFFFFFFFFFFFFFFFFFFFFFFFFFFFFFFFFFFFFFFFFFFFFFFFFFFF:FF:FFFFFFFFFFF
FFFFFFFFFFFFFFFFFFFFFFFFFFFFF::F:FFFFFFFFFFFFFFFFFFFFFFFFFFFFFFFFFF:
@A00155:342:HHGFNDSXY:1:2543:10511:12900 1:N:0:GAACCTAG+TCCGCATA
CTTCTCTCGGGCCCACAGCCTTACAGCTGTGGAGCACGGTATCCTCTGCCGAAACAGAGGTTGGACAA
GACCGGAGGGGTCTCCTAGTTCCAAAGGAGATGTACTCCGGGCTTGTTCACGACCTACCGTGTAAGTC
+
FFFFFFFFFFFFFFFFFFFFFFFFFFFFFFFFFFFFFFFFFFFFFFFFFFFFFFFFFFFFFFFFFFFF
FFFFFFFFFFFFFFFFFFFFFFFFFFFFFFFFFFFFFFFFFFFFFFFFFFFFFFFFFFFFFFFFFFFF
@A00155:342:HHGFNDSXY:1:2543:18376:18756 1:N:0:GAACCTAG+TCCGCATA
CTCAATGCCGAGAGGGATGTGGAGGGCGAGAGAGAGCGACTTCTCTCGGGCCCACAGCCTTACAGCTG
TGGAGCACGGTATCCTCTGCCGAAACAGAGGTTGGACAAGACCGGAGGGGTCTCCTAGTTCCAAAGGA
+
FFFFFFFFFFFFFFFFFFF:FFFFFFFFFFFFFFFFFFFFFFFFFFFFFFFFFFFFFFFFFFFFFFFF
FFFFFFFFFFFFFFFFFFFFFFFFFFFFFFFFFFFFFFFFFFFFFFFFFFFFFFFFFFFFFFFFFFFF
@A00155:342:HHGFNDSXY:1:2543:25482:27336 1:N:0:GAACCTAG+TCCGCATA
ATCTCAATGCCGAGAGGGATGTAGAGGGCGAGAGAGAGCGACTTCTCTCGGGCCCACAGCCTTACAGC
TGTGGAGCACGGTATCCTCTGCCGAAACAGAGGTTGGACAAGACCGGAGGGGTCTCCTAGTTCCAAAG
+
FFFFFFFFFFFFFFFFFF:FFFFFFFFFFFFFFFFFFFFFFFFFFFFFFFFFFFFFFFFFFFFFFFFF
FFFFFFFFFFFFFFFFFFFFFFFFFFFFFFFFF:FFFFFFFFFFFFFFFFFFFFFFFFFFFFFFFFFF
@A00155:342:HHGFNDSXY:1:2543:8657:34491 1:N:0:GAACCTAG+TCCGCATA
CCTCTGTTTCGGCAGAGGATACCGTGCTCCACAGCTGTAAGGCTGTGGGCCCGAGAGAAGTCGCTCTC
TCTCGCCCTCCACATCCCTCTCGGCATTGAGATGACCATAGGCCAGAGGAGAGTCCTTAAGTGGACAC
+
FFFFFFFFFFFFFFFFFFFFFFFFFFFFFFFFFFFFFFFFFFFFFFFFFFFFFFFFFFFFFFFFFFFF
FFFFFFFFFFFF::FFFFFFFFFFFFFFFFFFFF:FFFFFFFFFFFFFFFFFFFFFFFFFFFFFFFFF
@A00155:342:HHGFNDSXY:1:2544:17219:3035 1:N:0:GAACCTAG+TCCGCATA
ATCTCAATGCCGAGAGGGATGTGGAGGGCGAGAGAGAGCGACTTCTCTCGGGCCCACAGCCTTACAGC
TGTGGAGCACGGTATCCTCTGCCGAAACAGAGGTTGGACAAGACCGGAGGGGTCTCCTAGTTCCAAAG
+
FFFFFFFFFFFFFFFFFFFFFFFFFFFFFFFFFFFFFFFFFFFFFFFFFFFFFFFFFFFFFFFFFFFF
FFFFFFFFFFFFFFFFFFFFFFFFFFFFFFFFFFFFFFFFFFFFFFFFFFFFFFFFFFFFFFFFFFFF
@A00155:342:HHGFNDSXY:1:2544:28637:22279 1:N:0:GAACCTAG+TCCGCATA
ACTTCTCTCGGGCCCACAGCCTTACAGCTGTGGAGCACGGTATCCTCTGCCGAAACAGAGGTTGGACA
AGACCGGAGGGGTCTCCTGGTTCCAAAGGAGATGTACTCCGGGCTTGTTCACGACCTACCGTGTAAGT
+
FFFFFFFFFFFFF:FFFFFFFF:FFFFFFFFFFF:FFFFFFFFFFFFFFFFF:FFFFFFFFFFFFFFF
FFFFFFFFFF:FFFFF:FFFFFFFFFFFFFFFFFFFFFFFFFFF:F:FFFFFFFFFFFF:FFFFFFFF
@A00155:342:HHGFNDSXY:1:2544:16405:35258 1:N:0:GAACCTAG+TCCGCATA
GGACTCTCCTCTGGCCTATGGTCATCTCAATGCCGAGAGGGATGTGGAGGGCGAGAGAGAGCGACTTC
TCTCGGGCCCACAGCCTTACAGCTGTGGAGCACGGTATCCTCTGCCGAAACAGAGGTTGGACAAGACC
+
```

FFFFFFFFFFFFFFFFFFFFFFFFFFFFFFFFFFFFFFFFFF,FFFFFFFFFFFFFFFFFFFFFFFFF
FFFFFFFFFFFFFFFFF:FFFFFFFFFFFFFFFFFFFFFFFFFFFFFFFFFFFFFFF:FFFFFFFFFF
@A00155:342:HHGFNDSXY:1:2545:21151:4679 1:N:0:GAACCTAG+TCCGCATA
ATCTCAATGCCGAGAGGGATGTGGAGGGCGAGAGAGAGCGACTTCTCTCGGGCCCACAGCCTTACAGC
TGTGGAGCACGGTATCCTCTGCCGAAACAGAGGTTGGACTAGACCGGAGGGGTCTCCTAGTTCCAAAG
+
FFFFFFFFFFFFFFFFFFFFFFFFFFFFFFFFFF:FFF:FFFFFFFFFFFFFFFFFFFFFFFFFFFFF
FFFFFFFFFFFFFFFFFFFFFFFFFFFFFFFFFFFFFFF,FFFFFFFFFFFFFFFFFFFFFFFFFFFF
@A00155:342:HHGFNDSXY:1:2545:25174:14967 1:N:0:GAACCTAG+TCCGCATA
CTTCTCTCGGGCCCACAGCCTTACAGCTGTGGAGCACGGTATCCTCTGCCGAAACAGAGGTTGGACAA
GACCGGAGGGGTCTCCTAGTTCCAAAGGAGATGTACTCCGGGCTTGTTCACGACCTACCGTGTAAGTC
+
FFFFFFFFFFFFFFFFFFFFFFFFFFFFFFFFFFFFFFFFFFFFFFFFFFFFFFFFFFFFFFFFFFFF
FFFFFFFFFFFFFFFFFFFFFFFFFFFFFFFFFFFFFFFFFFFFFFFFFFFFFFFFFFFFFFFFFFFF
@A00155:342:HHGFNDSXY:1:2545:23158:16141 1:N:0:GAACCTAG+TCCGCATA
GCGACTTCTCTCGGGCCCACAGCCTTACAGCTGTGGAGCACGGTATCCTCTGCCGAAACAGAGGTTGG
ACAAGACCGGAGGGGTCTCCTAGTTCCAAAGGAGATGTACTCCGGGCTTGTTCACGACCTACCGTGTA
+
FFFFFFFFFFFFFFFFFFFFFFFFFFFFFFFFFFFFFFFFFFFFFFFFFFFFFFFFFFFFFFFFFFFF
FFFFFFFFFFFFFFFFFFFFFFFFFFFFFFFFFFFFFFFFFFFFFFFFFFFFFFFFFFFFFFFFFFFF
@A00155:342:HHGFNDSXY:1:2545:27859:35994 1:N:0:GAACCTAG+TCCGCATA
GGCGAGAGAGAGCGACTTCTCTCGGGCCCACAGCCTTACAGCTGTGGAGCACGGTATCCTCTGCCGAA
ACAGAGGTTGGACAAGACCGGAGAGGTCTCCTAGTTCCAAAGGAGATGTACTCCGGGCTTGTTCACGA
+
FFFFFFFFFFFF:FFFFFFFFFFFFFFFFFFFFF:FFFFFFFFFFFFFFFFFFFFFFFFFFFFFFFFF
FFFFFFFFFFFFFFFFFF:FFFFFFFFFFFFF:FFFFFFFFFFFFFFFFFFFFFF:FFFFFFFFFFFF
@A00155:342:HHGFNDSXY:1:2546:32859:26396 1:N:0:GAACCTAG+TCCGCATA
TACACGGTAGGTCGTGAACAAGCCCGGAGTACATCTCCTTTGGAACTAGGA
+
FFFFFF:FFFF:FFFFFFFFFFFFFFFFFFFFFFFFFFFFFFFFFFFFFFF
@A00155:342:HHGFNDSXY:1:2547:20600:14497 1:N:0:GAACCTAG+TCCGCATA
GATGTAGAGGGCGAGAGAGAGCGACTTCTCTCGGGCCCACAGCCTTACAGCTGTGGAGCACGGTATCC
TCTGCCGAAACAGAGGTTGGACAAGACCGGAGGGGTCTCCTAGTTCCAAAGGAGATGTACTCCGGGCT
+
FFFFFFFFFFFFFFFFFFFFFFFFFFFFFFFFFFFFFFFFFFFFFFFFFFFFFFFFFFFFFFFFFFFF
FFFFFFFFFFFFFFFFFFFFFFFFFFFFFFFFFFFFFFFFFFFFFFFFFFFFFFFFFFFFFFFFFFFF
@A00155:342:HHGFNDSXY:1:2547:26015:19680 1:N:0:GAACCTAG+TCCGCATA
GAGCGACTTCTCTCGGGCCCACAGCCTTACAGCTGTGGAGCACGGTATCCTCTGCCGAAACAGAGGTT
GGACAAGACCGGAGGGGTCTCCTAGTTCCAAAGGAGATGTACTCCGGGCTTGTTCACGACCTACCGTG
+
FFFFFFFFFFFFFFFFFFFFFFFFFFFFFFFFFFFFFFFFFFFFFFFFFF:FFFFFFFFFFFFFFFFF
FFFFFFFFFFFFFFFFFFFFFFFFFFFFFFFFFFFFFFFFFFFFFFFFFFFFFFFFFFFFFFFFFFFF
@A00155:342:HHGFNDSXY:1:2547:1371:29262 1:N:0:GAACCTAG+TCCGCATA
ACTTCTCTCGGGCCCACAGCCTTACAGCTGTGGAGCACGGTATCCTCTGCCGAAACAGAGGTTGGACA
AGACCGGAGGGGTCTCCTAGTTCCAAAGGAGATGTACTCAGGGCTTGTTCACGACCTACCGTGTAAGT
+
,:FFFF:FFFFFFF:,F:FFFFFF:F::FFFFFFFF:FFFFFFFFF:FFFFFFFF:,F,FFFFFF:FF
F:,,FFF:FFFFFFFFFF:FFF,FF,:FF:F,FFFF:F:,FFF,:FFFF,:FFF,FFF,:FFFF,,FF
@A00155:342:HHGFNDSXY:1:2548:3784:9111 1:N:0:GAACCTAG+TCCGCATA
AATGCCGAGAGGGATGTGGAGGGCGAGAGAGAGCGACTTCTCTCGGGCCCACAGCCTTACAGCTGTGG
AGCACGGTATCCTCTGCCGAAACAGAGGTTGGACAAGACCGGAGGGGTCTCCTGGTTCCAAAGG
+
FFFFFFFFFFFFFFFFFFFFFFFFFFFFFFFFFFFFFFFFFFFFFFFFFFFFFFFFFFFFFFFFFFFF
FFFFFFFFFFFFFFFFFFFFFFFFFFFFFFFFFFFFFFFFFFFFFFFFFFFFFFFFFFFFFFFF

```
@A00155:342:HHGFNDSXY:1:2548:26323:22247 1:N:0:GAACCTAG+TCCGCATA
GGGCGAGAGAGAGCGACTTCTCTCGGGCCCACAGCCTTACAGCTGTGGAGCACGGTATCCTCTGCCGA
AACAGAGGTTGGACAAGACCGGAGGGGTCTCCTAGTTCCAAAGGAGATGTACTCCGGGC
+
FFFFFFFFFFFFFFFFFFFFFFFFFFFFFFFFFFFFFFFFFFFFFFFFFFFFFFFFFFFFFFFFFFFF
FFFFFFFFFFFFFFFFFFFFFFFFFFFFFFFFFFFFFFFFFFFFFFFFFFFFFFFFFFF
@A00155:342:HHGFNDSXY:1:2548:12038:23343 1:N:0:GAACCTAG+TCCGCATA
GTATCCTCTGCCGAAACAGAGGTTGGACAAGACCGGAGGGGTCTCCTAGTTCCAAAGGAGATGTACTC
CGGGCTTGTTCACGACCTACCGTGTAAGTCGTAGTCTAGTAGGCTACCTGACGAGTCCTTTTTAGGAC
+
FFFFFFFFFFFFFFFFFFFFFFFFFFFFFFFFFFFFFFFFFFFFFFFFFFFFFFFFFFFFFFFFFFFF
FFFFFFFFFFFFFFFFFFFFFFFFFFFFFFFFFFFFFFFFFFFFFFFFFFFFFFFFFFFFF,FFFFFF
@A00155:342:HHGFNDSXY:1:2548:18710:28103 1:N:0:GAACCTAG+TCCGCATA
CTTCTCTCGGGCCCACAGCCTTACAGCTGTGGAGCACGGTATCCTCTGCCGAAACAGAGGTTGGACAA
GACCGGAGGGGTCTCCTAGTTCCAAAGGAGATGTACTCCGGGCTTGTTCACGACCTACCGTGTAAGTC
+
FFFFFFFFFFFFFFFFFFFFFFFFFF:FFFFFFFFFFFFFFFFFFFFFFFFFFFFFFFFFFF:FFFFFFF
FFFFFFFFFFFFFFFFFFFFFFFFFFFFFFFFF:FFFFFFFFFFFFFFFFFFFFFFFFF:FFFFFF:FFFF
@A00155:342:HHGFNDSXY:1:2548:6668:31266 1:N:0:GAACCTAG+TCCGCATA
ACTTCTCTCGGGCCCACAGCCTTACAGCTGTGGAGCACGGTATCCTCTGCCGAAACAGAGGTTGGACA
AGACCGGAGGGGTCTCCTAGTTCCAAAGGAGATGTACTCCGGACTTGTTCACGACCTACCGTGTAAGT
+
FFFFFFFFF,FFFFFFFFFFF:FFFFFFFFFFFFFFFFFFFFFFFFFFFFFFFFFFFFFFFFFFF:FFFF
FFFFFFFFFFFFFFFFFFFFFFFFFFFFFFFFFFFFFFFFFFFFFFFFFFFFFF:FFFFFFFFF,FF
@A00155:342:HHGFNDSXY:1:2548:17110:35603 1:N:0:GAACCTAG+TCCGCATA
AGGGCGAGAGAGAGCGACTTCTCTCGGGCCCACAGCCTTACAGCTGTGGAGCACGGTATCCTCTGCCG
AAACAGAGGTTGGACAAGACCGGAGGGGTCTCCTAGTTCCAAAGGAGATGTACTCCGAGCTTGTTCAC
+
FFFFFFFFFFFFFFFFFFFFFFFFFFFFFFFFFFFFFFFFFFFFFFFFFFFFFFFFFFFFFFFFFFFF
FFFFFFFFFFFFFFFFFFFFFFFFFFFFFFFFFFFFFFFFFFFFFFFFFFFFFFFFFFFFFFFFFFFF
@A00155:342:HHGFNDSXY:1:2549:16025:11209 1:N:0:GAACCTAG+TCCGCATA
GAGCGACTTCTCTCGGGCCCACAGCCTTACAGCTGTGGAGCACGGTATCCTCTGCCGAAACAGAGGTT
GGACAAGACCGGAGGGGTCTCCTAGTTCCAAAGGAGATGTACTCCGGGCTTGTTCACGACCTACCGTG
+
FFFFFFFFFFFFFFFFFFFFFFFFFFFFFFFFFFFFFFFFFFF:FFFFFFFFFFF:FFFFFFFFF
FFFFFFFFFFFFFFFFFFFFFF,FFFFFFFFFFFFFF,FFFFFFFFFFFFFFFFFFFFFFFFFFFFFF
@A00155:342:HHGFNDSXY:1:2549:7690:15029 1:N:0:GAACCTAG+TCCGCATA
GCGACTTCTCTCGGGCCCACAGCCTTACAGCTGTGGAGCACGGTATCCTCTGCCGAAACAGAGGTTGG
ACAAGACCGGAGGGGTCTCCTAGTTCCAAAGGAGATGTACTCCGGGCTTGTTCACGACCTACCGTGTA
+
FFFFFFFFFFFFFFFFFFFFFFFFFFFFFFFFFFFFFFFFFFFFFFFFFFFFFFFFFFFFFFFFFFFF
FFFFFFFFFFFFFFFFFFFFFFFFFFFFFFFFFFFFFFFFFFFFFFFFFF:FFFFFFFFFFFFFFFFF
@A00155:342:HHGFNDSXY:1:2549:15980:20118 1:N:0:GAACCTAG+TCCGCATA
AGAGAGAGCGACTTCTCTCGGGCCCACAGCCTTACAGCTGTGGAGCACGGTATCCTCTGCCGAAACAG
AGGTTGGACAAGACCGGAGGGGTCTCCTAGTTCCAAAGGAGATGTACTCCGGACTTGTTCACGACCTA
+
FFFFFFF:FFFFFFFFFFFFFFFFFFFFFFFFFFFFFFFFFFFFFFFFFFFFFFFFFFFFFFFFFFFF
FFFFFFFFFFFFFFFFFFFFFFFFFFFFFFFFFFFFFFFFFFFFFFFFFFFFFFFFFFFFFF:FFFFF:
@A00155:342:HHGFNDSXY:1:2550:8431:3004 1:N:0:GAACCTAG+TCCGCATA
CTTCTCTCGGGCCCACAGCCTTACAGCTGTGGAGCACGGTATCCTCTGCCGAAACAGAGGTTGGACAA
GACCGGAGGGGTCTCCTAGTTCCAAAGGAGATGTACTCCGGGCTTGTTCACGACCTACCGT
+
FFFFFFFFFFFFFFFFFFFFFFFFFFFFFFFFF:FFFFFFFFFFFFFFFFFFFFFFFFFFFFFFFFFF
FFFFFFFFFFFFFFFFFFFFFFFFFFFFFFFFFFFFFFFFFFFFFFFFFFFFFFFFFFFFFF
```

```
@A00155:342:HHGFNDSXY:1:2550:29713:13275 1:N:0:GAACCTAG+TCCGCATA
CACAGCCTTACAGCTGTGGAGCACGGTATCCTCTGCCGAAACAGAGGTTGGACAAGACCGGAGGGGTC
TCCTGGTTCCAAAGGAGATGTACTCCGGGCTTGTTCACGACCTACCGTGTAAGTCGTAGTCTAGT
+
FFFFFFFFFFFFFFFFFFFFFFFFFFFF:FFFFFFFFFFFFFFFFFFFFFFFFFFF:FFFFFFFFFFF
FFFFFFFFFFFFFFFFFFFFFFFFFFFFFFFFFFFFFFFFFFFFFFFFFFFFFFFFFFFFFF:FF
@A00155:342:HHGFNDSXY:1:2550:6885:19179 1:N:0:GAACCTAG+TCCGCATA
GAGCGACTTCTCTCGGGCCCACAGCCTTACAGCTGTGGAGCACGGTATCCTCTGCCGAAACAGAGGTT
GGACAAGACCGGAGGGGTCTCCTGGTTCCAAAGGAGATGTACTCCGGGCTTGTTCACGACCTACCGTG
+
FFFFFFFFFFFFFFFFFFFFFFFFFFFFFFFFFFFFFFFFFFFFFFFFFFFFF,FFFFFFFFFFFFFF
FFFFFFFFFFFF:FFFFFFFFFFFFFFFFFFFFFFFFFFFFFFFFFFFFFFFFFFFFFFFFFFFFFF
@A00155:342:HHGFNDSXY:1:2550:19831:25911 1:N:0:GAACCTAG+TCCGCATA
ATGCCGAGAGGGATGTGGAGGGCGAGAGAGAGCGACTTCTCTCGGGCCCACAGCCTTACAGCTGTGGA
GCACGGTATCCTCTGCCGAAACAGAGGTTGGACAAGACCGGAGGGGTCTCCTGGTTCCAAAGGAGATG
+
FFFFFFFFFFFFFFFFFFFFFFFFFFFFFFFFFFFFFFFFFFFFFFFFFFFFFFFFFFFFFFFFFFFF
FFFFFFFFFFFFFFFFFFFFFFFFFFFFFFFFFFFFFFFFFFFFFFFFFFFFFFFFFFFFFFFFFFFF
@A00155:342:HHGFNDSXY:1:2551:19623:5228 1:N:0:GAACCTAG+TCCGCATA
GGGGCATGGGTTTGAACCCCCATGACGGTCGGAGTCTAGTAGGCTCCCTGATGAGTCCGTTCCAAGGA
CGACACCGTCGTTACTCTTCGAGTTGTGTAAGTTTCGTCCTAAAAAGGACTCGTCAGGT
+
FFFFFFFFFFFFFFFFFFFFFFFFFFFFFFFFFFFFFFFFFFFFFFFFFFFFFFFFFFFFFFFFFFFF
FF,FFFFFFFFFFFFFFFFFFFFF:FFFFFFFFFFFFFFFFFFFFFFFFFFFFFFFFFF
@A00155:342:HHGFNDSXY:1:2551:2682:9392 1:N:0:GAACCTAG+TCCGCATA
CTTACAGCTGTGGAGCACGGTATCCTCTGCCGAAACAGAGGTTGGACAAGACCGGAGGGGTCTCCTAG
TTCCAAAGGAGATGTACTCCGGGCTTGTTCACGACCTACCGTGTAAGTCGTAGTCTAGTAGGCTACCT
+
FFFFFFFFFFFFFFFFFFFFF:FFF:FFFFFFFFFFFFFFF,FFFFFFFFFFFFFFFFFFFFFFFFFF
FFFFFFFFFFFFFFF:FFFFFFFFF:FFFFFFFFFFFFFFF:FFF:F:FFF:FFFFFFFFFFFF:F:F
@A00155:342:HHGFNDSXY:1:2551:17517:33896 1:N:0:GAACCTAG+TCCGCATA
ATCTCAATGCCGAGAGGGATGTGGAGGGCGAGAGAGAGCGACTTCTCTCGGGCCCACAGCCTTACAGC
TGTGGAGCACGGTATCCTCTGCCGAAACAGAGGTTGGACAAGACCGGAGGGGTCTCCTAGTTCCAAAG
+
FFFFFFFFFFFFFFFFFF,FFFFFFFFFFFFFFFFFFFFFFFFFFFFFFFFFFFFFFFFFFFFFFFFF
FFFFFFFFFFFFFFFFFFFFFFFFFFFFFFFFFFFFFFFFF:FFFFFFFFFFFFFFFFFFFFFFFFFF
@A00155:342:HHGFNDSXY:1:2552:26386:3098 1:N:0:GAACCTAG+TCCGCATA
CCCCGCCACACCGCCTTAGATCAGCTGTGTCCACTTAAGGACTCACCTCTGGCCTATGGTCATCTCAA
TGCCGAGAGGGATGTGGAGGGCGAGAGAGAGCGACTTCTCTCGGGCCCACAGCCTTACAGCTGTGGAG
+
FFFFFFFFFFF:FFFFFFFFFFFFFFFFFFFFFFF:FFFFFFFFFFFFFFFFFFFFFFFFFFFFFFFF
FFFFFFFFFFFF:FFFFFFFFFFFFFFFFFFFFFFFFFFFFFFFFFFFFFFFFFFFFFFFFFFFFFF
@A00155:342:HHGFNDSXY:1:2552:9543:5462 1:N:0:GAACCTAG+TCCGCATA
AGCGACTTCTCTCGGGCCCACAGCCTTACAGCTGTGGAGCACGGTATCCTCTGCCGAAACAGAGGTTG
GACAAGACCGGAGGGGTCTCCTAGTTCCAAAGGAGATGTACTCCGGGCTTGTTCACGACCTACCGTGT
+
FFFFFFFF:FFFFFFFFFFFFFFFFFFFFFFFFFFFFFFFFFFFFFFFFFFFFFFFFFFFFFFFFFF
FFFFFFFFFFFFFFFFFFFFFFFFFFFFFFFFFFFFFFFFFFFFFFFFFFFFFFFFFFFFFFFFFFFF
@A00155:342:HHGFNDSXY:1:2552:27389:5713 1:N:0:GAACCTAG+TCCGCATA
GGGCGAGAGAGAGCGACTTCTCTCGGGCCCACAGCCTTACAGCTGTGGAGCACGGTATCCTCTGCCGA
AACAGAGGTTGGACAAGACCGGAGGGGTCTCCTAGTTCCAAAGGAGATGTACTCCGGGCTTGTTCACG
+
FFFFFFFFFFFFFFFFFFFFFFFFFFFFFFFFFFFFFFFFFFFFFFFFFFFFFFFFFFFFFFFFFFFF
FFFFF:FFFFFFFFFFFFFFFFFFFFFFFFFFFFFFFFFFFFFFFFFFFFFFFFFFFFFFFFFF
```

```
@A00155:342:HHGFNDSXY:1:2552:18738:19977 1:N:0:GAACCTAG+TCCGCATA
GCGACTTCTCTCGGGCCCACAGCCTTACAGCTGTGGAGCACGGTATCCTCTGCCGAAACAGAGGTTGG
ACAAGACCGGAGGGGTCTCCTAGTTCCAAAGGAGATGTACTCCGGGCTTGTTCACGACCTACCGTGTA
+
FFFFFFFFFFFFFFFFFFFFFFFF:FFFFFFFFFFFFFFFFFFFFFFFFF:FFFFFFFFFFFFFFFFF
FFFFFFFFFFFFFFFFFFFFF:FFFFFFF,:FFFFFFFFFFFFFFFF::FFFFF,FFFFFFFFFFFFFF
@A00155:342:HHGFNDSXY:1:2552:18710:32236 1:N:0:GAACCTAG+TCCGCATA
GCGACTTCTCTCGGGCCCACAGCCTTACAGCTGTGGAGCACGGTATCCTCTGCCGAAACAGAGGTTGG
ACAAGACCGGAGGGGTCTCCTAGTTCCAAAGGAGATGTACTCCGGGCTTGTTCACGACCTACCGTGTA
+
FFFFFFFFFFFFFFFFFFFFFFFFFFFFFFFFFFFFFFFFFFFFFFFFFFFFFFFFFFFFFFFFFFFF
FFFFFFFFFFFFFFFFFFFFFFFFFFFFFFFFFFFFFFFFFFFFFFFFFFFFFFFFFFFFFFFFFFFF
@A00155:342:HHGFNDSXY:1:2553:30553:4773 1:N:0:GAACCTAG+TCCGCATA
GAGCGACTTCTCTCGGGCCCACAGCCTTACAGCTGTGGAGCACGGTATCCTCTGCCGAAACAGAGGTT
GGACAAGACCGGAGGGGTCTCCTAGTTCCAAAGGAGATGTACTCCGGGCTTGTTCACGACCTACCGTG
+
FFFFFFFFFFFFFFFFFFFFFFFFFFFFFFFFFFFFFFFFFFFFFFFFFFFFFFFFFFFFFFFFFFFF
FFFFFFFFFFFFFFFFFFFFFFFFFFFFFFFFFFFFFFFFFFFFFFFFFFFFFFFFFFFFFFFFFFFF
@A00155:342:HHGFNDSXY:1:2553:27380:9111 1:N:0:GAACCTAG+TCCGCATA
AGCGACTTCTCTCGGGCCCACAGCCTTACAGCTGTGGAGCACGGTATCCTCTGCCGAAACAGAGGTTG
GACAAGACCGGAGGGGTCTCCTAGTTCCAAAGGAGATGTACTCCGGGCTTGTTCACGACCTACCGTGT
+
FFFFFFFFFFFFFFFFFFFFFFFFFFFFFFFFFFFFFFFFFFFFFFFFFFFFFFFFFFFFFFFFFFFF
FFFFFFFFFFFFFFFFFFFFFFFFFFFFFFFFFFFFFFFFFFFFFFFFF:FFFFFFFFFFFFFFFFFF
@A00155:342:HHGFNDSXY:1:2553:29523:18396 1:N:0:GAACCTAG+TCCGCATA
ACCTCTGTTTCGGCAGAGGATACCGTGCTCCACAGCTGTAAGGCTGTGGGCCCGAGAGAAGTCGCTCT
CTCTCGCCCTCTACATCCCTCTCGGCATTGAGATGACCATAGGCCAGAGGTGAGTCCTTAAGTGGACA
+
FFFFFFFFFFFFFFFFFFFF:F::FFFFFFFFF:FFFFFFF,FFFFFFFFFF:FFFFFFFFFFFFFF:
:FFFFF:FFFFFFFFFFFFFF,FFFFFF:F:FFFFFFFF,FFF:FFFFFFFFFF:FF,:F:F,F,FFF
@A00155:342:HHGFNDSXY:1:2553:27796:21104 1:N:0:GAACCTAG+TCCGCATA
TCGGCAGAGGATACCGTGCTCCACAGCTGTAAGGCTGTGGGCCCGAGAGAAGTCGCTCTCTCTCGCCC
TCCACATCCCTCTCGGCATTGAGATGACCATAGGCCAGAGGTGAGTCCTTAAGTGGACACA
+
FFFFFFFFFFFFFFFFFFFFFFFFFFFFFFFFFFFFFFFFFFFFFFFFFFFFFFFFFFFFFFFFFFFF
FFFFFFFFFFFFFFFFFFFFFFFFFFFFFFFFFFFFFFFFFFFFFFFFFFFFFFFFFFFFF
@A00155:342:HHGFNDSXY:1:2553:1181:23234 1:N:0:GAACCTAG+TCCGCATA
CACATCCCTCTCGGCATTGAGATGACCATAGGCCAGAGGTGAGTCCTTAAGTGGACACAGCTGATCTA
AGGCGGTGTGGCGGGGCATGGGTTTGAACCCCCATGACGGTCGGAGTCTATTAGGCTCCCTGATGAGT
+
FFFFFFFFFFFFFFFFFFFFFF:FFF:FFFFFF:FF:FF:FF::F:FFF:FF:::FFFFF,FF:FF:F
FF:F,FFFFF:FFFFFFFFFFFFFFFF:F:FF:FFFF:FFFFFFFFFFFFFFFFFFFFFFFF:FF,:FF
@A00155:342:HHGFNDSXY:1:2553:14100:35775 1:N:0:GAACCTAG+TCCGCATA
TTCTCTCGGGCCCACAGCCTTACAGCTGTGGAGCACGGTATCCTCTGCCGAAACAGAGGTTGGACAAG
ACCGGAGGGGTCTCCTAGTTCCAAAGGAGATGTACTCCGGGCTTGTTCACGACCTACCGTGTAAGTCG
+
FFFFFFFFFFFFFFFFFFFFFFFFFFFFFFFFFFFFFFF:FFFFFFFFFFFFFFFFFFFFFFFFFFFF
FFFFFFFFFFFFFFFFFFFFFFFFFFFFFFFFFFFFFFFFFFFFFFFFFFFFFFFFFFFFFFFFFFFF
@A00155:342:HHGFNDSXY:1:2554:13801:2660 1:N:0:GAACCTAG+TCCGCATA
CAGCCTTACAGCTGTGGAGCACGGTATCCTCTGCCGAAACAGAGGTTGGACAAGACCGGAGGGGTCTC
CTAGTTCCAAAGGAGATGTACTCCGGGCTTGTTCACGACCTACCGTGTAAGTCGTAGTCTAGTAGGC
+
FFFFFFFFFFFFFFFFFFFFFFFFFFFFFFFFFFFFFFFFFFFFFFFFFFFFFFFFFFFFFFFFFFFF
FFFFFFFFFFFFFFFFFFFFFFFFFFFFFFFFFFFFFFFFFFFFFFFFFFFFFFFFFFFFFFFFFFF
```

```
@A00155:342:HHGFNDSXY:1:2554:24487:2660 1:N:0:GAACCTAG+TCCGCATA
GCCCACAGCCTTACAGCTGTGGAGCACGGTATCCTCTGCCGAAACAGAGGTTGGACAAGACCGGAGGG
GTCTCCTAGTTCCAAAGGAGATGTACTCCGGGCTTGTTCACGACCTACCGTGTAAGTCGTAGTCTAGT
+
FFFFFFFFFFFFFFFFFFFFFFFFFFFFFFFFFFFFFFFFFFFFFFFFFFFFFFFFFFFFFFFFFFFF
FFFFFFFFFFFFFFFFFFFFFFFFFFFFFFFFFFFFFFFFFFFFFFFFFFFFFFFFFFFFFFFFFFFF
@A00155:342:HHGFNDSXY:1:2554:9724:3333 1:N:0:GAACCTAG+TCCGCATA
ACTTCTCTCGGGCCCACAGCCTTACAGCTGTGGAGCACGGTATCCTCTGCCGAAACAGAGGTTGGACA
AGACCGGAGGGGTCTCCTAGTTCCAAAGGAGATGTACTCCGGGCTTGTTCACGACCTAC
+
FFFFFFFFFFFFFFFFFFFFFFFFFFFFFFFFFFFFFFFFFFFFFFFFFFFFFFFFFFFF:FFFFFFF
FFFFFFFFFFFFFFFFFFFFFFFFFFFFFFFFFFFFFFFFFFFFFFFFFFFFF,FFFFF
@A00155:342:HHGFNDSXY:1:2554:26910:16313 1:N:0:GAACCTAG+TCCGCATA
TGTTTCGGCAGAGGATACCGTGCTCCACAGCTGTAAGGCTGTGGGCCCGAGAGAAGTCGCTCTCTCTC
GCCCTTCACATCCCTCTCGGCATTGAGATGACCATAGGCCAGAGGTGAGTCCTTAAGTGGACACAGCT
+
FFFFFFFFFFFFFFFFFFFFFFFFFFFFFFFFFFFFFFFFFFFFFFFFFFFFFFFFFFFFFFFFFFFF
FFFFFFFFFFFFFFFFFFFFFF:FFFFFFFFFFFFFFFFFFFFFFFFFFFFFFFFFFFFFF:FFFFFF
@A00155:342:HHGFNDSXY:1:2554:3595:22592 1:N:0:GAACCTAG+TCCGCATA
ACAGCTGTGGAGCACGGTATCCTCTGCCGAAACAGAGGTTGGACAAGACCGGAGGGGTCTCCTGGTTC
CAAAGGAGATGTACTCCGGGCTTGTTCACGACCTACCGTGTAAGTCGTAGTCTAGTAGGCTACCTGAC
+
FFFFFFFFFFFFFFFFFFFFFFFFFFFFFFFFFFFFFFFFFFFFFFFFFFFFFFFFFFFFFFFFFFFF
FFFFFFFFFFFFFFFFFF,FFF:FFFFFF::FFFFFFFFFFFFFFFFFFFFFFFF:FFFFFF::FFFF
@A00155:342:HHGFNDSXY:1:2554:7826:24471 1:N:0:GAACCTAG+TCCGCATA
ATGCCGAGAGGGATGTAGAGGGCGAGAGAGAGCGACTTCTCTCGGGCCCACAGCCTTACAGCTGTGGA
GCACGGTATCCTCTGCCGAAACAGAGGTTGGACAAGACCGGAGGGGTCTCCTAGTTCCAAAGGAGATG
+
FFFFFFFFFFFFFFFFFFFFFFFFFFFFFFFFFFFFFFFFFFFFFFFFFFFFFFFFFFFFFFFFFFFF
FFFFFFFFFFFFFFFFF::FFFFFFFFFFFFFFFFFFFFFFFF:FFFFFFFFFFFFFFFFFFFFFFFF
@A00155:342:HHGFNDSXY:1:2555:12943:4053 1:N:0:GAACCTAG+TCCGCATA
CAATGCCGAGAGGGATGTGGAGGGCGAGAGAGAGCGACTTCTCTCGGGCCCACAGCCTTACAGCTGTG
GAGCACGGTATCCTCTGCCGAAACAGAGGTTGGACAAGACCGGAGGGGTCTCCTGGTTCCAAAGGAGA
+
FFFFFFFFFFFFFFF:FFFFFFFFFFFFFFFFFFFFFFFFFFFFFFFFFFFFFFFFFFFFFFFFFFFF
FFFFFFFFFFFFFFFFFFFFFFFFFFFFFFFFFFFFFFFFFFFFFFFFFFFFFFFFFFFFFF:FFFFF
@A00155:342:HHGFNDSXY:1:2555:30987:30827 1:N:0:GAACCTAG+TCCGCATA
GAGCGACTTCTCTCGGGCCCACAGCCTTACAGCTGTGGAGCACGGTATCCTCTGCCGAAACAGAGGTT
GGACAAGACCGGAGGGGTCTCCTAGTTCCAAAGGAGATGTACTCCGGGCTTGTTCACGACCTACCGTG
+
FFFFFFFFFFFFFFFFFFFFFFFFFFFFFFFFFFFFFFFFFFFFFFFFF:FFFFFFFFFFFFFFFFFF
FFFFF:FFFFFFFFFFFFFFFFFFFFFFFFFFFFFFFFFFFFFFFFFFFF:FFFFFFFFFFFFFFFFF
@A00155:342:HHGFNDSXY:1:2556:25446:8672 1:N:0:GAACCTAG+TCCGCATA
AGGGATGTGGAGGGCGAGAGAGAGCGACTTCTCTCGGGCCCACAGCCTTACAGCTGTGGAGCACGGTA
TCCTCTGCCGAAACAGAGGTTGGACAAGACCGGAGGGGTCTCCTGGTTCCAAAGGAGATGTACTCCGG
+
FF:FF:FFFFFF::FFFFFFFFFFFFFFFFFFFFFFFF,FFFFFFFF:FFF:FFFFFFFFFFFFFFFF
FFFFFFF:FFFFF:,::FF:FFFFFFFFFFFFFFFFFFF:FFFFFFFF,FFFFFFFFFFFFFFFFFFF
@A00155:342:HHGFNDSXY:1:2556:5575:10708 1:N:0:GAACCTAG+TCCGCATA
GAGGGCGAGAGAGAGCGACTTCTCTCGGGCCCACAGCCTTACAGCTGTGGAGCACGGTATCCTCTGCC
GAAACAGAGGTTGGACAAGACCGGAGGGGTCTCCTAGTTCCAAAGGAGATGTACTCCGGGCTTGTTCA
+
FFFFFFFFFFFFFFFFFFFFFFFFFFFFFFFFFFFFFFFFFFFFFFFFFFFFFFFFFFFF:FFF:FFF
FFFFF,F:FFFFFFFF:FFFFFFFFFFFFFFFFFFFFFFFFFFFFF::FFFFFFFFFFFFFFFF,FFF
```

```
@A00155:342:HHGFNDSXY:1:2556:22372:11209 1:N:0:GAACCTAG+TCCGCATA
AAGGACTCTCCTCTGGCCTATGGTCATCTCAATGCCGAGAGGGATGTGGAGGGCGAGAGAGAGCGACT
TCTCTCGGGCCCACAGCCTTACAGCTGTGGAGCACGGTATCCTCTGCCGAAACAGAGGTTGGACAAGA
+
FFFFFFFFFFFFFFFFFFFFFFFFFFFFFFFFFFFFFFFFFFFF:FFFFFFFFFFFFFFFFFFFFFFF
FFFFFFFFFFFFFFFFFF:FFFFFFFFFFFFFFFFFFFFFFFFFFFFFFFF:FFFFFFFFFFFFFFFF
@A00155:342:HHGFNDSXY:1:2556:28140:26897 1:N:0:GAACCTAG+TCCGCATA
GCAGAGGATACCGTGCTCCACAGCTGTAAGGCTGTGGGCCCGAGAGAAGTCGCTCTCTCTCGCCCTTC
ACATCCCTCTCGGCATTGAGATGACCATAGGCCAGAGGTGAGTCCTTAAGTGGACACAGCTGAT
+
FFFFFF:FFFFFFFFFFFFFFFFF:FFFFFF:FFFFFFFF:FFFFFFFF:FFFFFFFFFFFFFF:FFF
FFFFFFF:,FFFFF,:,:FFFFFFFFFFFFFFFFFF:FFF:FFFFFFFFFFFFFFFFFFFFFFFF
@A00155:342:HHGFNDSXY:1:2556:14045:27821 1:N:0:GAACCTAG+TCCGCATA
TGTTTCGGCAGAGGATACCGTGCTCCACAGCTGTAAGGCTGTGGGCCCGAGAGAAGTCGCTCTCTCTC
GCCCTTCACATCCCTCTCGGCATTGAGATGACCATAGGCCAGAGGTGAGTCCTTAAGTGGACACAGCT
+
FFFFFFFFFFFFFFFFFFFFFFFFFFFFFFFFFFFFFFFFFFFFFFFFFFFFFFFFFFFFFFFFFFFF
FFFFFFFFFFFFFFFFFF:FFFFFFFFFFFFFFFFFFFFFFFFFFFFFFFFFFFFFFFFFFFFFFFFF
@A00155:342:HHGFNDSXY:1:2556:32786:35509 1:N:0:GAACCTAG+TCCGCATA
ACTTCTCTCGGGCCCACAGCCTTACAGCTGTGGAGCACGGTATCCTCTGCCGAAACAGAGGTTGGACA
AGACCGGAGGGGTCTCCTAGTTCCAAAGGAGATGTACTCCGGGCTTGTTCACGACCTACCGTGTAAGT
+
FFFFFFFFFFFFFFFFFFFFFFFFFFFFFFFFF:FFFFFFFFFFFFFFFFFFFFFFFFFFFFFFFFFF
FF:FFFFFFFFFFFFFFFFFFFFFFFFFFFFFF:FFFFFFFFFFFFFFFFFFFFFFFFFFFFFFFFFF
@A00155:342:HHGFNDSXY:1:2557:17065:11506 1:N:0:GAACCTAG+TCCGCATA
CGACTTCTCTCGGGCCCACAGCCTTACAGCTGTGGAGCACGGTATCCTCTGCCGAAACAGAGGTTGGA
CAAGACCGGAGGGGTCTCCTAGTTCCAAAGGAGATGTACTCCGGGCTTGTTCACGACCTACCGTGTAA
+
FFFFFFFFFFFFFFFFFFFFFFFFFFFFFFFFFFFFFFFFFFFFFFFFFFFFFFFFFFFFFFFFFFFF
FFFFFFFFFFFFFFFFFFFFFFFFFFFFFFFFFFFFFFFFFFFFFFFFFFFFFFFFFFFFFFFFFFFF
@A00155:342:HHGFNDSXY:1:2557:16423:17033 1:N:0:GAACCTAG+TCCGCATA
AGGGATGTGGAGGGCGAGAGAGAGCGACTTCTCTCGGGCCCACAGCCTTACAGCTGTGGAGCACGGTA
TCCTCTGCCGAAACAGAGGTTGGACAAGACCGGAGGGGTCTCCTAGTTCCAAAGGAGATGTACTCCGG
+
FF:FFF,FF:FFFFF:F,FFFFFFFFFF:FFF:FFFFFFFFFFFFFFFFF,F,F::FF:F::FF,FFF
FFFFFF::FFFFFFFFFFFFFFFFFFFFFFFF:F:FFF:FFFFF:F,:FFFFFFFF,FFFFFFFFFFF:
@A00155:342:HHGFNDSXY:1:2557:31801:24784 1:N:0:GAACCTAG+TCCGCATA
ACCTCTGTTTCGGCAGAGGATACCGTGCTCCACAGCTGTAAGGCTGTGGGCCCGAGAGAAGTCGCTCT
CTCTCGCCCTTCACATCCCTCTCGGCATTGAGATGACCATAGGCCAGAGGTGAGTCCTTAAGTGGAC
+
FFFFFFFF,FFFFFFF:FFFFFFFFFFFFFFFF:FFFFF,F:FF:FFFFFFF:FF,FFFF,F::FFFF
FFFFFFF:,:FFF:FFF,FFFFFF:FFF,,,FFFFFF,FFFFFFFF:F:F,FFFFFFF,FFFFFFFF
@A00155:342:HHGFNDSXY:1:2558:13747:8453 1:N:0:GAACCTAG+TCCGCATA
ACCTCTGTTTCGGCAGAGGATACCGTGCTCCACAGCTGTAAGGCTGTGGGCCCGAGAGAAGTCGCTCT
CTCTCGCCCTTCACATCCCTCTCGGCATTGAGATGACCATAGGCCAGAGGTGAGTCCTTAAGTGGACA
+
FFFFFFFFFFFFFFFF:FFFFFFFFFF:FFFFF:FFFFFF:FFFFFFFFFFFFFFFFFFF:FF,FFF
FFFFFFFFF:F:F:FF::FFFFFFFFFFFFFFFFFF,FF:F:FFFFFFFF:FFFF:FF:FF:FFFFFF
@A00155:342:HHGFNDSXY:1:2558:28158:19413 1:N:0:GAACCTAG+TCCGCATA
TTGTCCAACCTCTGTTTCGGCAGAGGATACCGTGCTCCACAGCTGTAAGGCTGTGGGCCCGAGAGAAG
TCGCTCTCTCTCGCCCTTCACATCCCTCTCGGCATTGAGATGACCATAGGCCAGAGGTGAGTCCTTAA
+
FFFFFFFFFFFFFFFFFFFFFFFFFFFFFFFFFFFFFFFFFFFFFFFFFFFFFFFFFFFFFFFFFFFF
FFFFFFFFFFFFFFFFFFFFFFFFFFFFFFFFFF,FFFFFFFFFFFFFFFFFFFFFFFFFFFFFFFFF
```

```
@A00155:342:HHGFNDSXY:1:2558:23176:26506 1:N:0:GAACCTAG+TCCGCATA
CTCTATGCCGAGAGGGATGTGGAGGGCGAGAGAGAGCGACTTCTCTCGGGCCCACAGCCTTACAGCTG
TGGAGCACGGTATCCTCTGCCGAAACAGAGGTTGGACAAGACCGGAGGGGTCTCCTAGTTCCAAAGGA
+
FFFFFFFFFFFFFFFFFFFFFFFFFFF:FFFFFFFFFFFFFFFFFFFFFFFFFFFFFFFFFFFFFFFF
FFFFFFFFFFFFFFFFFFFFFFFFFFFFFFFFFFFFFFFFFFFFFFFFFFFFFFF:FFFFFFFFFFFF
@A00155:342:HHGFNDSXY:1:2558:10050:30358 1:N:0:GAACCTAG+TCCGCATA
ATCTCAATGCCGAGAGGGATGTGGAGGGCGAGAGAGAGCGACTTCTCTCGGGCCCACAGCCTTACAGC
TGTGGAGCACGGTATCCTCTGCCGAAACAGAGGTTGGACAAGACCGGAGGGGTCTCCTAGTTCCAAAG
+
FFFFFFFFFFFFFFFFFFFFFFFFFFFFFFFFFFFFFFFFFFFFFFFFFFFFFFFFFFFFFFFFFFFF
FFFFFFFFFFFFFFFFFFFFFFFFFFFFFFFFFFFFFFFFFFFFFFFFFFFFFFFFF:FFFFFFFFFF
@A00155:342:HHGFNDSXY:1:2559:22670:2018 1:N:0:GAACCTAG+TCCGCATA
CAAGCCCGGAGTACATCTCCTTTGGAACTAGGAGACCCCTCCGGTCTTGTCCAACCTCTGTTTCGGCA
GAGGATACCGTGCTCCACAGCTGTAAGGCTGTGGGCCCGAGAGAAGTCGCTCTCTCTCGCCCTCCACA
+
FFFFFFFFFFFFFFFFFFFFFFFFFFFFFFFFFFFFFFFFFFFFFFFFFFFFFFFFFFFFFFFFFFFF
FFFFFFFFFFFFFFFFFFFFFFFFFFFFFFFFFFFFFFFFFFFFFFFFFFFFFFFFFFFFFFFFFFFF
@A00155:342:HHGFNDSXY:1:2559:11171:6965 1:N:0:GAACCTAG+TCCGCATA
GCGACTTCTCTCGGGCCCACAGCCTTACAGCTGTGGAGCACGGTATCCTCTGCCGAAACAGAGGTTGG
ACAAGACCGGAGGGGTCTCCTAGTTCCAAAGGAGATGTACTCCGGGCTTGTTCACGACCTACCGTGTA
+
FFFFFFFFFFFFFFFFFFFFFFFFFFFFFFFFFFFFFFFFFFFFFFFFFFFFFFFFFFFFFFFFFFFF
FFFFFFFFFFFFFFFFFFFFFFFFFFFFFFFFFFFFFFFFFFFFFFFFFFFFFFF,FFFFFFFFFFFF
@A00155:342:HHGFNDSXY:1:2559:26521:32800 1:N:0:GAACCTAG+TCCGCATA
GGGCGAGAGAGAGCGACTTCTCTCGGGCCCACAGCCTTACAGCTGTGGAGCACGGTATCCTCTGCCGA
AACAGAGGTTGGACAAGACCGGAGGGGTCTCCTAGTTCCAAAGGAGATGTACTCCGGGCTTGTTCACG
+
FFFFFFFFFFFFFFFFFFFFFFFFFFFFFFFFFFFFFFFFFFFFFFFFFFFFFFFFFFFFFFFFFFFF
FFFFFFFFFFFFFFFFFFFFFFFFFFFFFFFFFFFFFFFFFFFFFFFFFFFFFFFFFFFFFFFFFFFF
@A00155:342:HHGFNDSXY:1:2560:30942:6136 1:N:0:GAACCTAG+TCCGCATA
GGGCGAGAGAGAGCGACTTCTCTCGGGCCCACAGCCTTACAGCTGTGGAGCACGGTATCCTCTGCCGA
AACAGAGGTTGGACAAGACCGGAGGGGTCTCCTGGTTCCAAAGGAGATGTACTCCGGGCTTGTTCACG
+
FFFFFFFFFFFFFFFFFFFFFFFFFFFFFFFFFFFFFFFFFFFFFFFFFFFFFFFFFFFFFFFFFFFF
FFFFFFFFFFFFFFFFFFFFFFFFFFFFFFFFFFFFFFFFFFFFFFFFFFFFFFFFFFFFFFFFFFF:
@A00155:342:HHGFNDSXY:1:2560:13584:23015 1:N:0:GAACCTAG+TCCGCATA
GAGGGCGAGAGAGAGCGACTTCTCTCGGGCCCACAGCCTTACAGCTGTGGAGCACGGTATCCTCTGCC
GAAACAGAGGTTGGACAAGACCGGAGGGGTCTCCTAGTTCCAAAGGAGATGTACTCCGGGCTTGTTCA
+
FFFFFFFFFFFFFFFFFFFFFFFFFFFFFFFFFFFFFFFFFFFFFFFFFFFFFFFFFFFFFFFFFFFF
FFFFFFFFFFFFFFFFFFFFFFFFFFFFFFFFFFFFFFFFFFFFFFFFFFFFFFFFFFFFFFFFFFFF
@A00155:342:HHGFNDSXY:1:2560:30445:26349 1:N:0:GAACCTAG+TCCGCATA
GAGAGAGCGACTTCTCTCGGGCCCACAGCCTTACAGCTGTGGAGCACGGTATCCTCTGCCGAAACAGA
GGTTGGACAAGACCGGAGGGGTCTCCTAGTTCCAAAGGAGATGTACTCCGGGCTTGTTCACGACCTAC
+
FFFFFFFFFFFFFFFFFFFFFFFFFFFFFFFFFFFFFFFFFFFFFFFFFFFFFFFFFFFFFFFFFFFF
FFFFFFFFFFFFFFFFFFFFFFFFFFFFFFFFFFFFFFFFFFFFFFFFFFFFFFFFFFFFFFFFFFFF
@A00155:342:HHGFNDSXY:1:2560:8811:31062 1:N:0:GAACCTAG+TCCGCATA
CTTCTCTCGGGCCCACAGCCTTACAGCTGTGGAGCACGGTATCCTCTGCCGAAACAGAGGTTGGACAA
GACCGGAGGGGTCTCCTGGTTCCAAAGGAGATGTACTCCGGGCTTGTTCACGACCTACCGTGT
+
FFFFFFFFFFFFFFFFFFFFFFFFFFFFFFFFFFFFFFFFFFFFFFFFFFFFFFFFFFFFFFFFFFFF
FFFFFFFFFFFFFFFFFFFFFFFFFFFFFFFFFFFFFFFFFFFFFFFFFFFFFFFFFFFFFFF
```

```
@A00155:342:HHGFNDSXY:1:2561:1805:7811 1:N:0:GAACCTAG+TCCGCATA
GAGAGGGATGTGGAGGGCGAGAGAGAGCGACTTCTCTCGGGCCCACAGCCTTACAGCTGTGGAGCACG
GTATCCTCTGCCGAAACAGAGGTTGGACAAGACCGGAGGGGTCTCCTAGTTCCAAAGGAGATGTACTC
+
F,FFFFF,:F,FF,F:FFFFF:F:FFFFF,FFFFFFFFFFFFFFFFFF:,FFF,:F:FFFFFFFFFF,FF
,,,:FFFFFFFFFFFF:FFFF:F:,FF:FF,:FF:FF,FFFFFF:FF,:FF:F::FFFFFFFF:FF:FFF
@A00155:342:HHGFNDSXY:1:2561:4182:7983 1:N:0:GAACCTAG+TCCGCATA
CGACTTCTCTCGGGCCCACAGCCTTACAGCTGTGGAGCACGGTATCCTCTGCCGAAACAGAGGTTGGA
CAAGACCGGAGGGGTCTCCTAGTTCCAAAGGAGATGTACTCCGGGCTTGTTCACGACCTACCGTGTA
+
FFFFFFF:FFF,FFFFFFFFFF:FF:FFF:FF:FFFFF:FFFF,FFFFFFFFFF:FFFFFFFFFFF,FFF
F,FFFFF:FFFFFFFFF,FFFFFFFFFF:FF:FF:FF,F,FFFFFFFFFFF:FFFFFFFFFF,FFFF:F
@A00155:342:HHGFNDSXY:1:2562:27570:9972 1:N:0:GAACCTAG+TCCGCATA
GAGCGACTTCTCTCGGGCCCACAGCCTTACAGCTGTGGAGCACGGTATCCTCTGCCGAAACAGAGGTT
GGACAAGACCGGAGGGGTCTCCTAGTTCCAAAGGAGATGTACTCCGGGCTTGTTCACGACCTACCGTG
+
FFFFFFFFFFFFFFFFFFFFFFFFFFFFFFFFFFFFFFFFFFFFFFFFFFFFFFFFFFFFFFFFFFFFFF
FFFFFFFFFFFFFFFFFFFFFFFFFFFFFFFFFFFFFFFFFFFFFFFFFFFFFFFFFFFFFFFFFFFFFF
@A00155:342:HHGFNDSXY:1:2562:3278:12743 1:N:0:GAACCTAG+TCCGCATA
CGACTTCTCTCGGGCCCACAGCCTTACAGCTGTGGAGCACGGTATCCTCTGCCGAAACAGAGGTTGGA
CAAGACCGGAGGGGTCACCTAGTTCCAAAGGAGATGTACTCCGGGCTTGTTCACGACCTACCGTGTAA
+
FFFFFFF:FFF:FFFFFFFFFFFFFFFFFFFFFFFFFFFFFFFFFFFFF:FFFFFFFFFFFFFFFFFFFF
FFFFFFFFFFF,FFFFFFFFFFFFFFFFFFFFFFFFFFFFFFFFFFFFFFFFFFFFFFFFFFFFF:FFFF
@A00155:342:HHGFNDSXY:1:2562:27805:16297 1:N:0:GAACCTAG+TCCGCATA
GAGCGACTTCTCTCGGGCCCACAGCCTTACAGCTGTGGAGCACGGTATCCTCTGCCGAAACAGAGGTT
GGACAAGACCGGAGGGGTCTCCTAGTTCCAAAGGAGATGTACTCCGGGCTTGTTCACGACCTACCGTG
+
FFFFFFF:FFFFFFFF:FFFFFFFFFFFFFFFFFFFFFFFFFFFFFFFFFFFFFFFFFFFFFFFFFFFFF
FFFFFFFFFFFFFF:FFFF:FFFFF:FFFFFFFFFFFFFFFF,FFFFFFFFFFFFFFFFFFFFFFFFFFF
@A00155:342:HHGFNDSXY:1:2562:23176:21997 1:N:0:GAACCTAG+TCCGCATA
CCCACAGCCTTACAGCTGTGGAGCACGGTATCCTCTGCCGAAACAGAGGTTGGACAAGACCGGAGGGG
TCTCCTAGTTCCAATGGAGATGTACTCCGGGCTTGTTCACGACCTACCGTGTAAGTCGTAGTCTAGTA
+
FFFFFFFFFF:,FFFFFFFFFFFFFFFFFFFFFFFFFFFFFFFFFF:FFFFFFFFFFFFFFFFFFFFFFF
FFFFFFFFFFFFFFFFFFFFFFFFFFFFFFFFFFFFFFFFFFFFFFFFFFFFFFFFFFFFFFFFFFFFFF
@A00155:342:HHGFNDSXY:1:2562:17363:31876 1:N:0:GAACCTAG+TCCGCATA
GAGCGACTTCTCTCGGGCCCACAGCCTTACAGCTGTGGAGCACGGTATCCTCTGCCGAAACAGAGGTT
GGACAAGACCGGAGGGGTCTCCTAGTTCCAAAGGAGATGTACTCCGGGCTTGTTCACGACCTACCGTG
+
FFFFFFFFFFFFFFFFFFFFFFFFFFFFFFFFFFFFFFFFFFFFFFFFFFFFFFFFFFFFFFFFFFFFFF
FFFFFFFFFFFFFFFFFFFFFFFFFFFFFFFFFFFFFFFFFFFFFFFFFFFFFFFFFFFFFFFFFFFFFF
@A00155:342:HHGFNDSXY:1:2563:26033:14418 1:N:0:GAACCTAG+TCCGCATA
CTTCTCTCGGGCCCACAGCCTTACAGCTGTGGAGCACGGTATCCTCTGCCGAAACAGAGGTTGGACAA
GACCGGAGGGGTCTCCTGGTTCCAAAGGAGATGTACTCCGGGCTTGTTCACGACCTACCGTGTAAGTC
+
FFFFFFFFFFFFFFFFFFFFFFFFFFFFFFF:FFFFFFFFFFFFFFFFFFFFFFFFFFFFFFFFFFFFFF
FFFFFFFFFFFFFFFFFFFFFFFFFFFFFFFFFFFFFFFFFFFFFFFFFFFFFFFFFFFFFFFFFF:FFF
@A00155:342:HHGFNDSXY:1:2563:7627:26193 1:N:0:GAACCTAG+TCCGCATA
AGAGAGAGCGACTTCTCTCGGGCCCACAGCCTTACAGCTGTGGAGCACGGTATCCTCTGCCGAAACAG
AGGTTGGACAAGACCGGAGGGGTCTCCTAGTTCCAAAGGAGATGTACTCCGGGCTTGTTCACGACCTA
+
FFFFFFF:FFFFFFFFFFFFFFFFFFFFF:FFFFFFFFFFFFFFFFFFFFFFFF:FFFFFFFFFFFFFFF
FFFFFFFFFFFFFFFFFFFFFFFFF:FFFFFFFF:FFF:FFFFFFFF,FFFFFFFFFFFFFFF:FFFFFF
```

```
@A00155:342:HHGFNDSXY:1:2563:5620:30608 1:N:0:GAACCTAG+TCCGCATA
AGAGAGAGCGACTTCTCTCGGGCCCACAGCCTTACAGCTGTGGAGCACGGTATCCTCTGCCGAAACAG
AGGTTGGACAAGACCGGAGGGGTCTCCTTGTTCCAAAGGAGATGTACTCCGGGCTTGTTCACGAC
+
FFFFFFFFFFFFFFFFFFFFFFFFFFFFF,FFFFF:FFFFFFF::FFF:FFFFFF,FFFFFFFFFF
FFFFFFFFFFF,FFFFFFF:FFFFFFFF,FFFFFFFFFFFFFFFFF,FFF,FFFF::FFFFF,FF
@A00155:342:HHGFNDSXY:1:2564:16993:23187 1:N:0:GAACCTAG+TCCGCATA
GTCCTAAAAAGGACTCGTCAGGTAGCCTACTAGACTACGACTTACACGGTA
+
FFFFFFFFFFFFFFFFFFFFFFFFFFFFFFFFFFFFFFFFFFFFFFFFFFF
@A00155:342:HHGFNDSXY:1:2564:9272:33489 1:N:0:GAACCTAG+TCCGCATA
ATGCCGAGAGGGATGTGGAGGGCGAGAGAGAGCGACTTCTCTCGGGCCCACAGCCTTACAGCTGTGGA
GCACGGTATCCTCTGCCGAAACAGAGGTTGGACAAGACCGGAGGGGTCTCCCAGTTCCAAAGGAGATG
+
FFFFFFFFFFFFFFFFFFFFFFFFFFFFFFFFFFFFFFFFFFFFFFFFFFFFFFFFFFFFFFFFFFFF
FFFFFFFFFFFFFFFFFFFFFFFFFFFFFFFFFFFFFFFFFFFFFFFFFFFFFFFFFFFFFFFFFFFF
@A00155:342:HHGFNDSXY:1:2565:10330:5635 1:N:0:GAACCTAG+TCCGCATA
TGCCGAGAGGGATGTGGAGGGCGAGAGAGAGCGACTTCTCTCGGGCCCACAGCCTTACAGCTGTGGAG
CACGGTATCCTCTGCCGAAACAGAGGTTGGACAAGACCGGAGGGGTCTCCTAGTTCCAAAGGAGATGT
+
FFFFFFFFFFFFFFFFFFFFFFFFFFFFFFFFFFFFFFFFFFFFFFFFFFFFFFFFFFFFFFFFFFFF
FFFFFFFFFFFFFFFFFFFFFFFFFFFFFFFFFFFFFFFFFFFFFFFFFFFFFFFFFFFFFFFFFFFF
@A00155:342:HHGFNDSXY:1:2565:3088:23500 1:N:0:GAACCTAG+TCCGCATA
ATCTCAATGCCGAGAGGGATGTAGAGGGCGAGAGAGAGCGACTTCTCTCGGGCCCACAGCCTTACAGC
TGTGGAGCACGGTATCCTCTGCCGAAACAGAGGTTGGACAAGACCGGAGGGGTCTCCTAGTTCCAAAG
+
FFFFFFFFFFFFFF,F:FFFF:FFFFFFFFFFFFFFFFFFFFFFFFFFFFFFFFFFFFFFFFFFFFFF
FFFFFFFFFFFFFFFFFFFFFFFFFFFFFFFFFFFFFFFFFFFFFFFFFFFFFFFFFFFFFFFFFFFF
@A00155:342:HHGFNDSXY:1:2565:25816:24846 1:N:0:GAACCTAG+TCCGCATA
TCCTCTGCCGAAACAGAGGTTGGACAAGACCGGAGGGGTCTCCTAGTTCCAAAGGAGATGTACTCCGG
GCTTGTTCACGACCTACCGTGTAAGTCGTAGTCTAGTAGGCTACCTGACGAGTCCTTTTTAGGACGAA
+
FFFFFFFFFFFFFFFFFFFFFFFFFFFFFFFFFFFFFFFFFFFFFFFFFFFFFFFFFFFFFFFFFFFF
FFFFFFFFFFFFFFFFFFFFFFFFFFFFFFFFFFFFFFFFFFFFFFFFFFFFFFFFFFFFFFFFFFFF
@A00155:342:HHGFNDSXY:1:2565:13539:28197 1:N:0:GAACCTAG+TCCGCATA
GGATGTGGAGGGCGAGAGAGAGCGACTTCTCTCGGGCCCACAGCCTTACAGCTGTGGAGCACGGTATT
CTCTGCCGAAACAGAGGTTGGACAAGACCGGAGGGGTCTCCTAGTTCCAAAGGAGATGTACTCC
+
FFFFFFFFFFFFFFFFFFFFFFFFFFFFFFFFFFFFFFFFFFFFFFFFFFFFFFFFFFFFFFFFFFFF
FFFFFFFFFFFFFFFFFFFFFFFFFFFFFFFFFFFFFFFFFFFFFFFFFFFF:FFFFFFFFFF
@A00155:342:HHGFNDSXY:1:2565:19985:34194 1:N:0:GAACCTAG+TCCGCATA
ATGCCGAGAGGGATGTGGAGGGCGAGAGAGAGCGACTTCTCTCGGGCCCACAGCCTTACAGCTGTGGA
GCACGGTATCCTCTGCCGAAACAGAGGTTGGACAAGACCGGAGGGGTCTCCTGGTTCCAAAGGAGATG
+
FFFFFFFFFFFFF:FFFFFFFFFFFFFFFFFFFFFFFFFFFFFFFFFFFFFFFFF:FFFFFFFFFF
FFFFFFFFFFFFFFFFFFFFFFFFFFFFFFFFFFFFFFFFFFFFFFFFFFFFFFFFFFFFFFFFFFFF
@A00155:342:HHGFNDSXY:1:2565:15465:34350 1:N:0:GAACCTAG+TCCGCATA
TATCCTCTGCCGAAACAGAGGTTGGACAAGACCGGAGGGGTCTCCTAGTTCCAAAGGAGATGTACTCC
GGGCTTGTTCACGACCTACCGTGTAAGTCGTAGTCTAGTAGGCTACCTGACGAGTCCTTTTTAGGACG
+
FFFFFFFFFFFFFFFFFFFFFFFFFFFFFFFFFFFFFFFFFFFFFFFFFFFFFFFFFFFFFFFFFFFF
FFFFFFFFFFFFFFFFFFFFFFFFFFFFFFFFFFFFFFFFFFFFFFFFFFFFFFFFFFFFFFFFFFFF
@A00155:342:HHGFNDSXY:1:2566:32253:8124 1:N:0:GAACCTAG+TCCGCATA
AGAGAGAGCGACTTCTCTCGGGCCCACAGCCTTACAGCTGTGGAGCACGGTATCCTCTGCCGAAACAG
```

```
AGGTTGGACAAGACCGGAGGGGTCTCCTAGTTCCAAAGGAGATGTACTCCGGGCTTGTTCACGACCTA
+
FFFFFFFFFFFFFFFFFFFFFFFFFFFFFFFFFFFFFFFFFFFFFFFFFFFFFFFFFFFFFFF:FFFF
FFFFFFFFFFFFFFFFFFFFFFFFFFFFFFFFFFFFFFFFFFFF::FFFFFFFFFFFFFF:FFFFFFF
@A00155:342:HHGFNDSXY:1:2567:19045:3223 1:N:0:GAACCTAG+TCCGCATA
GGATGTGGAGGGCGAGAGAGAGCGACTTCTCTCGGGCCCACAGCCTTACAGCTGTGGAGCACGGTATC
CTCTGCCGAAACAGAGGTTGGACAAGACCGGAGGGGTCTCCTGGTTCCAAAGGAGATGTACTCC
+
FFFFFFFFFFFFFFFFFFFF,FFFFFFFFFFFFFFFFFFFFFFFFFFFFFFFFFFFFFFFFFFFF:FF
FFFFFFFFFFFFFFFFFFFFFFFFFFFFFFFFFFFFFFFFFFFFFFFFFFFFFFFFFFFFFFFF
@A00155:342:HHGFNDSXY:1:2567:27272:8641 1:N:0:GAACCTAG+TCCGCATA
ATCTCAATGCCGAGAGGGATGTAGAGGGCGAGAGAGAGCGACTTCTCTCGGGCCCACAGCCTTACAGC
TGTGGAGCACGGTATCCTCTGCCGAAACAGAGGTTGGACAAGACCGGAGGGGTCTCCTAGTTCCAAAG
+
FFFFFFFFFFFFFFFFFFFFFFFFFFFFFFFFFFFFFFFFFFFFFFFFFFFFFF:FFFFFFFF:FFFF
FFFFFFFFFFFFFFFFFFFFFFFFFFFFFFFFFFFFFFFFFFF,FFFFFFFFFFFFFFFFFFFFFFFF
@A00155:342:HHGFNDSXY:1:2567:20184:9392 1:N:0:GAACCTAG+TCCGCATA
AGGAGATGTACTCCGGGCTTGTTCACGACCTACCGTGTAAGTCGTAGTCTAGTAGGCTACCTGACGAG
TCCTTTTTAGGACGAAACTTACACAACTCAAAGAGTAACGACGGTTTCGTCCTTGGAAC
+
FFFFFFFFFFFFFFFFFFFFFFFFFFFFFFFFFFFFFFFFFFFFFFFFFFFFFFFFFFFFFFFFFFFF
FFFFFFFFFFFFFFFFFFFFFFFFFFFFFFFFFFFFFFFFFFFFFFFFFFFF:FFFFFF
@A00155:342:HHGFNDSXY:1:2567:14226:15608 1:N:0:GAACCTAG+TCCGCATA
GAGAGAGAGCGACTTCTCTCGGGCCCACAGCCTTACAGCTGTGGAGCACGGTATCCTCTGCCGAAACA
GAGGTTGGACAAGACCGGAGGGGTCTCCTAGTTCCAAAGGAGATGTACTCCGGGCTTGTTCACGACCT
+
FFFFFFFFFFFFFFFFFFFFFFFFFFFFFFFFFFFFFFFFFFFFFFFFFFFFFFFFFFFFFFFFFFFF
FFFFFFFFFFFFFFFFFFFFFFFFFFFFFFFFFFFFFFFFFFFFFFFFFFFFFFFFFFFFFFFFFFFF
@A00155:342:HHGFNDSXY:1:2567:8703:29684 1:N:0:GAACCTAG+TCCGCATA
GCCCCACCACACCGCCTTAGATCAGCTGTGTCCACTTAAGGACTCACCTCTGGCCTATGGTCATCTCA
ATGCCGAGAGGGATGTGGAGGGCGAGAGAGAGCGACTTCTCTCGGGCCCACAGCCTTACAGCTGTGGA
+
FFFFFFFFFFFFFFFFFFFF::FFFFFFFFFFFFFFFFFFFFFFFFFFFFFFFFFFFFFFFFFFFFFF
FFFFFFFF:FFFFFFFFFFFFFFFFFFFFFFFFFFFFF:FFFFFFFFFFFFFFFFFFF:FFFFFFFFFF
@A00155:342:HHGFNDSXY:1:2567:11632:32127 1:N:0:GAACCTAG+TCCGCATA
ACCTCTGTTTCGGCAGAGGATACCGTGCTCCACAGCTGTAAGGCTGTGGGCCCGAGAGAAGTCGCTCT
CTCTCGCCCTCCACATCCCTCTCGGCATTGAGATGACCATAGGCCAGAGGAGAGTCCTTAAGTGGACA
+
FFFFFFFFFFFFFFFFFFFFFFFFFFFFFFFFFFFFFFFFFFFFFFFFFFFFFFFFFFFFFFFFFFFF
FFFFFFFFFFFFFFFFFFFFFFFFFFFFFFFFFFFFFFFFFFFFFFFFFFFFFFFFFFFFFFFFFFFF
@A00155:342:HHGFNDSXY:1:2567:12066:33348 1:N:0:GAACCTAG+TCCGCATA
ACCTCTGTTTCGGCAGAGGATACCGTGCTCCACAGCTGTAAGGCTGTGGGCCCGAGAGAAGTCGCTCT
CTCTCGCCCTCCACATCCCTCTCGGCATTGAGATGACCATAGGCCAGAGGAGAGTCCTTAAGTGGACA
+
FFFFFFFFFFFFFFFFFFFFFFFFFFFFFFFFFFFFFFFFFFFFFFFFFFFFFFFFFFFFFFFFFFFF
FFFFFFFFFFFFFFFFFFFFFFFFFFFFF:FFFFFF:FFFFFFFFFFFFFFFFFFFFFFFFFFFFFFF
@A00155:342:HHGFNDSXY:1:2568:20853:4413 1:N:0:GAACCTAG+TCCGCATA
GAGAGGGATGTGGAGGGCGAGAGAGAGCGACTTCTCTCGGGCCCACAGCCTTACAGCTGTGGAGCACG
GTATCCTCTGCCGAAACAGAGGTTGGACAAGACCGGAGGGGTCTCCTAGTTCCAAAGGAGATGTACTC
+
FFF:FFFFFFFFFFFFFFFFFFFFFFFFFFFFFFFFFFFFFFFFFFFFFFFFFFFFFFFFFFFFFFFF
FFFFFFFFFFFFFFFFFFFFFFFFFFFFFFFFFFFFFFFFFFFFFFFFFFFFFFFFFFFFFFF:FFFF
@A00155:342:HHGFNDSXY:1:2568:29758:8030 1:N:0:GAACCTAG+TCCGCATA
ATCTCAATGCCGAGAGGGATGTGGAGGGCGAGAGAGAGCGACTTCTCTCGGGCCCACAGCCTTACAGC
```

```
TGTGGAGCACGGTATCCTCTGCCGAAACAGAGGTTGGACAAGACCGGAGGGGTCTCCTAGTTCCAAAG
+
FFFFFFFFFFFFFFFFFFFFFFFFFFFFFFFFFFFFFFFFFFFFFFFFFFFFFFFFFFFFFFFFFFFF
FFFFFFFFFFFFFFFFFFFFFFFFFFFFFFFFFFFFFFFFFFFFFFF:FFFFFFFFFFFFFFFFFFFF
@A00155:342:HHGFNDSXY:1:2568:25834:11945 1:N:0:GAACCTAG+TCCGCATA
CTTCTCTCGGGCCCACAGCCTTACAGCTGTGGAGCACGGTATCCTCTGCCGAAACAGAAGTTGGACAA
GACCGGAGGGGTCTCCTAGTTCCAAAGGAGATGTACTCCGGGCTTGTTCACGACCTACCGTGTAAGTC
+
FFFFFFFFFFFFFFFFFFFFFFFFFFFFFFFFFFFFFFFFFFFFFFFFFFFFFFFFFFFF:FFFFFFF
FFFFFFFFFFFFFFFFFFFFFFFFFFFFFFFFFFFFFFFFFFFFFFFFFFFFFFFFFFFFFFFFFFFF
@A00155:342:HHGFNDSXY:1:2568:11107:15749 1:N:0:GAACCTAG+TCCGCATA
CAGCCTTACAGCTGTGGAGCACGGTATCCTCTGCCGAAACAGAGGTTGGACAAGACCGGAGGGGTCTC
CTAGTTCCAAAGGAGATGTACTCCGGGCTTGTTCACGACCTACCGTGTAAGTCGTAGTCTAGTAGGCT
+
FFFFFFFFFFFFFFFFFFFFFFFFF:FFFFFFFFFF:FFFFFFFFF:::FFFFF,F:FFFFFFFFFFF
FFFFFFFFFFF:FFFFFFFFFFFFFFFFFFFFFFFFFFF:FFFFFFFFFFFFFFFFFFF,FFFFFFFFF
@A00155:342:HHGFNDSXY:1:2568:31024:29481 1:N:0:GAACCTAG+TCCGCATA
GACTTCTCTCGGGCCCACAGCCTTACAGCTGTGGAGCACGGTATCCTCTGCCGAAACAGAGGTTGGAC
AAGATCGGAGGGGTCTCCTAGTTCCAAAGGAGATGTACTCCGGGCTTGTTCACGACCTACCGTGTAAG
+
FFFFFFFFFFFFFFFFFFFFFFFFFFFFFFFFFFFFFFFFFFFFFFFFFFFFFFFFFFFFFFFFFFFF
FFFFFFFFFFFFFFFFFFFFFFFFFFFFFFFFFFFFFFFFFFFFFFFFFFFFFFFFFFFFFFFFF:FF
@A00155:342:HHGFNDSXY:1:2568:25762:32675 1:N:0:GAACCTAG+TCCGCATA
GCTGATCTAAGGCGGTGTGGCGGGGCATGGGTTTGAACCCCCATGACGGTCGGAGTCTAGTAGGCTCC
CTGATGAGTCCGTTCCAAGGACGAAACCGTCGTTACTCTACGAGTTGTGTAAGTTTCGTC
+
FFFFFFFFFFFFFFFFFFFFFFFFFFFFFFFFFFFFFFFFFFFFFFFFFFFFFFFFFFFFFFFFFFFF
FFFF:FFFFFFFFFFFFFFFFFFFFFFFFFFFFFFFFFFFFFFFFFFFFFFFFFFFFFF
@A00155:342:HHGFNDSXY:1:2569:16821:3443 1:N:0:GAACCTAG+TCCGCATA
GATGTAGAGGGCGAGAGAGAGCGACTTCTCTCGGGCCCACAGCCTTACAGCTGTGGAGCACGGTATCC
TCTGCCGAAACAGAGGTTGGACAAGACCGGAGGGGTCTCCTAGTTCCAAAGGAGATGTACTCCGGGCT
+
FFFFFFFFFFFFFFFFFFFFFFFFFFFFFFFFFFFFFFFFFFFFFFFFFFFFFFFFFFFFFFFFFFFF
FFFFFFFFFFFFFFFFFFFFFFFFFFFFFFFFFFFFFFFFFFFFFFFFFFFFFFFFFFFFFFFFFFFF
@A00155:342:HHGFNDSXY:1:2569:11505:17096 1:N:0:GAACCTAG+TCCGCATA
TCGGCAGAGGATACCGTGCTCCACAGCTGTAAGGCTGTGGGCCCGAGAGAAGTCGCTCTCTCTCGCCC
TTCACATCCCTCTCGGCATTGAGATGACCATAGGCCAGAGGTGAGTCCTTAAGTGGACACA
+
FFFFFFFFFFFFFFFFFFFFFFFFFFFFFFFFFFFFFFFFFFFFFFFFFFFFFFFFFFFFFFFFFFFF
FFFFFFFFFFFFFFFFFFFFFFFFFFFFFFFFFFFFFFFFFFFFFFFFFFFFFFFFFFFFF
@A00155:342:HHGFNDSXY:1:2569:29677:26209 1:N:0:GAACCTAG+TCCGCATA
GAGAGAGCGACTTCTCTCGGGCCCACAGCCTTACAGCTGTGGAGCACGGTATCCTCTGCCGAAACAGA
GGTTGGACAAGACCGGAGGGGTCTCCTAGTTCCAAAGGAGATGTACTCCGGGCTTGTTCACGAC
+
FFFFFFFFFFFFFFFFFFFFFFFFFFFFFFFFFFFFFFFFFFFFFFFFFFFFFFFFFFFFFFFFFFFF
FFFFFFFFFFFFFFFFFFFFFFFFFFFFFFFFFFFFFFFFFFFFFFFFFFFFFFFFFFFFFFFF
@A00155:342:HHGFNDSXY:1:2569:29884:26600 1:N:0:GAACCTAG+TCCGCATA
GAGAGAGCGACTTCTCTCGGGCCCACAGCCTTACAGCTGTGGAGCACGGTATCCTCTGCCGAAACAGA
GGTTGGACAAGACCGGAGGGGTCTCCTAGTTCCAAAGGAGATGTACTCCGGGCTTGTTCACGAC
+
FFFFFFFFFFFFFFFFFFFFFFFFFFFFFFFFFFFFFFFFFFFFFFFFFFFFFFFFFFFF:FFFFFFF
FFFFFFFFFFFFFFFFFFFFFFFFFFFFFFFFFFFFFFFFFFFFFFFFFFFFFFFFFFFFFFFF
@A00155:342:HHGFNDSXY:1:2569:28800:32831 1:N:0:GAACCTAG+TCCGCATA
TGGTCATCTCAATGCCGAGAGGGATGTGGAGGGCGAGAGAGAGCGACTTCTCTCGGGCCCACAGCCTT
```

ACAGCTGTGGAGCACGGTATCCTCTGCCGAAACAGAGGTTGGACAAGACCGGAGGGGTCTCCTAGTTC
+
FFFFFFFFFFFFFFFFFFFFFFFFF:F:FFFFFFFFFFFFFFFFFFFFFFFFFFFFFFFFFFFFFFFFF
FFFFFFFFFFFFFFFFFFFFFFFFFFFFFFFFFFFFFFFFFFFFFFFFFFFFFFFFFFFFFFFFFFFFF
@A00155:342:HHGFNDSXY:1:2570:30038:15311 1:N:0:GAACCTAG+TCCGCATA
TCGGCAGAGGATACCGTGCTCCACAGCTGTAAGGCTGTGGGCCCGAGAGAAGTCGCTCTCTCTCGCCC
TTCACATCCCTCTCGGCATTGAGATGACCATAGGCCAGAGGTGAGTCCTTAAGTGGACACA
+
FFFFFFFFFFFFFFFFFFFFFFFFFFFFFFFFFFFFFFFFFFFFFFFFFFFFFFFFFFFFF:FFFFFFF
FFFFFFFFFFFFFFFFFFFFFFFFFFFFFFFFFFFFFFFFFFFFFFFFFFFFFFFFFFFFFF
@A00155:342:HHGFNDSXY:1:2570:22779:17675 1:N:0:GAACCTAG+TCCGCATA
CGACTTCTCTCGGGCCCACAGCCTTACAGCTGTGGAGCACGGTATCCTCTGCCGAAACAGAGGTTGGA
CAAGACCGGAGGGGTCTCCTAGTTCCAAAGGAGATGTACTCCGGGCTTGTTCACGACCTACCGTGTAA
+
FFFFFFFFFFFFFFFFFFFFFFFFFFFFFFFFFFFFFFFFFFFFFFFFFFFFFF:FFFFFFFFFFFFF
FFF:FFFFFFFFFFFFFFFFFFFFFFFF:FFFFFFFFFFFFFFFFFFFFFFFFFFFFFFFFFFFFFFFF
@A00155:342:HHGFNDSXY:1:2570:10872:21324 1:N:0:GAACCTAG+TCCGCATA
AATGCCGAGAGGGATGTAGAGGGCGAGAGAGAGCGACTTCTCTCGGGCCCACAGCCTTACAGCTGTGG
AGCACGGTATCCTCTGCCGAAACAGAGGTTGGACAAGACCGGAGGGGTCTCCTAGTTCCAAAGGAGAT
+
FFFFFFFFFFFFFFFFFFFFFFFFFFFFFFFFFFFFFFFFFFFFFFFFFFFFFFFFFFFFFFFFFFFFF
FFFFFFFFFFFFFFFFFFFFFFFFFFFFFFFFFFFFFFFFFFFFFFFFFFFFFFFFFFFFFFFFFFFFF
@A00155:342:HHGFNDSXY:1:2570:10493:23046 1:N:0:GAACCTAG+TCCGCATA
AATGCCGAGAGGGATGTAGAGGGCGAGAGAGAGCGACTTCTCTCGGGCCCACAGCCTTACAGCTGTGG
AGCACGGTATCCTCTGCCGAAACAGAGGTTGGACAAGACCGGAGGGGTCTCCTAGTTCCAAAGGAGAT
+
FFFFFFFFFFFFFFFFFFFFFFFFFFFFFFFFFFFFFF:FFFF:FFFFFFFFFFFFFFFFFFFFFFFFFF
FFFF:FFFFFFFFFFFFFFFFFF:FFFFFFFFFFFFFFFFFFFFFFFFFFFFFFFFFFFFFFFFFFFFF
@A00155:342:HHGFNDSXY:1:2570:24297:30389 1:N:0:GAACCTAG+TCCGCATA
GCGACTTCTCTCGGGCCCACAGCCTTACAGCTGTGGAGCACGGTATCCTCTGCCGAAACAGAGGTTGG
ACAAGACCGGAGGGGTCTCCTAGTTCCAAAGGAGATGTACTCCGGGCTTGTTCACGACCTACCGTGTA
+
FFFFFFFFFFFFFFFFFFFFFFFFFFFFFFFFFFFFFFFFFFFFFFFFFFFFFFFFFFFFFFFFFFFFF
FFFFFFFFFFFFFFFFFFFFFFFFFFFFFFFFFFFF:FFFFFFFFFFFFFFFFFFFFFFFFFFFFFFFF
@A00155:342:HHGFNDSXY:1:2570:32362:34741 1:N:0:GAACCTAG+TCCGCATA
CTTCTCTCGGGCCCACAGCCTTACAGCTGTGGAGCACGGTATCCTCTGCCGAAACAGAGGTTGGACAA
GACCGGAGGGGTCTCCTAGTTCCAAAGGAGATGTACTCCGGGCTTGTTCACGACCTACCGTGTAAGTC
+
FFFFFFFFFFFFFFFFFFFFFFFFFFFFFFFFFFFFFFFFFFFFFFFFFFFFFFFFFFFFFFFFFFFFF
FFFFFFFFFFFFFFFFFFFFFFFFFFFFFFFFFFFFFFFFFFF:FFFFFFFFFFFFFFFFFFFFFFFFF
@A00155:342:HHGFNDSXY:1:2570:19298:37043 1:N:0:GAACCTAG+TCCGCATA
CTTCTCTCGGGCCCACAGCCTTACAGCTGTGGAGCACGGTATCCTCTGCCGAAACAGAGGTTGGACAA
GACCGGAGGGGTCTCCTGGTTCCAAAGGAGATGTACTCCGGGCTTGTTCACGACCTACCGTGTAAGTC
+
FFFFFFFFFFFFFFFFFFFFFFFFFFFFFFFFFFFFFFFFFFFFFFFFFFFFFFFFFFFFFFFFFFFFF
FFFFFFFFFFFFFFFFFFFFFFFFFFFFFFFFFFFFFFFFFFFFFFFFFFFFFFFFFFFFFFFFFFFFF
@A00155:342:HHGFNDSXY:1:2571:26377:1955 1:N:0:GAACCTAG+TCCGCATA
CTCTCTCGCCCTCCACATCCCTCTCGGCATTGAGATGACCATAGGCCAGAGGTGAGTCCTTAAGTGGA
CACAGCTGATCTAAGGCGGTGTGGCGGGGCATGGGTTTGAACCCCCATGACGGTCGGAGTCTAG
+
FFFFFFFFFFFFFFFFFFFFFFFFFFFFFF,FFFFFFFFFFFFFFFFFFFFFFFFFFFFFFFFFFFFFF
F,FFFFFFFFFFFFFFFFFFFFFFFFFFFFFFFFFFFFFFFFFFFFFF:FFF:FFFFFFFFFFFF
@A00155:342:HHGFNDSXY:1:2571:8802:10535 1:N:0:GAACCTAG+TCCGCATA
TTCTCTCGGGCCCACAGCCTTACAGCTGTGGAGCACGGTATCCTCTGCCGAAACAGAGGTTGGACAAG

```
ACCGGAGGGGTCTCCTAGTTCCAAAGGAGATGTACTCCGGGCTTGTTCACGACCTACCGTGTAAGTCG
+
FFFFFFFFFFFFFFFFFFFFFFFFFFFFFFFFFFFFFFFFFFFFFFFFFFFFFFFFFFFFFFFFFFFFFF
FFFFFFFFFFFFFFFFFFFFFFFFFFFFFFFFFFFFFFFFFFF,FFFFFFFFFFFFFFFFFFFFFFFFFF
@A00155:342:HHGFNDSXY:1:2571:22806:32816 1:N:0:GAACCTAG+TCCGCATA
TTACAGCTGTGGAGCACGGTATCCTCTGCCGAAACAGAGGTTGGACAAGACCGGAGGGGTCTCCTAGT
TCCAAAGGAGATGTACTCCGGGCTTGTTCACGACCTACCGTGTAAGTCGTAGTCTAGTAGGCTACCTG
+
FFFFFFFFFFFFFFFFFFFFFFFFFFFFFFFFFFFFFFFFFFFFFFFFFFFFFFFFFFFFFFFFFFFFFF
FFFFFFFFFFFFFFFFFFFFFFFFFFFFFFFFFFFFFFFFFFFFFFFFFFFFF:FFFFFFFFFFFFFFFF
@A00155:342:HHGFNDSXY:1:2572:10077:5196 1:N:0:GAACCTAG+TCCGCATA
TTCTCTCGGGCCCACAGCCTTACAGCTGTGGAGCACGGTATCCTCTGCCGAAACAGAGGTTGGACAAG
ACCGGAGGGGTCTCCTAGTTCCAAAGGAGATGTACTCCGGGCTTGTTCACGACCTACCGTGTAAGTC
+
:FFF:FFFFFFFFFFFFF,F,F:FF:FFFFFFFFFFFF::FFFFFFFFFFFFFFFFFFF:FFFFFFFFF:
F::FFFF,F::FFFFFFFFFFFFFFFFFFFFF:FFFFFFFFFF:FFFFFFFFFFFF:FFF,FF,FFFFF
@A00155:342:HHGFNDSXY:1:2572:4110:14340 1:N:0:GAACCTAG+TCCGCATA
CTCTGCCGAAACAGAGGTTGGACAAGACCGGAGGGGTCTCCTAGTTCCAAAGGAGATGTACTCCGGGC
TTGTTCACGACCTACCGTGTAAGTCGTAGTCTAGTAGGCTACCTGACGAGTCCTTTTTAGGACGAAAC
+
FFFFFFFFFFFF:FFFFFFFFFFFFFFFFFFFFFFFFFFFF:FFFFFFFFFFFFFFFFFFFFFFFF:FF,
F:FFFFFFFFFFFFF:FFFFFFFFFFFFFFFFFFFFFFFFFFFFFFFF:FFFFFF:FFFFFFFFFFFFFF
@A00155:342:HHGFNDSXY:1:2572:4227:14418 1:N:0:GAACCTAG+TCCGCATA
CTCTGCCGAAACAGAGGTTGGACAAGACCGGAGGGGTCTCCTAGTTCCAAAGGAGATGTACTCCGGGC
TTGTTCACGACCTACCGTGTAAGTCGTAGTCTAGTAGGCTACCTGACGAGTCCTTTTTAGGACGAAAC
+
FFFFFFFFFFFFFFFFFFFFFFFFFFFFFFFFFFFFFFFFFFFFFFFFFFFFFFFFFFFFFFFFFFFFFF
FFFFFFFFFFFFFFFFFFFFFFFFFFFFFFFFF,FFFFFFFFFFFFFFFFFFFFFF:FFFF,FFFFFFFF
@A00155:342:HHGFNDSXY:1:2572:6696:16658 1:N:0:GAACCTAG+TCCGCATA
CTCTGCCGAAACAGAGGTTGGACAAGACCGGAGGGGTCTCCTAGTTCCAAAGGAGATGTACTCCGGGC
TTGTTCACGACCTACCGTGTAAGTCGTAGTCTAGTAGGCTACCTGACGAGTCCTTTTTAGGACGAAAC
+
:FFF::FFFFFFF,F:FFFFFFFFFFFF:FF::FFFFFFFFFFFF:FFFFFFFFFFFFF,:FFFFFFFFF
FFFFFFF:FFFFFFFF,FFFFFFFF,:FF,FFFF,F,F,FFFF:::FFFFFF:F,F,FFFF:FFFFFFFF
@A00155:342:HHGFNDSXY:1:2572:8802:18114 1:N:0:GAACCTAG+TCCGCATA
TCTCTCGCCCTCCACATCCCTCTCGGCATTGAGATGACCATAGGCCAGAGGTGAGTCCTTAAGTGGAC
ACAGCTGATCTAAGGCGGTGTGGTGGGGCATGGGTTTGAACCCCCATGACGGTCGGAGTCTAGTAGGC
+
FFFFFFFFFFFFFFFFFFFFFFFFFFFFFFFFFFFFFFFFFFFFFFFFFFFFFFFFFFFFFFFFFFFFFF
FFFFFFFFFFFFFFFFFFFFFFFFFFFFFFFFFFFFFFFFFFFFFFFFFFFFFFFFFFFFFFFFFFFFFF
@A00155:342:HHGFNDSXY:1:2572:21504:24518 1:N:0:GAACCTAG+TCCGCATA
CTTAGATCAGCTGTGTCCACTTAAGGACTCACCTCTGGCCTATGGTCATCTCAATGCCGAGAGGGATG
TGAAGGGCGAGAGAGAGCGACTTCTCTCGGGCCCACAGCCTTACAGCTGTGGAGCACGGTATCCTCTG
+
FFFFFFFFFFFFFFFFFFFFFFFFFFFFFFFFFFFFFFFFFFFFF:FFFFFFFFFFFF::FFFFFF::FF
FFFFFFFFFFFFFFFFFF:FFFFFFFFFFFFFF:FFFFFFFFFFFFF:FFFFFFFFFFFFFFFFFFFFFF
@A00155:342:HHGFNDSXY:1:2572:15374:33160 1:N:0:GAACCTAG+TCCGCATA
CTCAATGCCGAGAGGGATGTGGAGGGCGAGAGAGAGCGACTTCTCTCGGGCCCACAGCCTTACAGCTG
TGGAGCACGGTATCCTCTGCCGAAACAGAGGTTGGACAAGACCGGAGGGGTTTCCTAGTTCCAAAGGA
+
FFFFFFFFFFFFFFFFFF:FF:FFFFFFFFFFFFFFFFFFFFFFFFFFFFFFFFFFFFFFFFFFFFFFFF
FFFFFFFFFFFFFFFFFFFFFFFFFFFFFFFFFFFFFFFFFFFFFFFFFFFFFFFFFFFFF:FFF:FFFF
@A00155:342:HHGFNDSXY:1:2573:29188:7200 1:N:0:GAACCTAG+TCCGCATA
TCAATGCCGAGAGGGATGTGGAGGGCGAGAGAGAGCGACTTCTCTCGGGCCCACAGCCTTACAGCTGT
```

GGAGCACGGTATCCTCTGCCGAAACAGAGGTTGGACAAGACCGGAGGGGTCTCCTAGTTCCAAAGGAG
+
FFFFFFFFFFFFFFFFFFFFFFFFFFFFFFFFFFFFFFFFFFFFFFFFFFFFFFFFFFFFFFFFFFFFFF
FFFFFFFFFFFFFFFFFFFFFFFFFFFFFFFFFFFFFFF:FFFFFFFFFFFFFFFF:FFFFFFFFFFFF
@A00155:342:HHGFNDSXY:1:2573:13801:34444 1:N:0:GAACCTAG+TCCGCATA
GGATGTAGAGGGCGAGAGAGAGCGACTTCTCTCGGGCCCACAGCCTTACAGCTGTGGAGCACGGTATC
CTCTGCCGAAACAGAGGTTGGACAAGACCGGAGGGGTCTCCTAGTTCCAAAGGAGATGTACTCC
+
FFFFFFFFFFFFFFFFFFFFFFFFFFFFFFFFFFFFFFFFFFFFFFFFFFFFFFFFFFFFFFFFFFFFFF
FFFFFFFFFFFFFFFFFFFFFFFFFFFFFFFFFFFFFFFFFFFFFFFFFFFFFFFFFFFFFFF
@A00155:342:HHGFNDSXY:1:2573:26196:36119 1:N:0:GAACCTAG+TCCGCATA
CTTCTCTCGGGCCCACAGCCTTACAGCTGTGGAGCACGGTATCCTCTGCCGAAACAGAGGTTGGACAA
GACCGGAGGGGTCTCCTAGTTCCAAAGGAGATGTACTCCGGGCTTGTTCACGACCTACCGTGTAAGTC
+
FFFFFFFFFFFFFFFFFFFFFFFFFFFFFFFFFFFFFFFFFFFFFFFFFFFFF:FFFFFFFFFFFFFF
FFFFFFFFFFFFFFFFFFFFFFFFFFFFFFFFFFFFFFFFFFFFFFFFFFFFFFFFFFFFFFFFFFFF
@A00155:342:HHGFNDSXY:1:2574:12563:3082 1:N:0:GAACCTAG+TCCGCATA
GAGAGGGATGTGGAGGGCGAGAGAGAGCGACTTCTCTCGGGCCCACAGCCTTACAGCTGTGGAGCACG
GTATCCTCTGCCGAAACAGAGGTTGGACAAGACCGGAGGGGTCTCCTAGTTCCAAAGGAGATGTACTC
+
FFFFFFFFFFFFFFFFFFFFFFFFFFFFFFFFFFFFFFFFFFFFFFFFFFFFFFFFFFFFFFFFFFFF
FFFFFFFFFFFFFFFFFFFFFFFFFFFFFFFFFFFFFFFFFFFFFFFFFFFFFFFFFFFFFFFFFFFF
@A00155:342:HHGFNDSXY:1:2574:25102:34788 1:N:0:GAACCTAG+TCCGCATA
GCTGTGGGCCCGAGAGAAGTCGCTCTCTCTCGCCCTCCACATCCCTCTCGGCATTGAGATGACCATAG
GCCAGAGGTGAGTCCTTAAGTGGACACAGCTGATCTAAGGCGGTGTGGCGGGGCATGGGTTTGAACCC
+
FFFFFFFFFFFFFFFFFFFFFFFFFFFFFFFFFFFFFFFFFFFFFFFFFFFFF:FFFFFFFFFFFFFF
FFFFFFFFFFFFFFFFFFFFFFFFFFFFFFFFFFFFFFFFFFFFFFFFFFFFFF,FFFFFFFFFFFFF
@A00155:342:HHGFNDSXY:1:2575:27679:10755 1:N:0:GAACCTAG+TCCGCATA
GAGGGCGAGAGAGAGCGACTTCTCTCGGGCCCACAGCCTTACAGCTGTGGAGCACGGTATCCTCTGCC
GAAACAGAGGTTGGACAAGACCGAAGGGGTCTCCTAGTTCCAAAGGAGATGTACTCCGGGCTTGTTCA
+
FFFFFFFFFFFFFFF:FFFFFFFFFFFFFFFFFFFFFFFFFFFFFFFFFFFFFFFFFFFFFFFFFFFF
:FFFFFFFFFFFFFFFFFFFFFFFFFFFFFFFFF:FFFFFFFFFFFFFFFF:FFF:FFFFFFFF,FFF
@A00155:342:HHGFNDSXY:1:2575:11650:15374 1:N:0:GAACCTAG+TCCGCATA
TAGACTACGACTTACACGGTAGGTCGTGAACAAGCCCGGAGTACATCTCCTTTGGAACTAGGAGACCC
CTCCGGTCTTGTCCAACCTCTGTTTCGGCAGAGGATACCGTGCTCCACAGCTGTAAGGCTGTGGGCCC
+
FFFFFFFFFFFFFFFFFFFFFFFFFFFFFFFFFFFFFFFFFFFFFFFFFFFFFFFFF:FFFFFFFFFF
FFFFFFFFFFFFFFFFFFFFFFF:FFFFFFFFFFFFFFFFFFFFFFFF:FFFFFFFFFFFFFFFFFFF
@A00155:342:HHGFNDSXY:1:2575:31539:21418 1:N:0:GAACCTAG+TCCGCATA
GTCATCTCAATGCCGAGAGGGATGTGGAGGGCGAGAGAGAGCGACTTCTCTCGGGCCCACAGCCTTAC
AGCTGTGGAGCACGGTATCCTCTGCCGAAACAGAGGTTGGACAAGACCGGAGGGGTCTCCTGGTTCCA
+
FFFFFFFFFFFFFFFFFFFFFFFFFFFFFFFFFFFFFFFFFFFFFFFFFFFFFFFFFFFFFFFFFFFF
FFFFFFFFFFFFFFFFFFFFFFFFFFFFFFFF:FFFFFFFFFFFFFFFFFFFFFFFFFFFFFFFFFFF
@A00155:342:HHGFNDSXY:1:2575:15176:31407 1:N:0:GAACCTAG+TCCGCATA
GGGCGAGAGAGAGCGACTTCTCTCGGGCCCACAGCCTTACAGCTGTGGAGCACGGTATCCTCTGCCGA
AACAGAGGTTGGACAAGACCGGAGGGGTCTCCTGGTTCCAAAGGAGATGTACTCCGGGCTTGTTCACG
+
FFFFFFFFFFFFFFFFFFFFFFFFFFFFFFFFFFFFFFFFFFFFFFFFFFFFFFFFFFFFFFFFFFFF
FFFFFFFFFFFFFFFF:FFFFFFFFFFFFFFFFFFFFFFFFFFFFFFFFFFFFFFFFFFFFFFFFFFF
@A00155:342:HHGFNDSXY:1:2575:3224:35039 1:N:0:GAACCTAG+TCCGCATA
TTCTCTCGGGCCCACAGCCTTACAGCTGTGGAGCACGGTATCCTCTGCCGAAACAGAGGTTGGACAAG

ACCGGAGGGGTCTCCTAGTTCCAAAGGAGATGTACTCCGGGCTTGTTCACGACCTACCGTGTAAGTCG
+
FFFFFFFFFFFFFFFFFFFFFFFFFFFFFFFFFFFFFFFFFFFFFFFFFFFFFFFFFFFFFFFFFFFF
FFFFFFFFFFFFFFFFFF:FFFFFFFFFFFFFFFFFFFFFFFFFFFFFF:FFFFFFFFFFFFFFFF:FF
@A00155:342:HHGFNDSXY:1:2575:28085:35916 1:N:0:GAACCTAG+TCCGCATA
GTCCACTTAAGGACTCACCTCTGGCCTATGGTCATCTCAATGCCGAGAGGGATGTGGAGGGCGAGAGA
GAGCGACTTCTCTCGGGCCCACAGCCTTACAGCTGTGGAGCACGGTATCCTCTGCCGAAACAGAGGTT
+
FFFFFFFFFFFFFFFFFFFFFFFFFFFFFFFFFFFFFFFFFFFFFFFFFFFFFFFFFFFFFFFFFFFF
FFFFFFFFFFFFFFFFFFFFFFFFFFFFFFFFFFFFFFFFFFFFFFFFFFFFFFFFFF:FFFFFFFFF
@A00155:342:HHGFNDSXY:1:2576:23176:27790 1:N:0:GAACCTAG+TCCGCATA
CAATGCCGAGAGGGATGTGGAGGGCGAGAGAGAGCGACTTCTCTCGGGCCCACAGCCTTACAGCTGTG
GAGCACGGTATCCTCTGCCGAAACAGAGGTTGGACAAGACCGGAGGGGTCTCCTAGTTCCAAAGGAGA
+
FFFFFFFFFFFFFFFFFFFFFFFFFFFFFFFFFFFFFFFFFFFFFFFFFFFFFFFFFFFFFFFFFFFF
FFFFFFFFFFFFFFFFFFFFFFFFFFFFFFFFFFFFFFFFFFFFFFFFFFFFFFFFFFFFFFFFFFFF
@A00155:342:HHGFNDSXY:1:2576:12283:31501 1:N:0:GAACCTAG+TCCGCATA
CTTCTCTCGGGCCCACAGCCTTACAGCTGTGGAGCACGGTATCCTCTGCCGAAACAGAGGTTGGACAA
GACCGGAGGGGTCTCCTAGTTCCAAAGGAGATGTACTCCGGGCTTGTTCACGACCTACCGTGTAAGTC
+
FFFFFFFFFFFFFFFFFFFFFFFFFFFFFFFFFFFFFFFFFFFFFFFFFFFFFFFFFFFFFFFFFFFF
FFFFFFFFFFFFFFFFFFFFFFFFFFFFFFFFFFFFFFFFFFFFFFFFFFFFFFFFFFFFFFFFFFFF
@A00155:342:HHGFNDSXY:1:2577:15031:6981 1:N:0:GAACCTAG+TCCGCATA
GAGCGACTTCTCTCGGGCCCACAGCCTTACAGCTGTGGAGCACGGTATCCTCTGCCGAAACAGAGGTT
GGACAAGACCGGAGGGGTCTCCTAGTTCCAAAGGAGATGTACTCCGGGCTTGTTCACGACCTACCGTG
+
FFFFFFFFFFFFFFFFFFFFFFFFFFFFFFFFFFFFFFFFFFFFFFFFFFFFFFFFFFFFFFFFFFFF
FFFFFFFFFFFFFFFFFFFFFFFFFFFFFFFFFFFFFFFFFFFFFFFFFFFFFFFFFFFFFFFFFFFF
@A00155:342:HHGFNDSXY:1:2577:18249:21198 1:N:0:GAACCTAG+TCCGCATA
GAGCGACTTCTCTCGGGCCCACAGCCTTACAGCTGTGGAGCACGGTATCCTCTGCCGAAACAGAGGTT
GGACAAGACCGGAGGGGTCTCCTAGTTCCAAAGGAGATGTACTCCGGGCTTGTTCACGACCTACCGTG
+
FFFFFFFFFFFFFFFFFFFFFFFFFFFFFFFFFFFFFFFFFFFFFFFFFFFFFFFFFFFFFFFFFFFF
FFFFFFFFFFFFFFFFFFFFFFFFFFFFFFFFFFFFFFFFFFFFFFFFFFFFFFFFFFFFFFFFFFFF
@A00155:342:HHGFNDSXY:1:2577:31611:27367 1:N:0:GAACCTAG+TCCGCATA
CGAGAGGGATGTAGAGGGCGAGAGAGAGCGACTTCTCTCGGGCCCACAGCCTTACAGCTGTGGAGCAC
GGTATCCTCTGCCGAAACAGAGGTTGGACAAGACCGGAGGGGTCTCCTAGTTCCAAAGGAGATGTACT
+
FFFFFFFFF,FF:FFFFFFFFFFFFFFFF,F:FFFFFFFFFFFFFFFFFFFFFFFFFFFFFFFFFF,F
FFFFFFFFFFFFFFFFFFFFFFF:FFFFFFFF,FFFFFFFFFFFFFFFF:FFFFFFFFFFFFFFFFFF
@A00155:342:HHGFNDSXY:1:2578:16188:7326 1:N:0:GAACCTAG+TCCGCATA
CTATGGTCATCTCAATGCCGAGAGGGATGTGGAGGGCGAGAGAGAGCGACTTCTCTCGGGCCCACAGC
CTTACAGCTGTGGAGCACGGTATCCTCTGCCGAAACAGAGGTTGGACAAGACCGGAGGGGTCTCCTAG
+
FFFFFFFFFFFFFFFFFFFFFFFFFFFFFFFFFFFFFFFFFFFFFFFFFFFFFFFFFFFFFFFFFFFF
FFFFFFFFFFFFFFFFFFFFFFFFFFFFFFFFFFFFFFFFFFFFFFFFFFFFFFFFFFFFFFFFFFF:
@A00155:342:HHGFNDSXY:1:2578:32127:14074 1:N:0:GAACCTAG+TCCGCATA
AGAGAGAGCGACTTCTCTCGGGCCCACAGCCTTACAGCTGTGGAGCACGGTATCCTCTGCCGAAACAG
AGGTTGGACAAGACCGGAGGGGTCTCCTAGTTCCAAAGGAGATGTACTCCGGGCTTGTTCACGACCTA
+
FFFFFFFFFFFFFFFFFFFFFFFFFFFFFFFFFFFFFFFFFFFFFFFFFFFFFFFFFFFFFFFFFFFF
FFFFFFFFFFF,FFFFFFFFFF:FFFFFFFFFFFFF:FFFFFFFFFFFFFFFFFFFFFFFFFFFFFFF
@A00155:342:HHGFNDSXY:1:2578:16459:24706 1:N:0:GAACCTAG+TCCGCATA
GGATGTAGAGGGCGAGAGAGAGCGACTTCTCTCGGGCCCACAGCCTTACAGCTGTGGAGCACGGTATC

CTCTGCCGAAACAGAGGTTGGACAAGACCGGAGGGGTCTCCTAGTTCCAAAGGAGATGTACTCC
+
FFFFFFFFFFFFFFFFFFFFFFFFFFFFFFFFFFFFFFFFFFFFFFFFFFFFFFFFFFFFFFFFFFFF
FFFFFFFFFFFFFFFFFFFFFFFFF:FFFFFFFFFFFFFFFFFFFFFFFFFFFFFFFFFFFFFF
@A00155:342:HHGFNDSXY:1:2578:2139:25394 1:N:0:GAACCTAG+TCCGCATA
TTACAGCTGTGGAGCACGGTATCCTCTGCCGAAACAGAGGTTGGACAAGACCGGAGGGGTCTCCTAGT
TCCAAAGGAGATGTACTCCGGGCTTGTTCACGACCTACCGTGTAAGTCGTAGTCTAGTAGGCTACCT
+
FFFFFFFFFFFFFFFFFFFFFFFFFFFFFF:FFFFFFFFFFFFFFFFFF:FFFFFFFFFFFFFFFFFF
FFFFFFFFFFFFFFFFFFFFFFFFFFFFFFFFF,FFFFFFFFFFFFFFFFFFFFFFFFFFFFFFFFF
@A00155:342:HHGFNDSXY:1:2578:4092:35415 1:N:0:GAACCTAG+TCCGCATA
ATGCCGAGAGGGATGTGGAGGGCGAGAGAGAGCGACTTCTCTCGGGCCCACAGCCTTACAGCTGTGGA
GCACGGTATCCTCTGCCGAAACAGAGGTTGGACAAGACCGGAGGGGTCTCCTAGTTCCAAAGGAGATG
+
FFFFFFFFFFFFFFFFFFFFFFFFFFFFFF,FFFFFF:FFFFFFFFFFFFFFFFFFFFFFF:FFFFFF
FFFFFFFFFFFFFFFFFFFFFFFFFFFFFFFFFFFFFFF:FFF,FFFFFFFFF::FFF:FFFFFFFFF
@A00155:342:HHGFNDSXY:1:2578:14299:36338 1:N:0:GAACCTAG+TCCGCATA
AGAGAGAGCGACTTCTCTCGGGCCCACAGCCTTACAGCTGTGGAGCACGGTATCCTCTGCCGAAACAG
AGGTTGGACAAGACCGGAGGGGTCTCCTAGTTCCAAAGGAGATGTACTCCGGGCTTGTTCACGACCTA
+
FFFFFFFFFFFFFFF:FFFFFFFFFFFFFFFFFFFFFFFFFFFFFFFFFFF:FFFFFFFFFFFFFFFF
FFFFFFFFFFF,FFFFFFFFFFFFFFFFFFFFFFFFFFFFFFFFFFFFFFFFFFFFFFFFFFFFFFF:
@A00155:342:HHGFNDSXY:1:2601:32108:14544 1:N:0:GAACCTAG+TCCGCATA
GGATGTAGAGGGCGAGAGAGAGCGACTTCTCTCGGGCCCACAGCCTTACAGCTGTGGAGCACGGTATC
CTCTGCCGAAACAGAGGTTGGACAAGACCGGAGGGGTCTCCTAGTTCCAAAGGAGATGTACTCC
+
F::FFFFFFFFFFFFFF:FFFFFFFF,FFFFFFFFFFFFFFFFFFFFFFFFFFFFFFF:FFF:F:FFF
,,FF:F:FFFFFFFFFFFFFFFFFFFFF:FFF:FFFFFFF:FFF::FFFFFF:FFFFFFFFFF:
@A00155:342:HHGFNDSXY:1:2601:16776:21903 1:N:0:GAACCTAG+TCCGCATA
TGTGGAGGGCGAGAGAGAGCGACTTCTCTCGGGCCCACAGCCTTACAGCTGTGGAGCACGGTATCCTC
TGCCGAAACAGAGGTTGGACAAGACCGGAGGGGTCTCCTAGTTCCAAAGGAGATGTACTCCGGG
+
F:FFFFFFFFFFFFFFFFFFFFFFFFFFFFFFFFFFFFFFFFFFFFFFFFFFFFFFFFFFFFFFFFFF
FFFFFFFFFFFFFFFFFFFFFFFFFFFFFFFFFFFFFFFFFFFFFFFFFFFFFFFFFFFFFFFF
@A00155:342:HHGFNDSXY:1:2601:14064:31046 1:N:0:GAACCTAG+TCCGCATA
GGATGTGGAGGGCGAGAGAGAGCGACTTCTCTCGGGCCCACAGCCTTACAGCTGTGGAGCACGGTATC
CTCTGCCGAAACAGAGGTTGGACAAGACCGGAGGGGTCTCCTAGTTCCAAAGGAGATGTACTCC
+
FFFFFFFFFFFFFFFFFFFFFFFFFFFFFFFFFFFFFFFFFFFFFFFFFFFFFFFFFFFFFFFFFFFF
FFFFFFFFFFFFFFFFFFFFFFFFFFFFFFFFFFFFFFFFFFFFFFFFFFFFFFFFFFFFFFFF
@A00155:342:HHGFNDSXY:1:2601:2392:31626 1:N:0:GAACCTAG+TCCGCATA
GTCATCTCAATGCCGAGAGGGATGTGGAGGGCGAGAGAGAGCGACTTCTCTCGGGCCCACAGCCTTAC
AGCTGTGGAGCACGGTATCCTCTGCCGAAACAGAGGTTGGACAAGACCGGAGGGGTCTCCAAGTTCCA
+
FFFFFFFFFFFFFFFFFFFFFFFFFFFFFFFFFFFFFFFFFFF:FFFFFFFFFFFFFFFFFF:FFFFF
:FFFFFFFFFFFFFFFFFF:FFFFFFFFFFFFFFFFFFFFFFFFFFFFFFFFFFF:FFFFF:FFFFFF
@A00155:342:HHGFNDSXY:1:2601:16468:31704 1:N:0:GAACCTAG+TCCGCATA
CAGCCTTACAGCTGTGGAGCACGGTATCCTCTGCCGAAACAGAGGTTGGACAAGACCGGAGGGGTCTC
CTAGTTCCAAAGGAGATGTACTCCGGGCTTGTTCACGACCTACCGTGTAAGTCGTAGTCTAGTAGGCT
+
FFFFFFFFFFFFFFFFFFFF,FFFFFFFFFFFFFFFFFFFFFFFFFFFFFFFFFFFFFFFFFFFFFFF
FFFFFFFFFFFFFFFFFFFFFFFFFFFFFFFFFFFFFFFFFFFFFFFFFFFFFFFFFFFFFFFFFFFF
@A00155:342:HHGFNDSXY:1:2602:28944:14888 1:N:0:GAACCTAG+TCCGCATA
TTAAGGACTCTCCTCTGGCCTATGGTCATCTCAATGCCGAGAGGGATGTGGAGGGCGAGAGAGAGCGA

CTTCTCTCGGGCCCACAGCCTTACAGCTGTGGAGCACGGTATCCTCTGCCGAAACAGAGGTTGGACAA
+
FFFFFFFFFFFFFFFFFFFFFFFFFFFFFFFFFFFFFFFFFFFFFFFFFFFFFFFFFFFFFFFFFFFF
FFFFFFFFFFFFFFFFFFFFFFFFFFFFFFFFFFFFFFFFFFFFFFFFFFFFFFFFFFFFFFFFFFFF
@A00155:342:HHGFNDSXY:1:2602:29884:15264 1:N:0:GAACCTAG+TCCGCATA
GAGCGACTTCTCTCGGGCCCACAGCCTTACAGCTGTGGAGCACGGTATCCTCTGCCGAAACAGAGGTT
GGACAAGACCGGAGGGGTCTCCTAGTTCCAAAGGAGATGTACTCCGGGCTTGTTCACGACCTACCGTG
+
FFFFFFF:FFFFFFFFFFFFFFFFFFFFFFFFFFFFFFFFFFFFFFFFFFFFFFFFFFFFFFFFFFFF
FFFFFFFFFFFFFFFFFFFFFFFFFFFFFFFFFFFFFFFFFFFFFFFFFFFFFFFFFFFFFFFFFFFF
@A00155:342:HHGFNDSXY:1:2602:2862:16157 1:N:0:GAACCTAG+TCCGCATA
GTATCCTCTGCCGAAACAGAGGTTGGACAAGACCGGAGGGGTCTCCTAGTTCCAAAGGAGATGTACTC
CGGGCTTGTTCACGACCTACCGTGTAAGTCGTAGTCTAGTAGGCTACCTGACGAGTCCTTTTTAGGAC
+
FFFFFFFFFFFFFFFFFFFFFFFFF,FFFF,FFFFFFFFFFFFFFFFFFFFFFFFFFFFFF:FFFFFF
FFF,FFFFFFFFFF:FFFFFFFFFFFFFFFFFFFFFFFFFFFFFFFF:FFFFFFFFF,FFFFFFFF:F
@A00155:342:HHGFNDSXY:1:2603:15700:2127 1:N:0:GAACCTAG+TCCGCATA
AGAGAGAGCGACTTCTCTCGGGCCCACAGCCTTACAGCTGTGGAGCACGGTATCCTCTGCCGAAACAG
AGGTTGGACAAGACCGGAGGGGTCTCCTAGTTCCAAAGGAGATGTACTCCGGGCTTGTTCACGACCTA
+
FFFFFFFFFFFFFFFFFFFFFFFFFFFFFFFFFFFFFFFFFFFFFFFFFFFFFFFFFFFFFFFFFFFF
FFFFFFFFFFFFFFFFFFFFFFFFFFFFFFFFFFFFFFFFFFFFFFFFFFFFFFFFFFFFFFFFFFF:
@A00155:342:HHGFNDSXY:1:2603:28302:7795 1:N:0:GAACCTAG+TCCGCATA
GGACTCACCTCTGGCCTATGGTCATCTCAATGCCGAGAGGGATGTGAAGGGCGAGAGAGAGCGACTTC
TCTCGGGCCCACAGCCTTACAGCTGTGGAGCACGGTATCCTCTGCCGAAACAGAGGTTGGACAAGACC
+
FFFFFFFFFFFFFFFFFFFFFFFFFFFFFFFFFFFFFFFFFFFFFFFFFFFFFFFFFFFFFFFFFFFF
FFFFFFFFFFFFFFFFFFFFFFFFFFFFFFFFFFFF,FFFFFFFFFFFFFF:FFFFFFFFFFFFFFFF
@A00155:342:HHGFNDSXY:1:2603:8169:8844 1:N:0:GAACCTAG+TCCGCATA
CTTCTCTCGGGCCCACAGCCTTACAGCTGTGGAGCACGGTATCCTCTGCCGAAACAGAGGTTGGACAA
GACCGGAGGGGTCTCCTAGTTCCAAAGGAGATGTACTCCGGGCTTGTTCACGACCTACCGTGTAAGTC
+
FFFFFFFFFFFFFFFFFFFFFFFFFFFFFFFFFFFFFFFFFFFFFFFFFFFFFFFFFFFFFFFFFFFF
FFFFFFFFFFFFFFFFFFFFFFFFFFFFFFFFFFFFFFFFFFFFFFFFFFFFFFFFFFFFFFFFFFFF
@A00155:342:HHGFNDSXY:1:2603:15465:14967 1:N:0:GAACCTAG+TCCGCATA
CTCAATGCCGAGAGGGATGTGGAGGGCGAGAGAGAGCGACTTCTCTCGGGCCCACAGCCTTACAGCTG
TGGAGCACGGTATCCTCTGCCGAAACAGAGGTTGGACAAGACCGGAGGGGTCTCCTAGTTCCAAAGGA
+
FFFFFFFFFFFFFFFF:FFFFFFFFFFFFFFFFFFFFFFFFFFFFFFFFFFFFFFFFFFFFFFFFFF
FFFFFFFFFFF:FFFFFFFFFFFFFFFFFFFFFFFFFFFF:FFFFFFFFFFFFFFFFFFFFFFFFFFF
@A00155:342:HHGFNDSXY:1:2604:2519:5259 1:N:0:GAACCTAG+TCCGCATA
AGAGAGAGCGACTTCTCTCGGGCCCACAGCCTTACAGCTGTGGAGCACGGTATACTCTGCCGAAACAG
AGGTTGGACAAGACCGGAGGGGTCTCCTAGTTCCAAAGGAGATGTACTCCGGGCTTGTTCACGACCTA
+
FFFFF,FFFFF,FFFFFFFFFF:F:FFFFFFFFFFFFFFFFFFF:FFFF:FFF,FFFFFFFFFFFFFF
FFFFFFFFFFF:FFFFFF:,FFFFFFFFFFFFFFFFFFFF,FFFFFFFFFF,FFFFFFFFFFFFFFFF
@A00155:342:HHGFNDSXY:1:2604:24777:7858 1:N:0:GAACCTAG+TCCGCATA
TCTGTTTCGGCAGAGGATACCGTGCTCCACAGCTGTAAGGCTGTGGGCCCGAGAGAAGTCGCTCTCTC
TCGCCCTCCACATCCCTCTCGGCATTGAGATGACCATAGGCCAGAGGAGAGTCCTTAAGTG
+
FFFFFFFFFFFFFFFFFFFFFFFFFFFFFFFFFFFFFFFFFFF:FFFFFFFFFFFFFFFFFFFFFFFF
FFFFFFFFFFFFFFFFFFFFFFF:FFFFFFFF,FFFFFFFFFFFFFF:FFFFFFFFFFFF:
@A00155:342:HHGFNDSXY:1:2604:23520:9251 1:N:0:GAACCTAG+TCCGCATA
TGCCGAGAGGGATGTAGAGGGCGAGAGAGAGCGACTTCTCTCGGGCCCACAGCCTTACAGCTGTGGAG

CACGGTATCCTCTGCCGAAACAGAGGTTGGACAAGACCGGAGGGGTCTCCTAGTTCCAAAGGAGATGT
+
FFFFFFFFFFFFFFFFFFFFFFFFFFFFFFFFFFFFFFFFFFFFFFFFFFFFFFFFFFFFFFFFFFFF
FFFFFFFFFFFFFFFFFFFFFFFFFFFFFFFFFFFFFFFFFFFFFFFFFFFFFFFFFFFFFFFFFFFF
@A00155:342:HHGFNDSXY:1:2604:11225:13103 1:N:0:GAACCTAG+TCCGCATA
GATCAGCTGTGTCCACTTAAGGACTCACCTCTGGCCTATGGTCATCTCAATGCCGAGAGGGATGTGGA
GGGCGAGAGAGAGCGACTTCTCTCGGGCCCACAGCCTTACAGCTGTGGAGCACGGTATCCTCTGCCGA
+
FF:FFFFFFFFFFFFFFFFFFFFFFFFFFFFFFFFFFFFFFFFFFFFFFFFFFFFFFFFFFFFFFFFF
FFFFFFFFFFFFFFFFFFFFFFFFFFFFFFFFFFFFFFFFFFFFFFFFFFFFFFFF:FFFFFFFFFFF
@A00155:342:HHGFNDSXY:1:2604:11722:13213 1:N:0:GAACCTAG+TCCGCATA
GATCAGCTGTGTCCACTTAAGGACTCACCTCTGGCCTATGGTCATCTCAATGCCGAGAGGGATGTGGA
GGGCGAGAGAGAGCGACTTCTCTCGGGCCCACAGCCTTACAGCTGTGGAGCACGGTATCCTCTGCCGA
+
FFFFF:FFFFFF:FFFFFFFFFF:FFFF,FFFFFFFFFFFFFFFFFFFFFFFFFFFF,FFFFFF,FFF
FFFFFFF:FF:FFFFF:FFFFFFF,F:FF:FFFF:F:FFFFFFFFFFFFFFFFFFFFFFFFFFF:FF:
@A00155:342:HHGFNDSXY:1:2604:15266:18944 1:N:0:GAACCTAG+TCCGCATA
GTCATCTCAATGCCGAGAGGGATGTGGAGGGCGAGAGAGAGCGACTTCTCTCGGGCCCACAGCCTTAC
AGCTGTGGAGCACGGTATCCTCTGCCGAAACAGAGGTTGGACAAGACCGGAGGGGTCTCCTAGTTCCA
+
FFFFFFFFFFFFFFFFFFFFFFFFFFFFFFFFFFFFFFFFFFFFFF:FFFFFFFFFFFFFFFFF:FFF
FFFFFFFFFFFFFFFFFFFFFFFFFFFFFFFFFFFFFFFFFFFF:FFFFFFFFFFFFFFFFFFFFFFF
@A00155:342:HHGFNDSXY:1:2604:14742:22326 1:N:0:GAACCTAG+TCCGCATA
GCCGAGAGGGATGTAGAGGGCGAGAGAGAGCGACTTCTCTCGGGCCCACAGCCTTACAGCTGTGGAGC
ACGGTATCCTCTGCCGAAACAGAGGTTGGACAAGACCGGAGGGGTCTCCTAGTTCCAAAGGAGATGT
+
:FFFFF,F::F,:FFFF:FFFFFFFFFFFFFFFFFFFFFFFF:::FFFFF,FFF:FFFFF:F,:FFFF
FF::FFF:F:FF:FFFFFFF::FFFFFF:FFFF,FFF,FF:::,FFFF,,FFFFF,FFF:,FFFFFF
@A00155:342:HHGFNDSXY:1:2604:13982:23954 1:N:0:GAACCTAG+TCCGCATA
TGCCGAGAGGGATGTAGAGGGCGAGAGAGAGCGACTTCTCTCGGGCCCACAGCCTTACAGCTGTGGAG
CACGGTATCCTCTGCCGAAACAGAGGTTGGACAAGACCGGAGGGGTCTCCTAGTTCCAAAGGAGATGT
+
FFFFFFFFFFFFFFFFFFFFFFFFFFFFFFFFFFFFFFFFFFFFFFFFFFFFFFFFFFFFFFFFFFFF
FFFFFFFFFFFFFFFFFFFFFFFFFFFFFFFFFFFFFFFFFFFFFFFFFFFFFFFFFFFFFFFFFFFF
@A00155:342:HHGFNDSXY:1:2604:18783:27915 1:N:0:GAACCTAG+TCCGCATA
GAGCGACTTCTCTCGGGCCCACAGCCTTACAGCTGTGGAGCACGGTATCCTCTGCCGAAACAGAGGTT
GGACAAGACCGGAGGGGTCTCCTAGTTCCAAAGGAGATGTACTCCGGGCTTGTTCACGACCTACCGTG
+
FFFFFFFFFFFFFFFFFFFFFFFFFFFFFFFFFFFFFFFFFFFFFFFFFFFFFFFFFFFFFFFFFFFF
FFFFFFFFFFFFFFFFFFFFFFFFFFFFFFF:FFFFFFFFFFFFFFFFFFFFFFFFFFFFFFFFFFFF
@A00155:342:HHGFNDSXY:1:2604:29405:33912 1:N:0:GAACCTAG+TCCGCATA
AAGGACTCACCTCTGGCCTATGGTCATCTCAATGCCGAGAGGGATGTGAAGGGCGAGAGAGAGCGACT
TCTCTCGGGCCCACAGCCTTACAGCTGTGGAGCACGGTATCCTCTGCCGAAACAGAGGTTGGACAAGA
+
FFFFFFFFFFFFFFFFFFFFFFFFFFFFFFFFFFFFFFFFFFFF:FFFFFFFFFFFFFFFFFFFFFFF
FFFFFFFFFFFFFFFFFFFFFFFFFFFFFFFFFFFFFFFFFFFFFFFFFFFFFFFFFFFFFFFFFFFF
@A00155:342:HHGFNDSXY:1:2604:26729:35603 1:N:0:GAACCTAG+TCCGCATA
GGTTGGACAAGACCGGAGGGGTCTCCTAGTTCCAAAGGAGATGTACTCCGGGCTTGTTCACGACCTAC
CGTGTAAGTCGTAGTCTAGTAGGCTACCTGACGAGTCCTTTTTAGGACGAAACTTACACAACTCGAAG
+
FFFFFFFFFFFFFFFFFFFFFFFFFFFFFFFFFFFFFFFFFFFFFFFFFFFFFFFFFFFFFFFFFFFF
FFFFFFFFFFFFFFFFFFFFFFFFFFFFFFFFFFFFFFFFFFFFF:FFFFFFFFFFFFFFFF:FFFFF
@A00155:342:HHGFNDSXY:1:2605:15329:10441 1:N:0:GAACCTAG+TCCGCATA
CTTCTCTCGGGCCCACAGCCTTACAGCTGTGGAGCACGGTATCCTCTGCCGAAACAGAGGTTGGACAA

```
GACCGGAGGGGTCTCCTAGTTCCAAAGGAGATGTACTCCGGGCTTGTTCACGACCTACCGTGTAAGTC
+
F,FFFFFFFFFFFFFFFFFFFFFFFFFFFFFFFFFFFFFFFFFFFFFFFFFFFFFFFFFFFFFFFFFFFF
FFFFFFFFFFFFFFFFFFFFFFFFFFFFFFFFFFFFFFFFFFFFFFFFFFFFFFFFFFFFFFFFFFFFFF
@A00155:342:HHGFNDSXY:1:2605:21739:15906 1:N:0:GAACCTAG+TCCGCATA
CTTCTCTCGGGCCCACAGCCTTACAGCTGTGGAGCACGGTATCCTCTGCCGAAACAGAGGTTGGACAA
GACCGGAGGGGTCTCCTAGTTCCAAAGGAGATGTACTCCGGGCTTGTTCACGACCTACCGTGTAAGTC
+
FFFFFFFFFFFFFFFFFFFFFFFFFFFFFFFFFFFFFFFFFFFFFFFFFFFFFFFFFFFFFFFFFFFFFF
FFFFFFFFFFFFFFFFFFFFFFFFFFFFFFFFFFFFFFFFFFFFFFFFFFFFFFFFFFFFFFFFFFFFFF
@A00155:342:HHGFNDSXY:1:2605:18954:24298 1:N:0:GAACCTAG+TCCGCATA
GAGCGACTTCTCTCGGGCCCACAGCCTTACAGCTGTGGAGCACGGTATCCTCTGCCGAAACAGAGGTT
GGACAAGACCGGAGGGGTCTCCTAGTTCCAAAGGAGATGTACTCCGGGCTTGTTCACGAC
+
FFFFFFFFFFFFFFFF:FFFFFFFF:FFFFFFFFFFFFFFFFFFFFFFFFFFFFFFFFFFFFFFFFFFFF
FFFFFFFFFFFFF:FFFFFFFFFFFFFFFFFFFFFFFFFFFFFFFFFFFFFFFFFFFF
@A00155:342:HHGFNDSXY:1:2605:20356:33802 1:N:0:GAACCTAG+TCCGCATA
GAGAGGGATGTGGAGGGCGAGAGAGAGCGACTTCTCTCGGGCCCACAGCCTTACAGCTGTGGAGCACG
GTATCCTCTGCCGAAACAGAGGTTGGACAAGACCGGAGGGGTCTCCTAGTTCCAAAGGAGATGTACTC
+
FFFFFFFFFFFFFFFFFFFFFFFFFFFFFFFFFFFFFFFFFFFFFFFFFFFFFFFFFFFFFFFFFFFFFF
FFFFFFFFFFFFFFFFFFF:FFFFFFFFFFFFFFFFFFFFFFFFFFFF:FFFFFFFFFFFFFFFFFFFFF
@A00155:342:HHGFNDSXY:1:2606:27281:2237 1:N:0:GAACCTAG+TCCGCATA
ATCTCAATGCCGAGAGGGATGTGGAGGGCGAGAGAGAGCGACTTCTCTCGGGCCCACAGCCTTACAGC
TGTGGAGCACGGTATCCTCTGCCGAAACAGAGGTTGGACAAGATCGGAGGGGTCTCCTAGTTCCAAAG
+
FFFFFFFFFFFFFFFFFFFFFFFFFFFFFFFFFFFFFFFFFFFFFFFFFFFFFFFFFFFFFFFFFFFFFF
FFFFFFFFFFFFFFFFFFFFFFFFFFFFFFFFFFFFFFFFFFFFFFFFFFFFFFFFFFF:FFFFFFFFF
@A00155:342:HHGFNDSXY:1:2607:11035:29403 1:N:0:GAACCTAG+TCCGCATA
TGTGGAGGGCAAGAGAGAGCGACTTCTCTCGGGCCCACAGCCTTACAGCTGTGGAGCACGGTATCCTC
TGCCGAAACAGAGGTTGGACAAGACCGGAGGGGTCTCCTAGTTCCAAAGGAGATGTACT
+
FFFFFFFFFFFFFFFFFFFFFFFFFFFFFFFFFFFFFFFFFFFFFFFFFFFFFFFFFFFFFFFFFFFFFF
FFFFFFFFFFFFFFFFFFFFFFFFFFFFFFFFFFFFFFFFFFFFFFFFFFFFFFFFFFF
@A00155:342:HHGFNDSXY:1:2608:9995:6809 1:N:0:GAACCTAG+TCCGCATA
CTTCTCTCGGGCCCACAGCCTTACAGCTGTGGAGCACGGTATCCTCTGCCGAAACAGAGGTTGGACAA
GACCGGAGGGGTCTCCTAGTTCCAAAGGAGATGTACTCCGGGCTTGTTCACGACCTACCGTGTAAGTC
+
FFFFFFFFFFFFFFFFFFFFF:FFFFFFFFFFFFFFFFFFFFFFFFFFFFFFFFFFFFFFFFFFFFFFFF
FFFFFFFFFFFFFFFFFFFFFFFFFFFFFFFFFF:FFFFFFFFFFFFFFFFFFFFFFFFFFFFFF:FFF
@A00155:342:HHGFNDSXY:1:2608:18050:22482 1:N:0:GAACCTAG+TCCGCATA
GAGAGGGATGTGGAGGGCGAGAGAGAGCGACTTCTCTCGGGCCCACAGCCTTACAGCTGTGGAGCACG
GTATCCTCTGCCGAAACAGAGGTTGGACAAGACCGGAGGGGTCTCCTAGTTCCAAAGGAGATGTACTC
+
FFF:FFFF:FFFFFFFFFFFFFFFFFFFFFFFFFFFFFFFFFFFFFFFFFFFFFFFFFFFFFFFFFFFFF
FFFFFFFFFFFFFFFFFFFFFFFFFFFFFFFFF:FFFFFFFFFFFFFFFFFFFFFFFFFFFFFFFFFFFF
@A00155:342:HHGFNDSXY:1:2608:25328:31767 1:N:0:GAACCTAG+TCCGCATA
AATGCCGAGAGGGATGTGGAGGGCGAGAGAGAGCGACTTCTCTCGGGCCCACAGCCTTACAGCTGTGG
AGCACGGTATCCTCTGCCGAAACAGAGGTTGGACAAGACCGGAGGGGTCTCCTGGTTCCAAAG
+
FFFFFFFFFFFFFF:FFFFFFFFFFFFFFFFFFFFFFFFFFFFFFFFFFFFFFFFFFFFFFFFFFFFFFF
FFFFFFFFFFFFFFFFFFFFFFFFFFFFFFFFFFFFFFFFFFFFFFFFFFFFFFFFFFFFFFFF
@A00155:342:HHGFNDSXY:1:2610:32786:1720 1:N:0:GAACCTAG+TCCGCATA
TCCACTTAAGGACTCACCTCTGGCCTATGGTCATCTCAATGCCGAGAGGGATGTGGAGGGCGAGAGAG
```

```
AGCGACTTCTCTCGGGCCCACAGCCTTACAGCTGTGGAGCACGGTATCCTCTGCCGAAACAGAGGTTG
+
FFFFFFFFFFF:FFFFFFFFFFFFFFFFFF:FFFF:FFFFFFFFFFFFFFFFFFFFFFFF:FF:FFFFF
FFFFFFFFFFFFFFFFFFFF:FFFFF:FFFFFF:FFFFFFFFFFFFFFFF,FFF:FFFFFFFFFFFFFF
@A00155:342:HHGFNDSXY:1:2610:22019:3333 1:N:0:GAACCTAG+TCCGCATA
TTCTCTCGGGCCCACAGCCTTACAGCTGTGGAGCACGGTATCCTCTGCCGAAACAGAGGTTGGACAAG
ACCGGAGGGGTCTCCTAGTTCCAAAGGAGATGTACTCCGGGCTTGTTCACGACCTACCGTGTAAGTCG
+
FFFFFFFFFFFFFFFFFFFFFFFFFFFFFFFFFFFFFFFFFFFFFFFFFFFFFFFFF:FFFFFFFFFF
FFFFFFFFFFFFFFFFFFFFFFFFFFFFFFFFFFFFFFFFFF:F:FFFFFFFFFFFFFFFFFFFFFFFF
@A00155:342:HHGFNDSXY:1:2610:22770:11710 1:N:0:GAACCTAG+TCCGCATA
TTCTCTCGGGCCCACAGCCTTACAGCTGTGGAGCACGGTATCCTCTGCCGAAACAGAGGTTGGACAAG
ACCGGAGGGGTCTCCTAGTTCCAAAGGAGATGTACTCCGGGCTTGTTCACGACCTACCGTGTAAGTCG
+
:FFFFFFFFFFFFFFFFFFFFFFFFFFFFFFFFFFFFFFFFFFFFFFFFFFFFFFFFFFFFFFFFFFF
FFFFFFFFFFFFFFFFFFFFFFFFFFFFFFFFFFFFFFFFFFFFFFFFFFF:FFFF:FFFFFFFFFF
@A00155:342:HHGFNDSXY:1:2611:7817:19445 1:N:0:GAACCTAG+TCCGCATA
GAGCGACTTCTCTCGGGCCCACAGCCTTACAGCTGTGGAGCACGGTATCCTCTGCCGAAACAGAGGTT
GGACAAGACCGGAGGGGTCTCCTAGTTCCAAAGGAGATGTACTCCGGGCTTGTTCACGACCTACCGTG
+
FFFFFFFFFFFFFFFFFFFFFFFFFFF:F:FFFFFFFFFFFFFFFFFFFFFFFFFFFFFFFFFFFFFFF
FFFFFFFFFFFFFFFFFFFFFFFFFFFFFFFFFFFFFFFFFFFFFFFFFFFFFFFFFFFFFFF,FFF:F
@A00155:342:HHGFNDSXY:1:2613:31792:8390 1:N:0:GAACCTAG+TCCGCATA
ATCTCAATGCCGAGAGGGATGTAGAGGGCGAGAGAGAGCGACTTCTCTCGGGCCCACAGCCTTACAGC
TGTGGAGCACGGTATCCTCTGCCGAAACAGAGGTTGGACAAGACCGGAGGGGTCTCCTAGTTCCAAAG
+
FFFFFFFFFFFFFFFFFFFFFFF:FFFFFFFFFFFFFFFFFFF:FFFFFFFFFFFFFFFFFF:,FFFFF
FFFFFFFFFFFF:FFFFFFFFFFFFFFFFFFFFFFFFFFFFFFFFFFFFFFFFFFFFFFFFFFFF,FFF
@A00155:342:HHGFNDSXY:1:2613:15754:13683 1:N:0:GAACCTAG+TCCGCATA
ACCTCTGTTTCGGCAGAGGATACCGTGCTCCACAGCTGTAAGGCTGTGGGCCCGAGAGAAGTCGCTCT
CTCTCGCCCTTCACATCCCTCTCGGCATTGAGATGACCATAGGCCAGAGGTGAGTCCTTAAGTGGACA
+
FFFFF:FFFFFFFFFF:FFFFFFFFFFFFFFFFFFFFFFFFFFFFFFFFFFFFFFFFFFFFFFFFFFFF
F:FFFFFFFFFFFFFFFFFFFFFFFFFFFFFFFFFFFFFFFFFFFFF:FFFFFFFF:FFFFFFFFFFF
@A00155:342:HHGFNDSXY:1:2613:16432:27633 1:N:0:GAACCTAG+TCCGCATA
CCCACAGCCTTACAGCTGTGGAGCACGGTATCCTCTGCCGAAACAGAGGTTGGACAAGACCGGAGGGG
TCTCCTAGTTCCAAAGGAGATGTACTCCGGGCTTGTTCACGACCTACCGTGTAAGTCGTAGTCTAGTA
+
FFFFFFFFF:FFFFFF:FFFFFFFFFFFFFFFFFFFFFFFFFFFFFFFFFFFFFFFFFFFFFFFFFFFF
FFFFFFFFFFFFFFFFFFFFFFFFFFFFFFFFFFF:FFFFFFFFFFFFFFFFFFFFFFFFFFFFFFFFFF
@A00155:342:HHGFNDSXY:1:2613:13322:27853 1:N:0:GAACCTAG+TCCGCATA
GAGCGACTTCTCTCGGGCCCACAGCCTTACAGCTGTGGAGCACGGTATCCTCTGCCGAAACAGAGGTT
GGACAAGACCGGAGGGGTCTCCTAGTTCCAAAGGAGATGTACTCCGGGCTTGTTCACGACCTACCGTG
+
FFFFFFFFFFFFFFFFFFFFFFFFFFFFFFFFFFFFFFFFFFFFFFFFFFFFFFFFFFFFFFFFFFFF
FFFFFFFFFFFFFFFFFFFFFFFFFFFFFFFFFFFFFFFFFFFFFFFFFFFFFFFFFFFFFFFFFFFF
@A00155:342:HHGFNDSXY:1:2613:13458:28056 1:N:0:GAACCTAG+TCCGCATA
GAGCGACTTCTCTCGGGCCCACAGCCTTACAGCTGTGGAGCACGGTATCCTCTGCCGAAACAGAGGTT
GGACAAGACCGGAGGGGTCTCCTAGTTCCAAAGGAGATGTACTCCGGGCTTGTTCACGACCTACCGTG
+
FFFFFFFFFFFFFFFFFFFFFFFFFFFFFFFFFFFFFFFFFFFFFFFFFFFFFFFFFFFFFFFFFFFF
FFFFFFFFFFFFFFFFFFFFFFFFFF:FFFFFFFFFFFFFFFFFFFFFFFF:FFFFFFFFFFFFFFFFFF
@A00155:342:HHGFNDSXY:1:2614:6497:9486 1:N:0:GAACCTAG+TCCGCATA
ATCTCAATGCCGAGAGGGATGTGGAGGGCGAGAGAGAGCGACTTCTCTCGGGCCCACAGCCTTACAGC
```

```
TGTGGAGCACGGTATCCTCTGCCGAAACAGAGGTTGGACAAGACCGGAGGGGTCTCCTAGTTCCAAAG
+
FFFFFFFFFFFFFFFFFFFFFFFFFFFFFFFFFFFFFFFFFFF:FFFFFFFFFFFFFFFFFFFF::FFFF
FFFFFFFFFFFFFFFFFFFFFFFFFFFFF,FFFFFFFFFFFFFFFFFFFFFFFFFFFFFFFFFFFFFF:F
@A00155:342:HHGFNDSXY:1:2614:8404:21778 1:N:0:GAACCTAG+TCCGCATA
TGCTCCACAGCTGTAAGGCTGTGGGCCCGAGAGAAGTCGCTCTCTCTCGCCCTCCACATCCCTCTCGG
CATTGAGATGACCATAGGCCAGAGGTGAGTCCTTAAGTGGACACAGCTGATCTAAGGCGGTGTGGCGG
+
FFFFFFFFFFFFFFFFFFFFFFFFFFFFFFFFFFFFFFFFFFFFFFFFFFFFFFFFFFFFFFFFFFFFFF
FFFFFFFFFFFFFFFFFFFFFFFFFFFFFFFFFFFFFFFFFFFFFFFFFFFFFFFFFFFFFFFFFFFFFF
@A00155:342:HHGFNDSXY:1:2614:19813:22091 1:N:0:GAACCTAG+TCCGCATA
ACTTCTCTCGGGCCCACAGCCTTACAGCTGTGGAGCACGGTATCCTCTGCCGAAACAGAGGTTGGACA
AGACCGGAGGGGTCTCCTAGTTCCAAAGGAGATGTACTCCGGGCTTGTTCACGACCTACCGTGTAAGT
+
FFFFFFFFFFFFFFFFFFFFFFFFFFFFFFFFFFFFFFFFFFFFFFFFFFFFFFFFFFFFFFFFFFFFFF
FFFFFFFFFFFFFFFFFFFFFFFFFFFFFFFFFFFFFFFFFFFFFFFFFFFFFFFFFFFFFFFFFFFFF:
@A00155:342:HHGFNDSXY:1:2614:4273:25363 1:N:0:GAACCTAG+TCCGCATA
GTATCCTCTGCCGAAACAGAGGTTGGACAAGACCGGAGGGGTCTCCTAGTTCCAAAGGAGATGTACTC
CGGGCTTGTTCACGACCTACCGTGTAAGTCGTAGTCTAGTAGGCTACCTGACGAGTCCTTTTTAGGAC
+
FFFFFFFFFFFFFF:FFFFFFFFFFFFFFFFFFFFFFFFFFFFFFFFFFFFFFFFFFFFFFFFFFFFFFF
FFFFFFFFFFFFFFFFFFFFFFFFFFFFFFFFFFFFFFFFFFFFFFFFFFFFFFFFFFFFFFF:FFF:FF
@A00155:342:HHGFNDSXY:1:2614:4508:25739 1:N:0:GAACCTAG+TCCGCATA
GTATCCTCTGCCGAAACAGAGGTTGGACAAGACCGGAGGGGTCTCCTAGTTCCAAAGGAGATGTACTC
CGGGCTTGTTCACGACCTACCGTGTAAGTCGTAGTCTAGTAGGCTACCTGACGAGTCCTTTTTAGGAC
+
FFFFFFFFFFFFFFFFFFFFFFFFFFFFFFFFFFFFFFFFFFFFFFFFFFFFFFFFFFFFFFFFFFFFFF
FFFFF:FFFFFFFFFFFFFFFF:FFFFFFFFFF:FFFFFFFFFFFFFFFFFFFFFFFFFFF:FFFFFFFF
@A00155:342:HHGFNDSXY:1:2614:1551:29356 1:N:0:GAACCTAG+TCCGCATA
CTTCTCTCGGGCCCACAGCCTTACAGCTGTGGAGCACGGTATCCTCTGCCGAAACAGAGGTTGGACAA
GACCGGAGGGGTCACCTAGTTCCAAAGGAGATGTACTCCGGGCTTGTTCACGACCTACCGTGTAAGTC
+
FFFFFFFFFFFFFFFFFFFFFFFFFFFFFFFFFFFFFFFFFFFFFFFFFFFFFFFFFFFFFFFFFFFFFF
FFFFFFFFFFFFFFFFFFFFFFFFFFFFFFFFFFFFFFFFFFFFFFFFFFFFFFFFFFFFFFF:FFFFFF
@A00155:342:HHGFNDSXY:1:2614:17327:30843 1:N:0:GAACCTAG+TCCGCATA
ATGCCGAGAGGGATGTAGAGGGCGAGAGAGAGCGACTTCTCTCGGGCCCACAGCCTTACAGCTGTGGA
GCACGGTATCCTCTGCCGAAACAGAGGTTGGACAAGACCGGAGGGGTCTCCTAGTTCCAAAGGAGATG
+
FFFFFFFFFFFFFFFFFFFFFFFFFFFFFFFFFFFFFFFFFFFFFFFFFFFFFFFFFFFFFFFFFFFFFF
FFFFFFFFFFFFFFFFFFFFFFFFFFFFFFFFFFFFFFFFFFFFFFFFFFFFFFFFFFFFFFFFFFFFFF
@A00155:342:HHGFNDSXY:1:2615:28293:6590 1:N:0:GAACCTAG+TCCGCATA
CTTCTCTCGGGCCCACAGCCTTACAGCTGTGGAGCACGGTATCCTCTGCCGAAACAGAGGTTGGACAA
GACCGGAGGGGTCTCCTAGTTCCAAAGGAGATGTACTCCGGGCTTGTTCACGACCTACCGTGTAAGTC
+
FFFFFFFFFFFFFFFFFFFFFFFFFFFFFFFFFFFFFFFFFF:FFFFFFFFFFFFFFFF:FFFFFFFFFF
FFFFFFFFFFFFFFFFFFFFFFFFFFFFFFFFFFFFFFFFFFFFFFFFFFFFFFFFFFFFFFFFFFFFFF
@A00155:342:HHGFNDSXY:1:2615:9552:9893 1:N:0:GAACCTAG+TCCGCATA
GGATGTGGAGGGCGAGAGAGAGCGACTTCTCTCGGGCCCACAGCCTTACAGCTGTGGAGCACGGTATC
CTCTGCCGAAACAGAGGTTGGACAAGACCGGAGGGGTCTCCTGGTTCCAAAGGAGATGTACTCCGGGC
+
FFFFFFFFFFFFFFFFFFFFFFFFFFFFFFFFFFFFFFFFFFFFFFFFFFFFFFFFFFFFFFFFFFFFFF
FFFFFFFFFFFFFFFFFFFFFFFFFF:FFFFFFFFFFFFFFFFFFFFFFFFFFF:FFFFFFFFFFFFFFF
@A00155:342:HHGFNDSXY:1:2615:4444:22122 1:N:0:GAACCTAG+TCCGCATA
GGGCGAGAGAGAGCGACTTCTCTCGGGCCCACAGCCTTACAGCTGTGGAGCACGGTATCCTCTGCCGA
```

```
AACAGAGGTTGGACAAGACCGGAGGGGTCTCCTAGTTCCAAAGGAGATGTACTCCGGGCTTGTTCACG
+
FFFFFFFFFFFFFFFFFFFFFFFFFFFFFFFFFFFFFFFFFFFFFFFFFFFFFFFFFFFFFFFFFFFF
FFFFFFFFFFFFFFFFFFFFFFFFFFFFFFF:FFFFFFFFFFFFFFFFFFFFFFFFFFFFFFFFFFFF
@A00155:342:HHGFNDSXY:1:2615:29270:24314 1:N:0:GAACCTAG+TCCGCATA
AGAGAGAGCGACTTCTCTCGGGCCCACAGCCTTACAGCTGTGGAGCACGGTATCCTCTGCCGAAACAG
AGGTTGGACAAGACCGGAGGGGTCTCCTAGTTCCAAAGGAGATGTACTCCGGGCTTGTTCACGACCTA
+
FFFFFFFFFFFFFFFFFFFFFFFFFFFFFFFFFFFFFFF,FFFFFFFFFFF:FFFFFFFFFFFFFFFF
FF:FFFFFFFFFFFFFFFFFFFFFFFFFFFFFFFFFFFFFFFF:FFFFFF:FFFFFFFF:FFFFFFFF
@A00155:342:HHGFNDSXY:1:2615:15926:25817 1:N:0:GAACCTAG+TCCGCATA
CTTCTCTCGGGCCCACAGCCTTACAGCTGTGGAGCACGGTATCCTCTGCCGAAACAGAGGTTGGACAA
GACCGGAGGGGTCTCCTAGTTCCAAAGGAGATGTACTCCGGGCTTGTTCACGACCTACCGTGTAAGTC
+
FFFFFFFFFFFFFFFFFFFFFFFFFFFFFFFFFFFFFFFFFFFFFFFFFFFFFFFFFFFFFFFFFFFF
FFFFFFFFFFFFFFFFFFFFFFFFFFFFFFFFFFFF:FFFFFFFFFFFFFFFFFFFFFFFFFFF:FFF
@A00155:342:HHGFNDSXY:1:2615:10013:26412 1:N:0:GAACCTAG+TCCGCATA
CTTCTCTCGGGCCCACAGCCTTACAGCTGTGGAGCACGGTATCCTCTGCCGAAACAGAGGTTGGACAA
GACCGGAGGGGTCTCCTAGTTCCAAAGGAGATGTACTCCGGGCTTGTTCACGACCTACCGTGTAAGTC
+
FFFFFFFFFFFFFFFFFFFFFFFFFFFFFFFFFFFFFFFFFFFFFFFFFFFFFFFFFFFFFFFFFFFF
FFFFFFFFFFFFFFFFFFFFFFFFFFFFFFFFFFFFFFFFFFFFFFFFFFFFFFFF:FFF:FFFFFFF
@A00155:342:HHGFNDSXY:1:2615:10212:26475 1:N:0:GAACCTAG+TCCGCATA
CTTCTCTCGGGCCCACAGCCTTACAGCTGTGGAGCACGGTATCCTCTGCCGAAACAGAGGTTGGACAA
GACCGGAGGGGTCTCCTAGTTCCAAAGGAGATGTACTCCGGGCTTGTTCACGACCTACCGTGTAAGTC
+
FFFFFFFFFFFFFFFFFFFFFFFFFFFFFFFFFFFFFFFFFFFFFFFFFFFFFFFFFFFFFFFFFFFF
FFFFFFFFFFFFFFFFFFFFFFFFFFFFFFFFFFFFFFFFFFFFFFFFF:FF:FFFFFFFFFFFFFFF
@A00155:342:HHGFNDSXY:1:2615:8703:31876 1:N:0:GAACCTAG+TCCGCATA
CAGCCTTACAGCTGTGGAGCACGGTATCCTCTGCCGAAACAGAGGTTGGACAAGACCGGAGGGGTCTC
CTAGTTCCAAAGGAGATGTACTCCGGGCTTGTTCACGACCTACCGTGTAAGTCGTAGTCTAGTAGGCT
+
FFFFFFFFFFFFFFFFFFFFFFFFFFFFFFFFFFFFFFFFFFFFFFFFFFFFFFFFFFFFFFFFFFFF
FFFFFFFFFFFFFFFFFFFFFFFFFFFFFFFFFF:FFFFFFFFFFFFFFFFFFFFFFFFFFFFFFFFF
@A00155:342:HHGFNDSXY:1:2615:7798:32941 1:N:0:GAACCTAG+TCCGCATA
GGACTCTCCTCTGGCCTATGGTCATCTCAATGCCGAGAGGGATGTGGAGGGCGAGAGAGAGCGACTTC
TCTCGGGCCCACAGCCTTACAGCTGTGGAGCACGGTATCCTCTGCCGAAACAGAGGTTGGACAAGACC
+
FFFFFFFFFFFF:FFFFFFFFFFFFFFFFFFFFFFFFFFFFFFFFFFFFFFFFFFFFFFFFFFFFFFF
FFFF:FFFFFFFFFFFF:FFFFFFFFFFFFFFFFFFFFFFFFFFFFFFFFF:FFFFFFF,FFFFFFFF
@A00155:342:HHGFNDSXY:1:2616:6605:7827 1:N:0:GAACCTAG+TCCGCATA
CTTCTCTCGGGCCCACAGCCTTACAGCTGTGGAGCACGGTATCCTCTGCCGAAACAGAGGTTGGACAA
GACCGGAGGGGTCTCCTAGTTCCAAAGGAGATGTACTCCGGGCTTGTTCACGACCTACCGTGTAAGTC
+
F:FFFFFFFFFFFFFFFFFFFFFFFFFFFFFFFFFFFFFFFFFFFFFFFFFFFFFFFFFFFFFFFFFF
FFFFFFFFFFFFFFFFFFFFFFFFFFFF:FFFFFFFFFF:FFFFFFFFFFFFFFF:FFFFF::FFFFF
@A00155:342:HHGFNDSXY:1:2616:17264:13980 1:N:0:GAACCTAG+TCCGCATA
CTCAATGCCGAGAGGGATGTGGAGGGCGAGAGAGAGCGACTTCTCTCGGGCCCACAGCCTTACAGCTG
TGGAGCACGGTATCCTCTGCCGAAACAGAGGTTGGACAAGACCGGAGGGGTCTCCTAGTTCCAAAGGA
+
FFFFFFFFFFFFFFFFFFFFFFFFFFFFFFFFFFFFFFFFFFFFFFFFFFFFFFFFFFFF:FFFFFFF
FFFFFFFFFFFFFFFFFFFFFFFFFFFF:FFFFFFFFFFFFFFFFFFFFFFFFFFFFFFFFFFFFFFF
@A00155:342:HHGFNDSXY:1:2616:4453:16094 1:N:0:GAACCTAG+TCCGCATA
TCTCAATGCCGAGAGGGATGTGGAGGGCGAGAGAGAGCGACTTCTCTCGGGCCCACAGCCTTACAGCT
```

```
GTGGAGCACGGTATCCTCTGCCGAAACAGAGGTTGGACAAGACCGGAGGGGTCTCCTGGTTCCAAAGG
+
FFFFFFFFFFFFFFFFFFFFFFFFFFFFFFFFFFFFFFFFFFFFFFFFFFFFFFFFFFFFFFFFFFFF
FFFFFFFFFFFFFFFFFFFFFFFFFFFFFFFF:FFFFFFFFFFFFFFFFFFFFFFFFFFFFFFFFFFF
@A00155:342:HHGFNDSXY:1:2616:16523:20369 1:N:0:GAACCTAG+TCCGCATA
CTCAATGCCGAGAGGGATGTGGAGGGCGAGAGAGAGCGACTTCTCTCGGGCCCACAGCCTTACAGCTG
TGGAGCACGGTATCCTCTGCCGAAACAGAGGTTGGACAAGACCGGAGGGGTCTCCTAGTTCCAAAGGA
+
FFFFFF:FFFFFFFFFF,F:FFFFFFFFFFFFFFFFFFFFFFFFFFFFFFFFFFFFFFFFFFFF:FFF
FFFFFFFFFFFFFFFFFFFFFFFF:FFFFFFFFFFFFFFFFFFFFFFFFFFFFFFFFFFFFFFFFFF:
@A00155:342:HHGFNDSXY:1:2616:30228:29356 1:N:0:GAACCTAG+TCCGCATA
GAGAGCGACTTCTCTCGGGCCCACAGCCTTACAGCTGTGGAGCACGGTATCCTCTGCCGAAACAGAGG
TTGGACAAGACCGGAGGGGTCTCCTAGTTCCAAAGGAGATGTACTCCGGGCTTGTTCACGACCTACC
+
FFFFFFFFF:FFFFFFFFFFFFFFFFFFFFFFFFFFFFFFFFFFFFFFFFFFFFFFFFFFF:FFFFFFF
FFFFFFFFFFFFFFFFFFFFFFFFFF,FFFFFFFFFFFFFFFFFFFFFFFFFFFFFFFFFFFFFFFFF
@A00155:342:HHGFNDSXY:1:2616:14344:36479 1:N:0:GAACCTAG+TCCGCATA
GGGCGAGAGAGAGCGACTTCTCTCGGGCCCACAGCCTTACAGCTGTGGAGCACGGTATCCTCTGCCGA
AACAGAGGTTGGACAAGACCGGAGGGGTCTCCTAGTTCCAAAGGAGATGTACTCCGGGCTTGTTTACG
+
FFFFFFFFFFFFFFFFFFFFFFFFFFFFFFFFFFFFFFFFFFFFFFFFFFFFFFFFFFFFFFFFFFFF
FFFFFFFFFFFFFFFFFFFFFFFFFFFFFFFFFFFFFFFFFFFFFFFFFFFFFFFFFFFF:FFFFFFF
@A00155:342:HHGFNDSXY:1:2617:2962:4586 1:N:0:GAACCTAG+TCCGCATA
AGTACATCTCCTTTGGAACTAGGAGACCCCTCCGGTCTTGTCCAACCTCTGTTTCGGCAGAGGATACC
GTGCTCCACAGCTGTAAGGCTGTGGGCCCGAGAGAAGTCGCTCTCTCTCGCCCTCCACATCCCTCTCG
+
FFFFFFFFFFFFFFFFFFFFFFFFFFFFFFFFFFFFFFFF:FF,FFFFFFFFFFFFFFFFFFFFFFFFF
FFFFFFFFFFF:FFF:FFFFFFFFF,FFFFFFFFFFFFFFFFFFFFFFFFF:FFFFFFF:FFFFFF:FF
@A00155:342:HHGFNDSXY:1:2617:28465:21699 1:N:0:GAACCTAG+TCCGCATA
GTCCACTTAAGGACTCACCTCTGGCCTATGGTCATCTCAATGCCGAGAGGGATGTGGAGGGCGAGAGA
GAGCGACTTCTCTCGGGCCCACAGCCTTACAGCTGTGGAGCACGGTATCCTCTGCCGAAACAGAGGTT
+
FFFFFFFFFFFFFFFFFFFFFFFFFFFFFFFFFFFFFFFFFFFFFFFFFFFFFFFFFFFFF:FFFFFFF
FFFFFFFFFFFFF:FFFFFFFFFFFFFFFFFFFFFFFFFFFFFFFFFFFFFFFFFF:FFFFFFFFFFFF
@A00155:342:HHGFNDSXY:1:2617:5828:33849 1:N:0:GAACCTAG+TCCGCATA
GTATCCTCTGCCGAAACAGAGGTTGGACAAGACCGGAGGGGTCTCCTAGTTCCAAAGGAGATGTACTC
CGGGCTTGTTCACGACCTACCGTGTAAGTCGTAGTCTAGTAGGCTACCTGACGAGTCCTTTTTAGGAC
+
FF:FFFFFFFFFFFFFFFFFFFFFFFFFFFFFFFFFFFFFFFFFFFFFFFFFFFFFFFFFF:FFFFFFF
FFFFFFFFFFFFFFFF:FFFFFFFFFFFFFFFFFFFFFFFFFFFFFFFFF:FFFFFFFFFFF:FFFFF:FF
@A00155:342:HHGFNDSXY:1:2618:5113:26036 1:N:0:GAACCTAG+TCCGCATA
CTTCTCTCGGGCCCACAGCCTTACAGCTGTGGAGCACGGTATCCTCTGCCGAAACAGAGGTTGGACAA
GACCGGAGGGGTCTCCTGGTTCCAAAGGAGATGTACTCCGGGCTTGTTCACGACCTACCGTGTAAGTC
+
FFFFFFFFFFFFFFFFFFFFFFF:FFFFFFFFFFFFFFFFFFFFFFFFFFFFFFFFFFFFFFFFFFFF
F:FFFFFFFFFFFFFFFFFFFFFFFFF:FFFFFFFFF:FFFFFFFFFFFFFFFFFFFFFFFFFFFFFFF
@A00155:342:HHGFNDSXY:1:2618:3965:27492 1:N:0:GAACCTAG+TCCGCATA
TCTCTCGGGCCCACAGCCTTACAGCTGTGGAGCACGGTATCCTCTGCCGAAACAGAGGTTGGACAAGA
CCGGAGGGGTCTCCTAGTTCCAAAGGAGATGTACTCCGGGCTTGTTCACGACCTACCGTGTAAGTCGT
+
FFFFFFFFFFFFFFFFFFFFFFFFFFFFFFF:FFFFFFFFFFFFFFFFFFFFFFFFFFFFFFFFFFFF
FFFFFFFFFFFFFFFFFFFFFFFFFFFFFFFFFFFFFFFFFFFFFFFFFFFFFFFFFFFFFFFFFFFF
@A00155:342:HHGFNDSXY:1:2619:18123:10739 1:N:0:GAACCTAG+TCCGCATA
ATCCTCTGCCGAAACAGAGGTTGGACAAGACCGGAGGGGTCTCCTTGTTCCAAAGGAGATGTACTCCG
```

```
GGCTTGTTCACGACCTACCGTGTAAGTCGTAGTCTAGTAGGCTACCTGACGAGTCCTTTTTAGGACG
+
FFFFF:FFFFFFFF:FF:F:FFFFF:FFFFFFFFFF:FFFFFFFFFF:FFFFF,FFFFFFF:FFFFFF
FFF:FFFFFFFFFFFFFFFFF:F:,:FFFFFF,FFFFFFF,FFFFF:FFFFFFFF::,FFF::FFFF
@A00155:342:HHGFNDSXY:1:2619:22761:18865 1:N:0:GAACCTAG+TCCGCATA
ATGCCGAGAGGGATGTGGAGGGCGAGAGAGAGCGACTTCTCTCGGGCCCACAGCCTTACAGCTGTGGA
GCACGGTATCCTCTGCCGAAACAGAGGTTGGACAAGACCGGAGGGGTCTCCTAGTTCCAAAGGAGATG
+
FFFFFFFFFFFFFFFFFFFFFFFFFFFFFFFFFFFFFFFFFFFFFFFFFFFFFFFFFFFFFFFFFFFF
FFFFFFFFFFFFFFFFFFFF:FFFFFFFFFFFFFFFFFFFFFFFFFFFFFFFFFFFFFFFFFFFFFF
@A00155:342:HHGFNDSXY:1:2619:27362:29997 1:N:0:GAACCTAG+TCCGCATA
AGAGAGAGCGACTTCTCTCGGGCCCACAGCCTTACAGCTGTGGAGCACGGTATCCTCTGCCGAAACAG
AGGTTGGACAAGACCGGAGGGGTCTCCTGGTTCCAAAGGAGATGTACTCCGGGCTTGTTCACG
+
FFFFFFFFF:FFFFFFFFFFFFFFFFFFFFFFFFFFFF:FFFFFFFFFFFFFFFFFFFFFF:FFFFF:F
FF,FFFFFFFFF:,FFFFFFFFFFFFFFFFFFFFFFFFFFFFFFFFFFFFF:FFFFFFFFFFF
@A00155:342:HHGFNDSXY:1:2619:10782:30780 1:N:0:GAACCTAG+TCCGCATA
CTTCTCTCGGGCCCACAGCCTTACAGCTGTGGAGCACGGTATCCTCTGCCGAAACAGAGGTTGGACAA
GACCGGAGGGGTCTCCTGGTTCCAAAGGAGATGTACTCCGGGCTTGTTCACGACCTACCGTGTAAGTC
+
FFFFFFFFFFFFFFFFFFFFFFFFFFFFFFFFFFFFFFFFFFFFFFFFFFFFFFFFFFFF:FFFFFFF
FFFFFFFFFFFFFFFFFFFFFFFFF:FFFFFFFFFFFFFFFFFFFFFFFFFFFFFFFFFF,FF:FFFF
@A00155:342:HHGFNDSXY:1:2619:10520:33051 1:N:0:GAACCTAG+TCCGCATA
CTTCTCTCGGGCCCACAGCCTTACAGCTGTGGAGCACGGTATCCTCTGCCGAAACAGAGGTTGGACAA
GACCGGAGGGGTCTCCTGGTTCCAAAGGAGATGTACTCCGGGCTTGTTCACGACCTACCGTGTAAGTC
+
FFFFFFFFFFFFFFFFFFFFFFFFFFFFFFFFFFFFFFFFFFFFFFFFFFFFFFFFFFFFFFFFFFFF
FFFFFFFFFFFFFFFFFFFFFFFFFFFFFFFFFFFFFFFFFFFFFFFFFFFFFFFFFFFFFFFFFFFF
@A00155:342:HHGFNDSXY:1:2619:29053:34366 1:N:0:GAACCTAG+TCCGCATA
CTCAATGCCGAGAGGGATGTGGAGGGCGAGAGAGAGCGACTTCTCTCGGGCCCACAGCCTTACAGCTG
TGGAGCACGGTATCCTCTGCCGAAACAGAGGTTGGACAAGACCGGAGGGGTCTCCTAGTTCCAAAGGA
+
FFFFFFFFFFFFFFFFFFFFFFFFFFFFFFFFFFFFFFFFFFFFFFFFFFFFFFFFFFFFFFFFFFFF
FFFFFFFFFFFFFFFFFFFFFFFFFFFFFFFFFFFFFFFFF:F:FFFFFFFFFFFFFFFFFFFFFFFF
@A00155:342:HHGFNDSXY:1:2621:32452:12320 1:N:0:GAACCTAG+TCCGCATA
AGATCAGCTGTGTCCACTTAAGGACTCACCTCTGGCCTATGGTCATCTCAATGCCGAGAGGGATGTGA
AGGGCGAGAGAGAGCGACTTCTCTCGGGCCCACAGCCTTACAGCTGTGGAGCACGGTATCCTCTGCCG
+
FFF:F:FFFFFFFFFFF:FFFFFFFFFFFF:FFFFFF,,FFFFF,FFFFFFFFFFFFFFFFF,,FFFF
:FFFFF:FFFFFFFFF,FF:FFF:FFFFFFFFF:FFF:FFFFFFFFFFFFFFFFFFF,:FF:FFFFFF
@A00155:342:HHGFNDSXY:1:2621:10131:28870 1:N:0:GAACCTAG+TCCGCATA
ATCTCAATGCCGAGAGGGATGTGGAGGGCGAGAGAGAGCGACTTCTCTCGGGCCCACAGCCTTACAGC
TGTGGAGCACGGTATCCTCTGCCGAAACAGAGGTTGGACAAGACCGGAGGGGTCTCCTAGTTCCAAAG
+
FFFFFFFFFFFFFFFFFFFFFFFFFFFFFFFFFFFFFFFFFFFFFFFFFFFFFFFFFFFFFFFFFFFF
FFFFFFFFFFFFFFFFFFFFFFFFFFFFFFFFFFFFFFFFFFFFFFFFFFFFFFFFFFFFFFFFFFFF
@A00155:342:HHGFNDSXY:1:2621:26259:36229 1:N:0:GAACCTAG+TCCGCATA
GGGATGTGGAGGGCGAGAGAGAGCGACTTCTCTCGGGCCCACAGCCTTACAGCTGTGGAGCACGGTAT
CCTCTGCCGAAACAGAGGTTGGACAAGACCGGAGGGGTCTCCTAGTTCCAAAGGAGATGTACTCCGGG
+
FFFFFFFFF:FFFFFFFFFFFFFFFFFFFFFFFFFFFFFFFFFFFFFFFFFFFFFFFFFFFFFFFFFF
FFFFFFFFFFFFFFFFFFFFFFFFFFFFFFFFFFFFFFFFFFFFFFFFFFFFFFFFFFFFFFFFFFFF
@A00155:342:HHGFNDSXY:1:2622:7735:1266 1:N:0:GAACCTAG+TCCGCATA
TGCCGAGAGGGATGTGGAGGGCGAGAGAGAGCGACTTCTCTCGGGCCCACAGCCTTACAGATGTGGAG
```

```
CACGGTATCCTCTGCCGAAACAGAGGTTGGACAAGACCGGAGGGGTCTCCTAGTTCCAAAGGAGATGT
+
FFFFFFFFFFFFFFFFFFFFFFFFFFFFFFFFFFFFFFFFFFFFFFFFFFFFFFFFFFFFFFFFFFFFF
FFFFFFFFFFFFFFFFFFFFFFFFFFFFFFFFFFFFFFFFFFFFFFFFFFFFFFFFFF:FFFFFF,FF
@A00155:342:HHGFNDSXY:1:2622:29839:6355 1:N:0:GAACCTAG+TCCGCATA
TTACAGCTGTGGAGCACGGTATCCTCTGCCGAAACAGAGGTTGGACAAGACCGGAGGGGTCTCCTAGT
TCCAAAGGAGATGTACTCCGGGCTAGTTCACGACCTACCGTGTAAGTCGTAGTCTAGTAGGCTACCTG
+
FFFFFFFFFFFFFFFFFFFFFFFFFFFFFFFFFFFFFFFFFFFFFFFFFFFFFFFFFFFFFFFFFF:FF
FFFFFFFFFFFFFFFFFFFFFFFFFF:FFFFFFFFFFFFFFFFF,FFFFFFFFFFFFFF:FFF:FFF:
@A00155:342:HHGFNDSXY:1:2622:9091:6872 1:N:0:GAACCTAG+TCCGCATA
GCGAGAGAGAGCGACTTCTCTCGGGCCCACAGCCTTACAGCTGTGGAGCACGGTATCCTCTGCCGAAA
CAGAGGTTGGACAAGACCGGAGGGGTCTCCTGGTTCCAAAGGAGATGTACTCCGGGCTTGTTCACGAC
+
FFFFFFFFFFFFFFFFFFFFFFFFFFFFFFFFFFFFFFFFFFFFFFFFFFFF:FFFFFFFFFF:,F
FFFFFFFFFFFF::FFFFFFFFFFFFFFFFFFFFFFFFFFFFFFFFFFFFFFFFFFFFFFFFFFF:FF
@A00155:342:HHGFNDSXY:1:2622:23357:8155 1:N:0:GAACCTAG+TCCGCATA
TTAGATCAGCTGTGTCCACTTAAGGACTCACCTCTGGCCTATGGTCATCTCAATGCCGAGAGGGATGT
GGAGGGCGAGAGAGAGCGACTTCTCTCGGGCCCACAGCCTTACAGCTGTGGAGCACGGTATCCTCTGC
+
FFFFFFFFFFFFFFFFFFFFFFFFFFFFFFFFFFFFFFFFFFFFFFFFFFFFFFFFFFFFFFFFFFFFF
FFFFFFFFFFFFFFFFFFFFFFFFFFFFFFFFFFFFFFFFFFFFFFFFFFFFFFFFFFFFFFFFFFFFF
@A00155:342:HHGFNDSXY:1:2622:11080:12226 1:N:0:GAACCTAG+TCCGCATA
GCGACTTCTCTCGGGCCCACAGCCTTACAGCTGTGGAGCACGGTATCCTCTGCCGAAACAGAGGTTGG
ACAAGACCGGAGGGGTCTCCAAGTTCCAAAGGAGATGTACTCCGGGCTTGTTCACGACCTACCGTGTA
+
FFFFFFFFFFFFFFFFFFFFFFFFFFFFFFFFFFFFFFFFFFFFFFFFFFFFFFFFFFFFFFFFFFFFF
FFFFFFFFFFFFFFFFFFFFFFFFFFFFFFFFFFFFFFFFFFFFFFFFFFFFFFFFFFFFFFFFFFFFF
@A00155:342:HHGFNDSXY:1:2622:19244:20729 1:N:0:GAACCTAG+TCCGCATA
GAGAGAGAGCGACTTCTCTCGGGCCCACAGCCTTACAGCTGTGGAGCACGGTATCCTCTGCCGAAACA
GAGGTTGGACAAGACCGGAGGGGTCTCCTAGTTCCAAAGGAGATGTACTCCGGGCTTGTTCACGACCT
+
FFFFFFFFFFFFFFFFFFFFFFFFFFFFFFFFFFFFFFFFFFFFFFFFFFFFFFFFFFFFFFFFFFFFF
FFFFFFFFFFFFFFFFFFFFFFFFFFFFFFFFFFFFFFFFFFFFFFFFFFFFFFFFFFFFFFFFFFFFF
@A00155:342:HHGFNDSXY:1:2622:30192:26725 1:N:0:GAACCTAG+TCCGCATA
GGATGTAGAGGGCGAGAGAGAGCGACTTCTCTCGGGCCCACAGCCTTACAGCTGTGGAGCACGGTATC
CTCTGCCGAAACAGAGGTTGGACAAGACCGGAGGGGTCTCCTAGTTCCAAAGGAGATGTACTCC
+
FFF:FFFFFFFFFFFFFFFFFFFFFFFFFFFFFFFFFFFFFFFFFFFFFFFFFFFFFFFFFFFFFFFFF
FFFFFFFFFFFFFFFFFFFFFFFFFFFFFFFFFFFFFFFFFFFFFFFFFFFFFFFFFFFFFFFFF
@A00155:342:HHGFNDSXY:1:2622:19660:37043 1:N:0:GAACCTAG+TCCGCATA
TCAATGCCGAGAGGGATGTGGAGGGCGAGAGAGAGCGACTTCTCTCGGGCCCACAGCCTTACAGCTGT
GGAGCACGGTATCCTCTGCCGAAACAGAGGTTGGACAAGACCGGAGGGGTCTCCTAGTTCCAAAGGAG
+
FFFFFFFFF:F:FFFFFFFFFFFFFFFFFFFFFFFFFFFFFFFFFFFFFFFFFFFFFFFFFFFFFFFFF
FFFFFFFFFFFFFFFFFFFFFFFFFFFFFFFFFFFFFFF:FFFFFFFFFFFFFFFFFFFFFFFFFFFFF
@A00155:342:HHGFNDSXY:1:2623:31015:19163 1:N:0:GAACCTAG+TCCGCATA
GTAGAGGGCGAGAGAGAGCGACTTCTCTCGGGCCCACAGCCTTACAGCTGTGGAGCACGGTATCCTCT
GCCGAAACAGAGGTTGGACAAGACCGGAGGGGTCTCCTAGTTCCAAAGGAGATGTACTCCGGGC
+
FFFFFFFFFFFFFFFFFFFFFFFFFFFFFFFFFFFFFFFFFFFFFFFFFFFFFFFFFFFFFFFFFFFFF
FFFFFFFFFFFFFFFFFFFFFFFFFFFFFFFFFFFFFFFFFFFFFFFFFFFFFFFFFFFFFFFFF
@A00155:342:HHGFNDSXY:1:2623:10484:22686 1:N:0:GAACCTAG+TCCGCATA
TTACAGCTGTGGAGCACGGTATCCTCTGCCGAAACAGAGGTTGGACAAGACCGGAGGGGTCTCCTAGT
```

TCCAAAGGAGATGTACTCCGGGCTTGTTCACGACCTACCGTGTAAGTCGTAGTCTAGTAGGCTACCTG
+
FFFFFFFFFFFFFFFFFFFFFFFFFFFFFFFFFFFFFFFFFFFFFFFFFFFFFFFFFFFFFFFFFFFF
FFFFFFFFFFFFFFFFFFFFFFFFFFFFFFFFFFFFFFFFFFFFFFFFFFFFFFFFFFFFFFFFFFFF
@A00155:342:HHGFNDSXY:1:2623:31584:23500 1:N:0:GAACCTAG+TCCGCATA
GAGCGACTTCTCTCGGGCCCACAGCCTTACAGCTGTGGAGCACGGTATCCTCTGCCGAAACAGAGGTT
GGACAAGACCGGAGGGGTCTCCTAGTTCCAAAGGAGATGTACTCCGGGCTTGTTCACGACCTACCGTG
+
FFFFFFFFFFFFFFFFFFFFFFFFFFFFFFFFFFFFFFFFFFFFFFFFFFFFFFFFFFFFFFFFFFFF
FFFFFFFFFFFFFFFFFFFFFFFFFFFFFFFFFFFFFFFFFFFFFFFFFFFFFFFFFFFFFFFFFFFF
@A00155:342:HHGFNDSXY:1:2623:11424:24251 1:N:0:GAACCTAG+TCCGCATA
ATCTCAATGCCGAGAGGGATGTGGAGGGCGAGAGAGAGCGACTTCTCTCGGGCCCACAGCCTTACAGC
TGTGGAGCACGGTATCCTCTGCCGAAACAGAGGTTGGACAAGACCGGAGGGGTCTCCTGGTTCCAAAG
+
FFFFFFFFFFFFFFFFFFF:FFFFFFFFFFFFFFFFFFFFFFFFFFFFFFFFFFFFFFFFFFFFFFFF
FFFFFFFFFFFFFFFFFFFFFFFFFFFFFFFFFFFFFFFFFFFFFFFFFFFFFFFFFFFFFFFFFFFF
@A00155:342:HHGFNDSXY:1:2623:28040:33520 1:N:0:GAACCTAG+TCCGCATA
GCGAGAGAGAGCGACTTCTCTCGGGCCCACAGCCTTACAGCTGTGGAGCACGGTATCCTCTGCCGAAA
CAGAGGTTGGACAAGACCGGAGGGGTCTCCTGGTTCCAAAGGAGATGTACTCCGGGCTTGTTC
+
FFFFFFFFFFFFFFFFFFFFFFFFFFFFFFFFFFFFFFFFFFFFF:FFFFFFFFFFFFFFFFFFFFFF
FFFFFFFFFFFFFFFFFFFFFFFFFFFFFFFFFFFFFFFFFFFFFFFFFFFFFFFFFFFFFFF
@A00155:342:HHGFNDSXY:1:2624:21341:8453 1:N:0:GAACCTAG+TCCGCATA
TCCTCTGCCGAAACAGAGGTTGGACAAGACCGGAGGGGTCTCCTAGTTCCAAAGGAGATGTACTCCGG
GCTTGTTCACGACCTACCGTGTAAGTCGTAGTCTAGTAGGCTACCTGACGAGTCCTTTTTAGGACGAA
+
FFFFFFFFFFFFFFFFFFFFFFFFFFFFFFFFFFFFFFFFFFFFFFFFFFFFFFFFFFFFFFFFFFFF
FFFFFFFFFFFFFFFFFFFFFFFFFFFFFFFFF:FF:FFFFFFFFFFFFFFFFFFFFFFFFFFFFFFF
@A00155:342:HHGFNDSXY:1:2624:2293:13354 1:N:0:GAACCTAG+TCCGCATA
TCCTCTGCCGAAACAGAGGTTGGACAAGACCGGAGGGGTCTCCTAGTTCCAAAGGAGATGTACTCCGG
GCTTGTTCACGACCTACCGTGTAAGTCGTAGTCTAGTAGGCTACCTGACGAGTCCTTTTTAGGACGAA
+
FFFFFFFFFFFFF:FF:FFFFFFFFFFFF:FFFFFFFFFFFFFFFFFFFFFFFFFFFFFFFFFFFFFF
F:FFFFFFFFFFFFFF:FFFFFFFFFFFFFFF:FFFFFFFFFFF:FFFFFFFFF,FFFFFFFF:FFFF
@A00155:342:HHGFNDSXY:1:2624:26883:34460 1:N:0:GAACCTAG+TCCGCATA
CATCTCAATGCCGAGAGGGATGTAGAGGGCGAGAGAGAGCGACTTCTCTCGGGCCCACAGCCTTACAG
CTGTGGAGCACGGTATCCTCTGCCGAAACAGAGGTTGGACAAGACCGGAGGGGTCTCCTAGTTCCAAA
+
FFFFFFFFFFFFFFFFFFFFFFFFFFFFFFFFFFFF,FFFFFFFFFFFFFF,F,FFFFFFFFFFF:F:
FFFFFFFF:FFF,FFFF:FFFFF:FFFFFFFFFFFFFFFFFFFFFFFFFF:FF:FFFFFFFFF:FFFF
@A00155:342:HHGFNDSXY:1:2625:17508:4038 1:N:0:GAACCTAG+TCCGCATA
CGACTTCTCTCGGGCCCACAGCCTTACAGCTGTGGAGCACGGTATCCTCTGCCGAAACAGAGGTTGGA
CAAGACCGGAGGGGTCTCCTAGTTCCAAAGGAGATGTACTCCGGGCTTGTTCACGACCTACCGTGTAA
+
F:F,FFFFFFFF:FFFFFFFFFF,F:FFFFFF:FFFFF:FFF:FFFFFFFFFFFF::FFF,F:FFFF,
FFFF:FFFFFFFFFFFFF:FFFFFFFF:FFF:FF,FFFFF:FFFF:F,FFF,FFF,FF:F:FF,FFFF
@A00155:342:HHGFNDSXY:1:2625:13630:20306 1:N:0:GAACCTAG+TCCGCATA
GGCCTATGGTCATCTCAATGCCGAGAGGGATGTGGAGGGCGAGAGAGAGCGACTTCTCTCGGGCCCAC
AGCCTTACAGCTGTGGAGCACGGTATCCTCTGCCGAAACAGAGGTTGGACAAGACCGGAGGGGTCTCC
+
FFFFFFFFFFFFFFFFFFFFFFFFFFFFFFFFFFFFFFFFFFFFFFFFFFFFFFFFFFFFFFFFFFFF
FFFFFFFFFFFFFFFFFFFFFFFFFFFFFFFFFFFFFFFFFFFFFFFFFFFFFFFFFFFFFFFFFFFF
@A00155:342:HHGFNDSXY:1:2625:27624:30044 1:N:0:GAACCTAG+TCCGCATA
TGCCCCACCACACCGCCTTAGATCAGCTGTGTCCACTTAAGGACTCACCTCTGGCCTATGGTCATCTC

AATGCCGAGAGGGATGTGGAGGGCGAGAGAGAGCGACTTCTCTCGGGCCCACAGCCTTACAGCTGTGG
+
F:FFFFFFFFFFF:FFF:FFFFFFFFFFFFFFFFF:FFFFFFFFFF:FFFFFFFFFFFFF:FFF::FF
FFFFFFF:FFFFFF:FFFFFFFFFFF:FFFFFFFFFFFFFFFFF:FFFFFFFFFFFFFFFFFFFF:FF
@A00155:342:HHGFNDSXY:1:2625:1344:35164 1:N:0:GAACCTAG+TCCGCATA
CTTACAGCTGTGGAGCACGGTATCCTCTGCCGAAACAGAGGTTGGACAAGACCGGAGGGGTCTCCTAG
TTCCAAAGGAGATGTACTCCGGGCTTGTTCACGACCTACCGTGTAAGTCGTAGTCTAGTAGGCTACCT
+
FFF:FFFFFFFFF:FFFFFFFFFF,FFFFFFF:FF::FFFFFFFF:FFFF,FFFFFF:FFFFFFFF,F
FFFFF::F::FFFFF,FF::FFF:,FFFFFFFF,,FF:FFFFFF:F,FFFF:FFFFF,FFFF,FF:,F
@A00155:342:HHGFNDSXY:1:2626:1036:4977 1:N:0:GAACCTAG+TCCGCATA
CTTACAGCTGTGGAGCACGGTATCCTCTGCCGAAACAGAGGTTGGACAAGACCGGAGGGGTCTCCTAG
TTCCAAAGGAGATGTACTCCGGGCTTGTTCACGACCTACCGTGTAAGTCGTAGTCTAGTAGGCTACCT
+
FFFFFFF:FFFFFFFFFFFFFFFFFFFFFFFFF:FFFFFFFFFFFFFFFFF:FFFFFFFFFFFFFFFFF
FFFFF::FFFFFFFFF:FF:FFFFFFFFFF,F:FFFFFFF:FFFFFFFFFFFFFFF:FFF,FFFF,FF
@A00155:342:HHGFNDSXY:1:2626:20166:11397 1:N:0:GAACCTAG+TCCGCATA
AATGCCGAGAGGGATGTAGAGGGCGAGAGAGAGCGACTTCTCTCGGGCCCACAGCCTTACAGCTGTGG
AGCACGGTATCCTCTGCCGAAACAGAGGTTGGACAAGACCGGAGGGGTCTCCTAGTTCCAAAGGAGAT
+
FFFFFFFFFFFFFFFFFFFFFFFFFFFFFFFFFFFFFFFFFFFFFFFFFFFFFFFF:FFFFFFFFFF
FFFFFFFFFFFFFFFFFFFFFFFFFFFFFFFFFFFFFFFFFFFFFFFFFFFFFFFFFFFFFFFFFFFF
@A00155:342:HHGFNDSXY:1:2626:3360:16579 1:N:0:GAACCTAG+TCCGCATA
GACTTCTCTCGGGCCCACAGCCTTACAGCTGTGGAGCACGGTATCCTCTGCCGAAACAGAGGTTGGAC
AAGACCGGAGGGGTCTCCTAGTTCCAAAGGAGATGTACTCCGGGCTTGTTCACGACCTACCGTGTAAG
+
FFFFFFFFFFFFFFFFFFFFFFFFFFFFFFFF:FFFFFFFFFFFFFFFFFFFFFFFFFFFFFFFFFFF
FF:FFFFFFFFFFFFFFFFFFFFFF:FFFFFFFFFFFFFFF::FFFFFFFFFFFFFFFFFFFF:FFFF:
@A00155:342:HHGFNDSXY:1:2626:13982:18724 1:N:0:GAACCTAG+TCCGCATA
AGAGGGATGTGGAGGGCGAGAGAGAGCGACTTCTCTCGGGCCCACAGCCTTACAGCTGTGGAGCACGG
TATCCTCTGCCGAAACAGAGGTTGGACAAGACCGGAGGGGTCTCCTAGTTCCAAAGGAGATGTACTCC
+
FFFFFFFFFFFFFFFFFFFFFFFFFFFFFFFFFFFFFFFFFFFFFFFFFFFFFFFFFFFFFFFFFFFF
FFFFFFFFFFFFFFFFFFFFFFFFFFFFFFFFFFFFFFFFFFFFFFFFFFFFFFFFFFFFFFFFFFFF
@A00155:342:HHGFNDSXY:1:2626:14036:18756 1:N:0:GAACCTAG+TCCGCATA
AGAGGGATGTGGAGGGCGAGAGAGAGCGACTTCTCTCGGGCCCACAGCCTTACAGCTGTGGAGCACGG
TATCCTCTGCCGAAACAGAGGTTGGACAAGACCGGAGGGGTCTCCTAGTTCCAAAGGAGATGTACTCC
+
FFFFFFFFFFFFFFFFFFFFFFFFFFFFFFFFFFFFFFFFFFFFFFFFFFFFFFFFFFFFFFFFFFFF
FFFFFFFFFFFFFFFFFFFFFFFFFFFFFFFFFFFFFFFFFFFFFFFFFFFFFFFFFFFFFFFFFFFF
@A00155:342:HHGFNDSXY:1:2626:32271:24502 1:N:0:GAACCTAG+TCCGCATA
GTATCCTCTGCCGAAACAGAGGTTGGACAAGACCGGAGGGGTCTCCTAGTTCCAAAGGAGATGTACTC
CGGGCTTGTTCACGACCTACCGTGTAAGTCGTAGTCTAGTAGGCTACCTGACGAGTCCTTTTTAGGAC
+
FFFFFFFFFFFFFFFFFFFFFFFFFFFFFFFFFFFFFFFFFFFFFFFFFFFFFFFFFFFFFFFFFFFF
FFFFFFFFFFFFFFFFFFFFFFFFFFF,FFFFFFFFFFFFFFFFFF:FFFFFFFFFFFFFFFFFFFFFF
@A00155:342:HHGFNDSXY:1:2626:29532:28119 1:N:0:GAACCTAG+TCCGCATA
TGTGGAGGGCGAGAGAGAGCGACTTCTCTCGGGCCCACAGCCTTACAGCTGTGGAGCACGGTATCCTC
TGCCGAAACAGAGGTTGGACAAGACCGGAGGGGTCTCCTAGTTCCAAAGGAGATGTACTCCGGG
+
FFFFFFFFFFFFFFFFFFFFFFFFFFFFFFFFFFFFFFFFFFFFF:FFFFFFFFFFFFFFFFFFFFFF
FFFFFFFFFFFFFFFFFFFFFFFFFFFFFFFFFFFFFFFFFFFFFFFFFFFF:FFFFF,FFFFFFF
@A00155:342:HHGFNDSXY:1:2627:11993:5196 1:N:0:GAACCTAG+TCCGCATA
GAGAGGGATGTGGAGGGCGAGAGAGAGCGACTTCTCTCGGGCCCACAGCCTTACAGCTGTGGAGCACG

```
GTATCCTCTGCCGAAACAGAGGTTGGACAAGACCGGAGGGGTCTCCTAGTTCCAAAGGAGATGTACTC
+
FFFFFFFFFFFFFFFFFF:FFFFFFFFFFFFFFFFFFFFFFFFFFFFFFFFFFFFFFFFFFFFFFFFF
FFFFF:FFFFFFFFFFF:FFFFFFFFFFFFFFFFFFFFFFFFFFFFFFFFFFFFFFFFFFFFFFFFFF
@A00155:342:HHGFNDSXY:1:2627:31186:31328 1:N:0:GAACCTAG+TCCGCATA
AGGTCATCTCAATGCCGAGAGGGATGTGGAGGGCGAGAGAGAGCGACTTCTCTCGGGCCCACAGCCTT
ACAGCTGTGGAGCACGGTATCCTCTGCCGAAACAGAGGTTGGACAAGACCGGAGGGGTCTCC
+
FFFFFFFFFF:FFFFFFFFFFFFFFF:FFFFFFFFFFFFFFFFFFFFFFFFFFFFFFFFFFFFFFFFF
FFFFFFFFFFFFFFFFFF:FFFFFFFFFFFFFFFFFFFFFFFFF:FFFFFFFFFFFFFFFFF
@A00155:342:HHGFNDSXY:1:2627:28348:36370 1:N:0:GAACCTAG+TCCGCATA
ACTTCTCTCGGGCCCACAGCCTTACAGCTGTGGAGCACGGTATCCTCTGCCGAAACAGAGGTTGGACA
AGACCGGAGGGGTCTCCTAGTTCCAAAGGAGATGTACTCCGGGCTTTTTCACGACCTACCGTGTAAG
+
FFFFFFFFFFFFFFFFFFFFF:FFFFFF:FFFFFFFFFFFFFFFFFFFFFFFFFFFFFFFFFFFFFFF
FFFFFFFFFFFFFFFFFFFFFFFFFFFFFFFFFFF,FFFFFFFFFFFFFFFFFFFFFFFFFFFF:FF
@A00155:342:HHGFNDSXY:1:2628:21287:6887 1:N:0:GAACCTAG+TCCGCATA
AGATCAGCTGTGTCCACTTAAGGACTCACCTCTGGCCTATGGTCTTCTCAATGCCGAGAGGGATGTGG
AGGGCGAGAGAGAGCGACTTCTCTCGGGCCCACAGCCTTACAGCTGTGGAGCACGGTATCCTCTGCCG
+
FFFFFFFFFFFFFFFFFFFFFFFFFFFFFFFFFFFFFFFFFFFFFFFFFFFFFFFFFFFFFFFFFFFF
FFFFFFFFFFFFFFFFFFFFFFFFFFFFFFFFFFFFFFFFFFFFFFFFFFFF,FFFF,FFFFFFFFFF
@A00155:342:HHGFNDSXY:1:2628:8458:33677 1:N:0:GAACCTAG+TCCGCATA
AGGGATGTGGAGGGCGAGAGAGAGCGACTTCTCTCGGGCCCACAGCCTTACAGCTGTGGAGCACGGTA
TCCTCTGCCGAAACAGAGGTTGGACAAGACCGGAGGGGTCTCCTGGTTCCAAAGGAGATGTACT
+
FFFFFFFFFFFFFFFFFFFFFFFFFFFFFFFFFFFFFFFFFFFFFFFFFFFFFFFFFFFFFFFFFFFF
FFFFFFFFFFFFFFFFFFFFFFFFFFFFFFFFFFFFFFFFFFFFFFFFFFF:FFFFFFFFFFFF
@A00155:342:HHGFNDSXY:1:2628:1524:35759 1:N:0:GAACCTAG+TCCGCATA
CTTCTCTCGGGCCCACAGCCTTACAGCTGTGGAGCACGGTATCCTCTGCCGAAACAGAGGTTGGACAA
GACCGGAGGGGTCTCCTAGTTCCAAAGGAGATGTACTCCGGGCTTGTTCACGACCTACCGTGTAAGTC
+
FFFFFFFFFFFFFFFFFFFFFFFFFFFFFFFFFFFFF:FFFFFFFFFFFFFFFFFFFFFFFFFFFFFF
FFFFFFFFFFFFFFFFFFFFFFF:FFFFFF:FFFFFFFFFFFFFFFFFFFFFFF,FFFFFFF:FFFFF
@A00155:342:HHGFNDSXY:1:2629:14434:17722 1:N:0:GAACCTAG+TCCGCATA
CTCTATGCCGAGAGGGATGTGGAGGGCGAGAGAGAGCGACTTCTCTCGGGCCCACAGCCTTACAGCTG
TGGAGCACGGTATCCTCTGCCGAAACAGAGGTTGGACAAGACCGGAGGGGTCTCCTAGTTCCAAAGGA
+
FFFFFFFFFFFFFFFF,FFFFFFFFFFFFFFFFFFFFFFFFFFFFFFFFFFFFFFFFFFFFFFFFFFF
FFFFFFFFFFFFFFFFFFFFFFFFFFFFFFFFFFFFFFFFFFFFFFFFFFFFFFFFFFFFFFFFFFFF
@A00155:342:HHGFNDSXY:1:2629:32768:19664 1:N:0:GAACCTAG+TCCGCATA
AGGGATGTGGAGGGCGAGAGAGAGCGACTTCTCTCGGGCCCACAGCCTTACAGCTGTGGAGCACGGTA
TCCTCTGCCGAAACAGAGGTTGGACAAGACCGGAGGGGTCTCCTAGTTCCAAAGGAGATGTACTCCGG
+
FFFFFFFFFFFFFFFFFFFFFFFFFFFFFFFFFFFFFFFFFFFFFFFFFFFFFFFFFFFFFFFFFFFF
FFFFFFFFFFFFF:FFFFFFFFF:FFFF:F:FFFFFFFFF:FFFFFFFFFFF:FFFF:FFF:FFFFFF
@A00155:342:HHGFNDSXY:1:2629:9299:22576 1:N:0:GAACCTAG+TCCGCATA
CTATGGTCATCTCAATGCCGAGAGGGATGTGGAGGGCGAGAGAGAGCGACTTCTCTCGGGCCCACAGC
CTTACAGCTGTGGAGCACGGTATCCTCTGCCGAAACAGAGGTTGGACAAGACCGGAGGGGTCTCCTAG
+
FFFFFFFFFFFFFFFFFFFFFFFFFFFFFFFFFFFFFFFFFFFFFFFFFFFFFFFFFFFFFFFFFFFF
FFFFFFFFFFFFFFFFFFFFFFFFFFFFFFFFFFFFFFFFFFFFFFFFFFFFFFFFFFFFFFFFFFFF
@A00155:342:HHGFNDSXY:1:2629:1398:36855 1:N:0:GAACCTAG+TCCGCATA
ACCATAGGCCAGAGGTGAGTCCTTAAGTGGACACAGCTGATCTAAGGCGGTGTGGTGGGGCATGGGTT
```

```
TGAACCCCCATGACGGTCGGAGTCTAGTAGGCTCCCTGATGAGTCCGTTCCAAGGACGAAACCGTCGT
+
:FFFF,FFFFFF:FFFFFFFFFFFF:FFFFFFFFFFFFFFFFF,:FFFFFFFFFFFFFFFFFFFFFFF
FFF:FFFFFFFFFFFFFFFF:FF:FFFFF:FFFFF:FFFFFFFFF:FFFFF,FFF,:FF,,:::FFFF
@A00155:342:HHGFNDSXY:1:2630:15926:2707 1:N:0:GAACCTAG+TCCGCATA
GCGAGAGAGAGCGACTTCTCTCGGGCCCACAGCCTTACAGCTGTGGAGCACGGTATCCTCTGCCGAAA
CAGAGGTTGGACAAGACCGGAGGGGTCTCCTAGTTCCAAAGGAGATGTACTCCGGGCTTGTTCACGAC
+
FFFFFFFFFFFFFFFFFFFFFFFFFFFFFFFFFFFFFFFFFFFFFFFFFFFFFFFFFFFFFFFFFFFF
FFFFFFFFFFFFFFFFFFFFFFFFFFFFFFFFFFFFFFFFFFFFFFFFFFFFFFFFFFFFFFFFFFFF
@A00155:342:HHGFNDSXY:1:2630:9173:8171 1:N:0:GAACCTAG+TCCGCATA
TGCCGAGAGGGATGTAGAGGGCGAGAGAGAGCGACTTCTCTCGGGCCCACAGCCTTACAGCTGTGGAG
CACGGTATCCTCTGCCGAAACAGAGGTTGGACAAGACCGGAGGGGTCTCCTAGTTCCAAAGGAGATGT
+
FFFFFFFFFFFFFFFFFFFFF:FFFFFFFFFFFFFFFFFFFFFFFFFFFFFFFFFFFFFFFFFFFFFF
FFFFFFFFFFFFFFFFFFFFFFFFFFFFFFFFFFFFFFFFFFFFFFFFFFFFFFFFFFFFFFFFFFFF
@A00155:342:HHGFNDSXY:1:2630:20184:9674 1:N:0:GAACCTAG+TCCGCATA
GTGTCCACTTAAGGACTCACCTCTGGCCTATGGTCATCTCAATGCCGAGAGGGATGTAGAGGGCGAGA
GAGAGCGACTTCTCTCGGGCCCACAGCCTTACAGCTGTGGAGCACGGTATCCTCTGCCGAAACAGAGG
+
FFFFFFFFFFFFFFFFFFFFFFFFFFFFFFFFFFFFFFFFFFFFFFFFFFFFFFFFFFFFFFFFFFFF
FFFFFFFFFFFFFFFFFFFFFFFFFFFFFFFFFFFFFFFFFFFFFFFFFFFFF:FFFFFFFFFFFFFF
@A00155:342:HHGFNDSXY:1:2630:25165:16047 1:N:0:GAACCTAG+TCCGCATA
GGACAAGACCGGAGGGGTCTCCTAGTTCCAAAGGAGATGTACTCCGGGCTTGTTCACGACCTACCGTG
TAAGTCGTAGTCTAGTAGGCTACCTGACGAGTCCTTTTTAGGACGAAACTTACACAACTCAAAGAGTA
+
FFFFFFFFFFFFFFFFFFFFFFFFFFFFFFFFFFFFFFFFFFFFFFFFFFFFFFFFFFFFFFFFFFFF
FFFFFFFFFFFFFFFFFFFFFFFFFFFFFFFFFFFFFFFFFFFFFF:FFFFFFFFFFFFFFFFFFFFF
@A00155:342:HHGFNDSXY:1:2630:25599:16203 1:N:0:GAACCTAG+TCCGCATA
GGACAAGACCGGAGGGGTCTCCTAGTTCCAAAGGAGATGTACTCCGGGCTTGTTCACGACCTACCGTG
TAAGTCGTAGTCTAGTAGGCTACCTGACGAGTCCTTTTTAGGACGAAACTTACACAACTCAAAGAGTA
+
FFFFFFFFFFFFFFFFFFFFFFFFFFFFFFFFFFFFFFFFFFFFFFFFFFFFFFFFFFFFFFFFFFFF
FFFFFFFFFFFFFFFFFFFFFFFFFFFFFFFFFFFFFFFFFFFFFFFFFFFFFFFFFFFFFFFFFFFF
@A00155:342:HHGFNDSXY:1:2630:17680:21371 1:N:0:GAACCTAG+TCCGCATA
CTCAATGCCGAGAGGGATGTGGAGGGCGAGAGAGAGCGACTTCTCTCGGGCCCACAGCCTTACAGCTG
TGGAGCACGGTATCCTCTGCCGAAACAGAGGTTGGACAAGACCGGAGGGGTCTCCTAGTTCCAAAGGA
+
FFFFFFFFFFFFFFFFFFFFFFFFFFFFFFFFFFFFFFFFFFFFFFFFFFFFFFFFFFFFFFFFFFFF
FFFFFFFFFFFFFFFFFFFFFFFFFFFFFFFFFFFF:FFFFFFFFFFFFFFFFFFFFFFFFFF:FFFF
@A00155:342:HHGFNDSXY:1:2630:17445:22028 1:N:0:GAACCTAG+TCCGCATA
CTCAATGCCGAGAGGGATGTGGAGGGCGAGAGAGAGCGACTTCTCTCGGGCCCACAGCCTTACAGCTG
TGGAGCACGGTATCCTCTGCCGAAACAGAGGTTGGACAAGACCGGAGGGGTCTCCTAGTTCCAAAGGA
+
FFFFFFFFFF,FFFFF:FFFFFFFFFFFFFFFFF,FFFFFFFFFFFFFFFFFFFFFFFFFFFFFFFFF
FFFFFFFFFFFFFFFFFFFFFFFFFF:FFFFFFFFFFFFFFFFFFFFFFFFFFFFFFFFFFFFFFFFF
@A00155:342:HHGFNDSXY:1:2630:11541:30718 1:N:0:GAACCTAG+TCCGCATA
CGAGAGAGAGCGACTTCTCTCGGGCCCACAGCCTTACAGCTGTGGAGCACGGTATCCTCTGCCGAAAC
AGAGGTTGGACAAGACCGGAGGGGTCTCCTAGTTCCAAAGGAGATGTACTCCGGGCTTGTTCACGACC
+
FFFFFFFFFFFFFFFFFFFFFFFFFFFFFFFFFFFFFFFFFFFFFFFFFFFFFFFFFFFFFFFFFFFF
FFFFFFFFFFFFFFFFFFFFFFFFFFFFFFFFFFFFFFFFFFFFFFFFFFFFFFFFFFFFFFFFFFFF
@A00155:342:HHGFNDSXY:1:2630:31665:31219 1:N:0:GAACCTAG+TCCGCATA
TGTCCACTTAAGGACTCACCTCTGGCCTATGGTCATCTCAATGCCGAGAGGGATGTGGAGGGCGAGAG
```

```
AGAGCGACTTCTCTCGGGCCCACAGCCTTACAGCTGTGGAGCACGGTATCCTCTGCCGAAACAGAGGT
+
FFFFFFFF:FFFFFFFFFFFFFFFFFFFFFFFFFFFFFFFFFFFFFFFFFF:FFFFFFFFFFFFFF
FFFFFFFFFFFFFFFFFFFFFFFFFFFFFFFFFFFFFFFFFFFFFFFFFFFFFFFFFFFFFFFFFFFF
@A00155:342:HHGFNDSXY:1:2630:11641:31641 1:N:0:GAACCTAG+TCCGCATA
CGAGAGAGAGCGACTTCTCTCGGGCCCACAGCCTTACAGCTGTGGAGCACGGTATCCTCTGCCGAAAC
AGAGGTTGGACAAGACCGGAGGGGTCTCCTAGTTCCAAAGGAGATGTACTCCGGGCTTGTTCACGACC
+
FFFFFFFFFFFFFFFFFFFFFFFFFFFFFFFFFFFFFFFFFFFFFFFFFFFFFFFFFFFFFFFFFFFF
FFFFFFFFFFFFFFFFFFFFFFFFFFFFFFFFFFFFFFFFFFFFFFFFFFFFFFFFFFFFFFFFFFF:
@A00155:342:HHGFNDSXY:1:2630:8196:35634 1:N:0:GAACCTAG+TCCGCATA
CTGGCCTATGGTCATCTCAATGCCGAGAGGGATGTGGAGGGCGAGAGAGAGCGACTTCTCTCGGGCCC
ACAGCCTTACAGCTGTGGAGCACGGTATCCTCTGCCGAAACAGAGGTTGGACAAGACCGGAGGGGTCT
+
FFFFFFFFFFFFFFFFFFFFFFFFFFFFFFFF:FFFFFFFFFFFFFFFFFFFFFFFFFFFFFFFFFFF
FFFFFFFFFFFFFFFFFFFFFFFFFFFF:FFFFFFFFFFFFFFFFFFFFFFFFFFFFFFFFFFFFFFF
@A00155:342:HHGFNDSXY:1:2631:27914:15201 1:N:0:GAACCTAG+TCCGCATA
GGATGTGGAGGGCGAGAGAGAGCGACTTCTCTCGGGCCCACAGCCTTACAGCTGTGGAGCACGGTATC
CTCTGCCGAAACAGAGGTTGGACAAGACCGGAGGGGTCTCCTAGTTCCAAAGGAGATGTACTCC
+
FFFFFFFFFFFFFFFFFFFFFFFFFFFFFFFFFFFFFFFFFFFFFFFFFFF:FFFFFFFFFFFFFF:FF
FFFFFFFFFFFFFFFFFFFFFFFFFFFFFFFFFFFFFFFFFF:FFFFFFFFF,FFFFFFFFFFF
@A00155:342:HHGFNDSXY:1:2631:7084:21872 1:N:0:GAACCTAG+TCCGCATA
CTTCTCTCGGGCCCACAGCCTTACAGCTGTGGAGCACGGTATCCTCTGCCGAAACAGAGGTTGGACAA
GACCGGAGGGGTCTCCTAGTTCCAAAGGAGATGTACTCCGGGCTTGTTCACGACCTACCG
+
FFFFFFFFFFFFFFFFFFFFFFFFFFFFFFFFFFFFFFFFFFFFFFFFFFFFFFF:FFFFFFFF:FFF
FFFFFFFFFFFFFFFFFFFFFFFFFFFFFFFFFFFF:FFFFFF::FFFFFFFFFFFFFFFF
@A00155:342:HHGFNDSXY:1:2631:13946:24236 1:N:0:GAACCTAG+TCCGCATA
TGTAGAGGGCGAGAGAGAGCGACTTCTCTCGGGCCCACAGCCTTACAGCTGTGGAGCACGGTATCCTC
TGCCGAAACAGAGGTTGGACAAGACCGGAGGGGTCTCCTAGTTCCAAAGGAGATGTACT
+
FFFFFFFFFFFFFFFFFFFFFFFFFFFFFFFFFFFFFFFFFFFFFFFFFFFFFFFFFFFFFFFFFFFF
FFFFFFFFFFFFFFFFFFFFFFFFFFFFFFFFFFFFFFFFFFFFFFFFFFFFFFFFFFFF
@A00155:342:HHGFNDSXY:1:2631:32913:30686 1:N:0:GAACCTAG+TCCGCATA
TGGAGGGCGAGAGAGAGCGACTTCTCTCGGGCCCACAGCCTTACAGCTGTGGAGCACGGTATCCTCTG
CCGAAACAGAGGTTGGACAAGACCGGAGGGGTCTCCTAGTTCCAAAGGAGATGTACTCCGGGCTTGTT
+
FFFFFFFFFFFFFFF:FFFFFFFFFFFFFFFFFFFFFFFFFFFFFFFFFFFFFFFFFFFFFFFFFFFF
FFFFFFFFFFFFFFFFFFFFFF:FFFFFFFFFFFFFFFFFFFFFFFFFFFFFFFFFFFFFFFFFFFFF
@A00155:342:HHGFNDSXY:1:2631:9751:36417 1:N:0:GAACCTAG+TCCGCATA
GTATCCTCTGCCGAAACAGAGGTTGGACAAGACCGGAGGGGTCTCCTAGTTCCAAAGGAGATGTACTC
CGGGCTTGTTCACGACCTACCGTGTAAGTCGTAGTCTAGTAGGCTACCTGACGAGTCCTTTTTAGGAC
+
FFFFFFFFFFFFFFFFFFFFFFFFFFFFFFFFFFFFFFFFFFFFFFFFFFFFFFFFFFFFFFFFFFFF
FFFFFFFFFFFFFFFFFFFFFFFFFFFFFFFFFFFFFFFF:FFFFFFFFFFFFFFFFFFFFFFFFFFF
@A00155:342:HHGFNDSXY:1:2632:27037:7607 1:N:0:GAACCTAG+TCCGCATA
AGAGAGAGCGACTTCTCTCGGGCCCACAGCCTTACAGCTGTGGAGCACGGTATCCTCTGCCGAAACAG
AGGTTGGACAAGACCGGAGGGGTCTCCTGGTTCCAAAGGAGATGTACTCCGGGCTTGTTCACGACCTA
+
FFFFFFFFFFFFFFFFFFFFFFFFFFFFFFFFFFFFFFFFFFFFFFFFFFFFFFFFFFFFFFFFFFFF
FFFFFFFFFFFFFFFFFFFFFFFFFFFFFFFFFFFFFFFFFFFFFFFFFFFFFFFFFFFFFFFFFFFF
@A00155:342:HHGFNDSXY:1:2632:31919:18286 1:N:0:GAACCTAG+TCCGCATA
AGAGAGAGCGACTTCTCTCGGGCCCACAGCCTTACAGCTGTGGAGCACGGTATCCTCTGCCGAAACAG
```

AGGTTGGACAAGACCGGAGGGGTCTCCTAGTTCCAAAGGAGATGTACTCCGGGCTTGTTCACGACCTA
+
FFFFFFFFFFFFFFFFFFFFFFFFFFFFFFFFFFFFFFFFFFFFFFFFFFFFFFFFFFFFFFFFFFFF
FFFFFFFFFFFFFFFFFFFFFFFFFFFFFFFFFFFFFFFFFFFFFFFFFFFFFFFFFFFFFFFFFFF:
@A00155:342:HHGFNDSXY:1:2633:2284:24455 1:N:0:GAACCTAG+TCCGCATA
GAGCGACTTCTCTCGGGCCCACAGCCTTACTGCTGTGGAGCACGGTATCCTCTGCCGAAACAGAGGTT
GGACAAGACCGGAGGGGTCTCCTAGTTCCAAAGGAGATGTACTCCGGGCTTGTTCACGACCTACCGTG
+
F:F:FF:FFFFFFFFFFF:FFFFFFFFFFFFFFFFFFFF:FF:FFFFFFFFFFFFFFFFFF:FFFFFF
:FFFFFF:FFF::FFFFFFFF:FFFFFFFF:FFFFFFFFF:,F:F:F::FFFFF,:FF:FFFFFFFF:
@A00155:342:HHGFNDSXY:1:2633:30472:24549 1:N:0:GAACCTAG+TCCGCATA
CCTCTGGCCTATGGTCATCTCTATGCCGAGAGGGATGTGGAGGGCGAGAGAGAGCGACTTCTCTCGGG
CCCACAGCCTTACAGCTGTGGAGCACGGTATCCTCTGCCGAAACAGAGGTTGGACAAGAC
+
FFFFFFFFFFFFFFFFFFFFFFFFFFFFFFFFFFFFFFFFFFFFFFFFFFFFFFFFFFFFFFFFFFFF
FFFFFFFFFFFFFFFFFFFFFFFFFFFFFFFFFFFFFFFFFFFFFFFFFFFFFFFFFFFF
@A00155:342:HHGFNDSXY:1:2633:11433:28839 1:N:0:GAACCTAG+TCCGCATA
CTTCTCTCGGGCCCACAGCCTTACAGCTGTGGAGCACGGTATCCTCTGCCGAAACAGAGGTTGGACAA
GACCGGAGGGGTCTCCTAGTTCCAAAGGAGATGTACTCAGGGCTTGTTCACGACCTACCGTGTAAGTC
+
FFFFFFFFFFF:FFFFFFF:FFFFFFFFFFFFFF,FF,,FFFFFFFFFFFFFFFFF:FFFFFFFFFFF
FFFFF:FFFFFF:FFFFFFFFFFFFFFFFFF,FFFFFF,F,FFF:FFFFFFFFFFFF:FFFFFFF,:F
@A00155:342:HHGFNDSXY:1:2633:31783:34178 1:N:0:GAACCTAG+TCCGCATA
AGAGAGAGCGACTTCTCTCGGGCCCACAGCCTTACAGCTGTGGAGCACGGTATCCTCTGCCGAAACAG
AGGTTGGACAAGACCGGAGGGGTCTCCTAGTTCCAAAGGAGATGTACTCCGGGCTTGTTCACGACCTA
+
FFFFFFFFFFFFFFFFFFFFFFFFFFFFFFFFFFFFFFFFFFFFFFFFFFFFFFFFFFFFFFFFFFFF
FFFFFFFFFFFFFFFFFFFFFFFFFFFFFFFFFFFFFFF,FFFFFFFFFFFFFFFFFFFFFFFFFFFF
@A00155:342:HHGFNDSXY:1:2633:11469:34256 1:N:0:GAACCTAG+TCCGCATA
GAGCGACTTCTCTCGGGCCCACAGCCTTACAGCTGTGGAGCACGGTATCCTCTGCCGAAACAGAGGTT
GGACAAGACCGGAGGGGTCTCCTAGTTCCAAAGGAGATGTACTCCGGGCTTGTTCACGACCTACCGTG
+
FFFFFFFFFFFFFFFFFFFFFFF:FFFFFFFFFFFFFFFFFFFFFF:FFFFFFFFFFFFFFFFFFFFF
FFFFF:FFFFFFFFFFFFFFFFFFFFFFFFFFFFFFFFFFFFFFFFFFFFFFFFFFFFFFFFFFFFFF
@A00155:342:HHGFNDSXY:1:2633:31430:34475 1:N:0:GAACCTAG+TCCGCATA
AGAGAGAGCGACTTCTCTCGGGCCCACAGCCTTACAGCTGTGGAGCACGGTATCCTCTGCCGAAACAG
AGGTTGGACAAGACCGGAGGGGTCTCCTAGATCCAAAGGAGATGTACTCCGGGCTTGTTCACGACCTA
+
FFFFFFFF:FFFFFFFFFFFFFFFFFFFF:FFFFFF:FFF:FFFFFF:FFFFFFFFFFFFFFFFFFFF
FFFFFFFFFFF,FFFFFFFFF,FFFFFFFF,FF:FF::FFFFF:FFFF:FFFFFF:FFFFFF:FFF:F
@A00155:342:HHGFNDSXY:1:2634:16007:1219 1:N:0:GAACCTAG+TCCGCATA
CTGGCCTATGGTCATCTCAATGCCGAGAGGGATGTGAAGGGCGAGAGAGAGCGACTTCTCTCGGGCCC
ACAGCCTTACAGCTGTGGAGCACGGTATCCTCTGCCGAAACAGAGGTTGGACAAGACCGGAG
+
FFFFFFFFFFFFFFFFFFFFFFFFFFFFFFFFFFFFFFFFFFFFFFFFFFFFFFFFFFFFFFFFFFFF
FFFFFFFFFFFFFFFFFFFFFFFFFFFFFFFFFFFFFFFFFFFFFFFFFFFFFFFFFFFFFF
@A00155:342:HHGFNDSXY:1:2634:3242:14340 1:N:0:GAACCTAG+TCCGCATA
GAGCGACTTCTCTCGGGCCCACAGCCTTACAGCTGTGGAGCACGGTATCCTCTGCCGAAACAGAGGTT
GGACAAGACCGGAGGGGTCTCCTAGTTCCAAAGGAGATGTACTCCGGGCTTGTTCACGACCTACCGTG
+
FFFFFFFFFFFFFFFFFFFFFFFFFFFFFFFFFFFFFFFFFFFFFFFFFFFFFFFFFFFFFFFFFFFF
FFFFFFF:FFFFFFFFFFFFFFFFFFFFFFFFFFFFFFFFFFFFFFFFFFFFFFFFFFFFFFFFFFFF
@A00155:342:HHGFNDSXY:1:2634:28989:26804 1:N:0:GAACCTAG+TCCGCATA
GTCATCTCAATGCCGAGAGGGATGTGGAGGGCGAGAGAGAGCGACTTCTCTCGGGCCCACAGCCTTAC

```
AGCTGTGGAGCACGGTATCCTCTGCCGAAACAGAGGTTGGACAAGACCGGAGGGGTCTCCTAGTTCCA
+
FFFFFFFFFFFFFFFFFFFFFFFFFFFFFFFFFFFFFFFFFFFFFFFFFFFFF:FFFFFFFFFFFFFF
FFFFFFFFFFFFFFFFFFFFFFFFFFFFFFFFFFFFFFFFFFFFFFFFFFFFFFFFFFFFFFFFFFFF
@A00155:342:HHGFNDSXY:1:2634:31693:27226 1:N:0:GAACCTAG+TCCGCATA
TGTGGAGGGCGAGAGAGAGCGACTTCTCTCGGGCCCACAGCCTTACAGCTGTGGAGCACGGTATCCTC
TGCCGAAACAGAGGTTGGACAAGACCGGAGGGGTCTCCTAGTTCCAAAGGAGATGTACTCCGGG
+
FFFFFFFFFFFFFFFFFFFFFFFFFFFFFFFFFFFFFFFFFFFFFFFFFFFFFFFFFFFFFFFFFFFF
FFFFFFFF:FF:FFFFFFFFFFFFFFFFFFFFFFFFFFF,FFFFFFFFFFFFFFFFFFFFFFFF
@A00155:342:HHGFNDSXY:1:2634:29677:27492 1:N:0:GAACCTAG+TCCGCATA
GTCATCTCAATGCCGAGAGGGATGTGGAGGGCGAGAGAGAGCGACTTCTCTCGGGCCCACAGCCTTAC
AGCTGTGGAGCACGGTATCCTCTGCCGAAACAGAGGTTGGACAAGACCGGAGGGGTCTCCTAGTTCCA
+
FFFFFFFFFFFFFFFFFFFFFFFFFFFFFFFFFFFFFFFFFFFFFFFF:FFFFFFFFFFFFFFFFF,F
FFFFFFFFFFFFFFF:FFFFFFFFFFFFFFFFFFFFFFFFFFFFFFFFFFFFFFFFFFFFFFFFFFFF
@A00155:342:HHGFNDSXY:1:2634:2492:29042 1:N:0:GAACCTAG+TCCGCATA
GCTGTGTCCACTTAAGGACTCACCTCTGGCCTATGGTCATCTCAATGCCGAGAGGGATGTGAAGGGCG
AGAGAGAGCGACTTCTCTCGGGCCCACAGCCTTACAGCTGTGGAGCACGGTATCCTCTGCCGAAACAG
+
FFFFFFFFFFFFFFFFFFFFFFFFFFFFFFFFFFFFFFFFFFFFFFFFFFFFFFFFFFFFFFFFFFFF
FFFFFFFFFFFFFFFFFFFFFFFFFFFFFFFFFFFFFFFFFFFFFFFFFFFFFFFFFFF:FFFFFFFF
@A00155:342:HHGFNDSXY:1:2634:15338:30874 1:N:0:GAACCTAG+TCCGCATA
GTCATCTCAATGCCGAGAGGGATGTGGAGGGCGAGAGAGAGTGACTTCTCTCGGGCCCACAGCCTTAC
AGCTGTGGAGCACGGTATCCTCTGCCGAAACAGAGGTTGGACAAGACCGGAGGGGTCTCCTAGTTCCA
+
FFFFFFFFFFFFFFFFFFFFFFFFFFFFFFFFFFFFFFFFFFFFFFFFFFFFFFFFFFFFFFFFFFFF
FFFFFFFFFFFFFFFFF:FFFFFFFFFF:FFFFFFFFFFFFFFFFFFFFFFFFFFFFFFF:FFFFFFF
@A00155:342:HHGFNDSXY:1:2635:11153:8375 1:N:0:GAACCTAG+TCCGCATA
AGGGATGTGGAGGGCGAGAGAGAGCGACTTCTCTCGGGCCCACAGCCTTACAGCTGTGGAGCACGGTA
TCCTCTGCCGAAACAGAGGTTGGACAAGACCGGAGGGGTCTCCTGGTTCCAAAGGAGATGTACTCCGG
+
FFFFFFF,FFFFFFFFFFFFFFFFFFFFFFFFFFFFFFFF:FFFFFFFFFFFFFFFFFFFFFFFFFFF
FFFFFFFFFFFFFFFFFFFFFFFFFFFFFFFFFFFFFFFFFFFFFFFFFFFFFFFFFFFFFFFFFFFF
@A00155:342:HHGFNDSXY:1:2635:21079:19523 1:N:0:GAACCTAG+TCCGCATA
GGATGTGGAGGGCGAGAGAGAGCGACTTCTCTCGGGCCCACAGCCTTACAGCTGTGGAGCACGGTATC
CTCTGCCGAAACAGAGGTTGGACAAGACCGGAGGGGTCTCCTGGTTCCAAAGGAGATGTACTCC
+
FFFFFFFFFFFFFFFFFFFFFFFFFFFFFFFFFFFFFFFFFFFFFFFFFFFFFFFFFFFFFFFFFFFF
FFFFFFFFFFFFFFFFFFFFFFFFFFFFFFFFFFFFFFFFFFFFFFFFFFFFFFFFFFFFFFFF
@A00155:342:HHGFNDSXY:1:2635:5348:20087 1:N:0:GAACCTAG+TCCGCATA
AAGGACTCTCCTCTGGCCTATGGTCATCTCAATGCCGAGAGGGATGTGGAGGGCGAGAGAGAGCGACT
TCTCTCGGGCCCACAGCCTTACAGCTGTGGAGCACGGTATCCTCTGCCGAAACAGAGGTTGGACAAGA
+
FFFFFFFFFFFFFFFFFFFFFFFFFFFFFFFFFFFFFFFFFFFFFF,FFFFFFFFFFFFFFFFFFFFF
FFFFFFFFFFFFFFFFFF:FFFFFFFFFFFFFFFFFFFFFFFFFFFFFFFF,FFFFFFFFFFFFF:FF
@A00155:342:HHGFNDSXY:1:2636:30083:10222 1:N:0:GAACCTAG+TCCGCATA
CATCTCAATGCCGAGAGGGATGTGGAGGGCGAGAGAGAGCGACTTCTCTCGGGCCCACAGCCTTACAG
CTGTGGAGCACGGTATCCTCTGCCGAAACAGAGGTTGGACAAGACCGGAGGGGTCTCCTTGTTCCAAA
+
FFFFFFFFFFFFFFFFFFFFFFFFF:FFFFFFFFFFFFFFFFFFFFFFFFFFFFFFFF:FFFFFFFFF
FFFFFFFFFFFFFFFFFFFFFFFFFFFFFFFFFFFFFFFFFFFFFFFFFFFFFFFFFFFFFFFFFFFF
@A00155:342:HHGFNDSXY:1:2636:30020:14716 1:N:0:GAACCTAG+TCCGCATA
GGCATAGAGATGACCATAGGCCAGAGGTGAGTCCTTAAGTGGACACAGCTGATCTAAGGCGGTGTGGC
```

GGGGCATGGGTTTGAACCCCCATGACGGTCGGAGTCTAGTAGGCTCCCTGATGAGTCCGTTCCAAGGA
+
FFFF:FFFFFFFFFFFFFFFFFFFFFFFFFFFFFFFFFFFFFFFFFFFFFFFFFFFFFFFFFFFFFFFF
FFFFFFFFFFFFFFFFFFFFFFFFFFFFFFFFFFFFFFFFFFFFFFFFFFFFFFFFFFFFFFFFFFFFF
@A00155:342:HHGFNDSXY:1:2637:21856:24032 1:N:0:GAACCTAG+TCCGCATA
TGTGGAGGGCGAGAGAGAGCGACTTCTCTCGGGCCCACAGCCTTACAGCTGTGGAGCACGGTATCCTC
TGCCGAAACAGAGGTTGGACAAGACCGGAGGGGTCTCCTAGTTCCAAAGGAGATGTACTCCGGG
+
:FFFFFFFFFFFFFFFFFFFFFFFFFFFFFFFFFFFFFFFFFFFFFFFFFFFFFFFFFFFFFFFFFFF
FFFFFFFFFFFFFFFFFFFFFFFFFFFFFFFFFFF:FFFFFFFFFFFFFFFFF,FFFFFFFFFF
@A00155:342:HHGFNDSXY:1:2637:26711:24486 1:N:0:GAACCTAG+TCCGCATA
TCCTCTGCCGAAACAGAGGTTGGACAAGACCGGAGGGGTCTCCTAGTTCCAAAGGAGATGTACTCCGG
GCTTGTTCACGACCTACCGTGTAAGTCGTAGTCTAGTAGGCTACCTGACGAGTCCTTTTTAGGACGAA
+
F:FFFFFFFFFFF:FFFFFFFFFFFFFFFFFFFF,F:FFFFFF:FFFFFFFFFFF:FFFFFFFFFF:F
FFFFFFFFFF,FFFFFFFFFF:FFF,FF:FFFF:FFFFFFFFFFFFFFF:F:FFFF,::FFF,:FFFF
@A00155:342:HHGFNDSXY:1:2637:27389:24565 1:N:0:GAACCTAG+TCCGCATA
TCTCTCGCCCTCCACATCCCTCTCGGCATTGAGATGACCATAGGCCAGAGGTGAGTCCTTAAGTGGAC
ACAGCTGATCTAAGGCGGTGTGGTGGGGCATGGGTTTGAACCCCCATGACGGTCGGAGTCTAGTAGGC
+
FFFFFFFFFFFFFFFFFFFFFFFFFFFF:FFFFFFFFFFFFFFFFFFFFFFFFFFFFFFFFFFFFFFFF
FFFFFFFFFFFFFFFFFFFFF:FFFFFFFFFFFFFFFFFFFFFFFFFFFFFFFFFFFFFFFFFFFFFFF
@A00155:342:HHGFNDSXY:1:2637:5692:29450 1:N:0:GAACCTAG+TCCGCATA
ATGCCGAGAGGGATGTAGAGGGCGAGAGAGAGCGACTTCTCTCGGGCCCACAGCCTTACAGCTGTGGA
GCACGGTATCCTCTGCCGAAACAGAGGTTGGACAAGACCGGAGGGGTCTCCTAGTTCCAAAGGAGATG
+
FFFFFFFFFFFFFFFFFFFFFFFFFFFFFFFFFFFF:FFFFFFFFFFFFFFFFFFFFFFFFFFFFFFFF
FFFFFFFFFFFFFFFFFFFFFFFFF:F:FFFFFFFFFFFFFF:FFFFFFFFFFFFFFFFFFFFFFFFFF
@A00155:342:HHGFNDSXY:1:2638:10041:19507 1:N:0:GAACCTAG+TCCGCATA
CACCTCTGGCCTATGGTCATCTCAATGCCGAGAGGGATGTGAAGGGCGAGAGAGAGCGACTTCTCTCG
GGCCCACAGCCTTACAGCTGTGGAGCACGGTATCCTCTGCCGAAACAGAGGTTGGACAAGACCGGAG
+
FFFFFFFFFFFFFFFFFFFFFFFFFFFFFFFFFFFFFFFFFFFFFFFFFFFFFFFFFFFFFFFFFFFFF
FFFFFFFFFFFFFFFFFFFFFFFFFFFFFFFFFFFFFFFFFFFFFFFFFFFFFFFFFFFFFFFFFFFF
@A00155:342:HHGFNDSXY:1:2639:21314:2895 1:N:0:GAACCTAG+TCCGCATA
GGCCAGAGGTGAGTCCTTAAGTGGACACAGCTGATCTAAGGCGGTGTAGCGGGGCATGGGTTTGAACC
CCCATGACGGTCGGAGTCTAGTAGGCTCCCTGATGAGTCCGTTCCAAGGACGAAACCGTCGTTAC
+
FFFFFFFFFFFFFFFFFFFFFFFFFFF:FFFFFFFFFFFFFFFFFFFFFFFFFFFFFFFFFFFFFFFFF
FFFFFFFFFFFFFFFFFFFFFFFFFFFFFFFFFFFFFFFFFFFFFFFFFFFFFFFFFFFFFFFFFF
@A00155:342:HHGFNDSXY:1:2639:4743:15123 1:N:0:GAACCTAG+TCCGCATA
GAGAGAGCGACTTCTCTCGGGCCCACAGCCTTACAGCTGTGGAGCACGGTATCCTCTGCCGAAACAGA
GGTTGGACAAGACCGGAGGGGTCTCCTGGTTCCAAAGGAGATGTACTCCGGGCTTGTTCACGACCTAC
+
FFFFFFFFFFFFFFFFFFFFFFFFFFFFFFFFFFFFFF:FFFFFFFFFFFFFFFFFFFFFFFFFFFFFF
F:FFFFFFFFFFFFFFFFFFFFFFFFFFFFFFFFFFFFFFF:F:FFFFFFFFFFFFFFFFFFFFFFFFF
@A00155:342:HHGFNDSXY:1:2639:9182:16517 1:N:0:GAACCTAG+TCCGCATA
CTTCTCTCGGGCCCACAGCCTTACAGCTGTGGAGCACGGTATCCTCTGCCGAAACAGAGGTTGGACAA
GACCGGAGGGGTCTCCTAGTTCCAAAGGAGATGTACTCCGGGCTTGTTCACGACCTACCGTGTAAGTC
+
FFFFFFFFFFFFFFFFFFFFFFFFFFFFFFFFFFFFFFFFFFFFFFFFFFFFFFFFFFFFFFFFFFFFF
FFFFFFFFFFFFFFFFF,FFFFFFFFFFFFFFFFFFFFFFFFFFFFFFFFFFFFFFFFFFFFFFF:FFF
@A00155:342:HHGFNDSXY:1:2639:3378:18928 1:N:0:GAACCTAG+TCCGCATA
TCCTCTGCCGAAACAGAGGTTGGACAAGACCGGAGGGGTCTCCTAGTTCCAAAGGAGATGTACTCCGG

```
GCTTGTTCACGACCTACCGTGTAAGTCGTAGTCTAGTAGGCTACCTGACGAGTCCTTTTTAGGACGAA
+
FFFFFFFFFFFFFFFFFFFFFFFFFFFFFFFFF:FFFFFFFFFFFFFFFFFFFFFFFFFFFFFFFFFF
FF,FFFFFFFFFFFFFFFFFF:FFFFFFFFFFFFFFFFFFFFFFFFFFFFFFFFFFFFFFFFFFFF:F
@A00155:342:HHGFNDSXY:1:2640:14000:7138 1:N:0:GAACCTAG+TCCGCATA
AGAGAGAGCGACTTCTCTCGGGCCCACAGCCTTACAGCTGTGGAGCACGGTATCCTCTGCCGAAACAG
AGGTTGGACAAGACCGGAGGGGTCTCCTAGTTCCAAAGGAGATGTACTCCGGGCTTGTTCACGACCTA
+
FFFFFFFFFFFFFFFFFFFFFFFFFFFFFFFFFFFFFFFFFFFFFFFFFFFFFFFFFFFFFFFFFFFF
FFFFFFFFFFFFFFFFFFFFFFFFFFFFFFFFFFFFFFFFFFFFFFFFFFFFFFFFFFFFFFFFFFFF
@A00155:342:HHGFNDSXY:1:2640:12852:11475 1:N:0:GAACCTAG+TCCGCATA
GGATGTGGAGGGCGAGAGAGAGCGACTTCTCTCGGGCCCACAGCCTTACAGCTGTGGAGCACGGTATC
CTCTGCCGAAACAGAGGTTGGACAAGACCGGAGGGGTCTCCTAGTTCCAAAGGAGATGTACTCC
+
FFFFFFFFFFFFFFFFFFFFFFFFFFFFFFFFFFFFFFFFFFFFFFFFFF,FFFFFFFFFFFFFFFFF
FFFFFFFFFFFFFFFFFFFFFFFFFFFFFFFFFFFFFFFFFFFFFFFFFFFFFFFFFFFFFFFF
@A00155:342:HHGFNDSXY:1:2640:31503:11960 1:N:0:GAACCTAG+TCCGCATA
GACTTCTCTCGGGCCCACAGCCTTACAGCTGTGGAGCACGGTATCCTCTGCCGAAACAGAGGTTGGAC
AAGACCGGAGGGGTCTCCTAGTTCCAAAGGAGATGTACTCCGGGCTTGTTCACGACCTACCGTGTAAG
+
FFFFFFFFFFFFFFFFFFFFFFF:FFFFFFFFFFFFFFFFFFFFFFFFFFFFFFFFFFFFFFFFFFFF
FFFFFFFFFFFFFFFFFFFFFFFFFFFFFFFFFFFFFFFFFFFFFF:FFFFFFFFFFFFFFFFFFFF:F
@A00155:342:HHGFNDSXY:1:2640:17644:29168 1:N:0:GAACCTAG+TCCGCATA
TTCTCTCGGGCCCACAGCCTTACAGCTGTGGAGCACGGTATCCTCTGCCGAAACAGAGGTTGGACAAG
ACCGGAGGGGTCTCCTAGTTCCAAAGGAGATGTACTCCGGGCTTGTTCACGACCTACCGTGTAAG
+
FFFFFFFFFFFFFFFFFFFFF::FFFFFF,FFFFFFFFFFFFFFFFFFFFFFFFFFFFFFFFFFFFFF
FFFFFFFFFFFFFFFFFFFFFFFFFFFFFFFFFFFFFFFFFFF:FFFFFFFFFFFFFFFF,FFFFF
@A00155:342:HHGFNDSXY:1:2641:11107:2284 1:N:0:GAACCTAG+TCCGCATA
GGGCGAGAGAGAGCGACTTCTCTCGGGCCCACAGCCTTACAGCTGTGGAGCACGGTATCCTCTGCCGA
AACAGAGGTTGGACAAGACCGGAGGGGTCTCCTGGTTCCAAAGGAGATGTACTCCGGGCTTGTTCACG
+
FFFF:F:FFFFFFF:F::FF,F:F:FFFFFFFF:FFF:FFFFFF,F:FFF::,FFFFFFF,:FFF:FF
FFF:,F:F:FFFFFFFFFFFFF,,F::,F,FFFFFFFF,FF:,FFFFF:FFF,FFFF,FFFFFFFFF:
@A00155:342:HHGFNDSXY:1:2641:16071:18615 1:N:0:GAACCTAG+TCCGCATA
CTTCTCTCGGGCCCACAGCCTTACAGCTGTGGAGCACGGTATCCTCTGCCGAAACAGAGGTTGGACAA
GACCGGAGGGGTCTCCTAGTTCCAAAGGAGATGTACTCCGGGCTTGTTCACGACCTACCGTGTAAGTC
+
FFFFFFFFFFFFFFFFFFFFFFF:FFFFFFFFFFFFFFFFFFFFFFFFFFFFFFFFFFFFFFFFFFFF
FFFFFF:FFFFFFFFFFFFFFFFFFFFFFFFFFFFFFFFFFFFFFFFFFFFFFFFFFFFFFFFFFFFF
@A00155:342:HHGFNDSXY:1:2641:21748:20243 1:N:0:GAACCTAG+TCCGCATA
GAGAGCGACTTCTCTCGGGCCCACAGCCTTACAGCTGTGGAGCACGGTATCCTCTGCCGAAACAGAGG
TTGGACAAGACCGGAGGGGTCTCCTGGTTCCAAAGGAGATGTACTCCGGGCTTGTTCACGACCTACCG
+
FFFFFFFFFFFFFFFFFFFFFFFFFFFFFFFFFFFFFFFFFFFFFFFFFFFFFFFFFFFFFFFFFFFF
FFFFFFFFFFFFFFFFFFFFFFFFFFFFFFFFFFFFFFFFFFFFFFFFFFFFFFFF:FFFFFFFFFFF:F
@A00155:342:HHGFNDSXY:1:2641:8151:29262 1:N:0:GAACCTAG+TCCGCATA
GATGTGGAGGGCGAGAGAGAGCGACTTCTCTCGGGCCCACAGCCTTACAGCTGTGGAGCACGGTATCC
TCTGCCGAAACAGAGGTTGGACAAGACCGGAGGGGTCTCCTAGTTCCAAAGGAGATGTACTCCGGGCT
+
FFFFFFFFFFFFFFFFFFFFFFFFFF:FFFFFFFFFFFFFFFFFFFFFFFFFFFFFFFFFFFF:F:FFF
FF:FFFFFFFFFFFFFFFFFFFFFFFFFFFFFF,FFFFFFFFFFFFFFFFFFFFFFFF:FFFFF::FFFF
@A00155:342:HHGFNDSXY:1:2642:18783:8594 1:N:0:GAACCTAG+TCCGCATA
ATCTCAATGCCGAGAGGGATGTGGAGGGCGAGAGAGAGCGACTTCTCTCGGGCCCACAGCCTTACAGC
```

```
TGTGGAGCACGGTATCCTCTGCCGAAACAGAGGTTGGACAAGACCGGAGGGGTCTCCTAGTTCCAAAG
+
FFFFFFFFFF:FFFFFF:,FFFFFFFFFFFFFFFF::FFFFFFFF:FFFFFFFFFFFFFFF:FFFFFF
FFFFFFFFFFFFFFFFFFFFFFFFFFFFFFFF:FFFFFFFFF:FFFF:FFFFFFFFFFFFFFFFF:FF
@A00155:342:HHGFNDSXY:1:2642:32560:27602 1:N:0:GAACCTAG+TCCGCATA
ACTTCTCTCGGGCCCACAGCCTTACAGCTGTGGAGCACGGTATCCTCTGCCGAAACAGAGGTTGGACA
AGACCGGAGGGGTCTCCTAGTTCCAAAGGAGATGTACTCCGGGCTTGTTCACGACCTACCGTGTAAGT
+
FFFFFFFFFFFFFFFFFFFFF:FFFFFFFF:FF:FF,FFFFFFFFFFFFFFFFFFFFFFFFFFFFFFF
FFFFFFF,FFFFFFFFFF:F,FFFFFFFFFFFFFFFFFFFFFFFF:FFFFFFFFFFFFFFF:FFF:FF
@A00155:342:HHGFNDSXY:1:2642:32099:33223 1:N:0:GAACCTAG+TCCGCATA
GAGCGACTTCTCTCGGGCCCACAGCCTTACAGCTGTGGAGCACGGTATCCTCTGCCGAAACAGAGGTT
GGACAAGACCGGAGGGGTCTCCTAGTTCCAAAGGAGATGTACTCCGGGCTTGTTCACGACCTACCGTG
+
FFFFFFFFFFFFFFFFFFFFFFFFFFFFFFFFFFFFFFFFFFFFFFFFFFFFFFFFFFFFFFFFFFFF
FFFFFFFFF:FFFFFFFFFFFFFFFFFFFFFFFFFFFFFFFFFFFFFFF:FFFFFFFFFFFFFFFFFF
@A00155:342:HHGFNDSXY:1:2643:23665:24627 1:N:0:GAACCTAG+TCCGCATA
GGGATGTAGAGGGCGAGAGAGAGCGACTTCTCTCGGGCCCACAGCCTTACAGCTGTGGAGCACGGTAT
CCTCTGCCGAAACAGAGGTTGGACAAGACCGGAGGGGTCTCCTAGTTCCAAAGGAGATGTACTCCGG
+
,:FF,F,F,F:,,F,F:F,F:FFFFFF,:F:::F:,:FFF:,,::,::F,F:FFF:,,FF:::F,:FF
:FFFF,FF:FFF::F:F,FFFFFFFF:F:FFF:,F::FF,:F,FF:F::FFF,FFFF:,,FFF,F,:
@A00155:342:HHGFNDSXY:1:2643:12879:27774 1:N:0:GAACCTAG+TCCGCATA
CTTCTCTCGGGCCCACAGCCTTACAGCTGTGGAGCACGGTATCCTCTGCCGAAACAGAGGTTGGACAA
GACCGGAGGGGTCTCCTAGTTCCAAAGGAGATGTACTCCGGGCTTGTTCACGACCTACCGTGTA
+
FFFFFFFFFFFFFFFFFFFFFF:FFFFFF:FFFFFFFFFFFFFFFFFFFFFFFFFFFFFFFFFFFFFF
FFFFFFFFFFFFFFFFFFF:FF:FFF:FFFFFFFFFFFFFFFFF,FFFFFFFFFF:FFFFFFFF
@A00155:342:HHGFNDSXY:1:2644:14353:2644 1:N:0:GAACCTAG+TCCGCATA
TCCTCTGCCGAAACAGAGGTTGGACAAGACCGGAGGGGTCTCCTAGTTCCAAAGGAGATGTACTCCGG
GCTTGTTCACGACCTACCGTGTAAGTCGTAGTCTAGTAGGCTACCTGACGAGTCCTTTTTAG
+
FFFFFFFFFFFFFFFFFFFFFFFFFFFFFFFFFFFFFFFFFFFFFFFFFFFFFFFFFFFFFFFFFFFF
FFFFFFFFFFFFFFFFFFFFFFFFFFFFFFFFFFFFFFFFFFFFFFFFFFFFFFFFFFFFFF
@A00155:342:HHGFNDSXY:1:2644:13548:3317 1:N:0:GAACCTAG+TCCGCATA
TCCTCTGCCGAAACAGAGGTTGGACAAGACCGGAGGGGTCTCCTAGTTCCAAAGGAGATGTACTCCGG
GCTTGTTCACGACCTACCGTGTAAGTCGTAGTCTAGTAGGCTACCTGACGAGTCCTTTTTAG
+
FFFFFFFFFFFFFFFFFFFFFFFFFFFFFFFFFFFFFFFFFFFFFFFFFFFFFFFFFFFFFFFFFFFF
FFFFFFFFFFFFFFFFFFFFFFFFFFFFFFFFFFFFFFFFFFFFFFFFFFFFFFFFFFFFFF
@A00155:342:HHGFNDSXY:1:2645:4625:5024 1:N:0:GAACCTAG+TCCGCATA
CTCAATGCCGAGAGGGATGTGGAGGGCGAGAGAGAGCGACTTCTCTCGGGCCCACAGCCTTACAGCTG
TGGAGCACGGTATCCTCTGCCGAAACAGAGGTTGGACAAGACCGGAGGGGTCTCCTGGTTCCAAAGGA
+
FFFFFFFFFF:FFFFFFFFFFFFFFFFFFFFFFFFFFFFFFFFFFFFFFFFFFFFFFFFFFFFFFFFF
FFFFFFFFFFFFFFFFFFFFFFFFFF:FFFFFFFFFFFFFFFFFFFFFFFFFFFFFFFFFFFFFFFFF
@A00155:342:HHGFNDSXY:1:2645:16116:8015 1:N:0:GAACCTAG+TCCGCATA
ATGCCGAGAGGGATGTGGAGGGCGAGAGAGAGCGACTTCTCTCGGGCCCACAGCCTTACAGCTGTGGA
GCACGGTATCCTCTGCCGAAACAGAGGTTGGACAAGACCGGAGGGGTCTCCTAGTTCCAAAGGAGATG
+
FFFFFFFFFFFFFFFFFFFFFFFFFFFFFFFFFFFFFFFFFFFFFFFFFFFFFFFFF:FFFFFFFFFF
FFFFFFFFFFFFFFFFFFFFFFFFFFFFFFFFFFFFFFFFFFFFFFFFFFFFFFFFFFFFFFFFFFFF
@A00155:342:HHGFNDSXY:1:2645:26069:18020 1:N:0:GAACCTAG+TCCGCATA
ATCTCAATGCCGAGAGGGATGTGGAGGGCGAGAGAGAGCGACTTCTCTCGGGCCCACAGCCTTACAGC
```

```
TGTGGAGCACGGTATCCTCTGCCGAAACAGAGGTTGGACAAGACCGGAGGGGTCTCCTAGTTCCAAAG
+
FFFFFFFFFFFFFFFFFFFFFFFFFFFFFFFFFFFFFFFFFFFFFFFFFFFFFFFFFFFFF:FFFFFFF
FFFFFFFFFFFFFFFFFFFFFFFFFFFFFFFFFFFFFFFFF:FFFFFFFFFFFFFFFFFFFFFFFFFFFF
@A00155:342:HHGFNDSXY:1:2645:23610:24283 1:N:0:GAACCTAG+TCCGCATA
AGAGAGAGCGACTTCTCTCGGGCCCACAGCCTTACAGCTGTGGAGCACGGTATCCTCTGCCGAAACAG
AGGTTGGACAAGACCGGAGGGGTCTCCTAGTTCCAAAGGAGATGTACTCCGGGCTTGTTCACGACCTA
+
FFFFFFFFFFFFFFFFFFFFFFFFFFFFFFFFFFFFFFFFFFFFFFFFFFFFFFFFFFFFFFFFFFFF
FFFFFFFFFFFFFFFFFFFFFFFFFFFFFFFFFFFFFFFFFFFFFFFFFFFFFFFFFFFFFFFFFFFF
@A00155:342:HHGFNDSXY:1:2645:25825:25676 1:N:0:GAACCTAG+TCCGCATA
GAGACCCCTCCGGTCTTGTCCAACCTCTGTTTCGGCAGAGGATACCGTGCTCCACAGCTGTAAGGCTG
TGGGCCCGAGAGAAGTCTCTCTCTCTCGCCCTCCACATCCCTCTCGGCATTGAGATGAC
+
FFFFFFFFFFFFFFFFFFFFFFFFFFFFFFFFFFFFFFFFFFFFFFFFFFFFFFFFFFFFFFFFFFFF
FFFFFFFFFFFFFFFFFFFFFFFFFFFFFFFFFFFFFFFFFFFFFFFFFFFFFFFFFFF
@A00155:342:HHGFNDSXY:1:2646:31123:19351 1:N:0:GAACCTAG+TCCGCATA
CTTCTCTCGGGCCCACAGCCTTACAGCTGTGGAGCACGGTATCCTCTGCCGAAACAGAGGTTGGACAA
GACCGGAGGGGTCTCCTAGTTCCAAAGGAGATGTACTCCGGGCTTGTTCACGACCTACCGTGTAAGTC
+
FFFFFFFFFFFFFFFFFFFFFFFFFFFFFFFFFFFFFFFFFFFFFFFFFFFFFFFFFFFFFFFFFFFF
FFFFFFFFFFFFFFFFFFFFFFFFFFFFFFFFFFFFFFFFFFFFFFFFFFFFFFFFFFFFFFF:FFFF
@A00155:342:HHGFNDSXY:1:2646:29948:24173 1:N:0:GAACCTAG+TCCGCATA
ACCTCTGTTTCGGCAGAGGATACCGTGCTCCACAGCTGTAAGGCTGTGGGCCCGAGAGAAGTCGCTCT
CTCTCGCCCTCTACATCCCTCTCGGCATTGAGATGACCATAGGCCAGAGGTGAGTCCTTAAGTGGACA
+
FFFFFFFFFFFFFFFFFFFFFFFFFFFFFFFFFFFFFFFFFFFFFFFFFFFFFFFFFFFF:FFFFFFFF
FFFFFFFFFFFFFFFFFFFFFFFFFFFF:FFFFFFFFFFFFFFFFFFFFFFFFFFFFFFFFFFFFFFF:
@A00155:342:HHGFNDSXY:1:2646:6804:32753 1:N:0:GAACCTAG+TCCGCATA
CTCAATGCCGAGAGGGATGTGGAGGGCGAGAGAGAGCGACTTCTCTCGGGCCCACAGCCTTACAGCTG
TGGAGCACGGTATCCTCTGCCGAAACAGAGGTTGGACAAGACCGGAGGGGTCTCCTAGTTCCAAAGGA
+
FFFFFFFFFFFFFFFF:FF:FFFFFFFFFFFFFFFFFFF:FFFFFFFFFFFFFFFFFFFFFFFFFFFFF
FFFFFFFFFFFFFFFFFFFFFFFFFFFFFFFFFFFFFFFFFFFFFFFFFFFFFFFFFFFFFFFFFFFF
@A00155:342:HHGFNDSXY:1:2647:17228:11099 1:N:0:GAACCTAG+TCCGCATA
TCCACTTAAGGACTCTCCTCTGGCCTATGGTCATCTCAATGCCGAGAGGGATGTGGAGGGCGAGAGAG
AGCGACTTCTCTCGGGCCCACAGCCTTACAGCTGTGGAGCACGGTATCCTCTGCCGAAACAGAGGTTG
+
FFFFFFFFFFFFFFFFFFFFFFFFFFFFFFFFFFFFFFFFFFF:FFFFFFFFFFFFFFFFFFFFFFFF
FFFFFFFFFFFFFFFFFFFFFFFFFFFFFFFFFFFFFFFFFFFFFFFFFFFFFFFFFFFFFFFFFFFF
@A00155:342:HHGFNDSXY:1:2647:18701:13620 1:N:0:GAACCTAG+TCCGCATA
GTCATCTCAATGCCGAGAGGGATGTGGAGGGCGAGAGAGAGCGACTTCTCTCGGGCCCACAGCCTTAC
AGCTGTGGAGCACGGTATCCTCTGCCGAAACAGAGGTTGGACAAGACCGGAGGGGTCTCCTAGTTCCA
+
FFFFFFFFFFFFFFFFFFFFFFFFFFFFFFFFFFFFFFFFFFFFFFFFFFFFFFFFFFFFFFFFFFFF
FFFFFFFFFFFFFFFFFFFFFFFFFFFFFFFFFFFFFFFFFFFFFFFFFFFFFFFFFFFFFFFFFFFF
@A00155:342:HHGFNDSXY:1:2647:20193:20650 1:N:0:GAACCTAG+TCCGCATA
CTGGCCTTTGGTCATCTCAATGCCGAGAGGGATGTGGAGGGCGAGAGAGAGCGACTTCTCTCGGGCCC
ACAGCCTTACAGCTGTGGAGCACGGTATCCTCTGCCGAAACAGAGGTTGGACAAGACCGGAGGGGTCT
+
FFFFFFFFFFFFFFFFFFFFFFFFFFFFFFFFF,FFFFFFFFFFFFFFFFFFFFFFFFFFFFFFFFFF
FFFFFFFFFFFFFFFFFFFFFFFFFFFFFFFFFFFFFFFFFFFFFFFFFFFFFFFFFFFFFFFFFFFF
@A00155:342:HHGFNDSXY:1:2647:11867:30749 1:N:0:GAACCTAG+TCCGCATA
CACCTCTGGCCTATGGTCATCTCAATGCCGAGAGGGATGTGAAGGGCGAGAGAGAGCGACTTCTCTCG
```

```
GGCCCACAGCCTTACAGCTGTGGAGCACGGTATCCTCTGCCGAAACAGAGGTTGGACAAGACCGGAGG
+
FFFFFFFFFFFF:FFFFFFFFFFFFFFFFFFFFFFFF:FFFFFFFFFFFFFFFFFFFFFFFFFFFFFFF
FFFFFFFFFFFFFFFFFFFFFFFFFFFFFFFFFFFFFFFFFFFFFFFFFFF:FFFFFFFFFFFFFFFF
@A00155:342:HHGFNDSXY:1:2648:5692:3677 1:N:0:GAACCTAG+TCCGCATA
AGGGATGTGGAGGGCGAGAGAGAGCGACTTCTCTCGGGCCCACAGCCTTACAGCTGTGGAGCACGGTA
TCCTCTGCCGAAACAGAGGTTGGACAAGACCGGAGGGGTCTCCTAGTTCCAAAGGAGATGTACTCCGG
+
FFFFFFFFFFFFFFFFFFFFFFFFFFFFFFFFFFFFFFFFFFFFFFFFFFFFFFFFFFFFFFFFFFFF
FFFFFF:FFFFFFFFFFFFFFFFFFFFFFFFFFFFFFFFFFFFFFFFFFFFFFFFFFFFFFFFFFF:F
@A00155:342:HHGFNDSXY:1:2648:31973:11459 1:N:0:GAACCTAG+TCCGCATA
CTTCTCTCGGGCCCACAGCCTTACAGCTGTGGAGCACGGTATCCTCTGCCGAAACAGAGGTTGGACAA
GACCGGAGGGGTCTCCTAGTTCCAAAGGAGATGTACTCCGGGCTTGTTCACGACCTACCGTGTAAGTC
+
FFFFFFFFFFFFFFFFFFFFFFFFFFFFFFFFFFFFFFFFFFFFFFFFFFFFFFFFFFFFFFFFFFFF
FFFFFFFFFFFFFFFFFFFFFFFFFFFFFFFFFFFFFFFFFFFFFFFFFFFFFFFFFFFFFFFF:FFF
@A00155:342:HHGFNDSXY:1:2648:13313:17848 1:N:0:GAACCTAG+TCCGCATA
CACCTCTGGCCTATGGTCATCTCAATGCCGAGAGGGATGTGAAGGGCGAGAGAGAGCGACTTCTCTCG
GGCCCACAGCCTTACAGCTGTGGAGCACGGTATCCTCTGCCGAAACAGAGGTTGGACAAGACCGGAGG
+
FFFFFFFFFFFFFFFFFFFFFFFFFFFFFFFFFFFFFFFFFFFFFFFFF:FFFFFFFFFFFFFFFFFF
FFFFFFFFFFFFFFFFFFFFFFFF:FFFFFFF:FFFFFFFFFFFFFFFFFFFFFFFFFFFFFFFFFFF
@A00155:342:HHGFNDSXY:1:2648:12409:27899 1:N:0:GAACCTAG+TCCGCATA
GGGCGAGAGAGAGCGACTTCTCTCGGGCCCACAGCCTTACAGCTGTGGAGCACGGTATCCTCTGCCGA
AACAGAGGTTGGACAAGACCGGAGGGGTCTCCTGGTTCCAAAGGAGATGTACTCCGGGCTTGTTCACG
+
FFFFFFFFFFFFFFFFFFFFFFFFFFFFFFFFFFFFFFFFFFFFFFFFFFFFFFFFFFFFFFFFFFFF
FFFFFFFFFFFFFFFFFFFFFFFFFFFFFFFFFFFFFFFFFFF:FFFFFFFFFFFFFFFFFFFFFFFF
@A00155:342:HHGFNDSXY:1:2649:21296:1548 1:N:0:GAACCTAG+TCCGCATA
AGGGATGTGGAGGGCGAGAGAGAGCGACTTCTCTCGGGCCCACAGCCTTACAGCTGTGGAGCACGGTA
TCCTCTGCCGAAACAGAGGTTGGACAAGACCGGAGGGGTCTCCTGGTTCCAAAGGAGATGTACTCCGG
+
FFFFFFFFFFFFFFFFFFFFFFFFFFFFFFFFFFFFFFFFFFFFFFFFFFFFFFFFFFFFFFFFFFFF
FFFFFFFFFFFFFFFFFFFFFFFFFFFFFFFFFFFFFFFFFFFFFFFFFFFFFFFFFFFFFFFFFFFF
@A00155:342:HHGFNDSXY:1:2649:30355:13448 1:N:0:GAACCTAG+TCCGCATA
ACTTCTCTCGGGCCCACAGCCTTACAGCTGTGGAGCACGGTATCCTCTGCCGAAACAGAGGTTGGACA
AGACCGGAGGGGTCTCCTAGTTCCAAAGGAGATGTACTCCGGGCTTGTTCACGACCTACCGTGTAAGT
+
FFFFFFFFFFFFFFFFFFFFFFFFFFFFFFFFFFFFFFFFF:FFFF:FFFFFFFFFFFFFF:FFFFFF
FFFFFFFFFFFFFFFFFFFFFFFF:FFFFFFFFFFFFFFFFFFFFFFFFFFFFFFFFFFFFFFFFFFF
@A00155:342:HHGFNDSXY:1:2649:8956:21762 1:N:0:GAACCTAG+TCCGCATA
CTTCTCTCGGGCCCACAGCCTTACAGCTGTGGAGCACGGTATCCTCTGCCGAAACAGAGGTTGGACAA
GACCGGAGGGGTCTCCTAGTTCCAAAGGAGATGTACTCCGGGCTTGTTTACGACCTACCGTGTAAGTC
+
FFFFFFFFFFFFFFFFFFFFFFFFFFFFFFFFFFFFFFFFFFFFFFFFFFFFFFFFFFFFFFFFFFFF
FFFFFFFFFFFFFFFFFFFFFFFFFFFFFFFFFFFFFFFFFFFFFFFFFFFFFFFFFFFFFFFFFFFF
@A00155:342:HHGFNDSXY:1:2649:32922:35399 1:N:0:GAACCTAG+TCCGCATA
CTTCTCTCGGGCCCACAGCCTTACAGCTGTGGAGCACGGTATCCTCTGCCGAAACAGAGGTTGGACAA
GACCGGAGGGGTCTCCTAGTTCCAAAGGAGATGTACTCCGGGCTTGTTCACGACCTACCGTGTAAGTC
+
FFFFFFFFFFFFFFFFFFFFFFFFFFFFFFFFFFFFFFFFFFFFFFFFFFFFFFFFFFFFFFFFFFFF
F:FFFFFFFFFFFFFFFFFFFFFFFFFFFFFFFFFFFFFFFFFFFFFFF:FF:FFFFFFFFFFFFFFF
@A00155:342:HHGFNDSXY:1:2650:6777:18834 1:N:0:GAACCTAG+TCCGCATA
TGTCCACTTAAGGACTCACCTCTGGCCTATGGTCATCTCAATGCCGAGAGGGATGTGAAGGGCGAGAG
```

AGAGCGACTTCTCTCGGGCCCACAGCCTTACAGCTGTGGAGCACGGTATCCTCTGCCGAAACAGAGGT
+
FFFFFFFF:FFFFFFFFFFFFFFFFFFFFFFFFFF:FFFFFFFFFFFFFFFFF,FFFFFFFFFFFFFFF
FF:FFFFFFFFFFFFFFFFFFFFFFFFFFFFFFFFFFFFFFFFFFFFFFFFFFFFFFFFFFFFFFFFFF
@A00155:342:HHGFNDSXY:1:2650:15573:20478 1:N:0:GAACCTAG+TCCGCATA
GAGAGAGCGACTTCTCTCGGGCCCACAGCCTTACAGCTGTGGAGCACGGTATCCTCTGCCGAAACAGA
GGTTGGACAAGACCGGAGGGGTCTCCTAGTTCCAAAGGAGATGTACTCCGGGCTTGTTCACGACCTA
+
F:F:F,FFF,F:FFFFFFFFFFFFFFFF:FFFFF:FFFFFFF,F:F:FF,FF::FFFF,:FFFF::F,
F:F:FFFFFFF::FFFFFFFFFFFFF:FFFF,:F:,FFFF,:FFF,F:FFFF,:FFFF:FFF::FF:
@A00155:342:HHGFNDSXY:1:2650:32606:21198 1:N:0:GAACCTAG+TCCGCATA
AGGGATGTGGAGGGCGAGAGAGAGCGACTTCTCTCGGGCCCACAGCCTTACAGCTGTGGAGCACGGTA
TCCTCTGCCGAAACAGAGGTTGGACAAGACCGGAGGGGTCTCCTAGTTCCAAAGGAGATGTACTCCGG
+
FFFFFFFFFFFFFFFFFFFFFFFFFFFFFFFFFFFFFFFFFFFFFFFFFFFFFFFFFFFFFFFFFFFF
FFFFFFFFFFFFFFFFFFFFFFFFFFFFFFFFFFFFFFFFFFFFFFFFFFFFFFFFFFFFFFFFFFFF
@A00155:342:HHGFNDSXY:1:2650:12454:35963 1:N:0:GAACCTAG+TCCGCATA
TTACAGCTGTGGAGCACGGTATCCTCTGCCGAAACAGAGGTTGGACAAGACCGGAGGGGTCTCCTAGT
TCCAAAGGAGATGTACTCCGGGCTTGTTCACGACCTACCGTGTAAGTCGTAGTCTAGTAGGCTACCTG
+
FFFFFFFFFFFFFFFFFFFFFFFFFFFFFFFFFFFFFFFFFFFFFFFFFFFFFFFFFFFFFFFFFFFF
FFFFFFFFFFFFFFFFFFFF:FFFFFFFFFFFFFFFFFFFFFFFFFFFFFFFFFFFFFFFFFFFF:FF
@A00155:342:HHGFNDSXY:1:2651:10366:2534 1:N:0:GAACCTAG+TCCGCATA
CTCAATGCCGAGAGGGATGTAGAGGGCGAGAGAGAGCGACTTCTCTCGGGCCCACAGCCTTACAGCTG
TGGAGCACGGTATCCTCTGCCGAAACAGAGGTTGGACAAGACCGGAGGGGTCTCCTAGTTCCAAAGGA
+
FFFFFFFFFFFFFFFFFFFFFFFFFFFFFFFFFFFFFFFFFFFFFFFFFFFFFFFFFFFFFFFFFFFF
FFFFFFFFFFFFFFFFFFFFFFFFFFFFFFFFFFFFFFFFFFFFFFFFFFFFFFFFFFFFFFFFFFFF
@A00155:342:HHGFNDSXY:1:2651:18322:27367 1:N:0:GAACCTAG+TCCGCATA
TTAAGGACTCTCCTCTGGCCTATGGTCATCTCAATGCCGAGAGGGATGTGGAGGGCGAGAGAGAGCGA
CTTCTCTCGGGCCCACAGCCTTACAGCTGTGGAGCACGGTATCCTCTGCCGAAACAGAGGTTGGACAA
+
FFFFFFFFFFFFFFFFFFFFFFFFFFFFFFFFFFFFFFFFFFFFFFFFF:FFFFFFFFFFFFFFFFFF
FFFFFFFFFFFFFFFF,FFFFFFF:FFFFFFFFFFFFFFFFFFFFFFFFFFFFFFFFFFFFF:FFFFFF
@A00155:342:HHGFNDSXY:1:2651:21920:34726 1:N:0:GAACCTAG+TCCGCATA
TTAAAGACTCACCTCTGGCCTATGGTCATCTCAATGCCGAGAGGGATGTGGAGGGCGAGAGAGAGCGA
CTTCTCTCGGGCCCACAGCCTTACAGCTGTGGAGCACGGTATCCTCTGCCGAAACAGAGGTTGGACAA
+
FFFFFFFFFFFFFFFFFFFFFF,FFFFFFFFFFFFFFFFFFFFFFFFFFFFFFFFFFFFFFFFFFFFF
FFFF:FFFFFFFFFFFFFFFFFFF:FFFFFFFFFFFFFFFFFFFFFFFFFFFFFFFFFFFFFFFFFFFF
@A00155:342:HHGFNDSXY:1:2652:8404:2832 1:N:0:GAACCTAG+TCCGCATA
GTATCCTCTGCCGAAACAGAGGTTGGACAAGACCGGAGGGGTCTCCTAGTTCCAAAGGAGATGTACTC
CGGGCTTGTTCACGACCTACCGTGTAAGTCGTAGTCTAGTAGGCTACCTGACGAGTCCTTTTTAGGAC
+
FFFFFFFFFF:FFFFFFFF:FFFFFFFFFFFFFFFFFFFFFFFFFFFFFFFFFFFFFFFFFFFFFFFF
FFFFFFFFFFFFFFFFFFFFFFFFFFFFFFFFFFFFFFFFFFFFF:FFFFFFFFFFFFFFFFFFFFFF
@A00155:342:HHGFNDSXY:1:2652:9100:5509 1:N:0:GAACCTAG+TCCGCATA
TTCTCTCGGGCCCACAGCCTTACAGCTGTGGAGCACGGTATCCTCTGCCGAAACAGAGGTTGGACAAG
ACCGGAGGGGTCTCCTAGTTCCAAAGGAGATGTACTCCGGGCTTGTTCACGACCTACCGTGTAAGTCG
+
FFFFFFFFFFFFFFFFFFFFFFFFFFFFFFFFFFFFFFFFFFFFFFFFFFFFFFFFFFFFFFFFFFFF
FFFFFFFFFFFFFFFFFFFFFFFFFFFFFFFFFFFFFFFFFF:FFFFFFFFFFFFFFFFFFFFFFFFF
@A00155:342:HHGFNDSXY:1:2652:18267:16470 1:N:0:GAACCTAG+TCCGCATA
GAGAGGGATGTAGAGGGCGAGAGAGAGCGACTTCTCTCGGGCCCACAGCCTTACAGCTGTGGAGCACG

GTATCCTCTGCCGAAACAGAGGTTGGACAAGACCGGAGGGGTCTCCTAGTTCCAAAGGAGATGTACTC
+
FFFFFFFFFFFFFFFFFFFFFFFFFFFFFFFFFFFFFFFFFFFFFFFFFFFFFFFFFFFFFFFFFFFFFF
FFFFFFFFFFFFFFFFFFFFFFFFFFFFFFFFFFFFFFFFFFFFFFFFFFFFFFFFFFFFFFFFFFFFFF
@A00155:342:HHGFNDSXY:1:2652:8648:27085 1:N:0:GAACCTAG+TCCGCATA
CATAGGCCAGAGGTGAGTCCTTAAGTGGACACAGCTGATCTAAGGCGGTGTGGCGGGGCATGGGTTTG
AACCCCCATGACGGTCGGAGTCTAGTAGGCTCCCTGATGAGTCCGTTCCAAGGACGAAAC
+
FFFFFFFFFFFFFFFFFFFFFFFFFFFFFFFFFFFFFFFFFFFFFFFFFFFFFFFFFFFFFFFFFFFFFF
FFFFFFFFFFFFFFFFFFFFFFFFFFFFFFFFFFFFFFFFFFFFFFFFFFFFFFFFFFFF
@A00155:342:HHGFNDSXY:1:2652:22236:29168 1:N:0:GAACCTAG+TCCGCATA
AGAGAGAGCGACTTCTCTCGGGCCCACAGCCTTACAGCTGTGGAGCACGGTATCCTCTGCCGAAACAG
AGGTTGGACAAGACCGGAGGGGTCTCCTGGTTCCAAAGGAGATGTACTCCGGGCTTGTTCACGAC
+
FFFFFFFFFFFFFFFFFFFFFFFFFFFFFFFFFFFFFFFFFFFFFFFFFFFFFFFFFFFFFFFFFFFFFF
FFFFFFFFFFFFFFFFFFFFFFFFFFFFFFFFFFFFFFFFFFFFFFFFFFFFFFFFFFFFFFFFFFF
@A00155:342:HHGFNDSXY:1:2653:22083:3630 1:N:0:GAACCTAG+TCCGCATA
AGAGAGAGCGACTTCTCTCGGGCCCACAGCCTTACAGCTGTGGAGCACGGTATCCTCTGCCGAAACAG
AGGTTGGACAAGACCGGAGGGGTCTCCTAGTTCCAAAGGAGATGTACTCCGGGCTTGTTCACGACCTA
+
FFFFFFFFFFFFFFFFFFFFFFFFFFFFFFFFFFFFFFFFFFFFFFFFFFFFFFFFFFFFFFFFFFFFFF
FFFFFFFFFFFFFFFFFFFFFFFFFFFFFFFFFFFFFFFFFFFFFFFFFFFFFFFFFFFFFFFFFFFFFF
@A00155:342:HHGFNDSXY:1:2653:31295:17487 1:N:0:GAACCTAG+TCCGCATA
CTCAATGCCGAGAGGGATGTGGAGGGCGAGAGAGAGCGACTTCTCTCGGGCCCACAGCCTTACAGCTG
TGGAGCACGGTATCCTCTGCCGAAACAGAGGTTGGACAAGACCGGAGGGGTCTCCTAGTTCCAAAGGA
+
FFFFFFFFFFFFFFFFFFFFFFFFFFFFFFFFFFFFFFFFFFFFFFFFFFFFFFFFFFFFFFFFFFFFFF
FFFFFFFFFFFFFFFFFFFFFFFFFFFFFFFFFFFFFF:FFFFFFFFFFFFFFFFFFFFFFFFFFFFFF:
@A00155:342:HHGFNDSXY:1:2653:23176:20995 1:N:0:GAACCTAG+TCCGCATA
TGCCGAGAGGGATGTGGAGGGCGAGAGAGAGCGACTTCTCTCGGGCCCACAGCCTTACAGCTGTGGAG
CACGGTATCCTCTGCCGAAACAGAGGTTGGACAAGACCGGAGGGGTCTCCTGGTTCCAAAGGAGAT
+
FFFFFFFFFFFFFFF:FFFFFFFFFFFFFFFFFFFFFFFFFFFFFFFFFFFFFFFFFFFFFFFFFFFFFF
FFFFFFFFFFFFFFFFFFFFFFF:FFFFFFFFFFFFFFFFFFFFFFFFFFFFFFFFFFFFFFFFFFF
@A00155:342:HHGFNDSXY:1:2654:7789:2801 1:N:0:GAACCTAG+TCCGCATA
CTCAATGCCGAGAGGGATGTAGAGGGCGAGAGAGAGCGACTTCTCTCGGGCCCACAGCCTTACAGCTG
TGGAGCACGGTATCCTCTGCCGAAACAGAGGTTGGACAAGACCGGAGGGGTCTCCTAGTTCCAAAGGA
+
FFFFFFFFFFFFFFFFFFFFFFFFFFFFFFFFFFFFFFFFFFFFFFFFFFFFFFFFFFFFFFFFFFFFFF
FFFFFFFFFFFFFFFFFFFFFFFFFFFFFFFFFFFFFFFFFFFFFFFFFFFFFFFFFFFFFFFFFFFFFF
@A00155:342:HHGFNDSXY:1:2654:21965:20807 1:N:0:GAACCTAG+TCCGCATA
CTTCTCTCGGGCCCACAGCCTTACAGCTGTGGAGCACGGTATCCTCTGCCGAAACAGAGGTTGGACAA
GACCGGAGGGGTCTCCTAGTTCCAAAGGAGATGTACTCCGGGCTTGTTCACGACCTACCGTGTAAGTC
+
FFFFFFFFFFFFFFFFFFFFFFFFFFFFFFFFFFFFFFFFFFFFFFFFFFFFFFFFFFFFFFFFFFFFFF
FFFFFFFFFFFFFFFFFFFFFFFFFFFFFFFFFFFFFFFFFFFFFFFFFFFFFFFFFFFFFFFFFFFFFF
@A00155:342:HHGFNDSXY:1:2654:12057:30107 1:N:0:GAACCTAG+TCCGCATA
TGCCGAGAGGGATGTGGAGGGCGAGAGAGAGCGACTTCTCTCGGGCCCACAGCCTTACAGCTGTGGAG
CACGGTATCCTCTGCCGAAACAGAGGTTGGACAAGACCGGAGGGGTCTCCTAGTTCCAAAGGAGATGT
+
FFFFFFFFFFFFF:FFFFFFFFFFFFFFFFFFFFFFFFFFFFFFFFFFFFFFFFFFFFFFFFFFFFFFFF
FFFFFFFFFFFFFFFFFFFFF:FFFFFFFFFFFFFFFFFFFFFFFFFFFFFFFFFFFFFFFFFFFFFFFF
@A00155:342:HHGFNDSXY:1:2654:10719:30232 1:N:0:GAACCTAG+TCCGCATA
TGCCGAGAGGGATGTGGAGGGCGAGAGAGAGCGACTTCTCTCGGGCCCACAGCCTTACAGCTGTGGAG

```
CACGGTATCCTCTGCCGAAACAGAGGTTGGACAAGACCGGAGGGGTCTCCTAGTTCCAAAGGAGATGT
+
FFFFFFFFFFFF:FFFFFFFFFFFFFFFFFFFFFFFFFFFFFFFFFFFFFFFFFFFFFFFFFFFFFFF
FFFFFFFFFFFFFFFFFFFFFFFFFFFFFFFFFFFFFFFFFFFFFFFFFFF,FFFFFFFFFFFFFFFF
@A00155:342:HHGFNDSXY:1:2655:11885:2910 1:N:0:GAACCTAG+TCCGCATA
TGCCGAGAGGGATGTGGAGGGCGAGAGAGAGCGACTTCTCTCGGGCCCACAGCCTTACAGCTGTGGAG
CACGGTATCCTCTGCCGAAACAGAGGTTGGACAAGACCGGAGGGGTCTCCTAGTTCCAAAGGAGATGT
+
FFF:FFFFFFFFFFFFFFFFFFFFFFFFFFFFFFFFFFFFFFFFFFFFFFFFFFFFFF:FFFFFFFF
FFFFFFFFFFFFFFFFFFFFFFFFFFFF:FFFFFFFFFFFFFFFFFFFFFFFFFFFFFFFFFFFFFFF
@A00155:342:HHGFNDSXY:1:2655:7627:6339 1:N:0:GAACCTAG+TCCGCATA
ATCTCAATGCCGAGAGGGATGTGGAGGGCGAGAGAGAGCGACTTCTCTCGGGCCCACAGCCTTACAGC
TGTGGAGCACGGTATCCTCTGCCGAAACAGAGGTTGGACAAGACCGGAGGGGTCTCCTAGTTCCAAAG
+
FFFFFFFFFFFFFFFFFFFFFFFFFFFFFFFFFFFFFFFFFFFFFFFFFFFFFFFFFFFFFFF:FFFF
FFFFFFFFFFFFFFFFFFFFFFFFFFFFFFFFFF:FFFFFFFFFFFFFFFFFFFFFFFFFFFFFFFFF
@A00155:342:HHGFNDSXY:1:2655:30011:15953 1:N:0:GAACCTAG+TCCGCATA
GAGAGGGATGTAGAGGGCGAGAGAGAGCGACTTCTCTCGGGCCCACAGCCTTACAGCTGTGGAGCACG
GTATCCTCTGCCGAAACAGAGGTTGGACAAGACCGGAGGGGTCTCCTAGTTCCAAAGGAGATGTACTC
+
FFFFFFFFFF:FFFFFFFFFFFFFFFFFFFFFFFFFFFFFFFF:FFFFFFF::,FFFFFFFFFFFFFFF
FF:FFFFFFFFFFFFFFF:FF:FFFFFFFF:FFFFFFFFFFFFFFFFFFFFFFFFFF:FFFF:FFFFFF
@A00155:342:HHGFNDSXY:1:2655:16703:27696 1:N:0:GAACCTAG+TCCGCATA
GGGCGAGAGAGAGCGACTTCTCTCGGGCCCACAGCCTTACAGCTGTGGAGCACGGTATCCTCTGCCGA
AACAGAGGTTGGACAAGACCGGAGGGGTCTCCTAGTTCCAAAGGAGATGTACTCCGGGCTTGTTCACG
+
FFFFFFFFFFFFFFFFFFFFFFFFFFFFFFFFFFFFFFFFFFF:FFFFFFFFFFFFFFFFFFFFFFFFF
FFFFFFFFFFFFFFFFFFFFFFFFFFFFFFFFFF:FFFFFF:FFFFFFFFFFFFFFFFFFFFFFFFFFF
@A00155:342:HHGFNDSXY:1:2655:15682:35196 1:N:0:GAACCTAG+TCCGCATA
TGTTTCGGCAGAGGATACCGTGCTCCACAGCTGTAAGGCTGTGGGCCCGAGAGAAGTCGCTCTCTCTC
GCCCTTCACATCCCTCTCGGCATTGAGATGACCATAGGCCAGAGGTGAGTCCTTAAGTGGACACAGCT
+
FFFFFFFFFFFFFFFFFFFFFFFFFFFFFFFFFFFFFFFFFFFFFFFFFFFFFFFFFFFFFFFFFFFF
FFFFFFFFFFFFFFFFFFFFFFFFFFFFFFFFFFFFFFFFFFFFFFFFFFFFFFFFFFFFFFFFFFFF
@A00155:342:HHGFNDSXY:1:2656:10755:11725 1:N:0:GAACCTAG+TCCGCATA
GGATGTAGAGGGCGAGAGAGAGCGACTTCTCTCGGGCCCACAGCCTTACAGCTGTGGAGCACGGTATC
CTCTGCCGAAACAGAGGTTGGACAAGACCGGAGGGGTCTCCTAGTTCCAAAGGAGATGTACTCC
+
:FFFFFFFF,:FF,FFFFF:FFFFF:FF:FFFF:FFFFFFFF:FF,FFFFFF:FF,FF,FFFFFFFFF
F,FFFFF:FFFFFFFF,FF,FF,FFFFFF:FFFF::FFFFFFF:FFFFFF:,:FFFFFFFFFFF
@A00155:342:HHGFNDSXY:1:2656:12979:11851 1:N:0:GAACCTAG+TCCGCATA
TTCTCTCGGGCCCACAGCCTTACAGCTGTGGAGCACGGTATCCTCTGCCGAAACAGAGGTTGGACAAG
ACCGGAGGGGTCTCCTAGTTCCAAAGGAGATGTACTCCGGGCTTGTTCACGACCTACCGTGTAAGTCG
+
FFFFFFFFFFFFFFFFFFFFF:FFFFFFFFFFFFFFFFFFFFFFFFFFFFFFFFFFFFFFFFFFFFFF
FFFFFFFFFFFFFFFFFFFFFFFFFFFFF:FFFFFFFFFFFFFFFFFFFFFFFF:FFFFFFFFFFFFFFF
@A00155:342:HHGFNDSXY:1:2656:9860:12774 1:N:0:GAACCTAG+TCCGCATA
GAGGGCGAGAGAGAGCGACTTCTCTCGGGCCCACAGCCTTACAGCTGTGGAGCACGGTATCCTCTGCC
GAAACAGAGGTTGGACAAGACCGGAGGGGTCTCCTAGTTCCAAAGGAGATGTACTCCGGGCTTGTTCA
+
FFFFFFFFFFFFFFFFFFFFFFFFFFFFFFFFFFFFFFFFFFFFFFFFFFFFFFFFFFFFFFFFFFFF
FFFFFFFFFFFFFFFFFFFFFFFFFFFFFFFFFFFFFFFFFFFF,FFFFFFFFFFFFFFFFFFFFFFF
@A00155:342:HHGFNDSXY:1:2656:25093:20870 1:N:0:GAACCTAG+TCCGCATA
ATCTCAATGCCGAGAGGGATGTGGAGGGCGAGAGAGAGCGACTTCTCTCGGGCCCACAGCCTTACAGC
```

TGTGGAGCACGGTATCCTCTGCCGAAACAGAGGTTGGACAAGACCGGAGGGGTCTCCTAGTTCCAAAG
+
FFFFFFFFFFFFFFFFFFFFFFFFFFFFFFFFFFFFFFFFFFFFFFFFFFFFFFFFFFFFFFFFFFFF
FFFFFFFFFFFFFFFFFFFFFFFFFFFFFFFFFFFFFFFFFFFFFFFFFFFFFFFFFFFFFFFFFFFF
@A00155:342:HHGFNDSXY:1:2656:22182:25880 1:N:0:GAACCTAG+TCCGCATA
TATCCTCTGCCGAAACAGAGGTTGGACAAGACCGGAGGGGTCTCCTGGTTCCAAAGGAGATGTACTCC
GGGCTTGTTCACGACCTACCGTGTAAGTCGTAGTCTAGTAGGCTACCTGACGAGTCCTTTTTAGGACG
+
FFFFFFFFFFFFFFFFFFFFFFFFFFFFFFFFFFFFFFFFFFFFFFFFFFFFFFFFFFFFFFFFFFFF
FFFFFFFFFFFFFFFFFFFFFFFFFFFFFFFFFFFFFFFFFFFFFFFFFFFFFFFFFFFFFFFF:FFF
@A00155:342:HHGFNDSXY:1:2656:4761:33974 1:N:0:GAACCTAG+TCCGCATA
AGAGAGAGCGACTTCTCTCGGGCCCACAGCCTTACAGCTGTGGAGCACGGTATCCTCTGCCGAAACAG
AGGTTGGACAAGACCGGAGGGGTCTCCTGGTTCCAAAGGAGATGTACTCCGGGCTTGTTCACGACCTA
+
FFFFFFFFFFFFFFFFFFFFFFFFFFFFFF:FFFFFFFFFFFFFFFFFFFFFFFFFFFF:FFFFFFF
FFFFFFFFFFFFFFFFFFFFFFFFFFFFFFFFFFFFFFFFFFFFFFFF:FFFFFFFFFFFFFFFFFFF
@A00155:342:HHGFNDSXY:1:2657:19379:4523 1:N:0:GAACCTAG+TCCGCATA
CGAGAGAGAGCGACTTCTCTCGGGCCCACAGCCTTACAGCTGTGGAGCACGGTATCCTCTGCCGAAAC
AGAGGTTGGACAAGACCGGAGGGGTCTCCTGGTTCCAAAGGAGATGTACTCCGGGCTTGTTCACGACC
+
FFFFFFFFFFFFFFFFF:FFFFFFFFFFFFFFFFFFFFFFFFFFFFFFFFFFFFFFFFFFFFFFFFFF
FFFFFFFFFFFFFFFFFFFFFFFFFFFFFFFFFFFFFFFFFFFFFFFFFFFFFFFFFFFFFFFFFFFF
@A00155:342:HHGFNDSXY:1:2657:18005:11005 1:N:0:GAACCTAG+TCCGCATA
TGCCGAGAGGGATGTGGAGGGCGAGAGAGAGCGACTTCTCTCGGGCCCACAGCCTTACAGCTGTGGAG
CACGGTATCCTCTGCCGAAACAGAGGTTGGACAAGACCGGAGGGGTCTCCTGGTTCCAAAGGAGATGT
+
FFFFFFFFFFFFFFFFFFFFFFFFFFFFFFFFFFFFFFFFFFFFFFFFFFFFFFFFFF:FFFFFFFFF
FFFFFFFFFFFFFFFFFFFFF:FFFFFFFFFFFFFFFFFFFFFFFFFFFFFFFFFFF:F:FFFFFFFF
@A00155:342:HHGFNDSXY:1:2657:6668:13448 1:N:0:GAACCTAG+TCCGCATA
ACCTCTGTTTCGGCAGAGGATACCGTGCTCCACAGCTGTAAGGCTGTGGGCCCGAGAGAAGTCGCTCT
CTCTCGCCCTCTACATCCCTCTCGGCATTGAGATGACCATAGGCCAGAGGTGAGTCCTTAAG
+
FFFFFFFFFFFFFFFFFFFFFFFFFFFFFFFFFFFFFFFFFFFFFFFFFFFFFFFFFFFFFFFFFFFF
FFFFFFFFFFFFFFFFFFFFFFFFFFFFFFFFFFFFFFFFFFFFFFFFFFFFFFFFFFFFFF:
@A00155:342:HHGFNDSXY:1:2657:12364:29324 1:N:0:GAACCTAG+TCCGCATA
CGAGAGGGATGTAGAGGGCGAGAGAGAGCGACTTCTCTCGGGCCCACAGCCTTACAGCTGTGGAGCAC
GGTATCCTCTGCCGAAACAGAGGTTGGACAAGACCGGAGGGGTCTCCTAGTTCCAAAGGAGATGTACT
+
FFFFFFFFFFFFFFFFFFFFFFFFFFFFFFFFFFFFFFFFFFFFFFFFFFFFFFFFFFFFFFFFFFFF
FFFFFFFFFFFFFFFFFFFFFFFFFFFFFFFFFFFFFFFFFFFFFFFFFFFFFFFFFFFFFFFFFFFF
@A00155:342:HHGFNDSXY:1:2658:31467:11835 1:N:0:GAACCTAG+TCCGCATA
ACCTCTGTTTCGGCAGAGGATACCGTGCTCCACAGCTGTAAGGCTGTGGGCCCGAGAGAAGTCGCTCT
CTCTCGCCCTTCACATCCCTCTCGGCATTGAGATGACCATAGGCCAGAGGTGAGTCCTTAAGTGGAC
+
FFFFFFFFFFFFFFFFFFFFFFFFFFFFFFFFFFFFFFFF:FFFFFFFFFFFFFFFFFFFFFFFFFFF
FFFFFFFFFFFFFFFFFFFFFFFFFFFF:FFFFFFFFFFFFFFFFFFFFFFFFFFFFFFFFFFFFFFF
@A00155:342:HHGFNDSXY:1:2658:30906:13839 1:N:0:GAACCTAG+TCCGCATA
GATCAGCTGTGTCCACTTAAGGACTCACCTCTGGCCTATGGTCATCTCAATGCCGAGAGGGATGTGAA
GGGCGAGAGAGAGCGACTTCTCTCGGGCCCACAGCCTTACAGCTGTGGAGCACGGTATCCTCTGCCGA
+
FFFFFFFFFFFFFFFFFFFFFFFFFFFFFFFFFFFFF:FFFFFFFFFFFFFFFFFFFFFFFF:FFFFFF
FFFFFFFFFFFFFFFFFFFFFFFFFFFFFFFFFFFFFFFFFFF:FFFFFFFFFFFFFFFFFFFFFFFF
@A00155:342:HHGFNDSXY:1:2658:26946:29183 1:N:0:GAACCTAG+TCCGCATA
AATGCCGAGAGGGATGTGGAGGGCGAGAGAGAGCGACTTCTCTCGGGCCCACAGCCTTACAGCTGTGG

AGCACGGTATCCTCTGCCGAAACAGAGGTTGGACAAGACCGGAGGGGTCTCCTAGTTCCAAAGGAGAT
+
FFFFFFFFFFFFFFFFFFFFFFFFFFFFFFF:FFFFFFFFFFFFFFFFFFFFFFFFFFFFFFFFFFFFF
FFFFFFFFFFFFFFFFFFFFFFFFFFFFFFFFFFFFFFFFFFFFFFFFFFFFFFFFFFFFFFFFFFFFF
@A00155:342:HHGFNDSXY:1:2658:19199:31297 1:N:0:GAACCTAG+TCCGCATA
CGAGAGGGATGTAGAGGGCGAGAGAGAGCGACTTCTCTCGGGCCCACAGCCTTACAGCTGTGGAGCAC
GGTATCCTCTGCCGAAACAGAGGTTGGACAAGACCGGAGGGGTCTCCTAGTTCCAAAGGAGATGTACT
+
FF:FFFFFFFF:FFFFFFFFFFFFFFFFFFFFFFFFFFFFFFFFFFFFFFFFFFFFFFFFFFFFFFFFF
FFFFFFFFFFFFFFFFFFFFFFFFFFFFFFFFFFFFFFFFFFFFFFFFFFFFFFFFFFFFFFFFFFFFF
@A00155:342:HHGFNDSXY:1:2659:21522:7921 1:N:0:GAACCTAG+TCCGCATA
TCTGTTTCGGCAGAGGATACCGTGCTCCACAGCTGTAAGGCTGTGGGCCCGAGAGAAGTCGCTCTCTC
TCGCCCTCTACATCCCTCTCGGCATTGAGATGACCATAGGCCAGAGGTGAGTCCTTAAGTGGACACAG
+
FFFFFFFFFFFFF,FFFFFFFFFFFFFFFFFFFFFFFFFFFFFFFFFFFFFFFFFFFFFFFFFFFFFFF
FFFFFFFFFFFFFFFFFFFFFF:FFFFFFFFFFFFFFFFFFFFFFFFFF:FFFFFFFFFFFF:FFFF:FFF
@A00155:342:HHGFNDSXY:1:2659:1976:18505 1:N:0:GAACCTAG+TCCGCATA
GGGCGAGAGAGAGCGACTTCTCTCGGGCCCACAGCCTTACAGCTGTGGAGCACGGTATCCTCTGCCGA
AACAGAGGTTGGACAAGACCGGAGGGGTCTCCTAGTTCCAAAGGAGATGTACTCCGGGCTTGTTCACG
+
FFFFFFFFFFFFFFFFFFFFFFFFFFFFFFFFFFFFFFFFFFFFFFFFFFFFFFF:FFFFFFFFFFF
FFFFFFFFFFFFFFFFFFFFFFFFFFFFFFFFFFFFFFFFFFFFFFFFFFFFFFFFFFFFFFFFFFFFF
@A00155:342:HHGFNDSXY:1:2659:22742:28855 1:N:0:GAACCTAG+TCCGCATA
AGAGAGAGCGACTTCTCTCGGGCCCACAGCCTTACAGCTGTGGAGCACGGTATCCTCTGCCGAAACAG
AGGTTGGACAAGACCGGAGGGGTCTCCTAGTTCCAAAGGAGATGTACTCCGGGCTTGTTCACGACCTA
+
FFFFFFFFFFFFFFFFFFFFFFFFFFFFFFFFFFFFFFFFFFFFFFFFFFFFFFFFFFFFFFFFFFFFF
FFFFFFFFFFFFFFFFFFFFFFFFFFFFFFFFFFFFFFFFFFFFFFFFFFFFFFFFFFFFFFFFFFFFF
@A00155:342:HHGFNDSXY:1:2659:4869:31469 1:N:0:GAACCTAG+TCCGCATA
GAGCGACTTCTCTCGGGCCCACAGCCTTACAGCTGTGGAGCACGGTATCCTCTGCCGAAACAGAGGTT
GGACAAGACCGGAGGGGTCTCCTAGTTCCAAAGGAGATGTACTCCGGGCTTGTTCACGACCTACCGTG
+
FFFFFFFFFFFFFFFFFFFFFFFFFFFFFFFFFFFFFFFFFFFFFFFFFFFFFFFFFFF:FFFFFFFFF
FFFFFFFFFFFFFFFFFFFFFFFFFFFFFFFFFFFFFFFFFFFFFFFFFFFFFFFFFFFFFFFFFFFFF
@A00155:342:HHGFNDSXY:1:2659:31403:35712 1:N:0:GAACCTAG+TCCGCATA
CTTCTCTCGGGCCCACAGCCTTACAGCTGTGGAGCACGGTATCCTCTGCCGAAACAGAGGTTGGACAA
GACCGGAGGGGTCTCCTGGTTCCAAAGGAGATGTACTCCGGGCTTGTTCACGACCTACCGTGTAAGTC
+
FFFFFFFFFFFFFFFFFFFFFF:FFFFFFFFFFFFFFFFFFFFFFFFFFFFFFFFFFFFFFFFFFFFFF
FFFFFFFFFFFFFFFFFFFFFFFFFFFFFFFFFFFFFFFFFFFFFFFFFFFFFFFFFFFFFFFFFFFFF
@A00155:342:HHGFNDSXY:1:2659:31855:36746 1:N:0:GAACCTAG+TCCGCATA
CTTCTCTCGGGCCCACAGCCTTACAGCTGTGGAGCACGGTATCCTCTGCCGAAACAGAGGTTGGACAA
GACCGGAGGGGTCTCCTGGTTCCAAAGGAGATGTACTCCGGGCTTGTTCACGACCTACCGTGTAAGTC
+
FFFFFFFFFFFFFFFFFFFFFFFFFFFFFFFFFFFFFFFFFFFFFFFFFFFFFFFFFFFFFFFFFFFFF
FFFFFFFFFFFFFFFFFFFFFFFFFFFFFFFFFFFFFFFFFFFFFFFFFFFFFFFFFFFFFFFFF:FFF
@A00155:342:HHGFNDSXY:1:2660:2537:13964 1:N:0:GAACCTAG+TCCGCATA
ATCTCAATGCCGAGAGGGATGTGGAGGGCGAGAGAGAGCGACTTCTCTCGGGCCCACAGCCTTACAGC
TGTGGAGCACGGTATCCTCTGCCGAAACAGAGGTTGGACAAGACCGGAGGGGTCTCCTAGTTCCAAAG
+
FFFFFFFFFFFFFFFFFFFF:FFFFFFFFFFFFFFFFFFFFFFFFFFFFFFFFFFFFFFFFFFFFFFFF
F:FFFFFFFFFFFFFFFFFFFFFFFFFFFFFFFFFFFFFFFFFFFFFFFF:FFFFFFFFFFFFFFFFFF
@A00155:342:HHGFNDSXY:1:2660:26567:14184 1:N:0:GAACCTAG+TCCGCATA
CTCAATGCCGAGAGGGATGTGGAGGGCGAGAGAGAGCGACTTCTCTCGGGCCCACAGCCTTACAGCTG

```
TGGAGCACGGTATCCTCTGCCGAAACAGAGGTTGGACAAGACCGGAGGGGTCTCCTAGTTCCAAAGGA
+
FFFFFFFFFFFFFFFFF:FFFFFF:FFFFFFFFFFFFFFFFFFFFFFFFFFFFFFFFFFF:FFFFFFF
FFFFFFFFFFFFFFFFFFFFFFFFFFFFFFFFFFFFFFFFFFFFFFFFFFFFFFFF,FFFFFFFFFFF
@A00155:342:HHGFNDSXY:1:2660:18385:17644 1:N:0:GAACCTAG+TCCGCATA
GAGCGACTTCTCTCGGGCCCACAGCCTTACAGCTGTGGAGCACGGTATCCTCTGCCGAAACAGAGGTT
GGACAAGACCGGAGGGGTCTCCTGGTTCCAAAGGAGATGTACTCCGGGCTTGTTCACGACCTACCGTG
+
FFFFFFFFFFFFFFFFFFFFFFFFFFFFFFFFFFFFFFFFFFFFFFFFFFFFFFFFFFFFFFFFFFFF
FFFFFFFFFFFFFFFFFFFFFFFFFFFFFFFFFFFFFFFFFFFFFFFFFFFFFFFFFFFFFFFFFFFF
@A00155:342:HHGFNDSXY:1:2660:22806:19507 1:N:0:GAACCTAG+TCCGCATA
GGCCAGAGGTGAGTCCTTAAGTGGACACAGCTGATCTAAGGCGGTGTAGCGGGGCATGGGTTTGAACC
CCCATGACGGTCGGAGTCTAGTAGGCTCCCTGATGAGTCCGTTCCAAGGACGAAACCGTCGTTAC
+
FFFFFFFFFFFFFFFFFFFFFFFFFFFFFFFFFFFFFFFFFFFFFFFFFFFFFFFFFFFFFFFFFFFF
FFFFFFFFFFFFFFFFFFFFFFFFFFFFFFFFFFFFFFFFFFFFFFFFFFFFFFFFFFFFFFFFF
@A00155:342:HHGFNDSXY:1:2661:8223:3521 1:N:0:GAACCTAG+TCCGCATA
CTTCTCTCGGGCCCACAGCCTTACAGCTGTGGAGCACGGTATCCTCTGCCGAAACAGAGGTTGGACAA
GACCGGAGGGGTCTCCTAGTTCCAAAGGAGATGTACTCCGGGCTTGTTCACGACCTACCGTGTAAGTC
+
FFFFFFFFFFFFFFFFFFFFFFFFFFFFFFFFFFFFFFF:FFFFFFFFFFFFFFFFFFFFFFF,FFFF
FFFFFFFFFFFFFFFFFFFFFFFFFFFFFFFFFFFFFFFFFFFFFFFFFFFFFFFFFFFFFFFF::FF
@A00155:342:HHGFNDSXY:1:2661:32931:21261 1:N:0:GAACCTAG+TCCGCATA
GAGGGCGAGAGAGAGCGACTTCTCTCGGGCCCACAGCCTTACAGCTGTGGAGCACGGTATCCTCTGCC
GAAACAGAGGTTGGACAAGACCGGAGGGGTCTCCTAGTTCCATAGGAGATGTACTCCGGGCTTGTTCA
+
FFFFFFF:FFFFFFFFFFFFFFFFFFFFFFFFFFFFFFFFFFFFFFFFFF:FFFFFFFFFFFFFFFFF
F:FFFFFFFFFFFFFFFFF:FFFFFFFFFFFFFFFFFFFFFF:FFF:FFFFFFF:FFFFF:FFFFFFF
@A00155:342:HHGFNDSXY:1:2662:28899:14716 1:N:0:GAACCTAG+TCCGCATA
ATCAGCTGTGTCCACTTAAGGACTCACCTCTGGCCTATGGTCATCTCAATGCCGAGAGGGATGTGGAG
GGCGAGAGAGAGCGACTTCTCTCGGGCCCACAGCCTTACAGCTGTGGAGCACGGTATCCTCTGC
+
FFFFFFFF,FFFFFFFFFFFFFFFFFFFFFF:FFFFFFF:::FFFFFFF:,FFFF,F,FFFFFFFFFF
FFFFFFFFFFF,FFFFFFFF::FFFFFF,FF:FFFFFF:F:FFF:FFFFFFFFFFFFFFFFFFF
@A00155:342:HHGFNDSXY:1:2662:10836:21449 1:N:0:GAACCTAG+TCCGCATA
CTTCTCTCGGGCCCACAGCCTTACAGCTGTGGAGCACGGTATCCTCTGCCGAAACAGAGGTTGGACAA
GACCGGAGGGGTCTCCTAGTTCCAAAGGAGATGTACTCCGGGCTTGTTCACGACCTACCGTGTAAGTC
+
FFFFFFFFFFFFFFFFFFFFFF:FFFFFFFFFFFFFFFFFFFFFFFFFFFFFFFFFFFFFFFFFFFFF
FFFFFFFFFFFFFFFFFFFFFFFFFFFFFF:FFFFFFFFFFFFFFFFFFFFFFFFFFFFFFFFFFFFF
@A00155:342:HHGFNDSXY:1:2662:18132:21527 1:N:0:GAACCTAG+TCCGCATA
CTTCTCTCGGGCCCACAGCCTTACAGCTGTGGAGCACGGTATCCTCTGCCGAAACAGAGGTTGGACAA
GACCGGAGGGGTCTCCTGGTTCCAAAGGAGATGTACTCCGGGCTTGTTCACGACCTACCGTGTAAGTC
+
FFFFFFFFFFFFFFFFFFFFFFFFFFFFFFFFFFFFFFFFFFFFFFFFFFFFFFFFFFFFFFFFFFFF
F,FFFFFFF:FFFFFFFFFFFFFFFFFFFFFFFFFFFFFFFFFFFFFFFFFFFFFFFFFFFFFFFFFF
@A00155:342:HHGFNDSXY:1:2662:10773:21590 1:N:0:GAACCTAG+TCCGCATA
CTTCTCTCGGGCCCACAGCCTTACAGCTGTGGAGCACGGTATCCTCTGCCGAAACAGAGGTTGGACAA
GACCGGAGGGGTCTCCTAGTTCCAAAGGAGATGTACTCCGGGCTTGTTCACGACCTACCGTGTAAGTC
+
FFFFFFFFFFFFFFFFFFFFFFFFFFFFFFFFFFFFFFFFFFFFFFFFFFFFFFFFFFFFFFFFFFFF
FFFFFFFF:FFFFFFFFFFFFFFFFFFFFFFFFFFFFFFFFFFFFFFFFFFFFFFFFFFFFFF:FFF
@A00155:342:HHGFNDSXY:1:2662:9796:24283 1:N:0:GAACCTAG+TCCGCATA
CCCATGACGGTCGGAGTCTAGTAGGCTCCCTGATGAGTCCGTTCCAAGGACGAAACCGTCGTTACTCT
```

TTGAGTTGTGTAAGTTTCGTCCTAAAAAGGACTCGTCAGGTAGCCTACTAGACTACGAC
+
FFFFFFFFFFFFFFFFFFFFFFFFFFFFFFFFFFFFFFFFFFFFFFFFFFFFFFFFFFFFFFFFFFFF,
FFFFFFFF:FFFFFFFFFFFFFFFFFFFFFFFFFFFFFFFFFFFFFFFFFFFFFFFFFFF
@A00155:342:HHGFNDSXY:1:2663:30698:15076 1:N:0:GAACCTAG+TCCGCATA
TCGGCAGAGGATACCGTGCTCCACAGCTGTAAGGCTGTGGGCCCGAGAGAAGTCGCTCTCTCTCGCCC
TCCACATCCCTCTCGGCATTGAGATGACCATAGGCCAGAGGAGAGTCCTTAAGTGGAC
+
FFFFFFFFFFFFFFFFFFFFFFFFFFFFFFFFFFFFFFFFFFFFFFFFFFFFFFFFFFFFFFFFFFFFF
FFFFFFFFFFFFFFFFFFFFFFFFFFFFFFFFFFFFFFFFFFFFFFFFFFFFFFFFFF
@A00155:342:HHGFNDSXY:1:2663:23990:18051 1:N:0:GAACCTAG+TCCGCATA
GTCCACTTAAGGACTCACCTCTGGCCTATGGTCATCTCAATGCCGAGAGGGATGTGGAGGGCGAGAGA
GAGCGACTTCTCTCGGGCCCACAGCCTTACAGCTGTGGAGCACGGTATCCTCTGCCGAAACAGAGGTT
+
FFFFFFFFFFFFFFFFFFFFFFFFFFFFFFFFFFFFFFFFFFFFFFFFFFFFFFFFFFFFFFFFFFFFF
FFFFFFFFFFFFFFFFFFFFFFFFFFFFFFFFFF:FFFFFFFFFFFFFFFFFFFFFFFFFFFFFFFFFF
@A00155:342:HHGFNDSXY:1:2663:13232:26537 1:N:0:GAACCTAG+TCCGCATA
ATCCTCTGCCGAAACAGAGGTTGGACAAGACCGGAGGGGTCTCCTAGTTCCAAAGGAGATGTACTCCG
GGCTTGTTCACGACCTACCGTGTAAGTCGTAGTCTAGTAGGCTACCTGACGAGTCCTTTTTAGGACGA
+
FFFFFFFFFFFFFFFFFFFFFFFFFFFFFFFFFFFFFFFFFFFFFFFFFFFFFFFFFFFFFFFFFFFFF
FFFFFFFFFFFFFFFFFFFFFFFFFFFFFFFFFFFFFFFFFFFFFFFFFFFFFFFFFFFFFFFFFFFFF
@A00155:342:HHGFNDSXY:1:2663:5656:32487 1:N:0:GAACCTAG+TCCGCATA
AAGGACTCACCTCTGGCCTATGGTCATCTCAATGCCGAGAGGGATGTGAAGGGCGAGAGAGAGCGACT
TCTCTCGGGCCCACAGCCTTACAGCTGTGGAGCACGGTATCCTCTGCCGAAACAGAGGTTGG
+
FFFFFFFFFFFFFFFFFFFFFFFFFFFFFFFFFFFFF:FFFFFFFFFFFFFFFFFFFFFFFFFFFFFFF
FFFFFFFFFFFFFFFFFFFFFFFFFFFFFFFFFFFFFFFFFFFFFFFFFFFFFFFFFFF,FFF
@A00155:342:HHGFNDSXY:1:2664:4607:2801 1:N:0:GAACCTAG+TCCGCATA
GGATGTGGAGGGCGAGAGAGAGCGACTTCTCTCGGGCCCACAGCCTTACAGCTGTGGAGCACGGTATC
CTCTGCCGAAACAGAGGTTGGACAAGACCGGAGGGGTCTCCTAGTTCCAAAGGAGATGTACTCC
+
FFFFFFFFFFFFFFFFFFFFFFFFFFFFFFFFFFFFFFFFF:FFFFFFFFFFFFFFFFFFFFFFFFFFF
FFFFFFFFFFFFFFFFFFFFFFFFF:FFFFFFFFFFFFFFFFF:FFFFFFFFFFFFFF:FFFFF:
@A00155:342:HHGFNDSXY:1:2664:11143:18975 1:N:0:GAACCTAG+TCCGCATA
ATCTCAATGCCGAGAGGGATGTGGAGGGCGAGAGAGAGCGACTTCTCTCGGGCCCACAGCCTTACAGC
TGTGGAGCACGGTATCCTCTGCCGAAACAGAGGTTGGACAAGACCGGAGGGGTCTCCTAGTTCCAAAG
+
FFFFFFFFFFFFFFFFFFFFFFFFFFFFFFFFFFFFFFFFFFFFFFFFFFFFFFFFFFFFFFFFFFFFF
FFFFFFFFFFFFFFFFFFFFFFFFFFFFFFFFFFFFFFFFFFFFFFFFFFFFFFFFFFFFFFFFFFFFF
@A00155:342:HHGFNDSXY:1:2665:4535:17863 1:N:0:GAACCTAG+TCCGCATA
GAGCGACTTCTCTCGGGCCCACAGCCTTACAGCTGTGGAGCACGGTATCCTCTGCCGAAACAGAGGTT
GGACAAGACCGGAGGGGTCTCCTAGTTCCAAAGGAGATGTACTCCGGGCTTGTTCACGACCTACCGTG
+
FFFFFFFFFFFFFFFFFFFFFFFFFFFFFFFFFFF:FFFFFFFFFFFFFFFFFFFFFFFFFFFFFFFFF
FFFFFFFFFFFFFFFFFFFFFFFFFFFFFFFF:FFFFFFFFFFFFF:FFFFFFFFFFFFFFFFFFFFFFF
@A00155:342:HHGFNDSXY:1:2665:2537:18693 1:N:0:GAACCTAG+TCCGCATA
TCCTCTGCCGAAACAGAGGTTGGACAAGACCGGAGGGGTCTCCTAGTTCCAAAGGAGATGTACTCCGG
ACTTGTTCACGACCTACCGTGTAAGTCGTAGTCTAGTAGGCTACCTGACGAGTCCTTTTTAGGACGAA
+
FFFFFFFFFFFFFFFFFFFFFFFFFFFFFFFFFFFFFFFFFFFFFFFFFFFFFFFFFFFFFFF:FFFFF
FFFFFFFFFFFFFFFFFFFFFFFFF:FFFFFFFFFFFFFFFFFF:FFFFFFFFFFFFF:FFFF:FFFFFF
@A00155:342:HHGFNDSXY:1:2666:11803:4961 1:N:0:GAACCTAG+TCCGCATA
GGATGTGGAGGGCGAGAGAGAGCGACTTCTCTCGGGCCCACAGCCTTACAGCTGTGGAGCACGGTATC

```
CTCTGCCGAAACAGAGGTTGGACAAGACCGGAGGGGTCTCCTAGTTCCAAAGGAGATGTACTCCGGGC
+
FFFFFFFFFFFFFFFFFFFFFFFFFFFFFFFFFFFFFFFFFFFFFFFFFFFFFFFFFFFFFFFFFFFF
FFFFFFFFFFFFFFFFFFFFFFFFFFFFFFFFFFFFFFFFFFFFFFFFFFFFFFFFFFFFFFFFFFFF
@A00155:342:HHGFNDSXY:1:2666:15004:4993 1:N:0:GAACCTAG+TCCGCATA
ATCTCAATGCCGAGAGGGATGTAGAGGGCGAGAGAGAGCGACTTCTCTCGGGCCCACAGCCTTACAGC
TGTGGAGCACGGTATCCTCTGCCGAAACAGAGGTTGGACAAGACCGGAGGGGTCTCCTAGTTCCAAAG
+
FFFFFFFFFFFFFFFFFFFFFF:FFFFFFFFFFFFFFFFFF:FFFFFFFFFFFFFFFFFFFF,FFFFF
FFFFFFFFFFFFFFFFFFFFFFFFFFFFFFFFFFFFFFFFFFFFFFFFFFFFFFFFFFFFF:FFFFFF
@A00155:342:HHGFNDSXY:1:2666:10203:18161 1:N:0:GAACCTAG+TCCGCATA
TACACGGTAGGTCGTGAACAAGCCCGGAGTACATCTCCTTTGGAACTAGGA
+
FFFFFFFFFFFFFFFFFFFFFFFFFFFFFFFFFFFFFFFFFFFFFFFFFFF
@A00155:342:HHGFNDSXY:1:2666:1579:23547 1:N:0:GAACCTAG+TCCGCATA
ATCTCAATGCCGAGAGGGATGTGGAGGGCGAGAGAGAGCGACTTCTCTCGGGCCCACAGCCTTACAGC
TGTGGAGCACGGTATCCTCTGCCGAAACAGAGGTTGGACAAGACCGGAGGGGTCTCCTAGTTCCAAAG
+
FFFFFFFFFFFFFFFFFF:FFFFFFFFFFFFFFFFFFFFFFF:FFFFFFFFFFFFFFFFFFFFFFFFF
FFFFFFFF:FFFFFFF:FFFFFFFFFFFFFFFFFFFFFFFFFFFFFFFFFFFFFFFFFF:FFFFFFFF
@A00155:342:HHGFNDSXY:1:2667:21323:14340 1:N:0:GAACCTAG+TCCGCATA
CCTCCACATCCCTCTCGGCATTGAGATGACCATAGGCCAGAGGTGAGTCCTTAAGTGGACACAGCTGA
TCTAAGGCGGTGTGGTGGGGCATGGGTTTGAACCCCCATGACGGTCGGAGTCTAGTAGGCTCCCT
+
FFFFFFFFFFFFFFFFFFFFFFF:FFFFFFFFFFFFFFFFFFFFFFFFFFFFFFFFFFFFFFFFFFFF
FFFFFFFFFFFFFFFFFFFFFFFFFFFFFFFFFFFFFFFFFFFFFFFFFFFFFFFFFFFFFFFFF
@A00155:342:HHGFNDSXY:1:2667:3848:32174 1:N:0:GAACCTAG+TCCGCATA
CAATGCCGAGAGGGATGTGGAGGGCGAGAGAGAGCGACTTCTCTCGGGCCCACAGCCTTACAGCTGTG
GAGCACGGTATCCTCTGCCGAAACAGAGGTTGGACAAGACCGGAGGGGTCTCCTAGTTCCAAAGGAGA
+
FFFFFFFF:FFFFF,FF,FF:FFFFFFFF:F:FFF:FFFFF,F,,FFFFFFFFF,FFF:F:FFFFFFF
FFFFFFFFFFFFFFFFFFFFFFFFF,FFFFFFFFFFFFFFF:FFFFFFFFFFF,FFFFFFFFFFFFFF
@A00155:342:HHGFNDSXY:1:2667:18367:32236 1:N:0:GAACCTAG+TCCGCATA
GAGAGAGCGACTTCTCTCGGGCCCACAGCCTTACAGCTGTGGAGCACGGTATCCTCTGCCGAAACAGA
GGTTGGACAAGACCGGAGGGGTCTCCTGGTTCCAAAGGAGATGTACTCCGGGCTTGTTC
+
FFFFFFFFFFFFFFFFFFFFFFFFFFFFFFFFFFFFFFFFFFFFFFFFFFFFFFFFFFFFFFFFFFFF
FFFFFFFFFFFFFFFFFFFFFFFFFFFFFFFFFFFFFFFFFFFFFFFFFFFFFFFFFFF
@A00155:342:HHGFNDSXY:1:2668:6117:4131 1:N:0:GAACCTAG+TCCGCATA
TTCTCTCGGGCCCACAGCCTTACAGCTGTGGAGCACGGTATCCTCTGCCGAAACAGAGGTTGGACAAG
ACCGGAGGGGTCTCCTGGTTCCAAAGGAGATGTACTCCGGGCTTGTTCACGACCTACCGTGTAAGTCG
+
FF:FFFFFFFFFFFFFFFFFFFFFF:FFFFFFFFFFF:FFFFFFFFFFFFFFFFFFFFFFFFFFFFFF
FFFFFFFFFFFFFFFFFFFFF:FF,FFFF:FFFFFFF:FFFF,FFFFFFFFFFFF:F:FFFFFFFFFF
@A00155:342:HHGFNDSXY:1:2668:2953:7326 1:N:0:GAACCTAG+TCCGCATA
CTTCTCTCGGGCCCACAGCCTTACAGCTGTGGAGCACGGTATCCTCTGCCGAAACAGAGGTTGGACAA
GACCGGAGGGGTCTCCTAGTTCCAAAGGAGATGTACTCCGGGCTTGTTCACGACCTACCGTGTAAGTC
+
FFFFFFFFFFFFFFFFFFFFFFFFFFFFFFFFFFFFFFFFFFFFFFFFFFFFFFFFFFFFFFFFFFFF
:FFFFFFFFFFFFFFFFFFFFFFFFFFFFFFFFFFFFFFFFFFFFFFFFFFFFFFFFFFFFFFFFFFF
@A00155:342:HHGFNDSXY:1:2668:21251:14716 1:N:0:GAACCTAG+TCCGCATA
TTAAGGACTCACCTCTGGCCTATGGTCATCTCAATGCCGAGAGGGATGTGGAGGGCGAGAGAGAGCGA
CTTCTCTCGGGCCCACAGCCTTACAGCTGTGGAGCACGGTATCCTCTGCCGAAACAGAGGTTGGACAA
+
```

FFFFFFFFFFFFFFFFFFFFFFFFFFFFFFFFFFFFFFFFFFFFFFFFFFFFFFFFFFFFFFFFFFFF
FFFFFFFFFFFFFFFFFFFF:FFFFFFFFFFFFFFFFFFFFFFFFFFFFFFFFFFFFFFFFFFFFFFF
@A00155:342:HHGFNDSXY:1:2668:19931:27680 1:N:0:GAACCTAG+TCCGCATA
ACTTCTCTCGGGCCCACAGCCTTACAGCTGTGGAGCACGGTATCCTCTGCCGAAACAGAGGTTGGACA
AGACCGGAGGGGTCTCCTAGTTCCAAAGGAGATGTACTCCGGGCTTGTTCACGACCTACCGTGTAAGT
+
FFFFFFFFFFFFFFFFFFFFFFFFFFF:FF::FFFFFF::FFFFFFFF:FFFFFF:FFFF,FFFFFFF
FFFF:FF:FFFF:FFFFFFFFFFFFFFFFFF:FFFFFFFFFFFFF,FFFFFFFFFFFFFF:FFFFFFF
@A00155:342:HHGFNDSXY:1:2668:11071:32972 1:N:0:GAACCTAG+TCCGCATA
CTCAATGCCGAGAGGGATGTGGAGGGCGAGAGAGAGCGACTTCTCTCGGGCCCACAGCCTTACAGCTG
TGGAGCACGGTATCCTCTGCCGAAACAGAGGTTGGACAAGACCGGAGGGGTCTCCTAGTTCCAAAGG
+
FFFFFFFFFFFFFFFFFFFFFFFFFFFFFFFFFFFFFFFFFFFFFFFFFFFFFFFFFFFFFFFFFFFF
FFFFFFFFFFFFFFFFFFFFFFFFFFFFFFFFFFFFF:FFFFFFFFFFFFFFFFFFFFFFFFFFFFF
@A00155:342:HHGFNDSXY:1:2669:3115:8641 1:N:0:GAACCTAG+TCCGCATA
CCTTACAGCTGTGGAGCACGGTATCCTCTGCCGAAACAGAGGTTGGACAAGACCGGAGGGGTCTCCTA
GTTCCAAAGGAGATGTACTCCGGGCTTGTTCACGACCTACCGTGTAAGTCGTAGTCTAGTAGGCTACC
+
FFFFFFFFFFFFFFFFFFFFFFFFFFFFFFFFFFFFFFF:FFFFFFFFFFFFFFFFFFFFFFFFFFFF
FFFFFFFFFFFFFFFFFFFFFFFFFFFFFFFFFFFFFFFFFFFFFFFFFF:FFFFFFFFFFFFFFFFF
@A00155:342:HHGFNDSXY:1:2669:23701:12132 1:N:0:GAACCTAG+TCCGCATA
TGCCGAGAGGGATGTGGAGGGCGAGAGAGAGCGACTTCTCTCGGGCCCACAGCCTTACAACTGTGGAG
CACGGTATCCTCTGCCGAAACAGAGGTTGGACAAGACCGGAGGGGTCTCCTAGTTCCAAAGGAGATGT
+
FFFFFFFFFFF:FFFFFFFFFFFFFFFFFFFFFFFFFFFFFFFFFFFFFFFFFFFFFFFFFFFFFFFF
FFFFFFFFFFFFFFFFFFFFFFFFFFFFFFFFFFFFFFFFFFFFFFFFFFFFFFFFFFFFFFFFFFFF
@A00155:342:HHGFNDSXY:1:2669:3595:15890 1:N:0:GAACCTAG+TCCGCATA
CAGCCTTACAGCTGTGGAGCACGGTATCCTCTGCCGAAACAGAGGTTGGACAAGACCGGAGGGGTCTC
CTGGTTCCAAAGGAGATGTACTCCGGGCTTGTTCACGACCTACCGTGTAAGTCGTAGTCTAGTAGGCT
+
FFFFFFF,FFFFFFFFFFFFFFFFFFFFFFFFFFFFFFFFFFFFFFFFFFFFFFFFFFFFFFFFFFFF
FFFFFFFFFFFFFFFFFFFFFFFFFFFFFFFFFFFFFFFFF:FFFFFFFFFFFFFFFFFFFFFFFFFF
@A00155:342:HHGFNDSXY:1:2669:28004:21903 1:N:0:GAACCTAG+TCCGCATA
TGCCGAGAGGGATGTGGAGGGCGAGAGAGAGCGACTTCTCTCGGGCCCACAGCCTTACAGCTGTGGAG
CACGGTATCCTCTGCCGAAACAGAGGTTGGACAAGACCGGAGGGGTCTCCTAGTTCCAAAGGAGATGT
+
FFFFFFFFFFFFFFFFFFFFFFFFFFFFFFFFFFFFFFFFFFFFFFFFFFFFFFFFFFFFFFFFFFFF
FFFFFFFFFFFFFFFFFFFFFFFFFFFFFFFFFFFFFFFFFFFFFFFFFFFFFFFFFFFFFFFFFFFF
@A00155:342:HHGFNDSXY:1:2669:20826:22623 1:N:0:GAACCTAG+TCCGCATA
GCGAGAGAGAGCGACTTCTCTCGGGCCCACAGCCTTACAGCTGTGGAGCACGGTATCCTCTGCCGAAA
CAGAGGTTGGACAAGACCGGAGGGGTCTCCTAGTTCCAAAGGAGATGTACTCCGGGCTTGTTCACGAC
+
FFFFFFFFFFFFFFFFFFFFFFFFFFFFFFFFFFFFFFFFFFFFFFFFFFFFFFFFFFFFFFFFFFFF
FFFFFFFFFFFFFFFFFFFFFFFFFFFFFFFFFFFFFFFFFFFFFFFFFFFFFFFFFFFFFFFFFFFF
@A00155:342:HHGFNDSXY:1:2669:31439:25379 1:N:0:GAACCTAG+TCCGCATA
GCGACTTCTCTCGGGCCCACAGCCTTACAGCTGTGGAGCACGGTATCCTCTGCCGAAACAGAGGTTGG
ACAAGACCGGAGGGGTCTCCTAGTTCCAAAGGAGATGTACTCCGGGCTTGTTCACGACCTACCG
+
FFFFFFFFFFFFFFFFFFFFFFFFF:FFFFFFFFFFFFFFFFFFFFFFFFFFFFFFFFFFFFFFFFFF
FFFFFFFFFFFFFFFFFFFFFFFFFFFFFFFFFFFFFF:FFFFFFFFFFFFFFFFFFFFFFFFF
@A00155:342:HHGFNDSXY:1:2670:9173:1313 1:N:0:GAACCTAG+TCCGCATA
GAGCGACTTCTCTCGGGCCCACAGCCTTACAGCTGTGGAGCACGGTATCCTCTGCCGAAACAGAGGTT
GGACAAGACCGGAGGGGTCTCCTAGTTCCAAAGGAGATGTACTCCGGGCTTGTTCACGACCTACCGTG
+

```
FFFFFFFFFFFFFFFFFFFFFFFFFFFFFFFFFFFFFFFFFFFFFFFFFFFFFFFFFFFFFFFFFFFF
FFFFFFFFFFFFFFFFFFFFFFFFFFFF:FFFFFFFFFFFFFFFFFFFFFFFFFFFFFFFFFFFFFFF
@A00155:342:HHGFNDSXY:1:2670:23737:4711 1:N:0:GAACCTAG+TCCGCATA
GAGAGAGCGACTTCTCTCGGGCCCACAGCCTTACAGCTGTGGAGCACGGTATCCTCTGCCGAAACAGA
GGTTGGACAAGACCGGAGGGGTCTCCTAGTTCCATAGGAGATGTACTCCGGGCTTGTTCACGACCTAC
+
FFFFFFFFFF:F:FFFFFFFFFFFFFFFFFFFFFFFFFFFFFFFFFFFFFFFFFFFFFFFFFFFFFFF
FFFFFFFFFFFFFFFFFFFFFFF:FFFFFFFFFFFFFFFFFFFFFFFFFFFFFFFFFFFFFFFFFFFF
@A00155:342:HHGFNDSXY:1:2670:29658:5603 1:N:0:GAACCTAG+TCCGCATA
GAGCGACTTCTCTCGGGCCCACAGCCTTACAGCTGTGGAGCACGGTATCCTCTGCCGAAACAGAGGTT
GGACAAGACCGGAGGGGTCTCCTAGTTCCAAAGGAGATGTACTCCGGGCTTGTTCACGACCTACCGTG
+
FFFFFFFFFFFFFFFFFFFFFFFFFFFFFFFFFFFFFFFFFFFFFFFFFFFFFFFFFFFFFFFFFFFF
FFFFFFFFFFFFFFFFFFFFFFFFFFFFFFFFFFFFFFFFFFFFFFFFFFFFFFFFFFFFFFFFFFFF
@A00155:342:HHGFNDSXY:1:2670:9019:8469 1:N:0:GAACCTAG+TCCGCATA
CCATGCCCCGCCACACCGCCTTAGATCAGCTGTGTCCACTTAAGGACTCACCTCTGGCCTATGGTCAT
CTCAATGCCGAGAGGGATGTGGAGGGCGAGAGAGAGCGACTTCTCTCGGGCCCACAGCCTTACAGCTG
+
FFFFFFFFFFFFFFFFFFFFFFFFFFFFFFFFFFFFFFFFFFFFFFFFFFFFFFFFFFFFFFFFFFFF
FFFFFFFFFFFFFFFFFFFFFFFFFFFFFFFFFFFFFFFFFFFFFFFFFFFFFFFFFFFFFFFFFFFF
@A00155:342:HHGFNDSXY:1:2670:17463:9126 1:N:0:GAACCTAG+TCCGCATA
CTTCTCTCGGGCCCACAGCCTTACAGCTGTGGAGCACGGTATCCTCTGCCGAAACAGAGGTTGGACAA
GACCGGAGGGGTCTCCTCGTTCCAAAGGAGATGTACTCCGGGCTTGTTCACGACCTACCGTGTAAGTC
+
FFF:FFFFFF:FFFFFFFFF,FFFFFFFFF,:FFFFF,FFFFFFFF:FFFFFFFFFFFFFFFFFFFFF
FFFF::F:FFFFFF:FFF,FFFFFFFFFFFFFFFFFFFFFFFFFFFFFFFFFFF:FFF:FFFFFFFFF
@A00155:342:HHGFNDSXY:1:2670:20320:25410 1:N:0:GAACCTAG+TCCGCATA
TATCCTCTGCCGAAACAGAGGTTGGACAAGACCGGAGGGGTCTCCTGGTTCCAAAGGAGATGTACTCC
GGGCTTGTTCACGACCTACCGTGTAAGTCGTAGTCTAGTAGGCTACCTGACGAGTCCTTTTTAGGACG
+
FFFFFFFFFFFFFFFF:FFFFFFFFFFFF:FFFFFFFFFFFFFF:FFFFFFFFFFFFFFFFFFFFFFF
FFFFFFFFFFFFFFFFFFFFFFFFFFFFFFFFFFFFFFFFFFFF:FFFFFFFFFFFFFFFFFFFFFFF
@A00155:342:HHGFNDSXY:1:2670:32904:30358 1:N:0:GAACCTAG+TCCGCATA
GAGCGACTTCTCTCGGGCCCACAGCCTTACAGCTGTGGAGCACGGTATCCTCTGCCGAAACAGAGGTT
GGACAAGACCGGAGGGGTCTCCTGGTTCCAAAGGAGATGTACTCCGGGCTTGTTCACGACCTACCGTG
+
FFFFFFFFFFFFFFFFFFFFFFFFFFFFFFFFFFFFFFFFFFFFFFFFFF:FFFFFFFFFFFFFFFFF
FFFFFFFFFFFFFFFFFFFFFFFFFFFFFFF:FFFFFFFFFFFFFFFFFFFFFFFFFF:FFFFFFFFF
@A00155:342:HHGFNDSXY:1:2670:5005:31767 1:N:0:GAACCTAG+TCCGCATA
AGCGACTTCTCTCGGGCCCACAGCCTTACAGCTGTGGAGCACGGTATCCTCTGCCGAAACAGAGGTTG
GACAAGACCGGAGGGGTCTCCTAGTTCCAAAGGAGATGTACTCCGGGCTTGTTCACGACCTACCGTGT
+
FFFFFFFFFFFFFF:FFFFF:FFFFFFFFFFFFFFFFFFF:FFFFFFFFFFFFFFFFFFFFFFFFFFF
FFFFF,FFFFFFFFFFFFFFFFFFFFFFFFFFFFFFF,FFFFFFFFFFFFFFFFFFFFFFFFFFFFFF
@A00155:342:HHGFNDSXY:1:2671:26124:9972 1:N:0:GAACCTAG+TCCGCATA
GAGAGAGAGCGACTTCTCTCGGGCCCACAGCCTTACAGCTGTGGAGCACGGTATCCTCTGCCGAAACA
GAGGTTGGACAAGACCGGAGGGGTCTCCTAGTTCCAAAGGAGATGTACTCCGGGCTTGTTCACGACCT
+
FFFFFFFFFFFFFFFFFFFFFFFFFFFFFFFFFFFFFFFFFFFFFFFFFFFFFFFFFFFFFFFFFFFF
FFFFFFFFFFFFFFFFFFFFFFFFFFFFFFFFFFFFFFFFFFFFF,FFFFFFFFFFFFFFFFFFFFFF
@A00155:342:HHGFNDSXY:1:2671:14751:17112 1:N:0:GAACCTAG+TCCGCATA
GCGACTTCTCTCGGGCCCACAGCCTTACAGCTGTGGAGCACGGTATCCTCTGCCGAAACAGAGGTTGG
ACAAGACCGGAGGGGTCTCCTGGTTCCAAAGGAGATGTACTCCGGGCTTGTTCACGACCTACCG
+
```

```
FFFFFFFFFFFFFFFFFFFFFFFFFFFFFFFFFFFFFFFFFFFFFFFFFFFFFFFFFFFFFFFFFFFF
FF:FFFFFFFFFFFFFFFFFFFFFFFFFFFFFFFFFFFFFFFFFFFFFFFFFFFFF:FFFFFFF
@A00155:342:HHGFNDSXY:1:2671:28745:18959 1:N:0:GAACCTAG+TCCGCATA
GAGCGACTTCTCTCGGGCCCACAGCCTTACAGCTGTGGAGCACGGTATCCTCTGCCGAAACAGAGGTT
GGACAAGACCGGAGGGGTCTCCTGGTTCCAAAGGAGATGTACTCCGGGCTTGTTCACGACCTACCGTG
+
FFFFFFFFFFFFFFFFFFFFFFFFFFFF:FFFFFFFFFFFFFFFFFFFFFFFFFFFFFFFFFFFFFFF
FFFFFFFFFFFFFFFFFFFFFFFFFFFFFFFFFFFFFFFFFFFFFFFFFFFFFFFFFFFFFFFFFFFF
@A00155:342:HHGFNDSXY:1:2671:18575:34381 1:N:0:GAACCTAG+TCCGCATA
CAGCCTTACAGCTGTGGAGCACGGTATCCTCTGCCGAAACAGAGGTTGGACAAGACCGGAGGGGTCTC
CTAGTTCCAAAGGAGATGTACTCCGGGCTTGTTCACGACCTACCGTGTAAGTCGTAGTCTAGTAGGCT
+
FFFFFFF:FFFF:F:FFF:FFFFFFFFFFFF:FFFFFFFFFFFFFFFFFFFFFFFFFFFFFFFFFFFF
FFFF:FFFFFFFFF:F:FFFF:FFFFFF,FFFFFFFFFFF,F:FF:FFFF:FFFFFFFFFFFFFFFFF
@A00155:342:HHGFNDSXY:1:2672:14199:19789 1:N:0:GAACCTAG+TCCGCATA
GCGAGAGAGAGCGACTTCTCTCGGGCCCACAGCCTTACAGCTGTGGAGCACGGTATCCTCTGCCGAAA
CAGAGGTTGGACAAGACCGGAGGGGTCTCCTAGTTCCAAAGGAGATGTACTCCGGGCTTGTTCACGAC
+
FFFFFFFFFFFFFFFFFFFFFFFFFFFFFFFFFFFFFFFFFFFFFFFFFFFFFFFFFFFFFFFFFFFF
FFFFFFFFFFFFFFFFFFFFFFFFFFFFFFFFFFFFFFFFFFFFFFFFFFFFFFFFFFFFFFFFFFFF
@A00155:342:HHGFNDSXY:1:2672:20121:23907 1:N:0:GAACCTAG+TCCGCATA
GAGGGCGAGAGAGAGCGACTTCTCTCGGGCCCACAGCCTTACAGCTGTGGTGCACGGTATCCTCTGCC
GAAACAGAGGTTGGACAAGACCGGAGGGGTCTCCTAGTTCCAAAGGAGATGTACTCCGGGCTTGTTCA
+
FFFFFFFFFFFFFFFFFFFFFFFFFFFFFFFFFFFFFFFFFFFFFFFFFF,FFFFFFFFFFFFFFFFF
FFFFFFFFFFFFFFF:FFFFF:FFFFFFFFFFFFFFFFFFFFFFFFFFFFFFFFFFFFFFFFFF:FFF
@A00155:342:HHGFNDSXY:1:2672:14290:28463 1:N:0:GAACCTAG+TCCGCATA
TCCACATCCCTCTCGGCATTGAGATGACCATAGGCCAGAGGTGAGTCCTTAAGTGGACACAGCTGATC
TAAGGCGGTGTGGCGGGGCATGGGTTTGAACCCCCATGACGGTCGGAGTCTAGTAGGCTCCCTGATGA
+
FFFFFFFFFFFFFFFFFFFFFFFFFFFFFFFFFFFFFFFFFFFFFFFFFFFFFFFFFFFFFFFFFFFF
FF:FFFFFFFFFFFFFFFFFFFFFFFFFFFFFFFFFFFFFFFFFFFFFFFFFFFFFFFFFFFFFF:FF
@A00155:342:HHGFNDSXY:1:2672:20681:30264 1:N:0:GAACCTAG+TCCGCATA
GGGCGAGAGAGAGCGACTTCTCTCGGGCCCACAGCCTTACAGCTGTGGAGCACGGTATCCTCTGCCGA
AACAGAGGTTGGACAAGACCGGAGGGGTCTCCTGGTTCCAAAGGAGATGTACTCCGGGCTTGTTCACG
+
FFFFFFFFFFFFFFFFFFFFFFFFFFFFFFFFFFFFFFFFFFFFFFFFFFFFFFFFFFFFFFFFFFFF
FFFFFFFFFFFFFFFFFFFFFFFFFFFFFFFFFFFFFFFFFFFF:FFFFFFFFFFFFFFFFFFFFFFF
@A00155:342:HHGFNDSXY:1:2672:25771:31626 1:N:0:GAACCTAG+TCCGCATA
CGACTTCTCTCGGGCCCACAGCCTTACAGCTGTGGAGCACGGTATCCTCTGCCGAAACAGAGGTTGGA
CAAGACCGGAGGGGTCTCCTAGTTCCAAAGGAGATGTACTCCGGGCTTGTTCACGACCTACCGTGTAA
+
FFFFFFFFFFFFFFFFFFFFFFFFFFFFFFFFFFFFFFFFFFFFFFFFFFFFFFFFFFFFFFFFFFFF
FFFFFFFFFFFFFFFFFFFFFFFFFFFFFFFFFFFFFFFFFFFFFFFFFFFFFFFFFFFFFFFFFFF:
@A00155:342:HHGFNDSXY:1:2672:8079:33520 1:N:0:GAACCTAG+TCCGCATA
GCGACTTCTCTCGGGCCCACAGCCTTACAGCTGTGGAGCACGGTATCCTCTGCCGAAACAGAGGTTGG
ACAAGACCGGAGGGGTCTCCTGGTTCCAAAGGAGATGTACTCCGGGCTTGTTCACGACCTACCGTGTA
+
FFFFFFFFFFFFFFFFFFFFFFFFFFFFFFFFFFFFFFFFFFFFFFFFFFFFFFFFFFFFFFFFFFFF
FFFFFFFFFFFFFFFFFFFFF:FFFFFFFFFFFFFFFFFFFFFFFFFFFFFFFFFFFFFFFFF:FFFF
@A00155:342:HHGFNDSXY:1:2672:27787:35336 1:N:0:GAACCTAG+TCCGCATA
TCTCAATGCCGAGAGGGATGTGGAGGGCGAGAGAGAGCGACTTCTCTCGGGCCCACAGCCTTACAGCT
GTGGAGCACGGTATCCTCTGCCGAAACAGAGGTTGGACAAGACCGGAGGGGTCTCCTAGTTCCAAAGG
+
```

FFFFFFFFFFFFFFFFFFFFF:FFFFFFFFFFFFFFFFFFF:FFFFFFFFFFFFFFFFFF:FFFFFFF
FFFFFFFFFFFFFFFFFFFFFFFFF:FFFFFFFFFFFFFFFFFFF:FFFFFFFFFFFFFFFFFFFFF:
@A00155:342:HHGFNDSXY:1:2673:32642:14058 1:N:0:GAACCTAG+TCCGCATA
CTTCTCTCGGGCCCACAGCCTTACAGCTGTGGAGCACGGTATCCTCTGCCGAAACAGAGGTTGGACAA
GACCGGAGGGGTCTCCTAGTTCCAAAGGAGATGTACTCCGGGCTTGTTCACGACCTACCGTGTAAGTC
+
FFFFFFFFFFFFFFFFFFFFFFFFFFFFFFFFFFFFFFFFFFFFFFFFFFFFFFFFFFFFFFFFFFFF
FFFFFFFFFFFFFFFFFFFFFFFFFFFFFFFFFFFFFFFFFFFFFFFFFFFFFFFFFFFFFFFFFFFF
@A00155:342:HHGFNDSXY:1:2673:16685:14512 1:N:0:GAACCTAG+TCCGCATA
CTTCTCTCGGGCCCACAGCCTTACAGCTGTGGAGCACGGTATCCTCTGCCGAAACAGAGGTTGGACAA
GACCGGAGGGGTCTCCTAGTTCCAAAGGAGATGTACTCCGGGCTTGTTCACGACCTACCGTGTAAGTC
+
FF:FFF::FFF:FFFFFFFFF:FFFFFFFFFFFFFFF,FFFF:FFFFFF:FFFFFFFFFFFFFFFFFF
FFFFFFFFFFFFF:FF,FFFFF:FFF:FFFF:F:FFFFFFFFF:FFFFFFFFFFFFFFF:FFFFFFFF
@A00155:342:HHGFNDSXY:1:2673:27715:16767 1:N:0:GAACCTAG+TCCGCATA
AGCGACTTCTCTCGGGCCCACAGCCTTACAGCTGTGGAGCACGGTATCCTCTGCCGAAACAGAGGTTG
GACAAGACCGGAGGGGTCTCCTGGTTCCAAAGGAGATGTACTCCGGGCTTGTTCACGACCTACCGTGT
+
FFFFFFFFFFFFFFFFFFFFFFFFFF,FFFFFFFFFFFFFFFFFFFFFFFFFFFFFFFFFFFFFFFFF
FFFFF:FFFFFFFFFFFFFFFFFFFFFFFFFFFFFFFFFFFFFFFFFFFFFFFFFFFFFFFFFFFFFF
@A00155:342:HHGFNDSXY:1:2673:28754:31782 1:N:0:GAACCTAG+TCCGCATA
AGGGCGAGAGAGAGCGACTTCTCTCGGGCCCACAGCCTTACAGCTGTGGAGCACGGTATCCTCTGCCG
AAACAGAGGTTGGACAAGACCGGAGGGGTCTCCTAGTTCCAAAGGAGATGTACTCCGGGCTTGTTCAC
+
FFFFFFFFFFFFFFFFFFFFFFFFFFFFFFFFFFFFFFFFFFFFFFFFFFFFFFFFFFFFFFFFFFFF
FFFFFFFFFFFFFFFFFFFFFFFFFFFFFFFFFFFFFFFFFFFFFFFFFFFFFFFFFFFFFFFFFF:F
@A00155:342:HHGFNDSXY:1:2674:5032:1595 1:N:0:GAACCTAG+TCCGCATA
GAGGGCGAGAGAGAGCGACTTCTCTCGGGCCCACAGCCTTACAGCTGTGGAGCACGGTATCCTCTGCC
GAAACAGAGGTTGGACAAGACCGGAGGGGTCTCCTAGTTCCAAAGGAGATGTACTCCGGGCTTGTTCA
+
FFFFFFFFFFFFFFFFFFFFFF,FFFF,FFFFFFFFFFFFFFFFFFFFFFFFFFFFFFFFFFFFFFFF
FFFFFFFFFFFFFFFFFF:FFFFFF:FFFFFFFFFFF,FFFFFF:FFFFFFFFFFFFFFFF:FFFFFF
@A00155:342:HHGFNDSXY:1:2674:12409:2628 1:N:0:GAACCTAG+TCCGCATA
TTCTCTCGGGCCCACAGCCTTACAGCTGTGGAGCACGGTATCCTCTGCCGAAACAGAGGTTGGACAAG
ACCGGAGGGGTCTCCTAGTTCCAAAGGAGATGTACTCCGGGCTTGTTCACGACTTACCGTGTAAGTCG
+
FFFFFFFFFFFFFFFFFFFFFFFFFFFF:FFFFFFFFFFFFFFFFFFFFFFFFFFF:FF:FFFFFFFF
FFFFFFFFFFFFFFFF:FFFFFFFFFFFFF:FFFFFFFFFF:,FFFF:,FFFFFF,FFFFFF:FFFFF
@A00155:342:HHGFNDSXY:1:2674:18466:8359 1:N:0:GAACCTAG+TCCGCATA
TGTTTCGGCAGAGGATACCGTGCTCCACAGCTGTAAGGCTGTGGGCCCGAGAGAAGTCGCTCTCTCTC
GCCCTCCACATCCCTCTCGGCATTGAGATGACCATAGGCCAGAGGTGAGTCCTTAAGTGGACACAGCT
+
FFFFFFFFFFFFFFFFFFFFFFFFFFFFFFFFFFFFFFFFFFFFFFFFFFFFFFFFFFFFFFFFFFFF
FFFFFFFFFFFFFFFF:FFFFFFFFFFFFFFFFFFFFFFFFFFFFFFFFFFFFFFFFFFFFFFFFFF
@A00155:342:HHGFNDSXY:1:2674:22173:18161 1:N:0:GAACCTAG+TCCGCATA
CTCTCTCGCCCTCCACATCCCTCTCGGCATTGAGATGACCATAGGCCAGAGGTGAGTCCTTAAGTGGA
CACAGCTGATCTAAGGCGGTGTGGCGGGGCATGGGTTTGAACCCCCATGACGGTCGGAGTCTAGTAGG
+
FFFFFFFFFFFFFFFFFFFFFFFFFFFFFFFFFFFFFFFFFFFFFFFFFFFFFFFFFFFFFFFFFFFF
FFFFFFFFFFFFFFFFFFF:FFFFFFFFFFFFFFFFFFFFFFFFFFFFFFFFFFFFFFFFFFFFFFFF
@A00155:342:HHGFNDSXY:1:2674:16288:19085 1:N:0:GAACCTAG+TCCGCATA
CGAGAGGGATGTGGAGGGCGAGAGAGAGCGACTTCTCTCGGGCCCACAGCCTTACAGCTGTGGAGCAC
GGTATCCTCTGCCGAAACAGAGGTTGGACAAGACCGGAGGGGTCTCCTGGTTCCAAAGGAGATGTACT
+

```
FFFFFFFFFFFFFFFFFFFFFFFFFFFFFFFFFFFFFFFFFFFFFFFFFFFFFFFFFFFFFFFFFFFF
FFFFFFFFFFFFFFFFFFFFFFFFFFFFFFFFFFFFFFFFFFFFFFFFFFFFFFFFFFFFFFFFFFFF
@A00155:342:HHGFNDSXY:1:2674:15519:22232 1:N:0:GAACCTAG+TCCGCATA
GGGGCATGGGTTTGAACCCCCATGACGGTCGGAGTCTAGTAGGCTCCCTGATGAGTCCGTTCCAAGGA
CGACACCGTCGTTACTCTTCGAGTTGTGTAAGTTTCGTCCTAAAAAGGACTCGTCAGGT
+
FFFFFFFFFF:FFFFFFFFFFFFFFFFFFFFF:FFF:FFFFFFFFFFFFFFFFFFFFFFFFFFFFFFF
FF,F,FFFFFFFFFFFFFFFFFF::FFFFFFFFFFFFFFFFFFFF:FFFFFFFFFFFFF
@A00155:342:HHGFNDSXY:1:2674:15763:32268 1:N:0:GAACCTAG+TCCGCATA
GGACTCACCTCTGGCCTATGGTCATCTCAATGCCGAGAGGGATGTGAAGGGCGAGAGAGAGCGACTTC
TCTCGGGCCCACAGCCTTACAGCTGTGGAGCACGGTATCCTCTGCC
+
FFFFFF:FFFFFFFFFFFFFFFFFFFFFFFFFFFFFFFFFFFFFFFFFFFFFFFFFFFFFFFFFFFFF
FFFFFFFFFFFFFFFFFFFFFFFFFFFFFFFFF:FFFFFFFFFFFF
@A00155:342:HHGFNDSXY:1:2675:29197:2613 1:N:0:GAACCTAG+TCCGCATA
CTTCTCTCGGGCCCACAGCCTTACAGCTGTGGAGCACGGTATCCTCTGCCGAAACAGAGGTTGGACAA
GACCGGAGGGGTCTCCTGGTTCCAAAGGAGATGTACTCCGGGCTTGTTCACGACCTACCGTGTAAGTC
+
FFFFFFFFFFFFFFFFFFFFFFFFFFFFFFFFFFFFFFFFFFFFFFFFFFFFFFFFFFFFFFFFFFFF
FFFFFFFFFFFFFFFFFFFFFFFFFFFFFFFFFFFFFFFFFFF:FFFFFFFFFFFFFFFFFFFFFFFF
@A00155:342:HHGFNDSXY:1:2675:5249:10019 1:N:0:GAACCTAG+TCCGCATA
AGACTCACCTCTGGCCTATGGTCATCTCAATGCCGAGAGGGATGTGGAGGGCGAGAGAGAGCGACTTC
TCTCGGGCCCACAGCCTTACAGCTGTGGAGCACGGTATCCTCTGCCGAAACAGAGGTTGGACAAGACC
+
FFFFFFFFFFFFFFFFF:FFFFFFFFFFFFFFFFFFFFFFF:FFFFFFFFFF:FF:FFFFFFFFFFFF
FFFFFFFFF:FFFFFFFFFFFFFFFFFFFFFFFFFFFFFFFFFFFFFFF,F,FFFFFFFFFFFFFFFF
@A00155:342:HHGFNDSXY:1:2675:12346:14888 1:N:0:GAACCTAG+TCCGCATA
ACTTCTCTCGGGCCCACAGCCTTACAGCTGTGGAGCACGGTATCCTCTGCCGAAACAGAGGTTGGACA
AGACCGGAGGGGTCTCCTAGTTCCAAAGGAGATGTACTCCGGGCTTGTTCACGACCTACCGTGTAAGT
+
FFFFFFFFFFFFFFFFFFFFFFFFFFFFFFFFFFFFFFFFFFFFFFFFFFFFFFFFFFFFFFFFFFFF
FFFFFFFFFFFFFFFFFFFFFFFFFFFFFFFFFF:FFFFFFFFFFFFFFFFFFFFFFFFFFFFFFFFF
@A00155:342:HHGFNDSXY:1:2675:28067:24799 1:N:0:GAACCTAG+TCCGCATA
TGTTTCGGCAGAGGATACCGTGCTCCACAGCTGTAAGGCTGTGGGCCCGAGAGAAGTCGCTCTCTCTC
GCCCTCTACATCCCTCTCGGCATTGAGATGACCATAGGCCAGAGGTGAGTCCTTAAGTGGACACAGCT
+
FFFFFFFFFFFFFFFFFFFFFFFFFFFFFFFFFFFFFFFFFFFFFFFFFFFFFFFFFFFFFFFFFFFF
FFFFFFFFFFFFFFFFFFFFFFFFFFFFFFFFFFFFFFFFFFFFFFFFFFFFFFFFFFFFFF:FFFFF
@A00155:342:HHGFNDSXY:1:2676:5367:8124 1:N:0:GAACCTAG+TCCGCATA
TTCTCTCGGGCCCACAGCCTTACAGCTGTGGAGCACGGTATCCTCTGCCGAAACAGAGGTTGGACAAG
ACCGGAGGGGTCTCCTAGTTCCAAAGGAGATGTACTCCGGGCTTGTTCACGACCTACCGTGTAAGTC
+
FFFFFFFFFFFFFFFFFFFFFFFFFFFFFFFFFFFFFFFFFFFFFFFFFFFFFFFF:FFFFFFFFFF:
FFFFFFFFF:FFFFFFFFFFF,FFFFFFFFFFFFFFFFFFFFFFFFFFFFFFFFFFF,FFFFFFFFF
@A00155:342:HHGFNDSXY:1:2676:32199:21840 1:N:0:GAACCTAG+TCCGCATA
ACCTCTGTTTCGGCAGAGGATACCGTGCTCCACAGCTGTAAGGCTGTGGGCCCGAGAGAAGTCGCTCT
CTCTCGCCCTCCACATCCCTCTCGGCATTGAGATGACCATAGGCCAGAGGTGAGTCCTTAAGTGGACA
+
FFFFFFFFFFFFFFFFFFFFFFFFFFFFFFFFFFFFFFFFFFFFFFFFFFFFFFFFFFFFFFFFFFFF
FFFFFFFFFFFFFFFFFFFFFFFFFFF,:FFFFFFFFFFF,FFFFFFFFFFFFFFFFFFFFFFFFFF:
@A00155:342:HHGFNDSXY:1:2677:4779:5854 1:N:0:GAACCTAG+TCCGCATA
GTGTCCACTTAAGGACTCACCTCTGGCCTATGGTCATCTCAATGCCGAGAGGGATGTGGAGGGCGAGA
GAGAGCGACTTCTCTCGGGCCCACAGCCTTACAGCTGTGGAGCACGGTATCCTCTGCCGAAACAGAGG
+
```

FFFFFFFFFFFFFFFFFFFFFFFFFFFFFFFFFFFFFFFFFFFFFFFFFFFFFFFFFFFFFFFFFFFF
FFF:FFFFFFFFFFFFFFFFFF,FFFF:FFF:FF:FFFF:FFFFFFFFFFFFF:FFFFFFFFFF,FFF
@A00155:342:HHGFNDSXY:1:2677:5810:7733 1:N:0:GAACCTAG+TCCGCATA
GTGTCCACTTAAGGACTCACCTCTGGCCTATGGTCATCTCAATGCCGAGAGGGATGTGGAGGGCGAGA
GAGAGCGACTTCTCTCGGGCCCACAGCCTTACAGCTGTGGAGCACGGTATCCTCTGCCGAAACAGAGG
+
FFFFFFFFFFFFFFFFFFFFFFFFFFFFFFFFFFFFFFFFFFFFFFFFFFFFFFFFFFFFFFFFF:FF
FFFFFFFFFFFFFFFFFFFFFFFFFFFFFFFFF:FFFFFFFFFFFFFFFFFFFFFFFFFFFFFFFFFF
@A00155:342:HHGFNDSXY:1:2677:23827:17143 1:N:0:GAACCTAG+TCCGCATA
AGCGACTTCTCTCGGGCCCACAGCCTTACAGCTGTGGAGCACGGTATCCTCTGCCGAAACAGAGGTTG
GACAAGACCGGAGGGGTCTCCTAGTTCCAAAGGAGATGTACTCCGGGCTTGTTCACGACCTACCGTGT
+
FFFFFFFFFFFFFFFFFFFFFFFFFFFFFFFFFFFFFFFFFF:FFFFFFFFFFFFFFFFFFFFFFFFF
FFFFFFFFFFFFFFFFFFFFFFFFFFFFFFFFFFFFFFFFFFFFFFFFFFFFFFFFFFFFFFFFFFF:
@A00155:342:HHGFNDSXY:1:2677:25988:18724 1:N:0:GAACCTAG+TCCGCATA
AGCGACTTCTCTCGGGCCCACAGCCTTACAGCTGTGGAGCACGGTATCCTCTGCCGAAACAGAGGTTG
GACAAGACCGGAGGGGTCTCCTAGTTCCAAAGGAGATGTACTCCGGGCTTGTTCACGACCTACCGTGT
+
FFFFFFFFFFFFF:FF,FFFFFFFFFFFFFF:FFFFFFFFFFFFFFFFFFF,FFFFFFFFFFFFF:FF
FFFFFFFFFFFFFFFF:FFFFFFFFFFFFFFFFFFFFFFFFFFFF:FFFFFFFFFFFFFFFFFFFFFF
@A00155:342:HHGFNDSXY:1:1101:25382:14262 2:N:0:GAACCTAG+TCCGCATA
GACCCCTCCGGTCTTGTCCAACCTCTGTTTCGGCAGAGGATACCGTGCTCCACAGCTGTAAGGCTGTG
GGCCCGAGAGAAGTCGCTCTCTCTCGCCCTCCACATCCCTCTCGGCATTGAGATGACCATAGGC
+
,FF:FFFFFFFFFFFFFFFFFFF:FF:FFFF::FF:FF:FFFF:FF:FFFFFFF:FFFFFF,FFFF,F
FFF:FFFFFFFFFFFF:FFFFFFFFFFF:FFFF:FFF:FF:FFFFFFFFFFF:,FFFFFF::F
@A00155:342:HHGFNDSXY:1:1102:6081:1376 2:N:0:GAACCTAG+TCCGCATA
GTGAACAAGCCCGGAGTACATCTCCTTTGGAACTAGGAGACCCCTCCGGTCTTGTCCAACCTCTGTTT
CGGCAGAGGATACCGTGCTCCACAGCTGTAAGGCTGTGGGCCCGAGAGAAGTCGCTCTCTCTCGCCCT
+
FFFFFFF,F:FFFFFFFFFFFFFFFFFFFFFFFFFFFFFFFFFFFFFFFFF:FFFFFFFFFFF,FFFF
FFFFFFFFFFFFFFFFFFFFFFFFFFFFFFFFFFFFFFFFFFFFFFFFFFFFFFF:FFFFFFFFFFFF
@A00155:342:HHGFNDSXY:1:1102:10303:21840 2:N:0:GAACCTAG+TCCGCATA
GAACAAGCCCGGAGTACATCTCCTTTGGAACTAGGAGACCCCTCCGGTCTTGTCCAACCTCTGTTTCG
GCAGAGGATACCGTGCTCCACAGCTGTAAGGCTGTGGGCCCGAGAGAAGTCGCTCTCTCTCGCCCTCT
+
FFFFFFFFFFFFFFFFFFFFFFFFF,FFFFFFFFFFFFFFFFFFFFFFFFFFFFFFFFFFFFFFF:FFF
FFFFFFFFFFFFFFFFFFF:FFFF:FFFFFFFFFFFFFFFFFFFFF,FFFFFFFFFFFFFFFFFFFFF
@A00155:342:HHGFNDSXY:1:1103:29595:12289 2:N:0:GAACCTAG+TCCGCATA
GCCTACTAGACTCCGACCGTCATGGGGGTTCAAACCCATGCCCCACCACACCGCCTTAGATCAGCTGT
GTCCACTTAAGGACTCACCTCTGGCCTATGGTCATCTCAATGCCGAGAGGGATGTGGAGGGCGAGAGA
+
FFFFFFFFFF:FFFFFFFFFFFFFFFFFFFFFFFFFFFFFFFFFFFFFFFFFFFFFFFFFFF:FFFF
FFFFFFFFFFF:FFFFFF:FFFFFFFFFFFFFFFFFFFFFFFFFFFFFFFFFFFFFFFFFFFFFFFFF
@A00155:342:HHGFNDSXY:1:1103:8929:19492 2:N:0:GAACCTAG+TCCGCATA
GTCGTGAACAAGCCCGGAGTACATCTCCTTTGGAACTAGGAGACCCCTCCGGTCTTGTCCAACCTCTG
TTTCGGCAGAGGATACCGTGCTCCACAGCTGTAAGGCTGTGGGCCCGAGAGAAGTCGCTCTCTCTCGC
+
FFFFFFFFFFFFFFFFFFFFFFFFFFFF:FFFFFFFFFFFFFFFFFFFFFFFFFFFFFFFFFFFFFFF
FFFFFFFFFFFFFFFFFFFFFFFFFFFFFFFFFF:FFFFFFFFFFFFFFFFFFFFFFFFFFFFFFFFF
@A00155:342:HHGFNDSXY:1:1103:27715:28792 2:N:0:GAACCTAG+TCCGCATA
CCTACTAGACTACGACTTACACGGTAGGTCGTGAACAAGCCCGGAGTACATCTCCTTTGGAACTAGGA
GACCCCTCCGGTCTTGTCCAACCTCTGTTTCGGCAGAGGATACCGTGCTCCACAGCTGTAAGGCTGTG
+

```
FFFFFFFF:FFFFFFFFFFFFFFFFFFFFFFFFFFFFFFFFFFFFFFFFFFFFFFFFFF:FFFFFF
FFFFFFFFFFFFF,FFFFFFFFFFFFFFFFFFFFFFFFFFFFFFFFFFFFFFFFFFFFFFFFFFFF
@A00155:342:HHGFNDSXY:1:1104:7545:6198 2:N:0:GAACCTAG+TCCGCATA
ACTAGACTACGACTTACACGGTAGGTCGTGAACAAGCCCGGAGTACATCTCCTTTGGAACCAGGAGAC
CCCTCCGGTCTTGTCCAACCTCTGTTTCGGCAGAGGATACCGTGCTCCACAGCTGTAAGGCTGTGGGC
+
FFFFFFFFFFFFFFFFF:FFFFFFFFFFFFFFFFF,FFFFFFFFFFFFFFFFFFFFFFFFF:FFFFFF
FFFFFFFFFFFFFFF:FFFFFFFFFFFFFFFFFFFFFFFFFFFFFFFFFFFF:FFFFFFFFFFFFFFF
@A00155:342:HHGFNDSXY:1:1104:27769:31735 2:N:0:GAACCTAG+TCCGCATA
GGAGTACATCTCCTTTGGAACTAGGAGACCCCTCCGGTCTTGTCCAACCTCTGTTTCGGCAGAGGATA
CCGTGCTCCACAGCTGTAAGGCTGTGGGCCCGAGAGAAGTCGCTCTCTCTCGCCCTCTACATCCCTCT
+
FFFFFFFFFFFFFFFFFFFF:FFFFFFFFFFFFFFFFFFFFFFFFFFFFFFFFFFFFFFFFFFFFFFF
FFFFFFFFFFFFFFFFFF:FFFFFFFFFFFFFFFFFFF,FFFFFFFFFFFFFFFFFFFFF,:FFFFFFF
@A00155:342:HHGFNDSXY:1:1105:30517:6778 2:N:0:GAACCTAG+TCCGCATA
AAAAGGACTCGTCAGGTAGCCTACTAGACTACGACTTACACGGTAGGTCGTGAACAAGCCCGGAGTAC
ATCTCCTTTGGAACTAGGAGACCCCTCCGGTCTTGTCCAACCTCTGTTTCGGCAGAGGATACCGTGCT
+
FFFFFFFFFFFFFFFFFFFFFFFFFFFFFFFFFFFFFFFFFFFFFFFFFFFFFFFFFFFFFFFFFFFF
FFFFFFFFFFFFFFFFFFFFFFFFFFFFFFFFFFFFFFFFFFFFFFFFFFFFFFFFFFFFFFFFFFFF
@A00155:342:HHGFNDSXY:1:1105:2257:19366 2:N:0:GAACCTAG+TCCGCATA
ACACCGCCTTAGATCAGCTGTGTCCACTTAAGGACTCACCTCTGGCCTATGGTCATCTCAATGCCGAG
AGGGATGTGAAGGGCGAGAGAGAGCGACTTCTCTCGGGCCCACAGCCTTACAGCTGTGGAGCACGGTA
+
FFFFFFFFFFFFFFFFFFFFFFFFFFFF:FFFFFFFFFFFFFFFFFFFFFFFFFFFFFFFFFFF,FFFF
FFFFFFFFFFFFFFFFFFFFFFFFFFFFFFFFFFFFFFF:FFFFFF,FFFFFFFFFFFFFFFFFFFFF
@A00155:342:HHGFNDSXY:1:1105:32922:33739 2:N:0:GAACCTAG+TCCGCATA
AAACTTACACAACTCGAAGAGTAACGACGGTTTCGTCCTTGGAACGGACTCATCAGGGAGCCTACTAG
ACTCCGACCGTCATGGGGGTTCAAACCCATGCCCCGCCACACCGCCTTAGATCAGCTGTGTCTACTTA
+
FF:FFFFFFFFFFFFFFFFFFFFFFFFFFFFF,FFF,FF,FFFF:FFFFF,F:FFFFFFFFFF:FFFF
FFFFFFFFFFFFFFFFFFFF::FFFFFFFF,FFFFFFFFFFFFFFFFFFF:F,FFFF:F,FFF,FF,FF
@A00155:342:HHGFNDSXY:1:1106:31919:20384 2:N:0:GAACCTAG+TCCGCATA
GTCTTGTCCAACCTCTGTTTCGGCAGAGGATACCGTGCTCCACAGCTGTAAGGCTGTGGGCCCGAGAG
AAGTCGCTCTCTCTCGCCCTCCACATCCCTCTCGGCATTGAGATGACCATAGGCCAGGG
+
FFFF:F:FFFFFFFFFFFFFFFFFFFFFFFFFFFFFFFFFFFFFFFFF:FFFF,F,FFFFFFFFFF
FFFFFFFFFFFFF:FFFFFFFFFFFFFFFFFFFFFFFFF:FFFFFFFFFF:FFFFFFFFF
@A00155:342:HHGFNDSXY:1:1106:32850:23062 2:N:0:GAACCTAG+TCCGCATA
CCTACTAGACTACGACTTACACGGTAGGTCGTGAACAAGCCCGGAGTACATCTCCTTTGGAACTAGGA
GACCCCTCCGGTCTTGTCCAACCTCTGTTTCGGCAGAGGATACCGTGCTCCACAGCTGTAAGGCTGTG
+
FFFFFFFFFFFFFFFFFFFFFFFFFFFFFFFFFFFFFFFFFFFFFFFFFFFFFFF,FFFFFFFFFFF
FFFFFFFFFFFFFFFFFFFFFFFFFFFFFFFFFFFFFFFFFFFFFFFFFFFFFFFFFFFFFFFF:FFF
@A00155:342:HHGFNDSXY:1:1106:7636:27148 2:N:0:GAACCTAG+TCCGCATA
TTACACGGTAGGTCGTGAACAAGCCCGGAGTACATCTCCTTTGGAACTAGGAGACCCCTCCGGTCTTG
TCCAACCTCTGTTTCGGCAGAGGATACCGTGCTCCACAGCTGTAAGGCTGTGGGCCCGAGAGAAGTCG
+
:FFFFFFFFFFFFFFFFFFFFFFFFFFFFFFFFFFFFFFFFFFFFFFFFFFFFFFFFFFFFFFFFFFF
FFFFFFFFFFFFFFFFFFFFFFFFFFFFFFFFFFFFFF:FFFFFFFFFFFFFFFFFFFFFFFFFFFFF
@A00155:342:HHGFNDSXY:1:1106:12554:28369 2:N:0:GAACCTAG+TCCGCATA
CCGTTCCAAGGACGAAACCGTCGTTACTCTTTGAGTTGTGTAAGTTTCGTCCTAAAAAGGACTCGTCA
GGTAGCCTACTAGACTACGACTTACACGGTAGGTCGTGAACAAGCCCGGAGTACATCTCCTTTGGAAC
+
```

```
FFF:FFFFFFFFFFFFFFFF:FFFFFFFFFF:FFF:FF,FF:FFF,FFFFFFFFFFFFFFFFFFFFFF
FFFFFFFFFFFFFFFFFFFFFFFFFFFFFFFFFFFFFFFFFFFFFFFFFFFFFFFFFFFFFFFFF:FF
@A00155:342:HHGFNDSXY:1:1107:26775:8124 2:N:0:GAACCTAG+TCCGCATA
CGGTTTCGTCCTTGGAACGGACTCATCAGGGAGCCTACTAGACTCCGACCGTCATGGGGGTTCAAACC
CATGCCCCGCTACACCGCCTTAGATCAGCTGTGTCCACTTAAGGACTCACCTCTGGCCTATGGTCATC
+
FFFFFFFFFFF:FFFFFFFFFFFFFFFFFFFFFFFFFFFF,FFFFFFFFFFFFFFFFFFFFFFFFFFF
FF:FFFFFFFFFFFFFFFFFFFFFFFFFFFFFFFFFFFFFFFFFFFFFFFFFFFFFFFFFFFFFFFFF
@A00155:342:HHGFNDSXY:1:1107:19913:21762 2:N:0:GAACCTAG+TCCGCATA
TACACGGTAGGTCGTGAACAAGCCCGGAGTACATCTCCTTTGGAACTAGGAGACCCCTCCGGTCTTGT
CCAACCTCTGTTTCGGCAGAGGATACCGTGCTCCACAGCTGTAAGGCTGTGGGCCCGAGAGAAGTCGC
+
FFFFFFFFFFFFFFFFFFFFFFFFFFFFFFFFFFFFFFFFFFFFFFFFFFFFFFFFFFFFFFFFFFFF
FFFFFFFFFFFFFFFFFFFFFFFFFFFFFFFFFFFFFFFFFFFFFFFFFFFFFFFFFFFFFFFFFFFF
@A00155:342:HHGFNDSXY:1:1108:22299:2691 2:N:0:GAACCTAG+TCCGCATA
GGTCGTGAACAAGCCCGGAGTACATCTCCTTTGGAACTAGGAGACCCCTCCGGTCTTGTCCAACCTCT
GTTTCGGCAGAGGATACCGTGCTCCACAGCTGTAAGGCTGTGGGCCCGAGAGAAGTCGCTCTCTCTCG
+
FFFFFFFFFFFFFFFFFFFFFFFFFFFFFFFFFFFFFFFFFFFFFFFFFFFFFFFFFFFF:FFFFFFF
FFFFFFFFFFFFFFF:FFFFFFFFFFFFFFFFFFFFFF,FFFFFFFFFFFFFFFFFFFFFFFFFF:FF
@A00155:342:HHGFNDSXY:1:1108:23068:12007 2:N:0:GAACCTAG+TCCGCATA
GGACTCGTCAGGTAGCCTACTAGACTACGACTTACACGGTAGGTCGTGAACAAGCCCGGAGTACATCT
CCTTTGGAACTAGGAGACCCCTCCGGTCTTGTCCAACCTCTGTTTCGGCAGAGGATACCGTGCTCCAC
+
FFFFFFFFFFFFFFFFFFFFFFFFFF:FFFFFFFFFFFFFFFFFFFFFFFFFF:FFFFFFFFFFFFFF
FFFFFFFFFFFFFFFFFFFFFFFFFFFFFFFFFFFFFFFFFF:FFFFFFFFF:FFFFFFF:FFFFFFF
@A00155:342:HHGFNDSXY:1:1108:2718:21449 2:N:0:GAACCTAG+TCCGCATA
GAAACCGTCATTACTCTTCGAGTTGTGTAAGTTTCGTCCTAAAAAGGACTCGTCAGGTAGCCTACTAG
ACTACGACTTACACGGTAGGTCGTGAACAAGCCCGGAGTACATCTCCTTTGGAACTAGGAGACCCCTC
+
FFF:FF,FFFFFF,F:FFFF:FFFFFFFFFFFFFF:FF,FFFFFFFFFFFFFFFFFFFF:FFFFFFFF
FFFFFFFFFFF,FFFFF,FFFF:FFFFFFFFFFFFFFFFFFFFFFFFFFFF,:FFFFF,FFFFF,,FF
@A00155:342:HHGFNDSXY:1:1109:19370:2628 2:N:0:GAACCTAG+TCCGCATA
AGCCTACTAGACTACGACTTACACGGTAGGTCGTGAACAAGCCCGGAGTACATCTCCTTTGGAACTAG
GAGACCCCTCCGGTCTTGTCCAACCTATGTTTCGGCAGAGGATACCGTGCTCCACAGCTGTAAGGCTG
+
FFFFFFFFFFFFFFFFFFFFFFFFFFFFFFFFFFFFFFFFFFFFFFFFFFFFFFFFFFFFFFFFFFFF
FFFFFFFFFFFFFFFFFFFFFFFFFFFFFFFFF:FFFFFFFFFFFFFFFFFFFFFFFFFFFF:FFFFF
@A00155:342:HHGFNDSXY:1:1109:21549:7811 2:N:0:GAACCTAG+TCCGCATA
CCCCTCCGGTCTTGTCCAACCTCTGTTTCGGCAGAGGATACCGTGCTCCACAGCTGTAAGGCTGTGGG
CCCGAGAGAAGTCGCTCTCTCTCGCCCTTCACATCCCTCTCGGCATTGAGATGACCATAG
+
F:FFFFFFFFFFFFFFFFFFFFFFFFFFFFFFFFFFFFFFFFFFFFFFFFFFFFFFFFFFFFFFFFFF
FFFFFFFFFFFFFFFFFFFFFFFFFFFFFFFFFFFFFFFFFFFFFFFFFFFFFFFF:FFF
@A00155:342:HHGFNDSXY:1:1109:16161:24220 2:N:0:GAACCTAG+TCCGCATA
GTGTAAGTTTCGTCCTAAAAAGGACTCGTCAGGTAGCCTACTAGACTACGACTTACACGGTAGGTCGT
GAACAAGCCCGGAGTACATCTCCTTTGGAACTAGGAGACCCCTCCGGTCTTGTCCAACCTCTGTTTCG
+
FFFFFFFFFFFFFFFFFFFF:FFFFFFFFFFFFFFFFFFFFFFFFFFFFFFFFFFFFFFFFFFFFFFF
FFFFFF,FFFFFFFFFFFFFFFFFFFFFFFFFFFFFFFFFFFFFFFFFFFFFFFFFFFFFFFFFFFFF
@A00155:342:HHGFNDSXY:1:1109:16595:28729 2:N:0:GAACCTAG+TCCGCATA
GACTCGTCAGGTAGCCTACTAGACTACGACTTACACGGTAGGTCGTGAACAAGCCCGGAGTACATCTC
CTTTGGAACTAGGAGACCCCTCCGGTCTTGTCCAACCTCTGTTTCGGCAGAGGATACCGTGCTCCACA
+
```

```
FFFFFFFF,FFFFFFFFFFFFFFFFFFFFFFFFFFFFFFFFFFFFFFFFFFFFFFFFFFFFFFFFFFF
F:FFFFFFFFFFFFFFFFFFFFFFFFFFFFFFFFFFFFFFFFFFFFFFFFFFFFFFFFFFFFFFFFFF
@A00155:342:HHGFNDSXY:1:1110:16658:5196 2:N:0:GAACCTAG+TCCGCATA
TCCTCTGCCGAAACAGAGGTTGGACAAGACCGGAGGGGTCTCCTAGTTCCAAAGGAGATGTACTCCGG
GCTTGTTCACGACCTACCGTGTAAGTCGTAGTCTAGTAGGCTACCTGACGAGTCCTTTTTAGGACGAA
+
FFFFFFFFFFFFFFFFFFFFFFFFFFFFFFFFFFFFFFFFFFFFFFFFFFFFFFFFFFFFFFFFFFFF
FFFFFFFFFFFFFFFFFFFFFFFFFFFFFFFFFFFFFFFFFFFFFFFFFFFFFFFFFFFFFFFFFFFF
@A00155:342:HHGFNDSXY:1:1110:12771:36699 2:N:0:GAACCTAG+TCCGCATA
AGTTGTGTAAGTTTCGTCCTAAAAAGGACTCGTCAGGTAGCCTACTAGACTACGACTTACACGGTAGG
TCGTGAACAAGCCCGGAGTACATCTCCTTTGGAACTAGGAGACCCCTCCGGTCTTGTCCAACCTCTGT
+
FFFFFFFFFFFFFF:FFFFFFFFFFFFFFF:FFFFFFFFFFF:FFFFFFFFFFFFFFFFFFFFFFFFF
FFF:FFFFFFFF:FFFFFFFFFF:FFFFFF:FF:FFFF:FFFF:FF:F:FFFFFFFFFFFFFFFFFFF
@A00155:342:HHGFNDSXY:1:1111:18810:2785 2:N:0:GAACCTAG+TCCGCATA
AGCCCGGAGTACATCTCCTTTGGAACTAGGAGACCCCTCCGGTCTTGTCCAACCTCTGTTTCGGCAGA
GGATACCGTGCTCCACAGCTGTAAGGCTGTGGGCCCGAGAGAAGTCGCTCTCTCTCGCCCTC
+
FFFFFFFFFFFFFFFFFFFFFFFFFFFFFFFFFFFFFFFFFFFFFFFFFFFFFFFFFFFFFFFFFFFF
FFFFFFFFFFFFFFFFFFFFFFFFFFFFFFFFFFFFFFFFFFFFFFFFFFFFFFFFFFFFFF
@A00155:342:HHGFNDSXY:1:1111:4996:10238 2:N:0:GAACCTAG+TCCGCATA
CTGGCCTATGGTCATCTCAATGCCGAGAGGGATGTGAAGGGCGAGAGAGAGCGACTTCTCTCGGGCCC
ACAGCCTTACAGCTGTGGAGCACGGTATCCTCTGCCGAAACAGAGGTTGGACAAGACCGG
+
FFFFFFFFFFFFFFF:FFFFFFFFF:FFFFFFF:FFFFFFFFFFFFFFFF:FFFFFF:FFFFFFFFFF
FFFFFFFFFFFFFFFFFFFFFFFFFF:FF,FFFFF:FFFFFFFFFFFFFFFFF,FFFFFF
@A00155:342:HHGFNDSXY:1:1111:11026:15796 2:N:0:GAACCTAG+TCCGCATA
TCTTGTCCAACCTCTGTTTCGGCAGAGGATACCGTGCTCCACAGCTGTAAGGCTGTGGGCCCGAGAGA
AGTCGCTCTCTCTCGCCCTCCACATCCCTCTCGGCATTGAGATGACCATAGGCCAGAGGAGAGTCCTT
+
FFF,FFFFFFFFFFFFFFFFFFFFFFFFFFFFFFFFFFFFFFFFFFFFFFFFFFFFFFFFFFFFFFFF
FFFFFFFFFFFFFFFFFFFFFFFFFFFFFFFFFFFFFFFFFFFFFFFFFFFFFFFFFFFFFFFFFFFF
@A00155:342:HHGFNDSXY:1:1111:26892:22294 2:N:0:GAACCTAG+TCCGCATA
CGGCAGAGGATACCGTGCTCCACAGCTGTAAGGCTGTGGGCCCGAGAGAAGTCGCTCTCTCTCGCCCT
TCACATCCCTCTCGGCATTGAGATGACCATAGGCCAGAGGTGAGTCCTTAAGTGGACACAGCTGATCT
+
FFFFFFFFFFFFFFFFFFFFFFFFFFFFFFFFF:FFFFFFFFFFFFFFF:FFFFFFFFFFFFFFFFFF
FFFFFFFFFFFFFFFFFFFFFFFFFFFFFFFFFFFFFFFFFFFFFFFFFF:FFFFFFFFFFFFFFFFF
@A00155:342:HHGFNDSXY:1:1111:30553:24565 2:N:0:GAACCTAG+TCCGCATA
GAAACCGTCGTTACTCTTTGAGTTGTGTAAGTTTCGTCCTAAAAAGGACTCGTCAGGTAGCCTACTAG
ACTACGACTTACACGGTAGGTCGTGAACAAGCCCGGAGTACATCTCCTTTGGAACTAGGAGACCCCTC
+
FFFFFFFFFFFFFFFF,:FFFFFFFFFFFFFFFFFFFFFFFFFFFFFFFFFFFFFFFFFFFFFFFFFF
FFFFFFFFFFFFFFFFFFFFFFFFFFFFFFFFFFFFFFFFF:FFFFFFFFFFFFFFFFFFFFFFFFFF
@A00155:342:HHGFNDSXY:1:1112:4670:5791 2:N:0:GAACCTAG+TCCGCATA
TCGTGAACAAGCCCGGAGTACATCTCCTTTGGAACTAGGAGACCCCTCCGGTCTTGTCCAACCTCTGT
TTCGGCAGAGGATACCGTGCTCCACAGCTGTAAGGCTGTGGGCCCGAGAGAAGTCGCTCTCTCTCGCC
+
FFFFFFFFFFFF,FFFFFFFFFFFFFFFFFFFFFFFFFFFFFFFFFFFFFFFFFFFFFFFFFF,FFFF
FFFFFFFFFFFFFFFFFFFFFFFFFFFFFFFFFF:F,FFFF,FFFFFFFFFFFFFFFFFFFFFFFFF:F
@A00155:342:HHGFNDSXY:1:1112:16595:8500 2:N:0:GAACCTAG+TCCGCATA
TGAACAAGCCCGGAGTACATCTCCTTTGGAACTAGGAGACCCCTCCGGTCTTGTCCAACCTCTGTTTC
GGCAGAGGATACCGTGCTCCACAGCTGTAAGGCTGTGGGCCCGAGAGAAGTCGCTCTCTCTCGCC
+
```

```
FFFFFFFFFFFFFFFFFFFFFFFF:FFFFFFFFFFFFFFFFFFFFFFFFFFFFFFFFFFFFFFFFFFF
:FFFFFFFFFFFFFFFFFFFFFFFFFFFFFFF:FFFFFFFFFFFFFFFFFFFFFFFFFFFFFFFF
@A00155:342:HHGFNDSXY:1:1112:8757:21324 2:N:0:GAACCTAG+TCCGCATA
CCTACTAGACTACGACTTACACGGTAGGTCGTGAACAAGCCCGGAGTACATCTCCTTTGGAACCAGGA
GACCCCTCCGGTCTTGTCCAACCTCTGTTTCGGCAGAGGATACCGTGCTCCACAGCTGTAAGGCTGTG
+
FFFFFFFFFFFFFFFFFFFFFFFFFFFFFFFFFFFFFFFFFFFFFFFFFFFFFFFFFFFFFFFFFFFF
FFFFFFFFFFFFFFFFFFFFFFFFFFFFFFFFFFFFFFFFFFFFFFFFFFFFFFFFFFFFFFFFFFFF
@A00155:342:HHGFNDSXY:1:1112:25220:21746 2:N:0:GAACCTAG+TCCGCATA
AGTACATCTCCTTTGGAACTAGGAGACCCCTCCGGTCTTGTCCAACCTCTGTTTCGGCAGAGGATACC
GTGCTCCACAGCTGTAAGGCTGTGGGCCCGAGAGAAGTCGCTCTCTCTCGCCCTCCACATCCCTCTCG
+
FFFFF:FFFFFFFFFFFFFFF:FFFFFFFFFFFFFFF:FFFFFFFFFFFFFFFFFFFFFFFFFF:FFF
FFFFFFFFFFFFFF:FFFF:,FFFFFFFFFFFFFF:FFFFFFFFFFF:F:FFFFFF:FFFFFFFF:FF
@A00155:342:HHGFNDSXY:1:1112:16477:25238 2:N:0:GAACCTAG+TCCGCATA
TCGGCAGAGGATACCGTGCTCCACAGCTGTAAGGCTGTGGGCCCGAGAGAAGTCGCTCTCTCTCGCCC
TTCACATCCCTCTCGGCATTGAGATGACCATAGGCCAGAGGTGAGTCCTTAAGTGGACACA
+
FFFFFFFFFFFFFFFFFFFFFFFFFFFFFFFFFFFFFFFFFFFFFFFFFFFFFFFFFFFFFFFFFFFF
FFFFFFFFFFFFFFFFFFFFFFFFFFFFFFFFFFFFFFFFFFFFFFFFFFFFFFFFFFFFF
@A00155:342:HHGFNDSXY:1:1113:20609:25567 2:N:0:GAACCTAG+TCCGCATA
CGGTTTCGTCCTTGGAACGGACTCATCAGGGAGCCTACTAGACTCCGACCGTCATGGGGGTTCAAACC
CATGCCCCGCTACACCGCCTTAGATCAGCTGTGTCCACTTAAGGACTCACCTCTGGCCTATGGTCATC
+
FFFFFFFF,FFFFFFFFFFFFFFFFFFFFFFFFFFFFFF:FFFFFFFFFFFFFFFFFFFFFFFFFFFF
FFFFFFFFFFFFFFFFFFFFFFFFFFFFFFFFFFFFFFFFFFFFFFFFFFFFFFFFFFFFFFFFFFFF
@A00155:342:HHGFNDSXY:1:1113:17345:28150 2:N:0:GAACCTAG+TCCGCATA
GTTACTCTTCGAGTTGTGTAAGTTTCGTCCTAAAAAGGACTCGTCAGGTAGCCTACTAGACTACGACT
TACACGGTAGGTCGTGAACAAGCCCGGAGTACATCTCCTTTGGAACTAGGAGACCCCTCCGGTCTTGT
+
FFFFFFFFFFFFFFFFFFFFFFFFFFFFF:FFFFF,FFFFFFFFFFFFFFFFFFFFFFFFFFFFFFFF
FFFFFFFFFFFFFFFFFFFFFFFFFFFFFFFFFFFFFF:FFFFFF:F:FFFFFFFFFF:FFFFFFFFF
@A00155:342:HHGFNDSXY:1:1114:15646:16000 2:N:0:GAACCTAG+TCCGCATA
GTAGCCTACTAGACTACGACTTACACGGTAGGTCGTGAACAAGCCCGGAGTACATCTCCTTTGGAACC
AGGAGACCCCTCCGGTCTTGTCCAACCTCTGTTTCGGCAGAGGATACCGTGCTCCACAGCTGTAAGG
+
FFFFFFFFFFFFFFFFFFFFFFFFFFFFFFFFFFFFFFFFFFFFFFFFFFFFFFFFFFFFFFFFFFFF
FFFFFFFFFFFFFFFFFF:FFFFFFFFFFFFFFFFFFF:FFFFFFFFFFFFFFFFFFFFFFFFFFFF
@A00155:342:HHGFNDSXY:1:1114:25021:21371 2:N:0:GAACCTAG+TCCGCATA
TCCACTTAAGGACTCACCTCTGGCCTATGGTCATCTCAATGCCGAGAGGGATGTAGAGGGCGAGAGAG
AGCGACTTCTCTCGGGCCCACAGCCTTACAGCTGTGGAGCACGGTATCCTCTGCCGAAACAGAGGTTG
+
FFFFFFFFFFFFFFFFFFFFFFFFFF:FFFFFFFFFFFFFFF:FFFFFFFFFFFFFFFFFFFFFFFFF
,FFFFFFFFFFFFFFFFFFFFFFFF:FFFFFFFFFFFF:FFFFFFFFFFFFFFFFFFFFFFFFFFFFF
@A00155:342:HHGFNDSXY:1:1115:7229:21527 2:N:0:GAACCTAG+TCCGCATA
AGGACGAAACTTACACAACTCGAAGAGTAACGACGGTTTCGTCCTTGGAACGGACTCATCAGGGAGCC
TACTAGACTCCGACCGTCATGGGGGTTCAAACCCATGCCCCGCTACACCGCCTTAGATCAGCTGTGTC
+
FFFFFFFFFFFFFFFFFFFFFFFFFFFFFFFFFFFFFFFFFFFFFFFFFFFFFFFFFFFFFFFFFFFF
FFFFFFFFF:FFFFFFFFFFFFFFFFFFFFFFFFFFFFFFFFFFFFFFFFFFFFFFFFFFFFFFFFFF
@A00155:342:HHGFNDSXY:1:1115:17463:34992 2:N:0:GAACCTAG+TCCGCATA
GGTCTTGTCCAACCTCTGTTTCGGCAGAGGATACCGTGCTCCACAGCTGTAAGGCTGTGGGCCCGAGA
GAAGTCGCTCTCTCTCGCCCTCCACATCCCTCTCGGCATTGAGATGACCATAGGCCAGAGGAG
+
```

FFFF:FFFFFFFFFFFFFFFFFFFFFFFFFFFFFFFFFFFFFFF:FFFFFFFFFFFFFFFFFFF
FF,FFFFF:FFFFF,FFFFFFFFFF::FFFFFFFFFFF:,FFFFFFFFFFFFFFFFFF:FFFF
@A00155:342:HHGFNDSXY:1:1116:9091:9502 2:N:0:GAACCTAG+TCCGCATA
GGACTCGTCAGGTAGCCTACTAGACTACGACTTACACGGTAGGTCGTGAACAAGCCCGGAGTACATCT
CCTTTGGAACTAGGAGACCCCTCCGGTCTTGTCCAACCTCTGTTTCGGCAGAGGATACCGTGCTCCAC
+
FFFFFFFFFFFFFFFFFFFFFFFFFFFFFFFFFFFFFFFFFFFFFFFFFFFFFFFFFFFFFFFFFFFF
FFFFFFFFFF:FFFFFFFFFFFFFFFFFFFFFFFFFFFFFFFFFFFFFFFFFF:FFFFFFFFFFFFF
@A00155:342:HHGFNDSXY:1:1116:13630:12727 2:N:0:GAACCTAG+TCCGCATA
GGTCTTGTCCAACCTCTGTTTCGGCAGAGGATACCGTGCTCCACAGCTGTAAGGCTGTGGGCCCGAGA
GAAGTCGCTCTCTCTCGCCCTCCACATCCCTCTCGGCATTGAGATGACCATAGGCCAGAGGAG
+
FFFFFFFFFFFFFFFFFFFFFFFFFFFFFFFFFFFFFFFFFFFFFFFFFFFFFFFFFFFFFFFFFFFF
FFFFFFFFFFFFFFFFFFFFFFFFFFFFFFFFFFFFFF:FFFFFFFFFFFFFFFFFFFFFFFF
@A00155:342:HHGFNDSXY:1:1116:29225:21010 2:N:0:GAACCTAG+TCCGCATA
CCACACCGCCTTAGATCAGCTGTGTCCACTTAAGGACTCACCTCTGGCCTATGGTCATCTCAATGCCG
AGAGGGATGTGGAGGGCGAGAGAGAGCGACTTCTCTCGGGCCCACAGCCTTACAGCTGTGGAGCACGG
+
FFFFFFFFFFFFFFFFFFFFFFFFFFFFFF:FFFFFFFFFFFFFFFFFFFFFFFFFFFFFFFFFFFFF
FFFFFF:FFFFFFFFFFFFF:FFFFFFFFFFFFFFFFFFFFFFFFFFFFFF,,FFFF:FFFFFFFFFF
@A00155:342:HHGFNDSXY:1:1116:30897:24659 2:N:0:GAACCTAG+TCCGCATA
GTTTCGTCCTAACAAGGACTCGTCAGGTAGCCTACTAGACTACGACTTACACGGTAGGTCGTGAACAA
GCCCGGAGTACATCTCCTTTGGAACTAGGAGACCCCTCCGGTCTTGTCCAACCTCTGTTTCGGCAGAG
+
FFFFFFFFFFFFFFFFFFFFFFFFFFFFFFFFFFFFFFFFFFFFFFFFFFFFFFFFFFFFFFFFFFFF
FFFFFFFFFFFFFFFFFFFFFFFFFFFFFFFFFF:FFFFFFFFFFFFFFFFFFFFFFFFFFF:FFFFFF
@A00155:342:HHGFNDSXY:1:1116:30915:25066 2:N:0:GAACCTAG+TCCGCATA
GTTTCGTCCTAACAAGGACTCGTCAGGTAGCCTACTAGACTACGACTTACACGGTAGGTCGTGAACAA
GCCCGGAGTACATCTCCTTTGGAACTAGGAGACCCCTCCGGTCTTGTCCAACCTCTGTTTCGGCAGAG
+
FFFFFFFFFFFFFFFFFFFFFFFFFFFFFFFFFFFFFFFFFFFFFFFFFFF:FFFFFFF:FFFFFFF
:FF:FFFFFFFFFFFFFFFFFFFFFFFFFFFFFFFFFFFFFFFF,FFFFFFFFFFFFFFFFFFFFFFFF
@A00155:342:HHGFNDSXY:1:1118:28031:7232 2:N:0:GAACCTAG+TCCGCATA
GCTGTGTCCACTTAAGGACTCACCTCTGGCCTATGGTCATCTCAATGCCGAGAGGGATGTGGAGGGCG
AGAGAGAGCGACTTCTCTCGGGCCCACAGCCTTACAGCTGTGGAGCACGGTATCCTCTGC
+
FFFFFFFFFFFFFFFFFFFFFFFFFFFFFFFF:FFFFFFFFFFFFFFFFFFFFFFFFF:FFFFFFFFFF
FFFFFFFFFFFFFFFFFFFFFFFFFFFFFFFFF:FFFFFFFFFFFFFFFFFFFFFFFFFFFF
@A00155:342:HHGFNDSXY:1:1118:31792:18505 2:N:0:GAACCTAG+TCCGCATA
AAAAGGACTCGTCAGGTAGCCTACTAGACTACGACTTACACGGTAGGTCGTGAACAAGCCCGGAGTAC
ATCTCCTTTGGAACTAGGAGACCCCTCCGGTCTTGTCCAACCTCTGTTTCGGCAGAGGATACCGTGCT
+
FFFFFFFFFFFFFFFFF,FFFF:FFFFFFFFFFFFFFFFFFFFFFFFFFFFFFFFFFFFFFFFFFF:F
FFFFFFF:FFFF:FFFFFFFFFFFF:FFF:FF:FF:FFFFFFFFFFFFFFFFFFFFF:FFFFFFFFFF
@A00155:342:HHGFNDSXY:1:1119:22869:25410 2:N:0:GAACCTAG+TCCGCATA
ACGGTAGGTCGTGAACAAGCCCGGAGTACATCTCCTTTGGAACTAGGAGACCCCTCCGGTCTTGTCCA
ACCTCTGTTTCGGCAGAGGATACCGTGCTCCACAGCTGTAAGGCTGTGGGCCCGAGAGAAGTCGCTCT
+
F:FFFFFFFFFFFFFFFFFFFFFFFFFFFFFFFFFFFFFFFFF:FFFFFF:FFF:F:FFFFF:FFFFFF
FFF,FFFFFFFFFFFFFFFFFFFFFFFFFF:FFFFFFFFFFFFF:FFFFFFFFFFFFFF,:,FFFFFF
@A00155:342:HHGFNDSXY:1:1120:1814:3818 2:N:0:GAACCTAG+TCCGCATA
ACTTACACGGTAGGTCGTGAACAAGCCCGGAGTACATCTCCTTTGGAACCAGGAGACCCCTCCGGTCT
TGTCCAACCTCTGTTTCGGCAGAGGATACCGTGCTCCACAGCTGTAAGGCTGTGGGCCCGAGAGAAGT
+

FFFFFFFFFFFFFFFFFFFFFFFFFFFFFFFFFFFFFFFFFFFFFFFFFFFFFFFFFFFFFFFFFFFFFF
FFFFFFFFFFFFFFFFFFFFFFFFFFFF:FFFFFFFFFFFFFFFFFFFFFFFFFFFFFFFFFFFFFFFFF
@A00155:342:HHGFNDSXY:1:1120:11505:7764 2:N:0:GAACCTAG+TCCGCATA
GCCCACAGCCTTACAGCTGTGGAGCACGGTATCCTCTGCCGAAACAGAGGTTGGACAAGACCGGAGGG
GTCTCCTAGTTCCAAAGGAGATGTACTCCGGGCTTGTTCACGACCTACCGTGTAAGTCGTAGTCTAGT
+
FFFFFFFFFFFFFFFFFFFFFFFFFFFFFFFFFFFFFFFFFFFFFFFFFFFFFFFFFFFFFFFFFFF,FF
FFFFFFFFFFFFFFFFFFFFFFFFFFFFFFFFF:FFFFFFFFFFFFFFFFFFFFFFFFFFFFFFFFFFFF
@A00155:342:HHGFNDSXY:1:1120:15139:15405 2:N:0:GAACCTAG+TCCGCATA
GTTTCGTCCTAAAAAGGACTCGTCAGGTAGCCTACTAGACTACGACTTACACGGTAGGTCGTGAACAA
GCCCGGAGTACATCTCCTTTGGAACTAGGAGACCCCTCCGGTCTTGTCCAACCTCTGTTTCGGCAGAG
+
FFFFFFFFFFFFFFFFFFFFFFFFFFFFFFFFFFFFFFFFFFFFFFFFFFFFFFFFFFFFFFFFFFFFFF
FFFFFFFFFFFFFFFFFFFFFFFFFFFFFFFFFFFFFFFFFFFFFFFFFFFFFFFFFFFFFFFFFFFFFF
@A00155:342:HHGFNDSXY:1:1120:18575:18004 2:N:0:GAACCTAG+TCCGCATA
ACTAGACTACGACTTACACGGTAGGTCGTGAACAAGCCCGGAGTACATCTCCTTTGGAACCAGGAGAC
CCCTCCGGTCTTGTCCAACCTCTGTTTCGGCAGAGGATACCGTGCTCCACAGCTGTAAGGCTGTGGGC
+
FFFFFFFFFFFFFFF:FFFFFFFFFFFFFFFFFFFFFFFFFFFFFFF,FFFFFFFFFFFFFFFFFFFFFF
FFFFFFFFFFFFFFFFFFFFFFFFFFFFFFFFFFFFFFFFFFFFFFFFFFFFFFFFFFFFFFFFFFFFFF
@A00155:342:HHGFNDSXY:1:1120:24144:28494 2:N:0:GAACCTAG+TCCGCATA
GTGAACAAGCCCGGAGTACATCTCCTTTGGAACTAGGAGACCCCTCCGGTCTTGTCCAACCTCTGTTT
CGGCAGAGGATACCGTGCTCCACAGCTGTAAGGCTGTGGGCCCGAGAGAAGTCGCTCTCTCTCGCCCT
+
FFFFFFFFFFFFFFFFFFFFFFFFFFFFFFFF:FFFFFFFFFFFFFFFFFFFFF:FFFFFFFFFFFFFFF
FFFFFFFFFFFFFFFFFFFFFFFFFFFFFFFFFFFFFFFFFFFFFFFFFFFFFFFFFFFFFFFFFFFFFF
@A00155:342:HHGFNDSXY:1:1120:29197:32268 2:N:0:GAACCTAG+TCCGCATA
CAGCTGTGTCCACTTAAGGACTCACCTCTGGCCTATGGTCATCTCAATGCCGAGAGGGATGTGAAGGG
CGAGAGAGAGCGACTTCTCTCGGGCCCACAGCCTTACAGCTGTGGAGCACGGTATCCTCTGCCGAAAC
+
FFFFFFFFFFFFFFFF:FFFFFFFFFFFFFFFFFF:FFFFFFFFFFFFFFFFFFFFFFFF:FFFFF:FFF
FFFFFFFFFFFFFFF:FFFFFFFFF:FFFFFFFFFF:FFFFFFFFFFFFFFFFFFFFFFFFFFFFFFFFF
@A00155:342:HHGFNDSXY:1:1120:6985:33661 2:N:0:GAACCTAG+TCCGCATA
GAGACCCCTCCGGTCTTGTCCAACCTCTGTTTCGGCAGAGGATACCGTGCTCCACAGCTGTAAGGCTG
TGGGCCCGAGAGAAGTCGCTCTCTCTCGCCCTCCACATCCCTCTCGGCATTGAGATGAC
+
FFFFFFFFFFFFFFFFFFFFFFFFFFFFFFFFFFFFFFFFFFFFFFFFFFFFFFFFFFFFFFFFFFFF,F
FFFFFFFFFFFFFFFFFFFF:FFFFFFFFFFFFFFFFFFFFFFFFFFFFFFFFFFFFFF
@A00155:342:HHGFNDSXY:1:1121:32967:15499 2:N:0:GAACCTAG+TCCGCATA
GAGTAACGACGGTTTCGTCCTTGGAACGGACTCATCAGGGAGCCTACTAGACTCCGACCGTCATGGGG
GTTCAAACCCATGCCCCACCACACCGCCTTAGATCAGCTGTGTCCACTTAAGGACTCACCTCTGGCCT
+
FFFFFFFFFFFFFF::FFFFFFFFFF:FFFFFFFFFFFFFFFFFFFFFFFFFFFFFFFFF:FFFFFFFFF
FFFFFFFFFFFFFFFFFFFFFFFFFFFFFFFFFFFFFFFFFFFFFFFFF,FFFFF:F:FFFF:FFFFFFF
@A00155:342:HHGFNDSXY:1:1121:1217:27649 2:N:0:GAACCTAG+TCCGCATA
GGACTCGTCAGGTAGCCTACTAGACTACGACTTACACGGTAGGTCGTGAACAAGCCCGGAGTACATCT
CCTTTGGAACTAGGAGACCCCTCCGGTCTTGTCCAACCTCTGTTTCGGCAGAGGATACCGTGCTCCAC
+
FF,FFFFFF:FFFFFFFF:FFFFFFF:FFFFFF,FFFFFFFFFFFFFFFFFFFF:FFFFF::FF,FFFFF
FFF:FFF,,FF:FFFF:FFFFFFFFFFFFFF::::,FFFF:F:FFFFFF,FFFFFF:FFFFFFFFFFFFF
@A00155:342:HHGFNDSXY:1:1122:20609:7341 2:N:0:GAACCTAG+TCCGCATA
ACACCGCCTTAGATCAGCTGTGTCCACTTAAGGACTCACCTCTGGCCTATGGTCATCTCAATGCCGAG
AGGGATGTGAAGGGCGAGAGAGAGCGACTTCTCTCGGGCCCACAGCCTTACAGCTGTGGAGCACGGTA
+

```
FFFFFFFFFFFFFFFFFFFFFFFFFFFF::FFFFFFFFFFFFFFFFFFFFFFFFFFFFFFFFFFFFFF
FFFFFFFFFFFFFFFFFFFFFFFFFFFFFFFFFFFFFFFFFFFFFFF,FFFFFFFFFFFFFFFFFFF:
@A00155:342:HHGFNDSXY:1:1122:10710:8046 2:N:0:GAACCTAG+TCCGCATA
AAGGACGAAACCGTCGTTACTCTTAGAGTTGTGTAAGTTTCGTCCTAAAAAGGACTCGTCAGGTAGCC
TACTAGACTACGACTTACACGGTAGGTCGTGAACAAGCCCGGAGTACATCTCCTTTGGAACTAGGAGA
+
FFFFFFFFFFFFFFFFFFFFFFFFFFFFFFFFFFFFFFFFFFFFFFFFFFFFFFFFFFFFFFFFFFFF
FFFFFFFFFFFFFFFFFFFFFFFFFFFFFFFFFFFFFFFFFFFFFFFFFFFFFFFFFFFFF:FFF:FF
@A00155:342:HHGFNDSXY:1:1122:22553:34976 2:N:0:GAACCTAG+TCCGCATA
GGACGAAACTTACACAACTCGAAGAGTAACGACGGTTTCGTCCTTGGAACGGACTCATCAGGGAGCCT
ACTAGACTCCGACCGTCATGGGGGTTCAAACCCATGCCCCGCCACACCGCCTTAGATCAGCTGTGTCC
+
FFFFFFFFFFFFFFFFFFFFFFFFFFFFFFFFFFFFFFFFFFFFFFFFFFFFFFFFFFF:FFFFFFFF
FFFFFFFFFFFFFFFFFFFFFFFF,FFFF:FFFFFFFFFFFFFFFFFFFFFFFFFFFF:F:FFFFFFF
@A00155:342:HHGFNDSXY:1:1123:26015:7686 2:N:0:GAACCTAG+TCCGCATA
CACGGTAGGTCGTGAACAAGCCCGGAGTACATCTCCTTTGGAACTAGGAGACCCCTCCGGTCTTGTCC
AACCTCTGTTTCGGCAGAGGATACCGTGCTCCACAGCTGTAAGGCTGTGGGCCCGAGAGAAGTCGCTC
+
FFFFFFFFFFFFFFFFFFFFFFFFFFFFFFFFFFFFFFFFFF:FFFFFFFFFFFFFFFFFFFFFFFFF
FFFFFFFFFFFFFFFFFFFFFFFFF:FFFFFFFFFFFFFFFFFFFFFFFFFFFFFFFFFF:FFFFFFF
@A00155:342:HHGFNDSXY:1:1123:12653:28228 2:N:0:GAACCTAG+TCCGCATA
GGACTCGTCAGGTAGCCTACTAGACTACGACTTACACGGTAGGTCGTGAACAAGCCCGGAGTACATCT
CCTTTGGAACTAGGAGACCCCTCCGGTCTTGTCCAACCTCTGTTTCGGCAGAGGATACCGTGCTCCAC
+
FFFFFFFFFFFF:FFFFFFFFFFFFFFFFFFF:FFFFFFFFFFFFFFFFFFFFFFFFFFFFFFFFFFF
FFFFFFFFFFFFFFFFFFFFFFFFFFFFFFFFF:FFFFFFFFFFFFFFFFFFFFFFFFFFFFFFFFFF
@A00155:342:HHGFNDSXY:1:1123:16477:36605 2:N:0:GAACCTAG+TCCGCATA
GAACTAGGAGACCCCTCCGGTCTTGTCCAACCTCTGTTTCGGCAGAGGATACCGTGCTCCACAGCTGT
AAGGCTGTGGGCCCGAGAGAAGTCGCTCTCTCTCGCCCTCCACATCCCTCTCGGCATTGAGATGACCA
+
FF:FFFFFFFFFFFFFFFFFFFFFFFFFFFFFFFFFFFFFFFFFFFFFFFFFFFFFFFFFFFFFFFFF
FFFFFFFFFFFFFFFFFFFFFFFFFF:FFFFFFFFFFFFFFFFFFFFFFFFFFFFFFFFFFFFFFFFF
@A00155:342:HHGFNDSXY:1:1124:32090:10410 2:N:0:GAACCTAG+TCCGCATA
AAGGACGAAACCGTCGTTACTCTTTGAGTTGTGTAAGTTTCGTCCTAAAAAGGACTCGTCAGGTAGCC
TACTAGACTACGACTTACACGGTAGGTCGTGAACAAGCCCGGAGTACATCTCCTTTGGAACTAGGAGA
+
FFFFFFFFFFFFF,FFFFFFFF,,FFFF:FFFFFFFF:FFFFFFF:F,FFFFFFFFFFFFFFFFFFFF
FFFFFFFFFFFFFFFFFF,FFFFFFFFFFFFFFFFFFFFFFFFFFFFFFFFFFFFFFFFFFFFFFFFF
@A00155:342:HHGFNDSXY:1:1124:11785:26381 2:N:0:GAACCTAG+TCCGCATA
GTAGGTCGTGAACAAGCCCGGAGTACATCTCCTTTGGAACCAGGAGACCCCTCCGGTCTTGTCCAACC
TCTGTTTCGGCAGAGGATACCGTGCTCCACAGCTGTAAGGCTGTGGGCCCGAGAGAAGTCGCTCTCTC
+
FFFFFFFFFFFFFFFFFFFFFFFFFFFFFFFFFFFFFFFFFFFFFFFFFFFFFFFFFFFFFFFFFFFF
FFFFFFFFFFFFFFFFFFFFFFFFFFFFFFFFFFFFFFFFFFF:FFFFFFFFFFFFFFFFFFFFFFFF
@A00155:342:HHGFNDSXY:1:1124:19397:27226 2:N:0:GAACCTAG+TCCGCATA
GTTGTGTAAGTTTCGTCCTAAAAAGGACTCGTCAGGTAGCCTACTAGACTACGACTTACACGGTAGGT
CGTGAACAAGCCCGGAGTACATCTCCTTTGGAACTAGGAGACCCCTCCGGTCTTGTCCAACCTCTGTT
+
FFFFFFFFFFFFFFFFFFFFFFFFFFFFFFFFFFFFFFFFFFFFFFFFFFFFFFFFFFFFFFFFFFFF
FFFFFFFFFFFFFFFFFFFFFFFFFFFFFFFF:FFFFFFFFFFFFFFFFFFFFFFFFFFFFFFFFFFF
@A00155:342:HHGFNDSXY:1:1124:2166:28385 2:N:0:GAACCTAG+TCCGCATA
AGCCCGGAGTACATCTCCTTTGGAACTAGGAGACCCCTCCGGTCTTGTCCAACCTCTGTTTCGGCAGA
GGATACCGTGCTCCACAGCTGTAAGGCTGTGGGCCCGAGAGAAGTCGCTCTCTCTCGCCCTCCACATC
+
```

FFFFFFFFFFFFFFFFFFFFFFFFFFFFFFFFFFFFFFFFFFFFFFFFFFFFFFFFFFFFFFFFFFFFFFFF
FFFFFFFFFFFFFFFFFFFFFFFFFFFFFFFFFFFFFFFF:FFFFFFFFFFFFFFFFFFFFFFFFFFFFFFF
@A00155:342:HHGFNDSXY:1:1124:2492:31172 2:N:0:GAACCTAG+TCCGCATA
ACTACGACTTACACGGTAGGTCGTGAACAAGCCCGGAGTACATCTCCTTTGGAACTAGGAGACCCCTC
CGGTCTTGTCCAACCTCTGTTTCGGCAGAGGATACCGTGCTCCACAGCTGTAAGGCTGTGGGCCCGAG
+
FFFFFFFFFFFFFFFFFFFFFFFFFFFFFFFFFFFFFFFFFFFFFFFFF:FFFFFFFFFFFFFFFFFFFF
FFFFF:FFFFFFFFFFFFF:FFF:FFFFFFFFFFFFFFFFFFFFFFFFFFFFFFFFFFFFFFFFFFFFFFF
@A00155:342:HHGFNDSXY:1:1125:16179:2895 2:N:0:GAACCTAG+TCCGCATA
GGTAGGTCGTGAACAAGCCCGGAGTACATCTCCTTTGGAACTAGGAGACCCCTCCGGTCTTGTCCAAC
CTCTGTTTCGGCAGAGGATACCGTGCTCCACAGCTGTAAGGCTGTGGGCCCGAGAGAAGTCGCTCTCT
+
FFFFFFFFFFFFFFFFFFFFFFFFFFFFFFFFFFFFFFFFFFFFFFFFFFFFFFFFFFFFFF,FFFFFFFF
FFFFFFFFFFFFFFFFFFFFFFFFFFFFFFFFFFFFFFFFFFFFFFFFFFFFFFFFFFFFFFFFFFFFFFFF
@A00155:342:HHGFNDSXY:1:1125:26648:4053 2:N:0:GAACCTAG+TCCGCATA
AAGGACTCGTCAGGTAGCCTACTAGACTACGACTTACACGGTAGGTCGTGAACAAGCCCGGAGTACAT
CTCCTTTGGAACCAGGAGACCCCTCCGGTCTTGTCCAACCTCTGTTTCGGCAGAGGATACCGTG
+
FFFFFFFF:FFFFFFFFFFF:FFFFFFFFF:FFFFFFFFFFFFFFFFFFFFFFFFFFFFFFFFFFFFFFFF
FFFFFFFFFFFFFFFFFFFFFFFFFFFFFFFFFFFFFFFFFFFFFFFFFFFFFFFFFFFFFFFFFFFF
@A00155:342:HHGFNDSXY:1:1125:8730:13886 2:N:0:GAACCTAG+TCCGCATA
CCTACTAGACTACGACTTACACGGTAGGTCGTGAACAAGCCCGGAGTACATCTCCTTTGGAACCAGGA
GACCCCTCCGGTCTTGTCCAACCTCTGTTTCGGCAGAGGATACCGTGCTCCACAGCTGTAAGGCTGTG
+
FFFFFFFFFFFFFFFFFFFFFFFFFFFFFFFFFFFFFFFFFFFFFFFFFFF:FFFFFFFFFFFFFFFFFFF
FFFFFFFFFFFFFFFFFFFFFFFFFFFFFFFFFFFFFFFFF:FFFFFFFFFFFFFFFFFFFFFFFFFFFFF
@A00155:342:HHGFNDSXY:1:1125:23818:15749 2:N:0:GAACCTAG+TCCGCATA
CACACCGCCTTAGATCAGCTGTGTCCACTTAAGGACTCACCTCTGGCCTATGGTCATCTCAATGCCGA
GAGGGATGTGGAGGGCGAGAGAGAGCGACTTCTCTCGGGCCCACAGCCTTACAGCTGTGGAGCACGGT
+
FFFFFFFFFFFFFFFFFFFFFFFFFFFFFF,FFFFFFFFFFFFFFFFFFFFFFFFFFFFFFFFFFFFFFFFF
FFFFFF,FFFFFFFFFFFFFFFFFFFFFFFFFFFFFFFFFFFFFFFFFFFFFFFFFFFFFFFFFFFFFFFFF
@A00155:342:HHGFNDSXY:1:1125:17418:29246 2:N:0:GAACCTAG+TCCGCATA
CTTGTCCAACCTCTGTTTCGGCAGAGGATACCGTGCTCCACAGCTGTAAGGCTGTGGGCCCGAGAGAA
GTCGCTCTCTCTCGCCCTCCACATCCCTCTCGGCATTGAGATGACCATAGGCCAGAGGTGAGTCCTTA
+
FFFFFFFFFFFFFFFFFFFFFFFFFFFFFFFFFFFFFFFFFFFFFFFFFFFFFFFFFFFFFFFFFFFFFFFF
FFFFFFFFFFFFFFFFFFFFFFFFFFFFFFFFFFFF:FFFFFFFFFFFFFFFFFFFFFFFFFFFFFFFF:F
@A00155:342:HHGFNDSXY:1:1126:6596:30389 2:N:0:GAACCTAG+TCCGCATA
AGGACGAAACCGTCGTAACTCTTCGAGTTGTGTAAGTTTCGTCCTAAAAAGGACTCGTCAGGTAGCCT
ACTAGACTACGACTTACACGGTAGGTCGTGAACAAGCCCGGAGTACATCTCCTTTGGAACTAGGAGAC
+
FFFFFFFFFFFFFFFFFFFFFFFFFFFFFFFFFFFFFFFFFFFFFF,:FFFFFFFFFFFFF,FFFFFFFF
FFFF:FFFFFFFFFFFF:FFFFFFF:FFFFFFF:FFFFFFFFFF:FFFFFFFFFFFFFFFFFFFFFFFFF
@A00155:342:HHGFNDSXY:1:1126:13548:36417 2:N:0:GAACCTAG+TCCGCATA
GGACTCGTCAGGTAGCCTACTAGACTACGACTTACACGGTAGGTCGTGAACAAGCCCGGAGTACATCT
CCTTTGGAACTAGGAGACCCCTCCGGTCTTGTCCAACCTCTGTTTCGGCAGAGGATACCGTGCTCCAC
+
FFFFFFFFFFFF:FFFFFFFFFFFFFFFFFFFFFFFFFFFFFFFFFFF:FFFFFFFFFFFFFFFFFFFFF
FFFFFFFFFFFFFFFFFFFFFFFFF:FFFFFFFFFFFF:FFFFFFFFFF:FFFFFFFFFFFFF:FFFFF
@A00155:342:HHGFNDSXY:1:1126:7193:36558 2:N:0:GAACCTAG+TCCGCATA
GTTTCGTCCTAAAAAGGACTCGTCAGGTAGCCTACTAGACTACGACTTACACGGTAGGTCGTGAACAA
GCCCGGAGTACATCTCCTTTGGAACTAGGAGACCCCTCCGGTCTTGTCCAACCTCTGTTTCGGCAGAG
+

F:F:FFFFFFFFFFFFFFFFFFFFFFFFFFFFFFFF:FFFFFFFFFFFFFFFF:FFFFFFFFFFF
FFF:FFFFFFFFFFFFFFFFFFFFFFFFFFFFFF:FFFFFFFFFFFFFFFFFFFFFFFFFF:FFFFFF:F
@A00155:342:HHGFNDSXY:1:1127:2862:11490 2:N:0:GAACCTAG+TCCGCATA
TAGGAGACCCCTCCGGTCTTGTCCAACCTCTGTTTCGGCAGAGGATACCGTGCTCCACAGCTGTAAGG
CTGTGGGCCCGAGAGAAGTCGCTCTCTCTCGCCCTCCACATCCCTCTCGGCATTGAGATGACCATAGG
+
FFFFFFFFFFFFFFFFFFFFFFFFFFFFFFFFFFFFFFFFFFFFFFFFFFFFFFFFFFFFFFFFFFFF
FFFFFFFFFFFFFFFFFFFFFFFFFFFFFFFFFFFFFFFF:FFFFFFFFFFFFFFFFFFFFFFFFFFF
@A00155:342:HHGFNDSXY:1:1127:18349:19993 2:N:0:GAACCTAG+TCCGCATA
CAAGGACGAAACCGTCGTTACTCTTTGAGTTGTGTAAGTTTCGTCCTAAAAAGGACTCGTCAGGTAGC
CTACTAGACTACGACTTACACGGTAGGTCGTGAACAAGCCCGGAGTACATCTCCTTTGGAACTAGGAG
+
FFFFFFFFFFFFFFFFFFFFFFF:FFFFFFFFFFFFFFFFFFFFFFFF:FFFFFFFFFFFFFFFFFFF
FFFFFFFFFFFFFFFFFFFFFFFFFFFFFFFFFFFF,FFFFFFFFFFFFFFFF,FFFFFFFFFFFFF
@A00155:342:HHGFNDSXY:1:1127:28185:28354 2:N:0:GAACCTAG+TCCGCATA
GAGACCCCTCCGGTCTTGTCCAACCTCTGTTTCGGCAGAGGATACCGTGCTCCACAGCTGTAAGGCTG
TGGGCCCGAGAGAAGTCGCTCTCTCTCGCCCTCCACATCCCTCTCGGCATTGAGATGACCATAGGCCA
+
FFFFFFFFFFFFFFFFFFFFFFFFFFFFFFFFFFFFFFFFF:FFFFFFFFFFFFFFFFFFFFFFFFFF
FFFFFFFFFFFFFFFFFFFFFFFFFFFFFFFFF:FF:FFFFFFFFFFFFFFFFFFFFFFFFFFFFFFF
@A00155:342:HHGFNDSXY:1:1127:27877:28510 2:N:0:GAACCTAG+TCCGCATA
GAGACCCCTCCGGTCTTGTCCAACCTCTGTTTCGGCAGAGGATACCGTGCTCCACAGCTGTAAGGCTG
TGGGCCCGAGAGAAGTCGCTCTCTCTCGCCCTCCACATCCCTCTCGGCATTGAGATGACCATAGGCCA
+
FFFF:FFFFFFFFFFFFFFFFFFFFFFFFFFFFFFFFFFFFFFFFFFFFFFFFFFFFFFFFFFFFFFF
FFFFFFFFFF,FFFFFFFFFFFFFFFFFFFFFFFFFFFFFFFFFFFFFF:FFFFFFFFFFFFFFFFFF
@A00155:342:HHGFNDSXY:1:1127:28718:28682 2:N:0:GAACCTAG+TCCGCATA
GAGACCCCTCCGGTCTTGTCCAACCTCTGTTTCGGCAGAGGATACCGTGCTCCACAGCTGTAAGGCTG
TGGGCCCGAGAGAAGTCGCTCTCTCTCGCCCTCCACATCCCTCTCGGCATTGAGATGACCATAGGCCA
+
FF,FF,:F,FFFFF,,::FFFFF:FFFF:FFFFF:FFFFF,FFFF,FFFFFFFFFFFFFFFFFF:FFF
FFFFFFFFFFFFF,F:FFFFFFFFFF:F,FF,FFFFFF:F:FFFFFFFF:,FFFFFFFFFF,FFFFF:
@A00155:342:HHGFNDSXY:1:1127:8856:31015 2:N:0:GAACCTAG+TCCGCATA
GTTGTGTAAGTTTCGTCCTAAAAAGGACTCGTCAGGTAGCCTACTAGACTACGACTTACACGGTAGGT
CGTGAACAAGCCCGGAGTACATCTCCTTTGGAACTAGGAGACCCCTCCGGTCTTGTCCAACCTCTGGT
+
FFFFFFFFFFFFFFFFFFFFFFFFFFF:FFFFFFFFFFFFFFFFFFF:FFFFFFFFFFFFFFFFFFFF
FFF:FFFFF:FFFFFFFF:FFFFFFFF:FFFF:FFFFFFFFFFFFFFFFFFFFFFFFFFFFFFFFF,F
@A00155:342:HHGFNDSXY:1:1127:16875:31250 2:N:0:GAACCTAG+TCCGCATA
GGCTGTGGGCCCGAGAGAAGTCGCTCTCTCTCGCCCTCCACATCCCTCTCGGCATTGAGATGACCATA
GGCCAGAGGTGAGTCCTTAAGTGGACACAGCTGATCTAAGGCGGTGTGGTGGGGCATGGGTTTGAACC
+
FFFFFFFFFFFFFFFFFFFFFFFFFFFFFFFFFFFFFFFFFFFFFFFFFFF:FFFFFFFFFFFFFFFF
FFFFFFFFFFFFFFFFFFF:FFFFFFFFFFFFF:FFFFFFFFFFFFFFFFFFFFFFFFFFFFFFFFFF
@A00155:342:HHGFNDSXY:1:1127:24885:33599 2:N:0:GAACCTAG+TCCGCATA
CTGGCCTATGGTCATCTCAATGCCGAGAGGGATGTGAAGGGCGAGAGAGAGCGACTTCTCTCGGGCCC
ACAGCCTTACAGCTGTGGAGCACGGTATCCTCTGCCGAAACAGAGGTTGGACAAGACCGGAG
+
FFFFFFFFFFFFFFFFFFFFFFFFFF:F:FFFFFFFFFFFFFFFFFFFFFFFFFFFFFFFFFFFFFFF
FFFFFF,FFFFFFFFFFFFFFFFFFFFFFFFFFFFFFFFF:FFFFFFFFFFFFFFFFFFFFFF
@A00155:342:HHGFNDSXY:1:1128:21549:7091 2:N:0:GAACCTAG+TCCGCATA
ACTTACACGGTAGGTCGTGAACAAGCCCGGAGTACATCTCCTTTGGAACTAGGAGACCCCTCCGGTCT
TGTCCAACCTCTGTTTCGGCAGAGGATACCGTGCTCCACAGCTGTTAGGCTGTGGGCCCGAGAGAAGT
+

FFFFFFFFFFFFFFFFFFFFFFFF:FFFFFFFFFFFFFFFFFFFFFFFFFFFFFFFFFFFFFFFFFFF
FFFFFFFFFFFFFFFFFFFFFFFFFFFFFFFFFFFFFFFFFFFFFFFFFF:FFFFFFFFFFFFFFFFF
@A00155:342:HHGFNDSXY:1:1128:25753:15468 2:N:0:GAACCTAG+TCCGCATA
CTCTACGAGTTGTGTAAGTTTCGTCCTAAAAAGGACTCGTCAGGTAGCCTACTAGACTACGACTTACA
CGGTAGGTCGTGAACAAGCCCGGAGTACATCTCCTTTGGAACTAGGAGACCCCTCCGGTCTTGTCCAA
+
FFFFFFFFFFFFFFFFFFFFFFFFFFF:FFF:FFFFFFFFFFFFFFFFF,FFFFFFFFFFFFFFFFFF
FFFFFF:FFFFFFFFFF:FFFFFFFFFFFFFFFFFFFFFFFFFFFFF:FFFFFFF:FFFFFFFFFFFF
@A00155:342:HHGFNDSXY:1:1129:17282:15765 2:N:0:GAACCTAG+TCCGCATA
GGGCCCGAGAGAAGTCGCTCTCTCTCGCCCTCCACATCCCTCTCGGCATTGAGATGACCATAGGCCAG
AGGTGAGTCCTTAAGTGGACACAGCTGATCTAAGGCGGTGTGGCGGGGCATGGGTTTGAACCCCCATG
+
FFFFFFFFFFFFFFFFFFFFFFFF:FFFFFFFFFFFFFFFFFFFFFFFFFFFFFF:FFFFFFFFFFFF
FFFFFFFFF:FFFFFFFFFFFFFF:FFFFFFF:FFFFFFFFFFFFFFFFFFFFFFFFFFFFFFFFFFF
@A00155:342:HHGFNDSXY:1:1129:17264:24314 2:N:0:GAACCTAG+TCCGCATA
ACTACGACTTACACGGTAGGTCGTGAACAAGCCCGGAGTACATCTCCTTTGGAACTAGGAGACCCCTC
CGGTCTTGTCCAACCTCTGTTTCGGCAGAGGATACCGTGCTCCACAGCTGTAAGGCTGTGGGCCCGAG
+
FFFFFFFFFFFFFFFFFFFFFFFFFFFFFFFFFFFFFFFFFFFFFFFFFFFFFFFFFFFFFFFFFFFF
FFFFFFFFFFFFFFFFFFFFFFFFFFFFFFFFFFFFFFFFFFFFFFFFFF,FFFFFFFFFFFFFFFFF
@A00155:342:HHGFNDSXY:1:1130:11379:8453 2:N:0:GAACCTAG+TCCGCATA
CAGGTAGCCTACTAGACTACGACTTACACGGTAGGTCGTGAACAAGCCCGGAGTACATCTCCTTTGGA
ACTAGGAGACCCCTCCGGTCTTGTCCAACCTCTGTTTCGGCAGAGGATACCGTGCTCCACAGCTGTAA
+
FFFFFFFFFFFFFFFFFFFFFFFFFFFFFFFFFFFFFFFFFFFFFFFFFFFFFFFFF:FFFFF:FFFF
FFFFFFFFFFFFFFFFFFFFFFFFFFFFFFFFFFFFFFFFFFFFFFFFFFFFFFFFFFFFFFFFFFFF
@A00155:342:HHGFNDSXY:1:1130:29324:9408 2:N:0:GAACCTAG+TCCGCATA
AAAAGGACTCGTCAGGTAGCCTACTAGACTACGACTTACACGGTAGGTCGTGAACAAGCCCGGAGTAC
ATCTCCTTTGGAACTAGGAGACCCCTCCGGTCTTGTCCAACCTCTGTTTCGGCAGAGGATACCGTGCT
+
FFFFFFFFFFFFFFFFFFFFFFFFFFFFFFFFFFFFFFFFFFFFF,FFFFFFFFFFFFFFFFFFFFFF
FFFFFFFFFFFFFFFFFFFFFFFFFFFFFFFFFFFFFFFFFFFFFFFFFFFFFFFFFFFFFFFFFF:F
@A00155:342:HHGFNDSXY:1:1131:23023:16689 2:N:0:GAACCTAG+TCCGCATA
GTTGTGTAAGTTTCGTCCTAAAAAGGACTCGTCAGGTAGCCTACTAGACTACGACTTACACGGTAGGT
CGTGAACAAGCCCGGAGTACATCTCCTTTGGAACTAGGAGACCCCTCCGGTCTTGTCCAACCTCTGTT
+
FFFFFFFFFFFFFFFFFFF,FFF:FFFFFFFFF:FFFFFFFFFFFFFFFFFFFF:FFFFFFFFF:FFF
FFFFFFFFFFFFFFFFFFFFFFFFFFFFFFFF:FFFFFFFFFFFFFFFFFFFFFFFFFFFFFFFFFFF
@A00155:342:HHGFNDSXY:1:1132:11451:8171 2:N:0:GAACCTAG+TCCGCATA
GGACTCGTCAGGTAGCCTACTAGACTACGACTTACACGGTAGGTCGTGAACAAGCCCGGAGTACATCT
CCTTTGGAACTAGGAGACCCCTCCGGTCTTGTCCAACCTCTGTTTCGGCAGAGGATACCGTGCTCCAC
+
FFFFFFFFFFFFFFFFFFFFFFFFFFFFFFFFFFFFFFFFFFFFFFFFFFFFFFFFFFFFFFFFFFFF
FFFFFFFFFFFFFFFFFFFFFFFFFFFFFFFFFFFFFFFFFFFFFFFFFFFFF:FFFFFFFFFFFFFF
@A00155:342:HHGFNDSXY:1:1132:17897:27070 2:N:0:GAACCTAG+TCCGCATA
TTTCGTCCTAAAAAGGACTCGTCAGGTAGCCTACTAGACTACGACTTACACGGTAGGTCGTGAACAAG
CCCGGAGTACATCTCCTTTGGAACTAGGAGACCCCTCCGGTCTTGTCCAACCTCTGTTTCGGCAGAGG
+
FFFFFFFFFFFFFFFFFFFFFFFFFFFFFFFFFFFFFFFFFFFFFFFFFFFFFFFFFFFFFFFFFFF,
FFFFFFFFFFFFFFFFFFFFFFFFFFFFFFFFFFFFFFFFFFF,FFFFFFFFFFFFFFFFFFF:FFFF
@A00155:342:HHGFNDSXY:1:1132:4589:34366 2:N:0:GAACCTAG+TCCGCATA
TCTCCTTTGGAACTAGGAGACCCCTCCGGTCTTGTCCAACCTCTGTTACGGCAGAGGATACCGTGCTC
CACAGCTGTAAGGCTGTGGGCCCGAGAGAAGTCGCTCTCTCTCGCCCTCCACATCCCTCTCGGCATTG
+

```
FFFFFFFFFFFFFFFFFFFFFFFFFFFFFFF:FFFFFFFFFFFFFFFFFFFFFFFFFFFFFFFFFFFF
FFFFFFFF:FFFFF,FFFFFFFFFFFFFFFFFFFFFFFFFFFFFFFFFFFFFFFFFFFFFFFFFFFFF
@A00155:342:HHGFNDSXY:1:1132:8440:36965 2:N:0:GAACCTAG+TCCGCATA
ATGCCCCACCACACCGCCTTAGATCAGCTGTGTCCACTTAAGGACTCACCTCTGGCCTATGGTCATCT
CAATGCCGAGAGGGATGTGGAGGGCGAGAGAGAGCGACTTCTCTCGGGCCCACAGCCTTACAGCTGTG
+
FFFFFFFFFFFFFFFFFFFFFFFFF:FFFFFFFFFFFFFFFFFFFFFFFFFFFFFFFFFFFFFFFFFF
FFFFFFFF:FFFFFFFF:FFFFFFFFFFFFFFFFF:FFFFFFF:FFFFFFFFFFFFFFFFFFFFFF::
@A00155:342:HHGFNDSXY:1:1132:15221:37027 2:N:0:GAACCTAG+TCCGCATA
AAAGGGACTCGTCAGGTAGCCTACTAGACTACGACTTACACGGTAGGTCGTGAACAAGCCCGGAGTAC
ATCTCCTTTGGAACTAGGAGACCCCTCCGGTCTTGTCCAACCTCTGTTTCGGCAGAGGATACCGTGCT
+
FF:FFFF:FFFFFFFF:FFFFFFFFFFF:FFFF:FFFFFFF:FFFFF::FFFFFFFF:FF:F,FFFFF
FFFFFFFFFFFFFFFFFFFFFFFFFFFFFFFFFFFFFFFFF:FFFFFFFFFFF:FFFFFFFFFFF,FFF
@A00155:342:HHGFNDSXY:1:1133:31620:10786 2:N:0:GAACCTAG+TCCGCATA
TGAACAAGCCCGGAGTACATCTCCTTTGGAACTAGGAGACCCCTCCGGTCTTGTCCAACCTCTGTTTC
GGCAGAGGATACCGTGCTCCACAGCTGTAAGGCTGTGGGCCCGAGAGAAGTCGCTCTCTCTCGCCCTC
+
FFFFFFFFFFFFFFFFFFFFFFFFF,FFFF,FFFFFFFFFFFFFFFFFFFFFFFFFFFFFFFFFFFFF
FFFFFFFFFFFFFFFFFFFFFFFFFFFFFFFFFF:FFFFFFFFFFFFFFFFFFFFFFFFFFFFFFFFF
@A00155:342:HHGFNDSXY:1:1133:28791:19163 2:N:0:GAACCTAG+TCCGCATA
CCTACTAGACTACGACTTACACGGTAGGTCGTGAACAAGCCCGGAGTACATCTCCTTTGGAACTAGTA
GACCCCTCCGGTCTTGTCCAACCTCTGTTTCGGCAGAGGATACCGTGCTCCACAGCTGTAAGGCTGTG
+
FFFFFFFFFFFFFFFFFFF:FFF:F::FFF:FFFFFFFFFFF,FFFFFFFFFFFFFFF:FFFFFFFFF
FFF:FFFFFFFFFFFFFF,FFFFFFFFFFFFFFFFFFFFFFFF:FF:FFFFFFFFFFFFFFFFFFFFFF
@A00155:342:HHGFNDSXY:1:1134:25834:26005 2:N:0:GAACCTAG+TCCGCATA
AACTAGGAGACCCCTCCGGTCTTGTCCAACCTCTGTTTCGGCAGAGGATACCGTGCTCCACAGCTGTA
AGGCTGTGGGCCCGAGAGAAGTCGCTCTCTCTCGCCCTCCACATCCCTCTCGGCATTGAGATGACCAT
+
FFFFFFFFFFFFFFFFFFFFFF:FFFFFFFFFFFFFFFFF:FFFFFFFFFFFFFFFFFFFFFFFFFFFF
FFFFFFFFFFFFFFFFFFFFFFFFFFFFFFFFFFFFFFFFFF:FFFFFFFFFFFFFFFFFFFFFFFFF
@A00155:342:HHGFNDSXY:1:1135:13693:19351 2:N:0:GAACCTAG+TCCGCATA
GACCCCTCCGGTCTTGTCCAACCTCTGTTTCGGCAGAGGATACCGTGCTCCACAGCTGTAAGGCTGTG
GGCCCGAGAGAAGTCGCTCTCTCTCGCCCTCCACATCCCTCTCGGCATTGAGATGACCATAGGC
+
FFFFFFFFFFFFFFFFFFFFFFFFFFFFFFFFFFFFFFFFFFFFFFFFFFFFFFFFFFFFFFFFFFFF
FFFFFFFFF:FFFFFFFFFFFFFFFFFFFFFFFFFFFFFFFFFFFFF,FFFFFFFFFFFFF,FFF
@A00155:342:HHGFNDSXY:1:1136:4128:35728 2:N:0:GAACCTAG+TCCGCATA
GGAGTACATCTCCTTTGGAACTAGGAGACCCCTCCGGTCTTGTCCAACCTCTGTTTCGGCAGAGGATA
CCGTGCTCCACAGCTGTAAGGCTGTGGGCCCGAGAGAAGTCGCTCTCTCTCGCCCTCCACATCCCTCT
+
FFFF:FFFFFFFFFFFFFFFFFFFF:FFFFFFFFFFFFFFFFFFFFFFFFFFFFFFFFFFFFFFFFF:
FFFFFFFFF:FFFFFFFFFFFFFFFFFFFFFFFFFFFFFFFFFFFFFFFFFFFFFFFFFFFFFFFFFF
@A00155:342:HHGFNDSXY:1:1136:3803:35916 2:N:0:GAACCTAG+TCCGCATA
GGAGTACATCTCCTTTGGAACTAGGAGACCCCTCCGGTCTTGTCCAACCTCTGTTTCGGCAGAGGATA
CCGTGCTCCACAGCTGTAAGGCTGTGGGCCCGAGAGAAGTCGCTCTCTCTCGCCCTCCACATCCCTCT
+
FFFFFFFFFFFFFFFFFFFFFFFFFFFFFFFFFFFFFFFFFFFFFFFFFFF:FFFFFFFFFFFFFFFF
FFFFFFFFFFFFFFFFFFFFFFFFFFFFFFFFFFFFFFFFFFFFFFFFFFFFFFFFFFFFFFFFFFFF
@A00155:342:HHGFNDSXY:1:1137:4824:1485 2:N:0:GAACCTAG+TCCGCATA
GAGTACATCTCCTTTGGAACTATGAGACCCCTCCGGTCTTGTCCAACCTCTGTTTCGGCAGAGGATAC
CGTGCTCCACAGCTGTAAGGCTGTGGGCCCGAGAGAAGTCGCTCTCTCTCGCCCTCCACATCCCTCT
+
```

```
FFFFFFFFFFFF,FFFFFFF,F,FFFFFFFFFFFFFF:FFFFFFFFF,FFFFFFFFFFFFFFFFFFFF
FFFFFFFFFFFFFFFFFFFFFFFFFFFFFF:FFFFFFFFFF:FFFFFFFFFFFFFFFFFFFFFF:FF
@A00155:342:HHGFNDSXY:1:1137:21947:5181 2:N:0:GAACCTAG+TCCGCATA
GTTACTCTTCGAGTTGTGTAAGTTTCGTCCTAAAAAGGACTCGTCAGGTAGCCTACTAGACTACGACT
TACACGGTAGGTCGTGAACAAGCCCGGAGTACATCTCCTTTGGAACTAGGAGACCCCTCCGGTCTTGT
+
FFFFFFFFFFFFFFFFFFFFFFFFFFFFFFFFFFFFFFFFFFFFFFFFFFFFFFFFFF:FFFFFFFFF
FFFFFFFF:FFFFFFFFFFFFFFFFFFFFFFFF:FFFFF,FFFFFFFFFFFFFF:FFFFFFFFFFFFF
@A00155:342:HHGFNDSXY:1:1137:4788:28541 2:N:0:GAACCTAG+TCCGCATA
GAGACCCCTCCGGTCTTGTCCAACCTCTGTTTCGGCAGAGGATACCGTGCTCCACAGCTGTAAGGCTG
TGGGCCCGAGAGAAGTCGCTCTCTCTCGCCCTCTACATCCCTCTCGGCATTGAGATGACCATAGGCCA
+
FFFFFFFFFFFFFFFFFFFFFFFFFFFFFFFFFFFFFFFFFFFFFFFFFFFFFFFFFFFFFFFFFFFF
FFF:FFFFFFFFF:FFFFFFFFFFFFFFFFFFFFFFFFFFFFFFFFFFFFFFFFFFFFFF:FFFFFFF
@A00155:342:HHGFNDSXY:1:1137:31882:35070 2:N:0:GAACCTAG+TCCGCATA
TAGGACGAAACTTACACAACTCAAAGAGTAACGACGGTTTCGTCCTTGGAACGGACTCATCAGGGAGC
CTACTAGACTCCGACCGTCATGGGGGTTCAAACCCATGCCCCGCCACACCGCCTTAGATCAGCTGTGT
+
FFFFFFFFFFFFFFFFFFFFFFFFFFFFF:FFFFFFFFFFFF:FFFFFFF:FFFFFFFFFFFFFFF:F
FFFFFF:FFFFFFFFFFFFFFFFFFFFFFFFFFFFFFFFFF:FFFFFFFFFFFFFFFFFFFFFFFFFF
@A00155:342:HHGFNDSXY:1:1138:2094:6872 2:N:0:GAACCTAG+TCCGCATA
CACGGTAGGTCGTGAACAAGCCCGGAGTACATCTCCTTTGGAACTAGGAGACCCCTCCGGTCTTGTCC
AACCTCTGTTTCGGCAGAGGATACCGTGCTCCACAGCTGTAAGGCTGTGGGCCCGAGAGAAGTCGCTC
+
F:F:FFFFFFFFFFFFFFFFF:FFFFFFFFFFFFFFFFFFFFFFFFFFFF:FFFFFFFFFFFFFFFFF
F,FFF:FFFFFFFFFFFFFFFFFFFFFFFFF:FFFFF:FFFFFFFFFFFFFFFFFFFFFFF:FF:FFF
@A00155:342:HHGFNDSXY:1:1138:2383:13103 2:N:0:GAACCTAG+TCCGCATA
CCACACCGCCTTAGATCAGCTGTGTCCACTTAAGGACTCACCTCTGGCCTATGGTCATCTCAATGCCG
AGAGGGATGTGGAGGGCGAGAGAGAGCGACTTCTCTCGGGCCCACAGCCTTACAGCTGTGGAGCACGG
+
FFFFFFFFFFFFFFFFFFFFFFFFFF:F:FFFFFFFFFFFFFFFFFFFFFFFFFFFFFFFFFFFFFFF
FFFFFFFFFFFFFFFFFFFFFFFFFF:FFFF:FFFFFFFFFFFFFFF:FFF:FFFFFFFFFFFFFFFF
@A00155:342:HHGFNDSXY:1:1139:19081:9424 2:N:0:GAACCTAG+TCCGCATA
AAGACCGGAGTACATCTCCTTTGGAACTAGGAGACCCCTCCGGTCTTGTCCAACCTCTGTTTCGGCAG
AGGATACCGTGCTCCACAGCTGTAAGGCTGTGGGCCCGAGAGAAGTCGCTCTCTCTCGCCCTCCACAT
+
FF:,,FF:FFFFFFF,FFFFFFF:FFFFFFFFFFFFF:FFFFF,:,FFFFFFFFFFFFFFFF::FFFF
,FFFFFFF:FFFFFFFFFF:FFFFFFFFFFFFFFFF:F,FFFFFFFF,FFFFFFFFFFF,FFFF:FFF
@A00155:342:HHGFNDSXY:1:1139:21766:12446 2:N:0:GAACCTAG+TCCGCATA
TCCTAAAAAGGACTCGTCAGGTAGCCTTCTAGACTACGACTTACACGGTAGGTCGTGAACAAGCCCGG
AGTACATCTCCTTTGGAACTAGGAGACCCCTCCGGTCTTGTCCAACCTCTGTTTCGGCAGAGGATACC
+
FFFFFFFFFFFFFFFFFFFFFFFFFFFFFFFFFFFFFFFFFFFFFFFFFFFFFFFFFFFFFFFFFFFF
FFFFFFFFFFFFFFFFFFFFFFFFFFFFFFFFFFFFFFFFFFFFFFFFFFFFFFFFFFFFFFFFFFFF
@A00155:342:HHGFNDSXY:1:1140:4300:2738 2:N:0:GAACCTAG+TCCGCATA
GGACTCGTCAGGTAGCCTACTAGACTACGACTTACACGGTAGGTCGTGAACAAGCCCGGAGTACATCT
CCTTTGGAACTAGGAGACCCCTCCGGTCTTGTCCAACCTCTGTTTCGGCAGAGGATACCGTGCTCCAC
+
FFFFFFFFFFFFF:FFFFFFFFFFFFFFFFFFFFFFFFFFFFFFFFFFF:FFFFFFFFFFFFFFFFFF
FFFFFFFFFFFFFFFFFFFFFFFFFFFFFFFFFFFFFFFFFFFFFFFFFFFFFFFFF:FFFFFFFFFF
@A00155:342:HHGFNDSXY:1:1140:30834:5729 2:N:0:GAACCTAG+TCCGCATA
TACGACTTACACGGTAGGTCGTGAACAAGCCCGGAGTACATCTCCTTTGGAACTAGGAGACCCCTCCG
GTCTTGTCCAACCTCTGTTTCGGCAGAGGATACCGTGCTCCACAGCTGTAAGGCTGTGGGCCCG
+
```

FFFFFFFFFFFFFFFFFFFFFFFFFFFFFFFFFFFFFFFFFFFF:FFFFFFFFFFFFFFFFFFFFF
FFFF:FFFFFFFFFFFFFFFFF:FFFFFFFFFFFFFFFFFFFF:FFFFFFFFFFFFFFFFFFFF
@A00155:342:HHGFNDSXY:1:1140:31693:12759 2:N:0:GAACCTAG+TCCGCATA
CGTTACTCTTCGAGTTGTGTAAGTTTCGTCCTAAAAAGGACTCGTCAGGTAGCCTACTAGACTACGAC
TTACACGGTAGGTCGTGAACAAGCCCGGAGTACATCTCCTTTGGAACTAGGAGACCCCTCCGGTCTTG
+
FFFFFFFFFFFFFF:F:FFFF:FFFFFFFFFFFFFFFFFFFFFFFFFFFFFFFFFFFFFFFFFFFFF
FFFFFFFFFFFFFFFFFFFFFFF:FFFFFFFFFFFFFFFF:FFFFF:FFFFFFFFFFFFFFFFFFFFFF
@A00155:342:HHGFNDSXY:1:1140:24189:16203 2:N:0:GAACCTAG+TCCGCATA
CATCTCCTTTGGAACTAGGAGACCCCTCCGGTCTTGTCCAACCTCTGTTTCGGCAGAGGATACCGTGC
TCCACAGCTGTAAGGCTGTGGGCCCGAGAGAAGTCGCTCTCTCTCGCCCTCTACATCCCTCTCGGCAT
+
FFFFFFFFFFFFFFFFFF:FFFFFFFFFFFFFFFFFFFFFFFFFFFFFFFFFFF:FFFFFFFFFFFF
FFFFFFFFFFFFFFFFFFFFFFFFFFF:FFFFFFFFFFFFFFFFFFFFFFFFFFFFFFFFFFF:FFF:
@A00155:342:HHGFNDSXY:1:1140:30129:16971 2:N:0:GAACCTAG+TCCGCATA
AACCAGGAGACCCCTCCGGTCTTGTCCAACCTCTGTTTCGGCAGAGGATACCGTGCTCCACAGCTGTA
AGGCTGTGGGCCCGAGAGAAGTCGCTCTCTCTCGCCCTCCACATCCCTCTCGGCATTGAGATGACCAT
+
FFFFFFFFFFFFFFFFFFFFFF:,FFFFFFFFFFFFFFFFFFFFFFFFFFFFFFFFFFFFFFFFFFFF
FFFFFFFFFFFFFFFFFFFFFFFFFFFFFFFFFFFFFFFFFFFFFFFFFFFFFFF,FFFFFFFFFFFF
@A00155:342:HHGFNDSXY:1:1140:28510:20713 2:N:0:GAACCTAG+TCCGCATA
AAGGACGAAACCGTCGTTACTCTTGGAGTTGTGTAAGTTTCGTCCTAAAAAGGACTCGTCAGGTAGCC
TACTAGACTACGACTTACACGGTAGGTCGTGAACAAGCCCGGAGTACATCTCCTTTGGAACTAGGAGA
+
FFFFFFFFFFFFFFFFFFFFFFF:,FFFFFFFFFFFFFFFFFFFFF,FFFFFFFFFF:FFFFFFFFFFF
FFFFFFFFFFFFFFFFFFFFFFFFFFFFFFFFFFFFFFFFFFFFFFFFFFFFFFFFFFFFFFF:FFFFF
@A00155:342:HHGFNDSXY:1:1140:4508:23015 2:N:0:GAACCTAG+TCCGCATA
CTGGCCTATGGTCATCTCAATGCCGAGAGGGATGTGAAGGGCGAGAGAGAGCGACTTCTCTCGGGCCC
ACAGCCTTACAGCTGTGGAGCACGGTATCCTCTGCCGAAACAGAGGTTGGACAAGACCGG
+
FFFFFFFFFFFFFFFFFFFFFFF:FFFFFFFFFFFFFFFFFFFFFFFFFFFFFFFFFFFFFFFFFFFF
FFFFFFFFFFFFFFFFFF:FFFFFFFFFFFFFFFFFFFFFF:FFFFFFFFFFFFFFFFFFFF
@A00155:342:HHGFNDSXY:1:1141:4797:7106 2:N:0:GAACCTAG+TCCGCATA
AAAAGGACTCGTCAGGTAGCCTACTAGACTACGACTTACACGGTAGGTCGTGAACAAGCCCGGAGTAC
ATCTCCTTTGGAACCAGGAGACCCCTCCGGTCTTGTCCAACCTCTGTTTCGGCAGAGGATACCGTGCT
+
FFFFFFFFFFFFFFFFFFFFFFFFFFFFFF:FFFFFFFFFFFFFFFFFFFFFFFFFFFFFFFFFFFFF
FFFFFFFFFFFFFFFFFFFFFFFFFFFFFFFFFFFFFFFFFFFFFFFF:FF,FFFFFFFFFFFFFFFF
@A00155:342:HHGFNDSXY:1:1141:32624:10113 2:N:0:GAACCTAG+TCCGCATA
ACATCTCCTTTGGAACCAGGAGACCCCTCCGGTCTTGTCCAACCTCTGTTTCGGCAGAGGATACCGTG
CTCCACAGCTGTAAGGCTGTGGGCCCGAGAGAAGTCGCTCTCTCTCGCCCTCCACATCCCTCTCGGC
+
FFFFFFFFFFFFFF:FFFFFFFFFFFFFFFFFFFF:FFFFFFFFFFFFFFFFFFFF:FFFF:FFFFFFF
FFFFFFFFFFFFFFFFFF,FFFFFFFFFFFFFF,FFFFFFFFFFFFFFFFFFFFFFFFFFFFFFFFFFF
@A00155:342:HHGFNDSXY:1:1141:11288:10332 2:N:0:GAACCTAG+TCCGCATA
TAGACTACGACTTACACGGTAGGTCGTGAACAAGCCCGGAGTACATCTCCTTTGGAACTAGGAGACCC
CTCCGGTCTTGTCCAACCTCTGTTTCGGCAGAGGATACCGTGCTCCACAGCTGTAAGGCTGTGGGCCC
+
FFFFFFFFFFFFFFFFFFFFFFFFF:FFFFFFFFFFFFFFFFFFFFFFFFFFFFFFFFFFF,FFFFFFFFF
FFFFFFFFFFFFFF:FFFFFFFFFFFFFFFFF:FFFFF,FFFFFFFFFFFFFFFFFFFFFFFFFFFFFFFF
@A00155:342:HHGFNDSXY:1:1141:23692:26897 2:N:0:GAACCTAG+TCCGCATA
GAACCAGGAGACCCCTCCGGTCTTGTCCAACCTCTGTTTCGGCAGAGGATACCGTGCTCCACAGCTGT
AAGGCTGTGGGCCCGAGAGAAGTCGCTCTCTCTCGCCCTCCACATCCCTCTCGGCATTGAGATGACC
+

```
FFFFFFFFFFFFFFFFFFFFFFFFFFFFFFFFFFFFFFFFFFF:F:FFFFFFFFFFFFFFFFFFFFFF
FFFFFFFFFFFFFFFFFFFF,FFFFFFFFFFFFFFFFFFFFFFFFFFFFFFFFFFFFFFFFFFFFFF
@A00155:342:HHGFNDSXY:1:1141:12147:29669 2:N:0:GAACCTAG+TCCGCATA
AAAGGACTCGTCAGGTAGCCTACTAGACTACGACTTACACGGTAGGTCGTGAACAAGCCCGGAGTACA
TCTCCTTTGGAACTAGGAGACCCCTCCGGTCTTGTCCAACCTCTGTTTCGGCAGAGGATACCGTGCTC
+
FFFFFFFFFFFFFFFFFFFFFFFFFFFFFFFFFFFFFFFFFFFFFFFFFFFFFFFFFFFFFFFFFFFF
FFFFFFFFFFFFFFFFFFFFFFFFFFFFFFFFFFFFFFFFFFFFFFFFFFFFFFFFFFFFFFFFFFFF
@A00155:342:HHGFNDSXY:1:1141:25174:33160 2:N:0:GAACCTAG+TCCGCATA
AAAAGGACTCGTCAGGTAGCCTACTAGACTACGACTTACACGGTAGGTCGTGAACAAGCCCGGAGTAC
ATCTCCTTTGGAACTAGGAGACCCCTCCGGTCTTGTCCAACCTCTGTTTCGGCAGAGGATACCGTGCT
+
FFFFFFFFFFFFFFFFFFFFFFFFFFFFFFFFFFFFFFFFFFFFFFFFFFFFFFFFFFFFFFFFFFFF
FFFFFFFFFFFFFFFFFFFFFFFFFFFFFFFFFFFFFFFFFFFFFFFFFFFFFFFFFFFF:FFFFFFF
@A00155:342:HHGFNDSXY:1:1142:30391:29168 2:N:0:GAACCTAG+TCCGCATA
TAGACTACGACTTACACGGTAGGTCGTGAACAAGCCCGGAGTACATCTCCTTTGGAACTAGGAGACCC
CTCCGGTCTTGTCCAACCTCTGTTTCGGCAGAGGATACCGTGCTCCACAGCTGTAAGGCTGTGGGCCC
+
FFFFFF,FFFFFFFFFFFFFFFFFFFFFFFFFFFFFFFFFFFFFFFFFFFF:FFFFFFF,FFFFFFFF
FFFFFFFFFFFFFFF:FFFFFFFFFFFFFFFFFFFFFFFFFFFFFFFF:FFFFFFFFFF:FFFFFFFF
@A00155:342:HHGFNDSXY:1:1143:2311:4398 2:N:0:GAACCTAG+TCCGCATA
TACTCTTCGAGTTGTGTAAGTTTCGTCCTAAAAAGGACTCGTCAGGTAGCCTACTAGACTACGACTTA
CACGGTAGGTCGTGAACAAGCCCGGAGTACATCTCCTTTGGAACTAGGAGACCCCTCCGGTCTTGTCC
+
FFFFFFFF,FF::F,FFFFFFFFF:FFFFF:FFFFFFFF:FFFF::FFFFFFFFFF:FFFFFFFFFFF
FFFFFFFFFFFFFFFFF:F,FF,FFFFFFFFFFFFF::FFFF:,FFFFF:FF,FFFFFFFF:::FFFF
@A00155:342:HHGFNDSXY:1:1144:17734:4398 2:N:0:GAACCTAG+TCCGCATA
TACTCTTCGAGTTGTGTAAGTTTCGTCCTAAAAAGGACTCGTCAGGTAGCCTACTAGACTACGACTTA
CACGGTAGGTCGTGAACAAGCCCGGAGTACATCTCCTTTGGAACTAGGAGACCCCTCCGGTCTTGTCC
+
FFFFF:FFFFFFFFFFFFFFFFFFFFFFFFFFF,FFFFFFF:FFFFFFFFFFFFFFFFFFFFFFFFFF
FFFFFFFFFFFFFFFFFFFFFFFFFFFFF::FFFFFFFFFFFFFFFFFFFFFFFFFFFFFFF:FFFFF
@A00155:342:HHGFNDSXY:1:1144:15185:9095 2:N:0:GAACCTAG+TCCGCATA
TACGACTTACACGGTAGGTCGTGAACAAGCCCGGAGTACATCTCCTTTGGAACCAGGAGACCCCTCCG
GTCTTGTCCAACCTCTGTTTCGGCAGAGGATACCGTGCTCCACAGCTGTAAGGCTGTGGGCCCG
+
FFFFFFFFFFFFFFFFFFFFFFFFFFF:FFFFFFFFF,FFFFFFFFFFFFF:FFFFFFFFFFFFFFFF
FFF:FFFFFFFFFFFFFFFFFFFFFF:FFFF:FFFFFFFFFFFFFFFFFFFFFFFFFFFFFFFF
@A00155:342:HHGFNDSXY:1:1144:27543:27023 2:N:0:GAACCTAG+TCCGCATA
AGTACATCTCCTTTGGAACTAGGAGACCCCTCCGGTCTTGTCCAACCTCTGTTTCGGCAGAGGATACC
GTGCTCCACAGCTGTAAGGCTGTGGGCCCGAGAGAAGTCGCTCTCTCTCGCCCTCCACATCCCTCTCG
+
FFFFFFFFFFFFFFFFFFFFFFFFFFFFFFFFFFFFFFFFFFFFFFFFFFFFFFFFFFFFFFFFFFFF
FFFFFFFFFFFFFFFFFFFFFFFFFFFFFFFFFFFFFFFFFFFFFFFFFFFFFFFFFFFFFFFFFFFF
@A00155:342:HHGFNDSXY:1:1144:27661:27070 2:N:0:GAACCTAG+TCCGCATA
AGTACATCTCCTTTGGAACTAGGAGACCCCTCCGGTCTTGTCCAACCTCTGTTTCGGCAGAGGATACC
GTGCTCCACAGCTGTAAGGCTGTGGGCCCGAGAGAAGTCGCTCTCTCTCGCCCTCCACATCCCTCTCG
+
FFFFFFFFFFFFFFFFFFFFFFFFFFFFFFFFFFFFF:FFFFFFFFFFFFFFFFFFFF:FFFFFFFFF
FFFFFFFFFFFFFFFFFFFFFFFFFFFFFFFFFFFFFFFFFFFFFFFFFFFFFFFFFFFFFFFFFFFF
@A00155:342:HHGFNDSXY:1:1144:28727:31767 2:N:0:GAACCTAG+TCCGCATA
CCGTCGTTACTCTTTGAGTTGTGTAAGTTTCGTCCTAAAAAGGACTCGTCAGGTAGCCTACTAGACTA
CGACTTACACGGTAGGTCGTGAACAAGCCCGGAGTACATCTCCTTTGGAACTAGGAGACCCCTCCGGT
+
```

FFFFFFFFFFFF,:FFFFFFFFFFFFFFFFFFFFFFFFFFFFFFFFFFFFFFFFFFFFFFFFFFFFFF
FFFFFFFFFFFFFFFFFFFFFFFFFFFFFFFFFFFFFFFFFFF,FFFFFFFFFFFFFFFFFFFFFFFF
@A00155:342:HHGFNDSXY:1:1144:8187:33739 2:N:0:GAACCTAG+TCCGCATA
AAGGACGAAACCGTCGTTACTCTTCGAGTTGTGTAAGTTTCGTCCTAAAAAGGACTCGTCAGGTAGCA
TACTAGACTACGACTTACACGGTAGGTCGTGAACAAGCCCGGAGTACATCTCCTTTGGAACTAGGA
+
FFFFFFF,FFFFF,F:FFFFFF:F:F:F,:FFF:FFFFFFFFFFFFFFF,FFFFFFF:FFFFFFFFF,
FFFFFFF:FFFFFFFFF:FFFFFFFFFFFFFFFFFF,FFFFFFF,FFFF,FF:FFFFFFFFFFFFF
@A00155:342:HHGFNDSXY:1:1144:25979:35430 2:N:0:GAACCTAG+TCCGCATA
GCCTATGGTCATCTCAATGCCGAGAGGGATGTGGAGGGCGAGAGAGAGCGACTTCTCTCGGGCCCACA
GCCTTACAGCTGTGGAGCACGGTATCCTCTGCCGAAACAGAGGTTGGACAAGACCGGAG
+
FFFFFFFFFFFFFFFFFFFFFFFFFFFFFFFFFFFFFFFFFFFFFFFFFFFFFFFFFFFFFFFFFFFF
FFFFFFFFFFFFFFFFFFFFFFFFFFFFFFFFFFFFFFFFFFFFFFFFFFFFFFFFFFF
@A00155:342:HHGFNDSXY:1:1145:11939:1564 2:N:0:GAACCTAG+TCCGCATA
AGAGGATACCGTGCTCCACAGCTGTAAGGCTGTGGGCCCGAGAGAAGTCGCTCTCTCTCGCCCTTCAC
ATCCCTCTCGGCATTGAGATGACCATAGGCCAGAGGTGAGTCCTTAAGTGGACACAGCTG
+
FFFFFFFFFFFFFFFFFFFFFFFFFFFFFF:FFFFFFFFFFFFFFFFFFFFFFFFFFFFFFFFFFFFF
FFFFFFFFFFFFF:FFFFFFFFFFFFFFFFFFFFFFFFFFFFFFFFFFFFFFFFFFFFFF
@A00155:342:HHGFNDSXY:1:1145:3197:4742 2:N:0:GAACCTAG+TCCGCATA
CGAGTTGTGTAAGTTTCGTCCTAAAAAGGACTCGTCAGGTAGCCTACTAGACTACGACTTACACGGTA
GGTCGTGAACAAGCCCGGAGTACATCTCCTTTGGAACTAGGAGACCCCTCCGGTCTTGTCCAACCTCT
+
FFFFFFFFFFFFFFFFFFFFFFFFFFFFFFFFFFFFFFFFFFFFFFFFFFFFFFFF,FFFFFFFFFFF
FFFFFFFFFFFFFFFFFFFFFFFFFFF:FFFFFFFFFFFFFFFFFFFFFFFFFFFFFFFFFFFFFFFF
@A00155:342:HHGFNDSXY:1:1145:3106:4773 2:N:0:GAACCTAG+TCCGCATA
CGAGTTGTGTAAGTTTCGTCCTAAAAAGGACTCGTCAGGTAGCCTACTAGACTACGACTTACACGGTA
GGTCGTGAACAAGCCCGGAGTACATCTCCTTTGGAACTAGGAGACCCCTCCGGTCTTGTCCAACCTCT
+
FF:FFFFFFFFFFFFFFFFFFFFFFFFFFFF:FFFFFFFFFFFFFFFFFFFFFFFF:FFFFFFFFFFF
FFFFFFFFFFFFFFFFFFFFFFFFFFF::FFFFFFFFFFFFFFFFFFFFFFFFFFFFFFFFFFFFFFF
@A00155:342:HHGFNDSXY:1:1145:14154:8813 2:N:0:GAACCTAG+TCCGCATA
AAAAGGACTCGTCAGGTAGCCTACTAGACTACGACTTACACGGTAGGTCGTGAACAAGCCCGGAGTAC
ATCTCCTTTGGAACTAGGAGACCCCTCCGGTCTTGTCCAACCTCTGTTTCGGCAGAGGATACCGTGCT
+
FFFFFFFFFFFFFFFFFFFFFFFFFFFFFFFFFFFFFFFFFFFFFFFFFFFFFFFFFFFFFFFFFFFF
FFFFFFFFFFFFFFFFFFFFFFFFFFFFFFFF:FFFFFFFFFFFFFFFFFFFFFFFFFFFFFFFFFFF
@A00155:342:HHGFNDSXY:1:1145:21775:19225 2:N:0:GAACCTAG+TCCGCATA
CCTTTGGAACTAGGAGACCCCTCCGGTCTTGTCCAACCTCTGTTTCGGCAGAGGATACCGTGCTCCAC
AGCTGTAAGGCTGTGGGCCCGAGAGAAGTCGCTCTCTCTCGCCCTCCACATCCCTCTCAGC
+
FFFFFFFF,F,FFFFFFFFFFFFFFFFFFFFFFFFFFFFFFFFFFFFFF:FFFFFFFFFFFFFFFFFF
FFFFFFFFFFFFFFFFFFFFFFFFFFFFFFFFFFFFFFFFFFFFFFFFFFFFFFFFFFFFF
@A00155:342:HHGFNDSXY:1:1146:16721:4022 2:N:0:GAACCTAG+TCCGCATA
GGTAGCCTACTAGACTACGACTTACACGGTAGGTCGTGAACAAGCCCGGAGTACATCTCCTTTGGAAC
TAGGAGACCCCTCCGGTCTTGTCCAACCTCTGTTTCGGCAGAGGATACCGTGCTCCACAGCTGTAAGG
+
FFFFFFFFFFFFFFFFFFFFFFFFFFFFFFFFFFFFFFFFFFF:FFFFFFFFFFFFFFFFFFFFFFFF
FFFFFFFFFFFFFFFFFFFFFFFFFFFFFFFFFFFFFFFFFFFFFFFFFFFFFFFFFFFFFFFFFFFF
@A00155:342:HHGFNDSXY:1:1146:25229:20478 2:N:0:GAACCTAG+TCCGCATA
CCTACTAGACTACGACTTACACGGTAGGTCGTGAACAAGCCCGGAGTACATCTCCTTTGGAACCAGGA
GACCCCTCCGGTCTTGTCCAACCTCTGTTTCGGCAGAGGATACCGTGCTCCACAGCTGTAAGGCTGTG
+

```
FFFFFFFFFFFFFFFFFFFFFFFFFF:FFFFFFFFFFFFFFFFFFFFFFFF:FFFFFFFFFFFFFFFFFF
FFFFFFFFFFF:FFFFFFFFFFFFFFFFFFFFFFFFFFFFFFFFFFFFFFFFFFFFFFFFFFFFFFFFF
@A00155:342:HHGFNDSXY:1:1146:29740:31078 2:N:0:GAACCTAG+TCCGCATA
CAAGGACGAAACCGTCGTTACTCTTCGAGTTGTGTAAGTTTCGTCCTAAAAAGGACTCGTCAGGTAGC
CTACTAGACTACGACTTACACGGTAGGTCGTGAACAAGCCCGGAGTACATCTCCTTTGGAACTAGGAG
+
FFFFFFFFFFFFFFFFFFFF:F,FFFFFFFFFFFF:FFFFFFFF:FF:F:FF,:FFFFFFF:FFFF:F
FFFFFFFFFFFFFFFFFFFFFFFFFFFFFFFFFFFF,FFFFFFFFFFFFFFFFF,F:FF:FFFFFFF
@A00155:342:HHGFNDSXY:1:1146:30508:33974 2:N:0:GAACCTAG+TCCGCATA
GGAGTACATCTCCTTTGGAACTAGGAGACCCCTCCGGTCTTGTCCAACCTCTGTTTCGGCAGAGGATA
CCGTGCTCCACAGCTGTAAGGCTGTGGGCCCGAGAGAAGTCGCTCTCTCTCGCCCTCCACATCCCTCT
+
FFFFFFFFFFFFFFFFFFF:FFFFFFFFFFFFFFFFFFF,FFFFFFFFFFFFF,FFFFFFFFFFFFFF
FFFFFFFFFFFFFFFFFFFFFFFFFFFFFFFFFFFFF:,FFFFFFFFFFF:FFFFFFFFFFFFFFFFF
@A00155:342:HHGFNDSXY:1:1147:30391:27007 2:N:0:GAACCTAG+TCCGCATA
CTGGCCTATGGTCATCTCAATGCCGAGAGGGATGTGGAGGGCGAGAGAGAGCGACTTCTCTCGGGCCC
ACAGCCTTACAGCTGTGGAGCACGGTATCCTCTGCCGAAACAGAGGTTGGACAAGACCGGAG
+
FFFFFFFFFFFFFFFFFFFFFFFFFFFFFFFFFFFFFFFFFFFFFFFFFFFFFFFFFFFFFFFFFFFF
FFFFFF:FFFFFFFFFFFFFFFFFFFFFFFFFFFFFFFFFFFFFFFFFF:FFFFFFFFF:FFFF
@A00155:342:HHGFNDSXY:1:1147:31232:32221 2:N:0:GAACCTAG+TCCGCATA
ACTAGGAGACCCCTCCGGTCTTGTCCAACCTCTGTTTCGGCAGAGGATACCGTGCTCCACAGCTGTAA
GGCTGTGGGCCCGAGAGAAGTCGCTCTCTCTCGTCCTCCACATCCCTCTCGGCATTGAGATGAC
+
FFFFFFFFFFFFFFFFFFFF,FFFFFFFFFFFFFFFFFFFFFFFFFFF:FFFFFFFFFFFFFFF:FFFF
FFFFFFFFFFFFFFFFFFFFFFFFFFFFFFFFFFFFFFFFFFFFFFFFFFFFFFF,FFFFFFFFF
@A00155:342:HHGFNDSXY:1:1148:29098:3630 2:N:0:GAACCTAG+TCCGCATA
CACGGTAGGTCGTGAACAAGCCCGGAGTACATCTCCTTTGGAACCAGGAGACCCCTCCGGTCTTGTCC
AACCTCTGTTTCGGCAGAGGATACCGTGCTCCACAGCTGTAAGGCTGTGGGCCCGAGAGAAGTCGCTC
+
FFFFFFFFFFFFFFFFFFFFFFFFFFFFFFFFFFFFFFFFFFFFFFFFFFFFFFFFFFFFFFFFFFFF
FFFFFFFFFFFFFFFFFFFFFFFFFFFFFFFFFFFFFFFFFFFFF:FFFFFFFFFFF:FFF:FFFFFF
@A00155:342:HHGFNDSXY:1:1148:5692:6934 2:N:0:GAACCTAG+TCCGCATA
TTAGGACGAAACTTACACAACTCGAAGAGTAACGACGGTTTCGTCCTTGGAACGGACTCATCAGGGAG
CCTACTAGACTCCGACCGTCATGGGGGTTCAAACCCATGCCCCGCTACACCGCCTTAGAT
+
FFFFFFFFFFFFF,FFFFFFFFFFFFFFFFFFFFFFFFFFFFFFFFFFFFFFFFFFFFFFFFFFFFFF
FFFFFFFFFFF:FFFFFFFFFFFFFFFFF:FF:FFFFFFFFFFFFFFFFF:FFFFFFFFF
@A00155:342:HHGFNDSXY:1:1148:8947:27038 2:N:0:GAACCTAG+TCCGCATA
GGACTCGTCAGGTAGCCTACTAGACTACGACTTACACGGTAGGTCGTGAACAAGCCCGGAGTACATCT
CCTTTGGAACCAGGAGACCCCTCCGGTCTTGTCCAACCTCTGTTTCGGCAGAGGATACCGTGCTCCAC
+
FFFFFFFFFFFFFF:FFFFFFFFFFFFFFFFFFFFFFFFFFFFFFFFFFFFFFFFFFFFFFFFFFFFF
FFFFFFFFFFFFFFFFFFFFFFFFFFFFFFFFFFFFFFFFFFFFFFFFFFFFFFFFFFFFFFFFFFFF
@A00155:342:HHGFNDSXY:1:1148:6542:28260 2:N:0:GAACCTAG+TCCGCATA
CAGGTAGCCTACTAGACTACGACTTACACGGTAGGTCGTGAACAAGCCCGGAGTACATCTCCTTTGGA
ACTAGGAGACCCCTCCGGTTTTGTCCAACCTCTGTTTCGGCAGAGGATACCGTGCTCCACAGCTGTAA
+
FFFFFFFFFFFFFFFFFFFFFFFFFFFFFFFFFFFFFFFFFFFFFFFFFFFFFFFFFFFFFFFFFFFF
FFFFFFFFFFFFFFFFFFFFFFFFFFFFFFFFFFFFFFF:FFFFFFFFFFFFFFFFFFFFFFFFFFFF:
@A00155:342:HHGFNDSXY:1:1149:27398:2347 2:N:0:GAACCTAG+TCCGCATA
TAGCCTACTAGACTACGACTTACACGGTAGGTCGTGAACAAGCCCGGAGTACATCTCCTTTGGAACCA
GGAGACCCCTCCGGTCTTGTCCAACCTCTGTTTCGGCAGAGGATACCGTGCTCCACAGCTGTAAGGCT
+
```

```
FFFFFFFFFFFFFFFFFFFFFFFFFFFFFFF:FFFFFFFFF:FFFFFFFFFFFFFFFFFFFFFFFFFF
FFFFFFFFFFFFFFFFFFFFFFFFFFFFFF,FFFFFFFFFFFFFFFFFFFFFFFFFFFFFFFFFFFFF
@A00155:342:HHGFNDSXY:1:1150:23140:6934 2:N:0:GAACCTAG+TCCGCATA
GTTTCGTCCTAAAAAGGACTCGTCAGGTAGCCTACTAGACTACGACTTACACGGTAGGTCGTGAACAA
GCCCGGAGTACATCTCCTTTGGAACTAGGAGACCCCTCCGGTCTTGTCCAACCTCTGTTTCGGCAGAG
+
FFFFFFFFFFFFFFFFFFFFFFFFFFFFFFFFFFFFFFFFFFFFFFFFFFFF:FFFFFFFFFFFFFFF
FFFFFFFFFFFFFFFFF:FFFFFFFFFFFFFF:FFFFFFFFFFFFFFFFFFFFFFFFFFFFFFFFFFF
@A00155:342:HHGFNDSXY:1:1150:27353:10535 2:N:0:GAACCTAG+TCCGCATA
GTTTCGTCCTAAAAAGGACTCGTCAGGTAGCCTACTAGACTACGACTTACACGGTAGGTCGTGAACAA
GCCCGGAGTACATCTCCTTTGGAACTAGGAGACCCCTCCGGTCTTGTCCAACCTCTGTTTCGGCAGAG
+
FFFFFFFFFFFFFFFFFF:FFFFFFFFFFFFFF:FFFFFFFFFFFFFFFFF,FFFFFFFFFFFFF:FF
FFFFFFFFFFFFFFF:FFFFFFF,FFFF,FFFFFFFFFFFFFFF,FFFFFFFFFFFFFFFFFFFFFFF
@A00155:342:HHGFNDSXY:1:1150:26910:29747 2:N:0:GAACCTAG+TCCGCATA
CCGTCGTTACTCTTTGAGTTGTGTAAGTTTCGTCCTAAAAAGGACTCGTCAGGTAGCCTACTAGACTA
CGACTTACACGGTAGGTCGTGAACAAGCCCGGAGTACATCTCCTTTGGAACTAGGAGACCCCTCCGGT
+
FFFFFFFF:FFF,FFFFFFFFFFFFFFFFFFFFFFFFFFFFFFFFFFFFF,FFFFFFF,FFFF:FFFF
FFFFFFFFFFFFFFFFFFFFFFFFFF:FFFFFFFFFFFFFFFFFFFFF:,FFFFFFFFFFFFFFFFFF
@A00155:342:HHGFNDSXY:1:1150:22607:35352 2:N:0:GAACCTAG+TCCGCATA
CCGTCGTTACTCTTTGAGTTGTGTAAGTTTCGTCCTAAAAAGGACTCGTCAGGTAGCCTACTAGACTA
CGACTTACACGGTAGGTCGTGAACAAGCCCGGAGTACATCTCCTTTGGAACTAGGAGACCCCTCCGGT
+
FFFFFFFFFFFFFFFFFF:FFFF:FFFFFFFFFFFFFF::FFFFFFFFFF,:FFFFFFF,FFFFFFFF
FFFFFFFFFFFFFFFFFFFFFFFFFFFFFFFFFFFFFFFFFFFFFFFFFFFFFFFFFFFFFFFFFFFF
@A00155:342:HHGFNDSXY:1:1150:29713:37043 2:N:0:GAACCTAG+TCCGCATA
TGTTTCGGCAGAGGATACCGTGCTCCACAGCTGTAAGGCTGTGGGCCCGAGAGAAGTCGCTCTCTCTC
GCCCTCTACATCCCTCTCGGCATTGAGATGACCATAGGCCAGAGGTGAGTCCTTAAGTGGACACAGCT
+
FFFFFFFFFFFFFFFFFFFFFFFFFFFFFFF:FFFFFFFFFFFFFFFFFFFFFFFFFFFFFFFFFFFF
FFFFFFFFFFFFFFFFFFFFFFFFFFFFFFFFFFF,FFFFFFFFFFFFFFFFFF,FFFFFFFFFFFFF
@A00155:342:HHGFNDSXY:1:1151:26404:19006 2:N:0:GAACCTAG+TCCGCATA
TCAGCTGTGTCCACTTAAGGACTCACCTCTGGCCTATGGTCATCTCAATGCCGAGAGGGATGTAGAGG
GCGAGAGAGAGCGACTTCTCTCGGGCCCACAGCCTTACAGCTGTGGAGCACGGTATCCTCTGCCGAAA
+
FFFFFFFFFFFFFFFFFFFFFFFFFFFFFFFFFFFFFFFFFFFFFFFFFF:FFFFFFFFF:FFFFFFF
FFFFFFFFFFFFFFFFFFFFFFFFFFFFFFFFFF:FFFFFFFFFFFFFFFFFFF:FFFFFFFFFFFFF
@A00155:342:HHGFNDSXY:1:1152:3586:10050 2:N:0:GAACCTAG+TCCGCATA
GAACTAGGAGACCCCTCCGGTCTTGTCCAACCTCTGTTTCGGCAGAGGATACCGTGCTCCACAGCTGT
AAGGCTGTGGGCCCGAGAGAAGTCGCTCTCTCTCGCCCTCCACATCCCTCTCGGCAGTGAGATGACCA
+
FFFFFFFFFFFFFFF:FFFFFFFFFFFFFFFFFFFF:FFFFFFFFFFFFFFFFFFFFFF:F:FFFFFF
FFFFF:::FFFFFFFFFFFF,FFFFFFFFFFFFFFFFFFFFFFFFFFFFFFF:FF,FFFFFFFFFFFF
@A00155:342:HHGFNDSXY:1:1152:6171:15562 2:N:0:GAACCTAG+TCCGCATA
GAACTAGGAGACCCCTCCGGTCTTGTCCAACCTCTGTTTCGGCAGAGGATACCGTGCTCCACAGCTGT
AAGGCTGTGGGCCCGAGAGAAGTCGCTCTCTCTCGCCCTCCACATCCCTCTCGGCATTGAGATGACCA
+
FFFFFFFFFFFFFFFFFFFFFFFFFFFFFFFFFFFFFFFFFFFFFFFFFFFFFFFFFFFFFFFFFFFF
FFFFFFFFFFFFFFFFFFFFFFFFFFFFFFFFFFFFFFFFFFFFFFFFFFFFFFFFFF:F:FFFFFFF
@A00155:342:HHGFNDSXY:1:1152:18041:24032 2:N:0:GAACCTAG+TCCGCATA
GTGAACAAGCCCGGAGTACATCTCCTTTGGAACTAGGAGACCCCTCCGGTCTTGTCCAACCTCTGTTT
CGGCAGAGGATACCGTGCTCCACAGCTGTAAGGCTGTGGGCCCGAGAGAAGTCGCTCTCTCTCGCCCT
+
```

FFFFFFFFFFFFFFFFFFFFFFFFFFFFFFF:FFFFFFFFFFFFFFFFFFFFFFFFFFFFFFFFFFFF
FFFFFFFFFF:FFFFFFFFFFFFFFFFFFFFFFFFFFFFFFFFFFFFFFFFFFFFFFFFFFFFFFFFF
@A00155:342:HHGFNDSXY:1:1152:25111:31955 2:N:0:GAACCTAG+TCCGCATA
GGTAGCCTACTAGACTACGACTTACACGGTAGGTCGTGAACAAGCCCGGAGTACATCTCCTTTGGAAC
CAGGAGACCCCTCCGGTCTTGTCCAACCTCTGTTTCGGCAGAGGATACCGTGCTCCACAGCTGTAAGG
+
FFFFFFFFFFFFFFFF:FFFFFFFFFFFFFFFFFFFFFFFFFF:FFFFFFFFFFFFFFFFFFFFFFFF
FFFFFFFFFFFFFFFFFF:FF:FFFFFFFFFFFFFFFFFFFFFFFFFFFFFFFFFFFFFFFFFFFFFF
@A00155:342:HHGFNDSXY:1:1152:18123:33630 2:N:0:GAACCTAG+TCCGCATA
TCCAAGGACGAAACCGTCGTTACTCTTCGAGTTGTGTAAGTTTCGTCCTAAAAAGGACTCGTCAGGTA
GCCTACTAGACTACGACTTACACGGTAGGTCGTGAACAAGCCCGGAGTACATCTCCTTTGGAACTAGG
+
FFFFFFFFFFFF:FFFFFFFFFFFFFFFFFFFFFFF,:FFFFFFFFFF:FFF:,FFFFFFFFFFFFFF
FFFFFFFFFFFFFFFFFFFFFFFFFFFFFFFFFFFFFFFFFFFFFFFFFFFFFFFFFFFFFF:FFFFF
@A00155:342:HHGFNDSXY:1:1152:9173:34632 2:N:0:GAACCTAG+TCCGCATA
GTTGTGTAAGTTTCGTCCTAAAAAGGACTCGTCAGGTAGCCTACTAGACTACGACTTACACGGTAGGT
CGTGAACAAGCCCGGAGTACATCTCCTTTGGAACTAGGAGACCCCTCCGGTCTTGTCCAACCTCTGTT
+
FFFFFFFFFFFFF:FFFFFFFFFFFFFFFFFFFFFFFFFFFFFFFFFFFFFFF:FFFFFFFFFFFFFF
FFFFFFFFFFFFFFFFFFFFFFFFFFFFFFFFFFFFFF:F:FFFFFFFFFFFFFFFFFFFFFFFFFFF
@A00155:342:HHGFNDSXY:1:1153:27977:2879 2:N:0:GAACCTAG+TCCGCATA
CGTCAGGTAGCCTACTAGACTACGACTTACACGGTAGGTCGTGAACAAGCCCGGAGTACATCTCCTTT
GGAACTAGTAGACCCCTCCGGTCTTGTCCAACCTCTGTTTCGGCAGAGGATACCGTGCTCCACAGCTG
+
FFFFF:FFFF:FFFFFFF:FFFFFFFFFFFFFFFFFFFF:FFFFFF:FF,FFFFF:FFF,:FFF::FF
,FFF,FFF,FFFFFFFFFF,FFF,,F:FFFFFFFFFFFFFFFFFFFFF:FFF,FFF,FFF:FFFFFFF
@A00155:342:HHGFNDSXY:1:1153:25599:16172 2:N:0:GAACCTAG+TCCGCATA
ACCGCCTTAGATCAGCTGTGTCCACTTAAGGACTCACCTCTGGCCTATGGTCATCTCAATGCCGAGAG
GGATGTGAAGGGCGAGAGAGAGCGACTTCTCTCGGGCCCACAGCCTTACAGCTGTGGAGCACGGTATC
+
FFFFFFFFFFFFFFFFFFFF:FFFFF,FFFFFFFFFFFFFFFFFFF,FFFFFFFFFFFFFFFFFFF,F
FFF,FFFFFFFFFFFFFFFFFFFFFFFFFFFFFFFFFFFFFFFFFFF:FFFFFFFFFFFFFFFFFFFF
@A00155:342:HHGFNDSXY:1:1154:7672:26835 2:N:0:GAACCTAG+TCCGCATA
CTACTAGACTACGACTTACACGGTAGGTCGTGAACAAGCCCGGAGTACATCTCCTTTGGAACCAGGAG
ACCCCTCCGGTCTTGTCCAACCTCTGTTTCGGCAGAGGATACCGTGCTCCACAGCTGTAAGGCTGTGG
+
FFFFFFFFFFFFFFFFFFFFFFFFFFFFFFFFFFFFF:FFFFFFFFFFFFFFFFFFFFFFFFFFFFFF
:FFFFFFFFFFFFFFFFFFFFFFFFFFFFFFFF:FFF:FFFFFFFFFFFF:FFFFFFFFFFFFFFFFF
@A00155:342:HHGFNDSXY:1:1155:22462:16282 2:N:0:GAACCTAG+TCCGCATA
ACTTACACGGTAGGTCGTGAACAAGCCCGGAGTACATCTCCTTTGGAACTAGGAGACCCCTCCGGTCT
TGTCCAACCTCTGTTTCGGCAGAGGATACCGTGCTCCACAGCTGTAAGGCTGTGGGCCCGAGAGAAGT
+
:FFFFFFFFFFFFFFFFFFFFFFFFFFFFFFFFFFFFFFFFF:FFFFFFFFFFFFFFFFFFFFFFFF,
FFFFFFFFFFFFFFFFFFFFFFFFFFFFFFFFFFFFFFFFFFFFFFFFFFFFFFFFFFFFFFFFF:F:
@A00155:342:HHGFNDSXY:1:1155:16351:25488 2:N:0:GAACCTAG+TCCGCATA
TTAAGGACTCACCTCTGGCCTATGGTCATCTCAATGCCGAGAGGGATGTGAAGGGCGAGAGAGAGCGA
CTTCTCTCGGGCCCACAGCCTTACAGCTGTGGAGCACGGTATCCTCTGCCGAAACAGAGGTTGGACAA
+
FFFFFFFFFFFFFFFFFFFFFFFFFFFFFFFFFFFFFFFFFFFFFFFFFFFFFFFFFFF:F:FFFFFF
FFFFFFFFFFFFFFFFFF:FFFFFFFFFFFFFFFFFFFFFFFFFFFFFFFFFFFFF:FFFFFFFFFFFF
@A00155:342:HHGFNDSXY:1:1155:16821:26146 2:N:0:GAACCTAG+TCCGCATA
TTAAGGACTCACCTCTGGCCTATGGTCATCTCAATGCCGAGAGGGATGTGAAGGGCGAGAGAGAGCGA
CTTCTCTCGGGCCCACAGCCTTACAGCTGTGGAGCACGGTATCCTCTGCCGAAACAGAGGTTGGACAA
+

```
FFFFFFFFFFFFFFFFFFFFFFFFFFFFFFFFFFFFFFFFFFFFFFFFFFFFFFFFFFFFFFFFFFFFFF
FFFF:FFFFFFFFFFFFFFFF,FFFFFFFFFFFFFFFFFFFFFFFFFFFFFFFFFFFFFFFFFFFFFFFF
@A00155:342:HHGFNDSXY:1:1156:31792:11992 2:N:0:GAACCTAG+TCCGCATA
CAGAGGTGAGTCCTTAAGTGGACACAGCTGATCTAAGGCGGTGTGGCGGGGCATGGGTTTGAACCCCC
ATGACGGTCGGAGTCTAGTAGGCTCCCTGATGAGTCCGTTCCAAGAACGAAACCGTCGTTACTCTTCG
+
FFFFFFFFFFFFFF:FFFFFFFFFFFFFFFFFF:FFFFFFFFFFFFFFFFFFFFFFF::FFFFFFFFF
FFFFFFFFFFFFFFFFFFFFFFFFFFFFFFFFFFFFFFFFFFFFFFFFFFFFFFFFFF:FFFFFFFFF
@A00155:342:HHGFNDSXY:1:1156:13774:12446 2:N:0:GAACCTAG+TCCGCATA
AACTAGGAGACCCCTCCGGTCTTGTCCAACCTCTGTTTCGGCAGAGGATACCGTGCTCCACAGCTGTA
AGGCTGTGGGCCCGAGAGAAGTCGCTCTCTCTCGCCCTCCACATCCCTCTCGGCATTGAGATGACCAT
+
FFFFFFFFFFFFFFFFFFFFFFFFFFFFFFFFFFFFFFFFFFFFFFFFFFFFFFFFFFFFFFFFFFFF
FFFFFFFFFFFFFFFFFFFFFFFFFFFFFFFFFFFFFFFFFFFFFFFFFFFFFFFFFFFFFFFFFFFF
@A00155:342:HHGFNDSXY:1:1156:16866:20995 2:N:0:GAACCTAG+TCCGCATA
AAAAGGACTCGTCAGGTAGCCTACTAGACTACGACTTACACGGTAGGTCGTGAACAAGCCCGGAGTAC
ATCTCCTTTGGAACTAGGAGACCCCTCCGGTCTTGTCCAACCTCTGTTTCGGCAGAGGATACCGTGCT
+
FFFFFFFFFFFFFFFFFFFFFFFFFFFFFFFFFFFFFFFFFFFFFFFFFFFFFFFF:FF:F:FFFFF
FFFFFFFFFFFFFFFFFFFFFFFFFFFFFFFFFFFFFFFFFFFFFFFFFFFFFFFFFFFFFFFFFFFF
@A00155:342:HHGFNDSXY:1:1156:10538:34366 2:N:0:GAACCTAG+TCCGCATA
GGTAGCCTACTAGACTACGACTTACACGGTAGGTCGTGAACAAGCCCGGAGTACATCTCCTTTGGAAC
TAGGAGACCCCTCCGGTCTTGTCCAACCTCTGTTTCGGCAGAGGATACCGTGCTCCACAGCTGTAAGG
+
FFFFFFFFFFFFFFFFFFFFFFFFFFFFFFFFFFFFFFFFFFFFFFFFFFFFFFFFFFFFFFFFFFFF
FFFFFFFFFFFFFFFFFFFFFFFFFFFFFFFFFFFFFFFFFFFFFFFFFFFFFFFFFFFFFFFFF:F:
@A00155:342:HHGFNDSXY:1:1157:32054:7717 2:N:0:GAACCTAG+TCCGCATA
AAAAGGACTCGTCAGGTAGCCTACTAGACTACGACTTACACGGTAGGTCGTGAACAAGCCCGGAGTAC
ATCTCCTTTGGAACTAGGAGACCCCTCCGGTCTTGTCCAACCTCTGTTTCGGCAGAGGATACCGTGCT
+
FFFFFFFFFFFFFFFFFFFFFFFFF:FFFFFFFFFFFFFFFFFF:FFFFFFFFFFFFFFFFFFFFFFFF
FFFFFFFFFFFFFFFFFFFFFFFFFFFFFFFF::FFFFFFFFFFFFFFFFFFFFFFFFFFFFFFFFFF
@A00155:342:HHGFNDSXY:1:1158:24569:10254 2:N:0:GAACCTAG+TCCGCATA
TCCTAAAAAGGACTCGTCAGGTAGCCTACTAGACTACGACTTACACGGTAGGTCGTGAACAAGCCCGG
AGTACATCTCCTTTGGAACTAGGAGACCCCTCCGGTCTTGTCCAACCTCTGTTTCGGCAGAGGATACC
+
FFFFFFFFFFFFFFFFFFFFFFFFFFFFFFFFFFFFFFFF:FFFFFFFFFFFFFFFFFFFFFFFFFFF
FFFFFFFFFFFFFFFFFFFFFFFFFFFFFFFFFFFF:FF:F:FFFFFFFFFFFFFFFFFFFFFFFFFF
@A00155:342:HHGFNDSXY:1:1158:2889:20181 2:N:0:GAACCTAG+TCCGCATA
ACTTACACGGTAGGTCGTGAACAAGCCCGGAGTACATCTCCTTTGGAACTAGGAGACCCCTCCGGTCT
TGTCCAACCTCTGTTTCGGCAGAGGATACCGTGCTCCACAGCTGTAAGGCTGTGGGCCCGAGAGAAGT
+
FFFFFFFFFFFFFFFFFFFFFFFFFFFF:FFFFFFFFFFF:FFFFFFFFFFFFFFFFFFFFFFFFFFF
:FFFFFFFFFFFFFFFFFFFFFFFFFFFFFFFFFFFFFFFFFFFFFFFFFFFFFFFFFFFFFFFFFF:
@A00155:342:HHGFNDSXY:1:1158:2456:20275 2:N:0:GAACCTAG+TCCGCATA
ACTTACACGGTAGGTCGTGAACAAGCCCGGAGTACATCTCCTTTGGAACTAGGAGACCCCTCCGGTCT
TGTCCAACCTCTGTTTCGGCAGAGGATACCGTGCTCCACAGCTGTAAGGCTGTGGGCCCGAGAGAAGT
+
F,FFFFFFFFFFF:FFFFFFFFFFFFFFFFFFFFFFFFFFFFFFFFFFF:FFFFFFFFFFFFFFFF:F
FFFFFFFFFFFFFFFFF,FFFFFFFFFFFFFFFFF:FFF,FFFFFFFFFFFFFFFFFFFFFFF:FFFFF
@A00155:342:HHGFNDSXY:1:1159:12617:25191 2:N:0:GAACCTAG+TCCGCATA
ACTTACACGGTAGGTCGTGAACAAGCCCGGAGTACATCTCCTTTGGAACCAGGAGACCCCTCCGGTCT
TGTCCAACCTCTGTTTCGGCAGAGGATACCGTGCTCCACAGCTGTAAGGCTGTGGGCCCGAGAGAAGT
+
```

FFFFFFFFFFFFFFFFFFFFFFFFFFFFFFFFFFFFFFFFFFFFFFFFFFFFFFFFFFFFFFFFFFFF
FFFFFFFFFFFFFFFFFFFFFFFFFFFFFFFFFFFFFFFFFFFFFFFFFFFFFFFFFFFFFFFF:FFF
@A00155:342:HHGFNDSXY:1:1159:14443:32362 2:N:0:GAACCTAG+TCCGCATA
AAAAGGACTCGTCAGGTAGCCTACTAGACTACGACTTACACGGTAGGTCGTGAACAAGCCCGGAGTAC
ATCTCCTTTGGAACTAGGAGACCCCTCCGGTCTTGTCCAACCTCTGTTTCGGCAGAGGATACCGTGCT
+
FFFFFFFFFFFFFFFFFFFFFFFFFFFFFFFFFFFFFFFFFFFFFFFFFFFFFFFFFFF:FFFFFFFF
FFFFFFFFFFFFFFFFFFFFFF:FFFFFFFFFFFFFFFFFFFFFFFFFFFFFFFFFFFFFFFFFFFFF
@A00155:342:HHGFNDSXY:1:1159:4716:35994 2:N:0:GAACCTAG+TCCGCATA
ACATCTCCTTTGGAACTAGGAGACCCCTCCGGTCTTGTCCAACCTCTGTTTCGGCAGAGGATACCGTG
CTCCACAGCTGTAAGGCTGTGGGCCCGAGAGAAGTCGCTCTCTCTCGCCCTCCACATCCCTCTCGGCA
+
FFFFFFFFFFFFFFFFF:FFFFFFFFFFFFFFFFFFFFFFFFFFFFFFFFFFFFFFFFFFFFFFFFFF
FFFFFFFFFFFFFF:FFFFFFFFFFFFFFFFF:FFFFFFFFFFFFFFFFFFFFFFFFFFFFFFFFFFF
@A00155:342:HHGFNDSXY:1:1160:1497:36652 2:N:0:GAACCTAG+TCCGCATA
TAGGTCGTGAACAAGCCCGGAGTACATCTCCTTTGGAACCAGGAGACCCCTCCGGTCTTGTCCAACCT
CTGTTTCGGCAGAGGATACCGTGCTCCACAGCTGTAAGGCTGTGGGCCCGAGAGAAGTCGCTCTCTCT
+
FFFFFFFF,FFFFF,FFFFFFFFFFFFFFFFFFFFFFFF,FFFFF,FFFFFFFFFFFFFFFFFFFFFF
FFFFFFFFFFFFFFFFFFFFFFFFFFFFFFFFFFFFFFFFFFFFFFFFFF:FFFF,FFFFFFFFFFFF
@A00155:342:HHGFNDSXY:1:1161:8775:1814 2:N:0:GAACCTAG+TCCGCATA
ACTTACACGGTAGGTCGTGAACAAGCCCGGAGTACATCTCCTTTGGAACTAGGAGACCCCTCCGGTCT
TGTCCAACCTCTGTTTCGGCAGAGGATACCGTGCTCCACAGCTGTAAGGCTGTGGGCCCGAGAGAAG
+
FFFFFFFFFFFFFFFFFFFFF:FFFFFFFFFFFFFFFFFFF:FFFFF,FFFFFFFFFFFFFFFFFFF,
FFFFFFFFFFFFFFFFFFFFFFFFFFFFFFFFFFFFFFFFFFFFFFFFFFFFFFFFFFFFFFFFFF,F
@A00155:342:HHGFNDSXY:1:1161:28709:26193 2:N:0:GAACCTAG+TCCGCATA
TAGGTCGTGAACAAGCCCGGAGTACATCTCCTTTGGAACTAGGAGACCCCTCCGGTCTTGTCCAACCT
CTGTTTCGGCAGAGGATACCGTGCTCCACAGCTGTAAGGCTGTGGGCCCGAGAGAAGTCGCTCTCTCT
+
FFFFFFFFFFFFFFFFFFFFFFFFFFFFFFFFFFFFFFFFFFFFFFFFFFFFFFFFFF:FFFFFFFFF
FFFFFFFFFFFFFFFFFFFFFFFFFFFFFFFFFFFFFFFFFFFFFFFFFFFFFFF,FFFFFFFFFFFF
@A00155:342:HHGFNDSXY:1:1161:21739:30342 2:N:0:GAACCTAG+TCCGCATA
AAAAGGACTCGTCAGGTAGCCTACTAGACTACGACTTACACGGTAGGTCGTGAACAAGCCCGGAGTAC
ATCTCCTTTGGAACTAGGAGACCCCTCCGGTCTTGTCCAACCTCTGTTTCGGCAGAGGATACCGTGCT
+
FFFFFFFFFFFFFFFFFFFFFFFFFFFFFFFFFFFFFFFFF,FFFFFFFFFFFFFFFFFFFFFFFFFF
FFFFFFFFFFFFFFFFFFFFFFFFFFFFFFFFFFFFFFFFFFFFFFFFFFFFFFFFFFFFFFFFFFFF
@A00155:342:HHGFNDSXY:1:1161:23258:33254 2:N:0:GAACCTAG+TCCGCATA
CAAGGACGAAACCGTCGTTACTCTTGGAGTTGTGTAAGTTTCGTCCTAAAAAGGACTCGTCAGGTAGC
CTACTAGACTACGACTTACACGGTAGGTCGTGAACAAGCCCGGAGTACATCTCCTTTGGAACTAGGAG
+
FFFFFFFFFFFFFFFFFFFFFFF,:FFFFFFFFFFFFFF,FFFFFFFFFFFFFFFFFFFFFFFFFFFF
FFFFFFFFFFFFFFFFFFFFFFFFFFFFFFFFFFFFFFFFFFFFFFFFFFFFFFFFFFF:FF:FFFFF
@A00155:342:HHGFNDSXY:1:1161:3983:36636 2:N:0:GAACCTAG+TCCGCATA
CGACGGTTTCGTCCTTGGAACGGACTCATCAGGGAGCCTACTAGACTCCGACCGTCATGGGGGTTCAA
ACCCATGCCCCGCCACACCGCCTTAGATCAGCTGTGTCCACTTAAGGACTCACCTCTGGCCTATGGTC
+
FFFFFFFFFFFFFFFFFFFFFFFFF,FFFFFFFFFFFFFFFFF:FFFFFFFFFFFFFFFFFFFFFFFF
FFFFFFFFFFFFFFFFFFFFFFFFFFFFFFFFFFFFFFFFFFFFFFFFFFF:FFFFFFFFFFFFFFFF
@A00155:342:HHGFNDSXY:1:1162:7798:10864 2:N:0:GAACCTAG+TCCGCATA
TACGACTTACACGGTAGGTCGTGAACAAGCCCGGAGTACATCTCCTTTGGAACTAGGAGACCCCTCCG
GTCTTGTCCAACCTCTGTTTCGGCAGAGGATACCGTGCTCCACAGCTGTAAGGCTGTGGGCCCGAGAG
+

:FFFFFFFFFFFFFFFFFFFFFFFFFFFFFFFFFFFFFFFFFFFFFFFFFF:FFFFFFFFFFFFFFFF
FFFFFFFFFFFFFFFFFFFFFFFF:FFF,FFFFFFFFFFFFFFFFFFFFFFFFF:FFFFFFFFFFFFF
@A00155:342:HHGFNDSXY:1:1162:23827:21433 2:N:0:GAACCTAG+TCCGCATA
GTGAACAAGCCCGGAGTACATCTCCTTTGGAACCAGGAGACCCCTCCGGTCTTGTCCAACCTCTGTTT
CGGCAGAGGATACCGTGCTCCACAGCTGTAAGGCTGTGGGCCCGAGAGAAGTCGCTCTCTCTCGCCC
+
FFFFFFFFFFFFFFFFFFFFFFFFFFFFFFFFFFFF:FFFFFFFFFFFFF::FFFFFFFFFFFFFFFF
FFFFFF:F:FFFFFFFFFFFFFFFFFFFFFFFFFFFFFFFFFFFFFFFF:FFFFFFFFFFFFFFFFF
@A00155:342:HHGFNDSXY:1:1163:25943:3458 2:N:0:GAACCTAG+TCCGCATA
AAAAGGACTCGTCAGGTAGCCTACTAGACTACGACTTACACGGTAGGTCGTGAACAAGCCCGGAGTAC
ATCTCCTTTGGAACTAGGAGACCCCTCCGGTCTTGTCCAACCTCTGTTTCGGCAGAGGATACCGTGCT
+
FFFFFFFFFFFFFFFFFFFFFFFFFFFFFFFFFFFFFFFFFFFFFFFFFFFFFFFFFFFFFFFFFFFF
FFFFFFFFFFFFFFFFFFFFFFFFFFFFFFFFFFFFFFFFFFFFFFFFFFFFFFFFFFFFFF:FFFFF
@A00155:342:HHGFNDSXY:1:1163:7952:29637 2:N:0:GAACCTAG+TCCGCATA
TTTCGTCCTAAAAAGGACTCGTCAGGTAGCCTACTAGACTACGACTTACACGGTAGGTCGTGAACAAG
CCCGGAGTACATCTCCTTTGGAACCAGGAGACCCCTCCGGTCTTGTCCAACCTCTGTTTCGGCAGAGG
+
FFFFFFFF,FFFFFFFFFFFFFFFFFFFFFFFFFFFFFFFFFFFFFFFFFFFFFFFFFFFFFFFFFFF
FFFFFFFFFFFFFFFFFFFF:FFFFFF:FFFFFF:FFFFF:FFFFFFFFFF:FFFFFFFFFFFFFFFF
@A00155:342:HHGFNDSXY:1:1164:7355:29387 2:N:0:GAACCTAG+TCCGCATA
AAAAGGACTCGTCAGGTAGCCTACTAGACTACGACTTACACGGTAGGTCGTGAACAAGCCCGGAGTAC
ATCTCCTTTGGAACCAGGAGACCCCTCCGGTCTTGTCCAACCTCTGTTTCGGCAGAGGATACCGTGCT
+
FF:FFFFFFFFFFFFFF:FFFFFFFFFFFFFFFFFFFFFFFFFFFFFFFFFFFFFFF,FFFFFFFFFF
FFFFFFFFFFFFFFFFFFFFFFFFFFFFFFFFFFFFFFFFFFFFFFFFFFFFFFF:FFFFFFFFFFFF
@A00155:342:HHGFNDSXY:1:1165:31530:25598 2:N:0:GAACCTAG+TCCGCATA
GAGACCCCTCCGGTCTTGTCCAACCTCTGTTTCGGCAGAGGATACCGTGCTCCACAGCTGTAAGGCTG
TGGGCCCGAGAGAAGTCGCTCTCTCTCGCCCTCCACATCCCTCTCGGCATTGAGATGAC
+
FFFFFFFFFFFFFFFFFFFFFFFFFFFFFFFFFFFFFFFFFFFFFFFFFFFF:FFFFFFFFF:FFF:F
FFFFFFFFFFFFFFFFFFFFFFFFFFFFFFFFFFFF::FFFFFFFFFF,FFFFFFFFFF
@A00155:342:HHGFNDSXY:1:1167:3188:1282 2:N:0:GAACCTAG+TCCGCATA
AAAAGGACTCGTCAGGTAGCCTACTAGACTACGACTTACACGGTAGGTCGTGAACAAGCCCGGAGTAC
ATCTCCTTTGGAACTAGGAGACCCCTCCGGTCTTGTCCAACCTCTGTTTCGGCAGAGGATACCGTGCT
+
FFFFFFFFFFFFFFFFFFFFFFFFFFFFFFFFFFFFFF:FFFFFFFFFFFF,FFFFF,F:FFFFF:FF
FFFFF:FFFFFFFFFFFFFFFFFFFFFFFFFFFFF:FFFFFFFFFFFFFFFFFFFFFF:FFFFFFFFF
@A00155:342:HHGFNDSXY:1:1167:17445:3803 2:N:0:GAACCTAG+TCCGCATA
CGTCCTAAAAAGGACTCGTCAGGTAGCCTACTAGACTACGACTTACACGGTAGGTCGTGAACAAGCCC
GGAGTACATCTCCTTTGGAACTAGGAGACCCCTCCGGTCTTGTCCAACCTCTGTTTCGGCAGAGGATA
+
FFFFFFFFFFFFFF:FFFFFFFFFFFFF,:FFF::FFFFFFFFFFFFFFFFFFFFFFFFFFFF:FFFF
FFFFFFFFFFFFFFFFFFFFFFFFFFFFFFFFFFFFFFFFFFFFFFFFFFFFF:FFFFFFFF:FFFFF
@A00155:342:HHGFNDSXY:1:1167:15031:11835 2:N:0:GAACCTAG+TCCGCATA
AGGACGAAACCGTCGTTACTCTTTGAGTTGTGTAAGTTTCGTCCTAAAAAGGACTCGTCAGGTAGCCT
ACTAGACTACGACTTACACGGTAGGTCGTGAACAAGCCCGGAGTACATCTCCTTTGGAACTAGGAGAC
+
FFFFFFFFFFFFFFFFFFFF::,FFFFFFFFF:FFFF,FFFFFFFFFFFFFFFFFFFFFFFFFFFFFF
FFFFFFFFFFFFFFFFFFFFFFFF::FFFFFFFFFFFFFFFFFFFFFFFFFFFFFFFF,FFFFFFFFF
@A00155:342:HHGFNDSXY:1:1167:20546:14998 2:N:0:GAACCTAG+TCCGCATA
GGACTCGTCAGGTAGCCTACTAGACTACGACTTACACGGTAGGTCGTGAACAAGCCCGGAGTACATCT
CCTTTGGAACTAGGAGACCCCTCTGGTCTTGTCCAACCTCTGTTTCGGCAGAGGATACCGTGCTCCAC
+

FFFFFFFFFFFFFFFFFFFFFFFFFFFFFFFFFFFFFFFFFFFFFFFFFFF:FFFFFFFFF:FFFFF
FFFFFFFFFFFFFFFFFFFFFFFFFFFF:FFFFFFFFFFFFFFFFFFFFFFFFFFFFFFFFFFFFFFFF
@A00155:342:HHGFNDSXY:1:1167:14687:18474 2:N:0:GAACCTAG+TCCGCATA
AAAAGGACTCGTCAGGTAGCCTACTAGACTACGACTTACACGGTAGGTCGTGAACAAGCCCGGAGTAC
ATCTCCTTTGGAACTAGGAGACCCCTCCGGTCTTGTCCAACCTCTGTTTCGGCAGAGGATACCGTGCT
+
FFFFFFFFFFFFFFFF:FFFFF:FFFFFFFFFFFFFFFFFFFFFFFFFFFFFFFFFFFFFFFFFFFFF
FFFFFFFFFFFF,FFFFFFFFFFFFFFFFFFFFFFFFFF,FFFFF:F,FFFFFFFFFFFFFFFFFFFFF
@A00155:342:HHGFNDSXY:1:1167:21088:24017 2:N:0:GAACCTAG+TCCGCATA
ACTTACACGGTAGGTCGTGAACAAGCCCGGAGTACATCTCCTTTGGAACTAGGAGACCCCTCCGGTCT
TGTCCAACCTCTGTTTCGGCAGAGGATACCGTGCTCCACAGCTGTAAGGCTGTGGGCCCGAGAGAAGT
+
FFFFFFFFFFF,FFFFFFFFFFFFFFFFFFFFFFFFFFFFFFFFFFFFFFFFFFFFFFFFFFFFF:FF
FFFFFFFFFFFFF:FFFFFFFFFFFFFFFFFFFFFFFFFFFFFFFFFFFF:FFFFFFFFFFFFFFFFF
@A00155:342:HHGFNDSXY:1:1167:8829:24111 2:N:0:GAACCTAG+TCCGCATA
GTAGCCTACTAGACTACGACTTACACGGTAGGTCGTGAACAAGCCCGGAGTACATCTCCTTTGGAACT
AGGAGACCCCTCCGGTCTTGTCCAACCTCTGTTTCGGCAGAGGATACCGTGCTCCACAGCTGTAAGGC
+
FFFFFFFFFFFFFFFFFFFFFFFFFFFFFFFFFFFFFFFFFFFFFFFFFFFFFFFFFFFFFFFFFFFF
FFFFFFFFFFFFFFFFFFFFFFFFFFFFFFFFFFFFFFFFFFFFFFFFFFFFFFFFFFFFFFFFFFFF
@A00155:342:HHGFNDSXY:1:1167:20094:32534 2:N:0:GAACCTAG+TCCGCATA
ACTTACACGGTAGGTCGTGAACAAGCCCGGAGTACATCTCCTTTGGAACTAGGAGACCCCTCCGGTCT
TGTCCAACCTCTGTTTCGGCAGAGGATACCGTGCTCCACAGCTGTAAGGCTGTGGGCCCGAGAGAAGT
+
FFFFFFFFFFFFFFFFFFFFFFFFFFFFFFFFFFFFFFFFFFFFFFFFFFFFFFFFFFFFFFFFFFFF
FFFFFFFFFFFFFFFFFFFFFFFFFFF,FFFFFFFFFFFFFFFFF:FFFFF:FFFFFFFFFFFFFFFFF
@A00155:342:HHGFNDSXY:1:1167:17824:35462 2:N:0:GAACCTAG+TCCGCATA
ACTAGACTACGACTTACACGGTAGGTCGTGAACAAGCCCGGAGTACATCTCCTTTGGAACTAGGAGAC
CCCTCCGGTCTTGTCCAACCTCTGTTTCGGCAGAGGATACCGTGCTCCACAGCTGTAAGGCTGTGGGC
+
FFFFFFFFFFFFFFFFFFFFFFFFFFFFFFFFFFFFFFFFFFFFFFFFFFFFFFFFFFFFFFFFFFFF
FFFFFFFFFFFFFFFFFFF:FFFFFFFFFFFFFFFFF:FFFFFFFFFFFFFFFFFFFFFFFFFFFFFF
@A00155:342:HHGFNDSXY:1:1168:27887:5729 2:N:0:GAACCTAG+TCCGCATA
GACCCCTCCGGTCTTGTCCAACCTCTGTTTCGGCAGAGGATACCGTGCTCCACAGCTGTAAGGCTGTG
GGCCCGAGAGAAGTCGCTCTCTCTCGCCCTCCACATCCCTCTCGGCATTGAGATGACCATAGGC
+
FFFFFFFFFFFFFFFFFFFFFFFFFFFFFFFFFFFFFFFFF:FFFFFFFFFFFFFFFF,FFFFFFFFF
FFFFFFFFFFFFFFFFFFF:FFFFFFFFFFFFFFFFFFFFFFFFFFFF::FFFFFFFFF,FFFFF
@A00155:342:HHGFNDSXY:1:1169:26657:1689 2:N:0:GAACCTAG+TCCGCATA
GTCGTGAACAAGCCCGGAGTACATCTCCTTTGGAACCAGGAGACCCCTCCGGTCTTGTCCAACCTCTG
TTTCGGCAGAGGATACCGTGCTCCACAGCTGTAAGGCTGTGGGCCCGAGAGAAGTCGCTCTCTCTCGC
+
FFFFFFFFFFFFFFFFFFFFFFFFFFFFFFFFFFFFFFFFFFFFFFFFFFFFFFFFFFFF,FFFFFFF
FFFFFFFFFFFFFFFFFFFFFFFFFFFFFFFFFFFFFFF:FFFFFFFFFFFFFFFFFFFFFFFFFFFF:
@A00155:342:HHGFNDSXY:1:1169:29794:9032 2:N:0:GAACCTAG+TCCGCATA
TCCTTGGAACGGACTCATCAGGGAGCCTACTAGACTCCGACCGTCATGGGGGTTCAAACCCATGCCCC
GCCACACCGCCTTAGATCAGCTGTGTCCACTTAAGGACTCACCTCTGGCCTATGGTCATCTCAATGCC
+
,FFF:FFFFFFFFFFFFFFFFFFFFFFFFFF:FFFFFFFFFFFFFFFFFFFFFFFFFFFFFFFFFFFFF
FFFFF,FFFFF::FFFFFFFFFF::FFFFFFFFF:FFFFFFFFFF,FFFFFF:FFFFFFFFFFF:FFF
@A00155:342:HHGFNDSXY:1:1169:3441:15655 2:N:0:GAACCTAG+TCCGCATA
CCTTTTTAGGACGAAACTTACACAACTCGAAGAGTAACGACGGTTTCGTCCTTGGAACGGACTCATCA
GGGAGCCTACTAGACTCCGACCGTCATGGGGGTTCAAACCCATGCCCCGCCACACCGCCTTAGATCAG
+

FFFFFFFFFFFFFFFFFFFFFFFFFFFFFFFFFFFFFFFFFFFFFFFFFFFFFFFFFFFFFFFFFF:FFF
FFFFFFFFFFFFFFFFFFFFFFFFFFFFFFFFF:FFFFFFFFFFFFFFFFFFFFFFFFFFFFFFFFFFFF
@A00155:342:HHGFNDSXY:1:1170:15691:1611 2:N:0:GAACCTAG+TCCGCATA
GTGAACAAGCCCGGAGTACATCTCCTTTGGAACTAGGAGACCCCTCCGGTCTTGTCCAACCTCTGTTT
CGGCAGAGGATACCGTGCTCCACAGCTGTAAGGCTGTGGGCCCGAGAGAAGTCGCTCTCTCTCGCC
+
FFFFFFFFFFFFFFFFFFFFFFFFFFFFFFFFFFFFFF,FFFFFFFFFFFFFFFFFFFFFFFFFFFFF
FFFFFFFFFFFFFFFFFFFFFFFFFFFFFFFFFFFFFFFFFFFFFFFFFFFFFFFFFFFFFFFFFFF
@A00155:342:HHGFNDSXY:1:1170:19985:13088 2:N:0:GAACCTAG+TCCGCATA
GGACTCGTCAGGTAGCCTACTAGACTACGACTTACACGGTAGGTCGTGAACAAGCCCGGAGTACATCT
CCTTTGGAACTAGGAGACCCCTCCGGTCTTGTCCAACCTCTGTTTCGGCAGAGGATACCGTGCTCCAC
+
FFFFFFFFFFFFFFFFFFFFFFFFFFFFFFFFFFFFFFFFFFFFFFFFFFFFFFFFFFFFFFFFFFFF
FFFFFFFFFFFFFFFFFFFFFFFFFFFFF,FFFFFFFFFFFFFFF:FFFFFFFFFFFFFFFFFF:FFFF
@A00155:342:HHGFNDSXY:1:1170:2474:13730 2:N:0:GAACCTAG+TCCGCATA
GCTGTGTCCACTTAAGGACTCACCTCTGGCCTATGGTCATCTCAATGCCGAGAGGGATGTGGAGGGCG
AGAGAGAGCGACTTCTCTCGGGCCCACAGCCTTACAGCTGTGGAGCACGGTATCCTCTGCCGAAACAG
+
FFFFFFFFFFFFFFFFFFFFFFFFFFFFFFFFFFFFFFFFFFFFFFFFFFFFFFFFFFFFFFFFFFFF
FFFFFFFFFFFFFFFFFFFFFFFFFF:FFFFFFFFFFFFFFFFFFFFFFFFFFFFFFFFFFFFFFFFF
@A00155:342:HHGFNDSXY:1:1170:29478:22263 2:N:0:GAACCTAG+TCCGCATA
GGACTCGTCAGGTAGCCTACTAGACTACGACTTACACGGTAGGTCGTGAACAAGCCCGGAGTACATCT
CCTTTGGAACTAGGAGACCCCTCCGGTCTTGTCCAACCTCTGTTTCGGCAGAGGATACCGTGCTCCAC
+
FFFFFFFFFFFFFFFFFFFFFFFFFFFFFFFFFFFFFFFFFFFFF:FFFFFFF:FFFFFFFFFFFFFF
FFFFFFFFFFFFFFFFFFFFFFFFFFFFFFFFFFFFFFFFFFFFFFFFFFFFFFFFFFFFFFFFFFFF
@A00155:342:HHGFNDSXY:1:1170:7202:30655 2:N:0:GAACCTAG+TCCGCATA
CACGGTAGGTCGTGAACAAGCCCGGAGTACATCTCCTTTGGAACTAGGAGACCCCTCCGGTCTTGTCC
AACCTCTGTTTCGGCAGAGGATACCGTGCTCCACAGCTGTAAGGCTGTGGGCCCGAGAGAAGTCGCTC
+
F:FF,FFFFFFFFFFFFF:FFF,FFFFFFFFFFFFFFFFFFFF:FF:,:FFFFF,,:FFFFF,F:FFF
FFFFFF:FFFFFFFFFFFFFFF,FFFFFFFFFFF:FFFFFFFFFFFFFFFFFFFFFFFFFFFFFFFFF
@A00155:342:HHGFNDSXY:1:1171:31738:16626 2:N:0:GAACCTAG+TCCGCATA
ACTAGACTACGACTTACACGGTAGGTCGTGAACAAGCCCGGAGTACATCTCCTTTGGAACTAGGAGAC
CCCTCCGGTCTTGTCCAACCTCTGTTTCGGCAGAGGATACCGTGCTCCACAGCTGTAAGGCTGTGGGC
+
FFFFFFFFFFFFFFFFFFFFFFFFFFFFFFFFFFFFFFFFFFFFFFFFFFFFFFFFFFFFFFFFFFFF
FFFFFFFFFF,:FFFFFFFFFFFFFFFFFFFFFFFFFFFFFFF,FFFFFFF:FFFFF::FFFFFFFFF
@A00155:342:HHGFNDSXY:1:1171:30761:21950 2:N:0:GAACCTAG+TCCGCATA
TCTACGAGTTGTGTAAGTTTCGTCCTAAAAAGGACTCGTCAGGTAGCCTACTAGACTACGACTTACAC
GGTAGGTCGTGAACAAGCCCGGAGTACATCTCCTTTGGAACTAGGAGACCCCTCCGGTCTTGTCCAAC
+
FFF:FFFFFFFF:FFFFFFFFFFFFFFFFFFFFFFFFFF:FFFFFF:FFFFFFFFFFFFF:FFFFFFFF
FFFFFFFFFFFFFFFFFFFFFFFFFFFFFFFFFFFFFFFFFFFFFFFFFFFFFFFFFFFFFFFFFFFF
@A00155:342:HHGFNDSXY:1:1172:27850:6261 2:N:0:GAACCTAG+TCCGCATA
TGTGTAAGTTTCGTCCTAAAAAGGACTCGTCAGGTAGCCTACTAGACTACGACTTACACGGTAGGTCG
TGAACAAGCCCGGAGTACATCTCCTTTGGAACCAGGAGACCCCTCCGGTCTTGTCCAACCTCTGTTTC
+
FFFFFFFFF,FFFFFFFFFFFFFFFFFFFFFFFFFFFFFFFFFFFFFFFFFFFFFFFFFFFFFFFFFF
FFFFFFFFFFFFFFFFFFFFFFFFFFFFFFFFFFFFFFFFFFFFFFFFFFFFFFFFFFFFFFFFFFFF
@A00155:342:HHGFNDSXY:1:1173:12066:3599 2:N:0:GAACCTAG+TCCGCATA
GGACTCGTCAGGTAGCCTACTAGACTACGACTTACACGGTAGGTCGTGAACAAGCCCGGAGTACATCT
CCTTTGGAACCAGGAGACCCCTCCGGTCTTGTCCAACCTCTGTTTCGGCAGAGGATACCGTGCTCCAC
+

FFFFFFFFFFFFFFFFFFFFFFFFFFFFFFFFFFFFFFFFFFFFFFFFFFFFF:FFFFFFFFFFFFFF
FFFFFFFFFFFFFFF:FFFFFFFFFFFFFFFFFFF:FFFFFFFFFFFFFFFFFFFFFFFFFFFFFFFF
@A00155:342:HHGFNDSXY:1:1173:29975:11945 2:N:0:GAACCTAG+TCCGCATA
AAAAAGGACTCGTCAGGTAGCCTACTAGACTACGACTTACACGGTAGGTCGTGAACAAGCCCGGAGTA
CATCTCCTTTGGAACTAGGAGACCCCTCCGGTCTTGTCCAACCTCTGTTTCGGCAGAGGATACCGTGC
+
FFFF:FFFFFFFFFFFFFFFFFFFFFFFFFFFFFFFFFFFFFFFFFFFFFFFFFFFFFFFFFFFFFFF
F:FFFFFFFFFFFFFFFFFFFFFFFFFFFFFFFFFFFFFFFFFFFFFFFFF:FFF:FFFFFFFFFFFF
@A00155:342:HHGFNDSXY:1:1173:8314:15264 2:N:0:GAACCTAG+TCCGCATA
GGACTCGTCAGGTAGCCTACTAGACTACGACTTACACGGTAGGTCGTGAACAAGCCCGGAGTACATCT
CCTTTGGAACTAGGAGACCCCTCCGGTCTTGTCCAACCTCTGTTTCGGCAGAGGATACCGTGCTCCAC
+
FFFFFFFFFFFFFFFFFFFFFFFFFFFFFFFFFFFFFFFFFFFFFFFFFFFFFFFFFFFFFFFFFFFF
FFFFFFFFFFFFFFFFFFFFFFFFFFFFFFFFFFFFFFFFFFFFFFFFFFF:FFFFFFFFFFFFFF:F
@A00155:342:HHGFNDSXY:1:1173:16830:16892 2:N:0:GAACCTAG+TCCGCATA
TACACGGTAGGTCGTGAACAAGCCCGGAGTACATCTCCTTTGGAACTAGGAGACCCCTCCGGTCTTGT
CCAACCTCTGTTTCGGCAGAGGATACCGTGCTCCACAGCTGTAAGGCTGTGGGCCCGAGAGAAGTCGC
+
FFFFFFFFFFFFFFFFFFFFFFFFFFFFFFFFFFFFFFF,FFFFFFFFFFFFFFFFFFFFFFFFFFFF
FFFFFFFFFFFFFFFFFFFFFFFFFFFFFFFFFFFFFFFFFFFFFFFFFFFFFFFFFFFFFFFFFFFF
@A00155:342:HHGFNDSXY:1:1174:16324:3396 2:N:0:GAACCTAG+TCCGCATA
TACGACTTACACGGTAGGTCGTGAACAAGCCCGGAGTACATCTCCTTTGGAACCAGGAGACCCCTCCG
GTCTTGTCCAACCTCTGTTTCGGCAGAGGATACCGTGCTCCACAGCTGTAAGGCTGTGGGCCCG
+
FFFFFF:FFFFFFFFFF:FFFFFFFFFFFFFFFF,FFFF::FFFFFFFFFFFFFFFFFFFFFFFFFFF
FFF,FFFFFFFFFFFFFFFFFFFF:FFFFFFFFFFFFFFFFFFFFF:FFF:FFFFFFFF:FFFF
@A00155:342:HHGFNDSXY:1:1174:26865:8437 2:N:0:GAACCTAG+TCCGCATA
GCCCGGAGTACATCTCCTTTGGAACTAGGAGACCCCTCCGGTCTTGTCCAACCTCTGTTTCGGCAGAG
GATACCGTGCTCCACAGCTGTAAGGCTGTGGGCCCGAGAGAAGTCGCTCTCTCTCGCCCTCCACATCC
+
:FFFFFFFFFFFFFFFFFFFFFFFFFFFFFFFFFFFFFFFFFFFFFFFFFFFFFFFFFFFFFFFFFFF
FFFFFFFFFFFFFFFFFF:FFFFFFF:FFFFFFFFFFFFFFFFFFFFFFFFFFFFFFFFFFFFFFFFF
@A00155:342:HHGFNDSXY:1:1174:23936:21652 2:N:0:GAACCTAG+TCCGCATA
CTTCTCTCGGGCCCACAGCCTTACAGCTGTGGAGCACGGTATCCTCTGCCGAAACAGAGGTTGGACAA
GACCGGAGGGGTCTCCTAGTTCCAAAGGAGATGTACTCCGGGCTTGTTCACGACCTACCGTGTAAGTC
+
F:FFFFFFF:FFFFFFFF:FFF:FFFF:F:FFFFFFFFFFFFFFFFFFFFFF::F:FFFFFFFFFF:F
FFFFF:FFFFFFFF:F,FFFFFFF:F:FFFFFF:FFFFFFF:FFFFFFFFFFFFFFFFFF,FFFFF,F
@A00155:342:HHGFNDSXY:1:1174:28899:23891 2:N:0:GAACCTAG+TCCGCATA
CCACACCGCCTTAGATCAGCTGTGTCCACTTAAGGACTCACCTCTGGCCTATGGTCATCTCAATGCCG
AGAGGGATGTGGAGGGCGAGAGAGAGCGACTTCTCTCGGGCCCACAGCCTTACAGCTGTGGAGCACGG
+
FFFFFFFFFFFFFFFFFFFF:FFFFFFFFFFFFFFFFFF:FFFFFFFFFFFFFFFFFFFFFFF:FF:F
:FFFFFF:FFFFFFFFFFFFFFFF,FFFFFFFFFFFFFFFFFFFFFFFF,F,FFFFFF:FFFFFFFFF
@A00155:342:HHGFNDSXY:1:1176:1958:20791 2:N:0:GAACCTAG+TCCGCATA
GTTTCGTCCTAAAAAGGACTCGTCAGGTAGCCTACTAGACTACGACTTACACGGTAGGTCGTGAACAA
GCCCGGAGTACATCTCCTTTGGAACTAGGAGACCCCTCCGGTCTTGTCCAACCTCTGTTTCGGCAGAG
+
F:FFFFFFFFFFFFFFFFFFFFFFFFFFFFFFFFFFFFFFFFFFFFFFFFFFFFFFFFFFFFFFFFFF
,FFFFFFFFFFFFFFFFFFFFFFFFFFFFF:FFFFFFFFFFFFFFF:FFFFFFFFFFFFFFFFFFFFF
@A00155:342:HHGFNDSXY:1:1176:4788:20807 2:N:0:GAACCTAG+TCCGCATA
TACACGGTAGGTCGTGAACAAGTCCGGAGTACATCTCCTTTGGAACTAGGAGACCCCTCCGGTCTTGT
CCAACCTCTGTTTCGGCAGAGGATACCGTGCTCCACAGCTGTAAGGCTGTGGGCCCGAGAGAAGTCGC
+

FFFFFFFFFFFFFFFFFFFFFFFFFFFFFFFFFFFFFFFFFFFFFFFFFFFFFFFFFFFFFFFFFFFF
FFFFFFFFFFFFFFFFFFFFFFFFFFFFFFFFFFFFFFFFFFFFF:FFFFFFFFFFFFFFFFFFFFFF
@A00155:342:HHGFNDSXY:1:1176:19750:22169 2:N:0:GAACCTAG+TCCGCATA
ACGGTAGGTCGTGAACAAGCCCGGAGTACATCTCCTTTGGAACTAGGAGACCCCTCCGGTCTTGTCCA
ACCTCTGTTTCGGCAGAGGATACCGTGCTCCACAGCTGTAAGGCTGTGGGCCCGAGAGAAGTCGCTCT
+
FFFFFFFFFFFFFFFFFFFFFFFFFFFFFFFFFFFF:FFFFFFFFFFFFFFFFFFFFFFFFFFFFFFF
FFFFFFFFFFFFFFFFFFFFFFFFFFFFFFFFFFFFFFFFFFFFF:FFFFFFFFFFFFFFFFFFFFFF
@A00155:342:HHGFNDSXY:1:1176:25174:28995 2:N:0:GAACCTAG+TCCGCATA
TACACGGTAGGTCGTGAACAAGCCCGGAGTACATCTCCTTTGGAACTAGGAGACCCCTCCGGTCTTGT
CCAACCTCTGTTTCGGCAGAGGATACCGTGCTCCACAGCTGTAAGGCTGTGGGCCCGAGAGAAGTCGC
+
FFFFFFFFFFFFFFFFFFFFF:FFFFF:FFFFFFFFFFFFFFFFFFFFFFFFFFFFFFFFFFFFF:FFF
FFFFFFFFFFFFFFFFFFFFFFFFFFFF:FFFFFFFFFF:FFFFFFFFFFFFFFFFFFFFFFF:FFFFF
@A00155:342:HHGFNDSXY:1:1177:16288:12884 2:N:0:GAACCTAG+TCCGCATA
CCATGCCCCACCACACCGCCTTAGATCAGCTGTGTCCACTTAAGGACTCACCTCTGGCCTATGGTCAT
CTCAATGCCGAGAGGGATGTGGAGGGCGAGAGAGAGCGACTTCTCTCGGGCCCACAGCCTTACAGCTG
+
FFFFFFFFFFFFFFFFFFFFFF:FFFFFFFFFFF:FFFFFFFFFFFFFFFFFFFFFFFFFFFFFFFFF
FFFFFFFFFFFFFFFFFFF,FFFFFFFFFFFFF:FFFFFFFFFFFFFFFFFFFFFFFFFF,FFFFFFFF
@A00155:342:HHGFNDSXY:1:1177:31702:13714 2:N:0:GAACCTAG+TCCGCATA
AAAAGGACTCGTCAGGTAGCCTACTAGACTACGACTTACACGGTAGGTCGTGAACAAGCCCGGAGTAC
ATCTCCTTTGGAATTAGGAGACCCCTCCGGTCTTGTCCAACCTCTGTTTCGGCAGAGGATACCGTGCT
+
FFFFFFFFFFFFFFFF:FFFFFFFFFFFFFFFFFFF:FFFFFFFFFFFFFFFFFFFFFFFFFFFFFFFF
FFFFFFFFFFFF,FFFFFFFFFFFFFFFFFFF,FFFFF:FFFFF,FFFFFFFFFFFFFFFFFFFFFFFF
@A00155:342:HHGFNDSXY:1:1177:32850:24940 2:N:0:GAACCTAG+TCCGCATA
ACTACGACTTACACGGTAGGTCGTGAACAAGCCCGGAGTACATCTCCTTTGGAACCAGGAGACCCCTC
CGGTCTTGTCCAACCTCTGTTTCGGCAGAGGATACCGTGCTCCACAGCTGTAAGGCTGTGGGCCCGAG
+
FFFFFFFFFFFFFFFFFFFFF:FFFFFFFFFFFFFFFFFFFF:FFFFFFFFFFF,FFFFF:FFFFFFFF
FFFFF,:FFFFFFFFFFFFFFFFFFFFFFFFFFFFFFFF:FFFFFFFFFFFFFFFFFFFFFFFFFFFFFF
@A00155:342:HHGFNDSXY:1:1178:28619:9690 2:N:0:GAACCTAG+TCCGCATA
ACTTAAAGACTCACCTCTGGCCTATGGTCATCTCAATGCCGAGAGGGATGTGGAGGGCGAGAGAGAGC
GACTTCTCTCGGGCCCACAGCCTTACAGCTGTGGAGCACGGTATCCTCTGCCGAAACAGAGGTTGGAC
+
FFF:FFFFF:FFFFFF:FFFFFFF:FFFF:FFFFFFFFFFFF:FFFF,FF:FFFFFFFFFFFFFFFFF
FFFFFF:FFF,FFFFFFFFFFFFFFFFFFFFFFFFFFFF,F:FFFFFF:FFFFFFFFFFFFFFF,FFFF
@A00155:342:HHGFNDSXY:1:1178:11216:17566 2:N:0:GAACCTAG+TCCGCATA
GAACTAGGAGACCCCTCCGGTCTTGTCCAACCTCTGTTTCGGCAGAGGATACCGTGCTCCACAGCTGT
AAGGCTGTGGGCCCGAGAGAAGTCGCTCTCTCTCGCCCTCCACATCCCTCTCGGCATTGAGATGACCA
+
FFFFFFFFF:FFFFFFFFFFFFFFFFFFFFFFFFFFFFFFFFFFFFFFFFFFFFFFFFFFFFFFFFFF
FFFFF:FFFFFFFFFFFFFFFFFFFFFFFFFFFFFF:FFFFFFFFFFFFFFFFFFFFFFFFFFFFFFF
@A00155:342:HHGFNDSXY:1:1178:2790:30499 2:N:0:GAACCTAG+TCCGCATA
CACGGTAGGTCGTGAACAAGCCCGGAGTACATCTCCTTTGGAACTAGGAGACCCCTCCGGTCTTGTCC
AACCTCTGTTTCGGCAGAGGATACCGTGCTCCACAGCTGTAAGGCTGTGGGCCCGAGAGAAGTCGCTC
+
FFFFFFFFFFFFFFFFFFFFFFFFFFFFFFFFFFFFFFFFFFFFF:FFFFF,FFFFFFFFFFFFFFFFF
FFFFFFFFFFFFFFFFF,FFFFFFFF:FFFFFFFFFFFFFFFF:FFFFFFFFFFFFFFFF,FFFFFFF
@A00155:342:HHGFNDSXY:1:1201:20546:3474 2:N:0:GAACCTAG+TCCGCATA
AAAAGGACTCGTCAGGTAGCCTACTAGACTACGACTTACACGGTAGGTCGTGAACAAGCCCGGAGTAC
ATCTCCTTTGGAACTAGGAGACCCCTCCGGTCTTGTCCAACCTCTGTTTCGGCAGAGGATACCGTGCT
+

FFFFFFFFFF:FFFFFFFFFFFFFFFFFFFFFFFFFFFFFFFFFFFFFFFFFFFFFFFFFFFFFFFFFFF
FFFFFF:FFFFFFFFFFFFFF:FFFFFFFFFFFFFFFFFFFFFFFFFFFFFFFFFFFFFFFFFFFFFFFF
@A00155:342:HHGFNDSXY:1:1201:32280:5447 2:N:0:GAACCTAG+TCCGCATA
GTGTAAGTTTCGTCCTAAAAAGGACTCGTCAGGTAGCCTACTAGACTACGACTTACACGGTAGGTCGT
GAACAAGCCCGGAGTACATCTCCTTTGGAACTAGGAGACCCCTCCGGTCTTGTCCAACCTCTGTTTCG
+
FFFF:FFF,FF:FFFFFF:FFFFFFFFFFFFFFFFFFFFFFFFFFFFFFFFFFFFFFF:FFFF:FFFFFF
FFFFFFFFFFFFFFFFF:FFFFFFFFFFFFFFFFFFFFFFFFFFFFFFFFFF:FFFF,FFFFFFFFFFFF
@A00155:342:HHGFNDSXY:1:1201:18710:17362 2:N:0:GAACCTAG+TCCGCATA
GTCAGGTAGCCTACTAGACTACGACTTACACGGTAGGTCGTGAACAAGCCCGGAGTACATCTCCTTTG
GAACCAGGAGACCCCTCCGGTCTTGTCCAACCTCTGTTTCGGCAGAGGATACCGTGCTCCACAGCTG
+
,,FFF::FFFFFF,,F:FFF,F:FFFF,FFFF,,,FFF,,F,FF,FFF,,:,FFF,FF,FFF,,,::F,
FFF:F:FFFFFFFFF,F,,FFF,F::::FFF,FFF:F:F::,F,F,FFFFFFF:F,,FF:F::,:F:
@A00155:342:HHGFNDSXY:1:1201:26205:18662 2:N:0:GAACCTAG+TCCGCATA
GTCCTAAAAAGGACTCGTCAGGTAGCCTACTAGACTACGACTTACACGGTAGGTCGTGAACAAGCCCG
GAGTACATCTCCTTTGGAACTAGGAGACCCCTCCGGTCTTGTCCAACCTCTGTTTCGGCAGAGGATAC
+
FFFFFFFFFFFFFFFFFFFFFFFFFFFFFFFFFFFFFFFFFFFFFFFFFFFFFFFFFFFFFFFFFFFFFF
FFFFFFFFFFFFFFFFFF,FFFFFFFFFFFFFFFFFFFFFFFFFFFFFFFFFFFFFFFFFFFFFFFFFFF
@A00155:342:HHGFNDSXY:1:1201:10963:25269 2:N:0:GAACCTAG+TCCGCATA
CAAGGACGAAACCGTCGTTACTCTTCGAGTTGTGTAAGTTTCGTCCTAAAAAGGACTCGTCAGGTAGC
CTACTAGACTACGACTTACACGGTAGGTCGTGAACAAGCCCGGAGTACATCTCCTTTGGAACTAGGAG
+
FFFFFFFFFFFF:FFFFFFFFFFF:FFFFFFFFFFFFFFF:FFFFFFFFF:::FFFFFFF:F:FFFFFFF
FFFFFFFFFFFFFFFFFFFFFFFFFFFFFFFFFFFFFFFFFFFFFFFFF:F:FFFFFFFFFFFFFFFFFF
@A00155:342:HHGFNDSXY:1:1202:12147:25379 2:N:0:GAACCTAG+TCCGCATA
TCGTGAACAAGCCCGGAGTACATCTCCTTTGGAACTAGGAGACCCCTCCGGTCTTGTCCAACCTCTGT
TTCGGCAGAGGATACCGTGCTCCACAGCTGTAAGGCTGTGGGCCCGAGAGAAGTCGCTCTCTCTCGCC
+
FFFFFFFFFFFFFFFFFFFFFFFFFFFFFFFFFFFFFFFFFFFFFFFFFFFFFFFFFFFFFFFFFFFFFF
FFFFFFFFFFFFFFFFFFFFFFFFFFFFFFFFFFFFFFFFFFFFFFFFFFFFFFFFFFFFFFFFFFFFFF
@A00155:342:HHGFNDSXY:1:1202:2130:34272 2:N:0:GAACCTAG+TCCGCATA
ACATCTCCTTTGGAACTAGGAGACCCCTCCGGTCTTGTCCAACCTCTGTTTCGGCAGAGGATACCGTG
CTCCACAGCTGTAAGGCTGTGGGCCCGAGAGAAGTCGCTCTCTCTCGTCCTCCACATCCCTCTCGGCA
+
FFFFFFFFFFFFFFFFF:FFFFFFFFFFFFFFFFFFFFFFFFFFFFFFFFFFF:FFFF:FFFFFFFFFFF
FFFFFFFFFFFFFFFFFFFFFFFFFFFFFFFFFFFFFFFFFFFFFFFFFFFFFFFFFFFFFFFFFFFFFF
@A00155:342:HHGFNDSXY:1:1202:6207:34788 2:N:0:GAACCTAG+TCCGCATA
TCGTCCTAAAAAGGACTCGTCAGGTAGCCTACTAGACTACGACTTACACGGTAGGTCGTGAACAAGCC
CGGAGTACATCTCCTTTGGAACTAGGAGACCCCTCCGGTCTTGTCCAACCTCTGTTTCGGCAGAGGAT
+
FFFFFFFFFFFFFFFFFFFFFFFFFFFFFFFFFFFFFFFFFFFFFFFFFFFFFFFFFFFFFFFFFFFFFF
FFFFFFFFFFFFFFFFFFFFFFFFFFFFFFFFFFFFFFFFFFFFFFFFFFFFFFFFFFFFFFFFFFFFFF
@A00155:342:HHGFNDSXY:1:1202:6804:35509 2:N:0:GAACCTAG+TCCGCATA
GGACTCGTCAGGTAGCCTACTAGACTACGACTTACACGGTAGGTCGTGAACAAGCCCGGAGTACATCT
CCTTTGGAACTAGGAGACCCCTCCGGTCTTGTCCAACCTCTGTTTCGGCAGAGGATACCGTGCTCCAC
+
FFFFFFFFF:FFFFFFFFFFFFFFFFFFFFFFFFFFFFFFFFFFFFFFFFFFFFFFFFFFFFFFFFFFFF
FFFFFFFFFFFFFFFFFFFFFFFFFFFFFFFFFFFFFFFFFFFFFFFFFFFFFFFFFFFF,FFFFFFF,F
@A00155:342:HHGFNDSXY:1:1203:31629:9330 2:N:0:GAACCTAG+TCCGCATA
GAAACCGTCGTTACTCTTCGAGTTGTGTAAGTTTCGTCCTAAAAAGGACTCGTCAGGTAGCCTACTAG
ACTACGACTTACACGGTAGGTCGTGAACAAGCCCGGAGTACATCTCCTTTGGAACTAAGAGACCCCTC
+

FFFFFFFFFFFFFFFFFFFFFFFFFFFFFFF:F:FFFFFFFFFFFFFFFFFFF,FFFF:F:FFFFF:FF
FFFFFFFFFFFFFFFFFFFFFFFFFFFFFFFFFFFFFFFFFF,:FFFFFFFFFFFFFFFFFFFFFFFFFF
@A00155:342:HHGFNDSXY:1:1203:27208:15107 2:N:0:GAACCTAG+TCCGCATA
AGTCCGTTCCAAGGACGAAACCGTCGTTACTCTTTGAGTTGTGTAAGTTTCGTCCTAAAAAGGACTCG
TCAGGTAGCCTACTAGACTACGACTTACACGGTAGGTCGTGAACAAGCCCGGAGTACATCTCCTTTGG
+
FFFFFFFFFFF:FFFFFFFFFFFFFFFFFFFF,FFFFFFFFFFFFFFFFFFFFFFFFFFFFFFFFFFFF
FFFFFFF:FFFFFFFFFFF:FFFFFFFFFFFFFFFFFFFFFFFFFFFFFFFFFFFFFFFFFFFFF:FFF
@A00155:342:HHGFNDSXY:1:1203:26856:15186 2:N:0:GAACCTAG+TCCGCATA
AGTCCGTTCCAAGGACGAAACCGTCGTTACTCTTTGAGTTGTGTAAGTTTCGTCCTAAAAAGGACTCG
TCAGGTAGCCTACTAGACTACGACTTACACGGTAGGTCGTGAACAAGCCCGGAGTACATCTCCTTTG
+
FFFFFFFFFFFFFFFFFFFFFFF:FFFFFF:F:,FFFFFFFFFFF,F:,FFFFFF,FFFFFF:FFFF:
FFFFFFFFFFFFFFFFFFFFFFFFFFFFFFFFF:FFFFFFF:FFFFFFFFFFFFFFFFFFFFFFFFFF
@A00155:342:HHGFNDSXY:1:1203:7735:19335 2:N:0:GAACCTAG+TCCGCATA
AAGTTTCGTCCTAAAAAGGACTCGTCAGGTAGCCTACTAGACTACGACTTACACGGTAGGTCGTGAAC
AAGCCCGGAGTACATCTCCTTTGGAACCAGGAGACCCCTCCGGTCTTGTCCAACCTCTGTTTCGGCAG
+
FFFFFFFFF:FFF:FFF:FFFFFFFFFFFFFFFFFFFFFFFFFFFFFFFFFFFFFFFFFFFFFFFFFFF
FFFFFFFFFFFFFFFFFFFFFF:FFFFFFFFFFFFFFFFFFFFFFFFFFFFFFFFFFFFFFFFF:FFFF
@A00155:342:HHGFNDSXY:1:1204:17996:8234 2:N:0:GAACCTAG+TCCGCATA
AGTACATCTCCTTTGGAACTAGGAGACCCCTCCGGTCTTGTCCAACCTCTGTTTCGGCAGAGGATACC
GTGCTCCACAGCTGTAAGGCTGTGGGCCCGAGAGAAGTCGCTCTCTCTCGCCCTCCACATCCC
+
FFFFFFFFFFFF:FFF:FFFFFFFFFFFFFFFFFFFFFFFFFFFFFFFFFFFFFFFFFFFFFFFFFFFF
FFFFFFFFFFFFFFFFFFFFFFFFFFFFFFFFFFF:FFFFFFFFFFFFFFFFFFFFFFFF:F:F
@A00155:342:HHGFNDSXY:1:1204:4309:11271 2:N:0:GAACCTAG+TCCGCATA
GTTTCGGCAGAGGATACCGTGCTCCACAGCTGTAAGGCTGTGGGCCCGAGAGAAGTCGCTCTCTCTCG
CCCTCCACATCCCTCTCGGCATTGAGATGACCATAGGCCAGAGGTGAGTCCTTAAGTGGACACAGCTG
+
FFFFFFFFFFFFFFFFFFFFFFFFFFFFFFFFFFFFFFFFFFFFFFFFFFFFFFFFFFFFFFFFFFFFF
FFFFFFFFFFFFFFFFFFFFFF,FFFFFF:FFFFFFFFFFFFFFFFFFFFFFFFFFFFFFFFFFFFFF:F
@A00155:342:HHGFNDSXY:1:1204:19361:12038 2:N:0:GAACCTAG+TCCGCATA
GATCAGCTGTGTCCACTTAAGGACTCACCTCTGGCCTATGGTCATCTCAATGCCGAGAGGGATGTAGA
GGGCGAGAGAGAGCGACTTCTCTCGGGCCCACAGCCTTACAGCTGTGGAGCACGGTATCCTCTGCCGA
+
FFFFFFFFFFFFFFFFFFFFFFFFFFFFFFFFFFFFFFFFFFFFFFFFFFFFFFFFFFFFFFFF:F:FFF
FFFFFFFFFFFFFFFFFFFFFFF:FFFFFFFFFFFFFFFFFFFFFFFFFFFFFFFFFFFFFFFFFFFFFF
@A00155:342:HHGFNDSXY:1:1204:24831:34444 2:N:0:GAACCTAG+TCCGCATA
GGACTCGTCAGGTAGCCTACTAGACTACGACTTACACGGTAGGTCGTGAACAAGCCCGGAGTACATCT
CCTTTGGAACTAGGAGACCCCTCCGGTCTTGTCCAACCTCTGTTTCGGCAGAGGATACCGTGCTCCAC
+
FFFFFFFFFFFFFFFFFFFFFFFFFFFFFFFFFFFFFFFFFFFFFFFFFFFFFFFFFFFFFFFFFFFFFF
FFFFFFFFFFFFFF:FFF:FFFFFFFFFFFFFF:FFFFFFFFFFFFFFFFFFFFFFFFFFFFFFFFFFFF
@A00155:342:HHGFNDSXY:1:1205:21070:6167 2:N:0:GAACCTAG+TCCGCATA
ACTTACACGGTAGGTCGTGAACAAGCCCGGAGAACATCTCCTTTGGAACTAGGAGACCCCTCCGGTCT
TGTCCAACCTCTGTTTCGGCAGAGGATACCGTGCTCCACAGCTGTAAGGCTGTGGGCCCGAGAGAAGT
+
FFFFFFFFFFFFFFFFFFFFFFFFFFFFFFFFFFFFFFFFFFFFFFFFFFFFFFFFFFFFFFFFFFFFFF
FFFFFFFFFFFFFFFFFFFFFFFFFF:FFFFFFFFFFFFFFFFFFFFFFF:FFFFFFFFFFFFFFFFFF:FF
@A00155:342:HHGFNDSXY:1:1205:12156:14810 2:N:0:GAACCTAG+TCCGCATA
TCCTTGGAACGGACTCATCAGGGAGCCTACTAGACTCCGACCGTCATGGGGGTTCAAACCCATGCCCC
ACCACACCGCCTTAGATCAGCTGTGTCCACTTAAGGACTCACCTCTGGCCTATGGTCATCTCAATGCC
+

```
FFFFFFFF:FFFFFFFFFFFFFFFFFFFFFFFFFFFFFFFFFFFFF:FFFFFFFFF:FFFFFFFFFF
FFFFFFFFFFFFFFFFFFFFFFFFFFFFF,FFFF:FFFFFFFFFFFFFFFFFFFFFFFFFFFFFFFF
@A00155:342:HHGFNDSXY:1:1205:25798:16454 2:N:0:GAACCTAG+TCCGCATA
GAGTAACGACGGTTTCGTCCTTGGAACGGACTCATCAGGGAGCCTACTAGACTCCGACCGTCATGGGG
GTTCAAACCCATGCCCCGCTACACCGCCTTAGATCAGCTGTGTCCACTTAAGGACTCACCTCTGGCCT
+
F:FFF,FF:FFF,FFFFFF:FFFFFFFFFFFFFFFFFFFFFFFFFFFFFFFFFFFFFFFFFFFFFFFF
FFFFFFF:FFFFFFFFFFFF:FFFFFFFFFFFFFFFFFFFFFFFFFFFFFFFFFFFFF:FFFFFFF:
@A00155:342:HHGFNDSXY:1:1205:27272:20948 2:N:0:GAACCTAG+TCCGCATA
AAAAGGACTCGTCAGGTAGCCTACTAGACTACGACTTACACGGTAGGTCGTGAACAAGCCCGGAGTAC
ATCTCCTTTGGAACTAGGAGACCCCTCCGGTCTTGTCCAACCTCTGTTTCGACAGAGGATACCGTGCT
+
FFFFFFFFFFFFFF:FFFFFFFFFFFFFFFFFFFFFFFFFFF:FF,FFFFFFFFF:FFFFFFFFFFFF
FFFFFFFFFFFFFFFFFFFFFFFFFFFFFFFFFFFFFFFFFFFFFFFFFFFFFFFFFFFFF:FFFFFF
@A00155:342:HHGFNDSXY:1:1205:10881:24721 2:N:0:GAACCTAG+TCCGCATA
GAGACCCCTCCGGTCTTGTCCAACCTCTGTTTCGGCAGAGGATACCGTGCTCCACAGCTGTAAGGCTG
TGGGCCCGAGAGAAGTCGCTCTCTCTCGCCCTCCACATCCCTCTCGGCATAGAGATGACCATAGGC
+
FFFFFFFFFFFFFFFFFFFFFFFFFFFFFFFFFFFFFFFFFFFFFFFFFFFFFFFFFFFFFFFFFFFF
FFFFFFFF:FFFF:FFFFFFFFF:FFFFFFFFFFFFFFFFFFFFFFFFFFFFFF:FFFFF:FF:F:F
@A00155:342:HHGFNDSXY:1:1205:3938:34585 2:N:0:GAACCTAG+TCCGCATA
GGACTCGTCAGGTAGCCTACTAGACTACGACTTACACGGTAGGTCGTGAACAAGCCCGGAGTACATCT
CCTTTGGAACTAGGAGACCCCTCCGGTCTTGTCCAACCTCTGTTTCGGCAGAGGATACCGTGCTCCAC
+
FFFFFFFFFFFFFFFFFFFFFFFFFFFFFFFFFFFFFFFFFFFFFFFFFFFFFFFFFFFFFFFF:FFF
FFFFFFFFFFFFF:FFFFFFFFFFFF:FFFFFF:FFFFFFFFFFFFFFFFFFFFFFFFFFFFFFFFFF
@A00155:342:HHGFNDSXY:1:1206:26323:2018 2:N:0:GAACCTAG+TCCGCATA
CCTCCACATCCCTCTCGGCATTGAGATGACCATAGGCCAGAGGTGAGTCCTTAAGTGGACACAGCTGA
TCTAAGGCGGTGTGGCGGGGCATGGGTTTGAACCCCCATGACGGTCGGAGTCTAGTAGGCTCCCTGAT
+
FFFFFFFFFFFFFFFFFFFFFFFFFFFFFFFFFFFFFFFFFFFFFFFFFFFFFFFFFFFF,FFFFFFF
FF:::FFFFFFFFFFFFFFFFFFFFFFFFFFFFFFFFFFFFFFFFFFFFFFFFFFFFFFFFFFFFFFF
@A00155:342:HHGFNDSXY:1:1206:19714:2253 2:N:0:GAACCTAG+TCCGCATA
TACGACTTACACGGTAGGTCGTGAACAAGCCCGGAGTACATCTCCTTTGGAACTAGGAGACCCCTCCG
GTCTTGTCCAACCTCTGTTTCGGCAGAGGATACCGTGCTCCACAGCTGTAAGGCTGTGGGCCCG
+
FFFFFFFFFFFFFFFFFFFFFFFFFFFFFFFFFFFFFFFFFFFFFFFFFFF:FF:FFFFFFFFFFFFF
FFFF:FFFFFFFFFFFFFFFFFFFFFFFFFFFFFFFFFFFFFFFFFFFFFF,FFFFFFFFFFFF
@A00155:342:HHGFNDSXY:1:1206:20980:9392 2:N:0:GAACCTAG+TCCGCATA
AGTTTCGTCCTAAAAAGGACTCGTCAGGTAGCCTACTAGACTACGACTTACACGGTAGGTCGTGAACA
AGCCCGGAGTACATCTCCTTTGGAACCAGGAGACCCCTCCGGTCTTGTCCAACCTCTGTTTCGGCAGA
+
FFFFFFFFFFFFFF:FFFFFFFFFFFFFF:FFF:FFFFFFFFFFFFFFFFFFFFFFFFFFFFFFFFFF
FFFFFFFFFFFFFFFFFFFFFFFFFFFFFFFFFFFFFFFFFFFFFFFFFFFFFFFFFFFFFFFFFFFF
@A00155:342:HHGFNDSXY:1:1206:5665:14278 2:N:0:GAACCTAG+TCCGCATA
AAGCCCGGAGTACATCTCCTTTGGAACTAGGAGACCCCTCCGGTCTTGTCCAACCTCTGTTTCGGCAG
AGGATACCGTGCTCCACAGCTGTAAGGCTGTGGGCCCGAGAGAAGTCGCTCTCTCTCGCC
+
FFF:FFF,FFFFFF:FFFFFF:FF:FFFFFFFFF:F,FFFFFFFFFFFFFFFFFFFF:FFFFFFFF:F
FFFF,FFFFFFFFFFFFFFFFFFFFFFFF,F,FFFFFFFFFFFFFFFFFFFFFFFFFFFFFF
@A00155:342:HHGFNDSXY:1:1206:1940:21950 2:N:0:GAACCTAG+TCCGCATA
GTTTCGTCCTAAAAAGGACTCGTCAGGTAGCCTACTAGACTACGACTTACACGGTAGGTCGTGAACAA
GCCCGGAGTACATCTCCTTTGGAACTAGGAGACCCCTCCGGTCTTGTCCAACCTCTGTTTCGGCAGAG
+
```

FFFFFFFFFFFFFFFFFFFFFFFFFFFFFFFFFFF:FFFFFFFFFFFFFFFFFFFFFFFFFFFFFF
FFFFFFFFFFF:FFFFF:FFFFF:FFFFFFFFFFFFFFFFFFFFFFFFFFFFFFFFFFFFFFFFFFFF
@A00155:342:HHGFNDSXY:1:1206:26250:30796 2:N:0:GAACCTAG+TCCGCATA
ACAGCTGTAAGGCTGTGGGCCCGAGAGAAGTCGCTCTCTCTCGCCCTCCACATCCCTCTCGGCATTGA
GATGACCATAGGCCAGAGGTGAGTCCTTAAGTGGACACAGCTGATCTAAGGCGGTGTGGCGGG
+
FFFFFFFFFFFFFFFFFFFFFFFFFFF,FFFFFFFFFFFFFFFFFFFFFFFFFFFFFFFFFFFFFFFF
FFFFFFFFFFFFFFFFFFFFFFFFFFFFFFFFFFFFFFFFFFFFFFFFFFFFFFFFFFFFFFF
@A00155:342:HHGFNDSXY:1:1207:21016:17253 2:N:0:GAACCTAG+TCCGCATA
GGACTCGTCAGGTAGCCTACTAGACTACGACTTACACGGTAGGTCGTGAACAAGCCCGGAGTACATCT
CCTTTGGAACTAGGAGACCCCTCCGGTCTTGTCCAACCTCTGTTTCGGCAGAGGATACCGTGCTCCAC
+
FFFFFFFFFFFFF:FFFFFFFFFFFFFFFFFFFFFFFFFFFFFFFFFFFFFFFFFFFFFFFFFF:FFF
FFFFFFFFFFFFFFFFFFFFFFFFFFFF:FFFFFFFFFFFFFFFFFFFFFFFFFFFFFFFFFFFFFFF
@A00155:342:HHGFNDSXY:1:1207:10366:18944 2:N:0:GAACCTAG+TCCGCATA
ACGACTTACACGGTAGGTCGTGAACAAGCCCGGAGTACATCTCCTTTGGAACTAGGAGACCCCTCCGG
TCTTGTCCAACCTCTGTTTCGGCAGAGGATACCGTGCTCCACAGCAGTAAGGCTGTGGGCCCGAGAGA
+
FFFFFFFFFFFFFF,FFFFFFFFFFFFFFFFFFFFFFFFFFFFFFFFFFFFFFFFFFFFFFFFFFFFF
FFFFFFFFFFFFFFFFFFFFFFFFFFFFFFFFFFFFFFFFFFFFFFFFFFFFF:FFFFFFFFFFFFFF
@A00155:342:HHGFNDSXY:1:1208:32072:2550 2:N:0:GAACCTAG+TCCGCATA
CCGGAGTACATCTCCTTTGGAACTAGGAGACCCCTCCGGTCTTGTCCAACCTCTGTTTCGGCAGAGGA
TACCGTGCTCCACAGCTGTAAGGCTGTGGGCCCGAGAGAAGTCGCTCTCTCTCGCCCTCCACATCCCT
+
FFFFFFFFFFFFFFFFFFFFFFFFFFFFFFFFFFFFFFFFFFFFFFFFFFFFFFFFFFFFFFFFFFFF
,FFFFFFFFFFFFFFFFFFFFFFFF:FFFFFFFFFFFFFF:FFFFFFFFFFFFFFFFFFFFFFFFFFFF
@A00155:342:HHGFNDSXY:1:1208:4806:9784 2:N:0:GAACCTAG+TCCGCATA
GAAACCGTCGTTACTCTTAGAGTTGTGTAAGTTTCGTCCTAAAAAGGACTCGTCAGGTAGCCTACTAG
ACTACGACTTACACGGTAGGTCGTGAACAAGCCCGGAGTACATCTCCTTTGGAACTAGGAGACCCCTC
+
FFFFFFFFFF:FF,F:FFFFFFFFFFF,FFFFFFFFFFFFFFF::FFFF:FFFF:FFF:FFF,,FFF:
FFFFFFFFFFFFFFFFFFFFFFFFFF:FFFF,FFFFF:FFFFFFFFFFFFFFFFFFFFFFFFFFFFFF
@A00155:342:HHGFNDSXY:1:1208:19434:11162 2:N:0:GAACCTAG+TCCGCATA
TAGGAGACCCCTCCGGTCTTGTCCAACCTCTGTTTCGGCAGAGGATACCGTGCTCCACAGCTGTAAGG
CTGTGGGCCCGAGAGAAGTCGCTCTCTCTCGCCCTCCACATCCCTCTCGGCATTGAGATG
+
FFFFFFFFFFFFFFFFFFFFFFFFFFFFFFFFFFFFFFFFFFFFFFFFFFFFFFFFFFFFFFFFFFFF
FFFFFFFF:FFFFFFFFFFFFFFFFFFF:FFFFFFFFFFFFFFFFFFFFFFFFFFFFFFFF
@A00155:342:HHGFNDSXY:1:1209:2293:5932 2:N:0:GAACCTAG+TCCGCATA
ACGGTAGGTCGTGAACAAGCCCGGAGTACATCTCCTTTGGAACTAGGAGACCCCTCCGGTCTTGTCCA
ACCTCTGTTTCGGCAGAGGATACCGTGCTCCACAGCTGTAAGGCTGTGGGCCCGAGAGAAGTCGCTCT
+
FFFFFFFFFFFF::FFFFFFFFFFFFFFFFFFFFFF:FFFFFFFFFFFFFFFFFFFFFFFF:FFFFFFF
FF:FFFFFFFFFFFFFFFFFFFFFFFFFF:FFFFFFFFFFFFFFFFFFFFFFFFFFFFFFFFFFFFFF
@A00155:342:HHGFNDSXY:1:1209:4101:5963 2:N:0:GAACCTAG+TCCGCATA
AGTACATCTCCTTTGGAACCAGGAGACCCCTCCGGTCTTGTCCAACCTCTGTTTCGGCAGAGGATACC
GTGCTCCACAGCTGTAAGGCTGTGGGCCCGAGAGAAGTCGCTCTCTCTCGCCCTCCACATCCC
+
FFFFFFFFFFFFFFFFF:FFFFFFFFFFFFFFFFFFFFFFFFFF:FFFFFFFFFFFFFF:FFFFFFFFF
FFFFFFFFFFFFFFFFFFFFFFFFFFFFFFFFFFFFFFFFFFFFFFFFFFFFFFFFFFFFFFF
@A00155:342:HHGFNDSXY:1:1209:32723:16673 2:N:0:GAACCTAG+TCCGCATA
GAACAAGCCCGGAGTACATCTCCTTTGGAACTAGGAGACCCCTCCGGTCTTGTCCAACCTCTGTTTCG
GCAGAGGATACCGTGCTCCACAGCTGTAAGGCTGTGGGCCCGAGAGAAGTCGCTCTCTCTCGCCCTCC
+

FFFFFFFFFFFFFFFFFFFFFFFFFFFFFFFFF:FFFFFFFFFFFFFFFFFFF,FFFFFFFFFFFFFFF
FFFFFFF:F:FFFFFFFFFFFFFFF:FFFFFFF:FFFFFFFFFFFFFF:FFFFFFFFFFFFFFFFFFFF
@A00155:342:HHGFNDSXY:1:1209:24903:31501 2:N:0:GAACCTAG+TCCGCATA
GGACTCGTCAGGTAGCCTACTAGACTACGACTTACACGGTAGGTCGTGAACAAGCCCGGAGTACATCT
CCTTTGGAACTAGGAGACCCCTCCGGTCTTGTCCAACCTCTGTTTCGGCAGAGGATACCGTGCTCCAC
+
FFFFFFFFF:FFFFFFFFFFFFFFFFFFFFFFFFFFFFFFFFFFFFFFFFFFFFFFFFFFFFFFFFFF
FFFFFFFFFFFFFFFFFFFFFFFFFFFFFFFFFFFFFFFFFFFFFFFFFFFFFFFFF,FFFFFFFFFFF
@A00155:342:HHGFNDSXY:1:1209:32922:31735 2:N:0:GAACCTAG+TCCGCATA
AAAAGGACTCGTCAGGTAGCCTACTAGACTACGACTTACACGGTAGGTCGTGAACAAGCCCGGAGTAC
ATCTCCTTTGGAACTAGGAGACCCCTCCGGTCTTGTCCAACCTCTGTTTCGGCAGAGGATACCGTGCT
+
F:F:FFFFF:FFFFFFFFFFFFFFFFFFFFFFFFFFFFFFFFFFFFFFFFFFFFFFFFFFFFFFFFFF
FFFFFF:FFFFFFFFFFFFFFFFFFFFFFFFFFFFFFFF:FFFFFFFF,,FFFFFFFFFFF:FFFFFFFF
@A00155:342:HHGFNDSXY:1:1210:3369:16720 2:N:0:GAACCTAG+TCCGCATA
GGTCGTGAACAAGCCCGGAGTACATCTCCTTTGGAACTAGGAGACCCCTCCGGTCTTGTCCAACCTCT
GTTTCGGCAGAGGATACCGTGCTCCACAGCTGTAAGGCTGTGGGCCCGAGAGAAGTCGCTCTCTCTCG
+
FFFFFFFFFFFFFFFFFFFFFFFFFFFFFFFFFFFF:FFFFFFFFFFFFFFFFFFFFFFFFFFFFFFFF
F::FFFFFFFFFFFFFFFFFFFFFFFFFFFFFFFFFFFFFFFFFFFFFFFFFFF,FFFFFFFFFFFFFF
@A00155:342:HHGFNDSXY:1:1210:15537:20854 2:N:0:GAACCTAG+TCCGCATA
GGACTCGTCAGGTAGCCTACTAGACTACGACTTACACGGTAGGTCGTGAACAAGCCCGGAGTACATCT
CCTTTGGAACTAGGAGACCCCTCCGGTCTTGTCCAACCTCTGTTTCGGCAGAGGATACCGTGCTCCAC
+
FFFFFFFFFFFFFFFFFFFFFFFFFFFFFFFFFFFFFFFFFFFFFFFFFFFFFFFFFFFFFFFFF:FFF
FFFFFFFFFFFFFFFFFFFFFFFFFFFFFF:FFFFFFFFFFFFFFFF:FFFFFFFFFFFFFFFFFFFFF
@A00155:342:HHGFNDSXY:1:1210:11487:23359 2:N:0:GAACCTAG+TCCGCATA
AGGTCGTGAACAAGCCCGGAGTACATCTCCTTTGGAACTAGGAGACCCCTCCGGTCTTGTCCAACCTC
TGTTTCGGCAGAGGATACCGTGCTCCACAGCTGTAAGGCTGTGGGCCCGAGAGAAGTCGCTCTCTCTC
+
FFFFFFFFFFFFFFFFFFFFFFFFFFFFFFFFFFFFFFFFFFFFFFFFFFFFFFFFF:FFFFFFFFFFF
FFFFFFFFFFFFFFFFFFFFFFFFFFFFFFFFFFFFFFFFFFFFFFFFFFFFFFFFFFFFFFFFFFFFF
@A00155:342:HHGFNDSXY:1:1211:3631:35900 2:N:0:GAACCTAG+TCCGCATA
CAGCTGTGTCCACTTAAGGACTCACCTCTGGCCTATGGTCATCTCAATGCCGAGAGGGATGTGAAGGG
CGAGAGAGAGCGACTTCTCTCGGGCCCACAGCCTTACAGCTGTGGAGCACGGTATCCTCTGCCGAAAC
+
FFFFFFFFFFFFFFFFFFFFFFFFFFFFFFFFFFFFFFFFFFFFFFFFFFFFFFFFFFFFFFFFFFFFF
FFFFFFFFFFFFFFFFFFFFFFFFFFFFFFFFFFFFFFFFFFFFFFFFFFFFF:FFFFFFFFFFFFFF
@A00155:342:HHGFNDSXY:1:1212:19199:6778 2:N:0:GAACCTAG+TCCGCATA
AAGGACTCACCTCTGGCCTATGGTCATCTCAATGCCGAGAGGGATGTGGAGGGCGAGAGAGAGCGACT
TCTCTCGGGCCCACAGCCTTACAGCTGTGGAGCACGGTATCCTCTGCCGAAACAGAGGTTGGACAAGA
+
FFFFFFFFFFFFFFFFFFFFFFFFFFFFFFFFFFFFFFFFFFFF:FFFFFFFFFFFFFFFFFFFFF
FFFFFFFFFFFFFFFFFFFFFFFFFFFFFFFFFFFFFFFFFFFFFFFFFFFFFFFFFFFFFFFFFFFFF
@A00155:342:HHGFNDSXY:1:1212:29252:7185 2:N:0:GAACCTAG+TCCGCATA
GGCCTATGGTCATCTCAATGCCGAGAGGGATGTGAAGGGCGAGAGAGAGCGACTTCTCTCGGGCCCAC
AGCCTTACAGCTGTGGAGCACGGTATCCTCTGCCGAAACAGAGGTTGGACAAGACCGGAG
+
FFFFFFFFFFFFFFFFF:FFFFF:FFFFFFFF:FFFFFFFFFFFFFFFFFFFFFFFFFFFFFFFFFFF
FFFF:FFFFFFFF:FFFFFFFFFFFFFFFFFFFFFFFFFFFFFFFFFF:FFFFFFFFFFF
@A00155:342:HHGFNDSXY:1:1212:5493:15170 2:N:0:GAACCTAG+TCCGCATA
CCACACCGCCTTAGATCAGCTGTGTCCACTTAAGGACTCACCTCTGGCCTATGGTCATCTCAATGCCG
AGAGGGATGTGGAGGGCGAGAGAGAGCGACTTCTCTCGGGCCCACAGCCTTACAGCTGTGGAGCACGG
+

FFFFFFFFFFFFFFFFFFFFFFFFFFFFFF:FFFFFFFFFFFFFFFFFFFFFFFFFFFFFFFFFFFFF
FFFFFFFFFFFFFFFFFFFFFFFFFFFFFFFFFFFFFFFFFFFFFFFFFFF,FFFFFFFFFFFFFFFF
@A00155:342:HHGFNDSXY:1:1212:7925:17534 2:N:0:GAACCTAG+TCCGCATA
AGCCCGGAGTACATCTCCTTTGGAACTAGGAGACCCCTCCGGTCTTGTCCAACCTCTGTTTCGGCAGA
GGATACCGTGCTCCACAGCTGTAAGGCTGTGGGCCCGAGAGAAGTCGCTCTCTCTCGCCCTCCACATC
+
FFFFFFFFFFFFFFFFFFFFFFFFFFFFFFFFFFFFFFFFFFFFFFFFFFFFFFFFFFFFF:FFFFFF
FFFFFFFFFFFFFFFFFFFFFFFFFFFFFFFFFFFFFFFFFF:FFFFFFFFFFFFFFFFFFFFFFFFF
@A00155:342:HHGFNDSXY:1:1212:16242:23641 2:N:0:GAACCTAG+TCCGCATA
CAGGTAGCCTACTAGACTACGACTTACACGGTAGGTCGTGAACAAGCCCGGAGTACATCTCCTTTGGA
ACTAGGAGACCCCTCCGGTCTTGTCCAACCTCTGTTTCGGCAGAGGATACCGTGCTCCACAGCTGTAA
+
FFFFFFFFFFFFFFFFFFFFFFFFFFFFFFFFFFFFFFFFFFFFFFFFFFFFFFFFFFFFFFFFFFFF
FFFFFFFFFFFFFFFFFFFFFFFFFFFFFFFFFFFFFFFFFFFFFFFFFFFFFFFFFFFFFFFFFFFF
@A00155:342:HHGFNDSXY:1:1212:8187:28354 2:N:0:GAACCTAG+TCCGCATA
TTTCGTCCTAAAAAGGACTCGTCAGGTAGCCTACTAGACTACGACTTACACGGTAGGTCGTGAACAAG
CCCGGAGTACATCTCCTTTGGAACCAGGAGACCCCTCCGGTCTTGTCCAACCTCTGTTTCGGCAGAGG
+
F:FFFFFFFFFF:FFFFFFFFFF:FFFFFFFF,FFFFFFFFFFFFFFFFFFFFFFF:,FFFFF:FFFF
FFFFFFFFFFFFFFF:F,FFFF,FFFFFFFFFFFFFFFFFFF,,FFFFFFFFFFFFFFF:FFFFFFFF
@A00155:342:HHGFNDSXY:1:1212:5330:31454 2:N:0:GAACCTAG+TCCGCATA
AAACTTACACAACTCGAAGAGTAACGACGGTTTCGTCCTTGGAACGGACTCATCAGGGAGCCTACTAG
ACTCCGACCGTCATGGGGGTTCAAACCCATGCCCCGCCACACCGCCTTAGATCAGCTGTGTCTACTTA
+
FFFFFFFFFFFFFFFFFFFFFFFFFFFFFFFFFFFFFFFFFFFFFFFFFFFFFFFFFFFFFFFFFFFF
FFFFFFFFFFFFFFFFFFFFFFFF:FFFFFFFFFFFFFFFFFFFFFFFFFFFFFFF:FFFFFFFFFFF
@A00155:342:HHGFNDSXY:1:1212:24053:35446 2:N:0:GAACCTAG+TCCGCATA
GTGAACAAGCCCGGAGTACATCTCCTTTGGAACTAGGAGACCCCTCCGGTCTTGTCCAACCTCTGTTT
CGGCAGAGGATACCGTGCTCCACAGCTGTAAGGCTGTGGGCCCGAGAGAAGTCGCTCTCTCTCGCCCT
+
FFFFFFFFFFFFFFFFFFFFFFFFFFFFFFFFFFFFFFFFFFFFFFFFFFF,FFFFFFFFFFFFFFFF
FFFFFFFFFFFFFFFFFFFFFFFFFFFFFFFFFFFFFFFFFFFFFFF:FFFFFFFFFFFFFFFFFFFF
@A00155:342:HHGFNDSXY:1:1213:13964:1094 2:N:0:GAACCTAG+TCCGCATA
CACGGTAGGTCGTGAACAAGCCCGGAGTACATCTCCTTTGGAACCAGGAGACCCCTCCGGTCTTGTCC
AACCTCTGTTTCGGCAGAGGATACCGTGCTCCACAGCTGTAAGGCTGTGGGCCCGAGAGAAGTCGCTC
+
FFFFFFFFFFFFFFFFFFF:FFFFFFFFFFFFFFFFFFFFFFFFFFFFFFFFFFFFFFFFFFFFFFFF
FFFFFFFFFFFFFFFFFFFFFFFFFFFFFFFFFFFFF:FFFFFFF:FFFFFFFFFFFFFFFF:FFFFF
@A00155:342:HHGFNDSXY:1:1213:32805:3474 2:N:0:GAACCTAG+TCCGCATA
TGTGTAAGTTTCGTCCTAAAAAGGACTCGTCAGGTAGCCTACTAGACTACGACTTACACGGTAGGTCG
TGAACAAGCCCGGAGTACATCTCCTTTGGAACTAGGAGACCCCTCCGGTCTTGTCCAACCTCTGTTTC
+
FFFF,FFFFFF:FFFFF:FFFFFFFFFFFFFFFFFFFFFFFFF:FFFFFFFFFFFFFFFFFFFFFFFF
FFFFFFFFFFFFFFFFFFF:FFFFFFFFFFFFFFFFFFFFFFFFFFFFFFFFFFF,FFFFFFFFFFFF
@A00155:342:HHGFNDSXY:1:1213:30083:11631 2:N:0:GAACCTAG+TCCGCATA
GTGAACAAGCCCGGAGTACATCTCCTTTGGAACCAGGAGACCCCTCCGGTCTTGTCCAACCTCTGTTT
CGGCAGAGGATACCGTGCTCCACAGCTGTAAGGCTGTGGGCCCGAGAGAAGTCGCTCTCTCTCGC
+
FFFFFFFFFFFFFFFFFFFF:FFFFFFFFFFFFFFFFFFFFFFFFFFFFFFFFFFFFFFFFFF,FFFF
FFFFFFFFFFFFFFFFFFFFFFFFFFFFFFFFFF:FFFFFFFFFFFFFFFFFFFFFFFFFFFFFF
@A00155:342:HHGFNDSXY:1:1213:10285:20306 2:N:0:GAACCTAG+TCCGCATA
GGACTCGTCAGGTAGCCTACTAGACTACGACTTACACGGTAGGTCGTGAACAAGCCCGGAGTACATCT
CCTTTGGAACTAGGAGACCCCTCCGGTCTTGTCCAACCTCTGTTTCGGCAGAGGATACCGTGCTCCAC
+

```
FFFFFFFFFFFFFFFFFFFFFFF:FFFFFFFFFFFFFFFFFFFFFFFFFFFFF:FFFFFFFFFFFFFF
FFFFFFFFFFFFFFFFFFFFFFFFFFFFFFFFFFFFFFFFFFFFFFFFFFFFFFF:FFFFFFFFFFFF
@A00155:342:HHGFNDSXY:1:1213:19307:36808 2:N:0:GAACCTAG+TCCGCATA
ACTTACACGGTAGGTCGTGAACAAGCCCGGAGTACATCTCCTTTGGAACTAGGAGACCCCTCCGGTCT
TGTCCAACCTCTGTTTCGGCAGAGGATACCGTGCTCCACAGCTGTAAGGCTGTGGGCCCGAGAGAAGT
+
FFFFFFFFFFFFFFFFFFFFFFFFFFFFFFFFFFFFFFFFFFFFFFFFFFFFFFFFFFFFFFFFFFFF
FFFFFFFFF:FFFFFFFFFFFFFFFFFFFFFFFFFFFFFFFFFFFFFFFFFFFFFFFFFFFFFFFFFF
@A00155:342:HHGFNDSXY:1:1214:23936:11725 2:N:0:GAACCTAG+TCCGCATA
AAGGACTCGTCAGGTAGCCTACTAGACTACGACTTACACGGTAGGTCGTGAACAAGCCCGGAGTACAT
CTCCTTTGGAACTAGGAGACCCCTCCGGTCTTGTCCAACCTCTGTTTCGGCAGAGGATACCGTGCTCC
+
FFFFFFFFFFFFFFFFFFFFFFFF:FFFFFFFFFFFFFFFFFFFFFFFFFFFFFFFFF:FFFFFFFFF
FFFFFFFFFFFFFFFFFFFF:FFFFFFFFFFFFFFFFFFFFFFFFFFFFFFFFFFFFFFFFFFFFFF
@A00155:342:HHGFNDSXY:1:1214:25066:23766 2:N:0:GAACCTAG+TCCGCATA
AGGTAGCCTACTAGACTACGACTTACACGGTAGGTCGTGAACAAGCCCGGAGTACATCTCCTTTGGAA
CTAGGAGACCCCTCCGGTCTTGTCCAACCTCTGTTTCGGCAGAGGATACCGTGCTCCACAGCTGTAAG
+
:FFF:FFFFFFFFFFFFFFFFFFFFFFFFFF:FFFFFFFFFFFFFFFFFFFFFFFFFFFFFFFFFFFF
FF,FFFFFFFFFFFFFFFFFFF:FFFFFFFFFFFFFFFFFFFFFFFFFFFFFFFFFFFFFFFFFFFFF
@A00155:342:HHGFNDSXY:1:1214:28519:36229 2:N:0:GAACCTAG+TCCGCATA
GGACTCGTCAGGTAGCCTACTAGACTACGACTTACACGGTAGGTCGTGAACAAGCCCGGAGTACATCT
CCTTTGGAACTAGGAGACCCCTCCGGTCTTGTCCAACCTCTGTTTCGGCAGAGGATACCGTGCTCCAC
+
FFFFFFFFFFFFFFFFFFFFFFFFFFFFFFFFFFFFFFFF:FFFFFFFFFFFFFFFFFFFFFFFFFFF
FFF:FFFFFFFFF,FFFFFFFFFFFFFFFFFFF:FFFFFFFF:FFFFFFFFFFFFFFFFFFFFFFFFF
@A00155:342:HHGFNDSXY:1:1215:14543:11459 2:N:0:GAACCTAG+TCCGCATA
AGTACATCTCCTTTGGAACTAGGAGACCCCTCCGGTCTTGTCCAACCTCTGTTTCGGCAGAGGATACC
GTGCTCCACAGCTGTAAGGCTGTGGGCCCGAGAGAAGTCGCTCTCTCTCGCCCTCTACATCCCTCTCG
+
FFFFFFFFFFFFFFFFFFFFFFFFFFFFFFFFFFFFFFFFFFFFFFFFFFFFFFFFFFFFFFFFFFFF
FFFFFFFFFFFFFFFFFFFFFFFFFFFF:FFFFFFFFFFFFFFFFFFFFFFFFFFFFFFFFFFFFFFF
@A00155:342:HHGFNDSXY:1:1215:14000:30279 2:N:0:GAACCTAG+TCCGCATA
ACTTACACGGTAGGTCGTGAACAAGCCCGGAGTACATCTCCTTTGGAACTAGGAGACCCCTCCGGTCT
TGTCCAACCTCTGTTTCGGCAGAGGATACCGTGCTCCACAGCTGTAAGGCTGTGGGCCCGAGAGAAGT
+
FFFFFFFFFFFFFFFFFFFFFFFF:FFFFFFFFFFFFFFFFFFFFFF,FFFFFFFFFFFFFFFFFFF,
FFFFFFFFFFFFFFFFFFFFFFFFFFFFFFFFFFFFFFFFFFFFFFFFFFFFFFFFFFFFFFFFF,FF
@A00155:342:HHGFNDSXY:1:1215:24505:30874 2:N:0:GAACCTAG+TCCGCATA
GACCCCTCCGGTCTTGTCCAACCTCTGTTTCGGCAGAGGATACCGTGCTCCACAGCTGTAAGGCTGTG
GGCCCGAGAGAAGTCGCTCTCTCTCGCCCTCCACATCCCTCTCGGCATTGAGATGAC
+
FFFFFFFFFFFFFFFFFFFFFFFFFFF:FFFFFFFFFFFFFFFFFFFFFFFFFFFFFFFFFFFFFFFF
FFFFFFFFFFFFFFFFFFFFFFFFFFFFFFFFFFFFFFFFFFFFFFF::FFFFFFFF
@A00155:342:HHGFNDSXY:1:1216:29957:3427 2:N:0:GAACCTAG+TCCGCATA
GAAACCGTCGTTACTCTTTGAGTTGTGTAAGTTTCGTCCTAAAAAGGACTCGTCAGGTAGCCTACTAG
ACTACGACTTACACGGTAGGTCGTGAACAAGCCCGGAGTACATCTCCTTTGGAACTAGGAGACCCCTC
+
FFFFFFFFFFFFFFF:,:FFFFFFFFFFFFFFFFFFFFFF,:FFFFFFFFFFFF,FFFF:FFFFFFFF
FFFFFFFFFFF:FFFFFFFFFFFFFFFFFFFFFFFFFFFFFFFFF:FF:FFFFFFFFFFFFFFFFFFF
@A00155:342:HHGFNDSXY:1:1216:15338:6918 2:N:0:GAACCTAG+TCCGCATA
TACGACTTACACGGTAGGTCGTGAACAAGCCCGGAGTACATCTCCTTTGGAACTAGGAGACCCCTCCG
GTCTTGTCCAACCTCTGTTTCGGCAGAGGATACCGTGCTCCACAGCTGTAAGGCTGTGGGCCCG
+
```

```
FFFFFFFFFFFFFFFFFFFFFFFFFFFFFFFFFFFFFFFFFFFFFFFFFFFFFFFFFFFFFFFFFFFFFF
FFFFFFFFFFFFFFFFFFFFFFFFFFFFFFFFFFFFFFFFFFFFFFFFFFFFFFFFFFFFFFFFFFFF
@A00155:342:HHGFNDSXY:1:1216:12409:12837 2:N:0:GAACCTAG+TCCGCATA
AAACTTACACAACTCGAAGAGTAACGACGGTTTCGTCCTTGGAACGGACTCATCAGGGAGCCTACTAG
ACTCCGACCGTCATGGGGGTTCAAACCCATGCCCCGCCACACCGCCTTAGATCAGCTGTGTCCACTTA
+
FFFFFFFFFFFFFFFFFFFFFFFFFFFFFFFFFFFFFFFFFFF:FFFFFFFFFFFFFFFFFFFF:FFF:
FFFFFFFFFFFFFFFFFFFFFFFFFFFFFFFFFFFFFFFFFFFFFFFFFFFFFFFFFFFFFFFFFFF,FF
@A00155:342:HHGFNDSXY:1:1216:8061:30608 2:N:0:GAACCTAG+TCCGCATA
TCCTAAAAAGGACTCGTCAGGTAGCCTACTAGACTACGACTTACACGGTAGGTCGTGAACAAGCCCGG
AGTACATCTCCTTTGGAACTAGGAGACCCCTCCGGTCTTGTCCAACCTCTGTTTCGGCAGAGGATACC
+
FFFFFFFFFFFFFFFFFFFFFFFFFFFFFFFFFFFF:FFFFFFFFFFFFFFFFFFFFFFFFFFFFFFF:F
FFFFFF:FFFFFFFFFFFFFF:FF:FFFFFF:FFFFFFFFFFFFFFFFFFFFFFFFFFFFF:FFFF:FF
@A00155:342:HHGFNDSXY:1:1217:31647:13870 2:N:0:GAACCTAG+TCCGCATA
CTCTTCGAGTTGTGTAAGTTTCGTCCTAAAAAGGACTCGTCAGGTAGCCTACTAGACTACGACTTACA
CGGTAGGTCGTGAACAAGCCCGGAGTACATCTCCTTTGGAACTAGGAGACCCCTCCGGTCTTGTCCAA
+
FFF:FFFFFFFFFFFFFFFFFFFFFFFFFFFFFFFFFFFFFFFFFFFFFFFFFFFFFFFFFFFFFFFFFF
FFFFFFFFFFFFFFFFFFFFFFFFFFFFFFFFFFFFFFFFF:FFF:FFFFFFF:,FFFFFFFFFFFFFF
@A00155:342:HHGFNDSXY:1:1217:7835:15781 2:N:0:GAACCTAG+TCCGCATA
GGAGACCCCTCCGGTCTTGTCCAACCTCTGTTTCGGCAGAGGATACCGTGCTCCACAGCTGTAAGGCT
GTGGGCCCGAGAGAAGTCGCTCTCTCTCGCCCTCCACATCCCTCTCGGCATAGAGATGACCATAGGCC
+
FFFFFFFFFFFFFFFFFFFFFFFFFFFFFFFFFFFFFFF:FFFFFFFFFFFFFFFFFFFFFFFFFFFFFF
FFFFFFFFFFFFFF:FFFFFFFFFFFFFFFFFFFFFFFFFFFFFFFFFFFF::FFFFFFFFFFFFFFFF
@A00155:342:HHGFNDSXY:1:1217:24496:19742 2:N:0:GAACCTAG+TCCGCATA
GTTGTGTAAGTTTCGTCCTAAAAAGGACTCGTCAGGTAGCCTACTAGACTACGACTTACACGGTAGGT
CGTGAACAAGCCCGGAGTACATCTCCTTTGGAACTAGGAGACCCCTCCGGTCTTGTCCAACCTCTGTT
+
FFFFFFFFFFFFF:FFFFFFFFFFFFFFFFFFFFFFFFFFFFFFFFFFFFFFFFFFFFFFFFFFFFFFFF
FF:FFFF:FFFFFFFFFFFFFFFFFFFFFFFFF:FFFFFFFFFFFFFFFFFFFFFFFFF:FFFFFFFFFF
@A00155:342:HHGFNDSXY:1:1217:1651:30436 2:N:0:GAACCTAG+TCCGCATA
TCCAAGGACGAAACCGTCGTTACTCTTTGAGTTGTGTAAGTTTCGTCCTAAAAAGGACTCGTCAGGTA
GCCTACTAGACTACGACTTACACGGTAGGTCGTGAACAAGCCCGGAGTACATCTCCTTTGGAACTAGG
+
FFFFFFFFFFFFFFFFFFFFFFFFF:FFFFFFF:FFFFFFFFFFFFFFFF:FFF,FFFFFFFFF:FFFF
FFFFFFFFFFFFFFFFFFFFFFFFFFFFFFFFFFFFFFFFFFFFFFFFFFFFFFFFFFFFFFFFFFFFFF
@A00155:342:HHGFNDSXY:1:1217:3061:31908 2:N:0:GAACCTAG+TCCGCATA
GGAGACCCCTCCGGTCTTGTCCAACCTCTGTTTCGGCAGAGGATACCGTGCTCCACAGCTGTAAGGCT
GTGGGCCCGAGAGAAGTCGCTCTCTCTCGCCCTCTACATCCCTCTCGGCATTGAGATGACCATAGGCC
+
FFFFFFFFFFFFFFFF:FFFFFFFFFFFFFFFFFFFFFFFFFFFFFFFFFFFFFFFFFFFFFFFFFFF:
FFFFFFFFFFFFFFFFFFFFFFFFFFFFFFFFFFFFFFFFFFFFFFFFFFFF:FFFFFFFFFFFFFFFFF
@A00155:342:HHGFNDSXY:1:1218:18376:24674 2:N:0:GAACCTAG+TCCGCATA
AGGACGAAACTTACACAACTCGAAGAGTAACGACGGTTTCGTCCTTGGAACGGACTCATCAGGGAGCC
TACTAGACTCCGACCGTCATGGGGGTTCAAACCCATGCCCCGCTACACCGCCTTAGATCAGCTGTGTC
+
FFFFFFFFFFFFFFFFFFFFFFFFF:FFFFFFFFFFFFFFFFFFFFFFFFFFFFFFFFFFFFFFFFFFF
FFFFFFFFFFFFFFFFFFFFFFFFFFFFFFFFFFFFFFFFFFFFFFFFFFFFFFFFFFFFFFFFFFFFFF
@A00155:342:HHGFNDSXY:1:1218:28637:27602 2:N:0:GAACCTAG+TCCGCATA
CTGGCCTATGGTCATCTCAATGCCGAGAGGGATGTGAAGGGCGAGAGAGAGCGACTTCTCTCGGGCCC
ACAGCCTTACAGCTGTGGAGCACGGTATCCTCTGCCGAAACAGAGGTTGGACAAGACCGGAG
+
```

FFFFFFFFFFFFFFFFFFFFFFFFFFFFFFF:FFFFFFFFFFFFFFFFFFFFFFFFFFFFFFFFFF
FFFFFF:FFFFFFFFFFFFFFFFFFFFFFFFFFFFFFFFFFFFFFF:FFFFFFFFFFFFFFF
@A00155:342:HHGFNDSXY:1:1218:28094:28823 2:N:0:GAACCTAG+TCCGCATA
CTGGCCTATGGTCATCTCAATGCCGAGAGGGATGTGAAGGGCGAGAGAGAGCGACTTCTCTCGGGCCC
ACAGCCTTACAGCTGTGGAGCACGGTATCCTCTGCCGAAACAGAGGTTGGACAAGACCGGAG
+
FFFFFFFFFFFFFFFFFFFFFFFFFFFFFFFFFFFFFFFFFFFFFFFFFFFFFFFFFFFFFFFFFFFF
FFFFFF,FFFFFFFFFFFFFFFFFFF::FFFFFFFFFFFFFFFFFFFFFFFFFFFFFFFFFFF
@A00155:342:HHGFNDSXY:1:1218:23312:29371 2:N:0:GAACCTAG+TCCGCATA
ACATCTCCTTTGGAACTAGGAGACCCCTCCGGTCTTGTCCACCCTCTGTTTCGGCAGAGGATACCGTG
CTCCACAGCTGTAAGGCTGTGGGCCCGAGAGAAGTCGCTCTCTCTCGCCCTCCACATCCCTCTCGGCA
+
FFFFFFFFFFFF:FFFFFFFFFFFFFFFFFFFFFF,FFFFF,FFFFFFFFF:FFFFFFFFFFFFFFF
FFFFFFFF:FF:FFFFFFFFFFFFF:FFFFFFFFFFFFFFFFFFFFFFFFFFFFFF:FF,FFFFFFFFF
@A00155:342:HHGFNDSXY:1:1218:25229:36855 2:N:0:GAACCTAG+TCCGCATA
CGTCCTAAAAAGGACTCGTCAGGTAGCCTACTAGACTACGACTTACACGGTAGGTCGTGAACAAGCCC
GGAGTACATCTCCTTTGGAACTAGGAGACCCCTCCGGTCTTGTCCAACCTCTGTTTCGGCAGAGGATA
+
FFFFFFFFFFFFFFFFFFFFFFFFFFFFFFFFFFFFFFFFFFFFFFFFFFFFFFFFFFFF:FFFFFFF
FFFFFFFFFFFFFFFFFFF:FFFFFFFFFFFFFFFFFFFFFFFFFFFFFFFFFFFFFFFFFFFFFFFF
@A00155:342:HHGFNDSXY:1:1219:14850:16313 2:N:0:GAACCTAG+TCCGCATA
GTGAACAAGCCCGGAGTACATCTCCTTTGGAACCAGGAGACCCCTCCGGTCTTGTCCAACCTCTGTTT
CGGCAGAGGATACCGTGCTCCACAGCTGTAAGGCTGTGGGCCCGAGAGAAGTCGCTCTCTCTCGCCCT
+
FFFFFFFFFFFFFFFFFFFFFFFFFFFFFFFFFFFFFFFFFFFFFFFFFFFFFFFFFFFFFFFFFFFF
FFFFFFFFFFFFF:FFFFFFFFFFFF:F:FFFFFFFFFFFFFFFFFFFFFFFFFFFFFFFFFFFFFFF
@A00155:342:HHGFNDSXY:1:1219:15917:16313 2:N:0:GAACCTAG+TCCGCATA
GTGAACAAGCCCGGAGTACATCTCCTTTGGAACCAGGAGACCCCTCCGGTCTTGTCCAACCTCTGTTT
CGGCAGAGGATACCGTGCTCCACAGCTGTAAGGCTGTGGGCCCGAGAGAAGTCGCTCTCTCTCGCCCT
+
FFFFFFFFFFFFFFFFFFFFFFFFFFFFFFFFFFFFFFFFFFFFFFFFFFFFFFFFFFFFFFFFFFFF
FFFFFFFFFFFFFFFFFFFFFFFFFFFFFFFFFFFFFFFFFFFFFFFFFFFFFFFFFFFFFFFFFFFF
@A00155:342:HHGFNDSXY:1:1220:26811:3302 2:N:0:GAACCTAG+TCCGCATA
GACCCCTCCGGTCTTGTCCAACCTCTGTTTCGGCAGAGGATACCGTGCTCCACAGCTGTAAGGCTGTG
GGCCCGAGAGAAGTCGCTCTCTCTCGCCCTCTACATCCCTCTCGGCATTGAGATGACCATAGGC
+
FFFFFFFFFFFFF,FFFFFFFFFFFFFFFFFFFFFFFFFFFFFFF:FFFFFFFFFFFFFFFFFFFFFF
FFFFFFFFFFFFFFFFFFFFFFFFFFFFFFFFFFFFFFFFFFFFFFF:FFFFFFFFFFF:FFF
@A00155:342:HHGFNDSXY:1:1220:13666:16861 2:N:0:GAACCTAG+TCCGCATA
CACGGTAGGTCGTGAACAAGCCCGGAGTACATCTCCTTTGGAACTAGGAGACCCCTCCGGTCTTGTCC
AACCTCTGTTTCGGCAGAGGATACCGTGCTCCACAGCTGTAAGGCTGTGGGCCCGAGAGAAGTCGCTC
+
FFFFFFFFFFFFFFFFFFF:FFFFFFFFFFFFFFFF,FFFFFFF,FFFFF:FFFFFFFFFF::F:FF
FFFFFFFFFFFF:FFFF:FFFFFFFFFFFFFFFFFFFFFFFFFFFF:FFFFFFFFFFFFFF:F,FFFFF
@A00155:342:HHGFNDSXY:1:1220:26901:21684 2:N:0:GAACCTAG+TCCGCATA
GGACTCGTCAGGTAGCCTACTAGACTACGACTTACACGGTAGGTCGTGAACAAGCCCGGAGTACATCT
CCTTTGGAACTAGGAGACCCCTCCGGTCTTGTCCAACCTCTGTTTCGGCAGAGGATACCGTGCTCCAC
+
,FF,F,FFFFFFFF:FFFFFFFFFFFF,FFFFFFFFFF:FFFFF:FF:FFFFF,FFFFFFFFF,F,:F
F,,FFFFFFFFFFFFFF::FFFFFFF:FFFFFFFFFF:FFF:FFFFFF:FFFF,FFF,FF:F:FFFFF
@A00155:342:HHGFNDSXY:1:1220:8558:27993 2:N:0:GAACCTAG+TCCGCATA
AAAAGGACTCGTCAGGTAGCCTACTAGACTACGACTTACACGGTAGGTCGTGAACAAGCCCGGAGTAC
ATCTCCTTTGGAACTAGGAGACCCCTCCGGTCTTGTCCAACCTCTGTTTCGGCAGAGGATACCGTGCT
+

```
FFFFFFFFFFFFFFFFFFFFFF:FFF:FF:FFFFFFFFFFFFFFFFFFFFFFFFFFFF:FFFFFFFFF
FFFFFFFFFFFFFFFFFFFFFFFFFFFFFFFFFFFFFFFFFFFFFFFFFFFFFFFFFFFFFFFFFFFF
@A00155:342:HHGFNDSXY:1:1220:5828:36730 2:N:0:GAACCTAG+TCCGCATA
ACTCTTTGAGTTGTGTAAGTTTCGTCCTAAAAAGGACTCGTCAGGTAGCCTACTAGACTACGACTTAC
ACGGTAGGTCGTGAACAAGCCCGGAGTACATCTCCTTTGGAACTAGGAGACCCCTCCGGTCTTGTCCA
+
FFFF:FFFFFFFFFFFFFFFFFFFFFFFFFFFFFFFFFFFFFFFFFFFFFFFFFFFFFFFFFFFFFFF
FFFFFFFFFFFFFFFFFFFFFFFFFFFFF:FFFFFFFFFFF:FFFFFFFFF:FFFFFFFFFF,FFFFF
@A00155:342:HHGFNDSXY:1:1221:6207:19476 2:N:0:GAACCTAG+TCCGCATA
AACGGACTCATCAGGGAGCCTACTAGACTCCGACCGTCATGGGGGTTCAAACCCATGCCCCGCCACAC
CGCCTTAGATCAGCTGTGTCCACTTAAGGACTCACCTCTGGCCTATGGTCATCTCAATGCCGAGAGGG
+
FFFFF:F:FFFFFFFFFFFF:FFFFFFFFFFFFFFFFF,FFFFFFFFFFFFFFFFFFFFFFFFFFFFF
FFFFFFFFFFFFFFFFFFFFFFFFF:FFFFFFFFFFFFFFF:FFFFFFFFFFFFFFFFFFF:FFFFFFF
@A00155:342:HHGFNDSXY:1:1221:20726:27618 2:N:0:GAACCTAG+TCCGCATA
ACACGGTAGGTCGTGAACAAGCCCGGAGTACATCTCCTTTGGAACTAGGAGACCCCTCCGGTCTTGTC
CAACCTCTGTTTCGGCAGAGGATACCGTGCTCCACAGCTGTAAGGCTGTGGGCCCGAGAGAAGTCGCT
+
FFFFFFFFFFFFFFFFFFFFFFFFFFFFFFFF:FFFFFFFFFFFFFFFFFFFFFFFFFFFFFFF,FFF
FFFFFFFFFFFFFFFFFFFFFFFFFFFFFFFFFFFFFFFFFFFFFFFFFFFFFFFFFFFFFF:FFFFFF
@A00155:342:HHGFNDSXY:1:1222:6696:3662 2:N:0:GAACCTAG+TCCGCATA
GGAGTACATCTCCTTTGGAACAAGGAGACCCCTCCGGTCTTGTCCAACCTCTGTTTCGGCAGAGGATA
CCGTGCTCCACAGCTGTAAGGCTGTGGGCCCGAGAGAAGTCGCTCTCTCTCGCCCTCCACATCCC
+
FFFFFFFFFFFFFFFFFFFFFFFFFFFFFFFFFFFFFFFFFFFFFFFFFFFFFFFFFFFFFFFFFFFF
FFFFFFFFFFFFFFFFFFFFFFFFFFFFFFFFFFFFFFFFFFFFFFFFFFFFFFFFFFFFFFFFF
@A00155:342:HHGFNDSXY:1:1222:32136:17660 2:N:0:GAACCTAG+TCCGCATA
TAGACTACGACTTACACGGTAGGTCGTGAACAAGCCCGGAGTACATCTCCTTTGGAACTAGGAGACCC
CTCCGGTCTTGTCCAACCTCTGTTTCGGCAGAGGATACCGTGCTCCACAGCTGCAAGGCTGTGGGCCC
+
FFFFFFFFFFFFFFFFFFFFFFFF:FFFFFFFFFFFFFFFFFFFFFFFFFFFFFFFFFFFFFFFFFFF
FFFFFFF:FFFFFFFFFFFFFFFFFFFFFF,FFFFF:FFFFFFFFFFFFFFFFFFFFFFFF:FFFFFFFF
@A00155:342:HHGFNDSXY:1:1222:10095:19946 2:N:0:GAACCTAG+TCCGCATA
ACCTCTGTTTCGGCAGAGGATACCGTGCTCCACAGCTGTAAGGCTGTGGGCCCAAGAGAAGTCGCTCT
CTCTCGCCCTCCACATCCCTCTCGGCATTGAGATGACCATAGGCCAGAGGTGAGTCCTTAAGTGGACA
+
FFFFFFFFFFFFFFFFFFFFFFFFFFFFFFFF:FFFFFFFFFFFFFFFFFFFFFFFFFFFFFFFFFFF
FFFFFFFFFFFFFFFFFFFFFFFFFFFFFFFFFFFFFFFFFFFFFFFFFFFFFFFFFFFFFFFFFFFF
@A00155:342:HHGFNDSXY:1:1222:9236:20776 2:N:0:GAACCTAG+TCCGCATA
ACCTCTGTTTCGGCAGAGGATACCGTGCTCCACAGCTGTAAGGCTGTGGGCCCAAGAGAAGTCGCTCT
CTCTCGCCCTCCACATCCCTCTCGGCATTGAGATGACCATAGGCCAGAGGTGAGTCCTTAAGTGGACA
+
FFFFFFFFFFFFFFFFFFFFFFFFFFFFFFFFFFFFFFFFFFFFFFFFFFFFFFFFFFFFFFFFFFFF
FFFFFFFFFFFFFFFFFFFFFFFFFFFFF:FFFFFFFFFFFFFFFFFFFFFFFFFFFFFFFFFFFFFF
@A00155:342:HHGFNDSXY:1:1222:14208:22122 2:N:0:GAACCTAG+TCCGCATA
TAAGTTTCGTCCTAAAAAGGACTCGTCAGGTAGCCTACTAGACTACGACTTACACGGTAGGTCGTGAA
CAAGCCCGGAGTACATCTCCTTTGGAACGAGGAGACCCCTCCGGTCTTGTCCAACCTCTGTTTCGGCA
+
FFFFFFFFFFFFFFFFF:FFFFFFFFFFFFF,FFFFFFFFFFFFFFFFFFFFFFFFFFF:FFFFFFFFF
FFFFFFFFFFFFFFFFFFFFFFFFFFFFFFFFFFFFFFFFFFFFFFFFFFFFFFFFFFFFFFFFFFFF
@A00155:342:HHGFNDSXY:1:1223:14172:30076 2:N:0:GAACCTAG+TCCGCATA
TACGACTTACACGGTAGGTCGTGAACAAGCCCGGAGTACATCTCCTTTGGAACTAGGAGACCCCTCCG
GTCTTGTCCAACCTCTGTTTCGGCAGAGGATACCGTGCTCCACAGCTGTAAGGCTGTGGGCCCG
+
```

FFFFFFFFFFFFFFFFFFFFFFFFFFFFFFFFFFFFFFFFFFFFFFFFFFFFFFFFFFFFFFFFFFFF
FFFFFFFFFFFFFFFFFFFFFFFFFFFFFFFFFFFFFFFFFFFFFFFFFFFFFFFFFFFFFFFF
@A00155:342:HHGFNDSXY:1:1223:17824:31704 2:N:0:GAACCTAG+TCCGCATA
TTGTCCAACCTCTGTTTCGGCAGAGGATACCGTGCTCCACAGCTGTAAGGCTGTGGGCCCGAGAGAAG
TCGCTCTCTCTCGCCCTCTACATCCCTCTCGGCATTGAGATGACCATAGGCCAGAGGTGAGTCCTTAA
+
:FFFFFFFFFFFFFFFFFFFFFFFFFFFFFF,FFFFFFFFFFF:FFFFFFFFFFFFFFFFFFFFFF::
FF:FFFFFFFFF:FFFFFFF::FF,FFFF:FFF::F:FFFFFFFFF,::,FF,FFFFFFFF:FF:FFF
@A00155:342:HHGFNDSXY:1:1224:2537:11835 2:N:0:GAACCTAG+TCCGCATA
GGTCTTGTCCAACCTCTGTTTCGGCAGAGGATACCGTGCTCCACAGCTGTAAGGCTGTGGGCCCGAGA
GAAGTCGCTCTCTCTCGCCCTCCACATCCCTCTCGGCATTGAGATGACCATAGGCCAGAGGTGAGTCC
+
FFFFFFFFFFFFFFFFFFFFFFFFFFFFFFFFFFFFFFFFFFFFFFFFFFFFFFFFFFFFFFFFFFFF
FFFFFFFFFFFFFFFFFFFFFFFFFFFFFFFFFFFFFFFFFFFFFFFFFFFFFFFFFFFFFFFFFFFF
@A00155:342:HHGFNDSXY:1:1224:2889:19523 2:N:0:GAACCTAG+TCCGCATA
ACTAGGAGACCCCTCCGGTCTTGTCCAACCTCTGTTTCGGCAGAGGATACCGTGCTCCACAGCTGTAA
GGCTGTGGGCCCGAGAGAAGTCGCTCTCTCTCGCCCTCCACATCCCTCTCGGCATTGAGATGACCATA
+
FFFFFFFFFFFFFFFFFFFF:FFFFFFFFFFFFFFFFFFFFFFFFFFFFFFFFFFFFFFFFFFFFFFF
FFFFFFFFFFFFF:FFFFFFFFFFFFFFFFFFFFFFFFFFFFFFFFFFFFFFFFFFFFFFFFFFFFFF
@A00155:342:HHGFNDSXY:1:1224:27145:32690 2:N:0:GAACCTAG+TCCGCATA
AGCCCGGAGTACATCTCCTTTGGAACTAGGAGACCCCTCCGGTCTTGTCCAACCTCTGTTTCGGCAGA
GGATACCGTGCTCCACAGCTGTAAGGCTGTGGGCCCGAGAGAAGTCGCTCTCTCTCGCCC
+
FFFFFFFFFFFFFFFFFFFFFFFF,FFFFFFFFFFFFFFFFFFFFFFFFFFFFFFFFFFFFFFFFFFF
FFFFFFFFFFFFFFFFFFFFFFFFFFFFFFFFFFFFFFFFFFFFFFFFFFFFFFFFFFFF
@A00155:342:HHGFNDSXY:1:1224:1325:33254 2:N:0:GAACCTAG+TCCGCATA
AAAAGGACTCGTCAGGTAGCCTACTAGACTACGACTTACACGGTAGGTCGTGAACAAGCCCGGAGTAC
ATCTCCTTTGGAACCAGGAGACCCCTCCGGTCTTGTCCAACCTCTGTTTCGGCAGAGGATAC
+
FFFFFFFFFFFFFFFFFFFFFFFFFFFFFFFFFFFFFFFFFFFFFFFFFFFFFFFFFFFFFFFFFFFF
FFFFFFFFFFFFFFFFFFFFFFFFFFFFFFFFFFFFFFFFFFFFFFFFFFFFFFFFFFFFFF
@A00155:342:HHGFNDSXY:1:1225:15944:13698 2:N:0:GAACCTAG+TCCGCATA
GACCCCTCCGGTCTTGTCCAACCTCTGTTTCGGCAGAGGATACCGTGCTCCACAGCTGTAAGGCTGTG
GGCCCGAGAGAAGTCGCTCTCTCTCGCCCTCCACATCCCTCTCGGCATTGAGATGACCATAGGC
+
FFFFFFFFFFFFFFFFFFFFFFFFFFFFFFFFFFFFFFFFFFFFFFFFFFFFFFFFFFFFFFFFFFFF
FFFFFFFFFFFFFFFFFFFFFFFFFFFFFFFFFFFFFFFFFFFFFFF:FFFFFFFFFFFFFFFF
@A00155:342:HHGFNDSXY:1:1225:30743:18850 2:N:0:GAACCTAG+TCCGCATA
AGCCCGGAGTACATCTCCTTTGGAACTAGGAGACCCCTCCGGTCTTGTCCAACCTCTGTTTCGGCAGA
GGATACCGTGCTCCACAGCTGTAAGGCTGTGGGCCCGAGAGAAGTCGCTCTCTCTCGCCCTCCACATC
+
FFFFFFFFFFFFFFFFFFF:FFFFFFFFFFFFFFFFFFFFFFFFFFF:FFFFFFFFFFFFFFFFFFFF
FFFFFFFFFFFFFF:FFFFFFFFFFFFFFFFFFFFFFFFFF:FFFFFFFFFFFFFFFFFFFFFFFFFF
@A00155:342:HHGFNDSXY:1:1226:19786:30467 2:N:0:GAACCTAG+TCCGCATA
GGACGAAACTTACACAACTCGAAGAGTAACGACGGTTTCGTCCTTGGAACGGACTCATCAGGGAGCCT
ACTAGACTCCGACCGTCATGGGGGTTCAAACCCATGCCCCGCCACACCGCCTTAGATCAGCTGTGTCC
+
FFFFFFFFFFFFFFFFFFFFFFFFFFFFFFFFFFFFFFFFFFFFFFFFFFFFFFFFFFFFFFFFFFFF
FFFFFFFFFFFFFFFFFFFFFFFFFFFFFFFFFFFFFFFFFFFFFFFFFFFFFFFFFFFFFFFFFFFF
@A00155:342:HHGFNDSXY:1:1227:14841:3333 2:N:0:GAACCTAG+TCCGCATA
GTGAACAAGCCCGGAGTACATCTCCTTTGGAACTAGGAGACCCCTCCGGTCTTGTCCAACCTCTGTTT
CGGCAGAGGATACCGTGCTCCACAGCTGTAAGGCTGTGGGCCCGAGAGAAGTCGCTCTCTCTCGCCCT
+

FFFFFFFFFFFFFFFFFFFFFFFFFFFFFFFFFFFFFFFFFFFFFFFFFFFFFFFFFFFFFFFFFFF:
FFFFFFFFF:FFFFFFFFFFFFFFFFFFFFFFFFFFFFFFFFFFFFFFFFFFFFFFFFFFFFFFFFFFF
@A00155:342:HHGFNDSXY:1:1227:27208:17738 2:N:0:GAACCTAG+TCCGCATA
GAACTAGGAGACCCCTCCGGTCTTGTCCAACCTCTGTTTCGGCAGAGGATACCGTGCTCCACAGCTGT
AAGGCTGTGGGCCCGAGAGAAGTCGCTCTCTCTCGCCCTCCACATCCCTCTCGGCATTGAGATGACCA
+
FFFFFFFFFFFFFFFFFFFFFFFFFFFFFFFFFFFFFF:FFFF:FFFFFFFFFFFFFFFFFF,FFFFF
FFFFFFFFFFFFFFFFFFFFFF:FFFFFFFFFFFFFFFFFFFFFFFFFFFFFFFFFF,FFFFFFFFFF
@A00155:342:HHGFNDSXY:1:1227:18258:27289 2:N:0:GAACCTAG+TCCGCATA
TTTTAGGACGAAACTTACACAACTCGTAGAGTAACGACGGTTTCGTCCTTGGAACGGACTCATCAGGG
AGCCTACTAGACTCCGACCGTCATGGGGGTTCAAACCCATGCCCCGCCACACCGCCTTAGATCAGCTG
+
FFFFFFFFFFFFFFFFFFFFFFFFFFFFFFFFFFFFFFFFFFFFFFFFFFFFFFFFFFFFFFFFFFFF
FFFFFFFFFFFFFFFFFFFFFFFFFFFFFFFFFFFFFFF:FFFFFFFFFFFFFFFFFFFFFFFFFFFF
@A00155:342:HHGFNDSXY:1:1227:4327:27586 2:N:0:GAACCTAG+TCCGCATA
AAGGACGAAACCGTCGTTACTCTTCGAGTTGTGTAAGTTTCGTCCTAAAAAGGACTCGTCAGGTAGCC
TACTAGACTACGACTTACACGGTAGGTCGTGAACAAGCCCGGAGTACATCTCCTTTGGAACTAGGAGA
+
FFFFFFFFFFFFFFFFFFFFFFFFFFFF:FFFFFFFFFFFFFFFFFFFFFFFFFFFFFFFFFFFF,FF
FFFFFFFFFFFFFFF:FFFFFFFFFFFFFFFFFFFF:FFFFFFFFFFFFFFFFFFFFFFFFFFFFFF:
@A00155:342:HHGFNDSXY:1:1227:18620:28322 2:N:0:GAACCTAG+TCCGCATA
TTTTAGGACGAAACTTACACAACTCGTAGAGTAACGACGGTTTCGTCCTTGGAACGGACTCATCAGGG
AGCCTACTAGACTCCGACCGTCATGGGGGTTCAAACCCATGCCCCGCCACACCGCCTTAGATCAGCTG
+
FFFFFFFFFFFFFFFFFFFFFFFFFFFFFFFFFFFFFFFFFFFFFFFFFFFFFFFFFFFFFFFFFFFF
FFFFF:FFFFFFFFFFFFFFFFFFFFFFFFFFFFFFFFFFFFFFFFFFFFFFFFFFFFFFFFFFFFFF
@A00155:342:HHGFNDSXY:1:1227:22914:33411 2:N:0:GAACCTAG+TCCGCATA
CTGGCCTATGGTCATCTCAATGCCGAGAGGGATGTGAAGGGCGAGAGAGAGCGACTTCTCTCGGGCCC
ACAGCCTTACAGCTGTGGAGCACGGTATCCTCTGCCGAAACAGAGGTTGGACAAGACCGGAG
+
FFFFFFFFFFFFFFFFFFFFFFFFFFFFFFFFFFFFFFFFFFFFFFFFFFFFFFFFFFFFFFFFFFFF
FFFFFFFFFFFFFFFFFFFFFFFFFFFFFFFFFFFFFFFFFFFFFFFFFFFFFFFFFFFFFF
@A00155:342:HHGFNDSXY:1:1228:32262:14246 2:N:0:GAACCTAG+TCCGCATA
CGGCAGAGGATACCGTGCTCCACAGCTGTAAGGCTGTGGGCCCGAGAGAAGTCGCTCTCTCTCGCCCT
CCACATCCCTCTCGGCATTGAGATGACCATAGGCCAGAGGTGAGTCCTTAAGTGGACACAGCTGATCT
+
FFFFFFFFFFFFFFFFFFFFFFFFFFFFFFFFFFFFFFFFFFFFFFFF:FFFFFFFFFFFFFFFFFFF
FFFFFFFFFFFFF::FFF:FFFFFFF:FFFFF:FF,F,FFFFFFFFFF:FFFF:FFF,FFFFFFFFFF
@A00155:342:HHGFNDSXY:1:1228:17544:16814 2:N:0:GAACCTAG+TCCGCATA
GGACTCGTCAGGTAGCCTACTAGACTACGACTTACACGGTAGGTCGTGAACAAGCCCGGAGTACATCT
CCTTTGGAACTAGGAGACCCCTCCGGTCTTGTCCAACCTCTGTTTCGGCAGAGGATACCGTGCTCCAC
+
FFFFFFF:FFFFFFFFFFFFFFFFFFFFFFFFFFFFFFFFFFF:FFFFFFFFFFFFFFFFFFFF:FFF
FFFFFFFFFFFFFFFFFFFFFFFFFFFFFFFFFFFFFFFFFFFFFFFFFFFFFFFFFFFFFFFFFFFF
@A00155:342:HHGFNDSXY:1:1228:6108:17487 2:N:0:GAACCTAG+TCCGCATA
AGATCAGCTGTGTCCACTTAAGGACTCACCTCTGGCCTATGGTCATCTCAATGCCGAGAGGGATGTGA
AGGGCGAGAGAGAGCGACTTCTCTCGGGCCCACAGCCTTACAGCTGTGGAGCACGGTATCCTCTGCCG
+
FFFFFFFFFFFFFFF:FFFFFFF:FFF:FFFFFFFFFFFFFF:FFFFFFFFFFFFFFFFFFFFFFFFF
FFFFFFFFFFFFFFFFFFFFFFFFFFFFFFFFFFFFF:FFFFFFFFFFFFFFFFFFF:FFFFFFFFFF
@A00155:342:HHGFNDSXY:1:1228:10511:18223 2:N:0:GAACCTAG+TCCGCATA
GTGAACAAGCCCGGAGTACATCTCCTTTGGAACTAGGAGACCCCTCCGGTCTTGTCCAACCTCTGTTT
CGGCAGAGGATACCGTGCTCCACAGCTGTAAGGCTGTGGGCCCGAGAGAAGTCGCTCTCTCTCG
+

FFFFFFFFFFFFFFFFFFFFFF:FFFFFFFFFFFFFFFFFFFFFFFFFFFF:FFFFFFFFFFFFFFFF
FFFFFFFFFFFFFFFFFFFFFFFFFF:FFFFFFF:FFFFFFFFFFFFF:FFFFFFFFFFFFFFF
@A00155:342:HHGFNDSXY:1:1228:3794:23813 2:N:0:GAACCTAG+TCCGCATA
GTTGTGTAAGTTTCGTCCTAAAAAGGACTCGTCAGGTAGCCTACTAGACTACGACTTACACGGTAGGT
CGTGAACAAGCCCGGAGTACATCTCCTTTGGAACTAGGAGACCCCTCCGGTCTTGTCCAACCTCTGTT
+
FFFFFFFFFFF,FFFFFFFFFFFFFFFFFFFFFFFFFFFFFFFFFFFFFFFFFFFFFFFFFFFFFFFF
FFFFFFFFFFFFFFFFFFFFFFFFFFFFFFFFFFFFFFFFFFFFFFFFFFFFFFFFFFFFFFFFFF:F
@A00155:342:HHGFNDSXY:1:1228:27977:27054 2:N:0:GAACCTAG+TCCGCATA
CGGCAGAGGATACCGTGCTCCACAGCTGTAAGGCTGTGGGCCCGAGAGAAGTCGCTCTCTCTCGCCCT
CCACATCCCTCTCGGCATTGAGATGACCATAGGCCAGAGGTGAGTCCTTAAGTGGACACAGCTGATCT
+
FFFFFFFFFFFFFFFFFFFFFFFFFFFFFFFFFFFFFFFFFFFFFFFFF:FFFFFFFFFFFFFFFFFF
FFFFFFFFFFFFFFFFFFFFFFF:FFFFFFFFFFFFFFFFFFFFFFFFFFFFFFFFFFFFFFFFFFFF
@A00155:342:HHGFNDSXY:1:1228:4390:33270 2:N:0:GAACCTAG+TCCGCATA
TTACACGGTAGGTCGTGAACAAGCCCGGAGTACATCTCCTTTGGAACTAGGAGACCCCTCCGGTCTTG
TCCAACCTCTGTTTCGGCAGAGGATACCGTGCTCCACAGCTGTAAGGCTGTGGGCCCGAGAGAAGTCG
+
FFFFFFFFFFFFFFFFFFFFFFFFFFFFFFFFFFFFFFFFFFFFFFFFFFFFFFFFFFFFFFFFFFFF
FFFFFFFFFFFFFFFFFFFFFFFFFF:FFFFFFFFFFFFFFFFFFFFFFFFFFFFFFFFFFFFFFFFF
@A00155:342:HHGFNDSXY:1:1229:32127:13166 2:N:0:GAACCTAG+TCCGCATA
GACCCCTCCGGTCTTGTCCAACCTCTGTTTCGGCAGAGGATACCGTGCTCCACAGCTGTAAGGCTGTG
GGCCCGAGAGAAGTCGCTCTCTCTCGCCCTCCACATCCCTCTCGGCATTGAGATGACCATAGGCCAGA
+
FFFFFFFFFFFFFFFFFFFFFFFFFFF:FFFFFFFFFFFFFFFFFFFFFFFFFFFFFFFFFFFF:FFF
FFFFFFFFFFFFFFFFFFFFFFFFFFFFFFFFFFFFFFFFFFFFFFFFFFFFFFFFFF:FFF:FFFFF
@A00155:342:HHGFNDSXY:1:1229:26684:23218 2:N:0:GAACCTAG+TCCGCATA
AAAAGGACTCGTCAGGTAGCCTACTAGACTACGACTTACACGGTAGGTCGTGAACAAGCCCGGAGTAC
ATCTCCTTTGGAACCAGGAGACCCCTCCGGTCTTGTCCAACCTCTGTTTCGGCAGAGGATACCGTGCT
+
FFFFFFFFFFFFFFFFFFFFFFFFFFFFFFFFFFFFFFFFFFFFFFFFFFFFFFFFFFFFFFFFFFFF
FFFFFFFFFFFFFFFFFFFFFFFFFFFFFFFFFFFFFFFFFFFFFFFFFFFFFFFFFFFFFFFFFFFF
@A00155:342:HHGFNDSXY:1:1230:13955:3176 2:N:0:GAACCTAG+TCCGCATA
GGTCGTGAACAAGCCCGGAGTACATCTCCTTTGGAACTAGGAGACCCCTCCGGTCTTGTCCAACCTCT
GTTTCGGCAGAGGATACCGTGCTCCACAGCTGTAAGGCTGTGGGCCCGAGAGAAGTCGCTCTCTCTCG
+
FFFFFFFFFFFFFFFFFFFFFFFFFFFFFFFFFFFFFFFFFFFFFFFFFFFFFFFFFFFFFFFFFFFF
FFFFFFFFFFFFFFFFFFFFFFFFFFFFFFFFFFFFFFFFFFFFF:FFFFFFFFFFFFFFFFFFFFFF
@A00155:342:HHGFNDSXY:1:1230:26187:6417 2:N:0:GAACCTAG+TCCGCATA
ACGAAACCGTCGTTACTCTTGGAGTTGTGTAAGTTTCGTCCTAAAAAGGACTCGTCAGGTAGCCTACT
AGACTACGACTTACACGGTAGGTCGTGAACAAGCCCGGAGTACATCTCCTTTGGAACTAGGAGACCCC
+
FFFFFFFFFFFFFFFFFFFFFFFFFF:FFFFF:F:FFFFFFFFFF,,FF:FFFFFFFFFFFFFFFFFF
FFF:FFFFFFFFFFFFFFFFFFFFFFFFFFFFFFFFFFFFFFFFFFFFFF:FFFFFFFFFFFFFFFFF
@A00155:342:HHGFNDSXY:1:1230:1533:11412 2:N:0:GAACCTAG+TCCGCATA
TAGACTACGACTTACACGGTAGGTCGTGAACAAGCCCGGAGTACATCTCCTTTGGAACTAGGAGACCC
CTCCGGTCTTGTCCAACCTCTGTTTCGGCAGAGGATACCGTGCTCCACAGCTGTAAGGCTGTGGGCCC
+
FFFFFFFFFFFFFFFFFFFFFFFFFFFFFFFFFFFFFFFFFFFFFFFFFFFFFFFFFFFFFFFF:FFF
FFFFFFFFFFFFFFFFFFFFFFFFFFFFFFFFFFFF:FFFFFFFFFFFFFFFFFFFFFFFFFFFFFFF
@A00155:342:HHGFNDSXY:1:1230:11749:12571 2:N:0:GAACCTAG+TCCGCATA
TGTCCACTTAAGGACTCACCTCTGGCCTATGGTCATCTCAATGCCGAGAGGGATGTGAAGGGCGAGAG
AGAGCGACTTCTCTCGGGCCCACAGCCTTACAGCTGTGGAGCACGGTATCCTCTGCCGAAACAGAGGT
+

```
FFFFFFFFFFFFFFFFFFFFFFFFFFFFFFFFFFFFFFFFFFFFFFFFFFFFFFFFFFFFFFFFFFFFFF
FFFFFFFFFFFFFFFFFFFFFFFFFFFFFFFFFFFFFFFFFFFFFFFFFFFFFFFFFFFFFFFFFFFFFF
@A00155:342:HHGFNDSXY:1:1230:19325:13636 2:N:0:GAACCTAG+TCCGCATA
ACGACTTACACGGTAGGTCGTGAACAAGCCCGGAGTACATCTCCTTTGGAACTAGGAGACCCCTCCGT
TCTTGTCCAACCTCTGTTTCGGCAGAGGATACCGTGCTCCACAGCTGTAAGGCTGTGGGCCCGAGAGA
+
FFFFFFFFFFFFFF,FFFFFFFFFFFFFFFFFFFFFFFFFFFFFFFFFFFFFFFFFFFFFFFFFFFFFF
FFFFFFFFFFFFFFFFFFFFFFFFFFFFFFFFFFFFFFFFFFFFFFFFFFFFFFFFFFFFFFFFFFFFFF
@A00155:342:HHGFNDSXY:1:1230:29017:34773 2:N:0:GAACCTAG+TCCGCATA
GTTTCGTCCTAAAAAGGACTCGTCAGGTAGCCTACTAGACTACGACTTACACGGTAGGTCGTGAACAA
GCCCGGAGTACATCTCCTTTGGAACTAGGAGACCCCTCCGGTCTTGTCCAACCTCTGTTTCGGCAGAG
+
FFFF:FFFFFFFFFFFFFFFFFFFFFFFFFFFFFFFFFFFFFFFFFFFFFFFFFFFFFFFFFFFFFFFF
FFFFFFFFFFFFFFFFFFFFFFFFFFFFFFFFFFFFFFFFFFFFFFFFFFFFFFFFFFFFFFFFFFFFFF
@A00155:342:HHGFNDSXY:1:1231:18475:12790 2:N:0:GAACCTAG+TCCGCATA
AGTACATCTCCTTTGGAACTAGGAGACCCCTCCGGTCTTGTCCAACCTCTGTTTCGGCAGAGGATACC
GTGCTCCACAGCTGTAAGGCTGTGGGCCCGAGAGAAGTCGCTCTCTCTCGCCCTCCACATCCCTCTCG
+
FFFFFFFFFFFFFFFFFFFFFFFFFFFFFFFFFFFFFFFFFFFFFF,FFFFFFFFFFFFFFFFFFFFFFF
FFFFFFFFFFFFFFFFFFFFF,FFFFFFFFFFF:FF,FFFFFFFFFFFFFFFFFFFFFFFFFFFFFFFFF
@A00155:342:HHGFNDSXY:1:1231:26404:34162 2:N:0:GAACCTAG+TCCGCATA
CGGCAGAGGATACCGTGCTCCACAGCTGTAAGGCTGTGGGCCCGAGAGAAGTCGCTCTCTCTCGCCCT
TCACATCCCTCTCGGCATTGAGATGACCATAGGCCAGAGGTGAGTCCTTAAGTGGACACAGCTG
+
FFFFFFFFFFFFFFF,FFFFFFFFFFFFFFFFFFFFFFFFFFFFFFFFFFFFFFFFFFFFFFFFFFFFFF
FFFFFFFFFFFFFFFFF,FFFFFFFFFFFFFFFFFFFFFFFFFFFFFFFFFFFFFFFFFFFFFF
@A00155:342:HHGFNDSXY:1:1232:18014:1313 2:N:0:GAACCTAG+TCCGCATA
TACGACTTACACGGTAGGTCGTGAACAAGCCCGGAGTACATCTCCTTTGGAACCAGGAGACCCCTCCG
GTCTTGTCCAACCTCTGTTTCGGCAGAGGATACCGTGCTCCACAGCTGTAAGGCTGTGGGCCCG
+
FFFFFFFFFFFFFFF,FFFFFF:FFFFFFFFFFFFFFFFFFFFFFFFF,FFFF,FFFFFFFFFFFFFFFF
FFFF,FFFFFFFFFFFFFFFFFFFFFFFFFF:,FFFFFFFFFFFFFF:FFFFFFF:FFFFFFFFF
@A00155:342:HHGFNDSXY:1:1232:12409:19476 2:N:0:GAACCTAG+TCCGCATA
CTGGCCTATGGTCATCTCAATGCCGAGAGGGATGTGGAGGGCGAGAGAGAGCGACTTCTCTCGGGCCC
ACAGCCTTACAGCTGTGGAGCACGGTATCCTCTGCCGAAACAGAGGTTGGACAAGACCGGAG
+
FFFFF:FFFFFFFFFFFFFFFFFFFFFFFFFF:FF:FFFFFFFFFFFFFFFFFFFFFFFFFFFFFFFFFF
FFFFFFFF:FFFFFFFFFFFFFFFFFFFFFFFFFFFFFFFFFFFFFFFFFFFFFFFFFFF
@A00155:342:HHGFNDSXY:1:1232:31105:20353 2:N:0:GAACCTAG+TCCGCATA
CAGGTAGCCTACTAGACTACGACTTACACGGTAGGTCGTGAACAAGCCCGGAGTACATCTCCTTTGGA
ACTAGGAGACCCCTCCGGTCTTGTCCAACCTCTGTTTCGGCAGAGGATACCGTGCTCCACAGCTGTAA
+
FFFFFFFFFFFFFFFFFFFFFFFFFFFFFFFFFFFFFFFFFFFFFFFFFFFFFFFFFFFFFFFFFFFFFF
FFFFFFFFFFFFFFFFFFFFFFFFFFFFFFFFFFFFFFFFFFFFFFFFFFFFFFFFFFFFFFFFFFFFFF
@A00155:342:HHGFNDSXY:1:1232:3839:22576 2:N:0:GAACCTAG+TCCGCATA
CTCTTCGAGTTGTGTAAGTTTCGTCCTAAAAAGGACTCGTCAGGTAGCCTACTAGACTACGACTTACA
CGGTAGGTCGTGAACAAGCCCGGAGTACATCTCCTTTGGAACTAGGAGACCCCTCCGGTCTTGTCCAA
+
FFFFFFFFFFFFFFFFFFFFFFFFFFFFFFF:FFFFFFFFFFFFFFF,FFFFFFFFFFFFFFFFFFFFFF
FF:FFFFFFFFFF:FFFF,FFFFFFFFFFFFFFFFF:FF:FFF:F:FFFF,FFFFFFFFFFF:FFF:F
@A00155:342:HHGFNDSXY:1:1232:30391:31767 2:N:0:GAACCTAG+TCCGCATA
CCTACTAGACTACGACTTACACGGTAGGTCGTGAACAAGCCCGGAGTACATCTCCTTTGGAACCAGGA
GACCCCTCCGGTCTTGTCCAACCTCTGTTTCGGCAGAGGATACCGTGCTCCACAGCTGTAAGGCTGTG
+
```

```
FFFFFFFFFFFFFFFFFFFFFFFFFFFFFFFFFFFFFFFFFFFFFFFFFFFFFFFFFFF:FFFFFF
FFFFFFFFFFFFFFFFFFFFFFFFFFFFFFFFFFFFFFFFFFFFFFFFFFFFFFFFF:FFFFFFFF
@A00155:342:HHGFNDSXY:1:1233:2275:3333 2:N:0:GAACCTAG+TCCGCATA
GGAGTACATCTCCTTTGGAACTAGGAGACCCCTCCGGTCTTGTCCAACCTCTGTTTCGGCAGAGGATA
CCGTGCTCCACAGCTGTAAGGCTGTGGGCCCGAGAGAAGTCGCTCTCTCTCGCCCTCTACATCCCTCT
+
FFFFFFFFFFFFFFFFFFFFFFFFFFFFFFFFFFFFFFFFFFFFFFFFFFFFFFFFFFFFFFFFFFFF
FFFFFFFFFFFFFF::FFFFFFFFFFFFFFFFFFFFFF:FFFFFFFFFFFFFFFFFFFFFFFFFFFFF
@A00155:342:HHGFNDSXY:1:1233:12726:9909 2:N:0:GAACCTAG+TCCGCATA
GAACTAGGAGACCCCTCCGGTCTTGTCCAACCTCTGTTTCGGCAGAGGATACCGTGCTCCACAGCTGT
AAGGCTGTGGGCCCGAGAGAAGTCGCTCTCTCTCGCCCTCCACATCCCTCTCGGCATTGAGATGACGA
+
F:FFFFFFFFFFFFFFFFFF:FFFFFFFFFFFFF:F,:FFF:FFFFFFFFFFFFFFFFF::FFFF:FF
FFFFFFFFFF:FFFFFFFFF:FFFFFFFFFFFFFFFFFFFFFF:FFFFFFFFFFF,,:FFFF:FFFFF
@A00155:342:HHGFNDSXY:1:1233:4417:26647 2:N:0:GAACCTAG+TCCGCATA
GAAACCGTCGTTACTCTTCGAGTTGTGTAAGTTTCGTCCTAAAAAGGACTCGTCAGGTAGCCTACTAG
ACTACGACTTACACGGTAGGTCGTGAACAAGCCCGGAGTACATCTCTTTTGGAACTAGGAGACCCCTC
+
FFFFFFFFFFFFFFFFFFFFFFFFFFFFFFFFFFFFFFFFFFFF:FFFFFFFFFFFFFFFFFFFFFFF
FFFFFFFFFFFFFFFFFFFFFFFFFFFFFFFFFFFFFFFFFFFFFFFF:FFFFFFFFFFFFFFFFFFF
@A00155:342:HHGFNDSXY:1:1234:18656:28416 2:N:0:GAACCTAG+TCCGCATA
GGACTCGTCAGGTAGCCTACTAGACTACGACTTACACGGTAGGTCGTGAACAAGCCCGGAGTACATCT
CCTTTGGAACCAGGAGACCCCTCCGGTCTTGTCCAACCTCTGTTTCGGCAGAGGATACCGTGCTCCAC
+
FFFFFFFFFFFFF:FFFFFFFFFFFFFFFFFFFFFFFFFFF:FFFFFFFFFFFFFFFFFFFFFFFFFF
FFFFFFFFFFFFFFFFFFFFFFFFFFFFFFFFFFFFFFF:FFFFFFFFFFFFFFFFFFFFF:FFFFF:F
@A00155:342:HHGFNDSXY:1:1235:20491:13808 2:N:0:GAACCTAG+TCCGCATA
TTTCGTCCTAAAAAGGACTCGTCAGGTAGCCTACTAGACTACGACTTACACGGTAGGTCGTGAACAAG
CCCGGAGTACATCTCCTTTGGAACCAGGAGACCCCTCCGGTCTTGTCCAACCTCTGTTTCGGCAGAGG
+
FFFFFFFFFFFFF:FFFFFFFFFFFFFFFFFFFFFFFFFFFFFFFFFFFFFFFFF,FFFFFFFFFFFF
FFFFFFFFFFFFFFFFFFFFFFFFFFFFFFFFFFFFFFFFFFFFFFFFFFFFFFFFFFFFFFFFFFFF
@A00155:342:HHGFNDSXY:1:1236:16866:4836 2:N:0:GAACCTAG+TCCGCATA
AAAAAGGACTCGTCAGGTAGCCTACTAGACTACGACTTACACGGTAGGTCGTGAACAAGCCCGGAGTA
CATCTCCTTTGGAACTAGGAGACCCCTCCGGTCTTGTCCAACCTCTGTTTCGGCAGAGGATACCGTGC
+
FFFF:FFFFFFFFFFFFF:FFFFFFFFFFFFFFFFFFFFFFFFFFFFFFFFFFFFFFFFFFFFFFFFF
FFFFFFFFFFFFFFFFFFFFFFFFFFFFFFFFFFFFFFFFFFFFFFFFFFFFFFFFFFFFFFFFF:FF
@A00155:342:HHGFNDSXY:1:1236:26928:12962 2:N:0:GAACCTAG+TCCGCATA
AAACTTACACAACTCGAAGAGTAACGACGGTTTCGTCCTTGGAACGGACTCATCAGGGAGCCTACTAG
ACTCCGACCGTCATGGGGGTTCAAACCCATGCCCCGCCACACCGCCTTAGATCAGCTGTGTCCACTTA
+
FFFFFFFFFFFFFFFFFFFFFFFFFFFFFFFFFFFFFFFFFFFFFFFFFFFFFFFFFFFFFFFFFFFF
FFFFFFFFFF:FFFFFFFFFFFFFFFFFFFFFFFFFFFFFFFFFFFFF:FFFFFFFFFFFFFFF:F
@A00155:342:HHGFNDSXY:1:1236:28040:25567 2:N:0:GAACCTAG+TCCGCATA
GGTCGTGAACAAGCCCGGAGTACATCTCCTTTGGTACTAGGAGACCCCTCCGGTCTTGTCCAACCTCT
GTTTCGGCAGAGGATACCGTGCTCCACAGCTGTAAGGCTGTGGGCCCGAGAGAAGTCGCTCTCTCTCG
+
FFFFFFFFFFFF:F:FFFFFFFFFFFFFFFFFFFFFFFFFFFFFFFFFFFFFFFFFFFFFFFFFFFFF
FFFFFFFFFFFFFFFFFFFFFFFFFFFFFFFFFF:FFFFFFFFFFFFFFFFFFF,FFFF:FFFFFFFFF
@A00155:342:HHGFNDSXY:1:1236:2772:28369 2:N:0:GAACCTAG+TCCGCATA
CGTCCTAAAAAGGACTCGTCAGGTAGCCTACTAGACTACGACTTACACGGTAGGTCGTGAACAAGCCC
GGAGTACATCTCCTTTGGAACTAGGAGACCCCTCCGGTCTTGTCCAACCTCTGTTTCGGCAGAGGATA
+
```

FFFFFF:FFF,FFFFFFFFFFFFFFFFFFFFFFFFFFFFFFFFFFFFFFFFFFFFFFFFFFFFFFFFF
FFFFFFFFFFFFFFFFFFFFFFFFFFFFFFFFFFFFFFFFFFFFFFFFFFFFFFFFFFFFFFFFFFFF
@A00155:342:HHGFNDSXY:1:1237:15655:16329 2:N:0:GAACCTAG+TCCGCATA
TTGGAACTAGGAGACCCCTCCGGTCTTGTCCAACCTCTGTTTCGGCAGAGGATACCGTGCTCCACAGC
TGTAAGGCTGTGGGCCCGAGAGAAGTCGCTCTCTCTCGCCCTCTACATCCCTCTCGGCATTGAGATGA
+
,FFFFFFFFFFFFFFFFFFFFFFFF:FFFFFFFFFFFFFF:FFFFFFFFFFFFFFFFFFFFFFFFFFF
FFFFFFFFFFFFFFFFFFFFFFFFFFFFFFFFFFFFFFFFFFFFFFFFFFFFFF:FFF:FFFFFFFFF
@A00155:342:HHGFNDSXY:1:1237:17626:31548 2:N:0:GAACCTAG+TCCGCATA
GTGTAAGTTTCGTCCTAAAAAGGACTCGTCAGGTAGCCTACTAGACTACGACTTACACGGTAGGTCGT
GAACAAGCCCGGAGTACATCTCCTTTGGAACTAGGAGACCCCTCCGGTCTTGTCCAACCTCTGTTTCG
+
FF:FFFFFFF:FFFFFFFFFFFFFFFFFFFFFFFFFFF:FFFFFFFFFFFFFFFFFFFFFF:FFFFFF
FFFFFFFFFFFFFFF:FFFFFFFFFFFFFFFFFFFFFFFFFFFFFFFFFFFFFF:FFFFFFFFFFFFF
@A00155:342:HHGFNDSXY:1:1238:27434:9674 2:N:0:GAACCTAG+TCCGCATA
TCTCCTTTGGAACCAGGAGACCCCTCCGGTCTTGTCCAACCTCTGTTTCGGCAGAGGATACCGTGCTC
CACAGCTGTAAGGCTGTGGGCCCGAGAGAAGTCGCTCTCTCTCGCCCTCCACATCCCTCTCGGCATTG
+
FFFFFFFFFFF:FFFFFFFFFFFFFFFFFFFFFFFFFFFFFFFFFFFFFFFFFFFFFFFFFFFFFFFF
FFFFFFFFFFFFFFFFFFFFFFFFFF:FFFFFFFFFFFFFFFFFFFFFFFFFFFFFFFFFFFFF::FF
@A00155:342:HHGFNDSXY:1:1240:28230:4758 2:N:0:GAACCTAG+TCCGCATA
GTAGGTCGTGAACAAGCCCGGAGTACATCTCCTTTGGAACTAGGAGACCCCTCCGGTCTTGTCCAACC
TCTGTTTCGGCAGAGGATACCGTGCTCCACAGCTGTAAGGCTGTGGGCCCGAGAGAAGTCGCTCTCTC
+
FF:FFFFFFFFFFFFFFFFFFFFFFFFFFFFF:FFFFFFFFFFFFFFFFFFFFFFFFFFFFFFFFFFF
FFFFFFFFFFFFFFFFFFFFFFFFFFFFFFFFFFFFFFFFF:FFFFFFFFFFFFFFFFFFFFFFFFFF
@A00155:342:HHGFNDSXY:1:1240:16233:6934 2:N:0:GAACCTAG+TCCGCATA
CAAGGACGAAACCGTCGTTACTCTTTGAGTTGTGTAAGTTTCGTCCTAAAAAGGACTCGTCAGGTAGC
CTACTAGACTACGACTTACACGGTAGGTCGTGAACAAGCCCGGAGTACATCTCCTTTGGAACTAGGAG
+
FFFFFFFFFFFFFFFFFFFFFFFF,FFFFFFFFFFFFFFFFFFFFFFFFFFFFFFFFFFFFFFFFFFF
FFFFFFFFFFFFFFFFFFFFFFFFFFFFFFFFFFFFFFFFFFFFFFFF:FFFFF,FFFFFFFFFFFFF
@A00155:342:HHGFNDSXY:1:1240:4137:9408 2:N:0:GAACCTAG+TCCGCATA
TACGACTTACACGGTAGGTCGTGAACAAGCCCGGAGTACATCTCCTTTGGAACTAGGAGACCCCTCCG
GTCTTGTCCAACCTCTGTTTCGGCAGAGGATACCGTGCTCCACAGCTGTAAGGCTGTGGGCCCG
+
FFFFFFFFFFFFF:FFFFFFFFFFFFFFFFFFFFFFFFFFFFFFFFFFFFF:FFFFFF:FFFFFFFFF
FFFFFFFFFFFFFFFFFFF:FFFFFFFFFFFFFFF:FFFFFFFFFFFFFFFFFFFFFFFF:FFF
@A00155:342:HHGFNDSXY:1:1240:6370:10864 2:N:0:GAACCTAG+TCCGCATA
TACGACTTACACGGTAGGTCGTGAACAAGCCCGGAGTACATCTCCTTTGGAACTAGGAGACCCCTCCG
GTCTTGTCCAACCTCTGTTTCAGCAGAGGATACCGTGCTCCACAGCTGTAAGGCTGTGGGCCCG
+
FFFFFFFFFFFFFFFFFFFFFFFFFFFFFFFFFFFFFFFFFFFFFFFFFFFFFFFFFFF:FFFFFFFF
FFFFFFFFFFFFFFFFFFFFF:FFFFFFFFFFFFFFFFFFFFFFFFFFFFFFFFFFFFFFFFFFFF
@A00155:342:HHGFNDSXY:1:1240:4580:18881 2:N:0:GAACCTAG+TCCGCATA
ACTCTTGGAGTTGTGTAAGTTTCGTCCTAAAAAGGACTCGTCAGGTAGCCTACTAGACTACGACTTAC
ACGGTAGGTCGTGAACAAGCCCGGAGTACATCTCCTTTGGAACTAGGAGACCCCTCCGGTCTTGTCCA
+
FFFF:FFFFFFFFFFFFFF:FFFFFFFF:FFFFFFFFFFFFFFFFFF:FFFFF:FFFFFF,F:FF:FF
FFFFFFFFFF::FFF,FFFFFFFFFF,FFFFF:F:FFFF:FFFFFFFFFF,FFF:FFFFFFFFFFFFF
@A00155:342:HHGFNDSXY:1:1241:6361:3114 2:N:0:GAACCTAG+TCCGCATA
AGCCCGGAGTACATCTCCTTTGGAACTAGGAGACCCCTCCGGTCTTGTCCAACCTCTGTTTCGGCAGA
GGATACCGTGCTCCACAGCTGTAAGGCTGTGGGCCCGAGAGAAGTCGCTCTCTCTCGCCCTCTACATC
+

FFFFFFFFFFFFFFFFFFFFFFFFFFFFFFFFFFFFFFFFFFF:FFFFFFFFFFFFFFFFFFFFFFFF
FFFFFFFFFFFFFFFFFFFFFFFFFFFFFFF:FFFFFF:FFFFFFFFFFFF:FFFFFFFFFFFFFFFF
@A00155:342:HHGFNDSXY:1:1241:27416:11365 2:N:0:GAACCTAG+TCCGCATA
TCCTAAAAAGGACTCGTCAGGTAGCCTACTAGACTACGACTTACACGGTAGGTCGTGAACAAGCCCGG
AGTACATCTCCTTTGGAACTAGGAGACCCCTCCGGTCTTGTCCAACCTCTGTTTCGGCAGAGGATACC
+
FFFFFFFFFFFFFFFF:FFFFFFFFFFFFFFFFFFFFFFFFFFFFFFFFFFFFFFFFFFFFFF:FFFFF
FFFFFFFFFFFFFFFFFFFFFFFFFFFFFFFFFFFFFFFF:FFFFFFFFFFFFFFFFFFFFFFFFFFFF
@A00155:342:HHGFNDSXY:1:1241:3188:21010 2:N:0:GAACCTAG+TCCGCATA
GACCCCTCCGGTCTTGTCCAACCTCTGTATCGGCAGAGGATACCGTGCTCCACAGCTGTAAGGCTGTG
GGCCCGAGAGAAGTCGCTCTCTCTCGCCCTCCACATCCCTCTCGGCATTGAGATGACCATAGGC
+
FFFFFFFFFFFFFFFFFFFFFFFFFFFFFFFFFFFFFFFFFFFFFFFFFFFFFFFFF:FFFFFFFFFF
FFFFFF:FFFFF:FFFFFFFFFFFF:FFFFFFFFFFFFFFFFFFFFFFFFF:FFFFFFFFFFFF
@A00155:342:HHGFNDSXY:1:1241:9661:33379 2:N:0:GAACCTAG+TCCGCATA
CCGTTCCAAGGACGAAACCGTCGTTACTCTTTGAGTTGTGTAAGTTTCGTCCTAAAAAGGACTCGTCA
GGTAGCCTACTAGACTACGACTTACACGGTAGGTCGTGAACAAGCCCGGAGTACATCTCCTTTGGAAC
+
FFFFFFFFFFFFFFFFFFFF:FFFFFFF:FFFFFFFFFFFFFFFFFFFFFFF:F:FFFFFFFFFFFF
FF,FFFFFFFFFFFFFFFFFFFFFFFFFFFF,FFFFFFFFFFFFFFFFFFFFFFFFFFFFFFFFFFFFF
@A00155:342:HHGFNDSXY:1:1241:3992:35399 2:N:0:GAACCTAG+TCCGCATA
CCTTGGAACGGACTCATCAGGGAGCCTACTAGACTCCGACCGTCATGGGGGTTCAAACCCATGCCCCG
CTACACCGCCTTAGATCAGCTGTGTCCACTTAAGGACTCACCTCTGGCCTATGGTCATCTCAATGCCG
+
FFFFFFFFFFF,FFFFFFFFFFFFFFFFFFFFFFFFFFFFFFFF:FFFFFFFFFFFFFFFFFFFFFFFF
FFFFFFFFFFFFFFFFFFFFFFFFFFFFFFFFFFFFFFFFFFFFFFFFFFFFFFFFFFFFFFFFFFFF
@A00155:342:HHGFNDSXY:1:1242:6271:1141 2:N:0:GAACCTAG+TCCGCATA
GACCCCTCCGGTCTTGTCCAACCTCTGTTTCGGCAGAGGATACCGTGCTCCACAGCTGTAAGGCTGTG
GGCCCGAGAGAAGTCGCTCTCTCTCGCCCTCCACATCCCTCTCGGCATTGAGATGACCATAGGC
+
FFFFF:FFFFFF:F,FFFFFFFFFF,FFF,FFFFFFFFFFFFFFFF,FFFFFFFFF,FFF:FF:FFFF
FFFFFFFFFFFFFFFFFFFFFFFFFFFFFFFFFFF:FFFFFFFFFFFFF:FFFFFFFFFFFFF,F
@A00155:342:HHGFNDSXY:1:1243:31855:10347 2:N:0:GAACCTAG+TCCGCATA
GTGAACAAGCCCGGAGTACATCTCCTTTGGAACTATGAGACCCCTCCGGTCTTGTCCAACCTCTGTTT
CGGCAGAGGATACCGTGCTCCACAGCTGTAAGGCTGTGGGCCCGAGAGAAGTCGCTCTCTCTCGCCCT
+
FFFFFFFF:FFFFFFFFFFFFFFFFFFFFFF:FFFFFFFFFFFFFFFFFFF:FFFFFFFFFFF,FFFF
FFFFFFFFFF:FFFFFFFFFFFFFFF:FFFFFFFFFFFFFFFFF,FFFF,FFFFFFFFFFFFFF:FFF
@A00155:342:HHGFNDSXY:1:1243:8748:19272 2:N:0:GAACCTAG+TCCGCATA
AAAAGGACTCGTCAGGTAGCCTACTAGACTACGACTTACACGGTAGGTCGTGAACAAGCCCGGAGTAC
ATCTCCTTTGGAACTAGGAGACCCCTCCGGTCTTGTCCAACCTCTGTTTCGGCAGAGGATACCGTGCT
+
:FFFFFFFF,F::FFFFFF:FFF,F:::FFF:FFFFFFFFFFFFFF,FFFFFFFFFF,FFFFFFF,FF
FFFF:FFFFFFFFFF:FFFF:FFFFFFFF,FFFF:FFFFFFFFFF:FFFFFFFFFFFFFFFFFFFF:
@A00155:342:HHGFNDSXY:1:1243:18611:20384 2:N:0:GAACCTAG+TCCGCATA
AAGTCGCTCTCTCTCGCCCTCCACATCCCTCTCGGCATTGAGATGACCATAGGCCAGAGGTGAGTCCT
TAAGTGGACACAGCTGATCTAAGGCGGTGTGGTGGGGCATGGGTTTGAACCCCCATGACGGTCGGAGT
+
FFFFFFFFFFFFFFFFFFFFFFFFFFFFFFFFFFFFFFFFFFFFFFFFFFFFFFFFFFFFFFFFFFFF
FFFFFFFFFFFFFFFFFFFFFFFFFFFFFFFFFFFFFFFFFFFFFFFFFFFFFFFFFFFFFFFFFFFF
@A00155:342:HHGFNDSXY:1:1243:29451:30514 2:N:0:GAACCTAG+TCCGCATA
AAAAGGACTCGTCAGGTAGCCTACTAGACTACGACTTACACGGTAGGTCGTGAACAAGCCCGGAGTAC
ATCTCCTTTGGAACTAGGAGACCCCTCCGGTCTTGTCCAACCTCTGTTTCGGCAGAGGATACCGTGCT
+

```
FFFFFFFFFFFFFFFFFFFFFFF,FF,FFF:FFFFFFFFFFFFFFFFFFFFFFFFFFFFFFFFFFFFFFF
FFFFFFFFFFFFFFFFFFFFFFFFFFFFFFFFFF:FFFFFFFFFFF:FFFFFFFFFFFFFFFFF,FFFFF
@A00155:342:HHGFNDSXY:1:1244:26856:6167 2:N:0:GAACCTAG+TCCGCATA
ACTCTTCGAGTTGTGTAAGTTTCGTCCTAAAAAGGACTCGTCAGGTAGCCTACTAGACTACGACTTAC
ACGGTAGGTCGTGAACAAGCCCGGAGTACATCTCCTTTGGAACCAGGAGACCCCTCCGGTCTTGTCCA
+
FFFFFFFFFF,FFFFFFFFFFFFFFFFFFFFFFFFFFFFFFFFFFFFFFFFFFFFFFFFFFFFFFFFFFF
FFFFFFFFFFFFFFFFFFFFFFFFFFFFFFFFFFFFFFFFFFFFFFFFF:FF:FFFFFFFFFFFFFFFFF
@A00155:342:HHGFNDSXY:1:1244:32931:10300 2:N:0:GAACCTAG+TCCGCATA
CAAGGACGAAACCGTCGTTACTCTTTGAGTTGTGTAAGTTTCGTCCTAAAAAGGACTCGTCAGGTAGC
CTACTAGACTACGACTTACACGGTAGGTCGTGAACAAGCCCGGAGTACATCTCCTTTGGAACTAGGAG
+
FFFFFFFFFFFFFFFFFFFFFFF,FFFFF:FFFFFFFFF:FFFFFFFFFF:FFFFFFFFFFFFFFFFFFF
FFFFFFFFFFFFFFFFFF:FFFF,FFFFFFFFFFFFFFFFFFFFFFFFFFFFFFFF:FFFFFFFFFFFFF
@A00155:342:HHGFNDSXY:1:1244:5846:33536 2:N:0:GAACCTAG+TCCGCATA
TTACACGGTAGGTCGTGAACAAGCCCGGAGTACATCTCCTTTGGAACCAGGAGACCCCTCCGGTCTTG
TCCAACCTCTGTTTCGGCAGAGGATACCGTGCTCCACAGCTGTAAGGCTGTGGGCCCGAGAGAAGTCG
+
FFFFFFFFFFFFFFFFFFFFFFFFFFFFFFFFFFFFFFFFFFFFFFFFFFF:FFFFFFFFFFFFFFF:FF
FFFFFFFFFFFFFFFFFFFFF:FFFFFFFFFFFFFFFFFFFFFFFFFFFFFFFFFFFFFFFFFFFFF:FF
@A00155:342:HHGFNDSXY:1:1245:22788:4319 2:N:0:GAACCTAG+TCCGCATA
TAGGAGACCCCTCCGGTCTTGTCCAACCTCTGTTTCGGCAGAGGATACCGTGCTCCACAGCTGTAAGG
CTGTGGGCCCGAGAGAAGTCGCTCTCTCTCGCCCTCCACATCCCTCTCGGCATTGAGATGACCATAGG
+
FFFFFFFFFFFFFFFFFFFFFFFFFFFFFFFFFFFFFFFFFFFFFFFFFFFFFFFFFFFFFFFFFFFFFF
FFFFFFFFFFFFFFFFFFFFFFFFFFFFFFFFFFFFFFFFFFFFFFFFFFFFFFFFFFFFFFFFFFFFFF
@A00155:342:HHGFNDSXY:1:1245:14018:8015 2:N:0:GAACCTAG+TCCGCATA
TCTCCTTTGGAACCAGGAGACCCCTCCGGTCTTGTCCAACCTCTGTTTCGGCAGAGGATACCGTGCTC
CACAGCTGTAAGGCTGTGGGCCCGAGAGAAGTCGCTCTCTCTCGCCCTCCACATCCCTCTCGGCATTG
+
FFFFFFFFFFFFFFFFFFFFFFFFFFFFFFFFFFFFFFFFFFFFFFFFFFFFFFFFFFFFFFFFFFFFFF
FFFFFFFFFFFFFFFFFFFFFFFFFFFFFFFFFFFFFFFFFFFFFFFFFFFFFFFFFFFFFFFFFFFFFF
@A00155:342:HHGFNDSXY:1:1245:21558:27054 2:N:0:GAACCTAG+TCCGCATA
GTGTAAGTTTCGTCCTAAAAAGGACTCGTCAGGTAGCCTACTAGACTACGACTTACACGGTAGGTCGT
GAACAAGCCCGGAGTACATCTCCTTTGGAACTAGGAGACCCCTCCGGTCTTGTCCAACCTCTGTTTCG
+
FFFFFFFFFF::FFFFFFFF:FFFFFFFFFFFFFFFFFFFFFFFFFFF:FFFFFFFFFFFFFFF:FF:F,
FFFFFFFFFFFFFFFFFFFFFFFFFFFFFF,FF:FF:FF:FFFFFFFFFFFFFFFFF:FFFFF:FFF:FF
@A00155:342:HHGFNDSXY:1:1245:14986:30953 2:N:0:GAACCTAG+TCCGCATA
GGAACTAGGAGACCCCTCCGGTCTTGTCCAACCTCTGTTTCGGCAGAGGATACCGTGCTCCACAGCTG
TAAGGCTGTGGGCCCGAGAGAAGTCGCTCTCTCTCGCCCTCCACATCCCTCTCGGCATTGAGATGACC
+
FFFFFFFFFFFFFFFFFFFFFFFFFFFF,FFFFFFFFFFFFFFFFFFFFFFFFFFFFFFFFFFFFFFFFF
:FFFFFFFFFFFFFFFFFFFFFFFFFFFFFFFFFFFFFFFFFFFFFFFFFFFFFFFFF:,FFFF:FFFFF
@A00155:342:HHGFNDSXY:1:1246:11921:10457 2:N:0:GAACCTAG+TCCGCATA
GAAACCGTCGTTACTCTTTGAGTTGTGTAAGTTTCGTCCTAAAAAGGACTCGTCAGGTAGCCTACTAG
ACTACGACTTACACGGTAGGTCGTGAACAAGCCCGGAGTACATCTCCTTTGGAACTAGGAGACCCCTC
+
FFFFFFFFFFFFFFFFFFFFFFFFFFFFFFFFF,FFFFFF:,FFFFFFFFFFFFFFFFFFFFFFFFFF,F
FFFFFFFFFFFFFFFFFFFFFFFFFFFFFFFFFFFFFFFFFFFFFFFFFFFF:FF:FFFFFFFFFFFFFF
@A00155:342:HHGFNDSXY:1:1246:17363:14215 2:N:0:GAACCTAG+TCCGCATA
TACATCTCCTTTGGAACTAGGAGACCCCTCCGGTCTTGTCCAACCTCTGTTTCGGCAGAGGATACCGT
GCTCCACAGCTGTAAGGCTGTGGGCCCGAGAGAAGTCGCTCTCTCTCGCCCTCTACATCCCTCTCGGC
+
```

```
FFFFFFFFFFFFFFFFFFFFFFFFFFFFFFFFFFFF:FFFFFFFFFFFFF:FFFFFFFFFFFFFFFFF
FFFFFFFFFFFFFFFFFFFFFFFFFFFFFFFFFFFFFFFFFFFFFFFFFFFFFFFFFFFFFFFFFFFF
@A00155:342:HHGFNDSXY:1:1246:23999:14747 2:N:0:GAACCTAG+TCCGCATA
GTAGGTCGTGAACAAGCCCGGAGTACATCTCCTTTGGAACTAGGAGACCCCTCCGGTCTTGTCCAACC
TCTGTTTCGGCAGAGGATACCGTGCTCCACAGCTGTAAGGCTGTGGGCCCGAGAGAAGTCGCTCTCTC
+
FF:FFFFFFFFFFFFFFFFFFFFFFFFFFFFFFFFFFFFFFFFFFFFFFFFFFFF:FFFFFF:FFFF
FF:F:FFFF:FFFFF:FFFFFFF:FFFFFF:FFFFFFFFFFFFFFFFFFFFFFFFFFFFFFFFFFFFF
@A00155:342:HHGFNDSXY:1:1246:2582:15452 2:N:0:GAACCTAG+TCCGCATA
ACATCTCCTTTGGAACTAGGAGACCCCTCCGGTCTTGTCCAACCTCTGTTTCGGCAGAGGATACCGTG
CTCCACAGCTGTAAGGCTGTGGGCCCGAGAGAAGTCGCTCTCTCTCGCCCTCTACATCCCTCTCGGCA
+
FFFFFFFF:FFFFFFFFFFFFFFFFFFFFF::FFF,:FFFFFFF:F:,FFF,:FFFFF::F,FFF:FF
FFFFF:FF:F:FFFF,FFF:FFFFF,,FFFFFF,FF:F:F:F:FFFFFFFFF,FFFFFFF:FFF:F:F
@A00155:342:HHGFNDSXY:1:1247:30779:13463 2:N:0:GAACCTAG+TCCGCATA
CTGATCTAAGGCGGTGTGGCGGGGCATGGGTTTGAACCCCCATGACGGTCGGAGTCTAGTAGGCTCCC
TGATGAGTCCGTTCCAAGGACGAAACCGTCGTTACTCTTCGAGTTGTGTAAGTTTCGTCCTAAAAAGG
+
FFFFFFFFFFFFFFFFFF:FFFFFFFFFFFFFFFFFFFFFFFFFFFFFFFFFFFFFFFFFFFFFFFFF
FFFFFF:FFFFFFFFFFFFFFFFFFFFFFFFFFFFFFFFFFF:FFFFFFF:FFFFFFFFFFF:FFFFFF
@A00155:342:HHGFNDSXY:1:1247:31322:26303 2:N:0:GAACCTAG+TCCGCATA
GAGACCCCTCCGGTCTTGTCCAACCTCTGTTTCGGCAGAGGATACCGTGCTCCACAGCTGTAAGGCTG
TGGGCCCGAGAGAAGTCGCTCTCTCTCGCCCTCCACATCCCTCTCGGCATAGAGATGACCATAGGCCA
+
FFFFFFFFFFFFFFFFFFFFFFFFFFFFFFFFFFFFFFFFFFFFFFFFFFFFFFFFFFFFFFFFFF,F
F:FFFFFFFFFFFFFFFFFFFFFFFFFFFFFFFFF:FFFFFFFFFFFFFF:FFFFFFFFFFFFFFFFFF
@A00155:342:HHGFNDSXY:1:1247:23854:34194 2:N:0:GAACCTAG+TCCGCATA
AAGGACTCGTCAGGTAGCCTACTAGACTACGACTTACACGGTAGGTCGTGAACAAGCCCGGAGTACAT
CTCCTTTGGAACTAGGAGACCCCTCCGGTCTTGTCCAACCTCTGTTTCGGCAGAGGATACCGTG
+
FFFFFFFFFFFFFFFFFFFFFFFFFFFFFFFFFFFFFFFFFFFFFFFFFFFFFFFFFFFFFFFFFFFF
FFFFFFFFFFFFFFFFFFFFFFFFFFFFFFFFFFFFFFFFFFFFFFFFFFFFFFFFFFFFFFFF
@A00155:342:HHGFNDSXY:1:1248:19497:35321 2:N:0:GAACCTAG+TCCGCATA
GTAAGTTTCGTCCTAAAAAGGACTCGTCAGGTAGCCTACTAGACTACGACTTACACGGTAGGTCGTGA
ACAAGCCCGGAGTACATCTCCTTTGGAACTAGGAGACCCCTCCGGTCTTGTCCAACCTCTGTTTCGGC
+
FFFFFFFFFFFFFFFFFFFFFFFFFFFFF:FFFFFFFFFFFFFFFFFFFFFFFFFFFFFFFFFFFFFF
FFFF:FFFFFFFFFFFFF:FFFFFFFFFFFFFFFFFFFF:FF:FFFFFFFF:FFFFFFFFF,FFF:FF
@A00155:342:HHGFNDSXY:1:1249:18837:10817 2:N:0:GAACCTAG+TCCGCATA
ACTTACACGGTAGGTCGTGAACAAGCCCGGAGTACATCTCCTTTGGAACTAGGAGACCCCTCCGGTCT
TGTCCAACCTCTGTTTCGGCAGAGGATACCGTGCTCCACAGCTGTAAGGCTGTGGGCCCGAGAGAAGT
+
FFFFFFFFF,FFFFFFF:FFFFFF,FFFFFFFFFFFFFFFFFFFFFFFFFF:FFFF:F,FFFFFF,FFF
F,FF:FFFFFFFF,FFFFFFFFFFFFFFFF:FFFFFFFFFFFF:FFFFFFFFF,FFF:F:FFF,FFFF
@A00155:342:HHGFNDSXY:1:1249:22299:20415 2:N:0:GAACCTAG+TCCGCATA
AAGGACTCGTCAGGTAGCCTACTAGACTACGACTTACACGGTAGGTCGTGAACAAGCCCGGAGTACAT
CTCCTTTGGAACTAGGAGACCCCTCCGGTCTTGTCCAACCTCTGTTTCGGCAGAGGATACCGTGCTCC
+
FFFFFFFFFFFFFFFFFFFFFFFFFFFFFFFFFFFFFFFFFFFFFFFFFFFFFFFFFFF:FFFFFFFF
FFFFFFFFFFFFFFFFFFFFFFF:FFFFFFFFFFFFFFFFFFFFFFFFFFFFFFFFFFF:FFFFFFFFF
@A00155:342:HHGFNDSXY:1:1249:8006:26036 2:N:0:GAACCTAG+TCCGCATA
ACTAGACTACGACTTACACGGTAGGTCGTGAACAAGCCCGGAGTACATCTCCTTTGGAACTAGGAGAC
CCCTCCGGTCTTGTCCAACCTCTGTTTCGGCAGAGGATACCGTGCTCCACAGCTGTAAGGCTGTGGGC
+
```

```
FFFFFFFFFFFFFFFFFFFFFFF:FFFFFFFFFFFFFFFFFFFFFFFFFFFFFFFFFFFFFFFFFFFFFF
FFFFFFFFFFFFFFFFFFFFFFFFFFFFFFFFFFFFFFFFFFFFFFFFFFFFFFFFFFFF:FFFFFFFF
@A00155:342:HHGFNDSXY:1:1249:26964:35571 2:N:0:GAACCTAG+TCCGCATA
CCTACTAGACTACGACTTACACGGTAGGTCGTGAACAAGCCCGGAGTACATCTCCTTTGGAACCAGGA
GACCCCTCCGGTCTTGTCCAACCTCTGTTTCGGCAGAGGATACCGTGCTCCACAGCTGTAAGGCTGTG
+
:FFFFFF,FFFFF,FFFFFFFFFFFFFFFFFFFFFFFFF:F:FFFFFFFF:FFFFFFF:F:F:FFFFFF
FFFFFFFFFFFFFFFFFFFFFFF:FFFFFFFF,FFFFFFFFFFF:FFFFFFFFFFFFFFFFFFFFFFFFF
@A00155:342:HHGFNDSXY:1:1250:16224:14465 2:N:0:GAACCTAG+TCCGCATA
GAGACCCCTCCGGTCTTGTCCAACCTCTGTTTCGGCAGAGGATACCGTGCTCCACAGCTGTAAGGCTG
TGGGCCCGAGAGAAGTCGCTCTCTCTCGCCCTCCACATCCCTCTCGGCATTGAGATGACCATAGGCCA
+
:FFFFFFFFFF:FF:F:FFFFFFFFFF::F,FFFFF:FF:FFFFFFFFFFFFFF:F:F:FFFF::FF:
F:,F:F,FFFFFFF:F::FFFFF:F:,:FFFFFFF:FFFFFFFF:FF,F,FFFFFF:FFFFFFFFFFF
@A00155:342:HHGFNDSXY:1:1250:30553:27915 2:N:0:GAACCTAG+TCCGCATA
GGACTCGTCAGGTAGCCTACTAGACTACGACTTACACGGTAGGTCGTGAACAAGCCCGGAGTACATCT
CCTTTGGAACTAGGAGACCCCTCCGGTCTTGTCCAACCTCTGTTTCGGCAGAGGATACCGTGCTCCAC
+
FFFFFFFFFFFFF:FFFFFFFFFFFFFFFFFFFFFFFFFFFFFFFFFFFFFFFFFFFFFFFFFFFFFFF
FFF:FFFFFFFFFFFFFFFFFFFFF:FFF:FFFFFFFF:FF:FF:FFFFFFFFFFFFFFFFFFFFFFFF
@A00155:342:HHGFNDSXY:1:1251:30192:6433 2:N:0:GAACCTAG+TCCGCATA
AGGACGAAACCGTCGTAACTCTTCGAGTTGTGTAAGTTTCGTCCTAAAAAGGACTCGTCAGGTAGCCT
ACTAGACTACGACTTACACGGTAGGTCGTGAACAAGCCCGGAGTACATCTCCTTTGGAACTAGGAGAC
+
FFFFFFFFFFFFFFFFFFFFFF::F:FFFFFFFFFFFFFFFFFFFFF:FFFFFFFFFFFFFFFFFFFFFFF
FFFFFFFFFFFFFFFFFFFFFFFFFFFFFFFFFFFFFFFFFFFFFFFFFFFFFFFFFFFFFFFFFFFFFFFF
@A00155:342:HHGFNDSXY:1:1252:16550:4225 2:N:0:GAACCTAG+TCCGCATA
CACGGTAGGTCGTGAACAAGCCCGGAGTACATCTCCTTTGGAACTAGGAGACCCCTCCGGTCTTGTCC
AACCTCTGTTTCGGCAGAGGATACCGTGCTCCACAGCTGTAAGGCTGTGGGCCCGAGAGAAGTCGCTC
+
FFFFFFFFFFFFFFFFFFFFFFFFFFFFFFFFFFFFFF:FFFFFFFFFFFFFFFFFFFFFFFFF,FFFFF
FFFFFFFFFFFFFFFFFFFFFFFFFFFFFFFFFFFFFFFFFFFFFFFFFFFFFFFFFFFFF:FFFFFFF
@A00155:342:HHGFNDSXY:1:1252:14805:12164 2:N:0:GAACCTAG+TCCGCATA
CATGGGGGTTTAAACCCATGCCCCGCCACACCGCCTTAGATCAGCTGTGTCCACTTAAGGACTCACCT
CTGGCCTATGGTCATCTCAATGCCGAGAGGGATGTGGAGGGCGAGAGAGAGCGACTTCTCTCGGGCCC
+
FFFFFFFFFFFFFFFFFFFFFFFFFFFFFFFFFFFFFFFFFFFFFFFFFFFFFFFFFFFFFFF:FFFFF
FFFFFFF,FFFFFFFFFFFFFFFFFFFFFFFFFFFFFFFFFFFFFFFFFFFFFFFFFFFFFFFFFFFFFF
@A00155:342:HHGFNDSXY:1:1252:32145:24815 2:N:0:GAACCTAG+TCCGCATA
ACATCTCCTTTGGAACTAGGAGACCCCTCCGGTCTTGTCCAACCTCTGTTTCGGCAGAGGATACCGTG
CTCCACAGCTGTAAGGCTGTGGGCCCGAGAGAAGTCGCTCTCTCTCGCCCTCTACATCCCTCTCGGCA
+
FFFFFFFFFFFFFFFFFFFFFFFFFFFFFFFFFFFFFFFFFFFFFFFFFFFFFFFFFFFFFFFFFFFFFF
FFFFFFFFFFFFFF:FFFFFFFFFFFFFFFFFF:FFFFFFFFFFFFFFFFFFFFFFFFFFFFFFFFFFFF
@A00155:342:HHGFNDSXY:1:1252:7265:30264 2:N:0:GAACCTAG+TCCGCATA
ACTAGACTACGACTTACACGGTAGGTCGTGAACAAGCCCGGAGTACATCTCCTTTGGAACTAGGAGAC
CCCTCCGGTCTTGTCCAACCTCTGTTTCGGCAGAGGATACCGTGCTCCACAGCTGTAAGGCTGTGGGC
+
FFFF:FFFFFFFFFFFFFFFFFFFFFFFFFFFFFFFFFF:FFFFFFFFFFFFFFFFFFFFFFFFFFF,F
FFF:FFFFFFFFFFFFFFFFFFFFFFFFFFFFF:FFFFFFFFFFFFFFFFFFFFFFFFFFFFFFFFFFFF
@A00155:342:HHGFNDSXY:1:1253:26648:5306 2:N:0:GAACCTAG+TCCGCATA
GGTCGTGAACAAGCCCGGAGTACATCTCCTTTGGAACTAGGAGACCCCTCCGGTCTTGTCCAACCTCT
GTTTCGGCAGAGGATACCGTGCTCCACAGCTGTAAGGCTGTGGGCCCGAGAGAAGTCACTCTCTCTCG
+
```

```
FF:FFFFFFFFFFFFF:FFFFFF:FFFFFFFFFFFFFFFFFFFFFFFFFFFFFFFFFFFFFFFFFFFF
FFFFFFFFFFFFF::FFFFFFFFFFFFFFFFFFFFFFFFFFFFFFFFFFFFFFFFFFFFFFFFFFFFF
@A00155:342:HHGFNDSXY:1:1253:4589:27571 2:N:0:GAACCTAG+TCCGCATA
AGGACGAAACCGTCGTTACTCTTCGAGTTGTGTAAGTTTCGTCCTAAAAAGGACTCGTCAGGTAGCCT
ATTAGACTACGACTTACACGGTAGGTCGTGAACAAGCCCGGAGTACATCTCCTTTGGAACTAGGAGAC
+
FFFFFFFFFFFFFFFFFFFFF:FFFFFFFFFFFFFFFFFFFFFFFF:FFFFFFFFFFFFFFFF:FFFF
FFFFFFFFFFFFFFFFFFFFFFFFFFFFFFFFFFFFFFFFFFFFFFFFFFFFFFFFFFFFFFFFFFFF
@A00155:342:HHGFNDSXY:1:1254:5873:20776 2:N:0:GAACCTAG+TCCGCATA
TGTGTAAGTTTCGTCCTAAAAAGGACTCGTCAGGTAGCCTACTAGACTACGACTTACACGGTAGGTCG
TGAACAAGCCCGGAGTACATCTCCTTTGGAACTAGGAGACCCCTCCGGTCTTGTCCAACCTCTGTTTC
+
FFFFFFFFFFFF,FFFFFFFF:FFFFFFFFFFFFF,FFFFFFFFFFFFFF:F:FFFFFFFFFFFFFF:
FFF:FFF:FFFFFFFFFFFFFFFFFFFFFFFFF:FFFFFFFFFFFFFFFF,:FFFFFFFFFFFFFFFF
@A00155:342:HHGFNDSXY:1:1255:1922:3724 2:N:0:GAACCTAG+TCCGCATA
GTTGTGTAAGTTTCGTCCTAAAAAGGACTCGTCAGGTAGCCTACTAGACTACGACTTACACGGTAGGT
CGTGAACAAGCCCGGAGTACATCTCCTTTGGAACTAGGAGACCCCTCCGGTCTTGTCCAACCTCTGTT
+
FFFFFFFFFFFFFFFFFFFFFFFFFFFFFFFFFFFFFFFFFFFFFFFFFFFFFFFFFFFFFFF:FFF
FFFFFFFFFFFFFFFFFFFFFFFFFFFFFFFFFFFFFFFFFFFFFFFFFFFFFFFFFFFFFFFFFFFF
@A00155:342:HHGFNDSXY:1:1255:2220:6840 2:N:0:GAACCTAG+TCCGCATA
TCTCCTTTGGAACTAGGAGACCCCTCCGGTCTTGTCCAACCTCTGTTACGGCAGAGGATACCGTGCTC
CACAGCTGTAAGGCTGTGGGCCCGAGAGAAGTCGCTCTCTCTCGCCCTCCACATCCCTCTCGGCATTG
+
FFFFFFFFFFFFFFFF:FFFF:FFFF:FFFFFFFFFFFFFFFFFFFFFFFFFFFFFFFFFFFF:FFFF
FFFFFFFFFFFFFFFFFFFFFFFFFFFFFFFFFFFFFFFFFFFFFFFFFFFFFFFFF:FFFF:FFF:F
@A00155:342:HHGFNDSXY:1:1255:21667:17409 2:N:0:GAACCTAG+TCCGCATA
CACGGTAGGTCGTGAACAAGCCCGGAGTACATCTCCTTTGGAACCAGGAGACCCCTCCGGTCTTGTCC
AACCTCTGTTTCGGCAGAGGATACCGTGCTCCACAGCTGTAAGGCTGTGGGCCCGAGAGAAGTCGCTC
+
FFFFFFFFFFFFFFFFFFFFFFFFFFFFFFFFFFFFFFFFFF:FFFFFFFFFFFFFFFFFFF:FFFFF
FFFFFFFFFFFFFFFFFFFFFFFFFFFFFFFFFFFFFFFFFFFFF:FFFFFFFFF:FFF,FFFFFFFF
@A00155:342:HHGFNDSXY:1:1255:20980:24643 2:N:0:GAACCTAG+TCCGCATA
ACTTACACGGTAGGTCGTGAACAAGCCCGGAGTACATCTCCTTTGGAACCAGGAGACCCCTCCGGTCT
TGTCCAACCTCTGTTTCGGCAGAGGATACCGTGCTCCACAGCTGTAAGGCTGTGGGCCCGAGAGAAGT
+
FFFFFFFFFFFFFFFFFFFFFFFFFFFFFF:F:F::FFFFFFFF:FFFFFFFF,FFFFFFFFFFFFF:
FFFFFFFFF:FFF,F,F:FFFFFFFFFFFF:FFFFFFFFFFFFFFFFFFF,F:FFFFFFFFF:F::F:
@A00155:342:HHGFNDSXY:1:1258:23421:11992 2:N:0:GAACCTAG+TCCGCATA
CCGTTCCAAGGACGAAACCGTCGTTACTCTTCGAGTTGTGTAAGTTTCGTCCTAAAAAGGACTCGTCA
GGTAGCCTACTAGACTACGACTTACACGGTAGGTCGTGAACAAGCCCGGAGTACATCTCCTTTGGAAC
+
FFFFFFFFFFFFFFFFFFFFFFFFFFFFFFFFFFF:FFFF:FFFF:FFFFFF:FFFFFFFFFFFFFFF
FFF:FFFF:FFFFFFFFFFF:FFFFFFFFFFFF:FFFFFFFFFFFFFFFFFFFF:FFFFFFFFFFFFF
@A00155:342:HHGFNDSXY:1:1258:1958:29403 2:N:0:GAACCTAG+TCCGCATA
CAGGTAGCCTACTAGACTACGACTTACACGGTAGGTCGTGAACAAGCCCGGAGTACATCTCCTTTGGA
ACCAGGAGACCCCTCCGGTCTTGTCCAACCTCTGTTTCGGCAGAGGATACCGTGCTCCACAGCTGTAA
+
FFFFFFFFFFFFFFFFFFFFFFFFFFFFFFFFFFFFFFFFFFFFFFFFFFFFFFFFFFFFFFFFFFFF
FFFFFFFFFFFFFFFFFFFF:FFFFFFFFFFFFFFFFFFFFFFFFFFFFFFFFFFFFFFFFFFFFFFF
@A00155:342:HHGFNDSXY:1:1259:32678:15499 2:N:0:GAACCTAG+TCCGCATA
CGGAGTACATCTCCTTTGGAACTAGGAGACCCCTCCGGTCTTGTCCAACCTCTGTTTCGGCAGAGGAT
ACCGTGCTCCACAGCTGTAAGGCTGTGGGCCCGAGAGAAGTCGCTCTCTCTCGCCCTCTACATCCCTC
+
```

FFFFFFFFFFFFFFFFFFFFFFF:FFFFFFFFFFFFFFFF,FF:FFFFFFFFFFFF,FFFFFFFFFFF
FFFFFFFFFFFFFFFFFFFFFFFFFFFFFFFFFFFFFF:FFFFFFFFFFFFFFFFFFFFFFFFFFF:F
@A00155:342:HHGFNDSXY:1:1260:9118:30311 2:N:0:GAACCTAG+TCCGCATA
GAACTAGGAGACCCCTCCGGTCTTGTCCAACCTCTGTTTCGGCAGAGGATACCGTGCTCCACAGCTGT
AAGGCTGTGGGCCCGAGAGAAGTCACTCTCTCTCGCCCTCCACATCCCTCTCGGCATTGAGATGAC
+
FFFFFFFFFFFFFFFFFFFFFFFFFFFFFFFFFFFFFFFFFFFFFFFFFFFFFFFFFFFFFFFFFFFF
FFFFFFFFFFFFFFFFFFFFFFFFFFFFFFFFFFFFFFFFFFFFFFFFFFFFFFFFFFFFFFFFFF
@A00155:342:HHGFNDSXY:1:1261:30924:7764 2:N:0:GAACCTAG+TCCGCATA
ACGACTTACACGGTAGGTCGTGAACAAGCCCGGAGTACATCTCCTTTGGAACTAGGAGACCCCTCCGG
TCTTGTCCAACCTCTGTTTCGGCAGAGGATACCGTGCTCCACAGCTGTAAGGCTGTGGGCCCGAGAGA
+
FFFFFFFFFFFFFFFFF,FFFFFFFFFFFFFFFFFFFFFFFFFF:FFFFFFFFFFFFFFFFFFFFFFF
FFFFFFFFFFFFFF:FFFFFFFFFFFFFFFFFFFFFFFFFFFFFFFFFFFFFFFFFFFFFFFFFFFFF
@A00155:342:HHGFNDSXY:1:1261:5421:14105 2:N:0:GAACCTAG+TCCGCATA
CACGGTAGGTCGTGAACAAGCCCGGAGTACATCTCCTTTGGAACTAGGAGACCCCTCCGGTCTTGTCC
AACCTCTGTTTCGGCAGAGGATACCGTGCTCCACAGCTGTAAGGCTGTGGGCCCGAGAGAAGTCGCTC
+
FFFFFFFFFFFFFFFFFFF,FFFFFFFFFFFFFFFFFFFFFFFFFFFFFFFFFFFFFFFFFFFFFFFF
FFFFFFFFFFFFFFF:FFFFFFFFFFFFFFFFFFFFFFFFFFFFFFFFFFFFFFFFFFFF,FFFFFFF
@A00155:342:HHGFNDSXY:1:1261:15664:16000 2:N:0:GAACCTAG+TCCGCATA
AAGGACGAAACCGTCGTTACTCTTTGAGTTGTGTAAGTTTCGTCCTAAAAAGGACTCGTCAGGTAGCC
TACTAGACTACGACTTACACGGTAGGTCGTGAACAAGCCCGGAGTACATCTCCTTTGGAACTAGGAGA
+
FFFFFFFFFFFFFFFFFFFFFF::FFFFF:FFFFFFFFFFFFFFFFFFFFFFFFFFFFFFFFFFFFFF
FFFFFFFFFFFFFFFFFFFFFFFFFFFFFFFFFFFFFFFFFFFFFFFFFFFFFFFFFFFFFFFFFFFF
@A00155:342:HHGFNDSXY:1:1261:6388:27430 2:N:0:GAACCTAG+TCCGCATA
AAAAGGACTCGTCAGGTAGCCTACTAGACTACGACTTACACGGTAGGTCGTGAACAAGCCCGGAGTAC
ATCTCCTTTGGAACCAGGAGACCCCTCCGGTCTTGTCCAACCTCTGTTTCGGCAGAGGATACCGTGCT
+
FFFFF:FFFFFFFFFFFFFFFFFFFFFFFF:F,FFFFFFFFFFFF:FFFFFFFFFFFFFFF:FFFFFF
FFFFFFFFFFFFF,FFFFFFFFFFFFFFFFFFFFFFFFFFFFFFFFFFFFFFFFFFFFFFFFFF:FFF
@A00155:342:HHGFNDSXY:1:1261:32669:32863 2:N:0:GAACCTAG+TCCGCATA
CGTCAGGTAGCCTACTAGACTACGACTTACACGGTAGGTCGTGAACAAGCCCGGAGTACATCTCCTTT
GGAACTAGGAGACCCCTCCGGTCTTGTCCAACCTCTGTTTCGGCAGAGGATACCGTGCTCCACAG
+
FFFFFFFF:FFFFFFFFFFFFFFFFFFFFFFFFFFFFF:FFFFFFFFFFFFFFFFFFFFFFFFFFFFF
FFFFFFFFFFFFFFFFFFFFFFFFFFFFF:FFFFFFFFF:F:FFFFFFFFFFFFFFFFFFFFFFF
@A00155:342:HHGFNDSXY:1:1261:8594:34601 2:N:0:GAACCTAG+TCCGCATA
GAAACCGTCGTTACTCTTCGAGTTGTGTAAGTTTCGTCCTAAAAAGGACTCGTCAGGTAGCCTACTAG
ACTACGACTTACACGGTAGGTCGTGAACAAGCCCGGAGTACATCTCCTTTGGAACTAGGAGACCCCTC
+
FFFFFFFFFFFFFFFFFFFFFFFFFFFFFFFFFFFFFFF:FFFFFFFFFFFFFFFFFFFFFFFFFFFF
FFFFFFFFFFFFFFFFFFFFFFFFFFFFFFFFFFFFFFFFFFFFFFFFFFFFFFFFFFFFFFFFFFFF
@A00155:342:HHGFNDSXY:1:1262:23999:12117 2:N:0:GAACCTAG+TCCGCATA
AACTAGGAGACCCCTCCGGTCTTGTCCAACCTCTGTTTCGGCAGAGGATACCGTGCTCCACAGCTGTA
AGGCTGTGGGCCCGAGAGAAGTCGCTCTCTCTCGCCCTCCACATCCCTCTCGGCATTGAGATGACCC
+
FFFFFFFFFFFFFFFFFFFFFFFFFFFFFFFFFFFFFFFFFFFFFFFFFFFFFFFFFFFFFFFFFFFF
FFFFFFFFFFFFFFFFFFF:FFFFFFFFFFFFFFFFFFFFFFFFFFFFFFFFFFFFFFFFFFFFFFF
@A00155:342:HHGFNDSXY:1:1262:30671:32346 2:N:0:GAACCTAG+TCCGCATA
GAGACCCCTCCGGTCTTGTCCAACCTCTGTTTCGGCAGAGGATACCGTGCTCCACAGCTGTAAGGCTG
TGGGCCCGAGAGAAGTCGCTCTCTCTCGCCCTCCACATCCCTCTCGGCATTGAGATGACCATAGGCCA
+

```
FFFFFFFFFFFFF:FFFFFFFFFFFFFFFFFFFFFFFFFFFFFFFFFFFFFFFFFFFFFFFFFFFFFF
FFFFFFFFFFFFF,FFFFFFFFFFF:FFFFFFFFFFFFFFFFFFFFFFFFFFFFFFFFFFFFFFFFFF
@A00155:342:HHGFNDSXY:1:1264:15872:7153 2:N:0:GAACCTAG+TCCGCATA
GAGTACATCTCCTTTGGAACTAGGAGACCCCTCCGGTCTTGTCCAACCTCTGTTTCGGCAGAGGATAC
CGTGCTCCACAGCTGTAAGGCTGTGGGCCCGAGAGAAGTCGCTCTCTCTCGCCCTCCAC
+
FFFFFFFFFFFFFFFFFFFFFFFFFFFFFFFFFFFFFFFFFFFFFFFFFFFFFFFFFFFFFFFFFFFF
FFFFFFFFFFFFFFFFFFFFFFFFFFFFFFF:FFFFFFFFFFFFFFFFFFFFFFFFFFF
@A00155:342:HHGFNDSXY:1:1264:23249:22341 2:N:0:GAACCTAG+TCCGCATA
GGACTCGTCAGGTAGCCTACTAGACTACGACTTACACGGTAGGTCGTGAACAAGCCCGGAGTACATCT
CCTTTGGAACCAGGAGACCCCTCCGGTCTTGTCCAACCTCTGTTTCGGCAGAGGATACCGTGCTCCAC
+
FFFFFFFFFFFFFFFFFFFFFFFFFFFFFFFFFFFFFFFFFFFFF:FFFFFFFFFFFFFFFFFF:FFFFF
FFFFFFFF:FFFFFFFFFFFFFFFFFFFFFFFFFFFFFFFF:FFFFFFFFFFFFFFFFFFFFFFFFFFF
@A00155:342:HHGFNDSXY:1:1264:9941:29293 2:N:0:GAACCTAG+TCCGCATA
TGTCCACTTAAGGACTCACCTCTGGCCTATGGTCATCTCAATGCCGAGAGGGATGTGGAGGGCGAGAG
AGAGCGACTTCTCTCGGGCCCACAGCCTTACAGCTGTGGAGCACGGTATCCTCTGCCGAAACAGAGGT
+
FFFFFFFFFFFFFFFFFFFFFFFFFFFFFFFFFFFFFFFFFFFFFFFFFFFFFFFFFFFFFFFFFFFF
FFFFFFFFFFFFFFFFFFFFFFFFFFFFFFFFFFFFFFFFF:FFFFFFFFFFFFFFFFFFFFFFFFFF:
@A00155:342:HHGFNDSXY:1:1264:5367:36401 2:N:0:GAACCTAG+TCCGCATA
CTCCTTTGGAACTAGGAGACCCCTCCGGTCTTGTCCAACCTCTGTTTCGGCAGAGGATACCGTGCTCC
ACAGCTGTAAGGCTGTGGGCCCGAGAGAAGTCGCTCTCTCTCGCCCTCTACATCCCTCTCGGCATTGA
+
FFFFFFFFFFFFFFFFFFFFFFFFFFFFFFFFFFFFFFFFFFFFFFFFFFFFFFFFFFFFFFFF,FFF
FFFFFFFFFFFFFFFFFFFFFFFFFFFFFFFFFFFFFFFFF:FFFFFFFFFFFFFFFFF:FFFFFFF
@A00155:342:HHGFNDSXY:1:1265:16532:8234 2:N:0:GAACCTAG+TCCGCATA
CTTGGAACGGACTCATCAGGGAGCCTACTAGACTCCGACCGTCATGGGGGTTCAAACCCATGCCCCGC
TACACCGCCTTAGATCAGCTGTGTCCACTTAAGGACTCACCTCTGGCCTATGGTCATCTCAATGCCGA
+
FFFFFFFFFFFFFFFFFFFFFFFFFFFFFFFFFFFFFFFFFF:FFFFFFFFFFFFFFFFFFFFFFFFF
FFFFF:FFFFFFFFFFFFFFFFFFFFFFFFFFFFFFFFFFFFFFFFFFFFFFFFFFFFFFFFFFFFFF
@A00155:342:HHGFNDSXY:1:1265:3305:30827 2:N:0:GAACCTAG+TCCGCATA
GGCAGAGGATACCGTGCTCCACAGCTGTAAGGCTGTGGGCCCGAGAGAAGTCGCTCTCTCTCGCCCTC
CACATCCCTCTCGGCATTGAGATGACCATAGGCCAGAGGTGAGTCCTTAAGTGGACACAGCTGA
+
FFFFFFFFFFFFFFFFFFFFFFFFFFFFFFFFFFFFFFFFFFFFFFFFFFFFFFFFFFFFFFFFFFFF
FFFFFFFFFFFFFFFF:FFFFFFFFFFFFFFFFF:FFFFFFFFFFFFFFFFFFFFF:FFFFFF:
@A00155:342:HHGFNDSXY:1:1266:16260:2597 2:N:0:GAACCTAG+TCCGCATA
ACTTACACGGTAGGTCGTGAACAAGCCCGGAGTACATCTCCTTTGGAACCAGGAGACCCCTCCGGTCT
TGTCCAACCTCTGTTTCGGCAGAGGATACCGTGCTCCACAGCTGTAAGGCTGTGGGCCCGAGAGAAGT
+
FFFFFFFFFFFFFFFFFFFFFFFFFFFFFFFFFFFFFFFFF:FFFFF:FFFFFFFFFFFFFFFFFFF,
FFFFFFFFFFFFFFFFFFFFFFFFFFFFFFFFFFFFFFFFFFFFFFFFFFFFFFFFFFFFFFFFFF,F
@A00155:342:HHGFNDSXY:1:1266:16423:2973 2:N:0:GAACCTAG+TCCGCATA
ACTTACACGGTAGGTCGTGAACAAGCCCGGAGTACATCTCCTTTGGAACCAGGAGACCCCTCCGGTCT
TGTCCAACCTCTGTTTCGGCAGAGGATACCGTGCTCCACAGCTGTAAGGCTGTGGGCCCGAGAGAAGT
+
FFFFFFFFFFFFFF,FFFFFFFFF:FFFFFFFFFFFFFFFFFFFFFFFFFFFFFFFFFFFFFFFFFFF
FFFFFFFFFFF:FF:FFFFFFFFFFF:FFFFFFFFFF:FFFF::FFFFFF:FFFFFFFFFFFFFFFFF
@A00155:342:HHGFNDSXY:1:1267:10465:9502 2:N:0:GAACCTAG+TCCGCATA
AGCCTACTAGACTACGACTTACACGGTAGGTCGTGAACAAGCCCGGAGTACATCTCCTTTGGAACTAG
GAGACCCCTCCGGTCTTGTCCAACCTCTGTTTCGGCAGAGGATACCGTGCTCCACAGCTGTAAGGCTG
+
```

```
:FFFFFFFFFFFFFFFFFFFFFFFFFFFFFFFFFFFFFFFFFFFFFFFFFFFFFFFFFFFFFFFFFFF
FFFFFFFFFFFFFFFFFFFFFFFFFFFFFFFFFFFFFFFFFFFFFFFFFFFFFFFFFFFFFFFFFFFF
@A00155:342:HHGFNDSXY:1:1267:30572:30358 2:N:0:GAACCTAG+TCCGCATA
TAGGAGACCCCTCCGGTCTTGTCCAACCTCTGTTTCGGCAGAGGATACCGTGCTCCACAGCTGTAAGG
CTGTGGGCCCGAGAGAAGTCGCTCTCTCTCGCCCTCCACATCCCTCTCGGCATTGAGATGACCATAGG
+
FFFFFFFFFFFFFFFFFFFFFFFFFFFFFFFFFFFFFFFFFFFFFFFFFFFFFFFFFFFFFFFFFFFF
FFFFFFFFFFFFFFFFFFFFFFFFFFFFFFFFFFFFFFFFFFFFFFFFFFFF:FFFFFFFFFFFFFFF
@A00155:342:HHGFNDSXY:1:1267:16197:35837 2:N:0:GAACCTAG+TCCGCATA
CGGTAGGTCGTGAACAAGCCCGGAGTACATCTCCTTTGGAACTAGGAGACCCCTCCGGTCTTGTCCAA
CCTCTGTTTCGGCAGAGGATACCGTGCTCCACAGCTGTAAGGCTGTGGGCCCGAGAGAAGTCGCTCTC
+
FFFFFFFFFFFFFFFFFFFFFFFFFFFFFFFFFF:F:FFFFFFFFFFFFFFFFFFFFF,FFFFFFFFF
F,FFFFFFFFFFFFFFFFFFFFFFFFFFFFFFFFFFFFFFFFFFFFFFFFFFFFFFF:,FFFFFFFFF
@A00155:342:HHGFNDSXY:1:1268:11993:4413 2:N:0:GAACCTAG+TCCGCATA
AAGGACTCGTCAGGTAGCCTACTAGACTACGACTTACACGGTAGGTCGTGAACAAGCCCGGAGTACAT
CTCCTTTGGAACCAGGAGACCCCTCCGGTCTTGTCCAACCTCTGTTTCGGCAGAGGATACCGTG
+
FFFFFFFFFFFFFFF,FFFFFFFFFFFFFF:FFFFFFFFFFFFFFFFFFFFFF:FFFFFFFFFFFFFF
FFFFFFFFFFFFFFFFFFFFFFF:FFFFFFFFFFFFFFFFFFFFFFFFFFFFFFFFFFFFFFFF
@A00155:342:HHGFNDSXY:1:1268:13467:12540 2:N:0:GAACCTAG+TCCGCATA
GTTGTGTAAGTTTCGTCCTAAAAAGGACTCGTCAGGTAGCCTACTAGACTACGACTTACACGGTAGGT
CGTGAACAAGCCCGGAGTACATCTCCTTTGGAACCAGGAGACCCCTCCGGTCTTGTCCAACCTCTGTT
+
FFFFFFFFFFFFFFFFFFFFFF:FFFFFFFFFFFFFFFFFFFFFFFFFFFFFFFFFFFFFFFFFFFFF
FFFFFFFFFFFFFF:FFFFFFFFFFFFFFFFFFFFFFFFFFFFFFFFFFFFFF:FFFFFFFFFFFFFF
@A00155:342:HHGFNDSXY:1:1268:13024:18161 2:N:0:GAACCTAG+TCCGCATA
CCTACTAGACTACGACTTACACGGTAGGTCGTGAACAAGCCCGGAGTACATCTCCTTTGGAACTAGGA
GACCCCTCCGGTCTTGTCCAACCTCTGTTTCGGCAGAGGATACCGTGCTCCACAGCTGTAAGGCTGTG
+
FFFFFFFFFFFFFFFFFFFFFFFFFFFFFFFFFFFFFFFFFFFFFFFFFFFFFFFFFFFFFFFFFFFF
FFFFFFFFFFFFFFFFFFFFFFFFFFFFFFFFFFFFFFFFFFFFF:FFFFFFFFFFFFFFFF:FF:FF
@A00155:342:HHGFNDSXY:1:1268:5846:25989 2:N:0:GAACCTAG+TCCGCATA
TAGCCTACTAGACTACGACTTACACGGTAGGTCGTGAACAAGCCCGGAGTACATCTCCTTTGGAACTA
GGAGACCCCTCCGGTCTTGTCCAACCTCTGTTTCGGCAGAGGATACCGTGCTCCACAGCTGTAAGGCT
+
FFFFFFFFFFFFFFFFFFFFFFFFFFFFFFFFFFFFFFFF::FFFFFFFFFFFFFFFFFFFFFF:FFF
FFFFFFFFFFFFFFFFFFFFFFF:FFFFFFFFFFFFFFFFFFFFFFFFFFFFFFFFFFFFFFFFFFFF
@A00155:342:HHGFNDSXY:1:1268:3414:36401 2:N:0:GAACCTAG+TCCGCATA
CGTCAGGTAGCCTACTAGACTACGACTTACACGGTAGGTCGTGAACAAGCCCGGAGTACATCTCCTTT
GGAACCAGGAGACCCCTCCGGTCTTGTCCAACCTCTGTTTCGGCAGAGGATACCGTGCTCCACAGCTG
+
FFFFFFFFFFFFFFFFFFFFFFFFFFFFFFFFFFF:FFFFFFFFFFFFFFFFFFFFFFFFFFFFFFFF
FFF,FFFFFFFFFFFFFFFFFFFFFFFFFFFFFFFFFFFFFFFFFFFF:FFFF:FFFFFFFFFFFFFF
@A00155:342:HHGFNDSXY:1:1269:8223:10629 2:N:0:GAACCTAG+TCCGCATA
GAGACCCCTCCGGTCTTGTCCAACCTCTGTTTCGGCAGAGGATACCGTGCTCCACAGCTGTAAGGCTG
TGGGCCCGAGAGAAGTCGCTCTCTCTCGCCCTCTACATCCCTCTCGGCATTGAGATGACCATAG
+
FFFFFFFFFFFFFFFFFFFFFFFFFFFFFFFFFFFFFF:FFFFFFFFFFFFFFFFFFFFFFFFFFFFF
FFFFFFFFFF:FFFFFFFFFFFFFFFFFFFFFFFFFFFFFFFFFF:FFFFFFFFFFFFFFFFFF
@A00155:342:HHGFNDSXY:1:1269:9607:19727 2:N:0:GAACCTAG+TCCGCATA
GGTCGTGAACAAGCCCGGAGTACATCTCCTTTGGAACTAGGAGACCCCTCCGGTCTTGTCCAACCTCT
GTTTCGGCAGAGGATACCGTGCTCCACAGCTGTAAGGCTGTGGGCCCGAGAGAAGTCGCTCTCTCTCG
+
```

```
FFFFFFFFFFFFFFFFFFFFFFFFFFFFFFFFFFFFFFFFFFFFFFFFFFFFFFFFFFFFFFFFFFFF
FFFFFFFFFFFFFFFFFFFFFFFFFFFFFFFFFFFFFFFFFFFFFFFFFFFFFF:FFFF:FFFFFFFF
@A00155:342:HHGFNDSXY:1:1269:8594:22138 2:N:0:GAACCTAG+TCCGCATA
GGTCGTGAACAAGCCCGGAGTACATCTCCTTTGGAACTAGGAGACCCCTCCGGTCTTGTCCAACCTCT
GTTTCGGCAGAGGATACCGTGCTCCACAGCTGTAAGGCTGTGGGCCCGAGAGAAGTCGCTCTCTCTCG
+
FFFFFFFFFFFFFFFFFFFFFFFFFFFFFFFFFFFFF,FFFFFFFFFFFFFFFFFF:FFFFFFFFFFF
FFFFFFFFFFFFFFFFFFFFFFFFFFFFFF:FFFFFFFFFFFFFFFFFFFFFFFFFFFFFFFFFFFFF
@A00155:342:HHGFNDSXY:1:1269:31123:25175 2:N:0:GAACCTAG+TCCGCATA
TTACACGGTAGGTCGTGAACAAGCCCGGAGTACATCTCCTTTGGAACTAGGAGACCCCTCCGGTCTTG
TCCAACCTCTGTTTCGGCAGAGGATACCGTGCTCCACAGCTGTAAGGCTGTGGGCCCGAGAGAAGTCG
+
FFFFFFFFFFFFFFFFFFFFFFFFFFFFFFFFF:FFFFFFFFFFFFFFFFFFFFFFFFFFFFFFF,FF
FFFFFFFFFFFFFFFFFFFFFFFFFFFFFFFFFFFFFFFFFFFFFFFFFFFFFFFFFFFFFFFFFFFF
@A00155:342:HHGFNDSXY:1:1269:28004:32174 2:N:0:GAACCTAG+TCCGCATA
ACCCCTCCGGTCTTGTCCAACCTCTGTTTCGGCAGAGGATACCGTGCTCCACAGCTGTAAGGCTGTGG
GCCCGAGAGAAGTCGCTCTCTCTCGCCCTCCACATCCCTCTCGGCATTGAGATGACCATAGGCCAGAG
+
FFFFFFFFFFFFFFFFFFFFFFFFFFFFFFFFFFFFFFFFFFFFFFFFFFFFFFFFFFF:FFFFFFFF
FFFFFFFFFFFFFFFFFFFFFFFFFFFFFFFFFFFFFFFFFFFFFF,FFFFFFFFFFFFFFFFFFFFF
@A00155:342:HHGFNDSXY:1:1270:20735:4742 2:N:0:GAACCTAG+TCCGCATA
TACGACTTACACGGTAGGTCGTGAACAAGCCCGGAGTACATCTCCTTTGGAACCAGGAGACCCCTCCG
GTCTTGTCCAACCTCTGTTTCGGCAGAGGATACCGTGCTCCACAGCTGTAAGGCTGTGGGCCCG
+
FFFFFFFFFFFFFFFFFFFFFFFFFFFFFFFFFFFFFFFFFFFFFFFFFFFFFFFFFFFFFFFFFFFF
FFFFFFFFFFFFFFFFFFFFFFFFFFFFFFFFFFFFFFFFFFFFFFFFFFFFFFFFFFFFFFFF
@A00155:342:HHGFNDSXY:1:1270:15555:10833 2:N:0:GAACCTAG+TCCGCATA
GGGTTCAAACCCATGCCCCGCCACACCGCCTTAGATCAGCTGTGTCCACTTAAGGACTCACCTCTGGC
CTATGGTCATCTCAATGCCGAGAGGGATGTGGAGGGCGAGAGAGAGCGACTTCTCTCGGGCCCACAGC
+
FFFFFFFFFFFFFFFFFFFFFFFFFFFFFFFFFFFFFFFFFFFFFFFFFFFFFFFFFFFFFFFFFFFF
FF:FFFFFFFFFFFFFFFFFFFFFFF,FFFFFFFFFFFFFFFFF:FFFFFFFFFFFF:FFFFFFFFFF
@A00155:342:HHGFNDSXY:1:1270:9625:26459 2:N:0:GAACCTAG+TCCGCATA
ACTTACACGGTAGGTCGTGAACAAGCCCGGAGTACATCTCCTTTGGAACTAGGAGACCCCTCCGGTCT
TGTCCAACCTCTGTTTCGGCAGAGGATACCGTGCTCCACAGCTGTAAGGCTGTGGGCCCGAGAGAAGT
+
FFFFFFFFFFFFFFFFFFFFFFF:FFFFFFFFFFF:FFFFFFFFFFFFFFFFFFFFFFFFFFFFFFFF
FFFFFFFFFFFFFFFFFFFFFFFFFF:FFFFFFFFFFFFFFFFFF:FFFFFFFFFFFFFFFFFFFFF:
@A00155:342:HHGFNDSXY:1:1271:18430:2284 2:N:0:GAACCTAG+TCCGCATA
GGAGCACGGTATCCTCTGCCGAAACAGAGGTTGGACAAGACCGGAGGGGTCTCCTAGTTCCAAAGGAG
ATGTACTCCGGGCTTGTTCACGACCTACCGTGTAAGTCGTAGTCTAGTAGGCTACCTGAC
+
FFFFFFFFFFFFFFFFFFFFFFFFFFFFFFFFFFFFFFFFFFFFFFFFFFFFFFFFFFFFFFFFFFFF
FFFFFFFFFFFFFFFFFFFFFFFFFFFFFFFFFFFFFFFFFFFFFFFFFFFFFFFFFFFF
@A00155:342:HHGFNDSXY:1:1271:20374:6339 2:N:0:GAACCTAG+TCCGCATA
AGAGTTGTGTAAGTTTCGTCCTAAAAAGGACTCGTCAGGTAGCCTACTAGACTACGACTTACACGGTA
GGTCGTGAACAAGCCCGGAGTACATCTCCTTTGGAACTAGGAGACCCCTCCGGTCTTGTCCAACCTCT
+
FFFFFFFFFFFFFFFFFFFFFFFFFFFFFFFFF:FFFFFFFFFFFFFFFFFFFFF:FFFFFFFFFFFF
FFFFFFFFFFFF:FFFFFFFFFFFFFFFFFFFFFFFFFFFFF,FFFFFFFFFFFF:FFFFF:FFFFFF
@A00155:342:HHGFNDSXY:1:1271:21052:30499 2:N:0:GAACCTAG+TCCGCATA
AGATCAGCTGTGTCCACTTAAGGACTCACCTCTGGCCTATGGTCATCTCAATGCCGAGAGGGATGTGA
AGGGCGAGAGAGAGCGACTTCTCTCGGGCCCACAGCCTTACAGCTGTGGAGCACGGTATCCTCTGCCG
+
```

FFFFFFFFFFFFFFFFFFFFFFFFFFFFFFFFFFFFFFFFFFFFFFFFFFFFFFFFFFFFFFF:FFFF
,FFFFFFFFFFFFFFFFFFFFFFFFFFFFFFFFFFFFF:FFFFFFFFFFFFFFFFFFF:FFFFFFFFFF
@A00155:342:HHGFNDSXY:1:1271:14091:36542 2:N:0:GAACCTAG+TCCGCATA
TACGACTTACACGGTAGGTCGTGAACAAGCCCGGAGTACATCTCCTTTGGAACCAGGAGACCCCTCCG
GTCTTGTCCAACCTCTGTTTCGGCAGAGGATACCGTGCTCCACAGCTGTAAGGCTGTGGGCCCGAGAG
+
FFFFFFFFFFFFFFFFFFFFFFFFFFFFFFFFFFFFFFFFFFFFFFFFFFFFFFFFFFFFFFFFFFFF
FFFFFFFFFFFFFFFFFFFFFFFFFFFFFFFFFFFFFFFFFFFFFFFFFFFFFF:FFFFFFFFFFFFF
@A00155:342:HHGFNDSXY:1:1272:8974:2440 2:N:0:GAACCTAG+TCCGCATA
TAGGTCGTGAACAAGCCCGGAGTACATCTCCTTTGGAACTAGGAGACCCCTCCGGTCTTGTCCAACCT
CTGTTTCGGCAGAGGATACCGTGCTCCACAGCTGTAAGGCTGTGGGCCCGAGAGAAGTCGCTCTCTCT
+
FFFFFFFFFFFFFFFFFFFFFFFFFFFFFFFFFFFFFFFFFFFFF:FFFFFFFFFFFFFFFFFFFFFF
FFFFFFFFFFFFFFFFFFFFFFFFFFFFFFFFFFFFFFFFFFFFFFFFFFFFFFFFFFFFFFFFFFFF
@A00155:342:HHGFNDSXY:1:1272:8305:11397 2:N:0:GAACCTAG+TCCGCATA
TACGACTTACACGGTAGGTCGTGAACAAGCCCGGAGTACATCTCCTTTGGAACTAGGAGACCCCTCCG
GTCTTGTCCAACCTCTGTTTCGGCAGAGGATACCGTGCTCCACAGCTGTAAGGCTGTGGGCCCG
+
FFFFFFFFFFFFFFFFFFFFFFFFFFFFFFFFFFFFFFFFFFFFFFFFFFFFFFFFFFFFFFFFFFFF
FFFFFF,FFFFFFFFFFFF:FFFFFF:FFFFFFFFFFFFFFFFFFFFFFFFFFFFFFFFF:FFFF
@A00155:342:HHGFNDSXY:1:1272:20690:11553 2:N:0:GAACCTAG+TCCGCATA
GCCCGGAGTACATCTCCTTTGGAACTAGGAGACCCCTCCGGTCTTGTCCAACCTCTGTTTCGGCAGAG
GATACCGTGCTCCACAGCTGTAAGGCTGTGGGCCCGAGAGAAGTCGCTCTCTCTCGCCCTCCACATCC
+
FFFFFFFFFFFFFFFFFFFFFFFFFFFFFFFFFFFFFFFFFFFFFFFFFFFFFFFFFFFFFFFFFFFF
FFFFFFFFFFFFFFFFFF:FFFFFFFFFFFFFFFFFFFFFFFFFFFFFFFFFFFFFFFFFFFFFFFFF
@A00155:342:HHGFNDSXY:1:1272:17381:18161 2:N:0:GAACCTAG+TCCGCATA
TACTCTTCGAGTTGTGTAAGTTTCGTCCTAAAAAGGACTCGTCAGGTAGCCTACTAGACTACGACTTA
CACGGTAGGTCGTGAACAAGCCCGGAGTACATCTCCTTTGGAACTAGGAGACCCCTCCGGTCTTGTCC
+
FFFFFFFFFFFFFFFFFFFFFFFFFFFFFFFFFFFFFFFFFFFFFFFFFFFFFFFFFFFFFFFFFFFF
FFFFFFFFFFFFFFFFFFFFFFFFFF:FFFFFFFFFFFFFFFFFFFFFFFFFFFFFFFFFFFFFFFFF
@A00155:342:HHGFNDSXY:1:1272:16441:20729 2:N:0:GAACCTAG+TCCGCATA
TAGGAGACCCCTCCGGTCTTGTCCAACCTCTGTTTCGGCAGAGGATACCGTGCTCCACAGCTGTAAGG
CTGTGGGCCCGAGAGAAGTCGCTCTCTCTCGCCCTCCACATCCCTCTCGGCATTGAGATGACCATAGG
+
FFFFFFFFFFFFFFFFFFFFFFFFFFFFFFFFFFFFFFFFFFFFFFFFFFFFFFFFFFFFFFFFFFFF
FFFFFFF,FFFFFFFF,FFFFFFFFFFFFFFFFFFFFFFFFFFFFFFFFFFF:FFFFFFFFFFFFFF
@A00155:342:HHGFNDSXY:1:1272:12138:30405 2:N:0:GAACCTAG+TCCGCATA
GAAACCGTCGTTACTCTTCGAGTTGTGTAAGTTTCGTCCTAAAAAGGACTCGTCAGGTAGCCTACTAG
ACTACGACTTACACGGTAGGTCGTGAACAAGTCCGGAGTACATCTCCTTTGGAACTAGGAGACCCCTC
+
FFFFFFFFFFFFFFFFFFFFFFFFFFFFFFFFF:FFFFFF:F,FFFFFFFFFFFFFFFFFFFFFFFFFF
FFFFFFFFFFFFFFFFFFFFFFFFFFFFFFFFFFFFFFFFFFFFFFFFFFFFFFFFFFFFFFFFFFFF
@A00155:342:HHGFNDSXY:1:1272:10095:34820 2:N:0:GAACCTAG+TCCGCATA
GTTGTGTAAGTTTCGTCCTAAAAAGGACTCGTCAGGTAGCCTACTAGACTACGACTTACACGGTAGGT
CGTGAACAAGCCCGGAGTACATCTCCTTTGGAACCAGGAGACCCCTCCGGTCTTGTCCAACCTCTGTT
+
FFFFFFFFFFFFFFFFFFFFFFFFFFFFFFFFF:FFFFFFFFFFFFFFFFFFFFFFFFFFFFFFFFFF
FFFFFFFFFFFFFFFFFFFFFFFFFFFFFFFFFF:FFFFFFFFFFFFFFFFFFFFFFFFFF:FFFFFFFF
@A00155:342:HHGFNDSXY:1:1272:3369:34851 2:N:0:GAACCTAG+TCCGCATA
GGTCGTGAACAAGCCCGGAGTACATCTCCTTTGGAACTAGGAGACCCCTCCGGTCTTGTCCAACCTCT
GTTTCGGCAGAGGATACCGTGCTCCACAGCTGTAAGGCTGTGGGCCCGAGAGAAGTCGCTCTCTCTCG
+

FFFFFFFFFFFF,FFFF,FFFFFFFFFFFFFFFFFF,FF:FFFFFFFFFFFFFFFFFFFFFFFFFF:F
FFFFFFFFFFFFFFFFFFFFFFFFFFFFF,FFFFFFFFFFFFFFFFFFFFFFFFFFFFFFFFFFFFFF
@A00155:342:HHGFNDSXY:1:1272:29604:35164 2:N:0:GAACCTAG+TCCGCATA
GTACATCTCCTTTGGAACTAGGAGACCCCTCCGGTCTTGTCCAACCTCTGTTTCGGCAGAGGATACCG
TGCTCCACAGCTGTAAGGCTGTGGGCCCGAGAGAAGTCGCTCTCTCTCGCCCTCCACATCCCTCTCGG
+
FFFFFFFFFFFFFFFFFFFFFFFFFFFFFFFFFFFFFFFFFFFFFFFFFFFFFFFFFFF:FFFFFFFF
FFFFFFFFFFFFFFFFFFFFFFFFFFFFFFFFFFFFFFFFFFFFFFFFFFFFFFFFFFFFFFFFFFFF
@A00155:342:HHGFNDSXY:1:1273:10438:11804 2:N:0:GAACCTAG+TCCGCATA
GGTAGGTCGTGAACAAGCCCGGAGTACATCTCCTTTGGAACTAGGAGACCCCTCCGGTCTTGTCCAAC
CTCTGTTTCGGCAGAGGATACCGTGCTCCACAGCTGTAAGGCTGTGGGCCCGAGAGAAGTCGCTCTCT
+
FFFFFFFFFFFFFFFFFFFFFFFFFFFFFFFFFFFFFFFFFFFFFFFFFFFFFFFFFFFFFFFFFFFF
FFFFFFFFFFFFFF:FFFFFFFFFFFFFFFFFFFFFFFFFFFF,FFFFFFFFFFFFFFFFFFFFFFFF
@A00155:342:HHGFNDSXY:1:1273:6036:14043 2:N:0:GAACCTAG+TCCGCATA
ACCGCCTTAGATCAGCTGTGTCCACTTAAGGACTCACCTCTGGCCTATGGTCATCTCAATGCCGAGAG
GGATGTGGAGGGCGAGAGAGAGCGACTTCTCTCGGGCCCACAGCCTTACAGCTGTGGAGCACGGTATC
+
FFFFFFFFFFFFFFFFFFFFFFFFFFFFFFFFFFFFFFFFFFFFFFFFFFFFFFFFFFFFFFFFFFFF
FFF:FFFFFFFFFFFFFFFFFFFFFFFFFFFFFFFFFFFFFFFFFFFFFFFFFFFFFFFF:FFFFFFF
@A00155:342:HHGFNDSXY:1:1273:31349:16579 2:N:0:GAACCTAG+TCCGCATA
CAGGTAGCCTACTAGACTACGACTTACACGGTAGGTCGTGAACAAGCCCGGAGTACATCTCCTTTGGA
ACTAGGAGACCCCTCCGGTCTTGTCCAACCTCTGTTTCGGCAGAGGATACCGTGCTCCACAGCTGTAA
+
FFFFFFFFFF:FFFFFFFFFFFFFFFFFFFFFFFFFFFFFFFFFFFFFFFFFFFFFFFFFFFFFFFFF
FFFFFFFFFFFF:FFFFFFFFFFFFFFFFFFFFFFFFFFFFFFFFFFFFFF:FFFFFFFFFFFFFFFF
@A00155:342:HHGFNDSXY:1:1273:18548:23688 2:N:0:GAACCTAG+TCCGCATA
TCTCCTTTGGAACTAGGAGACCCCTCCGGTCTTGTCCAACCTCTGTTTCGGCAGAGGATACCGTGCTC
CACAGCTGTAAGGCTGTGGGCCCGAGAGAAGTCGCTCTCTCTCGCCCTCCACATCCCTCTCGGCATTG
+
FFFFFFFFFFFFFFFFFFFFFFFFFFFFFFFF:,FFFFFFFFFFFF:FFFFFFFFFFFFFFFFFFFFF
FFFFFFFFFFFFFFFFFFFFFFFFFFFFFFFFFFFFFFFFFFF:FFFFFFFFFFFFFFFFFFFFFFFF
@A00155:342:HHGFNDSXY:1:1273:19407:25394 2:N:0:GAACCTAG+TCCGCATA
GACTCGTCAGGTAGCCTACTAGACTACGACTTACACGGTAGGTCGTGAACAAGCCCGGAGTACATCTC
CTTTGGAACCAGGAGACCCCTCCGGTCTTGTCCAACCTCTGTTTCGGCAGAGGATACCGTGCTCCACA
+
FFFFFFFFFFFFFFFFFFFFFFFFFFFFFFFFFFFFF::FFFFFFFFFFFFFFFFFFFFFFFF:FFFF
FFFFFFFFFFFFFFFFFFFFFFFFFFFFFFFFFFFFFFFFFFFFFFFFFFFFFF:FFFFFFFFFFFFF
@A00155:342:HHGFNDSXY:1:1273:9182:26349 2:N:0:GAACCTAG+TCCGCATA
AAGGACTCGTCAGGTAGCCTACTAGACTACGACTTACACGGTAGGTCGTGAACAAGCTCGGAGTACAT
CTCCTTTGGAACTAGGAGACCCCTCCGGTCTTGTCCAACCTCTGTTTCGGCAGAGGATACCGTGCTCC
+
FF:FFFFF,,F,FF:FFF,FFFFF,FFF:FFFF,FFFFF,FF:FFFFFFFF,FFF:FFFFFFFF,FFF
F,FFFFF::FFFFFF:F,FFFF:FFF,FF:FF,FFFFFFF:FFFF,,F:FFF,FFFFFFFFFFFF:FF
@A00155:342:HHGFNDSXY:1:1274:6008:23672 2:N:0:GAACCTAG+TCCGCATA
TAGACTACGACTTACACGGTAGGTCGTGAACAAGCCCGGAGTACATCTCCTTTGGAACTAGGAGACCC
CTCCGGTCTTGTCCAACCTCTGTTTCGGCAGAGGATACCGTGCTCCACAGCTGTAAGGCTGTGGGCCC
+
FFFFFFFFFFFFFFFFFFFFFFFFFFFFFFFFFFFFFFFFFFFFFFFFFFFFFFFFFFFFFFFFFFFF
FFFFFFFFFFFFFFFFFFFFFFFFFFFFFFFFFFFFFFFFFFFFFFFFFFFFFFFFFFFFFFFFFFFF
@A00155:342:HHGFNDSXY:1:1275:24849:2378 2:N:0:GAACCTAG+TCCGCATA
CCTCCGGTCTTGTCCAACCTCTGTTTCGGCAGAGGATACCGTGCTCCACAGCTGTAAGGCTGTGGGCC
CGAGAGAAGTCGCTCTCTCTCGCCCTTCACATCCCTCTCGGCATTGAGATGACCATAGGCCAGAG
+

FFFFFFFFFFFFFFFFFFFFFFFFFFFFFFFFFFFFFFFFFFFFFFFFFFFFFFFFFFFFFFFFFFFF
FFFFFFFFFFFFFFFFFFFFFFFFFFFFFFFFFFFFFFFFFFF:FFFFFFFFFFFFFFFFFFFFF
@A00155:342:HHGFNDSXY:1:1275:10809:7279 2:N:0:GAACCTAG+TCCGCATA
TCAGGTAGCCTACTAGACTACGACTTACACGGTAGGTCGTGAACAAGCCCGGAGTACATCTCCTTTGG
AACTAGGAGACCCCTCCGGTCTTGTCCAACCTCTGTTTCGGCAGAGGATACCGTGCTCCACAGCT
+
FFFFFFFFFFFFFFFFFFFFFFFFFFFFFFFFFFFFFFFFFFFFFFFFFFFFFFFFFFFFFFFFFFFF
FFFFFFFFFFFFFFFFFFFFFFFFFFFFFFFFFFFFFFFFFFFFFFFFFFFFFFFFFFFFFFFFF
@A00155:342:HHGFNDSXY:1:1275:32190:27712 2:N:0:GAACCTAG+TCCGCATA
ACTCGAAGAGTAACGACGGTTTCGTCCTTGGAACGGACTCATCAGGGAGCCTACTAGACTCCGACCGT
CATGGGGGTTCAAACCCATGCCCCGCCACACCGCCTTAGATCAGCTGTGTCCACTTAAGGACTCACCT
+
FFFFFFFF:FFFFFFFFFFFFFFFFFFFFFFFFFFFFFFFFFFFFFFFFFFFFFFFFFFFFFFFFFF
FFFFFFFF:FFFFFFFFFFFFFFFFFFFFFFFFFF:FFFFFFFFFFFFFFFFFFFFFFFFFFFFFFFF
@A00155:342:HHGFNDSXY:1:1276:25247:2566 2:N:0:GAACCTAG+TCCGCATA
AAACTTACACAACTCGAAGAGTAACGACGGTTTCGTCCTTGGAACGGACTCATCAGGGAGCCTACTAG
ACTCCGACCGTCATGGGGGTTCAAACCCATGCCCCGCCACACCGCCTTAGATCAGCTGTGTCCACTTA
+
FFFFFFFFFFFFFFFFFFFFFFFFFFFFFFFFFFFFFFFFFFFFFFFFFFFFFFFFFFFFFFFFFFF:
FFFFFF:FFFFFFFFFFFFFFFFFFFFFFFFFFFFFFFFFFFFFFFFFFFFFFFFFFFFFFFFFFFFF
@A00155:342:HHGFNDSXY:1:1276:8594:14184 2:N:0:GAACCTAG+TCCGCATA
GTTTCGTCCTAAAAAGGACTCGTCAGGTAGCCTACTAGACTACGACTTACACGGTAGGTCGTGAACAA
GCCCGGAGTACATCTCCTTTGGAACTAGGAGACCCCTCCGGTCTTGTCCAACCTCTGTTTCGGCAGAG
+
FFFFFFFFFFFFFFFFFFFFFFFFFFFFFFFFFFFFFFFFFFFFFFFFFFFFFFFFFFFFFFFFFFFF
FFFFFFFFFFFFFFFFFF:FFFFFFFFFFFF:FFFFFFFFFFFFFFFFFFFFFFFFFFFFFFFFFFFF
@A00155:342:HHGFNDSXY:1:1276:4860:14512 2:N:0:GAACCTAG+TCCGCATA
GTACATCTCCTTTGGAACCAGGAGACCCCTCCGGTCTTGTCCAACCTCTGTTTCGGCAGAGGATACCG
TGCTCCACAGCTGTAAGGCTGTGGGCCCGAGAGAAGTCGCTCTCTCTCGCCCTCCACATCCCTCTCGG
+
FFFFFFFFFFFFFFFFFFFFFFFFFFFFFFFFFFFFFFFFFFFFFFFFFFFFFFFFFFFFFFF:FFFF
FFFFFFFFFFF:FFFFFFFFFFFFFFFFFFF:FFFFFFFFFFFFFFFFFFFFFFF:FFFFFFFFFFFF
@A00155:342:HHGFNDSXY:1:1276:9435:14763 2:N:0:GAACCTAG+TCCGCATA
GTTTCGTCCTAAAAAGGACTCGTCAGGTAGCCTACTAGACTACGACTTACACGGTAGGTCGTGAACAA
GCCCGGAGTACATCTCCTTTGGAACTAGGAGACCCCTCCGGTCTTGTCCAACCTCTGTTTCGGCAGAG
+
FFFFFFFFFFF:FFFFFFFFFFFFFFFFFFF:FFFFFFFFFFFF:FFFFFFFFFFFFFFFFFFFFFFF
FFFFFF:FFFFFFFFFFFFFFFFFFF:FFFFFFFFFFFFFFFFFFFFFFFFFFFFFFFFFFFFFFFFF
@A00155:342:HHGFNDSXY:1:1276:12301:21793 2:N:0:GAACCTAG+TCCGCATA
ACTAGACTACGACTTACACGGTAGGTCGTGAACAAGCCCGGAGTACATCTCCTTTGGAACCAGGAGAC
CCCTCCGGTCTTGTCCAACCTCTGTTTCGGCAGAGGATACCGTGCTCCACAGCTGTAAGGCTGTGGGC
+
FFFFFFFFFFFFFFFFFFFFFFFFF:FFFFFFFFFFFFFFFFFFFFFFFFFFFF:FFFFFFFFFFF:F
FFFFFFFFFFFFFFFFFFFFFFFFFFFFFFFFFFFFFFFFFFFFFFFFFFFFFFFFFFFFFFFFFFFF
@A00155:342:HHGFNDSXY:1:1276:15438:27602 2:N:0:GAACCTAG+TCCGCATA
TCTTGTCCAACCTCTGTTTCGGCAGAGGATACCGTGCTCCACAGCTGTAAGGCTGTGGGCCCGAGAGA
AGTCGCTCTCTCTCGCCCTTCACATCCCTCTCGGCATTGAGATGACCATAGGCCAGAGGTGAGTCCTT
+
FF:FFFFFFFFFFFFFFFFFFFFFFFFFFFFFFFFFFFFFFFFFFFFFFFFFF:FFFFFFFFFFFFFF
FFFFFFFFFFFF:FFFFFFFFFFFFFFFFFFFFFF::FFFFFFFFFFFFFFFFFFFFFFFFFFFFFFF
@A00155:342:HHGFNDSXY:1:1277:12138:2973 2:N:0:GAACCTAG+TCCGCATA
AAGCCCGGAGTACATCTCCTTTGGAACTAGGAGACCCCTCCGGTCTTGTCCAACCTCTGTTTCGGCAG
AGGATACCGTGCTCCACAGCTGTAAGGCTGTGGGCCCGAGAGAAGTCGCTCTCTCTCGCCCTCTACAT
+

```
FF:FFF::FFFFFFFFFFF:FFFFFFFFFFFFF:FFFFFFFFFFF,FF:FFFFFFFFFFFFFFFFFFF
FFFFFF:FFFFFFFFFFFFFFFF:FFFF:F:FFFFFFFFFFFFFFFFFFFFFFFFFFFFFFFFF,FFF
@A00155:342:HHGFNDSXY:1:1277:8974:4006 2:N:0:GAACCTAG+TCCGCATA
AGGACGAAACTTACACAACTCGAAGAGTAACGACGGTTTCGTCCTTGGAACGGACTCATCAGGGAGCC
TACTAGACTCCGACCGTCATGGGGGTTCAAACCCATGCCCCGCCACACCGCCTTAGATCAGCTGTGTC
+
FFFFFFFFFFFFFFFFFFFFFFFFFFFFFFFFFFFFFF:FFFFFFFFFF:FFFF:FFFFFFFFFFFFF
:FFFFFFFFFFFFFFFFFFFFFFFFF:FFFFFFFFFFFFFFFFFFFFFFFFFFFFF:FFFFF:FFFFF
@A00155:342:HHGFNDSXY:1:1277:18855:35462 2:N:0:GAACCTAG+TCCGCATA
TAAGTTTCGTCCTAAAAAGGACTCGTCAGGTAGCCTACTAGACTACGACTTACACGGTAGGTCGTGAA
CAAGCCCGGAGTACATCTCCTTTGGAACTAGGAGACCCCTCCGGTCTTGTCCAACCTCTGTTTCGGCA
+
:FFFFFFFFFFF:FFFFFFFFFFFFFFFFFFFFFFFFFFFFFFFFFFFFFFFFFFFFFFFFFFFFFFF
FFFFFFFFFFFFFF:FFFFFFFFFFFFF,FFFFFFFFFFFFFFFFFFFFFFFFFFFFF:FFFFFFFFF
@A00155:342:HHGFNDSXY:1:1278:3649:6558 2:N:0:GAACCTAG+TCCGCATA
GCCCCGCCACACCGCCTTAGATCAGCTGTGTCCACTTAAGGACTCACCTCTGGCCTATGGTCATCTCA
ATGCCGAGAGGGATGTGGAGGGCGAGAGAGAGCGACTTCTCTCGGGCCCACAGCCTTACAGCTGTGGA
+
FFFFFFFFFFFFFFFFFFFFFFFFFFFFFFFFFFFFFFFFFFFFFFFFFFFFFFFFFFFFFFFFFFFF
FFFFFFFFFFFFF:FFFFFFFFFFFFFFFFFFFFFFFFFFFFFFFFFFFFFFFFFFFFFFFFFFFFFF
@A00155:342:HHGFNDSXY:1:1278:12780:16579 2:N:0:GAACCTAG+TCCGCATA
GGAGTACATCTCCTTTGGAACTAGGAGACCCCTCCGGTCTTGTCCAACCTCTGTTTCGGCAGAGGATA
CCGTGCTCCACAGCTGTAAGGCTGTGGGCCCGAGAGAAGTCGCTCTCTCTCGCCCTCCACATCCCTCT
+
FFF:F,FFFF:FF:FFFFFFFFFFFF,FFFFFFFFFFFFFFF:FFFFFFFFFFFFFFFFFFF:FFFFF
FFFFFFFFFFF:FF::FFFFFFFFF:FFFFFFFFF:FF::FFFFFFFFFFF:FFFFFFFFFFFFFFFF
@A00155:342:HHGFNDSXY:1:1278:19397:36526 2:N:0:GAACCTAG+TCCGCATA
GTAGCCTACTAGACTACGACTTACACGGTAGGTCGTGAACAAGCCCGGAGTACATCTCCTTTGGAACT
AGGAGACCCCTCCGGTCTTGTCCAACCTCTGTTTCGGCAGAGGATACCGTGCTCCACAGCTGTTAGGC
+
FFF:FFFFFFFFFFFFFFFFFFFFFFFF:FFFFFFFFFFFFF:FFFFFFFFFFFFFFFFFFFFFFFFF
FFFFFFFFFFFFFFFFFFFFFFFFFFFFFFFFFFFFFFFFFFFFFFFFFFFFFFFFFFFFFFFFFFFF
@A00155:342:HHGFNDSXY:1:1301:5285:11835 2:N:0:GAACCTAG+TCCGCATA
GTTTCGTCCTAAAAAGGACTCGTCAGGTAGCCTACTAGACTACGACTTACACGGTAGGTCGTGAACAA
GCCCGGAGTACATCTCCTTTGGAACTAGGAGACCCCTCCGGTCTTGTCCAACCTCTGTTACGGC
+
FFFFFFFFFF:FFF:FFFFFF:FFFFFFFFFFFFFFF,FFFFFFFFFFFFFFFFFFFFFFFFFFFFFF
FFFFFFFFFFFFFFFFFFFFFFFFFFFFFFFFFFFFFFFFFFFFFFFFFF:FFFFFFFFF:FFF
@A00155:342:HHGFNDSXY:1:1301:5330:12038 2:N:0:GAACCTAG+TCCGCATA
GTTTCGTCCTAAAAAGGACTCGTCAGGTAGCCTACTAGACTACGACTTACACGGTAGGTCGTGAACAA
GCCCGGAGTACATCTCCTTTGGAACTAGGAGACCCCTCCGGTCTTGTCCAACCTCTGTTACGGC
+
:FFFFFFFFFFFFF,FFFFFFFFFFFFFFFFFFFFFFFFF:FFFFFFFFFFFFFFFFFFFFFFF:FFF
:FFFFFFFFFFFFFFFFFFFF::FFFF:FFFFF,FFFFFFFFFFF:FFFFFFFFFFFFFFF,FF
@A00155:342:HHGFNDSXY:1:1301:11758:25300 2:N:0:GAACCTAG+TCCGCATA
TCCAAGGACGAAACCGTCGTTACTCTTGGAGTTGTGTAAGTTTCGTCCTAAAAAGGACTCGTCAGGTA
GCCTACTAGACTACGACTTACACGGTAGGTCGTGAACAAGCCCGGAGTACATCTCCTTTGGAACTAGG
+
FFFFFFFFFFFFFFFFFFFFFFFFFF:FFFFFFFFFFFFF:FFFFFFFFFFFF,FFFFFFFFFFFFFF
FFFFFFFFFFFFFFFFFF:FFFFFFFFFF:FFFF:FFFFFFFFFFFFFFFFFFFFFF:FFFFFFFF:F
@A00155:342:HHGFNDSXY:1:1301:11632:25676 2:N:0:GAACCTAG+TCCGCATA
TCCAAGGACGAAACCGTCGTTACTCTTGGAGTTGTGTAAGTTTCGTCCTAAAAAGGACTCGTCAGGTA
GCCTACTAGACTACGACTTACACGGTAGGTCGTGAACAAGCCCGGAGTACATCTCCTTTGGAACTAGG
+
```

```
FFFFFFFFFFFFFFFFFFFFFFFFFF:FFFFFFFFFFFFFFFFFFFFFFF:FFFFFFFFFFFFFFFFFFF
FFFFFFFFFFFFFFFFFFFFFFFFFFFFFFFFFFFFFFFFFFFFFFFFFFFFFFFFFFFFFFFFFFFFF
@A00155:342:HHGFNDSXY:1:1301:25572:26835 2:N:0:GAACCTAG+TCCGCATA
CCGTCGTTACTCTTCGAGTTGTGTAAGTTTCGTCCTAAAAAGGACTCGTCAGGTAGCCTACTAGACTA
CGACTTACACGGTAGGTCGTGAACAAGCCCGGAGTACATCTCCTTTGGAACTAGGAGACCCCTCCGGT
+
FFFFFFFFFFFFFFFFFFFFFFFFFFFFFFFFFFFF:FFFFFFFFFF:FFFFFF:FFFFFFFFF:FF
FFFFFFFFFFFFFFFFFFFFFFFFFFFFFFFFFFFFFFFFFFFFFFFFFFFFF:FFFFF:FFFFFFFF
@A00155:342:HHGFNDSXY:1:1301:14181:35321 2:N:0:GAACCTAG+TCCGCATA
GTCGTGAACAAGCCCGGAGTACATCTCCTTTGGAACTAGGAGACCCCTCCGGTCTTGTCCAACCTCTG
TTTCGGCAGAGGATACCGTGCTCCACAGCTGTAAGGCTGTGGGCCCGAGAGAAGTCGCTCT
+
FFFFFFFFFFFFFFFFFFFFFFFFFFFFFFFFFFFFFFFFFFFFFFFFFFFFFFFFFFFFFFFFFFFFF
FFFFFFFFFFFFFFFFFFFFFFFFFFFFFFFFFFFFFFFFFFFFFFFFFFFFFFFFFFFFF
@A00155:342:HHGFNDSXY:1:1302:30346:22106 2:N:0:GAACCTAG+TCCGCATA
AAAAGGACTCGTCAGGTAGCCTACTAGACTACGACTTACACGGTAGGTCGTGAACAAGCCCGGAGTAC
ATCTCCTTTGGAACTAGGAGACCCCTCCGGTCTTGTCCAACCTCTGTTTCGGCAGAGGATACCGTGCT
+
FFFFFFFFFFFFFFFFF:FFFFFFFFFFFFFFFFFFFFFFFF:FFFFFFFFFFF,FFFFFFFFFFFFF
FFFFFFFFFFFFFFFFFFFFFFFFFFFFFFFFFF,FFFFFFFFFFFFFFFFFFFFFFFFFFFFFFFFFF
@A00155:342:HHGFNDSXY:1:1303:8585:8437 2:N:0:GAACCTAG+TCCGCATA
ACGGTAGGTCGTGAACAAGCCCGGAGTACATCTCCTTTGGAACTAGGAGACCCCTCCGGTCTTGTCCA
ACCTCTGTTTCGGCAGAGGATACCGTGCTCCACAGCTGTAAGGCTGTGGGCCCGAGAGAAGTCGCTCT
+
FFFFFFFFFFFFFFFFFFFFFFFFFFFFFFFFFFFFFFFFFFFFFFFFFFFFFFFFFFFFFFFFFFFFF
FFFFFFFFFFFFFFFFFFFFFFFFFFFFFFFFFFFFFFF:F:FFFFFFFFFFFFFFFFFFFFFFFFFFF
@A00155:342:HHGFNDSXY:1:1303:3341:9752 2:N:0:GAACCTAG+TCCGCATA
AGGACGAAACCGTCGTTACTCTTTGAGTTGTGTAAGTTTCGTCCTAAAAAGGACTCGTCAGGTAGCCT
ACTAGACTACGACTTACACGGTAGGTCGTGAACAAGCCCGGAGTACATCTCCTTTGGAACTAGGAGAC
+
FFFFFFFFFF:FFFFFFFFFF:FFFFFFFFFFFFFFFFFFFFFF:FF:FFFFFFFFFFFFFFFF,FFFF
F:FFFFFFFFF:F:FFFFFFFF:FFFFFFF:FFFFFFFFFFFFFFFFFFFFFFFFFFFF,FFFFFFFFF
@A00155:342:HHGFNDSXY:1:1303:20012:18364 2:N:0:GAACCTAG+TCCGCATA
CCTACTAGACTACGACTTACACGGTAGGTCGTGAACAAGCCCGGAGTACATCTCCTTTGGAACTAGTA
GACCCCTCCGGTCTTGTCCAACCTCTGTTTCGGCAGAGGATACCGTGCTCCACAGCTGTAAGGCTGTG
+
FFFFFFFFFFFFFFFFFFFFFFFFFFFFFFFFFFFFFFFFFFFFFFFFFFFFFFFFFFFFFFFFFFFFF
FFFFFFFFFFFFFFFFFFFFFFFFFFFFFFFFFFFFFFFFFFFFFFFFFFFFFFFFFFFFFFFFFFFFF
@A00155:342:HHGFNDSXY:1:1304:2889:11443 2:N:0:GAACCTAG+TCCGCATA
GTGAACAAGTCCGGAGTACATCTCCTTTGGAACTAGGAGACCCCTCCGGTCTTGTCCAACCTCTGTTT
CGGCAGAGGATACCGTGCTCCACAGCTGTAAGGCTGTGGGCCCGAGAGAAGTCGCTCTCTCTCGCCCT
+
FFFFFFFFFFFFFFFFFFFFFFFFFFFFFFFFFFFFFFFF:FFFFFFFFFFFFFFFFFFFFFFFFFFFF
FFFFFFFFFFFFFFFFFFFFFFFFFFFFFFFFFFFFFFFFFFFFFFFFFFFFFFFFFFFFFFFFF:FF:
@A00155:342:HHGFNDSXY:1:1304:19452:36464 2:N:0:GAACCTAG+TCCGCATA
ACCTCTGTTTCGGCAGAGGATACCGTGCTCCACAGCTGTAAGGCTGTGGGCCCGAGAGAAGTCGCTCT
CTCTCGCCCTTCACATCCCTCTCGGCATTGAGATGACCATAGGCCAGAGGTGAGTCCTTAAGTGGAC
+
FFFFFFF:FFFFFF:FFFFFFFFFFFFFFFFFFFFFFFFFF:FFFF:FFFFFFFFFFFFF:FFFFFFFFF
FFFFFFFFFFFFFFFFFF:FF,FFFF,,FFFFFFFFFF:FFFFFF,FFFF:F:FFFF:F,FF,FFFF
@A00155:342:HHGFNDSXY:1:1305:8684:13369 2:N:0:GAACCTAG+TCCGCATA
CGGTAGGTCGTGAACAAGCCCGGAGTACATCTCCTTTGGAACCAGGAGACCCCTCCGGTCTTGTCCAA
CCTCTGTTTCGGCAGAGGATACCGTGCTCCACAGCTGTAAGGCTGTGGGCCCGAGAGAAGTCGCTCTC
+
```

:FFFFFFFFFFFFFFFFFFFFFFFFFFFFFFFFFFF:FFFFFFFFFFFFFFFFFFFFFFF:FFFFFFF
FFFFFFFFFFFFFFFFFFFFFFFFFFFFFFFFFFF:FFFFFFFFFFFFFFFFFFFFFFFFFFFFFFFF
@A00155:342:HHGFNDSXY:1:1305:8793:15342 2:N:0:GAACCTAG+TCCGCATA
TACACGGTAGGTCGTGAACAAGCCCGGAGTACATCTCCTTTGGAACTAGGAGACCCCTCCGGTCTTGT
CCAACCTCTGTTTCGGCAGAGGATACCGTGCTCCACAGCTGTAAGGCTGTGGGCCCGAGAGAAGTCGC
+
FFF,FFFF:,FFFFFFFFFFFFFFFFFFFFFFFFFFFF:F:FFFFFFFFFFFFFFFFFFFFF:FF:FF
FFFFFFFFFFFFF,FFFFFF:F:FFFFFFFFFFFFFFFFFFFFFFFFFFFFFFFFFFFFFFFF,FFFF
@A00155:342:HHGFNDSXY:1:1305:16893:16783 2:N:0:GAACCTAG+TCCGCATA
ACTTACACGGTAGGTCGTGAACAAGCCCGGAGTACATCTCCTTTGGAACTAGGAGACCCCTCCGGTCT
TGTCCAACCTCTGTTTCGGCAGAGGATACCGTGCTCCACAGCTGTAAGGCTGTGGGCCCGAGAGAAG
+
FFFFFFFFFFF,FF,FFFFFFFFFFFFFFFFFFFFFFFFFFF:FFFFFFFFFFFFFFFFFFFFFFFFF
FFFFFFFFFFFFFFFFFFFFFF,FFFFFFFFFFFFFFFFFFFFFFFFFFF:FFFFFFFFFFFFFFFF
@A00155:342:HHGFNDSXY:1:1306:11379:4131 2:N:0:GAACCTAG+TCCGCATA
GAGTACATCTCCTTTGGAACCAGGAGACCCCTCCGGTCTTGTCCAACCTCTGTTTCGGCAGAGGATAC
CGTGCTCCACAGCTGTAAGGCTGTGGGCCCGAGAGAAGTCGCTCTCTCTCGCCCTCCACATCCCTC
+
FFFFFFFFFF:FFFFF:FFFFF:FFFFFFFFFFF:FFFFFFFFFFFFF:FFFFFF,FFFFFFFFFFFF
FFFFFFFFFFFFFFFFFFF:FF:F:FFFFFFFFFFFFF:FFFFFFFFFFFFFFF:FFF:F:FFFFF
@A00155:342:HHGFNDSXY:1:1306:9001:4899 2:N:0:GAACCTAG+TCCGCATA
GGAGTACATCTCCTTTGGAACCAGGAGACCCCTCCGGTCTTGTCCAACCTCTGTTTCGGCAGAGGATA
CCGTGCTCCACAGCTGTAAGGCTGTGGGCCCGAGAGAAGTCGCTCTCTCTCGCCCTCCACATCCCTCT
+
FFFFFFFFFFFFFFFFFFFFFFFFFFFFFFFFFFFFFFFFFFFFFFFFFFFFFFFFFFFFFFFFFFFF
FFFFFFFFFFFFFFFFFFFFF:FFFFFFFFFFFFFFF,FFFFFFFFFFFFFFFFFFFFFFFFFFFFFF
@A00155:342:HHGFNDSXY:1:1306:16938:6590 2:N:0:GAACCTAG+TCCGCATA
TGTGTAAGTTTCGTCCTAAAAAGGACTCGTCAGGTAGCCTACTAGACTACGACTTACACGGTAGGTCG
TGAACAAGCCCGGAGTACATCTCCTTTGGAACTAGGAGACCCCTCCGGTCTTGTCCAACCTCTGTTTC
+
FFFFFFFFFFFFFFFF,FFFFFFFFFFFFFFFFFFFFFFFFFFFFFFFFFFFFFFFFFFFFFFFFFFF
FF:FFFFFFFFFFFFFFFFFFFFFFFFFFFFFFFFFFFFFFFFFFFFFFFFFFFFFFFFF:FFFFFFF
@A00155:342:HHGFNDSXY:1:1306:2239:14575 2:N:0:GAACCTAG+TCCGCATA
TAGGTCGTGAACAAGCCCGGAGTACATCTCCTTTGGAACCAGGAGACCCCTCCGGTCTTGTCCAACCT
CTGTTTCGGCAGAGGATACCGTGCTCCACAGCTGTAAGGCTGTGGGCCCGAGAGAAGTCGCTCTCTCT
+
FFFFFFFFFFFFFFFFFFFFFFFFFFFFF:FFFFFFFFFFFFFFFFFFFFFFFFFFFFFFFFFFFFFF
FFFFFFFFFF:F,FFFFF:FFFFFFFFFFFFFFFFFFFFFFFFFFFFFFFFFF::FFFFFFFFFFFFF
@A00155:342:HHGFNDSXY:1:1306:5177:17503 2:N:0:GAACCTAG+TCCGCATA
CGAAACTTACACAACTCGAAGAGTAACGACGGTTTCGTCCTTGGAACGGACTCATCAGGGAGCCTACT
AGACTCCGACCGTCATGGGGGTTCAAACCCATGCCCCGCCACATCGCCTTAGATCAGCT
+
FFFFFFFFFFFFFFFFFFFFFFFFFFFFFFFFFFFFFFFFFFFF:FFFFFFFFFFFFFFFFFFFFFFF
FFFFFFFF:FFFFFFFFFFFFFFFFFFFFFFFFFFFFFFFFFFFFFFFFFFFFFFFFF
@A00155:342:HHGFNDSXY:1:1306:5041:17550 2:N:0:GAACCTAG+TCCGCATA
CGAAACTTACACAACTCGAAGAGTAACGACGGTTTCGTCCTTGGAACGGACTCATCAGGGAGCCTACT
AGACTCCGACCGTCATGGGGGTTCAAACCCATGCCCCGCCACATCGCCTTAGATCAGCT
+
FFFFFFF:FFFFFFFFFFFFFFFFFFFFFFFFFFFFFFFFFFFFFFFFFFFFFFFFFFFFFFFFFFFF
FFFFFFFFFFFFFFFFFFFFFFFFFFFFFFFFFFFFFFFFFF:FFFFFFFFFFFFFFF:
@A00155:342:HHGFNDSXY:1:1306:4842:17832 2:N:0:GAACCTAG+TCCGCATA
CGAAACTTACACAACTCGAAGAGTAACGACGGTTTCGTCCTTGGAACGGACTCATCAGGGAGCCTACT
AGACTCCGACCGTCATGGGGGTTCAAACCCATGCCCCGCCACATCGCCTTAGATCAGCT
+

```
FFFF:FFFFFFFFFFFFFFFF:FFF:FFFFFFF:FFFFFFFFFFFFFFFFFFF,FFFFFFFFFFFFF
:FFFFFFFFFFFFFFFFFFFFFFFFFFFFF:FFFFF:FFFFFFFFFFFFFF:FFFFFF
@A00155:342:HHGFNDSXY:1:1307:9308:29042 2:N:0:GAACCTAG+TCCGCATA
CAGGTAGCCTACTAGACTACGACTTACACGGTAGGTCGTGAACAAGCCCGGAGTACATCTCCTTTGGA
ACTAGGAGACCCCTCCGGTCTTGTCCAACCTCTGTTTCGGCAGAGGATACCGTGCTCCACAGCTGTAA
+
FFFFFFFFFFFFFFFFFFFFFFFFFFFFFFFFFFFF:FFFFFFFFFFFFFFFFFFFFFFFFFFFF:FFF
FFFF:FFFF:FFFFFFFFFFFFFF:FFF::FFFFFFFFFFFFFFFFFFF,FFFFFFFFFFFFFFFFF
@A00155:342:HHGFNDSXY:1:1307:5448:29371 2:N:0:GAACCTAG+TCCGCATA
ACTAGACTACGACTTACACGGTAGGTCGTGAACAAGCCCGGAGTACATCTCCTTTGGAACTAGGAGAC
CCCTCCGGTCTTGTCCAACCTCTGTTTCGGCAGAGGATACCGTGCTCCACAGCTGTAAGGCTGTGGGC
+
FFFFFFFFFFFFF:FFFFFFFFFFFFFFFFFFFFFFFFFFFFFFFFFFFFFFFFFFFFFFFFFFFFFFF
FFFFFFFFFFFFFFFFFFFFFFFF:FFFFFFFFFFFFFFFFFFFFFFFFFFFF,F:FFFFFFFFFFF
@A00155:342:HHGFNDSXY:1:1307:6325:34741 2:N:0:GAACCTAG+TCCGCATA
ACTAGACTACGACTTACACGGTAGGTCGTGAACAAGCCCGGAGTACATCTCCTTTGGAACTAGGAGAC
CCCTCCGGTCTTGTCCAACCTCTGTTTCGGCAGAGGATACCGTGCTCCACAGCTGTAAGGCTGTGGGC
+
FFFFFFFFFFFFFFFFFFFFFFFFFFFFFFFFFFFF:FFFFFFFFFFFFFFFFFFFFFF:FFFFFFFFF
FFFFFFFFFF:FFFFF:FFFFF,FFFFFFFFFFFFFFFFFFFFFFFFFFFFFFFFFFFFFFFFFFFFFF
@A00155:342:HHGFNDSXY:1:1307:9155:35790 2:N:0:GAACCTAG+TCCGCATA
CAGGTAGCCTACTAGACTACGACTTACACGGTAGGTCGTGAACAAGCCCGGAGTACATCTCCTTTGGA
ACTAGGAGACCCCTCCGGTCTTGTCCAACCTCTGTTTCGGCAGAGGATACCGTGCTCCACAGCTGTAA
+
FFFFFFF:FFFFFFFFFFFFFFFFFFFFFF:FFFFFFFFFFFFFFFFFFFFFFFFFFFFFFFFFFFFFF
FFFFFFFFFFFFFFFFFFFFFFFFFFFFFFFFFFFFFFFFFFFFFFFFFFFFFFFFFFFFFFFFFFFF:
@A00155:342:HHGFNDSXY:1:1308:30770:7216 2:N:0:GAACCTAG+TCCGCATA
GTAGCCTACTAGACTACGACTTACACGGTAGGTCGTGAACAAGCCCGGAGTACATCTTCTTTGGAACT
AGGAGACCCCTCCGGTCTTGTCCAACCTCTGTTTCGGCAGAGGATACCGTGCTCCACAGCTGTAAGGC
+
FFFFFFFFFFFFFFFFFFFFFFFFFFFFFFFFFFFFFFFF:FFFFFFFFFFFFFFFFFFFFFFFFFFFF
FFFFFFFFFFFFFFFFFFFFFFFFFFFFFFFFFFFFFFFFFFFFFFFFFFFFFFFFFFFFFFFF:FFFF
@A00155:342:HHGFNDSXY:1:1308:17824:25880 2:N:0:GAACCTAG+TCCGCATA
TCCTAAAAAGGACTCGTCAGGTAGCCTACTAGACTACGACTTACACGGTAGGTCGTGAACAAGCCCGG
AGTACATCTCCTTTGGAACTAGGAGACCCCTCCGGTCTTGTCCAACCTCTGTTTCGGCAGAGGATACC
+
FFFFFFFFFFFFFFFFFFFFFFF:FFFFFFFFFFFFFFFFFFFFFFFFFFFFFFFFFFFFFFFFFFFFF
FFFFFFFFFFFFFFFFFFFFFFFFFF:FFFFFFFFFFFFFFFFFFFFFFFFFFFFFFFFFFFFFFFFFF
@A00155:342:HHGFNDSXY:1:1309:19000:15702 2:N:0:GAACCTAG+TCCGCATA
TACATCTCCTTTGGAACTAGGAGACCCCTCCGGTCTTGTCCAACCTCTGTTTCGGCAGAGGATACCGT
GCTCCACAGCTGTAAGGCTGTGGGCCCGAGAGAAGTCGCTCTCTCTCGCCCTCCACATCCCTCTCGGC
+
FFFFFFFFFFF:FFFFFFFFF:FFFFFFFFFFFFFFFFFFFFFFFFFFFFFFFFFFF:FFFFFFFFFF
FFFFFFFFFFFFFFFFFF:FFFFFFFFFFFFFF,:FFFFFFFFFFFFFFFFFFFFFFFFFFFFFFFFFF
@A00155:342:HHGFNDSXY:1:1309:27317:20369 2:N:0:GAACCTAG+TCCGCATA
CTAGGAGACCCCTCCGGTCTTGTCCAACCTCTGTTTCGGCAGAGGATACCGTGCTCCACAGCTGTAAG
GCTGTGGGCCCGAGAGAAGTCGCTCTCTCTCGCCCTCTACATCCCTCTCGGCATTGAGATGACCATAG
+
FFFFFFFFFFFFFFFFFFFFFFFFFFFFFFFFFFFFFFFFF,FFFFFFFFFFF:FFFFFFFFFFFFF:F
:FFFFFFFFFFFFFFFFFFFFFFFFFFFFFFFFFFFFFFFFFFFFFFFFFF:FFFFFFFFFFFFFFF
@A00155:342:HHGFNDSXY:1:1310:32000:13416 2:N:0:GAACCTAG+TCCGCATA
AGTACATCTCCTTTGGAACTAGGAGACCCCTCCGGTCTTGTCCAACCTCTGTTTCGGCAGAGGATACC
GTGCTCCACAGCTGTAAGGCTGTGGGCCCGAGAGAAGTCGCTCTCTCTCGCCCTCTACATCCCTCTCG
+
```

```
FFFFF:FFFFFFFFFFF:FFFFFFFFFFFFFFFFFFF:FFFFFFFFFFF:FFFFFFFF:FFFFF,FFF
FFFFFFFFFFFFFFFFFFFF,FFFFFFFFFFFFF:FF,FFFFFFFFFFFFFFFFF:FFFFFFFFFFFF
@A00155:342:HHGFNDSXY:1:1310:20383:16658 2:N:0:GAACCTAG+TCCGCATA
CGCCCTCCACATCCCTCTCGGCATTGAGATGACCATAGGCCAGAGGTGAGTCCTTAAGTGGACACAGC
TGATCTAAGGCGGTGTGGTGGGGCATGGGTTTGAACCCCCATGACGGTCGGAGTCTAGTAGGCTCCCT
+
FFFFFFFFFFFFFFFFFFFFFFFF:FFFFFFFFFFFFFFFFFFFFFFFFFFFFFFFFF:FFFFFFFFF
FFFFFFFFFFFFFFFFFFFFFFFFFFFFFFFFFFFFFFFFFFFFFFFFFFFFFFFF:FFFFFFFFFFF
@A00155:342:HHGFNDSXY:1:1310:25183:18959 2:N:0:GAACCTAG+TCCGCATA
GGATGTGGAGGGCGAGAGAGAGCGACTTCTCTCGGGCCCACAGCCTTACAGCTGTGGAGCACGGTATC
CTCTGCCGAAACAGAGGTTGGACAAGACCGGAGGGGTCTCCTAGTTCCAAAGGAGATGTACTCC
+
,,FF:,:FFFFFFFFFF:F:FF:FF:F::FFF:FFFFFFFFFFFFFF,:FFFFFF:FFFFFFFFFFF:
FFFFFF:FFFFFFFF,F:,FFFFFFFFFFF,FF::FFFFFFFFF:FFFFFFFFF::FFF::FFF
@A00155:342:HHGFNDSXY:1:1310:9489:28385 2:N:0:GAACCTAG+TCCGCATA
CACGGTAGGTCGTGAACAAGCCCGGAGTACATCTCCTTTGGAACTAGGAGACCCCTCCGGTCTTGTCC
AACCTCTGTTTCGGCAGAGGATACCGTGCTCCACAGCTGTAAGGCTGTGGGCCCGAGAGAAGTCGCTC
+
FFFFFFFFFFFFFFFFFFFFFFFFFFFFFFFFFFFFFFFFFFFFFFFFFFFFFFFFFFFFFFFFFFFF
FFFFFFFFFFFFFFFFFFFFFFFFFFFFFFFFFFFFFFFFFFFFFFFFFFFFFFFFFFFFFFFFFFFF
@A00155:342:HHGFNDSXY:1:1310:30671:28995 2:N:0:GAACCTAG+TCCGCATA
CGTTCCAAGGACGAAACCGTCGTTACTCTACGAGTTGTGTAAGTTTCGTCCTAAAAAGGACTCGTCAG
GTAGCCTACTAGACTACGACTTACACGGTAGGTCGTGAACAAGCCCGGAGTACATCTCCTTTGGAAC
+
FFFFFFFFFFFFFFFFFFFFFFFFFFFFFFFFFFFFFFFFFFFF:FFFFFFFFFFFFFFFFFFFFFFF
FFFFFFFFFFFFFFFFFFFFFFFFFFFFFFFFFFFFFFFFFFFFFFFFFFFFFFFFFFF,FFFFFFF
@A00155:342:HHGFNDSXY:1:1311:18005:1517 2:N:0:GAACCTAG+TCCGCATA
AAAAGGACTCGTCAGGTAGCCTACTAGACTACGACTTACACGGTAGGTCGAGAACAAGCCCGGAGTAC
ATCTCCTTTGGAACTAGGAGACCCCTCCGGTCTTGTCCAACCTCTGTTTCGGCAGAGGATACCGTGCT
+
FFFFFFFFFFFF:FFFFFFFFFFFFFFFFFFFFF:FFFFFFFFFFFFFFF,FFFFFFFFFFFFFFFFF
FFFFFFFFFFFFF,FFFFFFFFFFFFF:FFFFFFFFF:FFFFFFFF:FFF:FF,FFFFFFFF:FFFFF
@A00155:342:HHGFNDSXY:1:1311:7328:9142 2:N:0:GAACCTAG+TCCGCATA
CCAAGGACGAAACCGTCGTTACTCTACGAGTTGTGTAAGTTTCGTCCTAAAAAGGACTCGTCAGGTAG
CCTACTAGACTACGACTTACACGGTAGGTCGTGAACAAGCCCGGAGTACATCTCCTTTGGAACTAGGA
+
FFFFFFFFFFFFFFFFFFFFFFFFFFFFFFFFFFFFFFFFFFFFFFFFFFFFFFFFFFFFFFFFFFFF
FFFFFFFFFFFFFFFFFFFFFFFFFFFFFFFFFFFFFFFFFFFFFFFFFFFFFFFFFFF:FFFFFFFF
@A00155:342:HHGFNDSXY:1:1311:26784:10426 2:N:0:GAACCTAG+TCCGCATA
GTGTAAGTTTCGTCCTAAAAAGGACTCGTCAGGTAGCCTACTAGACTACGACTTACACGGTAGGTCGT
GAACAAGCCCGGAGTACATCTCCTTTGGAACTAGGAGACCCCTCCGGTCTTGTCCAACCTCTGTTTCG
+
FFFFFFFFFFFFFFFFFFFFFFFFFFFFFFFFFFFFFFF:FFFFFFFFFFFFFFFFFFFFFFFFFFFF
FFFFFFFFFFFFFFFFF:FFFFFFFFF:FFFFFF:FFFFF:FFFFFFFFFFFFFFF:FFFFFFFFFFF
@A00155:342:HHGFNDSXY:1:1311:18412:29528 2:N:0:GAACCTAG+TCCGCATA
CTGTGTCCACTTAAGGACTCACCTCTGGCCTATGGTCATCTCAATGCCGAGAGGGATGTAGAGGGCGA
GAGAGAGCGACTTCTCTCGGGCCCACAGCCTTACAGCTGTGGAGCACGGTATCCTCTGCCG
+
FFFFFFFFFFFFFFFFFFFFFFFFFFFFFFFFFFFFFFFFFFFFFFFFFFFFFFFF:FFFFFFFFFFF
FFFFFFFFFFFFFFFFFFFFFFFFFFFFFFFFFFFFFFFFFFFFFFFFFFFFFFFFFFFFF
@A00155:342:HHGFNDSXY:1:1311:22959:31579 2:N:0:GAACCTAG+TCCGCATA
GTAGCCTACTAGACTACGACTTACACGGTAGGTCGTGAACAAGCCCGGAGTACATCTCCTTTGGAACC
AGGAGACCCCTCCGGTCTTGTCCAACCTCTGTTTCGGCAGAGGATACCGTGCTCCACAGCTGTAAGGC
+
```

```
FFFFFFFFFFFFFFFFFFFFFFFFFFFFFFFFFFFFFFFFFFFFFFFFFFFFFFFFFFFFFFFFFFFF
FFFFFFFF::FFFFFFFFFFFF,FFFFFFFFFFFFFFFFFFFFFFFFFFFFFFFFFFFFFFFFFFFFF
@A00155:342:HHGFNDSXY:1:1311:10022:32597 2:N:0:GAACCTAG+TCCGCATA
GGAGTACATCTCCTTTGGAACTAGGAGACCCCTCCGGTCTTGTCCAACCTCTGTTTCGGCAGAGGATA
CCGTGCTCCACAGCTGTAAGGCTGTGGGCCCGAGAGAAGTCGCTCTCTCTCGCCCTCCACATCCCTCT
+
FFF:FFFFFFFF:F:FFFF,F:FFFFFFF:F:FF:F,F:FFFFFFFF,FF,FFF::FFFFF:FFFFFF
FF:FFFFFFF:FFFFF:FFF:FFFF:FFFFFFFFFFFF:,FFFFFFFFFFFFF,FFFFFFFFFFFFFF
@A00155:342:HHGFNDSXY:1:1311:25418:36182 2:N:0:GAACCTAG+TCCGCATA
TTACACGGTAGGTCGTGAACAAGCCCGGAGTACATCTCCTTTGGAACTAGGAGACCCCTCCGGTCTTG
TCCAACCTCTGTTTCGGCAGAGGATACCGTGCTCCACAGCTGTAAGGCTGTGGGCCCGAGAGAAGTCG
+
FFFFFFFFFFFF,FFFFFFFFFFFF:FFFFFFFFFFFFFFFFFFFFFFF,FFFFFFFFFFFFFFFFFF
:FFFFFFFFFFFF:FFFFFFFF,F:FFFFFFFFFFFFFFFFF:FF:FFFFFFFFFFFF:FFF:,FFFF
@A00155:342:HHGFNDSXY:1:1312:14705:1720 2:N:0:GAACCTAG+TCCGCATA
CTACTAGACTACGACTTACACGGTAGGTCGTGAACAAGCCCGGAGTACATCTCCTTTGGAACTAGGAG
ACCCCTCCGGTCTTGTCCAACCTCTGTTTCGGCAGAGGATACCGTGCTCCACAGCTGTAAGGCTGTGG
+
FFFFFFFFFFFFFFFFFFFFFFFFFFFFFFFFFFFFFFFFFFFFFFFFFFFFFFFFFFFFFFFFFFFF
FFFFFFFFFFFFFFFFFFFFFFFFFFFFFFFFFFFFFFFFFFFFFFFFFFFFFFFFFF:FFFFFFFFF
@A00155:342:HHGFNDSXY:1:1312:3233:10629 2:N:0:GAACCTAG+TCCGCATA
TCTCCTTTGGAACTAGGAGACCCCTCCGGTCTTGTCCAACCTCTGTTTCGGCAGAGGATACCGTGCTC
CACAGCTGTAAGGCTGTGGGCCCGAGAGAAGTCGCTCTCTCTCGCCCACCACATCCCTCTCGGCATTG
+
FFFFFFFF:FFFFFFFFFFFFFFFFFFFFFFF:FFFFFFFFFFFFFFFFFFFFFFFFFFFFFFFFFFF
FFFFFFFFFFFFFFFFFFFFFFFFFFFFFFFFFFFFFFFFFFFFFFF,FFF:FFF,FFFFFF:FFFFF
@A00155:342:HHGFNDSXY:1:1312:14769:30452 2:N:0:GAACCTAG+TCCGCATA
GGACTCGTCAGGTAGCCTACTAGACTACGACTTACACGGTAGGTCGTGAACAAGCCCGGAGTACATCT
CCTTTGGAACTAGGAGACCCCTCCGGTCTTGTCCAACCTCTGTTTCGGCAGAGGATACCGTGCTCCAC
+
FFFFFFFFFFFFFFFFFFFF:FFFFFFFFFFFFFFFFFFFFFFFFFFFFFFFFFFFFFFFFFFFFFFF
FFFFFFFFFFFFFFFFFFFFFFFF:FFFFFFFF,FF:FFFFFFFFFFFFFFFFFFF:FFFFF,FFFFF
@A00155:342:HHGFNDSXY:1:1312:9579:36401 2:N:0:GAACCTAG+TCCGCATA
AGTTTCGTCCTAAAAAGGACTCGTCAGGTAGCCTACTAGACTACGACTTACACGGTAGGTCGTGAACA
AGCCCGGAGTACATCTCCTTTGGAACCAGGAGACCCCTCCGGTCTTGTCCAACCTCTGTTTCGGCAGA
+
F,,,FF:FFFF,FF:,,FF::F,:FF:F,FF:F:FF,FFFFFFFFF,FFFFF,FF:F::F,,,:FFFF
F,:FFF:F:FF,F,FF:FFF:FFFFF::,FF:FFFFF:FFF,:FFFFFF:FFFFFFF:FF:F,FFFFF
@A00155:342:HHGFNDSXY:1:1312:29414:36746 2:N:0:GAACCTAG+TCCGCATA
CAGGTAGCCTACTAGACTACGACTTACACGGTAGGTCGTGAACAAGCCCGGAGTACATCTCCTTTGGA
ACTAGGAGACCCCTCCGGTCTTGTCCAACCTCTGTTTCGGCAGAGGATACCGTGCTCCACAGCTGTAA
+
FFFFFFFFFFFFFFFFFFFFFFFFFFFFFFFFFFFFFFFFFFFFF:FFFFFFFFFFFFFFFFFFFFFF
FFFFFFFFFFFFFFFFFFFFFFFFFFFFFFFFFFFFFFFFFFFFFFFFFFFFFFFFFFFFFFFFFFFF
@A00155:342:HHGFNDSXY:1:1313:19759:13009 2:N:0:GAACCTAG+TCCGCATA
GGTCGTGAACAAGCCCGGAGTACATCTCCTTTGGAACTAGGAGACCCCTCCGGTCTTGTCCAACCTCT
GTTTCGGCAGAGGATACCGTGCTCCACAGCTGTAAGGCTGTGGGCCCGAGAGAAGTCGCTCTCTCTC
+
FFFFFFFFFFFFFFFFFFFFFFFFFFFFFFFFFFFFFFFFFFFFFFFFF:FFFFFFFFFFFFFFFFFF
FFFFFFFFFFFFFF,FFFF:FFF:FFFFFF:F:FFFFFFFFFFFFFFFFFFFFFFFFFFFFFFFFFF
@A00155:342:HHGFNDSXY:1:1313:9471:19304 2:N:0:GAACCTAG+TCCGCATA
GACCCCTCCGGTCTTGTCCAACCTCTGTTTCGGCAGAGGATACCGTGCTCCACAGCTGTAAGGCTGTG
GGCCCGAGAGAAGTCGCTCTCTCTCGCCCTCCACATCCCTCTCGGCATTGAGATGACCATAGGCCAGA
+
```

FFFFFFFFFFFFFFFFFFFFFFFFFFFFFFFFFFFFFFFFFFFFFFFFFFFFFFFFFF:FFFFFFFFFFF
FFFFFFFFFFFFFFFFFFFFFFFFFFFFFFFFFFFFFFFFFFFFFFFF:,FFFFFFFFFFFFFFFFFFFF
@A00155:342:HHGFNDSXY:1:1313:19018:19492 2:N:0:GAACCTAG+TCCGCATA
CACGGTAGGTCGTGAACAAGCCCGGAGTACATCTCCTTTGGAACCAGGAGACCCCTCCGGTCTTGTCC
AACCTCTGTTTCGGCAGAGGATACCGTGCTCCACAGCTGTAAGGCTGTGGGCCCGAGAGAAGTCGCTC
+
FFFFFF:FFFFFFFFFFFFFFFFFFFFFFFFFFFFFFFFFFFFFFFFFFFFFFFFFFFFFFFFFFF
FFFFFFFFFFFFFFFFFFFFFFF:FFFFFF:FFFFFFFFFFFFFFFFFFFFFFFFFFFFFFF:FFFFFFF
@A00155:342:HHGFNDSXY:1:1313:6596:34585 2:N:0:GAACCTAG+TCCGCATA
GTGAACAAGCCCGGAGTACATCTCCTTTGGAACTAGGAGACCCCTCCGGTCTTGTCCAACCTCTGTTT
CGGCAGAGGATACCGTGCTCCACAGCTGTAAGGCTGTGGGCCCGAGAGAAGTCGCTCTCTCTCGCCCT
+
FFFFFFFFFFFF:FFFFFFFFFFFF:FFFFFFFFFFFFFFFFFFFFFFFFFFFFFFFFFFFFFFFFFFFF
FFFFFFFFFFFFFFFFFFFFFFFFFFFFFFFFFFFFFFFFFFFFFFFFFFFFFFFFFFFFFFFFFFFFFF
@A00155:342:HHGFNDSXY:1:1314:9308:4617 2:N:0:GAACCTAG+TCCGCATA
AAACCGTCGTTACTCTTAGAGTTGTGTAAGTTTCGTCCTAAAAAGGACTCGTCAGGTAGCCTACTAGA
CTACGACTTACACGGTAGGTCGTGAACAAGCCCGGAGTACATCTCCTTTGGAACTAGGAGACCCCTC
+
FFFFFFFFFFFFF:FF:FFFF:FFFFFFFFFFFFFFFFFFFFF,FFFFFFFFFFF,FF:FFFFFFFFF,F
FFFF:FFFFFFFFFFF,FF:FFFFFFFFFFFFFFF:FF:FFF,FFFFFFFFFFFFFFFFFFFFFFFFFF
@A00155:342:HHGFNDSXY:1:1314:10257:6261 2:N:0:GAACCTAG+TCCGCATA
GAACTAGGAGACCCCTCCGGTCTTGTCCAACCTCTGTTTCGGCAGAGGATACCGTGCTCCACAGCTGT
AAGGCTGTGGGCCCGAGAGAAGTCGCTCTCTCTCGCCCTCCACATCCCTCTCGGCATTGAGATGACCA
+
FFFFFFFFFFFFFFFFFFFFFFFFFFFFFFFFFFFFFFFFFFFFFFFFFFFFFFFFFFFFFFFFFFFFFF
FFFFF:FFFFFFFFFFFFFF:FFFFFFFFFFFFFFFFFFFFFFFFFFFFFFFFFFFFFFFFFFFFFFFFF
@A00155:342:HHGFNDSXY:1:1314:4246:14638 2:N:0:GAACCTAG+TCCGCATA
AAAAGGACTCGTCAGGTAGCCTACTAGACTACGACTTACACGGTAGGTCGTGAACAAGCCCGGAGTAC
ATCTCCTTTGGAACTAGGAGACCCCTCCGGTCTTGTCCAACCTCTGTTTCGGCAGAGGATACCGTGCT
+
FFFFFFFFFFFFFF:FFFFFFFFFFFFFFFFFFFFFFFFFFFFFFFFFFFFFFFFFFFFFFFFFFFFFFF
FFFFFFFFFFFFFFFFFFFFFFFFFFFFFFFFFFFFFFFFFFFFFFFFFFFFFFFFFFFFFFFFFF:FFF
@A00155:342:HHGFNDSXY:1:1315:2871:6089 2:N:0:GAACCTAG+TCCGCATA
AAAAGGACTCGTCAGGTAGCCTACTAGACTACGACTTACACGGTAGGTCGTGAACAAGCCCGGAGTAC
ATCTCCTTTGGAACTAGGAGACCCCTCCGGTCTTGTCCAACCTCTGTTTCGGCAGAGGATACCGTGCT
+
FFF:FFFFFFFF:FFFFF:FFFF:FFFF:FFFFFFFFFFFFFFFFFFFFFF:FFFFFFF,FFFFFFFFFF
FFFFF:FFFFFFFFFFFFFFFFFFFFFFFFFFFFFFFFFFFFF:FFFFFFFF:FFFFFFFFFFFFFF:FF
@A00155:342:HHGFNDSXY:1:1315:11143:12211 2:N:0:GAACCTAG+TCCGCATA
CAAGGACGAAACCGTCGTTACTCTTCGAGTTGTGTAAGTTTCGTCCTAAAAAGGACTCGTCAGGTAGC
CTACTAGACTACGACTTACACGGTAGGTCGTGAACAAGCCCGGAGTACATCTCCTTTGGAACTAGGA
+
FFFFFFFFFFFFFF:FFFFFFFFFFFF:FFF:FFFFFFFFFFFFFFF::FFFFFFFFFFFFFFFF,FFFF
FFFFFFFFFFFFFFFFFFFFF,FFFFFFFFFFFFFFFFF:FFFFFFFFFFFFFF,FFFFFFFF,FFFFF
@A00155:342:HHGFNDSXY:1:1315:19524:15045 2:N:0:GAACCTAG+TCCGCATA
TCCAAGGACGAAACCGTCGTTACTCTTCGAGTTGTGTAAGTTTCGTCCTAAAAAGGACTCGTCAGGTA
GCCTACTAGACTACGACTTACACGGTAGGTCGTGAACAAGCCCGGAGTACATCTCCTTTGGAACCAGG
+
FFFFFFFFFFFFFFFFFFFFFFFFFFFFFFFFFFFFFFFFFF:,FFFFFFFFFFFFFFFFFFFFFFFFFF
FFFFFFFFFFFFFFFFFFFFFFFFFF:FFFFFFFFFFFFFFFFFFFFFFFFFFFFFFFFFFFF:FFFFFF
@A00155:342:HHGFNDSXY:1:1315:18285:26334 2:N:0:GAACCTAG+TCCGCATA
TACGACTTACACGGTAGGTCGTGAACAAGCCCGGAGTACATCTCCTTTGGAACTAGGAGACCCCTCCG
GTCTTGTCCAACCTCTGTTTCGGCAGAGGATACCGTGCTCCACAGCTGTAAGGCTGTGGGCCCGAGAG
+

```
FFFFFFFFFFFFFFF,FFFFFFFFFFFFFFFFFFFFFFFFFFFFFFF:FFFFFFFFFFFFFFFFFFFF
FFFFFFFFFFFFFFFFFFF:FFFFFFFFFF:FFFFFFFFFFFFFFFFFFFFFFFFFFFF:FFFFFFFF
@A00155:342:HHGFNDSXY:1:1315:23900:27790 2:N:0:GAACCTAG+TCCGCATA
AAAAGGACTCGTCAGGTAGCCTACTAGACTACGACTTACACGGTAGGTCGTGAACAAGCCCGGAGTAC
ATCTCCTTTGGAACTAGGAGACCCCTCCGGTCTTGTCCAACCTCTGTTTCGGCAGAGGATACCGTGCT
+
FFFFFFFF:FFFFFFFFFFFFFFFFF:FFFFFFFFFFFFFFF:FFFF:FFFFFFFFFFFFFFFFFFFF
FFFFFFFFFFFFFFFFFFFFFFFFFFFFFFFFFFFFFFFFFFFF,FFFFFFFF,FFFFFFFFFFFFFF
@A00155:342:HHGFNDSXY:1:1316:30418:2942 2:N:0:GAACCTAG+TCCGCATA
GCTCTTCGAGTTGTGTAAGTTTCGTCCTAAAAAGGACTCGTCAGGTAGCCTACTAGACTACGACTTAC
ACGGTAGGTCGTGAACAAGCCCGGAGTACATCTCCTTTGGAACTAGGAGACCCCTCCGGTCTTGTCCA
+
FFF:FFFFFFFFFFFFFFFF:F:FFFFFFFFFFFFFFFFFFFFFFFFF:FFFFFFFFFFFFFFFFFFF
FFFFF,FFFFFFFF:FFFFF:FFFF:FFFFFFFFFFFFFFFFFFFFFF:FFF:FFFFFFFFFFFFFFF
@A00155:342:HHGFNDSXY:1:1316:6759:35336 2:N:0:GAACCTAG+TCCGCATA
AAAAGGACTCGTCAGGTAGCCTACTAGACTACGACTTACACGGTAGGTCGTGAACAAGCCCGGAGTAC
ATCTCCTTTGGAACTAGGAGACCCCTCCGGTCTTGTCCAACCTCTGTTTCGGCAGAGGATACCGTGCT
+
FFF:FFFFFFFFFFFFFFFFFFFFFFFFFFFFFFFFFFFFFFFFFFFFFFFFF:FFFFFFFFFFFFFF
FFFFFFFFFFFFFFFFFFFFFFFFFFFFFFFFFFFFFFFFFFFFFFFFFFFFFFF:FF:FFFFF:FFF
@A00155:342:HHGFNDSXY:1:1317:17101:2550 2:N:0:GAACCTAG+TCCGCATA
AAGGACTCGTCAGGTAGCCTACTAGACGACGACTTACACGGTAGGTCGTGAACAAGCCCGGAGTACAT
CTCCTTTGGAACTAGGAGACCCCTCCGGTCTTGTCCAACCTCTGTTTCGGCAGAGGATACCGTG
+
FFFFFFFFFFFFFFFFFFFFFFFFFFF,FFFFFFFFFFFFFFFFFFFFFFFFFFFFFFFFFFFFFFFF
FFFFFFFFFFFFFFFFFFFFFFFFFFFFFF:FFFFFFFFFFFFFFFFFFF:FFFFFFFFFFFFF
@A00155:342:HHGFNDSXY:1:1317:29369:10645 2:N:0:GAACCTAG+TCCGCATA
CATCTCCTTTGGAACCAGGAGACCCCTCCGGTCTTGTCCAACCTCTGTTTCGGCAGAGGATACCGTGC
TCCACAGCTGTAAGGCTGTGGGCCCGAGAGAAGTCGCTCTCTCTCGCCCTCCACATCCCTCTCGGCAT
+
FFFFFFFFFFFFFFFFFFFFFFFFFFFFFFFFFFFFFFFFFFFFFFFFFFFFFFFFFFFFFFFFFFFF
FFFFFFFFFFFFFFFFFFFFFFFFFFFFFFFFFFFFFFFFFFFFFFFFFFFFFF:FFFFFFFFFFF,F
@A00155:342:HHGFNDSXY:1:1317:8983:14606 2:N:0:GAACCTAG+TCCGCATA
GTTTCGTCCTAAAAAGGACTCGTCAGGTAGCCTACTAGACTACGACTTACACGGTAGGTCGTGAACAA
GCCCGAAGTACATCTCCTTTGGAACTAGGAGACCCCTCCGGTCTTGTCCAACCTCTGTTTCGGCAGAG
+
FFFFFFFFFFF:FFFFF:FFFFFFFFFFFFFFFFFFFFFFFFFFFFFFFFFFFFFFFFFFFFFFFFFF
FFFFFFFFFFFFFFFFFFFFFFF:FFFFFFFFFFFFFFFFFFFFFFFFFFFFFFFFFFFFFFFFFFFF
@A00155:342:HHGFNDSXY:1:1317:10456:36918 2:N:0:GAACCTAG+TCCGCATA
TACTAGACTACGACTTACACGGTAGGTCGTGAACAAGCCCGGAGTACATCTCCTTTGGAACTAGGAGA
CCCCTCCGGTCTTGTCCAACCTCTGTTTCGGCAGAGGATACCGTGCTCCACAGCTGTAAGGCTGTGGG
+
FFFFFFFFFFFFFFFFFFFFFFFFFFFFFFFFFFFFFFFFFFFFFFFFFFFFFFFFFFFFFFFFFFFF
FFFFFFFFFFFFFF:FFFFFFFFFFFFFFFFFFFFFF:FFFFFFFFFFFFFFFFFFFFFFFFFFFFFF
@A00155:342:HHGFNDSXY:1:1319:21197:3349 2:N:0:GAACCTAG+TCCGCATA
GAAACCGTCGTTACTCTTCGAGTTGTGTAAGTTTCGTCCTAAAAAGGACTCGTCAGGTAGCCTACTAG
ACTACGACTTACACGGTAGGTCGTGAACAAGCCCGGAGTACATCTCCTTTGGAACTAGGAGACCCCTC
+
FFFFFFFFFFFFFFFFFFFFFFFFFFFFF:FFFFFFFFFFFFFF:FFFFFFFFFFFFFFFFFFFFFFF
FFFFFFFFFFFFFFFFFFFF:FFFFFFFFFFFFFFFFFFFFFFFFFFF:FFFFFFFFFFFFFFFFFFF
@A00155:342:HHGFNDSXY:1:1319:12807:8140 2:N:0:GAACCTAG+TCCGCATA
ACTTACACGGTAGGTCGTGAACAAGCCCGGAGTACATCTCCTTTGGAACTAGGAGACCCCTCCGGTCT
TGTCCAACCTCTGTTTCGGCAGAGGATACCGTGCTCCACAGCTGTAAGGCTGTGGGCCCGAGAGAAGT
+
```

FFFFFFFFFFFFFFFFFFFFFFFFFFFFFFFFFFFFFFFFFFFFFFFFFFFFFFFFFFFFFFFFFFFF
FFFFFFFFFFFFFF::FFFFFFFFFFFFFFFFFFFFFFFFFFF,FFFFF:FFFFFFFFFFFFFFF,FF
@A00155:342:HHGFNDSXY:1:1320:17734:17769 2:N:0:GAACCTAG+TCCGCATA
GTGAACAAGCCCGGAGTACATCTCCTTTGGAACTAGGAGACCCCTCCGGTCTTGTCCAACCTCTGTTT
CGGCAGAGGATACCGTGCTCCACAGCTGTAAGGCTGTGGGCCCGAGAGAAGTCGCTCTCTCTCGCC
+
FFFFFFFFFFFFFFFFFFFFFFFFF:FFFFFFFFFFFFFFFFFFFFFFFFFFFFFFFFFFFFFFFF:F
FFFFFFFFFFFFFFFFFFFFFFFFFFFFFFF:FFFFFFFFFFFFF:F:FFFFFFFFFFFF:FFFFFF
@A00155:342:HHGFNDSXY:1:1320:5023:18208 2:N:0:GAACCTAG+TCCGCATA
AAGGACTCGTCAGGTAGCCTACTAGACTACGACTTACACGGTAGGTCGTGAACAAGCCCGGAGTACAT
CTCCTTTGGAACTAGGAGACCCCTCCGGTCTTGTCCAACCTCTGTTTCGGCAGAGGATACCGTG
+
FFFFFFFFFFFFFFFFFFFFFFFFFF:FFFFFFFFFFFFFFFFFFFFFFFFFFFFFFFFFFFFFFFFF
FFFFF:FFFF:FFFFFFFFFFFFFFFFFFFFFFFFFFFFFFFFFFFFFF:FFFFFFFFFFF,FFF
@A00155:342:HHGFNDSXY:1:1320:31259:20525 2:N:0:GAACCTAG+TCCGCATA
CTGGCCTATGGTCATCTCAATGCCGAGAGGGATGTAGAGGGCGAGAGAGAGCGACTTCTCTCGGGCCC
ACAGCCTTACAGCTGTGGAGCACGGTATCCTCTGCCGAAACAGAGGTTGGACAAGACCGGAGGGGTCT
+
F:FFFFFFFFFFFFFFFFFFFFFFFFFFFFFFF,FF:FFFFFFF:FFFFFFFFFFFFFFFFFFFFFFFF
FFFFFFFFFFFFFFF,FFFFFFFF:F,FFFFF:FFFF:FFFF:FFFFFFFFFFF:FFFFFFFF:FFFF
@A00155:342:HHGFNDSXY:1:1320:17282:21778 2:N:0:GAACCTAG+TCCGCATA
TACTCTTCGAGTTGTGTAAGTTTCGTCCTAAAAAGGACTCGTCAGGTAGCCTACTAGACTACGACTTA
CACGGTAGGTCGTGAACAAGCCCGGAGTACATCTCCTTTGGAACCAGGAGACCCCTCCGGTCTTGTCC
+
FFFFF::FF:FFFFFFFFFFFFFFFFFFFFFFFFFFFFFFFFFFFFFFFFFFFFFFFFFFFFFFFFFF
FFFFFFFFFFFFFFFFFFFFFFFFFFFFFFFFFFFFFFFFFFFF,FFFFFFF:FFFFFFFF:FFFFFFF
@A00155:342:HHGFNDSXY:1:1320:23032:23437 2:N:0:GAACCTAG+TCCGCATA
GACTCGTCAGGTAGCCTACTAGACTACGACTTACACGGTAGGTCGTGAACAAGCCCGGAGTACATCTC
CTTAGGAACTAGGAGACCCCTCCGGTCTTGTCCAACCTCTGTTTCGGCAGAGGATACCGTGCTCCACA
+
FFFFFFFFFFFFFFFFFFFFFFFFFF:FFFF:FFFFFFFFFFF,FFFFFFFFFFFFFFFFFFFFFFFFF
FFFFF::FFFFFFF:FFFFFFFFFFFF,FFFFFFFFFFF:FFFFFFFF:FFFFFFFFFFF:FFFFFF:
@A00155:342:HHGFNDSXY:1:1320:23782:24205 2:N:0:GAACCTAG+TCCGCATA
GACTCGTCAGGTAGCCTACTAGACTACGACTTACACGGTAGGTCGTGAACAAGCCCGGAGTACATCTC
CTTAGGAACTAGGAGACCCCTCCGGTCTTGTCCAACCTCTGTTTCGGCAGAGGATACCGTGCTCCACA
+
FFFFFFFFFFFFFFFFFFFFFFFFFFFFFFFFFFFFFFFFFFFFFFFFFFFFFFFFFFFFFFFFFFFF
FFF::FFFFFFFFFFFF:FFFFFFFFFF:FFFFFFFFFFFFFFFFFFFFFFFFFFFFFFFFFFFFFFF
@A00155:342:HHGFNDSXY:1:1321:11333:7717 2:N:0:GAACCTAG+TCCGCATA
TTGTGTAAGTTTCGTCCTAAAAAGGACTCGTCAGGTAGCCTACTAGACTACGACTTACACGGTAGGTC
GTGAACAAGCCCGGAGTACATCTCCTTTGGAACTAGGAGACCCCTCCGGTCTTGTCCAACCTCTGTT
+
FFFFFFFFFFFFFFFFFFFFFFFFFFFFFFFFFFF,FFFFFFFFFF:FFFFFFFFFFFFFFFFFFFFF
FFFFFFFF:FFFFFFFFFFFFFFFFFFFFFFFFFFFFFFFFFF:FFFFF:FFFFFFFFFFFFFFFFFF
@A00155:342:HHGFNDSXY:1:1321:25373:16282 2:N:0:GAACCTAG+TCCGCATA
TAGACTACGACTTACACGGTAGGTCGTGAACAAGCCCGGAGTACATCTCCTTTGGAACTAGGAGACCC
CTCCGGTCTTGTCCAACCTCTGTTTCGGCAGAGGATACCGTGCTCCACAGCTGTAAGGCTGTGGGCCC
+
FFFFFFFFFFFFFFFFFFFFFFFFFFFFFFFFFFFFFFFFFFFFFFFFFFFFFFFFFFFFFFFFFFFF
FFFFFFFFFFFFFFFFFFFFFFFFFFFFFFFFFFFFFFFFFFFFFF:FFFFFFFFFFF:FFFFFFFFFF
@A00155:342:HHGFNDSXY:1:1321:19623:31688 2:N:0:GAACCTAG+TCCGCATA
AGGTAGCCTACTAGACTACGACTTACACGGTAGGTCGTGAACAAGCCCGGAGTACATCTCCTTTGGAA
CTAGGAGACCCCTCCGGTCTTGTCCAACCTCTGTTTCGGCAGAGGATACCGTGCTCCACAGCTGTAAG
+

```
FFFFFFFFFFFFFFFFFFFFFFFFFFFFFFFFFF:FFFFFFFFF:FFFFFFFFFFF:FFFFFFFFFFF
FFFFFFFFFFFFFFFFFFFFFFFFFFFFFFFFFFFFFFFFFFFFFFFFFFF:FFFFFFFFFFFFFFFF
@A00155:342:HHGFNDSXY:1:1322:11279:5462 2:N:0:GAACCTAG+TCCGCATA
TAGACTACGACTTACACGGTAGGTCGTGAACAAGCCCGGAGTACATCTCCTTTGGAACTAGGAGACCC
CTCCGGTCTTGTCCAACCTCTGTTTCGGCAGAGGATACCGTGCTCCACAGCTGTAAGGCTGTGGGCCC
+
FFFFFFFFFFFFFFFFFFFFFFFFFFFFFFFFFFFFFFFFFFFFFFFFFFFFFFFFFFFFFFFFFFFF
FFFFFFFFFFFFFFFFFFFFFFFFFFFFFFFFFFFFFFFFFFFFFFFFFFFFFFFFFFFFFFFFFFFF
@A00155:342:HHGFNDSXY:1:1322:25030:8797 2:N:0:GAACCTAG+TCCGCATA
ACCCCTCCGGTCTTGTCCAACCTCTGTTTCGGCAGAGGATACCGTGCTCCACAGCTGTAAGGCTGTGG
GCCCGAGAGAAGTCGCTCTCTCTCGCCCTTCACATCCCTCTCGGCATTGAGATGACCATAGGCCAGAG
+
FFFFFFFFFFFFFFFFFFFFFFFFFFFFFFFFFFFFFF:FFFFFFFFFFFFFFFFFFFFFFFFFFFFF
FFFFFFFFFFFFFFFFFF:FFFFFFFFFFFFFFFFFFFFFFFFFFF,:FFFFFFFFF:FFFFFF,FFF
@A00155:342:HHGFNDSXY:1:1322:15167:9784 2:N:0:GAACCTAG+TCCGCATA
ACTTACACGGTAGGTCGTGAACAAGCCCGGAGTACATCTCCTTTGGAACTAGGAGACCCCTCCGGTCT
TGTCCAACCTCTGTTTCGGCAGAGGATACCGTGCTCCACAGCTGTAAGGCTGTGGGCCCGAGAGAAGT
+
FFFFFFFFFFFFFFFFFFFF,FFFFFFFFFFFFFFFFFFFFFFFFFFFFFFFFFFFFFFFFFFFFFFF
,FFFFFFFFFFFF:F:FFFFFFFFFFFFFFFFFFFFFFFFFFFFFFFFFFFFFFFFFFFFFFFFFFFF
@A00155:342:HHGFNDSXY:1:1322:25211:21825 2:N:0:GAACCTAG+TCCGCATA
AAAAGGACTCGTCAGGTAGCCTACTAGACTACGACTTACACGGTAGGTCGTGAACAAGCCCGGAGTAC
ATCTCCTTTGGAACTAGGAGACCCCTCCGGTCTTGTCCAACCTCTGTTTCGGCAGAGGATACCGTGCT
+
FFFFFFFFFFFFFFFFFFFFFFFFFFFFFFFFFFFFFFFFFFFFFFFFFFFFFFFFFFFFFFFFFFFF
FFFFFFFFFFFFFFFFFFFFFFFFFFFFFFFFFFFFFFFFFFFF:FFFFFFFFFFFFFFFFFFFFFFF
@A00155:342:HHGFNDSXY:1:1322:27010:36370 2:N:0:GAACCTAG+TCCGCATA
CGGCAGAGGATACCGTGCTCCACAGCTGTAAGGCTGTGGGCCCGAGAGAAGTCGCTCTCTCTCGCCCT
CCACATCCCTCTCGGCATTGAGATGACCATAGGCCAGAGGTGAGTCCTTAAGTGGACACAGCTG
+
FFFFFFFFFFFFFFFFFFFFFFFFFFFFFFFFFFFFFFFFFFFFFFFFFFFFFFFFFFFFFFFFFFFF
FFFFFFFFFFFFFFFFFF:FFF:FFFFFFFFFFFFFFFFFFFFFFFFFFFFFFFF:F,FFFFFF
@A00155:342:HHGFNDSXY:1:1323:9516:1720 2:N:0:GAACCTAG+TCCGCATA
AAAAGGACTCGTCAGGTAGCCTACTAGACTACGACTTACACGGTAGGTCGTGAACAAGCCCGGAGTAC
ATCTCCTTTGGAACTAGGAGACCCCTCCGGTCTTGTCCAACCTCTGTTTCGGCAGAGGATACCGTGCT
+
FFF:FFFFFFFFFFFFFFFFFFFFFFFFFFFFFFFFFFFFFFFFFFFFFFFFFFFFF:FFFFFFFFFF
FFFFFFFFFFFFFFFFFFFFFFFFFFFFFFFFFFFFFFFFFFFFFFFFFFFFFFFFFFFFFFFFFFFF
@A00155:342:HHGFNDSXY:1:1323:23891:3411 2:N:0:GAACCTAG+TCCGCATA
CGAAACCGTCGTTACTCTTCGAGTTGTGTAAGTTTCGTCCTAAAAAGGACTCGTCAGGTAGCCTACTA
GACTACGACTTACACGGTAGGTCGTGAACAAGCCCGGAGTACATCTCCTTTGGAACTAGGAGACCCCT
+
FFFFFFFFFFFFFFFF:FFFFFFFFFFF:FFFFFFFFFFFFFFF:FFFFFFFFFFFFFFFFFF:FFFF
FFFFFFFFFFFFFFFFFF:F:,FFFFFFFFF:FFFFFFFFFFF:FFFFFFFFF:FFFFFFFFFFFFFF
@A00155:342:HHGFNDSXY:1:1323:10239:14559 2:N:0:GAACCTAG+TCCGCATA
AACTAGGAGACCCCTCCGGTCTTGTCCAACCTCTGTTTCGGCAGAGGATACCGTGCTCCACAGCTGTA
AGGCTGTGGGCCCGAGAGAAGTCGCTCTCTCTCGCCCTCCACATCCCTCTCGGCAGTGAGATGAC
+
FFFFFFFFFFFFFFFFFFFFF,FFFFFFFFFFFFFFFFFFFFFFFFFFFFFFFFFFFFFFFFFFFF:F
FFFFFFFFFFFFFFFFFFFFFFFFFFFFFFFFFFFFFFFFFFFFFFFFFFFFFFF,FFFFFFFFF
@A00155:342:HHGFNDSXY:1:1323:7184:28682 2:N:0:GAACCTAG+TCCGCATA
ACCGTGCTCCACAGCTGTAAGGCTGTGGGCCCGAGAGAAGTCGCTCTCTCTCGCCCTTCACATCCCTC
TCGGCATTGAGATGACCATAGGCCAGAGGTGAGTCCTTAAGTGGACACAGCTGATCTAAGGCGGTGTG
+
```

```
FFFFFFFFFFFFFFF:FFFFFFFFFFFFFFFFFFFFFFFFFFFFFFFFFFFFFFFFFFFFFFFFFFFFFFFF
FFFFFFFFFFFFFFFFFFFFFFFFFFFFFFFFF:FFFFFFFFFFFFFFFFFFFFFFFFFFFFFFFFFFFF:F
@A00155:342:HHGFNDSXY:1:1324:17047:4711 2:N:0:GAACCTAG+TCCGCATA
TCCTTTGGAACTAGGAGACCCCTCCGGTCTTGTCCAACCTCTGTTTCGGCAGAGGATACCGTGCTCCA
CAGCTGTAAGGCTGTGGGCCCGAGAGAAGTCGCTCTCTCTCGCCCTCCACATCCCTCTCGGCATTGAG
+
FFFFFFFFF:FFFFFFFFFFFFFFFFFFFFFFFFFFFFFF:FFFFFFFFFFFFFFFFFFFFFFFFFFFFF
FFFFFFFFFFFFFF:FFFFFFFFFFFFFFFFFFFFFFFFFFFFFFFFFFFFFFFFFFFFFFFFFFF,FFFF
@A00155:342:HHGFNDSXY:1:1324:2953:13933 2:N:0:GAACCTAG+TCCGCATA
TGAACAAGCCCGGAGTACATCTCCTTTGGAACTAGGAGACCCCTCCGGTCTTGTCCAACCTCTGTTTC
GGCAGAGGATACCGTGCTCCACAGCTGTAAGGCTGTGGGCCCGAGAGAAGTCGCTCTCTCTCGCCCT
+
FFFFFFFFFFFFFF:FFFF:FFF,FF:FFFFF::,FFF:F:F:FF,FF:F,FFF,:FFFFFFF,FF::
FFFFFFFFFFF:FFFFFF:FFFFFFFFFFF:FFFFFFF:FFFFFFFFF:FFFFFFFFFFFFF:FFFF
@A00155:342:HHGFNDSXY:1:1324:30924:15311 2:N:0:GAACCTAG+TCCGCATA
GAAACCGTCGTTACTCTTGGAGTTGTGTAAGTTTCGTCCTAAAAAGGACTCGTCAGGTAGCCTACTAG
ACTACGACTTACACGGTAGGTCGTGAACAAGCCCGGAGTACATCTCCTTTGGAACTAGGAGACCCCTC
+
FFFFFFFFFFFFFFFFF,FFFF,FFFFFFFFFFFFFFFFFFFFFFFFFFFFFFFFFFFFFFFFFFFFFFF
FFFFFFFFFFFFFFFFF:FFFFFFFFFFFFFFFFFFFF:FFFFFFFFFFFFFFFFFFFFFF:FFF:FF
@A00155:342:HHGFNDSXY:1:1324:31521:19633 2:N:0:GAACCTAG+TCCGCATA
AGCCTACTAGACTACGACTTACACGGTAGGTCGTGAACAAGCCCGGAGTACATCTCCTTTGGAACTAG
GAGACCCCTCCGGTCTTGTCCAACCTCTGTTTCGGCAGAGGATACCGTGCTCCACAGCTGTAAGGCTG
+
FFFFFFFFFFFFF:FFFFFFFFFFFFFF:FFFFFFFFFFFFFFFFFFFFFFFFFFFFFFFFFFFFFFFFF
FFFFFFFFFFFFFFF::FFFFFFFFFFFFFFFFFFFFFFFF:FFFFFFFFFFFFFFFFF:FFFFFFFFF
@A00155:342:HHGFNDSXY:1:1324:4300:22592 2:N:0:GAACCTAG+TCCGCATA
CGTTCCAAGGACGAAACCGTCGTTACTCTTTGAGTTGTGTAAGTTTCGTCCTAAAAAGGACTCGTCAG
GTAGCCTACTAGACTACGACTTACACGGTAGGTCGTGAACAAGCCCGGAGTACATCTCCTTTGGAACT
+
FFFFFFFFFFFFFFFFFFFFFFFFFF:FFF,FFFFFFFFFFFFFF:FFFFFFF:FFFFFFFFFFFFFFF
FFFFFFFFFFFFFFFFFFFFF:F:F:FFFFFFFFF:FFFFFFFF,,FFFFFFFFFFFFFFFFFFFF:FF
@A00155:342:HHGFNDSXY:1:1325:16541:20995 2:N:0:GAACCTAG+TCCGCATA
TTTCGTCCTAAAAAGGACTCGTCAGGTAGCCTACTAGACTACGACTTACACGGTAGGTCGTGAACAAG
CCCGGAGTACATCTCCTTTGGAACCAGGAGACCCCTCCGGTCTTGTCCAACCTCTGTTTCGGCAGAGG
+
F,FFFFFFFFFFFFFFFFFFFFF:FFFFFFFFFFFFFFFFFFFFFFFFFFFFFFFFFFFFFFFFFFFFFF
FFFFFFFFFFFFFFFFFFFFFFFFFFFFFFFFFFFFFFFFFFFFFFFFFFFFFFFFFFF:FFFFFFFFF
@A00155:342:HHGFNDSXY:1:1325:2121:34663 2:N:0:GAACCTAG+TCCGCATA
AAGTCGCTCTCTCTCGCTCTCCACATCCCTCTCGGCATTGAGATGACCATAGGCCAGAGGTGAGTCCT
TAAGTGGACACAGCTGATCTAAGGCGGTGTGGCGGGGCATGGGTTTGAACCCCCATGACGGTCGGAGT
+
FFFFFFFFFFFFFFFFFFFFFFF:FFFFFFFFFFFFFFFFFFFFFFFFFFFF:FFFFFFFFFFFFFF:FF
F::FFFFFFFFFFFFF,FFFFFFFFFFFFFFFFFF:FF:FFFFFFFFFFFFFFF:FF:FFFFFFFFFF
@A00155:342:HHGFNDSXY:1:1326:13792:3834 2:N:0:GAACCTAG+TCCGCATA
AAAAAGGACTCGTCAGGTAGCCTACTAGACTACGACTTACACGGTAGGTCGTGAACAAGCCCGGAGTA
CATCTCCTTTGGAACTAGGAGACCCCTCCGGTCTTGTCCAACCTCTGTTTCGGCAGAGGATACCGTGC
+
FFFFFFFFF:FFFFFFFFFFFFFFFFFFFFFFFFF:FFFFFFFF:FFFFFFF:FFFFFFF:FFFFFFFFF
:FFF:F:,FFFFFFFFFFFFFFFFFFFFFFFFF:FFFFFFFFFFFFF:FFFFFFFFFFFFFFFFFFFF:F:
@A00155:342:HHGFNDSXY:1:1326:13973:21778 2:N:0:GAACCTAG+TCCGCATA
GTGAACAAGCCCGGAGTACATCTCCTTTGGAACTAGGAGACCCCTCCGGTCTTGTCCAACCTCTGTTT
CGGCAGAGGATACCGTGCTCCACAGCTGTAAGGCTGTGGGCCCGAGAGAAGTCGCTCTCTCTCGCCCT
+
```

```
FFFFFFFFFFFFFFFFFFFFFFFFFFFFFFFFFFFFFFFFFFFFFFFFF::FFFFFFFFFFFFFFF
FFFFFFFFFFFFFFFFFFFFFFFFFFFFFFFFFFFFFFFFFFFFFFFFFFFFFFFFFFFFFFFFFF
@A00155:342:HHGFNDSXY:1:1326:29405:28588 2:N:0:GAACCTAG+TCCGCATA
TCCTTGGAACGGACTCATCAGGGAGCCTACTAGACTCCGACCGTCATGGGGGTTCAAACCCATGCCCC
GCTACACCGCCTTAGATCAGCTGTGTCCACTTAAGGACTCACCTCTGGCCTATGGTCATCTCAATGCC
+
FFFFFFFFFFFFFFFFFFFFFFFFFFFFFFFFFFFFFFFFFFFFFFFFFFFFFFFFFFFFFFFFFF
FFFFFFFFFFFFFFFF:FFFFFFFFFFFFFFFFFFFFFFFFFFFFFFFFFFFFFFFFFFFFFFFFF
@A00155:342:HHGFNDSXY:1:1326:10095:30436 2:N:0:GAACCTAG+TCCGCATA
TACACGGTAGGTCGTGAACAAGCCCGGAGTACATCTCCTTTGGAACTGGGAGACCCCTCCGGTCTTGT
CCAACCTCTGTTTCGGCAGAGGATACCGTGCTCCACAGCTGTAAGGCTGTGGGCCCGAGAGAAGTCGC
+
FFFFFFFFFFFFFFFFFFFFFFFFFFFFFFFFFFFFFFFFFFFFFFFFFFFF:FFFFFFFFFFFF
FFFFFFFFFFFFFF:FFFFFFFFFFFFFFFFFFFFFFFFFFFFFFFFFFFFFFFFFFFFFFFFFFF
@A00155:342:HHGFNDSXY:1:1326:4752:33896 2:N:0:GAACCTAG+TCCGCATA
GTACATCTCCTTTGGAACTAGGAGACCCCTCCGGTCTTGTCCAACCTCTGTTTCGGCAGAGGATACCG
TGCTCCACAGCTGTAAGGCTGTGGGCCCGAGAGAAGTCGCTCTCTCTCGCCCTCTACATCCCTCTCGG
+
FFFFFFFFFFFFFFFFFFFFFFFFFFFFFFFFFFFFFFFFFFFFFFFFFFFFFFFFF:FFFFFFFFF
FFFFFFFFFFFFFFFFFFFFFFFFFFFFFFFF:FFFFFFFFFFFFFFFFFFFFFFFF:FFFFFFFFFFF
@A00155:342:HHGFNDSXY:1:1327:11623:4429 2:N:0:GAACCTAG+TCCGCATA
CACGGTAGGTCGTGAACAAGCCCGGAGTACATCTCCTTTGGAACTAGGAGACCCCTCCGGTCTTGTCC
AACCTCTGTTTCGGCAGAGGATACCGTGCTCCACAGCTGTAAGGCTGTGGGCCCGAGAGAAGTCGCTC
+
FFFFFFFFFFFFFFFFFFFFFFFFFFFFFFFFFFFFFFFFFF:FFFFFFFFFFFFFFFFFFFFFFFFF
FFFFFFFFFFFFFFFFFFFFFFFFFFFFFFFFFFFFFFFFFFFFFFFFFFFFFFFFFFFFFFFFFFFF
@A00155:342:HHGFNDSXY:1:1327:5014:17848 2:N:0:GAACCTAG+TCCGCATA
TAGACTACGACTTACACGGTAGGTCGTGAACAAGCCCGGAGTACATCTCCTTTGGAACTAGGAGACCC
CTCCGGTCTTGTCCAACCTCTGTTTCGGCAGAGGATACCGTGCTCCACAGCTGTAAGGCTGTGGGCCC
+
FFFFFFFFFFFFFFFFFFFFFFFFFFFFFFFFFFFFFFFFFFFFFFFFFFFFFFFFFFFFFFFFFF:F
FFFFFFFFFFFFFFFFFFF:FFFFFFFFFFFFFFFF:FFFFFFFFFFFFFFFFFFFFFFFFFFFFFFF
@A00155:342:HHGFNDSXY:1:1327:30572:25504 2:N:0:GAACCTAG+TCCGCATA
TCAGGTAGCCTACTAGACTACGACTTACACGGTAGGTCGTGAACAAGCCCGGAGTACATCTCCTTTGG
AACTAGGAGACCCCTCCGGTCTTGTCCAACCTCTGTTTCGGCAGAGGATACCGTGCTCCACAGCTGTA
+
FFFFFFFFFFFFFFFFFFFF:FFFFFFFFFFFFFFFFFFFFFFFFFFFFFFFFFFFFFFFFFFFFFFF
FFFFFFFFFFFFFFFFFFFFFFFFFFFFFFFFFFFF:FFFFFFFFFFFFFFFFFFFFFF:FFFFFFF:
@A00155:342:HHGFNDSXY:1:1327:24813:32722 2:N:0:GAACCTAG+TCCGCATA
GTGTAAGTTTCGTCCTAAAAAGGACTCGTCAGGTAGCCTACTAGACTACGACTTACACGGTAGGTCGT
GAACAAGCCCGGAGTACATCTCCTTTGGAACTAGGAGACCCCTCCGGTCTTGTCCAACCTCTGTTTCG
+
FFFFFFFFFFFFFFFFF:FFFFFFFF:FFFFFFFFFFFFFFFFFFFFFF,FFFFFFFF:FFFFFFFF
FFF,FFFFFFFFFFFFFFFFFFFFFFFFFFFF:FFFFFFFFFFFFFFFFFFFFFF:F:FFFFFFFFFF
@A00155:342:HHGFNDSXY:1:1327:25446:32753 2:N:0:GAACCTAG+TCCGCATA
GTGTAAGTTTCGTCCTAAAAAGGACTCGTCAGGTAGCCTACTAGACTACGACTTACACGGTAGGTCGT
GAACAAGCCCGGAGTACATCTCCTTTGGAACTAGGAGACCCCTCCGGTCTTGTCCAACCTCTGTTTCG
+
FFFFFF,FFFFFFFFFFFFFFFFFFFFFFFFFFFFFFFFFFFFFFFFF:FFFFFFFFFFFFFFFFFFF
FFFFFFFFFFFFFFFFFFFFFFFFFFFFFFFFFFFFFFFF::F:FFF:FFFFFFFF:FFFFFFFFFFFF
@A00155:342:HHGFNDSXY:1:1327:26096:33348 2:N:0:GAACCTAG+TCCGCATA
ACTAGACTACGACTTACACGGTAGGTCGTGAAAAAGCCCGGAGTACATCTCCTTTGGAACTAGGAGAC
CCCTCCGGTCTTGTCCAACCTCTGTTTCGGCAGAGGATACCGTGCTCCACAGCTGTAAGGCTGTGGG
+
```

```
FFFF:FFFF,FFFFFFFFF:FFFF:F,F::FF,FFFFFFFF:FFFFFFFF:FFF,,:FFFF,FFFFF:
,FFFFFF:F,F:F,FFFFFFFF:F:F,:F:FF,F,::,F:,:FF:FF:FF,FFFFFFF,FFF:FFFF
@A00155:342:HHGFNDSXY:1:1328:22263:8077 2:N:0:GAACCTAG+TCCGCATA
GGATACCGTGCTCCACAGCTGTAAGGCTGTGGGCCCGAGAGAAGTCGCTCTCTCTCGCCCTCTACATC
CCTCTCGGCATTGAGATGACCATAGGCCAGAGGTGAGTCCTTAAGTGGACACAGCTGATCTAAGG
+
FFFFFFFFFFFFFFFFFFFF:FFFFFFFFFFFFFFFFFFFFFFFFFFFFFFFFFFFFFFFFF:FFFFF
F:FFFFFFFFFFFFFFFFFFFFFFFFFFF:FFFFFFF:FF::FFF:FFFFFFFFF,FFFFFFF,FF
@A00155:342:HHGFNDSXY:1:1328:5656:21590 2:N:0:GAACCTAG+TCCGCATA
AAGCCCGGAGTACATCTCCTTTGGAACTAGGAGACCCCTCCGGTCTTGTCCAACCTCTGTTTCGGCAG
AGGATACCGTGCTCCACAGCTGTAAGGCTGTGGGCCCGAGAGAAGTCGCTCTCTCTCGCCCTCCACAT
+
FFF::FFFFFFFFFFFFFFFFFFF:FFF:FFFFFFFFFFFFFFFF:FF:FF:FFFFFFFFFFFFFFFF
FFFF:,FFFFFFFFFFFFFFFFFFFFFFF,FFFFFFFFF:F:FFFFFFFF:FFFFFFFFFFFFFFFFFF
@A00155:342:HHGFNDSXY:1:1328:17300:33270 2:N:0:GAACCTAG+TCCGCATA
TCCTTGGAACGGACTCATCAGGGAGCCTACTAGACTCCGACCGTCATGGGGGTTCAAACCCATGCCCC
GCCACACCGCCTTAGATCAGCTGTGTCCACTTAAGGACTCACCTCTGGCCTATGGTCATCTCAATGCC
+
FFFFFFFFFFFFFFFF:FFFFFFFFFFFFFFFFFFFFFFFFFFFFFFFFFFFFFFFFFFFFFFFFFFF
FFFFFFFFFFFFFFFFFFFFFFFFFFFFF:FFFFFFFFFFFFFFFFFFFFFFFFFFFFF,FFFFFFF:FF
@A00155:342:HHGFNDSXY:1:1329:1759:7420 2:N:0:GAACCTAG+TCCGCATA
CTCTTTGAGTTGTGTAAGTTTCGTCCTAAAAAGGACTCGTCAGGTAGCCTACTAGACTACGACTTACA
CGGTAGGTCGTGAACAAGCCCGGAGTACATCTCCTTTGGAACTAGGAGACCCCTCCGGTCTTGTCCAA
+
FF::FFF:FFF:FF:FF:FFFFFFFFFFFFFFFF:FFFFFFFFFFF:FFFFFFFFFFFFFFFFFF:FF
FFFFFFFFFFFFFF,FFFFFFFFFFFFFFFFF:FFFFFFFFFFFFFFFF:FFFFFFFFFFF:FFFFF
@A00155:342:HHGFNDSXY:1:1329:24858:21371 2:N:0:GAACCTAG+TCCGCATA
GAACTAGGAGACCCCTCCGGTCTTGTCCAACCTCTGTTTCGGCAGAGGATACCGTGCTCCACAGCTGT
AAGGCTGTGGGCCCGAGAGAAGTCGCTCTCTCTCGCCCTCCACATCCCTCTCGGCATTGAG
+
FFF:FFFFFFFFF:FFFFFFFFFFFFF:FFFFFF:F:FFF::FF:FFFFF:F,::FFFFFFFF,FFFF
FF:F,:FF:F,FFFFFFF,:F:F:F:FFFFFFF,:FFFF:FFFFF,FFFFFF,:FFF,FFF
@A00155:342:HHGFNDSXY:1:1329:9399:22091 2:N:0:GAACCTAG+TCCGCATA
AGCCCGGAGTACATCTCCTTTGGAACTAGGAGACCCCTCCGGTCTTGTCCAACCTCTGTTTCGGCAGA
GGATACCGTGCTCCACAGCTGTAAGGCTGTGGGCCCGAGAGAAGTCGCTCTCTCTCGCCCTC
+
FFFFFFFFFFFFFFFFFFFFFFFFFFFFFFF:FFFFFFFFFFFFFFFFFFFFFFFFFFFFFFFFFFFF
FFFFFFFFFFFFFFFFFFFFFFFFFFFFFFFFFFFFFFFFF:FFFFFFFFFFFFFFFFFFF
@A00155:342:HHGFNDSXY:1:1329:9688:23312 2:N:0:GAACCTAG+TCCGCATA
AAAAGGACTCGTCAGGTAGCCTACTAGACTACGACTTACACGGTAGGTCGTGAACAAGCCCGGAGTAC
ATCTCCTTTGGAACTAGGAGACCCCTCCGGTCTTGTCCAACCTCTGTTTCGGCAGAGGATACCGTGCT
+
FFFFFFFFFFFFFFFFFFFFFFFFFFFFFFFFFFFFFFFFFFFFFFFFFFFFFFFFFFFFFFFFFFFF
FFFFFFFFFFFFFFFFFFFFFFFFFFFFFFFFFF:FFFFFFFFFFFFFFFFFFFFFFFFFFFFFFFFF
@A00155:342:HHGFNDSXY:1:1329:17065:33708 2:N:0:GAACCTAG+TCCGCATA
TAGGAGACCCCTCCGGTCTTGTCCAACCTCTGTTTCGGCAGAGGATACCGTGCTCCACAGCTGTAAGG
CTGTGGGCCCGAGAGAAGTCGCTCTCTCTCGCCCTCTACATCCCTCTCGGCATTGAGATGACCATAGG
+
FFFFFFFFFFFFFFFFF:FFFFFFFFFFFFFFFFFFFFFFFFFFFFFFFFFFFFFFFFFFFFFFFFFF
FFFFFFFFFFFFFFFFF:FFFFFFFFFFFFFFFFFFFFFFFFF:FFFFFFFFFFFFFFFFFFFFFFFFFF
@A00155:342:HHGFNDSXY:1:1329:17291:36292 2:N:0:GAACCTAG+TCCGCATA
TAGGAGACCCCTCCGGTCTTGTCCAACCTCTGTTTCGGCAGAGGATACCGTGCTCCACAGCTGTAAGG
CTGTGGGCCCGAGAGAAGTCGCTCTCTCTCGCCCTCTACATCCCTCTCGGCATTGAGATGACCATAGG
+
```

FFFFFFFFFFFFFFFFFFFFFFFFFFFFFFFFFFFFFFFFFFF:FFFFFFFFFFFFFFFFFFFFFFFF
FFFFFFFFFFFFFFFFFFFFFFFFFFFFFFFFFFFFFFFFFFFFFFFFFFFF:FFFFFFFFFF,FFFF
@A00155:342:HHGFNDSXY:1:1330:21043:2362 2:N:0:GAACCTAG+TCCGCATA
GAACTAGGAGACCCCTCCGGTCTTGTCCAACCTCTGTTTCGGCAGAGGATACCGTGCTCCACAGCTGT
AAGGCTGTGGGCCCGAGAGAAGTCGCTCTCTCTCGCCCTCCACATCCCTCTCGGCATTGAGATGACCA
+
FFFFFFFFFFFFFFFFFFFFFFFFFFFFFFFFFFFFFFFFFFF:FFFFFFFFFFFFFFFFFFFFFFFF
FFFFFFFFFFFFFFFFFFFF,FFFFFFFFFFFFFFFFFFFFFFFFFFFFFFFFFF:FFFFFFFFFFFF
@A00155:342:HHGFNDSXY:1:1330:2889:5243 2:N:0:GAACCTAG+TCCGCATA
TCGGCAGAGGATACCGTGCTCCACAGCTGTAAGGCTGTGGGCCCGAGAGAAGTCGCTCTCTCTCGCCC
TTCACATCCCTCTCGGCATTGAGATGACCATAGGCCAGAGGTGAGTCCTTAAGTGGAC
+
FFFFFFF:F:FFFFFFFFFFFFFFFFFFFFFFFFFFFFFFFFFFFFFFFFFF:FFFFFFFFFFF:FFF
FFFFFFFFFFFFFFFFFF,FFFFFFFFFFFFF:FFFFFFFFFFFFF::FFFFFFFFFF
@A00155:342:HHGFNDSXY:1:1330:24397:24173 2:N:0:GAACCTAG+TCCGCATA
AAAAGGACTCGTCAGGTAGCCTACTAGACTACGACTTACACGGTAGGTTGTGAACAAGCCCGGAGTAC
ATCTCCTTTGGAACTAGGAGACCCCTCCGGTCTTGTCCAACCTCTGTTTCGGCAGAGGATACCGTGCT
+
FFF:FFFFFFFFFFFFFFFFFF:FF,FFFFFF:FFFFFFFFFFFF:FFFFFFFFFFFFFFFFFFFFFF
FFFFFFFFFFFFFFFFFFF:FFFFFFFFFFFF::FFFFFFFFFFFFFFFFFFFFFFFFFFFFFFFFFF
@A00155:342:HHGFNDSXY:1:1330:5810:32064 2:N:0:GAACCTAG+TCCGCATA
CCCCTCCGGTCTTGTCCAACCTCTGTTTCGGCAGAGGATACCGTGCTCCACAGCTGTAAGGCTGTGGG
CCCGAGAGAAGTCGCTCTCTCTCGCCCTTCACATCCCTCTCGGCATTGAGATGACCATAGGCCAGAGG
+
FFFFFFFFFFFFFFFFFFFFFFFFFFFFFFFFFFFFFFFFFFFFFFFFF:FFFFFFFFFFFFFFFFFF
FFFFFFFF:FFFFFFF:FFFFFFFFFFFFFFFFFFFFFFFFFFFF,:FFF:FFFFFFFFFFFFFFFFF
@A00155:342:HHGFNDSXY:1:1330:5674:32330 2:N:0:GAACCTAG+TCCGCATA
CCCCTCCGGTCTTGTCCAACCTCTGTTTCGGCAGAGGATACCGTGCTCCACAGCTGTAAGGCTGTGGG
CCCGAGAGAAGTCGCTCTCTCTCGCCCTTCACATCCCTCTCGGCATTGAGATGACCATAGGCCAGAGG
+
FFFFFFFFFFFFFFFFFFFFFFFFFFFFFFFFFFFFFFFFFFFFFFFFFFFFFFFFFFFFFFFFFFFF
FFFFFFFFF:FFFFFFFFFFFFFFFFFFFFFFFFFFFFFFFFFFF,FFFFFFFFFFFFFFFFFFFFFF
@A00155:342:HHGFNDSXY:1:1330:5023:36495 2:N:0:GAACCTAG+TCCGCATA
GACGAAACCGTCGTTACTCTTTGAGTTGTGTAAGTTTCGTCCTAAAAAGGACTCGTCAGGTAGCCTAC
TAGACTACGACTTACACGGTAGGTCGTGAACAAGCCCGGAGTACATCTCCTTTGGAACTAGGAGACCC
+
FFFFFFFFFFFFFFFFFFF,F:FFFFFFFF:F:FFFFFFFF:FFFFFFFFFFFFFFFFFF:FFFFFFF
FFFFFFFFFFFFFFFFFFFFFFFFFFFFFFFF:FFFFFFFFFFFFFFFFFFFFFFFFFFFFFFFFFFF
@A00155:342:HHGFNDSXY:1:1331:11822:9408 2:N:0:GAACCTAG+TCCGCATA
AAGCCCGGAGTACATCTCCTTTGGAACTAGGAGACCCCTCCGGTCTTGTCCAACCTCTGTTTCGGCAG
AGGATACCGTGCTCCACAGCTGTAAGGCTGTGGGCCCGAGAGAAGTCGCTCTCTCTCGCCCTCCACAT
+
FFFFFFF:FFFFFFFFFFFFFFFFFFFFFFFFF:FFFFFFFFFFFFFFFFFFFFFFFFFFFFFFFFFF
FFFFFFFFFFFFFFFFFFFFFFFF:FFFFFFFFFFFFFFFF:,FFFFFFFFFFFFFFFFFFFFFFFFF
@A00155:342:HHGFNDSXY:1:1331:31783:10473 2:N:0:GAACCTAG+TCCGCATA
GGTCTTGTCCAACCTCTGTTTCGGCAGAGGATACCGTGCTCCACAGCTGTAAGGCTGTGGGCCCGAGA
GAAGTCGCTCTCTCTCGCCCTCCACATCCCTCTCGGCATTGAGATGACCATAGGCCAGAGGTGAGTCC
+
FFFF,FFFFF:FFFFFFF,FFFFFFFFFFFFFFFFFFFFFFFFFFFFFFFFFFFFFFFFFFFFFFFFF
FFFFFFFFFFFFFFFFFFFFFFFFFFFFFFFFFFFFF:F:FFFFFFFFFFFFFFFFFFFFFFFFFFF:
@A00155:342:HHGFNDSXY:1:1331:31919:10614 2:N:0:GAACCTAG+TCCGCATA
GGTCTTGTCCAACCTCTGTTTCGGCAGAGGATACCGTGCTCCACAGCTGTAAGGCTGTGGGCCCGAGA
GAAGTCGCTCTCTCTCGCCCTCCACATCCCTCTCGGCATTGAGATGACCATAGGCCAGAGGTGAGTCC
+

```
FFFFFFFFFFFFFFFFFFFFFFFFF:FFFF:FFFFFFFFFFFFFFFFFFFF,FFF:FFFFFFFFFFFF
FF,FFFFFFFFF:FFFFFFFFFFFFFFFFFFFFFFFF:,FFFFFFFFFFFFFFFFFFFFFFFFFFFFF
@A00155:342:HHGFNDSXY:1:1332:23656:9079 2:N:0:GAACCTAG+TCCGCATA
CTCCACAGCTGTAAGGCTGTGGGCCCGAGAGAAGTCGCTCTCTCTCGCCCTCCACATCCCTCTCGGCA
TTGAGATGACCATAGGCCAGAGGTGAGTCCTTAAGTGGACACAGCTGATCTAAGGCGGTGTGGCGGGG
+
FFFFFFFFFFFFFFFFFFFFFFFFFFFFFFFFFFFFFFFFFF:FFFFFFFFFFFFFFFFFFFFFFFFF
,FFFFFFFFFF:FFFFFFFFFFFFFFFFFFFFFFFFFFFF,FFFFFFFFFFFFFFFFFFFFFFFFFFF
@A00155:342:HHGFNDSXY:1:1332:25337:11647 2:N:0:GAACCTAG+TCCGCATA
CTCGAAGAGTAACGACGGTTTCGTCCTTGGAACGGACTCATCAGGGAGCCTACTAGACTCCGACCGTC
ATGGGGGTTCAAACCCATGCCCCGCCACACCGCCTTAGATCAGCTGTGTCCACTTAAGGACTCACCTC
+
FFFFFFFFFFFFFFFFFFFFFFFFFFFFFFFFFFFFFFFFFFFFFFFFFFFFFFFFFFFFFFFFFFFF
F:FFFFF:FFFFFFFFFFFFFFFFFFFFFFFFFFFFFFFFFFFFFFFFFFFFFFFFFFFFFFFFFFFF
@A00155:342:HHGFNDSXY:1:1332:24632:35540 2:N:0:GAACCTAG+TCCGCATA
CCTTTTTAGGACGAAACTTACACAACTCGAAGAGTAACGACGGTTTCGTCCTTGGAACGGACTCATCA
GGGAGCCTACTAGACTCCGACCGTCATGGGGGTTCAAACCCATGCCCCGCCACACCGCCTTAGATCAG
+
FFFF:FFFFFFFFF:FFFFFFFFFFFFFFFFFFFFFFFFFFFFFFFFFFFFFFFFF:FFFFFFFFFFF
FFFFFFFFFF:FFFFFFFFFF:FFFFFFFFFFFFFFFFFFFFFFFFFFFFFFFFFFFFFFFFFFFFFF
@A00155:342:HHGFNDSXY:1:1333:19849:2394 2:N:0:GAACCTAG+TCCGCATA
CGTTACTCTTCGAGTTGTGTAAGTTTCGTCCTAAAAAGGACTCGTCAGGTAGCCTACTAGACTACGAC
TTACACGGTAGGTCGTGAACAAGCCCGGAGTACATCTCCTTTGGAACTAGGAGACCCCTCCGGTCTTG
+
FFFFFFFFFFFF:FFFFFFF:FFF:FFFFFF:FFFFFFFFFFFFFFFFFFFFFFFFFFFFFFFFFFFF
FFFFFFFFFFFFFFFFF:FFFFFFFFFFFFFFFFFFFFF:FFFFFFFFFFFFFFFFFFFFFFF:FFFF
@A00155:342:HHGFNDSXY:1:1333:23312:9111 2:N:0:GAACCTAG+TCCGCATA
GGACTCGTCAGGTAGCCTACTAGACTACGACTTACACGGTAGGTCGTGAACAAGCCCGGAGTACATCT
CCTTTGGAACTAGGAGACCCCTCCGGTCTTGTCCAACCTCTGTTTCGGCAGAGGATACCGTGCTCCAC
+
:FFFFFFFFFFFF:FFFFFFFFFFFFFFFFFFFFFFFFFFFFF:FFFFFFFFFFFFFFFFFFFFFFFF
FFFFFFFFFFFFFFFFFFFFFFFFFFFF:FFFFFFFFFFF:F:FFFFFFFFFFFFFFFFFFFFFFFFF
@A00155:342:HHGFNDSXY:1:1333:18783:14544 2:N:0:GAACCTAG+TCCGCATA
AGACTACGACTTACACGGTAGGTCGTGAACAAGCCCGGAGTACATCTCCTTTGGAACTAGGAGACCCC
TCCGGTCTTGTCCAACCTCTGTTTCGGCAGAGGATACCGTGCTCCACAGCTGTAAGGCTGTGGGCCC
+
FFFFFFF:FFFFFFFFFF:FFFFFFFFFFFFF:FFFFFFFFFFFFFFFFFFFFFFFF,FFFFFFF:FF
FFFF:FF::F:FFFFFFFFFFFFFF:FFFFFFFF:FFFFFFFFF:FFFFFFFFFFFFFFFFFFFFFF
@A00155:342:HHGFNDSXY:1:1333:23285:17487 2:N:0:GAACCTAG+TCCGCATA
TTACACGGTAGGTCGTGAACAAGCCCGGAGTACATCTCCTTTGGAACTAGGAGACCCCTCCGGTCTTG
TCCAACCTCTGTTTCGGCAGAGGATACCGTGCTCCACAGCTGTAAGGCTGTGGGCCCGAGAGAAGTCG
+
FFFFFFFFFFFF:FFFFFFFFFFFFFFFFFFFFFFFFFFFFFFFFFFFFFFFFFFFFFFFFFFF::FF
FFFFFFFFFFFFFFFFFFFFFFFFFFFFFFFFFFFFFFFFFFFFFFFFFFFFFFFFFF:FFFF:FFFF
@A00155:342:HHGFNDSXY:1:1333:5150:20838 2:N:0:GAACCTAG+TCCGCATA
ACGAAACCGTCGTTACTCTTCGAGTTGTGTAAGTTTCGTCCTAAAAAGGACTCGTCAGGTAGCCTACT
AGACTACGACTTACACGGTAGGTCGTGAACAAGTCCGGAGTACATCTCCTTTGGAACTAGGAGACCCC
+
FFFFFFFFFFFFFFFFF:FFFFFFFFFFFFFFFFFFFFFFF:FFFFFFFFFFFFFFFFFFFFFFFFFF
FFFF:FFFFFFFFFFFFFFFFFFFFFFFFFFFFFFFFFFFFFFFFFFF:FFFFFFFFFFFFFFFFFF
@A00155:342:HHGFNDSXY:1:1334:31937:1282 2:N:0:GAACCTAG+TCCGCATA
ACTAGACTACGACTTACACGGTAGGTCGTGAACAAGCCCGGAGTACATCTCCTTTGGAACTAGGAGAC
CCCTCCGGTCTTGTCCAACCTCTGTTTCGGCAGAGGATACCGTGCTCCACAGCTGTAAGGCTGTGGGC
+
```

FFFFFFFF:FFFFFFFFFFFFFFFFFFFFFFFFFFFFFFFFFFFFFFFFFFFF,FFFFF:F,FFFFFFF
FFFFFFFFFFFFFFFFFFFFFFFFFFFFFFFFFFFFFF:FFFFFFFFFFFFFFFFFF,FFFFFFFFFF
@A00155:342:HHGFNDSXY:1:1335:30825:6652 2:N:0:GAACCTAG+TCCGCATA
GGACTCGTCAGGTAGCCTACTAGACTACGACTTACACGGTAGGTCGTGAACAAGCCCGGAGTACATCT
CCTTTGGAACCAGGAGACCCCTCCGGTCTTGTCCAACCTCTGTTTCGGCAGAGGATACCGTGCTCCAC
+
FFFF:FFFFFFFFFFFFFFFFFFFF:FFFFFFFFFFFFFFFFFFFFFFFFFFFFFFFFFFF:FFFFFFF
FFFFFFFF:FFFFFFFFFFFFFFFFFFFFFFFFFFFFFFFFFFFFFFFFFFF::FFFFFFFFFFF
@A00155:342:HHGFNDSXY:1:1335:25102:7983 2:N:0:GAACCTAG+TCCGCATA
AAAAGGACTCGTCAGGTAGCCTACTAGACTACGACTTACACGGTAGGTCGTGAACAAGCCCGGAGTAC
ATCTCCTTTGGAACTAGGAGACCCCTCCGGTCTTGTCCAACCTCTGTTTCGGCAGAGGATACCGTGCT
+
FFFFFFFFFFFFFFFFFFFFFFFFFFFFFFFFFFFFFFFFFFFFFFFFFFF:FFFFFFFFFFFFFFFF
:FFFFFFFFFFF,FFFFFFFFFFFFFFFFF:F:FFFFFFFFFFFFFFFFFFFFFFFF:FFF,FFFFFFF
@A00155:342:HHGFNDSXY:1:1335:10321:14325 2:N:0:GAACCTAG+TCCGCATA
GGTCGTGAACAAGCCCGGAGTACATCTCCTTTGGAACTAGGAGACCCCTCCGGTCTTGTCCAACCTCT
GTATCGGCAGAGGATACCGTGCTCCACAGCTGTAAGGCTGTGGGCCCGAGAGAAGTCGCTCTCTCTCG
+
FFFFFFFFFFFFFFFFFFFFFFFFFFFFFFF:FFFFFFFFFFFFFFFFFFFFFFFFFFFFFFFFFFFFF
FFFFFFFFFFFFFFFFFFFFFFFFFFFFFFFFFFFFFFF:FFFFFFFFFFFFFFFFFFFFFFFFFFFFF
@A00155:342:HHGFNDSXY:1:1336:14850:4946 2:N:0:GAACCTAG+TCCGCATA
GTTACTCTTCGAGTTGTGTAAGTTTCGTCCTAAAAAGGACTCGTCAGGTAGCCTACTAGACTACGACT
TACACGGTAGGTCGTGAACAAGCCCGGAGTACATCTCCTTTGGAACTAGGAGACCCCTCCGGTCTTGT
+
FFFFFFFFFFFFFFFFFFFFFFFFFFFFFFFFFFFFFFFFFFFFFFFFFFFFFFFFFFFFFFFFFFFF
FFFFFFFFFFFFFFFFF,FFF,FFFF:FFFFFFFFFFFFFFFFFFFFFFFFFFFFFFFFFFFFFFFFF
@A00155:342:HHGFNDSXY:1:1336:24062:9095 2:N:0:GAACCTAG+TCCGCATA
TTTGTGTAAGTTTCGTCCTAAAAAGGACTCGTCAGGTAGCCTACTAGACTACGACTTACACGGTAGGT
CGTGAACAAGCCCGGAGTACATCTCCTTTGGAACTAGGAGACCCCTCCGGTCTTGTCCAACCTCTGTT
+
FFFFFFFFFFFFFFFFFFF,FF:FFF:FF:FFFFFFF:FFFF:FFFFFFFFFFFFFFFFFFFFFFFFFF
FFFFFFFFFFFFFFFFFFFFFFFFFFFFFFFFFFFFFFFFFFFFF:FFFFFFFFFFFFFFFFFFFFF:F
@A00155:342:HHGFNDSXY:1:1337:22598:13322 2:N:0:GAACCTAG+TCCGCATA
ACCGCCTTAGATCAGCTGTGTCCACTTAAGGACTCACCTCTGGCCTATGGTCATCTCAATGCCGAGAG
GGATGTGAAGGGCGAGAGAGAGCGACTTCTCTCGGGCCCACAGCCTTACAGCTGTGGAGCACGGTATC
+
FFFFF:FFFFFFFFFFFFFFFFFFFF:FFFFFFFFFFFFFFFFFFF,FFFFFFFFFFFFFFFFFFFFFF
FFFFFFFFFFFFFF::FFFFFFFFFFFFFFFFFFFFFFFFFFFFFFFFFFFFFFFFFFFFFFFFFFFF
@A00155:342:HHGFNDSXY:1:1337:31403:15702 2:N:0:GAACCTAG+TCCGCATA
CCTTTTTAGGACGAAACTTACACAACTCGAAGAGTAACGACGGTTTCGTCCTTGGAACGGACTCATCA
GGGAGCCTACTAGACTCCGACCGTCATGGGGGTTCAAACCCATGCCCCACCACACCGCCTTAGATCAG
+
F:F:FFF:FFFFFFFFFF:FFFFFFFFFFFFFFFFFFFFFFFFF,FFFF:FFFF:FF:FF:FFFFFFFF
FFFFF:FFFFFFF:FFFFFFFFFFFFFF,FF:F,FFFFFFFF,FFFFFFFFFFFFFFFFFFF:FFFFF
@A00155:342:HHGFNDSXY:1:1337:19361:25504 2:N:0:GAACCTAG+TCCGCATA
ACTACGACTTACACGGTAGGTCGTGAACAAGCCCGGAGTACATCTCCTTTGGAACCAGGAGACCCCTC
CGGTCTTGTCCAACCTCTGTTTCGGCAGAGGATACCGTGCTCCACAGCTGTAAGGCTGTGGGCCCGAG
+
FFFFFFFFFFFFFFFFF:FFFFFF:FFFFFFFFFFFFFFFFFFFFFFFFFFFFFFFFFFFFFFFFFFF
FFFFFFFFFFFFFFFFFFFFFFFFFFFFFFFFFFFFFFFFFFFFFFFFFFFFFFFFFFFFFFFFFFFF
@A00155:342:HHGFNDSXY:1:1338:16016:13103 2:N:0:GAACCTAG+TCCGCATA
CCTACTAGACTACGACTTACACGGTAGGTCGTGAACAAGCCCGGAGTACATCTCCTTTGGAACCAGGA
GACCCCTCCGGTCTTGTCCAACCTCTGTTTCGGCAGAGGATACCGTGCTCCACAGCTGTAAGGCTGTG
+

FFFFFFFFFFFFFFFFFFFFFFFFFFFFFFFFFFFFFFFFFFFFFFFFFFFFFFFFFFFFFFFFFFFFFF
FFFFFFFFFFFFFFFFFFFFFFFFFFFFFFFFFFF,FFFFF,FFFFFFF:FFFFFFF:FFFFFFFFFFF
@A00155:342:HHGFNDSXY:1:1338:11388:30765 2:N:0:GAACCTAG+TCCGCATA
GAGACCCCTCCGGTCTTGTCCAACCTCTGTTTCGGCAGAGGATACCGTGCTCCACAGCTGTAAGGCTG
TGGGCCCGAGAGAAGTCGCTCTCTCTCGCCCTCCACATCCCTCTCGGCATTGAGATGACCATAGGCCA
+
FFFFFFFFFFFFFFFFFFFFFFFFFFFFFFFFFFFFFFFFFFFFFFFFFFFFFFFFFFFFFFFFFFFFFF
FFFFFFFFFFFFFFFFFFFFFFFFFFFFFFFFFFFFFFFFFFFFFFFFFFFFFFFFFFFFFFF,FFFFFF
@A00155:342:HHGFNDSXY:1:1339:19976:7560 2:N:0:GAACCTAG+TCCGCATA
ACTCTTCGAGTTGTGTAAGTTTCGTCCTAAAAAGGACTCGTCAGGTAGCCTACTAGACTACGACTTAC
ACGGTAGGTCGTGAACAAGCCCGGAGTACATCTCCTTTGGAACTAGGAGACCCCTCCGGTCTTGTCCA
+
FFFFFFFF:FFFFFF:FFFF,FFFFFFFFFFFFFFFFFFFFFFFFFFFFFFFFFFFFFFFFFFFFFFFFF
FFFFFFFFFFFFFFFFFFFFFFFFFFFFFFFFFFFFFFFFFFFFFFFFFF:FFFFFFFFFFFFFFF
@A00155:342:HHGFNDSXY:1:1339:23656:8703 2:N:0:GAACCTAG+TCCGCATA
TTACACGGTAGGTCGTGAACAAGCCCGGAGTACATCTCCTTTGGAACTAGGAGACCCCTCCGGTCTTG
TCCAACCTCTGTTTCGGCAGAGGATACCGTGCTCCACAGCTGTAAGGCTGTGGGCCCGAGAGAAGTCG
+
FFFFFFFFFFFFFFFFF:FFFFFFFFFFFFFFFFFFFFFFFFFFFF,FFFFFFFFFFFFFFFFFFFF:F
FFFFFFFFFFFFFFFFFFFFFFFFFFFFFFFFFFFFFFFFFFFFFFFFFFFFFFFFFFFFFFFF:FFFF
@A00155:342:HHGFNDSXY:1:1339:28980:8750 2:N:0:GAACCTAG+TCCGCATA
AGTACATCTCCTTTGGAACTAGGAGACCCCTCCGGTCTTGTCCAACCTCTGTTTCGGCAGAGGATACC
GTGCTCCACAGCTGAAAGGCTGTGGGCCCGAGAGAAGTCGCTCTCTCTCGCCCTCCACATCCCTCTCG
+
FFFFFFFFFFFFFFFFFFFFFFFFFFF:FFFFFFFFFFFFFFFFFFFFFFFFFFFFFFFFFFFFFFFFFF
FFFFFFFFFFF:FFFFFFFFFFFFFFFFFFFFFFFFFFFFFFFFFFFFFFFF:FFFFFFFF:FFF:FFFFF
@A00155:342:HHGFNDSXY:1:1339:30563:9142 2:N:0:GAACCTAG+TCCGCATA
AGTACATCTCCTTTGGAACTAGGAGACCCCTCCGGTCTTGTCCAACCTCTGTTTCGGCAGAGGATACC
GTGCTCCACAGCTGAAAGGCTGTGGGCCCGAGAGAAGTCGCTCTCTCTCGCCCTCCACATCCCTCTCG
+
FF:FFFFFFFFFFFFFFFFFF:FFFFFFFFFFFFFFFFFFFFFFFFFFFFFFFF,FFFFFF:FFFF:FFF
:FFFFFFFFFFFFFFFF:FFFFFFFFFFFF:FFFFF,FFFFFFFFFFFFFFFFFFFFFFFFF:FFFFFF
@A00155:342:HHGFNDSXY:1:1339:13557:12727 2:N:0:GAACCTAG+TCCGCATA
CCATGCCCCGCCACACCGCCTTAGATCAGCTGTGTCCACTTAAGGACTCACCTCTGGCCTATGGTCAT
CTCAATGCCGAGAGGGATGTGGAGGGCGAGAGAGAGCGACTTCTCTCGGGCCCACAGCCTTACAGCTG
+
FFFFFFFFFFFFFFFFFFFFFFFFFFFFFFFFFFFFFFFFFFFFFFFFFFFFFFFFFFFFFFFFFFFFFF
FFFFFFFFFFFFFFFFFF,FFFFFFFFFFFFFFFFFFFFFFFFFFFFFFFFFFFFFFFFF:FFFFFFFF
@A00155:342:HHGFNDSXY:1:1340:23900:9627 2:N:0:GAACCTAG+TCCGCATA
GTTGTGTAAGTTTCGTCCTAAAAAGGACTCGTCAGGTAGCCTACTAGACTACGACTTACACGGTAGGT
CGTGAACAAGCCCGGAGTACATCTCCTTTGGAACTAGGAGACCCCTCCGGTCTTGTCCAACCTCTGTT
+
FF:FFFFFFFFFFFFFFFFFFFFFFFFFFFFFFFFFFFFFFFFFFFFFFFFFFFFFFFFFFFFFFFFFFF
FFFFFFFFFFFFFFFFFFFFFFFFFFFFFFFFFFFFFFFFFF:FFFFFFFFF,FFFFFFFFFFFFFF
@A00155:342:HHGFNDSXY:1:1340:11758:31469 2:N:0:GAACCTAG+TCCGCATA
TAGGTCGTGAACAAGCCCGGAGTACATCTCCTTTGGAACTAGGAGACCCCTCCGGTCTTGTCCAACCT
CTGTTTCGGCAGAGGATACCGTGCTCCACAGCTGTAAGGCTGTGGGCCCGAGAGAAGTCGCTCTCTCT
+
FFFFFFFFFFFFFFFFFFFFFFFFFFF:FFFFFFFF,FFFFFFFFFFFFFFFFFFFFFFFFFFFFFFFF
FFFFFFFFF::FFFFFFFFFFFFFFFFFFFFFFFFFFFFFFFFFFFFFFFFFFFFFFFFFFFFFFFFFFF
@A00155:342:HHGFNDSXY:1:1341:17427:7216 2:N:0:GAACCTAG+TCCGCATA
AGTACATCTCCTTTGGAACTAGGAGACCCCTCCGGTCTTGTCCAACCTCTGTTTCGGCAGAGGATACC
GTGCTCCACAGCTGTAAGGCTGTGGGCCCGAGAGAAGTCGCTCTCTCTCGCCCTCTACATCCCTCTCG
+

```
FFFFFFFFFFFFFFFFF:FFFFFFFFFFFFFFFFFFF::FFFFFFFFFFFF:FFFFFFFFFFFFFFFF
FFFFFFFFFFFF:FFFFFFFFFFFFFFFFFFFFFFFFFFFFFFFFFFFFFFF:FFFFFFFFFFFFFFF
@A00155:342:HHGFNDSXY:1:1341:3134:22388 2:N:0:GAACCTAG+TCCGCATA
GAGACCCCTCCGGTCTTGTCCAACCTCTGTTTCGGCAGAGGATACCGTGCTCCACAGCTGTAAGGCTG
TGGGCCCGAGAGAAGTCGCTCTCTCTCGCCCTCTACATCCCTCTCGGCATTGAGATGACCATAG
+
FFFFFFFFFFFFFFFFFFFFFFFFFFFFFFFFFFFFFFFFFFFF:FFFFFFFFFFFFFFFF:FFFFFF
FFFFFFFFFFFF:FFFFFFFFFFFFFFFFFFFFFFFFFFFFFFFFFFFFFFFFFFFFFFF:FF
@A00155:342:HHGFNDSXY:1:1341:29279:23547 2:N:0:GAACCTAG+TCCGCATA
ACCGTGCTCCACAGCTGTAAGGCTGTGGGCCCGAGAGAAGTCGCTCTCTCTCGCCCTCCACATCCCTC
TCGGCATTGAGATGACCATAGGCCAGAGGTGAGTCCTTAAGTTGACACAGCTGATCTAAGGCGGTGTG
+
FFFFFFFFFFFFFFFFFFFFFFFFFFFFFFFFFFFFFFFFFFFFFFFFFFFFFFFFFFFFFFFFFFFF
FFFFFF:FFFFFFFFFFFFFFFFFFFFFFFFFFFFFFFFFFFFFFF:FFFFFFFFFFFFFFFFFFFFF
@A00155:342:HHGFNDSXY:1:1341:17662:26036 2:N:0:GAACCTAG+TCCGCATA
TTTAGGACGAAACTTACACAACTCGAAGAGTAACGACGGTTTCGTCCTTGGAACGGACTCATCAGGGA
GCCTACTAGACTCCGACCGTCATGGGGGTTCAAACCCATGCCCCACCACACCGCCTTAGATCAGCTGT
+
FFFFFFFFFFFFFFFFFFFFFFFFFFFFFFFFFFFFFFFFFFFFFFFFFFFFFFFFFFFFFFF:FFFF
FFFFFFFFFFFFFFFFFFFFFFFFFFFFFFFFFFFFF,FFFFFFFFFFFFFFFFFFFFFFFF:FFFFF
@A00155:342:HHGFNDSXY:1:1341:18602:29575 2:N:0:GAACCTAG+TCCGCATA
TTTAGGACGAAACTTACACAACTCGAAGAGTAACGACGGTTTCGTCCTTGGAACGGACTCATCAGGGA
GCCTACTAGACTCCGACCGTCATGGGGGTTCAAACCCATGCCCCACCACACCGCCTTAGATCAGCTGT
+
FFFFFFFF:FFFFFFFFFFFFFFFFFFFFFFFFFFFFFFFFFFFFFF:FFFFFFFFFFFFFFFFFFF,
FFFFFFFFFFFFFFFFFFFFFFFFFFFFFFFFFFFFFFFFFFFFFFFFFFFFFFF:FFFFFFFFFFFF
@A00155:342:HHGFNDSXY:1:1341:21630:30342 2:N:0:GAACCTAG+TCCGCATA
TAGGAGACCCCTCCGGTCTTGTCCAACCTCTGTTTCGGCAGAGGATACCGTGCTCCACAGCTGTAAGG
CTGTGGGCCCGAGAGAAGTCGCTCTCTCTCGCCCTCCACATCCCTCTCGGCATTGAGATGACCATAGG
+
FFFFFFFFFFFFFFFFFFFFFFFFFFFFFFFFFFFFFFFFFFFFFFFFFFFFFFFFFFFFFFFFFFFF
FFFFFFFFFFFFFFFFFFFFFFFFFFFFFFFFFFFFFFFFFFFFFFFFFFFFFFFFFFFFFFFF,:FF
@A00155:342:HHGFNDSXY:1:1342:21287:9799 2:N:0:GAACCTAG+TCCGCATA
ACTAGGAGACCCCTCCGGTCTTGTCCAACCTCTGTTTCGGCAGAGGATACCGTGCTCCACAGCTGTAA
GGCTGTGGGCCCGAGAGAAGTCGCTCTCTCTCGCCCTCCACATCCCTCTCGGCATTGAGATGAC
+
FFFFFFFFFFFFFFFFFFFFFFFFFFFFFFFFFFFFFFFFF:FFFFFFFFFFFFFFFFFFFFFFFFFF
FFFFFFFFFFFFFFFFFF:FFFFFFFFF:FFFFFFFFFFFF,FFFFFFFFFFF:FFFFFFFFFF
@A00155:342:HHGFNDSXY:1:1342:16342:10817 2:N:0:GAACCTAG+TCCGCATA
GAACTAGGAGACCCCTCCGGTCTTGTCCAACCTCTGTTTCGGCAGAGGATACCGTGCTCCACAGCTGT
AAGGCTGTGGGCCCGAGAGAAGTCGCTCTCTCTCGCCCTCCACATCCCTCTCGGCATTGAGATGAC
+
FFFFFFFFFFFF:FFFFFFFFFFFFFFFFFFFFFFFFFFFFFFFFFFFFFFFFFFFFFFFFFFFFFF
FFFFFFFF:FFFFFFFFFFFFFFFFFFFFFFFFFFF,FFFFFFFFFFFFFFFFFFFFFFFF:FFFF
@A00155:342:HHGFNDSXY:1:1342:22299:12868 2:N:0:GAACCTAG+TCCGCATA
CGTTACTCTTCGAGTTGTGTAAGTTTCGTCCTAAAAAGGACTCGTCAGGTAGCCTACTAGACTACGAC
TTACACGGTAGGTCGTGAACAAGCCCGGAGTACATCTCCTTTGGAACTAGGAGACCCCTCCGGTCTTG
+
FFFFFFF:FFFFFFFFFFFFF:FFFF:FFFFFF,FFFFFFFFFFFFFFFFFFFFFFFFFFFFFFFFFF
FFFFFFFFFFFF,FFFFFFFFFFFFFFFFFFFFFFFFFF:FFFFFFFFF::FFFFFF:FFFFFFFFFF
@A00155:342:HHGFNDSXY:1:1342:28149:13604 2:N:0:GAACCTAG+TCCGCATA
AAACTTACACAACTCAAAGAGTAACGACGGTTTCGTCCTTGGAACGGACTCATCAGGGAGCCTACTAG
ACTCCGACCGTCATGGGGGTTCAAACCCATGCCCCGCCACACCGCCTTAGATCAGCTGTGTCCACTTA
+
```

FFFFFFFFFFFFFFFFFFFFFFFFFFFFFFFFFFFFFFFFFFFFFF:F:FFFFFFFF,FF:FFFFFFF
FFFFFFFFFFF:FFFFFF,FFFFFFFFFFFFFFFFFFFFFFF,FFFFFFFFFFFFFFFFFFFFFFFFF
@A00155:342:HHGFNDSXY:1:1342:2293:18114 2:N:0:GAACCTAG+TCCGCATA
TACACGGTAGGTCGTGAACAAGCCCGGAGTACATCTCCTTTGGAACCAGGAGACCCCTCCGGTCTTGT
CCAACCTCTGTTTCGGCAGAGGATACCGTGCTCCACAGCTGTAAGGCTGTGGGCCCGAGAGAAGTCGC
+
FFFFFFFFFFFFFFFFFFFFFFFFFFFFFFFFFFFFFFFFFFFFFFFFFFFFFFFFFFFFFFFFFFFF
FFFFFFFFFFFFFFFFFFFFFF:FFFFFFFFFFFFFFFFFFFFFFFFFFFFFFF:FFFFFFFFFFFFF
@A00155:342:HHGFNDSXY:1:1342:8278:20055 2:N:0:GAACCTAG+TCCGCATA
ACTCGTCAGGTAGCCTACTAGACTACGACTTACACGGTAGGTCGTGAACAAGCCCGGAGTACATCTCC
TTTGGAACTAGGAGACCCCTCCGGTCTTGTCCAACCTCTGTTTCGGCAGAGGATACCGTGCTCCACAG
+
FFF,FFFFFFFF,FFFFFFFFFFFFFFFFFFFFFFF:FFFFFFFFFFFFFFFFFFFFFFFFFFFFFFF
FFFFFFFFFFFFFFFFFFFFFFF:FF,FFFFFF:FFFF,FFFFFFFFFFFFFFF:FFFFFF:F:FFFF
@A00155:342:HHGFNDSXY:1:1342:13286:24220 2:N:0:GAACCTAG+TCCGCATA
GGACTCTCCTCTGGCCTATGGTCATCTCAATGCCGAGAGGGATGTGGAGGGCGAGAGAGAGCGACTTC
TCTCGGGCCCACAGCCTTACAGCTGTGGAGCACGGTATCCTCTGCCGAAACAGAGGTTGGACAAGACC
+
FFFFFFFFFFFFFFFFFFFFFFFFFFFFFFFFFFFFFFFFFFFFFFFFFFFFFFFFFFFFFFFFFFFF
FFFFFFFFFFFFFFFFFFFFFFFFFFFFFFFFFFFFFFFFFFFFFFFFFFFFFFFFF:FF:FFFFFFF
@A00155:342:HHGFNDSXY:1:1343:12481:17973 2:N:0:GAACCTAG+TCCGCATA
ACATCTCCTTTGGAACTAGGAGACCCCTCCGGTCTTGTCCAACCTCTGTTTCGGCAGAGGATACCGTG
CTCCACAGCTGTAAGGCTGTGGGCCCGAGAGAAGTCGCTCTCTCTCGCCCTCCACATCCCTCTCGGCA
+
FFFFFFFFF:FFFFFFFFFFFFFFFFFFFFFFFFFFFFFFFFFFFFFFFFFFFFFFFFFFFFFFFFFF
FFFFFFFFFFFFFFF:FFFFFFFFFFFFFFFF:FFFFFFFFFFFFFFFFFFFFFFFFFFFFFFFFFFF
@A00155:342:HHGFNDSXY:1:1343:11831:18912 2:N:0:GAACCTAG+TCCGCATA
ACATCTCCTTTGGAACTAGGAGACCCCTCCGGTCTTGTCCAACCTCTGTTTCGGCAGAGGATACCGTG
CTCCACAGCTGTAAGGCTGTGGGCCCGAGAGAAGTCGCTCTCTCTCGCCCTCCACATCCCTCTCGGCA
+
FFFFFFFFFFFFFFFFFFFFFFFFFFFFFFFFFFFFFFFFFFFFFFFFFFFFFFFFFFFFFFFFFFFF
FFFFFFFFFFFFFFFFFFFFFFFFFFFFFFFFFFFFFFFFFFFFFFFFFFFFFFFFFFFFFFFFFFFF
@A00155:342:HHGFNDSXY:1:1344:30020:12743 2:N:0:GAACCTAG+TCCGCATA
GTGTAAGTTTCGTCCTAAAAAGGACTCGTCAGGTAGCCTACTAGACTACGACTTACACGGTAGGTCGT
GAACAAGCCCGGAGTACATCTCCTTTGGAACTAGGAGACCCCTCCGGTCTTGTCCAACCTCTGTTTCG
+
FFFFFFFFFFFFFFFFFFFF:FFFFFFFFFFFFFFFFFFFFFFFFFFFFFFFFFFFFFFFFFFFFFFF
:FFFFFFFFFFFFFFFFFFFFFFFFFFFFFFFFFFFFFF:FFFFFFFFFFFFFFFFFF:FFFF:FFFF
@A00155:342:HHGFNDSXY:1:1345:18548:3490 2:N:0:GAACCTAG+TCCGCATA
GACCGTCATGGGGGTTCAAACCCATGCCCCGCCACACCGCCTTAGATCAGCTGTGTCCACTTAAGGAC
TCACCTCTGGCCTATGGTCATCTCAATGCCGAGAGGGATGTGGAGGGCGAGAGAGAGCGACTTCTCTC
+
FFFFFFFFFFFFFFFFFFFFFFFFFFFFFFFFFFFFFFFFF:FFFFFFFFFFFFFFFFFFF:FFFF:F
FFFFF:FFFFFFF:FFFFF,FFFFFFFFFFFFFFFFFF:FFFFFFFFFFFFFFFFFFFFFF:FFFFFF
@A00155:342:HHGFNDSXY:1:1345:3748:4946 2:N:0:GAACCTAG+TCCGCATA
CCGTTCCAAGGACGAAACCGTCGTTACTCTACGAGTTGTGTAAGTTTCGTCCTAAAAAGGACTCGTCA
GGTAGCCTACTAGACTACGACTTACACGGTAGGTCGTGAACAAGCCCGGAGTACATCTCCTTTGGAA
+
FFFFFFFFFFFFFFFFFFFFFFFFFFFFFFFFFFFFFFFFFFFFFFFFFFFFFFFFFFFFFFFFFFFF
FFFF:FFFFFFFFFFFFFFFFFFFFFFFFFFFFFFFFFFFFFFFFFFFFFFFFFFFFFFFF,FFFF
@A00155:342:HHGFNDSXY:1:1345:3640:4977 2:N:0:GAACCTAG+TCCGCATA
CCGTTCCAAGGACGAAACCGTCGTTACTCTACGAGTTGTGTAAGTTTCGTCCTAAAAAGGACTCGTCA
GGTAGCCTACTAGACTACGACTTACACGGTAGGTCGTGAACAAGCCCGGAGTACATCTCCTTTGGAA
+

```
FFFFFFFFFFFFFFFFFFFFFFFFFFFFFFFFFFFFFFFFFFFFFFFFFFFFFFFFFFFFFFFFFFFF
FFFFFFFFFFFFFFFFFFFFFFFFFFFFF:FFFFFFFF:FFFFFFFFFFFFFFFFFFFFFFFFFFFF
@A00155:342:HHGFNDSXY:1:1345:23529:27618 2:N:0:GAACCTAG+TCCGCATA
ACCCCTCCGGTCTTGTCCAACCTCTGTTTCGGCAGAGGATACCGTGCTCCACAGCTGTAAGGCTGTGG
GCCCGAGAGAAGTCGCTCTCTCTCGCCCTCCACATCCCTCTCGGCATTGAGATGACCATAGGCCAGAG
+
FFFFFFFFFFFF:FFFFFFFFFFFFFFFFFFFFFFFFFFFFFFFFFFFFFFFFFFFFFFFFFFFFFF
FFFFFFFFFFFFFFFFFFFFFFFFFFFFFFFFFFFFFFFFFFFFFF,FFFFFFFFFFF:FFFFFFFFF
@A00155:342:HHGFNDSXY:1:1345:24288:28307 2:N:0:GAACCTAG+TCCGCATA
GTGAACAAGCCCGGAGTACATCTCCTTTGGAACTAGGAGACCCCTCCGGTCTTGTCCAACCTCTGTTT
CGGCAGAGGATACCGTGCTCCACAGCTGTAAGGCTGTGGGCCCGAGAGAAGTCGCTCTCTCTCGCCCT
+
FFFFFFFF:FFFFFFFFFFFFFFFF:FFFFFFFFFFFFFFFFFFFFFFFFF,FFFFFFFFFFFFFFFF
FFFFFFFFFFFFFFFFFFFFFFFFFFFFFFFFFFFFFFFFFFFFFF:FFFFFFFFFFFFFFFFFFFFF
@A00155:342:HHGFNDSXY:1:1345:22028:32440 2:N:0:GAACCTAG+TCCGCATA
GTGAACAAGCCCGGAGTACATCTCCTTTGGAACTAGGAGACCCCTCCGGTCTTGTCCAACCTCTGTTT
CGGCAGAGGATACCGTGCTCCACAGCTGTAAGGCTGTGGGCCCGAGAGAAGTCGCTCTCTCTCGCCCT
+
FFFFFFFFFFFFFFFFFFFFFFFFFFFFFFFFFFFFFFFFFFFFFFFFFFFFFFFFFFFFFFFFFFFF
FFFFFFFFFFFFFFFFFFFFFFFFFFFFFFFFFFFFFFFFFFFFFFFF:FFFFFFFFFFFFFFFFFFF
@A00155:342:HHGFNDSXY:1:1345:12156:36354 2:N:0:GAACCTAG+TCCGCATA
GGAGTACATCTCCTTTGGAACTAGGAGACCCCTCCGGTCTTGTCCAACCTCTGTTTCGGCAGAGGATA
CCGTGCTCCACAGCTGTAAGGCTGTGGGCCCGAGAGAAGTCGCTCTCTCTCGCCCTCCACATCCCTCT
+
FFFFFFFFFFFFFFFFFFFFFFFFFFFFFFFFFFFFFFFFFFFFFFFFFFFFFFFFFFFFFFFFFFFF
FFF:FFFFFFFFFFFFFFFFFFFFFFFFFFFFFFFFFFFFFFFFFFFFFFFFFFFFFFFFFFFFFFFF
@A00155:342:HHGFNDSXY:1:1346:19949:4664 2:N:0:GAACCTAG+TCCGCATA
ACCTCTGTTTCGGCAGAGGATACCGTGCTCCACAGCTGTAAGGCTGTGGGCCCGAGAGAAGTCGCTCT
CTCTCGCCCTCTACATCCCTCTCGGCATTGAGATGACCATAGGCCAGAGGTGAGTCCTTAAGTGGACA
+
FFFFFFFFFFFFFFFFFFFFFFFFFFFFFFFFFFFFFFFFFFFFFFFFFFFFFFFFFFFFFFFFFFFF
FFFFFFFFFFFFFFFFFFFFFFFFFFFFFFFFFFFFFFFFFFF:FFFFFFFFFFFFFF,FFFFFFFFF
@A00155:342:HHGFNDSXY:1:1346:14552:22216 2:N:0:GAACCTAG+TCCGCATA
GGTCGTGAACAAGCCCGGAGTACATCTCCTTTGGAACTAGGAGACCCCTCCGGTCTTGTCCAACCTCT
GTTTCGGCAGAGGATACCGTGCTCCACAGCTGTAAGGCTGTGGGCCCGAGAGAAGTCGCTCTCTCTCG
+
FFFFFFFFFFFFFFFFFFFFFFFFFFFFFFFFFFFFFFFFFFFFFFFFFFFFFFFF,FFFFFFFFFFF
FFFFFFFFFFFFFFFFFFFFFFFFF:FFFFF:FFFFFF:FFFFFFFFFFFFFFFFFFFF:FFFFFFFF
@A00155:342:HHGFNDSXY:1:1346:9977:30671 2:N:0:GAACCTAG+TCCGCATA
TTTCGTCCTAAAAAGGACTCGTCAGGTAGCCTACTAGACTACGACTTACACGGTAGGTCGTGAACAAG
CCCGGAGTACATCTCCTTTGGAACCAGGAGACCCCTCCGGTCTTGTCCAACCTCTGTTTCGGCAGAGG
+
FFFFFFFFFFFFFFFFFFFFFFFFFFFFFFFFFFFFFFFFFFFFFFFFF:FFFFFFFFFFFFFFFFFF
FFFFFFFFFFFFFFFFFFFFFF:FFFFFFFFFFFFFF,FFFFFFFFFFFFFFFFFFFFFFFFFFFFFF
@A00155:342:HHGFNDSXY:1:1347:22055:24345 2:N:0:GAACCTAG+TCCGCATA
GAAACCGTCGTTACTCTTCGAGTTGTGTAAGTTTCGTCCTAAAAAGGACTCGTCAGGTAGCCTACTAG
ACTACGACTTACACGGTAGGTCGTGAACAAGCCCGGAGTACATCTCCTTTGGAACTAGGAGACCCCTC
+
FFFFFFFFFFFFFFFFFFFFFFFFFFFFF:F::FFFFFF,:FFFFFFFFFFFFFFFF,FFFFFFFFFF
FFF:FFFFFFFFFFF:,FFF:FFFFF:FFF::FFFFFFFFFFFFFFF:FFFFFFFFFFFFFFF:FFFF
@A00155:342:HHGFNDSXY:1:1347:28004:28573 2:N:0:GAACCTAG+TCCGCATA
GAAACCGTCGTTACTCTTGGAGTTGTGTAAGTTTCGTCCTAAAAAGGACTCGTCAGGTAGCCTACTAG
ACTACGACTTACACGGTAGGTCGTGAACAAGCCCGGAGTACATCTCCTTTGGAACTAGGAGACCCCTC
+
```

FFFFFFFFFFFFFFF:,:FFFFFFFFFFFFFF:FFFFFFFFFF,:FFFFFFFFFFFFFFFFFFFFFFF
FFFFFFFFFFFFFFFFFFFFFFFFFFF,FFFFFFFFFFFFFFFFFFFF:FFFFFFFFFFFFFFFFFFFF
@A00155:342:HHGFNDSXY:1:1348:17716:8531 2:N:0:GAACCTAG+TCCGCATA
TGGAACTAGGAGACCCCTCCGGTCTTGTCCAACCTCTGTTTCGGCAGAGGATACCGTGCTCCACAGCT
GTAAGGCTGTGGGCCCGAGAGAAGTCGCTCTCTCTCGCCCTCCACATCCCTCTCGGCATTGAGATGAC
+
FFFFFFFFFFFFFFFFFFFFFFFFFFFFFFFFFFFFFFFFFFFFFFFFFFFFFFFFFFFFFFFFFFFF
FFFFFFF:FFFFFFFFFFFFFF,FFFFFFFFFFF:FFFFFFFFFFFFFFFFFFFFFFFFFFFFFFFFF
@A00155:342:HHGFNDSXY:1:1348:12228:18035 2:N:0:GAACCTAG+TCCGCATA
CTTTTTAGGACGAAACTTACACAACTCAAAGAGTAACGACGGTTTCGTCCTTGGAACGGACTCATCAG
GGAGCCTACTAGACTCCGACCGTCATGGGGGTTCAAACCCATGCCCCGCCACACCGCCTTAGATCAGC
+
FFFFFFFFFFFFFFFFFFFFFFFFFFFFFFFFFFFFFFFFFFFFFFFFFFFFFFFFFFFFFFFFFFFF
FFFFFFFFFFFFFFFFFFFFFFFFFFFFFFFFFFF:FFFFFFFFFFFFFFFFFFFFFFFFFFFFFFFF
@A00155:342:HHGFNDSXY:1:1348:22616:25504 2:N:0:GAACCTAG+TCCGCATA
ACTCTTTGAGTTGTGTAAGTTTCGTCCTAAAAAGGACTCGTCAGGTAGCCTACTAGACTACGACTTAC
ACGGTAGGTCGTGAACAAGCCCGGAGTACATCTCCTTTGGAACTAGGAGACCCCTCCGGTCTTGTCCA
+
FFFF:FFFFFFFFFF,FFFF:FFFFFFF,F,FFFFFFFFF:FFFFFFFFFFFFFFFFFFFFFFFFFFF
FFFFFFFFFFFFFFFFFFFFFFFFFFFFFFFFFFFFFFFFFFFFFFFFFFFFF:FFFF,FFFFFFFFFF
@A00155:342:HHGFNDSXY:1:1348:22544:25567 2:N:0:GAACCTAG+TCCGCATA
ACTCTTTGAGTTGTGTAAGTTTCGTCCTAAAAAGGACTCGTCAGGTAGCCTACTAGACTACGACTTAC
ACGGTAGGTCGTGAACAAGCCCGGAGTACATCTCCTTTGGAACTAGGAGACCCCTCCGGTCTTGTCCA
+
FFFFFF:FFFFFFFFFFFFF,FFFFFFFFFFFFFFFFFFFFFFFFFFFFFFFFFFFFFFFFFFFFFFF
FFFFF:FFFFFFFFFFFFFFFFFFFFFFFFFFFFFFFFFFF:FFFFFFFFF:FFFFFFFFFFFF:FF
@A00155:342:HHGFNDSXY:1:1350:28293:14137 2:N:0:GAACCTAG+TCCGCATA
CCACACCGCCTTAGATCAGCTGTGTCCACTTAAGGACTCACCTCTGGCCTATGGTCATCTCAATGCCG
AGAGGGATGTGGAGGGCGAGAGAGAGCGACTTCTCTCGGGCCCACAGCCTTACAGCTGTGGAGCACGG
+
FFFFFFFFFFFFFFFFFFFFFFFFFFFFFFFFFFFFFFFFFFFFFFFFFFFFFFFFFFFFFFFFFFFF
FFFFFFF,FFFFFFFFFFFFFFFFFFFFFFFFFFFFFFFFFFFFFFFFFFFF,FFFFFFFFFFFFFFF
@A00155:342:HHGFNDSXY:1:1350:11406:20149 2:N:0:GAACCTAG+TCCGCATA
GATCAGCTGTGTCCACTTAAGGACTCACCTCTGGCCTATGGTCATCTCAATGCCGAGAGGGATGTGGA
GGGCGAGAGAGAGCGACTTCTCTCGGGCCCACAGCCTTACAGCTGTGGAGCACGGTATCCTCTGCCGA
+
FFFFFFFFFFFFFFFFFFFFFFFFFFFFFFFFFFFFFFFFFFFFFFFFFFFFFFFFFFFFFFFFFFFF
FFFFFFFFFFFFFFFFFFFFFFFFFFFFFFFFFFF:FFFFFFFFFFFFFFFFFFFFFFFFFFFFFFFF
@A00155:342:HHGFNDSXY:1:1350:25916:28432 2:N:0:GAACCTAG+TCCGCATA
CCTTTGGAACTAGGAGACCCCTCCGGTCTTGTCCAACCTCTGTTTCGGCAGAGGATACCGTGCTCCAC
AGCTGTAAGGCTGTGGGCCCGAGAGAAGTCGCTCTCTCTCGCCCTCCACATCCCTCTCGGCATTGAGA
+
FFFFFFFFFFFFFFFFFFFFFFFFFFFFFFFFFFFFFFFFFFFFFFFFFFF:FFFFFFFFFFFFFFFF
FFFFFFFFFFFFFFFFFFFFFFFFFFFFFFFFFFFFFFFFFFFFFFFFFFFFFFFFFFFFFFFFFFFF
@A00155:342:HHGFNDSXY:1:1350:17987:36432 2:N:0:GAACCTAG+TCCGCATA
CCGGAGTACATCTCCTTTGGAACTAGGAGACCCCTCCGGTCTTGTCCAACCTCTGTTTCGGCAGAGGA
TACCGTGCTCCACAGCTGTAAGGCTGTGGGCCCGAGAGAAGTCGCTCTCTCTCGCCCTCCACATCCCT
+
FFFFFFFFFFFFFFFFFFFFFFFFFFFFFFFFFFFFFFFFF:FFFFFFFFFFFFFFFFFFFFFFFFFF
FFFFFFFFFFFFFFFFFFFFFFFFFFFFFFFFFFFFFFFFFFFFFFFFFFFFFFFFFFFFFFFFFFFF
@A00155:342:HHGFNDSXY:1:1351:10854:9048 2:N:0:GAACCTAG+TCCGCATA
TACGACTTACACGGTAGGTCGTGAACAAGCCCGGAGTACATCTCCTTTGGAACTAGGAGACCCCTCCG
GTCTTGTCCAACCTCTGTTTCGGCAGAGGATACCGTGCTCCACAGCTGTAAGGCTGTGGGCCCG
+

```
FFFFFFFFFFFFFFFFFFFFFFFFFFFFFFFFFFFFFFFFFFF:FFFFFFFFFFFFFFFFFFFFFF
FFFFFFFFFFFFFFFFFFFFFFFFFFFFFF:FFFFFFFFFFFFFFFF:FFFFFFFFFFFFFFFFF
@A00155:342:HHGFNDSXY:1:1351:9751:15468 2:N:0:GAACCTAG+TCCGCATA
AGGTAGCCTACTAGACTACGACTTACACGGTAGGTCGTGAACAAGCACGGAGTACATCTCCTTTGGAA
CTAGGAGACCCCTCCGGTCTTGTCCAACCTCTGTTTCGGCAGAGGATACCGTGCTCCACAGC
+
:FFFFFFFFFFFFFFFFFFFFFFFFFF:FFFFFFFFFFFFF:FFF,F,F:FFFFFFFFFFFF:FFFFFF
FFFFFFFFF:FFFFFFFFFF:FFFFFFFFFFFFFFF:FFFF::FFFFFF:FFFFFFFFFFFF
@A00155:342:HHGFNDSXY:1:1351:17852:26428 2:N:0:GAACCTAG+TCCGCATA
AAAAGGACTCGTCAGGTAGCCTACTAGACTACGACTTACACGGTAGGTCGTGAACAAGCCCGGAGTAC
ATCTCCTTTGGACCTAGGAGACCCCTCCGGTCTTGTCCAACCTCTGTTTCGGCAGAGGATACCGTGCT
+
FFFFFFFFFFFFFFFFFFFFFFFFFFFFFFFFFFFFFFFFFFFFFFFFFFFFFFFFFFFFFFFFFFFF
FFFFFFF,FFFF,FFFFFFFFFFFFFFFFFFFFFFFFFFFFFFFFFFFFFFFFFFFFFFFFFFFFFFF
@A00155:342:HHGFNDSXY:1:1351:7301:31234 2:N:0:GAACCTAG+TCCGCATA
TCGAAGAGTAACGACGGTTTCGTCCTTGGAACGGACTCATCAGGGAGCCTACTAGACTCCGACCGTCA
TGGGGGTTCAAACCCATGCCCCGCTACACCGCCTTAGATCAGCTGTGTCCACTTAAGGACTCACCTCT
+
FFFFFFFFFFFFFFFFFFFFFFFFFFFFFFFFFFFFFFFFFFFFFFFFFFFFFFFFFFFFFFFFFFFF
FFFFFFFFFFFFFFFFFFFFFFFFFFFFFFFFFFFFFFFFFFFFFFFFFFFFFFF:FFFFFFFFFFFF
@A00155:342:HHGFNDSXY:1:1351:26096:33035 2:N:0:GAACCTAG+TCCGCATA
TAGACTACGACTTACACGGTAGGTCGTGAACAAGCCCGGAGTACATCTCCTTTGGAACTAGGAGACCC
CTCCGGTCTTGTCCAACCTCTGTTTCGGCAGAGGATACCGTGCTCCACAGCTGTAAGGCTGTGGGCCC
+
FFFFFFFFFFFFFFFFFFFFFFFFFFFFFFFFFF:FFFFFFFFFFFFFFFFFFFFF,FFFFFFFFFFFFF
FFFFFFFFFFFFFFFFFFFFFFFFFFFFFFFF:FFFFFFFFFFFFFFFFFFFFFFFFFFFFFFFFFFF
@A00155:342:HHGFNDSXY:1:1352:24569:4586 2:N:0:GAACCTAG+TCCGCATA
AAAAGGACTCGTCAGGTAGCCTACTAGACTACGACTTACACGGTAGGTCGTGAACAAGCCCGGAGTAC
ATCTCCTTTGGAACTAGGAGACCCCTCCGGTCTTGTCCAACCTCTGTTTCGGCAGAGGATACCGTGCT
+
FFFFFFFFFFFFFFFFFFFFFF:FFFFFFFF:FFFFFFFFFFFFFFFFFFFFFFFFFFFFF:FFFFFFF
FF:FFFFFFF:FFFFFFFF:FFFFFFFFFFFFFFFFFFFFFFFFFFFFFFFFF:FFF:FFFFFFFFF:F
@A00155:342:HHGFNDSXY:1:1352:24505:4726 2:N:0:GAACCTAG+TCCGCATA
AAAAGGACTCGTCAGGTAGCCTACTAGACTACGACTTACACGGTAGGTCGTGAACAAGCCCGGAGTAC
ATCTCCTTTGGAACTAGGAGACCCCTCCGGTCTTGTCCAACCTCTGTTTCGGCAGAGGATACCGTGCT
+
FFFFFFFFFFFFFFFFFFFFFFFFFFFFFF:FFFFFFFFFFFFFFFFFFFFFFFFFFFF:FFFF,FFFFF
FFFFFFFFFF:FFFFFFFFFFFFFFFFFFFFF:FFFFFFFFFFFFFFFFFFFFFFFFFFFF:FFFFFFF
@A00155:342:HHGFNDSXY:1:1352:11568:5901 2:N:0:GAACCTAG+TCCGCATA
TAGCCTACTAGACTACGACTTACACGGTAGGTCGTGAACAAGCCCGGAGTACATCTCCTTTGGAACCA
GGAGACCCCTCCGGTCTTGTCCAACCTCTGTTTCGGCAGAGGATACCGTGCTCCACAGCTGTAAGGCT
+
FFFFFFFFFFFFFFFFFFFFFFFFFFFFFFFFFFFFFFFFFFFFFFFFFFFF:FFFFFFFFFFFFFF:
FFFFFFFFFFFFFFFFFFFFFFFFFFFFFFFFFFFFFFFFFFF:FFFFFFFFFFFFFFFFFFFFFFFFF
@A00155:342:HHGFNDSXY:1:1352:5068:9204 2:N:0:GAACCTAG+TCCGCATA
GAAACCGTCGTTACTCTTCGAGTTGTGTAAGTTTCGTCCTAAAAAGGACTCGTCAGGTAGCCTACTAG
ACTACGACTTACACGGTAGGTCGTGAACAAGCCCGGAGTACATCTCCTTTGGAACTATGAGACCCCTC
+
FFFFFFFFFFFFFFFFFFFFFFFFFFFFFFFFFF:FFFFFFFFFFFFFFFFFFFFFFFFFFFFFFFFFF
FFFFFFFFFFFFFFFFFFFFFFFFFFFFFFFFFFFFFFFFFFFFFFFFFFFFFFFF:FF,FFFFFFFFF
@A00155:342:HHGFNDSXY:1:1352:8386:18897 2:N:0:GAACCTAG+TCCGCATA
GAAACCGTCGTTACTCTTTGAGTTGTGTAAGTTTCGTCCTAAAAAGGACTCGTCAGGTAGCCTACTAG
ACTACGACTTACACGGTAGGTCGTGAACAAGCCCGGAGTACATCTCCTTTGGAACTAGGAGACCCCTC
+
```

```
FFFFFFFFFFFFFFFFF:FFFFFFFFFFFFFF,FFFFFF:FFFFFFFFFFFFFFFFFFFFFFFFFFFF
FFFFFFFFFFFFFFFFFFFFFFFFFFFF:FFFFFFFFFFFFFFFFFFFFFF:FFFFFFFFFFFFFFFF
@A00155:342:HHGFNDSXY:1:1352:8232:18944 2:N:0:GAACCTAG+TCCGCATA
GAAACCGTCGTTACTCTTTGAGTTGTGTAAGTTTCGTCCTAAAAAGGACTCGTCAGGTAGCCTACTAG
ACTACGACTTACACGGTAGGTCGTGAACAAGCCCGGAGTACATCTCCTTTGGAACTAGGAGACCCCTC
+
FFFFFFFFFFFFFFFF,:FFFFFFFFFFFFFFFFFFFFFFFFFFFFFFFFFFFFFFFFFFFFFFFFFF
FFFFFFFFFFFFFFFFF:FFFFFFFFFFFFFFFFFFFFFFFFFFFFFFFFFFFFFFFFFFFFFFFFFF
@A00155:342:HHGFNDSXY:1:1352:19795:19022 2:N:0:GAACCTAG+TCCGCATA
AGGTAGCCTACTAGACTACGACTTACACGGTAGGTCGTGAACAAGCCCGGAGTACATCTCCTTTGGAA
CCAGGAGACCCCTCCGGTCTTGTCCAACCTCTGTTTCGGCAGAGGATACCGTGCTCCACAGCTGTAAG
+
FFFFFFFFFFFFFFFFFFFFFFFFFFFFFFFFFFFFFFFFFFFFFFFF:FFFFFFFFFFFFFFFFFFF
FFFFFFFFFFFFFFFFFFFFFFFFFFFFFFFFFFFFFFFFFFFFFFFFFFFF:FFFFFFFFFFFFFFF
@A00155:342:HHGFNDSXY:1:1352:17309:27743 2:N:0:GAACCTAG+TCCGCATA
TCCGGTCTTGTCCAACCTCTGTTTCGGCAGAGGATACCGTGCTCCACAGCTGTAAGGCTGTGGGCCCG
AGAGAAGTCGCTCTCTCTCGCCCTCCACATCCCTCTCGGCATTGAGATGACCATAGGCCAGAGGAGAG
+
FFFFFFF:FFFFFFFFFFFFFFFFFFFFFFFFFFFFFFFFFFFFFFFFFFFFFFFFFFFFFFFFFFFF
FFFFF:FFFFFFFFFFFFFFFFFFFFFFFFFFFFFFFFFFFFFFFFFFFFFFFFFFFFFFFFFFFFFF
@A00155:342:HHGFNDSXY:1:1353:31304:32095 2:N:0:GAACCTAG+TCCGCATA
AAGCCCGGAGTACATCTCCTTTGGAACTAGGAGACCCCTCCGGTCTTGTCCAACCTCTGTTTCGGCAG
AGGATACCGTGCTCCACAGCTGTAAGGCTGTGGGCCCGAGAGAAGTCGCTCTCTCTCGCC
+
FFFFF:FFFFF,FFFFFFFF::FFF:FF,FFFFFFFFFFFFFFFFF,FFFFFFFFFFFF:FFFFFF,F
FFFF,F:FFFFFFFFFFFFF:FFFFFFF:FFFFFFFFF:FFFF,FFFFFFFFFFFFFFFF
@A00155:342:HHGFNDSXY:1:1354:7437:8077 2:N:0:GAACCTAG+TCCGCATA
GTTTCGTCCTTGGAACGGACTCATCAGGGAGCCTACTAGACTCCGACCGTCATGGGGGTTCAAACCCA
TGCCCCGCCACACCGCCTTAGATCAGCTGTGTCCACTTAAGGACTCACCTCTGGCCTATGGTCATCTC
+
FFFFFFFFFFFFFFFFFFFFFFFFFFFFFFFFFFFFFFFFFFFFFFFFFFFFFFFFFFFFFFFFFFFF
,FFFFFFFFFF:FFFFFFFFFFFFFFFFFFFFFFFFFFFFFFFFFFFFFFFFFFFFFFFFFFFFFFFF
@A00155:342:HHGFNDSXY:1:1355:14271:10457 2:N:0:GAACCTAG+TCCGCATA
ACGGTAGGTCGTGAACAAGCCCGGAGTACATCTCCTTTGGAACTAGGAGACCCCTCCGGTCTTGTCCA
ACCTCTGTTTCGGCAGAGGATACCGTGCTCCACAGCTGTAAGGCTGTGGGCCCGAGAGAAGTCGCTCT
+
FFFFFFFFFFFFFFFFFFFFFFFFFFFFFFFFFFFFFFFFFFFFFFFFFFFFFFFFFFFFFF:FFFFF
FFFFFFFFFFFFFFFFFFFFFFFFFFFFFFFFFFFFFFFFFFFFFFFFFFFFFFFFFFF:FFFFFFFF
@A00155:342:HHGFNDSXY:1:1355:21359:16094 2:N:0:GAACCTAG+TCCGCATA
CGGTCTTGTCCAACCTCTGTTTCGGCAGAGGATACCGTGCTCCACAGCTGTAAGGCTGTGGGCCCGAG
AGAAGTCGCTCTCTCTCGCCCTCCACATCCCTCTCGGCATTGAGATGACCAGAGGCCAGAGGTGAGTC
+
FFFFFFFFFFFFFFFFFFFFFFFFFFFFFFFFFFFFFFFFFFFFFFFF:FFFFFFFFFFFFFFFFFFF
FFFFFFFFFFFFF:FFFFFFFFFFFF:FFFFFFFFFFFF,,FFFFFFFFFF,FFFFFFFFFFFFFFFF
@A00155:342:HHGFNDSXY:1:1355:31774:29997 2:N:0:GAACCTAG+TCCGCATA
TACACGGTAGGTCGTGAACAAGCCCGGAGTACATCTCCTTTGGAACCAGGAGACCCCTCCGGTCTTGT
CCAACCTCTGTTTCGGCAGAGGATACCGTGCTCCACAGCTGTAAGGCTGTGGGCCCGAGAGAAGTCGC
+
FFFFFFF:FFF,FFFFFFFFFFFFFFFFFFFFFFFFFFFFFFFF,FFFFFFFFFFFF,FFFF,FFFFF
FFFFFFFFFF:FFFFFFFFFFFFF:FFFFF:FFFFFFFFFFFFFF,FFF:FFFFFFFF,FFF::FFFF
@A00155:342:HHGFNDSXY:1:1355:26765:31344 2:N:0:GAACCTAG+TCCGCATA
AGTACATCTCCTTTGGAACCAGGAGACCCCTCCGGTCTTGTCCAACCTCTGTTTCGGCAGAGGATACC
GTGCTCCACAGCTGTAAGGCTGTGGGCCCGAGAGAAGTCGCTCTCTCTCGCCCTCCACATCCCTCTCG
+
```

```
FFFFFFFFFFFFFFFFFFFFFFFFFFFFFFFFFFFFFFFFFFFFFFF:FFFFFFFFFFFFFFFFFFFF
FFFFFFFFFFFFFFFFFFFFFFFFFFFFFFFFFFF,FFFFFFFFFFFFFFFFFFFFFFFFFFFFFFFF
@A00155:342:HHGFNDSXY:1:1355:32597:31516 2:N:0:GAACCTAG+TCCGCATA
TGTAAGTTTCGTCCTAAAAAGGACTCGTCAGGTAGCCTACTAGACTACGACTTACACGGTAGGTCGTG
AACAAGCCCGGAGTACATCTCCTTTGGAACCAGGAGACCCCTCCGGTCTTGTCCAACCTCTGTT
+
FF:FFFF::FFFF,FF:F:FFFFFFFFF:FFFF,FFFFFFFFFFFFFFFFF:F,FFFFFF,FFFFFF:
F:FFFF:FFFF:FF,FFFFFFF:FFF:F:FF,FFFF:FFFFFF:FFFFF,FFFF:,FFFF::FF
@A00155:342:HHGFNDSXY:1:1356:9507:4679 2:N:0:GAACCTAG+TCCGCATA
CGGCAGAGGATACCGTGCTCCACAGCTGTAAGGCTGTGGGCCCGAGAGAAGTCGCTCTCTCTCGCCCT
TCACATCCCTCTCGGCATTGAGATGACCATAGGCCAGAGGTGAGTCCTTAAGTGGACACAGCTG
+
FFFFFFFFFFFFFFFFFFFFFFFFFFFFFFFFFFFFFFFFFFFFFFFFFFFFFFFFFFFFFFFFFFFF
FFFFFFFFFFFFFFFFFFFFFFFFFFFFFFFFFFFFFFFFFFFFFFFFFFFFFFFFFFFFFFFF
@A00155:342:HHGFNDSXY:1:1356:19533:11992 2:N:0:GAACCTAG+TCCGCATA
GGACTCGTCAGGTAGCCTACTAGACTACGACTTACACGGTAGGTCGTGAACAAGCCCGGAGTACATCT
CCTTTGGAACTAGGAGACCCCTCCGGTCTTGTCCAACCTCTGTTTCGGCAGAGGATACCGTGCTCCAC
+
:FFFFFFFFFFFFFFFFFFFFFFFFFFFFF:FFFFFFFFFFFFFFFF,FFFFFFFFFFFFFFFFFFFF
FFFFFFFFFFFFFFFFFFFFFFFFFFFFFFFFFFFFFFFFF:FFFFFFFFFFFFFF:F:FFFFFFFFF
@A00155:342:HHGFNDSXY:1:1356:24370:13197 2:N:0:GAACCTAG+TCCGCATA
CGTCCTAAAAAGGACTCGTCAGGTAGCCTACTAGACTACGACTTACACGGTAGGTCGTGAACAAGCCC
GGAGTACATCTCCTTTGGAACTAGGAGACCCCTCCGGTCTTGTCCAACCTCTGTTTCGGCAGAGGATA
+
FFFFFFFFFFFFFFFFFFFFFFFFFFFFFFFFFFFFFFFFFFFFFFFFFFFFFFFFFFFFFFFFFFFF
FFFFFFFFFFFFFFFFFFFFFFFFFFFFFFFF,FFFFFF,:FFFFFFFFFFFFFFFFFFFFFFFFFFF
@A00155:342:HHGFNDSXY:1:1356:24695:18865 2:N:0:GAACCTAG+TCCGCATA
ACTTACACGGTAGGTCGTGAACAAGCCCGGAGTACATCTCCTTTGGAACTAGGAGACCCCTCCGGTCT
TGTCCAACCTCTGTTTCGGCAGAGGATACCGTGCTCCACAGCTGTAAGGCTGTGGGCCCGAGAGAAGT
+
FFFFFFFFFFFFFFFFFFFFFFFFFFFFFFFFFFFFFFFFFFFFFFFFFFFFFFFFFFFFFFFFFFF:
FFFFFFFFFFFFFFFFFFFFFFFFFFFFFFFFFFFFFFFFFFFFFFFFFFFFFFFFFFFFFFFF:FFF
@A00155:342:HHGFNDSXY:1:1356:21296:23907 2:N:0:GAACCTAG+TCCGCATA
AAAAGGACTCGTCAGGTAGCCTACTAGACTACGACTTACACGGTAGGTCGTGAACAAGCCCGGAGTAC
ATCTCCTTTGGAACTAGGAGACCCCTCCGGTCTTGTCCAACCTCTGTTTCGGCAGAGGATACCGTGCT
+
FFFFFFFFFFFFFFFFFFFFFFFFFFFFFFFFFFFFFFFFFFFFFFFFFFFFFFFFFFFFFFFFFFFF
FFFFFFFFFFFFFFFFFFFFFFF:FFFFFFFFFFFFF:FFFFFFFFFFFFF:FF::FFFFFFFFFFFF
@A00155:342:HHGFNDSXY:1:1357:11216:7764 2:N:0:GAACCTAG+TCCGCATA
AGCCCGGAGTACATCTCCTTTGGAACTAGGAGACCCCTCCGGTCTTGTCCAACCTCTGTTTCGGCAGA
GGATACCGTGCTCCACAGCTGTAAGGCTGTGGGCCCGAGAGAAGTCGCTCTCTCTCGCCCTCCACATC
+
FFFFFFFFFFFFFFFFFF:FFFFF:FFFFFFFFFFFFFFFFFFFFFFFFFFFFFFFFFFFFFFFFFFF
FFFFFFFFFFFFFFFFFFFFFFFFFFFFFFFFFFFFFFFFFFFFFFFFFFFFFFFFFFFFFFFFFFFF
@A00155:342:HHGFNDSXY:1:1357:14633:29841 2:N:0:GAACCTAG+TCCGCATA
AGCCCGGAGTACATCTCCTTTGGAACTAGGAGACCCCTCCGGTCTTGTCCAACCTCTGTTTCGGCAGA
GGATACCGTGCTCCACAGCTGTAAGGCTGTGGGCCCGAGAGAAGTCGCTCTCTCTCGCCCTCCACATC
+
FFFFFFFFFFFFFFFFFFF:FFFFFFF,:FFFFFFFFFFFFFFFFFFFFFFFFFFFFFFFFFFFFFFF
FFFFFFFFFFFFFFFFFFFFFFFFFFFFFFFFFFFFFFFFFF:FFFFFFFFFFFFFFFFFFFFFFFFF
@A00155:342:HHGFNDSXY:1:1357:30924:34726 2:N:0:GAACCTAG+TCCGCATA
GTAAGTTTCGTCCTAAAAAGGACTCGTCAGGTAGCCTACTAGACTACGACTTACACGGTAGGTCGTGA
ACAAGCCCGGAGTACATCTCCTTTGGAACTAGGAGACCCCTCCGGTCTTGTCCAACCTCTGTTACGGC
+
```

```
FFFFFFFFFFFFFFFFFFFFFFFFFFFFFFFFFFFFFFFFFFFFFFFFFFFFFFFFFFF:FFFFFFF:
FFFF,FFFFFFFFFFFFFFF:FFFFFFFFFF,FFFFFFFFFFFFFFFFFFFFFFFFFFFFFFFFFFFF
@A00155:342:HHGFNDSXY:1:1358:14190:9502 2:N:0:GAACCTAG+TCCGCATA
TCCAAGGACGAAACCGTCGTTACTCTTCGAGTTGTGTAAGTTTCGTCCTAAAAAGGACTCGTCAGGTA
GCCTACTAGACTACGACTTACACGGTAGGTCGTGAACAAGCCCGGAGTACATCTCCTTTGGAACTAGG
+
FFFFFFFFFFFFFF:FFFFFFFFFF:FFFFFFFFFF:FFFFFFFFFFFFFF,F:FFFFFFFFF:FFFF
FFFFFFFFFFFFFFFFFFFFFFFFFFFFFFFFFF:FFFFFFFFFFFFFFFFFFFFF:FFFFFFFFFFF
@A00155:342:HHGFNDSXY:1:1358:26449:12258 2:N:0:GAACCTAG+TCCGCATA
GTTGTGTAAGTTTCGTCCTAAAAAGGACTCGTCAGGTAGCCTACTAGACTACGACTTACACGGTAGGT
CGTGAACAAGCCCGGAGTACATCTCCTTTGGAACTAGGAGACCCCTCCGGTCTTGTCCAACCTCTGTT
+
FFFFFFFFFFFFFFFFFFFFFFFFFFFFFFFFFFFFFFFFFFFFFFFFFFF:FFFFFFFFFFFFFFFF
FFFFFFFFFFFFFFFFFFF,FFFFFFFFFFFF:FFFFFFFFFFFFFFFFFFFFFFFFFFFFFFFFFFF
@A00155:342:HHGFNDSXY:1:1358:20844:18646 2:N:0:GAACCTAG+TCCGCATA
GGACTCGTCAGGTAGCCTACTAGACTACGACTTACACGGTAGGTCGTGAACAAGCCCGGAGTACATCT
CCTTTGGAACCAGGAGACCCCTCCGGTCTTGTCCAACCTCTGTTTCGGCAGAGGATACCGTGCTCCAC
+
FFFFFFFFFFFFFFFFFFFFFFFFFFFFFFFFFFFFFFFFFFFFFFFFFFFFFFFFFFFFFFFFFFFF
FFFFFFFFFFFFFFFFFFFFFFFFFFFFFFFFFFFFFFFFFFFFFFFFFFFFFFFFFFFFFFFFFFFF
@A00155:342:HHGFNDSXY:1:1358:15646:26678 2:N:0:GAACCTAG+TCCGCATA
GGTCTTGTCCAACCTCTGTTTCGGCAGAGGATACCGTGCTCCACAGCTGTAAGGCTGTGGGCCCGAGA
GAAGTCGCTCTCTCTCGCCCTCCACATCCCTCTCGGCATTGAGATGACCATAGGCCAGAGGTGAGTCC
+
FFFFFFFFFFFFFFFFFFFFFFFFFFFFFFFFFFFFFFFFFFFFFFF:FFFFFFFFFFFFFFFFFFFF
FFFFFFFFFFFFFFFFFFFFFFFFFFFFFFFFFFFFFF:FFFFFFFFFFFFFFFFFFF:FFFFFFFFF
@A00155:342:HHGFNDSXY:1:1358:29758:32643 2:N:0:GAACCTAG+TCCGCATA
TCCGTTCCAAGGACGAAACCGTCGTTACTCTTTGAGTTGTGTAAGTTTCGTCCTAAAAAGGACTCGTC
AGGTAGCCTACTAGACTACGACTTACACGGTAGGTCGTGAACAAGCCCGGAGTACATCTCCTTTGGAA
+
FFFFFFFFFFFFFFFFFFFFFFFFFFFFFFFFFFFFFFFFFFFFFFFFFFFFFFFFFFFFFFFFFFFF
FFFFFFFFFFF:FFFFFFFFFFFFFFFFFFFF:FFFFFFFFFFFFFFFFFFFFFFFFFFFFFFFFFFF
@A00155:342:HHGFNDSXY:1:1359:8594:5321 2:N:0:GAACCTAG+TCCGCATA
AGGACGAAACCGTCGTTACTCTTTGAGTTGTGTAAGTTTCGTCCTAAAAAGGACTCGTCAGGTAGCCT
ACTAGACTACGACTTACACGGTAGGTCGTGAACAAGCCCGGAGTACATCTCCTTTGGAACTAGGAGAC
+
FFFFFFFFFFFFFFF:FFFFFF:FFFFFFFFF:FFFFFFFFFFFFFF,FFFFFFFFFFFFFFFFFFFF
FFFFFFFFFFFFFFFFFFFFFFFFFFFFFFFFFFFFFFFFFFFFFFFFFFFFFFFFFFFFFFFFFFFF
@A00155:342:HHGFNDSXY:1:1359:21603:34209 2:N:0:GAACCTAG+TCCGCATA
TACGACTTACACGGTAGGTCGTGAACAAGCCCGGAGTACATCTCCTTTGGAACCAGGAGACCCCTCCG
GTCTTGTCCAACCTCTGTTTCGGCAGAGGATACCGTGCTCCACAGCTGTAAGGCTGTGGGCCCGAGAG
+
FFFFFFFFFFFFFFFFF:FFFFFFFFFFFFFFFFFFFFFFFFFFFFFFFFFFFFFFFFFFF:FFFFFF
FFFFFFFFFFFFFFFFFFFFFFFFFFFFFFFFFFFFFFFFFFFFFFFFFFFFFFFFFFFFFFFFFFFF
@A00155:342:HHGFNDSXY:1:1360:21866:1720 2:N:0:GAACCTAG+TCCGCATA
CCCTCCGGTCTTGTCCAACCTCTGTTTCGGCAGAGGATACCGTGCTCCACAGCTGTAAGGCTGTGGGC
CCGAGAGAAGTCGCTCTCTCTCGCCCTTCACATCCCTCTCGGCATTGAGATGACCATAGGCCAGAGG
+
FFFFFFFFFFFFFFF:FFFFFFFFFFFFFFFFFFFFFFFFFFFFFFFFFFFFFFFFFFFFFFFFFFFF
FFFFFFFFFFFFFFFFFFFFFFFFFFFFFFFFFFFFFFFFFFFFFFFF:FFF::FFFFFFFFFFFFF
@A00155:342:HHGFNDSXY:1:1360:25220:8406 2:N:0:GAACCTAG+TCCGCATA
GTGAACAAGCCCGGAGTACATCTCCTTTGGAACTAGGAGACCCCTCCGGTCTTGTCCAACCTCTGTTT
CGGCAGAGGATACCGTGCTCCACAGCTGTAAGGCTGTGGGCCCGAGAGAAGTCGCTCTCTCTCGCCCT
+
```

FFFFFFFFFFFFFFFFFFFFFFFFFFFFFFFFFFFFFFFFFFFFFFFFFF,FFFFFFFFFFFFFFF
FFFFFFFFFFFFFFFFFFFFFFFFFFFFFFFFF:FFFFFFFFFFFFFFFFFFFFFF:FFFFFFFFFFFF
@A00155:342:HHGFNDSXY:1:1360:13829:15483 2:N:0:GAACCTAG+TCCGCATA
TCGTGAACAAGCCCGGAGTACATCTCCTTTGGAACTAGGAGACCCCTCCGGTCTTGTCCAACCTCTGT
TTCGGCAGAGGATACCGTGCTCCACAGCTGTAAGGCTGTGGGCCCGAGAGAAGTCGCTCTCTCTCGCT
+
FFFFFFFFFFFFFFFFFF::FFFFFF:F:FFFFFFFFFFFFF:FFFFFFF:FFFFF:FF:FFFFF:FF
F:FF:FFF:FFFFFFFFF:FFFFFFFFFFFFFFFFFFFF,FF::FFFFFFFFFFFFFFFFFFFFFFFFF:
@A00155:342:HHGFNDSXY:1:1360:13431:36245 2:N:0:GAACCTAG+TCCGCATA
CTCGAAGAGTAACGACGGTTTCGTCCTTGGAACGGACTCATCAGGGAGCCTACTAGACTCCGACCGTC
ATGGGGGTTCAAACCCATGCCCCGCCACACCGCCTTAGATCAGCTGTGTCCACTTAAGGACTCACCTC
+
FFFFFFFFFFFFFFFFFFFFFFFFFFFFFFFFFFFFFFFFFFFFFFFFFFFFFFFFFFFFFFFF:FF:
FFFFFFFFFFFFFFFFFFFFFFFFFFFFFFFFFFFFFFFFFFFFFFFFFFFFF:FFFFFFFFFFFFFF
@A00155:342:HHGFNDSXY:1:1361:22137:1720 2:N:0:GAACCTAG+TCCGCATA
GGTAGCCTACTAGACTACGACTTACACGGTAGGTCGTGAACAAGCCCGGAGTACATCTCCTTTGGAAC
TAGGAGACCCCTCCGGTCTTGTCCAACCTCTGTTTCGGCAGAGGATACCGTGCTCCACAGCTGTAAGG
+
FFFFFFFFFFFFFFFFFFFFFFFFFFFFFFFFFFFFFFFFFFFFFFFFFFFFFFFFFFFFFFFFFF:F
FFFFFFFFFFFFFFFFFFFFFFFFFFFFFFFFFFFFFFFFFFFFFFFFFFFFFFF:FFFFFFFFFFFF
@A00155:342:HHGFNDSXY:1:1361:17788:12383 2:N:0:GAACCTAG+TCCGCATA
GTGTAAGTTTCGTCCTAAAAAGGACTCGTCAGGTAGCCTACTAGACTACGACTTACACGGTAGGTCGT
GAACAAGCCCGGAGTACATCTCCTTTGGAACTAGGAGACCCCTCCGGTCTTGTCCAACCTCTGTTTCG
+
FFF:FFFFFFFFFFFFFFFFFFFFFFFFFFFFFFFFFFFFFFFFFFFFFFFFFFFFFFFFFFFFFFFF
FFFFFFFFFFFFFFFFF:FFFFFFFFFFFFFFFFFFFFFFFFFFFFFFFFFF,FFFFFFFFFFFFFFFF
@A00155:342:HHGFNDSXY:1:1361:29152:13056 2:N:0:GAACCTAG+TCCGCATA
GTGAACAAGCCCGGAGTACATCTCCTTTGGAACTAGGAGACCCCTCCGGTCTTGTCCAACCTCTGTTT
CGGCAGAGGATACCGTGCTCCACAGCTGTAAGGCTGTGGGCCCGAGAGAAGTCGCTCTCTCTCGCCCT
+
FFFFFFFFFFFFFFFFFFFFFFFFFFFFFFFFFFFFFFFFFFFFFFFFFFFF:FFFFFFFFFFFFFFF
FFFFFFFFFFFFFFFFFFFFFFFFFFFFFFFFF,FFFFFFFFFFFFFFFFFFFFFFFFFFFFFFFFFF
@A00155:342:HHGFNDSXY:1:1361:29469:13228 2:N:0:GAACCTAG+TCCGCATA
GTGAACAAGCCCGGAGTACATCTCCTTTGGAACTAGGAGACCCCTCCGGTCTTGTCCAACCTCTGTTT
CGGCAGAGGATACCGTGCTCCACAGCTGTAAGGCTGTGGGCCCGAGAGAAGTCGCTCTCTCTCGCCCT
+
FFFFFFFFFFFFFFFFFFFFFFFFFFFFFFFFFFFFFFFFFFFFFFFFFFFFFFFFFFFFFFFFFFFF
FFFFFFFFFFFFFFFFFFFFFFFFFFFFF:FFFFFFFFFFFFFFFFFFFF,:FFFFFFFFFFFFFFFF
@A00155:342:HHGFNDSXY:1:1361:27959:19163 2:N:0:GAACCTAG+TCCGCATA
GTTGTGTAAGTTTCGTCCTAAAAAGGACTCGTCAGGTAGCCTACTAGACTACGACTTACACGGTAGGT
CGTGAACAAGCCCGGAGTACATCTCCTTTGGAACTAGGGGACCCCTCCGGTCTTGTCCAACCTCTGTT
+
FFFFFFFFFFFFFFFFF:FFFFF:FFFFFFFFFFFFFFFFFFFFFF,FFFFFFFFFFFFFFFFFFF::
FFFFFFFFFFFFFFFFFFFFFFFFFFFFFFFFFFFFFFFFFF:FFFFFFFFFFFFFFFFFFFFFFFFF
@A00155:342:HHGFNDSXY:1:1361:27742:19695 2:N:0:GAACCTAG+TCCGCATA
GTTGTGTAAGTTTCGTCCTAAAAAGGACTCGTCAGGTAGCCTACTAGACTACGACTTACACGGTAGGT
CGTGAACAAGCCCGGAGTACATCTCCTTTGGAACTAGGAGACCCCTCCGGTCTTGTCCAACCTCTGTT
+
FFFFFFFFFFFFFFFFFFFFFFFFFFFFFFFFFFFFFFFFFFFFFFFFFFFFFFFFFFFFFFFFFFFF
FFFFFFFFF:FFFFFFFFFFFFFFFFFFF:FFFFFFFFFFFFFFFFFFFFFFFFFFFFFFFFFFFFFF
@A00155:342:HHGFNDSXY:1:1361:9426:22138 2:N:0:GAACCTAG+TCCGCATA
ACCCCTCCGGTCTTGTCCAACCTCTGTTTCGGCAGAGGATACCGTGCTCCACAGCTGTAAGGCTGTGG
GCCCGAGAGAAGTCGCTCTCTCTCGCCCTTCACATCCCTCTCGGCATTGAGATGACCATAGGCCAGAG
+

```
FFFFFFFFFFFFFFFFFFFFFFFFFFFFF,,FFF:FFFFFFFFFFFFFFFFFFFFFFFFFFF:FFFFF
:FFFFFFFFFF:F:FFFFFFFFFFFFFFFFFFFFFFFF,FFFFF:FFFFFFFFFFFF,FFFFF,FFFFF
@A00155:342:HHGFNDSXY:1:1362:19795:11537 2:N:0:GAACCTAG+TCCGCATA
CCTACTAGACTACGACTTACACGGTAGGTCGTGAACAAGCCCGGAGTACATCTCCTTTGGAACTAGGA
GACCCCTCCGGTCTTGTCCAACCTCTGTTTCGGCAGAGGATACCGTGCTCCACAGCTGTAAGGCTGTG
+
FF,FFFFFFFFFFFFFFFFFFFFFFFFFFFFFFFFFF:FFFFF:F:FFFFFFFFFFFFFFFFFFFFF
FFFFFFFFFFFF:F,FFFFFFFFFFFF:FFF:FFFFFFF:F:FFFFFFFFFFFFFFFFFFF,FF:FFFF
@A00155:342:HHGFNDSXY:1:1362:1714:34303 2:N:0:GAACCTAG+TCCGCATA
GGACTCGTCAGGTAGCCTACTAGACTACGACTTACACGGTAGGTCGTGAACAAGCCCGGAGTACATCT
CCTTTGGAACTAGGAGACCCCTCCGGTCTTGTCCAACCTCTGTTTCGGCAGAGGATACCGTGCTCCAC
+
FFFFFFFFFFFFFFFFFFFFFFFFFFFFFFFFFFFF,FFFFFFFF:F:FFFFFFFFFFFFFFFFFFF
FFFFFFFFFFFFFFFFFFFFFFFFFFFFFFFFFFFFFFFFFFFFFFFFFFF,FFF:FF,FFFFFFFFF
@A00155:342:HHGFNDSXY:1:1362:24261:35274 2:N:0:GAACCTAG+TCCGCATA
GAGACCCCTCCGGTCTTGTCCAACCTCTGTTTCGGCAGAGGATACCGTGCTCCACAGCTGTAAGGCTG
TGGGCCCGAGAGAAGTCGCTCTCTCTCGCCCTCCACATCCCTCTCGGCATTGAGATGACCATAGGCCA
+
FFFFFFFFFFFFFFF:FFFFFF:FFFFFFFFFFFFFFFFFFFFFFFFFFFFFFFFF:FFFFFFFF:FFF
FFFFFFFFFFFFFFFFFFFFFFFFFFFFFFFFFFFFFFFFFFFFFFFFFFFFFFFFFFFFFF:FFF,FFF
@A00155:342:HHGFNDSXY:1:1363:26955:18176 2:N:0:GAACCTAG+TCCGCATA
GTGAACAAGCCCGGAGTACATCTCCTTTGGAACTAGGAGACCCCTCCGGTCTTGTCCAACCTCTGTTT
CGGCAGAGGATACCGTGCTCCACAGCTGTAAGGCTGTGGGCCCGAGAGAAGTCGCTCTCTCTCGCCCT
+
FFFFFFFFFFFFFFFFFFFFFFFFFFFFFFFFFFFFFFFFFFFFFFFFFFFFFFFFFFFFFFFF:FFF
FFFFFFFFFFFFFFFFFFFFFFFFFFFFFFFFFFFFFFFFFFFFFFFFF,:FFFFFFFFFFFFFFFFFF
@A00155:342:HHGFNDSXY:1:1363:24560:24643 2:N:0:GAACCTAG+TCCGCATA
ATGGAACTAGGAGACCCCTCCGGTCTTGTCCAACCTCTGTTTCGGCAGAGGATACCGTGCTCCACAGC
TGTAAGGCTGTGGGCCCGAGAGAAGTCGCTCTCTCTCGCCCTCCACATCCCTCTCGGCATTGAGATGA
+
FFFFFFFFFFFFFFFFFFFFFFFFFFFFFFFFFFFFFFFFFFFFFFFFF:FFFFFFFFFFFFFFFFFFF
,FFFFFFFFFFFFFFFFFFFFFFFFFFFFFFFFFFFFFFFFFFFFFFFFFFFFFFFFFFFFFFFFF:FF
@A00155:342:HHGFNDSXY:1:1363:6144:29262 2:N:0:GAACCTAG+TCCGCATA
GAAACCGTCGTTACTCTTCGAGTTGTGTAAGTTTCGTCCTAAAAAGGACTCGTCAGGTAGCCTACTAG
ACTACGACTTACACGGTAGGTCGTGAACAAGCCCGGAGTACATCTCCTTTGGAACTAGGAGACCCCTC
+
FFFFFFF,FF:FFFFF:FFFFFFFFFFFFFFF:FFFFFFFFFFFFFF:FFFFFFFFFFFFFFFFFFFFF
FFFFFFFFFFFFFFFFFFFFFFFFFFFFFFF:FFFFFFFFFFFFFFFFFFFFFFF,FFFFFFFFFFFFF
@A00155:342:HHGFNDSXY:1:1363:2880:33661 2:N:0:GAACCTAG+TCCGCATA
GTGAACAAGCCCGGAGTACATCTCCTTTGGAACTAGGAGACCCCTCCGGTCTTGTCCAACCTCTGTTT
CGGCAGAGGATACCGTGCTCCACAGCTGTAAGGCTGTGGGCCCGAGAGAAGTCGCTCTCTCTCGCCCT
+
FFF:FFFFFFFFFFFFFFFFFFFFFFFFFFFFFFFFFFFFFFFFFFFFFFFFFFFFFFFFFFFFFFFFF
FFFFFFFFFFFFFFFFFFFFFFFFFFFFFFFFFFFFFFFFFFFFFFFFFFFFFFFFFFFFFFFFFFF:FF
@A00155:342:HHGFNDSXY:1:1364:28782:18239 2:N:0:GAACCTAG+TCCGCATA
AAAAAGGACTCGTCAGGTAGCCTACTAGACTACGACTTACACGGTAGGTCGTGAACAAGCCCGGAGTA
CATCTCCTTTGGAACCAGGAGACCCCTCCGGTCTTGTCCAACCTCTGTTTCGGCAGAGGATACCGTGC
+
FFFFFFFFFFFFFFFFFFFFFFFFFFFFFFFFFFFFFFFFFFFFFFFFFFFFFFFFFFFFFFFFFFFFF
FFFFFFFFFFFFF:FFFFFFFFFFFFFFFFFFFFFFFFFFFFFFFFFFFFFFFFFFFFFFFFFFFFFFF
@A00155:342:HHGFNDSXY:1:1364:25328:35399 2:N:0:GAACCTAG+TCCGCATA
GGTCGTGAACAAGCCCGGAGTACATCTCCTTTGGAACCAGGAGACCCCTCCGGTCTTGTCCAACCTCT
GTTTCGGCAGAGGATACCGTGCTCCACAGCTGTAAGGCTGTGGGCCCGAGAGAAGTCGCTCTCTCTCG
+
```

FFFFFFFFFFFFFFFFFFFFFFFFFFFFFFFFFFFFFFFFFFFFFFFFFFFFFFFFFFFFFFFFFFFF
FFFFFFFFFFFFFFFFFFFFFFFFFFFFFFFFFFFFFFFFFFFFFFFFFFFFFFFFFFFFFFFFFFFF
@A00155:342:HHGFNDSXY:1:1365:4255:7983 2:N:0:GAACCTAG+TCCGCATA
GGACTCGTCAGGTAGCCTACTAGACTACGACTTACACGGTAGGTCGTGAACAAGCCCGGAGTACATCT
CCTTTGGAACTAGGAGACCCCTCCGGTCTTGTCCAACCTCTGTTTCGGCAGAGGATACCGTGCTCCAC
+
FFFFFFFFFFFFFFFFFFFFFF:FFFFFFFFFFFFFFFFFFFFFFFFFFFFFFFFFFFFFFFFFFFFF
FFFFFFFFFFFFFFF,FFFFFFFFFFFFFFFFFFFFFFFFFFFFFFFFFFFFF:FFFFFFFFFFFFFF
@A00155:342:HHGFNDSXY:1:1365:32723:15201 2:N:0:GAACCTAG+TCCGCATA
GTACATCTCCTTTGGAACTAGGAGACCCCTCCGGTCTTGTCCAACCTCTGTTTCGGCAGAGGATACCG
TGCTCCACAGCTGTAAGGCTGTGGGCCCGAGAGAAGTCGCTCTCTCTCGCCCTCCACATCCCTCTCGG
+
FFFFFFFFFFFFFFFFFFFFFFFFFFFFFFFFFFFFF:F,FFFFFFFF:FFFFFFFFFFFFFFFFFFF
FFFFFFFFFFFFFFFFFFF:FFFFFFFFFFFFFF,FFFFF:FFFFFFFFFFFFFFFFFFFFFFFFFFF
@A00155:342:HHGFNDSXY:1:1365:26521:22435 2:N:0:GAACCTAG+TCCGCATA
AAACTTACACAACTCGAAGAGTAACGACGGTTTCGTCCTTGGAACGGACTCATCAGGGAGCCTACTAG
ACTCCGACCGTCATGGGGGTTCAAACCCATGCCCCGCCACACCGCCTTAGATCAGCTGTGTCTACTTA
+
FFFFFFFFFFFFFFFFFFFFFFFFFFFFFFFFFFFFFFFFFFFFF:FFFFFFFFFFFFFFFFFFFFFF
FF:FFFFFFFFFFFFFFFFFFFFFF,FFFFFFFFFFFFFFFFFFFFFFFFFFFFFFFFFFFFFFF::F
@A00155:342:HHGFNDSXY:1:1365:18566:23876 2:N:0:GAACCTAG+TCCGCATA
CAGGTAGCCTACTAGACTACGACTTACACGGTAGGTCGTGAACAAGCCCGGAGTACATCTCCTTTGGA
ACTAGGAGACCCCTCCGGTCTTGTCCAACCTCTGTTTCGGCAGAGGATACCGTGCTCCACAGCTGTAA
+
FFFFFFFFFFFFFFFFFFFFFFFFFFFFFFFFFFFFFFFFFFFFFFFFFFFFFFFFFFFFFFFFFFFF
:FFFFFFFFFFFFFFFFFFFFFFFFFFFFFFFFFFFFFFFFFFFFFFFFFFFFFFFFFFFFFFFFFFF
@A00155:342:HHGFNDSXY:1:1366:31602:8844 2:N:0:GAACCTAG+TCCGCATA
GAAACCGTCGTTACTCTTCGAGTTGTGTAAGTTTCGTCCTAAAAAGGACTCGTCAGGTAGCCTACTAG
ACTACGACTTACACGGTAGGTCGTGAACAAGCCCGGAGTACATCTCCTTTGGAACTAGGAGACCCCTC
+
FFFFFFFFFFFFFFFFFFFFFFFFFFFFFFFF:FFFFFF,,FFFFFFFFFFF:FFFFFFFFFFFFFFF
FFF:FF:F:FFF:FFFFFF:FFFFFFFFFFFFFFFFFFFFFFFFFFFFFFFFFFFFFFFFFFFFFFFF
@A00155:342:HHGFNDSXY:1:1366:6488:8907 2:N:0:GAACCTAG+TCCGCATA
TAGACTACGACTTACACGGTAGGTCGTGAACAAGCCCGGAGTACATCTCCTTTGGAACTAGGAGACCC
CTCCGGTCTTGTCCAACCTCTGTTTCGGCAGAGGATACCGTGCTCCACAGCTGTAAGGCTGTGGGCCC
+
FFFFFFFFFFFFFFFFFFFFFFFFFFFFFFFFFFFFFFFFFFFFFFFFFFFFFFFFFFFFFFFFFFFF
FFFFFFFFFFFFFFFFFFFFFFFFFFFFFFFFFFFFFFFFFFFFFFFFFFFFFFFFFFFFFFFFFFFF
@A00155:342:HHGFNDSXY:1:1366:16848:9095 2:N:0:GAACCTAG+TCCGCATA
TACGACTTACACGGTAGGTCGTGAACAAGCCCGGAGTACATCTCCTTTGGAACCAGGAGACCCCTCCG
GTCTTGTCCAACCTCTGTTTCGGCAGAGGATACCGTGCTCCACAGCTGTAAGGCTGTGGGCCCG
+
FFFFFFFFFFFFFFFFFFFFFFF:FFFFFFFFFFFFFFF:FFFFFFFFFFFFFFFFFFFFFFFFFFFF
FFF:FFFFF,FFFFFFF:FFFFFFFFFFFFFFFFFFFFFFFFFFFFFFFFFFFFFFFFFFFFFF
@A00155:342:HHGFNDSXY:1:1366:12382:12947 2:N:0:GAACCTAG+TCCGCATA
GTGAACAAGCCCGGAGTACATCTCCTTTGGAACTAGGAGACCCCTCCGGTCTTGTCCAACCTCTGTTT
CGGCAGAGGATACCGTGCTCCACAGCTGTAAGGCTGTGGGCCCGAGAGAAGTCGCTCTCTCTCGCCCT
+
:FFFFFFFFFFFFFFFFFFFFFFFFF:FFFF:FFFFFF:FFF:FFFFFFFFFFFFFFFFFFFFFFFFF
FFFFFF,FFFF,FFFFFFFFFFFFFFFFFFFFFFFFFFFFFFFFFFFFFFFFFFFFFFFFFFFFFFFF
@A00155:342:HHGFNDSXY:1:1366:10827:13416 2:N:0:GAACCTAG+TCCGCATA
GGACTCGTCAGGTAGCCTACTAGACTACGACTTACACGGTAGGTCGTGAACAAGCCCGGAGTACATCT
CCTTTGGAACCAGGAGACCCCTCCGGTCTTGTCCAACCTCTGTTTCGGCAGAGGATACCGTGCTCCAC
+

```
FFFFFFFFFFFFFFFFFFFFFFFFFFFFFFFFFFFFFFFFFFFFFFFFFFFFFFFFFFFFFFFFFFFF
FFFFFFFFFFFFFFFFFFFFFFFFFFF:FFFFFFFFFFFFFFF:FFFFFFFFFFFFFFFFFFFFFFFFF
@A00155:342:HHGFNDSXY:1:1366:10655:20979 2:N:0:GAACCTAG+TCCGCATA
AGGGATGTAGAGGGCGAGAGAGAGCGACTTCTCTCGGGCCCACAGCCTTACAGCTGTGGAGCACGGTA
TCCTCTGCCGAAACAGAGGTTGGACAAGACCGGAGGGGTCTCCTAGTTCCAAAGGAGATGTACTCCGG
+
FFFFFFFFFFFFFFFFFFFFFFFFFFFFFFFFFFFFFFFFFFFFFFFF:FFFFFFFFFFFFFFFFFFF:
FFFFFFFFFFFFFFFFFFFFFFFFFF:FFFFFFFFFFFFFFFFFFFFFFFFFFFFFFFFFFF:FFFFFFF
@A00155:342:HHGFNDSXY:1:1366:7419:28651 2:N:0:GAACCTAG+TCCGCATA
TAGGAGACCCCTCCGGTCTTGTCCAACCTCTGTTTCGGCAGAGGATACCGTGCTCCACAGCTGTAAGG
CTGTGGGCCCGAGAGAAGTCGCTCTCTCTCGCCCTCCACATCCCTCTCGGCATTGAGATGACCATAGG
+
FFFFFFFFFFFFFFFFFFFFFFFFFFFFFFFFFFFFFFFFFFFFFFFFFFFFFFFFFFFFFFFFFFFF
FFFFFFFFFFFFFFFF,FFFFFFFFFFFFFFFFFFFFFFFFFFFFFFFFFFF,FFFFFFFFFFFFFF
@A00155:342:HHGFNDSXY:1:1366:9127:32174 2:N:0:GAACCTAG+TCCGCATA
CACCTCTGGCCTATGGTCATCTCAATGCCGAGAGGGATGTGGAGGGCGAGAGAGAGCGACTTCTCTCG
GGCCCACAGCCTTACAGCTGTGGAGCACGGTATCCTCTGCCGAAACAGAGGTTGGACAAGACCG
+
FFFFFFFF:FFFFFFFF:::FFFFFFFFFFFFFFFFFFF:FFFFFFFFFFFFFFFFFFFFFFFFFFFF
FFFFFFFFFFFFFFFFFFFFFFFFFFFFFFFFFFFFFFFFFFFFFFFFF,FFFFFFFFFFFFFFFF
@A00155:342:HHGFNDSXY:1:1367:32714:3724 2:N:0:GAACCTAG+TCCGCATA
CCTACTAGACTACGACTTACACGGTAGGTCGTGAACAAGCCCGGAGTACATCTCCTTTGGAACCAGGA
GACCCCTCCGGTCTTGTCCAACCTCTGTTTCGGCAGAGGATACCGTGCTCCACAGCTGTAAGGCTGTG
+
FFFFFFFFFFFFFFFFFFFFFFFFFFFFFFFFFFFFFFFFFFFFFFFFFFFFFFFFFFFFFFFFFFFF
FFFFFFFFFFFFFFFFFFFFFFFFFFFFFFFFFFFFFFFFFFFFFFFFFFFFFFFFF:FFFFFFFFFF
@A00155:342:HHGFNDSXY:1:1367:10881:6934 2:N:0:GAACCTAG+TCCGCATA
CTACGACTTACACGGTAGGTCGTGAACAAGCCCGGAGTACATCTCCTTTGGAACTAGGAGACCCCTCC
GGTCTTGTCCAACCTCTGTTTCGGCAGAGGATACCGTGCTCCACAGCTGTAAGGCTGTGGGCCCGAGA
+
FFFFFFFFFFFFFFFFFFFFFFFFFFFFFFFFFFFFFFFFFFFFFFFFFFFFFFFFFFFFFFFFFFFF
FFFFFFFFFFF:FFFFFFFFFFFFFFFFFFFFFFFFFFFFFFFFFFFFFFF:FFFFFFFFFFFFFFFF
@A00155:342:HHGFNDSXY:1:1367:23375:18991 2:N:0:GAACCTAG+TCCGCATA
ACTAGACTCCGACCGTCATGGGGGTTCAAACCCATGCCCCGCCACACCGCCTTAGATCAGCTGTGTCC
ACTTAAGGACTCACCTCTGGCCTATGGTCATCTCAATGCCGAGAGGGATGGGGAGGGCGAGAGAGAGC
+
FFFFFFFFFFFFFFFFFFFFFFFFFFFFFFFFFFFFFFFFFFFFFFFFFFFF:FFFF:FFFFF:FFFFF
:FFFF,FFFFFFFFFFFFFFFF:FFFFFFFFFFFFFFFFFFFFFFFFF:FF,FFFFFFFFFFF:FFFFF
@A00155:342:HHGFNDSXY:1:1367:20907:19977 2:N:0:GAACCTAG+TCCGCATA
GAGACCCCTCCGGTCTTGTCCAACCTCTGTTTCGGCAGAGGATACCGTGCTCCACAGCTGTAAGGCTG
TGGGCCCGAGAGAAGTCGCTCTCTCTCGCCTTCCACATCCCTCTCGGCATTGAGATGACCATAGGCCA
+
FFFFFFFFFFFFFFFFFFFFFFFFFFFFFFFFFFFFFFFFFFFFFFFFFFFFFFFFFFFFFFFFFFFF
FFFFFFFFFFFFFFFFFFFFFFFFFFFFFFFFFFFFFFFFFFFFFFFFFFFFFFFFFFFF,FFFFFFF
@A00155:342:HHGFNDSXY:1:1367:26024:22075 2:N:0:GAACCTAG+TCCGCATA
ACTAGACTCCGACCGTCATGGGGGTTCAAACCCATGCCCCGCCACACCGCCTTAGATCAGCTGTGTCC
ACTTAAGGACTCACCTCTGGCCTATGGTCATCTCAATGCCGAGAGGGATGTGGAGGGCGAGAGAGAGC
+
FFFFFFFFFFFFFFFFFFFFFFFFFFFFFFFFFFFFFFFFFFFFFFFFFFFFFFFFFFFFFFFFFFFF
FFFFFFFFFFFFFFFFFFFFFFFFFFFFFFFFFFFFFFFFFFFFFFFFFFFFFFFFFFFFFFF:FFFFF
@A00155:342:HHGFNDSXY:1:1367:27498:35493 2:N:0:GAACCTAG+TCCGCATA
CAGGTAGCCTACTAGACTACGACTTACACGGTAGGTCGTGAACAAGCCCGGAGTACATCTCCTTTGGA
ACTAGGAGACCCCTCCGGTCTTGTCCAACCTCTGTTTCGGCAGAGGATACCGTGCTCCACAGCTGTAA
+
```

```
F:FFF:FFFFFFFFFFFFFFFFFFFFFFFFFFFFFFFFFFFFFFFFFFFFFFFFFFFFFFFFFF
FFFFF:FFFFFFFFFFFFFF,FFFFFFFFFFFFFFFFFFFFFFFFFFFFFFFFFFFFFFFFFFFFFFF
@A00155:342:HHGFNDSXY:1:1368:3794:4335 2:N:0:GAACCTAG+TCCGCATA
ACTAGACTACGACTTACACGGTAGGTCGTGAACAAGCCCGGAGTACATCTCCTTTGGAACTAGGAGAC
CCCTCCGGTCTTGTCCAACCTCTGTTTCGGCAGAGGATACCGTGCTCCACAGCTGTAAGGCTGTGGGC
+
FFFFFFFFFFFFFFFFFFFFFFFFFFFFFFFFFFFFFFFFFFFFFFFFFFFFFFFFFFFFFFFFFFFF
FFFFFFFFFFF:FFFFFFFFFFFFFFFFFFFFFFFFFFFFFFFFFFFFFFFFFFFFFFFFFFFFFFFF
@A00155:342:HHGFNDSXY:1:1368:11822:7905 2:N:0:GAACCTAG+TCCGCATA
ACTAGGAGACCCCTCCGGTCTTGTCCAACCTCTGTTTCGGCAGAGGATACCGTGCTCCACAGCTGTAA
GGCTGTGGGCCCGAGAGAAGTCGCTCTCTCTCGCCCTCCACATCCCTCTCGGCATTGAGATGACCATA
+
FFFFFFFFFFFFFFFFFFFFFFFFFFFFFFFFFFFFFFFFFFF:FFFFFFFFFFFFFFFFFFFFFFFF
FFFFFFFFFFFFFFFFFFFFFFFFFFFFFFFFFFFFFFFFFFFFFFFFFFFFFFFFFFFFFFFFFFFF
@A00155:342:HHGFNDSXY:1:1368:11785:7936 2:N:0:GAACCTAG+TCCGCATA
ACTAGGAGACCCCTCCGGTCTTGTCCAACCTCTGTTTCGGCAGAGGATACCGTGCTCCACAGCTGTAA
GGCTGTGGGCCCGAGAGAAGTCGCTCTCTCTCGCCCTCCACATCCCTCTCGGCATTGAGATGACCATA
+
FFFFFFFFFFFFFFFFFFFFFFFFFFFFFFFFFFFFFFFFFFFFFFFFFFFFFFFFFFFFFFFFFFFF
FF:FFFFFFFFFFFFFFFFFFFFFFFFFFFFFFFFFFFFFFFFFFF:FFFFFFFFFFFFFFFFFFFFF
@A00155:342:HHGFNDSXY:1:1368:5710:11882 2:N:0:GAACCTAG+TCCGCATA
AGTTGTGTAAGTTTCGTCCTAAAAAGGACTCGTCAGGTAGCCTACTAGACTACGACTTACACGGTAGG
TCGTGAACAAGCCCGGAGTACATCTCCTTTGGAACTAGGAGACCCCTCCGGTCTTGTCCAACCTCTGT
+
FFFFFFFFF,FFFFFFFFFF,FFFFFFFFFFFFFFFFFFFFFFFFFF,FFFFFFFF:FFFFFFFFF:F
::FFFFFFFFFFFFFFFF:FFFFFF,:F:FFFFFFFFFFFFFFFFFF::FFFFF,FFFFFFFFFFF:F
@A00155:342:HHGFNDSXY:1:1368:7771:20118 2:N:0:GAACCTAG+TCCGCATA
GGACTCGTCAGGTAGCCTACTAGACTACGACTTACACGGTAGGTCGTGAACAAGCCCGGAGTACATCT
CCTTTGGAACTAGGAGACCCCTCCGGTCTTGTCCAACCTCTGTTTCGGCAGAGGATACCGTGCTCCAC
+
FFFFFFFFFFFFFF:FFFFFFFFFFFFFFFFFFFFFFFFFFFFFFFFFFFFFFFFFFFFFFFFF:FFF
FFFFFFFFFFFFFFFF:FFFFFFFFFFFFFFFFFFFFFFFFFFFFFFFFFFFFFFFFFFFFFFFFFF
@A00155:342:HHGFNDSXY:1:1368:10456:22326 2:N:0:GAACCTAG+TCCGCATA
GGACTCGTCAGGTAGCCTACTAGACTACGACTTACACGGTAGGTCGTGAACAAGCCCGGAGTACATCT
CCTTTGGAACTAGGAGACCCCTCCGGTCTTGTCCAACCTCTGTTTCGGCAGAGGATACCGTGCTCCAC
+
FFFFFFFFFFFFFFFFFFFFFFFFFF:FFFFFFFFFFFFFFFFFFFFFFFFFFFFFFFFFFFFFFFFF
FFFFFFFF,FFFFFF:FFFFFFFFFFFFFFFFFFFFFFFFFFFFFF:FFFFFFFFFFFFFFFFFFFFF
@A00155:342:HHGFNDSXY:1:1369:31448:1595 2:N:0:GAACCTAG+TCCGCATA
ACTAGGAGACCCCTCCGGTCTTGTCCAACCTCTGTTTCGGCAGAGGATACCGTGCTCCACAGCTGTAA
GGCTGTGGGCCCGAGAGAAGTCGCTCTCTCTCGCCCTCTACATCCCTCTCGGCATTGAGATGAC
+
FFFFFFFFFFFFFFFFFFFFFFFFFFFFFFFFFFFFFFFFFFFFFFFFFFFFFFFFFFFFFFF:FFFF
FFFFFFFFFFFFFFFFFFFFFFFFFFFFFFFFFFFFFFF:FFFFFFFFFFFFF:FFFFFFFFFF
@A00155:342:HHGFNDSXY:1:1369:9055:2832 2:N:0:GAACCTAG+TCCGCATA
ACATCTCCTTTGGAACTAGGAGACCCCTCCGGTCTTGTCCAACCTCTGTTTCGGCAGAGGATACCGTG
CTCCACAGCTGTAAGGCTGTGGGCCCGAGAGAAGTCGCTCTCTCTCGCCCTCTACATCCCTCTCGGCA
+
FFFFFFFFFFFFFFFFFFFFFFFFFFFFFFFFFFFFFFFFFFFFFFFFFFFFFFFFFFFFFFFFFFFF
FFFFFFFFFFFFFFFFFFFFFFFFFFFFFFFFFFFFFFFFFFFFFFFFFFFFFFFFFFFFFFFFFFFF
@A00155:342:HHGFNDSXY:1:1369:4128:8390 2:N:0:GAACCTAG+TCCGCATA
AAAAGGACTCGTCAGGTAGCCTACTAGACTACGACTTACACGGTAGGTCGTGAACAAGCCCGGAGTAC
ATCTCCTTTGGAACTAGGAGACCCCTCCGGTCTTGTCCAACCTCTGTTTCGGCAGAGGATACCGTGCT
+
```

FFFFFFFFFFFFFFFFFFFFFFFFFFFFFFFFFFFFFFFFFFFFFFFFFFFFFFFFFFFFFFFFFFFF
:FFFFFF:FFFFFFFFFFFFFFFFFFFFFFFFFFFFFFFFFFFFFFFFFFFFFFF:FFFFFFFFFFFF
@A00155:342:HHGFNDSXY:1:1369:14199:17127 2:N:0:GAACCTAG+TCCGCATA
TTTCGTCCTAAAAAGGACTCGTCAGGTAGCCTACTAGACTACGACTTACACGGTAGGTCGTGAACAAG
CCCGGAGTACATCTCCTTTGGAACTAGGAGACCCCTCCGGTCTTGTCCAACCTCTGTTTCGGCAGAGG
+
FFFFFFFFFFFFF,FFFFF:FFF,FFFFFF:FF:FFFFFFFFFFFFFFFFFFFFFFFFFFFFFFFFFF
,FFFFFFFFFFFFF,F,FFFFFFFFFFFFFFFFFFF:FFFFF:FFFFFFFFFFFF,:FFFF,FF:FF:
@A00155:342:HHGFNDSXY:1:1369:24198:19601 2:N:0:GAACCTAG+TCCGCATA
AGGTAGCCTACTAGACTACGACTTACACGGTAGGTCGTGAACAAGCCCGGAGTACATCTCCTTTGGAA
CTAGGAGACCCCTCCGGTCTTGTCCAACCTCTGTTTCGGCAGAGGATACCGTGCTCCACAGCTGTAAG
+
FFFFFFFFFFFFFFFFFFFFFFFFFFFFFFFFFFFFFFFFFFF:FFFFFFFFFFFFFFFFFFFFFFFF
FFFFFF:FFFFFFFFFFFFFFFFFFFFFFFFFFFFFFFFFFF:FFFFFFFFFFFFFFFFFFFFFFFFF
@A00155:342:HHGFNDSXY:1:1369:26992:22467 2:N:0:GAACCTAG+TCCGCATA
ACTCTTCGAGTTGTGTAAGTTTCGTCCTAAAAAGGACTCGTCAGGTAGCCTACTAGACTACGACTTAC
ACGGTAGGTCGTGAACAAGCCCGGAGTACATCTCCTTTGGAACTAGGAGACCCCTCCGGTCTTGTCCA
+
,FFFFFFFFFFFFFFFFFFF:FFFFFFFFFFFFFFFFFFFFFFF:FFFFFFFFFFFFFFFFFFFFFFF
FFFFF,FFFFFFFFFFFF:FFFFFFFFFFFFF:FFFFFFFFFFFFFFFFFFFFFFFFFFFFFFFFFFF
@A00155:342:HHGFNDSXY:1:1369:30544:29434 2:N:0:GAACCTAG+TCCGCATA
TAGGACGAAACTTACACAACTCGAAGAGTAACGACGGTTTCGTCCTTGGAACGGACTCATCAGGGAGC
CTACTAGACTCCGACCGTCATGGGGGTTCAAACCCATGCCCCGCTACACCGCCTTAGATCAGCTGTGT
+
FFFFFFFFFFFFFFFFFFFFFFFFFFFFFFFFFFFFFFFFFFFFFFFFFFFFFFFFFFFFFFFFFFFF
FFFFFFFFFFFFFFFFFFFFFFFFFFFFFFFFFFFFFFFFFFFFFFFFFFFFFFFFFFFFFFFFFFFF
@A00155:342:HHGFNDSXY:1:1369:30065:35274 2:N:0:GAACCTAG+TCCGCATA
TAGGACGAAACTTACACAACTCGAAGAGTAACGACGGTTTCGTCCTTGGAACGGACTCATCAGGGAGC
CTACTAGACTCCGACCGTCATGGGGGTTCAAACCCATGCCCCGCTACACCGCCTTAGATCAGCTGTGT
+
FFFFFFFFFFFFFFFFFFFFFFFFFFFFFFFFFFFFFFFFFFFFFFFFFFFFFFFFFFFFFFFFFFFF
FFFFFF:FFFFFFFFFFFFFFFFFFFFFFF:FFFF:FFFFFFFFFFFFFFFFFFFFF:FFFFFFFFFF
@A00155:342:HHGFNDSXY:1:1370:8603:3615 2:N:0:GAACCTAG+TCCGCATA
AGCCCGGAGTACATCTCCTTTGGAACTAGGAGACCCCTCCGGTCTTGTCCAACCTCTGTTTCGGCAGA
GGATACCGTGCTCCACAGCTGTAAGGCTGTGGGCCCGAGAGAAGTCGCTCTCTCTCGCCCTCCACATC
+
FFFFFFFFFFFFFFFFFFFFFFFFFFFFFFFFFFFFFFFFFFFFFFFFFFF,FFFFFF:FFFFFFFF:
FFFFFFFFFFFFFFFFFFFFFFFFF:FFFFFFFFFFFFFFF:FFFFFFFFFFFFFFFFFFF:FFF,FF
@A00155:342:HHGFNDSXY:1:1370:14913:6872 2:N:0:GAACCTAG+TCCGCATA
TAGACTACGACTTACACGGTAGGTCGTGAACAAGCCCGGAGTACATCTCCTTTGGAACTAGGAGACCC
CTCCGGTCTTGTCCAACCTCTGTTTCGGCAGAGGATACCGTGCTCCACAGCTGTAAGGCTGTGGGCCC
+
FFFFFFFFFFFFFFFFFFFFFFFFFFFF:FFFFFFFFFFFFFFFFFFFFFFFFFFFFFFFFFFFFFFF
FFFFFFFFFFF:FFFFFFFFFFFFFFFFFFFFFFFFFFFFFFFFFFFFFFFFFFFFFFFFF:FFFFFF
@A00155:342:HHGFNDSXY:1:1370:18828:20635 2:N:0:GAACCTAG+TCCGCATA
AGGACGAAACCGTCGTTACTCTTCGAGTTGTGTAAGTTTCGTCCTAAAAAGGACTCGTCAGGTAGCCT
ACTAGACTACGACTTACACGGTAGGTCGTGAACAAGCCCGGAGTACATCTCCTTTGGAACTAGGAGAC
+
FFFFFFFFFFFF:FFFFFFFFFFFFFFFFFFFFFFFFFFFFFFFFFFFFFFFFFFFFF:FFFFFFFFF
FFFFFFFFFFFFFFFFFFFFFFFFFFFFFFFFFFFFFFFFFFFFFFFFFFFFFFFFFFFFFFFFFFF:
@A00155:342:HHGFNDSXY:1:1371:20699:16391 2:N:0:GAACCTAG+TCCGCATA
TCCAAGGACGAAACCGTCGTTACTCTTCGAGTTGTGTAAGTTTCGTCCTAAAAAGGACTCGTCAGGTA
GCCTACTAGACTACGACTTACACGGTAGGTCGTGAACAAGCCCGGAGTACATCTCCTTTGGAACTAGG
+

FFFFFFFFFFFFFFFFFFFFFFFFF:FFFFFFFFFFFFFFFFFFFFFFFFFFFFFFFFFFF:FFFFFF
FFFFFFFFFFFFFFFFF:FFFFFFFFFFFFFFFFFFFFFFFFFFFFFFFFFFFFFFFFFFFF,FFFFF
@A00155:342:HHGFNDSXY:1:1371:30761:16532 2:N:0:GAACCTAG+TCCGCATA
GGAGTACATCTCCTTTGGAACTAGGAGACCCCTCCGGTCTTGTCCAACCTCTGTTTCGGCAGAGGATA
CCGTGCTCCACAGCTGTAAGGCTGTGGGCCCGAGAGAAGTCGCTCTCTCTCGCCCTCTACATCCCTCT
+
FFFFFFFFFFFFFFF:FFFFFFFFFFFFFFFFFFFFFFFFFFFFFFFFFFFFFFFFFFFF:F:FFFFF
FFFFFFFFFFF:FFFFF,FFFFFFFFFFFFFFFFFFFFFFFFFFFFFFFFFFFFFFFFFFFFFFFFFF
@A00155:342:HHGFNDSXY:1:1371:12698:21668 2:N:0:GAACCTAG+TCCGCATA
AAGCCCGGAGTACATCTCCTTTGGAACTAGGAGACCCCTCCGGTCTTGTCCAACCTCTGTTTCGGCAG
AGGATACCGTGCTCCACAGCTGTAAGGCTGTGGGCCCGAGAGAAGTCGCTCTCTCTCGCCCTCCA
+
FF,FF::FFFFFFFFFFFFFFFFFFFFFFFFFFFFFFFFFFFFFF,FFFFFFFFFFFFFFFFFFFFFF
FFFFFFFFFFFFFFFFFFFFFFFFFFFFFFFFFFFFFF:FFFF,FFFFFFFFFFFFFFFFFFFFF
@A00155:342:HHGFNDSXY:1:1371:29496:23109 2:N:0:GAACCTAG+TCCGCATA
TAGGAGACCCCTCCGGTCTTGTCCAACCTCTGTTTCGGCAGAGGATACCGTGCTCCACAGCTGTAAGG
CTGTGGGCCCGAGAGAAGTCGCTCTCTCTCGCCCTCTACATCCCTCTCGGCATTGAGATGACCATAGG
+
FFFFFFFFFFFFFFFFFFFFFFFFFFFFFFFFFFFFFFFFFFFFFFFFFFFFFFFFFFFFFFFFFFFF
FFFFFFFFFFFFFFFFFFFFFFFFFFFFFFFFFFFFFFFFFFFFFFFFFFFFFFFFFFFFFFFFFFFF
@A00155:342:HHGFNDSXY:1:1372:24044:10285 2:N:0:GAACCTAG+TCCGCATA
TGTCCAACCTCTGTTTCGGCAGAGGATACCGTGCTCCACAGCTGTAAGGCTGTGGGCCCGAGAGAAGT
CGCTCTCTCTCGCCCTCCACATCCCTCTCGGCATTGAGATGACCATAGGCCAGAGGAGAGTCCTTAAG
+
FFFFFFFFFFFFFF:FFFFFFFFFFF,FFFFFFFFFF:FFFFFFFF:FFFFFFFFFFFFF:FFFFFFF
FFFFFFFFFFFFFFFFFFFFFFFFFF,FFFF,FFFFFFFFFFFFF:FFFFFFFFF:FFFFFFFFFFF
@A00155:342:HHGFNDSXY:1:1372:5755:12367 2:N:0:GAACCTAG+TCCGCATA
ACTAGACTACGACTTACACGGTAGGTCGTGAACAAGCCCGGAGTACATCTCCTTTGGAACTAGGAGAC
CCCTCCGGTCTTGTCCAACCTCTGTTTCGGCAGAGGATACCGTGCTCCACAGCTGTAAGGCTGTGGGC
+
FFF:FFFFFFFFFFFFFFFFFFFFFFF:FFFFFFF:FFFFFFFFFFFFFFFFFFFFFFFFFFFFFFFF
FFFFFFFFFFFFFFFFFFFFFFFFFFFFFFFFFFFFFFFFFFFFFFFFFFFFF:FFFFFFF,FFFFFF
@A00155:342:HHGFNDSXY:1:1372:28004:15890 2:N:0:GAACCTAG+TCCGCATA
CATGGGGGTTCAAACCCATGCCCCGCCACACCGCCTTAGATCAGCTGTGTCCACTTAAGGACTCACCT
CTGGCCTATGGTCATCACAATGCCGAGAGGGATGTGGAGGGCGAGAGAGAGCGACTTCTCTCGGGCCC
+
FFFFFFFFFFFFFFFFFFFFFFFFFFFFFFFFFFF:FFFFFFFFFFFFFFFFFFFFFFFFFFFFFFFF
FFFFFFFFFFFFFFFFFFFFFFFFFFFFFFFFFFFFFFFFFFFFF:FFFFFFFFFFFFFFFFFFFFFF
@A00155:342:HHGFNDSXY:1:1372:22372:29559 2:N:0:GAACCTAG+TCCGCATA
CAGCTGTAAGGCTGTGGGCCCGAGAGAAGTCGCTCTCTCTCGCCCTCCACATCCCTCTCGGCATTGAG
ATGACCATAGGCCAGAGGTGAGTCCTTAAGTGGACACAGCTGATCTAAGGCGGTGTGGCGGGG
+
FFFFFFFFFFFFFFFFFFFFFFFFFFFFFFFFFFFFFFFFFFFFFFFFFFFFFFFFFFFFFFF:F:FF
FFFFFFFFFFFFFFFFFFFFFFFFFF:FFFFFFFFFFFFFFFFFFFFFFFFFFFFFFFFFFFF
@A00155:342:HHGFNDSXY:1:1372:16007:32972 2:N:0:GAACCTAG+TCCGCATA
TCCAAGGACGAAACCGTCGTTACTCTTGGAGTTGTGTAAGTTTCGTCCTAAAAAGGACTCGTCAGGTA
GCCTACTAGACTACGACTTACACGGTAGGTCGTGAACAAGCCCGGAGTACATCTCCTTTGGAACTAGG
+
FFFFFFFFFFF:FFFFFFFFFFFFFFFFFFFFFFFFFFFFFFFFFFFFFFFFFFFFFFFFFFFFFFFF
FFFFFFFFFFFFFFFFFFFFFFFFFFFFFFFFFF:FFFFFFFFFFFFFFFFFFFFFFFFFFFFFFFFF
@A00155:342:HHGFNDSXY:1:1373:12219:12070 2:N:0:GAACCTAG+TCCGCATA
AGTACATCTCCTTTGGAACTAGGAGACCCCTCCGGTCTTGTCCAACCTCTGTTTCGGCAGAGGATACC
GTGCTCCACAGCTGTAAGGCTGTGGGCCCGAGAGAAGTCGCTCTCTCTCGCCCTCCACATCC
+

```
FFF:FFFFFFFFFFFFFFFFFFFFFFFFFFFFFFFFFFFFFFFFFFFFFFFFFFFFFFFFFFFFFFFF
FFFFFFFFFFFFFFFFFFFFFFFFFFFFFFFFFFFFFFFFFFFFFFFFFFFFFFFFFFFFFF
@A00155:342:HHGFNDSXY:1:1373:9055:27759 2:N:0:GAACCTAG+TCCGCATA
ACTTACACGGTAGGTCGTGAACAAGCCCGGAGTACATCTCCTTTGGAACCAGGAGACCCCTCCGGTCT
TGTCCAACCTCTGTTTCGGCAGAGGATACCGTGCTCCACAGCTGTAAGGCTGTGGGCCCGAGAGAAGT
+
FFFFFFFFFFFFFFFFFFFFFFFFFFFFFFFFFFFFFFFFFFFFFFFFFFFFFFFFFFFFFFFFFFFF
FFFFFFFFFFFFFFFFFFFFFFFFFFFFFFFFFFFFFFFFFFFFFFFFFFFFFFFFFFFFFFFFFFFF
@A00155:342:HHGFNDSXY:1:1373:9435:28103 2:N:0:GAACCTAG+TCCGCATA
ACTTACACGGTAGGTCGTGAACAAGCCCGGAGTACATCTCCTTTGGAACCAGGAGACCCCTCCGGTCT
TGTCCAACCTCTGTTTCGGCAGAGGATACCGTGCTCCACAGCTGTAAGGCTGTGGGCCCGAGAGAAGT
+
FFFFFFFFFFFFFFFFFFFFFFFFFFFFFFFFFFFFFFFFFFFFFFFFFFFFFFFFFFFFFFFFFFFF
FFFFFF:FFFFFFFFFFFFF:FFFFFFFFFFFFFFFFFFFFF:FF:FFFFFFFFFFFFFFFFFFFFFF
@A00155:342:HHGFNDSXY:1:1373:28004:31861 2:N:0:GAACCTAG+TCCGCATA
TAGGAGACCCCTCCGGTCTTGTCCAACCTCTGTTTCGGCAGAGGATACCGTGCTCCACAGCTGTAAGG
CTGTGGGCCCGAGAGAAGTCGCTCTCTCTCGCCCTCCACATCCCTCTCGGCATTGAGATGACCATAGG
+
FFF:FFFFFF:FFFFFFFFFFFFFFFFFFFFFFFFFFFFFFFFFFFFFFF:FFFFFFFFFFFFFFFFF
FFFFFFFFFFFFFFFF:FFFFFFFFFFFFFFFFFFFFFFFFFFFFFFFFFF,,FFFFFFFFFFFFFFF
@A00155:342:HHGFNDSXY:1:1374:27308:15280 2:N:0:GAACCTAG+TCCGCATA
GTTGTGTAAGTTTCGTCCTAAAAAGGACTCGTCAGGTAGCCTACTAGACTACGACTTACACGGTAGGT
CGTGAACAAGCCCGGAGTACATCTCCTTTGGAACTAGGAGACCCCTCCGGTCTTGTCCAACCTCTGTT
+
FFFFFFFFFFFFFFFFFFFFFFFFFFFFFFFFFFFFFFFFFFFFFFFFFFFFFFFFFFFFFFFFFFFF
FFFFFFFFF:FFFFFFFFFFFFFFFFFFFFFFFFFFFFFFFFFFFFFFFFFFFFFFFFFFFFFFFFFF
@A00155:342:HHGFNDSXY:1:1374:5032:19476 2:N:0:GAACCTAG+TCCGCATA
GAAACCGTCGTTACTCTTGGAGTTGTGTAAGTTTCGTCCTAAAAAGGACTCGTCAGGTAGCCTACTAG
ACTACGACTTACACGGTAGGTCGTGAACAAGCCCGGAGTACATCTCCTTTGGAACTAGGAGACCCCTC
+
FFFFFFFFF:FFFFFF:FFFFFFFFFFFFFFFFFFFFFFFFFFFFF,FFFFFFFFFFFFFFFFFFFFF
FF:FFFF::FF:FFFFFFFFFFFFFFFFFFFFFFFFF,FFFFFFFFFFFFFFF::FFFFFFFFFFFFF
@A00155:342:HHGFNDSXY:1:1374:20193:21966 2:N:0:GAACCTAG+TCCGCATA
ACTCTTCGAGTTGTGTAAGTTTCGTCCTAAAAAGGACTCGTCAGGTAGCCTACTAGACTACGACTTAC
ACGGTAGGTCGTGAACAAGCCCGGAGTACATCTCCTTTGGAACTAGGAGACCCCTCCGGTCTTGTCCA
+
FFFFFFFFFFFFFFFFFFFFFFFFFFFFFFFF:FFFFFFFFFFFFFFFFFFFFFFFFF:FFFFFFFFF
FFFFFFFFFFFFFFFFFFFFFFFFFFFFF,FFFFF,FFFFFFFFFFFF:FFFFFFFFFFFFFFFFFFF
@A00155:342:HHGFNDSXY:1:1375:1036:36980 2:N:0:GAACCTAG+TCCGCATA
ACAAGCCCGGAGTACATCTCCTTTGGAACTAGGAGACCCCTCCGGTCTTGTCCAACCTCTGTTTCGGC
AGAGGATACCGTGCTCCACAGCTGTAAGGCTGTGGGCCCGAGAGAAGTCGCTCTCTCTCGCCCTCT
+
FFFFFFFFFFFFF:FFFF:FF::FFF:,F:FFFFFFFFFF:FFFFFF,,FFFFFFFFFFFF::FFFFF
::FFFF:FFFFFFFFFF,FFFF,FF:FFFF:FFFFFFFFFFF::::FFFFFFFFF:FFFFFFFFFF
@A00155:342:HHGFNDSXY:1:1376:28420:5963 2:N:0:GAACCTAG+TCCGCATA
TGTGTAAGTTTCGTCCTAAAAAGGACTCGTCAGGTAGCCTACTAGACTACGACTTACACGGTAGGTCG
TGAACAAGCCCGGAGTACATCTCCTTTGGAACTAGGAGACCCCTCCGGTCTTGTCCAACCTCTGTTTC
+
FFFFFFFFFFFFFFFFFFFFFFFFFFFFFFFFFFFFFFFFFFFFFFFFFFFFFFFFFFFFFFFFFFFF
FFFFFFF,FFFFFFFFFFFFFFFFFFFFFFFFFFFFFFFFFFFFFFFFFFFFFFFFFFFFFFFFFFFF
@A00155:342:HHGFNDSXY:1:1376:8603:6402 2:N:0:GAACCTAG+TCCGCATA
TCAGGTAGCCTACTAGACTACGACTTACACGGTAGGTCGTGAACAAGCCCGGAGTACATCTCCTTTGG
AACCAGGAGACCCCTCCGGTCTTGTCCAACCTCTGTTTCGGCAGAGGATACCGTGCTCCACAGCTGTA
+
```

FFFFFFFFFFFFFFFFFFFFFFFFFFFFFFFFFFFF:FFFFFFFFFFFFFFFFFFFF:FFFFFFFFFF
FFFFFFFFFFFFFFFFFFFFFFFFFFFFFFFFFFFFF:FFFFFFFFF,FFFFFFFFFFFFFFFF:FFF
@A00155:342:HHGFNDSXY:1:1376:8829:6574 2:N:0:GAACCTAG+TCCGCATA
TCAGGTAGCCTACTAGACTACGACTTACACGGTAGGTCGTGAACAAGCCCGGAGTACATCTCCTTTGG
AACCAGGAGACCCCTCCGGTCTTGTCCAACCTCTGTTTCGGCAGAGGATACCGTGCTCCACAGCTGTA
+
FFFFFFFFFFFFFFFFFFFFFFFFFFFFFFFFFFFFFFFFFFFFFF,FFFFFFFFFFFFFFFFFFFFF
FFFFFFFFFFFFFFFFFFFFFFFFFFFFFFFFFFFFFFFFFFFFFFFFFFFFFFFFFFFFFFFF:FFF
@A00155:342:HHGFNDSXY:1:1377:7898:6370 2:N:0:GAACCTAG+TCCGCATA
ACTTACACGGTAGGTCGTGAACAAGCCCGGAGTACATCTCCTTTGGAACTAGGAGACCCCTCCGGTCT
TGTCCAACCTCTGTTTCGGCAGAGGATACCGTGCTCCACAGCTGTAAGGCTGTGGGCCCGAGAGAAGT
+
FFFFFFFFFFFFFFFFFFF:FFFFFFFFFFFFFFFFFFFFFF:FFFFFFFFFFFFFFFFFFFFFFFFF
FFFFFFFFFFFFF:FFFFFFFFFFFFFFFFF:FFFFFFFFFFFFFFFFFFFFFFFFFFFFFFFFFFFF
@A00155:342:HHGFNDSXY:1:1377:3125:9283 2:N:0:GAACCTAG+TCCGCATA
TTAAGGACTCACCTCTGGCCTATGGTCATCTCAATGCCGAGAGGGATGTGGAGGGCGAGAGAGAGCGA
CTTCTCTCGGGCCCACAGCCTTACAGCTGTGGAGCACGGTATCCTCTGCCGAAACAGAGGTTGGACAA
+
FFFFFFFFFFFFFFFFFFFFFFFFFFFFFFFFFFFFFFFFFFFFFFFFFFFFFFFFFFFFFFFFFFFF
FFFFFFFFFFFFFFFFFFFFFFFFFFFFFFFFFFFFFFFFFFFFFFFFFFFFFFFFFFFFFFFFFFFF
@A00155:342:HHGFNDSXY:1:1377:4661:18521 2:N:0:GAACCTAG+TCCGCATA
CGGCAAAGGATACCGTGCTCCACAGCTGTAAGGCTGTGGGCCCGAGAGAAGTCGCTCTCTCTCGCCCT
CCACATCCCTCTCGGCATTGAGATGACCATAGGCCAGAGGTGAGTCCTTAAGTGGACACAG
+
FFFFFFFFFFFFFFFFFFFFFFF:FFF,FFFFFFFFFFFFFFFF:FFFFFFFFFFFFFFFFFFFFFFF
FFFFFFFFFFFFFFFFF:FFFFFFFFFFFFFFFFFFFFFFFFFFFFFFFFFFFFFFF:FFF
@A00155:342:HHGFNDSXY:1:1378:7699:10942 2:N:0:GAACCTAG+TCCGCATA
TAGACTACGACTTACACGGTAGGTCTTGAACAAGCCCGGAGTACATCTCCTTTGGAACTAGGAGACCC
CTCCGGTCTTGTCCAACCTCTGTTTCGGCAGAGGATACCGTGCTCCACAGCTGTAAGGCTGTGGGCCC
+
FFFFFF:FFFFFFFFFFFFF:FFFF,FFFFFF,,FFFFF:FFFFF,FFF:FFFFFFFFFFFFF,FFFF
FFFFFF:FFFFFFFFFFFFFF,FFFFF:FF:F:FFFF,FFFFFFFFFFFFFFFFFF,FFFFFFFFFF:
@A00155:342:HHGFNDSXY:1:1378:16857:11960 2:N:0:GAACCTAG+TCCGCATA
AGCCCGGAGTACATCTCCTTTGGAACTAGGAGACCCCTCCGGTCTTGTCCAACCTCTGTTTCGGCAGA
GGATACCGTGCTCCACAGCTGTAAGGCTGTGGGCCCGAGAGAAGTCACTCTCTCTCGCCCTCCACATC
+
FFFFFFFFFFFFFFFFFFF:FFFFFFFFFFFFFFFFFFFFFFFFF:FFFFFFFFFFFFFFFFFFFFF:
FFF:FFFFFFFFFFFFFFFFFFFFF:FFFFFFFFFFFFFFFF:FFFFFFFFFFFFFFFFFFFFFFF:F
@A00155:342:HHGFNDSXY:1:1378:15609:14058 2:N:0:GAACCTAG+TCCGCATA
ACATCTCCTTTGGAACTAGGAGACCCCTCCGGTCTTGTCCAACCTCTGTTTCGGCAGAGGATACCGTG
CTCCACAGCTGTAAGGCTGTGGGCCCGAGAGAAGTCGCTCTCTCTCGCCCTCCACATCCCTCTCGGCA
+
FFFFFFFFFFFFFFFFFFFFFFFFFFFFFFFFFFFFFFFFFFFFFFFFFFFFFFFFFFFFFFFFFFFF
FFFFFFFFFFFFFFFFFFFFFFFFFFFFF:FFFFFFFFFFFFFFFFFFFFFFFFFFFFFFFFFFFFFF
@A00155:342:HHGFNDSXY:1:1378:21694:32925 2:N:0:GAACCTAG+TCCGCATA
CCGGAGTACATCTCCTTTGGAACTAGGAGACCCCTCCGGTCTTGTCCAACCTCTGTTTCGGCAGAGGA
TACCGTGCTCCACAGCTGTAAGGCTGTGGGCCCGAGAGAAGTCGCTCTCTCTCGCCCTCCACATCCCT
+
FFFFFFFFFFFFFFFFFFFFFFFFFFFFFFFFFFFFFFFFFFFFFFFFFFFFFFFFFFFFFFFFFFFF
,FFFFFFFFFFFFFFFFFFFFFFF:FFFFFFFFF:FFFFFFFFFFFFFFFFFFFFFFFFFFFFFFFFF
@A00155:342:HHGFNDSXY:1:1401:28574:3317 2:N:0:GAACCTAG+TCCGCATA
AGGACGAAACCGTCGTTACTCTTTGAGTTGTGTAAGTTTCGTCCTAAAAAGGACTCGTCAGGTAGCCT
ACTAGACTACGACTTACACGGTAGGTCGTGAACAAGCCCGGAGTACATCTCCTTTGGAACTAGGAGAC
+

```
FFFFFFFFFFFF:FFFFFFFF,,,FFFFFFFF,FFFF,F:FFFF:FFFFFF:FFFFFFF:FFFFFFFF
FF:FFFFFFFF,F,FFFFFFFFFFFFFFFFFFFF:FFFFFFFFFFFFFFFFFFF:FFFFFFFFFFFFF
@A00155:342:HHGFNDSXY:1:1401:26205:17597 2:N:0:GAACCTAG+TCCGCATA
GGAACGGACTCATCAGGGAGCCTACTAGACTCCGACCGTCATGGGGGTTCAAACCCATGCCCCGCCAC
ACCGCCTTAGATCAGCTGTGTCCACTTAAGGACTCACCTCTGGCCTATGGTCATCTCAATGCCGAGAG
+
FFFFFFFFFFFFFFFFFFFFFFFFFFFFFFFFFFFFFFFFFFFFFFFFFFFF:FFFFFFFFFFFFFFF
FFFFFFFFFFFFFFFFFFFFFFFFFFF,FFFFFFFFFFFFFFFFFFFFFFFFFFFFFFFF:FFFFFF:FFF
@A00155:342:HHGFNDSXY:1:1402:28772:5697 2:N:0:GAACCTAG+TCCGCATA
GGAGCCTACTAGACTCCGACCGTCATGGGGGTTCAAACCCATGCCCCGCCACACCGCCTTAGATCAGC
TGTGTCCACTTAAGGACTCACCTCTGGCCTATGGTCATCTCTATGCCGAGAGGGATGTGGAGGG
+
FFF:FFFFFFFFFFFFFFFFFFF:FFFFFFFFFFFFFFFFFFF:FFFFFFFFFFFFFFF:FFFFFFFF
FFFFFFFFFFFFFFFFFFFFFFFFFFF:FF,,FFFF:FFFFFFFFFF:FFF:FFF:FFF:FFFFF
@A00155:342:HHGFNDSXY:1:1402:25464:5791 2:N:0:GAACCTAG+TCCGCATA
ACGACTTACACGGTAGGTCGTGAACAAGCCCGGAGTACATCTCCTTTGGAACTAGGAGACCCCTCCGG
TCTTGTCCAACCTCTGTTTCGGCAGAGGATACCGTGCTCCACAGCTGTAAGGCTGTGGGCCCGAGAGA
+
FFFFFFFFFFFFFFFFFFFFFFFFFFFFFFFFFFFFFFFFFFFFFFFFFFFFFFFFFFFFFFFFFFFF
FFFFFFFFFFFFFFFFFFFFFFFFFFFFFFFFFFFFFFFFFFFFFFFFFFFFFFF:F:FFFFFFFFFF
@A00155:342:HHGFNDSXY:1:1402:27453:17503 2:N:0:GAACCTAG+TCCGCATA
GACTCGTCAGGTAGCCTACTAGACTACGACTTACACGGTAGGTCGTGAACAAGCCCGGAGTACATCTC
CTTTGGAACTAGGAGACCCCTCCGGTCTTGTCCAACCTCTGTTTCGGCAGAGGATACCGTGCTCCACA
+
FFFFFFFFFFFFFFFFFFFFFFFFFFFFFFFFFFFFFFFFFFFFFFFFFFFFFFFFFFFFFFFFFFFF
FFFFFFFFF:FFFFFFFFFFFFFFFFFFFFFFFFFFFFFFFFFFFFFFFFFFFFFFFFFFFFFFFFFF
@A00155:342:HHGFNDSXY:1:1402:22110:30859 2:N:0:GAACCTAG+TCCGCATA
GACGAAACCGTCGTTACTCTTTGAGTTGTGTAAGTTTCGTCCTAAAAAGGACTCGTCAGGTAGCCTAC
TAGACTACGACTTACACGGTAGGTCGTGAACAAGCCCGGAGTACATCTCCTTTGGAACTAGGAGACCC
+
FFFFFFFFFFFFFFFFFFF:F:FFFFFFFF:F:FFFFFFFFF,FFFFFFFFFFFFFF:FFFFFFFFFF
FFFFFF,FFFFFFFFFFFFFFFFFFFFFFFFFFFFFFFFFFFFFFFFFFF,FFFFFFFF:FFFFFFFF
@A00155:342:HHGFNDSXY:1:1403:21630:6261 2:N:0:GAACCTAG+TCCGCATA
GGACTCGTCAGGTAGCCTACTAGACTACGACTTACACGGTAGGTCGTGAACAAGCCCGGAGTACATCT
CCTTTGGAACTAGGAGACCCCTCCGGTCTTGTCCAACCTCTGTTTCGGCAGAGGATACCGTGCTCCAC
+
FFFFFFFFFFFFFFFFFFFFFFFFFFFFFFFFFFFFFFFFF:FFFFFFFFFFF:FFFFFFFFFFFFFF
FFFFFFFFFFFFFFFFFF:FFFFFFFFFFFFFFFFFFFFFFFFFFFFFFFFFFFFFFFF:FFF:FFFFFF
@A00155:342:HHGFNDSXY:1:1403:3577:8844 2:N:0:GAACCTAG+TCCGCATA
ACACGGTAGGTCGTGAACAAGCCCGGAGTACATCTCCTTTGGAACTAGGAGACCCCTCCGGTCTTGTC
CAACCTCTGTTTCGGCAGAGGATACCGTGCTCCACAGCTGTAAGGCTGTGGGCCCGAGAGAAGTCGCT
+
FFFFFFFFFFFFFFFFFFFFFFFFFFFFFFFFFFFFFFFFFFFFFFFFFFFFFFFFFFFFFFFFFFFF
FFFFFFFFFFFFFFFFFFFFFFFFFFFFFFFFFFFFFFF:FFFFFFFFFFFFFFFFFFFFFFFFFFFFF
@A00155:342:HHGFNDSXY:1:1403:26982:15060 2:N:0:GAACCTAG+TCCGCATA
TAGACTACGACTTACACGGTAGGTCGTGAACAAGCCCGGAGTACATCTCCTTTGGAACTAGGAGACCC
CACCGGTCTTGTCCAACCTCTGTTTCGGCAGAGGATACCGTGCTCCACAGCTGTAAGGCTGTGGGCCC
+
FF:FFFFFFFFFFFFFFFFFFFFFFFFFFFFFFF:FFFFFFFFFFFFFFFFF,FFFFFFFFFFFFFFF
F,,FFF:FFFFFFFFFFFFFFFF,FFFFFFFFFFFFFFFFFFFFFFFFFFFFFFFFFFFFFFFFFFFF
@A00155:342:HHGFNDSXY:1:1403:27597:15092 2:N:0:GAACCTAG+TCCGCATA
GATACCGTGCTCCACAGCTGTAAGGCTGTGGGCCCGAGAGAAGTCGCTCTCTCTCGCCCTCTACATCC
CTCTCGGCATTGAGATGACCATAGGCCAGAGGTGAGTCCTTAAGTGGACACAGCTGATCTAAGGCGGT
+
```

```
FFFFFFFFFFFFFFFFFFFFFFFFFFFFFFFFFFFFFFFFFFFFFFFFFFFFFFFFFFFFFFFFFFFF
FFFFFFFFF:FFFFFFFFFFFFFFFFFFFFFFFFFFFFFFF:FFFFFFFFFFFFFFFFFFFFFFFFFFF
@A00155:342:HHGFNDSXY:1:1403:27272:15154 2:N:0:GAACCTAG+TCCGCATA
TAGACTACGACTTACACGGTAGGTCGTGAACAAGCCCGGAGTACATCTCCTTTGGAACTAGGAGACCC
CTCCGGTCTTGTCCAACCTCTGTTTCGGCAGAGGATACCGTGCTCCACAGCTGTAAGGCTGTGGGCCC
+
FFFF:FFFFFFFFFFFF:FFFFFFFFFFFFFF:FFFFFFFFFF:FFFFFFFFFFFFFFFFFFFFFFF
FFFFFFFFFFFFFFFFFFFFFFFFFFFFFFFFFFF,FFFFFFFFFFFFFFFFFFF:FFFFFFFFFFFF
@A00155:342:HHGFNDSXY:1:1403:18358:35227 2:N:0:GAACCTAG+TCCGCATA
ATGCCCCGCCACACCGCCTTAGATCAGCTGTGTCCACTTAAGGACTCACCTCTGGCCTATGGTCATCT
CAATGCCGAGAGGGATGTGGAGGGCGAGAGAGAGCGACTTCTCTCGGGCCCACAGCCTTACAGC
+
FFFFFFFFFFFFFFFFFFFFFFFFFFFFFFFFFFFFFFFFFFFFFFFFFFFFFFFFFFFFFFFFFFFF
FFFFFFFFFFFFFFF:FFFFFFFFFFFFFFFFFFFFFFFFFFFFFFFFFFF::FFFFFF::FFFF
@A00155:342:HHGFNDSXY:1:1403:18204:35274 2:N:0:GAACCTAG+TCCGCATA
ATGCCCCGCCACACCGCCTTAGATCAGCTGTGTCCACTTAAGGACTCACCTCTGGCCTATGGTCATCT
CAATGCCGAGAGGGATGTGGAGGGCGAGAGAGAGCGACTTCTCTCGGGCCCACAGCCTTACAGC
+
FFFFFFFFFFFFFFFFFFFFFFFFF:FFFFFFFFFFFFFFFFFFFFFFFFFFFFFFFFFFF:FFFFFFF
FFFFFFFFFFFFFFFFFFFFFFFFFFFFFFFFFFFFFFFFFFF:FFF:FFFFFFFFFFFFFFFF
@A00155:342:HHGFNDSXY:1:1404:6479:1501 2:N:0:GAACCTAG+TCCGCATA
CACGGTAGGTCGTGAACAAGCCCGGAGTACATCTCCTTTGGAACTAGGAGACCCCTCCGGTCTTGTCC
AACCTCTGTTTCGGCAGAGGATACCGTGCTCCACAGCTGTAAGGCTGTGGGCCCGAGAGAAGTCGCTC
+
FFFFFFFFFFFFFFFFFFFFFFFFFFFFFFFFFFFFFFFFFF:FFFFFFFFFFFFFFFFFFFFFFFFFF
FFFFFFFFFFFFFFFFFFFFFFFFFFFFFFFFFFFFFF,FFFFFFFFFFFFFFFFFFFFFF:FFFFFFF
@A00155:342:HHGFNDSXY:1:1404:7491:17096 2:N:0:GAACCTAG+TCCGCATA
TAGTAGGCTACCTGACGA
+
FFFFFFFFFFFFFFFFFF
@A00155:342:HHGFNDSXY:1:1404:3667:19774 2:N:0:GAACCTAG+TCCGCATA
TAGCCTACTAGACTACGACTTACACGGTAGGTCGTGAACAAGCCCGGAGTACATCTCCTTTGGAACTA
GGAGACCCCTCCGGTCTTGTCCAACCTCTGTTTCGGCAGAGGATACCGTGCTCCACAGCTGTAAGGCT
+
FFFFFFFFFFFFFFFFFFFFFFFFFFFFFFFFFFFFFFFFFFFFFFFFFFFFFFFFFFFFFFFFFFFF
FFFFFFFFFFFFFFFF:FFFFFFFF,FFFFFFFF:FFFFF:FFFFFFFFFFFFFFFFFFFFFFFFFFF
@A00155:342:HHGFNDSXY:1:1405:18005:18364 2:N:0:GAACCTAG+TCCGCATA
AGTTTCGTCCTAAAAAGGACTCGTCAGGTAGCCTACTAGACTACGACTTACACGGTAGGTCGTGAACA
AGCCCGGAGTACATCTCCTTTGGAACCAGGAGACCCCTCCGGTCTTGTCCAACCTCTGTTTCGGCAGA
+
FFFF:FFFFFFFFFFFFFFFFFFFFFFFFFFFFFFFFFFFFFFFFFFFFFFFF:FFFFFFFFFFFFF
F:FFFFFFFFFFFFFFFFFFFFFFFFFFFFFF:FF:FFFFFFFFF:FFFFFFFFFFFFFFFFFF:FFFF
@A00155:342:HHGFNDSXY:1:1405:10149:21261 2:N:0:GAACCTAG+TCCGCATA
ACTACGACTTACACGGTAGGTCGTGAACAAGCCCGGAGTACATCTCCTTTGGAACTAGGAGACCCCTC
CGGTCTTGTCCAACCTCTGTTTCGGCAGAGGATACCGTGCTCCACAGCTGTAAGGCTGTGGGCCCGAG
+
FFFFFFFFFFFFFFFFFFFFFFFFFFFFFFFFFFFFFFFFFFFFFFFFFFFFFF:FF:FFFFFFFFFFF
FFFFFFFFFFFFFFFFFFFFFFFFFFFFFFFFFFFFFFFFFFFFFFFFFFFFFFFFFFFFFFFFFFFF
@A00155:342:HHGFNDSXY:1:1405:20591:24721 2:N:0:GAACCTAG+TCCGCATA
CCCCTCCGGTCTTGTCCAACCTCTGTTTCGGCAGAGGATACCGTGCTCCACAGCTGTAAGGCTGTGGG
CCCGAGAGAAGTCGCTCTCTCTCGCCCTTCACATCCCTCTCGGCATTGAGATGACCATAGGCCAGAG
+
FFFFFFFFFFFFFFFFFFFFFFFFFFFFFFFFFFFFFFFFFFFFFFFFFFFFFFF:FFFFFFFFFFFFF
FFFF:FFFF:FFFFFFFFFFFFFFFFFFFFFFFFFFFFFFFFFFFFFF:FFFFFFFFF:FFFFFFFFFF
```

```
@A00155:342:HHGFNDSXY:1:1406:4065:2237 2:N:0:GAACCTAG+TCCGCATA
TAGGTCGTGAACAAGTCCGGAGTACATCTCCTTTGGAACTAGGAGACCCCTCCGGTCTTGTCCAACCT
CTGTTTCGGCAGAGGATACCGTGCTCCACAGCTGTAAGGCTGTGGGCCCGAGAGAAGTCGCTCTCTCT
+
FFFFFFFFFFFFFFFFFFFFFFFFFFFFFFFFFFFFFFFFFFFFFFFFFFFFFFFFFFFFFFFFFFFF
FFFFFFFFFFFFFFFFFFFFFFFFFFFFFFFFFFFFFFFFFFFFFFFFFFFFFFFFFFFFFFFFFFFF
@A00155:342:HHGFNDSXY:1:1407:3432:25191 2:N:0:GAACCTAG+TCCGCATA
AGTTGTGTAAGTTTCGTCCTAAAAAGGACTCGTCAGGTAGCCTACTAGACTACGACTTACACGGTAGG
TCGTGAACAAGCCCGGAGTACATCTCCTTTGGAACTAGGAGACCCCTCCGGTCTTGTCCAACCTCTGT
+
FFFFFFFFFFF::FFFFFFFFFFF:FFFFFFFFFFF:FFFFFFFFFFFFFFFFFFFFFF:F:FFFFFF
FFFFFFFFFFFFFFFFFFFFFFFFFFFFFFFFFFFFFFFFFFFFFFFFFFFFF:FFFFFFFFFFFFFF
@A00155:342:HHGFNDSXY:1:1408:17508:12680 2:N:0:GAACCTAG+TCCGCATA
GTTTCGTCCTAAAAAGGACTCGTCAGGTAGCCTACTAGACTACGACTTACACGGTAGGTCGTGAACAA
GCCCGGAGTACATCTCCTTTGGAACTAGGAGACCCCTCCGGTCTTGTCCAACCTCTGTTTCGGCAGAG
+
FFFFF:FFFFFFFFFFFFFFFFFFF,FFFFFFFFFFFFFFFFFFF,FFFFFFFFF,FF:FFFFFFFF
FF:FFFFFFFFF:FFFFF:FFFFFF:FF:F:FFF::FFFFFFF:FFFF,FFFFFF:FFFFF,F:FFFF
@A00155:342:HHGFNDSXY:1:1408:29523:14732 2:N:0:GAACCTAG+TCCGCATA
AAAAGGACTCGTCAGGTAGCCTACTAGACTACGACTTACACGGTAGGTCGTGAACAAGCCCGGAGTAC
ATCTCCTTTGGAACCAGGAGACCCCTCCGGTCTTGTCCAACCTCTGTTTCGGCAGAGGATACCGTGCT
+
FFFFFFFFFFFFFFFFFFFFFFFFFFFFFFFFFFFFFFFFFFFFFFFF:FFFFFFFFFFFFFFFFFFF
FFFFFFFFFFFFFFFFFFFFFFFFFFFFFFFFFFFFFFFFFFFFFFFFFFFFFFFFFFFFFFFFFFFF
@A00155:342:HHGFNDSXY:1:1408:17571:18897 2:N:0:GAACCTAG+TCCGCATA
AAAAGGACTCGTCAGGTAGCCTACTAGACTACGACTTACACGGTAGGTCGTGAACAAGCCCGGAGTAC
ATCTCCTTTGGAACTAGGAGACCCCTCCGGTCTTGTCCAACCTCTGTTTCGGCAGAGGATACCGTGCT
+
FFFFFFFFFFFFFFFFFFFFFFFFFFFFFFFFFFFFFFF:FFFFFFFFFFF:FFFFFFFFFFFFFFFF
:FFFFFFF:FFF,FFFFFFFFFFFFFFFFFFFFFFFFFFFFFFFFFFFFFFF:FFFFFFFFFFFFFFF
@A00155:342:HHGFNDSXY:1:1408:22815:25535 2:N:0:GAACCTAG+TCCGCATA
TACATCTCCTTTGGAACTAGGAGACCCCTCCGGTCTTGTCCAACCTCTGTTTCGGCAGAGGATACCGT
GCTCCACAGCTGTAAGGCTGTGGGCCCGAGAGAAGTCGCTCTCTCTCGCCCTCCACATCCCTCTCGGC
+
FFFFFFFFF:FFFFFFFFFFFFFFFFFFFFFFFFFFFFFFFFFFFFFFFFFFFFFFFFFFFFFFFFFF
FFFFFFFFFFFFFFFFFF:FFFFFFFFFFFFFF,FFFFFFFFFFFFFFF:FFFFFFFFFFFFFFFFFF
@A00155:342:HHGFNDSXY:1:1408:18891:29387 2:N:0:GAACCTAG+TCCGCATA
GTGAACAAGCCCGGAGTACATCTCCTTTGGAACTAGGAGACCCCTCCGGTCTTGTCCAACCTCTGTTT
CGGCAGAGGATACCGTGCTCCACAGCTGTAAGGCTGTGGGCCCGAGAGAAGTCGCTCTCTCTCGCCC
+
FFFFFFFF:FFFFFFFFFFFFFFFF:FFFFFFFFFFFFFFFFFFFFFFFFFF,FFFFFFFFFFFFFFF
FFFFFFFFFFFFFFFFFFFFFFFFF:FFFFFFFFFFFFFFFFFFFFFFFFFFFFFFFFFFFFFFFFF
@A00155:342:HHGFNDSXY:1:1408:19759:31923 2:N:0:GAACCTAG+TCCGCATA
ACGACTTACACGGTAGGTCGTGAACAAGCCCGGAGTACATCTCCTTTGGAACTAGGAGACCCCTCCGG
TCTTGTCCAACCTCTGTTTCGGCAGAGGATACCGTGCTCCACAGCTGTAAGGCTGTGGGCCCGAGAGA
+
:FFFFFFFFFFFFF:FFFFFFFFFFFFFFFFFFFFFFFFFFFFFFFFFFFFFFFFFFFFFFFFFFFFF
,FF:FFFFFFFFFFFFFFFFFFF,FFFFFFFFFFFFFFFFFFFFFFFFFFFFFFFFFFFFFFFFFFFF
@A00155:342:HHGFNDSXY:1:1409:20745:5259 2:N:0:GAACCTAG+TCCGCATA
CGTCCTAAAAAGGACTCGTCAGGTAGCCTACTAGACTACGACTTACACGGTAGGTCGTGAACAAGCCC
GGAGTACATCTCCTTTGGAACTAGGAGACCCCTCCGGTCTTGTCCAACCTCTGTTTCGGCAGAGGATA
+
FFFFFFFFFFFFFFFFFFFFFFFFFFFFFFFFFF,FFFFFFFFFFFFFFFFFFFFFFFFFFFFFFFFF
FFFFFFFFFFFFFFFFFFFFFFFFFFFFFFFFFFFFFFFFFFFFFFFFFFFFFFFFFFFFFFFFFFFF
```

```
@A00155:342:HHGFNDSXY:1:1409:13512:14935 2:N:0:GAACCTAG+TCCGCATA
AAAAGGACTCGTCAGGTAGCCTACTAGACTACGACTTACACGGTAGGTCGTGAACAAGCCCGGAGTAC
ATCTCCTTTGGAACTAGGAGACCCCTCCGGTCTTGTCCAACCTCTGTTTCGGCAGAGGATACCGTGCT
+
FF:FFFFFFFFFFFFFFFFFFFFFFFF:FFFFFFFFFFFFFFFFFFFFFFFFFFFFF,FFFFFFFFFF
FFFFFFFFFFFFFFFFFFFFFFFF:FFFFFFFFFFFFFFFFFF:FFFFFFFFFFFFFFFFFFFFFFFFF
@A00155:342:HHGFNDSXY:1:1409:28664:25144 2:N:0:GAACCTAG+TCCGCATA
AAAAGGACTCGTCAGGTAGCCTACTAGACTACGACTTACACGGTAGGTCGTGAACAAGCCCGGAGTAC
ATCTCCTTTGGAACTAGGAGACCCCTCCGGTCTTGTCCAACCTCTGTTTCGGCAGAGGATACCGTGCT
+
FFFFFFFFFFFFFFFFF:FFFFFFF:FFFFFFFFFFFFFFFFFFFFFFFFFFFFFFFF:FFFFFFFFFF
FFFFFFFFFFFFFFFFFFFFFFFFFFFFFFFFFFFFFFFFFFFFFFFFFFFFFFFFFFFFFFFFFFFF
@A00155:342:HHGFNDSXY:1:1409:29116:25426 2:N:0:GAACCTAG+TCCGCATA
AAAAGGACTCGTCAGGTAGCCTACTAGACTACGACTTACACGGTAGGTCGTGAACAAGCCCGGAGTAC
ATCTCCTTTGGAACTAGGAGACCCCTCCGGTCTTGTCCAACCTCTGTTTCGGCAGAGGATACCGTGCT
+
FFFFFFFFFFFFFFFFFFFFFFFFFFFFFFF:FFFFFFFFFFFFFFFFFFFFFFFFF:FFFFFFFFFF
FFFFFFFFFFFFFFFFFFFFFFFFFFFFFFFFFFFFFFFFFFFFFFFFFFFFFFFFFFFFFFFFFFFF
@A00155:342:HHGFNDSXY:1:1409:11614:29716 2:N:0:GAACCTAG+TCCGCATA
CAAGGACGAAACCGTCGTTACTCTTCGAGTTGTGTAAGTTTCGTCCTAAAAAGGACTCGTCAGGTAGC
CTACTAGACTACGACTTACACGGTAGGTCGTGAACAAGCCCGGAGTACATCTCCTTTGGAACTAGGAG
+
FFFFFFFFFFFFFFFFFFFFFFFFFFFFFFFFFFFFFFF:FFFFF:FFFFFF:FFFFFFFFFFFFFFF
FFFFFFFFFFFFFFF:FFFFFFFFFFFFFFFFFFFFFFFFFFFFFFFFFFFFFFFFFFFFFFFFFFFF
@A00155:342:HHGFNDSXY:1:1410:9805:8547 2:N:0:GAACCTAG+TCCGCATA
AAAAGGACTCGTCAGGTAGCCTACTAGACTACGACTTACACGGTAGGTCGTGAACAAGCCCGGAGTAC
ATCTCCTTTGGAACTAGGAGACCCCTCCGGTCTTGTCCAACCTCTGTTTCGGCAGAGGATACCGTGCT
+
FFF:FFFFFFFFFFFFFFFFFFFFFFFFFFFFFFFFFFFFFFFFFFFFFFFFFFFFFFFFFFFFFFFF
FFFFFFFFFFFFFFFFFFFFFFFFFFFFFFFFF,FFFFFFFFFFFFFFFFFFFFFFFFFFFF:FFFFFF
@A00155:342:HHGFNDSXY:1:1410:6696:22044 2:N:0:GAACCTAG+TCCGCATA
CACGGTAGGTCGTGAACAAGCCCGGAGTACATCTCCTTTGGAACTAGGAGACCCCTCCGGTCTTGTCC
AACCTCTGTTTCGGCAGAGGATACCGTGCTCCACAGCTGTAAGGCTGTGGGCCCGAGAGAAGTCGCTC
+
FFFFFFFFFFFFFFFFFFFFFFFFFFFFFFFFFFFFF,FFFFFFFFFFFFFFFFFFFFFFFFFFFFFFF
FFFFFFFFFFFFFFFFFFFFFFFFFFFFFFFFFFFFFFFFFFFFFF:FFFFFFFFFFFFFFFFFFFFFF
@A00155:342:HHGFNDSXY:1:1410:1597:26459 2:N:0:GAACCTAG+TCCGCATA
CGTTCCAAGGACGAAACCGTCGTTACTCTACGAGTTGTGTAAGTTTCGTCCTAAAAAGGACTCGTCAG
GTAGCCTACTAGACTACGACTTACACGGTAGGTCGTGAACAAGCCCGGAGTACATCTCCTTTGGAACT
+
FFFFFF::FFFFFFFFFFFFFFFFFFFFFFF:F:FFFFFFFFFFFFFFFFFF:FFFF,:FFFFFFFFF:F
FFFFFFFFFFFFFFFFFFFFFFFFFFFFFFFFFFFFFF:FFF:FFFFFFFFFFF:FFFF:FFFFFFF:F
@A00155:342:HHGFNDSXY:1:1410:30870:33473 2:N:0:GAACCTAG+TCCGCATA
GGCAGAGGATACCGTGCTCCACAGCTGTAAGGCTGTGGGCCCGAGAGAAGTCGCTCTCTCTCGCCCTC
TACATCCCTCTCGGCATTGAGATGACCATAGGCCAGAGGTGAGTCCTTAAGTGGACACAGCTGATCTA
+
FFFFFFFFFFFFFFFFFFFFFFFFFFFFFFFFFFF,FFFFFFFFFFFFFFFFFFFFFFFFFFFFFFFFF
FFFFFFFFFFFFFFFF,FFFFFFFFFFFFFFFFFFFFFFFFFFFFFFFFFFFFFFFFFFFFFFFFFFFF
@A00155:342:HHGFNDSXY:1:1410:25319:35697 2:N:0:GAACCTAG+TCCGCATA
CTACGACTTACACGGTAGGTCGTAAACAAGCCCGGAGTACATCTCCTTTGGAACTAGGAGACCCCTCC
GGTCTTGTCCAACCTCTGTTTCGGCAGAGGATACCGTGCTCCACAGCTGTAAGGCTGTGGGCCCGAG
+
FFFFF:FFFFFFFFFF,FF:FF,F:FFFFFFFFFFFFFFF:FFFFFFFFFFFFFFFFFFFFFFFFFFFF
FFFF:FFFFFFFFFFFFFFFFFFFFFFFFFFFFFFFFFFFFFFFF:FFFFFFFFFFFFFFFFFFFFFFF
```

```
@A00155:342:HHGFNDSXY:1:1411:10691:4069 2:N:0:GAACCTAG+TCCGCATA
CGGTAGGTCGTGAACAAGCCCGGAGTACATCTCCTTTGGAACTAGGAGACCCCTCCGGTCTTGTCCAA
CCTCTGTTTCGGCAGAGGATACCGTGCTCCACAGCTGTAAGGCTGTGGGCCCGAGAGAAGTCGCTCTC
+
FFFFFFFFFFFFFFFFFFFFFFFFFFF:FFFFFFFFFFF:FFFFFFFFFFFFFFFFFFFFFFFFFFFF
FFFFFFFF,FFFFFFFFFFFFFFFFFFFFFFFFFFFFFFFFFFFFF,FFFFFFFFFFFF:FFFFFFFFF
@A00155:342:HHGFNDSXY:1:1411:26756:11193 2:N:0:GAACCTAG+TCCGCATA
AACGGACTCATCAGGGAGCCTACTAGACTCCGACCGTCATGGGGGTTCAAACCCATGCCCCGCCACAC
CGCCTTAGATCAGCTGTGTCCACTTAAGGACTCACCTCTGGCCTATGGTCATCTCAATACCGAGAGGG
+
FFFFFFFFFFFFFFFFFFFFFFFFFFFFFFFFFFFFFFFF:FFFFFFFFFFFFFFFFFFFFFFFFFFFF
FFFFFFFFFFFFFFFFFFFFFFFFFFFFFFFFFFFFFFFFFFFFFFFFFFFFFFFFFFFFFFFFFFFF
@A00155:342:HHGFNDSXY:1:1411:28619:19022 2:N:0:GAACCTAG+TCCGCATA
GAAACCGTCGTTACTCTTGGAGTTGTGTAAGTTTCGTCCTAAAAAGGACTCGTCAGGTAGCCTACTAG
ACTACGACTTACACGGTAGGTCGTGAACAAGCCCGGAGTACATCTCCTTTGGAACTAGGAGACCCCTC
+
FFF:FFFFFFFFFFFFF:FFFFFFFFFFFFF::FFFFFFF:FFF:FFFFFFFFF,FFFFFFF:FF:FF
FFF:FFFFFFFFFFFF,FFFFFFFFFFFFFFFFFFFFFFFFFFFFFFFFFFFFFFFFFFFFFFFFFFF
@A00155:342:HHGFNDSXY:1:1412:10945:17033 2:N:0:GAACCTAG+TCCGCATA
GTGAACAAGCCCGGAGTACATCTCCTTTGGAACTAGGAGACCCCTCCGGTCTTGTCCAACCTCTGTTT
CGGCAGAGGATACCGTGCTCCACAGCTGTAAGGCTGTGGGCCCGAGAGAAGTCGCTCTCTCTCGCCCT
+
FFFFFFFFFFFFFFFFFFFFFFFFFFFFFFFFFFFFFFFFFFFFFFFFFFFFFFFFFFFFFFFFFFFF
FFFFFFFFFFFFFFFFFFFFFFFFFFFFF:FFFFFFFFFFFFFFFFFFFFFFFFFFFFFFFFFFFFFF
@A00155:342:HHGFNDSXY:1:1412:21649:17315 2:N:0:GAACCTAG+TCCGCATA
CCGTCGTTACTCTTTGAGTTGTGTAAGTTTCGTCCTAAAAAGGACTCGTCAGGTAGCCTACTAGACTA
CGACTTACACGGTAGGTCGTGAACAAGCCCGGAGTACATCTCCTTTGGAACTAGGAGACCCCTCCGGT
+
FFFFFFFFFFFFF:FFFFF:FFFFFF:FFFFFFFFFFFFFFFFFFFFFFFFFFFFFFFFFFFFFFFFF
FF:FFFFFFFFFFFFFFFFFFFFFFFFFFFFFFFFFFFFFFFFFFFFFFFFFFFFFFFFFFFFFFFFF
@A00155:342:HHGFNDSXY:1:1413:7943:1532 2:N:0:GAACCTAG+TCCGCATA
TTGTCCAACCTCTGTTTCGGCAGAGGATACCGTGCTCCACAGCTGTAAGGCTGTGGGCCCGAGAGAAG
TCGCTCTCTCTCGCCCTTCACATCCCTCTCGGCATTGAGATGACCATAGGCCAGAGGTGAGTCCTTAA
+
FFFFFFFFFFFFFFFFFFFFFFFFFFFFFFFFFFFFFFFFFFFFFFFFFFFFFFFFFFFFFFFFFFFF
FFFFFFFFFFFFFFFFFFFFFFFFFFFFFFFFFFFFFFFFFFF:FFFFFFFFFFFFFFFFFFFFFFFF
@A00155:342:HHGFNDSXY:1:1413:31512:2206 2:N:0:GAACCTAG+TCCGCATA
GGAGTACATCTCCTTTGGAACTAGGAGACCCCTCCGGTCTTGTCCAACCTCTGTTTCGGCAGAGGATA
CCGTGCTCCACAGCTGTAAGGCTGTGGGCCCGAGAGAAGTCGCTCTCTCTCGCCCTCCACATCCCTCT
+
FFFFFFF:FFFFFFFFFFFFFFFFFFFFFFFFFFFFFFFFFF,,FFFFFFFFFFFFFFFFFFFFFFFF,F
FFFFFFFFFFFFFFFFFFFFFFF:FFFFFFFFFFFFFFFFFFFFFFFFFFFFFFFFFFFFFF:FFFFFF
@A00155:342:HHGFNDSXY:1:1413:3739:21496 2:N:0:GAACCTAG+TCCGCATA
GCCCGGAGTACATCTCCTTTGGAACTAGGAGACCCCTCCGGTCTTGTCCAACCTCTGTTTCGGCAGAG
GATACCGTGCTCCACAGCTGTAAGGCTGTGGGCCCGAGAGAAGTCGCTCTCTCTCGCCCTCCACATCC
+
FFFFFFF:FFF:FFFFFFFFFFFFFFFFFFFFFFFFFFFFFFFFFFFFFFFFFFFFFFFFFFFFFFFF
FFFFFFFFFFFFFFFFFFFFFFFFFFFFFFFFFFFFFFFFFFFFFF:F:FFFFFFFFFFFFFFFFFFF
@A00155:342:HHGFNDSXY:1:1413:30183:34444 2:N:0:GAACCTAG+TCCGCATA
TGAACAAGCCCGGAGTACATCTCCTTTGGAACTAGGAGACCCCTCCGGTCTTGTCCAACCTCTGTTTC
GGCAGAGGATACCGTGCTCCACAGCTGTAAGGCTGTGGGCCCGAGAGAAGTCGCTCTCTCTCGCC
+
FFFFFFFFFFFFFFFFFFFFFFFFF:FFFFFFFFFFFFFFF:FFFFFFFFFF:FFFFFFFFFFFFFFFF
FFFFFFFFFFFFFFFFFFFFFFFFFFFFFFFFFFFFFFFFFFFFFFFFFFFFFFFFFFFFFFFFF
```

```
@A00155:342:HHGFNDSXY:1:1414:11017:2002 2:N:0:GAACCTAG+TCCGCATA
CGGTTTCGTCCTTGGAACGGACTCATCAGGGAGCCTACTAGACTCCGACCGTCATGGGGGTTCAAACC
CATGCCCCGCTACACCGCCTTAGATCAGCTGTGTCCACTTAAGGACTCACCTCTGGCCTATGGTCATC
+
FFF,FFFFFFFFFFFFFFFFFFFFFFFFFFFFFFFFFFFFFFFFFF:FFFFFFFFFFFFFFFFF
FFFFFFFFFFFFFFFFFFFFFFFFFFFFFFFFFFFFFFF,FFFFFFFFFFFFFFFFFF:FFFFFFFF
@A00155:342:HHGFNDSXY:1:1414:30002:4100 2:N:0:GAACCTAG+TCCGCATA
CCGGAGTACATCTCCTTTGGAACAAGGAGACCCCTCCGGTCTTGTCCAACCTCTGTTTCGGCAGAGGA
TACCGTGCTCCACAGCTGTAAGGCTGTGGGCCCGAGAGAAGTCGCTCTCTCTCGCCCTCCACATCCCT
+
FFFFFFFFFFFFFFFFFFFFFFFFFFFFFFFFFFFFFFFFFFFFFFFF:FFFFFFFFFFFFFFFFFFF
FFFFFFFFFFFFFFFFFFFF:FFFFFFFFFFFFF:FFFFFFFFFFFFFFFFFFFFFFFFFFFFFFFFF
@A00155:342:HHGFNDSXY:1:1414:7853:27242 2:N:0:GAACCTAG+TCCGCATA
TCGAAGAGTAACGACGGTTTCGTCCTTGGAACGGACTCATCAGGGAGCCTACTAGACTCCGACCGTCA
TGGGGGTTCAAACCCATGCCCCGCCACACCGCCTTAGATCAGCTGTGTCCACTTAAGGACTCACCTCT
+
FFFFFFFFFFFFFFFFFFFFFFFFFFFFFFFFFFFFFFFFFFFFFFFFFFFFFFFFFFFFFFFFFFFF
FFFFFFFFFFFFF:FFFFFF,FFFFFFFFFFFFFFFFFFFFFFFFFFFFFFFFF,FFFFFFFFFFFFF
@A00155:342:HHGFNDSXY:1:1415:6867:1047 2:N:0:GAACCTAG+TCCGCATA
CGGCAAAGGATACCGTGCTCCACAGCTGTAAGGCTGTGGGCCCGAGAGAAGTCGCTCTCTCTCGCCCT
CCACATCCCTCTCGGCATTGAGATGACCATAGGCCAGAGGTGAGTCCTTAAGTGGACACAG
+
FFFFFFFFFFFFFFFFFFFFFFFFFFFFFFFFFFFFFFFFFFFFFFFFFFFFFFFFFFFFFFFF:FFF
FFFFFFFFFFFFFFFFFFFFFFFFFFFF,FFFFFF:FFFFFFFFFFFF:FFFFFF:FFF:F
@A00155:342:HHGFNDSXY:1:1415:10791:17174 2:N:0:GAACCTAG+TCCGCATA
GAACTAGGAGACCCCTCCGGTCTTGTCCAACCTCTGTTTCGGCAGAGGATACCGTGCTCCACAGCTGT
AAGGCTGTGGGCCCGAGAGAAGTCGCTCTCTCTCGCCCTCCACATCCCTCTCGGCATTGAGATG
+
FF:FFFFFFFFFFFFFFFFFFFFFFFFFFFFFFFFFFFFFFFFFFFFFFFFF:FFFFFFFFFFFFFFF
FFFFFFFFFFFFFFFFFFFFFFFFFFFFFFFFFFFFFFFFFFF:FFFFFFFFFFF::FFFFFFF
@A00155:342:HHGFNDSXY:1:1415:20311:19351 2:N:0:GAACCTAG+TCCGCATA
CCAACCTCTGTTTCGGCAGAGGATACCGTGCTCCACAGCTGTAAGGCTGTGGGCCCGAGAGAAGTCGC
TCTCTCTCGCCCTCCACATCCCTCTCGGCATTGAGATGACCATAGGCCAGAGGAGAGTCCTTAAGTGG
+
FFFFFFFFFFFFFFFFFFFFFFFFFFFFFFFFFFFFFFFFFFFFFFFFFFFFFFFFFFFFFFFFFFFF
FFFFFFFFFFFFFFFFFFFFFFFFFFFFFFF,FFFFFFFFF:FFFFFF:FFFFFFFFFFFF,FFFFFF
@A00155:342:HHGFNDSXY:1:1416:1877:1297 2:N:0:GAACCTAG+TCCGCATA
CGGTAGGTCGTGAACAAGCCCGGAGTACATCTCCTTTGGAACTAGGAGACCCCTCCGGTCTTGTCCAA
CCTCTGTTTCGGCAGAGGATACCGTGCTCCACAGCTGTAAGGCTGTGGGCCCGAGAGAAGTCGCTCTC
+
FFFFFFFFFFFFFFFFFFFFFFFFFFFFFFFFFFFFFFFFFFFFFFFFFFFFFFFFFFFFFFFFFFFF
FFFFFFFFFFFFFFFFFFFFFFFFFFFFFFFFFFFFFF:FFFF,FFFFFFFFFFF:FFFFFF:FFFFF
@A00155:342:HHGFNDSXY:1:1416:15176:6699 2:N:0:GAACCTAG+TCCGCATA
ATCTCAATGCCGAGAGGGATGTAGAGGGCGAGAGAGAGCGACTTCTCTCGGGCCCACAGCCTTACAGC
TGTGGAGCACGGTATCCTCTGCCGAAACAGAGGTTGGACAAGACCGGAGGGGTCTCCTAGTTCCAAAG
+
FFFFFFFFFFFFFFFFFFFFFF,FF:FFFFFFFFFFFFFFFFFFFFFFFFFFFFFFFFFFFFFFFFFF
FFFFFFFFFFFFFFFFFFFFFFFFFFFFFFFFFFFFFFFFFFFFFFFFFFFFFFFFFFFFFFFFFFFF
@A00155:342:HHGFNDSXY:1:1416:19144:26256 2:N:0:GAACCTAG+TCCGCATA
AGGACGAAACTTACACAACTCGTAGAGTAACGACGGTTTCGTCCTTGGAACGGACTCATCAGGGAGCC
TACTAGACTCCGACCGTCATGGGGGTTCAAACCCATGCCCCGCCACACCGCCTTAGATCAGCTGTGTC
+
FFFFFFF:FFFFFFFFFFFFFFFFFF:FFFFFFFFFFF:FFFFFFFFFFFFFFFFFFFFFFFFFFFFF
F:FFFFFFFFFFFFFFFFFFFFFFFFF:FFFFFFFFFFFFFFFFFFFFFFFFFFFFFFFFFFFFFFFFFF
```

```
@A00155:342:HHGFNDSXY:1:1417:24243:8406 2:N:0:GAACCTAG+TCCGCATA
GACTTACACGGTAGGTCGTGAACAAGCCCGGAGTACATCTCCTTTGGAACTAGGAGACCCCTCCGGTC
TTGTCCAACCTCTGTTTCGGCAGAGGATACCGTGCTCCACAGCTGTAAGGCTGTGGGCCCGAGAGAAG
+
FFFFFFFFFFFFFFFFFFFFFFFFFFFFFFFFFFFFFFFFFFFFFFFFFFFFFFFFFFFFFFFFFFFFFF
FFFFFFFFFFFFFFFFFFFFFFFFFFFFFFFFFFFFFFFFFFFFFFFFFFFFFFFFFFFFFFFFFFFFFF
@A00155:342:HHGFNDSXY:1:1417:20347:19132 2:N:0:GAACCTAG+TCCGCATA
TAGGTCGTGAACAAGCCCGGAGTACATCTCCTTTGGAACCAGGAGACCCCTCCGGTCTTGTCCAACCT
CTGTTTCGGCAGAGGATACCGTGCTCCACAGCTGTAAGGCTGTGGGCCCGAGAGAAGTCGCTCTCTCT
+
FFFFFFFFFFFFFFFFFFFFFFFFFFFFFFFFFFFFFFF:FFFFFFFFFFFFFFFF:FFFFFFFFFFFFF
FFFFFFFFFFFFFFFFF:FFFFFFFFFFFFFFFFFFFFFFFFFFFFFFFFFFFFFFFFFFFFFFFFFFF:
@A00155:342:HHGFNDSXY:1:1417:20184:23077 2:N:0:GAACCTAG+TCCGCATA
TAGACTACGACTTACACGGTAGGTCGTGAACAAGCCCGGAGTACATCTCCTTTGGAACTAGGAGACCC
CTCCGGTCTTGTCCAACCTCTGTTTCGGCAGAGGATACCGTGCTCCACAGCTGTAAGGCTGTGGGCCC
+
FFFFFFFFFFFFFFFFFFFFFFFFFFFFFFFFFFFFFFFFFFFFFF:FFFFFFFFFFFFFFFFFFFFFFF
FFFFFFFFFFFFFFFFFFFFFFFFFFFFFFFFFFFFFFFFFFFFFFFFFFFFFFFFFFFFFFFFFFFFFF
@A00155:342:HHGFNDSXY:1:1418:10402:2096 2:N:0:GAACCTAG+TCCGCATA
ACCCCTCCGGTCTTGTCCAACCTCTGTTTCGGCAGAGGATACCGTGCTCCACAGCTGTAAGGCTGTGG
GCCCGAGAGAAGTCGCTCTCTCTCGCCCTCCACATCCCTCTCGGCATTGAGATGACCATAGGCCAGAG
+
FFFFFFFFFFFFFFFFFFFFFFFF:FFFFFFFFFFFFFFFFFFFFFFFFFFFFFFFFFFFFFFFFFFFF
FFFFFFFFFFFFFFFFFFFFFFFFFFFFFFFFFFFFFFFFFFFFFFFFF:FFFFFFFF:FFFFFFFFFF
@A00155:342:HHGFNDSXY:1:1418:5810:8641 2:N:0:GAACCTAG+TCCGCATA
CCGCCACACCGCCTTAGATCAGCTGTGTCCACTTAAGGACTCACCTCTGGCCTATGGTCATCTCAATG
CCGAGAGGGATGTGGAGGGCGAGAGAGAGCGACTTCTCTCGGGCCCACAGCCTTACAGCTGTGGAGCA
+
FFFFFFFFFFFFFFFFFFFFFFFFFFFFFFFFFFFFFFFFFFFF:FFFFFFFFFFFFFFFFFFFFFFFFF
FFFFF:FFFF,FFFFFF:FFFFFFFFFFFFFFFFFFFFFFFFFFFFFFFFFFFFFF:FFFFFFFF:FFFF
@A00155:342:HHGFNDSXY:1:1418:8558:15029 2:N:0:GAACCTAG+TCCGCATA
GAACTAGGAGACCCCTCCGGTCTTGTCCAACCTCTGTTTCGGCAGAGGATACCGTGCTCCACAGCTGT
AAGGCTGTGGGCCCGAGAGAAGTCGCTCTCTCTCGCCCTCCACATCCCTCTCGGCATTGAGATGACCA
+
FFFFFFFFFFFFFFFFFFFFFFFFFFFFFFFFFFFFFFFFFFFFFFFFFFF:FFFFFFFFFFFFFFFFFF
FFFFFFFFFFFFFFFFFFFFFFFFFFFFFFFFFFFFFFFFFFFFFFFFFFFFFFFFFF:FFFFFFFFFFF
@A00155:342:HHGFNDSXY:1:1418:5927:19179 2:N:0:GAACCTAG+TCCGCATA
GACCCCTCCGGTCTTGTCCAACCTCTGTTTCGGCAGAGGATACCGTGCTCCACAGCTGTAAGGCTGTG
GGCCCGAGAGAAGTCGCTCTCTCTCGCCCTCCACATCCCTCTCGGCATTGAGATGACCATAGGC
+
F:FFFFFFFFFFFFFFFFFFFFFFFFFFFFFFFFFFFF:FFFFFFFFFFFFFFFFFFFFFF:FFFFFF:F
FFFFFFFFFFFFFFFFFFFFFFFFFFFFFFFFFFFFFFFFFFFFFFFFF:FFFFFFFFFFF:FFFF
@A00155:342:HHGFNDSXY:1:1418:24704:23641 2:N:0:GAACCTAG+TCCGCATA
AAAAGGACTCGTCAGGTAGCCTACTAGACTACGACTTACACGGTAGGTCGTGAACAAGCCCGGAGTAC
ATCTCCTTTGGAACTAGGAGACCCCTCCGGTCTTGTCCAACCTCTGTTTCGGCAGAGGATACCGTGCT
+
FFFFFFFFFFFFFFFFFFFFFFFFFFFFFFFFFFFFFFFFFFFFFFFFFFFFFFFFFFFFFFFFFFFFFF
FFFFFFFFFFFFFFFFFFFFFF:FFFFFFFFFFFFFFFFFFFFFFFFFFFFFFFFFFFFFFFFFFFFFFF
@A00155:342:HHGFNDSXY:1:1418:16179:27790 2:N:0:GAACCTAG+TCCGCATA
GGACTCGTCAGGTAGCCTACTAGACTACGACTTACACGGTAGGTCGTGAACAAGCCCGGAGTACATCT
CCTTTGGAACTAGGAGACCCCTCCGGTCTTGTCCAACCTCTGTTTCGGCAGAGGATACCGTGCTCCAC
+
FFFFFFFFFFFFFFFFFFFFFFFFFFFFFFFFFFFFFFFFFF:FFFFFFFFFFFFFFFFFFFFFFFFFFF
F:,FFFFFFFFFF:FFFFFFFFFFFFFFFFFFFFFFFFFFFFFFFFFFFFFFFFFFFFFFFFFFFFFFFF
```

```
@A00155:342:HHGFNDSXY:1:1418:20952:28291 2:N:0:GAACCTAG+TCCGCATA
AAAAGGACTCGTCAGGTAGCCTACTAGACTACGACTTACACGGTAGGTCGTGAACAAGCCCGGAGTAC
ATCTCCTTTGGAACTAGGAGACCCCTCCGGTCTTGTCCAACCTCTGTTTCGGCAGAGGATACCGTGCT
+
FFFFFFFFFFFFFFFFFFFFFFFFFFFFFFFFFFFFFFFFFFFFFFFFFFFFFFFFFFFFFFFFFFFF
FFFFFFFFFFFFFF:FFFFFFFFFFFFFFFFFFFFFFFFFFFFFFFFFFFFFFFFFFFFFF:FFFFFF
@A00155:342:HHGFNDSXY:1:1419:14199:2628 2:N:0:GAACCTAG+TCCGCATA
ACTACGACTTACACGGTAGGTCGTGAACAAGCCCGGAGTACATCTCCTTTGGAACTAGGAGACCCCTC
CGGTCTTGTCCAACCTCTGTTTCGGCAGAGGATACCGTGCTCCACAGCTGTAAGGCTGTGGGCCCGAG
+
FFFFFFFFFFFFFFFFF,FFFFFFFFFFFFFFFFFFFFFFFFFFFFFFFFFFFFFFFFFFFFFFFFFF
FFFFFF:FFFFFFFFFFFFFFFFFFFFFFFFFFFFFFFFFFFFFFFFFFFFFFFFFFFFFFFFFFFFF
@A00155:342:HHGFNDSXY:1:1419:7310:14622 2:N:0:GAACCTAG+TCCGCATA
GAAACCGTCGTTACTCTTGGAGTTGTGTAAGTTTCGTCCTAAAAAGGACTCGTCAGGTAGCCTACTAG
ACTACGACTTACACGGTAGGTCGTGAACAAGCCCGGAGTACATCTCCTTTGGAACTAGGAGACCCCTC
+
FFFFFFFFFFFFFFFF,:FFFFFFFFFFFFFF,FFFFFFF:FFFFFFFFFFFFFFFFFFFFFFFFFFF
:FFFFFFFFFFFFFFFFFFFFFFFFFFFFFFFFFFFFFFFFFFFFFFF:FFFFFFFFFFFFFFFFFFF
@A00155:342:HHGFNDSXY:1:1419:24189:20494 2:N:0:GAACCTAG+TCCGCATA
CCGCCACACCGCCTTAGATCAGCTGTGTCCACTTAAGGACTCACCTCTGGCCTATGGTCATCTCAATG
CCGAGAGGGATGTGGAGGGCGAGAGAGAGCGACTTCTCTCGGGCCCACAGCCTTACAGCTGTGGAGCA
+
FFFFFFFFFFFFFFFFFFFFFFFFFFFFFFFFFF:FFFFFFFFFFFFFFFFFFFFFFFFFFFFFFFFF
FFFFFFFFF,FFFFFFFFFFFFFFFFFFFFFFFFFFFFFFFFFFFFFFFFFFFF:FFFFFFFFFFFFF
@A00155:342:HHGFNDSXY:1:1420:31946:21621 2:N:0:GAACCTAG+TCCGCATA
AGTACATCTCCTTTGGAACTAGGAGACCCCTCCGGTCTTGTCCAACCTCTGTTTCGGCAGAGGATACC
GTGCTCCACAGCTGTAAGGCTGTGGGCCCGAGAGAAGTCGCTCTCTCTCGCCCTCTACATCCCTCTCG
+
FFFFFFFFFFF,FFFFF,FFFFFFFFFFFFFFFFFFF,:F:FFF:FFFF,F,FFFFFF:FFFFFFFFF
FFFFFFFFFFFFFFFFFFFF:FFFFFFFFFFFFFFFFFFFFFFFFFF,FFFFFFF:FFF:FFFFF:FF
@A00155:342:HHGFNDSXY:1:1420:31684:25207 2:N:0:GAACCTAG+TCCGCATA
ACCCCTCCGGTCTTGTCCAACCTCTGTTTCGGCAGAGGATACCGTGCTCCACAGCTGTAAGGCTGTGG
GCCCGAGAGAAGTCGCTCTCTCTCGCCCTTCACATCCCTCTCGGCATTGAGATGACCATAGGCCAGAG
+
:FFFFFFFFF:FFFF:FFFFFFFFFFFFFFFFFFFFFFFFFFFFF:FFFFFFFFFFFFFFFFFFFF:F
FFFFF:FFFFFFFFFFFFFFFF:FFFFFFFF:FFFFFFFFFFFFFF,FFFFFFFFFF,::FFFFFFFF
@A00155:342:HHGFNDSXY:1:1420:32190:28307 2:N:0:GAACCTAG+TCCGCATA
TACATCTCCTTTGGAACTAGGAGACCCCTCCGGTCTTGTCCAACCTCTGTTTCGGCAGAGGATACCGT
GCTCCACAGCTGTAAGGCTGTGGGCCCGAGAGAAGTCGCTCTCTCTCGCCCTCCACATCCCTCTCGGC
+
FFFFFFFFFFFFFFFFFFFFFFFFFFFFFFFFFFFFFFFFFFFFFFFFFFFFFFFFFFFFFFFFFFFF
FFFFFFFFFFFFFFFFFF:FFFFFFFFFFFFFF:FFFFFFFFFFFFFFFFFFFFFFFFFFFFFFFFFF
@A00155:342:HHGFNDSXY:1:1421:4164:7044 2:N:0:GAACCTAG+TCCGCATA
AACAAGCCCGGAGTACATCTCCTTTGGAACTAGGAGACCCCTCCGGTCTTGTCCAACCTCTGTTTCGG
CAGAGGATACCGTGCTCCACAGCTGTAAGGCTGTGGGCCCGAGAGAAGTCGCTCTCTCTCGCCCTCCA
+
FFFFFFFFFFFFFFFFFFFFFFFFFFFFFF:FFFFFFFFFFFFFFFFFFFFFFFFFFFFFFFFFFFFF
FFFFFFFFFFFFFFFFFFFFFFFFFFFFFFFFFFFFFFFFFFFFFFFFFFFFFFFFFFFFFFFFFFFF
@A00155:342:HHGFNDSXY:1:1422:15826:34788 2:N:0:GAACCTAG+TCCGCATA
AACAAGCCCGGAGTACATCTCCTTTGGAACTAGGAGACCCCTCCGGTCTTGTCCAACCTCTGTTTCGG
CAGAGGATACCGTGCTCCACAGCTGTAAGGCTGTGGGCCCGAGAGAAGTCGCTCTCTCTCGCCCTCCA
+
FFFFFFFFFFFFFFFFFFFFFFFFFFFFFFFFFFFFFFFFFFFFFFFFFFFFFFFFFFFFFFFFFFFF
FFFFFFFFFFFFFFFFFFFFFFFFFFFFFFFFFFFFFFFFFFFFFFFFFFFFFFFFFFFFFFFFFFFF
```

@A00155:342:HHGFNDSXY:1:1422:23800:35603 2:N:0:GAACCTAG+TCCGCATA
AAGGACTCATCAGGTAGCCTACTAGACTACGACTTACACGGTAGGTCGTGAACAAGCCCGGAGTACAT
CTCCTTTGGAACTAGGAGACCCCTCCGGTCTTGTCCAACCTCTGTTTCGGCAGAGGATACCGTG
+
FFFFFFFFFFFFFFFFFFFFFFFFFFFFFFFFFFFFFFFFFFFFFFFFFFFFFFF:FFFFFFFFFFFF
FFFFFFFFFFFFFFFFFFFFFFFFFFFFFFF:FFFFFFFFFFFFFFFF:FFFFFFF,FFFFFFF
@A00155:342:HHGFNDSXY:1:1422:11749:35837 2:N:0:GAACCTAG+TCCGCATA
ACTAGGAGACCCCTCCGGTCTTGTCCAACCTCTGTTTCGGCAGAGGATACCGTGCTCCACAGCTGTAA
GGCTGTGGGCCCGAGAGAATTCGCTCTCTCTCGCCCTCCACATCCCTCTCGGCATTGAGATGACCATA
+
F,:FF,FFFFFF:FFF:FF,F,,FFFFFFFFFFFF::,FFF:FFFFF,FF,FFFFF:,FFFF:::,FF
:FFF::,,:,F:FFFFFFF,:F,,FFFFF:FF:FFFFFFF:F,FF:F::FFF::,:,,,F,F::F,::
@A00155:342:HHGFNDSXY:1:1423:3974:17581 2:N:0:GAACCTAG+TCCGCATA
GAGACCCCTCCGGTCTTGTCCAACCTCTGTTTCGGCAGAGGATACCGTGCTCCACAGCTGTAAGGCTG
TGGGCCCGAGAGAAGTCGCTCTCTCTCGCCCTCCACATCCCTCTCGGCATTGAGATGACCATAGGCCA
+
FFFFFFFFFFFFFFFFFFFFFFFFFFFFFFFFFFFFFFFFFFFFFFFFFFFFFFFFFFFFFFFFFFFF
FFFFFFFFFFFFFFFFFFFFFFFFFFFFFFFFFFFFFFFFFFFFFFFF:,FFFFFFFFFFFFFFFFFF
@A00155:342:HHGFNDSXY:1:1425:5954:5384 2:N:0:GAACCTAG+TCCGCATA
GAAACCGTCGTTACTCTTCGAGTTGTGTAAGTTTCGTCCTAAAAAGGACTCGTCAGGTAGCCTACTAG
ACTACGACTTACACGGTAGGTCGTGAACAAGCCCGGAGTACATCTCCTTTGGAACTAGGAGACCCCTC
+
FFFFF:FFFFFFFF:F:FFFFFFFFFFFF:F:FFFFFFFFFFFF:FFFFFFFFFFFFFFFFFFFFFFF
FFFFFFFFFFFFFFFFFFFFFFFFFFFFFF:FFFFFFFFFFFFFFFFFFFFFFFFF,FFFFFFFFFFF
@A00155:342:HHGFNDSXY:1:1425:5348:12477 2:N:0:GAACCTAG+TCCGCATA
GACCCCTCCGGTCTTGTCCAACCTCTGTTTCGGCAGAGGATACCGTGCTCCACAGCTGTAAGGCTGTG
GGCCCGAGAGAAGTCGCTCTCTCTCGCCCTCCACATCCCTCTCGGCATTGAGATGACCATAGG
+
FFFFFFFFFFFFFFFFFFFFFFFFFFFFFFFFFFFFFFFFFFFFFFFFFFFFFFFFFFFFFFFFFFFF
FFFFFFFFFFF:FFFFFFFFFFFFFFFFFFFFFFFFFFFFFFFFFFF:FFFFFFFFFFFFFFF
@A00155:342:HHGFNDSXY:1:1425:8115:28698 2:N:0:GAACCTAG+TCCGCATA
GAGACCCCTCCGGTCTTGTCCAACCTCTGTTTCGGCAGAGGATACCGTGCTCCACAGCTGTAAGGCTG
TGGGCCCGAGAGAAGTCGCTCTCTCTCGCCCTCCACATCCCTCTCGGCATTGAGATGAC
+
FFFFFFF:FFFFFFFFFFFFFFFFFFFFFFFFFFFFFFFFFFFFFFFFFFFFFFFFFFFFFFFFFFFF
FFFFFFFFFFFFFFFFFFFFFFFFFFFFFFFFFFFFFFFFFFFFF:FF,:FFFFFFF:F
@A00155:342:HHGFNDSXY:1:1425:24930:33552 2:N:0:GAACCTAG+TCCGCATA
ACTTACACGGTAGGTCGTGAACAAGCCCGGAGTACATCTCCTTTGGAACTAGGAGACCCCTCCGGTCT
TGTCCAACCTCTGTTTCGGCAGAGGATACCGTGCTCCACAGCTGTAAGGCTGTGGGCCCGAGAGAAG
+
FFFFFFFFFF:FFFFFFFFFFFFFFFFFFFFFFFFFFFFFFFFFFFFFF,FFFFFFFFFFFFFFFFFF
FFFFFFFFFFFFFFF:FFFFFFFFFFFFFFFFFFFFFFFFFFFF:FFFFF:FFFFFFFFFFFFFFFF
@A00155:342:HHGFNDSXY:1:1426:16920:21057 2:N:0:GAACCTAG+TCCGCATA
TGTCCACTTAAGGACTCTCCTCTGGCCTATGGTCATCTCAATGCCGAGAGGGATGTGGAGGGCGAGAG
AGAGCGACTTCTCTCGGGCCCACAGCCTTACAGCTGTGGAGCACGGTATCCTCTGCCGAAACAGAGGT
+
FFFFFFFFFFFFFFFFFFFFFFFFFFFF:FFFFFFFFFFFFFFFFFFFFFFFFFFFFFFFFFFFFFFF
FFFFFFFFFFFFFFFFFFF:FFFFFFF,FFFFFFFFFFFFFFFFFFF,FFFFFFFFFFFFFFFFFFFF
@A00155:342:HHGFNDSXY:1:1426:7771:29951 2:N:0:GAACCTAG+TCCGCATA
GGACTCGTCAGGTAGCCTACTAGACTACGACTTACACGGTAGGTCGTGAACAAGCCCGGAGTACATCT
CCTTTGGAACTAGGAGACCCCTCCGGTCTTGTCCAACCTCTGTTTCGGCAGAGGATACCGTGCTCCAC
+
FFFFFFFFFFFFFF:FFFFFFFFFFFFFFFFFFFFFFFFFF:FFFFFF:FFFFFFFFFFFFFFFFFFF
FFFFFFFFFFFFFFFFFFFFFFFFFFFFFFF:FFFFFFFFFFFFFFFFFFFFFFFFFFFFFFFFFFFF

```
@A00155:342:HHGFNDSXY:1:1426:8133:30483 2:N:0:GAACCTAG+TCCGCATA
GTAAGGCTGTGGGCCCGAGAGAAGTCGCTCTCTCTCGCCCTCCACATCCCTCTCGGCATTGAGATGAC
CATAGGCCAGAGGTGAGTCCTTAAGTGGACACAGCTGATCTAAGGCGGTGTGGCGGGGCATGGGTTTG
+
FFFFFFFFFFFFFFFFFFFFFFFFFFFFFFFFFFFFFFFFFFFFFFFFFFFFFFFFFFFFFFFFFFFF
FFFFFFFFFFFFFFFFFFFFFFFFFFFFFF,FFFF:FFFFFFFFFFFFFFFFFFFFFFFFFFFFFFFF
@A00155:342:HHGFNDSXY:1:1426:28438:31203 2:N:0:GAACCTAG+TCCGCATA
GGCAGAGGATACCGTGCTCCACAGCTGTAAGGCTGTGGGCCCGAGAGAAGTCGCTCTCTCTCGCCCTC
CACATCCCTCTCGGCATTGAGATGACCATAGGCCAGAGGTGAGTCCTTAAGTGGACACAGCTG
+
FFFFFFFFFFFFFFFFFFFFFFFFFFFFFFFFFFFFFFFFFFFFFFFF:FFFFFFFFFFFFFFFFFFF
FFFFFFFFFFFFFFFF:FFFFFFFFFF:FFFFFFFFFFFFFFFFFFFFFFFFFFFFFFFFFFF
@A00155:342:HHGFNDSXY:1:1426:8404:36370 2:N:0:GAACCTAG+TCCGCATA
GAGACCCCTCCGGTCTTGTCCAACCTCTGTTTCGGCAGAGGATACCGTGCTCCACAGCTGTAAGGCTG
TGGGCCCGAGAGAAGTCGCTCTCTCTCGCCCTCCACATCCCTCTCGGCATTGAGATGACCATAGGCC
+
FFFFFFFFFFFFFFFFFFFFFFFFFFFFFFFFFFFFFF:FFFFFFFFFFFFFFFFFFFFFFFFFFFFF
FFFFFFFFFFFFFFFFFFFFFFFFFFFFFFFFFFFFFFFFFFFFFFFFFFFFFFFFFFFF:FFFFFF
@A00155:342:HHGFNDSXY:1:1427:5385:14826 2:N:0:GAACCTAG+TCCGCATA
CCCCTCCGGTCTTGTCCAACCTCTGTTTCGGCAGAGGATACCGTGCTCCACAGCTGTAAGGCTGTGGG
CCCGAGAGAAGTCGCTCTCTCTCGCCCTTCACATCCCTCTCGGCATTGAGATGACCATAGGCCAGAG
+
FFFFFFFFFFFFFFFFFFFFFFFFFFFFFFFFFFFFFFFFFFFFFFFFFFFFFFFFFFFFFFFFFFFF
FFFFFFFFF:FFFFFFFFFFFFFFFFFFFF:FFFFFFFFFFFFFF,FFFFFFFFFFF:FFFFF:FFF
@A00155:342:HHGFNDSXY:1:1428:11324:8672 2:N:0:GAACCTAG+TCCGCATA
AGTACATCTCCTTTGGAACCAGGAGACCCCTCCGGTCTTGTCCAACCTCTGTTTCGGCAGAGGATACC
GTGCTCCACAGCTGTAAGGCTGTGGGCCCGAGAGAAGTCGCTCTCTCTCGCCCTCCACATCCCTCTCG
+
FFFFFFFF:FFFFFFFFFFFFFFFFFFFFFFFFFFFFFFFFFFFFFFFFFFFFFFFFFFFFFFFFFF
FFFFFFFFFFFFFFFFFFFFFFFFFFFFFFFFFFFFFFFFFFFFFFFFFFFFFFFFFFFFFFFFFFFF
@A00155:342:HHGFNDSXY:1:1428:11379:8985 2:N:0:GAACCTAG+TCCGCATA
AGTACATCTCCTTTGGAACCAGGAGACCCCTCCGGTCTTGTCCAACCTCTGTTTCGGCAGAGGATACC
GTGCTCCACAGCTGTAAGGCTGTGGGCCCGAGAGAAGTCGCTCTCTCTCGCCCTCCACATCCCTCTCG
+
FFFFFFFFFFFFFFFFFFFFFFFFFFFFFFFFFFFFFFFFFFFFFFFFFFFFFFFFFFFFFFFFFFFF
FFFFFFFFFFFF:FFFFFFFFFFFFFFFFFFFFFFFFFFFFFFFFFFFFFFFFFFFFFFFFFFFFFF
@A00155:342:HHGFNDSXY:1:1428:30156:13823 2:N:0:GAACCTAG+TCCGCATA
AAAAGGACTCGTCAGGTAGCCTACTAGACTACGACTTACACGGTAGGTCGTGAACAAGCCCGGAGTAC
ATCTCCTTTGGAACCAGGAGACCCCTCCGGTCTTGTCCAACCTCTGTTTCGGCAGAGGATACCGTGCT
+
FFFFFFFFFFFFFFFFFFFFFFFFFFFFFFFFFFFFFFFFFFFFFFFFFFFFFFFFF:FFFFFFFFFF
:FFFFFFFFFFFFFFFFFFFFFFFFFFFFFFFFFFFFFFFFFFFFFFFFFFFFF:FFFFFFFFFFFF
@A00155:342:HHGFNDSXY:1:1428:7572:29763 2:N:0:GAACCTAG+TCCGCATA
AGAGAGAGCGACTTCTCTCGGGCCCACAGCCTTACAGCTGTGGAGCACGGTATCCTCTGCCGAAACAG
AGGTTGGACAAGACCGGAGGGGTCTCCTGGTTCCAAAGGAGATGTACTCCGGGCTTGTTCACGACCTA
+
FFFFFFFFFFFFFFFFFFF,FFFFFFFFFFFFFFF:FFFFFFFFFFFFFFFFFFFFFFFFFFFFFFFF
FFFFFFFFFFFFFFFFFFFFFFFFFFF:F:FF:FFFFFFFFFFFFFFFFFFFFFFFFFFFFF,FFFFF
@A00155:342:HHGFNDSXY:1:1428:7581:29904 2:N:0:GAACCTAG+TCCGCATA
AGAGAGAGCGACTTCTCTCGGGCCCACAGCCTTACAGCTGTGGAGCACGGTATCCTCTGCCGAAACAG
AGGTTGGACAAGACCGGAGGGGTCTCCTGGTTCCAAAGGAGATGTACTCCGGGCTTGTTCACGACCTA
+
FFFFFFFFFFFFFFF,,FF:FFFFFFFF:FFFFFFFFFFFFF,FFFF,:,FF,FFFFF:F:FFFFF:F
F:FFFFFFFFF:FF:FFF:FFFFFFFFFFFFFFFFFFFFF:FFFF:FFFFFFFFFFF:::FFF:FFFF
```

@A00155:342:HHGFNDSXY:1:1429:9227:16094 2:N:0:GAACCTAG+TCCGCATA
ACGACTTACACGGTAGGTCGTGAACAAGCCCGGAGTACATCTCCTTTGGAACTAGGAGACCCCTCCGG
TCTTGTCCAACCTCTGTTTCGGCAGAGGATACCGTGCTCCACAGCTGTAAGGCTGTGGGCCCGAG
+
FFFFFFFFFFFFFFFFFFFFFFFFFFFFFFFFFFFFFFFFFFFFFFFFFFFFFFFFFFFFFFFFFFFF
FFFFFFFFFFFFFFFFFFFFFFFFFFFFFFFFFFFFFFFFFFFFFFFFFFFFFFFFFFFFFFFFF
@A00155:342:HHGFNDSXY:1:1429:3938:23907 2:N:0:GAACCTAG+TCCGCATA
GCCTACTAGACTACGACTTACACGGTAGGTCGTGAACAAGCCCGGAGTACATCTCCTTTGGAACTAGG
AGACCCCTCCGGTCTTGTCCAACCTCTGTTTCGGCAGAGGATACCGTGCTCCACAGCTGTAAGGCTGT
+
FFFFFFFFFFFFFFFFFFFFFFFFFFFFFFFFFFFFFFFFFFFFFFFFFFFFFFFFFFFFFFFFFFFF
FFFFFFFFFFFFFFFFFFFFFFFFFFFFFFFFFFFFFFFFFFFFFFFFFFFFFFFFFFFFF,FFFFFF
@A00155:342:HHGFNDSXY:1:1429:31078:26256 2:N:0:GAACCTAG+TCCGCATA
CGGCAGAGGATACCGTGCTCCACAGCTGTAAGGCTGTGGGCCCGAGAGAAGTCGCTCTCTCTCGCCCT
CCACATCCCTCTCGGCATTGAGATGACCATAGGCCAGAGGTGAGTCCTTAAGTGGACACAGCTGATCG
+
FFFFFFFFFFFFFFFFFFFFFFFFFFFFFFFF:FFFFFFFFFFFFFFFFFFFFFFFFFFFF:FFFFFF
FF:FFFFFFFFFFFFFF,FFFFFFFFFFFFFFFFFFFFFFFFFFFFFFFFFFFFF:FFFFFFFFFFFF
@A00155:342:HHGFNDSXY:1:1429:16233:28855 2:N:0:GAACCTAG+TCCGCATA
TAGACTACGACTTACACGGTAGGTCGTGAACAAGCCCGGAGTACATCTCCTTTGGAACTAGGAGACCC
CTCCGGTCTTGTCCAACCTCTGTTTCGGCAGAGGATACCGTGCTCCACAGCTGTAAGGCTGTTGGCCC
+
FFFFFFF:FFF:FFFFF:FFFFF,:FF::FFFF,:FFFFFFFFFFFFFFFFFFF:F,FFFFFFFF:FF
F:FFFFFF:F,FFFFF:FF,F:F:F,:F,F:FF,FFFFF,F:FFF,:FF:,,FFF,,:,F:F,F::,F
@A00155:342:HHGFNDSXY:1:1429:30752:36401 2:N:0:GAACCTAG+TCCGCATA
CCGGTCTTGTCCAACCTCTGTTTCGGCAGAGGATACCGTGCTCCACAGCTGTAAGGCTGTGGGCCCGA
GAGAAGTCGCTCTCTCTCGCCCTTCACATCCCTCTCGGCATTGAGATGACCATAGGCCAGAGGTGAGT
+
FFFFFFFFFFFFFFFFFFFFFFFFFFFFFFFFFFFFFFFFFFFFFFFFFFFFFFFFFFFFFFFFFFFF
FFFFFFFFFFFFFFFFFFFFFFFFFFFFFFFFFFFFFFFF:FFFFFFFFFFFF:FFFFFFFFFFFFFF
@A00155:342:HHGFNDSXY:1:1430:13901:21026 2:N:0:GAACCTAG+TCCGCATA
ACGACTTACACGGTAGGTCGTGAACAAGCCCGGAGTACATCTCCTTTGGAACTAGGAGACCCCTCCGG
TCTTGTCCAACCTCTGTTTCGGCAGAGGATACCGTGCTCCACAGCTGTAAGGCTGTGGGCCCGAGAGA
+
FFFF:FFFFFFFFFFFFFFFFFFFFFFFFFF:F:F:,F,FFFFFF,FFF,:FFF,FFFFFFFFFFFFF
FFFF:FFFF,FFFFFFF,FFFFFFFFFF:FFFFF,FFFFFFFF:FFFFFFF:FF:,:F,FFF:FFFF:
@A00155:342:HHGFNDSXY:1:1430:2609:29716 2:N:0:GAACCTAG+TCCGCATA
GGACTCGTCAGGTAGCCTACTAGACTACGACTTACACGGTAGGTCGTGAACAAGCCCGGAGTACATCT
CCTTTGGAACTAGGAGACCCCTCCGGTCTTGTCCAACCTCTGTTTCGGCAGAGGATACCGTGCTCCAC
+
FF:FFFFFFFFFFFFFFFFFFFFFFFFFFFFFFFFFFFFFFFFFFFFFFFFF:FFFFFFFFFFFFFFF
FFFFFFFFFFFFFFFFFFFFFFFFFFFFFFFFFFFFF:FFFFFFFFFFFFFFFFFFFFF,FFFFFFF:
@A00155:342:HHGFNDSXY:1:1430:20907:35728 2:N:0:GAACCTAG+TCCGCATA
GTTTCGTCCTAAAAAGGACTCGTCAGGTAGCCTACTAGACTACGACTTACACGGTAGGTCGTGAACAA
GCCCGGAGTACATCTCCTTTGGAACTAGGAGACCCCTCCGGTCTTGTCCAACCTCTGTTTCGGCAGAG
+
F:FFFFFFF:FFFFFFF:FF:FFFFFFFF:FF:FFFFFFFFFF,FFFFF:FFFFFFFFFFFFFFF:FF
FF,FFFFF:FFFFFFFFFFFF,FFFFF:FFF:F,F,FFF,F,FFFFF:F:FFFFFFFF,F:FF:F,FF
@A00155:342:HHGFNDSXY:1:1431:2899:3443 2:N:0:GAACCTAG+TCCGCATA
GGACGAAACCGTCGTTACTCTTCGAGTTGTGTAAGTTTCGTCCTAAAAAGGACTCGTCAGGTAGCCTA
CTAGACTACGACTTACACGGTAGGTCGTGAACAAGCCCGGAGTACATCTCCTTTGGAACTAGGAGACC
+
FFFFFFFFFFFFFFFFFFFFFFFFFFFFFFFFFFFF:FFFFFFFFFFF:FFFFFFFF:FFFFFFFFFF
FFFF,FFFFFFFFFFFFFFFFFFFFFFFFFFFFF:FFFFFFFFFFFFFFFFFFFFFFFFFFFFFFFFF

```
@A00155:342:HHGFNDSXY:1:1431:29821:10927 2:N:0:GAACCTAG+TCCGCATA
TCTCCTTTGGAACTAGGAGACCCCTCCGGTCTTGTCCAACCTCTGTTTCGGCAGAGGATACCGTGCTC
CACAGCTGTAAGGCTGTGGGCCCGAGAGAAGTCGCTCTCTCTCGCCCTCCACATCCCTCTCGGCATTG
+
FFFFFFFFFFFFFFFFFFFFFFFFFFFFFFFFFFFFFFFFFFFFFFF:FFFFFFFFFFFF,FFFFFFF
FFFFFFFFFFFFFF,FFFFFFFFFFFFFFFFFFFFFFFFFFFFFFFFFFFFFFFFFFFFFFFFFF:FF
@A00155:342:HHGFNDSXY:1:1431:32994:18270 2:N:0:GAACCTAG+TCCGCATA
GTAGCCTACTAGACTACGACTTACACGGTAGGTCGTGAACAAGCCCGGAGTACATCTCCTTTGGAACT
CGGAGACCCCTCCGGTCTTGTCCAACCTCTGTTTCGGCAGAGGATACCGTGCTCCACAGCTGTAAGGC
+
FFFFFFFFFFFFFFFFFFFF:FFFFFFF:FFFFFFFFFFFFFFFFFFFFFF:FFFFFFF::FFFF:FF
,FFF:FFFFFFFFFFFFF,F,FFFFFFFF:FFFFF:FFFFFFFFFFFFFFFFFFFFFFFF,F:FFFFF
@A00155:342:HHGFNDSXY:1:1431:17716:20369 2:N:0:GAACCTAG+TCCGCATA
AAAAAGGACTCGTCAGGTAGCCTACTAGACTACGACTTACACGGTAGGTCGTGAACAAGCCCGGAGTA
CATCTCCTTTGGAACTAGGAGACCCCTCCGGTCTTGTCCAACCTCTGTTTCGGCAGAGGATACCGTGC
+
FFFFFFFFFFFFFFFFFFFFFFFFFFFFFFFFFFFFFFFFFFFFFFFFFFFFFFFFFFFFFFFFFFFF
FFFFFFFFFFFFF,FFFFFFFFFFFFFFFFFFFFFFFFFFFFFFFFFFFFFFFFFFFFFFF:FFFFFF
@A00155:342:HHGFNDSXY:1:1431:18213:24956 2:N:0:GAACCTAG+TCCGCATA
AAAAAGGACTCGTCAGGTAGCCTACTAGACTACGACTTACACGGTAGGTCGTGAACAAGCCCGGAGTA
CATCTCCTTTGGAACTAGGAGACCCCTCCGGTCTTGTCCAACCTCTGTTTCGGCAGAGGATACCGTGC
+
F:FFFFFFFFFFFFFFFFFFFFFFFFFFFFFFFFFFFFFFFFFFFFFFFFFFFFFFFFFFFFFFFFFF
FFFF:FF:FFFFFFFFFFFFFFFFFFFFFFFFFFFFFFFFFFFFFFFFFFFFFFFFFFFFFFFFFFFF
@A00155:342:HHGFNDSXY:1:1432:30219:6793 2:N:0:GAACCTAG+TCCGCATA
ACACGGTAGGTCGTGAACAAGCCCGGAGTACATCTCCTTTGGAACTAGGAGACCCCTCCGGTCTTGTC
CAACCTCTGTTTCGGCAGAGGATACCGTGCTCCACAGCTGTAAGGCTGTGGGCCCGAGAGAAGTCGCT
+
FFFFFFFFFFFFFFFFFFFFFFFFFFFFFFFFFFFFFFFFFFFFFFFFFFFFFFFFFFFFFFFFFFFF
FFFFFFFFFFFFFFFFFFFFFFFFFFFFFFFFFFFFFFFFFFFFFFFFFFFFFFFFFFFFFFFFFFF:
@A00155:342:HHGFNDSXY:1:1432:1895:9784 2:N:0:GAACCTAG+TCCGCATA
AGCCCGGAGTACATCTCCTTTGGAACTAGGAGACCCCTCCGGTCTTGTCCAACCTCTGTTTCGGCAGA
GGATACCGTGCTCCACAGCTGTAAGGCTGTGGGCCCGAGAGAAGTCGCTCTCTCTCGCCCTCCACATC
+
FFFFFFFFFFFFFFFFFFFFFFFF:FFFFFFFFFFFFFFFFFFFFFFFFFFFFFFFFFFFFFFFFFF
FFFFFFFFFFFFFFFFFFFFFF,FFFFFFFFFFFFFFFFFFFFFFFFFFFFFFFFFFFFFFFFFFFFF
@A00155:342:HHGFNDSXY:1:1432:19904:22467 2:N:0:GAACCTAG+TCCGCATA
ACTACGACTTACACGGTAGGTCGTGAACAAGCCCGGAGTACATCTCCTTTGGAACTAGGAGACCCCTC
CGGTCTTGTCCAACCTCTGTTTCGGCAGAGGATACCGTGCTCCACAGCTGTAAGGCTGTGGGCCCGAG
+
FFFFFFFFFFFFFFFFFFFFFFFFFFFFFFFFFFFFFFFFFFFFFFFFFFFFFFFFFFFFFFFFFFFF
FFFFFF:FFFFF:FFFFFFFFFFFFF:FFFFFFFFFFFFFFFFFFFFFFFFF,FFFFFFFFFFFFFFF
@A00155:342:HHGFNDSXY:1:1433:9028:8234 2:N:0:GAACCTAG+TCCGCATA
GAACTAGGAGACCCCTCCGGTCTTGTCCAACCTCTGTTTCGGCAGAGGATACCGTGCTCCACAGCTGT
AAGGCTGTGGGCCCGAGAGAAGTCGCCCTCTCTCGCCCTCCACATCCCTCTCGGCATTGAGATGACCA
+
FFFFFFFFFFFFFFFFFFFFFFFFFFFFFFFFFFFFFFFFFFFFFFFFFFFFFFFFFFFFFFFFFFFF
FFFFFFFFFFFFFFFFFFFFFFFFFFFFFFFFFFFFFFFFFFFFFFFFFFFFFFFFFFFFFFFFFFFF
@A00155:342:HHGFNDSXY:1:1433:5638:27696 2:N:0:GAACCTAG+TCCGCATA
CTGTGTCCACTTAAGGACTCACCTCTGGCCTATGGTCATCTCAATGCCGAGAGGGATGTGAAGGGCGA
GAGAGAGCGACTTCTCTCGGGCCCACAGCCTTACAGCTGTGGAGCACGGTATCCTCTGCCGAAACAGA
+
FFFFFFFFFFFFFFFFFFFFFFFFFFFFFFFFFFFFFFFFFFFFFFFFFFFFFFFFFFFFFFFFFFFF
FFFFFFFFFFFFFFFFFFFFFFFFFFFFFFFFFFFFFFFFFFFFFFFFFF,FFFFFFFFFFFFFFFFF
```

```
@A00155:342:HHGFNDSXY:1:1433:7554:32017 2:N:0:GAACCTAG+TCCGCATA
CCTACTAGACTACGACTTACACGGTAGGTCGTGAACAAGCCCGGAGTACATCTCCTTTGGAACTAGGA
GACCCCTCCGGTCTTGTCCAACCTCTGTTTCGGCAGAGGATACCGTGCTCCACAGCTGTAAGGCTGTG
+
FFFFF:FFFFFFFFFF:,FFF::FFFFFFF::FFFFF:FFFFFFFFF:FFFF:FFFFFF:FFFFFFFF
FFF,FF:FF,F,FF,FFFFFFFF:FFFFFFFFFFF:FF:FFFFF,FF:FFFFFFFFFFFFFFFFFFFF
@A00155:342:HHGFNDSXY:1:1433:22336:35477 2:N:0:GAACCTAG+TCCGCATA
CGGACTCATCAGGGAGCCTACTAGACTCCGACCGTCATGGGGGTTCAAACCCATGCCCCGCCACACCG
CCTTAGATCAGCTGTGTCCACTTAAGGACTCACCTCTGGCCTATGGTCATCTCAATGCCGAGAGGGA
+
FFFFFFFFFFFFFFFFFFFFFFFFFFFFFFFFFFFFFFFFFFFFFFFFFFFFFFFFFFFFFFFFFFFF
FFFFFFFFFFFFFFFFFFFFFFFFFFFFFFFFFFFFFFFFFFFFFFFFFFFFFFFFFFFFFFFFFFF
@A00155:342:HHGFNDSXY:1:1433:22625:35603 2:N:0:GAACCTAG+TCCGCATA
CGGACTCATCAGGGAGCCTACTAGACTCCGACCGTCATGGGGGTTCAAACCCATGCCCCGCCACACCG
CCTTAGATCAGCTGTGTCCACTTAAGGACTCACCTCTGGCCTATGGTCATCTCAATGCCGAGAGGGAT
+
FFF::FFFFFFFFFFFFFFFFFFFFFFFFFFF:FFFFFFFFFFFFF:F,FFFFFFFFFFFFFFFFFF:
FFFFFFFFFFFFFFFFFFFFFFFFF:FFFFFFFFFFFFFFFFFFFFFFFFFFFFFFFFFFFFFFFFFF:
@A00155:342:HHGFNDSXY:1:1434:16089:13573 2:N:0:GAACCTAG+TCCGCATA
ACTAGGAGACCCCTCCGGTCTTGTCCAACCTCTGTTTCGGCAGAGGATACCGTGCTCCACAGCTGTAA
GGCTGTGGGCCCGAGAGAAGTCGCTCTCTCTCGCCCTCCACATCCCTCTCGGCATTGAGATGACCATA
+
FF,FFFFFFFFFFFFFFFFFFFFFFFFFFFFFFFF:FFFFFF:FFFFFFFFFFFFFFFFFFFFFFFFF:F
FFFFFFFFFFFFFFFFFFFFFFFFFFFFFFF,FFFFFFFFFF:FFFFFFFFFFFFFFFFFFFFFFFFFFF
@A00155:342:HHGFNDSXY:1:1434:13449:31454 2:N:0:GAACCTAG+TCCGCATA
TCCAAGGACGAAACCGTCGTTACTCTTCGAGTTGTGTAAGTTTCGTCCTAAAAAGGACTCGTCAGGTA
GCCTACTAGACTACGACTTACACGGTAGGTCGTGAACAAGCCCGGAGTACATCTCCTTTGGAACTAGG
+
FFFFFFFFFFFFFFFFFFFFFFFFFF:FFFFFFFFFFFFFFFFFFFFFFFFFFFFFFFFFFFFFFFFF
FFFFFFFFFFFFFFFFFFFFFFFFFFFFFFFFFFFFFFFFFFFFFFFFFFFFFFFFFFFFFFF:FFFFF
@A00155:342:HHGFNDSXY:1:1434:22607:31751 2:N:0:GAACCTAG+TCCGCATA
AAGGACTCACCTCTGGCCTATGGTCATCTCAATGCCGAGAGGGATGTGGAGGGCGAGAGAGAGCGACT
TCTCTCGGGCCCACAGCCTTACAGCTGTGGAGCACGGTATCCTCTGCCGAAACAGAGGTTGGACAAGA
+
FFFFFFFFFFFFFFFFFFF:FFFFFFFFFFFFFFFFFFFFFFFFFFFFFFFFFFFFFFFFFFFFFFFF
FFFFFFFFFFFFFFFFFFFFFFFFFFFFFFFFFFFFFFF:FFFFFFFFFFFFFF:FFFFFFFFFFFFFF
@A00155:342:HHGFNDSXY:1:1435:2121:2253 2:N:0:GAACCTAG+TCCGCATA
CCCCTCCGGTCTTGTCCAACCTCTGTTTCGGCAGAGGATACCGTGCTCCACAGCTGTAAGGCTGTGGG
CCCGAGAGAAGTCGCTCTCTCTCGCCCTTCACATCCCTCTCGGCATTGAGATGACCATAGGCCAGAGG
+
FFFFFFFFFFFFFFFFFFFFFFFFFFFFFFFFFF:FFFFFFFFFFFFFFFFFFFFFFFFFFFFFFFFFF
FFFFFFFF:FFFFF:FFFFFFFFFFFFFFFFFFFFFFFFFFFFFFF,FFFFFFFFFFFFFFFFFFFF,FF
@A00155:342:HHGFNDSXY:1:1435:3396:3521 2:N:0:GAACCTAG+TCCGCATA
CAGGTAGCCTACTAGACTACGACTTACACGGTAGGTCGTGAACAAGCCCGGAGTACATCTCCTTTGGA
ACTAGGAGACCCCTCCGGTCTTGTCCAACCTCTGTTTCGGCAGAGGATACCGTGCTCCACAGCTG
+
FFFFFFFFFFFFFFFFFFFFFFFFFFFFFFFFFFFFFFFFFFFFFFFFFFFFFFFFFFFFFFFFFFFF
FFFFFFFFFFFFFFFFFFFFFFFFFFFFFFFFFFFFFFFFFFFFFF:FFFFFFFFFFFFFFFFFFF
@A00155:342:HHGFNDSXY:1:1435:21893:5118 2:N:0:GAACCTAG+TCCGCATA
TACGACTTACACGGTAGGTCGTGAACAAGCCCGGAGTACATCTCCTTTGGAACCAGGAGACCCCTCCG
GTCTTGTCCAACCTCTGTTTCGGCAGAGGATACCGTGCTCCACAGCTGTAAGGCTGTGGGCCCG
+
F:FFFFFFFFFFFFFFFFFFFFFFFFFFFFFFFFFFFFFFFFFFFFFFFFFFFFFFFFFFFFFFFFFF
FFFFFFFFFFFFFFFFFFFFFFFFFFFFFFFFFF:FFFFFFFFFFFFFFFFFF:FFFFFFFFFFF
```

```
@A00155:342:HHGFNDSXY:1:1436:29767:2033 2:N:0:GAACCTAG+TCCGCATA
TGTGTAAGTTTCGTCCTAAAAAGGACTCGTCAGGTAGCCTACTAGACTACGACTTACACGGTAGGTCG
TGAACAAGCCCGGAGTACATCTCCTTTGGAACTAGGAGACCCCTCCGGTCTTGTCCAACCTCTGTTTC
+
FFFFFFFFFFFFFFFFFFFFFFFFFFFFFFFFFFFFFFFFFFFFFFFFFFFFFFFFFFFFFFFFFFFF
FFFFFFFFFFFFFFFFF:FFFFFFFFFFFFFFFFFFFFFFFFFFFFFFFFFFFFFFFFFFFFFF:FFF
@A00155:342:HHGFNDSXY:1:1436:6876:15687 2:N:0:GAACCTAG+TCCGCATA
GTGAACAAGCCCGGAGTACATCTCCTTTGGAACCAGGAGACCCCTCCGGTCTTGTCCAACCTCTGTTT
CGGCAGAGGATACCGTGCTCCACAGCTGTAAGGCTGTGGGCCCGAGAGAAGTCGCTCTCTCTCGCCCT
+
FFFFFFFFFFFFFFFFFFFFFFFFFFFFFFFFFFFFFFFFFFFFFFFFFFFFFFFFFFFFFFFFFFFF
FFFFFFFFFFFFFFFFFFFFFFFFFFFFFFFFFFFFFFFFFFFFFFFFFFFFFFFFFFFFFFFFFFFF
@A00155:342:HHGFNDSXY:1:1436:7392:17863 2:N:0:GAACCTAG+TCCGCATA
AAAAGGACTCGTCAGGTAGCCTACTAGACTACGACTTACACGGTAGGTCGTGAACAAGCCCGGAGTAC
ATCTCCTTTGGAACCAGGAGACCCCTCCGGTCTTGTCCAACCTCTGTTTCGGCAGAGGATACCGTGCT
+
:FF:FFFFFFFFFFFFFFFFFFFFFFFFFFFFFFFF:FFFFFFFFFFFFFFFFFFFF,FFFFFFFFF,
FFFFF,FFFFFF,FFFFFF:FFFFFFFFFFFFFFFF:FFFFFFFF,FFFFFFFFFFFFFFFFFFFFFF
@A00155:342:HHGFNDSXY:1:1436:17273:18787 2:N:0:GAACCTAG+TCCGCATA
GAAACCGTCGTTACTCTTCGAGTTGTGTAAGTTTCGTCCTAAAAAGGACTCGTCAGGTAGCCTACTAG
ACTACGACTTACACGGTAGGTCGTGAACAAGCCCGGAGTACATCTCCTTTGGAACTAGGAGACCCCTC
+
FFFFFFF,FFFFFFFFF::FFFFFF,F:FFFF,FFFFFF:,FFFFFFFFFFFFF:FFFFFFFFFFFFF
FF:,FFFFFFFFFFFFFFFFFFFFFFFF:FFFFFFFFFFFFFFFFFFFFF:F:FFFFFFFFFFFFFFF
@A00155:342:HHGFNDSXY:1:1436:10167:25614 2:N:0:GAACCTAG+TCCGCATA
CCTACTAGACTACGACTTACACGGTAGGTCGTGAACAAGCCCGGAGTACATCTCCTTTGGAACTAGGA
GACCCCTCCGGTCTTGTCCAACCTCTGTTTCGGCAGAGGATACCGTGCTCCACAGCTGTAAGGCTGTG
+
FFFFFFFFFFFFFFFFFFFFFFFFFFFFFFFFFFFFFFFFFFFFFFFFFFFFFFFFFFFFFFFFFFFF
FFFFFFFFFFFFFFFFFFFFFFFFFFFFFFFFFFFFFFFFFFFFFFFFFFFFFFFFFFFFFFFFFFFF
@A00155:342:HHGFNDSXY:1:1437:14353:9565 2:N:0:GAACCTAG+TCCGCATA
AAAAAGGACTCGTCAGGTAGCCTACTAGACTACGACTTACACGGTAGGTCGTGAACAAGCCCGGAGTA
CATCTCCTTTGGAACCAGGAGACCCCTCCGGTCTTGTCCAACCTCTGTTTCGGCAGAGGATACCGTGC
+
FFFF:FFFFFFFFFFFFFFFFFFFFFFFFFFFFFFFFFFFFFFFFFFFFFFFFFFFFFFFFFFFFFFF
FFFFFFFFFFFFFFFFFFFFFFFFFFFFFFFFFFFFFFFFFFFFFFFFFFFFFFFFFFFFFFFFFFFF
@A00155:342:HHGFNDSXY:1:1437:30174:22842 2:N:0:GAACCTAG+TCCGCATA
GTGAACAAGCCCGGAGTACATCTCCTTTGGAACTAGGAGACCCCTCCGGTCTTGTCCAACCTCTGTTT
CGGCAGAGGATACCGTGCTCCACAGCTGTCAGGCTGTGGGCCCGAGAGAAGTCGCTCTCTCTCGCCCT
+
FFFFFFFFFFFFFFFFFFFFFFFFFFFFFFFFFFFFF:FFFFFFFFFFFFFFFFFFFFFFFFFFFFFF
F:FFFFFFFFFFFFFFFFFFFFFFFFFFF,FFFFFFFFFFFFFFFFFFFFFFFFFFFFFFFFF:FFF:
@A00155:342:HHGFNDSXY:1:1438:31159:13432 2:N:0:GAACCTAG+TCCGCATA
AAGGACTCGTCAGGTAGCCTACTAGACTACGACTTACACGGTAGGTCGTGAACAAGCCCGGAGTACAT
CTCCTTTGGAACCAGGAGACCCCTCCGGTCTTGTCCAACCTCTGTTTCGGCAGAGGATACCGTG
+
FFF:FFFFFFFFFFFFFFFFFFFFFFFFFFFFFFFFFFFFFF:FFFFFFFF:FFFFFFFFFFFFFFFF
FFFFFFFFFFFFFFFFFFFFFFFFFFFFFFFFFFFFFFFFFFFFFFFFFFFFFFFFFFFFFFFF
@A00155:342:HHGFNDSXY:1:1438:9598:14199 2:N:0:GAACCTAG+TCCGCATA
AACTAGGAGACCCCTCCGGTCTTGTCCAACCTCTGTTTCGGCAGAGGATACCGTGCTCCACAGCTGTA
AGGCTGTGGGCCCGAGAGAAGTCGCTCTCTCTCGCCCTCTACATCCCTCTCGGCATTGAGATGACCAT
+
FFFFFFFFFFFFFFFFFFFFFFFFFFFFFFFFFFFFFFFFFFFFFFFFFFFFFFFFFFFFFFFFFFFF
FFFFFFFFFFFFFFFFFFFFFFFFFFFFFFFFFFFFFFFFFFFFFFFFFFFFFFF:FFFFFFFFFFFF
```

```
@A00155:342:HHGFNDSXY:1:1438:17264:20275 2:N:0:GAACCTAG+TCCGCATA
GCCTACTAGACTACGACTTACACGGTAGGTCGTGAACAAGCCCGGAGTACATCTCCTTTGGAACTAGG
AGACCCCTCCGGTCTTGTCCAACCTCTGTTTCGGCAGAGGATACCGTGCTCCACAGCTG
+
FFFFFFFFFFFFFFFFFFFFFFFFFFFFFFFFFFFFFFF:FFFFFFFFFFFFFFFFFFFFFFFF:FFF
FFFFFFFFFFFFFFFFFFFFFFFFFFFFFFFFFFFFFFFFFFFFFFFFFFFFFFFFFFF
@A00155:342:HHGFNDSXY:1:1438:29957:36933 2:N:0:GAACCTAG+TCCGCATA
GGAGTACATCTCCTTTGGAACTAGGAGACCCCTCCGGTCTTGTCCAACCTCTGTTTCGGCAGAGGATA
CCGTGCTCCACAGCTGTAAGGCTGTGGGCCCGAGAGAAGTCGCTCTCTCTCGCCCTCTACATCCCTCT
+
FFFFFFFFFFFFFFFFFFFFFFFFFFFFFFFFFFFFFFF:FFFFFFFFFFFFFFFFFFFFFFFFFFFF
FFFFFFFFFFFFFFFFFFFFFFFFFFFFFFFFFFFFF:FFFFFFFFFFFFFFFFFFFFFFFFFFFFFF
@A00155:342:HHGFNDSXY:1:1439:13639:2472 2:N:0:GAACCTAG+TCCGCATA
CAAGCCCGGAGTACATCTCCTTTGGAACTAGGAGACCCCTCCGGTCTTGTCCAACCTCTGTTTCGGCA
GAGGATACCGTGCTCCACAGCTGTAAGGCTGTGGGCCCGAGAGAAGTCGCTCTCTCTCGCCCTCCACA
+
FFFFFFFFFFFFFFFFF:FFFFFFFFFFFFFFF:FFFFFFFFFFFFFFFFFFFFFFFFFFFFFFFFFF
F:FFFFFFFFFFFFFFFFFFF:FFFFFFFFFFFFFFFFFFFFFFFFFFFFFFFFFFFFFFFFFFFFFF
@A00155:342:HHGFNDSXY:1:1439:18150:17237 2:N:0:GAACCTAG+TCCGCATA
GTTTCGTCCTAAAAAGGACTCGTCAGGTAGCCTACTAGACTACGACTTACACGGTAGGTCGTGAACAA
GCCCGGAGTACATCTCCTTTGGAACTAGGAGACCCCTCCGGTCTTGTCCAACCTCTGTTTCGGCAGAG
+
FFFFFFFFFFFFFFFFFFFFFFFFFFFFFFFFF:FFF:FFFF:FFFFFFFFFFFFFFFFFFFFFFFFF
FFF:FFFFFFFFFFFFFF:FFFFFFFFFFFFFFFFFFFFFFFFFFFFFFFF:FFFFFFFFFFFF:FFF
@A00155:342:HHGFNDSXY:1:1439:19443:23766 2:N:0:GAACCTAG+TCCGCATA
GTTGTGTAAGTTTCGTCCTAAAAAGGACTCGTCAGGTAGCCTACTAGACTACGACTTACACGGTAGGT
CGTGAACAAGCCCGGAGTACATCTCCTTTGGAACTAGGAGACCCCTCCGGTCTTGTCCAACCTCTGTT
+
FFFFFFFFFFFFFFFFFF:FFFFFFFFFFFFFFFFFFFFFFF:FF:FFFFFFFFFFFFFFFFFFFFFF
FFFFFFFFFFFFFFFFF:FFFFFFFFFFFFFFFFFFF:FFF:FFFFFFFFFFFFFFFFFFFFFFFFFF
@A00155:342:HHGFNDSXY:1:1439:19768:24768 2:N:0:GAACCTAG+TCCGCATA
GTTGTGTAAGTTTCGTCCTAAAAAGGACTCGTCAGGTAGCCTACTAGACTACGACTTACACGGTAGGT
CGTGAACAAGCCCGGAGTACATCTCCTTTGGAACTAGGAGACCCCTCCGGTCTTGTCCAACCTCTGTT
+
FFFFFF,FFFFFFFFFFFFFFFF,FFFFFFFFFFFFFFFFFFFFFFFFFFFFFFFFFFFFFFFFFFFF
FFFFFFFFFFFFFFFFF:FF,FFFFF:FFFFFFFFFFFFFFFFFFFFFFFFFFFFFFFFFF:FFFFFF
@A00155:342:HHGFNDSXY:1:1439:9444:27273 2:N:0:GAACCTAG+TCCGCATA
TCAGCTGTGTCCACTTAAGGACTCACCTCTGGCCTATGGTCATCTCAATGCCGAGAGGGATGTAGAGG
GCGAGAGAGAGCGACTTCTCTCGGGCCCACAGCCTTACAGCTGTGGAGCACGGTATCCTCTGCCGAAA
+
FFFFFFFFFFFFFFFFFFFFFFFFFFFFFFFFFFFFFFFFFFFFFFFFFFFFF:FFFFFF:FFFFFFF
FFFFFFFFFFFFFFFFFFFFFFFFFFFFFFFFF:FF:FFFFFFFFFFFFFFFFFFFFFFFFFFFFFFF
@A00155:342:HHGFNDSXY:1:1440:21866:22388 2:N:0:GAACCTAG+TCCGCATA
ACTTACACGGTAGGTCGTGAACAAGCCCGGAGTACATCTCCTTTGGAACTAGGAGACCCCTCCGGTCT
TGTCCAACCTCTGTTTCGGCAGAGGATACCGTGCTCCACAGCTGTAAGGCTGTGGGCCCGAGAGAAGT
+
FFFFFFFFFFFFFFFFFFFFFFFFFFFFFFFFFFFFFFFFFFFFFFFFF:FFFF:FFFFFFFFFFFFF
:FFFFFFFFFFFFFFFFFFFFFFFFFFFFFFFFFFFFFF:FF:FFFFFFF,FFFFFFFFFFFFFFFFF
@A00155:342:HHGFNDSXY:1:1440:24460:36088 2:N:0:GAACCTAG+TCCGCATA
TCGTGAACAAGCCCGGAGTACATCTCCTTTGGAACTAGGAGACCCCTCCGGTCTTGTCCAACCTCTGT
TTCGGCAGAGGATACCGTGCTCCACAGCTGTAAGGCTGTGGGCCCGAGAGAAGTCGCTCTCTCTCGCC
+
FFFFFFFFFFFFFFFFFFFFFFFFFFFFFFFFFFFFFFFFFFFFFFFFFFFFFFFFFFFFFFFFFFFF
FFFFFFFF:FFFFFFFFFFFFFFFFFFFFFF:FFFFFFFFFFFFFFFFFFFFFFFFFFFFFFFFFFFF
```

@A00155:342:HHGFNDSXY:1:1441:9362:14262 2:N:0:GAACCTAG+TCCGCATA
AAAAGGACTCGTCAGGTAGCCTACTAGACTACGACTTACACGGTAGGTCGTGAACAAGCCCGGAGTAC
ATCTCCTTTGGAACTAGGAGACCCCTCCGGTCTTGTCCAACCTCTGTTTCGGCAGAGGATACCGTGCT
+
FFF:FFFFFFFFFFFFFFFFFFFFFFFFFFFFFFFFFFFFFFFFFFFFFFFFFFFFFFFFFFFFF
FFFFFFFFFFFFFFFFFFFFFFFFFFFFFFFFF:FFFFFFFFFFFFFFFFFFFFFFFFF:FFFFFFFFF
@A00155:342:HHGFNDSXY:1:1441:31024:24596 2:N:0:GAACCTAG+TCCGCATA
ACGACTTACACGGTAGGTCGTGAACAAGCCCGGAGTACATCTCCTTTGGAACCAGGAGACCCCTCCGG
TCTTGTCCAACCTCTGTTTCGGCAGAGGATACCGTGCTCCACAGCTGTAAGGCTGTGGGCCCGAGAGA
+
FFFFFFFFFFFFFFFFFFF:FFFFFFFFFFFFFFFFFFFFFFFFFFFFFFFFFFFFFFFFFFFFFFFF
FFFFF:FFFFFFFFFF:FFFFFFFFFFFFFFFFFFFFFFF:FFFFFFFFFFFFF:FFFFFFFFFFFFFF
@A00155:342:HHGFNDSXY:1:1441:1515:28729 2:N:0:GAACCTAG+TCCGCATA
TACACGGTAGGTCGTGAACAAGCCCGGAGTACATCTCCTTTGGAACTAGGAGACCCCTCCGGTCTTGT
CCAACCTCTGTTTCGGCAGAGGATACCGTGCTCCACAGCTGTAAGGCTGTGGGCCCGAGAGAAGTCGC
+
FFFFFFFFFFFFFFFF:FFFFFFFFFF,FFFFFFFFFFFFFFF:FFFFFFFFFFFFFFFFFFFFFFFFF
FFF:FFFFFFFFFFFFFFFFFFFFFFFFFFFFFFFFFFFFF:FFFFFFFFFFFFFFFFFFFFF:FFFFF:
@A00155:342:HHGFNDSXY:1:1442:30391:2143 2:N:0:GAACCTAG+TCCGCATA
GGACTCGTCAGGTAGCCTACTAGACTACGACTTACACGGTAGGTCGTGAACAAGCCCGGAGTACATCT
CCTTTGGAACTAGGAGACCCCTCCGGTCTTGTCCAACCTCTGTTTCGGCAGAGGATACCGTGCTCCAC
+
FFFFFFFFFFFFF:FFFFFFFFFFFFFFFFFFFFFFFFFFFFFF:FFFFFFFFFFFFFFFFFFFF:FFF
FF:FFFFFFFFFFFFFFFFFFFFFFFFFFFFFFFFFFFFFFFFFFFFFFFFFF:FFFFFFFFFFFFFF
@A00155:342:HHGFNDSXY:1:1442:1976:3286 2:N:0:GAACCTAG+TCCGCATA
AACTAGGAGACCCCTCCGGTCTTGTCCAACCTCTGTTTCGGCAGAGGATACCGTGCTCCACAGCTGTA
AGGCTGTGGGCCCGAGAGAAGTCGCTCTCTCTCGCCCTCCACATCCCTCTCGGCATTGAGATGACCC
+
FFFFFFFFFFFFFFFFFFFFFF::FFFFFFFFFFFFFFFFFFFFFFFFFFF:FFFFFFFFFFFFFFFFFF
FFFFFFFFFFFFFFFFFFFFFFFFFFFFFFFFFFFFFFFFFFFFFFFFFFFFFFF,FFFFFFFFFFF
@A00155:342:HHGFNDSXY:1:1442:4065:13197 2:N:0:GAACCTAG+TCCGCATA
TTTCGTCCTAAAAAGGACTCGTCAGGTAGCCTACTAGACTACGACTTACACGGTAGGTCGTGAACAAG
CCCGGAGTACATCTCCTTTGGAACTAGGAGACCCCTCCGGTCTTGTCCAACCTCTGTTTCGGCAGAG
+
FFF:FFF:FFFFFFFFFFFFFFFFFFFFFFFFFFFFFFFFFFFFFFFFFFFFFFFFFFFFFF:FFFFF
FFFFFFFFFFFFFFFFFFFFFFF:FFFFFFFFFFF,F,FFFFFFFFFFFFFFFFFFFFFFFFFFFFFF
@A00155:342:HHGFNDSXY:1:1442:17318:14137 2:N:0:GAACCTAG+TCCGCATA
TCGTTACTCTTCGAGTTGTGTAAGTTTCGTCCTAAAAAGGACTCGTCAGGTAGCCTACTAGACTACGA
CTTACACGGTAGGTCGTGAACAAGCCCGGAGTACATCTCCTTTGGAACTAGGAGACCCCTCCGGTCTT
+
FFFFFFFFFFFFFFFFFFFFFFFFFFFFFFFFFFFFFFFFFFFFFFFFFFFFFFFFFFFF:FFFFFFF
FFFFFFFFFFFFFFFFFFFFFFFFFFFFFFFFFFFFFFFFFFFFFFFFFFFFFFFFFFFFFFFFFFFF
@A00155:342:HHGFNDSXY:1:1442:17481:14418 2:N:0:GAACCTAG+TCCGCATA
TCGTTACTCTTCGAGTTGTGTAAGTTTCGTCCTAAAAAGGACTCGTCAGGTAGCCTACTAGACTACGA
CTTACACGGTAGGTCGTGAACAAGCCCGGAGTACATCTCCTTTGGAACTAGGAGACCCCTCCGGTCTT
+
FFFFFFFFFFFFFFFFFFFFFFFFFFFFFFFFFFFFFFFFFFFFFFFFFFFFFFFFFFFFFFFFFFFF
FFFFFFFFFFFFFFFFFFFFFFFFFFFFFFFFFFFFFFFF:FFFFFFFFFFFFFFFFFFFFFFFFFFF
@A00155:342:HHGFNDSXY:1:1442:27299:23030 2:N:0:GAACCTAG+TCCGCATA
GTGTAAGTTTCGTCCTAAAAAGGACTCGTCAGGTAGCCTACTAGACTACGACTTACACGGTAGGTCGT
GAACAAGCCCGGAGTACATCTCCTTTGGAACTAGGAGACCCCTCCGGTCTTGTCCAACCTCTGTTTCG
+
:FFFFFFF:F,FFFFFFFFFF,:FFFFFFFFFFFFFFFFFFF::FFFFFFFFFFF:FF,FFF:FFFFF
FFFFFF,F:F:FFFFFF:FFF,:FFF:FFFFFFF,FFF,:F:FFFFFFFFFFFFFFFFF:FFFFFFF:

```
@A00155:342:HHGFNDSXY:1:1442:19425:33191 2:N:0:GAACCTAG+TCCGCATA
CACGGTAGGTCGTGAACAAGCCCGGAGTACATCTCCTTTGGAACTAGGAGACCCCTCCGGTCTTGTCC
AACCTCTGTTTCGGCAGAGGATACCGTGCTCCACAGCTGTAAGGCTGTGGGCCCGAGAGAAGTCGCTC
+
FFFFFFFFFFFFFFFFFFFFFFF:FFFFFFFFFFFFFFFFFF:FFFFFFFFFFFFFFFFFFF,FFFFF
FFFFFFFFFFFFFFFFFFFFFFFFFFFFFFFFFFFFFFFFFFFFFFFFFFFFFFFFFFFFFFFFFFFF
@A00155:342:HHGFNDSXY:1:1443:5918:14779 2:N:0:GAACCTAG+TCCGCATA
GACTCGTCAGGTAGCCTACTAGACTACGACTTACACGGTAGGTCGTGAACAAGCCCGGAGTACATCTC
CTTTGGAACTAGGAGACCCCTCCGGTCTTGTCCAACCTCTGTTTCGGCAGAGGATACCGTGCTCCACA
+
FFFFFFFFFFFFFFFFFFFFFFFF:FFFFFFFFFFFFFFFFFFFFFFFFFFFFFFFFFFFFFFFFFFF
FFFFFFFFFFFFFFFFFFFF:FFFFFFFFFFFFFFFFFFFFFFFFFFFFFFFFF:FFFFFFFFFFFFF
@A00155:342:HHGFNDSXY:1:1443:19479:20635 2:N:0:GAACCTAG+TCCGCATA
ACTCTTTGAGTTGTGTAAGTTTCGTCCTAAAAAGGACTCGTCAGGTAGCCTACTAGACTACGACTTAC
ACGGTAGGTCGTGAACAAGCCCGGAGTACATCTCCTTTGGAACTAGGAGACCCCTCCGGTCTTGTCCA
+
FFFFFFFFFFFFFFFFFFFFFFFFFFFFFFFFFFFFFFFFFFF:FFFFFFFF:FF:FFFFFFFFFFFF
FFFFFFFFFFFFFFFFFFF:FFFFFFFF:FF:FFFFFFFFFFFFFFFFFFFFFFFFFFFFFFFFFFFF
@A00155:342:HHGFNDSXY:1:1443:5593:35446 2:N:0:GAACCTAG+TCCGCATA
AGGTCGTGAACAAGCCCGGAGTACATCTCCATTGGAACTAGGAGACCCCTCCGGTCTTGTCCAACCTC
TGTTTCGGCAGAGGATACCGTGCTCCACAGCTGTAAGGCTGTGGGCCCGAGAGAAGTCGCTCTCTCTC
+
FFFFFFFFFFFFF:FFFFFFFFFFFFFFFFFFFFFFFFFFFFFFFFFFFFFFFFFFFFFFFF:FFFFF
FFFFFFFFFFFFFFFFFFFFFFFFFFFFFFFFFFFFFFF:FFFFFFFFFFFFFFFFFFFFFFFFFFFF
@A00155:342:HHGFNDSXY:1:1444:22218:1110 2:N:0:GAACCTAG+TCCGCATA
CTCTTTGAGTTGTGTAAGTTTCGTCCTAAAAAGGACTCGTCAGGTAGCCTACTAGACTACGACTTACA
CGGTAGGTCGTGAACAAGCCCGGAGTACATCTCCTTTGGAACTAGGAGACCCCTCCGGTCTTGTCCAA
+
FFFFFFFFFFFFFFFFFFFFFFFFFFFFFFFF:FFFFFFFFFFFFFFFFFFFFFFFFF:FFFFFFFFF
FFFFFFFFFFFFFFFFF:FFFFFFFFFFFFFFFFFFFFFFFFFFFFF:FFFFFFFFFFFFFFFFFFFF
@A00155:342:HHGFNDSXY:1:1444:29206:5666 2:N:0:GAACCTAG+TCCGCATA
CATCTCCTTTGGAACTAGGAGACCCCTCCGGTCTTGTCCAACCTCTGTTTCGGCAGAGGATACCGTGC
TCCACAGCTGTAAGGCTGTGGGCCCGAGAGAAGTCGCTCTCTCTCGCCCTCCACATCCCTCTCGGCAT
+
FFFFFFFFFFFFFFFFFFFFFFFFFFFFFFFFFFFFFFFFFFFFFFFFFFFFFFFF:,:FFFFFFF:F
FFFFFFF,FFFFF::FFFFFFF,FFFFFFFFFFFF:FFFF,FFFF:FFFFFFFFFFF:FFFF:FFFFF
@A00155:342:HHGFNDSXY:1:1444:14805:6120 2:N:0:GAACCTAG+TCCGCATA
GCCCGGAGTACATCTCCTTTGGAACTAGGAGACCCCTCCGGTCTTGTCCAACCTCTGTTTCGGCAGAG
GATACCGTGCTCCACAGCTGTAAGGCTGTGGGCCCGAGAGAAGTCGCTCTCTCTCGCCCTCCACATCC
+
FFFFFFFFFFFFFFFFFFFFFFFFFFFFFFFFFFFFFFFFFFFFFFFFFFFFFFFFFFFFFFFFFFFF
FFFFFFFFFFFFFFFFFFFFFFFFFFFFFFFFFFFFFFFFFFFFFFFFFFFFF:FFFFFFFFFFFFFF
@A00155:342:HHGFNDSXY:1:1444:31458:12007 2:N:0:GAACCTAG+TCCGCATA
CCTACTAGACTACGACTTACACGGTAGGTCGTGAACAAGCCCGGAGTACATCTCCTTTGGAACCAGGA
GACCCCTCCGGTCTTGTCCAACCTCTGTTTCGGCAGAGGATACCGTGCTCCACAGCTGTAAGGCTGTG
+
FFFFFFFFFFFFFFFFFFFFFFFFFFFFFFFFFFFFFFFFFFFFFFFFFFFFFFFFFFFFF:FFFFFF
FFFFFFFFFFFFFFFFFFFFFFFFFFFFFFFFFFFFFFFFFFFFFFFFFFFFFFFFFFFFFFFFFFFF
@A00155:342:HHGFNDSXY:1:1444:8965:24032 2:N:0:GAACCTAG+TCCGCATA
GAAACCGTCGTTACTCTTCGAGTTGTGTAAGTTTCGTCCTAAAAAGGACTCGTCAGGTAGCCTACTAG
ACTACGACTTACACGGTAGGTCGTGAACAAGCCCGGAGTACATCTCCTTTGGAACCAGGAGACCCCTC
+
FFFFFFFFFFFFFFFFFFFFFFFFFFFFFFFFFFFFFFFF:FFF:FFFFFFFFFFFFFFFFFFFFFFF
FFFFFFFFFFFFFFFFFFFFFFFFFFFFFFFFFFFFFFFFFFFFFFFFFFFFFFFFFFFFFFFFFFFF
```

@A00155:342:HHGFNDSXY:1:1444:8983:23970 2:N:0:GAACCTAG+TCCGCATA
GAAACCGTCGTTACTCTTCGAGTTGTGTAAGTTTCGTCCTAAAAAGGACTCGTCAGGTAGCCTACTAG
ACTACGACTTACACGGTAGGTCGTGAACAAGCCCGGAGTACATCTCCTTTGGAACCAGGAGACCCCTC
+
FFFFFFFFFFFFFFFFFFFFFFFFFFFFFFFFFFFFFF,:FFFFFFFFFFFFFFFFFFFFFFFFFFFF
FFFFFFFFFFFFFFFFFFFFFFFFFF:FFF,FFFFFFFFFF,FFFFFFFFFFFFFFF:FFFFFFFFFF
@A00155:342:HHGFNDSXY:1:1444:15664:24142 2:N:0:GAACCTAG+TCCGCATA
CCCGGAGTACATCTCCTTTGGAACTAGGAGACCCCTCCGGTCTTGTCCAACCTCTGTTTCGGCAGAGG
ATACCGTGCTCCACAGCTGTAAGGCTGTGGGCCCGAGAGAAGTCGCTCTCTCTCGCCCTCTACATCCC
+
FFFFFFFFFFFFFFFFFFFFFFFFFFFFFFFFFFFFFFFFFFFFFFFFFFFFFFFFFFFFFFFFFFFF
FFFFFFFFFFFFFFFFFFF:FFFFFFFFFFFFFFFFFFFFFFFFFFFFFFFFFFFFFFFFFFFFFFFF
@A00155:342:HHGFNDSXY:1:1444:8223:30671 2:N:0:GAACCTAG+TCCGCATA
AGTACATCTCCTTTGGAACTAGGAGACCCCTCCGGTCTTGTCCAACCTCTGTTTCGGCAGAGGATACC
GTGCTCCACAGCTGTAAGGCTGTGGGCCCGAGAGAAGTCGCTCTCTCTCGCCCTCCACAAC
+
FFFFFFFFFFF:FFFFFFFFFFFFFFFFFFFFFFFFFFFFFFFFFFFFFFFFFFFFFFFFFFFFFFFF
FFFFFFFFFFFFFFFFFFFFFFFFFFFFFFFF:F:FFFFFFFFFFFFFFFFFFFFFFFF:F
@A00155:342:HHGFNDSXY:1:1445:25816:13855 2:N:0:GAACCTAG+TCCGCATA
TACATCTCCTTTGGAACTAGGAGACCCCTCCGGTCTTGTCCAACCTCTGTTTCGGCAGAGGATACCGT
GCTCCACAGCTGTAAGGCTGTGGGCCCGAGAGAAGTCGCTCTCTCTCGCCCTCCACATCCCTCTCGGC
+
FFFFFFFF::FF,FFFF:FF,F,FF,F,FFFFF:FFF,FFFFFFF:FF,FFF,F,:FFFFFFFF,:FF
,FFFFFFFF::FFFFFFF,FFFFFFFFFFFFFFFFFF:F:FFFFFF:FF:FFF,FFFF:FF:FF:FFF
@A00155:342:HHGFNDSXY:1:1445:2636:21214 2:N:0:GAACCTAG+TCCGCATA
AGTACATCTCCTTTGGAACTAGGAGACCCCTCCGGTCTTGTCCAACCTCTGTTTCGGCAGAGGATACC
GTGCTCCACAGCTGTAAGGCTGTGGGCCCGAGAGAAGTCGCTCTCTCTCGCCCTCCACATCCCTCTCG
+
FFFFFFFFFFFFFFFFFFFFFFFFFFFFFFFFFFFFFFFF:FFFFFFFFFFFFFFFFFFFFFFFFFFF
FFFFFFFFFFFFFFFFFFFFFFFFFFFFFFFFFFFFFFFFFFFFFFFFFFFFFFFFFFFFFFFFFFFF
@A00155:342:HHGFNDSXY:1:1445:23701:28291 2:N:0:GAACCTAG+TCCGCATA
TGAACAAGCCCGGAGTACATCTCCTTTGGAACTAGGAGACCCCTCCGGTCTTGTCCAACCTCTGTTTC
GGCAGAGGATACCGTGCTCCACAGCTGTAAGGCTGTGGGCCCGAGAGAAGTCGCTCTCTCTCGCC
+
FFFFFFFFFFFFFFFFFFFFFFFFFFFFFFFFFFFFFFFFFFFFFFFFFFFFFFFFFFFFFFFFFFFF
FFFFFFFFFFFFFFFFFFFF:FFFFFFFFFFFFFFFFFFFFFFFFFFFFFFFFFFFFFFFFFFF
@A00155:342:HHGFNDSXY:1:1446:8603:12132 2:N:0:GAACCTAG+TCCGCATA
ACTAGGAGACCCCTCCGGTCTTGTCCAACCTCTGTTTCGGCAGAGGATACCGTGCTCCACAGCTGTAA
GGCTGTGGGCCCGAGAGAAGTCGCTCTCTCTCGCCCTCCACATCCCTCTCGGCATTGAGATGAC
+
FFFFF:FFFFFFFFFFFFFFFFFFFFFF:FFFFFFFFFFFFFFFFFFFFFFFFFFFFFFFFFFFFFFF
F,FFFFFFFFFFFFFFFFFFFFF:FFFFFFF:FFFFFFFFFFFF:FFFFFFFFF:FFFFFFFF:
@A00155:342:HHGFNDSXY:1:1446:12915:22044 2:N:0:GAACCTAG+TCCGCATA
ACCTCTGTTTCGGCAGAGGATACCGTGCTCCACAGCTGTAAGGCTGTGGGCCCGAGAGAAGTCGCTCT
CTCTCGCCCTCCACATCCCTCTCGGCATTGAGATGACCATAGGCCAGAGGTGAGTCCTTAAGTGGACA
+
FFFFFFFFFFFFFFFFFFFFFFFFFFFFFFFFFFFF,FFFFFFFFFFFFFFFFFFFFFFFFFFFFFFF
FFFFFFFFFFFFFFFFFFFFFFFFFFFFFFFFFFFFFFFFFFFFF:FFFFFFFFFFFFFFFFFFFFFF
@A00155:342:HHGFNDSXY:1:1447:8250:11522 2:N:0:GAACCTAG+TCCGCATA
CAGGTAGCCTACTAGACTACGACTTACACGGTAGGTCGTGAACAAGCCCGGAGTACATCTCCTTTGGA
ACTAGGAGACCCCTCCGGTCTTGTCCAACCTCTGTTTCGGCAGAGGATACCGTGCTCCACAGCTGTAA
+
FFFFFFFFFFFFFFFFFFFFFFFFFFFFFFFFFFFFFFFFFFFFFFFFFFFFFFFFFFFFFFFFFFFF
FFFFFFFFFFFFFFFFFFFFFFFFFFFFFFFFFFFFFFFFFFFFFFFFFFFFFFFFFFFFFFFFFFFF

```
@A00155:342:HHGFNDSXY:1:1447:6849:17206 2:N:0:GAACCTAG+TCCGCATA
TCCTAAAAAGGACTCGTCAGGTAGCCTACTAGACTACGACTTACACGGTAGGTCGTGAACAAGCCCGG
AGTACATCTCCTTTGGAACTAGGAGACCCCTCCGGTCTTGTCCAACCTCTGTTTCGGCAGAGGATACC
+
FFFFFFFFFFFFFFFFFFFFFFFFFFFFFFFFFFFFFFFFFFFFFFFFFFFFFFFFFFFFFFFFFFFF
FFFFFFFFFFFFFFFFF:FFFFFFFF:FFFFFFFFFFFFFFFFF:FF:FFFFFFFFFFFFFFFFFFFF
@A00155:342:HHGFNDSXY:1:1447:25500:26240 2:N:0:GAACCTAG+TCCGCATA
TGTTTCGGCAGAGGATACCGTGCTCCACAGCTGTAAGGCTGTGGGCCCGAGAGAAGTCGCTCTCTCTC
GCCCTCCACATCCCTCTCGGCATTGAGATGACCATAGGCCAGAGGTGAGTCCTTAAGTGGACACAGCT
+
FFFFFFFFFFFFFFFFFFFFFFFFFFFFFF:FFFF,FFFFFFFFFFFFFFFFFFFFFFFFFFFFFFFF
FFFFFFFFFFFFFFFFFFFFFFFFFFFFFFFFFFFFFFFFFFFFFFFFFFFFF,FFFFFFFF,FFFFF
@A00155:342:HHGFNDSXY:1:1448:17698:12790 2:N:0:GAACCTAG+TCCGCATA
GAGACCCCTCCGGTCTTGTCCAACCTCTGTTTCGGCAGAGGATACCGTGCTCCACAGCTGTAAGGCTG
TGGGCCCGAGAGAAGTCGCTCTCTCTCGCCCTCTACATCCCTCTCGGCATTGAGATGACCATAGGCCA
+
FFFF:FFFFFFFFFFFFFFFFFFFFFF:FFFFFFFFFFFFFF:FFFFFFFFFFFFFFFFFFFFFFFFF
FFFFFFFFFFFFFFFFFFFFFFFFFFFFFFFFFFFFFFFFFFFFFFFFFF,FFFFFFFFFFFFFFFFF
@A00155:342:HHGFNDSXY:1:1448:18005:13166 2:N:0:GAACCTAG+TCCGCATA
GAGACCCCTCCGGTCTTGTCCAACCTCTGTTTCGGCAGAGGATACCGTGCTCCACAGCTGTAAGGCTG
TGGGCCCGAGAGAAGTCGCTCTCTCTCGCCCTCTACATCCCTCTCGGCATTGAGATGACCATAGGCCA
+
FFFFFFFFFFFFFFFFFFFFFFFFFFFFF:FFFFFFFFFFFFFFFFFFFFFFFFFFFFFFFFFFFFFF
FFFFFFFFFFFFF:FFFFFFFFFFFFFFFFFFFFFFFFFFFFFFFFFFFFFFFFFFFFFFFFFFFFFF
@A00155:342:HHGFNDSXY:1:1449:8250:10081 2:N:0:GAACCTAG+TCCGCATA
TCTTCGAGTTGTGTAAGTTTCGTCCTAAAAAGGACTCGTCAGGTAGCCTACTAGACTACGACTTACAC
GGTAGGTCGTGAACAAGCACGGAGTACATCTCCTTTGGAACTAGGAGACCCCTCCGGTCTTGTCCAAC
+
FFFFFFFFFFFFFFFFFFFFFFFFF:FFFFFFFFFFFFFFFFFFFFFFFFFFFFFFFFFFFFFFFFFF
FFFFFFFFFFFFFFFFFF:FFFFFFFFFFFFF::FFFF:FFFFFFF:FFFFFFFFFFFFFFFFFFFFF
@A00155:342:HHGFNDSXY:1:1449:13304:36213 2:N:0:GAACCTAG+TCCGCATA
ACTACGACTTACACGGTAGGTCGTGAACAAGCCCGGAGTACATCTCCTTTGGAACCAGGAGACCCCTC
CGGTCTTGTCCAACCTCTGTTTCGGCAGAGGATACCGTGCTCCACAGCTGTAAGGCTGTGGGCCCGAG
+
FFFFFFFFFFFFFFFFFFFFFFFFFF:FFFFFFFFFFFFFFFFFFFFFFFFFFFFFFFFFFFFFFFFF
FFFFFFFFFFFFFFFFFFFFFFFFFFFFFFFFFFFFFFFFFFFFFFFFFFFFFFFFFFFFFFFFFFFF
@A00155:342:HHGFNDSXY:1:1450:7690:11553 2:N:0:GAACCTAG+TCCGCATA
TCTTGTCCAACCTCTGTTTCGGCAGAGGATACCGTGCTCCACAGCTGTAAGGCTGTGGGCCCGAGAGA
AGTCGCTCTCTCTCGCCCTCCACATCCCTCTCGGCATTGAGATGACCATAGGCCAGAGGAGAGTCCTT
+
FFFFFFFFFFFFFFFFFFFFFFFFFFFFFFFFFFFFFFFFFFFFFFFFF:FFFFFFFFFFFFFFFFFF
FFFFFFFFFFFFFFFFFFFFFFFFFFFFFFFFFFFFFFFFFFFFFFFFFFFFFFFFFFFFFFFFF:FF
@A00155:342:HHGFNDSXY:1:1450:8070:12117 2:N:0:GAACCTAG+TCCGCATA
TCTTGTCCAACCTCTGTTTCGGCAGAGGATACCGTGCTCCACAGCTGTAAGGCTGTGGGCCCGAGAGA
AGTCGCTCTCTCTCGCCCTCCACATCCCTCTCGGCATTGAGATGACCATAGGCCAGAGGAGAGTCCTT
+
FF,FFFFFFFFFFFFFFFFFFFFFFFF:FFFFFFFFFFFFFFFFFFFFFFFFFFFFF,FFFFFFF,F:
FFFFFFFFFFFFFFFFFFFFFFFFFFFFFFFFFFFFFFFFFFFFFFFFFFFFFFFFFFFFFF:FF,F:
@A00155:342:HHGFNDSXY:1:1450:12409:16376 2:N:0:GAACCTAG+TCCGCATA
TCTCCTTTGGAACTAGGAGACCCCTCCGGTCTTGTCCAACCTCTGTTTCGGCAGAGGATACCGTGCTC
CACAGCTGTAAGGCTGTGGGCCCGAGAGAAGTCGCTCTCTCTCGCCCTCCACATCCCTCTCGGCATTG
+
FFFFFF,FFFFFFFFFFFFFFFFFFFFFFF:FFFFFFFFFFFFFFFFFFFFF:FFFFFFFFFFFFF:F
FFFFFFFFFFFFFFFFFFFFFFFFFFFFFFFFFFFFFFFFFFFFFFFFFFFFFFFFFFFFFFFFFFFF
```

```
@A00155:342:HHGFNDSXY:1:1450:9100:16783 2:N:0:GAACCTAG+TCCGCATA
GTTGTGTAAGTTTCGTCCTAAAAAGGACTCGTCAGGTAGCCTACTAGACTACGACTTACACGGTAGGT
CGTGAACAAGCCCGGAGTACATCTCCTTTGGAACTAGGAGACCCCTCCGGTCTTGTCCAACCTCTGTT
+
FFFFFFFFFFFFFFFFFFFFFFFFFFFFFFFFFFFFFFFFFFFFFFFFFFFFFFFFFFFFFFFF:FFF
FFFFFFFFFFFFFFF,FFFFFFFFFFFFFFFFFFFFFFFFFFFFF:FFFFFFFFFFFFFFFFFFFFFF
@A00155:342:HHGFNDSXY:1:1450:5312:17362 2:N:0:GAACCTAG+TCCGCATA
CGAAACCGTCGTTACTCTTTGAGTTGTGTAAGTTTCGTCCTAAAAAGGACTCGTCAGGTAGCCTACTA
GACTACGACTTACACGGTAGGTCGTGAACAAGCCCGGAGTACATCTCCTTTGGAACTAGGAGACCCC
+
FFFFFFFFFFFFFFFFFFFFFFFFFFFF:F:FFFFFFFFFFF:FFF:FFFFFFFFFFFFFFFFFFFFF
FFFFFFFFFFFFFF,FFFFFFFF,FF:FFFFFFFFFFFFFFFFFFFFFF:FFFFFF,FFFFFFFFFF
@A00155:342:HHGFNDSXY:1:1451:10999:4100 2:N:0:GAACCTAG+TCCGCATA
CCTACTAGACTACGACTTACACGGTAGGTCGTGAACAAGCCCGGAGTACATCTCCTTTGGAACTAGGA
GACCCCTCCGGTCTTGTCCAACCTCTGTTTCGGCAGAGGATACCGTGCTCCACAGCTGTAAGGCTGTG
+
FF:FFFFFFFFFFFFFFFFFFFFFFFFFFFFFFFFFFFFFFFFFFFFFFFFFFFFFFFFFFFFFFFFF
FFFFFFFFFFFFFFFFFFFFFFFFFFFFFFFFFFFFFFFFFFFFFFFFFFFFFFFFFFFFFFFFFFFF
@A00155:342:HHGFNDSXY:1:1451:11812:5040 2:N:0:GAACCTAG+TCCGCATA
ACACAACTCGAAGAGTAACGACGGTTTCGTCCTTGGAACGGACTCATCAGGGAGCCTACTAGACTCCG
ACCGTCATGGGGGTTAAAACCCATGCCCCGCCACACCGCCTTAGATCAGCTGTGTCCACTTAAGGACT
+
FFFFFFFFFFFFFFFFFFFFFFFFFFFFFFFFFFFFFFFFFFFFFFFFFFFFFFFFFFFFFFFFFFFF
FFFFF:FFFFFFF,FFFFFFFFFFFFFFF:FFFFFFFF:FFFFFFFFF::FFFFFFFFFF,FFFFFF:
@A00155:342:HHGFNDSXY:1:1451:4390:11318 2:N:0:GAACCTAG+TCCGCATA
TTTGGAACTAGGAGACCCCTCCGGTCTTGTCCAACCTCTGTTTCGGCAGAGGATACCGTGCTCCACAG
CTGTAAGGCTGTGGGCCCGAGAGAAGTCGCTCTCTCTCGCCCTCCACATCCCTCTCGGCATTGAGATG
+
FFFFFFFFFFFFFFFFFFFFFFFFFFFFFFFFFFFFFFFF:FFFFFFFFFFFFF:FFFFFFFFFFFFF
FFFFFFFFFFFFFFFFFFFFFFFFFFFFFFFFFFFFFFFFFFFFFFFFFFFFFFFF:FFFFFFFFFFF
@A00155:342:HHGFNDSXY:1:1451:16179:17331 2:N:0:GAACCTAG+TCCGCATA
GGTAGCCTACTAGACTACGACTTACACGGTAGGTCGTGAACAAGCCCGGAGTACATCTCCTTTGGAAC
TAGGAGACCCCTCCGGTCTTGTCCAACCTCTGTTTCGGCAGAGGATACCGTGCTCCACAGCTGTAAGG
+
FFFFFFFFFFFFFFFFFFFFFFFFFFFFFFFFFFFFFFFFFFFFFFFFFFFFFFFFFFFFFFFFFFFF
FFFFFFFFFFFFFFFFFFFFFFFFFF:FFFFFFFFFFFFFFFFFFFFFFFFFFFFFFFFFFFFFFFFF
@A00155:342:HHGFNDSXY:1:1451:11840:29418 2:N:0:GAACCTAG+TCCGCATA
ACTTACACGGTAGGTCGTGAACAAGCCCGGAGTACATCTCCTTTGGAACTAGGAGACCCCTCCGGTCT
TGTCCAACCTCTGTTTCGGCAGAGGATACCGTGCTCCACAGCTGTAAGGCTGTGGGCCCGAGAGAAG
+
FFFFFFFFFFFFFFFFFFFFFFFF,FFFFFFFFFFFFFFFF,FFF:FFF,FFFFFFFFFFFFFFFF:F
FFFFFFFFF,FFFFF:FF:F:FFFFFFFFFFFFFFFFFFFFF:FF:FFFFF:FFFFFFFFF:FFF,F
@A00155:342:HHGFNDSXY:1:1451:18213:31657 2:N:0:GAACCTAG+TCCGCATA
CCGGAGTACATCTCCTTTGGAACTAGGAGACCCCTCCGGTCTTGTCCAACCTCTGTTTCGGCAGAGGA
TACCGTGCTCCACAGCTGTAAGGCTGTGGGCCCGAGAGAAGTCGCTCTCTCTCGCCCCCCAC
+
FFFFFFFFFFFFFFFFFFFFFFFFFFFFFFFFFFFFFFFFF,FFFFFF:FFFFFFFFFF:FFFFFFFF
FFFFFFFFFFFFFFFFFFFFFFFFFF:FFFFFFFFFFF:FFFFFFFFFFFFFFFFFFFFFFF
@A00155:342:HHGFNDSXY:1:1451:23647:35149 2:N:0:GAACCTAG+TCCGCATA
GGAGTACATCTCCTTTGGAACCAGGAGACCCCTCCGGTCTTGTCCAACCTCTGTTTCGGCAGAGGATA
CCGTGCTCCACAGCTGTAAGGCTGTGGGCCCGAGAGAAGTCGCTCTCTCTCGCCCTCCACATCCCTCT
+
FFFFFFFFFFFFFFFFFFFFFFFFFFFFFFFFFFFFFFFFFFFFFFFFFFFFFFFFFFFFFFFFFFFF
FFFFFFFFFFFFFFFFFFFFFFFFFFFFFFFFFFFFFFFFFFFFFFFFFFFFFFFFFFFFFFFFFFFF
```

```
@A00155:342:HHGFNDSXY:1:1452:30418:1031 2:N:0:GAACCTAG+TCCGCATA
GTACATCTCCTTTGGAACTAGGAGACCCCTCCGGTCTTGTCCAACCTCTGTTTCGGCAGAGGATACCG
TGCTCCACAGCTGTAAGGCTGTGGGCCCGAGAGAAGTCGCTCTCTCTCGCCCTCCACATCCCTCTCG
+
FFFFFFFFFFFFFFFFFFFFFFFFFFFFFFFFFFFFFFFFFFFFFFFFFFFFFFFFFFFFFFFFFFFF
FFFFFFFFFFFFFFFFFFFFFFFFFFFFFFFFFFFFFFFFFFFFFFFFFFFFFFFFFFFFFFFFFFF
@A00155:342:HHGFNDSXY:1:1452:28727:10097 2:N:0:GAACCTAG+TCCGCATA
GGACTCGTCAGGTAGCCTACTAGACTACGACTTACACGGTAGGTCGTGAACAAGCCCGGAGTACATCT
CCTTTGGAACCAGGAGACCCCTCCGGTCTTGTCCAACCTCTGTTTCGGCAGAGGATACCGTGCTCCAC
+
FFFFFFFFFFFFFFFFFFFFF,FFFF:FFFFFFFFFFFFFFFFFFFFFFFFFFFFFFFFFFFFFFFFF
FFFFFFFFFFFFFFFF:F:FFFF:FFFFFFFFFFF:FFFFFFFFFFFFF:FFFF:FFFFFFFFF:FFF
@A00155:342:HHGFNDSXY:1:1453:15176:11271 2:N:0:GAACCTAG+TCCGCATA
GGAGTACATCTCCTTTGGAACTAGGAGACCCCTCCGGTCTTGTCCAACCTCTGTTTCGGCAGAGGATA
CCGTGCTCCACAGCTGTAAGGCTGTGGGCCCGAGAGAAGTCGCTCTCTCTCGCCCTCCACATCCC
+
FFFFFFFFFFFFFFFFFFFFFFFFFFFFFFFFFFFFFFFFFFFFFFFFFFFFFFFFFFFFFFFFFF,F
FFFFFFFFFFFFFFFFFFFFFFF:FFFFFFFF:FFFFFFFFFFFFFFFFFFFFFFFFFFFFFFFFF
@A00155:342:HHGFNDSXY:1:1453:17571:12164 2:N:0:GAACCTAG+TCCGCATA
TAGCCTACTAGACTACGACTTACACGGTAGGTCGTGAACAAGCCCGGAGTACATCTCCTTTGGAACTA
GGAGACCCCTCCGGTCTTGTCCAACCTCTGTTTCGGCAGAGGATACCGTGCTCCACAGCTGTAAGGCT
+
FFFFFFFFFFFFFFFFFFFFFFFFFFFFFFFFFFFFFFFFFFFFFFFFFFFFFFFFFFFFFFFFFFFF
FFFFFFFFFFFFFFFFFFFFFFFFFFFFFFFFF:FFFFFFFFFFFFFFFFFFFFFFFFFFFFFFFFFF
@A00155:342:HHGFNDSXY:1:1454:11695:1172 2:N:0:GAACCTAG+TCCGCATA
CGGCAGAGGATACCGTGCTCCACAGCTGTAAGGCTGTGGGCCCGAGAGAAGTCGCTCTCTCTCGCCCT
TCACATCCCTCTCGGCATTGAGATGACCATAGGCCAGAGGTGAGTCCTTAAGTGGACACAGCTGATCT
+
F,::FFF:F,FFFFFFF:FF:FF:F:FFFFFF,FFFFF,FF:FFFF,FF:FFF,FFFF:FFFF:FFFF
FFFFFFFFFFFFFFF:F:FFFFFFFFFFF:FFFFF:FFFFFFFFFFFF:FF,FFFFF,FFFFFFFFFF
@A00155:342:HHGFNDSXY:1:1454:10926:34256 2:N:0:GAACCTAG+TCCGCATA
TAGACTACGACTTACACGGTAGGTCGTGAACAAGCCCGGAGTACATCTCCTTTGGAACTAGGAGACCC
CTCCGGTCTTGTCCAACCTCTGTTTCGGCAGAGGATACCGTGCTCCACAGCTGTAAGGCTGTGGGCCC
+
FFFFFFFFFFFFFFFFFFFFFFFFFFFFFFFFFFFFFFFFFFFFFFFFFFFFFFFFFFFFFFFFFFFF
FFFFFFFFFFFFFFFFFFFFFFFFFFFFFFFFFFFFFFFFFFFFFFFFFFFFFFF,FFFFFFFFFFFF
@A00155:342:HHGFNDSXY:1:1454:28122:36104 2:N:0:GAACCTAG+TCCGCATA
AAAAGGACTCGTCAGGTAGCCTACTAGACTACGACTTACACGGTAGGTCGTGAACAAGCCCGGAGTAC
ATCTCCTTTGGAACTAGGAGACCCCTCCGGTCTTGTCCAACCTCTGTTTCGGCAGAGGATACCGTGCT
+
FFFFFFFFFFFFFFFFFFFFFFFFFFFFFFFFFFFFFFFFFFFFFFFFFFF:FFFFFFFFFFFFFFFF
FFFFFF:FFFFFFFFFFFFFFFFFFFFFFFFFFFFFFFFFFFFFFFFFFFFFFFFFF:FFFF:FFFFF
@A00155:342:HHGFNDSXY:1:1455:15691:5494 2:N:0:GAACCTAG+TCCGCATA
GCTCCCTGATGAGTCCGTTCCAAGGACGAAACCGTCGTTACTCTTCGAGTTGTGTAAGTTTCGTCCTA
AAAAGGACTCGTCAGGTAGCCTACTAGACTACGACTTACACGGTAGGTCGTGAACAAGCCCGGAGTAC
+
FFFFFFFFFFFFFFFFFF:FFFFFFFFFFFFFFFFFF,FFFFFFFFF:FFFFFFFFFFFFFFFFFF::
FFF:FFFFFFFFFFFFFFFFFFFFFFFFFFFFFFFFFF:FFFFFFFFFFFFFFF:FFFFFFFFFFF,FF
@A00155:342:HHGFNDSXY:1:1455:15808:5603 2:N:0:GAACCTAG+TCCGCATA
GCTCCCTGATGAGTCCGTTCCAAGGACGAAACCGTCGTTACTCTTCGAGTTGTGTAAGTTTCGTCCTA
AAAAGGACTCGTCAGGTAGCCTACTAGACTACGACTTACACGGTAGGTCGTGAACAAGCCCGGAGTAC
+
FFFFFFFFFFFFFFFFFFFFFFFFFFFFFFFFFFFFFFFFFFFFFFFFFFFFFFF,FFF,FFFFFFFF
FFFFFFFFFFFFFFFFFFFFFFFFF:FFFF,FFFFFFFFFFFFF,FFFFFFFFFFFFFFFFF:FFFFF
```

```
@A00155:342:HHGFNDSXY:1:1455:28592:7858 2:N:0:GAACCTAG+TCCGCATA
TAGCCTACTAGACTACGACTTACACGGTAGGTCGTGAACAAGCCCGGAGTACATCTCCTTTGGAACCA
GGAGACCCCTCCGGTCTTGTCCAACCTCTGTTTCGGCAGAGGATACCGTGCTCCACAGCTGTAAGGC
+
FFFFFFFFFFFFFFFFFFFFFFFFFFFFFFFFFFFFFFFFFFFFFFFFFFFFFFFFFFFFFFFFFFFF
FFFFFFFFFFFFFFFFFFFFFFFFFFFFFFFFFFFFFFFFFFFFFFFFFFFFF:FFFFFFFFFFFFF
@A00155:342:HHGFNDSXY:1:1455:14705:25144 2:N:0:GAACCTAG+TCCGCATA
CACGGTAGGTCGTGAACAAGCCCGGAGTACATCTCCTTTGGAACTAGGAGACCCCTCCGGTCTTGTCC
AACCTCTGTTTCGGCAGAGGATACCGTGCTCCACAGCTGTAAGGCTGTGGGCCCGAGAGAAGTCGCTC
+
FFFFFFFFFFFFFFFFFFFFFFFFFFFFFFFFFFFFFFFFFFFF:FFFFFFFFFFFFFFFFF,FFFFF
FFFFFFFFFFFFFFFFFFFFF:FFFFFFFFFFFFFFFFFFFFFFFFFFFFFFFFFFFFFFFFFFFFFF
@A00155:342:HHGFNDSXY:1:1455:10339:26819 2:N:0:GAACCTAG+TCCGCATA
TAGGAGACCCCTCCGGTCTTGTCCAACCTCTGTTTCGGCAGAGGATACCGTGCTCCACAGCTGTAAGG
CTGTGGGCCCGAGAGAAGTCGCTCTCTCTCGCCCTCCACATCCCTCTCGGCATTGAGATGACCATAGG
+
FFFFFFFFFFFFFFFFFFFFFFFFFFFFFFFFFFFFFFFFFFFFFFFFFFFFFFFFFFFFFFFFFFFF
FFFFFFFFFFFFFFFFFFFFFFFFFFFFFFFFFFFFFFFFFFFFFFFFFFFFFFFFFFFFFFFFFFFF
@A00155:342:HHGFNDSXY:1:1455:23267:33927 2:N:0:GAACCTAG+TCCGCATA
GGACTCGTCAGGTAGCCTACTAGACTACGACTTACACGGTAGGTCGTGAACAAGCCCGGAGTACATCT
CCTTTGGAACTAGGAGACCCCTCCGGTCTTGTCCAACCTCTGTTTCGGCAGAGGATACCGTGCTCCAC
+
FFFFFFFFFFFFFFFFFFFFFFFFFFFFFFFFFFFFFFFFFFFFFFFFFFFFFFFFFFFFFFFFFFFF
FFFFFFFFFFFFFFFFFFFFFFFFFFFFF:FFFFFFFFFFFFFFFFFFFFFFFFFFFFFFFFFFFFFF
@A00155:342:HHGFNDSXY:1:1456:10601:14465 2:N:0:GAACCTAG+TCCGCATA
ACGACTTACACGGTAGGTCGTGAACAAGCCCGGAGTACATCTCCTTTGGAACTAGGAGACCCCTCCGG
TCTTGTCCAACCTCTGTTTCGGCAGAGGATACCGTGCTCCACAGCAGTAAGGCTGTGGGCCCGAGAGA
+
FFFFFFFFFFFFFFFFFFFFFFFFFFFFFFFFFFFFFFFFFFFFFFFFFFFFFFFFFFFFFFFFFFFF
FFFFFFFFFFFFFFFFFFFFFFFFFFFFFFFFFFFFFFFFFFFFFFFFFFFFFFFFFFFFFFFFFFFF
@A00155:342:HHGFNDSXY:1:1457:19922:8155 2:N:0:GAACCTAG+TCCGCATA
GAACTAGGAGACCCCTCCGGTCTTGTCCAACCTCTGTTTCGGCAGAGGATACCGTGCTCCACAGCTGT
AAGGCTGTGGGCCCGAGAGAAGTCGCTCTCTCTCGCCCTCTACATCCCTCTCGGCATTGAGATGACCA
+
FFFFFFFFFFFFFFFFFFFFFFFFFFFFFFFFFFFFFFFFFFFFFFFFFFFFFFFFFFFFFFFFFFFF
FFFFFFFFFFFFFFFFFFFFFFFFFFFFFFFFFFFFFFFFFFFFFFFFFFFFFFFF:FFFFFFFFFFF
@A00155:342:HHGFNDSXY:1:1457:27796:25927 2:N:0:GAACCTAG+TCCGCATA
ACCTCTGTTTCGGCAGAGGATACCGTGCTCCACAGCTGTAAGGCTGTGGGCCCGAGAGAAGTCGCTCT
CTCTCGCCCTTCACATCCCTCTCGGCATTGAGATGACCATAGGCCAGAGGTGAGTCCTTAAGTGGAC
+
FFFFFFFFFFFFFFFFFFFFFFFFFFFFFFFFFFFFFFFFFFFFFFFFFFFFFFFFFFFFFFFFFFF:
FFFFFFFFFFFFFFFFFFFFFFFFFFF:FFFFFFFFFFFFFFFF::FFFFFFFFFFFFFFFFFFFFF
@A00155:342:HHGFNDSXY:1:1458:1289:14215 2:N:0:GAACCTAG+TCCGCATA
ACTTACACGGTAGGTCGTGAACAAGCCCGGAGTACATCTCCTTTGGAACTAGGAGACCCCTCCGGTCT
TGTCCAACCTCTGTTTCGGCAGAGGATACCGTGCTCCACAGCTGTAAGGCTGTGGGCCCGAGAGAAGT
+
FFFFFFFFFF:FFFFFFFFFFFFFFFFFFFFFFFFFFFFFFF:FFFFFF:FFFFFFFFFFFFFFFFFF
FFFFFFFFFFFFFFF:FFFFFFFFFFFFFFFFFFFFFFFFFFFFFFFFFF:FFFFFFFFFFFFF::FF
@A00155:342:HHGFNDSXY:1:1458:10637:28087 2:N:0:GAACCTAG+TCCGCATA
ACACGGTAGGTCGTGAACAAGCCCGGAGTACATCTCCTTTGGAACTAGGAGACCCCTCCGGTCTTGTC
CAACCTCTGTTTCGGCAGAGGATACCGTGCTCCACAGCTGTAAGGCTGTGGGCCCGAGAGAAGTCGCT
+
FFFFFFFFFFFFFFFFFFFFFFFFFFFFFFFFFFFFFFFFFFFFFFFFFFFFFFFFFFFFFFFF:FFF
FFFFFFFFFFFFFFFFFFFFFFFFFFFFFFFFFFFFFFFFFFFFFFFFFFFFFFFFFFFFFFFFFFFF
```

@A00155:342:HHGFNDSXY:1:1459:3613:13823 2:N:0:GAACCTAG+TCCGCATA
GTTGTGTAAGTTTCGTCCTAAAAAGGACTCGTCAGGTAGCCTACTAGACTACGACTTACACGGTAGGT
CGTGAACAAGCCCGGAGTACATCTCCTTTGGAACCAGGAGACCCCTCCGGTCTTGTCCAACCTCTGTT
+
FFFFFFFF:FFFFFFFFFFFFFFFFFFFFFFFFFFFFFFFFFFFFFFFFFFFFFFFFFFFFFFFFFF
FFFFFFFFFFFFFFFFFFFFFFFFFFFF:FFFFFFFFFF,FF:FFFFFFFFFFFFFFFFFFFFFFFFF
@A00155:342:HHGFNDSXY:1:1459:6813:15327 2:N:0:GAACCTAG+TCCGCATA
TAGGTCGTGAACAAGCCCGGAGTACATCTCCTTTGGAACTAGGAGACCCCTCCGGTCTTGTCCAACCT
CTGTTTCGGCAGAGGATACCGTGCTCCACAGCTGTAAGGCTGTGGGCCCGAGAGAAGTCGCTCTCTCT
+
FFFFFFFFFFFFFFFFFFFFFFFFFFFFFF:FFFFFFFFFFFFFFFFFFFFFFFFFFFFFFFFFFFFF
FFFFFFFF:FFFFFFFFFFFFFFFFFFFFF:FFF:FFFFFFFFFFFFFFFFFFFFFFFFFFFFFFFFF
@A00155:342:HHGFNDSXY:1:1459:16857:26616 2:N:0:GAACCTAG+TCCGCATA
GGAGTACATCTCCTTTGGAACTAGGAGACCCCTCCGGTCTTGTCCAACCTCTGTTTCGGCAGAGGATA
CCGTGCTCCACAGCTGTAAGGCTGTGGGCCCGAGAGAAGTCGCTCTCTCTCGCCCTCCATATCCCTCT
+
FF,FFFFFFFFFFFFFFFFFFFFFFF:FFFFFFFFFFFFF:FFFF:FFFFFFFFFFFFFFFFFFFFFF
FFFFFFFFFFF:FFFFFFFFFFFFFFFFFFFFFFFFF:FFFFF,FFFFFFFFFFFFFFFFFFFFFFFF
@A00155:342:HHGFNDSXY:1:1459:7545:28839 2:N:0:GAACCTAG+TCCGCATA
CGGTAGGTCGTGAACAAGCCCGGAGTACATCTCCTTTGGAACCAGGAGACCCCTCCGGTCTTGTCCAA
CCTCTGTTTCGGCAGAGGATACCGTGCTCCACAGCTGTAAGGCTGTGGGCCCGAGAGAAGTCGCTCTC
+
FFF:FFFFF:::FFFFFFFFFF:F,:FFF:FFF:,FFFFFFFFFFFFFFFFFFF:FFFFFF:FFFFFF
FFFF,FFFFFFFFFFFFFFFF,F:::FFFFFFFFFFFFFFFFFFF:FFFFF,FFFFFFF,F,FFFFFF
@A00155:342:HHGFNDSXY:1:1459:12391:28902 2:N:0:GAACCTAG+TCCGCATA
GGAGTACATCTCCTTTGGAACTAGGAGACCCCTCCGGTCTTGTCCAACCTCTGTTTCGGCAGAGGATA
CCGTGCTCCACAGCTGTAAGGCTGTGGGCCCGAGAGAAGTCGCTCTCTCTCGCCCTCCACATCCCTCT
+
FFFFFFFFFFFFFFFFFFFFFFFFFFFFFFFFFFFFFFF:FFFFFFFFFFFFFFFFFFFFFFFFFFFF
FFFFFFFFFFFFFFFFFFFFFFFFFFFFFFFFFFFFFFFFFFFFFFFFFFFFFFFFFFFFFFFFFFF:
@A00155:342:HHGFNDSXY:1:1459:5918:31626 2:N:0:GAACCTAG+TCCGCATA
AGAGGATACCGTGCTCCACAGCTGTAAGGCTGTGGGCCCGAGAGAAGTCGCTCTCTCTCGCCCTCCAC
ATCCCTCTCGGCATTGAGATGACCATAGGCCAGAGGTGAGTCCTTAAGTGGACACAGCTGATCTAAGG
+
FFFFFFFFFFFFFFFFFFFFFFFFFFFFFF:FFFFFFFFFFFFFFFFFFFFFFFFFFFFFFFFFFFFF
FFFFFFFFFFFFFFFFFFFFFFFFFFFFFFFFFFFFFFFFFFFFFFFFFFFF:FFFFFFFFFFFFFFF
@A00155:342:HHGFNDSXY:1:1459:29903:35117 2:N:0:GAACCTAG+TCCGCATA
CTTGTCCAACCTCTGTTTCGGCAGAGGATACCGTGCTCCACAGCTGTAAGGCTGTGGGCCCGAGAGAA
GTCGCTCTCTCTCGCCCTTCACATCCCTCTCGGCATTGAGATGACCATAGGCCAGAGGTGAGTCCTTA
+
FFFFFFFFFFFFFFFFFFFFFFFFFFFFFFFFFFFFFFFFFFFFFFFFFFFFFFFFFFFFFF:FFFFF
FFFFFFFFFFFFFFFFFFFFFFFFFFFFFFFFFFFF,FFFFFFFFFFFFFFFFFFFFFFFFFFFFFFF
@A00155:342:HHGFNDSXY:1:1460:20645:32800 2:N:0:GAACCTAG+TCCGCATA
TCGTGAACAAGCCCGGAGTACATCTCCTTTGGAACTAGGAGACCCCTCCGGTCTTGTCCAACCTCTGT
TTCGGCAGAGGATACCGTGCTCCACAGCTGTAAGGCTGTGGGCCCGAGAGAAGTCGCTCTCTCTCGCC
+
FFFFFFFFFFFFFFFFFFFFFFFFFFFFFFFFFFF:FFFFFFFFFFFFFFF,FFFFFFFFFFFFFF:F
FFFFFFFFFFFFFFFFFFFFFFFFFFFFFFFFFFFFFFFFFFFFFFF:FFFFFFFFFFFFFFFFFFFF
@A00155:342:HHGFNDSXY:1:1461:1371:11443 2:N:0:GAACCTAG+TCCGCATA
TGTCCACTTAAGGACTCACCTCTGGCCTATGGTCATCTCAATGCCGAGAGGGATGTGAAGGGCGAGAG
AGAGCGACTTCTCTCGGGCCCACAGCCTTACAGCTGTGGAGCACGGTATCCTCTGCCGAAACAGAGGT
+
FFFFFFFFFFFFFFFFFFFFFFFFFFFFFFFFFFFFFFFFFFFFFFFFFFFFFFFFFFFFFFFFFFFF
FFFFFFFFFFFFFFFFFFFFFFFFFFFFF:FFFFFFFFFFFFFFFFFFFFFFFFFFFFFF:,FF:FF:

```
@A00155:342:HHGFNDSXY:1:1461:21070:20760 2:N:0:GAACCTAG+TCCGCATA
GGACTCGTCAGGTAGCCTACTAGACTACGACTTACACGGTAGGTCGTGAACAAGCCCGGAGTACATCT
CCTTTGGAACCAGGAGACCCCTCCGGTCTTGTCCAACCTCTGTTTCGGCAGAGGATACCGTGCTCCAC
+
FFFFFFFFFFFFFFFFFFFFFF:FFFFFFFFFFFFFFFFFFFFFFFFFFFFFFFFFFFFFFFFFFFFF
FFFFFFFF:FFFFFFFFFFFFFFFFFFFFFFFFFFFFFFFFFFFFFFFFFFFFFFFFFFFFFFFFFFF
@A00155:342:HHGFNDSXY:1:1461:28330:31673 2:N:0:GAACCTAG+TCCGCATA
GAGTTGTGTAAGTTTCGTCCTAAAAAGGACTCGTCAGGTAGCCTACTAGACTACGACTTACACGGTAG
GTCGTGAACAAGCCCGGAGTACATCTCCTTTGGAACTAGGAGACCCCTCCGGTCTTGTCCAACCTCTG
+
FFFFFFFFFFFFFFFFFFFFFFFFF:FFFFFFFFFFFFFFFFFFFFFFFFFFF:FFFFFFFFFFFF:F
FFFF:FFFFFF,FFFFFFFFFFFF:FFFFFFFFFFFFFFFF:FFFFFFFFFFFFFFFF:FFF:FFFFF
@A00155:342:HHGFNDSXY:1:1461:31060:31767 2:N:0:GAACCTAG+TCCGCATA
CACGGTAGGTCGTGAACAAGCCCGGAGTACATCTCCTTTGGAACTAGGAGACCCCTCCGGTCTTGTCC
AACCTCTGTTTCGGCAGAGGATACCGTGCTCCACAGCTGTAAGGCTGTGGGCCCGAGAGAAGTCGCTC
+
FFFFFFFFFFFFFFFFFFFFF:FFFFFFFFFF:FFF,FFF:FFFFFF:FFFFFFFFFFFFFFFFFFFF
FFFFFFFFFFFFFFFFFFFFFFFFFFFFFFFFFFFFFFFFFFFFF:FFFFFFFFFFFFFFFFFFFF:F
@A00155:342:HHGFNDSXY:1:1462:5593:3067 2:N:0:GAACCTAG+TCCGCATA
CTAGGAGACCCCTCCGGTCTTGTCCAACCTCTGTTTCGGCAGAGGATACCGTGCTCCACAGCTGTAAG
GCTGTGGGCCCGAGAGAAGTCGCTCTCTCTCGCCCTCCACATCCCTCTCGGCATTGAGATGACCATAG
+
FFFFFFFFFFFFFFFFFFF:FFFFFFFFFFFFFFFFFFFFFFFFFFFFFFFFFFFFFFFFFFFFFFFF:FFFFF
FFFFFFFFFFFFFFFFFFFFFFFFFFFFFF::FFFFFFFFFF:F,FFFFFFFFF:FFFFFFFFFF:FF
@A00155:342:HHGFNDSXY:1:1462:9986:24079 2:N:0:GAACCTAG+TCCGCATA
CGGTCTTGTCCAACCTCTGTTTCGGCAGAGGATACCGTGCTCCACAGCTGTAAGGCTGTGGGCCCGAG
AGAAGTCGCTCTCTCTCGCCCTCCACATCCCTCTCGGCATTGAGATGACCATAGGCCAGAGGAGAGTC
+
F:FFF::FF:FFFFFFFFF,FFFF:FFFF:FFFFFFF,F,:FFFF:FFFFF:FF:FFFFF,:F,F,FF
FFF:FFF,FFF:FFFFFFFF:FFFFFFF:FFFF:FFFFF,:FFFFFFF,FFFFFFF:FF:,FFFFFFF
@A00155:342:HHGFNDSXY:1:1463:5593:1877 2:N:0:GAACCTAG+TCCGCATA
ACTCTTCGAGTTGTGTAAGTTTCGTCCTAAAAAGGACTCGTCAGGTAGCCTACTAGACTACGACTTAC
ACGGTAGGTCGTGAACAAGCCCGGAGTACATCTCCTTTGGAACTAGGAGACCCCTCCGGCCTTGTCC
+
FFFFFFFFFFFFFFFFF:FFFFFFFFFFFFF:FFFFFFFFFF:FFFFFFFFFFFFFFFFFFFFFFFFF
FFFFFFFFFFFFFFFFFFFFFFFFFFFFF,FFFFF,FFFFFFFFFFFFFFFFFFFFFFF,FFFFFFF
@A00155:342:HHGFNDSXY:1:1464:15637:17613 2:N:0:GAACCTAG+TCCGCATA
AAGGACTCACCTCTGGCCTATGGTCATCTCAATGCCGAGAGGGATGTAGAGGGCGAGAGAGAGCGACT
TCTCTCGGGCCCACAGCCTTACAGCTGTGGAGCACGGTATCCTCTGCCGAAACAGAGGTTGGAC
+
FFFFFFFFFFFFFFFFFFFFFFFFFFFFFFFFFFFFFFFFFFFF:FFFFFFFFFFFFFFFFFFFFFFF
FFFFFFFFFFFFFFFFFFFFFFFFFFFFFFFFFFFFFFFFFFFFFFFFFFFFFFFFFFFFFFFF
@A00155:342:HHGFNDSXY:1:1464:25012:22514 2:N:0:GAACCTAG+TCCGCATA
GTTTCGTCCTAAAAAGGACTCGTCAGGTAGCCTACTAGACTACGACTTACACGGTAGGTCGTGAACAA
GCCCGGAGTACATCTCCTTTGGAACTAGGAGACCCCTCCGGTCTTGTCCAACCTCTGTTTCGGCAGAG
+
FFFFFFFFFFFFFFFFFFFFFFFFFFFFFFFFFFFFFFFFFFFFFFFFFFFFFFFFFFFFFFFFFFFF
FFFFFFFF,:FFFFFFFFFFFFF,FF:FFFFFFFFFFFFFF,F,FFFFF:FFFFF,FFFFFFFFFFFF
@A00155:342:HHGFNDSXY:1:1464:7346:33943 2:N:0:GAACCTAG+TCCGCATA
CAAGGACGAAACCGTCGTTACTCTACGAGTTGTGTAAGTTTCGTCCTAAAAAGGACTCGTCAGGTAGC
CTACTAGACTACGACTTACACGGTAGGTCGTGAACAAGCCCGGAGTACATCTCCTTTGGAACTAGGAG
+
FFFFFFFFFFFFFFFFFFFFFFFFFFFFFFFFFFFFFFFFFFFFFFFFFFFFFFFFFFFFFFFFFFF:
FFFFFFFFFFFFFFFFFFFFFFFFFFFFFF:FFFFFFFFFFFFFFFFFFFFFFF:FFFFFFFFFFFF:
```

@A00155:342:HHGFNDSXY:1:1464:28103:36198 2:N:0:GAACCTAG+TCCGCATA
GTACATCTCCTTTGGAACTAGGAGACCCCTCCGGTCTTGTCCAACCTCTGTTTCGGCAGAGGATACCG
TGCTCCACAGCTGTAAGGCTGTGGGCCCGAGAGAAGTCGCTCTCTCTCGCCCTCCACATCCCTCTCGG
+
FFFFFFFFFFFFFFFFFFFFFFFFFFFFFFFFFFFF:FFFFFFFFFFFFFFFFFFFFFFFFFF:FFFF
FFFFFFFFFFFFFFFFFFFFFFFFFFFFFFFFFFFFFFFFFF:FFFFFFFFFFFFFFFFFFFFFFFFF
@A00155:342:HHGFNDSXY:1:1465:25825:11334 2:N:0:GAACCTAG+TCCGCATA
ACGGTAGGTCGTGAACAAGCCCGGAGTACATCTCCTTTGGAACTAGGAGACCCCTCCGGTCTTGTCCA
ACCTCTGTTTCGGCAGAGGATACCGTGCTCCACAGCTGTAAGGCTGTGGGCCCGAGAGAAGTCGCTCT
+
FFFFFFFFFFFFFFFFFFFFFFFFFFFFFFFFFFFFFFFFFFFFFFFFFFF:FFFFFFFFF::FFFFF
FFFFFFFFFFFFFFF:FFFFFFFFFFFFFF:FFFFFFFFFFFFFFFFFFFFFFFFF::FFFFFFFFFF
@A00155:342:HHGFNDSXY:1:1465:14904:12900 2:N:0:GAACCTAG+TCCGCATA
GACTTACACGGTAGGTCGTGAACAAGCCCGGAGTACATCTCCTTTGGAACTAGGAGACCCCTCCGGTC
TTGTCCAACCTCTGTTTCGGCAGAGGATACCGTGCTCCACAGCTGTAAGGCTGTGGGCCCGAGAGAAG
+
FFFFFFFFFFFFFFFFFFFF:FFFFFFFFFFFFFFFFFFFFFFFFFFFFF:FFFFFFFFFFFFFFFFF
,FFFFFFFFFFFFFFF:FFFFFF:FFFFFFFFFFFFFFFFFFFFFFF:FFF:F:FFFFFFFFFFFFFF
@A00155:342:HHGFNDSXY:1:1465:29713:15906 2:N:0:GAACCTAG+TCCGCATA
ACGGTAGGTCGTGAACAAGCCCGGAGTACATCTCCTTTGGAACTAGGAGACCCCTCCGGTCTTGTCCA
ACCTCTGTTTCGGCAGAGGATACCGTGCTCCACAGCTGTAAGGCTGTGGGCCCGAGAGAAGTCGCTC
+
FFFFFFFFFFFFFFFFF:,FFFFFFFFFFFFFFFFFFFFFFFFFFFFFFFFFFFFFFFFF:FFFFFFF
FFFF:FF:FFFFF::FFFFFFFFFFFFFFFFFFFFFFFF,FF:F:FFFFFFF:FFFFFFFFFFFFFF
@A00155:342:HHGFNDSXY:1:1465:7473:21136 2:N:0:GAACCTAG+TCCGCATA
GTTGTGTAAGTTTCGTCCTAAAAAGGACTCGTCAGGTAGCCTACTAGACTACGACTTACACGGTAGGT
CGTGAACAAGCCCGGAGTACATCTCCTTTGGAACCAGGAGACCCCTCCGGTCTTGTCCAACCTCTGTT
+
FFFFFFF,FFFFFFFFFFF:FFFFFFFFF,FFFFFFFFF:FFFFFFFFFFFFFFFFFFFFFFFFFF:F
FFFFFFFFFFFFFFFFFFFFFFFFFFFF::FFFFFFFFFFFFFFFF,FFFFF:FFFFF:FFFFFF:FF
@A00155:342:HHGFNDSXY:1:1465:30364:21731 2:N:0:GAACCTAG+TCCGCATA
TAGGAGACCCCTCCGGTCTTGTCCAACCTCTGTTTCGGCAGAGGATACCGTGCTCCACAGCTGTAAGG
CTGTGGGCCCGAGAGAAGTCGCTCTCTCTCGCCCTCCACATCCCTCTCGGCATTGAGATGACCATAGG
+
FFFFFFFFFFFFFFFFFFFFFFFFFFFFFFFFFFFFFFFFFFFFFFFFFFFFFFFFFFFFFFFFFFFF
FFFFFFFFFFFFFFFFFFFFFFFFFFFFFFFFFFFFFFFFFFFFFFFFFFFF:FFFFFFFFF:FFFFF
@A00155:342:HHGFNDSXY:1:1465:3721:32769 2:N:0:GAACCTAG+TCCGCATA
TACTCTTCGAGTTGTGTAAGTTTCGTCCTAAAAAGGACTCGTCAGGTAGCCTACTAGACTACGACTTA
CACGGTAGGTCGTGAACAAGCCCGGAGTACATCTCCTTTGGAACTAGGAGACCCCTCCGGTCTTGTCC
+
FFFFFFFFFFFFFFFFFFFFF:FFFFFFFFFFFFFFFFFFFFFFFFF:FFFFFFFFFFFFFFFFFFFF
FFFFFFFFFFFFFF:FFFFFFFFFFFFF:FFFFFFF:F:FFFFFFFFFFFFFFFFFFFFFFFFFFFFF
@A00155:342:HHGFNDSXY:1:1466:3043:3286 2:N:0:GAACCTAG+TCCGCATA
ACGGTAGGTCGTGAACAAGCCCGGAGTACATCTCTTTTGGAACTAGGAGACCCCTCCGGTCTTGTCCA
ACCTCTGTTTCGGCAGAGGATACCGTGCTCCACAGCTGTAAGGCTGTGGGCCCGAGAGAAGTCGCTCT
+
FFFFFFFFFFFFFFFFFFFFFFFFFFFFFFFFFFFFFFFFFFFFFFFFFFFFFFFFFFFFF:FFFFFF
FFFFFFFFFFFFFFFFFFFFFFFFFFFFFFFFFFFF:FFFFFFFFFFFFFFFFFFFFFFFFFFFFFFF
@A00155:342:HHGFNDSXY:1:1466:28565:6151 2:N:0:GAACCTAG+TCCGCATA
TTCGTCCTTGGAACGGACTCATCAGGGAGCCTACTAGACTCCGACCGTCATGGGGGTTCAAACCCATG
CCCCACCACACCGCCTTAGATCAGCTGTGTCCACTTAAGGACTCACCTCTGGCCTATGGTCATCTCAA
+
:FFFFFFFFFFF:FFFFFFFF:FFFFFFFF:FFF:FFFFFF:FFFFFF:FFF:FFFFFFFFFFFFFF:
FFFFFFFFFFFFFFF,FFFFFFFFFFFFFFFFFF,FFFFFFFFFFFFFFFFFFFFFFFFFFFFFFFFF

```
@A00155:342:HHGFNDSXY:1:1466:28854:6214 2:N:0:GAACCTAG+TCCGCATA
TTCGTCCTTGGAACGGACTCATCAGGGAGCCTACTAGACTCCGACCGTCATGGGGGTTCAAACCCATG
CCCCACCACACCGCCTTAGATCAGCTGTGTCCACTTAAGGACTCACCTCTGGCCTATGGTCATCTCAA
+
FFFFFFFFFFFFFFFFFFFFFFFFFFFFFFFFFFFFFFFFFFFFFFFFFFFFFFFFFFFFFFFFFFFF
FFFFFFFFFFFFFFFFFFFFFFFFFFFFFFFFFFFFFFFFFFFFFFFFFFFFFFF:FFFFFFFFFFFF
@A00155:342:HHGFNDSXY:1:1466:17897:31422 2:N:0:GAACCTAG+TCCGCATA
CAAGGACGAAACCGTCGTTACTCTTCGAGTTGTGTAAGTTTCGTCCTAAAAAGGACTCGTCAGGTAGC
CTACTAGACTACGACTTACACGGTAGGTCGTGAACAAGCCCGGAGTACATCTCCTTTGGAACTAGGAG
+
FFFFFFFFFFFFFF:FFFFFFFF,FFFFFFFFFFFF:FF,FFFFFFFF,::FFFFFFFFFFFFFFFFF
FFFFFFFFFF:FF,FFFFFFFFF:FFFFFFFFFFFFFFFFFFFFFFFFFFFFFFFFFFFFFFFFFFFF
@A00155:342:HHGFNDSXY:1:1468:31566:5776 2:N:0:GAACCTAG+TCCGCATA
GTGAACAAGCCCGGAGTACATCTCCTTTGGAACCAGGAGACCCCTCCGGTCTTGTCCAACCTCTGTTT
CGGCAGAGGATACCGTGCTCCACAGCTGTAAGGCTGTGGGCCCGAGAGAAGTCGCTCTCTCTCGCCCT
+
FFFFFFFFFFFFFFFFFFF:FFFFFFFFFFF,FFFFFFFFFFFFFFFFFFFFFFFFFFFFFFFFFFFF
FFFFFFFFFFFFFFFFFFFFFFFFFFFFFF:FFF:FFFFFFFFFFFFFFFFFFFFFFFFFFFFFFFFF
@A00155:342:HHGFNDSXY:1:1468:27398:13213 2:N:0:GAACCTAG+TCCGCATA
ATCTCCTTTGGAACCAGGAGACCCCTCCGGTCTTGTCCAACCTCTGTTTCGGCAGAGGATACCGTGCT
CCACAGCTGTAAGGCTGTGGGCCCGAGAGAAGTCGCTCTCTCTCGCCCTCCACATCCCTCTCGGCA
+
FFFFFFFFFFFFFFFFFFFFFFFFFFFFFFFFFFF:FF:FFFFFFFFFFFFFFFFFFFFFFFFFFFFF
FFFFFFFFFFFFFFFFFFFFFFFFFFFFFFFFFFFF:FFFFFFFFFFFFFFFFFFFFFFFFFFFFF
@A00155:342:HHGFNDSXY:1:1468:30888:15405 2:N:0:GAACCTAG+TCCGCATA
TACATCTCCTTTGGAACTAGGAGACCCCTCCGGTCTTGTCCAACCTCTGTTTCGGCAGAGGATACCGT
GCTCCACAGCTGTAAGGCTGTGGGCCCGAGAGAAGTCGCTCTCTCTCGCCCCCCACATCCCTCTCGGC
+
FFFFFFFFFFF:FFF:FFFFFFFFFFFFFFFFFFFFFF:FFFFFFFFFFFFFFFFFFFFFFFFFFFFF
FFFFF:FFFFFFFFFFFF:FFFFFFFFF:FFFF:FFFFFFFFFFFFFFFFFFFFFFFFFFFFF,FFFF
@A00155:342:HHGFNDSXY:1:1468:30535:24565 2:N:0:GAACCTAG+TCCGCATA
GCCTATGGTCATCTCAATGCCGAGAGGGATGTGAAGGGCGAGAGAGAGCGACTTCTCTCGGGCCCACA
GCCTTACAGCTGTGGAGCACGGTATCCTCTGCCGAAACAGAGGTTGGACAAGACCGGAG
+
FFFFFFFFFFFFFFFFFFFFFF:FFFFFFFFFFFFFFFFFFFFFF:F:FFFFFFFFFFFFFFFFFFFF
FFFFF:FFFFFFFFFFFFFFFFFFFFFFFFFFFFFFFFFFFFFFFFFFFF:FFFFFFFF
@A00155:342:HHGFNDSXY:1:1469:15881:5760 2:N:0:GAACCTAG+TCCGCATA
CTACTAGACTACGACTTACACGGTAGGTCGTGAACAAGCCCGGAGTACATCTCCTTTGGAACTAGGAG
ACCCCTCCGGTCTTGTCCAACCTCTGTTTCGGCAGAGGATACCGTGCTCCACAGCTGTAAGGCTGTGG
+
FFFFFFFFFFFFFFFFFFFFFFFFFFFFFFFFFFFFFFFFFFFFFFFFFFFFFF,FFFFF:FFFFFFF
FFFFFFFFFFFFFFFFFFFFFFFFFFFFFFFFFFFFFFFFFFFFFFFFFFFFFFFFFFFFFFFFFFFF
@A00155:342:HHGFNDSXY:1:1469:17544:8891 2:N:0:GAACCTAG+TCCGCATA
GAAACCGTCGTTACTCTTTGAGTTGTGTAAGTTTCGTCCTAAAAAGGACTCGTCAGGTAGCCTACTAG
ACTACGACTTACACGGTAGGTCGTGAACAAGCCCGGAGTACATCTCCTTTGGAACTAGGAGACCCCTC
+
FFFFFFFFFFFFFFFFFFFFFFFFFFFFFFFFFFFFFFFFFFFFFFFFFFFFFFFFFF:FFFFFFFFF
FFFFFFFF:FFFFFFFFFFFFFFFFFFFFFFFFFFFFFFFFFFFFFFFFFFFFFFFFFFFFFFF:FFF
@A00155:342:HHGFNDSXY:1:1469:32678:12430 2:N:0:GAACCTAG+TCCGCATA
AGGTAGCCTACTAGACTACGACTTACACGGTAGGTCGTGAACAAGCCCGGAGTACATCTCCTTTGGAA
CTAGGAGACCCCTCCGGTCTTGTCCAACCTCTGTTTCGGCAGAGGATACCGTGCTCCACAGCTGTAAG
+
FFFFFFFFFFFFFFFFFFFFFFFFFFFFFFFFFFFFFFFFFFFFFFFFFFFFFFFFFFFFFFFFFFF,
FFFFFFFFF:FFFFFFFFFFFFFFFFFFFFFFFFFFFFFFFFFFFFFFFFFFFFFFFFFFFFFFF,FF
```

```
@A00155:342:HHGFNDSXY:1:1469:19777:28197 2:N:0:GAACCTAG+TCCGCATA
TGTAGAGGGCGAGAGAGAGCGACTTCTCTCGGGCCCACAGCCTTACAGCTGTGGAGCACGGTATCCTC
TGCCGAAACAGAGGTTGGACAAGACCGGAGGGGTCTCCTAGTTCCAAAGGAGATGTACTCCGGG
+
:FFFFFFF:FFFFFFFFFFFFFFFFFFFFFFFF,FFFFF:FFFFFFFFFFFFFFFFFFFFFFFFFF:F
FFF:FFFFFFFFF:FFFFFFFF:FFF:FFFFFFFFFFF:FFFFF:FFF:FFFFFFFFFFFF:FF
@A00155:342:HHGFNDSXY:1:1469:22516:30311 2:N:0:GAACCTAG+TCCGCATA
GGACTCGTCAGGTAGCCTACTAGACTACGACTTACACGGTAGGTCGTGAACAAGCCCGGAGTACATCT
CCTTTGGAACTAGGAGACCCCTCCGGTCTTGTCCAACCTCTGTTTCGGCAGAGGATACCGTGCTCCAC
+
FFFFFFFFFFFFFFFFFFFFFFFFFFFFFFFFFFFFFFFFFFFFFFFFFFFFFFFFFFFFFFFFFFFF
FFFFFFFFFFFFFFFFFFFFFFFFFFFFFFFFFFFFFFFFFFFFFFFFFFFFFFFFFFFFFFFFFFFF
@A00155:342:HHGFNDSXY:1:1470:20934:21652 2:N:0:GAACCTAG+TCCGCATA
CTGGCCTATGGTCATCTCAATGCCGAGAGGGATGTGAAGGGCGAGAGAGAGCGACTTCTCTCGGGCCC
ACAGCCTTACAGCTGTGGAGCACGGTATCCTCTGCCGAAACAGAGGTTGGACAAGACCGGAG
+
FFFFFFF:FFFFFFFFFFF,FFFFFFF:FFFF:FFFFFFFFFFFFFFFFFFFFFFFFFFFFFFFFFFF
FFFFFFFFFFFFFFFFFFFFFFFFFFFFFFFFFFFFFFFFFFFFFFFFFFFFFFFFFFFFFF
@A00155:342:HHGFNDSXY:1:1470:19741:26631 2:N:0:GAACCTAG+TCCGCATA
AGTACATCTCCTTTGGAACTAGGAGACCCCTCCGGTCTTGTCCAACCTCTGTTTCGGCAGAGGATACC
GTGCTCCACAGCTGTAAGGCTGTGGGCCCGAGAGAAGTCGCTCTCTCTCGCCCTCCACATCCCTCTCG
+
FFFFFFFFFFFFFFFFFFF:FFFFFFFFFFFFFFF:FFFFFFF:FFFFFFF:FFFFFFFFFFFFFFF
FFFFFFFFFFFFFFFF:FFF:FFFFFFFFFFFFFFFFFFFFFFFFFFF,FFFFFFFFFFFFFFFFFFFF
@A00155:342:HHGFNDSXY:1:1471:5701:9768 2:N:0:GAACCTAG+TCCGCATA
GGTAGCCTACTAGACTACGACTTACACGGTAGGTCGTGAACAAGCCCGGAGTACATCTCCTTTGGAAC
CAGGAGACCCCTCCGGTCTTGTCCAACCTCTGTTTCGGCAGAGGATACCGTGCTCCACAGCTGTAAGG
+
FFFFF:FFFFFFFFFFFF:FFFFFFF,FFFFFFFFFFFFFFFFFFFFFFFFFFFFFFFFFFFFFFFFF
FFFFFFFFFFFFFFF:FFFFFFFFFFFFFFFFF:FFFFFFFFFFFFFFFFFFFFFFFFFFF:FFFFFFF
@A00155:342:HHGFNDSXY:1:1471:28682:15530 2:N:0:GAACCTAG+TCCGCATA
ACGACTTACACGGTAGGTCGTGAACAAGCCCGGAGTACATCTCCTTTGGAACTAGGAGACCCCTCCGG
TCTTGTCCAACCTCTGTTTCGGCAGAGGATACCGTGCTCCACAGCTGTAAGGCTGTGGGCCCGAGAGA
+
FFFFFFFFFFFFFFFFFFFFFFFFFF,FFFFFFFFFFFFFFFFFFF:FFF,FFFFFFFFFFFFFFFFF
FFFFFFFFFFFFFFFFFFFFFFFFFFFFFFFFFFFFFFFFFFFFFFFFFFFFFFFFFFFFFFFFFFFF
@A00155:342:HHGFNDSXY:1:1471:22525:22341 2:N:0:GAACCTAG+TCCGCATA
GTGAACAAGCCCGGAGTACATCTCCTTTGGAACTAGGAGACCCCTCCGGTCTTGTCCAACCTCTGTTT
CGGCAGAGGATACCGTGCTCCACAGCTGTAAGGCTGTGGGCCCGAGAGAAGTCGCTCTCTCTCGCCCT
+
FFFFFFFFFFFFFFFFFFFFFFFFFFFFFFFFFFFFFFFF:FFFFFFFFFFFFFFFFFFFFFFFFFFFFF
FFFFFFFFFFFFFFFFFFFFFFFFFFFFFFFFFFFFFFFFFFFFFFFFFFFFFFFFFFFFFFFFFFFF
@A00155:342:HHGFNDSXY:1:1471:23954:23750 2:N:0:GAACCTAG+TCCGCATA
GTGAACAAGCCCGGAGTACATCTCCTTTGGAACTAGGAGACCCCTCCGGTCTTGTCCAACCTCTGTTT
CGGCAGAGGATACCGTGCTCCACAGCTGTAAGGCTGTGGGCCCGAGAGAAGTCGCTCTCTCTCGCCCT
+
FFFFFFFF:FFFFFFFFFFFFFFFFFFFFFFFFFFFFFFFF:FFFFFFFFFFFFFFFFFF:FFFFFFF
FFFFFFFFFFFFFFFFFFFFFFFFFFFFFFFFFFFFFFFFFFFFFFFFF:FFFFFFFFFFFFFFFFFF
@A00155:342:HHGFNDSXY:1:1471:13557:24377 2:N:0:GAACCTAG+TCCGCATA
GGTCGTGAACAAGCCCGGAGTACATCTCCTTTGGAACCAGGAGACCCCTCCGGTCTTGTCCAACCTCT
GTTTCGGCAGAGGATACCGTGCTCCACAGCTGTAAGGCTGTGGGCCCGAGAGAAGTCGCTCTCTCTCG
+
FFFFFFFFFFFFFFFFFFFFFFFFFFFFFFFFFFFFFFFFFFFFFFFFFFFFFFFFFFFFFFFFFFFF
FFFFFFFFFFFFFFFFF:F:FFFFFFFFFFFFFFFFFFFFFFFFFFFFFFFFFFFFFFFFFFFFFFFF
```

```
@A00155:342:HHGFNDSXY:1:1471:12228:24925 2:N:0:GAACCTAG+TCCGCATA
GGTCGTGAACAAGCCCGGAGTACATCTCCTTTGGAACCAGGAGACCCCTCCGGTCTTGTCCAACCTCT
GTTTCGGCAGAGGATACCGTGCTCCACAGCTGTAAGGCTGTGGGCCCGAGAGAAGTCGCTCTCTCTCG
+
FFFFFFFFFFFFFFFFFFFFFFFFFFFFFFFFFFFFFFFFFFFFFFFFFFFFFFFF:FFFFFFFFFFF
FFFFFFFFFFFFFFFFFFFFFFFFFFFFFFFFFFFFFFFFFFFFFFFFFFFFFFFFFFFFFFFFFFFF
@A00155:342:HHGFNDSXY:1:1471:25861:25457 2:N:0:GAACCTAG+TCCGCATA
GGACTCGTCAGGTAGCCTACTAGACTACGACTTACACGGTAGGTCGTGAACAAGCCCGGAGTACATCT
CCTTTGGAACTAGGAGACCCCTCTGGTCTTGTCCAACCTCTGTTTCGGCAGAGGATACCGTGCTCCAC
+
FFFF:FFFFFFFFF:FFFFFFFFFFFFFFFFFFFFF:FFFFFFFFFFFFFFFFFFFFFFFFFFFFFFF
FFFFFFFFFFFF,FFFFFFFFFFFFFFFFFFFFFFFFFFFFFFFFFFFFFFFFFFFF:FFFFFFFFFF
@A00155:342:HHGFNDSXY:1:1471:29197:26381 2:N:0:GAACCTAG+TCCGCATA
GAAACCGTCGTTACTCTTTGAGTTGTGTAAGTTTCGTCCTAAAAAGGACTCGTCAGGTAGCCTACTAG
ACTACGACTTACACGGTAGGTCGTGAACAAGCCCGGAGTACATCTCCTTTGGAACTAGGAGACCCCTC
+
FFFFFFFFFFFFF:F,FFFFFFFFFFFF:FFFFFF:FF,FFFFF,FFF,FF:FF,FFF:FFFFFFFFF
FFF:F:FFFFF,FF:FFFFFFFFFFFFFFFFFFFFFFFFFFFFFF,FFFFFFFF:F:F:FF:FFFFF:
@A00155:342:HHGFNDSXY:1:1471:9688:34272 2:N:0:GAACCTAG+TCCGCATA
AAAAGGACTCGTCAGGTAGCCTACTAGACTACGACTTACACGGTAGGTCGTGAACAAGCCCGGAGTAC
ATCTCCTTTGGAACCAGGAGACCCCTCCGGTCTTGTCCAACCTCTGTTTCGGCAGAGGATACCGTGCT
+
FFFFFFFFFFFFFFFFFFFFFFFFFFFFFFFFFFFFFFFFFFFFFFFFFFFFFFFFFFFFFFFFFFFF
FFFFFFFFFFFFFFFFFFFFFFFFFFFFFFFFFFFFFFFFFFFFFFFFFFFFFFFFFFFFFFFFFFFF
@A00155:342:HHGFNDSXY:1:1472:2076:4679 2:N:0:GAACCTAG+TCCGCATA
AAGCCCGGAGTACATCTCCTTTGGAACTAGGAGACCCCTCCGGTCTTGTCCAACCTCTGTTTCGGCAG
AGGATACCGTGCTCCACAGCTGTAAGGCTGTGGGCCCGAGAGAAGTCGCTCTCTCTCGCC
+
FF:FFFFFFFFFFFFFFFF:FFFFFFFFFFFFFFFFFFFFFFFFFFFF:FFFFFFFFFFF:FFFFF:F
FFF:F:FFFFFFFFFFF:FF:FFF:FFF,FFFFFFFFF,FFFFFFFFFFFFFFFFFFFFF
@A00155:342:HHGFNDSXY:1:1472:8567:23876 2:N:0:GAACCTAG+TCCGCATA
GTACATCTCCTTTGGAACCAGGAGACCCCTCCGGTCTTGTCCAACCTCTGTTTCGGCAGAGGATACCG
TGCTCCACAGCTGTAAGGCTGTGGGCCCGAGAGAAGTCGCTCTCTCTCGCCCTCCACATCCCTCTCGG
+
FFFFFFFFFFFFFFFFFFFFFFFFFFFFFFFFFFFFFFFFFFFFFFFFFFFFFFFFFFFFFFFFFFFF
FFFFFFFFFFFFFFFFFFFFFFFFFFFFFFFFFFFFFFFFFFFFFF:FFFFFFFFFFFFFFFFFFFFF
@A00155:342:HHGFNDSXY:1:1472:3016:25128 2:N:0:GAACCTAG+TCCGCATA
AGGACGAAACCGTCGTTACTCTTCGAGTTGTGTAAGTTTCGTCCTAAAAAGGACTCGTCAGGTAGCCT
ACTAGACTACGACTTACACGGTAGGTCGTGAACAAGCCCGGAGTACATCTCCTTTGGAACTAGGAGAC
+
FFFFFFFFFFFFFFFFFFFFFFFFFFFFFFFFFFFFFFFFFFFFFFFFFFFFFFF:FFFFFFFFFFFF
FFFF,FF:FFFFFFFFFFFFFFFFFF:FFFFFFFFFFFFFFFFFFFFFFFFFFFFFFFFFFFFFFFFF
@A00155:342:HHGFNDSXY:1:1472:19388:26490 2:N:0:GAACCTAG+TCCGCATA
GCCTACTAGACTCCGACCGTCATGGGGGTTCAAACCCATGCCCCGCCACACCGCCTTAGATCAGCTGT
GTCCACTTAAGGACTCACCTCTGGCCTATGGTCATCTCAATGCCGAGAGGGATGTGGAGGGCGAGAGA
+
FFFFFFFFF:FFFFFFFFFFFFFFFFFFFFFFFFFFFFFFFFFFFFFFFFFFFFFFFFFFFFFFFFFF
FFFFFFFFFFFFFFFFFFFFFFFFFFFFFFFFFFFFFFFFFF:FFFFFFFFF:FFFFFFFFFFFFFFF
@A00155:342:HHGFNDSXY:1:1472:28971:32064 2:N:0:GAACCTAG+TCCGCATA
TAACGACGGTTTCGTCCTTGGAACGGACTCATCAGGGAGCCTACTAGACTCCGACCGTCATGGGGGTT
CAAACCCATGCCCCGCCACACCGCCTTAGATCAGCTGTGTCCACTTAAGGACTCACCTCTGGCCTATG
+
FF,FFFFFFFFFFFFFFFFFFFFFFFFFFFFFFFFFFFFFFFFFFFFFFFFFFFFFFFF:FFFFFFF
FFFFFFFFFFFFFFFFFFFFFFFFF:FFFFFFFFFFFFFFFFFFFFFFFFFFFF:FFFFFFFFFFFFF
```

```
@A00155:342:HHGFNDSXY:1:1473:13521:12226 2:N:0:GAACCTAG+TCCGCATA
AGCCTACTAGACTACGACTTACACGGTAGGTCGTGAACAAGCCCGGAGTACATCTCCTTTGGAACTAG
GAGACCCCTCCGGTCTTGTCCAACCTCTGTTTCGGCAGAGGATACCGTGCTCCACAGCTGTAAGGCTG
+
FFFFFFFFFFFFFFFFFFFFFFFFFFFFFFFFFFFFFFFF:FFFFFFFFFFFFFFFFFFFFFFFFFFF
FFFFFFFFFFFFFFFFFFFFFFFFFFFFFFFFFFFFFFFFFFFFFFFFFFFFFFFFFFFFFFFFFFFF
@A00155:342:HHGFNDSXY:1:1473:14344:34757 2:N:0:GAACCTAG+TCCGCATA
CCTACTAGACTACGACTTACACGGTAGGTCGTGAACAAGCCCGGAGTACATCTCCTTTGGAACTAGGA
GACCCCTCCGGTCTTGTCCAACCTCTGTTTCGGCAGAGGATACCGTGCTCCACAGCTGTAAGGCTGTG
+
FFFFFFFFFFFFFFFFFFFFFFFFF:FFFFFFFFFFFFFFFFFFFFFFFFFFFFFFFFFFFFFFFFFF
FFFFFFFFFFFFFFFFFFFFFFFFFFF:FFFFFFFFFFFFFFFFFFFFFFFFFFFFFFFFFF::FFFFFF
@A00155:342:HHGFNDSXY:1:1474:10945:10457 2:N:0:GAACCTAG+TCCGCATA
ACTTACACGGTAGGTCGTGAACAAGCCCGGAGTACATCTCCTTTGGAACTAGGAGACCCCTCCGGTCT
TGTCCAACCTCTGTTTCGGCAGAGGATACCGTGCTCCACAGCTGTAAGGCTGTGGGCCCGAGAGAAGT
+
FF:FFFFFFFFFFFFFFFFFFFFFFFFFFFFFF:FFFFFFFFFFFFFFFFFFFFFFFFFFFFFFFFFF
FFFFFFFFFFFFFFFFFFFFFFFFFFFFFFFFFFFFFFFFFFFFFFFFFFFFFFFFFFFFFFFFF:FF
@A00155:342:HHGFNDSXY:1:1474:14271:12461 2:N:0:GAACCTAG+TCCGCATA
AGGACGAAACCGTCGTTACTCTTCGAGTTGTGTAAGTTTCGTCCTAAAAAGGACTCGTCAGGTAGCCT
ACTAGACTACGACTTACACGGTAGGTCGTGAACAAGCCCGGAGTACATCTCCTTTGGAACTAGGAGAC
+
FFFFFFFFFFFF:FF:FFFFFFFFFFFFFFFFFFFFFFFFFFFFFFFFFFFFFFFFFFFFFFFFFFFF
FFFFFFFFFFFFFFFFFFFFFFFFFFFFFFFFFFFFFFFFFFFFFFFFFFFFFFFFFFFFFFFFFFFF
@A00155:342:HHGFNDSXY:1:1474:19533:13808 2:N:0:GAACCTAG+TCCGCATA
AGTACATCTCCTTTGGAACTAGGAGACCCCTCCGGTCTTGTCCAACCTCTGTTTCGGCAGAGGATACC
GTGCTCCACAGCTGTAAGGCTGTGGGCCCGAGAGAAGTCGCTCTCTCTCGCCCTCCACATCCCTCTCG
+
FFFFFFFFFFFFFFFFFFFFFFFFFFFFFFFFFFFFFFFFFFFFFFFFFFFFFFFFFFFF:FFFFFFF
FFFFFFFFFFFFFFFFFFFFFFFFFFFFFFFFFFFFFFFFFFFFFFFFFFFFFFFFFFFFFFFFFFFF
@A00155:342:HHGFNDSXY:1:1474:28239:18458 2:N:0:GAACCTAG+TCCGCATA
GTGAACAAGCCCGGAGTACATCTCCTTTGGAACTATGAGACCCCTCCGGTCTTGTCCAACCTCTGTTT
CGGCAGAGGATACCGTGCTCCACAGCTGTAAGGCTGTGGGCCCGAGAGAAGTCGCTCTCTCTCGCCCT
+
FFFFFFFF:FFFFFFFFFFFFFFFFFFFFFFFFFFFFFFFFFFFFFFFFFFFFFFF:FFFFFFFFFFF
FF:FFFFFFFFFFFFFFFFFFFFFFFFFFFFFFFFFFFFFFFFFFFFFFFFF,FFFFFFFFFFFFFFFFF
@A00155:342:HHGFNDSXY:1:1474:16260:18474 2:N:0:GAACCTAG+TCCGCATA
AGTACATCTCCTTTGGAACTAGGAGACCCCTCCGGTCTTGTCCAACCTCTGTTTCGGCAGAGGATACC
GTGCTCCACAGCTGTAAGGCTGTGGGCCCGAGAGAAGTCGCTCTCTCTCGCCCTCCACATCCCTCTCG
+
FFFFFFFFFFFFFFFFFFFFFFFFFFFFFFFFFFFFFFFFFFFFFFFFFFFFFFFFFFFFFFFF,FFF
FFFFFFFFFFFFFFFFFFFFFFFFFFFFFFFFFFFFFFFFFFFFFFFFFFFFFFFFFFFFFFFFFFFF
@A00155:342:HHGFNDSXY:1:1475:3206:1063 2:N:0:GAACCTAG+TCCGCATA
GACTTACACGGTAGGTCGTGAACAAGCCCGGAGTACATCTCCTTTGGAACCAGGAGACCCCTCCGGTC
TTGTCCAACCTCTGTTTCGGCAGAGGATACCGTGCTCCACAGCTGTAAGGCTGTGGGCCCGAGAGAAG
+
FFFFFFFFFFFFFFFFFFFFFFFFFFFFFFFFFFFFFFFFFFFFFFFFFFFFFFFFFFFFFFFFFFFF
FFFFFFFFFFFFFFFFFFFFFFFFFFFFFFFFFFFFFFFFFFFFFFFFFFF:FFFFFFFFFF:FFFFFF
@A00155:342:HHGFNDSXY:1:1475:13096:11021 2:N:0:GAACCTAG+TCCGCATA
GGGCCCACAGCCTTACAGCTGTGGAGCACGGTATCCTCTGCCGAAACAGAGGTTGGACAAGACCGGAG
GGGTCTCCTAGTTCCAAAGGAGATGTACTCCGGGCTTGTTCACGACCTACCGTGTAAGTCGTAGTCTA
+
FFFFFFFFFFFFFFFFFFFFFFFFFFFFFFFFFFFFFFFFFFFFFFFFFFFFFFFFFFFFFFFFFFFF
FFFFFFFFFFFFFFFFFFFFFFFFFFFFFFFFFFFFFFFFFF,FFFFFFFF:F,FFF:FFFF:F:FFFF
```

```
@A00155:342:HHGFNDSXY:1:1475:23366:11522 2:N:0:GAACCTAG+TCCGCATA
ACGACTTACACGGTAGGTCGTGAACAAGCCCGGAGTACATCTCCTTTGGAACCAGGAGACCCCTCCGG
TCTTGTCCAACCTCTGTTTCGGCAGAGGATACCGTGCTCCACAGCTGTAAGGCTGTGGGCCCGAGAGA
+
FFFFFFFFFFFFFFFFFFFFFFFFFFFFFFFFFFFFFFFFFFFFFFFFFFFFFFFFFFFFFFFFFFFF
FFFFFFFFFFFFFFFFF:FFFFFFFFFFF:FFFFFFFFFFFFFFF:FFFFFFFFFFFFFFFFFFFFFF
@A00155:342:HHGFNDSXY:1:1475:4110:16282 2:N:0:GAACCTAG+TCCGCATA
TGGAACGGACTCATCAGGGAGCCTACTAGACTCCGACCGTCATGGGGGTTCAAACCCATGCCCCACCA
CACCGCCTTAGATCAGCTGTGTCCACTTAAGGACTCACCTCTGGCCTATGGTCATCTCAATGCCGAGA
+
FFFFFFFFFFFFFFFFFFFFFFFFFFFFFFFFFFFFFFFFFFFFFFFFFF:FFFFFFFFFFFFFFFFF
FFFFFFFFFFF:FFFFFFF:FFFFFFFFFFFFFFFFFFFFFFFFFFFF,FFF:FFFFFFFFFFFFFFF
@A00155:342:HHGFNDSXY:1:1476:22092:28823 2:N:0:GAACCTAG+TCCGCATA
TCCTAAAAAGGACTCGTCAGGTAGCCTACTAGACTACGACTTACACGGTAGGTCGTGAACAAGCCCGG
AGTACATCTCCTTTGGAACTAGGAGACCCCTCCGGTCTTGTCCAACCTCTGTTTCGGCAGAGGATACC
+
FFFFFFFFFFFFFFFFFFFFFFFFFFFFFFFFFFFFFFFFFFFFF:FFFFFFFFFFFFFFFFFFFFFF
FFFFFFFFFFF,FFFFFFFFFFFFFFFFFFFFFFFFFFFFFFFFFFFFFFFFFFFFFFFFFFFFFFFF
@A00155:342:HHGFNDSXY:1:1476:4218:29465 2:N:0:GAACCTAG+TCCGCATA
AGCCCGGAGTACATCTCCTTTGGAACTAGGAGACCCCTCCGGTCTTGTCCAACCTCTGTTTCGGCAGA
GGATACCGTGCTCCACAGCTGTAAGGCTGTGGGCCCGAGAGAAGTCGCTCTCTCTCGCCCTCCA
+
FFFFFFFFFFFFFFFFFFFFFFFFFFFFFFFFFFFFFFFFFFFFFFFFFFFFFFFFFFFFFFFFFFFF
FFFFFFFFFFFFFFFFFFFFFFFFFFFFFFFFFFFFFFFFFFFFFFFFFFFFFFFFFFFFFFFF
@A00155:342:HHGFNDSXY:1:1477:13006:1063 2:N:0:GAACCTAG+TCCGCATA
CGGACTCATCAGGGAGCCTACTAGACTCCGACCGTCATGGGGGTTCAAACCCATGCCCCGCTACACCG
CCTTAGATCAGCTGTGTCCACTTAAGGACTCACCTCTGGCCTATGGTCATCTCAATGCCGAGAGGGAT
+
FFFFFFFFFFFFFFFFFFFFFFFFFFFFFFFFFFFFFFFFFFFFFFFFFFFFFFFFFFFFFFFFFFFF
FFFFFFFFFFFFFFFFFFFFFFFFFFFFFFFFFFFFFFFFFFFFFFFFFFFFFFFFFFFFFFFFFFFF
@A00155:342:HHGFNDSXY:1:1477:24451:5196 2:N:0:GAACCTAG+TCCGCATA
GGACTCGTCAGGTAGCCTACTAGACTACGACTTACACGGTAGGTCGTGAACAAGCCCGGAGTACATCT
CCTTTGGAACTAGGAGACCCCTCCGGTCTTGTCCAACCTCTGTTTCGGCAGAGGATACCGTGCTCCAC
+
FFFFFFFFFF:FFFFFFFFFFFFFFFFFFFFF,FFFFFFFF:FFFFF,FFFFFFFFFFFFFFFFFF::
FF:FF:FF,FFFFFF:FFF,FFFF:F:FFFFFF:FFFFFFFFFFFFFFFFFFFFFFFFF::FFFFFFFF
@A00155:342:HHGFNDSXY:1:1477:4417:11240 2:N:0:GAACCTAG+TCCGCATA
TACGACTTACACGGTAGGTCGTGAACAAGCCCGGAGTACATCTCCTTTGGAACTAGGAGACCCCTCCG
GTCTTGTCCAACCTCTGTTTCGGCAGAGGATACCGTGCTCCACAGCTGTAAGGCTGTGGGCCCG
+
FFFFFFF:FFFFFFFFFFFFFFFFFFFF:FFFFFFFFFFFFFFFFFFFFFFFFFFFFFFFFFFFFFFF
FFFFFFFFFFFFFFFFFFFFFFFFFFFFFFFFFFFFFFFFFFFFFFFFFFFF,FFFFFFFFFFFF
@A00155:342:HHGFNDSXY:1:1477:32967:14027 2:N:0:GAACCTAG+TCCGCATA
CTACGACTTACACGGTAGGTCGTGAACAAGCCCGGAGTACATCTCCTTTGGAACTAGGAGACCCCTCC
GGTCTTGTCCAACCTCTGTTTCGGCAGAGGATACCGTGCTCCACAGCTGTAAGGCTGTGGGCCCGAGA
+
FFFFFFFFFFFFFFFFFFF:FFFFFFFFFFFFFFFFFFFFFFFF:FFFFFFFF,F:FFFFFFFFFFFF
FFFF:FFFFFFFFFFFFFFF,FFFFFFFFFFFFFFFF:FFFFFFFFF,FFFFFFFFFFFFFFFFFF:F
@A00155:342:HHGFNDSXY:1:1477:28302:23109 2:N:0:GAACCTAG+TCCGCATA
GACCCCTCCGGTCTTGTCCAACCTCTGTTTCGGCAGAGGATACCGTGCTCCACAGCTGTAAGGCTGTG
GGCCCGAGAGAAGTCGCTCTCTCTCGCCCTCCACATCCCTCTCGGCATAGAGATGACCATAGGC
+
FFFFFFFFFFFFFFFFFFFFFFFFFFFFFFFFFFFFFFFFFFFFFFFFFFFFFFFFFFFFFFFFFFFF
FFFFFFFFFFFFFFFFFFFFFFFFFFFFFFFFFFFFFFFFFFFFFFFFFFFFFFFFFF:FFFFF
```

```
@A00155:342:HHGFNDSXY:1:1477:27046:23249 2:N:0:GAACCTAG+TCCGCATA
GACCCCTCCGGTCTTGTCCAACCTCTGTTTCGGCAGAGGATACCGTGCTCCACAGCTGTAAGGCTGTG
GGCCCGAGAGAAGTCGCTCTCTCTCGCCCTCCACATCCCTCTCGGCATAGAGATGACCATAGGC
+
FFFFFFFFFFFFFF,F:FFFFFFFFFFFFFFFFFFFFFFFFFFFFFFFFFFFFFFFFFFFFFFFFFFF
FFFFFFFFFFFFFFFFFFFFFFFFFFFFFFFFFFFFFFFFFFFFFFFF:FFFFFFFFFFFFFFFF
@A00155:342:HHGFNDSXY:1:1477:16251:32831 2:N:0:GAACCTAG+TCCGCATA
GAAACCGTCATTACTCTTCGAGTTGTGTAAGTTTCGTCCTAAAAAGGACTCGTCAGGTAGCCTACTAG
ACTACGACTTACACGGTAGGTCGTGAACAAGCCCGGAGTACATCTCCTTTGGAACTAGGAGACCCCTC
+
FFFFFFFFFFFFFFFFFFFFFFFFFFFFFFFF,:FFFFFFFFFFFFFFFFFFFFFFFFFFFFFFFFFF
FFFFFFFFFFFFFFFFFFFFFFFFFFFFFFFFFFFFFFFFFFFFFFFFFFFFFFFFFFFFFFFFFFFF
@A00155:342:HHGFNDSXY:1:1478:27190:13103 2:N:0:GAACCTAG+TCCGCATA
CGGTAGGTCGTGAACAAGCCCGGAGTACATCTCCTTTGGAACTAGGAGACCCCTCCGGTCTTGTCCAA
CCTCTGTTTCGGCAGAGGATACCGTGCTCCACAGCTGTAAGGCTGTGGGCCCGAGAGAAGTCGCTCTC
+
FFFFFFFFFFFFFFFFFFFFFFFF:FFFFFFFFFFFFFFFFFFFFFFFFFFFFFFFFFFFFFFFFFFF
FFFFFFFFFFFFFFFFFFFFFFFFFFFFFFFFFFFFF:FFFFFFFFFFFFFFFFFFFFFFFFFFFFFF
@A00155:342:HHGFNDSXY:1:1501:32389:5447 2:N:0:GAACCTAG+TCCGCATA
AAAAGGACTCGTCAGGTAGCCTACTAGACTACGACTTACACGGTAGGTCGTGAACAAGCCCGGAGTAC
ATCTCCTTTGGAACTAGGAGACCCCTCCGGTCTTGTCCAACCTCTGTTTCGGCAGAGGATACCGTGCT
+
FFF:FFFFFFFFFFFFFFFFFFFFFFFFFFFFFFFFFFFFFFFFFFFFFFFFFFFFFFFFFFFFFFFF
FFFFFFFFFFFFFFFFFFFFFFFFFFFFFFFFFFFFFFFFFF:FFFFFFFFFFFFFFFFFF,FFFFFFF
@A00155:342:HHGFNDSXY:1:1501:10682:16673 2:N:0:GAACCTAG+TCCGCATA
AAGGACTCGTCAGGTAGCCTACTAGACTACGACTTACACGGTAGGTCGTGAACAAGCCCGGAGTACAT
CTCCTTTGGAACTAGGAGACCCCTCCGGTCTTGTCCAACCTCTGTTTCGGCAGAGGATACCGTGCTCC
+
F:FFFFFFFFFFFFFFFFFFFFFFFFFFFF,FFFFFFFFFFFFFFFFFFFFFFFFFFFFFFFFFFFFF
FFFFFFF:FFFFFFFFFFFFFFFFFFFFFFFFF:FFFFFFFFFFFFFFFFFFFFFFFFFFFFFFFFFF
@A00155:342:HHGFNDSXY:1:1501:15546:17519 2:N:0:GAACCTAG+TCCGCATA
TAGGAGACCCCTCCGGTCTTGTCCAACCTCTGTTTCGGCAGAGGATACCGTGCTCCACAGCTGTAAGG
CTGTGGGCCCGAGAGAAGTCGCTCTCTCTCGCCCTCCACATCCCTCTCGGCATTGAGATGACCATAGG
+
FFFFFFFFFFFFFFFFFF,FF:FFFFFFFFFFFFFFFFFFFFFFFFFFFFFFFFFFFFFFFFFFFFFF
FFFFFFFFFFFFFFFFF:FFFFFFFFFFFFFFFFFFFFFFFFFFFFFFFFFFFFFFFFFFFFFFFFFF
@A00155:342:HHGFNDSXY:1:1501:20030:34428 2:N:0:GAACCTAG+TCCGCATA
GCCCGGAGTACATCTCCTTTGGAACTAGGAGACCCCTCCGGTCTTGTCCAACCTCTGTTTCGGCAGAG
GATACCGTGCTCCACAGCTGTAAGGCTGTGGGCCCGAGAGAAGTCGCTCTCTCTCGCCCTCCACATCC
+
FFFFFFFFFFFFFFFFFFFFFFFFFFFFFFFFFFFFFFFFFFFFFFFFFFFFFFFFFFFFFFFFFFFF
FFFFFFFFFFFFFFFFFFFFFFFFFFFFFFFFFFFFFFFFFFFFFFFFFFFFFFFFFFFFFFFFFFFF
@A00155:342:HHGFNDSXY:1:1502:27796:2910 2:N:0:GAACCTAG+TCCGCATA
GTTGTGTAAGTTTCGTCCTAAAAAGGACTCGTCAGGTAGCCTACTAGACTACGACTTACACGGTAGGT
CGTGAACAAGCCCGGAGTACATCTCCTTTGGAACTAGGAGACCCCTCCGGTCTTGTCCAACCTCTGTT
+
FFFF:FFF:FFFFFFFFFFFFFF:FFFFFFFFF:FFFFFFFFFFFFFFFFFFFFFF:FFFFFFFFFFFF
FFFFFFFFFFFFFFFFFFFFFFFFFFFFFFFFFF:FFFFFFFF:FFFFFFFFFFFFFFFFFFFFFFFFF
@A00155:342:HHGFNDSXY:1:1503:26558:4492 2:N:0:GAACCTAG+TCCGCATA
GGACTCGTCAGGTAGCCTACTAGACTACGACTTACACGGTAGGTCGTGAACAAGCCCGGAGTACATCT
CCTTTGGAACTAGGAGACCCCTCCGGTCTTGTCCAACCTCTGTTTCGGCAGAGGATACCGTGCTCCAC
+
FFFFFFF:FFFFFFFFFFFFFFFFFF,FF:FFFFFFFFFFFFFFFFFFFFFFFFFFFFFFFFFFFFFF
FFFFFFFFFFFFF:F:FFF,FFFFFFFFF,:FFFFFFFFF,FFFFFF:FFFFF,FFFFFFFFFFFFFF
```

@A00155:342:HHGFNDSXY:1:1503:11080:7467 2:N:0:GAACCTAG+TCCGCATA
GGACTCGTCAGGTAGCCTACTAGACTACGACTTACACGGTAGGTCGTGAACAAGCCTGGAGTACATCT
CCTTTGGAACTAGGAGACCCCTCCGGTCTTGTCCAACCTCTGTTTCGGCAGAGGATACCGTGCTCCAC
+
FFFFFFFFFFFFFFFFFFFFFFFFFFFFFFF:FFFFFFFFFFFFFFFFFFFFFFFFFFFFFFFFFFFF
FF:FFFFFFFFFFFFF:FFFFFFFFFFFF:FFFF:FFFFFFFFFFFFFFFFFFFFFFFFFFFFFFFFF
@A00155:342:HHGFNDSXY:1:1503:14082:20713 2:N:0:GAACCTAG+TCCGCATA
AGTTTCGTCCTAAAAAGGACTCGTCAGGTAGCCTACTAGACTACGACTTACACGGTAGGTCGTGAACA
AGCCCGGAGTACATCTCCTTTGGAACTAGGAGACCCCTCCGGTCTTGTCCAACCTCTGTTTCGGCAGA
+
FFFFFFFFFF:FFFF,FFFFFFF:FFFFFFFFFFFFFFFFFFFFFFFFFFFFFFFFFFFFFFFFFFFF
FFFFFFFFFFFFFFFFFFFFFFFFFFFFFFFFFFFFFFFFFFFFFFFFFFFFFFFFFF:FFFFFFFFF
@A00155:342:HHGFNDSXY:1:1503:13476:29183 2:N:0:GAACCTAG+TCCGCATA
GGAGTACATCTCCTTTGGAACTAGGAGACCCCTCCGGTCTTGTCCAACCTCTGTTTCGGCAGAGGATA
CCGTGCTCCACAGCTGTAAGGCTGTGGGCCCGAGAGAAGTCGCTCTCTCTCGCCCTCTACATCCCTCT
+
FFFFFFFFFFFFFFFFFFFFFFFFFFFFFFFFFFFFFFF:FFFFFFFFFFFFFFFFFFFFFFFFFFFF
:FFFFFFFFFFFFFFFFFFFFFFFFFFFFFFFFFFFFFFFFFFFFFFFFFFFFFFFFFFFFFFFFFFF
@A00155:342:HHGFNDSXY:1:1503:5529:32142 2:N:0:GAACCTAG+TCCGCATA
ACGACTTACACGGTAGGTCGTGAACAAGCCCGGAGTACATCTCCTTTGGAACTAGGAGACCCCTCCGG
TCTTGTCCAACCTCTGTTTCGGCAGAGGATACCGTGCTCCACAGCTGTAAGGCTGTGGGCCCGAGAGA
+
FFFFFFFFFFFFFFFFFFFFFFFFFFFFFFFFFFFFFFFFFFFFFFFFFFFF:FFFFFFFFFFFFFFF
FFF:FFFFFFFFFFFFFFFFFFFFFFFFFFFFFFFFFFFFFF:FFFFFFFFFFFFFFFFFFFFFFFFF
@A00155:342:HHGFNDSXY:1:1503:22137:35258 2:N:0:GAACCTAG+TCCGCATA
CTACTAGACTACGACTTACACGGTAGGTCGTGAACAAGCCCGGAGTACATCTCCTTTGGAACTAGGAG
ACCCCTCCGGTCTTGTCCAACCTCTGTTTCGGCAGAGGATACCGTGCTCCACAGCTGTAAGGCTGTGG
+
FFFFFFF:FFFFFFFFFFFFFFFFFFFFFFFFFFFFF:FFFFFFFFFFFFFFFFFFFFFFFFFFFF:F
FFF:FFFFFFFF:FFFFFFFFFFFFFFFFFFFFFFFFFFFFFFFFFFFFFFFFFFFFFFFFFFFFFF
@A00155:342:HHGFNDSXY:1:1503:22318:35258 2:N:0:GAACCTAG+TCCGCATA
CTACTAGACTACGACTTACACGGTAGGTCGTGAACAAGCCCGGAGTACATCTCCTTTGGAACTAGGAG
ACCCCTCCGGTCTTGTCCAACCTCTGTTTCGGCAGAGGATACCGTGCTCCACAGCTGTAAGGCTGTGG
+
FFFFFFFFFFFFFFFFFFFFFFFFFFFFFFFFFFFFFFFFFFFFFFFFFFFFFFFFFFFFFFFFFFFF
FFFFFFFFFFFF::FFFFFFFFFFFFFFF:FFFFF:FFFFFFFFFFFFFFFFFFFFFFFFFFFFFFFF
@A00155:342:HHGFNDSXY:1:1504:6786:3035 2:N:0:GAACCTAG+TCCGCATA
ACATCTCCTTTGGAACTAGGAGACCCCTCCGGTCTTGTCCAACCTCTGTTTCGGCAGAGGATACCGTG
CTCCACAGCTGTAAGGCTGTGGGCCCGAGAGAAGTCGCTCTCTCTCGCCCTCCACATCCCTCTCGGCA
+
F::FFFFFFFFFFFFFFFFFFFFFFFFFFFFFFFFFFFFFFFFFFFFFFFF:FFFFFFFFFFFFFFFF
FFFFFFFFFFFFF:F:FFFFF:FFFFFFFFFF,FFFFFFFFFFFFFFFFFFFFFF:FFFFFFFFFFFF
@A00155:342:HHGFNDSXY:1:1504:24722:8703 2:N:0:GAACCTAG+TCCGCATA
GGAGTACATCTCCTTTGGAACTAGGAGACCCCTCCGGTCTTGTCCAACCTCTGTTTCGGCAGAGGATA
CCGTGCTCCACAGCTGTAAGGCTGTAGGCCCGAGAGAAGTCGCTCTCTCTCGCCCTCCACATCCCTCT
+
FFFFFFFFFFFFFFFFFFFFFFFFFFFFFFFFFFF:FFFFFFFFFFFFFFFFFF:FFFFFFFFFFFFF
FFF:FFFFFFFFFFFFFFFFFFFFFFFFFFFFFFFFFFFFFFFFFFFFF:FFFFFFFFFFF:FFFFFF
@A00155:342:HHGFNDSXY:1:1504:13910:9612 2:N:0:GAACCTAG+TCCGCATA
GAAACCGTCGTTACTCTTTGAGTTGTGTAAGTTTCGTCCTAAAAAGGACTCGTCAGGTAGCCTACTAG
ACTACGACTTACACGGTAGGTCGTGAACAAGCCCGGAGTACATCTCCTTTGGAACTAGGAGACCCCTC
+
FFFFFFFFFFFFFF,F,FFFFFFFFFFF:FFFFFFFFF:FFFFF:FFFFFFFFFFFFFFFFFFFFFFF
FFFFFFFFFFFFFFFFFFFFFFFFFFFFFFFFFFFFFFFFFFFFFFFFFFFFF:FFFFFFFFFFFF:F

```
@A00155:342:HHGFNDSXY:1:1505:17852:11177 2:N:0:GAACCTAG+TCCGCATA
GAGACCCCTCCGGTCTTGTCCAACCTCTGTTTCGGCAGAGGATACCGTGCTCCACAGCTGTAAGGCTG
TGGGCCCGAGAGAAGTCGCTCTCTCTCGCCCTCCACATCCCTCTCGGCATTGAGATGACCATAGGCCA
+
FFFFFFFFFFFFFFFFFFFFFFFFFFFFFFFFFFFFFFFFFFFFFFFFFFFFFFFFFFFFFFFFFFFF
FFFFFFFFFFFFFFFFFFFFFFFFFFFFFFFFFFFFFFFFFFFFFFFFFFFFFFFFFFFFFFFFFFFF
@A00155:342:HHGFNDSXY:1:1505:16857:24236 2:N:0:GAACCTAG+TCCGCATA
GGTAGCCTACTAGACTACGACTTACACGGTAGGTCGTGAACAAGCCCGGAGTACATCTCCTTTGGAAC
TAGGAGACCCCTCCGGTCTTGTCCAACCTCTGTTTCGGCAGAGGATACCGTGCTCCACAGCTGTAAG
+
F:FFF:FFFFFF,FFFFFFF,FFFFFFFFF:::FF::FFFFFF:,FF:,F:FF,FFFF:F:FF::FF,
FF:,FFFF:::FF:FFFFFFFFF,FF::,:FF:FFF,FFF:F,FF,F:F,:F,F:FFFF::F,FFFF
@A00155:342:HHGFNDSXY:1:1506:32018:35399 2:N:0:GAACCTAG+TCCGCATA
TAGGTCGTGAACAAGCCCGGAGTACATCTCCTTTGGAACCAGGAGACCCCTCCGGTCTTGTCCAACCT
CTGTTTCGGCAGAGGATACCGTGCTCCACAGCTGTAAGGCTGTGGGCCCGAGAGAAGTCGCTCTCTCT
+
F:FFFFFFFFFFFFFFFFFFFFFFFFFFFFFFFFFFFFFFFFFFFFFFFFFFFF:F,FF,FFFFFFF
FFFFFFFFFFFFFFFFFFFFFFFFFFFFF,FFFFFFFFFFFFF:FFFFF,FFFF:FFFFFFFFFFF:
@A00155:342:HHGFNDSXY:1:1507:22733:12242 2:N:0:GAACCTAG+TCCGCATA
GTGTAAGTTTCGTCCTAAAAAGGACTCGTCAGGTAGCCTACTAGACTACGACTTACACGGTAGGTCGT
GAACAAGCCCGGAGTACATCTCCTTTGGAACTAGGAGACCCCTCCGGTCTTGTCCAACCTCTGTTTCG
+
FFFFFFFFFFFFFFFFFFFFFFFFFFFFFFFFFFFFFFFFFF:FFFFFFFFFFFFFFFFFFFFFFFF:F
FF:FFF:FFFFFFFFFF:FFFFFFFFFFFFFFFFFFFFFFFFFFFFFFFF,FFFFFFFF:FFFFFFFFF
@A00155:342:HHGFNDSXY:1:1507:18593:30718 2:N:0:GAACCTAG+TCCGCATA
GGACTCGTCAGGTAGCCTACTAGACTACGACTTACACGGTAGGTCGTGAACAAGCCCGGAGTACATCT
CCTTTGGAACTAGGAGACCCCTCCGGTCTTGTCCAACCTCTGTTTCGGCAGAGGATACCGTGCTCCAC
+
FFFFFFFFFFFFFFFFFFFFFFFFFFFFFFFFFFFFFFFFFFFFFFFFFFFFFFFFFFFFFFFFFFFF
FFF:FFFFFF:FFFFFFFFFFFFFFFFFFFFFFFFFFFFFFFFFFFFFFFFFFFFFFFFFFFFFFFFF
@A00155:342:HHGFNDSXY:1:1508:3360:3959 2:N:0:GAACCTAG+TCCGCATA
GAACTAGGAGACCCCTCCGGTCTTGTCCAACCTCTGTTTCGGCAGAGGATACCGTGCTCCACAGCTGT
AAGGCTGTGGGCCCGAGAGAAGTCGCTCTCTCTCGCCCTCCACATCCCTCTCGGCATTGAGATGAC
+
FFFFFFFFFFFFFFFFFFFFFFFFFFFFFFFFFFFFFFFFFFFFFFFFFFFFFFFFFFFFFFFFFFFF
FFFFFFFFFFFFFFFFFFFF:FFFFFFFFFFFFFFFFFFFFFFFFFFFFFFFFFFFFFFFFFFFFFF
@A00155:342:HHGFNDSXY:1:1508:31530:23312 2:N:0:GAACCTAG+TCCGCATA
AGTTGTGTAAGTTTCGTCCTAAAAAGGACTCGTCAGGTAGCCTACTAGACTACGACTTACACGGTAGG
TCGTGAACAAGCCCGGAGTACATCTCCTTTGGAACTAGGAGACCCCTCCGGTCTTGTCCAACCTCTGT
+
FFFFFFFFFFFFFFFFFFFFFFFFFFF:FFFFFFFFFF:FFFFF:FFFFFFFFFFFFFFFFFFFFFFF
F:FFFFFFFF,:FFFFFFF,F:FFFFFFFFFFF,FFFFFFFF::FFF:FFFFFFFFFF::FF:FFFFF
@A00155:342:HHGFNDSXY:1:1508:5005:35900 2:N:0:GAACCTAG+TCCGCATA
GAACAAGTCCGGAGTACATCTCCTTTGGAACTAGGAGACCCCTCCGGTCTTGTCCAACCTCTGTTTCG
GCAGAGGATACCGTGCTCCACAGCTGTAAGGCTGTGGGCCCGAGAGAAGTCGCTCTCTCTCGCC
+
FFFFFFFFFFFFFFFFFFFFFFFFFFFFFFFFFFFFFFFFFFFFFFFFFFFFFFFFFFFFFFFFFFFF
FFFFFFFFFFFF:FFFFFFFFFFFFFFFFF:F,FFFFFFFFFFFFFFFFFFFFFFFFFFFFF,FF
@A00155:342:HHGFNDSXY:1:1510:9570:12242 2:N:0:GAACCTAG+TCCGCATA
GTGAACAAGCCCGGAGTACATTTCCTTTGGAACTAGGAGACCCCTCCGGTCTTGTCCAACCTCTGTTT
CGGCAGAGGATACCGTGCTCCACAGCTGTAAGGCTGTGGGCCCGAGAGAAGTCGCTCTCTCTCGCCCT
+
FFFFFFFFFFFFFFFFFFFFFFFFFFFFFFFFFFFFFFFFFFFFFFFFFFFFFFFFFFFFFFFFFFFF
FFFFFFFFFFFFFFFFFFFFFFFFFFFFFFFFFFF,FFFFFFFFFFFF:::FFFFFFFFFFFFFFFFF
```

```
@A00155:342:HHGFNDSXY:1:1510:2248:31657 2:N:0:GAACCTAG+TCCGCATA
GAAACCGTCGTTACTCTTCGAGTTGTGTAAGTTTCGTCCTAAAAAGGACTCGTCAGGTAGCCTACTAG
ACTACGACTTACACGGTAGGTCGTGAACAAGCCCGGAGTACATCTCCTTTGGAACTAGGAGACCCCTC
+
FFFFFFFFFFFFFFFFF:F:FFF:FFFFFFFFFFFFFFFFFFFFFFFFFFFFFFFFFFFFFFFFFFFF
FFFFFFFFFFFFFFFFFFFFFFFFFFFFFFFFFFFFFFFFFFFFFFFF:FFFFFFFFFFFFFFFFFFF
@A00155:342:HHGFNDSXY:1:1510:2926:31861 2:N:0:GAACCTAG+TCCGCATA
GAAACCGTCGTTACTCTTCGAGTTGTGTAAGTTTCGTCCTAAAAAGGACTCGTCAGGTAGCCTACTAG
ACTACGACTTACTCGGTAGGTCGTGAACAAGCCCGGAGTACATCTCCTTTGGAACTAGGAGACCCCTC
+
FFFFFFFFFFFFFF:FFFFF:FFFFFFFFF:FFFFFFFF,FFFFF:FFFF:F:FFFFFFFFFFFFFF
:FFF:FFFFFF:,FFFFFFFFFFFFFF::FFFFFFFFFFFFFFFF,FFFF:F:,FFFFFFFFFFFFFF
@A00155:342:HHGFNDSXY:1:1511:3477:11960 2:N:0:GAACCTAG+TCCGCATA
TAGGAGACCCCTCCGGTCTTGTCCAACCTCTGTTTCGGCAGAGGATACCGTGCTCCACAGCTGTAAGG
CTGTGGGCCCGAGAGAAGTCGCTCTCTCTCGCCCTCCACATCCCTCTCGGCATTGAGATGACCATAGG
+
FFFFFFFFFFFFFFFFFFFFFFFFFFFFFFFFFFFFFFFFFFFFFF:FFFFFFFFFFFFFFFFFFFFF
FFFFFFFFFFFFFFFFFF:FFFFF:FF,FFFFFFFFFFFF:FFFFFFFFFFFFFFFFFFFFFFFFFFF
@A00155:342:HHGFNDSXY:1:1511:32768:14810 2:N:0:GAACCTAG+TCCGCATA
CCCGGAGTACATCTCCTTTGGAACTAGGAGACCCCTCCGGTCTTGTCCAACCTCTGTTTCGGCAGAGG
ATACCGTGCTCCACAGCTGTAAGGCTGTGGGCCCGAGAGACGTCGCTCTCTCTCGCCCTCCACATCCC
+
FFFFFFFFFFFFFFFFFFFFFFFFFFFFFFFFFFFFFFFFFFF,FFFFFFFFFFFFFFFFFFFFFFFF
:FFFFFFFFFFFFFF:FFF:FFFFF:FFFFFFFFFFFFFF,FFFFFFF:FFFFFFFFFFFFFFFFFFF
@A00155:342:HHGFNDSXY:1:1511:27218:32910 2:N:0:GAACCTAG+TCCGCATA
AGGTAGCCTATTAGACTACGACTTACACGGTAGGTCGTGAACAAGCCCGGAGTACATCTCCTTTGGAA
CTAGGAGACCCCTCCGGTCTTGTCCAACCTCTGTTTCGGCAGAGGATACCGTGCTCCACAGCTGTAAG
+
FFFFFFFFFF:FFFFFFFFFFFFFFFFFF:FFFFFFFFFFFFFF:FFFFFFFFFFFFFFFFF:FFFFF
FFFFF:FFFFFFFFFFFFFFFFFFFFFFFFF:FFFFFF:FFFFFFFFFF:FFFFFFFFFF:FFFFFFFF
@A00155:342:HHGFNDSXY:1:1512:22001:5807 2:N:0:GAACCTAG+TCCGCATA
CGAAACTTACACAACTCGAAGAGTAACGACGGTTTCGTCCTTGGAACGGACTCATCAGGGAGCCTACT
AGACTCCGACCGTCATGGGGGTTCAAACCCATGCCCCGCCACACCGCCTTAGATCAGCTGTGTCTACT
+
FFFFFFFFFFFFFFFFFFFFFFFFFFFFFFFFFFFFFFFFFFFFFFFFFFFFFFFFFFFFFFFFFFFF
FFFFFFFFFF:FFFFFFFFFFFFFFFFFFFFFFFFFFFFFFFFFFFFFFF:FFFFFFFFFFFFFFFFF
@A00155:342:HHGFNDSXY:1:1513:8630:19257 2:N:0:GAACCTAG+TCCGCATA
GGACTCGTCAGGTAGCCTACTAGACTACGACTTACACGGTAGGTCGTGAACAAGCCCGGAGTACATCT
CCTTTGGAACCAGGAGACCCCTCCGGTCTTGTCCAACCTCTGTTTCGGCAGAGGATACCGTGCTCCAC
+
F:FFFF::FFFFFFFFFFFFFFFFFFFFFFFFFFFFFFFFFF:FFFFFFFFFFFFFFFFFFFFFFFFF
FFFFFFFFFFFFFFFF:FFFFFFFFFFFFFFFFFFFFFFFFFFFFFFFFFFFFFFFF:FFFFF:FFFFFF
@A00155:342:HHGFNDSXY:1:1513:17472:22983 2:N:0:GAACCTAG+TCCGCATA
AAAAGGACTCGTCAGGTAGCCTACTAGACTACGACTTACACGGTAGGTCGTGAACAAGCCCGGAGTAC
ATCTCCTTTGGAACTAGGAGACCCCTCCGGTCTTGTCCAACCTCTGTTTCGGCAGAGGATACCGTGCA
+
FFF,FFFFFFFFFFFFFFFFFFFFFFFFFFFFFFFFFFFFFFFFFFFFFFFFFFFFFFFFFFFFFFFF
FFFFFFFFFFFFFFFFFFFFFFFFFFFFFFFFF:FFFFFFFFFFFFFFFFFFFFFFFFFFFFFFF:FF:
@A00155:342:HHGFNDSXY:1:1513:11397:31469 2:N:0:GAACCTAG+TCCGCATA
ACCTCTGTTTCGGCAGAGGATACCGTGCTCCACAGCTGTAAGGCTGTGGGCCCGAGAGAAGTCGCTCT
CTCTCGCCCTTCACATCCCTCTCGGCATTGAGATGACCATAGGCCAGAGGTGAGTCCTTAAGTGGAC
+
FFFFFFFFFF:FFFFFFFFFFFFFFFFFFFFFFFFFFFFFFFFFFFFFFFFFFFFFFFFFFFFFFFFF
FFFFFFFFFFF:FFFFFFFFFFFFFFFF,:FFFFFFFFFFFFFFFFF:FFFFF:FF,FFFFFFFFFF
```

```
@A00155:342:HHGFNDSXY:1:1514:28863:20134 2:N:0:GAACCTAG+TCCGCATA
AGCCTACTAGACTCCGACCGTCATGGGGGTTCAAACCCATGCCCCACCACACCGCCTTAGATCAGCTG
TGTCCACTTAAGGACTCACCTCTGGCCTATGGTCATCTCAATGCCGAGAGGGATGTGGAGGGCGAGAG
+
,FFFFFFFFFFFFFFFFFFFFFFFFFFFFFFFFFFFFFFFFFFFFFFFFFFFFFFFFFFFFFFFFFFF
FFFFFFFF,FFFFFFFFFFFFFFFFFFFFFFFFFFFFFFFFFFFFFFFFFFFFFFFFFFFFFFFFFFF
@A00155:342:HHGFNDSXY:1:1514:7102:23124 2:N:0:GAACCTAG+TCCGCATA
GTGTAAGTTTCGTCCTAAAAAGGACTCGTCAGGTAGCCTACTAGACTACGACTTACACGGTAGGTCGT
GAACAAGCCCGGAGTACATCTCCTTTGGAACTAGGAGACCCCTCCGGTCTTGTCCAACCTCTGTTTC
+
FFFFFFFFFFFFFFFFFFFFFFFFFFFFFFFFFFFFFFFFFFFFFFFFFFFFFFFFFFFFFFFFFFFF
FFFFFFFFFFFFFFFFFFFFFFFFFFFFFFFFFFFFFFFFFFFFFFFFFFFFFFFFFF,FFFFFFFF
@A00155:342:HHGFNDSXY:1:1515:18747:14951 2:N:0:GAACCTAG+TCCGCATA
ACGACTTACACGGTAGGTCGTGAACAAGCCCGGAGTACATCTCCTTTGGAACTAGGAGACCCCTCCGG
TCTTGTCCAACCTCTGTTTCGGCAGAGGATACCGTGCTCCACAGCTGTAAGGCTGTGGGCCCGAGAGA
+
FFFFFFFFF:FFFFFFFFFFFFFFFFFFFFFFFFFFFFFFFFFFFFFFFFFFFFFFF:FFFFFFFFFF
:FF:FFFFFFFFFFFFFFFFFFFFFFFFFFFFFFFFFFFFFFFFFFFFFFFFFFFFFFFFFFFFFFFF
@A00155:342:HHGFNDSXY:1:1515:11659:18709 2:N:0:GAACCTAG+TCCGCATA
TAGGTCGTGAACAAGCCCGGAGTACATCTCCTTTGGAACCAGGAGACCCCTCCGGTCTTGTCCAACCT
CTGTTTCGGCAGAGGATACCGTGCTCCACAGCTGTAAGGCTGTGGGCCCGAGAGAAGTCGCTCTCTCT
+
FFFFFFFFFFFFFFFFFFFFFFFFFFFFFFFFFFFFFFFFFFFFFFFFFFFFFFFFFFFFFFFFFFFF
FFFFFFFFFFFFFFFFFFFFFFFFFFFFFFFFFFFFF:FFFFFFFFFFFFFFFFF,FFFFFFFFFFFF
@A00155:342:HHGFNDSXY:1:1515:19759:20588 2:N:0:GAACCTAG+TCCGCATA
ACGAAACCGTCGTTACTCTTTGAGTTGTGTAAGTTTCGTCCTAAAAAGGACTCGTCAGGTAGCCTACT
AGACTACGACTTACACGGTAGGTCGTGAACAAGCCCGGAGTACATCTCCTTTGGAACTAGGAGACCCC
+
FFFFFFFFFFFFFFFFFF,,FFFFFFFFFFFFFFFFFFFFF:FFFF:FFFFFFFFFFFFFF,FFFFFF
FFFFFFFFFFFFFFF:F:F:FFFFFFFFFFFFFFFFFFFFFFFFFFFFFF:FFFFFFF:FFFFFFFFF
@A00155:342:HHGFNDSXY:1:1517:12029:4413 2:N:0:GAACCTAG+TCCGCATA
GTCTTGTCCAACCTCTGTTTCGGCAGAGGATACCGTGCTCCACAGCTGTAAGGCTGTGGGCCCGAGAG
AAGTCGCTCTCTCTCGCCCTCTACATCCCTCTCGGCATTGAGATGACCATAGGCCAGAG
+
FFFFFFFFFFFFFFFFFFFFFFFFFFFFFFFF:FFFFFFFFFFFFFFFFFFFFFFFFFFFFFFFFFFF
FFFFFFFFFFFFFFFFFFFFFFFFFFFFFFFFFFFFFFFFFFFFFFFFFFFFFFF,FFF
@A00155:342:HHGFNDSXY:1:1517:26937:13542 2:N:0:GAACCTAG+TCCGCATA
GACTCGTCAGGTAGCCTACTAGACTACGACTTACACGGTAGGTCGTGAACAAGCCCGGAGTACATCTC
CTTTGGAACTAGGAGACCCCTCCGGTCTTGTCCAACCTCTGTTTCGGCAGAGGATACCGTGCTCCACA
+
FFFFFFFFFFFFFFFFFFFFFFFFFFFFFFFFFFFFFFFFFFFFFFFFFFFFFFFFFFFFFFFFFFFF
FFFFFFFFFFFFFFFFFFFFFFFFFFFFFFFFFFFFFFFFFFFFFFFFFFFFFFFFFFFFFFFFFFFF
@A00155:342:HHGFNDSXY:1:1517:12554:13745 2:N:0:GAACCTAG+TCCGCATA
GTTTCGTCCTAAAAAGGACTCGTCAGGTAGCCTACTAGACTACGACTTACACGGTAGGTCGTGAACAA
GCCCGGAGTACATCTCCTTTGGAACTAGGAGACCCCTCCGGTCTTGTCCAACCTCTGTTTCGGCAGAG
+
FFFFFFFFFFFFFFFFFFFFFFFFFFFFFFFFFFFFFFFFFFFFFFFFFFFFFFFFFFFFFFFFFFFF
FFFFFFFFFFF:FFFFFFFFFFF,FFFFFF:FFFFFFFFFFFFFFFFFFFFFFFFFFFFFFFFFFFFF
@A00155:342:HHGFNDSXY:1:1517:24867:18537 2:N:0:GAACCTAG+TCCGCATA
GTGTAAGTTTCGTCCTAAAAAGGACTCGTCAGGTAGCCTACTAGACTACGACTTACACGGTAGGTCGT
GAACAAGCCCGGAGTACATCTCCTTTGGAACTAGGAGACCCCTCCGGTCTTGTCCAACCTCTGTTTCG
+
FFFFFFFFFFFFFFFFFFFF,FFFFFFFFFFFFFF:FFFFFFF,FFFFFFFFFFFFFFFFFFFFFFFF
FFFFFF:FFFFFFFFFFFFFFFFFFFFFF:FFFFFFFFF:FFFFFFFFFFFFFFFFFFFFFFFFFFFF
```

```
@A00155:342:HHGFNDSXY:1:1518:6686:7435 2:N:0:GAACCTAG+TCCGCATA
CAGGTAGCCTACTAGACTACGACTTACACGGTAGGTCGTGAACAAGCTCGGAGTACATCTCCTTTGGA
ACTAGGAGACCCCTCCGGTCTTGTCCAACCTCTGTTTCGGCAGAGGATACCGTGCTCCACAGCTGTAA
+
FFFFFFFFFFFFFFF:FFFFFFFFFFFFFFFFFFFFFFFFFFFFFFFFFFFFFFFFFFFFFFFFFFFF
FFFFFFFFFFFFFFFFFFFFFFFFFFFFFFFFFFFFFFFFFFFFFFFFFFFFFFFFFFFFFFFFFF,F
@A00155:342:HHGFNDSXY:1:1518:23330:9142 2:N:0:GAACCTAG+TCCGCATA
TACGACTTACACGGTAGGTCGTGAACAAGCCCGGAGTACATCTCCTTTGGAACTAGGAGACCCCTCCG
GTCTTGTCCAACCTCTGTTTCGGCAGAGGATACCGTGCTCCACAGCTGTAAGGCTGTGGGCCCG
+
FFFFFFFFFFFFFFFFFFFFFFFFFFFFFFFFFFFFFFFFFFFFF:FFFFFFFFFFFFFFFFFFFFFF
FFFFFFFFFFFFFFFFFFFFFFFFFFFFFFFFFFFFFFFFFFFFFFFF,FFFFFFFFFFFFFFF
@A00155:342:HHGFNDSXY:1:1519:30400:2253 2:N:0:GAACCTAG+TCCGCATA
GGAACGGACTCATCAGGGAGCCTACTAGACTCCGACCGTCATGGGGGTTCAAACCCATGCCCCGCCAC
ACCGCCTTAGATCAGCTGTGTCCACTTAAGGACTCACCTCTGGCCTATGGTCATCTCAATGCCGAGAG
+
FFFF:FFFF:FFFFFFFFFFFFFF:FFFFFFFF:FFFFFFFFFF:FFFFFFF:F:FFFF,FFFFFFFF
FFFFFF,:FFFFFFFFFFF:FFFFFFF:F:FFFF:FFFFFFF:FFFFFF,FFFFFFFFFFF,FFFFFF
@A00155:342:HHGFNDSXY:1:1519:3305:27258 2:N:0:GAACCTAG+TCCGCATA
AAACTTACACAACTCGAAGAGTAACGACGGTTTCGTCCTTGGAACGGACTCATCAGGGAGCCTACTAG
ACTCCGACCGTCATGGGGGTTCAAACCCATGCCCCGCCACACCGCCTTAGATCAGCTGTGTCCACTTA
+
FFFFFFFFFFFFFFFFFFFFFFFFFFFFFFFFFFFFFFFFFFFFFFFFFFFFFFFFFFFFFFFFFFFF
FFFFFFFFFFFF::FFFFFF:FFFFFFFFFFFFFFFFFFFFFFFFFFFFFFFFFFFFFFFFFFFFFFF
@A00155:342:HHGFNDSXY:1:1519:15103:29872 2:N:0:GAACCTAG+TCCGCATA
CTTGTCCAACCTCTGTTTCGGCAGAGGATACCGTGCTCCACAGCTGTAAGGCTGTGGGCCCGAGAGAA
GTCGCTCTCTCTCGCCCTCCACATCCCTCTCGGCATTGAGATGACCATAGGCCAGAGGAGAGTCCTTA
+
FF,FFF,FFFF,FFFFF:FFFFFFF:FFFFFFFFFFFFFFF:,:FFFFFFF:FFF:FFF:FFFFF:FF
FFFFFFFFF:FFFF:F:F:FFFF,FFFFFFFFF,FFF:FFFF,FFF,FF,F:FF,F,FF::F,:,F,:
@A00155:342:HHGFNDSXY:1:1520:25120:2190 2:N:0:GAACCTAG+TCCGCATA
GCCTACTAGACTCCGACCGTCATGGGGGTTCAAACCCATGCCCCGCCACACCGCCTTAGATCAGCTGT
GTCCACTTAAGGACTCACCTCTGGCCTATGGTCATCTCAATGCCGAGAGGGATGTGGAGGGCGAGAGA
+
FFFFFFFFFFFFFFFFFFFFFFFFFFFFFFFFFFFFFFFFFFFFFFFFFFFFFFFFFFFF:FFFFFFF
FFFFFFF:FFFFFFFFFFFFFFFFFFFFFFFFFFFFFFFFFFFFFFFFFFFFFF:FFFFFFFFFFFFF
@A00155:342:HHGFNDSXY:1:1520:17879:6089 2:N:0:GAACCTAG+TCCGCATA
TCTGTTTCGGCAGAGGATACCGTGCTCCACAGCTGTAAGGCTGTGGGCCCGAGAGAAGTCGCTCTCTC
TCGCCCTTCACATCCCTCTCGGCATTGAGATGACCATAGGCCAGAGGTGAGTCCTTAAGTGGACACAG
+
FFFFFFFFFFFFFFFFFFFFFFFFFFFFFFFFFFFFFFFFFFFFFFFFFFFFFFFFFFFFFFFFFFFF
FFFFFFFFFFFFFFFFFFFFFFFFF:FFFFFFFFFFFFFFFFFFFFFFFFFFFFFFFFFFFFFFFFFF
@A00155:342:HHGFNDSXY:1:1520:15456:8187 2:N:0:GAACCTAG+TCCGCATA
TCTGTTTCGGCAGAGGATACCGTGCTCCACAGCTGTAAGGCTGTGGGCCCGAGAGAAGTCGCTCTCTC
TCGCCCTTCACATCCCTCTCGGCATTGAGATGACCATAGGCCAGAGGTGAGTCCTTAAGTGGACACAG
+
FFFFF,FF:FF:FFFFFFFFFFFFFFFFFFFFFFFFF:FFFFFFFFFFFFF:FFFF:FFFFFFFFFFF
FFFFFFFFFFFFFFFFFFFFFFFFF:FFFFFFF:FFFFFFFFFFFFFFFFFFFFF,FFFFFFFFFFFF
@A00155:342:HHGFNDSXY:1:1520:7563:34507 2:N:0:GAACCTAG+TCCGCATA
AGCCTACTAGACTACGACTTACACGGTAGGTCGTGAACAAGCCCGGAGTACATCTCCTTTGGAACTAG
GAGACCCCTCCGGTCTTGTCCAACCTCTGTTTCGGCAGAGGATACCGTGCTCCACAGCC
+
FFF:FFFFFFFFFFFFFFFFFFFFFFFFFFFFFFFF:FFFFFFFFFFFFFFFFFFFFFFFFFFFF:FF
FFFFFFFFFFFFFFFFFFFFFF:FFFFFFFFFFFFFFF::FFF:FF,FFFFFFFFFFFF
```

```
@A00155:342:HHGFNDSXY:1:1521:28863:11616 2:N:0:GAACCTAG+TCCGCATA
CTGGCCTATGGTCATCTCAATGCCGAGAGGGATGTGAAGGGCGAGAGAGAGCGACTTCTCTCGGGCCC
ACAGCCTTACAGCTGTGGAGCACGGTATCCTCTGCCGAAACAGAGGTTGGACAAGACCGGAG
+
FFFFFFFFFFFFFFFFFFFFFFFFFFFFFFFFFFFFFFFFFFF:FFFFFFFFFFFFFFFFFFFFFFFF
FFFFFFFFFFFFFFFFFFFFFFFFFF:FFFFFFFFFFFFFF:FFFF:FFFFFFFFFF:FFFF
@A00155:342:HHGFNDSXY:1:1521:23005:21324 2:N:0:GAACCTAG+TCCGCATA
ACCGTGCTCCACAGCTGTAAGGCTGTGGGCCCGAGAGAAGTCGCTCTCTCTCGCCCTCCACATCCCTC
TCGGCATTGAGATGACCATAGGCCAGAGGTGGGTCCTTAAGTGGACACAGCTGATCTAAGGCGGTGTG
+
FFFFFFFFFFFFFFFFFFFFFFF,FFFFFFFFFFFFFFFFFFFFFFFFFFFFFFFFFFFFFFFFFFFF
FFFFFF:FFFFFFFFFFFFFFFFF:FFFFFF:FFFFF:,FFFFFFFFFFFFFFFFFF:FFFFFFFFFF
@A00155:342:HHGFNDSXY:1:1522:32940:3740 2:N:0:GAACCTAG+TCCGCATA
GTGAACAAGCCCGGAGTACATCTCCTTTGGAACCAGGAGACCCCTCCGGTCTTGTCCAACCTCTGTTT
CGGCAGAGGATACCGTGCTCCACAGCTGTAAGGCTGTGGGCCCGAGAGAAGTCGCTCTCTCTCGCCC
+
FFFFFFF,FFFFFFFFFFFFFFFFF:FFFFF:FFFFFFFFFFFFFFFFFFF,FFFFFFFFFFF,FFFF
FFFFFFFFFFFFFFFFFFFFFFFFFF:F:F:FFFFFFFFFFFFFFF:FFFFFFFF:FFFFFFFFFFF
@A00155:342:HHGFNDSXY:1:1522:18249:23265 2:N:0:GAACCTAG+TCCGCATA
TCGTCAGGTAGCCTACTAGACTACGACTTACACGGTAGGTCGTGAACAAGCCCGGAGTACATCTCCTT
TGGAACTAGGAGACCCCTCCGGTCTTGTCCAACCTCTGTTTCGGCAGAGGATACCGTGCTCCACAGCT
+
FFFFFFFFFFFFFFFFFFFFFFFFFFFFFFFFFFFFFFFFFFFFFFFFFFFFFFFFFFFFFFFFFFFF
FFF:FFFFFFFFFFFFFFFFFFFFFFFFFFFFFFFFFFFFFFFFFFFFFFFFFFFFFFFFFFFFFFFF
@A00155:342:HHGFNDSXY:1:1522:16143:26725 2:N:0:GAACCTAG+TCCGCATA
GACCCCTCCGGTCTTGTCCAACCTCTGTTTCGGCAGAGGATACCGTGCTCCACAGCTGTAAGGCTGTG
GGCCCGAGAGAAGTCGCTCTCTCTCGCCCTCTACATCCCTCTCGGCATTGAGATGACCATAGGCCAGA
+
FFFFFFFFFFFFFFFFFFFFFFFFFFFFFFFFFFFFFFFFFFFFFFFFFFFFFFFFFFFFFFFFFFFF
FFFFFFFFFFFFFFFFFFFFFFFFFFFFFFFFFFFFFFFFFFFFFFF:FFFFFFFFFFF:FFFFFFFF
@A00155:342:HHGFNDSXY:1:1522:5656:32424 2:N:0:GAACCTAG+TCCGCATA
CACGGTAGGTCGTGAACAAGCCCGGAGTACATCTCCTTTGGAACTAGGAGACCCCTCCGGTCTTGTCC
AACCTCTGTTTCGGCAGAGGATACCGTGCTCCACAGCTGTAAGGCTGTGGGCCCGAGAG
+
FFFFFFFFFFFFFFFFFFFFFFFFFFFFFFFFFFFFF,FFFFFFFFFFFFFFFFFFFFF,FFFFFFFF
FFFFFFFFFFFF:FFFFFFFFFFFFFFFFFFFFFFFFFFFFFFFFFFFFFF:FFFFFFF
@A00155:342:HHGFNDSXY:1:1523:26503:20995 2:N:0:GAACCTAG+TCCGCATA
TCGTGAACAAGCCCGGAGTACATCTCCTTTGGAACTAGGAGACCCCTCCGGTCTTGTCCAACCTCTGT
TTCGGCAGAGGATACCGTGCTCCACAGCTGTAAGGCTGTGGGCCCGAGAGAAGTCGCTCTCTCTCGC
+
FFF:FFFFFFFFFFFFFFFFFFFFFFFFFFFFFFFFFFFFFFFFFFFFFFFFFFFFFF:FFFFFFF
FFFFFFFFFFFFFFFFFFFFFFFFFFFFFF:FFFFF,FFFFFFFFFFFFFFFFFFFFFFFFFFFFFF
@A00155:342:HHGFNDSXY:1:1523:1524:21731 2:N:0:GAACCTAG+TCCGCATA
CCTTTGGAACCAGGAGACCCCTCCGGTCTTGTCCAACCTCTGTTTCGGCAGAGGATACCGTGCTCCAC
AGCTGTAAGGCTGTGGGCCCGAGAGAAGTCGCTCTCTCTCGCCCTCCACATCCCTCTCGGCATTGAGA
+
::FFFFFFFFFFFFFF,FFFFFFFFF:FFFFF:F:FFFFFFFFFFFFFFFF:FFFFF:FFFFFFFFFF
FFFF,F,FFFFFFFFFFF:FFFFFF:FFFFFFF:FFFFFFFFFFFFFFFFFFFFFFFFFF:FFFF:FF
@A00155:342:HHGFNDSXY:1:1524:6126:24471 2:N:0:GAACCTAG+TCCGCATA
GGACTCGTCAGGTAGCCTACTAGACTACGACTTACACGGTAGGTCGTGAACAAGCCCGGAGTACATCT
CCTTTGGAACTAGGAGACCCCTCCGGTCTTGTCCAACCTCTGTTTCGGCAGAGGATACCGTGCTCCAC
+
FFFFFFFFFFFFFFFFFFFFFFFFFFFFFFFFFFFFFF:FFFFFFFFFFFFFFFFFFFFFFFF:FFFF
FFFFFFFFFFFFFFFFFFFFFFFFFFFFFFF:FFF,FFFFFFFFFFFFFFFFFFFFFFFFFFFFFFFF
```

```
@A00155:342:HHGFNDSXY:1:1524:1597:26647 2:N:0:GAACCTAG+TCCGCATA
GAAACCGTCGTTACTCTTTGAGTTGTGTAAGTTTCGTCCTAAAAAGGACTCGTCAGGTAGCCTACTAG
ACTACGACTTACACGGTAGGTCGTGAACAAGCCCGGAGTACATCTCCTTTGGAACTAGGAGACCCCTC
+
F,F:FFFFFFFFF:::FFFF,FFFFFFFFFFFFFFFF:F::F::FF::FFFF:FFFFFFF:FFFFFF
:FF:FFFFFFFF:FFFFFFFFFFFF:FFFF,FFFFFFFF,FFFFFFFFFFFFFF,FFF:FFFFFFFFF
@A00155:342:HHGFNDSXY:1:1525:23059:6668 2:N:0:GAACCTAG+TCCGCATA
AGGACGAAACCGTCGTTACTCTTCGAGTTGTGTAAGTTTCGTCCTAAAAAGGACTCGTCAGGTAGCCT
ACTAGACTACGACTTACACGGTAGGTCGTGAACAAGCCCGGAGTACATCTCCTTTGGAACTGGGAGAC
+
FFFFFFFFFFFFFFFFFFFFFFF:FFFFFFFFFFFF:FFFFFFF:,FFFFFFFFFFFFFFFF,FFFF
FFFFFFFFFFF:FFFFFFFFFFFFFFFFFFFFFFFFFFFFFFFFFFFFFFFFFF:FFFFF,FFFFF
@A00155:342:HHGFNDSXY:1:1525:23068:6872 2:N:0:GAACCTAG+TCCGCATA
AGGACGAAACCGTCGTTACTCTTCGAGTTGTGTAAGTTTCGTCCTAAAAAGGACTCGTCAGGTAGCCT
ACTAGACTACGACTTACACGGTAGGTCGTGAACAAGCCCGGAGTACATCTCCTTTGGAACTGGGAGAC
+
FFFFFFFFFFFFFF:FFFFFFFFFFF:FFF:FFFFF,F:FFFFFFFFFFFFFFFFFFFFFFFFFFFF
FFFFFFFFFFFFFFFFFFFFFFFFFF:FFFFF:FFFFFFFFFFFFFF:FFFFF:FFFFFFFF:FFFFF
@A00155:342:HHGFNDSXY:1:1525:23077:6887 2:N:0:GAACCTAG+TCCGCATA
AGGACGAAACCGTCGTTACTCTTCGAGTTGTGTAAGTTTCGTCCTAAAAAGGACTCGTCAGGTAGCCT
ACTAGACTACGACTTACACGGTAGGTCGTGAACAAGCCCGGAGTACATCTCCTTTGGAACTGGGAGAC
+
FFFFFFFFFFFFFFFFFFFFFF,FFFFFFFFFFFFFFFFFFFFFFF:FFFFFFFFFFFFF:FFF,FFFF
FFFFFFFFFFFFFFFFFFFFFFFFFFFFFFFFF:F:FFFFFFFF:FFFFFFF,FFFFF:FFFFFFFFF
@A00155:342:HHGFNDSXY:1:1525:29839:13526 2:N:0:GAACCTAG+TCCGCATA
ACATCTCCTTTGGAACTAGGAGACCCCTCCGGTCTTGTCCAACCTCTGTTTCGGCAGAGGATACCGTG
CTCCACAGCTGTAAGGCTGTGGGCCCGAGAGAAGTCGCTCTCTCTCGCCCTCCACATCCCTCTCGGCA
+
FFFFFFFFFFFFFFFFFFFFFFFFFFFFFFFFFFFFFFFFFFFFFFFFFFFFFFFFFFFFFFFFFFFF
FFFFFFFFFFFFFFFFF:FFFFFFFFF:FFFFFFFFFFFFFFFFFFFFFFFFFFFF:FFFFFFFFFFF
@A00155:342:HHGFNDSXY:1:1525:24569:22185 2:N:0:GAACCTAG+TCCGCATA
AAAAGGACTCGTCAGGTAGCCTACTAGACTACGACTTACACGGTAGGTCGTGAACAAGCCCGGAGTAC
ATCTCCTTTGGAACTAGGAGACCCCTCCGGTCTTGTCCAACCTCTGTTTCGGCAGAGGATACCGTGCT
+
FFFFFFFFFFFFFFFFFFFFFFFFFFFFFFFFFFFFFFFFFFFFF:FFFFFFFFFFFFFFFFFFFFFFF
FFFFFFFFFFFF:FFFFFFFFF:FFFFFFFFF:FFFFFFFFFFFFFFFFFFFFFFFFFF:F:FFFFFFF
@A00155:342:HHGFNDSXY:1:1525:24388:22279 2:N:0:GAACCTAG+TCCGCATA
AAAAGGACTCGTCAGGTAGCCTACTAGACTACGACTTACACGGTAGGTCGTGAACAAGCCCGGAGTAC
ATCTCCTTTGGAACTAGGAGACCCCTCCGGTCTTGTCCAACCTCTGTTTCGGCAGAGGATACCGTGCT
+
FF::FFFFFFFFFFFFFFFFFFFFFFFFFFFFFFFFFFFFFFFFFFFFFFFFFFFFFFFFFFFFFFFF
FFFFFFFFFFFFFFFFFFFFFFFFFFFFFFFFF,FFFFFFFFFFFFFFFFFFFFFFFFFFFFFFFFFF
@A00155:342:HHGFNDSXY:1:1526:26078:30499 2:N:0:GAACCTAG+TCCGCATA
TTACACGGTAGGTCGTGAACAAGCCCGGAGTACATCTCCTTTGGAACTAGGAGACCCCTCCGGTCTTG
TCCAACCTCTGTTTCGGCAGAGGATACCGTGCTCCACAGCTGTAAGGCTGTGGGCCCGAGAGAAGTCG
+
FFFFFFFFFF:F,FFFFFFFFFFFFFFFFFFFFFFFFFFFFFFFFFFFFFFF:FFFFFFFFFFFFFFF
FFFFFFFFF:FFFFFFFFFFFFFFFFFFFFFFFFFFFFFFFFFFFFFFFFFFFFFFFFFFFFFFFFFF
@A00155:342:HHGFNDSXY:1:1527:27760:5259 2:N:0:GAACCTAG+TCCGCATA
GAGACCCCTCCGGTCTTGTCCAACCTCTGTTTCGGCAGAGGATACCGTGCTCCACAGCTGTAAGGCTG
TGGGCCCGAGAGAAGTCGCTCTCTCTCGCCCTCCACATCCCTCTCGGCATTGAGATGAC
+
FFFFFFFFFFFFFFF:FFFFFFFFFFFFFFFFFFFFFFFFFFFFFFFFFFFFFFFFFFFFFFFFF:FF
FFFFFFFFFFFFF:FFFFFFFFFFFFFFFFFFFFFFFFFFFFFFFFFFFFFFFFFFFF
```

```
@A00155:342:HHGFNDSXY:1:1527:10881:15734 2:N:0:GAACCTAG+TCCGCATA
GGACTCGTCAGGTAGCCTACTAGACTACGACTTACACGGTAGGTCGTGAACAAGCCCGGAGTACATCT
CCTTTGGAACTAGGAGACCCCTCCGGTCTTGTCCAACCTCTGTTTCGGCAGAGGATACCGTGCTCCAC
+
FFFFFFFFFFFFFFFFFFFFFFF:FFFFFFFFFFFFFFFFFFFFFFFFFFFFFFFFFFFFFFFFFFFF
FFFFFFFFFFFFFFFFFFFFFFFFFFFFFFFFFFFFFFFFFFFFFFFFFFFFFFFFFFFFFFFFFFFF
@A00155:342:HHGFNDSXY:1:1527:3794:36652 2:N:0:GAACCTAG+TCCGCATA
GACGAAACCGTCGTTACTCTTCGAGTTGTGTAAGTTTCGTCCTAAAAAGGACTCGTCAGGTAGCCTAC
TAGACTACGACTTACACGGTAGGTCGTGAACAAGCCCGGAGTACATCTCCTTTGGAACCAGGAGACCC
+
FFFFFFFFFFFFFFFFFFF:FF::FFFFFFFFFFF:FFFFFFFFFFFFFFFFFFFFFFFFFFFFFFFF
FFFFFFFFFFFFFFF:FFFFFFFFFFFFFFFFFFFFFFFFFFFFFFFFFFFFFFFFFFFFFFFFFFFF
@A00155:342:HHGFNDSXY:1:1527:3812:36714 2:N:0:GAACCTAG+TCCGCATA
GACGAAACCGTCGTTACTCTTCGAGTTGTGTAAGTTTCGTCCTAAAAAGGACTCGTCAGGTAGCCTAC
TAGACTACGACTTACACGGTAGGTCGTGAACAAGCCCGGAGTACATCTCCTTTGGAACCAGGAGACCC
+
FFFFFFFFFF:FFFFFF:FF,FFFF,FFFFF:FFF,FFFFFFFFF,FFFFFFFFFFFFFFFF,FF,:FF
F,FF:FFFFFFFFFFFFFFFFFFFFFFFFFFFFFF:FFFFFFFFFFFFFFFFFF:FFFFFFFFFFFFF
@A00155:342:HHGFNDSXY:1:1527:3269:36746 2:N:0:GAACCTAG+TCCGCATA
GACGAAACCGTCGTTACTCTTCGAGTTGTGTAAGTTTCGTCCTAAAAAGGACTCGTCAGGTAGCCTAC
TAGACTACGACTTACACGGTAGGTCGTGAACAAGCCCGGAGTACATCTCCTTTGGAACCAGGAGACCC
+
FFFFFFFFFFFFFFFFFFFFFFFF:FFFFFFFFFF:FFFFFFFFFFF,FFFFFFFFFFFFFFFFFFFF
FFFFFFFFFFFFFFFFFFFFFFFFFFFFFFFFF:FFFFFFFFFFFFFFFFF:FFFFFFFFFFF,FFFF
@A00155:342:HHGFNDSXY:1:1528:3757:3302 2:N:0:GAACCTAG+TCCGCATA
GACGAAACCGTCGTTACTCTTCGAGTTGTGTAAGTTTCGTCCTAAAAAGGACTCGTCAGGTAGCCTAC
TAGACTACGACTTACACGGTAGGTCGTGAACAAGCCCGGAGTACATCTCCTTTGGAACCAGGAGACCC
+
FFFFFFF,FFFFFFFFFFF:F:FFFFFFFFFFFFFFFFFFFFFFFFF:FFFFFFF:F:F,FFFFFFFF
FF,FFFFFFFFFFFFFFFFFFFFFFFFF:FFFF,FFFFFFFFFFFFFFFFFFFFF:FFFFFFFFFFFF
@A00155:342:HHGFNDSXY:1:1528:5575:13557 2:N:0:GAACCTAG+TCCGCATA
AAAAGGACTCGTCAGGTAGCCTACTAGACTACGACTTACACGGTAGGTCGTGAACAAGCCCGGAGTAC
ATCTCCTTTGGAACTAGGAGACCCCTCCGGTCTTGTCCAACCTCTGTTTCGGCAGAGGATACCGTGCT
+
FF,F:FFFFFFFFFFFFFFF:FFFFF::FFFFFFFFFFFF:FFF:FFFFFFFF:FFFFFFFFFFFFFF
FFF:F,:FFFFF:FFFFFFFFFFFF,FFFFFF:FFFFFFFFFFFFF:FFF:,FFFF,FFFFFF::,FF
@A00155:342:HHGFNDSXY:1:1529:8006:5995 2:N:0:GAACCTAG+TCCGCATA
TCAGGTAGCCTACTAGACTACGACTTACACGGTAGGTCGTGAACAAGCCCGGAGTACATCTCCTTTGG
AACTAGGAGACCCCTCCGGTCTTGTCCAACCTCTGTTTCGGCAGAGGATACCGTGCTCCACAGCTG
+
FFFFFFFFFFFFFFFFFFFFFFFFFFFF,FFFFFFFFFFFFFFFFF:FFFFFFFFFFFFFFFFFFFFF
FFFFFFFFFFFFFFFFF:FFFFFFFFFFFFFFFFFFFFFFFFFFFFFFFFFFFFFFFFFFFFFFFF
@A00155:342:HHGFNDSXY:1:1529:29460:10895 2:N:0:GAACCTAG+TCCGCATA
CAAGGACGAAACCGTCGTTACTCTTCGAGTTGTGTAAGTTTCGTCCTAAAAAGGACTCGTCAGGTAGC
CTACTAGACTACGACTTACACGGTAGGTCGTGAACAAGCCCGGAGTACATCTCCTTTGGAACTAGGAG
+
FFFFFFFFFFFFFFFFFFFFFFFFFFFFFFFFFFFFFFFFFFFFFF:FFFFFFFFFFFFFFFFFFFFF
FFFFFFFFFFFFFFFFFFFFFFFFFFFFFFFFFFFFFFFFFFFFFFFFFFFFF:FFFFFFFFFFFFFF
@A00155:342:HHGFNDSXY:1:1529:31665:15781 2:N:0:GAACCTAG+TCCGCATA
GGTCGTGAACAAGCCCGGAGTACATCTCCTTTGGAACTAGGAGACCCCTCCGGTCTTGTCCAACCTCT
GTTTCGGCAGAGGATACCGTGCTCCACAGCTGTAAGGCTGTGGGCCCGAGAGAAGTCGCTCTCTCTCG
+
FFFFFFFFFFFFFFFFFFFFFFFFFFFFFFFFFFFFFFF:FFFF:FFFFFFFFFFFFF,FFFFFFFFF
FFFFFFFFFFFFFFFFFFFFFFFFFFFFFFFF,,FFFFFFFFFFFFFFFFFFFFFFFFFFFFFFFFFF
```

```
@A00155:342:HHGFNDSXY:1:1529:9227:35227 2:N:0:GAACCTAG+TCCGCATA
TACGACTTACACGGTAGGTCGTGAACAAGCCCGGAGTACATCTCCTTTGGAACTAGGAGACCCCTCCG
GTCTTGTCCAACCTCTGTTTCGGCAGAGGATACCGTGCTCCACAGCTGTAAGGCTGTGGGCCCG
+
FFFFFFFFFFFFFFFFFFFFFFFFFFFFFFFFFFFFFFFFFFFFFFFFFFFFFFFFFFFFFFFFFFFF
FFFFFFFFFFFFFFFFFFFFFFFFFFFFFFFFFFFFFFFFFFFFFFFFFFFFFFFFFFFFFFFF
@A00155:342:HHGFNDSXY:1:1530:25464:2221 2:N:0:GAACCTAG+TCCGCATA
ACCCCTCCGGTCTTGTCCAACCTCTGTTTCGGCAGAGGATACCGTGCTCCACAGCTGTAAGGCTGTGG
GCCCGAGAGAAGTCGCTCTCTCTCGCCCTCCACATCCCTCTCGGCATTGAGATGACCATAGGCCAG
+
,FFFFFFFFFFFFFFFFFFFFFFFFFFF,FFFFFFFFFFFFFFFFFFFFFFFFFFFFFFFFFFFFFFF
FFFFFFFFFFFFFFFFFFFFFFFFFFFFFFFFFFFFFFFFFFFFF,FFFFFFFFFFFF:FFFFFF
@A00155:342:HHGFNDSXY:1:1530:1841:22811 2:N:0:GAACCTAG+TCCGCATA
ACGACTTACACGGTAGGTCGTGAACAAGCCCGGAGTACATCTCCTTTGGAACTAGGAGACCCCTCCGG
TCTTGTCCAACCTCTGTTTCGGCAGAGGATACCGTGCTCCACAGCTGTAAGGCTGTGGGCCCGAGAGA
+
FFFFFFFFF:FFFF:FF:FFFFFFFF::FFFFFFFFFFFFFFFFFFFFFFFFFFFFFF:FFFFFF,FF
FFFFFFFFFFFFFFFFF:FFFFFFFFFFFFFFFFFFFFFFFFFFFFFFFFFFFFFFFFFFFFFFFFFF:
@A00155:342:HHGFNDSXY:1:1530:11559:26960 2:N:0:GAACCTAG+TCCGCATA
GAAACCGTCGTTACTCTTCGAGTTGTGTAAGTTTCGTCCTAAAAAGGACTCGTCAGGTAGCCTACTAG
ACTACGACTTACACGGTAGGTCGTGAACAAGCCCGGAGTACATCTCCATTGGAACTAGGAGACCCCTC
+
FFFFFFFFFFFFFFFFFFFFFFFFFFFFFFFFF,FF:F:FFFFFFFFFFFFFFFFFFFFFFFFFFFFF
FFFFFF:FFFFFFFFFFFF:FFFFFFFF,F,FFFFFFFFFFFFFFFF:FFFFFFFFFFFFFFFFFFFF
@A00155:342:HHGFNDSXY:1:1530:10673:27117 2:N:0:GAACCTAG+TCCGCATA
GCCTACTAGACTACGACTTACACGGTAGGTCGTGAACAAGCCCGGAGTACATCTCCTTTGGAACCAGG
AGACCCCTCCGGTCTTGTCCAACCTCTGTTTCGGCAGAGGATACCGTGCTCCACAGCTGTAAGGCTGT
+
FFFFFFF,FFFFFFFFFFFFFFFFFFFFFF,FFFFFFFFFFFFFFF:FFFFFFFFFFFFFFF:FFFFFF
FFFFFFFFFFFFFFFFF:FFFFFFFFFFFFFFFFFFFFFFFFFFFFFF:FFFFFFFFFFFFFF:FFFFFF
@A00155:342:HHGFNDSXY:1:1531:27127:21605 2:N:0:GAACCTAG+TCCGCATA
AAGCCCGGAGTACATCTCCTTTGGAACTAGGAGACCCCTCCGGTCTTGTCCAACCTCTGTTTCGGCAG
AGGATACCGTGCTCCACAGCTGAAAGGCTGTGGGCCCGAGAGAAGTCGCTCTCTCTCGCCCTCCACAT
+
FF,:FF,FFFFFFFFFFFFFFFFFFFFFFFFFFFFFFFFFFFFFFFFFFFFFFFFFFFFFFFFFFF:F
FFFFFFFFFFFFFFFFFFFFFFFFFFFFFF:FFFFFFFFFFFFFF,FFFFFFFFFFF:FFFFFFFFFF:F
@A00155:342:HHGFNDSXY:1:1531:29749:34788 2:N:0:GAACCTAG+TCCGCATA
GGACTCGTCAGGTAGCCTACTAGACTACGACTTACACGGTAGGTCGTGAACAAGCCCGGAGTACATCT
CCTTTGGAACTAGGAGACCCCTCCGGTCTTGTCCAACCTCTGTTTCGGCAGAGGATACCGTGCTCCAC
+
FFFFFFF,FFFFFFFFFFFFFFF:FFFFFFFFFFFFFFFFFFFFFFFFFFFFFFFFFFFFFFFFFFFF
FFFFFFFFFFFFFFFFFFFFFFFFFFFFF,F:FFFFF:FFFFFFFFFFFFFFFFFFFFFFFFFFFFFFF
@A00155:342:HHGFNDSXY:1:1532:31186:11318 2:N:0:GAACCTAG+TCCGCATA
ACCTCTGTTTCGGCAGAGGATACCGTGCTCCACAGCTGTAAGGCTGTGGGCCCGAGAGAAGTCGCTCT
CTCTCGCCCTTCACATCCCTCTCGGCATTGAGATGACCATAGGCCAGAGGTGAGTCCTTAAGTGGAC
+
FFFFFFFFFFFFFFFFFFFFFFFFFFFFFFFFFFFFFFFFFFFFFFFFFFFFFFFFFFFFFFFFFFFF
FFFFFFFFFF:FFFFFFFFFFFFFFFFFFFFFFF:FFFFFF,FFFF,FFFFFFFFFFFFFFFF:FFFF
@A00155:342:HHGFNDSXY:1:1532:31774:25614 2:N:0:GAACCTAG+TCCGCATA
ACATCTCCTTTGGAACTAGGAGACCCCTCCGGTCTTGTCCAACCTCTGTTTCGGCAGAGGATACCGTG
CTCCACAGCTGTAAGGCTGTGGGCCCGAGAGAAGTCGCTCTCTCTCGCCCTCCACATCCCTCTCGGC
+
FFFFFFFFFFFFFF:FFFFFFFFFFFFFFFFFFFF:FFFFFFFFFFFFFFFFFFFFFFFFFFFFFFFFF
FFFFFF:FFFFFF,FFFFF:FFFFFFFFFFFF:FFFFF:FFFFFFFFFFFFFFFFFFFFFFFFFFFFF
```

```
@A00155:342:HHGFNDSXY:1:1532:26449:30608 2:N:0:GAACCTAG+TCCGCATA
TTACTCTTCGAGTTGTGTAAGTTTCGTCCTAAAAAGGACTCGTCAGGTAGCCTACTAGACTACGACTT
ACACGGTAGGTCGTGAACAAGCCCGGAGTACATCTCCTTTGGAACTAGGAGACCCCTCCGGTCTTGTC
+
FFFFFFFFFFFFFFFFFFFFFF,FFFFFFFFFFF,FFFFFFFFFFFFFFFFFFFFFFFFFFFFFFFFF
FFFFFFFFFFFFFFFF:F:FFFFFFFFFFFFFFFFFFFFFFFFFFFFFFFFFFFFFFFFFFFFFFFFF
@A00155:342:HHGFNDSXY:1:1532:8712:33113 2:N:0:GAACCTAG+TCCGCATA
GAAACCGTCGTTACTCTTTGAGTTGTGTAAGTTTCGTCCTAAAAAGGACTCGTCAGGTAGCCTACTAG
ACTACGACTTACACGGTAGGTCGTGAACAAGCCCGGAGTACATCTCCTTTGGAACTAGGAGACCCCTC
+
FFFFFFFFFFFFFFFF:,FF:FFFFFFFFFF:FFFF:FFFF:,FFFFFFFFFFFFFFFFFFFFFFFFF
FFFFFFFFFFFFF:FFFFFFFFFFFFFFFFFFFFFFFFFFFFFFFFFFFFFFFFFFFFFFFFFFFFFF
@A00155:342:HHGFNDSXY:1:1534:13810:18584 2:N:0:GAACCTAG+TCCGCATA
GTTGTGTAAGTTTCGTCCTAAAAAGGACTCGTCAGGTAGCCTACTAGACTACGACTTACACGGTAGGT
CGTGAACAAGCCCGGAGTACATCTCCTTTGGAACCAGGAGACCCCTCCGGTCTTGTCCAACCTCTGTT
+
FFFFFFFFFFFFFFFFFFFFFFFFFFFFFFFFFFFFFFFFFFFFFFFFFFFFFFFFFFFFFFFFFFFF
FFFFFFFFFF:FFFFFFFFFFFFFFFFFFFFFFFFFFF:FFFF:FFFFFFFFFFFFFFFFFFFFFFFF
@A00155:342:HHGFNDSXY:1:1535:8079:9251 2:N:0:GAACCTAG+TCCGCATA
ACATCTCCTTTGGAACTAGGAGACCCCTCCGGTCTTGTCCAACCTCTGTTTCGGCAGAGGATACCGTG
CTCCACAGCTGTAAGGCTGTGGGCCCGAGAGAAGTCGCTCTCTCTCGCCCTCCACATCCCTCTCGGCA
+
FFFFFFFF:FFFFFFF,FFFFFFFFFFFFFFF:F:FF:,FFFFFFF,FFFFFFFF:F:FFFFFFFFFF
FF:FFF:F:,FFFFFFFFFFFFFFFF:FFF:FFFFFFFFFFF:FFFFFF:F:,FFFFFFF,FFFFFFF
@A00155:342:HHGFNDSXY:1:1535:25382:16485 2:N:0:GAACCTAG+TCCGCATA
CGTCAGGTAGCCTACTAGACTACGACTTACACGGTAGGTCGTGAACAAGCCCGGAGTACATCTCCTTT
GGAACTAGGAGACCCCTCCGGTCTTGTCCAACCTCTGTTTCGGCAGAGGATACCGTGCTCCACAGCTG
+
FFFFFFFF:FFFFFFFFFFFFFFFFFFFFFFFFFFFFFFFFFFFFFFFFFFFFFFFFFFFFFFFFFFF
FFFFFFFFFFFFF:FFFFFFFFFFFFFF:FFFFFFFFFFFFFFFFFFFFFFFFFFFFFFFFFFFFFFF
@A00155:342:HHGFNDSXY:1:1535:15727:24314 2:N:0:GAACCTAG+TCCGCATA
CGTGAACAAGCCCGGAGTACATCTCCTTTGGAACTAGGAGACCCCTCCGGTCTTGTCCAACCTCTGTT
TCGGCAGAGGATACCGTGCTCCACAGCTGTAAGGCTGTGGGCCCGAGAGAAGTCGCTCTCTCTCGCCC
+
FFFFFFFFFFFFFFFFFFFFFFFFFFFFFFFFF:FFFFFFFFFFFFFFFFFFF:FFFFFFFFFFFFFF
FFFFFFFFFFFF:FFFFFFFFFFFFFFFFFFFFFFFFFFFFFFFFFFFFFFFFFFFFFFFFFFFFFFF
@A00155:342:HHGFNDSXY:1:1535:6099:27993 2:N:0:GAACCTAG+TCCGCATA
AGTACATCTCCTTTGGAACTAGGAGACCCCTCCGGTCTTGTCCAACCTCTGTTTCGGCAGAGGATACC
GTGCTCCACAGCTGTAAGGCTGTGGGCCCGAGAGAAGTCGCTCTCTCTCGCCCTCCACATCCCTCTCG
+
FFFFFFFFFFFFFFFFFFFFFFFFFFFFFFFFFFFFFFFFFFFFFFFFFFFFFFFFFFFFFFFF:FFF
FFFFFFFFFFFFFFFFFFFFFFFFFFFFFFFFFFF::FFFFF:FFFFFFFFFFFFFFFFFFFFFFFFF
@A00155:342:HHGFNDSXY:1:1535:23213:28917 2:N:0:GAACCTAG+TCCGCATA
AGTACATCTCCTTTGGAACTAGGAGACCCCTCCGGTCTTGTCCAACCTCTGTTTCGGCAGAGGATACC
GTGCTCCACAGCTGTAAGGCTGTGGGCCCGAGAGAAGTCGCTCTCTCTCGCCCTCTACATCCCTCTCG
+
FFFFFFFFFFF:FFFFFFFFFFFFFFFFFFFFFFFFFF:FFFFFFFFFFFFFFFFFFFFFFFFFFFFF
FF:FFFFFFFFFFFFFFFFFFFF:FFFFFFFFFFFFFFFFFFFFFFFFFFFFFFFFFFFFF:FFFFFFFF
@A00155:342:HHGFNDSXY:1:1535:6632:29700 2:N:0:GAACCTAG+TCCGCATA
AGTACATCTCCTTTGGAACTAGGAGACCCCTCCGGTCTTGTCCAACCTCTGTTTCGGCAGAGGATACC
GTGCTCCACAGCTGTAAGGCTGTGGGCCCGAGAGAAGTCGCTCTCTCTCGCCCTCCACATCCCTCTCG
+
FFFFFFFFFFFFFFFFFFFFFFFFFFFFFFFFFFFFFFFF,FFFFFFFFFFFFFFFFFFFFFFFFF:FF
FFFFFFFFFFFFFFFFFFFFFFFFFFFFFFFFFFFFFFFFFFFFFFFFFFFFFFFFFFFFFFFFFFFF
```

```
@A00155:342:HHGFNDSXY:1:1535:17083:33552 2:N:0:GAACCTAG+TCCGCATA
AGTACATCTCCTTTGGAACTAGGAGACCCCTCCGGTCTTGTCCAACCTCTGTTTCGGCAGAGGATACC
GTGCTCCACAGCTGTAAGGCTGTGGGCCCGAGAGAAGTCGCTCTCTCTCGCCCTCCACATCCCTCTCG
+
FFFFFFFFFFF:FFFFFFFFFFFFFFFFFFFFFFFFFFFFFFFFFFFFFFFFFFFFFFFFFFFFFFFF
FFFFFFFFFFFFFFFFFFFFFFFFFFFFFFFFFFFF:FFFFFFFFFFFFFFFFFFFFFFFFFFFFFFF
@A00155:342:HHGFNDSXY:1:1536:20564:9392 2:N:0:GAACCTAG+TCCGCATA
TACGACTTACACGGTAGGTCGTGAACAAGCCCGGAGTACATCTCCTTTGGAACTAGGAGACCCCTCCG
GTCTTGTCCAACCTCTGTTTCGGCAGAGGATACCGTGCTCCACAGCTGTAAGGCTGTGGGCCCG
+
FFFFFFFFFFFFFFFFFFFFFFFFFFFFFFFFFFFFFFFFFFFFFFFFFFFFFFFFFFFFFFFFFFFF
FFF,:F:FFFFFFFFFFFF:FFFFFFFFFF,FFFFFFFFFFFFFFF:FFF:FFFF:FFFFFFFF
@A00155:342:HHGFNDSXY:1:1536:14841:29700 2:N:0:GAACCTAG+TCCGCATA
TTTCGTCCTAAAAAGGACTCGTCAGGTAGCCTACTAGACTACGACTTACACGGTAGGTCGTGAACAAG
CCCGGAGTACATCTCCTTTGGAACCAGGAGACCCCTCCGGTCTTGTCCAACCTCTGTTTCGGCAGAGG
+
FFFFFFFFFFF:FFFFFFFFFFFFFFFFFFFFFFFFFFFFFFFFFFFFFFFFFFFFFFFFFFFFFFFF
FFFFFFFFFF:FFFFFFF:FFFFFFFFFFFFFFFFFFFFFFFFFFFFFFFFFFFFFFFFFFFFFFFFF
@A00155:342:HHGFNDSXY:1:1537:6099:3787 2:N:0:GAACCTAG+TCCGCATA
GAAACCGTCGTTACTCTTTGAGTTGTGTAAGTTTCGTCCTAAAAAGGACTCGTCAGGTAGCCTACTAG
ACTACGACTTACACGGTAGGTCGTGAACAAGCCCGGAGTACATCTCCTTTGGAACTAGGAGACCCCTC
+
FFFFF:FFFFFFFFF:,FFFFFFFFFFFFFFFFFFFFFFFFFFF,:FFFFFFFFFFFFFFFFFFFFFF
FFFF,FFFFFFFFFFFFFFFFFFFFFFFF:FFFFFFFFFFFFFFFFFFFFFFFFF:FFFFFFFFFFFF
@A00155:342:HHGFNDSXY:1:1537:15194:5008 2:N:0:GAACCTAG+TCCGCATA
ACTACGACTTACACGGTAGGTCGTGAACAAGCCCGGAGTACATCTCCTTTGGAACCAGGAGACCCCTC
CGGTCTTGTCCAACCTCTGTTTCGGCAGAGGATACCGTGCTCCACAGCTGTAAGGCTGTGGGCCCGAG
+
FFFFFFFFFF:FFFFFFFFF,FFFFFFFFFFFFFFFFFFFF:FFFFFFFFFFFFF,FF,FFFF:FFFF
FFFFFFFFF:F:FFFFFF:FFFFFFFFF:FF:FFF:F:FFFFFFFFF:FFFFFFFFFFFFFFFFFFFF
@A00155:342:HHGFNDSXY:1:1537:7075:7294 2:N:0:GAACCTAG+TCCGCATA
TCTCCTTTGGAACTAGGAGACCCCTCCGGTCTTGTCCAACCTCTGTTTCGGCAGAGGATACCGTGCTC
CACAGCTGTAAGGCTGTGGGCCCGAGAGAAGTCGCTCTCTCTCGCCCTCCACATCCCTCTCGGCATTG
+
FFFFFFFFFFFFFFFFFFFFF:FFFFFFFFFF:FFFFFFFFFFFFFFFFFFF:FFFFFFFFFFFFFFF
FFFFFFFFFFFFFFFFFFFFFFFFFFFFFFFFFFFFFFFFF:FFFFFFFFFFFFFFFFFFFFFFFFFF
@A00155:342:HHGFNDSXY:1:1537:7527:13338 2:N:0:GAACCTAG+TCCGCATA
GGTCGTGAACAAGCCCGGAGTACATCTCCTTTGGAACTAGGAGACCCCTCCGGTCTTGTCCAACCTCT
GTTTCGGCAGAGGATACCGTGCTCCACAGCTGTAAGGCTGTGGGCCCGAGAGAAGTCGCTCTCTCTCG
+
FFFFFFFFFFFFFFFFFFFFFFFFF:FFFFFFFFFFF:FFFFFFFFFFFFFFFFFFFFFFFFFFFFFF
FFFFFF:FFFFFFFFFFFFFFFFFFFFFFFFF:FFFFFFFFFFFFFFFFFFFF:FFFFFFFFFFFFFFF
@A00155:342:HHGFNDSXY:1:1538:22426:3724 2:N:0:GAACCTAG+TCCGCATA
CTGGCCTATGGTCATCTCAATGCCGAGAGGGATGTGGAGGGCGAGAGAGAGCGACTTCTCTCGGGCCC
ACAGCCTTACAGCTGTGGAGCACGGTATCCTCTGCCGAAACAGAGGTTGGACAAGACCGGAGGGGTCT
+
FFFFFFFFFFFFFFFFFFFFFFFFFFFFFFFFFF:FFFF:FFFFFFFFFFFFFFFFFFFFFFFF:FFFFF
FFFFFF,FFFFFF:FFFFFFFFFFF:FFF:FFFFFFFFFFFFFFFFFFFFFFFFFFFFFFFFFF,FFFFF
@A00155:342:HHGFNDSXY:1:1538:1570:7404 2:N:0:GAACCTAG+TCCGCATA
AAAAGGACTCGTCAGGTAGCCTACTAGACTACGACTTACACGGTAGGTCGTGAACAAGCCCGGAGTAC
ATCTCCTTTGGAACCAGGAGACCCCTCCGGTCTTGTCCAACCTCTGTTTCGGCAGAGGATACCGTGCT
+
FFF,FFFFFFFFFFFFFFFFFFFFFFFFFFFFFFFFFFFFFFFFFFFFFFFF:FFFFFFFFFFFFFFF
FFFFFFFFFFFFFFFFFFFFFFFFFFFFFFF:FFFFFFFF,FFFFFFFFFFFF:FF:FFFFFFFFFFFF
```

@A00155:342:HHGFNDSXY:1:1538:2112:14638 2:N:0:GAACCTAG+TCCGCATA
GGAGTACATCTCCTTTGGAACTAGGAGACCCCTCCGGTCTTGTCCAACCTCTGTTTCGGCAGAGGATA
CCGTGCTCCACAGCTGTAAGGCTGTGGGCCCGAGAGAAGTCGCTCTCTCTCGCCCTCCAC
+
FFFFFFFFFFFFFFFFFFFFFFFFFFFFFFFFFFFFFFFFFFFFFFFFFFFFFFFFFFFF:F:FFFFF
FFFFFFFFF:FFFFFFFFFFFFFFFFFFFFFFFFFFF,FFFFFFFFFFFFFFFFFFFF:F
@A00155:342:HHGFNDSXY:1:1538:28727:34428 2:N:0:GAACCTAG+TCCGCATA
GGACGAAACTTACACAACTCGAAGAGTAACGACGGTTTCGTCCTTGGAACGGACTCATCAGGGAGCCT
ACTAGACTCCGACCGTCATGGGGGTTCAAACCCATGCCCCGCTACACCGCCTTAGATCAGCTGTGTCC
+
F::FFFFFFFFFFFF:FFFFFFFFFFFFFFFFFFFFFFFFFFFFFFFFFFFFFFFFFFFFFFFFFFFF
FFFFFFFFFFFFFFFFFFFFFFFFFFFFFFFFFFFFFFFFFFFFFFFFFFFFFFFFFFFFFFFFFFFF
@A00155:342:HHGFNDSXY:1:1538:5186:35243 2:N:0:GAACCTAG+TCCGCATA
ACGAAACTTACACAACTCGAAGAGTAACGACGGTTTCGTCCTTGGAACGGACTCATCAGGGAGCCTAC
TAGACTCCGACCGTCATGGGGGTTCAAACCCATGCCCCGCCACACCGCCTTAGATCAGCTGTGTCCAC
+
FFFFFFFFFFFFFFFFFFFFFFFFFF:FFFFFFFFFF:FFFFFFFFFFFFFFFFFFFFFFFFFFFFFF
FFFFFFFFFFFFFFFFFFFFFFFFFFF:FFFFFFFFFFF:FFFFFFFFF,FFF:FFFFFFFFFFFFFF
@A00155:342:HHGFNDSXY:1:1539:15664:20228 2:N:0:GAACCTAG+TCCGCATA
GGTCGTGAACAAGTCCGGAGTACATCTCCTTTGGAACTAGGAGACCCCTCCGGTCTTGTCCAACCTCT
GTTTCGGCAGAGGATACCGTGCTCCACAGCTGTAAGGCTGTGGGCCCGAGAGAAGTCGCTCTCTCTCG
+
FFFFFFFFFFFF:FFFFFFFFFFFFFFFFFFFFFFFFFFFFFFFFFFFFFFFFFFFFFFFFFFFFFF
FFFFFFFFFF,FFFFFFFFFFFFFFFFFFFFFFFFFFF:FFFFFFFFFFFFFFFFFFFFFF:FFFFFF
@A00155:342:HHGFNDSXY:1:1539:28357:21292 2:N:0:GAACCTAG+TCCGCATA
ACAAGCCCGGAGTACATCTCCTTTGGAACCAGGAGACCCCTCCGGTCTTGTCCAACCTCTGTTTCGGC
AGAGGATACCGTGCTCCACAGCTGTAAGGCTGTGGGCCCGAGAGAAGTCGCTCTCTCTCGCCCTC
+
FFFFFFFFFFFFFFFFFFFFFFFFFFFFFFFFFFFFFFFFFFFFFFFFFFFFFFFFFFFFFFFFFFFF
FFFFFFFFFFFFFFFFFFFFFFFFFFFFFFFFFFFFFFFF:FFFF:FFFFFFFFFFFFFFFFFF:
@A00155:342:HHGFNDSXY:1:1540:30680:2268 2:N:0:GAACCTAG+TCCGCATA
ATCAGCTGTGTCCACTTAAGGACTCACCTCTGGCCTATGGTCATCTCAATGCCGAGAGGGATGTGGAG
GGCGAGAGAGAGCGACTTCTCTCGGGCCCACAGCCTTACAGCTGTGGAGCACGGTATCCTCTGCCG
+
FFFFFFFFFFFFFFFFFFFFFFFFFFFFFFFFFFFFFFFFFFFFFFFFFFFF:FFFFFFFFFFFFFFF
FFFFFFFFFFFFFFFFFFFFFFFFFFFFFFFFFFFFF,FFFFFFFFFFFFFFFFFFFFF:FFFFFF
@A00155:342:HHGFNDSXY:1:1540:27100:4335 2:N:0:GAACCTAG+TCCGCATA
TACTCTTTGAGTTGTGTAAGTTTCGTCCTAAAAAGGACTCGTCAGGTAGCCTACTAGACTACGACTTA
CACGGTAGGTCGTGAACAAGCCCGGAGTACATCTCCTTTGGAACTAGGAGACCCCTCCGGTCTTGTCC
+
FFFFFFFFFFFFFFFFFFFFFFFFFFFFFF:FFFFFFFFFFFFFFFFF:FFFFFFFFFFFFFFFFFFF
FFFFFFFFFFFFFFFF:FFFFFFFFFFFFF,FFFFFFFFFFFFFFFFFFFFFFFFFF:,FFFFFFFFF
@A00155:342:HHGFNDSXY:1:1540:18728:6151 2:N:0:GAACCTAG+TCCGCATA
CTACGACTTACACGGTAGGTCGTGAACAAGCCCGGAGTACATCTCCTTTGGAACTAGGAGACCCCTCC
GGTCTTGTCCAACCTCTGTTTCGGCAGAGGATACCGTGCTCCACAGCTGTAAGGCTGTGGGCCCGAGA
+
FFFFFFFFFFFFFFFFFFF:FFFFFFFFFFFFFFFFFFFFFFFFFF:FFFFFFFF:FFFFFFFFFFFF
FFFFFFFFFFFFFFFFFFFFFF:FFFF:FFFFFFFFFFFFFFFFFFFFF:FFFFFFFFFFFFFFFFFF
@A00155:342:HHGFNDSXY:1:1540:18647:7952 2:N:0:GAACCTAG+TCCGCATA
CTACGACTTACACGGTAGGTCGTGAACAAGCCCGGAGTACATCTCCTTTGGAACTAGGAGACCCCTCC
GGTCTTGTCCAACCTCTGTTTCGGCAGAGGATACCGTGCTCCACAGCTGTAAGGCTGTGGGCCCGAGA
+
FFFFFFFFFFFFFFFFFFFFFFFFFFFFFFFFFFFFFF:FFFFFFFF:FFFFFFFFFFFFFFFFFFFF
FFFFFFFF:FFFFFFFFFFFFFFFFFFFFFFFFFFFFFFFFFFFFFFFFFFFFFFFFFFFFFFF:FF

```
@A00155:342:HHGFNDSXY:1:1540:16685:12790 2:N:0:GAACCTAG+TCCGCATA
ACTCTTCGAGTTGTGTAAGTTTCGTCCTAAAAAGGACTCGTCAGGTAGCCTACTAGACTACGACTTAC
ACGGTAGGTCGTGAACAAGCCCGGAGTACATCTCCTTTGGAACTAGGAGACCCCTCCGGTCTTGTCCA
+
FFFFFFFFFFFFFFFFFFFFFFFFFFFFFFFFFFFFFFFFFFFFFFFFFFFFFFFFFFFFFFFFFFF:
FFFFFFFFFFFFFFFFFFFFFFFFFFFFFFFFFFF:FFFFFFFFFFFFFFFFFFFFFFFFFFFFFFFF
@A00155:342:HHGFNDSXY:1:1540:5963:30201 2:N:0:GAACCTAG+TCCGCATA
CTACGACTTACACGGTAGGTCGTGAACAAGCCCGGAGTACATCTCCTTTGGAACCAGGAGACCCCTCC
GGTCTTGTCCAACCTCTGTTTCGGCAGAGGATACCGTGCTCCACAGCTGTAAGGCTGTGGGCCCGAGA
+
FFFFFFFFFFFFFFFFFFFFFFFFFFFFFFFFFFFFFFFFFFFFFFFFFFFFFFFFFFFFFFFFFFFF
FFFFFFFFFFFFFFFFF:FFFFFFFFFFFFFFFFFFFFFFFFFFFFFFFFFFFFFFFFFFFFFFFFFF
@A00155:342:HHGFNDSXY:1:1540:6596:33082 2:N:0:GAACCTAG+TCCGCATA
TAGGTCGTGAACAAGCCCGGAGTACATCTCCTTTGGAACTAGGAGACCCCTCCGGTCTTGTCCAACCT
CTGTTTCGGCAGAGGATACCGTGCTCCACAGCTGTAAGGCTGTGGGCCCGAGAGAAGTCGCTCTCTCT
+
FFFFFFFFFFFFFFFFFFFFFFFFFFFFFFFFFFFFFFFFFFFFFFFFFFFFFFFFFFFFFFFFFFFF
FFFFFFFFFFFFFFFFFFFFFFFFFFFFFFFFFFFFFFFFFFFFFFFFFFFFFFFFFFFFFFFFFFFF
@A00155:342:HHGFNDSXY:1:1541:12355:22013 2:N:0:GAACCTAG+TCCGCATA
AGAGAGAGCGACTTCTCTCGGGCCCACAGCCTTACAGCTGTGGAGCACGGTATCCTCTGCCGAAACAG
AGGTTGGACAAGACCGGAGGGGTCTCCTAGTTCCAAAGGAGATGTACTCCGGGCTTGTTCACGACCTA
+
FFFFFFFFFFFFFFFFFFFFFFFFFFFFFFFFFFFFFFFFFFFFFFFFFFFFFFFFFFFF:F:FFFFF
FFFFFFFFFFFFFFFFFFFFFFFFFFFFF:FFFF:FFFFFFFFFFF,FFFFFFFF:FFFF,FFFFFFFF
@A00155:342:HHGFNDSXY:1:1541:27172:28541 2:N:0:GAACCTAG+TCCGCATA
CAGGGAGCCTACTAGACTCCGACCGTCATGGGGGTTTAAACCCATGCCCCGCCACACCGCCTTAGATC
AGCTGTGTCCACTTAAGGACTCACCTCTGGCCTATGGTCATCTCAATGCCGAGAGGGATGTGGAGGGC
+
FFFFFFFFFFFFFFFFFFFFFFFFFFFFFFFFFFFFFFFFFFFFFFFFFFFFFFFFFFFFFFFFFFFF
FFFFFFFFFFFFFFFFFFFFFFFFFFFFFFFFFFFFFFFFFFFFFFFFFFFFFFFFFFFF:FFF:FFFFF
@A00155:342:HHGFNDSXY:1:1541:19569:30686 2:N:0:GAACCTAG+TCCGCATA
GCCTACTAGACTCCGACCGTCATGGGGGTTCAAACCCATGCCCCGCCACACCGCCTTAGATCAGCTGT
GTCCACTTAAGGACTCACCTCTGGCCTATGGTCATCTCAATGCCGAGAGGGATGTGGAGGGCGAGAGA
+
FFFFFFFF,FFFFFFFFFFFFFFFFFFFFFFFFFFFFFFFFFFFFFFFFFFFFFFFFFFFFFFFFFFF
FFFFFF:FF:FFFFFFFFFFFFFFFFF:FFFFFFFFFFFFF:F:FFFFFFF:FFFFFFFFFFFFFFFF
@A00155:342:HHGFNDSXY:1:1542:15121:14184 2:N:0:GAACCTAG+TCCGCATA
CACGGTAGGTCGTGAACAAGCCCGGAGTACATCTCCTTTGGAACCAGGAGACCCCTCCGGTCTTGTCC
AACCTCTGTTTCGGCAGAGGATACCGTGCTCCACAGCTGTAAGGCTGTGGGCCCGAGAGAAGTCGCTC
+
FFFFFFFFFFFFFFFFFFFFFFFFFFFFFFFFFFFFFFFFFFFFFFFFFFFFFFFFFFFFFFF,FF:FF
FFFFFFFFFFFFFFFFFFFFFFFFFFFFFFFFFFFFFFFFFFFFFFFFFFFFFFFFFFF:F:FFFFFFFF
@A00155:342:HHGFNDSXY:1:1542:12472:24878 2:N:0:GAACCTAG+TCCGCATA
CAGGTAGCCTACTAGACTACGACTTACACGGTAGGTCGTGAACAAGCCCGGAGTACATCTCCTTTGGA
ACTAGGAGACCCCTCCGGTCTTGTCCAACCTCTGTTTCGGCAGAGGATACCGTGCTCCACAGCTGTAA
+
FFFFF:FFFFFFFFFFFFFFFFFFFFFFFFFFFFFFFFFFFFFFFFFFFFFFFFFFFFFFFFFFFFFFF
FFFFFFFFFFFFFFFFFFFFFF:FFFFFFFFFFFFFFFFFFFFFFFFFFFFFFFFFFFFFFFFFFFF,F
@A00155:342:HHGFNDSXY:1:1542:26069:24972 2:N:0:GAACCTAG+TCCGCATA
GGAGTACATCTCCTTTGGAACTAGGAGACCCCTCCGGTCTTGTCCAACCTCTGTTTCGGCAGAGGATA
CCGTGCTCCACAGCTGTAAGGCTGTGGGCCCGAGAGAAGTCGCTCTCTCTCGCCCTCCACATCCCTCT
+
FFFFFFFFFFFFFFFFFFFFFFFFFFFFFFFFFFFFFFFFFFFFFFFFFFFFFFFFFFFFFFFFF:FFF
FFFFFFFFFFFFFFFFFF:FFFFFFFFFFFFF:FFFFFFFFFFFFFFFFFFFFFFFFFFFFFFFFFFFF
```

```
@A00155:342:HHGFNDSXY:1:1542:6497:27712 2:N:0:GAACCTAG+TCCGCATA
AGCCCGGAGTACATCTCCTTTGGAACTAGGAGACCCCTCCGGTCTTGTCCAACCTCTGTTTCGGCAGA
GGATACCGTGCTCCACAGCTGTAAGGCTGTGGGCCCGAGAGAAGTCGCTCTCTCTCGCCCTCCACATC
+
FFFFFFFFFFFFFFFFFFFFFFFFF:FFFFFFFFFFFFFFFFFFFFFFFFFFFFFF:FFFFFFFFFF:
FFFFFFFFFFF:FFFFFFFFFFF:FFFFFFFF:FFFFFFFFFFFFFFFFFFFFFFFFFFFFFFFFFFF
@A00155:342:HHGFNDSXY:1:1542:2266:27868 2:N:0:GAACCTAG+TCCGCATA
GTGAACAAGCCCGGAGTACATCTCCTTTGGAACTAGGAGACCCCTCCGGTCTTGTCCAACCTCTGTTT
CGGCAGAGGATACCGTGCTCCACAGCTGTAAGGCTGTGGGCCCGAGAGAAGTCGCTCTCTCTCGCC
+
FFFFFFFFFFFFFFFFFFFFFFFFFFFFFFFFFFFFFFFFFFFFFFFFFFFFFFFFFFFFFFFFFFFF
FFFFFFFFFFFFFFFFFFFFFFFFFFFFFFFFFFFFFFFFFFFFFFFFFFFFFFFFFFFFFFFFFF
@A00155:342:HHGFNDSXY:1:1542:2727:32330 2:N:0:GAACCTAG+TCCGCATA
TACGACTTACACGGTAGGTCGTGAACAAGCCCGGAGTACATCTCCTTTGGAACGAGGAGACCCCTCCG
GTCTTGTCCAACCTCTGTTTCGGCAGAGGATACCGTGCTCCACAGCTGTAAGGCTGTGGGCCCG
+
,FFFF,FFF:FFFFFFFFFFFF:FFFFF,FFFF,F,FFFFFFFF:F,FFFFFF:FF:FFFFFF:FFFF
FFFFF,FFFFFFF:F:FFFFFF,FFF:FFFFFFFFF,FFFFFFFFF::FFFFFFF,FFFFFFF:
@A00155:342:HHGFNDSXY:1:1543:13991:23156 2:N:0:GAACCTAG+TCCGCATA
AAGCCCGGAGTACATCTCCTTTGGAACTAGGAGACCCCCCCGGTCTTGTCCAACCTCTGTTTCGGCAG
AGGATACCGTGCTCCACAGCTGTAAGGCTGTGGGCCCGAGAGAAGTCGCTCTCTCTCGC
+
FF:FFFFFFFFFFFFFFF:FFFFF:FFFFFFFFFFFFFFFFFFFF:FFFFFFFFFFFFF:FFFFFFFF
:FFFF:FFFFFFFFFFFFFFFFFFFFFF,FFFFFFFFFFFFFFFFFFFFFFFFFFFFFF
@A00155:342:HHGFNDSXY:1:1543:7844:29324 2:N:0:GAACCTAG+TCCGCATA
TGGAACTAGGAGACCCCTCCGGTCTTGTCCAACCTCTGTTTCGGCAGAGGATACCGTGCTCCACAGCT
GTAAGGCTGTGGGCCCGAGAGAAGTCGCTCTCTCTCGCCCTCCACATCCCTCTCGGCATTGAGATGAC
+
FFFFFFFFFFFFFFFFFFFFFFFFF,FFFFFFFFFFFFFF:FFFF:FFFFFFFFFFFFFFFFFF:FFF
FFFFFFFFFFFFFFFFF,FFF,,FF:FFFFFFFFFFFFFFFFFFF,F,FFFFFFFFFFF,FFFFF:FF
@A00155:342:HHGFNDSXY:1:1544:8034:16313 2:N:0:GAACCTAG+TCCGCATA
GACGAAACCGTCGTAACTCTTCGAGTTGTGTAAGTTTCGTCCTAAAAAGGACTCGTCAGGTAGCCTAC
TAGACTACGACTTACACGGTAGGTCGTGAACAAGCCCGGAGTACATCTCCTTTGGAACTAGGAGACCC
+
FFFFFFFFFFFFFFFFFFFFFFFFFFFFFFFFFFFFFFFFFFFFFFFFFFFFFFFFFFFFFFFFFFFF
FFFFFFFFFFFFFFFFF:FFF:FFFFFFFFFFFFFFFFFFFFFFFFFFFFFFFFFFFFFFFFFF:FFF
@A00155:342:HHGFNDSXY:1:1544:27760:20290 2:N:0:GAACCTAG+TCCGCATA
GGACTCGTCAGGTAGCCTACTAGACTACGACTTACACGGTAGGTCGTGAACAAGCCCGGAGTACATCT
CCTTTGGAACCAGGAGACCCCTCCGGTCTTGTCCAACCTCTGTTTCGGCAGAGGATACCGTGCTCCAC
+
FFFFFFFFFFFFFFFFFFFFF:FFFFFFFFFFFFFFFFFFFFFFFFFFFFFFFFFFF:FFFFFFFFFF
FFFFFFFFFFFFFFFFFFFFFFFFFFFFFFFFFFFFFFFFFFFFFFFFFFFFFF:FFFFFFF:FFFFF
@A00155:342:HHGFNDSXY:1:1544:27868:28933 2:N:0:GAACCTAG+TCCGCATA
GGACTCGTCAGGTAGCCTACTAGACTACGACTTACACGGTAGGTCGTGAACAAGCCCGGAGTACATCT
CCTTTGGAACCAGGAGACCCCTCCGGTCTTGTCCAACCTCTGTTTCGGCAGAGGATACCGTGCTCCAC
+
FFFFFFFF:FFFF,FFFFFFFFFFFFFFF:FFFF:FFFFFFF:FFFFFFFFFFFFFFF:FFF:FFFFF
FFF:FFFFFF:,F:FFFFFFFFFFFFFFFFFFFF:FFFFFFF,FFFFFFFFFFFFFFFFFFFFFFFFF
@A00155:342:HHGFNDSXY:1:1544:12445:36761 2:N:0:GAACCTAG+TCCGCATA
GAGACCCCTCCGGTCTTGTCCAACCTCTGTTTCGGCAGAGGATACCGTGCTCCACAGCTGTAAGGCTG
TGGGCCCGAGAGAAGTCGCTCTCTCTCGCCCTCCACATCCCTCTCGGCATTGAGATGACCATAGGCCA
+
FFFFFFFFFFFFFFFFFFFFFFFFFFFFFFFFFFFFFFFFFF:FFFFFFFFFFFFFFFFFFF:FFF:F
:FFFFFFFFFFFFFFFFFFFFFFFFFFFFFF:FFFFFFFFF:FFFFFFFF,FFF:FFFFF,FFFFFFF
```

@A00155:342:HHGFNDSXY:1:1545:29496:8891 2:N:0:GAACCTAG+TCCGCATA
AAAAAGGACTCGTCAGGTAGCCTACTAGACTACGACTTACACGGTAGGTCGTGAACAAGCCCGGAGTA
CATCTCCTTTGGAACTAGGAGACCCCTCCGGTCTTGTCCAACCTCTGTTTCGGCAGAGGATACCGTGC
+
FFFFFFFFFFFFFFFFFFFFFFFFFFFFFFFFFFFFFFFFFFFFFFFFFFFFFFFFFFFFFFFFFFFF
FFFFFFFFFFFFFFFFFFFFFFFF:FFFFFFFFFFFFFFFFFFFFFFFFFFFFFFFFFFFF:FFFFFF
@A00155:342:HHGFNDSXY:1:1545:5520:10645 2:N:0:GAACCTAG+TCCGCATA
TACGACTTACACGGTAGGTCGTGAACAAGCCCGGAGTACATCTCCTTTGGAACTAGGAGACCCCTCCG
GTCTTGTCCAACCTCTGTTTCGGCAGAGGATACCGTGCTCCACAGCTGTAAGGCTGTGGGCCCG
+
FFFFFFFFFFFFFFFFFFFFFFFFFFFFFFFFFFFFFFFFFFFFFFFFFFF:FFFFFFFFF:FFFFFF
FFFFFFFFFFFFFFFFFFFFFFFFFFFFFFFFFFFFFFFFFF:FFFFFFFFFFFFFFFFFFFFF
@A00155:342:HHGFNDSXY:1:1545:17852:14904 2:N:0:GAACCTAG+TCCGCATA
ACTAGGAGACCCCTCCGGTCTTGTCCAACCTCTGTTTCGGCAGAGGATACCGTGCTCCACAGCTGTAA
GGCTGTGGGCCCGAGAGAAGTCGCTCTCTCTCGCCCACCACATCCCTCTCGGCATTGAGAT
+
FFFFFFFFFF::FFFFFFFF,FFFFF:FFF,FF:,F,FFFFFFFFFFFFFFFFF:F:F::F:FFFFF:
:FFF:,FFFFFFFFFFFFFFFF:FFF:FFFFFFFFF,FFFFFFFFFFFFFFFF:,:FFFF:
@A00155:342:HHGFNDSXY:1:1546:32696:10207 2:N:0:GAACCTAG+TCCGCATA
CAGGTAGCCTACTAGACTACGACTTACACGGTAGGTCGTGAACAAGTCCGGAGTACATCTCCTTTGGA
ACTAGGAGACCCCTCCGGTCTTGTCCAACCTCTGTTTCGGCAGAGGATACCGTGCTCCACAGCTGTAA
+
FFFFFFFFFFFFFFFFFFFFFFFFFFFFFFFFFFF:FFFFFFFFFFFFFFFFFFFF:FFFFFFFFFFF
FFF:FFFFFFFFFFFFFFFFFFFFFFFFF:FFFFFFFFFFFFFFFFFFFF:FFFFFFFFFFFFFFFFF
@A00155:342:HHGFNDSXY:1:1546:25681:21512 2:N:0:GAACCTAG+TCCGCATA
AGGACTCGTCAGGTAGCCTACTAGACTACGACTTACACGGTAGGTCGTGAACAAGCCCGGAGTACATC
TCCTTTGGAACCAGGAGACCCCTCCGGTCTTGTCCAACCTCTGTTTCGGCAGAGGATACCGTGCTCCA
+
FFFFFFFFFF:FFFFFFFFFFFFFFFFFFFFFFFFFFFFFF:FFFFFFFFFFFF:FFFFFFFFFFFFF
FFFFFFFFFFFFFFFFFFFFFFFFFFFFFFFFFFFFFFFFFFFFFFFFFFFFFFFFFFFFFFFFFFFF
@A00155:342:HHGFNDSXY:1:1546:9652:34648 2:N:0:GAACCTAG+TCCGCATA
CCTACTAGACTACGACTTACACGGTAGGTCGTGAACAAGCCCGGAGTACATCTCCTTTGGAACTAGGA
GACCCCTCCGGTCTTGTCCAACCTCTGTTTCGGCAGAGGATACCGTGCTCCACAGCTGTAAGGCTGTG
+
FFFFFFFFFFFFFFFFFFFFFFFFFFFFFFFFFFFFFFFFFFFFFFFFFFFFFFFFFFFFFFFF:FFF
FFFFFFFFFFFFFFFFFFFFFFFFFFF:FFFFFFFF,FFFFFF:FFFFFFFFFFFFFF:FFFFFFFFF
@A00155:342:HHGFNDSXY:1:1547:12789:14935 2:N:0:GAACCTAG+TCCGCATA
ACTTACACGGTAGGTCGTGAACAAGCCCGGAGTACATCTCCTTTGGAACTAGGAGACCCCTCCGGTCT
TGTCCAACCTCTGTTTCGGCAGAGGATACCGTGCTCCACAGCTGTAAGGCTGTGGGCCCGAGAGAAGT
+
FFFFFFFFFFFFFFFFFFFFFFFFFFFFFFF:FFFFFFFFFFFFFFFFFFFFFFFFFFFFFFFFFFFF
FFFFFFFFFFFFF:FFFFFFFFFFFFFFFFFFFFFFFFF:FFFFFF,FFFFFFF:FFFFFFFFFFFF:
@A00155:342:HHGFNDSXY:1:1548:20980:30060 2:N:0:GAACCTAG+TCCGCATA
GGACTCGTCAGGTAGCCTACTAGACTACGACTTACACGGTAGGTCGTGAACAAGCCCGGAGTACATCT
CCTTTGGAACTAGGAGACCCCTCCGGTCTTGTCCAACCTCTGTTTCGGCAGTGGATACCGTGCTCCAC
+
FFFFFFFFFFFFFFFFFFFFFFFFFFFFFFFFFFFFFFFFFFFFFFFFFFFFFFFFFFFFFFFFFFFF
FFFFFFFFFFFFFFFFFFFFFFFFFFFFFFFFFFFFFFFFFFFFFF:FFFF,FFFFFF:FFFFFFFFF
@A00155:342:HHGFNDSXY:1:1549:25482:8766 2:N:0:GAACCTAG+TCCGCATA
GGAGTACATCTCCTTTGGAACCAGGAGACCCCTCCGGTCTTGTCCAACCTCTGTTTCGGCAGAGGATA
CCGTGCTCCACAGCTGTAAGGCTGTGGGCCCGAGAGAAGTCGCTCTCTCTCGCCCTCCAC
+
FFFFFFFFFFF:FFFFFFFFFFFFFFFFFFFFFFFFFFFFFFFFFFFFFFFFFFFFF:FFFFFFFFFF
FFFFFFFFFFFFFFFFFFFFFFFFFFFFFFFFFF:FFFFFFFFFFFFFFFFFFFFFFFFF

```
@A00155:342:HHGFNDSXY:1:1550:13476:12680 2:N:0:GAACCTAG+TCCGCATA
AACTAGGAGACCCCTCCGGTCTTGTCCAACCTCTGTTTCGGCAGAGGATACCGTGCTCCACAGCTGTA
AGGCTGTGGGCCCGAGAGAAGTCGCTCTCTCTCGCCCTCCACATCCCTCTCGGCATTGAGATGAC
+
FFFFFFFFFFFFFFFFFFFFFFFFFFFFFFFFFFFFFFFFFFFFFFFFFFFFFFFFFFFFFFFFFFFF
FFFFFFFFFFFFFFFFFFFFFFFFFFFFFFFFFFFFFFFFFFFFFFFFFFFFFFFFFFFFFFFFF
@A00155:342:HHGFNDSXY:1:1550:25337:17221 2:N:0:GAACCTAG+TCCGCATA
GTTTCGTCCTAAAAAGGACTCGTCAGGTAGCCTACTAGACTACGACTTACACGGTAGGTCGTGAACAA
GCCCGGAGTACATCTCCTTTGGAACTAGGAGACCCCTCCGGTCTTGTCCAACCTCTGTTTCGGCAGAG
+
FFFF:FFFFFFFFFFFFFFFFFFFFF:FFFFF:F,FFFFFFFFF:FFFFFFFF:FFF:FFFFFFFFFF
:FFFFFFFFFFFFF:FFFFFFFFFFFFFFFFFFFF::FFFFFF::FFFFFFFFFFFFF:FFFFFF:FF
@A00155:342:HHGFNDSXY:1:1550:11903:25300 2:N:0:GAACCTAG+TCCGCATA
TACTCTTCGAGTTGTGTAAGTTTCGTCCTAAAAAGGACTCGTCAGGTAGCCTACTAGACTACGACTTA
CACGGTAGGTCGTGAACAAGCCCGGAGTACATCTCCTTTGGAACTAGGAGACCCCTCCGGTCTTGTC
+
FFFF:F,FFFFFFFFFFFFFFFFFFFFFFFFFFF:FFFFFFFFF,F::FFFF,FFFFFFF,FFFFF:F
FFFFF,FFFFFFFFFFFFFFFFFFFFFFFF:FFF:FFFFFFFFFFFFF,FFFFFFFFF:F:FFF:FFF
@A00155:342:HHGFNDSXY:1:1550:11767:26318 2:N:0:GAACCTAG+TCCGCATA
TACTCTTCGAGTTGTGTAAGTTTCGTCCTAAAAAGGACTCGTCAGGTAGCCTACTAGACTACGACTTA
CACGGTAGGTCGTGAACAAGCCCGGAGTACATCTCCTTTGGAACTAGGAGACCCCTCCGGTCTTGTCC
+
FFFFFFFFFFFFFFFFF:FFFFFFFFFFFFFFFFFFFFFFFFFFFFFFFFFFFFFFFFFFFFFFFFFF
FFFF:FFFFFFFFFFFFFFFFFFFFFFFFFFFFFFFFFFFFFFFFFFFFFFFFFFFFFFFFFFFFFFF
@A00155:342:HHGFNDSXY:1:1551:23122:2707 2:N:0:GAACCTAG+TCCGCATA
GGAGTACATCTCCTTTGGAACTAGGAGACCCCTCCGGTCTTGTCCAACCTCTGTTTCGGCAGAGGATA
CCGTGCTCCACAGCTGTAAGGCTGTGGGCCCGAGAGAAGTCGCTCTCTCTCGCCCTCCACATCCCTCT
+
FFFFFFFFFFFFFFFFFFFFFFFFFFFFFFFFFFFFFFFFFFFFFFFFFFFFFFFFFFFFFFFFFFFF
FFFFFFFFFFFFFFFFFFFFFFFFFFFFFFFFFFFFFFFFFFFFFFFFFFFFFFFFFFFFFFFFFFFF
@A00155:342:HHGFNDSXY:1:1551:31485:20290 2:N:0:GAACCTAG+TCCGCATA
GTACATCTCCTTTGGAACTAGGAGACCCCTCCGGTCTTGTCCAACCTCTGTTTCGGCAGAGGATACCG
TGCTCCACAGCTGTAAGGCTGTGGGCCCGAGAGAAGTCGCTCTCTCTCGCCCTCTACATCCCTCTCG
+
F:FFFFFFFFFFFFFF:FFFFFFFFFFFFFFFFFFF::F:FFFFFFFF:FFF,FFFF:FFFF:FFFFF
FFFFFFFF:FF,FF,,FFF:FFFFFFFFFFFFFF:F:FFFFFFFFFFFFFFFFFFFF:FFFF:FFFF
@A00155:342:HHGFNDSXY:1:1551:9381:30545 2:N:0:GAACCTAG+TCCGCATA
CTGGCCTATGGTCATCTCAATGCCGAGAGGGATGTGAAGGGCGAGAGAGAGCGACTTCTCTCGGGCCC
ACAGCCTTACAGCTGTGGAGCACGGTATCCTCTGCCGAAACAGAGGTTGGACAAGACCGG
+
FFFFFFFFFFFFFFFFFFFFFFFFFFFF:FFFFFFFFFF:FFFFFFFFFFFFFFFFFF:FFFFFFFFF
FFFFFFFFFFFFFFFFFFFFFFFFFFF:FFFFFFFFFFFFFFFFFFFFFFFFFFFFFFFFF
@A00155:342:HHGFNDSXY:1:1552:16260:3881 2:N:0:GAACCTAG+TCCGCATA
TCCTAAAAAGGACTCGTCAGGTAGCCTACTAGACTACGACTTACACGGTAGGTCGTGAACAAGCCCGG
AGTACATCTCCTTTGGAACTAGGAGACCCCTCCGGTCTTGTCCAACCTCTGTTTCGGCAGAGGATACC
+
FFFFFFFFFFFFFFFFFFFFFFFFFFFFFFFFFFFFFFFFFFFFFFFFFFFFFFFFFFFFFFFFFFFF
FFFFFFFFFFFFFFFFFFFFFFFFFFFFFFFFFFFFFFFFFFFFFFFFFFFFFFFFFFFFFFF:FFFF
@A00155:342:HHGFNDSXY:1:1552:22688:10723 2:N:0:GAACCTAG+TCCGCATA
CTAGTTCCAAAGGAGATGTACTCCGGGCTTGTTCACGACCTACCGTGTAAGTCGTAGTCTAGTAGGCT
ACCTGACGAGTCCTTTTTAGGACGAAACTTACACAACTCGAAGAGTAACGACGGTTTCGTCCTTGGAA
+
FFFFFFFFFFFFFFFFFFFFFFFFFFFFF,FFFFFFFFFFFFFFFFFFFFFFFFFFFFFFFFFFFFFF
FFFFFFFFFFFFFFFFFFFFFFFFFFFFFF:FFFFFFFFFFFFFFFFFFFFFFF:FF:FFF:FFFFFFF:
```

```
@A00155:342:HHGFNDSXY:1:1552:19180:20932 2:N:0:GAACCTAG+TCCGCATA
CAGGTAGCCTACTAGACTACGACTTACACGGTAGGTCGTGAACAAGCCCGGAGTACATCTCCTTTGGA
ACTAGGAGACCCCTCCGGTCTTGTCCAACCTCTGTTTCGGCAGAGGATACCGTGCTCCACAGCTGTAA
+
FFFFFFFFFFFFFFFFFFFFFFFFFFFFFFFFFFFFFFFFFFFF,FFFFFFFFFFFFFFF,FF:FF
:FFFFFFFFFFFFFFFFFFFFFFFFFFFFFFFFFFFF:FFFFFFFFFFF:FFF:FFFFFFFFFF,F:F:
@A00155:342:HHGFNDSXY:1:1552:32090:23249 2:N:0:GAACCTAG+TCCGCATA
GTTTCGTCCTAAAAAGGACTCGTCAGGTAGCCTACTAGACTACGACTTACACGGTAGGTCGTGAACAA
GCCCGGAGTACATCTCCTTTGGAACTAGGAGACCCCTCCGGTCTTGTCCAACCTCTGTTTCGGCAGAG
+
FFFF:FFFFFFFFFFFFFFFFFFFFFFFFFFFFFFFFFFFFFFFFFFFFFFFFFFFFFFFFFFFFFF
FFFFFFFFFFFFFFFFF:FFFFFFFFFFFFFFFFFFFFFFFFFFFFFFFFFFFF:FFFFFFFFFFFFFF
@A00155:342:HHGFNDSXY:1:1553:13810:9314 2:N:0:GAACCTAG+TCCGCATA
GCCCGGAGTACATCTCCTTTGGAACTAGGAGACCCCTCCGGTCTTGTCCAACCTCTGTTTCGGCAGAG
GATACCGTGCTCCACAGCTGTAAGGCTGTGGGCCCGAGAGAAGTCGCTCTCTCTCGCCC
+
FFFFFFFFFFFFFFFFFFFFFFFFFFFFFFFFFFFFFFFFFFFFFFFFFFFFFFFFFFFFFFFFFFFF
FFFFFFFFFFFFFFFFFFFFFFFFFFF:FFFFFFFFFFFFFFFFFFFFFFFFFFFFFFFFFFF
@A00155:342:HHGFNDSXY:1:1553:23113:19163 2:N:0:GAACCTAG+TCCGCATA
GGACTCACCTCTGGCCTATGGTCATCTCAATGCCGAGAGGGATGTAGAGGGCGAGAGAGAGCGACTTC
TCTCGGGCCCACAGCCTTACAGCTGTGGAGCACGGTATCCTCTGCCGAAACAGAGGTTGGACAAGACC
+
FFFFFFFFFFFFFFFFFFFFFFFFFFFFFF:FFFFFFFFFFFFFFFFFFFFFFFFFFFFFFFFFFFFF
FFFF:FFFFFFFFFFFFFFFFFFFFFFFFFFFFFFFFFFFFFFFFFFFFFFFFFFF:FFFFFFFFFF
@A00155:342:HHGFNDSXY:1:1553:7554:23563 2:N:0:GAACCTAG+TCCGCATA
CACGGTAGGTCGTGAACAAGCCCGGAGTACATCTCCTTTGGAACTAGGAGACCCCTCCGGTCTTGTCC
AACCTCTGTTTCGGCAGAGGATACCGTGCTCCACAGCTGTAAGGCTGTGGGCCCGAGAGAAGTCGCTC
+
FFFFFFFFFFFFFFFFFFFFFFFFFFFFFFFFFFFFFFFFFFFFFFFFFFFFFFFFFFFFFFF:FFFFF
:FFFFFFFFFFFFFFFFFFFFFFFFFFFFFFFFFFFFFFFFFFFFFFFFFFFFFFFFFFFFFFFFFFFF
@A00155:342:HHGFNDSXY:1:1553:24334:28604 2:N:0:GAACCTAG+TCCGCATA
CTACGACTTACACGGTAGGTCGTGAACAAGCCCGGAGTACATCTCCTTTGGAACTAGGAGACCCCTCC
GGTCTTGTCCAACCTCTGTTTCGGCAGAGGATACCGTGCTCCACAGCTGTAAGGCTGAGGGCCCGAGA
+
FFFFFFFFFFFFFFFFFFFFFFFFFFFFFFFFFFFFFFFFFFFFFFFFFFFFFFFFFFFFFFFFF:FFF
FFFFFFFFFFFFFFFFFFFFFFFFFFFFFFFFFFFFFFFFFFFFFFFF:FFFFFFF,FFFFF:FFFF
@A00155:342:HHGFNDSXY:1:1553:9073:34585 2:N:0:GAACCTAG+TCCGCATA
GTTTCGTCCTAAAAAGGACTCGTCAGGTAGCCTACTAGACTACGACTTACACGGTAGGTCGTGAACAA
GCTCGGAGTACATCTCCTTTGGAACTAGGAGACCCCTCCGGTCTTGTCCAACCTCTGTTTCGGCAGAG
+
FFFFFFFFFFFFFFFFFFFFFFFFFFFFFFFFFFFFFFFFFFFFFFFFFFFFFFFFFFFFFFFFFFFF
FFFFFFFFFFFFFFFFFFFFFFFFFFFFF,FFFFFFFFFFFFF:FFFFFFFFFFFFFFFFFFFFFFFF:F
@A00155:342:HHGFNDSXY:1:1554:29008:7200 2:N:0:GAACCTAG+TCCGCATA
GGAACGGACTCATCAGGGAGCCTACTAGACTCCGACCGTCATGGGGGTTCAAACCCATGCCCCGCCAC
ACCGCCTTAGATCAGCTGTGTCCACTTAAGGACTCACCTCTGGCCTATGGTCATCTCAATGCCGAGAG
+
FFFFFFFFFFFFFFFFFFFFFFFFFFFFFFFFFFFFFFF:FFFFFFFFFFFFFFFFFFFFFFFFFFFF
FFFFFFFFFFFFFFFFFFFFFFFFFFFFFFFFFFFFFFFFFFFFFFFFFFFFFFFFFFFFFFFFFFFF
@A00155:342:HHGFNDSXY:1:1554:17644:9314 2:N:0:GAACCTAG+TCCGCATA
CAGGTAGCCTACTAGACTACGACTTACACGGTAGGTCGTGAACAAGCCCGGAGTACATCTCCTTTGGA
ACTAGGAGACCCCTCCGGTCTTGTCCAACCTCTGTTTCGGCAGAGGATACCGTGCTCCACAGCTGTAA
+
FFFFFFFFFFFFFFFFFFFFFFFFFFFFFFFFFFFFFFFFFFFFF:FFFFFFFFFFFFFFFFFFFFFF
:F:FFFFFFFFFFFFFFFFF:FFFFFFFFFFFFF:FFFFFFFFFFF:FFFFFFFFFFFFFFFFFFFFFF
```

@A00155:342:HHGFNDSXY:1:1554:6008:13745 2:N:0:GAACCTAG+TCCGCATA
ACATCTCCTTTGGAACTAGGAGACCCCTCCGGTCTTGTCCAACCTCTGTTTCGGCAGAGGATACCGTG
CTCCACAGCTGTAAGGCTGTGGGCCCGAGAGAAGTCGCTCTCTCTCGCCCTCTACATCCC
+
FFFFFFFFFFFFFFFFFFF:FFFFFFFFFFFFFFFFFFFFFFFFFFFFFFFFFFFFFFFFFFFFFFFF
FFFFFF:FFFFFFFFFFFFFFFFFFFFFFFFFFFFFFFFFFFFFFFFFFFFFFFFF
@A00155:342:HHGFNDSXY:1:1554:13666:14606 2:N:0:GAACCTAG+TCCGCATA
ACACGGTAGGTCGTGAACAAGCCCGGAGTACATCTCCTTTGGAACTAGGAGACCCCTCCGGTCTTGTC
CAACCTCTGTTTCGGCAGAGGATACCGTGCTCCACAGCTGTAAGGCTGTGGGCCCGAGAGAAGTCGCT
+
FFFF:FFFFFFFFFFFFFFFFFFFFFFFFFFFFFFFFFFFFFFFFFFFFFFFFFFFFFFFFFFF:FFF
FFFFFFFFFFFFFFFF:FFFFFFFFFFFFFFFFFFFFFFFFFFFFFFFFFFFFFFFFF:FFFFFFFFF
@A00155:342:HHGFNDSXY:1:1554:17219:24079 2:N:0:GAACCTAG+TCCGCATA
GTCCTAAAAAGGACTCGTCAGGTAGCCTACTAGACTACGACTTACACGGTAGGTCGTGAACAAGCCCG
GAGTACATCTCCTTTGGAACTAGGAGACCCCTCCGGTCTTCTCCAACCTCTGTTTCGGCAGAGGATAC
+
FFFFFFFFFFFFFFFFFFFFFFFFFFFFFFFF:FFFFFFFFFFFFFFFFFFFFFFFFFFFFFF:FFFF
FFFFFFFFFFFFFFFFFFFFFFFFFFFFFFFFFFFFFFFFFFFFFFFFFFFFFFFFFFFFFFFFFFFF
@A00155:342:HHGFNDSXY:1:1554:17173:24659 2:N:0:GAACCTAG+TCCGCATA
GTCCTAAAAAGGACTCGTCAGGTAGCCTACTAGACTACGACTTACACGGTAGGTCGTGAACAAGCCCG
GAGTACATCTCCTTTGGAACTAGGAGACCCCTCCGGTCTTCTCCAACCTCTGTTTCGGCAGAGGATAC
+
FFFFFFFFFFFFFFFFFFFFFFFFFFFFFFFFFFFFFFFFFFFFFFFFFFFFFFFFFFFFFFFFFFFF
FFFFFFFFFFFFFFFFFFFFFFFFFFFFFF:FFFFFFFFFFFFFFFFFFFFFFFFF:FFFFFFFFFFF
@A00155:342:HHGFNDSXY:1:1554:12093:34648 2:N:0:GAACCTAG+TCCGCATA
TTCGAGTTGTGTAAGTTTCGTCCTAAAAAGGACTCGTCAGGTAGCCTACTAGACTACGACTTACACGG
TAGGTCGTGAACAAGCCCGGAGTACATCTCCTTTGGAACTAGGAGACCCCTCCGGTCTTGTCCAACCT
+
FFFFFFFFFFFFFFFFFF:FFFFFFFFFFFFFFFFF:FFFFFFFFFFFFFFFFFFFFFFFFFFFFFFF
FFFFFFFFFFFFFFFFFFFFFFFFFFFFFFFFFFFFFFFFFFFFFFFFFFF:FFFFFFFFFFFFFFFF
@A00155:342:HHGFNDSXY:1:1554:11677:35305 2:N:0:GAACCTAG+TCCGCATA
TTCGAGTTGTGTAAGTTTCGTCCTAAAAAGGACTCGTCAGGTAGCCTACTAGACTACGACTTACACGG
TAGGTCGTGAACAAGCCCGGAGTACATCTCCTTTGGAACTAGGAGACCCCTCCGGTCTTGTCCAACCT
+
FFFF,FFFFFFFFFFFFFFFFFFFFFFFFFFFFFFFFFF:FFFFFFFFFFF,FFFFFFFFFFFFFFFF
FFFFFFFFFFFFFFFFF:FFFFFFFFFFFFFFFFFFFFFFFFFFFFFFFFFFFFFFFFFFFFFFFFFF
@A00155:342:HHGFNDSXY:1:1554:13738:35336 2:N:0:GAACCTAG+TCCGCATA
TTCGAGTTGTGTAAGTTTCGTCCTAAAAAGGACTCGTCAGGTAGCCTACTAGACTACGACTTACACGG
TAGGTCGTGAACAAGCCCGGAGTACATCTCCTTTGGAACTAGGAGACCCCTCCGGTCTTGTCCAACCT
+
FFFFFFFFFFFFFFFFFFFF:FFFFFFFF:FFFFFFFFFFFFF,FFFFFFFFFFFFFFFF:FFFFFFF
FFFFFFFFFFFFFFFFFFFFFFFFFFFFFFFFFFFFFFFFFFFFFFF:FFFFFFFFFFFFFFFFFFFF
@A00155:342:HHGFNDSXY:1:1555:26476:4570 2:N:0:GAACCTAG+TCCGCATA
ACTAGACTACGACTTACACGGTAGGTCGTGAACAAGCCCGGAGTACATCTCCTTTGGAACTAGGAGAC
CCCTCCGGTCTTGTCCAACCTCTGTTTCGGCAGAGGATACCGTGCTCCACAGCTGTAAGGCTGTGGGC
+
FFFFFFFFFFFFFFFFFFFFFFFFFFFFFFFFFFFFFFFFFFFFFFFFFFFFFFFFFFFFF:FFF:FF
FFFFFFFFFF,FFFFFFFFFFFFFFFFFFFFFF:FF:FFFFFFFFFFFFFFFFFFFFFFFFFFFFFFF
@A00155:342:HHGFNDSXY:1:1555:15004:8625 2:N:0:GAACCTAG+TCCGCATA
GAACCAGGAGACCCCTCCGGTCTTGTCCAACCTCTGTTTCGGCAGAGGATACCGTGCTCCACAGCTGT
AAGGCTGTGGGCCCGAGAGAAGTCGCTCTCTCTCGCCCTCCACATCCCTCTCGGCATTGAGATGACCA
+
FFFFFFFFFFFFFFFFFFFFFFFFFFFFFFFFFFFFFFFFFFFFFFFFFFFFFFFFFFFFFFFFFFFF
FFFFFFFFFFFFFFFFFFFFFFFFFFFFFFFFFFFFFFFFFFFFFFFFFFFFFFFFFFFFFFFFFF:F

```
@A00155:342:HHGFNDSXY:1:1555:2772:34914 2:N:0:GAACCTAG+TCCGCATA
TCGTGAACAAGCCCGGAGTACATCTCCTTTGGAACTAGGAGACCCCTCCGGTCTTGTCCAACCTCTGT
TTCGGCAGAGGATACCGTGCTCCACAGCTGTAAGGCTGTGGGCCCGAGAGAAGTCGCTCTCTCTCGCC
+
FFFFFFFFFFFFFFFFFFFFFFFFFFFFFFFFFFFFFFFFFFFFFFFFFFFFFFFFFFFFFFFFFFFF
FFFFFFFFFFFFFFFFFFFFFFFFFFFFFFFFFFFFFFFFFFFFF:FFFFFFFFF:FFFFFFFFFFFF
@A00155:342:HHGFNDSXY:1:1556:4020:6981 2:N:0:GAACCTAG+TCCGCATA
CCTCTGGCCTATGGTCATCTCAATGCCGAGAGGGATGTGAAGGGCGAGAGAGAGCGACTTCTCTCGGG
CCCACAGCCTTACAGCTGTGGAGCACGGTATCCTCTGCCGAAACAGAGGTTGGACAAGACCGG
+
FFFFFFFFFFFFFFFFFFFFFFFFFFFFFFFFFFFFFFFFFFFFFFFFFFFFFFFFFFFFFFFFFFFF
FFFFFFFFFFFFF:FFFFFFFFFF:FFFFFFFFFFFFFFFFFFFFFFFFFFFFFFFFFFFFFFF
@A00155:342:HHGFNDSXY:1:1556:1823:12070 2:N:0:GAACCTAG+TCCGCATA
CCTTTTTAGGACGAAACTTACACAACTCAAAGAGTAACGACGGTTTCGTCCTTGGAACGGACTCATCA
GGGAGCCTACTAGACTCCGACCGTCATGGGGGTTCAAACCCATGCCCCGCCACACCGCCTTAGATCAG
+
FFFFFFFFFFFFF:FFFFFFFFFFFFFFFFFFFFFFFFFFFFFFFFFFFFFFFFFFFFFFFFFFFFFF
FFFFFFFFFFFFFFFFFFFFFFFFFFFFFFFFFFFFFFFFF:FFFFFFFFFFFFFFFFFFFFFFFFFF
@A00155:342:HHGFNDSXY:1:1556:7563:12336 2:N:0:GAACCTAG+TCCGCATA
GGTCTTGTCCAACCTCTGTTTCGGCAGAGGATACCGTGCTCCACAGCTGTAAGGCTGTGGGCCCGAGA
GAAGTCGCTCTCTCTCGCCCTCCACATCCCTCTCGGCATTGAGATGACCATAGGCCAGAGGAGAGTCC
+
FFFFFFFFFFFFFFFFFFFFFFFFFFFFFFFFFFFFFFFFFFFFFFFFFFFFFFFFFFFFFFFFFFFF
FFFFFFFFFFFFFFFFFFFFFFFFFFFFFFFFFFFFFFF:FFFFFFFFFFFFFFFFF::FFFFFFFFFF:
@A00155:342:HHGFNDSXY:1:1557:27914:3396 2:N:0:GAACCTAG+TCCGCATA
AGGACGAAACCGTCGTTACTCTTTGAGTTGTGTAAGTTTCGTCCTAAAAAGGACTCGTCAGGTAGCCT
ACTAGACTACGACTTACACGGTAGGTCGTGAACAAGCCCGGAGTACATCTCCTTTGGAACTAGGAGAC
+
FFFFFFFFFFFFFFFFFFFFFF,FFF:FFFFFFFFFFFFFFFFFFFFFFFFFFFFFFFFFFFFFFFFF
FFFFFFFFFFFFFFFFFFFFFFFFFFFFFFFFFFFFFFFFFFFFFFFFFFFFFFFFFFFFFFFFFFFF
@A00155:342:HHGFNDSXY:1:1557:8892:18427 2:N:0:GAACCTAG+TCCGCATA
TCCTTTGGAACTAGGAGACCCCTCCGGTCTTGTCCAACCTCTGTTTCGGCAGAGGATACCGTGCTCCA
CAGCTGTAAGGCTGTGGGCCCGAGAGAAGTCGCTCTCTCTCGCCCTCCACATCCCTCTCGG
+
FFFFFFF,F,FF:FF::F,:FFFFFFFFFFFFF,FFFF,FFFFFF,FFF:FFFFFFFFF,FFFFFFFF
FFFFFFFFFFFF,FFFFFFFFFFFFFF,,FFFFFFFFFFFFFFFFFFFFF:FFFFFFFFFFF
@A00155:342:HHGFNDSXY:1:1557:15881:32503 2:N:0:GAACCTAG+TCCGCATA
GAACTAGGAGACCCCTCCGGTCTTGTCCAACCTCTGTTTCGGCAGAGGATACCGTGCTCCACAGCTGT
AAGGCTGTGGGCCCGAGAGAAGTCGCTCTCTCTCGCCCTCCACATCCCTCTCGGCATTGAGATGAC
+
FFFFFFFFFFFFFFFFFFFFFFFFFFFFFFFFFFFFFFFFFFFFFFFFFFFFFFFFFFFFFFF:FFFFF
FFFFF:FFFFFFFFFFFFFF:FFFFFFFFFFFFFFFFFFFFFFFFFFFFFFFFFFFF:FFFFFFFFFF
@A00155:342:HHGFNDSXY:1:1557:29243:34319 2:N:0:GAACCTAG+TCCGCATA
CTTTTTAGGACGAAACTTACACAACTCGAAGAGTAACGACGGTTTCGTCCTTGGAACGGACTCATCAG
GGAGCCTACTAGACTCCGACCGTCATGGGGGTTCAAACCCATGCCCCGCTACACCGCCTTAGATCAGC
+
FFFFFFFFFFFFFFFF,FFFFFFFFFFFFFFFFFFFFFFFFFFFFFFFFFFFFFFFFF:FFFFFFFFFF
FFFFFFFFFFFFFFFFFFFFFFFFFFFFFFFFFFFFFFFFFFFFFFFFFFFFFFFFFFFFFFFFFFFF
@A00155:342:HHGFNDSXY:1:1558:10077:17722 2:N:0:GAACCTAG+TCCGCATA
GAAACCGTCGTTACTCTTTGAGTTGTGTAAGTTTCGTCCTAAAAAGGACTCGTCAGGTAGCCTACTAG
ACTACGACTTACACGGTAGGTCGTGAACAAGCCCGGAGTACATCTCCTTTGGAACTAGGAGACCCCTC
+
FFFFFFFFFFFFFFFF,::FFFFFFFFFFFFFFFFFFFFFFFFFFFFFFFFFFFFFFFFFFFFFFFFF
FFFFFFFFFFFFFFFFFFFFFFFFFFFFFFFFFFFFFFFFFFFFFFFFFFFFFFFFFFFFFFFF:FFFF
```

```
@A00155:342:HHGFNDSXY:1:1558:13006:28401 2:N:0:GAACCTAG+TCCGCATA
GGAGTACATCTCCTTTGGAACTAGGAGACCCCTCCGGTCTTGTCCAACCTCTGTTTCGGCAGAGGATA
CCGTGCTCCACAGCTGTAAGGCTGTGGGCCCGAGAGAAGTCGCTCTCTCTCGCCCTCCACATCCCTCT
+
FFFFFFFFFFFFFFFFFFFFFFFFFFFFFFFFFFFFFFFFFFFFFFFFFFFFFFFFFFFFFFFFFFFF
FFFFFFFFFFFFFFFFFFFFFFFFFFF:FFFFFFFFFFFFFFFFFFFFFFFFFFFFFFFFFFFFFFFF
@A00155:342:HHGFNDSXY:1:1558:4444:31516 2:N:0:GAACCTAG+TCCGCATA
AAGGACGAAACCGTCGTTACTCTTCGAGTTGTGTAAGTTTCGTCCTAAAAAGGACTCGTCAGGTAGCC
TACTAGACTACGACTTACACGGTAGGTCGTGAACAAGCCCGGAGTACATCTCCTTTGGAACTAGGAGA
+
FFFFFFFFFFF:FFFFFFFFFFFFFFFFFFFFFFFFFFFF:FFFFFFFFFFFFFFFFFFFFFFFFFFF
FFFFFFFFFFFFFFFFFFFFFFFFFFFFFFFFFFFFFFFF:FFFFFFFFFFFFFFFFFFFFFFFFF:FFF
@A00155:342:HHGFNDSXY:1:1558:23285:36996 2:N:0:GAACCTAG+TCCGCATA
GGAGTACATCTCCTTTGGAACTAGGAGACCCCTCCGGTCTTGTCCAACCTCTGTTTCGGCAGAGGATA
CCGTGCTCCACAGCTGTAAGGCTGTGGGCCCGAGAGAAGTCGCTCTCTCTCGCCCTCTACATCCC
+
FFFFFFFFFFFFFFFFFFFFFFFFFFFFFFFFFFFFFFFFFFFFFFFFFFFFFFFFFFFFFFFFFFFF
FFFFFFFFFFFFFFFFFFFFFFFFFFFFFFFFFFFFFFFFFFFFFFFFFFFFFFFFFFFFFFFFF
@A00155:342:HHGFNDSXY:1:1559:30246:6120 2:N:0:GAACCTAG+TCCGCATA
GTCATCTCAATGCCGAGAGGGATGTGGAGGGCGAGAGAGAGCGACTTCTCTCGGGCCCACAGCCTTAC
AGCTGTGGAGCACGGTATCCTCTGCCGAAACAGAGGTTGGACAAGACCGGAGGGGTCTCCTA
+
FFFFFFFFFFFFFFFFFFFFFFFFFFFFFFFFFFFFFFFFFFFFFFFFFFFFFFFFFFFFFFFFFFFF
FFFFFFFFFFFFFFFFFFFFFFFFFFFFFFFFFFFFFFFFFFFFFFFFFFFFFFFFFFFFFF:
@A00155:342:HHGFNDSXY:1:1559:11975:10269 2:N:0:GAACCTAG+TCCGCATA
TAGCCTACTAGACTACGACTTACACGGTAGGTCGTGAACAAGCCCGGAGTACATCTCCTTTGGAACTA
GGAGACCCCTCCGGTCTTGTCCAACCTCTGTTTCGGCAGAGGATACCGTGCTCCACAGCTGTAAGGCT
+
FFFFFFFFFFFFFFFFFFF:FFFFFFFFFFFFFFFFFFFFFFFFFFFFFFFFFFFFFFFFFFFFFFFF
FFFFFFFFFFFFFFFFF:FF:FFFFFFFFFF:FFFFFFFFFFFFFFFFFFFFFF:FFFF:FFFFFFFF
@A00155:342:HHGFNDSXY:1:1559:22236:12070 2:N:0:GAACCTAG+TCCGCATA
GGACTCGTCAGGTAGCCTACTAGACTACGACTTACACGGTAGGTCGTGAACAAGCCCGGAGTACATCT
CCTTTGGAACTAGGAGACCCCTCCGGTCTTGTCCAACCTCTGTTTCGGCAGAGGATACCGTGCTCCAC
+
FFFFFFFFFFFFFFFFFFFFFFFFFFFFFFFFFFFFFFFFFFFFFFFFFFFFFFFFFFFFFFFFFFFF
FFFFFFFFFFFFFFFF:F:FFFFFFFFFFFF:FFFFFFFFFFFF,FFFFFFFFFFFFFFFFFFFFFF
@A00155:342:HHGFNDSXY:1:1559:24894:17769 2:N:0:GAACCTAG+TCCGCATA
TGGTCATCTCAATGCCGAGAGGGATGTAGAGGGCGAGAGAGAGCGACTTCTCTCGGGCCCACAGCCTT
ACAGCTGTGGAGCACGGTATCCTCTGCCGAAACAGAGGTTGGACAAGACCGGAGGGGTCTCCTAGTTC
+
FFFFFFFFFFFFFFFFFFFFFFFFFFFFFFFFFFFFFFFFFFFFFFFFFFFFFFFFFFFFFFFFFFFF
FFFFFFFFFFFFFFFFFFFFFFFFFFFFFFFFFFFFFFFFFFFFFFFFFFFFFFFFFFFFFFFFFFFF
@A00155:342:HHGFNDSXY:1:1560:20274:6887 2:N:0:GAACCTAG+TCCGCATA
ACGGTAGGTCGTGAACAAGCCCGGAGTACATCTCCTTTGGAACCAGGAGACCCCTCCGGTCTTGTCCA
ACCTCTGTTTCGGCAGAGGATACCGTGCTCCACAGCTGTAAGGCTGTGGGCCCGAGAGAAGTCGCTCT
+
::FFFFFFFFFFFFFFFFFFFFFFFFFFFFFFFFFF,FFFFFFFFFFFFFFFFFFFFF:F,F:FFFFFF
FFFFFFFFFFFFFFFFFFF:FFFFFFFFFFF:F:FFFFFFFFFFFFFFFFFFFFFFFFF,FFFFFFFF
@A00155:342:HHGFNDSXY:1:1561:10619:2065 2:N:0:GAACCTAG+TCCGCATA
AACAAGCCCGGAGTACATCTCCTTTGGAACCAGGAGACCCCTCCGGTCTTGTCCAACCTCTGTTTCGG
CAGAGGATACCGTGCTCCACAGCTGTAAGGCTGTGGGCCCGAGAGAAGTCGCTCTCTCTCGCC
+
FFFFFFFFFFFFFFFFFFFFFFFFFFFFFFFFFFFFFFFFFFFFFFFFFFFFFFFFFFFFFFFFFFFF
FFFFFFFFFFFFFFFFFFFFFFFFFFFFFFFFFFFFFFFFFFFFFFFFFFFFFFFFFFFFFFF
```

@A00155:342:HHGFNDSXY:1:1561:32461:18912 2:N:0:GAACCTAG+TCCGCATA
GACTCGTCAGGTAGCCTACTAGACTACGACTTACACGGTAGGTCGTGAACAAGCCCGGAGTACATCTC
CTTTGGAACCAGGAGACCCCTCCGGTCTTGTCCAACCTCTGTTTCGGCAGAGGATACCGTGCTCCACA
+
FFFFFFFFFFFFFFFFFFFFFFFFFFFFFFFFFFFFFFFFFFFFFFFFFFFFFFFFFFFFFFFFFFFFFF
FFFFFFFFFFFFFFFFFFFFFFFFFFFFFFFFFFFFFFFFFFFFFFFFFFFFFFFFFFFFFFFFFFFFFF
@A00155:342:HHGFNDSXY:1:1561:28357:24424 2:N:0:GAACCTAG+TCCGCATA
AGGACGAAACCGTCGTTACTCTTTGAGTTGTGTAAGTTTCGTCCTAAAAAGGACTCGTCAGGTAGCCT
ACTAGACTACGACTTACACGGTAGGTCGTGAACAAGCCCGGAGTACATCTCCTTTGGAACTAGGAGAC
+
FFFFFF,F,FFF:FFFF::F:FF,:FF:FFFFFFF:FFFFFFFFFF:FFFFFFFFFFFF:FFFFFF:FFF
FF:FFFFFFFFFF:FFFFFFFFFFFF:FFFFFFFFFFFFFFFFFFFFF:FFFFF:F:FF:FFFFFFFFFF
@A00155:342:HHGFNDSXY:1:1563:15953:15843 2:N:0:GAACCTAG+TCCGCATA
CACGGTAGGTCGTGAACAAGCCCGGAGTACATCTCCTTTGGAACTAGGAGACCCCTCCGGTCTTGTCC
AACCTCTGTTTCGGCAGAGGATACCGTGCTCCACAGCTGTAAGGCTGTGGGCCCGAGAGAAGTCGCTC
+
FFFFFFFFFFFFFFFFFF:FFFFFFFFFFFFFFFFFF:FFFFFFFFFFFFFFFFFFFFFFFFFF:FFFFF
FFFFFFFFFFFFFFFFFFFFFFFFFFFFFFFFFFFFFFFFFFFFFFFFFFFFFFFFFFFFF:FFFFFFFF
@A00155:342:HHGFNDSXY:1:1563:2772:35008 2:N:0:GAACCTAG+TCCGCATA
TACATCTCCTTTGGAACTAGGAGACCCCTCCGGTCTTGTCCAACCTCTGTTTCGGCAGAGGATACCGT
GCACCACAGCTGTAAGGCTGTGGGCCCGAGAGAAGTCGCTCTCTCTCGCCCTCCACATCCCTCTCGGC
+
FFFFFFFFFFFFFFFFFFFF:FFFFFFFFFFFFFFFFFFFFFFFFFFFFFFFFFFFFFFF:FFFFFFFFFF
FFFFFFFFFFFFFFFFFFFFFFFFFFFFFFFFFFFFFF:FFFFFFFFFFFFFFFFFFFFFFFFFFFF:F:
@A00155:342:HHGFNDSXY:1:1564:27724:12649 2:N:0:GAACCTAG+TCCGCATA
AGCCTACTAGACTACGACTTACACGGTAGGTCGTGAACAAGCCCGGAGTACATCTCCTTTGGAACTAG
GAGACCCCTCCGGTCTTGTCCAACCTCTGTTTCGGCAGAGGATACCGTGCTCCACAGCTGTAAGGCTG
+
FFFFFFFFFFFFFFFFFFFFFFFFFFFFFFFFFFFFFFFF:FFFFFFFFFFFFFFF:FFF:FFFFFFFFF
FFFFFFFFFFFFFFFFFFFFFFFFFFFFFFFFFFFFFFFF:FFF:F:FFFFFFFFFFFFFFFFFFFFFFF
@A00155:342:HHGFNDSXY:1:1564:8486:34225 2:N:0:GAACCTAG+TCCGCATA
AGCCTACTAGACTACGACTTACACGGTAGGTCGTGAACAAGCCCGGAGTACATCTCCTTTGGAACTAG
GAGACCCCTCCGGTCTTGTCCAACCTCTGTTTCGGCAGAGGATACCGTGCTCCACAGCTGTAAGGCTG
+
FFFFFFFFFFFFFFFFFFFFFFFFFFFFFFFFFFFFFFFFFFFFFFFFFFFFFFFFFFFFFFFFF:FFFF
FFFFFFFFFFFFFFFFFFFFFFFFFFFFFFFFFFFFFFFFFFFFFFFFFFFFFFFFFFFFFF:F,FFFFF
@A00155:342:HHGFNDSXY:1:1565:30373:6277 2:N:0:GAACCTAG+TCCGCATA
ACTCTTCGAGTTGTGTAAGTTTCGTCCTAAAAAGGACTCGTCAGGTAGCCTACTAGACTACGACTTAC
ACGGTAGGTCGTGAACAAGCCCGGAGTACATCTCCTTTGGAACTAGGAGACCCCTCCGGTCTTGTCCA
+
FFFF:FF:F:FFFFFFFFFFFFFFFFF,FFFFFFFFFFFFFFFFFFFFFFFFFFFFF::FFFFFFF:,FF
FF:FFFFFFFFFFFFFFFFFFFFFFF:FFFF,FFF:,:F:FFFF:FFFFFFFFFFFFFFFFFFFFFFFFF
@A00155:342:HHGFNDSXY:1:1565:19434:12414 2:N:0:GAACCTAG+TCCGCATA
CGAAGAGTAACGACGGTTTCGTCCTTGGAACGGACTCATCAGGGAGCCTACTAGACTCCGACCGTCAT
GGGGGTTCAAACCCATGCCCCGCTACACCGCCTTAGATCAGCTGTGTCCACTTAAGGACTCACCTCTG
+
FFFFFFFFFFFFFFFFFFFFFFFFFFFFFFFFFFFFFFFFFFFFFFFFFFF:FFFFFFFFFF:FFF:FFF
FFFFFF:F:FFFFFFFFFFFFFFFFFFFFFFFFFFFFFFFFFFFFFFFFFFFFFFFFFFFFF,FFFFFF
@A00155:342:HHGFNDSXY:1:1565:18159:24674 2:N:0:GAACCTAG+TCCGCATA
GAGACCCCTCCGGTCTTGTCCAACCTCTGTTTCGGCAGAGGATACCGTGCTCCACAGCTGTAAGGCTG
TGGGCCCGAGAGAAGTCGCTCTCTCTCGCCCTCCACATCCCTCTCGGCATTGAGATGACCATAGGCCA
+
FFFFFFFFFFFFFFFFFFFFFFFFFFFFFFFFFFFFFFFF:FFFFFFFFFFFFFFFFFFFF,:FFFFFFF
FFFFFFFFFFFFFFFFFFFFFFFFFFFFFFFFFFF:FF,FFFFFFFFF:F::FF:FFFFFFFFF:FFF::

```
@A00155:342:HHGFNDSXY:1:1565:32307:26694 2:N:0:GAACCTAG+TCCGCATA
AGCCTACTAGACTACGACTTACACGGTAGGTCGTGAACAAGCCCGGAGTACATCTCCTTTGGAACTAG
GAGACCCCTCCGGTCTTGTCCAACCTCTGTTTCGGCAGAGGATACCGTGCTCCACAGCTGTAAGGCTG
+
FFFFFFFFFFFFFFFFFFFFFFFFFFFFFF,FFFFFFFFFFFFFFFFFFFFFFFFFFFFFFFF,FFFF
FFFFFFFFFFFFFFF:FFFFFFFFFFFFFFFFFFFFFFFFFFFFFFFFFF:FFFFFFFFFFFFFFFFF
@A00155:342:HHGFNDSXY:1:1565:21947:27352 2:N:0:GAACCTAG+TCCGCATA
GTCCTAAAAAGGACTCGTCAGGTAGCCTACTAGACTACGACTTACACGGTAGGTCGTGAACAAGCCCG
GAGTACATCTCCTTTGGAACCAGGAGACCCCTCCGGTCTTGTCCAACCTCTGTTTCGGCAGAGGATAC
+
FFFFFFFFFFFFFFFFFFFFFFFFFFFFFFFFFFFFFFFFFFFFFFFFFFFFFFFFFFF:FFFFFFFF
FFF:FF:FFFFFFFFFFFFFFFFFFFFFFFFFFFFFFFFF:F:FFFFFFFFFFFFFF:FFFFFFFFFF
@A00155:342:HHGFNDSXY:1:1565:29134:33473 2:N:0:GAACCTAG+TCCGCATA
AGGACGAAACCGTCGTTACTCTTTGAGTTGTGTAAGTTTCGTCCTAAAAAGGACTCGTCAGGTAGCCT
ACTAGACTACGACTTACACGGTAGGTCGTGAACAAGCCCGGAGTACATCTCCTTTGGAACTAGGAGAC
+
FFFFFFFFFFFFFFFFFFFFFF:FFFFFFFFFFFFFFFFFFFFFF:FFF:FFFFFFFFFFFFF:FFFF
FFFFFFFFFFFFFFFFFFFFFFFFFFFFFFFFFFFFFFFFFFFFFFFFFFFFFFFFFFFFFFFFFFFF
@A00155:342:HHGFNDSXY:1:1566:30445:1391 2:N:0:GAACCTAG+TCCGCATA
AAAGGACTCGTCAGGTAGCCTACTAGACTACGACTTACACGGTAGGTCGTGAACAAGCCCGGAGTACA
TCTCCTTTGGAACTAGGAGACCCCTCCGGTCTTGTCCAACCTCTGTTTCGGCAGAGGATACCGTGCTC
+
FFFFFFFFFFFFFFFFFFFFFFFFFFFFFFFFFFFFFFFFFFFFFFFFFFFFFFFFFFFFFFFFFFFF
FFFFFFFFFFFFFFFFFFFFFFFFFFFFFFFFFFFFFFFFFFFFFFFFFFFFFFFFFF:FFFFFFFFF
@A00155:342:HHGFNDSXY:1:1566:31991:2942 2:N:0:GAACCTAG+TCCGCATA
AAAGGACTCGTCAGGTAGCCTACTAGACTACGACTTACACGGTAGGTCGTGAACAAGCCCGGAGTACA
TCTCCTTTGGAACTAGGAGACCCCTCCGGTCTTGTCCAACCTCTGTTTCGGCAGAGGATACCGTGCTC
+
FFFFFFFFFFFFFFFFFFFFFFFF:FFFFFFFFFFFFFFFFFFFFFFFFFFFFF:FFFFFFFFFFFFF
FFFFFFFFFFFFFFFFFFFFF:FFFFFFF:FF:FFF:FFFFFFFFFFFFFFFFFFFF:FFFFFFFFFF
@A00155:342:HHGFNDSXY:1:1566:11288:8829 2:N:0:GAACCTAG+TCCGCATA
CTCCTTTGGAACTAGGAGACCCCTCCGGTCTTGTCCAACCTCTGTTTCGGCAGAGGATACCGTGCTCC
ACAGCTGTAAGGCTGTGGGCCCGAGAGAAGTCGCTCTCTCTCGCCCTCCACATCCCTCTCGGCATTGA
+
FF,FFFFFFFFFFFFFFFFFFFFFFFFFFFFFFFFFFFFFFFFFFFFFFFFFFFFFFFFFFFFFFFFF
FFFFFFFFFFFFFFFFFFFFFFFFFFFFFFFFFFFFFFFFFFFFFFFFFFFFFFFFFFFFFFFFF:F:
@A00155:342:HHGFNDSXY:1:1566:18258:13823 2:N:0:GAACCTAG+TCCGCATA
TACGACTTACACGGTAGGTCGTGAACAAGCCCGGAGTACATCTCCTTTGGAACTAGGAGACCCCTCCG
GTCTTGTCCAACCTCTGTTTCGGCAGAGGATACCGTGCTCCACAGCTGTAAGGCTGTGGGCCCG
+
FFFFFFFFFFFFFFFFFFFFFFFFFFFFFFFFFFFFFFFFFFFFFFFFFFFFFFFFFFFFFFFFFFFF
FFFFFFFFFFFFFFFFFFFFFFFFFFFFFFFFFFFFFFFFFFFFFFFFFFFFFFFFFFFFFFFF
@A00155:342:HHGFNDSXY:1:1566:31367:26663 2:N:0:GAACCTAG+TCCGCATA
GAACTAGGAGACCCCTCCGATCTTGTCCAACCTCTGTTTCGGCAGAGGATACCGTGCTCCACAGCTGT
AAGGCTGTGGGCCCGAGAGAAGTCGCTCTCTCTCGCCCTCCACATCCCTCTCGGCATTGAGATGACCA
+
FFFFF:FFFFFFFFFFFFFFFFFFFFFFFFFFFFFFFFFFFFFFFFFFFFFFFFFFFFFFFFFFFFFF
FFFFFFFFFFFFFFFFFFFFFFFFFFFFFFFFFFFFFFFFFFF:FFFFFFFFFFFFF:FFFFFFFFFF
@A00155:342:HHGFNDSXY:1:1566:28113:34961 2:N:0:GAACCTAG+TCCGCATA
GGACTCATCAGGTAGCCTACTAGACTACGACTTACACGGTAGGTCGTGAACAAGCCCGGAGTACATCT
CCTTTGGAACTAGGAGACCCCTCCGGTCTTGTCCAACCTCTGTTTCGGCAGAGGATACCGTGC
+
FFFFFFFFFFFFFFFFFFFFFFFFFFFFFFFFFFFFFFFFFFF:FFFFFFFFFFFFFFFFFFFFFFFF
FFFFFFFFFFF:FFFFFFFFFFFFFFFFFFFFFFFFFFFFFF:F:FFFFFFFFFFFFFFFFFF
```

```
@A00155:342:HHGFNDSXY:1:1566:6903:35556 2:N:0:GAACCTAG+TCCGCATA
CCTACTAGACTACGACTTACACGGTAGGTCGTGAACAAGCCCGGAGTACATCTCCTTTGGAACTAGGA
GACCCCTCCGGTCTTGTCCAACCTCTGTTTCGGCAGAGGATACCGTGCTCCACAGCTGTAAGGCTGTG
+
FFFFFFFFFFFFFFFFFFFFFFFFFFFFFFFFFFFFFFFFFFFFFF:FFFFFFFFFFF:FFFFFF
FFFFFFFFFFFFFFFFFFF:FFFFFFFFFFFF:FF:FFFFFFFFFFFFFFFFFFFFFFFFFFFFFFFF
@A00155:342:HHGFNDSXY:1:1567:4661:2394 2:N:0:GAACCTAG+TCCGCATA
GAAACCGTCGTTACTCTTCGAGTTGTGTAAGTTTCGTCCTAAAAAGGACTCGTCAGGTAGCCTACTAG
ACTACGACTTACACGGTAGGTCGTGAACAAGCCCGGAGTACATCTCCTTTGGAACTAGGAGACCCCTC
+
FFFFF:FFFFFFFFFFF:FFFFFFF:FFFFFFFFFFFFFFFFF:FFFFFFFFFFFFFFFFFFFFFFF:
FFFFFFFFFFFFFFFF:FFFFFFFFFFFFFFFFFFFFFFFFFFFFFFFFFFFFFFFFFFFFFFF:FFF
@A00155:342:HHGFNDSXY:1:1567:8467:10269 2:N:0:GAACCTAG+TCCGCATA
CCTACTAGACTACGACTTACACGGTAGGTCGTGAACAAGCCCGGAGTACATCTCCTTTGGAACTAGGA
GACCCCTCCGGTCTTGTCCAACCTCTGTTTCGGCAGAGGATACCGTGCTCCACAGCTGTAAGGCTGTG
+
FFFFFFFF:FFFFF:FFFFFFFFFFFFFFFFFFFFFFFFFFFFFFFFFFFFFFFFFFFFFFFFFFFFF
FFFFFFFFFFFFFFFFFFFFFFFFFFFFFFFFFFFFFFFF:FFFFFFFFFFFFFFFFFFFFFFFFFFF
@A00155:342:HHGFNDSXY:1:1567:23990:10911 2:N:0:GAACCTAG+TCCGCATA
ACTCTTCGAGTTGTGTAAGTTTCGTCCTAAAAAGGACTCGTCAGGTAGCCTACTAGACTACGACTTAC
ACGGTAGGTCGTGAACAAGCCCGGAGTACATCTCCTTTGGAACTAGGAGACCCCTCCGGTCTTGTCCA
+
FFFFFFFFFF:FFFFFF:FFFFFFFFFF:FFFFFFFFFFFFFFFFFFFFFFFFFFFFFFFFFFFFFFF
FFFFFFFFFFFFFFFFFFFFFFFFFFFFFFFFFFFFFFFFF:,FFFFFFFFFFFFFFFFFFFFFFFFF
@A00155:342:HHGFNDSXY:1:1567:9778:13792 2:N:0:GAACCTAG+TCCGCATA
GAAACCGTCGTTACTCTTCGAGTTGTGTAAGTTTCGTCCTAAAAAGGACTCGTCAGGTAGCCTACTAG
ACTACGACTTACACGGTAGGTCGTGAACAAGCCCGGAGTACATCTCCTTTGGAACTAGGAGACCCCTC
+
FFFFFFFFFFFFFFFFFFFFFFFFFFFFFFFFFFFFFFFFFFF,FFFFFFFFFFFFFFFFFFF:FFF
FFFFFFFFFFFFFFFFFFFFF:FFFFFFFFFFFFFFFFFFFFFFFFFFFFFFFFFFFFFFFFFFFFFF
@A00155:342:HHGFNDSXY:1:1567:7545:16626 2:N:0:GAACCTAG+TCCGCATA
CCTACTAGACTACGACTTACACGGTAGGTCGTGAACAAGCCCGGAGTACATCTCCTTTGGAACTAGGA
GACCCCTCCGGTCTTGTCCAACCTCTGTTTCGGCAGAGGATACCGTGCTCCACAGCTGTAAGGCTGTG
+
FFFFFFFFFFFFFFFFFFFFFFFFFFFFFF:FFFFFFFFFFFFFFFFFFFFFFFFFFFFFFFFFFFFF
FFFFFFFFFFFFFFFFFFFFFFFFFFFFFFFFFFFFF,FFFFFFFFFFFFFFFFFFFFFFFF,FFFFFFF
@A00155:342:HHGFNDSXY:1:1568:17065:8876 2:N:0:GAACCTAG+TCCGCATA
CCCCTCCGGTCTTGTCCAACCTCTGTTTCGGCAGAGGATACCGTGCTCCACAGCTGTAAGGCTGTGGG
CCCGAGAGAAGTCGCTCTCTCTCGCCCTTCACATCCCTCTCGGCATTGAGATGACCATAGG
+
FFFFFFFFFFFFFFFFFFFFFFFFFFFFFFFFFFFFFFFFFFFFFFFFFFFFFFFFFFFFFFFFFFFF
FFFFFFFFFFFFFFFFFFFFFFFFFFFFFFFFFFFFFFFFFFFFFFFFFFFFFFFFFFFFF
@A00155:342:HHGFNDSXY:1:1568:31575:29183 2:N:0:GAACCTAG+TCCGCATA
GAGACCCCTCCGGTCTTGTCCAACCTCTGTTTCGGCAGAGGATACCGTGCTCCACAGCTGTAAGGCTG
TGGGCCCGAGAGAAGTCGCTCTCTCTCGCCCTCCACATCCCTCTCGGCATTGAGATGACCATAGGCCA
+
FFFFFFFFFFFFFFFFFFFFFFFFFFFFFFFFFFFFFFFFFFFFFFFFFFFFFFF:FFFFFFFFFFFF
FFFFFFFFFFFFFFFFFFFFFFFFFFFFFFFFFFFFFFFFFFFFFFFFFF,FFFFFFFFFFFFFFFFFF
@A00155:342:HHGFNDSXY:1:1569:9046:7263 2:N:0:GAACCTAG+TCCGCATA
ACTTACACGGTAGGTCGTGAACAAGCCCGGAGTACATCTCCTTTGGAACTAGGAGACCCCTCCGGTCT
TGTCCAACCTCTGTTTCGGCAGAGGATACCGTGCTCCACAGCTGTAAGGCTGTGGGCCCGAGAGAAGT
+
:FFFFFFFFFFFFFFFFFFFFFFFFFFFFFFFFFFFFFFFFFFFFFFFFFFFFFFFFFFFFFFFFFFF
FFFFFFFFFFFFFFFFFFFFFFFFFFFFFFFFFFFFFFFFFFFF:FFFFFFFFFFFFFFFFFFFFFFF
```

```
@A00155:342:HHGFNDSXY:1:1569:4444:13855 2:N:0:GAACCTAG+TCCGCATA
CTGGCCTATGGTCATCTCAATGCCGAGAGGGATGTGAAGGGCGAGAGAGAGCGACTTCTCTCGGGCCC
ACAGCCTTACAGCTGTGGAGCACGGTATCCTCTGCCGAAACAGAGGTTGGACAAGACCGGAG
+
FFFFFFFFFF:FFFFFFFFFFFFFFFFFFFFFFFFFFFFFFFFFFFFFFFFFFFFFFFFFFFFFFFFF
FF,FFFFFFFFFFFFFFFFFFFFFFFFFFFFFFFFFFFFFFFFFFFFFFFFFFFFFFFFFFF
@A00155:342:HHGFNDSXY:1:1569:1542:17534 2:N:0:GAACCTAG+TCCGCATA
CTCCACAGCTGTAAGGCTGTGGGCCCGAGAGAAGTCGCTCTCTCTCGCCCTCCACATCCCTCTCGGCA
TTGAGATGACCATTGGCCAGAGGTGAGTCCTTAAGTGGACACAGCTGATCTAAGGCGGTGTGGCG
+
FFFFFFFFFFFFFFFFFFFFFFFFFFFFFFFFFFFFFFFFFFFFFFFFFFFFFFFFFFF:FFFFFFFF
FFFFFFFFFFFFFFFFFFFFFFFFFFFFFFFFFFFFFFF,:FFFFFFFFFFFF,FFFF:FFFFFF
@A00155:342:HHGFNDSXY:1:1569:21522:21073 2:N:0:GAACCTAG+TCCGCATA
CACGGTAGGTCGTGAACAAGCCCGGAGTACATCTCCTTTGGAACTAGGAGACCCCTCCGGTCTTGTCC
AACCTCTGTTTCGGCAGAGGATACCGTGCTCCACAGCTGTAAGGCTGTGGGCCCGAGAGAAGTCGCTC
+
FFFFFFFFFFFFFFFFFFFFFFFFFFFFFFFFFFFFFFFFFFFFFFFFFFFFFFFFFFFFFF,FFFFF
FFFFFFFFFFFFFFFFFFFFFFFFFFFFFFFFFFFFFFFFFFFFF:FFFFFFFFFFFFFFFFFFFFFF
@A00155:342:HHGFNDSXY:1:1569:29668:26882 2:N:0:GAACCTAG+TCCGCATA
CTAGGAGACCCCTCCGGTCTTGTCCAACCACTGTTTCGGCAGAGGATACCGTGCTCCACAGCTGTAAG
GCTGTGGGCCCGAGAGAAGTCGCTCTCTCTCGCCCTCCACATCCCTCTCGGCATTGAGATGACCATA
+
F,FFFFFFFF,FFFFF:FF,,FFF:FFF:,F:,F,,FFFFFFF:,FFFFFFF,,FFFFFFF::FFFFF
FFFFFFFFFFFFF:FFF:,FFFFFFFF:FFFFFFFFFFFFFFFFFFFFF,FFFFFFFFF,FFFFF:F
@A00155:342:HHGFNDSXY:1:1569:17183:35790 2:N:0:GAACCTAG+TCCGCATA
TCCTAAAAAGGACTCGTCAGGTAGCCTACTAGACTACGACTTACACGGTAGGTCGTGAACAAGCCCGG
AGTACATCTCCTTTGGAACCAGGAGACCCCTCCGGTCTTGTCCAACCTCTGTTTCGGCAGAGGATACC
+
FFFFFFFFFFFFFFFFFFFFFFFFFFFFFFFFFFFFFFFFFFFFFFFFFFFF:FFFFFFFFFFFFFFF
FFFFFFFFFFF,FFFFFFFFFFFFFFFFFFFFFFFFFF,FFFFFFFFFFFFFFFFFFFFFFFFFFFFF
@A00155:342:HHGFNDSXY:1:1569:17192:35806 2:N:0:GAACCTAG+TCCGCATA
TCCTAAAAAGGACTCGTCAGGTAGCCTACTAGACTACGACTTACACGGTAGGTCGTGAACAAGCCCGG
AGTACATCTCCTTTGGAACCAGGAGACCCCTCCGGTCTTGTCCAACCTCTGTTTCGGCAGAGGATACC
+
FFFFFFFFFFFFFFFFFFFFFFFFFFFFFFFFFFFFFFFFFFFFFFFFFFFFFFFFFFFFFFFFFFFF
FFFFFFFFFFF:FFFFFFFF:FFFFFFFFFFFFFFFFFFFFFFFFFFFFFFFFFFFFFFFFFFFFFF
@A00155:342:HHGFNDSXY:1:1570:10592:5525 2:N:0:GAACCTAG+TCCGCATA
GAACCAGGAGACCCCTCCGGTCTTGTCCAACCTCTGTTTCGGCAGAGGATACCGTGCTCCACAGCTGT
AAGGCTGTGGGCCCGAGAGAAGTCGCTCTCTCTCGCCCTCCACATCCCTCTCGGCATTGAGATGACCA
+
FFFFFFFFFFFFFFFFFFFFFFFFFFFFFFFFFF:F:FFFFFFFFFFFFFFFFFFFFFFFFFFFFFF:
F:FFFFFFFFFFFFFFFFFFFFFFFFFFFFFFFFFFFFFFFFFFFFFFFFFFFFFFFFFFFFFFFFFF
@A00155:342:HHGFNDSXY:1:1570:14253:8672 2:N:0:GAACCTAG+TCCGCATA
CTTCGAGTTGTGTAAGTTTCGTCCTAAAAAGGACTCGTCAGGTAGCCTACTAGACTACGACTTACACG
GTAGGTCGTGAACAAGCCCGGAGTACATCTCCTTTGGAACTAGGAGACCCCTCCGGTCTTGTCCAACC
+
FFFFFFFFFFFFFFFFFFFFFFFFFFFFFFFFFFFFFFFFFFFFFFFFFFFFFFFFFFFFFFFFFFFF
FFFFFFFFFFFFFFF,FFFFFFFFF:FFFFFFFFFFFFFFFFFFFFFF:FFFFFFFFFFFFFFFFFFF
@A00155:342:HHGFNDSXY:1:1571:14145:2942 2:N:0:GAACCTAG+TCCGCATA
GTAGCCTACTAGACTACGACTTACACGGTAGGTCGTGAACAAGCCCGGAGTACATCTCCTTTGGAACT
AGGAGACCCCTCCGGTCTTGTCCAACCTCTGTTTCGGCAGAGGATACCGTGCTCCACAGCTGTTAGGC
+
FFFFFFFFFFFFFFFFFFFFFFFFFFFFFFFFFFFFFFFFFFFFFFFFFFFFFFFFFFFFFFFFFFFF
FFFFFFFFFFFFFFFFF::FFFFFFFFFFFFFFFFFFFFF:FFFFFFFFFFFFFFFFFFFFFFFFFF:
```

```
@A00155:342:HHGFNDSXY:1:1571:22281:8453 2:N:0:GAACCTAG+TCCGCATA
TCCAAGGACGAAACCGTCGTTACTCTTCGAGTTGTGTAAGTTTCGTCCTAAAAAGGACTCGTCAGGTA
GCCTACTAGACTACGACTTACACGGTAGGTCGTGAACAAGCCCGGAGTACATCTCCTTTGGAACTAGG
+
FFFF:FFFFFFFFFFFFFFFFFFFFFFFFFFFFFFFFFFFFFFFFFFFFFFF:FFFFFFFFFFFFFFF
FFF:FFFF:FFFFFFFFFFFFFFFFF::F:FFFFFFFFFFFFFFFFFFFFFFFFFFFFFFFFFFFFFF
@A00155:342:HHGFNDSXY:1:1571:23113:9298 2:N:0:GAACCTAG+TCCGCATA
CATGACGGTCGGAGTCTAGTAGGCTCCCTGATGAGTCCGTTCCAAGGACGAAACCGTCGTTACTCTTC
GAGTTGTGTAAGTTTCGTCCTAAAAAGGACTCGTCAGGTAGCCTACTAGACTACGACTTACACGGTAG
+
:FFFFFFFFFFFFF::F:FFF:FF:FFFFFFFFFFFF::FFFFFFFFFFFFFF:FFFFF:FFFF,FF,
FFF:F:FFF:FF:FF,FF:FFFFFFFF:FFF,FFF:F:::F:FFFFFF,FFFF,:FFFFFFFF,F:FF
@A00155:342:HHGFNDSXY:1:1571:14443:11600 2:N:0:GAACCTAG+TCCGCATA
AAAAGGACTCGTCAGGTAGCCTACTAGACTACGACTTACACGGTAGGTCGTGAACAAGCCCGGAGTAC
ATCTCCTTTGGAACTAGGAGACCCCTCCGGTCTTGTCCAACCTCTGTTTCGGCAGAGGATACCGTGCT
+
FFFFFFFFFFFFFFFFFFFFFFFFFFFFFFFFFFFFFFFFFFFFFFFFFFFFFFFF:FFFFFFFFFFF
FFFFFFFFFFFFFFFFFFFFFFFFFFFFFFFFFFFFFFFFFFFFFFFFFFFF:FFFFFFFFFFFFFFF
@A00155:342:HHGFNDSXY:1:1572:21332:7905 2:N:0:GAACCTAG+TCCGCATA
GAGACCCCTCCGGTCTTGTCCAACCTCTGTTTCGGCAGAGGATACCGTGCTCCACAGCTGTAAGGCTG
TGGGCCCGAGAGAAGTCGCTCTCTCTCGCCCTCCACATCCCTCTCGGCATTGAGATGACCATAGGCC
+
FFFFFFFFFFFFFFFFFFFFFFFFFFFFFFF:FFFFFFFFFFFFFFFFFFFFFFFFFFFFFFFFFFFF
FFFFFFFFFFFFFFFFFFFFFFFFFFFFFFFFFFFFFFFFFFFFFFFFFFFFFFFFFFFFF,FFFFF
@A00155:342:HHGFNDSXY:1:1572:26937:19492 2:N:0:GAACCTAG+TCCGCATA
GTTGTGTAAGTTTCGTCCTAAAAAGGACTCGTCAGGTAGCCTACTAGACTACGACTTACACGGTAGGT
CGTGAACAAGCCCGGAGTACATCTCCTTTGGAACTAGGAGACCCCTCCGGTCTTGTCCAACCTCTGTT
+
FFFFFFFFFFFFFFFFFFFFFFFFFFFFFFFFFFFFFFFFFFFFFFFFFF:FFFFFFFFFFFFFFFFF
FFFFFFFFFFFFFFFFFFFFFFFFFFFFFFFFFFFFFFFFFFFFFFFFFFFFFFFFFFFFFFFFFFFF
@A00155:342:HHGFNDSXY:1:1572:22055:26725 2:N:0:GAACCTAG+TCCGCATA
CATGGGGGTTCAAACCCATGCCCCGCCACACCGCCTTAGATCAGCTGTGTCCACTTAAGGACTCACCT
CTGGCCTATGGTCATCTCAATGCCGAGAGGGATGTGGAGGGCGAGAGAGAGCGACTTCTCTCGGGCCC
+
FFFFFFFFFFFFFFFFFFFFFFFFFFFFFFFFFFFFFFFFFFFFFFFFFFFFFFFFFFFFFFFFFFFF
FFFFFFFFFFFFFFFFFFFFFFFFFFFFFFFFFF:FFFFFFFFFFFFFFFFFFFFFFFFFFFFFFFFF
@A00155:342:HHGFNDSXY:1:1573:9579:19930 2:N:0:GAACCTAG+TCCGCATA
GTTTCGTCCTAAAAAGGACTCGTCAGGTAGCCTACTAGACTACGACTTACACGGTAGGTCGTGAACAA
GCCCGGAGTACATCTCCTTTGGAACTAGGAGACCCCTCCGGTCTTGTCCAACCTCTGTTTCGGCAGAG
+
F:FFFFFFFFFFFFFFFFFFFFFFFFFFFFFFFFFFFFFFFFFF,FFFFFFFFFFFFFFFFFFFFFFF
FFFFFFFFFFFFFFFFFFFFFFFFFFFFFFFFFFFFFFFFFFF:FFFFFFFFFFFFFFFFFFFFFFFF
@A00155:342:HHGFNDSXY:1:1573:5746:21496 2:N:0:GAACCTAG+TCCGCATA
GTTTCGTCCTAAAAAGGACTCGTCAGGTAGCCTACTAGACTACGACTTACACGGTAGGTCGTGAACAA
GCCCGGAGTACATCTCCTTTGGAACTAGGAGACCCCTCCGGTCTTGTCCAACCTCTGTTTCGGCAGAG
+
F:FFFFFFFFFFFFFFFFFFFFFFFFFFFFFFFFFFFFFFFFFFFF:FFFFFFFFFFFFFFFFFF:FF
:FFFFFFFFFFFFFFFFFFFFFFFFFFFFFFFFFFFFFFFFFFFFFFFFFFFFFFFFFFFFFFFFFFF
@A00155:342:HHGFNDSXY:1:1573:26332:23641 2:N:0:GAACCTAG+TCCGCATA
GACCCCTCCGGTCTTGTCCAACCTCTGTTTCGGCAGAGGATACCGTGCTCCACAGCTGTAAGGCTGTG
GGCCCGAGAGAAGTCGCTCTCTCTCGCCCTCCACATCCCTCTCGGCATTGAGATGACCATAGG
+
FFFFFFFFFFFFFFFFFFFFFFFFFFFFFFFF,FFFFFFF,F:FFF,FFFFFFFFFFFFFFF,FFFFF
FFFFF:F,FFFF,FFFFFFFFFFFFFFFFFFFFFFFFF,:FFFFFFFF:FFFFF:FFFFFFFF
```

```
@A00155:342:HHGFNDSXY:1:1573:21305:28839 2:N:0:GAACCTAG+TCCGCATA
ACTCTTCGAGTTGTGTAAGTTTCGTCCTAAAAAGGACTCGTCAGGTAGCCTACTAGACTACGACTTAC
ACGGTAGGTCGTGAACAAGCCCGGAGTACATCTCCTTTGGAACTAGGAGACCCCTCCGGTCTTGTCCA
+
FFFFFFFFFFF:FFFFFFFFFFFFFFFFFFFFFFFFFFFFFFFFFFFFFFFFFFFFFFFFFFFFFFFF
FFFFFFFFFFFFFFFFFF:FFFFFFFFFF:FFFFFFFFFFFFFFFFFFFFFFFFFFFFFFFFFFFFFF
@A00155:342:HHGFNDSXY:1:1573:8504:33098 2:N:0:GAACCTAG+TCCGCATA
TAGACTACGACTTACACGGTAGGTCGTGAACAAGCCCGGAGTACATCTCCTTTGGAACTAGGAGACCC
CTCCGGTCTTGTCCAACCTCTGTTTCGGCAGAGGATACCGTGCTCCACAGCTGTAAGGCTGTGGGCCC
+
FFFFFFFFF:FFFFFFFFFFFFFFFFFFFFFFFFFFFFFFFFFFFFFFFFFFFFFFFF:FFFFFFFFF
FFFFFFFFFFFFF:FFFFFFFFFFFFFFFFFFFFFFFFFF:FFFFFFFFFFFFFFFFFFFFFFFFFFF,F
@A00155:342:HHGFNDSXY:1:1573:8250:33599 2:N:0:GAACCTAG+TCCGCATA
TAGACTACGACTTACACGGTAGGTCGTGAACAAGCCCGGAGTACATCTCCTTTGGAACTAGGAGACCC
CTCCGGTCTTGTCCAACCTCTGTTTCGGCAGAGGATACCGTGCTCCACAGCTGTAAGGCTGTGGGCCC
+
FFFFFFFFFFFFFFFFFFFFFFFFFFFFFFFFFFFFFFFFFFFFFFFFFFFFFFFFFFFFFFFFFFFF
FFFFFFFFFFFFFFFFFFFFFFFFFFFFFFFFFFFFFFFFFFFFFFFFFFFFFFFFFFFFFFFFFFFF
@A00155:342:HHGFNDSXY:1:1574:8684:29904 2:N:0:GAACCTAG+TCCGCATA
AGGACGAAACCGTCGTTACTCTTCGAGTTGTGTAAGTTTCGTCCTAAAAAGGACTCGTCAGGTAGCCT
ACTAGACTACGACTTACACGGTAGGTCGTGAACAAGCCCGGAGTACATCTCCTTTGGAACTAGGAGAC
+
FFFFFFFFFF:F,FF:FFFFF,FFFFFFFFFFFFFFF,FFFFFFFFFFFFFFFFF:FFF:FFF:FFFF
,FFF:FFFFFFFF,:FFFFFFFFF:FFF:FFFFFF,F:FFFFFF:FF:FFFF::FFFF,FFFFFFFFF
@A00155:342:HHGFNDSXY:1:1574:4001:33285 2:N:0:GAACCTAG+TCCGCATA
GTGAACAAGCCCGGAGTACATCTCCTTTGGAACTAGGAGACCCCTCCGGTCTTGTCCAACCTCTGTTT
CGGCAGAGGATACCGTGCTCCACAGCTGTAAGGCTGTGGGCCCGAGAGAAGTCGCTCTCTCTCGCCCT
+
FFFFFFFFFFFFFFFFFFFFFFFFFFFFFFFFFFFFFFFFFFFFFFFFFFFF,FFFFFFFFFFFFFFF
FFFFFFFFFFFFFFFFFFFFFFFFFFFFFFFFFFFFFFFFFFFFFFFFFFF:FFFFF:FFFFFFFFFF
@A00155:342:HHGFNDSXY:1:1575:1551:1485 2:N:0:GAACCTAG+TCCGCATA
GTTTCGTCCTAAAAAGGACTCGTCAGGTAGCCTACTAGACTACGACTTACACGGTAGGTCGTGAACAA
GCCCGGAGTACATCTCCTTTGGAACTAGGAGACCCCTCCGGTCTTGTCCAACCTCTGTTTCGGCAGAG
+
FFFFFFFFFFFFFFFFFFFFFFFFFFFFFFFFFFFFFFF:FFFFFFFFF:FFFFFFFFFFFF:FFFF:F
FFFFFFFFFFFFFFFFFFFFFFFFFFFFFF:FFFFFFFFFFFFFFFFFFFFFFFFFFFFFFFFFFFFFF
@A00155:342:HHGFNDSXY:1:1575:13928:26083 2:N:0:GAACCTAG+TCCGCATA
AAGGACGAAACCGTCGTTACTCTTCGAGTTGTGTAAGTTTCGTCCTAAAAAGGACTCGTCAGGTAGCC
TACTAGACTACGACTTACACGGTAGGTCGTGAACAAGCCCGGAGTACATCTCCTTTGGAACCAGGAG
+
FFFFFFFFFFFFFFFFFFFFFFFFFFFFFFFFF:FFFFFFFFFFFF:F:FFFFFFFFF:FFFFFFFFF
FFFFFFFFFFFFFFFFFFFFFFFFFFFFFFFFF,F,FFFFFFFFFFFFFFFFFFF,FFFFFFFFFFFF
@A00155:342:HHGFNDSXY:1:1576:6180:29951 2:N:0:GAACCTAG+TCCGCATA
GAACCAGGAGACCCCTCCGGTCTTGTCCAACCTCTGTTTCGGCAGAGGATACCGTGCTCCACAGCTGT
AAGGCTGTGGGCCCGAGAGAAGTCGCTCTCTCTCGCCCTCCACATCCCTCTCGGCATTGAGATGACCA
+
FFFFFFFFFFFFFFFFFFFFFFFF,FFF:FFFFFFFFFFFFFFFFFFFFFFFFFFFFFFFFFFFFFFFF
F:FFFFFFFFFFFFFFFFFFFFFFFFFFFFFFFFFFFFFFFFFFF:FFFFFFFFFF:FFFFFFFFFFFF
@A00155:342:HHGFNDSXY:1:1577:16721:11945 2:N:0:GAACCTAG+TCCGCATA
AACAAGCCCGGAGTACATCTCCTTTGGAACTAGGAGACCCCTCCGGTCTTGTCCAACCTCTGTTTCGG
CAGAGGATACCGTGCTCCACAGCTGTAAGGCTGTGGGCCCGAGAGAAGTCGCTCTCTCTCGCCCT
+
FFFFF:FFFFFFFFFFFFFFFFFFFFFFFFFFFFFFF:FFFFFFFFFFFFFFFFFFFFFFFFFFFFFF
FFFFFFFFFFFFFFFFFFFFFFFFFFFF:FFFFFFFFFFFFFFFFFFFFFFFFFFFFFFFFFFFFFF
```

```
@A00155:342:HHGFNDSXY:1:1577:24795:22420 2:N:0:GAACCTAG+TCCGCATA
CGTTCCAAGGACGAAACCGTCGTTACTCTTCGAGTTGTGTAAGTTTCGTCCTAAAAAGGACTCGTCAG
GTAGCCTACTAGACTACGACTTACACGGTAGGTCGTGAACAAGCCCGGAGTACATCTCCTTTGGAACT
+
FFFFFFFFFFFFFFFFFFFFFFFFFFFFF:FFFFFFFFF:FFF:FFFFFF,FFFFFFFFFFFFFFFFF
FFFFFFFFFF:FFFFFFFFFFFFFFFFFFFFFFFFFF:FFFFFF:FFFFFF:FFFFFFFFFFFFFFFF
@A00155:342:HHGFNDSXY:1:1577:12952:26584 2:N:0:GAACCTAG+TCCGCATA
GAACTAGGAGACCCCTCCGGTCTTGTCCAACCTCTGTTTCGGCAGAGGATACCGTGCTCCACAGCTGT
AAGGCTGTGGGCCCGAGAGAAGTCGCTCTCTCTCGCCCTCCACATCCCTCTCGGCATAGAGATGACCA
+
FFFFFFFFFFFFFFFFFFFFFF:FFFFFFFFFFFFF:FFFFFFFFFFFFFFFFFFFFFFFFFFFFFFF
FFFFFFFFFFFFFFFFFFFF:FFFFFFFFFFFFFFFFFFFFFFFFFFFFFFFFFFF,FFFFFFFFFFF
@A00155:342:HHGFNDSXY:1:1577:7835:30686 2:N:0:GAACCTAG+TCCGCATA
GGACTCGTCAGGTAGCCTACTAGACTACGACTTACACGGTAGGTCGTGAACAAGCCCGGAGTATATCT
CCTTTGGAACTAGGAGACCCCTCCGGTCTTGTCCAACCTCTGTTTCGGCAGAGGATACCGTGCTCCAC
+
FFFFFFFFFFFFFFFFFFFFFFFFFFFFFFF,FFFFFFFFFFFFF:FFFFFF:,FFFFFFFFFFFFFF
FFFFFFFFFFFFFFFFFFFFFFFFFFFFFFFFFFFFFFFFFFFFFFFFFFFFFFFFFFFFFFFFFFFF
@A00155:342:HHGFNDSXY:1:1578:2700:6918 2:N:0:GAACCTAG+TCCGCATA
ACCTCTGTTTCGGCAGAGGATACCGTGCTCCACAGCTGTAAGGCTGTGGGCCCGAGAGAAGTCGCTCT
CTCTCGCCCTTCACATCCCTCTCGGCATTGAGATGACCATAGGCCAGAGGTGAGTCCTTAAGTGGAC
+
:FFFFFFFFFFFFFFFFFFFFFFFFFFFFFFFFFFFFF:FFFFFFFFFFFFFFFFFFFFF:FFFFFFF
FFFFFFFFFFFFFFFFFFFFFFFFFFFFFFFFFFFFFFFFFF:F:F:FFFFFFFFFFF:FFFFFFFF
@A00155:342:HHGFNDSXY:1:1578:4056:8390 2:N:0:GAACCTAG+TCCGCATA
ACCTCTGTTTCGGCAGAGGATACCGTGCTCCACAGCTGTAAGGCTGTGGGCCCGAGAGAAGTCGCTCT
CTCTCGCCCTTCACATCCCTCTCGGCATTGAGATGACCATAGGCCAGAGGTGAGTCCTTAAGTGGAC
+
FFFFFFFFFFFFFF:FFFFFFFFFFFFFFFFFFFFF:FFFFFFFFFFFFFFFFFFFFFFFFFFFFFFF
FFFFFFFFFFFFFFFFFFFFFFFFFFF:FFFFFFFFFFFFFFFFFFFFFFFFFFFFFF:FF:FFFFF
@A00155:342:HHGFNDSXY:1:1578:23845:10535 2:N:0:GAACCTAG+TCCGCATA
CTACTAGACTACGACTTACACGGTAGGTCGTGAACAAGCCCGGAGTACATCTCCTTTGGAACTAGGAG
ACCCCTCCGGTCTTGTCCAACCTCTGTTTCGGCAGAGGATACCGTGCTCCACAGCTGTAAGGCTGTGG
+
FFFFFFFFFFFFFFFFFFFFFFFFFFFFFFFFFFFFFFFFFFFFFFFFFFFFFFFFFFFFFFFFFFFF
FFFFFFFFFFFFFFF,FFFFFFFFFFFFFFFFFFFFFFFFFFFFFFFFFFFFFFFFFF,FFFFFFFFF
@A00155:342:HHGFNDSXY:1:1578:29405:11960 2:N:0:GAACCTAG+TCCGCATA
TTACACGGTAGGTCGTGAACAAGCCCGGAGTACATCTCCTTTGGAACTAGGAGACCCCTCCGGTCTTG
TCCAACCTCTGTTTCGGCAGAGGATACCGTGCTCCACAGCTGTAAGGCTGTGGGCCCGAGAGAAGTCG
+
FFFFFFFF:FFFFFFFFFFFFFFFFFFFFFFFFFFFFFFFFFFFFFFFFFFFFFFFFFFFFFFF:FF
FFFFFFFFFFFFFFFFFFFFFFFFFFFFFFFFFFFFFFFFFFF:FFFFFFFFFFFFFFFFFFFFFFFF
@A00155:342:HHGFNDSXY:1:1578:28167:13166 2:N:0:GAACCTAG+TCCGCATA
ACTCTTCGAGTTGTGTAAGTTTCGTCCTAAAAAGGACTCGTCAGGTAGCCTACTAGACTACGACTTAC
ACGGTAGGTCGTGAACAAGCCCGGAGTACATCTCCTTTGGAACCAGGAGACCCCTCCGGTCTTGTCCA
+
FFFFFFFFFFFFFFFFFFFFFFF:FFFFFFFFFFFFFF:FFFFFFFFFFFFFFFFFFFFFFFFFFFFF
FFFFFFFFFFFFF:FFFF,FFFFFFFFFFFFFFFFFFFFFF:FFFF:FFFFFFFFFFFFFFFFFFFFF
@A00155:342:HHGFNDSXY:1:1578:22426:27242 2:N:0:GAACCTAG+TCCGCATA
GAACAAGCCCGGAGTACATCTCCTTTGGAACTAGGAGACCCCTCCGGTCTTGTCCAACCTCTGTTTCG
GCAGAGGATACCGTGCTCCACAGCTGTAAGGCTGTGGGCCCGAGAGAAGTCGCTCTCTCTCGCCCTCT
+
FFFFFFFFFFFFFFFFFFFFFFFFFFFFFFFFFFFFFFFFFFFFFFFFFFFFFFFFFFFFFFFFFFFF
FFFFFFFFFFFFFFFFFFFFFFFFFFFFFFFFFFFFFFFFFFFFFFFFFFFFFFFFFFFFFFFFFFFF
```

```
@A00155:342:HHGFNDSXY:1:1601:11216:23766 2:N:0:GAACCTAG+TCCGCATA
CACGGTAGGTCGTGAACAAGCCCGGAGTACATCTCCTTTGGAACCAGGAGACCCCTCCGGTCTTGTCC
AACCTCTGTTTCGGCAGAGGATACCGTGCTCCACAGCTGTAAGGCTGTGGGCCCGAGAGAAGTCGCTC
+
FFFFFFFFFFFFFFFFFFFFFFFFFFFFFFFFFFFFFFFFFFFFFFFFFFFFFFFFFFFFFFFFFFF
FFFFFFFFFFFFFFFFFFFFFFFF:FFFFFFFFFFFFFFFFFFFFFFFFFFFFFFFFFFFFFFFFFF
@A00155:342:HHGFNDSXY:1:1601:12373:25895 2:N:0:GAACCTAG+TCCGCATA
TAGCCTACTAGACTACGACTTACACGGTAGGTCGTGAACAAGCCCGGAGTACATCTCCTTTGGAACTA
GGAGACCCCTCCGGTCTTGTCCAACCTCTGTTTCGGCAGAGGATACCGTGCTCCACAGCTGTAAGGC
+
FFFFFFFFFFFFFFFFFFFFFFFFF:FFFFFFFFFFFFFFFFFFFFFFFFFFFFFFF,FFFFFFFFF
FFFFFFFFFFFFFFFF:FFFFFFFFFFFFFFF,FFFFFFFFFFFFFFFFFFFFFFFFFFFF:FFFF
@A00155:342:HHGFNDSXY:1:1601:16080:30655 2:N:0:GAACCTAG+TCCGCATA
ACTAGACTACGACTTACACGGTAGGTCGTGAACAAGCCCGGAGTACATCTCCTTTGGAACCAGGAGAC
CCCTCCGGTCTTGTCCAACCTCTGTTTCGGCAGAGGATACCGTGCTCCACAGCTGTAAGGCTGTGGGC
+
FFFFFFFFFFFFFFFFFFFFFFFFF:FFFFFFFFFFFFFFFFFFFFFFFFFFFFFFFFFFFFFFFF:FF
FFFFFFFFFFFFFFFFFFFFFFFFFFFFFFFFFFFFFFFFFFFFFFFFFFFFFFFFFFFFFFFFFFF
@A00155:342:HHGFNDSXY:1:1601:14570:31704 2:N:0:GAACCTAG+TCCGCATA
ACTAGACTACGACTTACACGGTAGGTCGTGAACAAGCCCGGAGTACATCTCCTTTGGAACCAGGAGAC
CCCTCCGGTCTTGTCCAACCTCTGTTTCGGCAGAGGATACCGTGCTCCACAGCTGTAAGGCTGTGGGC
+
FFFFFFFFFFFFFFFFFFFFFFFFFFFFFFFFFF,FF:,FFFFFF:FFFFFFFFF,FFFFF:FFFFF
FFFFFFFFF:FFFFFFFFFFFFF,FFFFFFFFFFFFFFFFFFFFFFFFFFFFFFFFFFFFFFFFFFF
@A00155:342:HHGFNDSXY:1:1602:6153:15060 2:N:0:GAACCTAG+TCCGCATA
GGACTCGTCAGGTAGCCTACTAGACTACGACTTACACGGTAGGTCGTGAACAAGCCCGGAGTACATCT
CCTTTGGAACTAGGAGACCCCTCCGGTCTTGTCCAACCTCTGTTTCGGCAGAGGATACCGTGCTCCAC
+
FFFFFFFFFFFFFFFFFFFFFFFFFFFFFFFFFFFFFFFFFFFFF:FF:FFFFFFFFFFFFFFFFFF
FFFFFFFFFFFFFFFF:FFFFFFFFFFFFFFFFFFFF:FFFFFFFFFFF::FFFFFFFFFFFFFFFF
@A00155:342:HHGFNDSXY:1:1603:10999:3881 2:N:0:GAACCTAG+TCCGCATA
AGTACATCTCCTTTGGAACTAGGAGACCCCTCCGGTCTTGTCCAACCTCTGTTTCGGCAGAGGATACC
GTGCTCCACAGCTGTAAGGCTGTGGGCCCGAGAGAAGTCGCTCTCTCTCGCCCTCCACATCCCTCTCG
+
FFFFFFFFFFF:FFFFFFFFFFFFF:FFFFFFFFF:FFFFFFFFFFFFFFFFFFFFFFFFFFFFFFF
FFFFFFFFFFFF:FFFFFFFFFFFFFFFFFFFFFFFFFFF,FFFFFFFFFFFFFFFFFFFFFFFFFF
@A00155:342:HHGFNDSXY:1:1603:1253:24987 2:N:0:GAACCTAG+TCCGCATA
AGTTGTGTAAGTTTCGTCCTAAAAAGGACTCGTCAGGTAGCCTACTAGACTACGACTTACACGGTAGG
TCGTGAACAAGCCCGGAGTACATCTCCTTTGGAACTAGGAGACCCCTCCGGTCTTGTCCAACCTCTGT
+
FFFFFFF:FFFFFF:FFFFF:FFFFFFFFFFFFFFFFFFFFFFFF,:FFF:FFF,FFFFFFFFF:FF
:FFFFFFFFFFFF:FFFFFFFFFFFFF,F:FFFFFFFFFF,FFFFFFFFF:F:FFFFFFFFFFFFFF
@A00155:342:HHGFNDSXY:1:1604:26241:18129 2:N:0:GAACCTAG+TCCGCATA
TAGGTCGTGAACAAGCCCGGAGTACATCTCCTTTGGAACTAGGAGACCCCTCCGGTCTTGTCCAACCT
CTGTTTCGGCAGAGGATACCGTGCTCCACAGCTGTAAGGCTGTGGGCCCGAGAGAAGTCGCTCTCTCT
+
FFFFFFFFFFFFFFFFFFFFFFFFFFFFFFFFFFFFFFFFFFFFFFFFFFFFFFFFFFFFFFFFFFF
FFFFFFFFFFFFFFFFFFFFFFFFFFFF:FFFFFFFFFFFFFFFFFFFFFFFFFFFFFFFFFFFFFF
@A00155:342:HHGFNDSXY:1:1604:17137:21214 2:N:0:GAACCTAG+TCCGCATA
TAGACTACGACTTACACGGTAGGTCGTGAACAAGCCCGGAGTACATCTCCTTTGGAACCAGGAGACCC
CTCCGGTCTTGTCCAACCTCTGTTTCGGCAGAGGATACCGTGCTCCACAGCTGTAAGGCTGTGGGCCC
+
FFFFFFFFFFFFFFFFFFFFFFFFFFFFFFFFFFFFFFFFFFFFFFFFFFFFFFFFFFFFFFFFFFF
FFFFFFFFFFFFFFFFFFFFFFFFFFFFFFFFFFFFFFFFFFFFF:FF:FFFFFFFFFFFFFFFFFF
```

@A00155:342:HHGFNDSXY:1:1605:15944:18239 2:N:0:GAACCTAG+TCCGCATA
GGACTCGTCAGGTAGCCTACTAGACTACGACTTACACGGTAGGTCGTGAACAAGCCCGGAGTACATCT
CCTTTGGAACCAGGAGACCCCTCCGGTCTTGTCCAACCTCTGTTTCGGCAGAGGATACCGTGCTCCAC
+
FFFFFFFFFFFFFFFFFFFFFFFFFFFFFFFFFFFFFFFF:FFFFFFFFFFFFFFFFFFFFFFFFFFF
FFFFFFFFFFFFFFFFFFFFFFFFFFFFFFFFFFFFFFFFFFFFFFFFFFFFFFF:FFFFFFFFFFFF
@A00155:342:HHGFNDSXY:1:1606:30418:11772 2:N:0:GAACCTAG+TCCGCATA
GTACATCTCCTTTGGAACTAGGAGACCCCTCCGGTCTTGTCCAACCTCTGTTTCGGCAGAGGATACCG
TGCTCCACAGCTGTAAGGCTGTGGGCCCGAGAGAAGTCGCTCTCTCTCGCCCTCTACATCCCTCTCGG
+
FFFFF:FFFFFFFFFFFFF::FFFFFFFFFFFFFFFFFF:FFFFFFFFFFFF:FFFFFFFFFF:,FFF
FFFFFFFFFFFFFFFFFFF:FFFFFFFFFF:FF:FFFFFFFFFFFF:FFFFFFFFFFFFFFFFFFFFF
@A00155:342:HHGFNDSXY:1:1606:15591:24486 2:N:0:GAACCTAG+TCCGCATA
TCTCCTTTGGAACTAGGAGACCCCTCCGGTCTTGTCCAACCTCTGTTTCGGCAGAGGATACCGTGCTC
CACAGCTGTAAGGCTGTGGGCCCGAGAGAAGTCGCTCTCTCTCGCCCTCCACATCCCTCTCGGCATTG
+
FFFFFF:FFFFFFFFFFFFFFFFFFFFFFFF:FFFFFFFFFFFFFFFFFFFFFFFFFFFFFFFFFFFF
FFFFFFFFFFFFFFFFFFFFFFFFFFFFFFFFFFFFFFFFFFFFFFFFFFFFF:FFFFFFFFFFF,FF
@A00155:342:HHGFNDSXY:1:1607:20175:13510 2:N:0:GAACCTAG+TCCGCATA
AAGGACGAAACCGTCGTTACTTTTCGAGTTGTGTAAGTTTCGTCCTAAAAAGGACTCGTCAGGTAGCC
TACTAGACTACGACTTACACGGTAGGTCGTGAACAAGCCCGGAGTACATCTCCTTTGGAACTAGGAGA
+
FFFFFFFFFFFFFFFFFFFFFFFFFFFFFFFFFFFFFFFFFFFFFFFFF:FFFFFFFFFFFFFFFFFF
:FFFFFFFFFFFFFFFFFFFFFFFFFFFFFFFFFFFFFFFFFFFFFFFFFFFFF:FFFF:FFFFFFFF
@A00155:342:HHGFNDSXY:1:1607:27787:22122 2:N:0:GAACCTAG+TCCGCATA
GAACTAGGAGACCCCTCCGGTCTTGTCCAACCTCTGTTTCGGCAGAGGATACCGTGCTCCACAGCTGT
AAGGCTGTGGGCCCGAGAGAAGTCGCTCTCTCTCGCCCTCCACATCCCTCTCGGCATTGAG
+
FFFF,FFFFFFFFFFFFFFFFFFFFFFFFFFFFFFFFFFFFFFFFFFFFFFFFFFFFFFFFFFFFFFF
FFFFFFFFFFFFFFFFFFFFFFFFFFFFFFFFFFFFFFFFFFFFFFFFFFFFFFFFFFFFF
@A00155:342:HHGFNDSXY:1:1607:13123:23124 2:N:0:GAACCTAG+TCCGCATA
GGTAGCCTACTAGACTACGACTTACACGGTAGGTCGTGAACAAGTCCGGAGTACATCTCCTTTGGAAC
TAGGAGACCCCTCCGGTCTTGTCCAACCTCTGTTTCGGCAGAGGATACCGTGCTCCACAGCTGTAAGG
+
FFFFFFFFFFFFFFFFFFFFFFFFFFFFFF:FFFFFFFFFFFFFFFFFFFFFFFFFFFFFFFFFFFFF
FFFFFFFFFFFFFFFFFFFFFFFFFFFFFFFFFFFFFFFFFFFFFFF:FFFFFFFFFFFFFFFFFFFF
@A00155:342:HHGFNDSXY:1:1607:23411:27508 2:N:0:GAACCTAG+TCCGCATA
TGGAACTAGGAGACCCCTCCGGTCTTGTCCAACCTCTGTTTCGGCAGAGGATACCGTGCTCCACAGCT
GTAAGGCTGTGGGCCCGAGAGAAGTCGCTCTCTCTCGCCCTCCACATCCCTCTCGGCAGTGAGATGAC
+
FF:FFFFFFFFFFFFF:FFFFFFFFFF:FFFF:FFFF:FFFFFFFFFFFFFFFFFFFFFFFFFFFFF,
FFFFFFF:FFFFFFFFFFF:F:FFFFFFFF:FFFFFFFFFFFFFFFFFFFFFFFFFFF,:FFF,FFFF
@A00155:342:HHGFNDSXY:1:1607:21124:36793 2:N:0:GAACCTAG+TCCGCATA
GAAACCGTCGTTACTCTACGAGTTGTGTAAGTTTCGTCCTAAAAAGGACTCGTCAGGTAGCCTACTAG
ACTACGACTTACACGGTAGGTCGTGAACAAGCCCGGAGTACATCTCCTTTGGAACTAGGAGACCCCTC
+
FFFFFFFFFFFF,FFFFF:FFFFFFFF,FFFF:FFFFFF:FFFF,FFFFFFFFFFFFFFFFF,FFFFF
FFFFFFFFFFFFFFFFFFFFFFFFFFFFFFFFFFFFFFFFFFFFFFFFFFFFFFFFFFFF:FFFFFFF
@A00155:342:HHGFNDSXY:1:1608:17716:5400 2:N:0:GAACCTAG+TCCGCATA
CCGTCGTTACTCTTCGAGTTGTGTAAGTTTCGTCCTAAAAAGGACTCGTCAGGTAGCCTACTAGACTA
CGACTTACACGGTAGGTCGTGAACAAGCCCGGAGTACATCTCCTTTGGAACTAGGAGACCCCTCCGGT
+
FFFFFFFF:FFFFFFFFFFFFFFFFFF:FFFFFFFFFFFFFFFFFFFFFFFFFFFFFFFFFFFFFFFF
FFFFFFFFFFFFFFFF,FFFFFFFFFFFFFFFFFFFFFFFFFFFFFFFFFFFFFFFFFFFFFFFFFF

```
@A00155:342:HHGFNDSXY:1:1608:25364:11537 2:N:0:GAACCTAG+TCCGCATA
AGGACGAAACCGTCGTTACTCTTGGAGTTGTGTAAGTTTCGTCCTAAAAAGGACTCGTCAGGTAGCCT
ACTAGACTACGACTTACACGGTAGGTCGTGAACAAGCCCGGAGTACATCTCCTTTGGAACTAGGAGAC
+
F:F:FFFFF:FFFFFFF::F,F,FFFFFFFFF:FFFFFFFFFFFF,FF:,F:FFFF:FFF:FFFFFFF
FFFFFFFFFFFF,FFFFFFFFFFFFFFFFFF:FFFFFFFFFFFFFF:FFFFFF,FFFFFF:FF,F,FF
@A00155:342:HHGFNDSXY:1:1608:31078:14544 2:N:0:GAACCTAG+TCCGCATA
ACTAGACTACGACTTACACGGTAGGTCGTGAACAAGCCCGGAGTACATCTCCTTTGGAACCAGGAGAC
CCCTCCGGTCTTGTCCAACCTCTGTTTCGGCAGAGGATACCGTGCTCCACAGCTGTAAGGCTGTGGGC
+
FFFFFFFFFFFFF:FFFFFFFFFFFFFFFFFFFFFFFFFFFFFFFFFFFFFFFFFFFFFFFFFFFFFF
FFFFFFFFFFF:FFFFFFFFFFFFFFFF:FFFFFFFFFFFFFFFFFFFFFFFFFFFFFFFFF:FFFFF
@A00155:342:HHGFNDSXY:1:1608:32217:27383 2:N:0:GAACCTAG+TCCGCATA
TTCGTCCTTGGAACGGACTCATCAGGGAGCCTACTAGACTCCGACCGTCATGGGGGTTCAAACCCATG
CCCCACCACACCGCCTTAGATCAGCTGTGTCCACTTAAGGACTCACCTCTGGCCTATGGTCATCTCAA
+
F:FFFFFFFFFFFFFFFFFFFFF,FFFFFFFFFFFFFFFFFFFFFFFFFFFFFFFFFFFFFFFFFFFF
FFFFFFFFFFFFFFFFFFFFFFFFFFFFFFFFFFFFFFFFFFFFFFFFFFFFFFFFFFFFF:FFFFFF
@A00155:342:HHGFNDSXY:1:1608:27154:32518 2:N:0:GAACCTAG+TCCGCATA
AGCCCGGAGTACATCTCCTTTGGAACTAGGAGACCCCTCCGGTCTTGTCCAACCTCTGTTTCGGCAGA
GGATACCGTGCTCCACAGCTGTAAGGCTGTGGGCCCGAGAGAAGTCGCTCTCTCTCGCCCTCCACATC
+
FFFFFFFFFFFFFFFFFFF,FFFFFFFFFFFFFFFFFFFFFFFFFFFFFFFFFFFFFFFFFFFFFFFF
FFFFFFFFFFFFFFFFFFFFFF:FFFFFFFFFFFFFFFFFF:FFFFFFFFFFFFFFFFFFFFFFFFFF
@A00155:342:HHGFNDSXY:1:1609:19443:2973 2:N:0:GAACCTAG+TCCGCATA
AAGTTTCGTCCTAAAAAGGACTCGTCAGGTAGCCTACTAGACTACGACTTACACGGTAGGTCGTGAAC
AAGCCCGGAGTACATCTCCTTTGGAACTAGGAGACCCCTCCGGTCTTGTCCAACCTCTGTTTCGGCAG
+
FFFFFFFFFFFFFFFFFFFFFFFF:FFFFFF:FFFFFFFFFFFFFFFFFFFFFFF:FF:FFFFFFFFF
FFFFFFFFFFFFFFF:FFFFFFF:FFFFFFFFFFF:FFFFFFFFFF:FFF:FFFFFFFFFFFFFFFFF
@A00155:342:HHGFNDSXY:1:1609:26992:18302 2:N:0:GAACCTAG+TCCGCATA
TAGGAGACCCCTCCGGTCTTGTCCAACCTCTGTTTCGGCAGAGGATACCGTGCTCCACAGCTGTAAGG
CTGTGGGCCCGAGAGAAGTCGCTCTCTCTCGCCCTCCACATCCCTCTCGGCATTGAGATGACCATAGG
+
FFFFFFFFFFFFFFFFFFFFFFFFFFFFFFFFFFFFFFFFFFFFFFFFFFFFFFFFFFFFFFFFFFFF
F:FFFFFFFFFFF:FFFFFFFFFFFFFFFFFFFFFFFFFFFFFFFFFFFFFFFFFFFFFFFFFFFF:F
@A00155:342:HHGFNDSXY:1:1609:13105:23249 2:N:0:GAACCTAG+TCCGCATA
CTACTAGACTACGACTTACACGGTAGGTCGTGAACAAGCCCGGAGTACATCTCCTTTGGAACTAGGAG
ACCCCTCCGGTCTTGTCCAACCTCTGTTTCGGCAGAGGATACCGTGCTCCACAGCTGTAAGGCTGTGG
+
FFFFFFFFFFFFFFFFFFFFFFFFFFFFFFFFFFFFFFFFFFFFFFFFFFFFFFFFFFFFFFFFFFFF
FFFFFFFFFFFFFFFFFFFFFFFFFFFFFFFFFFFFFF:FFFFFFFFFFFFFFFF,FFFFFFFFFFFF
@A00155:342:HHGFNDSXY:1:1609:26910:29121 2:N:0:GAACCTAG+TCCGCATA
TAGACTACGACTTACACGGTAGGTCGTGAACAAGCCCGGAGTACATCTCCTTTGGAACTAGGAGACCC
CTCCGGTCTTGTCCAACCTCTGTTTCGGCAGAGGATACCGTGCTCCACAGCTGTAAGGCTGTGGGCCC
+
FFFFFFFFFFFFFFFFFFFFFFFFFFFFF:FFFFFFFFFFFFFFFFFFFFFFFFFF:FFFFFFFFFFF
FFFFFFFFFFFFFFFFFFFFFFFFFFFFFFFFFFFFFFFF:FFFFFFFFFFFFF:FFFFFFFFFFF:F
@A00155:342:HHGFNDSXY:1:1609:26313:33411 2:N:0:GAACCTAG+TCCGCATA
GGACTCGTCAGGTAGCCTACTAGACTACGACTTACACGGTAGGTCGTGAACAAGCCCGGAGTACATCT
CCTTTGGAACCAGGAGACCCCTCCGGTCTTGTCCAACCTCTGTTTCGGCAGAGGATACCGTGCTCCAC
+
FFFFFFFFFFFFFFFFFFFFFFFFFFFFFFFFFFFFFFFFFFFFFFFFFFFFFFFFFFFFFFFFFFFF
FFFFFFFFFFFFFFFFFFFFFFFFFFFFFFFFFFFFFFFFFFFFFFFFFFFFFFFFFFFFFFFFFFFF
```

```
@A00155:342:HHGFNDSXY:1:1609:27326:34162 2:N:0:GAACCTAG+TCCGCATA
TAGACTACGACTTACACGGTAGGTCGTGAACAAGCCCGGAGTACATCTCCTTTGGAACTAGGAGACCC
CTCCGGTCTTGTCCAACCTCTGTTTCGGCAGAGGATACCGTGCTCCACAGCTGTAAGGCTGTGGGCCC
+
FFFFFFFFFFFFFFFFFFFF:FFFFFFFFFFF,FFFFFFFFFF:FFFFFFFFFFFFF:FFFFFFFFF
FFFFFFFF:FFFFFFFFFFFFFFFFF:FFFFFFFFFFFFFFFFFFFFFFFFFFFFF:FFFFFFFFFFFF
@A00155:342:HHGFNDSXY:1:1609:27263:34240 2:N:0:GAACCTAG+TCCGCATA
TAGACTACGACTTACACGGTAGGTCGTGAACAAGCCCGGAGTACATCTCCTTTGGAACTAGGAGACCC
CTCCGGTCTTGTCCAACCTCTGTTTCGGCAGAGGATACCGTGCTCCACAGCTGTAAGGCTGTGGGCCC
+
FFFFFFFFFFFFFFFFFFFFFFFFFFFFFFFFFFFFFFFFFFFFFFFFF:FFFF:FFFFFFFFFFF
FFFFFFFFFFFFFFFFFFFFFFFFFFFFF,FFFFFFFFFFFFFFFFFFFFFF:FFFFFFFFFFFFFFFF
@A00155:342:HHGFNDSXY:1:1609:12897:36260 2:N:0:GAACCTAG+TCCGCATA
GTGAACAAGCCCGGAGTACATCTCCTTTGGAACCAGGAGACCCCTCCGGTCTTGTCCAACCTCTGTTT
CGGCAGAGGATACCGTGCTCCACAGCTGTAAGGCTGTGGGCCCGAGAGAAGTCGCTCTCTCTCGCCCT
+
FFFFFFFFFFFFFFFFFFFFFFFFF:FFFFFFFFFFFFFFFFFFFFFFFFFFFFFFFFFFFFFFFFFF
FFFFFFFFFFFFFFFFFFFFFFFFFFFFFFFFFFFFFFFFFFFFFFFFFFFFFFFFFFFFFFFFFFFF
@A00155:342:HHGFNDSXY:1:1609:12807:36385 2:N:0:GAACCTAG+TCCGCATA
GTGAACAAGCCCGGAGTACATCTCCTTTGGAACCAGGAGACCCCTCCGGTCTTGTCCAACCTCTGTTT
CGGCAGAGGATACCGTGCTCCACAGCTGTAAGGCTGTGGGCCCGAGAGAAGTCGCTCTCTCTCGCCCT
+
FFFFFFFF,FFFFFFFFFFFFFFFFFFFFFF:FFFFFFFFFFFFFFFFFFFFFFFFFFFFFFF:FFFFF
FFFFFFFFFF:FFFFFFFFFFFFFFFFFF:FFFFFFFFFFFFFFFFFFFFFFFFFFFFFFFFFFFFFFF
@A00155:342:HHGFNDSXY:1:1610:26657:6261 2:N:0:GAACCTAG+TCCGCATA
TCCGTTCCAAGGACGAAACCGTCGTTACTCTTTGAGTTGTGTAAGTTTCGTCCTAAAAAGGACTCGTC
AGGTAGCCTACTAGACTACGACTTACACGGTAGGTCGTGAACAAGCCCGGAGTACATCTCCTTTGGAA
+
FFFFFFFFFFFF:FFFFFFFFFFFFFFFFFFFFFFFFFFFFFFFFFFFFFFFFFFF,FFFFFFFFF
FFFFFF:FFFFFFFFFFFFFFFFFFFFFFFFFFFFFFFFFFFFFFFFFF:FFFFFFFFFFFFFFFFFF:
@A00155:342:HHGFNDSXY:1:1610:15049:28776 2:N:0:GAACCTAG+TCCGCATA
TCGAAGAGTAACGACGGTTTCGTCCTTGGAACGGACTCATCAGGGAGCCTACTAGACTCCGACCGTCA
TGGGGGTTCAAACCCATGCCCCGCCACACCGCCTTAGATCAGCTGTGTCCACTTAAGGACTCACCTCT
+
FFFFFFFFFFFFFFFFFFFFFFFFFFFFFF:FFFFFFFFFFFFFFFFFFFFFFFFFFFFFFFFFFFFF
FFFFFFFFFFF,FFFFFFFFFFFFFFFFFFFFFFFFFFFFF:FFFFFFFFFFFFFFFFFFFFFFFFFF
@A00155:342:HHGFNDSXY:1:1610:14380:35070 2:N:0:GAACCTAG+TCCGCATA
GAGACCCCTCCGGTCTTGTCCAACCTCTGTTTCGGCAGAGGATACCGTGCTCCACAGCTGTAAGGCTG
TGGGCCCGAGAGAAGTCGCTCTCTCTCGCCCTCTACATCCCTCTCGGCATTGAGATGACCATAGGCCA
+
FFFFFFFFFFFFFFFFFFFFFFFFFFFFFFFFFFFFFFFFF,FFFFFFFFFFFFFFFFFFFFFFFFF,F
FFFFFFFFFFFFFFFFFFFFFFFFFFFFFFFFFFFFFFFFFFFFFFFFFFFFFFFFFFFFFFFFFFFFF
@A00155:342:HHGFNDSXY:1:1610:32461:35947 2:N:0:GAACCTAG+TCCGCATA
AGCCTACTAGACTACGACTTACACGGTAGGTCGTGAACAAGCCCGGAGTACATCTCCTTTGGAACTAG
GAGACCCCTCCGGTCTTGTCCAACCTCTGTTTCGGCAGAGGATACCGTGCTCCACAGCTGTAAGGCTG
+
FFFFFFFFFFFFFFFFFF:FFFFFFFFFFFFFFFFFFFFFFFFFFFFFFFFFFFFFFFFFFFFFFFFF
FFFFF:FFFFFFFFFFFFFFFFFFFFFFFFFFFFFFFFFFFFFFFF:FFFFFFFFFFFFFF:FF:FFF
@A00155:342:HHGFNDSXY:1:1611:15727:10504 2:N:0:GAACCTAG+TCCGCATA
TAGACTACGACTTACACGGTAGGTCGTGAACAAGCCCGGAGTACATCTCCTTTGGAACTAGGAGACCC
CTCCGGTCTTGTCCAACCTCTGTTTCGGCAGAGGATACCGTGCTCCACAGCTGTAAGGCTGTGGGCCC
+
FFFFFFFFFFFFFFFFFFFFFFFFFFFFFFFFFFFFFFFFFFFFFFFFFFFFFFFFFFFFFFFFFFFF
FFFFFFFFFFFFFFFFFFFFFFFFFFFFFFFFFFFFFFFFFFFFFFFFFFFFFFF:FFFFFFFFFFFF
```

```
@A00155:342:HHGFNDSXY:1:1611:12418:12383 2:N:0:GAACCTAG+TCCGCATA
CAGGTAGCCTACTAGACTACGACTTACACGGTAGGTCGTGAACAAGCCCGGAGTACATCTCCTTTGGA
ACCAGGAGACCCCTCCGGTCTTGTCCAACCTCTGTTTCGGCAGAGGATACCGTGCTCCACAGCTGTAA
+
FFFFFFFFFFFFFFFFFFFFFFFFFFFFFFFFFFFFFFFFFFFFFFFFFFFFFFFFFFFFFFFFFFFF
FFFF:FFFFFFFFFFFFFFFFFFFFFFFFFFFFFFFFFFFFFFFFFFFFFFFFFFFFFFFFFFFFFFF
@A00155:342:HHGFNDSXY:1:1611:7401:31125 2:N:0:GAACCTAG+TCCGCATA
TACGACTTACACGGTAGGTCGTGAACAAGCCCGGAGTACATCTCCTTTGGAACTAGGAGACCCCTCCG
GTCTTGTCCAACCTCTGTTTCGGCAGAGGATACCGTGCTCCACAGCTGTAAGGCTGTGGGCCCGAGAG
+
FFFFFFFFFFFFFFFFFF,FF:FFFFFFFFFFFFFFFFFFFFFFFF:FFFFFFFFFFFFFFFFFFFFF
FFFF:F:FFFFFFFFFFFFFFFFFFF:F:FFFFFFFFFFFFFFFFFFFFF:FFFFFFFFFFFFFFFFF
@A00155:342:HHGFNDSXY:1:1611:14742:34225 2:N:0:GAACCTAG+TCCGCATA
GTGAACAAGCCCGGAGTACATCTCCTTTGGAACTAGGAGACCCCTCCGGTCTTGTCCAACCTCTGTTT
CGGCAGAGGATACCGTGCTCCACAGCTGTAAGGCTGTGGGCCCGAGAGAAGTCGCTCTCTCTCGCCCT
+
FFFFFFFFFFFFFFFFFFFFFFFFFFFFFFFFFFFFFFFFFFFFFFFFFFFFFFFFFFFFFFFFFFFF
FFFF:FFFFFFFFFFFFFFFFFFFFFFFFFFFFFFFFFFFFFFFFFFFFFFFFFFFFFFFFFFFFFFF
@A00155:342:HHGFNDSXY:1:1612:29387:34663 2:N:0:GAACCTAG+TCCGCATA
CAGGTAGCCTACTAGACTACGACTTACACGGTAGGTCGTGAACAAGCCCGGAGTACATCTCCTTTGGA
ACTAGGAGACCCCTCCGGTCTTGTCCAACCTCTGTTTCGGCAGAGGATACCGTGCTCCACAGCTGTAA
+
FFFFFFFFFFFFFFFFFFFFFFFFFFFFFFFFFFFFFFFFFFFFFFFFFFFFFFFFFFFFFFFFFFFF
FFFFFFFFFFFFFFFFFFFFFFFFFFFFFFFFFFFFFFFFFFFFFFFFFFFFFFFFFFFFFFFFFFFF
@A00155:342:HHGFNDSXY:1:1612:22064:35227 2:N:0:GAACCTAG+TCCGCATA
GTGAACAAGCCCGGAGTACATCTCCTTTGGAACTAGGAGACCCCTCCGGTCTTGTCCAACCTCTGTTT
CGGCAGAGGATACCGTGCTCCACAGCGGTAAGGCTGTGGGCCCGAGAGAAGTCGCTCTCTCTCGCCC
+
FFFFFFF,:FFFFFFFFFFFFFFFF,FFFFFFFFFFFFFFFFFFFFFFF:F:FFFFFFFFFFFFF:FF
FFFFFFFFFFFFFFFFFFFFFFF,FF,FFFFFFFFFFFFFFFFFFFFFF,:FFFFFFFFFFFFFFFF
@A00155:342:HHGFNDSXY:1:1612:15022:36652 2:N:0:GAACCTAG+TCCGCATA
CAGGTAGCCTACTAGACTACGACTTACACGGTAGGTCGTGAACAAGCCCGGAGTACATCTCCTTTGGA
ACTAGGAGACCCCTCCGGTCTTGTCCAACCTCTGTTTCGGCAGAGGATACCGTGCTCCACAGCTGTAA
+
FFFFFFFFFFFFFFFFFFFFFFFFFFFFFFFFFFFFF:FFFFF:FFFFFFFFFFFFFFFFFFFFFFFF
FFFFFFFFFFFFFFFFFFFFFFFFFFFFFFFFFFFFFFFFFFFFFFFFFFFFFFFFFFFFFFFFFFFF
@A00155:342:HHGFNDSXY:1:1612:17888:36918 2:N:0:GAACCTAG+TCCGCATA
GTGAACAAGCCCGGAGTACATCTCCTTTGGAACTAGGAGACCCCTCCGGTCTTGTCCAACCTCTGTTT
CGGCAGAGGATACCGTGCTCCACAGCTGTAAGGCTGTGGGCCCGAGAGAAGTCGCTCTCTCTCGCCC
+
FFFFFFFFFFFFFFFFFFFFFFFFFF,FFFFFFFFFFFFFFFFFFFFFFFFFFFFFF,FFFFFFFFFF
FFFF:FFFFFFFFFFFFFFFFFFFFFFFFFFFFFFFFF:FFFFFFFFFFFFFF:FFFFFFFFFFFFF
@A00155:342:HHGFNDSXY:1:1613:11767:5149 2:N:0:GAACCTAG+TCCGCATA
GAGACCCCTCCGGTCTTGTCCAACCTCTGTTTCGGCAGAGGATACCGTGCTCCACAGCTGTAAGGCTG
TGGGCCCGAGAGAAGTCGCTCTCTCTCGCCCTCCACATCCCTCTCGGCATTGAGATGACCATAGGCCA
+
FFFFFFFFFFFFFFFFFFFFFFFFFFFFFFF:FFFFFFFFFFF:FFFFFFFFFFFFFFFFFFFFFFFF
FFFFFFFF:FFFF:FFFFFFFFFFFFFFFFFFFFFF:FFFFFFFFFFFFFFFFFFFFFFFFFFFFFF:
@A00155:342:HHGFNDSXY:1:1613:24334:21277 2:N:0:GAACCTAG+TCCGCATA
TACTCTTCGAGTTGTGTAAGTTTCGTCCTAAAAAGGACTCGTCAGGTAGCCTACTAGACTACGACTTA
CACGGTAGGTCGTGAACAAGCCCGGAGTACATCTCCTTTGGAACTAGGAGACCCCTCCGGTCTTGTCC
+
FFFFFFFFFFFFFFFFFF:FFFFFFFFFFFFFFFFFFFFFF:FFFFFFFFFFFFFFFFFFFFFFFFFF
FFFFFFFFFFFFFFFFFFFFFFFFFFFFFFFFFFFFFFFFF:FFFFFFFFFFFFFFFFFFFFFFFFF:
```

```
@A00155:342:HHGFNDSXY:1:1613:10013:24251 2:N:0:GAACCTAG+TCCGCATA
AACTAGGAGACCCCTCCGGTCTTGTCCAACCTCTGTTTCGGCAGAGGATACCGTGCTCCACAGCTGTA
AGGCTGTGGGCCCGAGAGAAGTCGCTCTCTCTCGCCCTCTACATCCCTCTCGGCATTGAGATGACCA
+
FFFFF:FFFFFFFFFFFFFFFFFFFFFFFFFFF,FFF,FFFFFFFFFFFFFFFFFFFFFFFFFFFFFF
FFFFFFFFFFFFFFFFFFFFFF:FFFFFFFFFFFFFFFFFFFFFFFFFFFFFFFFFFFFFFFFFFFF:
@A00155:342:HHGFNDSXY:1:1613:12988:29246 2:N:0:GAACCTAG+TCCGCATA
GGACTCGTCAGGTAGCCTACTAGACTACGACTTACACGGTAGGTCGTGAACAAGCCCGGAGTACATCT
CCTTTGGAACTAGGAGACCCCTCCGGTCTTGTCCAACCTCTGTTTCGGCAGAGGATACCGTGCTCCAC
+
FFFFFFFFFFFFFFFFFFFFFFFFFFFFFFFFFFFFFFFFFFFFFFFFFFFFFFFFFFFFFFFFFFFF
FFFFFFFFFFFFFFFFFFFFFFFFFFFFFFFFFFFFFFFFFFFFFFFFFFFFFF:FFFFFFFFFFFFF
@A00155:342:HHGFNDSXY:1:1614:4788:14418 2:N:0:GAACCTAG+TCCGCATA
CACGACCTACCGTGTAAG
+
FFFFFFFFFFFFFFFFFF
@A00155:342:HHGFNDSXY:1:1614:14796:30968 2:N:0:GAACCTAG+TCCGCATA
TACACGGTAGGTCGTGAACAAGCCCGGAGTACATCTCCTTTGGAACTAGGAGACCCCTCCGGTCTTGT
CCAACCTCTGTTTCGGCAGAGGATACCGTGCTCCACAGCTGTAAGGCTGTGGGCCCGAGAGAAGTCGC
+
FFFFFFFFFFFFFFFFFFFFFF:FFFFFFFFFFFFFFFFFFFFFFFFFFFFFFFFFFFFFFFFFFFFF
FFFFFFFFFFFFFFFFFFFFFFFFFFFFFFFFFFFFFFFFFFF:FFFFFFFFFFFFFFFFFFFFFFFF
@A00155:342:HHGFNDSXY:1:1614:21043:32174 2:N:0:GAACCTAG+TCCGCATA
AAAAGGACTCGTCAGGTAGCCTACTAGACTACGACTTACACGGTAGGTCGTGAACAAGCCCGGAGTAC
ATCTCCTTTGGAACTAGGAGACCCCTCCGGTCTTGTCCAACCTCTGTTTCGGCAGAGGATACCGTGCT
+
FFFFFFFFFFFFFFFFFFFFFFFFFFFFFFFFFFFFFFFFFFFFFFFFFFFFFFFFFFFFFFFFFF:F
FFFFFFFFFFFFFFFFFFFFFFFFFFFFFFFFFFFFFFFFFFFFFFFFFFFFFFFFFFFFFFFFFFFF
@A00155:342:HHGFNDSXY:1:1615:22923:14105 2:N:0:GAACCTAG+TCCGCATA
GGACTCGTCAGGTAGCCTACTAGACTACGACTTACACGGTAGGTCGTGAACAAGCCCGGAGTACATCT
CCTTTGGAACTAGGAGACCCCTCCGGTCTTGTCCAACCTCTGTTTCGGCAGAGGATACCGTGCTCCAC
+
FFFFFFFFFFFFF:FFFFFFFFFFFFFFFFFFFFFFFFFFFFFFFFFFFFFFFFFFFFFFFFFFFFFF
FFFFFFFFFFFFFFFFFFFFFFFFFFFFFF:FFFFFFFFFFFFFFFF:FFFFFFFFFFFFFFFFFFFF
@A00155:342:HHGFNDSXY:1:1615:18918:30060 2:N:0:GAACCTAG+TCCGCATA
GGACTCGTCAGGTAGCCTACTAGACTACGACTTACACGGTAGGTCGTGAACAAGCCCGGAGTACATCT
CCTTTGGAACCAGGAGACCCCTCCGGTCTTGTCCAACCTCTGTTTCGGCAGAGGATACCGTGCTCCAC
+
:FFF:FFF,FFF:FFFFFFFFFFFFF:F:FFFFFFF:,FFFFFFFFFFFFFFFFFF:FFFFFFFFFFF
FFFFFF:F:::FFFFFF:FF:FFFFFFF:FFF:::FFF,FFFF,FFFFFFFFFFFFFF::::FFFFF:
@A00155:342:HHGFNDSXY:1:1616:9299:25488 2:N:0:GAACCTAG+TCCGCATA
AGCCCGGAGTACATCTCCTTTGGAACTAGGAGACCCCTCCGGTCTTGTCCAACCTCTGTTTCGGCAGA
GGATACCGTGCTCCACAGCTGTAAGGCTGTGGGCCCGAGAGAAGTCGCTCTCTCTCGCCCTCTACATC
+
FFFFFFFFFFFFFF,FFFFFFFFFFFFFFFFFFFFFFFFFFFFFFFFF:FFFFFFFFFF:FFFFFFFFF
FFFFFFFFFFFFFFFFFFFFFF:FFFFFFFFFFFFFFFFFFFFFFFFFFFFFFFFFFFFFFFFFFFFF
@A00155:342:HHGFNDSXY:1:1616:20039:27586 2:N:0:GAACCTAG+TCCGCATA
TAGGACGAAACTTACACAACTCGAAGAGTAACGACGGTTTCGTCCTTGGAACGGACTCATCAGGGAGC
CTACTAGACTCCGACCGTCATGGGGGTTCAAACCCATGCCCCGCTACACCGCCTTAGATCAGCTGTGT
+
FFFFFFFFFFFFFFFFFFFFFFFFFFFFFFFFFFFFFFFFFFFFFFFFFFFFFFFFFFFFFFFFFFFF
FFFFFF,FFFFFFFFFFFFFFFFFFFFFFFFFFFFFFFFFFFFFFFFFFFFFFFFFFFFFFFFFFFFF
@A00155:342:HHGFNDSXY:1:1616:28339:30217 2:N:0:GAACCTAG+TCCGCATA
TCTTGTCCAACCTCTGTTTCGGCAGAGGATACCGTGCTCCACAGCTGTAAGGCTGTGGGCCCGAGAGA
```

AGTCGCTCTCTCTCGCCCTCCACATCCCTCTCGGCATTGAGATGACCATAGGCCAGAGGAGAGTCCT
+
F:FFFFFFFFFFFFFFF,:FFFFFFFFFFFFFFFFFFFFFFFFFFFFFFFFFF:FFFFFFFFFFFFFF
FFFFFFFFFFFFFFFFFF:FFFFFFFFFFFFFFFFFFFFFFFFFFFFFFFFFFF:FFFFFFFFFFFFF
@A00155:342:HHGFNDSXY:1:1617:30156:12164 2:N:0:GAACCTAG+TCCGCATA
ACATCTCCTTTGGAACTAGGAGACCCCTCCGGTCTTGTCCAACCTCTGTTTCGGCAGAGGATACCGTG
CTCCACAGCTGTAAGGCTGTGGGCCCGAGAGAAGTCGCTCTCTCTCGCCCTCTACATCCCTCTCGGC
+
FFFFFFFFFFFFFFFFFFFFFFFFFFFFFFFFFFFFFFFFFFFFFFFFFFFFFFFFFFFFFFFFFFFF
FFFFFF:FFFF:FFFFFFFFFFFFFFFFFFFF,F:FFFFFFFFFFFFFFFFFFFFFFFFFFFFFFFFF
@A00155:342:HHGFNDSXY:1:1619:10366:7232 2:N:0:GAACCTAG+TCCGCATA
GACCCCTCCGGTCTTGTCCAACCTCTGTTTCGGCAGAGGATACCGTGCTCCACAGCTGTAAGGCTGTG
GGCCCGAGAGAAGTCGCTCTCTCTCGCCCTCCACATCCCTCTCGGCATTGAGATGACCATAG
+
FFFFFFFFFFFFFFFFFFFFFFFFFFFFFFFFFFFFFFFFFFFFFFFFFFFFFFFFFFFFFFFFFFFF
FFFFFFFFFFF:FFFFFFFFFFFFFFFFFFFFFFFFFFFFFFFFFFFFFFFFFFFFF:FF,FF
@A00155:342:HHGFNDSXY:1:1619:10239:7326 2:N:0:GAACCTAG+TCCGCATA
GACCCCTCCGGTCTTGTCCAACCTCTGTTTCGGCAGAGGATACCGTGCTCCACAGCTGTAAGGCTGTG
GGCCCGAGAGAAGTCGCTCTCTCTCGCCCTCCACATCCCTCTCGGCATTGAGATGACCATAG
+
FFFFFFFFFFFFF:FFFFFFFFFFFFFFFFFFFFFFFFFFFFFFFFFFFFFFFFFFFFFFFFFFFFFF
FFFFFFFFFFFFFFFFFFFFFFFFFFFFFFFFFFFFFFFFFFFFFFFFFFFFFFFF,FFFFFF
@A00155:342:HHGFNDSXY:1:1619:12735:12680 2:N:0:GAACCTAG+TCCGCATA
AGCCCGGAGTACATCTCCTTTGGAACTAGGAGACCCCTCCGGTCTTGTCCAACCTCTGTTTCGGCAGA
GGATACCGTGCTCCACAGCTGTAAGGCTGTGGGCCCGAGAGAAGTCGCTCTCTCTCGCCCTCC
+
FFFFFFFFFFFFFFFFFF:FFFFFFFF:FFFFFFFFFFFFFFFFFFF:FFFFFFFFFFFFFFFFFFFF
FFFF:FFFFFFFFFFFFFFFFFF,FFFFFFFFFFFFFFFFFF,FFFFFFFFFFFFFFFFFFFF
@A00155:342:HHGFNDSXY:1:1620:8929:3458 2:N:0:GAACCTAG+TCCGCATA
GGAGTACATCTCCTTTGGAACTAGGAGACCCCTCCGGTCTTGTCCAACCTCTGTTTCGGCAGAGGATA
CCGTGCTCCACAGCTGTAAGGCTGTGGGCCCGAGAGAAGTCGCTCTCTCTCGCCCTCCACATCCCTCT
+
FFFFFFFFFFFFFFFFFFFFFFFFFFFFFFFFFFFFFFFFFFFFFFFFFFFFFFFFFFFFFFFFFFFF
FFFFFFFFFFFFFFFFFFFFFFFFFFFFFFFFFFFFFFFFFFFFFFFFFFFFFFFFFFFFFFFFFFFF
@A00155:342:HHGFNDSXY:1:1620:31177:21574 2:N:0:GAACCTAG+TCCGCATA
TACATCTCCTTTGGAACTAGGAGACCCCTCCGGTCTTGTCCAACCTCTGTTTCGGCAGAGGATACCGT
GCTCCACAGCTGTAAGGCTGTGGGCCCGAGAGAAGTCGCTCTCTCTCGCCCTCCACATCCCTCTCGGC
+
FFFFFFFFFFFFFFF:FFFFFFFFFFFFFFFFFFFFFFFFFFFFFFFFFFFFFFFFFFFFFFFFFFF:
FFFFFFFFFFFFFFFFFFFFFFFFFFFFFFFFFFFFFFFFFFFFFFFFFF:FFFFFFF:FFFFF:F
@A00155:342:HHGFNDSXY:1:1620:15157:22451 2:N:0:GAACCTAG+TCCGCATA
GACGAAACCGTCGTTACTCTTCGAGTTGTGTAAGTTTCGTCCTAAAAAGGACTCGTCAGGTAGCCTAC
TAGACTACGACTTACACGGTAGGTCGTGAACAAGCCCGGAGTACATCTCCTTTGGAACTAGGAGACCC
+
FFFFFFFF:FFFFFFFFFFFFFFFF:FFFFFFFFF:FFFFFFF:F:FFFFFFFFFFFFFFFF:FFFFF
FFFFFFFFFFFFFFFFFFFFFFFFFFFFFFFFFFFFFFFFFFFFFFFFFFFFFFFFFFFFFFFFFFFF
@A00155:342:HHGFNDSXY:1:1621:27769:3208 2:N:0:GAACCTAG+TCCGCATA
GCCCGGAGTACATCTCCTTTGGAACTAGGAGACCCCTCCGGTCTTGTCCAACCTCTGTTTCGGCAGAG
GATACCGTGCTCCACAGCTGTAAGGCTGTGGGCCCGAGAGAAGTCGCTCTCTCTCGCCCTCCACATCC
+
FFFFFFFFFFFF:FFFFFFFFFFFFFFFFF:FFFFFFFFFFFFFFFFFFFFF:FF:FFFF:FFFFFFF
FFFFFFFFFFFFFFFFFFFFFFFF::FFFFFFFFFFFFFFFFFFF::FFFFF:FFFF:FFFFFFFFFF
@A00155:342:HHGFNDSXY:1:1622:25156:7670 2:N:0:GAACCTAG+TCCGCATA
ACCTCTGTTTCGGCAGAGGATACCGTGCTCCACAGCTGTAAGGCTGTGGGCCCGAGAGAAGTCGCTCT

CTCTCGCCCTCCACATCCCTCTCGGCATTGAGATGACCATAGGCCAGAGGAGAGTCCTTAAGTGGAC
+
FFFFFFFFFFFFFFFFFFFFFFFFF:FFFFFFFFFF:FFFFFFFFFFFFFFFFFFFFFF,FFFFFFFF
FFFFFFFFFFFFFFFFFFFFFFFFFFFFFFFFFFFFFF:FFFFFFFF:FFFFFFFFFFF:FFFFFFF
@A00155:342:HHGFNDSXY:1:1622:18783:30577 2:N:0:GAACCTAG+TCCGCATA
AAGCCCGGAGTACATCTCCTTTGGAACTAGGAGACCCCTCCGGTCTTGTCCAACCTCTGTTTCGGCAG
AGGATACCGTGCTCCACAGCTGTAAGGCTGTGGGCCCGAGAGAAGTCGCTCTCTCTCGCCCTCCACAT
+
FFF:F:FFFFFFFFFFFFFFFFFFFFFFFFFFFFFFFF:FFFFFF,:FFFFFFFFFFFFFFFFFFFFF
FFF:FFFFFFFFFFF:FFFF:FFF:FFFFFFFF:FFFFFFFF:FFFFFFFFFFFFFFFFFFFFF:FFF
@A00155:342:HHGFNDSXY:1:1622:18747:30859 2:N:0:GAACCTAG+TCCGCATA
AAGCCCGGAGTACATCTCCTTTGGAACTAGGAGACCCCTCCGGTCTTGTCCAACCTCTGTTTCGGCAG
AGGATACCGTGCTCCACAGCTGTAAGGCGGTGGGCCCGAGAGAAGTCGCTCTCTCTCGCCCTCCACAT
+
FFFFFFFFFFFFFFFFFFFF:FFFFFFFFFFFFFFFFFFFFFFFFFFFF:FFFFFFFFFF,FFFFFFFF
FFFFFFFFFFFFFFFFFFFFFFF::,FFF,FFFFFFFFFFFFF,:FFFFFFFFFFFFFFFFFFFFFFF::
@A00155:342:HHGFNDSXY:1:1623:4915:2957 2:N:0:GAACCTAG+TCCGCATA
AGTACATCTCCTTTGGAACTAGGAGACCCCTCCGGTCTTGTCCAACCTCTGTTTCGGCAGAGGATACC
GTGCTCCACAGCTGTAAGGCTGTGGGCCCGAGAGAAGTCGCTCTCTCTCGCCCTCCACATCCCTCTCG
+
FFFFFFFFFFFFFFFFFFFFFFFFFFFFFFFFFFFFFFFFFFFFFFFFFFFFFFFFFFFFFFFF:FFF
FFFFFFFFFFFFFFFFFFFFFFFFFFFFFFFFFFFFFFFFFFFFFFFFFFFFFFFFFF:FFFFFFFFF
@A00155:342:HHGFNDSXY:1:1623:21504:5290 2:N:0:GAACCTAG+TCCGCATA
CCGTCGTTACTCTTGGAGTTGTGTAAGTTTCGTCCTAAAAAGGACTCGTCAGGTAGCCTACTAGACTA
CGACTTACACGGTAGGTCGTGAACAAGCCCGGAGTACATCTCCTTTGGAACTAGGAGACCCCTCCGGT
+
FFFFFF:FFFFFFFFFFFFFFFF:FFFFFFFFFFFFFFFFFFFFFFFFFFFFFFFFFFFFFFFFFFFF
FFFFFFFFFFFFFFFFFFFFFFFFFFFF:FFFFFFFFFFFFFFFFFFF,F,FFFFFFFFF:FFFFFFFF
@A00155:342:HHGFNDSXY:1:1623:27281:14888 2:N:0:GAACCTAG+TCCGCATA
CGGCAGAGGATACCGTGCTCCACAGCTGTAAGGCTGTGGGCCCGAGAGAAGTCGCTCTCTCTCGCCCT
CCACATCCCTCTCGGCATTGAGATGACCATAGGCCAGAGGTGAGTCCTTAAGTGGACACAGCTGATCT
+
FFFFFFFFFFFFFFFFFFFFFFFFFFFFFFFFFFFFFFFFFFFFFFFFFFF:FFFFFFFFFFFFFFFF
FFFFFFFFFFFFFFFFF,FFFFFFFFFFFFFFFFFFFFFFFFFFFFFFF:FFFFFFFFFFFFFFFFFF
@A00155:342:HHGFNDSXY:1:1623:5159:33974 2:N:0:GAACCTAG+TCCGCATA
CACGGTAGGTCGTGAACAAGCCCGGAGTACATCTCCTTTGGAACTAGGAGACCCCTCCGGTCTTGTCC
AACCTCTGTTTCGGCAGAGGATACCGTGCTCCACAGCTGTAAGGCTGTGGGCCCGAGAGAAGTCGCTC
+
FFFFFFFFFFFFFFFFFFFFFFFFFFFFFFFFFFF,FFFFFFFFFFFFFFFFFFFFFFFFFFFFFFFF
FFFFFFFF:FFFFFFFFFFFFFFFFFFFFFFFFFFFFFFFFFFF:FFFFFFFFFFFFFFFFFFFFFFF
@A00155:342:HHGFNDSXY:1:1623:2555:35383 2:N:0:GAACCTAG+TCCGCATA
GGAGTACATCTCCTTTGGAACTAGGAGACCCCTCCGGTCTTGTCCAACCTCTGTTTCGGCAGAGGATA
CCGTGCTCCACAGCTGTAAGGCTGTGGGCCCGAGAGAAGTCGCTCTCTCTCGCCCTCCACATCCCTCT
+
FFFFFFFFFFFFFFFFFFFFFFFFFFFF:FFFFFFFFFF::FFFFFFFFFFFFFFFFFFFFFF:FFFFF
FFFFFFFFFFFFFFFFFFFFFFFFFFFFFFFFF:FFFFFFFFFFFFFFFFFFFFFFFFFFFFFFFFFF
@A00155:342:HHGFNDSXY:1:1624:12472:27633 2:N:0:GAACCTAG+TCCGCATA
ACATCTCCTTTGGAACTAGGAGACCCCTCCGGTCTTGTCCAACCTCTGTTTCGGCAGAGGATACCGTG
CTCCACAGCTGTAAGGCTGTGGGCCCGAGAGAAGTCGCTCTCTCTCGCCCTCCACATCCCTCTCGGCA
+
FFFFFFFFFFFFFFFFFFFFFFFFFFFFFFFFFFFFFFFFFFFFFFFFFFFFFFFFFFFFFF:FFFFFF
FFFFFFFFFFFFFFFFFFFFFFFFFFFFFFFFFFFFFFFFFFFFFFFFFFFFFFFF:FFFFFFFFFFF
@A00155:342:HHGFNDSXY:1:1624:20573:34240 2:N:0:GAACCTAG+TCCGCATA
CAGCTGTAAGGCTGTGGGCCCGAGAGAAGTCGCTCTCTCTCGCCCTCCACATCCCTCTCGGCATTGAG

```
ATGACCATAGGCCAGAGGTGAGTTCTTAAGTGGACACAGCTGATCTAAGGCGGTGTGGCGGGGCATGG
+
FFFFFFFFFFFFFFFFFFFFFFFFFFFFFFFFFFFFFFFFFFFFFFFFFFFFFFFFFFFFFFFFFFFFF
FFFFFF:FFFFFF:FFFFFFFFFFFFFFFFFFFFF:FFFFFFFFFFF,FFFFFFFFFFFFFFFFFFFF
@A00155:342:HHGFNDSXY:1:1624:21269:37043 2:N:0:GAACCTAG+TCCGCATA
CAGCTGTAAGGCTGTGGGCCCGAGAGAAGTCGCTCTCTCTCGCCCTCCACATCCCTCTCGGCATTGAG
ATGACCATAGGCCAGAGGTGAGTTCTTAAGTGGACACAGCTGATCTAAGGCGGTGTGGCGGGGCATGG
+
FFFFFFFFFFFFFFFFFFFFFFFFFFFFFFFFFFFFFFFFF:FFFFFFFFFFFFFFFFFFFFFFFFFF
FFFFFF:FFFFFFFFFFFFFFFFFFFFFFFFFFFFF:FFFFFFFFFFFFFFFFFFFFFFFFFFFFFFFF
@A00155:342:HHGFNDSXY:1:1625:22110:1454 2:N:0:GAACCTAG+TCCGCATA
GGAACGGACTCATCAGGGAGCCTACTAGACTCCGACCGTCATGGGGGTTCAAACCCATGCCCCGCCAC
ACCGCCTTAGATCAGCTGTGTCCACTTAAGGACTCACCTCTGGCCTATGGTCATCTCAATGCCGAGAG
+
FFFFFFFFFFFFFFF:F:FFFF,FFFF:FFFFFFFFFFFFFFFFFFF,FFFFFFFFFF:FFFFF:FFF
FFFFFFF:FFFFFFFFFFFFFFFFFFFFFFFFFFFFFFFFFFFFFFFF:FF:FFFF,FFFFFFF:::FF
@A00155:342:HHGFNDSXY:1:1625:30752:4022 2:N:0:GAACCTAG+TCCGCATA
AAGGACTCTCCTCTGGCCTATGGTCATCTCAATGCCGAGAGGGATGTGGAGGGCGAGAGAGAGCGACT
TCTCTCGGGCCCACAGCCTTACAGCTGTGGAGCACGGTATCCTCTGCCGAAACAGAGGTTGGACAAGA
+
FFFFFFFFFFFFFFFFFFFFFFFFFFFFFFFFFFFFFFFFFFFFFFFFFFFFFFFFFFFFFFFFFFFFF
FFFFFFFFFFFFFFFFFFFF:FFFFFFFFFFFFFFFFFFFFFFFFFFFFFFFFFFFFFFFFFFFFFFFF
@A00155:342:HHGFNDSXY:1:1625:11134:5181 2:N:0:GAACCTAG+TCCGCATA
GAGTTGTGTAAGTTTCGTCCTAAAAAGGACTCGTCAGGTAGCCTACTAGACTACGACTTACACGGTAG
GTCGTGAACAAGCCCGGAGTACATCTCCTTTGGAACTAGGAGACCCCTCCGGTCTTGTCCAACCTCTG
+
FFFFFFFFFFFFFFFFFFFFFFFFF:F:FFFFFFFFFFFFFFFFFFFFFFFFFFFFFFFFFFFF,FFFF
FFFFFFFFFFF,FFFFFFFFFFFFFFFFFFFFFFFFFFFFFFFFF:FFFFFFFFFFFFFFFFFFFFFF
@A00155:342:HHGFNDSXY:1:1625:8684:5885 2:N:0:GAACCTAG+TCCGCATA
GTTTCGTCCTAAAAAGGACTCGTCAGGTAGCCTACTAGACTACGACTTACACGGTAGGTCGTGAACAA
GCCCGGAGTACATCTCCTTTGGAACTAGGAGACCCCTCCGGTCTTGTCCAACCTCTGTTTCGGCAGAG
+
FFFFFFFFFFFFFFFFFFFFFFFFFFFFFFFFFFFFFFFFFFFFFFFFFFFFFFFFFFFFFFFFFFFFF
FFFFFFFFFFFFFFFFFFFFFFFFFFFFFFFFFFFFFFFFFFFFFFFFFFFFFFFFFFFFFFFFFFFFF
@A00155:342:HHGFNDSXY:1:1625:16911:24643 2:N:0:GAACCTAG+TCCGCATA
CTACGACTTACACGGTAGGTCGTGAACAAGCCCGGAGTACATCTCCTTTGGATCTAGGAGACCCCTCC
GGTCTTGTCCAACCTCTGTTTCGGCAGAGGATACCGTGCTCCACAGCTGTAAGGCTGTGGGCCCGAGA
+
FFFFFFFFFFFFFFFFFFFFFFFFFFFFFFFFFFFFFFFFFFFFFFFFFFFFFFFFFFFFFFFFFFFFF
FFFFFFFFFFFFFFFFFFFFFFFF:F:FFFFFFFFFFFFFFFFFFFFFFFFFFFFFFFFFFFFFFFFFF
@A00155:342:HHGFNDSXY:1:1625:15239:26976 2:N:0:GAACCTAG+TCCGCATA
GTGAACAAGCCCGGAGTACATCTCCTTTGGAACTAGGAGACCCCTCCGGTCTTGTCCAACCTCTGTTT
CGGCAGAGGATACCGTGCTCCACAGCTGTAAGGCTGTGGGCCCGAGAGAAGTCGCTCTCTCTCGCCCT
+
FFFFFFFFFFFFFFFFFFFFFFFFFFFFFFFFFFFFFFFFFFFFFFFFFFFFFFFFFFFFFFFFFFFFF
FFFFFFFFFFFFFFFFFFFFFFFFFFFFFFFFFFFFFFFFFFFFFFFFFFFFFFFFFFFFFFFFFFFFF
@A00155:342:HHGFNDSXY:1:1626:14389:16391 2:N:0:GAACCTAG+TCCGCATA
TCCTAAAAAGGACTCGTCAGGTAGCCTACTAGACTACGACTTACACGGTAGGTCGTGAACAAGCCCGG
AGTACATCTCCTTTGGAACTAGGAGACCCCTCCGGTCTTGTCCAACCTCTGTTTCGGCAGAGGATACC
+
FFFFFFFFFFFFFFFFFFFFFFFFFFF,FFFFFFFFFFFFFFFFFFFFFFFFFFFFFFFFFFFFFFFFF
FFFFFF:FFFFFFFFFFFFFFFFFFFFFFFFFFFFFFFFFFFFFFFFFFFFFFF:FFFFFFFFFFFF
@A00155:342:HHGFNDSXY:1:1626:15799:24189 2:N:0:GAACCTAG+TCCGCATA
AGGACTCGTCAGGTAGCCTACTAGACTACGACTTACACGGTAGGTCGTGAACAAGCCCGGAGTACATC
```

```
TCCTTTGGAACCAGGAGACCCCTCCGGTCTTGTCCAACCTCTGTTTCGGCAGAGGATACCGTGCTCCA
+
FFFFFFFFFF:FFFFFFFFFFFFFFFFFFFFFFFFFFFFFFFFFFFFFFFFFFFFFFFFFFFFFFFFFFFF
FFFFFFFFFFFFFFFFFFFFFFFFFFFFFFFFFFFFFFFFFFFFFFFFFFFFFFFFFFFFFFFFFFFFFFFF
@A00155:342:HHGFNDSXY:1:1626:3983:26428 2:N:0:GAACCTAG+TCCGCATA
GTGTAAGTTTCGTCCTAAAAAGGACTCGTCAGGTAGCCTACTAGACTACGACTTACACGGTAGGTCGT
GAACAAGCCCGGAGTACATCTCCTTTGGAACTAGGAGACCCCTCCGGTCTTGTCCAACCTCTGTTTCG
+
FFFFFFF:FFFFFFFFFFFFFFFFFFFFFFFFFFFFFFFFFFFFFF:FFFFFFFFFFFFFFFFFFFF
FFFFFF,FFFFFFFFFF,:F:FFFFFFFFFFFFFFFFFF:F:FFF:FFFFFFFFFFFFF,FFFFFF:F
@A00155:342:HHGFNDSXY:1:1626:5547:29105 2:N:0:GAACCTAG+TCCGCATA
GGACTCGTCAGGTAGCCTACTAGACTACGACTTACACGGTAGGTCGTGAACAAGCCCGGAGTACATCT
CCTTTGGAACCAGGAGACCCCTCCGGTCTTGTCCAACCTCTGTTTCGGCAGAGGATACCGTGCTCCAC
+
FFFFFFFFFFFFFFFFFFFFFFFFFFFFFFFFFFFFFFFFFFFFFFFFFFFFF:FFFFFFFFFFFFFF
FFFFFFFFFFFFFFFFFF:FFFFFFFFFFFFFFFFFFFFFFFFFFFFFFFFFFFFF:FFFFFFFFFFFF
@A00155:342:HHGFNDSXY:1:1628:24343:5822 2:N:0:GAACCTAG+TCCGCATA
TAGGTCGTGAACAAGCCCGGAGTACATCTCCTTTGGAACTAGGAGACCCCTCCGGTCTTGTCCAACCT
CTGTTTCGGCAGAGGATACCGTGCTCCACAGCTGTAAGGCTGTGGGCCCGAGAGAAGTCGCTCTCTCT
+
FFFFFFFFFFFFFFFFFFFFFFFFFFFFFFFFFFFFFFFFFFFFFFFFFFFFFFFFFFFFFFFFFFFFFF
FFFFFFFFFFFFFFFFFFFFFFFFFFFFFFFFFFFFFFFFFFFFFFFFFFFFFFFFFFFFFFFFFFFFFF
@A00155:342:HHGFNDSXY:1:1629:25880:4288 2:N:0:GAACCTAG+TCCGCATA
CAGGTAGCCTACTAGACTACGACTTACACGGTAGGTCGTGAACAAGCCCGGAGTACATCTCCTTTGGA
ACCAGGAGACCCCTCCGGTCTTGTCCAACCTCTGTTTCGGCAGAGGATACCGTGCTCCACAGCTGTAA
+
FFFFFFFFFFFFFFFFFFFFFFFFFF:FFFFFFFFFFFFFFFFFFFFFFFFFFFFFFFFFFFFFFFFFF
FFFFFFFFFFFFFFFFFFFFFFFFFFFFF:FFFFFFFFFFFFFFF:FFFFFFF:FFFFFFFFFFFF
@A00155:342:HHGFNDSXY:1:1629:13711:12962 2:N:0:GAACCTAG+TCCGCATA
ACGACTTACACGGTAGGTCGTGAACAAGCCCGGAGTACATCTCCTTTGGAACTAGGAGACCCCTCCGG
TCTTGTCCAACCTCTGTTTCGGCAGAGGATACCGTGCTCCACAGCTGTAAGGCTGTGGGCCCGAGAGA
+
FFFFFFFFFFFFFF:FFFFFFFFFFFFFFFFFFFFFFFFFFFFFFFFFFFFFFFFFFFFFFFFFFFFFF
FFFFFFFFFFFFFFFFFFFFFFFFFFFFFFFFFFFFFFFFFFFFFFFFFFFFFFFFFFFFFFFFFFFFFF
@A00155:342:HHGFNDSXY:1:1629:23773:13385 2:N:0:GAACCTAG+TCCGCATA
AAGGACTCGTCAGGTAGCCTACTAGACTACGACTTACACGGTAGGTCGTGAACAAGCCCGGAGTACAT
CTCCTTTGGAACTAGGAGACCCCTCCGGTCGTGTCCAACCTCTGTTTCGGCAGAGGATACCGTG
+
FFFFFFFFFFF:FFFFFFF:FFFFFFFFFFFFFFFFFFFFFFFFF,FFFFFFFFFFFFFFFFFFFFFF
FFFFFFFFFFFFFFF:FFFFFFFFFFFFFF,FFFFFFFFFFFFFFFFFFFFF,FFFFFFFFFFFF
@A00155:342:HHGFNDSXY:1:1629:25789:14465 2:N:0:GAACCTAG+TCCGCATA
ACTACGACTTACACGGTAGGTCGTGAACAAGCCCGGAGTACATCTCCTTTGGAACTAGGAGACCCCTC
CGGTCTTGTCCAACCTCTGTTTCGGCAGAGGATACCGTGCTCCACAGCTGTAAGGCTGTGGGCCCGAG
+
FFF:FFFFFFFFFFFFFFFFFFFFFFFFFFFFFFFFFFFFFFFFFFFFFFFFFFFFFFFFFFFFFFFFFF
FFFFFFFFFFFFFFFFFFFFFFFFFFFFFFFFFFFFFFFFFFFFFFFFFFFFF,FFFFFFFFFFFFFFFF
@A00155:342:HHGFNDSXY:1:1629:26106:32111 2:N:0:GAACCTAG+TCCGCATA
GGACTCGTCAGGTAGCCTACTAGACTACGACTTACACGGTAGGTCGTGAACAAGCCCGGAGTACATCT
CCTTTGGAACTAGGAGACCCCTCCGGTCTTGTCCAACCTCTGTTTCGGCAGAGGATACCGTGCTCCAC
+
FFFFFFFFFFFFFFFFFFFFFFFFF:FFFFFFFFFFFFFFFFFFFFFFFFFFFFFFFFFFFFFFFFFFF
FFFFFFFFFFFFFFFFFFFFFFFFFFFFFFFFFFFFFFFFFFFFFFFFFFFFFFFFFFFFFFFFFFFFFF
@A00155:342:HHGFNDSXY:1:1629:11107:36949 2:N:0:GAACCTAG+TCCGCATA
AGCCTACTAGACTACGACTTACACGGTAGGTCGTGAACAAGCCCGGAGTACATCTCCTTTGGAACTAG
```

```
GAGACCCCACCGGTCTTGTCCAACCTCTGTTTCGGCAGAGGATACCGTGCTCCACAGCTGTAAGGCTG
+
FFFFFFFFFFFFFFFFFFFFFFFFFFFFFFFFFFFFFFFFFFFFFFFFFFFFFFFFFFFFFFFFFFFF
FFFFFFFFFFFFFFFFFFFF:FFFFFFFFFFFFFFFFFFFFFF:F:FFFFFFFFFFFF:FFFFFFFFF
@A00155:342:HHGFNDSXY:1:1630:8666:17910 2:N:0:GAACCTAG+TCCGCATA
AGTACATCTCCTTTGGAACTAGGAGACCCCTCCGGTCTTGTCCAACCTCTGTTTCGGCAGAGGATACC
GTGCTCCACAGCTGTAAGGCTGTGGGCCCGAGAGAAGTCGCTCTCTCTCGCCCTCCACATCCCTCTCG
+
FFFFFFFFFFFFFFFFFFFFFFFFFFFFFFFFFFFFF,FFFFFFFFFFFFFFFFFFFFFFFFFFFFFF
FFFFFFFFFFFFFFFFFFFFFFFFFFFFFFFFFFF:FFFFFFFFFFFFFFFFFFFFFFFFFFFFFFFF
@A00155:342:HHGFNDSXY:1:1630:18927:35274 2:N:0:GAACCTAG+TCCGCATA
TACACGGTAGGTCGTGAACAAGCCCGGAGTACATCTCCTTTGGAACTAGGAGACCCCTCCGGTCTTGT
CCAACCTCTGTTTCGGCAGAGGATACCGTGCTCCACAGCTGTAAGGCTGTGGGCCCGAGAGAAGTCGC
+
FFFFFFFFFFFFFFFFFFFFFFFFFFFFFFFFFFFFFFFFFFFF:FFFFFFFFFFFFFFFFFFFF:FF
FFFFFFFFFFFFFFFFFFFFFFFFFFFFFFFFFFFFFFFFFFFFFFFFFFFFFFFFFFFFFFFFFFFF
@A00155:342:HHGFNDSXY:1:1631:16975:5588 2:N:0:GAACCTAG+TCCGCATA
TTACACGGTAGGTCGTGAACAAGCCCGGAGTACATCTCCTTTGGAACTAGGAGACCCCTCCGGTCTTG
TCCAACCTCTGTTTCGGCAGAGGATACCGTGCTCCACAGCTGTAAGGCTGTGGGCCCGAGAGAAGTCG
+
FFFFFF::FF,FFFFFFFF:FF:FFFFFFFFFFFFFF:FF,FF,FF:FF:FF:FFF:FFFFF:,:FFF
FFFFF,:FFFF:FFFF,FF:FFFFFF::FF,FF,F:FFFFFFFFFFFF:F,FF:FFFFFFFFFFFFFF
@A00155:342:HHGFNDSXY:1:1631:15845:16094 2:N:0:GAACCTAG+TCCGCATA
ACCGCCTTAGATCAGCTGTGTCCACTTAAGGACTCACCTCTGGCCTATGGTCATCTCAATGCCGAGAG
GGATGTAGAGGGCGAGAGAGAGCGACTTCTCTCGGGCCCACAGCCTTACAGCTGTGGAGCACGGTATC
+
FFFFFFFFFFFFFFFFFFFFFFF:FFFFFFFFFFFFFFFFFFFFFFFFFFFFFFFFFF:FFFFF:F:F
FFFFFFFFFFFFFFFFFFFFFFFF:FFFFFFFFFFFFFFFFFFFFFFFFFFFFFFFFFFFFFFFFFFF
@A00155:342:HHGFNDSXY:1:1631:2908:16799 2:N:0:GAACCTAG+TCCGCATA
TAGGTCGTGAACAAGCCCGGAGTACATCTCCTTTGGAACCAGGAGACCCCTCCGGTCTTGTCCAACCT
CTGTTTCGGCAGAGGATACCGTGCTCCACAGCTGTAAGGCTGTGGGCCCGAGAGAAGTCGCTCTCTCT
+
FFFFFFFFFFFFFFFFFFFFFFFFFFFFFFFFFFFFFFFFFFFFFFFFFFFFFFFFFFFFFFFFFFFF
FFFFFFFFFFFFFFFFFFFFFF:FFFFFFFFFFFFFFFFFFFFFFFFFFFFFFFFFFFFFFFFFFFFF
@A00155:342:HHGFNDSXY:1:1631:23484:19711 2:N:0:GAACCTAG+TCCGCATA
GTACATCTCCTTTGGAACTAGGAGACCCCTCCGGTCTTGTCCAACCTCTGTTTCGGCAGAGGATACCG
TGCTCCACAGCTGTAAGGCTGTGGGCCCGAGAGAAGTCGCTCTCTCTCGCCCTCCACATCCCTCTCGG
+
FFFFFFFFFFFFFFFFFFFFFFFFFFFFFFFFFFFF:FFFFFFFFFFFFFFFFFFFFFFFFFF:FFFF
FFFFFFFFFFFFFFFFFFFFFFFFFFFFFFFFFF:FFFFFFFFFFFFFFFFFFFFFFFFFFFFFFFFF
@A00155:342:HHGFNDSXY:1:1631:29053:28447 2:N:0:GAACCTAG+TCCGCATA
GAGACCCCTCCGGTCTTGTCCAACCTCTGTTTCGGCAGAGGATACCGTGCTCCACAGCTGTAAGGCTG
TGGGCCCGAGAGAAGTCGCTCTCTCTCGCCCTCCACATCCCTCTCGGCATTGAGATGACCATAGGCC
+
FFFFFFFFFFFFFFF:FFFFFFFFFFFFFFFFFFFFFFFFFFFFFFFFFFFFFFFFFFFFFFFFFFFF
FFFFFFFFFFFFF:FFFFFFFFFFFFFFFFFFFFFFFFFFFFFFFFFFFFFFFFFFFFFF,FFFFFF
@A00155:342:HHGFNDSXY:1:1631:1452:33035 2:N:0:GAACCTAG+TCCGCATA
AGGTCGTGAACAAGCCCGGAGTACATCTCCTTTGGAACCAGGAGACCCCTCCGGTCTTGTCCAACCTC
TGTTTCGGCAGAGGATACCGTGCTCCACAGCTGTAAGGCTGTGGGCCCGAGAGAAGTCGCTCTCTCTC
+
FFFFFFFFFFF:FFFFFFFFFFFFFFFF:FFFF:FFFFFFFFFFFFFFFFFFFFF,FFFFFFFFFFFF
FFFFFFFFFFF:FFFFFFFFFFFFFFFFFFFFFFFFFFFFFFF:FF:FFFFFFFFFFF:FFFFFFFFF
@A00155:342:HHGFNDSXY:1:1632:27317:9471 2:N:0:GAACCTAG+TCCGCATA
AGTTGTGTAAGTTTCGTCCTAAAAAGGACTCGTCAGGTAGCCTACTAGACTACGACTTACACGGTAGG
```

TCGTGAACAAGCCCGGAGTACATCTCCTTTGGAACTAGGAGACCCCTCCGGTCTTGTCCAACCTCTGT
+
FFFFFFFFFFFFFFFFFFF,FFFFFFFFFFFFFFFFFFFFFFFFFFFFFFFFFFFFFFFFFFFFFFFFF
FFFFFFFFFFFFFFFFFFFFFFFFFFFFFFFFFFFFFFFFFFFFFFFFFFFFFFFFFFFFFFFFFFFFF
@A00155:342:HHGFNDSXY:1:1632:21721:19351 2:N:0:GAACCTAG+TCCGCATA
CCCCTCCGGTCTTGTCCAACCTCTGTTTCGGCAGAGGATACCGTGCTCCACAGCTGTAAGGCTGTGGG
CCCGAGAGAAGTCGCTCTCTCTCGCCCTTCACATCCCTCTCGGCATTGAGATGACCATAGGCCAGAGG
+
FFFFFFFFFFFFFFFFFFFFFFF,FFFFFFFFFFFFFFFFFFFFFFFFFFFFFFFFFF:FFFFFFFF
FFFF:F:FFFFFFFFFFFFFFFFFFFFFFFFFFFFFFFFFFFF::FFFFFFFFFF,:::FFFFFFF:
@A00155:342:HHGFNDSXY:1:1633:22842:22169 2:N:0:GAACCTAG+TCCGCATA
CCGGAGTACATCTCCTTTGGAACTAGGAGACCCCTCCGGTCTTGTCCAACCTCTGTTTCGGCAGAGGA
TACCGTGCTCCACAGCTGTAAGGCTGTGGGCCCGAGAGAAGTCGCTCTCTCTCGCCCTCTACATCCCT
+
FFFFFFFFFFFFFFF:FFFFFFFFFFFFFFFFFFFFFFFFFF,FFFFFFFFFFFFFFFFFFFFFFFFFF
F:FFFFFFFFFFFFFFFFFFFFFFFFFFFFFFFFFFFFF:FFFFFFFFFFFFFFFFFFFFFFFFFFFFF
@A00155:342:HHGFNDSXY:1:1633:30942:24925 2:N:0:GAACCTAG+TCCGCATA
GAACCAGGAGACCCCTCCGGTCTTGTCCAACCTCTGTTTCGGCAGAGGATACCGTGCTCCACAGCTGT
AAGGCTGTGGGCCCGAGAGAAGTCGCTCTCTCTCGCCCTCCACATCCCTCTCGGCATTGAGATGACCA
+
FFFFFFFFFFFFFFFFFFFFFFFFFFFFFFFFFFFFFFFFFFFFFFFFFFFFFFFFFFFFFFFFFFFFF
FFFFFFFFFFFFFFFFFFFFFFFFFFFFFFFFFFFFFFFFFFFFFFFFFFFFFFFF,FFFFFFFFFF
@A00155:342:HHGFNDSXY:1:1634:29451:10786 2:N:0:GAACCTAG+TCCGCATA
ACTTACACGGTAGGTCGTGAACAAGCCCGGAGTACATCTCCTTTGGAACTAGGAGACCCCTCCGGTCT
TGTCCAACCTCTGTTTCGGCAGAGGATACCGTGCTCCACAGCTGTAAGGCTGTGGGCCCGAGAGAAGT
+
FFFFFFFFFFFFFFFFFFFFFFFFFFFFFFFFFFFFFFFFFFFFFFFFFFFFFFFFFFFFFFFFFFFFF
FFFFFFFFFFFFFFFFFFFFFFFFFFFFFFFFFFFFFFFFFFFFFFFFFFFFFFFFFFFFFFFFFFFFF
@A00155:342:HHGFNDSXY:1:1634:19623:33974 2:N:0:GAACCTAG+TCCGCATA
GAGACCCCTCCGGTCTTGTCCAACCTCTGTTTCGGCAGAGGATACCGTGCACCACAGCTGTAAGGCTG
TGGGCCCGAGAGAAGTCGCTCTCTCTCGCCCTCCACATCCCTCTCGGCATTGAGATGACCATAGGCCA
+
FFFFFFFFFFFFFFFFFFFFFFFFFFFFFFFFFFFFFFFFFFFFFFFFFFFFFFFFFFFFFFFFFFFFF
FFFFFFFFFFFFFFFFFFFFFFFFFFFFFFFFFFFFFFFFFFFFFFFFFF:FFFFFFFFFFFFFFFFFF
@A00155:342:HHGFNDSXY:1:1634:19669:34021 2:N:0:GAACCTAG+TCCGCATA
GAGACCCCTCCGGTCTTGTCCAACCTCTGTTTCGGCAGAGGATACCGTGCACCACAGCTGTAAGGCTG
TGGGCCCGAGAGAAGTCGCTCTCTCTCGCCCTCCACATCCCTCTCGGCATTGAGATGACCATAGGCCA
+
FFFFFFFFFFFFFFF:FFFFF:FFFFFFFFFFFFFFFFFFFFFFFFFFFFFFFFFFFFFFFFFFFFFFF
FFFFFFFFFFFFFFFFF:FFFFFFFFFFFFFFFFFFFFFFFFFFFFFFFFF:FFFFFFFFFFFFFFFF
@A00155:342:HHGFNDSXY:1:1635:17788:13009 2:N:0:GAACCTAG+TCCGCATA
GTTTCGTCCTAAAAAGGACTCGTCAGGTAGCCTACTAGACTACGACTTACACGGTAGGTCGTGAACAA
GCCCGGAGTACATCTTCTTTGGAACTAGGAGACCCCTCCGGTCTTGTCCAACCTCTGTTTCGGCAGAG
+
FFFFFFFFFFFFFFFFFFFFFFFFFFFFFFFFFFFF:FFFFFFFFFFFFFFFFFFFFFFFFFFFFFFF
:FFFFFFFFFFFFFFFFFFFFFFFFFFFFFFFFFFFFFFFFFFFFFFFFFFF:FFFFFFFFFFFFFFF
@A00155:342:HHGFNDSXY:1:1635:27389:22153 2:N:0:GAACCTAG+TCCGCATA
TAGGTCGTGAACAAGCCCGGAGTACATCTCCTTTGGAACAAGGAGACCCCTCCGGTCTTGTCCAACCT
CTGTTTCGGCAGAGGATACCGTGCTCCACAGCTGTAAGGCTGTGGGCCCGAGAGAAGTCGCTCTCTCT
+
FFFFFFFFFFFFFFFFFFFFFFFFFFFFFFFFFFFFFFFFFFFFFFFFFFFFFFFFFFFFFFFFFFFFF
FFFFFFFFFFFFFFFFFFFFFFFFFFFFFFFFFFFF:FFFF,FFFFFFFFFFFFFFFFFFFFFFFFFFF
@A00155:342:HHGFNDSXY:1:1636:20980:16031 2:N:0:GAACCTAG+TCCGCATA
AGGACGAAACCGTCGTAACTCTTCGAGTTGTGTAAGTTTCGTCCTAAAAAGGACTCGTCAGGTAGCCT

```
ACTAGACTACGACTTACACGGTAGGTCGTGAACAAGCCCGGAGTACATCTCCTTTGGAACTAGGAGAC
+
FFFFFFFFFFFFFFFFFFFFFFFFFFF:FFFFFFFFF:FFFFFFFFFFFFFFFFFFFFFFFFFFFFFF
FFFFFFFFFFFFFFFFFFFFFFFFF:FFFFFFFFFFFFFFFFFFFFFFFFFF:FFFFFFFFFFFFFFF
@A00155:342:HHGFNDSXY:1:1636:24532:22748 2:N:0:GAACCTAG+TCCGCATA
GTTGTGTAAGTTTCGTCCTAAAAAGGACTCGTCAGGTAGCCTACTAGACTACGACTTACACGGTAGGT
CGTGAACAAGCCCGGAGTACATCTCCTTTGGAACTAGGAGACCCCTCCGGTCTTGTCCAACCTCTGTT
+
F:FFFFFFFFFFFFFFFF:FFFF::FFFFFFFFFFFFFFFFFFFFFFFFFFFFFFFFFFFFFFFFFFF
FFFFFFFFFFFFFFFFFFFFFFFFFF:FFFFFFFFFFFFFFFFFFFFFFFFFFFFFFFFFFFFFFFFF
@A00155:342:HHGFNDSXY:1:1636:17824:24565 2:N:0:GAACCTAG+TCCGCATA
GAACTAGGAGACCCCTCCGGTCTTGTCCAACCTCTGTTTCGGCAGAGGATACCGTGCTCCACAGCTGT
AAGGCTGTGGGCCCGAGAGAAGTCGCTCTCTCTCGCCCTCCACATCCCTCTCGGCAGTGAGATGACCA
+
FFFFFFFFFFFFFFFFFFFFFFF:FFFFFFFFFFFFFFFFFFF:FFFFFFFFFFFFFFFFFFFFFFFF
FFFFFFFFFFFFFFFFFFFFFFFFFFFFFFFFFFFFFFFFFFFFFFFFFFFFFFFF,FFFFFFFFFFF
@A00155:342:HHGFNDSXY:1:1636:22797:28260 2:N:0:GAACCTAG+TCCGCATA
TCCTTGGAACGGACTCATCAGGGAGCCTACTAGACTCCGACCGTCATGGGGGTTCAAACCCATGCCCC
GCCACACCGCCTTAGATCAGCTGTGTCCACTTAAGGACTCACCTCTGGCCTATGGTCATCTCAATACC
+
FFFF:FFFFFFFFFFFFFFFFFFFFFFFFFFFFFFFFFFFF:FFFFFFFFFFFFFFFFFFFFFFFF:F
FFFFFFFFFFFFFFFFFFFFFFFFFFFFFFFFF,FFFFFFFFFFFF,FFFFFFFFFFFFFF:FFFFFF
@A00155:342:HHGFNDSXY:1:1637:29731:13244 2:N:0:GAACCTAG+TCCGCATA
ACTAGACTACGACTTACACGGTAGGTCGTGAACAAGCCCGGAGTACATCTCCTTTGGAACTAGGAGAC
CCCTCCGGTCTTGTCCAACCTCTGTTTCGGCAGAGGATACCGTGCTCCACAGCTGTAAGGCTGTGGGC
+
FFFFFFFFFFFFFFFFFFFFFFFFF:FFFFFFFFFFFFFFFFFFFF:FFFFFFFFFFFFFFFFFFFFF
FFFFFFFFFFFFFFFFFF:FFFFFFFFFFFFFFFFFFFFFFFFF:F:FFFFFF,FFFFFFFFFFFFFF
@A00155:342:HHGFNDSXY:1:1637:24641:14199 2:N:0:GAACCTAG+TCCGCATA
GTTGTGTAAGTTTCGTCCTAAAAAGGACTCGTCAGGTAGCCTACTAGACTACGACTTACACGGTAGGT
CGTGAACAAGCCCGGAGTACATCTCCTTTGGAACCAGGAGACCCCTCCGGTCTTGTCCAACCTCTGTT
+
FFFFFFFFFF:FFFFFFFFFFFF:FFF:FFFFFF:FFFFFFFFFFFFFFFFFFFFFFFFFFFFFF,FF
FF:FFFFFFFF:FFFFFFFFFFFFF:FFFFFF,FFFFFFFF:,F:FFFFF:F:FFFF::F:FF:FFFF
@A00155:342:HHGFNDSXY:1:1637:8160:16877 2:N:0:GAACCTAG+TCCGCATA
GAAACCGTCGTTACTCTTCGAGTTGTGTAAGTTTCGTCCTAAAAAGGACTCGTCAGGTAGCCTACTAG
ACTACGACTTACACGGTAGGTCGTGAACAAGCCCGGAGTACATCTCCTTTGGAACTAGGAGACCCCTC
+
FFFFFFFFFFFFFFFFFF,FFFFFFFFFFFFFFFFFFFF:FFFFFFFFFFFFFF:FFFFFFFFFFFFF
FFFFFFFFFFFFFFFFFFFFFFFFFFFFFFFFFFFFFFFFFFFFFFFFFF:F:FFFFFFFFFF:FFFF
@A00155:342:HHGFNDSXY:1:1637:8467:17033 2:N:0:GAACCTAG+TCCGCATA
GAAACCGTCGTTACTCTTCGAGTTGTGTAAGTTTCGTCCTAAAAAGGACTCGTCAGGTAGCCTACTAG
ACTACGACTTACACGGTAGGTCGTGAACAAGCCCGGAGTACATCTCCTTTGGAACTAGGAGACCCCTC
+
FFFFFFFFFFFFFFFFFFFFFFFFFFFFFFFFFFFFFFFFFFFFFFFFFFFFFFFFFFFFFFFFFFFF
FFFFFFFF:FFFFFFFF:FFFFFFFFFFFFFFFFFFFFFFFFFFFFFFFFFFFFFFFFFFFFFF:FFF
@A00155:342:HHGFNDSXY:1:1637:32750:18380 2:N:0:GAACCTAG+TCCGCATA
ACGACTTACACGGTAGGTCGTGAACAAGCCCGGAGTACATCTCCTTTGGAACTAGGAGACCCCTCCGG
TCTTGTCCAACCTCTGTTTCGGCAGAGGATACCGTGCTCCACAGCTGTAAGGCTGTGGGCCCGAGAGA
+
FFFFFFFFFFFFFFFFFFFFFFFFFFFFFFFFFFFFFF:FFFFFF,FFFFFFFFFFFFFFFFFFFFFF
FFFFFFFFFFFFFF:FFFFFFFFFF:FFFFFFFFFFFFFFFFFFFFFFFFFFFFFFFFFFFFFFFFFFF
@A00155:342:HHGFNDSXY:1:1637:13259:18944 2:N:0:GAACCTAG+TCCGCATA
TCTTGTCCAACCTCTGTTTCGGCAGAGGATACCGTGCTCCACAGCTGTAAGGCTGTGGGCCCGAGAGA
```

```
AGTCGCTCTCTCTCGCCCTCCACATCCCTCTCGGCATTGAGATGACCATAGGCCAGAGGTGAGTCTTT
+
FFFFFFFFFFFFFFFFFFFFFFFFFFFFFFFFFFFFFFFFFFFFFFFFFFFFF:FFFFFFFFFFFFFF
FFFFFFFFFFFFFFFFFFFFFFFFFFFFFFFFFFFFF:FFFFFFFFFFFFFFFFFFFFFFFFFFFFFF
@A00155:342:HHGFNDSXY:1:1638:18656:18270 2:N:0:GAACCTAG+TCCGCATA
GAAACCGTCGTTACTCTTCGAGTTGTGTAAGTTTCGTCCTAAAAAGGACTCGTCAGGTAGCCTACTAG
ACTACGACTTACACGGTAGGTCGTGAACAAGCCCGGAGTACATCTCCTTTGGAACTAGGAGACCCCTC
+
FFFFFFFFFFFFFFFF:FFFFFFFFFFFFFFFFFFFFFFFFFFFFFFFFFFFFFFFFFFFFFFFFFFF
FFFFFFFFFFFFFFFFFFFFFFFFFFFFFFFFFFFFFFFFFFFFFFFFFFFFFFFFFFFFFFFFFFFF
@A00155:342:HHGFNDSXY:1:1638:17662:18584 2:N:0:GAACCTAG+TCCGCATA
GAAACCGTCGTTACTCTTCGAGTTGTGTAAGTTTCGTCCTAAAAAGGACTCGTCAGGTAGCCTACTAG
ACTACGACTTACACGGTAGGTCGTGAACAAGCCCGGAGTACATCTCCTTTGGAACTAGGAGACCCCTC
+
FFF:FFFFFFFFFF,FFFFFFF,FFFFFF:FFFFFFFF::FFFF:FFFFFFFFFFFFFFFFFFFFFFF
FFFFFFFFFFFFFFFFFFFF:FFFFFFF:FFFFFFFFFFFF:FFFFFFFFFFFFFFFFFFFFFFFFFF
@A00155:342:HHGFNDSXY:1:1638:12020:19335 2:N:0:GAACCTAG+TCCGCATA
TAGCCTACTAGACTACGACTTACACGGTAGGTCGTGAACAAGCCCGGAGTACATCTCCTTTGGAGCTA
GGAGACCCCTCCGGTCTTGTCCAACCTCTGTTTCGGCAGAGGATACCGTGCTCCACAGCTGTAAGGCT
+
FFFFFFFFFFFFFFFFFFFFFFFFFFFFFFFFFFFFF:FFF:FFFFFFFFFFFFFFFFFFFFFFFFFF
FFF:FFFFFFFFFF,FFFFFFFFFFFFFFFFFFFFFFFFFFFFFFFFFFFFFFFFFFFFFFFFFFFFF
@A00155:342:HHGFNDSXY:1:1639:4616:16282 2:N:0:GAACCTAG+TCCGCATA
AGGATACCGTGCTCCACAGCTGTAAGGCTGTGGGCCCGAGAGAAGTCGCTCTCTCTCGCCCTCCACAT
CCCTCTCGGCATTGAGATGACCATAGGCCAGAGGTGAGTCCTTAAGTGGACACAGCTGAT
+
:FFFFFFFFFFFFFFFFFFFFFFFFFFFFFFFFFFFFFFFFFFFFFFFFFFFFFFFFFFFFFFFFFFF
FFFFFFFFFFFFFFFFF:FFFFFF:FFFF:FFFFFFFF:FFFFFFFFFF:F,FFF:FFFF
@A00155:342:HHGFNDSXY:1:1639:4851:16407 2:N:0:GAACCTAG+TCCGCATA
AGGATACCGTGCTCCACAGCTGTAAGGCTGTGGGCCCGAGAGAAGTCGCTCTCTCTCGCCCCCCACAT
CCCTCTCGGCATTGAGATGACCATAGGCCAGAGGTGAGTCCTTAAGTGGACACAGCTGAT
+
FFFFF:FFFFFFFFFFFFFFFFFFFFFFFFFFFFFFFFFFFFFFFFFFF:FFFFFFFFFFFFF:FFFF
FFFFFFFFFFFFFFFFFFFFFFFFFFFFFFFFFFFFFFFFFFFFFFFFFFF:FFFFFFFF
@A00155:342:HHGFNDSXY:1:1639:8350:25692 2:N:0:GAACCTAG+TCCGCATA
AGCCCGGAGTACATCTCCTTTGGAACTAGGAGACCCCTCCGGTCTTGTCCAACCTCTGTTTCGGCAGA
GGATACCGTGCTCCACAGCTGTAAGGCTGTGGGCCCGAGAGAAGTCGCTCTCTCTCGCCCTCTACATC
+
FFFFFFFFFFFFFFFFFFFFFFFF:FFFFFFFFFFFFFFFFFFFFFFFFFFFFFFFFFFFFFFFFFFF
FFFFF:FFFFFFFFFFFFFFFFF:FFF:FFFFFFFFFFFFFFFFFFFFFFFFFFFFFFFFFFFFFFFF
@A00155:342:HHGFNDSXY:1:1639:29523:28635 2:N:0:GAACCTAG+TCCGCATA
TCCTAAAAAGGACTCGTCAGGTAGCCTTCTAGACTACGACTTACACGGTAGGTCGTGAACAAGCCCGG
AGTACATCTCCTTTGGAACTAGGAGACCCCTCCGGTCTTGTCCAACCTCTGTTTCGGCAGAGGATACC
+
FFFF,FFFFFFFFFFFFFFFFFFFFFFFFFFFFFFFFFFFFFFFFFFFFFFFFFFFFFF:FFFFFF:
FFF:FFFFFFFFFFFF:FFFFFFFFFF:FFFFFFFFFFFFFFFFFF:FFFFF:FFFFFFFFFFFFFFF
@A00155:342:HHGFNDSXY:1:1639:6135:29778 2:N:0:GAACCTAG+TCCGCATA
AGGATACCGTGCTCCACAGCTGTAAGGCTGTGGGCCCGAGAGAAGTCGCTCTCTCTCGCCCTCCACAT
CCCTCTCGGCATTGAGATGACCATAGGCCAGAGGTGAGTCCTTAAGTGGACACAGCTGAT
+
FFFFFFFFFFFFFFFFFFFFFFFFFFFFFFFFFFFFFFFFFFFFFFFFFFFFFFFFFFFFFFFFFFFF
FFFFFFFFFFFFFFFFFFFFFFFFFFFFFFFFFFFFFFFFFFFFFFFFFFF:FFFFFFF,
@A00155:342:HHGFNDSXY:1:1640:30761:6042 2:N:0:GAACCTAG+TCCGCATA
ACGACTTACACGGTAGGTCGTGAACAAGCCCGGAGTACATCTCCTTTGGAACTAGGAGACCCCTCCGG
```

TCTTGTCCAACCTCTGTTTCGGCAGAGGATACCGTGCTCCACAGCTGTAAGGCTGTGGGCCCGAGAGA
+
FFFFFFFFFFFFFFFFFFFFFFFFFFF:FFFFFFFFFFFFFFFFFFFFFFFFFFFFFFFFFFFFFFFFFFF
FF,FFFFFFFFFFFFFFFFFFFFFFFFFFFFFFFFFFFFFFFFFFFFFFFFFFFFFFFFFFFFFFFFFFF
@A00155:342:HHGFNDSXY:1:1640:19623:10426 2:N:0:GAACCTAG+TCCGCATA
TCTTCGAGTTGTGTAAGTTTCGTCCTAAAAAGGACTCGTCAGGTAGCCTACTAGACTACGACTTACAC
GGTAGGTCGTGAACAAGCCCGGAGTACATCTCCTTTGGAACTAGGAGACCCCTCCGGTCTTGTCCAAC
+
FFFFFFFFFFFFFFFFFFFFF:FFFFFFFFFFFFFFFFFFFFFFFFFFFFFFFFFFFFFFF:FFFFFFFFF
FFFFFFFFFFFFFFFFFFFFFFFFFFFFFFFFFFFFFFFFFFFFFFFFFFFFFFFF:FFFFFFFFFFFFF
@A00155:342:HHGFNDSXY:1:1640:25889:15107 2:N:0:GAACCTAG+TCCGCATA
ACCTCTGTTTCGGCAGAGGATACCGTGCTCCACAGCTGTAAGGCTGTGGGCCCGAGAGAAGTCGCTCT
CTCTCGCCCTCTACATCCCTCTCGGCATTGAGATGACCATAGGCCAGAGGTGAGTCCTTAAGTGGACA
+
FFFFFFFFFFFFF:FFFFFFFFFFFFFFFFFFFFFFFFFFFFFFFFFFFFFFFFFFFFFFFFFFFFFFFFF
FFFFFFFFFFFFFFFFFFFFFFFFFFFFFFFFFFFFFFFFFFFFFFFFFFFFFFFFFFFFFFFFFFFFFFF
@A00155:342:HHGFNDSXY:1:1640:3721:28510 2:N:0:GAACCTAG+TCCGCATA
GTCCAACCTCTGTTTCGGCAGAGGATACCGTGCTCCACAGCTGTAAGGCTGTGGGCCCGAGAGAAGTC
GCTCTCTCTCGCCCTCCACATCCCTCTCGGCATTGAGATGACCATAGGCCAGAGGTGAG
+
FFFFFFFFFFFFFFFFFFFFFFFFFFFFFFFFFFFFFFFFFFFFFFFFFFFFFFFFFFFFFFFFFFFFFFF
FFFFFFFFFFFFFFFFFFFFFFFFFFFFFFFFFFFFFFFFFFFF:FFFFFF:FFFFFFFF
@A00155:342:HHGFNDSXY:1:1641:7392:4335 2:N:0:GAACCTAG+TCCGCATA
GGACTCGTCAGGTAGCCTACTAGACTACGACTTACACGGTAGGTCGTGAACAAGCCCGGAGTACATCT
CCTTTGGAACTAGGAGACCCCTCCGGTCTTGTCCAACCTCTGTTTCGGCAGAGGATACCGTGCTCCAC
+
FFFFFFFFFFFFFFFFFFFFFFFFFFFFFFFFFFFFFFFFFFFFFFFFFFFFF:FFFFFFFFFFFFFF
FFFFFFFFFFFFFFFFFFFFFFFFFFFFFFFFFFFFFFFFFFFFFFFFFFFFFFFFFFFFFFFFFFFFFFF
@A00155:342:HHGFNDSXY:1:1642:7292:9956 2:N:0:GAACCTAG+TCCGCATA
GTAGCCTACTAGACTACGACTTACACGGTAGGTCGTGAACAAGCCCGGAGTACATCTCCTTTGGAACT
AGGAGACCCCTCCGGTCTTGTCCAACCTCTGTTTCGGCAGAGGATACCGTGCTCCACAGCTGTAAGGC
+
FFFFFFFFFFFFFFFFFFFFFFFFFFFFFFFFFFFFFFFFFFFFFFFFFFFFF:FFFFFFFFFFFFFF
FFFFFFFFFFFFFFFFFFFFFFFFFFFFFFFFFF:FFFFFFFFFF:FFFFFFFFFFFFFFF,FFFFFFFF
@A00155:342:HHGFNDSXY:1:1642:10393:11663 2:N:0:GAACCTAG+TCCGCATA
CTCTTTGAGTTGTGTAAGTTTCGTCCTAAAAAGGACTCGTCAGGTAGCCTACTAGACTACGACTTACA
CGGTAGGTCGTGAACAAGCCCGGAGTACATCTCCTTTGGAACTAGGAGACCCCTCCGGTCTTGTCCAA
+
FFFFFFFFFF:FFFFFFFFFFFFFFFFFFFFFFFFFFFFFFFFFFFFFFFFFFFFFFFFFFFFFFFFFFFF
FFFFFFFFFFFFFFFFFFFFF:FFFFFFF:FFFFFFFFFFFFFFFFFFFFFFFFFFFFFFFFFFFFFFF:FF
@A00155:342:HHGFNDSXY:1:1642:13105:14043 2:N:0:GAACCTAG+TCCGCATA
TACTCTTGGAGTTGTGTAAGTTTCGTCCTAAAAAGGACTCGTCAGGTAGCCTACTAGACTACGACTTA
CACGGTAGGTCGTGAACAAGCCCGGAGTACATCTCCTTTGGAACTAGGAGACCCCTCCGGTCTTGTCC
+
FFFFFFFFFFFFFFFFF:FFFFFFF:FFFFFFFFFFFFFFF:FFFFFFFFFFFFFFFFFFF:FFFFFFFF
FFFFFFFFFFFFFFFFFFFF:FFFFFFFFFFFFFFFFFFFFFFFFFFFFF:FFFFFFFFFFFFFFFFFFFF:
@A00155:342:HHGFNDSXY:1:1642:4426:23062 2:N:0:GAACCTAG+TCCGCATA
CGACGGTTTCGTCCTTGGAACGGACTCATCAGGGAGCCTACTAGACTCCGACCGTCATGGGGGTTCAA
ACCCATGCCCCGCCACACCGCCTTAGATCAGCTGTGTCCACTTAAGGACTCACCTCTGGCCTATGGTC
+
FFFFFFFFFFFFFFFFFFFFFFFFFFFFFFFFFFFFFFFFFFFFFFFFFFFFFFFFFFFFFFFFFFFFFFF
FFFFFFFFFFFFFFFFFFFFFFFFFFFFFFFFFFFFFFFFFFFFFFFFFFFFFFFFFFFFF:FFFFFFFFF
@A00155:342:HHGFNDSXY:1:1642:22923:34178 2:N:0:GAACCTAG+TCCGCATA
TAGCCTACTAGACTACGACTTACACGGTAGGTCGTGAACAAGCCCGGAGTACATCTCCTTTGGAACTA

```
GGAGACCCCTCCGGTCTTGTCCAACCTCTGTTTCGGCAGAGGATACCGTGCTCCACAGCTGTAAGGC
+
FFFFFFFFFFFFFFFFFFFFFFFFFFFFFFF,FFFFFFFFFFFFFFFFFF:FFFFFFFFFFFFFFFFF
FFFFFFFFFFFFFFFFFFFFFFFFFFFFFFFFFFFFFFFFFFF::FFFFFFFFFFFFFFF:FFFFFF
@A00155:342:HHGFNDSXY:1:1643:5801:3834 2:N:0:GAACCTAG+TCCGCATA
TCCAAGGACGAAACCGTCGTTACTCTTCGAGTTGTGTAAGTTTCGTCCTAAAAAGGACTCGTCAGGTA
GCCTACTAGACTACGACTTACACGGTAGGTCGTGAACAAGCCCGGAGTACATCTCCTTTGGAACTAGG
+
FFFFFFFFFFFFFFFFFFFFFFFFFFFFFFF,FFFFFFFFFFFFFFFF:FFFFFFFFFFFFFFFFFFF
FFFFFFFFFFFF:FFFFFFFFFFFFF:FFFFFFFFFFFF,FFFFFFFFFFFFFFFF,FFFFF,FFFFF
@A00155:342:HHGFNDSXY:1:1643:32570:6605 2:N:0:GAACCTAG+TCCGCATA
CCCCTCCGGTCTTGTCCAACCTCTGTTTCGGCAGAGGATACCGTGCTCCACAGCTGTAAGGCTGTGGG
CCCGAGAGAAGTCGCTCTCTCTCGCCCTTCACATCCCTCTCGGCATTGAGATGACCATAGGCCAGAGG
+
FFFFFFFFFFFFFFFFFFFFFFFFFFFFFFFFFFFFF:FFFFFFFFFFFFFFFFFFFF:FFFFF:FFF
FFFFFFFFF,FFFFFFFFFFFFFFFFFFFFFFFFFFFFFFFFFFF:FFFFFFFFFF::,FFFF:FFFF
@A00155:342:HHGFNDSXY:1:1643:32154:12680 2:N:0:GAACCTAG+TCCGCATA
ACGACTTACACGGTAGGTCGTGAACAAGCCCGGAGTACATCTCCTTTGGAACCAGGAGACCCCTCCGG
TCTTGTCCAACCTCTGTTTCGGCAGAGGATACCGTGCTCCACAGCTGTAAGGCTGTGGGCCCGAGAGA
+
:FFFF:FFFFFFF:FFFFFFFFFFFFFFFFFFFFFFFFFFFFFFFFFFFFFFFFFFFFFFFFFFFFFF
FF:FFFFFFFFFFFFFFFFFFFFFFFFFFF,FFFFFFFFFFFFFFFFFF,FFFFFFFFFFFFFFF:FF
@A00155:342:HHGFNDSXY:1:1643:14669:32002 2:N:0:GAACCTAG+TCCGCATA
GGACTCGTCAGGTAGCCTACTAGACTACGACTTACACGGTAGGTCGTGAACAAGCCCGGAGTACATCT
CCTTTGGAACTAGGAGACCCCTCCGGTCTTGTCCAACCTCTGTTTCGGCAGAGGATACCGTGCTCCAC
+
FFFFFFFFFFFFFFFFFFFFFFFFFFFFFFFFFFFFFFFFFFFFFFFFFFFFFFFFFFFFFFFFFFFF
FFFFFFFFFFFFFFFFF:FFFFFFFFFFFFFFF:FFFFFF:FFFFFFFFFFFFFFFFFFFFFFFFFFF
@A00155:342:HHGFNDSXY:1:1644:8278:7623 2:N:0:GAACCTAG+TCCGCATA
ACTTACACGGTAGGTCGTGAACAAGCCCGGAGTACATCTCCTTTGGAACTAGGAGACCCCTCCGGTCT
TGTCCAACCTCTGTTTCGGCAGAGGATACCGTGCTCCACAGCTGTAAGGCTGTGGGCCCGAGAGAAG
+
FFFFFFFFFFFFFFFFFFFFFFFF:FFFFFFFFFFFFFFFFFFFFFFFFFFFFFFFFFFFFFFFFFFF
FFFFFFFF:FFFFFFFFFFFFFFFFFFFFFFFFFFFFFFFFFFFFFF::FFFF:FFFFFFFFFFF:
@A00155:342:HHGFNDSXY:1:1644:27832:20509 2:N:0:GAACCTAG+TCCGCATA
GAAACCGTCGTTACTCTTCGAGTTGTGTAAGTTTCGTCCTAAAAAGGACTCGTCAGGTAGCCTACTAG
ACTACGACTTACACGGTAGGTCGTGAACAAGCCCGGAGTACATCTCTTTTGGAACTAGGAGACCCCTC
+
FFFFFFF:FFFFFFFFFFFFFFFFFFFFFFFF,:FFFFF:F:FF:FFFFFFFFFFFFFFFFFFFFFFF
FFFFFFFFFFFFFFFFFFFFFFFFFFFF:FFFFFFFFFFFFFFFFFFFFFFFFFFFFFFFFFFFFFFF
@A00155:342:HHGFNDSXY:1:1644:3712:33379 2:N:0:GAACCTAG+TCCGCATA
GTTGTGTAAGTTTCGTCCTAAAAAGGACTCGTCAGGTAGCCTACTAGACTACGACTTACACGGTAGGT
CGTGAACAAGCCCGGAGTACATCTCCTTTGGAACTAGGAGACCCCTCCGGTCTTGTCCAACCTCTGTT
+
FFFFFFFFFFFFFFFFFFFFFFFFFFFFFFFFFFFFFFFFFFFFFFFFFFFFFFFFFFFFFFFFFFFF
FFFFFFFFFFFFFFFFFFFFF:FFFFFFFFFFFFFFFFFFFFFFFFFFFFFFFFFFFFFFFFFFFFFF
@A00155:342:HHGFNDSXY:1:1644:10854:36417 2:N:0:GAACCTAG+TCCGCATA
GTCCTAAAAAGGACTCGTCAGGTAGCCTACTAGACTACGACTTACACGGTAGGTCGTGAACAAGCCCG
GAGTACATCTCCTTTGGAACTAGGAGACCCCTCCGGTCTTGTCCAACCTCTGTTTCGGCAGAGGATAC
+
FFFFFFFFFFFFFFFFFFFFFFFFFFFFFFFFFFFFFFFFFFFFFFFFFFFFFFFFFFFFFFFFFFFF
FFFFFFFFFFFFFFFF:FFFFFFFFFFFFFFFFFFFFF:FFFFFFFFFFFFFFFFFFFFFFFFFFFFF
@A00155:342:HHGFNDSXY:1:1645:6271:5619 2:N:0:GAACCTAG+TCCGCATA
ACTCAAAGAGTAACGACGGTTTCGTCCTTGGAACGGACTCATCAGGGAGCCTACTAGACTCCGACCGT
```

CATGGGGGTTCAAACCCATGCCCCGCCACACCGCCTTAGATCAGCTGTGTCCACTTAAGGACTCACCT
+
FFFFFFFFFFFFFFFFFFFFFFFFFFFFFFFFFFFFFFFFFFFFFFFFFFFFFFFF,FFFFFFFFFFF
FFFFFFFFFFFFFFFFFFFFFFFFFFFFFFFFFFFFFFFFFFFFFFFFFF:FFFFFFFFFF:FFFF:
@A00155:342:HHGFNDSXY:1:1645:12048:16971 2:N:0:GAACCTAG+TCCGCATA
GGAGTACATCTCCTTTGGAACTAGGAGACCCCTCCGGTCTTGTCCAACCTCTGTTTCGGCAGAGGATA
CCGTGCTCCACAGCTGTAAGGCTGTGGGCCCGAGAGAAGTCGCTCTCTCTCGCCCTCCACATCCCTCT
+
FFFFFFFFFFFFFFFFFFFFFFFF:FFFFFFFFFFF:FFFFFFFFFFFFFF::FF:FFFFF:FFFFFF
,FFFFFFFFFF,FFFFFFFFFFF:F:FFFFFFFFFFF,FFFFFFFFFFFFFFF:FFFFFFFF:FF,FF
@A00155:342:HHGFNDSXY:1:1645:18078:24471 2:N:0:GAACCTAG+TCCGCATA
CGTCAGGTAGCCTACTAGACTACGACTTACACGGTAGGTCGTGAACAAGCCCGGAGTACATCTCCTTT
GGAACCAGGAGACCCCTCCGGTCTTGTCCAACCTCTGTTTCGGCAGAGGATACCGTGCTCCACAGCTG
+
FFFFFFFFFFFFFFFFFFFFFFFFFFFFFFFFFFFFFFFFFFFF:FFFFFFFFFFFFFFFFFFFFFFF
FFF:FFFFFFFFFFFFFFFFFFFFFFFFFFFFFFFFFFFFF:FFFFFFFFFFFFFFFFFFF:FFFFFFF
@A00155:342:HHGFNDSXY:1:1646:11605:2895 2:N:0:GAACCTAG+TCCGCATA
AGTACATCTCCTTTGGAACTAGGAGACCCCTCCGGTCTTGTCCAACCTCTGTTTCGGCAGAGGATACC
GTGCTCCACAGCTGTAAGGCTGTGGGCCCGAGAGAAGTCGCTCTCTCTCGCCCTCTACATCCCTCTCG
+
FFFFFFFFFFFFFFFFFFFFFFFFFFFFFFFFFFFF,FFFFFFFFFFFFFFFFFFFFFF:FFFFFFF
FFFFFFFFFFF:FFFFFFFFFFFFFFFFFFFFFFFFFFFFFFFFFFFFF:FFFFFFFFFFFFFFFFFF
@A00155:342:HHGFNDSXY:1:1646:24957:9142 2:N:0:GAACCTAG+TCCGCATA
CTTCGAGTTGTGTAAGTTTCGTCCTAAAAAGGACTCGTCAGGTAGCCTACTAGACTACGACTTACACG
GTAGGTCGTGAACAAGCCCGGAGTACATCTCCTTTGGAACTAGGAGACCCCTCCGGTCTTGTCCAACC
+
FFFFFFFFFFFFFFFFFFFFFFFFFFFFFFFFFFFFFFFFFFFFFFFFFFFF:FFFFFFFFFFFFFF
FFFFFFFFFFFFFFFFFFFFFFFFFFFFFFFFF,FFFFFFFFFFFFFFFFFFFFFFFFF:FFFFFFFFF
@A00155:342:HHGFNDSXY:1:1646:12635:23625 2:N:0:GAACCTAG+TCCGCATA
TCCTAAAAAGGACTCGTCAGGTAGCCTACTAGACTACGACTTACACGGTAGGTCGTGAACAAGCCCGG
AGTACATCTCCTTTGGAACTAGGAGACCCCTCCGGTCTTGTCCAACCTCTGTTTCGGCAGAGGATACC
+
FFF:FFFFFFFFFFFFFFF:FFFFFFFFFFFFFFFFFFFFFFFFFFFFFFFFFFFFFFFFFFF,FFFFF
FFFFFFFFFFFFFFFFFFFFFFFFFFFFFFFFFFFFFFFFFFFFFFFFFFFFFFFFFFFFFFFFFFFF
@A00155:342:HHGFNDSXY:1:1647:32886:1924 2:N:0:GAACCTAG+TCCGCATA
ACATCTCCTTTGGAACTAGGAGACCCCTCCGGTCTTGTCCAACCTCTGTTTCGGCAGAGGATGCCGTG
CTCCACAGCTGTAAGGCTGTGGGCCCGAGAGAAGTCGCTCTCTCTCGCCCTCCACATCCCTCTCGGCA
+
FFFFFFFF::FFFFFF:FFFFFFFFFFFFFFFFFFFFFFFFFFFFFFFFFFFFFFFFF,FFFFFFFFF
FFFFFF:FFFFF,FFFF,FFFFFFFFFFFFFFFFFFFFFFFFF,FFFFFFFF:FFFF,FFFFFFFFFFF
@A00155:342:HHGFNDSXY:1:1647:9462:35133 2:N:0:GAACCTAG+TCCGCATA
TGTGTAAGTTTCGTCCTAAAAAGGACTCGTCAGGTAGCCTACTAGACTACGACTTACACGGTAGGTCG
TGAACAAGCCCGGAGTACATCTCCTTTGGAACTAGGAGACCCCTCCGGTCTTGTCCAACCTCTGTTTC
+
FFFFFFFFFFFFFFFFFFFFFF:F:FFFFFFFFFFFFFFFFFFFFFFFFFFFFFFFFFFFFFFFFFFF
FFFFFF:,FFF:FFFFFFFFFFFFFFFFF:FFFFFF:FFFFFFF:FFFFFFFFFFFFFFFFFFFFFFFF
@A00155:342:HHGFNDSXY:1:1648:29206:31563 2:N:0:GAACCTAG+TCCGCATA
ACGACTTACACGGTAGGTCGTGAACAAGCCCGGAGTACATCTCCTTTGGAACTAGGAGACCCCTCCGG
TCTTGTCCAACCTCTGTTTCGGCAGAGGATACCGTGCTCCACAGCTGTAAGGCTGTGGGCCCGAGAGA
+
FFFFFFFFFFFFFFFFFFFFFFFFFFFFFFFFFFFF:FFFFFFFFFFFFFFFFFFFF:FFFFFFFFFF
FFFFFFFFFFFFFFFFFFFFFFFFFFFFFFFFFFFFFFFFFFFFFFFFFFFFFF:FFFFFFFFFFFFFF
@A00155:342:HHGFNDSXY:1:1648:24695:32299 2:N:0:GAACCTAG+TCCGCATA
CATCTCCTTTGGAACTAGGAGACCCCTCCGGTCTTGTCCAACCTCTGTTTCGGCAGAGGATACCGTGC

TCCACAGCTGTAAGGCTGTGGGCCCGAGAGAAGTCGCTCTCTCTCGCCCTCCACATCCCTCTCGG
+
FFFFFFFFFFFFFFFFFFFFFFFFFFFFFFFFFFFFFFFFFFFFFFFFFFFFFFFFFFFFFFFFFFFF
FFFFFFFFFFFFFFFFFFFFFFFFFFFFFFFFFFFFFFFFFFFFFFFFFFFFFFFFFFFFFFFFF
@A00155:342:HHGFNDSXY:1:1648:7627:34366 2:N:0:GAACCTAG+TCCGCATA
ACACGGTAGGTCGTGAACAAGCCCGGAGTACATCTCCTTTGGAACTAGGAGACCCCTCCGGTCTTGTC
CAACCTCTGTTTCGGCAGAGGATACCGTGCTCCACAGCTGTAAGGCTGTGGGCCCGAGAGAAGTCGCT
+
FFFFFFFFFFFFFFFFFFFFFFFFFFFFFFFFFFFFFFFFFFFFFFFFFFFFFFFFFFFFFFFFFFFF
FFFFFFFFFFFFFFFFFFFFFFFFFFFFFFFFFFFFFFFFFFFFFF:FFFFFFFFFFFFFFFFFFFFF
@A00155:342:HHGFNDSXY:1:1648:17653:34632 2:N:0:GAACCTAG+TCCGCATA
GTTTCGTCCTAAAAAGGACTCGTCAGGTAGCCTACTAGACTACGACTTACACGGTAGGTCGTGAACAA
GCCCGGAGTACATCTCCTTTGGAACTAGGAGACCCCTCCGGTCTTGTCCAACCTCTGTTTCGGCAGAG
+
FFFFFFFFFF:FFFFFFFFFFFFFFFFFFFFFFFFFF:FFFFFFFFFFFFFFFFFFFFF:FFFFFFFFF
FFFFFFFFFFFFFFFFFFFFFFFFFFFFFFFFFFFFFFFFFFFFFFFFFFFFFFFFFFFFFFFFFFFF
@A00155:342:HHGFNDSXY:1:1649:8703:21261 2:N:0:GAACCTAG+TCCGCATA
AAAAGGACTCGTCAGGTAGCCTACTAGACTACGACTTACACGGTAGGTCGTGAACAAGCCCGGAGTAC
ATCTCCTTTGGAACTAGGAGACCCCTCCGGTCTTGTCCAACCTCTGTTTCGGCAGAGGATACCGTGCT
+
FFFFFFFFFFFFFFFFFFFFFFFFFFFFFFFFFFFFFFFFFFFFFFFFFFFFFFFFFFFFFFFFFFFF
FFFFFFFFFFFFFFFFFFFFFFFFFFFFFFFFFFFFFFFFFFFFFF:FFFFFFFFFFFFFFFFFFFFF
@A00155:342:HHGFNDSXY:1:1649:29939:24471 2:N:0:GAACCTAG+TCCGCATA
AAGGACGAAACCGTCGTTACTCTTCGAGTTGTGTAAGTTTCGTCCTAAAAAGGACTCGTCAGGTAGCC
TACTAGACTACGACTTACACGGTAGGTCGTGAACAAGCCCGGAGTACATCTCCTTTGGAACTAGGAGA
+
F,FFFFFFFFFFFFFFFFFFFFFFFFFFFFFFF:FFFFFFFFFFFFFFFFFFFFFFFFFFFFFFFFFF
FFFFFFFFFFFFFFFFFFFFFFFFFFFFFFFFFFFFFFFFFFFFFFF:FFFFF:FFFFF:FFFF:FFF
@A00155:342:HHGFNDSXY:1:1650:18078:2581 2:N:0:GAACCTAG+TCCGCATA
GTTGTGTAAGTTTCGTCCTAAAAAGGACTCGTCAGGTAGCCTACTAGACTACGACTTACACGGTAGGT
CGTGAACAAGCCCGGAGTACATCTCCTTTGGAACTAGGAGACCCCTCCGGTCTTGTCCAACCTCTGTT
+
FFFFFFFFFFFFF:FFFFFFFFFFFFFFFFFFFFFFFFFFFFFFFFFFF:FFFFFFFFFFFFFFFFFFF
FFFFFFFFFFFFFFFFFFF::FFFFFFFFFFF:FFFFFFFFFF:FFFFFFFFFFFFFFFFFFFFFF:FF
@A00155:342:HHGFNDSXY:1:1650:31702:26616 2:N:0:GAACCTAG+TCCGCATA
GAACAAGCCCGGAGTACATCTCCTTTGGAACTAGGAGACCCCTCCGGTCTTGTCCAACCTCTGTTTCG
GCAGAGGATACCGTGCTCCACAGCTGTAAGGCTGTGGGCCCGAGAGAAGTCGCTCTCTCTCGCC
+
FFFFFFFFFFFFFFFFFFFFFFFF:FFFFFFFFFFFFFFFFFFFFFFFFFFFFF:FFFFFFFFFFFFFF
FFFFFFFFF:FFFFFFFFFFFFFFFFF:FFFFFF:FFFFFFFFFFFFFFFFFFFFFFFFFFFFFF
@A00155:342:HHGFNDSXY:1:1651:21468:26804 2:N:0:GAACCTAG+TCCGCATA
GTGTAAGTTTCGTCCTAAAAAGGACTCGTCAGGTAGCCTACTAGACTACGACTTACACGGTAGGTCGT
GAACAAGCCCGGAGTACATCTCCTTTGGAACTAGGAGACCCCTCCGGTCTTGTCAAACCTCTGTTTCG
+
FFFFFFFFFFFFFFFFFFFF:FFFFFFFFFFFFFFFFFFFFFFFFFFFFFFFFFFFFFFFFFFFFFF:F
FFFFFFFFFFFFFFFFFFFFFFFFFFFFFFFFF:FF,FFFFFFF:FFFFFFFFFFFFFFFFFFFFFFFF
@A00155:342:HHGFNDSXY:1:1651:2329:30013 2:N:0:GAACCTAG+TCCGCATA
CGGCAGAGGATACCGTGCTCCACAGCTGTAAGGCTGTGGGCCCGAGAGAAGTCGCTCTCTCTCGCCCT
TCACATCCCTCTCGGCATTGAGATGACCATAGGCCAGAGGTGAGTCCTTAAGTGGACACAGCTGATCT
+
FFFFFFFFFFFFFFFFFFFFFFFFFFF:FFFFFFFFFFF:FFFFFFFFFFFFFFFFFFFFFFFFFFFFF
FFFFFFFFFFFFFFFFFFFFFFFFFFFFFFFFFFFFFFFFFFFFFFFFFFFFFFFFFFFFFFFFFFFF
@A00155:342:HHGFNDSXY:1:1651:32027:34914 2:N:0:GAACCTAG+TCCGCATA
TCCTAAAAAGGACTCGTCAGGTAGCCTACTAGACTACGACTTACACGGTAGGTCGTGAACAAGCCCGG

AGTACATCTCCTTTGGAACTAGGAGACCCCTCCGGTCTTGTCCAACCTCTGTTTCGGCAGAGGATACC
+
FFFFFFFF:FFFFFFFFFFFFFFFFFFFFFFFFFFFFFFFFFFFFFFFFF:FFFFFFFFFFFFFFFFFF
FFFFFFFFFFFFFFFFFFFFFFFFFFFFFF:FFFFFFFFFFFFFFFFFFFFFFFFF:FFFFFFFFFFFFFF:FF
@A00155:342:HHGFNDSXY:1:1652:25735:5259 2:N:0:GAACCTAG+TCCGCATA
AAAAGGACTCGTCAGGTAGCCTACTAGACTACGACTTACACGGTAGGTCGTGAACAAGCCCGGAGTAC
ATCTCCTTTGGAACTAGGAGACCCCTCCGGTCTTGTCCAACCTCTGTTTCGGCAGAGGATACCGTGCT
+
FFFFFFFFFFFFFFFFF:FFFFFFFFFFFFFFFFFFFFFFFFFFFFFFFFFFFFFFFFFFFFFFFFFFF
FFFFFFFFFFFFFFFFFFFFFFFFFFFFFFFFF:FF:FFFFFFFFFFFFFFFFFFFFFFFFF:FFFFFFFF
@A00155:342:HHGFNDSXY:1:1652:16080:9674 2:N:0:GAACCTAG+TCCGCATA
TTTCGTCCTAAAAAGGACTCGTCAGGTAGCCTACTAGACTACGACTTACACGGTAGGTCGTGAACAAG
CCCGGAGTACATCTCCTTTGGAACTAGGAGACCCCTCCGGTCTTGTCCAACCTCTGTTTCGGCAGAGG
+
F:FFFFFFFFFFF:FFFFFFFFFFFFFFFFFFFFFFFFFFFFF:FFFFFFFFFFFFFF:FFFFFFFFFFF
FFFFFFFFFFFFFFFFFFFFFFFFFFFFFFFFFF:FFFF:FFFFFFFFFFFFFFFFFFFFFFFFFFFFFF
@A00155:342:HHGFNDSXY:1:1652:8748:16297 2:N:0:GAACCTAG+TCCGCATA
GAACTAGGAGACCCCTCCGGTCTTGTCCAACCTCTGTTTCGGCAGAGGATACCGTGCTCCACAGCTGT
AAGGCTGTGGGCCCGAGAGAAGTCGCTCTCTCTCGCCCTCCACATCCCTCTCGGCATTGAGATGACCA
+
FFFFFFFFFFFFFFFFFFFFFFFFFFFFFFFFFFFFFFFFFFFFFFFFFFFFFFFFFFFFFFFFFFFFF
FFFFFFFFFFFFFFFFFFFFFFFFFFFFFFFFFFFFFFFFFFFFFFFFFFFFFFFFFFFFFFFFFFFF:F
@A00155:342:HHGFNDSXY:1:1652:19569:19601 2:N:0:GAACCTAG+TCCGCATA
GCCTACTAGACTACGACTTACACGGTAGGTCGTGAACAAGCCCGGAGTACATCTCCTTTGGAACTAGG
AGACCCCTCCGGTCTTGTCCAACCTCTGTTTCGGCAGAGGATACCGTGCTCCACAGCTGTAAGGCTGT
+
FFFFFFFFFFFFFFFFFFFFFFFFFFFFFFFFFFFFFFFFFFFFFFF:FFFFFFFFF:FFFFFFF:FFFFF
FFFF:FFFFFFFFFFFFFFFFFFFFFFFFFFFFFFFFFFF,FFFFFF:FFFFFFFFFFFFFFF:FFFFFFF
@A00155:342:HHGFNDSXY:1:1652:18539:32722 2:N:0:GAACCTAG+TCCGCATA
GTGAACAAGCCCGGAGTACATCTCCTTTGGAACTAGGAGACCCCTCCGGTCTTGTCCAACCTCTGTTT
CGGCAGAGGATACCGTGCTCCACAGCTGTAAGGCTGTGGGCCCGAGAGAAGTCGCTCTCTCTCGCCCT
+
FFFFFFFFFFFFFFFFFFFFFFFFFFFFFFFFFFFFFFFFFFFFFFFFFFF:FFFFFFFFFFFFFFFF
FFFFFFFFFF,FFFFFFFFFFFFFFFFFFFFFFFFFFFFFFFFFFFFFFF,FFFFFFFFFFFFFFFFFFF
@A00155:342:HHGFNDSXY:1:1653:15139:7451 2:N:0:GAACCTAG+TCCGCATA
GAACAAGCCCGGAGTACATCTCCTTTGGAACTAGGAGACCCCTCCGGTCTTGTCCAACCTCTGTTTCG
GCAGAGGATACCGTGCTCCACAGCTGTAAGGCTGTGGGCCCGAGAGAAGTCGCTCTCTCTCGCC
+
FFFFFFFFFFFFFFFFFFFFFFFFFFFFFFFFFFFFFFFFFFFFFFFFF:F:FFFFFFFFFFFFFFFF
FFFFFFFFFFFFFFFFFFFFFFFFFFFFF:FFFFFFFFFFFFFFFFFFFFFFFFFFFFFFFFFFFFFF
@A00155:342:HHGFNDSXY:1:1653:31060:24189 2:N:0:GAACCTAG+TCCGCATA
AGCCCGGAGTACATCTCCTTTGGAACCAGGAGACCCCTCCGGTCTTGTCCAACCTCTGTTTCGGCAGA
GGATACCGTGCTCCACAGCTGTAAGGCTGTGGGCCCGAGAGAAGTCGCTCTCTCTCGCCCTCCACATC
+
FFFFFFFFFFFFFFFFFFFFFFFFFFFFFFFFFFFFFFFFFFFFF::FFFFFFFFFFFFF:FFFFFFFFF
FFFFFFFF:FFFFF,FFFFFFFFFFFF:FFFFFFFFFFFFFF:FFFFFFFFFFFFFFFFFFFFFFFFFFF
@A00155:342:HHGFNDSXY:1:1653:20645:27821 2:N:0:GAACCTAG+TCCGCATA
GTACATCTCCTTTGGAACTAGGAGACCCCTCCGGTCTTGTCCAACCTCTGTTTCGGCAGAGGATACCG
TGCTCCACAGCTGTAAGGCTGTGGGCCCGAGAGAAGTTGCTCTCTCTCGCCCTCCACATCCCTCTCGG
+
FFFFFFFFFFFFFFFFFFFFFFFFFFFFFFFFFFFFF:FFFFFFFFFFFFFFFFFFFFFFFFFFFF,FFF
FFFFFFFFFFFFFF:FFFFFFFFFFFFFFFFFFFFFFFFFFFFFFFFFFFFFFFFFFFFFFFFFFFFFFF
@A00155:342:HHGFNDSXY:1:1653:21377:32377 2:N:0:GAACCTAG+TCCGCATA
GTACATCTCCTTTGGAACTAGGAGACCCCTCCGGTCTTGTCCAACCTCTGTTTCGGCAGAGGATACCG

```
TGCTCCACAGCTGTAAGGCTGTGGGCCCGAGAGAAGTCGCTCTCTCTCGCCCTCCACATCCCTCTCGG
+
FFFFFFFFFF,FFFFFFFFFFFFFFFFFFFFFFFF:F:,FFFFFFFFFFFFF:FFFFFFFFFFFFFFFFF
FFFFFFFFFFFFFFFFFFFFFFFFFFFFFF:FFFFFFFFFFFFFFFFF:FFFFFFFFFFFFFFFFFFFF:
@A00155:342:HHGFNDSXY:1:1654:4065:2769 2:N:0:GAACCTAG+TCCGCATA
ACCCCTCCGGTCTTGTCCAACCTCTGTTTCGGCAGAGGATACCGTGCTCCACAGCTGTAAGGCTGTGG
GCCCGAGAGAAGTCGCTCTCTCTCGCCCTCCACATCCCTCTCGGCATTGAGATGACCATAGGCCAGAG
+
FFFFFFFFFFFFFFFFFFFFFFFF,FFFFFFFF:FFFFFFFFFFFFFFFFFFFFFFFFFFFFFFFFFFFF
FFFFFFF:FF:,FFFFFFFFFFFFFFFFFFFFFFF:FF:FFFFFFFFFFFFFFFFFFFFFF:FF:FFFFF
@A00155:342:HHGFNDSXY:1:1654:14733:6527 2:N:0:GAACCTAG+TCCGCATA
TAGGAGACCCCTCCGGTCTTGTCCAACCTCTGTTTCGGCAGAGGATACCGTGCTCCACAGCTGTAAGG
CTGTGGGCCCGAGAGAAGTCGCTCTCTCTCGCCCTCCACATCCCTCTCGGCATTGAGATGACCATAGG
+
FFFFFFFFFFFFFFFFFFFFFFFFFFFFFFFFF:FFFFFFFFFFFFFFFFFFFFFFFFFFFFFFFFFFFF
FFFFFFFFFFFFFFFFFFFFFFFFFFFFFFFFFFFFFFFFFFFFFFFFFFFFFF:FF:FFFFFFFFF:FF
@A00155:342:HHGFNDSXY:1:1654:21721:11209 2:N:0:GAACCTAG+TCCGCATA
GTAGGTCGTGAACAAGCCCGGAGTACATCTCCTTTGGAACTAGGAGACCCCTCCGGTCTTGTCCAACC
TCTGTTTCGGCAGAGGATACCGTGCTCCACAGCTGTAAGGCTGTGGGCCCGAGAGAAGTCGCTCTCTC
+
FFFFF:FFFFFFFFFFFFFFFFFFFFFFFFFFFFFFFFFFFFFFFF:FFFFFFFFFFFFF:FFFFFFFFF
FFFFFFFFFFFFFFF:FFFFFFFFFFFFFFFFFFFFFFFFFFFFFFFFFFFFFFFFFFFFFFFFFFFFFF
@A00155:342:HHGFNDSXY:1:1654:21685:12430 2:N:0:GAACCTAG+TCCGCATA
GTAGGTCGTGAACAAGCCCGGAGTACATCTCCTTTGGAACTAGGAGACCCCTCCGGTCTTGTCCAACC
TCTGTTTCGGCAGAGGATACCGTGCTCCACAGCTGTAAGGCTGTGGGCCCGAGAGAAGTCGCTCTCTC
+
FFFFFFFFFFFFFFFFFFFFFFFFFFFFFFFFFFFFFFFFFFFFFFFF:FFFFFFFFFFFFFFFFFFFFF
FFFFFFFF:FFFFFFFFFFFFFFFFFFFFFFFFFFFFFFFFFFFFFFFFFFFFFFFFFFFFFFFFFFFFF
@A00155:342:HHGFNDSXY:1:1654:5737:21324 2:N:0:GAACCTAG+TCCGCATA
TCTTCGAGTTGTGTAAGTTTCGTCCTAAAAAGGACTCGTCAGGTAGCCTACTAGACTACGACTTACAC
GGTAGGTCGTGAACAAGCCCGGAGTACATCTCCTTTGGAACTAGGAGACCCCTCCGGTCTTGTCCAAC
+
FF,FFFFF,FFFFFFFFFFF:FFFFFFFFFFFFFFFFFFFFFFFF:FFFFFFFFFFFFFFFFFFFFFFFF
FFFFFFFFFFFFFF:FFFFFFFFFFFFFFFFFFFFFFFFF:FFFFFFFFFFFFFFFFF::FFFFFFFFFF
@A00155:342:HHGFNDSXY:1:1654:4580:22388 2:N:0:GAACCTAG+TCCGCATA
AAAAAGGACTCGTCAGGTAGCCTACTAGACTACGACTTACACGGTAGGTCGTGAACAAGCCCGGAGTA
CATCTCCTTTGGAACTAGGAGACCCCTCCGGTCTTGTCCAACCTCTGTTTCGGCAGAGGATACCGTGC
+
FFFFF:FFFFFFFFFFFFFF:FFFFFFFFF:FFFFFFFFFFFFFFFFFFFFFFFFFFFFFF:FFF:FFFF
:FFFFFFFFFFF:FFFFFFFFFF:FFFFFFFFFFFFFFFFFFFFFFFFFFFFFFFFFFFFF:FFFFFFFF
@A00155:342:HHGFNDSXY:1:1655:14570:6872 2:N:0:GAACCTAG+TCCGCATA
TTACACGGTAGGTCGTGAACAAGCCCGGAGTACATCTCCTTTGGAACTAGGAGACCCCTCCGGTCTTG
TCCAACCTCTGTTTCGGCAGAGGATACCGTGCTCCACAGCTGTAAGGCTGTGGGCCCGAGAGAAGTCG
+
FFFFFFFFFFFFFFFFFFFFFFFFFFFFFFFFFFFFFFFFFFFFFF,FFFFFFFFFF:FFFFFFFFFFFF
:FFFFFFFFFFFFFFFFFFFFFFFFFFFFFFFFFFFFFFFFFFFFFFFFFFFFFFFFFFFFFFFFFFFFF
@A00155:342:HHGFNDSXY:1:1655:8495:19116 2:N:0:GAACCTAG+TCCGCATA
AAGGACGAAACCGTCGTTACTCTTTGAGTTGTGTAAGTTTCGTCCTAAAAAGGACTCGTCAGGTAGCC
TACTAGACTACGACTTACACGGTAGGTCGTGAACAAGCCCGGAGTACATCTCCTTTGGAACTAGGAGA
+
FFFFFFFFFFFFFFFFFFFFFFFFFFFFFFFFFFFFFFFFFFFFFFF,FFFFFFFFFFFF:FFFFFFFFF
FFFFFFFFFFFFFFFFFFFFFFFFFFFFFFFFF:FFFFFFFFFFFFFFFFFFFFFFFFFFF:FFFFFFFF
@A00155:342:HHGFNDSXY:1:1656:6759:11224 2:N:0:GAACCTAG+TCCGCATA
TTTCGTCCTAAAAAGGACTCGTCAGGTAGCCTACTAGACTACGACTTACACGGTAGGTCGTGAACAAG
```

CCCGGAGTACATCTCCTTTGGAACCAGGAGACCCCTCCGGTCTTGTCCAACCTCTGTTTCGGCAGAGG
+
F:FFFF:FFFFFFF:FFFFFFFFFFFFFF,FFFFFFFFFFFFFFFF::,FFF:FFFFFFFFFFFFFFF
FFF::FFFFFFF:FFFFFFFFFF:FF,,FFFFFFF:F:,FFFFF,F::FFF,FFF:FFFF,F:FFFFF
@A00155:342:HHGFNDSXY:1:1656:15980:22091 2:N:0:GAACCTAG+TCCGCATA
GTCCTAAAAAGGACTCGTCAGGTAGCCTACTAGACTACGACTTACACGGTAGGTCGTGAACAAGCCCG
GAGTACATCTCCTTTGGAACTAGGAGACCCCTCCGGTCTTGTCCAACCTCTGTTTCGGCAGAGGATAC
+
FFFFFFFFF:FFFFFFFFFFFFF:FFF:FFFFFFFFFFFFFFFF:FFFFF:FFFFFFFFFFFFFFFFF
:FFFFF,FF:FFFFFFFF:F:FF:FFFFFFF:FFFFFFFFFFFFFFFFFFF:FF,FFFFFFFFFFFFF
@A00155:342:HHGFNDSXY:1:1656:21178:24142 2:N:0:GAACCTAG+TCCGCATA
ACGGTAGGTCGTGAACAAGCCCGGAGTACATCTCCTTTGGAACCAGGAGACCCCTCCGGTCTTGTCCA
ACCTCTGTTTCGGCAGAGGATACCGTGCTCCACAGCTGTAAGGCTGTGGGCCCGAGAGAAGTCGCTCT
+
FFFFFFFF:FFFFFFFFFFFFFFFFFFFFFFFFFFFFFFFFFFFFFFFFFFFFFFFFFFFFFFFFFFF
FFFFFFFFF:FFFFFFFFFFFFFFFFFFFFFFFFFFFFFFFFFFFFFFFFFFFFFFFFFFFFFFFFFF
@A00155:342:HHGFNDSXY:1:1656:19913:28369 2:N:0:GAACCTAG+TCCGCATA
GTTTCGTCCTAAAAAGGACTCGTCAGGTAGCCTACTAGACTACGACTTACACGGTAGGTCGTGAACAA
GCCCGGAGTACATCTCCTTTGGAACTAGGAGACCCCTCCGGTCTTGTCCAACCTCTGTTTCGGCAGAG
+
FFFFFFFFFFFFFFFFFFFFFFFFFFFFFFFFFFFFFFFFFF:FFFFFFF:FFFFFFFF:FFFFF:FF
:FFFFFFFFFFFFFFFFF:FFFFFFFFF::FFFFFFFFFFFFFFFFFFFFFFFFFFFFFF:FFFFFFF
@A00155:342:HHGFNDSXY:1:1656:27299:30326 2:N:0:GAACCTAG+TCCGCATA
ACTTACACGGTAGGTCGTGAACAAGCCCGGAGTACATCTCCTTTGGAACTAGGAGACCCCTCCGGTCT
TGTCCAACCTCTGTTTCGGCAGAGGATACCGTGCTCCACAGCTGTAAGGCTGTGGGCCCGAGAGAAG
+
::FFFFF:F::FFF:FFFFFFFFF:FF:FFFFFFFFFF:FFF:F:FFFFF,FFFFFFFFFFF::FFFF
FFFFFF,FF,F,F::FFF:FFFFF,FFFFFFFF,FFFFFFFFFF,F,F:FFFFFFFFFF:FFFFFFF
@A00155:342:HHGFNDSXY:1:1657:10782:7607 2:N:0:GAACCTAG+TCCGCATA
GTGAACAAGCCCGGAGTACATCTCCTTTGGAACTAGGAGACCCCTCCGGTCTTGTCCAACCTCTGTTT
CGGCAGAGGATACCGTGCTCCACAGCTGTAAGGCTGTGGGCCCGAGAGAAGTCGCTCTCTCTCGCCCT
+
F::FFFFF:FFFF:F:FFFF:FFFFFFF:FFFFFFFFFFFFFF:FFFF:,F,FFFFFFFF:FFFF::F
FFFFFFFF:FFFFFF:FFFFFFFFFFFF,FFFFFFF:FFFFFFFFFFFFFFFFFFFFFFFFFFFFFFF
@A00155:342:HHGFNDSXY:1:1657:20464:12853 2:N:0:GAACCTAG+TCCGCATA
GAGAGAGCGACTTCTCTC
+
FFFFFFFFFFFFFFFFFF
@A00155:342:HHGFNDSXY:1:1657:3387:19977 2:N:0:GAACCTAG+TCCGCATA
GACCCCTCCGGTCTTGTCCAACCTCTGTTTCGGCAGAGGATACCGTGCTCCACAGCTGTAAGGCTGTG
GGCCCGAGAGAAGTCGCTCTCTCTCGCCCTCCACATCCCTCTCGGCATTGAGATGACCATAGGC
+
FFFFFFFFFFFFFFFFFFFFFFFFF:FFFFFFFFFFFFFF,FFFFFFFFFFFFFFFFFFFF:FFFFFF
FFFFFFFFFFFFFFFFFFFFFFFFFFFFFFFFFFFFFFF,FFF:FFF,,FFFFFFFFFF:FFFF
@A00155:342:HHGFNDSXY:1:1658:29731:17848 2:N:0:GAACCTAG+TCCGCATA
ACCGTGCTCCACAGCTGTAAGGCTGTGGGCCCGAGAGAAGTCGCTCTCTCTCGCCCTTCACATCCCTC
TCGGCATTGAGATGACCATAGGCCAGAGGTGAGTCCTTAAGTGGACACAGCTGATCTAAGGCGGTGTG
+
FFFFFFFFFFFFFFFFFFFFFFFFFFFFFFFFFFFFFFFFFFFFFFFFFFFFFFFFFFFFFFFFFFFF
FFFFFF::FFFFFFFFF:FFFFFFFFFFFFFFFFFFFFFFFFFFFF:FFFFFFFFFFFFFFFFFFFFF
@A00155:342:HHGFNDSXY:1:1659:7383:7232 2:N:0:GAACCTAG+TCCGCATA
ACTTACACGGTAGGTCGTGAACAAGCCCGGAGTACATCTCCTTTGGAACTAGGAGACCCCTCCGGTCT
TGTCCAACCTCTGTTTCGGCAGAGGATACCGTGCTCCACAGCTGTAAGGCTGTGGGCCCGAGAGAAGT
+

FFFFFFFFFFF:FF:FFFFFFFFFFFFFFFFFFFFFFFFFFFFFFFFFFFFFFFFFFFFFFFFFFFFFF
FFFFFFFFFFFFFFFFFFFFFFFFFFFFFFFFFFFFFFFFFFFFFFFFFF:FFFFFFFFFFF:FF:FF
@A00155:342:HHGFNDSXY:1:1659:13458:18537 2:N:0:GAACCTAG+TCCGCATA
TTACACGGTAGGTCGTGAACAAGCCCGGAGTACATCTCCTTTGGAACTAGGAGACCCCTCCGGTCTTG
TCCAACCTCTGTTTCGGCAGAGGATACCGTGCTCCACAGCTGTAAGGCTGTGGGCCCGAGAGAAGTCG
+
FFFFFFFFFFFFFFFFFFFFFFFFFFFFFFFFFFFFFFFFFFFFFFFFFFFFFFF:FFFFFFF:FFF
FFFFFFFFFFFFFFFFFFFFFFFFFFFFFFFFFFFFFFFFFFFF,FFFFFFFFFFFFFFFFFFFFFFF
@A00155:342:HHGFNDSXY:1:1660:10574:19429 2:N:0:GAACCTAG+TCCGCATA
GAGACCCCTCCGGTCTTGTCCAACCTCTGTTTCGGCAGAGGATACCGTGCTCCACAGCTGTAAGGCTG
TGGGCCCGAGAGAAGTCGCTCTCTCTCGCCCTCTACATCCCTCTCGGCATTGAGATGACCATAGGCCA
+
FFFFFFFFFFFFFFFFFFFFFFFFFF:FFFFFFFFFFFFFFFFF:FFFFFFFFFFFFFFFFFFFFFFFF
FFFFFFFFFFFFFFFFFFFFFFFFFFFFFFFFFFFFFFFFFFFFFFFFF:FFFFFFFFF:F::FFFFF:
@A00155:342:HHGFNDSXY:1:1660:10999:20102 2:N:0:GAACCTAG+TCCGCATA
GAGACCCCTCCGGTCTTGTCCAACCTCTGTTTCGGCAGAGGATACCGTGCTCCACAGCTGTAAGGCTG
TGGGCCCGAGAGAAGTCGCTCTCTCTCGCCCTCTACATCCCTCTCGGCATTGAGATGACCATAGGCCA
+
FFFFF:FFFFFFF:FFFFFFFFFFFFFFF:FFFFFFFFFF:FFFFFFFFFFFFFFFFF,FFFF:FFFF
FFFFFFFFFFFFFFFFFFFFFFFFFFFFFFFFFFFFFFFFFFFFFFFF:FFFFFFFFFFFFFF,FFFFF
@A00155:342:HHGFNDSXY:1:1661:24849:8296 2:N:0:GAACCTAG+TCCGCATA
TAGACTACGACTTACACGGTAGGTCGTGAACAAGCCCGGAGTACATCTCCTTTGGAACTAGGAGACCC
CTCCGGTCTTGTCCAACCTCTGTTTCGGCAGAGGATACCGTGCTCCACAGCTGTAAGGCTGTGGGCCC
+
FFFFFFFFFFFFFFFFFFFFFFFFFFFFFFFFFFFFFFFFFFFFFFFFFFFFFFFFFFFFFFFFFFFF
FFFFFFFFFFFFFFFFFFFFFFFFFFFFFFFFFFFFFFFFFFFFFFFFFFFFFFFFFFFFFFFFFFFF
@A00155:342:HHGFNDSXY:1:1661:18376:17065 2:N:0:GAACCTAG+TCCGCATA
AACTAGGAGACCCCTCCGGTCTTGTCCAACCTCTGTTTCGGCAGAGGATACCGTGCTCCACAGCTGTA
AGGCTGTGGGCCCGAGAGAAGTCGCTCTCTCTCGCCCTCCACATCCCTCTCGGCATTGAGATGACCAT
+
FFFF:FFFFFFFFFFFFFFFFFFFFFFFFFFFFFFFFFFFFFFFFFFFFFFFFFFFFFFFFFFFFFFF
FFFFFFFFFFFFFFFFFFFFFFFFFFFFF:FFFFFFFFFFFFFFFFFFFFFFFFFFFFFFFFFFFFFF
@A00155:342:HHGFNDSXY:1:1661:22453:20807 2:N:0:GAACCTAG+TCCGCATA
GTTGTGTAAGTTTCGTCCTAAAAAGGACTCGTCAGGTAGCCTACTAGACTACGACTTACACGGTAGGT
CGTGAACAAGCCCGGAGTACATCTCCTTTGGAACTAGGAGACCCCTCCGGTCTTGTCCAACCTCTGTT
+
FFFFFFFFFFF::FFF:FFFFFFFFFFFFFFFFFFFFFFFFFFFFFFFFFFFFFFFFFFFFFFFFFFF
FFFFFFFFF:FFFFFFFFF:FFFFFFFFFFFFFFFFFFFFF:FFFFFFFFFFFFF,FFFFFFFFFFF:
@A00155:342:HHGFNDSXY:1:1661:13304:28322 2:N:0:GAACCTAG+TCCGCATA
TACGACTTACACGGTAGGTCGTGAACAAGCCCGGAGTACATCTCCTTTGGAACCAGGAGACCCCTCCG
GTCTTGTCCAACCTCTGTTTCGGCAGAGGATACCGTGCTCCACAGCTGTAAGGCTGTGGGCCCG
+
FFFF:FFFFFFFFFFFFFFFFFFFFFFFFFFFFFFFFFFFFFFFFFFFFFFFFFF:FFFFF:FFFFF
FFFFFFFFFFFFFFFFFFFFFFFFFFFFFFFFFFFFFFFFFFFFFFFFFFFFFFFFFFFFFFFF
@A00155:342:HHGFNDSXY:1:1663:6180:4867 2:N:0:GAACCTAG+TCCGCATA
TAACGACGGTTTCGTCCTTGGAACGGACTCATCAGGGAGCCTACTAGACTCCGACCGTCATGGGGGTT
CAAACCCATGCCCCGCCACACCGCCTTAGATCAGCTGTGTCCACTTAAGGACTCACCTCTGGCCGATG
+
FFFFFFFFF:FFFFFFFFFFFF,FFFFFFF:FFFFFFFFFFFFFF,FFFFFFFFFFFFFFFFFFFFFF
FFF:FFFFF:FFFFF:FFFFFFFFFFFFFFFFFFFFFFFFFFFFFFFFFFFFFFFFFFFFFFFF,FFF
@A00155:342:HHGFNDSXY:1:1663:11831:13150 2:N:0:GAACCTAG+TCCGCATA
ACTACGACTTACACGGTAGGTCGTGAACAAGCCCGGAGTACATCTCCTTTGGAACTAGGAGACCCCTC
CGGTCTTGTCCAACCTCTGTTTCGGCAGAGGATACCGTGCTCCACAGCTGTAAGGCTGTGGGCCCGAG
+

```
FFFFFFFFFFFFFFFFFFFFFFFFFFFFFF:FFFFFFFFFFFFFFFFFFFFFFFFFFFFFFFFFFF:F
FFFFF:FFFFFFFFFFFFFFFFFFFFFFFFFFFF:FFFFFFFFFFFFFFFFFFFFFFFFFFFFFFFFF
@A00155:342:HHGFNDSXY:1:1664:30364:34194 2:N:0:GAACCTAG+TCCGCATA
CCCCTCCGGTCTTGTCCAACCTCTGTTTCGGCAGAGGATACCGTGCTCCACAGCTGTAAGGCTGTGGG
CCCGAGAGAAGTCGCTCTCTCTCGCCCTTCACATCCCTCTCGGCATTGAGATGACCATAGGCCAGAG
+
FFFFFFFFFFFFFFFFFFFFFFFFFFFFFFFFFFFFFFFFFFFFFFFFFFFFFFFFFF:FFF,FFFFF
FFFFFFFFF,FFFFF,FFFFFFFFFFFFFFFFFFFFFFFFFFFFF:FFFFFFFFFFFF:FFFFFFFF
@A00155:342:HHGFNDSXY:1:1664:26323:36558 2:N:0:GAACCTAG+TCCGCATA
TAGGACGAAACTTACACAACTCGAAGAGTAACGACGGTTTCGTCCTTGGAACGGACTCATCAGGGAGC
CTACTAGACTCCGACCGTCATGGGGGTTCAAACCCATGCCCCGCCACACCGCCTTAGATCAGCG
+
FFFFFFFF:FFFFFFFFFFFFFFFFFFFFFFFFFFFFFFFFFFFFFFFFF,FFFFFFFFFFFFFFFFF
FFFF:FFFFFFFFFFFFFFF,FF:FFFFFFFFFFFFFFFFFFFFFFFFFFFFFFFFFFFFFFFF
@A00155:342:HHGFNDSXY:1:1665:18521:1313 2:N:0:GAACCTAG+TCCGCATA
CGGTCTTGTCCAACCTCTGTTTCGGCAGAGGATACCGTGCTCCACAGCTGTAAGGCTGTGGGCCCGAG
AGAAGTCGCTCTCTCTCGCCCTCCACATCCCTCTCGGCATTGAGATGACCATAGGCCAG
+
FFFFFF:FFFFFFFFFFFFFFFFFFFFF:FFF,FFFFFFFFFFFFFFFFFFFFFFFFFFFFFFFFFFF
FFFFFFFFFFFFFFFFFFFFFFFFFFFFFFFFFFFFFFFFFFFFFFFFFFFF,:FFFFF
@A00155:342:HHGFNDSXY:1:1665:22607:37043 2:N:0:GAACCTAG+TCCGCATA
CGGTAGGTCGTGAACAAGCCCGGAGTACATCTCCTTTGGAACCAGGAGACCCCTCCGGTCTTGTCCAA
CCTCTGTTTCGGCAGAGGATACCGTGCTCCACAGCTGTAAGGCTGTGGGCCCGAGAGAAGTCGCTCTC
+
FFF:FFFFFFFFFFFFFFFFFFFFFFFFFFFFFFFFFFFFFFFFFFFFFFFFFFFFFFFFFFFFFFFF
FFFFFFFFFFFF,FFFFFFFFFFFFFFFFFFFFFFFFFFFFFFFFFFFFFFFFFFFF:FFFFFFFFF
@A00155:342:HHGFNDSXY:1:1666:27914:4022 2:N:0:GAACCTAG+TCCGCATA
GAACTAGGAGACCCCTCCGGTCTTGTCCAACCTCTGTTTCGGCAGAGGATATCGTGCTCCACAGCTGT
AAGGCTGTGGGCCCGAGAGAAGTCGCTCTCTCTCGCCCTCCACATCCCTCTCGGCATTGAGATGACCA
+
FFFFFFFFFFFFFFFFFFFFFFFFFFFFFFFFFFFFFFFFFFFFFFFFFFFFFFFFFFFFFFFFFFFF
FFFFFFFFFFFFFFFFFFFFFFFFFFFFFFFFFFFFFFFFFFFFFFFFFFFFFFF:FFFFFFFFFFFF
@A00155:342:HHGFNDSXY:1:1666:6253:10441 2:N:0:GAACCTAG+TCCGCATA
ACTCTTCGAGTTGTTTAAGTTTCGTCCTAAAAAGGACTCGTCAGGTAGCCTACTAGACTACGACTTAC
ACGGTAGGTCGTGAACAAGCCCGGAGTACATCTCCTTTGGAACTAGGAGACCCCTCCGGTCTTGTC
+
FFF,FFFF,FFFFF:FFF:FFFFFFFF:FFFFFFFFFFFFFF,FFFFFFFFFFFFFFFFFFFFFFFFF
FFFFFFFFFFFFFFFFFFF,F,FF:,FFF:FFFFFFFFFFFFFFFFFFFFFFFFFFF:FFFFFFFF
@A00155:342:HHGFNDSXY:1:1666:17363:19038 2:N:0:GAACCTAG+TCCGCATA
CACGGTAGGTCGTGAACAAGCCCGGAGTACATCTCCTTTGGAACTAGGAGACCCCTCCGGTCTTGTCC
AACCTCTGTTTCGGCAGAGGATACCGTGCTCCACAGCTGTAAGGCTGTGGGCCCGAGAGAAGTCGCTC
+
FFFFFFFFFFFFFFFFFFFFFFFFFFFFFFFFFFFFFFFFFFFFFFFFFFFFFFFFFFFFFF::FFFF
FFFFFFFFFFFFFFFFFFFFFFFFFFFFFFFFFFFFFFFFFFFFFFFFFFFFFFFFFFFF:FFFFFFF
@A00155:342:HHGFNDSXY:1:1667:6126:1799 2:N:0:GAACCTAG+TCCGCATA
GGACTCGTCAGGTAGCCTACTAGACTACGACTTACACGGTAGGTCGTGAACAAGCCCGGAGTACATCT
CCTTTGGAACCAGGAGACCCCTCCGGTCTTGTCCAACCTCTGTTTCGGCAGAGGATACCGTGCTCCAC
+
FFFFFFFFFFFFFFFFFFFFFFFFF:FFFFFFFFFFFFFFFFFFFFFFFFFFFFFFFFFFFFFFFFFF
FFFFFFFFFFFFFFFFFFFFFFFFFFFFF:FFFFFFFFFFFFFFFFFFFFFFFFFFFFFFFFFFFFFF
@A00155:342:HHGFNDSXY:1:1667:3477:30467 2:N:0:GAACCTAG+TCCGCATA
ACTTACACGGTAGGTCGTGAACAAGCCCGGAGTACATCTCCTTTGGAACTAGGAGACCCCTCCGGTCT
TGTCCAACCTATGTTTCGGCAGAGGATACCGTGCTCCACAGCTGTAAGGCTGTGGGCCCGAGAGAAGT
+
```

FFFFFFFFFFFFFFFFFFFFFFFFFFFFFFFFFFFFFFFFFFFFFFFFFFFFFFFFFFFFFFFFFFFF
FFFFFFFFFFFFFFFFFFFFFFFFFFFFFFFFFFFFFFFFFFFFFF,FFFFFFFFFFFFFFFFFFFFF
@A00155:342:HHGFNDSXY:1:1668:26530:3004 2:N:0:GAACCTAG+TCCGCATA
CGTTACTCTTCGAGTTGTGTAAGTTTCGTCCTAAAAAGGACTCGTCAGGTAGCCTACTAGACTACGAC
TTACACGGTAGGTCGTGAACAAGCCCGGAGTACATCTCCTTTGGAACTAGGAGACCCCTCCGGTCTTG
+
FFFFFFFF:FFFFFFFFFFFFFFFFFFFFFFFFFFFFFFFFFFFFFFFFFFFFFFFFFFFFFFFFFF
FFFFFFFFFFFFFFFFFFFFFF:FFFFFFFFFFFFFFFFFFFFFFFFFFFFFFFFFFF:FFFFFFFFF
@A00155:342:HHGFNDSXY:1:1668:24632:16250 2:N:0:GAACCTAG+TCCGCATA
TACGACTTACACGGTAGGTCGTGAACAAGCCCGGAGTACATCTCCTTTGGAACTAGGAGACCCCTCCG
GTCTTGTCCAACCTCTGTTTCGGCAGAGGATACCGTGCTCCACAGCTGTAAGGCTGTGGGCCCG
+
FFFFFFFFFFFFFFFFFFFFFFFFFFFFFFFFFFFFFFF:FFFFFFFFFFFFFFFFFF:FFFFFFFFF
FFFFF:FFFFFFFFFFFFFFFFFFFFFFFFFFFFFFFFFFFFFF:FFFFFFF:FFFFFFFFFFF
@A00155:342:HHGFNDSXY:1:1668:18466:16752 2:N:0:GAACCTAG+TCCGCATA
GAACTAGGAGACCCCTCCGGTCTTGTCCAACCTCTGTTTCGGCAGAGGATACCGTGCTCCACAGCTGT
AAGGCTGTGGGCCCGAGAGAAGTCGCTCTCTCTCGCCCTCCACATCCCTCTCGGCATAGAGATGACCA
+
FFFFFFFFFFFFFFFFFFFFFFFFFFFFFFFFFFFFFFFFFFFFFFFFFFFFFFFFFFFFFFFFFFFF
FFFFFFFFFFFFFFFFF:FFFFFFFFFFFFFFFF:FFFFFFFFFFFFFFFFFFFFFFFFFFFFFFFFF
@A00155:342:HHGFNDSXY:1:1668:13584:18067 2:N:0:GAACCTAG+TCCGCATA
GAACTAGGAGACCCCTCCGGTCTTGTCCAACCTCTGTTTCGGCAGAGGATACCGTGCTCCACAGCTGT
AAGGCTGTGGGCCCGAGAGAAGTCGCTCTCTCTCGCCCTCCACATCCCTCTCGGCATTG
+
FFFFFFFFFFFFFFFFFFFFFFFFFFFFFFFFFFFFFFFFFFFFFFFFFFFFFFFFFFFFFFFFFFFF
FFFFFFFFFFFFFFFFFFFFFFFFFFFFFFFFFFFFFFFFFFFFFFFFFFFFFFFFFFF
@A00155:342:HHGFNDSXY:1:1668:13648:18082 2:N:0:GAACCTAG+TCCGCATA
GAACTAGGAGACCCCTCCGGTCTTGTCCAACCTCTGTTTCGGCAGAGGATACCGTGCTCCACAGCTGT
AAGGCTGTGGGCCCGAGAGAAGTCGCTCTCTCTCGCCCTCCACATCCCTCTCGGCATTG
+
FFFFFFFFFFFFFFFFFFFFFFFFFFFFFFFFFFFFFFFFFFFFFFFFFFFFFFFFFFFFFFFFFFFF
FFFFFFFFFFFFFFFFFFFFFFFFFFFFFFFFFFFFFFFFFFFFFFFFFFFFFFFFFFF
@A00155:342:HHGFNDSXY:1:1668:11758:19664 2:N:0:GAACCTAG+TCCGCATA
GAACTAGGAGACCCCTCCGGTCTTGTCCAACCTCTGTTTCGGCAGAGGATACCGTGCTCCACAGCTGT
AAGGCTGTGGGCCCGAGAGAAGTCGCTCTCTCTCGCCCTCCACATCCCTCTCGGCATTG
+
FFFFFFFFFFFFFFFFFFFFFFFFFFFFFFFFFFFFFFFFFFFFFFFFFFFFFFFFFFFFFF:FFFFF
FFFFFFFFFFFFFFFFFFFFFFFFFFFFFFFFFFFFFFFFFFFFFFFFFFFFFFFFFFF
@A00155:342:HHGFNDSXY:1:1668:3106:28197 2:N:0:GAACCTAG+TCCGCATA
AAGGACTCGTCAGGTAGCCTACTAGACTACGACTTACACGGTAGGTCGTGAACAAGCCCGGAGTACAT
CTCCTTTGGAACTAGGAGACCCCTCCGGTCTTGTCCAACCTCTGTTTCGGCAGAGGATACCGTGCTCC
+
FFFFFFFFFFFFFFFFFFFFFFFFFFFFFFFFFFFFFFFFFFFFFFFFFFFFFFF:FFFFFFFFFF:F
FFFFFFFFFF:FFFF:FFFFFFFFFFFFFFFFF:FFFFFFFFFFF:FFFFF:FF,FF,FFFFFFFFFF
@A00155:342:HHGFNDSXY:1:1669:29776:10723 2:N:0:GAACCTAG+TCCGCATA
GGACTCGTCAGGTAGCCTACTAGACTACGACTTACACGGTAGGTCGTGAACAAGCCCGGAGTACATCT
CCTTTGGAACCAGGAGACCCCTCCGGTCTTGTCCAACCTCTGTTTCGGCAGAGGATACCGTGCTCCAC
+
FFFFFFFFFFFFFFFFFFFFFFFFFFFFFFFFFFFFFFFFFFFFFFFFFFFFF,FFFFFFFFFFFFFF
FFFFFFFFFFFFFFF:FFFFFFFFFFFFFFFFFFFFFFFFFFFFFFFFFFFFFFFFFFFFFFFFFFFF
@A00155:342:HHGFNDSXY:1:1669:30960:21825 2:N:0:GAACCTAG+TCCGCATA
GGTAGCCTACTAGACTACGACTTACACGGTAGGTCGTGAACAAGCCCGGAGTACATCTCCTTTGGAAC
TAGGAGACCCCTCCGGTCTTGTCCAACCTCTGTTTCGGCAGAGGATACCGTGCTCCACAGCTGTAAGG
+

FFFFFFFFFFFFFFFFFFFFFFFFFFFFFFFFFFFFFFFFFFFFFFFFFFFFFFFFFFFFFFFFFFFF
FFFFFFFFFFFFFFFFFFFFFFFFFFFFFFFFFFFFFFFFFFFFFFFFFFFFFFFFFFFFFFFFFFFF
@A00155:342:HHGFNDSXY:1:1669:19714:22106 2:N:0:GAACCTAG+TCCGCATA
TACATCTCCTTTGGAACTAGGAGACCCCTCCGGTCTTGTCCAACCTCTGTTTCGGCAGAGGATACCGT
GCTCCACAGCTGTAAGGCTGTGGGCCCGAGAGAAGTCGCTCTCTCTCGCCCTCCACATCCCTCTCGGC
+
FFFFFFFFFFFFFFF,FFFFFFFFFFFFFFFFF,FFFFFFFFFFFFF:F,FFFFFFFFFFFFF:FFFF
FFFFFFFFFFFFFFFFFFFFFFFFFFFFFFFFFFFFFFFFFFFFFFFFFFFFFFFFFFFFFFF,FFFF
@A00155:342:HHGFNDSXY:1:1669:30165:24361 2:N:0:GAACCTAG+TCCGCATA
GGTAGCCTACTAGACTACGACTTACACGGTAGGTCGTGAACAAGCCCGGAGTACATCTCCTTTGGAAC
TAGGAGACCCCTCCGGTCTTGTCCAACCTCTGTTTCGGCAGAGGATACCGTGCTCCACAGCTGTAAGG
+
FFFFFFFFFFFFFFFF:FFFFFFFFFFFFFFFFFFFFFFFFFFFFFFFFFFFFFFFFFFFFFFFFFFF
FFFFFFFFFFFFFFFFFFFFFFFFFFFFFFFFFFFF:FFFFFF:FFFFFFFF:FFFFFFFFFFFFFFF
@A00155:342:HHGFNDSXY:1:1669:27733:29888 2:N:0:GAACCTAG+TCCGCATA
TGAACAAGCCCGGAGTACATCTCCTTTGGAACTAGGAGACCCCTCCGGTCTTGTCCAACCTCTGTTTC
GGCAGAGGATACCGTGCTCCACAGCTGTAAGGCTGTGGGCCCGAGAGAAGTCGCTCTCTCTCGCCCTC
+
FF:FFFF,FFF,FFFFFFFFFFF:F,FFFFF,,F:FF:FF:FFFFFF:FFFFFFFFF:FFFFF:FF::
F,FF:FF:FFFF,FFFF:FFF:FFF:FFFF:FFFFFFF:FFFFFFFFFFF:FFFF:FFFFFF:FFFFF
@A00155:342:HHGFNDSXY:1:1670:28546:23249 2:N:0:GAACCTAG+TCCGCATA
GTGTAAGTTTCGTCCTAAAAAGGACTCGTCAGGTAGCCTACTAGACTACGACTTACACGGTAGGTCGT
GAACAAGCCCGGAGTACATCTCCTTTGGAACTAGGAGACCCCTCCGGTCTTGTCCAACCTCTGTTTCG
+
FFFFFFFFFF:FFFFFFFFFFFFFFFFFFFFFFFFFFFFFFFFFFFFFFFFFFFFFFFFFFFFFFFFF
FFFFFFFFFFFFFFF:FFFFF:FFFFFFFFFFFFFFFFFFFFFFFFFFFFFFFFFFFFFFFFFFFFFF
@A00155:342:HHGFNDSXY:1:1670:12373:24893 2:N:0:GAACCTAG+TCCGCATA
TACGACTTACACGGTAGGTCGTGAACAAGCCCGGAGTACATCTCCTTTGGAACTAGGAGACCCCTCCG
GTCTTGTCCAACCTCTGTTTCGGCAGAGGATACCGTGCTCCACAGCTGTAAGGCTGTGGGCCCG
+
FFFFFFFFFFFFFFFFFFFFFFFFFFFFFFFFFFFFFFFFFFFFFFFFFFFFFFFFFFFFFFFFFFFF
FFFFFFFFFFFFFFFFFFFFFFFFFFFFFFFFFFFFFFFFFFFFFFFFFFFFFFFFFFFFFFFF
@A00155:342:HHGFNDSXY:1:1671:9878:2973 2:N:0:GAACCTAG+TCCGCATA
ACTTACACGGTAGGTCGTGAACAAGCCCGGAGTACATCTCCTTTGGAACTAGGAGACCCCTCCGGTCT
TGTCCAACCTCTGTTTCGGCAGAGGATACCGTGCTCCACAGCTGTAAGGCTGTGGGCCCGAGAGAAGT
+
FFFFFFFFFFFFFFFFFFFFFFFFFFFFFFFFFFFFFFFFFFFFFFFFFFFFFFFFFFFFFFFFFFFF
FFFFFF:FFFFFFFFFFFFFFFFFFFFFFFFFFFFFFFFFFFFFFFFFFFFFFFFFFFFFFFFFFFFF
@A00155:342:HHGFNDSXY:1:1671:3549:5165 2:N:0:GAACCTAG+TCCGCATA
CGTCCTAAAAAGGACTCGTCAGGTAGCCTACTAGACTACGACTTACACGGTAGGTCGTGAACAAGCCC
GGAGTACATCTCCTTTGGAACTAGGAGACCCCTCCGGTCTTGTCCAACCTCTGTTTCGGCAGAGGATA
+
FFFFFFFFFFFF:FFFFF:FFF,FFFFFF::FFFFFFFFFFFFFFFFFFFF:FFFFFFFFFFFFFFF:
F:FFFFFFF:F:FFFFFFF:FFFFF:FFFF,FF:FFF:FF,,FFFFFFFFF:FFFFFFFFFFFFFFFF
@A00155:342:HHGFNDSXY:1:1671:3712:5916 2:N:0:GAACCTAG+TCCGCATA
CGTCCTAAAAAGGACTCGTCAGGTAGCCTACTAGACTACGACTTACACGGTAGGTCGTGAACAAGCCC
GGAGTACATCTCCTTTGGAACTAGGAGACCCCTCCGGTCTTGTCCAACCTCTGTTTCGGCAGAGGAT
+
:FFFFFFFFFFFFFFFFFFFFF,FFFFFF,:FFFFFFFFFFFFFFFFFFFFFFFFFFFFFF:F:,FFF
FFFFFFFFFFFFFFF:::FFFFFFFFFFFFFFFFFFFFFF,F:FFFFFFFFFFF:FFFFFFFFFFFF
@A00155:342:HHGFNDSXY:1:1671:7482:13448 2:N:0:GAACCTAG+TCCGCATA
GGACTCGTCAGGTAGCCTACTAGACTACGACTTACACGGTAGGTCGTGAACAAGCCCGGAGTACATCT
CCTTTGGAACTAGGAGACCCCTCCGGTCTTGTCCAACCTCTGTTTCGGCAGAGGATACCGTGCTCCAC
+

FFFFFFFFFFFFFFFFFFFFFFFFFFFFFFFFFFFFFFFFFFFFFFFFFFFF:,FFFFFFFFFFFFFF
FFFFFFFFFFFFFFFFFFFFFFFFFFFFFFFFFFFFFFFFFFFFFFFFFFFFFFFFFFFFFFFFFFFF
@A00155:342:HHGFNDSXY:1:1671:22489:17707 2:N:0:GAACCTAG+TCCGCATA
CCGGTCTTGTCCAACCTCTGTTTCGGCAGAGGATACCGTGCTCCACAGCTGTAAGGCTGTGGGCCCGA
GAGAAGTCGCTCTCTCTCGCCCTCCACATCCCTCTCGGCATTGAGATGACCATAGGCCGGAG
+
FFFFFFFFFFFFFFFFFFFFFFFFFFFFFFFFFFFFFFFFFFFFF:FFFFFFFFFFFFFFFFFFFFFF
FFFF:FFFFFFFFFFFFFFFFFFFFFFFFFFFFFFFFFFFFFFFFFFFFFFFF:FFFFFFFF
@A00155:342:HHGFNDSXY:1:1671:15383:20932 2:N:0:GAACCTAG+TCCGCATA
TACATCTCCTTTGGAACTAGGAGACCCCTCCGGTCTTGTCCAACCTCTGTTTCGGCAGAGGATACCGT
GCTCCACAGCTGTAAGGCTGTGGGCCCGAGAGAAGTCGCTCTCTCTCGCCCTCCACATCCCTCTCGGC
+
FFFFFFFFF:FFFFF::FFFFFFFFFFFFFFFFFF,FFFFFFFFFFFFFFFFFFFFFFFFFFFFFFFF
FFFFFFFFFF:F,FFFFFFFFFFFFFFFFFFFFFFFFFFFFFFFFFFFFFFFFFFFFFF:FFFFFFFF
@A00155:342:HHGFNDSXY:1:1671:14850:30874 2:N:0:GAACCTAG+TCCGCATA
TACCGTGCTCCACAGCTGTAAGGCTGTGGGCCCGAGAGAAGTCGCTCTCTCTCGCCCTCCACATCCCT
CTCGGCATTGAGATGACCATAGGCCAGAGGTGAGTCCTTAAGTGGACACAGCTGATCTAAGGCGGG
+
FFFFFFFFFFFFFFFFFFFFFFFFFFFFFFFFFFFFFFFF:F,FFFFFFFFFFFFFFFFF:FFFFFFF
FFFFFFF,,FFFFFFFF:FFFFFFFFFFFFFFFFFF:FF:FFF:FFF,FF,FF:FFFFFFFFFFFF
@A00155:342:HHGFNDSXY:1:1671:17002:33724 2:N:0:GAACCTAG+TCCGCATA
GGAGTACATCTCCTTTGGAACTAGGAGACCCCTCCGGTCTTGTCCAACCTCTGTTACGGCAGAGGATA
CCGTGCTCCACAGCTGTAAGGCTGTGGGCCCGAGAGAAGTCGCTCTCTCTCGCCCTCCACATCCCTCT
+
FFFFFFFFFFFFFFFFFFFFF:FFFFFFFFFFFFFFFFFFFFFFFFFFFFFFFFFFF:FFFFFFFFFF
FFFFFFFFFFFFFFFFFFFFFFFFFFFFFFFFFFFFFFFFFFFFFFFFFFF:FFFFFFFFFFFFFFFF
@A00155:342:HHGFNDSXY:1:1671:3115:35352 2:N:0:GAACCTAG+TCCGCATA
AGCCCGGAGTACATCTCCTTTGGAACTAGGAGACCCCTCCGGTCTTGTCCAACCTCTGTTTCGGCAGA
GGATACCGTGCTCCACAGCTGTAAGGCTGTGGGCCCGCGAGAAGTCGCTCTCTCTCGCCC
+
FFFFFFFFFFFFFFFFFFFFFFF:FF:FFFFFFFFFFFFFFFFFF,FFFFFFFFFFFFFFFFFFFFF:
FFFFFFFFFFFFFFFFFFFFFFFFFFFFFFFFFFFFF,FFFFFF:FFFFFFFFFFFFF:F
@A00155:342:HHGFNDSXY:1:1672:14498:13322 2:N:0:GAACCTAG+TCCGCATA
ACTCTTCGAGTTGTTTAAGTTTCGTCCTAAAAAGGACTCGTCAGGTAGCCTACTAGACTACGACTTAC
ACGGTAGGTCGTGAACAAGCCCGGAGTACATCTCCTTTGGAACTAGGAGACCCCTCCGGTCTTGTCCA
+
FFFF:FFFFF:FFFFFFFFF:FFFFFFFFFFFFFFFFFFFFFFFFFFFFFFFFFF:FFFFFFFFFFFF
FFFFFFFFFFFFFFFFFFFFFFFFFFFFF:FFFFFFFFFFFFFFFFFFFFFFFFFFFFFFF,FFFFFF
@A00155:342:HHGFNDSXY:1:1672:20148:14465 2:N:0:GAACCTAG+TCCGCATA
CGGCAGAGGATACCGTGCTCCACAGCTGTAAGGCTGTGGGCCCGAGAGAAGTCGCTCTCTCTCGCCCT
CTACATCCCTCTCGGCATTGAGATGACCATAGGCCAGAGGTGAGTCCTTAAGTGGACACAGCTGATCT
+
FFFFFFFFFFFFFFFFFFFFFFFFFF:FFFFFFF:FFFFFFFFFFFFFFFFFFFFFFFFFFFF:FFFF
FFFFFFFFFFFFFFFFF:FFFFFFFFFFFFFFF:FFFFFFFFFFFFFFFFFFFFFFFFFFFFFFFFFF
@A00155:342:HHGFNDSXY:1:1672:31204:18302 2:N:0:GAACCTAG+TCCGCATA
GGACTCGTCAGGTAGCCTACTAGACTACGACTTACACGGTAGGTCGTGAACAAGCCCGGAGTACATCT
CCTTTGGAACCAGGAGACCCCTCCGGTCTTGTCCAACCTCTGTTTCGGCAGAGGATACCGTGCTCCAC
+
FFFFFFFFFFFFFFFFFF:FFFFFFFFFFFFFFFFFFFFFFFFFFFFFFFFF:FFFFFFFFFFFFFFF
FFFFFFFFFFFFFFFFFFFFFFFFFFFFFFFFFFFFFFFFFFFFFFFFFFFFFFFFFFFFFFFFFFFF
@A00155:342:HHGFNDSXY:1:1672:29704:20275 2:N:0:GAACCTAG+TCCGCATA
TCCAAGGACGAAACCGTCGTTACTCTTCGAGTTGTGTAAGTTTCGTCCTAAAAAGGACTCGTCAGGTA
GCCTACTAGACTACGACTTACACGGTAGGTCGTGAACAAGCCCGGAGTACATCTCCTTTGGAACTA
+

```
FFFFFFFFFFFFFFFFFFFFF:FFFFFFFFFFFFFFFFFFFF:FFFFFFFFFFFFFFFFFFF,FFFFFF
FFF,FFFFFFFFFFFFFFFFFFFFFFFFFFFFFFFFF:FFF,F:FFFFFFFFFFF:FFFFFFFFFFFF
@A00155:342:HHGFNDSXY:1:1672:25229:32878 2:N:0:GAACCTAG+TCCGCATA
GAGTAACGACGGTTTCGTCCTTGGAACGGACTCATCAGGAAGCCTACTAGACTCCGACCGTCATGGGG
GTTCAAACCCATGCCCCGCCACACCGCCTTAGATCAGCTGTGTCCACTTAAGGACTCACCTCTGGCCT
+
FFFFFFFFFFFFFFFFFFFFFFFFFFFFFFFFF:FFFFFFFFFFFFFFFFFFFFFFFFFFFFFFFFFF
FFFFFF:FFFFFFFFFFFFFFFFFFFFFF,FFFF,FFFFFFFFFFFFFFFFFFFFFFFFFFFFFFFFF
@A00155:342:HHGFNDSXY:1:1672:2971:33254 2:N:0:GAACCTAG+TCCGCATA
TTTCGTCCTTGGAACGGACTCATCAGGAAGCCTACTAGACTCCGACCGTCATGGGGGTTCAAACCCAT
GCCCCGCCACACCGCCTTAGATCAGCTGTGTCCACTTAAGGACTCACCTCTGGCCTATGGTCATCT
+
FFFFFFFFFFFFFFFFFFFFFFFFFFFFFF:FFFFFFFFFFFFFFFFFFFFFF:FFFFFFFFFFFFFFF:
FFFFFFFFFFFFFFFFFFFFF:FFFFFFF,:FF,FFFFFFFFFFFFFFFFFFFFFFFFFFFFFFFFFF
@A00155:342:HHGFNDSXY:1:1672:9390:35790 2:N:0:GAACCTAG+TCCGCATA
CTACGACTTACACGGTAGGTCGTGAACAAGCCCGGAGTACATCTCCTTTGGAACTAGGAGACCCCTCC
GGTCTTGTCCAACCTCTGTTTCGGCAGAGGATACCGTGCTCCACAGCTGTAAGGCTGTGGGCCCGAGA
+
FFFFFFFFFFFFFFFFFFFFFFFFFFFFFFFFFFFFFFFFFFFFFFFFFFFFFFFFFFFFFFFFFFFF
FFFFFFFFFFFFFFFFFFFFFFFFFFFFFFFFFFFFFFFFFFFFFFFFFFFFFFFFFFFFFFFFFFFF
@A00155:342:HHGFNDSXY:1:1673:9326:4460 2:N:0:GAACCTAG+TCCGCATA
AAGCCCGGAGTACATCTCCTTTGGAACTAGGAGACCCCTCCGGTCTTGTCCAACCTCTGTTTCGGCAG
AGGATACCGTGCTCCACAGCTGTAAGGCTGTGGGCCCGAGAGAAGTCGCTCTCTCTCGCCCTCTACAT
+
FFFFFFF:FF:FFFFFF:::FFFFF,F:FFFFF:FFFFFFFFFF:FFFFFFFFFFFFFFF:FFFFF:F
FFFFFFFFFFFFFFF,FFFFFFF,,FFFFFFFFF:FFFFFFF,,FFFFF,FFFFFFFFF:FFFFFFFF
@A00155:342:HHGFNDSXY:1:1673:18710:4930 2:N:0:GAACCTAG+TCCGCATA
GAACAAGCCCGGAGTACATCTCCTTTGGAACTAGGAGACCCCTCCGGTCTTGTCCAACCTCTGTTTCG
GCAGAGGATACCGTGCTCCACAGCTGTAAGGCTGTGGGCCCGAGAGAAGTCGCTCTCTCTCGCCCTCC
+
F:FFFFFFFFFFFFFFFFFFFFFFFFFFFFFFFFFFFFFFFFFFFFFFFFF:FFFFFFFFFFFFFFFFF
FFFFFFFFFFFFFFFFFFFFFFFFFFFFFFFFFFFFFFFFFFFFFFFFFFFFFFFFFFFFFFFFFFFF
@A00155:342:HHGFNDSXY:1:1673:32524:4993 2:N:0:GAACCTAG+TCCGCATA
CAAGGACGAAACCGTCGTTACTCTTTGAGTTGTGTAAGTTTCGTCCTAAAAAGGACTCGTCAGGTAGC
CTACTAGACTACGACTTACACGGTAGGTCGTGAACAAGCCCGGAGTACATCTCCTTTGGAACTAGGAG
+
FFFFF,FFFFFFFFF::FFFF,FF,FFFFFFFFFFFFF:::FFF,:FFF,:FFFFFFFFFFFFF::FF
FFFFF,FFFFFFFFF:FFFFFFFFFFFFFFFFFFFFFFFFFF,FF::FF:FF:FFFFFFFF,F,FFFFF
@A00155:342:HHGFNDSXY:1:1673:25238:6370 2:N:0:GAACCTAG+TCCGCATA
GAACTCGTCAGGTAGCCTACTAGACTACGACTTACACGGTAGGTCGTGAACAAGCCCGGAGTACATCT
CCTTTGGAACTAGGAGACCCCTCCGGTCTTGTCCAACCTCTGTTTCGGCAGAGGATACCGTGCTCCAC
+
FFFFFFFFFFFF,FFFFFFFFFFFFFF:FFFFFFFFFFFFFFFFFFFFFFFFFF:FFFFFFFFFFFFF:
FFFFFFFF:FFFFFFFFFFFFFFFFFFFF:FFFFFFFFFFFFFFFFF:FFFFFFFFFFFF:FFFFFFF
@A00155:342:HHGFNDSXY:1:1673:7048:31391 2:N:0:GAACCTAG+TCCGCATA
GTTGTGTAAGTTTCGTCCTAAAAAGGACTCGTCAGGTAGCCTACTAGACTACGACTTACACGGTAGGT
CGTGAACAAGCCCGGAGTACATCTCCTTTGGAACTAGGAGACCCCTCCGGTCTTGTCCAACCTCTGTT
+
FF:FFFFFFFFFFFFFFFFFFFFFFFFFFFFFFFFFFFFFFFFF,FF:FFFFF:FFFFF:FFFFF:FFF
FFFFFFFFFFFFFFFFFFFFFF:FFFFF:FF:FFFFFFFFFFFF,FF:FFF:FF:FF:FFFFFFFFFFF
@A00155:342:HHGFNDSXY:1:1674:7455:5635 2:N:0:GAACCTAG+TCCGCATA
GACGAAACCGTCGTTACTCTACGAGTTGTGTAAGTTTCGTCCTAAAAAGGACTCGTCAGGTAGCCTAC
TAGACTACGACTTACACGGTAGGTCGTGAACAAGCCCGGAGTACATCTCCTTTGGAACTAGGAGACCC
+
```

```
F:FFFFFFFFFFFFFFFFFFFFFFF,FFFFFFFFF,FFFFFFFFFFF:FFFFFFFFFF,FFFFFFFFFF
F:FFFFFFFFF:FFFFFFFF,FFFFFFFFFFFF:FF:FF:FF:FFFFFFFF,:FFF:FFFFFFFFFFF
@A00155:342:HHGFNDSXY:1:1674:25545:8375 2:N:0:GAACCTAG+TCCGCATA
AGGACGAAACCGTCGTTACTCTTCGAGTTGTGTAAGTTTCGTCCTAAAAAGGACTCGTCAGGTAGACT
ACTAGACTACGACTTACACGGTAGGTCGTGAACAAGCCCGGAGTACATCTCCTTTGGAACTAGGAGAC
+
FFFFFFFFFFFFFFFFFFFFFFFFFFFFFFFFFFFFFFFFFFFFFFFFFFFFFFFFFFF:FFFFFFFF
FFF:FFFFFFFFFFFFFFFFFFFFFFFFFFFFFFFFFFFFFFFFFFFFFFFFF:FFFFFFFFFFFFFF
@A00155:342:HHGFNDSXY:1:1674:29098:10488 2:N:0:GAACCTAG+TCCGCATA
TAGGACGAAACTTACACAACTCGAAGAGTAACGACGGTTTCGTCCTTGGAACGGACTCATCAGGGAGC
CTACTAGACTCCGACCGTCATGGGGGTTCAAACCCATGCCCCGCTACACCGCCTTAGATCAGCTGTGT
+
FFFFFFFFFFFFFFFFFFFFFFFFFFFFFFFFFFFFFFFFFFFFFFFFFFFFFFFFFFFFFFFFFFFF
FFFFFFFFFFFFFFFFFFFFFFFFFFFFFFFFFFFFFFFFFFFFFFFFFF:FFFFFFFFFFFFF:FFFF
@A00155:342:HHGFNDSXY:1:1674:23990:26099 2:N:0:GAACCTAG+TCCGCATA
ACCCCTCCGGTCTTGTCCAACCTCTGTTTCGGCAGAGGATACCGTGCTCCACAGCTGTAAGGCTGTGG
GCCCGAGAGAAGTCGCTCTCTCTCGCCCTCTACATCCCTCTCGGCATTGAGATGACCAT
+
FFFFFFFFFFFFF,FFFFFFFFFFFFFFFFFFFFFFFFFFFFFFFFFFFFFFFFFFFFFFFFFFF,FF
FFFFFFFFFFFFFFFFFFFFFFFFFFFFFFF:FFFFFFFFFFFFFFF,FFFFFFFF:FFF
@A00155:342:HHGFNDSXY:1:1674:25084:26490 2:N:0:GAACCTAG+TCCGCATA
TACGACTTACACGGTAGGTCGTGAACAAGCCCGGAGTACATCTCCTTTGGAACTAGGAGACCCCTCCG
GTCTTGTCCAACCTCTGTTTCGGCAGAGGATACCGTGCTCCACAGCTGTAAGGCTGTGGGCCCG
+
FFFFFFFFFFFFFFFFFFFFFFFFFFFFFFFFFFFFFFFFFFFFFFFFFFFF:FFFFFFFFFFFFFFF
FFF,FFFFFF:FFFFFFFFFFFFFFFFFFFF:FFFFFFFFFFFFFFF:FFFFFFFFFFFFFFFFF
@A00155:342:HHGFNDSXY:1:1674:15691:26663 2:N:0:GAACCTAG+TCCGCATA
TACGACTTACACGGTAGGTCGTGAACAAGCCCGGAGTACATCTCCTTTGGAACTAGGAGACCCCTCCG
GTCTTGTCCAACCTCTGTTTCGGCAGAGGATACCGTGCTCCACAGCTGTAAGGCTGTGGGCCCG
+
FFFFFFFFFFFFFFFFFFFFFFFFFFFFFFFFFFFFFFFFFFFFFFFFFFFFFFFFFFFFFFFFFFFF
FFFFFFFFFFFFFFFFFFFFFFFFFFFFFFFFFFFFFFFFFFFFFFFFFFFFFFFFFFFFFFFF
@A00155:342:HHGFNDSXY:1:1674:22652:29763 2:N:0:GAACCTAG+TCCGCATA
CCCCTCCGGTCTTGTCCAACCTCTGTTTCGGCAGAGGATACCGTGCTCCACAGCTGTAAGGCTGTGGG
CCCGAGAGAAGTCGCTCTCTCTCGCCCTTCACATCCCTCTCGGCATTGAGATGACCATAGGCCAG
+
F:FFFFFFFFFFFFF:FFFFFFFFFFFFFF:FFFFFFF::FFFFFFFFFFFFF::FFFFFFFFFFFF:
FFFFFFFFFFFFFFFFFFFFFFFFFFFFFFFFFFFFFFFFFFFFF:FFFFFFFFFFF,:FFFFFFF
@A00155:342:HHGFNDSXY:1:1674:7600:31219 2:N:0:GAACCTAG+TCCGCATA
GGAGTACATCTCCTTTGGAACTAGGAGACCCCTCCGGTCTTGTCCAACCTCTGTTTCGGCAGAGGATA
CCGTGCTCCACAGCTGTAAGGCTGTGGGCCCGAGAGAAGTCGCTCTCTCTCGCCCTCCACATCCCTCT
+
FFFFFFFFFFFFFFFFFFFFFFFFFFFFFFFFFFFFFFFFFFFFFFFFFFFFFFFFFFFFFFFFFFFF
FFFFFFFFFFFFFFFFFFFFFFFFFFFFFFFFFFFFFFFFFFFFFFFFFFFFFFFFFFFFFFF:FFFFF
@A00155:342:HHGFNDSXY:1:1674:4806:36683 2:N:0:GAACCTAG+TCCGCATA
ACCCCTCCGGTCTTGTCCAACCTCTGTTTCGGCAGAGGATACCGTGCTCCACAGCTGTAAGGCTGTGG
GCCCGAGAGAAGTCGCTCTCTCTCGCCCTCCACATCCCTCTCGGCATTGAGATGACCATAGGCCAGAG
+
FF:FFFFF:FFFF:FFFFFFFFFFFFFFFFFFFFFFFFFF:FFFFFFFFFFFFFFFFFFFFFFFFFF:FF
F:FFFFFF:F:FF:FFFFFFF,FFFFFFFFFFF:FFFFFFFFFFF,FFFFFFFFFFFFFFF,FFFFFF
@A00155:342:HHGFNDSXY:1:1675:28863:17942 2:N:0:GAACCTAG+TCCGCATA
ACTTACACGGTAGGTCGTGAACAAGCCCGGAGTACATCTCCTTTGGAACTAGGAGACCCCTCCGGTCT
TGTCCAACCTCTGTTTCGGCAGAGGATACCGTGCTCCACAGCTGTAAGGCTGTGGGCCCGAGAGAAGT
+
```

```
FFFFFFFFFFFFFFFF:FFFFFFFFFFFFFFFFFFFFFFFFF:FFFFFFFFFFFFFFFFFFFFFFFFF,
FFFFFFFFFFFFFFFFFFFFFFFFFFFFFFFFFFFFFFFFFFFFFFF:FFFFFFFFFFFFFFFFFFFF
@A00155:342:HHGFNDSXY:1:1675:12608:29716 2:N:0:GAACCTAG+TCCGCATA
GTAGGTCGTGAACAAGCCCGGAGTACATCTCCTTTGGAACTAGGAGACCCCTCCGGTCTTGTCCAACC
TCTGTTTCGGCAGAGGATACCGTGCTCCACAGCTGTAAGGCTGTGGGCCCGAGAGAAGTCGCTCTCTC
+
FFFFFFFFFFFFFFFFFFFFFFFFFFFFFFFFFFFFFFFFF:FFFFFFFFFFFFFFFFFFFFFFFFFF
FFFFFFFFFFFFFFFFFFFFFFFFFFFFFFFFFFFFFFFFFFFFFFFFFFFFFFF,,FFFFFFFFFFF
@A00155:342:HHGFNDSXY:1:1675:3558:35274 2:N:0:GAACCTAG+TCCGCATA
CTCTTCGAGTTGTGTAAGTTTCGTCCTAAAAAGGACTCGTCAGGTAGCCTACTAGACTACGACTTACA
CGGTAGGTCGTGAACAAGCCCGGAGTACATCTCCTTTGGAACTAGGAGACCCCTCCGGTCTTGTCCAA
+
FFFFFFFFF:FFFFFFFFFFFFFFFFFFFFFFFFFFFFFFFFFFFFF:FFFFFFFFFFFFFFFFFFFF
FFFFFFFFFFFFFFFFF:F:FFFFFF:FFFFFFFFFFFFF:FFFFFFFFFFFFFFFFFF:FFFFF:F:
@A00155:342:HHGFNDSXY:1:1675:8865:36605 2:N:0:GAACCTAG+TCCGCATA
ACATCTCCTTTGGAACTAGGAGACCCCTCCGGTCTTGTCCAACCTCTGTTTCGGCAGAGGATACCGTG
CTCCACAGCTGTAAGGCTGTGGGCCCGAGAGAAGTCGCTCTCTCTCGCCCTCCACATCCCTCTCGGCA
+
FFFFFFFFF:FFFF:FFFFFFFFFFFFFFF:FFF,FFFFFFFFFFFFFFFFFFFF:FFFFFFFFFFFF
FFFFFFFFF:FFFFF:FFFFFFFFFFFFFFFF:F:FFFFFFFFFFFFFFFFFFFFFFFFFFFFFFFFF
@A00155:342:HHGFNDSXY:1:1676:26549:6386 2:N:0:GAACCTAG+TCCGCATA
ATCTCCTTTGGAACCAGGAGACCCCTCCGGTCTTGTCCAACCTCTGTTTCGGCAGAGGATACCGTGCT
CCACAGCTGTAAGGCTGTGGGCCCGAGAGAAGTCGCTCTCTCTCGCCCTCCACATCCCTCTCGGCATT
+
FFFFFFFFFFFFFFFFFFFFFFFFFFFFFFFFFFFFFFFFFFFFFFFFF:FFFFFFFFFFFFFFFFFF
FFFFFFFFFFFFFFFFFFFFFFFFFFFFF,FFFFFFFFFFFFFFFFFFFFFFFFFFFFFFFFFFFFF::
@A00155:342:HHGFNDSXY:1:1676:7075:23484 2:N:0:GAACCTAG+TCCGCATA
AACTAGGAGACCCCTCCGGTCTTGTCCAACCTCTGTTTCGGCAGAGGATACCGTGCTCCACAGCTGTA
AGGCTGTGGGCCCGAGAGAAGTCGCTCTCTCTCGCCCTCCACATCCCTCTCGGCATTGAG
+
FFFFFFFFFFFFFFFFFFFFFFFFFFFFFFFFFFFFFFFFFFFFFFFFFFFFFFFFFFFFF,FFFFFF
FFFFFFFFFFFFFFFFFFFFFFFFFFFFFFFFFFFFFFFFFFFFFFFFFFFFFFFF,FFFF
@A00155:342:HHGFNDSXY:1:1677:2781:4899 2:N:0:GAACCTAG+TCCGCATA
CTACTAGACTACGACTTACACGGTAGGTCGTGAACAAGCCCGGAGTACATCTCCTTTGGAACTAGGAG
ACCCCTCCGGTCTTGTCCAACCTCTGTTTCGGCAGAGGATACCGTGCTCCACAGCTGTAAGGCTGTGG
+
:FFFFFFF:FFFFFFFFFFFF,:FFFFFFFFFFFFFF,FFF:FFFFFFFFFFFFFFFFFFFF,FF:F:,
FFFFFFFFFFFF:FFFFFFFFFF:FFFFFFFFFFFFFF:FFFFFFFFFFF::F:F,FFFFFFFFFFFF
@A00155:342:HHGFNDSXY:1:1677:16315:23422 2:N:0:GAACCTAG+TCCGCATA
TTAGGACGAAACTTACACAACTCGAAGAGTAACGACGGTTTCGTCCTTGGAACGGACTCATCAGGGAG
CCTACTAGACTCCGACCGTCATGGGGGTTCAAACCCATGCCCCGCCACACCGCCTTAGATCAGCTGTG
+
FFFFFFFFFFFFFFFFFFFFFFFFFFFFFFFFFFFFFFFFFFFFFFFFFFFFFFFFFFFFFFFFFFFF
FFFFFFF:FFFFFFFFFFFFFFFFFFFFFFFFFFFFFFFFFFFFFFFFFFFFFFFFFFFFFFFFFFFF
@A00155:342:HHGFNDSXY:1:1678:16206:13088 2:N:0:GAACCTAG+TCCGCATA
AAAAGGACTCGTCAGGTAGCCTACTAGACTACGACTTACACGGTAGGTCGTGAACAAGCCCGGAGTAC
ATCTCCTTTGGAACCAGGAGACCCCTCCGGTCTTGTCCAACCTCTGTTTCGGCAGAGGATACCGTGCT
+
FFFFFFFFFFFFFFFFFFFFFFFFFFFFFFFFFF,FFFFFFFFFFFFFFFFFFFFFFFF:FFFFF:FFFF
FFFFFFFFFFFFFFFFFFFFFFFFFFFFFFFFFFFFFFFFFFFFFFFFFFFFFFFFFFFFFFFFFFFF
@A00155:342:HHGFNDSXY:1:1678:25464:14904 2:N:0:GAACCTAG+TCCGCATA
AGCCCGGAGTACATCTCCTTTGGAACTAGGAGACCCCTCCGGTCTTGTCCAACCTCTGTTTCGGCAGA
GGATACCGTGCTCCACAGCTGTAAGGCTGTGGGCCCGAGAGAAGTCGCTCTCTCTCGCCC
+
```

```
FFFFFFFFFFFFFFFFFFFFFFFFFFFFFFFFFFFFFFFFFFFFFFFFFFFFFFFFFF:FFFFFFFF:
FFFFFFFFFFFFFFFFFFFFFFFFFFFFF:FFFFFFFFFFFFFFFFFFFFFFFFFFFFFF
@A00155:342:HHGFNDSXY:1:1678:27281:24565 2:N:0:GAACCTAG+TCCGCATA
TGGGTCGTGAACAAGCCCGGAGTACATCTCCTTTGGAACTAGGAGACCCCTCCGGTCTTGTCCAACCT
CTGTTTCGGCAGAGGATACCGTGCTCCACAGCTGTAAGGCTGTGGGCCCGAGAGAAGTCGCTCTCTCT
+
FFFF:FFFFFFFFFFFFFFFFFFFFFFFFFFFFFFFFFFFFFFFFFFFFFFFFFFFFF,FFFFFFFFF
FFFFFFFFFFFFFFFFFFFFFFFFFFFFFFFFFFFFFFFFFFFFFFFFFFFFFFFFFF:FFFFFFFFF
@A00155:342:HHGFNDSXY:1:2101:28465:29152 2:N:0:GAACCTAG+TCCGCATA
TAGGTCGTGAACAAGCCCGGAGTACATCTCCTTTGGAACTTGGAGACCCCTCCGGTCTTGTCCAACCT
CTGTTTCGGCAGAGGATACCGTGCTCCACAGCTGTAAGGCTGTGGGCCCGAGAGAAGTCGCTCTCTCT
+
FFFF:FFFFFFFFFFFFFFFFFFFFFFFFFFFFFFFFFFFFFFFFFFFFFFFFFFFFFFFFFFFFFFF
FFFFFFFFFFFFFFFFFFFFFFFFFFFFFFFFFFFFFFFFFFFFFFFFFFFFFFFFFFFFFFFFFFFF
@A00155:342:HHGFNDSXY:1:2101:7256:29684 2:N:0:GAACCTAG+TCCGCATA
CGTTACTCTTCGAGTTGTGTAAGTTTCGTCCTAAAAAGGACTCGTCAGGTAGCCTACTAGACTACGAC
TTACACGGTAGGTCGTGAACAAGCCCGGAGTACATCTCCTTTGGAACTAGGAGACCCCTCCGGTCTTG
+
FFFFFF:,FFFFFF,FF:,FF,FF:FFFF:FF,FFFFFFFFFFF:FFFFFF,FF,F:F:,FFFFFFFF
FFF,FFFFFFFFFFFFFFFFFFFFFFFFFFFFF:FF,F::,FFFFFFFFFFF:FFFFF::FFFFFFF:
@A00155:342:HHGFNDSXY:1:2101:32172:36448 2:N:0:GAACCTAG+TCCGCATA
ATGCCGAGAGGGATGTAGAGGGCGAGAGAGAGCGACTTCTCTCGGGCCCACAGCCTTACAGCTGTGGA
GCACGGTATCCTCTGCCGAAACAGAGGTTGGACAAGACCGGAGGGGTCTCCTAGTTCCAAAGGAGATG
+
FFFFFFFFFFFFFFFFFFFFFFFFFFFFFFFFFFFFFFFFFFFFFFFFFFFFFFFFFFFFFFFFFFFF
FFFFFFFFFFFFFFFFFFFFFFFFFFFFFFFFFFFFFFFFFFFFFFFFFFFF:FFFFFFFFFFFFFFF
@A00155:342:HHGFNDSXY:1:2102:29658:4789 2:N:0:GAACCTAG+TCCGCATA
TAGGTCGTGAACAAGCCCGGAGTACATCTCCTTTGGAACTTGGAGACCCCTCCGGTCTTGTCCAACCT
CTGTTTCGGCAGAGGATACCGTGCTCCACAGCTGTAAGGCTGTGGGCCCGAGAGAAGTCGCTCTCTCT
+
FFFFFFFFFFFFFFFFFFFFFFFFFFFFFFFFFFFFFFFFFFFFFFFFFFFFFFFFFF:FFFFFFFFF
FFFFFFFFFFFFFFFFFFFFFFFFFFFFFFFFFFFF:FFFFF,FFFFFFFFFFFF:FFFFFFFFFFFF
@A00155:342:HHGFNDSXY:1:2102:4616:8609 2:N:0:GAACCTAG+TCCGCATA
GGATACCGTGCTCCACAGCTGTAAGGCTGTGGGCCCGAGAGAAGTCGCTCTCTCTCGCCCTCCACATC
CCTCTCGGCATTGAGATGACCATAGGCCAGAGGTGAGTCCTTAAGTGGACACAGCTGATCTAAGGCGG
+
FFFFFFFFFFFFFFFFFFFFFFFFFFFFFFFFFFFFFFFFFFFFFFFFFFFFFFFFFFFFFFFFFFFF
FFFFFFFFFFFFFFFFFFFFFFFFFFFFFFFFFFFFFFFFF:FFFFFFFFFFFFFFFFFFFFFFFFFF
@A00155:342:HHGFNDSXY:1:2102:4300:11005 2:N:0:GAACCTAG+TCCGCATA
GGATACCGTGCTCCACAGCTGTAAGGCTGTGGGCCCGAGAGAAGTCGCTCTCTCTCGCCCTCCACATC
CCTCTCGGCATTGAGATGACCATAGGCCAGAGGTGAGTCCTTAAGTGGACACAGCTGATCTAAGGCGG
+
FFFFFFFFFFFFFFFFFFFFFFFFFFFFFFFFFFFFFFFF:F:FFFFFFFFFFFFFFFFFFFFFFFFF
FF:FFFFFFFF::FF:FFFFFFFFFFFFFFFFFFFFFFFFFFFF,FFFFFFFFFFFFFFFFFF:FFFF
@A00155:342:HHGFNDSXY:1:2102:11026:33708 2:N:0:GAACCTAG+TCCGCATA
TTTCGTCCTAAAAAGGACTCGTCAGGTAGCCTACTAGACTACGACTTACACGGTAGGTCGTGAACAAG
CCCGGAGTACATCTCCTTTGGAACTAGGAGACCCCTCCGGTCTTGTCCAACCTCTGTTTCGGCAGAGG
+
FFFFFFFFFFFFFFFFFFFFFFFFFFFFFFFFFFFFFFFFFFFFFFFFFFFFFFFFFFFFFFFFFFFF
FFFFFFFFFFFFFFFFFFFFFFFFFFFFFFFFFFFFFFFFFFFFFFFFFFFFFFFFFFFFFFFFFFFF
@A00155:342:HHGFNDSXY:1:2103:9525:14606 2:N:0:GAACCTAG+TCCGCATA
CTAAAAAGGACTCGTCAGGTAGCCTACTAGACTACGACTTACACGGTAGGTCGTGAACAAGCCCGGAG
TACATCTCCTTTGGAACTAGGAGACCCCTCCGGTCTTGTCCAACCTCTGTTTCGGCAGAGGATACCGT
+
```

FFFFFFFFFFFFFFFFFFFFFFFFFFFFFFFFFFFFFFF:FFFFFFFFFFFFFFFFFFFFFFFFFFFF
FFFFFFFFFFFFFFFFFFFFFFFFFFFFFFFFFFFFFFFFFFFFFFFFFFFFFFFFFF:FFFFFF,FF
@A00155:342:HHGFNDSXY:1:2103:22498:19977 2:N:0:GAACCTAG+TCCGCATA
TAGACTACGACTTACACGGTAGGTCGTGAACAAGCCCGGAGTACATCTCCTTTGGAACTAGGAGACCC
CTCCGGTCTTGTCCAACCTCTGTTTCGGCAGAGGATACCGTGCTCCACAGCTGTAAGGCTGTGGGCCC
+
FFFFFFFFFFFFFFFFFFFFFFFFFFFFFFFFFFFFFFFFFFFFFFFFFFFFFFFFFFFFFFFFFFFF
FFFFFFFFFFFFFFFFFFFFFFFFFFFFFFFFFFF:FFFFFFFFFFFFFFFFFFFFFFFFFFFFFFFF
@A00155:342:HHGFNDSXY:1:2103:22263:24361 2:N:0:GAACCTAG+TCCGCATA
GTGAACAAGCCCGGAGTACATCTCCTTTGGAACTAGGAGACCCCTCCGGTCTTGTCCAACCTCTGTTT
CGGCAGAGGATACCGTGCTCCACAGCTGTAAGGCTGTGGGCCCGAGAGAAGTCGCTCTCTCTCGCCCT
+
FFFFFFFFFFFFFFFFFFFFFFFFFF:FFFF:FFFFFFFFFFFFFFFFFFFFFFFFFFFFFFFFFFFF
FFFFFFFFFFFFFFFFFFFFFFFFFFFF:FFFFFFFFFFFFFFFFFFFFFFFFFFFFFFFFFFFFFFF
@A00155:342:HHGFNDSXY:1:2103:27389:28166 2:N:0:GAACCTAG+TCCGCATA
GAAACCGTCGTTACTCTTCGAGTTGTGTAAGTTTCGTCCTAAAAAGGACTCGTCAGGTAGCCTACTAG
ACTACGACTTACACGGTAGGTCGTGAACAAGCCCGGAGTACATCTCCTTTGGAACTAGGAGACCCCTC
+
FFFFFFFFFFFFFFFF:FFFFFFFFF:,FFFFFFFFFFFFFFFFFFFFFFFFFFFFFFFFFFFFFFFF
FFFFFFFFFFFFFFFFFFFFFFFFFFFFFFFFFFFFFFFFFFFFFF::FFFF:FFFFFFFFFF:FF:F
@A00155:342:HHGFNDSXY:1:2104:17390:6214 2:N:0:GAACCTAG+TCCGCATA
AGGACGAAACCGTCGTTACTCTTCGAGTTGTGTAAGTTTCGTCCTAAAAAGGACTCGTCAGGTAGCCT
ACTAGACTACGACTTACACGGTAGGTCGTGAACAAGCCCGGAGTACATCTCCTTTGGAACTAGGAGAC
+
FFFF:FFFFFFFFFFFFFFFFFFFFFFFFFFFFFFFF:FFFFFF:F:FF,:FFFFFFF:FFFF,FFFF
FFFFFFFFFFFFFFFFF:FFFFFFFFFFFFFFFFF:FFFFF,FFFFFFFFFFFFFF:FFFFFFFFF:F
@A00155:342:HHGFNDSXY:1:2104:25183:11851 2:N:0:GAACCTAG+TCCGCATA
GGACTCGTCAGGTAGCCTACTAGACTACGACTTACACGGTAGGTCGTGAACAAGCCCGGAGTACATCT
CCTTTGGAACTAGGAGACCCCTCCGGTCTTGTCCAACCTCTGTTTCGGCAGAGGATACCGTGCTCCAC
+
FFFFFFFFFFFFFFFFFFFFFF:FFFFFFFFFFFFFFFFFFFFFFFFFFFFFFFFFFFFFFFFFFFFF
FFF:FFFFFFFFFFFFFFFFFFFFFFFFFFFFFFFFFFFFFFFFFF:FFFFFFFF:FFFFFFFFFFFF
@A00155:342:HHGFNDSXY:1:2104:8133:17080 2:N:0:GAACCTAG+TCCGCATA
CAGGTAGCCTACTAGACTACGACTTACACGGTAGGTCGTGAACAAGCTCGGAGTACATCTCCTTTGGA
ACTAGGAGACCCCTCCGGTCTTGTCCAACCTCTGTTTCGGCAGAGGATACCGTGCTCCACAGCTGTAA
+
FFFFF,FFFFFFFFFFFFFFFFFFFFFFFFFFFFFFFFFFFFFFFFFFFFFFFFFFFFFFFFFFFFFF
:FFFFFFFFFFFFFFFFFFF,FFFFFFFFFFFFFFFFFFFFFFFFFFFFFFFFFFFFFFFFFFFFFFF
@A00155:342:HHGFNDSXY:1:2104:31060:24158 2:N:0:GAACCTAG+TCCGCATA
TACGACTTACACGGTAGGTCGTGAACAAGCCCGGAGTACATCTCCTTTGGAACTAGGAGACCCCTCCG
GTCTTGTCCAACCTCTGTTTCGGCAGAGGATACCGTGCTCCACAGCTGTAAGGCTGTGGGCCCG
+
FFFFFFFFFF:FFFFFFFFFFFFFFFFFFFFFFFFFFFFFFFFFFFFFF:F:FFFFFFFFFFFFFFFF
FFFFFFFFFFFFFFFFFFFFFFFFFFFFF:FFFFFFFFFFFFF:FFFFFFFFFFFFFFFFFFFF
@A00155:342:HHGFNDSXY:1:2104:26142:31203 2:N:0:GAACCTAG+TCCGCATA
GGACTCGTCAGGTAGCCTACTAGACTACGACTTACACGGTAGGTCGTGAACAAGCCCGGAGTACATCT
CCTTTGGAACTAGGAGACCCCTCCGGTCTTGTCCAACCTCTGTTTCGGCAGAGGATACCGTGCTCCAC
+
FFFFFFFFFFFFFFFFFFFFFFFFFFFFFFFFFFFFFFFFFFFFFFFFFFFFFFFFFFFFFFFFFFFF
FFFFFFFFFFFFFFFFFFFFFFFFFFFF:FFFFFFFFFFFFFFFFFFFFFFFFFFFFFFFFFFFFFFF
@A00155:342:HHGFNDSXY:1:2104:7780:32628 2:N:0:GAACCTAG+TCCGCATA
CTCTTCGAGTTGTGTAAGTTTCGTCCTAAAAAGGACTCGTCAGGTAGCCTACTAGACTACGACTTACA
CGGTAGGTCGTGAACAAGCCCGGAGTACATCTCCTTTGGAACTAGGAGACCCCTCCGGTCTTGTCCAA
+

FFF,FFF:FFFFFF:FFFFFFFFFFFFFFFFFFFFFFFFFFFFFFFFFFFFFFFFFFFFFFFFFFFF
FFFFFFFFFFFFFFFFFFFFFFFFFFFFFFFFFFFFFFFFF:FFFFFFFFFFFF:FFFFFFFFFFF,:F
@A00155:342:HHGFNDSXY:1:2105:25798:4272 2:N:0:GAACCTAG+TCCGCATA
CGTTACTCTTCGAGTTGTGTAAGTTTCGTCCTAAAAAGGACTCGTCAGGTAGCCTACTAGACTACGAC
TTACACGGTAGGTCGTGAACAAGCCCGGAGTACATCTCCTTTGGAACTAGGAGACCCCTCCGGTCTTG
+
FFFFFFFFFFFFFFFFFFFFFF:FFFFFFFF:FFFFFFFFFFFFFFFFFFFFFFFFFFFFFFFFF:FF
FFFFFFFFFFFF:FFFFFFFFFFFFFFFFFFFF:FFFFF:FFFFFFFF:FFFFF:F:FFFFFFFFFFF
@A00155:342:HHGFNDSXY:1:2106:22634:1423 2:N:0:GAACCTAG+TCCGCATA
CGTGAACAAGCCCGGAGTACATCTCCTTTGGAACTAGGAGACCCCTCCGGTCTTGTCCAACCTCTGTT
TCGGCAGAGGATACCGTGCTCCACAGCTGTAAGGCTGTGGGCCCGAGAGAAGTCGCTCTCTCTCGCC
+
FFFFFFFFF:FF:FFFFFFFFFFFFFFFFFFFFFF:FFFFFFFFFFFFFFFFFFFFFFFFFFFFFFFF
FFFFFFFFFFFFFF:FFFFFFFFFFFFFFFFF:FFFFFFFFFFFFFFFFFFFFFFFFFFFFFFFFFF
@A00155:342:HHGFNDSXY:1:2106:9959:5650 2:N:0:GAACCTAG+TCCGCATA
GAACTAGGAGACCCCTCCGGTCTTGTCCAACCTCTGTTTCGGCAGAGGATACCGTGCTCCACAGCTGT
AAGGCTGTGGGCCCGAGAGAAGTCGCTCTCTCTCGCCCTCTACATCCCTCTCGGCATTGAGATGACCA
+
FFFFFFFFFFFFFFFFFFFFFFFFFFFFFFFFFFFFFFFFFFFFFFFFFFFFFFFFFFFFFFFFFFFF
FFF:FFFFFFFFFFFFFFFFFFFFFFFFFFFFFFFFFFFFFFFFFFFFFFFFFFF:FFFFFFFFFFF
@A00155:342:HHGFNDSXY:1:2106:22435:15013 2:N:0:GAACCTAG+TCCGCATA
TACATCTCCTTTGGAACTAGGAGACCCCTCCGGTCTTGTCCAACCTCTGTTTCGGCAGAGGATACCGT
GCTCCACAGCTGTAAGGCTGTGGGCCCGAGAGAAGTCGCTCTCTCTCGCCCTCCACATCCCTCTCGGC
+
FFFFFFFFFF:FFFFFFFFFFFFFFFFFFFFFFFFF::FFFFFFFFFFFFFFFFFFFFFFFFFFFFFFF
FFFFFFFFFFFFFFFFFFFFFFFFFFFFFFFFFFFFFFFFFFFFFFFFFFFFFFFFFFFFFFFFFFFF
@A00155:342:HHGFNDSXY:1:2107:15989:6856 2:N:0:GAACCTAG+TCCGCATA
CCCCTCCGGTCTTGTCCAACCTCTGTTTCGGCAGAGGATACCGTGCTCCACAGCTGTAAGGCTGTGGG
CCCGAGAGAAGTCGCTCTCTCTCGCCCTTCACATCCCTCTCGGCATTGAGATGACCATAGGCCAGAG
+
FFFFFFFFFFF:FFFFFFFFFFFFFFFFFF:FFFFFFFFFFFFFFFFFFFFFFFFFFFFFFFFFFFFF
FFFFFFFFF,FFFFFFFFFFFFFFFFFFFFFFFFFFFFFFFFFFFF:FFFFFFF:FFFF:FFFFFFFF
@A00155:342:HHGFNDSXY:1:2107:25509:9878 2:N:0:GAACCTAG+TCCGCATA
AGCCCGGAGTACATCTCCTTTGGAACTAGGAGACCCCTCCGGTCTTGTCCAACCTCTGTTTCGGCAGA
GGATACCGTGCTCCACAGCTGTAAGGCTGTGGGCCCGAGAGAAGTCGCTCTCTCTCGCCCTCCACATC
+
FFFFFFFFFFFFFFFFFFFFFFFFF:FFFFFFFFFFFFFFFFFFFFFFFFFFFFFFFFFFFFFFFFFF
FFFFFFFFFFFFFFFFFFFFFFFFFFFFFFFFFFFFFFFFFFFFFFFFFFFFFFFFFFFFFFFFFFFF
@A00155:342:HHGFNDSXY:1:2107:10592:35712 2:N:0:GAACCTAG+TCCGCATA
AAGGACTCGTCAGGTAGCCTACTAGACTACGACTTACACGGTAGGTCGTGAACAAGCCCGGAGTACAT
CTCCTTTGGAACTAGGAGACCCCTCCGGTCTTGTCCAACCTCTGTTTCGGCAGAGGATACCGTGCTCC
+
FFFFFFFFFFFFFFFFFFFFFFFFFFFFFFFFFFFFFFFFFFFFFFFFFFFFFFFFFFFFFFFFFFFF
FFFFF:FFFFFFFFFFFFFFFFFFFFFFFFFFFFFFFFFFFFFFFFFFFFFFFFFFFFFFFFFFFFF:
@A00155:342:HHGFNDSXY:1:2107:2085:36198 2:N:0:GAACCTAG+TCCGCATA
TTAGGACGAAACTTACACAACTCAAAGAGTAACGACGGTTTCGTCCTTGGAACGGACTCATCAGGGAG
CCTACTAGACTCCGACCGTCATGGGGGTTCAAACCCATGCCCCGCCACACCGCCTTAGATCAGC
+
FFFFFFFFFFFFFFFFFFFFFFFFFFFFFFFFFFF:FFFFFFFFFFFFFFFFFFF:FFF:FFFFFFFF
FFFFFFFFFFFFFFFFFFFFFFFFFFFFFFFFFFFFFFFFFFFFFFFFFFFFFFFFFFFFFFFF
@A00155:342:HHGFNDSXY:1:2108:24144:30561 2:N:0:GAACCTAG+TCCGCATA
AGCCTACTAGACTACGACTTACACGGTAGGTCGTGAACAAGCCCGGAGTACATCTCCTTTGGAACTAG
GAGACCCCTCCGGTCTTGTCCAACCTCTGTTTCGGCAGAGGATACCGTGCTCCACAGCTGTAAGGCTG
+

FFFFFFFFFFFFFFFFFFFFFFFFFFFFFFFFFFFFFFFFFFFFFFFFFFFFFFFFFFFFFFFFFFFF:
FFFFFFFFFFFFFFFFFFFFFFFFFFFFFFFFFFFFFFFFFFFFFFFFFFFFFFFFFFFFFFFFFFFFF
@A00155:342:HHGFNDSXY:1:2109:18882:6699 2:N:0:GAACCTAG+TCCGCATA
GGGTTCAAACCCATGCCCCACCACACCGCCTTAGATCAGCTGTGTCCACTTAAGGACTCACCTCTGGC
CTATGGTCATCTCAATGCCGAGAGGGATGTGGAGGGCGAGAGAGAGCGACTTCTCTCGGGCCCACAGC
+
FFFFFFFFFFF:FFFFFFFFFFFFFFFFFFFFFFFFFFFFFFFFFFFFFFF:FFFFFFFFFFFFFFFF
FFF,FFFFFFFFFFFFFFFFFFFFFFFFFF:FFFFFFFFFFFFFFFFFFFFFFFFFFFFFFFFFFFFF
@A00155:342:HHGFNDSXY:1:2109:15863:13463 2:N:0:GAACCTAG+TCCGCATA
CACGGTAGGTCGTGAACAAGCCCGGAGTACATCTCCTTTGGAACTAGGAGACCCCTCCGGTCTTGTCC
AACCTCTGTTTCGGCAGAGGATACCGTGCTCCACAGCTGTAAGGCTGTGGGCCCGAGAGAAGTCGCTC
+
FFFFFFFFFFFFFFFFFFFFFFFFFFFFFFFFFFFFFFFFFF:FFFFFFFFFFFFFFFFFFFFFFFFF
FFFFFFFFFFFFFFFFFFFFFFFFFFFFFFFFFFFFFFFFFFFFFFFFFFFFFFFFFFFFFFFFFFFF
@A00155:342:HHGFNDSXY:1:2109:8431:23171 2:N:0:GAACCTAG+TCCGCATA
GGACTCGTCAGGTAGCCTACTAGACTACGACTTACACGGTAGGTCGTGAACAAGCCCGGAGTACATCT
CCTTTGGAACTAGGAGACCCCTCCGGTCTTGTCCAACCTCTGTTTCGGCAGAGGATACCGTGCTCCAC
+
FFFFFFFFFFFFFFFFFFFFFF:FFFFFFFFFFFFFFFFFFFFFFFF:FFFFFFFF:FFFFFFFFFFF
FFFFFFFFFFFFFFFFFFFFFFFFFFFFFFFFFFFFFFFFFFFFFFFFFFFFFFF:FFFFFFFFFFFF
@A00155:342:HHGFNDSXY:1:2111:7907:13463 2:N:0:GAACCTAG+TCCGCATA
ACACCGCCTTAGATCAGCTGTGTCCACTTAAGGACTCACCTCTGGCCTATGGTCATCTCAATGCCGAG
AGGGATGTGAAGGGCGAGAGAGAGCGACTTCTCTCGGGCCCACAGCCTTACAGCTGTGGAGCACGGTA
+
FFFFFFFFFFFFFFFFFFFFFFFFFFFFFFFFFFFFFFFFFFFFFFFFFFFFFFFFFFFFFFFFFFFF
FFFFFFFFFFFFFFFFFFFFFFFFFFFFFFFFFFFFFFFFFFFFFFF:FFFFFFFF:FFFFFFFFFFF
@A00155:342:HHGFNDSXY:1:2111:27136:31140 2:N:0:GAACCTAG+TCCGCATA
ACTACGACTTACACGGTAGGTCGTGAACAAGCCCGGAGTACATCTCCTTTGGAACTAGGAGACCCCTC
CGGTCTTGTCCAACCTCTGTTTCGGCAGAGGATACCGTGCTCCACAGCTGTAAGGCTGTGGGCCCGAG
+
FFFFFFFFFFFFFFFFFFFFFFFFFFFFFFFFFFFFFFFFFFFFFFFFFFFFFFFFFFFFFFFFFFFF
FFFFFFFFFFFFFFFFFFFF:FFFFFFFFFFFFFFFFFFFFF:FFFFFFFFFFFFFFF:FFFFFFFFF
@A00155:342:HHGFNDSXY:1:2112:3188:26146 2:N:0:GAACCTAG+TCCGCATA
TGTGTAAGTTTCGTCCTAAAAAGGACTCGTCAGGTAGCCTACTAGACTACGACTTACACGGTAGGTCG
TGAACAAGCCCGGAGTACATCTCCTTTGGAACTAGGAGACCCCTCCGGTCTTGTCCAACCTCTGTTTC
+
FFFFFFFFFFFFFFFFFFFFFF,FFFFFFFFFFFFFF:FFFFF:FFFF:FF:FFF,FFFFFFFFFFFF
FFFFFFF:FFFFFFFFFFFFFFFFFFFFFFFFFFFFFFFFFFFFFFFFFFFFFFFFFFFFFFFFFFFF
@A00155:342:HHGFNDSXY:1:2112:3378:33144 2:N:0:GAACCTAG+TCCGCATA
CCTACTAGACTACGACTTACACGGTAGGTCGTGAACAAGCCCGGAGTACATCTCCTTTGGAACCAGGA
GACCCCTCCGGTCTTGTCCAACCTCTGTTTCGGCAGAGGATACCGTGCTCCACAGCTGTAAGGCTGTG
+
FFFFFFFFFFFFFFFFFFFFFFFFFFFFFFFF:FFFFFFFFFFFFFFFFFFFFFFFF:FFFFFFFFFFF:
FFFFFFFFFFFFFFFFFFFFFFFFFFFFFFFFFFFFFFFFFFFFFFFFFFFFFFFFFFFFFFFFFFFF
@A00155:342:HHGFNDSXY:1:2112:10022:35603 2:N:0:GAACCTAG+TCCGCATA
AGTTTCGTCCTAAAAAGGACTCGTCAGGTAGCCTACTAGACTACGACTTACACGGTAGGTCGTGAACA
AGCCCGGAGTACATCTCCTTTGGAACTAGGAGACCCCTCCGGTCTTGTCCAACCTCTGTTTCGGCAGA
+
FFFFFFFFFFFFFFFFFFFFFFFFFFFFFFFFFFFFFFFFFFFFFFFFFFFFFFFFFFFFFF:FFFFF
FFFFFFFFFFFFFFFFFFFFFFFFF,FFFFFFFFFFFFFFFFFFFFFFFFFFFFFF:FFFFFFFFFFFFF
@A00155:342:HHGFNDSXY:1:2113:29306:6277 2:N:0:GAACCTAG+TCCGCATA
CTACTAGACTACGACTTACACGGTAGGTCGTGAACAAGCCCGGAGTACATCTCCTTTGGAACTAGGAG
ACCCCTCCGGTCTTGTCCAACCTCTGTTTCGGCAGAGGATACCGTGCTCCACAGCTGTAAGGCTGTGG
+

FFFFFFFFFFFFFFFFFFFFFFFFFFFFFFFFFFFFFFFFFFFFFFFFFFFFFFFFFFFFFFFFFFFF
FFFFFFFFFFFF:FFFFFFFFFFFFFFFFFFFFFFFFFFFFFFFFFFFFFFFFFFFFF:FFFFFFFF
@A00155:342:HHGFNDSXY:1:2113:13223:7513 2:N:0:GAACCTAG+TCCGCATA
CGTCCTAAAAAGGACTCGTCAGGTAGCCTACTAGACTACGACTTACACGGTAGGTCGTGAACAAGCCC
GGAGTACATCTCCTTTGGAACTAGGAGACCCCTCCGGTCTTGTCCAACCTCTGTTTCGGCAGAGGATA
+
FFFFFFFFFFFFFFFFFFFFFFFFFFFFFFFFFFFFFFFFFFFFFFFFFFFFFFFFFFFFFFFFFFFF
FFFFFFFFFFFFFFFFFFFFFFFFFFFFFFFFFFFFFFFFFFFFFFFFFFFFFFFFFFFFFFFFFFFF
@A00155:342:HHGFNDSXY:1:2113:23574:13479 2:N:0:GAACCTAG+TCCGCATA
CTCTTTGAGTTGTGTAAGTTTCGTCCTAAAAAGGACTCGTCAGGTAGCCTACTAGACTACGACTTACA
CGGTAGGTCGTGAACAAGCCCGGAGTACATCTCCTTTGGAACTAGGAGACCCCTCCGGTCTTGTCCA
+
FFF:FFFFFFFFFFFFFFFFFFFFFFFFFFFFFFFFFFFFFFFFFFFFFFFFFFFFFFFFFFFFFFFF
FFFFFFFFFFFFFFFFFF:FFFFFFF,FFFFFFFFFFFFFFFFFFFFFFFFFFFFFFFFFFFFFFFF
@A00155:342:HHGFNDSXY:1:2113:30834:16376 2:N:0:GAACCTAG+TCCGCATA
GGAACTAGGAGACCCCTCCGGTCTTGTCCAACCTCTGTTTCGGCAGAGGATACCGTGCTCCACAGCTG
TAAGGCTGTGGGCCCGAGAGAAGTCGCTCTCTCTCGCCCTCCACATCCCTCTCGGCAGTGAGATGACC
+
FFFFFFFFFFFFFFFFFFFFFFFFFFFFF:FFFFFFFFFFFFFFFFFFFFFFFFFFFFFFFFFFFFFFFF
FF:FFFFFFFFFFFFFFFFFFFFFFFFFFFFFFFF:FFFFFFFFFFFFFFFFFFFFF,FFFFFFFFFF
@A00155:342:HHGFNDSXY:1:2113:20844:23062 2:N:0:GAACCTAG+TCCGCATA
AGTACATCTCCTTTGGAACTAGGAGACCCCTCCGGTCTTGTCCAACCTCTGTTTCGGCAGAGGATACC
GTGCACCACAGCTGTAAGGCTGTGGGCCCGAGAGAAGTCGCTCTCTCTCGCCCTCCACATCCCTCTCG
+
FFFFFFFFFFFFFFFFFFFFFFFFFFFFFFFFFFFFFFFFFFFFFFFFFFFFFFFFFFFFFFFFFFFF
FFFFFFFFFFFFFFFFFFFFFFFFFFFFFFFFFFFFFFFFFFFFFFFFFFFFFFFFFFFFFFFFFFFF
@A00155:342:HHGFNDSXY:1:2113:16731:27367 2:N:0:GAACCTAG+TCCGCATA
CTTCTCTCGGGCCCACAGCCTTACAGCTGTGGAGCACGGTATCCTCTGCCGAAACAGAGGTTGGACAA
GACCGGAGGGGTCTCCTGGTTCCAAAGGAGATGTACTCCGGGCTTGTTCACGACCTACCGTGTAAGTC
+
FFFFFFFFFFFFFFFFFFFFFF:FFFFFFFFFFFFFFFFFFFFFFFFFFFFFFFFFFFFFFFFFFFFF
FFFFFFFFFFFFFFFFFFFFFFFFFFFFFFFFFFFFFFFFFFFFFFFFFFFFFFFFFFFFFFFFFFFF
@A00155:342:HHGFNDSXY:1:2113:26901:32831 2:N:0:GAACCTAG+TCCGCATA
GGAGTACATCTCCTTTGGAACTAGGAGACCCCTCCGGTCTTGTCCAACCTCTGTTTCGGCAGAGGATA
CCGTGCTCCACAGCTGTAAGGCTGTGGGCCCGAGAGAAGTCGCTCTCTCTCGCCCTCTACATCCCTCT
+
FFFFFFFFFFFFFFFFFFFFFFFFFFFFFFFFFFFFFFFF,FFFFFFFFFFFFFFFFFFFFFFFFFFF
FFFFFFFFFFFFFFFFFFFFFFFFFFFFFFFFFFFFFFFFFFFFFFFFFFF,FFFFFFF:FFFFFFFF
@A00155:342:HHGFNDSXY:1:2113:27724:35822 2:N:0:GAACCTAG+TCCGCATA
TCCAAGGACGAAACCGTCGTTACTCTTCGAGTTGTGTAAGTTTCGTCCTAAAAAGGACTCGTCAGGTA
GCCTACTAGACTACGACTTACACGGTAGGTCGTGAACAAGCCCGGAGTACATCTCCTTTGGAACTAGG
+
FFFFFFFFFFFFFFFF,FFFFFFF,:FFFFFFFFFFFFFFF::FFFFFFFFFFF:FFFFFFFFFFFFF
FFFFFFFF,F:FFFFF,FFFFFFFFFFFFFFFFFFFFFF:FFFFFFFFFFFF::FFF:FFFFFF,F:F
@A00155:342:HHGFNDSXY:1:2114:26341:8061 2:N:0:GAACCTAG+TCCGCATA
AAGCCCGGAGTACATCTCCTTTGGAACTAGGAGACCCCTCCGGTCTTGTCCAACCTCTGTTTCGGCAG
AGGATACCGTGCTCCACAGCTGTAAGGCTGTGGGCCCGAGAGAAGTCGCTCTCTCTCGCC
+
FFFFFFFFFFFFFFFFFFFFFFF:F:FFFFFFFFFFFFFFFFFFFFFF:FFFFFFFFFFFFFFFFFFF
FFFF,F:FFFFFFFF:FFFFFFFF,FFF:FFFFFFFFFFFFFFFFFFFFFFFFFFFFFFF
@A00155:342:HHGFNDSXY:1:2114:2284:20447 2:N:0:GAACCTAG+TCCGCATA
AAACCGTCGTTACTCTTCGAGTTGTGTAAGTTTCGTCCTAAAAAGGACTCGTCAGGTAGCCTACTAGA
ATACGACTTACACGGTAGGTCGTGAACAAGCCCGGAGTACATCTCCTTTGGAACTAGGAGACCCCTC
+

:FF:FFFFFFFFFFFFF:F:F:FFFFFFFFFFFFF,F,F:FFFF,:F:,FFFFFFFFFFFFFFF,FF,F
,FFF::FFFF:F,F:FFFFF:F,F:F,FFF:,FFFFFFFFF:FF:F:,F:FFFFFF:FFFFFFFFFF
@A00155:342:HHGFNDSXY:1:2114:9372:31845 2:N:0:GAACCTAG+TCCGCATA
GGCCTATGGTCATCTCAATGCCGAGAGGGATGTGAAGGGCGAGAGAGAGCGACTTCTCTCGGGCCCAC
AGCCTTACAGCTGTGGAGCACGGTATCCTCTGCCGAAACAGAGGTTGGACAAGACCGGAG
+
FFFF:FFFFFFFFFFFFFFFFFFFFFFFFFFFFFFFFFFFFFFFFFFFFFFFFFFFFFFFFFFFFFFFF
FFFFFFFFFFFFFFFFFFFFFFFFFFFFFFFFFFFFFFFF,FFFFF:FFFFFFFFF:FFFF
@A00155:342:HHGFNDSXY:1:2114:21441:32017 2:N:0:GAACCTAG+TCCGCATA
AGACCCCTCCGGTCTTGTCCAACCTCTGTTTCGGCAGAGGATACCGTGCTCCACAGCTGTAAGGCTGT
GGGCCCGAGAGAAGTCGCTCTCTCTCGCCCTCCACATCCCTCTCGGCATTGAGATGACCATAGGCCAG
+
FFFFFFFFFFFFFFFFFFFFFFFFFFFFFFFFFFFFFFFFFFFFFFFFFFFFFFFFF:FFFFFFFFFFF
FFFFFFFFFFFFFFFFFFFFFFFFFFFFFFFFFFFFFFFFFFFFFFFFF:FFFFFFFFFFFFFFFFFFF
@A00155:342:HHGFNDSXY:1:2115:5258:35744 2:N:0:GAACCTAG+TCCGCATA
GGACTCGTCAGGTAGCCTACTAGACTACGACTTACACGGTAGGTCGTGAACAAGCCCGGAGTACATCT
CCTTTGGAACCAGGAGACCCCTCCGGTCTTGTCCAACCTCTGTTTCGGCAGAGGATACCGTGCTCCAC
+
FFFFFFFFFFFFFFFFFFFFFFFFFFFFFFFFFFFFFFFFFFFFFFFFFFFFFFFFFFFFFFFFFFFFF
FFFFFFFF,FFFFFFFFFFFFFFFFFFFFFFFFFFFFFFFFFFFFFFF:FFFFFFFFFFFFFFFFFFFF
@A00155:342:HHGFNDSXY:1:2116:15094:19930 2:N:0:GAACCTAG+TCCGCATA
CTAGGAGACCCCTCCGGTCTTGTCCAACCTCTGTTTCGGCAGAGGATACCGTGCTCCACAGCTGTAAG
GCTGTGGGCCCGAGAGAAGTCGCTCTCTCTCGCCCTCTACATCCCTCTCGGCATTGAGATGACCATAG
+
FFFFFFFFFFFFFFFFFFFFFFFFFFFFFFFFFFFFFFFFFFFFFFFFFFFFFFFFFFFFFFFFFFFFF
FFFFFFFFFFFFFFFFFFFFFFFFFFFFFFFFFFFFFFFFFFFFFFFFFFFFFF,FFFFFFFFFFFFF
@A00155:342:HHGFNDSXY:1:2116:15383:22247 2:N:0:GAACCTAG+TCCGCATA
CCCCTCCGGTCTTGTCCAACCTCTGTTTCGGCAGAGGATACCGTGCTCCACAGCTGTAAGGCTGTGGG
CCCGAGAGAAGTCGCTCTCTCTCGCCCTTCACATCCCTCTCGGCATTGAGATGACCATAGGCCAGAGG
+
FFFFFFFFFFFFFFFFFFFFFFFFFFFFFFFFF:FFFFFFFFFFFFFFFFFFFFFFFFF,FFFFFFFFF
FFFF,FFFFFFFFFFFFFFFFF:FFFFFFFFFFFFFFFFFFFFFFF,FF:FFFFFFFFFFFFFFFFFFF
@A00155:342:HHGFNDSXY:1:2116:18150:33019 2:N:0:GAACCTAG+TCCGCATA
AGCCCGGAGTACATCTCCTTTGGAACTAGGAGACCCCTCCGGTCTTGTCCAACCTCTGTTTCGGCAGA
GGATACCGTGCTCCACAGCTGTAAGGCTGTGGGCCCGAGAGAAGTCGCTCTCTCTCGCC
+
FFFFFFFFFFFFFFFFFFFFFFFF:F:FFFFFFFFFFFFFFFFFFFFFFFFFFFFFFFFFFFFFFFFFF
FFFFFFFFFFFFFFFFFFFFFFFFFFFF,FFFFFFFFFFFFFFFFFFFFFFFFFFFFFFF
@A00155:342:HHGFNDSXY:1:2117:9444:8484 2:N:0:GAACCTAG+TCCGCATA
GGACGAAACTTACACAACTCGAAGAGTAACGACGGTTTCGTCCTTGGAACGGACTCATCAGGAAGCCT
ACTAGACTCCGACCGTCATGGGGGTTCAAACCCATGCCCCGCCACACCGCCTTAGATCAGCTGTGTCC
+
FFFFFFFFFFFFFFFFFFFFFFFFFFFFFFFFFFFFFFFFFFFFFFFFFFFFFFFFFFFFFFFFFFFFF
FFFFFFFFFFFFFFFFFFFFFFFFFFFFFFFFFFFFFFFFFFFFFFFFFFFFFFFFFFFFFFFFFFFFF
@A00155:342:HHGFNDSXY:1:2118:27778:6104 2:N:0:GAACCTAG+TCCGCATA
GGACTCGTCAGGTAGCCTACTAGACTACGACTTACACGGTAGGTCGTGAACAAGCCCGGAGTACATCT
CCTTTGGAACTAGGAGACCCCTCCGGTCTTGTCCAACCTCTGTTTCGGCAGAGGATACCGTGCTCCAC
+
FFFFFFFFFFFFFFFFFFFFFFFFFFFFFFFFFFFFFFFFFFFFFFFFFFFFFFFFFFFFFFFFFFFFF
FFFFFFFF:FFFFFFFFF::FFFFFFFFFFFFFF:FFFFFFFFFFFFFFFFFFFFFFFFFF:FFFFFFF
@A00155:342:HHGFNDSXY:1:2118:1533:6558 2:N:0:GAACCTAG+TCCGCATA
TCCACTTAAGGACTCACCTCTGGCCTATGGTCATCTCAATGCCGAGAGGGATGTAGAGGGCGAGAGAG
AGCGACTTCTCTCGGGCCCACAGCCTTACAGCTGTGGAGCACGGTATCCTCTGCCGAAACAGAGGTTG
+

```
FFFF:F:FFFFFFFFFFFFFFFFFFFFFFFFFF:FFFFFF:FFFFF:FFFFFFFFFFFFF:F:FFFFF
FFFFFFFFFFFFFF:FFFFFFFFFFFFFFFF:F:FFFFF:FFFFFFFFFFFFF,FFFFFF:FFFFFFF
@A00155:342:HHGFNDSXY:1:2118:16929:7733 2:N:0:GAACCTAG+TCCGCATA
AAAAGGACTCGTCAGGTAGCCTACTAGACTACGACTTACACGGTAGGTCGTGAACAAGCCCGGAGTAC
ATCTCCTTTGGAACCAGGAGACCCCTCCGGTCTTGTCCAACCTCTGTTTCGGCAGAGGATACCGTGCT
+
FFFFFFFFFFFFFFFFFFFFFFFFFFFFFFFFFFFFFFFFFFFFFFFFFFFFFFFFFFFFFFFFFFFF
FFFFFFFFFFFF,FFFFFF:FFFFFFFFFFFFFFFFFFFFFFFFFFFFFFFFFFFFFF:F:FFFFFFF
@A00155:342:HHGFNDSXY:1:2119:2130:5337 2:N:0:GAACCTAG+TCCGCATA
AGCCCGGAGTACATCTCCTTTGGAACTAGGAGACCCCTCCGGTCTTGTCCAACCTCTGTTTCGGCAGA
GGATACCGTGCTCCACAGCTGTAAGGCTGTGGGCCCGAGAGAAGTCGCTCTCTCTCGCC
+
FFFFFFFFFFFFFFFFFFFFFFFFFFFFFF:FFFFFFFFFFFFFFFFFFFFFFFFFFF,FFFFFFFFF
FFFFFFFFFF:FF:FF:FFFFFFFFFFFFFFFFF:FFFFFF:FFFFFFFFFFFFFF,FF
@A00155:342:HHGFNDSXY:1:2119:23918:12571 2:N:0:GAACCTAG+TCCGCATA
GCTGTGTCCACTTAAGGACTCACCTCTGGCCTATGGTCATCTCAATGCCGAGAGGGATGTGGAGGGCG
AGAGAGAGCGACTTCTCTCGGGCCCACAGCCTTACAGCTGTGGAGCACGGTATCCTCTGCCGAAACAG
+
FFFFFFFFF:FFFFFFFFFFFFFFFFFFFFFF:FFFFFFFFFFFFFFFFFFFFFFFFFFFFFFFFFFF
FFFFFFFFFFFFFFFFFFFFFFFFFFFFFFF:FFFFFFFFFFFFFFFFFFFFFFFFFFFFFFFFFFFF
@A00155:342:HHGFNDSXY:1:2120:27787:9596 2:N:0:GAACCTAG+TCCGCATA
AAAAGGACTCGTCAGGTAGCCTACTAGACTACGACTTACACGGTAGGTCGTGAACAAGCCCGGAGTAC
ATCTCCTTTGGAACTAGGAGACCCCTCCGGTCTTGTCCAACCTCTGTTTCGGCAGAGGATACCGTGCT
+
FFFFFFFFFFFFFFFFFFFFFFFFFFFFFFFFFFFFFFFFFFFFFFFFFFFFFFFFFFFFFFFFFFFF
FFFFFFFFFFFFFFFFFFFFF:FFFFFFFFFFFFFFFFFFFFFFFFFFFFFFFFFFFFFFFFFFFFFF
@A00155:342:HHGFNDSXY:1:2120:29279:12680 2:N:0:GAACCTAG+TCCGCATA
GGTAGGTCGTGAACAAGCCCGGAGTACATCTCCTTTGGAACTAGGAGACCCCTCCGGTCTTGTCCAAC
CTCTGTTTCGGCAGAGGATACCGTGCTCCACAGCTGTAAGGCTGTGGGCCCGAGAGAAGTCGCTCTCT
+
FFFFFFFFFFFFFFFFFFFFFFFFFFFFFFFFFFFFFFF,FFFFFFFFFFFFFFFFFFFFFFFFFFFF
FFFFFFFFFFFFFFFFFFFFFFFFFFFFFFFFFFFFFFFFFFFFFFFFFFFFFFFFF:FFFFFFFFFF
@A00155:342:HHGFNDSXY:1:2120:6958:20306 2:N:0:GAACCTAG+TCCGCATA
TTTCGTCCTAAAAAGGACTCGTCAGGTAGCCTACTAGACTACGACTTACACGGTAGGTCGTGAACAAG
CCCGGAGTACATCTCCTTTGGAACCAGGAGACCCCTCCGGTCTTGTCCAACCTCTGTTTCGGCAGAGG
+
FFFFFFFFFFFFFFFFFFFF:FFFFFFFFFFFFFFFFFFFFFFFFFFFFFFFFFFFFFFFFFFFFFFF
FFFFFFFFFFFFFFFFFFFFFFFFFFFFFFFFFFF:FFFFFFFFFFFFFFF,FFFFFFFF,FFFFFFF
@A00155:342:HHGFNDSXY:1:2120:25446:21324 2:N:0:GAACCTAG+TCCGCATA
TCGTGAACAAGCCCGGAGTACATCTCCTTTGGAACTAGGAGACCCCTCCCGTCTTGTCCAACCTCTGT
TTCGGCAGAGGATACCGTGCTCCACAGCTGTAAGGCTGTGGGCCCGAGAGAAGTCGCTCTCTCTCGCC
+
FFFFFFFFFFFFFFFFFFFFFFFFFFFFFFFFFFFFFFFFFFFFFFFFFFFFFF:FFFFFFFFFFFFF
FFFFFFFFFFFFFFFFFFFFFFFFFFFFFFFFFFFFFFFFFFFFFFFFFFFFFFFFFFFFFFFFFFFF
@A00155:342:HHGFNDSXY:1:2120:28212:23422 2:N:0:GAACCTAG+TCCGCATA
AAAAGGACTCGTCAGGTAGCCTACTAGACTACGACTTACACGGTAGGTCGTGAACAAGCCCGGAGTAC
ATCTCCTTTGGAACTAGGAGACCCCTCCGGTCTTGTCCAACCTCTGTTTCGGCAGAGGATACCGTGCT
+
FFFFFFFFFFFFFFFFFFFFFFFFFFFFFFFFFFFFFFFFFFFFFFFFFFFFFFFFFFFF:FFFFFFF
FFFFFF:FFFFFFFFFFFFFFFFFFFFFFFFFFFFFFFFFFFFFFFFFFFFFFFFFFFFFFFFFFFFF
@A00155:342:HHGFNDSXY:1:2120:31910:34522 2:N:0:GAACCTAG+TCCGCATA
AAAAGGACTCGTCAGGTAGCCTACTAGACTACGACTTACACGGTAGGTCGTGAACAAGCCCGGAGTAC
ATCTCCTTTGGAACTAGGAGACCCCTCCGGTCTTGTCCAACCTCTGTTTCGGCAGAGGATACCGTGCT
+
```

```
FFFFFFFFFFFFFFFFFFFFFFFFFFFFFFFFFFFFF:F:FFFFFF:,FFFFFFFFFF:FFFFFFFFF
FFFFFF:FFFFF,FFFF:FFFFFFFFFF:FFF,FFFFFFFFFFFFFFF,FFFFFFFFFFFFFFF,:FF
@A00155:342:HHGFNDSXY:1:2121:3522:34021 2:N:0:GAACCTAG+TCCGCATA
AACTAGGAGACCCCTCCGGTCTTGTCCAACCTCTGTTTCGGCAGAGGATACCGTGCTCCACAGCTGTA
AGGCTGTGGGCCCGAGAGAAGTCGCTCTCTCTCGCCCTCCACATCCCTCTCGGCATTGAGATGACCAT
+
FFFFFFFFFFFFFFFFFFFFFFFFFFFFFFFFFFFFFFFFFFFFFFFFFFFFFFFFFFFFFFFFFFFF
FFFFFFFFFFFFFFFFFFFFFFFFFFFFFFFFFFFFFFFFFFFFFFFFFFFFFFFFFFFFFFFFFFFF
@A00155:342:HHGFNDSXY:1:2122:18069:15562 2:N:0:GAACCTAG+TCCGCATA
AAGGACGAAACCGTCGTTACTCTTCGAGTTGTGTAAGTTTCGTCCTAAAAAGGACTCGTCAGGTAGCC
TACTAGACTACGACTTACACGGTAGGTCGTGAACAAGCCCGGAGTACATCTCCTTTGGAACTAGGAGA
+
FFFFFFFFFFFFFFFFFFFFFFFFFFFFFFFFFFFFFFFFFFFFFFFFFFFFFFFFFFFFFFFFFFFF
FFFFFFFFFFFFFFFFFFFFFFFFFF,FFFFFFFFFFFFFFFFFFFFFFFFFFF,FFFFFFFFFFFFF
@A00155:342:HHGFNDSXY:1:2122:29342:23782 2:N:0:GAACCTAG+TCCGCATA
TTACACGGTAGGTCGTGAACAAGCCCGGAGTACATCTCCTTTGGAACCAGGAGACCCCTCCGGTCTTG
TCCAACCTCTGTTTCGGCAGAGGATACCGTGCTCCACAGCTGTAAGGCTGTGGGCCCGAGAGAAGTCG
+
FFFFFFFFFFFFFFFFFFFFFFFFFFFFFFFFFFFFFFFFFFFFFFFFFFFFFFFFFFFFFFFFFFFF
FFFFFFFFFFFFFFFFFFFFFFFF:FFFFFFFFFFFF:FFFFFFFFFFFFFFFFFFFFFFFFFFFFFF
@A00155:342:HHGFNDSXY:1:2122:19199:33238 2:N:0:GAACCTAG+TCCGCATA
AGTTTCGTCCTAAAAAGAACTCGTCAGGTAGCCTACTAGACTACGACTTACACGGTAGGTCGTGAACA
AGCCCGGAGTACATCTCCTTTGGAACTAGGAGACCCCTCCGGTCTTGTCCAACCTCTGTTTCGGCAGA
+
FFFFFFFFFFFFFFFFFFFFFFFFFFFFFFFFFFFFFFFFF:FFFFFFFFFFFFFFFFFFFFFFFFFF
FFFFFFFFFFFF:FFFFFFFFFFFFFFFFFFFF:FFFFFFFFFFFFFFFFFFFFFFFFFFFFFFFFFF
@A00155:342:HHGFNDSXY:1:2122:11957:35540 2:N:0:GAACCTAG+TCCGCATA
ACTCGTCAGGTAGCCTACTAGACTACGACTTACACGGTAGGTCGTGAACAAGCCCGGAGTACATCTCC
TTTGGAACCAGGAGACCCCTCCGGTCTTGTCCAACCTCTGTTTCGGCAGAGGATACCGTGCTCCACAG
+
FFFFFFFFFFFFFFFFFFFFFFFFFFFFFFFFFFFFFFFFF:FFFFFFFFF:FFFFFFFFFFFFFFFF
FFFFFFFFFFFFFFFFFF:FFFFFFFFFFFFFFFFFFFFFFFFF:F:FFFFFFFFF:F:FFFFFFFFF
@A00155:342:HHGFNDSXY:1:2122:1362:36511 2:N:0:GAACCTAG+TCCGCATA
GTTTCGTCCTAAAAAGGACTCGTCAGGTAGCCTACTAGACTACGACTTACACGGTAGGTCGTGAACAA
GCCCGGAGTACATCTCCTTTGGAACTAGGAGACCCCTCCGGTCTTGTCCAACCTCTGTTTCGGCAGAG
+
FFFFFFFFFFFFFF:FFFFFFFF::FFFFF,:FFFFFFFFFFFF,FFFFF:FFFFFFFFFFFFFFFFF
FFFFFFFFFFFFFFFFFFFFFFFFFFFFFFF:FFFFFFFFFFFFFFFFFFFFFF::FFFFFFFFFF,F
@A00155:342:HHGFNDSXY:1:2123:12210:6574 2:N:0:GAACCTAG+TCCGCATA
TACGACTTACACGGTAGGTCGTGAACAAGCCCGGAGTACATCTCCTTTGGAACTAGGAGACCCCTCCG
GTCTTGTCCAACCTCTGTTTCGGCAGAGGATACCGTGCTCCACAGCTGTAAGGCTGTGGGCCCG
+
FFFFFFFFFFFFFFFFFFFFFFFFFFFFFFFFFFFFFFFFFFFFFFFFFFF,FFFFFFFFFFFFFFFF
FFFFFFFFFFFFFFFFFFFFFFFFFFFFFFFFFFFFFFFFFFFFFF:FFFFFFFFFFFFFFFFF
@A00155:342:HHGFNDSXY:1:2123:18954:9111 2:N:0:GAACCTAG+TCCGCATA
GGTAGCTTACTAGACTACGACTTACACGGTAGGTCGTGAACAAGCCCGGAGTACATCTCCTTTGGAAC
TAGGAGACCCCTCCGGTCTTGTCCAACCTCTGTTTCGGCAGAGGATACCGTGCTCCACAGCTGTAAGG
+
FFF:FFFFFFFFFFFFFFFFFFFFFFFFFFFFFFFFFFFFFFFFFFFFFFFFF:FFFFFFFFFFFF:F
FFFFFFFFFFFFFFFFFFFFFFFFFFFFFFFFFFFFFFFFFFFFFFFFFFFFF:FFFFFFFFFFFFFF
@A00155:342:HHGFNDSXY:1:2123:4544:20415 2:N:0:GAACCTAG+TCCGCATA
AGCCCGGAGTACATCTCCTTTGGAACTAGGAGACCCCTCCGGTCTTGTCCAACCTCTGTTTCGGCAGA
GGATACCGTGCTCCACAGCTGTAAGGCTGTGGGCCCGAGAGAAGTCGCTCTCTCTCGCCC
+
```

FFFFFFFFFFFFFFFFFFFFFFFFFFFFFFFFFFFFFFFFFFFFFFFFFFFFFFFFFFFFFFFFFFFF
FFFFFFFFFFFFFFFFFFFFFFFFFFFFFFFFFFFFFFFFFF:FFFFFFFFFFFFFFFFF
@A00155:342:HHGFNDSXY:1:2124:3450:28667 2:N:0:GAACCTAG+TCCGCATA
CAAGGACGAAACCGTCGTTACTCTTTGAGTTGTGTAAGTTTCGTCCTAAAAAGGACTCGTCAGGTAGC
CTACTAGACTACGACTTACACGGTAGGTCGTGAACAAGCCCGGAGTACATCTCCTTTGGAACTAGGAG
+
FFFFFFFFFFFF:FFFFFFFFFFFFF:F:FFFFFFFFFFFFFFFFFF,F:F,FFFFFFFF:FF:FFFF
FFF::FFFFFFF,:FFFFFFFFFFFFF:,FFF,FFFFFFFFFFFFFFFFFFF,FFFFF:FFFFFF,FF
@A00155:342:HHGFNDSXY:1:2124:28908:28917 2:N:0:GAACCTAG+TCCGCATA
GAGACCCCTCCGGTCTTGTCCAACCTCTGTTTCGGCAGAGGATACCGTGCTCCACAGCTGTAAGGCTG
TGGGCCCGAGAGAAGTCGCTCTCTCTCGCCCTCCACATCCCTCTCGGCATTGAGATGACCATAGGCC
+
FFFFFFFFFFFFFFFFFFFFFFFFFFFFFFFFFFFFFFFFFFFFFFFFFFFFFFFFFF:FFFFFFFFF
FFFFFF,FFFFFF:F:FFFFFFFFFFF,FFFFF,FFFFF:FFFFFFFFFF:FFFFFFFFFFFFFFFF
@A00155:342:HHGFNDSXY:1:2124:31882:31156 2:N:0:GAACCTAG+TCCGCATA
TACGACTTACACGGTGGGTCGTGAACAAGCCCGGAGTACATCTCCTTTGGAACTAGGAGACCCCTCCG
GTCTTGTCCAACCTCTGTTTCGGCAGAGGATACCGTGCTCCACAGCTGTAAGGCTGTGGGCCCG
+
FFFFFF:FFFFFFFF,FFFFFFFFFFFFFFFFFFFFFFF,:FFFFFFFFFFFFFFFFFFFFFF:FFFF
FFF,:FFFF:FFFFFFFFF:F:FF,FFFFF:,FFFFFFFF:FFFFFFF,FFFFFFFFFFFFFFF
@A00155:342:HHGFNDSXY:1:2125:23231:10755 2:N:0:GAACCTAG+TCCGCATA
GGACTCGTCAGGTAGCCTACTAGACTACGACTTACACGGTAGGTCGTGAACAAGCCCGGAGTACATCT
CCTTTGGAACTAGGAGACCCCTCCGGTCTTGTCCAACCTCTGTTTCGGCAGAGGATACCGTGCTCCAC
+
FFFFFFFFFFFFFFFFFFFFFFFFFFFFFFFFFFFFFFFFFFFFFFFFFFFFFFFFFFFFFFFFFFFF
FFFFFFFFFFFFFFFFFFFFFFF:FFFFFFFFFFFFFFFFFFFFFFFFFFFFFFFFFFFFFFFFFFFF
@A00155:342:HHGFNDSXY:1:2125:1940:18004 2:N:0:GAACCTAG+TCCGCATA
TAGGACGAAACTTACACAACTCGAAGAGTAACGACGGTTTCGTCCTTGGAACGGACTCATCAGGGAGC
CTACTAGACTCCGACCGTCATGGGGGTTCAAACCCATGCCCCGCCACACCGCCTTAGATCAGCTGTGT
+
FFFFFFFFFFFFFFFFF:FFFFFFFFFFFFFFFFFFFF,FFFFFFFFFFFFFFFFFFF,FF:FFFFFF
FFFFFFFFFFFFFFFFFFFFFFFFFFFFFF:FFFFFFFFFFFFFFFFFFFFFFFFFFF:FFFFFFFFF
@A00155:342:HHGFNDSXY:1:2125:7500:22247 2:N:0:GAACCTAG+TCCGCATA
GTGAACAAGCCCGGAGTACATCTCCTTTGGAACTAGGAGACCCCTCCGGTCTTGTCCAACCTCTGTTT
CGGCAGAGGATACCGTGCTCCACAGCTGTAAGGCTGTGGGCCCGAGAGAAGTCGCTCTCTCTCGCCCT
+
FFFFFFFFFFFFFFFFFFFFFFFFFFFFFFFFFFFFFFFFFFFFFFFFFFFFFFFFFFFFFFFFFFFF
FFFFFFFFFFFFFFFFFFFFFFFFFFFFFFFFFF:FFFFFFFFFFFFFF,F:FFFFFFFFFFFFFFFF
@A00155:342:HHGFNDSXY:1:2126:1994:5541 2:N:0:GAACCTAG+TCCGCATA
GGACTCGTCAGGTAGCCTACTAGACTACGACTTACACGGTAGGTCGTGAACAAGCCCGGAGTACATCT
CCTTTGGAACCAGGAGACCCCTCCGGTCTTGTCCAACCTCTGTTTCGGCAGAGGATACCGTGCTCCAC
+
F:FFFFFFFFFFFFFFFFFFFFFFFF,FFFFFFFFFFFFF:FFFFFFFFFFFF,FFFFFFFFFFFFFF
FFF,FFFFFFFFFFFFFFFFFFFFFFFFFFFFFF:FFFFFFFF:FFFFF:FFFFFFFFFFFFFFFFFF
@A00155:342:HHGFNDSXY:1:2126:30743:25394 2:N:0:GAACCTAG+TCCGCATA
GGTTTCGTCCTTGGAACGGACTCATCAGGGAGCCTACTAGACTCCGACCGTCATGGGGGTTCAAACCC
ATGCCCCGCTACACCGCCTTAGATCAGCTGTGTCCACTTAAGGACTCACCTCTGGCCTATGGTCATCT
+
FFFFFFFFFFFFFFFFFFFFFFFFFFFFFFFFFFFFFFFFFFFFFFFFFFFFFFFFFFFFFFFFFFFF
FFFFFFFFFFFFFFFFFFF:FFFFFFFFFFFFFFFFFFFFFFFFFFFFFFFFFFFFFFFFFFFFFFFF
@A00155:342:HHGFNDSXY:1:2127:22580:3082 2:N:0:GAACCTAG+TCCGCATA
GAACTAGGAGACCCCTCCGGTCTTGTCCAACCTCTGTTTCGGCAGAGGATACCGTGCTCCACAGCTGT
AAGGCTGTGGGCCCGAGAGAAGTCGCTCTCTCTCGCCCTCCACATCCCTCTCGGCATTGAGATGAC
+

FFFFFFFFFFFFFFFFFFFFFFFFFFFFFFFFFFFFFFFFFFFFFFFFFFFFFFFFFFFFFFFFFFFFFF
FFFFFFFFFFFFFFFFFFFFF,FFFFFFFFFFFFF:FFFFFFFFFFFFFFFFFFFFFF,FFFFFFFFF
@A00155:342:HHGFNDSXY:1:2127:30798:9549 2:N:0:GAACCTAG+TCCGCATA
GTGAACAAGCCCGGAGTACATCTCCTTTGGAACTAGGAGACCCCTCCGGTCTTGTCCAACCTCTGTTT
CGGCAGAGGATACCGTGCTCCACAGCTGTAAGGCTGTGGGCCCGAGAGAAGTCGCTCTCTCTCGCCCT
+
FFFFFFFFFFFFFFFFFFFFFFFFFFFFFFFFFFFFFFFFFFFFFFFFFFFFF,,FFFF,FFFFFFFFF:
FFFFFFFFFFFFFFFFFFFFFFFFFF,FFF,FFFFFFFFFFFFFFFFFFF,FFFF:FFFFFFFFFFFFFF
@A00155:342:HHGFNDSXY:1:2128:24243:15515 2:N:0:GAACCTAG+TCCGCATA
TAGACTACGACTTACACGGTAGGTCGTGAACAAGCCCGGAGTACATCTCCTTTGGAACCAGGAGACCC
CTCCGGTCTTGTCCAACCTCTGTTTCGGCAGAGGATACCGTGCTCCACAGCTGTAAGGCTGTGGGCCC
+
FFFFFFFFFFFFFFFFFFFFFFFFFFFFFFFFFFFFFFFFFFFFFFFFFFFFFFFFFF:FFFFFFFFFFF
FFFFFFFFFFFFFFFFFFFFFFFFFFFFFFFFFFFFFFFF:FFFFFFFFFFFFFFFFFFFFFFFFFFFF:
@A00155:342:HHGFNDSXY:1:2129:6497:2347 2:N:0:GAACCTAG+TCCGCATA
GTGTAAGTTTCGTCCTAAAAAGGACTCGTCAGGTAGCCTACTAGACTACGACTTACACGGTAGGTCGT
GAACAAGCCCGGAGTACATCTCCTTTGGAACCAGGAGACCCCTCCGGTCTTGTCCAACCTCTGTTTCG
+
FFFFFFFFFF:FFFF:FFFFFFFFFFFFFFF:,FFFFFFFFFF::FFFFFFFFFF:,FFFFFFFFFF,FF
FFFFFFFFFFFFFFF,FF:FFFFF:FFFFFFFFFFF,FFFFFFFFFFFFFFFFFF,FFFFFFF:FFFFFF
@A00155:342:HHGFNDSXY:1:2129:9489:18051 2:N:0:GAACCTAG+TCCGCATA
CCTCTGGCCTATGGTCATCTCAATGCCGAGAGGGATGTGGAGGGCGAGAGAGAGCGACTTCTCTCGGG
CCCACAGCCTTACAGCTGTGGAGCACGGTATCCTCTGCCGAAACAGAGGTTGGACAAGACCGGAG
+
FFFFF:FF,FFFFFFFFFFF:F:FFF::F,FFFFF::FFF:FFFFFFFFF:F:F:FFFFFFFFFFF:F:F
,:FFFF,FF,FFFFFFFFFFFFFFFF,FFFFF,FFFF:FFFFF:F:FFFFFF,FFFFFFFFFFF,:F
@A00155:342:HHGFNDSXY:1:2129:30879:20619 2:N:0:GAACCTAG+TCCGCATA
GTTGTGTAAGTTTCGTCCTAAAAAGGACTCGTCAGGTAGCCTACTAGACTACGACTTACACGGTAGGT
CGTGAACAAGCCCGGAGTACATCTCCTTTGGAACCAGGAGACCCCTCCGGTCTTGTCCAACCTCTGT
+
F:,FF:FFFFF:F,,FF:FFFFFFFFF:FFFFF,:F,:FF:,F:FFFF,F:FFF:FF:FF:F:F,F:F,,
FFF,FF:FF,F:FF,FFFF:F,FFFF:,:FFF:FF::,F,FFF:F,FFFFFFF:F,FFFF:,FFFFFFF
@A00155:342:HHGFNDSXY:1:2129:20772:24940 2:N:0:GAACCTAG+TCCGCATA
TCAGGTAGCCTACTAGACTACGACTTACACGGTAGGTCGTGAACAAGCCCGGAGTACATCTCCTTTGG
AACCAGGAGACCCCTCCGGTCTTGTCCAACCTCTGTTTCGGCAGAGGATACCGTGCTCCACAGCTGTA
+
FFFFFFFFFFFFFFFFFFFFFFFFFFFFFFFFFFFFFFFFFFFFFFFFFFFFFFFFFFFFFFFFFFFFFF
FFFFFFFFFFFFFFFFFFFFFF:FFFFFFFFFFFFFFFFFFFFFFFFFFF:FFFFFFFFFFFFFFFFFFF
@A00155:342:HHGFNDSXY:1:2129:12527:26349 2:N:0:GAACCTAG+TCCGCATA
TCCAAGGACGAAACCGTCGTTACTCTTTGAGTTGTGTAAGTTTCGTCCTAAAAAGGACTCGTCAGGTA
GCCTACTAGACTACGACTTACACGGTAGGTCGTGAACAAGCCCGGAGTACATCTCCTTTGGAACTAGG
+
FFFFFFFFFFFFFFFFFFFFFFFFFFFFFFFFFFFFFFFFFFFFFFFFFFFFFFFFFFFFFFFFFFFFFF
FFFFFFFFFFFFFFFFFFFFFFFFFFFFFFFFFFFFFFFFFFFFFFFFFFFFFFFFFFFF,FFFFFFFFF
@A00155:342:HHGFNDSXY:1:2130:18819:1767 2:N:0:GAACCTAG+TCCGCATA
GTGAACAAGCCCGGAGTACATCTCCTTTGGAACTAGGAGACCCCTCCGGTCTTGTCCAACCTCTGTTT
CGGCAGAGGATACCGTGCTCCACAGCTGTAAGGCTGTGGGCCCGAGAGAAGTCGCTCTCTCTCGCCCT
+
FFFFFFFFFFFFFFFFFFFFFFFFFFFFFFFFFFFFFFFFFFFFFFFFFFFFFFFFFFFFFFFFFFFFFF
FFFFFFFFFFFFFFFFFFFFFFFFFFFFFFFFFFFFFFFFFFFFFFFFFFFFFFFFFFFFFFFFF:FFFF
@A00155:342:HHGFNDSXY:1:2130:6054:12978 2:N:0:GAACCTAG+TCCGCATA
GAACTAGGAGACCCCTCCGGTCTTGTCCAACCTCTGTTTCGGCAGAGGATACCGTGCTCCACAGCTGT
AAGGCTGTGGGCCCGAGAGAAGTCGCTCTCTCTCGCCCTCCACATCCCTCTCGGCATTGAGATGAC
+

FFFFFFFFFFFFFFFFFFFFFFFFFFFF:FFFFFFFFFFFFFF,FFFFF:FFFFFFFFFFFFFFFFFF
FFFFFFFFFFFFFFFFFFFFF:FFFFF:FFFFFFFFFFFFFFFFFFFFFFFFFFFFFFFFFFFFFFFF
@A00155:342:HHGFNDSXY:1:2130:28302:14152 2:N:0:GAACCTAG+TCCGCATA
GATCAGCTGTGTCCACTTAAGGACTCACCTCTGGCCTATGGTCATCTCAATGCCGAGAGGGATGTAGA
GGGCGAGAGAGAGCGACTTCTCTCGGGCCCACAGCCTTACAGCTGTGGAGCACGGTATCCTCTGCCGA
+
FFFFFFFFFFFFFF:FFFFFFFFFFFFFFFFFFFFFFFFFFFFFFF,FFFFF:FF:FFFF:F::F,FFF
FF:FFFFFFFFF:FFFFFFFFF:FFF:FFFFFFF,FFFFFFFFF:FFFFF:FF,FF,FFFFFFFFF,F
@A00155:342:HHGFNDSXY:1:2130:5520:22420 2:N:0:GAACCTAG+TCCGCATA
GTCTTGTCCAACCTCTGTTTCGGCAGAGGATACCGTGCTCCACAGCTGTAAGGCTGTGGGCCCGAGAG
AAGTCGCTCTCTCTCGCCCTTCACATCCCTCTCGGCATTGAGATGACCATAGGCCAGAGGTGAGTCCT
+
FFFFFFFFFFFFFFFFFFFFFFFFFFFFFFFFFFFFFFFFFFFFFFFFFFFFFFFFFFFFFFFFFFFF
FFFFFFFFFFFFFFFFFFFFFFFFFFFFFFFFFFFFFFFFFFFFFFFFFFFFFFFFFFFFFFFFFFFF
@A00155:342:HHGFNDSXY:1:2130:25635:25128 2:N:0:GAACCTAG+TCCGCATA
TACACGGTAGGTCGTGAACAAGCCCGGAGTACATCTCCTTTGGAACTAGGAGACCCCTCCGGTCTTGT
CCAACCTCTGTTTCGGCAGAGGATACCGTGCTCCACAGCTGTAAGGCTGTGGGCCCGAGAGAAGTCGC
+
FFFFFFFFFFFFFFFFF:FFFFFFFFFFFFFFFFFFFFFFFFFFFF:FFFFF:FF:FFFFFFF,FFFFF
FFFFFFFFFFFFFFFFFFFF:FFFFFF::FFFFFFFF,FFFFFF:FFF,:FFFFF:FFFFFFFFFFFF
@A00155:342:HHGFNDSXY:1:2130:26612:25160 2:N:0:GAACCTAG+TCCGCATA
AAGCCTGGAGTACATCTCCTTTGGAACTAGGAGACCCCTCCGGTCTTGTCCAACCTCTGTTTCGGCAG
AGGATACCGTGCTCCACAGCTGTAAGGCTGTGGGCCCGAGAGAAGTCGCTCTCTCTCGCCCTCCACA
+
FFFFF:,FFFFFFFFFFFFFFFFFFFFFFFFFFFFFFFFFFFFFFFFFFFFFFFFFFFFFFFFFFFFFF
FFFFFFFFFFFFFFFFFFFF:FFFFFFFFFFFFFFFFFFFFFF:F:FFFFFFFFFFFFFFFFFFFFF
@A00155:342:HHGFNDSXY:1:2131:13295:7545 2:N:0:GAACCTAG+TCCGCATA
GAGACCCCTCCGGTCTTGTCCAACCTCTGTTTCGGCAGAGGATACCGTGCTCCACAGCTGTAAGGCTG
TGGGCCCGAGAGAAGTCGCTCTCTCTCGCCCTCCACATCCCTCTCGGCATTGAGATGACCATAGGCCA
+
FFFFFFFFFFFFFFFFFFFFFFFFFFFFFFFFFFFFFFFFFFFFFFFFFFFFFFFFFFFFFFFFFFFF
FFFFFFFFFF:FF:FFFFFFFFFFFFFFFFFFFFFFFFFFFFFFFFFFFFF:FFFFFFFFFFFFFFFF
@A00155:342:HHGFNDSXY:1:2132:24858:23249 2:N:0:GAACCTAG+TCCGCATA
AGCCCGGAGTACATCTCCTTTGGAACTAGGAGACCCCTCCGGTCTTGTCCAACCTCTGTTTCGGCAGA
GGATACCGTGCTCCACAGCTGTAAGGCTGTGGGCCCGAGAGAAGTCGCTCTCTCTCGCCCTC
+
FFFFFFFFFFFFFFFFFFFFFFFF:FFFFFFFFFFFFFFFFFFFFFFFFFFFFFFFFFFFFFFFFFFF
FFFF:FFFFFFFFFFFFFFFFF:FFFFFFFFFFFFFFFF:FF,FFFFFFFFFFFFFFFFFFF
@A00155:342:HHGFNDSXY:1:2133:32506:4429 2:N:0:GAACCTAG+TCCGCATA
TGAACAAGCCCGGAGTACATCTCCTTTGGAACTAGGAGACCCCTCCGGTCTTGTCCAACCTCTGTTTC
GGCAGAGGATACCGTGCTCCACAGCTGTAAGGCTGTGGGCCCGAGAGAAGTCGCTCTCTCTCGCCCTC
+
FFFFFFFFFFFFF:FFFF:FFFFF:F,FFF,FFFFFFFFFFFF,FFFFFFF,FFFF:FFF:FFF::FF
FFFFFFFF:::FFFFFF:FF:FFFFFFF:FFFF:F,FFFFFF:FFFFF,F:FFF:F:FF::F:FFFFF
@A00155:342:HHGFNDSXY:1:2133:6280:5008 2:N:0:GAACCTAG+TCCGCATA
GGAGTACATCTCCTTTGGAACTAGGAGACCCCTCCGGTCTTGTCCAACCTCTGTTTCGGCAGAGGATA
CCGTGCTCCACAGCTGTAAGGCTGTGGGCCCGAGAGAAGTCGCTCTCTCTCGCCCTCCACATCCCTCT
+
FFFFFFFFFFFFFFFFFFFFFFFFFFFFFFFFFFFFFFFFFFFFFF:FFFFFFFFFFFFFFFFFFFFFF
FFFFFFFFFFFFFFFFFFFFFFFFFFFFFFFFFFFFFFFFFFFFFFFFFFFFFFFFFFFFFFFFFFFF
@A00155:342:HHGFNDSXY:1:2133:20302:13510 2:N:0:GAACCTAG+TCCGCATA
CTACTAGACTACGACTTACACGGTAGGTCGTGAACAAGCCCGGAGTACATCTCCTTTGGAACCAGGAG
ACCCCTCCGGTCTTGTCCAACCTCTGTTTCGGCAGAGGATACCGTGCTCCACAGCTGTAAGGCTGTGG
+

```
FFFFFFFFFFFFFFFFFFFFFFFFFFFFFFFFFFFFFFFFFFFFFFFFFFFFFFFFFFFF:FFFFFFF
FFFFFFFFFFFFFFFFFFFFFFFFFFFFFFFFFFFFFFFFFFFFFFFFFFFFFFFFFFFFFFFFFFFF
@A00155:342:HHGFNDSXY:1:2133:20356:22279 2:N:0:GAACCTAG+TCCGCATA
CTACTAGACTACGACTTACACGGTAGGTCGTGAACAAGCCCGGAGTACATCTCCTTTGGAACCAGGAG
ACCCCTCCGGTCTTGTCCAACCTCTGTTTCGGCAGAGGATACCGTGCTCCACAGCTGTAAGGCTGTGG
+
FFFFFFFFFFFFFFFFFFFFFFFFFFFFFFFFFFFFF,FFFFFFFFFFFFFFFFFFFFFF:FFFFFFF
FFFFFFFFFFFFFFFFFF:FFFFFFFFFFF:FFFFFFFFFFFFFFFFFFFFFFFFFFFFFFFFFFFFF
@A00155:342:HHGFNDSXY:1:2134:15365:4930 2:N:0:GAACCTAG+TCCGCATA
CTTTGGAACTAGGAGACCCCTCCGGTCTTGTCCAACCTCTGTTTCGGCAGAGGATACCGTGCTCCACA
GCTGTAAGGCTGTGGGCCCGAGAGAAGTCGCTCTCTCTCGCCCTCTACATCCCTCTCGGCATTGAGAT
+
FFFFFFFFFFFFFFFFFFFFFFFFFFFFFFFFFFFFFFFFFFFFFFFFFFFFFFFFFFFFFFFFFFFF
FFFFFFFFFFFFFFFFFFFFFFFFFFFFFFFFFFFFFFFFFFFFFFFFFFFFFFFFFFFFFFFFFFFF
@A00155:342:HHGFNDSXY:1:2134:30798:8641 2:N:0:GAACCTAG+TCCGCATA
CCTACTAGACTACGACTTACACGGTAGGTCGTGAACAAGCCCGGAGTACATCTCCTTTGGAACTAGGA
GACCCCTCCGGTCTTGTCCAACCTCTGTTTCGGCAGAGGATACCGTGCTCCACAGCTGTAAGGCTGTG
+
FFFFFFFFFFFFFFFFFFFFFFFFFFFFFFFFFFFFFFFFFFFFFFFFFFFFFFFFFFFFFFFFFFFF
FFFFFFFFFFFFFFFFFFFFFFFFFFFFFFFFFF,FFFFFFFFFFFFFFFFF:FFFFFFF:FFFFFFF
@A00155:342:HHGFNDSXY:1:2134:15781:12258 2:N:0:GAACCTAG+TCCGCATA
AGCCCGGAGTACATCTCCTTTGGAACTAGGAGACCCCTCCGGTCTTGTCCAACCTCTGTTTCGGCAGA
GGATACCGTGCTCCACAGCTGTAAGGCTGTGGGCCCGAGAGAAGTCGCTCTCTCTCGCCCTCCACATC
+
FFFFFFFFFFFFFFFFFFFFFFFFFFFFFFFFFFFFFFFFFFFFFFFFFFFFFFFFFFFFFFFFFFFF
FFFFFFFFFFFFFFFFFFF:FFFFFFF:FFFFFFFFFFFFFFFFFFFFFF:FFFFFFFFFFFFFFFFF
@A00155:342:HHGFNDSXY:1:2134:8784:30452 2:N:0:GAACCTAG+TCCGCATA
GTTGTGTAAGTTTCGTCCTAAAAAGGACTCGTCAGGTAGCCTACTAGACTACGACTTACACGGTAGGT
CGTGAACAAGCCCGGAGTACATCTCCTTTGGAACTAGGAGACCCCTCCGGTCTTGTCCAACCTCTGTT
+
FFFFFFFFFFF:FFFFFFFFFFFFFFFFFFFFFFFFFFFFFF:FFFFFFFFFF:FFFFFFFFFFFFFF
FFFFFFFFFFFFFFFFFFFFFFFFFFF:FFF,FFF,FFFFFFFFFFFFFFF,:FFFFFFFFFFFFFF:
@A00155:342:HHGFNDSXY:1:2135:25599:1141 2:N:0:GAACCTAG+TCCGCATA
TAGCCTACTAGACTACGACTTACACGGTAGGTCGTGAACAAGCCCGGAGTACATCTCCTTTGGAACTA
GGAGACCCCTCCGGTCTTGTCCAACCTCTGTTTCGGCAGAGGATACCGTGCTCCACAGCTGTAAGGCT
+
FFFFFFFFFFFFFFFFFFFFFFFFFFFFFFFFFFFFFFFFF,F:FFFFFFFFFFFF:FFFFFFFFFFF
FFFFFFFFFFFFFFFFFFFFFFFFFFFFFFFFFFFFFFFFFFFF:FFFFFFFFFFFFFFF:FFFFFFF
@A00155:342:HHGFNDSXY:1:2135:26603:12900 2:N:0:GAACCTAG+TCCGCATA
AAAAGGACTCGTCAGGTAGCCTACTAGACTACGACTTACACGGTAGGTCGTGAACAAGCCCGGAGTAC
ATCTCCTTTGGAACCAGGAGACCCCTCCGGTCTTGTCCAACCTCTGTTTCGGCAGAGGATACCGTGCT
+
FFF:FFFFFFFFFFFFFFFFFFFFFFFFFFFFFFFFFFFFFFFFFFFFFFFFFFFFFFFFFFFFFFFF
FFFFFFFFFFFFFFFFFFFFFFFFFFFFFFFFFFFFFFFFFFFFFFFFFFFFFFFFFFF:FFFFFFFF
@A00155:342:HHGFNDSXY:1:2135:31521:30718 2:N:0:GAACCTAG+TCCGCATA
GGACGAAACTTACACAACTCGAAGAGTAACGACGGTTTCGTCCTTGGAACGGACTCATCAGGGAGCCT
ACTAGACTCCGACCGTCATGGGGGTTCAAACCCATGCCCCGCCACACCGCCTTAGATCAGCTGTGTCC
+
FFFFFFFFFFFFFFFFFFFFFFFFFFFFFFFFFFFFFFFFFFFFFFFFFFFFFFFFFFFFFFFFFFFF
FFFFFFFFFFFFFFFF:FFFFFFFFFFFFFFFFFFFFFFFFFFFFFFFFFFFFFFFFFF:FFFFFFF
@A00155:342:HHGFNDSXY:1:2135:21667:32033 2:N:0:GAACCTAG+TCCGCATA
GGTCGTGAACAAGCCCGGAGTACATCTCCTTTGGAACTAGGAGACCCCTCCGGTCTTGTCCAACCTCT
GTTTCGGCAGAGGATACCGTGCTCCACAGCTGTAAGGCTGTGGGCCCGAGAGAAGTCGCTCTCTCTCG
+
```

FF:FFFFFFFFFFFFFFFFFFFFFFFFFFFFFFFFFFFFFFFFFFFFFFFFFFFFFFFFFFFFFFFFF
F:FFFFFFFFFFFFF:FFFFFFFFFFFFFFFFFFFFFF:FFFFFFFFFFFFFF:FFFFFFFFFFFFFF
@A00155:342:HHGFNDSXY:1:2135:21314:33395 2:N:0:GAACCTAG+TCCGCATA
GGTCGTGAACAAGCCCGGAGTACATCTCCTTTGGAACTAGGAGACCCCTCCGGTCTTGTCCAACCTCT
GTTTCGGCAGAGGATACCGTGCTCCACAGCTGTAAGGCTGTGGGCCCGAGAGAAGTCGCTCTCTCTCG
+
FFFFFFFFFFFFFFFFFFFFFFFFFFFFFFFFFFFFFFFFFFFFFFFFFFFFFFFFFFFFFFFFFFFF
FFFFFFFFFFFFFFFFFFFFFFFFFFFFFFFFFFFFFF,FFFFFFFFFFFFFFFFFFFFFFFFFFFFF
@A00155:342:HHGFNDSXY:1:2136:23737:3239 2:N:0:GAACCTAG+TCCGCATA
GTTGTGTAAGTTTCGTCCTAAAAAGGACTCGTCAGGTAGCCTACTAGACTACGACTTACACGGTAGGT
CGTGAACAAGCCCGGAGTACATCTCCTTTGGAACTAGGAGACCCCTCCGGTCTTGTCCAACCTCTGTT
+
FFFFFF,:FF,FFFFFFFFFFFFFFFFFFFFFFFFFFFFFFFF:FFFFFFF:FFF:FFFFFFFFF:FF
FFFFFFFF:FFFFFFFFFFF:FFFFFFFF:FF:FF:FFFFFFFFFFFFF:FF:FFFFFFFFFFFFFFF
@A00155:342:HHGFNDSXY:1:2137:6153:3537 2:N:0:GAACCTAG+TCCGCATA
CACGGTAGGTCGTGAACAAGCCCGGAGTACATCTCCTTTGGAACTAGGAGACCCCTCCGGTCTTGTCC
AACCTCTGTTTCGGCAGAGGATACCGTGCTCCACAGCTGTAAGGCTGTGGGCCCGAGAGAAGTCGCTC
+
FFFFFFFFFFFFFFFFFFFFFFFFFFFFFFFFFFFFFFFFFFFFFFFFFFFFFFFFFFFFFFFFFFFF
FFFFFFFFFFFFFFFFFFFFFFFFFFFFFFFFFFFFFFFFFFFFFFFFFFFFFFFFFFFFFFFFFFFF
@A00155:342:HHGFNDSXY:1:2137:20238:6136 2:N:0:GAACCTAG+TCCGCATA
TGAACAATCCCGGAGTACATCTCCTTTGGAACTAGGAGACCCCTCCGGTCTTGTCCAACCTCTGTTTC
GGCAGAGGATACCGTGCTCCACAGCTGTAAGGCTGTGGGCCCGAGAGAAGTCGCTCTCTCTCGCC
+
F,FF,FF,:FF::FFFFFFFFF:,FFFF:FF,FF,FFFFFF,FFFFFF::FFF:F,FFFF:F:FF:FF
,::FFFF:FFF:,:FFF,FFFF::,FFFF:,:FFFFFF,,FFFFFFFFFFF,FFFF:F,,FF::F
@A00155:342:HHGFNDSXY:1:2137:16387:9361 2:N:0:GAACCTAG+TCCGCATA
ACTCTTTGAGTTGTGTAAGTTTCGTCCTAAAAAGGACTCGTCAGGTAGCCTACTAGACTACGACTTAC
ACGGTAGGTCGTGAACAAGCCCGGAGTACATCTCCTTTGGAACTAGGAGACCCCTCCGGTCTTGTCCA
+
FFFFFFFFFFFF:FFFF,FFFFFFFFFFFFFFFFFFFFFF:FFFFFFFFFFFFFFFFFFFFFFFFFFF
FFFFFF,FFFFFFFFFFFFFFFFFFFFFFFFFFFFFFFFFFFFFFFFFFFFFFFFFFFFFFFFFFFFF
@A00155:342:HHGFNDSXY:1:2137:10872:13463 2:N:0:GAACCTAG+TCCGCATA
AAAAGGACTCGTCAGGTAGCCTACTAGACTACGACTTACACGGTAGGTCGTGAACAAGCCCGGAGTAC
ATCTCCTTTGGAACTAGGAGACCCCTCCGGTCTTGTCCAACCTCTGTTTCGGCAGAGGATACCGTGCT
+
FFFFFFFFFFFFFFFFFFFFFFFFFFFFFFFFFFFFFFFFFFFFFFFFFFFFFFFFFFFFFFFFFFFF
FFFFFFFFFFFFFFFFFFFFFFFFFFFFFFFFFFFFFFFFFFFFFFFFFFFFFFFFFFFF:FFFFFF:
@A00155:342:HHGFNDSXY:1:2137:28836:22811 2:N:0:GAACCTAG+TCCGCATA
GAGTACATCTCCTTTGGAACTAGGAGACCCCTCCGGTCTTGTCCAACCTCTGTTTCGGCAGAGGATAC
CGTGCTCCACAGCTGTAAGGCTGTGGGCCCGAGAGAAGTCGCTCTCTCTCGCCCTCCAC
+
FFFF,FFFFFFFFFFFFFFFFFFFFFFFFFFFFFFFFFFFFFFFFFFFFFFFFFFFFFFFFFFFFFFF
FFFFFFFFFFFFFFFFF,FFFFFFFFFFFFFFFFFF:FFFFFFFFFFFFFFFFFFFFFF
@A00155:342:HHGFNDSXY:1:2138:11559:31250 2:N:0:GAACCTAG+TCCGCATA
AGTACATCTCCTTTGGAACTAGGAGACCCCTCCGGTCTTGTCCAACCTCTGTTTCGGCAGAGGATACC
GTGCTCCACAGCTGTAAGGCTGTGGGCCCGAGAGAAGTCGCTCTCTCTCGCCCTCTACATCCCTCTCG
+
FFFFFFFFFFFFFFFFFFFFFFFFFFFFFFFFFFFFFFFFFFFFFFFFFFFFFFFFFFFFFFFFFFFF
FFFFFFFFFFFFFFFFFFFFFFFFFFFFFFFFFFF:FFFFFFFFFFFFFFFFFFFFFFFFFFFFFFFF
@A00155:342:HHGFNDSXY:1:2139:7021:12900 2:N:0:GAACCTAG+TCCGCATA
AAACCGTCGTTACTCTTCGAGTTGTGTAAGTTTCGTCCTAAAAAGGACTCGTCAGGTAGCCTACTAGA
CTACGACTTACACGGTAGGTCGTGAACAAGCCCGGAGTACATCTCCTTTGGAACTAGGAGACCCCTCC
+

```
FFFFFFFFFFFFFFFFFFFFFFFFFFFFFFFFFFFFFFFFFFFFFFF:FFFFF,FFF,FFFFFFFFF
FFFFFFFFFFFFFFFFFFFFFFFFFFFFFF:FFFFFFFFFFFFFFF:FFF:FF:FFFFFFFFFFFF:FF
@A00155:342:HHGFNDSXY:1:2139:15004:27070 2:N:0:GAACCTAG+TCCGCATA
CCTTTTTAGGACGAAACTTACACAACTCGAAGAGTAACGACGGTTTCGTCCTTGGAACGGACTCATCA
GGGAGCCTACTAGACTCCGACCGTCATGGGGGTTCAAACCCATGCCCCGCCACACCGCCTTAGATCAG
+
FF::FFFFFFFFFFFFFFFFFFFFFFFFFFFFFFFFFFFFFFFFFFFFFFFFFFFFFFFFFFFFFFFF
FFFFFFFFFFFFFFFFFFFFFFFFFFFFFFFFFFFFFFFFFFFFFFFFFFF:FFF:FFFFFFFFFFFF
@A00155:342:HHGFNDSXY:1:2139:20021:31563 2:N:0:GAACCTAG+TCCGCATA
GAACTAGGAGACCCCTCCGGTCTTGTCCAACCTCTGTTTCGGCAGAGGATACCGTGCTCCACAGCTGT
AAGGCTGTTGGCCCGAGAGAAGTCGCTCTCTCTCGCCCTCCACATCCCTCTCGGCATTGAGATGACCA
+
FFFFFFFFFFFFFFFFFFFFFFFFFFFFFFFFFFFF:FFFFFFFFFFFFFFFFFFFFFFFFFFFFFFF
FFFFFFFFFFFFFFFFFFFFFFFFFFFFFFFFFFFFFFFFFFFFFFFFFFFFFFFFFFFFFFFFFFFF
@A00155:342:HHGFNDSXY:1:2139:27471:31563 2:N:0:GAACCTAG+TCCGCATA
CGTCAGGTAGCCTACTAGACTACGACTTACACGGTAGGTCGTGAACAAGCCCGGAGTACATCTCCTTT
GGAACTAGGAGACCCCTCCGGTCTTGTCCAACCTCTGTTTCGGCAGAGGATACCGTGCTCCACAG
+
FFFFFFFFFFFFFFFFFFFFFFFFFFFFFFFFFFFFFFFFFFFFFFF,:FFFFFFFFF:FFFFFFFF
FFF:FFFFFFFFFFFFFFFFFFFFFFFFFFFFFFFFFFFFFFFFFFFFFFF:FFFFFFFFFFFFF
@A00155:342:HHGFNDSXY:1:2140:17734:16078 2:N:0:GAACCTAG+TCCGCATA
AGTTTCGTCCTAAAAAGGACTCGTCAGGTAGCCTACTAGACTACGACTTACACGGTAGGTCGTGAACA
AGCCCGGAGTACATCTCCTTTGGAACTAGGAGACCCCTCCGGTCTTGTCCAACCTCTGTTTCGGCAGA
+
FFFFFFFFFFFFFFFFFFFFFFFFFFFFFFFFFFFFFFFFFFFFFFFFFFFFFFFFFFFFFFFFFFFF
FFFFFFFFFFFFFFFFFFFFFFFFFFFFFFFFFFFFFFFFFFFFFFFFFFFFFFFFFFFFFFFFFFFF
@A00155:342:HHGFNDSXY:1:2140:19669:29324 2:N:0:GAACCTAG+TCCGCATA
GTAAGTTTCGTCCTAAAAAGGACTCGTCAGGTAGCCTACTAGACTACGACTTACACGGTAGGTCGTGA
ACAAGCTCGGAGTACATCTCCTTTGGAACTAGGAGACCCCTCCGGTCTTGTCCAACCTCTGTTTCGGC
+
FFFFFF:FFFFFFFF:FFFFFFFFFFFFFFFFFFFFFFFFFFFFFFFFFFFFFFFFFFFFFFFFF,FF
FFFFFFFFFFFFFFFFFFFFFFFFF:FFFFFFFFFFFFFFFFFFFF:FFFFFFFF:FFFFFFFFF::FF
@A00155:342:HHGFNDSXY:1:2140:23665:30483 2:N:0:GAACCTAG+TCCGCATA
AAAAGGACTCGTCAGGTAGCCTACTAGACTACGACTTACACGGTAGGTCGTGAACAAGCCCGGAGTAC
ATCTCCTTTGGAACTAGGAGACCCCTCCGGTCTTGTCCAACCTCTGTTTCGGCAGAGGATACCGTGCT
+
FFFFFFFFFFFFFFFFFFFFFFFFFFFFFFFFFFFFFFFFFFFFF:FFFFFFFFFFFFFFFFFFFFFFFF
FFFFFFFFFFFF,FFFFFFFFFFFFFFFFFFFFFFFFFFFFFFFFFFFFFFFFFFFFFFF:FF:F:F
@A00155:342:HHGFNDSXY:1:2141:12183:1642 2:N:0:GAACCTAG+TCCGCATA
TAGACTACGACTTACACGGTAGGTCGTGAACAAGCCCGGAGTACATCTCCTTTGGAACTAGGAGACCC
CTCCGGTCTTGTCCAACCTCTGTTTCGGCAGAGGATACCGTGCTCCACAGCTGTAAGGCTGTGGGCCC
+
FFF:FFFFFFFFFFFFFFFFFFFFFFFFFFFFFFFFFFFFFFFFFFFFFFFFFFFFFFFFFFFFFFFF
FFFFFFFFFFFFFFFFFFFFFFFFFFFFFFFFFFFFF:FFFFFFFFFFFFFFFFFFFFFFFFFFFFFF
@A00155:342:HHGFNDSXY:1:2141:10872:2127 2:N:0:GAACCTAG+TCCGCATA
GTAAGTTTCGTCCTAAAAAGGACTCGTAAGGTAGCCTACTAGACTACGACTTACACGGTAGGTCGTGA
ACAAGCCCGGAGTACATCTCCTTTGGAACTAGGAGACCCCTCCGGTCTTGTCCAACCTCTGTTTCGGC
+
FFFFFFFFFFFFFFFFFFFFFFFFFFFFFFFFFFFFFFFFFFFFFFFFFFFFFFFFFFFFFFFFFFFF
FFFFFFFFFFFFFFFFFFFFFFFFFFFFFFFFFFF:FFFFFFFFFFFFFFFFFFFFFFFFFFFFFFFF
@A00155:342:HHGFNDSXY:1:2141:11397:7513 2:N:0:GAACCTAG+TCCGCATA
CCGAGAGAAGTCGCTCTCTCTCGCCCTCCACATCCCTCTCGGCATTGAGATGACCATAGGCCAGAGGT
GAGTCCTTAAGTGGACACAGCTGATCTAAGGCGGTGTGGTGGGGCATGGGTTTGAACCCC
+
```

```
FFFFFFFFFFFFFFFFFFFFFFFFFFFFFFFFFFFFFFFFFFFFF,FFFFFFFF:FFFFFFFFF:FFFF
FFFFFFFFFFFFFFFFFFFFFFFFFFFFFFFFFFF,FFFFFFFFFFFFFFFFFFFFFFFFFF
@A00155:342:HHGFNDSXY:1:2141:32805:9017 2:N:0:GAACCTAG+TCCGCATA
TCGGCAGAGGATACCGTGCTCCACAGCTGTAAGGCTGTGGGCCCGAGAGAAGTCGCTCTCTCTCGCCC
TCCACATCCCTCTCGGCATTGAGATGACCATAGGCCAGAGGTGAGTCCTTAAGTGGACACAGCTGATC
+
FFFFF:FFFFFFFFFFFFFFFFFFFFFFFFFFFFFFFFFFFFFFFFFF,FFFFFFFFFFFFFFFFF
FFFFFFFFFFFFFFFFFF,FFFFFFF:FFFFFFFFFFFFFFFFFFFFFFFFFFFF::FFFFFF:F,FF
@A00155:342:HHGFNDSXY:1:2141:6858:9799 2:N:0:GAACCTAG+TCCGCATA
AGTACATCTCCTTTGGAACTAGGAGACCCCTCCGGTCTTGTCCAACCTCTGTTTCGGCAGAGGATACC
GTGCTCCACAGCTGTAAGGCTGTGGGCCCGAGAGAAGTCGCTCTCTCTCGCCCTCCACATCCCTCTCG
+
FFFFF,::FFFFFFFFFFFFFFFFFFFFFFFFFFFFFFFFFFFFFFFFFFFFF:FF::FFFFFFFF
FFFFFFFFF:F:FFFFFFFFF:FFFFFFFFFFFFFFFFFFF,F:FFF:FFFFFFFFFFF:FFFFFFFF
@A00155:342:HHGFNDSXY:1:2141:19660:10238 2:N:0:GAACCTAG+TCCGCATA
ACTTACACGGTAGGTCGTGAACAAGCCCGGAGTACATCTCCTTTGGAACTAGGAGACCCCTCCGGTCT
TGTCCAACCTCTGTTTCGGCAGAGGATACCGTGCTCCACAGCTGTAAGGCTGTGGGCCCGAGAGAAGT
+
FFFFFFFFFFFFFFFFFFFFFFFFF:FFFFFFFFFFF:FFFFFFFFFFFFFFF:FFFFFFFFFFFFFF
FFFFFFFFFFFFFFFFFFFFFFFFFFFFFFFFFFFFFFFFFFFFFFFFFFFFFFFFFFFFFFFFFFFF
@A00155:342:HHGFNDSXY:1:2141:31295:24095 2:N:0:GAACCTAG+TCCGCATA
TACGACTTACACGGTAGGTCGTGAACAAGCCCGGAGTACATCTCCTTTGGAACTAGGAGACCCCTCCG
GTCTTGTCCAACCTCTGTTTCGGCAGAGGATACCGTGCTCCACAGCTGTAAGGCTGTGGGCCCG
+
FFFFFFFFFFFFFFFFFFFFFFFFFFFFFFFFFFFFF:FFFFFFF:FFFFF,FFFFFFFFFFFFFFFF
FFFFFFFFFFFFFFFFFFFFFFFFFFFFFFFFFFFF:FFFFFFFFFFFFFFFFFFFF:FFFFF:F
@A00155:342:HHGFNDSXY:1:2141:22571:25426 2:N:0:GAACCTAG+TCCGCATA
GCCTACTAGACTCCGACCGTCATGGGGGTTCAAACCCATGCCCCACCACACCGCCTTAGATCAGCTGT
GTCCACTTAAGGACTCACCTCTGGCCTATGGTCATCTCAATGCCGAGAGGGATGTGGAGGGCGAGAGA
+
FFFFFFFFFFFFFFFFFFFFFFFFFFFFFFFFFFFFFFFFFFFFFFFFFFFFFFFFFFFFFFFFFFFF
FFFFFFFFFFFFFFFFFFFFFFFFFFFFFFFFFFFFFFFFFFFFFFFFFFFFFFFFFFFFFFFFFFFF
@A00155:342:HHGFNDSXY:1:2142:16966:12524 2:N:0:GAACCTAG+TCCGCATA
TCCTAAAAAGGACTCGTCAGGTAGCCTACTAGACTACGACTTACACGGTAGGTCGTGAACAAGCCCGG
AGTACATCTCCTTTGGAACTAGGAGACCCCTCCGGTCTTGTCCAACCTCTGTTTCGGCAGAGGATACC
+
FFFFFFFFFFFFFFFFFFFFFFFFFFFFFFFFFFFFFFFFFFFFFFFFFFFFFFFFFFFFFFFFFFFF
FFFFFFFFFFFFFFFFF:FFFFFFFFFFFFFFFFFFFFFFFFFFFFFFFFFFFFFFFFFFFFFFFFFF
@A00155:342:HHGFNDSXY:1:2143:29966:1407 2:N:0:GAACCTAG+TCCGCATA
GTGAAAAAGCCCGGAGTACATCTCCTTTGGAACTAGGAGACCCCTCCGGTCTTGTCCAACCTCTGTTT
CGGCAGAGGATACCGTGCTCCACAGCTGTAAGGCTGTGGGCCCGAGAGAAGTCGCTCTCTCTCGCCC
+
FFF:FFFFFFFFFFFFFFFFFFFFFFFFFFFFFFFFFFFFFFFFFFFFFFFFFFFFF,FFFFFFFFFF
FFFFFFF:FFFFFFFFFFFFFFF:FF:F:FFFFFFFFFFFFFFFFFFFF:FFFFFFFFFFFFFFFF:
@A00155:342:HHGFNDSXY:1:2143:11812:9111 2:N:0:GAACCTAG+TCCGCATA
AGGACGAAACCGTCGTTACTCTTCGAGTTGTGTAAGTTTCGTCCTAAAAAGGACTCGTCAGGTAGCCT
ACTAGACTACGACTTACACGGTAGGTCGTGAACAAGCCCGGAGTACATCTCCTTTGGAACTAGGAGAC
+
FFFFFFFFFFFFFFFFFFFFF,FFFFFFFFFFFFFFFFFFFFFFF::FFFFFFF:FFFFFFFFFFF:F
:FFFFFFFFF:F:FFFFFFFF:FFFFFFFFFF,FFFFFFFFFFFFFFF:FFFF,F:FFFF:FFFF:FF
@A00155:342:HHGFNDSXY:1:2143:3992:9659 2:N:0:GAACCTAG+TCCGCATA
GGACTCGTCAGGTAGCCTACTAGACTACGACTTACACGGTAGGTCGTGAACAAGCCCGGAGTACATCT
CCTTTGGAACCAGGAGACCCCTCCGGTCTTGTCCAACCTCTGTTTCGGCAGAGGATACCGTGCTCCAC
+
```

FFFFFFFFFFFFFFFFFFFFFFFFFFFFFFFFFFFFFFFFFFFFFFFFFFFFFFFFFFFFFFFFFFFF
FFFFFFFFFFFFFFFFFFFFFFFFFFFFFFFFFFFFFFFFFFFFFFFFFFFFFFF,FFFFFFFFFFFF
@A00155:342:HHGFNDSXY:1:2143:7898:13886 2:N:0:GAACCTAG+TCCGCATA
TACTCTTCGAGTTGTGTAAGTTTCGTCCTAAAAAGGACTCGTCAGGTAGCCTACTAGACTACGACTTA
CACGGTAGGTCGTGAACAAGCCCGGAGTACATCTCCTTTGGAACTAGGAGACCCCTCCGGTCTTGTCC
+
FFFFFFFFFFFFFFFFFFFFFFFF:FFFFFFFFFFFFFFFFFFFFFFFFFFFFFFFFF:FFFFFF:FF
FFF:FFF,:FFFFFFFFFFFFF:FFFFFFFFFFFFFFFFFFFFFFFFF:FFFFFFFFFFFFFFFFFFFF:
@A00155:342:HHGFNDSXY:1:2143:4553:25786 2:N:0:GAACCTAG+TCCGCATA
TAGGAGACCCCTCCGGTCTTGTCCAACCTCTGTTTCGGCAGAGGATACCGTGCTCCACAGCTGTAAGG
CTGTGGGCCCGAGAGAAGTCGCTCTCTCTCGCCCTCCACATCCCTCTCGGCATTGAGATGAC
+
FFFFFFFFFFFFFFFFFFFFFFFFFFFFFFFFFFFFFFFFFFFFFFFFFFFFFFFFFFFFFFFFFFFF
FFFFFFFFFFFFFFFFFFFFFFFFFFFFFFFFFFFFFFFFFFFFFFFFFFFFFFFFFFFFFF
@A00155:342:HHGFNDSXY:1:2144:27579:9799 2:N:0:GAACCTAG+TCCGCATA
AGGATACCGTGCTCCACAGCTGTAAGGCTGTGGGCCCGAGAGAAGTCGCTCTCTCTCGCCCTCCACAT
CCCTCTCGGCATTGAGATGACCATAGGCCAGAGGTGAGTCCTTAAGTGGACACAGCTGAT
+
FFFFFFFFFFFFFFFFFFFFFFFFFFFFFFFFFFFFFFFFFFFFFFFFFFFF:FFFFF:FFFFFFFFF
FFFFFFFFFFFFFFFFFFFFFFFFFFFFFFFFFFFFFFFFFFFFFFFFFFF:FFFFFFFF
@A00155:342:HHGFNDSXY:1:2144:11577:33567 2:N:0:GAACCTAG+TCCGCATA
GGACTCGTCAGGTAGCCTACTAGACTACGACTTACACGGTAGGTCGTGAACAAGCCCGGAGTACATCT
CCTTTGGAACTAGGAGACCCCTCCGGTCTTGTCCAACCTCTGTTTCGGCAGAGGATACCGTGCTCCAC
+
FFFFFFFFFFFFFFFFFFFFFFFFFFFFFFFFFFFFFFFFFFFFFFFFFFFFFFFFFFFFFFFFFFFF
FFFFFFFFFFFFFFFFFFFFFFFFFFFFFFFFFFFFFFFFFFFFFFFFFFFFFFF:FFFFFFFFFFFF
@A00155:342:HHGFNDSXY:1:2145:12355:4319 2:N:0:GAACCTAG+TCCGCATA
AGTAACGACGGTTTCGTCCTTGGAACGGACTCATCAGGGAGCCTACTAGACTCCGACCGTCATGGGGG
TTCAAACCCATGCCCCACCACACCGCCTTAGATCAGCTGTGTCCACTTAAGGACTCACCTCTGGCCTA
+
FFFFFFFFFFFFFFFFFFFFFFFFFFFFFFFFFFFFFFFFFFFFFFFFFFF:FFFFFFFFFFFFFFFF
FFFFFFFFFFFFFFFFFFFFFFFFFFFFFFFFFFFFFFFFFFFFFFFFFFFFFFFFFFFFFFFFFFFF
@A00155:342:HHGFNDSXY:1:2145:13458:5102 2:N:0:GAACCTAG+TCCGCATA
ACCCCTCCGGTCTTGTCCAACCTCTGTTTCGGCAGAGGATACCGTGCTCCACAGCTGTAAGGCTGTGG
GCCCGAGAGAAGTCGCTCTCTCTCGCCCTCCACATCCCTCTCGGCATTGAGATGACCATAGGCCAGAG
+
FFFFFFFFF:F:FFFFFFFFFF:FFFFFF:F:FFFFFFFFFFFFFF:FFFF:F:FFFFFF:FF:F:FF
FFFFFFFF:FFFFFFFFF:FFFFF,FF:FFFF:F:FFF:FFFFF:F:FFFFFF::F,FFF,FFFF:FF
@A00155:342:HHGFNDSXY:1:2145:12002:7842 2:N:0:GAACCTAG+TCCGCATA
GAACTAGGAGACCCCTCCGGTCTTGTCCAACCTCTGTTTCGGCAGAGGATACCGTGCTCCACAGCTGT
AAGGCTGTGGGCCCGAGAGAAGTCGCTCTCTCTCGCCCTCCACATCCCTCTCGGCATTGAGATGACCA
+
FFFFFFFFFFFFFFFFFFFFFFFFFFFFFFFFFFFFFFFFFFFFFFFFFFFFFFFFFFFFFFFFFFFF
FFFFFFFFFFFFFFFFFFFFFFFFFFFFFFFFFFFFFFFFFFFFFFFFFFFFFFFFFFFFFFFFFFFF
@A00155:342:HHGFNDSXY:1:2145:2718:25363 2:N:0:GAACCTAG+TCCGCATA
GGACTCGTCAGGTAGCCTACTAGACTACGACTTACACGGTAGGTCGTGAACAAGCCCGGAGTACATCT
CCTTTGGAACTAGGAGACCCCTCCGGTCTTGTCCAACCTCTGTTTCGGCAGAGGATACCGTGCTCCAC
+
FFFFFFFFFFFFFFFFFFFFFFFFFFFFFFFFFFFFFFFFFFF:FFFFFFFFFFFFFFFFFFFFFFFF
FFFFFFFFFFFFFFFFFFFFFFFFFFFFFFF:FFFFFFFFFFFFFFFFFFFFFFFFFFFFFFFFFFFF
@A00155:342:HHGFNDSXY:1:2145:20989:32142 2:N:0:GAACCTAG+TCCGCATA
TAGGAGACCCCTCCGGTCTTGTCCAACCTCTGTTTCGGCAGAGGATACCGTGCTCCACAGCTGTAAGG
CTGTGGGCCCGAGAGAAGTCGCTCTCTCTCGCCCTCCACATCCCTCTCGGCATTGAGATGAC
+

FFFFFFFFFFFFFFFFF:FFFFFFFFFFFFF:FFFFFF:FFFFFFFFFFF:FFFFFFFFFFFFFFFFF
FFFFFF:F:FFFFFFFFFFF:FFFFFFFFFF,FF:F:FFFFFFF:FFFF::FFF:FFFFFFF
@A00155:342:HHGFNDSXY:1:2145:19985:36980 2:N:0:GAACCTAG+TCCGCATA
GTTGTGTAAGTTTCGTCCTAAAAAGGACTCGTCAGGTAGCCTACTAGACTACGACTTACACGGTAGGT
CGTGAACAAGCCCGGAGTACATCTCCTTTGGAACTAGGAGACCCCTCCGGTCTTGTCCAACCTCTGTT
+
FFFFFFFFFFFFFFFFFFFFFFFFFFFFFFFFFFFF::FFFFFFFFFFFFFF:FFFFFFFFFF:FFFF
FFFFFFFFFFFFFFFFFFFFFFFFFFFFFFFFFF:F:FF:FFFFFFFFFFFFFFF:FFFFFF:FFFFF
@A00155:342:HHGFNDSXY:1:2146:22896:5196 2:N:0:GAACCTAG+TCCGCATA
GTGAACAAGCCCGGAGTACATCTCCTTTGGAACTAGGAGACCCCTCCGGTCTTGTCCAACCTCTGTTT
CGGCAGAGGATACCGTGCTCCACAGCTGTAAGGCTGTGGGCCCGAGAGAAGTCGCTCTCTCTCGCCCT
+
:FFFFFFFFFFFFFFF:FFFF:FFFFFFFFFFFFFF:FFFF,F:FFFFFFFFF:FFFF,F:FFFFFFF
FFFFFFFFFFFFF:FFFFFFFFFFFFFFFFFFFFFFFFFFFFFFFFFFFFF:FFFFFFFFFFFFFFFF
@A00155:342:HHGFNDSXY:1:2146:30544:8641 2:N:0:GAACCTAG+TCCGCATA
GAACAAGCCCGGAGTACATCTCCTTTGGAACTAGGAGACCCCTCCGGTCTTGTCCAACCTCTGTTTCG
GCAGAGGATACCGTGCTCCACAGCTGTAAGGCTGTGGGCCCGAGAGAAGTCGCTCTCTCTCGCCCTCC
+
FFFFFFFFFFFFFFFFFFFFFFFFFFFFFFFFFFFFFFFFFFFFFFFFF:FFFFFFFFFFFFF:FFFF
FFFFFFFFFFFFFFFFFFFFFFFFFFFFFFFFFFFFFFFFFFFFFFFFFFFFFFFFFFFFFFFFFFFF
@A00155:342:HHGFNDSXY:1:2146:12084:12493 2:N:0:GAACCTAG+TCCGCATA
CCTACTAGACTACGACTTACACGGTAGGTCGTGAACAAGCCCGGAGTACATCTCCTTTGGAACCAGGA
GACCCCTCCGGTCTTGTCCAACCTCTGTTTCGGCAGAGGATACCGTGCTCCACAGCTGTAAGGCTGTG
+
FFFFFFFFFFFFFFFFFFFFFFFFF:FFFFFFFFFFFFFFFFFFFFFFFFFFFFFFFFFFFFFFFFFF
FFFFFFFFFFFFF:FFFFFFFFFFFFFFFF:FFFFF,FFFFFFFFFFFFFFFFFFFFFFFFFFFFFFF
@A00155:342:HHGFNDSXY:1:2146:3974:18427 2:N:0:GAACCTAG+TCCGCATA
ACTACGACTTACACGGTAGGTCGTGAACAAGCCCGGAGTACATCTCCTTTGGAACTAGGAGACCCCTC
CGGTCTTGTCCAACCTCTGTTTCGGCAGAGGATACCGTGCTCCACAGCTGTAAGGCTGTGGGCCCGAG
+
FFFFFFFFFFFFFFFFFFFFFFFFFFFFFFFFFFFFFFFFFFFFFFFFFFFFF:FFFFFFFFFFFFFF
FFFFFFFFFFFFFFFFFFFFFFFFFF:FFFFFFFFFFFFFFFFFFFFFFFFFFFFFFFFFFFFFFFFF
@A00155:342:HHGFNDSXY:1:2146:14181:21230 2:N:0:GAACCTAG+TCCGCATA
CAAGGACGAAACCGTCGTTACTCTTTGAGTTGTGTAAGTTTCGTCCTAAAAAGGACTCGTCAGGTAGC
CTACTAGACTACGACTTACACGGTAGGTCGTGAACAAGCCCGGAGTACATCTCCTTTGGAACTAGGAG
+
FFFFFFFFFFFFFFFFFFFFFFF,:FFFFFFFFFFFFFFFFFFFFFFFFFFFFFFFFFF,F:FFFFFF
FFFFFFFFFFFFFFFFFFFF:FFFFFFFFFFFFFFFFFFFFFFFFFFFFFFFFF,FF:FFFFFFFFFF
@A00155:342:HHGFNDSXY:1:2147:14823:7717 2:N:0:GAACCTAG+TCCGCATA
CAGGAGACCCCTCCGGTCTTGTCCAACCTCTGTTTCGGCAGAGGATACCGTGCTCCACAGCTGTAAGG
CTGTGGGCCCGAGAGAAGTCGCTCTCTCTCGCCCTCCACATCCCTCTCGGCATTGAGAT
+
FFFFFFFFFFFFFFFFFF,:FFFFFFFFFFFFFFFFFFFFFFFFFFFFFFFFFFFF,F,FFFFFFFFF
FFFFFFFFFFFFFFFFFF:FFFFFFFFFFFFFFFFFFFFFFFFFFFFFFFFFF:FFFFF
@A00155:342:HHGFNDSXY:1:2147:10465:8500 2:N:0:GAACCTAG+TCCGCATA
AGGTAGCCTACTAGACTACGACTTACACGGTAGGTCGTGAACAAGCCCGGAGTACATCTCCTTTGGAA
CCAGGAGACCCCTCCGGTCTTGTCCAACCTCTGTTTCGGCAGAGGATACCGTGCTCCACAGCTGTAAG
+
F:FFFFFFFFFFFFFFFFFFFFFFFFFFFFFFFFFFFFFFFFFF:FFFFFFFFFFFFFFFFFFFFFFF
FFFFFFFFF::FFFFFFFFFFFFFFFFFFFF:F:FFFFFFFFFFFFF:FFFFFFFF:F::F,FFFF:F
@A00155:342:HHGFNDSXY:1:2147:28944:8844 2:N:0:GAACCTAG+TCCGCATA
TACGACTTACACGGTAGGTCGTGAACAAGCCCGGAGTACATCTCCTTTGGAACTAGGAGACCCCTCCG
GTCTTGTCCAACCTCTGTTTCGGCAGAGGATACCGTGCTCCACAGCTGTAAGGCTGTGGGCCCG
+

FFFFFFFFFFFFFFF,FFFFFFFFFFF:FFFFFFFFFFFFFFFFFFFFFFFFFF:FFFFFFFFFFFF
FFFFFFFFFFFFFFFFFFFFFFFFFFFFFFFF:FFFFFFFFFFFFFFFF:FFFFFFFFFFFFFF
@A00155:342:HHGFNDSXY:1:2147:10285:18458 2:N:0:GAACCTAG+TCCGCATA
GGACTCGTCAGGTAGCCTACTAGACTACGACTTACACGGTAGGTCGTGAACAAGCCCGGAGTACATCT
CCTTTGGAACTAGGAGACCCCTCCGGTCTTGTCCAACCTCTGTTTCGGCAGAGGATACCGTGCTCCAC
+
FFFFFFFFF:FFFFFFFFFFFFFFFFFFFFFFFFFFFFFFFFFFFFFFFFFFFFFFFFFFFFFFFFFF
FFFFFFFFFFFFFFFFFFFFFFFFFFFFFFFFFFFFFFFFFFFFFFFFFFFFFFFFFFFFFFFFF::F
@A00155:342:HHGFNDSXY:1:2148:17933:24251 2:N:0:GAACCTAG+TCCGCATA
CGGCAGAGGATACCGTGCTCCACAGCTGTAAGGCTGTGGGCCCGAGAGAAGTCGCTCTCTCTCGCCCT
CTACATCCCTCTCGGCATTGAGATGACCATAGGCCAGAGGTGAGTCCTTAAGTGGACACAGCTGATCT
+
FFFFFFFFFFFFFFFFFFFFFFFFFFFFFFFFFFFFFFFFFFFFFFFFFFFFFFFFFFFFFFFFFFFF
FFFF:FFFFFFFFFFFF,FFFFFFFFFF:FFFFFFFFFFFFFFFFFFFFFFFFFFFFFFFFFFFFFFF
@A00155:342:HHGFNDSXY:1:2148:23303:32362 2:N:0:GAACCTAG+TCCGCATA
TAGACTACGACTTACACGGTAGGTCGTGAACAAGCCCGGAGTACATCTCCTTTGGAACTAGGAGACCC
CTCCGGTCTTGTCCAACCTCTGTTTCGGCAGAGGATACCGTGCTCCACAGCTGTAAGGCTGTGGGCCC
+
FFFFFFFFFFFFFFFF:FFFFFF:FFFFFFFFFFFFFFFFFFFFFFFFFFFFFFFFFFF:FFFFFFFFF
FFFFFFFFFFFFFFFFFFFFFFFFFFFFF:FFFFFFFFFFFFFFFFFFFFFFFFFFFFFFFFFFFFFFF
@A00155:342:HHGFNDSXY:1:2149:30318:18740 2:N:0:GAACCTAG+TCCGCATA
GTCCACTTAAGGACTCACCTCTGGCCTATGGTCATCTCAATGCCGAGAGGGATGTGGAGGGCGAGAGA
GAGCGACTTCTCTCGGGCCCACAGCCTTACAGCTGTGGAGCACGGTATCCTCTGCCGAAACAGAGGTT
+
FFFFFFFFFFFFFFFFFFFFFFFFFFFFFFFFFFFFFFFFFFFFFFFFFFF,FFFFFFFFFFFFFF
FFFFFFFFFFFFFFFFFFFFFFFFFFF:FFFFFFFFFFFFFFFFFFFFFFFFFFFFFFFFFFFFFFFF
@A00155:342:HHGFNDSXY:1:2151:20708:16219 2:N:0:GAACCTAG+TCCGCATA
GTTGTGTAAGTTTCGTCCTAAAAAGGACTCGTCAGGTAGCCTACTAGACTACGACTTACACGGTAGGT
CGTGAACAAGCCCGGAGTACATCTCCTTTGGAACTAGGAGACCCCTCCGGTCTTGTCCAACCTCTGTT
+
F:FFFFFFFFFFFFFFFFFFFFFFFFFFFFFFFFFFFFFFFFFFFFFFFFFFFFFFFFFFFFFFFFFF
FFFFFFFFFFFFFFFFFFFFFFFFFFFFFFFFF:FF:FFFFFFFFFFFFFFFFFFFFFFFFFFFFFFF
@A00155:342:HHGFNDSXY:1:2152:6569:15405 2:N:0:GAACCTAG+TCCGCATA
TAGGTCGTGAACAAGCCCGGAGTACATCTCCTTTGGAACTTGGAGACCCCTCCGGTCTTGTCCAACCT
CTGTTTCGGCAGAGGATACCGTGCTCCACAGCTGTAAGGCTGTGGGCCCGAGAGAAGTCGCTCTCTCT
+
FFFFFFFFFFFFFFFF:FFFFFFFFFFFFFFFFFFFFFFFFFFFFFFFFFF,FFFFFFFFFFFFFFFF
FFFFFFFFFFFFFFFFFFFFFF:FFFFFFFFFFFFFFFFFFFFFFFFFFFFFFFFFF:FFFFFFFFFF
@A00155:342:HHGFNDSXY:1:2152:3857:17096 2:N:0:GAACCTAG+TCCGCATA
ATCTCCTTTGGAACTAGGAGACCCCTCCGGTCTTGTCCAACCTCTGTTTCGGCAGAGGATACCGTGCT
CCACAGCTGTAAGGCTGTGGGCCCGAGAGAAGTCGCTCTCTCTCGCCCTCCACATCCCTCTCGGCATT
+
FFFFFFFFFFFFFFFFFFFFFFFFFFFFFFFFFF:FF:FFFFFFFFFFFFFFFFFFFFFFFFFF:FFFFF
FFFFFFFFFFFFFFFFFFFFFFFFFFFFFF:FFFFFFFFFFFFFFFFFFFFFFFFFFFFFFFFFFF,FF
@A00155:342:HHGFNDSXY:1:2152:32235:17801 2:N:0:GAACCTAG+TCCGCATA
AGTACATCTCCTTTGGAACTAGGAGACCCCTCCGGTCTTGTCCAACCTCTGTTTCGGCAGAGGATACC
GTGCTCCACAGCTGTAAGGCTGTGGGCCCGAGAGAAGTCGCTCTCTCTCGCCCTCCACATCCCTCTC
+
FFF,FFFFFFFFFFFFFFF:FFFFFFFFFFFFFFFFFFFFFFFFFFFFFFFFFFFFFF:F:FFFF,F:FF
FFFFFFFFFFFFFFFFFFFFFFFFFFFFFFFFFFFFFFFFFFFFFFFFF:FFFFFFFFFFFFFFFFF
@A00155:342:HHGFNDSXY:1:2152:4463:20369 2:N:0:GAACCTAG+TCCGCATA
AGTACATCTCCTTTGGAACTAGGAGACCCCTCCGGTCTTGTCCAACCTCTGTTTCGGCAGAGGATACC
GTGCTCCACAGCTGTAAGGCTGTGGGCCCGAGAGAAGTCGCTCTCTCTCGCCCTCTACATCCCTCTCG
+

FFFFFFFFFFFFFFFFF:FFFFFFFFFFFFFFFFFFF:FFFFFFF:FFFFFFFFFFFFF:FFFFFFFFFF
FFFFFFFFFFFFFFFFFFFFFFFFFFFFFFFFFFFF,FFFFFFFFFFFFFF:FFFFFFFFFFFF,FFFFF
@A00155:342:HHGFNDSXY:1:2152:11153:25692 2:N:0:GAACCTAG+TCCGCATA
TTTCGTCCTAAAAAGGACTCGTCAGGTAGCCTACTAGACTACGACTTACACGGTAGGTCGTGAACAAG
CCCGGAGTACATCTCCTTTGGAACCAGGAGACCCCTCCGGTCTTGTCCAACCTCTGTTTCGGCAGAGG
+
F::FFFF:FFFFFFFFFFFFFFFFF,FFFFFFFFFFFFFFFFFFFFFFFFFFFF,FFFFFFFFFFFFFFF
FFFF:F:FFFFFFFFFFFFFFFFFFFFFFFFFFFFFFFFFF,FFFF:FFFFF:F:F:FFFF:FFFFFFFF
@A00155:342:HHGFNDSXY:1:2153:32633:3521 2:N:0:GAACCTAG+TCCGCATA
TAGATCAGCTGTGTCCACTTAAGGACTCACCTCTGGCCTATGGTCATCTCAATGCCGAGAGGGATGTG
GAGGGCGAGAGAGAGCGACTTCTCTCGGGCCCACAGCCTTACAGCTGTGGAGCACGGTATCCTCTGCC
+
:FFFFFFFFFFFFFFFF:FF:FFFFF:F:FFFFFFFFFFFFFFFFFFFFFFFFFFFFFFFFFFFF,:F:F
FFFFFFFFFFFFFFFFFFFFFFFFFFFFFFFFFFFFFFFFFFFFFFF:FFFFFFFFFFFFFF:FFFFFFF
@A00155:342:HHGFNDSXY:1:2153:10818:15342 2:N:0:GAACCTAG+TCCGCATA
AAAAGGACTCGTCAGGTAGCCTACTAGACTACGACTTACACGGTAGGTCGTGAACAAGCCCGGAGTAC
ATCTCCTTTGGAACTAGGAGACCCCTCCGGTCTTGTCCAACCTCTGTTTCGGCAGAGGATACCGTGCT
+
FFFFFFFFFFFFFFFFFFFFFFFFFFFFFFFFFFFFFF:FFFFFFFFFFFFFFFFFFFFFFFFFFFFFFF
FFFFFFFFFFFF:FFFFFFFFFFFFFFFFFFFFFFFFFFFFFFFFFFFFFFFFFFFFFFFFFFFFFFFFF
@A00155:342:HHGFNDSXY:1:2153:24885:33818 2:N:0:GAACCTAG+TCCGCATA
GTGAACAAGCCCGGAGTACATCTCCTTTGGAACTAGGAGACCCCTCCGGTCTTGTCCAACCTCTGTTT
CGGCAGAGGATACCGTGCTCCACAGCTGTAAGGCTGTGGGCCCGAGAGAAGTCGCTCTCTCTCGCCCT
+
FFFFFFFFFFFFFFFFFFFFFFFFFFFFFFFFFFFFFFFFFFFFFFFFFFFFF:FFFFFFFFFFFFFFFF
FFFFFFFFFFFFFFFFFFFFFFFFFFFFFFFFFFFFFFFFFFFFFFFFFFF,FFFFFFFFFFFFFFFFFF
@A00155:342:HHGFNDSXY:1:2153:10854:36135 2:N:0:GAACCTAG+TCCGCATA
CAAGCCCGGAGTACATCTCCTTTGGAACTAGGAGACCCCTCCGGTCTTGTCCAACCTCTGTTTCGGCA
GAGGATACCGTGCTCCACAGCTGTAAGGCTGTGGGCCCGAGAGAAGTCGCTCTCTCTCGCCCTCCACA
+
FFFFFFFFFFFFFFFFFFFFFFFFFFFFFFFFFFFFFFFFFFFFFFFFFFFFFFFFFFFFFFFFFFFFFF
FFFFFFFFFFFFFFFFFFFFFFFFFFFFFFFFFFFFFFFFFFFFF:FFFFFFFFFFFFFFFFFFFFFFFF
@A00155:342:HHGFNDSXY:1:2154:29062:16438 2:N:0:GAACCTAG+TCCGCATA
AGGACGAAACCGTCGTTACTCTTCGAGTTGTGTAAGTTTCGTCCTAAAAAGGACTCGTCAGGTAGCCT
ACTAGACTACGACTTACACGGTAGGTCGTGAACAAGCCCGGAGTACATCTCCTTTGGAACCAGGAGAC
+
FFFFFFFFFFFFFFFFFFFFFFFFFFFFFFFFFFFFFF,FFFFFF,FFFF:FFFFFFFFFF:FFF:FFFF
FFFFFFFFFFFFFFFFFFFFFFFFFF:FFFFFFFFF:FFFFFFFFFFFFFFFFFFFFFFFFFFFFFFFFF
@A00155:342:HHGFNDSXY:1:2154:28899:21950 2:N:0:GAACCTAG+TCCGCATA
AAAAGGACTCGTCAGGTAGCCTACTAGACTACGACTTACACGGTAGGTCGTGAACAAGCCCGGAGTAC
ATCTCCTTTGGAACTAGGAGACCCCTCCGGTCTTGTCCAACCTCTGTTTCGGCAGAGGATACCGTGCT
+
FFFFFFFFFFFFFFFFFFFFFFFFFF:FFFFFFFFFFFFFFFF:FFFFFFFFFFFFFFFFFFFFFF:FFF
FFFFFFFFFFFFFFFFFFFFFFFFFFFFFFFFFFFFFF:FFFFFF,FFFFFFFF:FFFFFFFFFFF:FFF
@A00155:342:HHGFNDSXY:1:2155:1452:4069 2:N:0:GAACCTAG+TCCGCATA
GAACTAGGAGACCCCTCCGGTCTTGTCCAACCTCTGTTTCGGCAGAGGATACCGTGCTCCACAGCTGT
AAGGCTGTGGGCCCGAGAGAAGTCGCTCTCTCTCGCCCTCCACATCCCTCTCGGCATTGAGATGAC
+
:FFFF:FFFFFFFFFFFFFFFFFFFFFFF:FFFFFFFFFF:FFFFF,FFFF::FF:FFFFFFFFFF,FFF
FFFFFFFFFFFFFFFFFFFFFFFFFFFFFFFFFFFFFFFF:FFF:FFFFF:FFFFFF,FFFFFFFFFF
@A00155:342:HHGFNDSXY:1:2155:18376:27148 2:N:0:GAACCTAG+TCCGCATA
TTTAGGACGAAACTTACACAACTCGAAGAGTAACGACGGTTTCGTCCTTGGAACGGACTCATCAGGGA
GCCTACTAGACTCCGACCGTCATGGGGGTTCAAACCCATGCCCCGCCACACCGCCTTAGATCAGCTGT
+

```
FFFFFFFFFFFFFFFFFFFFFFFFFFFFFFFF:FFFFFF:FFFFFFFFFFFFFFFFFF:FFFF:FFFF
FFFFFFFFFFFFFFFFFFFFFF:FFFFFFFFFFFFFFFFFFFFFFFFF::FFFFFFFFFFFFFFFFFF
@A00155:342:HHGFNDSXY:1:2156:17580:4100 2:N:0:GAACCTAG+TCCGCATA
GAACTAGGAGACCCCTCCGGTCTTGTCCAACCTCTGTTTCGGCAGAGGATACCGTGCTCCACAGCTGT
AAGGCTGTGGGCCCGAGAGAAGTCGCTCTCTCTCGCCCTCTACATCCCTCTCGGCATTGAGATGACCA
+
FFFFFFFFFFFFFFFFFFFFFFFFFFFFFFFFFFFFFFFFFFFFFFFFFFFFFFFFFFFFFFFFFFFF
FFFFFFFFFFFFFFFFFFFFFFFFFFFFFFFFFFFFFFFFFFFFFFFFFFFFFFF:FFFFFFFFFFFF
@A00155:342:HHGFNDSXY:1:2156:11424:11757 2:N:0:GAACCTAG+TCCGCATA
GGACTCGTCAGGTAGCCTACTAGACTACGACTTACACGGTAGGTCGTGAACAAGCCCGGAGTACATCT
CCTTTGGAACCAGGAGACCCCTCCGGTCTTGTCCAACCTCTGTTTCGGCAGAGGATACCGTGCTCCAC
+
FFFFFFFFFFFFFFFFFFFFFFFFFFFFFFFFFFFFFFFFFFFFFFFFFFFFF:FFFFFFFFFFFFFF
FFFFFFFFFFFFFFFFFFFFFFFFFFFFFFFFFFFFFFFFFFFFFFFFFFFFFFFFFFFF:FFFFFFF
@A00155:342:HHGFNDSXY:1:2156:7726:20290 2:N:0:GAACCTAG+TCCGCATA
GTAAGTTTCGTCCTAAAAAGGACTCGTCAGGTAGCCTACTAGACTACGACTTACACGGTAGGTCGTGA
ACAAGCCCGGAGTACATCTCCTTTGGAACTAGGAGACCCCTCCGGTCTTGTCCAACCTCTGTTTCGGC
+
FFFFFFFFFFFFFFFFFF:FFFFFFFFFFFFFFFFFFFFFFFFFFFFFFFFFFFFFFFFFFFFFFFFF
FFFFFFFFFFFFFFFFFFFFFF:FFFFFFFFFFFFFFFFFFFFFFFFF:FFFFFFFFFFFFFFFFFFF
@A00155:342:HHGFNDSXY:1:2156:7120:21308 2:N:0:GAACCTAG+TCCGCATA
GTAAGTTTCGTCCTAAAAAGGACTCGTCAGGTAGCCTACTAGACTACGACTTACACGGTAGGTCGTGA
ACAAGCCCGGAGTACATCTCCTTTGGAACTAGGAGACCCCTCCGGTCTTGTCCAACCTCTGTTTCGTC
+
F:FFFFFFFFFFFF,:FF,FFFFFFF:FFF:::FFFFFFFF:FFFFFFFFFF:FFFFFFFFF:FFFF:
:FF::FF:F:FFF,FFFFFFFFFFFFFFFFFFFFFFFFFFFFFFFFFF:F,F,FFFFF,FF:FFFFF,F
@A00155:342:HHGFNDSXY:1:2156:5638:21903 2:N:0:GAACCTAG+TCCGCATA
ACAAGCCCGGAGTACATCTCCTTTGGAACTAGGAGACCCCTCCGGTCTTGTCCAACCTCTGTTTCGGC
AGAGGATACCGTGCTCCACAGCTGTAAGGCTGTGGGCCCGAGAGAAGTCGCTCTCTCTCGCCCTCTAC
+
FFFFFFFFFFFFFFFFFFFFFFFFFFFFFFFFFFFFFFFFFFFFFFFFFFFFF:FFFFFFFFFFFFFF
FFFFFFFFFFFFFFFFFFFFFFFFFFFFFFFFFFF:FFFFFFFFFFFFFFFFFFFFFFFFFFFFFFFF
@A00155:342:HHGFNDSXY:1:2156:7446:22874 2:N:0:GAACCTAG+TCCGCATA
AAAAGGACTCGTCAGGTAGCCTACTAGACTACGACTTACACGGTAGGTCGTGAACAAGCCCGGAGTAC
ATCTCCTTTGGAACTAGGAGACCCCTCCGGTCTTGTCCAACCTCTGTTTCGGCAGAGGATACCGTGC
+
FFFFFFFFFFFFFFFFFFFFFFFFFF:FFFFFFFFFFFFFFFFFFFFFFF:FFFFFFFFFFFFFFFFF
FFFFFFFFFFFFFFFFFFFFFFFFFFFFFFFFFFFFFFFFFFFFFFFFFFFFFFFFFFFFFFFF:FF
@A00155:342:HHGFNDSXY:1:2157:24279:14074 2:N:0:GAACCTAG+TCCGCATA
GTGAACAAGCCCGGAGTACATCTCCTTTGGAACTAGGAGACCCCTCCGGTCTTGTCCAACCTCTGTTT
CGGCAGAGGATACCGTGCTCCACAGCTGTAAGGCTGTGGGCCCGAGAGAAGTCGCTCTCTCTCGCCCT
+
FFFFFFFFFFFFFFFFFFFFFFFFFFFFFFFFFFFFFFFFFFFFFFFFFFFFFFFFFFFFFFFFFFFF
FFFFFFFFFFFFFFFFFFFFFFFFFFFFFFFFFFFFFFFFFFFFFFFFFFFFFFFFFFFFFFFFFFFF
@A00155:342:HHGFNDSXY:1:2157:9010:15875 2:N:0:GAACCTAG+TCCGCATA
CCTACTAGACTACGACTTACACGGTAGGTCGTGAACAAGCCCGGAGTACATCTCCTTTGGAACCAGGA
GACCCCTCCGGTCTTGTCCAACCTCTGTTTCGGCAGAGGATACCGTGCTCCACAGCTGTAAGGCTGTG
+
FFFFFFFFFFFFFFFFFFFFFFFFFFFFFFFFFFFFFFFFFFFFFFFFFFFFFFFFFFFFFFFFFFFF
FFFFFFFFFFFFFFFFFFFFFFFFFFFFFFFFF:FFFFFFFFFFFFFFFFFFFFFFFFFFFFFFFFFF
@A00155:342:HHGFNDSXY:1:2157:16034:22060 2:N:0:GAACCTAG+TCCGCATA
GTTGTGTAAGTTTCGTCCTAAAAAGGACTCGTCAGGTAGCCTACTAGACTACGACTTACACGGTAGGT
CGTGAACAAGCCCGGAGTACATCTCCTTTGGAACTAGGAGACCCCTCCGGTCTTGTCCAACCTCTGTT
+
```

FFFFFFFFFFFFFFFFFFFFFFFFFFFFFFFFFFFFF,FF:FFFFFFFFFFFFFF:FFFFFFFFFFFF
FFFFFFFFFFFFFFFFFFFFFFFFFFFFFFF,:FFFFFFFFFFFFFFFFFFFFFFFFFFFFFFFFFFF
@A00155:342:HHGFNDSXY:1:2158:11397:14152 2:N:0:GAACCTAG+TCCGCATA
GTACATCTCCTTTGGAACTAGGAGACCCCTCCGGTCTTGTCCAACCTCTGTTTCGGCAGAGGATACCG
TGCTCCACAGCTGTAAGGCTGTGGGCCCGAGAGAAGTCGCTCTCTCTCGCCCTCTACATCCCTCTCGG
+
FFFFFFFFFFFFFFFFFFFFFFFFFFFFFFFFFFFFFFFFFFFFFFFFFFFFFFFFFFFFFFFFFFFF
FFFFFFFFFFFFFFFFFFFFFFFFFFFFFFFFFFFFFFFFFFFFFFFFFFFFFFFFFFFFFFFFFFFF
@A00155:342:HHGFNDSXY:1:2158:10185:16407 2:N:0:GAACCTAG+TCCGCATA
GGAGTACATCTCCTTTGGAACTAGGAGACCCCTCCGGTCTTGTCCAACCTCTGTTTCGGCAGAGGATA
CCGTGCTCCACAGCTGTAAGGCTGTGGGCCCGAGAGAAGTCGCTCTCTCTCGCCCTCCAC
+
FFFFFFFFFFFFFFFFFFFFFFFFFFFFF:FFFFFFFFFFFFFFFFFFFFFFFFF:FFFFFFFFFFFF
FFFFFFFFFFFFFFFFFFFFFFFFFFFFFFFFFFFFFFFFFFFF:FFFFFFFF:FFFFFF
@A00155:342:HHGFNDSXY:1:2158:12111:26663 2:N:0:GAACCTAG+TCCGCATA
CCTACTAGACTACGACTTACACGGTAGGTCGTGAACAAGCCCGGAGTACATCTCCTTTGGAACTAGGA
GACCCCTCCGGTCTTGTCCAACCTCTGTTTCGGCAGAGGATACCGTGCTCCACAGCTGTAAGGCTGTG
+
FFFFFFFFFFFFFFFFFFFFFFFFFFFFFFFFFFFFFFFFFFFFFFFFFFF:FFFFFFFFFFFFFFFF
FFFFFFFFFFFFFFFFFFFFFFFFFFFFFF:FFFFFFFFFFFFFFFFFFFFFFFFFFFFFFFFFFFFF
@A00155:342:HHGFNDSXY:1:2159:27950:29168 2:N:0:GAACCTAG+TCCGCATA
ACTAGACTACGACTTACACGGTAGGTCGTGAACAAGCCCGGAGTACATCTCCTTTGGAACTAGGAGAC
CCCTCCGGTCTTGTCCAACCTCTGTTTCGGCAGAGGATACCGTGCTCCACAGCTGTAAGGCTGTGGGC
+
FFFFFFFFFFFFFFFFFFFF:FFFFFFFFFFFFFFFFFFFFFFFFFFFFFFFFFFFFF:FFFFFFFFF
FFFFFFFFFF:FFFFFFFFFFFFFFFFFFFFFFFFFFF:FFFFFFFFFFFFFFFFFFFFFFFFFFFFF
@A00155:342:HHGFNDSXY:1:2159:26982:30812 2:N:0:GAACCTAG+TCCGCATA
ACTAGACTACGACTTACACGGTAGGTCGTGAACAAGCCCGGAGTACATCTCCTTTGGAACTAGGAGAC
CCCTCCGGTCTTGTCCAACCTCTGTTTCGGCAGAGGATACCGTGCTCCACAGCTGTAAGGCTGTGGGC
+
FFFFFFFFFFFFFFFFFFFFFFFFFFFFFFFFFFFFFFFFFFFFFFFFFFFFFFFFFFFFFF:FFFFF
FFFFFFFFFFFFFFFFFFFFFFFFFFFFFFFFFFFFFFFFFFFFFFFFFFFF:FFFFFFFFFFFFFFF
@A00155:342:HHGFNDSXY:1:2159:20907:36385 2:N:0:GAACCTAG+TCCGCATA
GTACATCTCCTTTGGAACTAGGAGACCCCTCCGGTCTTGTCCAACCTCTGTTTCGGCAGAGGATACCG
TGCTCCACAGCTGTAAGGCTGTGGGCCCGAGAGAAGTCGCTCTCTCTCGCCCTCCACATCCATCTCG
+
:,F:FF:FF:FF,F,FFF,F:,:,::FFF:FFFF:,:::FF:F:F:,FF:F::,FFF:,F::F,FF,F
F,FF,FF:F::FF,FF::F:::FF,,FF,::FFF:,FFF,F,:,:FF,,F,FF:F:FF:,:,F,FFF
@A00155:342:HHGFNDSXY:1:2160:23918:12289 2:N:0:GAACCTAG+TCCGCATA
GGAGTACATCTCCTTTGGAACTAGGAGACCCCTCCGGTCTTGTCCAACCTCTGTTTCGGCAGAGGATA
CCGTGCTCCACAGCTGTAAGGCTGTGGGCCCGAGAGAAGTCGCTCTCTCTCGCCCTCCACATCCCTCT
+
FFFFFFFFFFFFFFFFFFF:FFFFFFFFFFF:FFFFFFFF:FFFFFFFFFFFFFFFFFFFFFFFFFFF
FFFFFFFFFFFFFFFFFFFFFFFFFFFFFFFFFFF:F:FFFFFFFFF:FFFFFFFFFFFFFFFFFFFF
@A00155:342:HHGFNDSXY:1:2160:23927:12367 2:N:0:GAACCTAG+TCCGCATA
GGAGTACATCTCCTTTGGAACTAGGAGACCCCTCCGGTCTTGTCCAACCTCTGTTTCGGCAGAGGATA
CCGTGCTCCACAGCTGTAAGGCTGTGGGCCCGAGAGAAGTCGCTCTCTCTCGCCCTCCACATCCCTCT
+
FFFFFFFFFFFFFF,FFFFFFFFFFFFFFFFFFFFFFFF::FFFFFFFFFFFFFFFFFFF:FFFFFFF
FFFFFFFFFFFFFFFFFFFFFFFFFFFFFFFF:FFFFFFFFFFFFFFFFFFFFFFFFFFFFFFFFFFF
@A00155:342:HHGFNDSXY:1:2160:26666:18490 2:N:0:GAACCTAG+TCCGCATA
GAACTAGGAGACCCCTCCGGTCTTGTCCAACCTCTGTTTCGGCAGAGGATACCGTGCTCCACAGCTGT
AAGGCTGTGGGCCCGAGAGAAGTCGCTCTCTCTCGCCCTCCACATCCCTCTCGGCATTGAGATGACCA
+

```
FF:FFFFFFFFFFFFFFFFFFFFFFFFFFFF::FFFFFFFFFFFFFFFFFFFFFFFFFFFFFFFFFFF
FFFFF:FFFFFFFFFFFFFFFFFFFFFFFFFFFFFFFFFFFFFFFFFFFFFFFFFFFFFFFFFF
@A00155:342:HHGFNDSXY:1:2160:23149:20854 2:N:0:GAACCTAG+TCCGCATA
TAGGAGACCCCTCCGGTCTTGTCCAACCTCTGTTTCGGCAGAGGATACCGTGCTCCACAGCTGTAAGG
CTGTGGGCCCGAGAGAAGTCGCTCTCTCTCGCCCTCCACATCCCTCTCGGCATTGAGATGACCATAGG
+
FFFFFFFFFFFFFFFFFF,FFFFFFFFFFFFFFFFFFFFFFFFFFFFFFFFFFFFFFFFFFFFFFFFF
F:FFFFFFFFFFFFFF,FFFFFFFFFFFFFFFFFFFFFFFFFFFFFFFFFFF:FFFFFFFFFFFFFF:
@A00155:342:HHGFNDSXY:1:2161:31051:3881 2:N:0:GAACCTAG+TCCGCATA
GACGAAACCGTCGTTACTCTTCGAGTTGTGTAAGTTTCGTCCTAAAAAGGACTCGTCAGGTAGCCTAC
TAGACTACGACTTACACGGTAGGTCGTGAACAAGCCCGGAGTACATCTCCTTTGGAACCAGGAGACCC
+
F:FFFFFFFFFFFFFFFFF:FFFFFFFFFFFFFFF:FFFFFFF:FFFFFFFFFFFFFFFFFFFFFFF:
FFFFFFFFFFFF:FFFFFFF::FFFFFFFFFFFF:FFFFFFFFFFFFFFFFFFFFFFFFFFFFFFFFF
@A00155:342:HHGFNDSXY:1:2161:24677:26099 2:N:0:GAACCTAG+TCCGCATA
AGCCCGGAGTACATCTCCTTTGGAACTAGGAGACCCCTCCGGTCTTGTCCAACCTCTGTTTCGGCAGA
GGATACCGTGCTCCACAGTTGTAAGGCTGTGGGCCCGAGAGAAGTCGCTCTCTCTCGCCCTCCACATC
+
FFFFFFFFFFFFFFFFFFFFFFFFFFFFFFFFFFFFFFFFFFFFFFFFFFFFFFFFFFFFFFFFFFFF
FFFFFFFFFFFFFFFFFF,FFFF:FFFFFFFFFFFFFFFFFFFFFFFFFFFFFFFFFFFFFFFFFFFF
@A00155:342:HHGFNDSXY:1:2161:14913:28260 2:N:0:GAACCTAG+TCCGCATA
GCTGTGTCCACTTAAGGACTCACCTCTGGCCTATGGTCATCTCAATGCCGAGAGGGATGTAGAGGGCG
AGAGAGAGCGACTTCTCTCGGGCCCACAGCCTTACAGCTGTGGAGCACGGTATCCTCTGCCGAAACAG
+
FFFFFFFFFFFFFFFFFFFFFFFFFFFFFFFFFFFFFFFFFFFFFFFFFFFFFFFFFFFFFFFFFFFF
FFFFFFFFFFFFFFFFFFFFFFFFFFFFFFFFFFFFFFFFFFFFFFFFFFFFFFFFFFFFFFFFFFFF
@A00155:342:HHGFNDSXY:1:2161:20193:30044 2:N:0:GAACCTAG+TCCGCATA
GGACTCGTCAGGTAGCCTACTAGACTACGACTTACACGGTAGGTCGTGAACAAGCCCGGAGTACATCT
CCTTTGGAACTAGGAGACCCCTCCGGTCTTGTCCAACCTCTGTTTCGGCAGAGGATACCGTGCTCCAC
+
FFFFFFFFFFFFFFFFFFFFFFFFFFFFFFFFFFFFFFFFFFFFFFFF:FFFFFFFFFFFFFFFFFFF
FFFFFFFFFFFFFFFFFFFFFFFFFFFF:FFFFFFFFFFFFFFFFFFFFFFFFFFFFFFFFFFFFFFF
@A00155:342:HHGFNDSXY:1:2162:4924:9893 2:N:0:GAACCTAG+TCCGCATA
TCCTAAAAAGGACTCGTCAGGTAGCCTACTAGACTACGACTTACACGGTAGGTCGTGAACAAGCCCGG
AGTACATCTCCTTTGGAACTAGGAGACCCCTCCGGTCTTGTCCAACCTCTGTTTCGGCAGAGGATACC
+
FFFFFFFFFFFFFFFFFFFFFFFFFFFFFFFFFFFFFFFFFFFFFFFFFFFFFF:FFFFFFFFFFFFF
FFFFFFFFFFFFFFFFFFFFFFFFFFFFFFFFFFFFF::FFFFFFFFFFFFFFFFFFFFFFFFFFFFF
@A00155:342:HHGFNDSXY:1:2162:2121:17472 2:N:0:GAACCTAG+TCCGCATA
GGACTCGTCAGGTAGCCTACTAGACTACGACTTACACGGTAGGTCGTGAACAAGCCCGGAGTACATCT
CCTTTGGAACTAGGAGACCCCTCCGGTCTTGTCCAACCTCTGTTTCGGCAGAGGATACCGTGCTCCAC
+
FFFFFFFFFFFFFFFFFFFFFFFFFFFFFFFFFFFFFFFFFFFFFFFFFFFFFFFFFFFFFFFFFFFF
FFFFFFFFFFFFFFFFFFFFFFFFFFFFFFFFFFF:FFFFFFFFFFFF,FFFFFFFFFFFFFFFFFFF
@A00155:342:HHGFNDSXY:1:2163:30147:6073 2:N:0:GAACCTAG+TCCGCATA
CGTCGTTACTCTTTGAGTTGTGTAAGTTTCGTCCTAAAAAGGACTCGTCAGGTAGCCTACTAGACTAC
GACTTACACGGTAGGTCGTGAACAAGCCCGGAGTACATCTCCTTTGGAACTAGGAGACCCCTCCGGTC
+
FFFFFFFFFFF::FF:FFFFFFFFFFFFFFFFFFFFFF:FFFFFFFFFFFFFFFFFFFFFFFFFFFFF
FFFFFFFFFFFFFFFFFFFFFFFFFFFFFFFFFFFFFFFFFFFFFFFFFFFFFFFFFFFFFFFFFFFF
@A00155:342:HHGFNDSXY:1:2163:6126:17613 2:N:0:GAACCTAG+TCCGCATA
ACACGGTAGGTCGTGAACAAGCCCGGAGTACATCTCCTTTGGAACCAGGAGACCCCTCCGGTCTTGTC
CAACCTCTGTTTCGGCAGAGGATACCGTGCTCCACAGCTGTAAGGCTGTGGGCCCGAGAGAAGTCGCT
+
```

FFFFFFFFFFFFFFFFFFFFFFFFFFFFFFFFFFFFFFFFFFF:FFFFFFFFFFFFFFFFFFFFFFFF
FFFFFFFFFFFFFFFFFFFFFFFFFFFFFFFFFFFF:FFFFFFFFFFFFFFFFFFFFFFFFFFFFFFF
@A00155:342:HHGFNDSXY:1:2163:30689:30561 2:N:0:GAACCTAG+TCCGCATA
AGCCTACTAGACTACGACTTACACGGTAGGTCGTGAACAAGCCCGGAGTACATCTCCTTTGGAACTAG
GAGACCCCTCCGGTCTTGTCCAACCTCTGTTTCGGCAGAGGATACCGTGCTCCACAGCTGTAAGGCTG
+
FFFFFFFFFFFFFFFFFFFFFFFFFFFFFFFFFFFFFFFFFFFFFFFFFFFFFFFFFFFFFFFFFFFF
FFFFFFFFFFFFFFF:FFFFFFFFFFFFFFFFFFFFFFFFFFFFFFFFFFFFFFFFFFFFFFFFFFFF
@A00155:342:HHGFNDSXY:1:2163:15230:33567 2:N:0:GAACCTAG+TCCGCATA
AGTACATCTCCTTTGGAACCAGGAGACCCCTCCGGTCTTGTCCAACCTCTGTTTCGGCAGAGGATACC
GTGCTCCACAGCTGTAAGGCTGTGGGCCCGAGAGAAGTCGCTCTCTCTCGCCCTCCACATCCCTCTCG
+
FFFFFFFFFFFFFFFFFFFFFFFFFFFFFFFFFFFFFFFFFFFFFFFFFFFFFFFFFFFFFFFFFFFF
FFFFFFFFFFFFFFFFFFFFFFFFFFFFFFFFFFFFFFFFFFFFFFFFFFFFFFFFFFFFFFFFFFFF
@A00155:342:HHGFNDSXY:1:2164:13765:32158 2:N:0:GAACCTAG+TCCGCATA
GGACTCGTCAGGTAGCCTACTAGACTACGACTTACACGGTAGGTCGTGAACAAGCCCGGAGTACATCT
CCTTTGGAACTAGGAGACCCCTCCGGTCTTGTCCAACCTCTGTTTCGGCAGAGGATACCGTGCTCCAC
+
FFFFFFFFFFFFFFFFFFFFFFFFFFFFFFFFFFFFFFFFFFFFFFFFFFFFFFFFFFFFFFFFFFFF
FFFFFFFFFFFFFFFFFFFFFFFFFFFFFF:FFFFFFFFFFFFFFFFFFFFFFFFFFFFFFFFFFFFF
@A00155:342:HHGFNDSXY:1:2165:21079:17018 2:N:0:GAACCTAG+TCCGCATA
TAGACTACGACTTACACGGTAGGTCGTGAACAAGCCCGGAGTACATCTCCTTTGGAACTAGGAGACCC
CTCCGGTCTTGTCCAACCTCTGTTTCGGCAGAGGATACCGTGCTCCACAGCTGTAAGGCTGTGGGCCC
+
FFFFFFFFFFFFFFFFFFFFFFFFFFFFFFFFFFFFFFFFFFFFFFFFFFFFFFFFFF:FFFFFFFFF
FFFFFFFFFFFFFFFFFFFFFFFFFFFFFFFFFFFFFFFFFFFFFFFFFFFFFFFFFFFFFFFFFFFF
@A00155:342:HHGFNDSXY:1:2165:18195:29152 2:N:0:GAACCTAG+TCCGCATA
GGTCGTGAACAAGCCCGGAGTACATCTCCTTTGGTACTAGGAGACCCCTCCGGTCTTGTCCAACCTCT
GTTTCGGCAGAGGATACCGTGCTCCACAGCTGTAAGGCTGTGGGCCCGAGAGAAGTCGCTCTCTCTCG
+
FFFFFFFFFFFFFFFFFFFFFFFFFFFFFFF:FFFFF:FFFFFFFFFFFFFFFFF:FFFFFFFFFFFF
FFFFFFFFFFFFFFFFFFFFFFFFFF:FFFFFFFFFFFFFFFFFFFFFFFFFFFFFFFFFFFFFFFFF
@A00155:342:HHGFNDSXY:1:2166:16125:21840 2:N:0:GAACCTAG+TCCGCATA
ACTTACACGGTAGGTCGTGAACAAGCCCGGAGTACATCTCCTTTGGAACTAGGAGACCCCTCCGGTCT
TGTCCAACCTCTGTTTCGGCAGAGGATACCGTGCTCCACAGCTGTAAGGCTGTGGGCCCGAGAGAAGT
+
FFFFFFFFFFFFFFFFFFFFFFFFFFFFFFFFFFFFFFFFFFFFFFFFFFFFFFFFFFFFFFFFFFFF
FFFFFFFFFFFFFFFFFFFFFFFFFF:FFFFFFFFFFFFFFFFFFFFFFFFFFFFFFFFFFFFFFFFF
@A00155:342:HHGFNDSXY:1:2166:30228:32174 2:N:0:GAACCTAG+TCCGCATA
CGACTTACACGGTAGGTCGTGAACAAGCCCGGAGTACATCTCCTTTGGAACTAGGAGACCCCTCCGGT
CTTGTCCAACCTCTGTTTCGGCAGAGGATACCGTGCTCCACAGCTGTAAGGCTGTGGGCCCGAGAGA
+
FFFFFFFFFFFFFFFF:FFFFFFFFFFFFFFFFFFFF:FFFFFFFFFFF:FFFFFFFFFFFFFFFFF:
F:FFFFFF:FFFFFFFFFFFFFFFF:FF:FFFFFFFFFFFFFFF,FFFFFFFFFFFFFFFFFFFFF
@A00155:342:HHGFNDSXY:1:2168:13060:25676 2:N:0:GAACCTAG+TCCGCATA
GGACTCGTCAGGTAGCCTACTAGACTACGACTTACACGGTAGGTCGTGAACAAGCCCGGAGTACATCT
CCTTTGGAACTAGGAGACCCCTCCGGTCTTGTCCAACCTCTGTTTCGGCAGAGGATACCGTGCTCCAC
+
FFFFFFFFFFFFFFFFFFFFFFFFFFFFFFFFFFFFFFFFFFFFFFFFFFFFFFF:FFFFFFFFFFFF
FFFFFF,F,FFFFFFFFF:FFFFFFFFFFFFFFFFFFFFFF:F:FFFFFFFFF:FFFFFFFFFFFFFF
@A00155:342:HHGFNDSXY:1:2168:28917:26553 2:N:0:GAACCTAG+TCCGCATA
AAGGACGAAACCGTCGTTACTCTTCGAGTTGTGTAAGTTTCGTCCTAAAAGGGACTCGTCAGGTAGCC
TACTAGACTACGACTTACACGGTAGGTCGTGAACAAGCCCGGAGTACATCTCCTTTGGAACTAGGAGA
+

FFFFFFFFFFFFFFFFFFFFFF:FFFFFFFFFFFFFFFFFFFFF:FFFFFFFFFFFFFFFFFFFFFFF
FFFFFFFFFFFFFFFFFFFFFFFFFFFFFFFFFFFFFFFFFFFFFFFFFFFFFFF:FFFFFFF:FFFF
@A00155:342:HHGFNDSXY:1:2168:19732:35540 2:N:0:GAACCTAG+TCCGCATA
ACATCTCCTTTGGAACTAGGAGACCCCTCCGGTCTTGTCCAACCTCTGTTTCGGCAGAGGATACCGTG
CTCCACAGCTGTAAGGCTGTGGGCCCGAGAGAAGTCGCTCTCTCTCGCCCTCCACATCCC
+
FFFFFFFFF:FFFFFFFFFFFFFFFFFFFFFFFFFFFFF:FFFFFFFFFFFFFFFFFFFFFFFF:FFF
FFFFFFFFFFFFFFF:F:FFFFFFFFFFFFFFFF:FFFFFFFFFFFFFFFFFFF:FFF:F
@A00155:342:HHGFNDSXY:1:2169:21314:11600 2:N:0:GAACCTAG+TCCGCATA
CTCGAAGAGTAACGACGGTTTCGTCCTTGGAACGGACTCATCAGGGAGCCTACTAGACTCCGACCGTC
ATGGGGGTTCAAACCCATGCCCCGCCACACCGCCTTAGATCAGCTGTGTCCACTTAAGGACTCACCTC
+
FFFFFFFFFFFFFFFFFFFFFFFFFFFFFFFFFFFFFFFFFFFFFFFFFFFFFFFFFFFFFFFFFFFF
,FFFFFFFFFFFFFFFFFFFFFFFFFFFFFFFFFFF:FFFFFFFFFFFFFF,FF:FFFFFFFFFFFF
@A00155:342:HHGFNDSXY:1:2169:4716:24001 2:N:0:GAACCTAG+TCCGCATA
GTGAACAAGCCCGGAGTACATCTCCTTTGGAACTAGGAGACCCCTCCGGTCTTGTCCAACCTCTGTTT
CGGCAGAGGATACCGTGCTCCACAGCTGTAAGGCTGTGGGCCCGAGAGAAGTCGCTCTCTCTCGCCC
+
FFFFFFFFFFFFFFFFFFFFFFFFFFFFFFFFFFFFFFFFFFFFFFFFFFFFFFFFFFFFFFFFFFFF
FFFFFFFFFFFFFFFFFFFFFFFFFFFFFFFFFFFFFFFFFFFFFFFFFFFFFFFFFFFFFFFFFFF
@A00155:342:HHGFNDSXY:1:2169:1145:28557 2:N:0:GAACCTAG+TCCGCATA
GGACTCGTCAGGTAGCCTACTAGACTACGACTTACACGGTAGGTCGTGAACAAGCCCGGAGTACATCT
CCTTTGGAACCAGGAGACCCCTCCGGTCTTGTCCAACCTCTGTTTCGGCAGAGGATACCGTGCTCCAC
+
FFFFF:FFF,FFFFFFFF:FF:FFFF,FFFFFF,FFFFFFFFFFFFFFFF,F,,F:FFF:FFF,,F:F
FF:FFFF:FF,FFF,:FF:FFF::FFFFFFFFFFF,::F:FFFFFFFFF,F,FFFFF,FFFFFFFF,F
@A00155:342:HHGFNDSXY:1:2169:3224:31908 2:N:0:GAACCTAG+TCCGCATA
GAGACCCCTCCGGTCTTGTCCAACCTCTGTTTCGGCAGAGGATACCGTGCTCCACAGCTGTAAGGCTG
TGGGCCCGAGAGAAGTCGCTCTCTCTCGCCCTCCACATCCCTCTCGGCATTGAGATGACCATAGG
+
FFFFFFFFFFFFFFFFFFFFFFFFFFFFFFFFFFFFFFFFFFFFFFFFF:FFFFFFFFFFFFFFFFFF
FFFFFFFFFFFFFFFFFFFFFFFFFFFFFFFF:FFFFFFFFFFFFFFFFF:FFFFFFFFFFFFFFF
@A00155:342:HHGFNDSXY:1:2169:26946:34914 2:N:0:GAACCTAG+TCCGCATA
GAAACCGTCGTTACTCTTCGAGTTGTGTAAGTTTCGTCCTAAAAAGGACTCGTCAGGTAGCCTACTAG
ACTACGACTTACACGGTAGGTCGTGAACAAGCCCGGAGTACATCTCCTTTGGAACTAGGAGACCCCTC
+
FFFFFFFFFFFFFFFFFFFFFFFFFFFFFFFFFFFFFFFFFFFFFFFFFFFFFFFFFFFFFFFFFFFF
FFFFFFFFFFFFFFFFFFFFFFFFFFFFFFFFFFFFFFFFFFFFFFFF:FFFFFFFFFFFFFFFFFFF
@A00155:342:HHGFNDSXY:1:2170:28402:6715 2:N:0:GAACCTAG+TCCGCATA
GAGACCCCTCCGGTCTTGTCCAACCTCTGTTTCGGCAGAGGATACCGTGCTCCACAGCTGTAAGGCTG
TGGGCCCGAGAGAAGTCGCTCTCTCTCGCCCTCTACATCCCTCTCGGCATTGAGATGACCATAG
+
FFFFFFFFFFFFFFFFFFFFFFFFFFFFFFFFFFFFFFFFFFFFFFFFFFFFFFFFFFFFFFFFFFFF
FFFFFFFFFFFFFFFFFFFFFFFFFFFFFFFFFFFFFFFFFFFFFFFFFFFFFFFFFFFFFFFF
@A00155:342:HHGFNDSXY:1:2170:2998:8750 2:N:0:GAACCTAG+TCCGCATA
CGTCCTAAAAAGGACTCGTCAGGTAGCCTACTAGACTACGACTTACACGGTAGGTCGTGAACAAGCCC
GGAGTACATCTCCTTTGGAACCAGGAGACCCCTCCGGTCTTGTCCAACCTCTGTTTCGGCAGAGGATA
+
FFFFFFFFFFFFFFFFFFFFFFFFFFFFFFFFFFFFFFFFFFFFFFFFFFFFFFFFFFFFFFFFFFFF
FFFFFFFFFFFFFFFFFFFFFFFFFFFFFFFFFFFFFFFFFFFFFFFFFFFFFFFFFFFFFFFFFFFF
@A00155:342:HHGFNDSXY:1:2170:3242:9361 2:N:0:GAACCTAG+TCCGCATA
CGTCCTAAAAAGGACTCGTCAGGTAGCCTACTAGACTACGACTTACACGGTAGGTCGTGAACAAGCCC
GGAGTACATCTCCTTTGGAACCAGGAGACCCCTCCGGTCTTGTCCAACCTCTGTTTCGGCAGAGGATA
+

FFFFFFFFFFFFFFFFFFFFFFFFFFFFFFFFFFFFFFFFFFFFFFFFFFFFFFFFFF:FFFFFFFF
FFFFFFFFFFFFFFFFFFFFFFFFFFFFFFFFFFFFFFFFFFFFFFFFFFFFFFFFFFFFFFFFFFF
@A00155:342:HHGFNDSXY:1:2170:28736:9893 2:N:0:GAACCTAG+TCCGCATA
AGGACGAAACTTACACAACTCGAAGAGTAACGACGGTTTCGTCCTTGGAACGGACTCATCAGGGAGCC
TACTAGACTCCGACCGTCATGGGGGTTTAAACCCATGCCCCGCCACACCGCCTTAGATCAGCT
+
FFFFFFFFFFFFFFFFFFFFFFFFFFFFFFFFFFFFFFFFFFFFFFFFFFFFFFFFFFFFFFFFFFF
FFFFFFFFFFFFFFFFFFFFFFFFFFFFFFFFFFFFFFFFFFFFFFFFFFFFFFFFFFFFFFF
@A00155:342:HHGFNDSXY:1:2170:16378:28134 2:N:0:GAACCTAG+TCCGCATA
GAAACCGTCGTTACTCTTTGAGTTGTGTAAGTTTCGTCCTAAAAAGGACTCGTCAGGTAGCCTACTAG
ACTACGACTTACACGGTAGGTCGTGAACAAGCCCGGAGTACATCTCCTTTGGAACTAGGAGACCCCTC
+
FFFFFFFFFFFFFFFFF,FFFF:FFFFFFFFF:FFFFFFFFFF::FFFFFFF,FFFFFFFFFFFFFF
FFFFFFFFFFFFFFFFFFFF,FFFFFFFFFFFFFFFFFFFFFFFF,FF:FFFFFFF:FFFFFFFFFFF
@A00155:342:HHGFNDSXY:1:2170:14064:29857 2:N:0:GAACCTAG+TCCGCATA
GAACTAGGAGACCCCTCCGGTCTTGTCCAACCTCTGTTTCGGCAGAGGATACCGTGCTCCACAGCTGT
AAGGCTGTGGGCCCGAGAGAAGTCGCTCTCTCTCGCCCTCCACATCCCTCTCGGCATTGAGATGACC
+
FFFFFFFFFFFFFFFFFFFFFF:FFFFFFFFFFFFFFFFFFFFFFFFFFFFFFFFFFFFFFFFFFFF
FFFFFFFFFFFFFFF:FFFFFFFFFFFFFFFFFFFFFFFFFFFFFFFFFFFFFFFFFFFFFFFFFFF
@A00155:342:HHGFNDSXY:1:2171:10728:7044 2:N:0:GAACCTAG+TCCGCATA
AAGGACGAAACCGTCGTTACTCTTGGAGTTGTGTAAGTTTCGTCCTAAAAAGGACTCGTCAGGTAGCC
TACTAGACTACGACTTACACGGTAGGTCGTGAACAAGCCCGGAGTACATCTCCTTTGGAACTAGGAGA
+
FFFFFFFFFFFFFFFFFFFF:FFFFFFFFFFFFFFFFFFFFFFFFFFFFFFFFFFFFFFFFFFFFFF
FFFFFFFFFFFFFFFFFFFFFFFFFFFFFFFFFFF:FFFFFFFFFFFFFFFFFFFFFFFF,FFFFFFFF
@A00155:342:HHGFNDSXY:1:2171:15826:18474 2:N:0:GAACCTAG+TCCGCATA
ATCTCCTTTGGAACTAGGAGACCCCTCCGGTCTTGTCCAACCTCTGTTTCGGCAGAGGATACCGTGCT
CCACAGCTGTAAGGCTGTGGGCCCGAGAGAAGTCGCTCTCTCTCGCCCTCTACATCCCTCTCGGCATT
+
:FFFFFF:FFFFFFF:FFFFFFFFFFFFFFFFFFFFFFFFFFFFFFFFFFFFFFFFFFFFFFFFFFF
FFFFFFFFFFFFFFFFFFFFFFFFFFFFFFFFFFFFFFFFFFFFFFFFFFFFFFFFFFFFFFFFFF,F
@A00155:342:HHGFNDSXY:1:2171:27190:21684 2:N:0:GAACCTAG+TCCGCATA
ACATCTCCTTTGGAACTAGGAGACCCCTCCGGTCTTGTCCAACCTCTGTTTCGGCAGAGGATACCGTG
CTCCACAGCTGTAAGGCTGTGGGCCCGAGAGAAGTCGCTCTCTCTCGCCCTCCACATCCCTCTCGGCA
+
FFFFFFFFFFFFFFFFFFFFFFFFFFFFFFFFFF:FFFFFFFFFFFFFFFFFFFFFFFFFFFFFFFFF
FFFFFFFFFFFFFFFFFFFFFFFFFFFFFFFFFFFFFFFFFFFFFFFFFFFFFFFFFFFFFFFFFFFF
@A00155:342:HHGFNDSXY:1:2172:29143:27383 2:N:0:GAACCTAG+TCCGCATA
CCTTTGGAACTAGGAGACCCCTCCGGTCTTGTCCAACCTCTGTTTCGGCAGAGGATACCGTGCTCCAC
AGCTGTAAGGCTGTGGGCCCGAGAGAAGTCGCTCTCTCTCGCCCTCCACATCCCTCTCG
+
FFFFF:FFFFFFFFFFFFFFFFFFFFFFFFFFFFFFFFFF:FFFFF:F,FFFFFFFFFF:FF:FFFFFFF
:FFF::FFFFFFFFFF:FFFFFFFFFFFF,F:FFFFFFF:FFFFFFFFFFF::FFFFFF
@A00155:342:HHGFNDSXY:1:2172:13910:33223 2:N:0:GAACCTAG+TCCGCATA
AGCCCGGAGTACATCTCCTTTGGAACTAGGAGACCCCTCCGGTCTTGTCCAACCTCTGTTTCGGCAGA
GGATACCGTGCTCCACAGCTGTAAGGCTGTGGGCCCGAGAGAAGTCGCTCTCTCTCGCC
+
FFFFFFFFFFFFFFFFFFFFFFFFFFFFFFFFFFFFFFFFFFFFFFFFFFFFFFFFFFFFFFFFFFFF
FFFFFFFFFFFFFFFFFFFFFFFFFFFFFFFFFFFFFFFFFFFFFFFFFFFFFFFFFFF
@A00155:342:HHGFNDSXY:1:2172:13566:33285 2:N:0:GAACCTAG+TCCGCATA
AGCCCGGAGTACATCTCCTTTGGAACTAGGAGACCCCTCCGGTCTTGTCCAACCTCTGTTTCGGCAGA
GGATACCGTGCTCCACAGCTGTAAGGCTGTGGGCCCGAGAGAAGTCGCTCTCTCTCGCC
+

FFFFFFFFFFFFFFFFFFFFFFFFFF:FFFFFFFFFFFFFFFFFFFFFFFFFFFFFFF:FFFFFFFFFFF
FFFFFFFFFFFFFFFFFFFFFFFF:FFFFFFFFFFFFFFFFFFFFFFFFFFFFFFFFFFFF
@A00155:342:HHGFNDSXY:1:2173:15600:1579 2:N:0:GAACCTAG+TCCGCATA
AAAAAGGACTCGTCAGGTAGCCTACTAGACTACGACTTACACGGTAGGTCGTGAACAAGCCCGGAGTA
CATCTCCTTTGGAACTAGGAGACCCCTCCGGTCTTGTCCAACCTCTGTTTCGGCAGAGGATACCGTGC
+
FFFFFFFFFFFFFFFFFFFFFFFFFFFFFFFFFFFFFFFFFFFFFFFFFFFFFFFFFFFFFFFFFFFFFF
FFFFFFFFFFFFFFFFFFFFF:FFFFFFFFFFFFFFFFFFFFFFFFFFFFFFFFFFFFFFFFFFFFFFFF
@A00155:342:HHGFNDSXY:1:2173:12608:9549 2:N:0:GAACCTAG+TCCGCATA
CGGTTTCGTCCTTGGAACGGACTCATCAGGGAGCCTACTAGACTCCGACCGTCATGGGGGTTCAAACC
CATGCCCCGCCACACCGCCTTAGATCAGCTGTGTCCACTTAAGGACTCACCTCTGGCCTATGGTCATC
+
FFFFFFFFFFFFFFFFFFFFFFFFFFFFFFFFFFFFFFFFFFFFFFFFFFFFFFFFFFFFFFFFFFF:FF
FFFFFFF:FFFFFFFFFFFFFFFFFFFFFFFFFFFFFFFFFFFFFFFFF:FFFFFFFFFFFFFFFFFFFF
@A00155:342:HHGFNDSXY:1:2173:5791:26866 2:N:0:GAACCTAG+TCCGCATA
GGACTCGTCAGGTAGCCTACTAGACTACGACTTACACGGTAGGTCGTGAACAAGCCCGGAGTACATCT
CCTTTGGAACTAGGAGACCCCTCCGGTCTTGTCCAACCTCTGTTTCGGCAGAGGATACCGTGCTCCAC
+
FFFFFFFFFFFFFFFFFFFFFFFFFFFFFFFFFFFFFFFFFFFFFFFFFFFFFFFFFFFFFFFFFFFFFF
FFFFFFFFFFFFFFFFF:FFFFFFFFFFFFFFFFFFFFFFFFFFFFFFFFFFFFFFFFFFFFFFFFFFFF
@A00155:342:HHGFNDSXY:1:2173:22435:28416 2:N:0:GAACCTAG+TCCGCATA
AGCCTACTAGACTACGACTTACACGGTAGGTCGTGAACAAGCCCGGAGTACATCTCCTTTGGAACTAG
GAGACCCCTCCGGTCTTGTCCAACCTCTGTTTCGGCAGAGGATACCGTGCTCCACAGCTG
+
FFFFFFFFFFFFFFFFFFFFFFFFFF:FFFFFFFFFFFFFFFFFFFFFFFFFFFFFFFFFF:FFFFFFFF
FFFFFFFFFFFFFFFFFFFFFFFFFFFFFFFFFFFFFFFFFFFFFFFFFFFFFFFFFFFF
@A00155:342:HHGFNDSXY:1:2174:5575:1188 2:N:0:GAACCTAG+TCCGCATA
AGTCCGTTCCAAGGACGAAACCGTCGTTACTCTTCGAGTTGTGTAAGTTTCGTCCTAAAAAGGACTCG
TCAGGTAGCCTACTAGACTACGACTTACACGGTAGGTCGTGAACAAGCCCGGAGTACATCTCCTTTGG
+
FFFFFFFFFFFFFFFFFFFFF:F,FFFFFFFFFFFFFFFFFFFFFFFFFF:FFFFFF:FFFFFFFFFFF:
FFFFF:FFFFFFFFFFFFFFFFFFFFFFFFFFFFFFFFFFFFFFFFFF:FFFFFFFFFFFF:FFFFFFFF
@A00155:342:HHGFNDSXY:1:2174:25934:30185 2:N:0:GAACCTAG+TCCGCATA
TGTTCACGACCTACCGTG
+
FFFFFFFFFFFFFFFFFF
@A00155:342:HHGFNDSXY:1:2175:13575:4961 2:N:0:GAACCTAG+TCCGCATA
AGTTGTGTAAGTTTCGTCCTAAAAAGGACTCGTCAGGTAGCCTACTAGACTACGACTTACACGGTAGG
TCGTGAACAAGCCCGGAGTACATCTCCTTTGGAACTAGGAGACCCCTCCGGTCTTGTCCAACCTCTGT
+
FFF:FFFFFFFFFFFFFFFFFFFFFFFFFFFFFFFFFFFFFFFFFFFFFFFFFFFFFFFFFFFFFFFFFF
FFFFF:FFFFFFFFFFFFFFF:FFFFFFFFFFFFFFFFFFFFFFFFFFFFFFFFFFFFFFFFFFFFFFFF
@A00155:342:HHGFNDSXY:1:2175:6244:9831 2:N:0:GAACCTAG+TCCGCATA
GTTGTGTAAGTTTCGTCCTAAAAAGGACTCGTCAGGTAGCCTACTAGACTACGACTTACACGGTAGGT
CGTGAACAAGCCCGGAGTACATCTCCTTTGGAACTAGGAGACCCCTCCGGTCTTGTCCAACCTCTGTT
+
FFFFFFFFFFFFFFFFFF,:FFF:FFFFFFFFFFFFFFFFFFFFFFFFFFFFFFFFFFFFFFFFFFFFFF
FFFFFFFFFFFFFFFFFFFFFFFFFFFFFFFFFFFF:FFFFFFFFFFFFFFFFF,F:FF:FFFFFFFFF:
@A00155:342:HHGFNDSXY:1:2175:9372:20071 2:N:0:GAACCTAG+TCCGCATA
AAGGACTCGTCAGGTAGCCTACTAGACTACGACTTACACGGTAGGTCGTGAACAAGCCCGGAGTACAT
CTCCTTTGGAACTAGGAGACCCCTCCGGTCTTGTCCAACCTCTGTTTCGGCAGAGGATACCGTG
+
FFFFFFFFFFFFFFFFFFFFFFFFFFFFFFFFFFFFFFFFFFFFFFFFFFFFFFFFF,FFFFFFFFFFFF
FFFFFFFFFFFFFFFFFFFFFFFFFFFFFFFFFFFFFFFFFFFFFFFFFFFFFFFFFFFFFFFF

```
@A00155:342:HHGFNDSXY:1:2175:19090:21214 2:N:0:GAACCTAG+TCCGCATA
CACGGTAGGTCGTGAACAAGCCCGGAGTACATCTCCTTTGGAACTAGGAGACCCCTCCGGTCTTGTCC
AACCTCTGTTTCGGCAGAGGATACCGTGCTCCACAGCTGTAAGGCTGTGGGCCCGAGAGAAGTCGCTC
+
FFFF:FF:FFFFFFFFFFFF,:F:FFFFFFFFFFFFFFFFFFFFFFFFF:FFFFFFFFFFF:FFFFFF
FFFFFFFFFFFFFFF:FFFFFFFFFFFF:F:FFFF:FFFFFFF,F:FFFF:FFFFFFFFFFFFFFFFF
@A00155:342:HHGFNDSXY:1:2175:6433:23437 2:N:0:GAACCTAG+TCCGCATA
TTTCGTCCTTGGAACGGACTCATCAGGAAGCCTACTAGACTCCGACCGTCATGGGGGTTCAAACCCAT
GCCCCGCCACACCGCCTTAGATCAGCTGTGTCCACTTAAGGACTCACCTCTGGCCTATGGTCATCT
+
:FFFFFFFFFFFFFFFFFFFFFFFFFFFFFFFFFFFFFFFFFFFFFFFFFFFFFFFFFFFFFFFFFFF
FFFFFFFFFFFFFFFFFFFFFFFF:FFFFFFFFFFFFFFFFFFFFFFFFFFFFFFFFFFFFFFF:FFF
@A00155:342:HHGFNDSXY:1:2175:22182:25097 2:N:0:GAACCTAG+TCCGCATA
AGTACATCTCCTTTGGAACTAGGAGACCCCTCCGGTCTTGTCCAACCTCTGTTTCGGCAGAGGATACC
GTGCTCCACAGCTGTAAGGCTGTGGGCCCGAGAGAAGTCGCTCTCTCTCGCCCTCCACATCCCTCTCG
+
FFFFFFFFFFFFFFFFFFFFFFFFFFFFFFFFFFFFFFFFFFFFFFFFFFFFFFFF:FFFFFFFFFF
FFFFFFFFFFFFFFFFFFFFFFFFFFFFFFFFFFFFFFFFFFFFFFFF:FFFFFFFFFFFFFFFFFFFF
@A00155:342:HHGFNDSXY:1:2175:25482:33473 2:N:0:GAACCTAG+TCCGCATA
GGACTCGTCAGGTAGCCTACTAGACTACGACTTACACGGTAGGTCGTGAACAAGCCCGGAGTACATCT
CCTTTGGAACTAGGAGACCCCTCCGGTCTTGTCCAACCTCTGTTTCGGCAGAGGATACCGTGCTCCAC
+
FFFFFFFFFFFFFFFFFFFFFFFFFFFFFFFFFFFFFFFFFFFFFFFFFFFFF:FFFFFFFFFFFFFF
FFFFFFFFFFFFFFFFFFFFFFFFFFFFFFFFFFFFFFFFFFFF:FFFF:FFFFFFFFFFFFFFFFFFF
@A00155:342:HHGFNDSXY:1:2176:16803:16501 2:N:0:GAACCTAG+TCCGCATA
GTTGTGTAAGTTTCGTCCTAAAAAGGACTCGTCAGGTAGCCTACTAGACTACGACTTACACGGTAGGT
CGTGAACAAGCCCGGAGTACATCTCCTTTGGAACTAGGAGACCCCTCCGGTCTTGTCCAACCTCTGTT
+
FFFFFFFFFFFFFFFFFFFFFFFFFFFFFFFFFFFFFFFFFFFFFFFFFFFFFFFFFFFFFFFF:FFF
FFFFFFFFF:FFFFFFFFFFFFFFFFFFFFFFFFFFFFFFFFFFFFFFFFFFFFFFFFFFFFFFFFFF
@A00155:342:HHGFNDSXY:1:2176:16957:16517 2:N:0:GAACCTAG+TCCGCATA
GTTGTGTAAGTTTCGTCCTAAAAAGGACTCGTCAGGTAGCCTACTAGACTACGACTTACACGGTAGGT
CGTGAACAAGCCCGGAGTACATCTCCTTTGGAACTAGGAGACCCCTCCGGTCTTGTCCAACCTCTGTT
+
FFFFFFFFFFFF::FFFFFFFFFFFFFFFFFFFFFFFFF:FFFFFFFFF:FFF:FFFFFFFFFFFFFF
FF:FFFFFF,FF:FFFFFFFFFF::FFFF:FFFFFFFFF,FF:F:FFFFFFFFF:FFFFFFFFFF,FF
@A00155:342:HHGFNDSXY:1:2176:20193:28761 2:N:0:GAACCTAG+TCCGCATA
CTGGCCTATGGTCATCTCAATGCCGAGAGGGATGTGGAGGGCGAGAGAGAGCGACTTCTCTCGGGCCC
ACAGCCTTACAGCTGTGGAGCACGGTATCCTCTGCCGAAACAGAGGTTGGACAAGACCGGAGGGGTCT
+
FFFFFFFFFFFFFFFFFFFFFFFFFFFFFFFFF:FFFFFFFFFFFFFFFFFFFFFFFFFFFFFFFFFF
FFFFFFFFFFFFFFFFFFFFFFFFFFFFFFFFFFFFFFFFFFFFFFFFFFFFFF:FFFFFFFFFFFFF
@A00155:342:HHGFNDSXY:1:2177:5683:8766 2:N:0:GAACCTAG+TCCGCATA
GAACTAGGAGACCCCTCCGGTCTTGTCCAACCTCTGTTTTGGCAGAGGATACCGTGCTCCACAGCTGT
AAGGCTGTGGGCCCGAGAGAAGTCGCTCTCTCTCGCCCTCCACATCCCTCTCGGCATTGAGATGACCA
+
FFFFFFFFFFFFFFFFFFFFFFFFFFFFFFFFFFFFFFFFFFFFFFFFFFFFFFFFFF:FFFFFFFFF
FFFFFFFFFFFFFFFFFFFFFFFFFFFFFFFFFFF:FFFFFFFFFFFFFFFFFFFFFFF:FFFFFFFFF
@A00155:342:HHGFNDSXY:1:2177:25536:13150 2:N:0:GAACCTAG+TCCGCATA
AAAGGACTCGTCAGGTAGCCTACTAGACTACGACTTACACGGTAGGTCGTGAACAAGCCCGGAGTACA
TCTCCTTTGGAACCAGGAGACCCCTCCGGTCTTGTCCAACCTCTGTTTCGGCAGAGGATACCGTGCTC
+
FFFFFFFFFFFFFFFF:FFFFFFFFFFFFFFFFFFFFFFFFFFFFFFFFFFFFFFFF:FFFFFFFFFFF
FFFFFFFFFFFFFFFFFFFFFFFFFFFFFFFFFFFFFFFFFFFFFF:FFFFFFFFFFFFFFFFFFFFF
```

```
@A00155:342:HHGFNDSXY:1:2177:5801:25661 2:N:0:GAACCTAG+TCCGCATA
AAGGACGAAACCGTCGTTACTCTTTGAGTTGTGTAAGTTTCGTCCTAAAAAGGACTCGTCAGGTAGCC
TACTAGACTACGACTTACACGGTAGGTCGTGAACAAGCCCGGAGTACATCTCCTTTGGAACTAGGAGA
+
FFFFFFFFFF:FFFFFFFFFFFFFFFFFFFFFFFFF,F:FFFFFFF:FFFFFFFFFFFFFFFFF:FFFF
FFFFF:FFFFFFFFFFFFFFF:FFFFFFFFFFFFFF:F:FFFFFFFFFFFFFFFFFFFFFFFFF:FFFF
@A00155:342:HHGFNDSXY:1:2177:29704:27383 2:N:0:GAACCTAG+TCCGCATA
GTTTCGTCCTAAAAAGGACTCGTCAGGTAGCCTACTAGACTACGACTTACACGGTAGGTCGTGAACAA
GCCCGGAGTACATCTCCTTTGGAACTAGGAGACCCCTCCGGTCTTGTCCAACCTCTGTTTCGGCAGAG
+
FFFFFFFFFFFFFFFFFFFFFFFFFFFFFFFFFFFFFFFF:FFFFFFFFFFFFFFFFFFFFFFFFFFF
:FFFFFFFFFF:FFFFFFFFFFFFFFFFFFFFFF:FFFFFFFFFFFFFFFFFFFFFFFFFFF:FFFFFFF
@A00155:342:HHGFNDSXY:1:2177:29514:27461 2:N:0:GAACCTAG+TCCGCATA
GTTTCGTCCTAAAAAGGACTCGTCAGGTAGCCTACTAGACTACGACTTACACGGTAGGTCGTGAACAA
GCCCGGAGTACATCTCCTTTGGAACTAGGAGACCCCTCCGGTCTTGTCCAACCTCTGTTTCGGCAGAG
+
FFFF:FFFFFFFFFFFFFFFFFFFFFFFFFFFFFFFFFFFFFFFFFFFFFFFFFFFFFFFFFFFFFFF
:FFFFFFFFFFFFFFFFFF:FFFFFFFFFFFFFFFFFFFFFFFFFFFFFFFFFFFFFFFFFFFFFFFFF
@A00155:342:HHGFNDSXY:1:2177:29668:27602 2:N:0:GAACCTAG+TCCGCATA
GTTTCGTCCTAAAAAGGACTCGTCAGGTAGCCTACTAGACTACGACTTACACGGTAGGTCGTGAACAA
GCCCGGAGTACATCTCCTTTGGAACTAGGAGACCCCTCCGGTCTTGTCCAACCTCTGTTTCGGCAGAG
+
FFFFFFFFFFFFFFFF:FFFFFFFFFFFFFFFFFFFFFFFFFFFFFFFFFFFFFFFF:FFFF:FFFFFFF
,FFFFFFF:FFFFFFFFFFFFFFFFFFFFFF:FFFFFFFFFFFFFFFFFFFFFF:FFFFFF:FFFFFFFF
@A00155:342:HHGFNDSXY:1:2178:7853:4100 2:N:0:GAACCTAG+TCCGCATA
GGTCGTGAACAAGCCCGGAGTACATCTCCTTTGGAACCAGGAGACCCCTCCGGTCTTGTCCAACCTCT
GTTTCGGCAGAGGATACCGTGCTCCACAGCTGTAAGGCTGTGGGCCCGAGAGAAGTCGCTCTCTCTCG
+
FFFFFFFFFFFFFFFFFFFFFFFFFFFFFFFFFFFFFFFFFFFFFFFFFFFFFFFFFFFFFFFFFFFF
FFFFFFFFFFFFFFFFFFFFFFFFFFFFFFFFF:FFFFFFFFFFFFFFFFFFFFFFFFFFFFFFFFFF
@A00155:342:HHGFNDSXY:1:2178:24532:8750 2:N:0:GAACCTAG+TCCGCATA
GGACTCGTCAGGTAGCCTACTAGACTACGACTTACACGGTAGGTCGTGAACAAGCCCGGAGTACATCT
CCTTTGGAACTAGGAGACCCCTCCGGTCTTGTCCAACCTCTGTTTCGGCAGAGGATACCGTGCTCCAC
+
FFFFFFFFFFFFFFFFFFFFFF:FFFFFFFFFFFFFFFFFFFFFFFFFFFFFFF:FFFFFFFFFFFFF
FF:FFFFFFFFFFFFFFFFFFFFFFFFFFFFFFFFFFFFFFFFFFFFFFFFFFFFFFFFFFFFFFFFFF
@A00155:342:HHGFNDSXY:1:2201:15212:9862 2:N:0:GAACCTAG+TCCGCATA
TCTTGTCCAACCTCTGTTTCGGCAGAGGATACCGTGCTCCACAGCTGTAAGGCTGTGGGCCCGAGAGA
AGTCGCTCTCTCTCGCCCTCCACATCCCTCTCGGCATTGAGATGACCATAGGCCAGAGGTGAGTCCTT
+
FFFFFFFFFFFFFFFFFFFFFFFFFFFFFFFFFFFFFFFF:FFFFFFFF:FFF,F:FFFFFFF,F:FF
:F:FFFFFF:FFFFFFFFFFFFFFFFFFFFFFFFF:F,FFFFFFFFFFF,FFFFFFFFFFFFFF:FFFF
@A00155:342:HHGFNDSXY:1:2201:18674:36088 2:N:0:GAACCTAG+TCCGCATA
ATCTCCTTTGGAACCAGGAGACCCCTCCGGTCTTGTCCAACCTCTGTTTCGGCAGAGGATACCGTGCT
CCACAGCTGTAAGGCTGTGGGCCCGAGAGAAGTCGCTCTCTCTCGCCCTCCACATCCCTCTCGGCATT
+
FFFFFFFFFFFF:FFFFFFFFFFFFFFFFF:F,FFFFFFFFFFF:FFFFFFFFFFFFFFFFFFFFFFFF
FFFFFFFFFFFFFFFFFFFFFFFFFFFFFFF:FFFFFFFFFFFFFF:FFFFFFFFFFFFFFFFFFFFFF
@A00155:342:HHGFNDSXY:1:2202:24903:9048 2:N:0:GAACCTAG+TCCGCATA
GGACTCGTCAGGTAGCCTACTAGACTACGACTTACACGGTAGGTCGTGAACAAGCCCGGAGTACATCT
CCTTTGGAACTAGGAGACCCCTCCGGTCTTGTCCAACCTCTGTTTCGGCAGAGGATACCGTGCTCCAC
+
FFFFFFFFFFFFFFFFFFFFFFFFFFFFFFFFFFFFFFFFFFFFFFFF:FFFFFFFFFFFFFFFFFFFF
FFFFFFFF:FFFFFFFFFFFFFFFFFFFFFFFFFFFFFFFFFFFF:FFFFFFFFFFFFFFFFFFFFFFFFF
```

```
@A00155:342:HHGFNDSXY:1:2202:27010:12258 2:N:0:GAACCTAG+TCCGCATA
ACTACGACTTACACGGTAGGTCGTGAACAAGCCCGGAGTACATCTCCTTTGGAACCAGGAGACCCCTC
CGGTCTTGTCCAACCTCTGTTTCGGCAGAGGATACCGTGCTCCACAGCTGTAAGGCTGTGGGCCCGAG
+
FFFFFFFFFFFFFFFFFFFF:FFFFFFFFF:FFF,FFFFFFFFFFFFFFFFFFFFFFFFFFFFFFFFF
:FFFFF,FFFFFFFFFFFF:FFF,FFFFFFFF:FFFFF:FFFFFFFFFFFFFFFFFFFFFFFFFFFFF
@A00155:342:HHGFNDSXY:1:2202:26621:17628 2:N:0:GAACCTAG+TCCGCATA
AAGGACTCACCTCTGGCCTATGGTCATCTCAATGCCGAGAGGGATGTGGAGGGCGAGAGAGAGCGACT
TCTCTCGGGCCCACAGCCTTACAGCTGTGGAGCACGGTATCCTCTGCCGAAACAGAGGTTGGACAAGA
+
FFFFFFFFFFFFFFFFFFFFFFFFFFFFFFFFFFFFFFFFFFFFFFFFFFFFFFFFFFFFFFFFFFFF
FFFFFFFFFFFFFFFFFFFFFFFFFFFFFFFFFFFFFFFFFFFFFFFFFFFFFFFFFFF:FFFFFFFF
@A00155:342:HHGFNDSXY:1:2202:25464:20979 2:N:0:GAACCTAG+TCCGCATA
ACATCTCCTTTGGAACTAGGAGACCCCTCCGGTCTTGTCCAACCTCTGTTTCGGCAGAGGATACCGTG
CTCCACAGCTGTAAGGCTGTGGGCCCGAGAGAAGTCGCTCTCTCTCGCCCTCTACATCCCTCTCGGCA
+
FFFFFFFFFFFFFFFFFFFFFFFFFFFFFFFFFFFFFFFFFFFFFFFFFFFFFFFFFFFFFFFFFFFF
F:FFFF:FFFFFFFFFF:FFFFFFFFFFFFFF,FFFFFFFFFFFFFFFFF:FFFFFFFFFFFFFFFFF
@A00155:342:HHGFNDSXY:1:2203:10402:3881 2:N:0:GAACCTAG+TCCGCATA
AGTACATCTCCTTTGGAACTAGGAGACCCCTCCGGTCTTGTCCAACCTCTGTTTCGGCAGAGGATACC
GTGCTCCACAGCTGTAAGGCTGTGGGCCCGAGAGAAGTCACTCTCTCTCGCCCTCCACATCCCTCTCG
+
FFFFFFFFFF,:FFFFFFFFF:FFFFFFFFFF,:F:F,,FF:FFFFFFFF:,F:FFFF:FFFF::FFF
FFFFFFFFFFFF,FFFFFFFFFFFFFFFFFF::,FFFFFFFFFFFFFFFFFFFFFFFFF:FFFFFFFF
@A00155:342:HHGFNDSXY:1:2203:23656:16219 2:N:0:GAACCTAG+TCCGCATA
TTTCGTCCTAAAAAGGACTCGTCAGGTAGCCTACTAGACTACGACTTACACGGTAGGTCGTGAACAAG
CCCGGAGTACATCTCCTTTGGAACTAGGAGACCCCTCCGGTCTTGTCCAACCTCTGTTTCGG
+
FFFFFFFFFFFFFFFFFFFFFFFFFFFFFFFFFFFFFFFFFFFFFFFFFFFFFFFFFFFFFFFFFFFF
FFFFFFFFFFFFFFFFFFFFFFFFFFFFFFFFFFFFFFFFFFFFFFFFFFFFFFFFFFFFFF
@A00155:342:HHGFNDSXY:1:2203:32090:23594 2:N:0:GAACCTAG+TCCGCATA
GAGACCCCTCCGGTCTTGTCCAACCTCTGTTTCGGCAGAGGATACCGTGCTCCACAGCTGTAAGGCTG
TGGGCCCGAGAGAAGTCGCTCTCTCTCGCCCTCCACATCCCTCTCGGCATTGAGATGACCATAGGCCA
+
FFFFFFFFFFFFFFFFFFFFFFFFFFFFFFFFFFFFFFFFFFFFFFFFFFFFFFFFFFFFFFFFFFFF
FFFFFFFFFFFFFFFFFFFFF:FFFFFFFFFFFFFFFFFFF:FFFFFFF,FFFFFFF:FFF,FFFFF:
@A00155:342:HHGFNDSXY:1:2204:2003:1391 2:N:0:GAACCTAG+TCCGCATA
GGACTCGTCAGGTAGCCTACTAGACTACGACTTACACGGTAGGTCGTGAACAAGCCCGGAGTACATCT
CCTTTGGAACTAGGAGACCCCTCCGGTCTTGTCCAACCTCTGTTTCGGCAGAGGATACCGTGCTCCAC
+
FFFFFFFFFFFFFFFFFFFFFFFFFFFFFFFFFFFFFFFF:FFFFFFFFFFFFFFFFFFFFFFFFFFF
FFFFFFFFFFFFFFFFFFFFFFFFFFFFFFFFFFFFFFFFFFFFFFFFFFFFFFFFF:FF:FFFFFFF
@A00155:342:HHGFNDSXY:1:2205:27624:4867 2:N:0:GAACCTAG+TCCGCATA
GAAACCGTCGTTACTCTTTGAGTTGTGTAAGTTTCGTCCTAAAAAGGACTCGTCAGGTAGCCTACTAG
ACTACGACTTACACGGTAGGTCGTGAACAAGCCCGGAGTACATCTCCTTTGGAACTAGGAGACCCCTC
+
FFFFFFFFFFFFFFF:FFFFFFFFFFFFFFFFFFFFFFFFFFFFFFFFFFFFFFFFFFFFFFFFFFFF
FFFFFFFFFFFFFFFFFFFFFFFFFFFFFF:FFFFFFFFFFFFFFFFFFFFFFFFFFFFFFFFFFFFF
@A00155:342:HHGFNDSXY:1:2205:17770:9157 2:N:0:GAACCTAG+TCCGCATA
ACTTACACGGTAGGTCGTGAACAAGCCCGGAGAACATCTCCTTTGGAACTAGGAGACCCCTCCGGTCT
TGTCCAACCTCTGTTTCGGCAGAGGATACCGTGCTCCACAGCTGTAAGGCTGTGGGCCCGAGAGAAGT
+
FFFFFFFFFFFFFFFFFFFFFFFFFFFFFFFFFFFFFFFFFFFFFFFFFFFFFFFFFFFFFFFFFFFF
FFFFFFFFFFFFFFFFFFFFFFFFFFF:FFFFFFFFFFFFFFFFFFFFFFFFFFFFFFFFFFFFF:F:
```

```
@A00155:342:HHGFNDSXY:1:2205:6334:13182 2:N:0:GAACCTAG+TCCGCATA
GGACTCGTCAGGTAGCCTACTAGACTACGACTTACACGGTAGGTCGTGAACAAGCCCGGAGTACATCT
CCTTTGGAACTAGGAGACCCCTCCGGTCTTGTCCAACCTCTGTTTCGGCAGAGGATACCGTGCTCCAC
+
FFFFFFFFFFFFFFFFFFFFFFFFFFFFFFFF:FFFFFFFFFFFFFFFFFFF:FFFFFFFFF:FFFFF
FFFFFF:FFFFFFFFF:FFFFFFFFFFFFFFFFFFFFFFFFFFFFFFFFFFFFFFFFFFFF:FFFFF
@A00155:342:HHGFNDSXY:1:2205:13774:21277 2:N:0:GAACCTAG+TCCGCATA
CAAGCCCGGAGTACATCTCCTTTGGAACTAGGAGACCCCTCCGGTCTTGTCCAACCTCTGTTTCGGCA
GAGGATACAGTGCTCCACAGCTGTAAGGCTGTGGGCCCGAGAGAAGTCGCTCTCTCTCGCCCTCCACA
+
FFFFFFFFFFFFFFFFF,FFF:FFFFF:FFFFF:FFFFFFFFFFFFF:FFFFFFFFFFFFFFFFF,FF
FFFFF:FF,FF:FFF:FFFFFFFFF,FFFFFFFFFFFFFFFFFF:FFFFFFFFFF:FFFF,FFFFFF:
@A00155:342:HHGFNDSXY:1:2205:6216:22467 2:N:0:GAACCTAG+TCCGCATA
GTGAACAAGCCCGGAGTACATCTCCTTTGGAACTAGGAGACCCCTCCGGTCTTGTCCAACCTCTGTTT
CGGCAGAGGATACCGTGCTCCACAGCTGTAAGGCTGTGGGCCCGAGAGAAGTCGCTCTCTCTCGCCCT
+
FFFFFFFFF,FFFFFFFFFFFFFFFF:FFFF,FFFFFFFFFFFFFFFFFFFF,F:FFFFFFFFFFFFF
FFFFFFFFFF,FFFFFFFFFFFFFFFFFFF,FFFFFFFFFFFFFFFFFFFF:FFFFFFFFFFFFFFFF
@A00155:342:HHGFNDSXY:1:2205:22544:22498 2:N:0:GAACCTAG+TCCGCATA
GGTCGTGAACAAGCCCGGAGTACATCTCCTTTGGAACTAGGAGACCCCTCCGGTCTTGTCCAACCTCT
GTTTCGGCAGAGGATACCGTGCTCCACAGCTGTAAGGCTGTGGGCCCGAGAGAAGTCGCTCTCTCTCG
+
FFFFFFFFFFFFFFFFFFFFFFFFFFFFFFFFFFFFFFFFFFFFFFFFFFFFFFFFFFFFFFFFFFFF
FFFFFFFFFFFFFF:FFFFFFFFFFFFFFFFFFFFFFFFF:FFFFFFFFFFFFFFFFFFFFFFFFFFF
@A00155:342:HHGFNDSXY:1:2205:7012:32925 2:N:0:GAACCTAG+TCCGCATA
GTACATCTCCTTTGGAACCAGGAGACCCCTCCGGTCTTGTCCAACCTCTGTTTCGGCAGAGGATACCG
TGCTCCACAGCTGTAAGGCTGTGGGCCCGAGAGAAGTCGCTCTCTCTCGCCCTCCACATCCCTCTCGG
+
FFFFFFFFFFFFFFFFFFFFFFFFFFFFFFFFFFFFFFFFFFFFFFFFFFFFFFFFFFFFFF,FFFFF
FFF:FFFFFFFFFFFFFFFFFFFFFFFFFFFFFFFF:FFFFFFFFFFFFFFFFFFFFFFFFFFFFFFF
@A00155:342:HHGFNDSXY:1:2206:30409:16892 2:N:0:GAACCTAG+TCCGCATA
TAGGACGAAACTTACACAACTCGAAGAGTAACGACGGTTTCGTCCTTGGAACGGACTCATCAGGGAGC
CTACTAGACTCCGACCGTCATGGGGGTTCAAACCCATGCCCCGCCACACCGCCTTAGATCAGCTGTGT
+
FFFFFFFFFFFFFFFFFFFFFFFFFFFFFFFFFFF:FF,FFFFFFFFF,FFF:FFFFFFFFFFFFFFF
:FFFFF,FFFFFFFFFFFF:FFFF,FFFFFFFFF,FFFFFFFFFFFFFF::FFFFFFFFFFFFFFF:F
@A00155:342:HHGFNDSXY:1:2206:9245:26052 2:N:0:GAACCTAG+TCCGCATA
GGACTCGTCAGGTAGCCTACTAGACTACGACTTACACGGTAGGTCGTGAACAAGCCCGGAGTACATCT
CCTTTGGAACTAGGAGACCCCTCCGGTCTTGTCCAACCTCTGTTTCGGCAGAGGATACCGTGCTCCAC
+
FFFFFFFFFFFFFFFFFFFFFF:FF:FFFFFFFFFFFFFFFFFFFFFF:FFFFFFFFFFFFFFFFFF:
FFFF:FFFFFFFFFFFFFFFF,FFFFFFFFFFFFFFFFFFFF,FFFFFFFFFFFFFFFFFFFFFFFFF
@A00155:342:HHGFNDSXY:1:2207:15700:5290 2:N:0:GAACCTAG+TCCGCATA
GAAACCGTCGTTACTCTTCGAGTTGTGTAAGTTTCGTCCTAAAAAGGACTCGTCAGGTAGCCTACTAG
ACTACGACTTACACGGTAGGTCGTGAACAAGCCCGGAGTACATCTCCTTTGGAACTAGGAGACCCCTC
+
FFFFFFFFFFFFFFFFFFFFFFFFFFFFFFFFFFFFFFF:FFFF:FFFFFFFFFFFFFFFFFFF,FFF
FFFFFF:FFF,FF:FFFFFFFFFFFFFFFF,FFFFFFFFFFFFFFFFFFFFFFFFFFFFFFFFFFFFF
@A00155:342:HHGFNDSXY:1:2207:20555:6308 2:N:0:GAACCTAG+TCCGCATA
TAGGACGAAACTTACACAACTCAAAGAGTAACGACGGTTTCGTCCTTGGAACGGACTCATCAGGGAGC
CTACTAGACTCCGACCGTCATGGGGGTTCAAACCCATGCCCCGCCACACCGCCTTAGATCAGCTGTGT
+
FFFFFFFFFFFFFFFFFFFFFFFFFFFFFFFFFFFFFFFFFFFFFFFFFFFFFFFFFFFFFFFFFFFF
FF:FFFFFFFFFFFFFFFF:FFFFFFFFFFFFFFFFFFFFFFFFFFFFFFFFF:FFFFFFFFFFFFFF
```

```
@A00155:342:HHGFNDSXY:1:2207:30183:17534 2:N:0:GAACCTAG+TCCGCATA
GGACTCGTCAGGTAGCCTACTAGACTACGACTTACACGGTAGGTCGTGAACAAGCCCGGAGTACATCT
CCTTTGGAACTAGGAGACCCCTCCGGTCTTGTCCAACCTCTGTTTCGGCAGAGGATACCGTGCTCCAC
+
FFFFFFFFFFFFFFFFFFFFFFFFFFFFFFFFFFFFFFFFFFF:FFFFFFFFFFFFFFFFFFFFFFFF
FFFFFFFF,F:FFFFFFFFFFFFFFFFF:FFFF:FFFFFFFFFFFFFFFFFFFFFFFFFFFFFFFFFF
@A00155:342:HHGFNDSXY:1:2207:10122:18082 2:N:0:GAACCTAG+TCCGCATA
TGTAAGGCTGTGGGCCCGAGAGAAGTCGCTCTCTCTCGCCCACCACATCCCTCTCGGCATTGAGATGA
CCATAGGCCAGAGGTGAGTCCTTAAGTGGACACAGCTGATCTAAGGCGGTGTGGCGGGGCATGGGTTT
+
FFFFFFFFFFFFFFFFFF:F:FFFFFFFFFFFFFFFFFFFFFFFFFFFFFFFFFFFFFF,:FFFFFFF
FFFFFFFFFFFFFFFFFFFFFFFFFFFFFFFFFFFFFF:FFFFFFFFFFFFFFFFFFFFFFFFFFFFF
@A00155:342:HHGFNDSXY:1:2207:10547:18724 2:N:0:GAACCTAG+TCCGCATA
TGTAAGGCTGTGGGCCCGAGAGAAGTCGCTCTCTCTCGCCCACCACATCCCTCTCGGCATTGAGATGA
CCATAGGCCAGAGGTGAGTCCTTAAGTGGACACAGCTGATCTAAGGCGGTGTGGCGGGGCATGGGTTT
+
FFFFFFFFFFFFFFFFFFFFFFFFFFFFFFFFFFFFFFFFFFFFFFFFFFFFFFFFFFF:FFFFFFFF
FFFFFFFFFFFFFFFFFFFFFFFFFFFFFFFFFFFFFFFFFFFFFFFFFFFFFFFFFFFF::FFFFFF
@A00155:342:HHGFNDSXY:1:2207:10393:19241 2:N:0:GAACCTAG+TCCGCATA
TGTAAGGCTGTGGGCCCGAGAGAAGTCGCTCTCTCTCGCCCACCACATCCCTCTCGGCATTGAGATGA
CCATAGGCCAGAGGTGAGTCCTTAAGTGGACACAGCTGATCTAAGGCGGTGTGGCGGGGCATGGGTTT
+
FFFFFFFFFFFFFFFFFFFFFFFFFFFFFFFFFFFFFFFFFFFFFFFFFFFFFFFFFFFFFFFFFFFF
FFFFFFFFFFFFFFFFFFFFFFFFFFFFFFFFFFFFFFFFFFFFFFFFFFFFFFFFFFFFF,FFFFFF
@A00155:342:HHGFNDSXY:1:2207:32190:27774 2:N:0:GAACCTAG+TCCGCATA
CCGGAGTACATCTCCTTTGGAACTAGGAGACCCCTCCGGTCTTGTCCAACCTCTGTTTCGGCAGAGGA
TACCGTGCTCCACAGCTGTAAGGCTGTGGGCCCGAGAGAAGTCGCTCTCTCTCGCCCTCCACATCCCT
+
FFFFFFFFFFFFFFFFFFFFFFFFFFFFFFFFFFFFFFFFFFFFFFFFFFFFFFFFFFFFFFFFFFFF
FFFFFFFFFFFFFFFFFFFFFFFFFFFFFFFFFFFFFFFFFFFFFFFFFFFFFFFFFFFFFF:FF:FF
@A00155:342:HHGFNDSXY:1:2207:2437:36902 2:N:0:GAACCTAG+TCCGCATA
GGAGTACATCTCCTTTGGAACTAGGAGACCCCTCCGGTCTTGTCCAACCTCTGTTTCGGCAGAGGATA
CCGTGCTCCACAGCTGTAAGGCTGTGGGCCCGAGAGAAGTCGCTCTCTCTCGCCCTCCACATCCCTCT
+
FFFFFFFFFFFFF:FFFFFFFFFFFFFFFFFFFFFFFFFFFFFFFFFFFFFFFFFFFFFFFFFFFFFF
FFFFFFFFFFFFFFFFFFFFFFFFFFFFFFFFFFFFFFFFFFFFFFFFFFFFFFFFFFFFFFFFF:FF
@A00155:342:HHGFNDSXY:1:2208:2808:3912 2:N:0:GAACCTAG+TCCGCATA
GGAACGGACTCATCAGGGAGCCTACTAGACTCCGACCGTCATGGGGGTTCAAACCCATGCCCCGCCAC
ACCGCCTTAGATCAGCTGTGTCCACTTAAGGACTCACCTCTGGCCTATGGTCATCTCAATGCCGAGAG
+
FFFFFFFFFFFFFFFFFFFFFFFFFFFFFFFFFFFFFFFFFFFFFFFFFFFF:FFFFFFFFFFFFFFF
FFFFFFFFFFFFFFFFFFFFFFFFFFFFFFFFFFF:FFFFFFFFFFFFFFFFFFFFFF:FFFFFFFFF
@A00155:342:HHGFNDSXY:1:2208:2790:4131 2:N:0:GAACCTAG+TCCGCATA
GGAACGGACTCATCAGGGAGCCTACTAGACTCCGACCGTCATGGGGGTTCAAACCCATGCCCCGCCAC
ACCGCCTTAGATCAGCTGTGTCCACTTAAGGACTCACCTCTGGCCTATGGTCATCTCAATGCCGAGAG
+
FFFFFFFFFFFFFFFFFFFFFFFFFFFFFFFFFFFFFFFFFFFFFFFFFFFF:FFFFFFFFFFFFFFF
FFFFFFFFFFFFFFFFFFFFFFFFFFFFFF:FFFFFFFF:FFFFFFFFFFFFFFFFFFFFFF:F:F:F
@A00155:342:HHGFNDSXY:1:2209:16134:4257 2:N:0:GAACCTAG+TCCGCATA
GAGACCCCTCCGGTCTTGTCCAACCTCTGTTTCGGCAGAGGATACCGTGCTCCACAGCTGTAAGGCTG
TGGGCCCGAGAGAAGTCGCTCTCTCTCGCCCTCCACATCCCTCTCGGCATTGAGATGACCATAGGCCG
+
FFFFFFFFFFFFFFFFFFFFFFFFFFFFFF,FFFFF:FFFF:FFFFFFFFFFFFF:FFFFFFFFFFFF
FFFFFFFFFFFFFFFFFFFFFF:FFFFFFFFFFFFFFFFFFFFFFFFFF:FFFFFFFFFFFFFFFFFF
```

@A00155:342:HHGFNDSXY:1:2209:26151:6230 2:N:0:GAACCTAG+TCCGCATA
GGTCGTGAACAAGCCCGGAGTACATCTCCTTTGGAACCAGGAGACCCCTCCGGTCTTGTCCAACCTCT
GTTTCGGCAGAGGATACCGTGCTCCACAGCTGTAAGGCTGTGGGCCCGAGAGAAGTCGCTCTCTCTCG
+
FF:FF:FFFFFF:FFFFFFFFFF:FFFFFFFFFFF:FFFFFFFFFFFFFFFFFFFFFFFFFFFFFFFF
FFFFFFFFFFFFFFFFFFFFFFF:FFFFFFFFFFFFFFF:FFFF:FFFFFFFFF,FFFFFFFFFFFFFF
@A00155:342:HHGFNDSXY:1:2209:4996:7044 2:N:0:GAACCTAG+TCCGCATA
AGCCCGGAGTACATCTCCTTTGGAACTAGGAGACCCCTCCGGTCTTGTCCAACCTCTGTTTCGGCAGA
GGATACCGTGCTCCACAGCTGTAAGGCTGTGGGCCCGAGAGAAGTCGCTCTCTCTCGCCCTCTA
+
FFFFFFF,FFFFFFFFFFF:FFFF,FF:FFFFFFFFFFFFFFFFF,FFFFFFFFFFFFFFFFFFFFFF
FFFFFFFFFFFFFFFFFFFFFFF:FFFFFFFFFFFFFFFFFFFF:FFFFFFFFFFFFFFFFFFFFF,
@A00155:342:HHGFNDSXY:1:2209:28628:10989 2:N:0:GAACCTAG+TCCGCATA
GGACTCGTCAGGTAGCCTACTAGACTACGACTTACACGGTAGGTCGTGAACAAGCCCGGAGTACATCT
CCTTTGGAACTAGGAGACCCCTCCGGTCTTGTCCAACCTCTGTTTCGGCAGAGGATACCGTGCTCCAC
+
FFFFF:FFFFFFFFFFF:FFFF:FFFFFFFFFFFFFFFFF:FFFFFFFFFF:FFFFFFFFFFFFFFFF
FF:FFFFF:FFFFFFFFFFFFFFFFFFFFF:FFFFFFFFFFFFFFFFFFFFFFFFFFFFFFFFFFFFF
@A00155:342:HHGFNDSXY:1:2209:14452:19069 2:N:0:GAACCTAG+TCCGCATA
TTTAGGACGAAACTTACACAACTCGAAGAGTAACGACGGTTTCGTCCTTGGAACGGACTCATCAGGGA
GCCTACTAGACTCCGACCGTCATGGGGGTTCAAACCCATGCCCCACCACACCGCCTTAGATCAGCTGT
+
FFFFFFFFFFFFFFFFFFFFFFFFFFFFFFFFFFFFFFFFFFFFFFFFFFFFFFFFFF:FFFFFFFFF
FFFFFFFFFFFFFFFFFFFFFFFFFFFFFFFFFFFFFFFFFFFFFFFFFFFFFFFFFFFF:FFFFFFF
@A00155:342:HHGFNDSXY:1:2209:19443:19319 2:N:0:GAACCTAG+TCCGCATA
ACTACGACTTACACGGTAGGTCGTGAACAAGCCCGGAGTACATCTCCTTTGGAACTAGGAGACCCCTC
CGGTCTTGTCCAACCTCTGTTTCGGCAGAGGATACCGTGCTCCACAGCTGTAAGGCTGTGGGCCCGAG
+
FFFFFFFFFFFFFFFFFFFFFFFFFFFFFFFFFFFFFFFFFFFFFFFFF:FF:F:FFFFFFFFFFFFF
FFFFFFFFFFFFFFFFFFFFFFFFFFFFFFFFFFFFFFFFFFFFFFFFF:FFFFFFFFF:FFFFFFFF
@A00155:342:HHGFNDSXY:1:2209:32154:20635 2:N:0:GAACCTAG+TCCGCATA
GTGTAAGTTTCGTCCTAAAAAGGACTCGTCAGGTAGCCTACTAGACTACGACTTACACGGTAGGTCGT
GAACAAGCCCGGAGTACATCTCCTTTGGAACTAGGAGACCCCTCCGGTCTTGTCCAACCTCTGTTTCG
+
FFFFF:FFFFFFFFFF:FFFFFFFFFFFFFFF:FFFFFFFFF:FFFFFFFFFFFFFFF,FF:FFFF
FFFFFFFFFFFFF:FFFFFFFFFFF:FF:FFFFFFFF:FFFFFFFFFFFFFFFFFFFFFFFFFFF:FF
@A00155:342:HHGFNDSXY:1:2210:23204:1564 2:N:0:GAACCTAG+TCCGCATA
CTGGCCTATGGTCATCTCAATGCCGAGAGGGATGTGGAGGGCGAGAGAGAGCGACTTCTCTCGGGCCC
ACAGCCTTACAGCTGTGGAGCACGGTATCCTCTGCCGAAACAGAGGTTGGACAAGACCGGAG
+
FFFFFFFFFFFFFFFFFFFFFFFFFFFFFFFF:,FFFFFFFFFFFFFFFFFFFFFFFFFFFFFFFFFF
FFFFFFFFFFFFFFFFFFFFFFFFFFFFFFFFFFFFFFFFFFFFFFFFFFFFFFFFFFFFFF
@A00155:342:HHGFNDSXY:1:2210:23466:7999 2:N:0:GAACCTAG+TCCGCATA
CACGGTAGGTCGTGAACAAGCCCGGAGTACATCTCCTTTGGAACTAGGAAACCCCTCCGGTCTTGTCC
AACCTCTGTTTCGGCAGAGGATACCGTGCTCCACAGCTGTAAGGCTGTGGGCCCGAGAGAAGTCGCTC
+
FFFFFFFFFFFFFFFFFFFFFFFFFFFFFFFFFFFFFFFFFFFFFFFFFFFFFFFFFFFFFFF:FFFF
:FFFFFFFFFFFFFFFFFFFFFFFFFFFFFFFFFFFFFFFF:FFFFFFFFFFFFFFFFFFFFFFFFFFF
@A00155:342:HHGFNDSXY:1:2210:32027:28401 2:N:0:GAACCTAG+TCCGCATA
GTTTCGTCCTAAAAAGGACTCGTCAGGTAGCCTACTAGACTACGACTTACACGGTAGGTCGTGAACAA
GCCCGGAGTACATCTCCTTTGGAACTAGGAGACCCCTCCGGTCGTGTCCAACCTCTGTTTCGGCAGAG
+
F:FFFFFFF:::FFFFFFFFFF,FFFFFFFFFFFFFFFFF,FFFFFFF:FFFFFF:FFFF:FFF,FFF
FFFFFFFFFFFFFFFFFFFFFFFFFFFFFFFFFF:FFFFFF:FF,F:FFFF:FFFFFFFFFFF:FF:F

```
@A00155:342:HHGFNDSXY:1:2211:30165:2628 2:N:0:GAACCTAG+TCCGCATA
CGGCAGAGGATACCGTGCTCCACAGCTGTAAGGCTGTGGGCCCGAGAGAAGTCGCTCTCTCTCGCCCT
CCACATCCCTCTCGGCATTGAGATGACCATAGGCCAGAGGTGAGTCCTTAAGTGGACACAGCTGATCT
+
FFFFFFFFFFFFFFFFFFFFFFFFFFFFFFFFFFFFFFFFFFFFFFFFFFFFFFFFFFFFFFFFFFFF
FFFFFFFFFFFFFFFFF:FFFFFFFFFFFFFFFFFFFFFFFFFFFFFFFFFFFFFFFFFFFFFFFFFF
@A00155:342:HHGFNDSXY:1:2212:18231:30499 2:N:0:GAACCTAG+TCCGCATA
ATCTCAATGCCGAGAGGGATGTGGAGGGCGAGAGAGAGCGACTTCTCTCGGGCCCACAGCCTTACAGC
TGTGGAGCACGGTATCCTCTGCCGAAACAGAGGTTGGACAAGACCGGAGGGGTCTCCTAGTTCCAAAG
+
FFFFFFFFFFFFFF,FFFFFFFFFFFFFFFFFFFFFFFFFFFFFFFFFFFFFFFFFFFFFFFF,FFFF
FFFFFFFFFFFFFFFFFFFFFFFFFFFFFFFFFFFFFFFFFFFFFFFFFFFFFFFFFFFFFFFF:FFF
@A00155:342:HHGFNDSXY:1:2212:16107:34679 2:N:0:GAACCTAG+TCCGCATA
AAGCCCGGAGTACATCTCCTTTGGAACTAGGAGACCCCTCCGGTCTTGTCCAACCTCTGTTTCGGCAG
AGGATACCGTGCTCCACAGCTGTAAGGCTGTGGGCCCGAGAGAAGTCGCTCTCTCTCGCCCTCCAC
+
FFFFFFFFFFFF:FFF,FFFFFFFFFFFFFFFFFFFFFFFFFFFFF:FFFFFFFFFFFF:FFFFFFFF
FFFFF:FFFFF:FF,FFFFFFF:FFFFFFFFFFFFFFFFFFFFFFFFFFFFFF:FFFFFFFFFF:FF
@A00155:342:HHGFNDSXY:1:2213:20066:12414 2:N:0:GAACCTAG+TCCGCATA
ACGGTAGGTCGTGAACAAGCCCGGAGTACATCTCCTTTGGAACTAGGAGACCCCTCCGGTCTTGTCCA
ACCTCTGTTTCGGCAGAGGATACCGTGCTCCACAGCTGTAAGGCTGTGGGCCCGAGAGAAGTCGCTCT
+
FFFFFFFFFFFFFFFFFFFFFFFFFFFFFFFFFFFFFFFFF:FFFFFFFFFFFFFFFFFFFFFFFFFF
FFFFFFFFFFFFFFFFFFFFFFFFFFFFFFFFFFFFFFFFFFFFFFFFFFFFFFFFFFFFFFFFFFFF
@A00155:342:HHGFNDSXY:1:2214:11116:25441 2:N:0:GAACCTAG+TCCGCATA
ACATCTCCTTTGGAACTAGGAGACCCCTCCGGTCTTGTCCAACCTCTGTTTCGGCAGAGGATACCGTG
CTCCACAGCTGTAAGGCTGTGGGCCCGAGAGAAGTCGCTCTCTCTCGCCCTCTACATCCCTCTCGGCA
+
FFFFFFFFFFFFFF:F:FFFFFFFFFFFFFFFFF,FFFFFFFFFFFFFFFFFFFFFFFFFFF:FFFFFF
FFFFFFFFFFFFFFFFF:FF:FFFFFFFFFFFFFF:FF:FFFFFFFFFFFFFFFFFFFFFFFFFFFFFF
@A00155:342:HHGFNDSXY:1:2214:27236:25676 2:N:0:GAACCTAG+TCCGCATA
AAAAGGACTCGTCAGGTAGCCTACTAGACTACGACTTACACGGTAGGTCGTGAACAAGCCCGGAGTAC
ATCTCCTTTGGAACTAGGAGACCCCTCCGGTCTTGTCCAACCTCTGTTTCGGCAGAGGATACCGTGCT
+
FFFFFFFFFFFFFFFFFFFFFFFFFFFFFFFFFFFFFFFFFFFFFFFFF:FFFFFFFFFFFFFFF:FF
FFFFFFF,FFFF:FFFFFFFFFFFFFFFFFFFFFFFFFFFFFFFFFFFFFF:FFFFFFFFFFFFFFFF
@A00155:342:HHGFNDSXY:1:2214:2067:30499 2:N:0:GAACCTAG+TCCGCATA
TACGACTTACACGGTAGGTCGTGAACAAGCCCGGAGTACATCTCCTTTGGAACTAGGAGACCCCTCCG
GTCTTGTCCAACCTCTGTTTCGGCAGAGGATACCGTGCTCCACAGCTGTAAGGCTGTGGGCCCG
+
FFFFFFFFFFFFFFFFFF:FFFFFFFFF::FFFFF:FFF:FFF:FFFFFFFFFFFFFFFFFFFFFFFF
FFF:FFFFFFFFFFFFFFFFF,FFFFFFFFF,FFFFFFFFFFFFFFFFFFFFFFF:FFF:FFFFF
@A00155:342:HHGFNDSXY:1:2215:25807:3724 2:N:0:GAACCTAG+TCCGCATA
CCACACCGCCTTAGATCAGCTGTGTCCACTTAAGGACTCACCTCTGGACTATGGTCATCTCAATGCCG
AGAGGGATGTGGAGGGCGAGAGAGAGCGACTTCTCTCGGGCCCACAGCCTTACAGCTGTGGAGCACGG
+
,F:FFF:,F,,,F,F,,F,F,,::F,FFFFFFFFFF:FFFFFFF:::,,,F:F,FFF,:F::F,:,,F
F,FF:FFFF,:FF:,,,FFFFFFFFFF,FFFF,,,F,,,:FF,FFFF:F,:FFFF,::F:,FFFF,:F
@A00155:342:HHGFNDSXY:1:2215:16315:34851 2:N:0:GAACCTAG+TCCGCATA
GTGAACAAGCCCGGAGTACATCTCCTTTGGAACTAGGAGACCCCTCCGGTCTTGTCCAACCTCTGTTT
CGGCAGAGGATACCGTGCTCCACAGCTGTAAGGCTGTGGGCCCGAGAGAAGTCGCTCTCTCTCGCCCT
+
FFFFFFFFFFFFFFFFFFFFFFFFFF:FFFFFFFFFFFFFFFFFFFFFFFFFFFFFFFFFFFFFFFFFF
FFFFFFFFFFFFFFFFFFFFFFFFFFFFFFFFFFFFFFFFFFFFFFFFFFFFFFFFFFFFFFFFFFF:
```

```
@A00155:342:HHGFNDSXY:1:2216:28339:2033 2:N:0:GAACCTAG+TCCGCATA
GAGACCCCTCCGGTCTTGTCCAACCTCTGTTTCGGCAGAGGATACCGTGCTCCACAGCTGTAAGGCTG
TGGGCCCGAGAGAAGTCGCTCTCTCTCGCCCTCCACATCCCTCTCGGCATTGAGATGAC
+
FFFFFFFFFFFFFFF,FFFFFFFFFFFFFFFFFFFFFFFFFFFFFFFFFFFFFFFFFFFFFFFFFFFF
FFFFFFFFFFFFFFFFFFFFFFFFFFFFFFFFFFFFFFFFFFFFFFFFFF:FFFFFFFF
@A00155:342:HHGFNDSXY:1:2216:20066:3082 2:N:0:GAACCTAG+TCCGCATA
ACTTACACGGTAGGTCGTGAACAAGCCCGGAGTACATCTCCTTTGGAACTAGGAGACCCCTCCGGTCT
TGTCCAACCTCTGTTTCGGCAGAGGATACCGTGCTCCACAGCTGTAAGGCTGTGGGCCCGAGAGAAGT
+
FFFFFFFFFFFFFFFFFFFFFFFFFFFFFFFFFFFFFFFFFFFFFFFFFF:FFFFFFFFFFFFFFFFF
FFFFFFFFFFFFFFFFFFFFFFFFFFFFFFFFFFFFF:FFFFFFFFFFFFFFFFFFFFFFFFFFFFFF
@A00155:342:HHGFNDSXY:1:2216:9263:7795 2:N:0:GAACCTAG+TCCGCATA
AAGGACGAAACCGTCGTTACTCTTCGAGTTGTGTAAGTTTCGTCCTAAAAAGGACTCGTCAGGTAGCC
TACTAGACTACGACTTACACGGTAGGTCGTGAACAAGCCCGGAGTACATCTCCTTTGGAACTAGGAGA
+
FFFFFFFFFFFFFFFFFFFFFFFFFF:FFFFFFFFFFFFFFFFFFFFFFF,FFFFFFFFFFFFFFFFF
FFFFFFFFFFFFFFFFFFFFFFFFFFFFFFFFFFFFFFFFFFFFFFFFFFFFFFFFFFFFFFFFFFFF
@A00155:342:HHGFNDSXY:1:2216:32072:28541 2:N:0:GAACCTAG+TCCGCATA
CTGGCCTATGGTCATCTCAATGCCGAGAGGGATGTGGAGGGCGAGAGAGAGCGACTTCTCTCGGGCCC
ACAGCCTTACAGCTGTGGAGCACGGTATCCTCTGCCGAAACAGAGGTTGGACAAGACCGGAG
+
FFFFFFFFFFFFFFFFFFFFFFFFFFFFFFFF:FFFFFFFFFFFFFFFFFFFFFFFFFFFFFFFFFFF
FFFFFFFFFFFFFFFFFFFFFFFFFF,FFFFFFFFFFF:FFFFFFFFFFFFFFFFFFFFFFF
@A00155:342:HHGFNDSXY:1:2217:1542:6042 2:N:0:GAACCTAG+TCCGCATA
TGCCCCACCACACCGCCTTAGATCAGCTGTGTCCACTTAAGGACTCACCTCTGGCCTATGGTCATCTC
AATGCCGAGAGGGATGTGGAGGGCGAGAGAGAGCGACTTCTCTCGGGCCCACAGCCTTACAGCTGTG
+
FFFFFFFFFFFFFFF,FFFFFF:FFFFFFF:FFFFFF:FFFFFFF:,F:FFF:FFFFFFFFFFFFF:F
FFFFFF::,FFF:FF,FFF:F,FFFFFF:F,FFFFF:FFFFFFFFFFF,FFFFFF:F:F:FFFFFFF
@A00155:342:HHGFNDSXY:1:2217:12988:13996 2:N:0:GAACCTAG+TCCGCATA
AAGCCCGGAGTACATCTCCTTTGGACCTAGGAGACCCCTCCGGTCTTGTCCAACCTCTGTTTCGGCAG
AGGATACCGTGCTCCACAGCTGTAAGGCTGTGGGCCCGAGAGAAGTCGCTCTCTCTCGCCCTCCACAT
+
FFF:FFFFFFFFFFFFFFFFFFFFFFFF:FFFFFFFFFFFFFFFFFFFFFFFFFFFFFFF:FFFFFFF
FFFFFFFFFFFFFFFFFFFFFFFFFFFFFFFF:FFFFFFFFFFFFFF:FFFFFF,FFFFFFFFFFFFF
@A00155:342:HHGFNDSXY:1:2217:7401:15436 2:N:0:GAACCTAG+TCCGCATA
AAGGACTCGTCAGGTAGCCTACTAGACTACGACTTACACGGTAGGTCGTGAACAAGCCCGGAGTACAT
CTCCTTTGGAACTAGGAGACCCCTCCGGTCTTGTCCAACCTCTGTTTCGGCAGAGGATACCGTG
+
F:FFFFFFFFFFF:FFFFFFFFFFFFFFFFFFFFFFFFFFFFFFFFFFFFF:FFFFFFFFFFFFFFFF
FFFFFFFFFF,FFFFFFFFFFF:FFFFFFFFFFFFF:FFFFFFFFFFFFFF:FFFFFFFFFFFF
@A00155:342:HHGFNDSXY:1:2217:27579:18537 2:N:0:GAACCTAG+TCCGCATA
ACTACGACTTACACGGTAGGTCGTGAACAAGCCCGGAGTACATCTCCTTTGGAACCAGGAGACCCCTC
CGGTCTTGTCCAACCTCTGTTTCGGCAGAGGATACCGTGCTCCACAGCTGTAAGGCTGTGGGCCCGAG
+
FFFFFFFFFFFFFFFFFFFFFFFFFFFFFFFFFFFFFFFFF:FFFFF:FFFFFFFFFFFFFFFFFFFF
FFFFFF:FFFFFFFFFFFFFFFFFFFFFFFFF,FFFFFFFFFFFFFFFFFFFFFFFFFFFFFFFFFFF
@A00155:342:HHGFNDSXY:1:2217:27651:21261 2:N:0:GAACCTAG+TCCGCATA
ACATCTCCTTTGGAACTAGGAGACCCCTCCGGTCTTGTCCAACCTCTGTTTCGGCAGAGGATACCGTG
CTCCACAGCTGTAAGGCTGTGGGCCCGAGAGAAGTCGCTCTCTCTCGCCCTCTACATCC
+
FFFFFFFFF:FFFFFFFFFFFFFFFFFFFFFFFFFFFFFFFFFFFFFFFFFFFFF:FFFFFFFFFFFF
FFFFFFFFFFFFFFFFFFFFFFFFFFFFFFFFFFFFFFFFFFFFFFFFFFFFFFFFFFF
```

```
@A00155:342:HHGFNDSXY:1:2217:20383:33912 2:N:0:GAACCTAG+TCCGCATA
CAAGGACGAAACCGTCGTTACTCTTCGAGTTGTGTAAGTTTCGTCCTAAAAAGGACTCGTCAGGTAGC
CTACTAGACTACGACTTACACGGTAGGTCGTGAACAAGCCCGGAGTACATCTCCTTTGGAACTAGGAG
+
FFFFFFFFFFFFFFFFFFFFFFFFFFFFFFFFFFFF:FFFFFFFFFFFFFFFFFFFFFFFFFFFFFFF
FFFFFFFFFFFFFFFFFFFFFFFFFFFFFFFFFFFFFFFFFFFFFFFFFFFFFFFFFFFFFFFFFFFF
@A00155:342:HHGFNDSXY:1:2217:26729:35196 2:N:0:GAACCTAG+TCCGCATA
AAGTTTCGTCCTAAAAAGGACTCGTCAGGTAGCCTACTAGACTACGACTTACACGGTAGGTCGTGAAC
AAGCCCGGAGTACATCTCCTTTGGAACTAGGAGACCCCTCCGGTCTTGTCCAACCTCTGTTTCGGCAG
+
FFFFFFFFFFFFFFFFFFFFFFFFFFFFFFFFF,FFFFFFFF,FFFFFFFFFFF:FF:FFFFFFFF:,
F:F:FFF:FFFFF:FFFFF::FFFF:FFFFFFFF::F:FFFFFFFFFFFF::FFFF:FFFFFFFFFFF
@A00155:342:HHGFNDSXY:1:2218:24252:9737 2:N:0:GAACCTAG+TCCGCATA
TAGGAGACCCCTCCGGTCTTGTCCAACCTCTGTTTCGGCAGAGGATACCGTGCTCCACAGCTGTAAGG
CTGTGGGCCCGAGAGAAGTCGCTCTCTCTCGCCCTCTACATCCCTCTCGGCATTGAGATGACCATAGG
+
FFFFFFFFFFFFFFFFFF:FFFFFFFFFFFFFFFFFFFFFFFFFF,FFFFFFFFFFFFFFFFFFFFFF
FFFFFFFFFFFFFFFF:FFFFFFFFFFFFFFFFFFFFFFFFFFFFFFFFFFF:FFFFFFFFFFFFF:F
@A00155:342:HHGFNDSXY:1:2218:4209:13291 2:N:0:GAACCTAG+TCCGCATA
GGAGTACATCTCCTTTGGAACTAGGAGACCCCTCCGGTCTTGTCCAACCTCTGTTTCGGCAGAGGATA
CCGTGCTCCACAGCTGTAAGGCTGTGGGCCCGAGAGAAGTCGCTCTCTCTCGCCCTCCACATCCCTC
+
F:FFFFFFFFFFF:FFFFFFF:FFFF:FF:FFFFFFFFF:F:F:F:FFFFF,FFFF:FFF,F:FF:,F
:FFF:FF,FFFFFFFFFFFF:,:,,FFFF:FFF:,,:,FF:F:FFFFFFFFF,F:,FF:FFFFFF:F
@A00155:342:HHGFNDSXY:1:2218:29559:17112 2:N:0:GAACCTAG+TCCGCATA
ACTAGGAGACCCCTCCGGTCTTGTCCAACCTCTGTTTCGGCAGAGGATACCGTGCTCCACAGCTGTAA
GGCTGTGGGCCCGAGAGAAGTCGCTCTCTCTCGTCCTCCACATCCCTCTCGGCATTGAGATGAC
+
:FFFFFFFFFFFFFFFFFFFFFFFFFFFFFFFFFFFFFFFFFFFFFFFFFFFFFFFFFFFFFFFFFFF
FFFFFFFFFFFFFFFFFFFF:FFFFFFFFFFFFFFFFFFFFFFFFFFFFFFFFFFFFFFFFFFF
@A00155:342:HHGFNDSXY:1:2219:4309:26960 2:N:0:GAACCTAG+TCCGCATA
TAAGTGGACACAGCTGATCTAAGGCGGTGTGGCGGGGCATGGGTTTGAACCCCCATGACGGTCGGAGT
CTAGTAGGCTCCCTGATGAGTCCGTTCCAAGGACGAAACCGTCGTTACTCTTCGAGTTGTGTAAGTTT
+
F:FFFFFFFFFFFFFFFFFFFFFFFFFFFFFFFFFFFFFFFFFFFFFFFFFFFFFFFFFFFFFFFFFF
FFFFFFFFFFFFFFFFFFFFFFFFFFFFFFFFFFFFF:FFF:FF:FFFFFFFFFFFFF:FF:FFFF:F
@A00155:342:HHGFNDSXY:1:2219:4689:33943 2:N:0:GAACCTAG+TCCGCATA
GGACTCGTCAGGTAGCCTACTAGACTACGACTTACACGGTAGGTCGTGAACAAGCCCGGAGTACATCT
CCTTTGGAACTAGGAGACCCCTCCGGTCTTGTCCAACCTCTGTTTCGGCAGAGGATACCGTGCTCCAC
+
FFFFFFFFFFFFFFFFFFFFFFFFFFFFFFFFFFFFFFFFFFFFFFFFFFFFFFF:FFFFFFFFFFFF
FFFFFFFF:F:FFFFFFFFFFFFFFFFFFFFFFFFFFF:FFFFFFFF,FFFFFFFFFFFFFFFFFFFF
@A00155:342:HHGFNDSXY:1:2220:1542:26271 2:N:0:GAACCTAG+TCCGCATA
ACACGGTAGGTCGTGAACAAGCCCGGAGTACATCTCCTTTGGAACTAGGAGACCCCTCCGGTCTTGTC
CAACCTCTGTTTCGGCAGAGGATACCGTGCTCCACAGCTGTAAGGCTGTGGGCCCGAGAGAAGTCGCT
+
FFFFFFFFFFFFFFFFFFFFFFFFFFFFFFFFFFFFF,FFFFFFFFFFFFFFFFFFFFFFFFFFFFFF
FFFFFFFFFFFFFFFFFFFFFFFFF:FFFFFFF,FFFFFFFFFF:FFFFFFFFFFFFFFFFFFFFFFF
@A00155:342:HHGFNDSXY:1:2220:16532:36636 2:N:0:GAACCTAG+TCCGCATA
GGAGTACATCTCCTTTGGAACCAGGAGACCCCTCCGGTCTTGTCCAACCTCTGTTTCGGCAGAGGATA
CCGTGCTCCACAGCTGTAAGGCTGTGGGCCCGAGAGAAGTCGCTCTCTCTCGCCCTCCACATCCCTCT
+
FFFFFFFFFFFFFFFFFFFFFFFFFFFFFFFFFFFFFFFFFFFFFFFFFFFFFFFFFFFFFFFFFF:F
FFFFFFFFFFFFFFFFFFFFFFFFFFFFFFFFFF:FFFFFFFFFFFFFFFFFFFFFFFFFFFFFFFFF
```

```
@A00155:342:HHGFNDSXY:1:2221:12852:3082 2:N:0:GAACCTAG+TCCGCATA
CAGGTAGCCTACTAGACTACGACTTACACGGTAGGTCGTGAACAAGCCCGGAGTACATCTCCTTTGGA
ACCAGGAGACCCCTCCGGTCTTGTCCAACCTCTGTTTCGGCAGAGGATACCGTGCTCCACAGCTGTAA
+
FFFFFF:F:FFFFFFFFFFFFF:FFFFFFFFFFFFFFFFFFFFFF::FFFFFFFFFFF:FFFFFFFFF
FFFFFFFFFFF,FFFFFFFFFFFFFFFFF,FF,FF:FFFFFFFFFFFFFFFF,FFFFFFFFFFF:FFF
@A00155:342:HHGFNDSXY:1:2221:30011:15045 2:N:0:GAACCTAG+TCCGCATA
AGTTTCGTCCTAAAAAGGACTCGTCAGGTAGCCTACTAGACTACGACTTACACGGTAGGTCGTGAACA
AGCCCGGAGTACATCTCCTTTGGAACTAGGAGACCCCTCCGGTCTTGTCCAACCTCTGTTTCGGCAGA
+
FFFFFFFFFFFFFFFFFFFFF:FFFFFFFFFFFFFFFFFFFFFFFFFFFFFFFFFFF:FFF:FFFFFFF
F::FFFFFFFFFFFFFFFF:FFFF:FFFFFFFFFFFFFFFFFFFFFFFFFFFFFFFFFFFFFFFFFFFF
@A00155:342:HHGFNDSXY:1:2221:28962:16016 2:N:0:GAACCTAG+TCCGCATA
AGTTTCGTCCTAAAAAGGACTCGTCAGGTAGCCTACTAGACTACGACTTACACGGTAGGTCGTGAACA
AGCCCGGAGTACATCTCCTTTGGAACTAGGAGACCCCTCCGGTCTTGTCCAACCTCTGTTTCGGCAGA
+
FFFFFFFFFFFFFFF:FFFFFFFFFFFFFFFFFFFFFFFFFFFFFFFFFFFFFFFFFFFF:FFFFFFF
FFFFFFFFFFFFFFFFFFFFFFFFFFFFFFFFFFF:FFFFFFFFFFFFFFFFFFFFFFFFFF:F:FFF
@A00155:342:HHGFNDSXY:1:2222:10791:5838 2:N:0:GAACCTAG+TCCGCATA
AAAAGGACTCGTCAGGTAGCCTACTAGACTACGACTTACACGGTAGGTCGTGAACAAGCCCGGAGTAC
ATCTCCTTTGGAACTAGGAGACCCCTCCGGTCTTGTCCAACCTCTGTTTCGGCAGAGGATACCGTGCT
+
::FF,FFFFFFFFFFF::FFFFFF::FFFFF,FFFFFFFF:FFFFF:FF:F:FF:FFFFFFFFFFFFF
FFFFFF,FF:FFFFFF:FFFFFFF:FFFFFFF:,FFFFFFFF:,F:F:FF,FFFFFFFFFFFFFFFFF
@A00155:342:HHGFNDSXY:1:2223:31711:11256 2:N:0:GAACCTAG+TCCGCATA
TAGGTCGTGAACAAGCCCGGAGTACATCTCCTTTGGAACTAGGAGACCCCTCCGGTCTTGTCCAACCT
CTGTTTCGGCAGAGGATACCGTGCTCCACAGCTGTAAGGCTGTGGGCCCGAGAGAAGTCGCTCTCTCT
+
FFFFFFFFFFFFFFFFFFFFFFFFFFFFFFFF:FFFFF:FF:FFFFFFFFFFFFFFFFF:FFFFFFFFF
FFFFFFFF:FFFFFF:FFFFFFFFFFFFFFFFFFFFFFFFFFFFFFFFFFFFFFFFFFFFFFFF:FFFF
@A00155:342:HHGFNDSXY:1:2223:32081:11835 2:N:0:GAACCTAG+TCCGCATA
TAGGTCGTGAACAAGCCCGGAGTACATCTCCTTTGGAACTAGGAGACCCCTCCGGTCTTGTCCAACCT
CTGTTTCGGCAGAGGATACCGTGCTCCACAGCTGTAAGGCTGTGGGCCCGAGAGAAGTCGCTCTCTCT
+
FFFFFFFFFFFFFFFFFFFFFFFFFFFFFFFFFFFFFFFFFFFFFFFFFFFFFFF:FFFFFFFFFFFF
FFFFFFFFFFFFFFFFFFFFFFFFFFFFFFFFFFF:FFFFF:FFFFFFFFFFFFFF,FFFFFFFFFFFF
@A00155:342:HHGFNDSXY:1:2224:20772:11099 2:N:0:GAACCTAG+TCCGCATA
AAAAGGACTCGTCAGGTAGCCTACTAGACTACGACTTACACGGTAGGTCGTGAACAAGCCCGGAGTAC
ATCTCCTTTGGAACTAGGAGACCCCTCCGGTCTTGTCCAACCTCTGTTTCGGCAGAGGATACCGTGCT
+
FFFFFFFFFFFFFFFFFFFFFFFFFFFFFFFFFFFFFFFF:FFFFFF:FFFFFFFFFFFFFFFFFFFF
FFFF:F,FFFFFFFFFFFFFFFFFFFFFFFFFFFFFFFFFFFFF,FFFFFFFFFFFFFF:FFFFFFF:
@A00155:342:HHGFNDSXY:1:2224:21775:11428 2:N:0:GAACCTAG+TCCGCATA
AAAAGGACTCGTCAGGTAGCCTACTAGACTACGACTTACACGGTAGGTCGTGAACAAGCCCGGAGTAC
ATCTCCTTTGGAACTAGGAGACCCCTCCGGTCTTGTCCAACCTCTGTTTCGGCAGAGGATACCGTGCT
+
FFFFFFFFFFFFFFFFFFFFFFFFFFFFFFFFFFFFFFFFFFFFFFFFFFFFFFFFFFF:FFFFFFFFF
FFFFFFFFFFFF,FFFFFFFF:FFFFFFFFFFFFFF::FFFFFFFFFFFF:FFFFFFFFF:FFFFF:F
@A00155:342:HHGFNDSXY:1:2224:12301:17848 2:N:0:GAACCTAG+TCCGCATA
ACATCTCCTTTGGAACTAGGAGACCCCTCCGGTCTTGTCCAACCTCTGTTTCGGCAGAGGATACCGTG
CTCCACAGCTGTAAGGCTGTGGGCCCGAGAGAAGTCGCTCTCTCTCGCCCTCCACATCCCTCTCGGCA
+
FFFFFFFFFFFFFF:FFFFFFFFFFFFFFFFFFFF:FFFFFFFFFFFFFFFFFFFFFFFFFFF::FFFFF
FFFFFFFFFFFFFFFFFFFFFFFFFFFF:FFFFFFFFFFFFFFFFFFFFFFFFFFFFFFFFFFFFFFFF
```

```
@A00155:342:HHGFNDSXY:1:2224:19452:30420 2:N:0:GAACCTAG+TCCGCATA
GTGAACAAGCCCGGAGTACATCTCCTTTGGAACTAGGAGACCCCTCCGGTCTTGTCCAACCTCTGTTT
CGGCAGAGGATACCGTGCTCCACAGCTGTAAGGCTGTGGGCCCGAGAGAAGTCGCTCTCTCTCGCCCT
+
FFFFFFFFFFFFFFFFFFFFFFFFFFFFFFFFFFFFFFFFFFFFFFFFFFF:FFFFFFFFFFFFFFFF
FFFFFFFFFFFFFFFFFFFFFFFFFFFFFFFFFF,FFFFFFFFFFFFFFFFFFFFFFFFFFFFFFFFF
@A00155:342:HHGFNDSXY:1:2225:27588:26506 2:N:0:GAACCTAG+TCCGCATA
AAAAGGACTCGTCAGGTAGCCTACTAGACTACGACTTACACGGTAGGTCGTGAACAAGCCCGGAGTAC
ATCTCCTTTGGAACTAGGAGACCCCTCCGGTCTTGTCCAACCTCTGTTTCGGCAGAGGATACCGTGCT
+
FFFFFFFFFFFFFFFFFFFF:FFFFFFFFFF:FFFFFFFFFFFFFFFFFFFFFFFFF:FFFFFFFFFF
:FFFFFFFFF:F,FFFFFFFFF:F:FFFFFFFFFFFFFFFFFFFFFFFFFFFFFFFFFFFFFFFFFFF
@A00155:342:HHGFNDSXY:1:2226:30689:17033 2:N:0:GAACCTAG+TCCGCATA
ACTAGACTCCGACCGTCATGGGGGTTCAAACCCATGCCCCGCCACACCGCCTTAGATCAGCTGTGTCC
ACTTAAGGACTCACCTCTGGCCTATGGTCATCTCAATGCCGAGAGGGATGTGGAGGGCGAGAGAGAGC
+
FFFFFFFFFFFFFFFFFFFFFFFFFFFFFFFFFFFFFFFFFFFFFFFFFFFFFFFFFF:FFFFFFFFF
FFFFF:FFFFFFFF:FFFFFFFFFFFFFFFFFFFFFFFFFFFFFFFFFFFF:FFFFF:FFFFFFFFF
@A00155:342:HHGFNDSXY:1:2226:1181:21324 2:N:0:GAACCTAG+TCCGCATA
AACTAGGAGACCCCTCCGGTCTTGTCCAACCTCTGTTTCGGCAGAGGATACCGTGCTCCACAGCTGTA
AGGCTGTGGGCCCGAGAGAAGTCGCTCTCTCTCGCCCTCCACATCCCTCTCGGCATTGAGATGAC
+
:FFF:FFFFFFFFFFFFFFF,FFF:FFFFFFFFFFFFFFFFFFF,FFFFFFFFFFFFFFFFFFFFFF:
FFF,FFFFFFFFFF:FFFFFFFF:FFF::FFFFFFFFFFFFF:FFFFFFFFFFFFFFF,F:FF::
@A00155:342:HHGFNDSXY:1:2226:19967:23453 2:N:0:GAACCTAG+TCCGCATA
ACGACTTACACGGTAGGTCGTGAACAAGCCCGGAGTACATCTCCTTTGGAACTAGGAGACCCCTCCGG
TCTTGTCCAACCTCAGTTTCGGCAGAGGATACCGTGCTCCACAGCTGTAAGGCTGTGGGCCCGAGAGA
+
FF:FF:FFFFFFFFFFFFFFFFFFFFFFFFFFFFFFFFFFFFFFFFFFFF,FFFFFFFFFFFFFFFFF
F:FFFFF:FFFFFF:FFF,F,FFFF:FFFFFFFF:FFFFFFFFFF,FFFFFFFFFFFFFFFFFFFFFF
@A00155:342:HHGFNDSXY:1:2226:12255:25160 2:N:0:GAACCTAG+TCCGCATA
CACGGTAGGTCGTGAACAAGCCCGGAGTACATCTCCTTTGGAACTAGGAGACCCCTCCGGTCTTGTCC
AACCTCTGTTTCGGCAGAGGATACCGTGCTCCACAGCTGTAAGGCTGTGGGCCCGAGAGAAGTCGCTC
+
FFFFFFFFFFFFFFFFFFFFFFFFFFFFFFFFFFFFFFFFFFFFFFFFFFFFFFFFFFFFFFFFFFFF
FFFFFFFFFFFFFFFFFFFFFFFFFFFFFFFFFFFFFFFFFFFFFFFFFFFFFFFFFFFFFFFFFFFF
@A00155:342:HHGFNDSXY:1:2226:13657:28620 2:N:0:GAACCTAG+TCCGCATA
GAAACCGTCGTTACTCTTCGAGTTGTGTAAGTTTCGTCCTAAAAAGGACTCGTCAGGTAGCCTACTAG
ACTACGACTTACACGGTAGGTCGTGAACAAGTCCGGAGTACATCTCCTTTGGAACTAGGAGACCCCTC
+
FFFFFFFFFFFFFFFFFFFFFFFFFFFFFFFFFFFFFFF:FFFFFFFFFFFFFFFFFFFFFFFFFFFF
FFFFFFFFFFFFFFFFFFFFFFFFFFFFFFFFFFFFFFFFFFFFFFFFFFFFFFFFFFFFFFFFFFFF
@A00155:342:HHGFNDSXY:1:2227:9019:13291 2:N:0:GAACCTAG+TCCGCATA
GGACTCGTCAGGTAGCCTACTAGACTACGACTTACACGGTAGGTCGTGAACAAGCCCGGAGTACATCT
CCTTTGGAACTAGGAGACCCCTCCGGTCTTGTCCAACCTCTGTTTCGGCAGAGGATACCGTGCTCCAC
+
,:FFFF:FFFFFFF,FFFF:FFFFFFFFFFFFFFFFFFF:FFFFFFFFFFFFFF,:FFFFFFFFFFFF
FFFFFFFFF:FFF:FF:FFFFFFFFFFFF:,F,FFFFFFFFFFFFFFF,FFFFFFFFF:FFFFFF:FF
@A00155:342:HHGFNDSXY:1:2227:22580:15295 2:N:0:GAACCTAG+TCCGCATA
GAGTCCGTTCCAAGGACGAAACCGTCGTTACTCTTTGAGTTGTGTAAGTTTCGTCCTAAAAAGGACTC
GTCAGGTAGCCTACTAGACTACGACTTACACGGTAGGTCGTGAACAAGCCCGGAGTACATCTCCTTTG
+
FFFFFFFFFFFFFFFFFFFFFFFFFFFFFFFFF,FFFFFFFFFFFFFFF:FFFFFFFFFFF:FFFFFF
F:FFFFFFFFF:FFFFFFFFFFFFF:FFFFF:FFFFFFFFFFFFFFFFFFFFFFFFFFFF:FFFF:FF
```

```
@A00155:342:HHGFNDSXY:1:2227:30662:20212 2:N:0:GAACCTAG+TCCGCATA
TACGACTTACACGGTAGGTCGTGAACAAGCCCGGAGTACATCTCCTTTGGAACTAGGAGACCCCTCCG
GTCTTGTCCAACCTCTGTTTCGGCAGAGGATACCGTGCTCCACAGCTGTAAGGCTGTGGGCCCG
+
FFFFFFFFFFFFFFFFFFFFFFFFFFFFFFFFFFFFFFFFFFFFFFFFFFFFFFFFFFFFFFFFFFFF
FFF:FFFFFFFFFFFFFFFFFFF:FFFFFFFFFFFFFFFFFFFFFFFFFFFFFFFFFFFFFFFF
@A00155:342:HHGFNDSXY:1:2228:12210:2566 2:N:0:GAACCTAG+TCCGCATA
ACGACTTACACGGTAGGTCGTGAACAAGCCCGGAGTACATCTCCTTTGGAACCAGGAGACCCCTCCGG
TCTTGTCCAACCTCTGTTTCGGCAGAGGATACCGTGCTCCACAGCTGTAAGGCTGTGGGCCCGAGAGA
+
FFFFFFFFFFFFFFFFF:FFFFFFFFFFFFFFFFFFFFFFFFFFFFFFFFFFFFFFFFFFFFFFFFFF
FFFFFFFFFFFFFFFFFFFFFFFFF:FF:FFFFFFFFFFFFFFFFFFFFF:FFFFFFFFFFFFFFFFF
@A00155:342:HHGFNDSXY:1:2228:22498:10551 2:N:0:GAACCTAG+TCCGCATA
ACTCTTCGAGTTGTGTAAGTTTCGTCCTAAAAAGGACTCGTCAGGTAGCCTACTAGACTACGACTTAC
ACGGTAGGTCGTGAACAAGCCCGGAGTACATCTCCTTTGGAACCAGGAGACCCCTCCGGTCTTGTCCA
+
FFFFFFFFFFFFFFFFFFFFFFFFFFFFF:FFFFFFFFFFFFFFFFFFFFFFFFFFFFFFFFFFFFFF
FFFFFFFFFFFFFF:FFF,FFFFFFFFFFFFFFFFFF:FFFFFFFFFFFFFFFFFF:FFFFFFFFFFF
@A00155:342:HHGFNDSXY:1:2228:8015:28181 2:N:0:GAACCTAG+TCCGCATA
AAAAGGACTCGTCAGGTAGCCTACTAGACTACGACTTACACGGTAGGTCGTGAACAAGCCCGGAGTAC
ATCTCCTTTGGAACTAGGAGACCCCTCCGGTCTTGTCCAACCTCTGTTTCGGCAGAGGATACCGTGCT
+
FFFF:FFFFFFFFFFFFFFFFFFFFFFFFFFFFFFFFFFFFFFFFFFFFFFFFFFFFF:FFFFFFFF
F:FFFF:FFFFFFFFFFFFFFFFFFFFFF:FFFFFFFFFFFFFFFFFFF:FFF:FFFFFFFFFFFFFF
@A00155:342:HHGFNDSXY:1:2228:21784:29262 2:N:0:GAACCTAG+TCCGCATA
TTACACAACTCGAAGAGTAACGACGGTATCGTCCTTGGAACGGACTCATCAGGGAGCCTACTAGACTC
CGACCGTCATGGGGGTTCAAACCCATGCCCCGCCACACCGCCTTAGATCAGCTGTGTCCACTTA
+
FFF:FFFFF,FFFF,F:FFFFFF,,:F,:F:FF,,F:,FF,::::F,FFFF,,:F,:,FFFFF,FFF:
,FF,:,F,,,:F,,:F,FFF:FFFF,FF,FF,,FFFF:FFF:,:FFF,FF:FF,,:,FFF,FFF
@A00155:342:HHGFNDSXY:1:2228:29903:31892 2:N:0:GAACCTAG+TCCGCATA
TCGTGAACAAGCCCGGAGTACATCTCCTTTGGAACTAGGAGACCCCTCCGGTCTTGTCCAACCTCTGT
TTCGGCAGAGGATACCGTGCTCCACAGCTGTAAGGCTGTGGGCCCGAGAGAAGTCGCTCTCTCTCGCC
+
:FFFFFFFFFFFFFF:FFFFFFFFFFFF:FFFF:FFFFFFFFFFFFFFFFFFFFFFFFFFFFFFFFFF
FFFFFFFFFFFF:,FFFFFFFFF,FFFF,FFFF,FFFFFFFFFFFFFFFFF,FFFFFFFFFFFFFFFF
@A00155:342:HHGFNDSXY:1:2228:10402:36761 2:N:0:GAACCTAG+TCCGCATA
CACGGTAGGTCGTGAACAAGCCCGGAGTACATCTCCTTTGGAACCAGGAGACCCCTCCGGTCTTGTCC
AACCTCTGTTTCGGCAGAGGATACCGTGCTCCACAGCTGTAAGGCTGTGGGCCCGAGAGAAGTCGCTC
+
FFFFFFFFFFFFFFFFFFFFFFFFFFFFFFFFFFFFFFFFFFFFFFFFFFFFFFFFFFFFF,FFFFFF
FFFFFFFFFFFFFFFFFFFFFFFFFFFFFFFFFFFFFFFFFFFFFFFFFFFF:FFFFFFFFFFFFFFF
@A00155:342:HHGFNDSXY:1:2229:5511:1235 2:N:0:GAACCTAG+TCCGCATA
AGATCAGCTGTGTCCACTTAAGGACTCACCTCTGGCCTATGGTCATCTCAATGCCGAGAGGGATGTGA
AGGGCGAGAGAGAGCGACTTCTCTCGGGCCCACAGCCTTACAGCTGTGGAGCACGGTATCCTCTGCCG
+
FFFFFFFFFFFFFFFFFFFF:FFFFFFFFFFFFFFFFFFFFFFFFFFFFFFFFFFFFFFFFFFFFFF
FFFFFFFFFFFFFFFFFFFFFFFFFFFFFFFFFFFFF,FFFFFFFFFFFFFFFFFFFFFFFFFFFFFF
@A00155:342:HHGFNDSXY:1:2229:14615:8547 2:N:0:GAACCTAG+TCCGCATA
CCTACTAGACTACGACTTACACGGTAGGTCGTGAACAAGCCCGGAGTACATCTCCTTTGGAACTAGGA
GACCCCTCCGGTCTTGTCCAACCTCTGTTTCGGCAGAGGATACCGTGCTCCACAGCTGTAAGGCTGTG
+
FFFFFFFFFFFFFFFFFFFFFFFFFFFFFFFFFFFFFFFFFFFFFFFFFFFFFFFFFFFFFFFFFFFF
FFFFFFFFFFFFFFFFFFFFFFFFFFFFFFFFFFFFFFFFFFFFFFFFFFFFFFFFFFFFFFFFFFFF
```

```
@A00155:342:HHGFNDSXY:1:2229:25889:8750 2:N:0:GAACCTAG+TCCGCATA
TGAACAAGCCCGGAGTACATCTCCTTTGGAACCAGGAGACCCCTCCGGTCTTGTCCAACCTCTGTTTC
GGCAGAGGATACCGTGCTCCACAGCTGTAAGGCTGTGGGCCCGAGAGAAGTCGCTCTCTCTCGCCCTC
+
FFFFFFFFFFFFFFFFFF:FFFFFF:FFFFFFFFFFFFFFFFFFFFFFFFFFFFFFFFFFFFFFFFFF
FFFFFFFFFFFFFFFFFFFFFFFFFFFFFFFFFFFFFFFFFFFFFFFFFFFFFFFFFFFFF:FFFFFFF
@A00155:342:HHGFNDSXY:1:2229:1515:16110 2:N:0:GAACCTAG+TCCGCATA
AAAAGGACTCGTCAGGTAGCCTACTAGACTACGACTTACACGGTAGGTCGTGAACAAGCCCGGAGTAC
ATCTCCTTTGGAACTAGGAGACCCCTACGGTCTTGTCCAACCTCTGTTTCGGCAGAGGATACCGTGCT
+
:FF,FFFFFFFF::FFFFFFFFFFFFFFFFFFFF,FFFFF,FFFFFF:FFFF,FFFF,FF,FF:FFFF
FFFF:FFFFFF:::FFFF,F::F:FF,:FFF:FFFFFFF,FFF::FF:F:FF,,FF::FFF,:FFFFF
@A00155:342:HHGFNDSXY:1:2229:11198:21355 2:N:0:GAACCTAG+TCCGCATA
CTACGACTTACACGGTAGGTCGTGAACAAGCCCGGAGTACATTTCCTTTGGAACTAGGAGACCCCTCC
GGTCTTGTCCAACCTCTGTTTCGGCAGAGGATACCGTGCTCCACAGCTGTAAGGCTGTGGGCCCGAGA
+
FFFFFFF:FFFFFFFFFFFFFFFF:FFFFFFFFFFFFFFFFFFFFFFFFFFFFFFFFFFFFFFFFFFF
FFFFFFFFFFFFFFFFFFFFFFFFFFFFFFFFFFFFFFFFFFFFFFFFFFFFFFFFFFFFFFFFFFFF
@A00155:342:HHGFNDSXY:1:2229:7636:34507 2:N:0:GAACCTAG+TCCGCATA
GTGAACAAGCCCGGAGTACATCTCCTTTGGAACTAGGAGACCCCTCCGGTCTTGTCCAACCTCTGTTT
CGGCAGAGGATACCGTGCTCCACAGCTGTAAGGCTGTGGGCCCGAGAGAAGTCGCTCTCTCTCGCC
+
FFFFFFFFFFFFFFFFFFFFFFFFFFFFFFFFFFFFFFFFFFFFFFFFFFFFFFFFFFFFFFFFFFFF
FFFFFFFFFFFFFFFFFFFFFFFFFFFFFFFFFFFFFFFFFFFFFFFFF:FFFFFFFFFFFFFFFF
@A00155:342:HHGFNDSXY:1:2229:5394:37043 2:N:0:GAACCTAG+TCCGCATA
GTGAACAAGCCCGGAGTACATCTCCTTTGGAACTAGGAGACCCCTCCGGTCTTGTCCAACCTCTGTTT
CGGCAGAGGATACCGTGCTCCACAGCTGTAAGGCTGTGGGCCCGAGAGAAGTCGCTCTCTCTCGCC
+
FFFFFFFFFFFFFF:FFFFFFFFFFF:FFFFFFFFFFFFFFFFFFFFFFFFFFFFFFFFFFFFFFFFF
:FFFFFFFFF:FFFFFFFFFFFFFFFFFFFFFFFFFFFFFFFFFFFFFFFFFFFFFFFFFFFFFFF
@A00155:342:HHGFNDSXY:1:2230:27823:23406 2:N:0:GAACCTAG+TCCGCATA
TCTTTGAGTTGTGTAAGTTTCGTCCTAAAAAGGACTCGTCAGGTAGCCTACTAGACTACGACTTACAC
GGTAGGTCGTGAACAAGCCCGGAGTACATCTCCTTTGGAACTAGGAGACCCCTCCGGTCTTGTCCAAC
+
:F:,FFFFFFFFFFFFFFFFFFFFFFF,FFFFFFFFFFFFFFFFF::FFF:FFF:F:FFFFFF,FFFFF
FFF,FFFF::FFFFFFFFF:FF::FFFF:FF:FFFFFFF::FF:FFFFFFFFFFF::FFFFFFFFFF
@A00155:342:HHGFNDSXY:1:2230:14733:33959 2:N:0:GAACCTAG+TCCGCATA
CTACTAGACTACGACTTACACGGTAGGTCGTGAACAAGTCCGGAGTACATCTCCTTTGGAACTAGGAG
ACCCCTCCGGTCTTGTCCAACCTCTGTTTCGGCAGAGGATACCGTGCTCCACAGCTGTAAGGCTGTGG
+
FFFFFFFFFFFFFFFFFFFFFFFFFFFFFFFFFFFFFFFFFFFFFFFFFFFFFFFFFFFFFFFFFFFF
FFFFFFFFFFFFFFFFFFFFFFFFFFFFFFFFFFFFFFFFFFFFFFFFFFFFFFFFFFF,FFFFFFFFF
@A00155:342:HHGFNDSXY:1:2231:8892:2550 2:N:0:GAACCTAG+TCCGCATA
TACGACTTACACGGTAGGTCGTGAACAAGCCCGGAGTACATCTCCTTTGGAACTAGGAGACCCCTCCG
GTCTTGTCCAACCTCTGTTTCGGCAGAGGATACCGTGCTCCACAGCTGTAAGGCTGTGGGCCCG
+
F:FFFFFFFFFFFFFFFFFFFFFFFFFFFFFFFFFFFFFFFFFF:FFFFFFF:FFFFFFFFFFFF:FFF
FFFFFFFFFFFFFFFFFFFFFFF:FFF:FFFFFFFFFFFFFFFFFFFFFFFFFFFFFFFFFFFFFF
@A00155:342:HHGFNDSXY:1:2231:9218:7529 2:N:0:GAACCTAG+TCCGCATA
TAGACTACGACTTACACGGTAGGTCGTGAACAAGCCCGGAGTACATCTCCTTTGGAACTAGGAGACCC
CTCCGGTCTTGTCCAACCTCTGTTTCGGCAGAGGATACCGTGCTCCACAGCTGTAAGGCTGTGGGCCC
+
FFFFFFFFFFFFFFFFFFFFFFFFFFFFFFFFFFFFFFFFFFFFFFFFFFFFFFFFFFFFFFFFFFFF
FFFFFFFFFFFFFFF:FFFFFFFFFFFFFFFFFFFFFFFFFFFFFFFFFFFFFFFFFFFFFFFFFFFF
```

```
@A00155:342:HHGFNDSXY:1:2231:20220:16720 2:N:0:GAACCTAG+TCCGCATA
TTTCGTCCTAAAAAGGACTCGTCAGGTAGCCTACTAGACTACGACTTACACGGTAGGTCGTGAACAAG
CCCGGAGTACATCTCCTTTGGAACCAGGAGACCCCTCCGGTCTTGTCCAACCTCTGTTTCGGCAGAGG
+
FFFFFFFFFFFFFFFFFFFFFFFFFFFFFFFFFFFFFFFFFFFFF:FFFFFFFFFFFFFFFFFFFFFFFFF
FFFFFFFFFFFFFFFFFFFFFFF:FFFFFFFFFFFFFFFFFFFFFFFFFFFFFFFFFFFFFFFFFFFFFFFF
@A00155:342:HHGFNDSXY:1:2231:16034:24126 2:N:0:GAACCTAG+TCCGCATA
GTGAACAAGCCCGGAGTACATCTCCTTTGGAACTAGGAGACCCCTCCGGTCTTGTCCAACCTCTGTTT
CGGCAGAGGATACCGTGCTCCACAGCTGTAAGGCTGTGGGCCCGAGAGAAGTCGCTCTCTCTCGCCCT
+
FFFFFFFFFFFFFFFFFFFFFFFFFFFFFFFFFFFFFFFFFFFFFFFFFFFFFFFFFFFFFFFFFFFFFFFF
FFFFFFFFFFFFFFFFFFFFFFFFFFFFFFFFFFFFFFFFFFFFFFFFFFFFFFFFFFFFFFFFFFFFFFFF
@A00155:342:HHGFNDSXY:1:2232:32353:18944 2:N:0:GAACCTAG+TCCGCATA
GTAGCCTACTAGACTACGACTTACACGGTAGGTCGTGAACAAGCCCGGAGTACATCTCCTTTGGAACT
AGGAGACCCCTCCGGTCTTGTCCAACCTCTGTTTCGGCAGAGGATACCGTGCTCCACAGCTGTAAGGC
+
FF:FFFFFFFFFFFFFFFFFFFFFFFFFFFFFFFF:FFFFFFFFFFFFFFF:FFFFF,FF:FFFFF,FF
FF,FFFFFFFFFFFFFFF,F,F:FFFFFF:F:FFFF:FF:::FFFFFFFFFFFFF,FFFFF:FFFFFF
@A00155:342:HHGFNDSXY:1:2232:13928:21292 2:N:0:GAACCTAG+TCCGCATA
CCTACTAGACTACGACTTACACGGTAGGTCGTGAACAAGCCCGGAGTACATCTCCTTTGGAACTAGGT
GACCCCTCCGGTCTTGTCCAACCTCTGTTTCGGCAGAGGATACCGTGCTCCACAGCTGTAAGGCTGTG
+
FFFFFFFFFFFFFFFFFFFFFFFFFFFFFFFFFFFFFFFFFFFFFFFFFFFFFFFFFFFFFFFFFFFFFFFF
FFFFFFFFFFFFFFFFFFFFFFFFFFFFFFFFFFFFFFFFFFFFF,FFFFFFFFFFFFFFFFFFFFFFFFFF
@A00155:342:HHGFNDSXY:1:2232:18511:22654 2:N:0:GAACCTAG+TCCGCATA
TCGTCCTAAAAAGGACTCGTCAGGTAGCCTACTAGACTACGACTTACACGGTAGGTCGTGAACAAGCC
CGGAGTACATCTCCTTTGGAACTAGGAGACCCCTCCGGTCTTGTCCAACCTCTGTTTCGGCAGAGGAT
+
FFFFFFF:FFFFFFFFFFFFFFFFFFFFFFFFFFFFFFFFFFFFFFFFFFFFFFFFFFFFFFFFFFFF:FF
FFFFFFFFFFFFFFFFFFFFFFFFFFFFFFFFFFFFFFFFFFFFFFFFFFFFFFFFFFFFFFFFFFFFFFFF
@A00155:342:HHGFNDSXY:1:2232:24288:31782 2:N:0:GAACCTAG+TCCGCATA
GGTCGTGAACAAGCCCGGAGTACATCTCCTTTGGAACTAGGAGACCCCTCCGGTCTTGTCCAACCTCT
GTTTCGGCAGAGGATACCGTGCTCCACAGCTGTAAGGCTGTGGGCCCGAGAGAAGTCGCTCTCTCTCG
+
FFFFFFFFFFFFFFFFFFFFFFFFFFFFFFFFFFFFFFFFFFFFFFFFFFFFFFFFFFFF:FFFFFFFFFF
FFFFFFFFFFFFFFFF:FFFFFFFFFFFFFFFFFFFFFFFFFFFFFFFFFFFFFFFFFFFFFFFFFFFFFFFF
@A00155:342:HHGFNDSXY:1:2232:19461:33786 2:N:0:GAACCTAG+TCCGCATA
TCGTGAACAAGCCCGGAGTACATCTCCTTTGGAACTAGGAGACCCCTCCGGTCTTGTCCAACCTCTGT
TTCGGCAGAGGATACCGTGCTCCACAGCTGTAAGGCTGTGGGCCCGAGAGAAGTCGCTCTCTCTCGCC
+
FFFFFFFFFFFFFFFFFFFFFFFFFFFFF,FFFFFFFFFFFFFFFFFFFFFFFFF::FFFFFFFFFFFFF
FFFFFFFFFFFF:FFFFFFFFFFFFFFFFFFFFFFFFFFFFFFFFFFFFFFFFFFFFFFFFFFFFFFFFFF
@A00155:342:HHGFNDSXY:1:2232:31313:36808 2:N:0:GAACCTAG+TCCGCATA
CCTACTAGACTACGACTTACACGGTAGGTCGTGAACAAGCCCGGAGTACATCTCCTTTGGAACCAGGA
GACCCCTCCGGTCTTGTCCAACCTCTGTTTCGGCAGAGGATACCGTGCTCCACAGCTGTAAGGCTGTG
+
FFFFFFFFFFFFFFFFFFFFFFFFFF,FFFFFFFFFFFF:FFFFFFFFFFFFFFFFFFFFFF:FFFFFF
FFFFFFFFFFFFFFFFFFFFFFFFFFFFF,FFFFFFFFFFF::FF:FFFFFFFFF,FFFFF,FFFFFFFF
@A00155:342:HHGFNDSXY:1:2233:6027:23578 2:N:0:GAACCTAG+TCCGCATA
GAAACCGTCGTTACTCTTTGAGTTGTGTAAGTTTCGTCCTAAAAAGGACTCGTCAGGTAGCCTACTAG
ACTACGACTTACACGGTAGGTCGTGAACAAGCCCGGAGTACATCTCCTTTGGAACTAGGAGACCCCTC
+
FFFFFFFFFFFFFFFFF:FFFFFFFFFFFFFFFFFFFFFF:FFFFFFFFFFFFFFF:FFFFFFFFFFFFF
FFFFFFFFFFFFFFFFFFFFF,FFFFFFFFFFFFFFFFFFFFFFFFFFFFFFFFFFFFFFFF:FFF:FFFFF
```

@A00155:342:HHGFNDSXY:1:2233:5990:24612 2:N:0:GAACCTAG+TCCGCATA
GAAACCGTCGTTACTCTTTGAGTTGTGTAAGTTTCGTCCTAAAAAGGACTCGTCAGGTAGCCTACTAG
ACTACGACTTACACGGTAGGTCGTGAACAAGCCCGGAGTACATCTCCTTTGGAACTAGGAGACCCCTC
+
FFFFFFFFFFFFFFFFFFFFFFFFFFFFFFFFFFFFFFFFFFF,FFFFFFFFFFFFFFFFFFFFFF,
FFFFFFFF,FFFFFFF:FFFFFFFFF:FFFFFFFFFFFFFFFFFFFFFFFFFFF:FFFFFFFFFFFFFF
@A00155:342:HHGFNDSXY:1:2233:22824:25081 2:N:0:GAACCTAG+TCCGCATA
ACATCTCCTTTGGAACTAGGAGACCCCTCCGGTCTTGTCCAACCTCTGTTTCGGCAGAGGATACCGTG
CTCCACAGCTGTAAGGCTGTGGGCCCGAGAGAAGTCGCTCTCTCTCGCCCTCCACATCCCTCTCGGCA
+
FFFFFFFFFFFFFFFFFFFFFFFFFFFFFFFFFF,FFFFFFFFFFFFFFFFFFFFFFFFFFFFFFFFF
FFFFFF:FFFFFFFFFFFFFFFFFFFFFFFFFFFFFFFFFFFFFFFFFFFFFFFFFFFFFFFFFFFFF
@A00155:342:HHGFNDSXY:1:2233:19578:32362 2:N:0:GAACCTAG+TCCGCATA
AAGGACGAAACCGTCGTTACTCTTTGAGTTGTGTAAGTTTCGTCCTAAAAAGGACTCGTCAGGTAGCC
TACTAGACTACGACTTACACGGTAGGTCGTGAACAAGCCCGGAGTACATCTCCTTTGGAACTAGGAGA
+
FFFFFFFFFFFFFFFFFFFFFFFFFFFFFFFFFFFFFFFFFFFFFFFFFF:FFFFFFFFFFFFFFFFF
FFFFFFFFFFFFFFFFFFFFFFFFFFFFFFFFFFFFFFF:FFFFFFFFFFFF:FFFFFFF,FFFFFF:F
@A00155:342:HHGFNDSXY:1:2234:23683:1767 2:N:0:GAACCTAG+TCCGCATA
ACCTCTGTTTCGGCAGAGGATACCGTGCTCCACAGCTGTAAGGCTGTGGGCCCGAGAGAAGTCGCTCT
CTCTCGCCCTCCACATCCCTCTCGGCATTGAGATGACCATAGGCCAGAGGTGAGTCCTTAAGTGGACA
+
FFFFFFFFFFFFFFFFFFFFFFFFFFFFFFFFFFFFFFF:FFFFFFFFFFFFFFFFFFFFFFFFFFFFF
:FFFFFFFFFFFFFFFFFFFFFFFFFFFFFFFFFFFFFFFFFFFFF,FFFFFFFFFFFFFFFFFFFFFF
@A00155:342:HHGFNDSXY:1:2234:22869:15546 2:N:0:GAACCTAG+TCCGCATA
GGAGTACATCTCCTTTGGAACTAGGAGACCCCTCCGGTCTTGTCCAACCTCTGTTTCGGCAGAGGATA
CCGTGCTCCACAGCTGTAAGGCTGTGGGCCCGAGAGAAGTCGCTCTCTCTCGCCCTCCACATCCCTCT
+
FFFFFFFFFFFFFFFFFFFFFF:FFFFFFFFFFFFFFFFFFFFFFFFFFFFFFFFFFFFFFFFFFFFF:
FFFFFFFFFFFFFFFFFFFFFFFFFFFFFFFFFFFFFF,FFFFFFFFFFFFFFFFFFFFFFFFFFFFFF
@A00155:342:HHGFNDSXY:1:2234:4218:33599 2:N:0:GAACCTAG+TCCGCATA
GACTCGTCAGGTAGCCTACTAGACTACGACTTACACGGTAGGTCGTGAACAAGCCCGGAGTACATCTC
CTTTGGAACTAGGAGACCCCTCCGGTCTTGTCCAACCTCTGTTTCGGCAGAGGATACCGTGCTCCACA
+
FFFFFFFFFFFFFFFFFFFFFFFFFFFFFFFFFFFFFFFFFFFFFFFFFFFFFFFFFFFFFFFFFFFF
FFFFFFF,FFFFFFFFFFFFFFFFFFFFFFFFFF:FFFFFFFFFFFFFFFFFFFFFFFFFFFFFFFFFF
@A00155:342:HHGFNDSXY:1:2234:20455:36292 2:N:0:GAACCTAG+TCCGCATA
CTGTTTCGGCAGAGGATACCGTGCTCCACAGCTGTAAGGCTGTGGGCCCGAGAGAAGTCGCTCTCTCT
CGCCCTCTACATCCCTCTCGGCATTGAGATGACCATAGGCCAGAGGTGAGTCCTTAAGTGGACACAGC
+
FFFFFFFFFF:FFFFFFFFFFFFFFFFFFFFFFFFFFFFFFFFFFFFFFFFFFFFFF:FFFFFFFFFF
FFFFFFFF:FFFFFFFFFFFFFFFFFFFFFFFFFFFFFFFFFFF:FFFFFFFFFF,F,FFFFFF:FFFF
@A00155:342:HHGFNDSXY:1:2235:4074:14278 2:N:0:GAACCTAG+TCCGCATA
GGACTCGTCAGGTAGCCTACTAGACTACGACTTACACGGTAGGTCGTGAACAAGCCCGGAGTACATCT
CCTTTGGAACTAGGAGACCCCTCCGGTCTTGTCCAACCTCTGTTTCGGCAGAGGATACCGTGCTCCAC
+
FFFFFFFFFFFFF,FFFFFFFFFFFF:FFFFFFFFFFFFFFFFFFFFFFFFFFFFFFFFFFFFFFFFF
FFF:FFFFFFFFFFFFFFFFFFFFF:FFF:FFFFFFFFF:FFFFFFFFFFFFFFFFFFFFFFFFFFF,F
@A00155:342:HHGFNDSXY:1:2235:21251:20509 2:N:0:GAACCTAG+TCCGCATA
CGTTCCAAGGACGAAACCGTCGTTACTCTTCTAGTTGTGTAAGTTTCGTCCTAAAAAGGACTCGTCAG
GTAGCCTACTAGACTACGACTTACACGGTAGGTCGTGAACAAGCCCGGAGTACATCTCCTTTGGAACT
+
FFFFFFFFFFFFFFFFFFFFFFFFFFFFFFFFFFFFFFFF:FFFFF:FFFFF:FFFFFFFFFFFFFF,F
FFFFFFFFFFFFFFFFFFFFFFFFFFFFFFFFFF:FFFFFFFFFFFFFFFFFFFFFFFFFFFFFFFFFF

```
@A00155:342:HHGFNDSXY:1:2236:25635:5901 2:N:0:GAACCTAG+TCCGCATA
GAGACCCCTCCGGTCTTGTCCAACCTCTGTTTCGGCAGAGGATACCGTGCTCCACAGCTGTAAGGCTG
TGGGCCCGAGAGAAGTCGCTCTCTCTCGCCCTCCACATCCCTCTCGGCATTGAGATGAC
+
FFFFFFFFFFFFFFFFFFFFFFFFFFFFFFFFFFFFFFFFFFFFFFFFFFFFFFFFFFFFF,FFFFFF
FFFFFFFFFFFFFFFFFFFFFFFFFFFFFFFFFFFFFFFFFFFFFFFFFFFFFFFFFFF
@A00155:342:HHGFNDSXY:1:2236:11930:9377 2:N:0:GAACCTAG+TCCGCATA
TCTCCTTTGGAACTAGGAGACCCCTCCGGTCTTGTCCAACCTCTGTTTCGGCAGAGGATACCGTGCTC
CACAGCTGTAAGGCTGTGGGCCCGAGAGAAGTCGCTCTCTCTCGCCCTCCACATCCCTCTCGGCATTG
+
FFFFFFFFFFFFFFFFFFFFFFFFFFFFFFF:FFFFFFFFFFF,F,FFFFFFFFFFFFFFFFFFFFF:
FFFFFFFFFFFFFFFFFFFFF:FF:F:FFFFFFFFFFFFFFFFFFFFFFFFFFFFFF:FFFFFF,:FF
@A00155:342:HHGFNDSXY:1:2236:2989:33254 2:N:0:GAACCTAG+TCCGCATA
ACTTACACGGTAGGTCGTGAACAAGCCCGGAGTACATCTCCTTTGGAACCAGGAGACCCCTCCGGTCT
TGTCCAACCTCTGTTTCGGCAGAGGATACCGTGCTCCACAGCTGTAAGGCTGTGGGCCCGAGAGAAGT
+
FFFFFFFFFFFFFFFFFFFFFFFFFFFFFFFFFFFFFFFFFFFFFFFFFFFFFFFFFFFFFFFFFFFF
FFFFFFFFFFFFFFFFFFFFFFFFFF::FFFFFFFFFFFFFFFFFFFFFF:FFFFFFFFFFF:FFFFF
@A00155:342:HHGFNDSXY:1:2237:17146:18662 2:N:0:GAACCTAG+TCCGCATA
GACTCGTCAGGTAGCCTACTAGACTACGACTTACACGGTAGGTCGTGAACAAGCCCGGAGTACATCTC
CTTTGGAACTAGGAGACCCCTCCGGTCTTGTCCAACCTCTGTTTCGGCAGAGGATACCGTGCTCCACA
+
FFFFFFFFFFFFFFFFFFFFFFFFFFFFFFFFFF,FFFFFFFFFFFFFFFFFFFFFFFFFFFFFFFFF
FFFFFFFFFFFFFFFFFFFFFFFFFFFFFFFFFFFFFFFFFFFFFFFFFFFFFFFFFFFFFFFFFFFF
@A00155:342:HHGFNDSXY:1:2238:21766:1423 2:N:0:GAACCTAG+TCCGCATA
GTGAACAAGCCCGGAGTACATCTCCTTTGGAACCAGGAGACCCCTCCGGTCTTGTCCAACCTCTGTTT
CGGCAGAGGATACCGTGCTCCACAGCTGTAAGGCTGTGGGCCCGAGAGAAGTCGCTCTCTCTCGCCCT
+
FFFFFFFFFFFFFFFFFFFFFFFFFFFFFFFFFFFFFFFFF:FFFFFFFFFF:FFFFFFFFFFFFFFF
FFFFFFFFFFFFFFFFFFFFFFFFFFFFFFFFFFFFFFFFFFFFFFFFFFFFFFFFFFFFFFFFFFFF
@A00155:342:HHGFNDSXY:1:2238:3278:15624 2:N:0:GAACCTAG+TCCGCATA
ACTCTTCGAGTTGTGTAAGTTTCGTCCTAAAAAGGACTCGTCAGGTAGCCTACTAGACTACGACTTAC
ACGGTAGGTCGTGAACAAGCCCGGAGTACATCTCCTTTGGAACTAGGAGACCCCTCCGGTCTTGTCC
+
FFFFFFFFFF:FFFFFFFFFFFFFFFFFFFFFFFFFFFFFFFFFFFFFFFF:FFFFFFFFFFFFFFFF
FFFFFFFFFFFFFFFFFFFFFFFFFFFFFFFFFFFFFFFFFFFFFFFFFFFFFFFFFFFFFFFFFFF
@A00155:342:HHGFNDSXY:1:2238:23583:31250 2:N:0:GAACCTAG+TCCGCATA
GTTTCGTCCTAAAAAGGACTCGTCAGGTAGCCTTCTAGACTACGACTTACACGGTAGGTCGTGAACAA
GCCCGGAGTACATCTCCTTTGGAACTAGGAGACCCCTCCGGTCTTGTCCAACCTCTGTTTCGGCAGAG
+
FFFF:FFFFFFFFFFFFFFFFFFFFFFFFFFFFFFFFFFFFFFFFFFF:FFFFFFFFFFFFFFFFF:F
:FFFFFFFFFFFFFFFFFFFFFFFFFFFFFFFFFFFFFFFFFF:FFFFFFFFFFFF:FFFFFFFFFFF
@A00155:342:HHGFNDSXY:1:2239:27778:9987 2:N:0:GAACCTAG+TCCGCATA
CCTACTAGACTACGACTTACACGGTAGGTCGTGAACAAGCCCGGAGTACATCTCCTTTGGAACTAGGA
GACCCCTCCGGTCTTGTCCAACCTCTGTTTCGGCAGAGGATACCGTGCTCCACAGCTGTAATGCTGTG
+
F:FFFFFF:FFFFFFFFFF:FFF,FFF:FFFFFFF,FF,FFF:FFFFFFF:FFFFFFFF:FFFFFFFF
FF,,FFFFFFFFFFF:F,FFFF,FFFFFFFFFFFF,F::FFFFFFFFFF,FFFFFFFFFFF,FFFFFF
@A00155:342:HHGFNDSXY:1:2239:30047:12696 2:N:0:GAACCTAG+TCCGCATA
CCTACTAGACTACGACTTACACGGTAGGTCGTGAACAAGCCCGGAGTACATCTCCTTTGGAACTAGGA
GACCCCTCCGGTCTTGTCCAACCTCTGTTTCGGCAGAGGATACCGTGCTCCACAGCTGTAAGGCTGTG
+
FFFFFFFFFFFFFFFFFFFFFFFFFFFFFFFFFFFFFFFFFFFFFFFFFFFFFFFFFFFFF:FFFFFF
FFFFFFFFFFFFFFFFFFFFFFFFFFFFFFFFFFFF:FFFFFFFFFFFFFFFF::FFFFFFFFFFFFF
```

```
@A00155:342:HHGFNDSXY:1:2239:10773:20807 2:N:0:GAACCTAG+TCCGCATA
TTTCGTCCTAAAAAGGACTCGTCAGGTAGCCTACTAGACTACGACTTACACGGTAGGTCGTGAACAAG
CCCGGAGTACATCTCCTTTGGAACTAGGAGACCCCTCCGGTCTTGTCCAACCTCTGTTTCGGCAGAGG
+
FF:FFFFFFFFFFFFFFFFFFFFFFFFFFFFFFFFFFFFFFFFFFFFFFFFFFFFFFFFFFFFFFFFF
FFFFFFFFFFFFFFFFFFFFFFFFFFFFFFFFFFFFFFFFFFFFFFFFFFFFFFFFFFFFFFFFFFFF
@A00155:342:HHGFNDSXY:1:2239:29984:35665 2:N:0:GAACCTAG+TCCGCATA
AGAGTTGTGTAAGTTTCGTCCTAAAAAGGACTCGTCAGGTAGCCTACTAGACTACGACTTACACGGTA
GGTCGTGAACAAGCCCGGAGTACATCTCCTTTGGAACTAGGAGACCCCTCCGGTCTTGTCCAACCTCT
+
FFFFFFFFFFFFFFFFFFFFF,FFFFFFFFFFFFFFFFFFFFFFFFFFFF:FFFFFFFFFFFFFFFFF
FFFFFFFFFFFF,FFFFFFFFFFFFFFFF:FFFFF:F:FFFFFFFF:FF:FFFFFFFFFFFFFFFFFF
@A00155:342:HHGFNDSXY:1:2240:29460:11678 2:N:0:GAACCTAG+TCCGCATA
TCGGCAGAGGATACCGTGCTCCACAGCTGTAAGGCTGTGGGCCCGAGAGAAGTCGCTCTCTCTCGCCC
TCCACATCCCTCTCGGCATTGAGATGACCATAGGCCAGAGGTGAGTCCTTAAGTGGACACAGCTGATC
+
FFFFFFFFFFFFFFFFFFFFFFFFFFFFFFFFFFFFFFFFFFFFFFFFFFFFFFFFFFFFFFFFFFFF
FFFFFFFFFFFFFFFFF,FFFFFFFFFFFFFFFFFFFFFFFFFFFFFFF:FFF:FFFFFF:FFFFFFF
@A00155:342:HHGFNDSXY:1:2240:15311:12038 2:N:0:GAACCTAG+TCCGCATA
TACGACTTACACGGTAGGTCGTGAACAAGCCCGGAGTACATCTCCTTTGGAACTAGGAGACCCCTCCG
GTCTTGTCCAACCTCTGTTTCGGCAGAGGATACCGTGCTCCACAGCTGTAAGGCTGTGGGCCCG
+
FFFFFFFFFFFFFFFFFFFFFFFFFFFFFFFFFFFFFFFFFFFFFFFFFFFFFFF:FFFFFFFFFFFF
FFF,FFFFFFFFFFFFFF:FFFFFFFFFFFFFFFFFFFFFFFFFFFFF:FFFFFFFFFFFFFFF
@A00155:342:HHGFNDSXY:1:2240:6216:25848 2:N:0:GAACCTAG+TCCGCATA
CACGGTAGGTCGTGAACAAGCCCGGAGTACATCTCCTTTGGAACTAGGAGACCCCTCCGGTCTTGTCC
AACCTCTGTTTCGGCAGAGGATACCGTGCTCCACAGCTGTAAGGCTGTGGGCCCGAGAGAAGTCGCTC
+
FFFFFFFFFFFFFFFFFFFFFFFFFFFFFFFFFFFFFFFFFFFFFFFFFFFFFFFFFFFFFFFFFFFF
FFFFFFFFFFFFFFFFFFFFFFFFFFFFFFFFFFFFFFFFFFFFFFFFFFFFFFFFFFFF:FFFFFFF
@A00155:342:HHGFNDSXY:1:2240:23900:27539 2:N:0:GAACCTAG+TCCGCATA
CTTACACGGTAGGTCGTGAACAAGCCCGGAGTACATCTCCTTTGGAACTAGGAGACCCCTCCGGTCTT
GTCCAACCTCTGTTTCGGCAGAGGATACCGTGCTCCACAGCTGTAAGGCTGTGGGCCCGAGAGAAGTC
+
FFFFFFFFFFFFFFFFFFFFFFFFFFFFFFFFFFFFFFFFFFFFFFFFFFFFFFFFFFFFFFFFFFFF
FFFFFFFFFFFFFFFFFFFFFFFFFFFFFFFFFFFFFFFFFFFFFFFFFFFFFFFFFFFFFFFFFFFF
@A00155:342:HHGFNDSXY:1:2240:16116:31626 2:N:0:GAACCTAG+TCCGCATA
CGTCAGGTAGCCTACTAGACTACGACTTACACGGTAGGTCGTGAACAAGCCCGGAGTACATCTCCTTT
GGAACCAGGAGACCCCTCCGGTCTTGTCCAACCTCTGTTTCGGCAGAGGATACCGTGCTCCACAGCTG
+
FFFFFFFFFFFF:FFFFFFFFFFFFFFFFFFFFFFFFFFFFFFFFFFFFFFFFFFFF:FFFFFFFFF
FFFFFFFFFFFFFFFFFFFFFFF,:FF:FFFFFFFFFFFFFFFFFFFFFFFFFFFFFFFFFFFFFFFF
@A00155:342:HHGFNDSXY:1:2240:12617:36839 2:N:0:GAACCTAG+TCCGCATA
CACGGTAGGTCGTGAACAAGCCCGGAGTACATCTCCTTTGGAACTAGGAGACCCCTCCGGTCTTGTCC
AACCTCTGTTTCGGCAGAGGATACCGTGCTCCACAGCTGTAAGGCTGTGGGCCCGAGAGAAGTCGCTC
+
FFFFFFFFFFFFFFFFFFFFFFFFFFFFFFFFFFFF::FFFFF:FFFFFFFFFFFFFFFFFFFFFFFF
FFFFFFFFFFFFFFFFFFFFFFFFFFFFFFFFFFFFFFFF,FFFFFFFFFFFFFFFFFFFFFFFFFFF
@A00155:342:HHGFNDSXY:1:2241:26377:1078 2:N:0:GAACCTAG+TCCGCATA
GTCCAACCTCTGTTTCGGCAGAGGATACCGTGCTCCACAGCTGTAAGGCTGTGGGCCCGAGAGAAGTC
GCTCTCTCTCGCCCTCCACATCCCTCTCGGCATTGAGATGACCATAGGCCAGAGGTGAG
+
FFFFFFFFFFFFFFFFFFFFFFFFFFFFFFFFFFFFFFFFFFFFFFFFF,FFFFFFFFFFFFFF,FFF
FFFFFFFFFFFFFFFFFFFFFFFFFFFFFFF:FFFFFFFFFFFFF,FFFFFFFFFFFFF
```

```
@A00155:342:HHGFNDSXY:1:2241:24939:16908 2:N:0:GAACCTAG+TCCGCATA
GGACTCGTCAGGTAGCCTACTAGACTACGACTTACACGGTAGGTCGTGAACAAGCCCGGAGTACATCT
CCTTTGGAACTAGGAGACCCCTCCGGTCTTGTCCAACCTCTGTTTCGGCAGAGGATACCGTGCTCCAC
+
FFFFFFFFFFFFFFFFF:FFFF:FFFFFFFFFFFFFFFFFFFFFFFFFFFFFF,FFFFFFFFFFFFFF
FFFFF,FFFFFFFFFFFFFFFFFFFF:F:FFFFFFFFFFFFFFFFFFFFFFFFFFFFFFFFFFFFFFF
@A00155:342:HHGFNDSXY:1:2241:31738:25269 2:N:0:GAACCTAG+TCCGCATA
TAGGAGACCCCTCCGGTCTTGTCCAACCTCTGTTTCGGCAGAGGATACCGTGCTCCACAGCTGTAAGG
CTGTGGGCCCGAGAGAAGTCGCTCTCTCTCGCCCTCCACATCCCTCTCGGCATTGAGATGACCATAG
+
FFFFFFFFFFFFFFFFFFFFFFFFFFFFFFFFFFFFFFFFFFFFFFFFFFFFFFFFFFFFFFFF::FF
FFFFFFFFFFFFFFFFF,FFFFF:FFFFFFFFFFFFFFFF:FFFFFFFFFFFFFFFFFFFFFFFF,FFF
@A00155:342:HHGFNDSXY:1:2241:29649:32800 2:N:0:GAACCTAG+TCCGCATA
ACTCTTCGAGTTGTGTAAGTTTCGTCCTAAAAAGGACTCGTCAGGTAGCCTACTAGACTACGACTTAC
ACGGTAGGTCGTGAACAAGCCCGGAGTACATCTCCTTTGGAACTAGGAGACCCCTCCGGTCTTGTCCA
+
FFFF,FFFFFFFFFFFF,FFFFFFFFFF::FFFFFFFFFFFFFFFFFFFFFFFFF,FFFFFFFFFFFFF
FFFFFFFFFFFFFFF:FFFFFFFFFF:FFFFFFFFF:F:FFFFFFFFFFF:FFFFFFFFFF::FFFF:F
@A00155:342:HHGFNDSXY:1:2242:6470:1078 2:N:0:GAACCTAG+TCCGCATA
ACTCTTCGAGTTGTGTAAGTTTCGTCCTAAAAAGGACTCGTCAGGTAGCCTACTAGACTACGACTTAC
ACGGTAGGTCGTGAACAAGCCTGGAGTACATCTCCTTTGGAACTAGGAGACCCCTCCGGTCTTGTCCA
+
FFFFFFFFFFFFFFFFFFFFFFFFFFFFFFFFFFFFFFFFFFFFFFFFFFFFFFFFFFFFFFFFFFFF
FFFFFFFFFFFFFF:FFFFFFFFFFFFFFFFFFFFFFFFFFFFFFFFFFFFFFFFFFFFFFFFFFFF
@A00155:342:HHGFNDSXY:1:2243:15501:4977 2:N:0:GAACCTAG+TCCGCATA
GTGTAAGTTTCGTCCTAAAAAGGACTCGTCAGGTAGCCTACTAGACTACGACTTACACGGTAGGTCGT
GAACAAGCCCGGAGTACATCTCCTTTGGAACTAGGAGACCCCTCCGGTCTTGTCCAACCTCTGTTTCG
+
FFFFFFFFFFFFFFFFFFFFFFFFFFFFFFFFFFFFF:FFFFFFFFFFFFFFFFFFFFFFFFFFFFFFFF
FFFFFFFFFFFFFFFFFFFFFFFFFFFFFFFFFFFFFFFFFFFFFFFFFF:FFFFFFFFFFFFFFFFF
@A00155:342:HHGFNDSXY:1:2243:12048:23202 2:N:0:GAACCTAG+TCCGCATA
AGCCCGGAGTACATCTCCTTTGGAACCAGGAGACCCCTCCGGTCTTGTCCAACCTCTGTTTCGGCAGA
GGATACCGTGCTCCACAGCTGTAAGGCTGTGGGCCCGAGAGAAGTCGCTCTCTCTCGCCCTCCACATC
+
FFFFFFFFFFFFFFFFFFFFFFFFFFFFFFFFFFFFFFFFFFFFF,FFFFFFFFFFFFFFFFFFFFFFFF
FFFFFFFFFFFFFFFFFFFFFFFFFFFFFFFFFFFFFFFFFFFFFFFFFFFFFFFFFFFFFFFFFFFFF
@A00155:342:HHGFNDSXY:1:2243:4237:23610 2:N:0:GAACCTAG+TCCGCATA
TCTCCTTTGGAACTAGGAGACCCCTCCGGTCTTGTCCAACCTCTGTTTCGGCAGAGGATACCGTGCTC
CACAGCTGTAAGGCTGTGGGCCCGAGAGAAGTCGCTCTCTCTCGCCCTCTACATCCCTCTCGGCATTG
+
FFFFFFFFF:FFFFFFFFFFFFFFFFFFFF:FFFFFFFFFFFFFFFFFFFFFFFFFFFFFFFFFFFFFF
FFFFFFFFFFFFFFFFFFFFFFFFFFFFFF:FFFFFFFFFFF:FFFFFFFFFF:FFFFFFFFFF:F:F
@A00155:342:HHGFNDSXY:1:2244:20166:11397 2:N:0:GAACCTAG+TCCGCATA
GACCTAGGAGACCCCTCCGGTCTTGTCCAACCTCTGTTTCGGCAGAGGATACCGTGCTCCACAGCTGT
AAGGCTGTGGGCCCGAGAGAAGTCGCTCTCTCTCGCCCTCCACATCCCTCTCGGCATTGAGATGACCA
+
FF,FFFFFFFFFFFFFFFFFFF:FFFFFFFFFFFFFFFFFFFFFFFFFFFFFFFFFFFFFFFFFFFFF
FFFFFFFFFFFFFFFFFFFFFFFFFFFFFFFFFFFFFFFFFFFFFFFFFFFFFFFFFFFFFFFFFFFFF
@A00155:342:HHGFNDSXY:1:2244:18638:29011 2:N:0:GAACCTAG+TCCGCATA
GGACTCGTCAGGTAGCCTACTAGACTACGACTTACACGGTAGGTCGTGAACAAGCCCGGAGTACATCT
CCTTTGGAACCAGGAGACCCCTCCGGTCTTGTCCAACCTCTGTTTCGGCAGAGGATACCGTGCTCCAC
+
FFFFFFFFFFFFFFFFFFFFFFFFFFFFFFFFFFFF:FFFFFFFFFFFFFFF,FFFFFFFFFFFFFFF
FFFFFFFFFFFFFFFF:FFFF,FFFFFF:FFFFFFFFFFF,FFFFF:FFFFFFFFFFFFFFFFFFFFF
```

```
@A00155:342:HHGFNDSXY:1:2244:19108:29450 2:N:0:GAACCTAG+TCCGCATA
GGACTCGTCAGGTAGCCTACTAGACTACGACTTACACGGTAGGTCGTGAACAAGCCCGGAGTACATCT
CCTTTGGAACCAGGAGACCCCTCCGGTCTTGTCCAACCTCTGTTTCGGCAGAGGATACCGTGCTCCAC
+
FFFFFFFFFFFFFFFFFFFFFFFFFFFFFFFFFFFFFFFF:FFFFFFFFFFFFFFFFFFFFFFFFFFF
FFFFFFFFFFFFFFFFFFFFFFFFFFFF,FFFFFFFFFFFFFFFFFFFF,FFFF:FFFFFFFFFFFFF
@A00155:342:HHGFNDSXY:1:2245:19117:23265 2:N:0:GAACCTAG+TCCGCATA
GTCTTGACCAACCTCTGTTTCGGCAGAGGATACCGTGCTCCACAGCTGTAAGGCTGTGGGCCCGAGAG
AAGTCGCTCTCTCTCGCCCTCCACATCCCTCTCGGCATTGAGATGACCATAGGCCAGAG
+
FFFFFFFFFFFFFFFFFFFFFFFFFFFFFFFFFFFFFFFFFFFFFFFF:FFFFF:F:FFFFFFFFFFF
FFFFFFFFFFFFFFFFFFFFFFFFFFFFFFFFFFFF:,FFFF:FFFFFF,FFFFFFFFF
@A00155:342:HHGFNDSXY:1:2245:5276:32894 2:N:0:GAACCTAG+TCCGCATA
GTGAACAAGCCCGGAGTACATCTCCTTTGGAACTAGGAGACCCCTCCGGTCTTGTCCAACCTCTGTTT
CGGCAGAGGATACCGTGCTCCACAGCTGTAAGGCTGTGGGCCCGAGAGACGTCGCTCTCTCTCGCCCT
+
FFFFFFFFFFFFFFFFFFFFFFFFFFFFFFF:FFFFFFFFFFFFFFFFFF:,FFFFFFFFFFFFFFF:
FFFFFFFFFFFFFFFFFFFFF,F,FFFFFFFFFFFFFFFFFFFF,FFFF,FFFFF:FFFFFFFFFFFF
@A00155:342:HHGFNDSXY:1:2245:5828:35665 2:N:0:GAACCTAG+TCCGCATA
TCGGCAGAGGATACCGTGCTCCACAGCTGTAAGGCTGTGGGCCCGAGAGAAGTCACTCTCTCTCGCCC
TCCACATCCCTCTCGGCATTGAGATGACCATAGGCCAGAGGTGAGTCCTTAAGTGGACACA
+
FFFFFFFFFFFFFFFFFFFFFFFFFFFFFFFFFFFFFFFFFFFFFFFFFFFFFFFFFFFFFFFFFFFF
FFFFFFFFFFFFFFFFFF,FFFFFFFFFFFFFFFFFFFFFFFFFFFFFFFFFF:FFFFFFF
@A00155:342:HHGFNDSXY:1:2245:20509:36542 2:N:0:GAACCTAG+TCCGCATA
ACTAGACTACGACTTACACGGTAGGTCGTGAACAAGCCCGGAGTACATCTCCTTTGGAACTAGGAGAC
CCCTCCGGTCTTGTCCAACCTCTGTTTCGGCAGAGGATACCGTGCTCCACAGCTGTAAGGCTGTGGGC
+
FFFFFFFFFFFFFFFFFFFFFFFFFFFFFFFFFFFFFFFFFFFFFFFFFFFFFFFFFFFFFFFFFFFF
FFFFFFFFFFFFFFFFFFFFFFFFFFFFFFFFFFFFFFFFFFFFFFFFFFFFFFFFFFFFF,FFFFFF
@A00155:342:HHGFNDSXY:1:2246:6741:12602 2:N:0:GAACCTAG+TCCGCATA
GTGAACAAGCCCGGAGTACATCTCCTTTGGAACTAGGAGACCCCTCCGGTCTTGTCCAACCTCTGTTT
CGGCAGAGGATACCGTGCTCCACAGCTGTAAGGCTGTGGGCCCGAGAGAAGTCGCTCTCTCTCGCCCT
+
FFFFFFFFFFFFFFFFFFFFFFFFFFFFFFFFFFFFFFFFFFFFFFFFFFFFFFFFFFFFFFFFFFFF
FFFFFFFFFFFFFFFFFFFFFFFFFFFFFFFFFFFFFFFFFFFFFFFFFFFFFFFFFFFFFFF:FFFF
@A00155:342:HHGFNDSXY:1:2246:18521:28714 2:N:0:GAACCTAG+TCCGCATA
GTCCTAAAAAGGACTCGTCAGGTAGCCTACTAGACTACGACTTACACGGTAGGTCGTGAACAAGCCCG
GAGTACATCTCCTTTGGAACTAGGAGACCCCTCCGGTCTTGTCCAACCTCTGTTTCGGCAGAGGATAC
+
FFFFFFFFFFFFFFFFFFFFFFFFFFFFFFFF:FFFFFFFFFFFFFFFFFFFFFFFFFFFFFFFFFFF
FFFFFFFFFFFFFFFFFF,FFFFFFF:FFFFFFFFFFFFFFFFFFFFFFFFFF:FFFFFFFFFFFFFF
@A00155:342:HHGFNDSXY:1:2246:13105:30295 2:N:0:GAACCTAG+TCCGCATA
GGGTTCAAACCCATGCCCCGCCACACCGCCTTAGATCAGCTGTGTCCACTTAAGGACTCACCTCTGGC
CTATGGTCATCTCAATGCCGAGAGGGATGTGGAGGGCGAGAGAGAGCGACTTCTCTCGGGCCCACAGC
+
FFFFFFFFFFFFFFFFFFFFFFFFFFFFFFFFFFFFFFFFFFFFFFFFFFFFFFFFFFF:FFFFFFFF
FFFFFFFFFFFFFFFFFFFFFFFFFFFFFFFFFFFFFFFFFFFFFFFFFFFFFFFFFFFFFFFFFFFF
@A00155:342:HHGFNDSXY:1:2247:15257:10504 2:N:0:GAACCTAG+TCCGCATA
TGAGTTGTGTAAGTTTCGTCCTAAAAAGGACTCGTCAGGTAGCCTACTAGACTACGACTTACACGGTA
GGTCGTGAACAAGCCCGGAGTACATCTCCTTTGGAACTAGGAGACCCCTCCGGTCTTGTCCAACCTCT
+
FF::FF:F:FFF:FFF,FF:F:FFFFF:FF:FF:FFF:FF:FFFFFFFFFFF:FF:F::FF:FFFFFF
F:FFFFFFFFFF:FFFFFFFFFFFF,FFF::FFFFFF,FFFF:FFFFF,:F:FFF,FFFFFFFFF:FF
```

```
@A00155:342:HHGFNDSXY:1:2247:10538:23218 2:N:0:GAACCTAG+TCCGCATA
CCTACTAGACTACGACTTACACGGTAGGTCGTGAACAAGCCCGGAGTACATCTCCTTTGGAACCAGGA
GACCCCTCCGGTCTTGTCCAACCTCTGTTTCGGCAGAGGATACCGTGCTCCACAGCTGTAAGGCTGTG
+
FFFFFFFFFFFFFFFFFFFFFFFFFFFFFFFFFFFFFFFFFFFFFFFFFFFFFFFFFFFFFFFFFFFF
FFFFFFFFFFFFFFFFFFFFFFFFFFFFFFFFFFFFFFFFFFFFFFFFFFFFFFFFFFFFFFFFFF:F
@A00155:342:HHGFNDSXY:1:2248:21287:30311 2:N:0:GAACCTAG+TCCGCATA
GTTGTGTAAGTTTCGTCCTAAAAAGGACTCGTCAGGTAGCCTACTAGACTACGACTTACACGGTAGGT
CGTGAACAAGCCCGGAGTACATCTCCTTTGGAACTAGGAGACCCCTCCGGTCTTGTCCAACCTCTGTT
+
FFFFFFFFFFFFFFFFFFFFFFFFFFFFFFFFFFFFFFFFFFFFFFFFFFFFFFFFFFFFFFFFFFFF
FFFFFFFFFFFFFFFFFFFFFFFFFFFFFFFFFFFFFFFFFFFFFFFFFFFFFFFFFFF:FFFFFFFF
@A00155:342:HHGFNDSXY:1:2248:27398:30906 2:N:0:GAACCTAG+TCCGCATA
AAGGACTCGTCAGGTAGCCTACTAGACTACGACTTACACGGTAGGTCGTGAACAAGCCCGGAGTACAT
CTCCTTTGGAACTGGGAGACCCCTCCGGTCTTGTCCAACCTCTGTTTCGGCAGAGGATACCGTG
+
FFFFFFFFFFFFFFFFFFFFFFFFFFFFFFFFFFFFFFFFFFFFFFFFFFFFFFFFFFFF:FFFFFFF
FFFFFFFFFFFFFFFFFFFFFFFFFFFFFF:FFFFFFFFFFFFF:FFF:FFFFFFFFFFFFFFF
@A00155:342:HHGFNDSXY:1:2248:23303:36746 2:N:0:GAACCTAG+TCCGCATA
GAGACCCCTCCGGTCTTGTCCAACCTCTGTTTCGGCAGAGGATACCGTGCTCCACAGCTGTAAGGCTG
TGGGCCCGAGAGAAGTCGCTCTCTCTCGCCCTCCACATCCCTCTCGGCATTGAGATGACCATAGGCCA
+
FFFFFFFFFFFFFFFFFFFFFFFFFFFFFFFFFFFFFFFFFFFFFFFFFFFFFFFFFFFFFFFFFFFF
FFFFFFFFFFFFF:FFFFFFFFFFFFFFFFFFFFFFFFFFFFFFFFFFF::FFFFFFFFFFFFFFFFF
@A00155:342:HHGFNDSXY:1:2249:11451:17628 2:N:0:GAACCTAG+TCCGCATA
GTTCAAACCCATGCCCCGCCACACCGCCTTAGATCAGCTGTGTCCACTTAAGGACTCACCTCTGGCCT
ATGGTCATCTCAATGCCGAGAGGGATGTGGAGGGCGAGAGAGAGCGACTTCTCTCGGGCCCACAGCCT
+
FFFFFF:FFFFFFFFFFFFFFFFFFFFFFFFFFFFFFFFFFFFFFFFFFFFFFFFFFFFFFFFFFFFF
:FFFFFFFFFFFFFFFFFFFFFFFFFFFFFFFFFFFFFFFFFFFFFFFFFFFFFFFFFFFFFFFFFFF
@A00155:342:HHGFNDSXY:1:2249:11487:18411 2:N:0:GAACCTAG+TCCGCATA
GTTCAAACCCATGCCCCGCCACACCGCCTTAGATCAGCTGTGTCCACTTAAGGACTCACCTCTGGCCT
ATGGTCATCTCAATGCCGAGAGGGATGTGGAGGGCGAGAGAGAGCGACTTCTCTCGGGCCCACAGCCT
+
FFFFFFFFFFFFFFFFFFFFFFFFFFFFFFFFFFFFFFFFFFFFF:FFFFFFFFFFFFFFFF:FFFFF
FFFFFFFFFFFFFFFFFFFFFFFFFFFFFFFFFFFFFFFFFFFFFFFFFFFFFFFFFFFFFFFFFFF:
@A00155:342:HHGFNDSXY:1:2249:16893:24612 2:N:0:GAACCTAG+TCCGCATA
TAGGAGACCCCTCCGGTCTTGTCCAACCTCTGTTTCGGCAGAGGATACCGTGCTCCACAGCTGTAAGG
CTGTGGGCCCGAGAGAAGTCGCTCTCTCTCGCCCTCCACATCCCTCTCGGCATTGAGATGACCATAGG
+
FFFFFFFFFFFFFFFFFFFFFFFFFFFFFFFFFFFFFFFFF:FFFFFFFFFFFFFFFFFFFFFFFFFF
FFFFFFFFFFFFFFFFFFFFFFFFFFFFFFFFFFFFFFFFFFFFFFFFFFFFFFFFFFFFFFFFFFFF
@A00155:342:HHGFNDSXY:1:2251:24505:20541 2:N:0:GAACCTAG+TCCGCATA
TCTCCTTTGGAACTAGGAGACCCCTCCGGTCTTGTCCAACCTCTGTTTCGGCAGAGGATACCGTGCTC
CACAGCTGTAAGGCTGTGGGCCCGAGAGAAGTCGCTCTCTCTCGCCCACCACATCCCTCTCGGCATTG
+
FFFFFFFFFFFFFFFFFFFFFFFFFFFFFFFFFFFFFFFFFFFFFFFFFFFFFFFFFFFFFFFFFFFF
FFFFFFFFFFFFFFFFFFFFFFFFFFFFFFFFFFFFFFFFFFFFFFFFFFFFFFFFFFFFFFFFFF,F
@A00155:342:HHGFNDSXY:1:2252:12662:32628 2:N:0:GAACCTAG+TCCGCATA
TTTCGTCCTAAAAAGGACTCGTCAGGTAGCCTACTAGACTACGACTTACACGGTAGGTCGTGAACAAG
CCCGGAGTACATCTCCTTTGGAACTAGGAGACCCCTCCGGTCTTGTCCAACCTCTGTTTCGGCAGAGG
+
FFFFFFFFFFFFFFFFFFFFFFFFFFFFFFFFFFFFFFFFFFFFFFFFFFFFFFFFFFFFFFFFFFFF
FFFFFFFFFFFFFFFFFFFFFFFFFFF::FFFF:FFFFFFF:F::,FFFFFFFF:FFFFFFFFFFFF:
```

```
@A00155:342:HHGFNDSXY:1:2253:26630:13636 2:N:0:GAACCTAG+TCCGCATA
TACATCTCCTTTGGAACCAGGAGACCCCTCCGGTCTTGTCCAACCTCTGTTTCGGCAGAGGATACCGT
GCTCCACAGCTGTAAGGCTGTGGGCCCGAGAGAAGTCGCTCTCTCTCGCCCTCCACATCCCTCTCGGC
+
FFFFFFFFFFFFFFFFFFFFFFFFFFFFFFFFFFF:FFFFFFFFFFFFFFFFFFFFFFFFFFFFFFFF
:FFFFFFFFFFFFFFFFFFFFFFFFFFFFFFFFFFFF:FFFFFFFFFFFFF:FFFFFFFFFFFFFFFF
@A00155:342:HHGFNDSXY:1:2253:4689:17879 2:N:0:GAACCTAG+TCCGCATA
CTTCGAGTTGTGTAAGTTTCGTCCTAAAAAGGACTCGTCAGGTAGCCTACTAGACTACGACTTACACG
GTAGGTCGTGAACAAGCCCGGAGTACATCTCCTTTGGAACTAGGAGACCCCTCCGGTCTTGTCCAACC
+
FFFFFFFFFFFFFFFFFFFFFFFFFFFFF:FFFFFFFFFFFFFFFFFFFFFFF:FFFFFFFFFFFFFF
FFFFFFFFFFFFFFFFFFFFFFFFFFFFFFF,FFFF:FFFFF:FFFFFFFFFFFFFF:FFFFFFFFFFF
@A00155:342:HHGFNDSXY:1:2254:28293:4993 2:N:0:GAACCTAG+TCCGCATA
ACATCTCCTTTGGAACTAGGAGACCCCTCCGGTCTTGTCCAACCTCTGTTTCGGCAGAGGATACCGTG
CTCCACAGCTGTAAGGCTGTGGGCCCGAGAGAAGTCGCTCTCTCTCGCCCTCTACATCCC
+
FFFFF:FFFFFFFFFFFFFFFFFFFFFFFFFFFFFFFFFFFFFFFFFFFFFFFFFFFFFFFFFFFFFF
FFFFFFFFFFFFFFFFFFFFFFFFFFFFFFFFFFFFFFFFFFFFFFFFFFFFFFFFFFFF
@A00155:342:HHGFNDSXY:1:2254:2691:12884 2:N:0:GAACCTAG+TCCGCATA
AGGACGAAACCGTCGTTACTCTTCGAGTTGTGTAAGTTTCGTCCTAAAAAGGACTCGTCAGGTAGCCT
ACTAGACTACGACTTACACGGTAGGTCGTGAACAAGCCCGGAGTACATCTCCTTTGGAACTAGGAGAC
+
FFFFFFFFFFFFFFFFFFFFFFFFFFF:FFFFFFFFFFFFFFFFFFFFFFFFFFFFFFFF:FFFFFFFF
FFFFFFFFFFFFFFFFFFFFFFFFFFFFFFFFF:FFFFFFFFFFFFFFFFFF:FFFFF,F::FFFFFF
@A00155:342:HHGFNDSXY:1:2254:25843:13432 2:N:0:GAACCTAG+TCCGCATA
TAGGACGAAACTTACACAACTCGAAGAGTAACGACGGTTTCGTCCTTGGAACGGACTCATCAGGGAGC
CTACTAGACTCCGACCGTCATGGGGGTTCAAACCCATGCCCCGCCACACCGCCTTAGATCAGCTGTGT
+
FFFFFFFFFFFFFFFFFFFFFFFFFFFFFFFFFFFFFFFFFFFFFFFFFFFFFFFFFFFFFFFFFFFF
FFFFFFFFFFFFFFFFFFFFFFFFFFFFFFFFFFFFFFFFFFFFFFFFFFFFFFFFFFFFFFFFFFFF
@A00155:342:HHGFNDSXY:1:2254:24596:22357 2:N:0:GAACCTAG+TCCGCATA
GGAACGGACTCATCAGGGAGCCTACTAGACTCCGACCGTCATGGGGGTTCAAACCCATGCCCCGCTAC
ACCGCCTTAGATCAGCTGTGTCCACTTAAGGACTCACCTCTGGCCTATGGTCATCTCAATGCCGAGAG
+
FFFFFFFFFFFFFFFFFFFFFFFFFFF:FFFFFFFFFFF:FFFF:FF:FFFFFFFFFFFFFFFFF:FF
FFFFFFFFFFFFFFFFFFFFFFFFFFFFF,F:FF:FFFF:FF,F:FFFFFFFF:FFFF,FFFFFFFF
@A00155:342:HHGFNDSXY:1:2254:28863:22764 2:N:0:GAACCTAG+TCCGCATA
CCCGGAGTACATCTCCTTTGGAACTAGGAGACCCCTCCGGTCTTGTCCAACCTCTGTTTCGGCAGAGG
ATACCGTGCTCCACAGCTGTAAGGCTGTGGGCCCGAGAGAAGTCGCTCTCTCTCGCCCTCCACATCCC
+
FFFFFFFFFFFFFFFFFFFFFFF:FFFFFFFFFFFFFFFFFFFFFFFFFFFFFFFFFFFFFFFFFFFF:
FF:FFFFF:FFFFFFFFFFFFFFFFFFFFF:FFFF:FFFFFFFFFFFFFFFFFFFFFFFF:F:FF,FF:
@A00155:342:HHGFNDSXY:1:2254:8639:26569 2:N:0:GAACCTAG+TCCGCATA
GCCTATGGTCATCTCAATGCCGAGAGGGATGTGAAGGGCGAGAGAGAGCGACTTCTCTCGGGCCCACA
GCCTTACAGCTGTGGAGCACGGTATCCTCTGCCGAAACAGAGGTTGGACAAGACCGGAG
+
FFFFFFFFFFFFFFFFFFFFFFFFFFFFFFFFFFFFFFFFFFFFFFFFFFFFFFFFFFFFFFFFFFFF
FFFFF:FFFFFFFFFFFFFFFFFFFFFFFFFFFFFFFFFFFFFF:FFFFFFFFFFFFFF
@A00155:342:HHGFNDSXY:1:2254:8241:27633 2:N:0:GAACCTAG+TCCGCATA
TACGACTTACACGGTAGGTCGTGAACAAGCCCGGAGTACATCTCCTTTGGAACTAGGAGACCCCTCCG
GTCTTGTCCAACCTCTGTTTCGGCAGAGGATACCGTGCTCCACAGCTGTAAGGCTGTGGGCCCG
+
FFFFFFFFFFFFFFFFFFFFFFFFFFFFFFFFFFFFFFFFFFFFFFFFFFFFFFFFFFFFFFFFFFFF
FFFFFFFFFFFFFFFFFFFFFFFFFFF:FFFFFFFFFFFFFFFFFFFFFFFFFFFFFFFFFFFFF
```

```
@A00155:342:HHGFNDSXY:1:2254:9805:27743 2:N:0:GAACCTAG+TCCGCATA
GACGAAACCGTCGTTACTCTTCGAGTTGTGTAAGTTTCGTCCTAAAAAGGACTCGTCAGGTAGCCTAC
TAGACTACGACTTACACGGTAGGTCGTGAACAAGCCCGGAGTACATCTCCTTTGGAACTAGGAGACCC
+
FFFFFFFFFFFFFFFFFFFFFFFFFFFFFFFFFFF,FFFFFFFFFFFFFFFFFFFFF:FFFFFFFFFF
FFFFFFFFFF:FFFFFFFFFFFFFFFFFFFFFFFFFFFFFFFFFFFFFFFFFFFFFFFFFFFFFFFFF
@A00155:342:HHGFNDSXY:1:2255:7401:12211 2:N:0:GAACCTAG+TCCGCATA
TAGGAGACCCCTCCGGTCTTGTCCAACCTCTGTTTCGGCAGAGGATACCGTGCTCCACAGCTGTAAGG
CTGTGGGCCCGAGAGAAGTCGCTCTCTCTCGCCCTCCACATCCCTCTCGGCATTGAGATGACCATAGG
+
FFFFFFFFFFFFFFFFFFFFFFFFFFFFFFFFFFFFFFFFFFFFFFFFFFFFFFFFFFFFFFFFFFFF
FFFFFFFFFFFFFFFFFFFFFFFFFFFFFFFFFFFFFFFFFFFFFFFFFFFFF,FFFFFFFFFFFFFF
@A00155:342:HHGFNDSXY:1:2255:22896:31782 2:N:0:GAACCTAG+TCCGCATA
GTTACTCTTCGAGTTGTGTAAGTTTCGTCCTAAAAAGGACTCGTCAGGTAGCCTACTAGACTACGACT
TACACGGTAGGTCGTGAACAAGCCCGGAGTACATCTCCTTTGGAACCAGGAGACCCCTCCGGTCTTGT
+
FFFFFFFFFFFFFFFFFFFFFF:FFFFFFFFFFFF:FFFFFFFFFFFFFFFFFFFFFFFFFFFFFFFF
FFFFFFFF:FFFFFFFFFFFF:FFFFFFFFFFFFFFFFFFFFFFFFFFFFFF:FFFFFFFFFFFFFFF
@A00155:342:HHGFNDSXY:1:2256:15998:1078 2:N:0:GAACCTAG+TCCGCATA
ACGACTTACACGGTAGGTCGTGAACAAGCCCGGAGTACATCTCCTTTGGAACTAGGAGACCCCTCCGG
TCTTGTCCAACCTCTGTTTCGGCAGAGGATACCGTGCTCCACAGCTGTAAGGCTGTGGGCCCGAGAGA
+
FFFFFFFFFFFFFFFFFFFFFFFFFFFFFFFFFFFFFFFFFFFFFFFFFFFFFFFFFFFFFFFFFFFF
FFFFFFFFFFFFFFFFF:FFFFFFFFFFFFFFFFFFFFFFFFFFFFFFF:FFFFFFFFFFFFFFFFFF
@A00155:342:HHGFNDSXY:1:2256:24849:1783 2:N:0:GAACCTAG+TCCGCATA
ACGACTTACACGGTAGGTCGTGAACAAGCCCGGAGTACATCTCCTTTGGAACTAGGAGACCCCTCCGG
TCTTGTCCAACCTCTGTTTCGGCAGAGGATACCGTGCTCCACAGCTGTAAGGCTGTGGGCCCGAGAGA
+
FFFFFFFFFFFFFFFFFFFFFFFFFFFFFFFFFFFFFF:FFFFFFFFFFFFFFFFFFFFFFFFFFFFF
FFFFFFFFFFFFFFFFFFFFFFFFFFFFFFFFFFFFFFFFFFFFFFFFFFFFFFFFFFFFFFFFFFFF
@A00155:342:HHGFNDSXY:1:2256:16333:3004 2:N:0:GAACCTAG+TCCGCATA
GTCCAACCTCTGTTTCGGCAGAGGATACCGTGCTCCACAGCTGTAAGGCTGTGGGCCCGAGAGAAGTC
GCTCTCTCTCGCCCTTCACATCCCTCTCGGCATTGAGATGACCATAGGCCAGAGGTGAGTCCTTAAGT
+
FFFFFFFFFFFFF:FFFFFFFFFFFFFFFFFFFFFFFF:FFFFFFFFFFFFFFFFFFFFFF:FFFFFF
FFFFFFFFFFFFFFF,FFFFFFFFFFFFFFFF::FFFFFFFFFFFFFFFFFFFFFFFFFFFFFFFFF:
@A00155:342:HHGFNDSXY:1:2256:15655:15201 2:N:0:GAACCTAG+TCCGCATA
TACGACTTACACGGTAGGTCGTGAACAAGCCCGGAGTACATCTCCTTTGGAACCAGGAGACCCCTCCG
GTCTTGTCCAACCTCTGTTTCGGCAGAGGATACCGTGCTCCACAGCTGTAAGGCTGTGGGCCCG
+
FFFFFFFFFFFFFFFFFFFFFFFFFFFFFFFFFFFFFFFFFFFFFFFFFFFFFFFFFFFFFFFFFFFF
FFFFFFFFFFFFFFFFFFFFFFFFFFFFFFFFFFFFFFFFFFFFFFFFFFFFFFFFFFFFFF
@A00155:342:HHGFNDSXY:1:2256:9995:16203 2:N:0:GAACCTAG+TCCGCATA
GCCTACTAGACTACGACTTACACGGTAGGTCGTGAACAAGCCCGGAGTACATCTCCTTTGGAACTAGG
AGACCCCTCCGGTCTTGTCCAACCTCTGTTTCGGCAGAGGATACCGTGCTCCACAGCTGTAAGGCTGT
+
FFFFFFFFFFFFFFFFFFFFFFFFFFFFFFFFFFFFFFF:FFFFFFFFFFFFFFFFFFFFFFFFFFFF
FFFFFFFFFFFFFFFFFFFFFFFF:FFFFFFFFFFFFFFFFF:FFFFFFFFFFFFFFFFF:FFFFFFF
@A00155:342:HHGFNDSXY:1:2256:32714:23766 2:N:0:GAACCTAG+TCCGCATA
GCCCGGAGTACATCTCCTTTGGAACTAGGAGACCCCTCCGGTCTTGTCCAACCTCTGTTTCGGCAGAG
GATACCGTGCTCCACAGCTGTAAGGCTGTGGGCCCGAGAGAAGTCGCTCTCTCTCGCCCTCCACATCC
+
FFFFFFFFFFFFFFFFFFFFFFFFFFFFFFFFFFFFFFFFFFFFFFFFFFFFFFFFFFFFFFFFFFFF
FFFFFFFFFFFFFFFFFFFFFFFFFFFFFFFFFFFFFFFFFFFFFFFFFFFFFFFFFFFFFFFFFFFF
```

@A00155:342:HHGFNDSXY:1:2257:18900:16282 2:N:0:GAACCTAG+TCCGCATA
ACTCGTCAGGTAGCCTACTAGACTACGACTTACACGGTAGGTCGTGAACAAGCCCGGAGTACATCTCC
TTTGGAACTAGGAGACCCCTCCGGTCTTGTCCAACCTCTGTTTCGGCAGAGGATACCGTGCTCCACAG
+
FFFFFFFFFFF:FFFFFFFFFFFFFFFFFFFFFFFFFF:FFFFFFFFFFFFFFFFFFFFFFFFFFFFF
FFFFFF:FFFFFFFFFFFFFFFFFFF:FFFFFFFFFFFFFFFFFFFF:FFFFFFFFFFFFFFFFFFFF
@A00155:342:HHGFNDSXY:1:2257:18032:17033 2:N:0:GAACCTAG+TCCGCATA
ACTCGTCAGGTAGCCTACTAGACTACGACTTACACGGTAGGTCGTGAACAAGCCCGGAGTACATCTCC
TTTGGAACTAGGAGACCCCTCCGGTCTTGTCCAACCTCTGTTTCGGCAGAGGATACCGTGCTCCACAG
+
FFFFFFFFFFFFFFFFFFFFFFFFFFFFFFFFFFFFFFFFFFFFFFFFFFFFFFFFFFFFFFFFFFFF
FFFFFFFFF:FFFFFFFFFFFFFFFFFFFFFFFFFFFFFFFFFFFFFFFFFFFFFFFFFFFFFFFFFF
@A00155:342:HHGFNDSXY:1:2258:7889:1564 2:N:0:GAACCTAG+TCCGCATA
TGTGTAAGTTTCGTCCTAAAAAGGACTCGTCAGGTAGCCTACTAGACTACGACTTACACGGTAGGTCG
TGAACAAGCCCGGAGTACATCTCCTTTGGAACTAGGAGACCCCTCCGGTCTTGTCCAACCTCTGTTTC
+
FFFFFFFFFFFFF:FFFFFFFFFFFFFFFFFFFFFFFFFFFFFFFFF:FFFFFFFFFFFFFFFFFFFF
FFFFFFFFFFFFFFFFFFFFFFFFFFFFFF,FFFFFFFFFFFFFFFFFFFF:FFFFFFFFFFFFFFFF
@A00155:342:HHGFNDSXY:1:2258:10963:24674 2:N:0:GAACCTAG+TCCGCATA
GAAACCGTCGTTACTCTTCGAGTTGTGTAAGTTTCGTCCTAAAAAGGACTCGTCAGGTAGCCTACTAG
ACTACGACTTACACGGTAGGTCGTGAACAAGCCCGGAGTACATCTCCTTTGGAACCAGGAGACCCCTC
+
FFFFFFFFFFFFFFFFFF:F:FFFFFFFFFFFFFFFFFFFFFFFFFFFFFFFFFFFFFFFFFFFFFFF
FFFFFF:F:FFFFFFFFFFFFFFFF:FFF:FFFFFFFFFFFFFFFFFFFFFFFFFFFFFFFFFFFFFF
@A00155:342:HHGFNDSXY:1:2259:22788:5321 2:N:0:GAACCTAG+TCCGCATA
AAGGACTCGTCAGGTAGCCTACTAGACTACGACTTACACGGTAGGTCGTGAACAAGCCCGGAGTACAT
CTCCTTTGGAACTAGGAGACCCCTCCGGTCTTGTCCAACCTCTGTTTCGGCAGAGGATACCGTGCTCC
+
FFFFFFFFFFFFFFFFFFFFFFFFFFFFFFFFFFFFFFFFFF:FFFFFFFFFFFFFFFFFFFFFFFFF
FFFFFFFFFFFFFFFFFFFFFFFFFFFFFFFFFFFFFFF:FFFFFFFFFFFFFFFF:FFFFFFFFFFF
@A00155:342:HHGFNDSXY:1:2259:23900:7404 2:N:0:GAACCTAG+TCCGCATA
AGGACTCGTCAGGTAGCCTACTAGACTACGACTTACACGGTAGGTCGTGAACAAGCCCGGAGTACATC
TCCTTTGGAACCAGGAGACCCCTCCGGTCTTGTCCAACCTCTGTTTCGGCAGAGGATACCGTGCTCCA
+
FFFFFFFFFFFFFFFFFFFFFFFFFFFFFFFFFFFFFFFFFFFFFFFFFFFFF:FFFFFFFFFFFFFF
FFFFFFFFFFFFFFFFFFFFFFFFFFFFFFFFFFFFFFFFFFFFFFFFFFFFFFFFFFFFFFFFFFFF
@A00155:342:HHGFNDSXY:1:2259:15953:29904 2:N:0:GAACCTAG+TCCGCATA
GGACTCACCTCTGGCCTATGGTCATCTCAATGCCGAGAGGGATGTGAAGGGCGAGAGAGAGCGACTTC
TCTCGGGCCCACAGCCTTACAGCTGTGGAGCACGGTATCCTCTGCC
+
FFFFFFFFFFFFFFFFFFFFFFFFFFFFFFFFFFFFFFFFFF:FFFFFFFFFFFFFFFFFFFFFFFFF
FFFFFFFFFFFFFFFFFFFFFFFFFFFFFFFFFFFFFFFFFFFFFF
@A00155:342:HHGFNDSXY:1:2260:2808:19695 2:N:0:GAACCTAG+TCCGCATA
GAAACCGTCGTTACTCTTCGAGTTGTGTAAGTTTCGTCCTAAAAAGGACTCGTCAGGTAGCCTACTAG
ACTACGACTTACACGGTAGGTCGTGAACAAGCCCGGAGTACATCTCCTTTGGAACTAGGAGACCCCTC
+
FFFFFFFFFFFFFFFFFFF::FFFFFFFFFFFFFFFFFFFFFFFFFFFFFFFFFFFFFFFFFFFFFFF
FFFFFFFFFFFFFFFFFFFFFF:FF::FFFFFFFFFFFFFFFFFFFFFFFFFFFFFF,FFFF:FFFF
@A00155:342:HHGFNDSXY:1:2260:16604:24424 2:N:0:GAACCTAG+TCCGCATA
CTGGCCTATGGTCATCTCAATGCCGAGAGGGATGTGAAGGGCGAGAGAGAGCGACTTCTCTCGGGCCC
ACAGCCTTACAGCTGTGGAGCACGGTATCCTCTGCCGAAACAGAGGTTGGACAAGACCGGAG
+
FFFFFFFFFFFFFFFFFFFFFFFFFFFFFFFFFFFFFFFFFFFFFFFFFFFFFFFFFFFFFFFFFFFF
FFFFFFFFFFFFFFFFFFFFFFFFFFFFFFFFFFFFFFFFFFFFFFF:FFFFFFFFFFFFFF

```
@A00155:342:HHGFNDSXY:1:2261:30092:9142 2:N:0:GAACCTAG+TCCGCATA
CGAGTTGTGTAAGTTTCGTCCTAAAAAGGACTCGTCAGGTAGCCTACTAGACTACGACTTACACGGTA
GGTCGTGAACAAGCCCGGAGTACATCTCCATTGGAACTAGGAGACCCCTCCGGTCTTGTCCAACCTCT
+
FFFFF:FFFFF:FFFFFFFFFFFFFFF:FFFFFFF::FFFFFFFFFFFFFFFFFFFFFFFF,FFFFFF
:FFFFF,FF::F,FF:,FF:FF,FF:FFF,FFFFF:F,FFFFFF:,F:,FF:FFF:F:FF:FFF,,::
@A00155:342:HHGFNDSXY:1:2261:3341:17926 2:N:0:GAACCTAG+TCCGCATA
GGTCTTGTCCAACCTCTGTTTCGGCAGAGGATACCGTGCTCCACAGCTGTAAGGCTGTGGGCCCGAGA
GAAGTCGCTCTCTCTCGCCCTTCACATCCCTCTCGGCATTGAGATGACCATAGGCCAGAG
+
FFFFFFFFFFFFFFFFFF:FFFFFFFFFFFFFFFFFFFFFFFFFFFFFFFFFFFFFFFFFF:FFFFFFF
FFFFFFFFFFFFFFFFFFFFFFFFFFFFFFFFFFFFFF:::FFF:FF:FFFFFFF,FFF:F
@A00155:342:HHGFNDSXY:1:2261:9588:27712 2:N:0:GAACCTAG+TCCGCATA
ACTCTTCGAGTTGTGTAAGTTTCGTCCTAAAAAGGACTCGTCAGGTAGCCTACTAGACTACGACTTAC
ACGGTAGGTCGTGAACAAGCCCGGAGTACATCTCCTTTGGAACTAGGAGACCCCTCCGGTCTTGTCCA
+
FFFFFFFFFF:FFFFFFFFF:FFFFFFFF:FFFFFFFFFFFFFFFF:FFFFFFFFFFFFFFFFFFFFF
FF:FFFFFFFFFFFFFFFFFFFFFFFFFFFFFFFFFFFFFFFFFFFFF:FFFFFFFFFFFFFFFFFFFF
@A00155:342:HHGFNDSXY:1:2262:22715:6104 2:N:0:GAACCTAG+TCCGCATA
TTTCGTCCTAAAAAGGACTCGTCAGGTAGCCTACTAGACTACGACTTACACGGTAGGTCGTGAACAAG
CCCGGAGTACATCTCCTTTGGAACTAGGAGACCCCTCCGGTCTTGTCCAACCTCTGTTTCGGCAGAGG
+
FFFFFFFFFFFFFFFFFFFFFFFFFFFFFFFFFFFFFFFFFFFFFFFFFFFF:FFFFFFFFFFFFFF:
FFFFFFFFFFFFFFFFFFFFFFFFFFFFFFF:FFFFFFFFFFFFFFFFFFFFFFFFFFFFFFFFFFFF
@A00155:342:HHGFNDSXY:1:2262:15962:6872 2:N:0:GAACCTAG+TCCGCATA
TCCTAAAAAGGACTCGTCAGGTAGCCTACTAGACTACGACTTACACGGTAGGTCGTGAACAAGCCCGG
AGTACATCTCCTTTGGAACTAGGAGACCCCTCCGGTCTTGTCCAACCTCTGTTTCGGCAGAGGATACC
+
FFFFFFFFFFFFFFFFFFFFFFFFFFFFFFFFFFFFFFFFFFFFFFFFFFFFF:FFFFFFFFFFFFFF
FFFFFFFFFFFFFFFFFFFFFFFFFFFFFFFFFFFFFFFFFFFFFFFFFFFFFFFFFFFFFFFFF,FF
@A00155:342:HHGFNDSXY:1:2262:9372:31000 2:N:0:GAACCTAG+TCCGCATA
GGACTCGTCAGGTAGCCTACTAGACTACGACTTACACGGTAGGTCGTGAACAAGCCCGGAGTACATCT
CCTTTGGAACCAGGAGACCCCTCCGGTCTTGTCCAACCTCTGTTTCGGCAGAGGATACCGTGCTCCAC
+
FFFFFFFFFFFFFFFFFFFFFF:FFFFFFFFFFFFFFFFFFFFFFFFFFFFFFFFFFFFFFFFFFFFF
FFFFFFFFFFFFFFFFFFFFFFFFFFFFFFFFFFFFFFFFFFFFFF:FFFF:FFFFFFFFFFFFFFFF
@A00155:342:HHGFNDSXY:1:2263:14326:16908 2:N:0:GAACCTAG+TCCGCATA
TCTTCGAGTTGTGTAAGTTTCGTCCTAAAAAGGACTCGTCAGGTAGCCTACTAGACTACGACTTACAC
GGTAGGTCGTGAACAAGCCCGGAGTACATCTCCTTTGGAACTAGGAGACCCCTCCGGTCTTGTCCAAC
+
FFFFFFFFF:FFFFFFFFFFFFFFFFFFFFFFFFFFFFFFFFFFFFFFFFFFFFFFFFFFFFFFFFFF
FFFFFFFFFFFFFFFFFFFFFFFFFF:FFFFFFFFFFFFFFFF,FFFFFFFFFFFFFFFFFFFFFFFFF
@A00155:342:HHGFNDSXY:1:2263:9815:18270 2:N:0:GAACCTAG+TCCGCATA
CAAGGACGAAACCGTCGTTACTCTTCGAGTTGTGTAAGTTTCGTCCTAAAAAGGACTCGTCAGGTAGC
CTACTAGACTACGACTTACACGGTAGGTCGTGAACAAGCCCGGAGTACATCTCCTTTGGAACCAGGAG
+
FFFFFFFFFFFFFFFFFFFFFFFFFFFFFFFFFFFFFFFFFFFFFF:F:FFFFFFFFF:FFFFFFFF
FFFFFFFFFFFFFFFFFFFFFFFFFFFFFFFFFFFFFFFFFFFFFF:F:FFFFFFFFFFFFFFFFFFF
@A00155:342:HHGFNDSXY:1:2263:30563:31313 2:N:0:GAACCTAG+TCCGCATA
GGACTCGTCAGGTAGCCTACTAGACTACGACTTACACGGTAGGTCGTGAACAAGCCCGGAGTACATCT
CCTTTGGAACCAGGAGACCCCTCCGGTCTTGTCCAACCTCTGTTTCGGCAGAGGATACCGTGCTCCAC
+
FFFFFFFFFFFFF:FFFFFFFFFFFFFFFFFFFFFFFFFFFFFFFFFFFFFFFFFFFFFFFFFFFFFF
FFFFFFFFFFFFFFFF:FFFFFFFF::FFFFFFFF:FFFFFF,F:F:FFFFFFFFFFFFFFFFFFFFF
```

```
@A00155:342:HHGFNDSXY:1:2263:30219:31657 2:N:0:GAACCTAG+TCCGCATA
GGACTCGTCAGGTAGCCTACTAGACTACGACTTACACGGTAGGTCGTGAACAAGCCCGGAGTACATCT
CCTTTGGAACCAGGAGACCCCTCCGGTCTTGTCCAACCTCTGTTTCGGCAGAGGATACCGTGCTCCAC
+
FFFFFFFFFFFFFFFFFFFFFFFFFFFFFFFFFFFFFFFFFFFFFFFFFFFFFFFFFFFFFFFFFFFF
FFFFFFFF,FFFFFFFFFFFFFFFFFFFFFFFFFFFFFFFFFFFFFFFFFFFFFFFFFFFFFFFFFFF
@A00155:342:HHGFNDSXY:1:2264:6677:3474 2:N:0:GAACCTAG+TCCGCATA
GGACTCGTCAGGTAGCCTACTAGACTACGACTTACACGGTAGGTCGTGAACAAGCCCGGAGTACATCT
CCTTTGGAACCAGGAGACCCCTCCGGTCTTGTCCAACCTCTGTTTCGGCAGAGGATACCGTGCTCCAC
+
FFFFFFFFFFFFFFFFFFFFFFFFFFFFFFFFFFFFFFFFFFFFFFFFFFFFF:FFFFFFFFFFFFFF
FFFFFFFF:FFFFFFFFFFFFFFFFFFF:FFFFFFF:FFFFFFFFFFFFFFFFF:FFFFFFFFFFFFF
@A00155:342:HHGFNDSXY:1:2264:6488:3677 2:N:0:GAACCTAG+TCCGCATA
GGACTCGTCAGGTAGCCTACTAGACTACGACTTACACGGTAGGTCGTGAACAAGCCCGGAGTACATCT
CCTTTGGAACCAGGAGACCCCTCCGGTCTTGTCCAACCTCTGTTTCGGCAGAGGATACCGTGCTCCAC
+
FFFFFFFFFFFFFFFFFFFFFFFFFFFFFFFFFFFFFFFFFFFFFFFFFFFFF,FFFFFFFFFFFFFF
FFFFFFFF:FFFFFFFFFFFFFFFFFFFFFFFFFFFFFFFFFFFFFFFFFFFFFFFFFFFFFFFFFFF
@A00155:342:HHGFNDSXY:1:2264:22535:18474 2:N:0:GAACCTAG+TCCGCATA
CAAGGACGAAACCGTCGTTACTCTTTGAGTTGTGTAAGTTTCGTCCTAAAAAGGACTCGTCAGGTAGC
CTACTAGACTACGACTTACACGGTAGGTCGTGAACAAGCCCGGAGTACATCTCCTTTGGAACTAGGAG
+
F:FFFFFFFFFFFFFFFFF:FF:,FFFFFFFFFFFFFFF,FFFFFFFFFF:FFFFFFFFFFFFFFFFF
FFFFFFFFFFFFFFFFFFFFFFFFFFFFFFFFF:FFFFFFFFFFFFFFFFFFFFFFFFFFFF,FFFFFFF
@A00155:342:HHGFNDSXY:1:2264:20482:25880 2:N:0:GAACCTAG+TCCGCATA
ATGCCCCGCCACACCGCCTTAGATCAGCTGTGTCCACTTAAGGACTCACCTCTGGCCTATGGTCATCT
CAATGCCGAGAGGGATGTGGAGGGCGAGAGAGAGCGACTTCTCTCGGGCCCACAGCCTTACAGC
+
FFFFFFFFFFFFFFFFFFFFF:FFFFFFFFFFFFFFFFFFFFFFFFFFFFFFFFFFFFF,FFFFFFFFF
FFFFFF:FFF,FFFF,FFFFFFF:FFFFFFFFFFFFFFFFFFFFFFFFFFFFFF,FFFFFFFFFF
@A00155:342:HHGFNDSXY:1:2265:6967:15029 2:N:0:GAACCTAG+TCCGCATA
GAACTAGGAGACCCCTCCGGTCTTGTCCAACCTCTGTTTCGGCAGAGGATACCGTGCTCCACAGCTGT
AAGGCTGTGGGCCCGAGAGAAGTCGCTCTCTCTCGCCCTCCACATCCCTCTCGGCATTGAGATGACCA
+
FFFFFFF:FFFFFFFFFFFFFFFFFFFFFFFFFFFFFFFFFFFFFFFFFFF:FFFF:FFFFFFFFFF
FFFFF:FFFFFFFFFFFFFFFFFFFFFFFFFFFFF:FFFFFFFFFF:FFFFFF:FFFFF,FFFFFFFF
@A00155:342:HHGFNDSXY:1:2265:19633:30671 2:N:0:GAACCTAG+TCCGCATA
GAGACCCCTCCGGTCTTGTCCAACCTCTGTTTCGGCAGAGGATACCGTGCTCCACAGCTGTAAGGCTG
TGGGCCCGAGAGAAGTCGCTCTCTCTCGCCCTCCACATCCCTCTCGGCATTGAGATGACCATAGGCCA
+
FFFFFFFFFFFFFFFFFFFFFFFFFFFFFFFFFFFFFFFFFFFFFFFFFFFFFFFFFFFFFFFFFFFF
FFFFFFFFFFFFFFFFFFFFFFFFFFFFFFFFFFFFFFFFFFFFFFFF:FFFFFFF,FFF:FFFFFF
@A00155:342:HHGFNDSXY:1:2265:7681:32550 2:N:0:GAACCTAG+TCCGCATA
TAGGACGAAACTTACACAACTCGAAGAGTAACGACGGTTTCGTCCTTGGAACGGACTCATCAGGGAGC
CTACTAGACTCCGACCGTCATGGGGGTTCAAACCCATGCCCCGCCACACCGCCTTAGATCAGCTGTGT
+
FFFFFFFFFFFFFFFFFFFFFFFF:FFFFFFFFFFFFFFFFFFFFFFFFFFFFFFFFFFFFFFFFFFF
FFFFFFFFFFFFFFFFFFFFFFFFFFFFFFFFFFFFFFFFFFFFFFFFFFFFFFFFFFFFFFFFF:FF
@A00155:342:HHGFNDSXY:1:2265:25437:35399 2:N:0:GAACCTAG+TCCGCATA
GAACTAGGAGACCCCTCCGGTCTTGTCCAACCTCTGTTTCGGCAGAGGATACCGTGCTCCACAGCTGT
AAGGCTGTGGGCCCGAGAGAAGTCGCTCTCTCTCGCCCTCCACATCCCTCTCGGCATTGAGATGACCA
+
FFFFFFFFFFFFFFFFFFFFFFF::FFFFFFFFFFFFFFFFFFF:FFFFFFFFFFFFFFFFFFFFFFFF
FFFFFFFFFFFFFFFFFFFFFFFFFFFFFFFFFFFFFFFFFFFFF:FFF:FFFFFFFFFFF:FFFFFF:
```

```
@A00155:342:HHGFNDSXY:1:2266:27480:18333 2:N:0:GAACCTAG+TCCGCATA
TCCTAAAAAGGACTCGTCAGGTAGCCTACTAGACTACGACTTACACGGTAGGTCGTGAACAATCCCGG
AGTACATCTCCTTTGGAACTAGGAGACCCCTCCGGTCTTGTCCAACCTCTGTTTCGGCAGAGGATACC
+
F:FFF,FFFFFFFFFFFFFFFFFFFFFF,FF,FFFFFFFFF:FFFFFFFFFFFFFF:FFFFF,FFFFF
FFFFFFFFF:FFFF::FFFFFF:F:F:F,FF:FFF:FFFFFFFFFF:FFFFFFFFFFFFFFFFFFFFF
@A00155:342:HHGFNDSXY:1:2266:18168:29763 2:N:0:GAACCTAG+TCCGCATA
TTTGGAACTAGGAGACCCCTCCGGTCTTGTCCAACCTCTGTTTCGGCAGAGGATACCGTGCTCCACAG
CTGTAAGGCTGTGGGCCCGAGAGAAGTCGCTCTCTCTCGCCCTCCACATCCCTCTCGGCATTGAGATG
+
FFFFFFFFFFFFFFFFFFFFFFFFFFFFFFFFFFFFFFFFFFFFFFFFFFFFFFFFFFFFFFFFFFF:
FFFFFFFFFFFFFFFFFFFFFFFFFFFFFFFFFFFFFFFFFFFFFFFFFFFFFFFFFFFF,:FFFFFF
@A00155:342:HHGFNDSXY:1:2267:25111:3427 2:N:0:GAACCTAG+TCCGCATA
TACACGGTAGGTCGTGAACAAGCCCGGAGTACATCTCCTTTGGAACTAGGAGACCCCTCCGGTCTTGT
CCAACCTCTGTTTCGGCAGAGGATACCGTGCTCCACAGCTGTAAGGCTGTGGGCCCGAGAGAAGTCGC
+
FFFFFFFFFFFFFFFFFFFFFFFFFFFFFFFFFFFFFFFFFFFFFFFFFFFFFFFFFFFFFFFF:FFF
FFFFFFFFFFFFFFFFFFFFFFFFFFFFFFFFFFFFFFFFFFFFFFFFFFFFFFFFFFFFFFFFFFFF
@A00155:342:HHGFNDSXY:1:2267:23909:5666 2:N:0:GAACCTAG+TCCGCATA
TACACGGTAGGTCGTGAACAAGCCCGGAGTACATCTCCTTTGGAACTAGGAGACCCCTCCGGTCTTGT
CCAACCTCTGTTTCGGCAGAGGATACCGTGCTCCACAGCTGTAAGGCTGTGGGCCCGAGAGAAGTCGC
+
FF:FFFFF,F,FF:F,FFFFF:FF::F:F,FF:,F:FFFFF:,FFFFF,,F,FFF:F:FF:F:FFFFF
FFFFFFF:,FF,FFFFFFFFF,F:FFFFF:,F:FF:FFFF,FFFF::FFF:,,FF:FFFFFFF,FFFF
@A00155:342:HHGFNDSXY:1:2267:4065:9502 2:N:0:GAACCTAG+TCCGCATA
GTGAACAAGCCCGGAGTACATTTCCTTTGGAACTAGGAGACCCCTCCGGTCTTGTCCAACCTCTGTTT
CGGCAGAGGATACCGTGCTCCACAGCTGTAAGGCTGTGGGCCCGAGAGAAGTCGCTCTCTCTCGCCCT
+
FFFFFFFFFFFFFFFFFFFFFFFFFFFFFFFFFFFFFFF:FFFFFFFFFFF,FFFFFFFFFFFFFFFF
FFFFFFFFFFFFFFFFFFFFFFFFFFFFFFFFFFFFFFFFFFFFFFF:,:FFFFFFFFFFFFFFFFFF
@A00155:342:HHGFNDSXY:1:2267:2419:10567 2:N:0:GAACCTAG+TCCGCATA
GACCCCTCCGGTCTTGTCCAACCTCTGTTTCGGCAGAGGATACCGTGCTCCACAGCTGTAAGGCTGTG
GGCCCGAGAGAAGTCGCTCTCTCTCGCCCTCTACATCCCTCTCGGCATTGAGATGACCATAGGC
+
FFFFFFFFFFFFFFFFFFFFFFFFFFFFFFFFFFFFFF:FFFFF:FFFFFFFFFFFFFFFFFFFFFFF
FFFFFFFFFFFFFFFFFFFFFFFFFFFFFFFFFFFFFFFFFFFFFFF:FFFFFFFFFFFFFFFF
@A00155:342:HHGFNDSXY:1:2268:28275:9345 2:N:0:GAACCTAG+TCCGCATA
ACTTACACGGTAGGTCGTGAACAAGCCCGGAGTACATCTCCTTTGGAACTAGGAGACCCCTCCGGTCT
TGTCCAACCTCTGTTTCGGCAGAGGATACCGTGCTCCACAGCTGTAAGGCTGTGGGCCCGAGAGAAGT
+
FFFFFF:FFFFFFFFFFFFFFFFFFFFFFFFFFFFFFFFFFFFFFFFFFFFFFFFFFFFFFFFFFFFF
FF:FFFFFFFFFFF:FFFFFF:FFFFFFFFFFFFFFFFFFFFFFFFFFFFFFFFFFFFFFFFFF:FFF
@A00155:342:HHGFNDSXY:1:2269:5683:4883 2:N:0:GAACCTAG+TCCGCATA
CCTACTAGACTACGACTTACACGGTAGGTCGTGAACAAGCCCGGAGTACATCTCCTTTGGAACTAGGA
GACCCCTCCGGTCTTGTCCAACCTCTGTTTCGGCAGAGGATACCGTGCTCCACAGCTGTAAGGCTGTG
+
FFFFFFFF:FFFFFFFFFFFFFFFFFFFFFFFF:FFFF:FFFFFFFFFFFFFFFFFFFFFFFFFFFFF
FFFFFFFFFFFFFFFFFFFFFFFFFFFFFFFFFFFF:FFFFFFFFFFFFFFFFFFFFFFFFFFFFFFF
@A00155:342:HHGFNDSXY:1:2269:8359:16063 2:N:0:GAACCTAG+TCCGCATA
GGACTCGTCAGGTAGCCTACTAGACTACGACTTACACGGTAGGTCGTGAACAAGCCCGGAGTACATCT
CCTTTGGAACTAGGAGACCCCTCCGGTCTTGTCCAACCTCTGTTTCGGCAGAGGATACCGTGCTCCAC
+
FFFFFFFFFFFFFFFFFFFFFFFFFFFFFFFFFFFFFFFFFFFFFFFFFFFFFFFFFFFFFFFFFFFF
FFFFFFFFFFFFFFFFFFFFFFFFFFFFFFFFFFFFFFFFFFFFFFFFFFFFFFFFFFFFFFFFFFFF
```

@A00155:342:HHGFNDSXY:1:2269:16622:32784 2:N:0:GAACCTAG+TCCGCATA
AGTACATCTCCTTTGGAACTAGGAGACCCCTCCGGTCTTGTCCAACCTCTGTTTCGGCAGAGGATACC
GTGCTCCACAGCTGTAAGGCTGTGGGCCCGAGAGAAGTCGCTCTCTCTCGCCCTCCACATCCCTCTCG
+
FFFFFFFFFFFFFFFFFFFFFFFFFFFFFFFFFFFFFFFFFFFFFFFFFFFF:FFFFFFFFFFFFFFFFF
FFFFFFFFFFFFFFFFFFFF:FFFFFFFFFFFFFF,FFFFFFFFFFFFFFFFFFFFFFFFFFFFFFF:FF
@A00155:342:HHGFNDSXY:1:2269:17526:37012 2:N:0:GAACCTAG+TCCGCATA
CCTACTAGACTACGACTTACACGGTAGGTCGTGAACAAGCCCGGAGTACATCTCCTTTGGAACTAGGA
GACCCCTCCGGTCTTGTCCAACCTCTGTTTCGGCAGAGGATACCGTGCTCCACAGCTGTAAGGCTGTG
+
FFFFFFFF:FFFFFFFFFFFFFFFFFFFFFFFFFFFFF,FFFFFFFFFFFFFFFFFFFFFFFFFFFFFFF
FFFFFFFFFFFFF:FFFFFFFFFFFFFF:FFFFFFFFFFFFFFFFFF:FFFFFFFFFFFFFFFFFFFFFF
@A00155:342:HHGFNDSXY:1:2270:25418:14669 2:N:0:GAACCTAG+TCCGCATA
CGGAGTACATCTCCTTTGGAACTAGGAGACCCCTCCGGTCTTGTCCAACCTCTGTTTCGGCAGAGGAT
ACCGTGCTCCACAGCTGTAAGGCTGTGGGCCCGAGAGAAGTCGCTCTCTCTCGCCCTCTACATCCCTC
+
FFFFFFFFFFFFFFFFFFFFFFFFFFFFFFFFFFFFFFFFFFFF:FFFFFFFFFFFFFFFFFFFFFFFFF
FFFFFFFFFFFFFFFFFFFFFFFFFFFFFFFFFFFFFFFFFFFFFFFFFFFFFFFFFFFFFFFFFFFFFF
@A00155:342:HHGFNDSXY:1:2270:30400:15154 2:N:0:GAACCTAG+TCCGCATA
ACCTCTGTTTCGGCAGAGGATACCGTGCTCCACAGCTGTAAGGCTGTGGGCCCGAGAGAAGTCGCTCT
CTCTCGCCCTTCACATCCCTCTCGGCATTGAGATGACCATAGGCCAGAGGTGAGTCCTTAAGTGGACA
+
FFFFFFFFFFFFFFFFFFFFFFFFFFFFFFFFFFFFFFFFFFFFFFFFFFFFFFFFFFFFFFFFFFF,FF
FFFFFFFFFFFFFFFFFFFFFFFFFFFFF:FFFFFFFFFFFFFFFFFFFFFFFFFFFFFFFFFFFF:FF:
@A00155:342:HHGFNDSXY:1:2270:30418:15374 2:N:0:GAACCTAG+TCCGCATA
ACCTCTGTTTCGGCAGAGGATACCGTGCTCCACAGCTGTAAGGCTGTGGGCCCGAGAGAAGTCGCTCT
CTCTCGCCCTTCACATCCCTCTCGGCATTGAGATGACCATAGGCCAGAGGTGAGTCCTTAAGTGGACA
+
FFFFFFFFFFFFFFFFFFFFFFFFFFFFFFFFFFFFFFFFFFFFFFFFFFFFFFFFFFFFFFFFFFFFFF
FFFFFFFFFFFFFFFFFFFFFFFFFFFF:FFFFFFFFFFFFFFFFFFFFFFFFFFFFFFFFFFFFFFFF:
@A00155:342:HHGFNDSXY:1:2270:10303:15765 2:N:0:GAACCTAG+TCCGCATA
AAACTTACACAACTCGAAGAGTAACGACGGTTTCGTCCTTGGAACGGACTCATCAGGGAGCCTACTAG
ACTCCGACCGTCATGGGGGTTCAAACCCATGCCCCGCTACACCGCCTTAGATCAGCTGTGTCCACTTA
+
FFFFFFFFFFFFFFFFFFFFFFFFFFFF:FFFFFFFFFFFFFFFFFFFFFFFFFFFFFFFFFFFFFFFFF
FFFFFFFFFFFFFFFFFFFFFFFFFFFFFFFFFFFFFFFFFFFFFFFFFFFFFFFFFFFFFFFFFFFFFF
@A00155:342:HHGFNDSXY:1:2271:10520:9721 2:N:0:GAACCTAG+TCCGCATA
ACGAAACCGTCGTTACTCTTCGAGTTGTGTAAGTTTCGTCCTAAAAAGGACTCGTCAGGTAGCCTACT
AGACTACGACTTACACGGTAGGTCGTGAACAAGCCCGGAGTACATCTCCTTTGGAACTAGGAGACCCC
+
F:FFFFFFFFFFFFFFFF,F:FFFFFFFFFFFFFFF:FFFF,FFFF,:FFFFFF:FFFFFF:FFFFFFFF
:F:FFFFFFFFFFFFFFFF:FF,FFFFFFFF:FFFFFF:FFFFFFFFFFFFFFFFFFFFFFFFFFFFFFF
@A00155:342:HHGFNDSXY:1:2271:27109:11898 2:N:0:GAACCTAG+TCCGCATA
ACTCTTCGAGTTGTGTAAGTTTCGTCCTAAAAAGGACTCGTCAGGTAGCCTACTAGACTACGACTTAC
ACGGTAGGTCGTGAACAAGCCCGGAGTACATCTCCTTTGGAACTAGGAGACCCCTCCGGTCTTGTCCA
+
FFFFFFFFFFFFFFFFFFFFFFFFFFFFFFFFFFFFFFFFFFFFFFFFFFFFFFFFFFFFFFFFFFFFFF
:FFFFFFFFFFFFFFFFF:FFFFFFFFFFFFFFFFFFFFFFFFFFFFFFFFFFFFFFFFFFFFFFFFFFF
@A00155:342:HHGFNDSXY:1:2271:27163:13338 2:N:0:GAACCTAG+TCCGCATA
ACTCTTCGAGTTGTGTAAGTTTCGTCCTAAAAAGGACTCGTCAGGTAGCCTACTAGACTACGACTTAC
ACGGTAGGTCGTGAACAAGCCCGGAGTACATCTCCTTTGGAACTAGGAGACCCCTCCGGTCTTGTCCA
+
FFFFFFFFFFFFFFFFFFFFFFFFFFFFFFFFFFFFFFFFFFFF:FFFFFFFFFFFF:FFFFFFFFFFFF
FFFFFFFFFFFFFFFFFF:F:FFFFFF:FFFFFFFFFFFFF:F:FFFFFFFFF:FFFF,FFFFFFFFFFF

```
@A00155:342:HHGFNDSXY:1:2271:31720:15374 2:N:0:GAACCTAG+TCCGCATA
GGACTCGTCAGGTAGCCTACTAGACTACGACTTACACGGTAGGTCGTGAACAAGCCCGGAGTACATCT
CCTTTGGAACTAGGAGACCCCTCCGGTCTTGTCCAACCTCTGTTTCGGCAGAGGATACCGTGCTCCAC
+
FFFFFFFFF:FFFFFFFFFFFFFFFFFFFFFFFFFFFFFFFFFFFFFFFFFFFFFFFFFFFF:FFFFF
FFFFFFFFFFFFFFF:F:FFFFFFFF,FFFFFFFF::FFFFFFFFFFFFFFFFFFFFF:FFFFFFFFF
@A00155:342:HHGFNDSXY:1:2271:5213:32221 2:N:0:GAACCTAG+TCCGCATA
GTTTCGTCCTAAAAAGGACTCGTCAGGTAGCCTACTAGACTACGACTTACACGGTAGGTCGTGAACAA
GCCCGGAGTACATCTCCTTTGGAACTAGGAGACCCCTCCGGTCTTGTCCAACCTCTGTTTCGGCAGAG
+
FFFFFFFFFFFFFFFFFFFFFFFFFFFFFFFFFFFFFFFFFFFFFFFFFFFFFFFFFFFFFFFFFFFF
FFFFFFFFFFFFFFFFFFFFFFFF:FFFFFFFFFFFFFFFFFFFFFFFFFFFFFFFFFFFFFFFFF:F
@A00155:342:HHGFNDSXY:1:2272:18041:3959 2:N:0:GAACCTAG+TCCGCATA
ACTAGACTACGACTTACACGGTAGGTCGTGAACAAGCCCGGAGTACATCTCCTTTGGAACTAGGAGAC
CCCTCCGGTCTTGTCCAACCTCTGTTTCGGCAGAGGATACCGTGCTCCACAGCTGTAAGGCTGTGGGC
+
FFFFFFFFFFFFFFFFFFFFFFFFFFFFFFFFFFFFFFFFFFFFFFFFFFFFFFFFFFFFFFFFFFFF
FFFFFFFFFFFFF:FFF:FFFFFFFFFFFFFFFFFFF:FFFFFFFFFFFFFFFFFFFFFFFFFFFFFF
@A00155:342:HHGFNDSXY:1:2272:29767:15499 2:N:0:GAACCTAG+TCCGCATA
ACTTACACGGTAGGTCGTGAACAAGCCCGGAGTACATCTCCTTTGGAACTAGGAGACCCCTCCGGTCT
TGTCCAACCTCTGTTTCGGCAGAGGATACCGTGCTCCACAGCTGTAAGGCTGTGGGCCCGAGAGAAGT
+
FFFFFFFFFFF:FFFFFFFFFFFFFFFFFFFFFFFFF:FFFFF:FFFFFF:FFFFFFFFFFFFFFFF:
FFFFFFFFFFFFFF::FFFFFFFFFF,,FF:FFFFFFFFFFF:FFFFFFF:FFFFFFFFFFFFFFFF:
@A00155:342:HHGFNDSXY:1:2272:14145:28745 2:N:0:GAACCTAG+TCCGCATA
GAGACCCCTCCGGTCTTGTCCAACCTCTGTTTCGGCAGAGGATACCGTGCTCCACAGCTGTAAGGCTG
TGGGCCCGAGAGAAGTCGCTCTCTCTCGCCCTCCACATCCCTCTCGGCATTGAGATGAC
+
FFFFFFFFFFFFFFFFFFFFFFFFFFFFFFFFFFFFFFFFFFFFFFFFFFFFFFFFFFFFFFFFFFFF
FFFFFFFFFFFFFFFFFFFFFFFFFFFFFFFFFFFFFFFFFFFFFFFFFF::FFFFFFFFF
@A00155:342:HHGFNDSXY:1:2272:11831:31532 2:N:0:GAACCTAG+TCCGCATA
TCTTCGAGTTGTGTAAGTTTCGTCCTAAAAAGGACTCGTCAGGTAGCCTACTAGACTACGACTTACAC
GGTAGGTCGTGAACAAGCCCGGAGTACATCTCCTTTGGAACTAGGAGACCCCTCCGGTCTTGTCCAAC
+
FFFFFFFFFFFFFFFFFFFFFFFFF:FFFF:FFFFFFFFFFFFFFFFFFFFFFF:FFFFFFFFFFFFF
FFFFFFF:FFFFFFFF,FFFFFFFFFFFFFFFFFF:FFFFFFFFFFFFF:FFFFF::FFFFFFFFFF
@A00155:342:HHGFNDSXY:1:2272:4942:35133 2:N:0:GAACCTAG+TCCGCATA
GAACGGACTCATCAGGGAGCCTACTAGACTCCGACCGTCATGGGGGTTCAAACCCATGCCCCACCACA
CCGCCTTAGATCAGCTGTGTCCACTTAAGGACTCACCTCTGGCCTATGGTCATCTCAATGCCGAGAGG
+
FFFFFFFFFFFFFFFFFFFFFFFFFFFFFFFFFFFFFFFFFFFFFFFFFFFFFFFFFFFFFFFFFFFF
FFFFFFFFF:FFFFFFFFFFFFFFFFFFFFFFFFF,FFFFFFFFFFFFFFFFFFFFFFFFFFF,FFF:FF
@A00155:342:HHGFNDSXY:1:2272:18484:36824 2:N:0:GAACCTAG+TCCGCATA
GGAGTACATCTCCTTTGGAACCAGGAGACCCCTCCGGTCTTGTCCAACCTCTGTTTCGGCAGAGGATA
CCGTGCTCCACAGCTGTAAGGCTGTGGGCCCGAGAGAAGTCGCTCTCTCTCGCCCTCCACATCCCTCT
+
FFFFFFFF:FFFFFFFFFFFFFFFFF:FFFFFFFFFFFF:FFFFFFFFFFFFFFFFFFFFFFFFFFFF
FFFFFFFFFFFFFFFFFFFFFFFFFFFFFFFFFFFFFFFFFFFFFFFFFFFFFFFFFFFFFFFFFFFF
@A00155:342:HHGFNDSXY:1:2273:5222:4899 2:N:0:GAACCTAG+TCCGCATA
GTGAACAAGCCCGGAGTACATCTCCTTTGGAACTAGGAGACCCCTCCGGTCTTGTCCAACCTCTGTTT
CGGCAGAGGATACCGTGCTCCACAGCTGTAAGGCTGTGGGCCCGAGAGAAGTCGCTCTCTCTCGCCCT
+
FFFFFFFFFFFFFFFFFFFFFF:FFFFFFFFFFFFFFFFFFFFFFFFFFFFFFFFFFFFFFFFFFFFF
FFFFFFFFFFFFFFFFFFFFFFFF:FFFFFFFFFF:FFFFFFFFFFFFFFFFFFFFFFFFFFFFFFFFF
```

@A00155:342:HHGFNDSXY:1:2273:19298:8829 2:N:0:GAACCTAG+TCCGCATA
GTACATCTCCTTTGGAACTAGGAGACCCCTCCGGTCTTGTCCAACCTCTGTTTCGGCAGAGGATACCG
TGCTCCACAGCTGTAAGGCTGTGGGCCCGAGAGAAGTCGCTCTCTCTCGCCCTCCACATCCCTCTCGG
+
FFFFFFFFFFFFFFFFFFFFFFFFFFFFFFFFFFFFFFFFFFFFFFFFFFFFFFFFFFFFFFFFFFFF
FFFFFFFFFFFFFFFFFFFFFFFFFFFFFFFFFFFFFFFFFFFFFFFFFFFFFFFFFFFFFFFFFFFF
@A00155:342:HHGFNDSXY:1:2273:19407:11083 2:N:0:GAACCTAG+TCCGCATA
GAGTACATCTCCTTTGGAACTAGGAGACCCCTCCGGTCTTGTCCAACCTCTGTTTCGGCAGAGGATAC
CGTGCTCCACAGCTGTAAGGCTGTGGGCCCGAGAGAAGTCGCTCTCTCTCGCCCTCCACATCCCTCTC
+
FFFFFFFFFFFFF:FFFFFFFFFFFFFFFFFFFFFFF:F:,FFFFFFFFFFFFFFFFFFFFFFFFF:FF
FFFFFFFFFFFFFFF:FFFFFFFFFFFFFFFFFFFFFFFFFFFFFF:FFF:FFFFFFFFFFFFFFFFFFF
@A00155:342:HHGFNDSXY:1:2273:21106:21104 2:N:0:GAACCTAG+TCCGCATA
GTCTTGTCCAACCTCTGTTTCGGCAGAGGATACCGTGCTCCACAGCTGTAAGGCTGTGGGCCCGAGAG
AAGTCGCTCTCTCTCGCCCTCCACATCCCTCTCGGCATTGAGATGACCATAGGCCAGAG
+
FFFFFFFFFFFFFFFFFFFFFFFFFFFFFFFFFFFFFFFFFFFFFF:FFFFFFFFFFFFFFFFFFFFF
F:FFFFFFFFFFFFFFFFFFFFFFFF:FFFFFFF:FFFFFFFFFFFFFF:F,FFFFFFFF
@A00155:342:HHGFNDSXY:1:2273:29432:31391 2:N:0:GAACCTAG+TCCGCATA
AAAAGGACTCGTCAGGTAGCCTACTAGACTACGACTTACACGGTAGGTCGTGAACAAGCCCGGAGTAC
ATCTCCTTTGGAACTAGGAGACCCCTCCGGTCTTGTCCAACCTCTGTTTCGGCAGAGGATACCGTGCT
+
FFFFFFFFFFFFFFFFFFFFFFFFFFFFFFFFFFFFFFFFFFFFFFFFFFFFFFFFFFFFFFF:FFFFF
FFFFFFFF:FFF:FFFFFFFFFFFFFFFFF:FF:FFFFFFFFFFFFFFFFFFFFFFFFFFFFFFF:FFF
@A00155:342:HHGFNDSXY:1:2274:19605:17691 2:N:0:GAACCTAG+TCCGCATA
TCTGGCCTATGGTCATCTCAATGCCGAGAGGGATGTGGAGGGCGAGAGAGAGCGACTTCTCTCGGGCC
CACAGCCTTACAGCTGTGGAGCACGGTATCCTCTGCCGAAACAGAGGTTGGACAAGACCGCAG
+
FFFFFFFFFFFFFFFFFFFFFFFFFFFFFFFFFF:FFFFFFFFFFFFFFFFFFFFFFFFFFFFFFFFFFF
FFFFFFF,FFFFFFFFFFFFFFFFFFFFFFFFFFFFFFF:FFFFFFFFFFFFFFFFFFFFFFFFF
@A00155:342:HHGFNDSXY:1:2275:18502:33098 2:N:0:GAACCTAG+TCCGCATA
GGACTCGTCAGATAGCCTACTAGACTACGACTTACACGGTAGGTCGTGAACAAGCCCGGAGTACATCT
CCTTTGGAACTAGGAGACCCCTCCGGTCTTGTCCAACCTCTGTTTCGGCAGAGGATACCGTGCTCCAC
+
FFFFFFFFFFFFFFFFFFFFFFFFFFFFFFFFFFFFFFFFFFFFFFFFFFFF:FFFFFFFFFFF:FF
FFFFFFFFFFFFFFFFFFFFFFFFFFFFFFFFFFFFFFFFFFFFFFFFFFFFFFFFFFFFFFFFFFFF
@A00155:342:HHGFNDSXY:1:2276:16839:3317 2:N:0:GAACCTAG+TCCGCATA
ACTCTTCGAGTTGTGTAAGTTTCGTCCTAAAAAGGACTCGTCAGGTAGCCTACTAGACTACGACTTAC
ACGGTAGGTCGTGAACAAGCCCGGAGTACATCTCCTTTGGAACTAGGAGACCCCTCCGGTCTTGTCCA
+
FFFFFFFFFFFFFFFFFFFFFFFFFFFFFFFFFFFFFFFFFFFFFFFFFFFFFFFFFFFFFFFFFFFF
FFFFFFFFFFFFFFFFFF:FFFFFFFFFFFFFFFFFFFFFFFFFFFFFFFFFFFFFFFFFFFFFFFFF
@A00155:342:HHGFNDSXY:1:2276:28438:9721 2:N:0:GAACCTAG+TCCGCATA
AGCCTACTAGACTACGACTTACACGGTAGGTCGTGAACAAGCCCGGAGTACATCTCCTTTGGAACTAG
GAGACCCCTCCGGTCTTGTCCAACCTCTGTTTCGGCAGAGGATACCGTGCTCCACAGCTGTAAGGCTG
+
FFFFFFFFFFFFFFFFFFFFFFFFFFFFFFFFFFFFFFFFFFFFFFFFFFFFFFFFFFFFFFFFFF:FF
FFFFFFFFFFFFFFFFFFF:FFFFFFFFFFF:FFFFFFFFFFFFFFFFFFFFFFFFFFFFFFFFFFFFF
@A00155:342:HHGFNDSXY:1:2276:18249:27680 2:N:0:GAACCTAG+TCCGCATA
AAAAGGACTCGTCAGGTAGCCTACTAGACTACGACTTACACGGTAGGTCGTGAACAAGCCCGGAGTAC
ATCTCCTTTGGAACCAGGAGACCCCTCCGGTCTTGTCCAACCTCTGTTTCGGCAGAGGATACCGTGCT
+
FFFFFFFFFFFFFFFFFF:FFFFF,FFFFFFFFFFFFFFFFFFFFFFFFFFFFFFFFF:FFFFFF:FF
FFF:FF:FFFFF:FFFFFFFFFFFFFFFFFFFFFFFFFFFFFFFFF:FF,FFFFFFFFFFF:FFFFFFFF

@A00155:342:HHGFNDSXY:1:2276:20148:33724 2:N:0:GAACCTAG+TCCGCATA
GGTCGTGAACAAGCCCGGAGTACATCTCCTTTGGAACTAGGAGACCCCTCCGGTCTTGTCCAACCTCT
GTTTCGGCAGAGGATACCGTGCTCCACAGCTGTAAGGCTGTGGGCCCGAGAGAAGTCGCTCTCTCTCG
+
FFFFFFFFFFFFFFFFFFFFFFFFFFFFFFFFFFFFFFFFFFFFFFFFFFFFFFFFFFFFFFFFFFFF
FFFFFFFFFFFFFFFFFFFFFFFFFFFFFFFFFFFFFFFFFFFFFFFFFFFFFFFFFFFFFFFFFFFF
@A00155:342:HHGFNDSXY:1:2277:25590:4038 2:N:0:GAACCTAG+TCCGCATA
AAAAGGACTCGTCAGGTAGCCTACTAGACTACGACTTACACGGTAGGTCGTGAACAAGCCCGGAGTAC
ATCTCCTTTGGAACTAGGAGACCCCTCCGGTCTTGTCCAACCTCTGTTTCGGCAGAGGATACCGTGCT
+
FFFFFFFFFFFFFFFFFFFFFFFFFFFFFFFFFFFFFFFFFFFFFFFFFFFFFFFFFFFFFFFFFFFF
FFFFFFFFFFFFFFFFFFFFFFFFFFFFFFFFFFFFFFFFFFFFFFFFFFFFFFFFFFFFFFFFFFFF
@A00155:342:HHGFNDSXY:1:2277:3468:10441 2:N:0:GAACCTAG+TCCGCATA
GTGAACAAGCCCGGAGTACATCTCCTTTGGAACCAGGAGACCCCTCCGGTCTTGTCCAACCTCTGTTT
CGGCAGAGGATACCGTGCTCCACAGCTGTAAGGCTGTGGGCCCGAGAGAAGTCGCTCTCTCTCGCCCT
+
F:FFFFFF:FFFFFFFFFFFFFFFFFFFFFFFFFFFFFFFFFFFFFFF:FFFFFFFFFFF:FFFFFFF
FFFFFFFFFFFFFFFFFFFFFFFFFFFF:FFFFFFFFFFFFFF:FFFFFF:FFFFFFFFFFFF,FFFF
@A00155:342:HHGFNDSXY:1:2277:2889:14450 2:N:0:GAACCTAG+TCCGCATA
AGTACATCTCCTTTGGAACTAGGAGACCCCTCCGGTCTTGTCCAACCTCTGTTTCGGCAGAGGATACC
GTGCTCCACAGCTGTAAGGCTGTGGGCCCGAGAGAAGTCGCTCTCTCTCGCCCTCTACATCCCTCTCG
+
FFFFFFFFFFFFFFFFFFFFFFFFFFFFFFFFFFFFFFFFFFF:FFFFFFFFFFFFFFFFFFFFFFFF
FFFFFFFFFFFFFFFFFFFFFFFFFFFFFF:FFFFFFFFFFFFFFFFFFFFFFFFFFFFFFFFFFFFF
@A00155:342:HHGFNDSXY:1:2278:5556:4539 2:N:0:GAACCTAG+TCCGCATA
ACTAGGAGACCCCTCCGGTCTTGTCCAACCTCTGTTTCGGCAGAGGATACCGTGCTCCACAGCTGTAA
GGCTGTGGGCCCGAGAGAAGTCGCTCTCTCTCGCCCTCCACATCCCTCTCGGCATTGAGATGACCAT
+
FFFFFFFFFFFFFF,FFFFFF,FFFFFFFFFFFFFFFFFFFFFFFFFFFFFFFFFFFFFFFFFFFFFF:
FFFFFFFFFF,F:FFFFFFFFFFFFFFFF:FFFFFFFFFFFFFFFFFFFFFFFFFFFFFFFFFFFFF
@A00155:342:HHGFNDSXY:1:2278:27199:15718 2:N:0:GAACCTAG+TCCGCATA
TACTCTTCGAGTTGTGTAAGTTTCGTCCTAAAAAGGACTCGTCAGGTAGCCTACTAGACTACGACTTA
CACGGTAGGTCGTGAACAAGCCCGGAGTACATCTCCTTTGGAACCAGGAGACCCCTCCGGTCTTGTCC
+
,FFFF,FFFFFFFFFFFFFFF:FFFFFFFFFFFFFFFFFFFFFFFFFFFFFFFFFFFFFFFFFFFFFF
FFFFFFF:FFF:FFFFFFF,FFFFFFFFFFFFFFFFFF,FFFFFFFFFFFFFFFF,FFFFFF:FFFF:
@A00155:342:HHGFNDSXY:1:2278:15673:17613 2:N:0:GAACCTAG+TCCGCATA
GTTTCGTCCTAAAAAGGACTCGTCAGGTAGCCTACTAGACTACGACTTACACGGTAGGTCGTAAACAA
GCCCGGAGTACATCTCCTTTGGAACTAGGAGACCCCTCCGGTCTTGTCCAACCTCTGTTTCGGCAGAG
+
FFFFFFFFFFFFFFFFFFFFFFFFFFFFFFFFFFFFFFFFFFFFFFFFFFFFFFFFFFFFFFFF:FFF
:FFFFFFFFFFFFFFFFFFFFFFFFFFFFF:FFFFFFFFFFFFFFFFFFFFFFFFFFFFFFFFFFFFF
@A00155:342:HHGFNDSXY:1:2278:20500:22404 2:N:0:GAACCTAG+TCCGCATA
CGAGTTGTGTAAGTTTCGTCCTAAAAAGGACTCGTCAGGTAGCCTACTAGACTACGACTTACACGGTA
GGTCGTGAACAAGCCCGGAGTACATCTCCTTTGGAACTAGGAGACCCCTCCGGTCTTGTCCAACCTCT
+
FFFFFFFFFFFFFFFF:FFFF,FFFFFFFFFFFFFFFFFFFFFFFF::F,FF,FFFFF,FFFFFFFFF
FFFF:FFFFFFF,FFFFFF:FFF:::FFF,FFFFFFFFFFFF:FF:FF,FFFFFFFFFFFFFF:F,FF
@A00155:342:HHGFNDSXY:1:2301:28085:1689 2:N:0:GAACCTAG+TCCGCATA
TGTCCACTTAAGGACTCACCTCTGGCCTATGGTCATCTCAATGCCGAGAGGGATGTAGAGGGCGAGAG
AGAGCGACTTCTCTCGGGCCCACAGCCTTACAGCTGTGGAGCACGGTATCCTCTGCCGAAACAGAGGT
+
FF:FFFFFFFFF:FFFFFFFFFFFFFFFFFFFFFFFFFFFFFFF:FFFFFFFF,FFFFFFF:FFFFFF
FFFFFFFFF:FFFFFFFFFFFFFFFFFFFFFFF:FF:FFFFFFFFFF:FFFFFFFFFFFFFFFFFFFF

```
@A00155:342:HHGFNDSXY:1:2301:26910:15749 2:N:0:GAACCTAG+TCCGCATA
ACTTACACGGTAGGTCGTGAACAAGCCCGGAGTACATCTCCTTTGGAACCAGGAGACCCCTCCGGTCT
TGTCCAACCTCTGTTTCGGCAGAGGATACCGTGCTCCACAGCTGTAAGGCTGTGGGCCCGAGAGAAGT
+
FFFFFF:FFFFFFF:FFFFFFFFFFFFFFFFFFFFFFFFFFFFFFFFFFFFFFFFFFFFFFFFFFFF
FFFFFFFFFFFFFFFFFFFFFFFFFFFFFFFFFFFFFFFFFFFFFFFFFFF:FFFFFFFFFFFFFFFF
@A00155:342:HHGFNDSXY:1:2301:20374:25285 2:N:0:GAACCTAG+TCCGCATA
```
[truncated: 393,679 more chars]
