# Supplementary material for: First Detection and Molecular Characterization of Apple Stem Grooving Virus, Apple Chlorotic Leaf Spot Virus, and Apple Hammerhead Viroid in Loquat in Spain
Source: Plants (Basel). 2021 Oct 25;10(11):2293. doi: 10.3390/plants10112293 (PMC8624106; doi:10.3390/plants10112293)
Supplement: Supplementary file 1 [file Mapping_reads_ACLSV.pdf › Mapping_reads_ACLSV.pdf]

@A00155:342:HHGFNDSXY:1:2220:26612:35399 1:N:0:GAACCTAG+TCCGCATA
TCCGATCTAGTATACACTCACGTCGTGAGTAAACAGATTGACGTAACGCCTCAATCGTGGTCAAGGAC
CAGCATTCAATGCTACCACATTAAATACAATCACATTGTATTTCAGAACTTCTTGTGTCCGCACATAG
AAGGTTAAAGATGGC
+
FF:FFFFFFFFFFFFFFFFFFFFFFFFFFFFFFFFFFFFFFFFFFFFFFFFFFFFFFFFFFFFF:FFF
FFFFFFFFFFFFFFFFFFFFFFFFFFFFFFFFFFF:FFFFFFFFFFFFFFFF:FFFFFFFFFFFFFFF
FFFFFFFFFFFFFFF
@A00155:342:HHGFNDSXY:1:2419:31177:16595 1:N:0:GAACCTAG+TCCGCATA
TTCCGATCTGTATACACTCACGTCGTGAGTAAACAGATTGACGTAACGCCTCAATCGTGGTCAAGGAC
CAGCATTCAATGCTACCACATTAAATACAATCACATTGTATTTCAGAACTTCTTGTGTCCGCACATAG
AAGGTTAAAGATGGC
+
FFFFFFFFFFFFFFFFFFFFFFFFFFFFFFFFFFFFF:FFFFFFF:FFFFFFFFFFFFFFFFFFFFFF
FFFFFFFFFFFFF,FFFFFFFFFFFFFFF:FF:FFFFFFFFFFFFFFFFFFF:FFFFFFFFFFFFFFF
FFFFFFFFFFFFFFF
@A00155:342:HHGFNDSXY:1:2674:13856:14685 1:N:0:GAACCTAG+TCCGCATA
TCCGATCTAGTATACACTCACGTCGTGAGTAAACAGATTGACGTAACGCCTCAATCGTGGTCAAGGAC
CAGCATTCAATGCTACCACATTAAATACAATCACATTGTATTTCAGAACTTCTTGTGTCCGCACATAG
AAGGTTAAAGATGGC
+
FFFFFFFFFFFF:FFFFFFFFFFFFFFFFFFFFFFFFFFFFFFFFFFFFFFF:FFFFFFFFFFFFFFF
FFFFFFFFFFFFFFFFFFFFFFFFFFFFF:FFFFFFFFFFFFFFFFFFFFFFFFFFFFFFFFFFFFFF
FFFFFFFFFFFFFFF
@A00155:342:HHGFNDSXY:1:1374:8947:21746 2:N:0:GAACCTAG+TCCGCATA
AGTATACACTCACGTCGTGAGTAAACAGATTGACGTAACGCCTCAATCGTGGTCAAGGACCAGCATTC
AATGCTACCACATTAAATACAATCACATTGTATTTCAGAACTTCTTGTGTCCGCACATAGAAGGTTAA
AGATGGCTTTCTCTT
+
F:FFFFFFFFFFFFFFFFFFFFFF:FFF:FFFFFFFFFFFFFFFFFFFFFFFFFFFFFFFFFFFFFFF
FFFFFFFFFFFFFFF:FFFFFFFFFFFFFFFFFFFFFFFFFFFFFFFFFFFFFFFFFFF,FFFFFFFF
FFFFFFFFFF:FFFF
@A00155:342:HHGFNDSXY:1:2577:10737:3427 2:N:0:GAACCTAG+TCCGCATA
AGTATACACTCACGTCGTGAGTAAACAGATTGACGTAACGCCTCAATCGTGGTCAAGGACCAGCATTC
AATGCTACTACATTAAATACAATCACATTGTATTTCAGAACTTCTTGTGTCCGCACATAGAAGGTTAA
AGATGGCTTTCTCTT
+
FFFFFFFFFFFFFFFFFFFFFFFF:FFFF,FF:FFFFFFFFFFFFFFFFFFFFFFFFFFFFFFFFFFF
F:FFFFFFFFFFFFFFFFFFFFFFF,F,FFFF,FFFFFFFFFFFFFFFFFFFFFFFFFFFFFFF:FFF
FFFFF:FFFF:FFFF
@A00155:342:HHGFNDSXY:1:2631:28845:31751 2:N:0:GAACCTAG+TCCGCATA
AGTATACACTCACGTCGTGAGTAAACAGATTGACGTAACGCCTCAATCGTGGTCAAGGACCAGCATTC
AATGCTACCACATTAAATACAATCACATTGTATTTCAGAACTTCTTGTGTCCGCACATAGAAGGTTAA
AGATGGCTTTCTCTT
+
FFFFFFFFFFFFFFFFFFFFFFFFFFFFFFFFFFFFFFFFFFFFFFFFF:FFFFFFFFFFF:FFFFFF
FFFFFFFFFFFFFFFFFFFFFFFFFFF:FFFFFFFFFFFFFFFFF,FFFFFFFFFFFFFFF:FFFFF:
FF::FFFFFFFFFFF
@A00155:342:HHGFNDSXY:1:1135:19180:29575 2:N:0:GAACCTAG+TCCGCATA
GTATACACTCACGTCGTGAGTAAACAGATTGACGTAACGCCTCAATCGTGGTCAAGGACCAGCATTCA
ATGCTACCACATTAAATACAATCACATTGTATTTCAGAACTTCTTGTGTCCGCACATAGAAGGTTAAA
GATGGCTTTCTCTTA
+
FFFFFFFFFFFFFFFF:FFFFFFFFFFFFFFFFFFFFF:FFFFFFF,FF,FFFFF,:FFFF:FFFFFF

```
FFFFFFFFFFFFFFFFFFFFF:FFFFFFFF,FFFFFFFFFFFF,FFFFFF,:FFFF,FFFFFFFFFFF
FFFFFFF,FFFFFFF
@A00155:342:HHGFNDSXY:1:1163:31756:27555 2:N:0:GAACCTAG+TCCGCATA
GTATACACTCACGTCGTGAGTAAACAGATTGACGTAACGCCTCAATCGTGGTCAAGGACCAGCATTCA
ATGCTACCACATTAAATACAATCACATTGGATTTCAGAACTTCTTGTGTCCGCACATAGAAGGTTAAA
GATGGCTTTCTCTTA
+
F:FFFFFFFFFFF:FF:FFFFFFFFFFFFFFFFF:FFFFFFFFFFFFFFFFFFFFFFFFFFFFFFFFF
FFFF:FFFFFFFFFFFFFFFFFFF:::FF,FFFFF:FFFFFFFFFFFFFFFFFFFFFF,FFFFFFF:F
FFFFFFFFFFFFFFF
@A00155:342:HHGFNDSXY:1:1415:31132:21339 2:N:0:GAACCTAG+TCCGCATA
GTATACACTCACGTCGTGAGTAAACAGATTGACGTAACGCCTCAATCGTGGTCAAGGACCAGCATTCA
ATGCTACCACATTAAATACAATCACATTGTATTTCAGAACTTCTTGTGTCCGTACATAGAAGGTTAAA
GATGGCTTTCTCTTA
+
FFFFFFFFFFFFF:FFFFFFFFFFFFFFFFFFFFFF:FFFFFFFFFF:FFFFFFFFFFFFFFFFFFFF
FFFFFFFFFFFFFFFF:FFFFFFFFFFFFFFFFFFFFF:FFFFFFF:FFFF::FFF:FFFFFF,FFFF
FFFFFFFFFFFFFFF
@A00155:342:HHGFNDSXY:1:1519:30608:30702 2:N:0:GAACCTAG+TCCGCATA
GTATACACTCACGTCGTGAGTAAACAGATTGACGTAACGCCTCAATCGTGGTCAAGGACCAGCATTCA
ATGCTACCACATTAAATACAATCACATTGTATTTCAGAACTTCTTGTGTCCGCACATAGAAGGTTAAA
GATGGCTTTCTCTTA
+
FFFFFFFFFFFFFFFFFFFFFF:FFFFFFFFFFFFFFFFFFFFFFFFFFFFFFFFFFF:FFFFFFFFF
FFFFFFFFFFFFFFFF:FFFFFFFFFFFFFFFFFFFFFFFFFFFFFFFFFF:FFFF:FFFF:F:FFFF
FFFFFFFFFFF:FFF
@A00155:342:HHGFNDSXY:1:1666:9127:16986 2:N:0:GAACCTAG+TCCGCATA
GTATACACTCACGTCGTGAGTAAACAGATTGACGTAACGCCTCAATCGTGGTCAAGGACCAGCATTCA
ATGCTACTACATTAAATACAATCACATTGTATTTCAGAACTTCTTGTGTCCGCACATAGAAGGTTAAA
GATGGCTTTCTCTTA
+
FFFFFFFFFFFFFFFFFFFFFFFFFFFFFFFFFFFFFFFFFFFFFFFFFFFFFFFFFFFFF:FFFFFF
FFFFFFFFFFFFFFFFFFFFFFFFFF:FFFFFFFFFFFFFFFFFFFFFFFFFFFFFFFFFFFFFF:FF
FFFFFFFFFFFFFFF
@A00155:342:HHGFNDSXY:1:2161:5520:12399 2:N:0:GAACCTAG+TCCGCATA
GTATACACTCACGTCGTGAGTAAACAGATTGACGTAACGCCTCAATCGTGGTCAAGGACCAGCATTCA
ATGCTACTACATTAAATACAATCACATTGTATTTCAGAACTTCTTGTGTCCGCACATAGAAGGTTAAA
GATGGCTTTCTCTTA
+
FFFFFFFFFFFFFFFFFFFFF,FFFFFFFFFFFFFFFFFFFFFFFFFFFFFFFFFFFFFFFFFFFFFF
FFFFFFFFFFFFFFFFFFFFFF,F,F:FFFFFFFFFFFFFFFFFFFF:FFFFFFFFFFFFFFFFFFF,
F:FFF:FFFFFFF::
@A00155:342:HHGFNDSXY:1:2153:11731:8437 2:N:0:GAACCTAG+TCCGCATA
GTATACACTCACGTCGTGAGTAAACAGATTGACGTAACGCCTCAATCGTGGTCAAGGACCAGCATTCA
ATGCTACCACATTAAATACAATCACATTGTATTTCAGAACTTCTTGTGTCCGCACATAGAAGGTTAAA
GATGGCTTTCTCTTA
+
FFFFFFFFFFFFFFFFFFFFFFFFFFFFFFFFFFFFFFFFFFFFFFFFFFFFFFFFFFFFFFFFFFFF
FFFFFFFFFFFFFFFFFFFFFFFF:FFFFFFFFFFFFFFFFFFFFFFFFFFFFFFFFFFFFFFFFFF
:FFF:FFFFFFFFFF
@A00155:342:HHGFNDSXY:1:2247:15908:28479 2:N:0:GAACCTAG+TCCGCATA
GTATACACTCACGTCGTGAGTAAACAGATTGACGTAACGCCTCAATCGTGGTCAAGGACCAGCATTCA
ATGCTACCACATTAAATACAATCACATTGTATTTCAGAACTTCTTGTGTCCGCACATAGAAGGTTAAA
GATGGCTTTCTCTTA
```

+
FFFFFFFFFFFFFFFFFFFFFFFFFFFFFFFFFFFFFFFFFFFFFFFFFFFFFFFFFFFFFFFF:FFF
FFFFFFFFFFFFFFFFFFFFFFFFFFF:FFFFFFFFFFFFFFFFFFFFFFFFFFFFFFFFFFFFFFF:F
FFFFFFFF:FFFFFF
@A00155:342:HHGFNDSXY:1:2331:31575:11177 2:N:0:GAACCTAG+TCCGCATA
GTATACACTCACGTCGTGAGTAAACAGATTGACGTAACGCCTCAATCGTGGTCAAGGACCAGCATTCA
ATGCTACCACATTAAATACAATCACATTGTATTTCAGAACTTCTTGTGTCCGCACATAGAAGGTTAAA
GATGGCTTTCTCTTA
+
FFFFFFFFFFFFFFFF:FFFFFFFFFFFFFFFFFFFFFFFFFFFFFFFFFFFF,FFFFFFFFFFFFFF
FFFFFFFFFFFFFFFFFFFFFFFFFFF,:FFFFFFFFFFFFFFFFFFFFFFFFFFFF:FFFFFFFFFFF
FFFFFFFFFFFFFFF
@A00155:342:HHGFNDSXY:1:2357:29857:20415 2:N:0:GAACCTAG+TCCGCATA
GTATACACTCACGTCGTGAGTAAACAGATTGACGTAACGCCTCAATCGTGGTCAAGGACCAGCATTCA
ATGCTACTACATTAAATACAATCACATTGTATTTCAGAACTTCTTGTGTCCGCACATAGAAGGTTAAA
GATGGCTTTCTCTTA
+
FFFFFFFFFFFFF,FFFFFFFFFFFFFF:FFFFFFFF:FFFFFFFFFFFFFFFFFFFFFFFFFFFFFF
FFFFFFFFFFFF:FFFFFFFFFFFFFF,FFFFF:FFF,FFF:FFFFFFFFFFFFFFF:FFF,FFFFFF:
FFFFFFFFFFFFFFF
@A00155:342:HHGFNDSXY:1:2503:32127:36025 2:N:0:GAACCTAG+TCCGCATA
GCATACACTCACGTCGTGAGTAAACAGATTGACGTAACGCCTCAATCGTGGTCAAGGACCAGCATTCA
ATGCTACCACATTAAATACAATCACATTGTATTTCAGAACTTCTTGTGTCCGCACATAGAAGGTTAAA
GATGGCTTTCTCTTA
+
:FFFFFFFFFFFFFFFFFFFFFFFFFFF,FFFFFFFFFFFFFFFFFFFFFF:FFFFFFFFFFFFFFFF
FFFFFFFFFFFFFFFFFFFFFFFFFFF,FFF:FFFFFF::FFFFFFFFFFFFFFFFFFFFF,FF:FFFF
FFFFFFF:FFFFFFF
@A00155:342:HHGFNDSXY:1:2504:32244:1188 2:N:0:GAACCTAG+TCCGCATA
GTATACACTCACGTCGTGAGTAAACAGATTGACGTAACGCCTCAATCGTGGTCAAGGACCAGCATTCA
ATGCTACCACATTAAATACAATCACATTGTATTTCAGAACTTCTTGTGTCCGCACATAGAAGGTTAAA
GATGGCTTTCTCTTA
+
FF:FFFFFFFFFFFFFFFFFFF::FFFFFFFFFFFFFFFFFFFFFFFFFFFFFFFFFFFFFFFFF:FFF
FFFFFFFFFFFFFFFFFFFFFFFFFFF:FFFFFFFFFFFFFFFFFFFFFFFFFFFFFFFFFFFFFFFFF
FFFFFFFFFF:FFFF
@A00155:342:HHGFNDSXY:1:2502:13575:4085 2:N:0:GAACCTAG+TCCGCATA
GTATACACTCACGTCGTGAGTAAACAGATTGACGTAACGCCTCAATCGTGGTCAAGGACCAGCATTCA
ATGCTACTACATTAAATACAATCACATTGTATTTCAGAACTTCTTGTGTCCGCACATAGAAGGTTAAA
GATGGCTTTCTCTTA
+
FFFFFFFFFFFFFFFFFFFFFFFFFFF:FFFFFFFFFFFFFFFFFFFFFFFFFFFFFFFFFFFFFFFFF
FFFFFFFFFFFFFFFFFFFFFFFFFFFFFFFFFFFFFFFFFFFFFFFFFFFFFFFFFFFFFFFFFFFFF
FFFFFFFFFFFFFFF
@A00155:342:HHGFNDSXY:1:2624:11071:14935 2:N:0:GAACCTAG+TCCGCATA
GTATACACTCACGTCGTGAGTAAACAGATTGACGTAACGCCTCAATCGTGGTCAAGGACCAGCATTCA
ATGCTACTACATTAAATACAATCACATTGTATTTCAGAACTTCTTGTGTCCGCACATAGAAGGTTAAA
GATGGCTTTCTCTTA
+
FFFFFFFFFFFFFFFFFFFFFFFFFFF:FFF:FFFFFFFFFFFFFFFFFF:FFFFFFFFFFFFFFFFF
FFFFFFFFFFFFFFFFFFFFFFFFFFFFFFFFFFFFFFF::FF:FFFFFFFFFF:FFFFFFFFFFFFFF
FFFFFFFFFFFFFFF
@A00155:342:HHGFNDSXY:1:2626:29044:27398 2:N:0:GAACCTAG+TCCGCATA
GTATACACTCACGTCGTGAGTAAACAGATTGACGTAACGCCTCAATCGTGGTCAAGGACCAGCATTCA

```
ATGCTACCACATTAAATACAATCACATTGTATTTCAGAACTTCTTGTGTCCGCACATAGAAGGTTAAA
GATGGCTTTCTCTTA
+
FFFFFFFFFFFFFFFFFFFFFFFFFFFFFFFFFFFFFFFFFFFFFFFFFFFFFFFFFFFFFFFFFFFF
FFFFFFFFFFFFFFFFFFFFFFFFFFFFFFFFFFFFFFFFFFFF:FFFFFFFFFFF:FFFFFFFF:FF
FFFFFFFFFFFFFFF
@A00155:342:HHGFNDSXY:1:2626:29062:27430 2:N:0:GAACCTAG+TCCGCATA
GTATACACTCACGTCGTGAGTAAACAGATTGACGTAACGCCTCAATCGTGGTCAAGGACCAGCATTCA
ATGCTACCACATTAAATACAATCACATTGTATTTCAGAACTTCTTGTGTCCGCACATAGAAGGTTAAA
GATGGATTTCTCTTA
+
FFFFFFFFFFFFFFFF:FFFFFFF:FFFFFFF:F:FF:FFFFFFFFF:FFFFFFFFFFFFF:FFFFFF
FFFFFFF::FF:FFFFFFFFF:,FFFFF,FFFFF,FFFFFFFFF,,F:FF,FFFFFFFFFF,:FFFFF
FFF,:,FFFFFFFFF
@A00155:342:HHGFNDSXY:1:1115:30264:28698 2:N:0:GAACCTAG+TCCGCATA
TATACACTCACGTCGTGAGTAAACAGATTGACGTAACGCCTCAATCGTGGTCAAGGACCAGCATTCAA
TGCTACCACATTAAATACAATCACATTGTATTTCAGAACTTCTTGTGTCCGCACATAGAAGGTTAAAG
ATGGCTTTCTCTTAT
+
:FFF,FFFFFFFFFFFFFFFF,FFFFF:F,F:F:FFFFFFFFFFFFFFFFFFFFFFFF:FF::FFFFF
FFFF,FFFF:FF:FFFF:FFFFFF,:FFF:FFFFFFFFFF,,FFFFFFFFFFFFF:FF:FFFFFFFF:
FFFFFFFFFFFFFFF
@A00155:342:HHGFNDSXY:1:1130:30318:4993 2:N:0:GAACCTAG+TCCGCATA
TATACACTCACGTCGTGAGTAAACAGATTGACGTAACGCCTCAATCGTGGTCAAGGACCAGCATTCAA
TGCTACCACATTAAATACAATCACATTGTATTTCAGAACTTCTTGTGTCCGCACATAGAAGGTTAAAG
ATGGCTTTCTCTTAT
+
FFFFFFFFFFFFFFFFFFFFFFFFFFF:FFFFFFFFFFFFFFFFFFFFFFFFFFFFFFFFFFFFFFFF
FFFFFFFFFFFFFFFFFFF:FFFFFFFFFFFFFFFFFFFFFFFFFFFFFFFFFFFFFFFFFFFF:FFF
FF,FFFFFFFFFFFF
@A00155:342:HHGFNDSXY:1:1166:23945:9737 2:N:0:GAACCTAG+TCCGCATA
TATACACTCACGTCGTGAGTAAACAGATTGACGTAACGCCTCAATCGTGGTCAAGGACCAGCATTCAA
TGCTACCACATTAAATACAATCACATTGTATTTCAGAACTTCTTGTGTCCGCACATAGAAGGTTAAAG
ATGGCTTTCTCTTAT
+
FFFFFFFFFFFFFFFFFFFFFFFFFFFFFFFFFFFFFFFFFFFFFFFFFFFFFFFFFFFFFFFFFFFF
FFFFFFFFFFFFFFFFFFFFFFF:FFFFFF,FFFFFFFFF:FFFFFFFFFFFFFFF:FFFFFFFFFFF
FFF:FFFFFFFF:FF
@A00155:342:HHGFNDSXY:1:1203:5041:11757 2:N:0:GAACCTAG+TCCGCATA
TATACACTCACGTCGTGAGTAAACAGATTGACGTAACGCCTCAATCGTGGTCAAGGACCAGCATTCAA
TGCTACCACATTAAATACAATCACATTGTATTTCAGAACTTCTTGTGTCCGCACATAGAAGGTTTAAG
ATGGCTTTCTCTTAT
+
FFFFFFFFFFFFFFFFFFFFF::FFFFFFFFFFFFFFFFFFFFFFFFFFFFFFFFFFFFFFFFFFFFF
FFFFFFFFFFFFFFFFFFFFFFF:FFFFFFFFF:FFFFFFFFFF,FFFFFFFFFFFFFFFFFFFFFF,
FFFFFFFF,FFFFFF
@A00155:342:HHGFNDSXY:1:1169:31232:29684 2:N:0:GAACCTAG+TCCGCATA
TATACACTCACGTCGTGAGTAAACAGATTGACGTAACGCCTCAATCGTGGTCAAGGACCAGCATTCAA
TGCTACTACATTAAATACAATCACATTGTATTTCAGAACTTCTTGTGTCCGCACATAGAAGGTTAAAG
ATGGCTTTCTCTTAT
+
FFFFFFFFFFFFFFFFFFFFFFFFFFFFFFFFFFFFFFFFFFFFFFFFFFFFFFFFFFFFFFFFFFFF
FFFFFFFFFFFFFFFFFFFFFFFFFFFFFFFFFFFFFFFFFFFFFFFFFFFFFFFFFFFFFFFFFFFF
FFF:FFFFFFFFFFF
```

```
@A00155:342:HHGFNDSXY:1:1206:28320:33755 2:N:0:GAACCTAG+TCCGCATA
TATACACTCACGTCGTGAGTAAACAGATTGACGTAACGCCTCAATCGTGGTCAAGGACCAGCATTCAA
TGCTACCACATTAAATACAATCACATTGTATTTCAGAACTTCTTGTGTCCGCACATAGAAGGTTAAAG
ATGGCTTTCTCTTAT
+
FFFFFFFFFFFFFFFFFFFFFFFFFFFFFFFFFFFFFFFFFFFFFFFFFFFFFFFFFFFFFFFFFFFF
F:FFFFFFFFFFFFFFFFFFFFFFF,:FFFFFFFFFFFFFFFFFFFFFFFFFFFF:FFFFFFFFFFFF
FFFFFFFFF:FFFFF
@A00155:342:HHGFNDSXY:1:1221:17363:29872 2:N:0:GAACCTAG+TCCGCATA
TATACACTCACGTCGTGAGTAAACAGATTGGCGTAACGCCTCAATCGTGGTCAAGGACCAGCATTCAA
TGCTACCACATTAAATACAATCACCTTGTATTGCAGAACTTCTTGTGTCCGCACATAGAAGGTTAAAG
ATGGCTTTCTCTTAT
+
FFFFFF:FFFFFFFFFFFFFFF:FFFF,F:,F,FFFFFF:::FFFFFFFFFFFFFFFFFFFF,:FFFF
FFFFFFFFFFFFFFFFF:F:FFFFF,FF,:FF,FFFF:FFF:FFFFF:FFFF:FFFF::FFF:FF:FF
FFFF:F:FFFFF:FF
@A00155:342:HHGFNDSXY:1:1219:12879:4069 2:N:0:GAACCTAG+TCCGCATA
TATACACTCACGTCGTGAGTAAACAGATTGACGCAACGCCTCAATCGTGGTCAAGGACCAGCATTCAA
TGCTACCACATTAAATACAATCACATTGTATTTCAGAACTTCTTGTGTCCGCACATAGAAGGTTAAAG
ATGGCTTTCTCTTAT
+
F,FFFFFFFFFFFFFFFFFFFFFFFFFFFFFFFFFFFFFFFFFFFFFFFFFFFFFFFFFFFFFFFFF:
FFFFFF:FFFFFFFFFFFF:FF:FF::FFFFFFFFFFFFFFFFFFFFFFFFFFFFFFFFFFFFFFFFF
FF:FFFFFFFFFFFF
@A00155:342:HHGFNDSXY:1:1261:3152:15530 2:N:0:GAACCTAG+TCCGCATA
TATACACTCACGTCGTGAGTAAACAGATTGACGTAACGCCTCAATCGTGGTCAAGGACCAGCATTCAA
TGCTACCACATTAAATACAATCACATTGTATTTCAGAACTTCTTGTGTCCGCACATAGAAGGTTAAAG
ATGGCTTTCTCTTAT
+
FFFFFFFFFFFF,FFFFFFFFFFFFF:FF::F:FFFF:FFFFFFFFFFFFFFFFFFFFFFFFF:FFFF
FFFFFFFFFFFFFFFFFFFFFFFFF:FFFFFFFFFFFFFFFFFFFFFFFFFFF:FFFFF,FFFFFFFF
FFF:FFFFFFFFFFF
@A00155:342:HHGFNDSXY:1:1335:16495:22827 2:N:0:GAACCTAG+TCCGCATA
GATACACTCACGTCGTGAGTAAACAGATTGACGTAACGCCTCAATCGTGGTCAAGGACCAGCATTCAA
TGCTCCTACATTAAATACAATCACAGTGTATTTCAGAACTTCTTGTGTCCGCACATAGAAGGTTAAAG
ATGGCTTTCTCTTAT
+
:FF:FFFFFFF,,FF,FFFF:FF,,FF:FFFFFFFFF::FFF,FFFF:FFFFFFF:FFFF:FF,FFF:
FFF:,FFFFFFF:FFFFFFF,FFFF,FFFF:::FFFFF,FFF:FFFFFF:FF:FF,FFFFFFFFFFFF
FFFF:FFF::,F:FF
@A00155:342:HHGFNDSXY:1:1332:29640:25488 2:N:0:GAACCTAG+TCCGCATA
TATACACTCACGTCGTGAGTAAACAGATTGACGTAACGCCTCAATCGTGGTCAAGGACCAGCATTCAA
TGCTACCACATTAAATACAATCACATTGTATTTCAGAACTTCTTGTGTCCGCACATAGAAGGTTAAAG
ATGGCTTTCTCTTAT
+
FFFFFFFFFFFFFFF:FFFFFFFFFFFFFFFFFFFFFFFFFFFFFFFFFFFFFFFFFFFFFFFFFFFF
FFFF:FFFFFFFFFFFFFFFFFFFFFFFFF:FFFFFFFFFFFFFFFFFF:FFFFFFFFFFFFFFFFFF
FFFFFFFFFFFFFFF
@A00155:342:HHGFNDSXY:1:1408:16568:29904 2:N:0:GAACCTAG+TCCGCATA
TATACACTCACGTCGTGAGTAAACAGATTGACGTAACGCCTCAATCGTGGTCAAGGACCAGCATTCAA
TGCTACCACATTAAATACAATCACATTGTATTTCAGAACTTCTTGTGTCCGCACATAGAAGGTTAAAG
ATGGCTTTCTCTTAT
+
FFFFFFFFFFFFFFFFFFFFFFFFFF::FFFFFFFFFFFFFFFFFFFFFFFFFFFFF:FFFFFFFFFF
```

```
FFFFFFFFFFFFFFFFFFFFFFFF:FFFFFFFFFFFFFFFFFFFFFFFFFFFFFFF:FFFFFFFFFFF
FFFFFFFF:FFFFFF
@A00155:342:HHGFNDSXY:1:1402:27670:23923 2:N:0:GAACCTAG+TCCGCATA
TATACACTCACGTCGTGAGTAAACAGATTGACGTAACGCCTCAATCGTGGTCAAGGACCAGCATTCAA
TGCTACCACATTAAATACAATCACATTGTATTTCAGAACTTCTTGTGTCCGCACATAGAAGGTTAAAG
ATGGCTTTCTCTTAT
+
FF:F:FFFFFFFFFF:FFFF:FFFFFFFFFFFFFFFFFFF:FFF,FF:F:FF:FFFFFFFFFF:FFFF
,FFFFFFF,F:FFFF:F,FFFFF,F,FFF::,:FFFFFFFFFFFF:FFFFFFFFF,F:F:FFF:FF:F
FF:F:,FFFF,FFFF
@A00155:342:HHGFNDSXY:1:1422:15221:29230 2:N:0:GAACCTAG+TCCGCATA
TATACACTCACGTCGTGAGTAAACAGATTGACGTAACGCCTCAATCGTGGTCAAGGACCAGCATTCAA
TGCTACCACATTAAATACAATCACATTGTATTTCAGAACTTCTTGTGTCCGCACATAGAAGGTTAAAG
ATGGCTTTCTCTTAT
+
FFFFFFFFFFFFFFFFFFFFFFFFFFFFFF:FFFFFFFFFFFFFFFFFFFFFFFFFFFFFFFFFFFFF
FFFFFFFFFFFFFFFFFFFFFFFF:FFFFFFFFFFFFFFFFFFFFFFFFF:FFFFFFFFFFFFFFFFF
FFFFFFFFFFFFFFF
@A00155:342:HHGFNDSXY:1:1461:14579:27242 2:N:0:GAACCTAG+TCCGCATA
TATACACTCACGTCGTGAGTAAACAGATTGACGTAACGCCTCAATCGTGGTCAAGGACCAGCATTCAA
TGCTACCACATTAAATACAATCACATTGTATTTCAGAACTTCTTGTGTCCGCACATAGAAGGTTAAAG
ATGGCTTTCTCTTAT
+
FFFFFFFFFFFFFFFFFF,FFFFFFFFFFFFFFFFFFFFFFFFFFFFFF:FFFFFFFFFFFFFFFFFF
FFFFFF:FFFFFFFFFFFFFFFF:FFFFFFFFFF:FFFFFFFFFFFFFFFFFFFFFFFFFF:FFFF:F
FF:FFFFFFFFF:FF
@A00155:342:HHGFNDSXY:1:1469:28754:35759 2:N:0:GAACCTAG+TCCGCATA
TATACACTCACGTCGTGAGTAAACAGATTGACGTAACGCCTCAATCGTGGTCAAGGACCAGCATTCAA
TGCTACCACATTAAATACAATCACATTGTATTTCAGAACTTCTTGTGTCCGCACATAGAAGGTTAAAG
ATGGCTTTCTCTTAT
+
FFFFFFFFFFFFFFFFFFFFFFFFFFFFFF:FFFFFFFFFFFFFFFFFFFFFFFFFFFFFFFFFFFFF
FFFF:FFFFFFFFFFFFFFFFFFFFFFF:FFFFFFFFFFFFFFFFFFFFFFFFF:FFFFFFFFFFFFF
FFF:FF,FFFFFFFF
@A00155:342:HHGFNDSXY:1:1466:12644:4507 2:N:0:GAACCTAG+TCCGCATA
TATACACTCACGTCGTGAGTAAACAGATTGACGTAACGCCTCAATCGTGGTCAAGGACCAGCATTCAA
TGCTACCACATTAAATACAATCACATTGTATTTCAGAACTTCTTGTGTCCGCACATAGAAGGTTAAAG
ATGGCTTTCTCTTAT
+
FFFFFFFFFF:FFFFFFFFFFFFFFFFFFFFFFFFFFFFFFFFFFFFFFFFFFFFFFFFFFFFFFFFF
FFFFFFFFFFFFFFFFFFFFFFFFFFFFFFFFFFFFFFFFFFFF:FFFFFFFFFFFF:FFFFFFFFF,
FFFFFFFFFFFFFFF
@A00155:342:HHGFNDSXY:1:1472:12834:28886 2:N:0:GAACCTAG+TCCGCATA
TATACACTCACGTCGTGAGTAAACAGATTGACGTAACGCCTCAATCGTGGTCAAGGACCAGCATTCAA
TGCTACCACATTAAATACAATCACATTGTATTTCAGAACTTCTTGTGTCCGCACATAGAAGGTTAAAG
ATGGCTTTCTCTTAT
+
FFFFFFFFFFFFFFFFFFFFFFFFFFFFFFFFFFFFFFFFFFFFFFFFFFFFFFFFFFFFFFFFFFFF
FFFFFFFFFFFFFFFFFFF:FFFFFFFFFFFFFFFFFFFFFFFFFFFFFFFFFFFFFFFFFFFFFFFF
FFFFFFFFFFFFFFF
@A00155:342:HHGFNDSXY:1:1478:24080:10504 2:N:0:GAACCTAG+TCCGCATA
TATACACTCACGTCGTGAGTAAACAGATTGACGTAACGCCTCAATCGTGGTCAAGGACCAGCATTCAA
TGCTACCACATTAAATACAATCACATTGTATTTCAGAACTTCTTGTGTCCGCACATAGAAGGTTAAAG
ATGGCTTTCTCTTAT
```

+
FFFFFFFFFFFFFFFFFFFFFFFFFF:FFFFFFFFFFFFFFFFFFFFFFFFFFFFFFFF:FFFFFFF
FFFFFFFFFFF:FFFF:FFFFFF:FFFFFFFFF:,FF:FFF:FFFFFFFFFFFFFFFFFF,FFF:F:
FFFFFFFFFFFFFFF
@A00155:342:HHGFNDSXY:1:1527:26901:13542 2:N:0:GAACCTAG+TCCGCATA
TATACACTCACGTCGTGAGTAAACAGATTGACGTAACGCCTCAATCGTGGTCAAGGACCAGCATTCAA
TGCTACCACATTAAATACAATCACATTGTATTTCAGAACTTCTTGTGTCCGCACATAGAAGGTTAAAG
ATGGCTTTCTCTTAT
+
FFFFFFFFFFFFFFFFFFFFFFFFFFF:FFFFFFFFFFFFFFFFFFFFFFFFFFFFFFFFFFFFFFF
FFFFFFFFFFFFFFFFFFF,FFFFF,FFFFFFFFFFFFFFFFFFFFFFFFFFFFFFFFFFFFFF:FF
FFFFFFFFFFFFFFF
@A00155:342:HHGFNDSXY:1:1537:20708:15593 2:N:0:GAACCTAG+TCCGCATA
TATACACTCACGTCGTGAGTAAACAGATTGACGTAACGCCTCAATCGTGGTCAAGGACCAGCATTCAA
TGCTACCACATTAAATACAATCACATTGTATTTCAGAACTTCTTGTGTCCGCACATAGAAGGTTAAAG
ATGGCTTTCTCTTAT
+
FFFFFFFFFFFFFFFFFFFFFFFFFFFFFFFFFFFFFFFFFFFFFFFFFFFFFFFFFFFFFFFFFFF
FFFFFFF:FFFFFFFFFFF:FFFFFFFF,FFFFFFFFFFFFFFFFFF:FFFFF,FFFFFFFFFFFFF
:F:FFFFFFFFFFFF
@A00155:342:HHGFNDSXY:1:1550:16857:5478 2:N:0:GAACCTAG+TCCGCATA
TATACACTCACGTCGTGAGTAAACAGATTGACGTAACGCCTCAATCGTGGTCAAGGACCAGCATTCAA
TGCTACTACATTAAATACAATCACATTGTATTTCAGAACTTCTTGTGTCCGCACATAGAAGGTTAAAG
ATGGCTTTCTCTTAT
+
FFFFFFFFFFFFFFFFFFFFFFFFFFF:FFFFFFFFFFFFFFFFFFFFFFFFFFFFFFFFFFFFFF:
FFFFFFF,FFFFFFFFFFFFFFFF,:FFFFFFFFFFFFFFF:FFFFFFFFFFFFF:FFFFFFFFFFF
FFFFFFFFFFFFFFF
@A00155:342:HHGFNDSXY:1:1548:7672:17628 2:N:0:GAACCTAG+TCCGCATA
TATACACTCACGTCGTGAGTAAACAGATTGACGTAACGCCTCAATCGTGGTCAAGGACCAGCGTTCAA
TGCTACCACATTAAATACAATCACATTGTATTTCAGAACTTCTTGTGTCCGCACATAGAAGGTTAAAG
ATGGCTTTCTCTTAT
+
FFFFFFFFFFFFFFFFFFFFFF,FFFFFFF:FFFFFFF:FF:FFFFFFFFFFFFFFFFFFF:FFFFF
F:FFFFFFFFFFFFFFFFFFFFFFFFFFFFF:FFFFFF:FFFFFFFFFFFFFFFFFFF:FFFF:F:F
:::FFFFFFFFFFFF
@A00155:342:HHGFNDSXY:1:1608:10981:8359 2:N:0:GAACCTAG+TCCGCATA
TATACACTCACGTCGTGAGTAAACAGATTGACGTAACGCCTCAATCGTGGTCAAGGACCAGCATTCAA
TGCTACCACATTAAATACAATCACATTGTATTTCAGAACTTCTTGTGTCCGCACATAGAAGGTTAAAG
ATGGCTTTCTCTTAT
+
FFFFFFFFFFFFFFFFFFFFFFFFFFFFFFFFFFFFFFFFFFFFFFFFFFFFFFF:FFFFF:FFFF:
FFFFFFF:FFFFFFFFFF,FFFFFFFFFFFFFFFFFFF::FF,F:FFFFFFFFF:FF:FFFFF,FFF
FFFFFF:FFFFFFFF
@A00155:342:HHGFNDSXY:1:1618:5457:24251 2:N:0:GAACCTAG+TCCGCATA
TATACACTCACGTCGTGAGTAAACAGATTGACGTAACGCCTCAATCGTGGTCAAGGACCAGCATTCAA
TGCTACTACATTAAATACAATCACATTGTATTTCAGAACTTCTTGTGTCCGCACATAGAAGGTTAAAG
ATGGCTTTCTCTTAT
+
FFFFFFFFFFFFFFFFFFFFFFFFFFFFFFFFFFFFFFFFFFFFFFFFFFFFFFFFFFFFFFFFFFF
FFFFFFFFFFFFFFFFFFFFFFFFFFFFFFFFFFFFFFFFFFFFFFFFFFFFFFFFFFFFFFFFFFF
FFF:FFFFFFFFFFF
@A00155:342:HHGFNDSXY:1:1619:12698:4977 2:N:0:GAACCTAG+TCCGCATA
TATACACTCACGTCGTGAGTAAACAGATTGACGTAACGCCTCAATCGTGGTCAAGGACCAGCATTCAA

```
TGCTACCACATTAAATACAATCACATTGTATTTCAGAACTTCTTGTGTCCGCACATAGAAGGTTAAAG
ATGGCTTTCTCTTAT
+
FFFFFFFFFFFFFFFFFFFFFFFFFFFFFFFFFFFFFFFFFFFFFFFFFFFFFFFFFFFFFFFFFFFF
FFFFFFFFFFFFFFF:FFFFFFFFFFFFFFFFFFFFFFFFFFFFFFFFFFFFFFFFFFFFFFFFFFFF
FFFFFFFFFFFFFFF
@A00155:342:HHGFNDSXY:1:2119:28330:5995 2:N:0:GAACCTAG+TCCGCATA
TATACACTCACGTCGTGAGTAAACAGATTGACGTAACGCCTCAATCGTGGTCAAGGACCAGCATTCAA
TGCTACCACATTAAATACAATCACATTGTATTTCAGAACTTCTTGTGTCCGCACATAGAAGGTTAAAG
ATGGCTTTCTCTTAT
+
FFFFFFFFFFFFFFFFFFFFFFFFFFF:FFFFFFFFFFFFFF::FFFFFFFFFFFFFFFFFFFFFFFF
:FFFFFFFFFFFFFFFFFFFFFFFF,:FFFFFFFFFFFFFFFFFFFFFFFFFF:FFFFFFFFFFFFFF
FFFFFF:FFFFFFFF
@A00155:342:HHGFNDSXY:1:2139:20094:16031 2:N:0:GAACCTAG+TCCGCATA
TATACACTCACGTCGTGAGTAAACAGATTGACGTAACGCCTCAATCGTGGTCAAGGACCAGCATTCAA
TGCTACTACATTAAATACAATCACATTGTATTTCAGAACTTCTTGTGTCCGCACATAGAAGGTTAAAG
ATGGCTTTCTCTTAT
+
FFFFFFFFFFFFFFFFFFFFFFFFFFFFFFFFFFFFFFFFFFFFFFFFFFFFFFFFFFFFFFFFFFFF
FFFFFFFFFFFFFFFFFFFFFFFFFFFFFFFFFFFFFFFFFFF:FFFFFFFFFFF:FFFFFFFFFFFF
FF,FF:FFFFFF:FF
@A00155:342:HHGFNDSXY:1:2136:10022:27430 2:N:0:GAACCTAG+TCCGCATA
TATACACTCACGTCGTGAGTAAACAGATTGACGTAACGCCTCAATCGTGGTCAAGGACCAGCATTCAA
TGCTACTACATTAAATACAATCACATTGTATTTCAGAACTTCTTGTGTCCGCACATAGAAGGTTAAAG
ATGGCTTTCTCTTAT
+
FFFFFFFFFFFFFFFFFFFFFFFFFFFFFFFFFFFFFFFFFFFFFFFFFFFFFFFFFFFFFFFFFFFF
FFFFFFFFFFFFFFFFFFFFFFFFFFF:FFFFFFFFFFFFFFFFFFFFFFFFFFFFFFF:FFFFFFFF
FFFFFF:FFFFFFFF
@A00155:342:HHGFNDSXY:1:2161:7735:19210 2:N:0:GAACCTAG+TCCGCATA
TATACACTCACGTCGTGAGTAAACAGATTGACGTAACGCCTCAATCGTGGTCAAGGACCAGCATTCAA
TGCTACCACATTAAATACAATCACATTGTATTTCAGAACTTCTTGTGTCCGCACATAGAAGGTTAAAG
ATGGCTTTCTCTTAT
+
FFFFFFFFFFFFFFFFFFFFFFF:FFFFFFFFFFFFFFFFFFFFFFFFFFFFFFFFFFFFFFFFFFFF
FFFFFFFFFFFFF:FFFFFFFFFFFFFFFFFFFFFFFFFFFFFFFFFFFFFFFFFFFFFFFFFFFFF:
FFFFFFFFFFFFFFF
@A00155:342:HHGFNDSXY:1:2226:8684:20384 2:N:0:GAACCTAG+TCCGCATA
TATACACTCACGTCGTGAGTAAACAGATTGACGTAACGCCTCAATCGTGGTCAAGGACCAGCATTCAA
TGCTACCACATTAAATACAATCACATTGTATTTCAGAACTTCTTGTGTCCGCACATAGAAGGTTAAAG
ATGGCTTTCTCTTAT
+
FFFFFFFFFFFFFFFFFFFFFFFFFFFFFFFFFFFFFFFFFF:FFFFFFFFFFFFFFFFFFFFFFFFF
FFFFFFFFFFFFFFFFFFFFFFFFFF:FFFFFFFFFFFFFFFFFFFFFFFFFF:FFFF:FFFFFFFF
FFFFFFFFFFFFFFF
@A00155:342:HHGFNDSXY:1:2239:6542:22686 2:N:0:GAACCTAG+TCCGCATA
TATACACTCACGTCGTGAGTAAACAGATTGACGTAACGCCTCAATCGTGGTCAAGGACCAGCATTCAA
TGCTACCACATTAAATACAATCACATTGTATTTCAGAACTTCTTGTGTCCGCACATAGAAGGTTAAAG
ATGGCTTTCTCTTAT
+
FFFFFFFFFFFFFFFFFFFFFFFFFFFFFFFFFFFFFFFFFFFFFFFFFFFFFFFFFFFFFFFFFFFF
FFFFFF:FFFFFFFFFFFFFFFFFFFFFFFFFFFFFFFFFFFFFFFFFFF:,FFFFFF:FFFFF
FFFFFFFFFFFFFFF
```

@A00155:342:HHGFNDSXY:1:2239:29640:30467 2:N:0:GAACCTAG+TCCGCATA
TATACACTCACGTCGTGAGTAAACAGATTGACGTAACGCCTCAATCGTGGTCAAGGACCAGCATTCAA
TGCTACTACATTAAATACAATCACATTGTATTTCAGAACTTCTTGTGTCCGCACATAGAAGGTTAAAG
ATGGCTTTCTCTTAT
+
FFFFFFFFFFFFFFFFFFFFFFFFFFFFFFFFFFFFFFFFFFFFFFFFFFFFFFFFFFFFFFFFFFFF
FFFFFFFFFFFFFFFFFFF:FFFFFFFFFFFFFFFFFFF::FFFFFFFFFFFFFFFFFFF:FFFFFFF
FFFFFFFFFFFFFFF
@A00155:342:HHGFNDSXY:1:2256:9480:11804 2:N:0:GAACCTAG+TCCGCATA
TATACACTCACGTCGTGAGTAAACAGATTGACGTAACGCCTCAATCGTGGTCAAGGACCAGCATTCAA
TGCTACCACATTAAATACAATCACATTGTATTTCAGAACTTCTTGTGTCCGCACATAGAAGGTTAAAG
ATGGCTTTCTCTTAT
+
FFFFFFFFF:FF:FFFFFFFFFFFFFFFFF:FFFFFFFFFFFFFFFFFFFFFFFFFFFFFFFFFFFFF
FFFFFFFFFFFFF,FFFFFFFFF::FFFFFFFFFFF:FFFFFFFFFFFF,FFFFFFFF:FFFFFFFFF
FF:FFFFFFFFFFFF
@A00155:342:HHGFNDSXY:1:2301:18918:22326 2:N:0:GAACCTAG+TCCGCATA
TATACACTCACGTCGTGAGTAAACAGATTGACGTAACGCCTCAATCGTGGGCAAGGACCAGCATTCAA
TGCTACCACATTAAATACAATCACATTGTATTTCAGAACTTCTTGTGTCCGCACCTAGAAGGTTAAAG
ATGGCTTTCTCTTAT
+
FF:FFFFFF:FFFFFFFFFFFFFFFFFFFFFFFFFF:FFFFFFF:F:FFF,FFFFFFFF,FF,FFFFF
FFFFFFFFFFFFFFFFFFFFFF,FF,,FF:,::FFFF,FFFFF:F:FFFFF:,F,F,FFFFFFF,FF:
,::F:FFF::FFFFF
@A00155:342:HHGFNDSXY:1:2327:27299:11130 2:N:0:GAACCTAG+TCCGCATA
TATACACTCACGTCGTGAGTAAACAGATTGACGTAACGCCTCAATCGTGGTCAAGGACCAGCATTCAA
TGCTACCACATTAAATACAATCACATTGTATTTCAGAACTTCTTGTGTCCGCACATAGAAGGTTAAAG
ATGGCTTTCTCTTAT
+
FFFFFFFFFFFFFFFFFFFFFFFFFFFFFFFFFFFFFFFFFFFFFFFFFFFFFFFFFFFFFFFFFFFF
FFFFFFFFFFFFFFFFFFFFFFFFF:FFFFFFFFFFFFFFFFFFFFFFFFFFFFFFFFFFFFF:FFFF
FF:FFFFFFFFFFFF
@A00155:342:HHGFNDSXY:1:2354:14597:26052 2:N:0:GAACCTAG+TCCGCATA
TATACACTCACGTCGTGAGTAAACAGATTGACGTAACGCCTCAATCGTGGTCAAGGACCAGCATTCAA
TGCTACCACATTAAATACAATCACATTGTATTTCAGAACTTCTTGTGTCCGCACATAGAAGGTTAAAG
ATGGCTTTCTCTTAT
+
FFFFFFFFFFFFFFFFFFFFFFFFFFFFFFFFFFFFFFFFFFFFFFFFFFFFFFFFFFFFFFFFFFFF
FFFFFFFFFFFFFFFFFFFFFFFFF,FFFFFFFFFFFFFFFFFFFFFFFFFFFF:FFFFF:FFFFF::
FFFFFF:FFFFFFFF
@A00155:342:HHGFNDSXY:1:2402:25735:28964 2:N:0:GAACCTAG+TCCGCATA
TATACACTCACGTCGTGAGTAAACAGATTGACGTAACGCCTCAATCGTGGTCAAGGACCAGCATTCAA
TGCTACCACATTAAATACAATCACATTGTATTTCAGAACTCCTTGTGTCCGCACATAGAAGGTTAAAG
ATGGCTTTCTCTTAT
+
FFFFFFFFFFFFFFFFFFFFFFFFFFFFFFFFFF:FFFFFFFFFFFFFFFFFFFFFFFFFFFFF:FFF
FFFFFFFFFFFFFFFFFFFFFFFFFF,FFFFFFFFFFFFFFFFFF:FFFFFFFFFFFFFFFFFFFFFF
FFFF,FFFFFFFFFF
@A00155:342:HHGFNDSXY:1:2421:24460:8876 2:N:0:GAACCTAG+TCCGCATA
TATACACTCACGTCGTGAGTAAACAGATTGACGTAACGCCTCAATCGTGGTCAAGGACCAGCATTCAA
TGCTACCACATTAAATACAATCACATTGTATTTCAGAACTTCTTGTGTCCGCACATAGAAGGTTAAAG
ATGGCTTTCTCTTAT
+
FFFFFFFFFFFFFFFFFFFFFFFFFFFFFFFFFFFFFFFFFFFFFFFFFFFFFFFFFFFFFFFFFFFF

FFFFFFFFFFFFFFFFFFFFFFFFFFFFFFFFFFFFFFFFFFFFFFFFFFF:FFFFFFFFFFFFFF:F
FFF:FFFFFFFFFFF
@A00155:342:HHGFNDSXY:1:2510:16586:24799 2:N:0:GAACCTAG+TCCGCATA
TATACACTCACGTCGTGAGTAAACAGATTGACGTAACGCCTCAATCGTGGTCAAGGACCAGCATTCAA
TGCTACTACATTAAATACAATCACATTGTATTTCAGAACTTCTTGTGTCCGCACATAGAAGGTTAAAG
ATGGCTTTCTCTTAT
+
FFFFFFFFFFFFFFFFFFFFFFFFFFFFFFFFFFFFFFFFFFFFFFFFFFFFFFFFFFFFFFFFFFFF
FFFFFFF:FFFFFFFFFFFFFFFFFFFFFFFFFFFFF:FFFFFFFFFFFFFFFFF:FFF:FFFFFF:F
FFFFFFFFFFFFFFF
@A00155:342:HHGFNDSXY:1:2578:2257:11851 2:N:0:GAACCTAG+TCCGCATA
TATACACTCACGTCGTGAGTAAACAGATTGACGTAACGCCTCAATCGTGGTCAAGGACCAGCATTCAA
TGCTACCACATTAAATACAATCACATTGTATTTCAGAACTTCTTGTGTCCGCACATAGAAGGTTAAAG
ATGGCTTTCTCTTAT
+
FFFFFFFFFFFFFFFFFFFFFFFFFFFFFFFFFFFFFFFFFFFFFFFFFFFFFFFFFFFFFFFFFFFF
F:FFFFFFFFFFFFFFFFFFFFF:FFFFFFFFFFFFFF:FF,FFFFFFFFFF:FFFF:FFFFF,FFFF
FF::,FFFFFFFFF:
@A00155:342:HHGFNDSXY:1:2639:8666:29403 2:N:0:GAACCTAG+TCCGCATA
TATACACTCACGTCGTGAGTAAACAGATTGACGTAACGCCTCAATCGTGGTCAAGGACCAGCATTCAA
TGCTACCACATTAAATACAATCACATTGTATTTCAGAACTTCTTGTGTCCGCACATAGAAGGTTAAAG
ATGGCTTTCTCTTAT
+
FFFFFFFFFFFFFFFFFFFFFFFFFFFFFFFFFFFFFFFFFFFFFFFFFFFFFFFFFFFFFFFFFFFF
FFFFFFFFFFFFFFFFFFFFFFFFFFFFF,FFFFFFFFFFFFFFFFFFFFFFFFF:FFFFFF:FFFFF
FFFFFF:F:FFFFFF
@A00155:342:HHGFNDSXY:1:2563:19162:29042 2:N:0:GAACCTAG+TCCGCATA
TATACACTCACGTCGTGAGTAAACAGATTGACGTAACGCCTCAATCGTGGTCAAGGACCAGCATTCAA
TGCTACTACATTAAATACAATCACATTGTATTTCAGAACTTCTTGTGTCCGCACATAGAAGGTTAAAG
ATGGCTTTCTCTTAT
+
FFFFFFFFFFFFFFFFFFFFFFFFFFF:FF:FFFFFF:FFFFF:FFFFFFFFFFFF:FFFFFFFFFFF
:FF:FFFFFFFFFFFFFFFFFFF::FFFFFFFFFFFF::FFFF:F,F:FFFFFF,:FFFFFFFFF:FF
:FFFFFFFFFFFFFF
@A00155:342:HHGFNDSXY:1:2655:13991:11475 2:N:0:GAACCTAG+TCCGCATA
TATACACTCACGTCGTGAGTAAACAGATTGACGTAACGCCTCAATCGTGGTCAAGGACCAGCATTCAA
TGCTACTACATTAAATACAATCACATTGTATTTCAGAACTTCTTGTGTCCGCACATAGAAGGTTAAAG
ATGGCTTTCTCTTAT
+
FFFFFFFFFFFFFFFFFFFFFFFFFFFFFFFFFFFFFFFFFFFFFFFFFFFFFFFFFFFFFFFFFFFF
FFFFFFFFFFF:FFFFFFFFF:FFF,FFFFFFFFFFFFFFFFFFFFFFFF:FFF:F,:FFFFFFFFFF
FFFFFFFFFFFFFFF
@A00155:342:HHGFNDSXY:1:2653:31801:22216 2:N:0:GAACCTAG+TCCGCATA
TATACACTCACGTCGTGAGTAAACAGATTGACGTAACGCCTCAATCGTGGTCAAGGACCAGCATTCAA
TGCTACCACATTAAATACAATCACATTGTATTTCAGAACTTCTTGTGTCCGCACAGAGAAGGTTAAAG
ATGGCTTTCTCTTAT
+
FFFFFFF:FFFFFFF,FFFFFFFFFFF,FFFFFFFFFFFFFFFFFFFFFF,F:FFF:FFFFFFFFFF:
FFFF:FFFFFFFFFFFFFFF:FFFF,FFFFF:FFFFFFFFFF,,F:FFFFFFFFF,FFFFFFFFFFFF
FFFFF,FFFFFFF::
@A00155:342:HHGFNDSXY:1:2667:4553:6496 2:N:0:GAACCTAG+TCCGCATA
TATACACTCACGTCGTGAGTAAACAGATTGACGTAACGCCTCAATCGTGGTCAAGGACCAGCATTCAA
TGCTACCACATTAAATACAATCACATTGTATTTCAGAACTTCTTGTGTCCGCACATAGAAGGTTAAAG
ATGGCTTTCTCTTAT

+
FFFFFFF:FFFFFFFFFFFFFFFFFFFFFFFFFFFFFFFFFFFFFFFFFFFFFFFFF,FFFFF:FFFF
FFFFFFFFFFFFF,FFFFFFFFF:FFFFFFFFFF,FFFFFF,FFFFFFF:FFFFFFFF,FFFFFFFFF
:FFFFFFFFFFFFFF
@A00155:342:HHGFNDSXY:1:2678:24659:12947 2:N:0:GAACCTAG+TCCGCATA
TATACACTCACGTCGTGAGTAAACAGATTGACGTAACGCCTCAATCGTGGTCAAGGACCAGCATTCAA
TGCTACCACATTAAATACAATCACATTGTATTTCAGAACTTCTTGTGTCCGCACATAGAAGGTTAAAG
ATGGCTTTCTCTTAT
+
FFFFFFFFFFFFFFFFFFF:FFFFFFF:FFFFFFFFFFF:FFFFFFFFFFFFF,FFFF:FFFF,F:FF
F::FFF,FFFF,FFF:FFFFFFFF:FFFFFFFFFFFFFFFFFFFFFF:FFFFFFFFF:FFF,FFFFFF
FFF,FFFFFF:F,FF
@A00155:342:HHGFNDSXY:1:1221:16893:29089 2:N:0:GAACCTAG+TCCGCATA
ATACACTCACGTCGTGAGTAAACAGATTGACGTAACGCCTCAATCGTGGTCAAGGACCAGCATTCAAT
GCTACCACATTAAATACAATCACATTGTATTTCAGAACTTCTAGTGTCCGCACATAGAAGGTTAAAGA
TGGCTTTCTCTTAT
+
FFF,F:FFFFFFF:::FFFFF::FFF,F:FF:FF:FF:F,FFFF,FFFFFF,FFFF:FF:,FFFFF,,
::F,,,:FFFF::,FFFF::FF,::FFFF,,FFFF:,FF,:F,FFFF,,FFFFF,FFFF:FFFF,FFF
:FFFF,FFFFF,FF
@A00155:342:HHGFNDSXY:1:2301:18900:22795 2:N:0:GAACCTAG+TCCGCATA
ATACACTCACGTCGTGAGTAAACAGATTGACGTAACGCCTCAATCGTGGTCAAGGACCAGCATTCAAT
GCTACCACATTAAATACAATCACATTGTATTTCAGAACTTCTTGTGTCCGCACATAGAAGGTTAAAGA
TGGCTTTCTCTTAT
+
FFFFFFFFFFFFFFFFFFFFFFFFFFFFFFFFFFFFFFFFFFFFFFFFFFFFFFFFFFFFFFFFFFFF
FFFFFFFFFFFFFFFFFFFFFF:FF,FFFF:FFFFFFFFFFFF:FFFFFFFFFFFFFFFFFFFFFFFFF
FFFFFFFFFFFFFF
@A00155:342:HHGFNDSXY:1:2511:16957:2738 2:N:0:GAACCTAG+TCCGCATA
ATACACTCACGTCGTGAGTAAACAGATTGACGTAACGCCTCAATCGTGGTCAAGGACCAGCATTCAAT
GCTACCACATTAAATACAATCACATTGTATTTCAGAACTTCTTGTGTCCGCACATAGAAGGTTAAAGA
TGGCTTTCTCTTAT
+
FFFFFFF:FFF,FFFFFF,FFFFFF:FF:FFFFFFFFFFF,FFFF:F:FFFFFFFFFFF:F:FFFFF
FF:F:FF:F:F,FFFF:FFF:FF,FF,FFF:FFFFFFFFF:F,F:F,FF:F,FFFFFFFFFFFFF,FF
:FFFFFFFF,FFFF
@A00155:342:HHGFNDSXY:1:2247:15908:28479 1:N:0:GAACCTAG+TCCGCATA
ATACACTCACGTCGTGAGTAAACAGATTGACGTAACGCCTCAATCGTGGTCAAGGACCAGCATTCAAT
GCTACCACATTAAATACAATCACATTGTATTTCAGAACTTCTTGTGTCCGCACATAGAAGGTTAAAGA
TGGCTTTCTCTTATA
+
FFFFFFFFFFFFFFFFFFFFFFFFFFFFFFFFFFFFFFFFFFFFFFFFFFFFFFFFFFFFFFFFFFFF
FFFFFFFFFFFFFFFFFFFFFFFFFFFFFFFFFFFFFFFFFFFFFFFFFFFFFFFFFFFFFFFFFFFF
FFFFFFFFFFFFFFF
@A00155:342:HHGFNDSXY:1:1138:23647:21057 2:N:0:GAACCTAG+TCCGCATA
ATACACTCACGTCGTGAGTAAACAGATTGACGTAACGCCTCAATCGTGGTCAAGGACCAGCATTCAAT
GCTACCACATTAAATACAATCACATTGTATTTCAGAACTTCTTGTGTCCGCACATAGAAGGTTAAAGA
TGGCTTTCTCTTATA
+
FFFFFFFFFFF,FFFFFF:FFFFFFFFFFFFFFFFFFFFFFFFFFFFFFFFFFFFFFFFFFFFFFFFF
FFFF:FFFFFFFF:FFF:FFFFFF:FFFFFFFFFFF,FFFFFFFFFFFFFFFFFFFFFFFFFFFF:FF
FFF:FFF:FFF:FF:
@A00155:342:HHGFNDSXY:1:1215:22770:16814 2:N:0:GAACCTAG+TCCGCATA
ATACACTCACGTCGTGAGTAAACAGATTGACGTAACGCCTCAATCGTGGTCAAGGACCAGCATTCAAT

```
GCTACCACAATAAATACAATCACATAGTATTTCAGAACTTATTGTGTCCGCACATAGAAGGTTAAAGA
TGGCTTTCTCTTATA
+
F:FFFF,,FF:F,F,:F,FFFFFF,:F::F,::F::::FFFFF,,F:FFF:FF,FF:FF,,FFFFF:F
::,FF,F,F,FFFF,FFF:FFFFF:,F,FFF,F:,FF:,,,:,F:,FF,F,FFF,F,FF::FFFFFFF
,,:::,,F:,,,FFF
@A00155:342:HHGFNDSXY:1:1301:16821:30968 2:N:0:GAACCTAG+TCCGCATA
ATACACTCACGTCGTGAGTAAACAGATTGACGTAACGCCTCAATCGTGGTCAAGGACCAGCATTCAAT
GCTACCACATTAAATACAATCACATTGTATTTCAGAACTTCTTGTGTCCGCACATAGAAGGTTAAAGA
TGGCTTTCTCTTATA
+
FFFFFFFFFFFFFF,FFF:FFFFFFFFF:FF:FFFFFFFFFFFFFFF:FFFFF:FFFFF:FFFFFFFF
FFFFF:F:FFFF:FFFFFFFFF:FFFFFFFFFFFFFFFF:F,FFFFFFFFFFFFFF:FFF:,FFFFFF
F,FFFFFFF:FFF:F
@A00155:342:HHGFNDSXY:1:1348:32407:23109 2:N:0:GAACCTAG+TCCGCATA
ATACACTCACGTCGTGAGTAAACAGATTGACGTAACGCCTCAATCGTGGTCAAGGACCAGCATTCAAT
GCTACCACATTAAATACAATCACATTGTATTTCAGAACTTCTTGTGTCCGCACATAGAAGGTTAAAGA
TGGCTTTCTCTTATA
+
FFFFFFFFFF::FFFFFFFFFFFFFFFFFFFFFFFFFFFFFF:FFFFFFFFFFFFFFFFFFFFFFFFF
FFFFFFFFFFFFFF,FFFFFFF:F,:FFF:,FFFFF:FF,FFFFFFFFFFFFFF,:FF:FFFFFFFF:
FFFFF:FF:FFF,FF
@A00155:342:HHGFNDSXY:1:1478:20121:17613 2:N:0:GAACCTAG+TCCGCATA
ATACACTCACGTCGTGAGTAAACAGATTGACGTAACGCCTCAATCGTGGTCAAGGACCAGCATTCAAT
GCTACCACATTAAATACAATCACATTGTATTTCAGAACTTCTTGTGTCCGCACATAGAAGGTTAAAGA
TGGCTTTCTCTTATA
+
FFFFFF:FFFFFFFFFFFFFFFFFF:FFFFFFFFFFFFFFFFFFFFFFFFFFFFFFFFFFFFFFFFFF
FFFFFFFFFFFF:FFFFFFFFFFFF,FFFFFFFFFFFFFFFFFFFFFFFFFFFFFF:FFFFFFFFFFF
FFFFFFFFFFFFFFF
@A00155:342:HHGFNDSXY:1:1513:1768:2926 2:N:0:GAACCTAG+TCCGCATA
ATACACTCACGTCGTGAGTAAACAGATTGACGTAACGCCTCAATCGTGGTCAAGGACCAGCATTCAAT
GCTACCACATTAAATACAATCACATTGTATTTCAGAACTTCTTGTGTCCGCACATAGAAGGTTAAAGA
TGGCTTTCTCTTATA
+
FFFFFFFFFFFFFFFFFFFFFFFFFFFFF:FFFFFFFFFFFFFFFFFFFFFFFFFFFFFFFFFFFFFF
FFFFFFFFFF:FFFFFFFFFFFFFFFFFFFFFFFFFFFFFFFFFFFFFFFFFFFFFFFFFFFFFF:FF
FFFFFFFFFFFFFFF
@A00155:342:HHGFNDSXY:1:1520:32036:35055 2:N:0:GAACCTAG+TCCGCATA
ATACACTCACGTCGTGAGTAAACAGATTGACGTAACGCCTCAATCGTGGTCAAGGACCAGCATTCAAT
GCTACCACATTAAATACAATCACATTGTATTTCAGAACTTCTTGTGTCCGCACATAGAAGGTTAAAGA
TGGCTTTCTCTTATA
+
FFFFFFFFFFFFFFFFFFFFFFFFFFFFFFFFFFFFFFFFFFFFFFFFFFFFFFFFFFFFFFFFFFFF
FFFFFFFFFFFFFFFFFFFFFFFFFFFFFFFFFFFFFFFFFFFF:FFFFFFFFFFFFFFFFFF:FFFF
FFFFFFFFFFFFFFF
@A00155:342:HHGFNDSXY:1:1620:19054:32424 2:N:0:GAACCTAG+TCCGCATA
ATACACTCACGTCGTGAGTAAACAGATTGACGTAACGCCTCAATCGTGGTCAAGGACCAGCATTCAAT
GCTACCACATTAAATACAATCACATTGTATTTCAGAACTTCTTGTGTCCGCACATAGAAGGTTAAAGA
TGGCTTTCTCTTATA
+
FFFFFFFFFFFFFFFFFFFFFFFFFFFFFFFFFFFFFFFFFFFFFFFFFFFFFFFFFFFFFFFFFFFF
FFFFFFFFFFFFFFFFFFFFFFFF:FFFFFFFFFFF:FFFFFFFFFFFFFFFFFFFFFFFFFFFFFFF
FFFFFFFFFFFFFFF
```

```
@A00155:342:HHGFNDSXY:1:1656:25482:11522 2:N:0:GAACCTAG+TCCGCATA
ATACACTCACGTCGTGAGTAAACAGATTGACGTAACGCCTCAATCGTGGTCAAGGACCAGCATTCAAT
GCTACCACATTAAATACAATCACATTGTATTTCAGAACTTCTTGTGTCCGCACATAGAAGGTTAAAGA
TGGCTTTCTCTTATA
+
FFFFFFFFFFFFFFFFFFFFFFFFFFFFFFFFFFFFFFFFFFFFFFFFFFFFFFFFFFFFFFFFFFFF
FFFFFFFFFFFFFFFFFFFFFFFFFFFFFFFFFFFFFFFFFFFFFFFFFFFFFFFFFFFFFFFFFFFF
FFFFFFFFFFFFFFF
@A00155:342:HHGFNDSXY:1:2509:32353:36104 2:N:0:GAACCTAG+TCCGCATA
ATACACTCACGTCGTGAGTAAACAGATTGACGTAACGCCTCAATCGTGGTCAAGGACCAGCATTCAAT
GCTACCACATTAAATACAATCACATTGTATTTCAGAACTTCTTGTGTCCGCACATAGAAGGTTAAAGA
TGGCTTTCTCTTATA
+
F:FFFFFFFFFFFFFFFFFF,FFFFFFFFFFFFFFFFFFFFFFFFFFFFFFFFFFFFFFFFFFFFFFF
FFFFFFFFFFFFFFFFFF,FFFFFF:FFFF,FFFFF:FFFFFFFFFFFFFFFFF,FFFFFFFFFFFFF
FFFFFFFFFFFFFFF
@A00155:342:HHGFNDSXY:1:2633:30454:30937 2:N:0:GAACCTAG+TCCGCATA
ATACACTCACGTCGTGAGTAAACAGATTGACGTAACGCCTCAATCGTGGTCAAGGACCAGCATTCAAT
GCTACCACATTAAATACAATCACATTGTATTTCAGAACTTCTTGTGTCCGCACATAGAAGGTTAAAGA
TGGCTTTCTCTTATA
+
:FFFFFFFFFFFFFFFFFFFF:FFFFFFFFFFFFFFFFFFFFFFFFFFFFFFFFFFFFFFFFFFFFFF
FFFFFFFFFF:FFFFF:FFFFFFF:FFFFFFFFFFFFFFFFFFFFFFFFFFFFFFFFFFFFFFFF:FF
FFFFFFFFFFFFF::
@A00155:342:HHGFNDSXY:1:2666:23484:31861 2:N:0:GAACCTAG+TCCGCATA
ATACACTCACGTCGTGAGTAAACAGATTGACGTAACGCCTCAATCGTGGTCAAGGACCAGCATTCAAT
GCTACCACATTAAATACAATCACATTGTATTTCAGAACTTCTTGTGTCCGCACATAGAAGGTTAAAGA
TGGCTTTCTCTTATA
+
FFFFFF:FFFFFFFFFFFFFFFFFFFFFFFFFFFFFFFFFFFFFFFFFFFF,FFFFFFFFFFFFFFF:
FF:FFFFF:F:F:FFFFFFFFFFFF:FFFFFFFFFFFFFFFFFFFFFFFFFFFFFF:FFF:FF:FFF:
FFFFFFF:FFFFFFF
@A00155:342:HHGFNDSXY:1:1323:18792:19633 2:N:0:GAACCTAG+TCCGCATA
ACACTCACGTCGTGAGTAAACAGATTGACGTAACGCCTCAATCGTGGTCAAGGACCAGCATTCAATGC
TACTACATTAAATACAATCACATTGTATTTCAGAACTTCTTGTGTCCGCACATAGAAGGTTAAAGATG
GCTTTCTCTTATAGG
+
FFFFFFFFFFFFFFFFFFFFFFFFFFFFFFFFFFFFFFFFFFFFFFFFFFFFFFFFFFFFFFFFFFFF
::FFFFFFFFFFFFFFFFFF:F,FFFF:FFFFFFFFFFFFFFFFFFFFFFFFFFFFFFFFFF:FFFFF
FFFFFFFFFFFFFFF
@A00155:342:HHGFNDSXY:1:2317:17499:8656 2:N:0:GAACCTAG+TCCGCATA
ACACTCACGTCGTGAGTAAACAGATTGACGTAACGCCTCAATCGTGGTCAAGGACCAGCATTCAATGC
TACCACATTAAATACAATCACATTGTATTTCAGAACTTCTTGTGTCCGCACATAGAAGGTTAAAGATG
GCTTTCTCTTATAGG
+
FFFFFFFFFFFF:FFFFFF,FFFF:FFFFFFFFFFFFFFFFFFFFFFFFFFFFFFFFFFFFF:FFFFF
FFFFFFFFFFFFFFFF:FFFFF:FF,F,FFFFFFFFFFFFFFFFFFFFF,FFFFFFFFFFFFFFFFFF
FFF,FFFFFFFFFFF
@A00155:342:HHGFNDSXY:1:2450:20573:33395 2:N:0:GAACCTAG+TCCGCATA
ACACTCACGTCGTGAGTAAACAGATTGACGTAACGCCTCAATCGTGGTCAAGGACCAGCATTCAATGC
TACCACATTAAATACAATCACATTGTATTTCAGAACTTCTTGTGTCCGCACATAGAAGGTTAAAGATG
GCTTTCTCTTATAGG
+
FFFFFFFFFFFFFFFFFFFFFFFFFFF,FFFFFFFFFFF:FFFFFFFFFFFFFFFFFFFFFFFFFFFF
```

FFFFFFFF::FFFFFFFFFFFFFFFFF:FFFFFF:FFFFFFFFFFFFFFFFFFFFFFFFFFFFFFFFF
FFFFFFFFFF:FF:F
@A00155:342:HHGFNDSXY:1:1647:13277:32941 2:N:0:GAACCTAG+TCCGCATA
CACTCACGTCGTGAGTAAACAGATTGACGTAACGCCTCAATCGTGGTCAAGGACCAGCATTCAATGCT
ACCACATTAAATACAATCACATTGTATTTCAGAACTTCTTGTGTCCGCACATAGAAGGTTAAAGATGG
CTTTCTCTTATAGGA
+
FFFFFFFFFFFFFFFFFFFFFFFFFF:FFFFFFFFFFFFFFFFFFFFFFFFFFFFFFFFFFFFFFFFF
FFFFFFFFFFFFFFFFFFF,FFFFFFFFFFFFFFF:FFFFFFF:FFFFFFFFFFFFFFFFFF:FFFFF
FFFFFFFFFFFFFFF
@A00155:342:HHGFNDSXY:1:2344:18376:6104 2:N:0:GAACCTAG+TCCGCATA
CACTCACGTCGTGAGTAAACAGATTGACGTAACGCCTCAATCGTGGTCAAGGACCAGCATTCAATGCT
ACCACATTAAATACAATCACATTGTATTTCAGAACTTCTTGTGTCCGCACATAGAAGGTTAAAGATGG
CTTTCTCTTATAGGA
+
FFFFFFFFFFFFFFFFFFFFFFFFFFFFFFFFFFFF:FFFFFFFFFFFFFFFFFFFFFFFFFFFFFFF
,FFFFFFFFFFFFFFFFFFFFFFFFFFFFFFFFFF:FFFFFFFFFFFFFFF:FFFFFFFFFFFFFFFF
FFFFFFFFFFFFFFF
@A00155:342:HHGFNDSXY:1:2506:21169:34867 2:N:0:GAACCTAG+TCCGCATA
CACTCACGTCGTGAGTAAACAGATTGACGTAACGCCTCAATCGTGGTCAAGGACCAGCATTCAATGCT
ACCACATTAAATACAATCACATTGTCTTTCAGAACTTCTTGTGTCCGCACCTAGAAGGTTAAAGATGG
CTTTCTCTTATAGGA
+
FFFFFFFFFFFFFFFFFFFFFFFFFFFFFFFFFFFFF:FFFFFFFFFFFFFFFFFFFFFFFFF,FFFF
FFFFFFFFFFFFFFFFFFFFFF:,F::FFFFFFFF:FFF,FF,FFFFFFFF,:,FF:FFFFF,FFF,FF
FFFFFFFFF:FFFF:
@A00155:342:HHGFNDSXY:1:1101:2600:5776 2:N:0:GAACCTAG+TCCGCATA
CTCACGTCGTGAGTAAACAGATTGACGTAACGCCTCAATCGTGGTCAAGGACCAGCATTCAATGCTAC
TACATTAAATACAATCACATTGTATTTCAGAACTTCTTGTGTCCGCACATAGAAGGTTAAAGATGGCT
TTCTCTTATAGGACG
+
FFFFFFFF:FFFFFFFFFFFFFFFFFFFFFFFFFFFFFFFFFFFFFFFFFFFFFFFFFFFFFFFFFFF
FFFFFFFFFFFFFFFFF:FFFFFFFFFFFFFFF:FFFFFFFFFFFFFFFFFFFF:FFFFF:FF:FFF
:FFFFFFFFFFFFF:
@A00155:342:HHGFNDSXY:1:1129:28474:36464 2:N:0:GAACCTAG+TCCGCATA
CTCACGTCGTGAGTAAACAGATTGACGTAACGCCTCAATCGTGGTCAAGGACTAGCATTCAATGCTAC
CACATTAAATACAATCACATTGTATTTCAGAACTTCTTGTGTCCGCACATAGAAGGTTAAAGATGGCT
TTCTCTTATAGGACG
+
FFFFFFFFFFFFFFFFFFFFFFFFFFFFFFFFFFFFFFFFFFFFFFFFF,FFFFFFFFFFFFFFFFFF
FFFFFFFFFFFFFFFFF:F:FFFFFFFFFFFFFFFF:FFF,FFFFFFFFFF,FFFFFFFFFFFFF:FF
FFFFFFFFF:FFFF:
@A00155:342:HHGFNDSXY:1:1270:12292:23375 2:N:0:GAACCTAG+TCCGCATA
CTCACGTCGTGAGTAAACAGATTGACGTAACGCCTCAATCGTGGTCAAGGACCAGCATTCAATGCTAC
CACATTAAATACAATCACATTGTATTTCAGAACTTCTTGTGTCCGCACATAGAAGGTTAAAGATGGCT
TTCTCTTATAGGACG
+
FFFFFFFFFFFFFFFF:FFFFFFFFFFFFFFFFFFFFFFFFFFFFFFFFFFFFFFFFFFFFFFFFFFF
FFFFFFFFFFFFFFFFFFFFFFFFF:FFFFFFFFFFFFFFFFFFFFFFFFFFFFFF:FFFFFFFFFFFFF
FFFFFFFFFFFFFFF
@A00155:342:HHGFNDSXY:1:1335:6786:19727 2:N:0:GAACCTAG+TCCGCATA
CTCACGTCGTGAGTAAACAGATTGACGTAACGCCTCAATCGTGGTCAAGGACCAGCATTCAATGCTAC
TACATTAAATACAATCACATTGTATTTCAGAACTTCTTGTGTCCGCACATAGAAGGTTAAAGATGGCT
TTCTCTTATAGGACG

```
+
FFFFFFFFFFFFFFFFFFFFFFFFFFFFFFFFFFFFFFFFFFFFFFFFFFFFFFFFFFFFFFFFFFFFFF
FFFFFFFFFFFFFFFFFFF:FFFFFFFFFFFFFFFFFFFFFFFFF:FFFFFFFFFFFFFFFFFFFFFFFF
FFFFFFFFFFFFFFF
@A00155:342:HHGFNDSXY:1:1410:13376:23062 2:N:0:GAACCTAG+TCCGCATA
CTCACGTCGTGAGTAAACAGATTGACGTAACGCCTCAATCGTGGTCAAGGACCAGCATTCAATGCTAC
CACATTAAATACAATCACATTGTATTTCAGAACTTCTTGTGTCCGCACATAGAAGGTTAAAGATGGCT
TTCTCTTATAGGACG
+
FFFFFFFFF:FFFFFFFFFFFFFFFFFFFFFFFFFFFFFFFFFFFFFFFFFFFFFFFFFFFFFFFFFFFF
FFFFFFFFFFFFFFFFFFFFFF,FFFFFFFFFFFFFFFFFFFFFFFFFF:F:FFFFFFFFFFFFFFFFFF
FFFFFFFFFFFFFFF
@A00155:342:HHGFNDSXY:1:1472:2356:31657 2:N:0:GAACCTAG+TCCGCATA
CTCACGTCGTGAGTAAACAGATTGACGTAACGCCTCAATCGTGGTCAAGGACCAGCATTCAATGCTAC
TACATTAAATACAATCACATTGTATTTCAGAACTTCTTGTGTCCGCACATAGAAGGTTAAAGATGGCT
TTCTCTTATAGGACG
+
FFFFFFFFFFFFFFFFFFFFFFFF:FFFFFFFFFFFFFFFFFFFFFFFFFFFFFFFFFFFFFFFFFFFFF
FFF:FFFFFFFFFFFFFFFFFFFFFFFFFFFFFFFFFFFFFFFFFF:F:FFFFFFFFFFFFFF,FF,FFF
FF:FFFFFFF,,F:F
@A00155:342:HHGFNDSXY:1:1611:7898:10347 2:N:0:GAACCTAG+TCCGCATA
CTCACGTCGTGAGTAAACAGATTGACGTAACGCCTCAATCGTGGTCAAGGACCAGCATTCAATGCTAC
CACATTAAATACAATCACATTGTATTTCAGAACTTCTTGTGTCCGCACATAGAAGGTTAAAGATGGCT
TTCTCTTATAGGACG
+
FFFFFFFFFFFFFFFFFFFFFFFFFFFFFFFFFFFFFFFFFFFFFFFFFFFFFFFFFFFFFFFFFFFFFF
FFFFFFFFFFFFFFFFF:F,FFFF:FFFFFFFFFFFFFFFFFFFFFFFFFFFFFFFFFFFFFFFFFFFFF
FFFFFFFFFFFFF:F
@A00155:342:HHGFNDSXY:1:2112:5231:31501 2:N:0:GAACCTAG+TCCGCATA
CTCACGTCGTGAGTAAACAGATTGACGTAACGCCTCAATCGTGGTCAAGGACCAGCATTCAATGCTAC
CACATTAAATACAATCACATTGTATTTCAGAACTTCTTGTGTCCGCACATAGAAGGTTAAAGATGGCT
TTCTCTTATAGGACG
+
FFFFFFFFFFFFFFFF:FFFFFFFFFFFFFFFFFFFFFFFFFFFFFFFFFFFFFFFFFFFFFFFFFFFF:
FFFFFFFFFFFFFFFFFFFF:FF:FFFFFFFFFFFFFFFFFFFF:FFFFFFFFFFF:FFFFFFFFFFF:F
FFFF,FFFFFFFFFF
@A00155:342:HHGFNDSXY:1:2139:8938:23891 2:N:0:GAACCTAG+TCCGCATA
CTCACGTCGTGAGTAAACAGATTGACGTAACGCCTCAATCGTGGTCAAGGACCAGCATTCAATGCTAC
TACATTAAATACAATCACATTGTATTTCAGAACTTCTTGTGTCCGCACATAGAAGGTTAAAGATGGCT
TTCTCTTATAGGACG
+
FFFFFFFFFFFFFFFFFFFFFFFFFFFFFFFFFFFFFFFFFFFFFFFFFFFFFFFFFFFFFFFFFFFF:F
FFFFF:FFFFF:::FFFFFF:FF:FFFFFFFFFFFFFFFFFFFFFFFF:FF:FFFFFFFFFFFFFFFFFF
FFFF:FF:FFFFFF:
@A00155:342:HHGFNDSXY:1:2122:26051:16517 2:N:0:GAACCTAG+TCCGCATA
CTCACGTCGTGAGTATACAGATTGACGTAACGCCTCAATCGTGGTCAAGGACCAGCATTCAATGCTAC
CACATTAAATACAATCACATTGTATTTCAGAACTTCTTGTGTCCGCACATAGAAGGTTAAAGATGGCT
TTCTCTTATAGGACG
+
FFFFFFFFF:FFFFFFFFFFFFFFFFFFFFFFFFFFFFFFFFFFFFFFFFFFFFFFFFFFFFFFFFFFFF
FFFFFFFFFFFFFFFFFFFFFFFFFFFFFFFFFFFFFFFFFFFFF:FFFFFFFFFFFFFFFF:FFF:FFF
FFFFFFFFFFFFFFF
@A00155:342:HHGFNDSXY:1:2123:25825:1094 2:N:0:GAACCTAG+TCCGCATA
CTCACGTCGTGAGTATACAGATTGACGTAACGCCTCAATCGTGGTCAAGGACCAGCATTCAATGCTAC
```

CACATTAAATACAATCACATTGTATTTCAGAACTTCTTGTGTCCGCACATAGAAGGTTAAAGATGGCT
TTCTCTTATAGGACG
+
FFFFFFFFFFFFFFFFFFFFFFFFFFFFFFFFFFFFFFFFFFFFFFFFFFFFFFFFFFFFFFFFFFFF
FFFFFFFFFFFFFFFFFFFFFFFFFFFFFFFFFFFFFFFFFFFFFFFF:FFFFFFFFFFFFFFFFF:F
FFFFFFFFFFFFFFF
@A00155:342:HHGFNDSXY:1:2237:31711:3490 2:N:0:GAACCTAG+TCCGCATA
CTCACGTCGTGAGTAAACAGATTGACGTAACGCCTCAATCGTGGTCAAGGACCAGCATTCAATGCTAC
CACATTAAATACAATCACATTGTATTTCAGAACTTCTTGTGTCCGCACATAGAAGGTTAAAGATGGCT
TTCTCTTATAGGACG
+
FFFFFFFFF:FFFFFFFFFFFFFFFFFFFFFFFFFFFFFFFFFFFFFFFFFFFFFFFFFFFFFFFFFF
FFFFFFFFFFFF:F:FFFF,FFFFFFFFFFFFFFFFFFFFFFFFFFFFF:FFFFFFFF,FFFFFFFFF
FFFFFFFFFFFFFFF
@A00155:342:HHGFNDSXY:1:2237:31485:3944 2:N:0:GAACCTAG+TCCGCATA
CTCACGTCGTGAGTAAACAGATTGACGTAACGCCTCAATCGTGGTCAAGGACCAGCATTCAACGCTAC
CACATTAAATACAATCACATTGTATTTCAGAACTTCTTGTGTCCGCACATAGAAGGTTAAAGATGGCT
TTCTCTTATAGGACG
+
FFFFFFFFFFFFFFFFFFFFFFFFFFFFFFFFFFFFFFFFFFFFFFFFFFFFFFFFFFFFFF,FFFFF
FFFFFFFFFFFFFFFFFFFFFFFFFFFFFFFFFFFFFFFFFFFFFFFFFFFFFFFFFFFFFFFFFFFF
FFFFFFFFFFFFFFF
@A00155:342:HHGFNDSXY:1:2247:30264:27915 2:N:0:GAACCTAG+TCCGCATA
CTCACGTCGTGAGTAAACAGATTGACGTAACGCCTCAATCGTGGTCAAGGACCAGCATTCAATGCTAC
TACATTAAATACAATCACATTGTATTTCAGAACTTCTTGTGTCCGCACATAGAAGGTTAAAGATGGCT
TTCTCTTATAGGACG
+
FFFFFFFFFFF:FFF::FFFFFFF:FFFFFFFFFFFFFFFFFFFFF:FFFFFFFFFFFFFFFFFFFFF
FFFFFFFFFFFFFFFFFFFFFFFFFFFF:FFFFFFFFFFFFFFFFFFFFFFFFFFFFFFFFFFFFFFF
FFFFFFFFFFFFFFF
@A00155:342:HHGFNDSXY:1:2366:15320:1313 2:N:0:GAACCTAG+TCCGCATA
CTCACGTCGTGAGTAAACAGATTGACGTAACGCCTCAATCGTGGTCAAGGACTAGCATTCAATGCTAC
CACATTAAATACAATCACATTGTATTTCAGAACTTCTTGTGTCCGCACATAGAAGGTTAAAGATGGCT
TTCTCTTATAGGACG
+
FFFFFFFFFFFFFFFFFFFFFFFFFFFFFFFFFFF:FFFFFFFFFFFFFFFFFFFFFFFFFFFFFFFF
FFFFFFFF:FFFFFFFFFFF:FFFFFFFFFFFFFF:FFFFFFFFFFFFFFFFFFFFFFFF,FFFFFFF
FFFFFFFFFFFFFFF
@A00155:342:HHGFNDSXY:1:2366:23656:28181 2:N:0:GAACCTAG+TCCGCATA
CTCACGTCGTGAGTAAACAGATTGACGTAACGCCTCAATCGTGGTCAAGGACCAGCATTCAATGCTAC
CACATTAAATACAATCACATTGTATTTCAGAACTTCTTGTGTCCGCACATAGAAGGTTAAAGATGGCT
TTCTCTTATAGGACG
+
FFFFFFFFFFFFFFFFFFFFFFFFFFFFFFFFFFFFFFFFFFFFFFFFFFFFFFFFFFFFFFFFFFFF
FFFFFFFFFFFFFFFFFFFFFFFFFFFFFFFFFFFFFFFFFFFFFFFFFFFFFFFFFFFFFFFFFFFF
FFFFF:FFFFFFFFF
@A00155:342:HHGFNDSXY:1:2452:29767:7138 2:N:0:GAACCTAG+TCCGCATA
CTCACGTCGTGAGTATACAGATTGACGTAACGCCTCAATCGTGGTCAAGGACCAGCATTCAATGCTAC
CACATTAAATACAATCACATTGTATTTCAGAACTTCTTGTGTCCGCACATAGAAGGTTAAAGATGGCT
TTCTCTTATAGGACG
+
FFFFFFFFFFFFFFFFFFFFFFFFFFFFFFFFFFFFFFFFFFFFFFFFFFFFFFFFFFFFFFFFFFFF
FFFFFFFFFFFF,F:FFFF:FF:FFFFFFFF:FFFFFFFFFFFF:FFFF,FFFFFFFFFFFFFFFFFF
F:FFFFFFFFFFFFF

```
@A00155:342:HHGFNDSXY:1:2567:9100:20823 2:N:0:GAACCTAG+TCCGCATA
CTCACGTCGTGAGTAAACAGATTGACGTAACGCCTCAATCGTGGTCAAGGACCAGCATTCAATGCTAC
CACATTAAATACAATCACATTGTATTTCAGAACTTCTTGTGTCCGCACATAGAAGGTTAAAGATGGCT
TTCTCTTATAGGACG
+
FFFFFFFFFFFFFFFFFFFFFFFFFFFFFFFFFFFFFFFFFFFFFFFFFFFFFFFFFFFFFFFFFFFF
FFFFFFFFFFFFFFFFFFFF:FFFFFFFFFFFFFF:FFFFFFFFFFFFFFFFFFFFFFFFFFFFFFFF
FFFFFF:FFFFFFFF
@A00155:342:HHGFNDSXY:1:2571:19958:6496 2:N:0:GAACCTAG+TCCGCATA
CTCACGTCGTGAGTAAACAGATTGACGTAACGCCTCAATCGTGGTCAAGGACCAGCATTCAATGCTAC
CACATTAAATACAATCACATTGTATTTCAGAACTTCTTGTGTCCGCACATAGAAGGTTAAAGATGGCT
TTCTCTTATAGGACG
+
FFFFFFFFFFFFFFFFFFFFFFFFFFFFFFFFFFFFFFFFFFFFFFFFFFFFFFFFFFFFFFFFFFFF
FFFFFFFFFFFFFFFFFFFFFFFFFFFFFFF:FFFFFFFFFFFFFFFFFFFFFFFFFFFFFF,FFFFFFF
FFFFFFFFFFFFFFF
@A00155:342:HHGFNDSXY:1:2631:28845:31751 1:N:0:GAACCTAG+TCCGCATA
TCACGTCGTGAGTAAACAGATTGACGTAACGCCTCAATCGTGGTCAAGGACCAGCATTCAATGCTACC
ACATTAAATACAATCACATTGTATTTCAGAACTTCTTGTGTCCGCACATAGAAGGTTAAAGATGGCTT
TCTCTTATAGGACGC
+
FFFFFFFF:FFFFFFFFFFFFFFFFFFFFFFFFFFFFFFFFFFFFFFFFFFFFFFFFFFFFFFFFFFF
FFFFFFFFFFFFFFFFFFFFFFFFFFFFFFFFFFFFFFFFFFFFFFFFFFFFFFFFFFFFFFFFFFFF
FFFFFFFFFFFFFFF
@A00155:342:HHGFNDSXY:1:2464:14687:10488 2:N:0:GAACCTAG+TCCGCATA
CACGTCGTGAGTAAACAGATTGACGTAACGCCTCAATCGTGGTCAAGGACCAGCATTCAATGCTACCA
CATTAAATACAATCACATTGTATTTCAGAACTTCTTGTGTCCGCACATAGAAGGTTAAAGATGGCTTT
CTCTTATAGGACGC
+
FF::F:FFFF,FFF::FFFFF,F::FFFFF,:F:FFFF,FFFFFFF:,FFFFFFFFFFFFFFFFF::F
FF:FFFFFFFFFF,F:FF,,FFF:FFF,FFFFFFFFFFFFF,FF:FFFF:,FF::FFFF:F:F::FFF
:FFFFFFF:FFFFF
@A00155:342:HHGFNDSXY:1:1519:30608:30702 1:N:0:GAACCTAG+TCCGCATA
CACGTCGTGAGTAAACAGATTGACGTAACGCCTCAATCGTGGTCAAGGACCAGCATTCAATGCTACCA
CATTAAATACAATCACATTGTATTTCAGAACTTCTTGTGTCCGCACATAGAAGGTTAAAGATGGCTTT
CTCTTATAGGACGCC
+
FFFFF:FFFFFFFFFFFFFFFFFFFFFFFFF,FFFFFFFFFFFFFFFFFFFFFFFFFFFFFFFFFFF:FF
FFFFFFFFFFFFFFFF:FFFFFFFFFFFFFFFFFFFFFFFFFFFFFFFFFFFFFFFFFFF:FFFFFFFF
FFFFFFFFFFFFFFF
@A00155:342:HHGFNDSXY:1:1442:6334:33755 2:N:0:GAACCTAG+TCCGCATA
CACGTCGTGAGTAAACAGATTGACGTAACGCCTCAATCGTGGTCAAGGACCAGCATTCAATGCTACCA
CATTAAATACAATCACATTGTATTTCAGAACTTCTTGTGTCCGCACATCGAAGGTTAAAGATGGCTTT
CTCTTATAGGACGCC
+
FFFFFFFFFFFFFFFFFFFFFFFFFFFFFFFFFFFFFFFFFFFFFFFFFFFFFFFFFFFFFFFFFFFF
FFFFFFFFFFFFFFFFFFFFFFFFFFFFFFFFF:FFFFFFFFFFFFFFF,:FFFFFFFFFFFFFFF:FF
FFFFFFFFFFFFFFF
@A00155:342:HHGFNDSXY:1:1676:9146:35430 2:N:0:GAACCTAG+TCCGCATA
CACGTCGTGAGTAAACAGATTGACGTAACGCCTCAATCGTGGTCAAGGACCAGCATTCAATGCTACCA
CATTAAATACAATCACATTGTATTTCAGAACTTCTTGTGTCCGCACATAGAAGGTTAAAGATGGCTTT
CTCTTATAGGACGCC
+
FFFFFFFFFFFFFFFFFFFFFFFFFFFFFFFFFFFFFFFFFFFFFFFFFFFF:FFFFFFFFFFFF:FFF
```

```
FFFFFFFFFFFFFFFFFFFFFFFFFFFFFFFFFFF:FFF:FFFFFFFFF:FFFFFFFFF,FFFFFF:F
FFFFFFFFFFFFF:F
@A00155:342:HHGFNDSXY:1:2510:28501:27273 2:N:0:GAACCTAG+TCCGCATA
CACGTCGTGAGTAAACAGATTGACGTAACGCCTCAATCGTGGTCAAGGACCAGCATTCAATGCTACCA
CATTAAATACAATCACATTGTATTTCAGAACTTCTTGTGTCCGCACATAGAAGGTTAAAGATGGCTTT
CTCTTATAGGACGCC
+
FFFFFFFFFFFFFFFFFFF:FFFFFF:FFFFFFF:,FFFFFFFF:FFFFFFFFFFFFFFFFFFFFFFF
FF:FFFFF:FFFFFF:,FFFF:FFFF,F:,F:FFFFFFFFFFFFFF,,FFFFF:FFFFFFF,:FFFF
:FF,F:F:FFFF,FF
@A00155:342:HHGFNDSXY:1:1278:18493:8719 2:N:0:GAACCTAG+TCCGCATA
GTCGTGAGTAAACAGATTGACGTAACGCCTCAATCGTGGTCAAGGACCAGCATTCAATGCTACCACAT
TAAATACAATCACATTGTATTTCAGAACTTCTTGTGTCCGCACATAGAAGGTTAAAGATGGCTTTCTC
TTATAGGACGCCAC
+
FFFFFFFFFFFFFFFFFFFFFFFFF:FFFFFFFFFFFFFFFFFFFFFFFFFFFFFFFFFFFFFFFFFF
FFFFFFFFFFFFFFF:FFFFFFFFFFFFFFFFFFFFFFFFFFFFFFFFFFFFFFFFFFFFFFFFFFFF
FFFFFFFFFFFFFF
@A00155:342:HHGFNDSXY:1:1323:5828:30624 2:N:0:GAACCTAG+TCCGCATA
GTCGTGAGTAAACAGATTGACGTAACGCCTCAATCGTGGTCAAGGACCAGCATTCAATGCTACCACAT
TAAATACAATCACATTGTATTTCAGAACTTCTTGTGTCCGCACATAGAAGGTTAAAGATGGCTTTCTC
TTATAGGACGCCAC
+
FFFFFFFFFFFFFFFFFFFFFFFFFFFFFFFFFFFFFFFFFFFFF:FFFFFFFFFFFFFFFFFFFFFF
FFFFFFFF:FFFFFFFFFFFFFF,FFFFFFFFFFFFFFFFFFFFFFFFFFF,FFFFFFFFFFFFFFFF
FFFFFFFFFFFFFF
@A00155:342:HHGFNDSXY:1:1227:27977:2065 2:N:0:GAACCTAG+TCCGCATA
GTCGTGAGTAAACAGATTGACGTAACGCCTCAATCGTGGTCAAGGACCAGCATTCAATGCTACCACAT
TAAATACAATCACATTGTATTTCAGAACTTCTTGTGTCCGCACATAGAAGGTTAAAGATGGCTTTCTC
TTATAGGACGCCACA
+
FFFFFFFFFFFFFFFFFFFFFFFFFFFFFFFFFFFFFFFFFFFFFFFFFFFFFFFFFFFF:FFFFFFF
FFFFFFFF:FFFFFFFF:FFFFFF:FFFFFFFFFFFFFFFFFFFFFFFFFF:FFFFFFFFF,FFFFFFF
FFFFFF:FFFFFFFF
@A00155:342:HHGFNDSXY:1:1233:12825:27085 2:N:0:GAACCTAG+TCCGCATA
GTCGTGAGTAAACAGATTGACGTAACGCCTCAATCGTGGTCAAGGACCAGCATTCAATGCTACCACAT
TAAATACAATCACATTGTATTTCAGAACTTCTTGTGTCCGCACATAGAAGGTTAAAGATGGCTTTCTC
TTATAGGACGCCACA
+
FFFFFFFFFFFFFFFFFFFFFFFFFFFFFFFFFFFFFFFFFFFFFFFFFFFFFFFFFFFFFFFFFFFF
FFFF:FFFFFFFFFFFFFFFFFFFFFFFFFFFFFFFFFFFFFFFFFFFFFFFF:FFFFFFFFFFFF
FFFFFFFFFFFFFFF
@A00155:342:HHGFNDSXY:1:1243:30942:6543 2:N:0:GAACCTAG+TCCGCATA
GTCGTGAGTAAACAGATTGACGTAACGCCTCAATCGTGGTCAAGGACCAGCATTCAATGCTACCACAT
TAAATACAATCACATTGTATTTCAGAACTTCTTGTGTCCGCACATAGAAGGTTAAAGATGGCTTTCTC
TTATAGGACGCCACA
+
FFFFFFFFFFFFFFFFFFFFFFFFFFFFFFFFFFFFFFFFFFFFFFFFFFFFFFFFFFFFFFFFFFFF
F:FF:FFF:FFFFFFFFFFFFFFFFFFFFFFF:FFFFFFFFFFFFFFFFFFFFFFFFFFFFFFFFFFF
FFFFFFFFFFFFFF:
@A00155:342:HHGFNDSXY:1:1507:17653:21418 2:N:0:GAACCTAG+TCCGCATA
GTCGTGAGTAAACAGATTGACGTAACGCCTCAATCGTGGTCAAGGACCAGCATTCAATGCTACCACAT
TAAATACAATCACATTGTATTTCAGAACTTCTTGTGTCCGCACATAGAAGGTTAAAGATGGCTTTCTC
TTATAGGACGCCACA
```

+
FFFFFFFFFFFFFFFFFFFFFFFFFFFFFFFFFFFFFFFFFFFFFFFFFFFFFFFFFFFFFFFFFFFF
FFFFFFFFFFFFFFFFFFFF:FFFFFFFFFFF:FFFFFFFFFFF:FFFFFFFFFFFFFFFFFFFFFFF
FFFFF:FFFFFF,F:
@A00155:342:HHGFNDSXY:1:1578:5638:22185 2:N:0:GAACCTAG+TCCGCATA
GTCGTGAGTAAACAGATTGACGTAACGCCTCAATCGTGGTCAAGGACCAGCATTCAATGCTACCACAT
TAAATACAATCACATTGTATTTCAGAACTTCTTGTGTCCGCACATAGAAGGTTAAAGATGGCTTTCTC
TTATAGGACGCCACA
+
FFFFFFFFFFFFFFFFFFFFFFFFFFFFFFFFFFFFFFFFFFFFFFFFFFFFFFFFFFFFFFFFFFFF
FFFFFFFFFFFFFFF:FFFFFFF:FFFFFFFFFF:FFFFFFFFFFFFFFFFFFFFFF,FFFFF:FFFF
:FFFFFFFFFFF,FF
@A00155:342:HHGFNDSXY:1:2259:8947:6026 2:N:0:GAACCTAG+TCCGCATA
GTCGTGAGTAAACAGATTGACGTAACGCCTCAATCGTGGTCAAGGACCAGCATTCAATGCTACCACAT
TAAATACAATCACATTGTATTTCAGAACTTCTTGTGTCCGCACATAGAAGGTTAAAGATGGCTTTCTC
TTATAGGACGCCACA
+
FFFFFFFFFFFFFFFFFFFFFFFFFFFFFFFFFFFFFFFFFFFFFFFFFFFFFFF:FFFFFFFFFFFF
FFFFFFFFFFFFFF,FFFFFFFFFFFFFFFFFFFFFFFFFFFFFFFFF:FFFFFFFFFFFFFFFFFFF
FFFFFFFFFFFFFFF
@A00155:342:HHGFNDSXY:1:2663:10511:30029 2:N:0:GAACCTAG+TCCGCATA
GTCGTGAGTAAACAGATTGACGTAACGCCTCAATCGTGGTCAAGGACCAGCATTCAATGCTACCACAT
TAAATACAATCACATTGTATTTCAGAACTTCTTGTGTCCGCACATAGAAGGTTAAAGATGGCTTTCTC
TTATAGGACGCCACA
+
FFFFFFFFFFFFFFFFFFFFFFFFFFFFFFFFFFFFFFFFFFFFFFFFFFFFFFFFFFFFFFFFFFFF
:F:FFFFFFFFFFFFFFFFFFFFFFFFFFFFFFFFFFFF:FFFFFFFFFFFFFFFFFFFFFFFFFFFF
FFFFFFFFFFFFFFF
@A00155:342:HHGFNDSXY:1:2675:32913:23923 2:N:0:GAACCTAG+TCCGCATA
GTCGTGAGTAAACAGATTGACGTAACGCCTCAATCGTGGTCAAGGACCAGCATTCAATGCTACCACAT
TAAATACAATCACATTGTATTTCAGAACTTCTTGTGTCCGCACATAGAAGGTTAAAGATGGCTTTCTC
TTATAGGACGCCACA
+
FFFFFFFFFFFFFFFFFFFFFFFFFFFFFFFF:FFFFFFFFFFFFFFFFFFFFFFFFFFFFFFFFFFF
:FFFFFFFFFFFFFFFFFFFFFFFF:FFFFFFFFFFFFFFFFFFFFFFFFFFFFFFFFFFFFFFFFFFFFFFFFFFFFFFFFFFFFFFFFFFFFFFFFFFFFFFFFF
FFFFF:F:FFFFFFF
@A00155:342:HHGFNDSXY:1:2678:20383:24361 2:N:0:GAACCTAG+TCCGCATA
GTCGTGAGTAAACAGATTGACGTAACGCCTCAATCGTGGTCAAGGACCAGCATTCAATGCTACTACAT
TAAATACAATCACATTGTATTTCAGAACTTCTTGTGTCCGCACATAGAAGGTTAAAGATGGCTTTCTC
TTATAGGACGCCACA
+
FFFFFFFFFFFFFFFFFFFFFFFFFFFFFFFFFFFFFFFFFFFFFFFFFFFFFFFFFFFFFFFFFFFF
FFFFFFFFFFFFFFFFFFFFFFFFFFFFFFFFFFFFFFFFFFFF,FFFFFFFFFFFFFFFFFFFFFFF
FFFFFFFFFFFFFFF
@A00155:342:HHGFNDSXY:1:1402:27670:23923 1:N:0:GAACCTAG+TCCGCATA
TAGTGAGTAAACAGATTGACGTAACGCCTCAATCGTGGTCAAGGACCAGCATTCAATGCTACCACATT
AAATACAATCACATTGTATTTCAGAACTTATTGTGTCCGCAAATAGAAGGTTAAAGATGGCTTTCTCT
TATAGGACGCCACAG
+
F,FFFFFFFFFFFFFFFFFFFFF,FF:FF:,,F,FF,FFF:FFFF:FF:,FF::F::F:F,FF,:,,F
F:,,,F,:FFFF:,F,FFFFFFF:FFF:F,:,FFF,FFFFF,FFFFF,FF:F,F,FFF,F,FFF,F,F
FFFFFFFFF,FFF,F
@A00155:342:HHGFNDSXY:1:1656:25482:11522 1:N:0:GAACCTAG+TCCGCATA
TCGTGAGTAAACAGATTGACGTAACGCCTCAATCGTGGTCAAGGACCAGCATTCAATGCTACCACATT

AAATACAATCACATTGTATTTCAGAACTTCTTGTGTCCGCACATAGAAGGTTAAAGATGGCTTTCTCT
TATAGGACGCCACAG
+
FFFFFFFFFFFFFFFFFFFFFFFFFFF,FFFFFFFFFFFFFFFFFFFFFFFFFFFFFFFFFFFFFFFF
FFFFFF:FFFFFFFFFF:FFFFFFFFFFFFFFFFFFFFF:FFFFFFFFFFFFFFFFFFFFFFFFFFFF
FFFFFFFFFF:FFFF
@A00155:342:HHGFNDSXY:1:2366:23656:28181 1:N:0:GAACCTAG+TCCGCATA
TCGTGAGTAAACAGATTGACGTAACGCCTCAATCGTGGTCAAGGACCAGCATTCAATGCTACCACATT
AAATACAATCACATTGTATTTCAGAACTTCTTGTGTCCGCACATAGAAGGTTAAAGATGGCTTTCTCT
TATAGGACGCCACAG
+
FFFFFFFFFFFFFF:FFFFFFFFFFFFFFFFFFFFFFFFFFFFFFFFFFFFFFF:FFFFFFFFFFFFF
FFFFFFFFFFFFFFFFFFFFFFFFFFFFFFFFFFFFFFFFFFFFFFFFFFFFFFFF:FFFFFFFFFFF
FFFFFFFFFFFFFFF
@A00155:342:HHGFNDSXY:1:2402:25735:28964 1:N:0:GAACCTAG+TCCGCATA
TCGTGAGTAAACAGATTGACGTAACGCCTCAATCGTGGTCAAGGACCAGCATTCAATGCTACCACATT
AAATACAATCACATTGTATTTCAGAACTCCTTGTGTCCGCACATAGAAGGTTAAAGATGGCTTTCTCT
TATAGGACGCCACAG
+
FFFFFFFFFFFFFFFFFFFFFFFFFFFFFFFFFFFFFFFFFFFFFFFFFFFFFFFFFFFFFFFFFFFF
FFFFFFFFFFFFFFFFFFFFFFFFFFFFFFFFFFFFFFFFFFFFFFFFFFFFFFFFFFFFFFFFFFFF
FFFFFFFFFFFFFFF
@A00155:342:HHGFNDSXY:1:2421:24460:8876 1:N:0:GAACCTAG+TCCGCATA
TCGTGAGTAAACAGATTGACGTAACGCCTCAATCGTGGTCAAGGACCAGCATTCAATGCTACCACATT
AAATACAATCACATTGTATTTCAGAACTTCTTGTGTCCGCACATAGAAGGTTAAAGATGGCTTTCTCT
TATAGGACGCCACAG
+
FFFFFFFFFFFFFFFFFFFFFFFFFFFFFFFFFFFFFFFFFFFFFFFFFFFFFFFFFFFFFFFFFFFF
FFFFFFFFFF:F::FFFFFFFFFFFFFFFFFFFFFFFFFFFFFFFFFFFFFFFFFFFFFFFFFFFFFF
FFFFFFFFFFFFFFF
@A00155:342:HHGFNDSXY:1:2667:4553:6496 1:N:0:GAACCTAG+TCCGCATA
TCGTGAGTAAACAGATTGACGTAACGCCTCAATCGTGGTCAAGGACCAGCATTCAATGCTACCACATT
AAATACAATCACATTGTATTTCAGAACTTCTTGTGTCCGCACATAGAAGGTTAAAGATGGCTTTCTCT
TATAGGACGCCACAG
+
:FFF:FFFFFFFFFFFFFFFFFFFFFFFFFFFFF:FFF:FFFFFFFFFFFFF:FFFFFFFFFFFFFFF
FFFFFFFFFFFFFFFFFFFFFFFFFFFF:FFFFFFFFFFFFFFFFFFFFFFFFFFFFFFFFFFFFFFF
FFFFFFFFFFFFFFF
@A00155:342:HHGFNDSXY:1:1374:8947:21746 1:N:0:GAACCTAG+TCCGCATA
CGTGAGTAAACAGATTGACGTAACGCCTCAATCGTGGTCAAGGACCAGCATTCAATGCTACCACATTA
AATACAATCACATTGTATTTCAGAACTTCTTGTGTCCGCACATAGAAGGTTAAAGATGGCTTTCTCTT
ATAGGACGCCACAGG
+
FFFFFFFFFFFFFFFFFFFFFFFFFFFFFFFFFFFFFFFFFFFFFFFFFFFF:FFFFFFFFFFFFFFF
FFFFFFFFF:FFFFFFFFFFFFFFFFFFFFFFFFFFFFFFFFFFFFFFFFFFFFFFFFFFFFFFFFFF
FFFFFFFFFFFFFFF
@A00155:342:HHGFNDSXY:1:1472:12834:28886 1:N:0:GAACCTAG+TCCGCATA
CGTGAGTAAACAGATTGACGTAACGCCTCAATCGTGGTCAAGGACCAGCATTCAATGCTACCACATTA
AATACAATCACATTGTATTTCAGAACTTCTTGTGTCCGCACATAGAAGGTTAAAGATGGCTTTCTCTT
ATAGGACGCCACAGG
+
:FFFFFFFFFFFFFFFFFFFFFFFFFFFFFFFFFFFFFFFFFFFFFFFFFFFFFFFFFFFFFFFFFFF
FFFFFF:FFFFFFFFFFFFFFFFFFFFFFFFFFFFFFFFFFFFFFFFFFFFFFFF:FFFFFFFFFFFF
FFFFFFFFFFFFFFF

```
@A00155:342:HHGFNDSXY:1:2118:32732:32440 1:N:0:GAACCTAG+TCCGCATA
TCCGATCTAACAGATTGACGTAACGCCTCAATCGTGGTCAAGGACCAGCATTCAATGCTACCACATTA
AATACAATCACATTGTATTTCAGAACTTCTTGTGTCCGCACATAGAAGGTTAAAGATGGCTTTCTCTT
ATAGGACGCCACAGG
+
FFFFFFFFFFFFFFFFFF:FFFFFFFF:FFFFFFFFFFFFFFFFFFFFFFFFFF:FFFFFFFFFFFFF
FFFFFFFFFFFFFFFFFFF:FFFFFFFFFFFFFFFFFFFFFFFFF:FFFFFFFFFFF:FFFFFFFFFF
FFFFFFFFFFFFFFF
@A00155:342:HHGFNDSXY:1:2234:4878:36871 1:N:0:GAACCTAG+TCCGCATA
TCCGATCTAACAGATTGACGTAACGCCTCAATCGTGGTCAAGGACCAGCATTCAATGCTACCACATTA
AATACAATCACATTGTATTTCAGAACTTCTTGTGTCCGCACATAGAAGGTTAAAGATGGCTTTCTCTT
ATAGGACGCCACAGG
+
FFFFF:FFFFFF:FFFFFFFFFFFFFFFFFFFFFFFFFFFFFFFFFFFFFFFFFFFFFFFFFFFFFFF
FFFFFFFFFFFFFFFFFFFFFFFFFFFFFFFFFFFFFFFFFFFFFFFFFFFFFFFFFFFFFFFFFFFF
FFFFFFFFFFFFFFF
@A00155:342:HHGFNDSXY:1:2575:4381:9298 1:N:0:GAACCTAG+TCCGCATA
GATCTGTAAACAGATTGAAGTAACGCCTCAATCGTGGTCAAGGACCAGCATTCAATGCTACCACATTA
AATACAATCACATTGTATTTCAGAACTTCTTGTGTCCGCACATAGAAGGTTAAAGATGGCTTTCTCTT
ATAGGACGCCACAGG
+
:F,FFFFF:FFFFF:FFF,FFFF,FFF,FFFFFFFFFFFFFFFF:FFFFFFFFFFFFF:F:FFFFFFF
FFF:FFFFFFFFFFFFFFFFFFFFFFFFFFFFFF::F:FF,FFFFFFFFFFFFFFFFFFFFFFFFFFF
FFFFFF:FFFFFFFF
@A00155:342:HHGFNDSXY:1:2578:2257:11851 1:N:0:GAACCTAG+TCCGCATA
CGTGAGTAAACAGATTGACGTAACGCCTCAATCGTGGTCAAGGACCAGCATTCAATGCTACCACATTA
AATACAATCACATTGTATTTCAGAACTTCTTGTGTCCGCACATAGAAGGTTAAAGATGGCTTTCTCTT
ATAGGACGCCACAGG
+
FFFFFFFFF:FFFFFFFFFFFFFFFFF:FFFFFFFFF:FFFFFFFF:F:FFFFFFFF:FFFFFFFFFF
FFFFFFFFFFFF:FFFFFF:FFFFFFFFFFFFFFFFFFFFFFFFFFFFF,FFFFFFFFFFFFFFFFFF
FFFFFFFFFFFFFFF
@A00155:342:HHGFNDSXY:1:1378:28257:23813 2:N:0:GAACCTAG+TCCGCATA
CGTGAGTAAACAGATTGACGTAACGCCTCAATCGTGGTCAAGGACCAGCATTCAATGCTACCACATTA
AATACAATCACATTGTATTTCAGAACTTCTTGTGTCCGCACATAGAAGGTTAAAGATGGCTTTCTCTT
ATAGGACGCCACAGG
+
FFFFFFFFFFFFFFFFFFFFFFFFFFFFFFFFFFFFFFFFFFFFFFFFFFFFFFFFFFFFFFFFFFFF
FFFFFFFFFFFFFFFFFFFFF:FFFFFFFFFFFFFFFFFFFFFFFFFFFFFFFFFFFFFFFFFFFFF:
FFFFFFFFFFFFFFF
@A00155:342:HHGFNDSXY:1:2266:29686:1454 2:N:0:GAACCTAG+TCCGCATA
CGTGAGTAAACAGATTGACGTAACGCCTCAATCGTGGTCAAGGACCAGCATTCAATGCTACTACATTA
AATACAATCACATTGTATTTCAGAACTTCTTGTGTCCGCACATAGAAGGTTAAAGATGGCTTTCTCTT
ATAGGACGCCACAGG
+
FFFFFFFFFFFFFFFFF:FFFFFFFFFFFFFFFFFFFFFFFFFFFFFFFFFFFFFFFFFFFFFFFFFF
FFFFFFFFFFFFFFFFFFFFFFFFFFFFFFFFFFFFFFFFFFFFFFFFFFFFFFFFFFFFFFFFFFFF
:FFFFFFFFFFFFFF
@A00155:342:HHGFNDSXY:1:2408:14443:20400 2:N:0:GAACCTAG+TCCGCATA
CGTGAGTAAACAGATTGACGTAACGCCTCAATCGTGGTCAAGGACCAGCATTCAATGCTACCACATTA
AATACAATCACATTGTATTTCAGAACTTCTTGTGTCCGCACATAGAAGGTTAAAGATGGCTTTCTCTT
ATAGGACGCCACAGG
+
FFFFFFFFFFFFFFFFFFFFFFFFFFFFFFFFFFFFF:FFFFFFFFFFFFFFFFFFFFFFFFFFFFFF
```

```
F,FFFFFFFFFFFFF:FFFFFFFFFFFFFFFFFFFFFFFFFFFFFFFFFFFFF,FFFFFFFFFFFFFF
FFFFFFFFFFFFFF:
@A00155:342:HHGFNDSXY:1:2447:27218:33567 2:N:0:GAACCTAG+TCCGCATA
CGTGAGTAAACAGATTGACGTAACGCCTCAATCGTGGTCAAGGACCAGCATTCAATGCTACCACATTA
AATACAATCACATTGTATTTCAGAACTTCTTGTGTCCGCACATAGAAGGTTAAAGATGGCTTTCTCTT
ATAGGACGCCACAGG
+
FFFFFFFFFFFFFFFFFFFFFFFFFFFFFFFFFFFFFFFFFFFFFFFFFFFFFFFFFFFFFFFFFFFF
FFFFFFFFFFFFFFFFFFFFFFFFFFFFFFFFFFFFFFFFFF,FFFFFFFFFFFFFFFFF:FFFFFFF
,FFFFFF:FFFFFFF
@A00155:342:HHGFNDSXY:1:2447:27950:35336 2:N:0:GAACCTAG+TCCGCATA
CGTGAGTAAACAGATTGACGTAACGCCTCAATCGTGGTCAAGGACCAGCATTCAATGCTACCACATTA
AATACAATCACATTGTATTTCAGAACTTCTTGTGTCCGCACATAGAAGGTTAAAGATGGCTTTCTCTT
ATAGGACGCCACAGG
+
FFFFFFFFFFFFFFFFFFFFFFFFFFFFFFFFFFFFFFFFFFFFFFFFFFFFFFFFFFFFFFFFFFFF
FFFFFFFFFFFFFFFFFFFFFFFFFFFFFFFFFFFFFFFFFFFFFFFFFFFFFFFFFFFFFFFFFFFF
FFFFFFFFFFFFFFF
@A00155:342:HHGFNDSXY:1:1367:10131:29403 2:N:0:GAACCTAG+TCCGCATA
GTGAGTAAACAGATTGACGTAACGCCTCAATCGTGGTCAAGGACCAGCATTCAATGCTACCACATTAA
ATACAATCACATTGTATTTCAGAACTTCTTGTGTCCGCACATAGAAGGTTAAAGATGGCTTTCTCTTA
TAGGACGCCACAGGA
+
FFFFFFFFFFFFFFFFFFFFFFFFFFFFFFFFFFFFFFFFFFFFFFFFFFFFFFFFFFFFFFFFFFFF
FFFFFFFFFFFFFFFFFFFFFFFFFFFFFFFFFFFFFFFFFFFFFFFFFFFFFFFFFFFFFFFFFFFF
FFFFFFFFFFFFFFF
@A00155:342:HHGFNDSXY:1:1502:4435:1219 2:N:0:GAACCTAG+TCCGCATA
GTGAGTAAACAGATTGACGTAACGCCTCAATCGTGGTCAAGGACCAGCATTCAATGCTACCACATTAA
ATACAATCACATTGTATTTCAGAACTTCTTGTGTCCGCACATAGAAGGTTAAAGATGGCTTTCTCTTA
TAGGACGCCACAGGA
+
F,FFFFFFFFF:F:FFFFF:FFFFFFFFF:FFFFFFFFFFFFFFFFFFFFFFFFFFFFFF:FFFFFFF
F:FFFFFFF:FFFFFFFFFFFFFFFFFFFFFFFFFFFFFF:FFFF:FFFFFFFFFFFFFFFFFFFFFF
FFF::,FFFFFFFFF
@A00155:342:HHGFNDSXY:1:2239:1479:13416 2:N:0:GAACCTAG+TCCGCATA
GTGAGTAAACAGATTGACGTAACGCCTCAATCGTGGTCAAGGACCAGCATTCAATGCTACCACATTAA
ATACAATCACATTGTATTTCAGAACTTCTTGTGTCCGCACATAGAAGGTTAAAGATGGCTTTCTCTTA
TAGGACGCCACAGGA
+
FFF:FFFF:FFFFFFFFFFFFFFFF:FFFFFFFFF,FF:FFFFFFFFFFFFF:FFFFFFFFFFFFFFF
FFFFFFF:F::FFFFFFFF::FFF:FFFFFFFFFFFFF::FF:F,:FFFFF:FFF:FF:FFF:F,FFF
F,FFF:::F,,FFF:
@A00155:342:HHGFNDSXY:1:2525:9887:29919 2:N:0:GAACCTAG+TCCGCATA
GTGAGTAAACAGATTGACGTAACGCCTCAATCGTGGTCAAGGACCAGCATTCAATGCTACCACATTAA
ATACAATCACATTGTATTTCAGAACTTCTTGTGTCCGCACATAGAAGGTTAAAGATGGCTTTCTCTTA
TAGGACGCCACAGGA
+
FFFFFFFFFFFFFFFFFFFFFFFFFFFFFFFFFFFFFFFFFFFFFFFFFFFFFFFFFFFFFFFFFFFF
FFFFFFFFFF,FFFFFFFFFFFFFFFFFFFFFFFFFFFFFF:FFFFFFFFFFFFFFF:FF:FFFFF,F
FFFFFFFFFFFFFFF
@A00155:342:HHGFNDSXY:1:2678:24659:12947 1:N:0:GAACCTAG+TCCGCATA
TGAGTAAACAGATTGACGTAACGCCTCAATCGTGGTCAAGGACCAGCATTCAATGCTACCACATTAAA
TACAATCACATTGTATTTCAGAACTTCTTGTGTCCGCACATAGAAGGTTAAAGATGGCTTTCTCTTAT
AGGACGCCACAG
```

+
F,FFFF,FFFF,FFFFFFFF:F:FFFF:,F,FF:F:,FFFFFF:F:FF:F:FFF,F:F:FF:FFFFF,
FFFFFFF,,FFFFF,FFFFFFFFFFF,:FFFFFFFFFF:FFFF:F,FFF,F,FFF,FFFFF:F:FFFF
FFFFFF:FFFFF
@A00155:342:HHGFNDSXY:1:1115:30264:28698 1:N:0:GAACCTAG+TCCGCATA
TGAGTAAACAGATTGACGTAACGCCTCAATCGTGGTCAAGGACCAGCATTCAATGCTACCACATTAAA
TACAATCACATTGTATTTCAGAACTTCTTGTGTCCGCACATAGAAGGTTAAAGATGGCTTTCTCTTAT
AGGACGCCACAGGAG
+
FFFFFFFFFFFFFFFFFFFF:FFFFF,F:FFFFFFFFFF:FFFFF:FFFFFFFFFFFFFFFFFFFFF
FFFFFFFF::FFFFFFFFFFFFFFF:F,FF:FFFFF,FFF:FFFF:FFFF:FF,FFFFFFFFFFFFF
FFFF:FFFFFFFFFF
@A00155:342:HHGFNDSXY:1:1126:14823:35900 1:N:0:GAACCTAG+TCCGCATA
CTAGTAAACAGATTGACGTAACGCCTCAATCGTGGTCAAGGACCAGCATTCAATGCTACCACATTAAA
TACAATCACATTGTATTTCAGAACTTCTTGTGTCCGCACATAGAAGGTTAAAGATGGCTTTCTCTTAT
AGGACGCCACAGGAG
+
FFFFFFFFFFFFFFFFFFFFFFFFFFFFFFFFFFFFFFFFFFFFFFFFFFFFFFFFFFFFFFFFFFF
:FFFFFF:FFF:FFFFFFFFFFFFFFFFFFFFFFFFFFFFFFFFFFF:FFFFFFFFFFFFFFFFFFF
FFFFFFFFFFFFFFF
@A00155:342:HHGFNDSXY:1:1203:5041:11757 1:N:0:GAACCTAG+TCCGCATA
TGAGTAAACAGATTGACGTAACGCCTCAATCGTGGTCAAGGACCAGCATTCAATGCTACCACATTAAA
TACAATCACATTGTATTTCAGAACTTCTTGTGTCCGCACATAGAAGGTTTAAGATGGCTTTCTCTTAT
AGGACGCCACAGGAG
+
FFFFFFFFFFFFFFFFFFFFFFFFFFFFFFFFFFFFFF:FFFFFFFFFFFFFFF,FFFFFFFFFFF,
FFFF:FF,FFFFFFF:FFFFFFFFFFFF:FFFFFFFFFFFFFFFF:FFFFFF:FFFFFFFFFFFFFF
FFFFFFFFFFFFFFF
@A00155:342:HHGFNDSXY:1:1238:24668:11772 1:N:0:GAACCTAG+TCCGCATA
TCCGATCTCAGATTGACGTAACGCCTCAATCGTGGTCAAGGACCAGCATTCAATGCTACCACATTAAA
TACAATCACATTGTATTTCAGAACTTCTTGTGTCCGCACATAGAAGGTTAAAGATGGCTTTCTCTTAT
AGGACGCCACAGGAG
+
FFFFFFFFFFFF,FFFFFFFFFFFFFF:FFFFFFFFFF:FFFFFFFFFFFF:FFFFFFFFFFFFFFF
FFFFFFFFF:FFFFFFFFFFFFFFFFF:F:FFFFFFFFFFFFFFFFFFFFFFFFFFFFFFFFFFFFF
FFFFFFFFFFFFFFF
@A00155:342:HHGFNDSXY:1:1243:30942:6543 1:N:0:GAACCTAG+TCCGCATA
TGAGTAAACAGATTGACGTAACGCCTCAATCGTGGTCAAGGACCAGCATTCAATGCTACCACATTAAA
TACAATCACATTGTATTTCAGAACTTCTTGTGTCCGCACATAGAAGGTTAAAGATGGCTTTCTCTTAT
AGGACGCCACAGGAG
+
FFFFFFFFFFFFFFFF,FFFFFF:FFFFFFFFFFFF:FFFFF:FFFFFFFFFFFFFFFFFFFFFFFF
FFFFFFFFFF:FFFFFFFFFFFFFFFFFFFFFFFFFFFFFFFFFFFFFFFFFFFFFFFFFFFFFFFF
FFFFFFFFFFFFFFF
@A00155:342:HHGFNDSXY:1:1278:18493:8719 1:N:0:GAACCTAG+TCCGCATA
TGAGTAAACAGATTGACGTAACGCCTCAATCGTGGTCAAGGACCAGCATTCAATGCTACCACATTAAA
TACAATCACATTGTATTTCAGAACTTCTTGTGTCCGCACATAGAAGGTTAAAGATGGCTTTCTCTTAT
AGGACGCCACAGGAG
+
FFFFFFFFFFFFFFFFFFFFFFFFFFFFFFFFFFFFFFFFFF:FFFFFFFFFFFFFFFFFFFFFFFF
FFFFFFFFFFFFFFFFFFFFFFFFFFFFFFFFFFFFFFFFFFFFFFFFFFFFFFFFFFFFFFFFFFF
FFFFFFFFFFFFFFF
@A00155:342:HHGFNDSXY:1:1332:29640:25488 1:N:0:GAACCTAG+TCCGCATA
TGAGTAAACAGATTGACGTAACGCCTCAATCGTGGTCAAGGACCAGCATTCAATGCTACCACATTAAA

TACAATCACATTGTATTTCAGAACTTCTTGTGTCCGCACATAGAAGGTTAAAGATGGCTTTCTCTTAT
AGGACGCCACAGGAG
+
FFFFFFFFFFFFFFFFFFFFFFFFFFFFFFFFFFFFFFFFFFFFFFFFFFFFFFFFFFFFFFFFFFFFFF
FFF:FFFFFFFFFFFFFFFFFFFFFFFFFFFFFFFFFFFFFFFFFFFFFFFFFFFFFFFFFFFFFFFFFF
FFFFFFFFFFFFFFF
@A00155:342:HHGFNDSXY:1:1461:14579:27242 1:N:0:GAACCTAG+TCCGCATA
TGAGTAATCAGATTGACGTAACGCCTCAATCGTGGTCAAGGACCAGCATTCAATGCTACCACATTAAA
TACAATCACATTGTATTTCAGAACTTCTTGTGTCCGCACATAGAAGGTTAAAGATGGCTTTCTCTTAT
AGGACGCCACAGGAG
+
FFFFFFF,:FFFFF:FFFF:FFFFFFFFFFFF:F:FFF,FFF:FFFF,FFF,,FFFFF,,FF:FFFFF
FF:,FFFFF,FFFFFFFFFFFFFFFFFF,FFF:FFFFFFFFFF,FFFF:FFFFFFFFFFFFFFFFFFF
F:FFF::::FFF,:F
@A00155:342:HHGFNDSXY:1:1472:2356:31657 1:N:0:GAACCTAG+TCCGCATA
TGAGTAAACAGATTGACGTAACGCCTCAATCGTGGTCAAGGACCAGCATTCAATGCTACTACATTAAA
TACAATCACATTGTATTTCAGAACTTCTTGTGTCCGCACATAGAAGGTTAAAGATGGCTTTCTCTTAT
AGGACGCCACAGGAG
+
FFFFFFFFFF:FFFFFFFFFFFFFFFFFFFFFFFFF:FFFFFFF:FFFFFFFFFFFFFFFFFFFFFFFF
FFFFF:FFF:FFFFFFFFFFF:FFFFF:FFFF:FFFFFFFFFFFFFFF,FFFFFFFF,FFFFFF:FFFFF
FFFFFFFFFFFFFFF
@A00155:342:HHGFNDSXY:1:1466:12644:4507 1:N:0:GAACCTAG+TCCGCATA
TGAGTAAACAGATTGACGTAACGCCTCAATCGTGGTCAAGGACCAGCATTCAATGCTACCACATTAAA
TACAATCACATTGTATTTCAGAACTTCTTGTGTCCGCACATAGAAGGTTAAAGATGGCTTTCTCTTAT
AGGACGCCACAGGAG
+
FFFFFFFFFFFFFFFFFFFFFFFFFFFFFFFFFFFFFFFFFFFFFFFFFF:FFFFFFFFFFFFFFFFFFF
FFFFFFF:FFFFFFFFFFFFFFFF::FFFFFFFFFFFFFFFFFFFFFFFFFFFFFFFFFFFFFFFFFFFF
FFFFFFFF:FFFFFF
@A00155:342:HHGFNDSXY:1:1478:20121:17613 1:N:0:GAACCTAG+TCCGCATA
TGAGTAAACAGATTGACGTAACGCCTCAATCGTGGTCAAGGACCAGCATTCAATGCTACCACATTAAA
TACAATCACATTGTATTTCAGAACTTCTTGTGTCCGCACATAGAAGGTTAAAGATGGCTTTCTCTTAT
AGGACGCCACAGGAG
+
FFFFFFFFFFFFF:FFFFFFFFFFFFFFFFFFFFFFFFFFFFFFFFFFFFFFFFFFFFFFFFFFFFFFFF
FFFFFFFFFFFFFFFFFFFFFFFFFFFFFFFFFFFFFFFFFFFFFFFFFFFFFFFFFFFFFFFFFFFFFF
FFFFFFFFFFFFFFF
@A00155:342:HHGFNDSXY:1:1548:7672:17628 1:N:0:GAACCTAG+TCCGCATA
TGAGTAAACAGATTGACGTAACGCCTCAATCGTGGTCAAGGACCAGCTTTCAATGCTACCACATTAAA
TACAATCACATTGTATTTCAGAACTTCTTGTGTCCGCACATAGAAGGTTAAAGATGGCTTTCTCTTAT
AGGACGCCACAGGAG
+
FFFFFFFFFFFFFFFFFFFFFFF:F:FFF:FFFFFFFFFF:FFFFFFFFFFF,FFF:FFFFFF:FFFF
FFFFFFFFFFFFFFFFFFFFFF:FFFF::FFFFFF:FFF:FFFF:FFFFFFFFFFFF,FFFFFFFFFFF
FFFFFFFFFFFFFFF
@A00155:342:HHGFNDSXY:1:1608:10981:8359 1:N:0:GAACCTAG+TCCGCATA
TGAGTAAACAGATTGACGTAACGCCTCAATCGTGGTCAAGGACCAGCATTCAATGCTACCACATTAAA
TACAATCACATTGTATTTCAGAACTTCTTGTGTCCGCACATAGAAGGTTAAAGATGGCTTTCTCTTAT
AGGACGCCACAGGAG
+
FFFFFFFFFFFFFFFFFFFFFFFFFFF:FFFFFFFFFFFFFFFFFFFF,FFFF:FFFFFF:FFFFFFF
:FFFFFF:FFFFFFFF:FFFFFFFFFFFFFFFFFFFFFFFFF:FFFFFFFFFFF,,FFFFFFFFFFFFF
FFFFFFFFFFFFFFF

```
@A00155:342:HHGFNDSXY:1:1619:12698:4977 1:N:0:GAACCTAG+TCCGCATA
TGAGTAAACAGATTGACGTAACGCCTCAATCGTGGTCAAGGACCAGCATTCAATGCTACCACATTAAA
TACAATCACATTGTATTTCAGAACTTCTTGTGTCCGCACATAGAAGGTTAAAGATGGCTTTCTCTTAT
AGGACGCCACAGGAG
+
FFFFFFFFFFFFFFFFFFFFFFFFFFFFFFFFFFFFFFFFFFFFFFFFFFFFFFFFFFFFFFFFFFFF
FFFFFFFFFFFFFFFFFFFFFFFFFFFFFF:FFFFFFFFFFFFFFFFFFFFFFFFFFFFFFFFFFFFF
FFFFFFFFFFFFFFF
@A00155:342:HHGFNDSXY:1:1618:5457:24251 1:N:0:GAACCTAG+TCCGCATA
TGAGTAAACAGATTGACGTAACGCCTCAATCGTGGTCAAGGACCAGCATTCAATGCTACTACATTAAA
TACAATCACATTGTATTTCAGAACTTCTTGTGTCCGCACATAGAAGGTTAAAGATGGCTTTCTCTTAT
AGGACGCCACAGGAG
+
FFFFFFFFFFFFFFFFFFFFFFFFFFFFFFFFFFFFFFFFFFFFFFFFFFFFFFFFFFFFFFFFFFFF
FFFFFFFFFFFFFFFFFFFFFFFFFFFFFFFFFFFFFFFFFFFFFFFFFFFFFFFFFFFFFFFFFFFF
FFFFFFFFFFFFFFF
@A00155:342:HHGFNDSXY:1:2119:28330:5995 1:N:0:GAACCTAG+TCCGCATA
TGAGTAAACAGATTGACGTAACGCCTCAATCGTGGTCAAGGACCAGCATTCAATGCTACCACATTAAA
TACAATCACATTGTATTTCAGAACTTCTTGTGTCCGCACATAGAAGGTTAAAGATGGCTTTCTCTTAT
AGGACGCCACAGGAG
+
FFFFFFFFFFF:FFFFF:FFFFFFFFFFFFFFFFFFFFFFFFFFFFFFFFFF:FFFFFFFFFFFFFFF
FFFF,FFFFFFFFFFFFFFFFFFFFFFFFFFFFFFFFFFFFFFFFFFFFFFFFFFFFFFFFFFFFFFF
FFFFFFFFFFFFFFF
@A00155:342:HHGFNDSXY:1:1676:9146:35430 1:N:0:GAACCTAG+TCCGCATA
TGAGTAAACAGATTGACGTAACGCCTCAATCGTGGTCAAGGACCAGCATTCAATGCTACCACATTAAA
TACAATCACATTGTATTTCAGAACTTCTTGTGTCCGCACATAGAAGGTTAAAGATGGCTTTCTCTTAT
AGGACGCCACAGGAG
+
FFFFFFFFFFFFFFFFFFF:FFFFF:F:FFFFFFFFFFFFFFFFFFFFFFFFFFFFFFFFFFFFFFFF
FFFFFFFFFFFFFFFFFFFFFFFFFFFFFFFFFFFFFFFFFFFFFFF:FFFFFFFFFFFFFFFFFFFF
FFFFFFFFFFFFFFF
@A00155:342:HHGFNDSXY:1:2161:7735:19210 1:N:0:GAACCTAG+TCCGCATA
TGAGTAAACAGATTGACGTAACGCCTCAATCGTGGTCAAGGACCAGCATTCAATGCTACCACATTAAA
TACAATCACATTGTATTTCAGAACTTCTTGTGTCCGCACATAGAAGGTTAAAGATGGCTTTCTCTTAT
AGGACGCCACAGGAG
+
FFFFFFFFFFFFFFFFFFFFFFFFFFFFFFFFFFFFFFFFFFFFFFFFFFFFFFFFFFFFFFFFFFFF
FFFFFFF:FFFFFFFFFFFFFFFFFFFFFFFFFFFFFFFFFFFFFFFFFFFFFFFFFFFFFFFFFFFF
FFFFFFFFFF:FFFF
@A00155:342:HHGFNDSXY:1:2344:18376:6104 1:N:0:GAACCTAG+TCCGCATA
TGAGTAAACAGAGTGACGTAACGCCTCAATCGTGGTCAAGGACCAGCATTCAATGCTACCACATTAAA
TACAATCACATTGTATTTCAGAACTTCTTGTGTCCGCACATAGAAGGTTAAAGATGGCTTTCTCTTAT
AGGACGCCACAGGAG
+
FFF:FFFFFFFF,FFFFFFFFFFFFFFFFFFFFFFFFFF:FF:FFFFFFFFFFFFFFFFF:FFFFFFF
FFFFFFFFF:FFF:FFFFFFFFFFFFFFFFFFFFFFFFFFFFFFFFFFFFF:F:FFFF,FFFFFFF:F
FFFFFFFFFFFFFFF
@A00155:342:HHGFNDSXY:1:2519:26503:18803 1:N:0:GAACCTAG+TCCGCATA
CTAGTAAACAGATTGACGTAACGCCTCAATCGTGGTCAAGGACCAGCATTCAATGCTACCACATTAAA
TACAATCACATTGTATTTCAGAACTTCTTGTGTCCGCACATAGAAGGTTAAAGATGGCTTTCTCTTAT
AGGACGCCACAGGAG
+
FFFFFFFFFFFFFFFFFFFFFFFFFFFFFFFFFFFFFFFFFFFFFFFFFFFFFFFFFFFFFFFFFFFF
```

FFFF,FFFF:FFFFFFFFFFFFFFFFF:FFFFFFFFFFFFFFFFFFFFFFFFFFFFFFFFFFFFFFFF
FFFFFFFFFFFFFFF
@A00155:342:HHGFNDSXY:1:2571:19958:6496 1:N:0:GAACCTAG+TCCGCATA
TGAGTAAACAGATTGACGTAACGCCTCAATCGTGGTCAAGGACCAGCATTCAATGCTACCACATTAAA
TACAATCACATTGTATTTCAGAACTTCTTGTGTCCGCACATAGAAGGTTAAAGATGGCTTTCTCTTAT
AGGACGCCACAGGAG
+
FFFFFFFFFFFFFFFFFFFFFFFFFFFFFFFFFFFFFFFFFFFFFFFFFFFFFFFFFFFFFFFFFFFF
FFFFFFFFFFFFFFFFFFFFFFFFF,FFFFFFFFFFFFFFFFFFFFFFFFFFFFFFFFFFFFFFFFFF
FFFFFFFFFFFFFFF
@A00155:342:HHGFNDSXY:1:2639:8666:29403 1:N:0:GAACCTAG+TCCGCATA
TGAGTAAACAGATTGACGTAACGCCTCAATCGTGGTCAAGGACCAGCATTCAATGCTACCACATTAAA
TACAATCACATTGTATTTCAGAACTTCTTGTGTCCGCACATAGAAGGTTAAAGATGGCTTTCTCTTAT
AGGACGCCACAGGAG
+
FFFFFFFFFFFFFFFFFFFFFFFFFFFFFFFFFFFFFFFFFFFFFFFFFFFFFFFFFFFFFFFFFFFF
FFFFFFF:FFFFFFFFFFFFFFFFFFFFFFFFFFFFFFFFFFFFFFFFFFFFFFFFFFFFFFFFFFFF
FFFFFFFFFFFFFFF
@A00155:342:HHGFNDSXY:1:2114:25907:22404 2:N:0:GAACCTAG+TCCGCATA
TGAGTAAACAGATTGACGTAACGCCTCAATCGTGGTCAAGGACCAGCATTCAATGCTACCACATTAAA
TACAATCACATTGTATTTCAGAACTTCTTGTGTCCGCACATAGAAGGTTAAAGATGGCTTTCTCTTAT
AGGACGCCACAGGAG
+
FFFFFFFFFFFFFFFFFFFFFFFFFFFFFFFFFFFFFFFFFFFFFFFFFFFFFFFFFFFFFFFFFFFF
FFFFFFFFFFFFFFFFFFFFFFFFFFFFFFFFFFFFFFFFFFFFFFFFFFFFFFFFFFFFFFFFFFFF
FFFFFFFFFFFFFFF
@A00155:342:HHGFNDSXY:1:2114:25925:22404 2:N:0:GAACCTAG+TCCGCATA
TGAGTAAACAGATTGACGTAACGCCTCAATCGTGGTCAAGGACCAGCATTCAATGCTACCACATTAAA
TACAATCACATTGTATTTCAGAACTTCTTGTGTCCGCACATAGAAGGTTAAAGATGGCTTTCTCTTAT
AGGACGCCACAGGAG
+
FFFFFFFFFFFFFFFFFFFFFFFFFFFFFFFFFFFFFFFFFFFFFFFFFFFFFFFFFFFFFFFFFFFF
FFFFFFFFFF,FF:F,FFFFFF:FFFFFFFFFFFFFFFF,FFFFFFFFFFFFFFFFFFFFFFFF,:,F
FFFFFFFFFFFFFFF
@A00155:342:HHGFNDSXY:1:2650:12400:36025 2:N:0:GAACCTAG+TCCGCATA
TGAGTAAACAGATTGACGTAACGCCTCAATCGTGGTCAAGGACCAGCATTCAATGCTACCACATTAAA
TACAATCACATTGTATTTCAGAACTTCTTGTGTCCGCACATAGAAGGTTAAAGATGGCTTTCTCTTAT
AGGACGCCACAGGAG
+
FFFFFFFFFFFFFFFFFFFFFFFFFFFFFFFFFFFFFFFFFFFFFFFFFFFFFFFFFFFFFFFFFFFF
FFFFFFFFFF:FFFFFFFF:FFFFFFFFFFFFFFFFFFFFFF:FFFFFFFFFFFFFFFFFFFFF:FFF
FFFFFFFFFFFFFFF
@A00155:342:HHGFNDSXY:1:2555:13006:31657 1:N:0:GAACCTAG+TCCGCATA
GAGTAAACAGATTGACGTAACGCCTCAATCGTGGTCAAGGACCAGCATTCAATGCTACCACATTAAAT
ACAATCACATTGTATTTCAGAACTTCTTGTGTCCGCACATAGAAGGTTAAAGATGGCTTTCTCTTATA
GGACGCCACAGGAG
+
FFFFFFFFFFFFFFFFFFFFFFFFFFFFFFFFFFFFFFFF:FFFFFFFFFFFFFFFFFFFFFFFFFFF
FFFFFFFFF:FFFFFFFFFFFFFFFFFFFFFFFFFFFFFFFFFFFFFFFFFFFFFFFFFFFFFFFFFF
FFFFFFFFFFFFFF
@A00155:342:HHGFNDSXY:1:2506:21169:34867 1:N:0:GAACCTAG+TCCGCATA
GAGTAAACAGATTGACGTAACGCCTCAATCGTGGTCAAGGACCAGCATTCAATGCTACCACATTAAAT
ACAATCACATTGTATTTCAGAACTTCTTGTGTCCGCACATAGAAGGTTAAAGATGGCTTTCTCTTATA
GGACGCCACAGGAGG

```
+
FFFFFFFFFFFFFFFFFFFFFFFFFFF:FFFFFFFFFFFFFFFFFFFFFF:FFFFFFFFFF:FFFFFF
FFF:FFFFFFFFFFFFFFFFFFFFFFFFFFFFFFFFFFFFFFFFFFFFFFF:,FFFFFFFFFFFFFF
FFFFFFFFFFFFFFF
@A00155:342:HHGFNDSXY:1:2128:16459:24267 2:N:0:GAACCTAG+TCCGCATA
GAGTAAACAGATTGACGTAACGCCTCAATCGTGGTCAAGGACCAGCATTCAATGCTACCACATTAAAT
ACAATCACATTGTATTTCAGAACTTCTTGTGTCCGCACATAGAAGGTTAAAGATGGCTTTCTCTTATA
GGACGCCACAGGAGG
+
FFFFFF:FFFFFFFFFFFFF:FFFFFFFFFFFFFFFFFFFF,:FFFFFFFFFFFFFFFFF:F:FFFFF
FFFFFFF,FFFFFFFFFFFFFF:FFF,F:FFFF,FFFFFF:FFFFFFFFFF:FFFFF:FFFFFFF:FF
FFFFFF:FFFFFFFF
@A00155:342:HHGFNDSXY:1:2555:13006:31657 2:N:0:GAACCTAG+TCCGCATA
GAGTAAACAGATTGACGTAACGCCTCAATCGTGGTCAAGGACCAGCATTCAATGCTACCACATTAAAT
ACAATCACATTGTATTTCAGAACTTCTTGTGTCCGCACATAGAAGGTTAAAGATGGCTTTCTCTTATA
GGACGCCACAGGAGA
+
FFFFFFFFFFFFFFFFFFFFFFFFFFFFFFFFFFFFFFFFFFFFFFFFFFFFFFFFFFFFFFFFFFFF
FFFFFFF:FFFFFFFFFFFFFFFFFFF,FFFFFFFFFFFFFFFFFFFFFFFF:F:FFFFFFFFFFFFF
FFFFFFF:FFFFFFF
@A00155:342:HHGFNDSXY:1:2224:1127:31219 1:N:0:GAACCTAG+TCCGCATA
ATTAAACAGATTGACGTAACGCCTCAATCTTGGTCAATGACCAGCATTCAATGCTACTACATTAAATA
CAATCACATTGTATTTCAGAACTTATTGTGTCCTCACATAGAAGGTTAAAGATGGCTTTCTCTTATAG
GACGACACAGGA
+
F,F:F:F:,FF,,F,FFF::F,,::,FF,,F,FF,,F,:,FF:FFFFF,,F:,,FF,FF,:FF,,FF:
,FFFFF::FF,F,FF::F,FF,F:,FF:FF,,:,FF,FFFFFF,:FFFFFFFF:,FFFFFF:F:FFFF
,FFF,:F::F::
@A00155:342:HHGFNDSXY:1:2501:17219:29403 1:N:0:GAACCTAG+TCCGCATA
ATCTAACAGATTGACGTAACGCCTCAATCGTGGTCAAGGACCAGCATTCAATGCTACTACATTAAATA
CAATCACATTGTATTTCAGAACTTCTTGTGTCCGCACATAGAAGGTTAAAGATGGCTTTCTCTTATAG
GACGCCACAGGAGGC
+
F:FFFFFFFFFFFFFFFFFFFFFFFFFFFFFFFFFFFFFFFFFFFFFFFFFFFFFFFFFFFFFFFFFF
FFFFFFFFFFFFFFFFFFFFFFFFFFFFFFFFFFFFFFFFFFFFFFFFFFFFFFFFFFFFFFFFFFFF
FFFFFFFFFFFFFFF
@A00155:342:HHGFNDSXY:1:1126:14823:35900 2:N:0:GAACCTAG+TCCGCATA
AGTAAACAGATTGACGTAACGCCTCAATCGTGGTCAAGGACCAGCATTCAATGCTACCACATTAAATA
CAATCACATTGTATTTCAGAACTTCTTGTGTCCGCACATAGAAGGTTAAAGATGGCTTTCTCTTATAG
GACGCCACAGGAGAG
+
FFFFFFFFFFFFFFFFFFFFFFFFFFFFFFFFFFFFFFFFFFFFFFFFFFFFFFFFFFFFFFFFFFFF
FFFFFFFFFFFFFFFFFFFFFFFFFF,FFFFFFFFFFFFFFFFFFFFFFFFFFFFFFFFFFFFFFFFF
FFFFFFFFFFFFFFF
@A00155:342:HHGFNDSXY:1:1146:22996:26381 2:N:0:GAACCTAG+TCCGCATA
AGTAAACAGATTGACGTAACGCCTCAATCGTGGTCAAGGACCAGCATTCAATGCTACTACATTAAATA
CAATCACATTGTATTTCAGAACTTCTTGTGTCCGCACATAGAAGGTTAAAGATGGCTTTCTCTTATAG
GACGCCACAGGAGGA
+
FFFFFFFFFFFFFFFFFFFFFFFFFFFFFFFFFFFFFFFFFFFFFFFFFFFFFFFFFFFFFFFFFFFF
FFFFFFFFFFFFFFFFFFFFFFFFFFFFFFFFFFFFFFFFFFFFFFFFFFFFFFFFFFFFFFFFFFFF
FFFFFFFFFFFFFFF
@A00155:342:HHGFNDSXY:1:1265:30219:25050 2:N:0:GAACCTAG+TCCGCATA
AGTAAACAGATTGACGTAACGCCTCAATCGTGGTCAAGGACCAGCATTCAATGCTACCACATTAAATA
```

CAATCACATTGTATTTCAGAACTTCTTGTGTCCGCACATAGAAGGTTAAAGATGGCTTTCTCTTATAG
GACGCCACAGGAGGA
+
FFFFFFFFFFFFFFFFFFFFFFFFFFFFFFFFFFFFFFFFFFFFFFFFFF:FFFFFFFFFFFFF:FFF
F:FFFFFF,FF,FF:FFFFFFFFFFFFFFFFFFFFFFFFFFFFFFFFFFFFFFFFFFFFFFFFFFFFF
FFFFFFFFFFFFFFF
@A00155:342:HHGFNDSXY:1:1415:12075:2832 2:N:0:GAACCTAG+TCCGCATA
AGTAAACAGATTGACGTAACGCCTCAATCGTGGTCAAGGACCAGCATTCAATGCTACCACATTAAATA
CAATCACATTGTATTTCAGAACTTCTTGTGTCCGCACATAGAAGGTTAAAGATGGCTTTCTCTTATAG
GACGCCACAGGAGGA
+
FFFFFFFFFFFFFFFFFFFFFFFFFFFFFFFFFFFFFFFFFFFFFFFFFFFFFFFFFFFFFFFFFFFF
FFFFFFFFF:FFFFFFFFFFFFFFFFFFFFFFFFFFFFFFFFFFFFFFFFFFFF:FFFFF
FFFFF:,FFFFFFFF
@A00155:342:HHGFNDSXY:1:2255:28022:11851 2:N:0:GAACCTAG+TCCGCATA
AGTAAACAGATTGACGTAACGCCTCAATCGTGGTCAAGGACCAGCATTCAATGCTACCACATTAAATA
CAATCACATTGTATTTCAGAACTTCTTGTGTCCGCACATAGAAGGTTAAAGATGGCTTTCTCTTATAG
GACGCCACAGGAGGA
+
FFFFFFFFFFFFFFFFFFFFFFFFFFFFFFFFFFFFFFFFFFFFFFFFFFFFFFFFFFFFFFFF:FFF
FFFFFFFFFFFFF:FFFFFFFFFFFFFFFFFFFFFFFFF:FFFFFFFFFFFFFFFFFFFFFFFFFFFF
FFFFFFFFFFFFFFF
@A00155:342:HHGFNDSXY:1:2519:26503:18803 2:N:0:GAACCTAG+TCCGCATA
AGTAAACAGATTGACGTAACGCCTCAATCGTGGTCAAGGACCAGCATTCAATGCTACCACATTAAATA
CAATCACATTGTATTTCAGAACTTCTTGTGTCCGCACATAGAAGGTTAAAGATGGCTTTCTCTTATAG
GACGCCACAGGAGAG
+
FFFFFFFFFFFFFFFFFFFFFFFFFFFFFFFFF:FFFFFFFFFFFFFFFFFFFFFFFFFFFFFFFFFF
FFFFFFFFFFFFFFFFFFFFFFFFFFFFFFFFFFFFFF:FFFFFFFFFFFFF:FFFFFFFFFFF,FFF
FFFFFFFF:FFFFFF
@A00155:342:HHGFNDSXY:1:2611:3974:31140 2:N:0:GAACCTAG+TCCGCATA
AGTAAACAGATTGACGTAACGCCTCAATCGTGGTCAAGGACTAGCATTCAATGCTACCACATTAAATA
CAATCACATTGTATTTCAGAACTTCTTGTGTCCGCACATAGAAGGTTAAAGATGGCTTTCTCTTATAG
GACGCCACAGGAGGA
+
FFFFFFFFFFFFFFFFFFFFFFFFFF::FFFFFFFFFFFFFF:FFFFFFFFFFFFFFFFFFFFFFFFF
FFFFFFFFFFFFFFFFFFFFF:FFFFFF:FFF:F:FFFFFFFFFFF:FFF,FFFFFFFFFFFFFFFF,
FFFFFFFFFFFFFFF
@A00155:342:HHGFNDSXY:1:2366:15320:1313 1:N:0:GAACCTAG+TCCGCATA
GTAAACAGATTGACGTAACGCCTCAATCGTGGTCAAGGACTAGCATTCAATGCTACCACATTAAATAC
AATCACATTGTATTTCAGAACTTCTTGTGTCCGCACATAGAAGGTTAAAGATGGCTTTCTCTTATAGG
ACGCCACAGGAGGAC
+
FFFFFFFFFFFFFFFFFFFFFFFFFFFFFFFFFFFFFFFFFFFFFFFFFFFFFFFFFFFFFFFFFFFF
FFFFFFF:FFFFFFFFFFFFFFFFFFFFFFFFFFFFFFFFFFFFFFFFFFFFFFFFFFFFFFFFFFFF
FFFFFFFFFFFFFFF
@A00155:342:HHGFNDSXY:1:2453:10710:6417 1:N:0:GAACCTAG+TCCGCATA
CTAAACAGATTGACGTAACGCCTCAATCGTGGTCAAGGACCAGCATTCAATGCTACCACATTAAATAC
AATCACATTGTATTTCAGAACTTCTTGTGTCCGCACATAGAAGGTTAAAGATGGCTTTCTCTTATAGG
ACGCCACAGGAGGAT
+
FFFFFFFFFFFFFFFFFFFFFFFFFFFFFFFFFFFFFFFFFFFFFFFFFFFFFFFFFFFFFFFFFFFF
FFFFFFFFFFFFFFFFFFFFFFFFFFFFFFFFFFFFFFFFFFFFFFFFFFFFFFFFFFFFFFFFFFFF
FFFFFFFFFFFFFFF

@A00155:342:HHGFNDSXY:1:1241:13160:8970 2:N:0:GAACCTAG+TCCGCATA
GTAAACAGATTGACGTAACGCCTCAATCGTGGTCAAGGACCAGCATTCAATGCTACCACATTAAATAC
AATCACATTGTATTTCAGAACTTCTTGTGTCCGCACATAGAAGGTTAAAGATGGCTTTCTCTTATAGG
ACGCCACAGGAGGAA
+
FFFF:FFFFFFFFFFFFFFFFFFFFFFFFFFFFFFFFFFFFFFFFFFFFFFFFFFFFFFFFFFFFFF
FFFFF:F:FF:F:FFFFF:FFFFFFFFFFFFFFFFFFFFFFFFFFFF::FFFFFFFFFFFFFFFFFFF
FFFFFFFFFFFFFFF
@A00155:342:HHGFNDSXY:1:2575:4381:9298 2:N:0:GAACCTAG+TCCGCATA
GTAAACAGATTGACGTAACGCCTCAATCGTGGTCAAGGACCAGCATTCAATGCTACCACATTAAATAC
AATCACATTGTATTTCAGAACTTCTTGTGTCCGCACATAGAAGGTTAAAGATGGCTTTCTCTTATAGG
ACGCCACAGGAGATC
+
FFFF:FFFFFFFFFFFFFFFFFFFFFFFFFFFFFF:FFFFFFFFFFFFFFFFFFFFFFFFFFF:FFFF
::FFF,F,FF:FFFFFFFFFFFFFFFFFFFFFFFFF:::FFFFFFFF:F:FFFFF:FF:FFFFFFFFF
FF:FFFFFFFFFFFF
@A00155:342:HHGFNDSXY:1:2409:1226:2487 2:N:0:GAACCTAG+TCCGCATA
TAAACAGATTGACGTAACGCCTCAATCGTGGTCAAGGACCAGCATTCAATGCTACCACATTAAATACA
ATCACATTGTATTTCAGAACTTCTTTTGTCCGCACATAGAAGGTTAAAGATGGCTTTCTCTTATAGTA
CTCAACAGGAGGAA
+
FF:::FF:FFF,FFFFFF:F:FFF:FF:F:FF,FF:F,FF:FFFFF,:FF,FFF:,FFFFF:::FFF:
:FFF,:FFFFFF:F::,F:FFF,FF,F,F,FF,F:FFF,F:FFFF:F,:,:,F:FFF,F:FF:F:,,:
:,F,,:,F,F,F,F
@A00155:342:HHGFNDSXY:1:2611:3974:31140 1:N:0:GAACCTAG+TCCGCATA
TAAACAGATTGACGTAACGCCTCAATCGTGGTCAAGGACTAGCATTCAATGCTACCACATTAAATACA
ATCACATTGTATTTCAGAACTTCTTGTGTCCGCACATAGAAGGTTAAAGATGGCTTTCTCTTATAGGA
CGCCACAGGAGGAAC
+
FF::FFFFF:F,FFFFFFFFFFF,FFFFFFFFF:FFFFF:FFFFFFFFFFFFFFFF,FFFFFFFFF:F
F:FFF::FFFFFFFFFFF:FFFF:FFFFFFFFFFFFFFFFFFFFFFFFFFFFF:FFFFFFFFFFFFFF
FFFFFFFFFFFFFFF
@A00155:342:HHGFNDSXY:1:1312:7916:15859 2:N:0:GAACCTAG+TCCGCATA
TAAACAGATTGACGTAACGCCTCAATCGTGGTCAAGGACCAGCATTCAATGCTACCACATTAAATACA
ATCACATTGTATTTCAGAACTTCTTGTGTCCGCACATAGAAGGTTAAAGATGGCTTTCTCTTATAGGA
CGCCACAGGAGGAAC
+
FFFFFFFFFFFFFFFFFFFFFFFFFFFFFFFFFFFFFFFFFFFFFFFFFFFFFFFFFFFFFFFFFFFF
FFFFFFFFFFF:FFFFFFFFFFFFFFFFFFFFFFFFFFFFFFFFFFFF:FFFFFFFFFFFFFFFFFFF
FF:FFFFFFFFFFF:
@A00155:342:HHGFNDSXY:1:2340:24216:34945 2:N:0:GAACCTAG+TCCGCATA
TAAACAGATTGACGTAACGCCTCAATCGTGGTCAAGGACCAGCATTCAATGCTACCACATTAAATACA
ATCACATTGTATTTCAGAACTTCTTGTGTCCGCACATAGAAGGTTAAAGATGGCTTTCTCTTATAGGA
CGCCACAGGAGGAAC
+
FFFFFFFFFFFFFFFFFFFFFFFFFFFFFFFFFFFFFFFFFFFFFFFFFFFFFFFFFFFFFFFFFF:F
FFFFFFFFFFFF:FFFFFFFFFFFFFFFFFFFFFFFFFFFFFFFFFFFFFFFFFFFFFFFFFFFFFF
FFFFFFFF:FFFFFF
@A00155:342:HHGFNDSXY:1:2120:2248:24236 2:N:0:GAACCTAG+TCCGCATA
AAACAGATTGACGTAACGCCTCAATCGTGGTCAAGGACCAGCATTCAATGCTACTACATTAAATACAA
TCACATTGTATTTCAGAACTTCTTGTGTCCGCACATAGAAGGTTAAAGATGGCTTTCTCTTATATGAC
GCCACAGGAGGAACT
+
FFFFFFFFFF:FFFFFFFFFFFFFFFFFFFFFFFFFFFFFFFFFFFFFFFFFFFFFFFFFFFFFFFFF

FFF,FFFFFFFFFFFFFF:FFFFF:FFFF:FFFFFFF::FF:FFFFF:FFF:FFFF:FFFFFFF,,FF
::FFF:FFF,FFFFF
@A00155:342:HHGFNDSXY:1:2453:10710:6417 2:N:0:GAACCTAG+TCCGCATA
AAACAGATTGACGTAACGCCTCAATCGTGGTCAAGGACCAGCATTCAATGCTACCACATTAAATACAA
TCACATTGTATTTCAGAACTTCTTGTGTCCGCACATAGAAGGTTAAAGATGGCTTTCTCTTATAGGAC
GCCACAGGAGGATAG
+
FFFFFFFFFFFFFFFFFFFFFFFFFFFFFFFFFFFFFFFFFFFFFFFFFFFFFFFFFFFFFFFFFFFF
FFFF:FFFFFFFFFFFFF:FFFFFFFFFFFFFFFFFFFFFFFFFFFFFFFFFFFFFFFFFFFFFFFFF
FFFFFFFFFFFFF:F
@A00155:342:HHGFNDSXY:1:1552:11849:36949 2:N:0:GAACCTAG+TCCGCATA
AACAGATTGACGTAACGCCTCAATCGTGGTCAAGGACCAGCATTCAATGCTACTACATTAAATACAAT
CACATTGTATTTCAGAACTTCTTGTGTCCGCACATAGAAGGTTAAAGATGGCTTTCTCTTATAGGACG
CCACAGGAGGAACT
+
FFFFFFFFF,FFFFFFFFFFFFFFFFFFFFFFFFFFFFFFFFFFFFF:FFFF:FFFFFFFFFFFFFF
FF:FFFFFFFFFFFFFFFFFFFFFFFFFFFF:FFFFFFFFFFFFFFFFFFFFFFFFFFFFFFFFFFFFF
FFFFFFFFFFFFFF
@A00155:342:HHGFNDSXY:1:1123:8024:4398 2:N:0:GAACCTAG+TCCGCATA
AACAGATTGACGTAACGCCTCAATCGTGGTCAAGGACCAGCATTCAATGCTACCACATTAAATACAAT
CACATTGTATTTCAGAACTTCTTGTGTCCGCACATAGAAGGTTAAAGATGGCTTTCTCTTATAGGACG
CCACAGGAGGAACTC
+
FFFFFFFFFFFFFFFFF:FFFFFFFFFFFFFFFFFFFFFFFFFFFFFFFFFFFFFFFFFFFFFFFFFF
FFFFFFFFFFFFFFFFFFFFFFFFFFFF:FFFFFFFFFFFFFFFFFFFFFFFFFFFFFFFFFFFFFFF:
FFFFFFFFFFF:FFF
@A00155:342:HHGFNDSXY:1:1123:8603:5713 2:N:0:GAACCTAG+TCCGCATA
AACAGATTGACGTAACGCCTCAATCGTGGTCAAGGACCAGCATTCAATGCTACCACATTAAATACAAT
CACATTGTATTTCAGAACTTCTTGTGTCCGCACATAGAAGGTTAAAGATGGCTTTCTCTTATAGGACG
CCACAGGAGGAACTC
+
FFFFFFFFFFFFFFFFFFFFFFFFFFFFFFFFFFFFFFFFFFFFFFFFFFFFFFFFFFFFFFFFFFFF
FFFFFFFFFFFFFFFFFFFFFFFFFFFFFFFFFFFFFFFFFFFFFFFFFFFFFFFFFFFFFFFFFFFF
FFF:FFFFFFF,FFF
@A00155:342:HHGFNDSXY:1:1118:4300:2362 2:N:0:GAACCTAG+TCCGCATA
AACAGATTGACGTAACGCCTCAATCGTGGTCAAGGACCAGCATTCAATGCTACCACATTAAATACAAT
CACATTGTATTTCAGAACTTCTTGTGTCCGCACATAGAAGGTTAAAGATGGCTTTCTCTTATAGGACG
CCACAGGAGGAACTC
+
FFFFFFFF:FFFFFFFFFFFFFFFFFFFFFFFFFFFFFFFFFFF,FFFFFFFFF:FFFFFFFFFFFFF:
FFFF:F,FFFF,FFFFF,FFF:F::FFFFFFF:FFFFFFFFFFFFFFFF:FFFFF:FFFFFFFFFFFF
FFFFFFFFF:F,,FF
@A00155:342:HHGFNDSXY:1:1118:18674:7842 2:N:0:GAACCTAG+TCCGCATA
AACAGATTGACGTAACGCCTCAATCGTGGTCAAGGACCAGCATTCAATGCTACTACATTAAATACAAT
CACATTGTATTTCAGAACTTCTTGTGTCCGCACATAGAAGGTTAAAGATGGCTTTCTCTTATAGGACG
CCACAGGAGGAACTC
+
F,FFFFFFFFFFFFFFFFFFFFFFFFFFFFFFFFFFFFFFFFFFFFFFFFFFFFFFFFFFFFFFFFFF
FFFF:FF:FFFFF:FFFFFFF,FFFFFFFFFFFFFFFFFFFFFFFFFFFFFFFFFFFFFFFFFFFFFF
FFFFFFFFFFFFFFF
@A00155:342:HHGFNDSXY:1:1216:13286:1924 2:N:0:GAACCTAG+TCCGCATA
AACAGATTGACGTAACGCCTCAATCGTGGTCAAGGACCAGCATTCAATGCTACCACATTAAATACAAT
CACATTGTATTTCAGAACTTCTTGTGTCCGCACATAGAAGGTTAAAGATGGCTTTCTCTTATAGGACG
CCACAGGAGGAACTC

+
FFFFFFFFFFFFFFFFFFFFFFFFFFFFFFFFFFFFFFFFFFFFFFFFFFFFFFFFFFFFFFFFFFFF
FFFFFFFFFFFFFFFFFFFFFFFFFFFFFFFFFFFFFFFFFFFFFFFFFFFFFFFFFFFF,FFFFFFF
FFFFFFFFFFFFFFF
@A00155:342:HHGFNDSXY:1:1243:7609:5055 2:N:0:GAACCTAG+TCCGCATA
AACAGATTGACGTAACGCCTCAATCGTGGTCAAGGACCAGCATTCAATGCTACCACATTAAATACAAT
CACATTGTATTTCAGAACTTCTTGTGTCCGCACATAGAAGGTTAAAGATGGCTTTCTCTTATAGGACG
CCACAGGAGGAACTC
+
FFFFFFFFFFFFFFFFFFFFFFFFFFFFFFFFFFFFFFFFFFFFFFFFFFFFFFFFFFFFFFFFFFFF
FFFFFFFFFFFFFFFFFFFFFFFFFFFFFFFFFFFFFFFFFFFFFFFFFFF:FFFFFFFFFFFFFFFF
FFFFFFFFFFFFFFF
@A00155:342:HHGFNDSXY:1:1329:32777:10441 2:N:0:GAACCTAG+TCCGCATA
AACAGATTGACGTAACGCCTCAATCGTGGTCAAGGACCAGCATTCAATGCTACCACATTAAATACAAT
CACATTGTATTTCAGAACTTCTTGTGTCCGCACATAGAAGGTTAAAGATGGCTTTCTCTTATATGACG
CCACAGGAGGAACTC
+
FF,FFFFFFFFFFFFF,F,FFFFFFFFFF:FFFF,FF:FF:FF:FFFFFFFFF,FFFFFFFFFF,FFF
FFFFFFF:FFFFFF,FFFFF:FF,FFFFF::FFF,:FFF,FFFFF:FFF:,FF,FFFFFF:FF,FFF:
FFFFFF:FF,FFFFF
@A00155:342:HHGFNDSXY:1:1363:27073:3912 2:N:0:GAACCTAG+TCCGCATA
AACAGATTGACGTAACGCCTCAATCGTGGTCAAGGACCAGCATTCAATGCTACCACATTAAATACAAT
CACATTGTATTTCAGAACTTCTTGTGTCCGCACATAGAAGGTTAAAGATGGCTTTCTCTTATAGGACG
CCACAGGAGGAACTC
+
FFFFFFFFFFFFFFFFFFFFFFFFFFFFFFFFFFFFFFFFFFFFFFFFFFFFFFFFFFFFF:FFFFFF
FFF:FFFFFFFFFFFFFFFFF,FFFFFFFFFFFF:FFFFFFFFFF::FFFFFFFFFFFF:FFFFFFFF
FFFFFFFFFFFFFFF
@A00155:342:HHGFNDSXY:1:1424:32479:16595 2:N:0:GAACCTAG+TCCGCATA
AACAGATTGACGTAACGCCTCAATCGTGGTCAAGGACCAGCATTCAATGCTACCACATTAAATACAAT
CACATTGTATTTCAGAACTTCTTGTGTCCGCACATAGAAGGTTAAAGATGGCTTTCTCTTATAGGACG
CCACAGGAGGAACTC
+
F:FFFFFFFFFFFFFFFFFF:FFFFFFFFFFFFFFFFFFFFF:FFFFFFFF,FFFFFFFFFFFFFFFF
FFFF,FF,FFF:FFFFFFFFFFFFFFFFFFFFFFFFFFFFFFFFFFFFFFFFFFFFFF::FFF:FF:F
FFFFFFFFFFFFFFF
@A00155:342:HHGFNDSXY:1:1439:16947:26052 2:N:0:GAACCTAG+TCCGCATA
AACAGATTGACGTAACGCCTCAATCGTGGTCAAGGACCAGCATTCAATGCTACCACATTAAATACAAT
CACATTGTATTTCAGAACTTCTTGTGTCCGCACATAGAAGGTTAAAGATGGCTTTCTCTTATAGGACG
CCACAGGAGGAACTC
+
FFFFFFFFFFFFFFFFFFFFFFFFFFFFFFFFFFFFFFFFFFFFFFFFFFFFFFFFFFFFFFFFFFFF
FFFFFFFFF:FFFFFFFF:FFF,FFFFFFFFFFFFF:FFFFFFFFFFFFFFFFFFFFFFF:FFFFFFF
FFFFFFFFFFFFFFF
@A00155:342:HHGFNDSXY:1:1442:29197:1204 2:N:0:GAACCTAG+TCCGCATA
AACAGATTGACGTAACGCCTCAATCGTGGTCAAGGACCAGCATTCAATGCTACCACATTAAATACAAT
CACATTGTATTTCAGAACTTCTTGTGTCCGCACATAGAAGGTTAAAGATGGCTTTCTCTTATAGGACG
CCACAGGAGGAACTC
+
FFFFFFFFFFFFFFFFFFFFFFFFFFFFFFFFFFFFFFFFFFFFFFFFFFFFFFFFFFFFFFFFFFFF
FFFFFFFFFFFFFFFFFFFFFFFFFFFFFFFFFFFFFFFFFFFFFFFFFFFFFFFFFFFFFFFFFFFF
:FFFFFFFFFFFFFF
@A00155:342:HHGFNDSXY:1:1470:9679:16939 2:N:0:GAACCTAG+TCCGCATA
AACAGATTGACGTAACGCCTCAATCGTGGTCAAGGACCAGCATTCAATGCTACCACATTAAATACAAT

CACATTGTATTTCAGAACTTCTTGTGTCCGCACATAGAAGGTTAAAGATGGCTTTCTCTTATAGGACG
CCACAGGAGGAACTC
+
FFFFF:FFFFFFFFFFFFFFFFFFFFFFFFFFFFFFFFFFFFFFFFFFFFFFFFFFFFFFFFFFFF
FFFFFFFFFFFFFFFFFFFFFFFFFFFFFFFFF,FFF:FFFFF:FFFFFF:FFFFFFFFFFFFFFF
F:FFFFFFFFFFFF:
@A00155:342:HHGFNDSXY:1:1567:5448:15374 2:N:0:GAACCTAG+TCCGCATA
AACAGATTGACGTAACGCCTCAATCGTGGTCAAGGACCAGCATTCAATGCTACCACATTAAATACAAT
CACATTGTATTTCAGAACTTCTTGTGTCCGCACATAGAAGGTTAAAGATGGCTTTCTCTTATAGGACG
CCACAGGAGGAACTC
+
FFFFFFFFFFFFFFFFFFFFFFFFFFFFFFFFFFFFFFFFFFFFFFFFFFFFFFFFFFFFFFFFFF
FF:FFFFFFFFFFFFFFFFFFFFFFFFFFFFFF:FFFFFFFFFFFFFFFFFFFFFFF:FFFFFFFF
FFFFFFFFFFF:FFF
@A00155:342:HHGFNDSXY:1:2118:32732:32440 2:N:0:GAACCTAG+TCCGCATA
AACAGATTGACGTAACGCCTCAATCGTGGTCAAGGACCAGCATTCAATGCTACCACATTAAATACAAT
CACATTGTATTTCAGAACTTCTTGTGTCCGCACATAGAAGGTTAAAGATGGCTTTCTCTTATAGGACG
CCACAGGAGATCGGA
+
FFFFFFFFFFFFFFFFFFFFFFFFFFFFFFFFFFFFFFFFFFFFFFFFFFFFFFFF:FFF:FFFFF
FF,FFFF:FFFFFFFFFFFFFFFFF:FFFFFFFFFF:FFFFFFF:FFFFFFFFFFFFFFFFFFFFF
FFFFFFFFFFFF:FF
@A00155:342:HHGFNDSXY:1:2112:6650:7185 2:N:0:GAACCTAG+TCCGCATA
AACAGATTGACGTAACGCCTCAATCGTGGTCAAGGACCAGCATTCAATGCTACCACATTAAATACAAT
CACATTGTATTTCAGAACTTCTTGTGTCCGCACATAGAAGGTTAAAGATGGCTTTCTCTTATAGGACG
CCACAGGAGGAACTC
+
FFFFFFFFFFFFFFFFFFFFFFFFFFFFFFFFFFFFFFFFFFFFFFFFFFFFFFFFFFFFFFFFFF
FFFFFFFFFFFFFFFFFFFFFFFFFFFFFFFFF,FFF,FFFFFFFFFFFFFF:FFFFFFFFFFFFF
FFFFFFFFFFFFFFF
@A00155:342:HHGFNDSXY:1:2234:4878:36871 2:N:0:GAACCTAG+TCCGCATA
AACAGATTGACGTAACGCCTCAATCGTGGTCAAGGACCAGCATTCAATGCTACCACATTAAATACAAT
CACATTGTATTTCAGAACTTCTTGTGTCCGCACATAGAAGGTTAAAGATGGCTTTCTCTTATAGGACG
CCACAGGAGATCGGA
+
FFFFFFFFFFFFFFFFFFFFFFFFFFFFFFFFFFFFFFFFFFFFFFFFFFFFFFFFFFFFFFFFFF
FFF:FFFFFFFFF:FFFFFFFFFFFFFFFFFFFFFFFFFFFFFFFFFFFFF:F:FFFF:FFFFFFF
FFFFFFFFFFFFFFF
@A00155:342:HHGFNDSXY:1:2236:22309:1924 2:N:0:GAACCTAG+TCCGCATA
AACAGATTGACGTAACGCCTCAATCGTGGTCAAGGACCAGCATTCAATGCTACCACATTAAATACAAT
CACATTGTATTTCAGAACTTCTTGTGTCCGCACATAGAAGGTTAAAGATGGCTTTCTCTTATAGGACG
CCACAGGAGGAACTC
+
FFFFFFFFFFFFFFFFFFFFFFFFFFFFFFFFFFFFFFFFFFFFFFFFFFFFFFFFFFFFFFFFFF
FFFFFFFF,FFFFFFFFFFFFFFFFFFFFFFFF:FFFFFFFFFFFFFFFFFFFFFFFFFFFFFFFF
FFFFFFFFFFF,FFF
@A00155:342:HHGFNDSXY:1:2417:32515:2315 2:N:0:GAACCTAG+TCCGCATA
AACAGATTGACGTAACGCCTCAATCGTGGTCAAGGACCAGCATTCAATGCTACCACATTAAATACAAT
CACATTGTATTTCAGAACTTCTTGTGTCCGCACATAGAAGGTTAAAGATGGCTTTCTCTTCTAGGACG
CCACAGGAGGACCTC
+
F,F,FF,FFFFFFFFFFFFFFFFFFFFF,F,FFFFFF,FF,:FFFFFF,:FF:FFFF,FFFFFF:F
FFF:::F,,,,FF:FF,FF,F,FF,FFFFFFF,,:FF,FF,,,FFFFF:FFF,FF,F,,,FFFFFF
FFFFFFF,FFF:F:F

```
@A00155:342:HHGFNDSXY:1:2473:15564:18051 2:N:0:GAACCTAG+TCCGCATA
AACAGATTGACGTAACGCCTCAATCGTGGTCAAGGACCAGCATTCAATGCTACCACATTAAATACAAT
CACATTGTATTTCAGAACTTCTTGTGTCCGCACATAGAAGGTTAAAGATGGCTTTCTCTTATAGGACG
CCACAGGAGGAACTC
+
FFFFFFFFFFFFFFFFFFFFFFFFFFFFFFFFFFFFFFFFFFFFFFFFFFFFFFFFFFFFFFFFFFFFFF
FF:FFFFFFFFF:FFFFFFFFFFFFFFF:FFFFFFF:FF:FFFFFF,FFFFFFFFFFFFFFFFFFFFFFF
FFFFFFFFFFF,FFF
@A00155:342:HHGFNDSXY:1:2550:29695:32659 2:N:0:GAACCTAG+TCCGCATA
AACAGATTGACGTAACGCCTCAATCGTGGTCAAGGACCAGCATTCAATGCTACCACATTAAATACAAT
CACATTGTATTTCAGAACTTCTTGTGTCCGCACATAGAAGGTTAAAGATGGCTTTCTCTTATAGGACG
CCACAGGAGGAACTC
+
:FFFFFFFFFFFFFFFFFFFFFFFFFFFFFFFFFFFFFFFFFFFFFFFFFFFFFFFFFFFFFFFFFFFFF
FFFFFFFFFFFFFFFFFFFFFFFFFFFFFFFFFFFFFFFFFFFFFFFFFFFFFFFFFFFFFFFFFFFFFF
FFFFFFFFFFFFFFF
@A00155:342:HHGFNDSXY:1:2501:17219:29403 2:N:0:GAACCTAG+TCCGCATA
AACAGATTGACGTAACGCCTCAATCGTGGTCAAGGACCAGCATTCAATGCTACTACATTAAATACAAT
CACATTGTATTTCAGAACTTCTTGTGTCCGCACATAGAAGGTTAAAGATGGCTTTCTCTTATAGGACG
CCACAGGAGGCAGAT
+
FFFFFFFFFFFFFFFFFFFFFFFFFFFFFFFFFFFFFFFFFFFFFFFFFFFFFFFFFFFFFFFFFFFFFF
FFFFFFFFFFFFFFFFFFFFFFFFFFFFFFFFFFFFFFFFFFFFFFFFFFFFFFFFFFFFFFFFFFFFFF
FFFFFFFFFFFFFFF
@A00155:342:HHGFNDSXY:1:2128:23656:8108 2:N:0:GAACCTAG+TCCGCATA
ACAGATTGACGTAACGCCTCAATCGTGGTCAAGGACCAGCATTCAATGCTACCACATTAAATACAATC
ACATTGTATTTCAGAACTTCTTGTGTCCGCACATAGAAGGTTAAAGATGGCTTTCTCTTATAGGACGC
CACAGGAGGAACTCT
+
FFFFFFFFFFFFFFFFFFFFFFFFFFFFFFFFFFFFFFFFFFFFFFFFFFFFFFFFFFFFFFFFFFFFFF
FFF:FFFFFFFFFFFFFFFFFFFFFFFFFFFFFFF::FFFFFFFFFFFFFFFFFFFFFFFFFFFF:FFFF
FFFFFFFFFFFFFFF
@A00155:342:HHGFNDSXY:1:1238:24668:11772 2:N:0:GAACCTAG+TCCGCATA
CAGATTGACGTAACGCCTCAATCGTGGTCAAGGACCAGCATTCAATGCTACCACATTAAATACAATCA
CATTGTATTTCAGAACTTCTTGTGTCCGCACATAGAAGGTTAAAGATGGCTTTCTCTTATAGGACGCC
ACAGGAGAGATCGGA
+
FFFFFFFFFFFFFFFFFFFFFFFFFFFFFFFFFFFFFFFFFFFFFFFFFFFFFFFFFFFFFFFFFF:FFF
FF:FFFFFFFFFFFFFFFFFFFFFFFFFFFFFFFFFFFFFFFFFFFFFFFFFFFFFFFFF:FFFFFFFF
FF:FFFFFFFFFFFF
@A00155:342:HHGFNDSXY:1:1326:20347:15687 2:N:0:GAACCTAG+TCCGCATA
AGATTGACGTAACGCCTCAATCGTGGTCAAGGACCAGCATTCAATGCTACCACATTAAATACAATCAC
ATTGTATTTCAGAACTTCTTGTGTCCGCACATAGAAGGTTAAAGATGGCTTTCTCTTATAGGACGCCA
CAGGAGGAACTCTTG
+
FFFFFFFFFFFFFFFFFFFFFFFFFFFFFFFFFFFFFFFFFFFFFFFFFFFFFFFFFFFFFFFFFFFFFF
FFFFFFFFFFFFFFFFFFFFFFFFFFFFFFFFFFFFFFFFFFFFFFFFFFFFFFFFFFFFFFFFFFFFFF
FFFFFFFF:FFFFFF
@A00155:342:HHGFNDSXY:1:1107:13187:1564 2:N:0:GAACCTAG+TCCGCATA
GATTGACGTAACGCCTCAATCGTGGTCAAGGACCAGCATTCAATGCTACCACATTAAATACAATCACA
TTGTATTTCAGAACTTCTTGTGTCCGCACATAGAAGGTTAAAGATGGCTTTCTCTTATAGGACGCCAC
AGGAGGAACTCTTGA
+
FFFFF:FFFFFFF,FFFFFFFFFF:FFFFFFFFFFFFFFFFFFFFFFFFFFFFFFFFFFFFFFFF:FF:F
```

F:FFF:FFFFFFFFFFFFF:::FF,FFFFFFFFFFFFFFFFFFFF:FF:FFFF:FFFFFFFFFF:FFF
FF,FFFFFFF:F:FF
@A00155:342:HHGFNDSXY:1:1527:20166:7576 2:N:0:GAACCTAG+TCCGCATA
GATTGACGTAACGCCTCAATCGTGGTCAAGGACCAGCATTCAATGCTACCACATTAAATACAATCACA
TTGTATTTCAGAACTTCTTGTGTCCGCACATAGAAGGTTAAAGATGGCTTTCTCTTATAGGACGCCAC
AGGAGGAACTCTTGA
+
FFFFFFFFFFFFFFFFFF:FFFFFFFFFFFFFFFFFFFFFFFFFFFFF,FFFFFFFFFFFFFFFFFFFF
FFF:FFFF:FFFFFFFFFFF:FFFFFFFFFFFFFFFF,FFFFFFFFFFF:FFFFFFFFFFF,FFFFFF
F:FFFFF,FFFFFF:
@A00155:342:HHGFNDSXY:1:1572:12093:32424 2:N:0:GAACCTAG+TCCGCATA
GATTGACGTAACGCCTCAATCGTGGTCAAGGACCAGCATTCAATGCTACCACATTAAATACAATCACA
TTGTATTTCAGAACTTCTTGTGTCCGCACATAGAAGGTTAAAGATGGCTTTCTCTTATAGGACGCCAC
AGGAGGAACTCTTGA
+
FFFFFFFFFFFFFFFFFFFFFFFFFFFFFFFFFFFFFFFFFFFFFFFFFFFFFFFFFFFFFFFFFFFF
:FFFFFFFF,FFFFFFFFFFFFFFFFFFFFFFFFFFFFFFFFFFFFFFFFFFFFFFFFFFFFFFFFFF
FFFFFFFFFFFFFFF
@A00155:342:HHGFNDSXY:1:2126:12102:23672 2:N:0:GAACCTAG+TCCGCATA
GATTGACGTAACGCCTCAATCGTGGTCAAGGACCAGCATTCAATGCTACCACATTAAATACAATCACA
TTGTATTTCAGAACTTCTTGTGTCCGCACATAGAAGGTTAAAGATGGCTTTCTCTTATAGGACGCCAC
AGGAGGAACTCTTAA
+
FFFFFFFFFFFFFFFFFFFFFFFFFFFFFFFFFFFFFFFFFFFFFFFFFFFFFFFFFFFFFFFFFFFF
FFFFFFFFFFFFFFFFFFFFFFFFFFFFFF:FFFFFFFFFFFF:FFFFFFFFFFFFFFFFF:FFFFFFF
FFFFFFF:FFFFFFF
@A00155:342:HHGFNDSXY:1:2468:14516:13542 2:N:0:GAACCTAG+TCCGCATA
GATTGACGTAACGCCTCAATCGTGGTCAAGGACCAGCATTCAATGCTACCACATTAAATACAATCACA
TTGTATTTCAGAACTTCTTGTGTCCGCACATAGAAGGTTAAAGATGGCTTTCTCTTATAGGACGCCAC
AGGAGGAACTCTTGA
+
FFFFFFFFFFFFFFFFFFFFFFFFFFFFFFFFFFFFFFFFFFFFFFFFFFFFFFFFFFFFFFFFFFFF
F,F,FFFFFFFFFFFFFFFFFFFFFFFFFFF,FFFFFFFFFFFFFFFFFFFFFFFFFFFFFFFFFFFFF
:FFFFFFFFFFFFF:
@A00155:342:HHGFNDSXY:1:2531:6623:24173 2:N:0:GAACCTAG+TCCGCATA
GATTGACGTAACGCCTCAATCGTGGTCAAGGACCAGCATTCAATGCTACCACATTAAATACAATCACA
TTGTATTTCAGAACTTCTTGTGTCCGCACATAGAAGGTTAAAGATGGCTTTCTCTTATAGGACGCCAC
AGGAGGAACTCTTGA
+
F:FFFFFFFFFFFFFFFFFFFFFFF:FFFFFFFFFFFFFFFFFFFFFFFFFFFFFFFFFFFFFFFFFFFF
FFFFFFFFFFFFFFFFFFFFFFFFFFFFFFFFFFFFFFFFFFF:FFFF,FFFFFFFFFFFFFFFFFFFFF
FF:FF:FFFFFF:FF
@A00155:342:HHGFNDSXY:1:1243:7609:5055 1:N:0:GAACCTAG+TCCGCATA
ATTGACGTAACGCCTCAATCGTGGTCAAGGACCAGCATTCAATGCTACCACATTAAATACAATCACAT
TGTATTTCAGAACTTCTTGTGTCCGCACATAGAAGGTTAAAGATGGCTTTCTCTTATAGGACGCCACA
GGAGGAACTCTTGAG
+
FF:FFFFFFFFF,FFFFFFFFFFFFFFFFFFFFFFFFFFFFFFFFFFFFFFFFFFFFFFFFFFFFFFFF
FFFFFFFFFFFFFFFFFFFFFFFFFFFFFFFFFFF:FFFFFFFFFF:FFFFFFFFFFFFFFFFFFFFFF
FFFFFFFFFFFFFFF
@A00155:342:HHGFNDSXY:1:1233:12825:27085 1:N:0:GAACCTAG+TCCGCATA
ATTGACGTAACGCCTCAATCGTGGTCAAGGACCAGCATTCAATGCTACCACATTAAATACAATCACAT
TGTATTTCAGAACTTCTTGTGTCCGCACATAGAAGGTTAAAGATGGCTTTCTCTTATAGGACGCCACA
GGAGGAACTCTTGAG

+
FFFFFFFFFFFFFFFFFFFFFFFFFFFFFFFFFFFFFFFFFFFFFFFFFFFFFFFFFFFFFFFFFFF:
FFFFFFFFFFFFFFFFFFFFFFFFFFFFFFFFFFFFFFFFFFFFFFFFFFFFFFFFFFFFFFFFFFFF
FFFFFFFFFFFFFFF
@A00155:342:HHGFNDSXY:1:1578:5638:22185 1:N:0:GAACCTAG+TCCGCATA
ATTGACGTAACGCCTCAATCGTGGTCAAGGACCAGCATTCAATGCTACCACATTAAATACAATCACAT
TGTATTTCAGAACTTCTTGTGTCCGCACATAGAAGGTTAAAGATGGCTTTCTCTTATAGGACGCCACA
GGAGGAACTCTTGAG
+
FFFFFFFFFFFFFFFFFFFFFFFFFFFFFFFFFFFFFFFFFFFFFFFFFFFFFFFFFFFFFFFFFFFF
FFFFFFFFFFFFFF,FFFF,FFFFFFFFFFFFFFFFFFFFFFFFFFFFFFFFFFFFFFFFFFFFFFFF
FFFFFFFFFFFFFFF
@A00155:342:HHGFNDSXY:1:1568:30337:5055 1:N:0:GAACCTAG+TCCGCATA
ATTGACGTAACGCCTCAATCGTGGTCAAGGACCAGCATTCAATGCTACCACATTAAATACAATCACAT
TGTATTTCAGAACTTCTTGTGTCCGCACATAGAAGGTTAAAGATGGCTTTCTCTTATAGGACGCCACA
GGAGGAACTCTTGAG
+
FFFFFFFFFFFFFFFFFFFFFFFFFFFFFFFFFFFFFFFFFFFFFFFFFFFFFFFFFFFFFFFFFFFF
FFFFFFFFFFFFFFFFFFFFFFFFFFFFFFFFFFFFFFFFFFFFFFFFFFFFFFFFFFFFFFFFFFFF
FFFFFFFFFFFFFFF
@A00155:342:HHGFNDSXY:1:1676:6858:8891 1:N:0:GAACCTAG+TCCGCATA
ATTGACGTAACGCCTCAATCGTGGTCAAGGACTAGCATTCAATGCTACCACATTAAATACAATCACAT
TGTATTTCAGAACTTCTTGTGTCCGCACATAGAAGGTTAAAGATGGCTTTCTCTTATAGGACGCCACA
GGAGGAACTCTTGAG
+
:FFFFFFFFFFFFFFFFFFFFFFFFFFFFFFFFFFFFFFFFFFFF:FFFFFFFFFFFFFFF:FFFFFF
FFFFFFFFFFFFFFFFFFFFFFFFFF,::F,FFFFF:FFF:FFFFFF:FFFFFFFFFFFFFFFFFFFF
FFFFFFFFFFFFFFF
@A00155:342:HHGFNDSXY:1:1436:3586:31845 2:N:0:GAACCTAG+TCCGCATA
ATTGACGTAACGCCTCAATCGTGGTCAAGGACCAGCATTCAATGCTACCACATTAAATACAATCACAT
TGTATTTCAGAACTTCTTGTGTCCGCACATAGAAGGTTAAAGATGGCTTTCTCTTATAGGACGCCACA
GGAGGAACTCTTGAG
+
FFFFFFFFFFFFFFFFFFFFFFFFFFF:FFFFFFFFFFFFFFFFFFFF:FFFFFFFFFFFFFFFFFFF
FFFFFFFFFFFFFFFFFFFFFFFF:FFFFFFFFFFFFFFFFFFFFFFFFFFFFFFFF:FFFF:FF:FF
FFFFFFFFFFFFFFF
@A00155:342:HHGFNDSXY:1:1554:7166:27712 2:N:0:GAACCTAG+TCCGCATA
ATTGACGTAACGCCTCAATCGTGGTCAAGGACCAGCATTCAATGCTACCACATTAAATACAATCACAT
TGTATTTCAGAACTTCTTGTGTCCGCACATAGAAGGTTAAAGATGGCTTTCTCTTATAGGACGCCACA
GGAGGAACTCTTGAG
+
FFFFFFFFFFFFFFFFFFFFFFFFFFFFFFFFFFFFFF:FFFFFFFFF:FFFF:FFFFFFFFFFFFFF
FFFFF,FFFFFFFFFFFFFFFFFFFFFF:FFFFFFFFFFFFFFFFFFFFFFFFFFFFFFFFFFFFFFF
FFFFFF:FFFFFFFF
@A00155:342:HHGFNDSXY:1:1568:30337:5055 2:N:0:GAACCTAG+TCCGCATA
ATTGACGTAACGCCTCAATCGTGGTCAAGGACCAGCATTCAATGCTACCACATTAAATACAATCACAT
TGTATTTCAGAACTTCTTGTGTCCGCACATAGAAGGTTAAAGATGGCTTTCTCTTATAGGACGCCACA
GGAGGAACTCTTGAG
+
FFFFFFFFFFFFFFFFFFFFFFFFFFFFFFFFFFFFFFFFFFFFFFFFFFFFFFF:FFFFFFFFFFFF
FF:FFFFFFFFFFFFFFFFFFFFFFFFFF,FFFFFFFFFFFFFFFFFFFFFFFFFFFFFFFFFFFFFF
FFFFFF:FFFFFFFF
@A00155:342:HHGFNDSXY:1:1619:11162:29997 2:N:0:GAACCTAG+TCCGCATA
ATTGACGTAACGCCTCAATCGTGGTCAAGGACTAGCATTCAATGCTACCACATTAAATACAATCACAT

TGTATTTCAGAACTTCTTGTGTCCGCACATAGAAGGTTAAAGATGGCTTTCTCTTATAGGACGCCACA
GGAGGAACTCTTGAG
+
FFFFFFFFFFFFFFFFFFFFFFFFFFFFFFFFFFFFFFFFFFFFFFFFFFFFFFFFFFFFFFFFFFFFFF
FFFFFFFFFFFFFFFFFFFFFFFFFFFFFFFFFFFFFFFFFFFFFFFFFFFFFFFFFFFFFFFFFFFFFF
FFFFFFFFFFFFF:F
@A00155:342:HHGFNDSXY:1:2252:17255:17942 2:N:0:GAACCTAG+TCCGCATA
ATTGACGTAACGCCTCAATCGTGGTCAAGGACCAGCATTCAATGCTACCACATTAAATACAATCACAT
TGTATTTCAGAACTTCTTGTGTCCGCACATAGAAGGTTAAAGATGGCTTTCTCTTATAGGACGCCACA
GGAGGAACTCTTGAG
+
FFFFFFFFFFFFFFFFFFFFFFFFFFFFFFFFFFFFFFFFFFFFFFFFFFFFFFFFFFFFFFFFFFFFFF
FFFFF,FFFFFFFFFF:FFFFFFFFFFFFF,FFFFFF:FFFFFFFF:FFFFFFFF:FFFFFFFFFFFFFF
FFFFFF:FFFFFFFF
@A00155:342:HHGFNDSXY:1:2259:22028:29496 2:N:0:GAACCTAG+TCCGCATA
ATTGACGTAACGCCTCAATCGTGGTCAAGGACCAGCATTCAATGCTACCACATTAAATACAATCACAT
TGTATTTCAGAACTTCTTGTGTCCGCACATAGAAGGTTAAAGATGGCTTTCTCTTATAGGACGCCACA
GGAGGAACTCTTGAG
+
FFFFFFFFFFFFFFFFFFFFFFFFFFFFFFFFFFFFFFFFFFFFFFFFFFFFFFFFFFFFFFFFFFFFFF
FFF:FFFFFFFFFFFFFFFFFFFFFFFFFFFFFFFFFFFFFFFFFFFFFFFFFF:FF:FFFFFFFFFFFF
FFFFFFFFFFFFFFF
@A00155:342:HHGFNDSXY:1:2415:2374:19852 2:N:0:GAACCTAG+TCCGCATA
ATTGACGTAACGCCTCAATCGTGGTCAAGGACCAGCATTCAATGCTACCACATTAAATACAATCACAT
TGTATTTCAGAACTTCTTGTGTCCGCACATAGAAGGTTAAAGATGGCTTTCTCTTATAGGACGCCACA
GGAGGAACTCTTGAG
+
FFFFFFFFFFFFFFFFFFFFFFFFFFFFFFFFFFFFFFFFFFFFFFFFFFFFFFFFFFFFFFF:FFFFFF
FFFFFFFFFFFFFFFFFFFFFFFFFFFFFFFFFFFFF:FFFF:FFFFFFFF:FFFFFFFFFFFFFFFFF:
FFFFFFF:FFFFFFF
@A00155:342:HHGFNDSXY:1:2547:15709:18928 2:N:0:GAACCTAG+TCCGCATA
ATTGACGTAACGCCTCAATCGTGGTCAAGGACCAGCATTCAATGCTACCACATTAAATACAATCACAT
TGTATTTCAGAACTTCTTGTGTCCGCACATAGAAGGTTAAAGATGGCTTTCTCTTATAGGACGCCACA
GGAGGAACTCTTGAG
+
FFFFFFFFFFFF,FFFFFFFFFFFFFFFF:FFFFFFFFFFFFFFFFFFFFFFFFFFFFFFFFFFFFFFF:
FF:FF:FFFFFFFF:FFFFFFF:,:FFFFFFFFFFFFF:FFF:FFFFFFFFFFF:,FF:FF,FF,FFFFF
FF:FFFFFFFFFFFF
@A00155:342:HHGFNDSXY:1:2616:18511:26757 2:N:0:GAACCTAG+TCCGCATA
CGATCTGTAACGCCTCAATCGTGGTCAAGGACCAGCATTCAATGCTACCACATTAAATACAATCACAT
TGTATTTCAGAACTTCTTGTGTCCGCACATAGAAGGTTAAAGATGGCTTTCTCTTATAGGACGCCACA
GGAGGAACTCTTGAG
+
FFFFFFFFFFFFFFFFFFFFFFFFFFFFFFFFFFFFFFFFFFFFFFFFFFFFFFFFFFFFFFFFFFFFFF
FFFFFF:FFFFFFFFFFFFFFFFFFFFFFFFFFFFFFFFFFFFFFFFFFFFFFFFFFFFFFFFFFFFFFF
FFFFFFFFFFFFFFF
@A00155:342:HHGFNDSXY:1:1268:23918:21559 2:N:0:GAACCTAG+TCCGCATA
GACGTAACGCCTCAATCGTGGTCAAGGACCAGCATTCAATGCTACCACATTAAATACAATCACATGGT
ATTTCAGAACTTCTTGTGTCCGCACATAGAAGGTTAAAGATGGCTTTCTCTTATAGGACGCCACAGGA
GGAACTCTTGAG
+
:FFFFFFFFF:,::F::F:::,,FFF,F,FF:FFFFFFFF::,::FFFFFF:FFFFFFFFFF:F,FF,,:
:FFFFF:FF:,FFFF,,FFF:F,FFF,:,FFF,F,F,FFFFFF,,FFFFFFFFFFFFF:F::FFF:FFFF
F,FF:::F,F:F

@A00155:342:HHGFNDSXY:1:1301:16821:30968 1:N:0:GAACCTAG+TCCGCATA
GACGTAACGCCTCAATCGTGGTCAAGGACCAGCATTCAATGCTACCACATTAAATACAATCACATTGT
ATTTCAGAACTTCTTGTGTCCGCACATAGAAGGTTAAAGATGGCTTTCTCTTATAGGACGCCACAGGA
GGAACTCTTGAGCAG
+
FFFFFFFF:FFFFF:FFFFFFFFFFFFF::FFFFF:FFFFF:FFF::FFFFFFFFFFFFFFFFFFFFF
FFFFFFFFFFFFF:FFFFFFFFFFFFFFFFFFFFFFFFFF:FFFFFFFFFFFFFFFFFFFFFFFFFFF
FFFFFFFFFFF,FFF
@A00155:342:HHGFNDSXY:1:1363:27073:3912 1:N:0:GAACCTAG+TCCGCATA
GACGTAACGCCTCAATCGTGGTCAAGGACCAGCATTCAATGCTACCACATTAAATACAATCACATTGT
ATTTCAGAACTTCTTGTGTCCGCACATAGAAGGTTAAAGATGGCTTTCTCTTATAGGACGCCACAGGA
GGAACTCTTGAGCAG
+
FFFFFFFFFFF:FFFFFFFFFFFF:FFFFFF:FFFFFFFFFFFFFFFFFFFFFFFFFFFFFFF,FFFF
FFFFFFFFFFFFFFFFFFFFFFFFFFFFFFFFFFFFFFFFF:FFFFFFFFFFFFFFFFFFFFFFFFFF
FFFFFFFFFFFFFFF
@A00155:342:HHGFNDSXY:1:1520:32036:35055 1:N:0:GAACCTAG+TCCGCATA
GACGTAACGCCTCAATCGTGGTCAAGGACCAGCATTCAATGCTACCACATTAAATACAATCACATTGT
ATTTCAGAACTTCTTGTGTCCGCACATAGAAGGTTAAAGATGGCTTTCTCTTATAGGACGCCACAGGA
GGAACTCTTGAGCAG
+
FFFFFFFFFFFFFFFFFFFFF:FFFFFFFFFFFFFFFFFFFFFFFFFFFFFFFFFFFF:FF:FFFFFF
FFFFFFFFFFFFFFFFFFFFFFFFFFFFFFFFFFFFFFFFFFFFFFFFFFFFFFFFFFFFFFFFFFFF
FFFFFFFFFFFFFFF
@A00155:342:HHGFNDSXY:1:1527:26901:13542 1:N:0:GAACCTAG+TCCGCATA
GACGTAACGCCTCAATCGTGGTCAAGGACCAGCATTCAATGCTACCACATTAAATACAATCACATTGT
ATTTCAGAACTTCTTGTGTCCGCACATAGAAGGTTAAAGATGGCTTTCTCTTATAGGACGCCACAGGA
GGAACTCTTGAGCAG
+
FFFFFFFFFFFFFFFFFFFFFFFFF:FFFFFFFFFFF:FFFFFFFFFFFFFFFFFFFFFF:FFFFFFF
FFFFFFFFFFFFFFFFFFFFFFFFFFFFFFFFFFFFFFFFFFFFFFFFFFFFFFFFFFFFFFFFFFFF
FFFFFFFFFFFFFFF
@A00155:342:HHGFNDSXY:1:2666:23484:31861 1:N:0:GAACCTAG+TCCGCATA
GACGTAACGCCTCAATCGTGGTCATGGACCAGCATTCAATGCTACCACATTAAATACAATCACATTGT
ATTTCAGAACTTCTTGTGTCCGCACATAGAAGGTTATAGATGGCTTTCTCTTATAGGACGCCACAGGA
GGAACTCTTGAGCAG
+
FFFFF,,:FFFFFFFF:FFF:F,F,F:F:F:FFFFF,:F:,,FFF:FF:FF:F:FFF:,F,:::FFFF
FFFF,:F,F:FFF:FFF:FFFFFF:FFFFF,FFFFF,F:FFFF,FFF,FFFF,F,FF,::F:FF:F::
,FF,FF:FFFFF,FF
@A00155:342:HHGFNDSXY:1:2567:9100:20823 1:N:0:GAACCTAG+TCCGCATA
ACGTAACGCCTCAATCGTGGTCAAGGACCAGCATTCAATGCTACCACATTAAATACAATCACATTGTA
TTTCAGAACTTCTTGTGTCCGCACATAGAAGGTTAAAGATGGCTTTCTCTTATAGGACGCCACAGGAG
GAACTCTTGAG
+
FFFFFFFFFF:FF::FFFFFFFFFFFFFFFFFFFFFF:FFFFFFFFFFFFFFF:FFF,FFFFFFFFFF
FFFFFFFFFFFFFFFFFFFFFFFFFFFFFFFFFFFFFFFFFFFFFFFFFFFFFFFFFFFFFFFFFFFF
FFFFFFFFFFF
@A00155:342:HHGFNDSXY:1:1537:20708:15593 1:N:0:GAACCTAG+TCCGCATA
ACGTAACGCCTCAATCGTGGTCAAGGACCAGCATTCAATGCTACCACATTAAATACAATCACATTGTA
TTTCAGAACTTCTTGTGTCCGCACATAGAAGGTTAAAGATGGCTTTCTCTTATAGGACGCCACAGGAG
GAACTCTTGAGCAGG
+
FFFFFFFFFFFFFFFFFFFFFFFFFFFFFFFFFFFFFFFFFFFFFFFFFFFFFFFFFFFFFFFFFFFF

```
FFFFFFFFFFFFFFFFFFF:FFFFFFFFFF:FFFFFFFFFFFFFFFFFFFFFFFFFFFFFFFFFFFFFF
FFFFFFFFFFFFFFF
@A00155:342:HHGFNDSXY:1:1415:31132:21339 1:N:0:GAACCTAG+TCCGCATA
CGTAACGCCTCAATCGTGGTCAAGGACCAGCATTCAATGCTACCACATTAAATACAATCACATTGTAT
TTCAGAACTTCTTGTGTCCGTACATAGAAGGTTAAAGATGGCTTTCTCTTATAGGACGCCACAGGAGG
AACTCTTGAG
+
FFFFFFFFFFFFFFFFFFFFFF:F:FFFFFFFFFFFFFFFFFFFFFFFFFFFFFFFFFFFFFFFFFFF
FFFFFF,FFF:FFFFFFFFFFFFFFFFFFFFFFFFFFFFFFFFFFFFFFFFFFFFFFF:FFFFFFFFFF
FFFFFFF:FF
@A00155:342:HHGFNDSXY:1:1408:16568:29904 1:N:0:GAACCTAG+TCCGCATA
CGTAACGCCTCAATCGTGGTCAAGGACCAGCATTCAATGCTACCACATTAAATACAATCACATTGTAT
TTCAGAACTTCTTGTGTCCGCACATAGAAGGTTAAAGATGGCTTTCTCTTATAGGACGCCACAGGAGG
AACTCTTGAGCAGGC
+
FFFFFFFFFFFFFF:FFFFFFFFFFF:FFFFFFFF,FFFFFFFFFF:FFFFFFFFFFFF,FFFFFFFF
FFFFFFFFFFFFFFFFFFFFFFFFFFFFFFFFFFFFFFFFFFFFFFFFFFFFFFFFFFFFFFFFFFFF
FFFFFFFFFFFFFFF
@A00155:342:HHGFNDSXY:1:1439:16947:26052 1:N:0:GAACCTAG+TCCGCATA
CGTAACGCCTCAATCGTGGTCAAGGACCAGCATTCAATGCTACCACATTAAATACAATCACATTGTAT
TTCAGAACTTCTTGTGTCCGCACATAGAAGGTTAAAGATGGCTTTCTCTTATAGGACGCCACAGGAGG
AACTCTTGAGCAGGC
+
FFFF:FF:FFFFFFFFFFFFFFFFFFFFFFFFFFFF:FFFFFFFFFFFFFFFFFFFFFF,FFF:FFFFFF
FFFFFF:FFFFFFFFFFFFFFFFFFFFFFFFFFFFFFFFFFFFFFFFFFFFFFFFFFFFFFFFFFF:FF
FFFFFFFFFFFFFFF
@A00155:342:HHGFNDSXY:1:1502:4435:1219 1:N:0:GAACCTAG+TCCGCATA
CGTAACGCCTCAATCGTGGTCAAGGACCAGCATTCAATGCTACCACATTAAATACAATCACATTGTAT
TTCAGAACTTCTTGTGTCCGCACATAGAAGGTTAAAGATGGCTTTCTCTTATAGGACGCCACAGGAGG
AACTCTTGAGCAGGC
+
FFFFFFFFFF:FFFFFFFFFFFFFFFFFF:F:FFFFFFFFFFFFFFFFFFF,FF,FFFFFFFFFFFFF
FFFFFF:FFF,:FFFFFFFF::FFFFFFFFFFFFFF,FF,FFF:FFFFFFFFFFFFFFFFFFFFFFFFF
F:FFFFFFFFFFFFF
@A00155:342:HHGFNDSXY:1:2511:16957:2738 1:N:0:GAACCTAG+TCCGCATA
CGTAACGCCTCAATCGTGGTCAAGGACCAGCATTCAATGCTACCACATTAAATACAATCACATGGTAT
TTCAGAACTTCTTGTGTCCGCACATAGAAGGTTAAAGATGGCTTTCTCTTATAGGACGCCACAGGAGG
AACTCTTGAGCAGGC
+
F,FF,F:FFFFFFFFFF,FFFFFF:FFFF::FFFFFF,FFFFFFFFFFFFF,,FF,,FFFF,,,FFFF
,FFFFFFFFFFF:FF::FFFFFFF,FFFFF,FFFFF:FFF,F,FF,F::,FFFFF:F:FF,FFFFFFF
FF,:FF:FF:FF:,F
@A00155:342:HHGFNDSXY:1:1118:24496:35900 2:N:0:GAACCTAG+TCCGCATA
CGTAACGCCTCAATCGTGGTCAAGGACCAGCATTCAATGCTACTACATTAAATACAATCACATTGTAT
TTCAGAACTTCTTGTGTCCGCACATAGAAGGTTAAAGATGGCTTTCTCTTATAGGACGCCACAGGAGG
AACTCTTGAGCAGGC
+
FFFFFFFFFFFFFFFFFFFFFFFFFFFFFFFFFFFFFFFFFFFFFFFFFFFFFFFF:FFFFFFFFFFF
:FFFFFFFFFFFFFFFFFFFFFFFFFFFFFFFFFFFFFFFFFFFFFFFF:FFFFFFFFFFFFFFFFFF
FFFFFFFFFFFFFFF
@A00155:342:HHGFNDSXY:1:1177:9896:20823 2:N:0:GAACCTAG+TCCGCATA
CGTAACGCCTCAATCGTGGTCAAGGACCAGCATTCAATGCTACCACATTAAATACAATCACATTGTAT
TTCAGAACTTCTTGTGTCCGCACATAGAAGGTTAAAGATGGCTTTCTCTTATAGGACGCCACAGGAGG
AACTCTTGAGCAGGC
```

```
+
FFFFFFFFFFFFFFFFFFFFFFFFFFFFFFFFFFFFFFFFFFFFFFFFFFFFFF:FFFFFFF:FFFFFFF
FFFF:FFFFFFFFFFFFFFFFFFFFFFFFFFFFFFFFFFFFFFFFFFFFFFFFFFFFFFFFFFFFFFFF
FF:FFFFFFFFFFFF
@A00155:342:HHGFNDSXY:1:1357:32759:21966 2:N:0:GAACCTAG+TCCGCATA
CGTAACGCCTCAATCGTGGTCAAGGACCAGCATTCAATGCTACCACATTAAATACAATCACATTGTAT
TTCAGAACTTCTTGTGTCCGCACATAGAAGGTTAAAGATGGCTTTCTCTTATAGGACGCCACAGGAGG
AACTCTTGAGCAGGC
+
FFFFFFFFFFFFFFFFFFFFFFFFFFFFFFFFFFFFFFFFFFFFFFFFFFFFFFFFFFFFFFF:F:FFF:
,FFFFFFFFFFFFFFFFFFFFFFFF,FFF:FF,FFFFFFFFFF:FFFFFFFFFFFFFFFFFFFFFFFFFF
FFFFFFFFFFFFFFF
@A00155:342:HHGFNDSXY:1:1570:13494:3004 2:N:0:GAACCTAG+TCCGCATA
CGTAACGCCTCAATCGTGGTCAAGGACCAGCATTCAATGCTACTACATTAAATACAATCACATTGTAT
TTCAGAACTTCTTGTGTCCGCACATAGAAGGTTAAAGATGGCTTTCTCTTATAGGACGCCACAGGAGG
AACTCTTGAGCAGGC
+
FFFFFFFFFFFFFFFFFFFFFFFFFFFFFFFFFFFFFFFFFFFFF:FFFFFFFFFFFFFFFFFFFFFFFF
FFFFFFFFFFF:FFFFFFFFFFFFFFFFFFFFFFFFFFFFFFFFFFFFFFFFFFFFFFFFFFFFFFFFFF
FFFFFFFFFFFFFFF
@A00155:342:HHGFNDSXY:1:2159:3134:17534 2:N:0:GAACCTAG+TCCGCATA
CGTAACGCCTCAATCGTGGTCAAGGACCAGCATTCAATGCTACCACATTAAATACAATCACATTGTAT
TTCAGAACTTCTTGTGTCCGCACATAGAAGGTTAAAGATGGCTTTCTCTTATAGGACGCCACAGGAGG
AACTCTTGAGCAGGC
+
FFFFFFFFFFFF:FFFFFFFFFFFFFFFFFFFFFFFFFFFFFFFFFFFFFFFFFFFFFFFFFFFFFFFF
FFF:F:FFFF:FFFFFFFFFFFFFFFFFFFFFFFFFFFFFFFFFFFFFFFFFFFF:FFFFFFFFFFFFFF
FFFFFFFFFFFF:FF
@A00155:342:HHGFNDSXY:1:2121:18502:7106 2:N:0:GAACCTAG+TCCGCATA
CGTAACGCCTCAATCGTGGTCAAGGACCAGCATTCAATGCTACTACATTAAATACAATCACATTGTAT
TTCAGAACTTCTTGTGTCCGCACATAGAAGGTTAAAGATGGCTTTCTCTTATAGGACGCCACAGGAGG
AACTCTTGAGCAGGC
+
FFFFFFFFFFFFFFFFFFFFFFFFFFFFFFFFFFFFFFFFFF:FFFFFFFFFFFFFFFFFFFF:FFFFFF
FFFFFFFFFFF:FFFFFFFFFFFFF,FFF,FFFFFFFFFFFFFFFFFFFFFFFFFFFFFFFFFFFFFFF:
FFFFFF::FFFFFFF
@A00155:342:HHGFNDSXY:1:2626:13006:11710 2:N:0:GAACCTAG+TCCGCATA
CGTAACGCCTCAATCGTGGTCAAGGACCAGCATTCAATGCTACCACATTAAATACAATCACATTGTAT
TTCAGAACTTCTTGTGTCCGCACATAGAAGGTTAAAGATGGCTTTCTCTTATAGGACGCCACAGGAGG
AACTCTTGAGCAGGC
+
FFFFFFFFFFFF,FFFFFFFFFFFF:,FFFFFFFFFFFFFFFFFFFFFFFFFFFFFFFF,F:,FF,FF:F
FFFFFFFFFFFFFFFFFFFF:FF:F,FFFFF,FFFFFFFFFFFFF,FFFFF,FFFF,FF:FFF,FFF:FF
FFFFF::FFFFFFFF
@A00155:342:HHGFNDSXY:1:2626:12617:12696 2:N:0:GAACCTAG+TCCGCATA
CGTAACGCCTCAATCGTGGTCAAGGACCAGCATTCAATGCTACCACATTAAATACAATCACATTGTAT
TTCAGAACTTCTTGTGTCCGCACATAGAAGGTTAAAGATGGCTTTCTCTTATAGGACGCCACAGGAGG
AACTCTTGAGCAGGC
+
FFFFFFFFFFFFFFFFFFFFFF:FFFFFFFFFFFFFFFFFFFFFFFFFFFFFFFFFFF:FFFFFFFFFFF
FFFFFFFFFFFFFFFFFFFFFFFFFFFFFFFFFFFFFFFFFFFFFFFFFFF:F:FFFFFFFFFFFFFFFF
F:FFFF:F:FFFFFF
@A00155:342:HHGFNDSXY:1:2605:22679:9674 2:N:0:GAACCTAG+TCCGCATA
CGTAACGCCTCAATCGTGGTCAAGGACCAGCATTCAATGCTACCACATTAAATACAATCACATTGTAT
```

```
TTCAGAACTTCTTGTGTCCGCACATAGAAGGTTAAAGATGGCTTTCTCTTATAGGACGCCACAGGAGG
AACTCTTGAGCAGGC
+
FFFFFFFFFFFFFFFFFFFFFFFFFFFF:FFFFFFFFFFFFFFFFFFFFFFFFFFFFFFFFF::FFFF
FFFFFFFFFFFFFFFFFFFFFFFFFFFFFFFFFFFFFFFFFFFFFFFFFFFFFFFFFFFFFFFFFFFF
F::FFFFFFFFFFFF
@A00155:342:HHGFNDSXY:1:1507:17653:21418 1:N:0:GAACCTAG+TCCGCATA
GTAACGCCTCAATCGTGGTCAAGGACCAGCATTCAATGCTACCACATTAAATACAATCACATTGTATT
TCAGAACTTCTTGTGTCCGCACATAGAAGGTTAAAGATGGCTTTCTCTTATAGGACGCCACAGGAGGA
ACTCTTGAGCAGGCG
+
FFFFFFFFFFFFFFFFFFFFFFFFFFFFFFFFFFFFFFFFFFFFFFFFFFFFFFFFFFFFFFFFFFFF
FFFFFFFFFFFFFFFFFFFFFFFFFFFFFFFFFFFFFFFFFFFFFFFFFFFFFFFFFFFFFFFFFFFF
FFFFFFFFFFFFFFF
@A00155:342:HHGFNDSXY:1:1513:1768:2926 1:N:0:GAACCTAG+TCCGCATA
GTAACGCCTCAATCGTGGTCAAGGACCAGCATTCAATGCTACCACATTAAATACAATCACATTGTATT
TCAGAACTTCTTGTGTCCGCACATAGAAGGTTAAAGATGGCTTTCTCTTATAGGACGCCACAGGAGGA
ACTCTTGAGCAAGCT
+
FFFFFFFFFFFFFFFFFFFFFFFFFFFFFFFFF:FFFFFFFFFFFFFFFFFFFFFFFFFFFFFFFFFF
FFFFFFFFFFFFF:FFFFF:FFFFFFFFF:FFFFFFFFFFFFFFFFFFFFFFFFFFFFFFFFFFFFFF
FFFFFFFFFFFFFFF
@A00155:342:HHGFNDSXY:1:2616:18511:26757 1:N:0:GAACCTAG+TCCGCATA
GTAACGCCTCAATCGTGGTCAAGGACCAGCATTCAATGCTACCACATTAAATACAATCACATTGTATT
TCAGAACTTCTTGTGTCCGCACATAGAAGGTTAAAGATGGCTTTCTCTTATAGGACGCCACAGGAGGA
ACTCTTGAGAGATCG
+
FFFFFFFFFFFFFFFFFFFFFFFFFFFFFFFFFFFFFFFFFFFFFFFFFFFFFFFFFFFFFFFFFFFF
FFFFFFFFFFFFFFFFFFFFFF,FFFFFFFFFFFFFFFFFFFFFFFFFFFFFFFFFFFFFFFFFFFFF
,FFFFFFFFFFFFFF
@A00155:342:HHGFNDSXY:1:2663:10511:30029 1:N:0:GAACCTAG+TCCGCATA
GTAACGCCTCAATCGTGGTCAAGGACCAGCATTCAATGCTACCACATTAAATACAATCACATTGTATT
TCAGAACTTCTTGTGTCCGCACATAGAAGGTTAAAGATGGCTTTCTCTTATAGGACGCCACAGGAGGA
ACTCTTGAGCAGGCG
+
FFFFFFFFFFFFFFFFFFFFFFFFFFFFFFFFFFFFFFFFFFFFFFFFFFFFFFF,FFFFFFFFFFFF
FFFFFFFFFFFFFFFFFFFFFFFFFFFFFFFFFFFFFFFFFFFFFFFFFFFFFFFFFFFFFFFFFFFF
FFFFFFFFFFFFFFF
@A00155:342:HHGFNDSXY:1:2546:30951:7874 2:N:0:GAACCTAG+TCCGCATA
GTAACGCCTCAATCGTGGTCAAGGACCAGCATTCAATGCTACCACATTAAATACAATCACATTGTATT
TCAGAACTTCTTGTGTCCGCACATAGAAGGTTAAAGATGGCTTTCTCTTATAGGACGCCACAGGAGGA
ACTCTTGAGCAGGCT
+
:::FFFFFFFFFFFFFFFFFFFFFFFFFFFFFFFFFFFFFFFFFFFFFFFFFFFFFFFFFF:FFFFFF
FFFFFFFFFFFFFFFFFFFFFFFFFFFFFFFFFFF:FFFFFFFFFFFFFFFFFFFFFFFFFFFFFFFF
FFFFFFFFFFFFFFF
@A00155:342:HHGFNDSXY:1:2226:8684:20384 1:N:0:GAACCTAG+TCCGCATA
AACGCCTCAATCGTGGTCAAGGACCAGCATTCAATGCTACCACATTAAATACAATCACATTGTATTTC
AGAACTTCTTGTGTCCGCACATAGAAGGTTAAAGATGGCTTTCTCTTATAGGACGCCACAGGAGGAAC
TCTTGAGCAGGCTCC
+
FFFFFFFFFFFFFFFFFFFFFFFFFFFFFFFFFFFFFFFFFFFFFFFF,FFFF:FFFFFFFFFFFF:F
FFFFFFFFFFFFFFFFFFFFFFFFFFFFFFFFFFFFFFFFFFFFFFFFFFFFFFFFFFFFFFFFFFFF
FFFFFFFFFFFFFFF
```

```
@A00155:342:HHGFNDSXY:1:1261:27091:14372 2:N:0:GAACCTAG+TCCGCATA
AACGCCTCAATCGTGGTCAAGGACCAGCATTCAATGCTACTACATTAAATACAATCACATTGTATTTC
AGAACTTCTTGTGTCCGCACATAGAAGGTTAAAGATGGCTTTCTCTTATAGGACGCCACAGGAGGAAC
TCTTGAGCAGGCTCC
+
FFFFFFFFFFFFFFFFFFFFFFFFFFFFFFFFFFFFFFFFFFFFFFFFFFFFFFFFFFFFFF:FFFFF
FFFFFFFFFFFFFFFFFFFFFFFFFFFFFFFFFFFFFFFFFFFFFFFF:FFFFFFFFFFFFFFFFFFF
FFFFFFFFFFFFFFF
@A00155:342:HHGFNDSXY:1:2163:2329:2456 2:N:0:GAACCTAG+TCCGCATA
AACGCCTCAATCGTGGTCAAGGACCAGCATTCAATGCTACCACATTAAATACAATCACATTGTATTTC
AGAACTTCTTGTGTCCGCACATAGAAGGTTAAAGATGGCTTTCTCTTATAGGACGCCACAGGAGGAAC
TCTTGAGCAGGCTCC
+
FFFFFFFFFF:FFFFFFFFF:FFFFFF,FFFFFFFFFFFFFF:FF:FFFFFF:FFFFFFFFFF:FFFF
FFFFFFFFFFFFFF:FFFF,FFFFFFFF,FF:FFF:::FFFF,F:FFFF:F,FFF,FFFFF,:FFFF:
FF,FF:FFFF,FFFF
@A00155:342:HHGFNDSXY:1:2457:32913:20885 2:N:0:GAACCTAG+TCCGCATA
AACGCCTCAATCGTGGTCAAGGACCAGCATTCAATGCTACCACATTAAATACAATCACATTGTATTTC
AGAACTTCTTGTGTCCGCACATAGAAGGTTAAAGATGGCTTTCTCTTATAGGACGCCACAGGAGGAAC
TCTTGAGCAGGCTCC
+
FFF:FFFFFFFFFFFFFFF:FF:FFFFFFFFF:FFFFFFFFFFFFFF:FFFFFFFFF:F:::FFFFFF
FFFFFFFFFFFFFF:FFFFFFFFFFFFFFFFF::FFFFFFFFFFFFFFFFFFFFF:F:F:FFFF,FF:
FFFFF:FF:F,FFFF
@A00155:342:HHGFNDSXY:1:2457:31114:24158 2:N:0:GAACCTAG+TCCGCATA
AACGCCTCAATCGTGGTCAAGGACCAGCATTCAATGCTACCACATTAAATACAATCACATTGTATTTC
AGAACTTCTTGTGTCCGCACATAGAAGGTTAAAGATGGCTTTCTCTTATAGGACGCCACAGGAGGAAC
TCTTGAGCAGGCTCC
+
FFFFFFFFFFFFFFFFFFFFFFFFFFFFFFFFFFFFFF:FFFFF::FFFFFFFFFFFFF:FFF,FFFF
FFFFFFFFF:FFFFFFFFFFFFFF:FFF:FFFFFFFFFFFFFFFFFFFF:FFFFFFFFFFFFFFF:,F
FFFFFFFFFFFFFFF
@A00155:342:HHGFNDSXY:1:1442:29197:1204 1:N:0:GAACCTAG+TCCGCATA
ACGCCTCAATCGTGGTCAAGGACCAGCATTCAATGCTACCACATTAAATACAATCACATTGTATTTCA
GAACTTCTTGTGTCCGCACATAGAAGGTTAAAGATGGCTTTCTCTTATAGGACGCCACAGGAGGAACT
CTTGAGCAGGCTCCC
+
F:FFFFFFFFFFFFFF:FFFFFFFFFFFFFFFF:FFFFFFFFFFFFFFFFF:FFF:FFFFFFFFFFFF
FFFFFFFFFFFFFFFFFFFFFFFFFFFFFFFFFFFFFFFFFFFFFFFFFFFFFFFFFFFFFFFFFFFF
FFFFFFFFFFFFFFF
@A00155:342:HHGFNDSXY:1:2626:29044:27398 1:N:0:GAACCTAG+TCCGCATA
ACGCCTCAATCGTGGTCAAGGACCAGCATTCAATGCTACCACATTAAATACAATCACATTGTATTTCA
GAACTTCTTGTGTCCGCACATAGAAGGTTAAAGATGGCTTTCTCTTATAGGACGCCACAGGAGGAACT
CTTGAGCAGGCTCCC
+
FFFF:FFFFFFFFFFFFFFFFFFFFFFFFFFFFFFFFFFFFFFFFFFFFFFFFFFFFFFFFFFFFFFF
FFFFFFFFFFFFFFFFFFFFFFFFFFFFFFFFFFFFFFFFFFFFFFFFFFFFFFFFFFFFFFFFFFFF
FFFFFFFFFFFFFFF
@A00155:342:HHGFNDSXY:1:2626:29062:27430 1:N:0:GAACCTAG+TCCGCATA
CGCCTCAATCGTGGTCAAGGACCAGCATTCAATGCTACCACATTAAATACAATCACATTGTATTTCAG
AACTTCTTGTGTCCGCACATAGAAGGTTAAAGATGGCTTTCTCTTATAGGACGACACAGGAGGAACTC
TTGAGCAGGCTCCC
+
FFFFFFF,F:FFFFF::,FFFFFFFFF:FF,FFFFFF,F,:FFFFFFFFF,FF:::,,:FFFFFFFF:
```

```
F,F:FFFFF,FFF:F,FFFF:FFF:FFFFFFF,FFFFFFFFFFFF:FFFFFFF,::FFF:F,,::FFF
FFFFFFFFF:FFFF
@A00155:342:HHGFNDSXY:1:2510:16586:24799 1:N:0:GAACCTAG+TCCGCATA
CGCCTCAATCGTGGTCAAGGACCAGCATTCAATGCTACTACATTAAATACAATCACATTGTATTTCAG
AACTTCTTGTGTCCGCACATAGAAGGTTAAAGATGGCTTTCTCTTATAGGACGCCACAGGAGGAACTC
TTGAGCAGGCTCCCG
+
FFFFFFFFFFFFF:FFFFFFFFFFFFFFFFFFFFFFFFFFFFFFFFFFFFF:FF,FFFFFFFFFFFF
FFFFFFFFFFFFFFFFFFFFFFFFFFFFFFFFFFFFFFFFFFFFFFFFFFFFFFFFFFFFFFFFFFFF
FFFFFFFFFFFFFFF
@A00155:342:HHGFNDSXY:1:2236:16586:22701 2:N:0:GAACCTAG+TCCGCATA
CGCCTCAATCGTGGTCAAGGACCAGCATTCAATGCTACTACATTAAATACAATCACATTGTATTTCAG
AACTTCTTGTGTCCGCACATAGAAGGTTAAAGATGGCTTTCTCTTATAGGACGCCACAGGAGGAACTC
TTGAGCAGGCTCCCA
+
FFFFFFFFFFFFFFFFFFFFFFFFFFFFFFF:FFFFFFF,FFFFFFFFFFFFFFFFFF:FFFF:FFFFF
FFFFFFFFFFFFFFFFFFFFFFFF:FFFFFFFFFFFFFFFFFFFFFFFFFFFFFFFFFFFFFFFF,FFF
F:FFFFFFFFFFFFF
@A00155:342:HHGFNDSXY:1:1325:18069:36104 1:N:0:GAACCTAG+TCCGCATA
TCCTCAATCGTGGTCAAGGACCAGCATTCAATGCTACCACATTAAATACAATCACATTGTATTTCAGA
ACTTCTTGTGTCCGCACATAGAAGGTTAAAGATGGCTTTCTCTTATAGGACGCCACAGGAGGAACTCT
TGAGCAGGCTCCCAC
+
FFFFFF,FFFFFFFFFFFFFFFFFFFFFFFFFFFFFFFFFFFFFFFFFFFFFFFFFFFFFFFFFFFFF
FFFFFFFFFFFFFFFFFFFFFFFFFFFFFFFFFFFFFFFFFFFFFFFFFFFFFFFFFFFFFFFFFFFF
FFFFFFFFFFFFFFF
@A00155:342:HHGFNDSXY:1:1326:20347:15687 1:N:0:GAACCTAG+TCCGCATA
GCCTCAATCGTGGTCAAGGACCAGCATTCAATGCTACCACATTAAATACAATCACATTGTATTTCAGA
ACTTCTTGTGTCCGCACATAGAAGGTTAAAGATGGCTTTCTCTTATAGGACGCCACAGGAGGAACTCT
TGAGCAGGCTCCCAC
+
FFFFFFFFFFFFFFFFFFFFFFFFFFFFFFFFFFFFFFFFFFFFFFF,FFFFFFFFFFFFFFFFFFFF
FFFFFFFFFFFFFFFFFFFFFFFFFFFFFFF,FFFFF:FFFFFFFFFFFFFFFFFFFFFFFFFFFFFF
FFFFFFFFFFFFFFF
@A00155:342:HHGFNDSXY:1:1330:23972:10097 1:N:0:GAACCTAG+TCCGCATA
GCCTCAATCGTGGTCAAGGACCAGCATTCAATGCTACCACATTAAATACAATCACATTGTATTTCAGA
ACTTCTTGTGTCCGCACATAGAAGGTTAAAGATGGCTTTCTCTTATAGGACGCCACAGGAGGAACTCT
TGAGCAGGCTCCCAC
+
FFFFFFFFFFFFFFFFFFFFFFFFFFFFFFFFFFFFFFFFFFFFFFFFFFFFFFF:FF:F:FFFFFFFF
FFFFFFFFFFFFFFFFFFFFF:FFFFFFFFFFFFFFFFFFF:FFFFFFFFFFFFFFFFFFFFFFFFF:FFFF
FFFFFFFFFFFFFFF
@A00155:342:HHGFNDSXY:1:2139:8938:23891 1:N:0:GAACCTAG+TCCGCATA
GCCTCAATCGTGGTCAAGGACCAGCATTCAATGCTACTACATTAAATACAATCACATTGTATTTCAGA
ACTTCTTGTGTCCGCACATAGAAGGTTAAAGATGGCTTTCTCTTATAGGACGCCACAGGAGGAACTCT
TGAGCAGGCTCCCAC
+
FFFFFF:FFFFFFFFFFFFFFFFFFFFFFFFFFFFFFFFFFFFFFFFFFFFFFFFFFFFFFFFFFFFFF
FFFFFFFFFFFFFFFFFFFFFFFFFFFFFFFFFFFFFFFFFFFFFFFFFFFFFFFFFFFFFFFFFFFF
FFFFFFFFFFFFFFF
@A00155:342:HHGFNDSXY:1:2239:1479:13416 1:N:0:GAACCTAG+TCCGCATA
GCCTCAATCGTGGTCAAGGACCAGCATTCAATGCTACCACATTAAATACAATCACATTGTATTTCAGA
ACTTCTTGTGTCCGCACATAGAAGGTTAAAGATGGCTTTCTCTTATAGGACGCCACAGGAGGAACTCT
TAAGCAGGCTCCCAC
```

```
+
:,F:,F,FFFF:F:FFF:FFFFFFFF:,FFFFF:FFFFFFFFFFFFFFFFF:FF:FFF:FFFFFF:FF
FFFFFFF,F:FFFFFFFFFFFFFF:FFFFFFFF:F:FFFF:FFFFFFFFFFFFFFFFFFFFFFFFFFF
FFFFFFFFFFFFFFF
@A00155:342:HHGFNDSXY:1:2301:18918:22326 1:N:0:GAACCTAG+TCCGCATA
GCCTCAATCGTGGTCAAGGACCAGCATTCAATGCTACCACATTAAATACAATCACATTGTATTTCAGA
ACTTCTTGTGTCCGCACATAGAAGGTTAAAGATGGCTTTCTCTTATAGGACGCCACAGGAGGAACTCT
TGAGCAGGCTCCCAC
+
FFF:FFFFFFFFFFFFFFFFFFFFFFF:F:F:FFFFFFFFFFFF,:FFFF,FFFFFFFFFFFFFFFFF
FFFFF:FFFFFFFFFFFFFFFFFFFFFFFFFFFFFFFFFFFFFFFFFFFFFFFFFFFFF:FFFFF:FF
FFFFFFFFFFFFFFF
@A00155:342:HHGFNDSXY:1:2301:18900:22795 1:N:0:GAACCTAG+TCCGCATA
GCCTCAATCGTGGTCAAGGACCAGCATTCAATGCTACCACATTAAATACAATCACATTGTATTTCAGA
ACTTCTTGTGTCCGCACATAGAAGGTTAAAGATGGCTTTCTCTTATAGGACGCCACAGGAGGAACTCT
TGAGCAGGCTCCCAC
+
FFFFFF,FFFFFFFFFFFF:FFFFFFFFFFF,FFF:FFFFFFFFFFFFFF,FFFFFFFFFFFFFFFFF
FF:FFFFFFFFFFFFFFFFFFFFFFFFFFFFFFF:FF,FFFF:FFF:FFFFFFFFFFFF::FFFFFFF
:FFFFFFFFFFFFFF
@A00155:342:HHGFNDSXY:1:2650:12400:36025 1:N:0:GAACCTAG+TCCGCATA
GCCTCAATCGTGGTCAAGGACCAGCATTCAATGCTACCACATTAAATACAATCACATTGTATTTCAGA
ACTTCTTGTGTCCGCACATAGAAGGTTAAAGATGGCTTTCTCTTATAGGACGCCACAGGAGGAACTCT
TGAGCAGGCTCCCAC
+
FF:FFFFFFFFFFFFFFFFFFFFFFFFFFFFFFFFFFFFFFFFFFFFFFFFFFFFFFFFFFFFFFFFF
FFFFFFFFFFFFFFFFFFFFFFFFFFFFFFFFFFFFFFFFFFFFFFFFFFFFFFFFFFFFFFFFFFFF
FFFFFFFFFFFFFFF
@A00155:342:HHGFNDSXY:1:2135:2871:12947 2:N:0:GAACCTAG+TCCGCATA
TCCTCAATCGTGGTCAAGGACCAGCATTCAATGCTACCACATTAAATACAATCACATTGTATTTCAGA
ACTTCTTGTGTCCGCACATAGAAGGTTAAAGATGGCTTTCTCTTATAGGACGCCACAGGAGGAACTCT
TGAGCAGGCTCCCAC
+
FFFFFFFFFFFFFFFFFFFFFFFFFFFFFFFFFFFFFFFFFFFFFFFFFFFFFFFFFFFFFFFFFFFF
FFFFFFFF:FFFFFFFFFFFFFFFFFFFFFFFFF:FFFFFFFFFFFFFFFFFFFFFFFFFFFFFFFFF
FFFFFFFFFFFFFFF
@A00155:342:HHGFNDSXY:1:2135:2871:12947 1:N:0:GAACCTAG+TCCGCATA
CCTCAATCGTGGTCAAGGACCAGCATTCAATGCTACCACATTAAATACAATCACATTGTATTTCAGAA
CTTCTTGTGTCCGCACATAGAAGGTTAAAGATGGCTTTCTCTTATAGGACGCCACAGGAGGAACTCTT
GAGCAGGCTCCCACA
+
FFFFFFFFFFFFFFFFFFFFFFFFFFFFFFFFFFFFFFFFFFFFFFFFFFFFFFFFFFFFFFFFFF:F
FFFFFFFFFFFFFFFFFFFFFFFFFFFFFFFFF:FFFF,FFFFFFFFFFF:FFFFFFFFFFF:FFFFF
FFFFFFFFFFFFFFF
@A00155:342:HHGFNDSXY:1:2331:31575:11177 1:N:0:GAACCTAG+TCCGCATA
CCTCAATCGTGGTCAAGGACCAGCATTCAATGCTACCACATTAAATACAATCACATTGTATTTCAGAA
CTTCTTGTGTCCGCACATAGAAGGTTAAAGATGGCTTTCTCTTATAGGACGCCACAGGAGGAACTCTT
GAGCAGGCTCCCACC
+
FFFFFFFFFFFFFFFFFFFFFFFFFFFFF:FFFFFF:FFFFFFFFFFFFFFFFFFFFFFFFFFFFFFF
FFFFFFFFFFFFFFFFFFFFFFFFFFFFFFFFFFFFFFFFFFFFFFFFFFFFFFFFFFFFFFFFFFFF
FFFFFFFFFFFFFFF
@A00155:342:HHGFNDSXY:1:1325:18069:36104 2:N:0:GAACCTAG+TCCGCATA
CCTCAATCGTGGTCAAGGACCAGCATTCAATGCTACCACATTAAATACAATCACATTGTATTTCAGAA
```

CTTCTTGTGTCCGCACATAGAAGGTTAAAGATGGCTTTCTCTTATAGGACGCCACAGGAGGACCTCTT
GAGCAGGCTCCCACA
+
FFFFFFFFFFFFFFFFFFFFFFFFFFFFFFFFFFFFFFFFFFFFFFFFFFFFFFFFFFFFFFFFFFFFFF
FFFFFFF:FFFFFFFFFFFFFFFFFFFFFFFFFFFFFFFFFFFFFFFFFFF:FFFFFFFF,FFFFF
FFFFFFFFFFFFFFF
@A00155:342:HHGFNDSXY:1:2307:6940:29324 2:N:0:GAACCTAG+TCCGCATA
CCTCAATCGTGGTCAAGGACCAGCATTCAATGCTACCACATTAAATACAATCACATTGTATTTCAGAA
CTTCTTGTGTCCGCACATAGAAGGTTAAAGATGGCTTTCTCTTATAGGACGCCACAGGAGGAACTCTT
GAGCAGGCTCCCACA
+
FFFFFFFFFFFFFFFFFFFFFFFFFFFFFFFFFFFFFFFFFFFFFFFFFFFFFFFF:FFFFFFFFF:F
FFFFFFFFFFFFFFFFFFFFFFFFFFFFF,FFFFFFFFFFFFFFFFF:FFFFFFFFFFFFFFFFFFFFFF
FFFFFFFFFFFFFFF
@A00155:342:HHGFNDSXY:1:1258:25301:24768 1:N:0:GAACCTAG+TCCGCATA
CTCAATCGTGGTCAAGGACCAGCATTCAATGCTACCACATTAAATACAATCACATTGTATTTCAGAAC
TTCTTGTGTCCGCACATAGAAGGTTAAAGATGGCTTTCTCTTATAGGACGCCACAGGAGGAACTCTTG
AGCAGGCTCCCACAG
+
FFFFFFFF,FFFFFFF:,FFFFFFFFFFFFFF,:FFFFFFFFFFFFFFFF:F:FFFFF,,,:FFFFFF
FFFFFFFFFF:FFFFF:FFFF:F,FFFFFFFF:FFFFFFFFFFFFFFFFF:FFFF,FFFFF:FFF:FF
FFFFFFFF,FF:FFF
@A00155:342:HHGFNDSXY:1:1367:10131:29403 1:N:0:GAACCTAG+TCCGCATA
CTCAATCGTGGTCAAGGACCAGCATTCAATGCTACCACATTAAATACAATCACATTGTATTTCAGAAC
TTCTTGTGTCCGCACATAGAAGGTTAAAGATGGCTTTCTCTTATAGGACGCCACAGGAGGAACTCTTG
AGCAGGCTCCCACAG
+
FFFFFFFFFFFFFFFFFFFFFFFFFFFFFFFFFFFFFFFFFFFFFFFFFFFFFFFFFFFFFFFFFFFFFF
F:FFFFFFFFFFFFFFFFFFFFFFFFFFFF::FFFFFFFFFFFFFFFFFFFFFFFFFFFFFFFFFFFFFF
FFFFFFFFFFFFFFF
@A00155:342:HHGFNDSXY:1:1473:26024:21167 1:N:0:GAACCTAG+TCCGCATA
CTCAATCGTGGTCAAGGACCAGCATTCAATGCTACCACATTAAATACAATCACATTGTATTTCAGAAC
TTCTTGTGTCCGCACATAGAAGGTTAAAGATGGCTTTCTCTAATAGGACGCCACAGGAGGAACTCTTG
AGCAGGCTCCCACAG
+
FFFFFFFFFFFFFFFFFFFFFFFFFFFFFFFFFFFFFFFFFFFFFFFFF:FFFF:FFFFFFFFFFFFFF
FFFFFFFFFFFFFFFFFFFFFFFFFFFFFFFFFFFFFFFFFFF,FFFFFFFFFFFFFFFFFFFFFFFFFFF
FFFFFFFFFFFFFFF
@A00155:342:HHGFNDSXY:1:2378:15546:28792 1:N:0:GAACCTAG+TCCGCATA
CTCAATCGTGGTCAAGGACCAGCATTCAATGCTACCACATTAAATACAATCACATTGTATTTCAGAAC
TTCTTGTGTCCGCACATAGAAGGTTAAAGATGGCTTTCTCTTATAGGACGCCACAGGAGGAACTCTTG
AGCAGGCTCCCACAG
+
FFFFFFFFFFFFFFFFFFFFFFFFFFFFFFFFFFFFFFFFFFFFFFFFF:FFFF:FFFFFFFFFFFFFF
FFFFFFFFFFFFFFFFFF:FFFF:FFFFFFFFFFF:FFFFFFFFFFFFFFFFFFFFFFFFFFFFFFFF:F
FFFFFFFFF:FFFFF
@A00155:342:HHGFNDSXY:1:2378:16504:28980 1:N:0:GAACCTAG+TCCGCATA
CTCAATCGTGGTCAAGGACCAGCATTCAATGCTACCACATTAAATACAATCACATTGTATTTCAGAAC
TTCTTGTGTCCGCACATAGAAGGTTAAAGATGGCTTTCTCTTATAGGACGCCACAGGAGGAACTCTTG
AGCAGGCTCCCACAG
+
FFFFFFFFFFFFFFFFFFFFFFFFFFFFFFFFFFFFFFFFFFFFFFFFFFFFFFF:FFFFFFFFFFFFF
FFFFFFFFFFFFFFFFFFFFFFFFFFFFFFFFFFFFFFFFFFFFFFFFFFFFFFFFFFFFFF,FFFFFF
FFFFFFF:FFF:FFF

```
@A00155:342:HHGFNDSXY:1:2525:9887:29919 1:N:0:GAACCTAG+TCCGCATA
CTCAATCGTGGTCAAGGACCAGCATTCAATGCTACCACATTAAATACAATCACATTGTATTTCAGAAC
TTCTTGTGTCCGCACATAGAAGGTTAAAGATGGCTTTCTCTTATAGGACGCCACAGGAGGAACTCTTG
AGCAGGCTCCCACAG
+
FFFFFFFFFFFFFFFFFFFFFFFFFFFFFFFFFFFFFFFFFFFFFFFFFFFFFFFFFFFFFFFFFFFF
FFFFFFFFFFFFFFFFFFFFFFFFFFFFFFFFFFFFFFFFFFFFFFFFFFFFFFFFFFFFFFFFFFFF
FFFFFFFFFFFFFFF
@A00155:342:HHGFNDSXY:1:1108:26051:23782 2:N:0:GAACCTAG+TCCGCATA
CTCAATCGTGGTCAAGGACCAGCATTCAATGCTACCACATTAAATACAATCACATTGTATTTCAGAAC
TTCTTGTGTCCGCACATAGAAGGTTAAAGATGGCTTTCTCTTATAGGACGCCACAGGAGGAACTCTTG
AGCAGGCTCCCACAG
+
FFFFFFFFFFFFFFFFFFFFFFFFFFFFFFFFFFFFFFFFFFFFFFFFFFFFFFFFFFFFFFFFFFFF
FFFFFFFFFFFFFFFFFFFF:FFFFFF:FFFFFFFFFFFFFFFFFFFFFFFFFFFFFFFFFFFFFFFF
FFFFFFFFFFFFF,F
@A00155:342:HHGFNDSXY:1:1501:1832:18568 2:N:0:GAACCTAG+TCCGCATA
TTCCGATCTGGTCAAGGACCAGCATTCAATGCTACCACATTAAATACAATCACATTGTATTTCAGAAC
TTCTTGTGTCCGCACATAGAAGGTTAAAGATGGCTTTCTCTTATAGGACGCCACAGGAGGAACTCTTG
AGCAGGCTCCCACAG
+
F,FF,FFFFF,:FFFFFF:FFF:FFFFF::FFFFFFFFFFFFF:FF,:FFFFFF:FFFFFFFFFFFFF
F:FFFF:FFFFFFFFFFFFFFFF:FFFFFF:::FFFFFFFFFFFFFFFFFFFFFFFFFFFFFFF,FFF
FF:FFFF:FFFFFFF
@A00155:342:HHGFNDSXY:1:2666:32316:21637 2:N:0:GAACCTAG+TCCGCATA
TTCCGATCTGGTCAAGGACCAGCATTCAATGCTACCACATTAAATACAATCACATTGTATTTCAGAAC
TTCTTGTGTCCGCACATAGAAGGTTAAAGATGGCTTTCTCTTATAGGACGCCACAGGAGGAACTCTTG
AGCAGGCTCCCACAG
+
FFFFFFFFFFFFFFFFFFFFFFF:FFFFFFFFFFFFFF,FFFFFFFFFFFF:FFFFFFFFFFFFFF:F
:FFF:FFFFFFFFFFFFFFFFFFFFFF,F,:FFF:FFFFF:::F,FFFFFFFFFFFFFFFF:FF:FFF
F:FFFFF:FFFFF,F
@A00155:342:HHGFNDSXY:1:2546:30951:7874 1:N:0:GAACCTAG+TCCGCATA
TCAATCGTGGTCAAGGACCAGCATTCAATGCTACCACATTAAATACAATCACATTGTATTTCAGAACT
TCTTGTGTCCGCACATAGAAGGTTAAAGATGGCTTTCTCTTATAGGACGCCACAGGAGGAACTCTTGA
GCAGGCTCCCACAGT
+
FFFFFFFFFFFFFFFFFF:FFFFFFFFFFFFFF:FFFFF:FFFFF:FF:FFFF,FFFFFFFFFFFFFF
FF:FFFFFFFFFFFF::FFFFFFFFF:::FFFFFFFFFFFFFFFFFFFFFFFFFFFFFFFFFFFFFFF
FFFFFFFFFFFFFFF
@A00155:342:HHGFNDSXY:1:1361:12771:11710 1:N:0:GAACCTAG+TCCGCATA
CAATCGTGGTCAAGGACCAGCATTCAATGCTACCACATTAAATACAATCACATTGTATTTCAGAACTT
CTTGTGTCCGCACATAGAAGGTTAAAGATGGCTTTCTCTTATAGGACGCCACAGGAGGAACTCTTGAA
CAGGCTCCCACAGTC
+
FFFFFFFFFFFFFFFFFFFFFFFFFFFFFFFFFFFFFFFFFFFFFFFFFFFFFFFFFF:FFFFFFFFF
FFFFFFFFFFFFFFFFFFFFFFFFFFFFFFFFFFFFFF::FFFF:FFFFFFFFFFFFFFFFFFFFF:F
FFFFFFFFFFFFFFF
@A00155:342:HHGFNDSXY:1:1469:28754:35759 1:N:0:GAACCTAG+TCCGCATA
CAATCGTGGTCAAGGACCAGCATTCAATGCTACCACATTAAATACAATCACATTGTATTTCAGAACTT
CTTGTGTCCGCACATAGAAGGTTAAAGATGGCTTTCTCTTATAGGACGCCACAGGAGGAACTCTTGAG
CAGGCTCCCACAGTC
+
FF:FFFFFFFFFFFFFFFFFFFFFF:FFFFFFF,FFFFFFFFF,FFFFFFFFFFFFFFFFFFFFFFFF
```

FFFF,FFFFFFFFFFF:,FFFFFFFFFFFFFFFFFFFFFFFFFFFFFFFFFFFFFFFFFFFFFFFFFF
FFFFFFFFFFFFFFF
@A00155:342:HHGFNDSXY:1:2610:31241:7936 1:N:0:GAACCTAG+TCCGCATA
CAATCGTGGTCAAGGACCAGCATTCAATGCTACCACATTAAATACAATCACATTGTATTTCAGAACTT
CTTGTGTCCGCACATAGAAGGTTAAAGATGGCTTTCTCTTATAGGACGCCACAGGAGGAACTCTTGAA
CAGGCTCCCACAGTC
+
FFF,FFFFFFF:FFFFFF:FFFFFFFFFFFF:FFFFFFFFFFFFFFF,FFFFFFF:FFFFFFFFFFF,
F,FFFF:FFFFFFF:FFFFFF:F,FFFFFFFFFFFFFFF:FFFFFFFFFFFFF:FF,FF,FFFFFFF:
FFFFF:FFFFF,FF:
@A00155:342:HHGFNDSXY:1:1119:23601:21261 2:N:0:GAACCTAG+TCCGCATA
CAATCGTGGTCAAGGACCAGCATTCAATGCTACCACATTAAATACAATCACATTGTATTTCAGAACTT
CTTGTGTCCGCACATAGAAGGTTAAAGATGGCTTTCTCTTATAGGACGCCACAGGAGGAACTCTTGAG
CAGGCTCCCACAGTC
+
FFFFFFFFFFFFFFFFFFFFFFFFFFFFFFFFFFFFFFFFFFFFFFFFFF,F,FF,FFFFFFFFF
FFFFFFFFFFFFFFFFFFFFFFFFFFFFFFFFFFFFFFFFFFFFFFFFFFFFFFFFFFFFFFFFFFFF
FFFFFFFFFFFFFFF
@A00155:342:HHGFNDSXY:1:2155:32985:16125 2:N:0:GAACCTAG+TCCGCATA
ATCGTGGTCAAGGACCAGCATTCAATGCTACCACATTAAATACAATCACATTGTATTTCAGAACTTCT
TGTGTCCGCACATAGAAGGTTAAAGATGGCTTTCTCTTATAGGACGCCACAGGAGGAACTCTTGAGCA
GGCTCCCACAGTCTC
+
FFFFFFFFFFFFFFFFFFFFFFFFFFFFFFFFFFFFFFFFF:FFFFFFFFF,FFFFFFFFFFFFFFFFFF
FFFFFFFFFFFFFFFFFFFFFFFFFFFFFFFFFFFFFFFFFFF:FFFFFFFFFFFFFFFFFFFFFFFFF
FFFFFFFFFFFFFFF
@A00155:342:HHGFNDSXY:1:2324:3351:2503 2:N:0:GAACCTAG+TCCGCATA
ATCGTGGTCAAGGACCAGCATTCAATGCTACCACATTAAATACAATCACATTGTATTTCAGAACTTCT
TGTGTCCGCACATAGAAGGTTAAAGATGGCTTTCTCTTATAGGACGCCACAGGAGGAACTCTTGAGCA
GGCTCCCACAGTCTC
+
FFFFFFFFFFFFFFFFFFFFFFFFFFFFFFFFFFFFFFFFFFFFFFFFFFFFFFFFFFFFFFFFFFFF
FFFFFFFFFFFFFFFFFFFFFFFFFFFFFFFFFFFFFFFFFFFFFFFFFFFFFFFFFFFFFFFFFFFF
FFFFFFFFFFFFFFF
@A00155:342:HHGFNDSXY:1:1258:25301:24768 2:N:0:GAACCTAG+TCCGCATA
TCGTGGTCAAGGACCAGCATTCAATGCTACCACATTAAATACAATCACATTGTATTTCAGAACTTCTT
GTGTCCGCACATAGAAGGTTAAAGATGGCTTTCTCTTATAGGACGCCACAGGAGGAACTCTTGAGCAG
GCTCCCACAGTCTCA
+
FFF,FFFFFFFFFFFFFFFFFFFFFFF:FFFFFFFFFF,FFFFFFF:FFFFFFF::FF:FFFFFFFFF
FFFFFFFFFFFFFFFFFFF,:FFFFFFFFFFFFFFFFFFFFFFFFFFFFFFFFFFFFFFFFFFFF:FFF
FF,FFFFFFFFFF:F
@A00155:342:HHGFNDSXY:1:1473:26024:21167 2:N:0:GAACCTAG+TCCGCATA
TCGTGGTCAAGGACCAGCATTCAATGCTACCACATTAAATACAATCACATTGTATTTCAGAACTTCTT
GTGTCCGCACATAGAAGGTTAAAGATGGCTTTCTCTTATAGGACGCCACAGGAGGAACTCTTGAGCAG
GCTCCCACAGTCTCA
+
FFFFFFFFFFFFFFFFFFFFFFFFFFFFFFFFFFFFFFFFFFFFFFFFF:FFFFFFFFFFFFFFFFFF
FFFFFFFFFFFFFFFFFFFFFFFFFFFFFFFFFFFF,FFFFFFFFFFFFFFFFFFFFFFFFFFFFFFF
:FFFFFFFFFFFFFF
@A00155:342:HHGFNDSXY:1:2378:15546:28792 2:N:0:GAACCTAG+TCCGCATA
TCGTGGTCAAGGACCAGCATTCAATGCTACCACATTAAATACAATCACATTGTATTTCAGAACTTCTT
GTGTCCGCACATAGAAGGTTAAAGATGGCTTTCTCTTATAGGACGCCACAGGAGGAACTCTTGAGCAG
GCTCCCACAGTCTCA

```
+
FFFFFFFFFFFFFFFFFFFFFFFFFFFFFFFFFFFFFFFFFFFFFFFF,FFFFFFFFFFFFFFFFFFFFF
FFFFFFFFFFFFFFFFFFFFFFFFFFFFFFFFFFFFFFFFFFFFFFFFFFFFF:FFFFFFFFFFFFFFF
FFFFFFFF:FFFFFF
@A00155:342:HHGFNDSXY:1:2378:16504:28980 2:N:0:GAACCTAG+TCCGCATA
TCGTGGTCAAGGACCAGCATTCAATGCTACCACATTAAATACAATCACATTGTATTTCAGAACTTCTT
GTGTCCGCACATAGAAGGTTAAAGATGGCTTTCTCTTATAGGACGCCACAGGAGGAACTCTTGAGCAG
GCTCCCACAGTCTCA
+
FFFFFFFFFFFFFFFFFFFFFFFFFFFFFFFFFFFFFFFFFFFFFFFFFFFFFFFFFFFFFFFFFFFF,F
FFFFFFFFFFFFFFFFFFFFFFFFFFFFFFFFFFFFFFFFFFFFFFFFFFFFFFFFFFFFFFFFFFFFFF
FFFFFFFFFFFFFFF
@A00155:342:HHGFNDSXY:1:1135:7301:9439 2:N:0:GAACCTAG+TCCGCATA
TGGTCAAGGACCAGCATTCAATGCTACCACATCAAATACAATCACATTGTATTTCAGAACTTCTTGTG
TCCGCACATAGAAGGTTAAAGATGGCTTTCTCTTATAGGACGCCACAGGAGGAACTCTTGAGCAGGCT
CCCACAGTCTCAAC
+
FFFFFFFFF:FFFFFFFFFFFFFFFFFFFFFFFFFFFFFFFFFFFFFFFFF:FFFFFFFFFFFFFFFFF
FFFFFF:FFFFFFFFFFFFFFFFFFFFFFFFFFFFFFFFFFFFFFFFFFFFFFFFFFFFFFFFFFFFFF
FFFFFFFFFFFFFF
@A00155:342:HHGFNDSXY:1:2114:25907:22404 1:N:0:GAACCTAG+TCCGCATA
GGTCAAGGACCAGCATTCAATGCTACCACATTAAATACAATCACATTGTATTTCAGAACTTCTTGTGT
CCGCACATAGAAGGTTAAAGATGGCTTTCTCTTATAGGACGCCACAGGAGGAACTCTTGAGCAGGCTC
CCACAGTCTCAACAG
+
FFFFFFFFFFFFFFFFFFFFFFFFFFFFFFFFFFFFFFFFFFFFFFFFFFFF:FFFFFFFFFFFFFFF
FFFFFFFFFFFFFFFFFFFFFFFFFFFFFFFFFFFFFFFFFFFFFFFFFFFFFFFFFFFFFFFFFFFF
FFFFFFFFFFFFFFF
@A00155:342:HHGFNDSXY:1:2114:25925:22404 1:N:0:GAACCTAG+TCCGCATA
GGTCAAGGACCAGCATTCACTGCTACCACATTAAATACAATCACATTGTATTTCAGAACTTCTTGTGT
CCGCACATAGAAGGTTAAAGATGGCTTTCTCTTATAGGACGCCACAGGAGGAACTCTTGAGCAGGCTC
CCACAGTCTCAACAG
+
FFFFFFFFFFFFFFFFFFF,FFFFFFFFFFFF:FFFFFFFFFFFFF:FFFFFFFFFFFFFFFFFFFFF
FFFFFFFFFFFFFFFFFFFFFFFFFFFFFFFFFFFFFFFFFFFFFFFFFFFFFFFFFFFFFFFFFFFF
FFFFFFFFFFFFFFF
@A00155:342:HHGFNDSXY:1:2551:17119:35462 2:N:0:GAACCTAG+TCCGCATA
GGTCAAGGACCAGCATTCAATGCTACCACATTAAATACAATCACATTGTATTTCAGAACTTCTTGTGT
CCGCACATAGAAGGTTAAAGATGGCTTTCTCTTATAGGACGCCACAGGAGGAACTCTTGAGCAGGCTC
CCACAGTCTCAACAG
+
FFFFFFFFFFFFFFFFFFFFFFFFFFFFFFFFFFFFFFFFFFFFF:FFFFFFFFFFFFFFFFFFFF:FF
FFFFFFFFFFFFFFFFFFFFFFFFFFFFFFFFFFFFFFFFFFFFFFFFF:FFF:FFFFFFFFFFFFFFF
FFFFFFFFFFF,FFF
@A00155:342:HHGFNDSXY:1:1619:11162:29997 1:N:0:GAACCTAG+TCCGCATA
CAAGGACTAGCATTCAATGCTACCACATTAAATACAATCACATTGTATTTCAGAACTTCTTGTGTCCG
CACATAGAAGGTTAAAGATGGCTTTCTCTTATAGGACGCCACAGGAGGAACTCTTGAGCAGGCTCCCA
CAGTCTCAACAGGAG
+
FFFF:FF:FFFFFFFFFFFFFFFFFFFFFFFFFFFFFFFFF:FFFFFFFFFFFFFFFFFFFFFFFFFFFFF
FFFFFFFFFFFFFFFFFFFFFFFFFFFFFFFFFFFFFFFFFFFFFFFFFFFFFFFFFFFFFFFFFFFFFF
FFFFFFFFFFFFFFF
@A00155:342:HHGFNDSXY:1:1353:24460:24752 2:N:0:GAACCTAG+TCCGCATA
CAAGGACCAGCATTCAATGCTACCACATTAAATACAATCACATTGTATTTCAGAACTTCTTGTGTCCG
```

```
CACATAGAAGGTTAAAGATGGCTTTCTCTTATAGGACGCCACAGGAGGAACTCTTGAGCAGGCTCCCA
CAGTCTCAACAGGAG
+
FFFFF:FF,FFFF,FF:F:FF::F,FFFFFFFFFFFF:FFF:,FFF,FFFFFFFFFFFFFFFFFFFFF
FFFF:,:F:,FFFFFFFFF:F:F,FFFF::F:FFFFFFFFFF::FF,FF:FFF:FFF:FFFFF:FFFF
::,FF:F:FFFFFFF
@A00155:342:HHGFNDSXY:1:2375:11388:15483 2:N:0:GAACCTAG+TCCGCATA
CAAGGACCAGCATTCAATGCTACCACATTAAATACAATCACATTGTATTTCAGAACTTCTTGTGTCCG
CACATAGAAGGTTAAAGATGGCTTTCTCTTATAGGACGCCACAGGAGGAACTCTTGAGCAGGCTCCCA
CAGTCTCAACAGGAG
+
FFFFFFFFFFFFFFFFFFFFFFFFFFFFFFFFFFFFFFFFFFFFFFF:FFFFFFFFFFFFFFFFFFFF
FFFFFFFFFFFFFFF:FFFFFFFFFFFFFFFFFFFFFFFFFFFFFFFFFFFFFFFFFFFFFFFFFFFF
FFFFFFF:FFFFFFF
@A00155:342:HHGFNDSXY:1:1659:3260:24298 2:N:0:GAACCTAG+TCCGCATA
AAGGACAAGCATTCAATGCTACCACATTAAATACAATCACATTGTATTTCAGAACTTCTTGTGTCCGC
ACATAGAAGGTTAAAGATGGCTTTCTCTTATAGGACGCCACAGGAGGAACTCTTGAGCAGGCTCCCAC
AGTCTCAACAGGAG
+
FF:FFFFF,FFFFFFFFFFFFFFFFFFF:FFFFFFFFFFFFFF:FFFFFFFFFF:FF:FF:FFFFFFF
FFFFFFFFFF:FFFFFF:FFFFFFFFFFFFFFFFFFFFFFFFFFFFFFFFFFFF,FFFFFFFFFFFFF
FFFFFFFFFFFFFF
@A00155:342:HHGFNDSXY:1:1407:8983:21120 2:N:0:GAACCTAG+TCCGCATA
AAGGACCAGCATTCAATGCTACTACATTAAATACAATCACATTGTATTTCAGAACTTCTTGTGTCCGC
ACATAGAAGGTTAAAGATGGCTTTCTCTTATAGGACGCCACAGGAGGAACTCTTGAGCAGGCTCCCAC
AGTCTCAACAGGAGG
+
FFFFFFFFFFFFFFFFFFFFFFFFFFF:FFFFFFFFFFFF:FFFFFFFFFFFFFFFFFFFFFFFFFFF
FF:FFFFFFFFFFFFFFFFFFFFFFFFFFFF,FFFFFFFFFFFFFFFFF:FFFFFF:FFFFFFFFFFFF
FFFFFFFFFFFFFFF
@A00155:342:HHGFNDSXY:1:1635:7265:5682 2:N:0:GAACCTAG+TCCGCATA
AAGGACCAGCATTCAATGCTACTACATTAAATACAATCACATTGTATTTCAGAACTTCTTGTGTCCGC
ACATAGAAGGTTAAAGATGGCTTTCTCTTATAGGACGCCACAGGAGGAACTCTTGAGCAGGCTCCCAC
AGTCTCAACAGGAGG
+
FFFFFFFFFFFFFFFFFFFFFFFFFFFFFFFFFFFFFFFFF:FFFFFFFFFFFFFFFFFFFFFFFFFF
FFFFFFFF:FFFFFFFFFFFFFFFFFFFFFFFFFFFFFFFFFFFFFFFF:FFFFFFFFFFFFFFFFFFF
FFFFFFFFFFFFFFF
@A00155:342:HHGFNDSXY:1:2635:1967:11130 2:N:0:GAACCTAG+TCCGCATA
AAGGACCAGCATTCAATGCTACTACATTAAATACAATCACATTGTATTTCAGAACTTCTTGTGTCCGC
ACATAGAAGGTTAAAGATGGCTTTCTCTTATAGGACGCCACAGGAGGAACTCTTGAGCCGGCTCCCAC
AGTCTCAACAGGAGG
+
FFFF:FFFFFFFFFFFFFFFFFFFFF:FFFFFFFF:FFFF:,FFF:FFFFFF,FFFFF:,FFF:FFFF
FFF,FF::FFFFFFFFFFFFFFFFFFFF::FFFFFFFFF,FFF:FFFF,F,FFFFFFF,:FFFFFFFF
:FFF,FFFF:FFFFF
@A00155:342:HHGFNDSXY:1:1215:12617:29481 1:N:0:GAACCTAG+TCCGCATA
AGGACCAGCATTCAATGCTACTACATTAAATACAATCACATTGTATTTCAGAACTTCTTGTGTCCGCA
CATAGAAGGTTAAAGATGGCTTTCTCTTATAGGACGCCACAGGAGGAACTCTTGAGCAGGCTCCCACA
GTCTCAACAGG
+
FFFFFFFFFFFFFFFFFFFFFFFFFFFFFFFFFFFFFFFFFFFFFFFFFFFFFFFFFFFFFFFFFFFF
F:FFFFFFFFFFFFFFFFFFFFFFFFFFFFFFFFFFFFFFFFFFFFF:FFFFFFFFFFFFFFFFFFFF
FFFFFFFFFFF
```

```
@A00155:342:HHGFNDSXY:1:2665:17300:17519 2:N:0:GAACCTAG+TCCGCATA
AGGACCAGCATTCAATGCTACCACATTAAATACAATCACATTGTATTTCAGAACTTCTTGTGTCCGCA
CATAGAAGGTTAAAGATGGCTTTCTCTTATAGGACGCCACAGGAGGAACTCTTGAGCAGGCTCCCACA
GTCTCAACAGG
+
FFFFFFFFFFFFFFFFFFFFFFFFFFFFFFFFFFFFFFFFFFFFFFFFFFFFFFFFFFFFFFFFFFFF
FFFFFFFFFFFFFFFFFFFFFFFFFFFFFFFFFFFFFFFFFFFFFFFFFFFFFFFFFFFFFFFFFFFF
FFFFFFFFFFF
@A00155:342:HHGFNDSXY:1:2669:30273:20384 2:N:0:GAACCTAG+TCCGCATA
AGGACCAGCATTCAATGCTACCACATTAAATACAATCACATTGTATTTCAGAACTTCTTGTGTCCGCA
CATAGAAGGTTAAAGATGGCTTTCTCTTATAGGACGCCACAGGAGGAACTCTTGAGCAGGCTCCCACA
GTCTCAACAGG
+
FFFFFFFFFFFFFFFFFFFFFFFFFFFFFFFFFFFFFFFFFFFFFFFFFFFFFFFFFFFFFFFFFFFF
FF:FFFFFFFFFFFFFFFFFFFFFFFFFFFFFFFFFFFFFFFFFFFFFFFFFFFFFFFFFFFFFFFFF
FFFFFFFFFFF
@A00155:342:HHGFNDSXY:1:2126:12102:23672 1:N:0:GAACCTAG+TCCGCATA
AGGACCAGCATTCAATGCTACCACATTAAATACAATCACATTGTATTTCAGAACTTCTTGTGTCCGCA
CATAGAAGGTTAAAGATGGCTTTCTCTTATAGGACGCCACAGGAGGAACTCTTAAGCAGGCTCCCACA
GTCTCAACAGGAGGC
+
FFFFFFFFFFFFFFFFFFFFFFFFFFFFFFFFFFFFFFFFFFFFFFFFFFFFFFFFFFFFFFFFFFFF
FFFFFFFFFFFFFFFFFFFFFFFFFFFFFFFFFFFFFFFFFFFFFFFFFFFFFFFFFFFFFFFFFFFF
FFFFFFFFFFFFFFF
@A00155:342:HHGFNDSXY:1:1313:21540:18505 2:N:0:GAACCTAG+TCCGCATA
AGGACCAGCATTCAATGCTACCACATTAAATACAATCACATTGTATTTCAGAACTTCTTGTGTCCGCA
CATAGAAGGTTAAAGATGGCTTTCTCTTATAGGACGCCACAGGAGGAACTCTTGAGCAGGCTCCCACA
GTCTCAACAGGAGGT
+
FFFFFFFFFFFFFFFFFFFFFFFFFFFFFFF:FFFFFFFFF:FFFFFFFFFFFFFFFFFFFFFFFFFF
FFFFFF:,FFFFFFFFFFFFFFFFFFFFFFFF::FFF:FFFFFF,FFFFFF,FFFFFFFFFFFFFFFF
FFFFFFFFFFFFFFF
@A00155:342:HHGFNDSXY:1:1457:24288:6574 2:N:0:GAACCTAG+TCCGCATA
AGGACCAGCATTCAATGCTACTACATTAAATACAATCACATTGTATTTCAGAACTTCTTGTGTCCGCA
CATAGAAGGTTAAAGATGGCTTTCTCTTATAGGACGCCACAGGAGGAACTCTTGAGCAGGCTCCCACA
GTCTCAACAGGAGGT
+
FFFFFFFFFFFFFFFFFFFFFFFFFFFFFFFFFFFFFFFFFFFFFFFFFFFFFFFFFFFFFFFFFFF:
FF:FFFFFFFFFFFFFFFFFFFFFFFFFFFFFFFFFFFFFFFFFFFFF,FFFFFFFFFFFFFFFFFFF
FFFFFFFFFFFFFFF
@A00155:342:HHGFNDSXY:1:1649:6144:18897 2:N:0:GAACCTAG+TCCGCATA
AGGACCAGCATTCAATGCTACCACATTAAATACAATCACATTGTATTTCAGAACTTCTTGTGTCCGCA
CATAGAAGGTTAAAGATGGCTTTCTCTTATAGGACGCCACAGGAGGAACTCTTGAGCAGGCTCCCACA
GTCTCAACAGGAGGT
+
FFFFFFFFFFFFFFFFFFFFFF,FFFFFFFFFFFFFFFFF::,FF:FFFFFFFFFFFFFFFF:FFFFFF
FFFFF:FFFFFFFFF:FFFFFFFFFFFFFFF:FFFFFFFFFFFFFFFFFFFFFF:FFFFFFFFFFFFFF
FFFFFFFFF:FFFFF
@A00155:342:HHGFNDSXY:1:2559:15040:28072 2:N:0:GAACCTAG+TCCGCATA
AGGACCAGCATTCAATGCTACCACATTAAATACAATCACATTGTATTTCAGAACTTCTTGGGTCCGCA
CATAGAAGGTTAAAGATGGCTTTCTCTTATAGGACGCCACAGGAGGAACTCTTGAGCAGGCTCCCACA
GTCTCAACAGGAGGT
+
FFFFFFF:FFFFFFFF:F,FFFFFFFFFFFFFFFF,:FFFFFFF:,FFFFF,FFFFF,FF,FFFFFFF
```

```
:FFFFFFFFFFFFFFFFFF,F,FFFF:FFFFFFFFF::FFFF:FFFFFFFFFFF:FF:FFFFFFFFF
FFFFFFFFFFFFFFF
@A00155:342:HHGFNDSXY:1:2563:21477:5713 2:N:0:GAACCTAG+TCCGCATA
AGGACCAGCATTCAATGCTACCACATTAAATACAATCACATTGTATTTCAGAACTTCTTGTGTCCGCA
CATAGAAGGTTAAAGATGGCTTTCTCTTATAGGACGCCACAGGAGGAACTCTTGAGCAGGCTCCCACA
GTCTCAACAGGAGGT
+
FFFFFFFFFFFFFFFFFFFFFFFFFFFFFFFFFFFFF:F:F,FFFFFFFFFF:FFFFFFFFFFFFF
F,FFFFFFFFFFFFFFFFFFF,:FFF:FFFFFFFFFFFFFFFFFFFFFFFFFFFFFFFFFFFFFFFFF
FFF:F:FFFFFFFFF
@A00155:342:HHGFNDSXY:1:1172:6379:2988 2:N:0:GAACCTAG+TCCGCATA
GACCAGCATTCAATGCTACCACATTAAATACAATCACATTGTATTTCAGAACTTCTTGTGTCCGCACA
TAGAAGGTTAAAGATGGCTTTCTCTTATAGGACGCCACAGGAGGAACTCTTGAGCAGGCTCCCACAGT
CTCAACAGGAGGTT
+
F:FFF:FFFFFFFFFFFFFFFFFFFFFFFFFFFFFFFFFFFFFFFFFFFFFFFFFFFFFFFFFFFFFF
FFFFFFFFFFFFFFFFFFFFFFFFFFFFFFFFFFFFFFFFFFFFFFFFFFFFFFFFFFFFFFFFFFFF
FFFFFFFFFFFFFF
@A00155:342:HHGFNDSXY:1:2261:20663:24565 2:N:0:GAACCTAG+TCCGCATA
GACCAGCATTCAATGCTACTACATTAAATACAATCACATTGTATTTCAGAACTTCTTGTGTCCGCACA
TAGAAGGTTAAAGATGGCTTTCTCTTATAGGACGCCACAGGAGGAACTCTTGAGCAGGCTCCCACAGT
CTCAACAGGAGGTT
+
FFFFFFFFFFFFFFFFFFFFFFFFFFFF:F:FFFFFFFFFFFF::FFFFFF:FFFFF::FFFF,:FFF
FF:FFFFFFFFFFF,FF:FF:F::F::FFFF::FFFFFFFFFF,FFFFF,FFFFFFF:FFF,FFFF::
FF:FFFFF:FFFFF
@A00155:342:HHGFNDSXY:1:1213:18087:37012 2:N:0:GAACCTAG+TCCGCATA
GACCAGCATTCAATGCTACCACATTAAATACAATCACATTGTATTTCAGAACTTCTTGTGTCCGCACA
TAGAAGGTTAAAGATGGCTTTCTCTTATAGGACGCCACAGGAGGAACTCTTGAGCAGGCTCCCACAGT
CTCAACAGGAGGTTA
+
FFFFFFFFFFFFFFFFFFFFFFFFFFFFFFFFFFFFFF:FFFFFFFFFFFFFFFFFFFFFFFFFFFFF
:FFFFFFFFFFFFFFF:FFFFFFFFFF:FFFFFFFFFFFFFFFFFFFFF:FFFFFFFFFFFFFFFFFF
FFFFFFFFFFFFFFF
@A00155:342:HHGFNDSXY:1:1403:32470:25410 2:N:0:GAACCTAG+TCCGCATA
CCAGCATTCAATGCTACCACATTAAATACAATCACAGTGTATTTCAGAACTTCTTGTGTCCGCACAGA
GAAGGTTAAAGATGGCTTTCTCTTATAGGACGCCACAGGAGGAACTCTTGAGCAGGCTCCCACAGTCT
CAACAGGAGGTTAT
+
FFFFFF:FFFFFFFFFFFFFFFFFF:FFFFFFFFF:,,FFF,FFFFFFFFFFFFFFFFFFFFFFFF,F
FFFFFF,FFFFFFFFFFFFFFFFFFFFFFFFFFFFF:FFFFFFFFFFF,FFFFFF:FFFFFFFFFFFF
FF,F:FFFFF::FF
@A00155:342:HHGFNDSXY:1:1102:20925:24549 2:N:0:GAACCTAG+TCCGCATA
CCAGCATTCAATGCTACCACATTAAATACAATCACATTGTATTTCAGAACTTCTTGTGTCCGCACATA
GAAGGTTAAAGATGGCTTTCTCTTATAGGACGCCACAGGAGGAACTCTTGAGCAGGCTCCCACAGTCT
CAACAGGAGGTTATA
+
FFFFF:FFFFFFFFFFFFFFFFFFFFFFFFFF:FFFF::FFFFFFFFFFFFFFFFFFFFFFFFFFFFF
FFFFFFFFFFFFFFFFFFFFFFFFFFFFFFFFFFF,F:FFFFFF,FFFFFFFFFFFFFFFFFFFFFFFF
FFFFFFF,FF:FF:F
@A00155:342:HHGFNDSXY:1:1230:23610:12352 2:N:0:GAACCTAG+TCCGCATA
CCAGCATTCAATGCTACCACATTAAATACAATCACATTGTATTTCAGAACTTCTTGTGTCCGCACATA
GAAGGTTAAAGATGGCTTTCTCTTATAGGACGCCACAGGAGGAACTCTTGAGCAGGCTCCCACAGTCT
CAACAGGAGGTTATA
```

+
FFFFFFFFFFFFFFFFFF:FFFFFFFFFFF:FFFFF,FFFFFFFFFFFFFFFFFFFFFFFFF:FFF:F
FFFFFFFFFFFFFFFFFFFFFFFFFFFFFFFFFFFFFFFFFFFFFFFFF:FFFFFFFF:FFFFFF
FFF,FFFFF:FFFFF
@A00155:342:HHGFNDSXY:1:1644:14651:7420 2:N:0:GAACCTAG+TCCGCATA
CCAGCATTCAATGCTACCACATTAAATACAATCACATTGTATTTCAGAACTTCTTGTGTCCGCACATA
GAAGGTTAAAGATGGCTTTCTCTTATAGGACGCCACAGGAGGAACTCTTGAGCAGGCTCCCACAGTCT
CAACAGGAGGTTATA
+
FFFFFFFFFFFFFFFFFFFFFFFFFFFFFFFFFFF:FFFFFF:FFFFFFFFFFFFFFFFFFFFFFFFFF
FFFFFFFFFFFFFFFFFFFFFFFFFFFFFFFFFFFFFFFFFFFF:FFFFFFFFFFFFFFFFFFFFFFFF
FFFFFFFFFFFF,FF
@A00155:342:HHGFNDSXY:1:2132:17797:24862 2:N:0:GAACCTAG+TCCGCATA
CCAGCATTCAATGCTACCACATTAAATACAATCACATTGTATTTCAGAACTTCTTGTGTCCGCACATA
GAAGGTTAAAGATGGCTTTCTCTTATAGGACGCCACAGGAGGAACTCTTGAGCAGGCTCCCACAGTCT
CAACAGGAGGTTATA
+
FFFFFFFFFFFFFFFFFFFFFFFFFFFFFFFFFFFFFFFFFFFFFFFFFFFFFFFFFFFFFFFFFFFF
FF:FFFFFFFFFFFFFF:FFFFFFFFFFFFFFFFFFFFFFFFFFFFFFFFFFFFFFFFFFFFF,FFFF
FFFFFFFFFFFFFFF
@A00155:342:HHGFNDSXY:1:2419:7428:18959 2:N:0:GAACCTAG+TCCGCATA
CCAGCATTCAATGCTACCACATTAAATACAATCACATTGTATTTCAGAACTTCTTGTGTCCGCACATA
GAAGGTTAAAGATGGCTTTCTCTTATAGGACGCCACAGGAGGAACTCTTGAGCAGGCTCCCACAGTCT
CAACAGGAGGTTATA
+
FFFFFFFFFFFFFFFFFFFFFFFFFFFFFFFFFFF:FFFFFFFFFFFFFFFFFFFFFFFFFFFFFFFFF
FFFFFFFFFFFFFFFFFFFFFFF,FFFFFFFFFFFFFFFFFFFF,FFFFFFFFFFFFFFFFFFFFFFFF
FFFFFFFFFFFFFFF
@A00155:342:HHGFNDSXY:1:2512:15546:3490 2:N:0:GAACCTAG+TCCGCATA
CCAGCATTCAATGCTACCACATTAAATACAATCACATTGTATTTCAGAACTTCTTGTGTCCGCACATA
GAAGGTTAAAGATGGCTTTCTCTTATAGGACGCCACAGGAGGAACTCTTGAGCAGGCTCCCACAGTCT
CAACAGGAGGTTATA
+
FFFFFFF:,F:FFFFFF:FF:FFFFFFFFFF:,F:FF:FFFFFF:F,:F::FFF,,FFFFF,FFFFFF
FF:F:F:FFFFFFFFFF,FFFFFFFFF::FFF::FF:FFFF,FFFFF:FFFFFFFFFFFFFFF:F:FF
::,,F:F,FF:FFFF
@A00155:342:HHGFNDSXY:1:2525:13051:9721 2:N:0:GAACCTAG+TCCGCATA
CCAGCATTCAATGCTACCACATTAAATACAATCACATTGTATTTCAGAACTTCTTGTGTCCGCACATA
GAAGGTTAAAGATGGCTTTCTCTTATAGGACGCCACAGGAGGAACTCTTAAGCAGGCTCCCACAGTCT
CAACAGGAGGTTATA
+
FFFFFFFFFFFFFFFFFFFFFFFFFFFFFFFFFFF::FFFFFFFFFFFFFFFFFFFFFFFFFFFFFF:F
FFFFFFFFFFFFFFFFFFFFFFFFFFFFFFFFFFFFFFFFFFFF:FFFFF:FFFFFFFFF:FFFFFFFF
F,FFFFFFFFFFFFF
@A00155:342:HHGFNDSXY:1:2527:1597:14058 2:N:0:GAACCTAG+TCCGCATA
CCAGCATTCAATGCTACCACATTAAATACAATCACATTGTATTTCAGAACTTCTTGTGTCCGCACATA
GAAGGTTAAAGATGGCTTTCTCTTATAGGACGCCACAGGAGGAACTCTTGAGCAGGCTCCCACAGTCT
CAACAGGAGGTTATA
+
FFFFFFFFFFFFFFFFFFFFFF:FFFFFFFFFFFFFFFFFFFFF:FFFFFFFFFFFFFFFFFFFFFFF,F
FFFFFFFFFFFFFFFFFFFFFFFFFFFFFFF:FFFFFFFFFF,FF:FF::FFFFFFF,FFFFFFFFFF
FFFFFFFF:FFFFFF
@A00155:342:HHGFNDSXY:1:2641:15709:15013 2:N:0:GAACCTAG+TCCGCATA
CCAGCATTCAATGCTACCACATTAAATACAATCACATTGTATTTCAGAACTTCTTGTGTCCGCACATA

GAAGGTTAAAGATGGCTTTCTCTTATAGGACGCCACAGGAGGAACTCTTGAGCAGGCTCCCACAGTCT
CAACAGGAGGTTATA
+
FFFFFFFFFFFFFFFFFFFFFFFFFFFFFFFFFFFFFFFFFFFFFFFFFFFFFFFFFFFFFFFFFFFF
FFFFFFFFFFFFFFFFFFFFFFFFFF:FFF:FFFFFFFFFFFFFFFFFFFFFFFFFFFFFFFFFFFFF
FFFFFFFFFF:FFFF
@A00155:342:HHGFNDSXY:1:1278:25708:12978 2:N:0:GAACCTAG+TCCGCATA
CAGCATTCAATGCTACCACATTAAATACAATCACATTGTATTTCAGAACTTCTTGTGTCCGCACATAG
AAGGTTAAAGATGGCTTTCTCTTATAGGACGCCACAGGAGGAACTCTTGAGCAGGCTCCCACAGTCTC
AACAGGAGGTTATAA
+
FFFFFFFFF::FF,FFFFFFFFF:FFFFFF:FF:F:,FFF:F:FFFFFFF:FFF:FFF:FFFFFFFF:
FFFFFFF:FFFFFFFFFFFFFFFFFFFFFFFFFFFFFFFF:FFF,FFFFFFFFFF:FFF:FFFFFFFF
FFFFFFFF:FFFFFF
@A00155:342:HHGFNDSXY:1:1441:27046:32957 2:N:0:GAACCTAG+TCCGCATA
CAGCATTCAATGCTACCACATTAAATACAATCACATTGTATTTCAGAACTTCTTGTGTCCGCACATAG
AAGGTTAAAGATGGCTTTCTCTTATAGGACGCCACAGGAGGAACTCTTGAGCAGGCTCCCACAGTCTC
AACAGGAGGTTATAA
+
FFFFFFFFFFFFFFFFFFFFFFFFFFFFFFFFFFF:FFFFFFFFFFFFFFFFFFFFFFFFFFFFFFFF
FFFFFFFFFFFFFFF:FFFFFFFFFFFFFFFFFFFFFFFFFFFFFFFFFFFFFFFFFFFFFFF:FFFF
FFFFFFFFF:F:FFF
@A00155:342:HHGFNDSXY:1:1441:26992:33207 2:N:0:GAACCTAG+TCCGCATA
CAGCATTCAATGCTACCACATTAAATACAATCACATTGTATTTCAGAACTTCTTGTGTCCGCACATAG
AAGGTTAAAGATGGCTTTCTCTTATAGGACGCCACAGGAGGAACTCTTGAGCAGGCTCCCACAGTCTC
AACAGGAGGTTATAA
+
FFFFFFFFFFFFFF:FFFFFFFFFFFFFFFFFFFFFFFFFFFFFFFFFFFFFFFFFFFFFFFFFFFFF
FFFFFFFFFFFFFFFFFFFFFFFFFFFFFF:FFFFFFFFFFFFFFFFFFFFFFFFFFFFFFFFFFFFF
FFFFFFFFFFFFFFF
@A00155:342:HHGFNDSXY:1:1463:24831:19382 2:N:0:GAACCTAG+TCCGCATA
TAGCATTCAATGCTACCACATTAAATACAATCACATTGTATTTCAGAACTTCTTGTGTCCGCACATAG
AAGGTTAAAGATGGCTTTCTCTTATAGGACGCCACAGGAGGAACTCTTGAGCAGGCTCCCACAGTCTC
AACAGGAGGTTATAA
+
F,FFFFFFFFFFFFFFFFFFFFFFFFFF:FFFFFFFFF:FFFFFFFFFFFFF:FFFFFFFFFFFFFFF
FFFFFFFF:FFF::FF,FFFF,:FFFFFFFFFFFFFFFFFFF::FFFFF:FFFFFFFFFFFFFFFFFF
FFFFFFFFFFFFF:F
@A00155:342:HHGFNDSXY:1:1632:12915:29121 2:N:0:GAACCTAG+TCCGCATA
CAGCATTCAATGCTACCACATTAAATACAATCACATTGTATTTCAGAACTTCTTGTGTCCGCACATAG
AAGGTTAAAGATGGCTTTCTCTTATAGGACGCCACAGGAGGAACTCTTGAGCAGGCTCCCACAGTCTC
AACAGGAGGTTATAA
+
FFFFFFFFFFFFFFFFFFFFFFFFFFFFF,FFFFFFFFFFFFFFFFF:FFFFFFFFFFFFFFFFFFFF
FFFF:FFF:FFFFFFFFFF:FFF:FFFFFFFFFFFFFFFFFF:FFFFFFFFFFFFF:FFFFFFFFFFF
FFFFFF:FFFFFFFF
@A00155:342:HHGFNDSXY:1:1632:12500:29434 2:N:0:GAACCTAG+TCCGCATA
CAGCATTCAATGCTACCACATTAAATACAATCACATTGTATTTCAGAACTTCTTGTGTCCGCACATAG
AAGGTTAAAGATGGCTTTCTCTTATAGGACGCCACAGGAGGAACTCTTGAGCAGGCTCCCACAGTCTC
AACAGGAGGTTATAA
+
FFFFFFFFFFFFFFFFFFFFFFF,FFFFFFFFFFFFFFFFFFFFFF:FFFFFF:F:FFFFFF::F::F
FFFFFFFFFFFFFFF:FFFFFFFFFFFFFFFFFFFFFFFFFFFFFFFFFFFFFFFFFFFFFFFFFFFF
:FFFFFFFFFFFFF:

@A00155:342:HHGFNDSXY:1:1664:25464:19382 2:N:0:GAACCTAG+TCCGCATA
CAGCATTCAATGCTACCACATTAAATACAATCACATTGTATTTCAGAACTTCTTGTGTCCGCACATAG
AAGGTTAAAGATGGCTTTCTCTTATAGGACGCCACAGGAGGAACTCTTGAGCAGGCTCCCACAGTCTC
AACAGGAGGTTATAA
+
FFFFFFFFFFFFFFFFFFFFFFFFFFFFFFF:FFFFFFFFFFFFFFFFFFFFFFFFFFFFFFFFFFFF
FFFFFFFFFFFFFFF:FFFFFFFFFFFFFFFFFFFFFFFFFFF,FFFFFFFFFFFFFFFFFFFFFFFF
:FFFFFFFFFF:F:F
@A00155:342:HHGFNDSXY:1:1227:21025:1924 1:N:0:GAACCTAG+TCCGCATA
AGCATTCAATGCTACTACATTAAATACAATCACATTGTATTTCAGAACTTCTTGTGTCCGCACATAGA
AGGTTAAAGATGGCTTTCTCTTATAGGACGCCACAGGAGGAACTCTTGAGCAGGCTCCCACAGTCTCA
ACAGGAGGTTATAAG
+
FFFF:FFFFFFFFFFFFFFFFFFF:FFFFFFFFFFFFFFFFFFFFFFFFFFFFFFFFFFFFFFFFFFF
FFFFFFFFF:FFFFFFFFFFFFFFFFFFFFFFFFFFFFFFFFFFFFFFFFFFFFFFFFFFFFFFFFFF
FFFFFFFFFFFFFFF
@A00155:342:HHGFNDSXY:1:1152:7337:10097 2:N:0:GAACCTAG+TCCGCATA
AGCATTCAATGCTACCACATTAAATACAATCACATTGTATTTCAGAACTTCTTGTGTCCGCACATAGA
AGGTTAAAGATGGCTTTCTCTTATAGGACGCCACAGGAGGAACTCTTGAGCAGGCTCCCACAGTCTCA
ACAGGAGGTTATAAG
+
FFFFFFFFFFFFFFFFFFFFFFFFFFFFFFFFFF,FFFFFFFFFFFFFFFFFFFFFFFFFFFFFFFFF
FFFFFFFFFFFFFFFFFFFFFF:FFFFFFFFFFFFFFFFFF:FFFFFFFFFFFFFFFFFFFFFFFFFF
FFFFF:FFF:FFFFF
@A00155:342:HHGFNDSXY:1:1215:12617:29481 2:N:0:GAACCTAG+TCCGCATA
AGCATTCAATGCTACTACATTAAATACAATCACATTGTATTTCAGAACTTCTTGTGTCCGCACATAGA
AGGTTAAAGATGGCTTTCTCTTATAGGACGCCACAGGAGGAACTCTTGAGCAGGCTCCCACAGTCTCA
ACAGGAGGTTATAAG
+
FFFFFFFFFFFFFFFFFFFFFF:FFFFFFFFFFFF:FFFFFFFFFFFFFFFFFFFFFFFFFFFFFFFF
FFFFFFFFFF,FFFFFFFFFF,FFFFFFFFFFFFFFFFFFFFFFFFFFFFFFFFFFFFF:FF:FFFFF
FFFFFFFFFFFFFFF
@A00155:342:HHGFNDSXY:1:2103:31647:18443 2:N:0:GAACCTAG+TCCGCATA
AGCATTCAATGCTACCACATTAAATACAATCACAGTGTATTTCAGAACTTCTTGTGTCCGCACATAGA
AGGTTAAAGATGGCTTTCTCTTATAGGACGCCACAGGAGGAACTCTTGAGCAGGCTCCCACAGTCTCA
ACAGGAGGTTATAAG
+
FFFF:FFFFFFFFFFFFF:FFFFFFFFFF:FFFF,FF,F,FFFFFFFF:FFFFFFF:FFFFFFFFFF:
,FF,FFFFFFFFFF:FFFFFFFFFFFFFFFFFFFFFF:FFF:FFF:FFFFFFFFFFFFFFFFFFFFFF
,F:FFFFFFFFFFFF
@A00155:342:HHGFNDSXY:1:2162:6985:19445 2:N:0:GAACCTAG+TCCGCATA
AGCATTCAATGCTACCACATTAAATACAATCACATTGTATTTCAGAACTTCTTGTGTCCGCACATAGA
AGGTTAAAGATGGCTTTCTCTTATAGGACGCCACAGGAGGAACTCTTGAGCAGGCTCCCACAGTCTCA
ACAGGAGGTTATAAG
+
FFFFFFFFFFFFFFFFFFFFFFFFFFFFFFFF::,FFFFFFFFFFFFF:F::F:FFFF:F:FFFFFF,
FFFFFFFFFFFFFFF:FFFFFF:FFFFF:FFFFFFFFFF:FFFFFFFFFFFFFFFFFFFFFFF:FFFF
FFFF:FF:FF,FFFF
@A00155:342:HHGFNDSXY:1:2515:18313:11569 2:N:0:GAACCTAG+TCCGCATA
AGCATTCAATGCTACCACATTAAATACAATCACATTGTATTTCAGAACTTCTTGTGTCCGCACATAGA
AGGTTAAAGATGGCTTTCTCTTATAGGACGCCACAGGAGGAACTCTTGAGCAGGCTCCCACAGTCTCA
ACAGGAGGTTATAAG
+
:F::FFF:FFFFF:FF:F:FF:FFF:FFFFF:FFFFFF:FFFF:FFFFFFFFFFFFFFFFFFFFFFFF

FFFFF::FF:FFFFFFFFFFFFFFFFFFFFFFFFFFFFFFFFFFFFFFFFFFFFFFFFFFFFFF:FF
FFFFFFFFFF:FFFF
@A00155:342:HHGFNDSXY:1:1209:4896:33332 2:N:0:GAACCTAG+TCCGCATA
GCATTCAATGCTACCACATTAAATACAATCACATTGTATTTCAGAACTTCTTGTGTCCGCACATAGAA
GGTTAAAGATGTCTTTCTCTTATAGGACGCCACAGGAGGAACTCTTAAGCAGGCTCCCACAGTCTCAA
CAGGAGTGTAGCA
+
FF:F,FFF:FFFFFFF,FFFF:FFFFFFF,FFF:F:FFFFF,FFFF,FF:FFF:FFFF:FF,F:FF,F
,F,,FFF,:F:,:FFFFFFFF,FF:FF:FFFFFF:FFFFFFFFFFFFFFF:F,FF:FFFFFF,FFFFF
FFF,F:,FFF:FF
@A00155:342:HHGFNDSXY:1:1277:17318:22028 2:N:0:GAACCTAG+TCCGCATA
GCATTCAATGCTACCACATTAAATACAATCACATTGGATTTCAGAACTTCTTGTGTCCGCACATAGAA
GGTTAAAGATGGCTTTCTCTTATAGGACGCCACAGGAGGAACTCTTGAGCAGGCTCCCACAGTCTCAA
CAGGAGGTTATAAGC
+
FFFFFFFFFFF:FFFFFFFFFFFFFFFFFFFFF,FF,FFFFFFFFFFFFFFFFFFFFFF:FFFFFFFF
FFFFFFFFFFFFFFFFFFFFFFFFFFFFFFFFFFFFFFF:FFFFFFFFFFFFFFFF,FFFFFFFFF
FFFFFFF,FF,FFFF
@A00155:342:HHGFNDSXY:1:1440:7247:7623 2:N:0:GAACCTAG+TCCGCATA
GCATTCAATGCTACCACATTAAATACAATCACATTGTATTTCAGAACTTCTTGTGTCCGCACATAGAA
GGTTAAAGATGGCTTTCTCTTATAGGACGCCACAGGAGGAACTCTTGAGCAGGCTCCCACAGTCTCAA
CAGGAGGTTATAAGC
+
FFFFFFF:FFFFFFFFFFFFFFFFFFFFFFF,:FFFFFFFFFFFFFFFFFFFFFFFFFFFFFF:FFFF,
FFFFFFFFFFFFF:FFFFF:FFFFFFFFFF:FFFFFFFFFFFFFFFFFFFFFFFFFFFFFFFFFFFFF
:FFFFFF,F:FF:FF
@A00155:342:HHGFNDSXY:1:1376:24623:15201 2:N:0:GAACCTAG+TCCGCATA
GCATTCAATGCTACCACATTAAATACAATCACATTGTATTTCAGAACTTCTTGTGTCCGCACATAGAA
GGTTAAAGATGGCTTTCTCTTATAGGACGCCACAGGAGGAACTCTTGAGCAGGCTCCCACAGTCTCAA
CAGGAGGTTATAAGC
+
FFFFFFFFFFFFFFFFFFFFFFFFFFFFFFFFFFFFFFFFFFFFFFFFFFFFFFFFFFFFFFF:FFFF
FFFFFFFFFFFFFFFFFFFFFFFFFFFFFFFF:FFFFFFFFFFFFFFFFFFFFFFFFFFFFFFFFFFF
FFFFFFF:FFFFFFF
@A00155:342:HHGFNDSXY:1:2417:5240:11600 2:N:0:GAACCTAG+TCCGCATA
GCATTCAATGCTACCACATTAAATACAATCACATTGTATTTCAGAACTTCTTGTGTCCGCACATAGAA
GGTTAAAGATGGCTTTCTCTTATAGGACGCCACAGGAGGAACTCTTGAGCAGGCTCCCACAGACTCAA
CAGGAGGTTATAAGC
+
FFFFFFFFFFFFFFFFFFFFFFFFFFFFFFFFFFFFFFFFF,FFFFFFFFFFFFFFFFFFFFFFF:FFF
FFFFFFFFFFFFFFFFFFFFFFFFFFFFF,:FFF,FFFFF:FFFFFF:FF:,FFFFFFFFFF,FFF:F
FFFFFFF:FFF,FFF
@A00155:342:HHGFNDSXY:1:2655:2446:24392 2:N:0:GAACCTAG+TCCGCATA
GCATTCAATGCTACCACATTAAATACAATCACATTGTATTTCAGAACTTCTTGTGTCCGCACATAGAA
GGTTAAAGATGGCTTTCTCTTATAGGACGCCACAGGAGGAACTCTTAAGCAGGCTCCCACAGTCTCAA
CAGGAGTGTAGCAAT
+
FFFFFFFFFFFFFF,FFFFFFFFFFFFFFFF:FFFFFFFFFFFFFFFFFFFFFFFFFFFF:FFFFFF,F
FFFFFFFFFFFF:FFFFFFFFFFFF:FFFFFFFFFF:FFF,FFFFFFFF,FF:FFFFFFFFFFFFFF:
FFFFFFFFFFFF:FF
@A00155:342:HHGFNDSXY:1:1101:2600:5776 1:N:0:GAACCTAG+TCCGCATA
CATTCAATGCTACTACATTAAATACAATCACATTGTATTTCAGAACTTCTTGTGTCCGCACATAGAAG
GTTAAAGATGGCTTTCTCTTATAGGACTCAACAGGAGTAACTCTTGAGCAGGCTCCCACAGTCTCAAC
AGGAGGTTATAAGTG

```
+
FFFFFFFFFFFFFFF,FFFFFFFF:FFF,FFFF,FFFFFFFFFFFFFF:FFFFFF:FFFF:FFFFFFF
FFFFFFFF:FFFFFF:F:FF:FF,,F,,F,F,FFFF,,:::F:FF,F:FF,FFFFF:FFFFFFFFFF:
F:FFFFFFFFFFFFF
@A00155:342:HHGFNDSXY:1:1206:28320:33755 1:N:0:GAACCTAG+TCCGCATA
CATTCAATGCTACCACATTAAATACAATCACATTGTATTTCAGAACTTCTTGTGTCCGCACATAGAAG
GTTAAAGATGGCTTTCTCTTATAGGACGCCACAGGAGGAACTCTTGAGCAGGCTCCCACAGTCTCAAC
AGGAGGTTATAAGCG
+
FFFFFFFFFFFFFF:FFFFFFFFFFFFFFFF:FFFFFFFFFFFFFFF:FFFFFFFFFFFFFFFFFFF:
FFFFFFFFFFFFFFFFFFFFFFFFFFFFFFFFFFFFFFFFFFFFFFFFFFFFFFFFFFFFFFFFFFFF
FFFFFFFFFFFFFFF
@A00155:342:HHGFNDSXY:1:1422:15221:29230 1:N:0:GAACCTAG+TCCGCATA
CATTCAATGCTACCACATTAAATACAATCACATTGTATTTCAGAACTTCTTGTGTCCGCACATAGAAG
GTTAAAGATGGCTTTCTCTTATAGGACGCCACAGGAGGAACTCTTGAGCAGGCTCCCACAGTCTCAAC
AGGAGGTTATAAGCG
+
FFFFFF:FFFFFFFFFFFFFFFFFFF:FFFF:FFFFFFFFFFFFFFFFFFFFFFFFFFFFFFFFFFFF
FFFFFFFFFFFFFFFFFFFFFFFFFFFFFFFFFFFFFFFFFFFFFFFFFFFFFFFFFFFFFFFFFFFF
FFFFFFFFFFFFFFF
@A00155:342:HHGFNDSXY:1:2428:16911:30843 2:N:0:GAACCTAG+TCCGCATA
CATTCAATGCTACCACATTAAATACAATCACATTGTATTTCAGAACTTCTTGTGTCCGCACATAGAAG
GTTAAAGATGGCTTTCTCTTATAGGACGCCACAGGAGGAACTCTTGAGCAGGCTCCCACAGTCTCAAC
AGGAGGTTATAAGCG
+
FFFFFFFFFFFFFFFFFFFFFFFFF:FFFF:FF:FFFFFFFFFF:FFFFFFFFFFFFFFFF,,:FFFF
FFFFFFFFFFFFFFFFFFFFFFFFFFFFFFFFFFFFFFFFFFFFFFF,FFFFFFFFFFFFFFFFFFFF
:FFFFFFFF,FFFF:
@A00155:342:HHGFNDSXY:1:1130:30318:4993 1:N:0:GAACCTAG+TCCGCATA
ATTCAATGCTACCACATTAAATACAATCACATTGTATTTCAGAACTTCTTGTGTCCGCACATAGAAGG
TTAAAGATGGCTTTCTCTTATAGGACGCCACAGGAGGAACTCTTGAGCAGGCTCCCACAGTCTCAACA
GGAGGTTATAAGCGG
+
FFFFFFFFFFFFFFFFFFFFFFFFFFFFFFFFFFFFFFFFFFFFFFFFFFFFFFFFFFFFFFFFFFFF
FFFFFFFFFFFFFFFFFFFF:FFF:FFFFFFFFFFFFFFFFFFFFFFFFFFFFFFFFFFFFFFFFFFF
FFFFFFFFFFFFFFF
@A00155:342:HHGFNDSXY:1:1216:13286:1924 1:N:0:GAACCTAG+TCCGCATA
ATTCAATGCTACCACATTAAATACAATCACATTGTATTTCAGAACTTCTTGTGTCCGCACATAGAAGG
TTAAAGATGGCTTTCTCTTTTAGGACGCCACAGGAGGAACTCTTGAGCAGGCTCCCACAGTCTCAACA
GGAGGTTATAAGCGG
+
FFFFFFFFFFFFFFFFFFFFFFFFFF:FFFFFFFFFFFFFFFFFFFFFFFFFFFFFFFFFFFFFFFFF
FFFFFFFFFFFFFFFFFFF,FFFFFFFFFFFFFFFFFFFFFFFFFFFFFFFFFFFFFFFFFFFFF:FF
FFFFFFFFFFFFFFF
@A00155:342:HHGFNDSXY:1:2132:17797:24862 1:N:0:GAACCTAG+TCCGCATA
ATTCAATGCTACCACATTAAATACAATCACATTGTATTTCAGAACTTCTTGTGTCCGCACATAGAAGG
TTAAAGATGGCTTTCTCTTATAGGACGCCACAGGAGGAACTCTTGAGCAGGCTCCCACAGTCTCAACA
GGAGGTTATAAGCGG
+
FFFFF:FFFFFFFFFFFFFF,FFFFFFF,FF,FFFFFFFFFFFFF:FFFFFFFFFFFFFFFFFFFFFFF
FFFFFF:FFFFFFFFFFFFFFFFFFFFFFFFFFFFFFFFFFFFFFFFFFFFFFFFFFFFFFFFFFFFF
FFFFFFFFFFFFFFF
@A00155:342:HHGFNDSXY:1:2473:15564:18051 1:N:0:GAACCTAG+TCCGCATA
ATTCAATGCTACCACATTAAATACAATCACATTGTATTTCAGAACTTCTTGTGTCCGCACATAGAAGG
```

```
TTAAAGATGGCTTTCTCTTATAGGACGCCACAGGAGGAACTCTTGAGCAGGCTCCCACAGTCTCAACA
GGAGGTTATAAGCGG
+
FFFFFFFFFFFF:FFFFFFF:FFFFFFFFFFFFFFFFFF,FFFFFFFFFFFFFFFFFFFFFFFFFFFFF
FFFFFF:FFF,FFFFFFFF:FFFFFFFFFFFFFFFFFFFFFFFFFFFFFFFFFFFFFFFFFFFFFFFFF
FFFFFFFFFFFFFFF
@A00155:342:HHGFNDSXY:1:1231:25220:2801 2:N:0:GAACCTAG+TCCGCATA
ATTCAATGCTACCACATTAAATACAATCACATTGTATTTCAGAACTTCTTGTGTCCGCACATAGAAGG
TTAAAGATGGCTTTCTCTTATAGGACGCCACAGGAGGAACTCTTGAGCAGGCTCCCACAGTCTCAACA
GGAGGTTATAAGCGG
+
F:FFFFFFFFFFFFFFFFFFFFFFFFFFFFF::FF::FFFFFFFFFFFF,FF:FFFFFFFFF,FFFFFF
FFFFFFFFFFFFFFFFFFFFFFFFFFFFFFFFFFFFFFFFFFFFFFFFFFFFFFFFFFFFFFFFFFFFF
FFFFF,FFFFFFFFF
@A00155:342:HHGFNDSXY:1:1537:27968:17581 2:N:0:GAACCTAG+TCCGCATA
ATTCAATGCTACCACATTAAATACAATCACATTGTATTTCAGAACTTCTTGTGTCCGCACATAGAAGG
TTAAAGATGGCTTTCTCTTATAGGACGCCACAGGAGGAACTCTTGAGCAGGCTCCCACAGTCTCAACA
GGAGGTTATAAGCGG
+
FFFFFFFFFFFFFFFFFFF:FFFFF:FFF:F::F,FFFFFFFFFFFFFFFFFFFFFFFFFF:FFF:FF
FFFFFFFFFFFFFFFFFFFFF:FFFFFFFFF:FFFF:FFFFFFFFFFFFFFFF::FFFF:FFFF:FFF
FFFFFFFFFFF,FFF
@A00155:342:HHGFNDSXY:1:1541:18918:19132 2:N:0:GAACCTAG+TCCGCATA
ATTCAATGCTACCACATTAAATACAATCACATTGTATTTCAGAACTTCTTGTGTCCGCACATAGAAGG
TTAAAGATGGCTTTCTCTTATAGGACGCCACAGGAGGAACTCTTGAGCAGGCTCCCACAGTCTCAACA
GGAGGTTATAAGCGG
+
FFFFFFFFFFFFFFFF:FFFF:FFFFFFFFFFFFFF,FFFFFFFFFFFFFFFFFFFFFFFF:FFFFFFF
FFFFF:FFFFFFFFFFFFFFFFFFFFFFFFFFFFFFFFFFFFFFF:FFFFFFFFFFFFFFFFFFFFFFFF:
FFFFFFFFFFFFFFF
@A00155:342:HHGFNDSXY:1:1550:15682:25989 2:N:0:GAACCTAG+TCCGCATA
ATTCAATGCTACTACATTAAATACAATCACATTGTATTTCAGAACTTCTTGTGTCCGCACATAGAAGG
TTAAAGATGGCTTTCTCTTATAGGACGCCACAGGAGGAACTCTTGAGCAGGCTCCCACAGTCTCAACA
GGAGGTTATAAGTGG
+
FFFFFFFFFFFFFFFFFFFFFFFFFFFFFFFF,FF:FFFFFFFFFFFFFFFFFFFFFFFFFF:FFFFFF
FFFFFFFFFFFFFFFFFFFFFFFFFFFFFFFFFFFFFFFFFFFFFFFFF:FFFFFFFFFFFFFFFFFFF
FFFFFF:FFFFFFFF
@A00155:342:HHGFNDSXY:1:1657:10167:5071 2:N:0:GAACCTAG+TCCGCATA
ATTCAATGCTACCACATTAAATACAATCACATTGTATTTCAGAACTTCTTGTGTCCGCACATAGAAGG
TTAAAGATGGCTTTCTCTTATAGGACGCCACAGGAGGAACTCTTGAGCAGGCTCCCACAGTCTCAACA
GGAGGTTATAAGCGG
+
FFFFFFFFFFFFFFFFFFFFFFFFFFFFFFFFFFFFFFFFFFFFFFFFFFFFFFFFFFFFFFFF::FFF
FFFFFFFFFFFFFFFFFFFFFFFFFFFFFFFFFF:FFFFFFFFFFF:FFFFFFFFFFFFFFFFFFF:FF
FF:FFFF,FFFF:FF
@A00155:342:HHGFNDSXY:1:2427:8983:35900 2:N:0:GAACCTAG+TCCGCATA
ATTCAATGCTACCACATTAAATACAATCACATTGTATTTCAGAACTTCTTGTGTCCGCACATAGAAGG
TTAAAGATGGCTTTCTCTTATAGGACGCCACAGGAGGAACTCTTGAGCAGGCTCCCACAGTCTCAACA
GGAGGTTATAAGCGG
+
FFFFFFFFFF:FFFFFFFFFFFFFFFFFFFF:FFFFFFFFFFFFFFFFFFFFFFFFFFFFFF:FF:FFF
FFFFFFFFFFFFFFFFFFFFFFFFFFFFFFFFFFFFFF,FFFFFFFFFFFFFFF,FFFFFFFFFFFFF
FFFFFFFFFFFFFFF
```

```
@A00155:342:HHGFNDSXY:1:2649:24117:29168 2:N:0:GAACCTAG+TCCGCATA
ATTCAATGCTACCACATTAAATACAATCACATTGTATTTCAGAACTTCTTGTGTCCGCACATAGAAGG
TTAAAGATGGCTTTCTCTTATAGGACGCCACAGGAGGAACTCTTGAGCAGGCTCCCACAGTCTCAACA
GGAGGTTATAAGCGG
+
FFFFFFFFFFFFFFFFFFFFFFFFF,FFFFF:FFFFFFFFFFFFFFFFFFFFFFFFFFFFF:FFFFFFF
FFFFFFFFFFFFFFFFFFFFFF:FFFFFFFFF:FFFFFFFF:FFFFFFFFFFFFFFFFFFFFFFFFFFF
FFFFFFFFFFFFFFF
@A00155:342:HHGFNDSXY:1:1102:20925:24549 1:N:0:GAACCTAG+TCCGCATA
TTCAATGCTACCACATTAAATACAATCACATTGTATTTCAGAACTTCTTGTGTCCGCACATAGAAGGT
TAAAGATGGCTTTCTCTTATAGGACGCCACAGGAGGAACTCTTGAGCAGGCTCCCACAGTCTCAACAG
GAGGTTATAAGCGGA
+
FFFFFFFFFFFFFFFFFFF:FFFF:FF,FFF:FFFFFFFFFFFFFFFFFFFFFFFFFFFFFFFFFFFFF
FFFFFFFFFFFFFFFFFFFFFFFFFFFFFFFFFFFFFFFFFFFFFFFFFFFFFFFFFFFFFFFFF:FFF
FFFFFFFFFFFFFFF
@A00155:342:HHGFNDSXY:1:1129:28474:36464 1:N:0:GAACCTAG+TCCGCATA
TTCAATGCTACCACATTAAATACAATCACATTGTATTTCAGAACTTCTTGTGTCCGCACATAGAAGGT
TAAAGATGGCTTTCTCTTATAGGACGCCACAGGAGGAACTCTTGAGCAGGCTCCCACAGTCTCAACAG
GAGGTTATAAGCGGT
+
FFFFFF:FFFFFFFFFFFFFFFFFFFFFFFFFFFFFFFFFFFFF,FFFFFFFFFFFFFFFFFFFFFFFF
FFFFFFFFFFFFFFFFFFFFFFFFFFFFFFF:FFFFFFFFFFFFFFFFFFFFFFFFFFFFFFFFF:FFF
FFFFFFFFFFFFFFF
@A00155:342:HHGFNDSXY:1:1271:11632:35822 1:N:0:GAACCTAG+TCCGCATA
TTCAATGCTACCACATTAAATACAATCACATTGTATTTCAGAACTTCTTGTGTCCGCACATAGAAGGT
TAAAGATGGCTTTCTCTTATAGGACGCCACAGGAGGAACTCTTGAGCAGGCTCCCACAGTCTCAACAG
GAGGTTATAAGCGGG
+
FFFF:FFFFFFFFFFFFFFFFFFFFFFF:FFFFFFFFFFFFFFFFFFFFFFFFFFFFFFFFFFFFFFFF,
FFFFFFFFFFFFFFF:FFFFFFFFFFFFFFFFFFFFFFFFFFFFFFFFFFFFFFFFFFFFFFFFF:FFF
FFFFFFFFFFFFFFF
@A00155:342:HHGFNDSXY:1:1478:24080:10504 1:N:0:GAACCTAG+TCCGCATA
TTCAATGCTACCACATTAAATACAATCACATTGTATTTCAGAACTTCTTGTGTCCGCACATAGAAGGT
TAAAGATGGCTTTCTCTTATAGGACGCCACAGGAGGAACTCTTGAGCAGGCTCCCACAGTCTCAACAG
GAGGTTATAAGCGGC
+
FFFFFFFFFFFFF:FFFFFF:FFFFFFFF,FFFFFFFFFFFFFFFFF,FFFFFFFFFFFFF:F::FF:
FFF,FFFFFFFFFFF,FFFF,FF:FFF:F,FFFF:FFFFF,,FFF:FFFFFFFFFFFFFFFFFFFFFFF
FFFFFFFFFFFF:F:
@A00155:342:HHGFNDSXY:1:1611:7898:10347 1:N:0:GAACCTAG+TCCGCATA
TTCAATGCTACCACATTAAATACAATCACATTGTATTTCAGAACTTCTTGTGTCCGCACATAGAAGGT
TAAAGATGGCTTTCTCTTATAGGACGCCACAGGAGGAACTCTTGAGCAGGCTCCCACAGTCTCAACAG
GAGGTTATAAGCGGG
+
FFFFFF:FFFFFFFFFFFFFFFFFFFF:FFFFFFFFFFFFFFFFFFFFFFFFFFFFFFFFFFFFFFFFF
FFFFFFFFFFFFFFFFFFFFFFFFFFFFFFFFF:FFFFFFFFFFFFFFFFFFFFFFFFFFFFFFFFFFF
FFFFFFFFFFFFFFF
@A00155:342:HHGFNDSXY:1:2139:20094:16031 1:N:0:GAACCTAG+TCCGCATA
TTCAATGCTACTACATTAAATACAATCACATTGTATTTCAGAACTTCTTGTGTCCGCACATAGAAGGT
TAAAGATGGCTTTCTCTTATAGGACGCCACAGGAGGAACTCTTGAGCAGGCTCCCACAGTCTCAACAG
GAGGTTATAAGTGGC
+
FFFF,FFFFFFFFFFFFFFFFFFFFFFFFFFFFFFFFFFFFFFFFFFFFFFFFFFFFFFFFFFFFFFFF
```

```
FFF:FFFFFFFFFFFFFFFFFFFFFFFFFFFFF:FFFFFFFFFFFFFFFFFFFFFFFFFFFFFFFFFF
FFFFFFFFFFFFFFF
@A00155:342:HHGFNDSXY:1:2239:29640:30467 1:N:0:GAACCTAG+TCCGCATA
TTCAATGCTACTACATTAAATACAATCACATTGTATTTCAGAACTTCTTGTGTCCGCACATAGAAGGT
TAAAGATGGCTTTCTCTTATAGGACGCCACAGGAGGAACTCTTGAGCAGGCTCCCACAGTCTCAACAG
GAGGTTATAAGTGGC
+
FFFFFFFFFFFFFFFFFFFFFF:FFFF:FFFFFFFFFFFFFFFFFFFFFFFFFFFFFFFFFFFFFFFF
FFFFFFFFFFFFF:FFFFFFFFFFFFFFFFFFFFFFFFFFFFFFFFFFFFFFFFFFFFFFFFFFFFFF
FFFFFFFFFFFFFFF
@A00155:342:HHGFNDSXY:1:2375:11388:15483 1:N:0:GAACCTAG+TCCGCATA
TTCAATGCTACCACATTAAATACAATCACATTGTATTTCAGAACTTCTTGTGTCCGCACATAGAAGGT
TAAAGATGGCTTTCTCTTATAGGACGCCACAGGAGGAACTCTTGAGCAGGCTCCCACAGTCTCAACAG
GAGGTTATAAGCGGA
+
FFFFFFFFFFFFFFFFFFFFFFFFFFFFFFFFFFFFFFFFFFFFFFFFFFFFFFFFFFFFFFFFFFF:
FFFFFFFFFFFFFFFFFFFFFFFFFFFFFFFFFFFFFFFFFFFFFFFFFFFFFFFFFFFFFFFFFFFF
FFFFFFFFF:FFFFF
@A00155:342:HHGFNDSXY:1:2531:6623:24173 1:N:0:GAACCTAG+TCCGCATA
TTCAATGCTACCACATTAAATACAATCACATTGTATTTCAGAACTTCTTGTGTCCGCACATAGAAGGT
TAAAGATGGCTTTCTCTTATAGGACGCCACAGGAGGAACTCTTGAGCAGGCTCCCACAGTCTCAACAG
GAGGTTATAAGCGGC
+
FFFFFFFFFFFFFFFFFFFFFFFFFFFFFFFFFFFFFFF:FFFFFFFFFFFFFFFFFFFFFFFFFFFF
FFFFFFFFFFFFFFFFFFFFFFFFFFFFFFFFFFFFFFFFFFFFFFFFFFFFFFFFFFFFFFFFFFFF
FFFFFFFFFFFFFFF
@A00155:342:HHGFNDSXY:1:2563:19162:29042 1:N:0:GAACCTAG+TCCGCATA
TTCAATGCTACTACATTAAATACAATCACATTGTATTTCAGAACTTCTTGTGTCCGCACATAGAAGGT
TAAAGATGGCTTTCTCTTATAGGACGCCACAGGAGGAACTCTTGAGCAGGCTCCCACAGTCTCAACAG
GAGGTTATAAGTGGC
+
FFFFFFFFFFFFFFFFFFFFFFFFFFFFFFFFFFFFFFFFFFFFFFFFFFFFFFFFFFFFFFFFFFFF
FFFFFF,FFFFFFFFFFFFFFFF:FFFFFFFFFFFFFFFFFFFFFFFFFFFFFFFFFFFFFFFFFFFF
FFFFFFFFFFFFFFF
@A00155:342:HHGFNDSXY:1:2665:17300:17519 1:N:0:GAACCTAG+TCCGCATA
TTCAATGCTACCACATTAAATACAATCACATTGTATTTCAGAACTTCTTGTGTCCGCACATAGAAGGT
TAAAGATGGCTTTCTCTTATAGGACGCCACAGGAGGAACTCTTGAGCAGGCTCCCACAGTCTCAACAG
GAGGTTATAAGCGGA
+
FFFFFFFFFFFFFFFFFFFF:FFF:FFFFFFFFFFFFFFFFFFFFFFFFFFFFFFFFFFFFFFFFFFF
FFFFF:FFFFFFFFFFFFFFFFFFFFFFFFFFFFFFFFFFFFFFFFFFFFFFFFFFFFFFFFFFFFFF
FFFFFFFFFFFFFFF
@A00155:342:HHGFNDSXY:1:1353:24460:24752 1:N:0:GAACCTAG+TCCGCATA
TCTATGCTACCACATTATATACATTCACATTGTATTTCAGAACTTCTTGTGTCCGCACTTAGAAGGTT
AAAGATGGCTTTCTCTTATAGGACGCCACAGGAGGAACTCTTGAGCAGGCTCCCACTGTCTCAACAGG
AGGTTATAAGCGGAC
+
F:,FF::,:FFFF:F,F,,:,,:,F:,:F,FFFFFFF:F:F,:FF:FFFFFF,FFFFF,FF:F,,FFF
:FFFFFFF:F:FFF::F:FFFFFFF,,,FFFF,:F,,,F:FFFFF:,F,FFFF:FF,FFFF::,F:FF
,FFFF:,:,FFF,F:
@A00155:342:HHGFNDSXY:1:2120:2248:24236 1:N:0:GAACCTAG+TCCGCATA
TCAATGCTACTACATTAAATACAATCACATTGTATTTCAGAACTTCTTGTGTCCGCACATAGAAGGTT
AAAGATGGCTTTCTCTTATAGGACGCCACAGGAGGAACTCTTGAGCAGGCTCCCACAGTCTCAACAGG
AGGTTATAAGTGGAC
```

+
F:FFF:,FF:FFFFFFFFFFFFFFFFFF:FFFFFFFF:FFFFFFFFFFFFFFFFFFFFFFFFFFFFFFFF
FFF:FFFFFFFFFFFFFFFFFFFFFFFFFFFFFFFFFFFFFFFFFFFFFFFFFFFFFFFF,FFFFFFFF
FFFFFFFFFFFFFFF
@A00155:342:HHGFNDSXY:1:2123:25825:1094 1:N:0:GAACCTAG+TCCGCATA
TCAATGCTACCACATTAAATACAATCACATTGTATTTCAGAACTTCTTGTGTCCGCACATAGAAGGTT
AAAGATGGCTTTCTCTTATAGGACGCCACAGGAGGAACTCTTGAGCAGGCTCCCACAGTCTCAACAGG
AGGTTATAAGCGGAC
+
FFFFFFFFF:FFFFFFFFFFFFF,FFF,FF:FFFFFFFFFFFFFFFFFFFFFFFFFFFFFFFFFFFFFFF
FFFFFFFFFFFFFFFFFFFFFFFFFFFFFFFFFFFFFFFFFFFFFFFFFFFFFFFFFFFFFFFFFFFFFF
FFFFFFFFFFFFFFF
@A00155:342:HHGFNDSXY:1:2122:26051:16517 1:N:0:GAACCTAG+TCCGCATA
TCAATGCTACCACATTAAATACAATCACATTGTATTTCAGAACTTCTTGTGTCCGCACATAGAAGGTT
AAAGATGGCTTTCTCTTATAGGACGCCACAGGAGGAACTCTTGAGCAGGCTCCCACAGTCTCAACAGG
AGGTTATAAGCGGAC
+
FFFFFFF:FFFFFFFFFFF:FFFFFFFFFFFFFFFFFFFFFFFFFFFFFFFFFFFFFFFFFFFFFFFFFF
FFFFFFFFFFFFFFFFFFFFFFFFFFFFFFFFFFFFFFFFFFFFFFFFFFFFFFFFFFFFFFFFFFFFFF
FFFFFFFFFFFFFFF
@A00155:342:HHGFNDSXY:1:2155:32985:16125 1:N:0:GAACCTAG+TCCGCATA
TCAATGCTACCACATTAAATACAATCACATTGTATTTCAGAACTTCTTGTGTCCGCACATAGAAGGTT
AAAGATGGCTTTCTCTTATAGGACGCCACAGGAGGAACTCTTGAGCAGGCTCCCACAGTCTCAACAGG
AGGTTATAAGCGGAT
+
FFFFFFFFFFFFFFFFFFFFFFFFFFFFFFFFFFFFFFFFFFFFFFFFFFFFFFFFFFFFFFFFFFFFFF
FFFFFFFFFFF:FFFFFFFFFFFFFFFFFFFFFFFFFFFFFFFFFFFFFFFFFFFFFFFFFFFFFFFFFF
FFFFFFFFFFFFFFF
@A00155:342:HHGFNDSXY:1:2239:6542:22686 1:N:0:GAACCTAG+TCCGCATA
TCAATGCTACCACATTAAATACAATCACATTGTATTTCAGAACTTCTTGTGTCCGCACATAGAAGGTT
AAAGATGGCTTTCTCTTATAGGACGCCACAGGAGGAACTCTTGAGCAGGCTCCCACAGTCTCAACAGG
AGGTTATAAGCGGAC
+
:FFFFFFFFFFFFFFFFFFFFFFFFFFFFFFFFFFFFFFFFFFFFFFFFFFFFFFFFFFFF:FFFFFFFF
FFFF:FFFFFFFFFFFFFFFFFFFFFFFFFFFFFFFFFFFFFFFFFFFFFFFFFFFFFFFFFFFFF
FFFF:FFFFFFFFF:
@A00155:342:HHGFNDSXY:1:2354:14597:26052 1:N:0:GAACCTAG+TCCGCATA
TCAATGCTACCACATTAAATACAATCACATTGTATTTCAGAACTTCTTGTGTCCGCACATAGAAGGTT
AAAGATGGCTTTCTCTTATAGGACGCCACAGGAGGAACTCTTGAGCAGGCTCCCACAGTCTCAACAGG
AGGTTATAAGCGGAT
+
FFFFFFFFFFFFFFFFFFFFFFFFFFFFFF:FFFFFFFFFFFFFFFFFFFFFFFFFFFFFFFFFFFFF:F
FFFFFFFFFFFFFFFFFFFFFFFFFFFFFFFFFFFFFFFFFFFFFFFFFFFFFFFFFFFFFFFFFFFFFF
FFFFFFFFFFFFFFF
@A00155:342:HHGFNDSXY:1:2447:27218:33567 1:N:0:GAACCTAG+TCCGCATA
TCAATGCTACCACATTAAATACAATCACATTGTATTTCAGAACTTCTTGTGTCCGCACATAGAAGGTT
AAAGATGGCTTTCTCTTATAGGACGCCACAGGAGGAACTCTTGAGCAGGCTCCCACAGTCTCAACAGG
AGGTTATAAGCGGAT
+
FFFFFFFFFFFFFFFFFFFFFFFFFFFFFFFFFFFFFFFFFFFFFFFFFFFFFFFFFFFFFFFFFFFFFF
FFFFFFFFFFFFFFFFFFFFFFFFFFFFFFFFFFFFFFFFFFFFFFFFFFFFFFFFFFFFFFFFFFFFFF
FFFFFFFFFFFFFFF
@A00155:342:HHGFNDSXY:1:2447:27950:35336 1:N:0:GAACCTAG+TCCGCATA
TCAATGCTACCACATTAAATACAATCACATTGTATTTCAGAACTTCTTGTGTCCGCACATAGAAGGTT

AAAGATGGCTTTCTCTTATAGGACGCCACAGGAGGAACTCTTGAGCAGGCTCCCACAGTCTCAACAGG
AGGTTATAAGCGGAT
+
FFFFFFFFFFFFFFFFFFFFFFFFFFFFFFFFFFFFFFFFFFFFFFFFFFFFFFFFFFFFFFFFFFFF
FFFFFFFFFFFFFFFFFFFFFFFFFFFFFFFFFFFFFFFFFFFFFFFFFFFFFFFFFFFFFFFFFFFF
FFFFFFFFFFFFFFF
@A00155:342:HHGFNDSXY:1:2452:29767:7138 1:N:0:GAACCTAG+TCCGCATA
TCAATGCTACCACATTAAATACAATCACATTGTATTTCAGAACTTCTTGTGTCCGCACATAGAAGGTT
AAAGATGGCTTTCTCTTATAGGACGCCACAGGAGGAACTCTTGAGCAGGCTCCCACAGTCTCAACAGG
AGGTTATAAGCGGAC
+
FFFFFFFFFFFF:FFFFFFFFFFFF:FFFFFFFFFFFFFFFFFFFFFFFFFFFFFFFFFFFFFFFFFF
FFFFFFFFFFFFFFFFFFFFFFFFFFFFFFFFFFFFFFFFFFFFFFFFFFFFFFFFFFFFFFFFFFFF
FFFFFFFFFFFFFFF
@A00155:342:HHGFNDSXY:1:2502:13575:4085 1:N:0:GAACCTAG+TCCGCATA
TCAATGCTACTACATTAAATACAATCACATTGTATTTCAGAACTTCTTGTGTCCGCACATAGAAGGTT
AAAGATGGCTTTCTCTTATAGGACGCCACAGGAGGAACTCTTGAGCAGGCTCCCACAGTCTCAACAGG
AGGTTATAAGTGGAT
+
FFFFFFFFFFFFFFFFFFFFFFFFFFFFFFFFFFFFFFFFFFFFFFFFFFFFFFFFFFFFFFFFFFFF
FFFFF:FFFFFFFFFFFFFFFFFFFFFFFFFFFFFFFFFFFFFFFFFFFFFFFFFFFFFFFFFFFFFF
FFFFFFFFFFFFFFF
@A00155:342:HHGFNDSXY:1:2525:13051:9721 1:N:0:GAACCTAG+TCCGCATA
TCAATGCTACCACATTAAATACAATCACATTGTATTTCAGAACTTCTTGTGTCCGCACATAGAAGGTT
AAAGATGGCTTTCTCTTATAGGACGCCACAGGAGGAACTCTTAAGCAGGCTCCCACAGTCTCAACAGG
AGGTTATAAGCGGAC
+
FFFFFFFFFFFFFFFFFFFFFFFFFFFFFFFFFFFFFFFFFFFFFFFFFFFFFFFFFFFFFFFFFFFF
FFFFFFFFFFFFFFFFFFFFFFFFFFFFFFFFFFFFFFFFFFFFFFFFFFFFFFFFFFFFFFFFFFFF
FFFFFFFFFFFFFFF
@A00155:342:HHGFNDSXY:1:1247:25907:17989 2:N:0:GAACCTAG+TCCGCATA
CAATGCTACCACATTAAATACAATCACATTGTATTTCAGAACTTCTTGTGTCCGCACATAGAAGGTTA
AAGATGGCTTTCTCTTATAGGACGCCACAGGAGGAACTCTTGAGCAGGCTCCCACAGTCTCAACAGGA
GGTTATAAGCGGATT
+
FFFFFFFFFFFFFFFFFFFFFFFFFFFFF:FFFFFFFFFFFFFFFFFFFFFFFFFFFFFFFFFFFFFF
FFFFFFFFFFFFFFFFFFFFFFFFFFFFFFFFFFFFFFFFFFFFFFFFFFFFFFFFFFFFFFFFFFFF
FFFFFFFFFFFFF:F
@A00155:342:HHGFNDSXY:1:1469:9896:13056 2:N:0:GAACCTAG+TCCGCATA
ATGCTACTACATTAAATACAATCACATTGTATTTCAGAACTTCTTGTGTCCGCACATAGAAGGTTAAA
GATGGCTTTCTCTTATAGGACGCCACAGGAGGAACTCTTGAGCAGGCTCCCACAGTCTCAACAGGAGG
TTATAAGTGGATTTC
+
FFFFFFFFFFFFFFFFFFFF:FFFFFFFFFFFFFFFFFFFFFFF:FFFFFFFFFFFF:FFFFFFFFFF
FFFFFFFFFFFFFFFFFFFFFFFFFFFFFFFFF:FFFFFF:FFFFFFFFFFFFFFFFFFFFFFFFFFF
:FFFFFFFFFFFFFF
@A00155:342:HHGFNDSXY:1:2515:27670:10770 2:N:0:GAACCTAG+TCCGCATA
TGCTACCACATTAAATACAATCACATTGTATTTCAGAACTTCTTGTGTCCGCACATAGAAGGTTAAAG
ATGGCTTTCTCTTATAGGACGCCACAGGAGGAACTCTTGAGCAGGCTCCCACAGTCTCAACAGGAGGT
TATAAGCGGATTTC
+
FFFFFFFFFFFFFFFFFFF:FFFFFFFFFFFFFFFFFFFFFFFFFFFFFFFFFFFFFFFFFFFFF:FF
FFFFFFFFFFFFFFFFFFFFFFFFFFFFFFFF:FFFFFFFFFFFFFFFFFFFFFFFFFF:FFFFFFFF
FFFFFFFFFFFFFF

```
@A00155:342:HHGFNDSXY:1:2466:4417:17754 2:N:0:GAACCTAG+TCCGCATA
TGCTACTACATTAAATACAATCACATTGTATTTCAGAACTTCTTGTGTCCGCACATAGAAGGTTAAAG
ATGGCTTTCTCTTATAGGACGCCACAGGAGGAACTCTTGAGCAGGCTCCCACAGTCTCAACAGGAGGT
TATAAGTGGATTTCA
+
FFFFFFFFFFFFFFFFFFFFFFFFFF:FFFFFFFFF:FFFFFFFFFFFFFFFFFFFFFFFFFFFFFFFFF
FFFFFFFFFFFFFFFFFFFFFFFFF,FFFFFFFFFFFFF:FFFFFFFFFFFFFFFFFFFFFFFFFFFFFF
FF:FFFFFFFFFFFF
@A00155:342:HHGFNDSXY:1:2655:13991:11475 1:N:0:GAACCTAG+TCCGCATA
GCTACTACATTAAATACAATCACATTGTATTTCAGAACTTCTTGTGTCCGCACATAGAAGGTTAAAGA
TGGCTTTCTCTTATAGGACGCCACAGGAGGAACTCTTGAGCAGGCTCCCACAGTCTCAACAGGAGGTT
A
+
FFFFFFFFFF:FFFFFFFFFFFFFFFFFFFFFFFFFFFFFFFFFFFFFFFFFFFFFFFFFFFFFFFFFF,
FFFF:FFFFFFFFFFFFFFFFFFFFFFFFFFFFFFFFFFFFFFFFFFFFFFFFFFFFFFFFFFFFFFFFF
F
@A00155:342:HHGFNDSXY:1:1108:26051:23782 1:N:0:GAACCTAG+TCCGCATA
GCTACCACATTAAATACAATCACATTGTATTTCAGAACTTCTTGTGTCCGCACATAGAAGGTTAAAGA
TGGCTTTCTCTTATAGGACGCCACAGGAGGAACTCTTGAGCAGGCTCCCACAGTCTCAACAGGAGGTT
ATAAGCGGATTTCAG
+
FFFFFFFFFFFFFFFFFFFFFFFFFFFFFFFFFFFFFFFFFFFFFFFFFFFFFFFFFFFFFFFFFFFFFF
FFFFFFFFFFFFFFFFFFFFFFFFFFFFFFFFFFFFFFFFFFFF:FFFFFFFFFFFFFFFFFFFFFFFFF
FFFFFFFFFFFFFFF
@A00155:342:HHGFNDSXY:1:2274:31620:24940 1:N:0:GAACCTAG+TCCGCATA
GCTACCACATTAAATACAATCACATTGTATTTCAGAACTTCTTGTGTCCGCACATAGAAGGTTAAAGA
TGGCTTTCTCTTATAGGACGCCACAGGAGGAACTCTTGAGCAGGCTCCCACAGTCTCAACAGGAGGTT
ATAAGCGGATTTCAG
+
FF:FFFFFFFFFFFFFF:FFF:FFFFFFFFFFFFFFFFFFFFFFF:FFFFFFFFFFFFFFFFF:FFFFF:
FFFFFFFFFFFFFFFFFFFFFFFFFFFFFFFFFFFFFFFFFFFFFFFFFFFFFFFFFFF:FFFFFFFFFF
FFFFFFFFFFFFFFF
@A00155:342:HHGFNDSXY:1:2327:27299:11130 1:N:0:GAACCTAG+TCCGCATA
GCTACCACATTAAATACAATCACATTGTATTTCAGAACTTCTTGTGTCCGCACATAGAAGGTTAAAGA
TGGCTTTCTCTTATAGGACGCCACAGGAGGAACTCTTGAGCAGGCTCCCACAGTCTCAACAGGAGGTT
ATAAGCGGATTTCAG
+
FFFFFFFFFFFFFFFFFFFFFFFFFFFFFFFFFFFFFFFFFF:FFFFFFFFFFFFFFFFFFFFFFFFFFF
FFFFFFFFFFFFFFFFFFFFFFFFFFFFFFFFFFFFFFFFFFFFFFFFFFFFFFFFFFFFFFFFFFFFFF
FFFFFFFFFFFFFFF
@A00155:342:HHGFNDSXY:1:2516:21540:3505 1:N:0:GAACCTAG+TCCGCATA
TCCGATCTATTAAATACAATCACATTGTATTTCAGAACTTCTTGTGTCCGCACATAGAAGGTTAAAGA
TGGCTTTCTCTTATAGGACGCCACAGGAGGAACTCTTGAGCAGGCTCCCACAGTCTCAACAGGAGGTT
ATAAGCGGATTTCAG
+
FFFFFF:FFFFFFFF:FFFFF:FFFFFFFFFFFFFFFFFFFFFFFFFFFFFFFFFFFFFFFFFFFFFFFF
FFFFFFFFFFFFF,FFF:FFFFFFFFF:FFFFFFFFFFFFFFFF:FFFFFFFFFFFFFF:FFFFFFFFFF
F:F:FFFFFFFFFFF
@A00155:342:HHGFNDSXY:1:2605:22679:9674 1:N:0:GAACCTAG+TCCGCATA
GCTACCACATTAAATACAATCACATTGTATTTCAGAACTTCTTGTGTCCGCACATAGAAGGTTAAAGA
TGGCTTTCTCTTATAGGACGCCACAGGAGGAACTCTTGAGCAGGCTCCCACAGTCTCAACAGGAGGTT
ATAAGCGGATTTCAG
+
FFFFFFFFFFFFFFFFFFFFFFFFFFFFFFFFFFFFFFFFFFFFFFFFFFFFFFFFFFFFFFFFFFFFF,
```

:FFFFFFFFFFFFFFFF:FFFFFFFFFFFFFFFFFFFFFFFFFFFFFFFFFFFFFFFFFFFFFFFFFF
FFFFFFFFFFFFFFF
@A00155:342:HHGFNDSXY:1:2626:13006:11710 1:N:0:GAACCTAG+TCCGCATA
GCTACCACATTAAATACAATCACATTGTATTTCAGAACTTCTTGTGTCCGCACATAGAAGGTTAAAGA
TGGCTTTCTCTTATAGGACGCCACAGGAGGAACTCTTGAGCAGGCTCCCACAGTCTCAACAGGAGGTT
ATAAGCGGATTTCAG
+
:FFFFFFFFFFFFFFF:FFFFFFFFFFFFFFFFFFFFFF,FFFFFFFF,FFFFF,FF:FFFFFFFFFF
FFFFFFFFFFFFFFFFFFFF:FFFFFFFFFFFFFFFFFFFFFFFFFFFFFFFFFFFFFFFFFFFFFFF
FFFFFFFFFFFFFFF
@A00155:342:HHGFNDSXY:1:2626:12617:12696 1:N:0:GAACCTAG+TCCGCATA
GCTACCACATTAAATACAATCACATTGTATTTCAGAACTTCTTGTGTCCGCACATAGAAGGTTAAAGA
TGGCTTTCTCTTATAGGACGCCACAGGAGGAACTCTTGAGCAGGCTCCCACAGTCTCAACAGGAGGTT
ATAAGCGGATTTCAG
+
FFFFFFFFFFFFFFFFF,F:FFFFFFFFFFFFFFFFFFFFFFFFFFFFFFFFFFF:FFFFFFFFFFFF
FFFFFFFFFFFFFFFFFFFFFFFFFFFFFFFFFFFFFFFFFFFFFFFFFFFFFFFFFFFFFFFFFFFF
FFFFFFFFFFFFFFF
@A00155:342:HHGFNDSXY:1:1361:12771:11710 2:N:0:GAACCTAG+TCCGCATA
GCTACCACATTAAATACAATCACATTGTATTTCAGAACTTCTTGTGTCCGCACATAGAAGGTTAAAGA
TGGCTTTCTCTTATAGGACGCCACAGGAGGAACTCTTGAACAGGCTCCCACAGTCTCAACAGGAGGTT
ATAAGCGGATTTCAG
+
FFFFFFFFFFFFFFFFF:FFFFFFFFFFFFFFFFFFFFFFFFFFFFFFFFFFFFFFFFFFFFFF:FFF
FFFFFFFFF:FFFFFFFFFFFFFFFFF:FFFFFFFFFF:FFFFFFFFFFFFFFFFFFFFFFFFFFFFF
FFFFFFFFFFFFFFF
@A00155:342:HHGFNDSXY:1:2239:32145:9659 2:N:0:GAACCTAG+TCCGCATA
GCTACCACATTAAATACAATCACAGTGTATTTCAGAACTTCTTGTGTCCGCACATAGAAGGTTAAAGA
TGGCTTTCTCTTATAGGACGCCACAGGAGGAACTCTTGAGCAGGCTCCCACAGTCTCAACAGGAGGTT
ATAAGCGGATTTCAG
+
FFFFFFFFFFFFFFFFFFFFFFFF,,FF:FFFFFFFFF:FFF:F:FFFFF:FF,,FFFFFFF,FFFFF
FFFFF:FFFFFFF:FFFFFFFFFFFFF:FFF,FFFFFFFFFFFFFFFFFFF:FFFFFFFFFFFFFFFF
,F:FFFFFF,FF:FF
@A00155:342:HHGFNDSXY:1:2274:31620:24940 2:N:0:GAACCTAG+TCCGCATA
GCTACCACATTAAATACAATCACATTGTATTTCAGAACTTCTTGTGTCCGCACATAGAAGGTTAAAGA
TGGCTTTCTCTTATAGGACGCCACAGGAGGAACTCTTGAGCAGGCTCCCACAGTCTCAACAGGAGGTT
ATAAGCGGATTTCAG
+
FFFFFFFFFFFFFFFFFFFFFFFF:FFFF:FFFFFFFFFFFFFFFFFFFFFFFFFFFFFFFFFFFFFF
FFFFFFFFFFFFFFFFFFFFFFFFFFFFFFFFFFFFFFFFFFFFFFFFFFFFFFFFFFFFFFFFFFFF
F,FFFFFFFFFFFFF
@A00155:342:HHGFNDSXY:1:2610:31241:7936 2:N:0:GAACCTAG+TCCGCATA
GCTACCACATTAAATACAATCACATTGTATTTCAGAACTTCTTGTGTCCGCACATAGAAGGTTAAAGA
TGGCTTTCTCTTATAGGACGCCACAGGAGGAACTCTTGAACAGGCTCCCACAGTCTCAACAGGAGGTT
ATAAGCGGATTTCAG
+
:FFFFFFFFFFFFFFFFF:FFFF,FFF:FFFFFFFF:FFFFFFFFFFFFFFFFFFFFFFFFFFFFFF:
FFFFFFFF:FFFFFFFFFFFFFFFFFF,FFFFFFFFFFFFFFFFFFFFFFFFFFFFFFFFFFFFFFFF
FFFFFFFFFFFF:FF
@A00155:342:HHGFNDSXY:1:2675:18078:35587 2:N:0:GAACCTAG+TCCGCATA
GCTACCACATTAAATACAATCACATTGTATTTCAGAACTTCTTGTGTCCGCACATAGAAGGTTAAAGA
TGGCTTTCTCTTATAGGACGCCACAGGAGGAACTCTTGAGCAGGCTCCCACAGTCTCAACAGGAGGTT
ATAAGCGGATTTCAG

```
+
FFFFFFFFFFFFFFFFFF:FFFFF:FFFFFFFFFFFFFFFFFFFFFFFFFFFFFFFFFF::FFFFFFFFF
FFFFFFFFFFFFFFFFFFFFFFFFFFFFFFFFFFFFFFFFFFFFFFFFFFFFFFFFFFF:FF:FFFFFFF
FFFFFFFFFFFFFFF
@A00155:342:HHGFNDSXY:1:1410:13376:23062 1:N:0:GAACCTAG+TCCGCATA
CTACCACATTAAATACAATCACATTGTATTTCAGAACTTCTTGTGTCCGCACATAGAAGGTTAAAGAT
GGCTTTCTCTTATAGGACGCCACAGGAGGAACTCTTGAGCAGGCTCCCACAGTCTCAACAGGAGGTTA
TAAGCGGATTTCAGT
+
FFFFFFFFFFFFFFFFFFFFFFFFFFFFFFFFFFFF:FFFFFFFFFFFFFFFFFFFFFFFFFFFFFFF:F
FFFFFFFFFFFFFFFFFFFFFFFFFFFFFFFFFFFFFFFFFFFFFFFFFFFFFFFFFFFFFFFFFFFFFF
FFFFFFFFFFFFFFF
@A00155:342:HHGFNDSXY:1:1527:20166:7576 1:N:0:GAACCTAG+TCCGCATA
CTACCACATTAAATACAATCACATTGTATTTCAGAACTTCTTGTGTCCGCACATAGAAGGTTAAAGAT
GGCTTTCTCTTATAGGACGCCACAGGAGGAACTCTTGAGCAGGCTCCCACAGTCTCAACAGGAGGTTA
TAAGCGGATTTCAGT
+
FFFFFFFFFFFFFFFFFFFF,FF:FFFFFFFFFFFFFFFFFFFFFFFFFFFFFFFFFFFFFFFFFFFF:F
FFFFFFFFFFFFFFFFFFFFFFFFFF,FFFFFFFFFFFFFFFFFFFFFFFFFFFFFFFFFFFFFFFFFFF
FFFFFFFFFFFFFFF
@A00155:342:HHGFNDSXY:1:2163:2329:2456 1:N:0:GAACCTAG+TCCGCATA
TACCACATTAAATACAATCACATTGTATTTCAGAACTTCTTGTGTCCGCACATAGAAGGTTAAAGATG
GCTTTCTCTTATAGGACGCCACAGGAGGAACTCTTGAGCAGGCTCCCACAGTCTCAACAGGAGGTTAT
AAGCGGATTTCAGT
+
FF:FFFFFFFFFFF:FFFF:FF:FFFFFFFFFFFFFFFFFFF:FFFF,FFFFFF,FFFFFFFFFF::F
F,FFFFFFFFFFFFFFF:FFFFFFFFFFFFFFFFF:,FFFFFFF:F,FFFF::FFFFF:FFFFFFFFF
:F:FFFFFFFFFFF
@A00155:342:HHGFNDSXY:1:1658:22227:27806 2:N:0:GAACCTAG+TCCGCATA
TACCACATTAAATACAATCACATTGTATTTCAGAACTTCTTGTGTCCGCACATAGAAGGTTAAAGATG
GCTTTCTCTTATAGGACGCCACAGGAGGAACTCTTGAGCAGGCTCCCACAGTCTCAACAGGAGGTTAT
AAGCGGATTTCAGTA
+
FFFFFFFFFFFFFFFFFFFFFFFFFFFFFFFFFFFFFFFFFFFFFFFFFFFFFFFFFFFFFFFFFFFF
FFFFFFFFFFFFFFFFFFFFFFFFFFFFFFFFFFFFFFFFFFFFFFFFFFFFFFFFFFFFFFFFFFF:F
FFFFFFFFFFFFFFF
@A00155:342:HHGFNDSXY:1:2178:25934:25582 2:N:0:GAACCTAG+TCCGCATA
TACCACATTAAATACAATCACATTGTATTTCAGAACTTCTTGTGTCCGCACATAGAAGGTTAAAGATG
GCTTTCTCTTATAGGACGCCACAGGAGGAACTCTTGAGCAGGCTCCCACAGTCTCAACAGGAGGTTAT
AAGCGGATTTCAGTA
+
FFFFFFFFFFFFFFFFFFFFFFFFFFFFFFFFFFFFFFFFFFFFFFFFFFFFFFFFFFFFFFFFFFFF
FFFFFFFFFFFFFFFFFFFFFFFFFFFFFFFFFFFFFFFFFFFFFFFFFFFFFFFFFFFFFFFFFFFF
FFFFFFFFFFFFFFF
@A00155:342:HHGFNDSXY:1:1118:24496:35900 1:N:0:GAACCTAG+TCCGCATA
ACTACATTAAATACAATCACATTGTATTTCAGAACTTCTTGTGTCCGCACATAGAAGGTTAAAGATGG
CTTTCTCTTATAGGACGCCACAGGAGGAACTCTTGAGCAGGCTCCCACAGTCTCAACAGGAGGTTATA
AGTGGATTTCAGTAT
+
FFFFFFFFFFFFFFFFF:FFFFFFFFFFFFFFFFFFFFFFFFFFFFFFFFFFFFFFFFFFFFFFFFFF
FFFFFFFFFFFFFFFFFFFFFFFFFFFFF:FF,FFFF,FFFFFFFF:FFFFFFFFFFFFFFFFFFFFFF
FFFFFFFFFFFFFFF
@A00155:342:HHGFNDSXY:1:1330:23972:10097 2:N:0:GAACCTAG+TCCGCATA
ACCACATTAAATACAATCACATTGTATTTCAGAACTTCTTGTGTCCGCACATAGAAGGTTAAAGATGG
```

```
CTTTCTCTTATAGGACGCCACAGGAGGAACTCTTGAGCAGGCTCCCACAGTCTCAACAGGAGGTTATA
AGCGGATTTCAGTAT
+
FFFFFFF:FFFFFFFFFF:F:FFFFFFFFFFFFFFFFFF:F:FFFFFFFFFFFFFFFFFFFFFFFFFFF
FFFFFFFFFFFFFFFFFFFFFFFFFFFFFFFFFFFFFFFFFFFFFFFFFFFFFFFFFFFFFFFFFFFFF
FFFFFFFFFFFFFFF
@A00155:342:HHGFNDSXY:1:1511:29333:34006 2:N:0:GAACCTAG+TCCGCATA
ACCACATTAAATACAATCACATTGTATTTCAGAACTTCTTGTGTCCGCACATAGAAGGTTAAAGATGG
CTTTCTCTTATAGGACGCCACAGGAGGAACTCTTGAGCAGGCTCCCACAGTCTCAACAGGAGGTTATA
AGCGGATTTCAGTAT
+
FFFFFFFFFF:FFF:FFFFFF:FFFFFFFFFFFFFFFFFFFFFFFFFFFFFFFFF,FFFFFFF:FFFFF
FFFF:FFFFFFFFFFFFFFFFFFFFFFFFFFFFFFFFFF:FFFFFFFF:FFFFFFFFFFFF:FFF:FFF
,FF:FF,FF::FFFF
@A00155:342:HHGFNDSXY:1:1127:18050:5603 2:N:0:GAACCTAG+TCCGCATA
CACATTAAATACAATCACATTGTATTTCAGAACTTCTTGTGTCCGCACATAGAAGGTTAAAGATGGCT
TTCTCTTATAGGACGCCACAGGAGGAACTCTTGAGCAGGCTCCCACAGTCTCAACAGGAGGTTATAAG
CGGATTTCAGTATGA
+
FFFFFFFFFFFF:FFFFFFF:FFFFFFF:FFFFFFF:FFFFFFFFFFFF:FFFFFFFFFF:FFFFFFF
FFFFFFFFFFFFFFFFFFFFFFFFFFFFFFFFFFFF:FFFFFFFFFFFFFFFFFFFFF:FF,:FFFFF
FFFFFFFFFFFFFFF
@A00155:342:HHGFNDSXY:1:1445:1344:6104 2:N:0:GAACCTAG+TCCGCATA
CACATTAAATACAATCACATTGTATTTCAGAACTTCTTGTGTCCGCACATAGAAGGTTAAAGATGGCT
TTCTCTTATAGGACGCCACAGGAGGAACTCTTGAGCAGGCTCCCACAGTCTCAACAGGAGGTTATAAG
CGGATTTCAGTATGA
+
FFFFFFFF:FFFFFF,FFFFFF:FFFFFFFFFFFFFFFFFFFFFFFFF:FFFF:FFFFFFFFFFFFFF
FFFFFFFFFFFFFF:FFFFFFFFFFF:FFF,FFFF:FFFFFFFFFFFFFFFFFFF:FFFF:FFF:FFF
FFFFFFFF:FFF:FF
@A00155:342:HHGFNDSXY:1:1270:12292:23375 1:N:0:GAACCTAG+TCCGCATA
ACATTAAATACAATCACATTGTATTTCAGAACTTCTTGTGTCCGCACATAGAAGGTTAAAGATGGCTT
TCTCTTATAGGACGCCACAGGAGGAACTCTTGAGCAGGCTCCCACAGTCTCAACAGGAGGTTATAAGC
GGATTTCAGTATGAA
+
FFFFFFFFFFFFFFFFFFFFFFFFFFFFFFFFFFFFFFFFFFFFFFFFFFFFFFFFFFFFFFFFFFFF
FFFFFFFFFFFFFFFFFFFFFFFFFFFFFFFFFFFFFFFFFFFFFFFFFFFFFFFFFFFFFFFFFFFF
FFFFFFFFFFFFFFF
@A00155:342:HHGFNDSXY:1:1424:32479:16595 1:N:0:GAACCTAG+TCCGCATA
ACATTAAATACAATCACATTGTATTTCAGAACTTCTTGTGTCCGCACATAGAAGGTTAAAGATGGCTT
TCTCTTATAGGACGCCACAGGAGGAACTCTTGAGCAGGCTCCCACAGTCTCAACAGGAGGTTATAAGC
GGATTTCAGTATGAA
+
FFFFFFFF:FF,FFFFFFFFFFFFFFF:FF:F:FFFFFFF:FFFFFF:FFF,FFFFFFFFF:FFFFFF
FFFF:FFFFFFFFFFFF:FFFFFFFFFFFFFFFFFFFFFFFFF:FFFFFFFFFF::FFFFF:FFFFFF:
FFFFFFF:FF:FF,F
@A00155:342:HHGFNDSXY:1:2413:17056:27649 1:N:0:GAACCTAG+TCCGCATA
ACATTAAATACAATCACATTGTATTTCAGAACTTCTTGTGTCCGCACATAGAAGGTTAAAGATGGCTT
TCTCTTATAGGACGCCACAGGAGGAACTCTTGAGCAGGCTCCCACAGTCTCAACAGGAGGTTATAAGC
GGATTTCAGTATGAA
+
FFFFFFFFFFFFFFFFFFFFFFFF:FFFFFFFFFFFFFFFFFFFFFFFFFFFFFFFFFFFFFFFFFFF
FFFFFFFFFFFFFF::FFFFFF:FFFFFFFFFFFFFFFFFFFFFFFFFFFFFFFFFFFFFFFFFFFFF
FFFFFFF:FFFFFFF
```

```
@A00155:342:HHGFNDSXY:1:2641:15709:15013 1:N:0:GAACCTAG+TCCGCATA
ACATTAAATACAATCACATTGTATTTCAGAACTTCTTGTGTCCGCACATAGAAGGTTAAAGATGGCTT
TCTCTTATAGGACGCCACAGGAGGAACTCTTGAGCAGGCTCCCACAGTCTCAACAGGAGGTTATAAGC
GGATTTCAGTATGAA
+
FFFFFFFFFFFFFFFFFFFFFFFFFFFFFFFFFFFFFFFFFFFFFFFFFFFFFFFFFFFFFFFFFFFF
FF:FFFFFFFFFFFFFFFFFFFFFFFFFFFFFFFFFFFFFFFFFF:FFFFFFFFFFFFFFFFFFFFFF
FFFFFFFFFFFFFFF
@A00155:342:HHGFNDSXY:1:1454:28962:22842 2:N:0:GAACCTAG+TCCGCATA
ACATTAAATACAATCACATTGTATTTCAGAACTTCTTGTGTCCGCACATAGAAGGTTAAAGATGGCTT
TCTCTTATAGGACGCCACAGGAGGAACTCTTGAGCAGGCTCCCACAGTCTCAACAGGAGGTTATAAGC
GGATTTCAGTATGAA
+
FFFFFFFFFFFFFFFFFFFFFFFF:FFFFFFFFFFFFFFFFFFFFFFFFFFFFFFFFFFFFFFFFFFF
FFFFFFFFFFFFFFFFFF:FFFFFFFFFFFFFFFFFFFFFFFFFFFFFFFFFFFFFFF:FFFF,FF:FF
FFF,FFFFFFFFFFF
@A00155:342:HHGFNDSXY:1:2559:15040:28072 1:N:0:GAACCTAG+TCCGCATA
CATTAAATACAATCACATTGTATTTCAGAACTTCTTGTGTCCGCACATAGAAGGTTAAAGATGGCTTT
CTCTTATAGGACGCCACAGGAGGAACTCTTGAGCAGGCTCCCACAGTCTCAACAGGAGGTTATAAGCG
GATTTCAGTATGAA
+
FFFFFFF:FFFFF:FF,FFFFFFFFFFFF,FFF,FFFFFFF:FFFFFFFF,F,F:FFF,F:FFF,FFF
FFFFF:FFFFF,FFFFFFF:FFF:F:FFFFFFF:FF,FF:,FFFFFFFFFFF,FFFFFFFF:FF,:FF
FF:FF:,FFFF,F:
@A00155:342:HHGFNDSXY:1:1127:18050:5603 1:N:0:GAACCTAG+TCCGCATA
CATTAAATACAATCACATTGTATTTCAGAACTTCTTGTGTCCGCACATAGAAGGTTAAAGATGGCTTT
CTCTTATAGGACGCCACAGGAGGAACTCTTGAGCAGGCTCCCACAGTCTCAACAGGAGGTTATAAGCG
GATTTCAGTATGAAC
+
FFFFF,FFFFF:FFFFFFFFFFFFFFFFFFFFFFFFFFFFF:FFFFFFFFFFFFFFFFFF:FFFFFFF
FFFFFFFFFFFFFFFFFFFFFFFFFFFFFFFFFFFFFFFFFFFFFFFFFFFFFFFFFFFFFFFFFFFF
FFFFFFFFFFFFFFF
@A00155:342:HHGFNDSXY:1:1146:22996:26381 1:N:0:GAACCTAG+TCCGCATA
CATTAAATACAATCACATTGTATTTCAGAACTTCTTGTGTCCGCACATAGAAGGTTAAAGATGGCTTT
CTCTTATAGGACGCCACAGGAGGAACTCTTGAGCAGGCTCCCACAGTCTCAACAGGAGGTTATAAGTG
GATTTCAGTATGAAC
+
:FFFFFF:FFFFFFFFFFFFF:FFFFFFFFFFFFFFFFFFFFFFFFFFFFF:FFFFFFF:FFFFFFFFF
FFFFFFFFFFFFFFFFFFFFFFFFFFFFFFFFFFFFF:FFFFFFFF:FFFFFFFFFFFFFFFFFFFFFF
FFFFFFFFFFFFFFF
@A00155:342:HHGFNDSXY:1:1172:6379:2988 1:N:0:GAACCTAG+TCCGCATA
CATTAAATACAATCACATTGTATTTCAGAACTTCTTGTGTCCGCACATAGAAGGTTAAAGATGGCTTT
CTCTTATAGGACGCCACAGGAGGAACTCTTGAGCAGGCTCCCACAGTCTCAACAGGAGGTTATAAGCG
GATTTCAGTATGAAC
+
FFFFFF:FFFFFFFFF:FFFFFF:FFFFFFFFFFFFFFFFFFFFFFFFFFFFFFFFFFFFFFFFFFFF
FFFFFFFFFFFFFFFFFFFFFFFFFFFFFFFFFFFFFF:FFFFFFFFFFFFFFFFFFFFFFFFFFFFF
FFFFFFFFFFFFFFF
@A00155:342:HHGFNDSXY:1:1335:6786:19727 1:N:0:GAACCTAG+TCCGCATA
CATTAAATACAATCACATTGTATTTCAGAACTTCTTGTGTCCGCACATAGAAGGTTAAAGATGGCTTT
CTCTTATAGGACGCCACAGGAGGAACTCTTGAGCAGGCTCCCACAGTCTCAACAGGAGGTTATAAGTG
GATTTCAGTATGAAC
+
:FFFFFFFFFFFFF:FFFFFFFFFFFFFFFFFFFFFFFFFFFFFFFFFFFFFFFFFFFFFFFFFFFFF
```

FFFFFFFFFFFFFFFFFFFFFFFFFFFFFFFFFFFFFFFFFFFFFFFFFFFFFFFFFFFFFFFFFFFF
FFFFFFFFFFFFFFF
@A00155:342:HHGFNDSXY:1:1376:24623:15201 1:N:0:GAACCTAG+TCCGCATA
CATTAAATACAATCACATTGTATTTCAGAACTTCTTGTGTCCGCACATAGAAGGTTAAAGATGGCTTT
CTCTTATAGGACGCCACAGGAGGAACTCTTGAGCAGGCTCCCACAGTCTCAACAGGAGGTTATAAGCG
GATTTCAGTATGAAC
+
FFFFFFFFFFFFFFFFFFFFFFFFFFFFFFFFFFFFFFFFFFFFFFFFFFFFFFFFFFFFFFFFFFFF
FFFFFFFFFFFFFFFFFFFFFFFFFFFFFFFFFFFFFFFFFFFFFFFFFFFFFFFFFFFFFFFF:FFF
FFFFFFFFFFFFFFF
@A00155:342:HHGFNDSXY:1:1644:14651:7420 1:N:0:GAACCTAG+TCCGCATA
CATTAAATACAATCACATTGTATTTCAGAACTTCTTGTGTCCGCACATAGAAGGTTAAAGATGGCTTT
CTCTTATAGGACGCCACAGGAGGAACTCTTGAGCAGGCTCCCACAGTCTCAACAGGAGGTTATAAGCG
GATTTCAGTATGAAC
+
FFFFFFFFFFFFFFFFFFFFFFFFFFFFFFFFFFFFFFFFFFFFFFFFFFFFFFFFF:FFFFFFFFFF
FFFFFFFFFFFFFFFFF:FFFFFFFFFFFFFFFFFFFFFFFFFFFFFFFFFFFFFFFFFFFFFFFFFF
FFFFFFFFFFFFFFF
@A00155:342:HHGFNDSXY:1:2128:23656:8108 1:N:0:GAACCTAG+TCCGCATA
CATTAAATACAATCACATTGTATTTCAGAACTTCTTGTGTCCGCACATAGAAGGTTAAAGATGGCTTT
CTCTTATAGGACGCCACAGGAGGAACTCTTGAGCAGGCTCCCACAGTCTCAACAGGAGGTTATAAGCG
GATTTCAGTATGAAC
+
FFFFFFFFFFFFFFFFFFFFFFFFFFFFFFFFFFFFFFFFFFFFFFFFFFFFFFFFFFFFFFFFFFFF
FFFFFFFFFFFFFFFFFFFFFFFFFFFFFFFFFFFFFFFFFFFFFFFFFFFFFFFFFFFFFFFFFFFF
FFFFFFFFFFFFFFF
@A00155:342:HHGFNDSXY:1:2247:30264:27915 1:N:0:GAACCTAG+TCCGCATA
CATTAAATACAATCACATTGTATTTCAGAACTTCTTGTGTCCGCACATAGAAGGTTAAAGATGGCTTT
CTCTTATAGGACGCCACAGGAGGAACTCTTGAGCAGGCTCCCACAGTCTCAACAGGAGGTTATAAGTG
GATTTCAGTATGAAC
+
FFFFFFFFFFFFFFFFFFFFFFFFFFFFFFFFFFFFFFFFFFFFFFFFFFFFFFFFFFFFFFFFFFFF
FFFFFFFFFFFFFFFFFFFFFFFFFFFFFFFFFFFFFFFFFFFFFFFFFFFFFFFFFFFFFFFFFFFF
FFFFFFFFFFFFFFF
@A00155:342:HHGFNDSXY:1:2408:14443:20400 1:N:0:GAACCTAG+TCCGCATA
CATTAAATACAATCACATTGTATTTCAGAACTTCTTGTGTCCGCACATAGAAGGTTAAAGATGGCTTT
CTCTTATAGGACGCCACAGGAGGAACTCTTGAGCAGGCTCCCACAGTCTCAACAGGAGGTTATAAGCG
GATTTCAGTATGAAC
+
FFFFFFFFFFFFFFFFFFFFFFFFFFFFFFFFFFFFFFFFFFFFFFFFFFFFFFFFFFFFFFFFFFFF
FFFFFFFFFFFFFFFFFFFFFFFFFFFFFFFFFFFFFFFFFFFFFFFFFFFFFFFFFFFFFFFFFFFF
FFFFFFFFFFFFFFF
@A00155:342:HHGFNDSXY:1:2509:32353:36104 1:N:0:GAACCTAG+TCCGCATA
CATTAAATACAATCACATTGTATTTCAGAACTTCTTGTGTCCGCACATAGAAGGTTAAAGATGGCTTT
CTCTTATAGGACGCCACAGGAGGAACTCTTGAGCAGGCTCCCACAGTCTCAACAGGAGGTTATAAGCG
GATTTCAGTATGAAC
+
FFFFFFFFFF:FFFFFFFFFFFFFFFFFFFFF:FFFF:FFFFFFFFFFFFFFFFFFFFFFF,FFFFFF
FFFFFFFFFFFFFFFFF:FFFFFFFFFFFFFFFFFFFFFFFFFFFFFFFFFFFFFFFFFFFFFF:FFF
FFFFFFFFFFFFF:F
@A00155:342:HHGFNDSXY:1:2551:17119:35462 1:N:0:GAACCTAG+TCCGCATA
CATTAAATACAATCACATTGTATTTCAGAACTTCTTGTGTCCGCACATAGAAGGTTAAAGATGGCTTT
CTCTTATAGGACGCCACAGGAGGAACTCTTGAGCAGGCTCCCACAGTCTCAACAGGAGGTTATAAGCG
GATTTCAGTATGAAC

+
FFFFFFFFFFFFFFFFFFFFFFFFFFFFFFFFFFFFFFFFFFFFFFFFFFFFFFFFFFFFFFFFFFFF
FFFFFFFFFF:FFFFFFFFFFFFFFFFFFFFFFFFFFFFFFFFFFFFFFFFFFFFFFFFFFFFFFFFF
FFFFFFFFFFFFFFF
@A00155:342:HHGFNDSXY:1:2633:30454:30937 1:N:0:GAACCTAG+TCCGCATA
CATTAAATACAATCACATTGTATTTCAGAACTTCTTGTGTCCGCACATAGAAGGTTAAAGATGGCTTT
CTCTTATAGGACGCCACAGGAGGAACTCTTGAGCAGGCTCCCACAGTCTCAACAGGAGGTTATAAGCG
GATTTCAGTATGAAC
+
FFFFFFFFFFFFFFFFFFFFFFFFFFFFFFFFFFFFFFFFFFFFFFFFFFFFFFFFFFFFFFFFFFFF
FFFFFFFFFFFFFFFFFFFF,FFFFFFFFFFFFFFFFFFFFFFFFFFFFFFFFFFFFFFFFFFFFFFF
FFFFFFFFFFFFFFF
@A00155:342:HHGFNDSXY:1:1277:17318:22028 1:N:0:GAACCTAG+TCCGCATA
ATTAAATACAATCACATTGTATTTCAGAACTTCTTGTGTCCGCACATAGAAGGTTAAAGATGGCTTTC
TCTTATAGGACGCCACAGGAGGAACTCTTGAGCAGGCTCCCACAGTCTCAACAGGAGGTTATAAGCGG
ATTTCAGTATGAACG
+
FFFFFFFFFFFFFFFFFFFFFFFFFFFFFFFFFFFFFFFFFFFFFFFFFFFFF:FFFFFFFFFFFFFF
FFFFFFFFFFFFFFFFFFF:FFFFFFFFFFFFFFFFFFFFFFFFFFFFFF:FFFFFFFFFFFFFFFFF
FFFFFFFFFFFFFFF
@A00155:342:HHGFNDSXY:1:1658:22227:27806 1:N:0:GAACCTAG+TCCGCATA
ATTAAATACAATCACATTGTATTTCAGAACTTCTTGTGTCCGCACATAGAAGGTTAAAGATGGCTTTC
TCTTATAGGACGCCACAGGAGGAACTCTTGAGCAGGCTCCCACAGTCTCAACAGGAGGTTATAAGCGG
ATTTCAGTATGAACG
+
FFFFFFF:FFFFFFFFFFFFFFFFFFFFFFFFF:FFFFFFFFFFFFFFFFFFFFFFFFFFFFFFFFFF
FFFFFFFFFFFFFFFFFFFFFFFFFFFFFFFFFFFFFFFFFFFFFFFFFF:FFFFFFFFFFFFFFFFF
FFFFFFFFFFFFFFF
@A00155:342:HHGFNDSXY:1:2149:22761:22968 1:N:0:GAACCTAG+TCCGCATA
ATCTATTACAATCACATTGTATTTCAGAACTTCTTGTGTCCGCACATAGAAGGTTAAAGATGGCTTTC
TCTTATAGGACGCCACAGGAGGAACTCTTGAGCAGGCTCCCACAGTCTCAACAGGAGGTTATAAGCGG
ATTTCAGTATGAACG
+
FFFFFF:FFF,FFFFFFFFFFFFFFFFFFFFFFFFFFFFFFFFFFFFFFFFFFFFFFFFFFFFFFFFF
FF:FFFFFFFFFFFFFFFFFFFFFFFFFFFFFFFFFFFFFFFFFFFFFFFFFFFFFFFFFFFFFFFFF
FFFFFFFFFFFFFFF
@A00155:342:HHGFNDSXY:1:1545:27145:20979 2:N:0:GAACCTAG+TCCGCATA
ATTAAATACAATCACATTGTATTTCAGAACTTCTTGTGTCCGCACATAGAAGGTTAAAGATGGCTTTC
TCTTATAGGACGCCACAGGAGGAACTCTTGAGCAGGCTCCCACAGTCTCAACAGGAGGTTATAAGCGG
ATTTCAGTATGAACG
+
FFFFFFFFFF:FFFF:,FF:FFFFFFFFFFFFFFFFFFFFFFFFFF:FFFFFFFFFFFFFFFFFFFFF
FFFFFFFFFFFFFFFFFFFFFFFFFFFFFFFFFFFFFFFFFFFFFFFFFFFFFFFFFFFFFFFFFFFF
F,FFFFFFFFFFFFF
@A00155:342:HHGFNDSXY:1:1671:31647:8077 2:N:0:GAACCTAG+TCCGCATA
ATTAAATACAATCACATTGTATTTCAGAACTTCTTGTGTCCGCACATAGAAGGTTAAAGATGGCTTTC
TCTTATAGGACGCCACAGGAGGAACTCTTGAGCAGGCTCCCACAGTCTCAACAGGAGGTTATAAGCGG
ATTTCAGTATGAACG
+
FFFFFFFFF:FFFFFFFFFFFFFFFFFFFFFFFFFFFFFFFFFFFFFFFFFFFFFFFFFFFFFFFF:F
:FFFFFFFFFFFFFFFFFFFFFFFFFFFFFFFFFFFFFFFF:FFFFFFFFFFFFFFFFFFFFF:FFFF
F:FFFFF:FFFFFFF
@A00155:342:HHGFNDSXY:1:2115:26747:25520 2:N:0:GAACCTAG+TCCGCATA
ATTAAATACAATCACATTGTATTTCAGAACTTCTTGTGTCCGCACATAGAAGGTTAAAGATGGCTTTC

TCTTATAGGACGCCACAGGAGGAACTCTTGAGCAGGCTCCCACAGTCTCAACAGGAGGTTATAAGCGG
ATTTCAGTATGAACG
+
FFFFFFFFFFFFFFFFFFFFFFFFFFFFFFFFFFFFFFFFFFFFFFFFFFFFFFFFFFFFFFFFFFFF
FFFFFFFFFFF:FFFFFFFFFFF,FFFF:FFFFFFFFFFFFFFFFFFFFFFFFFFFFFFFFFFFFFFF
FFFFFFFFFFFFFFF
@A00155:342:HHGFNDSXY:1:2413:28999:28134 2:N:0:GAACCTAG+TCCGCATA
ATTAAATACAATCACATTGTATTTCATAACTTCTTGTGTCCGCACATAGAAGGTTAAAGATGGCTTTC
TCTTATAGGACGCCACAGGAGGAACTCTTGAGCAGGCTCCCACAGTCTCAACAGGAGGTTATAAGCGG
ATTTCAGTATGAACG
+
FFFFFFFFF,FFFF:FFFF:FFFFF,,FFFFFFF,F:FF,FFFF,FF,FFFFFF:FFFFF:,F::FFF
FFF,FFFFFFFFFFFFFFFFFFF,FFFFFFFF:F,FFFFFFF,FFFFFFF,,,:F,,,::F,FF,FFF
FF,,::FFFFFFFFF
@A00155:342:HHGFNDSXY:1:2413:28375:30311 2:N:0:GAACCTAG+TCCGCATA
ATTAAATACAATCACATTGTATTTCAGAACTTCTTGTGTCCGCACATAGAAGGTTAAAGATGGCTTTC
TCTTATAGTACGCCACAGGAGGAACTCTTGAGCAGGCTCCCACAGACTCAACAGGAGGTTATAAGAGG
ATTTAAGTATGAACG
+
FFFF:FFFFF:FFFF,F,FFFFFFFFFFFFFFFF,:FFFF,FFF,FFF:FFFFFFFFFFFFFF,F:FF
FFFFFFFF,FFFF:FFF,FF,FFF,FFFFFFFFF,FFF,FFFFFF,F:FFFFFF,FFFF:FFFF,,FF
F:FF,FFFFFFFFFF
@A00155:342:HHGFNDSXY:1:2564:32913:31125 2:N:0:GAACCTAG+TCCGCATA
ATTAAATACAATCACATTGTATTTCAGAACTTCTTGTGTCCGCACATAGAAGGTTAAAGATGGCTTTC
TCTTATAGGACGCCACAGGAGGAACTCTTGAGCAGGCTCCCACAGTCTCAACAGGAGGTTATAAGCGG
ATTTCAGTATGAACG
+
FFF::FFFFFFFF::FFFFFFFFFFFF,FFFFFFFF:FFFFFFFFFFFFF,FFFFFF:FFFFFFFFFF
FFFFFF:F:FFFFFF:FFF:FFFFFFFF:FFF:FFFFFFFFF:FFFFFFF:FFFFFFFFFFFFF,F:F
FFFFF,FFFFFF::F
@A00155:342:HHGFNDSXY:1:2237:31711:3490 1:N:0:GAACCTAG+TCCGCATA
TTAAATACAATCACATTGTATTTCAGAACTTCTTGTGTCCGCACATAGAAGGTTAAAGATGGCTTTCT
CTTATAGGACGCCACAGGAGGAACTCTTGAGCAGGCTCCCACAGTCTCAACAGGAGGTTATAAGCGGA
TTTCAGTATAAACGC
+
FFFFFFF:F,FFFFFFFFFFFFFFFFFFFFFFFFFFFFFFFFFFFFFFFFFFFFFFFFFFFFFFFFFF
FFFF:FFFFFFFFFFFFFFFFFFFFFFFFFFFFFFFFFFFFFFFFFFFFFFFFFFFFFFFFFFFFFFF
FFFFFFFFFFFFFFF
@A00155:342:HHGFNDSXY:1:2237:31485:3944 1:N:0:GAACCTAG+TCCGCATA
TTAAATACAATCACATTGTATTTCAGAACTTCTTGTGTCCGCACATAGAAGGTTAAAGATGGCTTTCT
CTTATAGGACGCCACAGGAGGAACTCTTGAGCAGGCTCCCACAGTCTCAACAGGAGGTTATAAGCGGA
TTTCAGTATAAACGC
+
FFFFFFFFFFFFFFFFFFFF:FFFFFFFF:FFFFFFFFFFFFFFFFFFFFFF:FFFF:FFFFFFFFFF
FFFFFFFFFFFFFFFFFF:FFFFFFF:FFFFFFFFFFFFFFFFFFFFFFFFFFFFFFFFFF:FFFFFF
FFFFFFFFFFFFFFF
@A00155:342:HHGFNDSXY:1:2653:31801:22216 1:N:0:GAACCTAG+TCCGCATA
TTAAATACAATCACATTGTATTTCAGAACTTCTTGTGTCCGCACATAGAAGGTTAAAGATGGCTTTCT
CTTATAGGACGCCACAGGAGGAACTCTTGAGCAGGCTCCCACAGTCTCAACAGGAGGTTATAAGCGGA
TTTCAGTATGAACGC
+
FFFFFFFFF:FFFFFFFFFFFFFFFFFFFFFFFFFFFFFFFFFFFFFFFFFFFFFF:F:FFFFF:FFF
FFFF:FFFFFFFFFFFFFFFFFFFFFFFFFFFFFFFFFFFFF,FFFFF:FFFFFFFFFFFFFFFFFFF
FFFFFFFFFFFFFFF

```
@A00155:342:HHGFNDSXY:1:2314:13331:30749 2:N:0:GAACCTAG+TCCGCATA
TTAAATACAATCACATTGTATTTCAGAACTTCTTGTGTCCGCACATAGAAGGTTAAAGATGGCTTTCT
CTTATAGGACGCCACAGGAGGAACTCTTGAGCAGGCTCCCACAGTCTCAACAGGAGGTTATAAGCGGA
TTTCAGTATGAACGC
+
FFFFFFFFFFFFF:FFFFFFFFFFFFFFFFFFFFFFFFFFFFFFFFFFFFFFFFFFFFFFFFFFFFFF
FFF::FFFFFFFFFFFFFFFFFFFFFFFFFFF:FFFFFFFFFFFFFFFFFFFFFFFFFFFFFFF:FFFFF
,FF:FFFFFFFFFFF
@A00155:342:HHGFNDSXY:1:1118:4300:2362 1:N:0:GAACCTAG+TCCGCATA
TAAATACAATCACATTGTATTTCAGAACTTCTTGTGTCCGCACATATAAGGTTAAAGATGGCTTTCTC
TTATAGGACGCCACAGGAGGAACTCTTGAGCAGGCTCCCACAGTCTCAACAGGAGGTTATAAGCGGAT
TTCAGTATGAACGCG
+
FFFFFFFFFFFFFFF:FFFFFFFFFF:FFF,FFFF:FFF:FFFFFF,:FFFFFF,FFFFF:F:FFFF,
FFFFFFFFFF:F:,FFFF,FF,FFF:FFFFFF,FFFF:FF,FFF:,F,FF:F,FF,F:FFF,FFFFFF
FFFF,:FFFFFFF:F
@A00155:342:HHGFNDSXY:1:1268:23918:21559 1:N:0:GAACCTAG+TCCGCATA
TAAATACAATCACATTGTATTTCAGAACTTCTTGTGTCCGCACATAGAAGGTTAAAGATGGCTTTCTC
TTATAGGACGCCACAGGAGGAACTCTTGAGCAGGCTCCCACAGTCTCAACAGGAGGTTATAAGCGGAT
TTCAGTATGAACGCA
+
FFFFFFF:FFF,:,FFFF:FFFFFFFF:FF,:F,FFFF,F,FFFFF::FFFFF::F,,FF,:FF:FFF
FFFFFF:FFF:FFF:FFFFFFFF::FFFFFF:FF:F,,:FFF:FFF,FF:FF:FF:FF,FFFFF:FFF
FFF:F::F:FFFFF:
@A00155:342:HHGFNDSXY:1:2239:32145:9659 1:N:0:GAACCTAG+TCCGCATA
TAAATACAATCACATTGTATTTCAGAACTTCTTGTGTCCGCACATAGAAGGTTAAAGATGGCTTTCTC
TTATAGGACGCCACAGGAGGAACTCTTGAGCAGGCTCCCACAGTCTCAACAGGAGGTTATAAGCGGAT
TTCAGTATGAACGCT
+
FFFFFFFFFFFFFFFFFFFFFFFFFFFFFFFFFFFFFFFFFFFFFF:FFFFFFFFFFFFFFFFFFFFF
FFFFFFFFFFFFFFFFFFFFFFFFFFFFFFFFFFFFFFFFFFFFFFFFFFFFFFFFF:FFFFFFFFFF
FFFFFFFFFFFFFFF
@A00155:342:HHGFNDSXY:1:2510:28501:27273 1:N:0:GAACCTAG+TCCGCATA
TAAATACAATCACATTGTATTTCAGAACTTCTTGTGTCCGCACATAGAAGGTTAAAGATGGCTTTCTC
TTATAGGACGCCACAGGAGGAACTCTTGAGCAGGCTCCCACAGTCTCAACAGGAGGTTATAAGCGGAT
TTCAGTATGAACGCT
+
FF,FFFFFFFFFFFFFFFFFF:FFFFFFF:FFFFFFFFFFFFFFFFFFFFFFFFFFFFFFFFFFFFFF
FFFFFFF,FFFFFFFFFFFFFFFFFFFFFFFFFFFFFFFFFFFFFFFFFFFFFFFFFFFFFFFFFFFF
FFFFFFFFFFFFFFF
@A00155:342:HHGFNDSXY:1:2675:18078:35587 1:N:0:GAACCTAG+TCCGCATA
TAAATACAATCACATTGTATTTCAGAACTTCTTGTGTCCGCACATAGAAGGTTAAAGATGGCTTTCTC
TTATAGGACGCCACAGGAGGAACTCTTGAGCAGGCTCCCACAGTCTCAACAGGAGGTTATAAGCGGAT
TTCAGTATGAACGCT
+
FFFFFFFFFFFFFFFFFFFFFF:FFFFFFFFFFFFFFFFFFFFFFFFFFFFFFFFFFFFFFFFFFFFF
FFFFFFFFFFFFFFFFFFFFFFFFFFFFFFFFFFFFFFFFFFFFFFFFFFFFFFFFFFFFFFFFFFFF
FFFFFFFFFFFFFFF
@A00155:342:HHGFNDSXY:1:1630:17047:31516 2:N:0:GAACCTAG+TCCGCATA
TAAATACAATCACATTGTATTTCAGAACTTCTTGTGTCCGCACATAGAAGGTTAAAGATGGCTTTCTC
TTATAGGACGCCACAGGAGGAACTCTTGAGCAGGCTCCCACAGTCTCAACAGGAGGTTATAAGCGGAT
TTCAGTATGAACGCA
+
FFFFFFFF,FFFFFFFFFFFFFFFFFFFFFFFFFFFFFFFFFFFFFFFFFFFFFFFFFFFFFFFFFFF
```

```
FFFFFFFFFFFFFFFFFFFFFFFFFFFFFFFFFFFFFFFFFFFFFFFFFFFFFFFFFFFFFFFFFFFF
FFFFFFFFFFFFFFF
@A00155:342:HHGFNDSXY:1:1162:8684:22921 1:N:0:GAACCTAG+TCCGCATA
GATCTCACTCACATTGTATTTCAGAACTTCTTGTGTCCGCACATAGAAGGTTAAAGATGGCTTTCTCT
TATAGGACGCCACAGGAGGAACTCTTGAGCAGGCTCCCACAGTCTCAACAGGAGGTTATAAGCGGATT
TCAGTATGAACGCAT
+
FFFFFFF,FFFFFFFFFFFFFFFFFFFFFFFFFFFFFFFFFFFFFFFFFFFFFFFFFFFFFFFFFFFF
FFFFFFFFFFFFFFFFFFFFFFFFFFFFFFFFFFFFFFFFFFFFFFFFFFFFFFFFFFFFFFFFFFFF
:FFFFFFFFFFFFFF
@A00155:342:HHGFNDSXY:1:2236:16586:22701 1:N:0:GAACCTAG+TCCGCATA
AAATACAATCACATTGTATTTCAGAACTTCTTGTGTCCGCACATAGAAGGTTAAAGATGGCTTTCTCT
TATAGGACGCCACAGGAGGAACTCTTGAGCAGGCTCCCACAGTCTCAACAGGAGGTTATAAGTGGATT
TCAGTATGAACGCAT
+
FFFFFFFFFFFFFFFFFFFFFFFFFFFFFFFFFFFFFFFFFFFFFFFFFFFFFFFF::FFFFFFFFFF
FFFFFFFFFFFFFFFFFFFFFFFFFFFFFFFFFFFFFFFFFFFFFFFFFFFFFFFFFFFFFFFFFFFF
FFFFFFFFFFFFFFF
@A00155:342:HHGFNDSXY:1:2259:8947:6026 1:N:0:GAACCTAG+TCCGCATA
AAATACAATCACATTGTATTTCAGAACTTCTTGTGTCCGCACATAGAAGGTTAAAGATGGCTTTCTCT
TATAGGACGCCACAGGAGGAACTCTTGAGCAGGCTCCCACAGTCTCAACAGGAGGTTATAAGCGGATT
TCAGTATGAACGCAT
+
FFFFFFFFFFFFFFFFFFFFFFFFFFFFFFFFFFFFFFFFFFFFFFFFFFFFFFFFFFFFFFFFFFFF
FFFFFFFFFFFFFFFFFFFFFFFFFFFFFFFFFFFFFFFFFFFFFFFFFFFFFFFFFFFF:FFFFFFF
FFFFFFFFFFFFFFF
@A00155:342:HHGFNDSXY:1:2144:18032:3161 2:N:0:GAACCTAG+TCCGCATA
AAATACAATCACATTGTATTTCAGAACTTCTTGTGTCCGCACATAGAAGGTTAAAGATGGCTTTCTCT
TATAGGACGCCACAGGAGGAACTCTTGAGCAGGCTCCCACAGTCTCAACAGGAGGTTATAAGCGGATT
TCAGTATGAACGCAT
+
FFFFFFFFFFFFFFFFFFFFFFFFFFFFFFFFFFFFFFFFFFFFFFFFFF:FFFFFFF:FFFFFFFFF
FFFF:FFFFFFFFFFFFFFFFFFFFFFFFFFFFFFFFFFFFFFFFFFFFFFFFFFFFFFFFFFFFFFF
FFFFF:FFFFFFFFF
@A00155:342:HHGFNDSXY:1:2654:7401:24674 2:N:0:GAACCTAG+TCCGCATA
AATACAATCACATTGTATTTCAGAACTTCTTGTGTCCGCACATAGAAGGTTAAAGATGGCTTTCTCTT
ATAGGACGCCACAGGAGGAACTCTTGAGCAGGCTCCCACAGTCTCAACAGGAGGTTATAAGCGGATTT
CAGTATGAACGCAT
+
:FFFF::FFFFFF,FFF:FFFFFFFFFFFFFFFFFFFFFFFFFFFFFFFFFFFFFFFFFFFFFFFFFF
FFFFFFFFFFFFFFFFFFFF:FFFFFFFFFFFFFFFFFFFFFFFFFFFFFFFF,FF::FFF:FFFFFFFF
FFFFFFFFFFFFFF
@A00155:342:HHGFNDSXY:1:1141:2663:6480 2:N:0:GAACCTAG+TCCGCATA
AATACAATCACATTGTATTTCAGAACTTCTTGTGTCCGCACATAGAAGGTTAAAGATGGCTTTCTCTT
ATAGGACGCCACAGGAGGAACTCTTGAGCAGGCTCCCACAGTCTCAACAGGAGGTTATAAGCGGATTT
CAGTATGAACGCATT
+
FFFFFFFFFFFFFFFFFF:FFFFFFFFFFFFFFFFFFFFFFFFFFFFFFFFFFFFF,FFFFFFFFFFFFFF
FFFFFFFFFFFFFFFFFFFFFFFFFFFFFFFF:FFFFFFFFFFFFFFFFFFFFFFFFFFFFFFFFFFFF
FFFFFFFFFFFFFFF
@A00155:342:HHGFNDSXY:1:2149:22761:22968 2:N:0:GAACCTAG+TCCGCATA
ATTACAATCACATTGTATTTCAGAACTTCTTGTGTCCGCACATAGAAGGTTAAAGATGGCTTTCTCTT
ATAGGACGCCACAGGAGGAACTCTTGAGCAGGCTCCCACAGTCTCAACAGGAGGTTATAAGCGGATTT
CAGTATGAACGAGAT
```

+
FFFFFFFFFFFFFFFFFFFFFFFFFFFFFFFFFFFFFFFFFFFFFFFFFFFFFFFFFFFFFFFFFFFF
FFFFFFF:FFFFFFFFFFFFFFFFFFFFFFFFFFFFFFF:FFFFFFFFFFFFFFFFFFFFFFFFFFFF
FFFFFFFFFFFFFFF
@A00155:342:HHGFNDSXY:1:2545:31910:11381 2:N:0:GAACCTAG+TCCGCATA
AATACAATCACATTGTATTTCAGAACTTCTTGTGTCCGCACATAGAAGGTTAAAGATGGCTTTCTCTT
ATAGGACGCCACAGGAGGAACTCTTGAGCAGGCTCCCACAGTCTCAACAGGAGGTTATAAGCGGATTT
CAGTATGAACGCATT
+
:FFFFFFFFFFFFFFF:FFFFFFFFFFFFFFFFFFFFFFFFFFFFFFFFFFFFFFFFFFFFFFFFFFF
FFFFFFFFFFFFFFFFFFF:FFFFFFFFFFFFFFFFFFFFFFFFFFFFFFFFFFFFFFFFF:FFFFFF
FFFF:FFFF:FFFFF
@A00155:342:HHGFNDSXY:1:2675:21866:6511 2:N:0:GAACCTAG+TCCGCATA
AATACAATCACATTGTATTTCAGAACTTCTTGTGTCCGCACATAGAAGGTTAAAGATGGCTTTCTCTT
ATAGGACGCCACAGGAGGAACTCTTGAGCAGGCTCCCACAGTCTCAACAGGAGGTTATAAGCGGATTT
CAGTATGAACGCATT
+
FFFFFF:FFFFFFFFFFFFFFFFFFFFFFFFFFFFFFFFFFFFF:FFFFFFFFFFFFFFFFFFFFFFF
FFFFFFFFFFFFFFFFFF:FFFFFFFFFFFFFFFFFFFFFFFFFFFFFFFFFFFFF:FFFFFFFFFFF
FFF:FFFFFFFFFFF
@A00155:342:HHGFNDSXY:1:2635:14714:19961 2:N:0:GAACCTAG+TCCGCATA
AATACAATCACATTGTATTTCAGAACTTCTTGTGTCCGCACATAGAAGGTTAAAGATGGCTTTCTCTT
ATAGGACGCCACAGGAGGAACTCTTGAGCAGGCTCCCACAGTCTCAACAGGAGGTTATAAGCGGATTT
CAGTATGAACGCATT
+
F:FFFFFFFFFF:FFFF:FFFFFFFFFFFFFFFFFFFFFFFFFFFFFFFFFFFFFFFFFFFFFFFFFF
FFFFFFFFFFFFFFFFFFF,FFFFFFFFFFFFFFFFFFFFFFFFFFFFFFFFFFFFFFF::FFFFFFF
FFFFFFFFFFFFF,F
@A00155:342:HHGFNDSXY:1:1230:6207:35634 2:N:0:GAACCTAG+TCCGCATA
ATACAATCACATTGTATTTCAGAACTTCTTGTGTCCGCACATAGAAGGTTAAAGATGGCTTTCTCTTA
TAGGACGCCACAGGAGGAACTCTTGAGCAGGCTCCCACAGTCTCAACAGGAGGTTATAAGTGGATTTC
AGTATGAACGCATTC
+
FFFFF,FFFFFFFFFFFFFFFFFFFFFFFFFFFFFFFFFFFFFFFFFFFFFFFFFFFFFFFFFFFFFF
FFFFFFFFFFFFFFFFFF,FFFFFFFFFFFFFFFFFFFFFFFFFFFFFFFFFFFFFFFFFFFF::FFF
FFFFFFFFFFFFFFF
@A00155:342:HHGFNDSXY:1:1340:18322:28244 2:N:0:GAACCTAG+TCCGCATA
ATACAATCACATTGTATTTCAGAACTTCTTGTGTCCGCACATAGAAGGTTAAAGATGGCTTTCTCTTA
TAGGACGCCACAGGAGGAACTCTTGAGCAGGCTCCCACAGTCTCAACAGGAGGTTATAAGTGGATTTC
AGTATGAACGCATTC
+
FFFFFFFFFFF:FF:FFFFFFFFFFFFFFFFFFFFFFFFFFFFFFFFFFFFFFFFFF:FFFFFFFFF:
FFFFFFFFFFFFFFFFFF:FFFFFFFFFFFFFFFF:FFFFFFFFFFFF,FFFFFFFFF::FFF:FFFF
FF:FFFFFFFFFFFF
@A00155:342:HHGFNDSXY:1:1508:25138:14716 2:N:0:GAACCTAG+TCCGCATA
ATACAATCACATTGTATTTCAGAACTTCTTGTGTCCGCACATAGAAGGTTAAAGATGGCTTTCTCTTA
TAGGACGCCACAGGAGGAACTCTTGAGCAGGCTCCCACAGTCTCAACAGGAGGTTATAAGTGGATTTC
AGTATGAACGCATTC
+
FF:FFFFFFFFFFFFFFFFFFFFFFFFFFFF,FFFFFFFFFFFFFFFF:FFFFFFFFFFFFFFFFFFF
FFFFFFFFFFFFFFFFFFFFFFF:FFFFF:FFFFFFFFFFFFFFFFFFFFFFF:FFFFFFFFFFF,FF
FFF::FFFFFFFFFF
@A00155:342:HHGFNDSXY:1:2247:11541:8140 2:N:0:GAACCTAG+TCCGCATA
ATACAATCACATTGTATTTCAGAACTTCTTGTGTCCGCACATAGAAGGTTAAAGATGGCTTTCTCTTA

```
TAGGACGCCACAGGAGGAACTCTTGAGCAGGCTCCCACAGTCTCAACAGGAGGTTATAAGCGGATTTC
AGTATGAACGCATTC
+
FFFFFFFFFFFFFFFFFFFFFFFFFFFFFFFFFFFFFFFFFFFFFFFFFFFFFFFFFFFFFFFFFFFF
FFFFFFFFFFFFFFFFFFFFFFFFFFFFFFFFFFFFFFFFFFFFFFFFFFFFFFFFFFFFFFFFFFFF
FFFFFFFFFFFFFFF
@A00155:342:HHGFNDSXY:1:2247:8983:9721 2:N:0:GAACCTAG+TCCGCATA
ATACAATCACATTGTATTTCAGAACTTCTTGTGTCCGCACATAGAAGGTTAAAGATGGCTTTCTCTTA
TAGGACGCCACAGGAGGAACTCTTGAGCAGGCTCCCACAGTCTCAACAGGAGGTTATAAGCGGATTTC
AGTATGAACGCATTC
+
FFFFFFFFF,FFFFFFFFFFFFFFFFFFFFFFFFFFFFFFFF:FFFFFFFFFFFFFFFFFFFFFFFFFF
FFFFFF:FFFFFFFFFFFFFFFFFFFFFFFFFFFFFFFFFFFF:FFFFFF:FFFFFFFFFFFFFFFF
FFFFFF:FFFFFFFF
@A00155:342:HHGFNDSXY:1:2362:18873:31140 2:N:0:GAACCTAG+TCCGCATA
ATACAATCACATTGTATTTCAGAACTTCTTGTGTCCGCACATAGAAGGTTAAAGATGGCTTTCTCTTA
TAGGACGCCACAGGAGGAACTCTTGAGCAGGCTCCCACAGTCTCAACAGGAGGTTATAAGCGGATTTC
AGTATGAACGCATTC
+
FFFFFFFFFFFFFFFFFFFFFFFFFFFFFFFFFFFFFFFFFFFFFFFFFFFFFFFFFFFFFFFFFFFF
FFFFFFFFFFFFFFFFFF,FFFFFFFFFFFFFFFFFFFFFFFFFFFFFFFFFFFFFFFFFFFFFFFFF
FFFFFFFFFF:FFFF
@A00155:342:HHGFNDSXY:1:1440:7247:7623 1:N:0:GAACCTAG+TCCGCATA
CAATCACATTGTATTTCAGAACTTCTTGTGTCCGCACATAGAAGGTTAAAGATGGCTTTCTCTTATAG
GACGCCACAGGAGGAACTCTTGAGCAGGCTCCCACAGTCTCAACAGGAGGTTATAAGCGGATTTCAGT
ATGAACGCATTCAAA
+
FFFFFFFFFFFFFFFFFFFFFFF:FFF,:FFFFFFFFFFFFFFFF:FFFFFF::FFFFFFFF:FFFFF
:FFFFFFFFFFF:FFF:FF:FFFFFFFFFFFFFFFFFFFFFFF:FFFFFFFFFFFFFFFFFFFFFFFFF
FFFF:FFFFFFFFFF
@A00155:342:HHGFNDSXY:1:1109:7581:29277 2:N:0:GAACCTAG+TCCGCATA
CAATCACATTGTATTTCAGAACTTCTTGTGTCCGCACATAGAAGGTTAAAGATGGCTTTCTCTTATAG
GACGCCACAGGAGGAACTCTTGAGCAGGCTCCCACAGTCTCAACAGGAGGTTATAAGCGGATTTCAGT
ATGAACAGATCGGAA
+
FFFFFFFFFFFFFFFFFFFFFFFFFFFFFFFFFFFFFFFF,FFFFFFFFFFFFFFFFFFFFFFFFFFF
FFFFF:FFFFFFFFFFFFFFFFFFFFFFFFFF:FFFFFFFFFFFFFFFF:FFFFFFFFFFFFFFFFFFF
FFFFFFFFFFFF:F:
@A00155:342:HHGFNDSXY:1:1140:29080:26021 2:N:0:GAACCTAG+TCCGCATA
CAATCACATTGTATTTCAGAACTTCTTGTGTCCGCACAGAGAAGGTTAAAGATGGCTTTCTCTTATAG
GACGCCACAGGAGGAACTCTTGAGCAGGCTCCCACATTCTCAACAGGAGTTTATAATCGGATTTCAGT
ATGAACGCATTCAAA
+
FFF:FFFFFFFF:::FFF:FF,,FFFFFF,FFFFFFFF,F:FF,:,FFFF:FF,FFFF:F,:FFFFF:
:FF,:F:FFF:F:FF,F:FF,FFFF:FF:FFF::,F,,FFFFFFF::FF,FFFFFF,::,F:FFFFF:
FFFFF:,,F,FFFFF
@A00155:342:HHGFNDSXY:1:1162:8684:22921 2:N:0:GAACCTAG+TCCGCATA
CAATCACATTGTATTTCAGAACTTCTTGTGTCCGCACATAGAAGGTTAAAGATGGCTTTCTCTTATAG
GACGCCACAGGAGGAACTCTTGAGCAGGCTCCCACAGTCTCAACAGGAGGTTATAAGCGGATTTCAGT
ATGAACGCATAGATC
+
FFFFFFFF:FFFFFFFFFFFFFFFFFFFFFFFFFFFFFFFFFFFFFFFFFFFFFFFFFFFFFFFFFFF
FFFFFFFFFFFFFFFFFFFFFFFFFFFFFFFFFFFFFFFFFFFFFFFFFF:FFFFFFFFFFFFFFFFF
FFFFFFFFFFFFFFF
```

@A00155:342:HHGFNDSXY:1:1432:25211:34882 2:N:0:GAACCTAG+TCCGCATA
CAATCACATTGTATTTCAGAACTTCTTGTGTCCGCACATAGAAGGTTAAAGATGGCTTTCTCTTATAG
GACGCCACAGGAGGAACTCTTGAGCAGGCTCCCACAGTCTCAACAGGAGGTTATAAGCGGATTTCAGT
ATGAACGCATTCAAA
+
FF:FFF:F,FF:FFFFFFFFFFFFFFFFF:FFFFFFFFFF:FFFFFFF:FFFFFF::FFF::FFFFFFF:
:F:FFFFFFFFFF::FFFFF:FFFFFFFFFFFFFFFFFF:FF:F::FFFFFFFF,,FF:FFF:FFFFFFF
:F:FF::FFF:FFFF
@A00155:342:HHGFNDSXY:1:1421:4824:23813 2:N:0:GAACCTAG+TCCGCATA
CAATCACATTGTATTTCAGAACTTCTTGTGTCCGCACATAGAAGGTTAAAGATGGCTTTCTCTTATAG
GACGCCACAGGAGGAACTCTTGAGCAGGCTCCCACAGTCTCAACAGGAGGTTATAAGCGGATTTCAGT
ATGAACGCATTCAAA
+
FFFFFFF,FFFFFFFFFFFFFFFFFFFFFFFFFFF:FFFFFF:FFFF:FFFFFFFFFFFFFFFFFFF:FF
FFF:FFFFFFFFFFFF:FFFFFFFFF:FFFFFFFFFFFFFFFFFFFFFFFFFFFFFFFFFFFFFFFFFFF
FFFFFFFFF:FFFFF
@A00155:342:HHGFNDSXY:1:2206:4246:23813 2:N:0:GAACCTAG+TCCGCATA
CAATCACATTGTATTTCAGAACTTCTTGTGTCCGCACATAGAAGGTTAAAGATGGCTTTCTCTTATAG
GACGCCACAGGAGGAACTCTTGAGCAGGCTCCCACAGTCTCAACAGGAGGTTATAAGCGGATTTCAGT
ATGAACGCATTCAAA
+
F,FFFFFF:FFFFFFFFFFFFFFFFFFFFFFFFFFFFFFFFFFFFFFFFFFFFFFFFFFFFFFFFFFFFF
FFFFF:FFFFFFFFFFFFFFF:FFFFFFFFFFFFFFFFFFFFF:FFFFFFF:FF:FFFFFFFFFFFFFFF
FFFFFFFFFFFFFFF
@A00155:342:HHGFNDSXY:1:2221:7627:29794 2:N:0:GAACCTAG+TCCGCATA
CAATCACATTGTATTTCAGAACTTCTTGTGTCCGCACATAGAAGGTTAAAGATGGCTTTCTCTTATAG
GACGCCACAGGAGGAACTCTTGAGCAGGCTCCCACAGTCTCAACAGGAGGTTCTAAGCGGATTTCAGT
ATGAAAGCATTCAAA
+
:FFFFFFFFFFFFFFFFFFFFFFFFFFFFFFFFFF:FFFF:F:FFF,,:FFFFFF:FFFFFFFFFFFF:F
FFFFFFFFFFF:,FFFFFFFFF:FFF,FFFFFFF:F:FFFFF:FFFFFFFFFFF,FFFFFF:FFFFFFFF
FFFFF,FFFFF:FF:
@A00155:342:HHGFNDSXY:1:2246:23213:14888 2:N:0:GAACCTAG+TCCGCATA
CAATCACATTGTATTTCAGAACTTCTTGTGTCCGCACATAGAAGGTTAAAGATGGCTTTCTCTTATAG
GACGCCACAGGAGGAACTCTTGAGCAGGCTCCCACAGTCTCAACAGGAGGTTATAAGCGGATTTCAGT
ATGAACAGATCGGAA
+
FFFFFFFFFFFFFFFFFFFFFFFFFFFFFFFFFFFFFFFFFFFFFFFFFFFFFFFFFFFFFFFFFFFFFF
FFFFFFFFFFFFFFFFFFFFFFFFFFFFFFFFFFFFFFFFFFFFFFFFFFF:FFFFFFFFFFFFFFFFFF
FF:FFFFFFFFFFFF
@A00155:342:HHGFNDSXY:1:2607:25726:35149 2:N:0:GAACCTAG+TCCGCATA
CAATCACATTGTATTTCAGAACTTCTTGTGTCCGCACATAGAAGGTTAAAGATGGCTTTCTCTTATAG
GACGCCACAGGAGGAACTCTTGAGCAGGCTCCCACAGTCTCAACAGGAGGTTATAAGCGGATTTCAGT
ATGAACGCATTCAAA
+
FFFFFFFFFFFFFFFFFFFFFFFFFFFFFFFF:FFFFFFFFFFFFFFFFFFFFFFFFFFFFFFFFFFFFF
FFFFFFFFFFFFFFFFFFFFF:FFFFFFFFFFFFFFFFFFFF:FFFFFFFFFFFFFFFFFFFFFFFFFFF
FFFFFFFFFFFFFFF
@A00155:342:HHGFNDSXY:1:2644:22055:14199 2:N:0:GAACCTAG+TCCGCATA
CAATCACATTGTATTTCAGAACTTCTTGTGTCCGCACATAGAAGGTTAAAGATGGCTTTCTCTTATAG
GACGCCACAGGAGGAACTCTTGAGCAGGCTCCCACAGTCTCAACAGGAGGTTATAAGCGGATTTCAGT
ATGAACGCATTCAAA
+
FF,FFFFF,FFFFFFFFFFFFFFFFFFFFFFFFFFFFFFFFFFFFFFFFFF:FFFFFFFFFFFFFFFFFF

FFFFFFFFFF:FFFF:FFFFFFFFFFFFFF:FFFFFFFFFFFFFFFFFFFFFFFFFFFFFFFFFFFFF
FFFFFFFFFFFFFFF
@A00155:342:HHGFNDSXY:1:1622:6623:17253 2:N:0:GAACCTAG+TCCGCATA
AATCACATTGTATTTCAGAACTTCTTGTGTCCGCACATAGAAGGTTAAAGATGGCTTTCTCTTATAGG
ACGCCACAGGAGGAACTCTTGAGCAGGCTCCCACAGTCTCAACAGGAGGTTATAAGCGGATTTCAGTA
TGAACGCATTCAAAA
+
FFFFFFFFFFFFFFFFFFFFFFFFFFFFFFFFFFFFFFFFFFFFFFFFFFFFFFFFFFFFFFFFFFFF
FFFFFFFFFFFFFFFFFFFFFFFFFFFFFFFFFFFFFFFFFFFFFFFFFFFFFFFFFFFFFFFFFFFF
FFFFFFFFFFFFFFF
@A00155:342:HHGFNDSXY:1:2543:2302:6793 2:N:0:GAACCTAG+TCCGCATA
AATCACATTGTATTTCAGAACTTCTTGTGTCCGCACATAGAAGGTTAAAGATGGCTTTCTCTTATAGG
ACGCCACAGGAGGAACTCTTGAGCAGGCTCCCACAGTCTCAACAGGAGGTTATAAGCGGATTTCAGTA
TGAACGCATTCAAAA
+
FFFFF:FFFFFFFFFFFF:FFFFFFFFFFFFFFFFFFFFFFFFFFFFF::FFFF:FFFFFFFFFFFFF
FFFFFFFFFFFFFF:FFFFFFFFF:F,FFFFFFFFFFFFF:FFFF:FFFFFFFFF:FFFFFFFFFFFF
FFFF,:FFFFFFFF:
@A00155:342:HHGFNDSXY:1:2543:2220:6840 2:N:0:GAACCTAG+TCCGCATA
AATCACATTGTATTTCAGAACTTCTTGTGTCCGCACATAGAAGGTTAAAGATGGCTTTCTCTTATAGG
ACGCCACAGGAGGAACTCTTGAGCAGGCTCCCACAGTCTCAACAGGAGGTTATAAGCGGATTTCAGTA
TGAACGCATTCAAAA
+
FFFFFFFFFFFFFFFFFF:FFFFFFFFFFFFFFFFFFFFFFFFFFFF:,F:FFFFFFFFFFFFFFFFF
FFFFFFF:FFFFFFFFFFFFFFFFFFFFFFFFFFFFFFFFFFFFFFFF:FFFFFF:FFFFFFF:FFFF
F,FFFF,FFF,F:FF
@A00155:342:HHGFNDSXY:1:1247:25907:17989 1:N:0:GAACCTAG+TCCGCATA
ATCACATTGTATTTCAGAACTTCTTGTGTCCGCACATAGAAGGTTAAAGATGGCTTTCTCTTATAGGA
CGCCACAGGAGGAACTCTTGAGCAGGCTCCCACAGTCTCAACAGGAGGTTATAAGCGGATTTCAGTAT
GAACGCATTCAAAAG
+
FFFFFFFFFFFFFFFFFFFFFFFFFFFFFFFFFFFFFFFFFF:FFFFFFFFFFFFFFFFFFFFFFFFF
FF:FFFFFFFFFFFFFFFFFFFFFF:FF:FFFFFFFFF:FFFFF:FFFFFFFFFFFFFFFFFFFFFFF
FFFFFFFFFFFFFFF
@A00155:342:HHGFNDSXY:1:1312:7916:15859 1:N:0:GAACCTAG+TCCGCATA
ATCACATTGTATTTCAGAACTTCTTGTGTCCGCACATAGAAGGTTAAAGATGGCTTTCTCTTATAGGA
CGCCACAGGAGGAACTCTTGAGCAGGCTCCCACAGTCTCAACAGGAGGTTATAAGCGGATTTCAGTAT
GAACGCATTCAAAAG
+
FFFFF:FFFFFFFFFFFFFFFFFFFFFFFF:FFFFFFFFFFFFFFFFFFFFFFFFFFFFFFFFFFFFF
FFFFFFFFFFFFFFFFFFFFFFFFFFFFFFFFFFFFFFFFFFFFFFFFFFFFF,FFFFFFFFFFFFFF
FFFFFFFFFFFFFFF
@A00155:342:HHGFNDSXY:1:1344:18240:24001 1:N:0:GAACCTAG+TCCGCATA
GATCTATTGTATTTCAGAACTTCTTGTGTCCGCACATAGAAGGTTAAAGATGGCTTTCTCTTATAGGA
CGCCACAGGAGGAACTCTTGAGCAGGCTCCCACAGTCTCAACAGGAGGTTATAAGCGGATTTCAGTAT
GAACGCATTCAAAAG
+
FFFFFF:FFFFFFFFFFFFFFFFFFFFFFFFFFFFFFFFFFFFFFFFFFFFFFFFFFFFFFFFFFFFF
FFFFFFFFFFFFFFFFFFFFFFFFFFFFFFFFFFFFFFFF:FFFFFFFFFFFFFFFFFFFFFFFFFFF
FFFFFFFFFFFFFFF
@A00155:342:HHGFNDSXY:1:1378:28257:23813 1:N:0:GAACCTAG+TCCGCATA
ATCACATTGTATTTCAGAACTTCTTGTGTCCGCACATAGAAGGTTAAAGATGGCTTTCTCTTATAGGA
CGCCACAGGAGGAACTCTTGAGCAGGCTCCCACAGTCTCAACAGGAGGTTATAAGCGGATTTCAGTAT
GAACGCATTCAAAAG

```
+
FFF:FFFFFFFFFFFFFFFFFFFFFFFFFFFFFFFFFFFFFFFFFFFFFFFFFFFFFFFFFFFFFFFF
FFFFFFFFFFFFFFFFFFFFFFFFFFFFFFFFFFFFFFFFFFFFFFFFFFFFFFFFFFFFFFFFFFFF
FFFFFFFFFFFFFFF
@A00155:342:HHGFNDSXY:1:1451:7762:8390 1:N:0:GAACCTAG+TCCGCATA
ATCACATTGTATTTCAGAACTTCTTGTGTCCGCACATAGAAGGTTAAAGATGGCTTTCTCTTATAGGA
CGCCACAGGAGGAACTCTTGAGCAGGCTCCCACAGTCTCAACAGGAGGTTATAAGCGGATTTCAGTAT
GAACGCATTCAAAAG
+
FFFFFFFFFFF:FFFFFFFFFFFFFFFFFFFFFFFFFFFFFFFFFFFFFF:FFFFFFFFFFFFFFFF:
FFFFFFFFFFFFFFF:FFFFFFFFFFFFFFFFFFFFFFFFFFFFFFFFFFFFFFFFFFFFFFFFFFFF
FFFFFFFFFFFFFFF
@A00155:342:HHGFNDSXY:1:1470:9679:16939 1:N:0:GAACCTAG+TCCGCATA
ATCACATTGTATTTCAGAACTTCTTGTGTCCGCACATAGAAGGTTAAAGATGGCTTTCTCTTATAGGA
CGCCACAGGAGGAACTCTTGAGCAGGCTCCCACAGTCTCAACAGGAGGTTATAAGCGGATTTCAGTAT
GAACGCATTCAAAAG
+
FFFFFFFFFFFFFFFFFFFFFF:FFFFFFF:FFFFFFFFFFFFFFFFFFFF:FFFFF:FFFFFFFFFF
FFFFFFFFFFFFF:FFFFFFFFFFFFFF:FFFFFFFFFFFFFFFFFFFFFFFFFFFFFFFFFFFFFFF
FFFFFFFFFFFFFFF
@A00155:342:HHGFNDSXY:1:1552:11849:36949 1:N:0:GAACCTAG+TCCGCATA
ATCACATTGTATTTCAGAACTTCTTGTGTCCGCACATAGAAGGTTAAAGATGGCTTTCTCTTATAGGA
CGCCACAGGAGGAACTCTTGAGCAGGCTCCCACAGTCTCAACAGGAGGTTATAAGTGGATTTCAGTAT
GAACGCATTCAAAAG
+
F:FFFFFFFFFFFFFFFFFFFFFFFFFFFFFFFFFFFFFFFFFFFFFFFFFFFFFFFFFFFFFFFFFF
FFFFFFFFFFFFFFFFFFFFFFFFFFFFFFFFFFFFFFFFFFFFFFFF:FFFFFFFFF:FFFFFFFFF
FFFFFFFFFFFFFFF
@A00155:342:HHGFNDSXY:1:1572:12093:32424 1:N:0:GAACCTAG+TCCGCATA
ATCACATTGTATTTCAGAACTTCTTGTGTCCGCACATAGAAGGTTAAAGATGGCTTTCTCTTATAGGA
CGCCACAGGAGGAACTCTTGAGCAGGCTCCCACAGTCTCAACAGGAGGTTATAAGCGGATTTCAGTAT
GAACGCATTCAAAAG
+
FFFFF:FFFFFFF:FFFFFFFFFFFFFFFFFFFFFFFFFFFFF,FFFFFFFFFFFFFFFFFFFFFFFF
FFFFFFFFFFFFFFFFFFFFFFFFFFFFFFFFF:FFFFF:FFFFFFFFFFFFFFFFFFFFFFFFFFFF
FFFFFFFFFFFFFFF
@A00155:342:HHGFNDSXY:1:1632:12915:29121 1:N:0:GAACCTAG+TCCGCATA
ATCACATTGTATTTCAGAACTTCTTGTGTCCGCACATAGAAGGTTAAAGATGGCTTTCTCTTATAGGA
CGCCACAGGAGGAACTCTTGAGCAGGCTCCCACAGTCTCAACAGGAGGTTATAAGCGGATTTCAGTAT
GAACGCATTCAAAAG
+
FFF:FFFFFFFFFFFFFFFFFFFFFF:FFFFFFFFFFFFFFFFFFFF,FFFFFFFFFFFFFFFFFFFF
FFFFFFFFFFFFFFFFFFFFFFFFFFFFFFFFFFFFFFFFFFFFFFFFFFFFFFFFFFFFFFFFFFFF
FFFFFFFFFFFFFFF
@A00155:342:HHGFNDSXY:1:1632:12500:29434 1:N:0:GAACCTAG+TCCGCATA
ATCACATTGTATTTCAGAACTTCTTGTGTCCGCACATAGAAGGTTAAAGATGGCTTTCTCTTATAGGA
CGCCACAGGAGGAACTCTTGAGCAGGCTCCCACAGTCTCAACAGGAGGTTATAAGCGGATTTCAGTAT
GAACGCATTCAAAAG
+
:FFFFFFFFFFFFFFFFFFFFFFFFFFFFFFFFFFFFFFFFFFFFFFFFFFFFFFFFFFFFFFFFFFF
FFFFFFFFFFFFFFFFFFFFFFFFFFFFF:FFFFFFFFF:FFFFFFFFFFFFFFFFFFFFFFFFFFFF
FFFFFFFFFFFFFFF
@A00155:342:HHGFNDSXY:1:2112:6650:7185 1:N:0:GAACCTAG+TCCGCATA
ATCACATTGTATTTCAGAACTTCTTGTGTCCGCACATAGAAGGTTAAAGATGGCTTTCTCTTATAGGA
```

```
CGCCACAGGAGGAACTCTTGAGCAGGCTCCCACAGTCTCAACAGGAGGTTATAAGCGGATTTCAGTAT
GAACGCATTCAAAAG
+
FFFF:::FFFFFFFFFFFFFFFFFFFFFFFFFFFFFFFFFFFFFFFFFFFFFFFFFFFFFFFFFFFFF
FFFFFFFFFFFFFFFFFFFFFFFFFFFFFFFFFFFFFFFFFFFFFFFFFFFFF:FFFFFFFFFFFFFF
FFFFFFFFFFFFFFF
@A00155:342:HHGFNDSXY:1:2103:31647:18443 1:N:0:GAACCTAG+TCCGCATA
ATCACATTGTATTTCAGAACTTCTTGTGTCCGCACATAGAAGGTTAAAGATGGCTTTCTCTTATAGGA
CGCCACAGGAGGAACTCTTGAGCAGGCTCCCACAGTCTCAACAGGAGGTTATAAGCGGATTTCAGTAT
GAACGCATTCAAAAG
+
:FF:F:,FFFFFFFFFFFFFFFFFFF:FFFFFFFFFFFFFFFFFFFFFFFFFFFFFFFFFFFFFFFFF
FFFFFFFFFFFF:FFFFFFFFFFFFFFFFFFFFFFFFFFFFFFFFFFFFFFFFFFFFFFFFFFFFFFF
FFFFFFFFFFFFFFF
@A00155:342:HHGFNDSXY:1:2236:22309:1924 1:N:0:GAACCTAG+TCCGCATA
ATCACATTGTATTTCAGAACTTCTTGTGTCCGCACATAGAAGGTTAAAGATGGCTTTCTCTTATAGGA
CGCCACAGGAGGAACTCTTGAGCAGGCTCCCACAGTCTCAACAGGAGGTTATAAGCGGATTTCAGTAT
GAACGCATTCAAAAG
+
FFFFFFFFFFFFFFFFFFFFFFFFFFFFFFFFFFFFFFFFFFFFFFFFFF,FFFFFFFFFFFFFFFFFF
FFFFFFFFFFFFFFFFFFFFFFFFFFFFFFFFFFFFFFFFFFFFFFFFFFFFFFFFFFFFFFFFFFFFF
FFFFFFFFFFFFFFF
@A00155:342:HHGFNDSXY:1:2266:29686:1454 1:N:0:GAACCTAG+TCCGCATA
ATCACATTGTATTTCAGAACTTCTTGTGTCCGCACATAGAAGGTTAAAGATGGCTTTCTCTTATAGGA
CGCCACAGGAGGAACTCTTGAGCAGGCTCCCACAGTCTCAACAGGAGGTTATAAGTGGATTTCAGTAT
GAACGCATTCAAAAG
+
FFFFFFFFFFFFFFFFFFFFFFFFFFFFFFFFFFFFFFFFFFFFFFFFFFFFFFFFFFFFFFFFFFFF
FFFFFFFFFFFFFFFFFFFFFFFFFFFFFFFFFFFFFFFFFFFFFFFFFFFFF:FFFFFFFFFFFFFF
FFFFFFFFFFFFFFF
@A00155:342:HHGFNDSXY:1:2247:11541:8140 1:N:0:GAACCTAG+TCCGCATA
ATCACATTGTATTTCAGAACTTCTTGTGTCCGCACATAGAAGGTTAAAGATGGCTTTCTCTTATAGGA
CGCCACAGGAGGAACTCTTGAGCAGGCTCCCACAGTCTCAACAGGAGGTTATAAGCGGATTTCAGTAT
GAACGCATTCAAAAG
+
FFFFFFFFFFFFFFFFFFFFFFFFFFFFFFFFFFFFFFFFFFFFFFFFFFFFFFFFFFFFFFFFFFFF
FFFFFFFFFFFFFFFFFFFFFFFFFFFFFFFFFFFFFFFFFFFFFFFFFFFFFFFFFFFFFFFFFFFF
FFFFFFFFFFFFFFF
@A00155:342:HHGFNDSXY:1:2247:8983:9721 1:N:0:GAACCTAG+TCCGCATA
ATCACATTGTATTTCAGAACTTCTTGTGTCCGCACATAGAAGGTTAAAGATGGCTTTCTCTTATAGGA
CGCCACAGGAGGAACTCTTGAGCAGGCTCCCACAGTCTCAACAGGAGGTTATAAGCGGATTTCAGTAT
GAACGCATTCAAAAG
+
FFFFFF:FFFFFFFFFFFF:FFFFFFFFFFFFFFFFFFF:FFFFFFFFFFFFFFFFFFFFFFFFFFFF
FFFFFFFFFFFFFFFFFFFFFFFFFFFFFFFFFF:FFFFFFFFFFFFFFFFFFFFFFFFFFFFFFFFF
FFFFFFFFFFFFFFF
@A00155:342:HHGFNDSXY:1:2314:13331:30749 1:N:0:GAACCTAG+TCCGCATA
ATCACATTGTATTTCAGAACTTCTTGTGTCCGCACATAGAAGGTTAAAGATGGCTTTCTCTTATAGGA
CGCCACAGGAGGAACTCTTGAGCAGGCTCCCACAGTCTCAACAGGAGGTTATAAGCGGATTTCAGTAT
GAACGCATTCAAAAG
+
::FF:FFFFFFFFFFFF:FFFF:FFFFFFFFF,F,FFFFFF:FFFFFFFF,FFFFFFFFFFFFFFF:F
FFFFFFFFFFFFFF:FFFF::FFFF:FFFFFFFFFFFFFF,FFFFFFFFFFFFFFFFFFFFFFFFF,FF
FFFFFFFFFFFFFFF
```

```
@A00155:342:HHGFNDSXY:1:2451:6045:22482 1:N:0:GAACCTAG+TCCGCATA
ATCACATTGTATTTCAGAACTTCTTGTGTCCGCACATAGAAGGTTAAAGATGGCTTTCTCTTATAGGA
CGCCACAGGAGGAACTCTTGAGCAGGCTCCCACAGTCTCAACAGGAGGTTATAAGCGGATTTCAGTAT
GAACGCATTCAAAAG
+
FFFFFF:FFFFFFFFFFFFF,F:FFFFFFFFFFF:FFF:FFFFFF:FFFF:FFFF:FFFFFFFFF,FF
F:FFFFFFFFFFFFF:FFFFFF,:FFFFFFFF:FFFFFFF:FFFFFFFFFFFFF:FFFFFF:FFFFF
FFFFFFFFFFF,FFF
@A00155:342:HHGFNDSXY:1:2428:16911:30843 1:N:0:GAACCTAG+TCCGCATA
ATCACATTGTATTTCAGAACTTCTTGTGTCCGCACATAGAAGGTTAAAGATGGCTTTCTCTTATAGGA
CGCCACAGGAGGAACTCTTGAGCAGGCTCCCACAGTCTCAACAGGAGGTTATAAGCGGATTTCAGTAT
GAACGCATTCAAAAG
+
FFFF::FFFFFFFFFFFFFFFFFFFFFFFFFFFFFFFFFFFFFFFFFFFFFFFFFFFFFFFFFFFFFF
FFFFFFFFFFFFFFFFFFFFFFFFFFFFFFFFFFFFFFFFFFFFFFFFFFFFFFFFFFFFFFFFFFFF
FFFFFFFFFFFFFFF
@A00155:342:HHGFNDSXY:1:2468:14516:13542 1:N:0:GAACCTAG+TCCGCATA
ATCACATTGTATTTCAGAACTTCTTGTGTCCGCACATAGAAGGTTAAAGATGGCTTTCTCTTATAGGA
CGCCACAGGAGGAACTCTTGAGCAGGCTCCCACAGTCTCAACAGGAGGTTATAAGCGGATTTCAGTAT
GAACGCATTCAAAAG
+
:FFFFF,FFFFFFFFFFFFF:FFF,FFFFFFFFFFFFFFFFFFFFFF:FF:FFFFFFFFFFFFFFFF:
FFFFFFFFFFFFFFFFFFFFFFFFFFFFFFFFFFFFFFFFFFFFFFFFFFFFFFFFFFFFFFFFFFFF
FFFFFFFFFFFFFFF
@A00155:342:HHGFNDSXY:1:2526:1542:9204 1:N:0:GAACCTAG+TCCGCATA
GATCTATTGTATTTCAGAACTTCTTGTGTCCGCACATAGAAGGTTAAAGATGGCTTTCTCTTATAGGA
CGCCACAGGAGGAACTCTTGAGCAGGCTCCCACAGTCTCAACAGGAGGTTATAAGCGGATTTCAGTAT
GAACGCATTCAAAAG
+
FFFFFFFF:FFFFFFF:FFFFFFFFFFFFFFFFFFFFF:FFFF:FFFFFFFFFFFFFFFFFFFFFFFF
FFFFFFFFFFF:FFFFFFFFFFFFFFFFFFFFFFF::FFFFFFFFFFFFFFFFFFFFFFFFFFFFFFF
FFFFFFFFFFFFFFF
@A00155:342:HHGFNDSXY:1:2530:5041:13761 1:N:0:GAACCTAG+TCCGCATA
TCCGATCTGTATTTCAGAACTTCTTGTGTCCGCACATAGAAGGTTAAAGATGGCTTTCTCTTATAGGA
CGCCACAGGAGGAACTCTTGAGCAGGCTCCCACAGTCTCAACAGGAGGTTATAAGCGGATTTCAGTAT
GAACGCATTCAAAAG
+
FFFFFFFFFFFF:FFFFFFFFFFFFFFFFFFFFFFFFFFFFFFFFFFFFFFF,FFFFFFFFFFFFFFFF
FFFFFFFFFFFFFFFFFFFFFFFFFFFFFFFFFFFFFFFFFFFFFFFFFFFFFFFFFFFFFFFFFFFF
FFFFFFFFFFFFFFF
@A00155:342:HHGFNDSXY:1:2607:25726:35149 1:N:0:GAACCTAG+TCCGCATA
ATCACATTGTATTTCAGAACTTCTTGTGTCCGCACATAGAAGGTTAAAGATGGCTTTCTCTTATAGGA
CGCCACAGGAGGAACTCTTGAGCAGGCTCCCACAGTCTCAACAGGAGGTTATAAGCGGATTTCAGTAT
GAACGCATTCAAAAG
+
FFFFFFFFFFFFFFFFFF:FFFFFFF:FFFFFF:FFFFFFFFFFFFFFFFFFFFFFFFFFFFFFFFFF
FFFFFFFFFFFFFFFFFFFFFFFFFFFFFFFFFFFFFFFFFFFFFFFFFFFFFFFFFFFFFFFFFFFF
FFFFFFFFFFFFFFF
@A00155:342:HHGFNDSXY:1:2635:14714:19961 1:N:0:GAACCTAG+TCCGCATA
ATCACATTGTATTTCAGAACTTCTTGTGTCCGCACATAGAAGGTTAAAGATGGCTTTCTCTTATAGGA
CGCCACAGGAGGAACTCTTGAGCAGGCTCCCACAGTCTCAACAGGAGGTTATAAGCGGATTTCAGTAT
GAACGCATTCAAAAG
+
FFFFFFFFFFFFFFFFFFFFFFFFFFFFFFFFFFFFFFFFFFFFFFFFFFFFFFFFFFFFFFFFFFF:
```

FFFFFFFFFFFFFFF:FFFFFFFFFFFFFFFFFFFFFFF,FFFFFFFFFFFFFFFFFFFFFFFFFFFF
FFFFFFFFFFFFFFF
@A00155:342:HHGFNDSXY:1:2644:22055:14199 1:N:0:GAACCTAG+TCCGCATA
ATCACATTGTATTTCAGAACTTCTTGTGTCCGCACATAGAAGGTTAAAGATGGCTTTCTCTTATAGGA
CGCCACAGGAGGAACTCTTGAGCAGGCTCCCACAGTCTCAACAGGAGGTTATAAGCGGATTTCAGTAT
GAACGCATTCAAAAG
+
:FFFFFFFFFF:FFFFFFFFFFFFFFFFFFFFFFFFFFFFFFFFFFFFFFFFFFFFFFFFFFFFFFFF
FFFFFFFFFFFFFFFFFFFFFFFFFFFFFFFFFFFFFFFFFFFFFFFFFFFFF,FFFFFFFFFFFFFF
FFFFFFFFFFFFFFF
@A00155:342:HHGFNDSXY:1:1130:2763:15734 2:N:0:GAACCTAG+TCCGCATA
ATCACATTGTATTTCAGAACTTCTTGTGTCCGCACATAGAAGGTTAAAGATGGCTTTCTCTTATAGGA
CGCCACAGGAGGAACTCTTGAGCAGGCTCCCACAGTCTCAACAGGAGGTTATAAGCGGATTTCAGTAT
GAACGCATTCAAAAG
+
FFFFFFFFFFFFFFFFFFFFFFFFFFFFFFFFFFFFFFFFFFFFFFFFFFFFFFFFFFFFFFFFFFFF
FF:FFFFFFFF:FFFFFFFFFFFFFFFFFFFFFFFFFFFFFFFFFFFF:FFFFFFFFFFFFFFFFFFF
FFFFFFFFF:FFFF:
@A00155:342:HHGFNDSXY:1:1261:26096:9768 2:N:0:GAACCTAG+TCCGCATA
ATCACATTGTATTTCAGAACTTCTTGTGTCCGCACATAGAAGGTTAAAGATGGCTTTCTCTTATAGGA
CGCCACAGGAGGAACTCTTGAGCAGGCTCCCACAGTCTCAACAGGAGGTTATAAGCGGATTTCAGTAT
GAACGCATTCAAAAG
+
FFFFFFFFFFFFFFFFF:FFFFFFFFFFFFFFFFFFFFFFFFFFFFFFF:FFFFFFFFFFFFFFFFFF
FFFFFFFFFFFFFFFFFFFFFFFFFFFFFFFFF:FFFFFFFFFFFFFFFFFFFF::FFFFFFFFFFFF
FFFFFFFFFFFFFFF
@A00155:342:HHGFNDSXY:1:1231:12246:8171 2:N:0:GAACCTAG+TCCGCATA
ATCACATTGTATTTCAGAACTTCTTGTGTCCGCACATAGAAGGTTAAAGATGGCTTTCTCTTATAGGA
CGCCACAGGAGGAACTCTTGAGCAGGCTCCCACAGTCTCAACAGGAGGTTATAAGCGGATTTCAGTAT
GAACGCATTCAAAAG
+
:FFF:FFFFFFFFFFFFFFFFFFFFFFFFFFFFFFFFFFFFFFFFFFF:FFF,FFFFFFFFFFFF:FF
FFFFFFF,FFF,FFF:FFFFFFFFFFFFF:FFFFFFFFFFFFFFFFFFFFFFFFFFFFFF:FFFF:FF
FFFFF:FFFF:FFFF
@A00155:342:HHGFNDSXY:1:1311:10276:26866 2:N:0:GAACCTAG+TCCGCATA
ATCACATTGTATTTCAGAACTTCTTGTGTCCGCACATAGAAGGTTAAAGATGGCTTTCTCTTATAGGA
CGCCACAGGAGGAACTCTTGAGCAGGCTCCCACAGTCTCAACAGGAGGTTATAAGCGGATTTCAGTAT
GAACGCATTCAAAAG
+
FFFFFFFFFFFFFFFFFFFFFFFFFFFFFFFFFFFFFFFFFFFFFFFFFFFFFFFFFFFFFFFFFFFF
FFFFFFFFFFFFFFFFFFFFFFFFFFFFFFFFFFFFFFFFFFFFFFFFFFFFFFFFFFFFFFF:FFFF
FFFFFFFFFFFFFFF
@A00155:342:HHGFNDSXY:1:1645:30309:7388 2:N:0:GAACCTAG+TCCGCATA
ATCACATTGTATTTCAGAACTTCTTGTGTCCGCACATAGAAGGTTAAAGATGGCTTTCTCTTATAGGA
CGCCACAGGAGGAACTCTTGAGCAGGCTCCCACAGTCTCAACAGGAGGTTATAAGCGGATTTCAGTAT
GAACGCATTCAAAAG
+
FFFFFFFFFFFFFFFFFFFFFFFFFFFFFFFFFFFFFFFFFFFFFFFFFFFFFFFFFFFFFFFFFFFF
FF,FFFFFFFFFFFFFFFFFFFFFFFFFFFFFFFFFFFFFFFFFFF:FFFFFFFFFFFFFFFFFFF:F
FFFFFFFFFFFFFFF
@A00155:342:HHGFNDSXY:1:2157:29776:28792 2:N:0:GAACCTAG+TCCGCATA
ATCACATTGTATTTCAGAACTTCTTGTGTCCGCACATAGAAGGTTAAAGATGGCTTTCTCTTATAGGA
CGCCACAGGAGGAACTCTTGAGCAGGCTCCCACAGTCTCAACAGGAGGTTATAAGCGGATTTCAGTAT
GAACGCATTCAAAAG

```
+
FFFFFF:,FFFFFFFFFFFF,FFFFFFFFFFFFFFFFFFFFFFFFFFFFF:FFFFFFFFFFFF:FFFF
FFFFFFFFFFFF::FFFFFFF:FFF::FFFFFFFF,FFFFFFFFFFFF:FFFFF:FFFFFFFFFFFFF
,F:F:FFFFFFFFFF
@A00155:342:HHGFNDSXY:1:2222:17897:7999 2:N:0:GAACCTAG+TCCGCATA
ATCACATTGTATATCAGAACTTCTTGTGTCCGCACATAGAAGGTTAAAGATGGCTTTCTCTTATAGGA
CGCCACAGGAGGAACTCTTGAGCAGGCTCCCACAGTCTCAACAGGAGGTTATAAGCGGATTTCAGTAT
GAACGCATTCAAAAG
+
FFFFFFFFFFFF,FFFFFFFFFFFFFFFFF:FFFFFFFFFFFFFFFF:FFFFFFFFFFFFFFFFFFFF
FFFFFFFFFFFFFFFFFFFFFFFFFFFFFF:FFFFFFFF:FFFFFFFFFFFFFFFFFFF:FFFFFFFF
FFFFFFFFFFFFFFF
@A00155:342:HHGFNDSXY:1:2353:24722:23359 2:N:0:GAACCTAG+TCCGCATA
ATCACATTGTATTTCAGAACTTCTTGTGTCCGCACATAGAAGGTTAAAGATGGCTTTCTCTTATAGGA
CGCCACAGGAGGAACTCTTGAGCAGGCTCCCACAGTCTCAACAGGAGGTTATAAGCGGATTTCAGTAT
GAACGCATTCAAAAG
+
FFFFFFFFFFFFFFFFFFFFFFFFFFFFFFFFFFFFFFFFFFFFFFFFFFFFFFFFFFFFFFFFFFFF
FFFFFFFFFFFFFFFFFFFFFFFF:FFF,FFFFFFFFFFFFFFFFFFFFFFFFFFFFFFFFFFFFFFF
FFFFFFFF:FFFFFF
@A00155:342:HHGFNDSXY:1:2609:3016:18677 2:N:0:GAACCTAG+TCCGCATA
ATCACATTGTATTTCAGAACTTCTTGTGTCCGCACATAGAAGGTTAAAGATGGCTTTCTCTTATAGGA
CGCCACAGGAGGAACTCTTGAGCAGGCTCCCACAGTCTCAACAGGAGGTTATAAGCGGATTTCAGTAT
GAACGCATTCAAAAG
+
FFFFF:FF:F,FFFFFFFFFFFFFF:FFFFFFFFFFFFFFFFFFFFF:FFFF:FFFFFFFFFFFFFFF
FF:FFFF,FFFFF:FFFFFFF:FFFFFFFFFFFFFFFFFF:FFFFFFFFFFFFFF:FFFFFFFFFFFF
:FF,FFF,,FFFFFF
@A00155:342:HHGFNDSXY:1:1152:7337:10097 1:N:0:GAACCTAG+TCCGCATA
TCACATTGTATTTCAGAACTTCTTGTGTCCGCACATAGAAGGTTAAAGATGGCTTTCTCTTATAGGAC
GCCACAGGAGGAACTCTTGAGCAGGCTCCCACAGTCTCAACAGGAGGTTATAAGCGGATTTCAGTATG
AACGCATTCAAAAG
+
FFFF:FFFFFFFFFFFFFFFFFFFFFFF:FFFFFFFFFFFFF:FFFFFFFFFFFFFFFFFFFFFFFFF
FFFFFFFFFFFFFFFFFFFFFFFFFFFFFFFFF:FFFF:FFFFFFFFFFFFFFFFFFFFFFFFFFFFF
FFFFFFFFFFFFFF
@A00155:342:HHGFNDSXY:1:1316:3314:7983 1:N:0:GAACCTAG+TCCGCATA
TTCCGATCTATTTCAGAACTTCTTGTGTCCGCACATAGAAGGTTAAAGATGGCTTTCTCTTATAGGAC
GCCACAGGAGGAACTCTTGAGCAGGCTCCCACAGTCTCAACAGGAGGTTATAAGCGGATTTCAGTATG
AACGCATTCAAAAGG
+
FFFFFFFFFFFFFFFFFFFFFFFFFFFFFFFFFFFFFFFFFFFFFFFFFFFFFFFFFFFFFFFFFFFF
FFFFFFFFFFFFFFFFFFFFFFFFFFFFFFFFFFFF:FFFFFFFFFFFFFFFFFFFFFFFFFFFFFFF
FFFFFFFFFFFFFFF
@A00155:342:HHGFNDSXY:1:1441:27046:32957 1:N:0:GAACCTAG+TCCGCATA
TCACATTGTATTTCAGAACTTCTTGTGTCCGCACATAGAAGGTTAAAGCTGGCTTTCTCTTATAGGAC
GCCACAGGAGGAACTCTTGAGCAGGCTCCCACAGTCTCAACAGGAGGTTATAAGCGGATTTCAGTATG
AACGCATTCAAAAGG
+
FFFF:FFFFFFFFFFFF:FFFFFF::FF:FFFFFFFFFFFF:FFFFFF,FFFFFFFFFFFFFFFFFFF
FFFFFFFFF:FFFFFFFFFFFFFF:FF:FFFFFFFFFFFFFF:FF:FFFFFFF:FFFFFFFFFFFFFF
FFFFFFFFFFFFFFF
@A00155:342:HHGFNDSXY:1:1441:26992:33207 1:N:0:GAACCTAG+TCCGCATA
TCACATTGTATTTCAGAACTTCTTGTGTCCGCACATAGAAGGTTAAAGATGGCTTTCTCTTATAGGAC
```

GCCACAGGAGGAACTCTTGAGCAGGCTCCCACAGTCTCAACAGGAGGTTATAAGCGGATTTCAGTATG
AACGCATTCAAAAGG
+
FFFF:FFF:FFFFFFFFFFFFFFFFFFFFFFFFFFFFFFFFFFFFFFFFFFFFFFFFFFFFFFFFFFFFFF
FFFFFFFFFFFFFFFFFFFFFFFFFF:FFFFFFFFFFFFFFFFFFFFFFFFFFFFFFFFFFFFFFFFF
FFFFFFFFFFFFFFF
@A00155:342:HHGFNDSXY:1:1620:19054:32424 1:N:0:GAACCTAG+TCCGCATA
TCACATTGTATTTCAGAACTTCTTGTGTCCGCACATAGAAGGTTAAAGATGGCTTTCTCTTATAGGAC
GCCACAGGAGGAACTCTTGAGCAGGCTCCCACAGTCTCAACAGGAGGTTATAAGCGGATTTCAGTATG
AACGCATTCAAAAGG
+
FFFFF,FFFFFFFFFFFFFFFFFFFFFFFFFFFFFFFFFFFFFFFFFFFFFFFF:FFFFFFFFFFFFF
FFFFFFFFFFFFF:FFFFFFFFFFFFFFFFFFFFFFFFFFFFFFFFFFFFFFFF:FFFFFFFFFFFFF
FFFFFFFFFFFFFFF
@A00155:342:HHGFNDSXY:1:1664:25464:19382 1:N:0:GAACCTAG+TCCGCATA
TCACATTGTATTTCAGAACTTCTTGTGTCCGCACATAGAAGGTTAAAGATGGCTTTCTCTTATAGGAC
GCCACAGGAGGAACTCTTGAGCAGGCTCCCACAGTCTCAACAGGAGGTTATAAGCGGATTTCAGTATG
AACGCATTCAAAAGG
+
FFFFFFFFFFFFFFFFFFFFFFFFFFFFFFFFFFFFFFFFFFFFFFFFFFFFFFFFFFFFFFFFFFFF
FFFFFFFFFFFFFFFFFFFFFFFFFFFFFFFFFFFFFFFFFFFFFFFFFFFFFFFFFFFFFFFFFFFF
FFFFFFFFFFFFFFF
@A00155:342:HHGFNDSXY:1:1657:10167:5071 1:N:0:GAACCTAG+TCCGCATA
TCACATTGTATTTCAGAACTTCTTGTGTCCGCACATAGAAGGTTAAAGATGGCTTTCTCTTATAGGAC
GCCACAGGAGGAACTCTTGAGCAGGCTCCCACAGTCTCAACAGGAGGTTATAAGCGGATTTCAGTATG
AACGCATTCAAAAGG
+
FFFFFFFFFFFFFFFFFFFFFFFFFFFFFFFFFFFFFFFFFFFFFFFFFFFFFFFFFFFFFFFFFF:F
FFFFFFFFFFFFFFFFFFFFFFFFFFFFFFFFFFFFFFFFFFFFFFFFFFFFFFFFFFFFFFFFFFFF
FFFFFFFFFFFFFFF
@A00155:342:HHGFNDSXY:1:1247:15881:25958 2:N:0:GAACCTAG+TCCGCATA
TCACATTGTATTTCAGAACTTCTTGTGTCCGCACATAGAAGGTTAAAGATGGCTTTCTCTTATAGGAC
GCCACAGGAGGAACTCTTGAGCAGGCTCCCACAGTCTCAACAGGAGGTTATAAGCGGATTTCAGTATG
AACGCATTCAAAAGG
+
FFFFFFFFFFFFFFFFFFFFFFFFFFFFFFFFFFFFFFFFFFFFFFFFFFFFFFFFFFFFFFFFFFFF
FFFFFFFFFFFFFFFFFFFF:FFFFFFFFFFFFFFFFFFFFFFFFFFFFFFFFFFFFFFFFFFFFFF
FFFFFFFFFFFFFFF
@A00155:342:HHGFNDSXY:1:2233:8278:22185 1:N:0:GAACCTAG+TCCGCATA
TCTATTGTATTTCAGAACTTCTTGTGTCCGCACATAGAAGGTTAAAGATGGCTTTCTCTTATAGGACG
CCACAGGAGGAACTCTTGAGCAGGCTCCCACAGTCTCAACAGGAGGTTATAAGCGGATTTCAGTATGA
ACGCATTCAAAAGGA
+
FFFFFFFFFFFFFFFFFFFFFFFFFFFFFFFFFFFFFFFFFFFFFFFFFFFFFFFFFFFFFFFFFFFF
FFFFFFFFFFFFFFFFFFFFFFFFFFFFFFFFFFFFFFFFFFFFFFFFFFFFFFFFFFFFFFFFFFFF
FFFFFFFFFFFFFFF
@A00155:342:HHGFNDSXY:1:2250:4363:32440 1:N:0:GAACCTAG+TCCGCATA
TCTATTGTATTTCAGAACTTCTTGTGTCCGCACATAGAAGGTTAAAGATGGCTTTCTCTTATAGGACG
CCACAGGAGGAACTCTTGAGCAGGCTCCCACAGTCTCAACAGGAGGTTATAAGCGGATTTCAGTATGA
ACGCATTCAAAAGGA
+
FFFFFFFFFFFFFFFFFFFFFFFFFFFFFFFF:FFFFFFFFFFFFFFFFFFFFFFFFFFFFFFFFFFF
FFFFFFFFFFFFFFFFFFFFFFFFFFFFFFFFFFFFFFFFF:FFFFFFFFFFFFFFFFFFFFFFFFFF
FFFFFFFFFFFFFFF

```
@A00155:342:HHGFNDSXY:1:2415:2374:19852 1:N:0:GAACCTAG+TCCGCATA
CACATTGTATTTCAGAACTTCTTGTGTCCGCACATAGAAGGTTAAAGATGGCTTTCTCTTATAGGACG
CCACAGGAGGAACTCTTGAGCAGGCTCCCACAGTCTCAACAGGAGGTTATAAGCGGATTTCAGTATGA
ACGCATTCAAAAGGA
+
FFF::FFFFFFFFFFFFFFFFFFFFFFFFFFFFFFFFFFFFFFFFFFFFFFFFFFFFFFFFFFFFFFF
FFFFFFFF:FFFFFFFFFFFFFFFFFFFFFFFFFFFFFFFFFFFFFFFFFFFFFFFFFFFFFFFFFFF
FFFFFFFFFFFFFFF
@A00155:342:HHGFNDSXY:1:2169:20564:28119 2:N:0:GAACCTAG+TCCGCATA
CACATTGTATTTCAGAACTTCTTGTGTCCGCACATAGAAGGTTAAAGATGGCTTTCTCTTATAGGACG
CCACAGGAGGAACTCTTAAGCAGGCTCCCACAGTCTCAACAGGAGGTTATAAGCGGATTTCAGTATGA
ACGCATTCAAAAGGA
+
FFFFFFFFFFFFFF:FFFFFFFFFFFFFFFFFFFFFFFFFFFFFFFFFFFFFFFFFFFFFFFFFFFFF
FFFFFFFFFFFFFFFFFFFFFFFFFFFFFFFFFFFFFFFFFFFFFFFFFFFF,FFFFFFFFFFFFFFF
FFFFF:FFFFFFFFF
@A00155:342:HHGFNDSXY:1:2238:17092:12931 2:N:0:GAACCTAG+TCCGCATA
CACATTGTATTTCAGAACTTCTTGTGTCCGCACATAGAAGGTTAAAGATGGCTTTCTCTTATAGGACG
CCACAGGAGGAACTCTTGAGCAGGCTCCCACAGTCTCAACAGGAGGTTATAAGCGGATTTCAGTATGA
ACGCATTCAAAAGGA
+
FFFFFFFFFFFFFFFFFFFFFFFFFFFFFFFFFFFFFFFFFFFFFFFFFFFFFFFFFFFFFFFFFFFF
FFFFFFFFFFFFFFFFFFFFFFFFFFFFFFFFFFFFFFFFFFFFFFFFFFFFFFFFFFFFFFFFFFFF
FFFFFFFF:FFFFFF
@A00155:342:HHGFNDSXY:1:1140:29080:26021 1:N:0:GAACCTAG+TCCGCATA
ACATTGTATTTCAGAACTTCTTGTGTCCGCACATAGAAGGTTAAAGATGGCTTTCTCTTATAGGACGC
CACAGGAGGAACTCTTGAGATGGCTCCCACAGTCTCCACAGGAGGTTATAAGCGGATTTCAGTATGAA
CGCATTCATAA
+
FF:FFFFFFFFFFF,::,F,FFF::F,,F,,:,FFFFF::FF,,FFFFF:FFFFF:,FFFFFF:FFF,
FF,FF:F:,,,:FFFFFF:,,FF,F:F:,,FFF,FF,FFFF:FF:FF:FF,F:F:,FFF:FFFFFFF,
FFFFFFFF,:F
@A00155:342:HHGFNDSXY:1:2419:32371:12555 1:N:0:GAACCTAG+TCCGCATA
TCCGATCTTTTCAGAACTTCTTGTGTCCGCACATAGAAGGTTAAAGATGGCTTTCTCTTATAGGACGC
CACAGGAGGAACTCTTGAGCAGGCTCCCACAGTCTCAACAGGAGGTTATAAGCGGATTTCAGTATGAA
CGCATTCAAAAGGAA
+
:FFFFFFFFFFFFFFFFFFFFFF:FFFFFFFFFFFFFFFFFFFFFFFFFFFFFFFFFF:::FFFFFFF
FFF:FF:FFFFFFFFFFFFFFF:FFFFFFFFFFFFF,FFFFFFFFFFFFF:FFFFFFFFFFFFFFFFF
FFFFFFFFFFFFFFF
@A00155:342:HHGFNDSXY:1:2128:16459:24267 1:N:0:GAACCTAG+TCCGCATA
CATTGTATTTCAGAACTTCTTGTGTCCGCACATAGAAGGTTAAAGATGGCTTTCTCTTATAGGACGCC
ACAGGAGGAACTCTTGAGCAGGCTCCCACAGTCTCAACAGGAGGTTATAAGCGGATTTCAGTATGAAC
GCATTCAAAAGGAAG
+
F:FFFFF,FFFFFFFFFFFFFF,FFFFFFFFFFFFFFFF:FFF:FFF:F:FFFFFFFFFFFFFFFF:F
FFFFFFFFFFFFFFFFFFFFFFFFFFFFFFFF:FFF,FFFFFFFFF:FFFFFFFFFFFFFFFFFFFFF
FFFFF:FFFFFFFFF
@A00155:342:HHGFNDSXY:1:2577:10737:3427 1:N:0:GAACCTAG+TCCGCATA
ATTGTATTTCAGAACTTCTTGTGTCCGCACATAGAAGGTTAAAGATGGCTTTCTCTTATAGGACGCCA
CAGGAGGAACTCTTGAGCAGGCTCCCACAGTCTCAACAGGAGGTTATAAGTGGATTTCAGTATGAACG
CATTCAAAAGGAAC
+
FFFFFFFFFFFFFFFFFFFFFFFFFFFFFFFFFFFFFFFFFFFFFFFFFFFFFFFFFFFFFFFFFFFF
```

```
FFFFFFFFFFFFFFFFFFFFFFFFFFFFFFFFFFFFFFFFFFFFFFFFFFFFFFFFFFFFFFFFFFFFFF
FFFFFFFFFFFFFF
@A00155:342:HHGFNDSXY:1:1309:20437:12054 2:N:0:GAACCTAG+TCCGCATA
ATTGTATTTCAGAACTTCTTGTGTCCGCACATAGAAGGTTAAAGATGGCTTTCTCTTATAGGACGCCA
CAGGAGGAACTCTTGAGCAGGCTCCCACAGTCTCAACAGGAGGTTATAAGCGGATTTCAGTATGAACG
CATTCAAAAGGAAGA
+
FFFFFFFFFFFFFFFFFFFFFFFFFFFFFFFFFFFFFFFFFFFFFFFFFFFFFFFFFFFFFFFFFFFFFF
FFFFFFFFFFFFFFFFFFFFFFFFFFFFFFFFFFFFFFFFFFFFFFFFFFFFFFFFFFFFFFFFFFFFFF
FFFFFFFFFF:FFFF
@A00155:342:HHGFNDSXY:1:1255:7355:14732 2:N:0:GAACCTAG+TCCGCATA
ATTGTATTTCAGAACTTCTTGTGTCCGCACATAGAAGGTTAAAGATGGCTTTCTCTTATAGGACGCCA
CAGGAGGAACTCTTGAGCAGGCTCCCACAGTCTCAACAGGAGGTTATAAGCGGATTTCAGTATGAACG
CATCCAAAAGGAAGA
+
FFFFFFFFFFF:FFFFFFFFFFFFF,FFFFFFFFFFF:FFFFF:FFFFFFFFFFFFFFFFFFFFFFFFF,
FFFFFFFFFFFFFFFFFFFFFF:FFFFFFFFFFFF:FFFFFFFFFFFFFFFFF:FFFFFFFFFFFFFF:
FF:FFFFFFF:FFF:
@A00155:342:HHGFNDSXY:1:1344:18240:24001 2:N:0:GAACCTAG+TCCGCATA
ATTGTATTTCAGAACTTCTTGTGTCCGCACATAGAAGGTTAAAGATGGCTTTCTCTTATAGGACGCCA
CAGGAGGAACTCTTGAGCAGGCTCCCACAGTCTCAACAGGAGGTTATAAGCGGATTTCAGTATGAACG
CATTCAAAAGAGATC
+
FFFFFFFFFFFFFFFFFFFFFFFFFFFFFFF:FFFFFFFFFFFFFFFFFFFFFFFFFFFFFFFFFFFFFF
FFFFFFFFFFFFFFFFFFFFFFFFFFFFFFFFFFFFFFFFFFFFFFFFFFF,FFFFFFFFFFFFFFFFFF
FFFFFFFFFFFFFFF
@A00155:342:HHGFNDSXY:1:1502:29912:12649 2:N:0:GAACCTAG+TCCGCATA
ATTGTATTTCAGAACTTCTTGTGTCCGCACATAGAAGGTTAAAGATGGCTTTCTCTTATAGGACGCCA
CAGGAGGAACTCTTGAGCAGGCTCCCACAGTCTCAACAGGAGGTTATAAGCGGTTTTCAGTATGAACG
CATTCAAAAGGAAGA
+
FFF:FFFFFFFFFFFFFFFFFFFFFFFFFFFFFFFFFFFFFFFFFFFFFFFFFFFFFFFFFFFFFFFFFF
FFF:FFFFFFFFFFFFFFFFFFFFFFFFFFFFFFFF:FFFFFFFFFFFFF:FFFFFFFFFFFFFFFFFF:
FFFFFFFFFFFFFFF
@A00155:342:HHGFNDSXY:1:2218:10465:14231 2:N:0:GAACCTAG+TCCGCATA
ATTGTATTTCAGAACTTCTTGTGTCCGCACATAGAAGGTTAAAGATGGCTTTCTCTTATAGGACGCCA
CAGGAGGAACTCTTGAGCAGGCTCCCACAGTCTCAACAGGAGGTTATAAGCGGATTTCAGTATGAACG
CATTCAAAAGGAAGA
+
FFFFFFFFFFFFFFFFFFFFFFFFFFFFFFFFFFFFFFFFFFFFFFFFFFFFFFFFFFFFFFFFFFFFFF
FFFFFFFFFFFFFFFFFFFFFFFFFFFFFFFFFFFFFFFFFFFFFFFFFFFFFFFFFFFFFFFFFFFFFF
FFFFFFFFFFFFFFF
@A00155:342:HHGFNDSXY:1:2233:8278:22185 2:N:0:GAACCTAG+TCCGCATA
ATTGTATTTCAGAACTTCTTGTGTCCGCACATAGAAGGTTAAAGATGGCTTTCTCTTATAGGACGCCA
CAGGAGGAACTCTTGAGCAGGCTCCCACAGTCTCAACAGGAGGTTATAAGCGGATTTCAGTATGAACG
CATTCAAAAGGAAGA
+
FFFFFFFFFFFFFFFFFFFFFFFFFFFFFFFFFFFFFFFFFFFFFFFFFFFFFFFFFFFFFFFFFFFFFF
FFFFFFFFFFFFFFFFFFFFFFFFFFFFF:FFFFFFFFFFFFFFF:FFFFFFFFFFFFFFFFFFFFFFFF
FFFFFFFFFFFF,FF
@A00155:342:HHGFNDSXY:1:2250:4363:32440 2:N:0:GAACCTAG+TCCGCATA
ATTGTATTTCAGAACTTCTTGTGTCCGCACATAGAAGGTTAAAGATGGCTTTCTCTTATAGGACGCCA
CAGGAGGAACTCTTGAGCAGGCTCCCACAGTCTCAACAGGAGGTTATAAGCGGATTTCAGTATGAACG
CATTCAAAAGGAAGA
```

```
+
FFFFFFFFFFFF:FFFFFFFFFFFFFFFFFFFFFFFFFFFFFF:FFFFFFFFFFFFFFFFF:FFFFFFF
FFFFFFFFFFFFFFFFFFFFFFFFFFFFFFFFFFFFFFFFFFFFFFF:FFFFFFFFFFFFFFFFFFFF,
FF:FFFF:FFFF:FF
@A00155:342:HHGFNDSXY:1:2313:12500:10520 2:N:0:GAACCTAG+TCCGCATA
ATTGTATTTCAGAACTTCTTGTGTCCGCACATAGAAGGTTAAAGATGGCTTTCTCTTATAGGACGCCA
CAGGAGGAACTCTTGAGCAGGCTCCCACAGTCTCAACAGGAGGTTATAAGCGGATTTCAGTATGAACG
CATTCAAAAGGAAGA
+
FFFFFFFFFFFFFFFFFFFFFFFFFFFFFFFFFFFFFFFFFFF:FFFFFFFFFFFFFFFFFFFFFFFFF
FFFFFFFFFFFFFFFFFFFFFFFFFFFFFFFFFFFFFFFFFFFFFFFFFFFFFFFFFFFFFFFFFFFFF
FFFFFFFFFFFFFF:
@A00155:342:HHGFNDSXY:1:2526:1542:9204 2:N:0:GAACCTAG+TCCGCATA
ATTGTATTTCAGAACTTCTTGTGTCCGCACATAGAAGGTTAAAGATGGCTTTCTCTTATAGGACGCCA
CAGGAGGAACTCTTGAGCAGGCTCCCACAGTCTCAACAGGAGGTTATAAGCGGATTTCAGTATGAACG
CATTCAAAAGAGATC
+
FFFFFFFFFFFFFFFFFFFFFFFFFFFFFFFFFFFFFFF:FFFFFFFFFFFFF,FFFFFFFFFFFFFFF
F::FFFFFFFFFFFFFFFFFFFFFFFFFFFFFFFFF::::FFFFFFFFFFFFF:FFFF:,F,FFFFF:F
FFFFFFF:FF:FFF:
@A00155:342:HHGFNDSXY:1:2616:18186:33708 2:N:0:GAACCTAG+TCCGCATA
ATTGTATTTCAGAACTTCTTGTGTCCGCACATAGAAGGTTAAAGATGGCTTTCTCTTATAGGACGCCA
CAGGAGGAACTCTTGAGCAGGCTCCCACAGTCTCAACAGGAGGTTATAAGCGGATTTCAGTATGAACG
CATTCAAAGGAAGAA
+
FFFF:FFFFFFFFFFFFFFFFFFFFFFFFFFFFFFFFFFFFFFFFFFFFFFFFFFFFFFFFFFFFFFFF
FFFFFFFFFFFFF,FFF,FFFFFFFFFFFFFFFFFFFF:FFFFFFFFFFFF:FFFFFFFFFFFF:FFF
FFFFFFFFFFF:FFF
@A00155:342:HHGFNDSXY:1:1313:21540:18505 1:N:0:GAACCTAG+TCCGCATA
TGTATTTCAGAACTTCTTGTGTCCGCACATAGAAGGTTAAAGATGGCTTTCTCTTATAGGACGCCACA
GGAGGAACTCTTGAGCAGGCTCCCACAGTCTCAACAGGAGGTTATAAGCGGATTTCAGTATGAACGCA
TTCAAAAGGAAGAAG
+
:FF::FFFFFFFFFFFFFFFFFFFFFFFFFFFFFFF:FFF,FFF,FFFFFFFFFFF:FFFF:FFFFFFF
FFFF:FFFF,FFFFFFFFFFFFFFF:FFFFFFFFFFFFFFFFFFFFFFFFFFFFFFFFFFFFFFFFFFF
FF:FFFFFFFFFFFF
@A00155:342:HHGFNDSXY:1:2255:28022:11851 1:N:0:GAACCTAG+TCCGCATA
TGTATTTCAGAACTTCTTGTGTCCGCACATAGAAGGTTAAAGATGGCTTTCTCTTATAGGACGCCACA
GGAGGAACTCTTGAGCAGGCTCCCACAGTCTCAACAGGAGGTTATAAGCGGATTTCAGTATGAACGCA
TTCAAAAGGAAGAAG
+
FFFFFFFFFFF:FF:F:FFFFFFFFFFFFFF,FFFFFFFFFFFF:FFFFFFFFFFFFFFFFFFFFFFFF
FFFF,FFFFFFFFFFFFFFFFFFFFFFFFFFFFFFFFFFFFFFFFFFFFFFFFFFFFFFFFFFFFFFFF
FFFFFFFFFFFFFFF
@A00155:342:HHGFNDSXY:1:1241:3468:17268 2:N:0:GAACCTAG+TCCGCATA
TGTATTTCAGAACTTCTTGTGTCCGCACATAGAAGGTTAAAGATGGCTTTCTCTTATAGGACGCCACA
GGAGGAACTCTTGAGCAGGCTCCCACAGTCTCAACAGGAGGTTATAAGCGGATTTCAGTATGAACGCA
TTCAAAAGGAAGAAG
+
FFFFFFFFFFFFFFFFFFF,FFFFFFFFFFFFFFFFFFFFF:FFFFFFFFFFFFFFFFFFFFFFFFFFF
FFFFFFFFFFFFFFFFFFFFFFFFFFFFFFFFFF:FFFF:FF:F:FFFFFFFFFFFFFFFFFFFFFFFF
FFFF:FFFFFFFFFF
@A00155:342:HHGFNDSXY:1:2377:28691:10003 2:N:0:GAACCTAG+TCCGCATA
TGTATTTCAGAACTTCTTGTGTCCGCACATAGAAGGTTAAAGATGGCTTTCTCTTATAGGACGCCACA
```

GGAGGAACTCTTGAGCAGGCTCCCACAGTCTCAACAGGAGGTTATAAGCGGATTTCAGTATGAACGCA
TTCAAAAGGAAGAAG
+
FFFFFFFFFFFFFFFFFFFFFFFFFFFFFFFFFFF:FFFFFFFFFFFFFFFFFFFFFFFFFFFFFFFF
:FFFFFFFFFFFFFFFFFFFFFFFFFFFFFFFFFFFFFFFFFFFFFF:FFFFFFFFFFFFFFFFFFFF
FFFFFFFFFFFFFFF
@A00155:342:HHGFNDSXY:1:1178:15953:27430 2:N:0:GAACCTAG+TCCGCATA
GTATTTCAGAACTTCTTGTGTCCGCACATAGAAGGTTAAAGATGGCTTTCTCTTATAGGACGCCACAG
GAGGAACTCTTGAGCAGGCTCCCACAGTCTCAACAGGAGGTTATAAGCGGATTTCAGTATGAACGCAT
TCAAAAGGAAGAAGA
+
FFFFFFFFFFFFFF:FFFFFFFFFFFFFF,FF:FFFFFFFFFFFFFFFFFFFFFFFFFFFFFFFFFFF
FFFFFF,FFFFF:FFFFFFFFF:FFF:FF,FFFFFF:FFFFFFFFFFFFFFFFFFFFFFFFFFFFFF:
FFFFFFFFFF::FFF
@A00155:342:HHGFNDSXY:1:1264:2718:35603 2:N:0:GAACCTAG+TCCGCATA
GTATTTCAGAACTTCTTGTGTCCGCACATAGAAGGTTAAAGATGGCTTTCTCTTATAGGACGCCACAG
GAGGAACTCTTGAGCAGGCTCCCACAGTCTCAACAGGAGGTTATAAGCGGATTTCAGTATGAACGCAT
TCAAAAGGAAGAAGA
+
FFFFFFFF:FFFFF:FFFFFFF:FFFFFFF:FF:FFFFFF,FFFFFFFFFFFFFFFF:FFFFFFFFFF
FFFFFF:F,FFFFFFFFFFF:FFFFFF:FF,FFFFFFFFFFFFFFF,F:FFF:FFFFFFFFFFF,:FF
FFFFFFFFFF::FFF
@A00155:342:HHGFNDSXY:1:1305:27751:35587 2:N:0:GAACCTAG+TCCGCATA
GTATTTCAGAACTTCTTGTGTCCGCACATAGAAGGTTAAAGATGGCTTTCTCTTATAGGACGCCACAG
GAGGAACTCTTGAGCAGGCTCCCACAGTCTCAACAGGAGGTTATAAGCGGATTTCAGTATGAACGCAT
TCAAAAGGAAGAAGA
+
FFFFFFFFFFFFFFFFFFFFFFFFFFFFFFFFFFFFFFFFFFFFFFFFFFFFFFFFFFFFFFFFFFFF
F:FFFFFFFFFFFFFFFFFFFFFFFFFFFFFFFFFFFFFFFFFFFFFFFFFFFFFFFFFFFFFFF:FFF
FFFFFFF,FFFFFFF
@A00155:342:HHGFNDSXY:1:1313:26341:31829 2:N:0:GAACCTAG+TCCGCATA
GTATTTCAGAACTTCTTGTGTCCGCACATAGAAGGTTAAAGATGGCTTTCTCTTATAGGACGCCACAG
GAGGAACTCTTGAGCAGGCTCCCACAGTCTCAACAGGAGGTTATAAGTGGATTTCAGTATGAACGCAT
TCAAAAGGAAGAAGA
+
FF:FFFFFFFFFFFFFFFFFFFFFFFF:FFFFFFFFFFFFFFFFFFF:FFFFFFFFFFFFFFFFFFFF
FFFFF,FFFFFFFFFFFFFFFFFFFFFFFF:FFFFFFFFF:FFFFFF:FFFFFFFFFFFFFFFFFFFF
FFFFFFFFF:FFFFF
@A00155:342:HHGFNDSXY:1:1269:6732:18349 2:N:0:GAACCTAG+TCCGCATA
GTATTTCAGAACTTCTTGTGTCCGCACATAGAAGGTTAAAGATGGCTTTCTCTTATAGGACGCCACAG
GAGGAACTCTTGAGCAGGCTCCCACAGTCTCAACAGGAGGTTATAAGCGGATTTCAGTATGAACGCAT
TCAAAAGGAAGAAGA
+
FFFFFFFFFFFFFFFFFFFFFFFFFFFFFFFFFFFFFFFFFFFFFFFFFFFFFFFFFFFFF:FFFFFF
FFFFFFFFFFFFFFFFFFFFFFFFFFFFFFFFFFFFFFFFFFFFFFFFFFFFFFFFFFFFFFFFFFFF
FFFFFFFFFFFFFFF
@A00155:342:HHGFNDSXY:1:1670:24270:26772 2:N:0:GAACCTAG+TCCGCATA
GTATTTCAGAACTTCTTGTGTCCGCACATAGAAGGTTAAAGATGGCTTTCTCTTATAGGACGCCACAG
GAGGAACTCTTGAGCAGGCTCCCACAGTCTCAACAGGAGGTTATAAGCGGATTTCAGTATGAACGCAT
TCAAAAGGAAGAAGA
+
FF:FF:FFFFFFFFFFFFFFFFFFFFFFFFFFFFFFFFFFFFFFFFFFFFFFFFFFFFFFFFFFFFFF
FFFFFFFFFFFFFFF:FFFFFFFFFF:FFFFFFFFFF:FFFFFFFFFFFFFFF:FF:FFFFFFFFFFF
FFFFFFFFFFFF:FF

@A00155:342:HHGFNDSXY:1:1670:25970:31156 2:N:0:GAACCTAG+TCCGCATA
GTATTTCAGAACTTCTTGTGTCCGCACATAGAAGGTTAAAGATGGCTTTCTCTTATAGGACGCCACAG
GAGGAACTCTTGAGCAGGCTCCCACAGTCTCAACAGGAGGTTATAAGCGGATTTCAGTATGAACGCAT
TCAAAAGGAAGAAGA
+
F,FFFFFFFFFFFFFFFFFFFFFFFFFF:FFFFFFFFFFFFFFFF:FF,FF:FFFFF,FFFFFFFFFF
FFFFF,FF:FFFFFFFFFFFF,FFFFFFFFFFFFFF:FFFFFFFFFFF,FFFFFFFFF:FFF:FF:F:
FFFFFF,FFFFFFFF
@A00155:342:HHGFNDSXY:1:2245:3332:14841 2:N:0:GAACCTAG+TCCGCATA
GTATTTCAGAACTTCTTGTGTCCGCACATAGAAGGTTAAAGATGGCTTTCTCTTATAGAACGCCACAG
GAGGAACTCTTGAGCAGGCTCCCACAGTCTCAACAGGAGGTTATAAGCGGATTTCAGTATGAACGCAT
TCAAAAGGAAGAAGA
+
FFFFFFFFFFFFFFFFFFFFFFFFFFFFFFFF:FFFFFF:FFFFFFFFFFFFFFFFF,FFFFFFFFFF
FFFFFFFFFFFFFFFFFFFFFFFFFFFFFFFF,FFFFFFFFFFFFFFFFFFFFFFFFFFFFFFFFFFF
FFF:FFFFF:F:FFF
@A00155:342:HHGFNDSXY:1:2512:19551:17065 2:N:0:GAACCTAG+TCCGCATA
GTATTTCAGAACTTCTTGTGTCCGCACATAGAAGGTTAAAGATGGCTTTCTCTTATAGGACGCCACAG
GAGGAACTCTTGAGCAGGCTCCCACAGTCTCAACAGGAGGTTATAAGCGGATTTCAGTATGAACGCAT
TCAAAAGGAAGAAGA
+
FFFFFFFFFFFFFFFF:FFFFFFFFFFFFFFFFFFFFFFFFFFFFFFFFFFFF:FFFF:FFFFFFFFF
FFFFF,FFFFFFFFFFFFFFFFFFFFFFFFFFFFF:FFFF:F:FFFFFFFF:FFFFFF:FFFFF,FFF
FFFFFFFFFFFFFFF
@A00155:342:HHGFNDSXY:1:2512:20320:19680 2:N:0:GAACCTAG+TCCGCATA
GTATTTCAGAACTTCTTGTGTCCGCACATAGAAGGTTAAAGATGGCTTTCTCTTATAGGACGCCACAG
GAGGAACTCTTGAGCAGGCTCCCACAGTCTCAACAGGAGGTTATAAGCGGATTTCAGTATGAACGCAT
TCAAAAGGAAGAAGA
+
:F:FFFFFFFFFFFFFFFFFFFFFFFFFFFFFFFFFFFFFFFFFFFFFFFFFFFFFFFFFFFFFFFF
FFFFFFFFFFFFFFFFFFFFFFFFFFFFFFFFFFFFFFFFFFFFFFFFFFFFFFFFFFFFFFFFFFFF
FFFFFFFFFFFFFFF
@A00155:342:HHGFNDSXY:1:2530:5041:13761 2:N:0:GAACCTAG+TCCGCATA
GTATTTCAGAACTTCTTGTGTCCGCACATAGAAGGTTAAAGATGGCTTTCTCTTATAGGACGCCACAG
GAGGAACTCTTGAGCAGGCTCCCACAGTCTCAACAGGAGGTTATAAGCGGATTTCAGTATGAACGCAT
TCAAAAGAGATCGGA
+
FF:FFFFFFFFFFFFFFFFFFFFFFFFFFFFFFFFFFFFFFFFFFFFFFFFFFFFFFFFFFFFFFFFF
FFFFFFFFFFFFFFFFFFFFFFFFFFFFFFFFFFFFFFFFFFFFFFFFFFFFFFFFFF,FFFFFFFFF
FFFFFFFF,FFFFF:
@A00155:342:HHGFNDSXY:1:2573:7880:28510 2:N:0:GAACCTAG+TCCGCATA
GTATTTCAGAACTTCTTGTGTCCGCACATAGAAGGTTAAAGATGGCTTTCTCTTATAGGACGCCACAG
GAGGAACTCTTGAGCAGGCTCCCACAGTCTCAACAGGAGGTTATAAGCGGATTTCAGTATGAACGCAT
TCAAAAGGAAGAAGA
+
FFFFFFFFFFFF:FFFFFFFFFFFFFFFFFFFFFFFFFFFFFFFFFFFFFFFFFFFFFFFFFFFFFF
FFFFFFFFFFFFF,FFFFFFFFFFFFFFFFFFF:FFFFF,FFFFFF:FFFFFFFFFFFFFFFFFFFFF
:FFFFF,FFF,FFFF
@A00155:342:HHGFNDSXY:1:2409:1226:2487 1:N:0:GAACCTAG+TCCGCATA
TATTTCAGAACTTCTTGTGTCCGCACATAGAAGGTTAAAGATGGCTTTCTCTTATAGGACGCCACAGG
AGGAACTCTTGAGCAGGCTCCCACAGTCTCAACAGGAGGTTATAAGCGGATTTCAGTATGAACGCATT
CAAAAGGAAGAAGA
+
FF::,,F,FF,,,F:F,F::,F::F:FFF,FF,,,,FFF,FF,FF:,FF:F:FF:FF,FFF:,FFFF:

```
F:,FFF:F,,FFFFFF:F:FFFF:F:FF:FFFFFF,F:,F:FFFF,::FFFFF:FF,FF,FFF,:FFF
FFFFF,:FF:FFFF
@A00155:342:HHGFNDSXY:1:1231:25220:2801 1:N:0:GAACCTAG+TCCGCATA
TATTTCAGAACTTCTTGTGTCCGCACATAGAAGGTTAAAGATGGCTTTCTCTTATAGGACGCCACAGG
AGGAACTCTTGAGCAGGCTCCCACAGTCTCAACAGGAGGTTATAAGCGGATTTCAGTATGAACGCATT
CAAAAGGAAGAAGAG
+
FFFFFFFFFFFFFFFFFFFFFFFFFFFFFFFFFFFFFFFFFFF:FFFFFFFFFFF:FFFFFFFFFFFFFF
FFFFFFFFFFFFFFFFFFFFFF:FFFFFFFFFFFFFFFFFFFFFFFFFFFFFFFFFFFFFFFFFFFFFFF
FFFFFFFFFFFFFFF
@A00155:342:HHGFNDSXY:1:1454:28962:22842 1:N:0:GAACCTAG+TCCGCATA
TATTTCAGAACTTCTTGTGTCCGCACATAGAAGGTTAAAGATGGCTTTCTCTTATAGGACGCCACAGG
AGGAACTCTTGAGCAGGCTCCCACAGTCTCAACAGGAGGTTATAAGCGGATTTCAGTATGAACGCATT
CAAAAGGAAGAAGAG
+
FFFFFFFFF:FFFFFFFFFFFFFFFFFFFFFFFFFFFFFFFFFFFFFFFFFFFFFFFFFFFFFFFFFFFF
FFFFFFFFFFFFFFFFFFFFFFFFFFFFFFFFFFFFFFFFFFFFFFFFFFFFFFFFFFFFFFFFFFFFFF
FFFFFFFFFFFFFFF
@A00155:342:HHGFNDSXY:1:1525:32759:22780 1:N:0:GAACCTAG+TCCGCATA
TATTTCAGAACTTCTTGTGTCCGCACATAGAAGGTTAAAGATGGCTTTCTCTTATAGGACGCCACAGG
AGGAACTCTTGAGCAGGCTCCCACAGTCTCAACAGGAGGTTATAAGCGGATTTCAGTATGAACGCATT
CAAAAGGAAGAAGAG
+
FFFFFFFFFFFFFFFFFFFFFFFFFFFFFFFFFFFFFFFFFFFFFFFFFFFFFFFFFFFFFFFFFFFFFF
FFFFFFFFFFFFFFFFFFFFFFFFFFFFFFFFFFFFFFFFFFFFF,FFFFFFFFFFFFFFFFFFFFFFFF
FFFFFFF:FFFFFFF
@A00155:342:HHGFNDSXY:1:1545:27145:20979 1:N:0:GAACCTAG+TCCGCATA
TATTTCAGAACTTCTTGTGTCCGCACATAGAAGGTTAAAGATGGCTTTCTCTTATAGGACGCCACAGG
AGGAACTCTTGAGCAGGCTCCCACAGTCTCAACAGGAGGTTATAAGCGGATTTCAGTATGAACGCATT
CAAAAGGAAGAAGAG
+
FFFFFFFFFFFFFFFFFFFFFFFFFFFFFFFFFFFFFFFFFFFFFFFFFFFFFFFFFFFFFFFFFFFFFF
FFFFFFFFFFFFFFFFFFFFFF:FF:FFFFF,FFFFFF:FFFFFFFFFFFFFFFFFFFFFFFFFFFFFFF
FFFFFFFFFFFFFFF
@A00155:342:HHGFNDSXY:1:1671:31647:8077 1:N:0:GAACCTAG+TCCGCATA
TATTTCAGAACTTCTTGTGTCCGCACATAGAAGGTTAAAGATGGCTTTCTCTTATAGGACGCCACAGG
AGGAACTCTTGAGCAGGCTCCCACAGTCTCAACAGGAGGTTATAAGCGGATTTCAGTATGAACGCATT
CAAAAGGAAGAAGAG
+
FFFFFF:FFFFFFFFFFFFFFFFFFFFFFFFFFFFFFFFFFFFFFFFFFFFFFFFFFFFFFFFFFFFFFF
FFFFFFFFFFFFFFFFFFFFFFFFFFFFFF:FFFFFFFFFFFFFF:FFFFFFFFFFFFFFFFFFFFFFFF
FFFFFFFFFFFFFFF
@A00155:342:HHGFNDSXY:1:2206:4246:23813 1:N:0:GAACCTAG+TCCGCATA
TATTTCAGAACTTCTTGTGTCCGCACATAGAAGGTTAAAGATGGCTTTCTCTTATAGGACGCCACAGG
AGGAACTCTTGAGCAGGCTCCCACAGTCTCAACAGGAGGTTATAAGCGGATTTCAGTATGAACGCATT
CAAAAGGAAGAAGAG
+
FFF:FFFFF:FFFF:FF,FFFFFFFFFFFFFF,F:FFFFFFFFFFF,FFFFFFFFFFFFFFFFFFFFFFF
FFFFFFFFFFFFFFFFFFFFFFFFFFFFFFFFFFFFFFFFFFFF,FFFFFFFFFFFFFFFFFFFFFFFFF
FFFFFFFFFFFFFFF
@A00155:342:HHGFNDSXY:1:2238:17092:12931 1:N:0:GAACCTAG+TCCGCATA
TATTTCAGAACTTCTTGTGTCCGCACATAGAAGGTTAAAGATGGCTTTCTCTTATAGGACGCCACAGG
AGGAACTCTTGAGCAGGCTCCCACAGTCTCAACAGGAGGTTATAAGCGGATTTCAGTATGAACGCATT
CAAAAGGAAGAAGAG
```

```
+
FFFFFFFFFFFFFFFFFFFFFFFFFFFFFFFFFFFFFFFFFFFFFFFFFFFFFFFFFFFFFFFFFFFFFF
FFFFFFFFFFFFFFFFFFFFFFFFFFFFFFFFFFFFFFFFFFFFFFFFFFFFFFFFFFFFFFFFFFFFFF
FFFFFFFFFFFFFFF
@A00155:342:HHGFNDSXY:1:2340:24216:34945 1:N:0:GAACCTAG+TCCGCATA
TATTTCAGAACTTCTTGTGTCCGCACATAGAAGGTTAAAGATGGCTTTCTCTTATAGGACGCCACAGG
AGGAACTCTTGAGCAGGCTCCCACAGTCTCAACAGGAGGTTATAAGCGGATTTCAGTATGAACGCATT
CAAAAGGAAGAAGAG
+
FFFFFFFFFFFFFFFFFFFFFFFFFFFFFFFFFFFFFFFFF,FFFFFFFFFFF:FFFFFFFFFFFFFFF
FFFFFFFFFFFFFFFFFFFFFFFFFFFFFFFFFFFFFFFFFFFFFFFFFFFFFFFFFFFFFFFFFFFFFF
FFFFFFFFFFFFFFF
@A00155:342:HHGFNDSXY:1:2550:29695:32659 1:N:0:GAACCTAG+TCCGCATA
TATTTCAGAACTTCTTGTGTCCGCACATAGAAGGTTAAAGATGGCTTTCTCTTATAGGACGCCACAGG
AGGAACTCTTGAGCAGGCTCCCACAGTCTCAACAGGAGGTTATAAGCGGATTTCAGTATGAACGCATT
CAAAAGGAAGAAGAG
+
FFFFFFFFFFFFFFFFFFFFFFFFFFF,FFFFFFFFFFFFFFFFFFFFFFFFFFFFFFFFFFFFFFFFFF
FFFFFFFFFFFFFFFFFFFFFFFFFFFFFFFFFFF:FFFFFFFFF:FFFFFFFFFFFFFFFFF:FFFFFF
FFFFFF:FFF:FFFF
@A00155:342:HHGFNDSXY:1:1460:13639:11772 1:N:0:GAACCTAG+TCCGCATA
ATTTCAGAACTTCTTGTGTCCGCACATAGAAGGTTAAAGATGGCTTTCTCTTATAGGACGCCACAGGA
GGAACTCTTGAGCAGGCTCCCACAGTCTCAACAGGAGGTTATAAGCGGATTTCAGTATGAACGCATTC
AAAAGGAAGAAGAGA
+
FFFFFFFFFFFFFFFFFFFFFFFFFFFFFFFFFFFFFFFFFFFFFFF:FFFFFFFFFFFFFFFFFFFFFF
FFFFFFFFFFFFFFFFFFFFFFFFFFFFFFFFFFFFFFF:FFFFFFFFFFFFFFFFFFFFFFFFFFFFFF
FFFFFFFFFFFFFFF
@A00155:342:HHGFNDSXY:1:2515:24758:19382 1:N:0:GAACCTAG+TCCGCATA
ATTTCAGAACTTCTTGTGTCCGCACATAGAAGGTTAAAGATGGCTTTCTCTTATAGGACGCCACAGGA
GGAACTCTTGAGCAGGCTCCCACAGTCTCAACAGGAGGTTATAAGCGGATTTCAGTATGAACGCATTC
AAAAGGAAGAAGAGA
+
FFFFFFFFFFFFFFFFFFFFFFFFFFFFFFFFFFFFFFFFFFFFFFFFFFF:FFFFFFFFFFFFFFFFFF
FFFFFFFFFFFFFFFFFFFFFFFFFFFFFFFFFFFFFFFFFFFFFFFFFFFFFFFFFFFFFFFFFFFFFF
FFFFFFFFFFFFFFF
@A00155:342:HHGFNDSXY:1:1316:3314:7983 2:N:0:GAACCTAG+TCCGCATA
ATTTCAGAACTTCTTGTGTCCGCACATAGAAGGTTAAAGATGGCTTTCTCTTATAGGACGCCACAGGA
GGAACTCTTGAGCAGGCTCCCACAGTCTCAACAGGAGGTTATAAGCGGATTTCAGTATGAACGCATTC
AAAAGGAGATCGGAA
+
FFFFFFFFFFFFF:FFFFFFFFFFFFFFFFFFFFFFFFFFFFFFFFFFFFFFFFFF:FFFFFFFFFFFFF
FFFFFFFFFFFF:FFFFFFFFFFFFFFFFFF:FFFFFFFF:FFFFFFFFFFFFFFFFFFFFFFFFFF:FF
FFFFFFFFFFFFFFF
@A00155:342:HHGFNDSXY:1:1513:11577:23516 2:N:0:GAACCTAG+TCCGCATA
ATTTCAGAACTTCTTGTGTCCGCACATAGAAGGTTAAAGATGGCTTTCTCTTATAGGACGCCACAGGA
GGAACTCTTGAGCAGGCTCCCACAGTCTCAACAGGAGGTTATAAGTGGATTTCAGTATGAACGCATTC
AAAAGGAAGAAGAGA
+
FFFFFFFFF:FFFFFFFFFFFFFFFFFFF,FFFFFFFFFFFFFFFFFFFFFFFFFFFFFFFFFFFFFFFF
FFF:FFFFFFFFFFFFFFFFFFFFFFFFFFFFFFFFFFFFFFFFFFFFFFFFFFFFFFFFFFFFFFFFFF
FFFFF:FFFFFF:FF
@A00155:342:HHGFNDSXY:1:1525:32759:22780 2:N:0:GAACCTAG+TCCGCATA
ATTTCAGAACTTCTTGTGTCCGCACATAGAAGGTTAAAGATGGCTTTCTCTTATAGGACGCCACAGGA
```

GGAACTCTTGAGCAGGCTCCCACAGTCTCAACAGGAGGTTATAAGCGGATTTCAGTATGAACGCATTC
AAAAGGAAGAAGAGA
+
FFFFFFFFFFFFFFFFFFFFFFFFFFFFFFFFFFFFFFFFFFFFFFFFFFFFFFFFFFFFFFFFFFFFFF
FFFFFFFFFFFF:FFFFFFFFFFFFFFFFFFFF,FFFFFFFFFFFFFFFFFFFFFFFFFFFFFFFFFFFF
,FFFFFFFFF,F:FF
@A00155:342:HHGFNDSXY:1:2139:14895:12226 2:N:0:GAACCTAG+TCCGCATA
ATTTCAGAACTTCTTGTGTCCGCACATAGAAGGTTAAAGATGGCTTTCTCTTATAGGACGCCACAGGA
GGAACTCTTGAGCAGGCTCCCACAGTCTCAACAGGAGGTTATAAGCGGATTTCAGTATGAACGCATTC
AAAAGGAAGAAGAGA
+
FFFFFFFFFFFFFFFFFFFFFFFFFFFFFFFFFFFFFFFFFFF:FFFFFFF:FFFFFF,FFFFFFFFFFF
FFF,FFFFFFFFFF:FFFF:FFFFFFFFFFFFFFFFFFFFFFFFFFFFFFFFFFFFFFFFFFFF:FFFFF
FFFFFFFFFFFFFFF
@A00155:342:HHGFNDSXY:1:2369:3757:34679 2:N:0:GAACCTAG+TCCGCATA
ATTTCAGAACTTCTTGTGTCCGCACATAGAAGGTTAAAGATGGCTTTCTCTTATAGGACGCCACAGGA
GGAACTCTTGAGCAGGCTCCCACAGTCTCAACAGGAGGTTATAAGCGGATTTCAGTATGAACGCATTC
AAAAGGAAGAAGAGA
+
FFFFFFFFFFFFFFFFFFFFFFFFFFFFFFFFFFFFFFFFFFFFFFFFFFFFFFFFF:FFFFFFFFFFFF
FFFFFFFFFFFFFFFFFFFFFFFFFFFFFFFFFFFFFFFFFFFFFF:FFFF,FFFFFFFFFFFFF,F:FF
FFFFFFFF:FFFFFF
@A00155:342:HHGFNDSXY:1:2132:7374:3646 1:N:0:GAACCTAG+TCCGCATA
TCTCAGAACTTCTTGTGTCCGCACATAGAAGGTTAAAGATGGCTTTCTCTTATAGGACGCCACAGGAG
GAAATCTTGAGCAGGCTCCCACATTCTCAACAGGAGGTTATAAGCGGATTTCAGTATGAACGCATTCA
AAAGGAAGAAGAG
+
FF,,F::FFFFFFFF,FF:FFF:F:FFFFFFFF:,FFFFF,F,F,FFF,FFFF,FF:F:FF::FF:F:FF
FF,,F:FFFF,FF,FF:::FFFF,F:FF,,:F:FFFFFFF:F,,FF:FFFFFFFF:F:FF,:F,,FFF:F
,F,FFFFF:F::F
@A00155:342:HHGFNDSXY:1:2307:6940:29324 1:N:0:GAACCTAG+TCCGCATA
TTTCAGAACTTCTTGTGTCCGCACATAGAAGGTTAAAGATGGCTTTCTCTTATAGGACGCCACAGGAG
GAACTCTTGAGCAGGCTCCCACAGTCTCAACAGGAGGTTATAAGCGGATTTCAGTATGAACGCATTCA
AAAGGAAGAAGAGAA
+
FFFFFFFFFFFFFFFFFFFFFFFFFFFFFFFFFFF:FFFFFFFFFFF:FFFFFFFFFFFFFFFFFFFFFF
FFFFFFFFFFFFFFFFFFFFFFFFFFFFFFFFFFFFFFFFFFFFF:FFFFFFFFFFFFFFFFFFFFFFFF
FFFFFFFFFFFFFFF
@A00155:342:HHGFNDSXY:1:2512:15546:3490 1:N:0:GAACCTAG+TCCGCATA
TTTCAGAACTTCTTGTGTCCGCACATAGAAGGTTAAAGTTGGCTTTCTCTTATAGGACGCCACAGGAG
GAACTCTTGAGCAGGCTCCCACAGTCTCAACAGGAGGTTATAAGCGGATTTCAGTATGAACGCATTCA
AAAGGAAGAATAGAA
+
FFF:FFF:F:FFF:FFFF,F,:F,FFF,,F:FFFF:FF,F:,F:FF,FFFFFFFFFFFFF,F:FFFFF
FF:F,:FFFFFFF,FF:F:F:FFFFFFFFF,FFFFF,FFFF::FFFFF:FF:FFFFF:,FFFFFFF:,
FFFFFFFFF,,FF,F
@A00155:342:HHGFNDSXY:1:2612:27254:6887 1:N:0:GAACCTAG+TCCGCATA
ATTCAGAACTTCTTGTGTCCGCACATAGAAGGTTAAAGATGGCTTTCTCTTATAGGACGCCACAGGAG
GAACTCTTGAGCAGGCTCCCACAGTCTCAACAGGAGGTTATAAGCGGATTTCAGTATGAACGCATTCA
AAAGGAAGAAGAGAA
+
FFFFFFFFFFFFFFFFFFFFFFFFFFFFFFFFFFFFFFFFFFFFFFFFFFFFFFFFFFFFFFFFFFFFFF
FFFFFFFFFFFFFFFFFFFFFFFFFFFFFFFFFFFFFFFFFFFFFFFFFFFFFFFFFFFFFFFFFFFFFF
FFFFFFFFFFFFFFF

```
@A00155:342:HHGFNDSXY:1:2419:32371:12555 2:N:0:GAACCTAG+TCCGCATA
TTTCAGAACTTCTTGTGTCCGCACATAGAAGGTTAAAGATGGCTTTCTCTTATAGGACGCCACAGGAG
GAACTCTTGAGCAGGCTCCCACAGTCTCAACAGGAGGTTATAAGCGGATTTCAGTATGAACGCATTCA
AAAGGAAAGATCGGA
+
:FFFFFFFFFFF:FFFFFFFFFFF,:FFFFFFFFFFFFFFFFFFFFFFFF:FFFFFFFFFFFFFFFFFF
FF,FFFFFFFFFFFFFFFFFFFFFFFFFF:FFFFFFFFFFF:FFFFFFF,:FFFFF:FF,FFFFFFFFF
:FFFFF,FFF:FFF:
@A00155:342:HHGFNDSXY:1:1241:13160:8970 1:N:0:GAACCTAG+TCCGCATA
TTCAGAACTTCTTGTGTCCGCACATAGAAGGTTAAAGATGGCTTTCTCTTATAGGACGCCACAGGAGG
AACTCTTGAGCAGGCTCCCACAGTCTCAACAGGAGGTTATAAGCGGATTTCAGTATGAACGCATTCAA
AAGGAAGAAGAGAAG
+
FFFFFFFFFFFFFFFFFFFFFFFFFFFFFFFF:FFFFFFFFFFFFFFFFFFFFFFFFFFFFFFFFFFF
FFFFFFFFFFFFFF:FFFFFFFFFFFFFFFFFFFFFFFFFFFFFFFFFFFFFFFFFFFFFFFFFFFFF
FFFFFFFFFFFFFFF
@A00155:342:HHGFNDSXY:1:1232:15103:18881 1:N:0:GAACCTAG+TCCGCATA
TTCAGAACTTCTTGTGTCCGCACATAGAAGGTTAAAGATGGCTTTCTCTTATAGGACGCCACAGGAGG
AACTCTTAAGCAGGCTCCCACAGTCTCAACAGGAGGTTATAAGCGGATTTCAGTATGAACGCATTCAA
AAGGAAGAAGAGGAG
+
FFFFFFFFFFFFFFFFFFFFFF:FF:FFFFFFFFFFFFF:F:FFFFFFFFFFFFFF:FFFFFFFFFFF
FFF:FFFFFFFFFFFFFFFFFFFFFFFFFFFFFFFFFFFFFFF:FFFFFFFFFFFFFFF:FFFFFFFFFF
FFFFFFFFFFFFFFF
@A00155:342:HHGFNDSXY:1:1357:32759:21966 1:N:0:GAACCTAG+TCCGCATA
TTCAGAACTTCTTGTGTCCGCACATAGAAGGTTAAAGATGGCTTTCTCTTATAGGACGCCACAGGAGG
AACTCTTGAGCAGGCTCCCACAGTCTCAACAGGAGGTTATAAGCGGATTTCAGTATGAACGCATTCAA
AAGGAAGAAGAGAAG
+
FFFFFFFFFFFFFFFFFFFFFFFFFFFFFFFFFFFF,FFFFFFFFFFFFFFFFFFFFFFFFFFFFFFF
:FFFFFFFFFFFFFFFFFFFFFFFFFFFFFFFFFFFFFFFFFFFFFFFFFFFFFFFFFFFFFFFFFFF
FFFFFFFFFFFFFFF
@A00155:342:HHGFNDSXY:1:1511:29333:34006 1:N:0:GAACCTAG+TCCGCATA
TTCAGAACTTCTTGTGTCCGCACATAGAAGGTTAAAGATGGCTTTCTCTTATAGGACGCCACAGGAGG
AACTCTTGAGCAGGCTCCCACAGTCTCAACAGGAGGTTATAAGCGGATTTCAGTATGAACGCATTCAA
AAGGAAGAAGAGAAG
+
FFFFFFFF:FFFFFFFFF:FFF:FFF:FFFFFFFFFFFFFFFFFFFFFFFFFFFFFFFFFFF:FFFFF
FFFFFFFFFFFFFF:FF:FFFFFFFFFFFFFFFFFFFFFFFFFFFFFFFFFFFFFFFFFFFFFFFFFF
FFFFFFFFFFFFFFF
@A00155:342:HHGFNDSXY:1:1502:29912:12649 1:N:0:GAACCTAG+TCCGCATA
TTCAGAACTTCTTGTGTCCGCACATAGAAGGTTAAAGATGGCTTTCTCTTATAGGACGCCACAGGAGG
AACTCTTGAGCAGGCTCCCACAGTCTCAACAGGAGGTTATAAGCGGTTTTCAGTATGAACGCATTCAA
AAGGAAGAAGAGAAG
+
FFFFFFFFFFFFFFFFFFFFFFFFFFFFFFFFFFFFFFFFFFFFFFFFFFFFFFFFFFFFFFFFFFFF
FFFFFFFFFFFFFFFFFFFFFFFFFFF:FFFFFFFFFFFFFFFFFFFFFFFFFFFFFFFFFFFFFFFFF
FFFFFFFFFFFFFFF
@A00155:342:HHGFNDSXY:1:2159:3134:17534 1:N:0:GAACCTAG+TCCGCATA
TTCAGAACTTCTTGTGTCCGCACATAGAAGGTTAAAGATGGCTTTCTCTTATAGGACGCCACAGGAGG
AACTCTTGAGCAGGCTCCCACAGTCTCAACAGGAGGTTATAAGCGGATTTCAGTATGAACGCATTCAA
AAGGAAGAAGAGAAG
+
FFFFFFFFFFFFFFFFFFFFFFFFFFFFFFFFFFFFFFFFFFFFFFFFFFFFFFFFFFFFFFFFFFFF
```

FFFFFFFFFFFFFFFFFFFFFFFFFFFFFFFFFFFFFFFFFFFFFFFFFFFFFFFFFFFFFFFFFFFF
FFFFFFFFFFFFFFF
@A00155:342:HHGFNDSXY:1:2419:7428:18959 1:N:0:GAACCTAG+TCCGCATA
TTCAGAACTTCTTGTGTCCGCACATAGAAGGTTAAAGATGGCTTTCTCTTATAGGACGCCACAGGAGG
AACTCTTGAGCAGGCTCCCACAGTCTCAACAGGAGGTTATAAGCGGATTTCAGTATGAACGCATTCAA
AAGGAAGAAGAGAAG
+
FFFFFFFFFFFFFFFFFFFFFFFFFFFFFFFFFFFFFFFFFFF:FFFFFFFFFFFFFFFFFFFFFFFF
FFFFFFFFFFFFFFFFFFFFFFFFFFFFFFFFFFFFFFFFFFFFFFFFFFFFFFFFFFFFFFFFFFFF
FFFFFFFFFFFFFFF
@A00155:342:HHGFNDSXY:1:1578:32479:19069 2:N:0:GAACCTAG+TCCGCATA
TTCAGAACTTCTTGTGTCCGCACATAGAAGGTTAAAGATGGCTTTCTCTTATAGGACGCCACAGGAGG
AACTCTTGAGCAGGCTCCCACAGTCTCAACAGGAGGTTATAAGCGGATTTCAGTATGAACGCATTCAA
AAGGAAGAAGAGAAG
+
FFFFFFFFFFFFFFFFFFFFFFFF:FFFFFFFFFFFFFFFFFFFFFFFFFFFFFFFFFFFFFFFFFFF
FFF,FF:FFFFFFFFFFFFFFFFFFFFFFFFFFFFFFFFFF:FFFFFFFFFFFFFFFFFF:FFFFF:F
FFFFFFFFFFFFFFF
@A00155:342:HHGFNDSXY:1:2132:7374:3646 2:N:0:GAACCTAG+TCCGCATA
CAGAACTTCTTGTGTCCGCACATAGAAGGTTAAAGATGGCTTTCTCTTATAGGACGCCACAGGAGGAA
CTCTTGAGCAGGCTCCCACAGTCTCAACAGGAGGTTATAAGCGGATTTCAGGATGAACGCATTCAAAA
GGACGAAGAGAGA
+
FF,:FFFFFFFFFFFFFFFFFFFFFFFFF,FF,FFFFFFFFF:FFFFFFF:,FF,FF,FFFFFF,FF,
,F,::FF:F:F:,FF,FFFFFFFF:FF,FFFFF:,F,FF:F:F:FF:F:FF,F,:F,F,,F:,,FFF:
:FF,,FF,FF:FF
@A00155:342:HHGFNDSXY:1:1241:3468:17268 1:N:0:GAACCTAG+TCCGCATA
CAGAACTTCTTGTGTCCGCACATAGAAGGTTAAAGATGGCTTTCTCTTATAGGACGCCACAGGAGGAA
CTCTTGAGCAGGCTCCCACAGTCTCAACAGGAGGTTATAAGCGGATTTCAGTATGAACGCATTCAAAA
GGAAGAAGAGAAGAA
+
FFFFFFFFFFFFFFFFFFFFFFFFFFFFFFFFFFFFFFFFFFFFFFFFFFFFFFFFFFFFFFFFFFFF
FFFFFFFFFFFFFFFFFFFFFFFFFF:FFFFFFFFFFFFFFFFFFFFFFFFFFFFFFFFFFFFFFFFF
FFFFFFFFFFFFFFF
@A00155:342:HHGFNDSXY:1:2427:8983:35900 1:N:0:GAACCTAG+TCCGCATA
CAGAACTTCTTGTGTCCGCACATAGAAGGTTAAAGATGGCTTTCTCTTATAGGACGCCACAGGAGGAA
CTCTTGAGCAGGCTCCCACAGTCTCAACAGGAGGTTATAAGCGGATTTCAGTATGAACGCATTCAAAA
GGAAGAAGAGAAGAA
+
FFFFFFFFFFFFFFFFFFFFFFFFFFFFFFFFFFFFFFFFFFFFFFFFFFFFFFFFFFFFFFFFFFFF
FFFFFFFFFFFFFFFFFFFFFFFFF:FFFFFFFFFFFFFFFFFFFF:FFFFFFFFFFFFFFFFFFFFF
FFFFFFFFFFFF:FF
@A00155:342:HHGFNDSXY:1:1232:13340:31422 2:N:0:GAACCTAG+TCCGCATA
CAGAACTTCTTGTGTCCGCACATAGAAGGTTAAAGATGGCTTTCTCTTATAGGACGCCACAGGAGGAA
CTCTTGAGCAGGCTCCCACAGTCTCAACAGGAGGTTATAAGCGGATTTCAGTATGAACGCATTCAAAA
GGAAGAAGAGAAGAA
+
FFFFFFFFFFFFFFFFFFFFFFFFF:FFFFFFF:FFFFFFF:FFFFFFFFF,FFFFF:FFFFFFFFF:
,FFFFFFFFFFFFFFFFFFFFFFFFFFFFFFFFFF:F:FFFFFFFFFFFFFFFFFFFFFFFFFFFFFF
FFFFF:FFF:FFFFF
@A00155:342:HHGFNDSXY:1:1271:18991:9142 2:N:0:GAACCTAG+TCCGCATA
CAGAACTTCTTGTGTCCGCACATAGAAGGTTAAAGATGGCTTTCTCTTATAGGACGCCACAGGAGGAA
CTCTTGAGCAGGCTCCCACAGTCTCAACAGGAGGTTATAAGCGGATTTCAGTATGAACGCATTCAAAA
GGAAGAAGAGAAGAA

+
FFFFFFFFFFFFFFFFFFFFFFFFFFFFFFF,FFFFFFFFFFFFFFFFFFFFFFFFFFFFFFFFFFFF
FFFFFFFFFFFFFFFFFFFFFFFFF:FFFFFFFFFFFFFF,FFFFFFFFFFFFFFFFFFFFFFFFFFF
FFFFFFFFFFF:FFF
@A00155:342:HHGFNDSXY:1:1509:10321:32205 2:N:0:GAACCTAG+TCCGCATA
CAGAACTTCTTGTGTCCGCACATAGAAGGTTAAAGATGGCTTTCTCTTATAGGACGCCACAGGAGGAA
CTCTTGAGCAGGCTCCCACAGTCTCAACAGGAGGTTATAAGCGGATTTCAGTATGAACGCATTCAAAA
GGAAGAAGAGAAGAA
+
FFFFFFFFFFFFFFFFFFFFFFFFFFFFFFFFFFFFFFFFFFFFFFFFFFFFFFFFFFFFFFFFFFFF
FFF:FFFFFFFFFFFFFFFFFFFFFFFFFFFFFFFFFFFFFFFFFFFFFFFFFFFFFFFFFFFFFF:F
FFFFFFFFFFF:F:F
@A00155:342:HHGFNDSXY:1:1626:14588:20337 2:N:0:GAACCTAG+TCCGCATA
CAGAACTTCTTGTGTCCGCACATAGAAGGTTAAAGATGGCTTTCTCTTATAGGACGCCACAGGAGGAA
CTCTTGAGCAGGCTCCCACAGTCTCAACAGGAGGTTATAAGCGGATTTCAGTATGAACGCATTCAAAA
GGAAGAAGAGAAGAA
+
FFFFFFFFFFFFFFFFFFFFFFFFFFFFFFFFFFFFFFFFFFFFFFFFFFFFFFFFFFFFFFFFFFFF
FFFFFFFFFFFFFFFFFFFFFFFFFFFFFFFFFFFFFFFFFFFFFFFFFFFFFFFFFFFFFFFFFFFF
FFFFFFFFFFFFFFF
@A00155:342:HHGFNDSXY:1:2116:26214:36276 2:N:0:GAACCTAG+TCCGCATA
CAGAACTTCTTGTGTCCGCACATAGAAGGTTAAAGATGGCTTTCTCTTATAGGACGCCACAGGAGGAA
CTCTTGAGCAGGCTCCCACAGTCTCAACAGGAGGTTATAAGCGGATTTCAGTATGAACGCATTCAAAA
GGAAGAAGAGAAGAA
+
FFFFFFFFFFFFFFFFFFFFFFFFFFFFF:FFFFFFFFFFFFFFFFFFFFFFFFFFFFFFFFFFFFFF
FFFFFFFFFFFFFFFFFFFFFFFFFFFFFFFFFFFF,FFFFFFFFFFFFFF:FFFFFFFFFFFFFFFF
FFFFFFFFFFFFFFF
@A00155:342:HHGFNDSXY:1:2678:20383:24361 1:N:0:GAACCTAG+TCCGCATA
AGAACTTCTTGTGTCCGCACATAGAAGGTTAAAGATGGCTTTCTCTTATAGGACGCCACAGGAGGAAC
TCTTGAGCAGGCTCCCACAGTCTCAACAGGAGGTTATAAGTGGATTTCAGTATGAACGCATTCAAAGG
AA
+
FFFFFFFFFFFFFFFFFFFFFFFFFFFFFFFFFFFFFFFFFFFFFFFFFFFFFFFFFFFFFFFFFFFF
FFFFFFFFFFFFFFFFFFFFFFFFFFFFFFFFFFFFFFFFFFFFFFFFFFFFFFFFFFFFFFFFFFFF
FF
@A00155:342:HHGFNDSXY:1:1439:3179:18490 1:N:0:GAACCTAG+TCCGCATA
CCGATCTCTTGTGTCCGCACATAGAAGGTTAAAGATGGCTTTCTCTTATAGGACGCCACAGGAGGAAC
TCTTGAGCAGGCTCCCACAGTCTCAACAGGAGGTTATAAGCGGATTTCAGTATGAACGCATTCAAAAG
GAAGAAGAGAAGAAG
+
FFFFFFFFFFFFFFFFFFFFFFFFFFFFFFFFFFFFFFFFFFFFFFFFFFFFFFFFFFFFFFFFFFFF
FFFFFFFFFFFFFFFFFFFFFFFFFFFFFFFFFFFFFFFFFFFFFFFFFFFFFFFFFFFFFFFFFFFF
FFFFFFFFFFFFFFF
@A00155:342:HHGFNDSXY:1:2144:18032:3161 1:N:0:GAACCTAG+TCCGCATA
AGAACTTCTTGTGTCCGCACATAGAAGGTTAAAGATGGCTTTCTCTTATAGGACGCCACAGGAGGAAC
TCTTGAGCAGGCTCCCACAGTCTCAACAGGAGGTTATAAGCGGATTTCAGTATGAACGCATTCAAAAG
GAAGAAGAGAAGAAG
+
FFFFFFFFFFFFFFFFFFFFFFFFFFFFFFFFFFFFFFFFFFFFFFFFFFFFFFFFFFFFFFFFFFFF
FFFFFFFFFFFFFFFFFFFFFFFFFFFFFFF:FFFFFFFFFFFF:FFFFFFFFFFFFFFFFFFFFFFF
FFFFFFFFFFFFFFF
@A00155:342:HHGFNDSXY:1:2256:9480:11804 1:N:0:GAACCTAG+TCCGCATA
AGAACTTCTTGTGTCCGCACATAGAAGGTTAAAGATGGCTTTCTCTTATAGGACGCCACAGGAGGAAC

TCTTGAGCAGGCTCCCACAGTCTCAACAGGAGGTTATAAGCGGATTTCAGTATGAACGCATTCAAAAG
GAAGAAGAGAAGAAG
+
FFF,FFF,:FFFFFFFFFFFFFFF:FFFFFFFFFFFFFFFFF:FFFFFFFFFFFFFFFFFFFFFFFFFF
FFFFFFFFFFFFFFFFFFFFFFFFFFFFFFFFFFF:FFFF,FFFFFFFFFFFFFFFF:FFFFFFFFFF
:FFFFFF,FFFFFFF
@A00155:342:HHGFNDSXY:1:2662:28890:30702 1:N:0:GAACCTAG+TCCGCATA
CCGATCTCTTGTGTCCGCACATAGAAGGTTAAAGATGGCTTTCTCTTATAGGACGCCACAGGAGGAAC
TCTTGAGCAGGCTCCCACAGTCTCAACAGGAGGTTATAAGCGGATTTCAGTATGAACGCATTCAAAAG
GAAGAAGAGAAGAAG
+
FFFFFFFFFFFFFFFFFFFFFFFFFFFFFFFFFFFFFFFFFFFFFFFFFFFFFFFFFFFFFFFFFFFF
FFFFFFFFFFFFFFFFFFFFFFFFFFFFFFFFFFFFFFFFFFFFFFFFFFFFFFFFFFFFFFFFFFFF
FFFFFFFFFFFFFFF
@A00155:342:HHGFNDSXY:1:1103:32850:18897 2:N:0:GAACCTAG+TCCGCATA
AGAACTTCTTGTGTCCGCACATAGAAGGTTAAAGATGGCTTTCTCTTATAGGACGCCACAGGAGGAAC
TCTTGAGCAGGCTCCCACAGTCTCAACAGGAGGTTATAAGCGGATTTCAGTATGAACGCATTCAAAAG
GAAGAAGAGAAGAAG
+
FFFFFFFFFFFFFFFFFFFFF:FFFFFFF:FFFFFFFFFFFFFFF,:FFFFFFFFFFFFFFFFFFF,F
FFFFFFFFFFFFFFFFFFFFFFFFFFFFFFF:FF:FF,FFFFFFFFFFFFFFFFFFFF:FF:FFFFF:F
F:FFFFFFFFFFFFF
@A00155:342:HHGFNDSXY:1:1103:32307:20118 2:N:0:GAACCTAG+TCCGCATA
AGAACTTCTTGTGTCCGCACATAGAAGGTTAAAGATGGCTTTCTCTTATAGGACGCCACAGGAGGAAC
TCTTGAGCAGGCTCCCACAGTCTCAACAGGAGGTTATAAGCGGATTTCAGTATGAACGCATTCAAAAG
GAAGAAGAGAAGAAG
+
:FF:FFF:FF:FFFFFFFFFFFFFFFFFFF:FFFFFFFFFF:FFFFFF:FFFFFFFFFFFFFFFFFFF
FFFFFFFFFFFF:FFFFFFFF:FFFFFFFFFFF:FFFF:FFF,FF,F:FFFFFFFFFFFFFFFFFFFF
FFF:FFFFFFFFFFF
@A00155:342:HHGFNDSXY:1:1136:30761:11647 2:N:0:GAACCTAG+TCCGCATA
AGAACTTCTTGTGTCCGCACATAGAAGGTTAAAGATGGCTTTCTCTTATAGGACGCCACAGGAGGAAC
TCTTGAGCAGGCTCCCACAGTCTCAACAGGAGGTTATAAGCGGATTTCAGTATGAACGCATTCAAAAG
GAAGAAGAGAAGAAG
+
FFFFFFFFFFFFFFFFFFFFFFFFFFFFFFFFFFFFFFFFF:FFFFFFFFFFFFFFFFFFFFF:FFF,F
FFFFFFFFFFFFFFFFFFFFFFFFFFFFFF:FFFFFFFFFFFFFFFFFFFF:FFFFFFFFFFF:FFFF
FF:FFFFFFFFFFFF
@A00155:342:HHGFNDSXY:1:2130:14977:35258 2:N:0:GAACCTAG+TCCGCATA
AGAACTTCTTGTGTCCGCACATAGAAGGTTAAAGATGGCTTTCTCTTATAGGACGCCACAGGAGGAAC
TCTTGAGCAGGCTCCCACAGTCTCAACAGGAGGTTATAAGCGGATTTCAGTATGAACGCATTCAAAAG
GAAGAAGAGAAGAAG
+
FFFFFFFF:FFFFFFFFFFFFFFFFFFFFFFFFFFFFFFF:,FFFF:FFFFFFFFFFFFFFFFFFFFFF
FF,FFFFFFFFFFFFFFFFFFFFFFFFFF:FFFFFFFFF,FFFFFFFFFFFFFFFFF:FFFFFFFFFF
FF,FFFFFFFFFFFF
@A00155:342:HHGFNDSXY:1:2131:14398:2159 2:N:0:GAACCTAG+TCCGCATA
AGAACTTCTTGTGTCCGCACATAGAAGGTTAAAGATGGCTTTCTCTTATAGGACGCCACAGGAGGAAC
TCTTGAGCAGGCTCCCACAGTCTCAACAGGAGGTTATAAGCGGATTTCAGTATGAACGCATTCAAAAG
GAAGAAGAGAAGAAG
+
FF:FFFFFFFFFFFFFFFFFFFFFFFFFFFFFFFFFFFFFFFFFFFFFFFFFFFFFFFFFFFFFFFFF
FFFFFFFFFFFFFFFFFFFFFFFFF,FFFFFFFFFFFFFFFFFFFFFFFFFFFFFFFFFFFFFFFFFF
FFFFFFFFFF:FFFF

@A00155:342:HHGFNDSXY:1:2402:27245:4241 2:N:0:GAACCTAG+TCCGCATA
AGAACTTCTTGTGTCCGCACATAGAAGGTTAAAGATGGCTTTCTCTTATAGGACGCCACAGGAGGAAC
TCTTGAGCAGGCTCCCACAGTCTCAACAGGAGGTTATAAGCGGATTTCAGTATGAACGCATTCAAAAG
GAAGAAGAGAAGAAG
+
FFFFFFFFFF:FFFFFFFFF:FFFFFFFFFFFFFFFFFFFFFFFFFFFFFFFFFFFFFFFFFFFFFFFF
FFFFFFFFFFFFFFFFFFFFFFFFFFFFFFFFFFFF:FFFFFF:FFFFFFFFFFFFFFFFFFFFFFFFF
FFFFFFFFFFF,FFF
@A00155:342:HHGFNDSXY:1:2571:16071:4210 2:N:0:GAACCTAG+TCCGCATA
AGAACTTCTTGTGTCCGCACATAGAAGGTTAAAGATGGCTTTCTCTTATAGGACGCCACAGGAGGAAC
TCTTGAGCAGGCTCCCACAGTCTCAACAGGAGGTTATAAGTGGATTTCAGTATGAACGCATTCAAAAG
GAAGAAGAGAAGAAG
+
FFFFFFFFFFFFFFFFFFFFFFFF:FFFFFFFFFFFFFFFFFFFFFFFFFFFFFFFFFFFFFFFFFFFF
FFFFFFFFFFFFFFFFFFFFFFFFFFFFFFFFFFFFFFFFFFFFFFFFFFFFFFFFFFFFFFFF,FFFF
FFFFFFFFFFFFFFF
@A00155:342:HHGFNDSXY:1:1554:7166:27712 1:N:0:GAACCTAG+TCCGCATA
GAACTTCTTGTGTCCGCACATAGAAGGTTAAAGATGGCTTTCTCTTATAGGACGCCACAGGAGGAACT
CTTGAGCAGGCTCCCACAGTCTCAACAGGAGGTTATAAGCGGATTTCAGTATGAACGCATTCAAAAGG
AA
+
FFFFFFFFFFFFFFFFFFFFFFFFFFFFFFFFFFFFFFFFFFFFFFFFFFFFFFF:FFFFFFFFFFFFF
FFFFFFF:FFFFFFFFFFFFFFFFFFFFFFFFFFFFFFFFFFFFFFFFFFFFFFFFFFFFFFFFFFFFF
FF
@A00155:342:HHGFNDSXY:1:2252:17255:17942 1:N:0:GAACCTAG+TCCGCATA
GAACTTCTTGTGTCCGCACATAGAAGGTTAAAGATGGCTTTCTCTTATAGGACGCCACAGGAGGAACT
CTTGAGCAGGCTCCCACAGTCTCAACAGGAGGTTATAAGCGGATTTCAGTATGAACGCATTCAAAAGG
AA
+
FFFFFFFFFFFFFFFFFFFFFFFFFFFFFFFFFFFFFFFFFFFFFFFFFFFFFFF:FFFFFFFFFFFFF
FFFFFFFFFFFFFFFFFFFFFFFFFFFFFFFFFFFFFFFFFFFFFFFFFFFFFFFFFFFFFFFFFFFFF
FF
@A00155:342:HHGFNDSXY:1:2466:4417:17754 1:N:0:GAACCTAG+TCCGCATA
GAACTTCTTGTGTCCGCACATAGAAGGTTAAAGATGGCTTTCTCTTATAGGACGCCACAGGAGGAACT
CTTGAGCAGGCTCCCACAGTCTCAACAGGAGGTTATAAGTGGATTTCAGTATGAACGCATTCAAAAGG
AA
+
FFF:FFFFFFFFFFFFFFFFFFFFF:FFFFFFFFFFFFFFFFFFFFFFFFFFFFFF:FFFFFFFF:FFFF
FFFFFFFFFFFFFFFFFFFFFFFFFFFFFFFFFFFFFFFFFFFFFFFFFFFFFFFFFFFFFFFFFFFFF
FF
@A00155:342:HHGFNDSXY:1:2675:32913:23923 1:N:0:GAACCTAG+TCCGCATA
GAACTTCTTGTGTCCGCACATAGAAGGTTAAAGATGGCTTTCTCTTATAGGACGCCACAGGAGGAACT
CTTGAGCAGGCTCCCACAGTCTCAACAGGAGGTTATAAGCGGATTTCAGTATGAACGCATTCAAAAGG
AA
+
FFFFFF,F,FFFFF:FFFFFFFFFFFFFFFFFFFFFFFFFFFFFFFFFFFFFFFFFFFFFFFFFFFFF:
FFFFFFFFFFFFFFFFFFFFFFFFFFFFFFFFFFFFFFFFFFFFFFFFFFFFFFFFFFFFFFFFFFFFF
FF
@A00155:342:HHGFNDSXY:1:1306:23430:26381 2:N:0:GAACCTAG+TCCGCATA
GAACTTCTTGTGTCCGCACATAGAAGGTTAAAGATGGCTTTCTCTTATAGGACGCCACAGGAGGAACT
CTTAAGCAGGCTCCCACAGTCTCAACAGGAGGTTATAAGCGGATTTCAGTATGAACGCATTCAGAAGG
AAGAAGAGAAGAAGG
+
FFFFFFFFFFFFFFFFFFFFFFFFFFFF:FFFFFFFFFFFFFFFFFFFFFFFFFFFFFFFFFFFFFFFF

```
FFFFFFFFFFFFFFFFFFFFFFFFFFFFFFFFFFF:FFFFFFFFF,FFFFF,FFFFFFFFFFFF,FFFF
FFFFFFFFF:FFFFF
@A00155:342:HHGFNDSXY:1:2337:31774:11835 2:N:0:GAACCTAG+TCCGCATA
GAACTTCTTGTGTCCGCACATAGAAGGTTAAAGATGGCTTTCTCTTATAGGACGCCACAGGAGGAACT
CTTGAGCAGGCTCCCACAGTCTCAACAGGAGGTTATAAGCGGATTTCAGTATGAACGCATTCAAAAGG
AAGAAGAGAAGAAGG
+
FFFFFFFFFFFFFFFFFFFFFFFFFFFFFFFFFFFFFFFF:FFFFFFF:FFFFFFFFFFF:FFFFF:FF
FFFFFFFFFFFFFFFFFFFFFFFFFFFFFFFFFFF:FFFFFFFFFFFFFFFFFFFFFFFF:FFFFFFF
FFFFFFFFFFFFFFF
@A00155:342:HHGFNDSXY:1:2503:32127:36025 1:N:0:GAACCTAG+TCCGCATA
AACTTCTTGTGTCCGCACATAGAAGGTTAAAGATGGCTTTCTCTTATAGGACGCCACAGGAGGAACTC
TTGAGCAGGCTCCCACAGTCTCAACAGGAGGTTATAAGCGGATTTCAGTATGAACGCATTCAAAAGGA
A
+
FFFFFFFFFFFFFFFFFFFFFFFFFFFFFFFFFFFFFFFFFFFFFF:FFFFFFFFFFFFFFFFFFFFF
FFFFFFFFFFFFFFFFFFFFFFFFFFFFFFFFFFFFFFFFFFFFFFFFFFFFFFFFFFFFFFFFFFFF
F
@A00155:342:HHGFNDSXY:1:2504:32244:1188 1:N:0:GAACCTAG+TCCGCATA
AACTTCTTGTGTCCGCACATAGAAGGTTAAAGATGGCTTTCTCTTATAGGACGCCACAGGAGGAACTC
TTGAGCAGGCTCCCACAGTCTCAACAGGAGGTTATAAGCGGATTTCAGTATGAACGCATTCAAAAGGA
A
+
F,FFFFFFF:F:FF:FFFF:FF:FFFF:FFFFFFFFFFFFFFFF:FFFFFFFFFFFFFFFFFFFFFFF
FFFF:FFF:FFFFFFFFFFFFFFFFFFFFFFFFF:FFFFFFFFFFFFFFFFFFFFFFFFFFFFFFFFF
F
@A00155:342:HHGFNDSXY:1:1230:23610:12352 1:N:0:GAACCTAG+TCCGCATA
AACTTCTTGTGTCCGCACATAGAAGGTTAAAGATGGCTTTCTCTTATAGGACGCCACAGGAGGAACTC
TTGAGCAGGCTCCCACAGTCTCAACAGGAGGTTATAAGCGGATTTCAGTATGAACGCATTCAAAAGGA
AGAAGAGAAGAAGGT
+
FFFFFFFF,FFF:F,::FFFFFFF:FFFFFFFFFFFFFFFFFFFFFFFFF,FF,F:FFFFFFFFF:FFF
FFFFFFFF:FF:FFFFFFFFFFFFFFFFF,FFFFFFFF:FFFFFFFFFFFFFFFFFFFFFFFFFFFFFF
FFFF,FFF:FFFFFF
@A00155:342:HHGFNDSXY:1:1261:27091:14372 1:N:0:GAACCTAG+TCCGCATA
AACTTCTTGTGTCCGCACATAGAAGGTTAAAGATGGCTTTCTCTTATAGGACGCCACAGGAGGAACTC
TTGAGCAGGCTCCCACAGTCTCAACAGGAGGTTATAAGTGGATTTCAGTATGAACGCATTCAAAAGGA
AGAAGAGAAGAAGGT
+
FFFFFFFFFFFFF:FFFFFFFFFFFF:FFFFFFFFFFFFFFFFFFFFFFFFFFFFFFFFFFFFF:FFF:
FFFFFFFF:FFFFFFFFFFFFFFFFFFFFFFFFFFFFFFFFFFFFFFFFFFFFFFFFFFFFFFFFFFF
FFFFFFFFFFFFFFF
@A00155:342:HHGFNDSXY:1:2609:3016:18677 1:N:0:GAACCTAG+TCCGCATA
AACTTCTTGTGTCCGCACATAGAAGGTTAAAGATGGCTTTCTCTTATAGGACGCCACAGGAGGAACTC
TTGAGCAGGCTCCCACAGTCTCAACAGGAGGTTATAAGCGGATTTCAGTATGAACGCATTCAAAAGGA
AGAAGAGAAGAAGGG
+
FFFFFFFFFFFF,F,FFFFFFFFFFFFFF:FFFFFFFFFFFFFFFFFFFFFFFF:FF:FFFFF:FFFFF
FFFFF:FF:FFFFFFFFFFFF::FFFFFFFFFFFFFFFFFFFFFFFFFFFFFFFFFFFFFFFFFFFFF
FFFFFFFFFFFFFFF
@A00155:342:HHGFNDSXY:1:2564:32913:31125 1:N:0:GAACCTAG+TCCGCATA
AACTTCTTGTGTCCGCACATAGAAGGTTAAAGATGGCTTTCTCTTATAGGACGCCACAGGAGGAACTC
TTGAGCAGGCTCCCACAGTCTCAACAGGAGGTTATAAGCGGATTTCAGTATGAACGCATTCAAAAGGA
AGAAGAGAAGAAGGT
```

```
+
:FFF:FFFFFFFF:F:FFFFFFF:FF,FFFFFFFFF:FFFF:FFFFFFFFFFFFFFFFFFFFFFFFF:F
FFFFFFFFFFFFFFFFF:FFFFFFFFFFFFFFF,FFFFFFFFFFFFFFFFFFFFFFFFFF:FF:FFFFF
FFFFFFFFFFFFFFF
@A00155:342:HHGFNDSXY:1:1666:9127:16986 1:N:0:GAACCTAG+TCCGCATA
ACTTCTTGTGTCCGCACATAGAAGGTTAAAGATGGCTTTCTCTTATAGGACGCCACAGGAGGAACTCT
TGAGCAGGCTCCCACAGTCTCAACAGGAGGTTATAAGTGGATTTCAGTATGAACGCATTCAAAAGGAA
GAAGAGAAGAAGGTG
+
FF,FFFFFFFFFFFFFFFFF:FFFFFF:FFFFFFFFFFFFFFFFFFFFFFFFFFFFFFFFFFFFFFFFF
FFFFFFFFFFFFFFFFFFFFFFFFFFFFF:FFFFFFFFFFFFFFFFFFFFFFFFFFFFFFFFFFFFFFF
FFFFFFFFFFFFFFF
@A00155:342:HHGFNDSXY:1:2117:28040:8876 1:N:0:GAACCTAG+TCCGCATA
ATCTCTTGTGTCCGCACATAGAAGGTTAAAGATGGCTTTCTCTTATAGGACGCCACAGGAGGAACTCT
TGAGCAGGCTCCCACAGTCTCAACAGGAGGTTATAAGCGGATTTCAGTATGAACGCATTCAAAAGGAA
GAAGAGAAGAAGGTG
+
FFFFFFFFFFFFFF:FFFFFFFFFFFFFFFFFFFFFFFFFFFFFF:FFFFFFFFFFFFFFFFFFFFFFF
FFFFFFFFFFFFFFFFFFFFFFFFFFFFFFFFFFFFFFFFFFFFFFFFFFFFFFFFFFFFFFFFFFFFF
FFFFFFFFFFFFFFF
@A00155:342:HHGFNDSXY:1:2129:28420:10974 1:N:0:GAACCTAG+TCCGCATA
CGATCTTGTGTCCGCACATAGAAGGTTAAAGATGGCTTTCTCTTATAGGACGCCACAGGAGGAACTCT
TGAGCAGGCTCCCACAGTCTCAACAGGAGGTTATAAGCGGATTTCAGTATGAACGCATTCAAAAGGAA
GAAGAGAAGAAGGTG
+
FFFFFFFF:FFFFFFFFFFFF:FFFFFFFFFFFFFFFFFF:FFFFFFFFF:FFFFFFFFFFFFFFFF
FFFFFFFFFFFFFFFFFFFFFFFFFFFFFFFFFFFFFFFFFFFFFFFFFFFFFFFFFFFFFFFFFFFFF
FFFFFFFFFFFFFFF
@A00155:342:HHGFNDSXY:1:2157:29776:28792 1:N:0:GAACCTAG+TCCGCATA
ACTTCTTGTGTCCGCACATAGAAGGTTAAAGATGGCTTTCTCTTATAGGACGCCACAGGAGGAACTCT
TGAGCAGGCTCCCACAGTCTCAACAGGAGGTTATAAGCGGATTTCAGTATGAACGCATTCAAAAGGAA
GAAGAGAAGAAGGTG
+
FFFFFFFFFFFFFFFF:F:F:FFF:FFF:FFFFFFFFFFFFFFFFFFFFFFFFFFFFFFFFFFF:FFFF
FFFFFFF:FFFFFF:FFFFFFFFFF:FFFFFFFFFFFFFFFFFFFFFFFFFFFFFFFFFFFFFFFFFFF
FFFFFFFFFFFFFFF
@A00155:342:HHGFNDSXY:1:2624:11071:14935 1:N:0:GAACCTAG+TCCGCATA
ACTTCTTGTGTCCGCACATAGAAGGTTAAAGATGGCTTTCTCTTATAGGACGCCACAGGAGGAACTCT
TGAGCAGGCTCCCACAGTCTCAACAGGAGGTTATAAGTGGATTTCAGTATGAACGCATTCAAAAGGAA
GAAGAGAAGAAGGTG
+
FF:FFFFFFFFFFFFFFFFFF:FFFFFFFFFFFFFFFFFFFFFFFFFFFFFFFFFFFFFFFFFFFFFFF
FFFFFFFFFFFFFFFFFFFFFFFFFFFFFFFFFFFFFFFFFFFFFFFFFFFFFF:FFFFFFFFFFFFF
FFFFFFFFFFFFFFF
@A00155:342:HHGFNDSXY:1:1118:18674:7842 1:N:0:GAACCTAG+TCCGCATA
CTTCTTGTGTCCGCACATAGAAGGTTAAAGATGGCTTTCTCTTATAGGACGCCACAGGAGGAACTCTT
GAGCAGGCTCCCACAGTCTCAACAGGAGGTTATAAGTGGATTTCAGTATGAACGCATTCAAAAGGAAG
AAGAGAAGAAGGTGG
+
FFFFFFFFFFFFFFFFFFFFFFFFFFFFFFFFFFFFF:FFFFFFFFFFFFFFFFFFFFFFFFFFFFFFF
FFFFFF:FFFFFFFFFFFFFFFFFFFFFFFFFFFFFFFFFFFFFFFFFFFFFFFFFFFFFFFFFFFFFF
FFFFFFFFFFFFFFF
@A00155:342:HHGFNDSXY:1:1160:30427:10723 1:N:0:GAACCTAG+TCCGCATA
CTTCTTGTGTCCGCACATAGAAGGTTAAAGATGGCTTTCTCTTATAGGACGCCACAGGAGGAACTCTT
```

GAGCAGGCTCCCACAGTCTCAACAGGAGGTTATAAGCGGATTTCAGTATGAACGCATTCAAAAGGAAG
AAGAGAAGAAGGTGG
+
:FFFFFFFFFFFFFFFFFFFFFFFFFFFFFFFFFFFFFFFFFFFFFFFFFFFFFFFFFFFFFFFFFFF
FFFFFFFFFFFFFFFFFFFFFFFFFFFFFFFFFFFFFFFFFFFFFFFFFFFFFFFFFFFFFFFFFFFF
FFFFFFFFFFFFFFF
@A00155:342:HHGFNDSXY:1:1313:26341:31829 1:N:0:GAACCTAG+TCCGCATA
CTTCTTGTGTCCGCACATAGAAGGTTAAAGATGGCTTTCTCTTATAGGACGCCACAGGAGGAACTCTT
GAGCAGGCTCCCACAGTCTCAACAGGAGGTTATAAGTGGATTTCAGTATGAACGCATTCAAAAGGAAG
AAGAGAAGAAGGTGG
+
FFFFFFF:FFFFFF:FFFFFF:FFFFFFFFFFFFFFFFFFFFFFFFFFFFFFFFFFFFFFFFFFFFFF
FFFFFFFFFFFFFFFFFFFFFFFFFFFFF:FFFFFFFFFFFFFFFFFFFFFFFFFFFFFFFFFFFFFF
FFFFFFFFFFFFFFF
@A00155:342:HHGFNDSXY:1:1578:32479:19069 1:N:0:GAACCTAG+TCCGCATA
CTTCTTGTGTCCGCACATAGAAGGTTAAAGATGGCTTTCTCTTATAGGACGCCACAGGAGGAACTCTT
GAGCAGGCTCCCACAGTCTCAACAGGAGGTTATAAGCGGATTTCAGTATGAACGCATTCAAAAGGAAG
AAGAGAAGAAGGTGG
+
FFFFFFF:FFFFFFFFFFFFFFFFFFFFFF:FFFFFFFFFFFFFFFFFFFFFFFFFFFFFFFFFFFFF
FFFFFF:FFFFFFFFFFFFF:FFFFFFFFFFFFFFFFFFFFFFFFFFFFFFFFFFFFFFFFFFFFFFF
FFFFFF:FFFFFFF:
@A00155:342:HHGFNDSXY:1:2218:10465:14231 1:N:0:GAACCTAG+TCCGCATA
CTTCTTGTGTCCGCACATAGAAGGTTAAAGATGGCTTTCTCTTATAGGACGCCACAGGAGGAACTCTT
GAGCAGGCTCCCACAGTCTCAACAGGAGGTTATAAGCGGATTTCAGTATGAACGCATTCAAAAGGAAG
AAGAGAAGAAGGTGG
+
:FFFFFFFFFFFFFFFFFFFFFFFFFFFFFFFFFFF:FFFFFFFFFFFFFFFFFFFFFFFFFFFFFFF
FFFFFFFFFFFFFFFFFFFFFFFFFFFFFFFFFFFFFFFFFFFFFFFFFFFFFFFFFFFFFFFFFFFF
FFFFFFFFFFFFFFF
@A00155:342:HHGFNDSXY:1:2324:3351:2503 1:N:0:GAACCTAG+TCCGCATA
CTTCTTGTGTCCGCACATAGAAGGTTAAAGATGGCTTTCTCTTATAGGACGCCACAGGAGGAACTCTT
GAGCAGGCTCCCACAGTCTCAACAGGAGGTTATAAGCGGATTTCAGTATGAACGCATTCAAAAGGAAG
AAGAGAAGAAGGTGG
+
FFFFFFFFFFF:FFFFFFFFFFFFFFFFFFFFFFFFFFFFFFFFFFFFFFFFFFFFFFFFFFFFFFFF
FFFF:FFFFFFFFFFFFFFFFFFFFFFFFFFFFFFFFFFFFFFFFFFFFFFFFFFFFFFFFFFFFFFF
FFFFFFFFFFFFFFF
@A00155:342:HHGFNDSXY:1:2538:32940:14544 1:N:0:GAACCTAG+TCCGCATA
GATCTTGTGTCCGCACATAGAAGGTTAAAGATGGCTTTCTCTTATAGGACGCCACAGGAGGAACTCTT
GAGCAGGCTCCCACAGTCTCAACAGGAGGTTATAAGCGGATTTCAGTATGAACGCATTCAAAAGGAAG
AAGAGAAGAAGGTGG
+
FFFFFFFFFFFF,FFFFFFFFFFFFFFFFFFFFFFFFFFFFFFFFFFFFFFFFFFFFFFFF:FFFFFF
FFFFFFFFFFFFFFFFFFFFFFFFFFFFFFFFFFFFFFFFFFFFFFFFFFFFFFFFFFFFFFFFFFFF
FFFFFFFFFFFFFFF
@A00155:342:HHGFNDSXY:1:1227:27977:2065 1:N:0:GAACCTAG+TCCGCATA
TTCTTGTGTCCGCACATAGAAGGTTAAAGATGGCTTTCTCTTATAGGACGCCACAGGAGGAACTCTTG
AGCAGGCTCCCACAGTCTCAACAGGAGGTTATAAGCGGATTTCAGTATGAACGCATTCAAAAGGAAGA
AGAGAAGAAGGTGGA
+
FFFFFFFFF:FFFFFFFFFFFFF:FFFFFF:FFFFFFFFFFFFFFFFFFFFFFFFFFFFFFFFFFFFF
FFFFFFFFFFFFFFFFFFFFFFFF:FFFFFFFFFFFF:FFFFFFFFFFFFF:FFFFFFFFFFFFFFFF
FFFFFFFFFFFFFFF

@A00155:342:HHGFNDSXY:1:1457:24288:6574 1:N:0:GAACCTAG+TCCGCATA
TTCTTGTGTCCGCACATAGAAGGTTAAAGATGGCTTTCTCTTATAGGACGCCACAGGAGGAACTCTTG
AGCAGGCTCCCACAGTCTCAACAGGAGGTTATAAGTGGATTTCAGTATGAACGCATTCAAAAGGAAGA
AGAGAAGAAGGTGGG
+
FFFFFFFFFF:FFFFFFFFFFFF:FFFFFFFFF:FFFFFFFFFFFFFFFFFFFFFFF,FFFFFFFFFF
FFFFFFFFFFFFFFFFFFFFFFFFFFFFFFFFFFFFFFFFFFFFFFFFFFFFFFFFFFFFFFFFFFFF
FFFFFFFFFFFFFFF
@A00155:342:HHGFNDSXY:1:2112:5231:31501 1:N:0:GAACCTAG+TCCGCATA
TTCTTGTGTCCGCACATAGAAGGTTAAAGATGGCTTTCTCTTATAGGACGCCACAGGAGGAACTCTTG
AGCAGGCTCCCACAGTCTCAACAGGAGGTTATAAGCGGATTTCAGTATGAACGCATTCAAAAGGAAGA
AGAGAAGAAGGTGGA
+
FF,FFFFFFFFFFF,FFFF:FFFFFFFFFFFFFFFFFFFFFFFFFFFFFFFFFFFFFFFFF:FFFFFF
FFFFFFFFFFFF:FFFFFFFFFFFFFFFFFFFFFFFFFFFFFFFF:FFFFFFFFFFFFFFFFFFFFFF
FFFFFFFFFFFFFFF
@A00155:342:HHGFNDSXY:1:1219:12879:4069 1:N:0:GAACCTAG+TCCGCATA
TCTTGTGTCCGCACATAGAAGGTTAAAGATGGCTTTCTCTTATAGGACGCCACAGGAGGAACTCTTGA
GCAGGCTCCCACAGTCTCAACAGGAGGTTATAAGCGGATTTCAGTATGAACGCATTCAAAAGGAAGAA
GAGAAGAAGGTGGGG
+
FFFFFFFFFFFFFFFFFFFFFFFFFFFF::FFFFFFFFFFF:FFFFFFFFFFFFFFFFFFFFFFFFFF
FFFF:FFFFFFFFFFFFFFFFFFFFFFFFFFFFFFFFFFFFFFFFFFFFFFFFFFFFFF:FFFFFFF
FFFFFFFFFFFFFFF
@A00155:342:HHGFNDSXY:1:2178:25934:25582 1:N:0:GAACCTAG+TCCGCATA
TCTTGTGTCCGCACATAGAAGGTTAAAGATGGCTTTCTCTTATAGGACGCCACAGGAGGAACTCTTGA
GCAGGCTCCCACAGTCTCAACAGGAGGTTATAAGCGGATTTCAGTATGAACGCATTCAAAAGGAAGAA
GAGAAGAAGGTGGAC
+
F:FFFFFFFFFFFFFFFF:FFF:FFFFFFFFFFFFFFFFFFFFFFFFFFFFFFFFFFFFFFFFFFFFF
FFFF:FFFFFFFFFFFFFFFFFFFFFFFFFFFFFFFFFFFFFFFFF:FFFFFFFFFFFFFFFFFFFFFF
F:F:FFFFFFFFFFF
@A00155:342:HHGFNDSXY:1:2129:28420:10974 2:N:0:GAACCTAG+TCCGCATA
TGTGTCCGCACATAGAAGGTTAAAGATGGCTTTCTCTTATAGGACGCCACAGGAGGAACTCTTGAGCA
GGCTCCCACAGTCTCAACAGGAGGTTATAAGCGGATTTCAGTATGAACGCATTCAAAAGGAAGAAGAG
AAGAAGGTGAGATCG
+
FFFFFFFFFFFFFFFFFFFFFFFFFFFFFFFFFFFFFFFFFFFFFFFFFFFFFFFFF:FFFFFFFFFF
FFFFFFFFFFFFFFFFFFFFFFFFFFF:FFFFFFFFFFFFFFFFFFFFFFFFFFFFFFFFFFFFFFFF
FFFFFFFFFFFFFFF
@A00155:342:HHGFNDSXY:1:2538:32940:14544 2:N:0:GAACCTAG+TCCGCATA
TGTGTCCGCACATAGAAGGTTAAAGATGGCTTTCTCTTATAGGACGCCACAGGAGGAACTCTTGAGCA
GGCTCCCACAGTCTCAACAGGAGGTTATAAGCGGATTTCAGTATGAACGCATTCAAAAGGAAGAAGAG
AAGAAGGTGGAGATC
+
FFFFFFFFFFFFFFFFFFFFFFFFFFFFFFFFFFFFFFFFFFFFFFFFFFFFFFFFFFFFFFF,FFFFF
FFFFFFFFFFFFFFFFFFFFFFFFFFFFFFFF:FFFFFFFFFFFFFFFFFFFFFFFFFFFFFFF:FFFFF
FFFFFFFFFF:FFFF
@A00155:342:HHGFNDSXY:1:2367:19217:3865 2:N:0:GAACCTAG+TCCGCATA
TGTCCGCACATAGAAGGTTAAAGATGGCTTTCTCTTATAGGACGCCACAGGAGGAACTCTTGAGCAGG
CTCCCACAGTCTCAACAGGAGGTTATAAGTGGATTTCAGTATGAACGCATTCAAAAGGAAGAAGAGAA
GAAGGTGG
+
FFFFFFFFFFFFFFFFFFFFFFFFFFFFFFFFFFFFFFFFFFFFFFFFFFFFFFFFFFFFFFFFFFFF

FFFFFFFFFFFFFFFFFFFFFFFFFFFFFFFFFFFFFFFFFFFFFFFFFFFFFFFFFFFFFFFFFFFF
FFFFFFFF
@A00155:342:HHGFNDSXY:1:1109:20437:13338 2:N:0:GAACCTAG+TCCGCATA
TGTCCGCACATAGAAGGTTAAAGATGGCTTTCTCTTATAGGACGCCACAGGAGGAACTCTTGAGCAGG
CTCCCACAGTCTCAACAGGAGGTTATAAGCGGATTTCAGTATGAACGCATTCAAAAGGAAGAAGAGAA
GAAGGTGGAAA
+
FFFFFFFFF:FFFFFFFFFFFFFFFFFFFFFFFFFFFFFFFFFFFFFFFFFFFFF:FFFFFFFFFFFF
FFFFFFFFFFFFFFFFFFFFFFFFFFFFFFFFFFFFFFFFFFFFFFFFFFFFFFFFFFFFF:FFFFFF
FFFFFFFFFFF
@A00155:342:HHGFNDSXY:1:1232:10511:7138 2:N:0:GAACCTAG+TCCGCATA
TGTCCGCACATAGAAGGTTAAAGATGGCTTTCTCTTATAGGACGCCACAGGAGGAACTCTTGAGCAGG
CTCCCACAGTCTCAACAGGAGGTTATAAGTGGATTTCAGTATGAACGCATTCAAAAGGAAGAAGAGAA
GAAGGTGGAAA
+
FFFFFFFFFFFFFFFFFFFFFFFFFFFFFFFFFFFFFFFFFFFFFFFFFFFFFFFFFFFFFFFFFFFF
FFFFFFFFFFFFFFFFFFFFFFFFFF,:FFFFFFFFFFFFFFFFFFFFFFFFFFFFF:FFFFFFFFFF
FFFFFFFF,FF
@A00155:342:HHGFNDSXY:1:1262:20627:21997 2:N:0:GAACCTAG+TCCGCATA
TGTCCGCACATAGAAGGTTAAAGATGGCTTTCTCTTATAGGACGCCACAGGAGGAACTCTTGAGCAGG
CTCCCACAGTCTCAACAGGAGGTTATAAGCGGATTTCAGTATGAACGCATTCAAAAGGAAGAAGAGAA
GAAGGTGGAAA
+
FFFFFFFFFFFFFFFFF:FFFFFFFFFFFFFFFFFFFFFFFFFFFFFFFFFFFFFFFFFFFFFFF:FF
FFFFFFFFFFFFFFFFFFFFFF,FFF:FF,FFFF:FFFFFFFFFFFFFFFFFFFFFFFFFF::FFFF:
:FFFFFFFFFF
@A00155:342:HHGFNDSXY:1:1262:20627:21840 2:N:0:GAACCTAG+TCCGCATA
TGTCCGCACATAGAAGGTTAAAGATGGCTTTCTCTTATAGGACGCCACAGGAGGAACTCTTGAGCAGG
CTCCCACAGTCTCAACAGGAGGTTATAAGCGGATTTCAGTATGAACGCATTCAAAAGGAAGAAGAGAA
GAAGGTGGAAA
+
FFFFFFFFFFFFFFFFFFFFFFFFFFFFFFFFFFFFFFFFFFFFFFFFFFFFFFF:FFFFFFFFFFFF
FFFFFFFFFFFFFFFFFFFFFF::FFFFFFFFFFFFFFFF:FFFFFF:FFFFFFFFFFF:FFFFFFFF
FFFFFFFFFFF
@A00155:342:HHGFNDSXY:1:1439:3179:18490 2:N:0:GAACCTAG+TCCGCATA
TGTCCGCACATAGAAGGTTAAAGATGGCTTTCTCTTATAGGACGCCACAGGAGGAACTCTTGAGCAGG
CTCCCACAGTCTCAACAGGAGGTTATAAGCGGATTTCAGTATGAACGCATTCAAAAGGAAGAAGAGAA
GAAGAGATCGG
+
FFFFFFFFFFFFFF:FFFFFF:FFFFFFFFFFFFFFFFFFFFFFFFFFFFFFFFFFFFFFFF:FFFFF
FFFFFFFFFFFFF:FFFFFFFFFFFFFF:FFFFFFFFFFFFFFF:FFFFFFF:FF:FF:,F,FFFF,F
FFFF,FFFF:F
@A00155:342:HHGFNDSXY:1:2117:28040:8876 2:N:0:GAACCTAG+TCCGCATA
TGTCCGCACATAGAAGGTTAAAGATGGCTTTCTCTTATAGGACGCCACAGGAGGAACTCTTGAGCAGG
CTCCCACAGTCTCAACAGGAGGTTATAAGCGGATTTCAGTATGAACGCATTCAAAAGGAAGAAGAGAA
GAAGGTGAGAT
+
FFFFFFFFFF:FFFFFFFFFFFFFFFFFFFFFFFFFFFFFFFFFFFFFFFFFFFF:FFFFFFFFFFFF
FFFFFFFFFFFFFFFFFFFFFFF:FFFFFFFFFFFFFFFFFFFFFFFF:FFFFFFFFFF,FFFFFFFF
FFFFFFFFFFF
@A00155:342:HHGFNDSXY:1:2662:28890:30702 2:N:0:GAACCTAG+TCCGCATA
TGTCCGCACATAGAAGGTTAAAGATGGCTTTCTCTTATAGGACGCCACAGGAGGAACTCTTGAGCAGG
CTCCCACAGTCTCAACAGGAGGTTATAAGCGGATTTCAGTATGAACGCATTCAAAAGGAAGAAGAGAA
GAAGAGATCGG

```
+
FFFFFFFFF:FFFFFFFFFFFFFFFFFFFFFFFFFFFFFFFFFFFFFFFFFFFFFF:FFFFFFFFFFFF
F:FFFFFFFFFF:FFFFFFFFFFFFFFFFFFFFFFFFFFFFFFFFFFFFFFFFFFFF:FFFFFFF
FFFFFFFFFFF
@A00155:342:HHGFNDSXY:1:1608:1823:16047 1:N:0:GAACCTAG+TCCGCATA
GTCCGCACATAGAAGGTTAAAGATGGCTTTCTCTTATAGGACGCCACAGGAGGAACTCTTGAGCAGGC
TCCCACAGTCTCAACAGGAGGTTATAAGCGGATTTCAGTATGAACGCATTCAAAAGGAAGAAGAGAAG
AAGGTGGAAAACTTT
+
FFFFFFFFFFFFFFFFFFFFFFFFFFFFFFFFFFFFFFFFFFFF:FFFF:FFFFFFFFFFFFFFFFFF
FFF:FFFFFFFFFFFFFFFFFFFFFFFFFFFFFFFFFFFFFFFFFFFFFF:FFFFFFF::FFFFFFFF
FFFFFFFFFFFFFFF
@A00155:342:HHGFNDSXY:1:2175:13313:18004 1:N:0:GAACCTAG+TCCGCATA
GTCCGCACATAGAAGGTTAAAGATGGCTTTCTCTTATAGGACGCCACAGGAGGAACTCTTGAGCAGGC
TCCCACAGTCTCAACAGGAGGTTATAAGCGGATTTCAGTATGAACGCATTCAAAAGGAAGAAGAGAAG
AAGGTGGAAAACTTT
+
FFFFFFFFF,FFFFFFFFFFFFFFFFFFFFFFFFFFFFFFFFFFFFFFFFFFFFFFFFFFFFFFFFFF
FFFFFFFFFFFFFFFFFFFFFFFFFFFFFFFFFFFFFFFFFFFFFFFFFFFFFFFFFFFFFFFFFFF:
FFFFFFFFFFFFFFF
@A00155:342:HHGFNDSXY:1:2261:1235:16094 1:N:0:GAACCTAG+TCCGCATA
GTCCGCACATAGAAGGTTAAAGATGGCTTTCTCTTATAGGACGCCACAGGAGGAACTCTTGAGCAGGC
TCCCACAGTCTCAACAGGAGGTTATAAGCGGATTTCAGTATGAACGCATTCAAAAGGAAGAAGAGAAG
AAGGTGGAAAACTTT
+
FFFFFF,FFFFF:FFFFFFFFF:FFF:FFFFF,FFFFFFFFFFFFFFFFFFFFFFFFFFFF:FFFFFF
FFFFF:FFF:FF:FFFFFFFFFFFFFFFFFF:FFFF,FFFFF::FFFFFFF,F,,FF:FF:,F:F:FF
:FFFFFFFFF,,FFF
@A00155:342:HHGFNDSXY:1:1323:9082:4539 2:N:0:GAACCTAG+TCCGCATA
GTCCGCACATAGAAGGTTAAAGATGGCTTTCTCTTATAGGACGCCACAGGAGGAACTCTTAAGCAGGC
TCCCACAGTCTCAACAGGAGGTTATAAGCGGATTTCAGTATGAACGCATTCAAAAGGAAGAAGAGAAG
AAGGTGGAAAACTTT
+
FFFFFFFF:FFFFFFFFFFFFFFFFFFFFFFFFFFFFFFFFFFFFFFFFFFFFFFFFFFFFFFFFFFF
FFFFFFFFFFFFFFFFFFFFF:FFFFFFFFFFFFFFFFFFFFFFFFFFFFFFFFFFFFFFFFFFFFF:
FFFFFFFFFFFFF:F
@A00155:342:HHGFNDSXY:1:1575:2347:21496 2:N:0:GAACCTAG+TCCGCATA
CCGCACATAGAAGGTTAAAGATGGCTTTCTCTTATAGGACGCCACAGGAGGAACTCTTGAGCAGGCTC
CCACAGTCTCAACAGGAGGTTATAAGTGGATTTCAGTATGAACGCATTCAAAAGGAAGAAGAGAAGAA
GGTGGAAAACTTTT
+
:FFFF:FFFFFFFFF:FFFFFFFF:FFF,FFFFFFFFFFFF:FFFFFFF:F,FFF:FFFFFFFFFFFF
FF:FF,F,FFFFFFFFFF:FFF:FFFFFFFFFFFFF::FFFF,:FFFFFFFF:F:FFF:F:F::FFFF
,FFF,:FFFFFFFF
@A00155:342:HHGFNDSXY:1:1575:4318:19210 2:N:0:GAACCTAG+TCCGCATA
CCGCACATAGAAGGTTAAAGATGGCTTTCTCTTATAGGACGCCACAGGAGGAACTCTTGAGCAGGCTC
CCACAGTCTCAACAGGAGGTTATAAGTGGATTTCAGTATGAACGCATTCAAAAGGAAGAAGAGAAGAA
GGTGGAAAACTTTTC
+
FFFFFFFFF:F:,FFFF::FFFFFFFFFFFFFFFFFFFF:FFFFFFF:FFFF:FFFFFFFFFFFFFFF
FFFFFFFFFFFFFFFFFFF:FFFFFFFFFFFFFFFFFFFFFFFFFFFFFFFFF,FFFFFFFFFFFFFF
:FFFFFFFFFFFFFF
@A00155:342:HHGFNDSXY:1:2617:17653:1814 2:N:0:GAACCTAG+TCCGCATA
CCGCACATAGAAGGTTAAAGATGGCTTTCTCTTATAGGACGCCACAGGAGGAACTCTTGAGCAGGCTC
```

```
CCACAGTCTCAACAGGAGGTTATAAGTGGATTTCAGTATGAACGCATTCAAAAGGAAGAAGAGAAGAA
GGTGGAAAACTTTTC
+
FFFFFFFFFFFFFFFFFFFFFFFFFFFFFFFFFFFFFFFFFFFF,FFFFFFFF,FFFFFFFFFF:FFF
FFFF,:FFFFFFFFF:,F,FFFFFFFFFFF:FF:FFFFFFFF::FFFFFF,FFFFFFFFFFFFFFF
FFFFFFFFF:FFF:F
@A00155:342:HHGFNDSXY:1:1509:13413:27727 2:N:0:GAACCTAG+TCCGCATA
CGCACATAGAAGGTTAAAGATGGCTTTCTCTTATAGGACGCCACAGGAGGAACTCTTGAGCAGGCTCC
CACAGTCTCAACAGGAGGTTATAAGCGGATTTCAGTATGAACGCATTCAAAAGGAAGAAGAGAAGAAG
GTGGAAAACTTTTCT
+
FFFFFFFFFFFFFFFFFFFFFFFFFFFFFFFFFFFFFFFFFFFFFFFFFFFFFFFFFFFFFFFFFFFF
FFFFFFFFFFFFFFFFFFFFFF:FFFFFFFFFFFFFFFFFFFFFFFFFFFFFFFFFFFFFFFFFFFFF
FFFFFFF:FFFFFFF
@A00155:342:HHGFNDSXY:1:2255:14696:27571 2:N:0:GAACCTAG+TCCGCATA
CGCACATAGAAGGTTAAAGATGGCTTTCTCTTATAGGACGCCACAGGAGGAACTCTTGAGCAGGCTCC
CACAGTCTCAACAGGAGGTTATAAGTGGATTTCAGTATGAACGCATTCAAAAGGAAGAAGAGAAGAAG
GTGAAAAACTTTTCT
+
FFFFFFFFFFFFFFFFFFFFFFFFFFFFFFFFFFFFFFFFFFFFFFFFFFFFFFFFFFFFFFFFFFFF
FFFFFFFFFFFFFFFFFFFFFFFFFFFFFFFFFFFFFFFFFFF:FFFFFFFFFFFFFFFF,FFFFFFF
FFFFFF::FFFFFFF
@A00155:342:HHGFNDSXY:1:2412:21133:36401 2:N:0:GAACCTAG+TCCGCATA
CGCACATAGAAGGTTAAAGATGGCTTTCTCTTATAGGACGCCACAGGAGGAACTCTTGAGCAGGCTCC
CACAGTCTCAACAGGAGGTTATAAGCGGATTTCAGTATGAACGCATTCAAAAGGAAGAAGAGAAGAAG
GTGGAAAACTTTTCT
+
FFFFFF:FFFFFFFFFFFFFFFFFFFFFFFFFFFFFFFFFFFF:FFFFFFFFFFFFFFFFFFFFFFFFF
FFFFFFFFFFFFFFFFFFFFFFFFFFFFFFFFFFFFFFFFFFF:FFFFFFFFFFFFFFFF:FFFFFFF
FFFFFFF:FFFFFFF
@A00155:342:HHGFNDSXY:1:2440:12246:23265 2:N:0:GAACCTAG+TCCGCATA
CGCACATAGAAGGTTAAAGATGGCTTTCTCTTATAGGACGCCACAGGAGGAACTCTTGAGCAGGCTCC
CACAGTCTCAACAGGAGGTTATAAGCGGATTTCAGTATGAACGCATTCAAAAGGAAGAAGAGAAGAAG
GTGGAAAACTTTTCT
+
:FFFFFFFFFFFFFFFFFFFFFFFFFFFFFFFFFFFFFFFFFFFFFFFFFFFFFFFFFFFFFFFFFFF
FFFFFFFFFFFFFFFFFFFFFFFFF:FFFFFFFF:FFFFFFFFFFFFFFFFFFFFFFFFFFFFFFFFF
FFFFFFFFFFFFFFF
@A00155:342:HHGFNDSXY:1:1515:18078:9596 2:N:0:GAACCTAG+TCCGCATA
GCACATAGAAGGTTAAAGATGGCTTTCTCTTATAGGACGCCACAGGAGGAACTCTTGAGCAGGCTCCC
ACAGTCTCAACAGGAGGTTATAAGCGGATTTCAGTATGAACGCATTCAAAAGGAAGAAGAGAAGAAGG
TGGAAAACTTTTCTT
+
FFFFFFFFFFFFFFFFFFFFFFFFFFFFFFF:FFFFFFFFFFFFFFFFFF:FFFFFFFFFF:FFFFFF
FFFF:FFFFF,FFFFFFFFFFFFFFFFFFFFFFF:FFFFFFFFFFFFFFFFF:FFFFFFFFFFFFFFF
FFFFFFFFFFFFFFF
@A00155:342:HHGFNDSXY:1:1431:29008:12962 2:N:0:GAACCTAG+TCCGCATA
GCACATAGAAGGTTAAAGATGGCTTTCTCTTATAGGACGCCACAGGAGGAACTCTTGAGCAGGCTCCC
ACAGTCTCAACAGGAGGTTATAAGCGGATTTCAGTATGAACGCATTCAAAAGGAAGAAGAGAAGAAGG
TGGAAAACTTTTCTT
+
FFFFFFFFFFFFFFFFFFFFFFFFFFFFFFFFFFFFFFFFFFFFFFFFFFFFFFFFFFFFFFFFFF:FF
FFFFFFFFFFFFFFFFFFFFFFFFFFFFFFFFFFFFFFFFFFFFFFFFFFFFFFFFFFFF:FFFFFFF
FFFFFFFFFFFFFFF
```

```
@A00155:342:HHGFNDSXY:1:1431:29206:13777 2:N:0:GAACCTAG+TCCGCATA
GCACATAGAAGGTTAAAGATGGCTTTCTCTTATAGGACGCCACAGGAGGAACTCTTGAGCAGGCTCCC
ACAGTCTCAACAGGAGGTTATAAGCGGATTTCAGTATGAACGCATTCAAAAGGAAGAAGAGAAGAAGG
TGGAAAACTTTTCTT
+
FFFFFFFFFFFFFFFFFFFFFFFFFFFFFFFFFFFFFFFFFFFFFFFFFFFFFFFFFFFFFFFFFFFFFF
FFFFFFFFFFFFFFFFFFFFFFFFFFFFFFFFFFFFFFFFFFFFFFFFFFFFFFFFFFFFFFFF:FFFFF
FFFFFFFFFFF:FFF
@A00155:342:HHGFNDSXY:1:1542:13358:23093 2:N:0:GAACCTAG+TCCGCATA
GCACATAGAAGGTTAAAGATGGCTTTCTCTTATAGGACGCCACAGGAGGAACTCTTGAGCAGGCTCCC
ACAGTCTCAACAGGAGGTTATAAGCGGATTTCAGTATGAACGCATTCAAAAGGAAGAAGAGAAGAAGG
TGGAAAACTTTTCTT
+
FFFFFFFFFFFFFFFF:FFFFFFFFFFFFFFFFFFFFFFFFFFFFFFFFFFFFFFFFFFFFFFFFFFFFF
FFFFFFFFFFFFFFFFF:FFFFFFFFFFFFFFFFFFFFFFFFFFFFFFFFFFFFFFFFFFFFFFFFFFFF
FFFFFFFFFFFFFFF
@A00155:342:HHGFNDSXY:1:1550:11641:31109 2:N:0:GAACCTAG+TCCGCATA
GCACATAGAAGGTTAAAGATGGCTTTCTCTTATAGGACGCCACAGGAGGAACTCTTGAGCAGGCTCCC
ACAGTCTCAACAGGAGGTTATAAGCGGATTTCAGTATGAACGCATTCAAAAGGAAGAAGAGAAGAAGG
TGGAAAACTTTTCTT
+
FFF:FFFFFFFFFFFFFFFFFFFFFFFFFFFFFFFFFFFFFFFFFFFFFFFFFFFFFFFFFFF:FFFFFF
FFFFFFFFFFFF:FFFFFFFFFFFFFFFFFFFFFFFFFFFFFFFFFFFFFFFFFFFFFFFFFFFFFFFFF
FFFFFFFFFFFFFFF
@A00155:342:HHGFNDSXY:1:1618:19614:17581 2:N:0:GAACCTAG+TCCGCATA
GCACATAGAAGGTTAAAGATGGCTTTCTCTTATAGGACGCCACAGGAGGAACTCTTGAGCAGGCTCCC
ACAGTCTCAACAGGAGGTTATAAGCGGATTTCAGTATGAACGCATTCAAAAGGAAGAAGAGAAGAAGG
TGGAAAACTTTTCTT
+
FF:FFFFFFFFFFFFFFFFFFFFFFFFFFFFFFFFFFFFFFFFFFFFFFFFFFFFFFFFFFFFFFFFFFF
FFFFFFFFFFFFFFFFFFFFFFFFFFF:FFFFFFFFFFFFFFFFFFFFFFFFFFFFFFFFFF:FFFFFFF
FFFFFFFFFFFFFFF
@A00155:342:HHGFNDSXY:1:2413:17056:27649 2:N:0:GAACCTAG+TCCGCATA
GCACATAGAAGGTTAAAGATGGCTTTCTCTTATAGGACGCCACAGGAGGAACTCTTGAGCAGGCTCCC
ACAGTCTCAACAGGAGGTTATAAGCGGATTTCAGTATGAACGCATTCAAAAGGAAGAAGAGAAGAAGG
TGGAAAACTTTTCTT
+
FFFFFFFFF:FFFFFFFFFF:FFFFFFFFFFFFFFFFFFFFF,F,FFFF,FF:FFFFFFFFF:FFFFFFF
FFFFFFFFFFFFFFFFFFFFFFFFFFFFFFFFFFFFFFFFFFFFFFFFFFFFFF:FFFFFFFFFFFFFFF
FFFFFF:FFFFFFFF
@A00155:342:HHGFNDSXY:1:2610:10420:29434 2:N:0:GAACCTAG+TCCGCATA
GCACATAGAAGGTTAAAGATGGCTTTCTCTTATAGGACGCCACAGGAGGAACTCTTGAGCAGGCTCCC
ACAGTCTCAACAGGAGGTTATAAGTGGATTTCAGTATGAACGCATTCAAAAGGAAGAAGAGAAGAAGG
TGGAAAACTTTTCTT
+
FFFFFFFFFFFF:FFFFFFFFFFFFFFFFFFFFFFFFFFFFFFFFFFFFFFF,FFFFFFFFFFFFFFFFF
FFFFFFFFFFFFFFFFFFFFFFFFFFFFFFFFFFFFFFFF:FFFFFFFFFFFFFFF:F:FFFFFFFFFFF
FFFFFFFFFFFFFFF
@A00155:342:HHGFNDSXY:1:2610:10511:29496 2:N:0:GAACCTAG+TCCGCATA
GCACATAGAAGGTTAAAGATGGCTTTCTCTTATAGGACGCCACAGGAGGAACTCTTGAGCAGGCTCCC
ACAGTCTCAACAGGAGGTTATAAGTGGATTTCAGTATGAACGCATTCAAAAGGAAGAAGAGAAGAAGG
TGGAAAACTTTTCTT
+
FFFFFFFFFFFFFFFFFFFFFFFFFFFFFFFFFFFFFFFFFFFFFFFFFFFFFFFFFFFFFFFFFFFFFF
```

FFFFFFFFF:FFFFFFFFFFFFFFFFFFFFFFFFFFF:FFFFFFFFFFFFFFFFFFFFFFFFFFFFFFFF
FF,FFFFFFFFFFFF
@A00155:342:HHGFNDSXY:1:2619:26883:20901 2:N:0:GAACCTAG+TCCGCATA
GCACATAGAAGGTTAAAGATGGCTTTCTCTTATAGGACGCCACAGGAGGAACTCTTGAGCAGGCTCCC
ACAGTCTCAACAGGAGGTTATAAGCGGATTTCAGTATGAACGCATTCAAAAGGAAGAAGAGAAGAAGG
TGGAAAACTTTTCTT
+
FF:FFFF:FFFFFFFFFFFFFFFFFFFFFFFFFFFFFFFFFFFFFFF,FFFFFFFFFFFFFFFFF:FFFF
FFFFFFFFFFFFFFFF,FFFFFF,FF,FF:F,FFFFFFFFFFFFFFFFFFF:FF,FF,FFFF:FFFFFF:
FFFFFFFFF:FFFFF
@A00155:342:HHGFNDSXY:1:2619:26241:26897 2:N:0:GAACCTAG+TCCGCATA
GCACATAGAAGGTTAAAGATGGCTTTCTCTTATAGGACGCCACAGGAGGAACTCTTGAGCAGGCTCCC
ACAGTCTCAACAGGAGGTTATAAGCGGATTTCAGTATGAACGCATTCAAAAGGAAGAAGAGAAGAAGG
TGGAAAACTTTTCTT
+
FFFFFFFFFFFFFFFFFFFFFFFFFFFFFFFFFFFFFFFFFFFFFFFFFFFFFFFFFFFFFFFFFFFFFF
FFFFFFFFFFFFFFFFFFFFFFFFFFFFFFFFFFFFFFFFFFFFFFFFFFFFFFFFFFFFFFFFFFFFFF
FFFFFFFFF:FFFFF
@A00155:342:HHGFNDSXY:1:1572:29378:29982 2:N:0:GAACCTAG+TCCGCATA
CACATAGAAGGTTAAAGATGGCTTTCTCTTATAGGACGCCACAGGAGGAACTCTTGAGCAGGCTCCCA
CAGTCTCAACAGGAGGTTATAAGCGGATTTCAGTATGAACGCATTCAAAAGGAAGAAGAGAAGAAGGT
GGAAAACTTTTCTTT
+
FFFFFFFFFFFFFFFFFFFFFFFFFFFFFFFFFFFFFFFFFFFFFFFFFFFFFFFFFFFFFFFFFFFFFF
FFFFFFFFFFFFFFFFFFFFFFFFFFFFFFFFFFFFFFF:FFFFFFFFFFF:FFFFF:FFFFFFFFFFFF
FFFFFFFFFFFFFFF
@A00155:342:HHGFNDSXY:1:2318:3351:34006 2:N:0:GAACCTAG+TCCGCATA
CACATAGAAGGTTAAAGATGGCTTTCTCTTATAGGACGCCACAGGAGGAACTCTTGAGCAGGCTCCCA
CAGTCTCAACAGGAGGTTATAAGCGGATTTCAGTATGAACGCATTCAAAAGGAAGAAGAGAAGAAGGT
GGAAAACTTTTCTTT
+
FFFFFFFFFFFFFFFFFFFFFFFFFFFFFFFFFFFFFFFFFFFFFFFFFFFFFFFFFFFFFFFFFFFFFF
FFFFFFFFFFFFFFFFFFFFFFFFFFFFFFFFFFFFFFFFFFFFFFFFFF:FFFFFFFFFFFFFFFFFFF
FFFFF,FF:FFFFFF
@A00155:342:HHGFNDSXY:1:2562:28465:20603 2:N:0:GAACCTAG+TCCGCATA
CACATAGAAGGTTAAAGATGGCTTTCTCTTATAGGACGCCACAGGAGGAACTCTTGAGCAGGCTCCCA
CAGTCTCAACAGGAGGTTATAAGCGGATTTCAGTAAGAACGCATTCAAAAGGAAGAAGAGAAGAAGGT
GGAAAACTTTTCTTT
+
FFFFFFF,FF:FF:FF,F,FFFFFF,F:,:F,F,,F:,F,:FFFF::FF,F:FFFFFF,FFFF,:FF:F
FFF,F:FFFFF::FF,:FF:FFF,FFFFF,,F,FF,:FF,FFFF,FFFFF:FF,F:FFFF:,::F:FF
,:FF:FF,F,,FF:F
@A00155:342:HHGFNDSXY:1:1520:22589:1000 2:N:0:GAACCTAG+TCCGCATA
ACATAGAAGGTTAAAGATGGCTTTCTCTTATAGGACGCCACAGGAGGAACTCTTGAGCAGGCTCCCAC
AGTCTCAACAGGAGGTTATAAGTGGATTTCAGTATGAACGCATTCAAAAGGAAGAAGAGAAGAAGGTG
GAAAACTTTTCTTT
+
:FFFFFFFFFFFFFFFFFFFFFFFFFFFFFFFFFFFFFFF:FFFFFFFFFFFFFFFFFFFFFFFFFFFFF
FFFFFFFFFFFFFFFFFFFFFFFFFFFFFFFFFFFFFFFFFFFFFFFFFFFFFFFFFFFFFFFFFFFFFF
FF:FFFFFFFFFFF
@A00155:342:HHGFNDSXY:1:1604:24777:3787 2:N:0:GAACCTAG+TCCGCATA
CATAGAAGGTTAAAGATGGCTTTCTCTTATAGGACGCCACAGGAGGAACTCTTGAGCAGGCTCCCACA
GTCTCAACAGGAGGTTATAAGCGGATTTCAGTATGAACGCATTCAAAAGGAAGAAGAGAAGAAGGTGG
AAAACTTTTCTTTTT

+
:FFFFFFFFFFFFF:FF::FF,,FF,FFFFFFFFFF,FFFFFFF:FFF::FFF:F::FFFFFFF,FFF
FF:,F,F,FFFFFFFFFFFFFFFFFFFFFFFFFFFFFFF:FFF,FF,FF,FFFFF:FFFFFFFFFF::
F:FF:FFFFFFFFFF
@A00155:342:HHGFNDSXY:1:1330:32551:27743 2:N:0:GAACCTAG+TCCGCATA
ATAGAAGGTTAAAGATGGCTTTCTCTTATAGGACGCCACAGGAGGAACTCTTGAGCAGGCTCCCACAG
TCTCAACAGGAGGTTATAAGCGGATTTCAGTATGAACGCATTCAAAAGGAAGAAGAGAAGAAGGTGGA
AAACTTTTCTTTTTA
+
F::FFFFFFFFFFFFFFFFFFFFFFFFFFFFFFFFFFFFFFFFFF:FFFFFFFFFFFFFF:FFFFFF
FFFFFFFFFFFFFFF,FFF,FFFFFFFFFFFFFFFFFFF:FFF:FFFFFFFFFFFFFFFFFFFFFFFF
:FFFFFFFFFFFFFF
@A00155:342:HHGFNDSXY:1:1366:28592:8703 2:N:0:GAACCTAG+TCCGCATA
ATAGAAGGTTAAAGATGGCTTTCTCTTATAGGACGCCACAGGAGGAACTCTTGAGCAGGCTCCCACAG
TCTCAACAGGAGGTTATAAGCGGATTTCAGTATGAACGCATTCAAAAGGAAGAAGAGAAGAAGGTGGA
AAACTTTTCTTTTTA
+
FFFFFFFFFFFFFFFFFFFFFFFFFFFFFFFFFFFFFFFFFFFFFFFFFFFFFFFFFFFFFFFFFFFF
FFFFFFFFFFFFFFF:FFFFFFFFF:FFFFF,FFFFFFFFFFFFFFFFFFFFFFFFFFFFFFFFFFFF
FFFFFFFFF:FFFFF
@A00155:342:HHGFNDSXY:1:1261:26096:9768 1:N:0:GAACCTAG+TCCGCATA
TAGAAGGTTAAAGATGGCTTTCTCTTATAGGACGCCACAGGAGGAACTCTTGAGCAGGCTCCCACAGT
CTCAACAGGAGGTTATAAGCGGATTTCAGTATGAACGCATTCAAAAGGAAGAAGAGAAGAAGGTGGAA
AACTTTTCTTTTTAC
+
FFFFFFFFFFFFFFFFFFFFFFFFFFFFFFFFFFFFFFFFFFFFFFFFFF:FFFFF:FFFFFFFFFFF
FFFFFFFFFFFFFFFFFFFFFFFFFFFFFFFFFFFFFFFFFFFFFFFFFFFFFFFFFFFFFFFF:FFF
FFFFFFFFFFFFFFF
@A00155:342:HHGFNDSXY:1:2235:31521:32878 1:N:0:GAACCTAG+TCCGCATA
TAGAAGGTTAAAGATGGCTTTCTCTTATAGGACGCCACAGGAGGAACTCTTGAGCAGGCTCCCACAGT
CTCAACAGGAGGTTATAAGCGGATTTCAGTATGAACGCATTCAAAAGGAAGAAGAGAAGAAGGTGGAA
AACTTTTCTTTTTAC
+
F:FFFFFFFFF:FFFFFFFFFFFFFFF,FFFFFFFF,FFFFFFFFFFFFFFFFFFFF:FFFFFFFFFF
FFFFFFFFFFFFFFFFFFFFFFFFFFFFFFFFFFFFFFFFFFFFFFFFFFFFFFFFFFF::FFFFF,F
FFFFFFFFFFFFFFF
@A00155:342:HHGFNDSXY:1:2527:1597:14058 1:N:0:GAACCTAG+TCCGCATA
TAGAAGGTTAAAGATGGCTTTCTCTTATAGGACGCCACAGGAGGAACTCTTGAGCAGGCTCCCACAGT
CTCAACAGGAGGTTATAAGCGGATTTCAGTATGAACGCATTCAAAAGGAAGAAGAGAAGAAGGTGGAA
AACTTTTCTTTTTAC
+
FF:FFFF,FFFFFFFF,FFFFF:FFFFFFFFFFFFF::,FF::FF:FF,FFFFFFFFF:FFFFFF,FF
:FFFFFFFFF::FFFFFFFF:FFFFF:FFFFFFFFFFFFFFFFFFFFFFFFFFFFFF::FFFFFFFFF
FFFFFFFFFFFFFFF
@A00155:342:HHGFNDSXY:1:2216:23999:19664 2:N:0:GAACCTAG+TCCGCATA
TAGAAGGTTAAAGATGGCTTTCTCTTATAGGACGCCACAGGAGGAACTCTTGAGCAGGCTCCCACAGT
CTCAACAGGAGGTTATAAGCGGATTTCAGTATGAACGCATTCAAAAGGAAGAAGAGAAGAAGGTGGAA
AACTTTTCTTTTTAC
+
FFFFFFFFFFFFFFFFFFFFFFFFFFFFFFFFFFFFFFFFFFFFFFFFFFFFFFFFFFFFFFFFFFFF
FFFFFFFFFFFFFFFFFFFFFFFFFFFFFFFFFFFFFFFFFFFFF:FFFFF:FFFFFFFFFFFFFFFF:
FFFFFFFFFFFFFFF
@A00155:342:HHGFNDSXY:1:2506:3323:11663 2:N:0:GAACCTAG+TCCGCATA
TAGAAGGTTAAAGATGGCTTTCTCTTATAGGACGCCACAGGAGGAACTCTTAAGCAGGCTCCCACAGT

CTCAACAGGAGGTTATAAGCGGATTTCAGTATGAACGCATTCAAAAGGAAGAAGAGAAGAAGGTGGAA
AACTTTTCTTTTTAC
+
FF,:FFFFFFFFFFFFFFFFFFFFFFFFFFFFFFFFFFFFFFFFFFFFFFFFF:FFFFFFFFFFFFFF
FFFFF:F:FFFFFFFFFFF,FFFFFFFFFFFFFFFFFFFFFFFFFFFFFFFFFF:FFF:FFFFFF:FF
FFFFFFFFFFFFF:F
@A00155:342:HHGFNDSXY:1:2546:22978:27445 2:N:0:GAACCTAG+TCCGCATA
TAGAAGGTTAAAGATGGCTTTCTCTTATAGGACGCCACAGGAGGAACTCTTGAGCAGGCTCCCACAGT
CTCAACAGGAGGTTATAAGCGGATTTCAGTATGAACGCATTCAAAAGGAAGAAGAGAAGAAGGTGGAA
AACTTTTCTTTTTAC
+
FFF:FFFFFFFFFFFFFFFFFFFFFFFFFFFFFFFFFFFFFFFFF::FFFFFFF:FFFFFFFFFFFF:
FFFFFFFFFFFF:F:FFF:FFFFFFFFFFFFFFFFFFFF:FFFFFFFFFFFFFFFFFFFFFFFFFFFF
FFFF:FFFF,FFFFF
@A00155:342:HHGFNDSXY:1:2526:28619:15796 2:N:0:GAACCTAG+TCCGCATA
AGAAGGTTAAAGATGGCTTTCTCTTATAGGACGCCACAGGAGGAACTCTTGAGCAGGCTCCCACAGTC
TCAACAGGAGGTTATAAGCGGATTTCAGTATGAACGCATTCAAAAGGAAGAAGAGAAGAAGGTGGAAA
ACTTTTCTTTTTAC
+
FFFFFFFFFFFFFFFFFFFFFFF,FFFFFFFFFFFFFFFFFFFFFFFFFFFFFFFFFFFFFFFFFFFF
FFFFF:FFFFFFFFFFF:FFFFFFFFFFFFFFFFFFFFFFFFFFFFF:FFFFFFFFFFFFFFFFFFF:
FFFFFFFFFF:FFF
@A00155:342:HHGFNDSXY:1:2612:13241:31093 2:N:0:GAACCTAG+TCCGCATA
AGAAGGTTAAAGATGGCTTTCTCTTATAGGACGCCACAGGAGGAACTCTTGAGCAGGCTCCCACAGTC
TCAACAGGAGGTTATAAGCGGATTTCAGTATGAACGCATTCAAAAGGAAGAAGAGAAGAAGGTGGAAA
ACTTTTCTTTTTAC
+
FFFFFFFFFFFFFFFFFFFFFFFFFFFFFFFFFFFFFFFFFFFFFFFFFFFFFFFFFFFFF:FFFFFF
FFFFFFFFFFFFFFFFFFFFFFFFFFFFFFFF:FFFFFFFF,F,FFF::F:FF:FFFFFFFF:FFF,:
:,F,FFFF:FFF,:
@A00155:342:HHGFNDSXY:1:1567:5448:15374 1:N:0:GAACCTAG+TCCGCATA
AGAAGGTTAAAGATGGCTTTCTCTTATAGGACGCCACAGGAGGAACTCTTGAGCAGGCTCCCACAGTC
TCAACAGGAGGTTATAAGCGGATTTCAGTATGAACGCATTCAAAAGGAAGAAGAGAAGAAGGTGGAAA
ACTTTTCTTTTTACC
+
FFFFFFFFFFFFFFFFFFFFF:FFFFFFFFFFFFFFFFFFFFFFFFFFFFFFFFFFFFFFFFFFFFFF
FFFFFFFFFFFF:FFFFFFFFFFFFFFFFFFFFFFFFFFFFFFFFF:FFFFFFFFFFFFFFFFFFFFF
FFFFFFFFFFFFFFF
@A00155:342:HHGFNDSXY:1:2116:26214:36276 1:N:0:GAACCTAG+TCCGCATA
AGAAGGTTAAAGATGGCTTTCTCTTATAGGACGCCACAGGAGGAACTCTTGAGCAGGCTCCCACAGTC
TCAACAGGAGGTTATAAGCGGATTTCAGTATGAACGCATTCAAAAGGAAGAAGAGAAGAAGGTGGAAA
ACTTTTCTTTTTACC
+
FFFFFFFFFFFFFFFFFFFFFFFFFFFFFFFFFFF:FFFFFFFFFFFFFFFFFFFFFFFFFFFFFFFF
FFF:FFFFFFFFFFFFFFFFFFFFFFFFFFFFFFFFFFFFFFFFFFFFFFFFFFFFFFFFFFFFFFFF
FFFFFFFFFFFFFFF
@A00155:342:HHGFNDSXY:1:2319:25211:28526 1:N:0:GAACCTAG+TCCGCATA
AGAAGGTTAAAGATGGCTTTCTCTTATAGGACGCCACAGGAGGAACTCTTGAGCAGGCTCCCACAGTC
TCAACAGGAGGTTATAAGCGGATTTCAGTATGAACGCATTCAAAAGGAAGAAGAGAAGAAGGTGGAAA
ACTTTTCTTTTTACC
+
FFF:FFFFFFFFFFFFFFFFFFFFFFFFFFFFFFFFFFFFFFFFFFFFFFFFFFFFFFFFFFFFFFFF
FFFFFFFFFFFFFFFFFFFFFFFFFFFFFFFFFFFFFFFFFFFFFFFFFFFFFFFFFFFFFFFFFFFF
FFFFFFFFFFFFFFF

```
@A00155:342:HHGFNDSXY:1:2622:19840:4069 1:N:0:GAACCTAG+TCCGCATA
AGAAGGTTAAAGATGGCTTTCTCTTATAGGACGCCACAGGAGGAACTCTTGAGCAGGCTCCCACAGTC
TCAACAGGAGGTTATAAGTGGATTTCAGTATGAACGCATTCAAAAGGAAGAAGAGAAGAAGGTGGAAA
ACTTTTCTTTTTACC
+
FFFFFFFFFFFFFFFFFF:FFFFFFFFFFFFFFFFFFFFFFFFFFFFFFFFFFFFFFFFFFFFFFFFF
FFFFFFFFFFF:FFFFFFFFFFFFFFFFFFFFFFFFFFFFFFFFFFFFFFFFFFFFFFFFFFFFFF,F
FFFFFFFFFFFF,FF
@A00155:342:HHGFNDSXY:1:1231:2528:33708 2:N:0:GAACCTAG+TCCGCATA
AGAAGGTTAAAGATGGCTTTCTCTTATAGGACGCCACAGGAGGAACTCTTGAGCAGGCTCCCACAGTC
TCAACAGGAGGTTATAAGCGGATTTCAGTATGAACGCATTCAAAAGGAAGAAGAGAAGAAGGTGGAAA
ACTTTTCTTTTTACC
+
FFFFFFFFFFFFFFFFFFFFFFFFFFFFFFFFFFFFFFFFFFFFFFFFFFFFFFFFFFFF,FFFFFFF
FFFFFFFFFFF,FFFFFFFFFF:FFFFFFFFFFFFFFFF:F,FFFFFFFFFFFFFFFFFFFFFFFFFF
FFFFFFFFFFFFFFF
@A00155:342:HHGFNDSXY:1:1320:9588:14935 2:N:0:GAACCTAG+TCCGCATA
AGAAGGTTAAAGATGGCTTTCTCTTATAGGACGCCACAGGAGGAACTCTTGAGCAGGCTCCCACAGTC
TCAACAGGAGGTTATAAGCGGATTTCAGTATGAACGCATTCAAAAGGAAGAAGAGAAGAAGGTGGAAA
ACTTTTCTTTTTACC
+
F:FFFFFFFFFFFFFFFFFFFFFFFFFFFFFFFFFFFFFFFFFFFFFFFFFFFFFFFFFFFFFFFFFF
FFFFFFFFFFFFFFFFFFFFFFFFFF:FFFFF:FFFFFFFFFFFFFFFFFFFFFFFFFFFFF:FFFFF
:FFFFFFFFFFF:F:
@A00155:342:HHGFNDSXY:1:1320:8775:15593 2:N:0:GAACCTAG+TCCGCATA
AGAAGGTTAAAGATGGCTTTCTCTTATAGGACGCCACAGGAGGAACTCTTGAGCAGGCTCCCACAGTC
TCAACAGGAGGTTATAAGCGGATTTCAGTATGAACGCATTCAAAAGGAAGAAGAGAAGAAGGTGGAAA
ACTTTTCTTTTTACC
+
FFFFFFFF:FFFFFFFFFFFFFFFFFFFFFFFFFFFFFFFFFFFFFFFFFFFFFFFFFFFFFFFFFFF
FFFFFFFFFFFFFFFF:FFFFFFFFFFFFFFFFFFFFFFFFFFFFFFFFFFFF:F:,FFFFFFFFFFF
FFFFFFFFFFFFFFF
@A00155:342:HHGFNDSXY:1:1339:14443:17832 2:N:0:GAACCTAG+TCCGCATA
AGAAGGTTAAAGATGGCTTTCTCTTATAGGACGCCACAGGAGGAACTCTTGAGCAGGCTCCCACAGTC
TCAACAGGAGGTTATAAGCGGATTTCAGTATGAACGCATTCAAAAGGAAGAAGAGAAGAAGGTGGAAA
ACTTTTCTTTTTACC
+
FFFFFFFFFFFFFFFFFFFFFFFFFFFFFFFFFFFFFFFFFFFFFFFFFFFFFFFFFFFFFFFFFFFF
FFFFFFFFFFFFFFFFF:FFFFFFFFFFFFFFFFFFFFFFFFFFFFFFFF:FFFFFFFFFFFFFFFFF
FFFFFF:FFFFFFF:
@A00155:342:HHGFNDSXY:1:1645:15338:35415 2:N:0:GAACCTAG+TCCGCATA
AGAAGGTTAAAGATGGCTTTCTCTTATAGGACGCCACAGGAGGAACTCTTGAGCAGGCTCCCACAGTC
TCAACAGGAGGTTATAAGCGGATTTCAGTATGAACGCATTCAAAAGGAAGAAGAGAAGAAGGTGGAAA
ACTTTTCTTTTTACC
+
FFFFFFFFFFFFFFFFFFFFFFFFFFFFFFFFFFFFFFFFFFFFFFFFFFFFFFFFFFFFFFFFFFFF
FFFFFFFFFFFFFFFFFFFFFFFFFFFFFFFFFFFFFFFFFFFFFF:FFFFFFFFFFFFFFFFFFFFF
FFFFFFFFFFFFFF:
@A00155:342:HHGFNDSXY:1:2235:31521:32878 2:N:0:GAACCTAG+TCCGCATA
AGAAGGTTAAAGATGGCTTTCTCTTATAGGACGCCACAGGAGGAACTCTTGAGCAGGCTCCCACAGTC
TCAACAGGAGGTTATAAGCGGATTTCAGTATGAACGCATTCAAAAGGAAGAAGAGAAGAAGGTGGAAA
ACTTTTCTTTTTACA
+
FFFFFFFFFFFFFFFFFFFFFFFFFFFFFFFFFFFFFFFFFFFFFFFF:FFFFFFFFFFFFFFFFFFF
```

FFFFFFFFFFF:FFFFFFFFFFFFFFFFFFFFFFFFFFF:FF:FF:FFFFFFFFFFFFFFFFFFFFFFFF
FFFFFFFFFFFFFFF
@A00155:342:HHGFNDSXY:1:2319:25211:28526 2:N:0:GAACCTAG+TCCGCATA
AGAAGGTTAAAGATGGCTTTCTCTTATAGGACGCCACAGGAGGAACTCTTGAGCAGGCTCCCACAGTC
TCAACAGGAGGTTATAAGCGGATTTCAGTATGAACGCATTCAAAAGGAAGAAGAGAAGAAGGTGGAAA
ACTTTTCTTTTTACC
+
FFF:FFFFFFFFFFFFFFFFFFFFFFFFFFFFFFFFFFFFFFFFFFFFFFFFFFFFFFFFFFFFFFFFFF
FFFFFFFF:FF:FFFFFFFFFF:FFFFFFFFFFFFFFFFFFFFFFFFFFFFFFFFFFFFFFFFFFFFF:F
FFFFFFFF:FFFFFF
@A00155:342:HHGFNDSXY:1:1310:4661:27602 2:N:0:GAACCTAG+TCCGCATA
GAAGGTTAAAGATGGCTTTCTCTTATAGGACGCCACAGGAGGAACTCTTGAGCAGGCTCCCACAGTCT
CAACAGGAGGTTATAAGCGGATTTCAGTATGAACGCATTCAAAAGGAAGAAGAGAAGAAGGTGGAAAA
CTTTTCTTTTTACCT
+
FFFFFFFFFFFFFFFFFFFFFFFFFFFFFFFFFFFFFFFFFFFFFFFFFFFFFFFFFFFFFFFFFFFFFF
FFFFFFFFFFFFFFFF:FFFFFFFFFFFFFFFFFFFFFFFFFFFFFFFFFFFFFFFFFFFFFFFFFFFFF
FFFFFFFFFFFF:FF
@A00155:342:HHGFNDSXY:1:1608:32208:8140 2:N:0:GAACCTAG+TCCGCATA
GAAGGTTAAAGATGGCTTTCTCTTATAGGACGCCACAGGAGGAACTCTTGAGCAGGCTCCCACAGTCT
CAACAGGAGGTTATAAGCGGATTTCAGTATGAACGCATTCAAAAGGAAGAAGAGAAGAAGGTGGAAAA
CTTTTCTTTTTACCT
+
:::FFFFFFFFFFFFFF,:FFFFFFFFFF:FFFFFFFFFFFFFFFFFFFFFFFFFFF:FFFFFFFFFFFF
FFFF::FFFFFFF:FFFFF:FF:FFF::,:FFFFFFFFFFFFFFFFFFF:FFFFFFFFFF,FFF,FFFFF
FFFFFFFF,:FFFFF
@A00155:342:HHGFNDSXY:1:1216:10502:20682 2:N:0:GAACCTAG+TCCGCATA
AAGGTTAAAGATGGCTTTCTCTTATAGGACGCCACAGGAGGAACTCTTGAGCAGGCTCCCACAGTCTC
AACAGGAGGTTATAAGTGGATTTCAGTATGAACGCATTCAAAAGGAAGAAGAGAAGAAGGTGGAAAAC
TTTTCTTTTTACCTA
+
FFFFFFFFFFFFFFFFFFFFFFFFFFFFFFFFFFFF:FFFFFF:FFFFFFFFFFFFFFFFFFFFFFFFFF
FFFFFFFFFFFFF:FFFFFFFFFFFFFFFFFFFFFFFFFFFFFFF:FF:FFFFFFFFFFFFFFFFFFFFF
FFFFFFFF:FFFFFF
@A00155:342:HHGFNDSXY:1:1135:19180:29575 1:N:0:GAACCTAG+TCCGCATA
AGGTTAAAGATGGCTTTCTCTTATAGGACGCCACAGGAGGAACTCTTGAGCAGGCTCCCACAGTCTCA
ACAGGAGGTTATAAGCGGATTTCAGTATGAACGCATTCAAAAGGAAGAAGAGAAGAAGGTGGAAAACT
TTTCTTTTTACCTAC
+
FFF:FFFFFFF,:FFFF:FFFFFFFFFFFFF:FFFFF:FF:FFFFFFFFFFFFFFFFFFFFFFFFFFFFF
FFFF:F,FFFFFFF:F:FFFFF,FFF:FFFFFFFFFFFFF:F:FFFFFFFFFFFFFFF,::FFF:FFFFF
FFFFFF:FFFFFF:F
@A00155:342:HHGFNDSXY:1:1274:29315:27868 1:N:0:GAACCTAG+TCCGCATA
CTGTTAAAGATGGCTTTCTCTTATAGGACGCCACAGGAGGAACTCTTGAGCAGGCTCCCACAGTCTCA
ACAGGAGGTTATAAGCGGATTTCAGTATGAACGCATTCAAAAGGAAGAAGAGAAGAAGGTGGAAAACT
TTTCTTTTTACCTAC
+
FFFFFFF:FFFFFFFFFFFFFFFFFFFFFFFFFFF:FFFFFFFFFFFFFFFFFFFFFFFFFFFFFFFFFF
FFFFFFFFFFFFFFFFFFFFFFFFFFFFFFFFFFFFFFFFFFFFFFFFFFFFFFFFFF:FFFFFFFFFFF
FFFFFFFFFFFFFFF
@A00155:342:HHGFNDSXY:1:1366:28592:8703 1:N:0:GAACCTAG+TCCGCATA
AGGTTAAAGATGGCTTTCTCTTATAGGACGCCACAGGAGGAACTCTTGAGCAGGCTCCCACAGTCTCA
ACAGGAGGTTATAAGCGGATTTCAGTATGAACGCATTCAAAAGGAAGAAGAGAAGAAGGTGGAAAACT
TTTCTTTTTACCTAC

+
FFFFFFFFFFFFFFFFFFFFFFFFFFFFFFFFFFFFFFFFFFFFFFFFFFFFFFFFFFFFF:FFFFFF
FFFFF:FFFFFFFFFFFFFFFFFFFFFFFFFFFFFFFFFF,FFFFFFFFFFFFFFFFFF:FFFFFFFF
FFFFFFFFFFFFFFF
@A00155:342:HHGFNDSXY:1:1509:10321:32205 1:N:0:GAACCTAG+TCCGCATA
AGGTTAAAGATGGCTTTCTCTTATAGGACGCCACAGGAGGAACTCTTGAGCAGGCTCCCACAGTCTCA
ACAGGAGGTTATAAGCGGATTTCAGTATGAACGCATTCAAAAGGAAGAAGAGAAGAAGGTGGAAAACT
TTTCTTTTTACCTAC
+
FFFFFF:FFFFFFFFFFFFFFFFF,FF:FFFFFFFFFFFFFFFFFFFFFFFFFFFFFFFFFFFFFFF,
FFFFFFFFFFFFF,F:FFFFFFFFFFFFFFFFFFFFFFFFFFFFFFFFFFFFFFFFFFFFFFF:FFFF
FFFFFFFFFFFFFFF
@A00155:342:HHGFNDSXY:1:1216:10502:20682 1:N:0:GAACCTAG+TCCGCATA
GGTTAAAGATGGCTTTCTCTTATAGGACGCCACAGGAGGAACTCTTGAGCAGGCTCCCACAGTCTCAA
CAGGAGGTTATAAGTGGATTTCAGTATGAACGCATTCAAAAGGAAGAAGAGAAGAAGGTGGAAAACTT
TTCTTTTTACCTACC
+
FFFFFF,FFFFFFFFFFFFFFFFFFFFFFFFFFFFFFFFFFFFFFFFFFFFFFFFFFFFF,FFFFFFF
FFFFFFFFFFF:FFFFFFFFFFF:FFFFFFFFFFFFFFFFFFFFFFFFFFFFFF,FFFFFFFFFFFFF
FFFFFFFFFFFFFFF
@A00155:342:HHGFNDSXY:1:1264:2718:35603 1:N:0:GAACCTAG+TCCGCATA
GGTTAAAGATGGCTTTCTCTTATAGGACGCCACATGAGGAACTCTTGAGCAGGCTCCCACAGTCTCAA
CAGGAGGTTATAAGCGGATTTCAGTATGAACGCATTCAAAAGGAAGAAGAGAAGAAGGTGGAAAACTT
TTCTTTTTACCTACC
+
F:FFFFFFFFFFFFFFFF,FFFFFFF,,:F,:FF,FF:,FFFFFFFFFFFFFFFFF:FFFFFFFFFFF
:FFFFFFFFFFFFF,:FFFFFFF:FFFFF:,F:FFFFFF:F:,FF::FFFFFFFFFFFFFFFFFFFFF
FFFFFFFFFFFFF:F
@A00155:342:HHGFNDSXY:1:1269:6732:18349 1:N:0:GAACCTAG+TCCGCATA
GGTTAAAGATGGCTTTCTCTTATAGGACGCCACAGGAGGAACTCTTGAGCAGGCTCCCACAGTCTCAA
CAGGAGGTTATAAGCGGATTTCAGTATGAACGCATTCAAAAGGAAGAAGAGAAGAAGGTGGAAAACTT
TTCTTTTTACCTACC
+
FFFFFFFFFFFFFFFF:FFFFFFFFF:FFFFFFFFFFFFFFFFFFFFFFFFFFFFFFFFFFFFFFFFF
FFFFFFFFFFFFFFFFFFFFFFFFFFFFFFFFFFFFFFFFFFFFF:FFFFFFFF:F,,FFFFF:FFFF
FFFFFFFFFFFFFFF
@A00155:342:HHGFNDSXY:1:1348:32407:23109 1:N:0:GAACCTAG+TCCGCATA
GGTTAAAGATGGCTTTCTCTTATAGGACGCCACAGGAGGAACTCTTGAGCAGGCTCCCACAGTCTCAA
CAGGAGGTTATAAGCGGATTTCAGTATGAACGCATTCAAAAGGAAGAAGAGAAGAAGGTGGAAAACTT
TTCTTTTTACCTACC
+
FFFFF:FF,FFFFFFFFFFFFFF:FFFFFFFFFFFF,FF:FFFFFFF:FF:FFFFFFFFF:F:F:FFF
FFFFFFFFFFF:,FFFFFF:FFFFFF:F,FFFFFF:FFF::FF:FFFFFFFFFFFFFFFFFFFFFFFF
FFFFFFFFFFFFFFF
@A00155:342:HHGFNDSXY:1:1516:24487:33191 1:N:0:GAACCTAG+TCCGCATA
GGTTAAAGATGGCTTTCTCTTATAGGACGCCACAGGAGGAACTCTTGAGCAGGCTCCCACAGTCTCAA
CAGGAGGTTATAAGCGGATTTCAGTATGAACGCATTCAAAAGGAAGAAGAGAAGAAGGTGGAAAACTT
TTCTTTTTACCTACC
+
FFFFFFFFFFFFFFFFFFFFFFFFFFFFFFFFFFFFFFFFFFFFFFFFFFFFFFFFFFFFFFFFFFFF
FFFFFFFFFFFF,FFFFFFFF:FFFFFFFFFFFFFFFFF,FFFFFFFFFFFFFFFF::FF:F:FFFFF
FFFFFFFFFFFFFFF
@A00155:342:HHGFNDSXY:1:1542:13358:23093 1:N:0:GAACCTAG+TCCGCATA
GGTTAAAGATGGCTTTCTCTTATAGGACGCCACAGGAGGAACTCTTGAGCAGGCTCCCACAGTCTCAA

CAGGAGGTTATAAGCGGATTTCAGTATGAACGCATTCAAAAGGAAGAAGAGAAGAAGGTGGAAAACTT
TTCTTTTTACCTACC
+
FFF:FFFF:FFFFFFFFFFFFFFFFFFFFFFFFFFFFFFFFFFFFFFFFFFFFFFFFFFFFF:FFFFF
FFFFFFFF:FFFFFFFFFFFFFFFFFFFFFFFFFFFFFFFFFFFFFFFFFFFFFFFFFFFFF:FFFFF
FFFFFFFFFFFFFFF
@A00155:342:HHGFNDSXY:1:2245:3332:14841 1:N:0:GAACCTAG+TCCGCATA
GGTTAAAGATGGCTTTCTCTTATAGAACGCCACAGGAGGAACTCTTGAGCAGGCTCCCACAGTCTCAA
CAGGAGGTTATAAGCGGATTTCAGTATGAACGCATTCAAAAGGAAGAAGAGAAGAAGGTGGAAAACTT
TTCTTTTTACCTACC
+
FFFFFFFFFFFFFFFFFFFFFFFFFFFFFF:FFFFFFFFFFFFFFFFFFFFFFFFFFFFFFFFFFFFF
FFFFFFFFFFFF:FFFFFF::FFFFFFFFFFFFFFFFFFFFFFFFFFFFFF:F:F:FF,:FFF:FFFF
FFFFFFFFFFFFFFF
@A00155:342:HHGFNDSXY:1:1117:30725:3505 2:N:0:GAACCTAG+TCCGCATA
GGCTAAAGATGGCTTTCTCTTATAGGACGCCACAGGAGGAACTCTTGAGCAGGCTCCCACAGTCTCAA
CAGGAGGTTATAAGCGGATTTCAGTATGAACGCATTCAAAAGGAAGAAGAGAAGAAGGTGGAAAACTT
TTCTTTTTACCTACC
+
FFFFFFFFFFFFFFFFFFFFF:FFFFFFFFFFFFFFFFFF:FFFFFFFFFFFFFFFFFFFFFFFFFFF
F:FFFFFFFFFFFFFFFFFFFFFFFFFFFFFFFF:FFFFFFFFFFFFFFFFFFFFFFFFFFFFFFFFF
FFFFFFFFFFFFFFF
@A00155:342:HHGFNDSXY:1:1264:17752:8218 2:N:0:GAACCTAG+TCCGCATA
GGTTAAAGATGGCTTTCTCTTATAGGACGCCACAGGAGGAACTCTTGAGCAGGCTCCCACAGTCTCAA
CAGGAGGTTATAAGCGGATTTCAGTATGAACGCATTCAAAAGGAAGAAGAGAAGAAGGTGGAAAACTT
TTCTTTTTACCTACC
+
FFFFFFFFFFFFFFFFFFFFFFFFFFFFFFFFFFFFFFFFFFFFFFFFFFFFFFFFFFFFFFFFFFFF
FFFFFF:FFFFFFFFFFFFFFFFFFFFFFFFFFFFFFFFFFFFFFFFFFFFFFFFFFFFFFF:F:FFF
FFFFFFFFFFFFFFF
@A00155:342:HHGFNDSXY:1:1516:24487:33191 2:N:0:GAACCTAG+TCCGCATA
GGTTAAAGATGGCTTTCTCTTATAGGACGCCACAGGAGGAACTCTTGAGCAGGCTCCCACAGTCTCAA
CAGGAGGTTATAAGCGGATTTCAGTATGAACGCATTCAAAAGGAAGAAGAGAAGAAGGTGGAAAACTT
TTCTTTTTACCTACC
+
FFFFFFFFFFFFFFFFFFFFFFFFFFFFFFFFFFFFFFFF:FFFFFFFFFFFFFFFFFFFFFFFFFFF
FFFFFFFFFFFFFFFFFFFFFFFFFFFFFFF,FFFFFFFFF:FFFFFFFFFFFFFFFFFFFFFFFFFF
FFFFFFFFFFFFFFF
@A00155:342:HHGFNDSXY:1:1113:5032:22357 2:N:0:GAACCTAG+TCCGCATA
GTTAAAGATGGCTTTCTCTTATAGGACGCCACAGGAGGAACTCTTGAGCAGGCTCCCACAGTCTCAAC
AGGAGGTTATAAGTGGATTTCAGTATGAACGCATTCAAAAGGAAGAAGAGAAGAAGGTGGAAAACTTT
TCTTTTTACCTACCA
+
FFFFFFFFFFFFFFFFFFFFFFFFFFFFFFFFFFFF:FFFFFFFFFFFFFFFFFFFFFFFFFFFFFFF
FFFFFFFFFFFFFFFFFFFFFFFFFFFFFFFFFFFFFFFFFFFFFFFFFFFFFFFFFFFFFFFFF:FF
FFFFFFFFFFFFFFF
@A00155:342:HHGFNDSXY:1:1115:13720:12602 2:N:0:GAACCTAG+TCCGCATA
GTTAAAGATGGCTTTCTCTTATAGGACGCCACAGGAGGAACTCTTGAGCAGGCTCCCACAGTCTCAAC
AGGAGGTTATAAGCGGATTTCAGTATGAACGCATTCAAAAGGAAGAAGAGAAGAAGGTGGAAAACTTT
TCTTTTTACCTACCA
+
FFFFFF:FFFFFFFFFFFFFFFFFFFFFFFFFFFFFFFFFFFFFFFFFFFFFFFFFFFFFFFFFFFFF
FFFFFFFFFFFFFFFFFFFFFFFFFFFFFFFFFFFFFFFFFFFFFFFFFFFFFFFFFFFFFFFFFFFF
FFFFFFFFFFFFFFF

```
@A00155:342:HHGFNDSXY:1:1242:1741:8390 2:N:0:GAACCTAG+TCCGCATA
GTTAAAGATGGCTTTCTCTTATAGGACGCCACAGGAGGAACTCTTGAGCAGGCTCCCACAGTCTCAAC
AGGAGGTTATAAGCGGATTTCAGTATGAACGCATTCAAAAGGAAGAAGAGAAGAAGGTGGAAAACTTT
TCTTTTTACCTACCA
+
FFFF,:FFFFFFFFFFFFFFFFFFFFFFFFFF:FF:FF:FFFFFFF:FFFFFFFFFFFFFFFFFF:FF
FFFFFFFFFFFFFFFFFFFFF:FFFFFFFFFF:FFFFF:,F:FFF,FFFFFFFF:FFFFFFFF,:FFF
F:FFFF:FFFFFFF:
@A00155:342:HHGFNDSXY:1:1274:29315:27868 2:N:0:GAACCTAG+TCCGCATA
GTTAAAGATGGCTTTCTCTTATAGGACGCCACAGGAGGAACTCTTGAGCAGGCTCCCACAGTCTCAAC
AGGAGGTTATAAGCGGATTTCAGTATGAACGCATTCAAAAGGAAGAAGAGAAGAAGGTGGAAAACTTT
TCTTTTTACCTACAG
+
FFFFFFFFFFFFFFFFFFFFFFFFFFFFFFFFFFFFFFF:FFFFFFFFFFFFFFFFFFFFFFFFFFFF
FFFFFFFFFFFFFFFFFFFFFFFFFFFFFFFFFFFFFFFFFFFFFFFFFFF:FFFFFFFFFFFFFFFF
FFFFFFFFFF:FFFF
@A00155:342:HHGFNDSXY:1:1410:27543:32346 2:N:0:GAACCTAG+TCCGCATA
GTTAAAGATGGCTTTCTCTTATAGGACGCCACAGGAGGAACTCTTGAGCAGGCTCCCACAGTCTCAAC
AGGAGGTTATAAGCGGATTTCAGTATGAACGCATTCAAAAGGAAGAAGAGAAGAAGGTGGAAAACTTT
TCTTTTTACCTACCA
+
:FFFFFFFFFFFFFFFFFFFFFFFFFFFFFFFFFFFFFFFFFFFFFFFFF:FFFFFFFFFFFFFFFFF
FFFFFFFFFFFFFFFFFFFFFFFF:FFFFFFFFFFFFFFFFFFFFFFFFFFFFFFFFFFFFFF:FFFF
FFFFFFFFFFFFFFF
@A00155:342:HHGFNDSXY:1:1414:8323:5415 2:N:0:GAACCTAG+TCCGCATA
GTTAAAGATGGCTTTCTCTTATAGGACGCCACAGGAGGAACTCTTGAGCAGGCTCCCACAGTCTCAAC
AGGAGGTTATAAGCGGATTTCAGTATGAACGCATTCAAAAGGAAGAAGAGAAGAAGGTGGAAAACTTT
TCTTTTTACCTACCA
+
FFFFFFFFFFFFFFFFFFFFFFFFFFFFFFFFFFFFFFFFFFFFFFFFFFFFFFFFFFFFFFFFF:FF
FFF:FFFFFFFFFFFFFFFFFFFFFFFFFFFFFFFFFFFFFFFFF:FFFFFFFF:FFFFFFFF,FFFF
F:FFFFFFFFFFF,F
@A00155:342:HHGFNDSXY:1:1468:13693:18161 2:N:0:GAACCTAG+TCCGCATA
GTTAAAGATGGCTTTCTCTTATAGGACGCCACAGGAGGAACTCTTAAGCAGGCTCCCACAGTCTCAAC
AGGAGGTTATAAGCGGATTTCAGTATGAACGCATTCAAAAGGAAGAAGAGAAGAAGGTGGAAAACTTT
TCTTTTTACCTACCA
+
FFFFFFFFFFFFFFFFFFFFFFFFFFFFFFFFFFFFFFFFFFFFFFFFFFFFFFFFFFFFFFFFFFFF
FFFFFFFFFFFFFFFFFFFFFFFFFFFFFFFFFFFFFFFFFFFFFFFFFFFFFFFFFFFFFFF:FFFF
FFFFFFFFFFFFFFF
@A00155:342:HHGFNDSXY:1:1504:15564:23563 2:N:0:GAACCTAG+TCCGCATA
GTTAAAGATGGCTTTCTCTTATAGGACGCCACAGGAGGAACTCTTGAGCAGGCTCCCACAGTCTCAAC
AGGAGGTTATAAGTGGATTTCAGTATGAACGCATTCAAAAGGAAGAAGAGAAGAAGGTGGAAAACTTT
TCTTTTTACCTACCA
+
FFFFFFFFFFFFFFFFFFFFFFFFFFFFFFFFFFFFFFFFFFFFFFFFFFFFFFFFFFFFFFFFFFFF
FFFFFFFFFFFFFFFFFFFFFFFFFFFFFF:FFFFFFFFFFFFFFFFFFFFFFFFFFFFFFFFFFFFF
FFFFFFFFFFFFFFF
@A00155:342:HHGFNDSXY:1:1532:25889:17675 2:N:0:GAACCTAG+TCCGCATA
GTTAAAGATGGCTTTCTCTTATAGGACGCCACAGGAGGAACTCTTGAGCAGGCTCCCACAGTCTCAAC
AGGAGGTTATAAGTGGATTTCAGTATGAACGCATTCAAAAGGAAGAAGAGAAGAAGGTGGAAAACTTT
TCTTTTTACCTACCA
+
FFFFFFFFFFFFFFFFFFFFFFFFFFFFFFFFFFFFFFFFFFFFFFFFFFFFFFFFFFFFF:FFFFFF
```

FFFFFFFFFFFFF:FFFFFFFFFF:FFFFFFFFFFF:FFFFFFFFF:FFFFFFFFFFFFF:FFFFFF
FFFFFFFFFFFFFFF
@A00155:342:HHGFNDSXY:1:1562:28149:32957 2:N:0:GAACCTAG+TCCGCATA
GTTAAAGATGGCTTTCTCTTATAGGACGCCACAGGAGGAACTCTTGAGCAGGCTCCCACAGTCTCAAC
AGGAGGTTATAAGCGGATTTCAGTATGAACGCATTCAAAAGGAAGAAGAGAAGAAGGTGGAAAACTTT
TCTTTTTACCTACCA
+
FFFFFFFFFFFFFFFFFFFFFFF:FFFFFFFFFFFFFFFFFFFFFFFFFFFFFFF:::FFFFFFF,FF
FF:FFFFFFFFF:FFFFFFFFFFFFFFFFF:F:FFFFFFFFFFF,FFFFF,FFFFFFFFFFFFFFF
FFFFFFFFF,FFFFF
@A00155:342:HHGFNDSXY:1:1656:27552:13260 2:N:0:GAACCTAG+TCCGCATA
GTTAAAGATGGCTTTCTCTTATAGGACGCCACAGGAGGAACTCTTGAGCAGGCTCCCACAGTCTCAAC
AGGAGGTTATAAGCGGATTTCAGTATGAACGCATTCAAAAGGAAGAAGAGAAGAAGGTGGAAAACTTT
TCTTTTTACCTACCA
+
FFFFFFFFFFFFFFFFFFFFFFFFFFFFFFFFFFFFF,FFFFFFFFFFFFFFFFFFFFFFFFFFFFFF
FFFFFF:FFFFFFFFFFFFFFFFFFFFFFFFFFFFFFFFFFFFFFFFFF:FFFFFFFFFFFFFFFFF
FFFFFFFFFFFFFFF
@A00155:342:HHGFNDSXY:1:1658:19813:14011 2:N:0:GAACCTAG+TCCGCATA
GTTAAAGATGGCTTTCTCTTATAGGACGCCACAGGAGGAACTCTTGAGCAGGCTCCCACAGTCTCAAC
AGGAGGTTATAAGCGGATTTCAGTATGAACGCATTCAAAAGGAAGAAGAGAAGAAGGTGGAAAACTTT
TCTTTTTACCTACCA
+
FFFFFFFFFFFFFFFFFFFFFFFFFFFFFFFFFFFFF:FFFFFFFFFFFFFFFFFFFFFFFFFFFFF:
FFFFFFFFFFFFFFFFFFFFFFFFFFFFFFFFFFFFFFFFFFFFFFFFFFFFFFFFFFFFFFFFFFFF
F:FFFFFFFFFFFFF
@A00155:342:HHGFNDSXY:1:2137:18087:4977 2:N:0:GAACCTAG+TCCGCATA
GTTAAAGATGGCTTTCTCTTATAGGACGCCACAGGAGGAACTCTTGAGCAGGCTCCCACAGTCTCAAC
AGGAGGTTATAAGCGGATTTCAGTATGAACGCATTCAAAAGGAAGAAGAGAAGAAGGTGGAAAACTTT
TCTTTTTACCTACCA
+
FFFFFFFFFFFFFFFFFFFFFFFFFFFFFFFFFFFFFFFFFFFFFFFFFFFFFFFFFFFFFFFFFFF:
FFFFFFF:FFFFFFFFF,FFFFFFFFFFFFFFFFFFFFFFFFFFFFFFFFF:FFFFFFFFFFFFFFF:F
FFFFFFFFFFFF::F
@A00155:342:HHGFNDSXY:1:2240:19714:12994 2:N:0:GAACCTAG+TCCGCATA
GTTAAAGATGGCTTTCTCTTATAGGACGCCACAGGAGGAACTCTTGAGCAGGCTCCCACAGTCTCAAC
AGGAGGTTATAAGCGGATTTCAGTATGAACGCATTCAAAAGGAAGAAGAGAAGAAGGTGGAAAACTTT
TCTTTTTACCTACCA
+
FFFFFFFFFFFFFFFFFF,FFFFFFFFFFFFFFFFFFFFFFFFFFFFFFFFFFFFFFFFFFFFFFFFFF
:FFFFFFF:FFFFFFFFFFFFFFFFFFFFFFFFFFFFFFFFFFFFFFFFFFFFFFFFFFFFFFFFFFF
FFFFFFFFFFFFFFF
@A00155:342:HHGFNDSXY:1:2307:4047:3709 2:N:0:GAACCTAG+TCCGCATA
GTTAAAGATGGCTTTCTCTTATAGGACGCCACAGGAGGAACTCTTGAGCAGGCTCCCACAGTCTCAAC
AGGAGGTTATAAGCGGATTTCAGTATGAACGCATTCAAAAGGAAGAAGAGAAGAAGGTGGAAAACTTT
TCTTTTTACCTACCA
+
FFFFFFFFFFFFFFFFFFFFFFFFFFFFFFFFFFF:FFFFFFFFFFFFFFFFFFFFFFFFFFFFFFFF
FFFFFFF:FFFFFFFFFFFFFFFFFFFFFFFFFFFFFFFFFFFFFFFFFFFFFFFFFFFFFFFFFFFF
FFF:FFFFFFFFFFF
@A00155:342:HHGFNDSXY:1:2261:19180:27665 2:N:0:GAACCTAG+TCCGCATA
GTTAAAGATGGCTTTCTCTTATAGGACGCCACAGGAGGAACTCTTGAGCAGGCTCCCACAGTCTCAAC
AGGAGGTTATAAGCGGATTTCAGTATGAACGCATTCAAAAGGAAGAAGAGAAGAAGGTGGAAAACTTT
TCTTTTTACCTACCA

+
FFFFFFFFFFFFFFFFFFFFFFFFFFFFFF:FFFFFFFF,FFFFFFFFFFFFFFFFFFFFFFFFF,FF
FFFFFFFFFFFFFFFFFFFFFFFF:FFFFFFFFFFFFFFFFFFFFFFFFFFFFFFFFFFFFFFFFFFF
FFFFFFFFFFFFFFF
@A00155:342:HHGFNDSXY:1:2356:20627:36902 2:N:0:GAACCTAG+TCCGCATA
GTTAAAGATGGCTTTCTCTTATAGGACGCCACAGGAGGAACTCTTGAGCAGGCTCCCACAGTCTCAAC
AGGAGGTTATAAGCGGATTTCAGTATGAACGCATTCAAAAGGAAGAAGAGAAGAAGGTGGAAAACTTT
TCTTTTTACCTACCA
+
FFFFFFFFFFFFFFFFFFF:FFFFFFFFFFFFFFFFFFF:FFFFFFFFFFFFFFFFFFFFFFFFFFFF
FFFFFFFFFFFFFFFFF,FFFFFF:FFFFFFFFFFFFFFFFFFFFFFFFFFFFFFFFFFFFFFFFFFF
FFFFFFFFFFFFFFF
@A00155:342:HHGFNDSXY:1:2362:29794:28291 2:N:0:GAACCTAG+TCCGCATA
GTTAAAGATGGCTTTCTCTTATAGGACGCCACAGGAGGAACTCTTGAGCAGGCTCCCACAGTCTCAAC
AGGAGGTTATAAGCGGATTTCAGTATGAACGCATTCAAAAGGAAGAAGAGAAGAAGGTGGAAAACTTT
TCTTTTTACCTACCA
+
FFFFFFFFFFFFFFFFFF:FF:FFFFFFFFFFF:FFFFFFFFFFFFFFFFFFFFFFFFFFFFFF:FFF
FFFFFFFFFFFFFFFFFFFFFFFFFFFFFFFFFFFFFFFFFFFFFFFFFFFFFF,FFFFFFFFFFF,F
FFFFFFFFFFFFFFF
@A00155:342:HHGFNDSXY:1:2402:27624:22153 2:N:0:GAACCTAG+TCCGCATA
GTTAAAGATGGCTTTCTCTTATAGGACGCCACAGGAGGAACTCTTGAGCAGGCTCCCACAGTCTCAAC
AGGAGGTTATAAGCGGATTTCAGTATGAACGCATTCAAAAGGACGAAGAGAAGAAGGTGGAAAACTTT
TCTTTTTACCTACCA
+
:FFFFFFFFFFFFFFFFFFFFFF:FFF,FFFFFFFFFFFFFFF:FFFFFFFFFFF:FFFFFFFFFFFF
F:FFFFFFFFFFFFFFF,FFFFFF:FFFFFFFFFF:FFFFFFF,FFFFFF:FFFFFFFFFFFF::FF:
FFFFFFFFF,FFFFF
@A00155:342:HHGFNDSXY:1:2402:27679:22216 2:N:0:GAACCTAG+TCCGCATA
GTTAAAGATGGCTTTCTCTTATAGGACGCCACAGGAGGAACTCTTGAGCAGGCTCCCACAGTCTCAAC
AGGAGGTTATAAGCGGATTTCAGTATGAACGCATTCAAAAGGAAGAAGAGAAGAAGGTGGAAAACTTT
TCTTTTTACCTACCA
+
FFFFFFFFFFFFFFFFFFFFFFFFFFFFFF:FFFFFFFFFFFFFFFFFFFFFFFFFFFFFFFFFFFFF
FFFFFFFFFFFFFFFFFFFFFFFFFFFFFFFFFFFFFFFFF:FFFF:FFFFFFFF:FFFFFFFFFFFF
FFFFFF:FFFFFFFF
@A00155:342:HHGFNDSXY:1:2613:8820:14043 2:N:0:GAACCTAG+TCCGCATA
GTTAAAGATGGCTTTCTCTTATAGGACGCCACAGGAGGAACTCTTGAGCAGGCTCCCACAGTCTCAAC
AGGAGGTTATAAGCGGATTTCAGTATGAACGCATTCAAAAGGAAGAAGAGAAGAAGGTGGAAAACTTT
TCTTTTTACCTACCA
+
:FFFFFFFFFFFFFFFFFFFFFFFFFF:FFFFFFFFFFFFFFFFFFFFFFFFFFFFFFFFFFFFFFFF
FFFFFFFFFF:FFFFFFFFFFFFFFFFFFFFFFFFFFFFFFFFFFFFFFFFFFFFFFF:FFFFFFFFF
FFFFFFF:FFFFF,F
@A00155:342:HHGFNDSXY:1:2573:14145:25207 2:N:0:GAACCTAG+TCCGCATA
GTTAAAGATGGCTTTCTCTTATAGGACGCCACAGGAGGAACTCTTGAGCAGGCTCCCACAGTCTCAAC
AGGAGGTTATAAGCGGATTTCAGTATGAACGCATTCAAAAGGAAGAAGAGAAGAAGGTGGAAAACTTT
TCTTTTTACCTACCA
+
FFFFFFFFFFFFFFFFFFFFFFFFFFFFFFFFFFFFFFFFFFFFFFFFFFFFFFFFFFFFFFFFFFFF
FFFFFFFF:FFFFFFFFFFFFFFFFFFFFFFFFFFFFFFFFFFFFFFFFFFFFFFFFFFFFF:FFFF
FFFFFFFFFFFFFFF
@A00155:342:HHGFNDSXY:1:2601:12599:3427 2:N:0:GAACCTAG+TCCGCATA
GTTAAAGATGGCTTTCTCTTATAGGACGCCACAGGAGGAACTCTTGAGCAGGCTCCCACAGTCTCAAC

AGGAGGTTATAAGCGGATTTCAGTATGAACGCATTCAAAAGGAAGAAGAGAAGAAGGTGGAAAACTTT
TCTTTTTACCTACCA
+
FF:FFFFFFFFFFFFFFFFFFFFFFFFFFFFFFFFFFFFFFFFFFFFFFFFFFFFFFFFFFFFFFFFF
FFFFFFFFFFFFFFFFFFFFFFFF:FFFFFFFFFFFFFF:FFFFFFFFFFFFFFFFFFFFFFFFFFFF
FFFFFFFFFFFFFFF
@A00155:342:HHGFNDSXY:1:2663:9896:14090 2:N:0:GAACCTAG+TCCGCATA
GTTAAAGATGGCTTTCTCTTATAGGACGCCACAGGAGGAACTCTTGAGCAGGCTCCCACAGTCTCAAC
AGGAGGTTATAAGCGGATTTCAGTATGAACGCATTCAAAAGGAAGAAGAGAAGAAGGTGGAAAACTTT
TCTTTTTACCTACCA
+
FF:FFFFFFFFFFFFFFFFFFFFFFFFFF,FF,FFFF,F,FFFFFFFF:FFFFFF:FFFFFFFFFFFF
,FFFFFFFFFFFFFF:FF:FFFFFFF,FF:FFFFFFFFFFFFFFFFFFFF::FFFFFF,FFFF:FFFF
FFF,FFFFFFFF:FF
@A00155:342:HHGFNDSXY:1:1303:2211:4820 2:N:0:GAACCTAG+TCCGCATA
TTAAAGATGGCTTTCTCTTATAGGACGCCACAGGAGGAACTCTTGAGCAGGCTCCCACAGTCTCAACA
GGAGGTTATAAGTGGATTTCAGTATGAACGCATTCAAAAGGAAGAAGAGAAGAAGGTGGAAAACTTTT
CTTTTTACCTACCA
+
FFFFFFFFFFFFFFFFFFFFFFFFFF:FFFFFFFFF,FFFFFFFF:FFF,FFFFFFFFFFFFFF:::F
FFFFFFFFFFFFFFFFFFFFFFFFFFFFFF:FFFFFFF,FFFFF:FFF,FF:F:FFFFFFFFFFFFFF
FFFFFFFFFFF:,F
@A00155:342:HHGFNDSXY:1:1107:13187:1564 1:N:0:GAACCTAG+TCCGCATA
TTAAAGATGGCTTTCTCTTATAGGACGCCACAGGAGGAACTCTTGAGCAGGCTCCCACAGTCTCAACA
GGAGGTTATAAGCGGATTTCAGTATGAACGCATTCAAAAGGAAGAAGAGAAGAAGGTGGAAAATTTTT
CTTTTTACCTACCAG
+
FFF:F:,F:FFFFFFFFFFFFFFFF:FFFFFF:FFF,::FFFFFFFFF:FF:FF,,FFF:F,FFF:F:
FFFFFF,F:F:F,FFFFFFFFFF:FFFF:::FFF:FF::FFFFFFFFFFFFFFF::FF:FFFFFFF:F
FFFFFF:FFFFF:FF
@A00155:342:HHGFNDSXY:1:1115:13720:12602 1:N:0:GAACCTAG+TCCGCATA
TTAAAGATGGCTTTCTCTTATAGGACGCCACAGGAGGAACTCTTGAGCAGGCTCCCACAGTCTCAACA
GGAGGTTATAAGCGGATTTCAGTATGAACGCATTCAAAAGGAAGAAGAGAAGAAGGTGGAAAACTTTT
CTTTTTACCTACCAG
+
FFFFFFFFFFFFFFFFFFFFFFFFFFFFFFFFFFFFFFFFFFFFFFFFFFFFFFFFFFFFFFFF:FFF
FFFFFFFFFFFFFFFFFFFFFFFFFFFFFFFFFFFFFFFFFFFFFFFFFFFF,F:FFFFF,FFFFFFF
FFFFFFFFFFFFFFF
@A00155:342:HHGFNDSXY:1:1124:1832:28588 1:N:0:GAACCTAG+TCCGCATA
TCCGATCTGGCTTTCTCTTATAGGACGCCACAGGAGGAACTCTTGAGCAGGCTCCCACAGTCTCAACA
GGAGGTTATAAGCGGATTTCAGTATGAACGCATTCAAAAGGAAGAAGAGAAGAAGGTGGAAAACTTTT
CTTTTTACCTACCAG
+
FFFFFFFFF:F,FFFFFFFFF:FFFFFFFFFFFFFFFFFFFFFFFFFFFFFFFFFFFFFFFFFFFFFF
FFFFFFFFFF:FFFFFFFFFFF:FFFFFFFFFFFFFF:FFFFFFFFFFFFFF,:,FF:FF,FFFFFF:
FFFFFFFFFFFFFFF
@A00155:342:HHGFNDSXY:1:1136:30761:11647 1:N:0:GAACCTAG+TCCGCATA
TTAAAGATGGCTTTCTCTTATAGGACGCCACAGGAGGAACTCTTGAGCAGGCTCCCACAGTCTCAACA
GGAGGTTATAAGCGGATTTCAGTATGAACGCATTCAAAAGGAAGAAGAGAAGAAGGTGGAAAACTTTT
CTTTTTACCTACCAG
+
FFFFFFFFFFFFFFFFFFFFFFFFFFFFFFFFFFFFFFFFFFFFFFFFFFFFFFFFFFFFFFFF,FFF
FFFFFFFFFFFFFFFFFFFFFFFFFFFFFFFFFFFFFFFFFFFFFFFFFFFF,:F:FFFFFFFFFFFF
FFFFFFFFFFFFFFF

```
@A00155:342:HHGFNDSXY:1:1271:18991:9142 1:N:0:GAACCTAG+TCCGCATA
TTAAAGATGGCTTTCTCTTATAGGACGCCACAGGAGGAACTCTTGAGCAGGCTCCCACAGTCTCAACA
GGAGGTTATAAGCGGATTTCAGTATGAACGCATTCAAAAGGAAGAAGAGAAGAAGGTGGAAAACTTTT
CTTTTTACCTACCAG
+
:FFFFFFFFF:FFFFFFFFFFFFFFFFFFFFFFFF:FFF:FFFFFFFFFFFFFFFFFFFFFFFFFF::FF
FFFFFFFFFF,FFFFFFFFFFFFFFFFFFFFFFFFFFFFFFF:FFFFFFFFFFF::,:FF:FFFFFFF
FFFFFFFFFFFFFFF
@A00155:342:HHGFNDSXY:1:1445:1344:6104 1:N:0:GAACCTAG+TCCGCATA
TTAAAGATGGCTTTCTCTTATAGGACGCCACAGGAGGAACTCTTGAGCAGGCTCCCACAGTCTCAACA
GGAGGTTATAAGCGGATTTCAGTATGAACGCATTCAAAAGGAAGAAGAGAAGAAGGTGGAAAACTTTT
CTTTTTACCTACCAG
+
FFFFFF,FFFFF:FFFF::FFFFFFF,:FFF:,:FF:FFFFFF:FFFFFFFFFFFFF:FF:FFF::FF
FFFFF,FFFFFFFF:F::FFFF:FFF,FFFFFFFFFFFF:FFFFFFFFFFFF,F:,FFFFFFFFFFFF
FFFFFFFFFFFFFFF
@A00155:342:HHGFNDSXY:1:2318:3351:34006 1:N:0:GAACCTAG+TCCGCATA
TTAAAGATGGCTTTCTCTTATAGGACGCCACAGGAGGAACTCTTGAGCAGGCTCCCACAGTCTCAACA
GGAGGTTATAAGCGGATTTCAGTATGAACGCATTCAAAAGGAAGAAGAGAAGAAGGTGGAAAACTTTT
CTTTTTACCTACCAG
+
FFFF:FFFFFFFFFFFFFFFFFFFFFFFFF:FFFFFFFFFF:FFFFFFFFFFFFFFFFFFF,FFFFFFF
FFFFFFFFFFFFFFFFFFFFFFFFFFFFFFFFFF:FFFFFFFFFFFFFFFFFFFF,F,FFFF:FFFFFF
FFFFFFFFFFFFFFF
@A00155:342:HHGFNDSXY:1:2317:17499:8656 1:N:0:GAACCTAG+TCCGCATA
TTAAAGATGGCTTTCTCTTATAGGACGCCACAGGAGGAACTCTTGAGCAGGCTCCCACAGTCTCAACA
GGAGGTTATAAGCGGATTTCAGTATGAACGCATTCAAAAGGAAGAAGAGAAGAAGGTGGAAAACTTTT
CTTTTTACCTACCAG
+
FFFFFFFFFF::FFFFFFFFFFFFFFFFFFFFFFFFFFFFFFFFFFFFFFFFFFFFFFFFFFFFFFFF
FFFFFFFFFFFFFFFFFFFFFFFFFFFFFFFFFFFFFFFFFFFFFFFFFFFFFFFFFFFFFFFFFFFF
FFFFFFFFFFFFFFF
@A00155:342:HHGFNDSXY:1:2367:19217:3865 1:N:0:GAACCTAG+TCCGCATA
TTAAAGATGGCTTTCTCTTATAGGACGCCACAGGAGGAACTCTTGAGCAGGCTCCCACAGTCTCAACA
GGAGGTTATAAGTGGATTTCAGTATGAACGCATTCAAAAGGAAGAAGAGAAGAAGGTGGAAAACTTTT
CTTTTTACCTACCAG
+
FFF:FF:FFFFFFFFFFFFFFFFFFFFFFFFFFFFFFFFF:FFFFFFFFFFFFFFFFFFFFFFFFFFFF
FFFFFFFFFFF:FFFFFFFFFFFFFFFFFFFFFFFFFFFFFFFFFFFFFFFFF:,::F:FFFFFFFFFF
FFFFFFFFFFFFFFF
@A00155:342:HHGFNDSXY:1:2412:21133:36401 1:N:0:GAACCTAG+TCCGCATA
TTAAAGATGGCTTTCTCTTATAGGACGCCACAGGAGGAACTCTTGAGCAGGCTCCCACAGTCTCAACA
GGAGGTTATAAGCGGATTTCAGTATGAACGCATTCAAAAGGAAGAAGAGAAGAAGGTGGAAAACTTTT
CTTTTTACCTACCAG
+
FFFFFFFFFFFFFFFFFFFFFFFFFFFFFFFFFFFFFFFFFFFFFFFFFFFFFFFFFFFFFFFFFFFF
FFFFFFFFFFFFFFFFFFFFFFFFFFFFFFFFFFFFFFFFFFFFFFFFFFFFFF,::FFFFFFFFFF:
FFFFFFFFFFFFFFF
@A00155:342:HHGFNDSXY:1:2450:20573:33395 1:N:0:GAACCTAG+TCCGCATA
TTAAAGATGGCTTTCTCTTATAGGACGCCACAGGAGGAACTCTTGAGCAGGCTCCCACAGTCTCAACA
GGAGGTTATAAGCGGATTTCAGTATGAACGCATTCAAAAGGAAGAAGAGAAGAAGGTGGAAAACTTTT
CTTTTTACCTACCAG
+
FFFF:F:F::FFFFFFFFFFFFFFFFFFFFFFFFFFFFFFFFFFFFFFFFFFFFFFFFFFFFF:FFFFF
```

```
FFFFFFFFFFFFFFFFFFFFFFFFFFFFFFFFFFFFFFFFFFFFFFFFFFFFFFFFFFFF:FFFFFF
FFFFF,FFFFF,FFF
@A00155:342:HHGFNDSXY:1:2473:4743:18004 1:N:0:GAACCTAG+TCCGCATA
CCGATCTTGGCTTTCTCTTATAGGACGCCACAGGAGGAACTCTTGAGCAGGCTCCCACAGTCTCAACA
GGAGGTTATAAGCGGATTTCAGTATGAACGCATTCAAAAGGAAGAAGAGAAGAAGGTGGAAAACTTTT
CTTTTTACCTACCAG
+
FFFFF,FFFFFF:FFFFFFFFFFFFFFFFFFFFFFFFFFFFFFFFFFFFFFFFFFFFFFFFFFFFFFF
FFFFFFFFFFFFFFFFFFFFFFFFFFFFFFFFFFFFFFFFFFFFFFFFFFF::,FF:F:FFFFFFF
FFFFFFFFFFFFFFF
@A00155:342:HHGFNDSXY:1:2512:19551:17065 1:N:0:GAACCTAG+TCCGCATA
TTAAAGATGGCTTTCTCTTATAGGACGCCACAGGAGGAACTCTTGAGCAGGCTCCCACAGTCTCAACA
GGAGGTTATAAGCGGATTTCAGTATGAACGCATTCAAAAGGAAGAAGAGAAGAAGGTGGAAAACTTTT
CTTTTTACCTACCAG
+
FFFFFFFFFFFFFFFFFFFFFFFFFFFFFFFFFFFFFFFFFFFFFFFFFFFFFFFFFFFFFFFFFFFF
FFFFFF:FF:FFFFFFFFFFFFFFFFFFFFFFFFFFFFFFFFFFFFFFFFF:F:FFF,F:FFFFFFF
FFFFFFFFFFFFFFF
@A00155:342:HHGFNDSXY:1:2512:20320:19680 1:N:0:GAACCTAG+TCCGCATA
TTAAAGATGGCTTTCTCTTATAGGACGCCACAGGAGGAACTCTTGAGCAGGCTCCCACAGTCTCAACA
GGAGGTTATAAGCGGATTTCAGTATGAACGCATTCAAAAGGAAGAAGAGAAGAAGGTGGAAAACTTTT
CTTTTTACCTACCAG
+
FFFFFFFFFFFFFFFFFFFFFFFFFFFFFFFFFFFFFFFFFFFFFFFFFFFFFFFFFFFFFFFFFFFF
FFFFFFFFFFFFFFFFFF:FFFFFFFFFFFFFFFFFF:FFFFFFFFFFFF:F:::F,FFF:FFFFF:F
FFFFFFFFFFFFFFF
@A00155:342:HHGFNDSXY:1:1303:1524:6981 2:N:0:GAACCTAG+TCCGCATA
TTAAAGATGGCTTTCTCTTATAGGACGCCACAGGAGGAACTCTTGAGCAGGCTCCCACAGTCTCAACA
GGAGGTTATAAGTGGATTTCAGTATGAACGCATTCAAAAGGAAGAAGAGAAGAAGGTGGAAAACTTTT
CTTTTTACCTACCAG
+
FFFF:F:FFFFFFF:FFFFFFFFFFFFFFFFFFFFFFFFFFFF:FFFFFFFFFFFFFFFFFFF:FFFFF:
FFFFF:FFFFFFFFFFFFFFF:FFFFFFFFFFFFF:FFFF:::F,F,FF::F:F,FF,F:FF:FFFFF
:FFFFF,:,F::FFF
@A00155:342:HHGFNDSXY:1:2162:6985:19445 1:N:0:GAACCTAG+TCCGCATA
AAAGATGGCTTTCTCTTATAGGACGCCACAGGAGGAACTCTTGAGCAGGCTCCCACAGTCTCAACAGG
AGGTTATAAGCGGATTTCAGTATGAACGCATTCAAAAGGAAGAAGAGAAGAAGGTGGAAAACTTTTCT
TTTTACCTACCAGA
+
FFFFFFFFFFFFFFFFFFFFFFFFFFFFFFFFFFFFFFFFFF:FFFFFFFFFFFFFFFFFFFFFFFFF
FFFFFFFFFFFFFFFFFFFFFFFFFFFFFFFFFFFFFFFFFFFFFFFFFFFF:FFFFFFFFFFFFFF
FFFFFF:FF,FFFF
@A00155:342:HHGFNDSXY:1:1130:2763:15734 1:N:0:GAACCTAG+TCCGCATA
AAAGATGGCTTTCTCTTATAGGACGCCACAGGAGGAACTCTTGAGCAGGCTCCCACAGTCTCAACAGG
AGGTTATAAGCGGATTTCAGTATGAACGCATTCAAAAGGAAGAAGAGAAGAAGGTGGAAAACTTTTCT
TTTTACCTACCAGAG
+
FFFFFFFF:FFFFFFFFFFFFFFF:FFFFFFFF:FFFFFFFFFFFFFFFFFFFFFFFFFFFFFFFFFF
FFFFFFFFFFFFFFFFFFFFFFFFFFFFFFFFFFFF,FFFFFFFFFFFFFFF,FFFFFFFFFFFFFF
FFFFFFFFFFFFFF:
@A00155:342:HHGFNDSXY:1:1163:31756:27555 1:N:0:GAACCTAG+TCCGCATA
AAAGATGGCTTTCTCTTATAGGACGCCACAGGAGGAACTCTTGAGCAGGCTCCCACAGTCTCAACAGG
AGGTTATAAGCGGATTTCAGTATGAACGCATTCAAAAGGAAGAAGAGAAGAAGGTGGAAAACTTTTCT
TTTTACCTACCAGAG
```

```
+
FFFFFFFFFFFFFFFFFFFFFFFFFFFFFFFFFFFFFFFFFFFFFFFFFFFFFFFFFFFFFFFFFFFF
FFFFFFFFFFFFFFFFFFFFFFFFFFFFFFFFFFFFFFFFFFFFFFFF:FFF:FFFFFF:FFFFFFFF
FFFFFFFFFFFFFFF
@A00155:342:HHGFNDSXY:1:1177:9896:20823 1:N:0:GAACCTAG+TCCGCATA
AAAGATGGCTTTCTCTTATAGGACGCCACAGGAGGAACTCTTGAGCAGGCTCCCACAGTCTCAACAGG
AGGTTATAAGCGGATTTCAGTATGAACGCATTCAAAAGGAAGAAGAGAAGAAGGTGGAAAACTTTTCT
TTTTACCTACCAGAG
+
FFFFFFFFFFFFFFFFFFFFFFFFFFFFFFFFFFFFFFFFFFFFFFF:FFFFFFFFFFFFFF,FFFFF
FFFFFFFFFFFFFFFFFFFFFFFFFFFFFFFFFFFFFFFFFFFFFFFFFFFFFFFFFFFFFFFFFFFF
FFFFFFFFFFFFFFF
@A00155:342:HHGFNDSXY:1:1403:32470:25410 1:N:0:GAACCTAG+TCCGCATA
AAAGATGGCTTTCTCTTATAGGACGCCACAGGAGGAACTCTTGAGCAGGCTCCCACAGTCTCAACAGG
AGGTTATAAGCGGATTTCAGTATGAACGCATTCAAAAGGAAGAAGAGAAGAAGGTGGAAAACTTTTCT
TTTTACCTACCAGAG
+
FFFF,FFFFFFFFF:FFFFFFFFFFFFFFFFF:FFFFFFFFFFFFFFF:FFF:FFFFFFFFFFFFFFF
FFFFFFFF:FFFFFFFFFFFF:FFFFFFFFFFFFF:FFFFFFFFFFFFFFFFFFFFFFFFFFFFFFFF
FFFFFFFFFFFFF,F
@A00155:342:HHGFNDSXY:1:1436:3586:31845 1:N:0:GAACCTAG+TCCGCATA
AAAGATGGCTTTCTCTTATAGGACGCCACAGGAGGAACTCTTGAGCAGGCTCCCACAGTCTCAACAGG
AGGTTATAAGCGGATTTCAGTATGAACGCATTCAAAAGGAAGAAGAGAAGAAGGTGGAAAACTTTTCT
TTTTACCTACCAGAG
+
FFFFFFFFFFFFFFFFFFFFFFFFFFFFFFFFFFFFFFFFFFFFFFFFFFF:FFFF:FFFFF:FFFFF
FFFFFFFF,FFFFFF:FFFFFFFFFFFFFFFFFFFFFFFFFFFFFFFFFFFFF:FFFFFFFFFFFFFF
FFFFFFF,FFFFFFF
@A00155:342:HHGFNDSXY:1:1509:13413:27727 1:N:0:GAACCTAG+TCCGCATA
AAAGATGGCTTTCTCTTATAGGACGCCACAGGAGGAACTCTTGAGCAGGCTCCCACAGTCTCAACAGG
AGGTTATAAGCGGATTTCAGTATGAACGCATTCAAAAGGAAGAAGAGAAGAAGGTGGAAAACTTTTCT
TTTTACCTACCAGAG
+
FF:FFFFFFFFFFFFFFFFFFFFFFFFFFFFFFFFFFFFFFFFFFFFFFFFFFFFFFFFFFFFFFFFF
FFFFFFFFFFFFFFFFFFFFFFFFFFFFFFFFFFF:FFFFFFFFFFFFFFFF::F:FFFFFFFFFFFF
FFFFFFFFFFFFFFF
@A00155:342:HHGFNDSXY:1:1570:13494:3004 1:N:0:GAACCTAG+TCCGCATA
AAAGATGGCTTTCTCTTATAGGACGCCACAGGAGGAACTCTTGAGCAGGCTCCCACAGTCTCAACAGG
AGGTTATAAGTGGATTTCAGTATGAACGCATTCAAAAGGAAGAAGAGAAGAAGGTGGAAAACTTTTCT
TTTTACCTACCAGAG
+
FFFFFFFFFFFFFFFFFFFFFFFFFFFFF:FFFFFFFFFFFFFFFFFFFFFFFFFFFFFFFFFFFFFF
FFFFFFFFFFFFFFFFFFFFFFFFFFFFFFFFFFFFFFFFFFFFFFFFFFFFFFFFFFFFFFFFFFFF
FFFFFFFFFFFFFFF
@A00155:342:HHGFNDSXY:1:2216:23999:19664 1:N:0:GAACCTAG+TCCGCATA
AAAGATGGCTTTCTCTTATAGGACGCCACAGGAGGAACTCTTGAGCAGGCTCCCACAGTCTCAACAGG
AGGTTATAAGCGGATTTCAGTATGAACGCATTCAAAAGGAAGAAGAGAAGAAGGTGGAAAACTTTTCT
TTTTACCTACCAGAG
+
FFFFFFFFFFFFFFFFFFFFFFFFFFFFFFFFFFFFFFFFFFFFFFFFFFFFFFFFFFFFFF:FFFFF
FFFFFFFFFFFFFFFFFFFFFFFFFFFFFFFFFFFFFFFFFFFFFFFF:FFFFFFFFFFFFFFFFFFF
FFFFFFFFFFFFFFF
@A00155:342:HHGFNDSXY:1:2515:18313:11569 1:N:0:GAACCTAG+TCCGCATA
AAAGATGGCTTTCTCTTATAGGACGCCACAGGAGGAACTCTTGAGCAGGCTCCCACAGTCTCAACAGG
```

AGGTTATAAGCGGATTTCAGTATGAACGCATTCAAAAGGAAGAAGAGAAGAAGGTGGAAAACTTTTCT
TTTTACCTACCAGAG
+
FFFFFFFFFFFFFFFFFFFFFFFFFFFFFFFFFFFFFFFFFFFFFFFFFFFFFFFFFFFFFFFFFFFF
FFFFFFFF:FFFFFFFFFFFFFFFFFFFFFFFFFFFFFFFFFFFFFFFFFFFFFFFFFFFFFFFFFF
FFFFFFFFFFFFFFF
@A00155:342:HHGFNDSXY:1:2654:7401:24674 1:N:0:GAACCTAG+TCCGCATA
AAAGATGGCTTTCTCTTATAGGACGCCACAGGAGGAACTCTTGAGCAGGCTCCCACAGTCTCAACAGG
AGGTTATAAGCGGATTTCAGTATGAACGCATTCAAAAGGAAGAAGAGAAGAAGGTGGAAAACTTTTCT
TTTTACCTACCAGAG
+
F:FFF:FFF::FFFFF:F:,FFFFFFFFFFFFFFFF,FFFFFFFFFFFFFFFFFFFFFFFFFFFFFFF
FFFFFFFFFFFFFFFFFFFFFFFFFFFFFFFFFF:FFFFFF:FFFFFFFFFF:,FFFFFFFFFFFFFF
FFFFFFFFFFFFFFF
@A00155:342:HHGFNDSXY:1:2675:21866:6511 1:N:0:GAACCTAG+TCCGCATA
AAAGATGGCTTTCTCTTATAGGACGCCACAGGAGGAACTCTTGAGCAGGCTCCCACAGTCTCAACAGG
AGGTTATAAGCGGATTTCAGTATGAACGCATTCAAAAGGAAGAAGAGAAGAAGGTGGAAAACTTTTCT
TTTTACCTACCAGAG
+
FFFFFFFF:FFFFFFFFFFFFFFFFFFFFFFFFFFFFFFFFFFFFFFFFFFFFFFFFFFFFFFFFFFF
FFFFFFFFFFFFFFFFFFFFFFFFFFFFFFFFFFFFFFFFFFFFFFFFFFFFFFFFFFFFFFFFFFFF
FFFFFFFFFFFFFFF
@A00155:342:HHGFNDSXY:1:2261:20663:24565 1:N:0:GAACCTAG+TCCGCATA
AATATGGCTTTCTCTTATTGGACGCAACAGGAGGAACTCTTGAGCAGGCTCCCACAGTCTCAACAGTA
GGTTATAAGTGGATTTCAGTATGAACGCATTCAAAAGGAAGAAGAGAAGAAGGTGTAAAACTTTTCTT
TTTACCTACCAGA
+
FF,,F,FFFFFFF,:FFF,FF,,F:,F,,:F,FFF,F,,FFF:F,,:F,F,,:F,:FFFFF,,FFF,,
FFFF,FF,,F,FF:FFFFFFFFFFFF,FFFFFFFFF,FFFF,,FFF:FFF:F::F,,,FF:FFF::FF
F,F,,FFF::FFF
@A00155:342:HHGFNDSXY:1:2259:22028:29496 1:N:0:GAACCTAG+TCCGCATA
AAGATGGCTTTCTCTTATAGGACGCCACAGGAGGAACTCTTGAGCAGGCTCCCACAGTCTCAACAGGA
GGTTATAAGCGGATTTCAGTATGAACGCATTCAAAAGGAAGAAGAGAAGAAGGTGGAAAACTTTTCTT
TTTACCTACCAGAGG
+
FFFFFFFFFFFFFFFFFFFFFFFFFFFFFFFFFFFFFFFFFFFFFFFFFFFFFFFFFFFFFFFFFFFF
FFFFFFFFFFFFFFFFFFFFFFFFFFFFFFFFFFFFFFFFFFFFFFFFFFFFFFFFFFFFFFFFFFFF
FFFFFFFFFFFFFFF
@A00155:342:HHGFNDSXY:1:1231:12246:8171 1:N:0:GAACCTAG+TCCGCATA
AGATGGCTTTCTCTTATAGGACGCCACAGGAGGAACTCTTGAGCAGGCTCCCACAGTCTCAACAGGAG
GTTATAAGCGGATTTCAGTATGAACGCATTCAAAAGGAAGAAGAGAAGAAGGTGGAAAACTTTTCTTT
TTACCTACCAGAGAA
+
FFFFFF:FFFFFFFFFF:FFFFFFFFFFFFFFFFFFFFFFFFFFFFF::FFFFFFFFFF:::FFFFFF
FFFFFF::FFFFFFFFF,FFFFFFFFFFF:FFFFF,:FFFFFFFFFF,F:,:FFFFFFFFFFFFFFFF
FFFFF:FFFFFF:FF
@A00155:342:HHGFNDSXY:1:1323:5828:30624 1:N:0:GAACCTAG+TCCGCATA
AGATGGCTTTCTCTTATAGGACGCCACAGGAGGAACTCTTGAGCAGGCTCCCACAGTCTCAACAGGAG
GTTATAAGCGGATTTCAGTATGAACGCATTCAAAAGGAAGAAGAGAAGAAGGTGGAAAACTTTTCTTT
TTACCTACCAGAGAC
+
FF,FFF:FFFFFFFF:FFFFFF,FFFFFFF,FFFFFFFFFFFFFFF:FFFF:FFFFFFFFFFFFFFFF
FFFFF::FFF:FFFFFF::FFFFFFFFFFFFFFFFFFFFFFFFFFFF:FFFFFF,FFFFFFFFFFFFF
FFFFFFFFFFFFFFF

```
@A00155:342:HHGFNDSXY:1:1303:2844:32189 2:N:0:GAACCTAG+TCCGCATA
AGATGGCTTTCTCTTATAGGACGCCACAGGAGGAACTCTTGAGCAGGCTCCCACAGTCTCAACAGGAG
GTTATAAGCGGATTTCAGTATGAACGCATTCAAAAGGAAGAAGAGAAGAAGGTGGAAAACTTTTCTTT
TTACCTACCAGAGAA
+
F:FFFFFFFFFFFFFFFFFFFFFFFFFFFFFFFFFFFFFFFFFFFFFFFFFFFFFFFFFFFFFFFFFFFF
FFFFFFFFFFFFFFFFFFFFFFFFFFF:FFFFFF:FFFFFFFFFFFFFFFFFFFFFFFFFFFFFFFFFFF
FFF:FFFFFFFFFFF
@A00155:342:HHGFNDSXY:1:1467:16749:35571 2:N:0:GAACCTAG+TCCGCATA
AGATGGCTTTCTCTTATAGGACGCCACAGGAGGAACTCTTGAGCAGGCTCCCACAGTCTCAACAGGAG
GTTATAAGCGGATTTCAGTATGAACGCATTCAAAAGGAAGAAGAGAAGAAGGTGGAAAACTTTTCTTT
TTACCTACCAGAGAA
+
FFFFFFFFFFFFF:FFFFFFFFFFFFFFFFFFFFFFFFFFFFFFFFFFFFFFFFFFFFFFFFFFFFFFFF
FFFF,FFFFFFFFFFFFFFFFFFFFFFFFFFFFFFFFFFFFFFFFFFFFFFFFFFFFFFFFFFFFFFFF
FFFF:FFFFFFFFFF
@A00155:342:HHGFNDSXY:1:2513:20564:10708 2:N:0:GAACCTAG+TCCGCATA
GATGGCTTTCTCTTATAGGACGCCACAGGAGGAACTCTTGAGCAGGCTCCCACAGTCTCAACAGGAGG
TTATAAGCGGATTTCAGTATGAACGCATTCAAAAGGAAGAAGAGAAGAAGGTGGAAAACTTTTCTTTT
TACCTACAGATCGGA
+
FFFFFFFFFFFFFFFFFFFFFFFFFFFFFFFFFFFFFFFFFFFFFFFFFFFFFFFFFFFFFFFFFFFFFF
FF:FFFFFFFFFFFFFFF:FFFFFFFFFFFFFFFFFFFFFFFFFFF,FFFFFFFFFFFFFFFFFFFFFFF
FFFFFFFFFFFFFFF
@A00155:342:HHGFNDSXY:1:2513:20699:23469 2:N:0:GAACCTAG+TCCGCATA
GATGGCTTTCTCTTATAGGACGCCACAGGAGGAACTCTTGAGCAGGCTCCCACAGTCTCAACAGGAGG
TTATAAGCGGATTTCAGTATGAACGCATTCAAAAGGAAGAAGAGAAGAAGGTGGAAAACTTTTCTTTT
TACCTACAGATCGGA
+
FFFFFFFFFFFFFFFF:FFFFFFFFFFFFFFFFFFFFFFFFFFFFFFFFFFFFFFFFFFFFFFFFFFFFF
FFFFFFFFFFFFFFFFFFFFFFFFFFFFFFFFFFFFFFFFFFFFFFFFFFFFFFFFFFFFFFFFFF:FFF
FFF:FFFFFFFFFFF
@A00155:342:HHGFNDSXY:1:1474:19913:13275 1:N:0:GAACCTAG+TCCGCATA
ATGGCTTTCTCTTATAGGACGCCACAGGAGGAACTCTTGAGCAGGCTCCCACAGTCTCAACAGGAGGT
TATAAGCGGATTTCAGTATGAACGCATTCAAAAGGAAGAAGAGAAGAAGGTGGAAAACTTTTCTTTTT
ACCTACCAGAGAAAA
+
FF:,FFF:FFFFFFFFFFFFFFFFFFFFFFFFFFFFFFFFFFFFFFFFFFFFFFFFFFFFFFF:FFFFFF
F,FFF:F:FFF:FFFFFFFFFFFFFFFFFFFFFFFFFF,FFFFFFF::FF,FFFFFFFFFFFFFFFFFFF
FFFFF:FFFFFFF:F
@A00155:342:HHGFNDSXY:1:2470:10294:14403 2:N:0:GAACCTAG+TCCGCATA
ATGGCTTTCTCTTATAGGACGCCACAGGAGGAACTCTTGAGCAGGCTCCCACAGTCTCAACAGGAGGT
TATAAGCGGATTTCAGTATGAACGCATTCAAAAGGAAGAAGAGAAGAAGGTGGAAAACTTTTCTTTTT
ACCTACCAGAGAAAA
+
FFFFFFFFFFFFFFFFFFFFFFFFFFFFFFFFFFFFFFFFFFFFFFFFFFFFFFFFFFFFF:FFFFFFFF
FFFFFFFFFFFFFFFFFFFFFFFFFFFFFFFFFF:FFFFFFFFFFFFFFFFFFFFFFFFFFFFFFFFFFF
FFFFFFFFFFFFFFF
@A00155:342:HHGFNDSXY:1:2313:12500:10520 1:N:0:GAACCTAG+TCCGCATA
TGGCTTTCTCTTATAGGACGCCACAGGAGGAACTCTTGAGCAGGCTCCCACAGTCTCAACAGGAGGTT
ATAAGCGGATTTCAGTATGAACGCATTCAAAAGGAAGAAGAGAAGAAGGTGGAAAACTTTTCTTTTTA
CCTACCAGAGAAAAC
+
FFFFFFFFFFFFFFFFFFFFFFFFFFFFFFFFFF:FFFFFFFFFFFFFFFFFFFFFFFFFFFFFFFFFFF
```

FFFFFFFFFFFFFFFFFFFFFFFFFFFFFFFFFFFFFFFFFFFFFF:FF:FFFFFFFFFFFFFFFFFF
FFFFFFFFFFFFFFF
@A00155:342:HHGFNDSXY:1:1608:1823:16047 2:N:0:GAACCTAG+TCCGCATA
TGGCTTTCTCTTATAGGACGCCACAGGAGGAACTCTTGAGCAGGCTCCCACAGTCTCAACAGGAGGTT
ATAAGCGGATTTCAGTATGAACGCATTCAAAAGGAAGAAGAGAAGAAGGTGGAAAACTTTTCTTTTTA
CCTACCAGAGAAAAC
+
FF::FFFFFFFFFFFFFFFFFFFFFFFFFFFFFFFFFFFFFFFFFFFFFFFFFFFFFFFFFFFFFFFF
FFFFFFFFFFFFFFFFFFFFFFFFFFFFFFFFFFFFFFFFFFFFFFFFFFFFFFFFFFFFFFFFFFFF
FFFFFFFFFFFFFFF
@A00155:342:HHGFNDSXY:1:2473:4743:18004 2:N:0:GAACCTAG+TCCGCATA
TGGCTTTCTCTTATAGGACGCCACAGGAGGAACTCTTGAGCAGGCTCCCACAGTCTCAACAGGAGGTT
ATAAGCGGATTTCAGTATGAACGCATTCAAAAGGAAGAAGAGAAGAAGGTGGAAAACTTTTCTTTTTA
CCTACCAGAGATCGG
+
FFFFFFFFFFFFFFFFFFFFFFFFFFFFFFF:FFFFFFFFFFFFFFFFFFFFFFFFFFFFFFFFFFFF
FFFFFFFFFFFFFFFFFFFFFFFFFFFFFFFFFFFFFFFFFFFFFFFFFFFFFFFFFFFFFFFFFFFF
:FFFFFFFFFFFFFF
@A00155:342:HHGFNDSXY:1:1124:1832:28588 2:N:0:GAACCTAG+TCCGCATA
GGCTTTCTCTTATAGGACGCCACAGGAGGAACTCTTGAGCAGGCTCCCACAGTCTCAACAGGAGGTTA
TAAGCGGATTTCAGTATGAACGCATTCAAAAGGAAGAAGAGAAGAAGGTGGAAAACTTTTCTTTTTAC
CTACCAGAGATCGGA
+
FFFFFFFFFFFFFFFFFFFFFFFFFFFFFFFFFFFFFFFFFFFFFFFFFFFFFFFFFFFFFFFFF:FF
FFF:FFFFFFFFFFFFFF:FFFFFFFFFFFFFFFFFF:F:FF:FFFFFFFFFFFFFFFFFFFFFFFFF
FFFFFFFF:FFFFFF
@A00155:342:HHGFNDSXY:1:1567:19660:23547 2:N:0:GAACCTAG+TCCGCATA
GGCTTTCTCTTATAGGACGCCACAGGAGGAACTCTTGAGCAGGCTCCCACAGTCTCAACAGGAGGTTA
TAAGTGGATTTCAGTATGAACGCATTCAAAAGGAAGAAGAGAAGAAGGTGGAAAACTTTTCTTTTTAC
CTACCAGAGAAAACT
+
FFFFFFFFFFFFFFFFFFFFFFF:FFFFFFFFFFFFFFFFFFFFFFFFFFFFFFFFFFFFFFFFFFF:
:FFFFFFFFFFFFFFFFFFFFFFFFFFFFFFFFFFFF:FFFFFFF:FFFFFFFFFF:FFFFFFFFFFF
:FFFFFFFFFFFFFF
@A00155:342:HHGFNDSXY:1:2303:12653:16705 2:N:0:GAACCTAG+TCCGCATA
GGCTTTCTCTTATAGGACGCCACAGGAGGAACTCTTGAGCAGGCTCCCACAGTCTCAACAGGAGGTTA
TAAGCGGATTTCAGTATGAACGCATTCAAAAGGAAGAAGAGAAGAAGGTGGAAAACTTTTCTTTTTAC
CTACCAGAGAAAACT
+
FFFFFFFFFFFFFFFFFFFFFFFFFF:FFF:FFF:FFFFFFFFFFFFFFFFFFFFFFFFFFFFFF,F:
FFFFFFFFFFFF,FF,FFFFFFFFFFFFFFFFFFFFFFFFFF:FF:FFFFFFF,F:F:FFF:FFFFFF
FFFFFFFFFFFFFFF
@A00155:342:HHGFNDSXY:1:1508:4146:26710 2:N:0:GAACCTAG+TCCGCATA
GCTTTCTCTTATAGGACGCCACAGGAGGAACTCTTGAGCAGGCTCCCACAGTCTCAACAGGAGGTTAT
AAGCGGATTTCAGTATGAACGCATTCAAAAGGAAGAAGAGAAGAAGGTGGAAAACTTTTCTTTTTACC
TACCAGAGAAAACT
+
FFFFF:FFFFFFFFFFFFFFFFFFFFFFFFFFFFFFFFFFFFFFFFFFFFFFFFFFFFFFFFFFFFFF
FFFFFFFFFFFFFF,FFFFFFFFFFFFFFFFFFFFFFFFFFFFFFFFFFFFFFFFFFFFFF:FFFFFF
FFFFFFFFFFFFFF
@A00155:342:HHGFNDSXY:1:1320:16957:30733 1:N:0:GAACCTAG+TCCGCATA
GCTTTCTCTTATAGGACGCCACAGGAGGAACTCTTGAGCAGGCTCCCACAGTCTCAACAGGAGGTTAT
AAGTGGATTTCAGTATGAACGCATTCAAAAGGAAGAAGAGAAGAAGGTGGAAAACTTTTCTTTTTACC
TACCAGAGAAAACTC

+
FFFFFFFFFFFFFFFFFFF:FFFFFFFFFFFFFFFFFFFFFFFFFFFFFFFFFFFFFFFFFFFFF:FF
:F:FFFFFFFF:FFFFFFFF:FFFFFFFFFFFFFFFF::FFFFFFF:FFFFFFFFF:FFFF:FFFFFF
FFFF,FFFF:FFFFF
@A00155:342:HHGFNDSXY:1:1376:9010:35008 1:N:0:GAACCTAG+TCCGCATA
TCTTTCTCTTATAGGACGCCACAGGAGGAACTCTTGAGCAGGCTCCCACAGTCTCAACAGGAGGTTAT
AAGCGGATTTCAGTATGAACGCATTCAAAAGGAAGAAGAGAAGAAGGTGGAAAACTTTTCTTTTTACC
TACCAGAGAAAACTC
+
FFFFFFFFFFFFFFFFFFFFFF:FFFFFFFFFFFFFFFFFFFFFFFFFFFFFFFF,FFFFFFFFFFFF
:FFFFFFFFFFFFFFFFFFFFFFFFFFFF:FFFFFFFFFFFFF:FF:FFFFF,FFFFFFFFFFFFFFF
FFFFFFFFFFF:FFF
@A00155:342:HHGFNDSXY:1:2338:13160:3709 1:N:0:GAACCTAG+TCCGCATA
GCTTTCTCTTATAGGACGCCACAGGAGGAACTCTTGAGCAGGCTCCCACAGTCTCAACAGGAGGTTAT
AAGCGGATTTCAGTATGAACGCATTCAAAAGGAAGAAGAGAAGAAGGTGGAAAACCTTTCTTTTTACC
TACCAGAGAAAACTC
+
FFFFFFFFFFFFFFFFFFFFFFFFFFFFFFFFFFFFFFFFFFFFFFFFFFFFFFFFFFFFFFFF:FFF
FFFFFFFFFFFFFFFFFFFFFFFFFFFFFFFFFFFFFFFFFFFFFFFFFFFFFFFFFFFFFFFFFFFF
FFFFFFFFFFFFFFF
@A00155:342:HHGFNDSXY:1:1222:32678:3944 2:N:0:GAACCTAG+TCCGCATA
GCTTTCTCTTATAGGACGCCACAGGAGGAACTCTTGAGCAGGCTCCCACAGTCTCAACAGGAGGTTAT
AAGCGGATTTCAGTATGAACGCATTCAAAAGGAAGAAGAGAAGAAGGTGGAAAACTTTTCTTTTTACC
TACCAGAGAAAACTC
+
FFFFFFFFFFFFFFFFFFFFFFFFFFFFF,FFFFFFFFFFFFFFFFFFFFFFFFFFFFFFFFFF:FFF
FFFFFFFFFFFFFF:FFFFFFFFFFFFFFFFFFFFFFFFFFFFFFFFFFFFFFFFFFFFFFFFFFFFF
FFFFFFFFFF:FFFF
@A00155:342:HHGFNDSXY:1:2243:2691:32831 2:N:0:GAACCTAG+TCCGCATA
GCTTTCTCTTATAGGACGCCACAGGAGGAACTCTTGAGCAGGCTCCCACAGTCTCAACAGGAGGTTAT
AAGCGGATTTCAGTATGAACGCATTCAAAAGGAAGAAGAGAAGAAGGTGGAAAACTTTTCTTTTTACC
TACCAGAGAAAACTC
+
FFFFFFFFFFFFFFFFFFFFFFFFFFFFFFFFFFFFF:FFFFF:FFFFFFFFFFFFFFFFF:F,:FFF
FFFFFFFF,FFFFFF:FFFFFFFF,FFFFFFF:FFFFFFFFFFFF:FFFFFFFFFFFFFFF::F,FFF
FFF:FFFFFFF:FFF
@A00155:342:HHGFNDSXY:1:2640:2727:14888 2:N:0:GAACCTAG+TCCGCATA
GCTTTCTCTTATAGGACGCCACAGGAGGAACTCTTGAGCAGGCTCCCACAGTCTCAACAGGAGGTTAT
AAGCGGATTTCAGTATGAACGCATTCAAAAGGAAGAAGAGAAGAAGGTGGAAAACTTTTCTTTTTACC
TACCAGAGAAAACTC
+
FFFFFFFFFFFF:FFFFFFFFFFFFFFFFFFFFFFFFFFFFFFFFFFFFFFFFFFFFFFFFFFFFFF
FFFF,FFFFFFFFFFFFFFFFF:FFFFF,FFFFFFFF:FFFFFFFFFFFFFFFFFFFFFFFFFFFFFF
FFFFFFFFF::FFFF
@A00155:342:HHGFNDSXY:1:2657:10700:4147 2:N:0:GAACCTAG+TCCGCATA
GCTTTCTCTTATAGGACGCCACAGGAGGAACTCTTGAGCAGGCTCCCACAGTCTCAACAGGAGGTTAT
AAGTGGATTTCAGTATGAACGCATTCAAAAGGAAGAAGAGAAGAAGGTGGAAAACTTTTCTTTTTACC
TACCAGAGAAAACCC
+
FFFFFFFFFFFFFFFFFFFFFFFFFFFFFFFFFFFFFFFFFFFFFFFFFFFFFFFFFFFFFFFF:FF:
F,FFFFFFFFFFFFFFFFFFFFFFFF:FF,FFFFFFFFFFF:FF:FFFFFFFFFFFFFFFFFFFFFF:
FFFFFFFFFFFFF:F
@A00155:342:HHGFNDSXY:1:1232:10511:7138 1:N:0:GAACCTAG+TCCGCATA
CTTTCTCTTATAGGACGCCACAGGAGGAACTCTTGAGCAGGCTCCCACAGTCTCAACAGGAGGTTATA

AGTGGATTTCAGTATGAACGCATTCAAAAGGAAGAAGAGAAGAAGGTGGAAAACTTTTCTTTTTACCT
ACCAGAGAAAACTCG
+
FFFFFFFFFFFFFFFFFFFFFFFFFFFFFFFFFFFFFFFF:FFFFFFFFFFFFFFFFFFFFFFFFFFF
:FFFFFFFFFFFFFFFFFFFFFFFFFF:FFFFFFFFFFFFFF,:FFF:F:FFFFFFFFFFFFFFFFF
FFFFFFFFFFFFFFF
@A00155:342:HHGFNDSXY:1:1303:2211:4820 1:N:0:GAACCTAG+TCCGCATA
CTTTCTCTTATAGGACGCCACAGGAGGAACTCTTGAGCAGGCTCCCACAGTCTCAACAGGAGGTTATA
AGTGGATTTCAGTATGAACGCATTCAAAAGGAAGAAGAGAAGAAGGTGGAAAACTTTTCTTTTTACCT
ACCAGAGAAAACTCG
+
FFFFFFFF:FFFFFFF:FFFFFFFF,FFFFF,FF:FFFFFFFFFFFF,FFFFFFFFF:FFF:FFF:FF
FFFF,FFFFFFFFFFFFFF,:FFFFFFFF,F:FFFFFFFFFFF::,::FFFFFFFFFFFFFFFFF:FF
FFFFFFFFFFFFFFF
@A00155:342:HHGFNDSXY:1:1303:1524:6981 1:N:0:GAACCTAG+TCCGCATA
CTTTCTCTTATAGGACGCCACAGGAGGAACTCTTGAGCAGGCTCCCACAGTCTCAACAGGAGGTTATA
AGTGGATTTCAGTATGAACGCATTCAAAAGGAAGAAGAGAAGAAGGTGGAAAACTTTTCTTTTTACCT
ACCAGAGAAAACTCG
+
F::FFFFFFFFFF:FFFFFFFFFFFFFFFFFF:FFFFFFFFFFFFFFFF:FFF::FFFFFFF:FFFFF
FFFFFFFFFFF::F:FFF,:FFF,FFFFF:,FFFFFFF,FF:FF,,F:FF:FFF,FF,FFFFFFFFFF
FFFFFFFFFFF::FF
@A00155:342:HHGFNDSXY:1:1645:15338:35415 1:N:0:GAACCTAG+TCCGCATA
CTTTCTCTTATAGGACGCCACAGGAGGAACTCTTGAGCAGGCTCCCACAGTCTCAACAGGAGGTTATA
AGCGGATTTCAGTATGAACGCATTCAAAAGGAAGAAGAGAAGAAGGTGGAAAACTTTTCTTTTTACCT
ACCAGAGAAAACTCG
+
FFFFFFFFFFFFFFFFFFFFFFFFFFFFFFFFFFFFFFFFF:FFFFFFFFFFFFFFFFFFFFFFFFFF
FFFFFFFFFFFFFFFFFFFFFFFFFFFFFFFFFFFFFFFFFFFFF:FFFFFFFFFFFFFFFFFFFFFF
FFFFFFFFFFFFFFF
@A00155:342:HHGFNDSXY:1:1653:10556:23813 1:N:0:GAACCTAG+TCCGCATA
CTTTCTCTTATAGGACGCCACAGGAGGAACTCTTGAGCAGGCTCCCACAGTCTCAACAGGAGGTTATA
AGCGGATTTCAGTATGAACGCATTCAAAAGGAAGAAGAGAAGAAGGTGGAAAACTTTTCTTTTTACCT
ACCAGAGAAAACTCG
+
FFFFFFFFFFFFFFFFFFFFFFFFFFFFFFFFFFFFFFFFFFFFFFFFFFFFFFFFFFFFFFFFFFFF
FFFFFFFFFFFFFFFFFFFFFFFFFFFFFFFFFFFFFFFFFFFFFFFFFFFFFFFFFFFFFFFFFFFF
FFFFFFFFFFFFFFF
@A00155:342:HHGFNDSXY:1:1656:27552:13260 1:N:0:GAACCTAG+TCCGCATA
CTTTCTCTTATAGGACGCCACAGGAGGAACTCTTGAGCAGGCTCCCACAGTCTCAACAGGAGGTTATA
AGCGGATTTCAGTATGAACGCATTCAAAAGGAAGAAGAGAAGAAGGTGGAAAACTTTTCTTTTTACCT
ACCAGAGAAAACTCG
+
FFFFFFFFFFFFFFFFFFFFFFFFFFFFFFFFFFFFFFFFFFFFFFFFFFFFFFFFFFFFFFFFFFFF
FFFFFFFFFFFFFFFFFFFFFFFFFFFFFFFFFFFFFFFFFFFFFF:FFFFFFFFFFFFFFFFFFFFF
FFFFFFFFFFFFFFF
@A00155:342:HHGFNDSXY:1:1667:31946:25974 1:N:0:GAACCTAG+TCCGCATA
CTTTCTCTTATAGGACGCCACAGGAGGAACTCTTGAGCAGGCTCCCACAGTCTCAACAGGAGGTTATA
AGCGGATTTCAGTATGAACGCATTCAAAAGGAAGAAGAGAAGAAGGTGGAAAACTTTTCTTTTTACCT
ACCAGAGAAAACTCG
+
FFFFFF,:FF:FFFFF:FFFF,FFFFFFFFFFFFF:FFFFFFFFFFF:FFFFFF,:FFFFFFFF:FF:
FFFFFF:FFFFFFFFFFFF:FFFFFF:F:FFFFFFFFFFF:::FFF:FF,FFFFFFFFF,FFFFFFFF
FFFFFFFFFFFFFFF

```
@A00155:342:HHGFNDSXY:1:2153:11731:8437 1:N:0:GAACCTAG+TCCGCATA
CTTTCTCTTATAGGACGCCACAGGAGGAACTCTTGAGCAGGCTCCCACAGTCTCAACAGGAGGTTATA
AGCGGATTTCAGTATGAACGCATTCAAAAGGAAGAAGAGAAGAAGGTGGAAAACTTTTCTTTTTACCT
ACCAGAGAAAACTCG
+
FFFFFFFFFFFFFFFFFFFFFFFFFFFF,FFFFFFFFFFFFFFFFFFFFFFFFFFFFFFFFFFFFFFF
FFFFFFFFFFFFFFFFFFFFFFFFFFFFFFFFFFFFFFFFFFFFF:FFFFFFFFFFFFFFFFFFFFFF
FFFFFFFFFFFFFFF
@A00155:342:HHGFNDSXY:1:2402:27245:4241 1:N:0:GAACCTAG+TCCGCATA
CTTTCTCTTATAGGACGCCACAGGAGGAACTCTTGAGCAGGCTCCCACAGTCTCAACAGGAGGTTATA
AGCGGATTTCAGTATGAACGCATTCAAAAGGAAGAAGAGAAGAAGGTGGAAAACTTTTCTTTTTACCT
ACCAGAGAAAACTCG
+
:FFFFF,FFFFFFFFFFFFFFFFFFFFFFFFFFFFFFFFFFFFFFFFFFFFFFFFFFFFFFFFFFFFF
FFFFFFFFFFFFFFFFFFFFFFFFFFFFFFFFFFFFFFFFFFFFF:FFFFFFFFFFFFFFFFFFFFFF
FFFFFFFFFFFFFFF
@A00155:342:HHGFNDSXY:1:2417:32515:2315 1:N:0:GAACCTAG+TCCGCATA
CTTTCTCTTATAGGACGCCACAGGAGGAACTCTTGAGCAGGCTCCCACAGTCTCAACAGGAGGTTATA
AGCGGATTTCAGTATGAACGCATTCAAAAGGAAGAAGAGAAGAAGGTGGAAAACTTTTCTTTTTACCT
ACCAGAGAAAACTCG
+
FF,FFFFFFF:F:F,FFFF:FFFF,FFF,FFFFFFFFFFFFFFFFFFF,FFFFF,:F:FFFFF:FFFF
FFFFFFF::FFFFFFFFFFFFFFFFF,F:FF:FFFFFFFFFFF:FFFFFFFFFFFFFFFFFFFFFFFF
FFFFFFFFF,FFFF:
@A00155:342:HHGFNDSXY:1:2543:2302:6793 1:N:0:GAACCTAG+TCCGCATA
CTTTCTCTTATAGGACGCCACAGGAGGAACTCTTGAGCAGGCTCCCACAGTCTCAACAGGAGGTTATA
AGCGGATTTCAGTATGAACGCATTCAAAAGGAAGAAGAGAAGAAGGTGGAAAACTTTTCTTTTTACCT
ACCAGAGAAAACTCG
+
FFFF,FFFFFFF,FFFF,FFFFFFFFFFFFFFFFFFFFFFFFFFFFFFFFFF:FFFFFFFF:,FFFFF
FFFFFFFFFFFFFFFFFF,FFFF:FFF:FF:FFFFFFFFFF:FFFF:FFFFFFFFFFFFFFFFFFFFF
FFFFFFFFFFFFFFF
@A00155:342:HHGFNDSXY:1:2543:2220:6840 1:N:0:GAACCTAG+TCCGCATA
CTTTCTCTTATAGGACGCCACAGGAGGAACTCTTGAGCAGGCTCCCACAGTCTCAACAGGAGGTTATA
AGCGGATTTCAGTATGAACGCATTCAAAAGGAAGAAGAGAAGAAGGTGGAAAACTTTTCTTTTTACCT
ACCAGAGAAAACTCG
+
FFFFFF:FFFFF,FFF:FFFFFFFFF:FFFFFFFFFFFFFFFFFFFFFFFFF:FFFFFFFFFFFFFFF
F:FFFFFFFFFFFFFFFFF:FF::FFFF:FFFFFFFFFFFFFFFF,::FFFFFFFFFFFFFFFFFFFF
FFFFFFFFFFFFFFF
@A00155:342:HHGFNDSXY:1:2563:21477:5713 1:N:0:GAACCTAG+TCCGCATA
CTTTCTCTTATAGGACGCCACAGGAGGAACTCTTGAGCAGGCTCCCACAGTCTCAACAGGAGGTTATA
AGCGGATTTCAGTATGAACGCATTCAAAAGGAAGAAGAGAAGAAGGTGGAAAACTTTTCTTTTTACCT
ACCAGAGAAAACTCG
+
FFFFFFFFFFFFFFFFFFFFFFFFFFFFFFFFFFFFFFFF:FFFFFFFFFF:FFFFFFFFFFFFFFFF
FFFFFFFFFFFFFFFFFFFFFFFFFFF,FFFFFFFFFFFFFFFFFFFFFFFFFFFFFFFFFFFFFFFF
FFFFFFFFFFFFFFF
@A00155:342:HHGFNDSXY:1:2649:24117:29168 1:N:0:GAACCTAG+TCCGCATA
CTTTCTCTTATAGGACGCCACAGGAGGAACTCTTGAGCAGGCTCCCACAGTCTCAACAGGAGGTTATA
AGCGGATTTCAGTATGAACGCATTCAAAAGGAAGAAGAGAAGAAGGTGGAAAACTTTTCTTTTTACCT
ACCAGAGAAAACTCG
+
FFFFFFFFFFFFFFFFFFFFFFFFFFFFFFFFFFFFFFFF:FFFFFFFFFFFFFFFFFFFFFFFFFFF
```

FFFFFFFFFFFFFFFFFFFFFFFFFFFFFFFFFFFFFFFFFFFFFFFFFFFFFFFFFFFFFFFFFFFF
FFFFFFFFFFFFFFF
@A00155:342:HHGFNDSXY:1:1135:7301:9439 1:N:0:GAACCTAG+TCCGCATA
TTTCTCTTATAGGACGCCACAGGAGGAACTCTTGAGCAGGCTCCCACAGTCTCAACAGGAGGTTATAA
GCGGATTTCAGTATGAACGCATTCAAAAGGAAGAAGAGAAGAAGGTGGAAAACTTTTCTTTTTACCTA
CCAGAGAAAACTCGG
+
FFF:FFFFFFFFFFFFFFFFFFFFFFFFFFFFFFF:FF:FFF:FFFFFFFFFFFFFFFFFFFFFFFFF
FFFFFFFFFFFFFFFFFFFFFFFFFFFF:FFFFFFFFF:F,FFF,:FFFFFFFFFFFFFFFFFFFFFF
FFFFFFF:FFFFFFF
@A00155:342:HHGFNDSXY:1:1166:23945:9737 1:N:0:GAACCTAG+TCCGCATA
TTTCTCTTATAGGACGCCACAGGAGGAACTCTTGAGCAGGCTCCCACAGTCTCAACAGGAGGTTATAA
GCGGATTTCAGTATGAACGCATTCAAAAGGAAGAAGAGAAGAAGGTGGAAAACTTTTCTTTTTACCTA
CCAGAGAAAACTCAG
+
FFFFFFFFFFFFFFFFFFFFFFFFFFFFFFFFFFFFFFFFFFFFFFFFFFFFF:FFFFFFFFFFFFFF
FFFFFFFFFFFFFFFFFFFFFFFFFFFFFFFFFFFFFF,FFFFF:FFFFFFFFFFFFFFFFFFFFFFF
FFFFFFFFFFFFFFF
@A00155:342:HHGFNDSXY:1:1209:12617:10066 1:N:0:GAACCTAG+TCCGCATA
TTTCTCTTATAGGACGCCACAGGAGGAACTCTTGAGCAGGCTCCCACAGTCTCAACAGGAGGTTATAA
GTGGATTTCAGTATGAACGCATTCAAAAGGAAGAAGAGAAGAAGGTGGAAAACTTTTCTTTTTACCTA
CCAGAGAAAACTCGG
+
FFFFFFFFFFFFFFFFFFFFFFFFFFFFFFFFFFFFFFFFFFFFFFFFFFFFFFFFFFFFFFFFFFFF
FFFFFFFFFFFFFFFFFFFFFFFFFFFFFFFFFFFFFFFFFF:F:FFFFFFFFFFFFFFFFFFFFFFF
FFFFFFFFFFFFFFF
@A00155:342:HHGFNDSXY:1:1213:18087:37012 1:N:0:GAACCTAG+TCCGCATA
TTTCTCTTATAGGACGCCACAGGAGGAACTCTTGAGCAGGCTCCCACAGTCTCAACAGGAGGTTATAA
GCGGATTTCAGTATGAACGCATTCAAAAGGAAGAAGAGAAGAAGGTGGAAAACTTTTCTTTTTACCTA
CCAGAGAAAACTCGG
+
FFFFFFFF:FFFFFFFFFFFFFFFFFFFFFFFFFFFFFFFFFFFFFFFFFFFFFFFFFFFFFFFFFFF
FFFFFFFFFFFFFFFFFFFFFFFFFFFFFFFFFFFFFFFFFF:FFFFFFFFFFFFFFFFFF:FFFF:F
FFFFFFFFFFFFFFF
@A00155:342:HHGFNDSXY:1:1407:8983:21120 1:N:0:GAACCTAG+TCCGCATA
TTTCTCTTATAGGACGCCACAGGAGGAACTCTTGAGCAGGCTCCCACAGTCTCAACAGGAGGTTATAA
GTGGATTTCAGTATGAACGCATTCAAAAGGAAGAAGAGAAGAAGGTGGAAAACTTTTCTTTTTACCTA
CCAGAGAAAACTCGG
+
FFFFFFFFFFFFFFFFFFFFFFFFFFFFFFFFFFFFFFF:FFFFFFFFFFFFF:FFFFF:FF:FFFFF
FFFFFFFFFFFFFFFFFFFFFFFFFF:FFFFFFFFFFFFF:,,F:,FFFFFFFFFFFF:FFFFFFFFF
FFFFFFFFFFFFFFF
@A00155:342:HHGFNDSXY:1:1508:4146:26710 1:N:0:GAACCTAG+TCCGCATA
TTTCTCTTATAGGACGCCACAGGAGGAACTCTTGAGCAGGCTCCCACAGTCTCAACAGGAGGTTATAA
GCGGATTTCAGTATGAACGCATTCAAAAGGAAGAAGAGAAGAAGGTGGAAAACTTTTCTTTTTACCTA
CCAGAGAAAACTCGG
+
FFFFFFFFFFF:FFFFFFFFFF:FFFF,:F:FFFFFFFFFFFFFFFFFFFFFFFFFFFFFFFFFFFFF
FFFFFFFFFFFFFFFFFFF:FFFFFFFFFFFFFFFFFFFFFFFF:FFFFFFFFFFFFFFFFFFFFFFF
FFFFFFFFFFFFFFF
@A00155:342:HHGFNDSXY:1:1622:6623:17253 1:N:0:GAACCTAG+TCCGCATA
TTTCTCTTATAGGACGCCACAGGAGGAACTCTTGAGCAGGCTCCCACAGTCTCAACAGGAGGTTATAA
GCGGATTTCAGTATGAACGCATTCAAAAGGAAGAAGAGAAGAAGGTGGAAAACTTTTCTTTTTACCTA
CCAGAGAAAACTCGG

```
+
:FFFFFFFFFFFFFFFFFFFFFFFFFFFFFFFFFFFFFFFFFFFFFFFFFFFFFFFFFFFFFFFFFFFFFF:
FFFFFFFFFFFFFFFFFFFFFFFFFFFFFFFFFFFFFFFFF:FF:F:FF:FFFFFFFFFFFFFFFFFFF
FFFFFFFFFFFFFFF
@A00155:342:HHGFNDSXY:1:1645:30309:7388 1:N:0:GAACCTAG+TCCGCATA
TTTCTCTTATAGGACGCCACAGGAGGAACTCTTGAGCAGGCTCCCACAGTCTCAACAGGAGGTTATAA
GCGGATTTCAGTATGAACGCATTCAAAAGGAAGAAGAGAAGAAGGTGGAAAACTTTTCTTTTTACCTA
CCAGAGAAAACTCGG
+
FFFFFFFFFFFFFFFFFFFFFFFFFFFFFFFFFFFFFFFFFFFFFFFFFFFFFFFFFFFFFFFFFFFFF
FFFFFFFFFFFFFFFFFFFFFFFFFFFFFFFFFFFFFFF:FFFFFFFFFFFFFFFFFFFFFFFFFFFFF
FFFF:FFFFFFFFFF
@A00155:342:HHGFNDSXY:1:1635:7265:5682 1:N:0:GAACCTAG+TCCGCATA
TTTCTCTTATAGGACGCCACAGGAGGAACTCTTGAGCAGGCTCCCACAGTCTCAACAGGAGGTTATAA
GTGGATTTCAGTATGAACGCATTCAAAAGGAAGAAGAGAAGAAGGTGGAAAACTTTTCTTTTTACCTA
CCAGAGAAAACTCGG
+
FFFFFFFFFFFFFFFFFFFFFFFFFFFFF:FFFFFFFFFF:FFFFFFFFFFFFFFFFFFFFFFFFFFFF
FF:FFFFFFFFFFFFFFF,FFFFFFFFF,FFFFFFFFFFFFFFF:FFFF:FFFFFFFFFFFFFFFFFFF
FFFFFFFFFFFFFFF
@A00155:342:HHGFNDSXY:1:2320:19768:17973 1:N:0:GAACCTAG+TCCGCATA
CGATCTTTATAGGACGCCACAGGAGGAACTCTTGAGCAGGCTCCCACAGTCTCAACAGGAGGTTATAA
GCGGATTTCAGTATGAACGCATTCAAAAGGAAGAAGAGAAGAAGGTGGAAAACTTTTCTTTTTACCTA
CCAGAGAAAACTCGG
+
FFFFFFFFFFFFFFFFFFFFFFFFFFFFFFFFFFFFFFFFFFFFFFFFFFFFFFFFFFFFFFFFFFFFF
FFFFFFFFFFFFFFFFFFFFFFFFFFFFFFFFFFFFFFFFFFFFFF:FFF:FFFFFFFFFFFFFFFFFF
FFFFFFFFFFFFFFF
@A00155:342:HHGFNDSXY:1:2635:1967:11130 1:N:0:GAACCTAG+TCCGCATA
TTTCTCTTATAGGACGCCACAGGAGGAACTCTTGAGCAGGCTCCCACAGTCTCAACAGGAGGTTATAA
GTGGATTTCAGTATGAACGCATTCAAAAGGAAGAAGAGAAGAAGGTGGAAAACTTTTCTTTTTACCTA
CCAGAGAAAACTCGG
+
F,FFFFFFFFFFFFFFFFFFFF:FFFFFFFF:FFFFFFFF:FFFFFFFFFFFFFFF,FFFFFFF:F:
FFFFFFFFFFFFFFFF:FFFFFFFFFFFFFFFFFFFFFFFFFFFFFFFFFFFFFFFFFFFFFFFFFFF
FFFFFFFFFFFFFFF
@A00155:342:HHGFNDSXY:1:2657:10700:4147 1:N:0:GAACCTAG+TCCGCATA
TTTCTCTTATAGGACGCCACAGGAGGAACTCTTGAGCAGGCTCCCACAGTCTCAACAGGAGGTTATAA
GTGGATTTCAGTATGAACGCATTCAAAAGGAAGAAGAGAAGAAGGTGGAAAACTTTTCTTTTTACCTA
CCAGAGAAAACCCGG
+
:FFFFFFFFFFFFFFFFFFFFFFFFFFFFFFFFFFFFFF:FFFFFFFFFFFFFFFFFFFFFFFFFFFFF
FFFFFFFFFFFFFFFFFFFFFFFFFFFFFFFFFFFFFFF:FF,FFFFFFFFFF:FFFFFFFFFFFFFF
FFFFFFFFFFFFFF:
@A00155:342:HHGFNDSXY:1:2676:10176:11663 1:N:0:GAACCTAG+TCCGCATA
TTTCTCTTATAGGACGCCACAGGAGGAACTCTTGAGCAGGCTCCCACAGTCTCAACAGGAGGTTATAA
GTGGATTTCAGTATGAACGCATTCAAAAGGAAGAAGAGAAGAAGGTGGAAAACTTTTCTTTTTACCTA
CCAGAGAAAACTCGG
+
F:FFF,FFFFFFF:FFFFF:FFF:F,FFFFFFFF:FFFF:FFFFFFFFFFFFFFFFF::FF,FFFFFF
:F:F:FFFFFFFFFFFFFF:FFFFFFFFFFF:FFF,FFFFFFF::F:FFFF,FFFFFFFFFFFF,FFF
FFFFFFFFFFFFFFF
@A00155:342:HHGFNDSXY:1:1460:13639:11772 2:N:0:GAACCTAG+TCCGCATA
TTTCTCTTATAGGACGCCACAGGAGGAACTCTTGAGCAGGCTCCCACAGTCTCAACAGGAGGTTATAA
```

GCGGATTTCAGTATGAACGCATTCAAAAGGAAGAAGAGAAGAAGGTGGAAAACTTTTCTTTTTACCTA
CCAGAGAAAACTCGG
+
FFFF:FFFFFFFFFFFFFFF:FFFFFFFFFFFFFFFFFFFFF:FFFF,FFFFFFFFFFFFFFFFFFFF
FFFFFFFFFFFFFFFFFFFFFFFFFFFFFFFFFFFFFFFFFFFFFFFFFFFFFFFFFFFF:FFFFFFF
FFFFFFFFFFFFFFF
@A00155:342:HHGFNDSXY:1:2515:24758:19382 2:N:0:GAACCTAG+TCCGCATA
TTTCTCTTATAGGACGCCACAGGAGGAACTCTTGAGCAGGCTCCCACAGTCTCAACAGGAGGTTATAA
GCGGATTTCAGTATGAACGCATTCAAAAGGAAGAAGAGAAGAAGGTGGAAAACTTTTCTTTTTACCTA
CCAGAGAAAACTCGG
+
FFFFFFFFFFFFF:FFFFFFFFFFFFFFFF:FFFFFFFFFFFFFFFF,FFFFFFFFFFFFFFFFFF:F
FFFFFFFFFFF:FFFFFFFFFFFFFFFFFFFFFFFFFFFFFFFFFFFFFFFFFFFFFFFFFFFFFFFF
FFFF:FFFFFFFFFF
@A00155:342:HHGFNDSXY:1:2663:30798:26929 2:N:0:GAACCTAG+TCCGCATA
TTTCTCTTATAGGACGCCACAGGAGGAACTCTTAAGCAGGCTCCCACAGTCTCAACAGGAGGTTATAA
GCGGATTTCAGTATGAACGCATTCAAAAGGAAGAAGAGAAGAAGGTGGAAAACTTTTCTTTTTACCTA
CCAGAGAAAACTCGG
+
FFFFFFFFFFFFFFFFFFFFFFFFFFFFFFFFFFFFFFFFFFFFFFFFFFFFFFFFFFFFFFFFFFFF
FFFFFFFFFFFFFFFFFFFFFFFFFFFFFFFFFFFFFFFFFFFFFFFFFFFFFFFFFFFFFFFFFFFF
FFFFFFFFFFFFFFF
@A00155:342:HHGFNDSXY:1:1141:2663:6480 1:N:0:GAACCTAG+TCCGCATA
TTCTCTTATAGGACGCCACAGGAGGAACTCTTGAGCAGGCTCCCACAGTCTCAACAGGAGGTTATAAG
CGGATTTCAGTATGAACGCATTCAAAAGGAAGAAGAGAAGAAGGTGGAAAACTTTTCTTTTTACCTAC
CAGAGAAAACTCGGG
+
FFFFFFFFFFFFFFF:FFFFFFFFFFFFFFFFFFFFFFFFFFFFFFFFFFFFFFFFFFFFFFFFFFFF
FFFFFFFFFFFFFFFFFFFFFFFFFFFFFFFFFFFFFFFFFFF:::FFFFFFFFFFFFFFFFFFFFFF
FFFFFFFFFFFFFFF
@A00155:342:HHGFNDSXY:1:1265:30219:25050 1:N:0:GAACCTAG+TCCGCATA
TTCTCTTATAGGACGCCACAGGAGGAACTCTTGAGCAGGCTCCCACAGTCTCAACAGGAGGTTATAAG
CGGATTTCAGTATGAACGCATTCAAAAGGAAGAAGAGAAGAAGGTGGAAAACTTTTCTTTTTACCTAC
CAGAGAAAACTCGGG
+
FFFFFFFFFFFFFFFFFFFFFFFFFFFFFFFFFFFFFFFFFFFFFFFFFFFF:FFFFFFFFFFFFFFF
FFFFFFFFFFFFFFFFFFFFFFFFFFFFFFFFFFFFFFFFFFFFFFFFFFFFFFFFFFFFFFFFFFFF
FFFFFFFFFFFFFFF
@A00155:342:HHGFNDSXY:1:1339:14443:17832 1:N:0:GAACCTAG+TCCGCATA
TTCTCTTATAGGACGCCACAGGAGGAACTCTTGAGCAGGCTCCCACAGTCTCAACAGGAGGTTATAAG
CGGATTTCAGTATGAACGCATTCAAAAGGAAGAAGAGAAGAAGGTGGAAAACTTTTCTTTTTACCTAC
CAGAGAAAACTCGGG
+
FFFFFFFFFFFFFFFFFFFFFFFFFFFFFFFFFFFFFFFFFFFFFFFFFFFF,FFFFFFFFFFFFF:F
FFFFFFFFFFFFFFFFFFFFFFFFFFFFFFFFFFFFF:FFFFFFFFFFFFFFFFFFFFFFFFFFFFFF
FFFFFFFFFFFFFFF
@A00155:342:HHGFNDSXY:1:1415:12075:2832 1:N:0:GAACCTAG+TCCGCATA
TTCTCTTATAGGACGCCACAGGAGGAACTCTTGAGCAGGCTCCCACAGTCTCAACAGGAGGTTATAAG
CGGATTTCAGTATGAACGCATTCAAAAGGAAGAAGAGAAGAAGGTGGAAAACTTTTCTTTTTACCTAC
CAGAGAAAACTCGGG
+
FFFFFFFFFFFFFFFFFFFFFFFFFFFFFFFFFFFFFFFFFFFFFFFFFFFFFFFFFFFFFFFFFFFF
FFFFFFFFFFFFFFFFFFFFFFFFFFFFFFFFFFFFFFFFFFFFFFFFFFFFFFFFFFFFFFFFFFFF
FFFFFFFFFFFFFFF

```
@A00155:342:HHGFNDSXY:1:1567:19660:23547 1:N:0:GAACCTAG+TCCGCATA
TTCTCTTATAGGACGCCACAGGAGGAACTCTTGAGCAGGCTCCCACAGTCTCAACAGGAGGTTATAAG
TGGATTTCAGTATGAACGCATTCAAAAGGAAGAAGAGAAGAAGGTGGAAAACTTTTCTTTTTACCTAC
CAGAGAAAACTCGGG
+
FFFFFFFFFFFFFFFFFFFFFFFFFFFFFFFFFFFFFFFFFFFFFFFFFFFFFFFFFFFFFFFFFFFF
FFFFFFFFFFFFFFFFFFFFFFFFFFFFFFFFFFFFFFFF,FFFFFFFFFFFFFF:FFFFFFFFFFFF
FFFFFFFFFFFFFFF
@A00155:342:HHGFNDSXY:1:2169:20564:28119 1:N:0:GAACCTAG+TCCGCATA
TTCTCTTATAGGACGCCACAGGAGGAACTCTTAAGCAGGCTCCCACAGTCTCAACAGGAGGTTATAAG
CGGATTTCAGTATGAACGCATTCAAAAGGAAGAAGAGAAGAAGGTGGAAAACTTTTCTTTTTACCTAC
CAGAGAAAACTCGGG
+
FFFFFFFFFFFFFFFFFFFFFFFFFFFFFFFFFFFFFFFFFFFFFFFFFFFFFFFFFFFFFFFFFF:F
FFFFFFFFFFFFFFFFFFFFFFFFFFFFF:FFFFFFFFFF:FFFF:FFFFFFFFFFFFFF:FFFFFFF
FFFFFFFFFFFFFFF
@A00155:342:HHGFNDSXY:1:2545:31910:11381 1:N:0:GAACCTAG+TCCGCATA
TTCTCTTATAGGACGCCACAGGAGGAACTCTTGAGCAGGCTCCCACAGTCTCAACAGGAGGTTATAAG
CGGATTTCAGTATGAACGCATTCAAAAGGAAGAAGAGAAGAAGGTGGAAAACTTTTCTTTTTACCTAC
CAGAGAAAACTCGGG
+
FFFFFFFF:FFFFFFFFFFFFFFFFFFFFFFFFFFFFF:FFFFFFFFFFFFF,FFFFFFFFFFFFFFF
FFFFFFFFFFFFFFFFFFFFFFFFFFFFFFFFFFFFFFFFF:FF,FFFFFFFFFFFFFFFF:FFFFFF
FFFFFFFFFFFFFFF
@A00155:342:HHGFNDSXY:1:2547:15709:18928 1:N:0:GAACCTAG+TCCGCATA
TTCTCTTATAGGACGCCACAGGAGGAACTCTTGAGCAGGCTCCCACAGTCTCAACAGGAGGTTATAAG
CGGATTTCAGTATGAACGCATTCAAAAGGAAGAAGAGAAGAAGGTGGAAAACTTTTCTTTTTACCTAC
CAGAGAAAACTCGGG
+
FFFFFFFF:F:FFFFFFFFF:F:FFFFFFFF:FFFFFFFFFFFFFFF:FFFFFFFFFFFFFFFFFFFF
FFFFF:FFFFFFFFFFFFFF:FFFFFFFFFFFFFFFFFFFFFFFFFFFFFFFFFFFFFFFFFFFFFFF
FFFFFFFFFFFFFFF
@A00155:342:HHGFNDSXY:1:2571:16071:4210 1:N:0:GAACCTAG+TCCGCATA
TTCTCTTATAGGACGCCACAGGAGGAACTCTTGAGCAGGCTCCCACAGTCTCAACAGGAGGTTATAAG
TGGATTTCAGTATGAACGCATTCAAAAGGAAGAAGAGAAGAAGGTGGAAAACTTTTCTTTTTACCTAC
CAGAGAAAACTCGGG
+
FFFFFFFFFFFFFFFFFFFFFFFFFFFFFFFFFFFFFFFFFFFFFFFFFFFFFFFFFFFFFFFFFFFF
FFFFFFFFFFFFFFFFFFFFFFFFFFFFFFFFFFFFFFFFFFFFFFFFFFFFFFFFFFFFFFFFFFFF
FFFFFFFFFFFFFFF
@A00155:342:HHGFNDSXY:1:1209:12617:10066 2:N:0:GAACCTAG+TCCGCATA
TTCTCTTATAGGACGCCACAGGAGGAACTCTTGAGCAGGCTCCCACAGTCTCAACAGGAGGTTATAAG
TGGATTTCAGTATGAACGCATTCAAAAGGAAGAAGAGAAGAAGGTGGAAAACTTTTCTTTTTACCTAC
CAGAGAAAACTCGGA
+
FFFFFFFFFFFFFFFFFFFFFFFFFFFFFFFFFFFFFFFFFFFFFFFFFFFFFFFFFFFFFF,FFFFF
FFFFFFFFFFFFFFFFFFFFFFFFFF:FFFFFFFFFFFFFFFFFFFFFFF:FFFFFFFFFFFFFFFFF
FFFFFFFFFFFFFFF
@A00155:342:HHGFNDSXY:1:1376:9010:35008 2:N:0:GAACCTAG+TCCGCATA
TTCTCTTATAGGACGCCACAGGAGGAACTCTTGAGCAGGCTCCCACAGTCTCAACAGGAGGTTATAAG
CGGATTTCAGTATGAACGCATTCAAAAGGAAGAAGAGAAGAAGGTGGAAAACTTTTCTTTTTACCTAC
CAGAGAAAACTCAGA
+
FFFFFFFFFFFFFFFFFFFFFFFFFFFFFFFFFFFFFFFFFFFFFFFFFFFFFFFFFFFFFFFFFFFF
```

FFFFFFFFFFFFFFFFFFFFFFFFFFFFFFFFFFFFFFFFFFFFFFFFFF:FFFFFFFFFFFFFFFFF
FFFFFFFFFFFFFFF
@A00155:342:HHGFNDSXY:1:2676:10176:11663 2:N:0:GAACCTAG+TCCGCATA
TTCTCTTATAGGACGCCACAGGAGGAACTCTTGAGCAGGCTCCCACAGTCTCAACAGGAGGTTATAAG
TGGATTTCAGTATGAACGCATTCAAAAGGAAGAAGAGAAGAAGGTGGAAAACTTTTCTTTTTACCTAC
CAGAGAAAACTCGGA
+
FFFFFFF,FFFFF:FFFFFFFFFF:FFFFFFFFFFFFFFFFFFFFFFFFFFFFFFFFFFFFFFFF:FF
FFFFFFFFFFFFFFFFFFFF:FFFFFFFFFFFFF:FFFFFFFF:FFFFFF:FFFFFFFFFFFFF:FFF
FFFFFFFFFF,FFFF
@A00155:342:HHGFNDSXY:1:1138:23647:21057 1:N:0:GAACCTAG+TCCGCATA
CTCTTATAGGACGCCACAGGAGGAACTCTTGAGCAGGCTCCCACAGTCTCAACAGGAGGTTATAAGCG
GATTTCAGTATGAACGCATTCAAAAGGAAGAAGAGAAGAAGGTGGAAAACTTTTCTTTTTACCTACCA
GAGAAAACTCGGGAC
+
FFFFFFFFFFFFFFFFFFFFFF:FFFFFFFFFFFFFFFFFFFFFFFFFFFF:FFFFFFFFFFF:FFFF
FFFFFFFFFFFFFFFFFFFFFFFFFFFFFFFFFFFF:FFFFFFFFFFFFFFFFFFFFFFFFFFFFFFF
FFFFFFFFFFFFFFF
@A00155:342:HHGFNDSXY:1:2377:28691:10003 1:N:0:GAACCTAG+TCCGCATA
CTCTTATAGGACGCCACAGGAGGAACTCTTGAGCAGGCTCCCACAGTCTCAACAGGAGGTTATAAGCG
GATTTCAGTATGAACGCATTCAAAAGGAAGAAGAGAAGAAGGTGGAAAACTTTTCTTTTTACCTACCA
GAGAAAACTCGGGAG
+
FFFFFFFFFFFFFFFFFFFFFFFFFFFFFFFFFFFFFFFFFFFFFFFFFFFFFFFFFFFFFFFFFFFF
FFFFFFFFFFFFFFFFFFF:FFFFFFFFFFFFFF:FFFFF:FFFFFFFFFFFFFFFFFFFFFFFFFFF
FFFFFFFFFFFFFFF
@A00155:342:HHGFNDSXY:1:1139:29821:23453 2:N:0:GAACCTAG+TCCGCATA
CTTATAGGACGCCACAGGAGGAACTCTTGAGCAGGCTCCCACAGTCTCAACAGGAGGTTATAAGCGGA
TTTCAGTATGAACGCATTCAAAAGGAAGAAGAGAAGAAGGTGGAAAACTTTTCTTTTTACCTACCAGA
GAAAACTCGGGAATG
+
FFFFFFFFFFFFFFFFFFFFFFFFFFFFFFFFFFFFFFFFFFFFFFFFFFFFFFFFFFFFFFFFFFFF
FFFFFFFFFFFFFFFFFFFFFFFFFFFFFFFFFFFFFF:FFFFFFFFFFFFFFFFFFFFFFFFFFFFF
FFFFFFFFFFFF,FF
@A00155:342:HHGFNDSXY:1:2305:22815:18771 2:N:0:GAACCTAG+TCCGCATA
CTTATAGGACGCCACAGGAGGAACTCTTGAGCAGGCTCCCACAGTCTCAACAGGAGGTTATAAGCGGA
TTTCAGTATGAACGCATTCAAAAGGAAGAAGAGAAGAAGGTGGAAAACTTTTCTTTTTACCTACCAGA
GAAAACTCGGGAATG
+
FFFFFFFFFFFFFFFFFFFFFF:FFFFFFFFF:FFFFFFFFFFFFFFFFFFFFFFFFFFFFFFFFFFF
F,FFFFFFFFFFFFFFFFFFFFFFFFFFFFFFFF:FFFFFFFFFFFFFFFFFFFFFFFFFFFFFFFFF
FFFFFFFFFFFF,FF
@A00155:342:HHGFNDSXY:1:1320:16957:30733 2:N:0:GAACCTAG+TCCGCATA
TTATAGGACGCCACAGGAGGAACTCTTGAGCAGGCTCCCACAGTCTCAACAGGAGGTTATAAGTGGAT
TTCAGTATGAACGCATTCAAAAGGAAGAAGAGAAGAAGGTGGAAAACTTTTCTTTTTACCTACCAGAG
AAAACTCGGGAATGG
+
F::::FFFFFFFFFF:FFFFFFFFFFFFFFFFFFFFFFFFFFFFFFFFFFFFFFFFFFFFFFFFFFFF
FFFFFFFFFFFFFFFFFFFFFFFFFFFFFFFFFFFFFFFFFFFFFFFFFFFF:FFFFFFFFFFFFFFF
FFFFFFFFFFFFFFF
@A00155:342:HHGFNDSXY:1:2175:13313:18004 2:N:0:GAACCTAG+TCCGCATA
TTATAGGACGCCACAGGAGGAACTCTTGAGCAGGCTCCCACAGTCTCAACAGGAGGTTATAAGCGGAT
TTCAGTATGAACGCATTCAAAAGGAAGAAGAGAAGAAGGTGGAAAACTTTTCTTTTTACCTACCAGAG
AAAGCTCGGGAATGG

```
+
FFF:FFFFFFFFFFFFFFFFFFFFFFFFFFFFFFFFFFFFFFFFFFFFFFFFFFFFFFFFFFFFFFFFF
FFFFFFFFFFFFFFFFFFFFFFFFFFFFFFFFFFFFFFFFFFFFFFFFFFFFFFFFFFFFFFFFFFFFF
FFFFFFFFFFFFFFF
@A00155:342:HHGFNDSXY:1:2320:19768:17973 2:N:0:GAACCTAG+TCCGCATA
TTATAGGACGCCACAGGAGGAACTCTTGAGCAGGCTCCCACAGTCTCAACAGGAGGTTATAAGCGGAT
TTCAGTATGAACGCATTCAAAAGGAAGAAGAGAAGAAGGTGGAAAACTTTTCTTTTTACCTACCAGAG
AAAACTCGGAGATCG
+
FFFFFFFFFFFFFFFFFFFFFFFFFFFFFFFFFFFFFFFFFFFFFFFFFFFFFFFFFFFFFFFFFFFFF
FFF:FFFFFFFFFFF::FFFFFFFF:FFFFFFF:FFFFFFFFFFFF:FFFFFFFFFFFFFF:FFFFFF
FFFFF:FFFFFFFFF
@A00155:342:HHGFNDSXY:1:1139:29821:23453 1:N:0:GAACCTAG+TCCGCATA
TATAGGACGCCACAGGAGGAACTCTTGAGCAGGCTCCCACAGTCTCAACAGGAGGTTATAAGCGGATT
TCAGTATGAACGCATTCAAAAGGAAGAAGAGAAGAAGGTGGAAAACTTTTCTTTTTACCTACCAGAGA
AAACTCGGGAATGGT
+
FFFFFFFFFFFFFFFFFFFFFFFFFFFFFFFFFF:FFFFFFFFFFFFFFFFFFFFFFFFFFFFFFFFFF
FFFFFFFFFFFFFFFFFFFFFFFFFFFFFFFFF:FFF:F::FFFF:FFFFFFFFFFFFFFFFFFFFFF
FFFFFFFFFFFFFFF
@A00155:342:HHGFNDSXY:1:2158:18159:9392 2:N:0:GAACCTAG+TCCGCATA
TATAGGACGCCACAGGAGGAACTCTTGAGCAGGCTCCCACAGTCTCAACAGGAGGTTATAAGTGGATT
TCAGTATGAACGCATTCAAAAGGAAGAAGAGAAGAAGGTGGAAAACTTTTCTTTTTACCTACCAGAGA
AAACTCGGGAATGGT
+
F,FFFFFFFFFFFFFFFFFFFFF,FFFFFFFFFFFFFFFFFF:FFFFFFFFFFFF,FFFFFFFFFFFF
F:FFFFFFFFFF,FF,FFFFF,F:FFFFFFFFFFFFF,FFFFFFF,FFFFF::FFFF:FFFFFFFFFF
:FFF:FF::FFFF:F
@A00155:342:HHGFNDSXY:1:2250:4237:16157 2:N:0:GAACCTAG+TCCGCATA
TATAGGACGCCACAGGAGGAACTCTTGAGCAGGCTCCCACAGTCTCAACAGGAGGTTATAAGCGGATT
TCAGTATGAACGCATTCAAAAGGAAGAAGAGAAGAAGGTGGAAAACTTTTCTTTTTACCTACCAGAGA
AAACTCGGGAATGGT
+
F:FFFFFFFFFFFFFFFFFF:FFFFFFFFFFFFFFFFFFFFFFFFFFFFFFFFFFFFF:FFFFFFFFFF
FFFFFFFFFFFFFFFFFFFFFFFFFFFFFFFFFFFFFFFFFFFFFFFFFFFFFFFFFFFFFFFFFFFFF
FFFFFFFFFF,FFFF
@A00155:342:HHGFNDSXY:1:2338:13160:3709 2:N:0:GAACCTAG+TCCGCATA
ATAGGACGCCACAGGAGGAACTCTTGAGCAGGCTCCCACAGTCTCAACAGGAGGTTATAAGCGGATTT
CAGTATGAACGCATTCAAAAGGAAGAAGAGAAGAAGGTGGAAAACCTTTCTTTTTACCTACCAGAGAA
AACTCGGGAATGGTT
+
FF:FFFFFFFFFFFFFFFFFFFFFFFFFFFFFFFFFFFFFFFFFFFFFFFFFFFFFFFFFFFFFFFFFF
FFFFFFFFFFFFFFFFFFFFFFFFFFFFFFFFFFFFFFFFFFFFFFFFFFFFFFFFFFFFFFFFFFFFF
FFFFFFFFFFFFFFF
@A00155:342:HHGFNDSXY:1:1309:20437:12054 1:N:0:GAACCTAG+TCCGCATA
TAGGACGCCACAGGAGGAACTCTTGAGCAGGCTCCCACAGTCTCAACAGGAGGTTATAAGCGGATTTC
AGTATGAACGCATTCAAAAGGAAGAAGAGAAGAAGGTGGAAAACTTTTCTTTTTACCTACCAGAGAAA
ACTCGGGAATGGTTC
+
FFFFFFFFFFFFFFFF,FFFFFFFFFFFFFF:FFFFFFF,F:FFFFFFFFFF,FFFFF:FFFFFFFFF
FFFFFFFFFFFFFFFFFFFFFFFFFFFFFFF:FFF:,FFFFF:FFFFFFFFFFFFFFFFFFFFFFFFFF
FFFFFFFFFFFFFFF
@A00155:342:HHGFNDSXY:1:1468:13693:18161 1:N:0:GAACCTAG+TCCGCATA
TAGGACGCCACAGGAGGAACTCTTAAGCAGGCTCCCACAGTCTCAACAGGAGGTTATAAGCGGATTTC
```

```
AGTATGAACGCATTCAAAAGGAAGAAGAGAAGAAGGTGGAAAACTTTTCTTTTTACCTACCAGAGAAA
ACTCGGGAATGGTTC
+
FFFFFFFFFFFFFFFFFFFFFFFFFFFFFFFFFFFFFFFFFFFF:FFFFFFFFFFFFFFFFFFFFFFF
FFFFFFFFFFFFFFFFFFFFFFFFFFFFFFFF::F,,,FFFFFFFFFFFFFFFFFFFFFFFFFFFFFF
FFFFFFFFFFFFFFF
@A00155:342:HHGFNDSXY:1:2307:4047:3709 1:N:0:GAACCTAG+TCCGCATA
TAGGACGCCACAGGAGGAACTCTTGAGCAGGCTCCCACAGTCTCAACAGGAGGTTATAAGCGGATTTC
AGTATGAACGCATTCAAAAGGAAGAAGAGAAGAAGGTGGAAAACTTTTCTTTTTACCTACCAGAGAAA
ACTCGGGAATGGTTC
+
FFFFFFFFFFF:FFFFFFFFFFFFFFFFFFFFFFFFFFFFFFFFF:FFFFFFFFFFFFFFFFFFFFFF
FFFFFFF:FFFFFFFFFFFFFFFFFFFFFFF:,:F,F::FFFFFFFFFFFFFFFF:FFFFFFFFFFFF
FFFFFFFFFFFFF:F
@A00155:342:HHGFNDSXY:1:2663:9896:14090 1:N:0:GAACCTAG+TCCGCATA
TAGGACGCCACAGGAGGAACTCTTGAGCAGGCTCCCACAGTCTCAACAGGAGGTTATAAGCGGATTTC
AGTATGAACGCATTCAAAAGGAAGAAGAGAAGAAGGTGGAAAACTTTTCTTTTTACCTACCAGAGAAA
ACTCGGGAATGGTTC
+
FF,,FFF:,FFFFFFFFF,FFFFFF::F:FFFF:FFFFFFFFFF,::FF:FF:FFF:::,,FFFFFFF
FFF,FFFFFFFFFFFFFFF:F:FFFFFFFF:FF,F:F,FF:FF,FFFF:FFFFF:FFFFFF:FFFFFF
,:FFFFF,:F:FFFF
@A00155:342:HHGFNDSXY:1:1311:19325:15577 2:N:0:GAACCTAG+TCCGCATA
TAGGACGCCACAGGAGGAACTCTTGAGCAGGCTCCCACAGTCTCAACAGGAGGTTATAAGTGGATTTC
AGTATGAACGCATTCAAAAGGAAGAAGAGAAGAAGGTGGAAAACTTTTCTTTTTACCTACCAGAGAAA
ACTCGGGAATGGTTC
+
FFFFFFFFFFFFFFFFFFFFFFFFFFFFFFFFFFFFFFFFFFFFFFFFFFFFFFFFFFFFFFFFFFFF
FFFFFFFFFFFFFFFFFFFFFF:FFFFFFFFFFFFFFFFFFFFFFFFFFFFFFFFFFFFFFFFFFFFF
FFFFFFFFFFFFFFF
@A00155:342:HHGFNDSXY:1:2228:20030:24659 2:N:0:GAACCTAG+TCCGCATA
AGGACGCCACAGGAGGAACTCTTGAGCAGGCTCCCACAGTCTCAACAGGAGGTTATAAGTGGATTTCA
GTATGAACGCATTCAAAAGGAAGAAGAGAAGAAGGTGGAAAACTTTTCTTTTTACCTACCAGAGAAAA
CTCGGGAATGGTTCA
+
FFFFFFFFFFFFFFFFFFFFFFFFFFFFFFFFFFFFFFFFFFF:FFFFFFFFFFFFFFFFFFFFFFFF
FFFFFFFFFFFFFFFFFFFFFFFFFFFFFFFFFFFFFFFFFFFFFFFFFFFFFFFFFFFFFFFFFFFF
FFFFFFFFFFFFFFF
@A00155:342:HHGFNDSXY:1:1343:4381:2065 2:N:0:GAACCTAG+TCCGCATA
GGACGCCACAGGAGGAACTCTTGAGCAGGCTCCCACAGTCTCAACAGGAGGTTATAAGCGGATTTCAG
TATGAACGCATTCAAAAGGAAGAAGAGAAGAAGGTGGAAAACTTTTCTTTTTACCTACCAGAGAAAAC
TCGGGAATGGTTCAC
+
FF:FFFFFFFFFFFFFFFFFFFFFFFFFFFFFFFFFFFFFFFFFFFFFFFFFFFFFFFFFFFFFFFFF
FFFFFFFFFFFFFFFFFFFFFFFFFFFFFFFFFFFFFFFFFFFFFFFFFFFFFFFFFFFFFFFFFFFF
FFFFFF,FFFFFFFF
@A00155:342:HHGFNDSXY:1:1476:20681:24659 2:N:0:GAACCTAG+TCCGCATA
GGACGCCACAGGAGGAACTCTTGAGCAGGCTCCCACAGTCTCAACAGGAGGTTATAAGCGGATTTCAG
TATGAACGCATTCAAAAGGAAGAAGAGAAGAAGGTGGAAAACTTTTCTTTTTACCTACCAGAGAAAAC
TCGGGAATGGTTCAC
+
FFFFFFFFFFFFFFFFFFFFFFFFFFFFFFFFFFFFFFFFFFFFFFFFFFFFFFFFFFFFFFFFF:FF
FFFFFFFFFFFFFFFFFFFF:FFFFFFFFFFFFFF:FFFFFFFFFFFFFFFFFFFFFFFFFFFF:FFF
FFFFFF,FF:FFFFF
```

@A00155:342:HHGFNDSXY:1:2108:16830:10034 2:N:0:GAACCTAG+TCCGCATA
GGACGCCACAGGAGGAACTCTTGAGCAGGCTCCCACAGTCTCAACAGGAGGTTATAAGCGGATTTCAG
TATGAACGCATTCAAAAGGAAGAAGAGAAGAAGGTGGAAAACTTTTCTTTTTACCTACCAGAGAAAAC
TCGGGAATGGTTCAC
+
FFFFFFFFFFFFFFFFFFFFFFFFFFFFFFFFFFFFFFFFFFFFFFFFFFFFFFFFFFFFFFFFFFFF
FFFFFFFFFFFFFFFFFFFFFFFFFFFFFFFFFFFFFFFFFFFFFFFFFFFFFFFFFFFFFFFFFFFF
FFFFFF,FFFFFFFF
@A00155:342:HHGFNDSXY:1:2541:3766:33285 2:N:0:GAACCTAG+TCCGCATA
GGACGCCACAGGAGGAACTCTTGAGCAGGCTCCCACAGTCTCAACAGGAGGTTATAAGCGGATTTCAG
TATGAACGCATTCAAAAGGAAGAAGAGAAGAAGGTGGAAAACTTTTCTTTTTACCTACCAGAGAAAAC
TCGGGAATGGTTCAC
+
FFFFFFFFFFFFFFFFFFFF:FFFFFFFFFFFFFFFFFFFFFFFFFFFFFFFFFFFFFFFFFFFFFFF
FFFFFFFFFFFFFFFFFFFFFF,FFFFFFFFFFFFFFFFFFFFFFFFFFFFFFFFFFFFFFFFFFFFF
FFFFFF,FFFFFFFF
@A00155:342:HHGFNDSXY:1:2609:8675:35587 1:N:0:GAACCTAG+TCCGCATA
CGCCACAGGAGGAAATCTTGAGCAGGCTCCCACAGTCTCAACAGGAGGTTATAAGCGGA
+
FFFFFFF,FFF,FF,F,FFFF,F:FFFFFFFFFFFFFFFFFFFFFFFFFFFFFFFFF:FF
@A00155:342:HHGFNDSXY:1:2446:9028:10457 2:N:0:GAACCTAG+TCCGCATA
CGCCACAGGAGGAACTCTTGAGCAGGCTCCCACAGTCTCAACAGGAGGTTATAAGCGGATTTCAGTAT
GAACGCATTCAAAAGGAAGAAGAGAAGAAGGTGGAAAACTTTTCTTTTTACCTACCAGAGAAAACTCG
GGAATGGTTCACAAA
+
FFFFFFFFFFFFFFFFFFFFFFFFFFFFFFFFFFFFFFFFFF:FFFFFFFFFFFFFFFFFFFFFFF:F
F:FFFFFFFFFFFFFFFF::FFFFFFFFFFFFFFFFF:FFFFFFFFFFFFFFFFFFFFFFFF,FFFFF
FFFFFFFFFFFFFFF
@A00155:342:HHGFNDSXY:1:1504:15564:23563 1:N:0:GAACCTAG+TCCGCATA
GCCACAGGAGGAACTCTTGAGCAGGCTCCCACAGTCTCAACAGGAGGTTATAAGTGGATTTCAGTATG
AACGCATTCAAAAGGAAGAAGAGAAGAAGGTGGAAAACTTTTCTTTTTACCTACCAGAGAAAACTCGG
GAATGGTTCACAAAG
+
FFFFFFFFFFFFFFFFFFFFFFFF:FFFFFFFFFFFFFFFFFFFFFFFFFFFFFFFFFFFFFFFFFFF
FFFFFFFFFFFFFFFFFFFFFFFFFFF::::FFFFFFFFFFFFFFFFFFFFFFFFFFFFFF:FFFFFF
FFFFFFFFFFFFFFF
@A00155:342:HHGFNDSXY:1:2270:2121:5102 1:N:0:GAACCTAG+TCCGCATA
CGATCTGGAGGAACTCTTGAGCAGGCTCCCACAGTCTCAACAGGAGGTTATAAGTGGATTTCAGTATG
AACGCATTCAAAAGGAAGAAGAGAAGAAGGTGGAAAACTTTTCTTTTTACCTACCAGAGAAAACTCGG
GAATGGTTCACAAAG
+
FFFFFFFFFFFFFFFFFFFFFFFFFFFF:FFFFFFFFFFFFFFFFFFFFFFFFFFFFFFF:FF:FFFF
FFFFFFFFFFFFFFFFFFFFFFFFFFFFFFF::FFFFFFFFFFFFFFFFFFFFFFFFFFFFFFFFFFF
FFFFFFFFFFFFFFF
@A00155:342:HHGFNDSXY:1:1244:31204:31704 2:N:0:GAACCTAG+TCCGCATA
GCCACAGGAGGAACTCTTGAGCAGGCTCCCACAGTCTCAACAGGAGGTTATAAGCGGATTTCAGTATG
AACGCATTCAAAAGGAAGAAGAGAAGAAGGTGGAAAACTTTTCTTTTTACCTACCAGAGAAAACTCGG
GAATGGTTCACAAAG
+
F:FFFFFFFFFFFFFFFFFFFFFFFFFFFFFFFFFFFFFFFFFFFFFFFFFFFFFFFFFFFFFFFFFF
FFFFFFFFFFFFFFFFFFFFFFFFFFFFFFFFFFFFFFFFFFFFFFFFFFFFFFFFFFFFFFFFFFFF
FF:FFFFFFFFFFFF
@A00155:342:HHGFNDSXY:1:2245:29125:5431 2:N:0:GAACCTAG+TCCGCATA
GCCACAGGAGGAACTCTTGAGCAGGCTCCCACAGTCTCAACAGGAGGTTATAAGCGGATTTCAGTATG

```
AACGCATTCAAAAGGAAGAAGAGAAGAAGGTGGAAAACTTTTCTTTTTACCTACCAGAGAAAACTCGG
GAATGGTTCACAAAG
+
FFFFFFFFFFFF:FFFFFFFFFFFFFFFFFFFFFFFFFFFFFFFFFFFFFFFFFFFFFFFFFFFFFFF
FFFFFFFFFFFFFFFFFFFFFFFFFFF:FFFFFF:FFFFFFFFFFFFFFFFFFFFFFFFFFFFFFFFF
FF,FFFFFFFFFFFF
@A00155:342:HHGFNDSXY:1:2422:28610:13401 1:N:0:GAACCTAG+TCCGCATA
CACAGGAGGAACTCTTGAGCAGGCTCCCACAGTCTCAACAGGAGGTTATAAGCGGATTTCAGTATGAA
CGCATTCAAAAGGAAGAAGAGAAGAAGGTGGAAAACTTTTCTTTTTACCTACCAGAGAAAACTCGGGA
ATGGTTCACAAAGTC
+
FFFFFFFFFFFFFFFFFFFFFFFFFFFFFFFFFFFFFFFFFFFFFFFFFFFFFFFFFFFFFFFFFFFF
FFFFFFFFFFFFFFFFFFFFFFFFFFFFFFFFFFFFFFFFFFFFFFFFFFFFFFFFFFFFFFFFFFFF
FFFFFFFFF,FF,FF
@A00155:342:HHGFNDSXY:1:1416:19759:5963 2:N:0:GAACCTAG+TCCGCATA
CACAGGAGGAACTCTTGAGCAGGCTCCCACAGTCTCAACAGGAGGTTATAAGCGGATTTCAGTATGAA
CGCATTCAAAAGGAAGAAGAGAAGAAGGTGGAAAACTTTTCTTTTTACCTACCAGAGAAAACTCGGGA
ATGGTTCACAAAGTC
+
FFFFFFFFFFFFFFFFFFFFFFFFFFFFFFFFFFFFFFFFFFFFFFF:FFFFFFFFFFFFFFFFFFFF
FFFFFFFFFFFFFFFFFFFFFFFFFFFFFFF:FFFFFFFFFFFFFFFFFFFFFFFFFFFFFFFFFFFF
:FFFFF:FFFFFFFF
@A00155:342:HHGFNDSXY:1:1460:12933:4069 2:N:0:GAACCTAG+TCCGCATA
CACAGGAGGAACTCTTGAGCAGGCTCCCACAGTCTCAACAGGAGGTTATAAGCGGATTTCAGTATGAA
CGCATTCAAAAGGAAGAAGAGAAGAAGGTGGAAAACTTTTCTTTTTACCTACCAGAGAAAACTCGGGA
ATGGTTCACAAAGTC
+
FFFFFFFFFFFFFFFFFFFFFFFFFFFFFFFFFFFFFFFFFFFFFFFFFFFFFFFFFFFFFFFFFF:F
FFF::FFFFFFFFFFFFFFFFFFFFFFFFFFFFFFFFFFFFFFFFFFFFFFFFFFFFFFFFFFFFFFF
FFFF:FFFFFFFFFF
@A00155:342:HHGFNDSXY:1:2169:20401:29528 2:N:0:GAACCTAG+TCCGCATA
CACAGGAGGAACTCTTGAGCAGGCTCCCACAGTCTCAACAGGAGGTTATAAGCGGATTTCAGTATGAA
CGCATTCAAAAGGAAGAAGAGAAGAAGGTGGAAAACTTTTCTTTTTACCTACCAGAGAAAACTCGGGA
ATGGTTCACAAAGTC
+
FFFFFFFFFF:FFFFFFFFFFFFFFFFFFFFFFFFFFFFFFFFFFFFFFFFFFFFFFFFFFFFFFFFF
FFFFFFFFFFFFFFFFFFFFFFFFFF:FFFFFFFFFFFFFFFFFFFFFFFFFFFFFFFFF,:FFFFFFFF
,FFFFFFFFFFFFFF
@A00155:342:HHGFNDSXY:1:2174:10447:11788 2:N:0:GAACCTAG+TCCGCATA
CACAGGAGGAACTCTTGAGCAGGCTCCCACAGTCTCAACAGGAGGTTATAAGCGGATTTCAGTATGAA
CGCATTCAAAAGGAAGAAGAGAAGAAGGTGGAAAACTTTTCTTTTTACCTACCAGAGAAAACTCGGGA
ATGGTTCACAAAGTC
+
FFFFFFFFFFFFFFFFFFFFFFFFFFFFFFFFFFFFFFFFFFFFFFFFFFF:FFFFFFFFFFFFFFFF
FFFFFFFFFFFFFFFFFFFFFFFF:FFFFFFFFFFFFFFFFFFFFFFFFFFFFFFFFFFFF,FFFFFFFF
,FFFFFFFFFFFFF:
@A00155:342:HHGFNDSXY:1:2206:27498:18677 2:N:0:GAACCTAG+TCCGCATA
CACAGGAGGAACTCTTGAGCAGGCTCCCACAGTCTCAACAGGAGGTTATAAGCGGATTTCAGTATGAA
CGCATTCAAAAGGAAGAAGAGAAGAAGGTGGAAAACTTTTCTTTTTACCTACCAGAGAAAACTCGGGA
ATGGTTCACAAAGTC
+
FFFFFFFFFFFFFFFFFFFFFFFFFFFFFFFFFFFFFFFFFFFFFFF,FFFFFFFFFFFFFFFFFFFF
FFFFFFFFFFFFFFFFFFFFFFFFFFFFFFFFFFFFFFFFFFFFFFFFFFFFFFFFFFFFFFFFFFFF
,FFFFFFFFFF:FFF
```

```
@A00155:342:HHGFNDSXY:1:2347:2519:23672 1:N:0:GAACCTAG+TCCGCATA
ACAGGAGGAACTCTTGAGCAGGCTCCCACAGTCTCAACAGGAGGTTATAAGCGGATTTCAGTATGAAC
GCATTCAAAAGGAAGAAGAGAAGAAGGTGGAAAACTTTTCTTTTTACCTACCAGAGAAAACTCGGGAA
TGGTTCACAAAGTCT
+
FFFFFF:FF:FFFFFFFFFF,FFFFFFFFFFFF,,FFFF:,FFFFFF:F:,FFFFF,FFFFFFFFFF:
F:FFFFFFFFFFFF,FFFFFFFFF:FFFFFFFFFFFFFFFFFFFFFFFFFFFFFFFFFFFFFFFFFFF
F:FFFFFFFFFFF,F
@A00155:342:HHGFNDSXY:1:1654:15447:8296 2:N:0:GAACCTAG+TCCGCATA
ACAGGAGGAACTCTTGAGCAGGCTCCCACAGTCTCAACAGGAGGTTATAAGCGGATTTCAGTATGAAC
GCATTCAAAAGGAAGAAGAGAAGAAGGTGGAAAACTTTTCTTTTTACCTACCAGAGAAAACTCGGGAA
TGGTTCACAAAGTCT
+
FFFFFFFFFFFFFFFFFFFFFFFFFFFFFFFFFFFFFFFFFFFFFFFFFFFFFFFFFFFFFFFFFFFF
FFFFFFFFFFFFFFFFFFFFFFFFFFF,FFFFFFFFFFFFFFFFFFFFFFFFFFFFFFFFFFFFFFFF
FFFFFFFFFFFFFFF
@A00155:342:HHGFNDSXY:1:1303:2844:32189 1:N:0:GAACCTAG+TCCGCATA
CAGGAGGAACTCTTGAGCAGGCTCCCACAGTCTCAACAGGAGGTTATAAGCGGATTTCAGTATGAACG
CATTCAAAAGGAAGAAGAGAAGAAGGTGGAAAACTTTTCTTTTTACCTACCAGAGAAAACTCGGGAAT
GGTTCACAAAGTCTG
+
FFFFF:FFFFF:FFFFFFFFFFFFFFFFFFF,F:FFFFFFFF,FFFFFFF:FFFFF:FFFFFF:FFFF
:FFF,FFFF,FFFFFFFFFF,,F::,F:,FFFF,FFFFFFFFFFFF,FFFFFFFFFFFF:FFFFFFFF
FFFFFFFFFFFFFFF
@A00155:342:HHGFNDSXY:1:1310:4661:27602 1:N:0:GAACCTAG+TCCGCATA
CAGGAGGAACTCTTGAGCAGGCTCCCACAGTCTCAACAGGAGGTTATAAGCGGATTTCAGTATGAACG
CATTCAAAAGGAAGAAGAGAAGAAGGTGGAAAACTTTTATTTTTACCTACCAGAGAAAACTCGGGAAT
GGTTCACAAAGTCTG
+
FFFFFFFFFFFFFFFFFFFFFFFFF:F,FFFFF,:FFFFFFF,FFFFFFFFFF:FFFFFFFFFFFFFF
FFFFFFFFFFFFFFFFFFFF,:FFF:FF,FF,::FFFF:FFFFFFFFFFFFFFFFFFFFFFFFF:FFF
FFFFFFFFFFFFFFF
@A00155:342:HHGFNDSXY:1:1349:18936:12618 1:N:0:GAACCTAG+TCCGCATA
ATCTAGGAACTCTTGAGCAGGCTCCCACAGTCTCAACAGGAGGTTATAAGCGGATTTCAGTATGAACG
CATTCAAAAGGAAGAAGAGAAGAAGGTGGAAAACTTTTCTTTTTACCTACCAGAGAAAACTCGGGAAT
GGTTCACAAAGTCTG
+
FFFFFFFFFFFFFFFFFFFFFFFFFFFFFFFFFFFFFFFFFFFFFFFFFFFFFFFFFFFFFFFFFFFF
:FFFFFFFFFFFFFFFFFF:FFF:FFFFFFFFFFFFFFFFFFFFFFFFFFFFFFFFFFFFFFFFFFFF
FFFFFFFFFFFFFFF
@A00155:342:HHGFNDSXY:1:1572:29378:29982 1:N:0:GAACCTAG+TCCGCATA
CAGGAGGAACTCTTGAGCAGGCTCCCACAGTCTCAACAGGAGGTTATAAGCGGATTTCAGTATGAACG
CATTCAAAAGGAAGAAGAGAAGAAGGTGGAAAACTTTTCTTTTTACCTACCAGAGAAAACTCGGGAAT
GGTTCACAAAGTCTG
+
FFFFFFFFFFFFFFFFFFFFF:FFFFFFFFFF:FFFFFFFFFFFFFFFFFFFFFFFFFFFFFFFFFFF
FFFFFFFFFFFFFFFFFFFFFFFFFFF:FFFFFFFFFFFFFFFFFFFFFFFFFFFFFFFFFFFFFFFF
FFFFFFFFFFFFFFF
@A00155:342:HHGFNDSXY:1:1562:28149:32957 1:N:0:GAACCTAG+TCCGCATA
CAGGAGGAACTCTTGAGCAGGCTCCCACAGTCTCAACAGGAGGTTATAAGCGGATTTCAGTATGAACG
CATTCAAAAGGAAGAAGAGAAGAAGGTGGAAAACTTTTCTTTTTACCTACCAGAGAAAACTCGGGAAT
GGTTCACAAAGTCTG
+
FFFFFFFFFFFFFFFFFFFFFFFFFFFFFFF,FF::FFFFF,FFFFFFFFFFFFFFFFFFFFFFFFFF
```

:FFFFFFFFFFFFFFFFFFFFFFF:FFFFFFFFF:FFFFFFFFFFFFFFFFFFFFFFFFFFFFFFFFFF
FFFFFFFFFFFFFFF
@A00155:342:HHGFNDSXY:1:1630:17047:31516 1:N:0:GAACCTAG+TCCGCATA
CAGGAGGAACTCTTGAGCAGGCTCCCACAGTCTCAACAGGAGGTTATAAGCGGATTTCAGTATGAACG
CATTCAAAAGGAAGAAGAGAAGAAGGTGGAAAACTTTTCTTTTTACCTACCAGAGAAAACTCGGGAAT
GGTTCACAAAGTCTG
+
FFFFFFFFFFFFFFFFFFFFFFFFFFFFFFFFFFFFFFFFFFFFFFF::FFFFF:FFFFFFFFFFFFF
FFFFFFFFFFFFFFFFFFFFFFF:FFFFFFFFFFFFFFFFFFFFFFFFFFFFFFFFFFFFFFFFFFFF
FFFFFFFFFFFFFFF
@A00155:342:HHGFNDSXY:1:2137:18087:4977 1:N:0:GAACCTAG+TCCGCATA
CAGGAGGAACTCTTGAGCAGGCTCCCACAGTCTCAACAGGAGGTTATAAGCGGATTTCAGTATGAACG
CATTCAAAAGGAAGAAGAGAAGAAGGTGGAAAACTTTTCTTTTTACCTACCAGAGAAAACTCGGGAAT
GGTTCACAAAGTCTG
+
FFFFFFFFFFFFFFFFFFFFFFFFFFFF:FFFFF,FFFFFFFFFFFFFFFFFFFFFFFFFFFFFFFF:
FFFFFFFFFFFFFFFFFFFF,FFFF::FFFFFFFFFFFFF:FFFF:FFFFFFFFFFFFFFFFFFFFF
FFFFFFFFFFFF:FF
@A00155:342:HHGFNDSXY:1:2158:21856:29481 1:N:0:GAACCTAG+TCCGCATA
CGATCTGAACTCTTGAGCAGGCTCCCACAGTCTCAACAGGAGGTTATAAGCGGATTTCAGTATGAACG
CATTCAAAAGGAAGAAGAGAAGCAGGTGGAAAACTTTTCTTTTTACCTACCAGAGAAAACTCGGGAAT
GGTTCACAAAGTCTG
+
FFFFFFFFFFFFFFFFFFFFF:FFFFFFFFFFFFFFFFFFFFFFFFFFF:,FFFFFFFFFFFFFFFFFF
FFFFFFFFFFFFFFFFFFFFFF:,::F:FFFFFFFF:FFFFFFFFFFFFFFFFFFFFF:FFFFFFFFFF
FFFFFFFFFFFFFFF
@A00155:342:HHGFNDSXY:1:2206:27498:18677 1:N:0:GAACCTAG+TCCGCATA
CAGGAGGAACTCTTGAGCAGGCTCCCACAGTCTCAACAGGAGGTTATAAGCGGATTTCAGTATGAACG
CATTCAAAAGGAAGAAGAGAAGAAGGTGGAAAACTTTTCTTTTTACCTACCAGAGAAAACTCGGGAAT
GGTTCACAAAGTCTG
+
FFFFFFFFFFFFFFFFFFFFFFFFFFFFFFFFFFFFFFFFFFFFFFFFF:FFFFFFFFFFFFFFFFFFF
FFFFFFFFFFFFFFFFFFFFFFFFF:::FFFFFFFFFFFFFFFFFFFFFFFFFFFFFFFFFFFFFFFFF
FFFFFFFFFFFFFFF
@A00155:342:HHGFNDSXY:1:2240:19714:12994 1:N:0:GAACCTAG+TCCGCATA
CAGGAGGAACTCTTGAGCAGGCTCCCACAGTCTCAACAGGAGGTTATAAGCGGATTTCAGTATGAACG
CATTCAAAAGGAAGAAGAGAAGAAGGTGGAAAACTTTTCTTTTTACCTACCAGAGAAAACTCGGGAAT
GGTTCACAAAGTCTG
+
F:FFFFFFFFFFFFFFFFFFFFFFFFFFFFFFFFFFFFFFFFFFFFFFF,:FFFFFFFFFFF:FFFFFF
FFFFFFFFFFF:FFFFFFFFFFFFF:,:FFFFFFFFFFFFFFFFFFFFFFFFFFFFFFFFFFFFFFFFF
FFFFFFFFFFFFFFF
@A00155:342:HHGFNDSXY:1:2356:20627:36902 1:N:0:GAACCTAG+TCCGCATA
CAGGAGGAACTCTTGAGCAGGCTCCCACAGTCTCAACAGGAGGTTATAAGCGGATTTCAGTATGAACG
CATTCAAAAGGAAGAAGAGAAGAAGGTGGAAAACTTTTCTTTTTACCTACCAGAGAAAACTCGGGATT
GGTTCACAAAGTCTG
+
FFFFFFFFFFFFFFFFFFFFFFFFFFFFFFFFFFFFFFFFFFFFFFFFF:FFFFFFFFFFFFFFFFFFF
FF:FFFFFFFFFFFFFFFFF:F:F:::FFFFFFFFFFFFFFFFFFFFFFFFFFFFFFFFFFFFFFFF,F
FFFFFFFFFFFFFFF
@A00155:342:HHGFNDSXY:1:2459:8576:9768 1:N:0:GAACCTAG+TCCGCATA
ATCTAGGAACTCTTGAGCAGGCTCCCACAGTCTCAACAGGAGGTTATAAGCGGATTTCAGTATGAACG
CATTCAAAAGGAAGAAGAGAAGAAGGTGGAAAACTTTTCTTTTTACCTACCAGAGAAAACTCGGGAAT
GGTTCACAAAGTCTG

+
FFFFF,FFFFFFFFFFFFFFFFFFFFFFFFFFFFFFFFFFFFFF:FFFFFFFFFFFFF:FFFFFFFFFFF
FFFFFFFFFFFFFFFFFFFFF:FFFF:F::F:FFFFFFFFFFFFFFFFFFFFFFFFFFFFFFFFFFFFFF
FFFFFFFFFFFFFFF
@A00155:342:HHGFNDSXY:1:2440:12246:23265 1:N:0:GAACCTAG+TCCGCATA
CAGGAGGAACTCTTGAGCAGGCTCCCACAGTCTCAACAGGAGGTTATAAGCGGATTTCAGTATGAACG
CATTCAAAAGGAAGAAGAGAAGAAGGTGGAAAACTTTTCTTTTTACCTACCAGAGAAAACTCGGGAAT
GGTTCACAAAGTCTG
+
FFFFFFFFFFFFFFFFFFFFFFFFFFFFFFFFFFFFFFFFFFFFFFFFFFFFF:FFFF:FFFFFFFFFFF
FFFFFFFFFFFFFFFFFFFFFFFFFF:FFFFFFFFFFFFFFFFFFFFFFFFFFFFFFFFFFFFFFFFFFF
FFFFFFFFFFFFFFF
@A00155:342:HHGFNDSXY:1:2541:3766:33285 1:N:0:GAACCTAG+TCCGCATA
CAGGAGGAACTCTTGAGCAGGCTCCCACAGTCTCAACAGGAGGTTATAAGCGGATTTCAGTATGAACG
CATTCAAAAGGAAGAAGAGAAGAAGGTGGAAAACTTTTCTTTTTACCTACCAGAGAAAACTCGGGAAT
GGTTCACAAAGTCTG
+
FFFFFFFFFFFFFFFFFFFFFFFFFFFFFFFFFF:FFFFFFFFFFFFFFFFFFFFFFFFFFFFFFFFFFF
FFF:FFFFFFFFFFFFFFFFFFF:F:F:FFFFFFFFFFFFFFFFFFFFFFFFFFFFFFFFFFFFFFFFFF
FFFFFFFFFFFFFFF
@A00155:342:HHGFNDSXY:1:2610:10420:29434 1:N:0:GAACCTAG+TCCGCATA
CAGGAGGAACTCTTGAGCAGGCTCCCACAGTCTCAACAGGAGGTTATAAGTGGATTTCAGTATGAACG
CATTCAAAAGGAAGAAGAGAAGAAGGTGGAAAACTTTTCTTTTTACCTACCAGAGAAAACTCGGGAAT
GGTTCACAAAGTCTG
+
FFFFF:FFFFFFFFFFFFFFFFFFFFFFFFFFFFFFFFFFFFFFFFFFFFFFFFFFFFFFFFFFFFFFFF
FFFFFFFFFFFFFFFFFFFFFFFFFF:FFFFFFFFFFFFF:F:FFFFFFFFFFFFFFFFFFFFFFFFFFF
FFFFFFFFFFFFFFF
@A00155:342:HHGFNDSXY:1:2610:10511:29496 1:N:0:GAACCTAG+TCCGCATA
CAGGAGGAACTCTTGAGCAGGCTCCCACAGTCTCAACAGGAGGTTATAAGTGGATTTCAGTATGAACG
CATTCAAAAGGAAGAAGAGAAGAAGGTGGAAAACTTTTCTTTTTACCTACCAGAGAAAACTCGGGAAT
GGTTCACAAAGTCTG
+
FFFFFF:FFFFFFFFFFFFFFFFFFFFFFFFFFFFFFFFFFFFFFFFFFFFFFFFFFFFFFFFFFFFFFF
FFFFFFFFFFFFFFFFFFFFFFFFF:F::FFFFFFFFFFFFFFFFFFFFFFFFFFFFFFFFFFFFFFFFF
FFFFFFFFFFFFFFF
@A00155:342:HHGFNDSXY:1:1204:26693:9267 2:N:0:GAACCTAG+TCCGCATA
CAGGAGGAACTCTTGAGCAGGCTCCCACAGTCTCAACAGGAGGTTATAAGCGGATTTCAGTATGAACG
CATTCAAAAGGAAGAAGAGAAGAAGGTGGAAAACTTTTCTTTTTACCTACCAGAGAAAACTCGGGAAT
GGTTCACAAAGTCTG
+
FFFFFFFF:FFFFFFFFFFFFFFFFFFFFFFFFFFFFFFFFFFFFFFFFFFFFFFFFFFFFFFFFFFFF
FFFFFFFFFFFFFFFFFFFFFFFFFFFFFFFFFFFFFFFFFFFFFFFFFFFFFFFFFFFFFFFFFFFF,F
FFFFFFFFFFFFFFF
@A00155:342:HHGFNDSXY:1:1373:30752:10066 2:N:0:GAACCTAG+TCCGCATA
CAGGAGGAACTCTTGAGCAGGCTCCCACAGTCTCAACAGGAGGTTATAAGTGGATTTCAGTATGAACG
CATTCAAAAGGAAGAAGAGAAGAAGGTGGAAAACTTTTCTTTTTACCTACCAGAGAAAACTCGGGAAT
GGTTCACAAAGTCTG
+
FFFFFFFFFFFFFFFFFFFFFFFFFFFFFFFFFFFFFFFFFFFFFFFFFFFFFFFFFFFFFFF:FFFFFF
FFFFFFFFFFFFFFFFFFFFFFFF:FFFFFFFFFFFFFFFFFFFFFFFFFFFFFFFFFFFFFFFFFFF,F
FFFFFFFFFFFFFFF
@A00155:342:HHGFNDSXY:1:1422:25599:33364 2:N:0:GAACCTAG+TCCGCATA
CAGGAGGAACTCTTGAGCAGGCTCCCACAGTCTCAACAGGAGGTTATAAGCGGATTTCAGTATGAACG

CATTCAAAAGGAAGAAGAGAAGAAGGTGGAAAACTTTTCTTTTTACCTACCAGAGAAAACTCGGGAAT
GGTTCACAAAGTCTG
+
FFFFFFF::FFF::FFFFFFFFFFFFFFFFFFFFFFFFFFFFFFFFFFFFFFFFF,FFFFFFF:FFF:
FFFFFFFFFFFFFFFFFFFFFFFFFFFFFFFFFFFFFFFFFFFFFFFFFFFFFFFFFFFFFFFFFF,F
FFFFFFFFFFFFFFF
@A00155:342:HHGFNDSXY:1:1563:14082:33896 2:N:0:GAACCTAG+TCCGCATA
CAGGAGGAACTCTTGAGCAGGCTCCCACAGTCTCAACAGGAGGTTATAAGCGGATTTCAGTATGAACG
CATTCAAAAGGAAGAAGAGAAGAAGGTGGAAAACTTTTCTTTTTACCTACCAGAGAAAACTCGGGAAT
GGTTCACAAAGTCTG
+
FFFFFFFFFFFFFFFFFFFFFFFFFFFFFFFFFFFFFFFFFFFFFFFFFFFFFFFFFFFFFFFFFFFF
FFFFFFFFFFFFFFFFFFFFFFFFFFFF:FFFFFFFFFFFFFFFFFFFFFFFFFFFFF:FFFFFF,,F
FFF:FFFFFFFFFFF
@A00155:342:HHGFNDSXY:1:1619:23755:13667 2:N:0:GAACCTAG+TCCGCATA
CAGGAGGAACTCTTGAGCAGGCTCCCACAGTCTCAACAGGAGGTTATAAGCGGATTTCAGTATGAACG
CATTCAAAAGGAAGAAGAGAAGAAGGTGGAAAACTTTTCTTTTTACCTACCAGAGAAAACTCGGGAAT
GGTTCACAAAGTCTG
+
FFFFFFFFFFFFFFFFFFFFFFFFFFFFFFFFFFFFFFFFFFFFFFFFFFF:FF:FFFFFFFFFFFFF
FFFFFFFF:FFFFFFFFFFFF,FFFFFF:FFFFFFFFFFFFFFFFFFFFFFFFFFFFFFFFFFFFFFF
FFFFFFFFFFFF:FF
@A00155:342:HHGFNDSXY:1:1619:23448:13792 2:N:0:GAACCTAG+TCCGCATA
CAGGAGGAACTCTTGAGCAGGCTCCCACAGTCTCAACAGGAGGTTATAAGCGGATTTCAGTATGAACG
CATTCAAAAGGAAGAAGAGAAGAAGGTGGAAAACTTTTCTTTTTACCTACCAGAGAAAACTCGGGAAT
GGTTCACAAAGTCTG
+
F:FFFFFF,FFFFFFFFFFFFFFFFFFFFFFFFFFFFFFFFFFFFFFFFFFFFFF:FFFFFFFFFFFF
FFFFFFFF:FFFFFFFFFFFFFFFFFFFFFFFFFFFFFFFFFFFFFFF,FFFFFFFFF,FFFFFFF,F
FFFFFFFFFF:FFFF
@A00155:342:HHGFNDSXY:1:1166:7084:3270 1:N:0:GAACCTAG+TCCGCATA
ATCTGGAACTCTTGAGCAGGCTCCCACAGTCTCAACAGGAGGTTATAAGCGGATTTCAGTATGAACGC
ATTCAAAAGGAAGAAGAGAAGAAGGTGGAAAACTTTTCTTTTTACCTACCAGAGAAAACTCGGGAATG
GTTCACAAAGTCTGG
+
FFFFFFFFFFFFFFFFFFFFFFFFFFF:FFFFFFFFFFFFFFFFFFFFFFFFFFF:FFFFFFFFFFFF
FFFFFFFFFFFFFFFFFFFFFFFF::::FFFFF:FFFFFFFFFFFFFFFFFFFFFFFFFFFFFFFFFF
FFFFFFFF,FFFFFF
@A00155:342:HHGFNDSXY:1:1202:28257:20275 1:N:0:GAACCTAG+TCCGCATA
CTGAGGAACTCTTGAGCAGGCTCCCACAGTCTCAACAGGAGGTTATAAGCGGATTTCAGTATGAACGC
ATTCAAAAGGAAGAAGAGAAGAAGGTGGAAAACTTTTCTTTTTACCTACCAGAGAAAACTCGGGAATG
GTTCACAAAGTCTGG
+
FFFFFFFFFFFFFFFFFFFFFFFFFFFFFFFFFFFFFFFF:FFFFFFFFFFFFFFFFFFFFFFFFFFF
FFFFFFFFFFFFFFFFFFFFFF,FFF:FFFFFFFFFFFFFFFFFFFFFFFFFFFFFFFFFFFFFFFFF
FFFFFFFF,F:FFFF
@A00155:342:HHGFNDSXY:1:1323:18792:19633 1:N:0:GAACCTAG+TCCGCATA
AGGAGGAACTCTTGAGCAGGCTCCCACAGTCTCAACAGGAGGTTATAAGTGGATTTCAGTATGAACGC
ATTCAAAAGGAAGAAGAGAAGAAGGTGGAAAACTTTTCTTTTTACCTACCAGAGAAAACTCGGGAATG
GTTCACAAAGTCTGG
+
FFFFFFFFFFFFFFF:FFFFFFFFFFFFFFFFFFFFFFFFFFF:FFFFFFFFFFFFFF:FFFFFFFFF
FFFFFFFFFFFFFFFFFFFFF:FFFF:FF:FFFFFFFFFFFFFFFFFFFFFFFFFFFFFFFFFFFFFF
FFFFFFFFFFFFFFF

```
@A00155:342:HHGFNDSXY:1:1364:19470:23876 1:N:0:GAACCTAG+TCCGCATA
CTGAGGAACTCTTGAGCAGGCTCCCACAGTCTCAACAGGAGGTTATAAGCGGATTTCAGTATGAACGC
ATTCAAAAGGAAGAAGAGAAGAAGGTGGAAAACTTTTCTTTTTACCTACCAGAGAAAACTCGGGAATG
GTTCACAAAGTCTGG
+
FFFFFFFFFFFFFFFFFFFFFFFFFFFFFFFFFFFFFFFFFFFFFFFFFFFFFFFFFFFFFFFFFFFF
FFFFFFFFFFFFFFFFFFFFFFFFFFFFFFFFFFFFFFFFFFFFFFFFFFFFFFFFFFFFFFFFFFFF
FFFFFFFFFFFFF:F
@A00155:342:HHGFNDSXY:1:1469:9896:13056 1:N:0:GAACCTAG+TCCGCATA
AGGAGGAACTCTTGAGCAGGCTCCCACAGTCTCAACAGGAGGTTATAAGTGGATTTCAGTATGAACGC
ATTCAAAAGGAAGAAGAGAAGAAGGTGGAAAACTTTTCTTTTTACCTACCAGAGAAAACTCGGGAATG
GTTCACAAAGTCTGG
+
FFFFFFFFFFFFFFFFFFFFFFFFFFFFFFFFFF,FFFFFFFFFFFFFFFFFFFFFFFFFFFFFFFFF
FFFFFFFFFFFFFFFFFFF:FFF,,FFFF:FFFFFFFFFFFFF:FFFFFFFFFFFFFFFFFFFFFFFF
FFFFFFFFFFFFFFF
@A00155:342:HHGFNDSXY:1:1618:19614:17581 1:N:0:GAACCTAG+TCCGCATA
AGGAGGAACTCTTGAGCAGGCTCCCACAGTCTCAACAGGAGGTTATAAGCGGATTTCAGTATGAACGC
ATTCAAAAGGAAGAAGAGAAGAAGGTGGAAAACTTTTCTTTTTACCTACCAGAGAAAACTCGGGAATG
GTTCACAAAGTCTGG
+
FFFF:FFFFFFFFFFFFFFFFFFFFFFFFFFFF:FFFFFFF,,FFFFFFFFFFFFFFFFFFFFFFFFF
FFFFFFFFFFFFFFFFFFFFFFFFFFFF:FFFFFFFFFFFFFFFFF:FFFFFFFFFFFFFFFFFFFFF
FFFFFFFFFFFF:FF
@A00155:342:HHGFNDSXY:1:2108:16830:10034 1:N:0:GAACCTAG+TCCGCATA
AGGAGGAACTCTTGAGCAGGCTCCCACAGTCTCAACAGGAGGTTATAAGCGGATTTCAGTATGAACGC
ATTCAAAAGGAAGAAGAGAAGAAGGTGGAAAACTTTTCTTTTTACCTACCAGAGAAAACTCGGGAATG
GTTCACAAAGTCTGG
+
FFFFFFFFFFFFFFFFFFFFFFFFFFFFFFFFFFFFFFFFFFFFFFFFFFFFFFFFFFFFFFFFFFFF
FFFFFFFFFFFFFFFFFFFFFFF:FFFFFFFFFFFFFFFFFFFFFFFFFFFFFFFFFFFFFFFFFFFF
FFFFFFFFFFFFFFF
@A00155:342:HHGFNDSXY:1:2243:2691:32831 1:N:0:GAACCTAG+TCCGCATA
AGGAGGAACTCTTGAGCAGGCTCCCACAGTCTCAACAGGAGGTTATAAGCGGATTTCAGTATGAACGC
ATTCAAAAGGAAGAAGAGAAGAAGGTGGAAAACTTTTCTTTTTACCTACCAGAGAAAACTCGGGAATG
GTTCACAAAGTCTGG
+
FFFFF:F:FFFFFFFFFFFFFFFFFFFFFFFFFFFFFFFFF:FFFFFFFFFFFFFFFFFFFFFFFFFF
FFFFFFFFFFFFFFFFFFFFFF,FFFFFFFFFFFFFFFFFFFFFFFFFFFFFFFFFFFFFFFFFFFFF
FFFFFFFFFFFFFFF
@A00155:342:HHGFNDSXY:1:2255:14696:27571 1:N:0:GAACCTAG+TCCGCATA
AGGAGGAACTCTTGAGCAGGCTCCCACAGTCTCAACAGGAGGTTATAAGTGGATTTCAGTATGAACGC
ATTCAAAAGGAAGAAGAGAAGAAGGTGAAAAACTTTTCTTTTTACCTACCAGAGAAAACTCGGGAATG
GTTCACAAAGTCTGG
+
FFFFF:FFFFFFFFFFFFFFFFFFFFFFFFFFFFFFFF:FFFFFFFFFFFFFFFFFFFFFFFFFFFFF
FFFFFFFFFFFFFFFFFF:FFF:::FFFFFFFFFFFFFFFFFFFFFFFFFFFFFFFFFFFFFFFFFFF
FFFFFFFFFFFFFFF
@A00155:342:HHGFNDSXY:1:2457:32913:20885 1:N:0:GAACCTAG+TCCGCATA
AGGAGGAACTCTTGAGCAGGCTCCCACAGTCTCAACAGGAGGTTATAAGCGGATTTCAGTATGAACGC
ATTCAAAAGGAAGAAGAGAAGAAGGTGGAAAACTTTTCTTTTTACCTACCAGAGAAAACTCGGGAATG
GTTCACAAAGTCTGG
+
FFFFFFF:F:FF:FFFF,FFFFF:FFFFFFFF:FFFFFFFFFFFFFFFFFFFFFFFFFFFFFFFFF:F
```

```
FF:FFFFFFFF:FFFFFFFFFFFF:FF:FFFFFFFFFFFF:FFFFFFFFFFFFFFFFFFFFFFFFFFF
FF:FFFF:FFFFFFF
@A00155:342:HHGFNDSXY:1:2457:31114:24158 1:N:0:GAACCTAG+TCCGCATA
AGGAGGAACTCTTGAGCAGGCTCCCACAGTCTCAACAGGAGGTTATAAGCGGATTTCAGTATGAACGC
ATTCAAAAGGAAGAAGAGAAGAAGGTGGAAAACTTTTCTTTTTACCTACCAGAGAAAACTCGGGAATG
GTTCACAAAGTCTGG
+
FFFFFFFFFFFFFFFFFFFFFFFFFFFFFFFFFFFFFFFFFFFFFFFFFFFFFFFFFFFFFFF:FFFF
FFFFFFFFFFFFFFFFFFFFFF:FFFFFFF:FFFFFFFFFFF:FFFFFFFFFFFFFFFFFFFFFFFFF
FFFFFFFFFFFFFFF
@A00155:342:HHGFNDSXY:1:2640:2727:14888 1:N:0:GAACCTAG+TCCGCATA
AGGAGGAACTCTTGAGCAGGCTCCCACAGTCTCAACAGGAGGTTATAAGCGGATTTCAGTATGAACGC
ATTCAAAAGGAAGAAGAGAAGAAGGTGGAAAACTTTTCTTTTTACCTACCAGAGAAAACTCGGGAATG
GTTCACAAAGTCTGG
+
FFFFFFFFFFFFFFF:FFFFFFFFFFFFFFFFFFFFF:,FFFFFFF:FFFFFFFFFFFFFFFFFFFFFF
FFFFFFFFFFFFFFFFFFFFFFFFFFFFFFFFFFFFFF:FFFFFFFFFFFFFFFFFFFFFFFFFFFFF
FFFFFFFFFFFFFFF
@A00155:342:HHGFNDSXY:1:1474:19913:13275 2:N:0:GAACCTAG+TCCGCATA
AGGAGGAACTCTTGAGCAGGCTCCCACAGTCTCAACAGGAGGTTATAAGCGGATTTCAGTATGAACGC
ATTCAAAAGGAAGAAGAGAAGAAGGTGGAAAACTTTTCTTTTTACCTACCAGAGAAAACTCGGGAATG
GTTCACAAAGTCTGG
+
FFFFFFF:FF:FFFFFFFFFFFFFFFFFFFFFFFFFFFFFFFFFFFFFFF::FFFFFFFFFF:F:,:
FFFFFFFFFFFFF:FFF:F,F::FFFFFFFFFFFFFFFFFFFFFFFFFFFFFFFFFFFFFFFFFFFFF
FFFFF:FFFFF:FF:
@A00155:342:HHGFNDSXY:1:2505:25581:9533 2:N:0:GAACCTAG+TCCGCATA
AGGAGGAACTCTTGAGCAGGCTCCCACAGTCTCAACAGGAGGTTATAAGCGGATTTCAGTATGAACGC
ATTCAAAAGGAAGAAGAGAAGAAGGTGGAAAACTTTTCTTTTTACCTACCAGAGAAAACTCGGGAATG
GTTCACAAAGTCTGG
+
FFFFFFFFFFFFFFFFFFFFFFFFFFFFFFFFFFFFFFFFFFFFFFFFFFFFFFFFFFFFFFFFFFFF
FFFF:F:FFFFFFFFFFFFFFFFFFFFFFFFFFFFFFFF:FFFFFFFFFFFFFFFFFFFFFFFFFFFF:
FFFFFFFFFFFFFFF
@A00155:342:HHGFNDSXY:1:1628:5791:10927 1:N:0:GAACCTAG+TCCGCATA
TCTGGAACTCTTGAGCAGGCTCCCACAGTCTCAACAGGAGGTTATAAGCGGATTTCAGTATGAACGCA
TTCAAAAGGAAGAAGAGAAGAAGGTGGAAAACTTTTCTTTTTACCTACCAGAGAAAACTCGGGAATGG
TTCACAAAGTCTGG
+
FFFFFFFFF:FFFFFFFF,FFFFFFFFFF:FFF::FFF:,FFFFFF:FFFF:FFF:::FFFFFFFFFF
FFFFFFF,FFFFFFFFFF:FF:FFFF,F,,:FFFFFFFFFF:,FFFFFF:FFFF:FFFFFFFFFFFFF
FFFFF,,F:FFFFF
@A00155:342:HHGFNDSXY:1:1103:32850:18897 1:N:0:GAACCTAG+TCCGCATA
GGAGGAACTCTTGAGCAGGCTCCCACAGTCTCAACAGGAGGTTATAAGCGGATTTCAGTATGAACGCA
TTCAAAAGGAAGAAGAGAAGAAGGTGGAAAACTTTTCTTTTTACCTACCAGAGAAAACTCGGGAATGG
TTCACAAAGTCTGGT
+
FFFFFFFFFFFFFFFFFFFFFFFFFF,FFFFF,FFFFFFFFFFF:F,FFFFFFFFFFFFFFFFFFFFF
F:FFFFFFFFFFF,FFF:FFF,:FF:FFFFFFFFFFF:FF,FFFFFFFFFFFFFFFFFFFFFFFFFFF
FFFFFFFFFFFFFFF
@A00155:342:HHGFNDSXY:1:1109:20437:13338 1:N:0:GAACCTAG+TCCGCATA
GGAGGAACTCTTGAGCAGGCTCCCACAGTCTCAACAGGAGGTTATAAGCGGATTTCAGTATGAACGCA
TTCAAAAGGAAGAAGAGAAGAAGGTGGAAAACTTTTCTTTTTACCTACCAGAGAAAACTCGGGAATGG
TTCACAAAGTCTGGT
```

```
+
FFFFFFFFFFFFFFFFFFFFFFFFFFFFFFFFFFFFFFFFFFFFFFFFFFFFFFFFFFFFFFFFFFFFFF
FFFFFFFFFFFFFFFFF:FFF,FF:F:FFFFFF:FFFFFF:FFFFFFFFFFFFFFFFFFFFFFFFFFFFF
FFFFFFFFFFFFFFF
@A00155:342:HHGFNDSXY:1:1117:30725:3505 1:N:0:GAACCTAG+TCCGCATA
GGAGGAACTCTTGAGCAGGCTCCCACAGTCTCAACAGGAGGTTATAAGCGGATTTCAGTATGAACGCA
TTCAAAAGGAAGAAGAGAAGAAGGTGGAAAACTTTTCTTTTTACCTACCAGAGAAAACTCGGGAATGG
TTCACAAAGTCTGGT
+
FFFFFFFFFFFFFFFFFFFFFFFFFFFFFF:FFFFFFFFFFFFFFFFFFFFFFFFFFFFFFFFFFFFFFF
FFFFFFFFFFFFFFFFFFFFFFFF:F:FF:FFFFFFFFFFFFFFFFFFFFFFFFFFFFFFFFFFFFFFFF
FFFFFFFFFFFFFFF
@A00155:342:HHGFNDSXY:1:1222:32678:3944 1:N:0:GAACCTAG+TCCGCATA
GGAGGAACTCTTGAGCAGGCTCCCACAGTCTCAACAGGAGGTTATAAGCGGATTTCAGTATGAACGCA
TTCAAAAGGAAGAAGAGAAGAAGGTGGAAAACTTTTCTTTTTACCTACCAGAGAAAACTCGGGAATGG
TTCACAAAGTCTGGT
+
FFFFFFFFFFFFFFFFFFFFFFFFFFFFFFFFFFFFFFFFFFFFFF:FFFFFFFFFFFFFFFFFF,FFFF
FFFFFFFFFFFFFFFFFFFFFFFFFFFFFFFFFFFFFFFFFFFFFFFFFFFFFFFFFFFFFFFFFFFFFF
FFFFFFFFFFFFFFF
@A00155:342:HHGFNDSXY:1:1247:15881:25958 1:N:0:GAACCTAG+TCCGCATA
GGAGGAACTCTTGAGCAGGCTCCCACAGTCTCAACAGGAGGTTATAAGCGGATTTCAGTATGAACGCA
TTCAAAAGGAAGAAGAGAAGAAGGTGGAAAACTTTTCTTTTTACCTACCAGAGAAAACTCGGGAATGG
TTCACAAAGTCTGGC
+
FFFFFFFFFFFFFFFFFFFFFFFFFFFFFFFFFFFFFFFFFFFFFFFFFFFFFFFFFFFFFFFFFFFFFF
FFFFFFFFFFFFFFFFFFFFFFFFFFFFFFFFFFFFFFFFFFFFFF:FFFFFFFFFFFFFFFFFFFFFFF
FFFFFFFFFFFFFFF
@A00155:342:HHGFNDSXY:1:1264:17752:8218 1:N:0:GAACCTAG+TCCGCATA
GGAGGAACTCTTGAGCAGGCTCCCACAGTCTCAACAGGAGGTTATAAGCGGATTTCAGTATGAACGCA
TTCAAAAGGAAGAAGAGAAGAAGGTGGAAAACTTTTCTTTTTACCTACCAGAGAAAACTCGGGAATGG
TTCACAAAGTCTGGT
+
FFFFFFFFFFFFFFFFFFFFFFFFFFFFFFFFFFFFFFFFFFFFFFFFFFFFFFFFFFFFFFFFFFFFFF
FFFFFFFFFFFFFFFFFFFFFF:FFFFFF:FFFFFFFFFFFFFFFFFFFFFFFFFFFFFFFFFFFFFFFF
FFFFFFFFFFFFFFF
@A00155:342:HHGFNDSXY:1:1422:25599:33364 1:N:0:GAACCTAG+TCCGCATA
GGAGGAACTCTTGAGCAGGCTCCCACAGTCTCAACAGGAGGTTATAAGCGGATTTCAGTATGAACGCA
TTCAAAAGGAAGAAGAGAAGAAGGTGGAAAACTTTTCTTTTTACCTACCAGAGAAAACTCGGGAATGG
TTCACAAAGTCTGGT
+
FFFFFFFFFFFFFFFFF,FFFFFFFFFFFFFFFFFFFFF,FFFFFFFFFFF:FFFFFFFFFFFFFFF:FF
FFFFFFFFF::FFFFFF:F,FF,,:,:FF:F:FFFFFFFFFFF,FFFFFFFFFFFF:FFFFFF,FF:FFF
FFFFFFFFFFFFFFF
@A00155:342:HHGFNDSXY:1:1532:25889:17675 1:N:0:GAACCTAG+TCCGCATA
GGAGGAACTCTTGAGCAGGCTCCCACAGTCTCAACAGGAGGTTATAAGTGGATTTCAGTATGAACGCA
TTCAAAAGGAAGAAGAGAAGAAGGTGGAAAACTTTTCTTTTTACCTACCAGAGAAAACTCGGGAATGG
TTCACAAAGTCTGGT
+
FFF,FFFFFFFFFFFFFFFFFFFFFFFFFFFFFFFFFFFFFFFFFFFFFFFFFFFFFFFFFFFFFFFFFF
FFFFFFFFFFFFFFFFFFF,:,F::FFFF:FFFFFFFFFFFFFFFFFFFFFFFFFFFFFFFFFFFFFFFF
FFFFFFFFFFFFFFF
@A00155:342:HHGFNDSXY:1:1649:6144:18897 1:N:0:GAACCTAG+TCCGCATA
GGAGGAACTCTTGAGCAGGCTCCCACAGTCTCAACAGGAGGTTATAAGCGGATTTCAGTATGAACGCA
```

TTCAAAAGGAAGAAGAGAAGAAGGTGGAAAACTTTTCTTTTTACCTACCAGAGAAAACTCGGGAATGG
TTCACAAAGTCTGGT
+
FFFF,FFFFFFFFFFFF:FFFFFFFFF:FFF:,,FFFFF:FFFFFF,FFFFF:FFFFFFFFFFFFFFF
FFF:F,FFFFFFF:FF:F:F,FFF::F:F:FFFFFFFF:FFFF:FF::FFFFFF,,:FF::FFFFFFF
FF,:FF:FFFFFFFF
@A00155:342:HHGFNDSXY:1:1658:19813:14011 1:N:0:GAACCTAG+TCCGCATA
GGAGGAACTCTTGAGCAGGCTCCCACAGTCTCAACAGGAGGTTATAAGCGGATTTCAGTATGAACGCA
TTCAAAAGGAAGAAGAGAAGAAGGTGGAAAACTTTTCTTTTTACCTACCAGAGAAAACTCGGGAATGG
TTCACAAAGTCTGGT
+
FFFFFFFFFFFFFFFFFFF:FFFFFFFFFFFFFFFFFFFFFFFFFF:FFFFFFFFFFFFFFFFFFFFF
FFFFFFFFFFFFFFFFFFFFFFFFF::FF:FFFFFFFFFFFFFFFFFFFFFFFFFFFFFFFFFFFFFF
FFFFFFFFFFFFFFF
@A00155:342:HHGFNDSXY:1:2130:14977:35258 1:N:0:GAACCTAG+TCCGCATA
GGAGGAACTCTTGAGCAGGCTCCCACAGTCTCAACAGGAGGTTATAAGCGGATTTCAGTATGAACGCA
TTCAAAAGGAAGAAGAGAAGAAGGTGGAAAACTTTTCTTTTTACCTACCAGAGAAAACTCGGGAATGG
TTCACAAAGTCTAGT
+
FFFFFFFFFFFFFFFFFFFFFFFFFFFFFFFFFFFFFFFFFFFFFFFFFFFFFFFFFFFFFFFFFFFF
FFFFFF:FFFFFFFFFFFFFF::FF:FFFFFFFFFFFFFFFFFFFFFFFFFFFFFFFFFFFFFFFFFF
FFFFFFFFFFFFFFF
@A00155:342:HHGFNDSXY:1:2131:14398:2159 1:N:0:GAACCTAG+TCCGCATA
GGAGGAACTCTTGAGCAGGCTCCCACAGTCTCAACAGGAGGTTATAAGCGGATTTCAGTATGAACGCA
TTCAAAAGGAAGAAGAGAAGAAGGTGGAAAACTTTTCTTTTTACCTACCAGAGAAAACTCGGGAATGG
TTCACAAAGTCTGGT
+
FFFFFFFFFFFFFFFFFFFFFFFFFFFFFFFFFFFFFFFFFFFFF,FFFFFFFFFFFFF:FF:FFFFF
FFFFFFFFFFFFFFFFFFFFFFFFFFFFFFFFFFFFFFFFFFFFFFFFFFFFFFF:FFFFFFFFFFFF
FFFFFFFFFFFF,FF
@A00155:342:HHGFNDSXY:1:2161:5520:12399 1:N:0:GAACCTAG+TCCGCATA
GGAGGAACTCTTGAGCAGGCTCCCACAGTCTCAACAGGAGGTTATAAGTGGATTTCAGTATGAACGCA
TTCAAAAGGAAGAAGAGAAGAAGGTGGAAAACTTTTCTTTTTACCTACCAGAGAAAACTCGGGAATGG
TTCACAAAGTCTGGT
+
:FFFFFF,FFFFFFFFFFFFFFFFFF:FFFFFFFFFFFFFFFFFFFFFFFFFFFFFFFFFFFFFFFFF
FFFFFFFF:FFFFFFFFFFFFFF,FFFFFFFFFFF:FFFFFFFFFFFFFFFFFFFFFF:FFFFFFFF:
FFFFFFFFFFFFFFF
@A00155:342:HHGFNDSXY:1:2357:29857:20415 1:N:0:GAACCTAG+TCCGCATA
GGAGGAACTCTTGAGCAGGCTCCCACAGTCTCAACAGGAGGTTATAAGTGGATTTCAGTATGAACGCA
TTCAAAAGGAAGAAGAGAAGAAGGTGGAAAACTTTTCTTTTTACCTACCAGAGAAAACTCGGGAATGG
TTCACAAAGTCTGGT
+
FFFFFFFFFFFFF:FFFFFFFFFFFFFFFFFFFFFFFFFFFFFFFFF:FFFFFFFFFFFFFFFFFFFF
FFFFFFFFFFFFFFFFFFFFFFFFFFFFFFFFFFFFFFFFFFFFFF:FFFFFFFFFFFFFFFFFFFFF
FFFFFFFFFFFFFFF
@A00155:342:HHGFNDSXY:1:2526:28619:15796 1:N:0:GAACCTAG+TCCGCATA
GGAGGAACTCTTGAGCAGGCTCCCACAGTCTCAACAGGAGGTTATAAGCGGATTTCAGTATGAACGCA
TTCAAAAGGAAGAAGAGAAGAAGGTGGAAAACTTTTCTTTTTACCTACCAGAGAAAACTCGGGAATGG
TTCACAAAGTCTGGT
+
FFFFFFFFFFFFFFFFFF:FFF:FFFFFFFFFFFFFFFFFFFFFFFFFF:FFFFFFFFFFFFFFFFFF
FFFFFFFFFFFFFFFFFF,FFFFFFFFFF,FFFFFFFFFFFFFFFFFFFFFFFFFFFFFFFFFFFFFF
FFFFFFFFFFFFFFF

```
@A00155:342:HHGFNDSXY:1:2613:8820:14043 1:N:0:GAACCTAG+TCCGCATA
GGAGGAACTCTTGAGCAGGCTCCCACAGTCTCAACAGGAGGTTATAAGCGGATTTCAGTATGAACGCA
TTCAAAAGGAAGAAGAGAAGAAGGTGGAAAACTTTTCTTTTTACCTACCAGAGAAAACTCGGGAATGG
TTCACAAAGTCTGGG
+
FFFF:FFFFFFFFFFFFF:FFFFFFFFFFF:F:FFF,F::FFFFFFFFF,:FFFFFFFFFFFFFFFFF
FFFFFFFFFFFFFFFF,:FFFFFFF,:FFFFFFFFFFFFFFFFFFFFFFFFFFFFFFFFFFFFFFFFF
FFFFFFFFFFFFFFF
@A00155:342:HHGFNDSXY:1:2573:14145:25207 1:N:0:GAACCTAG+TCCGCATA
GGAGGAACTCTTGAGCAGGCTCCCACAGTCTCAACAGGAGGTTATAAGCGGATTTCAGTATGAACGCA
TTCAAAAGGAAGAAGAGAAGAAGGTGGAAAACTTTTCTTTTTACCTACCAGAGAAAACTCGGGAATGG
TTCACAAAGTCTGGG
+
FFFFFFFFFFFFFFFFFFFFFFFFFFFFFFFFFFFFFFFFFFFFFFFFFFFFFFFFFFFFFFFFFFFF
FFFFFFFFFFFFFFFFFF,F:FF:,:FFFFFFFFFFFFFFFFFFFFFFFFFFFFFFFFFFFFFFFFFF
FFFFFFFFFFFFFFF
@A00155:342:HHGFNDSXY:1:1401:20645:8437 2:N:0:GAACCTAG+TCCGCATA
GGAGGAACTCTTGAGCAGGCTCCCACAGTCTCAACAGGAGGTTATAAGCGGATTTCAGTATGAACGCA
TTCAAAAGGAAGAAGAGAAGAAGGTGGAAAACTTTTCTTTTTACCTACCAGAGAAAACTCGGGAATGG
TTCACAAAGTCTGGT
+
FFFFFFFFFFFFFFFFFFFFFFFFFF:FFFFFFFFFFFFFFFF:FFFFFFFFFFFFFFF:FFFFFFFF
FFFFFFFFFFFFFFFFFFFFFFFFFFFFFFFFFFFFFFFFFFFFFFFFFFFFF:FFFFFFFFFF,FFF
FFFFFFFFFFFFFFF
@A00155:342:HHGFNDSXY:1:1502:5719:4695 2:N:0:GAACCTAG+TCCGCATA
GGAGGAACTCTTGAGCAGGCTCCCACAGTCTCAACAGGAGGTTATAAGTGGATTTCAGTATGAACGCA
TTCAAAAGGAAGAAGAGAAGAAGGTGGAAAACTTTTCTTTTTACCTACCAGAGAAAACTCGGGAATGG
TTCACAAAGTCTGGT
+
FFFFFFFFFFFFFFFFFFFFFFFFFFFFFFFFFFFFFFFFFFFFFFFFFFFFFFFFFFFFFFFFFFFF
FFFFFF:FFFFFFFFFFF:FFFFFFFFFFFFFFFFFFFFFFFFFFFFFFFFFFFFF:FFFFFF:,FFF
FFFFFFFFFFFFFFF
@A00155:342:HHGFNDSXY:1:1662:17842:25911 2:N:0:GAACCTAG+TCCGCATA
GGAGGAACTCTTGAGCAGGCTCCCACAGTCTCAACAGGAGGTTATAAGCGGATTTCAGTATGAACGCA
TTCAAAAGGAAGAAGAGAAGAAGGTGGAAAACTTTTCTTTTTACCTACCAGAGAAAACTCGGGAATGG
TTCACAAAGTCTGGT
+
FFFFFFFFFFFFFFFFFFFFFFFFFF:FFFFFFFFFFFFFFFFFFFFFFFFFFFFFFFFFFFFFFFFF
FFFFFFFFFFFFFFFFFFFFFFFFFFFFFFFFFFFFFFFFFFFF,FFFFFFFFFFFFFFF:FFF:FFF
FFFFFFFFFFFFFFF
@A00155:342:HHGFNDSXY:1:1670:27552:28166 2:N:0:GAACCTAG+TCCGCATA
GGAGGAACTCTTGAGCAGGCTCCCACAGTCTCAACAGGAGGTTATAAGCGGATTTCAGTATGAACGCA
TTCAAAAGGAAGAAGAGAAGAAGGTGGAAAACTTTTCTTTTTACCTACCAGAGAAAACTCGGGAATGG
TTCACAAAGTCTGGT
+
FFFFFFFFFFFFFFFFFFFFFFFFFFFFFFFFFFFFFFFFFFFFFFFFFFFFFFFFFFF:FFFFFFFF
FFFFFFFFFF:FFFFFFFFFFFFFFFFFFF::FFFFFFFFFFFFFFFFFFFFFFFFFFFF:FFF,F:F
FFFFFFFFFFF:F,F
@A00155:342:HHGFNDSXY:1:2249:19208:13307 2:N:0:GAACCTAG+TCCGCATA
GGAGGAACTCTTGAGCAGGCTCCCACAGTCTCAACAGGAGGTTATAAGTGGATTTCAGTATGAACGCA
TTCAAAAGGAAGAAGAGAAGAAGGTGGAAAACTTTTCTTTTTACCTACCAGAGAAAACTCGGGAATGG
TTCACAAAGTCTGGT
+
FFFFFFFFFFFFFFFFFFFFFFFFFFFFFFFFFFFFFFFFFFFFFFFFFFFFFFFFFFFFFFFFFFFF
```

FFFFFFFFFFFFFFFFFFFFFFFFFFFFFFFFFFFFFFFFFFFFFFFFFFFFFFFFFFFFFFFFFFFFFF
FFFFFFFFFFFFFFF
@A00155:342:HHGFNDSXY:1:2270:2121:5102 2:N:0:GAACCTAG+TCCGCATA
GGAGGAACTCTTGAGCAGGCTCCCACAGTCTCAACAGGAGGTTATAAGTGGATTTCAGTATGAACGCA
TTCAAAAGGAAGAAGAGAAGAAGGTGGAAAACTTTTCTTTTTACCTACCAGAGAAAACTCGGGAATGG
TTCACAAAGAGATCG
+
FFFFFFFFFFFFFFFFFFFFFFFFFFFFFFFFFFFFFFFFFFFFFFFFFFFFFFFFFFFFFFFFFFFFFF
FFFFFFFFFFF:FFFFFFFFFFFFFFFFFFF:FFFFFFFFFFFFFFFFFFFFFFFFFFFFFFFFFFFFFF
FFF:FF:F,FFFFFF
@A00155:342:HHGFNDSXY:1:2669:4426:26475 2:N:0:GAACCTAG+TCCGCATA
GGAGGAACTCTTGAGCAGGCTCCCACAGTCTCAACAGGAGGTTATAAGCGGATTTCAGTATGAACGCA
TTCAAAAGGAAGAAGAGAAGAAGGTGGAAAACTTTTCTTTTTACCTACCAGAGAAAACTCGGGAATGG
TTCACAAAGTCTGGT
+
FFFFFFFFFFFFFFFFFFFFFFFFFFFFFFFFF:FFFFFFFFFFFFFFFFFFFFFFFFFFFFF:FFFFFF
F:FFFFFF,FFFFFFFFFFFFFFFFFFFFFFF:FFFFF:FFFFFFFFFFFFFFFFF:F,,FFFFFFFF:,
FFF:FFFFFFFFFFF
@A00155:342:HHGFNDSXY:1:1140:14452:8703 1:N:0:GAACCTAG+TCCGCATA
CGATCTCTCTTGAGCAGGCTCCCACAGTCTCAACAGGAGGTTATAAGTGGATTTCAGTATGAACGCAT
TCAAAAGGAAGAAGAGAAGAAGGTGGAAAACTTTTCTTTTTACCTACCAGAGAAAACTCGGGAATGGT
TCACAAAGTCTGGTG
+
FFFFFFFFFFFFFFFFFFFFFFFFFFFFFFFFFFFFFFFFFFFFFFFFFFFFFFFFFFFFFFFFFFFFFF
FFFF:FFFFFFFFFFFF:FFFFFFFFFFFFFFFFFFFFFFFFFFFFFFFFFFFFFFFFFFFFFFFFFFFF
FFFFFFFFFFFFFFF
@A00155:342:HHGFNDSXY:1:1220:14805:21433 1:N:0:GAACCTAG+TCCGCATA
TCCGATCTCTTGAGCAGGCTCCCACAGTCTCAACAGGAGGTTATAAGCGGATTTCAGTATGAACGCAT
TCAAAAGGAAGAAGAGAAGAAGGTGGAAAACTTTTCTTTTTACCTACCAGAGAAAACTCGGGAATGGT
TCACAAAGTCTGGTG
+
FFF:FFFF:,FFFFF,F,FFFFFFFF,::FFFFFFFFFFF:::FFF::,:F:FFFFFFFFFFFF:FF:FF
:FFFF:,FFFF:FFFF:::FF,:F:FFFF:FFFFFFFFFFFFF::FFFFFF::FF:,FFF::FFFFFFFF
FFFFFFFFF:FFFFF
@A00155:342:HHGFNDSXY:1:1146:4598:32471 2:N:0:GAACCTAG+TCCGCATA
GAGGAACTCTTGAGCAGGCTCCCACAGTCTCAACAGGAGGTTATAAGCGGATTTCAGTATGAACGCAT
TCAAAAGGAAGAAGAGAAGAAGGTGGAAAACTTTTCTTTTTACCTACCAGAGAAAACTCGGGAATGGT
TCACAAAGTCTGGTG
+
FFFFFFFFFFFFFFFFFFFFFFFFFFFFFFFFFFFFFFFFFFFFFFFFFFFFFFFFFFFFFFFFFFFFFF
F:FFFFFFFFFFFFFFFFF:FFFFFFFFFFFFFFFFFFFFFFFF:FFFFFFFFFFFFFFFFFFFFFFFFF
F:F:FFFFFFFFFFF
@A00155:342:HHGFNDSXY:1:1202:28257:20275 2:N:0:GAACCTAG+TCCGCATA
GAGGAACTCTTGAGCAGGCTCCCACAGTCTCAACAGGAGGTTATAAGCGGATTTCAGTATGAACGCAT
TCAAAAGGAAGAAGAGAAGAAGGTGGAAAACTTTTCTTTTTACCTACCAGAGAAAACTCGGGAATGGT
TCACAAAGTCTGGAG
+
FFFFF,FFFFFFFFFFFFFFFFFFFFFFFFFFFFFFFF:FFFFFFF,FFFFFFFFFFFFFFFFFFFFFFF
FFFFFFFFFFFFFFFFFFFFF:FFFFFFF::FFFFFFFFFFFFFFFFFFFFFFFFFFFFFFFFFF,FFFF
FFFFFFFFFFFFFFF
@A00155:342:HHGFNDSXY:1:1364:19470:23876 2:N:0:GAACCTAG+TCCGCATA
GAGGAACTCTTGAGCAGGCTCCCACAGTCTCAACAGGAGGTTATAAGCGGATTTCAGTATGAACGCAT
TCAAAAGGAAGAAGAGAAGAAGGTGGAAAACTTTTCTTTTTACCTACCAGAGAAAACTCGGGAATGGT
TCACAAAGTCTGGAG

```
+
FFFFFFFFFFFFFFFFFFFFFFFFFFFFFFFFFFFFFFFFFFFFFFFFFFFFFFFFFFFFFFFFFFFF
FFFFFFFFFFFFF:FFFFFFFFFFFFFFF:FFFFFFFFFFFFFFFFFFFFFFFFFF:FFFFFFF:FFFF
FFFFFFFFFFFFFFF
@A00155:342:HHGFNDSXY:1:1501:5023:6308 2:N:0:GAACCTAG+TCCGCATA
GAGGAACTCTTGAGCAGGCTCCCACAGTCTCAACAGGAGGTTATAAGCGGATTTCAGTATGAACGCAT
TCAAAAGGAAGAAGAGAAGAAGGTGGAAAACTTTTCTTTTTACCTACCAGAGAAAACTCGGGAATGGT
TCACAAAGTCTGGTG
+
FFFFFFFFFFFFFFFFFFFFFFFFFFFFFFFFFFFFFFFFFFFFFFFFFFFFFFFFFFFFFFFFFFFF
FFFFFFFFFFFFFFFFFFFFFFFFFFFFFFFFFFFFFFFFFFFFFFFFFFFFFFFFFFFFFFFFFFFF
F,FFFFFFFFFFFFF
@A00155:342:HHGFNDSXY:1:1506:21079:30389 2:N:0:GAACCTAG+TCCGCATA
GAGGAACTCTTGAGCAGGCTCCCACAGTCTCAACAGGAGGTTATAAGCGGATTTCAGTATGAACGCAT
TCAAAAGGAAGAAGAGAAGAAGGTGGAAAACTTTTCTTTTTACCTACCAGAGAAAACTCGGGAATGGT
TCACAAAGTCTGGTG
+
FFF:FFFFFFFFF::F:FF,FFFFFFFFFFFFF:FFFFFFFFFFFF::FFF:FFFFFFFFFFFFFF:F
FFFFFFF,FFFFFFFFFF:FFFFF::FFFF:FFFFFFFFFFFFFFFFFF,:FFFFFFFF:F,FFFF,FF
FFFFFFFF,FFFFFF
@A00155:342:HHGFNDSXY:1:1453:11162:11209 2:N:0:GAACCTAG+TCCGCATA
GAGGAACTCTTGAGCAGGCTCCCACAGTCTCAACAGGAGGTTATAAGCGGATTTCAGTATGAACGCAT
TCAAAAGGAAGAAGAGAAGAAGGTGGAAAACTTTTCTTTTTACCTACCAGAGAAAACTCGGGAATGGT
TCACAAAGTCTGGTG
+
FFFFF:FFFFFFFFFFFFFFFFFFFFFFFFFFFFFFFFFFFFFFFFFFFFFFFFFFFFFFFFFFFFFF
FFFFFFFFFFFFFFFFFFFFFFFFFFFFFFFFFFFFFFFFFFFFFFFFFFFFFFFFFFFFFFFFFFFF
FFFFFFFFFFFFFFF
@A00155:342:HHGFNDSXY:1:1103:32307:20118 1:N:0:GAACCTAG+TCCGCATA
AGGAACTCTTGAGCAGGCTGCCACTGTCTCAACAGGAGGTTATAAGCGGATTTCAGTATGAACGAATT
CAAAAGGAAGAATAGAAGAAGGTGGACAACTTTTCTTTTTACCTACCAGAGAAAAATCGGGAATGGTT
CACAAAGTCTGGT
+
FF,FF,F,FFF:FF,FF,F,,FFF,FF,:FF,FFFFFF,FF:F,FFF,F:FFFF:FF::FFF:F,,F:
F:F,::FFF,FF,FFF,F:,,,,F:F,,,,F:FFFF:FF,:,F,,F:FF,FFF::,FF,F:,:FF:FF
:FF::FFF,F,FF
@A00155:342:HHGFNDSXY:1:1245:27805:11256 2:N:0:GAACCTAG+TCCGCATA
AGGAACTCTTGAGCAGGCTCCCACAGTCTCAACAGGAGGTTATAAGCGGATTTCAGTATGAACGCATT
CAAAAGGAAGAAGAGAAGAAGGTGGAAAACTTTTCTTTTTACCTACCAGAGAAAACTCGGGAATGGTT
CACAAAGTCTGGTGT
+
FFFFFFFFFFFFFFFFFFFFFFFFFFFFFFFFFFFFFFFFFFFFFFFFFFFFFFFFFFFFFFFFFFFF
FFFF,FFFFFFFFFFFFFFFFFFFFFFFFFFFFFFFFFFFFFFFFFF,FFFFF:FFFFFFFFF:FFFFF
FFF:FFFF:F,FFFF
@A00155:342:HHGFNDSXY:1:1349:18936:12618 2:N:0:GAACCTAG+TCCGCATA
AGGAACTCTTGAGCAGGCTCCCACAGTCTCAACAGGAGGTTATAAGCGGATTTCAGTATGAACGCATT
CAAAAGGAAGAAGAGAAGAAGGTGGAAAACTTTTCTTTTTACCTACCAGAGAAAACTCGGGAATGGTT
CACAAAGTCTGAGAT
+
FFFFFFFFFFFFFFFFFFFFFFFFFFFFFFFFFFF:FFFFFFFFFFFFFFFFFFFFFFFFFFFFFFFF
FFFFFFFFFFFFFFFFFFFFFFFFFFFFFFFFFFFFFFFFFFFFFFFFFFFFFFFFFFFFFFFFFFFF
FFFFF:FFFFFFFFF
@A00155:342:HHGFNDSXY:1:2227:29812:13730 2:N:0:GAACCTAG+TCCGCATA
AGGAACTCTTGAGCAGGCTCCCACAGTCTCAACAGGAGGTTATAAGCGGATTTCAGTATGAACGCATT
```

CAAAAGGAAGAAGAGAAGAAGGTGGAAAACTTTTCTTTTTACCTACCAGAGAAAACTCGGGAATGGTT
CACAAAGTCTGGTGT
+
FFFFFFFFFFFFFFFFFFFFFFFFFFFFFFFFFFFFFFFFFFFFF:FFFFFFFFFFFFFFFFFFFFFFFF
FFF:FFFFFFFFF:FFFFFFFFFFFFFFFF:FFFFFFFFFFFFFFFFFFFFFF:FFFFFFFFFFFFFF
FFFF::FFFFFFFFF
@A00155:342:HHGFNDSXY:1:2459:8576:9768 2:N:0:GAACCTAG+TCCGCATA
AGGAACTCTTGAGCAGGCTCCCACAGTCTCAACAGGAGGTTATAAGCGGATTTCAGTATGAACGCATT
CAAAAGGAAGAAGAGAAGAAGGTGGAAAACTTTTCTTTTTACCTACCAGAGAAAACTCGGGAATGGTT
CACAAAGTCTGAGAT
+
FFFF,FFFFFFFFFFFF:FFFFFFFFFFFFFFFFFFFFFFFFFFF,FFFFFFFFFFFFFFFFFFFFFFFF
FFFFFFFFFFFFFFFFFFFFFFFFFFFFFFFFFF:FFFFFFFFFF:FFFFFFFFFFFFFFFFFFFFFF
FFFFF:FFFFFFFFF
@A00155:342:HHGFNDSXY:1:1628:5791:10927 2:N:0:GAACCTAG+TCCGCATA
GGAACTCTTGAGCAGGCTCCCACAGTCTCAACAGGAGGTTATAAGCGGATTTCAGTATGAACGCATTC
AAAAGGAAGAAGAGAAGAAGGTGGAAAACTTTTCTTTTTACCTACCAGAGAAAACTCGGGAATGGTTC
ACAAAGTCTGGTAG
+
FFFFFF:FF:F:FFFFFFFFFFFFFFFFFFFFFFFFFFF:FFFFFFFFFFFFFFFFFF:FFFFF:FFFFF
FF:FF:FFFFFFFFFFFFFFFF:FFFFFFFFFF:FFFFFF:FFFFFFF:FFFFFFFFFFFF,FFFFFF
FFFFF:FFFFFFFF
@A00155:342:HHGFNDSXY:1:1166:7084:3270 2:N:0:GAACCTAG+TCCGCATA
GGAACTCTTGAGCAGGCTCCCACAGTCTCAACAGGAGGTTATAAGCGGATTTCAGTATGAACGCATTC
AAAAGGAAGAAGAGAAGAAGGTGGAAAACTTTTCTTTTTACCTACCAGAGAAAACTCGGGAATGGTTC
ACAAAGTCTGGAGAT
+
FFFFFFFFFFFFFFFFF:FFFFFFFFFFFFFFFFFFFFFFFFFFFFFFFFFFFFFFFFFFFFFFFFFFFF
FFF:FFFFFFFFFFFFF:F:FFFFFFF:FFFFFFFFFFFFFFFFFFFFFFFF:FFFFFFF:F,FFFFFF
F:F:FFFFFFFFFFF
@A00155:342:HHGFNDSXY:1:1237:29451:24690 2:N:0:GAACCTAG+TCCGCATA
GGAACTCTTGAGCAGGCTCCCACAGTCTCAACAGGAGGTTATAAGCGGATTTCAGTATGAACGCATTC
AAAAGGAAGAAGAGAAGAAGGTGGAAAACTTTTCTTTTTACCTACCAGAGAAAACTCGGGAATGGTTC
ACAAAGTCTGGTGTG
+
FFFFFFFFFFFFF:FFFFFFFFFFFFFFFFFFFFFFFFF:F:FFFFFFFFFFFFFFFFFFFFFFFFF:FF
FFF:FFFFFFFFFFFFFFFFFFFFFFFF:FFFFFFFFFFFFFFFFFFFFFFFFFFFFFFFFFF,FFFFFF
F:,FFFFFFFFFFFF
@A00155:342:HHGFNDSXY:1:1266:2058:18490 2:N:0:GAACCTAG+TCCGCATA
GGAACTCTTGAGCAGGCTCCCACAGTCTCAACAGGAGGTTATAAGCGGATTTCAGTATGAACGCATTC
AAAAGGAAGAAGAGAAGAAGGTGGAAAACTTTTCTTTTTACCTACCAGAGAAAACTCGGGAATGGTTC
ACAAAGTCTGGTGTG
+
FFFFFFFFFFFFFFFFFFFFFFFFFFFFFFFFFFFFFFFFFFFFF,FFFFFFFFFFFFFFFFFFFFFFFF
FF::F:FFFFFFFFFF:FFFFFFFFFF:FFFFFFFFFFFFFFFFFFFFFFFFFFFFFFF,:,FFFFFFFF
:FFFFFFFFFF:FFF
@A00155:342:HHGFNDSXY:1:2176:20880:2331 2:N:0:GAACCTAG+TCCGCATA
GGAACTCTTGAGCAGGCTCCCACAGTCTCAACAGGAGGTTATAAGCGGATTTCAGTATGAACGCATTC
AAAAGGAAGAAGAGAAGAAGGTGGAAAACTTTTCTTTTTACCTACCAGAGAAAACTCGGGAATGGTTC
ACAAAGTCTGGTGTG
+
FFFF:FF:FFFF:F,FFFFFFFFF,F:FFFFFFFFFFF:FFFFFF:FFFFF:FFF:FF:FFFFFFF,F
FFFFF:FFFFFFFFFFFFF:FFFFF:F:FFFF:F:F:,FFFFFFFFFFFFFFFFFFFFF,F,:FF,FF
F:FFFFFFFFF:FF:

@A00155:342:HHGFNDSXY:1:2415:28872:32017 2:N:0:GAACCTAG+TCCGCATA
GGAACTCTTGAGCAGGCTCCCACAGTCTCAACAGGAGGTTATAAGCGGATTTCAGTATGAACGCATTC
AAAAGGAAGAAGAGAAGAAGGTGGAAAACTTTTCTTTTTACCTACCAGAGAAAACTCGGGAATGGTTC
ACAAAGTCTGGTGTG
+
FFF:FFFFFFFFFFFFFFFFFFFFFFFFFF:FFFFFFFFFFFFFFFFFFFFFFFFFFFFFFFFFFFFF
FFFFFFFFFFFFFFFFFFFFFFFFFFFFFFF:FFFFFFFFFFFFFFFFFFFFFFFFFFFFFFFFFFFF
FFFFFFFFFFFFFFF
@A00155:342:HHGFNDSXY:1:2513:31973:27492 2:N:0:GAACCTAG+TCCGCATA
GGAACTCTTGAGCAGGCTCCCACAGTCTCAACAGGAGGTTATAAGTGGATTTCAGTATGAACGCATTC
AAAAGGAAGAAGAGAAGAAGGTGGAAAACTTTTCTTTTTACCTACCAGAGAAAACTCGGGAATGGTTC
ACAAAGTCTGGTGTG
+
FFFFFFFFFFFFFFFFFFFFFFFFFFFFFFFF:FF:FF:FFFFFFFFFFF:FFFFFFFFFFFFFFFFF
FFFFFFFFFFFFFFFFFFFFF:FFFFFFFFFFFFFFFFFFFFFFFFFFFFFFFFFFFF:FFF:FF:FF
FFFF:FFFFFFFFFF
@A00155:342:HHGFNDSXY:1:2158:21856:29481 2:N:0:GAACCTAG+TCCGCATA
GAACTCTTGAGCAGGCTCCCACAGTCTCAACAGGAGGTTATAAGCGGATTTCAGTATGAACGCATTCA
AAAGGAAGAAGAGAAGAAGGTGGAAAACTTTTCTTTTTACCTACCAGAGAAAACTCGGGACTGGTTCA
CAAAGTCTGAGATCG
+
FF:FFFFFFFFFFFFFFFFFFFFFFFFFFFFFFF:FFFFFFFFFFFF:FFFFFFFFFFFFFFFF:FFF
FFFFFFFFFFFFFF:FFFFFFFFFFFFFFFFFFFFFFFFFFFFFFFFFFFFFFFFFFFFF,,FFFFFF
F,F:FFFFFFFFFFF
@A00155:342:HHGFNDSXY:1:2178:26928:35916 2:N:0:GAACCTAG+TCCGCATA
GAACTCTTGAGCAGGCTCCCACAGTCTCAACAGGAGGTTATAAGCGGATTTCAGTATGAACGCATTCA
AAAGGAAGAAGAGAAGAAGGTGGAAAACTTTTCTTTTTACCTACCAGAGAAAACTCGGGAATGGTTCA
CAAAGTCTGGTGTGT
+
FFFFFFFFFFFFFFFFFFFFFFFFFFFFFFFFFFFFFFFFFFFFFFFFFFFFFFFFFFFFFFFFFFFF
FF:FFFFFFFFFFFFFFFFFFFFFFFF:F:FFFFFFFFFFFFFFFFFFFFF:FFFFFFFF,F:FFF:,
,FFF,FFFFFFFFFF
@A00155:342:HHGFNDSXY:1:2246:23954:28948 2:N:0:GAACCTAG+TCCGCATA
GAACTCTTGAGCAGGCTCCCACAGTCTCAACAGGAGGTTATAAGCGGATTTCAGTATGAACGCATTCA
AAAGGAAGAAGAGAAGAAGGTGGAAAACTTTTCTTTTTACCTACCAGAGAAAACTCGGGAATGGTTCA
CAAAGTCTGGTGTGT
+
FFFFFFFFFFFFFFFFFFFFFF:FFFFFFFFFFFFFFFFFFFFFFFFFFFFFFFFFFFFFF:FFFFFF
FFFFFFFFFFFFFFFFFFFFFF:FFFFFFFFFFFFFFFFFFFFFFFFFFFFF,FFFFF:F,FFFFFFF
:FFFFFFFFFFFFFF
@A00155:342:HHGFNDSXY:1:2261:1235:16094 2:N:0:GAACCTAG+TCCGCATA
GAACTCTTGAGCAGGCTCCCACAGTCTCAACAGGAGGTTATAAGCGGATTTCAGTATGAACGCATTCA
AAAGGAAGAAGAGAAGAAGGTGGAAAACTTTTCTTTTTACCTACCAGAGAAAACTCGGGAATGGTTCA
CAAAGTCTGGTGTGT
+
FFFFFFF:FF:FF:F:,:FFFFF:,F:FFFFF:F:,:F:F:FFFFFFFFFF:F:FF,FFFF,FFF::F
FFF,FFFFFF:FFFF:FF:F:F:FFFFFF::,FF::FFFFFFFFFFFFFFFFFFFFFF:FFFFFF:F:
FFFFF:FFF:FFFF:
@A00155:342:HHGFNDSXY:1:2540:6460:27899 2:N:0:GAACCTAG+TCCGCATA
GAACTCTTGAGCAGGCTCCCACAGTCTCAACAGGAGGTTATAAGCGGATTTCAGTATGAACGCATTCA
AAAGGAAGAAGAGAAGAAGGTGGAAAACTTTTCTTTTTACCTACCAGAGAAAACTCGGGAATGGTTCA
CAAAGTCTGGTGTGT
+
FFFFFFFFFFFFFFFFFFFFFFFFFFFFFFFFFFFFFFFFFFFFFFFFFFFFFFFFFFFFFFFFFFFF

FFFFFFFFFFFFFFFFFFFFFFFFFFFFFFFFFFFFFFFFFFFFFFFFFFFFFFFFFFF:FFFFFFFF
FFFFFFFFFFFFFFF
@A00155:342:HHGFNDSXY:1:1140:14452:8703 2:N:0:GAACCTAG+TCCGCATA
CTCTTGAGCAGGCTCCCACAGTCTCAACAGGAGGTTATAAGTGGATTTCAGTATGAACGCATTCAAAA
GGAAGAAGAGAAGAAGGTGGAAAACTTTTCTTTTTACCTACCAGAGAAAACTCGGGAATGGTTCACAA
AGTCTGGTGAGATC
+
FFFFFFFFFFFFFFFFFFFFFFFFFFFFFFFFFFFFFFFFFFFFFFFFFFFFFFFFFFFFFFFFFFFF
FFFFFF:FFFFFFFFFFFFFFFFFFFFFFFFFFFFFFFFFFFFF,FFFFFFFFFFFFFFFFFFFFFFF
:FFFFFFFFFFF:F
@A00155:342:HHGFNDSXY:1:1228:28501:3067 2:N:0:GAACCTAG+TCCGCATA
CTCTTGAGCAGGCTCCCACAGTCTCAACAGGAGGTTATAAGCGGATTTCAGTATGAACGCATTCAAAA
GGAAGAAGAGAAGAAGGTGGAAAACTTTTCTTTTTACCTACCAGAGAAAACTCGGGAATGGTTCACAA
AGTCTGGTGTGTATC
+
F,FFF::,FFF:FFFFFFF::FF,,FF:FF,F::FFF,F:FF,FF,FF:FF,,FFFFFF,FF,FFFF:
FFFF:FFF:FFF:FFFF,FFF:FFFF,FFFFF::FFF,FF:FF,FF:FFFFFF,:F:FFFFF,F,FFF
FFFF:FFFFF:FF,F
@A00155:342:HHGFNDSXY:1:1427:8567:35931 2:N:0:GAACCTAG+TCCGCATA
CTCTTGAGCAGGCTCCCACAGTCTCAACAGGAGGTTATAAGCGGATTTCAGTATGAACGCATTCAAAA
GGAAGAAGAGAAGAAGGTGGAAAACTTTTCTTTTTACCTACCAGAGAAAACTCGGGAATGGTTCACAA
AGTCTGGTGTGTATC
+
FFFFFFFFFFFFFFFFFFFFFFFFFFFFFFF:FFFFF::FFFFFFFFFFFFF:FFFFFFFF:FFFFFF
FFFFFFFFFFF,FF:FFFFFFFFFFFFFFFFFFFFFFF:FFFFF,FFFFFFFFFFFFF:FFFFFFFFF
FFFF:FF:F:FFFFF
@A00155:342:HHGFNDSXY:1:1563:4038:9017 2:N:0:GAACCTAG+TCCGCATA
CTCTTGAGCAGGCTCCCACAGTCTCAACAGGAGGTTATAAGCGGATTTCAGTATGAACGCATTCAAAA
GGAAGAAGAGAAGAAGGTGGAAAACTTTTCTTTTTACCTACCAGAGAAAACTCGGGAATGGTTCACAA
AGTCTGGTGTGTATC
+
FFFFFFFFFFFFFFFFFFFFFFFFFFFFFFFFFFFFFFFFFFFFFFFFFFFFFFFFFFFFFFFFFFFF
FFFFFFFFFFF:F::F:F:FFFFFFFFFFFFFFFFFFFFFFFFFF,FFF::FFFFFFFFFFFFF:FFF
FFFFFFFFFFFFFFF
@A00155:342:HHGFNDSXY:1:2119:4652:21887 2:N:0:GAACCTAG+TCCGCATA
CTCTTGAGCAGGCTCCCACAGTCTCAACAGGAGGTTATAAGCGGATTTCAGTATGAACGCATTCAAAA
GGAAGAAGAGAAGAAGGTGGAAAACTTTTCTTTTTACCTACCAGAGAAAACTCGGGAATGGTTCACAA
AGTCTGGAGATCGGA
+
FFFFFFFFFFFFFFFFFFFFFFFFF:FFFFFFFFFFFFFFFFFFFFFFFFFFFFFFFFFFFFFFFFFF
FFFFFFFFFFFFFFFFFFFFFFFFFFFFFFFFFFFFFFFFFFFFFFFFF:FFFFFFF,:FFF:FFFFF
FFFFFFF:F:FFFFF
@A00155:342:HHGFNDSXY:1:2374:27272:18787 2:N:0:GAACCTAG+TCCGCATA
CTCTTGAGCAGGCTCCCACAGTCTCAACAGGAGGTTATAAGCGGATTTCAGTATGAACGCATTCAAAA
GGAAGAAGAGAAGAAGGTGGAAAACTTTTCTTTTTACCTACCAGAGAAAACTCGGGAATGGTTCACAA
AGTCTGGTGTGTATC
+
FFFFFFFFFFFFFFFFFFFFFFFFFFFFFFFFFFFFFFFFFFFFFF,FF:FFFFFFFFFFF,FFFFFF
FFF,FFFFFFFFFFFFFFFFFFFFFFFFFFFFFFFFFFFFFFFFFFFFF:FFFFFFFFFF,FFFFFFF
FFFFFFFFFFFFFF:
@A00155:342:HHGFNDSXY:1:2416:9697:6292 2:N:0:GAACCTAG+TCCGCATA
CTCTTGAGCAGGCTCCCACAGTCTCAACAGGAGGTTATAAGTGGATTTCAGTATGAACGCATTCAAAA
GGAAGAAGAGAAGAAGGTGGAAAACTTTTCTTTTTACCTACCAGAGAAAACTCGGGAATGGTTCACAA
AGTCTGGTGTGTATC

```
+
FFFFFF:FFFFFFFFFFFFFFFFFFFFFFFFFFFFFFFFFFF:FFFFFFFFFFFFFFFFFFFFFFFFFFFF
FFFFFFFFFFFFFFFFFFFFFFFFFFFFFFFFFFFFFFFFFFFFFFFFFFFFFFFFFFFFFFFFFFFFFF
FFFFFFFFFFFFFFF
@A00155:342:HHGFNDSXY:1:1245:4996:17159 2:N:0:GAACCTAG+TCCGCATA
GAGCAGGCTCCCACAGTCTCAACAGGAGGTTATAAGCGGATTTCAGTATGAACGCATTCAAAAGGAAG
AAGAGAAGAAGGTGGAAAACTTTTCTTTTTACCTACCAGAGAAAACTCGGGAATGGTTCACAAAGTCT
GGTGTGTATCTTTCA
+
FFFFFFFFFFFF:FFFFFFFFFFFFFFFFFFFFFF:FFFFFFFFFFFFFFFFFFFFF:FFFFFFFFFFF
,FFFFFFFFFFFFFFFFFFFFFFFFFFFFFFFFFFFFFFFFFFFFFFFFFFFF:FFFFFFFFFFFFFFFF
FFFFFFFFFFFFF:F
@A00155:342:HHGFNDSXY:1:1245:5159:17879 2:N:0:GAACCTAG+TCCGCATA
GAGCAGGCTCCCACAGTCTCAACAGGAGGTTATAAGCGGATTTCAGTATGAACGCATTCAAAAGGAAG
AAGAGAAGAAGGTGGAAAACTTTTCTTTTTACCTACCAGAGAAAACTCGGGAATGGTTCACAAAGTCT
GGTGTGTATCTTTCA
+
F,FFFFFFFFFFFFFFFFFFFFFFFFFFFF,FFFFFFFFFFFFFFFFFFFFFFFFFFFFFFFFF:FFFFF
F:FFFFFFFFFFFFFFFFF,FFFFFFFFFFFF:FFFFFFFFFFFFFFFFFFFFFFFFFFFFFFFFF:FFFF
FFFFFFFFFFFFFFF
@A00155:342:HHGFNDSXY:1:1220:14805:21433 2:N:0:GAACCTAG+TCCGCATA
AGCAGGCTCCCACAGTCTCAACAGGAGGTTATAAGCGGATTTCAGTATGAACGCATTCAAAAGGAAGA
AGAGAAGAAGGTGGAAAACTTTTCTTTTTACCTACCAGAGAAAACTCGGGAATGGTTCACAAAGTCTG
GTGAGATCGGA
+
FFFFFFFFFFFFFFFFFFFFFFFF:FF:F:FFFFFFFFFF:FFFF:FFFFFFFF:FFFFFFFFFF,,,
FFFFFFFFFFFFFFFFFFFFFFFFFFFFFFFFFFFFFFFFFFFFFFFFFFFFF,F::F:FFFFF:FFFF:
FFFFFFFF:FF
@A00155:342:HHGFNDSXY:1:2118:32823:34851 2:N:0:GAACCTAG+TCCGCATA
AGCAGGCTCCCACAGTCTCAACAGGAGGTTATAAGCGGATTTCAGTATGAACGCATTCAAAAGGAAGA
AGAGAAGAAGGTGGAAAACTTTTCTTTTTACCTACCAGAGAAAACTCGGGAATCGTTCACAAAGTCTG
GTGTGTATCTT
+
FFFFFFFFFFFFFFFF:FFFFFFFFFFFFFFFFF:FFFFFFFFFFFFFFFFFFFFFFFF:FF,FFFFF:
FFFFFFF,:F:FFFFFFF:FFFFFFFFF::F:FFFFFF:FFFFF:FFFFFFFFF:FFF:FFFFFFFFF
FFFFFFFF:FF
@A00155:342:HHGFNDSXY:1:2339:22724:32957 2:N:0:GAACCTAG+TCCGCATA
AGCAGGCTCCCACAGTCTCAACAGGAGGTTATAAGCGGATTTCAGTATGAACGCATTCAAAAGGAAGA
AGAGAAGAAGGTGGAAAACTTTTCTTTTTACTTACCAGAGAAAACTCGGGAATGGTTCACAAAGTCTG
GTGTGTATCTT
+
FFFFFFFFFFFFFFFFFFFFFFFFFFFFFF:FFFFFFFFFFFFFF:FFFFFFFFFFFFFFFFFFFFFFF
FFFFFFFFFFFFFFFFF:FF:FFFFFFFFF,FFF,FFFFFFFFFFFFFFFF:FFFFFFFFFFFFFFFF
FFFFFFFFFFF
@A00155:342:HHGFNDSXY:1:2671:1063:6684 2:N:0:GAACCTAG+TCCGCATA
AGCAGGCTCCCACAGTCTCAACAGGAGGTTATAAGCGGATTTCAGTATGAACGCATTCAAAAGGAAGA
AGAGAAGAAGGTGGAAAACTTTTCTTTTTACCTACCAGAGAAAACTCGGGAATGGTTCACAAAGTCTG
GTGTGTATCTT
+
:FFFFFF:FFFFF:FFFFF,FFFFFF,FFFFFFFFFFFFFFFFFFFF:FFFFFFFFFFF,F,FFF:F,
FFFF,FF:FFFF:FFF:,,FFFFF:FFFF::FF::,:,FF:FF,:FF,FFFFFFFFFF,:FF,FFF:F
FFFFFF:F::F
@A00155:342:HHGFNDSXY:1:1311:10276:26866 1:N:0:GAACCTAG+TCCGCATA
AGCAGGCTCCCACAGTCTCAACAGGAGGTTATAAGCGGATTTCAGTATGAACGCATTCAAAAGGAAGA
```

```
AGAGAAGAAGGTGGAAAACTTTTCTTTTTACCTACCAGAGAAAACTCGGGAATGGTTCACAAAGTCTG
GTGTGTATCTTTCAC
+
FFFFFFFFFFFFFFFFFFFFFFFFFFFFFFFF:FFFFFFFFFFFFFFFFFFFFFFFFFFFFFF:FFFF
FFFFFFFFFFF:F:FFFFFFFFFFFFFFFFFFFFFFFFFFFF:FFFFFFFFFFFFFFFFFFFFFFFFF
FFFFFFFFFFFFFFF
@A00155:342:HHGFNDSXY:1:1142:26187:22576 2:N:0:GAACCTAG+TCCGCATA
GCAGGCTCCCACAGTCTCAACAGGAGGTTATAAGTGGATTTCAGTATGAACGCATTCAAAAGGAAGAA
GAGAAGAAGGTGGAAAACTTTTCTTTTTACCTACCAGAGAAAACTCGGGAATGGTTCACAAAGTCTGG
TGTGTATCTTTCACC
+
FFFFFFFFFFFFFFFFFFFFFFFFFFFFFFFFFFFFFFFFFFFFFFFFFFFFFFFFFFFFFFFFFF:F
FFFFFFFFFFFFFFFFFFFFFFFFFFFFFFFFFFFFFFFFFFFFFFFFFFFFFFFFFFFFFFFFFFFF
FFFFFFFFFFFFFFF
@A00155:342:HHGFNDSXY:1:2512:22480:19695 2:N:0:GAACCTAG+TCCGCATA
GCAGGCTCCCACAGTCTCAACAGGAGGTTATAAGTGGATTTCAGTATGAACGCATTCAAAAGGAAGAA
GAGAAGAAGGTGGAAAACTTTTCTTTTTACCTACCAGAGAAAACTCGGGAATGGTTCACAAAGTCTGG
TGTGTATCTTTCACC
+
FFFFFFFFFFFFFFFFFFFFFFFFFFFFFFFFFFFFFFFFFFFFFFFFFFFFFFFFFFFFFFFFFFFF
FFFFFFFFFFFFFFFFFFFFFFFFFFFFFFFFFFFFFFFFFFFFFFFFFF,FFF:FFFFF:FFFFFFF
FFFFFFFFFFFFFFF
@A00155:342:HHGFNDSXY:1:2453:1262:5995 2:N:0:GAACCTAG+TCCGCATA
GGCTCCCACAGTCTCAACAGGAGGTTATAAGCGGATTTCAGTATGAACGCATTCAAAAGGAAGAAGAG
AAGAAGGTGGAAAACTTTTCTTTTTACCTACCAGAGAAAACTCGGGAATGGTTCACAAAGTCTGGTGT
GTATCTTTC
+
FFF:FFFFF:FFF::,,F,FF,FFF:F:FFFFFF:FF:F,FFFFF,:FFFFFFF:,,FFF::F,:F,F
:FF,F:FFFF,F,:,FF:F::FF:F:FFFFF:FF,F,F,F:FFFFFF:FFFFF,:,F:,FF,FFFFFF
,F,F,FFF:
@A00155:342:HHGFNDSXY:1:1478:7654:24486 2:N:0:GAACCTAG+TCCGCATA
GGCTCCCACAGTCTCAACAGGAGGTTATAAGCGGATTTCAGTATGAACGCATTCAAAAGGAAGAAGAG
AAGAAGGTGGAAAACTTTTCTTTTTACCTACCAGAGAAAACTCGGGAATGGTTCACAAAGTCTGGTGT
GTATCTTTCACCATT
+
FFFFFFFFFFFFFFFFFFFFFFFFFFFFFFFFFFFFFFFFFFFFFFFFFFFFFFFFF:FFF:FFFFFF
FFFF:F:FFFFFFFFFFFFFFFFFFFFFFFFFFFFFFFFFFFFFFFFFFFFFFFFFFFFFFFFFFFFF
FFFFFFFFFFFFFFF
@A00155:342:HHGFNDSXY:1:1541:2790:19194 2:N:0:GAACCTAG+TCCGCATA
GGCTCCCACAGTCTCAACAGGAGGTTATAAGTGGATTTCAGTATGAACGCATTCAAAAGGAAGAAGAG
AAGAAGGTGGAAAACTTTTCTTTTTACCTACCAGAGAAAACTCGGGAATGGTTCACAAAGTCTGGTGT
GTATCTTTCACCATT
+
FFFFFFFFFFFFFFFFFFFFFFFFFFFFFFFFFFFFFFFFFFFFFFFFFFFFFFFFF,FFFFFFFFFF
FFFFFFFFFFFFFFFFFFFFFFFFFFFFFFFFFFFFFFFFFFFFFFF,FFFFFFFFFFFFFFFFFFFF
FF:FFFFFF:FFFFF
@A00155:342:HHGFNDSXY:1:1628:31891:18270 2:N:0:GAACCTAG+TCCGCATA
GGCTCCCACAGTCTCAACAGGAGGTTATAAGCGGATTTCAGTATGAACGCATTCAAAAGGAAGAAGAG
AAGAAGGTGGAAAACTTTTCTTTTTACCTACCAGAGAAAACTCGGGAATGGTTCACAAAGTCTGGTGT
GTATCTTTCACCATT
+
FF,FFFFFFFFFFFFFFFFFFFFFFFFFFFFFFFFFFFFFFF:FFFFFFFFFFFFFFFFFFFFFFFFF
F:FFFFFFFFFFFFFFFFFFFFFFFFFFFFFFFFFFFFFFFFFFFFF:FFFFFFFFFFFFFFFFFFFF
FFFFFFFFFFFF:FF
```

```
@A00155:342:HHGFNDSXY:1:1559:23411:11757 2:N:0:GAACCTAG+TCCGCATA
GGCTCCCACAGTCTCAACAGGAGGTTATAAGCGGATTTCAGTATGAACGCATTCAAAAGGAAGAAGAG
AAGAAGGTGGAAAACTTTTCTTTTTACCTACCAGAGAAAACTCGGGAATGGTTCACAAAGTCTGGTGT
GTATCTTTCACCATT
+
FFFFFFFFFFFFFFFFFFFFFFFFFFFFFFFFFFFFFFFFFFFFFFFFFFFFFFFFF:FFFFFF:FFF
FFFFFFFFFFFFFFFFFFFFFFFFFFFFFFFFFFFFFFFFFFFFFFFFFFFFFFFFFFFFFFFFFFFF
FF:FFFFFFFFFFFF
@A00155:342:HHGFNDSXY:1:2244:28429:22764 2:N:0:GAACCTAG+TCCGCATA
GGCTCCCACAGTCTCAACAGGAGGTTATAAGCGGATTTCAGTATGAACGCATTCAAAAGGAAGAAGAG
AAGAAGGTGGAAAACTTTTCTTTTTACCTACCAGAGAAAACTCGGGAATGGTTCACAAAGTCTGGTGT
GTATCTTTCACCATT
+
:F,:::FFFFFFFFFFFFFFFFFFFFFFFFFFFFFFFFFFFFFFFFFFFFFFFFFFFFFFFFFFFFFF
FFFFFFFFFFFFFFFFFFFFFFFFFFFFFFFFFFFFFFFFFFFFFFFFFFF:FFFFFFFFFFFFFFFF
FFFFFFFFFFFFFFF
@A00155:342:HHGFNDSXY:1:2629:18349:24283 2:N:0:GAACCTAG+TCCGCATA
GGCTCCCACAGTCTCAACAGGAGGTTATAAGTGGATTTCAGTATGAACGCATTCAAAAGGAAGAAGAG
AAGAAGGTGGAAAACTTTTCTTTTTACCTACCAGAGAAAACTCGGGAATGGTTCACAAAGTCTGGTGT
GTATCTTTCACCATT
+
FFFFFFF:FFFFFFFFFFFFFFFFFF:FFFFFFFFFFFFFFFFFFFFFFFFFFFFFFFFFFFFFFFFF:
FFFFF:FFFFFFFFFFFFFFFF:FFFFFFFFFFFFFFFFFFFFFFFFFFF,FFFFFFFFFFFFFFFFFFF
FFFFFF:FFF:FFF:
@A00155:342:HHGFNDSXY:1:2627:23276:23985 2:N:0:GAACCTAG+TCCGCATA
CTCCCACAGTCTCAACAGGAGGTTATAAGCGGATTTCAGTATGAACGCATTCAAAAGGAAGAAGAGAA
GAAGGTGGAAAACATTTCTTTTTACCTACCAGAGAAAACTCGGGAATGGTTCACAAAGTCTGGTGTGT
ATCTTTCACCATTTG
+
FFFFFFFFFFFFFFFFFFFFF::,FFFFFFFFFFFFFFF:FFFFFFFFFFFFF,FFFF,FFFF:FFF
FFFFFFFFFFFFF,FFFFF:FFFFFFFFFFFFFFFFFFFFFF:FFF::FFFFFFFFFFFFF,FFFFFFF
FFFFFFFFF:FF:FF
@A00155:342:HHGFNDSXY:1:2339:22724:32957 1:N:0:GAACCTAG+TCCGCATA
CCCACAGTCTCAACAGGAGGTTATAAGCGGATTTCAGTATGAACGCATTCAAAAGGAAGAAGAGAAGA
AGGTGGAAAACTTTTCTTTTTACTTACCAGAGAAAACTCGGGAATGGTTCACAAAGTCTGGTGTGTAT
CTTTCACCATTTGCT
+
FFFFFFFFFF:F:FFFFFFFFFF:F::FFFFFFFFFFFFFFFFF:FFFFFFFFFFFFFFFFFFF:F:,
F:FF,:FFFFFFFFFF:FFFFFFFFFFFFFFFFFFFFFFFFFFFFFFFFFFFFFFFFFFFFFFFFFF
FFFFFFFFFFFFF:FF
@A00155:342:HHGFNDSXY:1:1513:6705:27821 2:N:0:GAACCTAG+TCCGCATA
CCCACAGTCTCAACAGGAGGTTATAAGCGGATTTCAGTATGAACGCATTCAAAAGGAAGAAGAGAAGA
AGGTGGAAAACTTTTCTTTTTACCTACCAGAGAAAACTCGGGAATGGTTCACAAAGTCTGGTGTGTAT
CTTTCACCATTTGCT
+
FFFFFFFFFFFFFFFFFFFFFFFFFFFFFFFFFFFFFFFFFFFFFFFFFFFFFFFFFFFFFFFFFFFF
FFFFFFFFF:FFFFFFFFFFFFFFFFFFFFFFFFFFFFFFFFFFFFFFFFFF,FFFFFFFFFFFF
FFFFFFFF,F:FFFF
@A00155:342:HHGFNDSXY:1:2362:18873:31140 1:N:0:GAACCTAG+TCCGCATA
CCACAGTCTCAACAGGAGGTTATAAGCGGATTTCAGTATGAACGCATTCAAAAGGAAGAAGAGAAGAA
GGTGGAAAACTTTTCTTTTTACCTACCAGAGAAAACTCGGGAATGGTTCACAAAGTCTGGTGTGTATC
TTTCACCATTTGCTT
+
FFFF:FFFFFFFFFFFFFFFFFFFFFFFFFFFFFFFFFFFFFFFFFFFFFFFFFFFFFFFFFFFFFF:
```

```
FFFFFFFFFF:FFFFFFFFFFFFFFFFFFFFFFFFFFFFFFFFFFFFFFFFFFFFFFFFFFFFFFFFF
FFFFFFFFFFFFFFF
@A00155:342:HHGFNDSXY:1:2513:31973:27492 1:N:0:GAACCTAG+TCCGCATA
CCACAGTCTCAACAGGAGGTTATAAGTGGATTTCAGTATGAACGCATTCAAAAGGAAGAAGAGAAGAA
GGTGGAAAACTTTTCTTTTTACCTACCAGAGAAAACTCGGGAATGGTTCACAAAGTCTGGTGTGTATC
TTTCACCATTTGCTT
+
FFFFFFFFFFFFFFFFFFFFFFFFFFFFFFFFFFFFFFFFF:FFFFFFFFFFFFFFFFFFFFFFFFF:
:F,FFFFFFFFFFFFFFFFFFFFFFFFFFFFFFFFFFFFFFFFFFFFFFFFFFFFFFFFFFFFFFFFF
FFFFFFFFFFFFFFF
@A00155:342:HHGFNDSXY:1:1402:9724:11569 2:N:0:GAACCTAG+TCCGCATA
CCACAGTCTCAACAGGAGGTTATAAGCGGATTTCAGTATGAACGCATTCAAAAGGAAGAAGAGAAGAA
GGTGGAAAACTTTTCTTTTTACCTACCAGAGAAAACTCGGGAATGGTTCACAAAGTCTGGTGTGTATC
TTTCACCATTTGCTT
+
FFFFFFFFFFFFFFFFFFFFFFFFFFFFFFFFFFFFFFFFFFFFFFFFFFFFFFFFFFFFFFFFFFFF
FFFFFFFFFFFFFFFFFFFFFFFFFFFFFFFFFFFFFFFFFFFFFFFFFFFFFFFFFFFFFFFFFFFF
FFFFFFFFFFFFFFF
@A00155:342:HHGFNDSXY:1:2512:27932:18897 2:N:0:GAACCTAG+TCCGCATA
CCACAGTCTCAACAGGAGGTTATAAGCGGATTTCAGTATGAACGCATTCAAAAGGAAGAAGAGAAGAA
GGTGGAAAACTTTTCTTTTTACCTACCAGAGAAAACTCGGGAATGGTTCACAAAGTCTGGTGTGTATC
TTTCACCATTTGCTT
+
FFFFFFFFFFFFFFFFFFFFFFFFFFFFFFF:FFFFFFFFFFFFFFFFFFFFFFFFFFFFFFFFFFF:
FFFFFF,FFF:FFFFFFFFFFFFFFFFFFFFFFFFFFFF:FFFFFFFFFFFFFFFFFFFFFFFFFFFF
FFF::FFFF,FFFFF
@A00155:342:HHGFNDSXY:1:2512:28013:19038 2:N:0:GAACCTAG+TCCGCATA
CCACAGTCTCAACAGGAGGTTATAAGCGGATTTCAGTATGAACGCATTCAAAAGGAAGAAGAGAAGAA
GGTGGAAAACTTTTCTTTTTACCTACCAGAGAAAACTCGGGAATGGTTCACAAAGTCTGGTGTGTATC
TTTCACCATTTGCTT
+
FFFFFFFFFFF:FFFFFFFF:FFFFFFFFFFFFFFFFFFFFFFFFFFFFFFFFFFFFF:FFFFFFFFF
FFFFFFFF:FFFFFFFFFFFFFFFFFFFFFFFFFFFFFF:FFFFFFFFFFFF:FFFFFFFFFFFFFFF
FFFFFFFFFFFF:FF
@A00155:342:HHGFNDSXY:1:1109:23231:27508 2:N:0:GAACCTAG+TCCGCATA
CACAGTCTCAACAGGAGGTTATAAGTGGATTTCAGTATGAACGCATTCAAAAGGAAGAAGAGAAGAAG
GTGGAAAACTTTTCTTTTTACCTACCAGAGAAAACTCGGGAATGGTTCACAAAGTCTGGTGTGTATCT
TTCACCATTTGCTTA
+
FFFFFFFFFFFFFFFFFFFFFFFFFFFFFFFFFFFFFFFFFFFFFFFFFFFF:FFFFFFFFFFFFFFF
F:FFFFFF:FFFFF:FFFFFFFFFFFFFFFFFFFFF:FFFFFF,FFFFFFFFFFFFFFFFFFFFFFFF
FFFFFFFFFFFFFFF
@A00155:342:HHGFNDSXY:1:1158:15221:31767 2:N:0:GAACCTAG+TCCGCATA
CACAGTCTCAACAGGAGGTTATAAGCGGATTTCAGTATGAACGCATTCAAAAGGAAGAAGAGAAGAAG
GTGGAAAACTTTTCTTTTTACCTACCAGAGAAAACTCGGGAATGGTTCACAAAGTCTGGTGTGTATCT
TTCACCATTTGCTTA
+
FFFFFFFFFFFFFFFFFFFFFFFFFFFFFFFFFFFFFFFFFFFFFFFFFFFFFFFFFFFFFFFFFFFF
FFFFFFFFFFFFFFFFFFFFFFFFFFFFFFFFFFFFFFFFF:FFFFFFFFFFFFFFFFFFFFFFFFFF
FFFFFFFFFFFFFFF
@A00155:342:HHGFNDSXY:1:1248:10737:24471 2:N:0:GAACCTAG+TCCGCATA
CACAGTCTCAACAGGAGGTTATAAGCGGATTTCAGTATGAACGCATTCAAAAGGAAGAAGAGAAGAAG
GTGGAAAACTTTTCTTTTTACCTACCAGAGAAAACTCGGGAATGGTTCACAAAGTCTGGTGTGTATCT
TTCACCATTTGCTTA
```

```
+
FFFFFFFFFFFFFFF:FFFFFF,FFFFFFFFFFFFFFFFFFFFFFFFFFFFFFFFFFFFFFFFFFFFFF
FFFFFFFFFFFFFFFFFFFFFFFFFFFFFFFFFFFFFFFFF,FFFFFFFFFF:FFFFFFFFFFFFFFF
,FFFFFFFFFFFFFF
@A00155:342:HHGFNDSXY:1:1251:6723:13260 2:N:0:GAACCTAG+TCCGCATA
CACAGTCTCAACAGGAGGTTATAAGCGGATTTCAGTATGAACGCACTCAAAAGGAAGAAGAGAAGAAT
GTGGAAAACTTTTCTTTTTACCTACCAGAGAAAACTCGGGAATGGTTCACAAAGTCTGGTGTGTATCT
TTCACCATTTGCTTA
+
FFFFFFF:F:FFFF:F,:FFF:FFFF:FF:FF:FFFFFFFFFFFFFFFFFFFF::FFFFFFFFF,F:,
FFF,FFFFF,FF:FFF:FFF,FFFF,FFFFFFFFFFFF:,FF,:,FFFFFFFFF,FF::F:FFFFFFF
FF,F:FFF,FF,F:F
@A00155:342:HHGFNDSXY:1:1310:20907:18411 2:N:0:GAACCTAG+TCCGCATA
CACAGTCTCAACAGGAGGTTATAAGCGGATTTCAGTATGAACGCATTCAAAAGGAAGAAGAGAAGAAG
GTGGAAAACTTTTCTTTTTACCTACCAGAGAAAACTCGGGAATGGTTCACAAAGTCTGGTGTGTATCT
TTCACCATTTGCTTA
+
FFFFFFFFFFFFFFFFFFFFFFFFFFFFFFFFFF:FFFFFFFFFFFFFFFFFFFFFFFFFFFFFFFFF
FFFFFFFFFFFFFFFFFFFFFFFFFFFFFFFFFFFFFFFFF:FFFFFFFFFFFFFFFFFFFFFFFF,F
FFFFFFFFFFFFFFF
@A00155:342:HHGFNDSXY:1:1353:13738:26976 2:N:0:GAACCTAG+TCCGCATA
CACAGTCTCAACAGGAGGTTATAAGCGGATTTCAGTATGAACGCATTCAAAAGGAAGAAGAGAAGAAG
GTGGAAAACTTTTCTTTTTACCTACCAGAGAAAACTCGGGAATGGTTCACAAAGTCTGGTGTGTATCT
TTCACCATTTGCTTA
+
FFFFFFFFFFFFFFFFFFFFFFFFFFFFFFFFFFFFFFFFFFFFFFFFFFFFFFFFFFFFFFFFFFFF
FFFFFFFFFFFFFFFFFFFFFFFFFFFFFFFFFFFFF:FFF:FFFFFFFFFFFFFFFFFFFFFFFFFF
FF:FFFFFFFFFFFF
@A00155:342:HHGFNDSXY:1:1419:16504:25755 2:N:0:GAACCTAG+TCCGCATA
CACAGTCTCAACAGGAGGTTATAAGCGGATTTCAGTATGAACGCATTCAAAAGGAAGAAGAGAAGAAG
GTGGAAAACTTTTCTTTTTACCTACCAGAGAAAACTCGGGAATGGTTCACAAAGTCTGGTGTGTATCT
TTCACCATTTGCTTA
+
FFFFFFFFFFFFFFFFFFFFFFFFFFFFFFFFFFFFFFFFFFFFFFFFFFFFFFFFFFFFFFFFFFFF
FFFFFFFFFFFFFFFFFFFFFFFFFFFFFFFFFFFFFFFFFFFFFFFFFFFFFFFFFFFFFFFFFFFF
FFFFFFFFFFFFFFF
@A00155:342:HHGFNDSXY:1:1650:8422:12978 2:N:0:GAACCTAG+TCCGCATA
CACAGTCTCAACAGGAGGTTATAAGCGGATTTCAGTATGAACGCATTCAAAAGGAAGAAGAGAAGAAG
GTGGAAAACTTTTCTTTTTACCTACCAGAGAAAACTCGGGAATGGTTCACAAAGTCTGGTGTGTATCT
TTCACCATTTGCTTA
+
FFFFFFFFFFFFFFFFFFFFFFFFFFFFF,FFFFFFFFFFFFFFFFFFFFFFFFFFFFFFFFFFFFFF
FFFFF:FFFFFFFFFFFFFFFFFFFFFFFFFFFFFFFFFFFFFFFFFFFFFFFFFF:FFF:FFFFFF
FFFFFFFFFFFFFFF
@A00155:342:HHGFNDSXY:1:2161:7916:17018 2:N:0:GAACCTAG+TCCGCATA
CACAGTCTCAACAGGAGGTTATAAGTGGATTTCAGTATGAACGCATTCAAAAGGAAGAAGAGAAGAAG
GTGGAAAACTTTTCTTTTTACCTACCAGAGAAAACTCGGGAATGGTTCACAAAGTCTGGTGTGTATCT
TTCACCATTTGCTTA
+
FFFFFFFFFFFFFFFFFFFFFFFFF:FFFFFFFFFFFFFFFFFFFFFFFFFFFFFFFFFFFFFFFFFF
FFFFFFFFFFFF:FFFFFFFFFFFFF:FFFFFFFFFFFFFFFFFFFFFF:FFFFFFFFFFFFFFFFFF
FFFFF:FFFFFFFFF
@A00155:342:HHGFNDSXY:1:2231:19994:13416 2:N:0:GAACCTAG+TCCGCATA
CACAGTCTCAACAGGAGGTTATAAGTGGATTTCAGTATGAACGCATTCAAAAGGAAGAAGAGAAGAAG
```

GTGGAAAACTTTTCTTTTTACCTACCAGAGAAAACTCGGGAATGGTTCACAAAGTCTGGTGTGTATCT
TTCACCATTTGCTTA
+
:FFFFFFFFFFFFFFFFFFFFFFFFFF:FFFFFFFFF:FFFFFFFFF::FFFFFFFFFFFFF:FFF:FFF
FFFFFFFFFFFFF,FFFFFF::FF:,F::,FFFF:,F:F,FFF::FF,FFFFFF:::,,:FF:::::F
FF,F::F::F,:FF:
@A00155:342:HHGFNDSXY:1:2433:4463:14763 2:N:0:GAACCTAG+TCCGCATA
CACAGTCTCAACAGGAGGTTATAAGTGGATTTCAGTATGAACGCATTCAAAAGGAAGAAGAGAAGAAG
GTGGAAAACTTTTCTTTTTACCTACCAGAGAAAACTCGGGAATGGTTCACAAAGTCTGGTGTGTATCT
TTCACCAGTTGCTTA
+
FFFFFFFFFFFF,:FFF:FFFFFFFFFFFFFFFF,FFFFFFFF:FFFFFFFF:FFFFFFFFFFFFFFF
FFFFFFFFFFFFFFFFFFFF:FFFFFFFFFFFFFFFF:FFFFFFFFFF:FFFFFFFFFFFFFFF,FFF
FFF:FF,,FF:FFFF
@A00155:342:HHGFNDSXY:1:2504:16884:24596 2:N:0:GAACCTAG+TCCGCATA
CACAGTCTCAACAGGAGGTTATAAGTGGATTTCAGTATGAACGCATTCACAAGGAAGAAGAGAAGAAG
GTGGAAAACTTTTCTTTTTACCTACCAGAGAAAACTCGGGAATGGTTCACAAAGTCTGGTGTGTATCT
TTCACCATTTGCTTA
+
FFFFFFFFFFFFFFFFFF:FFFFFFFF:FFFFFFFFFFFFFFFFFFFFF,FFFFFFFFFFFFFFFFFF
F,FFFFF:FFF:FFF:FFFF:F::FFFFFFFFFFFFFFF::F:FF:FFFF:FFFFFFFFFFFFFFFFF
FFFFFFFFFF::FFF
@A00155:342:HHGFNDSXY:1:2504:17092:25520 2:N:0:GAACCTAG+TCCGCATA
CACAGTCTCAACAGGAGGTTATAAGTGGATTTCAGTATGAACGCATTCAAAAGGAAGAAGAGAAGAAG
GTGGAAAACTTTTCTTTTTACCTACCAGAGAAAACTCGGGAATGGTTCACAAAGTCTGGTGTGTATCT
TTCACCATTTGCTTA
+
FFFFFFFFFFFFFFFFFF:FFFFFFFFFFFFFF:FFFFFFFFFFFFFFFFFFFFFFFFFF:FFFFFFF
FFFFFFFFFFF:FFFFFFFFFFFFFFFFFF:FFFFFFFFFFFFFFFFFFF,FFFFFFFFFFFFFFFFF
FFF,FFFFFF:FFFF
@A00155:342:HHGFNDSXY:1:2537:10972:12352 2:N:0:GAACCTAG+TCCGCATA
CACAGTCTCAACAGGAGGTTATAAGCGGATTTCAGTATGAACGCATTCAAAAGGAAGAAGAGAAGAAG
GTGGAAAACTTTTCTTTTTACCTACCAGAGAAAACTCGGGAATGGTTCACAAAGTCTGGTGTGTATCT
TTCACCATTTGCTTA
+
FFFFFFFFFFFFFFFFFF,FFFFFFFFFFFFFFFFF,FFFFFFFFFFFFFFFFFFFFFFFFFFFFFFF
FFFFFFFFFF,FFFFFFFFF:FFFFFFFFFFFFFFFFFFFFFFFFFFFFFFFFFFFFFFFFFFFFFFF
FFFFFF:FFF:FFFF
@A00155:342:HHGFNDSXY:1:1427:8567:35931 1:N:0:GAACCTAG+TCCGCATA
ACAGTCTCAACAGGAGGTTATAAGCGGATTTCAGTATGAACGCATTCAAAAGGAAGAAGAGAAGAAGG
TGGAAAACTTTTCTTTTTACCTACCAGAGAAAACTCGGGAATGGTTCACAAAGTCTGGTGTGTATCTT
TCACCATTTGCTTA
+
FFFFFFF,FFFFFFFFFFFFFF::FFFFFFFFFFFFFFFFFFFFFFFFFFFFFFFFFFFFFFFF,:,FF:
FFFFFFFF:FFFFFFFFFFFFFFFFFFFFFFFFFFFFFFFFFFFFFFFF:FFFFFFFFFFFFFFFFF
FFFFFFFFF:FFFF
@A00155:342:HHGFNDSXY:1:1207:2808:29997 2:N:0:GAACCTAG+TCCGCATA
ACAGTCTCAACAGGAGGTTATAAGCGGATTTCAGTATGAACGCATTCAAAAGGAAGAAGAGAAGAAGG
TGGAAAACTTTTCTTTTTACCTACCAGAGAAAACTCGGGAATGGTTCACAAAGTCTGGTGTGTATCTT
TCACCATTTGCTTAC
+
FFFFFFFFFFFFFFFFFFFFFFFFFFFFF:FFFFFFFFFFFFFFFFFFFFF:FFFFFFFFFFFFFFFFF
FFFFFFF,FFFFFFFFFFFFFFFFFFFFFFFFFFFFFFFFFFFF:FFFFFFFFFF:FF:FFFFFFFFFF
FFFF:FFFFF:F:FF

@A00155:342:HHGFNDSXY:1:1207:2871:30013 2:N:0:GAACCTAG+TCCGCATA
ACAGTCTCAACAGGAGGTTATAAGCGGATTTCAGTATGAACGCATTCAAAAGGAAGAAGAGAAGAAGG
TGGAAAACTTTTCTTTTTACCTACCAGAGAAAACTCGGGAATGGTTCACAAAGTCTGGTGTGTATCTT
TCACCATTTGCTTAC
+
FFFFFFFFFFFFFFFFF:FFFFFFFFFFFFFFFFFFFFFFFFFFFFFFFFFFFFFF:FFFFFFFFFFF
FFFFFFFFFFFFFFFFFFFFFFFFFFFFFFFFFFFFFFFF:FFFFFFFFFFFFFFFFFFFFFFFFFFF
FF:FFFFFFFFFFFF
@A00155:342:HHGFNDSXY:1:1245:28583:11005 2:N:0:GAACCTAG+TCCGCATA
ACAGTCTCAACAGGAGGTTATAAGCGGATTTCAGTATGAACGCATTCAAAAGGAAGAAGAGAAGAAGG
TGGAAAAATTTTCTTTTTACCTACCAGAGAAAACTCGGGAATGGTTCACAAAGTCTGGTGTGTATCTT
TAACCATTTGCTTAC
+
FFFFFFFFFFFFFFFFFFFFFFFF:FFFF:FFFF:FFFFFF:FFF,:FFFFFFFFFFF:F:FFFFFFF
:FFFFFF,FFFFF:F,FFF::FFF:F:F:FFFFFF:FF,F:FFFFFFFFFFF,FFF:FFFFFFFFFF,
:,FFFFFFFFF:FF:
@A00155:342:HHGFNDSXY:1:1354:28935:35759 2:N:0:GAACCTAG+TCCGCATA
ACAGTCTCAACAGGAGGTTATAAGCGGATTTCAGTATGAACGCATTCAAAAGGAAGAAGAGAAGAAGG
TGGAAAACTTTTCTTTTTACCTACCAGAGAAAACTCGGGAATGGTTCACAAAGTCTGGTGTGTATCTT
TCACCATTTGCTTAC
+
:FFFFFFFFFFFFFFFFFFFFFFFFFFFFFFFFFFFFFFFFFFFFFFFFFFFFFFFFFFFFFFFFFFF
FFFFFFFFFFFFFFFFFFFFFFFFFFFFFFFFFFFFFFFFFFFFFFFFFFFFFFFFFFFFFFFFFFFF
FFFFFFFFFFFFFFF
@A00155:342:HHGFNDSXY:1:1355:28709:1266 2:N:0:GAACCTAG+TCCGCATA
ACAGTCTCAACAGGAGGTTATAAGCGGATTTCAGTATGAACGCATTCAAAAGGAAGAAGAGAAGAAGG
TGGAAAACTTTTCTTTTTACCTACCAGAGAAAACTCGGGAATGGTTCACAAAGTCTGGTGTGTATCTT
TCACCATTTGCTTAC
+
:FFFFFFFFFFFFFFFFFF:FFFFFFFFFFFFFFFFFFFFFFFFFFFF:FFFFFFFFFFFFFFFFFFF
FFFFFFFFFFFFFFFFFFFFFFFFFFFFFFFFFFFFFFFF,FFFFFFFFFFFFFFFFFFFFFFFFFFF
FFFFFFFFFFFFFFF
@A00155:342:HHGFNDSXY:1:2551:9905:35430 2:N:0:GAACCTAG+TCCGCATA
CAGTCTCAACAGGAGGTTATAAGCGGATTTCAGTATGAACGCATTCAAAAGGAAGAAGAGAAGAAGGT
GGAAAACTTTTCTTTTTACCTACCAGAGAAAACTCGGGAATGGTTCACAAAGTCTGGTGTGTATCTTT
CACCATTTGCTTACG
+
FFFFFFFFFFFFFFFFFFFFFFFFFFFF:FFFFFFFFFFFFFFFFFFFFFFFFFFFFFFF:FF:FFFF
FFFFFFFFFFFF:FFFF:FFF:FFFFFFFF:FFFFFFFF,FFFFFFFFF:FFFFFFFFFFFF:FFFFF
FFFF:FFFFFFFFFF
@A00155:342:HHGFNDSXY:1:2111:17155:13573 2:N:0:GAACCTAG+TCCGCATA
AGTCTCAACAGGAGGTTATAAGCGGATTTCAGTATGAACGCATTCAAAAGGAAGAAGAGAAGAAGGTG
GAAAACTTTTCTTTTTACCTACCAGAGAAAACTCGGGAATGGTTCACAAAGTCTGGTGTGTATCTTTC
ACCATTTGCTTACGT
+
FFFFFFFFFFFFFFFFFFFFFFFFFFFFFFFFFFFFFFFFFFFFFFFFFFFFFFFFFFFFFFFFFFFF
FFFFFFFFFFFFFFFFFFFFFFFFFFFFFFFFFFFFFF:FFFFFFFFFFFFFFFFFFFFFF,FFFFFF
FFFFFFFFFFFFFFF
@A00155:342:HHGFNDSXY:1:1169:31232:29684 1:N:0:GAACCTAG+TCCGCATA
GTCTCAACAGGAGGTTATAAGTGGATTTCAGTATGAACGCATTCAAAAGGAAGAAGAGAAGAAGGTGG
AAAACTTTTCTTTTTACCTACCAGAGAAAACTCGGGAATGGTTCACAAAGTCTGGTGTGTATCTTTCA
CCATTTGCTTACGTG
+
FFFFFFFFFFFFFFFFFFFFFFFFFFFFFFFFFFF:FFFFFFFFFFFFFFFFFFFFFF:FFF:FFFF:

FFFFFFFFFFFFFFFFFFFFFFFFFFFFFFFFFFFFFFFFF:FFFFFFFFFFFFFFFFFFFFFFFFFFFFF
FFFFFFFFFFFFFFF
@A00155:342:HHGFNDSXY:1:1608:32208:8140 1:N:0:GAACCTAG+TCCGCATA
GTCTCAACAGGAGGTTATAAGCGGATTTCAGTATGAACGCATTCAAAAGGAAGAAGAGAAGAAGGTGG
AAAACTTTTCTTTTTACCTACCAGAGAAAACTCGGGAATGGTTCACAAAGTCTGGTGTGTATCTTTCA
CCATTTGCTTACGTG
+
FF:FF:FFFFF,:FFFFFFFFFFFFFF:FFF,FFFFFFFFFFFFFFFFFFFFFFFFF,:FFF,F:,::F
F:FFF:FFFFFFFFFF:FFFFFFFFFFF:FFFFFFFFFFFFFFFF:FFFFFFFFFFFFFFFFF:FFFF
FFFFFFFFF:FFFFF
@A00155:342:HHGFNDSXY:1:1416:15302:29810 2:N:0:GAACCTAG+TCCGCATA
GTCTCAACAGGAGGTTATAAGTGGATTTCAGTATGAACGCATTCAAAAGGAAGAAGAGAAGAAGGTGG
AAAACTTTTCTTTTTACCTACCAGAGAAAACTCGGGAATGGTTCACAAAGTCTGGTGTGTATCTTTCA
CCATTTGCTTACGTG
+
FFFFFFFFFFFFFFFFFFFFFFFFFFFFFFFFFFFFFFFFFFFFFFFFFFFFFFFFFFFFFFFFFFFF
FFFFFFFFFFFFFFFFFFFFFFFFFFFFFFFFFF:FFF,FFFFFFFFFFF,FFFFFFFFFFFFFFFFFF
:F:FF:FFFFFFFFF
@A00155:342:HHGFNDSXY:1:2101:23511:3349 2:N:0:GAACCTAG+TCCGCATA
GTCTCAACAGGAGGTTATAAGCGGATTTCAGTATGAACGCATTCAAAAGGAAGAAGAGAAGAAGGTGG
AAAACTTTTCTTTTTACCTACCAGAGAAAACTCGGGAATGGTTCACAAAGTCTGGTGTGTATCTTTCA
CCATTTGCTTACGTG
+
FFFFFFFFFFFFFFFFFFFFFFFFFFFFFFFFFFFFFFFFFFFFFFFFFFF:FF:FFFFFFFFFFFFFFF
FFFFFFFFFFFFFFFFF:FFFFFFFFFFFFFFFFFFFFFFFFFFFFFFFFFF,FFFFFFFFFFFFFFFFFF
,FFFFFFFFFFFFFF
@A00155:342:HHGFNDSXY:1:1528:18204:34397 1:N:0:GAACCTAG+TCCGCATA
CTCAACAGGAGGTTATAAGCGGATTTCAGTATGAACGCATTCAAAAGGAAGAAGAGAAGAAGGTAGAA
AACTTTTCTTTTTACCTACCAGAGAAAACTCGGGAATGGTTCACAAAGTCTGGTGTGTATCTTTCACC
ATTTGCTTACGTGAA
+
FFFFFFFFFFFFFFFFFFFFFF:FFFFFFFFFFFFFFFFFFFF:FFFFFFFFFFFFFFFFFFFFF:FFF
FFFFFFF:FFFFFFFFFFFFFFFFFFFFFF:FFFFFF:FFFFFFFF:FFFF:F:FFFFFFFFFFFFFFF
FF,FFFFFFFF:FFF
@A00155:342:HHGFNDSXY:1:1477:5014:31000 2:N:0:GAACCTAG+TCCGCATA
CTCAACAGGAGGTTATAAGCGGATTTCAGTATGAACGCATTCAAAAGGAAGAAGAGAAGAAGGTGGAA
AACTTTTCTTTTTACCTACCAGAGAAAACTCGGGAATGGTTCACAAAGTCTGGTGTGTATCTTTCACC
ATTTGCTTACGTGAA
+
FFFFFFFFFFFFFFFFFFFFFFFFFFFFFFFFFFFFFFFFFFFFFFFFFFF:FFFFFF:FFFFFFFFFFF
FFFFFFFFFFFFFFFFFFFFFFFFFFFFFFFFFFFFF:FFFFFFFFFFFFFFFFFFFFFFFFFFFFFFFFF
FFFFFFFFFFFFFFF
@A00155:342:HHGFNDSXY:1:1625:8323:16595 2:N:0:GAACCTAG+TCCGCATA
CTCAACAGGAGGTTATAAGCGGATTTCAGTATGAACGCATTCAAAAGGAAGAAGAGAAGAAGGTGGAA
AACTTTTCTTTTTACCTACCAGAGAAAACTCGGGAATGGTTCACAAAGTCTGGTGTGTATCTTTCACC
ATTTGCTTACGTGAA
+
FFFFFFFFFFFFFFFFFFFFFFFFFFFFFFFFFFFFFFFFFFFFFFFFFFFFFFFFFFFFFFFFFFFFFFF
FFFFFFFFFFFFFFFF:FFF,FFFFFFFFFFFFFFFFFF:FFFFFFFFFFFFFFFFFFFFFFFFFFF:FFF
FFFF:,F:FFFFFFF
@A00155:342:HHGFNDSXY:1:2531:18900:35603 2:N:0:GAACCTAG+TCCGCATA
CTCAACAGGAGGTTATAAGCGGATTTCAGTATGAACGCATTCAAAAGGAAGAAGAGAAGAAGGTGGAA
AACTTTTCTTTTTACCTACCAGAGAAAACTCGGGAATGGTTCACAAAGTCTGGTGTGTATCTTTCACC
ATTTGCTTACGTGAA

```
+
FFFFFFFFFFFFFFFFFFFFFFFFFFFFFFFFFFFFFFFFFFFFFFFFFFFFFFFFFFFFFFFFFFFFFF
:FFFFFFFFFFFFFFFFFFFFFFFFFFFFFFFFFFF:FFFFFFFFF:FFFFFFFFFFFFFFFFFFFFFF
F:FFFFFFFFFFFF:
@A00155:342:HHGFNDSXY:1:1528:18204:34397 2:N:0:GAACCTAG+TCCGCATA
TCAACAGGAGGTTATAAGCGGATTTCAGTATGAACGCATTCAAAAGGAAGAAGAGAAGAAGGTAGAAA
ACTTTTCTTTTTACCTACCAGAGAAAACTCGGGAATGGTTCACAAAGTCTGGTGTGTATCTTTCACCA
TTTGCTTACGTGAAT
+
FFFFFFFFFFFFFFFFFFFFFFFFFFFFFFFFFFFFFFFFFFFFFFFFFFFFFFFFFFFFFFFFFFFFFF
FFFFFFFFFFFFFFFFFFFFFFFFFFFFFFFFFFFFFFFFFFFFFFFFFFFFFFFFFFFFFFFFFFFFFF
FFFFFFFFFFFFFFF
@A00155:342:HHGFNDSXY:1:1132:19786:1564 2:N:0:GAACCTAG+TCCGCATA
CAACAGGAGGTTATAAGTGGATTTCAGTATGAACGCATTCAAAAGGAAGAAGAGAAGAAGGTGGAAAA
CTTTTCTTTTTACCTACCAGAGAAAACTCGGGAATGGTTCACAAAGTCTGGTGTGTATCTTTCACCAT
TTGCTTACGTGAATC
+
FFFFFFFFFFFFFFFFFFFFFFFFFFFFFFFFFFFFFFFFFFFFFFFFFFFFFFFF:FFFFFFFFFFFF
FFFFFFFFFFFFFFFFFFFFFFFFFFFFFFFFFFFFFFFFFFFFFFFFFFFFFFFFFFFFFFFFFFFFFF
FFFFFFFFFFFFFFF
@A00155:342:HHGFNDSXY:1:2244:7536:23813 2:N:0:GAACCTAG+TCCGCATA
CAACAGGAGGTTATAAGCGGATTTCAGTATGAACGCATTCAAAAGGAAGAAGAGAAGAAGGTGGAAAA
CTTTTCTTTTTACCTACCAGAGAAAACTCGGGAATGGTTCACAAAGTCTGGTGTGTATCTTTCACCAT
TTGCTTACGTGAATC
+
FFFFFFFFFFFFFFFFFFFFFFFFFFFFFFFFFFFFFFFFFFFFF:FFFFFFFFFFFFFFFFFFFFFFFF
FFFFFFFFFFFFFFFFFFFFFFFFF:FFFFFFFFFF:FFFFFFFFFFFFFFFFFFFFFFFFFFFFFFFFF
FFFFFFFFFFFFFFF
@A00155:342:HHGFNDSXY:1:1215:15655:28291 2:N:0:GAACCTAG+TCCGCATA
AACAGGAGGTTATAAGTGGATTTCAGTATGAACGCATTCAAAAGGAAGAAGAGAAGAAGGTGGAAAAC
TTTTCTTTTTACCTACCAGAGAAAACTCGGGAATGGTTCACAAAGTCTGGTGTGTATCTTTCACCATT
TGCTTACGTGAGA
+
FFFFFFF,FFFF:FF:F,:F:F::F::FFFFF:,FFF:,FFFFF,FF:FFFFFFF:FFFFF:,FFFF:
FFFFFFF:FFFFFFFFFFFFFFFFF:FFFFFFF:,FF::FFFFF,FFFFFFFFFFFFFF::FF,FFF:
FF:FFF,,FFFFF
@A00155:342:HHGFNDSXY:1:1308:14922:5071 2:N:0:GAACCTAG+TCCGCATA
AACAGGAGGTTATAAGCGGATTTCAGTATGAACGCATTCAAAAGGAAGAAGAGAAGAAGGTGGAAAAC
TTTTCTTTTTACCTACCAGAGAAAACTCGGGAATGGTTCACAAAGTCTGGTGTGTATCTTTCACCATT
TGCTTACGTGAATCA
+
FFFFFFFFFFFFFFFFFFFFFFFFFFFFFFFFFFFFFFFFFFFFFFFFFFFFFFFFFFFFFFFFFFFFFF
FFFFFFFFFFFFFFFFFFFFFFFFFFFFFFFFF,FFFFFFFFFFFFFFFFFFFFFFFFFFFFFFFFFFF
FFFFFFFFFFFFFFF
@A00155:342:HHGFNDSXY:1:1305:3007:30091 2:N:0:GAACCTAG+TCCGCATA
AACAGGAGGTTATAAGCGGATTTCAGTATGAACGCATTCAAAAGGAAGAAGAGAAGAAGGTGGAAAAC
TTTTCTTTTTACCTACCAGAGAAAACTCGGGAATGGTTCACAAAGTCTGGTGTGTATCTTTCACCATT
TGCTTACGTGAATCA
+
FFFFFFFFFFFFFFFFFFFFFFFFFFFFFFFFFFFFFFFFFFFFFFFFFFFFFFFFFFFFFFFFFFFFFF
FFFFFFFFFFFFFFFFFFFFFFFFFFFFFFFFFFFF:FFFFFFFFFFFFF,FFFFFFFFFFFFFFFFFF
F:FFFFFFFFFFFFF
@A00155:342:HHGFNDSXY:1:1664:20220:35227 2:N:0:GAACCTAG+TCCGCATA
AACAGGAGGTTATAAGCGGATTTCAGTATGAACGCATTCAAAAGGAAGAAGAGAAGAAGGTGGAAAAC
```

TTTTCTTTTTACCTACCAGAGAAAACTCGGGAATGGTTCACAAAGTCTGGTGTGTATCTTTCACCATT
TGCTTACGTGAATCA
+
FFFFFFFFF:FFFFFFFFFFFFFFFFFFFFFFFFFFFFFFFFFFFFFFFFFFFFF,FFFFFFFFFF:F
FFFF:FF:FFFFFFFFFFFF:FFFFFF:FFFFFFFFFF:FFFFFFFFFF:FFFFFFF:F:FFFFFFFF
FFF:FFF:::FFFFF
@A00155:342:HHGFNDSXY:1:2451:11966:35524 2:N:0:GAACCTAG+TCCGCATA
AACAGGAGGTTATAAGCGGATTTCAGTATGAACGCATTCAAAAGGAAGAAGAGAAGAAGGTGGAAAAC
TTTTCTTTTTACCTACCAGAGAAAACTCGGGAATGGTTCACAAAGTCTGGTGTGTATCTTTCACCATT
TGCTTACGTGAATCA
+
FFFFFFFFFFFFFFFFFFFFFFFFFFFFFFFFFFFFFFFFFFFFFFFFFFFFFFFFFFFFFFFFFFFF
F:FFFFFFFFFFFFF:FFF:FFFFFFFFFFFFFFFFFFFFFFF:FFFFFFFFFFFFFFFFFFFFF:FF
FFFFFFFFFFFFFFF
@A00155:342:HHGFNDSXY:1:2443:18466:20635 2:N:0:GAACCTAG+TCCGCATA
ACAGGAGGTTATAAGCGGATTTCAGTATGAACGCATTCAAAAGGAAGAAGAGAAGAAGGTGGAAAACT
TTTCTTTTTACCTACCAGAGAAAACTCGGGAATGGTTCACAAAGTCTGGTGTGTATCTTTCACCATTT
GCTTACGTGAATCA
+
::FFFFFFFFFFFFFFFFFFFFFFFFFFFFFFFFFFFFFFFFFFFFFFFFFFFFFFFFFFF:FFFFFF
FFF:FFFFFFF:FFFFFFFFFFFFF:F,::FFFF,FFFFFFFF,,FFFFFF:FF:F:FF:FF:FFF:F
F,FFF:,:FFF::F
@A00155:342:HHGFNDSXY:1:1237:29451:24690 1:N:0:GAACCTAG+TCCGCATA
ACAGGAGGTTATAAGCGGATTTCAGTATGAACGCATTCAAAAGGAAGAAGAGAAGAAGGTGGAAAACT
TTTCTTTTTACCTACCAGAGAAAACTCGGGAATGGTTCACAAAGTCTGGTGTGTATCTTTCACCATTT
GCTTACGTGAATCAC
+
FFFFFFFFFFFF:FFFFFFFFFFFFFFFFFFFFFFF:FFFFFFFFFFFFFFFF:FF:FF,FFFFFF:F
FFFFFFFFFFFFFFFFFF:FFFFFFFFFFFFFFFFFFFFFFFFFFFFFFFFFFFFFFFFFFFFFFFFF
FFFFFFFFFFFFFFF
@A00155:342:HHGFNDSXY:1:1421:20735:30796 2:N:0:GAACCTAG+TCCGCATA
CAGGAGGTTATAAGCGGATTTCAGTATGAACGCATTCAAAAGGAAGAAGAGAAGAAGGTGGAAAACTT
TTCTTTTTACCTACCAGAGAAAACTCGGGAATGGTTCACAAAGTCTGGTGTGTATCTTTCACCATTTG
CTTACGTGAATCACT
+
FFFFFFFFFFFFFFFFFFFFFFFFFFFFFFFFFFFFFFFFFFFFFFFFFFFFFFFFFFFFFFFFFFFF
FFFFFFFFFFFFFFFFFFFFFFFFFFFF:FFFFFFFFFFFFFFFFFFFFFFFFFFFFFFFFFFFFFFF
FFFFFFFFFFF:FFF
@A00155:342:HHGFNDSXY:1:1421:20600:31187 2:N:0:GAACCTAG+TCCGCATA
CAGGAGGTTATAAGCGGATTTCAGTATGAACGCATTCAAAAGGAAGAAGAGAAGAAGGTGGAAAACTT
TTCTTTTTACCTACCAGAGAAAACTCGGGAATGGTTCACAAAGTCTGGTGTGTATCTTTCACCATTTG
CTTACGTGAATCACT
+
FFFFFFFFF:FFFFFFF:FFFFFFF:FFFFFFFFFFFFFFFFFFFFFFFFFFFFFFFFFFFF,FFFFF
FFFFFFFFFFFFFFFFFFFF,FFFFFFFF::FFFFFFFFFFFFFFFFFFFFFFFFFFFFFFFFFFFFF
FFFFFF:FFFFFFFF
@A00155:342:HHGFNDSXY:1:1435:7871:7921 2:N:0:GAACCTAG+TCCGCATA
CAGGAGGTTATAAGCGGATTTCAGTATGAACGCATTCAAAAGGAAGAAGAGAAGAAGGTGGAAAACTT
TTCTTTTTACCTACCAGAGAAAACTCGGGAATGGTTCACAAAGTCTGGTGTGTATCTTTCACCATTTG
CTTACGTGAATCACT
+
FFFFFFFFFFFFFFFFFFFFFFFFFF:FFFFFFFF:FFFFF,FFF:FFFFFFFFFFFFFFFFFFFFFF
FFFFFFFFFFFFFFFFFFFFFF:FFFFFFF,FF:FFFFFFF:FFFFFFFFFFFFFFFFFF,FFFFFFF
FFFFFFFFFFFFFFF

@A00155:342:HHGFNDSXY:1:1509:22037:24220 2:N:0:GAACCTAG+TCCGCATA
CAGGAGGTTATAAGCGGATTTCAGTATGAACGCATTCAAAAGGAAGAAGAGAAGAAGGTGGAAAACTT
TTCTTTTTACCTACCAGAGAAAACTCGGGAATGGTTCACAAAGTCTGGTGTGTATCTTTCACCATTTG
CTTACGTGAATCACT
+
FFFFFFFFFFFFFFFFFFFFFFFFFFFFFFFFFFFFFFFFFFFF:FFFFFFFFFFFFFFFFFFFFFFF
FFFFFFFFFFFFFFFFFFFFFFFFFFFFFF,FFFFFFFFFFFFFFFFFFFFFF:FFFFFFFFFFFFFF
FFFFFFFFFFFF:FF
@A00155:342:HHGFNDSXY:1:2264:3667:25254 2:N:0:GAACCTAG+TCCGCATA
CAGGAGGTTATAAGCGGATTTCAGTATGAACGCATTCAAAAGGAAGAAGAGAAGAAGGTGGAAAACTT
TTCTTTTTACCTACCAGAGAAAACTCGGGAATGGTTCACAAAGTCTGGTGTGTATCTTTCACCATTTG
CTTACGTGAATCACT
+
FFFFFFFFFFFFFFFFFFFF:FFFFFFFFFFFFFFFFFFFFFFFFFFFFFFFFFFFFFFFFFFFFF:F
FFFFFFFFFFFFFFFFFFF:FFFFFFFFFFFFFFFFFFFFFFFFFFFFFFFFFFFFFFFFFFFFFFF:
FFFFF:FFFFFFFF:
@A00155:342:HHGFNDSXY:1:2331:19461:24831 2:N:0:GAACCTAG+TCCGCATA
CAGGAGGTTATAAGCGGATTTCAGTATGAACGCATTCAAAAGGAAGAAGAGAAGAAGGTGGAAAACTT
TTCTTTTTACCTACCAGAGAAAACTCGGGAATGGTTCACAAAGTCTGGTGTGTATCTTTCACCATTTG
CTTACGTGAATCACT
+
FFFFFFFFFFFFFFFFFFFFFFFFFFFFFFFFFFFFFFFFFFFFFFFFFFFFFFFFFFFFFFFFFFFF
FFFFFFFFF:FFFFFFFFFFFFFFFFFFFF,FFFFFFFFFFFFFFFFFFFFFFFFFFFFFFFFFFFFF
FFFFFFFFFFFFFFF
@A00155:342:HHGFNDSXY:1:2643:23601:12868 2:N:0:GAACCTAG+TCCGCATA
CAGGAGGTTATAAGCGGATTTCAGTATGAACGCATTCAAAAGGAAGAAGAGAAGAAGGTGGAAAACTT
TTCTTTTTACCTACCAGAGAAAACTCGGGAATGGTTCACAAAGTCTGGTGTGTATCTTTCACCATTTG
CTTACGTGAATCACT
+
FFFFFFFFFFFFFFFFFFFFFFFFFFFFFFFFFFFFFFFFFFFFFF:FFFFFFFFFFFFFFF:FFFFF
FFFFFFFFFFFFFFFFFFFFFFFFFFFFFF:FFFFFFFFFFFFFFFFFFFFFFFFFFFFFFFFFFFFF
FFFFFFFFFFFFFFF
@A00155:342:HHGFNDSXY:1:2446:9028:10457 1:N:0:GAACCTAG+TCCGCATA
AGGAGGTTATAAGCGGATTTCAGTATGAACGCATTCAAAAGGAAGAAGAGAAGAAGGTGGAAAACTTT
TCTTTTTACCTACCAGAGAAAACTCGGGAATGGTTCACAAAGTCTGGTGTGTATCTTTCACCATTTGC
TTACGTGAATCACTC
+
FFFFFFFFF,F:FFFFF:FFFFFFF,FFFFFFFFFFFFFF:FFFFFFFFFFFFF,,FF:F:FFFFF,F
FFFFFFFFFFFFFFFFFFFFFFFFFFFFF:FFFFFFFFFFFFFFFFFFFFFFFFFFFFFFFFFFFFFF
FFFFFFFFFFFFFFF
@A00155:342:HHGFNDSXY:1:2402:31611:23766 2:N:0:GAACCTAG+TCCGCATA
AGGAGGTTATAAGCGGATTTCAGTATGAACGCATTCAAAAGGAAGAAGAGAAGAAGGTGGAAAACTTT
TCTTTTTACCTACCAGAGAAAACTCGGGAATGGTTCACAAAGTCTGGTGTGTATCTTTCACCATTTGC
TTACGTGAATCACTC
+
FFF:FFFFFFFFFFFFF:FFFFFF:FFFFFFFFFFFFFFFFFF:FFFFFFFFFFFFFFFFF:FFFFFF
FFFFFFFFFFF:FFFFFFFFFFFF:FFFF,FFFFFFFFFFFFFFFFFFFFFFFFFFFFFFFF:FFFFF
FFFFFFFF:FFFFFF
@A00155:342:HHGFNDSXY:1:1365:4788:11600 2:N:0:GAACCTAG+TCCGCATA
GGAGGTTATAAGCGGATTTCAGTATGAACGCATTCAAAAGGAAGAAGAGAAGAAGGTGGAAAACTTTT
CTTTTTACCTACCAGAGAAAACTCGGGAATGGTTCACAAAGTCTGGTGTGTATCTTTCACCATTTGCT
TACGTGAATCACTCA
+
FFFFFFFFFFFFFFFFFFFFFFFFFFFFFFFFFFFFFFFFFFFFFFFFFFFFFFFFFFFFFFFFFFFF

FFFFFFFF:FFFFFFFFFFF,FFFFF:F,FFFFFFFFFFFFFFFFFFFFFFFFFFFFFFFFFFFFFF
FFFFFFFFFFFFFFF
@A00155:342:HHGFNDSXY:1:1456:9173:29810 2:N:0:GAACCTAG+TCCGCATA
GGAGGTTATAAGTGGATTTCAGTATGAACGCATTCAAAAGGAAGAAGAGAAGAAGGTGGAAAACTTTT
CTTTTTACCTACCAGAGAAAACTCGGGAATGGTTCACAAAGTCTGGTGTGTATCTTTCACCATTTGCT
TACGTGAATCACTCA
+
FFFFFFFFFFFFFFFFFFFFFFFFFFFFFFFFFFFFFFFFFFFFFFFFFFF::FFFFFFFFFFFFFFF
FFFF:FFFFFFFFFFFFFFFFFFFF:FFFFFFFFFFFF:FFFFFFFFFFFFFFFFFFFFFFFFFFFFFF
FFFFFFFFFFFFFFF
@A00155:342:HHGFNDSXY:1:2258:19551:16532 2:N:0:GAACCTAG+TCCGCATA
GGAGGTTATAAGCGGATTTCAGTATGAACGCATTCAAAAGGGAGAAGAGAAGAAGGTGGAAAACTTTT
CTTTTTACCTACCAGAGAAAACTCGGGAATGGTTCACAAAGTCTGGTGTGTATCTTTCACCATTTGCT
TACGTGAATCACTCA
+
FFFFFFFFFFFFFFFFFFFFFFFFFFFFFFFFFFFFFFFFFFFFFFFFFFFFFFFFF:FFFFFFFFFFF
FFFFFFFFFFFFFFFFFFFFFFFFFFFFFFFFFFFFFFFF:FFFFFFFFFFFFFFFFFFFF:FFFFFFF
FFFFFFFFFFFFFFF
@A00155:342:HHGFNDSXY:1:1330:32551:27743 1:N:0:GAACCTAG+TCCGCATA
GAGGTTATAAGCGGATTTCAGTATGAACGCATTCAAAAGGAAGAAGAGAAGAAGGTGGAAAACTTTTC
TTTTTACCTACCAGAGAAAACTCGGGAATGGTTCACAAAGTCTGGTGTGTATCTTTCACCATTTGCTT
ACGTGAATCACTCAC
+
FFFFFFF:FFFFFFFFFFFFFFFFFFFFFFFFFFFFFFFFFFFFFFFF,FF,:F:FFFFFFFFFFFF
FFFFFFFFFFFFFFFFFF:FFFFFFFFFFFFFFFFFFFFFFFFFFFFFFFFFFFFFFFFFFFFFFFFF
FFFFFFFFFFFFFFF
@A00155:342:HHGFNDSXY:1:1414:8323:5415 1:N:0:GAACCTAG+TCCGCATA
GAGGTTATAAGCGGATTTCAGTATGAACGCATTCAAAAGGAAGAAGAGAAGAAGGTGGAAAACTTTTC
TTTTTACCTACCAGAGAAAACTCGGGAATGGTTCACAAAGTCTGGTGTGTATCTTTCACCATTTGCTT
ACGTGAATCACTCAC
+
FFFFFFFFFFFFFFFFFFFFFFFFFFFFFFFFFFFFFFFFFFFFFFFFFFFFF,FFFFFFFFFFFFFF
FFFFFFFFFFFFFFFFFFFFFFFFFFFFFFFFFFFFFFFFFFFFFFFFFFFFFFFFFFFFFFFFFFFF
FFFFFFFFFFFFFFF
@A00155:342:HHGFNDSXY:1:1440:20781:16344 2:N:0:GAACCTAG+TCCGCATA
GAGGTTATAAGCGGATTTCAGTATGAACGCATTCAAAAGGAAGAAGAGAAGAAGGTGGAAAACTTTTC
TTTTTACCTACCAGAGAAAACTCGGGAATGGTTCACAAAGTCTGGTGTGTATCTTTCACCATTTGCTT
ACGTGAATCACTCAC
+
FFFFFFFFFFFFFFFFFFFFFFFFFFFFFFFFFFFFFFFFFFFFFFFFFFFFFFFFFFFFFFFFFFFF
FFFF:FFFFFFFFFFFFFFFFFFFFFFFFFFFFFFFFFFFFFFFFFFFFFFFFFFFFFFFFFFFFFFF
FFFFFFFFFFFFFFF
@A00155:342:HHGFNDSXY:1:1505:17743:16344 2:N:0:GAACCTAG+TCCGCATA
GAGGTTATAAGCGGATTTCAGTATGAACGCATTCAAAAGGAAGAAGAGAAGAAGGTGGAAAACTTTTC
TTTTTACCTACCAGAGAAAACTCGGGAATGGTTCACAAAGTCTGGTGTGTATCTTTCACCATTTGCTT
ACGTGAATCACTCAC
+
FFFFFFFFFFFFFFFFFFFFFFFFFFFFFFFFFFFFF:FFFFFFFFFFFFFFFFFFFFFFFFFFFFFF
FFFFFFFFFFFFFFFFFFFFFFFFFFFFFFFFFFFFFFFFFFFFFFFFFFFFFFFFFFFFFFFFFFFF
FFFFFFFFFFFFFFF
@A00155:342:HHGFNDSXY:1:2343:22652:7623 2:N:0:GAACCTAG+TCCGCATA
GAGGTTATAAGCGGATTTCAGTATGAACGCATTCAAAAGGAAGAAGAGAAGAAGGTGGAAAACTTTTC
TTTTTACCTACCAGAGAAAACTCGGGAATGGTTCACAAAGTCTGGTGTGTATCTTTCACCATTTGCTT
ACGTGAATCACTCAC

```
+
FFFFFFFFFFFFFFFFFFFFFFFFFFFFFFFFFFFFFFFFFFFFFFFFFFFFFFFFFFFFFFFFFFFF
FFFFFFFFFFFF::FFFFFFFFFFFFFFFFFFFFFFFFF,FFFFFFFFFFFFFFFFFFFFFFFFFFFF
FFF:FFFFFFFFFFF
@A00155:342:HHGFNDSXY:1:1205:3296:19914 2:N:0:GAACCTAG+TCCGCATA
AGGTTATAAGCGGATTTCAGTATGAACGCATTCAAAAGGAAGAAGAGAAGAAGGTGGAAAACTTTTCT
TTTTACCTACCAGAGAAAACTCGGGAATGGTTCACAAAGTCTGGTGTGTATCTTTCACCATTTGCTTA
CGTGAATCACTCACA
+
FFFFFFFFFFFFFFFFFFFFFFFFFFFFFFFFFFFF:FFFFFFF:FFFFFFFFF:,FFFFFFFFFFFF
FFFFFFFFFFFF,FFFFFFFFFFF,FFFFFFFFFFF:FFFFFFFFFFFFFFFFFFFF,FFFFF,FFFF
F,FFFFF::FFFFF:
@A00155:342:HHGFNDSXY:1:1205:3197:19993 2:N:0:GAACCTAG+TCCGCATA
AGGTTATAAGCGGATTTCAGTATGAACGCATTCAAAAGGAAGAAGAGAAGAAGGTGGAAAAATTTTCT
TTTTACCTACCAGAGAAAACTCGGGAATGGTTCACAAAGTCTGGTGTGTATCTTTCACCATTTGCTTA
CGTGAATCACTCACA
+
:FF:FFFFF:FFFFFFF:FFFFFFFFF,FFFFFFFFFFFFFFFFFF:FF:FFFFF,FFFFF,FFFFFF
F:FFF:FFFFFFFF:FFF:FF::FFF:FFFFFFFFF:F:FFFF,F,F:FFFFFF:FFFFFF:F:FF:F
F,FFFFFFFFFF:F:
@A00155:342:HHGFNDSXY:1:2171:29179:36057 2:N:0:GAACCTAG+TCCGCATA
AGGTTATAAGCGGATTTCAGTATGAACGCATTCAAAAGGAAGAAGAGAAGAAGGTGGAAAACTTTTCT
TTTTACCTACCAGAGAAAACTCGGGAATGGTTCACAAAGTCTGGTGTGTATCTTTCACCATTTGCTTA
CGTGAATCACTCACA
+
FFF:FFFF:FFFFFF,:FFFFFFFFF:FFFFFF::FFFFF::FFFFFFFFFFFF:FFFFFFFFFFFFF
FFFFFFFFFFFFFFFFFFFFFFFFF,FFFFFFFF:FF,FFFFFFFF::,:FFFFFFF:F:F,FFFF::F
FFFFF:F:FFF:FFF
@A00155:342:HHGFNDSXY:1:2255:19117:29528 2:N:0:GAACCTAG+TCCGCATA
AGGTTATAAGCGGATTTCAGTATGAACGCATTCAAAAGGAAGAAGAGAAGAAGGTGGAAAACTTTTCT
TTTTACCTACCAGAGAAAACTCGGGAATGGTTCACAAAGTCTGGTGTGTATCTTTCACCATTTGCTTA
CGTGAATCACTCACA
+
:FFFFFFFFFFFFFFFFFFFFFFFFFFFFFFFFFFF:FFFFFFFFFFFFFFFFFFFFFFFFFFFFFFF
FFFFFFFFFFFFFFFFFFFF,FFFF:FFFFFFFFFFFFFFFFFFFFFFFFFFFFFFFFFFFFFFFFFFF
FFFFF:FFFFFFFFF
@A00155:342:HHGFNDSXY:1:2165:10113:7827 1:N:0:GAACCTAG+TCCGCATA
CCGATCTAGCGGATTTCAGTATGAACGCATTCAAAAGGAAGAAGAGAAGAAGGTGGAAAACTTTTCTT
TTTACCTACCAGAGAAAACTCGGGAATGGTTCACAAAGTCTGGTGTGTATCTTTCACCATTTGCTTAC
GTGAATCACTC
+
FFFFFFFFFFFFFFFFFFFFFFFFFFFFFFFFFFFFFFFFFFFFFFFFFFFFFFFFFFFFFFFFFFFF
FFFFFFFFFFFFFFFF:FFFFFFFFFFFFFFFFFFFFFFFFFFFFFFFFFFFFFFFFFFFFFFFFFF
FFFFFFFFFFF
@A00155:342:HHGFNDSXY:1:2228:20030:24659 1:N:0:GAACCTAG+TCCGCATA
GGTTATAAGTGGATTTCAGTATGAACGCATTCAAAAGGAAGAAGAGAAGAAGGTGGAAAACTTTTCTT
TTTACCTACCAGAGAAAACTCGGGAATGGTTCACAAAGTCTGGTGTGTATCTTTCACCATTTGCTTAC
GTGAATCACTC
+
F:FFFFFFFFFFFFFFFFFFF:FFFFFFFFFFFFFFFFFFFFFFFFFFFFF:F::F:FFF:FFFFFFFFF
FFFFFFFFFFFFFFFFFFFFFFFFFFFFFFFFFFFFFFFFFFFFFFFFFFFFFFFFFFFFFFFFFFFF
FFFFFFFFFFF
@A00155:342:HHGFNDSXY:1:1653:10556:23813 2:N:0:GAACCTAG+TCCGCATA
GGTTATAAGCGGATTTCAGTATGAACGCATTCAAAAGGAAGAAGAGAAGAAGGTGGAAAACTTTTCTT
```

TTTACCTACCAGAGAAAACTCGGGAATGGTTCACAAAGTCTGGTGTGTATCTTTCACCATTTGCTTAC
GTGAATCACTC
+
F:FFFFFFFFFFFFFFFFFFFFFFFFFFFFFFFFFFF:FFFFFFFFFFFFFFFFFFFFFFF:FFFFFF
FFFFFFFFFFFFFFFFFFFFFFFFFFFFFFFFFFFFFFFFFFFFFFFFFFFFFFFFFFFFFFFFFFFF
FFFFFFFFFFF
@A00155:342:HHGFNDSXY:1:2502:11993:29496 2:N:0:GAACCTAG+TCCGCATA
GGTTATAAGCGGATTTCAGTATGAACGCATTCAAAAGGAAGAAGAGAAGAAGGTGGAAAACTTTTCTT
TTTACCTACCAGAGAAAACTCGGGAATGGTTCACAAAGTCTGGTGTGTATCTTTCACCATTTGCTTAC
GTGAATCACTCACAC
+
FFFFFFFFFFFFFFFFFFFFFFFFFFFFFFFFFFFFFFFFFFFFFFFFFFFFFFFFFFFFFFFFFFFF
FFFFFFFFFFFFFFFFFFFFFFFFFFFFFFFFFFFFFFFFFFFFFFFFFFFFFFF:FFF:FFFFFFFF
FF:FFFFFFFFFFFF
@A00155:342:HHGFNDSXY:1:1255:8214:6042 2:N:0:GAACCTAG+TCCGCATA
GTTATAAGTGGATTTCAGTATGAACGCATTCAAAAGGAAGAAGAGAAGAAGGTGGAAAACTTTTCTTT
TTACCTACCAGAGAAAACTCGGGAATGGTTCACAAAGTCTGGTGTGTATCTTTCACCATTTGCTTACG
TGAATCACTCACACC
+
FFFFFFFFFFFFFFFFFFFFFFFFFFFFFFFFFFFFFFFFFFFFFFFFFFFFFFFFFFFFFFFFFFFF
FFFFFFFFFFFFFFFFFFFFFFFFFFFFFFFFFFFFFFFFFFFFFFFFFFFFFFFFFFFFFFFFFFFF
FFFFFF:FFFFFFFF
@A00155:342:HHGFNDSXY:1:1336:22562:4867 2:N:0:GAACCTAG+TCCGCATA
GTTATAAGCGGATTTCAGTATGAACGCATTCAAAAGGAAGAAGAGAAGAAGGTGGAAAACTTTTCTTT
TTACCTACCAGAGAAAACTCGGGAATGGTTCACAAAGTCTGGTGTGTATCTTTCACCATTTGCTTACG
TGAATCACTCACACC
+
FFFFFFFFFFFFFFFFFFFFFFFFFFFFFFFFFFFFFFFFFFFFFFFFFFF:FFFFFFFFFFFFFFFF
FFFFFFFFFFFFFFFFFFFFFFF:FFFFFFFFFFFFFFFFFFFFFFFFF:FFFFFFFFF,FFFFFFFF
FFFFFFFFFFFFFFF
@A00155:342:HHGFNDSXY:1:1364:10140:6026 2:N:0:GAACCTAG+TCCGCATA
GTTATAAGCGGATTTCAGTATGAACGCATTCAAAAGGAAGAAGAGAAGAAGGTGGAAAACTTTTCTTT
TTACCTACCAGAGAAAACTCGGGAATGGTTCACAAAGTCTGGTGTGTATCTTTCACCATTTGCTTACG
TGAATCACTCACACC
+
FFFFFFFFFFFFFFFFFFFFFFFFFFFFFFFFFFFFFFFFFFFFFFFFFFFFFFFFFF:FFFFFFFFF
FFFFFFFFFFFFFFFFFFFFFFFFFFFFFFFFFFFFFFFFFFFFFFF:FF:FFFFFFFFFFFFFFFFF
FF:FFFFFFFFFFFF
@A00155:342:HHGFNDSXY:1:2610:20708:33285 2:N:0:GAACCTAG+TCCGCATA
TATAAGCGGATTTCAGTATGAACGCATTCAAAAGGAAGAAGAGAAGAAGGTGGAAAACTTTTCTTTTT
ACCTACCAGAGAAAACTCGGGAATGGTTCACAAAGTCTGGTGTGTATCTTTCACCATTTGCTTACGTG
AATCACTCACACCCT
+
F:FFFFFFFFFFFFFFF:FFFFFFFFFFFFFFFFFFFFFFFFFFFFFFFFFFFFFFFFFFFFFFFFFF
FFFF:FFFFFFFFFFFFFFF::FFFFFFFFFFFFFFFFFFFFFFFFFFFFFFFFFFFFFFFFFFFFFF
F:FFFFFFFFFFFFF
@A00155:342:HHGFNDSXY:1:1149:32425:13150 1:N:0:GAACCTAG+TCCGCATA
ATCTGCGGATTTCAGTATGAACGCATTCAAAAGGAAGAAGAGAAGAAGGTGGAAAACTTTTCTTTTTA
CCTACCAGAGAAAACTCGGGAATGGTTCACAAAGTCTGGTGTGTATCTTTCACCATTTGCTTACGTGA
ATCACTCACACCCTG
+
FFF,FF:F,FFFFFFFFFF,F:FFFFFFF,,:FFFFFFFFFFFFFFFFFF:F:F:FFFFFFFFFFFFF
FFFFFF:FFFFFFFFFFFFFFFFFFFFF:::,FFFFFFFFF:FF,FFFFFFF:FFFFFFFFFFFFFFF
FFF:FFF:FFFF:FF

```
@A00155:342:HHGFNDSXY:1:1149:32461:13182 1:N:0:GAACCTAG+TCCGCATA
ATCTGCGGATTTCAGTATGAACGCATTCAAAAGGAAGAAGAGAAGAAGGTGGAAAACTTTTCTTTTTA
CCTACCAGAGAAAACTCGGGAATGGTTCACAAAGTCTGGTGTGTATCTTTCACCATTTGCTTACGTGA
ATCACTCACACCCTG
+
FFFFFFFFFFFFFFFF:FFFFFFFFFFFFF:FFFFFFFFFFFFFFFFFFFFFFFFFFFFFFFFFFFFF
FFFFFFFFFFFFFFFFFFFFFFFFFFFFFFFFFFFFFFFFFFFFFFFFFFFFFFFFFFFFFFFFFFFF
FFFFFFFFFFFFFFF
@A00155:342:HHGFNDSXY:1:1407:23411:22811 1:N:0:GAACCTAG+TCCGCATA
ATCTGCGGATTTCAGTATGAACGCATTCAAAAGGAAGAAGAGAAGCAGGTGGAAAACTTTTCTTTTTA
CCTACCAGAGAAAACTCGGGAATGGTTCACAAAGTCTGGTGTGTATCTTTCACCATTTGCTTACGTGA
ATCACTCACACCCTG
+
FFFFFFFFFFFFF:FFFFFFFFFFFFFFFFFF:FFFFFFFFFFFF,FFFFFFFFFFFFFFFFFFFFFF
FFFFFFFFFFFFFFFFFFFFFFF:FFFFFFFFFFFFFFFFFFFFFFFFF:FFFFFFFFFFFFFFFFFF
FFFFFFFFFFFFFFF
@A00155:342:HHGFNDSXY:1:2415:28872:32017 1:N:0:GAACCTAG+TCCGCATA
ATAAGCGGATTTCAGTATGAACGCATTCAAAAGGAAGAAGAGAAGAAGGTGGAAAACTTTTCTTTTTA
CCTACCAGAGAAAACTCGGGAATGGTTCACAAAGTCTGGTGTGTATCTTTCACCATTTGCTTACGTGA
ATCACTCACACCCTG
+
FFF:FFFFFFFFFFFFFFFFFFFFFFFFFF:F,FFFFFFFFFFF::,:FF,:FFFFFFFFFFFFFFFF
FFFFFFFFFFFFFFFFFFFFFFFFFFFFFFFFFFFFFFFFFFFFFFFFFFFFFFFFFFFFFFFFFFFF
FFFFFFFFFFFFFFF
@A00155:342:HHGFNDSXY:1:1230:6207:35634 1:N:0:GAACCTAG+TCCGCATA
TAAGTGGATTTCAGTATGAACGCATTCAAAAGGAAGAAGAGAAGAAGGTGGAAAACTTTTCTTTTTAC
CTACCAGAGAAAACTCGGGAATGGTTCACAAAGTCTGGTGTGTATCTTTCACCATTTGCTTACGTGAA
TCACTCACACCCTGG
+
FF,FFFFFFFFFFF:FFFFFFFFFFFFFFFFFFFFFFFFFFFFFFFF,FFF::FFFFFFFFFFFFFFF
FFFFFFFFFF:FFFFFFFFFFFFFFFFFFFFFF:FFFF:FFFFFFFFFFFFFFFFFFFFFFFFFFFFF
FFFFFFFFFFFFFFF
@A00155:342:HHGFNDSXY:1:1453:11162:11209 1:N:0:GAACCTAG+TCCGCATA
TAAGCGGATTTCAGTATGAACGCATTCAAAAGGAAGAAGAGAAGAAGGTGGAAAACTTTTCTTTTTAC
CTACCAGAGAAAACTCGGGAATGGTTCACAAAGTCTGGTGTGTATCTTTCACCATTTGCTTACGTGAA
TCACTCACACCCTGG
+
F:FFFFFFFFFFFFFFFFFFFFFFFFFFFFFFFFFFFFFFFF:FF,FF:FFFFFFFFFFFFFFFFFFF
FFFFFFFFFFFFFFFFFFFFFFFFFFFFFFFFFFFF:FFFFFFFFFFFFFFFFFFFFFFFFFFFFFFF
FFFFFFFFFFFFFFF
@A00155:342:HHGFNDSXY:1:1508:25138:14716 1:N:0:GAACCTAG+TCCGCATA
TAAGTGGATTTCAGTATGAACGCATTCAAAAGGAAGAAGAGAAGAAGGTGGAAAACTTTTCTTTTTAC
CTACCAGAGAAAACTCGGGAATGGTTCACAAAGTCTGGTGTGTATCTTTCACCATTTGCTTACGTGAA
TCACTCACACCCTGG
+
FFFFFFFFFFFFFFFFFFFFF::FF:FFFFFFFFFFFFFFFFFF:FFFFFFFFFFFFFFFFFFFFFFF
FFFFFFFFF:FFFFFF:FFFFFFFFFFFFFF:FFFFFFFFFFFFFFFFFFFFFFFFFFFFFFFFFFFF
FFFFFFFFFFFFFFF
@A00155:342:HHGFNDSXY:1:2261:19180:27665 1:N:0:GAACCTAG+TCCGCATA
TAAGCGGATTTCAGTATGAACGCATTCAAAAGGAAGAAGAGAAGAAGGTGGAAAACTTTTCTTTTTAC
CTACCAGAGAAAACTCGGGAATGGTTCACAAAGTCTGGTGTGTATCTTTCACCATTTGCTTACGTGAA
TCACTCACACCCTGG
+
FF:FF:FFFFFFFFFFFFFFFFFFFFFFFFFFFFFFFFFFF,:F,:F:,:FFFFFFFFF:FFFFFFF
```

```
FFFFFFFFFFF:FFFFFFFFFFFFFFFFFFFFFFFFFFFFFFFFFFFFFFFFFFFFFFFFFFFFFFFF
FFFFFFFFFFFFFFF
@A00155:342:HHGFNDSXY:1:2344:4743:20979 1:N:0:GAACCTAG+TCCGCATA
TCTGCGGATTTCAGTATGAACGCATTCAAAAGGAAGAAGAGAAGAAGGTGGAAAACTTTTCTTTTTAC
CTACCAGAGAAAACTCGGGAATGGTTCACAAAGTCTGGTGTGTATCTTTCACCATTTGCTTACGTGAA
TCACTCACACCCTGG
+
FF:F,FF:FFF:FFFF::FFFFFFFFFFFFFFFFFFFFFFFFFFFFFFFFFFFFFFFFFFF:FFFFFF
FFFFFFFFFFFFFFFFFFFF:FFFFFFFFFFFFFFFFFFFFFFFFFFFFFFF:FFFFFFFFFFFFFFF
FFFFFFFFFF:FFFF
@A00155:342:HHGFNDSXY:1:2516:12120:13996 1:N:0:GAACCTAG+TCCGCATA
TCTGCGGATTTCAGTATGAACGCATTCAAAAGGAAGAAGAGAAGAAGGTGGAAAACTTTTCTTTTTAC
CTACCAGAGAAAACTCGGGAATGGTTCACAAAGTCTGGTGTGTATCTTTCACCATTTGCTTACGTGAA
TCACTCACACCCTGG
+
FFFFFFFFFFFFFFFFFFFFFFFFFFFFFFFFFFFFFFFFFFFFFFFFFFFFFFFFFFFFFFFFFFFF
FFFFFFFFFFFFFFFFFFFFFFFFFFFFFFFFFFFFFFFFFFFFFFFFFFFFFFFFFFFFFFFFFFFF
FFFFFFFFFFFFFFF
@A00155:342:HHGFNDSXY:1:2516:12192:14058 1:N:0:GAACCTAG+TCCGCATA
TCTGCGGATTTCAGTATGAACGCATTCAAAAGGAAGAAGAGAAGAAGGTGGAAAACTTTTCTTTTTAC
CTACCAGAGAAAACTCGGGAATGGTTCACAAAGTCTGGTGTGTATCTTTCACCATTTGCTTACGTGAA
TCACTCACACCCTGG
+
FFFFFFFFFFFFFFFFFFFFFFFFFFFFFFFFFFFFFFFFFFFFFFFFFFFFFFFFFFFFFFFFFFFF
FFFFFFFFFFFFFFFFFFFFFFFFFFFFFFFFFFFFFFFFFFFFFFFFFFFFFFFFFFFFFFFFFFFF
FFFFFFFFFFFFFFF
@A00155:342:HHGFNDSXY:1:2516:12201:14074 1:N:0:GAACCTAG+TCCGCATA
TCTGCGGATTTCAGTATGAACGCATTCAAAAGGAAGAAGAGAAGAAGGTGGAAAACTTTTCTTTTTAC
CTACCAGAGAAAACTCGGGAATGGTTCACAAAGTCTGGTGTGTATCTTTCACCATTTGCTTACGTGAA
TCACTCACACCCTGG
+
FFFFFFFFFFFFFFFFFFFFFFFFFFFFFFFFFFFFFFFFFFFFFFFFFFFFFFFFFFFFFFFFF,FF
FFFFFFFFFFFFFFFFFFFFFFFFFFFFFFFFFFFFFFFFFFFFFFFFFFFFFFFFFFFFFFFFFFFF
FFFFFFFFFFFFFFF
@A00155:342:HHGFNDSXY:1:2305:21667:32315 2:N:0:GAACCTAG+TCCGCATA
TAAGCGGATTTCAGTATGAACGCATTCAAAAGGAAGAAGAGAAGAAGGTGGAAAACTTTTCTTTTTAC
CTACCAGAGAAAACTCGGGAATGGTTCACAAAGTCTGGTGTGTATCTTTCACCATTTGCTTACGTGAA
TCACTCACACCCTGG
+
FFFFFFFFFFFFFFFFF:FFF:FFFFFFFFFFFF:FFFFFFF:FFFFFFFFFFFFFFFFFFFFFFFF:
FFFF,FFFFFFFFFFF,:F,FFF:FFFFFF:FFFFF:,F,FFFFFFF,FFFFFFF,FFFF:FF,FFFF
FFFFFFFFF,FFFFF
@A00155:342:HHGFNDSXY:1:2520:11532:19398 2:N:0:GAACCTAG+TCCGCATA
TAAGCGGATTTCAGTATGAACGCATTCAAAAGGAAGAAGAGAAGAAGGTGGAAAACTTTTCTTTTTAC
CTACCAGAGAAAACTCGGGAATGGTTCACAAAGTCTGGTGTGTATCTTTCACCATTTGCTTACGTGAA
TCACTCACACCCTGG
+
FFFFFFFFFFFFFFFFFFFFFFFF:FFFFFFFFFFF:FFFFFFFFFFFFFFFFFFFFFFFFFFFFFFF
FFFFF:FFFFFFFFFFFFFFFFFFFFFFFFFFFFFFFFFF:FFFFF:FFF:FFF:FFFFFFFFFFFFF
FFFFFFFFFFF:FFF
@A00155:342:HHGFNDSXY:1:1320:9588:14935 1:N:0:GAACCTAG+TCCGCATA
AAGCGGATTTCAGTATGAACGCATTCAAAAGGAAGAAGAGAAGAAGGTGGAAAACTTTTCTTTTTACC
TACCAGAGAAAACTCGGGAATGGTTCACAAAGTCTGGTGTGTATCTTTCACCATTTGCTTACGTGAAT
CACTCACACCCTGGC
```

+
F:F:::FFFFFFFFF:FFFF,,FFFFFFFF:FFFFFFFFFFFFF,:F:FFFFF,FFFFFFFFFFFFFF
FFFFFFFFFFFFFFFFFFFFFFFFFFFFFFFFFFFFFFFFFFFFFFFFF:FFFFFFFFFFFFFFFF
FFFFFFFFFFFFF:F
@A00155:342:HHGFNDSXY:1:1320:8775:15593 1:N:0:GAACCTAG+TCCGCATA
AAGCGGATTTCAGTATGAACGCATTCAAAAGGAAGAAGAGAAGAAGGTGGAAAACTTTTCTTTTTACC
TACCAGAGAAAACTCGGGAATGGTTCACAAAGTCTGGTGTGTATCTTTCACCATTTGCTTACGTGAAT
CACTCACACCCTGGC
+
FFFFFFFFFFFFFFFFFFFFFFFFFFFFFFFFFFFFFFFFFFFFFFFF:F:FFFFFFFFFFFFFFFFFF
FFFFFFFFFFFFFFFFFFFFFFFFFFFFF::FFFFFFFFFFFFFFFFFFFFFFFFFFFFFFFFFFFFF
FFFFFFFFFFFFFFF
@A00155:342:HHGFNDSXY:1:2416:9697:6292 1:N:0:GAACCTAG+TCCGCATA
AAGTGGATTTCAGTATGAACGCATTCAAAAGGAAGAAGAGAAGAAGGTGGAAAACTTTTCTTTTTACC
TACCAGAGAAAACTCGGGAATGGTTCACAAAGTCTGGTGTGTATCTTTCACCATTTGCTTACGTGAAT
CACTCACACCCTGGT
+
F:FF:FFFF:FFFFF:FFFFFFFF:FFFFFF:FFFFFFFFFFF::FFF::FFFFFFFFFFFFFFFFFF
FFFFFFFFFFFFFFFFFFFFFFFFFFFFFFFFFFFFFFFFFFFFFFFFFFFFFFFFFFFFFFFFFFFF
FFFFFFFFFFFFFFF
@A00155:342:HHGFNDSXY:1:1577:18204:34021 2:N:0:GAACCTAG+TCCGCATA
AAGCGGATTTCAGTATGAACGCATTCAAAAGGAAGAAGAGAAGAAGGTGGAAAACTTTTCTTTTTACC
TACCAGAGAAAACTCGGGAATGGTTCACAAAGTCTGGTGTGTATCTTTCACCATTTGCTTACGTGAAT
CACTCACACCCTGGT
+
FFFFFFFFFFFFFFFFFFFFFFFFFFFFFFFFFFFFFFFFFFFFFFFFFFFFFFFFFFFFFFFFFFFF
FFFFFFFFFFFFFFFFFFFFFFFFFFFFFFFFFFFFFFFFFFFFFFFFFFFFFFF:FFFFFFFFFFFF
FFFFFFFFFFFFFFF
@A00155:342:HHGFNDSXY:1:2525:20654:1094 2:N:0:GAACCTAG+TCCGCATA
AAGCGGATTTCAGTATGAACGCATTCAAAAGGAAGAAGAGAAGAAGGTGGAAAACTTTTCTTTTTACC
TACCAGAGAAAACTCGGGAATGGTTCACAAAGTCTGGTGTGTATCTTTCACCATTTGCTTACGTGAAT
CACTCACACCCTGGT
+
FF:FFFFF,FFFFFFFFFFFFFFFFFFFFFFFFFFFFFFFFFFFFFFF:FFFFFFFFFFFFFFFFFF:F
FFFFFFFFFFFFFFFFFFF:FFFFFFFFFFFFFFFFF,FFFFFFFFFFFFFFFFFFFFFFFFFFFFFF
FFFFFFFFFFF:FFF
@A00155:342:HHGFNDSXY:1:2665:11966:23813 2:N:0:GAACCTAG+TCCGCATA
AAGCGGATTTCAGTATGAACGCATTCAAAAGGAAGAAGAGAAGAAGGTGGAAAACTTTTCTTTTTACC
TACCAGAGAAAACTCGGGAATGGTTCACAAAGTCTGGTGTGTATCTTTCACCATTTGCTTACGTGAAT
CACTCACACCCTGGT
+
FFFFFFFFFFFFFFFFFFFFFFFFFFFFF:FFFFFFFF:FFF:FFFFF:FFFFFFFFFFFFFFFFFFF
FFFFFFFFFFFFFFFFFFFFFFF:FFFFFFFF:FFFFFFFFFFFF:FFFFFFFFFFFFFFFFFFFF:F
FFFFFFFFFFFFFFF
@A00155:342:HHGFNDSXY:1:2525:31421:8375 2:N:0:GAACCTAG+TCCGCATA
AGCGGATTTCAGTATGAACGCATTCAAAAGGAAGAAGAGAAGAAGGTGGAAAACTTTTCTTTTTACCT
ACCAGAGAAAACTCGGGAATGGTTCACAAAGTCTGGTGTGTATCTTTCACCATTTGCTTACGTGAATC
ACTCACACCCTGGT
+
FFFFFF:FFFFFFFFFF:FFFF:,FF,FFFFFFFFFFFFF,FFFFFFFFFFFFFFF,:FFFFFFFFFF
FFFFFFFFFFFFFFFFFF:FFFFFFFFFFFFFFFFFFFFFFFFFFF,FFFFFF::FFFFFF:FF:FF::F
FFFFFFFF:FFFFF
@A00155:342:HHGFNDSXY:1:2602:29957:25347 2:N:0:GAACCTAG+TCCGCATA
AGCGGATTTCAGTATGAACGCATTCAAAAGGAAGAAGAGAAGAAGGTGGAAAACTTTTCTTTTTACCT

ACCAGAGAAAACTCGGGAATGGTTCACAAAGTCTGGTGTGTATCTTTCACCATTTGCTTACGTGAATC
ACTCACAGATCGGA
+
FFFFFFFFFFFFFFFFFFFFFFFFFFFFFFFFFFFFFFFFFFFFFFFFFFFF:FFFFFFFFFFFFFFFFF
FFF:FFFFFFFFFFFFFFF:FFFFFFFFFFFFFFFFFFFFFFFFFFFFFFFFFFFFFFFFFFFFFFFFFF
FFFFFFFFFFFFFF
@A00155:342:HHGFNDSXY:1:1416:11089:31187 1:N:0:GAACCTAG+TCCGCATA
AGCGGATTTCAGTATGAACGCATTCAAAAGGAAGAAGAGAAGAAGGTGGAAAACTTTTCTTTTTACCT
ACCAGAGAAAACTCGGGAATGGTTCACAAAGTCTGGTGTGTATCTTTCACCATTTGCTTACGTGAATC
ACTCACACCCTGGTT
+
FFFFFFFFFFFFFFFFFFFFFFFFFFFF:FFFFFFFFFFFFFFFFFFFFFFFFFFFFFFFFFFFFFFFFF
:FFFFFFFFFFFFFFFFFFFFFFFFFFFFFFFFFFFFFFFFFFFFFFFFFFFFFFFFFF,FFFFFFFFFF
FFFFFFFFFFFFFFF
@A00155:342:HHGFNDSXY:1:1407:21224:22404 1:N:0:GAACCTAG+TCCGCATA
AGCGGATTTCAGTATGAACGCATTCAAAAGGAAGAAGAGAAGAAGGTGGAAAACTTTTCTTTTTACCT
ACCAGAGAAAACTCGGGAATGGTTCACAAAGTCTGGTGTGTATCTTTCACCATTTGCTTACGTGAATC
ACTCACACCCTGGTT
+
FFFFFFFFFFFFFFFFFFFFFFFFFFFFFFFFFFFFFFFFFFFFFFFFFFFFFFFFFFFFFFFFFFFFFF
FFFFFFFFFFFFFFFFFFFFFFFFFFFFFFFFFFFFFFFFFFFFFFFFFFFFFFFFFFFFFFFFFFFFFF
FFFFFFFFFFFFFFF
@A00155:342:HHGFNDSXY:1:1102:9191:3912 2:N:0:GAACCTAG+TCCGCATA
AGCGGATTTCAGTATGAACGCATTCAAAAGGAAGAAGAGAAGAAGGTGGAAAACTTTTCTTTTTACCT
ACCAGAGAAAACTCGGGAATGGTTCACAATGTCTGGTGTGTATCTTTCACCATTTGCTTACGTGAATC
ACTCACACCCTGGTT
+
F,FFFFFFFFFFFFFFFFFFFFFFFFFFFF:FFFFFFFFF:FFF:F,FFFFFFFF:FFFFFFFFFFFFFF
FFFFFFFFFFFFFF,FFFFFFFFFFFFFFF,:FFF:FFFFFFFFF,:FFFF,FFFFFFFFFFF:FFFFFF
FFFFFFFF:FF:FFF
@A00155:342:HHGFNDSXY:1:1236:7609:29450 2:N:0:GAACCTAG+TCCGCATA
AGCGGATTTCAGTATGAACGCATTCAAAAGGAAGAAGAGAAGAAGGTGGAAAACTTTTCTTTTTACCT
ACCAGAGAAAACTCGGGAATGGTTCACAAAGTCTGGTGTGTATCTTTCACCATTTGCTTACGTGAATC
ACTCACACCCTGGTT
+
FFFFFFFFFFFFFFFFFFFFFFFF,:FFFFFFFFFFFFFFFF:FFFFFFFFFFFFFFFFFFFFFFFFFFF
FFFFFFFFFFFFFFFFFFFFFFFFFFFFFFFFFFFFFFFFFFFFFFFFFFFF:FFF:FFFFFFFFFFFFF
FFFFFFFFFFFFFFF
@A00155:342:HHGFNDSXY:1:1252:12111:31829 2:N:0:GAACCTAG+TCCGCATA
AGCGGATTTCAGTATGAACGCATTCAAAAGGAAGAAGAGAAGAAGGTGGAAAACTTTTCTTTTTACCT
ACCAGAGAAAACTCGGGAATGGTTCACAAAGTCTGGTGTGTATCTTTCACCATTTGCTTACGTGAATC
ACTCACACCCTGGTT
+
FFFFFFFFFFFFFFFFFFFFFFFFFFFFFFFFFFFFFFFFFFFFFFFF:FFFFFFFFFFFFFFFFFFFFF
FFFFFFFFFFFFFFFFFFF:FFFFFFFFFFFFFFFFF:FFFFFFFFFFFFFFF:FFFFFFFFFFFFFFFF
FFFFFFFFFFFFFF:
@A00155:342:HHGFNDSXY:1:1407:31096:10097 2:N:0:GAACCTAG+TCCGCATA
AGCGGATTTCAGTATGAACGCATTCAAAAGGAAGAAGAGAAGAAGGTGGAAAACTTTTCTTTTTACCT
ACCAGAGAAAACTCGGGAATGGTTCACAAAGTCTGGTGTGTATCTTTCACCATTTGCTTACGTGAATC
ACTCACACCCTGGTT
+
FFFFFF:FFFFF:FFFFFFFFFFFFFFFFFFFFF,FFFFFFFFFFFFFFFFFFFFFFFFFFFFFF,FFFF
FF:FFFFFFFFFFFFFFF,FFFFFFFFFFFFFFFFFFFFF:FFFFFF:FFFFF:FFFFFFFFFF:FFFFF
:FFFFFFFFFFFFFF

```
@A00155:342:HHGFNDSXY:1:1373:27905:17096 2:N:0:GAACCTAG+TCCGCATA
AGCGGATTTCAGTATGAACGCATTCAAAAGGAAGAAGAGAAGAAGGTGGAAAACTTTTCTTTTTACCT
ACCAGAGAAAACTCGGGAATGGTTCACAAAGTCTGGTGTGTATCTTTCACCATTTGCTTACGTGAATC
ACTCACACCCTGGTT
+
FFFFFFFFFFFFFFFFF:FFFFFFFFFFFFFFFFFFFFFFFFFFFFFFF:FFFF:FFFFFFFFFFFFF
FFFFFFFFF:F:FFFFFFFFFFFFFF:FFFFFFFFFFFFFFFFF:,FFFFFF,FFFFFF,FF,FFFFF
:FFFFFFFFFFFFFF
@A00155:342:HHGFNDSXY:1:1422:8214:12806 2:N:0:GAACCTAG+TCCGCATA
AGCGGATTTCAGTATGAACGCATTCAAAAGGAAGAAGAGAAGAAGGTGGAAAACTTTTCTTTTTACCT
ACCAGAGAAAACTCGGGAATGGTTCACAAAGTCTGGTGTGTATCTTTCACCATTTGCTTACGTGAATC
ACTCACACCCTGGTT
+
FFFFFFF:FF:FFFFFFFFFFFFFFFFFFFFFFFFFFFFFFFFFFFFFFFFFFFF:FFFFFFFFFFFF
FFFFFFFF,F,:FFFFFFFFFFFFFFFFFFFFFFFFFF:FFFFFFFFFFFFFFFFFFFFFFFFFFFFF
FFFFFFFFFFFFF:F
@A00155:342:HHGFNDSXY:1:1437:6460:33160 2:N:0:GAACCTAG+TCCGCATA
AGCGGATTTCAGTATGAACGCATTCAAAAGGAAGAAGAGAAGAAGGTGGAAAACTTTTCTTTTTACCT
ACCAGAGAAAACTCGGGAATGGTTCACAAAGTCTGGTGTGTATCTTTCACCATTTGCTTACGTGAATC
ACTCACACCCTGGTT
+
FFFFFFFFFFFFFFFFFFFFFFFFFFFFFFFFFFFFFFFFFFFFFFFFFFFFFFFFFFFFFFFFFFFF
FFFFFFFFFFFFFFFFFFFFFFFFFFFFFFFFFFFFFFFFFFFFFFFFFFFFFFFFFFF:FFFFFFFF
FFFFFFFF:FFFFFF
@A00155:342:HHGFNDSXY:1:1574:8115:28917 2:N:0:GAACCTAG+TCCGCATA
AGCGGATTTCAGTATGAACGCATTCAAAAGGAAGAAGAGAAGAAGGTGGAAAACTTTTCTTTTTACCT
ACCAGAGAAAACTCGGGAATGGTTCACAAAGTCTGGTGTGTATCTTTCACCATTTGCTTACGTGAATC
ACTCACACCCTGGTT
+
FFFFFFFFFFFFFFFFFFFFFFFFFFFFFFFFFFFFFFFFFFFF:FFFFFFFFFFFFFFFFFFFFFFF
FFFFFFFFFFFFFFFFFFFFFFFFFFFF:FFFFFFFFF:FFFFFFFFFFFFFFFFFFFFFFFFFFFFF
FFFFFFFFFFFFFFF
@A00155:342:HHGFNDSXY:1:1628:20338:36182 2:N:0:GAACCTAG+TCCGCATA
AGCGGATTTCAGTATGAACGCATTCAAAAGGAAGAAGAGAAGAAGGTGGAAAACTTTTCTTTTTACCT
ACCAGAGAAAACTCGGGAATGGTTCACAAAGTCTGGTGTGTATCTTTCACCATTTGCTTACGTGAATC
ACTCACACCCTGGTT
+
FFFFFFFFFFFFFFFFFFFFFFFFFFFFFFFFFFFFFFFFFFFFFFFFFFFFFFFFFFFFFFFFFFFF
FFFFFFFFFFFFFFFFFFFFFFFFFFFFFFFFFFFFFFFFFFFFFFFFFFFFFFFFFFFFFFFFFFFF
FFFFFFFFFFFFFFF
@A00155:342:HHGFNDSXY:1:2121:22959:17456 2:N:0:GAACCTAG+TCCGCATA
AGCGGATTTCAGTATGAACGCATTCAAAAGGAAGAAGAGAAGAAGGTGGAAAACTTTTCTTTTTACCT
ACCAGAGAAAACTCGGGAATGGTTCACAAAGTCTGGTGTGTATCTTTCACCATTTGCTTACGTGAATC
ACTCACACCCTGGTT
+
FFFFFFFFFFFFFFFFFFFFFFFFFFFFFFFFFFFFFFFFFFFFFFFFFFFFFFFFFFFFFFFFFFFF
FFFFFFFFFFFFFFFFFFFFFFFFFFFFF:FFFFFFFFFFFFFFFFFFFFFFFFFFFFFFFFFFFFFF
FFFFFFF,:FFF,FF
@A00155:342:HHGFNDSXY:1:2164:16568:4288 2:N:0:GAACCTAG+TCCGCATA
AGCGGATTTCAGTATGAACGCATTCAAAAGGAAGAAGAGAAGAAGGTGGAAAACTTTTCTTTTTACCT
ACCAGAGAAAACTCGGGAATGGTTCACAAAGTCTGGTGTGTATCTTTCACCATTTGCTTACGTGAATC
ACTCACACCCTGGTT
+
FFFFFFFFFFFFFFFFFFFFFFFFFFFFFFFFFFFFFFFFFFFFFFFFFFFFFFFFFFFFFFFFFFFF
```

FFFFFFFFFFFFFFFFFFFFFFFFFFFFFFFFFFFFFFFFFFFFFFFFFFFFFFFFFFFFFFFFFFFF
FFFFFFFFFFFFFFF
@A00155:342:HHGFNDSXY:1:2165:10113:7827 2:N:0:GAACCTAG+TCCGCATA
AGCGGATTTCAGTATGAACGCATTCAAAAGGAAGAAGAGAAGAAGGTGGAAAACTTTTCTTTTTACCT
ACCAGAGAAAACTCGGGAATGGTTCACAAAGTCTGGTGTGTATCTTTCACCATTTGCTTACGTGAATC
ACTCACACAGATCGG
+
FFFFFFFFFFFFFFFFFFFFFFFFFFFFFFFFFFFFFFFFFFFFFFFFFFFFFFFFFFFFFFFFFFFF
FFF:FFFFFFFFFFFFF:FFFFFFFFFFFFFFFFFFFFFFFFFFFFFFFF:FFFFFFFFFFFFFFFFF
FFFFFFFFFFFFF,F
@A00155:342:HHGFNDSXY:1:2173:7618:31720 2:N:0:GAACCTAG+TCCGCATA
AGCGGATTTCAGTATGAACGCATTCAAAAGGAAGAAGAGAAGAAGGTGGAAAACTTTTCTTTTTACCT
ACCAGAGAAAACTCGGGAATGGTTCACAAAGTCTGGTGTGTATCTTTCACCATTTGCTTACGTGAATC
ACTCACACCCTGGTT
+
FFFFFFFFFFFFFFFFFFFFFFFFFFFF:FFFFFFFFFFFFFFFFFFFFFFFFFFFFFFFFFFFFFFF
FFFFFFFFFFFFFFFFFFFFFFFFFFFFFFFFFFFFFFFFFFFFFFFFFFFFFFFFFFFFFFFFFFFF
FFFFFFFFFFFFFFF
@A00155:342:HHGFNDSXY:1:2246:20256:16470 2:N:0:GAACCTAG+TCCGCATA
AGCGGATTTCAGTATGAACGCATTCAAAAGGAAGAAGAGAAGAAGGTGGAAAACTTTTCTTTTTACCT
ACCAGAGAAAACTCGGGAATGGTTCACAAAGTCTGGTGTGTATCTTTCACCATTTGCTTACGTGAATC
ACTCACACCCTGGTT
+
FFFFFFFFFF:FFFFFFFFFFFFFFFFFFFFFFFFFFFFFFFFFFFFFFFFFFFFFFFFFFFFFFFF
FFF:FFFF,FFFFFFFFF:FF:FFFFFFFFFFFFFFFFFFFFFFFFFFFFFFFFFFFFFFFFFFFFFF
FFFFFFFFFFFFF:F
@A00155:342:HHGFNDSXY:1:2336:19434:24314 2:N:0:GAACCTAG+TCCGCATA
AGTGGATTTCAGTATGAACGCATTCAAAAGGAAGAAGAGAAGAAGGTGGAAAACTTTTCTTTTTACCT
ACCAGAGAAAACTCGGGAATGGTTCACAAAGTCTGGTGTGTATCTTTCACCATTTGCTTACGTGAATC
ACTCACACCCTGGTT
+
F::FFFFFFFFFFFFFFFFFFFF:FFFFFFFFFFFFFFFFFFFFFFFFFFFFFFFFFFFFFFFFFFFF
FFFFFF:FFFFFFFFFFFFFFFFFFFFFF,FF,:FFFF:FFFFFFFFFFF:FFFFFFFFFF,FFFFF:
FF:FFF::FFFFFFF
@A00155:342:HHGFNDSXY:1:2340:10637:17190 2:N:0:GAACCTAG+TCCGCATA
AGCGGATTTCAGTATGAACGCATTCAAAAGGAAGAAGAGAAGAAGGTGGAAAACTTTTCTTTTTACCT
ACCAGAGAAAACTCGGGAATGGTTCACAAAGTCTGGTGTGTATCTTTCACCATTTGCTTACGTGAATC
ACTCACACCCTGGTT
+
FFFFFFFFFFFFFFFFFFFFFFFFFFFFFFFFFFFFFFFFFFFFFFFFFFFFFFFFFFFFFFFFFFFF
FFFFFFFFFFFFFFFFFFFFFFFFFFFFFFFFFFFFFFFFFFFFFFFFFFFFFFFFFFFFFFFFFFFF
FFFFFFFFFFFFFFF
@A00155:342:HHGFNDSXY:1:2617:4381:26741 2:N:0:GAACCTAG+TCCGCATA
AGCGGATTTCAGTATGAACGCATTCAAAAGGAAGAAGAGAAGAAGGTGGAAAACTTTTCTTTTTACCT
ACCAGAGAAAACTCGGGAATGGTTCACAAAGTCTGGTGTGTATCTTTCACCATTTGCTTACGTGAATC
ACTCACACCCTGGTT
+
:FFFFFFFFFFFFFFFFFFFFFFFFFFFFFFFFF:FFFFFFFFFFFFFFFFFFFFFFFFFFFFFFFFF
FFFFFFFFFFFFFFFFFFFFFFFFFFFFFFFFFFFFFFFFFFFFFFFFFFFFFFFFFFFFFFFFFFFF
FFFFFFFF:FFFFFF
@A00155:342:HHGFNDSXY:1:1112:30590:5807 2:N:0:GAACCTAG+TCCGCATA
GCGGATTTCAGTATGAACGCATTCAAAAGGAAGAAGAGAAGAAGGTGGAAAACTTTTCTTTTTACCTA
CCAGAGAAAACTCGGGAATGGTTCACAAAGTCTGGTGTGTATCTTTCACCATTTGCTTACGTGAATCA
CTCACACCCTGGTT

+
F:FFF,F:FFFFFFF:FFFFF:FFFFF:FFFF:F,FFFFFFFFFF:FFFFFFFFFFFFF:FFFFFFFF
FFFF,FFFFFFFF:FF:FFF:FFFFFFFFFFFFFFFFFFF:FFF,FFFFFFFFFFFFFFFFFFFF,FF
FFFFFFFFFFFFFFF
@A00155:342:HHGFNDSXY:1:1115:32560:28823 2:N:0:GAACCTAG+TCCGCATA
GCGGATTTCAGTATGAACGCATTCAAAAGGAAGAAGAGAAGAAGGTGGAAAACTTTTCTTTTTACCTA
CCAGAGAAAACTCGGGAATGGTTCACAAAGTCTGGTGTGTATCTTTCACCATTTGCTTACGTGAATCA
CTCACACCCTGGTTG
+
FFFFF,FFFFFFFFFF,FFFFFFFF,FFFF:FFFFFFFF,FF,FF:FFF:FFFFFFFFFFFFF,FFFF
FFFFFFFFFFFFFFFFF::FFFFFFF:,FFFFFFFF:FF:FFFFFFFFFFF,FFFFFFFF:::F,FFF
F,FFFFFFF:FFFFF
@A00155:342:HHGFNDSXY:1:1149:32461:13182 2:N:0:GAACCTAG+TCCGCATA
GCGGATTTCAGTATGAACGCATTCAAAAGGAAGAAGAGAAGAAGGTGGAAAACTTTTCTTTTTACCTA
CCAGAGAAAACTCGGGAATGGTTCACAAAGTCTGGTGTGTATCTTTCACCATTTGCTTACGTGAATCA
CTCACACCCTGAGAT
+
FFFFFFFFFF:FFFFFFFFFFFFFFFFFFFFFFFFFFFFFFFFFFFFFFFFFFFFFFFFFF:FFFFFF
FFFFFFFFFFFFFFFFFFFFFFFFFFFFFFFFFFFFFFFFFFFFFFFFFFFFFFFFFFFFFFFFFFFF
FFFFFFFFFFFFFFF
@A00155:342:HHGFNDSXY:1:1149:32425:13150 2:N:0:GAACCTAG+TCCGCATA
GCGGATTTCAGTATGAACGCATTCAAAAGGAAGAAGAGAAGAAGGTGGAAAACTTTTCTTTTTACCTA
CCAGAGAAAACTCGGGAATGGTTCACAAAGTCTGGTGTGTATCTTTCACCATTTGCTTACGTGAATCA
CTCACACCCTGAGAT
+
F:F:,:F:FFFFFFFFFFFFFFFFFFFFFFFFFFFFFFFFFFFFFFFFFFFFFFF:FFFFFFFFFFFF
FFFFFFFFFFFFFFFFF:FFFFFFFFFFFF:FFFFFFFF:FFFF,FFFFFFFFFFFFFFFFFFFFFFF
FFFFFFFFFFFFFFF
@A00155:342:HHGFNDSXY:1:1242:27606:9471 2:N:0:GAACCTAG+TCCGCATA
GCGGATTTCAGTATGAACGCATTCAAAAGGAAGAAGAGAAGAAGGTGGAAAACTTTTCTTTTTACCTA
CCAGAGAAAACTCGGGAATGGTTCACAAAGTCTGGTGTGTATCTTTCACCATTTGCTTACGTGAATCA
CTCACACCCTGGTTG
+
FFFFFFFFFFFF:FFFFFFFFFFFFFFFFFFFFFFFFFF:FFFFFFFFFFFFFFFFFFFFFFFFFFFF
FFFFFFFFFFFFFFFFF:FFFFFFFFFF:FFFFFFFFFFFFFFFFFFFFFF:FFFFFFFFFFFFFFFF
FFFFFFFFFFFFFFF
@A00155:342:HHGFNDSXY:1:1238:9127:15953 2:N:0:GAACCTAG+TCCGCATA
GCGTATTTCAGTATGAACGCATTCAAAAGGAAGAAGAGAAGAAGGTGGAAAACTTTTCTTTTTACCTA
CCAGAGAAAACTCGGGAATGGTTCACAAAGTCTGGTGTGTATCTTTCACCATTTGCTTACGTGAATCA
CTCACACCCTGGTTG
+
FFFFFFFFFFFFFFFFFFFFFFFFFFFFFFFFFFFFFFFFFFFFFFFFFFFFFFFFFFFFFFFFFFFF
FFFFFFFFFFFFFFFFFFFFFFFFFFFF:FFFFFFFFFFFFFFFFFFFFFFFFFFFFFFFFFFFFFFF
FFFFFFFFFFFFFFF
@A00155:342:HHGFNDSXY:1:1306:23692:4758 2:N:0:GAACCTAG+TCCGCATA
GCGGATTTCAGTATGAACGCATTCAAAAGGAAGAAGAGAAGAAGGTGGAAAACTTTTCTTTTTACCTA
CCAGAGAAAACTCGGGAATGGTTCACAAAGTCTGGTGTGTATCTTTCACCATTTGCTTACGTGAATCA
CTCACACCCTGGTTG
+
FFFFFFFFFFFFFFFFFFFFFFFFFFFFFFFFFFFFFFFFFFFFFFFFFFFFFFFFFFFFFFFFFFFF
:FFFFFFFFFFFFFFFFFFFFFFFFFFFFFFFFFFF:FFFFFFFFFFFFFFFFF:FFFFFFFFFFFFF
FFFFFFFFFFFFFFF
@A00155:342:HHGFNDSXY:1:1407:23411:22811 2:N:0:GAACCTAG+TCCGCATA
GCGGATTTCAGTATGAACGCATTCAAAAGGAAGAAGAGAAGAAGGTGGAAAACTTTTCTTTTTACCTA

```
CCAGAGAAAACTCGGGAATGGTTCACAAAGTCTGGTGTGTATCTTTCACCATTTGCTTACGTGAATCA
CTCACACCCTGAGAT
+
FFFFFFFFFFFFFFFFFFFFFFFFFFFFFFFFFFFF:,F,FFFFFFFFFFFFFF:FFFFFFFFFF
FFFFFFFFFFFFFFFFFFFFFFFFFFF:FFFFF:F,FFFFFFFFFFFFF:FFFFF:FFFFFFFFFFF
FFFFFFFFFFFFFFF
@A00155:342:HHGFNDSXY:1:2104:18548:11256 2:N:0:GAACCTAG+TCCGCATA
GCGTATTTCAGTATGAACGCATTCAAAAGGAAGAAGAGAAGAAGGTGGAAAACTTTTCTTTTTACCTA
CCAGAGAAAACTCGGGAATGGTTCACAAAGTCTGGTGTGTATCTTTCACCATTTGCTTACGTGAATCA
CTCACACCCTGGTTG
+
F:FFFFFFFFFFFFFFFFFFFFFFFFFFFFFFFFFFFFFFFFFFFFFFFFFFFFFFFFFFFFFFFFFF
FFFFFFFFFFFFFFFFF:FFFFFFFFFFFFFFFFFFFFFFFFFFFFFFFFFFFFFFFFFFFFFFFFF
FFFFFFF:FFFFFFF
@A00155:342:HHGFNDSXY:1:2120:2582:30044 2:N:0:GAACCTAG+TCCGCATA
GCGGATTTCAGTATGAACGCATTCAAAAGGAAGAAGAGAAGAAGGTGGAAAACTTTTCTTTTTACCTA
CCAGAGAAAACTCGGGAATGGTTCACAAAGTCTGGTGTGTATCTTTCACCATTTGCTTACGTGAATCA
CTCACACCCTGGTTG
+
FFFFFFFFFFFFFFFFFFFFFFFFFFFFFFFFFFFFFFFFFFFFFFFFFFFFFFF:FFF:FF,FF:FF
F:FFFFFFFFFFFFFFFFFFFFFFFFFFFF::FFF,FF::FF:FFFFFFFFFFF:FF:FF:FFF,FFF
FFFFFFFFF,FFFFF
@A00155:342:HHGFNDSXY:1:2204:16568:8015 2:N:0:GAACCTAG+TCCGCATA
GTGGATTTCAGTATGAACGCATTCAAAAGGAAGAAGAGAAGAAGGTGGAAAACTTTTCTTTTTACCTA
CCAGAGAAAACTCGGGAATGGTTCACAAAGTCTGGTGTGTATCTTTCACCATTTGCTTACGTGAATCA
CTCACACCCTGGTTG
+
FFFFFFFFFFFFFFFFFFFFFFFFFFFFFFFFFFFFFFFFFFFFFFFFFFFFFFFFFFFFFFFFFFFF
FFFFFFFFFFFFFFFFFFFFFFF:FFFFFFFFFFFFFFFFFFFFFFFFFFFFFFFFFFFFFFFFFFFF
FFFFFFFFFFFF:FF
@A00155:342:HHGFNDSXY:1:2344:4743:20979 2:N:0:GAACCTAG+TCCGCATA
GCGGATTTCAGTATGAACGCATTCAAAAGGAAGAAGAGAAGAAGGTGGAAAACTTTTCTTTTTACCTA
CCAGAGAAAACTCGGGAATGGTTCACAAAGTCTGGTGTGTATCTTTCACCATTTGCTTACGTGAATCA
CTCACACCCTGGAGA
+
FFFFFFFFFFFFFFFFFFFFFFFFFFFFFFFFFFFF,FFFFFFFFFFFFFFFFFFFFFFFFFFFFFFF
FFFFFFFFFFFFFFFFF:FFFFFFFFFFFFFFFFFFFFFFFFFFFFFFF:FFFF:FFF:FFFFFFFFFFF
:FFFFF:FFFFFFFF
@A00155:342:HHGFNDSXY:1:2516:12120:13996 2:N:0:GAACCTAG+TCCGCATA
GCGGATTTCAGTATGAACGCATTCAAAAGGAAGAAGAGAAGAAGGTGGAAAACTTTTCTTTTTACCTA
CCAGAGAAAACTCGGGAATGGTTCACAAAGTCTGGTGTGTATCTTTCACCATTTGCTTACGTGAATCA
CTCACACCCTGGAGA
+
FFFFFFFFFFFFFFFFFFFFFFFFFFF:FFFFFFFFFFFFFFFFFFFFFFFFFFFFFFFFFFFFFFFF
FFFFFFFFFFFFFFFFFF,FFFFFFFFFFFFFFFF:FFFFFFFFFFF:FF,FFFFFFFFFFFFFFFFF
FFFFFFFFFFFFFFF
@A00155:342:HHGFNDSXY:1:2516:12192:14058 2:N:0:GAACCTAG+TCCGCATA
GCGGATTTCAGTATGAACGCATTCAAAAGGAAGAAGAGAAGAAGGTGGAAAACTTTTCTTTTTACCTA
CCAGAGAAAACTCGGGAATGGTTCACAAAGTCTGGTGTGTATCTTTCACCATTTGCTTACGTGAATCA
CTCACACCCTGGAGA
+
FFFFFFFFFFFFFFFFFFFFFFFFFFFFFFFFFFFFFFFFFFFFFFFFFFFFFFFFFFFFFFFFFFFF
FFFFFFFFFFFFFFFFF:FFFFFFFFFFFFFFFFFFFFFFFFFFFFFFFFFFFFFFFFFFFFFFFFF
FFFFFFFFFFFFFFF
```

```
@A00155:342:HHGFNDSXY:1:2516:12201:14074 2:N:0:GAACCTAG+TCCGCATA
GCGGATTTCAGTATGAACGCATTCAAAAGGAAGAAGAGAAGAAGGTGGAAAACTTTTCTTTTTACCTA
CCAGAGAAAACTCGGGAATGGTTCACAAAGTCTGGTGTGTATCTTTCACCATTTGCTTACGTGAATCA
CTCACACCCTGGAGA
+
FFFFFFFFFFFFFFFFFFFFFFFFFFF:FFFFFFFFFFFFFFFFFFFFFFFFFFFFFFFFFFFFFFFF
FFFFFFFFFFFFFFFFFFFFFFFFFFFFFFFFFFFFFFFFFFFFFFFFFFFFFFFFFFFFFFFFFFFF
FFFFFFFFFFFFFFF
@A00155:342:HHGFNDSXY:1:2672:31882:5760 2:N:0:GAACCTAG+TCCGCATA
GCGTATTTCAGTATGAACGCATTCAAAAGGAAGAAGAGAAGAAGGTGGAAAACTTTTCTTTTTACCTA
CCAGAGAAAACTCGGGAATGGTTCACAAAGTCTGGTGTGTATCTTTCACCATTTGCTTACGTGAATCA
CTCACACCCTGGTTG
+
FFFFFFFFFFFFF:FFFFFFFFFFFFFFFFFFFFFFFFFFFFFFFFFFFFFFFFFFFFFFFFFFFFFF
FFFFFFFFFFFFFFFFF:FFFFFFFFFFFFFFFFFFFFFFFFFFFFF:FFFFFFF:FFFFF:FFFFFFFFF
FFFFFFFFFFFFFFF
@A00155:342:HHGFNDSXY:1:2445:6605:17409 2:N:0:GAACCTAG+TCCGCATA
CGGATTTCAGTATGAACGCATTCAAAAGGAAGAAGAGAAGAAGGTGGAAAACTTTTCTTTTTACCTAC
CAGAGAAAACTCGGGAATGGTTCACAAAGTCTGGTGTGTATCTTTCACCATTTGCTTACGTGAATCAC
TCACACCCTGGTTG
+
FFFFFFFFFFFFFFFFFFFFFFFFFFFFFFFFF:FFFFFFFF,FFF,FFFFFFFFF:FFFFFFFFFF:F
FF,FFFFFFFFFF:FFFFF:FFFFFFF:FFFFFFFFFFFFFFFF,FFFFFFFFFFFFFFFFFFFFF,F
F:FFFFFFFF::FF
@A00155:342:HHGFNDSXY:1:1149:11198:28056 2:N:0:GAACCTAG+TCCGCATA
CGGATTTCAGTATGAACGCATTCAAAAGGAAGAAGAGAAGAAGGTGGAAAACTTTTCTTTTTACCTAC
CAGAGAAAACTCGGGAATGGTTCACAAAGTCTGGTGTGTATCTTTCACCATTTGCTTACGTGAATCAC
TCACACCCTGGTTGT
+
FFFF,FFFFFFFFFFFFFFFFFFFFFFFFFFFFFFFFFFFFFFFFFFFFFFFFFFFFFFFFFFFFFFF
FFFFFFFFFFFFFFF,FFFFFFFFFF:FFFFFFFFFFFFFFFFFFFFFFFFFFFFFFFFFFFFFFFFF
FFFFFF:FFFFFFFF
@A00155:342:HHGFNDSXY:1:2520:18403:12978 2:N:0:GAACCTAG+TCCGCATA
GGATTTCAGTATGAACGCATTCAAAAGGAAGAAGAGAAGAAGGTGGAAAACTTTTCTTTTTACCTACC
AGAGAAAACTCGGGAATGGTTCACAAAGTCTGGTGTGTATCTTTCACCATTTGCTTACGTGAATCACT
CACACCCTGGTTGTA
+
FFFFFFFFFFFFFFFFFFFFFFFFFF:FFFFFFFFFFFFFFFFFFFFFFFFFFFFFFFFFFFFFFFF:FF
FFFFFFFFFFFFF:FFFFFFFFFFFF:FFFFFFFF:F:FFFFFFFFFFFFFFFFFFFFFFFFF:FFFFF
FFFFFFFFFFFFFFF
@A00155:342:HHGFNDSXY:1:2526:4182:35509 2:N:0:GAACCTAG+TCCGCATA
GATTTCAGTATGAACGCATTCAAAAGGAAGAAGAGAAGAAGGTGGAAAACTTTTCTTTTTACCTACCA
GAGAAAACTCGGGAATGGTTCACAAAGTCTGGTGTGTATCTTTCACCATTTGCTTACGTGAATCACTC
ACACCCTGGTTGTAA
+
FFFFFFFFFFFFFFFFFFFFFFFFFFFFFFFFFFFFFFFFFFFFFFFFFFFFFFFFFFFFFFFFFFFF
FFFFFFFFFFFFFF:FFFFFFFFFFFFFFFFFFFFFFFFF:FF:FFFFFF,FFFFFFFFFFFF,FFFFFF
FFFFFF:FFFFFFFF
@A00155:342:HHGFNDSXY:1:2526:4264:35681 2:N:0:GAACCTAG+TCCGCATA
GATTTCAGTATGAACGCATTCAAAAGGAAGAAGAGAAGAAGGTGGAAAACTTTTCTTTTTACCTACCA
GAGAAAACTCGGGAATGGTTCACAAAGTCTGGTGTGTATCTTTCACCATTTGCTTACGTGAATCACTC
ACACCCTGGTTGTAA
+
FF:FFFFFFFFFFFFFFFFFFFFFFFFFFFFFFFFFFFFFFFFFFFFFFFFFFFFFFFFFFFFFFFFF
```

```
FFFFFFFFFFFFFF:FFFFFFFFFFFFFFFFFFF,FFFFFF:FFFF:FFFF:FF,F:FFFFFFFFFFF
FFFFFFFFFFFFFFF
@A00155:342:HHGFNDSXY:1:2351:23791:22780 2:N:0:GAACCTAG+TCCGCATA
CAGTATGAACGCATTCAAAAGGAAGAAGAGAAGAAGGTGGAAAACTTTTCTTTTTACCTACCAGAGAA
AACTCGGGAATGGTTCACAAAGTCTGGTGTGTATCTTTCACCATGTGCTTACGTGAATCACTCACACC
CTGGTTGTAAAACT
+
FFFFFFFFFFFFFFFFFFFFFFFFFFFFFF:FFFFFF:FFFFFFFFFFFF:FFFFFF:,F,:FFFFFF
FFFFFFF:F:FFFFFFFFFFFFFFFFFFF:FFFF,FFFFFFFF,,FFFFFFFFFFFFF:FFFF:FFFF
FFFFFFFFFFFFFF
@A00155:342:HHGFNDSXY:1:1139:27064:14794 2:N:0:GAACCTAG+TCCGCATA
CAGTATGAACGCATTCAAAAGGAAGAAGAGAAGAAGGTGGAAAACTTTTCTTTTTACCTACCAGAGAA
AACTCGGGAATGGTTCACAAAGTCTGGTGTGTATCTTTCACCATTTGCTTACGTGAATCACTCACACC
CTGGTTGTAAAACTC
+
FFFFFFFFFFFFFFFFFFFFFFFFFFFFFFFFFFFFFFFFFFFFFFFFFFFFFFFFFFFFFFFFFFFF
FFFFFFFFFFFF:FFFFFFFFFFFFFFFFFFFFFFFFFFFFFFFFFFFFFFFFFFFFFFFFFFFFFF:
::FFFFFFFFFFFFF
@A00155:342:HHGFNDSXY:1:1507:11632:10551 2:N:0:GAACCTAG+TCCGCATA
CAGTATGAACGCATTCAAAAGGAAGAAGAGAAGAAGGTGGAAAACTTTTCTTTTTACCTACCAGAGAA
AACTCGGGAATGGTTCACAAAGTCTGGTGTGTATCTTTCACCATTTGCTTACGTGAATCACTCACACC
CTGGTTGTAAAACTC
+
FF:FFFFFFFFFFFFFFFFF:FFFFFFF:FFFFFFFFFFFFFFFFFFFFFFFFFFFFFFFFFFFFFFF
FFFFFFFFF,FFFFFF,FFFFFFFFFFFFFFFFFFFFFFFFFFFFFFF:FFF:FFFFFFFFFFFFFFF
FFFFFFFFFFF:FFF
@A00155:342:HHGFNDSXY:1:2271:3586:27492 2:N:0:GAACCTAG+TCCGCATA
CAGTATGAACGCATTCAAAAGGAAGAAGAGAAGAAGGTGGAAAACTTTTCTTTTTACCTACCAGAGAA
AACTCGGGAATGGTTCACAAAGTCTGGTGTGTATCTTTCACCATTTGCTTACGTGAATCACTCACACC
CTGGTTGTAAAACTC
+
FFFFFF:FFFFFFFFFFFFFFFFFFFFFFFFFFFFFFFFFFFFFFFFFFFFFFFFFFFFFFFFFFFFF
FFFFFFFFFFFFFFFFFFFFFFFFF::FFFFFFFFFFFFFFFFFFFFFFFFFFFFFFFFFFFFFFFFF
FFFFFFFF,FFFFFF
@A00155:342:HHGFNDSXY:1:2351:24722:23171 2:N:0:GAACCTAG+TCCGCATA
CAGTATGAACGCATTCAAAAGGAAGAAGAGAAGAAGGTGGAAAACTTTTCTTTTTACCTACCAGAGAA
AACTCGGGAATGGTTCACAAAGTCTGGTGTGTATCTTTCACCATTTGCTTACGTGAATCACTCACACC
CTGGTTGTAAAACTC
+
FFFFFFFFFFFFFFFFFFFFFFF:FFFFFFFFFFFFFFFFFFFFFFFFFFFFFFFFFFFFFFFFFFFF
FFFFFFFFFFFFFFFFFFFFFFFFFFFFFFFFFFFFFFFFFFFFFFFFFFFFFFFFFFFFF:FFFFFF
FFFFFFFFFFFFFFF
@A00155:342:HHGFNDSXY:1:2627:8024:3928 2:N:0:GAACCTAG+TCCGCATA
CAGTATGAACGCATTCAAAAGGAAGAAGAGAAGAAGGTGGAAAACTTTTCTTTTTACCTACCAGAGAA
AACTCGGGAATGGTTCACAAAGTCTGGTGTGTATCTTTCACCATTTGCTTACGTGAATCACTCACACC
CTGGTTGTAAAACTC
+
FFFFFFFFFFFFFFFFFFF:FFFFFFFFFFFFFFFFFFFFFFFFFFFFFFFFFFFF:FFFFFFFFFFF
FFFFFFFFF,FFFFFFFFFFFFFFFFFFFFFFFFFFFFFFF:F:FFFFFFFFFFFFFFFFFFFFFFFF
FFFFFFFFFFFFFFF
@A00155:342:HHGFNDSXY:1:1271:20672:24424 2:N:0:GAACCTAG+TCCGCATA
AGTATGAACGCATTCAAAAGGAAGAAGAGAAGAAGGTGGAAAACTTTTCTTTTTACCTACCAGAGAAA
ACTCGGGAATGGTTCACAAAGTCTGGTGTGTATCTTTCACCATTTGCTTACGTGAATCACTCACACCC
TGGTTGTAAAACTCT
```

+
FFFFFFFFFFFFFFFFFFFFFFFFFFFFFFFFFFFFFFFFFFFFFFFFFFFFFFFFFF:FFFFFFFFF
FFFFFFFFFFFFFFFFF,,,FFFFFF,FFFFFFFFFFFFFF:FFF:FFFFFFFFFFFFFFFFFFFFFF
FFFFFFFFFFFFFFF
@A00155:342:HHGFNDSXY:1:1102:29243:4633 2:N:0:GAACCTAG+TCCGCATA
GTATGAACGCATTCAAAAGGAAGAAGAGAAGAAGGTGGAAAACTTTTCTTTTTACCTACCAGAGAAAA
CTCGGGAATGGTTCACAAAGTCTGGTGTGTATCTTTCACCATTTGCTTACGTGAATCACTCACACCCT
GGTTGTAAAACTCTG
+
FF:FFFFFFFFFFFFFFFFFFFFFFFFFFFFFFFFFFFFFFFFFFFFFFFFFFFFFFFFFFFFFFFFF
FFF:FF::FFFFFFFFFFFFF:FFF:FFFFFFFFFFFF:FFFFF:FFFFFFFFFFFFFFFFFFFFFFF
FFFFFFFFFFFFFFF
@A00155:342:HHGFNDSXY:1:1243:3613:17707 2:N:0:GAACCTAG+TCCGCATA
GTATGAACGCATTCAAAAGGAAGAAGAGAAGAAGGTGGAAAACTTTTCTTTTTACCTACCAGAGAAAA
CTCGGGAATGGTTCACAAAGTCTGGTGTGTATCTTTCACCATTTGCTTACGTGAATCACTCACACCCT
GGTTGTAAAACTCTG
+
FFFFF:FFFFFFFF:F:FFFFFFFFFFFFFFFFFFFFFFFFFFFFFFFF:FFFFFFFFFFFFFFFFFF
FFFFFFFFFFFFFFFFF:FFFFFFFFFFFFFFFFFFFFFFFFFFFFFFFFFFFFFFFFFFFFFFFFFF
FFFFFFFFFFFFFFF
@A00155:342:HHGFNDSXY:1:1228:26151:33442 2:N:0:GAACCTAG+TCCGCATA
GTATGAACGCATTCAAAAGGAAGAAGAGAAGAAGGTGGAAAACTTTTCTTTTTACCTACCAGAGAAAA
CTCGGGAATGGTTCACAAAGTCTGGTGTGTATCTTTCACCATTTGCTTACGTGAATCACTCACACCCT
GGTTGTAAAACTCTG
+
FFFFFFFFFFFFFFFFF:FFFFFFFFFFFFFFFFFFFFFFFF:,FFFFFFFFFFFFFFFFFFFFFFFF
FFFFFFFFFFFFFFFFFFFFFFFFFFFFFFFFFFFFFFFFFFFFFFF:FFFFFFFFFFFFFFFFFFFF
FFFFFF:FFFFFFFF
@A00155:342:HHGFNDSXY:1:1228:26992:34209 2:N:0:GAACCTAG+TCCGCATA
GTATGAACGCATTCAAAAGGAAGAAGAGAAGAAGGTGGAAAACTTTTCTTTTTACCTACCAGAGAAAA
CTCGGGAATGGTTCACAAAGTCTGGTGTGTATCTTTCACCATTTGCTTACGTGAATCACTCACACCCT
GGTTGTAAAACTCTG
+
FFFFFFFFFFFFFFFFF:FFF:FF:FFFFFFFFFFFFFFFFFFFFFFFFFFFFFFFFFFFFFFFFFFF
FFFFFFF,FFFFFFFFFFFFFFFFFFFFFFFFFFFFFFFFFFFFFF:FFFFFFFFFFFFFFFFFFFFF
FFFFFFFFFFFFFFF
@A00155:342:HHGFNDSXY:1:1229:21495:4304 2:N:0:GAACCTAG+TCCGCATA
GTATGAACGCATTCAAAAGGAAGAAGAGAAGAAGGTGGAAAACTTTTCTTTTTACCTACCAGAGAAAA
CTCGGGAATGGTTCACAAAGTCTGGTGTGTATCTTTCACCATTTGCTTACGTGAATCACTCACACCCT
GGTTGTAAAACTCTG
+
FFFFFFFFFF:FFFFFF:FFF:FFFFFFFFFFFFFFFFFFF,FFFFFFFFFFFFFFFFFFFFFFFFFF
FFFFFFF:FFFFFFFFFFFFFFFFFFFFFFFFFFFFFFFFFF:FFFFFFFFFFF:FFFFFFFFFFFFF
FFFFFFFF:F:FFFF
@A00155:342:HHGFNDSXY:1:1404:20518:32769 2:N:0:GAACCTAG+TCCGCATA
GTATGAACGCATTCAAAAGGAAGAAGAGAAGAAGGTGGAAAACTTTTCTTTTTACCTACCAGAGATAA
CTCGGGAATGGTTCACAAAGTCTGGTGTGTATCTTTCACCATTTGCTTACGTGAATCACTCACACCCT
GGTTGTAAAACTCTG
+
FF:FFFFFFFFF:F:FF:FFFFFFFFFFFFFFFFFFFFFFFFFFFFFFFFFFFFFFFFFFFFFFFFFF
FFFFFFFFFFFFFFFFFFFFFFFFFFFFFFFFFFFFFFFFFFFFFFFFFFFFF:F:FFFFFFFFFFFF
FF:FFFF:FFFFFFF
@A00155:342:HHGFNDSXY:1:1404:20627:32863 2:N:0:GAACCTAG+TCCGCATA
GTATGAACGCATTCAAAAGGAAGAAGAGAAGAAGGTGGAAAACTTTTCTTTTTACCTACCAGAGATAA

CTCGGGAATGGTTCACAAAGTCTGGTGTGTATCTTTCACCATTTGCTTACGTGAATCACTCACACCCT
GGTTGTAAAACTCTG
+
FFFFFFFFFFFFFFFF:FFFFFFFFFFFFFFFFFFFFFFFFFFFFFFFFFFFFFFFFFFFFFFFFFFF
FFFFFFFFFFFFFFFFFFFFFFF:FFFFFFFFFFFFFFFFF:FFFFFFFFFFFFFFFFFF:FFFFFFF
FFFFFFFFFFFFFFF
@A00155:342:HHGFNDSXY:1:1439:2510:30358 2:N:0:GAACCTAG+TCCGCATA
GTATGAACGCATTCAAAAGGAAGAAGAGAAGAAGGTGGAAAACTTTTCTTTTTACCTACCAGAGAAAA
CTCGGGAATGGTTCACAAAGTCTGGTGTGTATCTTTCACCATTTGCTTACGTGAATCACTCACACCCT
GGTTGTAAAACTCTG
+
FFFFFFFFFFFFFFFF:FFFFFFFFFFFFFFFFFFFFFFFFFFFFFFFFFFFFFFFFF:FFFF:FFF:
FF:FFFFFFFFFFF,FFFFFFFFFFFFFFFFFFFFFFFF,FFFFFFFFFFFFFFFFFFFFFFFFFFFF
F,FFFFFFF:FFFFF
@A00155:342:HHGFNDSXY:1:1463:28122:29716 2:N:0:GAACCTAG+TCCGCATA
GTATGTACGCATTCAAAAGGAAGAAGAGAAGAAGGTGGAAAACTTTTCTTTTTACCTACCAGAGAAAA
CTCGGGAATGGTTCACAAAGTCTGGTGTGTATCTTTCACCATTTGCTTACGTGAATCACTCACACCCT
GGTTGTAAAACTCTG
+
FFFFFFF:FFFFFFFFFFFFFFFFFFFFFFFFF,F:FFFFFF:,FFFFFFFFFFFFFFFFFFFFFFF:
F:F:FF,FFFFFF,FFFFFFFFFFFF:FFFFFFFFFFFF,FFF::FFFFFFF:FF:FFFFFFFFFFFF
F,F:F:FFF,FFFFF
@A00155:342:HHGFNDSXY:1:2109:3956:25128 2:N:0:GAACCTAG+TCCGCATA
GTATGAACGCATTCAAAAGGAAGAAGAGAAGAAGGTGGAAAACTTTTCTTTTTACCTACCAGAGAAAA
CTCGGGAATGGTTCACAAAGTCTGGTGTGTATCTTTCACCATTTGCTTACGTGAATCACTCACACCCT
GGTTGTAAAACTCTG
+
F:FFFFFFFFFFFFFFFFFFFFFFFFFFFFFFFFFFFFFFFFFFFFFFFFFFFFFFFFFFFFFFFFFF
FFFFFFF:FFFFFFFFFFFFFFFFFFFFFFFFFFFFFFFFF:FFFFFFFFFFFFFFFFFFFFFFFFFF
FFFFFFFFFFFFFFF
@A00155:342:HHGFNDSXY:1:2174:25265:1063 2:N:0:GAACCTAG+TCCGCATA
GTATGAACGCATTCAAAAGGAAGAAGAGAAGAAGGTGGAAAACTTTTCTTTTTACCTACCAGAGAAAA
CTCGGGAATGGTTCACAAAGTCTGGTGTGTATCTTTCACCATTTGCTTACGTGAATCACTCACACCCT
GGTTGTAAAACTCTG
+
FFFFFFFFFFFFFFFFFFFFFFFFFFFFFFFFFFFFFFFFFFFFFFFFFFFFFFFFFFFFFFFFFFFF
FFFFFFFFFFFFFFFFFFFFFFFFFFFFFFFFFFFFF:FFFFFFFFFFFFFFFFFFFFFFFFFFFFFF
FFFFFFFFFFFFFFF
@A00155:342:HHGFNDSXY:1:2430:32380:4492 2:N:0:GAACCTAG+TCCGCATA
GTATGAACGCATTCAAAAGGAAGAAGAGAAGAAGGTGGAAAACTTTTCTTTTTACCTACCAGAGAAAA
CTCGGGAATGGTTCACAAAGTCTGGTGTGTATCTTTCACCATTTGCTTACGTGAATCACTCACACCCT
GGTTGTAAAACTCTG
+
FF:FFF:FFFFFFFFFFFFFF:FFFFFFFFFFFFFFFFFFFFFFFFFFFFFFFF:FFFFFFFFFFFFF
FFFFFFF:FFFFFFFFFFFFFFFFFFFFFFFFFFFFFFFFFFFFFFFF,FF:FFFFFFFFFFFFFFF:
FF,FFFFFFFFFFFF
@A00155:342:HHGFNDSXY:1:2450:20320:9940 2:N:0:GAACCTAG+TCCGCATA
GTATGAACGCATTCAAAAGGAAGAAGAGAAGAAGGTGGAAAACTTTTCTTTTTACCTACCAGAGAAAA
CTCGGGAATGGTTCACAAAGTCTGGTGTGTATCTTTCACCATTTGCTTACGTGAATCACTCACACCCT
GGTTGTAAAACTCTG
+
FFFFFF:FFFF:FFFFFFFFFFFFFFFFFFFFFFFFFFFFFFFFFFFFFFFFFFFFFFFFFFFFF:FF
FFFFFFFFFFFFFFFF:FFFFFFFFFFFFFFFFFFFFFFFFFFFFFFFFF:FFFFFFFFFFFFFFFFF
FFFFFFFFFFFFFFF

```
@A00155:342:HHGFNDSXY:1:2470:1344:6574 2:N:0:GAACCTAG+TCCGCATA
GTATGAACGCATTCAAAAGGAAGAAGAGAAGAAGGTGGAAAACTTTTCTTTTTACCTACCAGAGAAAA
CTCGGGAATGGTTCACAAAGTCTGGTGTGTATCTTTCACCATTTGCTTAAGTGAATCAATCACACCCT
GGTTGTAAAAATCTG
+
FF:FF,:F:FFFF::FF:FFFFFFFF,:,FFFF:FFFF,F:,FFFFFFFFFF::FFFFFF:F,F:F::
:F,F,FFFF,FFFF:FF,FFFFF:FF,FFFFF:FFF,,,::FFFF:FF,,FF,FFFF,,FF,,:,,:F
FFFFFF,F:,,F,FF
@A00155:342:HHGFNDSXY:1:2534:10312:3192 2:N:0:GAACCTAG+TCCGCATA
GTATGAACGCATTCAAAAGGAAGAAGAGAAGAAGGTGGAAAACTTTTCTTTTTACCTACCAGAGAAAA
CTCGGGAATGGTTCACAAAGTCTGGTGTGTATCTTTCACCATTTGCTTACGTGAATCACTCACACCCT
GGTTGTAAAACTCTG
+
FFF,FFFF,F:,F:FF:FFFFFF,F,FFFF,FF::F,:F:FF,FF:FFFF,:FFF:FFF:F,FFFFFF
,FF,,:F,,,,F:FF,FFFF:FFF:,FFF:FFFF::FFFFF:,,,::FF,::::FFFF:,FFFFF:,:
F,F,FFFF,F,:FFF
@A00155:342:HHGFNDSXY:1:1505:17743:16344 1:N:0:GAACCTAG+TCCGCATA
TATGAACGCATTCAAAAGGAAGAAGAGAAGAAGGTGGAAAACTTTTCTTTTTACCTACCAGAGAAAAC
TCGGGAATGGTTCACAAAGTCTGGTGTGTATCTTTCACCATTTGCTTACGTGAATCACTCACACCCTG
GTTGTAAAACTCTGG
+
FFFFFFFFFFFFFFFFFFFFFFFFFFFFFF:FFFFFFFFFFFFFFFFFFFFFFFFFFFFFFFFFFFFF
FFF:FFFFFFFFFFFFFFFFFFFFFFFFFFFFFFFFFFFFFFFF:FFFF:FFFFFFFFFFFFFFFFFF
FFFFFFFFFFFFFFF
@A00155:342:HHGFNDSXY:1:1574:8115:28917 1:N:0:GAACCTAG+TCCGCATA
TATGAACGCATTCAAAAGGAAGAAGAGAAGAAGGTGGAAAACTTTTCTTTTTACCTACCAGAGAAAAC
TCGGGAATGGTTCACAAAGTCTGGTGTGTATCTTTCACCATTTGCTTACGTGAATCACTCACACCCTG
GTTGTAAAACTCTGG
+
FFFFFFFFFFF:FFFFFFFFFFFFFFFFFF:FFF:F:FFFFFFFF:FFFFF:FFFFFFFFFFFFFFFF
FFFFFFFFFFFFFFFFFFFFFFFFFFFFFFFFFFFFFFFFF:FFFFFFFFFFFFFFFF:FFFFFFFFF
FFFFFFFFFFFFFFF
@A00155:342:HHGFNDSXY:1:1374:10230:16047 2:N:0:GAACCTAG+TCCGCATA
ATGAACGCATTCAAAAGGAAGAAGAGAAGAAGGTGGAAAACTTTTCTTTTTACCTACCAGAGAAAACT
CGGGAATGGTTCACAAAGTCTGGTGTGTATCTTTCACCATTTGCTTACGTGAATCACTCACACCCTGG
TTGTAAAACTCTGGA
+
FFFFFFFFFFFFFFF:FFFFFFFFFFFFFFFFFFFFFFFFFFFFFFFFFFFFFFFFFFFFFFFFFFFF
FFFFF:FFFFFFFFFFFFFFFFFFFFFFFFFFFFFFFFFFFFFFFFFF:FFFFFFFFFFFFFFFFFFF
FFFFF,::FFFFFFF
@A00155:342:HHGFNDSXY:1:1553:6705:34272 1:N:0:GAACCTAG+TCCGCATA
CGATCTCATTCAAAAGGAAGAAGAGAAGAAGGTGGAAAACTTTTCTTTTTACCTACCAGAGAAAACTC
GGGAATGGTTCACAAAGTCTGGTGTGTATCTTTCACCATTTGCTTACGTGAATCACTCACACCCTGGT
TGTAAAACTCTGGAG
+
:FFFFFFFFFFFF:FFFFFFFFFFFFFFFFFFFFFFFFFFFFFFFFFFFFFFFFFFFFFFFF:FFFFF
FFFFFFFFFFFFFFFFFFFFFFFFFFFFFFFFFFFFFFFFFFFFFFFFFFFFFFFFFFFFFFFFFFFF
FFFFFFFFFFFFFFF
@A00155:342:HHGFNDSXY:1:1149:12590:30217 2:N:0:GAACCTAG+TCCGCATA
TGAACGCATTCAAAAGGAAGAAGAGAAGAAGGTGGAAAACTTTTCTTTTTACCTACCAGAGAAAACTC
GGGAATGGTTCACAAAGTCTGGTGTGTATCTTTCACCATTTGCTTACGTGAATCACTCACACCCTGGT
TGTAAAACTCTGGAG
+
FFFFFFFFFFFFFFFFFFFFFFFFFFFFFFFF,FFFFFFFFFFFFFFFFFFFFFFFFFFFFFFFFFFF
```

FFFFFFFFFFFFFFFFFFFFFFFFFFFFFFFFFFFFFFFFFFFFFFFFFFFFFFFFFFFFFFFFFFFFFF
FFFFF:FFFFFFFFF
@A00155:342:HHGFNDSXY:1:1165:1714:8938 2:N:0:GAACCTAG+TCCGCATA
TGAACGCATTCAAAAGGAAGAAGAGAAGAAGGTGGAAAACTTTTCTTTTTACCTACCAGAGAAAACTC
GGGAATGGTTCACAAAGTCTGGTGTGTATCTTTCACCATTTGCTTACGTGAATCACTCACACCCTGGT
TGTAAAACTCTGGAG
+
FF,FFFFFFF:FF::FFFFFFFFFFFF:FFFFFFFFFFFFFFFFFFFFFFFFFFFFFFFF::FF,:FFFF
FF:FFFF:FFFFFFFFFFFF::FFFFFF:FFFFFFFFFFFFFF,:FFFFFFFFFFF:FFFFF:FFFFFFF
FFF,F,FFFFFFFFF
@A00155:342:HHGFNDSXY:1:1137:7247:12571 2:N:0:GAACCTAG+TCCGCATA
TGAACGCATTCAAAAGGAAGAAGAGAAGAAGGTGGAAAACTTTTCTTTTTACCTACCAGAGAAAACTC
GGGAATGGTTCACAAAGTCTGGTGTGTATCTTTCACCATTTGCTTACGTGAATCACTCACACCCTGGT
TGTAAAACTCTGGAG
+
FFFFFFFFFFFFFF:FFFFFFFFFFFFFFFFFFFFFFFFFFFFFFFFFFFFFFFFFFFFFF:FFFFFFFF
FFFFFFFFFFFFFFFFFFFFFF:FFFFFFFFFFFFFFFFFFFFFFFFFFFFFFFFFFFFFFFF,FFFFFF
FFFFFF:F:FFFFFF
@A00155:342:HHGFNDSXY:1:1137:7220:12806 2:N:0:GAACCTAG+TCCGCATA
TGAACGCATTCAAAAGGAAGAAGAGAAGAAGGTGGAAAACTTTTCTTTTTACCTACCAGAGAAAACTC
GGGAATGGTTCACAAAGTCTGGTGTGTATCTTTCACCATTTGCTTACGTGAATCACTCACACCCTGGT
TGTAAAACTCTGGAG
+
FF,FFFFFFFFFFFFFFFFFFFFFFFFFFFFFF:FFFFFFFFFFFFFFFFFFFFFFFFFFFFFFFF:FFF
FFFFFFFFFFFFFFFFFFFFFFFFFFFFFFFFFFFFFFFFFFFFFFFFFFFFFFFFFFFFFFFFFFFF:F
FFFFF:FFFFFFFFF
@A00155:342:HHGFNDSXY:1:1319:4137:12289 2:N:0:GAACCTAG+TCCGCATA
TGAACGCATTCAAAAGGAAGAAGAGAAGAAGGTGGAAAACTTTTCTTTTTACCTACCAGAGAAAACTC
GGGAATGGTTCACAAAGTCTGGTGTGTATCTTTCACCATTTGCTTACGTGAATCACTCACACCCTGGT
TGTAAAACTCTGGAG
+
FFFFFFFFFFFFFFFFFFFFFFFFFFFFFFFFFFFFFFFFFFFFFFFFFFFFFFFFFFFFFFFFFFFFFF
FFFFFFFFFFFFFFFFFFFFFFFFFFFFFFFFFFFFFFFFFFFFFFFFFFFFFFFFFFFFFFFFFFFFFF
FFFFFFFFFFFF:FF
@A00155:342:HHGFNDSXY:1:1661:7102:23657 2:N:0:GAACCTAG+TCCGCATA
TGAACGCATTCAAAAGGAAGAAGAGAAGAAGGTGGAAAACTTTTCTTTTTACCTACCAGAGAAAACTC
GGGAATGGTTCACAAAGTCTGGTGTGTATCTTTCACCATTTGCTTACGTGAATCACTCACACCCTGGT
TGTAAAACTCTGGAG
+
FFFFFFFFFFFFFFFFFFFFFF:FFFFFFFFFFFFFFFFFFFFFFFFFFFFFFFFFFFFFFFFFFFFFFF
FFFF:FFFFFFFFFFFFFFFFFFFFF:FFFFFFFFFFFFFFFFFFFFFFFFFFFFFFFFFFFFFFFFFFFF
FFFFFFFFFFFFFFF
@A00155:342:HHGFNDSXY:1:1672:5231:22733 2:N:0:GAACCTAG+TCCGCATA
TGAACGCATTCAAAAGGAAGAAGAGAAGAAGGTGGAAAACTTTTCTTTTTACCCACCAGAGAAAACTC
GGGAATGGTTCACAAAGTCTGGTGTGTATCTTTCACCATTTGCTTACGTGAATCACTCACACCCTGGT
TGTAAAACTCTGGAG
+
FFFFFFFFFFFFFF,FFFFFFFFFFFFFFFFFFFFFFFFFFFFFFFFFFFFFFFFFFFFFFFFFFFFFFF
FFFFFFFFFFFFFFFFFFFFFFFFFFFFFFFFFFFFFFFFFFFFFFFFF:FFFFFFFFFFFFFFFFFFFF
FFFFFFF,F:FF:FF
@A00155:342:HHGFNDSXY:1:2423:32136:9079 2:N:0:GAACCTAG+TCCGCATA
TGAACGCATTCAAAAGGAAGAAGAGAAGAAGGTGGAAAACTTTTCTTTTTACCTACCAGAGAAAACAC
GGGAATGGTTCACAAAGTCTGGTGTGTATCTTTCACCAATAGCTTACGTGAATCACTCACACACTCGT
TGTAAAACTCAGGAG

```
+
::FF,F:::FF:FFFF:FFFFF,:,FFFFFF:FFFFFF,,,FFFFF,,F:F,FFFF:FFFFF:FFF,F
,,FFF,FF::FFFFFF:,FFF:FF::FFFF,FF,FFF:,F,::FFFFF,FFFFFFFFFFF:F,FF,F,
F::FFFFFFF,F,F:
@A00155:342:HHGFNDSXY:1:2333:4137:13166 2:N:0:GAACCTAG+TCCGCATA
TGAACGCATTCAAAAGGAAGAAGAGAAGAAGGTGGAAAACTTTTCTTTTTACCTACCAGAGAAAACTC
GGGAATGGTTCACAAAGTCTGGTGTGTATCTTTCACCATTTGCTTACGTGAATCACTCACACCCTGGT
TGTAAAACTCTGGAG
+
FF,FFFFF:FFFFFFFFFFFF:FFFFFFF,:FFFFFFFFFFFFFFFFFFFFFFFFFFFFFFFFF:FFF
FFFF:FF:FFFFFFF:FFFFFFF:FFFFFFFFFFFFFFFFFFFFFFFFFFF,FFFFFFFFFFFFFFFF
FFFFFFFFFFFF:FF
@A00155:342:HHGFNDSXY:1:2614:14009:24064 2:N:0:GAACCTAG+TCCGCATA
TGAACGCATTCAAAAGGAAGAAGAGAAGAAGGTGGAAAACTTTTCTTTTTACCTACCAGAGAAAACTC
GGGAATGGTTCACAAAGTCTGGTGTGTATCTTTCACCATTTGCTTACGTGAATCACTCACACCCTGGT
TGTAAAACTCTGGAG
+
FFFFFFFFFFFFFFFFFFFFFFFFFFFFFFFFFFFFFFFFFFFFF:FFFFFFFFFFFFFFFFFFFFFF
FFFF:FFFFFFFFFF,F:FFFFFFFFFFFFFFFFFFFFF:FFFFFFFFFFF:FFFFFFFFFFFFF:FF
FFFFFFFFFFFFFFF
@A00155:342:HHGFNDSXY:1:1315:17607:6464 2:N:0:GAACCTAG+TCCGCATA
GAACGCATTCAAAAGGAAGAAGAGAAGAAGGTGGAAAACTTTTCTTTTTACCTACCAGAGAAAACTCG
GGAATGGTTCACAAAGTCTGGTGTGTATCTTTCACCATTTGCTTACGTGAATCACTCACACCCTGGTT
GTAAAACTCTGGAGA
+
FFFFFFF::FFFFFFFFFFFFFFFFFFFFFFFFFFFFFFFFFFFFFFFFFFFFFFFFFFFFFFFFFFF
FFFFFFFFFFFFFFFFFFFFFFFFFFFFFFFFFFFFFFFFFFFFFFFFFFFFFFFFFFFFFFFFFFFF
FFFFFFFFFFF:FFF
@A00155:342:HHGFNDSXY:1:1315:17155:6558 2:N:0:GAACCTAG+TCCGCATA
GAACGCATTCAAAAGGAAGAAGAGAAGAAGGTGGAAAACTTTTCTTTTTACCTACCAGAGAAAACTCG
GGAATGGTTCACAAAGTCTGGTGTGTATCTTTCACCATTTGCTTACGTGAATCACTCACACCCTGGTT
GTAAAACTCTGGAGA
+
FFFFFFFFFFF::FFFFFFFFFFFFFFFFFFFFFFFFFFFFFFFFFFFFFFFFFFFFFFFFFFFFFFF
:FFFFFFFFFFFFFF:FFFFFFFFFFFFFFFFFFF:FFFFFFFFFFFFFFF:FFFFFFFFF:FFFFF
FF:FFFF:FFFFFF:
@A00155:342:HHGFNDSXY:1:1608:14443:25222 2:N:0:GAACCTAG+TCCGCATA
GAACGCATTCAAAAGGAAGAAGAGAAGAAGGTGGAAAACTTTTCTTTTTACCTACCAGAGAAAACTCG
GGAATGGTTCACAAAGTCTGGTGTGTATCTTTCACCATTTGCTTACGTGAATCACTCACACCCTGGTT
GTAAAACTCTGGAGA
+
FFFFFFFFFFFFFFFFFFFFFFFFFFFFFFFFFFFFFFFF:FFFFFFFFFFFFFFFFFFFFFFFF:FFFF
FFFFFFFFFFFFFF:FFFFFFFFFFFFFFFFFFFFFFFFF:FFFFFFFFFFFFFFFFFFFFFFFFFFFFF
FFFFF:FFFFFFFFF
@A00155:342:HHGFNDSXY:1:2632:7545:5666 2:N:0:GAACCTAG+TCCGCATA
GAACGCATTCAAAAGGAAGAAGAGAAGAAGGTGGAAAACTTTTCTTTTTACCTACCAGAGAAAACTCG
GGAATGGTTCACAAAGTCTGGTGTGTATCTTTCACCATTTGCTTACGTGAATCACTCACACCCTGGTT
GTAAAACTCTGGAGA
+
FFFFFFFFFFFFFFFFFFFFFFFFFFFFFFFFFFFFFFFFFFFFFFFFFFFFFFFFFFFFFF:FF,FF:F
FFF:FFFFFFFFFFFFFFFFFFFFFFFFF:FFFFF:FFFFFFFFFFF:FF,FFFFFFFFFFF,FFFFFF
FFFF:FFFFFFFFFF
@A00155:342:HHGFNDSXY:1:1227:17806:20776 2:N:0:GAACCTAG+TCCGCATA
AACGCATTCAAAAGGAAGAAGAGAAGAAGGTGGAAAACTTTTCTTTTTACCTACCAGAGAAAACTCGG
```

```
GAATGGTTCACAAAGTCTGGTGTGTATCTTTCACCATTTGCTTACGTGAATCACTCACACCCTGGTTG
TAAAACTCTGGAGAA
+
FFFFFFFFFFFFFFFFFFFFFFFFFFFFFFFFFF:FFFFFFFFFFFFFFFFFFFFFFFFF:FFFFFFFF
FF,FFFFFFFFFFFFFFFFFFFFFFFFFFFFFFFFFFFFFFFFFFFFFFFFFFFFFFFFFFFFFFFFFF
FFFFFFFFFFFFFFF
@A00155:342:HHGFNDSXY:1:1227:16893:21449 2:N:0:GAACCTAG+TCCGCATA
AACGCATTCAAAAGGAAGAAGAGAAGAAGGTGGAAAACTTTTCTTTTTACCTACCAGAGAAAACTCGG
GAATGGTTCACAAAGTCTGGTGTGTATCTTTCACCATTTGCTTACGTGAATCACTCACACCCTGGTTG
TAAAACTCTGGAGAA
+
FFFFFFFFFFFFFFFFF:FFFFFFFFFFFFFFFFFFFFFFFFFFFFFFFFFFFFFFFFFFFFFFFFFF
FF,FFFFFFFFFFFFFFFFFFFFFFFFFFFFFFFFFFFFFFFFFFFFFFFFFFFFFFFFFFFFFFFFFF
FFF,FFFFFFFFFFF
@A00155:342:HHGFNDSXY:1:1255:12029:31438 2:N:0:GAACCTAG+TCCGCATA
AACGCATTCAAAAGGAAGAAGAGAAGAAGGTGGAAAACTTTTCTTTTTACCTACCAGAGAAAACTCGG
GAATGGTTCACAAAGTCTGGTGTGTATCTTTCACCATTTGCTTACGTGAATCACTCACACCCTGGTTG
TAAAACTCTGGAGAA
+
FFFFFFFFFFFFF:FFFFFFFFF:FFFFFFFFFFFF,FFFFFFFFFFFFFFFFFFFFFFFFFFFFFFF
FF:FFFFFFFFFFFFFFFFFFFFFFFF:FFFFFFFF,FFFFFFFFFFFFFFFF:FFFFFFFFFFFFFF
FFFFFFFFFFFFFFF
@A00155:342:HHGFNDSXY:1:2222:6705:8876 2:N:0:GAACCTAG+TCCGCATA
AACGCATTCAAAAGGAAGAAGAGAAGAAGGTGGAAAACTTTTCTTTTTACCTACCAGAGAAAACTCGG
GAATGGTTCACAAAGTCTGGTGTGTATCTTTCACCATTTGCTTACGTGAATCACTCACACCCTGGTTG
TAAAACTCTGGAGAA
+
FFFFFFFFFFFFFFFFFFFFFFFFFFFFFFFFFFFFFFFF:FFFFFFFFFFFFFFFFFFFFFFFFFFF
FF:FFFFFFFFFF:FFFFFFFFFFFFFFFFFFFFFFFFFFFFFFFFFFFFFFFFFFFFFFFFFFFFFF
FFFF::FFFFFFFFF
@A00155:342:HHGFNDSXY:1:2404:8856:17675 2:N:0:GAACCTAG+TCCGCATA
AACGCATTCAAAAGGAAGAAGAGAAGAAGGTGGAAAACTTTTCTTTTTACCTACCAGAGAAAACTCGG
GAATGGTTCACAAAGTCTGGTGTGTATCTTTCACCATTTGCTTACGTGAATCACTCACACCCTGGTTG
TAAAACTCTGGAGAA
+
FFFFFFFFFFFFFFFFFFFFFFFFFFFFFFFFFFFFFFFFFFFFFFFFFFFFFFFFFFFFFFFFFFFF
FF:FFFFFFFFFFFFFFFFFFFFFFFFFFFF:F:FFFFFFFFFFFFFFFFFFFFFFFFFFFFFFFFFF
FF:FFFFFFFFFFFF
@A00155:342:HHGFNDSXY:1:2503:28745:14826 2:N:0:GAACCTAG+TCCGCATA
AACGCATTCAAAAGGAAGAAGAGAAGAAGGTGGAAAACTTTTCTTTTTACCTACCAGAGAAAACTCGG
GAATGGTTCACAAAGTCTGGTGTGTATCTTTCACCATTTGCTTACGTGAATCACTCACACCCTGGTTG
TAAAACTCTGGAGAA
+
FFFFFFFFFFFFFFFFFFFFFFFFFFFFFFFFFFFF:FFFFFFFFFFFFFFFFFFFFFFFFFFFFFFF
FFFFFFFFFFFFFFFFFFFFFFFFFFFFFFFFFF:FFFFFFFFFF:FFFFF:FFF:FFFFFF:FFFFF
FFFFFFFFFFFFFFF
@A00155:342:HHGFNDSXY:1:2414:1353:7686 2:N:0:GAACCTAG+TCCGCATA
ACGCATTCAAAAGGAAGAAGAGAAGAAGGTGGAAAACTTTTCTTTTTACCTACCAGAGAAAACTCGGG
AATGGTTCACAAAGTCTGGTGTGTATCTTTCACCATTTGCTTACGTGAATCACTCTCACCCTGGTTGT
AATTCTCTGGAGAA
+
FFFF:FFFFF,:FF,FF,FF:F::FF:FFFFF,FF,:FFF:FFFFFF:F:FFFFFF,FFF:FFFFFFF
FFFFFF:FFF,F,FF:FFF:FFFFFFFFFFF,,::FFFF:FF,FFFFFFFF,,FF,,F,:FFFFFFFF
,F,,FFF:FF,:F:
```

```
@A00155:342:HHGFNDSXY:1:1373:30752:10066 1:N:0:GAACCTAG+TCCGCATA
ACGCATTCAAAAGGAAGAAGAGAAGAAGGTGGAAAACTTTTCTTTTTACCTACCAGAGAAAACTCGGG
AATGGTTCACAAAGTCTGGTGTGTATCTTTCACCATTTGCTTACGTGAATCACTCACACCCTGGTTGT
AAAACTCTGGAGAAC
+
FFFFFFFFFFFFFFFFFFFFFFFFFF,FFFF:FFF:FFFF:FFFFFFFFFFFFFFFFFFFFFFFFFFFF
FFFFFFFFFFFFFFFFFFFFFFFFFFFFFFFFFFFFFFFFFFFFFFFFFFFFFFFFFFFFFFFFFFFF
FFFFFFFFFFFFFFF
@A00155:342:HHGFNDSXY:1:2305:22815:18771 1:N:0:GAACCTAG+TCCGCATA
ACGCATTCAAAAGGAAGAAGAGAAGAAGGTGGAAAACTTTTCTTTTTACCTACCAGAGAAAACTCGGG
AATGGTTCACAAAGTCTGGTGTGTATCTTTCACCATTTGCTTACGTGAATCACTCACACCCTGGTTGT
AAAACTCTGGAGAAC
+
FFFFFFFFFFFFFFFFFFFF:F,FFFFFF:FFF::F,FFFFFFFFFFFFFFFFFFFFFFFFFFFFFFFF
FFFFFFFFFFFF:FFFFFFFFFFFFFFFFFFFFFFFFFFFFFFFFFFFFFFFFFFFFFFFFFFFFFFF
FFFFFFFFFFFFFFF
@A00155:342:HHGFNDSXY:1:2305:21667:32315 1:N:0:GAACCTAG+TCCGCATA
ACGCATTCAAAATGAAGAAGAGAAGAAGGTGGAAAACTTTTCTTTTTACCTACCAGAGAAAACTCGGG
AATGGTTCACAAAGTCTGGTGTGTATCTTTCACCATTTGCTTACGTGAATCACTCACACCCTGGTTGT
AAAACTCTGGAGAAC
+
FF:F:FFFFFF,,F:F:FFFF:FF::F:FFFFFFFF:FFFFFFF:FFF:FFF:FFFFFF:FFFFFF,F
:F,:FFFFF:,FF:FFFFFFFFFFFFFFFFFFFFFFF:FFFFF:FFFF:FFFFFFFFFFFFFFFFFFFF
FFFFFFFFFFF:FFF
@A00155:342:HHGFNDSXY:1:2472:24813:11397 1:N:0:GAACCTAG+TCCGCATA
ACGCATTCAAAAGGAAGAAGAGAAGAAGGTGGAAAACTTTTCTTTTTACCTACCAGAGAAAACTCGGG
AATGGTTCACAAAGTCTGGTGTGTATCTTTCACCATTTGCTTACGTGAATCACTCACACCCTGGTTGT
AAAACTCTGGAGAAC
+
FFFFFFFFFFFFFFFFFFFFFFFFFFFFFFFFFFFFFFFFFFFFFFFFFFFFFFFFFFFFFFFFFFFF
FFFFFFFFFFFFFFFFFFFFFFFFFFFFFFFFFFFFF:FFFFFFFFFFFFFFFFFFFFFFFFFFFFFFF
FFFFFFFFFFFFFFF
@A00155:342:HHGFNDSXY:1:2520:11532:19398 1:N:0:GAACCTAG+TCCGCATA
ACGCATTCAAAAGGAAGAAGAGAAGAAGGTGGAAAACTTTTCTTTTTACCTACCAGAGAAAACTCGGG
AATGGTTCACAAAGTCTGGTGTGTATCTTTCACCATTTGCTTACGTGAATCACTCACACCCTGGTTGT
AAAACTCTGGAGAAC
+
FF:FFFFFFFFF:FFFFFFFFFFFFFFF,FFFFFFFFFFFFF:FFFFFFFFFFFFFFFFFFFFFFFFFF
FFFFFFFFFFFFFFFFFFFFFFFFFFFFFFFFFFFFFF:FFFFFFFFFFFFFFFFFFFFFFFFFFFFFF
FFFFFFFFFFFFFFF
@A00155:342:HHGFNDSXY:1:1214:29912:17221 2:N:0:GAACCTAG+TCCGCATA
ACGCATTCAAAAGGAAGAAGAGAAGAAGGTGGAAAACTTTTCTTTTTACCTACCAGAGAAAACTCGGG
AATGGTTCACAAAGTCTGGTGTGTATCTTTCACCATTTGCTTACGTGAATCACTCACACCCTGGTTGT
AAAACTCTGGAGAAC
+
FFFFFFFFFFFFFFFFFFFFFFFF::FFFFFFFFFFFFFFFFFFFFFFFFFFFFFFFFFFFFFFFFFF
FFF:FFFFFFFFF:FFFFFFFFFFFFFFFFFFFFFFFFF:F,FFFFFFF:FFFFFFFFFF:FFFFF,FF
FFFFFFFFFFFFFFF
@A00155:342:HHGFNDSXY:1:1414:11713:7811 2:N:0:GAACCTAG+TCCGCATA
ACGCATTCAAAAGGAAGAAGAGAAGAAGGTGGAAAACTTTTCTTTTTACCTACCAGAGAAAACTCGGG
AATGGTTCACAAAGTCTGGTGTGTATCTTTCACCATTTGCTTACGTGAATCACTCACACCCTGGTTGT
AAAACTCTGGAGAAC
+
FFFFFFFFFFFFFFFFFFFFFFFFFFFFFFFFFFFFFFFFFFFFFFFFFFFFFFFF:FFFFFFFFFFF
```

FFFFFFFFFFFFFFFFFFFFFFFFFFFFFFFFFFFFFFFFFFFFFFFFFFFFFFFFFFFFFF:FFFFF
FF,FFFFFFFFFFFF
@A00155:342:HHGFNDSXY:1:1664:12120:9392 2:N:0:GAACCTAG+TCCGCATA
ACGCATTCAAAAGGAAGAAGAGAAGAAGGTGGAAAACTTTTCTTTTTACCTACCAGAGAAAACTCGGG
AATGGTTCACAAAGTCTGGTGTGTATCTTTCACCATTTGCTTACGTGAATCACTCACACCCTGGTTGT
AAAACTCTGGAGAAC
+
FFFFFFFF:FFFFFF:FFFFFFFFFFFFFFFFFFFFFFFFFFFFFF:FFFFFFFFFFFFFFFFFFFFF
:,FFFFFFFFFFFFFFFFFFFFFFFFFFFFFFFFFFFFFFFFFFFFFFFFFFFFFFFFFFFFFFFFFF
FFFFFFFFFFFF::F
@A00155:342:HHGFNDSXY:1:2133:2248:24643 2:N:0:GAACCTAG+TCCGCATA
ACGCATTCAAAAGGAAGAAGAGAAGAAGGTGGAAAACTTTTCTTTTTACCTACCAGAGAAAACTCGGG
AATGGTTCACAAATTATGGTGTGTATCTTTCACCATGTGCTTACGTGAATCACTCACACCCTGGTTGT
AAATCTCTGGAGAAC
+
FFFF:FF:FF,FF::FFFFFFFF,F,FFF:F:,F:FFFFFF,FFFFF,FFFFFFFF:F:F:,:FFF::
FFFF,FF,FFFFF,F,FFFFFFFFFF:FFFF::F,,,F:F,FF,:F:FFFFFFFFFFF,F:FF::FFF
:F:,FFFFF:,,FF:
@A00155:342:HHGFNDSXY:1:2415:21432:4601 2:N:0:GAACCTAG+TCCGCATA
TCGCATTCAAAAGGAAGAAGAGAAGAAGATGGAAAACTTTTCTTTTTACCTACCAGAGAAAACTCGGG
AATGGTTCACAAAGTCTGGTGTGTATCTTTCACCATTTGCTTACGTGAATCACTCACACCCTGGTTGT
AAAACTCTGGAGAAC
+
F:FFFFFFF,:FFFFFF:FF:FFFFFFFF:FF,:F,FFF,,F::FF:FFFF:FF,:FFFFFFFFFFFF
F,F,F,FFFFF,FFFFF:FFFFFFFFFFFF:FFFFFFFFFFF,FF,FF:FFFFFFFFFFFFFFFFFFF
FFFFFFF,F::FFFF
@A00155:342:HHGFNDSXY:1:1573:29695:11271 2:N:0:GAACCTAG+TCCGCATA
CGCATTCAAAAGGAAGAAGAGAATAAGGTGGAAAACTTTTCTTTTTACCTACCAGAGAAAACTCGGGA
ATGGTTCACAAAGTCTGGTGTGTATCTTTCACCATTTGCTTACGAGAATCACTCACACCCTGGTTGTA
AAACTCTGGAGAAC
+
FFFFFFFFFF:,FFFFFFFF:FF,FFF,FFFFFFFFFFFFFFFFF:FFFF:FFFFFFFFF:FFFFFFF
:FFFFFFFFFFF:FFF,FF,F,FFFFFFF:F::FF:F,:FFFF,,FFFFFFFFF:FF::F:F,F:FFF
FFFF:FFF,FFFFF
@A00155:342:HHGFNDSXY:1:2415:21432:4601 1:N:0:GAACCTAG+TCCGCATA
CGCATTCAAAAGGAAGAAGAGAAGAAGATGGAAAACTTTTCTTTTTACCTACCAGAGAAAACTCGGGA
ATGGTTCACAAAGTCTGGTGTGTATCTTTCACCATTTGCTTACGTGAATCACTCACACCCTGGTTGTA
AAACTCTGGAGAACA
+
FFFFFFFFFFFFFFFFFFFFFFFFFFFFFFFF,FFFFFFFFFFFFFFFFFFFFFFFFFFFFFFFFFFF
,FFFFFFFFFFFFFFF:FFF:FFFFFFFFFFFF:FFFFFFFFFFFFFFFFFFFFFFFFFFFFFFFFFF
FFFFFFFFFFFF,F:
@A00155:342:HHGFNDSXY:1:1114:11912:29168 2:N:0:GAACCTAG+TCCGCATA
CGCATTCAAAAGGAAGAAGAGAAGAAGGTGGAAAACTTTTCTTTTTACCTACCAGAGAAAACTCGGGA
ATGGTTCACAAAGTCTGGTGTGTATCTTTCACCATTTGCTTACGTGAATCACTCACACCCTGGTTGTA
AAACTCTGGAGAACC
+
FFFFFFFFFF:FFFFFFFFFFFFFFFFFFFFFFF,FFF:F:FFFFFFFFFFFFFFFFFFFFFFFFFFF
FFFFFFFFFFFFFFFFFFFFFFFFFFFFFFFFFFFFFFFFFFFFFFFFFFFFFFFFFFFFFFFFFFFF
FF:FFFFFFFFFF,F
@A00155:342:HHGFNDSXY:1:1114:11921:29121 2:N:0:GAACCTAG+TCCGCATA
CGCATTCAAAAGGAAGAAGAGAAGAAGGTGGAAAACTTTTCTTTTTACCTACCAGAGAAAACTCGGGA
ATGGTTCACAAAGTCTGGTGTGTATCTTTCACCATTTGCTTACGTGAATCACTCACACCCTGGTTGTA
AAACTCTGGAGAACC

```
+
FFFFF:FFFFFFFFFFFFFFFFFFFFFFF:FFFFFFFFFFFFFFFFFFFFFFFFFFFFFFFFFFFFFFFFF
FFFFFFFFFFFFFFFFFFFFFFFFFFFFFFFFFFFFFFFFFFFFFFFFFFFFFFFFFFFFFFFFFFFFFFF
FFFFFFFFFFFF:FF
@A00155:342:HHGFNDSXY:1:1348:14009:32643 2:N:0:GAACCTAG+TCCGCATA
CGCATTCAAAAGGAAGAAGAGAAGAAGGTGGAAAACTTTTCTTTTTACCTACCAGAGAAAACTCGGGA
ATGGTTCACAAAGTCTGGTGTGTATCTTTCACCATTTGCTTACGTGAATCACTCACACCCTGGTTGTA
AAACTCTGGAGAACC
+
FFFFFFFFFFFFFFFFFFFFFFFFFFFFFFFFFFFFFFFFFFFFFFFFFFFFFFFFF::FFFFFFFFF
FFFFFFFFFFFFFFFFFFFFFFFFFFFFFFFFFFFFF,FFFFFFFFFFFFFFFFFFFFFFFFFFFFFFFF
FFFFFFFFFFFFFFF
@A00155:342:HHGFNDSXY:1:1423:29279:10833 2:N:0:GAACCTAG+TCCGCATA
CGCATTCAAAAGGAAGAAGAGAAGAAGGTGGAAAACTTTTCTTTTTACCTACCAGAGAAAACTCGGGA
ATGGTTCACAAAGTCTGGTGTGTATCTTTCACCATTTGCTTACGTGAATCACTCACACCCTGGTTGTA
AAACTCTGGAGAACC
+
FFFFFFFFFFFFFFFFFFFFFFF:,FFFFFFFFFFFFFFFFFFFFFFFF:FFFFFFFFFFFFFFFFFFFF
:FFFFFFFFFFFFFFFFFFFFFFFFFFFFFFFFFFFFFFFFF:FFFFFFFFFFFFFFFFFFFFFF,FFFF
FFFFFFFFFFFFFFF
@A00155:342:HHGFNDSXY:1:1521:2419:33833 2:N:0:GAACCTAG+TCCGCATA
CGCATTCAAAAGGAAGAAGAGAAGAAGGTGGAAAACTTTTCTTTTTACCTACCAGAGAAAACTCGGGA
ATGGTTCACAAAGTCTGGTGTGTATCTTTCACCATTTGCTTACGTGAATCACTCACACCCTGGTTGTA
AAACTCTGGAGAACC
+
FFFFFFFFFF:FFFFFFFFFFFFFFFFFFFFFFFFFFFFFFFFFFFFFFFFFFFFFFFFFFFFFFFFFFF
FFFFFFFFFFFFFFFFFFFFFFFFFFFFFFFFFFFFFFFFFFFFFFFFFFFFFFFFF:FFFFFFFFF
F,:FFFFF,FFFFFF
@A00155:342:HHGFNDSXY:1:2161:10520:1517 2:N:0:GAACCTAG+TCCGCATA
CGCATTCAAAAGGAAGAAGAGAAGAAGGTGGAAAACTTTTCTTTTTACCTACCAGAGAAAACTCGGGA
ATGGTTCACAAAGTCTGGTGTGTATCTTTCACCATTTGCTTACGTGAATCACTCACACCCTGGTTGTA
AAACTCTGGAGAACC
+
FFFFFFFFFFFFFFFFFFFFFFFFFFFFFFFFFFFFFFFFFFFFFFFFFFFFFFFFFFFFFFFFFFFFFF
FFFFFFFFFFFFFFFFFFFFFFFFFFFFFFFFFFFFFFFFFFFFFFFFFFFFFFFFFFFFFFFFFFFFFF
F,FFFFFFFF:FFFF
@A00155:342:HHGFNDSXY:1:2161:10529:1564 2:N:0:GAACCTAG+TCCGCATA
CGCATTCAAAAGGAAGAAGAGAAGAAGGTGGAAAACTTTTCTTTTTACCTACCAGAGAAAACTCGGGA
ATGGTTCACAAAGTCTGGTGTGTATCTTTCACCATTTGCTTACGTGAATCACTCACACCCTGGTTGTA
AAACTCTGGAGAACC
+
FFF:FFFFFFFFFFFFFFFFFFFFFFFFFFFFFF,FFFFFFFFFFFFFFFFFFFFFFFFFFFFFFFFFFF
FFFFFFFFFFFFFFFFFFFFFFFFFFFFFFFFFFF:FFFFFFFFFFFFFFFFFFFFFFFFFFFFFFFFFF
FF:FFFFFFFFFFFF
@A00155:342:HHGFNDSXY:1:2340:19533:36260 2:N:0:GAACCTAG+TCCGCATA
CGCATTCAAAAGGAAGAAGAGAAGAAGGTGGAAAACTTTTCTTTTTACCTACCAGAGAAAACTCGGGA
ATGGTTCACAAAGTCTGGTGTGTATCTTTCACCATTTGCTTACGTGAATCACTCACACCCTGGTTGTA
AAACTCTGGAGAACC
+
FFF:FFFFFFFFFF:FFFFFFFFFFFFFFFFFFFFFFFF:FFFFFFFFFFFFFFFFFFFFFFFFFFFFFFFF
FFFF::FFFFFFFFFFFFFFFFFFFFFFFFFFFFFFFFFFFFFFFFFFFFFFFFFFFFFFFFFFFFFFFF
FF,FFFFFFFFFFFF
@A00155:342:HHGFNDSXY:1:2668:18918:23860 2:N:0:GAACCTAG+TCCGCATA
CGCATTCAAAAGGAAGAAGAGAAGAAGGTGGAAAACTTTTCTTTTTACCTACCAGAGAAAACTCGGGA
```

ATGGTTCACAAAGTCTGGTGTGTATCTTTCACCATTTGCTTACGTGAATCACTCACACCCTGGTTGTA
AAACTCTGGAGAACC
+
FFFFFFFF:FFFFFFF:FFFFFFFFFFFFFFFFFFFF:,FFFFF:FFF:FFFF,FFFFFFFFFFFFFFFF
FFFF:FF:FFFFFF:FFFFFFFFFFFFFFFFFFFFFFFFF:FFFFFFFFF:FFFFFFFFFFFFFF:FFF:
FFFFFFFF:FFFFFF
@A00155:342:HHGFNDSXY:1:1158:15221:31767 1:N:0:GAACCTAG+TCCGCATA
GCATTCAAAAGGAAGAAGAGAAGAAGGTGGAAAACTTTTCTTTTTACCTACCAGAGAAAACTCGGGAA
TGGTTCACAAAGTCTGGTGTGTATCTTTCACCATTTGCTTACGTGAATCACTCACACCCTGGTTGTAA
AACTCTGGAGAACCA
+
FFF,FFFFFF:FFFFFFFFFFFFFF:,F::FFFFFFFFFFFFFFFFFFFFFFFFFFFF:FFFFFFFFFF,
FF:FFFFFFFFFFFFFFFFFFFFFFFFFFFFFFFFFFFFFFFFFFFFFFFFFFFFFFFFFFFFFFFFFFF
FFFFFFFFFFFFFFF
@A00155:342:HHGFNDSXY:1:1511:11686:17722 1:N:0:GAACCTAG+TCCGCATA
GCATTCAAAAGGAAGAAGAGAAGAAGGTGGAAAACTTTTCTTTTTACCTACCAGAGAAAACTCGGGAA
TGGTTCACAAAGTCTGGTGTGTATCTTTCACCATTTGCTTACGTGAATCACTCACACCCTGGTTGTAA
AACTCTGGAGAACCA
+
FFFF:FFFFFFFFFFFFFFFFFFFFFFFF:FFFFF:FFFFFFF:FFFFFF,FFF:FF,FFFFFFF:FFFF
F::F,FFFFFFFFF:F:FFFFFFFFFFFFF:FFFF:FFFFFFFFFFFFFFFFFFFF:FFFFF:FFFFFFF
,FFFFFFFF:FFF:F
@A00155:342:HHGFNDSXY:1:2340:10637:17190 1:N:0:GAACCTAG+TCCGCATA
GCATTCAAAAGGAAGAAGAGAAGAAGGTGGAAAACTTTTCTTTTTACCTACCAGAGAAAACTCGGGAA
TGGTTCACAAAGTCTGGTGTGTATCTTTCACCATTTGCTTACGTGAATCACTCACACCCTGGTTGTAA
AACTCTGGAGAACCG
+
FFFFFFFF::FFFFFFFFFFF,F:FF:FFFF:FFFFFFFFFFFFFFFFFFFFFFFFFFFFFFFFFFFFFF
FFFFFFFFFFFFFFFFFFFFFFFFF,FFF:FFFFFFFFFFFFFFFFFFFFFFFFFFFFFFF:FFFFFFFF
FFFFFFFFFFFFFFF
@A00155:342:HHGFNDSXY:1:2369:3757:34679 1:N:0:GAACCTAG+TCCGCATA
CATTCAAAAGGAAGAAGAGAAGAAGGTGGAAAACTTTTCTTTTTACCTACCAGAGAAAACTCGGGAAT
GGTTCACAAAGTCTGGTGTGTATCTTTCACCATTTGCTTACGTGAATCACTCACACCCTGGTTGTAAA
ACTCTGGAGAACCA
+
FF:FFFFFFFFFFFF:FFF:FFF,:,,FFFFFFF:FFFFFFFF,FFFFFFFFFFFFFFFFFFFFFFFFFF
FFFFFFFFFFFFFFFFFFFFFFFFFFFFFFFFFFFFFFFFFFFFFFFFFFFFFFFFFFFFFFFFFFFFFF
FFFFFFFFFFFFFF
@A00155:342:HHGFNDSXY:1:1238:9127:15953 1:N:0:GAACCTAG+TCCGCATA
CATTCAAAAGGAAGAAGAGAAGAAGGTGGAAAACTTTTCTTTTTACCTACCAGAGAAAACTCGGGAAT
GGTTCACAAAGTCTGGTGTGTATCTTTCACCATTTGCTTACGTGAATCACTCACACCCTGGTTGTAAA
ACTCTGGAGAACCAC
+
FFFFFFFFF:FFFFFFFFFFFFFFFFFFFFFFFFFFFFFFFFFFFFFFFFFFFFFFFFFFFFFFFFFFFF
FF:FFFFFF,FFFFFFFFFFFFFFFFFFFFFFFF:FF:FFFFFFFFFFFFFFFFFFFFFFFFFFFFFFFF
FFFFFFFFFFFFFFF
@A00155:342:HHGFNDSXY:1:1608:14443:25222 1:N:0:GAACCTAG+TCCGCATA
CATTCAAAAGGAAGAAGAGAAGAAGGTGGAAAACTTTTCTTTTTACCTACCAGAGAAAACTCGGGAAT
GGTTCACAAAGTCTGGTGTGTATCTTTCACCATTTGCTTACGTGAATCACTCACACCCTGGTTGTAAA
ACTCTGGAGAACCAC
+
FFFFFFFFFFFFFFFFFFFFFFFFFFFFFFFFFFFFFFFFFFFFFFFFFFFFFFFFFFFFFFFFFFFFFF
FFFFFFFFFFFFFFFFFFFFFFFFFFFFFFFFFFFFFFFFFFFFFFFFFFFFFFFFFFFFFFFFFFFFFF
FFFFFFFFFFFFFFF

```
@A00155:342:HHGFNDSXY:1:1628:31891:18270 1:N:0:GAACCTAG+TCCGCATA
CATTCAAAAGGAAGAAGAGAAGAAGGTGGAAAACTTTTCTTTTTACCTACCAGAGAAAACTCGGGAAT
GGTTCACAAAGTCTGGTGTGTATCTTTCACCATTTGCTTACGTGAATCACTCACACCCTGGTTGTAAA
ACTCTGGAGAACCAC
+
FFFFFFFFFFFFFFFFFFFFFFFFFFFFFFF:FFFFFFFFFFF:FFFFFFFFFFFFFFFFFFFFFFFFFF
FFFFFFFFF:FFFFFFFFFF:FFFFFFFFFFFF:FFFFFFFFFFFFFFFFFFFFFFFFFFFFFFFFFFFF
FFFFFFFFFFFFFFF
@A00155:342:HHGFNDSXY:1:1661:7102:23657 1:N:0:GAACCTAG+TCCGCATA
CATTCAAAAGGAAGAAGAGAAGAAGGTGGAAAACTTTTCTTTTTACCTACCAGAGAAAACTCGGGAAT
GGTTCACAAAGTCTGGTGTGTATCTTTCACCATTTGCTTACGTGAATCACTCACACCCTGGTTGTAAA
ACTCTGGAGAACCAC
+
FFFF:FFFFFFFFFFFFFFFFFFFFFFFFFF,FFFFFFFFFFFFFFFFFFFFFFFFF:FFFFFFFFFFFF
FFFFFFFFFFFFFFFFFFFFFFFFFFFFFF,FF:FFF:FFFFFFFFFFFFFFFFFFFFFFFFFFFFFFFF
FFFFFFFFFFFFFFF
@A00155:342:HHGFNDSXY:1:2104:18548:11256 1:N:0:GAACCTAG+TCCGCATA
CATTCAAAAGGAAGAAGAGAAGAAGGTGGAAAACTTTTCTTTTTACCTACCAGAGAAAACTCGGGAAT
GGTTCACAAAGTCTGGTGTGTATCTTTCACCATTTGCTTACGTGAATCACTCACACCCTGGTTGTAAA
ACTCTGGAGAACCAC
+
FFFFFFFFFFFFFFFFFFFFFFFFFFFFFFFFFFFFFFFFFFF:FFFFFFFFFFFFFFFFFFFFFFFFFF
FFFFFFFF:FFFFFFFFFFFFFFFFFFFFFFFFFFFFFFFFF,FFFFFFFFFFFFFFFFFFFFFFFFFFF
FFFFFFFFFFFFFFF
@A00155:342:HHGFNDSXY:1:2139:14895:12226 1:N:0:GAACCTAG+TCCGCATA
CATTCAAAAGGAAGAAGAGAAGAAGGTGGAAAACTTTTCTTTTTACCTACCAGAGAAAACTCGGGAAT
GGTTCACAAAGTCTGGTGTGTATCTTTCACCATTTGCTTACGTGAATCACTCACACCCTGGTTGTACA
ACTCTGGAGAACCAC
+
FFFFFFFFF::FFFFFFFFFF,,::::,FFFFFFFFFFFFFFFFFFFFFFFFFFFFFFFFFFFFFFFFFF
FF:FFFFFFFF:FFFFFFFFFFFFFFFFFFFFFFFFFFFFFFFFFFFFFFFFFFFFFFFFFFFFFFFF,F
FFFFFFFFFFFFFFF
@A00155:342:HHGFNDSXY:1:2255:19117:29528 1:N:0:GAACCTAG+TCCGCATA
CATTCAAAAGGAAGAAGAGAAGACGGTGGAAAACTTTTCTTTTTACCTACCAGAGAAAACTCGGGAAT
GGTTCACAAAGGCTGGTGTGTATCTTTCACCATTTGCTTACGTGAATCACTCACACCCTGGTTGTAAA
ACTCTGGAGAACCAC
+
FF,FFFF:,:,FFFFFFF,FFFF,:,FFFFFFFFFFFFFFFFF:F:FFFFFF:FFF:F,FFFFFF::FFF
FFFFFFFF,,F,FFF,FFFFFFFFFFFFFFF:FFFF::FFFFFFFFFFFF:FFF:F:FFF:FFFFFF:,,
F,,F,FFFFFFFFFF
@A00155:342:HHGFNDSXY:1:2632:7545:5666 1:N:0:GAACCTAG+TCCGCATA
CATTCAAAAGGAAGAAGAGAAGAAGGTGGAAAACTTTTCTTTTTACCTACCAGAGAAAACTCGGGAAT
GGTTCACAAAGTCTGGTGTGTATCTTTCACCATTTGCTTACGTGAATCACTCACACCCTGGTTGTAAA
ACTCTGGAGAACCAC
+
FFFFFFF,FFF:FFFFFFFFFF:FF:FFFFFFFFF:FFFFF:FFFFFFFFFFFFFFFFFFFFFFFFFFFF
FFFFFFFFFFFFFFFFFFFFFFFFFFFFFFFFFFFFFFFFFF:FFFFFFFFFFFFFFFFFFFFFFFFFFF
FFFFFFFFFFFFFFF
@A00155:342:HHGFNDSXY:1:2672:31882:5760 1:N:0:GAACCTAG+TCCGCATA
CATTCAAAAGGAAGAAGAGAAGAAGGTGGAAAACTTTTCTTTTTACCTACCAGAGAAAACTCGGGAAT
GGTTCACAAAGTCTGGTGTGTATCTTTCACCATTTGCTTACGTGAATCACTCACACCCTGGTTGTAAA
ACTCTGGAGAACCAC
+
FFFFFFFFFFFFFFFFFFFFFFFFFFFFFFFFFFFFFFFFFFFFFFFFFFFFFFFFFFFFFFFFFFFFFF
```

FFFFFFFFFFFFFFFFFFFFFFFFFFFFFFFF:FFFFFFFFFFFFFFFFFFFFFFFFFFFFFFFFFF
FFFFFFFFFFFFFFF
@A00155:342:HHGFNDSXY:1:1553:6705:34272 2:N:0:GAACCTAG+TCCGCATA
CATTCAAAAGGAAGAAGAGAAGAAGGTGGAAAACTTTTCTTTTTACCTACCAGAGAAAACTCGGGAAT
GGTTCACAAAGTCTGGTGTGTATCTTTCACCATTTGCTTACGTGAATCACTCACACCCTGGTTGTAAA
ACTCTGGAGAGATCG
+
FFFFFFFFFFFFFFFFFFFFFFFFFFF:FFFFFFFFFFFFFFFFFFFFFFFFFFFFFFFFFFFFFFFF
FFFFFFFFFFFFFFFFFFFFFFF:FFFFFFFFFFFF:FF:FFFFFFFFFFFFFFFF,FFFFFFFFFF,
FFFFFFFFFFFF:FF
@A00155:342:HHGFNDSXY:1:2506:3323:11663 1:N:0:GAACCTAG+TCCGCATA
ATTCAAAAGGAAGAAGAGAAGAAGGTGGAAAACTTTTCTTTTTACCTACCAGAGAAAACTCGGGAATG
GTTCACAAAGTCTGGTGTGTATCTTTCACCATTTGCTTACGTGAATCACTCACACCCTGGTTGTAAAA
CTCTGGAGAACCAC
+
FF:FFFFF:FFF:FFFFFF:F::F:F,F:FFFFFFFFFFFFFFFFFFFFFFFFFFFFFFFFFF:FF:FF
FFF:F,FFFFFFFFFFFFFFFFFFFFFFFFFFFFFFFFFFFFFFFFFFFFFFFFF,FFFFFFFFFFF
FFFFFFFFFFF,FF
@A00155:342:HHGFNDSXY:1:1143:13847:16767 1:N:0:GAACCTAG+TCCGCATA
TCCGATCTGGAAGAAGAGAAGAAGGTGGAAAACTTTTCTTTTTACCTACCAGAGAAAACTCGGGAATG
GTTCACAAAGTCTGGTGTGTATCTTTCACCATTTGCTTACGTGAATCACTCACACCCTGGTTGTAAAA
CTCTGGAGAACCACC
+
FFFFFFFFFFFFFFFFFFFFFFFFFFFFFFFFF:FFFFFFFFFFFFFFFFFFFFFFFFFFFFFFFFFF
FFFFFFFFFFFFFFFFFFFFFFFFFFFFFFFFFFFFFFFFFFFFFFFFFFFFFFFFFFFFFFFFFFFF
FFFFFFFFFFFFFFF
@A00155:342:HHGFNDSXY:1:1252:12111:31829 1:N:0:GAACCTAG+TCCGCATA
ATTCAAAAGGAAGAAGAGAAGAAGGTGGAAAACTTTTCTTTTTACCTACCAGAGAAAACTCGGGAATG
GTTCACAAAGTCTGGTGTGTATCTTTCACCATTTGCTTACGTGAATCACTCACACCCTGGTTGTAAAA
CTCTGGAGAACCACC
+
FFFFFFFF,FFFFFFFFFFFFFF:FFFFFFFFFFFFFFFFFFFFFFFFFFFFFFFFFFFFFFFFFFFFF
FFFFFFFFFFFFFFFFFFFFFFFFFFFFFFFFFFFFFFFFFFFFFFFFFFFFFFFFFFFFFFFFFFFF
FFFFFFFFFFFFFFF
@A00155:342:HHGFNDSXY:1:1431:29008:12962 1:N:0:GAACCTAG+TCCGCATA
ATTCAAAAGGAAGAAGAGAAGAAGGTGGAAAACTTTTCTTTTTACCTACCAGAGAAGACTCGGGAATG
GTTCACAAAGTCTGGTGTGTATCTTTCACCATTTGCTTACGTGAATCACTCACACCCTGGTTGTAAAA
CTCTGGAGAACCACC
+
FFFFFFFFFFFFFFFFFFFFFFFF:F:FFFFFFFFFFFFFFFFFFFFFFFFFFFFFFFF,FFFFFFFFFFF
FFFFFFFFFFFFFFFFFFFFFFFFFFFFFFFFFFFFFFFFFFFFFFFFFFFFFFFFFFFFFFFFFFFF
FFFFFFFFFFFFFFF
@A00155:342:HHGFNDSXY:1:1431:29206:13777 1:N:0:GAACCTAG+TCCGCATA
ATTCAAAAGGAAGAAGAGAAGAAGGTGGAAAACTTTTCTTTTTACCTACCAGAGAAAACTCGGGAATG
GTTCACAAAGTCTGGTGTGTATCTTTCACCATTTGCTTACGTGAATCACTCACACCCTGGTTGTAAAA
CTCTGGAGAACCACC
+
F:FFFFFFFFFFFFFFFFF,FF::FF:FFFFFFFFFFFFFFFFFFFFFFFFFFFF:F:FFFFFFFFFFFF
FFFFFFFFFFFFFFFFFFFFFFFFFFFFFFFFFFFFFFFFFFFFFFFFFFFFFFFFFFFFFFFFFFFF
FFFFFFFFFFFFFFF
@A00155:342:HHGFNDSXY:1:1550:16857:5478 1:N:0:GAACCTAG+TCCGCATA
ATTCAAAAGGAAGAAGAGAAGAAGGTGGAAAACTTTTCTTTTTACCTACCAGAGAAAACTCGGGAATG
GTTCACAAAGTCTGGTGTGTATCTTTCACCATTTGCTTACGTGAATCACTCACACCCTGGTTGTAAAA
CTCTGGAGAACCACC

```
+
F:FFFFFFFFFFFFFFFFFFFFF,:FFFFFFF:FFFFFFFFFFFFFFFFFFFFFFFFF,FFFFFFFFFFF
FFFFFFFFFFFFFFFFFFFFFFFFFFFFFFFFFFFFFFFFFFFFFFFFFFFFFFFFFFFFFFFFFFFFFF
FFFFFFFFFFFFFFF
@A00155:342:HHGFNDSXY:1:1628:20338:36182 1:N:0:GAACCTAG+TCCGCATA
ATTCAAAAGGAAGAAGAGAAGAAGGTGGAAAACTTTTCTTTTTACCTACCAGAGAAAACTCGGGAATG
GTTCACAAAGTCTGGTGTGTATCTTTCACCATTTGCTTACGTGAATCACTCACACCCTGGTTGTAAAA
CTCTGGAGAACCACC
+
FFFFFFFFFFFFFFFFFFFFFFFFFF,,FFFFFFFFFFF:FFFFFFF:FFFFFFFFFFFFFFFFFFFFFF
FFFFFFFFFFFFFFFFFFFFFFFFFFFFFFFFFFFFFFFFFFFFFFFFFFFFFFFFFFFFFFFFFFFFFF
FFFFFFFFFFFFFFF
@A00155:342:HHGFNDSXY:1:2136:10022:27430 1:N:0:GAACCTAG+TCCGCATA
ATTCAAAAGGAAGAAGAGAAGAAGGTGGAAAACTCTTCTTTTTACCTACCAGAGAAAACTCGGGAATG
GTTCACAAAGTCTGGTGTGTATCTTTCACCATTTGCTTACGTGAATCACTCACACCCTGGTTGTAAAA
CTCTGGAGAACCACC
+
FFFFFFFFFFFFFFFFFFFFFFFFFFFFFFFFFF,FFFFFFFFFFFFFFFFFFFFFFFFFFFFFFFFFFF
FFFFFF:FFFFFFFFFFFFFFFFFFFFFFFFFFFFFFFFFFFFFFFFFFFFFFFFFFFFFFFFFFFFFFF
FFFFFFFFFFFFFFF
@A00155:342:HHGFNDSXY:1:2303:12653:16705 1:N:0:GAACCTAG+TCCGCATA
ATTCAAAAGGAAGAAGAGAAGAAGGTGGAAAACTTTTCTTTTTACCTACCAGAGAAAACTCGGGAATG
GTTCACAAAGTCTGGTGTGTATCTTTCACCATTTGCTTACGTGAATCACTCACACCCTGGTTGTAAAA
CTCTGGAGAACCACC
+
F:FFFFFF:FFFF:FFFF,F,,,:::FFFFFFF:FF:FFFF:FFFFFFFFFFFFFFFFFFFFFFFFFFFF
FFFFFFFFFFFFFFFFFFFFFFFFFFFFFFFFFFFFFFFFFFFFFFFFFFFFFFFFFFFFFFFFFFFFFF
FFFFFFFFFFFFFFF
@A00155:342:HHGFNDSXY:1:2443:18466:20635 1:N:0:GAACCTAG+TCCGCATA
ATTCAAAAGGAAGAAGAGAAGAAGGTGGAAAACTTTTCTTTTTACCTACCAGAGAAAACTCGGGAATG
GTTCACAAAGTCTGGTGTGTATCTTTCACCATTTGCTTACGTGAATCACTCACACCCTGGTTGTAAAA
CTCTGGAGAACCACC
+
FFFFFFFFFFFFFFFFFF:FFF:FFFFFFFFFFFFFFFFFFFFFFFFFFFFFFFFFFFFFFFFFFFFFFF
FFFFFFFF:FFFFFFFFFFFFFFFFFFFFFFFF:FFFFFFFFFFFFFFFFFFFFFFFFFFFFFFFFFFFF
FFFFFFFFFFFFFFF
@A00155:342:HHGFNDSXY:1:1570:27688:7451 2:N:0:GAACCTAG+TCCGCATA
ATTCAAAAGGAAGAAGAGAAGAAGGTGGAAAACTTTTCTTTTTACCTACCAGAGAAAACTCGGGAATG
GTTCACAAAGTCTGGTGTGTATCTTTCACCATTTGCTTACGTGAATCACTCACACCCTGGTTGTAAAA
CTCTGGAGAACCACC
+
FFFFFFFFFFFFFFFF:FFFFFFFFFFFFFF:FFFFFFFFFFFFFFFFFFFFFFFFFFFF:FFFFFFFFF
FFFFFFFFFFFFFFFF:FFFFFFFFFFFFFFFFFFFFFFFFFFFFFFFFFFFFFFFFFFFFFFFFFFFFF
FFFFFFFFFFFFFFF
@A00155:342:HHGFNDSXY:1:2472:24813:11397 2:N:0:GAACCTAG+TCCGCATA
ATTCAAAAGGAAGAAGAGAAGAAGGTGGAAAACTTTTCTTTTTACCTACCAGAGAAAACTCGGGAATG
GTTCACAAAGTCTGGTGTGTATCTTTCACCATTTGCTTACGTGAATCACTCACACCCTGGTTGTAAAA
CTCTGGAGAACCACC
+
FFFFFFFF:FFFFFFFFFFFFFFFFFFFFFFFFFFFFFFFFFFFFFFFF:FFFFFFFFFFFFFFFFFFFF
FFFFFFFF:FFFFFFFFFFFFFFFFFFFFFFFFFFFFF:FFFFFFFFFFFFFFFFFFFFFFFFFFFFFFF
FFFFFFFFFFFF:FF
@A00155:342:HHGFNDSXY:1:1550:15682:25989 1:N:0:GAACCTAG+TCCGCATA
TTCAAAAGGAAGAAGAGAAGAAGGTGGAAAACTTTTCTTTTTACCTACCAGAGAAAACTCGGGAATGG
```

```
TTCACAAAGTCTGGTGTGTATCTTTCACCATTTGCTTACGTGAATCACTCACACCCTGGTTGTAAAAC
TCTGGAGAACCACCT
+
FFFFFFFFFFFFFF:FFF:F:F,::,F:FFFFFFFFF:FFFF::F:FFFFFF,:FFFFFFFFFFFF:F
FFFFF:FF:FF:FFFFFFFFF::FFF:FFFFFFF:FFF:FFFFFFFFF:,F:F:FF,,FFF:FFFFFF
FFFFFFFFFFFFFFF
@A00155:342:HHGFNDSXY:1:1626:14588:20337 1:N:0:GAACCTAG+TCCGCATA
TTCAAAAGGAAGAAGAGAAGAAGGTGTAAAACTTTTCTTTTTACCTACCAGAGAAAACTCGGGAATGG
TTCACAAAGTCTGGTGTGTATCTTTCACCATTTGCTTACGTGAATCACTCACACCCTGGTTGTAAAAC
TCTGGAGAACCACCA
+
FFFFFFFFFFFFFFFF:FFFF:FF,:FFFFFF,FFFFFFFFFFFFFFFFFFF:FFFFFFFFFFFFFFF
FFFFFFFFFFFFFFFFFFFFFFFFFFFFFFFFFFFFFFFFFFFFFFFFFFFFFFFFFFFFFFFFFFFF
FFFFFFFFFFFFFFF
@A00155:342:HHGFNDSXY:1:2111:17155:13573 1:N:0:GAACCTAG+TCCGCATA
TTCAAAAGGAAGAAGAGAAGAAGGTGGAAAACTTTTCTTTTTACCTACCAGAGAAAACTCGGGAATGG
TTCACAAAGTCTGGTGTGTATCTTTCACCATTTGCTTACGTGAATCACTCACACCCTGGTTGTAAAAC
TCTGGAGAACCACCC
+
FFFFF:FFFFFFFFFFFFFFFF:FF:FF:FFFF:FFFFFFFFFFFFFFFFFFFFFFFFFFFFFFFFFF
FFFFFFFFFFFFFFFFFFFFFF:FFFFFFFFFFFFFFFFFFFFFFFFFFFFFFFFFFFFFFFFFFFFF
FFFFFFFFFFFFFFF
@A00155:342:HHGFNDSXY:1:2417:15619:26412 2:N:0:GAACCTAG+TCCGCATA
TTCAAAAGGAAGAAGAGAAGAAGGTGGAAAACTTTTCTTTTTACCTACCAGAGAAAACTCGGGAATGG
TTCACAAAGTCTGGTGTGTATCTTTCACCATTTGCTTACGTGAATCACTCACACCCTGGTTGTAAAAC
TCTGGAGAACCACCT
+
FFFFFFFFFFFFFFFFFFFF:FFFFFFFFFFFFFF:FFFFFFFFFFFFFFFFFFFFFFFFFFFFF:FFF
FFFFFFFFFFFFFFFFFFFFFFFFFFFFFFFFFFFFFFFFFFFFFFFFFFFFFF:FFFFFFFFFFFFF
FFFFFFFFFFFFFFF
@A00155:342:HHGFNDSXY:1:1335:10710:19946 2:N:0:GAACCTAG+TCCGCATA
CAAAAGGAAGAAGAGAAGAAGGTGGAAAACTTTTCTTTTTACCTACCAGAGAAAACTCGGGAATGGTT
CACAAAGTCTGGTGTGTATCTTTCACCATTTGCTTACGTGAATCACTCACACCCTGGTTGTAAAACTC
TGGAGAACCACCTAT
+
FFFFFFFFFFFFFFFFFFFFFFFFFFFFFFFFFFFFFFFFFFFFFFFFFFFFFFFFFFFFFFFFFFFF
FFFFFFFFFFFFFFFFFFFFFFFFFFFFFFFFFFFFFFFFFFFFFFFFFFFFFFFFFFFFF:FFFFFF
FFFFFFFF:FFFFFF
@A00155:342:HHGFNDSXY:1:1335:11541:36072 2:N:0:GAACCTAG+TCCGCATA
CAAAAGGAAGAAGAGAAGAAGGTGGAAAACTTTTCTTTTTACCTACCAGAGAAAACTCGGGAATGGTT
CACAAAGTCTGGTGTGTATCTTTCACCATTTGCTTACGTGAATCACTCACACCCTGGTTGTAAAACTC
TGGAGAACCACCTAT
+
FFFFFFFFFFFFFFFFFFFFFFFFFFFFFFFFFFFFFFFFFFFFFFFFFFFFFFFFFFFFFFFFFFFF
FFFFFFFFFFFFFFFFFFFFFFFFFFFFFFFFFFFFFFFFFFFFFFFFFFFFFFFFFFFFFFFFFFFF
FFFFFFFFFFFFFFF
@A00155:342:HHGFNDSXY:1:1350:12689:5556 2:N:0:GAACCTAG+TCCGCATA
CAAAAGGAAGAAGAGAAGAAGGTGGAAAACTTTTCTTTTTACCTACCAGAGAAAACTCGGGAATGGTT
CACAAAGTCTGGTGTGTATCTTTCACCATTTGCTTACGTGAATCACTCACACCCTGGTTGTAAAACTC
TGGAGAACCACCTAT
+
FFFFFFFFFFFFFFFFFFFFFFFFFFFFFFFFFFFFFFFFFFFFFFFF:FFFFFFFFFFFFFF:FFF:F
FFFFFF::FFF:,F::FFFFFFFFFFFFF:FFF:FFFFF:FFFFFFFFFFFFFFFFFFFFFFFFFFFFF
:FFFFF:FFFFFFFF
```

```
@A00155:342:HHGFNDSXY:1:2117:31982:3646 2:N:0:GAACCTAG+TCCGCATA
CAAAAGGAAGAAGAGAAGAAGGTGGAAAACTTTTCTTTTTACCTACCAGAGAAAACTCGGGAATGGTT
CACAAAGTCTGGTGTGTATCTTTCACCATTTGCTTACGTGAATCACTCACACCCTGGTTGTAAAACTC
TGGAGAACCACCTAT
+
FFFFFFFFFFFFFFFFFFFFFFFFFFFFFFFFFFFFFFFFFFFFFFFF:FFFF:FFFFFFFFFF:FFFFF
FFFFFFFFFFFF,FFFFFFFFFFFFFFFFFFFFFFFFFFFFFFFFFFFFFFFFFFFFFFFFFFF:FFFF
FFFFFFFFFFFFFFF
@A00155:342:HHGFNDSXY:1:2120:27868:16282 2:N:0:GAACCTAG+TCCGCATA
AAAAGGAAGAAGAGAAGAAGGTGGAAAACTTTTCTTTTTACCTACCAGAGAAAACTCGGGAATGGTTC
ACAAAGTCTGGTGTGTATCTTTCACCATTTGCTTACGTGAATCACTCACACCCTGGTTGTAAAACTCT
GGAGAACCACCTATT
+
FFFFFFFFFFFFFFFFFFFFFFFFFFFFFFFFFFFFFFFFFFFFFFFFFFFFFFFFFFFFFFF:FFFFFF
FFF:FFFFFFFFFFFFFFFFFFFFFFFFFFFFFFFFFFFFFFFFFFFFFFFFFFFFFFFFFFFFFFFFFF
FFFFFFFFFFFF:FF
@A00155:342:HHGFNDSXY:1:2360:27317:17707 2:N:0:GAACCTAG+TCCGCATA
AAAAGGAAGAAGAGAAGAAGGTGGAAAACTTTTCTTTTTACCTACCAGAGAAAACTCGGGAATGGTTC
ACAAAGTCTGGTGTGTATCTTTCACCATTTGCTTACGTGAATCACTCACACCCTGGTTGTAAAACTCT
GGAGAACCACCTATT
+
FFFFFFFFFFFFFFFFFFFFFFFFFFFFFFFFFFFFFFF:FFFFFFFFFFFFFFFFFFFFFFFFFFFFFF
FFFFFFFFFFFFFFFFFFFFFFFFFFFFFFFFFFFFFFFFFFFFFFFFFFFF,FFFFFFFFFFFFFFFFF
FFFFFFFFFF:FFFF
@A00155:342:HHGFNDSXY:1:2632:28574:22827 2:N:0:GAACCTAG+TCCGCATA
AAAAGGAAGAAGAGAAGAAGGTGGAAAACTTTTCTTTTTACCTACCAGAGAAAACTCGGGAATGGTTC
ACAAAGTCTGGTGTGTATCTTTCACCATTTGCTTACGTGAATCACTCACACCCTGGTTGTAAAACTCT
GGAGAACCACCTATT
+
F:F:FFFFF:FFFFFFFFFFF:FFFFFFFFFFFFFFFFFFFFFFFFFFFFFFFFFFFFFFFFFFFFFFFF
FFFFFFFFFF:FFFFFFFFFFFFFFFFFFFFFFFFFFFFFFFFFFFFFFFFFFFFFFFFFFFFFFFFFFF
FFFFFFFFFFFFFFF
@A00155:342:HHGFNDSXY:1:1171:16441:31970 2:N:0:GAACCTAG+TCCGCATA
AAGGAAGAAGAGAAGAAGGTGGAAAACTTTTCTTTTTACCTACCAGAGAAAACTCGGGAATGGTTCAC
AAAGTCTGGTGTGTATCTTTCACCATTTGCTTACGTGAATCACTCACACCCTGGTTGTAAAACTCTGG
AGAACCACCTATTAT
+
FFFFFFFFFFFFFFFFFFFFFFFFFFFFFFFFFFFFFFFFFFFFFFFFFF:FFFFFFFFFFFFFFFFFFF
FFFFFFFFFFFFFFFFFFFFFFFFFFFFFFFFFFFFFFFFFFFFFFFFFFFFFFFFFFFFFFFFFFFFFF
FFFFFFFFFFFFFFF
@A00155:342:HHGFNDSXY:1:1325:17508:16000 2:N:0:GAACCTAG+TCCGCATA
AAGGAAGAAGAGAAGAAGGTGGAAAACTTTTCTTTTTACCTACCAGAGAAAACTCGGGAATGGTTCAC
AAAGTCTGGTGTGTATCTTTCACCATTTGCTTACGTGAATCACTCACACCCTGGTTGTAAAACTCTGG
AGAACCACCTATTAT
+
FFFFFFFFFFFFFFFFFFFFFFFFFFFFFFFFFFFFFFFFFFFFFFFFFFFFFFFFFFFFF,FFFFFFFF
FF:FFFFFFFFFFFFFFFFFFFFFFFFFFFFFFFFFFFFFFFFFFFFFFFFFFFFFFFFFFF:FFFFFFF
FFFFFFFFFFFFF,F
@A00155:342:HHGFNDSXY:1:2649:8395:9455 2:N:0:GAACCTAG+TCCGCATA
AAGGAAGAAGAGAAGAAGGTGGAAAACTTTTCTTTTTACCTACCAGAGAAAACTCGGGAATGGTTCAC
AAAGTCTGGTGTGTATCTTTCACCATTTGCTTACGTGAATCACTCACACCCTGGTTGTAAAACTCTGG
AGAACCACCTATTAT
+
FFFFFFFFFFFFFFFFFFFFFFFFFFFFFFFFFFFFFFFFFFFFFFFFFFFFFFFFFFFFFFFFFFFFFF
```

FFFFFFFFFFFFFFFFFFFFFFFFFFFFFFFFFFFFFFFFFFFFFFFFFFFFFFFFFFFFFFFFFFFF,
FFFFFFFFFFFF:FF
@A00155:342:HHGFNDSXY:1:1143:13847:16767 2:N:0:GAACCTAG+TCCGCATA
GGAAGAAGAGAAGAAGGTGGAAAACTTTTCTTTTTACCTACCAGAGAAAACTCGGGAATGGTTCACAA
AGTCTGGTGTGTATCTTTCACCATTTGCTTACGTGAATCACTCACACCCTGGTTGTAAAACTCTGGAG
AACCACCAGATCGGA
+
FFFFFFFFFFFFFFFFFFFFFFFFFFFFFFFFFFFFFFFFFFFFFFFFFFFFFFFFFFFFFFFFFFFFF
FFFFFFF,FFFFFFFFFFFFFFFF,FFFFFFFF:FFFFFFFFFFFFFFFFFFFFFFFF:FFFFFFF:FF
FFFFFFFFFFFFFFF
@A00155:342:HHGFNDSXY:1:1416:22155:14027 2:N:0:GAACCTAG+TCCGCATA
GGAAGAAGAGAAGAAGGTGGAAAACTTTTCTTTTTACCTACCAGAGAAAACTCGGGAATGGTTCACAA
AGTCTGGTGTGTATCTTTCACCATTTGCTTACGTGAATCACTCACACCCTGGTTGTAAAACTCTGGAG
AACCACCTATTATTT
+
FFFFFFFFFFFFFFFFFFFFFFFFFFFFFFFFFFFFFFFFFFFFFFFFFFFFFFFFFFFFFFFFFFFFF
,FFFFFFFFFFFFF:FFFFFFFFFFFFFFFFFFFFFFFFFFFFFFFFFFFFFFFFFFFFFFFFFFFFFF
FFFFFFFFFFFFFF:
@A00155:342:HHGFNDSXY:1:1655:5945:1579 2:N:0:GAACCTAG+TCCGCATA
GGAAGAAGAGAAGAAGGTGGAAAACTTTTCTTTTTACCTACCAGAGAAAACTCGGGAATGGTTCACAA
AGTCTGGTGTGTATCTTTCACCATTTGCTTACGTGAATCACTCACACCCTGGTTGTAAAACTCTGGAG
AACCACCTATTATTT
+
F:FFFFFFF:FFFFFFFFFFFFFFFFFFFFFFFFFFF:FFFF,:F:FFFFFFFFFFFF:FFFFFFFFFF
FFFFFFFFFFFFFFFFFFFFFFFFFFFFFFFFF:,FFFFFFFFFFFFFFFFFFFFFFFFFFFFFFFFF:
FFFFFFFF:FF:FFF
@A00155:342:HHGFNDSXY:1:2621:10474:13964 2:N:0:GAACCTAG+TCCGCATA
GGAAGAAGAGAAGAAGGTGGAAAACTTTTCTTTTTACCTACCAGAGAAAACTCGGGAATGGTTCACAA
AGTCTGGTGTGTATCTTTCACCATTTGCTTACGTGAATCACTCACACCCTGGTTGTAAAACTCTGGAG
AACCACCAGATCGGA
+
FFF,,FFFFFF:FFFFFFFFFFFF:FF,FFFFFFFFFFFFFFF:,FFFFFFFFF,FFFF:FFFF:FFFF
FFFFFFFF:FFFF:FFFFFFFF,:FFF:,FFF:FFFF:FFFF:F:F,FFFFFF:FFFFF,F,FFFF,FF
FFF,FF,,FFF,:,F
@A00155:342:HHGFNDSXY:1:1132:3224:20353 2:N:0:GAACCTAG+TCCGCATA
GAAGAAGAGAAGAAGGTGGAAAACTTTTCTTTTTACCTACCAGAGAAAACTCGGGAATGGTTCACAAA
GTCTGGTGTGTATCTTTCACCATTTGCTTACGTGAATCACTCACACCCTGGTTGTAAAACTCTGGAGA
ACCACCTATTATTTA
+
FFFFFFFFFFFFFF:,FFFFFFFFFFFFFFFFFFF:FFFFFFFFF,FFFFFFFFFFFFFFFFFFFF:FF
FFF:FFFFFFFFF,FFFF:FFFF:FFFFFFFFFFFF,FFFFF:FFFFFF:FFFFFFFF:F:FFFFFFF,
FFFFFFFFF:FFFFF
@A00155:342:HHGFNDSXY:1:1177:6867:7247 2:N:0:GAACCTAG+TCCGCATA
GAAGAAGAGAAGAAGGTGGAAAACTTTTCTTTTTACCTACCAGAGAAAACTCGGGAATGGTTCACAAA
GTCTGGTGTGTATCTTTCACCATTTGCTTACGTGAATCACTCACACCCTGGTTGTAAAACTCTGGAGA
ACCACCTATTATTTA
+
FFF:,FFF:FFFFFFFFFFFFF:FFF,F,F,FFF,FFF,FFFFFFFFF:FFFFFF:FFFFF,FF:FFFF
,,FF:FFF:FFFF,FFFFFFFFFFF:F:F,FF,:FF:F:FFFFFFFF:F:FFF:FFFF:F,FFFFFFFF
,FF:FF:F:::FFFF
@A00155:342:HHGFNDSXY:1:1441:27814:36229 2:N:0:GAACCTAG+TCCGCATA
GAAGAAGAGAAGAAGGTGGAAAACTTTTCTTTTTACCTACCAGAGAAAACTCGGGAATGGTTCACAAA
GTCTGGTGTGTATCTTTCACCATTTGCTTACGTGAATCACTCACACCCTGGTTGTAAAACTCTGGAGA
ACCACCTATTATTTA

+
FFFFFFFFFFFFFFFFFFFFFFFFFFFFFFFFFFFFFFFFFFFFFFFFFFFFFFFFF,FFFFFFFFFFF
FFFFFFFFFFFFFFFFFFFFFFFFFFF:FFFFFFFFFFFFFFFFFFFFFFFFFFFFFFFFFFFFFFFFF
FFFFFFFFFFFFFFF
@A00155:342:HHGFNDSXY:1:1454:8160:14904 2:N:0:GAACCTAG+TCCGCATA
GAAGAAGAGAAGAAGGTGGAAAACTTTTCTTTTTACCTACCAGAGAAAACTCGGGAATGGTTCACAAA
GTCTGGTGTGTATCTTTCACCATTTGCTTACGTGAATCACTCACACCCTGGTTGTAAAACTCTGGAGA
ACCACCTATTATTTA
+
FFFFFFFFFFFFFFFFFFFFFFFFFFFFFFFFFFFFFFFFFFFFFFFFFFFFFFFFF:FFFFFFFFFFF
FFFFFFFFFFFFFFFFFFFFFFFFFFFFFFFFFFFFFFFFFFFFFFFFFFFFFFFFF:FFFFFFFFFFF
FFFFFFFFFFFFFFF
@A00155:342:HHGFNDSXY:1:2139:27380:20823 2:N:0:GAACCTAG+TCCGCATA
GAAGAAGAGAAGAAGGTGGAAAACTTTTCTTTTTACCTACCAGAGAAAACTCGGGAATGGTTCACAAA
GTCTGGTGTGTATCTTTCACCATTTGCTTACGTGAATCACTCACACCCTGGTTGTAAAACTCTGGAGA
ACCACCTATTATTTA
+
FFFFFFFFFFFFFFFF:FFFFFFFFFFFFFFFFFFFFFFFFFFFFFFFFFFFFFFFF:FFFFFFFFFFF
FFFFFFFFFFF:FFFFFFFFFFFFFFFFFFFFFFFFF,FFFFFFFFFF:FF:FFFFFFFFF:FFFFFFFF
FFFFFFFFF:FFFFF
@A00155:342:HHGFNDSXY:1:2169:32850:2487 2:N:0:GAACCTAG+TCCGCATA
GAAGAAGAGAAGAAGGTGGAAAACTTTTCTTTTTACCTACCAGAGAAAACTCGGGAATGGTTCACAAA
GTCTGGTGTGTATCTTTCACCATTTGCTTACGTGAATCACTCACACCCTGGTTGTAAAACTCTGGAGA
ACCACCTATTATTTA
+
F:FF,FF:FFFF:FFFFF:FFF:::FFFFFFFFFFFFFFFFFF:FFFFFFFFFFFFFFFFFFFFFFFFF
FFFFFFFFFFF:FFFF:FFF,FFF:FFFFFFFFF:FFFFFFFFFFFFFFF,F:F:FFF:FFFF::FFF
FFF,FFFFFFFFFFF
@A00155:342:HHGFNDSXY:1:2371:8314:24471 2:N:0:GAACCTAG+TCCGCATA
GAAGAAGAGAAGAAGGTGGAAAACTTTTCTTTTTACCTACCAGAGAAAACTCGGGAATGGTTCACAAA
GTCTGGTGTGTATCTTTCACCATTTGCTTACGTGAATCACTCACACCCTGGTTGTAAAACTCTGGAGA
ACCACCTATTATTTA
+
FFFFFFFFFFFFFFFFFFFFFFF:FFFFFFFFFFFFFFFFFFFFFFFFFF,FFFFFFFFFFFFFFFFFFF
FFFFFFFFFFFFFFFFFFFFFFFFFFFFFFFFFFFFFFF:FFFFFFFFFFFFFFFFFFFFFFFFFFFFF
FF:F:FFFF,FFFFF
@A00155:342:HHGFNDSXY:1:2548:25012:14622 2:N:0:GAACCTAG+TCCGCATA
GAAGAAGAGAAGAAGGTGGAAAACTTTTCTTTTTACCTACCAGAGAAAACTCGGGAATGGTTCACAAA
GTCTGGTGTGTATCTTTCACCATTTGCTTACGTGAATCACTCACACCCTGGTTGTAAAACTCTGGAGA
ACCACCTATTATTTA
+
FFFFFFFFFFFFFFFFFFFFFFFFFFFFFFFFFFFFFFFFFFFFFFFFFFFFFFFFF:FFFFFFFFFFF
FFFFFFFFFFFFFFFFFFFF:FFFFF:FFFFFF:FFFFFFF:FFFF:FFFFFFFFFFFFFFFFFFFFF
FF:FFFF:FFFFFFF
@A00155:342:HHGFNDSXY:1:2651:30002:5854 2:N:0:GAACCTAG+TCCGCATA
GAAGAAGAGAAGAAGGTGGAAAACTTTTCTTTTTACCTACCAGAGAAAACTCGGGAATGGTTCACAAA
GTCTGGTGTGTATCTTTCACCATTTGCTTACGTGAATCACTCACACCCTGGTTGTAAAACTCTGGAGA
ACCACCTATTATTTA
+
FFFFFFFFFFFFFFFFFFFFFFFFFFFFFFFFFFFFFFFFFFFFFFFFFFFFFFFFF,FFFFFFFFFFF
FFFFFF:FFFFFFFFFFFFFFFFFFFFFFFFFFFFFFFFFFFFFFFFFFFFFFFFFF:FFFFFFFFFF
FFFFFFFFFFFFFFF
@A00155:342:HHGFNDSXY:1:2651:29912:5948 2:N:0:GAACCTAG+TCCGCATA
GAAGAAGAGAAGAAGGTGGAAAACTTTTCTTTTTACCTACCAGAGAAAACTCGGGAATGGTTCACAAA

GTCTGGTGTGTATCTTTCACCATTTGCTTACGTGAATCACTCACACCCTGGTTGTAAAACTCTGGAGA
ACCACCTATTATTTA
+
:FFFFFFFFFFFFFFFFFFF:FFFFFFFFFFFFFFFFF,FFFFFFFFFFFFFFFF::FFFFFFFFFFF
FFFFFFFFFFFFFFFFFFFFF:FFFFFFFFFFFFFFF:FFFFFFFFFF:FFFFFFFFFFFFFFF:FFF
FFFFFFFFFF:FFFF
@A00155:342:HHGFNDSXY:1:1121:20943:18912 2:N:0:GAACCTAG+TCCGCATA
AAGAAGAGAAGAAGGTGGAAAACTTTTCTTTTTACCTACCAGAGAAAACTCGGGAATGGTTCACAAAG
TCTGGTGTGTATCTTTCACCATTTGCTTACGTGAATCACTCACACCCTGGTTGTAAAACTCTGGAGAA
CCACCTATTATTTAA
+
F,FFFFFFFFFFFFFFFFFFFFFFFFFFFFFFFF:FFFFFFFFF:FFFFFFFFFF,FFFFFFFF:FFF
FFFFFFFFFFFFFFFFFFFFFFFFFFFFFFFFFFFFFFFFFFFFFFFFFFFFFFFFFFFFFFFFFFFF
FFFFFFFFFFFFFFF
@A00155:342:HHGFNDSXY:1:1274:28284:8923 2:N:0:GAACCTAG+TCCGCATA
AAGAAGAGAAGAAGGTGGAAAACTTTTCTTTTTACCTACCAGAGAAAACTCGGGAATGGTTCACAAAG
TCTGGTGTGTATCTTTCACCATTTGCTTACGTGAATCACTCACACCCTGGTTGTAAAACTCTGGAGAA
CCACCTATTATTTAA
+
FFFFFFFFFFFFFFFFFFFFFFFFFFFFFFFFFFFFFFFFFFFFFFFFFFFFFFFFFFFFFFFFFFFF
FFFFFFFFFFFFFFFFFFFFFFFFFFFFFFFFFFFFFFFFFFFFFFFFFF:FFFFF:FFFFFFFFFFF
FFFFFFFF:FFFFFF
@A00155:342:HHGFNDSXY:1:1575:13946:17534 2:N:0:GAACCTAG+TCCGCATA
AAGAAGAGAAGAAGGTGGAAAACTTTTCTTTTTACCTACCAGAGAAAACTCGGGAATGGTTCACAAAG
TCTGGTGTGTATCTTTCACCATTTGCTTACGTGAATCACTCACACCCTGGTTGTAAAACTCTGGAGAA
CCACCTATTATTTAA
+
FFF:FFFFFFFFFFFFFFFFFFFFFFFFFFFFFFFFFFFFFFFFFFFFFFFFFFFFFFFFFFFFFFFF
FFFFFFFFFFFFFFFFFFFFFFFFFFFFFFFFFFFFFFFFFFFFFFFFFFFFFFFFFFFFFFFFFFFF
FFFFFFFFFFFF:FF
@A00155:342:HHGFNDSXY:1:2137:16007:25300 2:N:0:GAACCTAG+TCCGCATA
AAGAAGAGAAGAAGGTGGAAAACTTTTCTTTTTACCTACCAGAGAAAACTCGGGAATGGTTCACAAAG
TCTGGTGTGTATCTTTCACCATTTGCTTACGTGAATCACTCACACCCTGGTTGTAAAACTCTGGAGAA
CCACCTATTATTTAA
+
FFFFFFFFFFFFFFFF:FFFFFFFFFFFFFFFFFFFFFFFFFFFFFFFFFFFFFFFFFFFFFFFFFFF
FFFFFFFFFFFFFFFFFFFFFFFFF:FFFF:FFFFFFFFFFFFFFFFF:FFFFFFF:FFFFFFFFFFF
:FFFFFFFFF:FFFF
@A00155:342:HHGFNDSXY:1:2624:20428:7185 2:N:0:GAACCTAG+TCCGCATA
AAGAAGAGAAGAAGGTGGAAAACTTTTCTTTTTACCTACCAGAGAAAACTCGGGAATGGTTCACAAAG
TCTGGTGTGTATCTTTCACCATTTGCTTACGTGAATCACTCACACCCTGGTTGTAAAACTCTGGAGAA
CCACCTATTATTTAA
+
FFFFFFFFFFFFFFFFFFFFFFFFFFFFFFFFFFFFFFFFFFFFFFFFFFFFFFF:FFFFFFFFFFFF
FFFFFFFFFFFFFFFFFFFFFFFFFFFFFFFFFFFFFFFFFFFFFFFFFFFFFFFFFFFFFFFFFFFF
FFFFFFFFFFFFFFF
@A00155:342:HHGFNDSXY:1:1261:3152:15530 1:N:0:GAACCTAG+TCCGCATA
AGAAGAGAAGAAGGTGGAAAACTTTTCTTTTTACCTACCAGAGAAAACTCGGGAATGGTTCACAAAGT
CTGGTGTGTATCTTTCACCATTTGCTTACGTGAATCACTCACACCCTGGTTGTAAAACTCTGGAGAAC
CACCTATTATTTAAT
+
FFF:FFFFFFFFF:F:FFFFFFFFFFFFFFF:FFFFFFFF,FFFF:FFFFFFFFFFFFFFFFFFFFFFF
FFFFFFFFFFFFFFFFFFFFFFFF,FFFFFFFFFFFFFFF,FFFFF:FFFFFFFFFFF:FFFFFFFFFF
FFFFFFFFFF:FFFF

```
@A00155:342:HHGFNDSXY:1:1244:28971:22357 2:N:0:GAACCTAG+TCCGCATA
AGAAGAGAAGAAGGTGGAAAACTTTTCTTTTTACCTACCAGAGAAAACTCGGGAATGGTTCACAAAGT
CTGGTGTGTATCTTTCACCATTTGCTTACGTGAATCACTCACACCCTGGTTGTAAAACTCTGGAGAAC
CACCTATTATTTAAT
+
FFFFFFFFFFFFFFFFFFFFFFFFFFFFFFFFFFFFFFFFFFFFFFFFFFFFFFFFFFFFFFFFFFFF
FFFFFFFFFFFFFFFFFFFFFFFFFFFFFFFFFFFFFFFFFFFFFFFFFFFFFFFFFFFFFFFFFFFF
FFFFFFFFFFFFFFF
@A00155:342:HHGFNDSXY:1:1511:11686:17722 2:N:0:GAACCTAG+TCCGCATA
AGAAGAGAAGAAGGTGGAAAACTTTTCTTTTTACCTACCAGAGAAAACTCGGGAATGGTTCACAAAGT
CTGGTGTGTATCTTTCACCATTTGCTTACGTGAATCACTCACACCCTGGTTGTAAAACTCTGGAGAAC
CACCTATTATTTAAT
+
F:FFFFFFFFFFF,::FFFFFFFFFFFF:FFFFFFFFFFFFFFFFFFFFFFFFFFFFFFFFFFFFFFF
FFFFFFFFFFFFFFFFFFFFFFFFFFFFFF,FFFFFFFFFFFFFFFFFFFFFFFFFFFFFFFFFFFFF
FFFFFFFFF:FFFFF
@A00155:342:HHGFNDSXY:1:1565:18692:32080 2:N:0:GAACCTAG+TCCGCATA
AGAAGAGAAGAAGGTGGAAAACTTTTCTTTTTACCTACCAGAGAAAACTCGGGAATGGTTCACAAAGT
CTGGTGTGTATCTTTCACCATTTGCTTACGTGAATCACTCACACCCTGGTTGTAAAACTCTGGAGAAC
CACCTATTATTTAAT
+
F:FFFFFFFFFFFFFFFFFFFFF:FFFFFFFFFFFFFFFFFFFFFFFFFFFFFFFFFFFFFFFFFFFF
:FF:,FFFFFFFFFFFFFFF:FFFFFFFFFFFFFFFFFFFFFFFF:FFFFFFFFF:FFFFFFFFF:FF
FFFFFFFFFFF:F:F
@A00155:342:HHGFNDSXY:1:2237:24406:24189 2:N:0:GAACCTAG+TCCGCATA
AGAAGAGAAGAAGGTGGAAAACTTTTCTTTTTACCTACCAGAGAAAACTCGGGAATGGTTCACAAAGT
CTGGTGTGTATCTTTCACCATTTGCTTACGTGAATCACTCACACCCTGGTTGTAAAACTCTGGAGAAC
CACCTATTATTTAAT
+
FFFFFF:FFFFFFF:FFFFFFFFFFFFFFFFFF:FFFFFFFFFFFFFF:F:FFF,F::FFFFFFFFFF
FFFFFFFFFFF:FFFFFF,FFFFFFFFFFFFFFFFF:FFFFFFF:FFF,:FFFFFFFFFFFFFF:FF,
FFFFFFFFFFFFFFF
@A00155:342:HHGFNDSXY:1:2461:13494:34820 2:N:0:GAACCTAG+TCCGCATA
AGAAGAGAAGAAGGTGGAAAACTTTTCTTTTTACCTACCAGAGAAAACTCGGGAATGGTTCACAAAGT
CTGGTGTGTATCTTTCACCATTTGCTTACGTGAATCACTCACACCCTGGTTGTAAAACTCTGGAGAAC
CACCTATTATTTAAT
+
FFFFFFFFFF:FFFFFFFFFFFFFFFFFFFFFFFFFFFFFFFFFFFFFFFFFFFFFFFFFFFFFFFFF
FFFFFFFFFFFFFFFFFFFFFFFFFFFFFFFFFFFFFFFFFFFFFFFF::FFFFFFFFFFFFFFFFFF
FFFFFFFFFFFFFFF
@A00155:342:HHGFNDSXY:1:2567:6714:20259 2:N:0:GAACCTAG+TCCGCATA
AGAAGAGAAGAAGGTGGAAAACTTTTCTTTTTACCTACCAGAGAAAACTCGGGAATGGTTCACAAAGT
CTGGTGTGTATCTTTCACCATTTGCTTACGTGAATCACTCACACCCTGGTTGTAAAACTCTGGAGAAC
CACCTATTATTTAAT
+
FFFFFFFFFFFFFFF:FFFFFFFFFF:FFFFFFFFFF:FF:FFFFF,FFF:FFFFFFFF:FFFFFFFF
:FF,FFFFFFFFFFFFF:FFFFFFFFFFF:F:FFFFFF::F,F,F:FFFFFFFFF:FFFFFFFFFFFF
FFFFFFFFFFFFFFF
@A00155:342:HHGFNDSXY:1:2433:13060:13119 2:N:0:GAACCTAG+TCCGCATA
GAAGAGAAGAAGGTGGAAAACTTTTCTTTTTACCTACCAGAGAAAACTCGGGAATGGTTCACAAAGTC
TGGTGTGTATCTTTCACCATTTGCTTACGTGAATCACTCACACCCTGGTTGTAAAACTCTGGAGAACC
ACCTATTATTTAATG
+
FFFFFFFFFFFFFFFFFFFFFFFFFFFFFFFFFFFFFFFFFFFFFFFFFFFFF:FFFFFFFFFFFFFF
```

FFFFFFFFFFFFFFFFFFFFFFFFFFFFFFFFFF:FFFFFFFFFFFFFFFFFF:FFFFFFFFFFFFFF
FFFFFF:FFFFFFF:
@A00155:342:HHGFNDSXY:1:1174:19217:7717 2:N:0:GAACCTAG+TCCGCATA
AAGAGAAGAAGGTGGAAAACTTTTCTTTTTACCTACCAGAGAAAACTCGGGAATGGTTCACAAAGTCT
GGTGTGTATCTTTCACCATTTGCTTACGTGAATCACTCACACCCTGGTTGTAAAACTCTGGAGAACCA
CCTATTATTTAATGT
+
FFFFFFFFFFFFFFFFFFFFFFFFFFFFFFFFFFFFFFFFFFFFFFFFFFFFFFFFFFFFFFFFFFFF
FFF:FFFFFFFFF:FFFFFFFFFFFFFFFFFFFFFFFFFFFFFFFFFFFFFFFF,FFFF:FFFFFFFF
FFF:FFFFFFFFFFF
@A00155:342:HHGFNDSXY:1:1432:10438:35258 2:N:0:GAACCTAG+TCCGCATA
AAGAGAAGAAGGTGGAAAACTTTTCTTTTTACCTACCAGAGAAAACTCGGGAATGGTTCACAAAGTCT
GGTGTGTATCTTTCACCATTTGCTTACGTGAATCACTCACACCCTGGTTGTAAAACTCTGGAGAACCA
CCTATTATTTAATGT
+
FFFFFFFFFFFFFFFFFFFFFFFFFFFFFFFFFFFFFFFFFFFFFFFFFFFF::FFFFFFFFF,FFFF
FFFFFFFFFFFFFFFFFFFFFFFFFFFFFFFFFFFFFFFFFF,:FFFFFFFFFFF,FFFF:FFF:FFF
FFFFFFFFFFFF:FF
@A00155:342:HHGFNDSXY:1:2222:6641:3724 2:N:0:GAACCTAG+TCCGCATA
AAGAGAAGAAGGTGGAAAACTTTTCTTTTTACCTACCAGAGAAAACTCGGGAATGGTTCACAAAGTCT
GGTGTGTATCTTTCACCATTTGCTTACGTGAATCACTCACACCCTGGTTGTAAAACTCTGGAGAACCA
CCTATTATTTAATGT
+
FFFFFFFFFFFFFFFFFFFFFFFFFFFFFFFFFFFFFFFFFFFFFFFFFFFFFFFFFFFFFFFFFFFF
FFFFFFFFFFFFFFFFFF,FFFFFFFFFFFFFFFFFFFFFFF:FFFFFFFFFFFF:FFFFFFFFF:FF
FFFFFFFF:FFFFFF
@A00155:342:HHGFNDSXY:1:1358:24234:7795 2:N:0:GAACCTAG+TCCGCATA
AGAGAAGAAGGTGGAAAACTTTTCTTTTTACCTACCAGAGAAAACTCGGGAATGGTTCACAAAGTCTG
GTGTGTATCTTTCACCATTTGCTTACGTGAATCACTCACACCCTGGTAGTAAAACTCTGGAGAACAAC
CTATTATTTAATGT
+
FFFFF,:FF:F:FFFFFF,FF:,::,FF:FFF:FF,F,F:FFFFF:,,F,FFF,FFF,FFFFFFFFFF
FF:FFF:F,FF,:F,,FFFFF:FFFFF:FFFF,FF:F:FF,FFFFFF,:FFFFF,,FFFF:FF:F,:F
FFFFF:FFF,F:,F
@A00155:342:HHGFNDSXY:1:1375:15899:27179 1:N:0:GAACCTAG+TCCGCATA
AGAGAAGAAGGTGGAAAACTTTTCTTTTTACCTACCAGAGAAAACTCGGGAATGGTTCACAAAGTCTG
GTGTGTATCTTTCACCATTTGCTTACGTGAATCACTCACACCCTGGTTGTAAAACTCTGGAGAACCAC
CTATTATTTAATGTT
+
FFFFFFFFFFFFFFFFFFFFFFFFFFFFFFFFFFFFFFFFFFFFFFFFFFFFFFFFFFFFFFFFFFFF
FFFFFFFFFFFFFFFFFFFFFFFFFFF:FFFFFFFFFFFFFFFFFFFFFFFFFFFFFFFFFFFFFFFF
FFFFFFFFFFFFFFF
@A00155:342:HHGFNDSXY:1:1119:21703:23891 2:N:0:GAACCTAG+TCCGCATA
AGAGAAGAAGGTGGAAAACTTTTCTTTTTACCTACCAGAGAAAACTCGGGAATGGTTCACAAAGTCTG
GTGTGTATCTTTCACCATTTGCTTACGTGAATCACTCACACCCTGGTTGTAAAACTCTGGAGAACCAC
CTATTATTTAATGTT
+
FFFFFFFFFFFFFFFFFFFFFFFFFFFFFFFFFFFFFFFFFFFFFFFFFF,:FFFFFFFFFFFFFFFF
FFFFFFFFFFFFFFFFFFFFFFFFFFFFFFFFFFFFFFFFFFFF:FFFFFFFFFFFFFFFFFFFFFFF
FFFFFFFFFFFF,:F
@A00155:342:HHGFNDSXY:1:1375:15899:27179 2:N:0:GAACCTAG+TCCGCATA
AGAGAAGAAGGTGGAAAACTTTTCTTTTTACCTACCAGAGAAAACTCGGGAATGGTTCACAAAGTCTG
GTGTGTATCTTTCACCATTTGCTTACGTGAATCACTCACACCCTGGTTGTAAAACTCTGGAGAACCAC
CTATTATTTAATGTT

+
FFFFFFFFFFFFFFF:FFFFFFFFFFFFFFFFFFFFFFFFFFFFFFFFFFFFFFFFFFFFFFFFFFFF
FFFFFFFFFFFFFFFFFFFFFFFF:FFFFFFFFFFFFFFFFFFFFFFFFFFFFFFFFFFFFFFFFFFF
FF:FFFFFFFFFFFF
@A00155:342:HHGFNDSXY:1:2161:23863:14356 2:N:0:GAACCTAG+TCCGCATA
AGAGAAGAAGGTGGAAAACTTTTCTTTTTACCTACCAGAGAAAACTCGGGAATGGTTCACAAAGTCTG
GTGTGTATCTTTCACCATTTGCTTACGTGAATCACTCACACCCTGGTTGTAAAACTCTGGAGAACCAC
CTATTATTTAATGTT
+
:FFFFFFFFFFFF:F:F:F:FFFFF:FFFFFFFFFFF:FFFFFFFFFFFFFFFFF,FFFFFFFFF:FF
:FFF:FFFFFFFFFF:FFFFFFFFFFFF:FFFFFFFFFFFFFF::FF::FFFFF:FFFFFFFF:FFFF
FFFF:FF:FF,FFF:
@A00155:342:HHGFNDSXY:1:2161:25825:16658 2:N:0:GAACCTAG+TCCGCATA
AGAGAAGAAGGTGGAAAACTTTTCTTTTTACCTACCAGAGAAAACTCGGGAATGGTTCACAAAGTCTG
GTGTGTATCTTTCACCATTTGCTTACGTGAATCACTCACACCCTGGTTGTAAAACTCTGGAGAACCAC
CTATTATTTAATGTT
+
FFFFFFFFFFFFFFFFFFFFFFFFFFFFFFFFFFFFFFFFFFFFFFFFFFFFFFFFFFFFFFFFF,FF
FFFFFFFFFFFFFFFFFFFFFFFFFFFFFFFFFFFFFFFFFFFFFFF:FFFFF:FFFFFFFFFFFFFF
FFFFFFFFFFF:FFF
@A00155:342:HHGFNDSXY:1:2159:1172:15702 2:N:0:GAACCTAG+TCCGCATA
AGAGAAGAAGGTGGAAAACTTTTCTTTTTACCTACCAGAGAAAACTCGGGAATGGTTCACAAAGTCTG
GTGTGTATCTTTCACCATTTGCTTACGTGAATCACTCACACCCTGGTTGTAAAACTCTGGAGAACCAC
CTATTATTTAATGTT
+
:F:F:FFFFFFFFFFFF,:FFFFF:FFF:,FFFFFFFF:FFFF::F:FFFF:F:FFFF,FFF:FF,FF
FFFFFF:FFFFFF,:,FFFFF:FFF:FFF:FFFF,FFF:FF,FFFFFF,F:F,,:FFFF,:FFFF:,F
FF:FFFFFFF:FFFF
@A00155:342:HHGFNDSXY:1:2271:28067:3693 2:N:0:GAACCTAG+TCCGCATA
AGAGAAGAAGGTGGAAAACTTTTCTTTTTACCTACCAGAGAAAACTCGGGAATGGTTCACAAAGTCTG
GTGTGTATCTTTCACCGTTTGCTTACGTGAATCACTCACACCCTGGTTGTAAAACTCTGGAGAACCAC
CTATTATTTAATGTT
+
FFFFFFFFFFFFFFFFF:FFFFFFFFFFFFFFFFFFFFFFFFFFFFFFFFFFFFFFFFFFFFFFFFFF
FFFFFFFFFFFFFFFF:FFFFFFFFFFFFFFFFFFFFFFFFFFFFFFFFFFFFFFFFFFFFFFFFFFF
FFFFFFFFFFF:FFF
@A00155:342:HHGFNDSXY:1:2662:23366:17347 2:N:0:GAACCTAG+TCCGCATA
AGAGAAGAAGGTGGAAAACTTTTCTTTTTACCTACCAGAGAAAACTCGGGAATGGTTCACAAAGTCTG
GTGTGTATCTTTCACCATTTGCTTACGTGAATCACTCACACCCTGGTTGTAAAACTCTGGAGAACCAC
CTATTATTTAATGTT
+
F:FFF:FFFFF:,FFFFFFF:FFF:FFFFFFFFFFFFFFFFFFFFFFFF:FFFF:FFFFFF:FFFFFF
FFF,:FFFFF:FF,FFFFFFFFFFFFFFFFFFFFFFFFFFFFFFFFFFFFFFFFFFFFFFFFFFFFF
:FFFFFFFFF,FFFF
@A00155:342:HHGFNDSXY:1:2673:12744:17425 2:N:0:GAACCTAG+TCCGCATA
AGAGAAGAAGGTGGAAAACTTTTCTTTTTACCTACCAGAGAAAACTCGGGAATGGTTCACAAAGTCTG
GTGTGTATCTTTCACCATTTGCTTACGTGAATCACTCACACCCTGGTTGTAAAACTCTGGAGAACCAC
CTATTATTTAATGTT
+
FFFFFFFFFFFFFFFFFFFFFFFFFFFFFFFFFFFFFFFFFFFFFFFFFFFFFFFFFFFFFFFFFFFF
FFFFFFFFFFFFFFFFFFFFFFFFFFFFFFFFFFFFFFFFFFFFFFFFFFFFFFFFFFF:F:FFF:FF
FFFFFFFFFF,FFFF
@A00155:342:HHGFNDSXY:1:1621:30074:26490 2:N:0:GAACCTAG+TCCGCATA
GAGAAGAAGGTGGAAAACTTTTCTTTTTACCTACCAGAGAAAACTCGGGAATGGTTCACAAAGTCTGG

TGTGTATCTTTCACCATTTGCTTACGTGAATCACTCACACCCTGGTTGTAAAACTCTGGAGAACCACC
TATTATTTAATGTT
+
FFFFFFFFFFFFFFFFFFFFFFF:FFFFFFFFFFFFFFFFFFFFFFFFFFFFFFFFFFFFFFFFFFFFFF
FFFFFFFFFFFFFFFFFFFFFFFFFFFFFFFFFFFFFFFFFFFFFFFFFFFFF:FFFFFFFFFFFFFFFF
FFFFFFFFFFFFFF
@A00155:342:HHGFNDSXY:1:1149:12590:30217 1:N:0:GAACCTAG+TCCGCATA
GAGAAGAAGGTGGAAAACTTTTCTTTTTACCTACCAGAGAAAACTCGGGAATGGTTCACAAAGTCTGG
TGTGTATCTTTCACCATTTGCTTACGTGAATCACTCACACCCTGGTTGTAAAACTCTGGAGAACCACC
TATTATTTAATGTTG
+
FFF:FFFFFFFFFFFFFF:FFFFFFFFFFFFFFFFFFFFFFFFFFFFFFFFFFFFFFFFFFFFFFFFFFF
FFFFFFFFFFFFFFFFFFFFFFFFFFFFFFFFFFFFFFFFFFFFFFFFFFFFFFFFFFFFFFFFFFFFFF
FFFFFFFFFFFFFFF
@A00155:342:HHGFNDSXY:1:1174:23972:26349 1:N:0:GAACCTAG+TCCGCATA
ATCTAGAAGGTGGAAAACTTTTCTTTTTACCTACCAGAGAAAACTCGGGAATGGTTCACAAAGTCTGG
TGTGTATCTTTCACCATTTGCTTACGTGAATCACTCACACCCTGGTTGTAAAACTCTGGAGAACCACC
TATTATTTAATGTTG
+
FFFFFFFFFFFFFFFFFFFFFFFFFFFFFFFFFFFFFFFFFFFFFFFFFFFFFFFFFFFFFFFFFFFFFF
FFFFFF:FFFFFFFFFF:FFFFFFFFFFFFFFFFFFFFFFFFFFFFFFFFFFFFFFFFFFFFFFFFFFFF
FFFFFFFFFFFFFFF
@A00155:342:HHGFNDSXY:1:1227:17806:20776 1:N:0:GAACCTAG+TCCGCATA
GAGAAGAAGGTGGAAAACTTTTCTTTTTACCTACCAGAGAAAACTCGGGAATGGTTCACAAAGTCTGG
TGTGTATCTTTCACCATTTGCTTACGTGAATCACTCACACCCTGGTTGTAAAACTCTGGAGAACCACC
TATTATTTAATGTTG
+
FFFFFFFFFFFFFFFFFFFFFFFFFFFFFFFFFFFFFFFFFFFFFFFFFFFFFFFFFFFFFFFFFFFFFF
FFFFFFFFFFFFFFFFFFFFFFFFFFFFFFFFFFFFFFFFFFFFFFFFFFFFFFFFFFFFFFFFFFFFFF
FFFFF:FFFFFFFFF
@A00155:342:HHGFNDSXY:1:1227:16893:21449 1:N:0:GAACCTAG+TCCGCATA
GAGAAGAAGGTGGAAAACTTTTCTTTTTACCTACCAGAGAAAACTCGGGAATGGTTCACAAAGTCTGG
TGTGTATCTTTCACCATTTGCTTACGTGAATCACTCACACCCTGGTTGTAAAACTCTGGAGAACCACC
TATTATTTAATGTTG
+
FFFFFFFFFFFFFFFFFFFFFFFFFFFFFFFFFFFFFFFFFFFFFFFFFFFFFFFFFFFFFFFFFFFFFF
FFFFFFFFFFFFFFFFFFFFFFFFFFFFFFFFFF:FFFFFFFFFFFFFFFFFFFFFFFFFFFFFFFFFFF
FFFFFFFFFFFFFFF
@A00155:342:HHGFNDSXY:1:1245:28583:11005 1:N:0:GAACCTAG+TCCGCATA
GAGAAGAAGGTGGAAAACTTTTCTTTTTACCTACCAGAGACAACTCGGGAATGGTTCACAAAGTCTGG
TGTGTATCTTTCACCATTTGCTTACGTGAATCACTCACACCCTGGTTGTAAAACTCTGGAGAACCACC
TATTATTTAATGTTG
+
FFF,F,FFF:FFF:::FFFFFF,FFFFFF,FFF,FFFFFF,:F:FFFFFFF:,FFF:,:::F:F:F,F
FFFFF,F,FFFF,,FFFFFFFFF:,FFFFFFFFFFF:::FFFFFF::FF:,:,FF,FFFFF:,F,FF,
FFFFFFF:::FFFFF
@A00155:342:HHGFNDSXY:1:1255:12029:31438 1:N:0:GAACCTAG+TCCGCATA
GAGAAGAAGGTGGAAAACTTTTCTTTTTACCTACCAGAGAAAACTCGGGAATGGTTCACAAAGTCTGG
TGTGTATCTTTCACCATTTGCTTACGTGAATCACTCACACCCTGGTTGTAAAACTCTGGAGAACCACC
TATTATTTAATGTTG
+
FFFFFFFFFFFFFFFFFFFFFFFFFFF:FFFFFFFFFFFFFFFFF,FFFFFFFFFFFFFFFFFFFFFFFF
FFFFFFFFFFFFFFFFFFFFFFFFFFFFFFFFFFFFFFFFFFFFFFFFFFFFFFFFFFFFFFFFFFFFFF
FFFFFFFFFFFFFFF

```
@A00155:342:HHGFNDSXY:1:1308:14922:5071 1:N:0:GAACCTAG+TCCGCATA
GAGAAGAAGGTGGAAAACTTTTCTTTTTACCTACCAGAGAAAACTCGGGAATGGTTCACAAAGTCTGG
TGTGTATCTTTCACCATTTGCTTACGTGAATCACTCACACCCTGGTTGTAAAACTCTGGAGAACCACC
TATTATTTAATGTTG
+
FFFFFFF,FFFFFFFFFFFFFFFFFFFFFFFFFFFFFFFFFFFFFFFFFFFFFFFFFFFFFFFFFFFF
FFFFFFFFFFFFFFFFFFFFFFFFFFFFFFFFFFFFFFFFFFFFFFFFFFFFFFFFFFFFFFFFFFFF
FFFFFFFFFFFFFFF
@A00155:342:HHGFNDSXY:1:1365:4788:11600 1:N:0:GAACCTAG+TCCGCATA
GAGAAGAAGGTGGAAAACTTTTCTTTTTACCTACCAGAGAAAACTCGGGAATGGTTCACAAAGTCTGG
TGTGTATCTTTCACCATTTGCTTACGTGAATCACTCACACCCTGGTTGTAAAACTCTGGAGAACCACC
TATTATTTAATGTTG
+
FFFFFFFFFFFFFFFFFFFFFFF:FFFFFFFFFFFFFFFFFFFFFFFFFFFFFFFFFFFFFFFFFFFF
FFFFFFFFFFFFFFFFFFFFFFFFFFFFFFFFFFFFFFFFFFFFFFFFFFFFFFFFFFFFFFFFFFFF
FFFFF:FFFFFFFFF
@A00155:342:HHGFNDSXY:1:1423:29279:10833 1:N:0:GAACCTAG+TCCGCATA
GAGAAGAAGGTGGAAAACTTTTCTTTTTACCTACCAGAGAAAACTCGGGAATGGTTCACAAAGTCTGG
TGTGTATCTTTCACCATTTGCTTACGTGAATCACTCACACCCTGGTTGTAAAACTCTGGAGAACCACC
TATTATTTAATGTTG
+
FFFFFFFFFFFFFFFFFFFFFFFFFFFFFFFFFFFFFFFFFFFFFFFFFFFFFFFFFFFFF:FFFFFF
FFFFFFFFFFFFFFFFFFFFFFFFFFFFFFFFFFFFFFFFFF::FFFFFFFFFFFFF:FFFFFFFFFF
FFFFFFFFF:FFFFF
@A00155:342:HHGFNDSXY:1:1435:9372:19727 1:N:0:GAACCTAG+TCCGCATA
GAGAAGAAGGTGGAAAACTTTTCTTTTTACCTACCAGAGAAAACTCGGGAATGGTTCACAAAGTCTGG
TGTGTATCTTTCACCATTTGCTTACGTGAATCACTCACACCCTGGTTGTAAAACTCTGGAGAACCACC
TATTATTTAATGTTG
+
FFFFFFFFFFFFFFFFFFFFFFFFFFFFFFFFFFFFFFFFFFFFFFFFFFFFFFFFFFFFFFFFFFFF
FFFFFFFFFFFFFFFFFFFFFFFFFFFFFFFFFFFFFFFFFFFFFFFFFFFFFFFFFFFFFFFFFFFF
FFFFFFFFFFFFFFF
@A00155:342:HHGFNDSXY:1:1463:28122:29716 1:N:0:GAACCTAG+TCCGCATA
GAGAAGAAGGTGGAAAACTTTTCTTTTTACCTACCAGAGAAAACTCGGGAATGTTTCACAAAGTCTGG
TGTGTATCTTTCACCATTTGCTTACGTGAATCACTCACACCCTGGTTGTAAAACTCTGGAGAACCACC
TATTATTTAATGTTG
+
F:F,FF,FFFF:FFFFFFFFFF,FFFFFF,,FFFFFFFF,:,FF::FFFFFFF,FF:::FF,FF,FF:
F:F:FF,FFFFFFF,F:FFF:FFFFFFFFFFFFFFFFFF,,::FFFFFFFFF,FFFFFFFFF:,FF:F
FFFFFFFF:FFFFFF
@A00155:342:HHGFNDSXY:1:1478:7654:24486 1:N:0:GAACCTAG+TCCGCATA
GAGAAGAAGGTGGAAAACTTTTCTTTTTACCTACCAGAGAAAACTCGGGAATGGTTCACAAAGTCTGG
TGTGTATCTTTCACCATTTGCTTACGTGAATCACTCACACCCTGGTTGTAAAACTCTGGAGAACCACC
TATTATTTAATGTTG
+
FFFFFFFFF:FFFFFFFFFFFFFFFFF:FFFFFFFFFFFFFFFFFF:FFFFFFFFFFFFFF:FFFFFF
FFFFFFFFFFFFFFFFFFFF:FFFFFFFFFFFFFFFFFFFFFFFFFFFFFFFFFFFFFFFFFFFFFFF
FFFFFF:FFFFFFFF
@A00155:342:HHGFNDSXY:1:1502:5719:4695 1:N:0:GAACCTAG+TCCGCATA
GAGAAGAAGGTGGAAAACTTTTCTTTTTACCTACCAGAGAAAACTCGGGAATGGTTCACAAAGTCTGG
TGTGTATCTTTCACCATTTGCTTACGTGAATCACTCACACCCTGGTTGTAAAACTCTGGAGAACCACC
TATTATTTAATGTTG
+
FFFFFFFF::,::FFFFFFFFFFFFFFFFFFFFFFFFFFFFFFFFFFFFFFFFFFFFFFF:FFFFFFF
```

FFFFFFFFFFFFFFFFF:F:FFFFFFFFFFFFFFFFFFFFFFFFFFFFFFFFFFFFFFFFFFFFFF:
FFFFFFFFFFFFFFF
@A00155:342:HHGFNDSXY:1:1513:11577:23516 1:N:0:GAACCTAG+TCCGCATA
GAGAAGAAGGTGGAAAACTTTTCTTTTTACCTACCAGAGAAAACTCGGGAATGGTTCACAAAGTCTGG
TGTGTATCTTTCACCATTTGCTTACGTGAATCACTCACACCCTGGTTGTAAAACTCTGGAGAACCACC
TATTATTTAATGTTG
+
:FF,:F::F:F:FFFFF:FFF::FFFFFFFFFFFFFFFFFF,FFFFFFFFFFFFFFFFFFFFFFFFFFF
FFFFFFFFFFFFFFFFFFFFFFFFFFFFFFFFFFFFF:FFFFF:,FFFFFFFFF:FFFFFF:FFFFFF
FFFFFFFFFFFFFFF
@A00155:342:HHGFNDSXY:1:1521:2419:33833 1:N:0:GAACCTAG+TCCGCATA
GAGAAGAAGGTGGAAAACTTTTCTTTTTACCTACCAGAGAAAACTCGGGAATGGTTCACAAAGTCTGG
TGTGTATCTTTCACCATTTGCTTACGTGAATCACTCACACCCTGGTTGTAAAACTCTGGAGAACCACC
TATTATTTAATGTTG
+
FFFFFFFF:FFFFFFFFFFFFFFFFFFFFFFFFF:FFF:FFFFFFFFFFFFFFFFFFFFFFFFFFFFF
FFFFFFFFFFFFFFFFFFFFFFFFFFFFFFFFFFFFFFFFFFFFFFFFFFFFFFFFFFFFFFFFFFFF
FFFFFFFFFFFFFFF
@A00155:342:HHGFNDSXY:1:1559:23411:11757 1:N:0:GAACCTAG+TCCGCATA
GAGAAGAAGGTGGAAAACTTTTCTTTTTACCTACCAGAGAAAACTCGGGAATGGTTCACAAAGTCTGG
TGTGTATCTTTCACCATTTGCTTACGTGAATCACTCACACCCTGGTTGTAAAACTCTGGAGAACCACC
TATTATTTAATGTTG
+
FFFFF:,FFFFFFFFFFFFFFFFFFFFFFFFFFFFFFFFFFFFFFFFFFFFFFFFFFFFFFFFFFFFF
FFFFFFFFFFFFFFFFFFFFFFFFFFFFFFFFFFFFFFFFFFFFFFFFFFFFFFFFFFFFFFFFFFFF
FFFFFFFFFFFFFFF
@A00155:342:HHGFNDSXY:1:2204:16568:8015 1:N:0:GAACCTAG+TCCGCATA
GAGAAGAAGGTGGAAAACTTTTCTTTTTACCTACCAGAGAAAACTCGGGAATGGTTCACAAAGTCTGG
TGTGTATCTTTCACCATTTGCTTACGTGAATCACTCACACCCTGGTTGTAAAACTCTGGAGAACCACC
TATTATTTAATGTTG
+
FFFFFFFFFFFFFFFFFFFFFF:FFFFFFFFFFFFFFFFFFFFFFFFFFFFFFFFFFFFFFFFFFFFF
FFFFFFFFFFFFFFFFFFFFFFFFFFFFFFFFFFFFFFFFFFFFFFFFFFFFFFFFFFFFFFFFFFFF
FFFFFFFFFFFFFFF
@A00155:342:HHGFNDSXY:1:2249:19208:13307 1:N:0:GAACCTAG+TCCGCATA
GAGAAGAAGGTGGAAAACTTTTCTTTTTACCTACCAGAGAAAACTCGGGAATGGTTCACAAAGTCTGG
TGTGTATCTTTCACCATTTGCTTACGTGAATCACTCACACCCTGGTTGTAAAACTCTGGAGAACCACC
TATTATTTAATGTTG
+
FFF::F:FFFFFFFF:FFFFFFFFFFFFFFFFFFFFFFFFFFFFFFFFFFFFFFFFFFFFFFFFFFFF
FFFFFFFF:FFFFFFFFFFFFFFFFFFFFFFFFFFFFFFFFFFFFFFFFFFFFFFFFFFFFFFFFFFF
FFFFFFFFFFFFFFF
@A00155:342:HHGFNDSXY:1:1118:8621:5932 2:N:0:GAACCTAG+TCCGCATA
GAGAAGAAGGTGGAAAACTTTTCTTTTTACCTACCAGAGAAAACTCGGGAATGGTTCACAAAGTCTGG
TGTGTATCTTTCACCATTTGCTTACGTGAATCACTCACACCCTGGTTGTAAAACTCTGGGGAACCACC
TATTATTTAATGTTG
+
FFFFFFFFFFFFFFFFFFFFFFFFFFFFFFFFFFFFFFFFFFFFFFFFFFFFFFFFFFF:FFFFFFFF
FFFFFFFFFFFFFFFFFFFFFFFFFFFFFFFFFFFFFFFFFFFFFFFFFFFFFFFFFFF,FF,F:FFF
FFFFFFFFF,FFFFF
@A00155:342:HHGFNDSXY:1:1473:32777:2832 2:N:0:GAACCTAG+TCCGCATA
GAGAAGAAGGTGGAAAACTTTTCTTTTTACCTACCAGAGAAAACTCGGGAATGGTTCACAAAGTCTGG
TGTGTATCTTTCACCATTTGCTTACGTGAATCACTCACACCCTGGTTGTAAAACTCTGGAGAACCACC
TATTATTTAATGTTG

```
+
FFFFFFFFFFFFFFFFFFFFFFFFFFFFFFFFFFFFFFFFFFFFFFFFFFFFFFFFFFFFFFFFFFFF
FFFFFFFFFFFFFFFFFFFFFFFFFFFFFFFFF:FFFFFFFFFFFFFFFFFFFFFFFFFFFFFFFFFF
FFFFFFFFFFFFFFF
@A00155:342:HHGFNDSXY:1:2549:26268:19930 2:N:0:GAACCTAG+TCCGCATA
GAGAAGAAGGTGGAAAACTTTTCTTTTTACCTACCAGAGAAAACTCGGGAATGGTTCACAAAGTCTGG
TGTGTATCTTTCACCATTTGCTTACGTGAATCACTCACACCCTGGTTGTAAAACTCTGGAGAACCACC
TATTATTTAATGTTG
+
FFFFFFFFFFFFFFFFFFF,FFFFFFFFFFFFFFFFFFFFFF,FFFFFFFFFFFFFFFFF,FFFFFFF
FFFFFFFFFFFFFFFFFFFFFFFFFFFFFFFFFFFFFFFFFFFFFFFFFFFFFFFFFFFFF::FFFFF
FFFFFFFFFFFFFFF
@A00155:342:HHGFNDSXY:1:2428:2076:13949 2:N:0:GAACCTAG+TCCGCATA
AGAAGAAGGTGGAAAACTTTTCTTTTTACCTACCAGAGAAAACTCGGGAATGGTTCACAAAGTCTGGT
GTGTATCTTTCACCATTTGCTTACGTGAATAAATCACACCCTGGTTGTAAAACTCTGGAGAACCACCT
ATTATTTAATGTTG
+
FFFFFFFFFFFF:FFFFFFF:FFFFFFF,:FFFFFFF,FFF:FF:FFFF,F::,FFF,FF,FFFF,:F
:F:FFFFFFF,::,F:F,,FFF:FF:::FF,F,,:F,FFF:F,:FFFFFFFF:FF:,,FF,:::,F,F
F:,F,FFFFF:FF:
@A00155:342:HHGFNDSXY:1:1142:20094:14935 1:N:0:GAACCTAG+TCCGCATA
TGAAGAAGGTGGAAAACTTTTCTTTTTACCTACCAGAGAAAACTCGGGAATGGTTCACAAAGTCTGGT
GTGTATCTTTCACCATTTGCTTACGTGAATCACTCACACCCTGGTTGTAAAACTCTGGAGAACCACCT
ATTATTTAATGTTGT
+
FFFFFF:FFFF:FFFFFFFFFFFFFFFFFFFFFFFFFFFFFFFFFFFFFFFFFFFFFFFFFFFFFFFF
FFFFFFFFFFFFFFFFFFFFFFFFFFFFFFFFFFFFFFFFFFFFFFFFFFFFFFFFFFFFFFFFFFFF
FFFFFFFFFFFFFFF
@A00155:342:HHGFNDSXY:1:1228:26151:33442 1:N:0:GAACCTAG+TCCGCATA
AGAAGAAGGTGGAAAACTTTTCTTTTTACCTACCAGAGAAAACTCGGGAATGGTTCACAAAGTCTGGT
GTGTATCTTTCACCATTTGCTTACGTGAATCACTCACACCCTGGTTGTAAAACTCTGGAGAACCACCT
ATTATTTAATGTTGT
+
FFFFF:FFFFFFFFFFFFFFFFFFFFFFFFFFFFFFFFFFFFFF:FFFFFFFFFFFFFFFFFFFFFFF
FFFFFFFFFFFFF:FFFFFFFF:F:FFFFFFFFFFFFFFFFFFFFFFFFFFFFFFFFFFFFFFFFFFF
FFFFFFFFFFFFFFF
@A00155:342:HHGFNDSXY:1:1228:26992:34209 1:N:0:GAACCTAG+TCCGCATA
AGAAGAAGGTGGAAAACTTTTCTTTTTACCTACCAGAGAAAACTCGGGAATGGTTCACAAAGTCTGGT
GTGTATCTTTCACCATTTGCTTACGTGAATCACTCACACCCTGGTTGTAAAACTCTGGAGAACCACCT
ATTATTTAATGTTGT
+
FFFFFFFFFFFFFFFFFFFFFFFFFFFFFFFFFFF:FFFFFFFFFFFFFFFFFFFFFFFFFFFFFFFF
FFFFFF:FFFFFFFFFFFFFFFFFFFF::FFFFFFFFFFFFFFFFFFFFFFFFFFFFFFFFFFFFFFF
FFFFFFFFFFFFFFF
@A00155:342:HHGFNDSXY:1:1229:21495:4304 1:N:0:GAACCTAG+TCCGCATA
AGAAGAAGGTGGAAAACTTTTCTTTTTACCTACCAGAGAAAACTCGGGAATGGTTCACAAAGTCTGGT
GTGTATCTTTCACCATTTGCTTACGTGAATCACTCACACCCTGGTTGTAAAACTCTGGAGAACCACCT
ATTATTTAATGTTGT
+
FFFFFFFFFFFFFFFFFFFFFFFFFFFFFFFFFFFFFFFFFFFFFFFFFFFFFFFFFF:FFFFFFFFF
FFFFFFFFFFFFFFFFFFF:FFFFFFFFFFFFFFFFFFFFFFFFFFFFFFFFFFFFFFFFFFFFFFFF
FFFFFFFFFFFFFFF
@A00155:342:HHGFNDSXY:1:1245:27805:11256 1:N:0:GAACCTAG+TCCGCATA
AGAAGAAGGTGGAAAACTTTTCTTTTTACCTACCAGAGAAAACTCGGGAATGGTTCACAAAGTCTGGT
```

GTGTATCTTTCACCATTTGCTTACGTGAATCACTCACACCCTGGTTGTAAAACTCTGGAGAACCACCT
ATTATTTAATGTTGT
+
FFF:FFFF:FFFFFFFFFFFFFFFFFFFFFFFFFFFFFFFFFFFFFFFFFFFFFFFFFFFFFFFFFFF
FFFFFFFFFFFFFFFFFFFFFFFFFFFFFFFFFFFFFFFFFFFFFFFFFFFFFFFFFFFFFFFFFFFF
FFFFFFFFFFFFFFF
@A00155:342:HHGFNDSXY:1:1325:17508:16000 1:N:0:GAACCTAG+TCCGCATA
AGAAGAAGGTGGAAAACTTTTCTTTTTACCTACCAGAGAAAACTCGGGAATGGTTCACAAAGTCTGGT
GTGTATCTTTCACCATTTGCTTACGTGAATCACTCACACCCTGGTTGTAAAACTCTGGAGAACCACCT
ATTATTTAATGTTGT
+
FFFFFFFFFFFFFFFFFFFFFFFFFFFFFFFFFFFFFFFFFFF:FFFFFFFFFFFFFFFFFFFFFFFF
FFFFFFFFFFFFFFFFFFFFFFFFFFFFFFFFFFFFFFFFFFFFFFFFFFFFFFFFFFFFFFFFFFFF
FFFFFFFFFFFFFFF
@A00155:342:HHGFNDSXY:1:1332:27380:30185 1:N:0:GAACCTAG+TCCGCATA
TCTAGAAGGTGGAAAACTTTTCTTTTTACCTACCAGAGAAAACTCGGGAATGGTTCACAAAGTCTGGT
GTGTATCTTTCACCATTTGCTTACGTGAATCACTCACACCCTGGTTGTAAAACTCTGGAGAACCACCT
ATTATTTAATGTTGT
+
FFFFFFFFFFFFFFFFF:FFFFFFF,FFFFFFFF:FFFFFFFFFFFFFFFFFFFFFFFFFFFFFFFFF
FFFFFFFFFFFFFFFFFFFFFFFFFFFFFFFFFFFFFFFFFFFFFFFFFFFFFFFFFFFFFFFFFFFF
FFFFFFFFFFFFFFF
@A00155:342:HHGFNDSXY:1:1336:22562:4867 1:N:0:GAACCTAG+TCCGCATA
AGAAGAAGGTGGAAAACTTTTCTTTTTACCTACCAGAGAAAACTCGGGAATGGTTCACAAAGTCTGGT
GTGTATCTTTCACCATTTGCTTACGTGAATCACTCACACCCTGGTTGTAAAACTCTGGAGAACCACCT
ATTATTTAATGTTGT
+
FF::FFFFFFFFFFFFFFFFFFFFFFFFFFFFFFFFFFFFFFFFFFFFFFFFFFFFFFFFFFFFFFFF
FFFFFFFFFFFFFFFFFFFFFFFFFFFFFFFFFFFFFFFFFFFFFFFFFFFFFFFFFFFFFFFFFFFF
FFFFFFFFFFFFFFF
@A00155:342:HHGFNDSXY:1:1364:10140:6026 1:N:0:GAACCTAG+TCCGCATA
AGAAGAAGGTGGAAAACTTTTCTTTTTACCTACCAGAGAAAACTCGGGAATGGTTCACAAAGTCTGGT
GTGTATCTTTCACCATTTGCTTACGTGAATCACTCACACCCTGGTTGTAAAACTCTGGAGAACCACCT
ATTATTTAATGTTGT
+
FFFFFFFFFF:FFFFFFFFFFFFFFFFFFFFFFFFFFFFFFFFFFFFFFFFFFFFFFFFFFFFFFFFF
FFFFFFFFFFFFFFFFFFFFFF:FFFFFFFFFFFFFFFFFFFFFFFFFFFFFFFFFFFFFFFFFFFFF
FFFF:FFFFFFFFFF
@A00155:342:HHGFNDSXY:1:1439:2510:30358 1:N:0:GAACCTAG+TCCGCATA
AGAAGAAGGTGGAAAACTTTTCTTTTTACCTACCAGAGAAAACTCGGGAATGGTTCACAAAGTCTGGT
GTGTATCTTTCACCATTTGCTTACGTGAATCACTCACACCCTGGTTGTAAAACTCTGGAGAACCACCT
ATTATTTAATGTTGT
+
FFFFFFFF:FFFFFFFFFFFFFFFFFFFFFFFFFFFFFFFFFFFFFFFFFFFFFFFFFFFFF:FFFF
FFFFFFFFFFFFFFFFFFFFFFFF:FFFFFFFFFFFFFFFFFFFFFFFFFFFFFFFFFFFFFFFFFFF
FFFFFFFFFFFFFFF
@A00155:342:HHGFNDSXY:1:2178:26928:35916 1:N:0:GAACCTAG+TCCGCATA
AGAAGAAGGTGGAAAACTTTTCTTTTTACCTACCAGAGAAAACTCGGGAATGGTTCACAAAGTCTGGT
GTGTATCTTTCACCATTTGCTTACGTGAATCACTCACACCCTGGTTGTAAAACTCTGGAGAACCACCT
ATTATTTAATGTTGC
+
FFF,:,::,,:::,F,FFFFFFFFFFFFFFFFFFFFFFFFFFFFFFFFFFFFFFFFFFFFFFFFFFFF
FFFFFFFFFFFFFFFFFFFFFFFFFFFFFFFFFFFFFFFFFFFFFFFFFFFFFFFF:FFFFFFFFFFF
FFFFFFFFFFFFFFF

@A00155:342:HHGFNDSXY:1:2176:20880:2331 1:N:0:GAACCTAG+TCCGCATA
AGAAGAAGGTGGAAAACTTTTCTTTTTACCTACCAGAGAAAACTCGGGAATGGTTCACAAAGTCTGGT
GTGTATCTTTCACCATTTGCTTACGTGAATCACTCACACCCTGGTTGTAAAACTCTGGAGAACCACCT
ATTATTTAATGTTGT
+
F::,:F,FFFF:FFF::FFFFFFFFFFFF,:FFFFFFF:FFF,FFFFFFFFFFFFFF:FFF:F:FFFF
FF:FFF:FFFFF:,FFFFFFFFFFF:FFFFFF:FFF,FFFFFFFFFFFF,,F:FFFFF:FFFFFF:FF
FFFF:FF,,FFFFF:
@A00155:342:HHGFNDSXY:1:2173:7618:31720 1:N:0:GAACCTAG+TCCGCATA
AGAAGAAGGTGGAAAACTTTTCTTTTTACCTACCAGAGAAAACTCGGGAATGGTTCACAAAGTCTGGT
GTGTATCTTTCACCATTTGCTTACGTGAATCACTCACACCCTGGTTGTAAAACTCTGGAGAACCACCT
ATTATTTAATGTTGC
+
F:FFFFFFFFFFFFFFFFF:FF:FFFFFFFFFFFFFFFFFFFFFFFFFFFFFFFFFFFFFFFFFFFFF
FFFFFFFFFFFF:FFFFFFF,FFFFFFFFFFFFFFFFFFFFFFFFFFFFFFFFFFFFFFFFFFFFFFF
FFFFFFFFFFFFFFF
@A00155:342:HHGFNDSXY:1:2222:6641:3724 1:N:0:GAACCTAG+TCCGCATA
AGAAGAAGGTGGAAAACTTTTCTTTTTACCTACCAGAGAAAACTCGGGAATGGTTCACAAAGTCTGGT
GTGTATCTTTCACCATTTGCTTACGTGAATCACTCACACCCTGGTTGTAAAACTCTGGAGAACCACCT
ATTATTTAATGTTGT
+
FFFFFFFFFFFFFFFFFFFFFFFFFF:FFFFFFFFFFFFFFFFFFFFFFFFFFFFFFFFFFFFFFFFF
FFFFFFFFFFFFFFFFFFFF:FF:FFFFFFFFFFFFFFFFFFFFFFFFFFFFFFFFFFFFFFFFFFFF
FFFFFFFFFFFFFFF
@A00155:342:HHGFNDSXY:1:2231:19994:13416 1:N:0:GAACCTAG+TCCGCATA
AGAAGAAGGTGGAAAACTTTTCTTTTTACCTACCAGAGAAAACTCGGGAATGGTTCACAAAGTCTGGT
GTGTATCTTTCACCATTTGCTTACGTGAATCACTCACACCCTGGTTGTAAAACTCTGGAGAACCACCT
ATTATTTAATGTTGT
+
FFFFFFF::,FFFFFFFFF:FFFFFFFFFFFFFFFFFFFFFFFFFFFFFFFFFFFFFFFFFFFFFFFF
FFFFFFFFFFFFFFFFFFFFFFFFFFFFFFFFFFFFFFFFFFFFFFFFFFFFFFFFFFFFFFFFFFFF
FFFFFFFFFFFFFFF
@A00155:342:HHGFNDSXY:1:2258:19551:16532 1:N:0:GAACCTAG+TCCGCATA
AGAAGAAGGTGGAAAACTTTTCTTTTTACCTACCAGAGAAAACTCGGGAATGGTTCACAAAGTCTGGT
GTGTATCTTTCACCATTTGCTTACGTGAATCACTCACACCCTGGTTGTAAAACTCTGGAGAACCACCT
ATTATTTAATGTTGT
+
FFFFFFFFFFFFFFFFFFFFFFFFFFFFFFFFFFFFFFFFFFFFFFFFFFFFFFFFFFFFFFFFFFFF
FFFFFFFFFFFFFFFFFFFFFFFFFFFFFFFFFFFFFFFFFFFFFFFFFFFFFFFFFFFFFFFFFFFF
FFFFFFFFFFFFFFF
@A00155:342:HHGFNDSXY:1:2373:10411:2519 1:N:0:GAACCTAG+TCCGCATA
TTCCGATCTTGGAAAACTTTTCTTTTTACCTACCAGAGAAAACTCGGGAATGGTTCACAAAGTCTGGT
GTGTATCTTTCACCATTTGCTTACGTGAATCACTCACACCCTGGTTGTAAAACTCTGGAGAACCACCT
ATTATTTAATGTTGT
+
FFFFFFFFFFFFFFFFFFFFFFFFFFFFFFFFFFFFFFFFFFFFFFFFFFFFFFFFFFFFFFFFFFFF
FFFFFFFFFFFFFFFFFFFFFFFFFFFFFFFFFFF:F:FFFFFFFFFFFFFFFFFFFFFFFFFFFFFF
FFFFFFFFFFFFFFF
@A00155:342:HHGFNDSXY:1:2450:20320:9940 1:N:0:GAACCTAG+TCCGCATA
AGAAGAAGGTGGAAAACTTTTCTTTTTACCTACCAGAGAAAACTCGGGAATGGTTCACAAAGTCTGGT
GTGTATCTTTCACCATTTGCTTACGTGAATCACTCACACCCTGGTTGTAAAACTCTGGAGAACCACCT
ATTATTTAATGTTGT
+
FFFFFFFFFFFFFFFFFFFFFFFFFFFFFFFFFFFFFFFFFFFFFFFFFFFFFFFFFFFFFFFFFFFF

```
FFFFFFFFFFFFFFFFFFFFFFFFFFFFFFFFFFFFFFFFFFFFFFFFFFFFFFFFFFFFFFFFFFFF
FFFFFFFFFFFFFFF
@A00155:342:HHGFNDSXY:1:2433:4463:14763 1:N:0:GAACCTAG+TCCGCATA
AGAAGAAGGTGGAAAACTTTTCTTTTTACCTACCAGAGAAAACTCGGGAATGGTTCACAAAGTCTGGT
GTGTATCTTTCACCATTTGCTTACGTGAATCACTCACACCCTGGTTGTAAAACTCTGGAGAACCACCT
ATTATTTAATGTTGT
+
FFFF:FFFFFF:FFF,::FFFFFFFF,FF:FFFFFFFFFFFFFFFFFFFFFFFFFFFF:FFF:FFF:FF
FFFFFFFFFFFF,FFFFFFFFFFFFFFFFFFFFFFFFFFFFFFFFFFFFFFFFFFFFFF:FFFFFF
FFFFFFFFFF:FFFF
@A00155:342:HHGFNDSXY:1:2573:7880:28510 1:N:0:GAACCTAG+TCCGCATA
AGAAGAAGGTGGAAAACTTTTCTTTTTACCTACCAGAGAAAACTCGGGAATGGTTCACAAAGTCTGGT
GTGTATCTTTCACCATTTGCTTACGTGAATCACTCACACCCTGGTTGTAAAACTCTGGAGAACCACCT
ATTATTTAATGTTGT
+
FFF,FF:,:F,FFFFFFFFFF:FFFFFFFFFFFFFFFFFFFFFFFFFFFFFF,FFFFF,FFFFFFFFFF
FFFFFFFFFFFF,FFFFFFFFFFFFFFFFFFFFFFFFFFFFFFFFFFFFFFFF:FFFFF:FFFFFFFFF
FFFFFFFFFFFFFFF
@A00155:342:HHGFNDSXY:1:2627:23276:23985 1:N:0:GAACCTAG+TCCGCATA
AGAAGAAGGTGGAAAACCTTTCTTTTTACCTACCAGAGAAAACTCGGGAATGGTTCACAAAGTCTGGT
GTGTATCTTTCACCATTTGCTTACGTGAATCACTCACACCCTGGTTGTAAAACTCTGGAGAACCACCT
ATTATTTAATGTTGT
+
FFFFF:FFFFFFFFFFF,FFFF:FFFFFFFFFFFFFFFFFFFFFFFFFFFFFFFFFFFFFFFFFFFFFF
FFFFFFFFFFFFFFFFFFFFFFFFFFFFFFFFFFFFFFFFFFFFFFFFFFFFFFFFFFFFFFFFFFFF
FFFFFFFFFFFFFFF
@A00155:342:HHGFNDSXY:1:1110:26151:33473 2:N:0:GAACCTAG+TCCGCATA
AGAAGAAGGTGGAAAACTTTTCTTTTTACCTACCAGAGAAAACTCGGGAATGGTTCACAAAGTCTGGT
GTGTATCTTTCACCATTTGCTTACGTGAATCACTCACACCCTGGTTGTAAAACTCTGGAGAACCACCT
ATTATTTAATGTTGT
+
FFFFFFFFFFFFFFFFFFFFFFFFFFFFFFFFFFFFFFFFFFFFFFFFFFFFFFFFFFFFFFFFFFFF
FFFFFFFFFFFFFFFFFFFFFFFFFFFFFFFFFFFFFFFFFFFFFFFFFFF:FFFFFFFFFFFFFFFFF
:FFFFFFFFF,FFFF
@A00155:342:HHGFNDSXY:1:2673:12744:17425 1:N:0:GAACCTAG+TCCGCATA
AGAAGAAGGTGGAAAACTTTTCTTTTTACCTACCAGAGAAAACTCGGGAATGGTTCACAAAGTCTGGT
GTGTATCTTTCACCATTTGCTTACGTGAATCACTCACACCCTGGTTGTAAAACTCTGGAGAACCACCT
ATTATTTAATGTTGT
+
FFFFFFF:FFFFFFFFFFFFFFFFFFFFFFFFFFFFFFFFFFFFFFFFFFFFFFFFFFF:FFFFFFFF
FFFFFFFFFFFFFFFFFFFFFFFFFFFFFFFFFFFFFFFFFFFFFFFFFFFFFFFFFFFFFFFFFFFF
FFFFFFFFFFFFFFF
@A00155:342:HHGFNDSXY:1:1237:3016:4460 2:N:0:GAACCTAG+TCCGCATA
AGAAGAAGGTGGAAAACTTTTCTTTTTACCTACCAGAGAAAACTCGGGAATGGTTCACAAAGTCTGGT
GTGTATCTTTCACCATTTGCTTACGTGAATCACTCACACCCTGGTTGTAAAACTCTGGAGAACCACCT
ATTATTTAATGTTGT
+
FFFFFFFFFFFFFFFFFFFFFFFFFFFFFFFFFFFFFFFFFFFFF:FF:FFFFFFFFFFFFFFFFF:FF
FFFFFFFFFFFFFFFFFFFF:F:FFFFFFFFFFFFFFFFFFF:FFFFFFFFF:FFFFF:FFFFF:F:FF
FFFFF:FFFFFF:FF
@A00155:342:HHGFNDSXY:1:1337:10836:21292 2:N:0:GAACCTAG+TCCGCATA
AGAAGAAGGTGGAAAACTTTTCTTTTTACCTACCAGAGAAAACTCGGGAATGGTTCACAAAGTCTGGT
GTGTATCTTTCACCATTTGCTTACGTGAATCACTCACACCCTGGTTGTAAAACTCTGGAGAACCACCT
ATTATTTAATGTTGT
```

+
FFFFFFFFFFFFFFFFFFFFFFFFFFFFFFFFFFFFFFFFFFFFFFFFFFFFFFFFFFFFFFFFFFFF
FFFFFFFFFFFFFFFFFFFFFFFFFFFFFFFFFFFFFFFFFFFFFFFFFFFFFFFFFFFFFFFFFFFF
FFFFFFFFFFFFFFF
@A00155:342:HHGFNDSXY:1:1402:22435:7936 2:N:0:GAACCTAG+TCCGCATA
AGAAGAAGGTGGAAAACTTTTCTTTTTACCTACCAGAGAAAACTCGGGAATGGTTCACAAAGTCTGGT
GTGTATCTTTCACCATTTGCTTACGTGAATCACTCACACCCTGGTTGTAAAACTCTGGAGAACCACCT
ATTATTTAATGTTGT
+
FFF:FFFFF,FFFFFFFFFFFFFFFFFFFFFFFFFF:FFFFFFFFFFFF:FFFFFFFFFFFFFFFFFF
FFFFFFFFFFFFFFFFFFFFFFFFFFFFFFFFFFFFFFFFFFFFFFF:FFFFFFFFFFF:FFFFFFFF
FFF,FFFF:F:FFFF
@A00155:342:HHGFNDSXY:1:1438:20437:26991 2:N:0:GAACCTAG+TCCGCATA
AGAAGAAGGTGGAAAACTTTTCTTTTTACCTACCAGAGAAAACTCGGGAATGGTTCACAAAGTCTGGT
GTGTATCTTTCACCATTTGCTTACGTGAATCACTCACACCCTGGTTGTAAAACTCTGGAGAACCACCT
ATTATTTAATGTTGT
+
F:FF:FFF,:FFFFFFFFFF,:FFF,:FFFFFFFF,F:FFFFFFF::::,F:FFFFFFFFFFFFFF,,
F:,,FFF,FF:FFF,FFFFFFFFFF:FFFFFFFFFF,FFFFF:FFFFFFFF:FFFFFFFF:F:FFFFF
:FF:FF:FF:F:F:F
@A00155:342:HHGFNDSXY:1:1468:19379:3583 2:N:0:GAACCTAG+TCCGCATA
AGAAGAAGGTGGAAAACTTTTCTTTTTACCTACCAGAGAAAACTCGGGAATGGTTCACAAAGTCTGGT
GTGTATCTTTCACCATTTGCTTACGTGAATCACTCACACCCTGGTTGTAAAACTCTGGAGAACCACCT
ATTATTTAATGTTGT
+
FFFFFFFFFFFFFFFFFFFFFFFFFFFFFFFFFFFFFFFFFFFFFFFFFFFFFFFFFFFFFFFFFFFF
FFFFFFFFFFFFFFFFFFFFFFFFFFFFFFFFFFFFFFFFFFFFFFFFFFFFFFFFFFF:FFFFFFFF
FFFFFFFFFFFFFFF
@A00155:342:HHGFNDSXY:1:1565:14461:21089 2:N:0:GAACCTAG+TCCGCATA
AGAAGAAGGTGGAAAACTTTTCTTTTTACCTACCAGAGAAAACTCGGGAATGGTTCACAAAGTCTGGT
GTGTATCTTTCACCATTTGCTTACGTGAATCACTCACACCCTGGTTGTAAAACTCTGGAGAACCACCT
ATTATTTAATGTTGT
+
FFFFFFFFFFFFFFFFFF:FFFFFFFFFFFFFFFFFFFFFFFFFFFFFF:FFFFFFFFF::FFF:FFF
F,FFFFFFFFFFFFFFFFFFFFFFFFFF,FFFF:FFFFFFFFFFFFFFFFFFFFFFFFFFFFFFFFFF
FFFFFFFFFFFFFFF
@A00155:342:HHGFNDSXY:1:2103:29930:23296 2:N:0:GAACCTAG+TCCGCATA
AGAAGAAGGTGGAAAACTTTTCTTTTTACCTACCAGAGAAAACTCGGGAATGGTTCACAAAGTCTGGT
GTGTATCTTTCACCATTTGCTTACGTGAATCACTCACACCCTGGTTGTAAAACTCTGGAGAACCACCT
ATTATTTCATGTTGT
+
FFFF:FFFFFFFFFFFFFFFFFFFFFFFFFFF:FFFFFFFFFFF:FFFFFFFFFFFFFFFFFFFFFFF
FFFFFFFFFFFFFFFFFFFFFFFFFFFFFFFFFFFFFF:FF:F,FFFFFFFFFFFFFFFFFFFFFFFF
FFFFFFF,FFFF:FF
@A00155:342:HHGFNDSXY:1:2165:5159:12179 2:N:0:GAACCTAG+TCCGCATA
AGAAGAAGGTGGAAAACTTTTCTTTTTACCTACCAGAGAAAACTCGGGAATGGTTCACAAAGTCTGGT
GTGTATCTTTCACCATTTGCTTACGTGAATCACTCACACCCTGGTTGTAAAACTCTGGAGAACCACCT
ATTATTTAATGTTGT
+
FFFFFFFF,FFFFFFFFFFFFFFFFFFFFFFFFFFFFFFFF:FFFFFFFFFFFFFFFFFF:FFF:FFF
FFFFFF:FFF:FFFFFFFFFFFFFFFFFFFF:FFFFFFF,FF,FFFFFFFFFFFFFF:FFFFFFFFFF
FFFF,FFFFFFFFFF
@A00155:342:HHGFNDSXY:1:2343:10610:35023 2:N:0:GAACCTAG+TCCGCATA
AGAAGAAGGTGGAAAACTTTTCTTTTTACCTACCAGAGAAAACTCGGGAATGGTTCACAAAGTCTGGT

GTGTATCTTTCACCATTTGCTTACGTGAATCACTCACACCCTGGTTGTAAAACTCTGGAGAACCACCT
ATTATTTAATGTTGT
+
FFFFFFFFFFFFFFFFFFFFFFFFFFFFFFFFFFFFFFFFFFFFFFFFFFFFFFFFFF:FFFFFFFFF
FFFFFFFFFFFFFFFFFFFFFFFFFFFFFFFFFFFFFFFFFFFFFFFFFFF:FFFFFFFFFFFFFFFF
FFFFFFFFFFFFFFF
@A00155:342:HHGFNDSXY:1:2443:24306:19539 2:N:0:GAACCTAG+TCCGCATA
AGAAGAAGGTGGAAAACTTTTCTTTTTACCTACCAGAGAAAACTCGGGAATGGTTCACAAAGTCTGGT
GTGTATCTTTCACCATTTGCTTACGTGAATCACTCACACCCTGGTTGTAAAACTCTGGAGAACCACCT
ATTATTTAATGTTGT
+
FFFFFFFFFFFFFFFFFFFFFFFFFFFFFFFFFFFFFFFFFFFFFFFF:FFFFFFFFFFFFFFFFFFF
FFFFFFFFFFFFFFFFFFFFFFFF:FFF:FFFFFFFFFFFFFFFFFFFF:FFFFFFFFFFFFFFFFFF
FFF:FF:FFFFFFFF
@A00155:342:HHGFNDSXY:1:2454:25220:7028 2:N:0:GAACCTAG+TCCGCATA
AGAAGAAGGTGGAAAACTTTTCTTTTTACCTACCAGAGAAAACTCGGGAATGGTTCACAAAGTCTGGT
GTGTATCTTTCACCATTTGCTTACGTGAATCACTCACACCCTGGTTGTAAAACTCTGGAGAACCACCT
ATTATTTAATGTTGT
+
FFFFFFFFFFFFFFFFFFFFFFFFFFFFFFFFFFFFFFFFFFFFFFFFFFFFFFFFFFFFFFFFFFFF
FFFFFFFFFFFFFFFFFFFFFFFFFFFFFFFFFFFFFFFFFFF,,FFFF:FFFFFFFFFFFFFFFFFF
FFFFFFFFFFFFFFF
@A00155:342:HHGFNDSXY:1:2468:20699:17300 2:N:0:GAACCTAG+TCCGCATA
AGAAGAAGGTGGAAAACTTTTCTTTTTACCTACCAGAGAAAACTCGGGAATGGTTCACAAAGTCTGGT
GTGTATCTTTCACCATTTGCTTACGTGAATCACTCACACCCTGGTTGTAAAACTCTGGAGAACCACCT
ATTATTTAATGTTGT
+
FFFFFFFFFFFFFFFFFFFFFFFFFFFFFFFFFFFFFFFFFFFFFFFFFFFFFFFFFFFFFFFFFFFF
FFFFFFFFFFFFFFFFFFFFFFFFFFFFFFFFFFFFFFFFFFFFFFFFFFFFFFFFFFFFFFFFFFFF
FFFFFFFFFF:FFFF
@A00155:342:HHGFNDSXY:1:2428:2031:13996 2:N:0:GAACCTAG+TCCGCATA
GAAGAAGGTGGAAAACTTTTCTTTTTACCTACCAGAGAAAACTCGGGAATGGTTCACAAAGTCTGGTG
TGTATCTTTCACCATTTGCTTACGTGAATCACTCACACCCTGGTTGTAAAACTCTGGAGAACCACCTA
TTATTTAATGTTGT
+
FFFFFFFF:FFF:FFFFFFFFFFFFFFF:FFFFF:FFFFFFFFFFFFFFFFFFFFFFFFFFFFFF:FF
FFFFFFFFFFFFF,FFFFFFFFFFFFF:FFFFFFFFFFFFFFFFFFFFFFF:FFFFFFFFFFFFFFF:
FFFFFF::F,FFFF
@A00155:342:HHGFNDSXY:1:1109:23231:27508 1:N:0:GAACCTAG+TCCGCATA
GAAGAAGGTGGAAAACTTTTCTTTTTACCTACCAGAGAAAACTCGGGAATGGTTCACAAAGTCTGGTG
TGTATCTTTCACCATTTGCTTACGTGAATCACTCACACCCTGGTTGTAAAACTCTGGAGAACCACCTA
TTATTTAATGTTGTA
+
F:FF,FFFF,FFFFFFFFFFFFF,:FFFFFFFFFFFFFFFFFFFFFFFFFFFFFFFFFFFFFFFFFFF
FFFFFFFFFFFFFF:FFFFFFFFFFFFFFFFFFFFFFFFFFFFFFFFFFFFFFFFFFFFFFFF:FFFF
FFFFFFFFFFFFFFF
@A00155:342:HHGFNDSXY:1:1231:2528:33708 1:N:0:GAACCTAG+TCCGCATA
GAAGAAGGTGGAAAACTTTTCTTTTTACCTACCAGAGAAAACTCGGGAATGGTTCACAAAGTCTGGTG
TGTATCTTTCACCATTTGCTTACGTGAATCACTCACACCCTGGTTGTAAAACTCTGGAGAACCACCTA
TTATTTAATGTTGTA
+
:FFFF:FF,::FFFFF:FFFFFFFFFF:,FFFFFFF:FFFFFFFF,FFFFFFF:FFFFFFFF:FFFFF
FFFFFFFFFFFFFFFFFFFFFFFFFFFFFFFFFFFFFFFFFFFFFFFFFFFFFFFFFFFFFFFFFFFF
FFFFFFFFFFFFFFF

```
@A00155:342:HHGFNDSXY:1:1277:10429:3865 1:N:0:GAACCTAG+TCCGCATA
TTCCGATCTGGAAAACTTTTCTTTTTACCTACCAGAGAAAACTCGGGAATGGTTCACAAAGTCTGGTG
TGTATCTTTCACCATTTGCTTACGTGAATCACTCACACCCTGGTTGTAAAACTCTGGAGAACCACCTA
TTATTTAATGTTGTA
+
FFFFFFFFFFFFFFFFFFFFFFFFFFFFFFFFFFFFFFFFFFFFFFFFFFFFFFFFFFFFFFFFFFFFFF
FFFFFFFFFFFFFFFFFFFFFFFFFFFFFFFFFFFFFFFFFFFFFFFFFFFFFFFFFFFFFFFFFFFFFF
FFFFFFFFFFFFFFF
@A00155:342:HHGFNDSXY:1:1337:10836:21292 1:N:0:GAACCTAG+TCCGCATA
GAAGAAGGTGGAAAACTTTTCTTTTTACCTACCAGAGAAAACTCGGGAATGGTTCACAAAGTCTGGTG
TGTATCTTTCACCATTTGCTTACGTGAATCACTCACACCCTGGTTGTAAAACTCTGGAGAACCACCTA
TTATTTAATGTTGTA
+
FFFFFFFFFFFFFFFFFFFFFFFFFFFFFFFFFFFFFFFFFFFFFFFFFFFFFFFFFFFFFFFFFFFFFF
FFFFFFFFFFFFFFFFFFFFFFFFFFFFFFFFFFFFFFFFFFFFFFFFFFFFFFFFFFFFFFFFFFFFFF
FFFFFFFFFFFFFFF
@A00155:342:HHGFNDSXY:1:1354:28935:35759 1:N:0:GAACCTAG+TCCGCATA
GAAGAAGGTGGAAAACTTTTCTTTTTACCTACCAGAGAAAACTCGGGAATGGTTCACAAAGTCTGGTG
TGTATCTTTCACCATTTGCTTACGTGAATCACTCACACCCTGGTTGTAAAACTCTGGAGAACCACCTA
TTATTTAATGTTGTA
+
FFFF::FFFFFFFFF:FFFFFFFFFFFFFFFFFFFFFFF,FFFFFFFFFFFFFFFFFFFFFFFFFFFFFF
FFFFFFFFFFFFFFFFFFFFFFFFFFFFFFFFFFFFFFFFFFFFFFF:FFFFFFFFFFFFFFFFFFFFFF
FFFFFFFFFFFFFFF
@A00155:342:HHGFNDSXY:1:1355:28709:1266 1:N:0:GAACCTAG+TCCGCATA
GAAGAAGGTGGAAAACTTTTCTTTTTACCTACCAGAGAAAACTCGGGAATGGTTCACAAAGTCTGGTG
TGTATCTTTCACCATTTGCTTACGTGAATCACTCACACCCTGGTTGTAAAACTCTGGAGAACCACCTA
TTATTTAATGTTGTA
+
FFFFF,FFFFFFFFFFFFFFFFFFFFFFFFFFFFFFFFFFFFFFFFFFFFFFFFFFFFFFFFFFFFFFFF
FFFFFFFFFFFFFFFFFFFFFFFFFFFFFFFFFFFFFFFFFFFFFFFFFFFFFFFFFFFFFFFFFFFFFF
FFFFFFFFFFFFFFF
@A00155:342:HHGFNDSXY:1:1577:16351:35477 1:N:0:GAACCTAG+TCCGCATA
CTAGAAGGTGGAAAACTTTTCTTTTTACCTACCAGAGAAAACTCGGGAATGGTTCACAAAGTCTGGTG
TGTATCTTTCACCATTTGCTTACGTGAATCACTCACACCCTGGTTGTAAAACTCTGGAGAACCACCTA
TTATTTAATGTTGTA
+
:FFFFFFFFFFFFFFFFFFFFFFFFFFFFFFFFFFFFFFFFFFFFFFFFFFFFFFFFFFFFFFFFFFFFF
FFFFFFFFFFFFFFFFFFFFFFFFFFFFFFFFFFFFFFFFFFFFFFFFFFFFFFFFFFFFFFFFFFFFFF
FFFFFFFFFFFFFFF
@A00155:342:HHGFNDSXY:1:1616:5439:9377 1:N:0:GAACCTAG+TCCGCATA
TCTGAAGGTGGAAAACTTTTCTTTTTACCTACCAGAGAAAACTCGGGAATGGTTCACAAAGTCTGGTG
TGTATCTTTCACCATTTGCTTACGTGAATCACTCACACCCTGGTTTTAAAACTCTGGAGAACCACCTA
TTATTTAATGTTGTA
+
FFFFFFFFFFFFFFFFFFFF,FFF,FFFF:F,:FFFFFFFFFFFFFFFFFFFFFFFFFFF:FFFFFFFFF
FFFFFFFFFFF,FFFFFFFFFFFF:FFFFFF:FFFFFF:F:FFFF,FFFFFFFFFFFFFFFFFFFFFFFF
FFFFFFFFFFFFFFF
@A00155:342:HHGFNDSXY:1:2101:23511:3349 1:N:0:GAACCTAG+TCCGCATA
GAAGAAGGTGGAAAACTTTTCTTTTTACCTACCAGAGAAAACTCGGGAATGGTTCACAAAGTCTGGTG
TGTATCTTTCACCATTTGCTTACGTGAATCACTCACACCCTGGTTGTAAAACTCTGGAGAACCACCTA
TTATTTAATGTTGTA
+
FFFF:FFFF:FFFFFFFFFFFFFFFFFFFFFFFFFFFFFFFFFFFFFFFFFFFFFFFFFFFFFFFFFFFF
```

FFFFFFFFFFFFFFFFFFFFFFFFFFFFFFFFFFFFFFFFFFFFFFFFFFFFFFFFFFFFFFFFFFFF
FFFFFFFFFFFFFFF
@A00155:342:HHGFNDSXY:1:2109:3956:25128 1:N:0:GAACCTAG+TCCGCATA
GAAGAAGGTGGAAAACTTTTCTTTTTACCTACCAGAGAAAACTCGGGAATGGTTCACAAAGTCTGGTG
TGTATCTTTCACCATTTGCTTACGTGAATCACTCACACCCTGGTTGTAAAACTCTGGAGAACCACCTA
TTATTTAATGTTGTA
+
FFFFFFFFFFFFFFFFFFFFFFFFFFFFF:FF,FFFFFFFFFFFFFFFFFFFFFFFFFFFFFFFFFFF
FFFFFFF:FFFFFFFFFFFFFFFFFFF:FFFFFFFFFFFFFFFFFFFFFFFFFFFFFFFFFFFFFFFF
FFFFFFFFFFFFFFF
@A00155:342:HHGFNDSXY:1:2161:7916:17018 1:N:0:GAACCTAG+TCCGCATA
GAAGAAGGTGGAAAACTTTTCTTTTTACCTACCAGAGAAAACTCGGGAATGGTTCACAAAGTCTGGTG
TGTATCTTTCACCATTTGCTTACGTGAATCACTCACACCCTGGTTGTAAAACTCTGGAGAACCACCTA
TTATTTAATGTTGTA
+
FFFFFF:FF::FFFFFFFFFFF:FFFFFFFFFFFFFFFFFFFFFFF::FFFFFFFFFFFFF:FFFFFFF
FFFFFFFFFFFFFFFFFFFFFFFFFFFFFFFFFFFFFFFFFFFFFFFFFFFFFFFFFFFFFFFFFFFF
FFFFFFFFFFFFFFF
@A00155:342:HHGFNDSXY:1:2244:7536:23813 1:N:0:GAACCTAG+TCCGCATA
GAAGAAGGTGGAAAACTTTTCTTTTTACCTACCAGAGAAAACTCGGGAATGGTTCACAAAGTCTGGTG
TGTATCTTTCACCATTTGCTTACGTGAATCACTCACACCCTGGTTGTAAAACTCTGGAGAACCACCTA
TTATTTAATGTTGTA
+
FFFFFFFFFF:FFFFF:FFFFFFFFFFFFFFFFFFFFFFFFFFFFFFFFFFFFFFFFFFFFFFFFFFF
FFFFFFFFFFFFFFFFFFFFFFFFF:FFFFFFFFFFFFFFFFFFFFFFFFFFFFFFFFFFFFFFFFFF
FFFFFFFFFFFFFFF
@A00155:342:HHGFNDSXY:1:2222:6705:8876 1:N:0:GAACCTAG+TCCGCATA
GAAGAAGGTGGAAAACTTTTCTTTTTACCTACCAGAGAAAACTCGGGAATGGTTCACAAAGTCTGGTG
TGTATCTTTCACCATTTGCTTACGTGAATCACTCACACCCTGGTTGTAAAACTCTGGAGAACCACCTA
TTATTTAATGTTGTA
+
FFFFFFFFFFFFFFF:FF:FFFFFF:FFFFFFFFFFFFF:FFFFFFFFFFFFFFFFFFFFFFFFF,FFF
FFFFFFFFFFFFFFFFFFFFFFF:FFFFFFF:FFFFFFFFFFFFFFFFFFFFFFFFFFFFFFFFFFFF
FFFFFFFFFFFFFFF
@A00155:342:HHGFNDSXY:1:2277:9308:14763 1:N:0:GAACCTAG+TCCGCATA
TTCCGATCTGGAAAACTTTTCTTTTTACCTACCAGAGAAAACTCGGGAATGGTTCACAAAGTCTGGTG
TGTATCTTTCACCATTTGCTTACGTGAATCACTCACACCCTGGTTGTAAAACTCTGGAGAACCACCTA
TTATTTAATGTTGTA
+
FF::FFF:FFFFFFFFFFFFFFFFFFFFFFFFFFFFFFFFFFFFFFFFFFFFFFFFFFFFFFFFFFFF
FFFFFFFFFFFFFFFFFFFFFFFFFFFFFFFFFFFFFFFFFFFFFFFFFFFFFFFFFFFFFFFFFFFF
FFFFFFFFFFFFFFF
@A00155:342:HHGFNDSXY:1:2502:11993:29496 1:N:0:GAACCTAG+TCCGCATA
GAAGAAGGTGGAAAACTTTTCTTTTTACCTACCAGAGAAAACTCGGGAATGGTTCACAAAGTCTGGTG
TGTATCTTTCACCATTTGCTTACGTGAATCACTCACACCCTGGTTGTAAAACTCTGGAGAACCACCTA
TTATTTAATGTTGTA
+
FFFFFFFFFF:FFFFFFFFFFFFF:FFFFFFFFFFFFFFFFFFFFFFFFFFFFFFFFFFFF:FFFFFFFF
FFFFFFFFFFFFFFFFFFFFFFFFFFFFFFFFFFFFFFFFFFFFFFFFFFFFFFFFFFFFFFFFFFFF
FFFFFFFFFFFFFFF
@A00155:342:HHGFNDSXY:1:2540:6460:27899 1:N:0:GAACCTAG+TCCGCATA
GAAGAAGGTGGAAAACTTTTCTTTTTACCTACCAGAGAAAACTCGGGAATGGTTCACAAAGTCTGGTG
TGTATCTTTCACCATTTGCTTACGTGAATCACTCACACCCTGGTTGTAAAACTCTGGAGAACCACCTA
TTATTTAATGTTGTA

```
+
FF:F:FFFFFFFFFFFFFFFFFFFFFFFFFFFFFFFFFFFFFFFFFFFFFFFFFFFFFFFFFFFFFFFF
FFFFFFFFFFFFFFFFFF:FFFFFFFFFFFFFFFFFFFFFFFFFFFFFFFFFFFFFFFFFFFFFFFFFF
FFFFFFFFFFFFFFF
@A00155:342:HHGFNDSXY:1:2663:30798:26929 1:N:0:GAACCTAG+TCCGCATA
GAAGAAGGTGGAAAACTTTTCTTTTTACCTACCAGAGAAAACTCGGGAATGGTTCACAAAGTCTGGTG
TGTATCTTTCACCATTTGCTTACGTGAATCACTCACACCCTGGTTGTAAAACTCTGGAGAACCACCTA
TTATTTAATGTTGTA
+
FFFFFFFFFFFFF:FFF:FFFFFFFFFFFFFFFFF:FFFFFFFFFFFFFFFFFFFFFFFFFFFFFFFFF
FFFFF:FFFFFFFFFFFFFFFFFFFFFFFFFFFFFFFFFFFFFFFFFFFFFFFFFFFFFFFFFFFFFFF
FFFFFFFFFFFFFFF
@A00155:342:HHGFNDSXY:1:1142:20094:14935 2:N:0:GAACCTAG+TCCGCATA
GAAGAAGGTGGAAAACTTTTCTTTTTACCTACCAGAGAAAACTCGGGAATGGTTCACAAAGTCTGGTG
TGTATCTTTCACCATTTGCTTACGTGAATCACTCACACCCTGGTTGTAAAACTCTGGAGAACCACCTA
TTATTTAATGTTGTA
+
FFFFFFFFFFFFFFFFFFFFFFFFFFFFFFFFFFFFFFFFFFFFFFFFFFFFFFFFFFFFFFFFFFFFF
FFFFFFFFFFFFFFFFFFFFFFFFFFFFFFFFFFFFFFFFFFFFFFFFFFFFFFFFFFFFFFFFFFF:F
FFFFFFFFFFFFFFF
@A00155:342:HHGFNDSXY:1:1473:13087:18803 2:N:0:GAACCTAG+TCCGCATA
GAAGAAGGTGGAAAACTTTTCTTTTTACCTACCAGAGAAAACTCGGGAATGGTTCACAAAGTCTGGTG
TGTATCTTTCACCATTTGCTTACGTGAATCACTCACACCCTGGTTGTAAAACTCTGGAGAACCACCTA
TTATTTAATGTTGTA
+
FFFFFFFFF,FFFFFFFFFFFFFFFFFFFFFFFFFFFFFFFFFFFFFFFFFFFFFFFFFFFFFF:FFFF
FFFFFFFF:FFFFFFFF::FFFFFF:FFFFF:FFFFFFFFFFFFFFFFF:FFFFFFFFFFFFFFFFFF
FFFFFFFFF,FF:FF
@A00155:342:HHGFNDSXY:1:1472:2564:32142 2:N:0:GAACCTAG+TCCGCATA
GAAGAAGGTGGAAAACTTTTCTTTTTACCTACCAGAGAAAACTCGGGAATGGTTCACAAAGTCTGGTG
TGTATCTTTCACCATTTGCTTACGTGAATCACTCACACCCTGGTTGTAAAACTCTGGAGAACCACCTA
TTATTTAATGTTGTA
+
FFFFFFFFFFFFFFFFFFFFFFFFFFFF:FFFFFFFFFFF,FFFFFFFFFFFFFFFFFF:FFFFFF:F
FFFFFFFFFFFFFFFFFFFFFFFFFFFFFFFFFFFFFFFFFFFFFFFFF,FFFFF,F:FFFFFFFFF
FFFFFFFFFFFFFFF
@A00155:342:HHGFNDSXY:1:1514:3549:13745 2:N:0:GAACCTAG+TCCGCATA
GAAGAAGGTGGAAAACTTTTCTTTTTACCTACCAGAGAAAACTCGGGAATGGTTCACAAAGTCTGGTG
TGTATCTTTCACCATTTGCTTACGTGAATCACTCACACCCTGGTTGTAAAACTCTGGAGAACCACCTA
TTATTTAATGTTGTA
+
FFFFFFFFFFFFFFFFFFFFFFFFFFFFFFFFFFFFFFFFFFFFFFFFFFFFFFFFFFFFFFFFFFFFF
FFFFFFFFFFFFFFFFFFFFFFFFFFFFFFFFFFFFFFFFFFFFFFFFFFFFFFFFFFFFFFFFFFFFF
FFFFFFFFFFFFFFF
@A00155:342:HHGFNDSXY:1:2419:27877:29293 2:N:0:GAACCTAG+TCCGCATA
GAAGAAGGTGGAAAACTTTTCTTTTTACCTACCAGAGAAAACTCGGGAATGGTTCACAAAGTCTGGTG
TGTATCTTTCACCATTTGCTTACGTGAATCACTCACACCCTGGTTGTAAAACTCTGGAGAACCACCTA
TTATTTAATGTTGTA
+
FFFFFFFFFFFFFFFFFFFFFFF:FFFFFFFFFFFFFFFFFFFF:FFFFF:FFFFFFFFFFFFFFFFFFF
FFFFFFFFFFFFFFFFFFFFFFFFFFFFF:FFFFFFFFFFFFFFFFFFFFFFFF::FFFFFFFFFFFFF
FF:F:FFFFFFF:FF
@A00155:342:HHGFNDSXY:1:2147:6849:26913 1:N:0:GAACCTAG+TCCGCATA
TCCGATCTGGAAAACTTTTCTTTTTACCTACCAGAGAAAACTCGGGAATGGTTCACAAAGTCTGGTGT
```

GTATCTTTCACCATTTGCTTACGTGAATCACTCACACCCTGGTTGTAAAACTCTGGAGAACCACCTAT
TATTTAATGTTGTA
+
FFFFFFFFFFFFFFF:FFFFFFFF:FFFF,F:FFFFFFFFFF:F,FFFFFFFFFFF:FFFFFFF:FFF
FFFFFFFFFFFFFFFFFFFFFF:FFFFFFFFFFFFFFFFFFFFFFFFF,FFFFFFFFFFFFFFFFFFF
FFFFFFFFFFFFFF
@A00155:342:HHGFNDSXY:1:1118:8621:5932 1:N:0:GAACCTAG+TCCGCATA
AAGAAGGTGGAAAACTTTTCTTTTTACCTACCAGAGAAAACTCGGGAATGGTTCACAAAGTCTGGTGT
GTATCTTTCACCATTTGCTTACGTGAATCACTCACACCCTGGTTGTAAAACTCTGGTGAACCACCTAT
TATTTAATGTTGTAG
+
FFFFFFFFF:FFFFFFFFFFFFFFFFFF:FFFFFFFFFFFFFFFFFFFFFFFFFFFFFFFFFFFFFFF
FFFFFFFFFFFFFFFFFFFFFFF,FFFFFFFFFFFFFFFFFFFFFFFFFFFFFFFF,FFFFFFFFFFF
FFFFFFFFFFFFFFF
@A00155:342:HHGFNDSXY:1:1123:8024:4398 1:N:0:GAACCTAG+TCCGCATA
AAGAAGGTGGAAAACTTTTCTTTTTACCTACCAGAGAAAACTCGGGAATGGTTCACAAAGTCTGGTGT
GTATCTTTCACCATTTGCTTACGTGAATCACTCACACCCTGGTTGTAAAACTCTGGAGAACCACCTAT
TATTTAATGTTGTAG
+
FFFFFFFF::FFFFFFFFFFFFFFFFFFFFFFFFFFFFFFFFFFFFFFFFFF:F:FFFFFFFFFFFFF
FFFFFFFFFFFFFF:FFFFFFFFFFFFFFFFFFFFFFFFFFFFFFFFFFFFFFFFFFFFFFFFFFFFF
FFFFFFFFFFFFFFF
@A00155:342:HHGFNDSXY:1:1123:8603:5713 1:N:0:GAACCTAG+TCCGCATA
AAGAAGGTGGAAAACTTTTCTTTTTACCTACCAGAGAAAACTCGGGAATGGTTCACAAAGTCTGGTGT
GTATCTTTCACCATTTGCTTACGTGAATCACTCACACCCTGGTTGTAAAACTCTGGAGAACCACCTAT
TATTTAATGTTGTAG
+
:FFFFFFFFFFFFFFFFFFFFF:FFFFFFFFFFFFF:FFFFFFFFFFFFFFFFFFFFFFFFFFFFFFF
FFFFFFFFFFFFFFFFFFFFFFFFFFFFFFFFFFFFFFFFFFFFFFFFFFFFFFFFFFFFFFFFFFFF
FFFFFFFFFFFFFFF
@A00155:342:HHGFNDSXY:1:1142:26187:22576 1:N:0:GAACCTAG+TCCGCATA
AAGAAGGTGGAAAACTTTTCTTTTTACCTACCAGAGAAAACTCGGGAATGGTTCACAAAGTCTGGTGT
GTATCTTTCACCATTTGCTTACGTGAATCACTCACACCCTGGTTGTAAAACTCTGGAGAACCACCTAT
TATTTAATGTTGTAG
+
FFFFFFF:FFFFFFFFFFFFFFFFFFFFFFFFFFFFFFFFFFFFFFFFFFFFFFFFFFFFFFFFFFFF
FFFFFFFFFFFFFFFFFFFFFFFFFFFFFFFFFFFFFFFFFFFFFFFFFFFFFFFFFFFFFFFFFFFF
FFFFFFFFFFFFFFF
@A00155:342:HHGFNDSXY:1:1204:26693:9267 1:N:0:GAACCTAG+TCCGCATA
AAGAAGGTGGAAAACTTTTCTTTTTACCTACCAGAGAAAACTCGGGAATGGTTCACAAAGTCTGGTGT
GTATCTTTCACCATTTGCTTACGTGAATCACTCACACCCTGGTTGTAAAACTCTGGAGAACCACCTAT
TATTTAATGTTGTAG
+
FFFFFFFF,:FFFFFFFFFFFFFFFFFFFFFFFFFFFF:FFFFFFFFFFFFFFFFFFFFFFFFFFFFF
FFFFFFFFFFFFFFFFFFFFFFFFFFFFFFFFFFFFFFFFFFFFFFFFFFFFFFFFFFFFFFFFFFFF
FFFFFFFFFFFFFFF
@A00155:342:HHGFNDSXY:1:1236:7609:29450 1:N:0:GAACCTAG+TCCGCATA
AAGAAGGTGGAAAACTTTTCTTTTTACCTACCAGAGAAAACTCGGGAATGGTTCACAAAGTCTGGTGT
GTATCTTTCACCATTTGCTTACGTGAATCACTCACACCCTGGTTGTAAAACTCTGGAGAACCACCTAT
TATTTAATGTTGTAG
+
FFFFFFFFFFFFFFFFFFFFFFFFFFFFFFFFFFFFFFFFFFFFFFFFFFFFFFFFFFFFFFF:FFFF
FFF:FFFFFFFFFFFFF::F:FFFFFFFFFFFFFFFFFFFFFFFFFFFFFFFFFFFFFFFFFFFFFFF
FFFFFFFFFFFFFFF

```
@A00155:342:HHGFNDSXY:1:1244:31204:31704 1:N:0:GAACCTAG+TCCGCATA
AAGAAGGTGGAAAACTTTTCTTTTTACCTACCAGAGAAAACTCGGGAATGGTTCACAAAGTCTGGTGT
GTATCTTTCACCATTTGCTTACGTGAATCACTCACACCCTGGTTGTAAAACTCTGGAGAACCACCTAT
TATTTAATGTTGTAG
+
::FF,FFFFFFFFFFFFFFFFFFFFFFFFFFFFFFFFFFFFFFFFFFFFFFFFFFFFFFFFFFFFFFFFF
FFFFFFFFFFFFFFFFFFFFFFFFFFFFFFFFFFFFFFFFFFFFFF:FFFFFFFFFFFFFFFFFFFFFFF
FFFFFFFFFFFFFFF
@A00155:342:HHGFNDSXY:1:1245:4996:17159 1:N:0:GAACCTAG+TCCGCATA
AAGAAGGTGGAAAACTTTTCTTTTTACCTACCAGAGAAAACTCGGGAATGGTTCACAAAGTCTGGTGT
GTATCTTTCACCATTTGCTTACGTGAATCACTCACACCCTGGTTGTAAAACTCTGGAGAACCACCTAT
TATTTAATGTTGTAG
+
:FFF:FFFFFFFFFFFFFFFFFFFFFF:FFFFFFFFFFFFFFFFFFFFFFFFFFFFFFFFFFFFFFFFFF
FFFFFFFFFFFFFFFFFFF,FFFF,FFFFFF:FF:FFFFFFFFFFFFFFFFFFFFFFFFFFFFFFFFFFF
FFFFFFFFFFFFFFF
@A00155:342:HHGFNDSXY:1:1245:5159:17879 1:N:0:GAACCTAG+TCCGCATA
AAGAAGGTGGAAAACTTTTCTTTTTACCTACCAGAGAAAACTCGGGAATGGTTCACAAAGTCTGGTGT
GTATCTTTCACCATTTGCTTACGTGAATCACTCACACCCTGGTTGTAAAACTCTGGAGAACCACCTAT
TATTTAATGTTGTAG
+
FFFFFF:,:,FFFFF:FFFFF:FFFFF:FFFFFFFFFFFFFFFFFFFFFFFFFFFFFFFFFFFFFFFFFF
FFFFFFFFFFFFFFFFFFFFFFFFFFFFFFFFFFFFFFFFFFFFFFFFFFFFFFFFFFFFFFFFFFFFFF
FFFFFFFFFFFFFFF
@A00155:342:HHGFNDSXY:1:1242:27606:9471 1:N:0:GAACCTAG+TCCGCATA
AAGAAGGTGGAAAACTTTTCTTTTTACCTACCAGAGAAAACTCGGGAATGGTTCACAAAGTCTGGTGT
GTATCTTTCACCATTTGCTTACGTGAATCACTCACACCCTGGTTGTAAAACTCTGGAGAACCACCTAT
TATTTAATGTTGTAG
+
FFFFFFFFFFFFFFFFFFFFFFFFFFFFFFFFFFFFFF:FFFFFFFFFFFFFFFFFFFFFFFFFFFFFFF
FFFFFFFFFFFFFFFFFFFFFFFFFFFFFFFFFFFFFFFFFFFFFFFFFFFFFFFFFFFFFFFFFFFFFF
FFFFFFFFFFFFFFF
@A00155:342:HHGFNDSXY:1:1421:4824:23813 1:N:0:GAACCTAG+TCCGCATA
AAGAAGGTGGAAAACTTTTCTTTTTACCTACCAGAGAAAACTCGGGAATGGTTCACAAAGTCTGGTGT
GTATCTTTCACCATTTGCTTACGTGAATCACTCACACCCTGGTTGTAAAACTCTGGAGAACCACCTAT
TATTTAATGTTGTAG
+
FF::FF,:,:FFFFFFFFFFFFFFFFFF,F:FFFFFFFFFFFFFFFFFFFFFFFFFFFFFFFFFFFFFFF
FFFFFFFFFFFFFFFFFFFFFFF:FFFFFFFFFF:FFFFFFFFFFFFFFFFFFFFFFFFFFFFFFFFFFF
FFFFFFFFFFFFFFF
@A00155:342:HHGFNDSXY:1:1432:10438:35258 1:N:0:GAACCTAG+TCCGCATA
AAGAAGGTGGAAAACTTTTCTTTTTACCTACCAGAGAAAACTCGGGAATGGTTCACAAAGTCTGGTGT
GTATCTTTCACCATTTGCTTACGTGAATCACTCACACCCTGGTTGTAAAACTCTGGAGAACCACCTAT
TATTTAATGTTGTAG
+
FFFFFFFFFFFFFFFFFFFF:FFFFFFFFFFFFFFFFFFFFFF:FFFFFF,FFFFFFFFFFFFFFFFF,F
FFFFFFFFFFFFFFFFFFFFFFFFFFFFFFFFF:FFFFF:FFFFFFFFFFFFFFFFFFFFFFFFFFFFFF
FFFFFFFFFFFFFFF
@A00155:342:HHGFNDSXY:1:1440:20781:16344 1:N:0:GAACCTAG+TCCGCATA
AAGAAGGTGGAAAACTTTTCTTTTTACCTACCAGAGAAAACTCGGGAATGGTTCACAAAGTCTGGTGT
GTATCTTTCACCATTTGCTTACGTGAATCACTCACACCCTGGTTGTAAAACTCTGGAGAACCACCTAT
TATTTAATGTTGTAG
+
FFFFFFFFFFFFFFFFFFFFFFFF,FFFFFFFFFFFFFFF:FFFFFFFFFFFFFFFFFFFFFFFFFFFFF
```

FFFFFFFFFFFFFFFFFFFFFFFFFFFFFFFFFFFFFFFFFFFFFFFFFFFFFFFFFFFFFFFFFFFF
FFFFFFFFFFFFFFF
@A00155:342:HHGFNDSXY:1:1515:18078:9596 1:N:0:GAACCTAG+TCCGCATA
AAGAAGGTGGAAAACTTTTCTTTTTACCTACCAGAGAAAACTCGGGAATGGTTCACAAAGTCTGGTGT
GTATCTTTCACCATTTGCTTACGTGAATCACTCACACCCTGGTTGTAAAACTCTGGAGAACCACCTAT
TATTTAATGTTGTAG
+
FFF::FF,,FF:FFFFFFFFFFFFFFFFFFFFFFFFFFFFFFFFFFFFFFFFFFFFFFFFFFFFFFFF
FFFFFFFFFFFFFFFFFFFFFFFFFFFFFFFFFFFFFFFFFFFFFFFFFFFFFFFFFFFFFFFFFFFF
FFFFFFFFFFFFFFF
@A00155:342:HHGFNDSXY:1:1555:29197:3208 1:N:0:GAACCTAG+TCCGCATA
TAGAAGGTGGAAAACTTTTCTTTTTACCTACCAGAGAAAACTCGGGAATGGTTCACAAAGTCTGGTGT
GTATCTTTCACCATTTGCTTACGTGAATCACTCACACCCTGGTTGTAAAACTCTGGAGAACCACCTAT
TATTTAATGTTGTAG
+
FFFFFFFFFFFFFFFFFFFFF:FFFFFFFFFFFFFFFFFFFFFFFFFFFFFFFFFFFFFFFFFFFFFF
FFFFFFFFFFFFFFFFFFFFF:FFFFFFFFF:FFFFFFFFFFFFFFFFFFFFFFFFFFFFFFFFFFFF
FFFFFFFFFFFFFFF
@A00155:342:HHGFNDSXY:1:2121:22959:17456 1:N:0:GAACCTAG+TCCGCATA
AAGAAGGTGGAAAACTTTTCTTTTTACCTACCAGAGAAAACTCGGGAATGGTTCACAAAGTCTGGTGT
GTATCTTTCACCATTTGCTTACGTGAATCACTCACACCCTGGTTGTAAAACTCTGGAGAACCACCTAT
TATTTAATGTTGTAG
+
FFFFFFFF,FFFFFFFFFFFFFFFFFFFFFFFFFFFFFFFFFFFFFFFFFFFFFFFFFFFFFFFFFFF
FFFFFFFFFFF:FFFFFFFFFF:FFFFFFFFFFFFFFFFFFF:FFFFFFFFFFFFFFFFFFFFFFFFFF
FFFFFFFFFFFFFFF
@A00155:342:HHGFNDSXY:1:2245:29125:5431 1:N:0:GAACCTAG+TCCGCATA
AAGAAGGTGGAAAACTTTTCTTTTTACCTACCAGAGAAAACTCGGGAATGGTTCACAAAGTCTGGTGT
GTATCTTTCACCATTTGCTTACGTGAATCACTCACACCCTGGTTGTAAAACTCTGGAGAACCACCTAT
TATTTAATGTTGTAG
+
FFFFFFFFFFFFFFFFFFFFFFFF:FFFFFFFFFFFFFFFFFFFFFFFFFFFFFFFFFFFFFFFFFFF
FFFFFFFFFF:FFFFFFFFFFFFFFFFFFFFFFFFFFFFFFFFFFFFFFFFFFFFFFFFFFFFFFFFF
FFFFFFFFFFFFFFF
@A00155:342:HHGFNDSXY:1:2362:19642:13432 1:N:0:GAACCTAG+TCCGCATA
CTGAAGGTGGAAAACTTTTCTTTTTACCTACCAGAGAAAACTCGGGAATGGTTCACAAAGTCTGGTGT
GTATCTTTCACCATTTGCTTACGTGAATCACTCACACCCTGGTTGTAAAACTCTGGAGAACCACCTAT
TATTTAATGTTGTAG
+
FFFF:FFFFFFFFFFFFFFFFFF:FFFFFFFFFFFFFFFFFFFFFFFFFFFFFFFFFFFFFFFFFFFF
FFFFFFFFFFFFFFFFFFFFFFFFFFFFFFFFFFFFFFFFFFFFFFFFFFFFFFFFFFFFFFFFFFFF
F,FFFFFFFFFFFF:
@A00155:342:HHGFNDSXY:1:2453:1262:5995 1:N:0:GAACCTAG+TCCGCATA
AAGAACGATGAAAACTTTTCTTTTTACCTACCAGAGAAAACTCGGGAATGGTTCACAAAGTCTGGTGT
GTATCTTTCACCATTTGCTTACGTGAATCACTCACACCCTGGTTGTAAAACTCTGGAGAACCACCTAT
TATTTAATGTTGTAG
+
FF,FF,,,,,FFFFF,:F::::,FFFFF:FFFF:F,FFFFF:FF,F:FFFFFF,FFFFFF:FFF,FFF
:FFFFFFFFFFFFFF:FFF,FFFFFFFFFF::FFFFFFFFF::FFFFFFFFFFFFFFFFFFFFFFFFF:
FF,FFFFFFFF:FFF
@A00155:342:HHGFNDSXY:1:2461:13494:34820 1:N:0:GAACCTAG+TCCGCATA
AAGAAGGTGGAAAACTTTTCTTTTTACCTACCAGAGAAAACTCGGGAATGGTTCACAAAGTCTGGTGT
GTATCTTTCACCATTTGCTTACGTGAATCACTCACACCCTGGTTGTAAAACTCTGGAGAACCACCTAT
TATTTAATGTTGTAG

```
+
FFFFFFFFFFFFFFFFFFFF:FFFFFFFFFFFFFFFFFFFFFFFFFFFFFFFFFFFFFFFFFFFFFFFF
FFFFFFFFFFFFFFFFFFFFFFFFFFFFFFFFFFFFFFFFFFFFFFFFFFFFFFFFFFFFFFFFFFFFF
FFFFFFFFFFFFFFF
@A00155:342:HHGFNDSXY:1:2512:22480:19695 1:N:0:GAACCTAG+TCCGCATA
AAGAAGGTGGAAAACTTTTCTTTTTACCTACCAGAGAAAACTCGGGAATGGTTCACAAAGTCTGGTGT
GTATCTTTCACCATTTGCTTACGTGAATCACTCACACCCTGGTTGTAAAACTCTGGAGAACCACCTAT
TATTTAATGTTGTAG
+
FFFF:FFFFFFFFFFFFFFFFFFFFFFFF:FFFFFFFFFFFFFFFFFFFFFFFFFFFFFFFFFFFFFFFF
FFFFFFFFFFFFFFFFFFFFFF:FFFFFFFFFFFFFFFFFFFFFFFFFF:FFFFFFFFFFFFFFFFFFFFF
FFFFFFFFFFFFFFF
@A00155:342:HHGFNDSXY:1:2110:1452:22044 2:N:0:GAACCTAG+TCCGCATA
TAGAAGGTGGAAAACTTTTCTTTTTACCTACCAGAGAAAACTCGGGAATGGTTCACAAAGTCTGGTGT
GTATCTTTCACCATTTGCTTACGTGAATCACTCACACCCTGGTTGTAAAACTCTGGAGAACCACCTAT
TATTTAATGTTGTAG
+
FFFF:FF,FFFFFFF::F,F,:,FFF:,FFF,FF:FFFFFF:FF:FFFFFFF,F:FFFFFFFFFFFFF
FFFFFFFFFFFFFFFFFFFFFFF,FFFFFFFFFFFFFFFFFFF:F:FFFFFFFFFFFFFFFFFFFFFF
FFFFFFFFFFFFFFF
@A00155:342:HHGFNDSXY:1:2160:29505:30389 2:N:0:GAACCTAG+TCCGCATA
AAGAAGGTGGAAAACTTTTCTTTTTACCTACCAGAGAAAACTCGGGAATGGTTCACAAAGTCTGGTGT
GTATCTTTCACCATTTGCTTACGTGAATCACTCACACCCTGGATGTAAAACTCTGGAGAACCACCTAT
TATTTAATGTTGTAG
+
FFFFFFFFFFFFFFFFFFFFFFFFFFFFFFFFFFFFFFFFFFFFFFFF:FFFFFFFFFFFFFFFFFFFF
FFFFFFFFFFFF:FFFFFFFFFFFFFFFFFFFFFFFFFFFF:FF,FFFF:FFFFFFFFFFFFFFFFFFFF
FF:FFFFFFFFFFFF
@A00155:342:HHGFNDSXY:1:2638:22806:35947 2:N:0:GAACCTAG+TCCGCATA
TAGAAGGTGGAAAACTTTTCTTTTTACCTACCAGAGAAAACTCGGGAATGGTTCACAAAGTCTGGTGT
GTATCTTTCACCATTTGCTTACGTGAATCACTCACACCCTGGTTGTAAAACTCTGGAGAACCACCTAT
TATTTAATGTTGTAG
+
FFFFFFFFFFFFFFFFFFFFFFFFFFFFFFFFFFFFFFFFFFFFFFFFFFFF:FFFFFFFFFFFFFFFF
FFFFFFFFFFFFFFFFFFFFFFFFF:FFFFFFFFFFFFFFFFFFFFFFFFFFFF:FFFFFFFFFFFFFF
FFFFFFFFFFFFFFF
@A00155:342:HHGFNDSXY:1:1215:22770:16814 1:N:0:GAACCTAG+TCCGCATA
AGAAGGTGGAAAACTTTTCTTTTTACCTACCAGAGAAAACTCGGGAATGGTTCACAAAGTCTGGTGTG
TATCTTTCACCATTTGCTTACGTGAATCACTCACACCCTGGTTGTAAAACTCTGGAGAACCACCTATT
ATTTAATGTTGTAG
+
FF:,FF:F:FFFFFFFFFFFFFF,F,FFFF,:FF:FF,,:F::,FF:F:FFFFF:FFF,F,FFFF,F:
FFFFFFFFFF:FFFF::FFFFFFFFFF:FFFFFFFFFFF::FFFFFFFFFFFFFFF:,:FF:FFFFFF
,FFF,:F:FFFFF:
@A00155:342:HHGFNDSXY:1:1248:10737:24471 1:N:0:GAACCTAG+TCCGCATA
AGAAGGTGGAAAACTTTTCTTTTTACCTACCAGAGAAAACTCGGGAATGGTTCACAAAGTCTGGTGTG
TATCTTTCACCATTTGCTTACGTGAATCACTCACACCCTGGTTGTAAAACTCTGGAGAACCACCTATT
ATTTAATGTTGTAG
+
:F::FFF:FFFFFFFFFFF:FFF:FFF,FFFFFFFFFFFFFFFFFFFFFFFFFFFFFFFFFFFFFFFFF
FFFFF:FFFFFFFFFFFFFFFFFFFFFFFFFFFFFFFFFFFFFFFFFF:FFFFFFFFFFFFFFFFFFFF
FFFFFFFFFFFFFF
@A00155:342:HHGFNDSXY:1:1555:29197:3208 2:N:0:GAACCTAG+TCCGCATA
AGAAGGTGGAAAACTTTTCTTTTTACCTACCAGAGAAAACTCGGGAATGGTTCACAAAGTCTGGTGTG
```

TATCTTTCACCATTTGCTTACGTGAATCACTCACACCCTGGTTGTAAAACTCTGGAGAACCACCTATT
ATTTAATGTTGTAG
+
FFFFFFFFFFFFFFFFFFFFFFFFFFFFFFFFFFFFFF:FFFFFFFFFFFFFFFFFFFFFFFFFFFFF
FFFFFFFFFFFFFFFFFFFFFFFFFFFFFFFFFFFFFFFFFFFFFFFFFFFFFFFFFFFFFFFFFFFF
FFF,F:FFF:FFFF
@A00155:342:HHGFNDSXY:1:1112:30590:5807 1:N:0:GAACCTAG+TCCGCATA
AGAAGGTGGAAAACTTTTCTTTTTACCTACCAGAGAAAACTCGGGAATGGTTCACAAAGTCTGGTGTG
TATCTTTCACCATTTGCTTACGTGAATCACTCACACCCTGGTTGTAAAACTCTGGAGAACCACCTATT
ATTTAATGTTGTAGC
+
FFFFFFF:FFFFFFFFF:FFFFF,FFFFFFFFFFF,F:FFFFFFFFF:FF:FFFFFFFFFFFFFFF:F
FFFFFFFFFFFFF,FFFFFFFFFFFF:FF,FFFFFFFFFFFFFFFFF:FF:FFFF:FF:FFFFFFFFF
FFFFFFFFFFFFF,FF
@A00155:342:HHGFNDSXY:1:1305:3007:30091 1:N:0:GAACCTAG+TCCGCATA
AGAAGGTGGAAAACTTTTCTTTTTACCTACCAGAGAAAACTCGGGAATGGTTCACAAAGTCTGGTGTG
TATCTTTCACCATTTGCTTACGTGAATCACTCAAACCCTGGTTGTAAAACTCTGGAGAACCACCTATT
ATTTAATGTTGTAGC
+
FFF:::F,:FFFFFFFFFFFFFFFFFFFFFFFFF:FFFFFF,FFFFFFFFFFFFFFFFFF:FFFFFFF
FFFFFFFFFFFFFFFFFFF:FFF,FFFFFF:F:,F,,FFFFFFFF:FF::FFF:FFFFFFFFFFFFFF
F:FFFFFFFFFFFFF
@A00155:342:HHGFNDSXY:1:1507:21097:34741 1:N:0:GAACCTAG+TCCGCATA
CCGATCTGGAAAACTTTTCTTTTTACCTACCAGAGAAAACTCGGGAATGGTTCACAAAGTCTGGTGTG
TATCTTTCACCATTTGCTTACGTGAATCACTCACACCCTGGTTGTAAAACTCTGGAGAACCACCTATT
ATTTAATGTTGTAGC
+
FFFFFFFFFFFFFFFFFFF:FFFFFFFF:FFFFFFFFFFFFFFFFFFFFFFFFFFFFFFFFFFFFFF
FF:FFFFFFFFFFFFFFFFFFFFFFFFFFFFFFFFFFFFFFFFFFFFFFFFFFFFFFFFFFFFFFFF
FFFFFFFFFFFFFFF
@A00155:342:HHGFNDSXY:1:1550:11641:31109 1:N:0:GAACCTAG+TCCGCATA
AGAAGGTGGAAAACTTTTCTTTTTACCTACCAGAGAAAACTCGGGAATGGTTCACAAAGTCTGGTGTG
TATCTTTCACCATTTGCTTACGTGAATCACTCACACCCTGGTTGTAAAACTCTGGAGAACCACCTATT
ATTTAATGTTGTAGC
+
F:,:F:F:FFFFFF,FFFFFFFFFFF:FFFFFFFFFFFFFFFFFFFFFFFFFFFFFFFFFFFFFFFFF
FFFFFFFFFFFFFFFFFFFFFFFFFFFFFFFFFFFFFFFFFFFFFFFFFFFFFFFFFFFFFFFFFFFF
FFFFFFFFFFFFFFF
@A00155:342:HHGFNDSXY:1:2121:18502:7106 1:N:0:GAACCTAG+TCCGCATA
AGAAGGTGGAAAACTTTTCTTTTTACCTACCAGAGAAAACTCGGGAATGGTTCACAAAGTCTGGTGTG
TATCTTTCACCATTTGCTTACGTGAATCACTCACACCCTGGTTGTAAAACTCTGGAGAACCACCTATT
ATTTAATGTTGTAGC
+
FFFFFFFFFFFFFF:FFFFFFFFFFFFFFFFFFFFFFFFFFFFFFFFFFFFFFFFFFFFFFFFFFFFF
FFFFFFFFFFFFFFFFFFFFFFFFFFFFFFFFFFFFFFFFFFFFFFFFFFFFFFFFFFFFFFFFFFFF
FFFFFFFFFFFFFFF
@A00155:342:HHGFNDSXY:1:2110:1452:22044 1:N:0:GAACCTAG+TCCGCATA
AGAAGGTGGAAAACTTTTCTTTTTACCTACCAGAGAAAACTCGGGAATGGTTCACAAAGTCTGGTGTG
TATCTTTCACCATTTGCTTACGTGAATCACTCACACCCTGGTTGTAAAACTCTGGAGAACCACCTATT
ATTTAATGTTGTAGA
+
FFFFFFFFFFFFFFFFFFFFFFFFFFFFFFFFFFFFFFFFFFFFFFFFFFFFFFFFFF:FFFFFFFFF
FFFFFFFFF:FFFFFFFFFFFFFFFFFFFFFFFFF:FFFFFFFFF:FF:FFFFFFFFFF,FFF,FFFF
FFFFF,,,FFFF:F:

@A00155:342:HHGFNDSXY:1:2246:20256:16470 1:N:0:GAACCTAG+TCCGCATA
AGAAGGTGGAAAACTTTTCTTTTTACCTACCAGAGAAAACTCGGGAATGGTTCACAAAGTCTGGTGTG
TATCTTTCACCATTTGCTTACGTGAATCACTCACACCCTGGTTGTAAAACTCTGGAGAACCACCTATT
ATTTAATGTTGTAGC
+
FFF,FFFFFFFFFFFFFFFFFFFFFFFFFFFFFFFFFFFFFFFFFFFFFF:FFFF:FFFFFFFFFFFF
FFFFFFFFFFFFFFFFFFFFFFFFFFFFFFFFFFFFFFFFFFFFFFFFFFFFFFFFFFFFFFFFFFFF
FFFFFFFFFFFFFFF
@A00155:342:HHGFNDSXY:1:2362:29794:28291 1:N:0:GAACCTAG+TCCGCATA
AGAAGGTGGAAAACTTTTCTTTTTACCTACCAGAGAAAACTCGGGAATGGTTCACAAAGTCTGGTGTG
TATCTTTCACCATTTGCTTACGTGAATCACTCACACCCTGGTTGTAAAACTCTGGAGAACCACCTATT
ATTTAATGTTGTAGC
+
FFF::FFFFFF:FFFFFFFF:FFFFFFFFFFFFFFFF:FFFFFFFFFFFFFFFFFFFFFFFFFFFFFF
FFFFFFFFFFFFFFFFFFFFFFFFFFFFFFFFFFFFFFFFFFFFFF:FFFFFFFFFFFFFFFFFFFFF
FFFF:FFFF:FFFFF
@A00155:342:HHGFNDSXY:1:2638:22806:35947 1:N:0:GAACCTAG+TCCGCATA
AGAAGGTGGAAAACTTTTCTTTTTACCTACCAGAGAAAACTCGGGAATGGTTCACAAAGTCTGGTGTG
TATCTTTCACCATTTGCTTACGTGAATCACTCACACCCTGGTTGTAAAACTCTGGAGAACCACCTATT
ATTTAATGTTGTAGA
+
FFFFFFFFFFFFFFFFFFFFFFFFFFFFFFFFFFFFFFFFFFFFFFFFFFFFFFFFFFFFFFFFFFFF
FFFFFFFFFFFFFFFFFFFFFFFFFFFFFFFFFFFFFFFFFFFFFFF:FFFFFFFFFFFFFFFFFFFF
FFFFFFFFFFFFFFF
@A00155:342:HHGFNDSXY:1:2619:26883:20901 1:N:0:GAACCTAG+TCCGCATA
AGAAGGTGGAAAACTTTTCTTTTTACCTACCAGAGAAAACTCGGGAATGGTTCACAAAGTCTGGTGTG
TATCTTTCACCATTTGCTTACGTGAATCACTCACACCCTGGTTGTAAAACTCTGGAGAACCACCTATT
ATTTAATGTTGTAGC
+
FFFFFFF,FFFFF:FFFF:FFFF:FFFFFFFFFFFFFFFFFFF:FFFFFFFFFFFFFFFFFFFFFFFF
FFFFFFFFFFFFFFFFFFFFFFFFFFFFFFFFFFFFFFFFFFFFFFFFFFFFFFF:FFFF:FFFFFFF
FFFFFFFFFFFFFFF
@A00155:342:HHGFNDSXY:1:2619:26241:26897 1:N:0:GAACCTAG+TCCGCATA
AGAAGGTGGAAAACTTTTCTTTTTACCTACCAGAGAAAACTCGGGAATGGTTCACAAAGTCTGGTGTG
TATCTTTCACCATTTGCTTACGTGAATCACTCACACCCTGGTTGTAAAACTCTGGAGAACCACCTATT
ATTTAATGTTGTAGC
+
FFFF:::FFFFFFFFFFFFFFFFFFFFFFFFFFFFFFFFFFFF:FFFFFFFFFFFFFFFFFFFFFFFF
FFFFFFF:FFFFFFFFFFFFFFFFFFFFFFFFFFFFFFFFFFFFFFFFFFFFFFFFFFFFFFFFFFFF
FFFFFFFFFFFFFFF
@A00155:342:HHGFNDSXY:1:1174:9019:25911 2:N:0:GAACCTAG+TCCGCATA
AGAAGGTGGAAAACTTTTCTTTTTACCTACCAGAGAAAACTCGGGAATGGTTCACAAAGTCTGGTGTG
TATCTTTCACCATTTGCTTACGTGAATCACTCACACCCTGGTTGTAAAACTCTGGAGAACCACCTATT
ATTTAATGTTGTAGC
+
FFFFFF:FFFFFFFFFFFFFFFFFF:FFFFFFFFFFFFFFFFFFFFFFFFFFFFFFFFFFFFFFFFFF
FFFFFFFFFFF,FFFFFFFFFFFFFFFFFFFFFFFFFFFFFFFFFFF:FFFFFFFFFFFFFFFFFFF:
FFFFF,FFFFFFFFF
@A00155:342:HHGFNDSXY:1:1174:23972:26349 2:N:0:GAACCTAG+TCCGCATA
AGAAGGTGGAAAACTTTTCTTTTTACCTACCAGAGAAAACTCGGGAATGGTTCACAAAGTCTGGTGTG
TATCTTTCACCATTTGCTTACGTGAATCACTCACACCCTGGTTGTAAAACTCTGGAGAACCACCTATT
ATTTAATGTTGAGAT
+
FFFFFFFFFFFFFFFFFFFFFFFFFFFFFFFFFFFFFFFFFFFFFFFFFFFFFFFFFFFFFFFFFFFF

FFFFFFFFFFFFFFFFFFFFFFFFFFFFFFFFFFFFFFFFFFFFFFFFFFFFFFFFFFFFF:FFFFFFFFF
FFFFFF:FFF:FFFF
@A00155:342:HHGFNDSXY:1:1208:20618:18192 2:N:0:GAACCTAG+TCCGCATA
AGAAGGTGGAAAACTTTTCTTTTTACCTACCAGAGAAAACTCGGGAATGGTTCACAAAGTCTGGTGTG
TATCTTTCACCATTTGCTTACGTGAATCACTCACACCCTGGTTGTAAAACTCTGGAGAACCACCTATT
ATTTAATGTTGTAGC
+
FFFFFFFFFFFFFFFFFFFFFFFFFFFFFFFFFFFFFFFFFFFFFFFFFFFFFFFFFFFFFFFFFFFFFF
FFFF:FFFFFFFFFFFFFFFFFFFFFFFFFFFFFFFFFFFFFFFFFFFFFFFFFFFFFFFFFFFFF:F:
FFFFFFFFFFFFFFF
@A00155:342:HHGFNDSXY:1:1332:27380:30185 2:N:0:GAACCTAG+TCCGCATA
AGAAGGTGGAAAACTTTTCTTTTTACCTACCAGAGAAAACTCGGGAATGGTTCACAAAGTCTGGTGTG
TATCTTTCACCATTTGCTTACGTGAATCACTCACACCCTGGTTGTAAAACTCTGGAGAACCACCTATT
ATTTAATGTTGTAGA
+
FFFFFF:FFFFF:F:FFFFFFFFFFFFFFFFFFFFFFFFFFFFFFFFFFFFFFFFFFFFFFFFFFFFFF
FFFFFFFFFFFFFFFFF:FFFFFFFFFFFFFFFFFFFFFFFFFFFFFFFFFFFFFFFFFFFFFFFF,FFF
FFFFF:FF,FFFFF:
@A00155:342:HHGFNDSXY:1:1376:26431:27727 2:N:0:GAACCTAG+TCCGCATA
AGAAGGTGGAAAACTTTTCTTTTTACCTACCAGAGAAAACTCGGGAATGGTTCACAAAGTCTGGTGTG
TATCTTTCACCATTTGCTTACGTGAATCACTCACACCCTGGTTGTAAAACTCTGGAGAACCACCTATT
ATTTAATGTTGTAGC
+
FFFFFFFFFFFFFFFFFFFFFFFFFFFFFFF,FFFFFFFFFFFFFF:FFFFFFFFFFFFFFFFFFFFFF
FFFFFFFFFFFFFFFFFFFFFFFFFFFFFFFFFFFFFFFFFFFFFFFFFFFFFFFFFFFFF:FFFFFFFFF
FFFFFFFFFFFFFFF
@A00155:342:HHGFNDSXY:1:1574:21414:3223 2:N:0:GAACCTAG+TCCGCATA
AGAAGGTGGAAAACTTTTCTTTTTACCTACCAGAGAAAACTCGGGAATGGTTCACAAAGTCTGGTGTG
TATCTTTCACCATTTGCTTACGTGAATCACTCACACCCTGGTTGTAAAACTCTGGAGAACCACCTATT
ATTTAATGTTGTAGC
+
FFFFFF:FFFFFFFFFFFFFFFFFFFFFFFFFFFFFFFFFF:FFFFFFFFFFFFFFFFFFFFFFFF,FFF
FFFFFFF:FFFFFFFFFFFFFFFFFFFFFFFFFFFFFFFFFFFFFFFFFF:FFFFFFFFFFFFFFFFFFF
FFFFFFFFFFFFFFF
@A00155:342:HHGFNDSXY:1:1577:16351:35477 2:N:0:GAACCTAG+TCCGCATA
AGAAGGTGGAAAACTTTTCTTTTTACCTACCAGAGAAAACTCGGGAATGGTTCACAAAGTCTGGTGTG
TATCTTTCACCATTTGCTTACGTGAATCACTCACACCCTGGTTGTAAAACTCTGGAGAACCACCTATT
ATTTAATGTTGTAAG
+
FFFFFFFFFFFFFFFFFFFFFFFFFFFFFFFFFFFFFFFFFFFFFFFFFFFFFFFFFFFFFFFFFFFFFF
FFFFFFFFFFFFFFFFFFFFFFFFFFFFFFFFFFFFFFFFFFFFFFFFFFFFFFFFFFFFFFFFFFFFFF
FFFFFF::FFFFFFF
@A00155:342:HHGFNDSXY:1:2336:28908:5619 2:N:0:GAACCTAG+TCCGCATA
AGAAGGTGGAAAACTTTTCTTTTTACCTACCAGAGAAAACTCGGGAATGGTTCACAAAGTCTGGTGTG
TATCTTTCACCATTTGCTTACGTGAATCACTCACACCCTGGTTGTAAAACTCTGGAGAACCACCTATT
ATTTAATGTTGTAGC
+
FFFFFFFFFFFFFFFFFFFFFFFFFFFFFFFFFFFFFFFFFFFFFFFFFFFFFFFFFFFFFFFFFFFFFF
FFFFFFFFFFFFFFFFFFFFFFFFFFFFFFFFFFFFFFFFFFFFFFFFFFFFFFFFFFFFFFFFFFFFFF
FF:FF:FFFFFFFFF
@A00155:342:HHGFNDSXY:1:2652:31720:11303 2:N:0:GAACCTAG+TCCGCATA
AGAAGGTGGAAAACTTTTCTTTTTACCTACCAGAGAAAACTCGGGAATGGTTCACAAAGTCTGGTGTG
TATCTTTCACCATTTGCTTACGTGAATCACTCACACCCTGGTTGTAAAACTCTGGAGAACCACCTATT
ATTTAATGTTGTAGC

+
FFFFFF:FFFFFFFFFFFFFFFFFFFFFFFFFFFFFFF:FFFFFFFFFF:FFFFFFFFFFFFFFFF:FFF
FFFFFFFFFFFF:FFFFFFFFFFFFF:FFFFFFFFFFFFFFFFFFFFFFFFFFFFFFFFFFFFFFF:FF
FFFFFFFFFFFFFF:
@A00155:342:HHGFNDSXY:1:2652:31213:11365 2:N:0:GAACCTAG+TCCGCATA
AGAAGGTGGAAAACTTTTCTTTTTACCTACCAGAGAAAACTCGGGAATGGTTCACAAAGTCTGGTGTG
TATCTTTCACCATTTGCTTACGTGAATCACTCACACCCTGGTTGTAAAACTCTGGAGAACCACCTATT
ATTTAATGTTGTAGC
+
FFF,FFFFFF,FFFFFFFFFFFFFFFFFFFFFFFFFFFFFFFFFFFF:FFFFFFFFFFFFFFFFFFFFF
FFFFFFFF:FFFFFFFFFFFFFFFFFFFFFFFFFFFFFFFFFFFFFFF:FFF:FFFFFFFFFFFFF:FFF
:FFFFFFFFFFFFFF
@A00155:342:HHGFNDSXY:1:1171:16441:31970 1:N:0:GAACCTAG+TCCGCATA
GAAGGTGGAAAACTTTTCTTTTTACCTACCAGAGAAAACTCGGGAATGGTTCACAAAGTCTGGTGTGT
ATCTTTCACCATTTGCTTACGTGAATCACTCACACCCTGGTTGTAAAACTCTGGAGAACCACCTATTA
TTTAATGTTGTAGCT
+
FFFFFFFFFF:F:FFF:FFFF:FFFFFFFFFFFFFFFFFFFFFFFFFFFFFFFFFFFFFFFFFFFFFFF
FFFFFFFFFFFFFFFFFFFFFFFFFFFFFFFFFFFFFFFFFFFFFFFFFFFFFFF:FFFFFFFFFFFFF
FFFFFFFFFFFFFFF
@A00155:342:HHGFNDSXY:1:1414:11713:7811 1:N:0:GAACCTAG+TCCGCATA
GAAGGTGGAAAACTTTTCTTTTTACCTACCAGAGAAAACTCGGGAATGGTTCACAAAGTCTGGTGTGT
ATCTTTCACCATTTGCTTACGTGAATCACTCACACCCTGGTTGTAAAACTCTGGAGAACCACCTATTA
TTTAATGTTGTAGCT
+
FFFFFFFFFFFFFFFFFFFFFFFFFFFFFFFFF:FF:FFFFFFFFFFF:FFFFFFFFFFFFFFFFFFF
FFFFFFFFFFFFFFFFFFFFFFFFFFFFFFFFFFFFFFFFFFFFFFFFFFFFFFFFFFFFFFFFFFFF
FFFFFFFFFFFFFFF
@A00155:342:HHGFNDSXY:1:1563:14082:33896 1:N:0:GAACCTAG+TCCGCATA
GAAGGTGGAAAACTTTTCTTTTTACCTACCAGAGAAAACTCGGGAATGGTTCACAAAGTCTGGTGTGT
ATCTTTCACCATTTGCTTACGTGAATCACTCACACCCTGGTTGTAAAACTCTGGAGAACCACCTATTA
TTTAATGTTGTAGCT
+
:F,:FF,FF:FFFFFFFFFFFFFFFFFFFFFFFFFFFFFFFFFFFFFFFFFFFFFFFFFFFFFFFFFFF
FFFFFFFFFFFFFFFFFFF:FFFFFFFFFFFFFFFFFFFFFFFFFFFFFFFFFFFFFFFFFFFFFFFF
F:FFFFFFFFFFFFF
@A00155:342:HHGFNDSXY:1:2454:30038:34225 1:N:0:GAACCTAG+TCCGCATA
GAAGGTGGAAAACTTTTCTTTTTACCTACCAGAGAAAACTCGGGAATGGTTCACAAAGTCTGGTGTGT
ATCTTTCACCATTTGCTTACGTGAATCACTCACACCCTGGTTGTAAAACTCTGGAGAACCACCTATTA
TTTAATGTTGTAGCG
+
:FFFFF:FFFFFFFFFF:FFFFFFFFFFFFFFFFFFFFFFFFFFFFFFFFFFFFFFFFFFFFFFFFFFF
FF:FFFFFFFFFFFFFFFFFFFFFFFFFFFFFFFFFFFFFFFFFFFFFFFFFFFFFFFFFFFFFFFFFF
FFFFFFFFFFFFF:F
@A00155:342:HHGFNDSXY:1:2634:27841:25880 1:N:0:GAACCTAG+TCCGCATA
GAAGGTGGAAAACTTTTCTTTTTACCTACCAGAGAAAACTCGGGAATGGTTCACAAAGTCTGGTGTGT
ATCTTTCACCATTTGCTTACGTGAATCACTCACACCCTGGTTGTAAAACTCTGGAGAACCACCTATTA
TTTAATGTTGTAGCG
+
FFF,FFFFFFFFFFFFFFFFFFF:F:F:F:FFFFFFFFFFFF,FFFFFFFFFF:FFFF:FF:FFFFFF
:FFFFFFFFFFFFFFFFFFFFFFFFFFFFFFFFFFFFFFFFFFFFFFFFFF:FFFFFFFFFFFFFFFFF
FFFFFFFFFFFFFFF
@A00155:342:HHGFNDSXY:1:2624:19307:15734 1:N:0:GAACCTAG+TCCGCATA
ATCTGTGGAAAACTTTTCTTTTTACCTACCAGAGAAAACTCGGGAATGGTTCACAAAGTCTGGTGTGT

```
ATCTTTCACCATTTGCTTACGTGAATCACTCACACCCTGGTTGTAAAACTCTGGAGAACCACCTATTA
TTTAATGTTGTAGCT
+
FFFFFFFFFFFFF:FFFFFF:F,FFFFFFFFFFFFFFFFFFFFFFFFFFFFFFFFFFFFFFFFFFFFF
FFFFFFFFFFFFFFFFFFFFFFFFFFFFFFFFFFFFFFFFFFFFFFFFFFFFFFFFFFFFFFFFFFFF
FFFFFFFFFFFFFFF
@A00155:342:HHGFNDSXY:1:2669:30273:20384 1:N:0:GAACCTAG+TCCGCATA
GAAGGTGGAAAACTTTTCTTTTTACCTACCAGAGAAAACTCGGGAATGGTTCACAAAGTCTGGTGTGT
ATCTTTCACCATTTGCTTACGTGAATCACTCACACCCTGGTTGTAAAACTCTGGAGAACCACCTATTA
TTTAATGTTGTAGCT
+
FFFFFF:FFFFFFFFFFFFFFFFFFFFFFFFFFF,FFFFFFFFFFFFFFFFFFFFFFFFFFFFFFFFF
FFFFFFFFFFFFFFFFFFFFFFFFFFFFFFFFFFFFFFFFFFFFFFFFFFFFFFFFFFFFFFFFFFFF
FFFFFFFFFFFFFFF
@A00155:342:HHGFNDSXY:1:1616:5439:9377 2:N:0:GAACCTAG+TCCGCATA
GAAGGTGGAAAACTTTTCTTTTTACCTACCAGAGAAAACTCGGGAATGGTTCACAAAGTCTGGTGTGT
ATCTTTCACCATTTGCTTACGTGAATCACTCACACCCTGGTTGTAAAACTCTGGAGAACCACCTATTA
TTTAATGTTGTAAGA
+
FFFFFFFFFFFFFFFFFFFFFFFFFFFFFFFFFFFFFFFFFFFFFFF,FFFFFFFFFFFFFFFFFFFF
FFFFFFFFFFFFFFFFFFFFFFFFFFFFFFFFFFFFFFFFFFFFFFF:FFFFFFFFFFFFFFF,FFFFF
F,F:FF,FFFFFFFF
@A00155:342:HHGFNDSXY:1:2267:32416:34272 2:N:0:GAACCTAG+TCCGCATA
GAAGGTGGAAAACTTTTCTTTTTACCTACCAGAGAAAACTCGGGAATGGTTCACAAAGTCTGGTGTGT
ATCTTTCACCATTTGCTTACGTGAATCACTCACACCCTGGTTGTAAAACTCTGGAGAACCACCTATTA
TTTAATGTTGTAGCT
+
FF:FFFFFFFFFFFFFFFFFFFFFF:FFFFFFFFFFFFFFFFFFF:,FFFF:FFFF:FFFFFFFFFFF
FFFFFFFFFF:,FFFFFFFFFFFF:F:FFFFFFFFFFFFFFFFF:,FFFFFFFF:FF:FFFF::,FF,
FFF,FFFF:FFFFFF
@A00155:342:HHGFNDSXY:1:2362:19642:13432 2:N:0:GAACCTAG+TCCGCATA
GAAGGTGGAAAACTTTTCTTTTTACCTACCAGAGAAAACTCGGGAATGGTTCACAAAGTCTGGTGTGT
ATCTTTCACCATTTGCTTACGTGAATCACTCACACCCTGGTTGTAAAACTCTGGAGAACCACCTATTA
TTTAATGTTGTAGAG
+
FF:FF,FFFFFFFFFFFFFFFFFFFFFFFFFFFFFFFFFFFFFFFF:FFFFFFFFFFFFFFFFFFFFF
FFFFFFFFFFFFFFFFFFFFFFFFFFFFFFFFFFFFFFFF:FFF,FFFFFFFFFFFFFFFFFFFFFFFF
FFFFFFFFFFFFFFF
@A00155:342:HHGFNDSXY:1:1262:20627:21840 1:N:0:GAACCTAG+TCCGCATA
AAGGTGGAAAACTTTTCTTTTTACCTACCAGAGAAAACTCGGGAATGGTTCACAAAGTCTGGTGTGTA
TCTTTCACCATTTGCTTACGTGAATCACTCACACCCTGGTTGTAAAACTCTGGAGAACCACCTATTAT
TTAATGTTGTAGCCC
+
F:FF,F,FFFFFFFFFFFFFFFFFFFFFFFFF:FFFFFFFFFFFFFFFFFFFFFFFFFFFFFFFFFFF
FFFF:FFFFFFFFFFFFFFFFFFFFFFFFFFFFFFFFFFFFFFFFFFFFFFFFFFFFFFFFFF:FFFFF
FFFFFFFFFFFFFFF
@A00155:342:HHGFNDSXY:1:1412:18792:33317 2:N:0:GAACCTAG+TCCGCATA
AAGGTGGAAAACTTTTCTTTTTACCTACCAGAGAAAACTCGGGAATGGTTCACAAAGTCTGGTGTGTA
TCTTTCACCATTTGCTTACGTGAATCACTCACACCCTGGTTGTAAAACTCTGGAGAACCACCTATTAT
TTAATGTTGTAGCTA
+
FFFFF::FFFFFF,FFFF:F,FF,FFFFFFFFF:FFFFFF:F,F:FF:,F:F,FFF,::FFF:F::FF
FFFFF:FFFFFFF:FFFFF:FFFFFFFFFF,FFFF::FFF,FFFF:F:FFFF,,F:FFF,FFFFFFFFF
:FFFFF:F:FF:FFF
```

```
@A00155:342:HHGFNDSXY:1:2250:4010:3145 2:N:0:GAACCTAG+TCCGCATA
GAAGGTGAAAACTTTTCTTTTTACCTACCAGAGAAAACTCGGGAATGGTTCACAAAGACTGGTGTGTA
TCTTTCACCATTTGCTTACGTGAATCACTCACACCCTGGTTGTAAAACTCTGGAGAACCACCTATTAT
TTAATGTTGTAGCTA
+
FFF,FFFFFFFF,FFFFF:FF:FFFFFFFFFFFF,FFFF,F:FFF,FFF:FFFFFFFFFFFFFFFFFF
FFFFFFFFFFFFFFFFF:F:F,FFFFFFFFFFFFFF:FFFFFFFFFFFFFFFFFFFFFFFFFFFF:FF
FFFFFFFFFFFFFFF
@A00155:342:HHGFNDSXY:1:2250:3486:3646 2:N:0:GAACCTAG+TCCGCATA
GAAGGTGAAAACTTTTCTTTTTACCTACCAGAGAAAACTCGGGAATGGTTCACAAAGACTGGTGTGTA
TCTTTCACCATTTGCTTACGTGAATCACTCACACCCTGGTTGTAAAACTCTGGAGAACCACCTATTAT
TTAATGTTGTAGCTA
+
FFFFFF:FFFFFFFFFFFF:F:FFFFFFFFFF,FFFFFFFF:FFFFF,FFFFFFFFF:FF:FFFFFF:
FFFFFFFFFFFFFFFFFFFFFFFFFFFFFFFFFFFFFFFFF,FFFFFFFF:FFF:FFFF,FFFFFFF:
:FFFFFFFFFFFFFF
@A00155:342:HHGFNDSXY:1:2614:9100:10269 2:N:0:GAACCTAG+TCCGCATA
AAGGTGGAAAACTTTTCTTTTTACCTACCAGAGAAAACTCGGGAATGGTTCACAAAGTCTGGTGTGTA
TCTTTCACCATTTGCTTACGTGAATCACTCACACCCTGGTTGTAAAACTCTGGAGAACCACCTATTAT
TTAATGTTTTAGCTA
+
FFFFFFFFFFFFFFFFFFFFFFFFFFFFFFFFFFFFFFFFFFFFF:FFFFFFFFFFFFFFFFFFFFFFF
FFFFFFFFFFFFFFFFFFFFFFFFFFFFFFFFFFFF:FFFFFFFFFFFFFFFFFFFFFFFFF:FFFFFFF
FFFFFFFFFFF:,FF
@A00155:342:HHGFNDSXY:1:1358:24234:7795 1:N:0:GAACCTAG+TCCGCATA
AGGTGGAAAAATTTTCTTTTTACCTACCAGAGAAAACTCGGGAATGGTTCACAAAGTCTGTTGTGTAT
CTTTCACCATTTGCTTACGTGAATCACTCACACCCTGGTTGTAAAACTCTGGAGAACCACCTATTATT
TAATGTTTTAG
+
::FFFFF::,,FFFF,FFFFFFF,FF:FFFF:,F,F:F,:FF::FF:F:FFFFFFFFFF,,F,FFF,F
FFFF:,,,,:FFF,,FFFFF,FFFFFFFF,:F:F:F,,FF,F,,FF:FFFFF:F::F,:F:FFFFFFF
FFFFFFF,F:F
@A00155:342:HHGFNDSXY:1:1262:20627:21997 1:N:0:GAACCTAG+TCCGCATA
AGGTGGAAAACTTTTCTTTTTACCTACCAGAGAAAACTCGGGAATGGTTCACAAAGTCTGGTGTGTAT
CTTTCACCATTTGCTTACGTGAATCACTCACACCCTGGTTGTAAAACTCTGGAGAACCACCTATTATT
TAATGTTGTAGCCC
+
FF:F:FFF:FFFFFFF:FFFFFFFFFFFFFFF:FFFFFFFFFFFFFFFFFFFF:FFFFFFFFFFFFFF
FFFFFFFFFFFFFFFFFFFFFFFFFFFFFFFFFFFFFFFFFFFFFFFFFFFFFFFFFF:FFFFFFFFF
FFFFFFFFFFFFFF
@A00155:342:HHGFNDSXY:1:2414:1353:7686 1:N:0:GAACCTAG+TCCGCATA
AGGTGGAAAACTTTTCTTTTTACCTACCAGAGAAAACTCGGGAATGGTTCACAAAGTCTGGTGTGTAT
CTTTCACCATTTGCTTACGTGAATCACTCACACCCTGGTTGTAAAACTCTGGAGAACCACCTATTATT
TAATGTTGTAGCTA
+
FF,:,:FFFFFF:::F:,FFFF,F:FFFF:F:FFFFFFFF:FFFF,::FFFFFFFF::FF:FFFF,FF
::FFFFFFF,FFF,FFFFF,:FF,FFFFFF:FFFFFFFF,FFFFFFFFF:FF:FFFFFF,FFFFFFF:
FFF:FFFFFFFF:F
@A00155:342:HHGFNDSXY:1:1402:9724:11569 1:N:0:GAACCTAG+TCCGCATA
AGGTGGAAAACTTTTCTTTTTACCTACCAGAGAAAACTCGGGAATGGTTCACAAAGTCTGGTGTGTAT
CTTTCACCATTTGCTTACGTGAATCACTCACACCCTGGTTGTAAAACTCTGGAGAACCACCTATTATT
TAATGTTGTAGCTAG
+
FFFFFFFFFFFFFFFFFFFFFFFFFFFFFFFFFFFFFFFFF:FFFFFFFFFFFFFFFFFFFFFFFFFF
```

```
FFFFFFFFFFFFFFFFFFFFFFFFFFFFFFFFFFFFFFFFFFFFFFFFFFFFFFFFFFFFFFFFFFFF
FFFFFFFF:FFFFFF
@A00155:342:HHGFNDSXY:1:1473:13087:18803 1:N:0:GAACCTAG+TCCGCATA
AGGTGGAAAACTTTTCTTTTTACCTACCAGAGAAAACTCGGGAATGGTTCACAAAGTCTGGTGTGTAT
CTTTCACCATTTGCTTACGTGAATCACTCACACCCTGGTTGTAAAACTCTGGAGAACCACCTATTATT
TAATGTTGTAGCTAG
+
FFFFFFFFF,FFFF,FFFF::F::FF,:,FFFFFFFFFFFFFFFFFFFFFFF,FFFFFFFFF:FFFFF
FFFFFFF:FFFFF:FFFFFFFFFFF::FF,:F:F:FFFFFFFFFFFFFFFF:FFFFFF::FFFFFFFF
FFFF:FFFFF,FFFF
@A00155:342:HHGFNDSXY:1:1476:20681:24659 1:N:0:GAACCTAG+TCCGCATA
AGGTGGAAAACTTTTCTTTTTACCTACCAGAGAAAACTCGGGAATGGTTCACAAAGTCTGGTGTGTAT
CTTTCACCATTTGCTTACGTGAATCACTCACACCCTGGTTGTAAAACTCTGGAGAACCACCTATTATT
TAATGTTGTAGCTAG
+
FFFFFFFFFFFFFFFFFFFF,FFFFFFFFFFFFFFFFFFFFFFFFFFFFFFFFFFFFFFFFFFFFFF:
FFFFFFFFFFFFFFFFFFFFFFFFFFFFFFFFFFFFFFFFFFFFFFFFFFFFFFFFFFFFFFFFFFFF
FFFFFFFFFFFFFFF
@A00155:342:HHGFNDSXY:1:2222:17897:7999 1:N:0:GAACCTAG+TCCGCATA
AGGTGGAAAACTTTTCTTTTTACCTACCAGAGAAAACTCGGGAATGGTTCACAAAGTCTGGTGTGTAT
CTTTCACCATTTGCTTACGTGAATCACTCACACCCTGGTTGTAAAACTCTGGAGAACCACCTATTATT
TAATGTTGTAGCTAG
+
F:FFFFFFFFFFFFF:FFFFFFFFFFFFFFFFFFFFFFFFFFFFFFFFFFFFFF:FFFFFFFFFFFFF
FFFFFFFFFFFFFFFFFFFFFFFFFFFFFFFFFFFFFFFFFFFFFFFFFFFFFFFFFFFFFFFFFFFF
FFFFFFFFFFFFFFF
@A00155:342:HHGFNDSXY:1:2404:8856:17675 1:N:0:GAACCTAG+TCCGCATA
AGGTGGAAAACTTTTCTTTTTACCTACCAGAGAAAACTCGGGAATGGTTCACAAAGTCTGGTGTGTAT
CTTTCACCATTTGCTTACGTGAATCACTCACACCCTGGTTGTAAAACTCTGGAGAACCACCTATTATT
TAATGTTGTAGCTAG
+
FFFFFFFFFFFFF:FFFFFFFFFFFFFFFFFFFFFFFFFFFFFFFFFFFFFFFFFFFFFFFFFFFFFF
FFFFFFFFFFFFFFFFFFF:FFFFFFFFFFFFFFFFFFFFFFFFFFFFFFFFFF:FFFFFFFFFFFFF
FFFFFFFFFFFFFFF
@A00155:342:HHGFNDSXY:1:2504:16884:24596 1:N:0:GAACCTAG+TCCGCATA
AGGTGGAAAACTTTTCTTTTTACCTACCAGAGAAAACTCGGGAATGGTTCACAAAGTCTGGTGTGTAT
CTTTCACCATTTGCTTACGTGAATCACTCACACCCTGGTTGTAAAACTCTGGAGAACCACCTATTATT
TAATGTTGTAGCTAG
+
FFFFFFFFFFFFFFFFFFFFFFFFFFFFFFFFFFFFFFFFFFFFFFFFFFFFFFFFFFFFFFFFFFFF
FFFFFFFFFFFFFFFFFFFFFFFFFFFFFFFFFFFFFFFFFFFFFFFFFFFFFFFFFFFFFFFFFFFF
FFFFFFFFFFFFFFF
@A00155:342:HHGFNDSXY:1:2504:17092:25520 1:N:0:GAACCTAG+TCCGCATA
AGGTGGAAAACTTTTCTTTTTACCTACCAGAGAAAACTCGGGAATGGTTCACAAAGTCTGGTGTGTAT
CTTTCACCATTTGCTTACGTGAATCACTCACACCCTGGTTGTAAAACTCTGGAGAACCACCTATTATT
TAATGTTGTAGCTAG
+
FF:FFF:FFFFFFFFFFF,F:FFFFFFFFFFFFFFFFFFFFFFFFFFFFFFFFFFFFFFFFFFFFFFF
FFFFFFFFFFFFFFFFFFFFFFFFFFFFFFFFFFFFFFFFFFFFFFFFFFFF:FFFFFFFFFFFFFFF
FF:FFFFFFFFFFFF
@A00155:342:HHGFNDSXY:1:1344:21477:31673 2:N:0:GAACCTAG+TCCGCATA
AGGTGGAAAACTTTTCTTTTTACCTACCAGAGAAAACTCGGGAATGGTTCACAAAGTCTGGTGTGTAT
CTTTCACCATTTGCTTACGTGAATCACTCACACCCTGGTTGTAAAACTCTGGAGAACCACCTATTATT
TAATGTTGTAGCTAG
```

+
FFFFFFFFFFFFFFFFFFFFFFFFFFFFFFFFFFFFFFFFFFFF:FFFFFFFFFFFFFFFFFFFFFFFFF
FFFFFFFFFFFFFFFFFFFFFFFFFFFFFFFFFFFFFFFFFFFFFFFFFFFFFFFFFFFFFFFFFFFFFF
FFFFFFFFFFFFFFF
@A00155:342:HHGFNDSXY:1:2444:5312:21371 2:N:0:GAACCTAG+TCCGCATA
AGGTGGAAAACTTTTCTTTTTACCTACCAGAGAAAACTCGGGAATGGTTCACAAAGTCTGGTGTGTAT
CTTTCACCATTTGCTTACGTGAATCACTCACACCCTGGTTGTAAAACTCTGGAGAACCACCTATTATT
TAATGTTGTAGCTAG
+
FFFFFFFFFFFFFFFFFFFFFFFFFFFFFFFFFFFFFFFFFFFF:FFFFFFFFFFFFFFFFFFFFFFFFF
FFFFFFFFFFFFF:FFFFFFFFFFFFFFFFFFFFFFFFFFFFFFFFFFFFFFFFFFFFFFFFFFFFFFFF
FFFFFFFFFFFFFFF
@A00155:342:HHGFNDSXY:1:1353:13738:26976 1:N:0:GAACCTAG+TCCGCATA
GGTGGAAAACTTTTCTTTTTACCTACCAGAGAAAACTCGGGAATGGTTCACAAAGTCTGGTGTGTATC
TTTCACCATTTGCTTACGTGAATCACTCACACCCTGGTTGTAAAACTCTGGAGAACCACCTATTATTT
AATGTTGTAGCTAGT
+
FFFFFFFFFFFFFFFFFFFFFFFFFFFFFFFFFFFFFFFFFFFFFFFFFFFFFFFFFFFFFFFFFFFFFF
FFFFFFFFFFFFFFFFFFFFFFFFFFFFFFFFFFFFFFFFFFFFFFFFFFFFFFFFFFFFFFFFFFFFFF
FFFFFFFFFFFFFFF
@A00155:342:HHGFNDSXY:1:1419:16504:25755 1:N:0:GAACCTAG+TCCGCATA
GGTGGAAAACTTTTCTTTTTACCTACCAGAGAAAACTCGGGAATGGTTCACAAAGTCTGGTGTGTATC
TTTCACCATTTGCTTACGTGAATCACTCACACCCTGGTTGTAAAACTCTGGAGAACCACCTATTATTT
AATGTTGTAGCTAGT
+
::F,FFFFFFFFFFFFFFFFFFFFFFFFFFFFFFFFFFFFFFFFFFFFFFFFFFFFFFFFFFFFFFFFFF
FFFFFFFFFFFFFFFFFFFFFFFFFFFFFFFFFFFFFFFFFFFFFFFFFFFFFFFFFFFFFFFFFFFFFF
FFFFFFFFFFFFFFF
@A00155:342:HHGFNDSXY:1:1520:22589:1000 1:N:0:GAACCTAG+TCCGCATA
GGTGGAAAACTTTTCTTTTTACCTACCAGAGAAAACTCGGGAATGGTTCACAAAGTCTGGTGTGTATC
TTTCACCATTTGCTTACGTGAATCACTCACACCCTGGTTGTAAAACTCTGGAGAACCACCTATTATTT
AATGTTGTAGCTAGG
+
:FFF:FFFF:FFFFFFFFFFFFFFFFFFFFFFFFFFFFFFFFFFFFFFFFFFFFFFFFFFFFFFFFFFFF
FFFFFFFFFFFFFFFFFFFFFFFFFFFFFFFFFFFFFFFFFFFFFFFFFFFFFFFFFFFFFFFFFFFFFF
FFFFFFFFFFFFFFF
@A00155:342:HHGFNDSXY:1:2402:27624:22153 1:N:0:GAACCTAG+TCCGCATA
GGTGGAAAACTTTTCTTTTTACCTACCAGAGAAAACTCGGGAATGGTTCACAAAGTCTGGTGTGTATC
TTTCACCATTTGCTTACGTGAATCACTCACACCCTGGTTGTAAAACTCTGGAGAACCACCTATTATTT
AATGTTGTAGCTAGT
+
FFFFFFFFFFFF:FFFFFFFFFFFFFFFFFFF:FFFFFFFFFFFFFFF:FFFF:FFFFFFFFFFFFFFF
FFFFFF:FFFFFFFFF:FFF:FFFFFFFFFFFFFFFFFFFFFFF:FFFFFFFFFFFFFFF:F:FFFFFF
,FFFFFFFFFFFFFF
@A00155:342:HHGFNDSXY:1:2402:27679:22216 1:N:0:GAACCTAG+TCCGCATA
GGTGGAAAACTTTTCTTTTTACCTACCAGAGAAAACTCGGGAATGGTTCACAAAGTCTGGTGTGTATC
TTTCACCATTTGCTTACGTGAATCACTCACACCCTGGTTGTAAAACTCTGGAGAACCACCTATTATTT
AATGTTGTAGCTAGT
+
FFFFFFFFFFFFF:FFFFFFFFFFFFFFFFFFF:FFFFFFFFFFFFFFFFFFFFFFFFFFFFFFFFFFFF
FFFFFFFFFFFFFFFFFFFFFFFFFFFFFFFFFFFFFFFFFFFFFFFFFFFFFFFFFFFFFFFFFFFFFF
FFFFFFFFFFFFFFF
@A00155:342:HHGFNDSXY:1:2505:25581:9533 1:N:0:GAACCTAG+TCCGCATA
GGTGGAAAACTTTTCTTTTTACCTACCAGAGAAAACTCGGGAATGGTTCACAAAGTCTGGTGTGTATC

```
TTTCACCATTTGCTTACGTGAATCACTCACACCCTGGTTGTAAAACTCTGGAGAACCACCTATTATTT
AATGTTGTAGCTAGC
+
F:F:FFFFF:FFFFFFFFFFFFFFFFFFFFFFFFFFFFFF:FFFFFFFFFFFFFFFFFFFFFFFFFFFF
FFFFFFFFFFFFFFFFFFFFFFFFFFFFFFFFFFFFFFFF:FFFFFFFFFFFFFFFFFFFFFFFFFFF
FFFFFFFFFFFFFFF
@A00155:342:HHGFNDSXY:1:2551:9905:35430 1:N:0:GAACCTAG+TCCGCATA
GGTGGAAAACTTTTCTTTTTACCTACCAGAGAAAACTCGGGAATGGTTCACAAAGTCTGGTGTGTATC
TTTCACCATTTGCTTACGTGAATCACTCACACCCTGGTTGTAAAACTCTGGAGAACCACCTATTATTT
AATGTTGTAGCTAGT
+
FFFFFFFFFFFFF,F:FFFFFFFFFFFFFFFFFFF:FFFFFFFFFFFFFFFFFF:FFFFFFFFFFFFFF
FFFFFFFFFFFFFFFFFFFFFFFFFFFFFFFFFFFFFFF:FFFFFFFFFFFFFFFFFFFFFFFFFFFF
FFFFFFFFFFFFFFF
@A00155:342:HHGFNDSXY:1:1404:17309:25144 2:N:0:GAACCTAG+TCCGCATA
GGTGGAAAACTTTTCTTTTTACCTACCAGAGAAAACTCGGGAATGGTTCACAAAGTCTGGTGTGTATC
TTTCACCATTTGCTTACGTGAATCACTCACACCCTGGTTGTAAAACTCTGGAGAACCACCTATTATTT
AATGTTGTAGCTAGT
+
FFFFFFFFFFFFFFFFFFFFFFFFFFFFFFFFFFFFFFFFFFFFFFFFFFFFF:FFFFFFFFFFFFFF
FFFFFFFF:FFFF:FFFFFFFFFFFFFFFFFFF:FFFFFFFFFFFFFFFFFFFFFFFFFFFFFFFFFF
FFFFFFFFF:FFFFF
@A00155:342:HHGFNDSXY:1:1404:16586:25426 2:N:0:GAACCTAG+TCCGCATA
GGTGGAAAACTTTTCTTTTTACCTACCAGAGAAAACTCGGGAATGGTTCACAAAGTCTGGTGTGTATC
TTTCACCATTTGCTTACGTGAATCACTCACACCCTGGTTGTAAAACTCTGGAGAACCACCTATTATTT
AATGTTGTAGCTAGT
+
FFFFFFFFFFFFFFFFFFFFFFFFFFFFFFFFFFFFFFFFFFFFFFFFFFFFFFFFFFFFFFFFFFFF
FFFFFFFF:FFFFFFFF::FFFFFFFFFFFFFFFFFFFFFFFFFFFFFFFFFFFFFFFFF:FFFFFF
FFFFFFFFFFFFFFF
@A00155:342:HHGFNDSXY:1:1605:25807:3286 2:N:0:GAACCTAG+TCCGCATA
GGTGGAAAACTTTTCTTTTTACCTACCAGAGAAAACTCGGGAATGGTTCACAAAGTCTGGTGTGTATC
TTTCACCATTTGCTTACGTGAATCACTCACACCCTGGTTGTAAAACTCTGGAGAACCACCTATTATTT
AATGTTGTAGCTAGT
+
FFFFFFFFFFFFFFFFFFFFFFFFFFFFFFFFFFFFFFFFFFFFFFFFFFFFFFFFFFFFFFFFFFFF
FFFFFFFFFFFFFFFFFFFFFFFFFFFFFFFFFFFFFFFFFFFFFFFFFFFFFFFFFFFFFFFFFFFF
FFFFFFFF,FFFFFF
@A00155:342:HHGFNDSXY:1:1338:2808:15374 2:N:0:GAACCTAG+TCCGCATA
GTGGAAAACTTTTCTTTTTACCTACCAGAGAAAACTCGGGAATGGTTCACAAAGTCTGGTGTGTATCT
TTCACCATTTGCTTACGTGAATCACTCACACCCTGGTTGTAAAACTCTGGAGAACCACCTATTATTTA
ATGTTGTAGCTAGTT
+
FFFFFFFFFFFFFFFFFFFFFFFFFFFFFFFFFFFFFFFFFFFFFFFFFFFFFFFFFFFFFFFFFFFF
FFFFFFFFFFFFFFFFFFFFFFFFFFFFFFFFFFFFFFFFFFF:,FFFF:FF,FFFFFFFFFFFFFFF:
FFFFFFFFF:FFFFF
@A00155:342:HHGFNDSXY:1:1425:19280:4977 2:N:0:GAACCTAG+TCCGCATA
GTGGAAAACTTTTCTTTTTACCTACCAGAGAAAACTCGGGAATGGTTCACAAAGTCTGGTGTGTATCT
TTCACCATTTGCTTACGTGAATCACTCACACCCTGGTTGTAAAACTCTGGAGAACCACCTATTATTTA
ATGTTGTAGCTAGTT
+
FFFFFFFFFFFFFFFFFFFFFFFFFFFFFFFFFFFFFFFFFF:FFFFFFF:FFFFFFFFFFFFFFFFFF
FFFFFFFFFFFFFFFFFFFFFFFFFFFFF:FFFFFF:FFFFFFFFFFFFFFFFFFFFFFFFFFFFFFFF
FFFFFFFFF:FFFFF
```

```
@A00155:342:HHGFNDSXY:1:1642:24596:35790 2:N:0:GAACCTAG+TCCGCATA
GTGGAAAACTTTTCTTTTTACCTACCAGAGAAAACTCGGGAATGGTTCACAAAGTCTGGTGTGTATCT
TTCACCATTTGCTTACGTGAATCACTCACACCCTGGTTGTAAAACTCTGGAGAACCACCTATTATTTA
ATGTTGTAGATCGGA
+
FFFFFFFFFFFFFFFFFFFFFFFFFFFFFFFFFFFFFFFFFFFFFFFFFFFFFFFFFFFFFFFFFFFF
FFFFFFFFFFFFFFFFFFFFFFFFFFFFFFFFFFFFFFFFFFFFFFFFFFFFF:FFFFFF:FFFFFFF
F:F:FFFFFFFFFFF
@A00155:342:HHGFNDSXY:1:2271:10746:12837 2:N:0:GAACCTAG+TCCGCATA
GTGGAAAACTTTTCTTTTTACCTACCAGAGAAAACTCGGGAATGGTTCACAAAGTCTGGTGTGTATCT
TTCACCATTTGCTTACGTGAATCACTCACACCCTGGTTGTAAAACTCTGGAGAACCACCTATTATTTA
ATGTTGTAGCTAGTT
+
FFFFFFFFFFFF:FFFFFFFFFFFFFFFFFFFFFFFFFFFF:FFFFFFFFFFFFFFFFFFFFFFFFFF
FFFFFFFFFFFFFFFFFFFFFFFFFFFFFFFFFFFFFFFFFFFFFFFFFFFFFFFFFFFFFFFFFFFF
FFFFFFFFFFFFFFF
@A00155:342:HHGFNDSXY:1:2422:26585:35571 2:N:0:GAACCTAG+TCCGCATA
GTGGAAAACTTTTCTTTTTACCTACCAGAGAAAACTCGGGAATGGTTCACAAAGTCTGGTGTGTATCT
TTCACCATTTGCTTACGTGAATCACTCACACCCTGGTTGTAAAACTCTGGAGAACCACCTATTATTTA
ATGTTGTAGCTAGTT
+
FFFFFFFFFFFFFFF:FFFFFFFFFFFFFFFFFFFFFFFFF:FFFFFFFFFFFFFFFFFFFFFFFFFF
FFFFFFFFFFFF:FFFFFFFFFFFFFFFFFFFFFFFF:FFFFFFFFFFFFFFFFFFFFFFFFFFFFFF
FFFFFFFF:FFFFFF
@A00155:342:HHGFNDSXY:1:2624:19307:15734 2:N:0:GAACCTAG+TCCGCATA
GTGGAAAACTTTTCTTTTTACCTACCAGAGAAAACTCGGGAATGGTTCACAAAGTCTGGTGTGTATCT
TTCACCATTTGCTTACGTGAATCACTCACACCCTGGTTGTAAAACTCTGGAGAACCACCTATTATTTA
ATGTTGTAGCTAGAT
+
FFFFF:FF:FFFFFFFFFFFFFFFFFFFFFFFF:FFFFFFF:FFFFFFFFFFFFFFFFFFFFFFFFFF
FFFFFFFFFFF:FFFFFFFFFFFFFFFFFFFFFFFFFFFFFFFFFFFFFFFFFFFFFFFFFFFFFFFF
FFFFFFFF:FFFFFF
@A00155:342:HHGFNDSXY:1:1661:1642:15734 2:N:0:GAACCTAG+TCCGCATA
TGGAAAACTTTTCTTTTTACCTACCAGAGAAAACTCGGGAATGGTTCACAAAGTCTGGTGTGTATCTT
TCACCATTTGCTTACGTGAATCACTCACACCCTGGTTGTAAAACTCTGGAGAACCACCTATTATTTAA
TGTTGTAGCTAGTT
+
FFFFFF,FFFFFFFFFFF:FFFFFF:F:::FF:FFFFFFFFFFFFF:,:F,FFFFFFFFFFFFFFFFF
FF:FFFFFFFFFF:FFFFFFFFFFFF,F,F::FFF:FFFFF,,:FFFFF:FF:FF:::FFFFF:FF::
FFFFFFFF:FFFFF
@A00155:342:HHGFNDSXY:1:2373:10411:2519 2:N:0:GAACCTAG+TCCGCATA
TGGAAAACTTTTCTTTTTACCTACCAGAGAAAACTCGGGAATGGTTCACAAAGTCTGGTGTGTATCTT
TCACCATTTGCTTACGTGAATCACTCACACCCTGGTTGTAAAACTCTGGAGAACCACCTATTATTTAA
TGTTGTAGATCGGA
+
FFFFFFFFFFFFFFFFFFFFFFFFFFFFFFFFFFFFFFFFFFFFFFFFFFF,FFFFFFFFFFF,FFFF
FFFFFFFFFFFFFFFFFFFFFFFFFF:FFFFFFFFFFFFFFFFFFFFFFFFFF::FFF:FFFFFF,F:
FFFFFFFFFFFFFF
@A00155:342:HHGFNDSXY:1:1404:1687:11052 2:N:0:GAACCTAG+TCCGCATA
GGAAAACTTTTCTTTTTACCTACCAGAGAAAACTCGGGAATGGTTCACAAAGTCTGGTGTGTATCTTT
CACCATTTGCTTACGTGAATCACTCACACCCTGGTTGTAAAACTCTGGAGAACCACCTATTATTTAAT
GTTGTAGCTAGTTA
+
FFFFF:FFFFFFFFFFFFFFFFF:FFFF,FF:FFFFFFFFFFFFFFFFF::FFFFFFFFFFFFFFFFF
```

```
FF:FFF:FFFFFFFFFFFFFF:FFFFFFFFFFFFFFFFF:,F:F:FF:FF,FF:FFFFFFFFFFFFFF
FFFFFFFFFFFFFF
@A00155:342:HHGFNDSXY:1:1606:11767:25661 2:N:0:GAACCTAG+TCCGCATA
GGAAAACTTTTCTTTTTACCTACCAGAGAAAACTCGGGAATGGTTCACAAAGTCTGGTGTGTATCTTT
CACCATTTGCTTACGTGAATCACTCACACCCTGGTTGTAAAACTCTGGAGAACCACCTATTATTTAAT
GTTGTAGCTAGTTA
+
F:,::FFFFFFFFFFFFFFFFFFFFFFFFFFFFFFFFFFFFFFFFFFFFFFFFFFFFFFFFFFFFFFF
FFFFFFFFFFFFFFFFFFFFFFFFFFFFFFFFFFFFFFFFFFFFFFFFFFFFF:FF:FFFFFFFFFFF
FFF:FFF:FF::FF
@A00155:342:HHGFNDSXY:1:2269:9905:10066 2:N:0:GAACCTAG+TCCGCATA
GGAAAACTTTTCTTTTTACCTACCAGAGAAAACTCGGGAATGGTTCACAAAGTCTGGTGTGTATCTTT
CACCATTTGCTTACGTGAATCACTCACACCCTGGTTGTAAAACTCTGGAGAACCACCTATTATTTAAT
GTTGTAGCTAGTTA
+
:,:FFF:FFFFFFFFF:FF:FFFFF,F,FFFFFFF::FFFFFFFFF::FFFF::F::::FFFFFFFFF
FF::FF,FFFFFF:FFFFFF:FFFFF,FF:FF,FFFFFFFFFFFF::,F:FF:FFFFFFF,FFF:FFF
,F:FFF,:FFF,FF
@A00155:342:HHGFNDSXY:1:1210:29722:34272 2:N:0:GAACCTAG+TCCGCATA
GGAAAACTTTTCTTTTTACCTACCAGAGAAAACTCGGGAATGGTTCACAAAGTCTGGTGTGTATCTTT
CACCATTTGCTTACGTGAATCACTCACACCCTGGTTGTAAAACTCTGGAGAACCACCTATTATTTAAT
GTTGTAGCTAGTTAC
+
FFFFFFFFFFFFFFFFFFFFFFFFFFFFFFFFFFFFFFFFFFFFFFFFFFFFFFFFFFFFFFFFFFFF
FFFFFFFFFFFFFFFFFFFFFFFFFFFFFFFFFFFFFFFF:FFFFFFFFFFFFFFFFFFFFFFFFFFF
FFFFFFFFFFFFFFF
@A00155:342:HHGFNDSXY:1:1436:5710:2613 2:N:0:GAACCTAG+TCCGCATA
GGAAAACTTTTCTTTTTACCTACCAGAGAAAACTCGGGAATGGTTCACAAAGTCTGGTGTGTATCTTT
CACCATTTGCTTACGTGAATCACTCACACCCTGGTTGTAAAACTCTGGAGAACCACCTATTATTTAAT
GTTGTAGATAGTTAC
+
FFF,FFFFFFFFFFFFFFF:FFFFFFFFFFFFFFFF,FFFFFFFFFFFFF:F,FFF:F,FFFFFFFFF
FFF,FFFF:FFFFFFFFFFFF:FFFFF:FFFF:FFFFFFFF,:FFFFFFFF:,FFFFFFFFFFFFFFF
,FFFFF,,FFF:FFF
@A00155:342:HHGFNDSXY:1:1471:24478:17613 2:N:0:GAACCTAG+TCCGCATA
GGAAAACTTTTCTTTTTACCTACCAGAGAAAACTCGGGAATGGTTCACAAAGTCTGGTGTGTATCTTT
CACCATTTGCTTACGTGAATCACTCACACCCTGGTTGTAAAACTCTGGAGAACCACCTATTATTTAAT
GTTGTAGCTAGTTAC
+
FFFF:FFFFFFFFFFFFFFFFFFFFFFFFFFFFFFFFFFFFFFFFFFFFFFFFFFFFFFFFFFFFFFF
FFFFFFFFFFFFFFFFFFFFFFFFFFFFFFFFFFFFFFFF:FFFFFFFFFFFFFFFFFFFFFFFFFFF
FFFFFFFFFFFFFFF
@A00155:342:HHGFNDSXY:1:1507:21097:34741 2:N:0:GAACCTAG+TCCGCATA
GGAAAACTTTTCTTTTTACCTACCAGAGAAAACTCGGGAATGGTTCACAAAGTCTGGTGTGTATCTTT
CACCATTTGCTTACGTGAATCACTCACACCCTGGTTGTAAAACTCTGGAGAACCACCTATTATTTAAT
GTTGTAGCAGATCGG
+
FFFFFFFFFFFFFFFFFFFFFFFFFFFFFFFFFFFFFFFFFFFFFFFFFFFFFFFFFFF:FFFFFFFF
FFFFFFFFFFFFFFFFFFFFFFFFFFFFFFFFFFFFFFFF:FFFFFFFFFFF:FFFFFFFFFFFFFF:
FFFFFFFFFFFFFFF
@A00155:342:HHGFNDSXY:1:1518:27208:2331 2:N:0:GAACCTAG+TCCGCATA
GGAAAACTTTTCTTTTTACCTACCAGAGAAAACTCGGGAATGGTTCACAAAGTCTGGTGTGTATCTTT
CACCATTTGCTTACGTGAATCACTCACACCCTGGTTGTAAAACTCTGGAGAACCACCTATTATTTAAT
GTTGTAGCTAGTTAC
```

```
+
FFFFFFFFFFFFFFFF:FFFFF,FFFF,FFFFFFFFFFF:FF:FFFFFFFFFF:FFFFFF:FFF:FFF
FFFFFFFFFFFFFFFFFFFFFFFFF:FFFF::FFF,FFFFFFF:FFFFFFFFFFFFFFFFFFFFFFF:FF
FFFFFFFFFFFFFFF
@A00155:342:HHGFNDSXY:1:1548:29613:34428 2:N:0:GAACCTAG+TCCGCATA
GGAAAACTTTTCTTTTTACCTACCAGAGAAAACTCGGGAATGGTTCACAAAGTCTGGTGTGTATCTTT
CACCATTTGCTTACGTGAATCACTCACACCCTGGTTGTAAAACTCTGGAGAACCACCTATTATTTAAT
GTTGTAGCTAGTTAC
+
FFFFFFFFFFFFFFFFFFFFFFFFFFFFFFFFFFFFFFF,FFFFFFFFFFFFFFFFFFFFFFFFFFFF
FFFFFFFFFFFFFFFFFF,FFFFFFFFFFFFFFFFFFFFFFFFF:FFFFFFFFFFFFFFFFFFFFFFFF
FFFFFFFFFFFFFFF
@A00155:342:HHGFNDSXY:1:2147:6849:26913 2:N:0:GAACCTAG+TCCGCATA
GGAAAACTTTTCTTTTTACCTACCAGAGAAAACTCGGGAATGGTTCACAAAGTCTGGTGTGTATCTTT
CACCATTTGCTTACGTGAATCACTCACACCCTGGTTGTAAAACTCTGGAGAACCACCTATTATTTAAT
GTTGTAGAGATCGGA
+
FFFFFFFFFFFFFFFFFFFFFFFFFFFFFFFFFFFFFFFFFFFFFFFFF:FFFFFFFFFFFFFFFFF:F
FFFFF:FFFFFFFFFFFFFFFFFFFFF:,F:FFFFFFFFFF:FFFFFFFFFFFFFFFFF:FFFFFFF:F
FF:FFFFFFFFFFFF
@A00155:342:HHGFNDSXY:1:2209:15429:33599 2:N:0:GAACCTAG+TCCGCATA
GGAAAACTTTTCTTTTTACCTACCAGAGAAAACTCGGGAATGGTTCACAAAGTCTGGTGTGTATCTTT
CACCATTTGCTTACGTGAATCACTCACACCCTGGTTGTAAAACTCTGGAGAACCACCTATTATTTAAT
GTTGTAGCTAGTTAC
+
FFFFFFFFFFFFFFFFFFFFFFFFFFFFFFFFFFFFFFFFFFFFFFFFFFFFFFFFFFFFFFFFFFFF
FFFFFFFFFFFFFFFFFFFFFFFFFFFFFFFFFFF,FFFFFF:FFFFFFFFFFFFFFFFFFFFFFFFFF:
FFFFFFFFFFFFFF:
@A00155:342:HHGFNDSXY:1:2210:8513:19993 2:N:0:GAACCTAG+TCCGCATA
GGAAAACTTTTCTCTTTACCTACCAGAGAAAACTCGGGAATGGTTCACAAAGTCTGGTGTGTATCTTT
CACCATTTGCTTACGTGAATCACTCACACCCTGGTTGTAAAACTCTGGAGAACCACCTATTATTTAAT
GTTGTAGCTAGTTAC
+
FFFFFFFFFFFFFFFFFFFFFFFFFFFFFFFFFFFFF:FFFFFFFFFFFFFFFFFFFFFFFFFFFFFF
FFFFFFFFFFFFFFFFFFFFFFFFFFFFFFFFFFFFFFF:FFFFFFFFFFFFFFFFFFFFFFFFFFF:F
FFFFFFFFFFFFFFF
@A00155:342:HHGFNDSXY:1:2347:27670:24612 2:N:0:GAACCTAG+TCCGCATA
GGAAAACTTTTCTTTTTACCTACCAGAGAAAACTCGGGAATGGTTCACAAAGTCTGGTGTGTATCTTT
CACCATTTGCTTACGTGAATCACTCACACCCTGGTTGTAAAACTCTGGAGAACCACCTATTATTTAAT
GTTGTAGCTAGTTAC
+
FFFFFFFFFFFFFFFFFFFFFFF:FF:FFFF,FFFFFFFFFF:FFFFFF:,FFFFFFF:FFFFFFFFF
FFFFFFFFFFFFFF:FFFFFF:FFFFFF,FFFF,:FFFFFF,FFFF,FFFFFFFFFF:FFFFFFFFF:
FFFFFFFF:F:FFFF
@A00155:342:HHGFNDSXY:1:2520:15230:30091 2:N:0:GAACCTAG+TCCGCATA
GGAAAACTTTTCTTTTTACCTACCAGAGAAAACTCGGGAATGGTTCACAAAGTCTGGTGTGTATCTTT
CACCATTTGCTTACGTGAATCACTCACACCCTGGTTGTAAAACTCTGGAGAACCACCTATTATTTAAT
GTTGTAGCTAGTTAC
+
FF:FFFFFFFFFFFFFFFFFFFFFFFFFFFFFFFFFFFFFFFFFFFFFFFFFFFFFFFFFFFFFFFFF
FFFFFFFFFFFFFFFFFFFFFFFFFFFFFFFFFFFFFFFFFFFFFFFFFFFFFFFFFFFFF:FFFFFFF
FFFFFFFFFFFFFFF
@A00155:342:HHGFNDSXY:1:2570:22851:28447 2:N:0:GAACCTAG+TCCGCATA
GGAAAACTTTTCTTTTTACCTACCAGAGAAAACTCGGGAATGGTTCACAAAGTCTGGTGTGTATCTTT
```

```
CACCATTTGCTTACGTGAATCACTCACACCCTGGTTGTAAAACTCTGGAGAACCACCTATTATTTAAT
GTTGTAGTAAGAATT
+
FFFFFFFFFFFFFFFFFFFFFFFFFFFFFFFFFFFFFFF,FFFFFFFFFFFFFFFFFFFFF:FFFFFF
FFFFFFFFFFFFFFFFFFFFFFFFFFFFFFFFFF:FFF:FF,FFFFFFF:FFFFFF:FFFFFFFFFF:
FFFFFFFFFFFFFFF
@A00155:342:HHGFNDSXY:1:2639:4435:27023 2:N:0:GAACCTAG+TCCGCATA
GGAAAACTTTTCTTTTTACCTACCAGAGAAAACTCGGGCATGGTTCACAAAGTCTGGTGTGTATCTTT
CACCATTTGCTTACGTGAATCACTCACACCCTGGTTGTAAAACTCTGGAGAACCACCTATTATTTAAT
GTTGTAGCTAGTTAC
+
FFFFFFFFFFFFFFFFFFFFFFFFFFFFFFFFFFFFFF,FFFFFFFFFFFFFFFFFFFFFFFFFFFFF
FFFFFFFFFFFFFFFFFFFFFFFFFFFFFF,FFF:FFFFFF:FFFFFFFFFFFFFFFFFFFFFF:F:FF
FFFFFFFFF:FFFFF
@A00155:342:HHGFNDSXY:1:1269:9326:4961 2:N:0:GAACCTAG+TCCGCATA
GAAAACTTTTCTTTTTACCTACCAGAGAAAACTCGGGAATGGTTCACAAAGTCTGGTGTGTATCTTTC
ACCATTTGCTTACGTGAATCACTCACACCCTGGTTGTAAAACTCTGGAGAACCACCTATTATTTAATG
TTGTAGCTAGTTAC
+
FFFF:FFFFFFFFFFFFFFFFF:FFFFFFFFFFFFFFF,FFFFFFFFFFF,FFFF:,FFFFFFFF:FF
FFFFFFFFFFFFF:FFFFFFFFFFFFFFFFFFFFFFFFFFF:FFFFFFFFFFFFFF:FFFFFFFFFFF
F:FFFFFFFFFFFF
@A00155:342:HHGFNDSXY:1:1349:12364:16986 2:N:0:GAACCTAG+TCCGCATA
GAAAACTTTTCTTTTTACCTACCAGAGAAAACTCGGGAATGGTTCACAAAGTCTGGTGTGTATCTTTC
ACCATTTGCTTACGTGAATCACTCACACCCTGGTTGTAAAACTCTGGAGAACCACCTATTATTTAATG
TTGTAGCTAGTTACA
+
FFFF:FFFFFFFFFFFFFFFFFFFFFFFFFFFFFFFFFFFFFFFFFFFFFFFFFFFFFFFFFFFFFFF
FFFFFFFFFFFFFFFFFFFFFFFFFFFFFFFFFFFFFFFFFFFFFFFFFFFFFFFFFFFFFFFFFFFF
FFFFFFFFF:FFFFF
@A00155:342:HHGFNDSXY:1:1349:11559:17284 2:N:0:GAACCTAG+TCCGCATA
GAAAACTTTTCTTTTTACCTACCAGAGAAAACTCGGGAATGGTTCACAAAGTCTGGTGTGTATCTTTC
ACCATTTGCTTACGTGAATCACTCACACCCTGGTTGTAAAACTCTGGAGAACCACCTATTATTTAATG
TTGTAGCTAGTTACA
+
FFFFFFFFFFFFFFFFFFFFFFFFFFFFFFFFFFFFFFFFFFFFFFFFFFFFFFFFFFFFFFFFFFFF
FFFF:FFFFFFFFFFFFFFFFFFFFFFFFFFFFFFFFFFFFFFFFFFFFFFFFFFFFFFFFFFFFFFF
FFFFFFFFFFFFFFF
@A00155:342:HHGFNDSXY:1:1637:21938:31688 2:N:0:GAACCTAG+TCCGCATA
GAAAACTTTTCTTTTTACCTACCAGAGAAAACTCGGGAATGGTTCACAAAGTCTGGTGTGTATCTTTC
ACCATTTGCTTACGTGAATCACTCACACCCTGGTTGTAAAACTCTGGAGAACCACCTATTATTTAATG
TTGTAGCTAGTTACA
+
FFFFFFFFFFFFFFFFFFFFFFFFFFFFFFFFFFFFFFFFFFFFFFFFFFFFFFFFFFFFF:FFFFFFF
FFFFFFFFFFFFFFFFFFFFFFFFFFFFFFFFFFFFFFFFFFFFFFFFFFFFFFFFFFFFFFFFFFFF
FFFFFFFFFFFFFFF
@A00155:342:HHGFNDSXY:1:2239:21079:19053 2:N:0:GAACCTAG+TCCGCATA
GAAAACTTTTCTTTTTACCTACCAGAGAAAACTCGGGAATGGTTCACAAAGTCTGGTGTGTATCTTTC
ACCATTTGCTTACGTGAATCACTCACACCCTGGTTGTAAAACTCTGGAGAACCACCTATTATTTAATG
TTGTAGCTAGTTACA
+
FFFFFFFFF:FFFFFFFFFFFFFFFFFFFFFFFFFFFFFFFFFFFFFFFFF:FFFFFFFFFFFFFFFFFF
FFFFFFFFFFFFFFFFFFFFFFFFFFFFFFFFFFFFFFFFFFFFFFFFFFFFFFFFFFFFFFFFFFFF
FFFFFFFFF:FFFFF
```

@A00155:342:HHGFNDSXY:1:2318:27534:19147 2:N:0:GAACCTAG+TCCGCATA
GAAAACTTTTCTTTTTACCTACCAGAGAAAACTCGGGAATGGTTCACAAAGTCTGGTGTGTATCTTTC
ACCATTTGCTTACGTGAATCACTCACACCCTGGTTGTAAAACTCTGGAGAACCACCTATTATTTAATG
TTGTAGCTAGTTACA
+
FFFFFFFFFFFFFFFFFFFFFFFFFFFFFFFFFFFFFFFFFFFFFFFFFFFFFFFFFFFFFFFFFFFF
FFFFFFFFFFFFFFFFFFFFFFFFFFFFFFFFFFFFFFF:FFFFFFFFFFFFFFFFFFFFFFFFFFFF
F:,FFFFFFFFFFFF
@A00155:342:HHGFNDSXY:1:2318:27389:19648 2:N:0:GAACCTAG+TCCGCATA
GAAAACTTTTCTTTTTACCTACCAGAGAAAACTCGGGAATGGTTCACAAAGTCTGGTGTGTATCTTTC
ACCATTTGCTTACGTGAATCACTCACACCCTGGTTGTAAAACTCTGGAGAACCACCTATTATTTAATG
TTGTAGCTAGTTACA
+
FFFFFFFFFFFFFFFFFFFFFFFFFFFFFFFFFFFFFFFFFFFFFFFFF:FFFFFFFFFFFFFFFFFF
FFFFFFFFFFFFFFFFFFFFFFFFFFFFFFFFFFFFFFFFFFFFFFFFFFFFFFFFFFFFFFFFFFFF
FFFFFFFFFFFFFFF
@A00155:342:HHGFNDSXY:1:2318:28384:19867 2:N:0:GAACCTAG+TCCGCATA
GAAAACTTTTCTTTTTACCTACCAGAGAAAACTCGGGAATGGTTCACAAAGTCTGGTGTGTATCTTTC
ACCATTTGCTTACGTGAATCACTCACACCCTGGTTGTAAAACTCTGGAGAACCACCTATTATTTAATG
TTGTAGCTAGTTACA
+
FFFFFFFFFFFFFFFFFFFFFFFFFFFFFFFFFFFFFFFFFFFFFFF:FFFFFFFFFFFFFFFFFFFF
FFFFFFFFFFFFFFFFFFFFFFFFFFFFFFFFFFFFFFFFFFFFFFFFFFFFFFFFFFFFFFFFFFFF
FFFFFFFFFFFFFFF
@A00155:342:HHGFNDSXY:1:2676:20826:21590 2:N:0:GAACCTAG+TCCGCATA
GAAAACTTTTCTTTTTACCTACCAGAGAAAACTCGGGAATGGTTCACAAAGTCTGGTGTGTATCTTTC
ACCATTTGCTTACGTGAATCACTCACACCCTGGTTGTAAAACTCTGGAGAACCACCTATTATTTAATG
TTGTAGCTAGTTACA
+
FFFFFFFFFFFFFFFFFFFFFFFFFFFFFFFFFFFFFFFFFFFFFFFFFFFFFFFFFFFFFFFFFFFF
FFFFFFFFFFFFFFFFFFFFFFFFFFFFFFFFFFFFFFFFFFFFFFFFFFFFFFFFFFFFFFFFFFFF
FFFFFFFFFFFFFFF
@A00155:342:HHGFNDSXY:1:2612:13241:31093 1:N:0:GAACCTAG+TCCGCATA
AAAACTTTTCTTTTTACCTACCAGAGAAAACTCGGGAATGGTTCACAAAGTCTGGTGTGTATCTTTCA
CCATTTGCTTACGTGAATCACTCACACCCTGGTTGTAAAACTCTGGAGAACCACCTATTATTTAATGT
TGTA
+
F:F:FF:FFF:F,FFFFFFFFFFFFFFF:FFFFFFFFFFFFFFFFFFFFFFFFFFFFFFFFFFFFFFF
FFFFFFFFFFFFFFFFFFFFFFFFFFFFFFFFFFFFFFFFFFFFFFFFFFFFFFFFFFFFFFFFFFFF
FFFF
@A00155:342:HHGFNDSXY:1:2671:1063:6684 1:N:0:GAACCTAG+TCCGCATA
AAAACATTTCTTTTTACCTACCAGAGAAAACTCGGGAATGGTTCACAAAGTCTGGTGTGTATCTTTCA
CCATTTGCTTACGTGAATCACTCACACCCTGGTTGTAAAACTCTGGAGAACCACCTATTATTTAATGT
TGTAGCTAGC
+
FFFFF,::,F,:FFFFFFFF:FF:F:FFF:F:F:FFFFF:FF:F:FFFFFFFFFFFFFFFFFFFF,FF
FFFFFF:FFF:F,:FFFFFFFFFFFFFF:FFF::FFF:FFF,FFFFFFFFFFFFFFF:FF,,FFFFFF
FFFFFFFFFF
@A00155:342:HHGFNDSXY:1:2450:4689:33317 2:N:0:GAACCTAG+TCCGCATA
AAAACTTTTCTTTTTACCTACCAGAGAAAACTCGGGAATGGTTCACAAAGTCTGGTGTGTATCTTTCA
CCATTTGCTTACGTGAATCACTCACACCCTGGTTGTAAAACTCTGGAGAACCACCTATTATTTAATGT
TGTAGCTAGTTACA
+
FFFFFFFFFFFFFFFFFFFFFFFFFFFFFFF:FFFFFFFFFFFFFFF:FFFFFFFFFFFFFFFFFFFF

```
FFFFFFFFFFFFFFFFFFF:FFFFFFFF:FFFFFFFFF:F:FFF,FFFFFF,FFFFFFFFFFFFFFFF
:FFFF:FFFFF:F:
@A00155:342:HHGFNDSXY:1:1477:10357:18270 2:N:0:GAACCTAG+TCCGCATA
AAAACTTTTCTTTTTACCTACCAGAGAAAACTCGGGAATGGTTCACAAAGTCTGGTGTGTATCTTTCA
CCATTTGCTTACGTGAATCACTCACACCCTGGTTGTAAAACTCTGGAGAACCACCTATTATTTAATGT
TGTAGCTAGTTACAT
+
FFFFFFFFFFFFFFFFFFFFFFFFFFFFFFFFFFFFFFFFFFFFFFF:FFFFFFFF:FFFFFFFFFFF:
FFFFFFFFFFFFFFFFFFF,FFFFFFFFFFFFFFFFFFF:FFFFFFFFFFFFFFFFFFFFFFFFFFFFF
FFFFFFFFFFFFFF:
@A00155:342:HHGFNDSXY:1:1571:19144:20713 2:N:0:GAACCTAG+TCCGCATA
AAAACTTTTCTTTTTACCTACCAGAGAAAACTCGGGAATGGTTCACAAAGTCTGGTGTGTATCTTTCA
CCATTTGCTTACGTGAATCACTCACACCCTGGTTGTAAAACTCTGGAGAACCACCTATTATTTAATGT
TGTAGCTAGTTACAT
+
FFFFFFFFFFFFFFFFFFFFFFFFFFFFFFFFFFFF,FFFFFF:FFF,FFFFFFFFF:FFFFFFFF:
FFFFFFFFFF:FFFFFFFFFFFFFFFFFFFFFFFFFFFFF,FFFFFFFFFFFFFFFFFFFFFFFF:FFF
FFFFFFFFFFFFFFF
@A00155:342:HHGFNDSXY:1:2243:27588:22592 2:N:0:GAACCTAG+TCCGCATA
AAAACTTTTCTTTTTACCTACCAGAGAAAACTCGGGAATGGTTCACAAAGTCTGGTGTGTATCTTTCA
CCATTTGCTTACGTGAATCACTCACACCCTGGTTGTAAAACTCTGGAGAACCACCTATTATTTAATGT
TGTAGCTAGTTACAT
+
FFFFFFFFFFFFFFFFFFFFFFFFFFFFFFFFFFFFFFFFFFFFFFFF:FFFFFFFFFFFFFFFFFFFF
FFFFFFFFFFFFFFFFFFFFFFFFFFFFFFFFFFFFFFFFF:FFFFFFFFFFFFFFFFFFFFFFF:FFFF
FFFFFF:FFFFFFFF
@A00155:342:HHGFNDSXY:1:1218:18710:12759 2:N:0:GAACCTAG+TCCGCATA
AAACTTTTCTTTTTACCTACCAGAGAAAACTCGGGAATGGTTCACAAAGTCTGGTGTGTATCTTTCAC
CATTTGCTTACGTGAATCACTCACACCCTGGTTGTAAAACTCTGGAGAACCACCTATTATTTAATGTT
GTAGCTAGTTACATT
+
FFFFFFFFFFFFFFFFFFFFFFFFFFFFFFFFFFFFFFFFFFFFFFFF:FFFFFFFFFFFFFFFFFFFF
FFFFFFFFFFFFFFFFFFFFFFFFFFFFFFFFFFFFFFFFFFFFFFFFFFFFFFFF:FF:FFFFF,F,FF
:FFF,FF:FFFF,F:
@A00155:342:HHGFNDSXY:1:1255:14371:25661 2:N:0:GAACCTAG+TCCGCATA
AAACTTTTCTTTTTACCTACCAGAGAAAACTCGGGAATGGTTCACAAAGTCTGGTGTGTATCTTTCAC
CATTTGCTTACGTGAATCACTCACACCCTGGTTGTAAAACTCTGGAGAACCACCTATTATTTAATGTT
TTAGCTAGTTACATT
+
FFFFFFFFFFFFFFFFFFFFFFFFFFFFFFFFFFFF:FFFFFFFFFFFFFFFFFFFFFFFFFFFFFFFF
FFFFFFFFFFFFFFFFFFFFFFFFFFFFFFFFFFF:FFFF::FFFFFFFFFFFFFFFFFFFFFFFFFFFF
:FF,FFFF:FFF:FF
@A00155:342:HHGFNDSXY:1:1335:28881:28369 2:N:0:GAACCTAG+TCCGCATA
AAACTTTTCTTTTTACCTACCAGAGAAAACTCGGGAATGGTTCACAAAGTCTGGTGTGTATCTTTCAC
CATTTGCTTACGTGAATCACTCACACCCTGGTTGTAAAACTCTGGAGAACCACCTATTATTTAATGTT
GTAGCTAGTTACATT
+
FFFFFFFFFFFFFFF:FFFFFFFFFFFFFFFFFFFFFFFFFFFFFFFFFFFFFFFFFFFFFFFFFFFFF
FFFFFFFFFFFFFFFFFFFFFFFFFFFFFFFFFFFFFFFFFFFFFFFFFFFFFFFFFFFFFFFFFFFFFF
FF:FFFF:FFFFF,F
@A00155:342:HHGFNDSXY:1:1565:16685:16642 2:N:0:GAACCTAG+TCCGCATA
AAACTTTTCTTTTTACCTACCAGAGAAAACTCGGGAATGGTTCACAAAGTCTGGTGTGTATCTTTCAC
CATTTGCTTACGTGAATCACTCACACCCTGGTTGTAAAACTCTGAAGAACCACCTATTATTTAATGTT
GTAGCTAGTTACATT
```

```
+
FFFFFFF,FFFFFFFFFFFFFFFFFFFFF:FFF:FFFFFFFFFF:FF:FFFFFFFFFFFFFFFFFFFF
FF:FFFFFFFFFFFFFFFFFFFFFFF:FFFFFFFFFFFFFFFFFF,FF:FFFFFFFFFF:F:FFFFFFF
FFFFFF:FFFFFFFF
@A00155:342:HHGFNDSXY:1:2619:28610:23202 2:N:0:GAACCTAG+TCCGCATA
AAACTTTTCTTTTTACCTACCAGAGAAAACTCGGGAATGGTTCACAAAGTCTGGTGTGTATCTTTCAC
CATTTGCTTACGTGAATCACTCACACCCTGGTTGTAAAACTCTGGAGAACCACCTATTATTTAATGTT
GTAGCTAGTTACATT
+
:FFFFFFFFFFFFFFFFFFFFFFFFFFFFFFFFFFFF:FFFFFFFFFFFFFFFFFFFFFFFFFFFFFFF
FFFFFFFFFFFFFFFFFFFFFFFFFFFFFFFFFFFFFFFFFFFFFFFFFFFFFFFFFFFFFFFFFF:FF
FFFFFFFFFFFFFFF
@A00155:342:HHGFNDSXY:1:2114:9679:24518 2:N:0:GAACCTAG+TCCGCATA
AACTTTTCTTTTTACCTACCAGAGAAAACTCGGGAATGGTTCACAAAGTCTGGTGTGTATCTTTCACC
ATTTGCTTACGTGAATCACTCACACCCTGGTTGTAAAACTCTGGAGAACCACCTATTATTTAATGTTG
TAGCTAGTTACATTA
+
FFFFFFFFFFFFFFFFFFFFFFFFFFFFFFFFFFFF:FFFFFFFFFFFFFFF:FFFFFFFFFFFFFFFF
FFFFFFFFFFFFFFFFFFFFFFFFFFF,,FFFFFFFFF:F:FFFFFFFFFFFFFFFFFFFFFF:F,::FF
FFFFFFF:FFFF,FF
@A00155:342:HHGFNDSXY:1:2442:11930:22153 2:N:0:GAACCTAG+TCCGCATA
ACTTTTCTTTTTACCTACCAGAGAAAACTCGGGAATGGTTCACAAAGTCTGGTGTGTATCTTTCACCA
TTTGCTTACGTGAATCACTCACACCCTGGTTGTAAAACTCTGGAGAACCACCTATTATTTAATGTTGT
AGCTAGTTACATTAG
+
FFFFFFFFFFFFFFFFFFFFF:FFFFFFFFFFFFFFFFFFFFFFFFFFFFFFFFFFF:FFFFFFFFFFF
FFFFFFFFFFFFFFFFFFFFFFFFFFFFFFFFFFFFF,FFFFFFFFFFFFFFFFFFFFFFFFFFFFFFF
FFFF:FFFFFFFFFF
@A00155:342:HHGFNDSXY:1:1139:9417:7842 2:N:0:GAACCTAG+TCCGCATA
CTTTTCTTTTTACCTACCAGAGAAAACTCGGGAATGGTTCACAAAGTCTGGTGTGTATCTTTCACCTT
TTGCTTACGTGAATCACTCACACCCTGGTTGTAAAACTCTGGAGAACCACCTATTATTTAATGTTGTA
GCTAGTTACATTAGT
+
FFFFFFFFFFFFFFFFFFFFFFFFFFFFFFFFFFFFF:FFFF::FFFFFFFFFFFFFF,FFFFFF:FFF
FFFFFFFFFFFFFFFFFFFFFFFFFFFFFFFFFF,F,FFFFFFFFFFF:FFFFFFFFFFFF:FFFFFFF
FFFFFFFFFFFFFFF
@A00155:342:HHGFNDSXY:1:1408:26313:23171 2:N:0:GAACCTAG+TCCGCATA
CTTTTCTTTTTACCTACCAGAGAAAACTCGGGAATGGTTCACAAAGTCTGGTGTGTATCTTTCACCAT
TTGCTTACGTGAATCACTCACACCCTGGTTGTAAAACTCTGGAGAACCACCTATTATTTAATGTTGTA
GCTAGTTACATTAGT
+
FFFFFFFFFFFFFFFFFFFFFFFFFFFFFFFFFFFFFFFFFFFFFFFFFFFF:FFFFFFFFFFFFFFFF
FFFFF:FFFFFF:FFFFFFFFFFFF:FFFFFFFFFFFFFFFFFFFFFFFFFFFFFFFFFFFFFFFFFFF
FFFFFFFFFF:FFFF
@A00155:342:HHGFNDSXY:1:1445:3079:5228 2:N:0:GAACCTAG+TCCGCATA
CTTTTCTTTTTACCTACCAGAGAAAACTCGGGAATGGTTCACAAAGTCTGGTGTGTATCTTTCACCAT
TTGCTTACGTGAATCACTCACACCCTGGTTGTAAAACTCTGGAGAACCACCTATTATTTAATGTTGTA
GCTAGTTACATTAGT
+
FFFFFFFFFFFFFFFFFFFFFFFFFFFFFFFFFFFFFFFFFFFFFFFFFFFFFFFFFFFFFFFFFFFFF
FFFFFFFFFFFFFFFFFFFFFFFF:FFF:FFFFFFFFFFFFFFFFFFF,F:FFFFFFFFFF:FFFFFFF
FFFFFFFFFF,FFFF
@A00155:342:HHGFNDSXY:1:1508:1714:32769 2:N:0:GAACCTAG+TCCGCATA
CTTTTCTTTTTACCTACCAGAGAAAACTCGGGAATGGTTCACAAAGTCTGGTGTGTATCTTTCACCAT
```

```
TTGCTTACGTGAATCACTCACACCCTGGTTGTAAAACTCTGGAGAACCACCTATTATTTAATGTTGTA
GCTAGTTAAATTAGT
+
FFFFFFFF:F:F:FFFFFFFF:FFF:FFFFFFFFFFFFFFFFFFFFFFFFFFFFFFFFFFFFFF:FFF
FFF:FFFFFFFFFFF:FFFFFFFFFFF:FFFF:F,:FFFFFFFFFF,FFFFFFFFFFFFFFFFFFFFF
FFFFFFFF,FFF::F
@A00155:342:HHGFNDSXY:1:1546:10321:1329 2:N:0:GAACCTAG+TCCGCATA
CTTTTCTTTTTACCTACCAGAGAAAACTCGGGAATGGTTCACAAAGTCTGGTGTGTATCTTTCACCAT
TTGCTTACGTGAATCACTCACACCCTGGTTGTAAAACTCTGGAGAACCACCTATTATTTAATGTTGTA
GCTAGTTACATTAGT
+
FFFFFFFFFFFFFFFFFFFFFFFFFFFFFFFFF,FFFFFFFFFFFFFFFFFFFFFFFFFFFFFFFFFF
FFFFFFFFFFFFFFFFFFFFFFFFFFFFFFFFFFFFFFFFFFFF:FF:FFFFFFFFFFFFFFFFFFFF
FFFFFFFFFFFFFFF
@A00155:342:HHGFNDSXY:1:1623:2908:33270 2:N:0:GAACCTAG+TCCGCATA
CTTTTCTTTTTACCTACCAGAGAAAACTCGGGAATGGTTCACAAAGTCTGGTGTGTATCTTTCACCAT
TTGCTTACGTGAATCACTCACACCCTGGTTGTAAAACTCTGGAGAACCACCTATTATTTAATGTTGTA
GCTAGTTACATTAGT
+
FFFFFFFFFFFFFFFFFFFFFFFFFFFFFFFFFFFFFFFFFFFF:FFFFFFFFFF:FFFFFFFFFF,F
FFFFFFF::FFFFFFFFFFFFFFFFFFFFFFF:F:FFFFFFFFFFFFFFFFFFFFFFFFFFFFFFFFF
FFFFF:FFFFF,FFF
@A00155:342:HHGFNDSXY:1:1634:2691:18114 2:N:0:GAACCTAG+TCCGCATA
CTTTTCTTTTTACCTACCAGAGAAAACTCGGGAATGGTTCACAAAGTCTGGTGTGTATCTTTCACCAT
TTGCTTACGTGAATCACTCACACCCTGGTTGTAAAACTCTGGAGAACCACCTATTATTTAATGTTGTA
GCTAGTTACATTAGT
+
FFFFFFFFFFFFFFFFFFFFFFFFFFFFFF:FFFFF,FFFFFFFFFFFFFFFFFFFFFFFFFFF:FF:
FFFFFFFFFFFF,FFFFFFFFFFFFFFFFFFFFFFFFFFFFFFFFFFFFFFFFFFFFFFFFFFFFFFF
FFFFFFFFFFFFFFF
@A00155:342:HHGFNDSXY:1:1645:9588:7012 2:N:0:GAACCTAG+TCCGCATA
CTTTTCTTTTTACCTACCAGAGAAAACTCGGGAATGGTTCACAAAGTCTGGTGTGTATCTTTCACCTT
TTGCTTACGTGAATCACTCACACCCTGGTTGTAAAACTCTGGAGAACCACCTATTATTTAATGTTGTA
GCTAGTTACATTAGT
+
FFFFFFFFFFFFFFFFFFFFFFFFFFFFFFFFFFFFFFFFFFFF:FFFFFFFFFFFFFFFFFFFFFFF
FFF:FFFFFFFFFFFFFFFFFFFFFFFFFFFFFF,FFFFFFFFFFFFFFFFFFFFFFFFF:FFFFFFF
F:FFFFFFFFFFFFF
@A00155:342:HHGFNDSXY:1:2335:19497:11459 2:N:0:GAACCTAG+TCCGCATA
CTTTTCTTTTTACCTACCAGAGAAAACTCGGGAATGGTTCACAAAGTCTGGTGTGTATCTTTCACCAT
TTGCTTACGTGAATCACTCACACCCTGGTTGTAAAACTCTGGAGAACCACCTATTATTTAATGTTGTA
GCTAGTTACATTAGT
+
FFFFFFFFFFFFFFFFFF,FFFFFFFFFF:FFFFFFFFFFFFFFFFFFFFFFFFFFFFFFFFFFFFFF
FFFFFFFFFFFFFFFFFFFFFFFF,FFFFFF:FFF,F::FF:FFFFF:FFFFFFFFFFF:FFFFFFFF
FFFFFFFFFF:FFFF
@A00155:342:HHGFNDSXY:1:2518:15655:36307 2:N:0:GAACCTAG+TCCGCATA
CTTTTCTTTTTACCTACCAGAGAAAACTCGGGAATGGTTCACAAAGTCTGGTGTGTATCTTTCACCAT
TTGCTTACGTGAATCACTCACACCCTGGTTGTAAAACTCTGGAGAACCACCTATTATTTAATGTTGTA
GCTAGTTACATTAGT
+
FFFFFFFFFFFFFFFFFFFFFFFFFFFFFFFFF:FFFFFFFFFF:FFFFFFFFFFFFFFFFFFFF:FF
FFFFFFFFFFFFFFFFFFFFFFFFF:FFFFFFFFFFFFFFFFFFFFFFFFFFFFFFFFFFFFFFFFFF
FFFFFFFFFFFFF:F
```

```
@A00155:342:HHGFNDSXY:1:2518:15664:36323 2:N:0:GAACCTAG+TCCGCATA
CTTTTCTTTTTACCTACCAGAGAAAACTCGGGAATGGTTCACAAAGTCTGGTGTGTATCTTTCACCAT
TTGCTTACGTGAATCACTCACACCCTGGTTGTAAAACTCTGGAGAACCACCTATTATTTAATGTTGTA
GCTAGTTACATTAGT
+
FFFFFFFFFFFFFFFFFFFFFFFFFFFFFFFF:,FFFFFFFFF:FFFFFFFFFFFFFFFFFFFFFFFFF
FFFFFFFFFFFFFFFFFFFFFFFFFFFFFFFFFFFFFFFFFFFFFFF:FFFFFFFFFFFFFFFFFFFF
FFF,FFFFFF,FFFF
@A00155:342:HHGFNDSXY:1:2518:16034:36746 2:N:0:GAACCTAG+TCCGCATA
CTTTTCTTTTTACCTACCAGAGAAAACTCGGGAATGGTTCACAAAGTCTGGTGTGTATCTTTCACCAT
TTGCTTACGTGAATCACTCACACCCTGGTTGTAAAACTCTGGAGAACCACCTATTATTTAATGTTGTA
GCTAGTTACATTAGT
+
FFFFFFFFFFFFFFFFFFFFFFFFFFFFFFFFFFFFFFFFFFFFFFFFFFFFFFFFFFFFFFFFFFFF
FFFFFFFFFFFFFF:FFFFFFFFFFFFFFFFFFFFFFFFFFFF:FFFFFFFFFFFFFFFFFFFFFFFFF
FFFFFFFFFFFFFFF
@A00155:342:HHGFNDSXY:1:2537:32958:24815 2:N:0:GAACCTAG+TCCGCATA
CTTTTCTTTTTACCTACCAGAGAAAACTCGGGAATGGTTCACAAAGTCTGGTGTGTATCTTTCACCAT
TTGCTTACGTGAATCACTCACACCCTGGTTGTAAAACTCTGGAGAACCACCTATTATTTAATGTTGTA
GCTAGTTACATTAGT
+
FFFFFF::F:FFFFFFFF,FFFFFF:::FFFFFFFFFF,FFF:F::FFFFFFFFFFF:FFF:FFF::FF
FFFFFFFFFF:FFF:FFFFFFFFF:FFFFFFFFFFF:FFFF:FFF:FFFFF:F:FFFFFF:FFF:F:F
FFFFFFFFFFFFFFF
@A00155:342:HHGFNDSXY:1:2601:10384:18693 2:N:0:GAACCTAG+TCCGCATA
TTTTCTTTTTACCTACCAGAGAAAACTCGGGAATGGTTCACAAAGTCTGGTGTGTATCTTTCACCATT
TGCTTACGTGAATCACTCACACCCTGGTTGTAAAACTCTGGAGAACCACCTATTATTTAATGTTGTAG
CTAGTTACATTAGTA
+
FFFFFFFFFFFFFFFFFFFFFFFF:FFFFFFFFFFFFFFFFFFFFFFFFFFFFFFFFFFFFFFFFFFF
FFFFFFFFFFF,FFFFFFFFFFFFFFFFFFFFFF,FFFFFFFFF:FFFFFFFFFFFFFFFFFFFFFFFF:
FFFFFFF,FFF:FFF
@A00155:342:HHGFNDSXY:1:1524:12337:26021 2:N:0:GAACCTAG+TCCGCATA
TTTCTTTTTACCTACCAGAGAAAACTCGGGAATGGTTCACAAAGTCTGGTGTGTATCTTTCACCATTT
GCTTACGTGAATCACTCACACCCTGGTTGTAAAACTCTGGAGAACCACCTATTATTTAATGTTGTAGC
TAGTTACATTAGTAA
+
FFFFFFFFFFFFFFFFFFFFFFFFFFFFFFFFFFFFFFFFFF:FFFFFFFFFFFFFFFFFFFFFFFFF
FFFFFFFFFFFFFFFFFFFFFFFFFFFFFFFFFFFFFFFFFFFFFFFF:FFFFFFFFFFFFFFFFFFF
FFFFFFFFFFFFFFF
@A00155:342:HHGFNDSXY:1:2257:26295:26710 2:N:0:GAACCTAG+TCCGCATA
TTTCTTTTTACCTACCAGAGAAAACTCGGGAATGGTTCACAAAGTCTGGTGTGTATCTTTCACCATTT
GCTTACGTGAATCACTCACACCCTGGTTGTAAAACTCTGGAGAACCACCTATTATTTAATGTTGTAGC
TAGTTACATTAGTAA
+
FFFFFFFFFFFFFFFFFFFFFFFFFFFFFFFFFFFFFFFFFFFFFFFFFFF,FFFFFFFFFFFFFFFF
FFFFFFFFFFFFFFFFFFFFFFFF:FF:FFFFFFFFFFFFFFFFFFFFFFFFFFFFFFFFFFFF,FFFFF
FFFFFFFF:FFFFFF
@A00155:342:HHGFNDSXY:1:2526:32262:24142 2:N:0:GAACCTAG+TCCGCATA
TTTCTTTTTACCTACCAGAGAAAACTCGGGAATGGTTCACAAAGTCTGGTGTGTATCTTTCACCATTT
GCTTACGTGAATCACTCACACCCTGGTTGTAAAACTCTGGAGAACCACCTATTATTTAATGTTGTAGC
TAGTTACATTAGTAA
+
FFFFFFFFFFFFFFFFFFFFFFFFFFFFFFFFF:FFFFFFFFFFFFFFFFFFFFFFFFFFFFFFFFFFF
```

FFFFFFFFFFFFFFFFFFFFFFFFFFFFF:FFFFFFFFFFFFFFFFFFFFFFFFFFFFFFF:FFFF:F
FFFFFFFF,FFFFFF
@A00155:342:HHGFNDSXY:1:2631:14000:13996 2:N:0:GAACCTAG+TCCGCATA
TTTCTTTTTACCTACCAGAGAAAACTCGGGAATGGTTCACAAAGTCTGGTGTGTATCTTTCACCATTT
GCTTACGTGAATCACTCACACCCTGGTTGTAAAACTCTGGAGAACCACCTATTATTTAATGTTGTAGC
TAGTTACATTAGTAA
+
FF,FFFFF,FFFFFFFFFFF:FFFFF,,:FFF,FF:FF,FFFFFFFFFFFFF::FFFFFF,FFF:FF,
:F,:FFFFFF,:F,FF:FFF:F:FFF,FFF:FFFF:F,F,FFFFFFFFFFFFF,F,F::,::FFFF:F
FFFFFF,:FFFF,FF
@A00155:342:HHGFNDSXY:1:1355:8124:25801 2:N:0:GAACCTAG+TCCGCATA
TTCTTTTTACCTACCAGAGAAAACTCGGGAATGGTTCACAAAGTCTGGTGTGTATCTTTCACCATTTG
CTTACGTGAATCACTCACACCCTGGTTGTAAAACTCTGGAGAACCACCTATTATTTAATGTTGTAGCT
AGTTACATTAGTAAA
+
FFFFFFFFFFFFFFFFFFFFFFFFFFFFFF:FFFFFFFFFFFFFFFFFFFFFFFFFFFFFFFFFFFFF
FFFFFFFFFFFFFFFFFFFFFFFFFFFFFFF:FFFFFFFFFFFFFFFFFFFFFFFFFFFFFFFFFFFF
:FFFFFFFFFFFFFF
@A00155:342:HHGFNDSXY:1:1160:4444:34710 2:N:0:GAACCTAG+TCCGCATA
TTTTACCTACCAGAGAAAACTCGGGAATGGTTCACAAAGTCTGGTGTGTATCTTTCACCATTTGCTTA
CGTGAATCACTCACACCCTGGTTGTAAAACTCTGGAGAACCACCTATTATTTAATGTTGTAGCTAGTT
ACATTAGTAAATATT
+
FFFFFFFFFFFFFFFF,FFFFF:F:FFF:FFFFFFFFFFFFF,FFF,FFFFFFF:FFF:FF,FF,FFF
FF,FFFFFFFF,FFF:F,FFFFF,FFFFFFF,FFFFF:FFFFFFFFFFF,FFF::FFFFFFF:FF,:F
F:FFF,FFFFFFFFF
@A00155:342:HHGFNDSXY:1:1139:9417:7842 1:N:0:GAACCTAG+TCCGCATA
TTACCTACCAGAGAAAACTCGGGAATGGTTCACAAAGTCTGGTGTGTATCTTTCACCTTTTGCTTACG
TGAATCACTCACACCCTGGTTGTAAAACTCTGGAGAACCACCTATTATTTAATGTTGTAGCTAGTTAC
ATTAGTAAATATTCA
+
FFFFFF,FF:FFFFF:FFFFFFFFFFFFFFFFFFFFFFFFFFFFFFFFFFFFFFFFFFFFFFFFFFFF
FFFFFFFFFFFFFFFFFFFFFFFFFFFFFFFFFFFFFFFFFFFFFFFFFFFFFFFFFFFFFFFFFFFF
FFFFFFFFFFFFF:F
@A00155:342:HHGFNDSXY:1:1645:9588:7012 1:N:0:GAACCTAG+TCCGCATA
TTACCTACCAGAGAAAACTCGGGAATGGTTCACAAAGTCTGGTGTGTATCTTTCACCTTTTGCTTACG
TGAATCACTCACACCCTGGTTGTAAAACTCTGGAGAACCACCTATTATTTAATGTTGTAGCTAGTTAC
ATTAGTAAATATTCA
+
FFFFFFFFFFFFFFFFFFFFFFFFFFFFFFFFFFFFFFFFFFFFFFFFFFFFFFFFFFFFFFFFFFFF
FFFFFFFFFFFFFFFFFFFFFFFFFFFFF:FFFFFFFFFFFFFFFFFFFFFFFFFFFFFFFFFFFFFF
FFFFFFFFFFFFFFF
@A00155:342:HHGFNDSXY:1:1410:27543:32346 1:N:0:GAACCTAG+TCCGCATA
TACCAGAGAAAACTCGGGAATGGTTCACAAAGTCTGGTGTGTATCTTTCACCATTTGCTTACGTGAAT
CACTCACACCCTGGTTGTAAAACTCTGGAGAACCACCTATTATTTAATGTTGTAGCTAGTTACATTAG
TAAATATTCATATGT
+
FFFFFFFFFFFFFF:FFFFFFFFFFFFFFFFFFFFFFF:FFFFFFFF:FFFFFF:FF:FFFFF:FFFF
FFFFFFFFFFFFFFFFFFFFFFFFFFFFFFFFFF:FFFFFFFFFFF:FFFFFF:FFFFFFF:FFFFFF
FFFFFFFFFFFFFFF
@A00155:342:HHGFNDSXY:1:2352:16685:33959 2:N:0:GAACCTAG+TCCGCATA
TACCAGAGAAAACTCGGGAATGGTTCACAAAGTCTGGTGTGTATCTTTCACCATTTGCTTACGTGAAT
CACTCACACCCTGGTTGTAAAACTCTGGAGAACCACCTATTATTTAATGTTGTAGCTAGTTACATTAG
TAAATATTCATATGT

```
+
FFFFFFFFFFF:FFFFFFFFFFFFFFFF:FFFFFFFFFFFFFFFFFFFFFFFFF,FFFFFFFFFFFFF
F:FFFFFFFFFFFFFFFFFF:FFFFFF:FFFFFFFFFFFFFFFFFFFFFFFFFFFFFFF:FFFFFFFF:
FFFFFFFFFFFFFFF
@A00155:342:HHGFNDSXY:1:2148:25934:34851 1:N:0:GAACCTAG+TCCGCATA
ACCAGAGAAAACTCGGGAATGGTTCACAAAGTCTGGTGTGTATCTTTCACCATTTGCTTACGTGAATC
ACTCACACCCTGGTTGTAAAACTCTGGAGAACCACCTATTATTTAATGTTGTAGCTAGTTACATTAGT
AAATATTCATATGTA
+
FFFFFFFFFFFFFFFFFFFFFFFFFFFF:FFFFFFFF:FFFFFFFFFFF:FFFFFFFFFFFFFFFFFF
FFFFFFFFFFFFFFFFFFFFFFFFFFFFFFFF:FFFFFFFFFFFFFFFFFFFFFFFFFFFFFFFFFFF
FFFFFFF:FFFFFFF
@A00155:342:HHGFNDSXY:1:2450:9923:31704 1:N:0:GAACCTAG+TCCGCATA
ACCAGAGAAAACTCGGGAATGGTTCACAAAGTCTGGTGTGTATCTTTCACCATTTGCTTACGTGAATC
AATCACACCCTGGTTGTAAAACTCTGGAGAACCACCTATTATTTTATGTTGTAGCTAGTTACATTAGT
AAATATTCATATGTA
+
F::FFFFFFF,F:F:F:FFF,:FF:FF,,FFF:F:,:,FFFF:FFFF:FF:,F:FFFFFFFFFFFFFF
,,FF:,FF:,FFFFF:FFF:FFF:FF::FF:FF,:FFFFFFF:F,FFFFFFF,,:FFFFFFFFFFFFF
FF:F:FF::FFFFF:
@A00155:342:HHGFNDSXY:1:2515:9136:2190 1:N:0:GAACCTAG+TCCGCATA
ACCAGAGAAAACTCGGGAATGGTTCACAAAGTCTGGTGTGTATCTTTCACCATTTGCTTACGTGAATC
ACTCACACCCTGGTTGTAAAACTCTGGAGAACCACCTATTATTTAATGTTGTAGCTAGTTACATTAGT
AAATATTCATATGTA
+
FFFFFFFFFFFFFFFFFFFFFFFFFFFFFFFFFFFFFFFFFFFFFFFFFFFFFFFFFFFFFFF:FFFFF
FFFFFFFFFFFFFFFFFFFFFFFFFFFFFFFFFFFFFFFFFFFFFFFFFFFFFFFFFFFFFFFFFFFF
FFFFFFFFFFFFFFF
@A00155:342:HHGFNDSXY:1:2531:18900:35603 1:N:0:GAACCTAG+TCCGCATA
ACCAGAGAAAACTCGGGAATGGTTCACAAAGTCTGGTGTGTATCTTTCACCATTTGCTTACGTGAATC
ACTCACACCCTGGTTGTAAAACTCTGGAGAACCACCTATTATTTAATGTTGTAGCTAGTTACATTAGT
AAATATTCATATGTA
+
FFFFFFFFFFFFFFFFFFFFFFFFFFFFFFFFFFF:FFFFFFFFFFFFFFFFFF:FFFFFFFFFFFFFF
FFFFFFFFFFFFFFFFFFFFFFFFFFFFFFFFFFFFFFFFFFFFFFFFFFFFFFFFFF,FFFFFFFFF
FFFFFFFFFFFFFFF
@A00155:342:HHGFNDSXY:1:1335:28881:28369 1:N:0:GAACCTAG+TCCGCATA
CCAGAGAAAACTCGGGAATGGTTCACAAAGTCTGGTGTGTATCTTTCACCATTTGCTTACGTGAATCA
CTCACACCCTGGTTGTAAAACTCTGGAGAACCACCTATTATTTAATGTTGTAGCTAGTTACATTAGTA
AATATTCATATGTAG
+
FFFFFFFFFFFFFFFFFFFFFFFFFFFFFFFFFFFFFFFFFFFFFFFFFFFFFFFFFFFFFFF:FFFFFF
FFFFFFFFFFFFFFFFFFFFFFFFFFFFFFFFFFFFFFFFFF:F:FFFFFFFFFFFFFFFFFFFFFFFF
FFFFFFFFFFFFFFF
@A00155:342:HHGFNDSXY:1:1477:5014:31000 1:N:0:GAACCTAG+TCCGCATA
CCAGAGAAAACTCGGGAATGGTTCACAAAGTCTGGTGTGTATCTTTCACCATTTGCTTACGTGAATCA
CTCACACCCTGGTTGTAAAACTCTGGAGAACCACCTATTATTTAATGTTGTAGCTAGTTACATTAGTA
AATATTCATATGTAG
+
:FFFFFFFFFFFFFFFFFFFFFFFFFFFFF:F:FFFFFFFFFFFFFFFFFFFFFFFFFFFFFFFFFFFF
FFFFFFFFFFFFFFFFF::FFFFFFFFF:FFFFFFFFFF:F,FFFF:FFFFFFFFFF:FFFFFFFFF
FFFFFFFFFFFFFFF
@A00155:342:HHGFNDSXY:1:2235:31955:6637 1:N:0:GAACCTAG+TCCGCATA
TCTGAGAAAACTCGGGAATGGTTCACAAAGTCTGGTGTGTATCTTTCACCATTTGCTTACGTGAATCA
```

```
CTCACACCCTGGTTGTAAAACTCTGGAGAACCACCTATTATTTAATGTTGTAGCTAGTTACATTAGTA
AATATTCATATGTAG
+
FFFFFFFFFFFFFFFFF,FFFFFFFFFFFFFFFFFFFFF:FFFFFFFFFFFFFFFFFFFFFFFFFFFF
FFFFFFFFFFFF:FFFFFFFFFFFFFFFFFFFFFFFFFFF:FFFFFFFFF:FFFFFFFFFFFFFFFFF
FFFFFFFFFFFFFFF
@A00155:342:HHGFNDSXY:1:1514:3549:13745 1:N:0:GAACCTAG+TCCGCATA
CAGAGAAAACTCGGGAATGGTTCACAAAGTCTGGTGTGTATCTTTCACCATTTGCTTACGTGAATCAC
TCACACCCTGGTTGTAAAACTCTGGAGAACCACCTATTATTTAATGTTGTAGCTAGTTACATTAGTAA
ATATTCATATGTGGC
+
FFFFFFFFFFFFFFFFFFFFFFFFFFFFFFFFFFFFFFFFFFFFFFFFFFFFFF:FFFFFFFFFFFFF
FFFFFFFFFFFFFFFFFFFFFFFFFFFFFFFFFFFFFFFFFFFFFFFFFFFFFFFF:FFFFFFFFFFF
FFFFFFFFFFF:FFF
@A00155:342:HHGFNDSXY:1:1647:13277:32941 1:N:0:GAACCTAG+TCCGCATA
CAGAGAAAACTCGGGAATGGTTCACAAAGTCTGGTGTGTATCTTTCACCATTTGCTTACGTGAATCAC
TCACACCCTGGTTGTAAAACTCTGGAGAACCACCTATTATTTAATGTTGTAGCTAGTTACATTAGTAA
ATATTCATATGCAGC
+
FFFFFFFFFFFFFFFFFFFFFFFFFFFFFFFFFFFFFFFFFFFFFFFFFFFFFFFFFFFF,FFFFFFF
FFFFFFFFFFFFFFFFFFFFFFFFFFFFFFFFFFFFFFFFFFFFFFFFFFFFFFFFFFFFFFFFFFFF
FFFFFFFFFFFFFFF
@A00155:342:HHGFNDSXY:1:1404:17309:25144 1:N:0:GAACCTAG+TCCGCATA
AGAGAAAACTCGGGAATGGTTCACAAAGTCTGGTGTGTATCTTTCACCATTTGCTTACGTGAATCACT
CACACCCTGGTTGTAAAACTCTGGAGAACCACCTATTATTTAATGTTGTAGCTAGTTACATTAGTAAA
TATTCATATGTAGCA
+
FFFFFFFFFFFFFFFFFFFF:FFFFFFFFFFFFFFFFFFFFFFFFFFFFFFFFFFFFFFFFFFFFFF
FFFFFFFFFFFFFFFFFFFFFFFFFFFFFFFFFFFFFFFFFFFFFFFFFFFFFFFFFFFFFFFFFFFF
FFFFFFFFFFFFFFF
@A00155:342:HHGFNDSXY:1:1404:16586:25426 1:N:0:GAACCTAG+TCCGCATA
AGAGAAAACTCGGGAATGGTTCACAAAGTCTGGTGTGTATCTTTCACCATTTGCTTACGTGAATCACT
CACACCCTGGTTGTAAAACTCTGGAGAACCACCTATTATTTAATGTTGTAGCTAGTTACATTAGTAAA
TATTCATATGTAGCA
+
FFFFFFFFFFFFFFFFFFFFFFFFFFFFFFFFFFFFFFFFFFFFFFFFFFFFFFFFFFFFFFFFFFFF
FFFFFFFFFFFFFFFFFFFFFFFFFFFFFFFFFFFFFFFFFFFFFFFFFFFFFFFFFFFFFFFFFFFF
FFFFFFFFFFFFFFF
@A00155:342:HHGFNDSXY:1:1605:25807:3286 1:N:0:GAACCTAG+TCCGCATA
AGAGAAAACTCGGGAATGGTTCACAAAGTCTGGTGTGTATCTTTCACCATTTGCTTACGTGAATCACT
CACACCCTGGTTGTAAAACTCTGGAGAACCACCTATTATTTAATGTTGTAGCTAGTTACATTAGTAAA
TATTCATATGTAGCA
+
FFFFFFFFFFFFFFFFFFFFFFFFFFFFFFFFFFFFFFFFFFFFFF:FFFFFFFFFFFFFFFFFFFFF
FFFFFFFFFFFFFFFFFFFFFFFFFFFFFFFFFFFFFFFFFFFFFFFFFFFFFFFFFFFFFFFFFFFF
FFFFFFFFFFFFFFF
@A00155:342:HHGFNDSXY:1:2110:14127:21762 2:N:0:GAACCTAG+TCCGCATA
AGAGAAAACTCGGGAATGGTTCACAAAGTCTGGTGTGTATCTTTCACCATTTGCTTACGTGAATCACT
CACACCCTGGTTGTAAAACTCTGGAGAACCACCTATTATTTAATGTTGTAGCTAGTTACATTAGTAAA
TATTCATATGTAGCA
+
FFFFFFFFFFFFFFFFFFFFFFFFFFFFFFFFFFFFFFFFFFFFFFFFFFFFFFFFFFFFFFFFFFFF
FFFFFFFFFFFFFFFFFFFFFFFFFFFFFFFFFFFFFFFFFFFFFFFFFFFFFFFFFFFF:FFFFFFF
FFFFFFFFFFFFFFF
```

```
@A00155:342:HHGFNDSXY:1:2615:8947:29356 2:N:0:GAACCTAG+TCCGCATA
AGAGAAAACTCGGGAATGGTTCACAAAGTCTGGTGTGTATCTTTCACCATTTGCTTACGTGAATCACT
CACACCCTGGTTGTAAAACTCTGGAGAACCACCTATTATTTAATGTTGTAGCTAGTTACATTAGTAAA
TATTCATATGTAGCA
+
FFFFFFFFFFFFFFFFFFFFFFFFFFFFFFFFFFFFFFFFFFFFFFFFFFFFFFFFFFFFFFFFFFFF
FFFFF:FFFFFFFFFFFFFFFFFFFFFFFFFFFFFFFFFFFFFFFFFFFFFFFFFFFFFFFFFFFFFF
FFFFFFFFFFFF,FF
@A00155:342:HHGFNDSXY:1:2615:8892:29512 2:N:0:GAACCTAG+TCCGCATA
AGAGAAAACTCGGGAATGGTTCACAAAGTCTGGTGTGTATCTTTCACCATTTGCTTACGTGAATCACT
CACACCCTGGTTGTAAAACTCTGGAGAACCACCTATTATTTAATGTTGTAGCTAGTTACATTAGTAAA
TATTCATATGTAGCA
+
FFFFF,FFFFFFFFF:FFF:FFFFFFFFFFFFFFFFFFFFFFFFFFFFFFFFFF:FFFFFFFFFFFFF
FFFFFF,FFFFFFFF::FFFF:F,F:FFFFFFFFFF:FFFFFFFFFFFFFFFFFFFFFFFF:FFFFFFF
FFFFFFFFFFFFFF:
@A00155:342:HHGFNDSXY:1:2146:23375:26193 1:N:0:GAACCTAG+TCCGCATA
GAGAAAACTCGGGAATGGTTCACAAAGTCTGGTGTGTATCTTTCACCATTTGCTTACGTGAATCACTC
ACACCCTGGTTGTAAAACTCTGGAGAACCACCTATTATTTAATGTTGTAGCTAGTTACATTAGTAAAT
ATTCATATGTAGCAT
+
:FFFFFFFFFFFFFFFFFFFFFFF:FFFFFFFFFFFFFFFFFFFFFFFFFFFFFFFFF:FFFFFFFFF
FFFFFFFF:FFFFFFFFFFFFFFFFFFFFFFFFFFFFFFFFF:FFFFFFFFFFFFFFFFFFFFFFFFFF
FFFFFFFFFFFFFFF
@A00155:342:HHGFNDSXY:1:2444:5312:21371 1:N:0:GAACCTAG+TCCGCATA
GAGAAAACTCGGGAATGGTTCACAAAGTCTGGTGTGTATCTTTCACCATTTGCTTACGTGAATCACTC
ACACCCTGGTTGTAAAACTCTGGAGAACCACCTATTATTTAATGTTGTAGCTAGTTACATTAGTAAAT
ATTCATATGTAGCAT
+
FFFFFFFFFFFFFFFFFFFFFFFFFFFFFFFFFFFFFFFFFFFFFFFFFFFFFFFFFFFFFFFFFFFF
FFFFFFFFFFFFFFFFFFFFFFFFFFFFFFFFFFFFFFFFFFFFFFFFFFFFFFFFFFFFFFFFFFFF
FFFFFFFFFFFFFFF
@A00155:342:HHGFNDSXY:1:1155:22363:31548 2:N:0:GAACCTAG+TCCGCATA
GAGAAAACTCGGGAATGGTTCACAAAGTCTGGTGTGTATCTTTCACCATTTGCTTACGTGAATCACTC
ACACCCTGGTTGTAAAACTCTGGAGAACCACCTATTATTTAATGTTGTAGCTAGTTACATTAGTAAAT
ATTCATATGTAGCAT
+
FFFFFFFFFFFFFFFFFFFFFFF:FFFFFFFF,FFFFFFFFFFFFFFFFFFFFFFFFFFFFFFFFFFF
FFFFFFFFF,FFFFFFFFFFFFF:FFFFFFFFF:FFFFFFFFFFFFFFFFFFFFFFFFFFFFFFFFFF
FFFFFFFFFFFFFFF
@A00155:342:HHGFNDSXY:1:1346:24885:5729 2:N:0:GAACCTAG+TCCGCATA
GAGAAAACTCGGGAATGGTTCACAAAGTCTGGTGTGTATCTTTCACCATTTGCTTACGTGAATCACTC
ACACCCTGGTTGTAAAACTCTGGAGAACCACCTATTATTTAATGTTGTAGCTAGTTACATTAGTAAAT
ATTCATATGTAGCAT
+
FFFFFFFFFFFFF:FFFFFFFFFFFFFFFFFFFFF:FFFFFFFFFFFFFFFFFFFFFFFFFFFFFFFF
FFFFFFFFFF:FFFFFFFFFFFFFFFFFFFFFFFFFFFFFFFFFFFFF:FFFFFFFFFFFF,FFFFFFF
FFFF:FFFF:FFFFF
@A00155:342:HHGFNDSXY:1:1649:21621:9377 2:N:0:GAACCTAG+TCCGCATA
GAGAAAACTCGGGAATGGTTCACAAAGTCTGGTGTGTATCTTTCACCATTTGCTTACGTGAATCACTC
ACACCCTGGTTGTAAAACTCTGGAGAACCACCTATTATTTAATGTTGTAGCTAGTTACATTAGTAAAT
ATTCATATGTAGCAT
+
FFFFFFFFFFFFFF:FFFFFFFFFFFFFFFFFFFFFFFFFFFFFFFFFFF:FFFFFFFFFFFFFFFFFFF
```

FFFFFFFFFFFFFFFFFFFFFF:FFFFFFFFFFFFFFFFFFFFFFFFFFFFFFFFFFFFFFFFFFFFFFF
FFFFFFFFFFFFFFF
@A00155:342:HHGFNDSXY:1:2206:21395:23422 2:N:0:GAACCTAG+TCCGCATA
GAGAAAACTCGGGAATGGTTCACAAAGTCTGGTGTGTATCTTTCACCATTTGCTTACGTGAATCACTC
ACACCCTGGTTGTAAAACTCTGGAGAACCACCTATTATTTAATGTTGTAGCTAGTTACATTAGTAAAT
ATTCATATGTAGCAT
+
F:FFFFFFFFFFFFFFFFFFFFFFFFFFFFFFFFFFFFFFFFFFFFFFFFFFFFFFFFFFFFFFFFFFFF
FFFFFFFFFFFFFFFFFFFFFFFFFFFFFFFFFFFFFFFFFFFFFFFFFFFFFFFFFFFFFFFFFFFFFF
FFFFFFFFFFFFFFF
@A00155:342:HHGFNDSXY:1:2235:31955:6637 2:N:0:GAACCTAG+TCCGCATA
GAGAAAACTCGGGAATGGTTCACAAAGTCTGGTGTGTATCTTTCACCATTTGCTTACGTGAATCACTC
ACACCCTGGTTGTAAAACTCTGGAGAACCACCTATTATTTAATGTTGTAGCTAGTTACATTAGTAAAT
ATTCATATGTAGAGA
+
FFFFFFFFFFFFFF,FFFFFFFFFFFFFFFFFFFFFFFFFFFFFFFFFF,FFFFFFFFFFFFFFFFFFFF
FFFFFFFFFFFFFF:FFFFFFFFFFFFFFFFFFFFFFFFFFFFFFFFFFFFFFFF:FFFFFFFFFFFFFF
F:FFFFFFFFFFFFF
@A00155:342:HHGFNDSXY:1:2123:18864:14622 2:N:0:GAACCTAG+TCCGCATA
GAGAAAACTCGGGAATGGTTCACAAAGTCTGGTGTGTATCTTTCACCATTTGCTTACGTGAATCACTC
ACACCCTGGTTGTAAAACTCTGGAGAACCACCTATTATTTAATGTTGTAGCTAGTTACATTAGTAAAT
ATTCATATGTAGCAT
+
FFFFFFFFFFFFFF,FFFFFFFFFFFFFFFFFFFFFFFFFFFFFFFFFFFFFFFFFFFFFFFFFFFFFFF
FFFFFFFFFFFFFFFFFFFFFFFFFFFFFFFFFFFFFFFFFFFFFFFFFFFFFFFFFFFFFFFF:FFFFF
FFFFFFFFFFFFF:F
@A00155:342:HHGFNDSXY:1:2247:28330:23970 2:N:0:GAACCTAG+TCCGCATA
GAGAAAACTCGGGAATGGTTCACAAAGTCTGGTGTGTATCTTTCACCATTTGCTTACGTGAATCACTC
ACACCCTGGTTGTAAAACTCTGGAGAACCACCTATTATTTAATGTTGTAGCTAGTTACATTAGTAAAT
ATTCATATGTAGCAT
+
FFFFFFFFFFFFFFFFFFFFFFFFFFFFFFFFFFFFFFFFFFFFFFFFFFFFFFFFFFFFFFFFFFFFFF
FFFF:FFFF:FFFFFF:FFFFFFFFFFFF:FFFFFFFFF:FFFFFF,FFFFFFFFFFFFFF:FFFFFFFF
FF,FFFFFFFFF:FF
@A00155:342:HHGFNDSXY:1:2406:25518:8422 2:N:0:GAACCTAG+TCCGCATA
GAGAAAACTCGGGAATGGTTCACAAAGTCTGGTGTGTATCTTTCACCATTTGCTTACGTGAATCACTC
ACACCCTGGTTGTAAAACTCTGGAGAACCACCTATTATTTAATGTTGTAGCTAGTTACATTAGTAAAT
ATTCATATGTAGCAT
+
FFFFFFFFFFFFFF:FFFFFFFFFFFFFFFFFFFFFFFFFFFFFFFFFFFFFFFFFFFFFFFF:FFFFFF
FFFFFFFFFFFFFFFFFFFFFFFFFFFFFFFFFFFFFFFFFFF:FFFFFFFFFFFFFFFFF:F:FFFFFF
F::FFFFFFF::FFF
@A00155:342:HHGFNDSXY:1:2513:6822:21637 2:N:0:GAACCTAG+TCCGCATA
GAGAAAACTCGGGAATGGTTCACAAAGTCTGGTGTGTATCTTTCACCATTTGCTTACGTGAATCACTC
ACACCCTGGTTGTAAAACTCTGGAGAACCACCTATTATTTAATGTTGTAGCTAGTTACATTAGTAAAT
ATTCATATGTAGCAT
+
FFFFFFFFFFFFFFFFFFFFFFFFFFFFFFFFFFFFFFFFFFFFFFFFFFFF:FFFFFFFFFFFFFFFFF
FFFFFFFFFFFFFFF,FFFFFFFFFFF:FFFFFFFFFF:F:F:FFFFFFFF:FF::FFFFFFFFFFFFFF
FFFFFFFFFFFFFF:
@A00155:342:HHGFNDSXY:1:2304:7681:29951 2:N:0:GAACCTAG+TCCGCATA
AGAAAACTCGGGAATGGTTCACAAAGTCTGGTGTGTATCTTTCACCATTTGCTTACGTGAATCACTCA
CACCCTGGTTGTAAAACTCTGGAGAACCACCTATTATTTAATGTTGTAGCTAGTTACATTAGTAAATA
TTCATATGTAGCAT

```
+
:F:::FFFFFFF::FFFFFF:F::FFFFFFFFFFFF:F:FFFF:F::FFFFFFF:FF:F::FFFFFF:
F:FFFFFFFFFF:FFFF::FFF:F:F:F:F,::FF:FFF:FFF:F,F:,,F::FF:::FF:FF:FFF:
F,,:F:F:F:::FF
@A00155:342:HHGFNDSXY:1:2322:3170:27211 2:N:0:GAACCTAG+TCCGCATA
AGAAAACTCGGGAATGGTTCACAAAGTCTTGTGTGTATCTTTCACCATTTGCTTACGTGAATCACTCA
CACCCTGGTTGTAAAACTCTGGAGAACCACCTATTATTTAATGTTGTATCTAGTTACATTAGTAAATA
TTCATATGTAGCAT
+
F,FFF:,:::F:F::FFF::FFFFF,FFF,,,FFFFF::FF:FFF,,,FF,F:FFFF:FFF:FFFFFF
FF,FF,FF:FF:FFFFFFFF:FFFFFFFFF,FFF:FFFFFFF,,F,FF,FFFF,FF:F:,F,FFFFFF
FF:FFFFFFFFFFF
@A00155:342:HHGFNDSXY:1:1121:20943:18912 1:N:0:GAACCTAG+TCCGCATA
AGAAAACTCGGGAATGGTTCACAAAGTCTGGTGTGTATCTTTCACCATTTGCTTACGTGAATCACTCA
CACCCTGGTTGTAAAACTCTGGAGAACCACCTATTATTTAATGTTGTAGCTAGTTACATTAGTAAATA
TTCATATGTAGCATG
+
FFFFFFFFFFFFFFFFFFFFFFFFFFFFFFFFFFFFFFFFFFFFFFFFFFFFF:FFFFFFFFFFFFFF
FFFFFFFFFFFFFFFFFFFFFFFFFFFFFFFFFFFFFFFFFFFFFFFFFFF:FFFFFFFFFFFFFFFF
FFFFFFFFFFFFFFF
@A00155:342:HHGFNDSXY:1:1139:21124:23234 2:N:0:GAACCTAG+TCCGCATA
AGAAAACTCGGGAATGGTTCACAAAGTCTGGTGTGTATCTTTCACCATTTGCTTACGTGAATCACTCA
CACCCTGGTTGTAAAACTCTGGAGAACCACCTATTATTTAATGTTGTAGCTAGTTACATTAGTAAATA
TTCATATGTAGCATG
+
FFFFFFFFFFFFFFFFFFFFF:FFFFF:FFFFFF:FFFFF:FFFFFFF:FFFFFFFF,F:FFFFFFFF
F,FFFFFFFFFFFF,FFFFFFFF:FFF,FFFFF:,:FFFFFFFFFFFFF:FFFFFFFFFFFFFFF:F
FFFFFFFFFFFFFFF
@A00155:342:HHGFNDSXY:1:1430:10737:10254 2:N:0:GAACCTAG+TCCGCATA
AGAAAACTCGGGAATGGTTCACAAAGTCTGGTGTGTATCTTTCACCATTTGCTTACGTGAATCACTCA
CACCCTGGTTGTAAAACTCTGGAGAACCACCTATTATTTAATGTTGTAGCTAGTTACATTAGTAAATA
TTCATATGTAGCATG
+
FFFFFFFFFFFFF:FFFFFFFFFFFFFFFFFFFFFFFFFF,FFFFFFFFFFFFFFFFFFFFFFFFFFF
FFFFFFFFFFFFFFFFFFFFFFFFFFFFFF:FFFFFFFFFFFFFFFFFFFFFFFFFFF:FFFF:FFFF
FFFFFFFFFFFFFFF
@A00155:342:HHGFNDSXY:1:1376:17707:23171 2:N:0:GAACCTAG+TCCGCATA
AGAAAACTCGGGAATGGTTCACAAAGTCTGGTGTGTATCTTTCACCATTTGCTTACGTGAATCACTCA
CACCCTGGTTGTAAAACTCTGGAGAACCACCTATTATTTAATGTTGTAGCTAGTTACATTAGTAAATA
TTCATATGTAGCATG
+
FFFFFFFFF:FFFFFFFFFFFFFFFFFFFFF:FFFFFFFFFFFFFFFFFFF:FFFFFFFFFFFFFFFF
FFFFFFFFFFFFFFFFFFFFFFFFFFFFFFFFFFFFFFFFFFFFFFFFFFFFFFFFFFFFFFFFFFFF
FFFFFFFFFFFFFFF
@A00155:342:HHGFNDSXY:1:1476:4372:36808 2:N:0:GAACCTAG+TCCGCATA
AGAAAACTCGGGAATGGTTCACAAAGTCTGGTGTGTATCTTTCACCATTTGCTTACGTGAATCACTCA
CACCCTGGTTGTAAAACTCTGGAGAACCACCTATTATTTAATGTTGTAGCTAGTTACATTAGTAAATA
TTCATATGTAGCATG
+
FFFFFFFFFFFFFFFFFFFFFFFFFFFFFFFFFFFFFFFFFFFFFFFFFFFFFFFFFFFFFFFFFFFF
FFFFFFFFFF,FFFFFFFFFFFFFFFFFFFFFFFFFFF:FFFFFFFFFFFFFFFFFFFFFFFFFFFF:F
FFFFFFFFFFFFFF:
@A00155:342:HHGFNDSXY:1:1677:31765:34929 2:N:0:GAACCTAG+TCCGCATA
AGAAAACTCGGGAATGGTTCACAAAGTCTGGTGTGTATCTTTCACCATTTGCTTACGTGAATCACTCA
```

```
CACCCTGGTTGTAAAACTCTGGAGAACCACCTATTATTTAATGTTGTAGCTAGTTACATTAGTAAATA
TTCATATGTAGCATG
+
FFFFFFFFFFFFFFFFFFFFFFFFFFFFFFFFFFFFFFFFFFFFFFFFFFFFFFFFFFFFFFFFFFFF
FFFFFFFFFFFFFFFFFFFFFFFFFFFFFFFFFFFFFFFFFFFFFFFFFFFFFFFFFFFFFFFFFFFF
FFFFFFFFFFFFFFF
@A00155:342:HHGFNDSXY:1:2333:8594:14403 2:N:0:GAACCTAG+TCCGCATA
AGAAAACTCGGGAATGGTTCACAAAGTCTGGTGTGTATCTTTCACCATTTGCTTACGTGAATCACTCA
CACCCTGGTTGTAAAACTCTGGAGAACCACCTATTATTTAATGTTGTAGCTAGTTACATTAGTAAATA
TTCATATGTAGCATG
+
FFFFFFFFFFFFFFFFFFFFFFFFFFFFFFFFFFFFFFFFFFFFFFFFFFFFFFFFFFFFFFFFFFFF
FFFFFFFFFFFFFFFFFFFFFFFFFFFFFFFFFFF:FFFFFFF,FFFFFFFFFFFFFFFFFFFFFFFF
FFFFFFFFFFFF:FF
@A00155:342:HHGFNDSXY:1:2571:17671:16376 2:N:0:GAACCTAG+TCCGCATA
AGAAAACTCGGGAATGGTTCACAAAGTCTGGTGTGTATCTTTCACCATTTGCTTACGTGAATCACTCA
CACCCTGGTTGTAAAACTCTGGAGAACCACCTATTATTTAATGTTGTAGCTAGTTACATTAGTAAATA
TTCATATGTAGCATG
+
FFFFFFFFFFFFFFFFFFFFFFFFFFFFFFFFFFFFF:F:FFFF:FFFFF,FFFF::FFFF:FFFFF
FFFF::F:FFFFFF:FFFFFFFFFFFFFFFFFFFFFFFFFFFFFFFFFFFFFFF:FFFFFF,FFFFFFFF
FFFFFFFFFFFF:F:
@A00155:342:HHGFNDSXY:1:2571:15709:22999 2:N:0:GAACCTAG+TCCGCATA
AGAAAACTCGGGAATGGTTCACAAAGTCTGGTGTGTATCTTTCACCATTTGCTTACGTGAATCACTCA
CACCCTGGTTGTAAAACTCTGGAGAACCACCTATTATTTAATGTTGTAGCTAGTTACATTAGTAAATA
TTCATATGTAGCATG
+
FFFFFFFFFFFFFFFFFFFFFFFFFFFFFFFFFFFFFFFFFFFFFFFFFFFFFFFFFFFFFFFFFFFF
FFFFFFFFFFFFFFF:FFFFFFFFFFFFFFFFFFFFFFFFFFFFFFFFFFFFFFFFFFFFFFFFFFFF
FFFFFFFFFFFFFFF
@A00155:342:HHGFNDSXY:1:2622:21079:28166 2:N:0:GAACCTAG+TCCGCATA
AGAAAACTCGGGAATGGTTCACAAAGTCTGGTGTGTATCTTTCACCATTTGCTTACGTGAATCACTCA
CACCCTGGTTGTAAAACTCTGGAGAACCACCTATTATTTAATGTTGTAGCTAGTTACATTAGTAAATA
TTCATATGTAGCATG
+
FFFFFFFFFFFFFFFFFFFFFFFFFFFFFFFFFFFFFFFFFFFFFFFFFFFFFFFFFFFFFFFFFFFF
FFFFFFFFFFFFFFF:FFFFFFFFFFFFFFFFFFFFFFFFFFFFFFFFFFFFFFFFFFFFFFFFFFFF
FFFFFFFFFFFFFFF
@A00155:342:HHGFNDSXY:1:1404:20518:32769 1:N:0:GAACCTAG+TCCGCATA
GATAACTCGGGAATGGTTCACAAAGTCTGGTGTGTATCTTTCACCATTTGCTTACGTGAATCACTCAC
ACCCTGGTTGTAAAACTCTGGAGAACCACCTATTATTTAATGTTGTAGCTAGTTACATTAGTAAATAT
TCATATGTAGCATGC
+
FFFFFFFFFFFFFFFFFFFFFFFFFFFFFFFFFFFFFFFFFFFFFFFFFFFFFFFFFFFFFFFFFFFF
FFFFFFFFFFFFFFFFFFFFFFFFFFFFFFFFFFF:FFFFFFFFFFF:FFFFFFFFFFFFFFFFFFFF
FFFFFFFFFFFFFFF
@A00155:342:HHGFNDSXY:1:1404:20627:32863 1:N:0:GAACCTAG+TCCGCATA
GATAACTCGGGAATGGTTCACAAAGTCTGGTGTGTATCTTTCACCATTTGCTTACGTGAATCACTCAC
ACCCTGGTTGTAAAACTCTGGAGAACCACCTATTATTTAATGTTGTAGCTAGTTACATTAGTAAATAT
TCATATGTAGCATGC
+
FFFFFFFFFFFFFFFFFFFFFF:FFFFFFFFFFFFFFFFFFFFFFFF:FFFFFFFFFFFFFFFFFFFFF
FFFFFFFFFFFFFFFFFFFFFFFFFFFFFFFFFFFFFFFFFFFFFFFFFFFF,FFFFFFFFFFFFFFF
FFFFFFFFFFFFFFF
```

```
@A00155:342:HHGFNDSXY:1:2271:28067:3693 1:N:0:GAACCTAG+TCCGCATA
GAAAACTCGGGAATGGTTCACAAAGTCTGGTGTGTATCTTTCACCGTTTGCTTACGTGAATCACTCAC
ACCCTGGTTGTAAAACTCTGGAGAACCACCTATTATTTAATGTTGTAGCTAGTTACATTAGTAAATAT
TCATATGTAGCATGT
+
FFF:FFFFFFFFFFFFFFFFFFFFFFFFFFFFFFFFFFFFFFFFFFFFFFFFFFFFFFFFFFFFFFFFFF
FFFFFFFFFFFFFFFFFFFFFFFFFFFFFFFFFFFFFFFFFFFFFFFFFFFFFFFFFFFFFFFFFFFFFF
FFFFFFFFFFFFFFF
@A00155:342:HHGFNDSXY:1:1221:5466:33661 2:N:0:GAACCTAG+TCCGCATA
GAAAACTCGGGAATGGTTCACAAAGTCTGGTGTGTATCTTTCACCATTTGCTTACGTGAATCACTCAC
ACCCTGGTTGTAAAACTCTGGAGAACCACCTATTATTTAATGTTGTAGCTAGTTACATTAGTAAATAT
TCATATGTAGCATGT
+
FFFFFFFFFFFF:FFFFFFFFFF:FFFFFFFFFFFFFFFFFFFFFFFFFFFFFFFFFFFFFFFFFFFFF:
FF:FFFFFFFFFFF:FFFFFFFFF:FFFFFFFFFFFFFFFFFFF,FFFFFFFFFFFFFFFFFFFFF:F:F
FFFFFFFFFFFFFFF
@A00155:342:HHGFNDSXY:1:1333:29017:14763 2:N:0:GAACCTAG+TCCGCATA
GAAAACTCGGGAATGGTTCACAAAGTCTGGTGTGTATCTTTCACCATTTGCTTACGTGAATCACTCAC
ACCCTGGTTGTAAAACTCTGGAGAACCACCTATTATTTAATGTTGTAGCTAGTTACATTAGTAAATAT
TCATATGTAGCATGT
+
FFFFFFFFFFFF,FFFFFFFFFFFFFFFFFFFFFFFFFFFFFFFFFFFFFFFFFFFFFFFFFFFFFFFFF
FFFFFFFFFFFFFFFFFFFFFFFFFFFFFFFF:FFFFFFFFFFFFFFFFFFFFFFFFFFFFFFFFFFFFF
FFFFFFFFFFFFFFF
@A00155:342:HHGFNDSXY:1:1333:28872:15389 2:N:0:GAACCTAG+TCCGCATA
GAAAACTCGGGAATGGTTCACAAAGTCTGGTGTGTATCTTTCACCATTTGCTTACGTGAATCACTCAC
ACCCTGGTTGTAAAACTCTGGAGAACCACCTATTATTTAATGTTGTAGCTAGTTACATTAGTAAATAT
TCATATGTAGCATGT
+
FFFFFFFFFFFFFFFFFFFFFFFFFFFFFFFFFFFFFFFFFFFFFFFFFFFFFFFFFFFFFFFFFFFFFF
FFFFFFFFFFFFF:FFFFFFFFFFFFFFFFFFFFFF:FFFFFFFFFFFFFFF:FFFFFF:FFFFFFFFFF
FFFFFFFFFFFFFFF
@A00155:342:HHGFNDSXY:1:1333:28131:30953 2:N:0:GAACCTAG+TCCGCATA
GAAAACTCGGGAATGGTTCACAAAGTCTGGTGTGTATCTTTCACCATTTGCTTACGTGAATCACTCAC
ACCCTGGTTGTAAAACTCTGGAGAACCACCTATTATTTAATGTTGTAGCTAGTTACATTAGTAAATAT
TCATATGTAGCATGT
+
:FFFFFFFFFFFFFFFFFFFFFFFFFFFFFFFFFFFFFFFFFFFFFFFFFFFFFFFFFFFFFFFFF:FFF
FFFFFFFFFF:FFFFFFFFFFFFFFFFFFFFFFFFFFFFFFFFFFFFFFFFFFFFFFFFFFFFFFFFFFF
FFFFFFFFFFFFFFF
@A00155:342:HHGFNDSXY:1:1369:23339:34522 2:N:0:GAACCTAG+TCCGCATA
GAAAACTCGGGAATGGTTCACAAAGTCTGGTGTGTATCTTTCACCATTTGCTTACGTGAATCACTCAC
ACCCTGGTTGTAAAACTCTGGAGAACCACCTATTATTTAATGTTGTAGCTAGTTACATTAGTAAATAT
TCATATGTAGCATGT
+
FFFFFF:FFFFFFFFFFFFFFFFFFFFFFFFFFFFFFFFFFFFFFFFF,FFFFFFFFFFFFFFFFFFFFF
FFFFFFF:FFFFFFFFFFFFFFFFFFFFFFFFFFFFFFFFFFFF:FFFFFFFFFFFFFF,:FFFFFFFFF
FFFFFFFFFFFFFFF
@A00155:342:HHGFNDSXY:1:1530:12129:29763 2:N:0:GAACCTAG+TCCGCATA
GAAAACTCGGGAATGGTTCACAAAGTCTGGTGTGTATCTTTCACCATTTGCTTACGTGAATCACTCAC
ACCCTGGTTGTAAAACTCTGGAGAACCACCTATTATTTAATGTTGTAGCTAGTTACATTAGTAAATAT
TCATATGTAGCATGT
+
FFFFFFFFFFFFFFFFFFFFFFFFFFFFFFFFFFFFFFFFFFFFFFFFFFFFFFFFFFFFFF:FFFFFFF
```

FFFFFFFFFFFFFFFFFFFFFFFFFFFFFFFFFFFFFFFFFFFFFFFFFFFFFFFFFFFFFFFFFFFF
FFFFFFFF:FFFFFF
@A00155:342:HHGFNDSXY:1:2322:15845:4601 2:N:0:GAACCTAG+TCCGCATA
GAAAACTCGGGAATGGTTCACAAAGTCTGGTGTGTATCTTTCACCATTTGCTTACGTGAATCACTCAC
ACCCTGGTTGTAAAACTCTGGAGAACCACCTATTATTTAATGTTGTAGCTAGTTACATTAGTAAATAT
TCATATGTAGCATGT
+
FFFFFFFFFFFFFFFFFFFFFF,FFFFFFFFFFFFFFFFFFFFFFFFFFFFFFFFFFFFFFFFFFFFF
FFFFFFFFFFFFFF:FFFFFFFFFFFFFFFFFFFFFFFFFFFFFFFF:FFFFFFFFFFFFFFFFFFFF
FFFFFFFFFFF:FFF
@A00155:342:HHGFNDSXY:1:2448:26585:7232 2:N:0:GAACCTAG+TCCGCATA
GAAAACTCGGGAATGGTTCACAAAGTCTGGTGTGTATCTTTCACCATTTGCTTACGTGAATCACTCAC
ACCCTGGTTGTAAAACTCTGGAGAACCACCTATTATTTAATGTTGTAGCTAGTTACATTAGTAAATAT
TCATATGTAGCATGT
+
FFFFFFFFFFFF,FFFFFFFFFFFFFFFFFFFFFFFFFFFFFFFFFFFFFFFFFFFFFFFFF:FFFFF
FFFFFFFFFFFFFFFFFFFFFFFF,FFFFFFFFFFFFFF:FFFFFFFFFFFFFFFFFFFFFFFFFFFF
FFFFFFFF:FFFFF:
@A00155:342:HHGFNDSXY:1:2530:12156:30279 2:N:0:GAACCTAG+TCCGCATA
GAAAACTCGGGAATGGTTCACAAAGTCTGGTGTGTATCTTTCACCATTTGCTTACGTGAATCACTCAC
ACCCTGGTTGTAAAACTCTGGAGAACCACCTATTATTTAATGTTGTAGCTAGTTACATTAGTAAATAT
TCATATGTAGCATGT
+
FFFFFFFFFFFFFFFFFFFFFFFFFFFFFFFFFFFFFFFFFFFFFFFFFFFFFFFFFFFFFFFFFFFF
FFFFFFFFFFFFFFFFFFFFFFFFFFFFFFFFFFFFFFFFFFFFFFFFFFFFFFFFF:FFFFFFFFFF
FFFFFFFFFFFFFFF
@A00155:342:HHGFNDSXY:1:2537:14290:34475 2:N:0:GAACCTAG+TCCGCATA
GAAAACTCGGGAATGGTTCACAAAGTCTGGTGTGTATCTTTCACCATTTGCTTACGTGAATCACTCAC
ACCCTGGTTGTAAAACTCTGGAGAACCACCTATTATTTAATGTTGTAGCTAGTTACATTAGTAAATAT
TCATATGTAGCATGT
+
FFFFFFFFFFFFFFFFFFFFFFFFFFFFFFFFFFFFFFFFFFFFFFFFFFFFFFFFFFFFFFFFFFFF
FFFF:FFFFFFFFF:FFFFFFFFFFFF:FFFFFFFFFFFFFFFF,FFFFFFFFFFFFFFFFFFFFFFF
FFFFFFFFFFF:FFF
@A00155:342:HHGFNDSXY:1:2623:30092:8484 2:N:0:GAACCTAG+TCCGCATA
GAAAACTCGGGAATGGTTCACAAAGTCTGGTGTGTATCTTTCACCATTTGCTTACGTGAATCACTCAC
ACCCTGGTTGTAAAACTCTGGAGAACCACCTATTATTTAATGTTGTAGCTAGTTACATTAGTAAATAT
TCATATGTAGCATGT
+
FFFFFFF:FFFFFFFFFFFFFFFFFFFFFFFFFFFFFFFFFFFFFFFFFFFFFFFFFFFFFFFFFFFF
FFFFFFFFFFFFFFFFFFFFFFFFFFFFFFFFFFFFFFFFFFFF:FFFFFFF:FFFFF:F:F:FFFFF
FFFFFFFFFFFFFF:
@A00155:342:HHGFNDSXY:1:2639:18873:11506 2:N:0:GAACCTAG+TCCGCATA
GAAAACTCGGGAATGGTTCACAAAGTCTGGTGTGTATCTTTCACCATTTGCTTACGTGAATCACTCAC
ACCCTGGTTGTAAAACTCTGGAGAACCACCTATTATTTAATGTTGTAGCTAGTTACATTAGTAAATAG
TCATATGTAGCATGT
+
FFFFFFFFFFFFFFFFFFFFFFFFFFFFFFFFFFFFFFFFFFFFFFFFFFFFFFFFFFFFFFFFFFFF
FFFFFFFFFFFFF,FFFFFFFFF:FFFFFFFFF:FFFF:FFFFFFFFFFFFFF:FFFFFFFFFFFFF,
FFFF,FFFFFFF:FF
@A00155:342:HHGFNDSXY:1:2576:32949:29277 2:N:0:GAACCTAG+TCCGCATA
GAAAACTCGGGAATGGTTCACAAAGTCTGGTGTGTATCTTTCACCATTTGCTTACGTGAATCACTCAC
ACCCTGGTTGTAAAACTCTGGAGAACCACCTATTATTTAATGTTGTAGCTAGTTACATTAGTAAATAT
TCATATGTAGCATGT

+
FFFF,FFFFFFFFFFFFFFFFFFFFFFFFFFFFFFFFFFFFFFFFFFFFFFFFFFFFFFFFFFFFFFFFF
FFFFFFFFFFFFF:FFFFFFFFFFFFFFFFFFFFFFFFFFFFFFFFFFFFFFFFFFFFFFFFFFFFFFFF
FFFFFFFFFFF:FFF
@A00155:342:HHGFNDSXY:1:2614:22932:30217 2:N:0:GAACCTAG+TCCGCATA
AAACTCGGGAATGGTTCACAAAGTCTGGTGTGTATCTTTCACCATTTGCTTACGTGAATCACTCACAC
CCTGGTTGTAAAACTCTGGAGAACCACCTATTATTTAATGTTGTAGCTAGTTACATTAGTAAATATTC
ATATGTAGCATGTTT
+
FFFFFFFFFFFFFFFFFFFFFFFFFFFFFFFFFFFFFFFFFFFFFFFFFFFFFFFFFFFFFFFFFFFFFF
FFFFFFFFFFFFFFFFFFFFFFFFFFFFFFFFFFFFFFFFFFFFFFFFFFFFFFFFFFFF:FFFFFFFFF
FFFFFFFFFFFFFFF
@A00155:342:HHGFNDSXY:1:1114:18954:28307 2:N:0:GAACCTAG+TCCGCATA
AACTCGGGAATGGTTCACAAAGTCTGGTGTGTATCTTTCACCATTTGCTTACGTGAATCACTCACACC
CTGGTTGTAAAACTCTGGAGAACCACCTATTATTTAATGTTGTAGCTAGTTACATTAGTAAATATTCA
TATGTAGCATGTTTA
+
FFFFFFFFF:FFFFFFFFFFFFFFFFFFFFFFFFFFFFFFFFFFFFFFFFFFFFFFFFFFFFFFFFFFFF
FFFFFFFFFFFFFFFFFFFFFFFFFFFFFFFFFFFF,FFF:FFFFFFFFFFFFFFFFFFFFF:FFFFFFF
FFFFFFFFFFFFFFF
@A00155:342:HHGFNDSXY:1:2213:21088:21512 2:N:0:GAACCTAG+TCCGCATA
AACTCGGGAATGGTTCACAAAGTCTGGTGTGTATCTTTCACCATTTGCTTACGTGAATCACTCACACC
CTGGTTGTAAAACTCTGGAGAACCACCTATTATTTAATGTTGTAGCTAGTTACATTAGTAAATATTCA
TATGTAGCATGTTTA
+
FFFFFFFFFFFFFFFFFFFF:FFFFFFFFFFFFFFFFFFFFFFFFFFFFFFFFFFFFFFFFFFFFFFFFF
FFFFFFFFFFFFFFFFFFFFFFFFFFFFFFFFFFFFFFFFFFFFFF:FF:FFFFFFFFFFFFFFFFFFFF
FFFFFFFFFFFFFFF
@A00155:342:HHGFNDSXY:1:2673:24686:20040 2:N:0:GAACCTAG+TCCGCATA
AACTCGGGAATGGTTCACAAAGTCTGGTGTGTATCTTTCACCATTTGCTTACGTGAATCACTCACACC
CTGGTTGTAAAACTCTGGAGAACCACCTATTATTTAATGTTGTAGCTAGTTACATTAGTAAATATTCA
TATGTAGCATGTTTA
+
FFFFFFFFFFFFFFFFFFFFFFFFFFFFFFFFFFFFFFFFFFFFFFFFFFFFFFFFFFFFFFFFFFFFFF
FFFFFFFFFFFFFFFFFFFFFFFFFFFFFFFFFFFFFFFFFFFFFFFFFFFFFFF:F:FFFFFFFFFFFF
FFFFFF,FFFFFFFF
@A00155:342:HHGFNDSXY:1:1108:28863:32690 2:N:0:GAACCTAG+TCCGCATA
CTCGGGAATGGTTCACAAAGTCTGGTGTGTATCTTTCACCATTTGCTTACGTGAATCACTCACACCCT
GGTTGTAAAACTCTGGAGAACCACCTATTATTTAATGTTGTAGCTAGTTACATTAGTAAATATTCATA
TGTAGCATGTTTAAG
+
FFFFFFF,FFFFFFFFFFFFFFFFFFFFFFFFFFFFFFFFFFFFFFFFFFFFFFFFFFFFFFFFFFF,FF
FFFF:FFFFFFFFFFFFFFFFFFFFF,FFFFFFFFFF:F,FFFFFFFFFFFFF:FFFFFFFFFFFFFFFF
FFFFFFF:FFFFFFF
@A00155:342:HHGFNDSXY:1:1106:8106:31532 2:N:0:GAACCTAG+TCCGCATA
CTCGGGAATGGTTCACAAAGTCTGGTGTGTATCTTTCACCATTTGCTTACGTGAATCACTCACACCCT
GGTTGTAAAACTCTGGAGAACCACCTATTATTTAATGTTGTAGCTAGTTACATTAGTAAATATTCATA
TGTAGCATGTTTAAG
+
FFFFFFFFFFFFFFFFFFFFFFFFFFFFFFFFFFFFFFFFFFFFFFFFFFFFFFFFFFFFFFFFFFFFFF
FFFFFFFFFFFFFFFFFFFFFFFFFFFFFFFFFFFFFFFFFFFFFFF:FFFFFFFFFFFFFFFFFFFFFF
FFFFFFFFFFFFFFF
@A00155:342:HHGFNDSXY:1:1176:13304:16986 2:N:0:GAACCTAG+TCCGCATA
CTCGGGAATGGTTCACAAAGTCTGGTGTGTATCTTTCACCATTTGCTTACGTGAATCACTCACACCCT

```
GGTTGTAAAACTCTGGAGAACCACCTATTATTTAATGTTGTAGCTAGTTACATTAGTAAATATTCATA
TGTAGCATGTTTAAG
+
FFFFFFFFFFFFFFFFFFFFFFFFFFFFFFFFFFFFFFFFFFFFFFFFFFFFFFFFFFFFFFFFFF:FF
FFFFFFFFF:FFFFFFFFFFFFFFFFFFFFFFFFFFFFF:FFFFFFFFFFFF:FFFFFFFFFFFFF
FFFFFFFFFFFFFFF
@A00155:342:HHGFNDSXY:1:1235:13937:19554 2:N:0:GAACCTAG+TCCGCATA
CTCGGGAATGGTTCACAAAGTCTGGTGTGTATCTTTCACCATTTGCTTACGTGAATCACTCACACCCT
GGTTGTAAAACTCTGGAGAACCACCTATTATTTAATGTTGTAGCTAGTTACATTAGTAAATATTCATA
TGTAGCATGTTTAAG
+
FFFFFF::FFFFFFFFFFFFFFFFFFFFFFFFFFFFFFFFFFFFFFFFFFFFFFFFFFFFFFFFFFFF
FFFFFFFFFFFFF:FFFFFFFF:FFFFFFFFFFFFFFFFFFFFFFFFFFFFFFFFFFFFFFFFFFFFF
FFFFFFFFFFFFFFF
@A00155:342:HHGFNDSXY:1:1230:16929:24048 2:N:0:GAACCTAG+TCCGCATA
CTCGGGAATGGTTCACAAAGTCTGGTGTGTATCTTTCACCATTTGCTTACGTGAATCACTCACACCCT
GGTTGTAAAACTCTGGAGAACCACCTATTATTTAATGTTGTAGCTAGTTACATTAGTAAATATTCATA
TGTAGCATGTTTAAG
+
FFFFFFFFFFFFFFFFFFFFFFFFFFFFFFFFFFFFFFFFFFFFFFFFFFFFFFFFFFFFFFFFFFFF
FFFFFFFF:FFFFFFFFFFFFFFFFFFFFFFFFF:FFFFFFFFFFFFFFFFFFFFFFFFFFFF:FFFFF
FFF:FFF:FFFFFFF
@A00155:342:HHGFNDSXY:1:1339:13729:12806 2:N:0:GAACCTAG+TCCGCATA
CTCGGGAATGGTTCACAAAGTCTGGTGTGTATCTTTCACCATTTGCTTACGTGAATCACTCACACCCT
GGTTGTAAAACTCTGGAGAACCACCTATTATTTAATGATGTAGCTAGTTACATTAGTAAATATTCATA
TGTAGCATGTTTAAG
+
FFFF::F:FFFFFFFFFFFFFF:FFFF:FFFFF::FFF:FFFFFFFFFFFFFFFFF:FFFFFFF::FF
FF:FFFFFFFFF,FF:FFFFF::FFFF:FFF,FFFFF,F:,FFF:FFFFFFFFFFF:FFFFFF:FFF:
FFFFFF:,:FFFFFF
@A00155:342:HHGFNDSXY:1:1374:22083:29528 2:N:0:GAACCTAG+TCCGCATA
CTCGGGAATGGTTCACAAAGTCTGGTGTGTATCTTTCACCATTTGCTTACGTGAATCACTCACACCCT
GGTTGTAAAACTCTGGAGAACCACCTATTATTTAATGTTGTAGCTAGTTACATTAGTAAATATTCATA
GGTAGCATGTTTAAG
+
FFFFFFF:FFFFFFFFF:FFFFFFFFFFFFFFFFFFFFFFFFFFFFFFFFFFFFFFFFFFFFFFFFFF
FFFFFFFFFFFFFFFFFFFFFFF:FFFFFFFFFFFFFFFFFFFFFFFFFFFFFFFFFFFFFFFFFFFF
,FFFFFFFFFFFFFF
@A00155:342:HHGFNDSXY:1:1634:18358:3975 2:N:0:GAACCTAG+TCCGCATA
CTCGGGAATGGTTCACAAAGTCTGGTGTGTATCTTTCACCATTTGCTTACGTGAATCACTCACACCCT
GGTTGTAAAACTCTGGAGAACCACCTATTATTTAATGTTGTAGCTAGTTACATTAGTAAATATTCATA
TGTAGCATGTTTAAG
+
FFFFFFFFFFFFFFFFF,FFFFF:FFFFFFFFFFFFFFFFFFFFFFFFFFFFFFF,FFFFFFFFFF:FF
FFFFFFFFF:FFFFFFFFFFFFFFFFFFFFFFFFFFFF:FFFFFFF,FFFFFFFFFFFFFFFFFFFFFFF
FFFFFF:FFFFFFFF
@A00155:342:HHGFNDSXY:1:1665:24740:17002 2:N:0:GAACCTAG+TCCGCATA
CTCGGGAATGGTTCACAAAGTCTGGTGTGTATCTTTCACCATTTGCTTACGTGAATCACTCACACCCT
GGTTGTAAAACTCTGGAGAACCACCTATTATTTAATGTTGTAGCTAGTTACATTAGTAAATATTCATA
TGTAGCATGTTTAAG
+
FFFFFFFFFFFFFFFFFFFFFFFFFFFFFFFFFFFFFFFFFFFFFFFFFFFFFFFFFFFFFFFFFFF:
FFFFFFFFFFFFFFFFFFFFFFFFFFFFFFFFFFFFFFFFFFFFFFFFF:FFFFFF:FFFFFFFFFFFFFF:
FFFFFFFFFFFFF:F
```

```
@A00155:342:HHGFNDSXY:1:2142:18683:15562 2:N:0:GAACCTAG+TCCGCATA
CTCGGGAATGGTTCACAAAGTCTGGTGTGTATCTTTCACCATTTGCTTACGTGAATCACTCACACCCT
GGTTGTAAAACTCTGGAGAACCACCTATTATTTAATGTTGTAGCTAGTTACATTAGTAAATATTCATA
TGTAGCATGTTTAAG
+
FFFFFF:,FFFFFFFFFFFFFFFFFFFFFFFFFFFFFFF:FFFFFFFFFFFFFFFFFFFFFFFFFFFF
FFFFFFFFFFFFFFFFFFFFFFFFFFFFFFFFFFFFFFFFFFFFFFFFFFFFFF:FFFFFFFFFFFFF
FFFFFFFFFFFFFFF
@A00155:342:HHGFNDSXY:1:2142:18240:15577 2:N:0:GAACCTAG+TCCGCATA
CTCGGGAATGGTTCACAAAGTCTGGTGTGTATCTTTCACCATTTGCTTACGTGAATCACTCACACCCT
GGTTGTAAAACTCTGGAGAACCACCTATTATTTAATGTTGTAGCTAGTTACATTAGTAAATATTCATA
TGTAGCATGTTTAAG
+
FFF:FFFFFFFFFFFFFFFFFFFFFFFFFFFFFFFFFFFFFFFFFFFFFFFFFFFFFFFFFFFFFFFF
FFF:FFFFFFFFFF:FFFFFFFFFFFFFFFFFFFFFFFFFFFFFFFFFFFFFFFFFFFFFFFFFFFFF
:FFF,FF:F:FFF,F
@A00155:342:HHGFNDSXY:1:2154:11849:4695 2:N:0:GAACCTAG+TCCGCATA
CTCGGGAATGGTTCACAAAGTCTGGTGTGTATCTTTCACCATTTGCTTACGTGAATCACTCACACCCT
GGTTGTAAAACTCTGGAGAACCACCTATTATTTAATGTTGTAGCTAGTTACATTAGTAAATATTCATA
TGTAGCATGTTTAAG
+
FFFFFFFFFFFFFFFFFFFFFFFFFFF,FFFFFFFFFFFFF:FFFFFFFFFFFFFFFFFFFFFFFFFF
FFFFFFFFFFFFFFFFFFFFFFFFFFFFFFFFFFFFFFFFFFFF:FFFFFFFF:FFFFFFFF:FFFFF
FFFFFFF:FFFFFFF
@A00155:342:HHGFNDSXY:1:2439:28637:35900 2:N:0:GAACCTAG+TCCGCATA
CTCGGGAATGGTTCACAAAGTCTGGTGTGTATCTTTCACCATTTGCTTACGTGAATCACTCACACCCT
GGTTGTAAAACTCTGGAGAACCACCTATTATTTAATGTTGTAGCTAGTTACATTAGTAAATATTCATA
TGTAGCATGTTTAAG
+
FFFFFFFFFFFFFFFFFFFFFFFFFFFFFFFFFFFFFFFFFFFFF:FFFFFFFFFFFFFFFFFFFFFF
FFFFFFFFFFFFFFFFFFFFFFFFFFFFFFFFFFFFF:FFFFF:FFFFFFFFFFFFFFFFFFFFFFFF
FFFF:FF:FFFFFFF
@A00155:342:HHGFNDSXY:1:2468:9616:11224 2:N:0:GAACCTAG+TCCGCATA
CTCGGGAATGGTTCACAAAGTCTGGTGTGTATCTTTCACCATTTGCTTACGTGAATCACTCACACCCT
GGTTGTAAAACTCTGGAGAACCACCTATTATTTAATGTTGTAGCTAGTTACATTAGTAAATATTCATA
TGTAGCATGTTTAAG
+
FFFFFFF:FFFFFFFFFF::FFFFFFFFFFFFFFFFFFFFFFFFFFFFFFFFFFFFFFFFFFFFFFFF
FFFFFFFF:FFFFFFFFFFFFFFFFFFFFFFFF,FFFFFFFFF::FFFFFFF,FFFFFFFFFFFFFFF
FFFF:FFFFFFFFFF
@A00155:342:HHGFNDSXY:1:2476:19262:29434 2:N:0:GAACCTAG+TCCGCATA
CTCGGGAATGGTTCACAAAGTCTGGTGTGTATCTTTCACCATTTGCTTACGTGAATCACTCACACCCT
GGTTGTAAAACTCTGGAGAACCACCTATTATTTAATGTTGTAGCTAGTTACATTAGTAAATATTCATA
TGTAGCATGTTTAAG
+
FFFFFFFFFFFFFFFFFFFFFFFFFFFFFFFFFFFFFFFFFFFFFFFFFFFFFFFFFFFFFFFFFFFF
FFFFFFFFFFFFFFFFFFFFFFFFFFFFFFFFFFFFFFFFFFFFFFFFFFFF:FFFFFFFFFFFFFFF
FFFFFFFFFFFFFFF
@A00155:342:HHGFNDSXY:1:2539:30481:8437 2:N:0:GAACCTAG+TCCGCATA
CTCGGGAATGGTTCACAAAGTCTGGTGTGTATCTTTCACCATTTGCTTACGTGAATCACTCACACCCT
GGTTGTAAAACTCTGGAGAACCACCTATTATTTAATGTTGTAGCTAGTTACATTAGTAAATATTCATA
TGTAGCATGTTTAAG
+
FFFFFFFFFFFFFFFFFFFFFFFFFFFFFFFFFFFFFFFFFFFFFFFFFFFFFFFFFFFFFFFFFFFF
```

```
FFFFFFFFFFFFFFFFFFFFFFFFFFFFFFFFFFFFFFFFFFFFFFFFFFFFFFFFFFF:FFFFFFF
FFFFFFFFFFFFFFF
@A00155:342:HHGFNDSXY:1:2625:16450:29637 2:N:0:GAACCTAG+TCCGCATA
CTCGGGAATGGTTCACAAAGTCTGGTGTGTATCTTTCACCATTTGCTTACGTGAATCACTCACACCCT
GGTTGTAAAACTCTGGAGAACCACCTATTATTTAATGTTGTAGCTAGTTACATTAGTAAATATTCATA
TGTAGCATGTTTAAG
+
FFFFFFF:FFFFFFFFFFFFFFFFFFFFFFFFFFFFFFFFFFFFFFFFFFFFFFFFFFFFFFFFFFFF
FFFFFFFFFFFFFFFFFFFFFFFFFFFFFFFFFFFFFFFFFFFFFFFFFFFFFFFFFFFFFFFFFFFF
FFFF:FFFFFFFFF:
@A00155:342:HHGFNDSXY:1:2659:9335:30968 2:N:0:GAACCTAG+TCCGCATA
CTCGGGAATGGTTCACAAAGTCTGGTGTGTATCTTTCACCATTTGCTTACGTGAATCACTCACACCCT
GGTTGTAAAACTCTGGAGAACCACCTATTATTTAATGTTGTAGCTAGTTACATTAGTAAATATTCATA
TGTAGCATGTTTAAG
+
FFFFFFFFFFFFFFFFFFFFFFFFFFFFFFFFFFFFFFFFFFFFFFFFFFFFFFFFFFFFFFFFFFFF
FFFFFFFFFFFFFFFFFFFFFFFFFFFFFFFFFFFFFFFFFFFFFFFFFFFFFFFFFFFFFFFFFFFF
FFFFFFFFFFFFFFF
@A00155:342:HHGFNDSXY:1:1325:29261:3662 2:N:0:GAACCTAG+TCCGCATA
TCGGGAATGGTTCACAAAGTCTGGTGTGTATCTTTCACCATTTGCTTACGTGAATCACTCACACCCTG
GTTGTAAAACTCTGGAGAACCACCTATTATTTAATGTTGTAGCTAGTTACATTAGTAAATATTCATAT
GTAGCATGTTTAAGC
+
FFFFFF:FFFFFFFFFFFFFFFFFFFFFFF:FFFFFFFFFFFFFFFFFFFFFFFFFFFF:FFFFF:FF:F
FFFFFFFFFFFFFFFFFFFFFFFFFFFFFFFFFFFFFFFFF:FFFFFFFFFFFFFFFFFFFFFFFFFF:FF
FFFFFFFFFFFFFFF
@A00155:342:HHGFNDSXY:1:2506:8856:14888 2:N:0:GAACCTAG+TCCGCATA
TCGGGAATGGTTCACAAAGTCTGGTGTGTATCTTTCACCATTTGCTTACGTGAATCACTCACACCCTG
GTTGTAAAACTCTGGAGAACCACCTATTATTTAATGTTGTAGCTAGTTACATTAGTAAATATTCATAT
GTAGCATGTTTAAGC
+
FFFFFFFFFFFFFFFFFFFFFFFFFFFFFFFFFFFFF:FFFFFFFFFFFFFFFFFFFFFFFFFFFFFFF
FFFFFFFFFFFFFFFFFFFFFFFFFFFFFFFFFF,FFFFFFFFFFFFFFFFFFFFFFFFFFFFFFFFFF
FFFFF:FFFFFFFFF
@A00155:342:HHGFNDSXY:1:1131:9742:2362 2:N:0:GAACCTAG+TCCGCATA
CGGGAATGGTTCACAAAGTCTGGTGTGTATCTTTCACCATTTGCTTACGTGAATCACTCACACCCTGG
TTGTAAAACTCTGGAGAACCACCTATTATTTAATGTTGTAGCTAGTTACATTAGTAAATATTCATATG
TAGCATGTTTAAGCA
+
FFFFF:FFFFFFFF:FFFFFFFFFFFFFFFFFFFFFFFFFFFFFFFFFFFFFFFFFFFFFFFFFFFFF
FFFFFFF:FFFFFFFFFFFFFFFFFFFFFFFFFFFFFFFFFFFFF,F:FFF:FFFFFFFFFFFFFFFFF:
FFFFFFFFFFFFFFF
@A00155:342:HHGFNDSXY:1:1137:21242:30514 2:N:0:GAACCTAG+TCCGCATA
CGGGAATGGTTCACAAAGTCTGGTGTGTATCTTTCACCATTTGCTTACGTGAATCACTCACACCCTGG
TTGTAAAACTCTGGAGAACCACCTATTATTTAATGTTGTAGCTAGTTACATTAGTAAATATTCATATG
TAGCATGTTTAAGCA
+
FFFFFFFFFFFFFFFFFFFFFFFFFFFFFFFFFFFFFFFFFFFFFFFFFFFFFFFFFFFFFFFFFFFF
FFFFFFFFFFFFFFFFFFFFFFFFFFFFFFFFFFFFFFFFFFFFFFFFFFFFFFFFFFFFFFFFFFFF
FF:FFFFFFFFFFFF
@A00155:342:HHGFNDSXY:1:1247:32958:8375 2:N:0:GAACCTAG+TCCGCATA
CGGGAATGGTTCACAAAGTCTGGTGTGTATCTTTCACCATTTGCTTACGTGAATCACTCACACCCTGG
TTGTAAAACTCTGGAGAACCACCTATTATTTAATGTTGTAGCTAGTTACATTAGTAAATATTCATATG
TAGCATGTTTAAGCA
```

+
FFFFF:FFF:FFFFFFFFFFFFFFFFFF,FFFFFFFFFF:FFFFFFFFFFFFFFFFFFFFFFFFFFFF
FFFFFFFFFFFFFFFFFFFFFFFFFFFFFFFFFFF:FFFFFF:FFFFFFFFFFF:F,FFFFFFFFF:F
FFFFF,FFFFFFFFF
@A00155:342:HHGFNDSXY:1:1332:29017:27633 2:N:0:GAACCTAG+TCCGCATA
CGGGAATGGTTCACAAAGTCTGGTGTGTATCTTTCACCATTTGCTTACGTGAATCACTCACACCCTGG
TTGTAAAACTCTGGAGAACCACCTATTATTTAATGTTGTAGCTAGTTACATTAGTAAATATTCATATG
TAGCATGTTTAAGCA
+
FFFFF,FFFFFFFFFFFFFFFFFFF,FFFFFFFFFFFFFFFFFFFFFFFFFF:FFFFFFFFFFFFFFF
FFFFFFFFFFFFFFFFFFFFFFFFFFFFFFFFFFFFFFFFFFFFFFFFFFFFFFFFFF,FF:FFFFFF
FFFFFFFF:FFFFFF
@A00155:342:HHGFNDSXY:1:1503:21052:27618 2:N:0:GAACCTAG+TCCGCATA
CGGGAATGGTTCACAAAGTCTGGTGTGTATCTTTCACCATTTGCTTACGTGAATCACTCACACCCTGG
TTGTAAAACTCTGGAGAACCACCTATTATTTAATGTTGTAGCTAGTTACATTAGTAAATATTCATATG
TAGCATGTTTAAGCA
+
FFFFFFFFFFFFFFFFFFFFFFFFFFFFFFFFFFFFFFFFFFFFFFFFFFFFFFFFFFFFFFFFFFFF
FFFFFFFFFFFFFFFF:FFFFFFFFFFFF:FFFFFF:FFFFFFFFFFFFFF:FFFFFFFFFFFFFFFF
FFFFFFFFFFFFFFF
@A00155:342:HHGFNDSXY:1:1530:6325:24878 2:N:0:GAACCTAG+TCCGCATA
CGGGAATGGTTCACAAAGTCTGGTGTGTATCTTTCACCATTTGCTTACGTGAATCACTCACACCCTGG
TTGTAAAACTCTGGAGAACCACCTATTATTTAATGTTGTAGCTAGTTACATTAGTAAATATTCATATG
TAGCATGTTTAAGCA
+
FFFFFFFFFFFFFFFFFFFFFFFFFFFFFFFFFFFFFF:FFFFF:FFFFFFFFFFFFFFFFFFFFFFF
FFFFFFFFFFFFFFFFFFFFFFFFFFFFFFFFFFFF,FFFF:FFFFFF:FFFF:FF,FFFFFFFFF:FF
F:FFFFFFFFFFFFF
@A00155:342:HHGFNDSXY:1:1535:29604:18756 2:N:0:GAACCTAG+TCCGCATA
CGGGAATGGTTCACAAAGTCTGGTGTGTATCTTTCACCATTTGCTTACGTGAATCACTCACACCCTGG
TTGTAAAACTCTGGAGAACCACCTATTATTTAATGTTGTAGCTAGTTACATTAGTAAATATTCATATG
TAGCATGTTTAAGAA
+
FFFFFFFFFFFFFFFFFFFFFF:FFFFFFFFFFFFF::FFFFFFFF:FFFFFFFFFFFFFFFFFFFFF
FFFFFFFFFFFFFFFFFFFFFFFFFFFFFFFF:FFFFFFFFFFFFFFFFFFFFFFFFFFFFFFFFFFF
F:,FFFFFFFFFF,F
@A00155:342:HHGFNDSXY:1:1676:20383:9987 2:N:0:GAACCTAG+TCCGCATA
CGGGAATGGTTCACAAAGTCTGGTGTGTATCTTTCACCATTTGCTTACGTGAATCACTCACACCCTGG
TTGTAAAACTCTGGAGAACCACCTATTATTTAATGTTGTAGCTAGTTACATTAGTAAATATTCATATG
TAGCATGTTTAAGCA
+
FFFFF:FFFFFFFFFFFFFFFFFFFFFFFFFFFFFFFFFFFFFFFF:FFFFFFFFFFFFFFFFFFFFF
FFFFFFFFFFFFFFFFF:FFFFFFFFFFFFFFFFFFFFFFFFFFFFFFFFFFFFFF:FFFF:FFFFFFFF
FFFFFFFFFFFFFFF
@A00155:342:HHGFNDSXY:1:2172:30273:24831 2:N:0:GAACCTAG+TCCGCATA
CGGGAATGGTTCACAAAGTCTGGTGTGTATCTTTCACCATTTGCTTACGTGAATCACTCACACCCTGG
TTGTAAAACTCTGGAGAACCACCTATTATTTAATGTTGTAGCTAGTTACATTAGTAAATATTCATATG
TAGCATGTTTAAGCA
+
FFFFF:FFFFFFFFFFFFFFFFFFFFFFFFFFFFFFFFFFFFFFFFFFFFFFFFFFFFFFFFFFFFFF
FFFFFFFFFFFFFFFFFFFFFFFFFFFFFFFFFFFFFFFFFFFFFFFFFFF:FFFFFFFFFFFFFFFF
FFFFFFFFFFFFFFF
@A00155:342:HHGFNDSXY:1:2209:14208:11725 2:N:0:GAACCTAG+TCCGCATA
CGGGAATGGTTCACAAAGTCTGGTGTGTATCTTTCACCATTTGCTTACGTGAATCACTCACACCCTGG

TTGTAAAACTCTGGAGAACCACCTATTATTTAATGTTGTAGCTAGTTACATTAGTAAATATTCATATG
TAGCATGTTTAAGCA
+
FFFFFFFFFFFFFFFFFFFFFFFFFFFFFFFFFFFFFFFFFFFFFFFFFFFFFFFFFFFFFFFFFFFF
FFFFFFF:FFFFF:FFFFF:FFFFFFFFFFFFFFFFFFFFFFFFFFFFF::FFFFFF:FFFFFFFFFF
FFFFFFFFFFFF,::
@A00155:342:HHGFNDSXY:1:2251:18123:14810 2:N:0:GAACCTAG+TCCGCATA
CGGGAATGGTTCACAAAGTCTGGTGTGTATCTTTCACCATTTGCTTACGTGAATCACTCACACCCTGG
TTGTAAAACTCTGGAGAACCACCTATTATTTAATGTTGTAGCTAGTTACATTAGTAAATATTCATATG
TAGCATGTTTAAGCA
+
FFFF:FFFFFFFFFFFFFFFFFFFFFFFFFFFFFFFFFFFFFFFFFFFFFFFFFFFFFFFFFFFF:FF
FFFFFFFFFFF:FFFFFFFFFFFFFFFFFFFFF,FFFFFFFFFFFFFFFF,FFFFFFFFFFFFFFFFF
FFFF:FFFFFFFFFF
@A00155:342:HHGFNDSXY:1:2262:15022:34804 2:N:0:GAACCTAG+TCCGCATA
CGGGAATGGTTCACAAAGTCTGGTGTGTATCTTTCACCATTTGCTTACGTGAATCACTCACACCCTGG
TTGTAAAACTCTGGAGAACCACCTATTATTTAATGTTGTAGCTAGTTACATTAGTAAATATTCATATG
TAGCATGTTTAAGCA
+
FFFFF:FFFFFFFFFFFFFFFFFFFFFFFFFFFFFF:FFFFFFFFFFFFFFFFFFFFFFFF:FF:FFF
FFFFFFFF,FFFFFFFFFFFFFFFFFFFF:,FFFFFF:FFFFFFFFFFFFFFFFFFFF,FF:FFFFFF
F:F:FFFFFFFFFF:
@A00155:342:HHGFNDSXY:1:2349:29957:33489 2:N:0:GAACCTAG+TCCGCATA
CGGGAATGGTTCACAAAGTCTGGTGTGTATCTTTCACCATTTGCTTACGTGAATCACTCACACCCTGG
TTGTAAAACTCTGGAGAACCACCTATTATTTAATGTTGTAGCTAGTTACATTAGTAAATATTCATATG
TAGCATGTTTAAGCA
+
:FFFFFFFFFFFFFFFFFFFFFFFFFF:FFFFFFFFFFFFFFFFFFFFFFFFFFFFFFFFFFFFFFFF
:FFFFFFFFFFFFFFFFFFFFFFFFFFFFFFFFFF:FFFFFFF,FFFFFF:FFFFFFFFFFFFFFFFF
FFFFFFFFFFFFFFF
@A00155:342:HHGFNDSXY:1:2529:14244:29512 2:N:0:GAACCTAG+TCCGCATA
CGGGAATGGTTCACAAAGTCTGGTGTGTATCTTTCACCATTTGCTTACGTGAATCACTCACACCCTGG
TTGTAAAACTCTGGAGAACCACCTATTATTTAATGTTGTAGCTAGTTACATTAGTAAATATTCATATG
TAGCATGTTTAAGCA
+
FFFFF:FFFFFFFFFFFFFFFFF:FFFFFFFFFFFFFF:FFFFFFFFFFFFFFFFFFFFFFFFFFFFF
FFFFFFFFFFFFFFFF:FFFFFFFFFFFFFFFFFFFFFFFFFFFFFFFFFFFFFF:FF:FFFFFFFFF
FFFFFFFFFFFFFFF
@A00155:342:HHGFNDSXY:1:2542:28745:24690 2:N:0:GAACCTAG+TCCGCATA
CGGGAATGGTTCACAAAGTCTGGTGTGTATCTTTCACCATTTGCTTACGTGAATCACTCACACCCTGG
TTGTAAAACTCTGGAGAACCACCTATTATTTAATGTTGTAGCTAGTTACATTAGTAAATATTCATATG
TAGCATGTTTAAGCA
+
:FFFF:FFFFFFFFFFFFFFFFFFFFFFFFFFFFFFFFFFFFFFFFFFFFFFFFFFFFFFFFFFFFFF
FFFFFFFFFFFFFFFFFFFFFFFFFFFFFFFFFFFFFFFFFFFFFFFFFFFFFFFFFFFFFFFFFFFF
FFFFFFFFFFFFFFF
@A00155:342:HHGFNDSXY:1:2562:23005:31125 2:N:0:GAACCTAG+TCCGCATA
CGGGAATGGTTCACAAAGTCTGGTGTGTATCTTTCACCATTTGCTTACGTGAATCACTCACACCCTGG
TTGTAAAACTCTGGAGAACCACCTATTATTTAATGTTGTAGCTAGTTACATTAGTAAATATTCATATG
TAGCATGTTTAAGCA
+
FFFFF,FFFFFFFFFFFFFFFFFFFFFFFFFFFFFFFFFFFFFFFFFFFFFFFFFFFFFFFFFFFFFF
FFFFFFF:FFFF:FFFFFFFFFFFFFFFFFFFFFFFFFFFFF:FFFFFFFFFFFFFFFFFFFFFFFFF
FFFFFFFFFFFFFFF

```
@A00155:342:HHGFNDSXY:1:1576:24415:27085 2:N:0:GAACCTAG+TCCGCATA
GGGAATGGTTCACAAAGTCTGGTGTGTATCTTTCACCATTTGCTTACGTGAATCACTCACACCCTGGT
TGTAAAACTCTGGAGAACCACCTATTATTTAATGTTGTAGCTAGTTACATTAGTAAATATACATATGT
AGCATGTTTAAGCAT
+
:FFF,FF:FFFFFFFFFFFF:FFFF,FFFFFF,FFFFFFFFFFFF:FF:FFFFFFFF:FFFFFFFFF::
FFFFFFFFFFFFFFFFFFFFFFF,FF,FFFFFFF:FFFFF:,:FFFFFFF:FFF:FFFFFF,FFFF:::
FFFF:F,FFFFF::F
@A00155:342:HHGFNDSXY:1:1645:30798:15812 2:N:0:GAACCTAG+TCCGCATA
GGGAATGGTTCACAAAGTCTGGTGTGTATCTTTCACCATTTGCTTACGTGAATCACTCACACCCTGGT
TGTAAAACTCTGGAGAACCACCTATTATTTAATGTTGTAGCTAGTTACATTAGTAAATATTCATATGT
AGCATGTTTAAGCAT
+
FFF,,FFFFFFFFFFFFFFFFFFFFFFFFFFFFFFFFFFFFFFFFFFFFFFFFFFFFFFFFFFFFFFFF
FFFFFFFFFFFFFFFFFFFFFFFFFFFFFFFFFFFFFFFFFFFFFFFFFFFFFFFFFFFFFFFFFFFFF
FFFFFFFFFFFFFFF
@A00155:342:HHGFNDSXY:1:2319:8838:6089 2:N:0:GAACCTAG+TCCGCATA
GGGAATGGTTCACAAAGTCTGGTGTGTATCTTTCACCATTTGCTTACGTGAATCACTCACACCCTGGT
TGTAAAACTCTGGAGAACCACCTATTATTTAATGTTGTAGCTAGTTACATTAGTAAATATTCATATGT
AGCATGTTTAAGCAT
+
FFFF,FFFFFFFFFFFFF:FFFFFF:FFFFFFFFFFFFFFFFFFFFFFFFFFFF:FFFFFFFFFFFF:F
FFFFF:,FFFFFFFFFFFFFFFFFFFFFFFFFF:FFFFF,FFFFFFFFFFFFFFFFF:FFFFFFF:FFF
F:F:FFFFFFFF:FF
@A00155:342:HHGFNDSXY:1:1664:24505:35383 2:N:0:GAACCTAG+TCCGCATA
GGAATGGTTCACAAAGTCTGGTGTGTATCTTTCACCATTTGCTTACGTGAATCACTCACACCCTGGTT
GTAAAACTCTGGAGAACCACCTATTATTTAATGTTGTAGCTAGTTACATTAGTAAATATTCATATGTA
GCATGTTTAAGCATT
+
FFFFFFFFFFFFFFFFFFFFFFFFFFFFFFFFFFFFFFFFFFFFFFFFFFF:FFFFFFFFFFFFFFFFF
FFFFFFFFFFFFFFFFF:FFFFFFFFFFFFFFFFF:FFFFFFFFFFFFFFFFFFFFFFFFFFFFFFFFF
FFFFF:FFF,F,FF:
@A00155:342:HHGFNDSXY:1:2107:25690:1454 2:N:0:GAACCTAG+TCCGCATA
GGAATGGTTCACAAAGTCTGGTGTGTATCTTTCACCATTTGCTTACGTGAATCACTCACACCCTGGTT
GTAAAACTCTGGAGAACCACCTATTATTTAATGTTGTAGCTAGTTACATTAGTAAATATTCATATGTA
GCATGTTTAAGCATT
+
FFFFFFFFFFFFFFFFFFFFFFFFFFFFFFFFFFFFFFFFFFFFFFFFFFFFFFFFFFFFFFFFFFFFF
FFFFFFFFFFFFFFFFFFFFFFFFFFFFFFFFFFFFFFFFFFFFFFFFFFFFFFFFFFFFFFFFFFFFF
FFFFFFFFFFF:FFF
@A00155:342:HHGFNDSXY:1:2665:24288:5729 2:N:0:GAACCTAG+TCCGCATA
GGAATGGTTCACAAAGTCTGGTGTGTATCTTTCACCATTTGCTTACGTGAATCACTCACACCCTGGTT
GTAAAACTCTGGAGAACCACCTATTATTTAATGTTGTAGCTAGTTACATTAGTAAATATTCATATGTA
GCATGTTTAAGCATT
+
FFF,FFFFFFFFFFFFFFFFFFFFFFFFFFFFFFFFFFFFFFFFFFFFFFFFFFFFFFFFFFFFFFFFF
FFFFFFFFFFFFFFFFFFFFFFFFFFFF:FFFFFFFFFFFFFFFFFFFFFFFFFFFFFFF:FFFFFFFF
FFFFFFFFFFFFFFF
@A00155:342:HHGFNDSXY:1:2265:27570:27727 2:N:0:GAACCTAG+TCCGCATA
GAATGGTTCACAAAGTCTGGTGTGTATCTTTCACCATTTGCTTACGTGAATCACTCACACCCTGGTTG
TAAAACTCTGGAGAACCACCTATTATTTAATGTTGTAGCTAGTTACATTAGTAAATATTCATATGTAG
CATGTTTAAGCATTA
+
FFFFFFFFFFFFF:FFFFFF:FFFFFFFFFFFFFFFFFFFFFFFFFFFFF:FFFFFFFFFFFFFFFFFF
```

```
FFFF:FFFFF:FFFFFFFFFFFFFFFFFFFFF,FFFF:FFFFFFFFFFFFFFF:FFFFFFFFFFFFFF
FFFFFFFF:FFFFFF
@A00155:342:HHGFNDSXY:1:2162:23818:8766 2:N:0:GAACCTAG+TCCGCATA
AATGGTTCACAAAGTCTGGTGTGTATCTTTCACCATTTGCTTACGTGAATCACTCACACCCTGGTTGT
AAAACTCTGGAGAACCACCTATTATTTAATGTTGTAGCTAGTTACATTAGTAAATATTCATATGTAGC
ATGTTTAAGCATTAA
+
FFFFFFFFFFFFFFFFFF:FFFFFFFFFFFFF:FFFFFFFFFFFFFFFF:FFFFFFFFFFF:FFFFF
F:FFFFFFFFFFFFF,FFFFFF:FFF::FFFFFFFFFFFFFFFFFFFFFFFFFFFFFFFFFFFFFFF
FFFFFFFF:FFFFFF
@A00155:342:HHGFNDSXY:1:1558:1741:33599 2:N:0:GAACCTAG+TCCGCATA
TGGTTCACAAAGTCTGGTGTGTATCTTTCACCATTTGCTTACGTGAATCACTCACACCCTGGTTGTAA
AACTCTGGAGAACCACCTATTATTTAATGTTGTAGCTAGTTACATTAGTAAATATTCATATGTAGCAT
GTTTAAGCATTAAAT
+
FFFFFFFFFF:FFFFFFFFFFFFFFFFFFFFFFFFFFFFF:FFFFFFFFFFFFFFFFFFFF:FFFFFF
:F:FFFFFFFFFFF:FFFFFFFFFFFFFFFFFFFFFFFFFFFFF:FF:FFFFFFFFF:FFF:FFFFFF
FFFF::FF:FFFFFF
@A00155:342:HHGFNDSXY:1:1663:26973:29418 2:N:0:GAACCTAG+TCCGCATA
GGTTCACAAAGTCTGGTGTGTATCCTTCACCATTTGCTTACGTGAATCACTCACACCCTGGTTGTAAA
ACTCTGGAGAACCACCTATTATTTAATGTTGTAGCTAGTTACATTAGTAAATATTCATATGTAGCATG
TTTAAGCATTAAAT
+
FFFFFFF,FFFFFFF:FF:FFFFFF:FFFFFFFFFFFFFFFFFFFFFFFFFFFFFFFFFFFFFFFFFF
FFFFFFFFFFFFFFFFFFFFFFFFF:FFFFFFFFFFFFFFFF:FFFFFFFFFFFFFFFFFFFF:FFFF
FFFFFFFFFFFFFF
@A00155:342:HHGFNDSXY:1:2338:32913:5979 2:N:0:GAACCTAG+TCCGCATA
GGTTCACAAAGTCTGGTGTGTATCTTTCACCATTTGCTTACGTGAATCACTCACACCCTGGTTGTAAA
ACTCTGGAGAACCACCTATTATTTAATGTTGTAGCTAGTTACATTAGTAAATATTCATATGTAGCATG
TTTAAGCATTAAAT
+
FFFFFFFF::FF:FFFFFFFFFFFFFFFFF:FFFFFFFFFFFFFFFFF:FFFFFFF::FFFFFFFFF,
,:FFFFFFFFFFFFFFFFFFFFFF:FFFFFFFFFFFFFFFFFFFFFFFFFFFFFFFFF:FFFFFFFFF,FF
FFFF:FFFFFFFFF
@A00155:342:HHGFNDSXY:1:2505:4806:4773 2:N:0:GAACCTAG+TCCGCATA
GGTTCACAAAGTCTGGTGTGTATCTTTCACCATTTGCTTACGTGAATCACTCACACCCTGGTTGTAAA
ACTCTGGAGAACCACCTATTATTTAATGTTGTAGCTAGTTACATTAGTAAATATTCATATGTAGCATG
TTTAAGAATTAAAT
+
FFFFFFFFFFF:F,FFFFFFFFFFFFFFFFFFFFFFFFFFFFFFFFFFFFFFFFFFFF,:FFFFFF,
FFFFFFFFFFFFF:FFFFFFFFFFFFFFFFFFF:,FFFFFFF,FFFF::FFFFF:F,FFFF,FFFF,:,
FFFFF,,F:FFFFF
@A00155:342:HHGFNDSXY:1:1127:16125:5995 2:N:0:GAACCTAG+TCCGCATA
GGTTCACAAAGTCTGGTGTGTATCTTTCACCATTTGCTTACGTGAATCACTCACACCCTGGTTGTAAA
ACTCTGGAGAACCACCTATTATTTAATGTTGTAGCTAGTTACATTAGTAAATATTCATATGTAGCATG
TTTAAGCATTAAATC
+
FFFFFFFFFFFFFFFFFFFFFFFFFFFFFFFFFFFFFFFFFFFFFFFFFFFFFFFFFFFFFFFFFFFF
,FFFFFFFFFFF:FFFFFFFFFFFFF:F:FFFFFFFFFFFFFFFFFFFFFFFFFFFFFFF,:FF,FF:F
FFFFFFFFFFFFFFF
@A00155:342:HHGFNDSXY:1:1127:16224:12273 2:N:0:GAACCTAG+TCCGCATA
GGTTCACAAAGTCTGGTGTGTATCTTTCACCATTTGCTTACGTGAATCACTCACACCCTGGTTGTAAA
ACTCTGGAGAACCACCTATTATTTAATGTTGTAGCTAGTTACATTAGTAAATATTCATATGTAGCATG
TTTAAGCATTAAATC
```

```
+
FFFFFFF:FFFFFFFFFFFFFFFFFFFFFFFFFFFFFFFFFFFFFFFFFFFFFFFFFFFFFFFFFFFFFF
FFFFFFFFFFFFFFFFFFFFFFFFFFFFFFFFFFFFFFFFFFFFFFFFFFFFFFFFFFFFFFFFFFFFFF
FFFFFFF:FFF::FF
@A00155:342:HHGFNDSXY:1:1324:10339:14199 2:N:0:GAACCTAG+TCCGCATA
GGTTCACAAAGTCTGGTGTGTATCTTTCACCATTTGCTTACGTGAATCACTCACACCCTGGTTGTAAA
ACTCTGGAGAACCACCTATTATTTAATGTTGTAGCTAGTTACATTAGTAAATATTCATATGTAGCATG
TTTAAGCATTAAATC
+
FF:FFFFFFFFFFFFFFFFFFFFFFFFFFFFFFFFFFFFFFFFFFFFFFFFFFFFFFFFFFFFFFFFFFF
FFFFFFFFFFFFFFFFFFFFFFFFFFFFFFFFFFFFFFFFFFFFFFFFFF:FFFFFFFFFFFFFFFFFFF
FFFFFFFFFFFFFFF
@A00155:342:HHGFNDSXY:1:1462:9769:21136 2:N:0:GAACCTAG+TCCGCATA
GGTTCACAAAGTCTGGTGTGTATCTTTCACCATTTGCTTACGTGAATCACTCACACCCTGGTTGTAAA
ACTCTGGAGAACCACCTATTATTTAATGTTGTAGCTAGTTACATTAGTAAATATTCATATGTAGCATG
TTTAAGCATTAAATC
+
FFFFFFFFFFFFFFFFFFFFFFFFFFFFFFFFFFFFFFFFFFFFFFFFFFFFFFFFFFFFFFFFFFFFFF
FFFFFFFFFF,FFFFFFFFFFFFFFFFFFFFFFFFF:FFFFFFFFFFF:FFF:,FFFFFF:FFFFFFFFF
FFFFFFFFFFFFFFF
@A00155:342:HHGFNDSXY:1:1630:2410:25394 2:N:0:GAACCTAG+TCCGCATA
GGTTCACAAAGTCTGGTGTGTATCTTTCACCATTTGCTTACGTGAATCACTCACACCCTGGTTGTAAA
ACTCTGGAGAACCACCTATTATTTAATGTTGTAGCTAGTTACATTAGTAAATATTCATATGTAGCATG
TTTAAGCATTAAATC
+
FFFFFFFFFFFFFFF:FFFFFFFFFFFF:FFFFFF:FFF:FFFFFFFFFFFFFFFFFFFFFFFFFFFFFF
:FF:FF,F:FFFF:FFFFFFFFFFFFFFFFFFFFF,FF,FFFFFFFF:FFFFFFFFFFFFFFFFF:FFF:
FFFF,FF:FFFFFFF
@A00155:342:HHGFNDSXY:1:1638:11424:29543 2:N:0:GAACCTAG+TCCGCATA
GGTTCACAAAGTCTGGTGTGTATCTTTCACCATTTGCTTACGTGAATCACTCACACCCTGGTTGTAAA
ACTCTGGAGAACCACCTATTATTTAATGTTGTAGCTAGTTACATTAGTAAATATTCATATGTAGCATG
TTTAAGCATTAAATC
+
FFFFFFFFFFFFFFFFFFFFFFFFFFFFFFFFFFFFFFFFFFFFFFFFFFFFFFFFFFFFFFFFFFFFFF
FFFFFFFFFFFFFFFFFFFFFFFFFFFFFFFFFFFFFFFFFFFF:FFFFFFFFFFFFFFFFFFFFFFFFF
FFFFFFFFFFFFFFF
@A00155:342:HHGFNDSXY:1:1669:21558:12023 2:N:0:GAACCTAG+TCCGCATA
GGTTCACAAAGTCTGGTGTGTATCTTTCACCATTTGCTTACGTGAATCACTCACACCCTGGTTGTAAA
ACTCTGGAGAACCACCTATTATTTAATGTTGTAGCTAGTTACATTAGTAAATATTCATATGTAGCATG
TTTAAGCATTAAATC
+
FFFFFFFFFFFFFFFFFFFFFFFFFFFFFFFFFFFFFFFFFFFFFFFFFFFFFFFFFFFFFFFFFFFFFF
FFFFFFFFFFFFFFFFF:FFFFFFFFFFFFFFFFFFFFFFFFFF:FFFFFFFFFFFFFFFFFFFFFFFFF
FFFFFFFFFFFFFFF
@A00155:342:HHGFNDSXY:1:2150:32551:6887 2:N:0:GAACCTAG+TCCGCATA
GGTTCACAAAGTCTGGTGTGTATCTTTCACCATTTGCTTACGTGAATCACTCACACCCTGGTTGTAAA
ACTCTGGAGAACCACCTATTATTTAATGTTGTAGCTAGTTACATTAGTAAATATTCATATGTAGCATG
TTTAAGCATTAAATC
+
FF,FFFFFFFFFFFFFFF:FFFFFFFFFFFFFFFFFFFFFFFFFFFFFFFFFFFFFFFFFFFFFFFFFFF
FFFFFF:FFF:FFFFFFFFFFFFFFFFFFFFFF:FFFFFFF:FFF:FFF:,FFFFFFFF,FFFFFFFFFF
F:FFFFFF:FFFF,F
@A00155:342:HHGFNDSXY:1:2128:4535:9690 2:N:0:GAACCTAG+TCCGCATA
GGTTCACAAAGTCTGGTGTGTATCTTTCACCATTTGCTTACGTGAATCACTCACACCCTGGTTGTAAA
```

```
ACTCTGGAGAACCACCTATTATTTAATGTTGTAGCTAGTTACATTAGTAAATATTCATATGTAGCATG
TTTAAGCATTAAATC
+
FFFFFFFFFFFFFFFFFFFFFFFFFFFFFFFFFFFFFFFFFFFFFFFFFFFFFFFFFFFFFFFFFFFF
FFFFFFFFFFFFFFFFFFFFFFFFFFFFFFFFFFFFFFFFFFFFFFF:FFFFFFFFFFFFFFFFFFFF
FFFFFFFFFFFFFF:
@A00155:342:HHGFNDSXY:1:2202:17861:13730 2:N:0:GAACCTAG+TCCGCATA
GGTTCACAAAGTCTGGTGTGTATCTTTCACCATTTGCTTACGTGAATCACTCACACCCTGGTTGTAAA
ACTCTGGAGAACCACCTATTATTTAATGTTGTAGCTAGTTACATTAGTAAATATTCATATGTAGCATG
TTTAAGCATTAAATC
+
FFFFFFFFFFFFFFFFFFFFFFFFFFFFFFFFFFFFFFFFFFFFFFFFFFFFFFFFFFFFFFFFFFFF
FFFFFFFFFFFFFFFFF:FFFFFFFFFFFFFFFFFFFFFFFFFFFFFFFFFFFFFFFFFFFFFFFFFF
FFFFFFFFF:FFFFF
@A00155:342:HHGFNDSXY:1:2412:25898:12211 2:N:0:GAACCTAG+TCCGCATA
GGTTCACAAAGTCTGGTGTGTATCTTTCACCATTTGCTTACGTGAATCACTCACACCCTGGTTGTAAA
ACTCTGGAGAACCACCTATTATTTAATGTTGTAGCTAGTTACATTAGTAAATATTCATATGTAGCATG
TTTAAGCATTAAATC
+
FFFFFFFFFFFFFFFFFFFFFFFFFFFFFFFFFFFFFFFFFFFFFFFFFFFFFFFFFFFFFFFFFFFF
FFFFFFFFFFFFFFFFFFFFFFFFFFFFFFFFFFFFFFFFFFFFFFFFFFFFFFFFFFFFFFF:FFF:
FF:FFF:FFFFFFFF
@A00155:342:HHGFNDSXY:1:2508:29577:19022 2:N:0:GAACCTAG+TCCGCATA
GGTTCACAAAGTCTGGTGTGTATCTTTCACCATTTGCTTACGTGAATCACTCACACCCTGGTTGTAAA
ACTCTGGAGAACCACCTATTATTTAATGTTGTAGCTAGTTACATTAGTAAATATTCATATGTAGCATG
TTTAAGCATTAAATC
+
FFFFFFFFFFFFFFFFFFFFFFFFFFFFFFFFFFFFFFFFFFFFFFFFFFFFFFFFFFFFFFFFFFFF
FFFFFFFFFFFFFFFFFFFFFFFFFFFFFFFFFFFFFFFFFFF:FFFFFFFFFFFFFFFFFFFFFFFF
FFFFFFFFFFFFFFF
@A00155:342:HHGFNDSXY:1:2603:27190:32550 2:N:0:GAACCTAG+TCCGCATA
GGTTCACAAAGTCTGGTGTGTATCTTTCACCATTTGCTTACGTGAATCACTCACACCCTGGTTGTAAA
ACTCTGGAGAACCACCTATTATTTAATGTTGTAGCTAGTTATATTAGTAAATATTCATATGTAGCATG
TTTAAGCATTAAATC
+
FFFFFFFFFFFFFFFFFFFFFFFFFFFFFFFFFFFFFFFFFFFFFFFFFFFFFFFFFFFFFFFFFFFF
FFFFFFFFFFFFFFFFFFFFFFFFFF,FFFFFFFFF:FFFFFFF:FFF:FFFFFFF:FFFFFFFFFFF
FFFFFFFFFFFFFFF
@A00155:342:HHGFNDSXY:1:2667:31177:15718 2:N:0:GAACCTAG+TCCGCATA
GGTTCACAAAGTCTGGTGTGTATCTTTCACCATTTGCTTACGTGAATCACTCACACCCTGGTTGTAAA
ACTCTGGAGAACCACCTATTATTTAATGTTGTAGCTAGTTACATTAGTAAATATTCATATGTAGCATG
TTTAAGCATTAAATC
+
FFFFFFFFFFFFFFFFFFFFFFFFFFFFFFF:FFFFFFFFFFFFFFFFFFFFFFFFFFFFFFFFFFFF
FFFFFFFFFFFFFFFFFFFFFFFFFF,FFFFFFFF:FFFFFFF:F:FFF:FF:FFFFFFFFF:FFFFF
FFFFFFFFFF:FFFF
@A00155:342:HHGFNDSXY:1:2667:13268:32612 2:N:0:GAACCTAG+TCCGCATA
GGTTCACAAAGTCTGGTGTGTATCTTTCACCATTTGCTTACGTGAATCACTCACACCCTGGTTGTAAA
ACTCTGGAGAACCACCTATTATTTAATGTTGTAGCTAGTTACATTAGTAAATATTCATATGTAGCATG
TTTAAGCATTAAATC
+
FFFFFFFFFFFFFFFFFFFFFFFFFFFFFFFFFFFFFFFFFFFFFFFFFFFFFFFFFFFFFFFFFFFF
FFFFFFFFFFFFFFFFFFFFFFF:FF:FFFFFFFFFFFFFFFFFFFFF:FFFFFFFFFFFFFFFFFFF
FFFF,FFFFF:FFFF
```

```
@A00155:342:HHGFNDSXY:1:2661:8730:33646 2:N:0:GAACCTAG+TCCGCATA
GGTTCACAAAGTCTGGTGTGTATCTTTCACCATTTGCTTACGTGAATCACTCACACCCTGGTTGTAAA
ACTCTGGAGAACCACCTATTATTTAATGTTGTAGCTAGTTACATTAGTAAATATTCATATGTAGCATG
TTTAAGCATTAAATC
+
FFFFFFFFFFFFFFFF,FFFFFFFFFFFFFFFFFF:FFFFFFFFFFFFFFFFFFFFFFFFFFFFFFFF
FFFFFFFFFF:F:FFFFFFFFFF:FFFFFFFFFFF,FFFFFFFFFFFFFFFFFFFFFFFFFFFFFFFF
FFFFFFFFFFFFFFF
@A00155:342:HHGFNDSXY:1:1147:24795:17409 2:N:0:GAACCTAG+TCCGCATA
GTTCACAAAGTCTGGTGTGTATCTTTCACCATTTGCTTACTTGAATCACTCACACCCTGGTTGTAAAA
CTCTGGAGAACCACCTATTATTTAATGTTGTAGCTAGTTACATTAGTAAATATTCATATGTAGCATGT
TTAAGCATTAAATCA
+
:,FFFFFFFFFFFFF,:,F:F,,:FFFF:FFFF,F,FFFF,F:FF:FFFFFF:FFFF,FFFF:FFFFF
F:FF,:F:F:FFFFFFFFFF::FFF,::FFFFF:F,FFFFFF::FFFFFFFF:FF::F::FFF:F,FF
FFFFF,F:FFFFFFF
@A00155:342:HHGFNDSXY:1:1245:2148:9377 2:N:0:GAACCTAG+TCCGCATA
GTTCACAAAGTCTGGTGTGTATCTTTCACCATTTGCTTACGTGAATCACTCACACCCTGGTTGTAAAA
CTCTGGAGAACCACCTATTATTTAATGTTGTAGCTAGTTACATTAGTAAATATTCATATGTAGCATGT
TTAAGCATTAAATCA
+
FFFFFFFFFFFFFFFFFFFFFFFFFFFFFFFFFFF:FFFFFFFFFFFFFFFFFFFFFFFFFFFF:FFF
FFFFF:FFFFFFFFFFFFFFFFFFFFFFFFFFFF:FFFFFFF:F,FFFFFFFFFFFFFFFFFFFF:FFFF
FFF:FFFFFFF:F:F
@A00155:342:HHGFNDSXY:1:1307:12011:36229 2:N:0:GAACCTAG+TCCGCATA
GTTCACAAAGTCTGGTGTGTATCTTTCACCATTTGCTTACGTGAATCACTCACACCCTGGTTGTAAAA
CTCTGGAGAACCACCTATTATTTAATGTTGTAGCTAGTTACATTAGTAAATATTCATATGTAGCATGT
TTAAGCATTAAATCA
+
FFFFFFFFFFFFFFFFFFFFFFFFFFFFFFFFFFFFFFFFFFFFFFFFFFFFFFFFFFFFF:FFFFFFF
FFFFFFFFFFFFFFFFFFFFFFFFFF,FFFFFFFFFFFFFFFFFFFFFFFFFF,FFFFFFFFFFFFFFF:
FFFFFFFFF,FFFFF
@A00155:342:HHGFNDSXY:1:1307:12897:37043 2:N:0:GAACCTAG+TCCGCATA
GTTCACAAAGTCTGGTGTGTATCTTTCACCATTTGCTTACGTGAATCACTCACACCCTGGTTGTAAAA
CTCTGGAGAACCACCTATTATTTAATGTTGTAGCTAGTTACATTAGTAAATATTCATATGTAGCATGT
TTAAGCATTAAATCA
+
FFFFFFFFFFFFFFFFFFFFFFFFFFFFFFFFFFFFFFFFFFFFFFFFFFFFFFFFF:FFFFFFFF:F
FFFFFFFFFFFFFFFFFFFFFFFFFFFFFFFFFFFF,FFFFFFFFFFFFFFFFFFFFFFFFFFFFFFFF
FFFFFFFFFFFFFFF
@A00155:342:HHGFNDSXY:1:1334:18304:18850 2:N:0:GAACCTAG+TCCGCATA
GTTCACAAAGTCTGGTGTGTATCTTTCACCATTTGCTTACGTGAATCACTCACACCCTGGTTGTAAAA
CTCTGGAGAACCACCTATTATTTAATGTTGTAGCTAGTTACATTAGTAAATATTCATATGTAGCATGT
TTAAGCATTAAATCA
+
FFFFFFFFFFFFFFFFFFFFFFFFFFFFFFFFFFFFFFFFFFFFFFFFFFFFFFFFFFFFFFFFFFFF
FFFFFFFFFFFFFFFFFFFFFFFFFFFFFFFFFFFFFFFFFFFFFFFFFFFFFFFFFFFFFFFFFFFF
FFFFFFFFFFFFFFF
@A00155:342:HHGFNDSXY:1:1334:24867:23046 2:N:0:GAACCTAG+TCCGCATA
GTTCACAAAGTCTGGTGTGTATCTTTCACCATTTGCTTACGTGAATCACTCACACCCTGGTTGTAAAA
CTCTGGAGAACCACCTATTATTTAATGTTGTAGCTAGTTACATTAGTAAATATTCATATGTAGCATGT
TTAAGCATTAAATCA
+
FFFFFFFFFFFFFFFFFFFFFFFFFFFFFFFFFFFFFFFFFFFFFFFFFFFFFFF:FFFFFFFFFFFF
```

FFFFFFFFFFFFFFFFFFFFFFFFF:FFFFFFFFFFFFFFFFFFFFFFFFFFFFFFFFFFFFFFF,FF
FFFFFFFFF:FFFFF
@A00155:342:HHGFNDSXY:1:1377:25771:34507 2:N:0:GAACCTAG+TCCGCATA
GTTCACAAAGTCTGGTGTGTATCTTTCACCATTTGCTTACGTGAATCACTCACACCCTGGTTGTAAAA
CTCTGGAGAACCACCTATTATTTAATGTTGTAGCTAGTTACATTAGTAAATATTCATATGTAGCATGT
TTAAGCATTAAATCA
+
FFFFFFFFFFFFFFFFFFFFFFFFFFFFFF:FFFFFFFFFFFFFFFFFFFFFFFFFFFFF:FFFFFFF
FFFFFFFFFFFFFFFFFFFFFFFFFFFF:FFFFFFFFFFFFF:FFFFFFFFFFFFFFFFFFFFFFFFF
FFFFFFF:FFFFFFF
@A00155:342:HHGFNDSXY:1:1517:27073:7983 2:N:0:GAACCTAG+TCCGCATA
GTTCACAAAGTCTGGTGTGTATCTTTCACCATTTGCTTACGTGAATCACTCACACCCTGGTTGTAAAA
CTCTGGAGAACCACCTATTATTTAATGTTGTAGCTAGTTACATTAGTAAATATTCATATGTAGCATGT
TTAAGCATTAAATCA
+
FFFFFFFFFFFFFFFFFFFFFFFFFFFFFFFFFFFFFFFFFFFFFFFFFFFFFFFFFFFFFFFFFFFF
FFFFFFFFFFFFFFFFFFFFFFFFFFFFFFFFFFFFFFFFFFFFFFFFFFFFFFFFFFFFFFFFFFFF
FFFF:FFFFFFFFFF
@A00155:342:HHGFNDSXY:1:1517:27019:8015 2:N:0:GAACCTAG+TCCGCATA
GTTCACAAAGTCTGGTGTGTATCTTTCACCATTTGCTTACGTGAATCACTCACACCCTGGTTGTAAAA
CTCTGGAGAACCACCTATTATTTAATGTTGTAGCTAGTTACATTAGTAAATATTCATATGTAGCATGT
TTAAGCATTAAATCA
+
FFFFFFFFFFFFFFFFFFFFFFFFFFFFFFFFFFFFFFFFFFFFFFFFFFFFFFFFFFFFFFFFFFFF
FFFFFFFFF:FFFFFF:FFFFFFFFFFFFFFFFFFFFFFFFFFFFFFFFFFFFFFFFFFFFFFFFFF
FFF:FFFFFFFFFFF
@A00155:342:HHGFNDSXY:1:1627:20998:16845 2:N:0:GAACCTAG+TCCGCATA
GTTCACAAAGTCTGGTGTGTATCTTTCACCATTTGCTTACGTGAATCACTCACACCCTGGTTGTAAAA
CTCTGGAGAACCACCTATTATTTAATGTTGTAGCTAGTTACATTAGTAAATATTCATATGTAGCATGT
TTAAGCATTAAATCA
+
FFF:FFFFFFFFFFF:FFFFFFFFFFFFFFFFFFFFFFFFFFFFFFFFFFFFFFFFFFFFFFFFFFFF
FFFFFFFFFFFFFFFFFFFFFFFFFFFFFFFFFFFFFFFFFF:FFFFFFFFFFFFFFF:FF:FFFFFF
,FFFFFFFFFF:FFF
@A00155:342:HHGFNDSXY:1:1669:11532:17581 2:N:0:GAACCTAG+TCCGCATA
GTTCACAAAGTCTGGTGTGTATCTTTCACCATTTGCTTACGTGAATCACTCACACCCTGGTTGTAAAA
CTCTGGAGAACCACCTATTATTTAATGTTGTAGCTAGTTACATTAGTAAATATTCATATGTAGCATGT
TTAAGCATTAAATCA
+
FFFFFFFFFFFFFFFFFFFFFFFFFFFFFFFFFFFFFFFFFFFFFFFFFFFFFFFFFFFFFFFFFFFF
FFFFFFFFFFFFFFFFFFFFFFF:F,FFFFFFFFFFFFFFFFFFFFFFFFFFFFFFFFFFFFFFFFFF
FFFFFFFFFF:FFFF
@A00155:342:HHGFNDSXY:1:1669:9860:20541 2:N:0:GAACCTAG+TCCGCATA
GTTCACAAAGTCTGGTGTGTATCTTTCACCATTTGCTTACGTGAATCACTCACACCCTGGTTGTAAAA
CTCTGGAGAACCACCTATTATTTAATGTTGTAGCTAGTTACATTAGTAAATATTCATATGTAGCATGT
TTAAGCATTAAATCA
+
FFFFFFFFFFFFFFFFFFFFFFFFFFFFFFFFFFFFFFFFFFF:FFFFFFFFFFFFFFFFFFFF:FFF
FFFFFFFFFFFFFFFFFFFFFFFFFFFFF,FFFFFFFF:FFFFFFFFFFFFFFFFFFFFFFFFFFFFF
FFFF::FFFFFFFFF
@A00155:342:HHGFNDSXY:1:2165:30807:25911 2:N:0:GAACCTAG+TCCGCATA
GTTCACAAAGTCTGGTGTGTATCTTTCACCATTTGCTTACGTGAATCACTCACACCCTGGTTGTAAAA
CTCTGGAGAACCACCTATTATTTAATGTTGTAGCTAGTTACATTAGTAAATATTCATATGTAGCATGT
TTAAGCATTAAATCA

```
+
FFFFFFFFFFFFFFFFFFFFFFFFFFFFFFFFFFFFFFFFF:FFFFFFFFFFFFFFFFFFF:FFFFFFFF
FFFFFFFFFFFFFFFFFFFFFFFFFFFFFFFFFFFF:FFFFFFFFFFFFFFFFFFFFFFFFFFF:FFFFF
FFFFFFFFFFFFFFF
@A00155:342:HHGFNDSXY:1:2175:15835:5682 2:N:0:GAACCTAG+TCCGCATA
GTTCACAAAGTCTGGTGTGTATCTTTCACCATTTGCTTACGTGAATCACTCACACCCTGGTTGTAAAA
CTCTGGAGAACCACCTATTATTTAATGTTGTAGCTAGTTACATTAGTAAATATTCATATGTAGCATGT
TTAAGCATTAAATCA
+
FFFFFFFFFFFFFFFFFFFFFFFFFFFFFFFFFFFFFFFFFFFFFFFFFFFFFFFFFFFFFFFFFFFFFF
FFFFFF:FFFFFFFFFFFFFFFFFFFFFFFFFFFFFFFFFFFFFFFFFFFFFFFFFFFFFFFFFFFFFFF
FFFF:FFFFF:FFFF
@A00155:342:HHGFNDSXY:1:2274:19135:4288 2:N:0:GAACCTAG+TCCGCATA
GTTCACAAAGTCTGGTGTGTATCTTTCACCATTTGCTTACGTGAATCACTCACACCCTGGTTGTAAAA
CTCTGGAGAACCACCTATTATTTAATGTTGTAGCTAGTTACATTAGTAAATATTCATATGTAGCATGT
TTAAGCATTAAATCA
+
FFFFFFFFFFFFFFFFF:FFFFFFFFFFFFFFFFFFFFFFFFFFFFFFFFFFFFFFFFFFFFFFFFFF:F
FFFFFFFFFFFFFFFFFFFFFFFFFFFFFFFFFFFFFFFFFFFFFFFFFFFFFFFFFFFFFFFFFFFFFF
FFFFFFFFFFFFFFF
@A00155:342:HHGFNDSXY:1:2255:11822:17550 2:N:0:GAACCTAG+TCCGCATA
GTTCACAAAGTCTGGTGTGTATCTTTCACCATTTGCTTACGTGAATCACTCACACCCTGGTTGTAAAA
CTCTGGAGAACCACCTATTATTTAATGTTGTAGCTAGTTACATTAGTAAATATTCATATGTAGCATGT
TTAAGCATTAAATCA
+
FFFFFFFFFFFFFFFFFFFFFFFFFFFFFFFFFFFFFFFFFFFFFFFFFFFFFFFFFFFFFFFFFFFFFF
FFFFFFFFFFFFFFFFFFFFFFFFFFFFFFFFF:FFFFFFFFFFFFFFFFFFFFFFFFFFF:FFF:FFFF
FFFFFFFFFFFFF,F
@A00155:342:HHGFNDSXY:1:2361:16785:17879 2:N:0:GAACCTAG+TCCGCATA
GTTCACAAAGTCTGGTGTGTATCTTTCACCATTTGCTTACGTGAATCACTCACACCCTGGTTGTAAAA
CTCTGGAGAACCACCTATTATTTAATGTTGTAGCTAGTTACATTAGTAAATATTCATATGTAGCATGT
TTAAGCATTAAATCA
+
FFFFFFFFFFFFFFFFFFFFFFFFFFFFFFFFFFFFFFFFFFFFFFFFFFFFFFFFFFFFFFFFFFFFFF
FFFFFFFFFFFFFFFFFFFFFFFFFFFFFFFFF:FFFFFFFFFFFFFFFFFFFFFFFFFFFFFFFFFFFF
FFFFFFFFFFFFFFF
@A00155:342:HHGFNDSXY:1:2361:15555:20384 2:N:0:GAACCTAG+TCCGCATA
GTTCACAAAGTCTGGTGTGTATCTTTCACCATTTGCTTACGTGAATCACTCACACCCTGGTTGTAAAA
CTCTGGAGAACCACCTATTATTTAATGTTGTAGCTAGTTACATTAGTAAATATTCATATGTAGCATGT
TTAAGCATTAAATCA
+
FFFFFFFFFFFFFFFFF,FFFFFFFFFFFFFFFFFFFFFFFFFFFFFFFFFFFFFFFFFFFFFFFFFF:F
FFFFFFFFFFFF:FFFFFFFFFFFFFFFFFFFFFFFFFFFFFFFFFFFFFFFFFFFFFFFFF:FFF:FFF
FFFFFFFFFFFFFFF
@A00155:342:HHGFNDSXY:1:2413:6650:17362 2:N:0:GAACCTAG+TCCGCATA
GTTCACAAAGTCTGGTGTGTATCTTTCACCATTTGCTTACGTGAATCACTCACACCCTGGTTGTAAAA
CTCTGGAGAACCACCTATTATTTAATGTTGTAGCTAGTTACATTAGTAAATATTCATATGTAGCATGT
TTAATCATTAAATCA
+
F:::F:F:FFFFFFFFFFF:FFF:FF:FF:FFFFFFF:FFFF::FF:F:FFF:FFF:FFFFFF:FF:
F:F:FF:F:FFFFFF::FF:FFF:FFF:FFF:::F:FFF:::FF::::F:F:FF::F:F,F:F,:F,F
FF::,:FFFFFFF,:
@A00155:342:HHGFNDSXY:1:2539:5674:24627 2:N:0:GAACCTAG+TCCGCATA
GTTCACAAAGTCTGGTGTGTATCTTTCACCATTTGCTTACGTGAATCACTCACACCCTGGTTGTAAAA
```

```
CTCTGGAGAACCACCTATTATTTAATGTTGTAGCTAGTTACATTAGTAAATATTCATATGTAGCATGT
TTAAGCATTAAATCA
+
FFFFFFFFFFFFFFFFFFFFFFFFFFFFFFFFFF::FFF:FFFFFFFFFFFF:,FFF:F:FFFFFFFF
FFFFF:FFFFFFFF,FFFFFFFF,FFFFFFFFFFFFFF,F:F,FFFFFFFFF:FF:FFFFFF:FFF::
:FFFF:FFFFFFF,F
@A00155:342:HHGFNDSXY:1:1642:24551:36151 2:N:0:GAACCTAG+TCCGCATA
TTCACAAAGTCTGGTGTGTATCTTTCACCATTTGCTTACGTGAATCACTCACACCCTGGTTGTAAAAC
TCTGGAGAACCACCTATTATTTAATGTTGTAGCTAGTTACATTAGTAAATATTCATATGTAGCATGTT
TAAGCATTAAATCA
+
FFFFFFFFFFF:FFFFFFFFFFFFFFFFFFFFFFFFFFFFFFFFFFFFFFFF:FFFFFFFFFFFFFFF
FFFFFFFFFFFFFFFFF:FFFFFFFFFFFFFFFFFFFFFFFF:FFFFFFFFFFFFFFFFFF:FFF:,F
FFFF:FFFFFFF:F
@A00155:342:HHGFNDSXY:1:1105:2103:14904 2:N:0:GAACCTAG+TCCGCATA
TTCACAAAGTCTGGTGTGTATCTTTCACCATTTGCTTACGTGAATCACTCACACCCTGGTTGTAAAAC
TCTGGAGAACCACCTATTATTTAATGTTGTAGCTAGTTACATTAGTAAATATTCATATGTAGCATGTT
TAAGCATTAAATCAA
+
FFFFFFFFFFFFFFFFFFFFFFFFFFFFF:FFFFFFFFF:FFFFFFFFFFFFFFFFFFFFFFFFFFFF
FFFFFFFFFFFFFFFFF:FFFFFFFFFF:FFFFFFFFFFFFFFFFFFFFFFF:FFFFFFFF,FFFF:F
FFFFFFFFFFFFFFF
@A00155:342:HHGFNDSXY:1:2150:32551:6887 1:N:0:GAACCTAG+TCCGCATA
CACAAAGTCTGGTGTGTATCTTTCACCATTTGCTTACGTGAATCACTCACACCCTGGTTGTAAAACTC
TGGAGAACCACCTATTATTTAATGTTGTAGCTAGTTACATTAGTAAATATTCATATGTAGCATGTTTA
AGCATTAAATCAAA
+
FFFF:FFFFF:FFFFFF:FF:FFFFFFFFFFFF:FFFFFFFFFFFFFFFFFFFFFFFFFFFFFFFFFF
FFFFFFFFFFFFFFFFFFFFFFFFFFFFFFFFFF,FFFFFFFFFFFFFFFFFFFFFF:FFFFFFFFFF
FFFFFFFFFFFFFF
@A00155:342:HHGFNDSXY:1:1102:29243:4633 1:N:0:GAACCTAG+TCCGCATA
CACAAAGTCTGGTGTGTATCTTTCACCATTTGCTTACGTGAATCACTCACACCCTGGTTGTAAAACTC
TGGAGAACCACCTATTATTTAATGTTGTAGCTAGTTACATTAGTAAATATTCATATGTAGCATGTTTA
AGCATTAAATCAAAT
+
FFFFFFFFFFFFFFFFFFFFFFFFF:FFFFFFFFFF:FFFFFFFFFFFFFFF:FFFFFFFFFFFFFF:
FFFFFFFFFFFFFFFFFFFFFFFFFFFFFFFFF:FF:FFFFFFFFFFFFFFFFFFFFFFFFFFFFFFF
FFFFFFFFFFFFFFF
@A00155:342:HHGFNDSXY:1:1114:11921:29121 1:N:0:GAACCTAG+TCCGCATA
CACAAAGTCTGGTGTGTATCTTTCACCATTTGCTTACGTGAATCACTCACACCCTGGTTGTAAAACTC
TGGAGAACCACCTATTATTTAATGTTGTAGCTAGTTACATTAGTAAATATTCATATGTAGCATGTTTA
AGCATTAAATCAAAT
+
FFFFFFFFFFFFFFFFFFFFFFFFFFFFF,FFFFFFFF:FFFFFFFFFFFFFFFFFFFFFFFFFFFFF
FFFFFFFFFFFFFFFFF::FFFFFFFFFFFFFFFFFFFFFFFFFFFFFFFFFFFFFFFFFFFFFFFFF
FFFFFFFFFFFFFFF
@A00155:342:HHGFNDSXY:1:1114:11912:29168 1:N:0:GAACCTAG+TCCGCATA
CACAAAGTCTGGTGTGTATCTTTCACCATTTGCTTACGTGAATCACTCACACCCTGGTTGTAAAACTC
TGGAGAACCACCTATTATTTAATGTTGTAGCTAGTTACATTAGTAAATATTCATATGTAGCATGTTTA
AGCATTAAATCAAAT
+
FFFFFFFFFFFFFFFFFFFFFFFFFFFFFFFFFFFFFFFFFFFFFFFFFFFFFFFFFFFFFFFFFFFF
FFFFFFFFFFFFFFFFFFFFFFFFFFFFFFFFFFFFFFFFFFFFFFFFFFFFFFFFFFFFFFFFFFFF
FFFFFFFFFFFFFFF
```

@A00155:342:HHGFNDSXY:1:1137:21242:30514 1:N:0:GAACCTAG+TCCGCATA
CACAAAGTCTGGTGTGTATCTTTCACCATTTGCTTACGTGAATCACTCACACCCTGGTTGTAAAACTC
TGGAGAACCACCTATTATTTAATGTTGTAGCTAGTTACATTAGTAAATATTCATATGTAGCATGTTTA
AGCATTAAATCAAAT
+
FFFFFFFFFFFFFFFFFFFFFFFFFFFFFFFFFFFFFFFFFFFFFFFFFFFFFFFFFFFFFFFFFFFF
FFFFFFFFFFFFFFFFFFFFFFFFFFFFFFFFFFFFFFFFFFFFFFFFFFFFFFFFFFFFFFFFFFFF
FFFFFFFFFFFFFFF
@A00155:342:HHGFNDSXY:1:1178:15953:27430 1:N:0:GAACCTAG+TCCGCATA
CACAAAGTCTGGTGTGTATCTTTCACCATTTGCTTACGTGAATCACTCACACCCTGGTTGTAAAACTC
TGGAGAACCACCTATTATTTAATGTTGTAGCTAGTTACATTAGTAAATATTCATATGTAGCATGTTTA
AGCATTAAATCAAAT
+
FFFFFF:FFFFFFFFFFF:FFFFFFFFFFFFFFFFFFF,FFFFFFFFFFFFF:FFF,FFFFFFFFFFF
:FFFF:FFFFFFFFFFFFFFFFFFFFFFFFFFF,FFFFFFFFFFFFFF:FFFFFFFFFFFFFFFFFFF
FFFFFFFFFFFFFFF
@A00155:342:HHGFNDSXY:1:1247:32958:8375 1:N:0:GAACCTAG+TCCGCATA
CACAAAGTCTGGTGTGTATCTTTCACCATTTGCTTACGTGAATCACTCACACCCTGGTTGTAAAACTC
TGGAGAACCACCTATTATTTAATGTTGTAGCTAGTTACATTAGTAAATATTCATATGTAGCATGTTTA
AGCATTAAATCAAAT
+
FFFFFFFFFFF:FFFFFFFFFFFFFFFFF:FFFFFFFFFFFFFFFFFFFFFFFFFFFFFFFFFFFFFF
FFFFFFFFFFFFFFFF:FF:F,FFFFFF:FFFFF:FFFFFFFFFFFFFFFFFFFFFFFFFFF:FFFFF
FFFFFFFFFFFFFFF
@A00155:342:HHGFNDSXY:1:1332:29017:27633 1:N:0:GAACCTAG+TCCGCATA
CACAAAGTCTGGTGTGTATCTTTCACCATTTGCTTACGTGAATCACTCACACCCTGGTTGTAAAACTC
TGGAGAACCACCTATTATTTAATGTTGTAGCTAGTTACATTAGTAAATATTCATATGTAGCATGTTTA
AGCATTAAATCAAAT
+
FFFFFFFFFFFFFFFFFFFFFFFFFFFFFFFFFFFFFF:FFFF:FFFFFFFFFFFFFFFFFFFFFFFF
FFFFFFFFFFFFFFFFFFFFFFFFFFFFFFFFFFFFFFFFFFFFFFFFFFFFFFFFFF:FFFFFFFFF
FFFFFFFFFFFFFFF
@A00155:342:HHGFNDSXY:1:1344:21477:31673 1:N:0:GAACCTAG+TCCGCATA
CACAAAGTCTGGTGTGTATCTTTCACCATTTGCTTACGTGAATCACTCACACCCTGGTTGTAAAACTC
TGGAGAACCACCTATTATTTAATGTTGTAGCTAGTTACATTAGTAAATATTCATATGTAGCATGTTTA
AGCATTAAATCAAAT
+
FFFFFFFFFFFFFFFFFFFFFFFFFFFFFFFFFFFFFFFFFFFFFFFFFFFFFFFFFFFFFFFFFFFF
FFFFFFFFFFFFFFFFFF:FFFFFFFFFFFFFFFFFFFFFFFFFFFFFFFFF:FFFFFFFF:FFFFFF
FFFFFFFFFFFFFFF
@A00155:342:HHGFNDSXY:1:1518:27208:2331 1:N:0:GAACCTAG+TCCGCATA
CACAAAGTCTGGTGTGTATCTTTCACCATTTGCTTACGTGAATCACTCACACCCTGGTTGTAAAACTC
TGGAGAACCACCTATTATTTAATGTTGTAGCTAGTTACATTAGTAAATATTCATATGTAGCATGTTTA
AGCATTAAATCAAAT
+
FFFFFFFFFFFFFFFFFFFFFFFFFFFFFFFF:FFF,FF,FFFFFFFFFFFFFFFFFFFFFFFFFFFF
FFFF:FFFFFF:FFFFFFFFFFF:FFFFFF,FFF:FFFFFFFFFFFFFFFFFFFFFFFFFFFFFFFFF
FFFFFFFFFFFFFFF
@A00155:342:HHGFNDSXY:1:2402:31611:23766 1:N:0:GAACCTAG+TCCGCATA
CACAAAGTCTGGTGTGTATCTTTCACCATTTGCTTACGTGAATCACTCACACCCTGGTTGTAAAACTC
TGGAGAACCACCTATTATTTAATGTTGTAGCTAGTTACATTAGTAAATATTCATATGTAGCATGTTTA
AGCATTAAATCAAAT
+
FFFFF:FFFFFFFFFFFFFFFFFFFFFFFFFFFFFFFFFFFFFFFFFFFFFFFFFFFFFFFFFFFFFF

FFFFFFFFFFFFFFFFFFFFFFFFFFFFFFFFFFFF:FFFF:FFFFFFFFFFFFFFFFFFFFF:FFFFFF
FFFFFFFFFFFFFFF
@A00155:342:HHGFNDSXY:1:2406:25518:8422 1:N:0:GAACCTAG+TCCGCATA
CACAAAGTCTGGTGTGTATCTTTCACCATTTGCTTACGTGAATCACTCACACCCTGGTTGTAAAACTC
TGGAGAACCACCTATTATTTAATGTTGTAGCTAGTTACATTAGTAAATATTCATATGTAGCATGTTTA
AGCATTAAATCAAAT
+
FFFFFFFFFFFFFFFFFFFFFFFFFFFFFFFFFFFFFFFFFFFFFFFFFFFFFFFFFFFFFFFFFFFFFF
FFFFFFFFFFFFFFFFFFFFFFFFFFFFFFFFFFFFFFFFFFFFFFFFFFFFFFFFFFFFFFFFFFFFFF
FFFFFFFFFFFFFFF
@A00155:342:HHGFNDSXY:1:2430:32380:4492 1:N:0:GAACCTAG+TCCGCATA
CACAAAGTCTGGTGTGTATCTTTCACCATTTGCTTACGTGAATCACTCACACCCTGGTTGTAAAACTC
TGGAGAACCACCTATTATTTAATGTTGTAGCTAGTTACATTAGTAAATATTCATATGTAGCATGTTTA
AGCATTAAATCAAAT
+
FFFF,FFFFFFFF:FF:FFFFFFFFFFFFFFFFFFFFFFFFFFFFFFFFFFFFFFFFFFF:FFFFFFFFF
FFFF:FFFF:FFFF:FFFFFFFFFFFFFFFFFFFFFFFFFFFFFFFFFFFFFFFFF:FFFFFF:FFFFFF
FFFFFFFF:FFFFF:
@A00155:342:HHGFNDSXY:1:1116:8721:15186 2:N:0:GAACCTAG+TCCGCATA
CACAAAGTCTGGTGTGTATCTTTCACCATTTGCTTACGTGAATCACTCACACCCTGGTTGTAAAACTC
TGGAGAACCACCTATTATTTAATGTTGTAGCTAGTTACATTAGTCAATATTCATATGTAGCATGTTTA
AGCATTAAATCAAAT
+
FFFFFFFFFFFFFFFFFFFFFFFFFFFFFFFFFFFFFFFFFFFFFFFFFFFFFFFFFFFFFFFFFFFFFF
FFFFFF:FFFFFFFFFFFFFFFFFFFFFFFFFFFFFFFFFFF:FFF,FF:FFFFFFFFFFFFFFFFF:FF
FFFF,FFF:FFFFFF
@A00155:342:HHGFNDSXY:1:1261:3875:17409 2:N:0:GAACCTAG+TCCGCATA
CACAAAGTCTGGTGTGTATCTTTCACCATTTGCTTACGTGAATCACTCACACCCTGGTTGTAAAACTC
TGGAGAACCACCTATTATTTAATGTTGTAGCTAGTTACATTAGTAAATATTCATATGTAGCATGTTTA
AGCATTAAATCAAAT
+
FFFFFFFFFFFFFFFFFFFFFFFFFFFFFFFFFFFFFFFFFFFF:F:FFFFFFFFFFFFFFFFFFFFFFF
FFFFFFFFFFFFFFFFFFFFFFFF:FFFFFFFFFFFFFFFFFFFFFFFFFFFFFFFFFFFFFFFF:FFFF
:FFFFFFFFF:FFFF
@A00155:342:HHGFNDSXY:1:1403:15745:7780 2:N:0:GAACCTAG+TCCGCATA
CACAAAGTCTGGTGTGTATCTTTCACCATTTGCTTACGTGAATCACTCACACCCTGGTTGTAAAACTC
TGGAGAACCACCTATTATTTAATGTTGTAGCTAGTTACATTAGTAAATATTCATATGTAGCATGTTTA
AGCATTAAATCAAAT
+
:FFFFFFFFFFF:F:FFFFFFFFFFFFFFFFFFFFFFFFF:FFFFFFFF:FF:FFF:FF:FFFFFFFFFF
F::F:FFFFFFFFFFFFF,FFF:FF,FFFFFFF:FFFFF,FFF:FFFFFFFFFFFFFFFFFFFFFFFFF:
FFFFFFFF:FFFFFF
@A00155:342:HHGFNDSXY:1:1423:8341:4664 2:N:0:GAACCTAG+TCCGCATA
CACAAAGTCTGGTGTGTATCTTTCACCATTTGCTTACGTGAATCACTCACACCCTGGTTGTAAAACTC
TGGAGAACCACCTATTATTTAATGTTGTAGCTAGTTACATTAGTAAATATTCATATGTAGCATGTTTA
AGCATTAAATCAAAT
+
FFFFFFFFFFFFFFFFFFFFFFFFFFFFFFFFFFFFFFFFF:FFFFF:FFFFFFFFFFFFFFFFFF:FFF
FFFFFFFFFFFFFFFFFFFFFFFFFFFFFFFFFFFFFFFFFFFFFFFFFFFFFFFFFFFFFF:FFFFFFF
FFFFFFFFFFFFFFF
@A00155:342:HHGFNDSXY:1:1520:9055:7686 2:N:0:GAACCTAG+TCCGCATA
CACAAAGTCTGGTGTGTATCTTTCACCATTTGCTTACGTGAATCACTCACACCCTGGTTGTAAAACTC
TGGAGAACCACCTATTATTTAATGTTGTAGCTAGTTACATTAGTAAATATTCATATGTAGCATGTTTA
AGCATTAAATCAAAT

```
+
FFFFF:FFFFFF:FFFFFFFFFFFFFFFFFFFFFFFFFFFFFFFFFFFFFFFFFFFFFFFFFFFFFFF
FFFFFFFFFFFFFFFFFFFFFFFFFFFFFFFFFFFFFFFFFFFFF:FFF:FFFFFFFF::FFFF:FFF
:FFFFFFF,FF:FFF
@A00155:342:HHGFNDSXY:1:2113:4146:28494 2:N:0:GAACCTAG+TCCGCATA
CACAAAGTCTGGTGTGTATCTTTCACCATTTGCTTACGTGAATCACTCACACCCTGGTTGTAAAACTC
TGGAGAACCACCTATTATTTAATGTTGTAGCTAGTTACATTAGTAAATATTCATATGTAGCATGTTTA
AGCATTAAATCAAAT
+
FFFFFFFFFFFFFFFFF:FFFFFFFFFFFFFFFFFFFFFFFFFFFFFFFFFFFFFFFFFFFFFFFFFF
FF:FFFFFFFFFFFFFFFFFFFF:FFFFFFFFFF:FFFFFFFFFFFFFFFFFFFFFFFF,FFFFFFFF
FFFFFFFF:FFFFFF
@A00155:342:HHGFNDSXY:1:2152:15619:23594 2:N:0:GAACCTAG+TCCGCATA
CACAAAGTCTGGTGTGTATCTTTCACCATTTGCTTACGTGAATCACTCACACCCTGGTTGTAAAACTC
TGGAGAACCACCTATTATTTAATGTTGTAGCTAGTTACATTAGTAAATATTCATATGTAGCATGTTTA
AGCATTAAATCAAAT
+
FFFFFFFFFFFFFFFFFFFFFFFFFFFFFFFFFFFFFFFFFFFFFFFFFFFFFFFFFFFFFFFFFFFF
FFFFFFFFFFFFFFFFFFFFFFFFFFFFFFFFFFFFFFFFFFFFFFFFFFFFFFFFFFFFFFFFFFFF
FFFFFFFFFFFFFFF
@A00155:342:HHGFNDSXY:1:2127:15998:24533 2:N:0:GAACCTAG+TCCGCATA
CACAAAGTCTGGTGTGTATCTTTCACCATTTGCTTACGTGAATCACTCACACCCTGGTTGTAAAACTC
TGGAGAACCACCTATTATTTAATGTTGTAGCTAGTTACATTAGTAAATATTCATATGTAGCATGTTTA
AGCATTAAATCAAAT
+
FFFFFFFFFFFFFFFFFFFFFFFFFFFFFFFFFFFFFFFFFFFFFFFFFFFFFFFFFFFFFFFFFFFF
FFFFFFFFFFFFFFFFFFFFFFFFFFFFFFFFFFFFFFFFFFFFFFFFFFFFFFFFFFFFFFFFFFFF
FFFFFFFFFFFFFFF
@A00155:342:HHGFNDSXY:1:2162:4824:19742 2:N:0:GAACCTAG+TCCGCATA
CACAAAGTCTGGTGTGTATCTTTCACCATTTGCTTACGTGAATCACTCACACCCTGGTTGTAAAACTC
TGGAGAACCACCTATTATTTAATGTTGTAGCTAGTTACATTAGTAAATATTCATATGTAGCATGTTTA
AGCATTAAATCAAAT
+
FFFFFFFFFFFFFFFFFFFFFFFFFFFFFFFFFFFFFFFFFFFFFFFFFFFFFFFFFFFFFFFF:FFF
FFFFFFFFFFFFF:FFFFFFFFFFFFFFFF:FFFFFFFFFFFFFFFFFFFFFFFFFFFFFF:FFFFFF
F:FFFFF:FF,FF:F
@A00155:342:HHGFNDSXY:1:2157:13015:11005 2:N:0:GAACCTAG+TCCGCATA
CACAAAGTCTGGTGTGTATCTTTCACCATTTGCTTACGTGAATCACTCACACCCTGGTTGTAAAACTC
TGGAGAACCACCTATTATTTAATGTTGTAGCTAGTTACATTAGTAAATATTCATATGTAGCATGTTTA
AGCATTAAATCAAAT
+
FFFFFFFFFFFF:FFFFFFFFFFFFFFFFFFFFFFFFFFFFFFFFFFFFFFFFFFFFFFFFFFFFFFF
FFFFFFFFFFFFFFFFFFFFFFFFFFFFFFFFFFFFFFFFFFFFFFFFFFFF:FFFFFFF:FFFFFFF
FFFF,FFFFFFFFF:
@A00155:342:HHGFNDSXY:1:2208:11351:17832 2:N:0:GAACCTAG+TCCGCATA
CACAAAGTCTGGTGTGTATCTTTCACCATTTGCTTACGTGAATCACTCACACCCTGGTTGTAAAACTC
TGGAGAACCACCTATTATTTAATGTTGTAGCTAGTTACATTAGTAAATATTCATATGTAGCATGTTTA
AGCATTAAATCAAAT
+
FFFFFFFFFFFFFFFFFFFFFFFFFFFFFFFFFFFFFFFFFFFFFFFFFFFFFFFFFFFFFFF:FFFF
FFFFFFFFFFFFFFFFFFFFFFFFFFFFF:FFFFFFFFFFFFFFFFFFFFFFFFFFFFFFFFFFFFFF
FFFFFFFFFFFFFFF
@A00155:342:HHGFNDSXY:1:2208:11487:18380 2:N:0:GAACCTAG+TCCGCATA
CACAAAGTCTGGTGTGTATCTTTCACCATTTGCTTACGTGAATCACTCACACCCTGGTTGTAAAACTC
```

TGGAGAACCACCTATTATTTAATGTTGTAGCTAGTTACATTAGTAAATATTCATATGTAGCATGTTTA
AGCATTAAATCAAAT
+
FFFFFFFFFFFF:FFFFFFFFFFFFFFF:FFFFFFFFFFFFFFFFFFFFFFFFFFFFFFFFFFFFFFF
FFFFFFFFFFFFFFFFFFFFFFFFFFFFFFFFFFFFFFFFFFFFFFFFFFFFFFFFFFFFFFF,FFFF
FFFFFF:FFFFFFFF
@A00155:342:HHGFNDSXY:1:2418:13422:25895 2:N:0:GAACCTAG+TCCGCATA
CACAAAGTCTGGTGTGTATCTTTCACCATTTGCTTACGTGAATCACTCACACCCTGGTTGTAAAACTC
TGGAGAACCACCTATTATTTAATGTTGTAGCTAGTTACATTAGTAAATATTCATATGTAGCATGTTTA
AGCATTAAATCAAAT
+
FFFFFFFFFFFFFFFFFFFFFFFFFFFFFFFFFFFFFFFFFFFFFFFFFFFFFFFFFFFFFFFFFFFF
FFFFFFFFFFFFFFFFFFF:FFFFFFFFFFFFFFFFFFFFFFFFFF:FFFFF:FFFFFFFFFFFFFFF
FFFFFFFFFFFFFFF
@A00155:342:HHGFNDSXY:1:2418:13431:26162 2:N:0:GAACCTAG+TCCGCATA
CACAAAGTCTGGTGTGTATCTTTCACCATTTGCTTACGTGAATCACTCACACCCTGGTTGTAAAACTC
TGGAGAACCACCTATTATTTAATGTTGTAGCTAGTTACATTAGTAAATATTCATATGTAGCATGTTTA
AGCATTAAATCAAAT
+
FFFFFFFFFFFFFFFFFFFFFFFFFFFFFFFFFFFFFFFFFFFFFFFFFFFFFFFFFFFFFFFFFFFF
FFFFFFFFFFFFFFFFFFF:FFFFFFFFFFFFFFFFFFFFFFFFFFFFFFFFFFFFFFFFFFFFFFFF
FFFFFFFFFFFFFFF
@A00155:342:HHGFNDSXY:1:2263:7482:19993 2:N:0:GAACCTAG+TCCGCATA
ACAAAGTCTGGTGTGTATCTTTCACCATTTGCTTACGTGAATCACTCACACCCTGGTTGTAAAACTCT
GGAGAACCACCTATTATTTAATGTTGTAGCTAGTTACATTAGTAAATATTCATATGTAGCATGTTTAA
GCATTAAATCAAAT
+
:FFFFFFFFFFFFFFFFFFFFFFFFFFFFFFFFFF:FFFFFFFFFFFFFFFFFFFFFFFFFFFFFFFF
FFFFFFFFFFFFFFFFFFFFFFFFFFFFFFFFFFFFFFFFFF:FFFFFFFFFFFFFFFF,FF:FFFFF
:FFFFFFFFFFFF:
@A00155:342:HHGFNDSXY:1:2616:16224:13401 2:N:0:GAACCTAG+TCCGCATA
ACAAAGTCTGGTGTGTATCTTTCACCATTTGCTTACGTGAATCACTCACACCCTGGTTGTAAAACTCT
GGAGAACCACCTATTATTTAATGTTGTAGCTAGTTACATTAGTAAATATTCATATGTAGCATGTTTAA
GAATTAAATCAAAT
+
F::FF:F::F,FFFFFFF::FFFFFFF:FF::F:F:,F:FFFFFFFFFFFFFFF::,F:FFFFF:FFF
F,F,FFFFF,FFFFFFFFFFF::F:,FFF,FFF:FFFF,FFFFFFFFFFF,FFFFFFF:,FFF,FFFF
:,FF,FFFFFF,FF
@A00155:342:HHGFNDSXY:1:1273:3703:36683 2:N:0:GAACCTAG+TCCGCATA
ACAAAGTCTGGTGTGTATCTTTCACCATTTGCTTACGTGAATCACTCACACCCTGGTTGTAAAACTCT
GGAGAACCACCTATTATTTAATGTTGTAGCTAGTTACATTAGTAAATATTCATATGTAGCATGTTTAA
GCATTAAATCAAATA
+
FFFFFFF:FFFFFFFFFFFFFFFFFFFFFFFFFFFFFFFFFFFFFFFFFFFFFFFFFFFF:FFFFFFF
FFFFFFFFFFFFFFFFFFFFFFFFFFFFFFFFFFFFFFFFFFFFFF:FFFFFFFFFFFFFFFFFFFFF
,F:FFFFFFF:FFFF
@A00155:342:HHGFNDSXY:1:1368:26585:18818 2:N:0:GAACCTAG+TCCGCATA
ACAAAGTCTGGTGTGTATCTTTCACCATTTGCTTACGTGAATCACTCACACCCTGGTTGTAAAACTCT
GGAGAACCACCTATTATTTAATGTTGTAGCTAGTTACATTAGTAAATATTCATATGTAGCATGTTTAA
GCATTAAATCAAATA
+
FFFFFFFFFFFFFFFFFFFFFFFF:FFFFFFFFFFFFFFFFF:FFFFFFFFFFFFFFFFFFF:F,FFF
F:FFFFF,FFFFF:FFFFFF,FF,F::FFFFFFFFFFFFFFFFFFF,FFFF:FFFFFFFFFFFFFFFF
,FF,FF::F:FFFFF

```
@A00155:342:HHGFNDSXY:1:2521:22137:23578 2:N:0:GAACCTAG+TCCGCATA
ACAAAGTCTGGTGTGTATCTTTCACCATTTGCTTACGTGAATCACTCACACCCTGGTTGTAAAACTCT
GGAGAACCACCTATTATTTAATGTTGTAGCTAGTTACATTAGTAAATATTCATATGTAGCATGTTTAA
GCATTAAATCAAATA
+
FFFFFFFFFFFFFFFFFFFFFFFFFFFFFFFFFFFFFFFFFFFFFFFFFFFFFFFFFFFFFFFFFFFF
FFFFFFFFFFFFFFFFFFFFFFFFFFFFFFFFFFFFFFFFFFFFFFFFFFFFFF:FFFFFFFFFFFFF
FFFFFFFFFFFFFFF
@A00155:342:HHGFNDSXY:1:1127:1588:22748 2:N:0:GAACCTAG+TCCGCATA
AAAGTCTGGTGTGTATCTTTCACCATTTGCTTACGTGAATCACTCACACCCTGGTTGTAAAACTCTGG
AGAACCACCTATTATTTAATGTTGTAGCTAGTTACATTAGTAAATATTCATATGTAGCATGTTTAAGC
ATTAAATCAAATAA
+
FFFFFFFFFFFFFFFFFFFFFFFFFFFFFFFFFFFFFFFFFFFFFFFFFFFFFFFFFFFFFFFFFFFF
FFFFFFFFFFFFFFFFFFFFFFFFFFFFFFFFFFFFFFFFFFFFFFFF:FFFFFFFFFFFFFFFF:F:
FFFFFFFFFFFFFF
@A00155:342:HHGFNDSXY:1:1139:27064:14794 1:N:0:GAACCTAG+TCCGCATA
AAAGTCTGGTGTGTATCTTTCACCATTTGCTTACGTGAATCACTCACACCCTGGTTGTAAAACTCTGG
AGAACCACCTATTATTTAATGTTGTAGCTAGTTACATTAGTAAATATTCATATGTAGCATGTTTAAGC
ATTAAATCAAATAAG
+
FFFFFFFFFFFFFFFFFFFFFFFFFFFFFFFFFFFFFFFFFFFFFFFFFFFFFFFFFFFFFFFFFFFF
FFFFFFFFFFFFFFFFFFFFFFFFFF:FFFFFFFFFFFFFFFFFFFFFFFF:FFF:FFFFFFFFFFFF
FFFFFFFFFFFFFFF
@A00155:342:HHGFNDSXY:1:1205:3296:19914 1:N:0:GAACCTAG+TCCGCATA
AAAGTCTGGTGTGTATCTTTCACCATGTGCTTACGTGAATCACTCACACCCTGGTTGTAAAACTCTGG
AGAACCACCTATTATTTAATGTTGTAGCTAGTTACATTAGTAAATATTCATATGTAGCATGTTTAAGC
ATTAAATCAAATAAG
+
FF:FFFFFFFFFFFFFFFFFFFFFFF,FF:FFFFFFFFFFFFFFFFFFFFFFFFFFFFFFFFF:FFFF
FFFFFFFFFFFFFFFFFF,:FFFF:FFFFFF,FFFFFFFFFFFFFFFFFFFFFFFFFFFFFFFFFFFF
FFFFFFFFFFFFFFF
@A00155:342:HHGFNDSXY:1:1205:3197:19993 1:N:0:GAACCTAG+TCCGCATA
AAAGTCTGGTGTGTATCTTTCACCATTTGCTTACGTGAATCACTCACACCCTGGTTGTAAAACTCTGG
AGAACCACCTATTATTTAATGTTGTAGCTAGGTACATTAGTAAATATTCATATGTAGCATGTTTAAGC
ATTAAATCAAATAAG
+
FFF:FF:FFFFFFFFFFF:FFFFFFFFFFFFFFFFFFFFFFFFFFFFFFFFFFFFFFFFFFFFFFFFF
F:FFFFFFFFFF:FFFFF,FFFFFF,FF,FF,FFF,,FFFFFFFFFFFFFFFFFFFFFFFFFFFFFFF
FFFFFFFFFFFFFFF
@A00155:342:HHGFNDSXY:1:1319:4137:12289 1:N:0:GAACCTAG+TCCGCATA
AAAGTCTGGTGTGTATCTTTCACCATTTGCTTACGTGAATCACTCACACCCTGGTTGTAAAACTCTGG
AGAACCACCTATTATTTAATGTTGTAGCTAGTTACATTAGTAAATATTCATATGTAGCATGTTTAAGC
ATTAAATCAAATAAG
+
FFFFFFFF,FFFFFFFFFFFFFF:FFFFFFFFFFFFFFFFFFFF:FFFFFFFFFFFFFFFFFFFFFFF
FFFFFFFFFFFFFF:FFF,FFFFFFFFFFFFFFFFFFFFFFFFFFFFFFFFFFFFFFFFFFFFFFFFF
FFFFFFFFFFFFFFF
@A00155:342:HHGFNDSXY:1:1471:24478:17613 1:N:0:GAACCTAG+TCCGCATA
AAAGTCTGGTGTGTATCTTTCACCATTTGCTTACGTGAATCACTCACACCCTGGTTGTAAAACTCTGG
AGAACCACCTATTATTTAATGTTGTAGCTAGTTACATTAGTAAATATTCATATGTAGCATGTTTAAGC
ATTAAATCAAATAAG
+
FFFFFFFFFFFFFFFFFFFFFFFFFFFFFFFFF:FFFFFFFFFFFFFFFFFFFFFFFFFFFFFFFFFF
```

FFFFFFFFFFFFFFFF:F:FFFFFFFFFFFFFFFFFFFFFFFFFFFFFFFFFFFFFFFFFFFFFFFFF
FFFFFFFFFFFFFFF
@A00155:342:HHGFNDSXY:1:2118:32823:34851 1:N:0:GAACCTAG+TCCGCATA
AAAGTCTGGTGTGTATCTTTCACCATTTGCTTACGTGAATCACTCACACCCTGGTTGTAAAACTCTGG
AGAACCACCTATTATTTAATGTTGTAGCTAGTTACATTAGTAAATATTCATATGTAGCATGTTTAAGC
ATTAAATCAAATAAG
+
FFF:FFFF:FFF:FF,FF:,FFFFF,F,FFFFFF:,:FFFFFFFFFFFFFFFF,FFF,FFFFF:FF:F
FFFFFFFF:FFFFF:,FFFFF:F:FFFFFF:FFFFFFFF:FFFFFFFFFFFF,FFF:FFF:,FFFFFF
FFFFFFFFFFFFFFF
@A00155:342:HHGFNDSXY:1:2171:29179:36057 1:N:0:GAACCTAG+TCCGCATA
AAAGTCTGGTGTGTATCTTTCACCATTTGCTTACGTGAATCACTCACACCCTGGTTGTAAAACTCTGG
AGAACCACCTATTATTTAATGTTGTAGCTAGTTACATTAGTAAATATTCATATGTAGCATGTTTAAGC
ATTAAATCAAATAAG
+
F,F:FFFF:FFFFFFFFFFFFFF,FF:FF:FFFFF:FFFFF:FFFFFFFFFFFFFFFFFFFFFFFFFF
FFF:FFFF:FFFFFFFFFFFFFFFFFFFFFFFFFFFFFFFFFFFFFFFFFFFFFFFFFFFFFFFFFFF
FFFFFFFFFF:FFFF
@A00155:342:HHGFNDSXY:1:2333:4137:13166 1:N:0:GAACCTAG+TCCGCATA
AAAGTCTGGTGTGTATCTTTCACCATTTGCTTACGTGAATCACTCACACCCTGGTTGTAAAACTCTGG
AGAACCACCTATTATTTAATGTTGTAGCTAGTTACATTAGTAAATATTCATATGTAGCATGTTTAAGC
ATTAAATCAAATAAG
+
FFFFFFFFFFFFFFFFFFFFFFFFFFFFFFFFFFFFFFFFFFFFFFFFFFF:FFFFFFFFFFFFFFFF
FFFFFFFFFFFFFFFFFF:FFFFFFFFFFFFFFFFFFFFFFFFFFFFFFFFFFFF,FFFFFFFFFFFF
FFFFFFFFFFFFFFF
@A00155:342:HHGFNDSXY:1:2537:32958:24815 1:N:0:GAACCTAG+TCCGCATA
AAAGTCTGGTGTGTATCTTTCACCATTTGCTTACGTGAATCACTCACACCCTGGTTGTAAAACTCTGG
AGAACCACCTATTATTTAATGTTGTAGCTAGTTACATTAGTAAATATTCATATGTAGCATGTTTAAGC
ATTAAATCAAATAAG
+
FFF:FFFFFFFFFFF:FFFFFFFFFFFFFFFFFF,FFFFFFFFFFFFFFFFFFFFFFFFFFFFFFFFF
FFFFFFFFFFFFFFFF:FFFFFFFFF:FFFFFFFFFFFFFFFFFFFFFFFFFFFFFFFFFFF:FFFFFF
FFFFFFFFFFFFFFF
@A00155:342:HHGFNDSXY:1:2567:6714:20259 1:N:0:GAACCTAG+TCCGCATA
AAAGTCTGGTGTGTATCTTTCACCATTTGCTTACGTGAATCACTCACACCCTGGTTGTAAAACTCTGG
AGAACCACCTATTATTTAATGTTGTAGCTAGTTACATTAGTAAATATTCATATGTAGCATGTTTAAGC
ATTAAATCAAATAAG
+
F:FFFFFFFFFFFFFFFFFFFF:FFF:FFFF:F:FFFFFFFFFFFFFFFF,FFFFFFFFFFFFFFFFF
FF:F:FF,FFFFFFFFFFFFFFFFFFFFFFFF,FFFFFFFFFFFFFFFFF,FFFFFFF:FFFFFFFFFF
:FFFFFFFFFFFFFF
@A00155:342:HHGFNDSXY:1:2614:14009:24064 1:N:0:GAACCTAG+TCCGCATA
AAAGTCTGGTGTGTATCTTTCACCATTTGCTTACGTGAATCACTCACACCCTGGTTGTAAAACTCTGG
AGAACCACCTATTATTTAATGTTGTAGCTAGTTACATTAGTAAATATTCATATGTAGCATGTTTAAGC
ATTAAATCAAATAAG
+
:F,FFFFFFFFFFFFFFFFFFF,FFFFFFFFF:FFFFF:FFFFFFFF:FFFF::FFFFFFF:,FFFFF
FFF,,F,:FFFFFFFF,F:FFF:FFFFFFFFFFF,FFFFFFFFFFFFFFFFFFFFFFF:FFFFFFFFFF
FFFFFFFFF::FFFF
@A00155:342:HHGFNDSXY:1:2623:30092:8484 1:N:0:GAACCTAG+TCCGCATA
AAAGTCTGGTGTGTATCTTTCACCATTTGCTTACGTGAATCACTCACACCCTGGTTGTAAAACTCTGG
AGAACCACCTATTATTTAATGTTGTAGCTAGTTACATTAGTAAATATTCATATGTAGCATGTTTAAGC
ATTAAATCAAATAAG

```
+
FFFFFFFFFFFFFFFFFFFFFFFFFFFFFFFFFFFFFFFFFFFFFFFFFFFFFFFFFFFFFFFFFFFF
:FFFFF:FFFFFFFFFFFFFFFFFFFFFFFFFFFFFFFFFFFFFFFFFFFFFFFFFFFFFFFFFFFFF
FFFF:FFFFFFFFFF
@A00155:342:HHGFNDSXY:1:1334:18304:18850 1:N:0:GAACCTAG+TCCGCATA
AAGTCTGGTGTGTATCTTTCACCATTTGCTTACGTGAATCACTCACACCCTGGTTGTAAAACTCTGGA
GAACCACCTATTATTTAATGTTGTAGCTAGTTACATTAGTAAATATTCATATGTAGCATGTTTAAGCA
TTAAATCAAATAAGA
+
FFF:FFFFFFFFFFFFFFFFFFFFFFFFFFFFFFFFFFFFFFFFFFFFFFFFFFFFFFFFFFFFFFFF
FFFFFFFFFFFFFFFFFFFFFFFFFFFFFFFFFFFFFFFFFFFFFFFFFFFFFFFFFFFFFFFFFFFF
FFFFFFFFFFFFFFF
@A00155:342:HHGFNDSXY:1:1334:24867:23046 1:N:0:GAACCTAG+TCCGCATA
AAGTCTGGTGTGTATCTTTCACCATTTGCTTACGTGAATCACTCACACCCTGGTTGTAAAACTCTGGA
GAACCACCTATTATTTAATGTTGTAGCTAGTTACATTAGTAAATATTCATATGTAGCATGTTTAAGCA
TTAAATCAAATAAGA
+
FFFFFFFFFFFFFFFFFFFFFFFFFFFFFFFFFFFFFFFFFFFFFFFFFFFFFFFFFFFFFFFFFFFF
FFFFFFFFFFFF:FFFF:FFFFFFF:FFFFFFFFFFFFFFFFFFFFFFF:FFFFFFFFFFFFFFFFFF
FFFFFFFFFFFFFFF
@A00155:342:HHGFNDSXY:1:2202:17861:13730 1:N:0:GAACCTAG+TCCGCATA
AAGTCTGGTGTGTATCTTTCACCATTTGCTTACGTGAATCACTCACACCCTGGTTGTAAAACTCTGGA
GAACCACCTATTATTTAATGTTGTAGCTAGTTACATTAGTAAATATTCATATGTAGCATGTTTAAGCA
TTAAATCAAATAAGA
+
FFFFFFFFFFFFFFFFFFFFFFFFFFFFFFFFFF:FFFFFFFFFFFFFFFFFFFFFFFFFFFFFFFFF
FFFFFFFFFFFFFFFFFFFFFFFFFFFFFFFFFFFFFFFFFFFFFFFFFFFFFFFFFFFFFFFFFFFF
FFFFFFFFFFFFFFF
@A00155:342:HHGFNDSXY:1:2336:28908:5619 1:N:0:GAACCTAG+TCCGCATA
AAGTCTGGTGTGTATCTTTCACCATTTGCTTACGTGAATCACTCACACCCTGGTTGTAAAACTCTGGA
GAACCACCTATTATTTAATGTTGTAGCTAGTTACATTAGTAAATATTCATATGTAGCATGTTTAAGCA
TTAAATCAAATAAGA
+
FFFFFFFFFFFFFFFFFFFFFFFFFFFFFFFFFF:FFFFFFFFFFFFFFFFFFFFFFFFFFFFFFFFF
FFFFFFFFFFFFFFFF:FFFFFFFFFFFFFFFFFFFFFFFFFFFFFFFFFFFFFFFFFFFFFFFFFF
FFFFFFFFFFFFFFF
@A00155:342:HHGFNDSXY:1:2632:28574:22827 1:N:0:GAACCTAG+TCCGCATA
AAGTCTGGTGTGTATCTTTCACCATTTGCTTACGTGAATCACTCACACCCTGGTTGTAAAACTCTGGA
GAACCACCTATTATTTAATGTTGTAGCTAGTTACATTAGTAAATATTCATATGTAGCATGTTTAAGCA
TTAAATCAAATAAGA
+
FFFFFFFFFFFFFFFFFFFFFFFFFFFFFFFFFFFFFFFFFFFFFFFFFFFFFFFFFFFFFFFFFFFF
FFFFFFFFFFFFFFFFFFFFFFFFFFFFFFFFFFFFFFFFFFFFFFFFFFFFFFFF:FFF:FFFFFFF
FFFFFFFFFFFFFFF
@A00155:342:HHGFNDSXY:1:1230:7355:15076 2:N:0:GAACCTAG+TCCGCATA
AAGTCTGGTGTGTATCTTTCACCATTTGCTTACGTGAATCACTCACACCCTGGTTGTAAAACTCTGGA
GAACCACCTATTATTTAATGTTGTAGCTAGTTACATTAGTAAATATTCATATGTAGCATGTTTAAGCA
TTAAATCAAATAAGA
+
FFFFFFFFFFFFFFFFFFFFFFFFFFFFFFFFFFFFFFFFFFFFFFFFFFFFFFFFFFFFFFFFFFFF
FFFFFFFFFFFFFFFFFFFFFFFFFFFFFFFFFFFFFFFFFFFFFFFFFFFFFFFF,F:FFFFF,FFF
FFFFFFFFFFFFFF:
@A00155:342:HHGFNDSXY:1:2338:25952:27054 2:N:0:GAACCTAG+TCCGCATA
AAGTCTGGTGTGTATCTTTCACCATTTGCTTACGTGAATCACTCACACCCTGGTTGTAAAACTCTGGA
```

```
GAACCACCTATTATTTAATGTTGTAGCTAGTTACATTAGTAAATATTCATATGTAGCATGTTTAAGCA
TTAAATCAAATAAGA
+
FFFFFFFFFFFFFFFFFFFFFFFFFFFFFFFFFFFFFFFFFFFFFFFFFFFFFFFFFFFF,FFFFFFF
FFFFFFFFFFF:FFFFFF,FFFFFFFFFFFFFFFFFFFFFFFFFF:FFFFFFFF:FFFFFFFFF:FF
FFFFFFFFFFFFFF::
@A00155:342:HHGFNDSXY:1:1106:8106:31532 1:N:0:GAACCTAG+TCCGCATA
AGTCTGGTGTGTATCTTTCACCATTTGCTTACGTGAATCACTCACACCCTGGTTGTAAAACTCTGGAG
AACCACCTATTATTTAATGTTGTAGCTAGTTACATTAGTAAATATTCATATGTAGCATGTTTAAGCAT
TAAATCAAATA
+
FFFF,FFFFFFFFFFFFFFFFFFF:FFFFFFFF:FFFFFFFFFFFFFFFFFFFFFFFFFFFFFFFFFF
F:FFFFFFFFFFF:FFFFFFFFFFFFFFFFFFFFFFFFFFFFFFFFFFFFFFF:FFFFFF,FFFFFFFF
FFFFFFFFFFF
@A00155:342:HHGFNDSXY:1:1132:19786:1564 1:N:0:GAACCTAG+TCCGCATA
AGTCTGGTGTGTATCTTTCACCATTTGCTTACGTGAATCACTCACACCCTGGTTGTAAAACTCTGGAG
AACCACCTATTATTTAATGTTGTAGCTAGTTACATTAGTAAATATTCATATGTAGCATGTTTAAGCAT
TAAATCAAATA
+
FFFFFFFFFFFFFFFFFF,FFFFFFFFFFFFFF:FFFFFFFFFFFFFFFFFFFFFFFFFFFFFFFFFF
FFFFFFFFFFFFFFFFFFFFFFFFFFFFFFFFFFFFFFFFFFFFFFFFFFFFFFFFFFFFFFFFFFFF
FFFFFFFFFFF
@A00155:342:HHGFNDSXY:1:1173:13539:21026 1:N:0:GAACCTAG+TCCGCATA
TCCGATCTGTGTATCTTTCACCATTTGCTTACGTGAATCACTCACACCCTGGTTGTAAAACTCTGGAG
AACCACCTATTATTTAATGTTGTAGCTAGTTACATTAGTAAATATTCATATGTAGCATGTTTAAGCAT
TAAATCAAATA
+
FFFFFFFFFFFFFFFFFFFFFFFFFFFFFFFFFFFFFFFFFFFFFFFFFFFFFFFFFFFFFFFFFFFF
FFFFFFFFFFFFFFFFFFFFFFFFFFFFFFFFFFFFFFFFFFFFFFFFFFFFFFFFFFFFFFFFFFFF
FFFFFFFFFFF
@A00155:342:HHGFNDSXY:1:1221:5466:33661 1:N:0:GAACCTAG+TCCGCATA
AGTCTGGTGTGTATCTTTCACCATTTGCTTACGTGAATCACTCACACCCTGGTTGTAAAACTCTGGAG
AACCACCTATTATTTAATGTTGTAGCTAGTTACATTAGTAAATATTCATATGTAGCATGTTTAAGCAT
TAAATCAAATA
+
FFFFFFFFFFFFFFFFFFFFFFFFFFFFFFFFFFFFFFFF:FFFFFFFFFFFFFFFFFFFFFFFFFFF
FFFFFFFFFFFFFFFFFFFFFFFF:FFFFFFFFFFFFFFFFFFFFFFFFFFFFFFFFFFFFFFFFFFF
FFFFFFFFFFF
@A00155:342:HHGFNDSXY:1:1210:29722:34272 1:N:0:GAACCTAG+TCCGCATA
AGTCTGGTGTGTATCTTTCACCATTTGCTTACGTGAATCACTCACACCCTGGTTGTAAAACTCTGGAG
AACCACCTATTATTTAATGTTGTAGCTAGTTACATTAGTAAATATTCATATGTAGCATGTTTAAGCAT
TAAATCAAATA
+
FFFFFFFFFFFFFFFFFFFFFFFF:F:FFFFFFFFFFFFFFFFFFFFFFFFFFFFFFFFFFFFFFFFF
FFFFFFFFFFFFFFFFFFFFFFFFFFFFFFFFFFFFFFFFFFFFFFFFFFFFFFFFFFFFFFFFFFFF
FFFFFFFFFFF
@A00155:342:HHGFNDSXY:1:1535:29604:18756 1:N:0:GAACCTAG+TCCGCATA
AGTCTGGTGTGTATCTTTCACCATTTGCTTACGTGAATCACTCACACCCTGGTTGTAAAACTCTGGAG
AACCACCTATTATTTAATGTTGTAGCTAGTTACATTAGTAAATATTCATATGTAGCATGTTTAAGCAT
TAAATCAAATA
+
FFFFFFFFFFFFFFFFFFFF,FFFFFFFFFFFFFFFFFFFFFFFFFFF:FFFFFFFFFFFFFFFFFFF
FFFFFFFFFF:FF,FFFFFFFFFFF:FFFFFFFFFF:FFFFFFFFFFFF:FFFFFFFFFFFFFFFFFF
FFFFF:FFFFF
```

@A00155:342:HHGFNDSXY:1:1571:19144:20713 1:N:0:GAACCTAG+TCCGCATA
AGTCTGGTGTGTATCTTTCACCATTTGCTTACGTGAATCACTCACACCCTGGTTGTAAAACTCTGGAG
AACCACCTATTATTTAATGTTGTAGCTAGTTACATTAGTAAATATTCATATGTAGCATGTTTAAGCAT
TAAATCAAATA
+
FFFFFFFFFFFFFFFFFFFFFFFFFFFFFFFFFFFFFFFFFFFFFFFFFFFFFFFFFFFFFFFFFFFFFF
FFFFFFFFFFFFFFFFFFFFFFFFFFFFFFFFFFFFFFFFFFFFFFFFFFFFFFFFFFFFFFFFFFFFFF
FFFFFFFFFFF
@A00155:342:HHGFNDSXY:1:1663:26973:29418 1:N:0:GAACCTAG+TCCGCATA
AGTCTGGTGTGTATCCTTCACCATTTGCTTACGTGAATCACTCACACCCTGGTTGTAAAACTCTGGAG
AACCACCTATTATTTAATGTTGTAGCTAGTTACATTAGTAAATATTCATATGGAGCATGTTTAAGCAT
TAAATCAAATA
+
FFFFFFFFFFFFFFF:,FFFF:FF:FFFFF:FFFFFFFFFFFFF,FFFFFFF:::FFFFFFFFFF:FFFF
,FFF:FFF::F:FFF,FFFFFFFFF:F,FFFFFFFFFFFFFFFFFFFFFFF:FF,FF:FF,:FF:FF,FF
FFFFFF:FFF,
@A00155:342:HHGFNDSXY:1:1669:21558:12023 1:N:0:GAACCTAG+TCCGCATA
AGTCTGGTGTGTATCTTTCACCATTTGCTTACGTGAATCACTCACACCCTGGTTGTAAAACTCTGGAG
AACCACCTATTATTTAATGTTGTAGCTAGTTACATTAGTAAATATTCATATGTAGCATGTTTAAGCAT
TAAATCAAATA
+
FFFFFFFFFFFFFFFFFFFFFFFFFFFFFFFFFFFFFFFFFFFF:FFFFFFFFFFFFFFFFFFFFFFFFF
FFFFFFFFFFFF:FFFFFFFFFFFFFFFFFFFFFFFFFFFFFF:FFFFFFFFFF:FFFFFFFFFFFFFFF
FFFFFFFFFFF
@A00155:342:HHGFNDSXY:1:2154:11849:4695 1:N:0:GAACCTAG+TCCGCATA
AGTCTGGTGTGTATCTTTCACCATTTGCTTACGTGAATCACTCACACCCTGGTTGTAAAACTCTGGAG
AACCACCTATTATTTAATGTTGTAGCTAGTTACATTAGTAAATATTCATATGTAGCATGTTTAAGCAT
TAAATCAAATA
+
FFFFFFFFFFFFFFFFFFFFFFFFFFFFFFFFFFFFFFFFFFFFFFFFFFFFFFFFFFFFFFFFFFFFFF
FFFFFFFFFFFFFFFFFFFFFFFFFFFFFFFFFFFFFFFFFFFFFFFFFFFFFFFFFFFFFFFFFFFFFF
FFFFFFFFFFF
@A00155:342:HHGFNDSXY:1:2139:27380:20823 1:N:0:GAACCTAG+TCCGCATA
AGTCTGGTGTGTATCTTTCACCATTTGCTTACGTGAATCACTCACACCCTGGTTGTAAAACTCTGGAG
AACCACCTATTATTTAATGTTGTAGCTAGTTACATTAGTAAATATTCATATGTAGCATGTTTAAGCAT
TAAATCAAATA
+
FFFFFFFFFF:FFF,FFFFFFFFFFFFFFFFFFFFFFFFFFFFFFFFFFFFFFFFFFFFFFFFFFFFFFF
FFFF:FFFFFFFFFFFFFFFFFFFFFFFFFFFFFFFFFFFFFFFFFFFFFFFFFF:FFFFF:FFFFFFFF
FFFFFFFFFFF
@A00155:342:HHGFNDSXY:1:2159:1172:15702 1:N:0:GAACCTAG+TCCGCATA
AGTCTGGTGTGTATCTTTCACCATTTGCTTACGTGAATCACTCACACCCTGGTTGTAAAACTCTGGAG
AACCACCTATTATTTAATGTTGTAGCTAGTTACATTAGTAAATATTCATATGTAGCATGTTTAAGCAT
TAAATCAAATA
+
FFF:F,,:F::FF,FF,:FFFFF:F:FF:FFF,,:FFFFFF:FFFFFFFF,::::FFFFFF:F,::FF
FFFFFFFFFFFFF,,FFFFF,FFFF,FFF,FFFFF:FF::FFFFFF:FF,FFFFFFFFFF:F:FF:FF:
FFFFFFFFFFF
@A00155:342:HHGFNDSXY:1:2243:27588:22592 1:N:0:GAACCTAG+TCCGCATA
AGTCTGGTGTGTATCTTTCACCATTTGCTTACGTGAATCACTCACACCCTGGTTGTAAAACTCTGGAG
AACCACCTATTATTTAATGTTGTAGCTAGTTACATTAGTAAATATTCATATGTAGCATGTTTAAGCAT
TAAATCAAATA
+
FFFFFFFFFFFFFFFFFFFFFFFFFFFFFFFFFFFFFFFFFFFFFFFFFFFFFFFFFFFFFFFFFFFFFF

FFFFFFFFFFFFFFFFFFFFFFFFFFFFFFFFFFFFFFFFFFFFFFFFFFFFFFFFFFFFFFFFFFFF
FFFFFFFFFFF
@A00155:342:HHGFNDSXY:1:2246:23954:28948 1:N:0:GAACCTAG+TCCGCATA
AGTCTGGTGTGTATCTTTCACCATTTGCTTACGTGAATCACTCACACCCTGGTTGTAAAACTCTGGAG
AACCACCTATTATTTAATGTTGTAGCTAGTTACATTAGTAAATATTCATATGTAGCATGTTTAAGCAT
TAAATCAAATA
+
FFFFFFFFFFFFFFFFFFFFF:FFFFFFFFFFFFFFFFFFFFFFFFFFFFFFFFFFFFFFFFFFFFFF
FFFFFFFFFFFFFFFFFFFFFFFFFFFFFFFFFFFFFFFFFFFFFFFFFFFFF:FFFFF:FFFFFFFF
FFFFFFFFFFF
@A00155:342:HHGFNDSXY:1:2331:19461:24831 1:N:0:GAACCTAG+TCCGCATA
AGTCTGGTGTGTATCTTTCACCATTTGCTTACGTGAATCACTCACACCCTGGTTGTAAAACTCTGGAG
AACCACCTATTATTTAATGTTGTAGCTAGTTACATTAGTAAATATTCATATGTAGCATGTTTAAGCAT
TAAATCAAATA
+
FFFFFFFFFFFFFFFFFFFFFFFFFFFFFFFFFFFFFFFFFFFFFFFFFFFFFFFFFFFFFFFFFFFF
FFFFFFFFFFFFFFFFFFFFFFFFFFFFFFFFFFFFFFFFFFFFFFFFFFFFFFFFFFFFFFFFFFFF
FFFFFFFFFFF
@A00155:342:HHGFNDSXY:1:2412:25898:12211 1:N:0:GAACCTAG+TCCGCATA
AGTCTGGTGTGTATCTTTCACCATTTGCTTACGTGAATCACTCACACCCTGGTTGTAAAACTCTGGAG
AACCACCTATTATTTAATGTTGTAGCTAGTTACATTAGTAAATATTCATATGTAGCATGTTTAAGCAT
TAAATCAAATA
+
FFFFFFFFFFFFFFFFFFFFFFFFFFFFFFFFFFFFFFFFFFFFFFFFFF:FFFFFFFFFFFFF:FFF
FFFFFFFFFFFFFFFFFFFFFFFFFFFFFFFFFFFFFFFFFFFFFFFFFFFFFFFFFF::FFFFFFFF
FFFFFFFFFFF
@A00155:342:HHGFNDSXY:1:2451:11966:35524 1:N:0:GAACCTAG+TCCGCATA
AGTCTGGTGTGTATCTTTCACCATTTGCTTACGTGAATCACTCACACCCTGGTTGTAAAACTCTGGAG
AACCACCTATTATTTAATGTTGTAGCTAGTTACATTAGTAAATATTCATATGTAGCATGTTTAAGCAT
TAAATCAAATA
+
FFFFFFFFFFFFF:::FFFFFFFFFFF:FFFFF:FFFFFFFFFFFFF:FFFFFFFFFFFFFFFF:FFF
FFFFFFFFFFFF:FFFFFFFFFFFFFFFFFFFFFFFFFF::FFFFFFFF:FFFFFFFFFFFFFFFFFF
FFFFFFFFFFF
@A00155:342:HHGFNDSXY:1:2529:14244:29512 1:N:0:GAACCTAG+TCCGCATA
AGTCTGGTGTGTATCTTTCACCATTTGCTTACGTGAATCACTCACACCCTGGTTGTAAAACTCTGGAG
AACCACCTATTATTTAATGTTGTAGCTAGTTACATTAGTAAATATTCATATGTAGCATGTTTAAGCAT
TAAATCAAATA
+
FFFFFFFFFFFFF:FFFFFFFFFFFFFFFFFFFFFFFFFFFFFFFFFFFFFFFFFFFFFFFFFFFFFF
F:FFFFFFFFFF:FFFFFFFFFFFFFFFFFFFFFFFFFFFFFFFFFFFFFFFFFFFFFFFFFFFFFFF
FFFFFFFFFFF
@A00155:342:HHGFNDSXY:1:2639:4435:27023 1:N:0:GAACCTAG+TCCGCATA
AGTCTGGTGTGTATCTTTCACCATTTGCTTACGTGAATCACTCACACCCTGGTTGTAAAACTCTGGAG
AACCACCTATTATTTAATGTTGTAGCTAGTTACATTAGTAAATATTCATATGTAGCATGTTTAAGCAT
TAAATCAAATA
+
FFFFFFFFFFFFFFFFFFFFFF:FFF:FFFFFFFFFFFFFFFFFFFFFFFFFFFFFFFFFFFFFFFFF
FFFFFFFFFFFFFFFFFFFFFFFFFFFFFFFFFFFFFFFFFFFFFFFFFFFFFFFFFFFFFFFFFFFF
FFFFFFFFFFF
@A00155:342:HHGFNDSXY:1:2668:18918:23860 1:N:0:GAACCTAG+TCCGCATA
AGTCTGGTGTGTATCTTTCACCATTTGCTTACGTGAATCACTCACACCCTGGTTGTAAAACTCTGGAG
AACCACCTATTATTTAATGTTGTAGCTAGTTACATTAGTAAATATTCATATGTAGCATGTTTAAGCAT
TAAATCAAATA

+
FFFFFFFFFFFFFFFFFFFF::FFFFFFFFFFFFFFFFFFFFFFFFFFFFFFFFFFFFFFFFFFFFFF
FFFFFFFFFFFFFFFFFFFFFFFFFFFFFFFFFFFFFFFFFFFFFFFFFFFFFFFFFFFFFFFFFFFF
FFFFFFFFFFF
@A00155:342:HHGFNDSXY:1:2520:18403:12978 1:N:0:GAACCTAG+TCCGCATA
AGTCTGGTGTGTATCTTTCACCATTTGCTTACGTGAATCACTCACACCCTGGTTGTAAAACTCTGGAG
AACCACCTATTATTTAATGTTGTAGCTAGTTACATTAGTAAATATTCATATGTAGCATGTTTAAGCAT
TAAATCAAATAAGA
+
:FFFFFFFFFFFFF:FFFFFFFFF,FFFFFFFFFFFFFFFFFFFFFFFFFFFFFFFFFFFFFFFFFFF
FFFFFFFFFFFFF:FFFFFFFFFFFFFFFFFFFFFFFFFFFFFFFFFFFFFFFFFFFFFFFFFFFFFF
FFFFFFFFFFFFFF
@A00155:342:HHGFNDSXY:1:1127:16125:5995 1:N:0:GAACCTAG+TCCGCATA
AGTCTGGTGTGTATCTTTCACCATTTGCTTACGTGAATCACTCACACCCTGGTTGTAAAACTCTGGAG
AACCACCTATTATTTAATGTTGTAGCTAGTTACATTAGTAAATATTCATATGTAGCATGTTTAAGCAT
TAAATCAAATAAGAG
+
FFFF,FFFFFFFFFFFFFFF::FFFFFFFFF:F:FFFFFFFFFFF::FFFFFFF:FFFFF:FF:FFFF
FFF:FFFFFFF:F,FFFFFFFFFFFFFFFFF:FFFFFFFFF:FFFF::FFFF:FFFFFF,FFFFFFFF
FFFFFFFFFFFFFFF
@A00155:342:HHGFNDSXY:1:1127:16224:12273 1:N:0:GAACCTAG+TCCGCATA
AGTCTGGTGTGTATCTTTCACCATTTGCTTACGTGAATCACTCACACCCTGGTTGTAAAACTCTGGAG
AACCACCTATTATTTAATGTTGTAGCTAGTTACATTAGTAAATATTCATATGTAGCATGTTTAAGCAT
TAAATCAAATAAGAG
+
FFFFFFFFFFFFFFFFFFFFFFF:F:FFFFFFFFFFFFFFFFFFFFFFFFFFFFFFFFFFFFFFFFFF
FFFFF:FFFFFFF:FFFFFFFFFFFFFFFFFFFFFFFFFFFFFFFFFFFFFFFFFFFFFFFFFFFFFF
FFFFFFFFFFFFFFF
@A00155:342:HHGFNDSXY:1:2127:15998:24533 1:N:0:GAACCTAG+TCCGCATA
AGTCTGGTGTGTATCTTTCACCATTTGCTTACGTGAATCACTCACACCCTGGTTGTAAAACTCTGGAG
AACCACCTATTATTTAATGTTGTAGCTAGTTACATTAGTAAATATTCATATGTAGCATGTTTAAGCAT
TAAATCAAATAAGAC
+
FFFFFFFFFFFFFFF,FFFFFFFFFFFFFFFFFFFFFFFFFFFFFFFFFFFFFFFFFFFFFFFFFFFF
FFFFFFFFFFFFFFFFFFFFFFFFFFFFFFFFFFFFFFFFF:FFFFFFFFFFFFFFFFFFFFFFFFFF
FFFFFFFFFFFFFFF
@A00155:342:HHGFNDSXY:1:2353:24722:23359 1:N:0:GAACCTAG+TCCGCATA
AGTCTGGTGTGTATCTTTCACCATTTGCTTACGTGAATCACTCACACCCTGGTTGTAAAACTCTGGAG
AACCACCTATTATTTAATGTTGTAGCTAGTTACATTAGTAAATATTCATATGTAGCATGTTTAAGCAT
TAAATCAAATAAGAC
+
FFFFFFFF:FFFFFFFFFFFFFFFFFFFFFFFFFFFFFFFFFFFFFFFFFFFFFFFFFFFFFFFFFFF
FFFFFFFFFFFFFFFFFFFFFFFFFFFFFFFFFFFFFFFFFFFFFFFFFFFFFFFFFFFFFFFFFFFF
FFFFFFFFFFFFFFF
@A00155:342:HHGFNDSXY:1:1368:26585:18818 1:N:0:GAACCTAG+TCCGCATA
GTCTGGTGTGTATCTTTCACCATTTGCTTACGTGAATCACTCACACCCTGGTTGTAAAACTCTGGAGA
ACCACCTATTATTTAATGTTGTAGCTAGTTACATTAGTAAATATTCATATGTAGCATGTTTAAGCATT
AAATCAAATA
+
FFF:FFFFFFFFFFFFFF:FFF:FFFFFFFFFFFFFFFFF:::FF:,FFFFFF:FFFF::F,F,FFFF
F,::FFFFFF:FFFF:FFFFFFFFFFF:FFFFFFFFFFFFFFFFF,:F:FFFF:FFFFFFFFFF,FFF
FFFFFF::F:
@A00155:342:HHGFNDSXY:1:1174:9019:25911 1:N:0:GAACCTAG+TCCGCATA
GTCTGGTGTGTATCTTTCACCATTTGCTTACGTGAATCACTCACACCCTGGTTGTAAAACTCTGGAGA

ACCACCTATTATTTAATGTTGTAGCTAGTTACATTAGTAAATATTCATATGTAGCATGTTTTAGCATT
AAATCAAATAAGATG
+
FFFFFFFFFFFFFFFFFFFFFFFFFFFFF,FFFFFFFFFFFFFFFF:FF,FFFFFF:F:FFFFFFFFF
FFFFFFFFFFFFFFFFFFFFFFFFFFFFFFFFFFFFFFFFFFFFFFFFFFFF,FF:FFFFF,FFFFFF
FFFFFFFF::FFF:F
@A00155:342:HHGFNDSXY:1:1654:15447:8296 1:N:0:GAACCTAG+TCCGCATA
GTCTGGTGTGTATCTTTCACCATTTGCTTACGTGAATCACTCACACCCTGGTTGTAAAACTCTGGAGA
ACCACCTATTATTTAATGTTGTAGCTAGTTACATTAGTAAATATTCATATGTAGCATGTTTAAGCATT
AAATCAAATAAGATG
+
FFFFFFFFFFFFFFFFFFFFFFFFFFFFFFFFFFFFFFFFFFFFFFFFFFFFFFFFFFFFFFFFFFFF
FFFFF:FFFFFFFFFFFFFFFFFFFFFFFFFFFFFFFFFFFFFFFFFFFFFFFFFF:FFFFFFFFF
FFFFFFFFFFFFFFF
@A00155:342:HHGFNDSXY:1:2153:2754:1783 2:N:0:GAACCTAG+TCCGCATA
GTCTGGTGTGTATCTTTCACCATTTGCTTACGTGAATCACTCACACCCTGGTTGTAAAACTCTGGAGA
ACCACCTATTATTTAATGTTGTAGCTAGTTACATTAGTAAATATTCATATGTAGCATGTTTAAGCATT
AAATCAAATAAGATG
+
FFFFFF,FFFFFFFFFFFFFF:FFFFFFFFFFFFFFFFFFFFFFFF:FFFFFFFFFFFFFFFFFFFFF
FFFFFFFFFFFFFFFFFFFFFFFF:FFFFFFFFFFFFFFFFFFFFFFFFFFFFFFFFFFFFFFFFFFF
FF:F:FFFFFFF:F:
@A00155:342:HHGFNDSXY:1:2230:7563:15123 2:N:0:GAACCTAG+TCCGCATA
GTCTGGTGTGTATCTTTCACCATTTGCTTACGTGAATCACTCACACCCTGGTTGTAAAACTCTGGAGA
ACCACCTATTATTTAATGTTGTAGCTAGTTACATTAGTAAATATTCATATGTAGCATGTTTAAGCATT
AAATCAAATAAGATG
+
FFFFFFFFFFFFFFFFFFFFFFFFFFFFFFFFFFFFFFFFFFFFFFFFFFFFFFFFFFFFFFFFFFFF
FFFFFFFFFFFFFFFFFFFFFFFFFFFFFFFFFFFFFFFFFFFFFF:FFFFFFFFFFF:FFFF:FFFF
FFFFF:FFFFFF,FF
@A00155:342:HHGFNDSXY:1:2302:21766:18740 2:N:0:GAACCTAG+TCCGCATA
GTCTGGTGTGTATCTTTCACCATTTGCTTACGTGAATCACTCACACCCTGGTTGTAAAACTCTGGAGA
ACCACCTATTATTTAATGTTGTAGCTAGTTACATTAGTAAATATTCATATGTAGCATGTTTAAGCATT
AAATCAAATAAGATG
+
F,::FFFFFFFFFFFFFFFFFFFFFFFFFFFFFFFFFFFFFFFFFFFFFFFFFFFFFFFFFFFFFFFF
FFFFFFFFFFFFFFFFFFFFFFFFFFFFFFFFFFFFFFFFFFFFFFFFFFFFFFFFFFFFFFFF:FFF
FFFFFFFFFFFFFF:
@A00155:342:HHGFNDSXY:1:2302:20889:19382 2:N:0:GAACCTAG+TCCGCATA
GTCTGGTGTGTATCTTTCACCATTTGCTTACGTGAATCACTCACACCCTGGTTGTAAAACTCTGGAGA
ACCACCTATTATTTAATGTTGTAGCTAGTTACATTAGTAAATATTCATATGTAGCATGTTTAAGCATT
AAATCAAATAAGATG
+
F:F:FFFFFFFFFFFFFFFFFFFFFFFFFFFFFFFFF:FFFFFFFFFFFFFFFFFFFFFFFFFFFFFF
FFFFFFFFFF:FFFFFFFFFFFFFFFFFFFFFFFFFFFFFFFFFFFFFFFFFFFFFFFFFFFF:FFFF
FFFFFFFFFFFFFFF
@A00155:342:HHGFNDSXY:1:2554:1922:8578 2:N:0:GAACCTAG+TCCGCATA
GTCTGGTGTGTGTCTTTCACCATTTGCTTACGTGAATCACTCACACCCTGGTTGTAAAACTCTGGAGA
ACCACCTATTATTTAATGTTGTAGCTAGTTACATTAGTAAATATTCATATGTAGCATGTTTAAGCATT
AAATCAAATAAGATG
+
FFFFFFFFFFFFFFFFFFF:FFF:FF::FFFFFFFFFFFFFFFFFFFF,FFFFFFFFFFFFFFFFFFF
FFFFFFF:FFFFFFFFFFFFF,FFFFFFFF,FFF,FFFFFFFFFFFFF:FFF,FFFFFF,FFFF,,FF
FFF:FFFFF:FFFFF

@A00155:342:HHGFNDSXY:1:2255:11822:17550 1:N:0:GAACCTAG+TCCGCATA
CTGGTGTGTATCTTTCACCATTTGCTTACGTGAATCACTCACACCCTGGTTGTAAAACTCTGGAGAAC
CACCTATTATTTAATGTTGTAGCTAGTTACATTAGTAAATATTCATATGTAGCATGTTTAAGCATTAA
ATCAAATAAGA
+
FFFFFFFFFFFFFFFFFFFFFFFFFFFFFFFFFFFFFFFFFFFFFFFFFFFFFFFFFFFFFFFFFFFFFF
FFFFFFFFFFFFFFFFFFFFFFFFFFFFFFFFFFFFFFFFFFFFFFFFFFFFFFFFFFFFFFFFFFFFFF
FFFFFFFFFFF
@A00155:342:HHGFNDSXY:1:1266:2058:18490 1:N:0:GAACCTAG+TCCGCATA
CTGGTGTGTATCTTTCACCATTTGCTTACGTGAATCACTCACACCCTGGTTGTAAAACTCTGGAGAAC
CACCTATTATTTAATGTTGTAGCTAGTTACATTAGTAAATATTCATATGTAGCATGTTTAAGCATTAA
ATCAAATAAGATGAG
+
F:FFFFFFFFFFFFFFFFFFFFFF:FFF:,FFFFFFFFFFFFFFF,FFFF,FFFFFFFFFFFF,FFFFFF
FFFFFFFFF::FFFFFFFFFFFFFFFFFFFFFFFFFFFFF:FFFFFFFFFFFF:FFFFFFFFFFFFFFFF
FFFFFFFFFFFFFFF
@A00155:342:HHGFNDSXY:1:1271:20672:24424 1:N:0:GAACCTAG+TCCGCATA
CTGGTGTGTATCTTTCACCATTTGCTTACGTGAATCACTCACACCCTGGTTGTAAAACTCTGGAGAAC
CACCTATTATTTAATGTTGTAGCTAGTTACATTAGTAAATATTCATATGTAGCATGTTTAAGCATTAA
ATCAAATAAGATGAG
+
FFFFFFFFFFFFFFFFFFFFFFFFFFFFFFFFFFFFFFFFFFFFFFFFFFFFFFFFFFFFFFFFFFFFFF
FFFFFFFFFF:FFFFFFFFFFFFFFFF:F:FFFFFFFFFFFFFFFFFFFFFFFFFFFFFFFFFFFFFFFF
FFFFFFFFFFFFFFF
@A00155:342:HHGFNDSXY:1:1324:10339:14199 1:N:0:GAACCTAG+TCCGCATA
CTGGTGTGTATCTTTCACCATTTGCTTACGTGAATCACTCACACCCTGGTTGTAAAACTCTGGAGAAC
CACCTATTATTTAATGTTGTAGCTAGTTACATTAGTAAATATTCATATGTAGCATGTTTAAGCATTAA
ATCAAATAAGATGAG
+
FFFFFFFFFFFFFFFFFFFFFFFFFFFFFF:FFFFFFFFFFFFFFFFFFFFFFFFFFFFFFFFFFFFFFF
FFFFFFFF:FFFFFFFFFFFFFFFFFFFFFFFFFFFFFFFFFFFFFFFFFFFFFFFFFFFFFFFFFFFFF
FFFFFFFFFFFFFFF
@A00155:342:HHGFNDSXY:1:1416:19759:5963 1:N:0:GAACCTAG+TCCGCATA
CTGGTGTGTATCTTTCACCATTTGCTTACGTGAATCACTCACACCCTGGTTGTAAAACTCTGGAGAAC
CACCTATTATTTAATGTTGTAGCTAGTTACATTAGTAAATATTCATATGTAGCATGTTTAAGCATTAA
ATCAAATAAGATGAG
+
FFFFFFFFFFFFFFFFFFFFFFFFFFFFFFFFFFFFFFFFFFFFFFFFFFFFFFFFFFFFFFFFFFFFFF
:FFFFFFFFFFFFFFFFFFFFFFFFFFFFFFFFFFFFFFFFFFFFFFFFFFFFFFFFF:FFFFFFFFFFF
FFFFFFFFFFFFFFF
@A00155:342:HHGFNDSXY:1:1423:8341:4664 1:N:0:GAACCTAG+TCCGCATA
CTGGTGTGTATCTTTCACCATTTGCTTACGTGAATCACTCACACCCTGGTTGTAAAACTCTGGAGAAC
CACCTATTATTTAATGTTGTAGCTAGTTACATTAGTAAATATTCATATGTAGCATGTTTAAGCATTAA
ATCAAATAAGATGAG
+
FFFFFFFFFFFFFFFFFFFFF:FFFFFFFF:FFFFFFFFFFFFFFFFFFFFFFFFFFFFFFFFFFFFFFF
FF:FFFF:FFFFFFFFFFFFFFFFFFFFFFFFFFFFFFFFFFFFFFFFFFF:FFFFFFFFFFFFFFFFFF
FFFFFFFFFFFFFFF
@A00155:342:HHGFNDSXY:1:1460:12933:4069 1:N:0:GAACCTAG+TCCGCATA
CTGGTGTGTATCTTTCACCATTTGCTTACGTGAATCACTCACACCCTGGTTGTAAAACTCTGGAGAAC
CACCTATTATTTAATGTTGTAGCTAGTTACATTAGTAAATATTCATATGTAGCATGTTTAAGCATTAA
ATCAAATAAGATGAG
+
FFFFFFFFFFF:FFFFFFFFFFFFFFFFFFFFFFFFFFFFFFFF:FFFFFFFFF:FFFFFFFFFFFFFFF

```
FFFFFFF:FFFFFFFFFFFFFFFFFFFFFFFF:FFFFFFFFFFFFFFFFFFFFFFFFFFFFFFFFFFF
FFFFFFFFFFFFFFF
@A00155:342:HHGFNDSXY:1:1524:12337:26021 1:N:0:GAACCTAG+TCCGCATA
CTGGTGTGTATCTTTCACCATTTGCTTACGTGAATCACTCACACCCTGGTTGTAAAACTCTGGAGAAC
CACCTATTATTTAATGTTGTAGCTAGTTACATTAGTAAATATTCATATGTAGCATGTTTAAGCATTAA
ATCAAATAAGATGAG
+
FFFFFFFFFFFFFFFFFFFFFFFFFFFFFF:FFFFFFFFFFFFFFFFFFFFFFFFFFFFFFFFFFFFF
FFFFFFFFFFFFFFFFFFFFFFFFFFFFFFFFFFFFFFFFFFFFFFFFFFFFFFFFFFFFFFFFFFFF
FFFFFFFFFFFFFFF
@A00155:342:HHGFNDSXY:1:1530:12129:29763 1:N:0:GAACCTAG+TCCGCATA
CTGGTGTGTATCTTTCACCATTTGCTTACGTGAATCACTCACACCCTGGTTGTAAAACTCTGGAGAAC
CACCTATTATTTAATGTTGTAGCTAGTTACATTAGTAAATATTCATATGTAGCATGTTTAAGCATTAA
ATCAAATAAGATGAG
+
FFFFFFFFFFFFFFFFFFFFF:FFFFFFFFFFFFFFFFFFFFFFFFFFFFFFFFFFFFFFFFFFFFFF
FFFFFFFFFFFFFFFFFFFFFFFFFFFFFFFFFFFFFFFFFFFFFFFFFFFFFFFFFFFFFFFFFFFF
FFFFFFFFFFFFFFF
@A00155:342:HHGFNDSXY:1:1546:10321:1329 1:N:0:GAACCTAG+TCCGCATA
CTGGTGTGTATCTTTCACCATTTGCTTACGTGAATCACTCACACCCTGGTTGTAAAACTCTGGAGAAC
CACCTATTATTTAATGTTGTAGCTAGTTACATTAGTAAATATTCATATGTAGCATGTTTAAGCATTAA
ATCAAATAAGATGAG
+
:FFFFFF:FFFFFFFFFFFFF:FFFFFFFFFFFFFFFFFFFFFFFFFFFFFFFFFFFFFFFFFFFFFF
FFFFFFFFFFFFFFFFFFFFFFFFFFFFFFFFFFFFFFFFFFFFFFFFFFFFFFFFFFFFFFFFFFFF
FFFFFFFFFFFFFFF
@A00155:342:HHGFNDSXY:1:1577:18204:34021 1:N:0:GAACCTAG+TCCGCATA
CTGGTGTGTATCTTTCACCATTTGCTTACGTGAATCACTCACACCCTGGTTGTAAAACTCTGGAGAAC
CACCTATTATTTAATGTTGTAGCTAGTTACATTAGTAAATATTCATATGTAGCATGTTTAAGCATTAA
ATCAAATAAGATGAG
+
FFFFFFFFFFFFFFFFFFFFFFFFFFFFFFFFFFFFFFFFFFFFFFFFFFFFFFFFFFF:FFFFFFFF
FFFFFFFFFF,FFFFFFFFFFFFFFFFFFFFFFFFFFFFFFFFFFFFFFFFFFFFFFFFFFFFFFF:F
FFFFFFFFFFFFFFF
@A00155:342:HHGFNDSXY:1:1638:11424:29543 1:N:0:GAACCTAG+TCCGCATA
CTGGTGTGTATCTTTCACCATTTGCTTACGTGAATCACTCACACCCTGGTTGTAAAACTCTGGAGAAC
CACCTATTATTTAATGTTGTAGCTAGTTACATTAGTAAATATTCATATGTAGCATGTTTAAGCATTAA
ATCAAATAAGATGAG
+
FFFFFFFFFFFFFFFFFFFFFFFFFFFFFFFFFFFFFFFFFFFFFFFFFFFFFFFFFFFFFFFFFFFF
FFFFFFFFFF:FFFFFFFFFFFFFFFFFFFFFFFFFFFFFFFFFFFFFFFFFFFFF:FFFFFFFFFFF
FFFFFFFFFFFFFFF
@A00155:342:HHGFNDSXY:1:1621:30074:26490 1:N:0:GAACCTAG+TCCGCATA
CTGGTGTGTATCTTTCACCATTTGCTTACGTGAATCACTCACACCCTGGTTGTAAAACTCTGGAGAAC
CACCTATTATTTAATGTTGTAGCTAGTTACATTAGTAAATATTCATATGTAGCATGTTTAAGCATTAA
ATCAAATAAGATGAG
+
FFFFFFFFFFFFFFFFFFFFFFFFFFFFFF,FFFFFFFFFFFFFFFFFFFFFFFFFFFFFFFFFFFFF
FFFFFFFFFF:FFFFFFFFFFFFFFF:FFFFFFFFFFFFFFFFFFFFFFFFFFFFFFFFFFFFFFFFF
FFFFFFFFFFFFFFF
@A00155:342:HHGFNDSXY:1:1642:24551:36151 1:N:0:GAACCTAG+TCCGCATA
CTGGTGTGTATCTTTCACCATTTGCTTACGTGAATCACTCACACCCTGGTTGTAAAACTCTGGAGAAC
CACCTATTATTTAATGTTGTAGCTAGTTACATTAGTAAATATTCATATGTAGCATGTTTAAGCATTAA
ATCAAATAAGATGAG
```

+
:FFFF::,FFF,FFFFFFFFF:FFFFFFFFFFFFFFF,FFFF::FFFFFFFFFFFFFFFFF,FFFFF,
:FFFFFFFFFFFFFFFFFFFF,FFFF:FF:FFFFFFFFFFFFFFFFFFFFFFFFFFFFFFFFFFFFFF
FFFFFFFFFFFFFF:
@A00155:342:HHGFNDSXY:1:1676:20383:9987 1:N:0:GAACCTAG+TCCGCATA
CTGGTGTGTATCTTTCACCATTTGCTTACGTGAATCACTCACACCCTGGTTGTAAAACTCTGGAGAAC
CACCTATTATTTAATGTTGTAGCTAGTTACATTAGTAAATATTCATATGTAGCATGTTTAAGCATTAA
ATCAAATAAGATGAG
+
FFFFFFFFFFFFFFFFFFFFFFFFFFF:FFFFFFFFFFFFFFFF:FFFFFFFFFFFFFFFFFFFFFFF
FFFFFFFFFFFFFFFFFFFFFFFFFFFFFFFFFFFFFFFFFFFFF:FFFFFFFFFFFFFFFFFFFFFF
FFFFFFFFFFFFFFF
@A00155:342:HHGFNDSXY:1:2120:27868:16282 1:N:0:GAACCTAG+TCCGCATA
CTGGTGTGTATCTTTCACCATTTGCTTACGTGAATCACTCACACCCTGGTTGTAAAACTCTGGAGAAC
CACCTATTATTTAATGTTGTAGCTAGTTACATTAGTAAATATTCATATGTAGCATGTTTAAGCATTAA
ATCAAATAAGATGAG
+
:FFFFFFFFFFFFFFFFFFFFFFFFFFFFFFFFFFFFFFFFFFFFFFFFFFFFFFFFFFFFFFFFFFF
FFFFFFFFFFFFFFFFFFFFFFFFFFFFFFFFFFFFFFFFFFFFFFFFFFFFFFFF:FFFFFFFFFFF
FFFFFFFFFFFFFFF
@A00155:342:HHGFNDSXY:1:2114:9679:24518 1:N:0:GAACCTAG+TCCGCATA
CTGGTGTGTATCTTTCACCATTTGCTTACGTGAATCACTCACACCCTGGTTGTAAAACTCTGGAGAAC
CACCTATTATTTAATGTTGTAGCTAGTTACATTAGTAAATATTCATATGTAGCATGTTTAAGCATTAA
ATCAAATAAGACGAG
+
FFFFFFFFFFFFFFFFFFFFF:FFFFFFFFFFFFFFFFFFFFFFFFFF:FFFFFFFFFFFFFFFFFFF
FFFFFFFFFFFFF,FFFFFFFFFFFFFFFFFFFFFFFFFFFFFFFFFFFFFFFFFFFFFFFFFFFFFF
FFFFFFFFFFFFFFF
@A00155:342:HHGFNDSXY:1:2160:29505:30389 1:N:0:GAACCTAG+TCCGCATA
CTGGTGTGTATCTTTCACCATTTGCTTACGTGAATCACTCACACCCTGGTTGTAAAACTCTGGAGAAC
CACCTATTATTTAATGTTGTAGCTAGTTACATTAGTAAATATTCATATGTAGCATGTTTAAGCATTAA
ATCAAATAAGATGAG
+
FFFFFFFFFFFFFFFFFFFFFFFFFFFFFF:FFFFFFFFFFFFFFFFFF,FFFFFFFFFFFFFF:FFF
FFFFFFFFFFFFFFFFFFFFFFFFFFFFFFFFFFFFFFFFFFFFFFFFFFFFFFFFFFFFFFFFFFFF
FFFFFFFFFFFFFFF
@A00155:342:HHGFNDSXY:1:2165:30807:25911 1:N:0:GAACCTAG+TCCGCATA
CTGGTGTGTATCTTTCACCATTTGCTTACGTGAATCACTCACACCCTGGTTGTAAAACTCTGGAGAAC
CACCTATTATTTAATGTTGTAGCTAGTTACATTAGTAAATATTCATATGTAGCATGTTTAAGCATTAA
ATCAAATAAGATGAG
+
:FFFFFFFFFFFFFFFFFFFFFFFFFFFFF,FFFFFFFFFFFFFFFFFFFFFFFFFFFFFFFFFFFFF
FFFFFFFFFFFFFFFFFFFFFFFFFFFFFFFFFFFFFFFFFFFFFFFFFFFFFFFF:FFFFFFFFFFF
FFFFFFFFFFFFFFF
@A00155:342:HHGFNDSXY:1:2322:15845:4601 1:N:0:GAACCTAG+TCCGCATA
CTGGTGTGTATCTTTCACCATTTGCTTACGTGAATCACTCACACCCTGGTTGTAAAACTCTGGAGAAC
CACCTATTATTTAATGTTGTAGCTAGTTACATTAGTAAATATTCATATGTAGCATGTTTAAGCATTAA
ATCAAATAAGATGAG
+
FFFFFFFFFFFFFFFFFF:FFFFFF:FFFFFFFFFFFFFFFFFFFFFFFFFFFFFFFFFFFFFFFFFF
FFFFFFFFFFFFFFFFFFFFFFF:FFFFFFFFFFFFFFFFFFFFFFFFFFFFFFFFFFFFFFFFFFFF
FFFFFFFFFFFFFFF
@A00155:342:HHGFNDSXY:1:2351:23791:22780 1:N:0:GAACCTAG+TCCGCATA
CTGGTGTGTATCTTTCACCATTTGCTTACGTGAATCACTCACACCCTGGTTGTAAAACTCTGGAGAAC

CACCTATTATTTAATGTTGTAGCTAGTTACATTAGTAAATATTCATATGTAGCATGTTTAAGCATTAA
ATCAAATAAGACGAG
+
FFFFFFFFF:FFFFF,F,FF:FF:FFFFF::F:FFFFFFFFFFFF::F,FFFFFFF:,FFFFFFFFFF
:F::FFFFF:FFF:FFFF:F,F::F:FFF:FFFFFFF,FF:FFF,FFFFF:,FFFFF:FFFFF,FFFF
:F:F,FFF:FFFF:F
@A00155:342:HHGFNDSXY:1:2351:24722:23171 1:N:0:GAACCTAG+TCCGCATA
CTGGTGTGTATCTTTCACCATTTGCTTACGTGAATCACTCACACCCTGGTTGTAAAACTCTGGAGAAC
CACCTATTATTTAATGTTGTAGCTAGTTACATTAGTAAATATTCATATGTAGCATGTTTAAGCATTAA
ATCAAATAAGACGAG
+
FFFFFFFFFFFFFFFFFFFFF:FFFFFFFFFFFFFFFFFFFFFFFFFFFFFFFFFFFFFFFFFFFFFF
FFFFFFFFFFFFFFFFFFFFFFFFFFFFFFFFFFFFFFFFFFFFFFFFFFFFFFFFFFFFFFFFFFFF
FFFFFFFFFFFFFFF
@A00155:342:HHGFNDSXY:1:2340:19533:36260 1:N:0:GAACCTAG+TCCGCATA
CTGGTGTGTATCTTTCACCATTTGCTTACGTGAATCACTCACACCCTGGTTGTAAAACTCTGGAGAAC
CACCTATTATTTAATGTTGTAGCTAGTTACATTAGTAAATATTCATATGTAGCATGTTTAAGCATTAA
ATCAAATAAGATGAG
+
FFFFFFFFFFFFFF:FFFFFFFFFFFFFFF:FFFFFFFFFFFFFFFFFFFFFFFFFFFFFFFFFFFFF
FFFFFFFFFFFFFFFFFFFFFFFFFFFFFFFFFFFFFFFFFFFFFFFFF,FFFFFFFFFFFFFFFFFF
FFFFFFFFFFFFFFF
@A00155:342:HHGFNDSXY:1:2418:13422:25895 1:N:0:GAACCTAG+TCCGCATA
CTGGTGTGTATCTTTCACCATTTGCTTACGTGAATCACTCACACCCTGGTTGTAAAACTCTGGAGAAC
CACCTATTATTTAATGTTGTAGCTAGTTACATTAGTAAATATTCATATGTAGCATGTTTAAGCATTAA
ATCAAATAAGATGAG
+
FFFFFFFFFFFFFFFFF:FFFFFFFFFFFFFFFFFFFFFFFFFFFFFFFFFFFFFFFFFFFFFFFFFF
FFFFFFFFFFFFFFFFFF:FFFFFFFFFFFFFFFFFFFFFF:FFFFFFFFFFFFFFFFFFFFFFFFFF
FFFFFFFFFFFFFFF
@A00155:342:HHGFNDSXY:1:2418:13431:26162 1:N:0:GAACCTAG+TCCGCATA
CTGGTGTGTATCTTTCACCATTTGCTTACGTGAATCACTCACACCCTGGTTGTAAAACTCTGGAGAAC
CACCTATTATTTAATGTTGTAGCTAGTTACATTAGTAAATATTCATATGTAGCATGTTTAAGCATTAA
ATCAAATAAGATGAG
+
:FFFFFFFFFFFFFFFFFFFFFFFFFFFFFFFFFFFFFFFFFFFFFFFFFFFFFFFFFFFFFFFFF:F
FFFFFFFFFFFFFFFFFFFFFFFFFFFFFFFFFFFFFFFFFFFFFFFFFFFFFFFFFFFFFFFFFFFF
FFFFFFFFFFFFFFF
@A00155:342:HHGFNDSXY:1:2448:26585:7232 1:N:0:GAACCTAG+TCCGCATA
CTGGTGTGTATCTTTCACCATTTGCTTACGTGAATCACTCACACCCTGGTTGTAAAACTCTGGAGAAC
CACCTATTATTTAATGTTGTAGCTAGTTACATTAGTAAATATTCATATGTAGCATGTTTAAGCATTAA
ATCAAATAAGATGAG
+
FFFFFFFFFFFFFFFFFFFFFFFFFFFFFFFFFFFFFFFFFFFFFFFFFFFFFFFFFFFFFFFFFFFF
FFFFFFFFFFF:FFFFFFFFFFFFFFFFFFFFFFFFFFFFFFFFFFFFFFFFFFFFFFFFFFFFFFFF
FFFFFFFFFFFFFFF
@A00155:342:HHGFNDSXY:1:2469:32678:18286 1:N:0:GAACCTAG+TCCGCATA
ATCTTGTGTATCTTTCACCATTTGCTTACGTGAATCACTCACACCCTGGTTGTAAAACTCTGGAGAAC
CACCTATTATTTAATGTTGTAGCTAGTTACATTAGTAAATATTCATATGTAGCATGTTTAAGCATTAA
ATCAAATAAGATGAG
+
FFFFFFFFFFFFFFFFFFFFFFFFFFFFFF:FFFFFFFFFFFFFFFFFFFFFFFFFFFFFFFFF:FF:F
FFFFFFFFFFFFFFFFFFFFFFFFFFFFFFFFFFFFFFFFFFFFFFFFFFFFFFFFFFFFFFFFFFFF
FFFFFFFFFFFFFF:

@A00155:342:HHGFNDSXY:1:2521:22137:23578 1:N:0:GAACCTAG+TCCGCATA
CTGGTGTGTATCTTTCACCATTTGCTTACGTGAATCACTCACACCCTGGTTGTAAAACTCTGGAGAAC
CACCTATTATTTAATGTTGTAGCTAGTTACATTAGTAAATATTCATATGTAGCATGTTTAAGCATTAA
ATCAAATAAGATGAG
+
FFFFFFFFFFFFFFFFFFFFFFFFFFFFFFFFFFFFFFFFFFFFFFFFFFFFFFFFFFFFFFFFFFFF
FFFFFFFFFFFFFFFFFFF:FFFFFFFFFFFFFFFFFFFFFFFFFFFFFFFFFFFFFFFFFFFFFFFF
FFFFFFFFFFFFFFF
@A00155:342:HHGFNDSXY:1:2526:32262:24142 1:N:0:GAACCTAG+TCCGCATA
CTGGTGTGTATCTTTCACCATTTGCTTACGTGAATCACTCACACCCTGGTTGTAAAACTCTGGAGAAC
CACCTATTATTTAATGTTGTAGCTAGTTACATTAGTAAATATTCATATGTAGCATGTTTAAGCATTAA
ATCAAATAAGATGAG
+
FFFFFFFFFFFFFFFFFFFFFFFFFFFFFFFFFFFFFFFFFFFFFFFFFFFFFFFFFFFFFFFFFFFF
FFFFFFFFFFFFFFFFFFFFFFF:FFFFFFFFFFFFFFFFFFFFFFFFFFFFFFFFFFFFFFFFFFFF
FFFFFFFFFFFFFFF
@A00155:342:HHGFNDSXY:1:2549:26268:19930 1:N:0:GAACCTAG+TCCGCATA
CTGGTGTGTATCTTTCACCATTTGCTTACGTGAATCACTCACACCCTGGTTGTAAAACTCTGGAGAAC
CACCTATTATTTAATGTTGTAGCTAGTTACATTAGTAAATATTCATATGTAGCATGTTTAAGCATTAA
ATCAAATAAGATGAG
+
FFFFFFFFFFFFFFFFFFFFFFFFFFFFFFFFFFFFFFFFFFFFFFFFFFFFFFFFFFFFFFFFFFFF
FFFFFFFFFFFFFFFFFFFFFFFFFFFFFFFFFFFFFFFFFFFFFFFFFFFFFFFFFFFFFFFFFFFF
FFFFFFFFFFFFFFF
@A00155:342:HHGFNDSXY:1:2530:12156:30279 1:N:0:GAACCTAG+TCCGCATA
CTGGTGTGTATCTTTCACCATTTGCTTACGTGAATCACTCACACCCTGGTTGTAAAACTCTGGAGAAC
CACCTATTATTTAATGTTGTAGCTAGTTACATTAGTAAATATTCATATGTAGCATGTTTAAGCATTAA
ATCAAATAAGATGAG
+
FFFFFFFFFFFFFFFFFFFFFFFFFFFFFF:FFFFFFFFFFFFFFFFFFFFFFFFFFFFFFFFFFFFF
FFFFFF:FFFFF::FFFFFFF:FFFFF:FFFFFFFFFFFFFFFFFFFFFFFFFFFFFFFFFFFFFFFF
FFFFFFFFFFFFFFF
@A00155:342:HHGFNDSXY:1:2614:22932:30217 1:N:0:GAACCTAG+TCCGCATA
CTGGTGTGTATCTTTCACCATTTGCTTACGTGAATCACTCACACCCTGGTTGTAAAACTCTGGAGAAC
CACCTATTATTTAATGTTGTAGCTAGTTACATTAGTAAATATTCATATGTAGCATGTTTAAGCATTAA
ATCAAATAAGATGAG
+
FFFFFFFFFFFFFFFFFFFFFFFFFFFFFFFFFFFFFFFFFFFFFFFFFFFFFFFFFFFFFFFFFFFF
FFFFFFFFFFFF:FFFFFFFFFFFFFFFFFFFFFFFFFFFFFFFFFFFFFFFFFFF:FFFFFFFFFFF
FFFFFFFFFFFFFFF
@A00155:342:HHGFNDSXY:1:2620:2564:25504 1:N:0:GAACCTAG+TCCGCATA
TCTTTGTGTATCTTTCACCATTTGCTTTCGTGAATCACTCACACCCTGGTTGTAAAACTCTGGAGAAC
CACCTATTATTTAATGTTGTAGCTAGTTACATTAGTAAATATTCATATGTAGCATGTTTAAGCATTAA
ATCAAATAAGATGAG
+
FFF,FFFFF,F,FFFFF,F,FFF,FFF,:FF,:FFF:,FF,F,,:FF,,FFFFFFF,FF:F,FF,FFF
FFF:F:FFFFFF,FF:FFFFFF,FF:FF:::FF:FF:,FFFFF,FF:FFFFF:FFFFFFF:FFFFF,F
FF:F,:FFFF:FFFF
@A00155:342:HHGFNDSXY:1:2569:3920:3051 2:N:0:GAACCTAG+TCCGCATA
CTGGTGTGTATCTTTCACCATTTGCTTACGTGAATCACTCACACCCTGGTTGTAAAACTCTGGAGAAC
CACCTATTATTTAATGTTGTAGCTAGTTACATTAGTAAATATTCATATGTAGCATGTTTAAGCATTAA
ATCAAATAAGATGAG
+
FFFFFFFFFFFFFFFFFFFFFFFFFFFFFFF:FFFFFFFFFFFFFFFFFFFFFFFFFFFFFFFFFFFF

```
FFFFFFFFFFFFFFFFFFFFFFFFFFFFFFFFFFFFFFFFFFFFFFFFF,FFFFF,FFFFFFFFF,FFFFF
FFFFFFFFF:FFFF:
@A00155:342:HHGFNDSXY:1:1228:28501:3067 1:N:0:GAACCTAG+TCCGCATA
TGGTGTGTATCTTTCACCATTTGCTTACGTGAATCACTCACACCCTGGTTGTAAAACTCTGGAGAACC
ACCTATTATTTAATGTTGTAGCTAGTTACATTAGTAAATATTCATATGTAGCATGTTTAAGCATTAAA
TCAAATAAGATGAG
+
FFFFF:FFFFFFFFFF::FFFFF:FFF:F,:FFF,FFF:F,FFF:FF:FFF:,FFFFFFFFFF::FF,
:FFFFFF,FFFF,FF:,:FFFFF:FFF:FFFF:FF:,FF,FFFFF,FFF::,:FF:FFFFF:FF:FFF
F:F:FF:,FFFF,F
@A00155:342:HHGFNDSXY:1:1403:15745:7780 1:N:0:GAACCTAG+TCCGCATA
TGGTGTGTATCTTTCACCATTTGCTTACGTGAATCACTCACACCCTGGTTGTAAAACTCTGGAGAACC
ACCTCTTATTTAATGTTGTAGCTAGTTACATTAGTAAATATTCATATGTAGCATGTTTAAGCATTAAA
TCAAATAAGATGAG
+
F,FFFF:FFFFFF:FFFF,FFFFF:F,:FFF,FFFFFFF,FFFFFFF:FFFFFFFFFFFFFFFFF:,F
:F::,F:F:F:F:,FFFFFF::FFFF:FFFF,FFFFFFFFFF:FFFFF:FFF,FFFFFF:FFF:FFFF
F:FFFFFFFFF:F:
@A00155:342:HHGFNDSXY:1:1472:2564:32142 1:N:0:GAACCTAG+TCCGCATA
TGGTGTGTATCTTTCACCATTTGCTTACGTGAATCACTCACACCCTGGTTGTAAAACTCTGGAGAACC
ACCTATTATTTAATGTTGTAGCTAGTTACATTAGTAAATATTCATATGTAGCATGTTTAAGCATTAAA
TCAAATAAGATGAG
+
FFFFFFFFFF:F:FFFFFFF,FFFFFFFF,FFFFFFFFFFFFFF:FFFFFFFFFFFFFFFFFFFFFFF
FFFFFFFFF:FFFFFFFFFF,FFFFFFFFFFFFFFFFFFFFF,FFFFFF:FFFFF:FFFFFFFFFFFF
FFFFFFFFFFFFFF
@A00155:342:HHGFNDSXY:1:2264:3667:25254 1:N:0:GAACCTAG+TCCGCATA
TGGTGTGTATCTTTCACCATTTGCTTACGTGAATCACTCACACCCTGGTTGTAAAACTCTGGAGAACC
ACCTATTATTTAATGTTGTAGCTAGTTACATTAGTAAATATTCATATGTAGCATGTTTAAGCATTAAA
TCAAATAAGATGAG
+
FFFFFFFFFFFFFFFFFFFF,FFFFFFFFFFFFFFFFFFFFFFFFFFFFFFFFFFFFFFFFFFFFFFF
FF:FFFFFFFFFF:FFFFFFFFFFFFFFFFFFFFFFFFFFFFFFFFFFFFF:FFFFFFFFFFFFFFFF
FFFFFFFFFFFFFF
@A00155:342:HHGFNDSXY:1:2335:19497:11459 1:N:0:GAACCTAG+TCCGCATA
TGGTTTGTATCTTTCACCATTTGCTTACGTGAATCACTCACACCCTGGTTGTAAAACTCTGGAGAACC
ACCTATTATTTAATGTTGTAGCTAGTTACATTAGTAAATATTCATATGTAGCATGTTTAAGCATTAAA
TCAAATAAGATGAG
+
FF:F,FFFFFFFFF,::FFF,F,FFFFFFFFFFFFF:FF:FF::FF,FFFFF::,FFFFFF::FF::
,F:FFFFFFFFFFFF:FFFFFFFFFFF,FFFFFFFFFFF,FFFFFFFFFFF:FFF:FF,FFFFFFF,F
:,FFF::FFFFFFF
@A00155:342:HHGFNDSXY:1:1110:26151:33473 1:N:0:GAACCTAG+TCCGCATA
TGGTGTGTATCTTTCACCATTTGCTTACGTGAATCACTCACACCCTGGTTGTAAAACTCTGGAGAACC
ACCTATTATTTAATGTTGTAGCTAGTTACATTAGTAAATATTCATATGTAGCATGTTTAAGCATTAAA
TCAAATAAGATGAGC
+
FFFFFFFFFFFFFFFFFFFFFFFFFFFFFFFFFFFFFFFFFFFFFFFFFFFFFFFFFFFFFFFFFFFF
FFFFFFFFFFFF:FFFF::FF:FFFFFFFFFFFFFFFFFFFFFFF:FFFFFFFFFF,FFFFFFFFFFF
FFFFFFFFFFFFFFF
@A00155:342:HHGFNDSXY:1:1114:18954:28307 1:N:0:GAACCTAG+TCCGCATA
TGGTGTGTATCTTTCACCATTTGCTTACGTGAATCACTCACACCCTGGTTGTAAAACTCTGGAGAACC
ACCTATTATTTAATGTTGTAGCTAGTTACATTAGTAAATATTCATATGTAGCATGTTTAAGCATTAAA
TCAAATAAGATAAGC
```

```
+
FFFFFFFFFFFFFFFFFFFF:FFFFFFFFFFFFF:FFFFFFFFFFFFFFFFFFFFFFFFFFFFFFFFF
FFFFFFFFFFFFFFFFFFFFFFFFFFFFFFFFFFFFFFFFFFFFFFFFFFFFFFFFFFFFFFFFFF:F
FFFFFFFFFFFFFFF
@A00155:342:HHGFNDSXY:1:1116:8721:15186 1:N:0:GAACCTAG+TCCGCATA
TGGTGTGTATCTTTCACCATTTGCTTACGTGAATCACTCACACCCTGGTTGTAAAACTCTGGAGAACC
ACCTATTATTTAATGTTGTAGCTAGTTACATTAGTAAATATTCATATGTAGCATGTTTAAGCATTAAA
TCAAATAAGATGAGC
+
FFFFFFF:FFFFFFFFFFFFFFFFFFF:FFFFFFFFFFFFF,FFFFFFFFFFFFFFFFFFFFFFFFFF
FFFFFFFFF,FF:FFFFFFFFFFFFFF:FFF:FFFFFFFFFFFFF:FFFFFFFFFFFFFFFFFFFFFF
FFFFFFFFFFFFFFF
@A00155:342:HHGFNDSXY:1:1119:21703:23891 1:N:0:GAACCTAG+TCCGCATA
TGGTGTGTATCTTTCACCATTTGCTTACGTGAATCACTCACACCCTGGTTGTAAAACTCTGGAGAACC
ACCTATTATTTAATGTTGTAGCTAGTTACATTAGTAAATATTCATATGTAGCATGTTTAAGCATTAAA
TCAAATAAGATGAGC
+
FFFFFFFFFF:FFFFFFFFFFFFFFFFFF:FFFFFFFFFFFFFFFFFFFFFFFFFFFFFFFFFFFFFF
FFFFFFFFFFFFFFFFFFFFFFFFFFFFFFFFFFFFFFFFFFFFFFFF:FFFFFFFFFFFFFFFFFFF
FFFFFFFFFFFFFFF
@A00155:342:HHGFNDSXY:1:1137:7247:12571 1:N:0:GAACCTAG+TCCGCATA
TGGTGTGTATCTTTCACCATTTGCTTACGTGAATCACTCACACCCTGGTTGTAAAACTCTGGAGAACC
ACCTATTATTTAATGTTGTAGCTAGTTACATTAGTAAATATTCATATGTAGCATGTTTAAGCATTAAA
TCAAATAAGATGAGC
+
FFFFFFFFFFFFFFFFFF:FFFFFFFF:FFFFFFFFFFFFFFFFFFFFFFFFFFFFFFFFFFFFFFFF
FFFFFFFFFFFFFFFFFFFFFFFFFFFFFFF:FFFFFFFFFFFFFFFFFFFFFFFFFFFFFFFFFFFF
FFFFFFFFFFFFFFF
@A00155:342:HHGFNDSXY:1:1137:7220:12806 1:N:0:GAACCTAG+TCCGCATA
TGGTGTGTATCTTTCACCATTTGCTTACGTGAATCACTCACACCCTGGTTGTAAAACTCTGGAGAACC
ACCTATTATTTAATGTTGTAGCTAGTTACATTAGTAAATATTCATATGTAGCATGTTTAAGCATTAAA
TCAAATAAGATGAGC
+
FFFFFFFFFFFFFFFFF:FFFFFFFFFFFF,FFFFFFFFFFFFFFFFFFFFFFFFFFFFFFFFFFFFF
FF:FFFFF:FFF:FFFF:FFFFFFFFFFFFFFFFFFFFFFFFFFFFFFFFFFFFFFFFFFFFFFFFFF
FFFFFF:FFFFFFFF
@A00155:342:HHGFNDSXY:1:1160:4444:34710 1:N:0:GAACCTAG+TCCGCATA
TGTTGTGTATCTTTCACCATTTGCTTACGTGAATCACTCACACCCTGGTTGTAAAACTCTGGAGAACC
ACCTATTATATAATGTTGTAGCTAGTTACATTAGTAAATATTCATATGTAGCATGTTTAAGCATTAAA
TCAAATAAGAGGAGC
+
FF,FFF,FF,FFF:FF,FFF,FF,,FF::,,F:FFF:FFFFF,:F:F,FFFFFFFFFF:FFFFFFFF:
FFFF:FFFF,FFFFFFFFFFF:FFFFF,,FFF:FF,FFFFFFFF:,FFF,FF:F,:FFFFF,,FFFFF
FFFFFFFFFFFFFFF
@A00155:342:HHGFNDSXY:1:1207:2808:29997 1:N:0:GAACCTAG+TCCGCATA
TGGTGTGTATCTTTCACCATTTGCTTACGTGAATCACTCACACCCTGGTTGTAAAACTCTGGAGAACC
ACCTATTATTTAATGTTGTAGCTAGTTAAATTAGTAAATATTCATATGTAGCATGTTTAAGCATTAAA
TCAAATAAGATGAGC
+
FFFFFFFFFFFFFFFFFFF::FFFFF:,FF:FFFFFFFFF:FFFFFFFFFFFFFF:FFFF:F:FFFFF
FFFFFFFFFFFFFFFFFFFFF,F:FFF,:FFFFFFF:FFFFF:FF,FFFFFFFFF:FFFFFFFFFFF:
FFFFFFFFFFFFFFF
@A00155:342:HHGFNDSXY:1:1207:2871:30013 1:N:0:GAACCTAG+TCCGCATA
TGGTGTGTATCTTTCACCATTTGCTTACGTGAATCACTCACACCCTGGTTGTAAAACTCTGGAGAACC
```

ACCTATTATTTAATGTTGTAGCTAGTTACATTAGTAAATATTCATATGTAGCATGTTTAAGCATTAAA
TCAAATAAGATGAGC
+
FF:FFF,FFFFFFFFFFFFFFFF:FFFFFFFFFFFFFFFFFFFFFFFFFFFFFFFFFFFFFFFFFFFFFF
FFFFFFFFFFFFFFFFFFF:,FFF,FFF:FFFFFFFFFFFFFFFFFFFFFF:FFFFFFFFFFFFFFFFFF
FFFFFFFFFFFFFFF
@A00155:342:HHGFNDSXY:1:1208:20618:18192 1:N:0:GAACCTAG+TCCGCATA
TGGTGTGTATCTTTCACCATTTGCTTACGTGAATCACTCACACCCTGGTTGTAAAACTCTGGAGAACC
ACCTATTATTTAATGTTGTAGCTAGTTACATTAGTAAATATTCATATGTAGCATGTTTAAGCATTAAA
TCAAATAAGATGAGC
+
FFFFFFFFFFFFFFFFFFFF,FFFFFFFFFFFFFFFFFFFFFFFFFFFFFFFFFFFFFFFFFFFFFFFFF
FFFFFFFFFFFFFFFFFFFFFFFFF,FFFFFFFFFFFFFFFFFFFFFFFFF:FFFFFFFFFFFFFFFFFF
FFFFFFFFF:FFFFF
@A00155:342:HHGFNDSXY:1:1242:1741:8390 1:N:0:GAACCTAG+TCCGCATA
TGGTGTGTATCTTTCACCATTTGCTTACGTGAATCACTCACACCCTGGTTGTAAAACTCTGGAGAACC
ACCTATTATTTAATGTTGTAGCTAGTTACATTAGTAAATATTCATATGTAGCATGTTTAAGCATTAAA
TCAAATAAGATGAGC
+
FFFFFFFFFFFF::FFFFFFFFFFFFFFF,FFFFFFFFFFFFFFFFF:FFFFFFFFFFFFFFFFFFFFFF
FFFFFFFFF:FFFF:FFFFFFFFFF:FFFFFFFFFFFFFFFF:FFFFFFF:FFFFF,FFFFFFFFFFFFF
FFFFFFFFFFFFFFF
@A00155:342:HHGFNDSXY:1:1306:23692:4758 1:N:0:GAACCTAG+TCCGCATA
TGGTGTGTATCTTTCACCATTTGCTTACGTGAATCACTCACACCCTGGTTGTAAAACTCTGGAGAACC
ACCTATTATTTAATGTTGTAGCTAGTTACATTAGTAAATATTCATATGTAGCATGTTTAAGCATTAAA
TCAAATAAGATGAGC
+
FFFFFFFFFFF:FFFFFFFFFFF:FFFFFFFFFFFFFFFFFFFFFFFFFFFFFFFFFFFFFFFFFFFFFF
FFFFFFFFF:FFFFFFFF:FF:FFFFFFFFFFFFFFFFFFFFFFFFFFF:FFFFFFFFFFFFFFFFFFFF
FFFFFFFFFFFFFFF
@A00155:342:HHGFNDSXY:1:1307:12011:36229 1:N:0:GAACCTAG+TCCGCATA
TGGTGTGTATCTTTCACCATTTGCTTACGTGAATCACTCACACCCTGGTTGTAAAACTCTGGAGAACC
ACCTATTATTTAATGTTGTAGCTAGTTACATTAGTAAATATTCATATGTAGCATGTTTAAGCATTAAA
TCAAATAAGATGAGC
+
FFFFFFFFFFFFFFFFFFFFFFFFFFFFFFFFFFFFFFFFFFFFFFFFFFFFFFFFFFFFFFFFFFFFFF
FFFFFFFFFF:FFFFFFFFFFFFFFFFFFFFFFFFFFFFFFFFFFFFFFFFFFFFFFFFFFFFFFFFFFF
FFFFFFFFFFFFFFF
@A00155:342:HHGFNDSXY:1:1307:12897:37043 1:N:0:GAACCTAG+TCCGCATA
TGGTGTGTATCTTTCACCATTTGCTTACGTGAATCACTCACACCCTGGTTGTAAAACTCTGGAGAACC
ACCTATTATTTAATGTTGTAGCTAGTTACATTAGTAAATATTCATATGTAGCATGTTTAAGCATTAAA
TCAAATAAGATGAGC
+
:FFFFFFFFFFFFFFFFFFFFFFFFFFFFFFFFFFFFFFFFFFFFFFFFFFFFFFFFFFFFFFFFFFFFF
FFFFFFFFF:FFFFFFFFFFFFFFFFFFFFFFFFFFFFFFFFFFFFFFFFFFFFFFFFFFFFFFFFFFFF
FFFFFFFFFFFFFFF
@A00155:342:HHGFNDSXY:1:1346:24885:5729 1:N:0:GAACCTAG+TCCGCATA
TGGTGTGTATCTTTCACCATTTGCTTACGTGAATCACTCACACCCTGGTTGTAAAACTCTGGAGAACC
ACCTATTATTTAATGTTGTAGCTAGTTACATTAGTAAATATTCATATGTAGCATGTTTAAGCATTAAA
TCAAATAAGATGAGC
+
FFFFFFFFFFFFFFFFFFFFFFFFFFFFFFFFFFFFFFFFFFFFFF:FFFFFFFFFFFFFFFFFFFFFFF
FFFFFFFF:FFFFFFFFFFFFFFFFFFFFFFFFFFFFFFFFFFFFFFFFFFFFFFF,FFFFFFFFFFFF
FFFFFFFFFFFFFFF

@A00155:342:HHGFNDSXY:1:1348:14009:32643 1:N:0:GAACCTAG+TCCGCATA
TGGTGTGTATCTTTCACCATTTGCTTACGTGAATCACTCACACCCTGGTTGTAAAACTCTGGAGAACC
ACCTATTATTTAATGTTGTAGCTAGTTACATTAGTAAATATTCATATGTAGCATGTTTAAGCATTAAA
TCAAATAAGATGAGC
+
FFFFFFFFFFFF:FFF:FFFFFFFFFFFFFFFFFFFFFFFFFFFFFFFFFFFFFFFFFFFFFFFFFFF
FFFFFFFFFFFF:FFFFF::FFFFF,FFFFFFFFFFFFFFFFFFFFFFFFFFFFFFFFFFFFFFFFFF
FFFFFFFFFFFFFFF
@A00155:342:HHGFNDSXY:1:1339:13729:12806 1:N:0:GAACCTAG+TCCGCATA
TGGTGTGTATCTTTCACCATTTGCTTACGTGAATCACTCACACCCTGGTTGTAAAACTCTGGAGAACC
ACCTATTATTTAATGTTGTAGCTAGTTACATTAGTAAATATTCATATGTAGCATGTTTAAGCATTAAA
TCAAATAAGATGAGC
+
:FFFFFFFFF:FFFFFFF:F:FFFFFFFFF,::FF,FFFF:FF:FFF:FFFF::FFF:,FF:FFF,:,
F::FFFFFF:F::F,:FFFF:FFFFFF,:FFFFFFFFFFFFFF::FF:FFF,FFFFFFFFFFFFFFFF
FFFFFFFF,FFFFFF
@A00155:342:HHGFNDSXY:1:1374:10230:16047 1:N:0:GAACCTAG+TCCGCATA
TGGTGTGTATCTTTCACCATTTGCTTACGTGAATCACTCACACCCTGGTTGTAAAACTCTGGAGAACC
ACCTATTATTTAATGTTGTAGCTAGTTACATTAGTAAATATTCATATGTAGCATGTTTAAGCATTAAA
TCAAATAAGATGAGC
+
FFFFFFFFFFFFFFFFFFFF,FFFFFFFFFFFFFFFFFFFFFFFFFFFFFFFFFFFFFFFFFFFFFFF
FFFFFFFFFFFF:FFFFFFFFFFFFFFFFFF:FFFFFFFFFFFFFFFFFFFFFFFFFFFFFFFFFFFF
FFFFFFF:FFFFFFF
@A00155:342:HHGFNDSXY:1:1376:26431:27727 1:N:0:GAACCTAG+TCCGCATA
TGGTGTGTATCTTTCACCATTTGCTTACGTGAATCACTCACACCCTGGTTGTAAAACTCTGGAGAACC
ACCTATTATTTAATGTTGTAGCTAGTTACATTAGTAAATATTCATATGTAGCATGTTTAAGCATTAAA
TCAAATAAGATGAGC
+
FFFFFFFFFFFFFFFFFFFFFF:FFFFFFFFFFFFFFFFFFFFFFFFFFFFFFFFFF:FFFFFFFFFF
FFFFFFFFFFFFFFFFFFFFFFFFFF:FFFFFFFFFFFFFFFFFFFFFFFFFFFFFFFFFFFFFFFFF
FFFFFFFFFFFFFFF
@A00155:342:HHGFNDSXY:1:1416:15302:29810 1:N:0:GAACCTAG+TCCGCATA
TGGTGTGTATCTTTCACCATTTGCTTACGTGAATCACTCACACCCTGGTTGTAAAACTCTGGAGAACC
ACCTATTATTTAATGTTGTAGCTAGTTACATTAGTAAATATTCATATGTAGCATGTTTAAGCATTAAA
TCAAATAAGATGAGC
+
FFFFFFFFFFFFFFFFFFFFFFFFFFFFFFFFFFFFFFFFFFFFFFFFFFFFFFFFFFFFFFFFFFFF
FFFFFFFFFFFFFFFFFFFFFFFFFFFFFFFFFFFFFFFFFFFFF:FFFFFFFFF:FFFFFFFFFFFF
FFFFFFFFFFFFFFF
@A00155:342:HHGFNDSXY:1:1422:8214:12806 1:N:0:GAACCTAG+TCCGCATA
TGGTGTGTATCTTTCACCATTTGCTTACGTGAATCACTCACACCCTGGTTGTAAAACTCTGGAGAACC
ACCTATTATTTAATGTTGTAGCTAGTTACATTAGTAAATATTCATATGTAGCATGTTTAAGCATTAAA
TCAAATAAGATGAGC
+
FFFFFFFFFFFFFFFFFFFFFFFFFFFFF,FFFFFFFFFFFFFFFFFFFFFFFFFFFFFFFFFFFFFF
FFFFFFFFFFFFFFFFF::FFFFFFFFFFFFFFFFFFFFFFFFFFFFFFFFFFFFFFFFFFFFFFFFF
FFFFFFFFFFFFFFF
@A00155:342:HHGFNDSXY:1:1445:3079:5228 1:N:0:GAACCTAG+TCCGCATA
TGGTGTGTATCTTTCACCATTTGCTTACGTGAATCACTCACACCCTGGTTGTAAAACTCTGGAGAACC
ACCTATTATTTAATGTTGTAGCTAGTTACATTAGTAAATATTCATATGTAGCATGTTTAAGCATTAAA
TCAAATAAGACGAGC
+
FFFFFFFFFFFFFFFFFFFFFFFFFFFFFFFFFFFFFFFFFFFFFF:FFFFFFFFFFFFFFFFFFFFF

FFFFFFFFFFFFFFFFFFFFFFFFFFFFFFFFFFFFFFFFFFFFFFFFFFFFFFFFFFFFFFFFFFFF
FFFFFFFFFFFFFFF
@A00155:342:HHGFNDSXY:1:1441:27814:36229 1:N:0:GAACCTAG+TCCGCATA
TGGTGTGTATCTTTCACCATTTGCTTACGTGAATCACTCACACCCTGGTTGTAAAACTCTGGAGAACC
ACCTATTATTTAATGTTGTAGCTAGTTACATTAGTAAATATTCATATGTAGCATGTTTAAGCATTAAA
TCAAATAAGATGAGC
+
FFFFFFFFFFFFFFFFFFFFFFFFFFFFFFFFFFFFFFFFFFFFFFFFFFFFFFFFFFFFFFFFFFFF
FFFFFFFFFFFFFFFFF,FFF,FFF:FFFFFFFFFFFFFFFFFFF:FFFFFFFFFFFFFFFFFFFFFF
FFFFFFFFFFFFFFF
@A00155:342:HHGFNDSXY:1:1456:9173:29810 1:N:0:GAACCTAG+TCCGCATA
TGGTGTGTATCTTTCACCATTTGCTTACGTGAATCACTCACACCCTGGTTGTAAAACTCTGGAGAACC
ACCTATTATTTAATGTTGTAGCTAGTTACATTAGTAAATATTCATATGTAGCATGTTTAAGCATTAAA
TCAAATAAGATGAGC
+
FFFFFFFFFFFFF:FFFFFFFFFFFFF,FFFFFFFFFFFFFFFFFFFFFFFFFFFFFFFFFFFFFFFF
FFFFFFFFFFFFFFFFFFFFFFFFFFFFFFFFFFFFFFFFFFFFFFFFFFFFFFFFFFFFFFFFFFFF
FFFFFFFFFFFFFFF
@A00155:342:HHGFNDSXY:1:1503:21052:27618 1:N:0:GAACCTAG+TCCGCATA
TGGTGTGTATCTTTCACCATTTGCTTACGTGAATCACTCACACCCTGGTTGTAAAACTCTGGAGAACC
ACCTATTATTTAATGTTGTAGCTAGTTACATTAGTAAATATTCATATGTAGCATGTTTAAGCATTAAA
TCAAATAAGATGAGC
+
FFFFFFFFFFFFFFFFFFFF:FFFFFFFF:FFFFFFFFFFFFFFFFFFFFFFFFF:FFF:FFFFFFFF
FFFFFFF:FFFFFFFFFF:FFFFFFFFF:FFFFFFFFFFFFFFFFFFFFFFFFFF:FFFFFFFFFFFF
FFFFFFFFFFFFFFF
@A00155:342:HHGFNDSXY:1:1501:5023:6308 1:N:0:GAACCTAG+TCCGCATA
TGGTGTGTATCTTTCACCATTTGCTTACGTGAATCACTCACACCCTGGTTGTAAAACTCTGGAGAACC
ACCTATTATTTAATGTTGTAGCTAGTTACATTAGTAAATATTCATATGTAGCATGTTTAAGCATTAAA
TCAAATAAGATGAGC
+
FFFFFFFFFFFFFFFF:FFFFF:FFFFFFFFFFFFFFFFFFFFFFFFFFFFFFFFFFFFFFFFFFFFF
FFFFFFFFFFFFFFFFFFF:FFFFFFFFFFFFFFFFFFFFFFFFFFFFFFFFFFFFFFFFFFFFFFFF
FFFFFFFFFFFFFFF
@A00155:342:HHGFNDSXY:1:1509:22037:24220 1:N:0:GAACCTAG+TCCGCATA
TGGTGTGTATCTTTCACCATTTGCTTACGTGAATCACTCACACCCTGGTTGTAAAACTCTGGAGAACC
ACCTATTATTTAATGTTGTAGCTAGTTACATTAGTAAATATTCATATGTAGCATGTTTAAGCATTAAA
TCAAATAAGATGAGC
+
FFFFFFFFFFFFFFFFFFFFFFFFFFFFFFFFFFFFFFFFFFFFFFFFFFFFFFFFFFFFFFFFFFFF
FFFFFFFFFFFFFFFFFFFFFFFFF:FFFFFFFFFFFFFFFFFFFFFF:FFFFFFFFFFFFFFFFFFF
FFFFFFFFFFFFFFF
@A00155:342:HHGFNDSXY:1:1506:21079:30389 1:N:0:GAACCTAG+TCCGCATA
TGGTGTGTATCTTTCACCATTTGCTTACGTGAATCACTCACACCCTGGTTGTAAAACTCTGGAGAACC
ACCTATTATTTAATGTTGTAGCTAGTTACATTAGTAAATATTCATATGTAGCATGTTTAAGCATTAAA
TCAAATAAGATGAGC
+
FFFFFFFFFFFFFFFFF,FFFFFFFFFFFFFF:FFFFFFFFFF:FFFFFFFFFFF:FFFFFFFFFFFF
FFFFFFFFF::F,FFFFFF,FFFFFFFF,FFFFFFFFFFFFFFFFF:FFFFFFFFFFFFFFFFFFFFF
FFFFFFFFFFFFFFF
@A00155:342:HHGFNDSXY:1:1520:9055:7686 1:N:0:GAACCTAG+TCCGCATA
TGGTGTGTATCTTTCACCATTTGCTTACGTGAATCACTCACACCCTGGTTGTAAAACTCTGGAGAACC
ACCTATTATTTAATGTTGTAGCTAGTTACATTAGTAAATATTCATATGTAGCATGTTTAAGCATTAAA
TCAAATAAGATGAGC

+
FFFFFFFFFFFFFFFFFFFFFFFFFFFFFFFFFFFFFFFFFFFFFFFFFFFFFFFFFFFFFFFFFFFF
FFFFFFFFFFFFFFFFFFFFFFFFFFFFFFFFFFFFFFFFFFFFFFFFFFFFFFFFFFFFFFFFFFFF
FFFFFFFFFFFFFFF
@A00155:342:HHGFNDSXY:1:1537:27968:17581 1:N:0:GAACCTAG+TCCGCATA
TGGTGTGTATCTTTCACCATTTGCTTACGTGAATCACTCACACCCTGGTTGTAAAACTCTGGAGAACC
ACCTATTATTTAATGTTGTAGCTAGTTACATTAGTAAATATTCATATGTAGCATGTTTAAGCATTAAA
TCAAATAAGATGAGC
+
FFFFFFFFFFFFFFFFFFFFFFFFFFFFF,FFFFFFFFFFFFF:FFFFFFFFFFFFFFFFFFFFFFFF
FF:FFFFFFFFFFFFFFFFFFFFFFFFFFFF:FFFFFFFFFFFFFFFFFFFFFFFFFFFFFFFFFFFFF
:FFF:FFFFFFFFFF
@A00155:342:HHGFNDSXY:1:1541:18918:19132 1:N:0:GAACCTAG+TCCGCATA
TGGTGTGTATCTTTCACCATTTGCTTACGTGAATCACTCACACCCTGGTTGTAAAACTCTGGAGAACC
ACCTATTATTTAATGTTGTAGCTAGTTACATTAGTAAATATTCATATGTAGCATGTTTAAGCATTAAA
TCAAATAAGATGAGC
+
FF:FFFFFFFFFFFFFFFFFFFFFFFFFFFFFFFFFFFFFFFFFFFFFFFFFFFFFFFFFFFFFFFFF
FFFFFFFF::FFFFFFFFF:FFFFF:FFFF:FFFFFFFFFFFFFFFFF:FFFFFFFFFFF:FFFFFFF
FFFFF:FFFFFFFFF
@A00155:342:HHGFNDSXY:1:1570:27688:7451 1:N:0:GAACCTAG+TCCGCATA
TGGTGTGTATCTTTCACCATTTGCTTACGTGAATCACTCACACCCTGGTTGTAAAACTCTGGAGAACC
ACCTATTATTTAATGTTGTAGCTAGTTACATTAGTAAATATTCATATGTAGCATGTTTAAGCATTAAA
TCAAATAAGATGAGC
+
FFFFFFFFFFFFFFFFFFFFFFFFFFFFF:FFFFFFFFFFFFFFFFFFFFFFFFFFFFFFFFFFFFFF
FFFFFFFFFFFFFFFFFFFFFFFFFFFFFFFFFFFFFFFFFFFFFFFFFFFFFFFFFFFFFFFFFFFF
FFFFFFFFFFFFFFF
@A00155:342:HHGFNDSXY:1:1575:13946:17534 1:N:0:GAACCTAG+TCCGCATA
TGGTGTGTATCTTTCACCATTTGCTTACGTGAATCACTCACACCCTGGTTGTAAAACTCTGGAGAACC
ACCTATTATTTAATGTTGTAGCTAGTTACATTAGTAAATATTCATATGTAGCATGTTTAAGCATTAAA
TCAAATAAGATGAGC
+
FFFFFFF:FFFFFFFFFFFFFFFFFFFFFFFFFFFFFFFFFFFFFFFFFFFFFFFFFFFFFFFFFFFF
FFFFFFFFFFFFF:FFFFFFFFFFFFFFFFFFFFFFFFFFFFFFFFFFFFFFFFFFFFFFFFFFFFFF
FFFFFFFFFFFFFFF
@A00155:342:HHGFNDSXY:1:1576:24415:27085 1:N:0:GAACCTAG+TCCGCATA
TGGTGTGTATCTTTCACCATTTGCTTACGTGAATCACTCACACCCTGGTTGTAAAACTCTGGAGAACC
ACCTATTATTTAATGTTGTAGCTAGTTACATTAGTAAATATTCATATGTAGCATGTTTAAGCATTAAA
TCAAATAAGATGAGC
+
FFFFFFFFFFFFFFF:F:FFFFFFFFFFFFF:FFFFFFFFFFF:FFFF:FFFFF:FFFFFFFFFFFFF
FFFFFFFFFFF:FFFFFFF:FFFFFFFFFFFFFFFFFFF:FFFFFFFF,FFFFFFFFFF:FFFFF:FF
FFFFFF::FFFFFFF
@A00155:342:HHGFNDSXY:1:1623:2908:33270 1:N:0:GAACCTAG+TCCGCATA
TGGTGTGTATCTTTCACCATTTGCTTACGTGAATCACTCACACCCTGGTTGTAAAACTCTGGAGAACC
ACCTATTATTTAATGTTGTAGCTAGTTACATTAGTAAATATTCATATGTAGCATGTTTAAGCATTAAA
TCAAATAAGACGAGC
+
FFFFFFFFFFFFFFFFFFFFFFFFFFFFF:FFFFFFF,FFFFFFFF,:FF:FFFFFFFF:FFFFFFFF
FFFFFFFFFFFFFFFFFFFFFFFFF:FFFFFFFFFFFFFFFFFFFFFFFFFFFFFFFFFFFFFFFFFF
FFFFFFFFFFFFFFF
@A00155:342:HHGFNDSXY:1:1645:30798:15812 1:N:0:GAACCTAG+TCCGCATA
TGGTGTGTATCTTTCACCATTTGCTTACGTGAATCACTCACACCCTGGTTGTAAAACTCTGGAGAACC

ACCTATTATTTAATGTTGTAGCTAGTTACATTAGTAAATATTCATATGTAGCATGTTTAAGCATTAAA
TCAAATAAGATGAGC
+
FFFFFFFFFFFFFFFFFFFF:FFFFFFFFFFFFFFFFFFFFFFFFFFFFFFFFFFFFFFFFFFFFFF
FFFFFFFFFFFFFFFFFFFFFFFFF:FFFFFFFFFFFFFFFFFFF,FFFFFFFFFFFFFFFFFFFFFF
FFFFFFFFFFFFFFF
@A00155:342:HHGFNDSXY:1:1634:2691:18114 1:N:0:GAACCTAG+TCCGCATA
TGGTGTGTATCTTTCACCATTTGCTTACGTGATTCACTCACACCCTGGTTGTAAAACTCTGGAGAACC
ACCTATTATTTAATGTTGTAGCTAGTTACATTAGTAAATATTCATATGTAGCATGTTTAAGCATTAAA
TCAAATAAGATGGGC
+
FFFFFFFFFF:FFFFFF:FFFFFFFFF:FF:F,FFFFFFFFFFFFFFFFFFFFFFFFFFFFFFFFFFF
FFFFFFFFFFFFFFFFFFFFFFFFFFFFFFFFFFFFFFFFFFFFFFFFFFFFFFFFFFFFFFFFFFFF
FFFFFFFFFFFFFFF
@A00155:342:HHGFNDSXY:1:1672:5231:22733 1:N:0:GAACCTAG+TCCGCATA
TGGTGTGTATCTTTCACCATTTGCTTACGTGAATCACTCACACCCTGGTTGTAAAACTCTGGAGAACC
ACCTATTATTTAATGTTGTAGCTAGTTACATTAGTAAATATTCATATGTAGCATGTTTAAGCATTAAA
TCAAATAAGATGAGC
+
FFFFFFFFFFFFFFFFFFFFFFFFFFFF:FFFFFFFFF:FFFFFFF:FFFFFF:FFFFFFFFFFFFFF
FFFFFFFFFFFFFF:FFFFF,,FFF,FFFFFFF:FFFFFFFF,FFFFFFFFFFFFFFFFFFFFFFFFF
FFFFFFFFFFFFFFF
@A00155:342:HHGFNDSXY:1:2162:23818:8766 1:N:0:GAACCTAG+TCCGCATA
TGGTGTGTATCTTTCACCATTTGCTTACGTGAATCACTCACACCCTGGTTGTAAAACTCTGGAGAACC
ACCTATTATTTAATGTTGTAGCTAGTTACATTAGTAAATATTCATATGTAGCATGTTTAAGCATTAAA
TCAAATAAGATGAGC
+
FFFFFFFFFFFFFFFFFFFFFFFFFFFFFFFFFFFFFFFFF:FFFFFFFFFFFFFFFFFF:FFFFFFF
FFFFFFFF:::FFFFFFFFFFF:FFFFFFFFFFFFFFFFFFFFFFFFFFFFFFFF:FFFFFFFFFFFF
FFFFFFFFFFFFFFF
@A00155:342:HHGFNDSXY:1:2157:13015:11005 1:N:0:GAACCTAG+TCCGCATA
TGGTGTGTATCTTTCACCATTTGCTTACGTGAATCACTCACACCCTGGTTGTAAAACTCTGGAGAACC
ACCTATTATTTAATGTTGTAGCTAGTTACATTAGTAAATATTCATATGTAGCATGTTTAAGCATTAAA
TCAAATAAGATGAGC
+
FFFFFFFF:FFFFFFFFFFFFFFFFFFFFFFFFFFFFFFFFFFFFFFFFFFFFFFFFFFFFFFFFFFF
FFFFFFFFFFFFFFFFFFFFFFFFFFFFFFFFFFFFFFFFFFFFFFFF:FFF:FFFFFFFFFFFFFFF
FFFFFFFFFFFFFFF
@A00155:342:HHGFNDSXY:1:2161:10520:1517 1:N:0:GAACCTAG+TCCGCATA
TGGTGTGTATCTTTCACCATTTGCTTACGTGAATCACTCACACCCTGGTTGTAAAACTCTGGAGAACC
ACCTATTATTTAATGTTGTAGCTAGTTACATTAGTAAATATTCATATGTAGCATGTTTAAGCATTAAA
TCAAATAAGATGAGC
+
FFFFFFFFFFFFFFFFFFFF:FFFFFFFF:FFFFFFFFFFFFFFFFFFFFFFFFFFFFFFFFFFFFFF
FFFFFFFFF:F:F:FFFFFFFFFFFFFFFFFFFFFFFFFFFFFFFFFFFFFFFFF,FFFFFFFFFFFF
FFFFFFFFFFFFFFF
@A00155:342:HHGFNDSXY:1:2161:10529:1564 1:N:0:GAACCTAG+TCCGCATA
TGGTGTGTATCTTTCACCATTTGCTTACGTGAATCACTCACACCCTGGTTGTAAAACTCTGGAGAACC
ACCTATTATTTAATGTTGTAGCTAGTTACATTAGTAAATATTCATATGTAGCATGTTTAAGCATTAAA
TCAAATAAGATGAGC
+
F:FFFFFFFFFF:FFFFFFFFFFFFFFFF,FF:FFFFFFFFFFFFFFFFFFFFFFFFFFFFFFFFFFF
FFFFFFFF:FFFFFFFFFFFFFFFFFFFFFFFFFFFFFFFFFFFFFFF,FFFFFFFFFFFFFFFFFFF
FFFFFFFFFFFFFFF

```
@A00155:342:HHGFNDSXY:1:2161:23863:14356 1:N:0:GAACCTAG+TCCGCATA
TGGTGTGTATCTTTCACCATTTGCTTACGTGAATCACTCACACCCTGGTTGTAAAACTCTGGAGAACC
ACCTATTATTTAATGTTGTAGCTAGTTACATTAGTAAATATTCATATGTAGCATGTTTAAGCATTAAA
TCAAATAAGACGAGC
+
FF:FFFFFFFFFFFFFF:FFFF:FFFFFFF:FFFFFFFFFF:FFF,F,F:FF:FFFF:FFFFFF:F,FFF
F::FFFFFFFFFFFFF,:FFFFFFFFF:FFFFFFFFFFFFFFFFFFFFFFFFF:FF:FFFF:FF:FFFFF
FF,,FFFF::,FFF:
@A00155:342:HHGFNDSXY:1:2161:25825:16658 1:N:0:GAACCTAG+TCCGCATA
TGGTGTGTATCTTTCACCATTTGCTTACGTGAATCACTCACACCCTGGTTGTAAAACTCTGGAGAACC
ACCTATTATTTAATGTTGTAGCTAGTTACATTAGTAAATATTCATATGTAGCATGTTTAAGCATTAAA
TCAAATAAGACGAGC
+
F:FFFFFFFFFFFFFFFFFFFFFFFFFFFF,FFFFFFFFFFFFFFFFFFFFFFFFFFFFFFFFFFFFFFF
FFFFFFFFFFFFFFFFFFFFFFFFFFFFFFFFFFFFFFFFFFFFFFFFFFFFFFFFFFFFFFFFFFFFFF
FFFFFFFFFFFFFFF
@A00155:342:HHGFNDSXY:1:2210:8513:19993 1:N:0:GAACCTAG+TCCGCATA
TGGTGTGTATCTTTCACCATTTGCTTACGTGAATCACTCACACCCTGGTTGTAAAACTCTGGAGAACC
ACCTATTATTTAATGTTGTAGCTAGTTACATTAGTAAATATTCATATGTAGCATGTTTAAGCATTAAA
TCAAATAAGACGAGC
+
FFFFFFFFFFFFFFFFFFFFFF:FFFFFFF:FFFFFFFFFFFFFFFFFFFFFFFFFFFFFFFFFFFFFFF
FFFFFFFFFFFFFFFFFFFFFFFF:FFFFFFFFFFFFFFFFFFFFFFFFFFFFFFFFFFF:FFFFFFFFF
FFFFFFFFFFFFFFF
@A00155:342:HHGFNDSXY:1:2213:21088:21512 1:N:0:GAACCTAG+TCCGCATA
TGGTGTGTATCTTTCACCATTTGCTTACGTGAATCACTCACACCCTGGTTGTAAAACTCTGGAGAACC
ACCTATTATTTAATGTTGTAGCTAGTTACATTAGTAAATATTCATATGTAGCATGTTTAAGCATTAAA
TCAAATAAGATAAGC
+
FFFFFFFFFFFFFFFFFFFFFFFFFFFFFF:FFFFFFFFFFFFFFFFFFFFFFFFFFFFFFFFFFFFFFF
FFFFFFFFF:FFFFFFFFFFFFFFFFFFFFFFFFFFFFFFFFFFFFFFFFFFFFFFFFFFFFFFFFFFFF
FFFFFFFFFFFFFFF
@A00155:342:HHGFNDSXY:1:2251:18123:14810 1:N:0:GAACCTAG+TCCGCATA
TGGTGTGTATCTTTCACCATTTGCTTACGTGAATCACTCACACCCTGGTTGTAAAACTCTGGAGAACC
ACCTATTATTTAATGTTGTAGCTAGTTACATTAGTAAATATTCATATGTAGCATGTTTAAGCATTAAA
TCAAATAAGATGAGC
+
FFFFF:FFFFFF:FFFFFFFFFFF:FF:F:FF:FFFFFFFFFFFFFFFFFFFFFFFFFFFFFFFFFF:FF
FFFFFFFFFFFFFFFFFFFFFFFFFFFFFFFFFFFFFFFFFFFFFFFFFFFFFFFFFFFFFFFFFFFFFF
FFFFFFFFFFFFFFF
@A00155:342:HHGFNDSXY:1:2239:21079:19053 1:N:0:GAACCTAG+TCCGCATA
TGGTGTGTATCTTTCACCATTTGCTTACGTGAATCACTCACACCCTGGTTGTAAAACTCTGGAGAACC
ACCTATTATTTAATGTTGTAGCTAGTTACATTAGTAAATATTCATATGTAGCATGTTTAAGCATTAAA
TCAAATAAGATGAGC
+
FFFFFFFFFFFFFFFFFFFFFFFFFFFFFF:FFFFFFFFFFFFFFFFFFFFFFFFFFFFFFFFFFFFFFF
FFFFFFFFFFFFFFFFFFFFFFFFFFFFFFFFFFFFFFFFFFFFFFFFFFFFFFF,FFFFFFFFFFFFFF
FFFFFFFFFFFFFFF
@A00155:342:HHGFNDSXY:1:2263:7482:19993 1:N:0:GAACCTAG+TCCGCATA
TGGTGTGTATCTTTCACCATTTGCTTACGTGAATCACTCACACCCTGGTTGTAAAACTCTGGAGAACC
ACCTATTATTTAATGTTGTAGCTAGTTACATTAGTAAATATTCATATGTAGCATGTTTAAGCATTAAA
TCAAATAAGATGAGC
+
FFFFFFFFFFFFF:FF,FFFFFFF:FFFF:FFFF:FFFFFFFFFF:FFFFFFFFFFFFFFFFFFF:FFFF
```

FFFFFFFFFFF,FFFFFFFFFFFFFFFFFFFFFFFFFFFF:FFFFFFF:FFFFFFFFFFFFFFFFFFFFF
FFFFFFFFFFFFFFF
@A00155:342:HHGFNDSXY:1:2322:3170:27211 1:N:0:GAACCTAG+TCCGCATA
TGGTGTGTATCTTTCACCATTTGCTTACGTGAATCACTCACACCCTGGTTGTAAAACTCTGGATAACC
ACCTATTATTTAATGTTGTAGCTAGTTACATTAGTAAATATTCATATGTAGCATGTTTAAGCATTAAA
TCAAATAAGATGAGC
+
FFFF:F:FF:F:::,,FFFFFF:,FFFFF:,FFFF:F,,F,FF,FF:FFFFFFFFFFFFFFF:FF,FFFF
F,:FFFFF,,F::FFFF::F:FF:FFFFF,FF,,:FFFFFFFFFFFFFFF,FFFFFF,FFFFFF:F:FF:
FFFFFFF:F:FFF,:
@A00155:342:HHGFNDSXY:1:2352:16685:33959 1:N:0:GAACCTAG+TCCGCATA
TGGTGTGTATCTTTCACCATTTGCTTACGTGAATCACTCACACCCTGGTTGTAAAACTCTGGAGAACC
ACCTATTATTTAATGTTGTAGCTAGTTACATTAGTAAATATTCATATGTAGCATGTTTAAGCATTAAA
TCAAATAAGATGAGC
+
FFFFFFFFFFFFFF,F,:FFFF,FFFFFFF,FFFFFFFFFFFFFFFFFFFFFFF:FF:FFFFF:FFFF:F
FFFFFFFFFFFFFFFFF:FFF:FFFFFFFFFFFFFFFFFFFFFFFFFF,FFFFFF:FFFFFFFFFFFFFF
FFFFFFFFFFFFFFF
@A00155:342:HHGFNDSXY:1:2343:10610:35023 1:N:0:GAACCTAG+TCCGCATA
TGGTGTGTATCTTTCACCATTTGCTTACGTGAATCACTCACACCCTGGTTGTAAAACTCTGGAGAACC
ACCTATTATTTAATGTTGTAGCTAGTTACATTAGTAAATATTCATATGTAGCATGTTTAAGCATTAAA
TCAAATAAGATGAGC
+
FFFFFFFFFFFFFFFFFFFFFFFFFFFFFFFFFFFFFFFFFFFFFFFFFFFFFFFFFFFFFFFFFFFFFF
FFFFFFFFF,FFFFFFFF:FFFFFFFFFFFFFFFFFFFFFFFFFFFFFFFFFFFFFFFFFFFFFFFFFFF
FFFFFFFFFFFFFFF
@A00155:342:HHGFNDSXY:1:2374:27272:18787 1:N:0:GAACCTAG+TCCGCATA
TGGTGTGTATCTTTCACCATTTGCTTACGTGAATCACTCACACCCTGGTTGTAAAACTCTGGAGAACC
ACCTATTATTTAATGTTGTAGCTAGTTACATTAGTAAATATTCATATGTAGCATGTTTAAGCATTAAA
TCAAATAAGAGGAGC
+
FFFFFFFFFFFFFFFFFFFFFFFFFFFFFF:FFFFFFFFFFFFFFFFFFFFFFFFFFFFFFFFFFFFFFF
FFFFFFFF:FFFFFFFFFFFFFFFFFFFFFFFFFFFFFFFFFFFFFFFFFFFFFFFFFFFFFFFFFFFFF
FFFFFFFFFFFFFFF
@A00155:342:HHGFNDSXY:1:2349:29957:33489 1:N:0:GAACCTAG+TCCGCATA
TGGTGTGTATCTTTCACCATTTGCTTACGTGAATCACTCACACCCTGGTTGTAAAACTCTGGAGAACC
ACCTATTATTTAATGTTGTAGCTAGTTACATTAGTAAATATTCATATGTAGCATGTTTAAGCATTAAA
TCAAATAAGATGAGC
+
FFFFFFFFFFFFFFFFFFFFFFFFFFFFFFFFFFFFFFFFFFFFFFF:FFFFFFFFFFFFFFFFFFFFFF
FFFFFFFFFFFFFFFFFFFFFFFFFFFFFFFFFFFFFFFFFFFFFFFFFFFFFFFFFFFFFFFFFFFFFF
FFFFFFFFFFFFFFF
@A00155:342:HHGFNDSXY:1:2371:8314:24471 1:N:0:GAACCTAG+TCCGCATA
TGGTGTGTATCTTTCACCATTTGCTTACGTGAATCACTCACACCCTGGTTGTAAAACTCTGGAGAACC
ACCTATTATTTAATGTTGTAGCTAGTTACATTAGTAAATATTCATATGTAGCATGTTTAAGCATTAAA
TCAAATAAGATGAGC
+
FFFFFFFFFFFFFFFFFFFFFFFFFFFFFFFFFFFFFFFFFFFFFFFFFFFFFFFFFFFFFFFFFFFFFF
FFFFFFFFF:FFFFFFFFFFFFFFFFFFFFFFFFFFFFFFFFFFFFFFFFFFFFFF:FFFFFFFFFFFFF
FFFFFFFFFFFFFFF
@A00155:342:HHGFNDSXY:1:2423:32136:9079 1:N:0:GAACCTAG+TCCGCATA
TGGTGTGTATCTTTCACCATTTGCTTACGTGAATCACTCACACCCTGGTTGTAAAACTCTGGAGAACC
ACCTATTATTTAATGTTGTAGCTAGTTACATTAGTAAATATTCATATGTAGCATGTTTAAGCATTAAA
TCAAATAAGATGAGC

+
FFFFFFF:FF:FFFFFF,,F,FFFFFF:FF:FF:F,:::F,,FFFFFFFF:FFFF:FF::F,FF,FF,
,,FF:FFFFFFF,,:FF:F,FFFFFF::,,FF:F,F,:F,FFFFF:FF,:FF,:,,:FF:FF:F::FF
FF,:FFFFFFFF::F
@A00155:342:HHGFNDSXY:1:2537:10972:12352 1:N:0:GAACCTAG+TCCGCATA
TGGTGTGTATCTTTCACCATTTGCTTACGTGAATCACTCACACCCTGGTTGTAAAACTCTGGAGAACC
ACCTATTATTTAATGTTGTAGCTAGTTACATTAGTAAATATTCATATGTAGCATGTTTAAGCATTAAA
TCAAATAAGATGAGC
+
FFFFFFFFFFFFFFFFFFFFFFFFFFFFFFFFFFFFFFFFFFFFFFFFFFFFFFFFFFFFFFFFFFFF
FFFFFFFFF:FF:FFFFFFFFFFFFFFFFFFFFFFFFFFFFFFFFFFFFFFFFFFFFFFFFFFFFFF
FFFFFFFFFFFFFFF
@A00155:342:HHGFNDSXY:1:2571:15709:22999 1:N:0:GAACCTAG+TCCGCATA
TGGTGTGTATCTTTCACCATTTGCTTACGTGAATCACTCACACCCTGGTTGTAAAACTCTGGAGAACC
ACCTATTATTTAATGTTGTAGCTAGTTACATTAGTAAATATTCATATGTAGCATGTTTAAGCATTAAA
TCAAATAAGATGAGC
+
FFF:FFFFFFFFFFFFFFFFFFFFFFFFF,FFFFFFFFFFFFFFFFFFFFFFFFFFFFFFFFFFFFFF
FFFFFFFFFFFFFFFFFFFFFFFFFFFFFFFFFFFFFFFFFFFFFFFFFFFFFFFFFFFFFFFFFFFF
FFFFFFFFFFFFFFF
@A00155:342:HHGFNDSXY:1:2576:32949:29277 1:N:0:GAACCTAG+TCCGCATA
TGGTGTGTATCTTTCACCATTTGCTTACGTGAATCACTCACACCCTGGTTGTAAAACTCTGGAGAACC
ACCTATTATTTAATGTTGTAGCTAGTTACATTAGTAAATATTCATATGTAGCATGTTTAAGCATTAAA
TCAAATAAGATGAGC
+
FFFFFFFFFFFFF:FFFFFFFFFFFFFFFFFFFFFFFFFFFFFFFFFFFFFFFFFFFFFFFFFFFFFF
FFFFFFFF::FFFFF:FFFFFFFFFFFFFFFFFFFFFFFFFFFFFFFFFFFFFFF:FFFFFFF:FFFF
FFFFFFFFFFFFFFF
@A00155:342:HHGFNDSXY:1:2610:20708:33285 1:N:0:GAACCTAG+TCCGCATA
TGGTGTGTATCTTTCACCATTTGCTTACGTGAATCACTCACACCCTGGTTGTAAAACTCTGGAGAACC
ACCTATTATTTAATGTTGTAGCTAGTTACATTAGTAAATATTCATATGTAGCATGTTTAAGCATTAAA
TCAAATAAGATGAGC
+
FFFFFFFFFFFFFFFFFFFFFFFFFFFFF:FFFFFFFFFFFFFFFFFFFFFFFFFFFFFFFFFFFFFF
FFFFFFFFFFFFFFFFFFFFFFFFFFFFFFFFFFFFFFFFFFFFFFFFFFFFFFFFFFFFFFFFFFFF
FFFFFFFFFFFFFFF
@A00155:342:HHGFNDSXY:1:2631:19831:27946 1:N:0:GAACCTAG+TCCGCATA
ATCTGTGTATCTTTCACCATTTGCTTACGTGAATCACTCACACCCTGGTTGTAAAACTCTGGAGAACC
ACCTATTATTTAATGTTGTAGCTAGTTACATTAGTAAATATTCATATGTAGCATGTTTAAGCATTAAA
TCAAATAAGATGAGC
+
FFFFFFF:FFFFFFFFFFFF:FFFFFFFF:FFFFFFFFFFFFFFFFFFFFFFFFFFFFFFFFFFFFFF
FFFFFFFFFFFFFFFFFFFFFFFFFFFFFFFFFFFFFFFFFFFFFFFFFFFFFFFFFFFFFFFFFFFF
FFFFFFFFFFFFFFF
@A00155:342:HHGFNDSXY:1:2631:20347:27962 1:N:0:GAACCTAG+TCCGCATA
ATCTGTGTATCTTTCACCATTTGCTTACGTGAATCACTCACACCCTGGTTGTAAAACTCTGGAGAACC
ACCTATTATTTAATGTTGTAGCTAGTTACATTAGTAAATATTCATATGTAGCATGTTTAAGCATTAAA
TCAAATAAGATGAGC
+
FFFFFFFFFFFFFFFFFFFFFFFFFFFFF,FFFFFFFFFFFFFFFFFFFFFFFFFFFFFFFFFFFFFF
FFFFFFFFFFFF:FFFFFFFFFFFFFFFFFFFFFFFFFFFFFFFFFFFFFFFFFFFF:FFFFFFFFFFFF
FFFFFFFFFFFFFFF
@A00155:342:HHGFNDSXY:1:2652:31720:11303 1:N:0:GAACCTAG+TCCGCATA
TGGTGTGTATCTTTCACCATTTGCTTACGTGAATCACTCACACCCTGGTTGTAAAACTCTGGAGAACC

ACCTATTATTTAATGTTGTAGCTAGTTACATTAGTAAATATTCATATGTAGCATGTTTAAGCATTAAA
TCAAATAAGATGAGC
+
FFFFFFFFFFFFFFFFFFFFFFFFFFFFFFFFFFFFFFFFFFFFF:FFFFFFFFFFFFFFFFFFFFFF
FFFFFFFFFFFFFFFFFFFFFFFFFFFFFFFFFFFFFFFFFFFFFFFFFFFFFFF:FFFFFFFFFFFF
FFFFFFFFFFFFFFF
@A00155:342:HHGFNDSXY:1:2652:31213:11365 1:N:0:GAACCTAG+TCCGCATA
TGGTGTGTATCTTTCACCATTTGCTTACGTGAATCACTCACACCCTGGTTGTAAAACTCTGGAGAACC
ACCTATTATTTAATGTTGTAGCTAGTTACATTAGTAAATATTCATATGTAGCATGTTTAAGCATTAAA
TCAAATAAGATGAGC
+
FFF:FFFFFFFFFFFFFFFF:F:F,FFFFFFFF:FFFFFFFFFFFF::FFFFFFFFFFFFFFFFF:FF
FFFFFFFFFFFF::FFFFFFFFFFFFFFFFFF:FFFFFFFFFFFFFFF:FFFFFFFFFFFFFFFFFFF
FFFFFFFFFFFFFFF
@A00155:342:HHGFNDSXY:1:2643:23601:12868 1:N:0:GAACCTAG+TCCGCATA
TGGTGTGTATCTTTCACCATTTGCTTACGTGAATCACTCACACCCTGGTTGTAAAACTCTGGAGAACC
ACCTATTATTTAATGTTGTAGCTAGTTACATTAGTAAATATTCATATGTAGCATGTTTAAGCATTAAA
TCAAATAAGATGAGC
+
FFFFFFFFFFFFFFFFFFFFFFFFFFFFF:FFFFFFFFFFFFFFFFFFFFFFFFFFFFFFFFFFFFFF
FFFFFFFFFFFFFFFFFFFFFFFFFFFFFFFFFFFFFFFFFFFFFFFFFFFFF:FFFFFFFFFFFFFF
FFFFFFFFFFFFFFF
@A00155:342:HHGFNDSXY:1:2659:9335:30968 1:N:0:GAACCTAG+TCCGCATA
TGGTGTGTATCTTTCACCATTTGCTTACGTGAATCACTCACACCCTGGTTGTAAAACTCTGGAGAACC
ACCTATTATTTAATGTTGTAGCTAGTTACATTAGTAAATATTCATATGTAGCATGTTTAAGCATTAAA
TCAAATAAGATGAGC
+
FFFFFFFF:FFFFFFFFFFFFFFFFFFFF,FF:FFF::FFFFFFFFFFFFFFFFFFFFFFFFFFFFFF
F:FFFFFF,:FFFFFFFFFFFFFFFFFF:FFFFFFFFFFFFFFFFFFF:FFFFFFFFFFFFFFFFFFF
FFFFFFFFFFFFFFF
@A00155:342:HHGFNDSXY:1:1251:6723:13260 1:N:0:GAACCTAG+TCCGCATA
GGTGTGTATCTTTCACAATTTGCTTAAGTGAATCAATCACACCCTGGTTGTAAAACTCTGGAGAACCA
CCTATTATTTAATGTTGTAGCTAGTTACATTAGTAAATATTCATATGTAGCATGTTTAAGCATTAAAT
CAAATAAGATGAGC
+
FF:FFFF,F:FFF:F,,FF:FFFFFF,FFF::FFF,FFF,F,,,F,FFF,FFFFFFF:F,:F,FFFFF
,FF,FF,FFF:F:FFF:F:FFF,:FFFFFFFF,:FF,FFFFFFFFFF:FFF:F:FFFFF::FFFF,FF
FFF:FF:,,F:FFF
@A00155:342:HHGFNDSXY:1:1467:16749:35571 1:N:0:GAACCTAG+TCCGCATA
GGTGTGTATCTTTCACCATTTGCTTACGTGAATCACTCACACCCTGGTTGTAAAACTCTGGAGAACCA
CCTATTATTTAATGTTGTAGCTAGTTACATTAGTAAATATTCATATGTAGCATGTTTAAGCATTAAAT
CAAATAAGATGAGC
+
FFFFFFFFFFFFFFFFFFFFFFF:FFFFFFFFFFFFFFFFFFFFFFFFFFFFFFFFFFFFFFFFFFFF
FFFFFFFFFFFFFFFFFFFFFF:FFFFFFFFFFFFFFFFFFFFFFFFF:F:FFFFFFFFFFFFFFFFFFFF
FFFFFFFFFFF:FF
@A00155:342:HHGFNDSXY:1:2422:28610:13401 2:N:0:GAACCTAG+TCCGCATA
GGTGTGTATCTTTCACCATTTGCTTACGTGAATCACTCACACCCTGGTTGTAAAACTCTGGAGAACCA
CCTATTATTTAATGTTGTAGCTAGTTACATTAGTAAATATTCATATGTAGCATGTTTAAGCATTAAAT
CAAATAAGATGAGC
+
:FFFF,FFFFFFFFFFFFFFF:F:FFFF:FFFFFFFFFFFFFFFFFFFFFFFFFFFFFFFFFFFFFFF
FFFFFFFFFFFFFFFFFFFFFF:FFFFFFFFFFFFFFFFFFFFFFFFFFFFFFFFFFFFFFFFFFFFF
FFFFFFFFF:FFFF

```
@A00155:342:HHGFNDSXY:1:1111:8956:22733 1:N:0:GAACCTAG+TCCGCATA
GATCTGTATCTTTCACCATTTGCTTACGTGAATCACTCACACCCTGGTTGTAAAACTCTGGAGAACCA
CCTATTATTTAATGTTGTAGCTAGTTACATTAGTAAATATTCATATGTAGCATGTTTAAGCATTAAAT
CAAATAAGATGAGCA
+
FFFFFFFFFFFFFFFFFFF:FFFFFFFFFF:FFFFFFFFFFFFFFFFFFFFFFFFFFF:FFFFFFFFF
FFFFFFFFFFFFFFFFF,FFFFFF:FFF::,FFFFFFFFFFFFF,:F,:FF::FFFFFFFFFFFFFFF
FFFFFFFFFFFFFFF
@A00155:342:HHGFNDSXY:1:1244:28971:22357 1:N:0:GAACCTAG+TCCGCATA
GGTGTGTATCTTTCACCATTTGCTTACGTGAATCACTCACACCCTGGTTGTAAAACTCTGGAGAACCA
CCTATTATTTAATGTTGTAGCTAGTTACATTAGTAAATATTCATATGTAGCATGTTTAAGCATTAAAT
CAAATAAGATGAGCG
+
FFFFFFFFFFFFFFFF,FFFFFFFFF:F:FFFFFFFFFFFF,FFFFFFFFFFFFFFFFFFFFFFFFFF
FFFFFFFFFFFFFFFFFFFFFFFFFFF:FFFFFFFFFFFFFFFF:FFFFF:FFFFFFFFFFFFFFFFF
FFFFFFFFFFFFFFF
@A00155:342:HHGFNDSXY:1:1250:18575:3568 1:N:0:GAACCTAG+TCCGCATA
GATCTGTATCTTTCACCATTTGCTTACGTGAATCACTCACACCCTGGTTGTAAAACTCTGGAGAACCA
CCTATTATTTAATGTTGTAGCTAGTTACATTAGTAAATATTCATATGTAGCATGTTTAAGCATTAAAT
CAAATAAGATGAGCA
+
FFFFFFFFFFFFFFFFFFFFFFFFFFFFFFFFFFFFFFFFFFFF:FFFFFFFFFFFFFFFFFFFFFFF
FFFFFFF:FFFFFFFFFFFFFFFFFFFFFFFFFFFFFFFFFFFFFFFFFFFFFFFFFFFFFFFFFFFF
FFFFFFFFFFFFFFF
@A00155:342:HHGFNDSXY:1:1250:18114:5118 1:N:0:GAACCTAG+TCCGCATA
GATCTGTATCTTTCACCATTTGCTTACGTGAATCACTCACACCCTGGTTGTAAAACTCTGGAGAACCA
CCTATTATTTAATGTTGTAGCTAGTTACATTAGTAAATATTCATATGTAGCATGTTTAAGCATTAAAT
CAAATAAGATGAGCA
+
FFFFFFFFFFFFFFFFFFFFFFFFFFFFFFFFFFFFFFFFFFFFFFFFFFFFFFFFFFFFFFFFFFFF
F:FFFFFFFFFFFFFFFFFFFFFFFFFFFFFFFFFFFFFFFFFFFFFFFFFFFFFFFF:FFFFFFFFF
FFFFFFFFFFFFFFF
@A00155:342:HHGFNDSXY:1:1305:27751:35587 1:N:0:GAACCTAG+TCCGCATA
GGTGTGTATCTTTCACCATTTGCTTACGTGAATCACTCACACCCTGGTTGTAAAACTCTGGAGAACCA
CCTATTATTTAATGTTGTAGCTAGTTACATTAGTAAATATTCATATGTAGCATGTTTAAGCATTAAAT
CAAATAAGATGAGCA
+
FFFFF:FFFFFFFFFFFFFFFFFFFFFFFFFFFFFFFFFFFFFFFFFFFFFFFFFFFFFFFFFFFFFF
FFFFFFFFFFFFFFFFFFFFFFFF:F:FFFFFFFFFFFFFFFFFFFFFFFFFFFFFFFFFFFFF:FFF
FFFFFFFFFFFFFFF
@A00155:342:HHGFNDSXY:1:1310:20907:18411 1:N:0:GAACCTAG+TCCGCATA
GGTGTGTATCTTTCACCATTTGCTTACGTGAATCACTCACACCCTGGTTGTAAAACTCTGGAGAACCA
CCTATTATTTAATGTTGTAGCTAGTTACATTAGTAAATATTCATATGTAGCATGTTTAAGCATTAAAT
CAAATAAGATGAGCC
+
FFFFFFFFFFFFFFFFFFF,FFFF,FFFFFFFFFFFFFFFFFFFFFFFFFFFFFFFFFFFFFF:FFFF
FFFFFFFFFFF:FFFFFFFFFF:FFFFFFFFFFFFFFFFFFFFFFF:FFFFFF:FFFFFFFFFFFFFF
FFFFFFFFFFFFFFF
@A00155:342:HHGFNDSXY:1:1349:12364:16986 1:N:0:GAACCTAG+TCCGCATA
GGTGTGTATCTTTCACCATTTGCTTACGTGAATCACTCACACCCTGGTTGTAAAACTCTGGAGAACCA
CCTATTATTTAATGTTGTAGCTAGTTACATTAGTAAATATTCATATGTAGCATGTTTAAGCATTAAAT
CAAATAAGATGAGCA
+
FFFFFFFFFFFFFFFFFFFFFFFFFFFFFFFFFFFFFFFFFFFFFFFFFFFFFFFFFFFFFFFFFFFF
```

FFFFFFFFFFFFFFFFFFFFFFFFFFFFFFFFFFFFFFFFFFFFFFFFFFFFFFFFFFFFFFFFFFFF
FFFFFF:FFFFFF:F
@A00155:342:HHGFNDSXY:1:1349:11559:17284 1:N:0:GAACCTAG+TCCGCATA
GGTGTGTATCTTTCACCATTTGCTTACGTGAATCACTCACACCCTGGTTGTAAAACTCTGGAGAACCA
CCTATTATTTAATGTTGTAGCTAGTTACATTAGTAAATATTCATATGTAGCATGTTTAAGCATTAAAT
CAAATAAGATGAGCA
+
FFFFFFFFFFFFFFFFFFFFFFFFFFFF:FFFFFFFFFFFFFFFFFFFFFFFFFFFFFFFFFFFFFFF
FFFFFFF:FFFFFFFFFFFFFFFFFFFFFFFFFFFFFFFFFFFFFFFFFFF:FF:FFFFFFFFFFFFF
FFFFFFFFFFFFFFF
@A00155:342:HHGFNDSXY:1:1338:2808:15374 1:N:0:GAACCTAG+TCCGCATA
GGTGTGTATCTTTCACCATTTGCTTACGTGAATCACTCACACCCTGGTTGTAAAACTCTGGAGAACCA
CCTATTATTTAATGTTGTAGCTAGTTACATTAGTAAATATTCATATGTAGCATGTTTAAGCATTAAAT
CAAATAAGATGAGCA
+
FFFFFFFF:FFF:FFFFF:FFFFFFFFFFFFFFFFFFFFFFFFFF:FFFFFFFFFFFFFFFFFFFFFF
FFFFFF:FFFFFFFFFFFFFFFFFFFFFFFFFFFFFFFFFFFFFFFFFFFFFFFFFFFFFFFFFFFFF
FFFFFFFFFFFFFFF
@A00155:342:HHGFNDSXY:1:1340:18322:28244 1:N:0:GAACCTAG+TCCGCATA
GGTGTGTATCTTTCACCATTTGCTTACGTGAATCACTCACACCCTGGTTGTAAAACTCTGGAGAACCA
CCTATTATTTAATGTTGTAGCTAGTTACATTAGTAAATATTCATATGTAGCATGTTTAAGCATTAAAT
CAAATAAGATGAGCA
+
FFFFFFFFFFFFFFFFFFFFFFFFFFFFFFFFFFFFFFFFFFFFFFFFFFFFFFFFFFFFFFFFFFFF
FFFFFFFFFFFFFFFFFFFFFFFFFFFFFFFFFFFFFFFFFFFFFFFFFFFFFFFFFFFFFFFFFFFF
FFFFFFFFFFFFFFF
@A00155:342:HHGFNDSXY:1:1411:19352:23985 1:N:0:GAACCTAG+TCCGCATA
ATCTTGTATCTTTCACCATTTGCTTACGTGAATCACTCACACCCTGGTTGTAAAACTCTGGAGAACCA
CCTATTATTTAATGTTGTAGCTAGTTACATTAGTAAATATTCATATGTAGCATGTTTAAGCATTAAAT
CAAATAAGATGAGCA
+
FFFFFFFFFF:FFFFFFFFFFFFFFFFFFFFFFFFF:FFFFFFFFFFFFFFFFFFFFFFFFFFFFFFF
FFFFFFFFFFFFFFFFFFFFFFFFFFFFFF,FFFFFFFFFFFFFFFFFFFFFFF:FFFFFFFFFFFFF
FFFFFFFFFFFFFFF
@A00155:342:HHGFNDSXY:1:1468:16667:17268 1:N:0:GAACCTAG+TCCGCATA
TCTGTGTATCTTTCACCATTTGCTTACGTGAATCACTCACACCCTGGTTGTAAAACTCTGGAGAACCA
CCTATTATTTAATGTTGTAGCTAGTTACATTAGTAAATATTCATATGTAGCATGTTTAAGCATTAAAT
CAAATAAGATGAGCG
+
FFFFFFFFFFFFFFFFFFFFFFFFFFFFFFFFFFFFFFFFFFFFFFFFFFFFFFFFFFFFFFFFFFFF
FF:FFFFF:FFFFFFFFFFFFFFFFFFFFFFFFFFFFFFFFFFFFFFFFFFFFFFFFFFFFFFFFFFF
FFFFFFFFFFFFFFF
@A00155:342:HHGFNDSXY:1:1461:27615:16501 1:N:0:GAACCTAG+TCCGCATA
TCTGTGTATCTTTCACCATTTGCTTACGTGAATCACTCACACCCTGGTTGTAAAACTCTGGAGAACCA
CCTATTATTTAATGTTGTAGCTAGTTACATTAGTAAATATTCATATGTAGCATGTTTAAGCATTAAAT
CAAATAAGATGAGCA
+
FFFFFF:FFFFFFFFFFFFFFFFF:F:FFFFFFFFFFFFFFFFFFFFFFFFFFFFFFFFFFFFFFFFF
FFFFFFFF,FFFFFFFFFFFFFFFFFFFFFFFFFFFFFFFFFFFFFFFFFFFFFFFFFFFFFFFFFFF
FFFFFFFFFFFFFFF
@A00155:342:HHGFNDSXY:1:1473:32777:2832 1:N:0:GAACCTAG+TCCGCATA
GGTGTGTATCTTTCACCATTTGCTTACGTGAATCACTCACACCCTGGTTGTAAAACTCTGGAGAACCA
CCTATTATTTAATGTTGTAGCTAGTTACATTAGTAAATATTCATATGTAGCATGTTTAAGCATTAAAT
CAAATAAGATGAGCG

```
+
FFFFFFFFFFFFFFFFFFFFFFFFFFFF,FFFFFFFFFFFFFFFFFFFFFFFFFFFFFFFFFFFFFFFFFF
FFFFFFFFFFFFFFFFFFFFFFFFFFFFFFFFFFFFFFFFFFFFFFFFFFFFFFFFFFFFFFFFFFFFFFF
FFFFFFFFFFFFFFF
@A00155:342:HHGFNDSXY:1:1477:10357:18270 1:N:0:GAACCTAG+TCCGCATA
GGTGTGTATCTTTCACCATTTGCTTACGTGAATCACTCACACCCTGGTTGTAAAACTCTGGAGAACCA
CCTATTATTTAATGTTGTAGCTAGTTACATTAGTAAATATTCATATGTAGCATGTTTAAGCATTAAAT
CAAATAAGATGAGCA
+
FFFFFFFFFFFFFFFFFFFFFFFFFFFF,FFFFFFFFFFFFFFFFFFFFFFFFFFFF:FFFFFFFFFFF
FFFFFFFFFFFFFFFFFFFFFFFFFFF:FFFFFFFFFFFFF:FFFFFFFFFFFFFFFFFFFFFFFFFFF
FFFFFFFFFFFFFFF
@A00155:342:HHGFNDSXY:1:1508:1714:32769 1:N:0:GAACCTAG+TCCGCATA
GGTGTGTATCTTTCACCATTTGCTTACGTGAATCACTCACACCCTGGTTGTAAAACTCTGGAGAACCA
CCTATTATTTAATGTTGTAGCTAGTTACATTAGTAAATATTCATATGTAGCATGTTTAAGCATTAAAT
CAAATAAGATGAGCC
+
FFF:FF:FFFFFFFFFFFFFFFFFFFFF:FFFFFFFFFFFFFFFFFFFFFFFFFFFFFFFFFFFFFFFF
FFFFFFFFFFFFFFFF:FFFFFFFF:FFFFFFFFFFFFFFFFF:FFFFFFFFFFFF:FFFFF:FFFFF
FFFFFFFFFFFFFFF
@A00155:342:HHGFNDSXY:1:1530:6325:24878 1:N:0:GAACCTAG+TCCGCATA
GGTGTGTATCTTTCACCATTTGCTTACGTGAATCACTCACACCCTGGTTGTAAAACTCTGGAGAACCA
CCTATTATTTAATGTTGTAGCTAGTTACATTAGTAAATATTCATATGTAGCATGTTTAAGCATTAAAT
CAAATAAGATGAGCG
+
FFFFFFFFFFFFFFFFFFF:FFFFFFFFFFFFFFFFFFFFFFFFF:FFFFFFFFFFFFFFFFFFFFFFF
FFFFFFFF:FFFFFFFFFFF:FFFFFFFF:FF:FFFFFFFFFFF:FFFFFFF:FFFFFFFFFFFFFFF
FFFFFFFFFFFFFFF
@A00155:342:HHGFNDSXY:1:1627:20998:16845 1:N:0:GAACCTAG+TCCGCATA
GGTGTGTATCTTTCACCATTTGCTTACGTGAATCACTCACACCCTGGTTGTAAAACTCTGGAGAACCA
CCTATTATTTAATGTTGTAGCTAGTTACATTAGTAAATATTCATATGTAGCATGTTTAAGCATTAAAT
CAAATAAGATGAGCA
+
FFFFFFFFFFFFFFFFFFFFFFFFFFFFFFFFFFFFFFFFFFFFFFFFFFFFFFFFFFFFFFFFFFFFFFF
FFFFFFFFFFFFFFFFFFFFFFFFFFFFFFFFFFFFFFFFFFFFFFFFFFFFFFFFFFFFFFFFFFFFFFF
FFFFFFFFFFFFFFF
@A00155:342:HHGFNDSXY:1:1650:8422:12978 1:N:0:GAACCTAG+TCCGCATA
GGTGTGTATCTTTCACCATTTGCTTACGTGAATCACTCACACCCTGGTTGTAAAACTCTGGAGAACCA
CCTATTATTTAATGTTGTAGCTAGTTACATTAGTAAATATTCATATGTAGCATGTTTAAGCATTAAAT
CAAATAAGATGAGCT
+
FFFFFFFFFFFFFFFFFF:FFFFFFFFFFFFF:FFFFFFFFFFFFFFFFFFFFFFFFFFFFFFFFFFFFF
FFFFFFFFFFFF:FFFFFFFFFFF:FFFFFFFFFFFFFFFFFFFFFFFFFFFFFFFFFFFFFFFFFFFFF
FFFFFFFFFFFFFFF
@A00155:342:HHGFNDSXY:1:1662:17842:25911 1:N:0:GAACCTAG+TCCGCATA
GGTGTGTATCTTTCACCATTTGCTTACGTGAATCACTCACACCCTGGTTGTAAAACTCTGGAGAACCA
CCTATTATTTAATGTTGTAGCTAGTTACATTAGTAAATATTCATATGTAGCATGTTTAAGCATTAAAT
CAAATAAGATGAGCA
+
FFFFFFFFFFFFFFFFFFFFFFFFF:FFF:FFFFFFFFFFFFFFFFFFFFFFFFFFFFFFFFFFFFFFFF
FFFFFFFFFFFFFFFFFFFFFFFFF:FFFFFFFFFFFFFFFFFFFFFFFFFFFFFFFFFFFFFFFFFFFFF
FFFFFFFFFFFFFFF
@A00155:342:HHGFNDSXY:1:1670:24270:26772 1:N:0:GAACCTAG+TCCGCATA
GGTGTGTATCTTTCACCATTTGCTTACGTGAATCACTCACACCCTGGTTGTAAAACTCTGGAGAACCA
```

CCTATTATTTAATGTTGTAGCTAGTTACATTAGTAAATATTCATATGTAGCATGTTTAAGCATTAAAT
CAAATAAGATGAGCA
+
FFFFFFFFFFFFFFFFFFFFFFFFFFFFFFFFFFFFFFFFFFFFFFFFFFFFFFF:FFFFFFFFFFFFFF
FFFFFFFFFFFFFFFFFFFFFFFFFFFFFFFFFFFFFFFFFFFFFFFFFFFFFFF:FFFFFFFFFFFFFF
FFFFFFFFFFFFFFF
@A00155:342:HHGFNDSXY:1:1670:27552:28166 1:N:0:GAACCTAG+TCCGCATA
GGTGTGTATCTTTCACCATTTGCTTACGTGAATCACTCACACCCTGGTTGTAAAACTCTGGAGAACCA
CCTATTATTTAATGTTGTAGCTAGTTACATTAGTAAATATTCATATGTAGCATGTTTAAGCATTAAAT
CAAATAAGATGAGCA
+
FFFFFFFFFFFFFFFFFFFFFFFFFFFFFFFFFFFFFFFFFFFFFFFFFFFFFFFFFFFFFFFFFFFFFF
FFFFFFFFFFFFFFFFF:FFFFFFF:FFFFFFFFFFFFFFFFFFFFFFFFFFFFFFFFFFFFFFFFFFFF
FFFFFFFFFFFFFFF
@A00155:342:HHGFNDSXY:1:1670:25970:31156 1:N:0:GAACCTAG+TCCGCATA
GGTGTGTATCTTTCACCATTTGCTTACGTGAATCACTCACACCCTGGTTGTAAAACTCTGGAGAACCA
CCTATTATTTAATGTTGTAGCTAGTTACATTAGTAAATATTCATATGTAGCATGTTTAAGCATTAATT
CAAATAAGATGAGCA
+
FFFFFFFFFFFFFFFFFFFFFFFFFFFFFFFFFFFFFFFFFFFFFFFFFFFFFFFFFFFFFFFFFFFFFF
FFFFFFFFFFFFFFFFFFFFFFFFFFFFFFFFFFF:FFFFFFFFFFFFFFFFFFFFFFFFFFFFFFFF,F
FFFFFFFFFFFFFFF
@A00155:342:HHGFNDSXY:1:2111:26106:4899 1:N:0:GAACCTAG+TCCGCATA
GATCTGTATCTTTCACCATTTGCTTACGTGAATCACTCACACCCTGGTTGTAAAACTCTGGAGAACCA
CCTATTATTTAATGTTGTAGCTAGTTACATTAGTAAATATTCATATGTAGCATGTTTAAGCATTAAAT
CAAATAAGATGAGCA
+
FFFFFFFFFFFFFFFFFFFFFFFFFFFF:FFFFFFFFFFFFFFFFFFFFFFFFFFFFFFFFFFFFFFFFF
F:FFFFFF:FF:FFFFFFFFFFFF:FFFFFFFFFFFFFFFFFFFFFFFFFFFFFF:FFFFFFFFFFFFFF
FFFFFFFFFFFFFFF
@A00155:342:HHGFNDSXY:1:2153:2754:1783 1:N:0:GAACCTAG+TCCGCATA
GGTGTGTATCTTTCACCATTTGCTTACGTGAATCACTCACACCCTGGTTGTAAAACTCTGGAGAACCA
CCTATTATTTAATGTTGTAGCTAGTTACATTAGTAAATATTCATATGTAGCATGTTTAAGCATTAAAT
CAAATAAGATGAGCG
+
FFFFFFF,F,FFFFFFFFF,FFFFFFFF:FFFFFFFFFFFFFFFFFFFFFFFFFFFFFFFFFFFFFFFFF
FFFF::FF,:FFFFFFFFFFFFFFFFFFFFFFFFFFFFFFFFFFFFFFFFFFFFF:FFFFFFFFFFFFFF
FFFFFFFFFFFFFFF
@A00155:342:HHGFNDSXY:1:2164:16568:4288 1:N:0:GAACCTAG+TCCGCATA
GGTGTGTATCTTTCACCATTTGCTTACGTGAATCACTCACACCCTGGTTGTAAAACTCTGGAGAACCA
CCTATTATTTAATGTTGTAGCTAGTTACATTAGTAAATATTCATATGTAGCATGTTTAAGCATTAAAT
CAAATAAGATGAGCG
+
FFFFFFFFF:FFFFFFFFF:FFFFFFFFFFFFFFFFFFFFFFFFFFFFFFFFFFFFFFFFFFFFFFFFFF
FFFFFFFFFFFFFFFFFFFFFFFFFFFFFFFFFFFFFFFFFFFFFFFFFFFFFFFFFFFFFFFFFFFFFF
FFFFFFFFFFFFFFF
@A00155:342:HHGFNDSXY:1:2174:10447:11788 1:N:0:GAACCTAG+TCCGCATA
GGTGTGTATCTTTCACCATTTGCTTACGTGAATCACTCACACCCTGGTTGTAAAACTCTGGAGAACCA
CCTATTATTTAATGTTGTAGCTAGTTACATTAGTAAATATTCATATGTAGCATGTTTAAGCATTAAAT
CAAATAAGATGAGCA
+
FFFFFFFFFFFFFFFFFFFFFFFFFFFF:FFFFFFFFFFFFFFFFFFFFFFFFFFFFFF:FFFFFFFFFF
FFFFFFFF:FFFFFFFFFFFFFFF,FFFFFFFFFFFFFFFFFFFFFFFFFFFFFF:FFFFFFFFFFFFFF
FFFFFFFFFFFFFFF

```
@A00155:342:HHGFNDSXY:1:2216:6442:11490 1:N:0:GAACCTAG+TCCGCATA
CCGATCTATCTTTCACCATTTGCTTACGTGAATCACTCACACCCTGGTTGTAAAACTCTGGAGAACCA
CCTATTATTTAATGTTGTAGCTAGTTACATTAGTAAATATTCATATGTAGCATGTTTAAGCATTAAAT
CAAATAAGATGAGCT
+
FFFFFFFFFFFFFFFFFFFFFFFFFFFFF:FFFFFFFFFFFFF,FF:FFFFFFFFFFFFFFFFFFFFFFF
FFFFFFFFF,FFFFFFFFFFFFFFFF:FFFFFFFFFFFFFFFFFFFFFFFFFFFFFFFFFFFFFFFFFFF
FFFFFFFFFFFFFFF
@A00155:342:HHGFNDSXY:1:2257:26295:26710 1:N:0:GAACCTAG+TCCGCATA
GGTGTGTATCTTTCACCATTTGCTTACGTGAATCACTCACACCCTGGTTGTAAAACTCTGGAGAACCA
CCTATTATTTAATGTTGTAGCTAGTTACATTAGTAAATATTCATATGTAGCATGTTTAAGCATTAAAT
CAAATAAGATGAGCT
+
FFFFFFFFFFFFFFFFFFFFFFFFFFFFF,FFFFFFFFFFFFFFFFFFFFFFFFFFFFFFFFFFFFFFFF
FFFFFFFFFFFFFFFFFFFFFFFFFFFFFFFFFFFFFFFFFFFFFFFFFFFFFFFFFFFFFFFFFFFFFF
FFFFFFFFFFFFFFF
@A00155:342:HHGFNDSXY:1:2319:8838:6089 1:N:0:GAACCTAG+TCCGCATA
GGTGTGTATCTTTCACCATTTGCTTACGTGAATCACTCACACCCTGGTTGTAAAACTCTGGAGAACCA
CCTATTATTTAATGTTGTAGCTAGTTACATTAGTAAATATTCATATGTAGCATGTTTAAGCATTAAAT
CAAATAAGATGAGCC
+
FFFFFFFFFFFFFFFFFFFFFFFFFFFFFFFFFFFFFFFFFFFFFFFFFFFFFFFFFFFFFFFFFFFFFF
FFFFFFF:FFFFFFFFFFFFFFFFFFFFFFFFFFFFFFFFFFFFFFFFFFFFFFFFFFFFFFFFFFFFFF
FFFFFFFFFFFFFFF
@A00155:342:HHGFNDSXY:1:2365:23945:36573 1:N:0:GAACCTAG+TCCGCATA
CCGATCTATCTTTCACCATTTGCTTACGTGAATCACTCACACCCTGGTTGTAAAACTCTGGAGAACCA
CCTATTATTTAATGTTGTAGCTAGTTACATTAGTAAATATTCATATGTAGCATGTTTAAGCATTAAAT
CAAATAAGATGAGCT
+
:FFFFFFFFFFFFFFFFFFFFFFFFFFFF:FFFFFFFFFFFFFFFFFFFFFFFFFFFFFF:FFFFFFFFF
FFFFFF:FFFFFFFFFFFFFFFFFFFFFFFFFFFFFFFFFFFFFFFFFFFFFFFFFFFFFFFFFFFFFFF
FFFFFFFFFFFFFFF
@A00155:342:HHGFNDSXY:1:2366:9263:7983 1:N:0:GAACCTAG+TCCGCATA
TCTGTGTATCTTTCACCATTTGCTTACGTGAATCACTCACACCCTGGTTGTAAAACTCTGGAGAACCA
CCTATTATTTAATGTTGTAGCTAGTTACATTAGTAAATATTCATATGTAGCATGTTTAAGCATTAAAT
CAAATAAGATGAGCA
+
FFFFFFFFFFFFFFFF,FFFFF:FFFFFFFFFFFFFFFFFFFFFFFFFFFFFFFFFFFFFFFFFFFFFFF
FFFFFFFFFFF,FFFFFFFFFFFFFFFFFFFFFFFFFFFFFFFFFFFFFFFFFFF:FFFFFFFFFFFFFF
FFFFFFFFFFFFFFF
@A00155:342:HHGFNDSXY:1:2361:16785:17879 1:N:0:GAACCTAG+TCCGCATA
GGTGTGTATCTTTCACCATTTGCTTACGTGAATCACTCACACCCTGGTTGTAAAACTCTGGAGAACCA
CCTATTATTTAATGTTGTAGCTAGTTACATTAGTAAATATTCATATGTAGCATGTTTAAGCATTAAAT
CAAATAAGATGAGCT
+
FFFFFFFFFFFFFFFFFFFFFFFFFFFFF:FFFFFFFFFFFFFFFFFFFFFFFFFFFFFFFFFFFFFFFF
FFFFFFF:FFFFFFFFFFFFFFFFFFFFFFFFFFFFFFFFFFFFFFFFFFFFFFF,FFFFFFFFFFFFFF
FFFFFFFFFFFFFFF
@A00155:342:HHGFNDSXY:1:2361:15555:20384 1:N:0:GAACCTAG+TCCGCATA
GGTGTGTATCTTTCACCATTTGCTTACGTGAATCACTCACACCCTGGTTGTAAAACTCTGGAGAACCA
CCTATTATTTAATGTTGTAGCTAGTTACATTAGTAAATATTCATATGTAGCATGTTTAAGCATTAAAT
CAAATAAGATGAGCT
+
FFFFFFFFFFFFFFFFFFFFFFFFFFFFFFFFFFFFFFFFFFFFFFFFFFFFFFFFFFFFFFFFFFFFFF
```

```
FFFFFFF::FFFFFFFFFFFFFFFFFFFF:FFFFFFFFFFFFFFFFFFFFFFFFFFFFFFFFFFFFFFFF
FFFFFFFFFFFFFFF
@A00155:342:HHGFNDSXY:1:2417:5240:11600 1:N:0:GAACCTAG+TCCGCATA
GGTGTGTATCTTTCACCATTTGCTTACGTGAATCACTCACACCCTGGTTGTAAAACTCTGGAGAACCA
CCTATTATTTAATGTTGTAGCTAGTTACATTAGTAAATATTCATATGTAGCATGTTTAAGCATTAAAT
CAAATAAGATGAACG
+
FFFFFFFFFFFFFFFFFFFF:FFFFFFF:FFFFFFFFFFFFFFFFFFFFFFFFFFFFFFFFFFFFFFFF
F,FFFFFFFFFFFFFFFFFFFFFFFFFFFFFFFFFFFFFFFFFFFFFFFFFFFFFFFFFFFFFFFFFFF
FFFFFFFFFFFFFFF
@A00155:342:HHGFNDSXY:1:2413:6650:17362 1:N:0:GAACCTAG+TCCGCATA
GGTGTGTATCTTTCACCATTTGCTTACGTGAATCACTCACACCCTGGTTGTAAAACTCTGGAGAACCA
CCTATTATTTAATGTTGTAGCTAGTTACATTAGTAAATATTCATATGTAGCATGTTTAAGCATTAAAT
CAAATAAGATGAGCG
+
F,FFFFFFFFFFFFFFFFFFFFF:FFFF:FFFFFFFFFFFFFFFFFFFFFFFFFFFFFFFFFFFF:FFF
FFFFF:FFF,FFFFFFFFFFFFFFFFFFFFFFFFFFFFFFFFFFFFFFFFFFFFFFFFFFFFF:FFFFF
FFFFFFFFFFFFFFF
@A00155:342:HHGFNDSXY:1:2419:27877:29293 1:N:0:GAACCTAG+TCCGCATA
GGTGTGTATCTTTCACCATTTGCTTACGTGAATCACTCACACCCTGGTTGTAAAACTCTGGAGAACCA
CCTATTATTTAATGTTGTAGCTAGTTACATTAGTAAATATTCATATGTAGCATGTTTAAGCATTAAAT
CAAATAAGATGAGCT
+
FFFFFFFFFFFFFFFFFFFFFFFFFFFFFFFFFFFFFFFFFFFFFFFFFFFFFFFFFFFFFFFFFFFF
FFFFFFFFFFFFFFFFFFFFFFFFF:FFFFFFFFFFFFFFFFFFFFFFFFFFFFFFFFFFFFFFFFFFF
FFFFFFFFFFFFFFF
@A00155:342:HHGFNDSXY:1:2470:10294:14403 1:N:0:GAACCTAG+TCCGCATA
GGTGTGTATCTTTCACCATTTGCTTACGTGAATCACTCACACCCTGGTTGTAAAACTCTGGAGAACCA
CCTATTATTTAATGTTGTAGCTAGTTACATTAGTAAATATTCATATGTAGCATGTTTAAGCATTAAAT
CAAATAAGATGAGCA
+
FFFFFFFFFFFFFFFFFFFFFFFFFFFFFFFFFFFFFFFFFFFFFFFFFFFFFFFFFFFFFFFFFFFF
FFFFFFFFF:FFFFFFFFFFFFFFFFFFFFFFFFFFFFFFFFFFFFFFFFFFFFFFFFFFFFFFFFFFF
FFFFFFFFFFFFFFF
@A00155:342:HHGFNDSXY:1:2539:5674:24627 1:N:0:GAACCTAG+TCCGCATA
GGTGTGTATATTTCACCATTTGCTTACGTGAATCACTCACACCCTGGTTGTAAAACTCTGGAGAACCA
CCTATTATTGAATGTTGTATCTAGTTACATTAGTAAATATTCATATGTAGCATGTTTAAGCATTAAAT
CAAATAAGATGAGCT
+
:,FFF,FFF,:FFFF,:FFFF:FFFFF:FFFFFFFFFF:::FF:F,,FFFFFFFFFFFF,FFFFF::F
F:F:FFFF,,FFFFFFFFF,FFFFFFF:FFFFFFF,FFF:FFFFFFF,FFFF:F,F:FFFF:FFFFFF
FFFFFFFF,FFFF,F
@A00155:342:HHGFNDSXY:1:2616:18186:33708 1:N:0:GAACCTAG+TCCGCATA
GGTGTGTATCTTTCACCATTTGCTTACGTGAATCACTCACACCCTGGTTGTAAAACTCTGGAGAACCA
CCTATTATTTAATGTTGTAGCTAGTTACATTAGTAAATATTCATATGTAGCATGTTTAAGCATTAAAT
CAAATAAGATGAGCG
+
:FFFFFFFFFFFFFFFFFFFFFFFFFFFF,FFFFFFFFFFFFFFFFFFFFFFFFFFFFFFFFFFFFFFF
FFFFFFFFFFFFFFFFFF:FFFFFFFFFFFFFFFFFFFFFFFFFFFFFFFFFFFFFFFFFFFFFFFFFF
FFFFFFFFFFFFFFF
@A00155:342:HHGFNDSXY:1:2629:18349:24283 1:N:0:GAACCTAG+TCCGCATA
GGTGTGTATCTTTCACCATTTGCTTACGAGAATCACTCACACCCTGGTTGTAAAACTCTGGAGAACCA
CCTATTATTTAATGTTGTAGCTAGTTACATTAGTAAATATTCATATGTAGCATGTTTAAGCATTAAAT
CAAATAAGATGAGCC
```

```
+
FFFFFFFFF:FFFFFFFF,FFFFFFFFF,FFFFFFFFFFFFFFFFFFFFFFFFFFFF:FFFFFFFFFF
FFFFFFF::FFFFFFFF:FFFFFFFFFFFFFFFFFFFF:FFF:FFFF:FFFFFFFF:FFFFF,FFFFF
FFFFFFFFFFFF,FF
@A00155:342:HHGFNDSXY:1:2619:28610:23202 1:N:0:GAACCTAG+TCCGCATA
GGTGTGTATCTTTCACCATTTGCTTACGTGAATCACTCACACCCTGGTTGTAAAACTCTGGAGAACCA
CCTATTATTTAATGTTGTAGCTAGTTACATTAGTAAATATTCATATGTAGCATGTTTAAGCATTAAAT
CAAATAAGATGAGCA
+
FFFFF:FFFFFFFFFFFFFFFFFFFFFFFFFFFFFFFFFFFFFFFFFFFFFFFFFFFFFFFFFFFFFF
FFFFFFFFFFF:FFFFFFFFFFFFFFFFFFFFFFFFFFFFFFFFFFFFFF:FF:FFFFFFFFFFFFFFF
FFFFFFFFFFFFFFF
@A00155:342:HHGFNDSXY:1:1216:32425:25770 2:N:0:GAACCTAG+TCCGCATA
GGTGTGTATCTTTCACCATTTGCTTACGTGAATCACTCACACCCTGGTTGTAAAACTCTGGAGAACCA
CCTATTATTTAATGTTGTAGCTAGTTACATTAGTAAATATTCATATGTAGCATGTTTAAGCATTAAAT
CAAATAAGATGAGCA
+
FF:F:FF:FFFFFFFFFFFFFFFFFFFFFFFFFFFFFFFFFFFFFFFFFFFFFFFFFFFFFFFFFFFF
FFFFFFFFFFFFFFFFFFFFFFFFFFFFFFFFFFFFFFFFFFFFFFFFFFFFFFFFFFFFFFF:FFFF
FFFFFFFFFFFFFFF
@A00155:342:HHGFNDSXY:1:1473:12933:28056 2:N:0:GAACCTAG+TCCGCATA
GGTGTGTATCTTTCACCATTTGCTTACGTGAATCACTCACACCCTGGTTGTAAAACTCTGGAGAACCA
CCTATTATTTAATGTTGTAGCTAGTTACATTAGTAAATATTCATATGTAGCATGTTTAAGCATTAAAT
CAAATAAGATGAGCA
+
FFFFFFFFFFFFFFFFFFFFFFFFFFFFFFFFFFFFFFFFFFFFF:FFFFFFFFF::FFFFFFFFFFF
FFFFFFFFFFFFFFFFFFFFFFFFFFFFFFFFFFFFFF,F:FFFFFFFFFFFFFFFFFFF::FFFFFFF
:FFFF::FFFFFFFF
@A00155:342:HHGFNDSXY:1:2571:17671:16376 1:N:0:GAACCTAG+TCCGCATA
GTGTGTATCTTTCACCATTTGCTTACGTGAATCACTCACACCCTGGTTGTAAAACTCTGGAGAACCAC
CTATTATTTAATGTTGTAGCTAGTTACATTAGTAAATATTCATATGTAGCATGTTTAAGCATTAAATC
AAATAAGATGAGC
+
:F:F,FFFFF,F:FF:FFFF,:FFF::FFFFF:FF::F:F::,,:,FF,FFFFF,F:FFFF,FFF:F:
:F:FFFFFF,FFFFFFFFFFFF,FFFF:FFFFFFFFF:FFF:FFF:FFFFFFF:FFFFFFFFFFFFF:
FFFF:F:FFFF::
@A00155:342:HHGFNDSXY:1:1147:24795:17409 1:N:0:GAACCTAG+TCCGCATA
GTGTGTATCTTTCACCATTTTCTTACGTGAATCACTCACACCCTGGTTGTAAAACTCTGGAGAACCAC
CTATTATTTAATGTTGTAGCTAGTTACATTAGTAAATATTCATATGTAGCATGTTTAAGCATTAAATC
AAATAAGATGAGCC
+
FFFFFFFFFFFFF:,,FFFF,:F,:F,:FFFFFF,FFF:FF::,F:FF:FF:FFFFFFF,FFFFFFF:
FF,FF:F:FF:FFFF,F:F:FFFFFFF:FFFFFF,FFFFF:,FFFFFF:FFFF,FF,FF:FFF:FFFF
F,FF::FFF::FF:
@A00155:342:HHGFNDSXY:1:1655:5945:1579 1:N:0:GAACCTAG+TCCGCATA
GTGTGTATCTTTCACCATTTGCTTACGTGAATCACTCACACCCTGGTTGTAAAACTCTGGAGAACCAC
CTATTATTTAATGTTGTAGCTAGTTACATTAGTAAATATTCATATGTAGCATGTTTAAGCATTAAATC
AAATAAGATGAGCC
+
FFFFFFFFFFF:FFF,FF:FFFFFFFFFFFFFFFFFFFFFFFFFFFFFFF:FFFFFFFFFFFFF:FFFF
,FFFFF:FFFFFFFFFFFFFFFFFFFFFFFFFFFFFFF:,FFFFFFFFFFFFF:FFFFFFFFFFFFFF
FFFFFFFFFFFFFF
@A00155:342:HHGFNDSXY:1:1255:7355:14732 1:N:0:GAACCTAG+TCCGCATA
TGTGTATCTTTCACCATTTGCTTACGTGAATCACTCACACCCTGGTTGTAAAACTCTGGAGAACCACC
```

TATTATTTAATGTTGTAGCTAGTTACATTAGTAAATATTCATATGTAGCATGTTTAAGCATTAAATCA
AATAAGATGAG
+
FFFFFF:FFFFFFFFFFFFFFFFFF:FFFFFFFFFFFFFFFFFFFFFFFFFFFFFFFFFFFFFFFFFF
FFFFFFF:FFFFFFFFFFFFFF,FFFFFFFFFFFFFF:FFFFFFFFFFFFFFFFFFFFFFFFFFFFFF
FFFFFFFFFFF
@A00155:342:HHGFNDSXY:1:2110:14127:21762 1:N:0:GAACCTAG+TCCGCATA
TGTGTATCTTTCACCATTTGCTTACGTGAATCACTCACACCCTGGTTGTAAAACTCTGGAGAACCACC
TATTATTTAATGTTGTAGCTAGTTACATTAGTAAATATTCATATGTAGCATGTTTAAGCATTAAATCA
AATAAGATGAG
+
FFFFFFFFFFFFFFFFFFFFFFFFFFFFFFFFFFFFFFFFFFFFFFFFFFFFFFFFFFFFFFFFFFFF
FFFFFFFFFFFFFFFFFFFFFFFFFFFFFFFFFFFFFFFFFFFFFFFFFFFFFFFFFFFFFFFFFFFF
FFFFFFFFFFF
@A00155:342:HHGFNDSXY:1:2244:28429:22764 1:N:0:GAACCTAG+TCCGCATA
TGTGTATCTTTCACCATTTGCTTACGTGAATCACTCACACCCTGGTTGTAAAACTCTGGAGAACCACC
TATTATTTAATGTTGTAGCTAGTTACATTAGTAAATATTCATATGTAGCATGTTTAAGCATTAAATCA
AATAAGATGAG
+
FFFFFFFFFFFFFFFFFFFFFFFFFFFFFFFFFFFFFFFF:FFFFFFFFFFFFFFFFFFFFFFFFFFF
FFFFFFFFFFFFFFFFFFFFFFFFFFFFFFFFFFFF:FFFFFFFFFFFFFFFFFFFFFFFFFFFFFFF
FFFFFFFFFFF
@A00155:342:HHGFNDSXY:1:1452:17083:32174 2:N:0:GAACCTAG+TCCGCATA
TGTGTATCTTTCACCATTTGCTTACGTGAATCACTCACACCCTGGTTGTAAAACTCTGGAGAACCACC
TATTATTTAATGTTGTAGCTAGTTACATTAGTAAATATTCATATGTAGCATGTTTAAGCATTAAATCA
AATAAGATGAGCAAG
+
FFFFFFFFFFFFFFFFF:FFFFFFFFFFFFFFFFFFFFFFFFFFFFFFFFFFFFFFFFFF:FFFFFFF
FFFFFFFFFFFFFFFFFFFFFFFFFFFF:FFFFF,FFFFFFF:FFFFFFFFFFFFFFFFFFFF:FFFF
FF:FFFFFFFFFFFF
@A00155:342:HHGFNDSXY:1:2469:32678:18286 2:N:0:GAACCTAG+TCCGCATA
TGTGTATCTTTCACCATTTGCTTACGTGAATCACTCACACCCTGGTTGTAAAACTCTGGAGAACCACC
TATTATTTAATGTTGTAGCTAGTTACATTAGTAAATATTCATATGTAGCATGTTTAAGCATTAAATCA
AATAAGATGAGAGAT
+
FFFFFFFFFFFFFFFFFFFFFFFFFFFFFFFFFFFFFFFFFFFFFFFFFFFFFFFFFFFFFFFFFFFF
FFFFFFFFFF:FFFFFFFFFFFFFFFFFFF:FFFFFFFFFFFFFFFFFFFFFFFFFFFFFFFFFFFFF
FFFFFFFFF:FFF:F
@A00155:342:HHGFNDSXY:1:2616:16224:13401 1:N:0:GAACCTAG+TCCGCATA
GTGTATCTTTCACCATTTGCTTACGTGAATCACTCACACCCTGGTTGTAAAACTCTGGAGAACCACCT
ATTATTTAATGTTGTAGCTAGTTACATTATTAAATATTCATATGTAGCATGTTTAAGCATTAAATCAA
ATAAGATGAG
+
F:FFFFFFFFFF,,FFFFF,FFFFFFFFFFFF,F:F:F,:,F,FFFFF:FFF,:,FFFF,FF:FF:FF
FFF:FFFF,F,FFFFFF:FFF,F:,:FFF,FFFFFFFF:FFFF:F,FF:F,FFFFFFF:FF:FFFFFF
FFFFFFF,::
@A00155:342:HHGFNDSXY:1:1513:6705:27821 1:N:0:GAACCTAG+TCCGCATA
GTGTATCTTTCACCATTTGCTTACGTGAATCACTCACACCCTGGTTGTAAAACTCTGGAGAACCACCT
ATTATTTAATGTTGTAGCTAGTTACATTAGTAAATATTCATATGTAGCATGTTTAAGCATTAAATCAA
ATAAGATGAGCAAGA
+
FFFFF:FFFFFFFFFFFFF,FFFFFFFFFFFFFFFFFFFFFF:FFFFFFFFFFFFFFFFFFFFFFFFF
FFFFFFFFFF:FFFFFFFFFFFFFFFFFFFFFFFFFFFFFFFFFFF:FFFFFFFFFFFFFFFFFFFFF
FFFFFFFFFFFFFFF

@A00155:342:HHGFNDSXY:1:2615:8947:29356 1:N:0:GAACCTAG+TCCGCATA
GTGTATCTTTCACCATTTGCTTACGTGAATCACTCACACCCTGGTTGTAAAACTCTGGAGAACCACCT
ATTATTTAATGTTGTAGCTAGTTACATTAGTAAATATTCATATGTAGCATGTTTAAGCATTAAATCAA
ATAAGATGAGCAAGA
+
FFFFFFFFFFFFFFFFFFFFFFFFFF:FFFFFFFFFFFFF:FFFFFFFFFFFFFFFFFFFFFFFFFFFFF
FFFFFFFFFFFFFFFFFFFFFFFFFFFFFFFFFFFFFFFFFFFFFFFFFFFFFFFFFFFFFFFFFFFFFF
FFFFFFFFFFFFFFF
@A00155:342:HHGFNDSXY:1:2615:8892:29512 1:N:0:GAACCTAG+TCCGCATA
GTGTATCTTTCACCATTTGCTTACGTGAATCACTCACACCCTGGTTGTAAAACTCTGGAGAACCACCT
ATTATTTAATGTTGTAGCTAGTTACATTAGTAAATATTCATATGTAGCATGTTTAAGCATTAAATCAA
ATAAGATGAGCAAGA
+
FF:FFFFFFFFF:FFFFF:FF:FFFFFFFFFF:FFFFFF:FFFFFFFFFFFFFFFFF:FFFFFFFFFFFF
FFFFFFFF:FFFFFFFFFFFFFFFFFFFFFFFFFFFFFFFFFFFFFFFFFFFFFFFFFFFFFFFFFFFF
FFFFFFFFFFFFFFF
@A00155:342:HHGFNDSXY:1:2667:31177:15718 1:N:0:GAACCTAG+TCCGCATA
GTGTATCTTTCACCATTTGCTTACGTGAATCACTCACACCCTGGTTGTAAAACTCTGGAGAACCACCT
ATTATTTAATGTTGTAGCTAGTTACATTAGTAAATATTCATATGTAGCATGTTTAAGCATTAAATCAA
ATAAGATGAGCAAGA
+
FFFFFFFFFFFFFFFFFFFFFFFFFFFFFFFFFFFFFFFFFFFFFFFFFFFFFFFFFFFFFFFFFFFFFF
FFFFF,FFFFFFFFFFFFFFFFFFFFFFFFFFFFFFFFFFFFFFFFFFFFFFFFFFFFFFFFFFFFFFFF
FFFFFFFFFFFFFFF
@A00155:342:HHGFNDSXY:1:2174:25265:1063 1:N:0:GAACCTAG+TCCGCATA
TGTATCTTTCACCATTTGCTTACGTGAATCACTCACACCCTGGTTGTAAAACTCTGGAGAACCACCTA
TTATTTAATGTTGTAGCTAGTTACATTAGTAAATATTCATATGTAGCATGTTTAAGCATTAAATCAAA
TAAGATGAGCA
+
FFFFFFF:FFFFFFFFFFFFFFFFF,FFFFFFFFFFFFFFFFFFFFFFFFFFFFFFFFFFFFFFFFFFFF
FFF:FFFFFFFFFFFFFFFFFFFFFFFFFFFFFFFFFFFFFFFFFFFFFFFFFFFFFFFFFFFFFFFFFF
FFFFFFFFFFF
@A00155:342:HHGFNDSXY:1:2468:9616:11224 1:N:0:GAACCTAG+TCCGCATA
TGTATCTTTCACCATTTGCTTACGTGAATCACTCACACCCTGGTTGTAAAACTCTGGAGAACCACCTA
TTATTTTATGTTGTAGCTAGTTACATTAGTAAATATTCATATGTAGCATGTTTAAGCATTAAATCAAA
TAAGATGAGCA
+
FFFFFFFFFFFFFFFFFFFFFFFFF:FFFFFFFFFFFFFFFFFFFFFFFFFFFFFFFFFFFFFFFFFFFF
FFFFFF,FFFFFFFFFFFFFFFFFFF:FFFFFFFFFFFFFFFFFFFFFFFFFFFFFFFFFFFFFFFFFFF
FFFFFFFFFFF
@A00155:342:HHGFNDSXY:1:2508:29577:19022 1:N:0:GAACCTAG+TCCGCATA
TGTATCTTTCACCATTTGCTTACGTGAATCACTCACACCCTGGTTGTAAAACTCTGGAGAACCACCTA
TTATTTAATGTTGTAGCTAGTTACATTAGTAAATATTCATATGTAGCATGTTTAAGCATTAAATCAAA
TAAGATGAGCA
+
FFFFFFFFFFFFFFFFFFFFFFFFFFFFFFFFFFFFFFFFFFFFFFFFFFFFFFFFFFFFFFFFFFFFFF
FFFFFFFFFFFFFFFFFFFFFFFFFFFFFFFFFFFFFFFFFFFFFFFFFFFFFFFFFFFFFFFFFFFFFF
FFFFFFFFFFF
@A00155:342:HHGFNDSXY:1:1411:19352:23985 2:N:0:GAACCTAG+TCCGCATA
TGTATCTTTCACCATTTGCTTACGTGAATCACTCACACCCTGGTTGTAAAACTCTGGAGAACCACCTA
TTATTTAATGTTGTAGCTAGTTACATTAGTAAATATTCATATGTAGCATGTTTAAGCATTAAATCAAA
TAAGATGAGCAAGAT
+
FFFFFFFFFFFFF:FFFFFFFFFFFFFFFFFFFFFFFFFFFFFFFFFFFFFFFFFFFFFFFFFFFFFFFFFF

```
FFFFFFF:FFFFF:FFFFFFFFFF::FFFFFFFFFFFFFF,FFFFFFFFFFFF:FFFFFFFFFFF:FF
FFFFFFFFF:FFFFF
@A00155:342:HHGFNDSXY:1:1477:5249:13964 2:N:0:GAACCTAG+TCCGCATA
TGTATCTTTCACCATTTGCTTACGTGAATCACTCACACCCTGGTTGTAAAACTCTGGAGAACCACCTA
TTATTTAATGTTGTAGCTAGTTACATTAGTAAATATTCATATGTAGCATGTTTAAGCATTAAATCAAA
TAAGATGAGCAAGAT
+
FFFFFFFFFFFFFFFFFFFFFFFFFFFFFFFFFFFFFFFFFFFFFFFFF:FFFFFFFFFFFFFFFFFF
FFFFFFFFFF:FFFFFFFFFFFFF::FFFFFFFFFFFFFFFFFFFFFFF:FFFFFFFFFFFF:FFFFFF
FFFFFFFFFFFFFFF
@A00155:342:HHGFNDSXY:1:1529:28420:31735 2:N:0:GAACCTAG+TCCGCATA
TGTATCTTTCACCATTTGCTTACGTGAATCACTCACACCCTGGTTGTAAAACTCTGGAGAACCACCTA
TTATTTAATGTTGTAGCTAGTTACATTAGTAAATATTCATATGTAGCATGTTTAAGCATTAAATCAAA
TAAGATGAGCAAGAT
+
FFFFFFFFFFFFFFFFFFFFFFFFFFFFFFFFFFFFFFFFFFFFFFFFFFFFFFFFFFFFFFFFFFFF
FFFFFFFFFFFFFFFFFFFFFFFFFFFFFFFFFFFFFFFFFFFFFFFFFFFFFFFFFFFFFFFFFFFF
FFFFFFFFFFFFFFF
@A00155:342:HHGFNDSXY:1:2146:19904:7905 2:N:0:GAACCTAG+TCCGCATA
TGTATCTTTCACCATTTGCTTACGTGAATCACTCACACCCTGGTTGTAAAACTCTGGAGAACCACCTA
TTATTTAATGTTGTAGCTAGTTACATTAGTAAATATTCATATGTAGCATGTTTAAGCATTAAATCAAA
TAAGATGAGCAAGAT
+
F:FFFFFFFFFFFFFFFFFFFFF:FFFFFFFFFFFFFFFFFFFFFFFFFFFFFFFFFFFFFFF,FFFFF
FFFFFFFFFFFFF,FFFFFFFFFFFFF:FFFFFFFFF:FFFF:FFFFFFFFFFFFFFFFFFFFFFFFFF
FFFFFFFFFFFFFFF
@A00155:342:HHGFNDSXY:1:2221:3224:15123 2:N:0:GAACCTAG+TCCGCATA
TGTATCTTTCACCATTTGCTTACGTGAATCACTCACACCCTGGTTGTAAAACTCTGGAGAACCACCTA
TTATTTAATGTTGTAGCTAGTTACATTAGTAAATATTCATATGTAGCATGTTTAAGCATTAAATCAAA
TAAGATGAGCAAGAT
+
FFFFFFFFFFFFFFFFFFFFFFFFFFFFFFF,FFFFFFFFFFFFFFFFF:,FFFFFFFFFFFFFF:FF
FFFFFFFFFFFFFFFF,FFFFFFFFFFFFFFF:FFFF,FFFFFFFFFFFFF:FFF,:FFFFFFFFFFF
FFFFF:::FFFFFFF
@A00155:342:HHGFNDSXY:1:2221:4833:18568 2:N:0:GAACCTAG+TCCGCATA
TGTATCTTTCACCATTTGCTTACGTGAATCACTCACACCCTGGTTGTAAAACTCTGGAGAACCACCTA
TTATTTAATGTTGTAGCTAGTTACATTAGTAAATATTCATATGTAGCATGTTTAAGCATTAAATCAAA
TAAGATGAGCAAGAT
+
FFFFFFFFFFFFF:FFFFFFFFFFFFFFFFFFFFFFFFFFFF:F,FFFFFFFFFFFF:FFFFFFFF:FFF
FFFFFFFFFFFFFFFFFFFFFFFFFF:FFFFF:FFFFFFFFFFFFFF:FFFFFFFF,FFFFFF:FFFFF
FFF:FF:FFF,,:FF
@A00155:342:HHGFNDSXY:1:2221:4842:18834 2:N:0:GAACCTAG+TCCGCATA
TGTATCTTTCACCATTTGCTTACGTGAATCACTCACAACATGGTTGTAAAACTCTGGAGAACCACCTA
TTATTTAATGTTGTATCTAGTTACAATAGTAAATATTCATATGTAGCATTTTTAAGCATTAAATCAAA
TAAGATTAGCAATAT
+
FFFFFFFFFFFFF:F::FFF,FFF::FFF:FFFFF::,:,F,F:FFFFFFFFFFFFFFF:F,:F,:FF
F,FFF:FFF,FFFFF,FFF,:FF,F,FFF,FF:FFF:FFFFF:FFF,F,:,FFFF::FFFFF,FFFFF
::FFFF,FFFFF,FF
@A00155:342:HHGFNDSXY:1:2247:1136:27665 2:N:0:GAACCTAG+TCCGCATA
TGTATCTTTCACCATTTGCTTACGTGAATCACTCACACCCTGGTTGTAAAACTCTGGAGAACCACCTA
TTATTTAATGTTGTAGCTAGTTACATTAGTAAATATTCATATGTAGCATGTTTAAGCATTAAATCAAA
TAAGATGAGCAAGAT
```

+
F:FFFFFFF:FFFFFFFF:FF:FFFF,FFFFFFFFFFFFFFF:FFFFFF,:FF:FFF:F:FF:F:FFF
FFFFF:F,FFFFFF,FFF,,FFF:FFFFFFFFFF:FF,FF:FFF:::,FFFFFF:F::FF:::F,F:F
FFF,FFF,:::FFFF
@A00155:342:HHGFNDSXY:1:2367:18747:17331 2:N:0:GAACCTAG+TCCGCATA
TGTATCTTTCACCATTTGCTTACGTGAATCACTCACACCCTGGTTGTAAAACTCTGGAGAACCACCTA
TTATTTAATGTTGTAGCTAGTTACATTAGTAAATATTCATATGTAGCATGTTTAAGCATTAAATCAAA
TAAGATGAGCAAGAT
+
FFFFFFFFFFFFFFFFFFFFFFFFFFFFFFFFFFFFFFFFFFFFFFFFFF:FFFFFFFFFFFFFFFFF
FFFFFFFFFFFFFFFFFFFFFFFFF:FFFFFFF:FFFFFFFFFFFFFFFFFFFFF:FFFFFFFFFFFFF
:FFFFFFFFFFFFFF
@A00155:342:HHGFNDSXY:1:2549:19958:28980 2:N:0:GAACCTAG+TCCGCATA
GTATCTTTCACCATTTGCTTACGTGAATCACTCACACCCTGGTTGTAAAACTCTGGAGAACCACCTAT
TATTTAATGTTGTAGCTAGTTACATTAGTAAATATTCATATGTAGCATGTTTAAGCATTAAATCAAAT
AAGATGAGCAA
+
FFFFFFFFFFFFFFFFFFFFFFFFFFFFFFFFFFFFFFFFFFFFFFFFFFFFFFFFFFFFFFFFFFFF
FFFFFFFFFFFFFFFFFFFFFFFFFFFFFFFFFFFFFFFFFFFFFFFFFFFFFFFFFFFFFFFFFFFF
FFFFFFFFFFF
@A00155:342:HHGFNDSXY:1:2620:2564:25504 2:N:0:GAACCTAG+TCCGCATA
GTATCTTTCACCATTTGCTTACGTGAATCACTCACACCCTGGTTGTAAAACTCTGGAGAACCACCTAT
TATTTAATGTTGTAGCTAGTTACATTAGTAAATATTCATATGTAGCATGTTTAAGCATTAAATCAAAT
AAGATGAGAGA
+
FFFFFFFFFFFFFFFFFFFFFF:FFFFFFFFFFFFFF,FFFFFFFFFFF:,FFFFFFFFFFFFFFFFF
:FF:FFFF:FFFFFFFF:FFFFFFFFFFFFFFFFFFFFFFFFFFFFFFFF:FFFFF,FFFFFFFFFFFFF
FFFFFFF,F,F
@A00155:342:HHGFNDSXY:1:2158:18231:19382 2:N:0:GAACCTAG+TCCGCATA
GTATATTTCACCATTTGCTTACGTGAATAACTCACACACTGGTTGTAAAACTCTGGAGAACCAACTAT
TATTAAATGTTGTAGCTAGTTACATTAGTAAATATTCATATGTAGCATGTTTAAGAATTAAATCAAAT
AACATGAGCAAGAT
+
::,F,FFFFFF,F:,F,F,FFF:,,FF,,F,FFF,FF,F,,F:F,::F,:FF:FF,:FFFFF:,FFF:
F:,F,F,F:,,:FFFF,:,F:FFFFFF,FF:FF,F,,:FF,,FF,FFFFFF,F,F,:FFFF:F:F:FF
FF,:F:F,FFF:F:
@A00155:342:HHGFNDSXY:1:1111:8956:22733 2:N:0:GAACCTAG+TCCGCATA
GTATCTTTCACCATTTGCTTACGTGAATCACTCACACCCTGGTTGTAAAACTCTGGAGAACCACCTAT
TATTTAATGTTGTAGCTAGTTACATTAGTAAATATTCATATGTAGCATGTTTAAGCATTAAATCAAAT
AAGATGAGCAAGATC
+
FFFFFFFFFFFFFFFFFFFFFFFFFFFFFFFFFFFFFFFFFFFFFFFFFFFFFFFFFFFFFFFFFFFF
FFFFFFFFFFFFFFFFFFFFFFFFFFFFFFFFFFFFFFFFFFFFFFFFFFFFFFFFFFFFFFFFFFFF
FFFFFFFFFFFFFFF
@A00155:342:HHGFNDSXY:1:1136:26060:32941 2:N:0:GAACCTAG+TCCGCATA
GTATCTTTCACCATTTGCTTACGTGAATCACTCACACCCTGTTTGTAAAACTCTGGAGAACCACCTAT
TATTTAATGTTGTAGCTAGTTACATTAGTAAATATTCATATGTAGCATGTTTAAGCATTAAATCAAAT
AAGATGAGCAAGATG
+
:F:FFFFFFFFFFFFFFFFFFFFFFFFFFFFFFFFFFFFFFFFFFFFFFFFFFFFFFFFFFFFFFFFF
FFFFFFFFFFFFFFFFFFFFFFFFFFFFFFFFFFFFFFFFFFFFFFFFFFFFFFFFFFFFFFFFFFFF
FFFFFFFFFFFFFFF
@A00155:342:HHGFNDSXY:1:1169:32805:16376 2:N:0:GAACCTAG+TCCGCATA
GTATCTTTCACCATTTGCTTACGTGAATCACTCACACCCTGGTTGTAAAACTCTGGAGAACCACCTAT

TATTTAATGTTGTAGCTAGTTACATTAGTAAATATTCATATGTAGCATGTTTAAGCATTAAATCAAAT
AAGATGAGCAAGATG
+
FFFFFFFFFFFFFFFFFF:FFFF:FFFFFFFFFFFFFFFFFFFFFFFFF,FFFFFFFFFFFFFFFFFF
FFFFFFF:FFFFFFFFFFFFFFFFFFFFFFFFFFFFFFFFFFFFFF:FFFFFFFFFFFFFFFFFFFFF
FFFFFF,FFF:,FFF
@A00155:342:HHGFNDSXY:1:1250:18575:3568 2:N:0:GAACCTAG+TCCGCATA
GTATCTTTCACCATTTGCTTACGTGAATCACTCACACCCTGGTTGTAAAACTCTGGAGAACCACCTAT
TATTTAATGTTGTAGCTAGTTACATTAGTAAATATTCATATGTAGCATGTTTAAGCATTAAATCAAAT
AAGATGAGCAAGATC
+
FFFFFFFFFFFFFFFFFFFFFFFFFFFFFFFFFFFFFFFFFFFFFFFFFFFFFFFFFFF,FFFFFFFF
FFFFFFFFFFFFFFFFFFFFF:FFFFFFFFFFFFFFFFFFFFFFFFFFFFFFFFFFFFFFF,FFFFFF
FFFFFFFFFFFFFFF
@A00155:342:HHGFNDSXY:1:1250:18114:5118 2:N:0:GAACCTAG+TCCGCATA
GTATCTTTCACCATTTGCTTACGTGAATCACTCACACCCTGGTTGTAAAACTCTGGAGAACCACCTAT
TATTTAATGTTGTAGCTAGTTACATTAGTAAATATTCATATGTAGCATGTTTAAGCATTAAATCAAAT
AAGATGAGCAAGATC
+
FFFFFFFFFFFFFFFFFFFFFFFFFFFFFFFFFFFFFFFFFFFFFFFFFFFFFFFFFFFFFFFFFFFF
FFFFFFFFFFFFFFFFFFFFFFFFFFFFFFFFFFFFFFFFFFFFFFFFFFFFFFFFFFFFFFFFFFFF
FFFFFFFFFFFFFFF
@A00155:342:HHGFNDSXY:1:1329:6298:32941 2:N:0:GAACCTAG+TCCGCATA
GTATCTTTCACCATTTGCTTACGTGAATCACTCACACCCTGGTTGTAAAACTCTGGAGAACCACCTAT
TATTTAATGTTGTAGCTAGTTACATTAGTAAATATTCATATGTAGCATGTTTAAGCATTAAATCAAAT
AAGATGAGCAAGATG
+
FFFFFFFFFFFFFFFFFFFFFFFFFFFFFFFFFFFFFFFFFFFFFFFFFFFFFFFFFFFFFF:FFFFF
FFFFFFFFFFFFFFFFFFFFFFFFFFFFFFFFFFF:FFFFFFFFFFFFFFFFFFFFFFFFFFFFFFFF
FFFFFFF:FFFFFFF
@A00155:342:HHGFNDSXY:1:1450:11125:29089 2:N:0:GAACCTAG+TCCGCATA
GTATCTTTCACCATTTGCTTACGTGAATCACTCACACCCTGGTTGTAAAACTCTGGAGAACCACCTAT
TATTTAATGTTGTAGCTAGTTACATTAGTAAATATTCATATGTAGCATGTTTAAGCATTAAATCAAAT
AAGATGAGCAAGATG
+
FFFFFFFFFFFFFFFFFFFFF:FFFFFFFFFFFFFFFFFFFFFFFFFFFFFFFFFFFFFFFFFFFFFF
FFFFFFFFFFFFFFFFFFFFFFFF:FFFFFFFFFFFFFFFFFFFFFFFFFFF:FFFFFFFFFFFFF:F
FFFFFFFFF:FFFFF
@A00155:342:HHGFNDSXY:1:1618:30987:10066 2:N:0:GAACCTAG+TCCGCATA
GTATCTTTCACCATTTGCTTACGTGAATCACTCACACCCTGGTTGTAAAACTCTGGAGAACCACCTAT
TATTTAATGTTGTAGCTAGTTACATTAGTAAATATTCATATGTAGCATGTTTAAGCATTAAATCAAAT
AAGATGAGCAAGATG
+
FFFFFFFFFFFFFFFFFFFFFFFFFFFFFFFFFFFFFFFFFFFFFFF:FFFFFFFFFFFFFFFFFFFF
FFFFFFFFFFFFFFFFFFFFFFFFFFFFFFFFFFFFFFFFFFFFFFFFFFFFF:FFFFFFFFFFFFFF
FFFFFFFFFFFFFFF
@A00155:342:HHGFNDSXY:1:1675:22896:24612 2:N:0:GAACCTAG+TCCGCATA
GTATCTTTCACCATTTGCTTACGTGAATCACTCACACCCTGGTTGTAAAACTCTGGAGAACCACCTAT
TATTTAATGTTGTAGCTAGTTACATTAGTAAATATTCATATGTAGCATGTTTAAGCATTAAATCAAAT
AAGATGAGCAAGATG
+
FFFFFFFFF:FFFFFFFFFFFFFFFFFFFFFFFFFFFFFFFFFFFFFF,FFFFFF:FFFFFFF:FFFF
FFFFFFFFFFFFFFF:FFFFFFFFFFFFFFFFFFFFFFFFFFFFFFF:FFFFFFF:FFFFF:FFFFFFF
FFF:FFFFFFFFFFF

```
@A00155:342:HHGFNDSXY:1:2111:26106:4899 2:N:0:GAACCTAG+TCCGCATA
GTATCTTTCACCATTTGCTTACGTGAATCACTCACACCCTGGTTGTAAAACTCTGGAGAACCACCTAT
TATTTAATGTTGTAGCTAGTTACATTAGTAAATATTCATATGTAGCATGTTTAAGCATTAAATCAAAT
AAGATGAGCAAGATC
+
FFFFFF:FFFFFFFFFFFFFFFFFFFFFFFFFFFFFFFFFFFFFFFFFFFFFFFFFFFF:FFFFFFFF
FFFFFFFFFFF:FFFFFFFFFFFFFFFFFFFFFFFFFFFFFFFFFFFFFFFFFFFFFFFFFFFFFFFF
FFFFFF:FFFFFFFF
@A00155:342:HHGFNDSXY:1:2154:9824:22701 2:N:0:GAACCTAG+TCCGCATA
GTATCTTTCACCATTTGCTTACGTGAATCACTCACACCCTGGTTGTAAAACTCTGGAGAACCACCTAT
TATTTAATGTTGTAGCTAGTTACATTAGTAAATATTCATATGTAGCATGTTTAAGCATTAAATCAAAT
AAGATGAGCAAGATG
+
F:FFFFFFFFFFFFFFFFFFFFFFFFFFFFFFFFFFFFFFFFFFFFFF:FFFFFFFFFFFFFFFFFFF
FFFFFFFFFFFFFFFFFFFFFFFFFFFFFFFFFFFFFFFFF,FFFFFFFFFFFFFFFFFFFFFFFFFF
FFFFFFFFFFFFFFF
@A00155:342:HHGFNDSXY:1:2232:32145:14857 2:N:0:GAACCTAG+TCCGCATA
GTATCTTTCACCATTTGCTTACGTGAATCACTCACACCCTGGTTGTAAAACTCTGGAGAACCACCTAT
TATTTAATGTTGTAGCTAGTTACATTAGTAAATATTCATATGTAGCATGTTTAAGCATTAAATCAAAT
AAGATGAGCAAGATG
+
FFFFFFFFFFFFFFFFFFFFFFFFFFFF:FFFFFFFFFFFFFFFF,FFFF:FFFFFFFFFFFFFFF:FF
FFFFFFFFFFFFFFFFFFFFFFFFFFFFFFFFFFFFFFFF:FFFFFFFFFFFFFFFFFFFFFFFFFFF
FFFFFFFFFFFFFF:
@A00155:342:HHGFNDSXY:1:2318:18249:7701 2:N:0:GAACCTAG+TCCGCATA
GTATCTTTCACCATTTGCTTACGTGAATCACTCACACCCTGGTTGTAAAACTCTGGAGAACCACCTAT
TATTTAATGTTGTAGCTAGTTACATTAGTAAATATTCATATGTAGCATGTTTAAGCATTAAATCAAAT
AAGATGAGCAAGATG
+
FFFFFFFFFFFFFFFFFFFFFFFFFFFFFFFFFFFFFFFFFFFFFFFFFFFFFFFFFFFFFFFFFFFF
FFFFFFFFFFFFFFFFFFFFFFFFFFFFFFFFFFFFFFFFFFFFFFFF:FFFFFFFFFFFFFFFFFFF
FFFFFFFFFFFFFFF
@A00155:342:HHGFNDSXY:1:2311:21585:11005 2:N:0:GAACCTAG+TCCGCATA
GTATCTTTCACCATTTGCTTACGTGAATCACTCACACCCTGGTTGTAAAACTCTGGAGAACCACCTAT
TATTTAATGTTGTAGCTAGTTACATTAGTAAATATTCATATGTAGCATGTTTAAGCATTAAATCAAAT
AAGATGAGCAAGATG
+
FFFFFFFFFFFFFFFFFFFFFFFFFFFFFFFFFFFFFFF:FFFFFFFFFFFFFFFFFFFFFFFFFFFF
FFFFFF,:FFFFFFFFFFF:FFFF:FFFFFFFFFFFFFFFFFFF:FFFFFFFFFFFFFFFFFFFFFFF
FFFFFFFFFFFFFFF
@A00155:342:HHGFNDSXY:1:2534:10212:12602 2:N:0:GAACCTAG+TCCGCATA
GTATCTTTCACCATTTGCTTACGTGAATCACTCACACCCTGGTTGTAAAACTCTGGAGAACCACCTAT
TATTTAATGTTGTAGCTAGTTACATTAGTAAATATTCATATGTAGCATGTTTAAGCATTAAATCAAAT
AAGATGAGCAAGATG
+
FFFFFFFFFFFFF:FFFFFFFFFFFFFFFFFFFFFFFFFFFFFFFFFFFFFFFFFFFFFFFFFFFFFF
FFFFFFFFFFFFFFFF:FFFFFFFFFFFFFFFFFFFFFFF:FFFFF:FFFFFFFFFFFFFF,FFFFFF
FFFFFFFFFF:F:F:
@A00155:342:HHGFNDSXY:1:2551:28203:10817 2:N:0:GAACCTAG+TCCGCATA
GTATCTTTCACCATTTGCTTACGTGAATCACTCACACCCTGGTTGTAAAACTCTGGAGAACCACCTAT
TATTTAATGTTGTAGCTAGTTACATTAGTAAATATTCATATGTAGCATGTTTAAGCATTAAATCAAAT
AAGATGAGCAAGATG
+
FFFFFFFFFFFFFFFFFFFFFFFFFFFFFFFFFFFFFFFFFFFFFFFFFFFFFFFFFFFFFFFFFF:F
```

FFFF:FFFFFFFFFFFFFFFFFFFFFFFFFFF:FFFFFFFFFFFFFFFFFFFFFFF:FFFFFF:F:FFFF
:FFFFFFF:FFFFFF
@A00155:342:HHGFNDSXY:1:2557:25283:34601 2:N:0:GAACCTAG+TCCGCATA
GTATCTTTCACCATTTGCTTACGTGAATCACTCACACCCTGGTTGTAAAACTCTGGAGAACCACCTAT
TATTTAATGTTGTAGCTAGTTACATTAGTAAATATTCATATGTAGCATGTTTAAGCATTAAATCAAAT
AAGATGAGCAAGATG
+
FFFFFFFFFFFFFFFFFFFFFFFFFFFFFFFFFFFFFFFFFFFFFFFFFFFFFFFFFFFFFFFFFFFFFF
FFFFFFFFFFFFFFFFFFFFFFFFF:FFFFFFFFFFFFFFFFFFFFFFFFFFFFFFF:FFFFFFFFFFFF
FFFFFFFFFF,FFFF
@A00155:342:HHGFNDSXY:1:2567:26729:16219 2:N:0:GAACCTAG+TCCGCATA
GTATCTTTCACCATTTGCTTACGTGAATCACTCACACCCTGGTTGTAAAACTCTGGAGAACCACCTAT
TATTTAATGTTGTAGCTAGTTACAGTAGTAAATATTCATATGTAGCATGTTTAAGCATTAAATCAAAT
AAGATGAGCAAGATG
+
FFFFFFFFFFFFFFFFFFFFFFFFFFFFFFF:FFFFFFFFFFFFFFFFFFFFFFFFFFFFFFFFFFFFFF
FFFFFFFFF,FFFFFFFFFFFFFFF,FFFFFFFFFFFFFFFFFF:FFFFFFFFFFFFFFFFFFFFFFFFF
FFFFFFFFF:FFF::
@A00155:342:HHGFNDSXY:1:2563:24713:33739 2:N:0:GAACCTAG+TCCGCATA
GTATCTTTCACCATTTGCTTACGTGAATCACTCACACCCTGGTTGTAAAACTCTGGAGAACCACCTAT
TATTTAATGTTGTAGCTAGTTACATTAGTAAATATTCATATGTAGCATGTTTAAGCATTAAATCAAAT
AAGATGAGCAAGATG
+
FFFFFFFFFFFFFFFFFFFFFFFFFFFFFFFFFFFFFFFFFFFFFFFFFFFFFFFFFFFFFFFFFFFFFF
FFFFFFFFFFFFFFFFFFFFFFFF:FFFFFFFFFFFFFFFFFFF:FF,FFFFFFFFFFFFFFFFFFFFFF
FFFFFFFFFFFFFFF
@A00155:342:HHGFNDSXY:1:2645:8296:24784 2:N:0:GAACCTAG+TCCGCATA
GTATCTTTCACCATTTGCTTACGTGAATCACTCACACCCTGGTTGTAAAACTCTGGAGAACCACCTAT
TATTTAATGTTGTAGCTAGTTACATTAGTAAATATTCATATGTAGCATGTTTAAGCATTAAATCAAAT
AAGATGAGCAAGATG
+
FFFFFFFFFFFFFFFFFFFFFFFFFFFFFFFFFFFFFFFFFFFFFFFFFFFFFFFFFFFFFFFFFFFFFF
FFFFFFFFFFFFFFFFFFFFFFFFFFFFFFFFFFFFFFFFFFFFFFFFFFFFFFFFF:FFFFFFFFFFFF
FFFFFF:FF:FFFFF
@A00155:342:HHGFNDSXY:1:2575:15194:30123 2:N:0:GAACCTAG+TCCGCATA
GTATCTTTCACCATTTGCTTACGTGAATCACTCACACCCTGGTTGTAAAACTCTGGAGAACCACCTAT
TATTTAATGTTGTAGCTAGTTACATTAGTAAATATTCATATGTAGCATGTTTAAGCATTAAATCAAAT
AAGATGAGCAAGATG
+
FFFFF:FFFFFFFFFFFFFFFFFFFFFFFFFFFFFFFFF:FFFFFFFFFFFFFFFFF:FFFFFFFFFFF:
FFFFFFFFF,FFFFFFFF:FFFFFF:FFFFFFFFF:FFFFFFFFF:FF:F:FFFFFFFF:FFFFFFFFFF
FFFFFFFFFFFFFFF
@A00155:342:HHGFNDSXY:1:2601:31765:11882 2:N:0:GAACCTAG+TCCGCATA
GTATCTTTCACCATTTGCTTACGTGAATCACTCACACCCTGGTTGTAAAACTCTGGAGAACCACCTAT
TATTTAATGTTGTAGCTAGTTACATTAGTAAATATTCATATGTAGCATGTTTAAGCATTAAATCAAAT
AAGATGAGCAAGATG
+
FFFFFFFFFFFFFFFFFFFFFFFFFFFFFFFFFFFFFFFF:FFFFFFFFFFFFFFFFFFFFFFFFFFF,F
FFFFFF::FFFFFFFFFFFFFFFFFFFFFFFFFFFFFFFFFFF,FFFFFFFFFFFFF:F:FFFFFFFFFF
FFFFFFFFFF:FFFF
@A00155:342:HHGFNDSXY:1:1274:28284:8923 1:N:0:GAACCTAG+TCCGCATA
TATCTTTCACCATTTGCTTACGTGAATCACTCACACCCTGGTTGTAAAACTCTGGAGAACCACCTATT
ATTTAATGTTGTAGCTAGTTACATTAGTAAATATTCATATGTAGCATGTTTAAGCATTAAATCAAATA
AGATGAGCAAGATGG

```
+
:FFFFFFFFFFFFFFFFFFFFF:FFFFFFFFFFFFFFFFFFFFFFFFFFFFFFFFFFFFFFFFFFFFF
FFFFFFF:FFFFFFFFFFFFFFFFFFFFFFFFFFFFFFFFFFFFFFFFFFFFFFFFFFFFFFFFFFFF
FFFFFFFFFFFFFFF
@A00155:342:HHGFNDSXY:1:1323:9082:4539 1:N:0:GAACCTAG+TCCGCATA
TATCTTTCACCATTTGCTTACGTGAATCACTCACACCCTGGTTGTAAAACTCTGGAGAACCACCTATT
ATTTAATGTTGTAGCTAGTTACATTAGTAAATATTCATATGTAGCATGTTTAAGCATTAAATCAAATA
AGATGAGCAAGATGG
+
FFFFFFF:FFFFFFFFFFFFFFFFFFFFFFFFFFFFFFFFFFFFFFFFFFFFFFFFFFFFFFFF:FFF
FFFFF,FFFFFFFFFFFF,FFFFFFFFFFFFFFFFFFFFFFFFFFFFFFFFFFFFFFFFFFFFFFFFF
FFF:FFFFFFFFFFF
@A00155:342:HHGFNDSXY:1:2265:27570:27727 1:N:0:GAACCTAG+TCCGCATA
TATCTTTCACCATTTGCTTACGTGAATCACTCACACCCTGGTTGTAAAACTCTGGAGAACCACCTATT
ATTTAATGTTGTAGCTAGTTACATTAGTAAATATTCATATGTAGCATGTTTAAGCATTAAATCAAATA
AGATGAGCAAGATGG
+
FFFFFFFFFFFFFFFFFFFFFFF:FFFFFFFFFFFFFFFFFFFFFFFFFFFFFFFFFFF:FFFFFFFF
FFFFFFFFFFFFFFFFFFFFFFFFFFFFFFFFFFFFFFFFFFFFFFFFFFFFFFFFFFFFFFFFFFFF
FFFFFFFFFFFFFFF
@A00155:342:HHGFNDSXY:1:2624:20428:7185 1:N:0:GAACCTAG+TCCGCATA
TATCTTTCACCATTTGCTTACGTGAATCACTCACACCCTGGTTGTAAAACTCTGGAGAACCACCTATT
ATTTAATGTTGTAGCTAGTTACATTAGTAAATATTCATATGTAGCATGTTTAAGCATTAAATCAAATA
AGATGAGCAAGATGG
+
FFFFF:FFFFFFFFFFFFFFFFFFFFFFFFFFFFFFFFFFFFFFFFFFFFFFFFFFFFFFFFFFFFFF
FFFFF,FFFFFFFFFFFFFFFFFFFFFFFFFFFFFFFFFFFFFFFFFFFFFFFFFFFF:FFFFFFFFF
FFFFFFFFFFFFFFF
@A00155:342:HHGFNDSXY:1:1173:13539:21026 2:N:0:GAACCTAG+TCCGCATA
ATCTTTCACCATTTGCTTACGTGAATCACTCACACCCTGGTTGTAAAACTCTGGAGAACCACCTATTA
TTTAATGTTGTAGCTAGTTACATTAGTAAATATTCATATGTAGCATGTTTAAGCATTAAATCAAATAA
GATAGATCGGA
+
FFFFFFFFFFFFFFFFFFFFFFFFFFFFFFFFFFFFFFFFFFFFFFFFFFFFFFFFFFFFFFFFFFFF
FFFFF:FFFFFFFFFFFFFFFF:FFFF:FFFFFFFFFFFFFFFFFFFFFFF:FFFFFFFFFFFFFFFF
FFFFFFFFFFF
@A00155:342:HHGFNDSXY:1:1411:3088:13886 2:N:0:GAACCTAG+TCCGCATA
ATCTTTCACCATTTGCTTACGTGAATCACTCACACCCTGGTTGTAAAACTCTGGAGAACCACCTATTA
TTTAATGTTGTAGCTAGTTACATTAGTAAATATTCATATGTAGCATGTTTAAGCATTAAATCAAATAA
GATGAGCAAGA
+
FFFFFFFFFFFFFFFFFFFFFFFFFFFFFFFFFFFFFFFFFFFFFFFFFFFFFFFFFFFFFFFFFFFF
FFFFFFFFFFFFFFFFFFFFFFFFFFFFFFFFFF:FFFFFFFFFFFFFFFF:FFFFFFFFFFFFFFFF
FFFFFFFFFFF
@A00155:342:HHGFNDSXY:1:1461:27615:16501 2:N:0:GAACCTAG+TCCGCATA
ATCTTTCACCATTTGCTTACGTGAATCACTCACACCCTGGTTGTAAAACTCTGGAGAACCACCTATTA
TTTAATGTTGTAGCTAGTTACATTAGTAAATATTCATATGTAGCATGTTTAAGCATTAAATCAAATAA
GATGAGCAAGA
+
FFFFFFFFFFFFFFFFFFFFFFFFFFFFFFFFFFFFFFFFFFFFF:FFFFFFFFFFFFF:FFFFFFFF
FFFFFFFFFFFFFFF:FFFFFFFFFFFFFFFFFFFFFFFFFFFFFFFFFFFF:FFFFFFFFFF,:FFF
FFFFF:FF:FF
@A00155:342:HHGFNDSXY:1:1468:16667:17268 2:N:0:GAACCTAG+TCCGCATA
ATCTTTCACCATTTGCTTACGTGAATCACTCACACCCTGGTTGTAAAACTCTGGAGAACCACCTATTA
```

```
TTTAATGTTGTAGCTAGTTACATTAGTAAATATTCATATGTAGCATGTTTAAGCATTAAATCAAATAA
GATGAGCGAGA
+
FFFFFFFFFFFFFFFFFFFFFFFFFFFFFFFFFFFFFFFFFFFFFFFFFFFFFFFFFFFFFFFFFFFF
FFFFFFFFFFFFFFFFFFFFFFFFFFFFF:FFFFFFFFFFFFFFFFFFFFFF:FFFFFFFFFFF:FFFF
FFFFFFFFFFF
@A00155:342:HHGFNDSXY:1:1528:32588:11584 2:N:0:GAACCTAG+TCCGCATA
ATCTTTCACCATTTGCTTACGTGAATCACTCACACCCTGGTTGTAAAACTCTGGAGAACCACCTATTA
TTTAATGTTGTAGCTAGTTACATTAGTAAATATTCATATGTAGCATGTTTAAGCATTAAATCAAATAA
GATGAGCAAGA
+
FFFFFFFFFFFFFFFFF:FFFFFFF:::FFFFFF:FFFFFFFFFFF:FFFFFFFFFF::FFFFFF:FFF
FFFFFFFFFFFFFFFFF:,FFFFFFFFF:FFFFFFFFFF,FFFFFFFFFFFFFFFF,FFFFFFFFFFF
F::FFFFFF:F
@A00155:342:HHGFNDSXY:1:2169:4806:3552 2:N:0:GAACCTAG+TCCGCATA
ATCTTTCACCATTTGCTTACGTGAATCACTCACACCCTGGTTGTAAAACTCTGGAGAACCACCTATTA
TTTAATGTTGTAGCTAGTTACATTAGTAAATATTCATATGTAGCATGTTTAAGCATTAAATCAAATAA
GATGAGCAAGA
+
FFFFFFFFFFFFFFFFFFFFFFFFFFFFFF:FFFFFFFFFFFFFFFFFFF,FF:FFFFFFFFFFFFF:F:
FFFFFFFFFFFF:FFFF,FFFFFFFFFFFFFFFFFFFFFFFFFF,FFFF:FFFFFFFFFFFFFF::FFF
FFFFFFFFFFF
@A00155:342:HHGFNDSXY:1:2160:10926:14184 2:N:0:GAACCTAG+TCCGCATA
ATCTTTCACCATTTGCTTACGTGAATCACTCACACCCTGGTTGTAAAACTCTGGAGAACCACCTATTA
TTTAATGTTGTAGCTAGTTACATTAGTAAATATTCATATGTAGCATGTTTAAGCATTAAATCAAATAA
GATGAGCAAGA
+
FFFFFFFFFFFFFFFFFFFFFFFFFFFFFFFFFFFFFFFFFFFFFFFFFFFFFFFFFFFFFFFFFFFF
FFFFFFFFFFFFFFFFFFFFFFFFFFFFFFFFFFFFFFFFFFFFFFFFFFFFFFFFFFFFFFFFFFFF
FFFFFFFFFF:
@A00155:342:HHGFNDSXY:1:2243:32732:25426 2:N:0:GAACCTAG+TCCGCATA
ATCTTTCACCATTTGCTTACGTGAATCACTCACACCCTGGTTGTAAAACTCTGGAGAACCACCTATTA
TTTAATGTTGTAGCTAGTTACATTAGTAAATATTCATATGTAGCATGTTTAAGCATTAAATCAAATAA
GATGAGCAAGA
+
FFFFFFFFFFFFFFFFFFFFFFFFFFFFFFFFFFFFFFFFFFFFFFFFFFFFFFFFFFFFFFFFFFFF
FFFFFFFFFFFFF:FFFFFFFFFFFFFFFFFF:FFFF:FFFFFFFFFFFFFFFFFFFFFFF:FFFFF:FFF
FFFFF:FFFF:
@A00155:342:HHGFNDSXY:1:2366:9263:7983 2:N:0:GAACCTAG+TCCGCATA
ATCTTTCACCATTTGCTTACGTGAATCACTCACACCCTGGTTGTAAAACTCTGGAGAACCACCTATTA
TTTAATGTTGTAGCTAGTTACATTAGTAAATATTCATATGTAGCATGTTTAAGCATTAAATCAAATAA
GATGAGCAAGA
+
FFFFFFFFFFFFFFFFFFFFFFFFFFFFFFFFFFFFFFFFFFFFFFF:FFFFFFFFFFFFFFFFFFFFF
FF:FFFFFFFFFFFFFFFFFF:FFFFFFFFFFFFFFFFFFFFFFFFFFFFFFFFFFFFFFFFFFFFFF
FFF,FFF:F:F
@A00155:342:HHGFNDSXY:1:2631:20347:27962 2:N:0:GAACCTAG+TCCGCATA
ATCTTTCACCATTTGCTTACGTGAATCACTCACACCCTGGTTGTAAAACTCTGGAGAACCACCTATTA
TTTAATGTTGTAGCTAGTTACATTAGTAAATATTCATATGTAGCATGTTTAAGCATTAAATCAAATAA
GATGAGCAGAT
+
FFFFFFFFFFFFFFFFFFFFFFFFFFFFFFFFFFFFFFFFFFFFFFFFFFFFFFFFFFFFFFFFFFFF
FFFFFFFFFFFFFFFFFFFFFFFFFFFFFFFFFFFFFFFFFFFFFFFFFFFF:FFFFFFFFFFFFFFF
FFFFFFFFFFF
```

```
@A00155:342:HHGFNDSXY:1:2631:19831:27946 2:N:0:GAACCTAG+TCCGCATA
ATCTTTCACCATTTGCTTACGTGAATCACTCACACCCTGGTTGTAAAACTCTGGAGAACCACCTATTA
TTTAATGTTGTAGCTAGTTACATTAGTAAATATTCATATGTAGCATGTTTAAGCATTAAATCAAATAA
GATGAGCAGAT
+
:F:FFFFFFF:FFFFFFF:FFFFFFFFFFFFFFFFFFFFFFFFFFFFFFFFFFFF:FFFFFFFFF:FF:
FFFFFFFFFFF:FFF:FFFFFFFF:FFFFFF:FFFFF:F:F:F:FFFFFFFFF:FFFFFFF:FFFFFF
,:F::F,FF:F
@A00155:342:HHGFNDSXY:1:2250:4237:16157 1:N:0:GAACCTAG+TCCGCATA
CTTTCACCATTTGCTTACGTGAATCACTCACACCCTGGTTGTAAAACTCTGGAGAACCACCTATTATT
TAATGTTGTAGCTAGTTACATTAGTAAATATTCATATGTAGCATGTTTAAGCATTAAATCAAATAAGA
TGAGCAAGATGGA
+
FFFFFF:::FFFFFFF:FFF:FFF:FFFFFFFFF:FF:FF:FFFFF:FFFFFF:FFFFFF,FFFFFFF
FF:FFFFFFF:,FFFFFFF,FFFFFFFFFFFFF:FFFF:F:FFFFFFFFFF,FFFF,:FFFFFFFFFF
FFFFFFFFFFFFF
@A00155:342:HHGFNDSXY:1:1369:23339:34522 1:N:0:GAACCTAG+TCCGCATA
CTTTCACCATTTGCTTACGTGAATCACTCACACCCTGGTTGTAAAACTCTGGAGAACCACCTATTATT
TAATGTTGTAGCTAGTTACATTAGTAAATATTCATATGTAGCATGTTTAAGCATTAAATCAAATAAGA
TGAGCAAGATGGAAC
+
FFFFFFFFFFFFFFFFFFFFFFFFFFFFFFFFFFFFFFFFFFFFFFFFFFFFFFFFFFFFFFFFFFFF
FFFFFFFFFFFFFFFFFFFFFFFFFFFFFFFFFFF:FFFFFFFFFFFFFFFFFFFFFFFFFFFFFFFF
FFFFFFFFFFFFFFF
@A00155:342:HHGFNDSXY:1:1376:17707:23171 1:N:0:GAACCTAG+TCCGCATA
CTTTCACCATTTGCTTACGTGAATCACTCACACCCTGGTTGTAAAACTCTGGAGAACCACCTATTATT
TAATGTTGTAGCTAGTTACATTAGTAAATATTCATATGTAGCATGTTTAAGCATTAAATCAAATAAGA
TGAGCAAGATGGAAC
+
FFFFFFFFFFFFFFFFFFFFFFFFFFFFFFFFFFFFFFFFFFFF:FFFFFFFFFFFFFFFFFFFFFFF:
FFFFFFFFFFFFFFFFFFFFFFFFFFFFFFFFFFFFFFFFFFFFFFFFFFFFFFFFFFFFFFFFFFFF
FFFFFFFFFFFFFFF
@A00155:342:HHGFNDSXY:1:1437:19949:7545 1:N:0:GAACCTAG+TCCGCATA
CTTTCACCATTTGCTTACGTGAATCACTCACACCCTGGTTGTAAAACTCTGGAGAACCACCTATTATT
TAATGTTGTAGCTAGTTACATTAGTAAATATTCATATGTAGCATGTTTAAGCATTAAATCAAATAAGA
TGAGCAAGATGGAAC
+
FF,FFFFFFFFFFFFFFFF:FFFFFFFFFFFFFFFFFFFFFFFFFFFFFFFFFFFFFFFFFFFFFFFF
,FFFFFFFFFFFFFFF,FFFF:FFFFFFFFFFFFFFFFFFFFFFFFFFFFFFFFFFFFFFFFFFFFFF
FFFFFFFFFFFFFF:
@A00155:342:HHGFNDSXY:1:1437:6460:33160 1:N:0:GAACCTAG+TCCGCATA
CTTTCACCATTTGCTTACGTGAATCACTCACACCCTGGTTGTAAAACTCTGGAGAACCACCTATTATT
TAATGTTGTAGCTAGTTACATTAGTAAATATTCATATGTAGCATGTTTAAGCATTAAATCAAATAAGA
TGAGCAAGATGGAAC
+
FFFFFFFFFFFFFFFFFFFFFFFFFFFFFFFFFFFFFFFFFFFFFFFFFFFFFFFFFFFFFFFFFFFF
FFFFFFFFFFFFFFFFFFFFFFFFF:FFFFFFFFFFFFFFFFFFFFFFFFFFFFFFFFFFFFFFFFFF
FFFFFFFFFFFFFFF
@A00155:342:HHGFNDSXY:1:2115:26747:25520 1:N:0:GAACCTAG+TCCGCATA
CTTTCACCATTTGCTTACGTGAATCACTCACACCCTGGTTGTAAAACTCTGGAGAACCACCTATTATT
TAATGTTGTAGCTAGTTACATTAGTAAATATTCATATGTAGCATGTTTAAGCATTAAATCAAATAAGA
TGAGCAAGATGGAAC
+
FFFFFFFFFFFFFFFFF:FFFFFFFFFFFFFFFFFFFFFFFFFFFFFFFFFFFFFFFFFFFFFFFFFF
```

FFFFFFFFFFFFFFFFFFF,FFFFFFFF:FFFFFFFFFFFFFFFFFFFFFFFFFFFFFFFFFFFFFFFFF
FFFFFFFFFFFFFFF
@A00155:342:HHGFNDSXY:1:2247:28330:23970 1:N:0:GAACCTAG+TCCGCATA
CTTTCACCATTTGCTTACGTGAATCACTCACACCCTGGTTGTAAAACTCTGGAGAACCACCTATTATT
TAATGTTGTAGCTAGTTACATTAGTAAATATTCATATGTAGCATGTTTAAGCATTAAATCAAATAAGA
TGAGCAAGATGGAAC
+
FFFFFFFFFFFFFFFFFFFFFFFFFFFFFFFFFFFFF:FFFFFFFFFFFFFFF:FFFFFFFFFFFFFFFF
FFFFFFF:FFFFFFFFFFFFFFFFFFFFFFFFFFFFFFFFF:FFFFFFFFFFFFFFFFFFFFFFFFFFFF
FFFFFFFFFFFFFFF
@A00155:342:HHGFNDSXY:1:2267:32416:34272 1:N:0:GAACCTAG+TCCGCATA
CTTTCACCATTTGCTTACGTGAATCACTCACACCCTGGTTGTAAAACTCTGGAGAACCACCTATTATT
TAATGTTGTAGCTAGTTACATTAGTAAATATTCATATGTAGCATGTTTAAGCATTAAATCAAATAAGA
TGAGCAAGATGGAAC
+
FFFFFFFFFF,FFFFFFFFFFFFFFFFFFFFFFFF:FFFFF:FFFFFFFFFFFFFFFFFFFFFFFFFFFF
F::FF::F:FFFFFFF,FFFF:FFFFF:FFFFFF:FFFFFFF,FFFFF:FFFFFFFFFFFFFFFFFFFFF
FFFFFFFFFFFFFFF
@A00155:342:HHGFNDSXY:1:2262:15022:34804 1:N:0:GAACCTAG+TCCGCATA
CTTTCACCATTTGCTTACGTGAATCACTCACACCCTGGTTGTAAAACTCTGGAGAACCACCTATTATT
TAATGTTGTAGCTAGTTACATTAGTAAATATTCATATGTAGCATGTTTAAGCATTAAATCAAATAAGA
TGAGCAAGATGGAAC
+
FFFF:FFFFFF:F,FFF:FFF:FFFFFFFFFFFFFFFFFFFFFFFFFFFFFF,FF,:F:FF,:FFF:FFF
FFFFFFFFFF:FFFFFFFFFFFFFFFFFFFFFFFFFFFFF:FF:FFFFFFF,FFFF:FFFFFFFFFFFFF
FF,F:FFFF:FFFFF
@A00155:342:HHGFNDSXY:1:2318:27534:19147 1:N:0:GAACCTAG+TCCGCATA
CTTTCACCATTTGCTTACGTGAATCACTCACACCCTGGTTGTAAAACTCTGGAGAACCACCTATTATT
TAATGTTGTAGCTAGTTACATTAGTAAATATTCATATGTAGCATGTTTAAGCATTAAATCAAATAAGA
TGAGCAAGATGGAAC
+
FFFFFFFFFFFF:FFFFFF,FFFFFFFFFFFFFFFFFFFFFFFFFFFFFFFFFFFFFFFFFFFFFFFFFF
FFFFFFFF:FFFFFFFFFFFFFFFFFFFFFFFFFFFFFFFFFFFFFFFFFFFFFFFFFFFFFFFFFFFFF
FFFFFFFFFFFFFFF
@A00155:342:HHGFNDSXY:1:2318:27389:19648 1:N:0:GAACCTAG+TCCGCATA
CTTTCACCATTTGCTTACGTGAATCACTCACACCCTGGTTGTAAAACTCTGGAGAACCACCTATTATT
TAATGTTGTAGCTAGTTACATTAGTAAATATTCATATGTAGCATGTTTAAGCATTAAATCAAATAAGA
TGAGCAAGATGGAAC
+
FFFFFFFFFFFFFFFFFFFFFFFFFFFFFFFFFFFFFFFFFFFFFFFFFFFFFFFFFFFFFFFFFFFFFF
FFFFFFFFFFFFFFFFFFFFFFFFFFFFFFFFFFFFFFFFFFFFFFFFFFFFFFFFFFFFFFFFFFFFFF
FFFFFFFFFFFFFFF
@A00155:342:HHGFNDSXY:1:2318:28384:19867 1:N:0:GAACCTAG+TCCGCATA
CTTTCACCATTTGCTTACGTGAATCACTCACACCCTGGTTGTAAAACTCTGGAGAACCACCTATTATT
TAATGTTGTAGCTAGTTACATTAGTAAATATTCATATGTAGCATGTTTAAGCATTAAATCAAATAAGA
TGAGCAAGATGGAAC
+
FFFFFFFFFFFF:FFFFFFFFF:FFFFFFFFFFF:FFFFFFFFFFFFFFFFFFFFFFFFFFFFFFFFFFF
FFFFFFFFFFFFFFFFFFFFFFFFFFFFFFFFFFFFFFFFFFFFFFFFFFFFFFFFFFFFFFFFFFFFFF
FFFFFFFFFFFF:FF
@A00155:342:HHGFNDSXY:1:2304:7681:29951 1:N:0:GAACCTAG+TCCGCATA
CTTTCACCATTTGCTTAAGTGAATCACTCACACCCTGGTTGTAAAACTCTGGAGAACCACCTATTATT
TAATGTTGTAGCTAGTTACATTAGTAAATATTCATATGTAGCATGTTTAAGCATTAAATCAAATAAGA
TGAGCAAGATGGAAC

+
FFFFFF,FFFFFFFFFF,F:FFFFFFFFFFFFFFFFFFFFF,FFFFFFFFFFFFFFFFFFFFFFFF:F
FFFFFFF:FFFFFFFFFFFFFFFFFFFFFFFFFFFFFFFFFFFFFFFFFFFFFFFFFFFFFFFFFFFF
FFFF:FFFFFFFFFF
@A00155:342:HHGFNDSXY:1:2343:22652:7623 1:N:0:GAACCTAG+TCCGCATA
CTTTCACCATTTGCTTACGTGAATCACTCACACCCTGGTTGTAAAACTCTGGAGAACCACCTATTATT
TAATGTTGTAGCTAGTTACATTAGTAAATATTCATATGTAGCATGTTTAAGCATTAAATCAAATAAGA
TGAGCAAGATGGAAC
+
:FFFFFFFFFFFFFFFFFFFFFFFFFFFFFFFFFFFFFFFFFFFFFFFFFFFFFFFFFFFFFFFFFFF
FFFFFFFFFFFFFFFFFFFFFFFFFFFFFFFFFFFFFFFFFFFFFFFFFFFFFFFFFFFFFFFFFFFF
FFFFFFFFFFFFFFF
@A00155:342:HHGFNDSXY:1:2442:11930:22153 1:N:0:GAACCTAG+TCCGCATA
CTTTCACCATTTGCTTACGTGAATCACTCACACCCTGGTTGTAAAACTCTGGAGAACCACCTATTATT
TAATGTTGTAGCTAGTTACATTAGTAAATATTCATATGTAGCATGTTTAAGCATTAAATCAAATAAGA
TGAGCAAGATGGAAC
+
FFFFFFFFFFF:FFFFFFFFFFFFFFFFFFFFFFFFFFFFFFFFFFFFFFFFFFFFFFFFFFFFFFFF
FFFFFFFFFFFFFFFFFFFFFFFFFFFFFFFFFFFFFFFFFFFFFF:FFFFFFFFFFFFFFFFFFFFF
FFFFFFFFFFFFFFF
@A00155:342:HHGFNDSXY:1:2450:4689:33317 1:N:0:GAACCTAG+TCCGCATA
CTTTCACCATTTGCTTACGTGAATCACTCACACCCTGGTTGTAAAACTCTGGAGAACCACCTATTATT
TAATGTTGTAGCTAGTTACATTAGTAAATATTCATATGTAGCATGTTTAAGCATTAAATCAAATAAGA
TGAGCAAGATGGAAC
+
FFFFFFF,FFFFFFFFFFF,FFFFFFFFFFFFFFFFFFFF:FFFFFFFFFFFFFFFFFF:FFFFFFFF
FFFFFFFFFFFFFFFFFFFFFFFFFFFFFFFFFFFFFFFFFFFFFF:FFFFFFFFFFFFFFFFFFFFF
FFFFFFFFFFFFFFF
@A00155:342:HHGFNDSXY:1:2468:20699:17300 1:N:0:GAACCTAG+TCCGCATA
CTTTCACCATTTGCTTACGTGAATCACTCACACCCTGGTTGTAAAACTCTGGAGAACCACCTATTATT
TAATGTTGTAGCTAGTTACATTAGTAAATATTCATATGTAGCATGTTTAAGCATTAAATCAAACAAGA
TGAGCAAGATGGAAC
+
FFFFFFFFFFFFFFFFFFFFFFFFFFFFFFFFFFFFFFFFFFFFFFFFFFFFFF:FF:FFFFFFFFFF
FFFFFFFFFFFFFFFFFFFFFFFFFFFFFFFFFFFFFFF:FFFFFFFFFFFFFFFFFFFFFFFFFFFF
FFFFFFFFFFFFFFF
@A00155:342:HHGFNDSXY:1:2562:23005:31125 1:N:0:GAACCTAG+TCCGCATA
CTTTCACCATTTGCTTACGTGAATCACTCACACCCTGGTTGTAAAACTCTGGAGAACCACCTATTATT
TAATGTTGTAGCTAGTTACATTAGTAAATATTCATATGTAGCATGTTTAAGCATTAAATCAAATAAGA
TGAGCAAGATGGAAC
+
FFFFFFFFFFFFFFFFFFFFFFFFFF:FFFFFFFFFFFFFFFFFFFF:FFFFFFFFFFFFFFFFFFFF:
FFFFFFFFFFFF:FFFFFFFFFFFFFFFFFFFFFFFFFFFFFFFFFFFFFFFFFFFFFFFFFFFFFFF
FFFFFFFFFFFFFFF
@A00155:342:HHGFNDSXY:1:2531:29731:11209 1:N:0:GAACCTAG+TCCGCATA
CTTTCACCATTTGCTTACGTGAATCACTCACACCCTGGTTGTAAAACTCTGGAGAACCACCTATTATT
TAATGTTGTAGCTAGTTACATTAGTAAATATTCATATGTAGCATGTTTAAGCATTAAATCAAATAAGA
TGAGCAAGATGGAAC
+
FFFFFFFFFFFF:FFFFFFFFFFFFFFFFFFFFFFFFFFFFFFFFFFFFFFFFFFFFFFFFFFFFFFF
FFFFFFFFFFFFFFFFFFFFFFFFFFFFFFFFFFFFFFFFFFFFFFFFFFFFFFFFFFFFFFFFFFFF
FFFFFFFFFFFFFFF
@A00155:342:HHGFNDSXY:1:2603:27190:32550 1:N:0:GAACCTAG+TCCGCATA
CTTTCACCATTTGCTTACGTGAATCACTCACACCCTGGTTGTAAAACTCTGGAGAACCACCTATTATT

```
TAATGTTGTAGCTAGTTATATTAGTAAATATTCATATGTAGCATGTTTAAGCATTAAATCAAATAAGA
TGAGCAAGATGGAAC
+
FFFFFFFFFFFFFFFFFFFFFFFFFFFFFFFFFFFFFFFFFFFFFFFFFFFFFFFFFFFFFFFFFFFF
FF:FFFFFFFFFFFFFFFFFFFFFFFFFFFFFFFFFFFFFFFFFFFFFFFFFFFFFFFFFFFFFFFFF
FFFFFFFFFFFFFFF
@A00155:342:HHGFNDSXY:1:1104:2492:35305 2:N:0:GAACCTAG+TCCGCATA
CTTTCACCATTTGCTTACGTGAATCACTCACACCCTGGTTGTAAAACTCTGGAGAACCACCTATTATT
TAATGTTGTAGCTAGTTACATTAGTAAATATTCATATGTAGCATGTTTAAGCATTAAATCAAATAAGA
TGAGCAAGATGGAAC
+
FFFFFFFFFFF:FFFFFFFFFFFF,FFFFFFFFFFFFFFFFFFFFFFFFFFFFFFFFFFFFFFFFFFF
FFFFFFFFFFFFFFFFFF:FFFF:FFFFFFFFFFFFFFFFFFFFFFFFFFFFFFFFF:FFFFFFFFFF:
FFFFFFFFFFF,FFF
@A00155:342:HHGFNDSXY:1:1226:29568:27649 2:N:0:GAACCTAG+TCCGCATA
CTTTCACCATTTGCTTACGTGAATCACTCACACCCTGGTTGTAAAACTCTGGAGAACCACCTATTATT
TAATGTTGTAGCTAGTTACATTAGTAAATATTCATATGTAGCATGTTTAAGCATTAAATCAAATAAGA
TGAGCAAGATGGAAC
+
FFFFFFFF:FFFFFFFFFFFFFFFFFFFFFFFFFFFFF,FFFFFFFFFFFFFFFFFFFFFFFFFFFFF
FFFFFFFFFFFFFFFFFFFFFFFFFFFFFFFFFF:FFFF,FFFFFFFFFFFFF:FFFFFFFFFFFFFFF
FFFFFFFFFFFFFFF
@A00155:342:HHGFNDSXY:1:1243:29803:25927 2:N:0:GAACCTAG+TCCGCATA
CTTTCACCATTTGCTTACGTGAATCACTCACACCCTGGTTGTAAAACTCTGGAGAACCACCTATTATT
TAATGTTGTAGCTAGTTACATTAGTAAATATTCATATGTAGCATGTTTAAGCATTAAATCAAATAAGA
TGAGCAAGATGGAAC
+
FFFFFFFFFFFFFFFFFFFFFFFFFFFFFFFFFFFFFFFFFFFFFFFFFFFFFFFFFFFFFFFFFFFF
FFFFFFFFFFFFFFFFFFFFFFFFFFFFFFFFFFFFFFFFFFFFFFFFFFFFFFFFFFFFFFFFFFFF
FFFFFFFFFFFFFFF
@A00155:342:HHGFNDSXY:1:1263:20464:11193 2:N:0:GAACCTAG+TCCGCATA
CTTTCACCATTTGCTTACGTGAATCACTCACACCCTGGTTGTAAAACTCTGGAGAACCACCTATTATT
TAATGTTGTAGCTAGTTACATTAGTAAATATTCATATGTAGCATGTTTAAGCATTAAATCAAATAAGA
TGAGCAAGATGGAAC
+
FFFFFFFFFFFFFFFFFFFFFFFFFFFFFFFFFFFFFFFFFFFFFFFFFFFFFFFFFFFFFFFFFF:F
FFFFFFFFFFFFFFF:FF:FFFFFFFFFFFFFFFFFFFFFFF:FFF:FFFFFFFFFFFFFFFFFFFFFFF
FFFFFFFFFFFFFFF
@A00155:342:HHGFNDSXY:1:1307:2257:30765 2:N:0:GAACCTAG+TCCGCATA
CTTTCACCATTTGCTTACGTGAATCACTCACACCCTGGTTGTAAAACTCTGGAGAACCACCTATTATT
TAATGTTGTAGCTAGTTACATTAGTAAATATTCATATGTAGCATGTTTAAGCATTAAATCAAATAAGA
TGAGCAAGATGGAAC
+
FFFFFFFFFFFFFFFFFFFFFFFFFFFFFFFFFFFFFFFFFFFFF:FFFFFFFFFFFFFFFFFFFFFFF
FFFFFFFFFFFFFFFFFFFF:FFFFFFFFFFFFFFFFFFFF:FFFFFFFFFFFFFFFFFFFFFFFFFFF
FFFF,FFFFFFFFFF
@A00155:342:HHGFNDSXY:1:1417:6813:25567 2:N:0:GAACCTAG+TCCGCATA
CTTTCACCATTTGCTTACGTGAATCACTCACACCCTGGTTGTAAAACTCTGGAGAACCACCTATTATT
TAATGTTGTAGCTAGTTACATTAGTAAATATTCATATGTAGCATGTTTAAGCATTAAATCAAATAAGA
TGAGCAAGATGGAAC
+
FFFFFFFFFFFFFFFFFFFFFFFFFFFFFFFFFFFFFFFFFFFFFFFFFFFFFFFFFFFFFFFFFFFF
FFFFFFFFFFFFFFFFFFFFF:FFFFFFFFFFFFFFF,FFFFFFFF:,FFFFFFFFFFFFF:FFFFFFF
F::FFF,FFFFFFFF
```

@A00155:342:HHGFNDSXY:1:1417:6967:25614 2:N:0:GAACCTAG+TCCGCATA
CTTTCACCATTTGCTTACGTGAATCACTCACACCCTGGTTGTAAAACTCTGGAGAACCACCTATTATT
TAATGTTGTAGCTAGTTACATTAGTAAATATTCATATGTAGCATGTTTAAGCATTAAATCAAATAAGA
TGAGCAAGATGGAAC
+
FFFFFFFFFFFFFFFFFFFFFFFFFFFFFFFFFFFFFFFFFFF:FF:FFFF,FFFFFFFFFFFFFFFF:FF
FF:FF:FFFFFF:FFFFFFFFFFFFFFF:FFFFFFFFFFFFFFFFFFFFFF:FFF:FFF:FFFFFFFF:FF
FFFFF:FFF:FFFFF
@A00155:342:HHGFNDSXY:1:1437:19949:7545 2:N:0:GAACCTAG+TCCGCATA
CTTTCACCATTTGCTTACGTGAATCACTCACACCCTGGTTGTAAAACTCTGGAGAACCACCTATTATT
TAATGTTGTAGCTAGTTACATTAGTAAATATTCATATGTAGCATGTTTAAGCATTAAATCAAATAAGA
TGAGCAAGATGGAAC
+
FFFFFFFFFFFFFFFFFFFFFFFFFFFF:FFFFFFFFFFFFFFFFFFFFFFFFFFFFFFFFFFFFFFF,FF
FFFFF:FFFFFFFFFFFFFF:FFFFFFFFFFFFFFFFFFFFFFFF:FFFFFFFFFFFFFFFFFFFFFFFFF
FF:FFFFFFFF,FFF
@A00155:342:HHGFNDSXY:1:1526:29812:33583 2:N:0:GAACCTAG+TCCGCATA
CTTTCACCATTTGCTTACGTGAATCACTCACACCCTGGTTGTAAAACTCTGGAGAACCACCTATTATT
TAATGTTGTAGCTAGTTACATTAGTAAATATTCATATGTAGCATGTTTAAGCATTAAATCAAATAAGA
TGAGCAAGATGGAAC
+
FFFFFFFFFFFFFFFFFFFFFFFFFFFFFFFFFFFFFFFFFFFFF,FFFFFFFFFFFFFFFFFFFFFFFFF
FFFFFFFFFFFFFFFFFFFFF:FFFFFFFFFFFFFFFFFFFFF:FFFFFFFFFFFFFFFFFFFFFFFFFFF
F:FF,FFFFF:FFFF
@A00155:342:HHGFNDSXY:1:1646:15510:30545 2:N:0:GAACCTAG+TCCGCATA
CTTTCACCATTTGCTTACGTGAATCACTCACACCCTGGTTGTAAAACTCTGGAGAACCACCTATTATT
TAATGTTGTAGCTAGTTACATTAGTAAATATTCATATGTAGCATGTTTAAGCATTAAATCAAATAAGA
TGAGCAAGATGGAAC
+
:FFFFFFFFFFFFFFFFFFFFFFFFFFFFFFFFFFFFFFFFFFFFFFFFFFFFFFFFFFFFFFFFFFFFFF
FFFFFFFFFFFFFFFFFFFFFFFFFFFFFFFFFFFFFFFFFFFFFFFFFFFFFFFFFFFFFFFFFFFFFFF
FFFFFFFFFFFFFFF
@A00155:342:HHGFNDSXY:1:2227:26042:3004 2:N:0:GAACCTAG+TCCGCATA
CTTTCACCATTTGCTTACGTGAATCACTCACACCCTGGTTGTAAAACTCTGGAGAACCACCTATTATT
TAATGTTGTAGCTAGTTACATTAGTAAATATTCATATGTAGCATGTTTAAGCATTAAATCAAATAAGA
TGAGCAAGATGGAAC
+
FFFFFFFFFFFFFFFFFFFFFFFFFFFFFFFFFFFFFFFFFFFFFF:FFFFFFFFFFFFF:FFFFFFFFFF
FFFFFFFFFFFFFFFFFFFF:FFFFFFFFFFFFFFFFFFFFFFFF:FFFFFFFFFFFFFFFFFFFFFFFFF
FFFF:F:FFFFFFFF
@A00155:342:HHGFNDSXY:1:2255:14895:36088 2:N:0:GAACCTAG+TCCGCATA
CTTTCACCATTTGCTTACGTGAATCACTCACACCCTGGTTGTAAAACTCTGGAGAACCACCTATTATT
TAATGTTGTAGCTAGTTACATTAGTAAATATTCATATGTAGCATGTTTAAGCATTAAATCAAATAAGA
TGAGCAAGATGGAAC
+
FFFFFFFFFFFFFFFFFFFFFFFFFFFFFFFFFFFFFFFFFFFFFF,FFFFFFFFFFFFFFFFFFFFFFFF
FFFFFFFFFFFFFFFFFFFFF:FFFFFFFFFFFFFFFFFFFFFFFFFFFFF,FFFFFFFFFFFFFF:FFFF
FFFFFFFFFFFFFFF
@A00155:342:HHGFNDSXY:1:1468:19379:3583 1:N:0:GAACCTAG+TCCGCATA
TTTCACCATTTGCTTACGTGAATCACTCACACCCTGGTTGTAAAACTCTGGAGAACCACCTATTATTT
AATGTTGTAGCTAGTTACATTAGTAAATATTCATATGTAGCATGTTTAAGCATTAAATCAAACAAGAT
GAGCAAGATGGAAC
+
FFF::FFFFFFFFF:FFFFFFFFFFFFFFFFFFFFFFFFFFFFFFFFFFFFFFF:FFFFFFFFFFFF:FFF

FFFFFFFFFFFFFFFFFFFFFFFFFFFFFFFFFFFFFFFFFFFFFFFFFFFFFFFFFFFFFFFFFFFF
FFFFFFFFFFFFFF
@A00155:342:HHGFNDSXY:1:2232:4453:6167 1:N:0:GAACCTAG+TCCGCATA
TTTCACCATTTGCTTACGTGAATCACTCACAACCTGGTTGTAAAACTCTGGAGAACCACCTATTATTT
AATGTTGTAGCTAGTTACATTAGTAAATATTCATATGTAGCATGTTTAAGCATTAAATCAAATAAGAT
GAGCAAGATGGAAC
+
FFFFFF,FFFFFFFF,FF:FFFFF:FFFFFF,FFFFFFFFF:FFFFFFF:FFFFF:F:,FFF,F,FFF
,,FFFFFFFFF,FFFFFFFFF:FFF,FFFFFFF:F:FFFFFFFF,FFFFFFFFFFFFFFFFFFFFFFF
FFFFFFFFFF::FF
@A00155:342:HHGFNDSXY:1:1149:17418:24925 2:N:0:GAACCTAG+TCCGCATA
TTTCACCATTTGCTTACGTGAATCACTCACACCCTGGTTGTAAAACTCTGGAGAACCACCTATTATTT
AATGTTGTAGCTAGTTACATTAGTAAATATTCATATGTAGCATGTTTAAGCATTAAATCAAATAAGAT
GAGCAAGATGGAACG
+
FFFFFFF:FFFFFFFFFFFFFFFFFFFFFFFFFFFFFFFFFFFFFFFFFFFFFFFFFFFFFFFFFFFF
FFFFFFFFFFFFFFFFFFFFFFFFFFFFFFFFFFFFFFFFFFFFFFFFFFFFFFFFFF,FFFF:FF:F
:FFFFFFFFFFFFFF
@A00155:342:HHGFNDSXY:1:1565:7925:28369 2:N:0:GAACCTAG+TCCGCATA
TTTCACCATTTGCTTACGTGAATCACTCACACCCTGGTTGTAAAACTCTGGAGAACCACCTATTATTT
AATGTTGAAGCTAGTTACATTAGTAAATATTCATATGTAGCATGTTTAAGCATTAAATCAAATAAGAT
GAGCAAGATGGAACG
+
FFFFFFFFFFFFFFFFFFFFFFFFFFFFFFFFFFFFFFFFFFF:FFFFFFFFFFFFFFFFFFFFFFFF
FFFFFFF,FFFFFFF,FFFFFFFFFFFFFFFFFFFFFFFFFFFFFFFF:FFFFFF:FFFFFFFFFFFF
F:FFFFFFFFFFFFF
@A00155:342:HHGFNDSXY:1:2232:4453:6167 2:N:0:GAACCTAG+TCCGCATA
TTCACCATTTGCTTACGTGAATCACTCACACCCTGGTTGTAAAACTCTGGAGAACCACCTATTATTTA
ATGTTGTAGCTAGTTACATTAGTAAATATTCATATGTAGCATGTTTAAGCATTAAATCAAATAAGATG
AGCAAGATGGA
+
FFFFFFFFFFFFFFFFFFFFFFFFFFFFFFFFFFFFFFFFFFFFFFFFFFFFFFFFFFFFFFFFFFFF
FFFFFFFFFFFFFFFFFFFFFFFFFFFFFFFFFFF:FFFFF::FFFFFFFFFFF:FFFFFFFFFFFFF
FFFFFFFFFFF
@A00155:342:HHGFNDSXY:1:2216:6442:11490 2:N:0:GAACCTAG+TCCGCATA
TTCACCATTTGCTTACGTGAATCACTCACACCCTGGTTGTAAAACTCTGGAGAACCACCTATTATTTA
ATGTTGTAGCTAGTTACATTAGTAAATATTCATATGTAGCATGTTTAAGCATTAAATCAAATAAGATG
AGCTAGATCGG
+
FFFFFFFFFFFFFFFFFFFFFFFFFFFFFFFFFFFFFFFFFFFFFFFFFFFFFFFFFFFFFFFFFFFF
FFFFFFFFFFFFFFFFFFFFFFFFFFFFFFFFFFFFFFFFFFFFFFFFF:FFFFFFFFFFFFF:FFFF
F:FFFFFFFFF
@A00155:342:HHGFNDSXY:1:2365:23945:36573 2:N:0:GAACCTAG+TCCGCATA
TTCACCATTTGCTTACGTGAATCACTCACACCCTGGTTGTAAAACTCTGGAGAACCACCTATTATTTA
ATGTTGTAGCTAGTTACATTAGTAAATATTCATATGTAGCATGTTTAAGCATTAAATCAAATAAGATG
AGCTAGATCGG
+
FFFFFFFFFFFFFFFFFFFFFFFFFFFFFFFFFFFFFFFFFFFFFFFFFFFFFFFFFFFFFFFFFFFF
F:FFFFFFFFFFFFFFFF:FFFFFFFFFFFFFFFFFFFFFFFFFFFFFFFFFFFFFFFFFFFFFFFF
FFFFFFFFFFF
@A00155:342:HHGFNDSXY:1:2519:6596:9815 1:N:0:GAACCTAG+TCCGCATA
CACCATTTGCTTACGTGAATCACTCACACCCTGGTTGTAAAACTCTGGAGAACCACCTATTATTTAAT
GTTGTAGCTAGTTACATTAGTAAATATTCATATGTAGCATGTTTAAGCATTAAATCAAATAAGATGAG
CAAGATGGAACGTCT

```
+
FFFFFFFFFFFFFFFFFFFFFFFFFFFFFFFFFFFFFFFFFFFFFFFFFFFFFFFFFFFFFFFFFFFF
FFFFFFFFFFFFFFFFF:FFF:FFFFFFFFFFFFFFFFFFF:FFFFFFFFFFFFFFFFF:FFFFFFFF
FFFFFFFFFFFF:F:
@A00155:342:HHGFNDSXY:1:1269:9326:4961 1:N:0:GAACCTAG+TCCGCATA
AACATTTGCTTACGTGAATCACTCACACCCTGGTTGTAAAACTCTGGAGAACCACCTATTATTTAATG
TTGTAGCTAGTTACATTAGTAAATATTCATATGTAGCATGTTTAAGCATTAAATCAAATAAGATGAGC
AAGATGGAACGTCTG
+
F,FFFFFFFFFFFFFFFFFF:FFFFFF,FFFFFFFFFFFFFFFFFFFFF:FFFFF,FFFF:FFFFFFF
F:,FFFFFFFFFFFFFFFFF,FFFFFF:FFFFFFFFFF:FFFFFFFFFFFFFFFFFFFFFFFFFFFFF
FFFFFFFFFFFFFFF
@A00155:342:HHGFNDSXY:1:2269:9905:10066 1:N:0:GAACCTAG+TCCGCATA
ACCATTTGCTTACGTGAATCACTCACACCCTGGTTGTAAAACTCTGGAGAACCACCTATTATTTAATG
TTGTAGCTAGTTACATTAGTAAATATTCATATGTAGCATGTTTAAGCATTAAATCAAATAAGATGAGC
AAGATGGAACGTCTG
+
FF,FFFFFFFFFFFFFFFFFFFFFFFF:FFF:FFFF:FFFFFF:FFFFF:FFFF,FFFFFF::FFFFF
FFFFFFFFFFFFF,FFFF:F:FFFFFFFFFFFFFFFFFFFFFFFFFFFFFFFFFFFFFFFFFFFFFF
FFFFFFFFFFFFFFF
@A00155:342:HHGFNDSXY:1:2519:6596:9815 2:N:0:GAACCTAG+TCCGCATA
ACCATTTGCTTACGTGAATCACTCACACCCTGGTTGTAAAACTCTGGAGAACCACCTATTATTTAATG
TTGTAGCTAGTTACATTAGTAAATATTCATATGTAGCATGTTTAAGCATTAAATCAAATAAGATGAGC
AAGATGGAACGTCTG
+
F:FFFF:,FFF,:F:FFFFFFFFFFFFFFFFFFFFFFFFFFFFFFFFF:FFFFFFFFFFFFFFF:FFF
FFFFFFFFFFFF,FFFFFFFFFFFFFFFFFFFFFFFFFFFF:FFFFFFFFFFFFFFFFFFFFFFFFFF
FFFFFFFFFFFFFFF
@A00155:342:HHGFNDSXY:1:1311:19325:15577 1:N:0:GAACCTAG+TCCGCATA
CATTTGCTTACGTGAATCACTCACACCCTGGTTGTAAAACTCTGGAGAACCACCTATTATTTAATGTT
GTAGCTAGTTACATTAGTAAATATTCATATGTAGCATGTTTAAGCATTAAATCAAATAAGATGAGCAA
GATGGAACGTCTGG
+
FFFFFFFFFFFFF:FFFFFFFFFFFFFFFFFFFFFFFFFF:FFFFFFFFFFFFFFFFFFFFFFFFFFF
FFFFFFFFFFFFFFFFFFFFFFFFFFFF:FF:FFFFFF:F:FFFFFFFFFFFFFFFFFFFFFFFFFFF
FFFFFFFFFFFFFF
@A00155:342:HHGFNDSXY:1:1634:18358:3975 1:N:0:GAACCTAG+TCCGCATA
CATTTGCTTACGTGAATCACTCACACCCTGGTTGTAAAACTCTGGAGAACCACCTATTATTTAATGTT
GTAGCTAGTTACATTAGTAAATATTCATATGTAGCATGTTTAAGCATTAAATCAAATAAGATGAGCAA
GATGGAACGTCTGG
+
FFFFFFFFFFFF,FFFFFFFFFFFFFFFFF:FFFFFFFFFFFFFFFFFFFFF:FFFFFFFFF:FFFFF
FFFFFFFFFFFFFFFFFFFFFFFFFFFFFFFFFFFFFFFFFFFFFFFFFFFFFFFFFFFFFFFFFFFF
FFFFFFFFFFFFFF
@A00155:342:HHGFNDSXY:1:1146:4598:32471 1:N:0:GAACCTAG+TCCGCATA
CATTTGCTTACGTGAATCACTCACACCCTGGTTGTAAAACTCTGGAGAACCACCTATTATTTAATGTT
GTAGCTAGTTACATTAGTAAATATTCATATGTAGCATGTTTAAGCATTAAATCAAATAAGATGAGCAA
GATGGAACGTCTGGG
+
FFFFFFFFF:FFFFFFFFFFFFFFF:F:F:FFFFFFFFFFFFFFFFFFFFFF:FFFFFFFFFFFFFFF
FFFFFFFFFFFFFFFFFFFFFFFFFFFFFFFFFFFFFF:FFFFFFFFFFFFFFFFFFFFFFFFFFFFF
FFFFFFFFFFFFFFF
@A00155:342:HHGFNDSXY:1:1430:10737:10254 1:N:0:GAACCTAG+TCCGCATA
CATTTGCTTACGTGAATCACTCACACCCTGGTTGTAAAACTCTGGAGAACCACCTATTATTTAATGTT
```

```
GTAGCTAGTTACATTAGTAAATATTCATATGTAGCATGTTTAAGCATTAAATCAAATAAGATGAGCAA
GATGGAACGTCTGGG
+
FFFFFFFFFFFFFFFFFFFFFFFFFFFFFFFFFFFFFFFFFFFFFFFFFFFFFFFFFFFFFFFFFFFF
FFFFFFFFFFFFFFFFFFFFFFFFFFFFFFFFFFFFFFFFFFFFFFFFFFFFFFFFFFFFFFFFFFFF
FFFFFFFFFFFFFFF
@A00155:342:HHGFNDSXY:1:2512:27932:18897 1:N:0:GAACCTAG+TCCGCATA
CATTTGCTTACGTGAATCACTCACACCCTGGTTGTAAAACTCTGGAGAACCACCTATTATTTAATGTT
GTAGCTAGTTACATTAGTAAATATTCATATGTAGCATGTTTAAGCATTAAATCAAATAAGATGAGCAA
GATGGAACGTCTGGG
+
FFF:FFFFFFF:FFFFFFFFFF:FFFF,FF:FFFFFFFFFFFFFFFFFFFFFFFF:FFFFFFFFFFFF
FFFFFFFFFFFFFFFFFFFFFFFFFFFFFFFFFFFFFF:FFFFF:FFFFFFFFFFFFFFFFFFFFFFF
FFFFFFFFFFFFFFF
@A00155:342:HHGFNDSXY:1:2512:28013:19038 1:N:0:GAACCTAG+TCCGCATA
CATTTGCTTACGTGAATCACTCACACCCTGGTTGTAAAACTCTGGAGAACCACCTATTATTTAATGTT
GTAGCTAGTTACATTAGTAAATATTCATATGTAGCATGTTTAAGCATTAAATCAAATAAGATGAGCAA
GATGGAACGTCTGGG
+
FF:FF,FFFFFFF,,FFFFFF,FFFFFFFFFFFFF:F:::FFFF:FFFFFFFFFF,FFFFFFF:F:FF
F:,FFFFFFFFFFFFFFFFFFF::FF,FFFFFF:FFFFFFFFFFFFFFFFF:FFFFFF,FFFFFFFF:
FFFFFFFFFFF:FFF
@A00155:342:HHGFNDSXY:1:1657:22896:17848 2:N:0:GAACCTAG+TCCGCATA
ATTTGCTTACGTGAATCACTCACACCCTGGTTGTAAAACTCTGGAGAACCACCTATTATTTAATGTTG
TAGCTAGTTACATTAGTAAATATTCATATGTAGCATGTTTAAGCATTAAATCAAATAAGATGAGCAAG
ATGGAACGTCTGGGT
+
FFFFFFFFFFFFFFFFFFFFFFFFFFFFFFFFFFFFFFFFFFFFFFFFFFFFFFFFFFFFFFFFFFFF
FFFFFFFFFFFF:FFFFFFFFFF:FFFF:FF,FFF:FFFFFFFFFFFFFFFFFFFFFFFFFFFFFFFF
FFFFFFFFFFFFFFF
@A00155:342:HHGFNDSXY:1:1436:8124:6167 2:N:0:GAACCTAG+TCCGCATA
TTTGCTTACGTGAATCACTCACACCCTGGTTGTAAAACTCTGGAGAACCACCTATTATTTAATGTTGT
AGCTAGTTACATTAGTAAATATTCATATGTAGCATGTTTAAGCATTAAATCAAATAAGATGAGCAAGA
TGGAACGTCTGGGTT
+
FFFFFFFFFFFFFFFFFFFFFFFFFFFFFFFFFFFF:FFFFFFFFFFFFFFFFFFFFFFFF,FFFFF:
FFFFFFFFFFFFFFFFFFFFFFFFFFFFFFFFFFFF:FFF:FFFFFF:FFF:FFFFFFFFFFFFFFFF
F:FFFFFFFFFF:FF
@A00155:342:HHGFNDSXY:1:1669:9860:20541 1:N:0:GAACCTAG+TCCGCATA
TGCTTACGTGAATCACTCACACCCTGGTTGTAAAACTCTGGAGAACCACCTATTATTTAATGTTGTAG
CTAGTTACATTAGTAAATATTCATATGTAGCATGTTTAAGCATTAAATCAAATAAGATGAGCAAGATG
GAACGTCTGGGTTCC
+
FFFFFFFF:FFFFFFFFFFFFFFFFF:FF:FFFFFFFFFFFFFFFFFFFFFFFFFFFFFFFFFFFFFF
FFFFFFFFFFFFFFFFFFFFFFFFFFFFFFFFFFFFFFFFFFFFFFFFFFFFFFFFFFFFFFFFFFFF
FFFFFFFFFFFFFFF
@A00155:342:HHGFNDSXY:1:1669:11532:17581 1:N:0:GAACCTAG+TCCGCATA
TGCTTACGTGAATCACTCACACCCTGGTTGTAAAACTCTGGAGAACCACCTATTATTTAATGTTGTAG
CTAGTTACATTAGTAAATATTCATATGTAGCATGTTTAAGCATTAAATCAAATAAGATGAGCAAGATG
GAACGTCTGGGTTCC
+
FFFFFF:F,FFFFFFFFFFFFFFFFFFFFFFFFFFFFFFFFFFFFFFFFFFFFFFFFFFFFFFFFFFF
FFFFFFFFFFFFFFFFFFFFFFFFFFFFFFFFFFFFFFFFFFFFFFFFFFFFFFFFFFFFFFFFFFFF
FFFFFFFFFFFFFFF
```

```
@A00155:342:HHGFNDSXY:1:1230:5348:22467 2:N:0:GAACCTAG+TCCGCATA
TACGTGAATCACTCACACCCTGGTTGTAAAACTCTGGAGAACCACCTATTATTTAATGTTGTAGCTAG
TTACATTAGTAAATATTCATATGTAGCATGTTTAAGCATTAAATCAAATAAGATGAGCAAGATGGAAC
GTCTGGGTTCCAATT
+
FFFFFFFFFFFFFFFFFFFFFFFF:FFFFFFFFFFFFFFFFFFFFFFFFFFFFFFFFFFFFFFFFFFF
FFFFFFFFFFFFFFFFFFFFFFFFFFFF:FFFFFFFFFFFFFFFFFFFFFFFFFFFFFFFFFFFFFFF
FFFFFFFFFFFFFFF
@A00155:342:HHGFNDSXY:1:1103:26910:30405 2:N:0:GAACCTAG+TCCGCATA
CGTGAATCACTCACACCCTGGTTGTAAAACTCTGGAGAACCACCTATTATTTAATGTTGTAGCTAGTT
ACATTAGTAAATATTCATATGTAGCATGTTTAAGCATTAAATCAAATAAGATGAGCAAGATGGAACGT
CTGGGTTCCAATTCT
+
FFFFFFFFFFFFFFFFFFFFFFFFFFFFFFFFFFFFFFFFFFFFFFFFFFFFFFFF:F:FFFFFFFFFF
FFF:FFFFFFFFFFFFFF:FFF:FFFFFFFFFFFFFFFFFFFFFFFFFFFFFFFFFFFFFFFFFFFFFF
:FFFFFFFFFFFFFF
@A00155:342:HHGFNDSXY:1:1267:6831:32643 2:N:0:GAACCTAG+TCCGCATA
CGTGAATCACTCACACCCTGGTTGTAAAACTCTGGAGAACCACCTATTATTTAATGTTGTAGCTAGTT
ACATTAGTAAATATTCATATGTAGCATGTTTAAGCATTAAATCAAATAAGATGAGCAAGATGGAACGT
CTGGGTTCCAATTCT
+
FF:FF:FFFFFFFFFFFFFFFFFFFFFFFFF,FFFFFFFFFFFFFFFFFFFFFFFFFFFFFFFFFFFFF
FF:FFFFFFFFFFF:FFF:FFF:FFFFFFFFFFFFFFFFF:FFFFFFFFFFFFFFFFFFFFFFFFFFFF
FFFFFF:F,F,FFFF
@A00155:342:HHGFNDSXY:1:1269:25183:8844 2:N:0:GAACCTAG+TCCGCATA
CGTGAATCACTCACACCCTGGTTGTAAAACTCTGGAGAACCACCTATTATTTAATGTTGTAGCTAGTT
ACATTAGTAAATATTCATATGTAGCATGTTTAAGCATTAAATCAAATAAGATGAGCAAGATGGAACGT
CTGGGTTCCAATTCT
+
FFFFFFFFFFFFFFFFFFFFF:FFFFFFFF:FFFFFFFFFFFFFFFFFFFFFFF:FFFFFFFFFFFFF
,FFFFFFFFFFFFFFFFF:FFFFFFFFFFFFFFF:FFFFFFFFFFFFFFFFFFFFFFFFFFFFFF,FFF
FFFFFFFFFFFF:FF
@A00155:342:HHGFNDSXY:1:1528:6804:23453 2:N:0:GAACCTAG+TCCGCATA
CGTGAATCACTCACACCCTGGTTGTAAAACTCTGGAGAACCACCTATTATTTAATGTTGTAGCTAGTT
ACATTAGTAAATATTCATATGTAGCATGTTTAAGCATTAAATCAAATAAGATGAGCAAGATGGAACGT
CTGGGTTCCAATTCT
+
FFFFFFFFFFFFFFFFFF:FFFFFFFFFFFFFFFFFFFFFFFFFFFFFFFFFF:FFFFFFFFFFFFFFF
FFFFFFFFF:FFF:FFFFFFFFFFFFFFFFFFFFFFFFFFFFFFFFFFFFFFFFFFFFFFFFFFFFFFF
FFFFFFFFFFFFFFF
@A00155:342:HHGFNDSXY:1:1562:4553:15201 2:N:0:GAACCTAG+TCCGCATA
CGTGAATCACTCACACCCTGGTTGTAAAACTCTGGAGAACCACCTATTATTTAATGTTGTAGCTAGTT
ACATTAGTAAATATTCATATGTAGCATGTTTAAGCATTAAATCAAATAAGATGAGCAAGATGGAACGT
CTGGGTTCCAATTCT
+
FFFFFFFFFFFFFFFFFFFFFFFFFFFFFFFFFFFFFFFFFFFFF:FFFFFFFFFFFFFFFFFFFFFFF
FFFF:FFFF:F:FFFFFFFFFFFFFFFFFFFFF:FFFFFFFFFFFFFFFFFFFFFFFFFFFFFFFFFF,F
FFF::FFFFFFFFFF
@A00155:342:HHGFNDSXY:1:1604:2076:8844 2:N:0:GAACCTAG+TCCGCATA
CGTGAATCACTCACACCCTGGTTGTAAAACTCTGGAGAACCACCTATTATTTAATGTTGTAGCTAGTT
ACATTAGTAAATATTCATATGTAGCATGTTTAAGCATTAAATCAAATAAGATGAGCAAGATGGAACGT
CTGGGTTCCAATTCT
+
F,FFFFFFF:FFFFFFFFFFFFFFFFF:F:FFFFFFFFFFFFFFFFFFFFFFFFFFFFFFFFFFFFFFF
```

FFFFFFFFFFFFFFFFFFFFFFFFFF,:FFFFFFFFFFFFFFFFFFFFFFFF:FFFFFFFF,FFFFFF
:FFFFFFF:FFFFFF
@A00155:342:HHGFNDSXY:1:1604:2130:8844 2:N:0:GAACCTAG+TCCGCATA
CGTGAATCACTCACACCCTGGTTGTAAAACTCTGGAGAACCACCTATTATTTAATGTTGTAGCTAGTT
ACATTAGTAAATATTCATATGTAGCATGTTTAAGCATTAAATCAAATAAGATGAGCAAGATGGAACGT
CTGGGTTCCAATTCT
+
FFFFFFFFFFFFFFFF:FFFFFFFFFFFFFFFF:FFFFFFFFFFFFFFFFFFFFFFFFFFFF::FFFFF
FFFFFFFFFFFFFFFF:FFFFFFFFFFFFFFFFFFFFFFFFFFFFFFFFFFFFFFFFF:FFFFFF:FF
FFFFFFFFFFFFFFF
@A00155:342:HHGFNDSXY:1:2532:1551:15139 2:N:0:GAACCTAG+TCCGCATA
GTGAATCACTCACACCCTGGTTGTAAAACTCTGGAGAACCACCTATTATTTAATGTTGTAGCTAGTTA
CATTAGTAAATATTCATATGTAGCATGTTTAAGCATTAAATCAAATAAGATGAGCAAGATGGAAAGTC
TGGGTTACAATTA
+
FFFFFFFFFF:FFFFFFFFFFF,FFF:FFFFFFFFFFFFF:FFFFFFFFFFFFFFFFFFFFFFFFFFF
FFFFF:FFFFFFFFFFFFF,FF:F:FFFFFFFFF,FFF,FFFFFFF:FFFFF:,FFF::F:,FF,FFF
FFFFFF,,:FFF:
@A00155:342:HHGFNDSXY:1:2127:2862:22733 2:N:0:GAACCTAG+TCCGCATA
GTGAATCACTCACACCCTGGTTGAAAAACTCTGGAGAACCACCTATTATTTAATGTTGTAGCTAGTTA
CATTAGTAAATATTCATATGTAGCATTTTTAAGCATTAAATCAAATAAGATGAGCAAGATGGAACGTA
TGGGTTCCAATTCT
+
FFFFF,,F,F:F,F,FFFF:F::,FF:::,FFF:FFF:F,FFFFF,FF:FFFFFFF,:FFF:FF::FF
FFF:FF,FFFFFFFFF,FF,FF:FFF,FF,F::FFFFFFFF,FFF:FF::F,F,,FF,::,:FFF,F,
::,FFFFFFFFF,F
@A00155:342:HHGFNDSXY:1:1132:3224:20353 1:N:0:GAACCTAG+TCCGCATA
GTGAATCACTCACACCCTGGTTGTAAAACTCTGGAGAACCACCTATTATTTAATGTTGTAGCTAGTTA
CATTAGTAAATATTCATATGTAGCATGTTTAAGCATTAAATCAAATAAGATGAGCAAGATGGAACGTC
TGGGTTCCAATTCTG
+
FFFFFFFFFFFFFFFFFFFFFFFFFFFFFFFFFFFFFFFFFFFFFFFFFFFFFFFFFFFFFFF:FFFFF
FFFFFFFFFFFFFFFFFFFFFF:FFFFFFFFFFFFFFFFFFFFFFF:FFFFFFFFFFFFFFFFFFFFFFFF
FFFFFFFFFFFFFFF
@A00155:342:HHGFNDSXY:1:1374:22083:29528 1:N:0:GAACCTAG+TCCGCATA
GTGAATCACTCACACCCTGGTTGTAAAACTCTGGAGAACCACCTATTATTTAATGTTGTAGCTAGTTA
CATTAGTAAATATTCATATGTAGCATGTTTAAGCATTAAATCAAATAAGATGAGCAAGATGGAACGTC
TGGGTTCCAATTCTG
+
FFFFFFFFFFFFFFFFFFFFFFFF,FFFFFFFFFFFFFFFFFFFFFFFFFFFFFFFFFFFFFFFFFFF
FFFFFFFFFFFFFFFFFFFFFFFFFFFFFFFFFFFFFFFFFFFFFFFFFFFFFFFFFFFFFFFFFFFF
FFFFFFFFFFFFFFF
@A00155:342:HHGFNDSXY:1:1150:22146:18771 2:N:0:GAACCTAG+TCCGCATA
GTGAATCACTCACACCCTGGTTGTAAAACTCTGGAGAACCACCTATTATTTAATGTTGTAGCTAGTTA
CATTAGTAAATATTCATATGTAGCATGTTTAAGCATTAAATCAAATAAGATGAGCAAGATGGAACGTC
TGGGTTCCAATTCTG
+
FFFF:FFFFFFFFFFFFFFFFFFFFFFFFFFFFFFFFFFFFFFFFFFFFFFFF:FFFFFFFFFFFFFFF
FFFFFFFFFFFFFFFFFFFFFFFFFF:FFFFFFFFFFFFF:FFFFFFFFFFFFFFFFFFFFFFFFFFFF
FFFFFFFFFFFFFFF
@A00155:342:HHGFNDSXY:1:1150:25301:30311 2:N:0:GAACCTAG+TCCGCATA
GTGAATCACTCACACCCTGGTTGTAAAACTCTGGAGAACCACCTATTATTTAATGTTGTAGCTAGTTA
CATTAGTAAATATTCATATGTAGCATGTTTAAGCATTAAATCAAATAAGATGAGCAAGATGGAACGTC
TGGGTTCCAATTCTG

+
FFFFFFFFFFFFFFFFFFFFFFFFFFFFFFFFFFFFFFFFFFFFFFFFF:FFFFFFF:FFF:FFFFFFFF
FFFFFFFFFFFFFFFFFFFFFFFFF:FFFFFFFFFFFFFFFFFFFFFFFFFFFF:FFFFFFFFFFFFFFF
FFFFFFFFFFFFFFF
@A00155:342:HHGFNDSXY:1:1413:20763:15718 2:N:0:GAACCTAG+TCCGCATA
GTGAATCACTCACACCCTGGTTGTAAAACTCTGGAGAACCACCTATTATTTAATGTTGTAGCTAGTTA
CATTAGTAAATATTCATATGTAGCATGTTTAAGCATTAAATCAAATAAGATGAGCAAGATGGAACGTC
TGGGTTCCAATTCTG
+
FFFFFFFFFFFFFFFFFFFFFFFFFFFFFFFFFFFFFFFFFFFFFFFFFFFFFFFFFFFFFFFFFFFFFF
FFFFFFFFFFFFFFFFFFFFFFFFFFFFFFFFFFFFFFFFFFFFFFFFFFFFFFFFFFFFFFFFFFFFFF
FFFFFFFFFFFFFFF
@A00155:342:HHGFNDSXY:1:1536:7627:13761 2:N:0:GAACCTAG+TCCGCATA
GTGAATCACTCACACCCTGGTTGTAAAACTCTGGAGAACCACCTATTATTTAATGTTGTAGCTAGTTA
CATTAGTAAATATTCATATGTAGCATGTTTAAGCATTAAATCAAATAAGATGAGCAAGATGGAACGTC
TGGGTTCCAATTCTG
+
FFFF:FFFFFFFFFFFFFFFFFFFFFFFFFFFFFFFFF:FFFFFFFFFFFFFFFFFFFFFFFFFFFFFFF
FFFFFFFFFFFFFFFFFFFFFFFFFFFFFFFFFFFFFFFFFFFFFFFFF:FFFFFFFFFFFFFFFFFFFF
FFFFFFFFFFFFFFF
@A00155:342:HHGFNDSXY:1:2449:3405:3568 2:N:0:GAACCTAG+TCCGCATA
GTGAATCACTCACACCCTGGTTGTAAAACTCTGGAGAACCACCTATTATTTAATGTTGTAGCTAGTTA
CATTAGTAAATATTCATATGTAGCATGTTTAAGCATTAAATCAAATAAGATGAGCAAGATGGAACGTC
TGGGTTCCAATTCTG
+
FFFFFFFFFFFFFFFFFFFFFFFFFFFFFFFFFFFFFFFFFFFFFFFFFFFFFFFFF:FFFFFFFFFFF:
FF:F:FFFFFFFFFFFF:FFFFF,FFFFFFF,FFFFFF::FFFFFF:FF:FF:FFFFFFFFFF,FFF,
F:FFFFFFFF:FFFF
@A00155:342:HHGFNDSXY:1:2532:2157:15405 2:N:0:GAACCTAG+TCCGCATA
GTGAATCACTCACACCCTGGTTGTAAAACTCTGGAGAACCACCTATTATTTAATGTTGTAGCTAGTTA
CATTAGTAAATATTCATATGTAGCATGTTTAAGCATTAAATCAAATAAGATGAGCAAGATGGAACGTC
TGGGTTCCAATTCTG
+
FFFFFFFFFFFFFFFFFFFFFFFFFFF:FFFFFFFF:FFFFFFFFFFFFFFFFFFFFFFFFFFFFFFFFF
FFFFFFFF:FFFFFFFFFFFFFF:,FFFFFFFFF:FFFFFFFFFFFFFFF:F:,F:FFFFFFFFFF:FF
FFF:FFFFFFFFFF:
@A00155:342:HHGFNDSXY:1:2676:20826:21590 1:N:0:GAACCTAG+TCCGCATA
TGAATCACTCACACCCTGGTTGTAAAACTCTGGAGAACCACCTATTATTTAATGTTGTAGCTAGTTAC
ATTAGTAAATATTCATATGTAGCATGTTTAAGCATTAAATCAAATAAGATGAGCAAGATGGAACGTCT
GGGTTCCAATTCTGT
+
:FFFFFFFFFFFFFFFFFFFFFFFFFFFFFFFFFFFFFFFFFFFFFFFFFFFFFFFFFFFFFFFFFFFFF
FFFFFFFFFFFFFFFF:FFFF:FFFFFFFFFFFFFFFFFFFFFFFFFFFFFFFFF:FFFFFFFFF:FF
FFFFFFFFFFFFFFF
@A00155:342:HHGFNDSXY:1:1261:19976:10316 2:N:0:GAACCTAG+TCCGCATA
GAATCACTCACACCCTGGTTGTAAAACTCTGGAGAACCACCTATTATTTAATGTTGTAGCTAGTTACA
TTAGTAAATATTCATATGTAGCATGTTTAAGCATTAAATCAAATAAGATGAGCAAGATGGAACGTCTG
GGTTCCAATTCTGTC
+
FF:FFFFFFFFFFFFFFFFFFFFFFFFFFFFFFFFFFFFFFFFFFFFFFFFFFFFFFFFFFFFFFFFFFF
FFFFFF:FFFFFFFFFFFFFFFFFFFFFFFFFFFFFFF:FFFFFFFFFFFFFFFFFFFFFFFF:FFFFFF
FFFFFFFFFFFFFFF
@A00155:342:HHGFNDSXY:1:1675:24008:16611 2:N:0:GAACCTAG+TCCGCATA
GAATCACTCACACCCTGGTTGTAAAACTCTGGAGAACCACCTATTATTTAATGTTGTAGCTAGTTACA

TTAGTAAATATTCATATGTAGCATGTTTAAGCATTAAATCAAATAAGATGAGCAAGATGGAACGTCTG
GGTTCCAATTCTGTC
+
FFFFFFFFFFFFFFFFFFFFFFFFF::FFFFFFFFFFFFFFFFFFFFFFFFF:FFFFFFFFFFFF,FF
FFFFFFFFFFFFFFFFFFFFFFFFFFFFFFFFFFFFFFFFFFFFFFFFFFFFFFFFFFFFFFFFFFFF
F:FFFFFFFFFFFFF
@A00155:342:HHGFNDSXY:1:2302:4544:28025 2:N:0:GAACCTAG+TCCGCATA
GAATCACTCACACCCTGGTTGTAAAACTCTGGAGAACCACCTATTATTTAATGTTGTAGCTAGTTACA
TTAGTAAATATTCATATGTAGCATGTTTAAGCATTAAATCAAATAAGATGAGCAAGATGGAACGTCTG
GGTTCCAATTCTGTC
+
F:FFFFFFFFFFFFFFFFFFFFFF:FFFFFFFFFFFFFFFFFFFFFFFFFFFFFFFFFFFFFFFFFFF
FFFFFFFFFFFFFFFFFFFFFFFF:FFFFFFFFFFFFFFFFFFFFFFFFFF:FFF,FF:FFF:FFFFF
FFFFF:FFFFFFFFF
@A00155:342:HHGFNDSXY:1:2261:18041:14074 2:N:0:GAACCTAG+TCCGCATA
GAATCACTCACACCCTGGATGTAAAACTCTGGAGAACCACCTATTATTTAATGTTGTAGCTAGTTACA
TTAGTAAATATTCATATGTAGCATGTTTAAGCATTAAATCAAATAAGATGAGCAAGATGGAACGTCTG
GGTTCCAATTCTGTC
+
FFFFFFFFFFFFFFFFFFFFFFFFFFFFFFFFFFFFFFFFFFFFFFFFFFFFFFFFFFFFFFFFFFFF
F:FF,FFFFFFFFFFFFFFFFFFFFFFFFFFFFFFFFFFFFFF:FFFFFFFFFFFFFFFFFF:FFFFF
FFFFFFF,FFFFFFF
@A00155:342:HHGFNDSXY:1:2330:17110:10676 2:N:0:GAACCTAG+TCCGCATA
GAATCACTCACACCCTGGTTGTAAAACTCTGGAGAACCACCTATTATTTAATGTTGTAGCTAGTTACA
TTAGTAAATATTCATATGTAGCATGTTTAAGCATTAAATCAAATAAGATGAGCAAGATGGAACGTCTG
GGTTCCAATTCTGTC
+
FFFFFFFFFFFFFFFFFFFFFFFFFFFFFFFFFFFFFFFFFFFFFFFFF,FFF:F:FFFFFFFFFFFF
F,FFFFFFFFFFFFFFFFFFFFFFFFFFFFFFFFFFFFFFFFFFFFFFFFFFFFFFFFFFFFFFFFFF
FFFFFF:FFFFFFFF
@A00155:342:HHGFNDSXY:1:2338:32913:5979 1:N:0:GAACCTAG+TCCGCATA
AATCACTCACACCCTGGTTGTAAAACTCTGGAGAACCACCTATTATTTAATGTTGTAGCTAGTTACAT
TAGTAAATATTCATATGTAGCATGTTTAAGCATTAAATCAAATAAGATGAGCAAGATGGAACGTCTGG
GTTCCAATTCTGTC
+
FFFFFFFFFFFFF:FFFFFFFFFFFFFFFFFFFFFFFFFFFF:FFF,FFFFFFFFFFFFFFFFFFFFF
F:FFFFFFFFFFFFFFFFFFFFFFFFFFFFFFFFFFFFFFFFFFFFFFFF:FFFFFFFFFFFFFFFFF
FFFFFFFFFFFFFF
@A00155:342:HHGFNDSXY:1:1541:2790:19194 1:N:0:GAACCTAG+TCCGCATA
AATCACTCACACCCTGGTTGTAAAACTCTGGAGAACCACCTATTATTTAATGTTGTAGCTAGTTACAT
TAGTAAATATTCATATGTAGCATGTTTAAGCATTAAATCAAATAAGATGAGCAAGATGGAACGTCTGG
GTTCCAATTCTGTCA
+
FFFFFFFFFFFFFF:FFFFFFFFFFFFFFFFFFFFFFFFFFFFFFFFFFFFFFFFFFFFFFFFFFFFF
FFFFFFFFFFFFFF:FFFFFFFFFFFFFFFFFFFFFFFFFFFFFFFFFFF:FFFFFFFFFFFFFFFFF
FFFFFFFFFFFFFFF
@A00155:342:HHGFNDSXY:1:2247:16848:3897 1:N:0:GAACCTAG+TCCGCATA
TATCACTCACACCCTGGTTGTAAAACTCTGGAGAACCACCTATTATTTAATGTTGTAGCTAGTTACAT
TAGTAAATATTCATATGTAGCATGTTTAAGCATTAAATCAAATAAGATGAGCAAGATGGAACGTCTGG
GTTCCAATTCTGTCA
+
FFFFFFFFFFFFFFFFFFFFFFFFFFFFFFFFFFFFFFFFFFFFFFFFF::FFFFFFFFFFFFF:FFF
FFFFFFFFFFFFFFFFFFFFFFFFFFFF,FFFFFFFFFFFFFFFFFFFFFFFFFFFFFFFFFFFFFFF
FFFFFFFFFFFFFFF

```
@A00155:342:HHGFNDSXY:1:1323:31611:1752 2:N:0:GAACCTAG+TCCGCATA
AATCACTCACACCCTGGTTGTAAAACTCTGGAGAACCACCTATTATTTAATGTTGTAGCTAGTTACAT
TAGTAAATATTCATATGTAGCATGTTTAAGCATTAAATCAAATAAGATGAGCAAGATGGAACGTCTGG
GTTCCAATTCTGTCA
+
FFFFFFFF:FFFFF:FF:FFFFFFFFFFFFFFFFFFFFFFFFFFFFFFFF:FFFFFFFFFFFFFFFF
FFFFFFFFFFFFFFFFFF:FFFFFFFFFFFFFFFFFFFFFFFFF:FFFFFFFFFFFFFFFFFFFFFFFF
FFFFFFFFFFFFFFF
@A00155:342:HHGFNDSXY:1:1121:17852:36260 2:N:0:GAACCTAG+TCCGCATA
ATCACTCACACCCTGGTTGTAAAACTCTGGAGAACCACCTATTATTTAATGTTGTAGCTAGTTACATT
AGTAAATATTCATATGTAGCATGTTTAAGCATTAAATCAAATAAGATGAGCAAGATGGAACGTCTGGG
TTCCAATTCTGTCAA
+
FFFFFFFFFFFFFFFFF:FF:FFFFFFFFFFFFFFFFFFFFF:,FFFFFFFFFFFFFFFFFFFFFFFF
FFFFFF:FFFFFFFFFFFFFFFFFFFFFFFFFFFFFF:FFFFFFFFFFFFFFFFFFFFFFF:FFFF:FF
FF:FFFFF:FFFFF:
@A00155:342:HHGFNDSXY:1:1114:25753:14779 2:N:0:GAACCTAG+TCCGCATA
ATCACTCACACCCTGGTTGTAAAACTCTGGAGAACCACCTATTATTTAATGTTGTAGCTAGTTACATT
AGTAAATATTCATATGTAGCATGTTTAAGCATTAAATCAAATAAGATGAGCAAGATGGAACGTCTGGG
TTCCAATTCTGTCAA
+
FFFFFFFFFFFFFFFFFFFFFFFFFFFFFFFFFFFFFFFFFFFFFFFFFFFFFFFFFFFFFFFFFFFF
FFFFFFFFFFFFFFFFFFFFFFFFFFFFFFFFFFFFFFFFFFFFFFFFFFFFFFFFFFFFFFFFFFFF
FFFFFFFFFFFFFFF
@A00155:342:HHGFNDSXY:1:1273:4408:7341 2:N:0:GAACCTAG+TCCGCATA
ATCACTCACACCCTGGTTGTAAAACTCTGGAGAACCACCTATTATTTAATGTTGTAGCTAGTTACATT
AGTAAATATTCATATGTAGCATGTTTAAGCATTAAATCAAATAAGATGAGCAAGATGGAACGTCTGGG
TTCCAATTCTGTCAA
+
FFFFFFFFFFFFFFFFFFFFFFFFFFFFFFFFFFFFFFFFFFFFFFFFFFFFFFFFFFFFFFFFFFFF
FFFFFFFFFFFFF:FFFFFFFFFFFFFF:FFFFFF,FFFFFFFFFFFFFF:FFF:FFFFF:FFFF:F:
FFFFFFFFFFFFFFF
@A00155:342:HHGFNDSXY:1:1407:3341:9283 2:N:0:GAACCTAG+TCCGCATA
ATCACTCACACCCTGGTTGTAAAACTCTGGAGAACCACCTATTATTTAATGTTGTAGCTAGTTACATT
AGTAAATATTCATATGTAGCATGTTTAAGCATTAAATCAAATAAGATGAGCAAGATGGAACGTCTGGG
TTCCAATTCTGTCAA
+
FFFFFFFFFFFFFFFFFFFFFFFFFFFFFFFFFFFFFFFFFFFFFFFFFFFFFFFFFFFFFFFFFFFF
FFFFFFFFFFFFFFFFFFFFFFFFFFFFFFFFFFFF:FFFFFFFFFFFFFFFFFFFFFFF:FFFFFFFF
FFFFFFFFFFFFF,F
@A00155:342:HHGFNDSXY:1:1671:31937:36573 2:N:0:GAACCTAG+TCCGCATA
ATCACTCACACCCTGGTTGTAAAACTCTGGAGAACCACCTATTATTTAATGTTGTAGCTAGTTACATT
AGTAAATATTCATATGTAGCATGTTTAAGCATTAAATCAAATAAGATGAGCAAGATGGAACGTCTGGG
TTCCAATTCTGTCAA
+
FFFFFFFFFFFFF,FFFFFFFFFFFFFFFFFFFFFFFFFFFFFFFFFFFFFFFFFFFFFFFFFFFFFF
FFFFFFFF:FFFFFFFFFFFFFFFFFFFFFFFFFFFFFFFFFFFFFFFFFFFFFFFF,FFFFFFFF
FFFFFFFFFFFFFFF
@A00155:342:HHGFNDSXY:1:2114:24849:9298 2:N:0:GAACCTAG+TCCGCATA
ATCACTCACACCCTGGTTGTAAAACTCTGGAGAACCACCTATTATTTAATGTTGTAGCTAGTTACATT
AGTAAATATTCATATGTAGCATGTTTAAGCATTAAATCAAATAAGATGAGCAAGATGGAACGTCTGGG
TTCCAATTCTGTCAA
+
FFFFFFFFFFFFFFFFFFFFFFFFFFFFFFFFFFFFFFFFFFFFFFFFFF:FFFFFFFFFFFFFFFFF
```

FFFFFFFFFFFFFF,FFF:FFFFFFFFFFFFFFFFFFFFFFFFFFFFFFFFFFFFFFFF,FFFFFFFF
:FFFFFFFFFFFFFF
@A00155:342:HHGFNDSXY:1:2235:3748:31845 2:N:0:GAACCTAG+TCCGCATA
ATCACTCACACCCTGGTTGTAAAACTCTGGAGAACCACCTATTATTTAATGTTGTAGCTAGTTACATT
AGTAAATATTCATATGTAGCATGTTTAAGCATTAAATCAAATAAGATGAGAAAGATGGAACGTCTGGG
TTCCAATTCTGTCAA
+
FFFFFFFFFFFFFFFFFFFFFFF:FFFFFF:FFFFF:FFFFFFFFFFFFFFFFFFFFFFFFFFFFFFF
FFFFFFFFFFFFFFF:FFFFFFFFFFFFFFFFFFF:FFF:FFFFFFFFFF,F,FFF,FFFFFFFFFF:
FFF,FFFFFF,F:FF
@A00155:342:HHGFNDSXY:1:2238:10176:33990 2:N:0:GAACCTAG+TCCGCATA
ATCACTCACACCCTGGTTGTAAAACTCTGGAGAACCACCTATTATTTAATGTTGTAGCTAGTTACATT
AGTAAATATTCATATGTAGCATGTTTAAGCATTAAATCAAATAAGATGAGCAAGATGGAACGTCTGGG
TTCCAATTCTGTCAA
+
FFFFFFFFFFFFFFFFFFFFFFFFFFFFFFFFFFFFFFFFFFFFFFFFF:FFFFFFFFFFFFFFFFFF
FFFFFFFFFFFFFFFFFFFFFFFFFFFFFFFFFFFFFFFFFFFFFFFFFFFFFFFFFFFFFFFFFFFF
FFFFFFFFFFFFFFF
@A00155:342:HHGFNDSXY:1:2247:16848:3897 2:N:0:GAACCTAG+TCCGCATA
ATCACTCACACCCTGGTTGTAAAACTCTGGAGAACCACCTATTATTTAATGTTGTAGCTAGTTACATT
AGTAAATATTCATATGTAGCATGTTTAAGCATTAAATCAAATAAGATGAGCAAGATGGAACGTCTGGG
TTCCAATTCTGTCAA
+
FFFFFFF:FFFFF:FF:FFFFF:FFFFFFF:FFFFFFFFFFFFFF:FFFFF,FFFFFFFFFFFFF:F:
FFFFFFFF,FFFFF:F,FFFFFF:FFFFFFFFFFFFFFFFFFFFFFFFFFFFFFFFFFF,FFFFFFFF
FFFFFFFFFFFFFFF
@A00155:342:HHGFNDSXY:1:2564:32398:25974 2:N:0:GAACCTAG+TCCGCATA
ATCACTCACACCCTGGTTGTAAAACTCTGGAGAACCACCTATTATTTAATGTTGTAGCTAGTTACATT
AGTAAATATTCATATGTAGCATGTTTAAGCATTAAATCAAATAAGATGAGCAAGATGGAACGTCTGGG
TTCCAATTCTGTCAA
+
FFF:F:FFFFFFF,FF:FFFF:FFFFF:FFFFF:FFFFFFFFFF,FFFFFFF:FFFFF,,FFFFF:,F
FFFFFFFFFFFF:F,FFFFFFFFF,:FFFFF:FFFFFFF:FF,FFF:FFFF:F:FFFFF,FFFFFFFF
FFF:FF,FFFFFFFF
@A00155:342:HHGFNDSXY:1:1208:24605:17456 1:N:0:GAACCTAG+TCCGCATA
GATCTCACACCCTGGTTGTAAAACTCTGGAGAACCACCTATTATTTAATGTTGTAGCTAGTTACATTA
GTAAATATTCATATGTAGCATGTTTAAGCATTAAATCAAATAAGATGAGCAAGATGGAACGTCTGGGT
TCCAATTCTGT
+
FFFFFF,FFFFFFFFFFFFFFFFFFFFFFFFFFFFFFFFFFFF:FFFFFFFFF:FFFFFFFFFFFFFF
FFFFFFFFFFFFFFFFFFFFFFFFFFFFFFFFFFFFFFFFFFFFFFFFFFFFFFFFFFFFFFFFFFFF
FFFFFFFFFFF
@A00155:342:HHGFNDSXY:1:1208:26051:18364 1:N:0:GAACCTAG+TCCGCATA
GATCTCACACCCTGGTTGTAAAACTCTGGAGAACCACCTATTATTTAATGTTGTAGCTAGTTACATTA
GTAAATATTCATATGTAGCATGTTTAAGCATTAAATCAAATAAGATGAGCAAGATGGAACGTCTGGGT
TCCAATTCTGT
+
FFFFFFFFFFFFFFFFFFFFFFFFFFFFFFFF:FFFFFFFFFFF:FFFFFFFFF::FFFFFFFFFFFF
FFFFFFFFFFFF,FFFFFFFFF:FFFFF:FFFFFFFFFFFFFFFFFFFFFFFFFFFFFFFFFFFFFFF
FFFFFFFFFFF
@A00155:342:HHGFNDSXY:1:1164:1976:16689 1:N:0:GAACCTAG+TCCGCATA
TCACTCACACCCTGGTTGTAAAACTCTGGAGAACCACCTATTATTTAATGTTGTAGCTAGTTACATTA
GTAAATATTCATATGTAGCATGTTTAAGCATTAAATCAAATAAGATGAGCAAGATGGAACGTCTGGGT
TCCAATTCTGT

+
FFFFFFFFFFFFFFFFFFFFFFFFFFF,F:FFFFFFFFFFFFFF,FFFFFFFFFFFFFFFFFF:FFFFFF
FFFFFFFFFF,FFFFFFFFFFFFFFFFFFFFFFFFFFFFFFFFFFF:FFFFFFFFFFFFFFFFFFFFFF,
FFFFFFFFFFF
@A00155:342:HHGFNDSXY:1:1420:7084:24314 1:N:0:GAACCTAG+TCCGCATA
GATCTCACACCCTGGTTGTAAAACTCTGGAGAACCACCTATTATTTAATGTTGTAGCTAGTTACATTA
GTAAATATTCATATGTAGCATGTTTAAGCATTAAATCAAATAAGATGAGCAAGATGGAACGTCTGGGT
TCCAATTCTGT
+
FFFFFFFFFFFFFFFFFFFFFFFFFFFFFFFFFFFFFFFFFFFFFFFFFFFFFFFFFFFFFFFFFFFFFF
FFFFFFFFFFFFFFFFFFFFFFFFFFFFFFFFFFFFFFFFFFFFFFFFFFFFFFFFFFFFFFFFFFFFFF
FFFFFFFFFFF
@A00155:342:HHGFNDSXY:1:2123:18864:14622 1:N:0:GAACCTAG+TCCGCATA
TCACTCACACCCTGGTTGTAAAACTCTGGAGAACCACCTATTATTTAATGTTGTAGCTAGTTACATTA
GTAAATATTCATATGTAGCATGTTTAAGCATTAAATCAAATAAGATGAGCAAGATGGAACGTCTGGGT
TCCAATTCTGT
+
FFFFFFFFFFFFFFF:FFFFFFFFFFFFFFFFFFFFFFFFF:FFFFFFFFFFFFFFFFFFFFFFFFFF:F
FFFFFFFFFFFFFFFFFFFFFFFFFFFFFFFFFFFFFFFF:FFFFFFFFFFFFFFFFFFFFFFFFFFFF:
FFFFFFFFFFF
@A00155:342:HHGFNDSXY:1:2169:32850:2487 1:N:0:GAACCTAG+TCCGCATA
TCACTCACACCCTGGTTGTAAAACTCTGGAGAACCACCTATTATTTAATGTTGTAGCTAGTTACATTA
GTAAATATTCATATGTAGCATGTTTAAGCATTAAATCAAATAAGATGAGCAAGATGGAACGTCTGGGT
TCCAATTCTGT
+
FFFFFFFFFFFFFFFFFFFFFFFFFFFFFFFFFFFFFFFFFFFF::FFFFFFFFFFFFFFFFF::FFFFF
FFFFFFFFFFFFFFFFFFFFF:,FFFFFFFFFFFFFFFFFFFFFFFFFF:FFFFFF:FFFFFFFFFFFFF
FFFFFFFFFFF
@A00155:342:HHGFNDSXY:1:2526:4182:35509 1:N:0:GAACCTAG+TCCGCATA
TCACTCACACCCTGGTTGTAAAACTCTGGAGAACCACCTATTATTTAATGTTGTAGCTAGTTACATTA
GTAAATATTCATATGTAGCATGTTTAAGCATTAAATCAAATAAGATGAGCAAGATGGAACGTCTGGGT
TCCAATTCTGT
+
FFFFFFFFFFFFFFFFF:FF:FFF:FFFFFFFFF:FFFFFFFFFFFFFF,FFFFFFF:FFFFF:FFFFFF
FFFFFFFFFFFFFFFFFFFFFFFFF:FFFFFFFFFFFFFFFFFFFFFFFFFFFFFFFFFFFFFFFFFFFF
FFFFF:FFFFF
@A00155:342:HHGFNDSXY:1:2526:4264:35681 1:N:0:GAACCTAG+TCCGCATA
TCACTCACACCCTGGTTGTAAAACTCTGGAGAACCACCTATTATTTAATGTTGTAGCTAGTTACATTA
GTAAATATTCATATGTAGCATGTTTAAGCATTAAATCAAATAAGATGAGCAAGATGGAACGTCTGGGT
TCCAATTCTGT
+
FFF:FFFFFFFFFF:FF:FFFFFFFFFFFFFFFFFFFFFFFFFFFF,F:FFFFFFFFFFFFFFFFFFFFF
FFFFFFF:FFFFFFFFFFFFFFFFFFFFFFFFFFFFFFFFFFFFFFFFFFFFF:FFFFFFFFFFFFFFFF
FFFFFFFFFFF
@A00155:342:HHGFNDSXY:1:2614:9100:10269 1:N:0:GAACCTAG+TCCGCATA
TCACTCACACCCTGGTTGTAAAACTCTGGAGAACCACCTATTATTTAATGTTTTAGCTAGTTACATTA
GTAAATATTCATATGTAGCATGTTTAAGCATTAAATCAAATAAGATGAGCAAGATGGAACGTCTGGGT
TCCAATTCTGT
+
FFFFFFFFFFFFFF,FFFFFFFFFFF:FFFFFF::FFFFFFFF:FFFFFFFFFFFFFFFFFFFFFFFFFF
:FFFFFFFFFFFFFFFFFFFFFFFFFFFF:FFFFFFFFFFFFFFFFFFFFFFFFFFFFFFFFFFFFFFFF
FFFFFFFFFFF
@A00155:342:HHGFNDSXY:1:2645:8296:24784 1:N:0:GAACCTAG+TCCGCATA
TCACTCACACCCTGGTTGTAAAACTCTGGAGAACCACCTATTATTTAATGTTGTAGCTAGTTACATTA

```
GTAAATATTCATATGTAGCATGTTTAAGCATTAAATCAAATAAGATGAGCAAGATGGAACGTCTGGGT
TCCAATTCTGT
+
FFFFFFFFFFFFFFFFF:FFFFFFFFFFFFFFFFFFFFFFFFF,::FFFFFFFFFFFFFFFFFFFFFF
FFFFFFFFFFFFFFFFFFFFFF:FFFFFFFFFFFFFFFFFFFFFFFFFFFFFFFFFFFFFFFFFFFF:
FFFFFFFFFFF
@A00155:342:HHGFNDSXY:1:1413:29297:25551 2:N:0:GAACCTAG+TCCGCATA
TCACTCACACCCTGGTTGTAAAACTCTGGAGAACCACCTATTATTTAATGTTGTAGCTAGTTACATTA
GTAAATATTCATATGTAGCATGTTTAAGCATTAAATCAAATAAGATGAGCAAGATGGAACGTCTGGGT
TCCAATTCTGTCAA
+
FFFFFFFFF:F,FFFFFFFF,FFF::FFFFFFFFFFFFFFFFFFFFFFFFFFFFFF,FFFFFFFFFFFF
FFFFFFFF:FFFFFFFFFFFFF,FFFFF,FFFFFFFFFF:FF:,FFFFFFF:FFFF:,FFFFFFF:FF
F,,FFFFFF:FFFF
@A00155:342:HHGFNDSXY:1:1164:1976:16689 2:N:0:GAACCTAG+TCCGCATA
TCACTCACACCCTGGTTGTAAAACTCTGGAGAACCACCTATTATTTAATGTTGTAGCTAGTTACATTA
GTAAATATTCATATGTAGCATGTTTAAGCATTAAATCAAATAAGATGAGCAAGATGGAACGTCTGGGT
TCCAATTCTGTCAAG
+
F:FFFFFFFFF:FFFFFFFFFF:FFFFFFF:FFFFFF:FFFFFFFFFFFFFFFFFFFFFFFFFFFF,F
FFFFFFFFFFFFFFFFFFFFFFFFFFFFFFFFFFFFFFFFFF,FFFF,FFFFFFFFFFFFFF,FFFF
FFF:F:FFFFFFFFF
@A00155:342:HHGFNDSXY:1:1565:16685:16642 1:N:0:GAACCTAG+TCCGCATA
CACTCACACCCTGGTTGTAAAACTCTGTAGAACCACCTATTATTTAATGTTGTAGCTAGTTACATTAG
TAAATATTCATATGTAGCATGTTTAAGCATTAAATCAAATAAGATGAGCAAGATGGAACGTCTGGGTT
CCAATTCTGTCAAGA
+
FFFFF:FFFFFFFFFFFFFFFFF:FFF,F,FFFFFF:FFFFFFFFFFFFFFFFFFFFFFFFF:FFFFF
FFFFF::FF,FFFFF:FFF:,FFFFFFF:FFFFFFFFFFFFFFFFFFFFFFFFFFFFF:FFF:FFFFFF
FFFFFFF,FFFFFFF
@A00155:342:HHGFNDSXY:1:1339:14425:13072 2:N:0:GAACCTAG+TCCGCATA
CACTCACACCCTGGTTGTAAAACTCTGGAGAACCACCTATTATTTAATGTTGTAGCTAGTTACATTAG
TAAATATTCATATGTAGCATGTTTAAGCATTAAATCAAATAAGATGAGCAAGATGGAACGTCTGGGTT
CCAATTCTGTCAAGA
+
FFFFFFFFFFFFFFFFFFF,,F,FFFFFFFF:FF:FFFFFFFFFFFF,F,FFFFFFFFFFFFFFFFFF
FFFFFFFFFFFFFFFFFF:FFFFFFFFFFFFFFF,FFFFF:FFFFFFFFFFFFFFFFFFFFFFFFFFFF
FFFF:FFF:FFFFFF
@A00155:342:HHGFNDSXY:1:1366:19434:32675 2:N:0:GAACCTAG+TCCGCATA
CACTCACACCCTGGTTGTAAAACTCTGGAGAACCACCTATTATTTAATGTTGTAGCTAGTTACATTAG
TAAATATTCATATGTAGCATGTTTAAGCATTAAATCAAATAAGATGAGCAAGATGGAACGTCTGGGTT
CCAATTCTGTCAAGA
+
FFFFFFF:FF:FFFFFFFFFFF:FFFFFFFFFFFFFFFFFFFFFFFFFFF::FFFF:FFFFFFFFFFF
FFFFFFFFFFFFFF:FFFFFFFFFFFFFFFFFFFFFFFFFFFFFFFFFF:FFFFFFFFFF:FFFFFFFF
FFFFFFFFFFFFF:F
@A00155:342:HHGFNDSXY:1:1457:23827:11193 2:N:0:GAACCTAG+TCCGCATA
CACTCACACCCTGGTTGTAAAACTCTGGAGAACCACCTATTATTTAATGTTGTAGCTAGTTACATTAG
TAAATATTCATATGTAGCATGTTTAAGCATTAAATCAAATAAGATGAGCAAGATGGAACGTCTGGGTT
CCAATTCTGTCAAGA
+
FFFFFFFFFFFFFFFFFFFFFFFFFFFFFFFFFFFFF:FFFFFFFFFFFFFFFFFFFFFFFFFFFFFF
FFFFFFFFFFFFFFFFFFFFFFFFFFFFFFFFFFFFFFF:FFFFFFFFFFFFFFFFFFFFFFFFFFFF
FFFFFFFFFFFFFFF
```

@A00155:342:HHGFNDSXY:1:1654:13494:28213 2:N:0:GAACCTAG+TCCGCATA
CACTCACACCCTGGTTGTAAAACTCTGGAGAACCACCTATTATTTAATGTTGTAGCTAGTTACATTAG
TAAATATTCATATGTAGCATGTTTAAGCATTAAATCAAATAAGATGAGCAAGATGGAACGTCTGGGTT
CCAATTCTGTCAAGA
+
:FFFFFFFFFFFFFFFFFFFFFFFFFFFFFFFFFFFFFFFFFFFFFFFFFFFFFFFFFFFFFFFFFFF
FFFFFFFFFFFFFFFFFFFFFFFFFFFFFFFFFFFFFFFFFFFFFFFFFFFFFFFFFFFFFFFFFFFF
FFFFFFFFFFFFFFF
@A00155:342:HHGFNDSXY:1:2103:2112:6903 2:N:0:GAACCTAG+TCCGCATA
CACTCACACCCTGGTTGTAAAACTCTGGAGAACCACCTATTATTTAATGTTGTAGCTAGTTACATTAG
TAAATATTCATATGTAGCATGTTTAAGCATTAAATCAAATAAGATGAGCAAGATGGAACGTCTGGGTT
CCAATTCTGTCAAGA
+
F:FFFFFFFFFFFFFFFFFFF:FFFFFFFFFFFFFFFFFFFFFFFFFFFFFFFFFFFFFFFFFFFFFF
FFF:FFFFFFFFFFFFFFFF,FFFFF::FFFFFFFFFF:FFFFFFF:FFFFFFFF,FFFFF:FFFFFF
:FFFFFFFFFFF,FF
@A00155:342:HHGFNDSXY:1:2238:2428:13808 2:N:0:GAACCTAG+TCCGCATA
CACTCACACCCTGGTTGTAAAACTCTGGAGAACCACCTATTATTTAATGTTGTAGCTAGTTACATTAG
TAAATATTCATATGTAGCATGTTTAAGCATTAAATCAAATAAGATGAGCAAGATGGAACGTCTGGGTT
CCAATTCTGTCAAGA
+
FFFFFFFFFFFFFF:FFFFFFFFFFFFFFFF:FFFFFFFFFFFFF:FFFFFFFFFFFFFFFFFF,:FF
F:FFF:FFFFFFFF:FFFF:FFFFFFFFFFFFFFFFFFFFFFFFFFFFFFFFFFFFFFFFFFFFFFFF
FFFFFFFFFFFFF:F
@A00155:342:HHGFNDSXY:1:2606:8341:35853 2:N:0:GAACCTAG+TCCGCATA
CACTCACACCCTGGTTGTAAAACTCTGGAGAACCACCTATTATTTAATGTTGTAGCTAGTTACATTAG
TAAATATTCATATGTAGCATGTTTAAGCATTAAATCAAATAAGATGAGCAAGATGGAACGTCTGGGTT
CCAATTCTGTCAAGA
+
FFFFFFFFFFFFFFFFFF:FFFFFFFFF:FFFFFFFFF:FF:FFFFFFFFFFF:FFF:FFF:F:FF:F
F:FFF:FF,FF:FFF:FFFFFFFFFFFFFFF:FFFFFFFF:FFFF,FF,FF:FFF,:FFFFFFFFFFF
FFFFFF:FFFFFF,F
@A00155:342:HHGFNDSXY:1:2612:24623:13416 2:N:0:GAACCTAG+TCCGCATA
CACTCACACCCTGGTTGTAAAACTCTGGAGAACCACCTATTATTTAATGTTGTAGCTAGTTACATTAG
TAAATATTCATATGTAGCATGTTTAAGCATTAAATCAAATAAGATGAGCAAGATGGAACGTCTGGGTT
CCAATTCTGTCAAGA
+
FFFFFFFFFFFFFFFFFFFFF:FFFFFFFFFFFFFFF:FFFFFF:FFFFFFFFFFFFFFF:FFFFFFF
,FFFFFFFFFFFFFFFFFFFFF:FFFF:FF,FFFFFFFFFFFFFF:F::FFFFFFFFFFFFFFFFF:F
FFFFFFFFFFFFFFF
@A00155:342:HHGFNDSXY:1:1104:2492:35305 1:N:0:GAACCTAG+TCCGCATA
ACTCACACCCTGGTTGTAAAACTCTGGAGAACCACCTATTATTTAATGTTGTAGCTAGTTACATTAGT
AAATATTCATATGTAGCATGTTTAAGCATTAAATCAAATAAGATGAGCAAGATGGAACGTCTGGGTTC
CAATTCTGTCAAGAC
+
FFF,FFFFFFFFFFFFFFFFFFFFF:FFFFFFFF,F:FFF::FFFFFFFFFFFFFFFFFFFFFFFFFF
FFFFFFF:FFFFFF:FFFFFFFF,FF::FFFFFF,F,,FFFFFF,F,FFFF:F:FFFFFFFFFFFF:
FFFFFFFFFFFFFFF
@A00155:342:HHGFNDSXY:1:1114:25753:14779 1:N:0:GAACCTAG+TCCGCATA
ACTCACACCCTGGTTGTAAAACTCTGGAGAACCACCTATTATTTAATGTTGTAGCTAGTTACATTAGT
AAATATTCATATGTAGCATGTTTAAGCATTAAATCAAATAAGATGAGCAAGATGGAACGTCTGGGTTC
CAATTCTGTCAAGAC
+
FFFFFFFFFFFFFFFFFFFFFFFFFFFFFFFFFFFFFFFFFFFFFFFFFFFFFFFFFF:FFFFFFFFF

FFFFFFFFFFFFFFFFFFFF:FFFFFFFFFFFFFFFFFFFFFFFFFFFFFFFFFFFFFFFFFFFFFFF
FFFFFFFFFFFFFFF
@A00155:342:HHGFNDSXY:1:1223:29731:25488 1:N:0:GAACCTAG+TCCGCATA
TCTCACACCCTGGTTGTAAAACTCTGGAGAACCACCTATTATTTAATGTTGTAGCTAGTTACATTAGT
AAATATTCATATGTAGCATGTTTAAGCATTAAATCAAATAAGATGAGCAAGATGGAACGTCTGGGTTC
CAATTCTGTCAAGAC
+
FFFFFFFFFFFFFFFFFFFFFFFFFFFFFFFFFFFFFFFFFFFFF:FFFFFFFFFFFFFFFFFFFFFF
FFFFFFFF:FFFF:FFFFFF,FFFFFFFFFFFFFFFFFFFFFFFFFFFFFFFFFFFFFFFFFFFFFFF
FFFFFFFFFFFFFFF
@A00155:342:HHGFNDSXY:1:1277:23059:28588 1:N:0:GAACCTAG+TCCGCATA
TCTCACACCCTGGTTGTAAAACTCTGGAGAACCACCTATTATTTAATGTTGTAGCTAGTTACATTAGT
AAATATTCATATGTAGCATGTTTAAGCATTAAATCAAATAAGATGAGCAAGATGGAACGTCTGGGTTC
CAATTCTGTCAAGAC
+
FFFFFFFFFFFFFFFFFFFFFFFFFFFFFFF:FFFFFFFFFFF,FFFFFFFFFFFFFFFFFFFFFFFF
FFFFFFFFFFFFFFFFFFFFFFFFFFFFFFFFFFFFFFFFFFFFFFFFFF:FFFFFFFFFFFFFFFFF
FFFFFFFFFFFFFFF
@A00155:342:HHGFNDSXY:1:1435:7871:7921 1:N:0:GAACCTAG+TCCGCATA
ACTCACACCCTGGTTGTAAAACTCTGGAGAACCACCTATTATTTAATGTTGTAGCTAGTTACATTAGT
AAATATTCATATGTAGCATGTTTAAGCATTAAATCAAATAAGATGAGCAAGATGGAACGTCTGGGTTC
CAATTCTGTCAAGAC
+
FFFFFFFF:FFFFFF:FFFFFFFFFFFFFFFF:FFFFFFFFFFFF:FFFFFFFFFFFFFFFFFFFFFF
FFFFFFFFFFFFFFFFFFFFFFFFFFFFFFFFFFFFFFFFFFFFFFFFFFFFFFFFFFFFFFFFF:FF
FFFFFFFFFFFFFFF
@A00155:342:HHGFNDSXY:1:2117:31982:3646 1:N:0:GAACCTAG+TCCGCATA
ACTCACACCCTGGTTGTAAAACTCTGGAGAACCACCTATTATTTAATGTTGTAGCTAGTTACATTAGT
AAATATTCATATGTAGCATGTTTAAGCATTAAATCAAATAAGATGAGCAAGATGGAACGTCTGGGTTC
CAATTCTGTCAAGAA
+
FFFFFFFFFFFFFFFFFFFFFFFFFFFFFFFFFFFFFFFFFFFFFFFFFFFFFFFFFFFFFFFFFFFF
FFFFFFFFFFFFFFFFFFFFFFFFFFFFFFFFFFFFFFFFFFFFFFFFFFFFFFFFFFFFFFFFFFFF
FFFFFFFFFFFFFFF
@A00155:342:HHGFNDSXY:1:2114:24849:9298 1:N:0:GAACCTAG+TCCGCATA
ACTCACACCCTGGTTGTAAAACTCTGGAGAACCACCTATTATTTAATGTTGTAGCTAGTTACATTAGT
AAATATTCATATGTAGCATGTTTAAGCATTAAATCAAATAAGATGAGCAAGATGGAACGTCTGGGTTC
CAATTCTGTCAAGAC
+
FFFFFFFFFFFFFFFFFFFFFFFFFFFFFFFFFFFFFFFFFFFFFFFFFFFFFFFFFFFFFFFFFFFF
FFFFFFFFFFFFFFFFFFFFFFFFFFFFFFFFFFFFFFFFFFFFFFFFFFFFFFFFFFFFFFFFFFFF
FFFFFFFFFFFFFFF
@A00155:342:HHGFNDSXY:1:2520:15230:30091 1:N:0:GAACCTAG+TCCGCATA
ACTCACACCCTGGTTGTAAAACTCTGGAGAACCACCTATTATTTAATGTTGTAGCTAGTTACATTAGT
AAATATTCATATGTAGCATGTTTAAGCATTAAATCAAATAAGATGAGCAAGATGGAACGTCTGGGTTC
CAATTCTGTCAAGAC
+
FFFFFFFFFFFF:FFFFFFFFFFFFFFFFFFFFFFFFFF:FFFFFFFFFFFFFFFFFF:FFFFFFFFFF
FFFFFFFFFFFFFFFFFFFF:FFFFFFFFFFFFFFFFFFFFFFFFFFFFFFFFFFFFFFFFFFFFFFF
FFFFFFFFFFFFFFF
@A00155:342:HHGFNDSXY:1:1545:15465:5228 2:N:0:GAACCTAG+TCCGCATA
ACTCACACCCTGGTTGTAAAACTCTGGAGAACCACCTATTATTTAATGTTGTAGCTAGTTACATTAGT
AAATATTCATATGTAGCATGTTTAAGCATTAAATCAAATAAGATGAGCAAGATGGAACGTCTGGGTTC
CAATTCTGTCAAGAC

+
FFFFFFFFFFFFFFFFFFFFFFFFFFFFFFFFFFFFFFFFFFFFFFFFFFFFFFFFFFFFFFF:FFFF
FFFFFFFFFFFFFFFFFFF::FFFFFFFFFFFFFFFFFFFFFFFFFFFFFFFFFFFFFFFFFFFFFFF
FFFFFFFFFFFFFFF
@A00155:342:HHGFNDSXY:1:1163:21576:29058 2:N:0:GAACCTAG+TCCGCATA
CTCACACCCTGGTTGTAAAACTCTGGAGAACCACCTATTATTTAATGTTGTAGCTAGTTACATTAGTA
AATATTCATATGTAGCATGTTTAAGCATTAAATCAAATAAGATGAGCAAGATGGAACGTCTGGGTTCC
AATTCTGTCAAGACT
+
FFFFFFFF:FF::FFF:FFFFF:,:FFFF,FFFFFFFFFFFF,FF:FFF:FFF:FFFF:FF:FFFFF,
:FFF,:F,,FFFFFFF::F,FF:FFF:FFF:F,FFFFFFF,FFFF,::FFFFFF:F,:FFFF:FFFF:
FFFFFFFFFFF:FF:
@A00155:342:HHGFNDSXY:1:1218:24867:4257 2:N:0:GAACCTAG+TCCGCATA
CTCACACCCTGGTTGTAAAACTCTGGAGAACCACCTATTATTTAATGTTGTAGCTAGTTACATTAGTA
AATATTCATATGTGGCATGTTTAAGCATTAAATCAAATAAGATGAGCAAGATGGAACGTCTGGGTTCC
AATTCTGTCAAGACT
+
FFFFFFFFFFFFFFFFFFFFFFFFFFFFFFFFFFFFFFFFFFFFFFFFFFFF:FFFFFFFFF:FFFFF
FFFFFFFFFFFFFFFFFFFFFFFFFFFFFFFFFFFFFFFFFFFFFFFFFFFFF:FFFFFFFFFFFF:F
F:FFFFFFFFFFFFF
@A00155:342:HHGFNDSXY:1:1223:29731:25488 2:N:0:GAACCTAG+TCCGCATA
CTCACACCCTGGTTGTAAAACTCTGGAGAACCACCTATTATTTAATGTTGTAGCTAGTTACATTAGTA
AATATTCATATGTAGCATGTTTAAGCATTAAATCAAATAAGATGAGCAAGATGGAACGTCTGGGTTCC
AATTCTGTCAAGACA
+
FFFFFFFFFFFFFFFFFFFFFFFFFFFFFFFFFFFFFFFFFFFFFFFFFFFFFFFFFFFFFFFFFFFF
FFFFFFFFFFFFFFFFFFFFFFFFFFFFFFFFFFFFFFFFFFFFFFFFFFFFFFFFFFFFFFFFFFFF
FFFFFFFFFFFFFFF
@A00155:342:HHGFNDSXY:1:1277:23059:28588 2:N:0:GAACCTAG+TCCGCATA
CTCACACCCTGGTTGTAAAACTCTGGAGAACCACCTATTATTTAAGGTTGTAGCTAGTTACATTAGTA
AATATTCATATGTAGCATGTTTAAGCATTAAATCAAATAAGATGAGCAAGATGGAACGTCTGGGTTCC
AATTCTGTCAAGACA
+
FFFFFFFFF:FFFFFFFFFFFFFFFFFFFFFFFFFFFFFFFFFFF,FFFFFFFFFFFFFFFF,FFFFF
FFFFFFFFFFFFFFFFFFFFFFFFFFFFFFFFFFFFFFFFFFFFFFFFFFFFFFFFFFFFFFFFFFFF
FFFFFFFFFFFFFFF
@A00155:342:HHGFNDSXY:1:1326:19605:2378 2:N:0:GAACCTAG+TCCGCATA
CTCACACCCTGGTTGTAAAACTCTGGAGAACCACCTATTATTTAATGTTGTAGCTAGTTACATTAGTA
AATATTCATATGTAGCATGTTTAAGCATTAAATCAAATAAGATGAGCAAGATGGAACGTCTGGGTTCC
AATTCTGTCAAGACT
+
FFFFFFFFFFFFFFFFFF,FFFFFFFFFFFFFFFFFFFFFFFFFFFFFFFFFFFFFFFFFFFFFFFFF
FFFFFFFFFFFFFFFFFFFFFFFFFFFFF:FFFFFFFFFFFFFFFFFFFFFFFFF,FFFFFFFFFFF,
FFFFFFFFFFF:FFF
@A00155:342:HHGFNDSXY:1:1336:8359:32377 2:N:0:GAACCTAG+TCCGCATA
CTCACACCCTGGTTGTAAAACTCTGGAGAACCACCTATTATTTAATGTTGTAGCTAGTTACATTAGTA
AATATTCATATGTAGCATGTTTAAGCATTAAATCAAATAAGATGAGCAAGATGGAACGTCTGGGTTCC
AATTCTGTCAAGACT
+
FFFFFFFFFFFFFFFFFFFFFFFFFFFFFFFFFFFFFFFFFFFFF:FFFFFFFFFFFFFFFFFFFFFF
FF:FFFFFFFFFFFFFFFFF:FFFFFFFFFFFFFFFFFFF:FFFF:FFFFFFFFFFFFFFFFFFFFFF
FFFFFFFFFFFFFFF
@A00155:342:HHGFNDSXY:1:1412:6162:1611 2:N:0:GAACCTAG+TCCGCATA
CTCACACCCTGGTTGTAAAACTCTGGAGAACCACCTATTATTTAATGTTGTAGCTAGTTACATTAGTA

AATATTCATATGTAGCATGTTTAAGCATTAAATCAAATAAGATGAGCAAGATGGAACGTCTGGGTTCC
AATTCTGTCAAGACT
+
FFFFFFFFFFFFFFFFFFF:FFFFFF,FFF:FFFFFFFFFFFFFFFFFFFFFFFFFFFFFFFFFFFF:F
FFFFFFFFFFFFFFFFFFFFFFFFFFFFFFFF:FFFFFFF,FFFFFFFFFFFFFFFFF:FF:FFFFFFF
FFFFFFFFFFFFFFF
@A00155:342:HHGFNDSXY:1:1412:6885:3333 2:N:0:GAACCTAG+TCCGCATA
CTCACACCCTGGTTGTAAAACTCTGGAGAACCACCTATTATTTAATGTTGTAGCTAGTTACATTAGTA
AATATTCATATGTAGCATGTTTAAGCATTAAATCAAATAAGATGAGCAAGATGGAACGTCTGGGTTCC
AATTCTGTCAAGACT
+
FFFFFFFFFFFFFFFFFFFFFFFFFFFFFFFFFFFFFFFFFFFFFFFFFFFFFFFF:FFFFFF:FFFFF
FFFFFFFFF:FFFF:FFFFFFFFFFFFFFFFFFFFFFFFFFFFFFFFFFFFFF,FFFFFFF:FFFFFFF
FFFFFFFFFFFFFFF
@A00155:342:HHGFNDSXY:1:1504:30174:18552 2:N:0:GAACCTAG+TCCGCATA
CTCACACCCTGGTTGTAAAACTCTGGAGAACCACCTATTATTTAATGTTGTAGCTAGTTACATTAGTA
AATATTCATATGTAGCATGTTTAAGCATTAAATCAAATAAGATGAGCAAGATGGAACGTCTGGGTTCC
AATTCTGTCAAGACT
+
FFFFFFFFFFFFFFFFFFFFFFFFFFFFFFFFFFFFFFFFFFFFFFFFFFFF,FFFFFFFF:F:FFFFF
FFFFFFFFFF:FFF:FFFFFFFFFFFFFFFFFFFFFFFFFFFFFFFFFFFFFFFFFFFFFFFFFFFFFF
FFFFFFFFFFFFFF:
@A00155:342:HHGFNDSXY:1:1511:28944:1799 2:N:0:GAACCTAG+TCCGCATA
CTCACACCCTGGTTGTAAAACTCTGGAGAACCACCTATTATTTAATGTTGTAGCTAGTTACATTAGTA
AATATTCATATGTAGCATGTTTAAGCATTAAATCAAATAAGATGAGCAAGATGGAACGTCTGGGTTCC
AATTCTGTCAAGACT
+
FFFFFFFFFFFFFFFFF:FFFFFFFFFFFFFFFFFFFFFFFFFFFFFFFFFFFFFFFFFFFFFFFFFFF
FFFFFFFFFFFFFFFFFFFFFFFFFFFFFFFFFFFFFFFFFFFFFFFFFFFFFFFFFFFFFFFFFFFFF
FFFFFFFFFFFFFFF
@A00155:342:HHGFNDSXY:1:1540:5728:37059 2:N:0:GAACCTAG+TCCGCATA
CTCACACCCTGGTTGTAAAACTCTGGAGAACCACCTATTATTTAATGTTGTAGCTAGTTACATTAGTA
AATATTCATATGTAGCATGTTTAAGCATTAAATCAAATAAGATGAGCAAGATGGAACGTCTGGGTTCC
AATTCTGTCAAGACT
+
FFFFFFFFFFFFFFFFFFF:FFFFFFFFFFFFFFFFFFFFFFFFFFFFFFFFFFFFFFFFFFF:FF:FF
FFFFFFFFFFFFFFFFFFFFFFFFFFFFFFFF:FFFFFFF:FFF:F,FF::FFFFFFF:F:FFFFFF:F
FFFFFFFFFFFFFFF
@A00155:342:HHGFNDSXY:1:1562:30789:29919 2:N:0:GAACCTAG+TCCGCATA
CTCACACCCTGGTTGTAAAACTCTGGAGAACCACCTATTATTTAATGTTGTAGCTAGTTACATTAGTA
AATATTCATATGTAGCATGTTTAAGCATTAAATCAAATAAGATGAGCAAGATGGAACGTCTGGGTTCC
AATTCTGTCAAGACT
+
FFFFFFFFFFFFFFFFFFFFFFFFFFFFFFFFFFFFFFFFFFFFFFFF:FFFFFFFFFFFFF:FFFFFF
FFFFFFFFFFFFFFFFFFFFFFFFFFFFFFFFFFFFFFFFFFFFFFFFFFFFFF,FFF:FFFFFFFFFF
FFFFFFFFFFFFFFF
@A00155:342:HHGFNDSXY:1:2143:30798:1344 2:N:0:GAACCTAG+TCCGCATA
CTCACACCCTGGTTGTAAAACTCTGGAGAACCACCTATTATTTAATGTTGTAGCTAGTTACATTAGTA
AATATTCATATGTAGCATGTTTAAGCATTAAATCAAATAAGATGGGCAAGATGGAACGTCTGGGTTCC
AATTCTGTCAAGACT
+
FFFFFFFFFFFFFFFFFFFFFFFFFFFFFFFFFFFFFFFFFFFFFFFFFFFFFFFFFFFFFFFFFFFFF
FFFFFFFFFFFFFFFFFFFF:FFFFFFFFFFFFFFFFFFFFFFF:FFFFFFFFFFFFFF:FFFFFFFFF
FFFFFFFFFFFFFFF

```
@A00155:342:HHGFNDSXY:1:2164:6207:35321 2:N:0:GAACCTAG+TCCGCATA
CTCACACCCTGGTTGTAAAACTCTGGAGAACCACCTATTATTTAATGTTGTAGCTAGTTACATTAGTA
AATATTCATATGTAGCATGTTTAAGCATTAAATCAAATAAGATGAGCAAGATGGAACGTCTGGGTTCC
AATTCTGTCAAGACT
+
FFFFFFFFFFFFFFFFFFFFFFFFFFFFFFFFFFFFFFFFFFFFF:FFFFFFFFFFFFFFFFFFFFFFF
FFFFFFFFFFFFFFFFFFFFFFFFFFFFFFFFFFFFFFFFFFFFFFFFFFFFFFFFFFFFFFFFFFFFF
:FFFFFFFFFFFF:F
@A00155:342:HHGFNDSXY:1:2446:24035:7482 2:N:0:GAACCTAG+TCCGCATA
CTCACACCCTGGTTGTAAAACTCTGGAGAACCACCTATTATTTAATGTTGTAGCTAGTTACATTAGTA
AATATTCATATGTAGCATGTTTAAGCATTAAATCAAATAAGATGAGCAAGATGGAACGTCTGGGTTCC
AATTCTGTCAAGACT
+
FFFFFFFFFFFFFFFFFFFFFFFFFFFFFFFFFFFFFFFFFFFFFFFFFFFFFFFFFFFFFFF:FFFFF
FFFFFFFFFFFFFFFFFFFFFFFFFFFFFFFFFFFFFFFFFFFFFFFFFFFFFFFFFFFFFFFFFFFFF
FFFFF:FFFFFFFFF
@A00155:342:HHGFNDSXY:1:2447:19443:35571 2:N:0:GAACCTAG+TCCGCATA
CTCACACCCTGGTTGTAAAACTCTGGAGAACCACCTATTATTTAATGTTGTAGCTAGTTACATTAGTA
AATATTCATATGTAGCATGTTTAAGCATTAAATCAAATAAGATGAGCAAGATGGAACGTCTGGGTTCC
AATTCTGTCAAGACT
+
FFFFFFFF:FFFFFFFFFF,FFFFFFFFFFFFFFFFFFFFFFFF:FFFFF:FFFFFFFFFFFF,FFFF
FFFFFFFFFFF:FF:FFFFFFFFFFFF:FFFFFFFFFFFFFFFFFFFFFFFFFFFF,FFFFFFFFFFFF
FFFFFFFFF,FFFFF
@A00155:342:HHGFNDSXY:1:2540:16821:13307 2:N:0:GAACCTAG+TCCGCATA
CTCACACCCTGGTTGTAAAACTCTGGAGAACCACCTATTATTTAATGTTGTAGCTAGTTACATTAGTA
AATATTCATATGTAGCATGTTTAAGCATTAAATCAAATAAGATGAGCAAGATGGAACGTCTGGGTTCC
AATTCTGTCAAGACT
+
FFFFFFFFFFFFFFFFFFFFFFFFFFFFFFFFFFFFFFFFFFFFFFFFFFFFFFF:FFFFFF:FFFFFF
FFFFFFFFFFFFFFFFFFFFFFFFFFFF:FFFFFFFFFFFFFFFFFFFFFFF:FFF:FFFFFFFFFFFF
FF:FFFFFFFFFFFF
@A00155:342:HHGFNDSXY:1:1377:25771:34507 1:N:0:GAACCTAG+TCCGCATA
TCACACCCTGGTTGTAAAACTCTGGAGAACCACCTATTATTTAATGTTGTAGCTAGTTACATTAGTAA
ATATTCATATGTAGCATGTTTAAGCATTAAATCAAATAAGATGAGCAAGATGGAACGTCTGGGTTCCA
ATTCTGTCAAGACTT
+
FFFFFFFFFFFFFFFFFFFFFFFFFFFFFFFFFFFFFFFFF,FFFFFFFFFFFF:FFFFFFFFFFFFFF
FFFFFFFF:FFFFFFFFFFFFFFFFFFFFFFFF:FFFFFFFFFFFFFFFFFFFFFFFFFFFFFFFFF:FF
FFFFFFFFF:FFFFF
@A00155:342:HHGFNDSXY:1:2567:26729:16219 1:N:0:GAACCTAG+TCCGCATA
TCACACCCTGGTTGTAAAACTCTGGAGAACCACCTATTATTTAATGTTGTAGCTAGTTACATTAGTAA
ATATTCATATGTAGCATGTTTAAGCATTAAATCAAATAAGATGAGCAAGATGGAACGTCTGGGTTCCA
ATTCTGTCAAGAGGG
+
FFFFF,FFF,FFF:FFFFFFFFFFFFFFFFFFFFFFFFFFFF:FFFFFFFFFFFFFFFFFFFFFFFFFFF
FFFFFFFFFFFFFF:FFFFFFFFFFFFFFFFFFFFFFFFFFFFFFFFFFFFFFFFF:FFFFFFFF:FFFF
FFFFFFFFFFFFFFF
@A00155:342:HHGFNDSXY:1:2137:16007:25300 1:N:0:GAACCTAG+TCCGCATA
CACACCCTGGTTGTAAAACTCTGGAGAACCACCTATTATTTAATGTTGTAGCTAGTTACATTAGTAAA
TATTCATATGTAGCATGTTTAAGCATTAAATCAAATAAGATGAGCAAGATGGAACGTCTGGGTTCCAA
TTCTGTCAAGACTTA
+
:FF,F,:F,:FFFFFFFF:F,F:FF,FF,FF:FFFFFFFFFFFFFFF:FF,:FFFFFF:FFFF:FFF:
```

FFFFFFFFFFFFF:FF:FFFFFFFFFFFF:F:FFFFFFF:F:FFFFFFFFFFFFFFF:FF:FFFFFFF
FFFFFFFFF:FFFFF
@A00155:342:HHGFNDSXY:1:1208:24605:17456 2:N:0:GAACCTAG+TCCGCATA
CACACCCTGGTTGTAAAACTCTGGAGAACCACCTATTATTTAATGTTGTAGCTAGTTACATTAGTAAA
TATTCATATGTAGCATGTTTAAGCATTAAATCAAATAAGATGAGCAAGATGGAACGTCTGGGTTCCAA
TTCTGTCAAGAGATC
+
FFFFFFFFFFFFFFFFFFFFFFFFFFFFFFFFFFFFFFFFFFFFFFFFFFFFFFFFFFFFFFFF:FFF
FFFFFFFFFFFFFFFFFFFFFFFFFFFFFFFFFFF:FFFFFFFFFFFFFFFFFFFFFFFFFFFFFFFF
FFFFFFFFFFFFFFF
@A00155:342:HHGFNDSXY:1:1208:26051:18364 2:N:0:GAACCTAG+TCCGCATA
CACACCCTGGTTGTAAAACTCTGGAGAACCACCTATTATTTAATGTTGTAGCTAGTTACATTAGTAAA
TATTCATATGTAGCATGTTTAAGCATTAAATCAAATAAGATGAGCAAGATGGAACGTCTGGGTTCCAA
TTCTGTCAAGAGATC
+
FFFFFFF:FFFFFFFFFFFFFFFFFFFFFFFFFFFFFFFFFFFFFFFFFFFFFFFFFFFFFFFFFFFF
FFFFFFFFFFFFFFFFFFFFFFFFF:FFFFFFFFFFFFFFFFFFFFFFFFFFFFFFFFFFFFFFFFFF
FFFFFFFFFFFFFFF
@A00155:342:HHGFNDSXY:1:1420:7084:24314 2:N:0:GAACCTAG+TCCGCATA
CACACCCTGGTTGTAAAACTCTGGAGAACCACCTATTATTTAATGTTGTAGCTAGTTACATTAGTAAA
TATTCATATGTAGCATGTTTAAGCATTAAATCAAATAAGATGAGCAAGATGGAACGTCTGGGTTCCAA
TTCTGTCAAGAGATC
+
:FFFFFFFFFFFFFFFFFFFFFFFFFFFFFFFFFFFFFFFFFFFFFFFFFFFFFFFFFFFFFFFFFFF
FFFFFFFFFFFFFFFFFFFFFFFFFFFFFFFFFFFFFFFFFFFFFFFFFFFFFFFFFFFFFFFFFFFF
FFFFFFFFFFFFFFF
@A00155:342:HHGFNDSXY:1:1466:6497:16971 2:N:0:GAACCTAG+TCCGCATA
CACACCCTGGTTGTAAAACTCTGGAGAACCACCTATTATTTAATGTTGTAGCTAGTTACATTAGTAAA
TATTCATATGTAGCATGTTTAAGCATTAAATCAAATAAGATGAGCAAGATGGAACGTCTGGGTTCCAA
TTCTGTCAAGACTTA
+
FFFFFFFFFFFFFFFF:FFFFFFFFFFFFFFFFFFFFFFFFFFF:FF:FFFFFFFFFFFFFFFFFFFF
FFFFFFFFFFFFFFFFFFFFFFF:FFFFFFFFFFFFFFF,FFFFFFFFFFFFFFFFFFFFFFFFFFFF
FFFFFFFFFFFFFFF
@A00155:342:HHGFNDSXY:1:2628:17852:2190 2:N:0:GAACCTAG+TCCGCATA
CACACCCTGGTTGTAAAACTCTGGAGAACCACCTATAATTTAATGTTGTAGCTAGTTACATTAGTAAA
TATTCATAAGTAGCATGTTTAAGCATTAAATCAAATAAGATGAGCAAGATGGAACGTCTGGGTTCCAA
TTCTGTCAAGACTTA
+
FFFF:,::::F:,FFFFFFF:F,,F,FFF,F:::FF,FFFFFFF,FF,FF,F:F,,,F,FFFF,FFFF
:::FFFFF,:FF,FF,,FF,FFF,FFFFFF,FFFF:FFFFF:F:,FF:FF,FFF,::F:FF:F,:FFF
F,F,F,FFF,FF:FF
@A00155:342:HHGFNDSXY:1:1214:29912:17221 1:N:0:GAACCTAG+TCCGCATA
ACACCCTGGTTGTAAAACTCTGGAGAACCACCTATTATTTAATGTTGTAGCTAGTTACATTAGTAAAT
ATTCATATGTAGCATGTTTAAGCATTAAATCAAATAAGATGAGCAAGATGGAACGTCTGGGTTCCAAT
TCTGTCAAGACTTAT
+
FF,F:FFF,FF:,F:FFFFFFFFFFFF:FF,F:FFFFFFFF:FF,FFFFF,FF,FFFFF:FFFFFFFF
FFFFFFFFFFF,::FF:FF,F,FFFF,FFFFFFFFF:F::FFFFFF:FFFFFF:F::FFFFFFF:FFF
FFFFFFFFFFFFFFF
@A00155:342:HHGFNDSXY:1:2433:13060:13119 1:N:0:GAACCTAG+TCCGCATA
ACACCCTGGTTGTAAAACTCTGGAGAACCACCTATTATTTAATGTTGTAGCTAGTTACATTAGTAAAT
ATTCATATGTAGCATGTTTAAGCATTAAATCAAATAAGATGAGCAAGATGGAACGTCTGGGTTCCAAT
TCTGTCAAGACTTAT

+
FFFFFFFFFFFFFFFFFFFFFF:FFFFFFFFFFFFFFFFFFFFFFFFFFFFFFFFFFFFFFFFFFFFF
FFFFFFFFFFFFFFFFF:FFFFFFFFFFFFFFFFFFFFFFFFFFFFF:FFFFFFFFFFFFFFFFFFFFFF
FFFFFFFFFFFFFFF
@A00155:342:HHGFNDSXY:1:1155:22254:34084 2:N:0:GAACCTAG+TCCGCATA
ACACCCTGGTTGTAAAACTCTGGAGAACCACCTATTATTTAATGTTGTAGCTAGTTACATTAGTAAAT
ATTCATATGTAGCATGTTTAAGCATTAAATCAAATAAGATGAGCAAGATGGAACGTCTGGGTTCCAAT
TCTGTCAAGACTTAT
+
FFFFFFFFFFFFFFFFFFFFFFFFFFFFFFFFFFFFFFFFFFFFFFFFFFFFFFFFFFFFFFF:FFFFF
:FFFFFFFFFFFFFFFFFFFFFFFFFFFFFFFFFFFFFFFFFFFFFFFFFFFF:FFFFFFFFFFFFFF
:F:FFFFF:FFFFFF
@A00155:342:HHGFNDSXY:1:2628:17852:2190 1:N:0:GAACCTAG+TCCGCATA
CACCCTGGTTGTAAAACTCTGGAGAACCACCTATTATTTAATGTTGTAGCTAGTTACATTAGTAAATA
TTCATATGTAGCATGTTTAAGCATTAAATCAAATAAGATGAGCAAGATGGAACGTCTGGGTTCCAATT
CTGTCAAGACTTAT
+
FFF,FF::FFFFFFFF:F:F:FFFFFFFF,F:FFFFFFFFFFFFF,FF,::FFFFFFFFFFFFFFFFF
FFFFFFFFFFFFFFFFFF:FFF:FFFFFF:F:FF:FFFFFFFFFF:FF,F:F:FFFFF:FFF:FFFFF
FFFFFFFFF,FFFF
@A00155:342:HHGFNDSXY:1:1306:23430:26381 1:N:0:GAACCTAG+TCCGCATA
CACCCTGGTTGTAAAACTCTGGAGAACCACCTATTATTTAATGTTGTAGCTAGTTACATTAGTAAATA
TTCATATGTAGCATGTTTAAGCATTAAATCAAATAAGATGAGCAAGATGGAACGTCTGGGTTCCAATT
CTGTCAAGACTTATG
+
FFFFFFFFFFFFFFFFFFFFFFFFFFFFFFFFFFFFFFFFFFFFFFFFF:FFFFFFFFFFFFFFFFFF
FFFFFFFFFFFFFFFFFFFFFFFFFFFFFFFFFFFFFFFFFFFFFFFFFFFFFFFFFFFFFFFFFFFF
FFFFFFFFFFFFFFF
@A00155:342:HHGFNDSXY:1:1476:4372:36808 1:N:0:GAACCTAG+TCCGCATA
CACCCTGGTTGTAAAACTCTGGAGAAACACCTATTATTTAATGTTGTAGCTAGTTACATTAGTAAATA
TTCATATGTAGCATGTTTAAGCATTAAATCAAATAAGATGAGCAAGATGGAACGTCTGGGTTCCAATT
CTGTCAAGACTTATG
+
FF,FFFFFFFFFFFFFFFFFFFF:FF,:FFFFFFF:FFFFFF:FFFFFFFFFFFFFFFFFFFFFFFFFF
FFFFFFFFFFFFFFF:FFFFF:FFFFFFFFFFFFFFFFFFFFFFFF:FFFFFFFFFFFFFFFFFFFFFF
FFFFFFFFFFFFFFF
@A00155:342:HHGFNDSXY:1:1619:23755:13667 1:N:0:GAACCTAG+TCCGCATA
CACCCTGGTTGTAAAACTCTGGAGAACCACCTATTATTTAATGTTGTAGCTAGTTACATTAGTAAATA
TTCATATGTAGCATGTTTAAGCATTAAATCAAATAAGATGAGCAAGATGGAACGTCTGGGTTCCAATT
CTGTCAAGACTTATG
+
FFFFFFF:FFFFFFFFFFFFFFFFF:FFFFFFFFFFFFFFFFFFFFFFFFFFFFFFFFFFFFFFFFFF
FFFFFFFFF:FFFFFFFFFFFFFFFFFFFFFFFFFFFFF,FFFFFFFFFFFFFFFFFFFFFFFFFFFF
FFFFFFFFFFFFFFF
@A00155:342:HHGFNDSXY:1:1619:23448:13792 1:N:0:GAACCTAG+TCCGCATA
CACCCTGGTTGTAAAACTCTGGAGAACCACCTATTATTTAATGTTGTAGCTAGTTACATTAGTAAATA
TTCATATGTAGCATGTTTAAGCATTAAATCAAATAAGATGAGCAAGATGGAACGTCTGGGTTCCAATT
CTGTCAAGACTTATG
+
FFFFFFFFFFFFFFFFFFFFFFFFFFFFFFFFFFFFFFFFFFFFFFFFF:FFFFFFFFFFFFFFFFFF
FFFFFFFFFFFFFFFFFFFFFFFFFFFFFFFFFFFFFFFFFFFFFFFFFFFFFFFFFFFFFFFFFFFF
FFFFFFFFFFFFFFF
@A00155:342:HHGFNDSXY:1:1654:13494:28213 1:N:0:GAACCTAG+TCCGCATA
CACCCTGGTTGTAAAACTCTGGAGAACCACCTATTATTTAATGTTGTAGCTAGTTACATTAGTAAATA

TTCATATGTAGCATGTTTAAGCATTAAATCAAATAAGATGAGCAAGATGGAACGTCTGGGTTCCAATT
CTGTCAAGACTTATG
+
FFFFFFFFFFFFFFFFFFFFFFFFFFFFFFFFFFFFFF:FFFFFFFFFFFFFFFFFFFFFFFFFFFFF
FFFFFFFFFFFFFFFFFFFFFFFFFFFFFFFFFFFFFFFFFFFFFFFFFFFFFFFFFFFFFFFFFFFF
FFFFFFFFFFFFFFF
@A00155:342:HHGFNDSXY:1:1677:31765:34929 1:N:0:GAACCTAG+TCCGCATA
CACCCTGGTTGTAAAACTCTGGAGAACCACCTATTATTTAATGTTGTAGCTAGTTACATTAGTAAATA
TTCATATGTAGCATGTTTAAGCATTAAATCAAATAAGATGAGCAAGATGGAACGTCTGGGTTCCAATT
CTGTCAAGACTTATG
+
FFFFFFFFFFFFFFFFFFFFFFFFFFFFFFFFFFFFFFFFFFFFFFFFFFFFF:FFFFFFFFFFFFFF
FFFFF:FFFFFFFFFFFFFFFFFFFFFFFFFFFFFFFFFFFFFFFFFFFFFFFFFFFFFFFFFFFFFF
FFFFFFFFFFFFFFF
@A00155:342:HHGFNDSXY:1:2639:18873:11506 1:N:0:GAACCTAG+TCCGCATA
CACCCTGGTTGTAAAACTCTGGAGAACCACCTATTATTTAATGTTGTAGCTAGTTACATTAGTAAATA
TTCATATGTAGCATGTTTAAGCATTAAATCAAATAAGATGAGCAAGATGGAACGTCTGGGTTCCAATT
CTGTCAAGACTTATG
+
FFFFFFFFFFFFFFFFFFFFFFFFFFFFFFFFFFFFFFFFFFFFFFFFFFFFFFFFFFFFFFFFFFFF
FFFFFFFFFFFFFFFFFFFFFFFFFFFFFFFFFFFFFFFFFFFFFFFFFFFFFFFFFFFFFFFFFFFF
FFFFFFFFFFFFFFF
@A00155:342:HHGFNDSXY:1:2112:29541:26224 2:N:0:GAACCTAG+TCCGCATA
CACCCTGGTTGTAAAACTCTGGAGAACCACCTATTATTTAATGTTGTAGCTAGTTACATTAGTAAATA
TTCATATGTAGCATGTTTAAGCATTAAATCAAATAAGATGAGCAAGATGGAACGTCTGGGTTCCAATT
CTGTCAAGACTTATG
+
FFFFFFFFFFFFFFFFFFFFFFFFFFFFFFFFFFFFFFFFFFFFFFFFFFFFFFFFFFFFFFFFFFFF
FFFFFFFFFFFFFFFFFFFFFFFFFFFFFFFFFFFFFFFFFF:FFFFFFFFF:FFFFFFFFFFFFFFF
FFFF:FFFFFFFFFF
@A00155:342:HHGFNDSXY:1:1102:9191:3912 1:N:0:GAACCTAG+TCCGCATA
CCCTGGTTGTAAAACTCTGGAGAACCACCTATTATTTAATGTTGTAGCTAGTTACATTAGTAAATATT
CATATGTAGCATGTTTAAGCATTAAATCAAATAAGATGAGCAAGATGGAACGTCTGGGTTCCAATTCT
GTCAAGACTTA
+
FFFFFFF:FFFFFFFFF:FFFFFFFFFFFFFFFFFF:FFFFFFFFFFFFFFFFFFFFFFFFFFFFFFF
FFFFFFFFFFFFFFFFFF:FFFFFFFFFF:FFFF:FFFF:,FFFF,FFFFFFFFFFFFFFFFFFFFFF
FFFFFFFFFFF
@A00155:342:HHGFNDSXY:1:1401:20645:8437 1:N:0:GAACCTAG+TCCGCATA
CCCTGGTTGTAAAACTCTGGAGAACCACCTATTATTTAATGTTGTAGCTAGTTACATTAGTAAATATT
CATATGTAGCATGTTTAAGCATTAAATCAAATAAGATGAGCAAGATGGAACGTCTGGGTTCCAATTCT
GTCAAGACTTA
+
FFFFFFFFFFFFFFFFFFFFFFFFFFFFFFFFFF:FFFFFFFFFFFFFFFFFFFFFFFFFFFFFFFFF
FFFFFFFFFFFFFFFFFFFFFFFFFFFFFFFFFFFFFFFFFFFFFFFFFFFFFFFFFFFFFFFFFFFF
FFFFFFFFFFF
@A00155:342:HHGFNDSXY:1:1412:6162:1611 1:N:0:GAACCTAG+TCCGCATA
CCCTGGTTGTAAAACTCTGGAGAACCACCTATTATTTAATGTTGTAGCTAGTTACATTAGTAAATATT
CATATGTAGCATGTTTAAGCATTAAATCAAATAAGATGAGCAAGATGGAACGTCTGGGTTCCAATTCT
GTCAAGACTTA
+
FFFFFFFFFFFFFFFFFFFFFFFFFFF:FFFFFFFFFF:F:FFF:FFFFFFFFFFFFFFFFFFFFFFF
FFFFFFFFFFFFFFFFFFFFFFFFFFFFFFFFFFFFFFF:FFFFFFFFFFFFFFFFFFFFFFFF:FFF
FFFFFFFFFFF

```
@A00155:342:HHGFNDSXY:1:1412:6885:3333 1:N:0:GAACCTAG+TCCGCATA
CCCTGGTTGTAAAACTCTGGAGAACCACCTATTATTTAATGTTGTAGCTAGTTACATTAGTAAATATT
CATATGTAGCATGTTTAAGCATTAAATCAAATAAGATGAGCAAGATGGAACGTCTGGGTTCCAATTCT
GTCAAGACTTA
+
FFFFFFFFFFFFFFFFFFFFFFFFFFFFFFFFFFFFFFFFFFFF:FFFFFFFFFFFFFFFFFFFFFFF
FFFFFF,FFFFFFFFFFFFFFFFFFFFFFFFFFF:FFFFFFFFFFFFFFFFFFFFFFFFFFFFF:FFF
FFFFFFFFFFF
@A00155:342:HHGFNDSXY:1:1637:21938:31688 1:N:0:GAACCTAG+TCCGCATA
CCCTGGTTGTAAAACTCTGGAGAACCACCTATTATTTAATGTTGTAGCTAGTTACATTAGTAAATATT
CATATGTAGCATGTTTAAGCATTAAATCAAATAAGATGAGCAAGATGGAACGTCTGGGTTCCAATTCT
GTCAAGACTTA
+
FFFFFFFFFFFFFFFFFFFFFFF:,FFFFFFFFFFFFFFFFFFFFFFFFFF:FFFFF:FFFFFFFFFF
FFFFFF,FFFFFFFFFFFFFFFFFFFFFFFFFFFFFFFFFFFFF,FFFFFFFFFFFFFFFFFFFFFFF
FFFFFFFFF:F
@A00155:342:HHGFNDSXY:1:1649:21621:9377 1:N:0:GAACCTAG+TCCGCATA
CCCTGGTTGTAAAACTCTGGAGAACCACCTATTATTTAATGTTGTAGCTAGTTACATTAGTAAATATT
CATATGTAGCATGTTTAAGCATTAAATCAAATAAGATGAGCAAGATGGAACGTCTGGGTTCCAATTCT
GTCAAGACTTA
+
FFFFFFFFFFFFFFFFFFFFFFFFFFFFFFFFFFF,FFFFFFFFFFFFFFFFFFFFFFFFFFF:FFFF
FFFFFFFFFFFFFFFFFFFFFFFFFFFFFFFFFFFFFFFFFFFFFFFFFFFFFFFFFFFFFFFFFFFF
FFFFFFFFFFF
@A00155:342:HHGFNDSXY:1:2128:4535:9690 1:N:0:GAACCTAG+TCCGCATA
CCCTGGTTGTAAAACTCTGGAGAACCACCTATTATTTAATGTTGTAGCTAGTTACATTAGTAAATATT
CATATGTAGCATGTTTAAGCATTAAATCAAATAAGATGAGCAAGATGGAACGTCTGGGTTCCAATTCT
GTCAAGACTTA
+
FFFFFFFFFFFFFFFFFF,FF:FFFFFFFFFFFFFFFFFFFFFFFFFFFFFFFFFFFFFFFFFFFFFF
FFFFFF:FFFFFFFFFFFFFFFFFFFFFFFFFFFFFFFFFFFFFFFFFFFFFFFFFFFFFFFFFFFFF
FFFFFFFFFFF
@A00155:342:HHGFNDSXY:1:2172:30273:24831 1:N:0:GAACCTAG+TCCGCATA
CCCTGGTTGTAAAACTCTGGAGAACCACCTATTATTTAATGTTGTAGCTAGTTACATTAGTAAATATT
CATATGTAGCATGTTTAAGCATTAAATCAAATAAGATGAGCAAGATGGAACGTCTGGGTTCCAATTCT
GTCAAGACTTA
+
FFFFFFFFFFFFFFFFFFFFFFFFFFFFFFFFFFFFFFFFFFFFFFFFFFFFFFFFFFFFFFFFFFFF
FFFFFFFFFFFFF:FFFFFFFFFFFFFFFFFFFFFFFFFFFFFFFFFFFFFFFFFFFFFFFFFFFFFF
FFFFFFFFFFF
@A00155:342:HHGFNDSXY:1:2238:2428:13808 1:N:0:GAACCTAG+TCCGCATA
CCCTGGTTGTAAAACTCTGGAGAACCACCTATTATTTAATGTTGTAGCTAGTTACATTAGTAAATATT
CATATGTAGCATGTTTAAGCATTAAATCAAATAAGATGAGCAAGATGGAACGTCTGGGTTCCAATTCT
GTCAAGACTTA
+
FFFF:FFFFFFFFFFFFF:FFFFFFFFF:FFFF,FFFFFFFFFFFFFFFFFFFFFFFFFFFFFFFFFF
FFFFFFFFFFFFF:FFFFFFFFFFFFFFFFFFFFFFFFFFFFFFFFFFFFFFFFFFFFFFFFFFFFFF
FFFFFFFFFFF
@A00155:342:HHGFNDSXY:1:2427:27525:14090 1:N:0:GAACCTAG+TCCGCATA
CCCTGGTTGTAAAACTCTGGAGAACCACCTATTATTTAATGTTGTAGCTAGTTACATTAGTAAATATT
CATATGGAGCATGTTTAAGCATTAAATCAAATAAGATGAGCAAGATGGAACGTCTGGGTTCCAATTCT
GTCAAGACTTA
+
FFFFFFFF:FFFFFF,FFF:FFFFF:::FFF:FFFF:F:FFFF:FFF::FFFFF:FFFFFFFFFFFFF
```

FFFFFF,FFFFFFFFFFFFFFFFFFFFFFFFFFFFFFFFFFFFFFFFFFFFFF:FFFFFFFFFFFFFF
FFFFF:FFFFF
@A00155:342:HHGFNDSXY:1:2454:25220:7028 1:N:0:GAACCTAG+TCCGCATA
CCCTGGTTGTAAAACTCTGGAGAACCACCTATTATTTAATGTTGTAGCTAGTTACATTAGTAAATATT
CATATGTAGCATGTTTAAGCATTAAATCAAATAAGATGAGCAAGATGGAACGTCTGGGTTCCAATTCT
GTCAAGACTTA
+
FFFFFFF:FFFFFFFFFFFFFFFFFFFFFFFFFFFFFFFFFFFFFFFFFFFFFFFFFFFFFFFFFFFF
FFFFFFFFFFFFF:FFFFFFFFFFFFFFFFFFFFFFFFFFFFFFFFFFFFFFFFFFFFFFFFFFFFFF
FFFFFFFFFFF
@A00155:342:HHGFNDSXY:1:2606:8341:35853 1:N:0:GAACCTAG+TCCGCATA
CCCTGGTTGTAAAACTCTGGAGAACCACCTATTATTTAATGTTGTAGCTAGTTACATTAGTAAATATT
CATATGTAGCATGTTTAAGCATTAAATCAAATAAGATGAGCAAGATGGAACGTCTGGGTTCCAATTCT
GTCAAGACTTA
+
FFF:,FFF:FFFFFFFFFFFFFFFFFFFF,FFFFFFFFFFFFFFFFFFFFF:FFFFFFFFFFFFFFFF
FFFFF:FFFFFFFFFFFFFFFFFFFFFFFFFFFFFFFFFFFFFFFFFFFFFFFFFFFFFFFFFFFFFF
FFFFFFFFFFF
@A00155:342:HHGFNDSXY:1:2136:3595:35994 1:N:0:GAACCTAG+TCCGCATA
CCTGGTTGTAAAACTCTGGAGAACCACCTATTATATAATGTTGTAGCTAGTTACATTAGTAAATATAC
ATATGTAGCATGTTTAAGCATTAAATCAAATAAGATGAGCAAGATGGAACGTCAGGGTACCAATTCTG
TCAAGACATATGACA
+
FF:,::FFFFFFFF:F,FFF,FFFFFFF:FF,FF,,FF::F:FFFFFFF,FFFFF:,F:FFFF:FF,F
FFFF,:FFFF,F,:,FFFFFF:FFFFFFFFFFFF:FFF:FFFFFF,FFFF:F,,F:F,,FFF:,FF:F
FFFF:FF,:F,:FFF
@A00155:342:HHGFNDSXY:1:1122:3125:6370 2:N:0:GAACCTAG+TCCGCATA
CCTGGTTGTAAAACTCTGGAGAACCACCTATTATTTAATGTTGTAGCTAGTTACATTAGTAAATATTC
ATATGTAGCATGTTTAAGCATTAAATCAAATAAGATGAGCAAGATGGAACGTCTGGGTTCCAATTCTG
TCAAGACTTATGACA
+
FFFFFFFFFFF:FFFFFFFFFFFFFFFFFFFFFFFFFFFFFFFFFFFFFFFFFFFFFFFFFFFFFFFF
FFFFFFFFFFFFFFFFFFFFFFFFFFFFFFFFFFFF:FFFFFFFFFFFFFFFFFFFFFFFFFFFFFFF
FFFFFFFFFFFF:FF
@A00155:342:HHGFNDSXY:1:2627:17598:8891 2:N:0:GAACCTAG+TCCGCATA
CCTGGTTGTAAAACTCTGGAGAACCACCTATTATTTAATGTTGTAGCTAGTTACATTAGTAAATATTC
ATATGTAGCATGTTTAAGCATTAAATCAAATAAGATGAGCAAGATGGAACGTCTGGGTTCCAATTCTG
TCAAGACTTATGACA
+
F,FFFFFFFFFF,FFFFFFFFFFFFF:FFFFFFFFFFFFFFFFFFFFFFFFFFFFFFFFFFFFFFFFF
FFFFFFFFFF:FFFFFFFFFFFF:FFFFFFFFFFFFFFFF:FFFFFFFFFFF:FF:FFFFFFFFF:FF
FFF:FFFFFFFFFFF
@A00155:342:HHGFNDSXY:1:2627:18078:9471 2:N:0:GAACCTAG+TCCGCATA
CCTGGTTGTAAAACTCTGGAGAACCACCTATTATTTAATGTTGTAGCTAGTTACATTAGTAAATATTC
ATATGTAGCATGTTTAAGCATTAAATCAAATAAGATGAGCAAGATGGAACGTCTGGGTTCCAATTCTG
TCAAGACTTATGACA
+
FFFFFFFFFFFFFFFFFFFFFFFFFFFFFFFFFFFFFFFFFFFFFFFFFFFFFFFFFFFFFFFFFFFF
FFFFFFFFFF,FFFFFFFFFFFFFFFFFFFFFFFFFFFF:F:FFFFFF::FFFFFFFFFFFFFFFFFF
FFFFFFFFFFFFFFF
@A00155:342:HHGFNDSXY:1:2574:26964:5697 2:N:0:GAACCTAG+TCCGCATA
CCTGGTTGTAAAACTCTGGAGAACCACCTATTATTTAATGTTGTAGCTAGTTACATTAGTAAATATTC
ATATGTAGCATGTTTAAGCATTAAATCAAATAAGATGAGCAAGATGGAACGTCTGGGTTCCAATTCTG
TCAAGACTTATGACA

+
FF:FFF:FFFFFFFFFFF:FFFFFFFF,FFFFFFF:FFFFFFFFFF:FFFFFFFF:FFFFFFFFFFFF
FFFFFFFFFFFFFFFFFFFFFFFFFFFFFFFFFFFF,FFFFFFFFF:FFFFFFF,FFFFFFFFFFFFF
FFFFFFFFFFFFFFF
@A00155:342:HHGFNDSXY:1:2634:7970:36620 2:N:0:GAACCTAG+TCCGCATA
CCTGGTTGTAAAACTCTGGAGAACCACCTATTATTTAATGTTGTAGCTAGTTACATTAGTAAATATTC
ATATGTAGCATGTTTAAGCATTAAATCAAATAAGATGAGCAAGATGGAACGTCTGGGTTCCAATTCTG
TCAAGACTTATGACA
+
FFFFFFFFFFFFFFFFFFFFFFFFFFFFFFFFFF:FFFFFFFFFF:FFFFFFFFFFFFFFFFFFFFFF
FFFFFFFFFFFFFFFF:FFFF:FFFF:FFFFFFFFFFFFFFFFFFFFFFFFFFFFFFFFFFFFFFFFF
FFFFFFFFFFFFFFF
@A00155:342:HHGFNDSXY:1:2617:3821:8923 2:N:0:GAACCTAG+TCCGCATA
TGGTTGTAAAACTCTGGAGAACCACCTATTATTTAATGTTGTAGCTAGTTACATTAGTAAATATTCAT
ATGTAGCATGTTTAAGCATTAAATCAAATAAGATGAGCAAGATGGAACGTATGGGTTCCAATTATGTC
AAGACTTATGACATA
+
FFFFFFFFF,FFFFFFFFFFFFFFFFFFFFFFFFFFFFFFF,FFFF:FFFFFFF:F:FFFFFFFFFFF
FF:FFFFFF,FFFF::FFFFFFFF:FFFFF:FFFFFFFFF,FF,FFFF,,,FF:FFFFFF,FF,F,FF
,,FFFFFFFFFFFFF
@A00155:342:HHGFNDSXY:1:2617:11026:2581 1:N:0:GAACCTAG+TCCGCATA
GGTTGTAAAACTCTGGAGAACCACCTATTATTTAATGTTGTAGCTAGTTACATTAGTAAATATTCATA
TGTAGCATGTTTAAGCATTAAATCAAATAAGATGAGCAAGATGGAACGTCTGGGTTCCAATTCTGTCA
AGACTTATGACATAT
+
FFFFFFFFFFFFFFFFFFFFFFFFFFFFFFFFFFFFFFFFFFFFFFFFFFFFFFFFFFFFFFFFFFFF
FF:FFFFFFFFFFFFFFFFFFFFFFFFFFFFFFFFFFFFFFFFFFFFFFFFFFFFFFFFFFFFFFFFF
FFFFFFFFFFFF:FF
@A00155:342:HHGFNDSXY:1:1124:31096:10817 2:N:0:GAACCTAG+TCCGCATA
GGTTGTAAAACTCTGGAGAACCACCTATTATTTAATGTTGTAGCTAGTTACATTAGTAAATATTCATA
TGTAGCATGTTTAAGCATTAAATCAAATAAGATGAGCAAGATGGAACGTCTGGGTTCCAATTCTGTCA
AGACTTATGACATAT
+
FFFFFFFFFFFFFFFFF:FFFFFFFFFFFFFFFFF:FFFFFFFFFFFFFFFFF:FFFFFFFFFFFFFF
FFFFFFFFFFFFFFFFFFFFFFFFFFFFFFFFFFFFFFFFFFFFF,FFFFFF,FFFF,FFFFFFFFFF
FFFFFFFFFFF:FFF
@A00155:342:HHGFNDSXY:1:1276:2022:21120 2:N:0:GAACCTAG+TCCGCATA
GGTTGTAAAACTCTGGAGAACCACCTATTATTTAATGTTGTAGCTAGTTACATTAGTAAATATTCATA
TGTAGCATGTTTAAGCATTAAATCAAATAAGATGAGCAAGATGGAACGTCTGGGTTCCAATTCTGTCA
AGACTTATGACATAT
+
FFFFFFFFFFFF,FFFFFFF:FFFFFFFFFFFFFFFFFFFFFFFFFFFFFFFFFFFFFFFFFFFFFFF
FFFFFFFFFFFFFFFFF:FF::FFFFFFFF,FFFFFF,FF:FFFFFFFF:FF,FFFFF:FFFFFFFFF
FFFFFFFFFFFFFFF
@A00155:342:HHGFNDSXY:1:1272:4679:13886 2:N:0:GAACCTAG+TCCGCATA
TGTAAAACTCTGGAGAACCACCTATTATTTAATGTTGTAGCTAGTTACATTAGTAAATATTCATATGT
AGCATGTTTAAGCATTAAATCAAATAAGATGAGCAAGATGGAACGTCTGGGTTCCAATTCTGTCAAGA
CTTATGACATATTAA
+
FFFFFFFFFFFFFFFFFFFFFFFFFFFFFFFFFFFFFFFFFFFFFFFFFFFFFFFFFFFFFFFFFFFF
FFFFFFFFFF,FFFFFFFFFFFFFFFFFFFFFFFFF:FFFFFFFFFFFFFFFFFFFFFFFFFFFFFFF
FFFFF:FFFFFFFFF
@A00155:342:HHGFNDSXY:1:2173:2049:19382 2:N:0:GAACCTAG+TCCGCATA
TGTAAAACTCTGGAGAACCACCTATTATTTAATGTTGTAGCTAGTTACATTAGTAAATATTCATATGT

```
AGCATGTTTAAGCATTAAATCAAATAAGATGAGCAAGATGGAACGTCTGGGTTCCAATTCTGTCAAGA
CTTATGACATATTAA
+
F::FFFFFFFF:FFFFFFFFFFFFF:FFFFFFFFFF,FFF:FFFFFFFFFFF,FFFFFFFFFFFFFFF
FFFFFFF:FFFFFFFFFFFFFFFFFFFF,FFFFFFF,FFF,FFF:FF:FF,,FFFFFFF:FFFF:F,F
,,FFFFF:F:F,,FF
@A00155:342:HHGFNDSXY:1:2361:2501:5979 2:N:0:GAACCTAG+TCCGCATA
GTAAAACTCTGGAGAACCACCTATTATTTAATGTTGTAGCTAGTTACATTAGTAAATATTCATATGTA
GCATGTTTAAGCATTAAATCAAATAAGATGAGCAAGATGGAACGTCTGGGTTCCAATTCTGTCAAGAC
TTATGACATATTA
+
FFFF:FFFFFFFFFFFFFFFFFFFFFFFFFFFFFFFFFFFFFFFFFFFFFFFFFFFFFFFFFFFFFFF
FFFFFFFFFFF:F:FF:FFFFFFFFFFFFFF:FFFFFFFFFFF:FFFFFFFFFFF:FFFFFFFFFFFF
FFFFFF:F,FFF:
@A00155:342:HHGFNDSXY:1:1231:23122:28072 2:N:0:GAACCTAG+TCCGCATA
GTAAAACTCTGGAGAACCACCTATTATTTAATGTTGTAGCTAGTTACATTAGTAAATATTCATATGTA
GCATGTTTAAGCATTAAATCAAATAAGATGAGCAAGATGGAACGTCTGGGTTCCAATTCTGTCAAGAC
TTATGACATATTAA
+
FFFFFFFFFFFFFFFFFFFFFFFFFFFFFFFFFFFFFFFFFFFFFFFF:FFFFFFFFFFFFFFFFFFF
FFFFFFFFFFFFFFFFFFFFFFFFFFFFFFFFFFFFFFFFFFFFFFFFFFFFFFFFFFFFFFFFFFFF
FFFFFFFFFFFFFF
@A00155:342:HHGFNDSXY:1:1527:19741:27696 2:N:0:GAACCTAG+TCCGCATA
GTAAAACTCTGGAGAACCACCTATTATTTAATGTTGTAGCTAGTTACATTAGTAAATATTCATATGTA
GCATGTTTAAGCATTAAATCAAATAAGATGAGCAAGATGGAACGTCTGGGTTCCAATTCTGTCAAGAC
TTATGACATATTAA
+
FFFFFFFFFFFFFFFFFFFFFFFFFFFFFFFFFFFFFFFFFFFFFFFFFFFFFFFFFFFFFFFFFFFF
FFFFFFFFFFFFFFFFFFFFFFFFFFFFFFFFFFFFFFFFFFFFFFFFFFF:FFFFFFFFFFFFFFFF
FFFFFFFFFFFFFF
@A00155:342:HHGFNDSXY:1:2257:15275:11663 2:N:0:GAACCTAG+TCCGCATA
GTAAAACTCTGGAGAACCACCTATTATTTAATGTTGTAGCTAGTTACATTAGTAAATATTCATATGTA
GCATGTTTAAGCATTAAATCAAATAAGATGAGCAAGATGGAACGTCTGGGTTCCAATTCTGTCAAGAC
TTATGACATATTAA
+
FFFF:FFFFFFFFFFFFFFFFFFFFFFFFFFFFFFFFFFFFFFFFFFFFFFFFFFFFFFFFFFFFFFF
FFFFFFFFFFFFFFFFF:FFFFFF:FFFFFFFFFFFFFFFFFFFFFFFFFFFFFFFFFFFFFFFFFFF
FFFFFFFFFFFFFF
@A00155:342:HHGFNDSXY:1:2361:2257:11788 2:N:0:GAACCTAG+TCCGCATA
GTAAAACTCTGGAGAACCACCTATTATTTAATGTTGTAGCTAGTTACATTAGTAAATATTCATATGTA
GCATGTTTAAGCATTAAATCAAATAAGATGAGCAAGATGGAACGTCTGGGTTCCAATTCTGTCAAGAC
TTATGACATATTAA
+
FFFFFFFFFFFFFFFFFFFFFFFFFFFFF:FFFFFFFFFFFFFFFFFFFFFFF:FFFFFFFF,FFFFFFF
FFFFFFFFFFFFFFFFFFFFFFF,FFF,,FF::FFFF,:F,FFF:FFFFF:FF::FFFFFFFF:FFFFF
FFFFFF,FF:FFF:
@A00155:342:HHGFNDSXY:1:1115:18521:4539 2:N:0:GAACCTAG+TCCGCATA
GTAAAACTCTGGAGAACCACCTATTATTTAATGTTGTAGCTAGTTACATTAGTAAATATTCATATGTA
GCATGTTTAAGCATTAAATCAAATAAGATGAGCAAGATGGAACGTCTGGGTTCCAATTCTGTCAAGAC
TTATGACATATTAAA
+
FFFFFFFFFFFFFFFFFFFFFFFFFFFFFFFFFFFFFFFFFFFFFFFFFFFFFFFFFFFFFFFFFFFF
FFFFF:FFFFFFFFFFFFFFFFFFFFFFFFFFFFFFFFFFFFFFFFFFFFFFFFFFFFFFFFFFFFFF
FFFFFFFFFFFFFFF
```

@A00155:342:HHGFNDSXY:1:1176:5801:35837 2:N:0:GAACCTAG+TCCGCATA
GTAAAACTCTGGAGAACCACCTATTATTTAATGTTGTAGCTAGTTACATTAGTAAATATTCATATGTA
GCATGTTTAAGCATTAAATCAAATAAGATGAGCAAGATGGAACGTCTGGGTTCCAATTCTGTCAAGAC
TTATGACATATTAAA
+
FFFFF:FFFFFFFFFFFFFFFFFFFFFFFF:FFFFFFFFFFFFFFFFFF:FFFFFFFFFFFFFFFFFF
FFFFFFFFFFFFFFFFFFFFFFFFFFFFFFFFFFFFF,FFFFFFFF,F:FFFFFFFFFFFFFFFFFFF
FFFFFFFFFFFFFF:
@A00155:342:HHGFNDSXY:1:1236:28682:7827 2:N:0:GAACCTAG+TCCGCATA
GTAAAACTCTGGAGAACCACCTATTATTTAATGTTGTAGCTAGTTACATTAGTAAATATTCATATGTA
GCATGTTTAAGCATTAAATCAAATAAGATGAGCAAGATGGAACGTCTGGGTTCCAATTCTGTCAAGAC
TTATGACATATTAAA
+
FFFFFFFFFFFFFFFFFFFFFFFFFFFFFFFFFFFFFFFFFFFFF:FFFFFFFFFFFFFFFFFFFFFF
FFFFFFFFFFFFFFFFFFFFFFFFFFFFFFFFFFFFFFFFFFF:FFFFFFFFFFFFFFFFFFFFFFFF
FFFFFFFFFFFFFFF
@A00155:342:HHGFNDSXY:1:1231:23312:27868 2:N:0:GAACCTAG+TCCGCATA
GTAAAACTCTGGAGAACCACCTATTATTTAATGTTGTAGCTAGTTACATTAGTAAATATTCATATGTA
GCATGTTTAAGCATTAAATCAAATAAGATGAGCAAGATGGAACGTCTGGGTTCCAATTCTGTCAAGAC
TTATGACATATTAAA
+
FFFFFFFFFFFFFFFFFFFFFFFFFFFFFFFFFFFFFFFFFFFFFFFFFFFFFFFFFFFFFFFFFFFF
FFFFFFFFFFFFFFFFFFFFFFFFFFFFFFFFFFFFFFFFFFFFFFFFFFFFFFFFFFFFFFFFFFFF
:FFFFFFFFFFFFFF
@A00155:342:HHGFNDSXY:1:1253:22245:30217 2:N:0:GAACCTAG+TCCGCATA
GTAAAACTCTGGAGAACCACCTATTATTTAATGTTGTAGCTAGTTACATTAGTAAATATTCATATGTA
GCATGTTTAAGCATTAAATCAAATAAGATGAGCAAGATGGAACGTCTGGGTTCCAATTCTGTCAAGAC
TTATGACATATTAAA
+
FFFFFFFFFFFFFFFFFFFFFFFFFFFFFFFFFFFFFFFFFFFFFFFFFFFFFFFFFFFFFFFFFFFF
FFFFFFFFFF:FFFFFFFFFFFFFFFFFFFFFFFFFFFFFFFFFFFFFFFFFFFFFFFFFFFFFFFFF
:FFF:FFFFFFFFFF
@A00155:342:HHGFNDSXY:1:1374:13530:6449 2:N:0:GAACCTAG+TCCGCATA
GTAAAACTCTGGAGAACCACCTATTATTTAATGTTGTAGCTAGTTACATTAGTAAATATTCATATGTA
GCATGTTTAAGCATTAAATCAAATAAGATGAGCAAGATGGAACGTCTGGGTTCCAATTCTGTCAAGAC
TTATGACATATTAAA
+
FFFF,FFFFFFFFFFFFFFFFFFFFFFFFFFFFFFFFFFFFFFFFFFFFFFFFFFFFFFFFFFFFFFF
FFFFFFFFFFFFFFFF:FFFFFFFFFFFFFFFFFFFFFFFFFF:FFFFFFFFFFFFFFFFFFFFFFFF
FFFFFFFFFFFFF:F
@A00155:342:HHGFNDSXY:1:1413:23674:33191 2:N:0:GAACCTAG+TCCGCATA
GTAAAACTCTGGAGAACCACCTATTATTTAATGTTGTAGCTAGTTACATTAGTAAATATTCATATGTA
GCATGTTTAAGCATTAAATCAAATAAGATGAGCAAGATGGAACGTCTGGGTTCCAATTCTGTCAAGAC
TTATGACATATTAAA
+
FFFFFFFFFFFFFFFFFFFFFFFFFFFFFFFFFFFFFFFFFFFFFFFFFFFFFFFFFFFFFFFFFFFF
FFFFFFFFFFFFFFFFFFFFFFFFFFFFFFFFFFFFFFFFFFFFFFFFFFFFFFFFFFFFFFFFFFFF
FFFFFFF:FFFFFF:
@A00155:342:HHGFNDSXY:1:1527:18954:25989 2:N:0:GAACCTAG+TCCGCATA
GTAAAACTCTGGAGAACCACCTATTATTTAATGTTGTAGCTAGTTACATTAGTAAATATTCATATGTA
GCATGTTTAAGCATTAAATCAAATAAGATGAGCAAGATGGAACGTCTGGGTTCCAATTCTGTCAAGAC
TTATGACATATTAAA
+
FFFFFFFFFFFFFFFFFFFFFFFFFFFFFFFFFFFFFFFFFFFFFFFFFFFFFFFFFFFFFFFFFF:F

FFFFFF:FFFFFFF:FFFFFFFFFFFFFFFFFFFFFFFFFFFFFFFFFFFFFFFFFFFFFFFFFFFFF
FFFFFFFFFFFFFFF
@A00155:342:HHGFNDSXY:1:1551:17635:9048 2:N:0:GAACCTAG+TCCGCATA
GTAAAACTCTGGAGAACCACCTATTATTTAATGTTGTAGCTAGTTACATTAGTAAATATTCATATGTA
GCATGTTTAAGCATTAAATCAAATAAGATGAGCAAGATGGAACGTCTGGGTTCCAATTCTGTCAAGAC
TTATGACATATTAAA
+
FFFFFFFFFFFFFFFFFFFFFFFFFFFFFFFFFFFFFFFFFFFFFFFFFFFFFFFFFFFFFFFFFFFF
FFFFFFFFFFFFFFFFFFFF:FFFFFFFFFFFFFFFFFFFF:FFFFFFFFFFFFFFFFFFFFFFFFFF
FFFFFFFFFFFF:FF
@A00155:342:HHGFNDSXY:1:1621:29080:11240 2:N:0:GAACCTAG+TCCGCATA
GTAAAACTCTGGAGAACCACCTATTATTTAATGTTGTAGCTAGTTACATTAGTAAATATTCATATGTA
GCATGTTTAAGCATTAAATCAAATAAGATGAGCAAGATGGAACGTCTGGGTTCCAATTCTGTCAAGAC
TTATGACATATTAAA
+
FFFFFFFFFFFFFFFFFFFFFFFFFFFFFFFFFFFFFFFFFFFFFFFFFFFFFFFFFFFFFFFFFFFF
FFFFFFFFFF:FFFFFFFFFFFFFFFF:FFFFFFFFFFFFFFFFFFFFFFFFFFFFFFFFFFFFF:F:
FFFF:FFFFFFFF:F
@A00155:342:HHGFNDSXY:1:1635:30662:16736 2:N:0:GAACCTAG+TCCGCATA
GTAAAACTCTGGAGAACCACCTATTATTTAATGTTGTAGCTAGTTACATTAGTAAATATTCATATGTA
GCATGTTTAAGCATTAAATCAAATAAGATGAGCAAGATGGAACGTCTGGGTTCCAATTCTGTCAAGAC
TTATGACATATTAAA
+
FFFFFFFFFFFFFFFFFFFFFFFFFFFFFFFFFFFFFFFFFFFFFFFFFFFFFFFFFFFFFFFFFFFF
FFFFFFFF:FFFFFFFFFFFFFFFFFFFFFFFFFFFFFFFFFFFFFFFFFFFFFFFFFFFFFFFFFF
FFFFFFFFFFFFFFF
@A00155:342:HHGFNDSXY:1:2413:17083:11162 2:N:0:GAACCTAG+TCCGCATA
GTAAAACTCTGGAGAACCACCTATTATTTAATGTTGTAGCTAGTTACATTAGTAAATATTCATATGTA
GCATGTTTAAGCATTAAATCAAATAAGATGAGCAAGATGGAACGTCTGGGTTCCAATTCTGTCAAGAC
TTATGACATATTAAA
+
FFFF:FFFFFFFFFFFFFFFFFFFFFFFFFFF,FFFFFFFFFFFFFFFFFFFFFFFFFFFFFFFFFFF
FFFFFF:FFFFFFFFFFFFFFFFFFFFFFFFF:FFFFFFFFFFFFFF:FFFFF,FFFFFFFFFFFFFF
FF:F:FFFFFFFF:F
@A00155:342:HHGFNDSXY:1:2471:31910:24001 2:N:0:GAACCTAG+TCCGCATA
GTAAAACTCTGGAGAACCACCTATTATTTAATGTTGTAGCTAGTTACATTAGTAAATATTCATATGTA
GCATGTTTAAGCATTAAATCAAATAAGATGAGCAAGATGGAACGTCTGGGTTCCAATTCTGTCAAGAC
TTATGACATATTAAA
+
FFFFFFFFFFFFFFFFFFFFFFFFF:FFFFF,FFFFFFFFFFFFFFFF,FFFFFFFFFFFFFFFFFFF
FFFFFFFFFFFFFFF,FFFFFFFFFFFFFFFFFFFFFFFFFFFFFFFFFFFFFFFFFFFFFFFFFFFF
FFFFFFFFFFFFFFF
@A00155:342:HHGFNDSXY:1:2453:26232:19867 2:N:0:GAACCTAG+TCCGCATA
GTAAAACTCTGGAGAACCACCTATTATTTAATGTTGTAGCTAGTTACATTAGTAAATATTCATATGTA
GCATGTTTAAGCATTAAATCAAATAAGATGAGCAAGATGGAACGTCTGGGTTCCAATTCTGTCAAGAC
TTATGACATATTAAA
+
F::FFFFFFFFFFFFFFFFFFFFFFFFFFFFFFFFFFFFFFFFFFFFFFFFFFFFFFFFFFFFFFFFF
FFFFFFFFFFFFFFFFFFFFFFFFFFFFF:FF:FFFFFF:FFF:FFFFF:FFFFFFFF,FFF:FFFFF
FFFFFF:FFFFFFFF
@A00155:342:HHGFNDSXY:1:2643:9155:25801 2:N:0:GAACCTAG+TCCGCATA
GTAAAACTCTGGAGAACCACCTATTATTTAATGTTGTAGCTAGTTACATTAGTAAATATTCATATGTA
GCATGTTTAAGCATTAAATCAAATAAGATGAGCAAGATGGAACGTCTGGGTTCCAATTCTGTCAAGAC
TTATGACATATTAAA

```
+
FFFFFFFFFFFFFFFFFFFFFFFFFFFFFFFFFFFFFFFFFFFFFFFFFFFFFFFFFFFFFFFFFFFF
FFFFFFFFFFFFFFFFFFFFFFFFFFFFFFF:FFFFFFFFFF:FFFFFFFFFFFFFFFFFFFFFFFFF
FFFFFFFFFFFFFF:
@A00155:342:HHGFNDSXY:1:2651:26946:25708 2:N:0:GAACCTAG+TCCGCATA
GTAAAACTCTGGAGAACCACCTATTATTTAATGTTGTAGCTAGTTACATTAGTAAATATTCATATGTA
GCATGTTTAAGCATTAAATCAAATAAGATGAGCAAGATGGAACGTCTGGGTTCCAATTCTGTCAAGAC
TTATGACATATTAAA
+
FFFFFFFFFFFFFFFFFFFFFFFFFFFFFFFFFFFFFFFFFFFFFFFFFFFFFFFFFFFFFFFFFFFF
FFFFFFFFFFF:FFFFF:FFFFFFFFFFFFFFFFFFFFFFFFFFFFFFFFFFF:FFFFFFFFFFFFFF
FFFFFFFFF:FFFFF
@A00155:342:HHGFNDSXY:1:1354:30680:22968 2:N:0:GAACCTAG+TCCGCATA
TAAAACTCTGGAGAACCACCTATTATTTAATGTTGTAGCTAGTTACATTAGTAAATATTCATATGTAG
CATGTTTAAGCATTAAATCAAATAAGATGAGCAAGATGGAACGTCTGGGTTCCAATTCTGTCAAGACT
TATGACAT
+
FFFFFFFFFF,FFFFF,FFFFFFFFF::FFFFFF,FFFFFFFFFFFFFFFFFFF:FFFFFFFF::FFF
FFFFFF:FFFFFFFFFF::F:FFFF:FFF:F,FFF:F:FFFFFF:FFFFFF,FFFFFFFFFFFFFFFF
FFF:FFF:
@A00155:342:HHGFNDSXY:1:1348:11406:2926 1:N:0:GAACCTAG+TCCGCATA
TAAAACTCTGGAGAACCACCTATTATTTAATGTTGTAGCTAGTTACATTAGTAAATATTCATATGTAG
CATGTTTAAGCATTAAATCAAATAAGATGAGCAAGATGGAACGTCTGGGTTCCAATTCTGTCAAGACT
TATGACATATTAAAT
+
FFFFFFFFFFFFFFFFFFFFFFFFFFFFFFFFFFFFFFFFFFFFFFFFFFFFFFFFFFFFFFFFF:FF
FFFFFFFFFFFFFFFFFFFFFFFFFFFFFFFFFFFFFFFFFFFFFFFFFFFFFFFFFFFFFFFFFFFF
FFFFFFFFFFFFFFF
@A00155:342:HHGFNDSXY:1:1173:10863:3521 2:N:0:GAACCTAG+TCCGCATA
TAAAACTCTGGAGAACCACCTATTATTTAATGTTGTAGCTAGTTACATTAGTAAATATTCATATGTAG
CATGTTTAAGCATTAAATCAAATAAGATGAGCAAGATGGAACGTCTGGGTTCCAATTCTGTCAAGACT
TATGACATATTAAAT
+
FFF,FFFFFFFFFF:FFFFFFFFFFFFFFFFFFF:FFFFFFFFFFFFFFFFFFFFFFFFFFFFFFFFF
FFF:FFFFFF::FFFFFFFFFFFFFFFFFFF,FFFFFFFFFFFFFFFFF:FFFFFFFFFFFFFFFFFF
FFFFFFFFFF:FFFF
@A00155:342:HHGFNDSXY:1:1410:24813:19570 2:N:0:GAACCTAG+TCCGCATA
TAAAACTCTGGAGAACCACCTATTATTTAATGTTGTAGCTAGTTACATTAGTAAATATTCATATGTAG
CATGTTTAAGCATTAAATCAAATAAGATGAGCAAGATGGAACGTCTGGGTTCCAATTCTGTCAAGACT
TATGACATATTAAAT
+
FFFFFFFFFFFFFFFFFFFFFFFFFFFFFFFFFFFFFFFFFFFFFFFFFFFFFFFFFFFFFFFFFFFF
FF:FFFFFFFFFFFFFFFFFFFFFFFFFFFFFFFFFFFFFFFFFFFFFFFFFFFFFFFFFFFFFFFFF
FFFFFFFFFFFFFFF
@A00155:342:HHGFNDSXY:1:1502:31955:3035 2:N:0:GAACCTAG+TCCGCATA
TAAAACTCTGGAGAACCACCTATTATTTAATGTTGTAGCTAGTTACATTAGTAAATATTCATATGTAG
CATGTTTAAGCATTAAATCAAATAAGATGAGCAAGATGGAACGTCTGGGTTCCAATTCTGTCAAGACT
TATGACATATTAAAT
+
FFFFFFFFFFFFFFFFFFFFFFFFFFFF:FFF:FFFFFFFFFFFFFF:FFF:FFFFFFFFFFFFFFFF
FFFF:FFFFFFFFFFFFFFFFFFFFFFFFFFFF:FFFFFFFFFFF:FFFFFFFFFFFFFFF:F,FFFFF
FFFFFFFFFFFFFFF
@A00155:342:HHGFNDSXY:1:1536:7238:32503 2:N:0:GAACCTAG+TCCGCATA
TAAAACTCTGGAGAACCACCTATTATTTAATGTTGTAGCTAGTTACATTAGTAAATATTCATATGTAG
```

```
CATGTTTAAGCATTAAATCAAATAAGATGAGCAAGATGGAACGTCTGGGTTCCAATTCTGTCAAGACT
TATGACATATTAAAT
+
FFF,FFFFFFFFFFFFFFFFFFFFFFFFFFFFFFFFFFFFFFFFFFFFFFFFFFFFFFFFFFFFFFFF
FFFFFFFF,FFFFFFFFFFFFFFFFFFFFFFFFFFFFFFFFFFFFFFFFFFFFFFFFFFFFFFFFFFF
FFFFFFFFFFFFFFF
@A00155:342:HHGFNDSXY:1:1535:31159:33599 2:N:0:GAACCTAG+TCCGCATA
TAAAACTCTGGAGAACCACCTATTATTTAATGTTGTAGCTAGTTACATTAGTAAATATTCATATGTAG
CATGTTTAAGCATTAAATCAAATAAGATGAGCAAGATGGAACGTCTGGGATCCAATTCTGTCAAGACT
TATGACATATTAAAT
+
FFFFFF:FFFF:FFFFFFF,FFFFF:FF::FFFFFFFFF,:FF:FFF:FFFFFFFFFF:FF:FFFFF:
FF:FF,FFFFFFF:FF:FFFFFFF,FFFFFFFFFFFFFFF:FFFFFF,F:FFFFFF:FFFFFFF:FFF
FFFFFFFF,F,FFFF
@A00155:342:HHGFNDSXY:1:1348:11406:2926 2:N:0:GAACCTAG+TCCGCATA
AAAACTCTGGAGAACCACCTATTATTTAATGTTGTAGCTAGTTACATTAGTAAATATTCATATGTAGC
ATGTTTAAGCATTAAATCAAATAAGATGAGCAAGATGGAACGTCTGGGTTCCAATTCTGTCAAGACTT
ATGACATATTAAAT
+
FFFFFFFFFFFFFFFFFFFFFFFFFFFFFFFFFFFFFFFFFFFFFF:FFFFFFFFFFFFFFFFFFFFF
F:FFFFF:FFFFFFFFFFFF:FFFFFFFFFFFFFFFFFFF:FFFFFFFFFF:FFFFFFFFFFFFFFFF
FFFFFFF:FFFFFF
@A00155:342:HHGFNDSXY:1:1450:9534:25269 2:N:0:GAACCTAG+TCCGCATA
AAAACTCTGGAGAACCACCTATTATTTAATGTTGTAGCTAGTTACATTAGTAAATATTCATATGTAGC
ATGTTTAAGCATTAAATCAAATAAGATGAGCAAGATGGAACGTCTGGTTTCCAATTCTGTCAAGACTT
ATGACATATTAAAT
+
FFFFFFFFFFFFFFFFFFFFFFFFF:FFFFFFFFF:FFFFFFFFFFFFFFFFFFFFFFFFFFFFFFFF
FFF:FFF:FFFFFFF::FFF:FFF,FFFFFFFFFFFFFFFFFFFFFFFFFFFFFFFFFFFFFFFFFFF
FFFF:FFFFFFFFF
@A00155:342:HHGFNDSXY:1:1127:29125:24095 2:N:0:GAACCTAG+TCCGCATA
AAAACTCTGGAGAACCACCTATTATTTAATGTTGTAGCTAGTTACATTAGTAAATATTCATATGTAGC
ATGTTTAAGCATTAAATCAAATAAGATGAGCAAGATGGAACGTCTGGGTTCCAATTCTGTCAAGACTT
ATGACATATTAAATA
+
FF:FFFFFFFFFFF:FFFFFFFFFFFFFF,FFFFFFFFFFFFFFFFFFFFFFFFFFFFFFFFFFFFFF
FFFFFFFFFFFFFFFFFFFFFFFFFFFFFFFFFFFFFFFFFFFFFFFFFFFFFFFFFFFFFFFFFFFF
FFFFFFFFFFFFFFF
@A00155:342:HHGFNDSXY:1:1415:27199:27430 2:N:0:GAACCTAG+TCCGCATA
AAAACTCTGGAGAACCACCTATTATTTAATGTTGTAGCTAGTTACATTAGTAAATATTCATATGTAGC
ATGTTTAAGCATTAAATCAAATAAGATGAGCAAGATGGAACGTCTGGGTTCCAATTCTGTCAAGACTT
ATGACATATTAAATA
+
FFFFFFFFFFFFFFFFFFFFFFFFFFFFFFFFFFFFFFFFFFFFFF:FFFFFFFFFFFFFFFFFFFFF
FFFFFFFFFFFFFFFFFFFFFFFFFFFFFFFFFFFFFFFF:FFFFFFF:FFFFFFFFFFFFFFFFFFF
:FFFFFFFFFFFFFF
@A00155:342:HHGFNDSXY:1:1450:9995:25285 2:N:0:GAACCTAG+TCCGCATA
AAAACTCTGGAGAACCACCTATTATTTAATGTTGTAGCTAGTTACATTAGTAAATATTCATATGTAGC
ATGTTTAAGCATTAAATCAAATAAGATGAGCAAGATGGAACGTCTGGTTTCCAATTCTGTCAAGACTT
ATGACATATTAAATA
+
FFFFFFFFFFFFFFFFFFFFFFFFFFFFFFFFFFFFFFFFFFFFFFFFFFFFFFFFFFFFFFFFFFFF
FFFFFFFFFFFFFFFFFFFFFFFFFFFFFFFFFFFFFFFFFFF:FFFFFFFFFFFFFFFFFFFFFFFF
FFFFFFFFFFFFFFF
```

```
@A00155:342:HHGFNDSXY:1:1509:21395:1313 2:N:0:GAACCTAG+TCCGCATA
AAAACTCTGGAGAACCACCTATTATTTAATGTTGTAGCTAGTTACATTAGTAAATATTCATATGTAGC
ATGTTTAAGCATTAAATCAAATAAGATGAGCAAGATGGAACGTCTGGGTTCCAATTCTGTCAAGACTT
ATGACATATTAAATA
+
FFFFFFFFFFFFFFFFFFFFFFFFFFFF:FFFFFF::FFFFFFFFF:FFFFFF,FFFFFFFF,FFFFF
FFF:FFF:FFFFFFFFFFFFFFFFFFFFFFFFFFFFFFFFFFFFFFFFFFFFFFFFFFFFFFF:FFFF
,FFFF:FFFFFFFFF
@A00155:342:HHGFNDSXY:1:1558:12789:4570 2:N:0:GAACCTAG+TCCGCATA
AAAACTCTGGAGAACCACCTATTATTTAATGTTGTAGCTAGTTACATTAGTAAATATTCATATGTAGC
ATGTTTAAGCATTAAATCAAATAAGATGAGCAAGATGGAACGTCTGGGTTCCAATTCTGTCAAGACTT
ATGACATATTAAATA
+
FFFFFFFFFFFFFFFFFFFFFFFFFFFFFFFFFFFFFFFFFFFFFFFFFFFFFFFFFFFFFFFFFFFF
FFFFFFFFFFFFFFFFFFFFFFFFFFFFFFFFFFFFFFFFFFFFFFFFFFFFFFFFFFFFFFFFFFFF
FFFFFFFFFFFFFFF
@A00155:342:HHGFNDSXY:1:1669:28076:29356 2:N:0:GAACCTAG+TCCGCATA
AAAACTCTGGAGAACCACCTATTATTTAATGTTGTAGCTAGTTACATTAGTAAATATTCATATGTAGC
ATGTTTAAGCATTAAATCAAATAAGATGAGCAAGATGGAACGTCTGGGTTCCAATTCTGTCAAGACTT
ATGACATATTAAATA
+
FFFFFFFFFFFFFFFFFFFFFFFFFFFF,FFFFFFFFFFFFFFFFFF:FFFFFFFFFFFFFFFFFFFF
F:FFFFFFFFFFFFFFFFFFFFFFFFFFFFFFFFFFFFFFFFFFFFFFFFFFFFFFFFFFFFFFFFFF
F:FFFFFF:FF:FFF
@A00155:342:HHGFNDSXY:1:2203:18186:35963 2:N:0:GAACCTAG+TCCGCATA
AAAACTCTGGAGAACCACCTATTATTTAATGTTGTAGCTAGTTACATTAGTAAATATTCATATGTAGC
ATGTTTAAGCATTAAATCAAATAAGATGAGCAAGATGGAACGTCTGGGTTCCAATTCTGTCAAGACTT
ATGACATATTAAATA
+
FFFFFFFFFFFFFFFFFFFFFFFFFFFFFFFFFFFFFFF:FFFFFF:FFFFFFFFFFFFFFFFFFFFF
FFFFFFFF::FFFFFFFFFFFFFF:FFFF:FFFFFF:FFFFFFFFFFFFFFFFFFFFFFF:FFFFFFF
FFFFFFFFFFFFFFF
@A00155:342:HHGFNDSXY:1:2330:21703:3505 2:N:0:GAACCTAG+TCCGCATA
AAAACTCTGGAGAACCACCTATTATTTAATGTTGTAGCTAGTTACATTAGTAAATATTCATATGTAGC
ATGTTTAAGCATTAAATCAAATAAGATGAGCAAGATGGAACGTCTGGGTTCCAATTCTGTCAAGACTT
ATGACATATTAAATA
+
FFFFFFFFFFFFFFFFFFFFFFFFFFFFFFFFFFFFFF:FFFFFFFFFFFFFFFFFFFFFFFFFF:FF
FFFFFFFFFFFFFFFFFFFFFFFFFFF,FFFFFFFFFFFFFFFFFFFFFFFFFFFFFFFFFFFFFFFF
FFFFFFFFFFFFFFF
@A00155:342:HHGFNDSXY:1:2557:28176:25864 2:N:0:GAACCTAG+TCCGCATA
AAAACTCTGGAGAACCACCTATTATTTAATGTTGTAGCTAGTTACATTAGTAAATATTCATATGTAGC
ATGTTTAAGCATTAAATCAAATAAGATGAGCAAGATGGAACGTCTGGGTTCCAATTCTGTCAAGACTT
ATGACATATTAAATA
+
FFFFFFFFFFFFFFFFFFFFFFFFFFFFFFFFFFFFFFFFFFFFFFFFFFFFFFFFFFFFFFFFFFFF
FFFFFFFFFFFFFFFFFFFF:FFFFFFFFFFFFFFFFFFFFFFFFFFFFFFFFFFFFFFFFFFFFFF
FFFFFF:FFFFFFFF
@A00155:342:HHGFNDSXY:1:2668:13810:36432 2:N:0:GAACCTAG+TCCGCATA
AAAACTCTGGAGAACCACCTATTATTTAATGTTGTAGCTAGTTACATTAGTAAATATTCATATGTAGC
ATGTTTAAGCATTAAATCAAATAAGATGAGCAAGATGGAACGTCTGGGTTCCAATTCTGTCAAGACTT
ATGACATATTAAATA
+
FFFFFFFFFFFFFFFFFFFFFFFFFFFFFFFFFFFFFFFFFFFFFF,FFFFFFFFFFFFFFFFFFFFF
```

FFFFFFFFFFFFFFF:FFFFFFFFFFFFFFFFFFFFFFFFFFFFFFFFFFFFFFFFFFFFFFFFFFFF
FFFFFFFFFFFFFFF
@A00155:342:HHGFNDSXY:1:1315:17607:6464 1:N:0:GAACCTAG+TCCGCATA
AAACTCTGGAGAACCACCTATTATTTAATGTTGTAGCTAGTTACATTAGTAAATATTCATATGTAGCA
TGTTTAAGCATTAAATCAAATAAGATGAGCAAGATGGAACGTCTGGGTTCCAATTCTGTCAAGACTTA
TGACATATTAAATAG
+
FFFFFFFFFF:FFFFFFFFFFFFFFFFFFFFFFFFFFFFFFFFFFFFFFFFFFFFFFFFFFFFFFFFF
FFFFFFFFFFFFFFFFFFFFFFFFFFFFFFFFFFFFFFFFFFFFFFFFFFFFFFFFFFFFFFFFFFFF
FFFFFFFFFFFFFFF
@A00155:342:HHGFNDSXY:1:1315:17155:6558 1:N:0:GAACCTAG+TCCGCATA
AAACTCTGGAGAACCACCTATTATTTAATGTTGTAGCTAGTTACATTAGTAAATATTCATATGTAGCA
TGTTTAAGCATTAAATCAAATAAGATGAGCAAGATGGAACGTCTGGGTTCCAATTCTGTCAAGACTTA
TGACATATTAAATAG
+
FFFFFFFFFFFFFFFFFFFFFFFFFF:FFFFFFFFFFFFFFFF:FFFFFFFFFFFFFFFFFFFFFFFF
FFFFFFFFFFFFFFFFFFFFFFFFFFFFFFFFFFFFFFFFFFFFFFFFFFFFF:FFFFFFFFFFFFFF
FFFFFFFFFFFFFFF
@A00155:342:HHGFNDSXY:1:2569:3920:3051 1:N:0:GAACCTAG+TCCGCATA
AAACTCTGGAGAACCACCTATTATTTAATGTTGTAGCTAGTTACATTAGTAAATATTCATATGTAGCA
TGTTTAAGCATTAAATCAAATAAGATGAGCAAGATGGAACGTCTGGGTTCCAATTCTGTCAAGACTTA
TGACATATTAAATAG
+
FFFFFFFFFFFFFFFF:FFFF:FF:FFFFFFFFFF,FFFFFFFFFFFFFFFFFFFFFFFFFFFFFFFF
FFFFFFFFFFFFFFFFFFFFFFFFFFFFFFFF,FFFFFFFFFFFFFFF:FFFFFFFFFFFFFFFFFFF
FFFFFFFFFFFFFFF
@A00155:342:HHGFNDSXY:1:1519:3414:35994 2:N:0:GAACCTAG+TCCGCATA
AAACTCTGGAGAACCACCTATTATTTAATGTTGTAGCTAGTTACATTAGTAAATATTCATATGTAGCA
TGTTTAAGCATTAAATCAAATAAGATGAGCAAGATGGAACGTCTGGGTTCCAATTCTGTCAAGACTTA
TGACATATTAAATAG
+
FFFFFFFFFFFFFF,FFFFFFFFFFFFFFFFFFFFFFFFFFFFFFFFFFFFFFFFFFFFFFFFFFFFF
FFFFFFFFFFFFFFFFFFFFFFFFFFFFFFF:FFFFFFFFFFFFFFFFFFFFFFFFFFFFFFF:FFFF
FFFFFFFFFFFFFFF
@A00155:342:HHGFNDSXY:1:2151:14136:14857 2:N:0:GAACCTAG+TCCGCATA
AAACTCTGGAGAACCACCTATTATTTAATGTTGTAGCTAGTTACATTAGTAAATATTCATATGTAGCA
TGTTTAAGCATTAAATCAAATAAGATGAGCAAGATGGAACGTCTGGGTTCCAATTCTGTCAAGACTTA
TGACATATTAAATAG
+
FFFFFFFFFFFFFFFFFFFFFFFFFFFFFFFF:FFFFFFFFFFFFFFFFFFFFFFFF:FFFFFFFFFF
FFFFFFFFFFFFFFFFFFFF:FFFFFFFFFFFFFFFFFFFFFFFFFFFFFFFFFFFFFFFFF:FFFFF
F:F:FFFF:FFFFFF
@A00155:342:HHGFNDSXY:1:2527:17002:30029 2:N:0:GAACCTAG+TCCGCATA
AAACTCTGGAGAACCACCTATTATTTAATGTTGTAGCTAGTTACATTAGTAAATATTCATATGTAGCA
TGTTTAAGCATTAAATCAAATAAGATGAGCAAGATGGAACGTCTGGGTTCCAATTCTGTCAAGACTTA
TGACATATTAAATAG
+
:FFFFFFFFFFFFFFFFFFFFFFFFFFFFFFFFFFFFFFFFFFF:,FFFFFFFFFFFFFFFFFFFFFF
FFFFFFFFFF:FFFFFFFFFFFFFFFFFFFFFFFFFFFF::FFFFFFFF:FFFFFFFFFFFFFFFFFF
:FFFFFFF:FFFFFF
@A00155:342:HHGFNDSXY:1:1355:8124:25801 1:N:0:GAACCTAG+TCCGCATA
ACTCTGGAGAACCACCTATTATTTAATGTTGTAGCTAGTTACATTAGTAAATATTCATATGTAGCATG
TTTAAGCATTAAATCAAATAAGATGAGCAAGATGGAACGTCTGGGTTCCAATTCTGTCAAGACTTATG
ACATATTAAAT

```
+
FFFFFFFFFFFFFFFFFFFFFF::FFFFFFFFFFFFFFFFFFFFFFFFFFFFFFFFFFFFFFFFFFFFFFF
:FFFFFFFFFFFFFFFFFFFFFFFFFFFFFFFFFFFFFFFFFFFFFFFFFFFFFFFFFFFFFFFFFFFFF
FFFFFF:FFFF
@A00155:342:HHGFNDSXY:1:2206:21395:23422 1:N:0:GAACCTAG+TCCGCATA
ACTCTGGAGAACCACCTATTATTTAATGTTGTAGCTAGTTACATTAGTAAATATTCATATGTAGCATG
TTTAAGCATTAAATCAAATAAGATGAGCAAGATGGAACGTCTGGGTTCCAATTCTGTCAAGACTTATG
ACATATTAAAT
+
FFFFFFFFFFFFFF:FFFFFFFFFFFFFFFFFFFFFFFFFFFFFFFFFFFFFFFFFFFFFFFFFFFFFF
FFFFFFFFFFFFFFFFFFFFFFFFFFFFFFFFFFFFFFFFFFFFFFFFFFFFFFFFFFFFFFFFFFFFFF
FFFFFFFFFFF
@A00155:342:HHGFNDSXY:1:2643:9155:25801 1:N:0:GAACCTAG+TCCGCATA
ACTCTGGAGAACCACCTATTATTTAATGTTGTAGCTAGTTACATTAGTAAATATTCATATGTAGCATG
TTTAAGCATTAAATCAAATAAGATGAGCAAGATGGAACGTCTGGGTTCCAATTCTGTCAAGACTTATG
ACATATTAAAT
+
FFFFFFFFFFFFFFFFFFFFFFFFFFFFFFFFFFFFFFFFFFFFFFFFFFFFFFFFFFFFFFFFFFFFFF
FFFFFFFFFFFFFFFFFFFFFFFFFFFFFFFFFFFFFFFFFFFFFFFFFFFFFFFFFFFFFFFFFFFFFF
FFFFFFFFFFF
@A00155:342:HHGFNDSXY:1:2437:15103:17440 2:N:0:GAACCTAG+TCCGCATA
ACTCTGGAGAACCACCTATTATTTAATGTTGTAGCTAGTTACATTAGTAAATATTCATATGTAGCATG
TTTAAGCATTAAATCAAATAAGATGAGCAAGATGGAACGTCTGGGTTCCAATTCTGTCAAGACTTATG
ACATATTAAATAGAT
+
:FFFFFFFFFFFFFFFFFFFFFFFFFFFFFFFFFFFFFFFFFFFFFFFFFFFFFFFFFFFFFFFF:FFFF
FFFFFFFFFFFFFFFFFFFFFFFFFFFFFFFF:FF:FFFFFFFFFFFFFFFFFF,FFFFFF:FFFFFFF
FFFFFFFFFFFF:FF
@A00155:342:HHGFNDSXY:1:2645:32615:1579 2:N:0:GAACCTAG+TCCGCATA
CTCTGGAGAACCACCTATTATTTAATGTTGTATCTAGTTACATTAGTAAATATTCATATGTAGCATGT
TTAAGCATTAAATCAAATAAGATGAGCAAGATGGAACGTCTGGGTTCCAATTCTGTCAAGACTTATGA
CATATTAAATAGAT
+
FFFFFFFFFFFFFFFFFFF:FFFFF:FFFFFFFFFFFFFFFFF:FFFF:FFFFFFFFF:FF:FFFFFF
FFFFFFF:FFFFFFFFFFFFFFFFFFF:FFFFFFF:FFFFFFFFF:FFFFFFFFFFFFFFFFFF:FFF
FFFFF:FFFFFFFF
@A00155:342:HHGFNDSXY:1:2574:8287:19100 2:N:0:GAACCTAG+TCCGCATA
CTCTGGAGAACCACCTATTATTTAATGTTGTAGCTAGTTACATTAGTAAATATTCATATGTAGCATGT
TTAAGCATTAAATCAAATAAGATGAGCAAGATGGAACGTCTGGGTTCCAATTCTGTCAAGACTTATGA
CATATTAAATAGAT
+
FFFFFFF,FFFF:FFFFFFFFFFFF:FFFFFFF,:FFFF:FFFFFFFFFFFFFFFFFFFFFFFFFFFFF
FFFF:FFF:FFFFFFFFFF:FFF,FF:FFF:FFFFFFFFFFFF,:FF:FFF:FFFFF:,FF::,FF,F
FFFFFFF,FFFFFF
@A00155:342:HHGFNDSXY:1:1218:18710:12759 1:N:0:GAACCTAG+TCCGCATA
CTCTGGAGAACCACCTATTATTTAATGTTGTAGCTAGTTACATTAGTAAATATTCATATGTAGCATGT
TTAAGCATTAAATCAAATAAGATGAGCAAGATGGAACGTCTGGGTTCCAATTCTGTCAAGACTTATGA
CATATTAAATAGATT
+
:F,F,FFFFFFFF:FFFF::FFFFFFFFFFFFFFFFFFFFFFFF:F:FFFFFFFFFFFFFFFFFFFFFFF
FFFFFFFFFFFFFFFFFFFFFFFFFFFFFFFFFFFFFFFFFFFFFFFFFFFFFFFFFFFFFFFFFFFFFF
FFFFFFFFFFFFFFF
@A00155:342:HHGFNDSXY:1:1255:14371:25661 1:N:0:GAACCTAG+TCCGCATA
CTCTGGAGAACCACCTATTATTTAATGTTTTAGCTAGTTACATTAGTAAATATTCATATGTAGCATGT
```

```
TTAAGCATTAAATCAAATAAGATGAGCAAGATGGAACGTCTGGGTTCCAATTCTGTCAAGACTTATGA
CATATTAAATAGATT
+
FFFFFFFFFFFFFFFFFFFFF,FFFFFFFFFFFFFFFFFFFFFFFFFFFFFFFFFFFFFFFFFFFFFFF:
FFFFFFFFFFFFFFFFFFFFFFFFFFFFFFFFFFFFFFFFFFFFFFFFFFFFFFFFFFFFFFFFFFFFFF
FFFFFFFFFFFFFFF
@A00155:342:HHGFNDSXY:1:1172:2564:27352 2:N:0:GAACCTAG+TCCGCATA
CTCTGGAGAACCACCTATTATTTAATGTTGTAGCTAGTTACATTAGTAAATATTCATATGTAGCATGT
TTAAGCATTAAATCAAATAAGATGAGCAAGATGGAACGTCTGGGTTCCAATTCTGTCAAGACTTATGA
CATATTAAATAGATT
+
FFFFFFFFFFFFFFFFFFFFFFFFFFFFFFFFFFFFFFFFFFFFFFFFFFFFFFFFFFFFFFFFFFFFFF
FFFFFFFFFFFFFFFFFFFFFFFF:FFFFFFFFFFFFFFFFFFFFFFFFFFFFFFFFFFF,FF:FFFFFF
FFFFFF:,FF:FFFF
@A00155:342:HHGFNDSXY:1:1305:11460:35900 2:N:0:GAACCTAG+TCCGCATA
CTCTGGAGAACCACCTATTATTTAATGTTGTAGCTAGTTACATTAGTAAATATTCATATGTAGCATGT
TTAAGCATTAAATCAAATAAGATGAGCAAGATGGAACGTCTGGGTTCCAATTCTGTCAAGACTTATGA
CATATTAAATAGATT
+
FFFFFFFFFFFFFFFFFFFFFFFFFFFFFFFFFFFFFFFFFFFFFFFFFFFFFFFFF:FFFFFFFFFFFF
FFFFFFFFFF:FFFFFFFFFFFFFFFFFFFFFFFFF:FFFFFFFFFFFFFFFFFFFFFFFFFFF:FFFFF
FFFFFFFFFFFFFFF
@A00155:342:HHGFNDSXY:1:1334:1642:8688 2:N:0:GAACCTAG+TCCGCATA
CTCTGGAGAACCACCTATTATTTAATGTTGTAGCTAGTTACATTAGTAAATATTCATATGTAGCATGT
TTAAGCATTAAATCAAATAAGATGAGCAAGATGGAACGTCTGGGTTCCAATTCTGTCAAGACTTATGA
CATATTAAATAGATT
+
FFFFFFFFFFFFFFFFFFFFFFFFFFFFFFFFFFFFFFFFFF:FFFFFFFFFFFFFFFFFFFFFFFFFFF
FFFFFFFFFFFFFFF:FFFF:FFFF:FFFFFFFFFFFFFFFFFFFF:FFFFFFFFFFFFFFFFFFFFFFF
:F,FFF:F,FFFFFF
@A00155:342:HHGFNDSXY:1:1326:9109:36871 2:N:0:GAACCTAG+TCCGCATA
CTCTGGAGAACCACCTATTATTTAATGTTGTAGCTAGTTACATTAGTAAATATTCATATGTAGCATGT
TTAAGCATTAAATCAAATAAGATGAGCAAGATGGAACGTCTGGGTTCCAATTCTGTCAAGACTTATGA
CATATTAAATAGATT
+
FFFFFFFFFFFFFFFF:FFFFFFFFFFFFFFFFFFFFFFFFFF,FFFFFFFFFFFFFFFFFFFFFFFFFF
FFFFFFFFFF:FFFFF:FFFFFFF::FFFFFFF:FFFFFFFFFFFFFFFFFFF:FFFFFFFFFFFFFFFF
FFFFFFFFFFFFFFF
@A00155:342:HHGFNDSXY:1:1451:18828:3474 2:N:0:GAACCTAG+TCCGCATA
CTCTGGAGAACCACCTATTATTTAATGTTGTAGCTAGTTACATTAGTAAATATTCATATGTAGCATGT
TTAAGCATTAAATCAAATAAGATGAGCAAGATGGAACGTCTGGGTTCCAATTCTGTCAAGACTTATGA
CATATTAAATAGATT
+
FFFFFFFFFFFFFFFFFFFFFFFFFFFFFFFFFFFFFFFFFF:FFFFFFFFFFFFFFFFF:FFFFFFFFF
FFFFF:FFFFFFFFFFFFFFFFFFFFFFFFFFFFFFFFFFFFFFFFFFFFFFFFFFFFFFFFFFFFFF:F
FFFFFFFF,FFFFFF
@A00155:342:HHGFNDSXY:1:1541:21983:12665 2:N:0:GAACCTAG+TCCGCATA
CTCTGGAGAACCACCTATTATTTAATGTTGTAGCTAGTTACATTAGTAAATATTCATATGTAGCATGT
TTAAGCATTAAATCAAATAAGATGAGCAAGATGGAACGTCTGGGTTCCAATTCTGTCAAGACTTATGA
CATATTAAATAGATT
+
FFFFFFFFFFFFFFFF:FFFFFFFFFFF,FFFFFF:FFFFFFFFFFFF::FFFFFFFFFFFFF:FFF:F,
FF:FFFFFFFFFFFFFFFFFFFFFFFFFFFFFFFFFFFFFFFFFFFFFFFFFFFFFFFF,FFFFFFFFFF
FFFF:FFFFFFFFF:
```

```
@A00155:342:HHGFNDSXY:1:1539:22661:24017 2:N:0:GAACCTAG+TCCGCATA
CTCTGGAGAACCACCTATTATTTAATGTTGTAGCTAGTTACATTAGTAAATATTCATATGTAGCATGT
TTAAGCATTAAATCAAATAAGATGAGCAAGATGGAACGTCTGGGTTCCAATTCTGTCAAGACTTATGA
CATATTAAATAGATT
+
FFFFFFFFFFFFFFFFFFFFFFFFFFFFFFFFFFFFFFFFFFFFFFFFFFFFFFFFF:FFFFFFFF
FFFFFFFFFFFFFFFFFFFFFFFFFFFFFFFFFFFFF:FFFFFFFFFFFFFF:FFF,FFFFFFFFF
:FF,FFFFFFFFFFF
@A00155:342:HHGFNDSXY:1:1670:31955:10770 2:N:0:GAACCTAG+TCCGCATA
CTCTGGAGAACCACCTATTATTTAATGTTGTAGCTAGTTACATTAGTAAATATTCATATGTAGCATGT
TTAAGCATTAAATCAAATAAGATGAGCAAGATGGAACGTCTGGGTTCCAATTCTGTCAAGACTTATGA
CATATTAAATAGATT
+
FFFFFFFFFFFFFFFFFFFFFFFFF:FFFFFFFFFFFFFF:FFFFFFFFF:FFFFF:FFFFFF:FF
FFFFFFFFFFFFFFFFFFFFFFFFFFFFF:FFFFFFFFFF,FFFFFFFFFFFFFFFFFFFFFFFFF
FFFFFFFFFFFFFFF
@A00155:342:HHGFNDSXY:1:2107:7925:28025 2:N:0:GAACCTAG+TCCGCATA
CTCTGGAGAACCACCTATTATTTAATGTTGTAGCTAGTTACATTAGTAAATATTCATATGTAGCATGT
TTAAGCATTAAATCAAATAAGATGAGCAAGATGGAACGTCTGGGTTCCAATTCTGTCAAGACTTATGA
CATATTAAATAGATT
+
FFFFFFFFFFFFFFFFFFFFFFFFFFFFFFFFFFFFFFFF:FFFFFFFFFFFFFFFFF:FFFFFFF
:FFFFFFFFFFFFFFFFFFFFFF:FFFFFFF::FFFFFFFFFFFFFFFFFFFFFFFFF::FFFFFF
FFFFFFF,FFFF:,F
@A00155:342:HHGFNDSXY:1:2135:23059:15029 2:N:0:GAACCTAG+TCCGCATA
CTCTGGAGAACCACCTATTATTTAATGTTGTAGCTAGTTACATTAGTAAATATTCATATGTAGCATGT
TTAAGCATTAAATCAAATAAGATGAGCAAGATGGAACGTCTGGGTTCCAATTCTGTCAAGACTTATGA
CATATTAAATAGATT
+
FFFFFFFFFFFFFFFFFFFFFFFFFFFFFFFFFFFFFFFFFFFFFFFFFFFFFFFFFFF:FFFFFF
FFFFFFFFFFFFFFFFFFFFFFFFFFFFF:FFFFFFFFFFFFFFFFFFFFFFFFFFFFFFFF:FFF
FFFFFFFFFFFFFFF
@A00155:342:HHGFNDSXY:1:2143:23737:22091 2:N:0:GAACCTAG+TCCGCATA
CTCTGGAGAACCACCTATTATTTAATGTTGTAGCTAGTTACATTAGTAAATATTCATATGTAGCATGT
TTAAGCATTAAATCAAATAAGATGAGCAAGATGGAACGTCTGGGTTCCAATTCTGTCAAGACTTATGA
CATATTAAATAGATT
+
FFFFFFFFFFFF:FFFFFFFFFFFFFFFFFFFFFFFFF:FFFFFFFFFFFFFFFFFFFFFFFFFFF
FFFFFFFFFFFFFFFFFFFFF:FFF:FFFFFFFFFFFFFFFFFFF:FFFFFFFFFFFFFFFFFFFF
FFFF:FFF:FFFFFF
@A00155:342:HHGFNDSXY:1:2116:3857:28244 2:N:0:GAACCTAG+TCCGCATA
CTCTGGAGAACCACCTATTATTTAATGTTGTAGCTAGTTACATTAGTAAATATTCATATGTAGCATGT
TTAAGCATTAAATCAAATAAGATGAGCAAGATGGAACGTCTGGGTTCCAATTCTGTCAAGACTTATGA
CATATTAAATAGATT
+
FFFFFFFFFFFFFFFFFFFFFFFF:FFFFFFFFFFFFFFFFFFFFFFFFFFFFFFFFFFFFFFFFF
FFFFFFFFFFFF:FFF:FFFFFFFFFFFFFFFFFFFFFFFFFFFFFFFFFFFFFF:FFFFFF:FFF
FFFFFFFFFF:FFF:
@A00155:342:HHGFNDSXY:1:2278:4670:25770 2:N:0:GAACCTAG+TCCGCATA
CTCTGGAGAACCACCTATTATTTAATGTTGTAGCTAGTTACATTAGTAAATATTCATATGTAGCATGT
TTAAGCATTAAATCAAATAAGATGAGCAAGATGGAACGTCTGGGTTCCAATTCTGTCAAGACTTATGA
CATATTAAATAGATT
+
FFFFFFFFFFFFFFFFFFFFFFFFFFFFFFFFF,F:FFFFFFFFFFFFFFFFFFFFFFFFFFFFFF
```

```
FFFFFFFFFFFFFFFFFFFFFFFFFFFFFFFFFFFFFFFFF:FFFFFFFFFFFFFFFFFF:FFFFFFFFF
:FFFFFFFFFF,,FF
@A00155:342:HHGFNDSXY:1:2534:3269:34397 2:N:0:GAACCTAG+TCCGCATA
CTCTGGAGAACCACCTATTATTTAATGTTGTAGCTAGTTACATTAGTAAATATTCATATGTAGCATGT
TTAAGCATTAAATCAAATAAGATGAGCAAGATGGAACGTCTGGGTTCCAATTCTGTCAAGACTTATGA
CATATTAAATAGATT
+
FFFFFFFFFFFFFFFFFFFFFFF::FF,FFFFFFFFFFFFFFFFFFFFFFFFFFFFFFF:FF,FFFFFF
FFFFFFFFFF:FFFFFFFFFFFFFFF:FFFFFFFFFF,FFFFFFFFFF:FFFFFFFF::FFFFF:F:F
F:FF,FF,F:FFFF:
@A00155:342:HHGFNDSXY:1:2676:3965:36761 2:N:0:GAACCTAG+TCCGCATA
CTCTGGAGAACCACCTATTATTTAATGTTGTAGCTAGTTACATTAGTAAATATTCATATGTAGCATGT
TTAAGCATTAAATCAAATAAGATGAGCAAGATGGAACGTCTGGGTTCCAATTCTGTCAAGACTTATGA
CATATTAAATAGATT
+
FFFFFFFFFFFFFFFFFFFFFFFFFFFFFFFFFFFFFFFFFFFFFFFFFFFFFFFFFFFFFFFFFFFF
FFFFFFFFFFFFFFFFFFFFFFFFFFF:FFFFFFFFFFFFFFFFFFFFFFFFFFFFFFFFFFFF:F:FF
FFFFFFFFFFFFFF:
@A00155:342:HHGFNDSXY:1:2612:7274:9111 2:N:0:GAACCTAG+TCCGCATA
TCTGGAGAACCACCTATTATTTAATGTTGTAGCTAGTTACATTAGTAAATATTCATATGTAGCATGTT
TAAGCATTAAATCAAATAAGATGAGCAAGATGGAACGTCTGGGTTCCAATTCTGTCAAGACTTATGAC
ATATTAAATAGATT
+
FFFFFFFFFFFFFFFFFFFFFFF:FFFFFFFFFFFFFFFFFFFFFFFFFFFFFFFFFFFFFFFFFFFF
FFFFFFFFFFFFFFFFFFFFFFFFFFFFFFFFFFFFF,:FFFFFFFF:FFFFFFFFF:FFFFFFFFF
FFFFFFFFFFFFFF
@A00155:342:HHGFNDSXY:1:1278:24198:32784 2:N:0:GAACCTAG+TCCGCATA
TCTGGAGAACCACCTATTATTTAATGTTGTAGCTAGTTACATTAGTAAATATTCATATGTAGCATGTT
TAAGCATTAAATCAAATAAGATGAGCAAGATGGAACGTCTGGGTTCCAATTCTGTCAAGACTTATGAC
ATATTAAATAGATTG
+
FFFFFFFFFFFFFFFFFFFFFFFFFFFFFFFFFFFFFFFFFFFFFFFFFFFFFFFFFFFFFFFFFFFF
FFFFFFFFFFFFFFFFFFFFFFFFFFFFFFFFFFFFFFFFFFFFFFFFFFFFFFFFFFFFFFFFFFFF
FFFFFFFFFF:FFFF
@A00155:342:HHGFNDSXY:1:1278:24207:32831 2:N:0:GAACCTAG+TCCGCATA
TCTGGAGAACCACCTATTATTTAATGTTGTAGCTAGTTACATTAGTAAATATTCATATGTAGCATGTT
TAAGCATTAAATCAAATAAGATGAGCAAGATGGAACGTCTGGGTTCCAATTCTGTCAAGACTTATGAC
ATATTAAATAGATTG
+
FFFFFFFFFFFFFFFFFFFFFFFFFFFFFFFFFFFFFFFFFFFFFFFFFFFFFFFFFFFFFFFFFFFF
FFFFFFFFFFFFFFFFFFFFFFFFFFFFFFFFFFFFFFFFFFFFFFFFFFFFFFFFFFFFFFFFFFFF
FFFFFFFFFFFFFFF
@A00155:342:HHGFNDSXY:1:1310:12472:17895 2:N:0:GAACCTAG+TCCGCATA
CTGGAGAACCACCTATTATTTAATGTTGTAGCTAGTTACATTAGTAAATATTCATATGTAGCATGTTT
AAGCATTAAATCAAATAAGATGAGCAAGATGGAACGTCTGGGTTCCAATTCTGTCAAGACTTATGACA
TATTAAATAGATTGG
+
FFFFFFFFFFFFFFFFFFFFFFFFFFFFFFFFFFFFFFFFFFFFFFFFFFFFFFFFFFFFFFFFFFFF
FFFFFFFFFFFFFFFFFFFFFFFFFFFFFFFFFFFFFFFFFFFFFFFFFFFFFFFFF:FFFFFFFFFF
FFFFFFFF:FFFFFF
@A00155:342:HHGFNDSXY:1:1319:24270:8140 2:N:0:GAACCTAG+TCCGCATA
CTGGAGAACCACCTATTATTTAATGTTGTATCTAGTTACATTAGTAAATATTCATATGTAGCATGTTT
AAGCATTAAATCAAATAAGATGAGCAAGATGGAACGTCTGGGTTCCAATTCTGTCAAGACTTATGACA
TATTAAATAGATTGG
```

+
FFFFFFFFFFFFFFFFFFFFFFF:FFFFFFFFFFFFFFFFFFFFFFFFFFFFFFFFFFF:FFFFFFFFFF
FFFFFFFFFFFFFFFFFFFFFFFFFFFFFFFF,FFFF:FFFFFFFFFFFF:FFFFFFFFFFFFFFFFFFF
:F:FF,FFFFFFFFF
@A00155:342:HHGFNDSXY:1:1408:16486:5337 2:N:0:GAACCTAG+TCCGCATA
CTGGAGAACCACCTATTATTTAATGTTGTAGCTAGTTACATTAGTAAATATTCATATGTAGCATGTTT
AAGCATTAAATCAAATAAGATGAGCAAGATGGAACGTCTGGGTTCCAATTCTGTCAAGACTTATGACA
TATTAAATAGATTGG
+
FFFFFFFFFFFFFFFFFFFFFFFFFFFFFFFFFFFFFF:FFFFFFFFFFFFFFFFFFFFFFF:FFFFFFF
FFFFFFFFFFFFFFFFFFFFFFFFFFFFFFFFFFFFFFFFFFFFFFFFFFFFFFFFFF:FFFFFF:FFFF
FFFFFFF:FFFFFFF
@A00155:342:HHGFNDSXY:1:2127:22887:5650 2:N:0:GAACCTAG+TCCGCATA
CTGGAGAACCACCTATTATTTAATGTTGTAGCTAGTTACATTAGTAAATATTCATATGTAGCATGTTT
AAGCATTAAATCAAATAAGATGAGCAAGATGGAACGTCTGGGTTCCAATTCTGTCAAGACTTATGACA
TATTAAATAGATTGG
+
FFFFFFFFFFFFFFFFFFFFFFFFFFFFFFFFFFFFFFFFFFFFFFFFFFFFFFFFFFFFFFFFFFFFFF
FFFFFFFFFFFFFFFFFFFFFFFFFFFFFFFFFFFFFFFFFFFFFFFFFFFFFFFFFFFFFFFFFFFFFF
FFFFFFFFFFFFFFF
@A00155:342:HHGFNDSXY:1:2162:10511:9768 2:N:0:GAACCTAG+TCCGCATA
CTGGAGAACCACCTATTATTTAATGTTGTAGCTAGTTACATTAGTAAATATTCATATGTAGCATGTTT
AAGCATTAAATCAAATAAGATGAGCAAGATGGAACGTCTGGGTTCCAATTCTGTCAAGACTTATGACA
TATTAAATAGATTGG
+
FFFFFFFFFFFFFFFFFFFFFFFFFFFFFFFFFFFFFFFFFFFFFFFFFFFFFFFFFFFFF,FFFFFFFF
FFFFFFFFFF:F,FFFFFFFFFFFFFFFFFFFFFFFF:FFFFFFFFFFFFFFFFFFFFFFFFFFFFFFFF
FFFFFFFFFFFFFFF
@A00155:342:HHGFNDSXY:1:2157:23800:27117 2:N:0:GAACCTAG+TCCGCATA
CTGGAGAACCACCTATTATTTAATGTTGTAGCTAGTTACATTAGTAAATATTCATATGTAGCATGTTT
AAGCATTAAATCAAATAAGATGAGCAAGATGGAACGTCTGGGTTCCAATTCTGTCAAGACTTATGACA
TATTAAATAGATTGG
+
FFFFFFFFFFFFFFFFFFFFFFFFFFFFFFFFFFFFFFFFFFFFFFFFFFFFFFFFF:FFFFFFFFFFFF
FF:FFFFFFFFFFFFFFFFFFFFFFFFFFFF:FFFFFFFFFFFFFFFFFFFFFFFFFFFFFFFFFFFFFF
FFFFFFFFFFFFFF:
@A00155:342:HHGFNDSXY:1:2272:25409:36949 2:N:0:GAACCTAG+TCCGCATA
CTGGAGAACCACCTATTATTTAATGTTGTAGCTAGTTACATTAGTAAATATTCATATGTAGCATGTTT
AAGCATTAAATCAAATAAGATGAGCAAGATGGAACGTCTGGGTTCCAATTCTGTCAAGACTTATGACA
TATTAAATAGATTGG
+
FFFFFFFFFFFFFFFFFFFFFFFFFFFFFFFFFFFFFFFFFFFFFFFFFFFFFFFFFFFFFFFFFFF:FF
FFFFFFFFFFFFFFFF:FFFFFFFFFFFFFFFFFFFFFFFFFFFFFFFFFFFFFFFFFFFFFFFFFFFFF
FFFFFFFFFFFFFF:
@A00155:342:HHGFNDSXY:1:1612:9914:33912 1:N:0:GAACCTAG+TCCGCATA
TGGAGAACCACCTATTATTTAATGTTGTAGCTAGTTACATTAGTAAATATTCATATGTAGCATGTTTA
AGCATTAAATCAAATAAGATGAGCAAGATGGAACGTCTGGGTTCCAATTCTGTCAAGACTTATGACAT
ATTAAATAGATTGGT
+
FFFFFFFFFFFFFFFFFFFFFFFFFFFFFFFFFFFFFFF:FFFFFFFFFFFFFFFFFFFFFFFFFFFFF:
FFFFFFFFFFFFFFFFFFFFFFFFFFFFFFFFF,FFFFFFFFFFFFFF,FFF:FFFFFFFFFFFFFF:FF
FFFFF:FF:FFF:FF
@A00155:342:HHGFNDSXY:1:1641:9335:8578 1:N:0:GAACCTAG+TCCGCATA
TGGAGAACCACCTATTATTTAATGTTGTAGCTAGTTACATTAGTAAATATTCATATGTAGCATGTTTA

```
AGCATTAAATCAAATAAGATGAGCAAGATGGAACGTCTGGGTTCCAATTCTGTCAAGACTTATGACAT
ATTAAATAGATTGGT
+
FFFFFFFFFFFFFFFFFFFFFFFFFFFFF:FFFFFFFFFFFFFFFFFFFFFFFFFFFFFFFFFFFFFFF
FFFFFFFFFFFFFFFFFFFFFFFF,FFFFFFFFFFFFFFF:FFFFFFFFFFFFFFF:FFFFFFFFFFF
F,FFFFFFF:FFFFF
@A00155:342:HHGFNDSXY:1:1229:27362:30780 2:N:0:GAACCTAG+TCCGCATA
TGGAGAACCACCTATTATTTAATGTTGTAGCTAGTTACATTAGTAAATATTCATATGTAGCATGTTTA
AGCATTAAATCAAATAAGATGAGCAAGATGGAACGTCTGGGTTCCAATTCTGTCAAGACTTATGACAT
ATTAAATAGATTGGT
+
FFFF:FFFFFFFFFFFFFFFFFFFFFFFFFFFFFFFFFFFFFFFFFFFFFFFFFFFFFFFFFFFFFFF
FFFFFFFFFFFFFFFFFFFFFF:FFFFFFFFFFFFFF:FFFFFFFFFFFFFFFFFFFFFFFFFFFFFF
F:FFF:FFFFFFF:F
@A00155:342:HHGFNDSXY:1:1448:18412:10989 2:N:0:GAACCTAG+TCCGCATA
TGGAGAACCACCTATTATTTAATGTTGTAGCTAGTTACATTAGTAAATATTCATATGTAGCATGTTTA
AGCATTAAATCAAATAAGATGAGCGAGATGGAACGTCTGGGTTCCAATTCTGTCAAGACTTATGACAT
ATTAAATAGATTGGT
+
:FF:FFFFFFFFFFF:FFFFFFFFFFFFFFFFFFFFFFFFFFFFFFFFFFFFFFFFFFFFFFFFFFFF
FFFFFFFFFFFFFFFFFFFFFFFFF,FFFFFFFFF:FFFFFFFFFFFFF::FFFFF:F:FFFFFF:FFF
FFFFFFFFFFFFFFF
@A00155:342:HHGFNDSXY:1:1459:27019:17002 2:N:0:GAACCTAG+TCCGCATA
TGGAGAACCACCTATTATTTAATGTTGTAGCTAGTTACATTAGTAAATATTCATATGTAGCATGTTTA
AGCATTAAATCAAATAAGATGAGCAAGATGGAACGTCTGGGTTCCAATTCTGTCAAGACTTATGACAT
ATTAAATAGATTGGT
+
FFFFFFFFFFFFFFFFFFFF:F:FFFFFFFFFFFFFFFF:FFFFFFFFFFFFFFF:FF:FFFFFFFFF
FFFFFFFFFFFFFFFFFFFFFFFFFFFFFFFFFFFFFFFFFFFFFFFFFFFFFFFFFFFFFFFFFFFF
FFFFFFFFFFFFFFF
@A00155:342:HHGFNDSXY:1:1551:3712:9674 2:N:0:GAACCTAG+TCCGCATA
TGGAGAACCACCTATTATTTAATGTTGTAGCTAGTTACATTAGTAAATATTCATATGTAGCATGTTTA
AGCATTAAATCAAATAAGATGAGCAAGATGGAACGTCTGGGTTCCAATTCTGTCAAGACTTATGACAT
ATTAAATAGATTGGT
+
FFFFFFFFFFFFFFFFFFFFFFFF:FFFFFFFFFFFFFFFFFFFFFF:FFFFFFFFFFFFFFFFFFFFFF
FFFFFFFF:FFFFFFFFFFFFFFFFFFFFFFFFFFFFFFFFFFFFFFFFFFFFFFFFFFFFFFFFFF:
FFFFFFFFFFFFFFF
@A00155:342:HHGFNDSXY:1:1555:14181:12649 2:N:0:GAACCTAG+TCCGCATA
TGGAGAACCACCTATTATTTAATGTTGTAGCTAGTTACATTAGTAAATATTCATATGTAGCATGTTTA
AGCATTAAATCAAATAAGATGAGCAAGATGGAACGTCTGGGTTCCAATTCTGTCAAGACTTATGACAT
ATTAAATAGATTGGT
+
FFFFFFFFFFFFFFFFFFFFFFFFFFFFFFFFFFFFFFFFFFFFFFFFFFFFFFFFFFFFFFFF,FF
FFFFFFFFFFFFFFFFF:FFF,FFF,FFFFFFFFFFFFFFFFFFFFFFFFFFFFFFF:FFF:FFFFFFF
FFFF,FFF,FFFFFF
@A00155:342:HHGFNDSXY:1:1660:19904:6057 2:N:0:GAACCTAG+TCCGCATA
TGGAGAACCACCTATTATTTAATGTTGTAGCTAGTTACATTAGTAAATATTCATATGTAGCAGGTTTA
AGCATTAAATCAAATAAGATGAGCAAGATGGAACGTCTGGGTTCCAATTCTGTCAAGACTTATGACAT
ATTAAATAGATTGGT
+
FFFFFFFFF:FFFFFFFFFFF:FFFFFFFFFFFFFFFFFF:FFFFFFFFFFFFFFFFFFFFFF,FF:FF
FFFFFFFFFFFFFFFFFFFFFFFFFF:FFFFFFFFFFF:FFFFFFFFFFFFFFFFFFFFFFF:FFFFFF:
FFFFFFFFF:FFFFF
```

@A00155:342:HHGFNDSXY:1:2231:18511:12414 2:N:0:GAACCTAG+TCCGCATA
TGGAGAACCACCTATTATTTAATGTTGTAGCTAGTTACATTAGTAAATATTCATATGTAGCATGTTTA
AGCATTAAATCAAATAAGATGAGCAAGATGGAACGTCTGGGTTCCAATTCTGTCAAGACTTATGACAT
ATTAAATAGATTGGT
+
FFFFFF:FFFFFFFFFFFFFFFFFFFFFFFFFFFFFFFFFFFFFFFFFFFFFFFFFFFFFFF:FFFFF
FFFFFFFFFFFFFFFFFFFFFFFFFFFFFF::FFFFFFFFFFFFFFFFFFFFFFFFFFFFFFFFF:FF
FFFFFFFFFFFFFFF
@A00155:342:HHGFNDSXY:1:2231:19560:13573 2:N:0:GAACCTAG+TCCGCATA
TGGAGAACCACCTATTATTTAATGTTGTAGCTAGTTACATTAGTAAATATTCATATGTAGCATGTTTA
AGCATTAAATCAAATAAGATGAGCAAGATGGAACGTCTGGGTTCCAATTCTGTCAAGACTTATGACAT
ATTAAATAGATTGGT
+
FFFFFFFFFFFFFFFFFFFF::FFFFFFFFFFFFFFFFFFFFFFFFFFFFFFFFFFFFFFFFFFFFFF
FFFFFFFFFFFFFFFFFFF:FFFFFFFFFFFFFFFFFFFFFFFFFFFFFFFFFFFFFFFFFFFFFFFF
FFFFFFF:FFFFFF:
@A00155:342:HHGFNDSXY:1:2509:7021:21793 2:N:0:GAACCTAG+TCCGCATA
TGGAGAACCACCTATTATTTAATGTTGTAGCTAGTTACATTAGTAAATATTCATATGTAGCATGTTTA
AGCATTAAATCAAATAAGATGAGCAAGATGGAACGTCTGGGTTCCAATTCTGTCAAGACTTATGACAT
ATTAAATAGATTGGT
+
FFFFFFFFFFFFFFFFFFFFFFFFFFFFFFFFFFFFFFFF:FFFFF:FFFFFFFFFFFFFFFFFFFFF
:FFFFFFF:FFFFFFFFFFFFFFFFFFFFFFFF,FFFFF:FFFFFFFFFFFFFFFFFFFFFFFFF:FF
FFFF::F:FFFFF:F
@A00155:342:HHGFNDSXY:1:2550:4752:29042 2:N:0:GAACCTAG+TCCGCATA
TGGAGAACCACCTATTATTTAATGTTGTAGCTAGTTACATTAGTAAATATTCATATGTAGCATGTTTA
AGCATTAAATCAAATAAGATGAGCAAGATGGAACGTCTGGGTTCCAATTCTGTCAAGACTTATGACAT
ATTAAATAGATTGGT
+
FFFFFFFFFFFFFFFFFFFFFFFFFFFFFFFF:FFFFFFFFFFFFFFFFFFFFFFFFFFFFFFFFFFF
FFFFFFFFFFFFFFF:FFFFFFFFFFFFFF:FFFFFFF:FFFFF,FF:FFFFFFF::FFFF:FFFF:F
F,FF:FFFFF:FFFF
@A00155:342:HHGFNDSXY:1:2648:31928:30358 2:N:0:GAACCTAG+TCCGCATA
TGGAGAACCACCTATTATTTAATGTTGTAGCTAGTTACATTAGTAAATATTCATATGTAGCATGTTTA
AGCATTAAATCAAATAAGATGAGCAAGATGGAACGTCTGGGTTCCAATTCTGTCAAGACTTATGACAT
ATTAAATAGATTGGT
+
FFFFFFFFFFFFFFFFFFFFFFFFFFFFFFFFFFFFFFF:FFFFFFFFFFFFFFFFFFFFFFFFFFFF
FFFFFFFFFFFFFFFFFFFFFFFFFFFFFFFF:FFFFFFFFFFFFFFFFFFFFFFFFFFFFFFFFFFF
FFFFFFFFFFFFFFF
@A00155:342:HHGFNDSXY:1:1235:13937:19554 1:N:0:GAACCTAG+TCCGCATA
GGAGAACCACCTATTATTTAATGTTGTAGCTAGTTACATTAGTAAATATTCATATGTAGCATGTTTAA
GCATTAAATCAAATAAGATGAGCAAGATGGAACGTCTGGGTTCCAATTCTGTCAAGACTTATGACATA
TTAAATAGATTGGTT
+
FFFFFFFFFFFFFFFF:FFFFFFFFFFFFFFFFFFFFFFFFFFFFFFFFFFFFFFFFFFFFFFFFFFF
FFFFFFFFFFFFFFFFFFFFFFFFFFFFFFFFFFFFFFFFFFFFFF:FFFFFFFFFFFFFFFFFFFFF
FFFFFFFFFFFFFFF
@A00155:342:HHGFNDSXY:1:1558:12789:4570 1:N:0:GAACCTAG+TCCGCATA
GGAGAACCACCTATTATTTAATGTTGTAGCTAGTTACATTAGTAAATATTCATATGTAGCATGTTTAA
GCATTAAATCAAATAAGATGAGCAAGATGGAACGTCTGGGTTCCAATTCTGTCAAGACTTATGACATA
TTAAATAGATTGGTT
+
FFFFFFFFFFFFFFFFFFFFFFFFFFFFFFFFFFFFFFFFFFFFFFFFFFFFFFFFFFFFFFFFFFFF

FFFFFFFFFFFFFFFFFFFFFFFFFFFFFFFFFFFFFFFFFFFFFFFFFFFFFFFFFFFFFFFFFFFF
FFFFFFFFFFFFFFF
@A00155:342:HHGFNDSXY:1:1665:24740:17002 1:N:0:GAACCTAG+TCCGCATA
GGAGAACCACCTATTATTTAATGTTGTAGCTAGTTACATTAGTAAATATTCATATGTAGCATGTTTAA
GCATTAAATCAAATAAGATGAGCAAGATGGAACGTCTGGGTTCCAATTCTGTCAAGACTTATGACATA
TTAAATAGATTGGTT
+
FFFFFFFFFFFFFFFFF:FFFFFFFFFFFFFFFFFFFFFFFFFFFFFFFFFFFFFFFFFFFFF,FFFF
FF:FFFFFFFFFFFFFFFFFFFFFFFFFFFFFFFFFFFFFFFFFFFFFFFFFFFFFFFFFFFFFFFFF
FFFFFFFFFFFFFFF
@A00155:342:HHGFNDSXY:1:2160:10926:14184 1:N:0:GAACCTAG+TCCGCATA
GGAGAACCACCTATTATTTAATGTTGTAGCTAGTTACATTAGTAAATATTCATATGTAGCATGTTTAA
GCATTAAATCAAATAAGATGAGCAAGATGGAACGTCTGGGTTCCAATTCTGTCAAGACTTATGACATA
TTAAATAGATTGGTT
+
FFFFFFFFFFFFFFFFFFFFFFFFFFFFFFFFFFFFF:FFFFFFFFFFFFFFFFFFFFFFFFFFFFFF
FFFFFFFFFFFFFFFFFFFFFFFFFFFFFFFFFFFFFFFFFFFFFFFFFFFFFFFFFFFFFFFFFFFF
FFFFFFFFFFFFFFF
@A00155:342:HHGFNDSXY:1:2232:32145:14857 1:N:0:GAACCTAG+TCCGCATA
GGAGAACCACCTATTATTTAATGTTGTAGCTAGTTACATTAGTAAATATTCATATGTAGCATGTTTAA
GCATTAAATCAAATAAGATGAGCAAGATGGAACGTCTGGGTTCCAATTCTGTCAAGACTTATGACATA
TTAAATAGATTGGTT
+
:FFFF,:F:FFFFFFFFFFF,FFFFFFFFFF:FFFFFFFFFFFFFFFFFFFFFFFFFFFFFFFFFFF:
FFFFFFFFFFFFFFFFFFFFFFFFFFFFFFFFFFFFFFFFFFFFFFFFFFFFFFFFFFFFFFFFFFFF
FFFFFFFFFFFFFFF
@A00155:342:HHGFNDSXY:1:2302:21766:18740 1:N:0:GAACCTAG+TCCGCATA
GGAGAACCACCTATTATTTAATGTTGTAGCTAGTTACATTAGTAAATATTCATATGTAGCATGTTTAA
GCATTAAATCAAATAAGATGAGCAAGATGGAACGTCTGGGTTCCAATTCTGTCAAGACTTATGACATA
TTAAATAGATTGGTT
+
FFFFFFFFF:FFFFFFFFFFFFFFFFFFFFFFFFFFFFFFFFFFFFFFFFFFFFFFFFFFFFFFFFFF
F:FFFFFFFFFFFFFFFFFFFFFFFFFFFFFFFFFFFFFFFFFFFFFFFFFFFF:FFFFFFFFFFFF
FFFFFFFFFFFFFFF
@A00155:342:HHGFNDSXY:1:2302:20889:19382 1:N:0:GAACCTAG+TCCGCATA
GGAGAACCACCTATTATTTAATGTTGTAGCTAGTTACATTAGTAAATATTCATATGTAGCATGTTTAA
GCATTAAATCAAATAAGATGAGCAAGATGGAACGTCTGGGTTCCAATTCTGTCAAGACTTATGACATA
TTAAATAGATTGGTT
+
FFFFFFFFFFFFFFFFFFFFFFFFFFFFFFFFFFFFFFFFFFFFFFFFFFFFFFFFFFFFFFFFFFFF
FFFFFFFFFFFFFFFFFFFFFFFFFFFFFFFFFFFFFFFFFFFFFFFFFFFFFFFFFFFFFFFFFFFF
FFF::FFFFFFFFFF
@A00155:342:HHGFNDSXY:1:2449:3405:3568 1:N:0:GAACCTAG+TCCGCATA
GGAGAACCACCTATTATTTAATGTTGTAGCTAGTTACATTAGTAAATATTCATATGTAGCATGTTTAA
GCATTAAATCAAATAAGATGAGCAAGATGGAACGTCTGGGTTCCAATTCTGTCAAGACTTATGACATA
TTAAATAGATTGGTT
+
FFFFFFFFFFFF:FFFFFFFFFFFFFFFFFFFFFFFFFFFFFFFFFFFFFFFFFFFFFFFFFF:FFFF
FFFFFFFFFFFFFFFFFFFFFFFFFFFFFFFFFFFFFFFFFFFFFFF:FFFFFFFFFFFFFFFFFFFF
FFFFFFFFFFFFFFF
@A00155:342:HHGFNDSXY:1:2625:16450:29637 1:N:0:GAACCTAG+TCCGCATA
GGAGAACCACCTATTATTTAATGTTGTAGCTAGTTACATTAGTAAATATTCATATGTAGCATGTTTAA
GCATTAAATCAAATAAGATGAGCAAGATGGAACGTCTGGGTTCCAATTCTGTCAAGACTTATGACATA
TTAAATAGATTGGTT

```
+
FFFFFFFFFFFFFFFFFFFFFFFFFFFFFFFFFFFFFF:FFFFFFFFFFFFFFFFFFFFFFFFFFFFFF
FFFFFFFFFFFFFFFFFFFFFFFFFFFFFFFFFFFFFFFF:FFFFFFFFFFFFFFFFFFFFFFFFFFFF
FFFFFFFFFFFFFFF
@A00155:342:HHGFNDSXY:1:2669:4426:26475 1:N:0:GAACCTAG+TCCGCATA
GGAGAACCACCTATTATTTAATGTTGTAGCTAGTTACATTAGTAAATATTCATATGTAGCATGTTTAA
GCATTAAATCAAATAAGATGAGCAAGATGGAACGTCTGGGTTCCAATTCTGTCAAGACTTATGACATA
TTAAATAGATTGGTT
+
FFFFFFFFFFFFFFFFFFFFFFFFF,FFFF:FFFFFFFFFFFFFFFFFFFFFFFFFFFFFFFFFFFFFF
,FFFFFFFFFFFFFFFFFFFFFFFFFFFFFFFFFFFFFFFFFFFFFFFFFFFFFFFF:FFFFFFFFFF
FFFFFFFFFFFFFFF
@A00155:342:HHGFNDSXY:1:1424:11008:7780 2:N:0:GAACCTAG+TCCGCATA
GGAGAACCACCTATTATTTAATGTTGTAGCTAGTTACATTAGTAAATATTCATATGTAGCATGTTTAA
GCATTAAATCAAATAAGATGAGCAAGATGGAACGTCTGGGTTCCAATTCTGTCAAGACTTATGACATA
TTAAATAGATTGGTT
+
FFFFFFFFFFFFFFFFFFFFFFFFFFFFFFFFFFFFFFFF:FFFFFFFFFFFFFFFFFFFFFFFFFFFF
FFFFFFFFFFFFFFFFFFFFFFFFFFFFFFFFFF,FFFFFFFF:FFFFFFFFFFFFFF,FFFFFFFFFF
FFFFFFFFFFFFFFF
@A00155:342:HHGFNDSXY:1:2321:10719:27821 2:N:0:GAACCTAG+TCCGCATA
GGAGAACCACCTATTATTTAATGTTGTAGCTAGTTACATTAGTAAATATTCATATGTAGCATGTTTAA
GCATTAAATCAAATAAGATGAGCAAGATGGAACGTCTGGGTTCCAATTCTGTCAAGACTTATGACATA
TTAAATAGATTGGTT
+
FFFFFFFFFFFFFFFFFFFFF:FFFFFFFFFFFFFFFFFFFFFFFFFFFFFFF:FFFFFFF:FFFFFF
FFFFFFFFFFFFFFFFFFFFFFFFFFFFFFFFFFFFFFFFFFFFFFFFFFFFFFFFFFFF,FFFFFFF
FFF:FF:FFFFFFFF
@A00155:342:HHGFNDSXY:1:2501:30563:30091 2:N:0:GAACCTAG+TCCGCATA
GGAGAACCACCTATTATTTAATGTTGTAGCTAGTTACATTAGTAAATATTCATATGTAGCATGTTTAA
GCATTAAATCAAATAAGATGAGCAAGATGGAACGTCTGGGTTCCAATTCTGTCAAGACTTATGACATA
TTAAATAGATTGGTT
+
F:FFFFFFFFFFFF:FFFF:F:FFFFFFFFFFFFFFFF:FFFFFFFFFFFFFFFFFFFF::,:,FFFF
F:FF,FFFFFFF:FFF:FFFFFFF::FFFFFF:FFFFF,FF,:FFFFF,F:FFFFFFFFFF,:F,FF,
F,FF:FFFFF,FFFF
@A00155:342:HHGFNDSXY:1:2443:24306:19539 1:N:0:GAACCTAG+TCCGCATA
GAGAACCACCTATTATTTAATGTTGTAGCTAGTTACATTAGTAAATATTCATATGTAGCATGTTTAAG
CATTAAATCAAATAAGATGAGCAAGATGGAACGTCTGGGTTCCAATTCTGTCAAGACTTATGACATAT
TAAATAGATTGGTTA
+
F:FFFFFFF:FFFFFF,FFFFFFFFFFFFFFFFFFFFFFF:FFFFFFFFFFFFFFFFFFFFFFFFFFFF
FFFFFFFFFFFFFFFFFFFFFFFF:FFFFFFFFFFFFFFFFFFFFFFFFFFFFFFFFFFFFFFFFFFF
FFFFFFFFFFFFFFF
@A00155:342:HHGFNDSXY:1:1416:22155:14027 1:N:0:GAACCTAG+TCCGCATA
AGAACCACCTATTATTTAATGTTGTAGCTAGTTACATTAGTAAATATTCATATGTAGCATGTTTAAGC
ATTAAATCAAATAAGATGAGCAAGATGGAACGTCTGGGTTCCAATTCTGTCAAGACTTATGACATATT
AAATAGATTGGTTAC
+
FFFFFFFFFFFFFFFFFFFFFFFFFFFF,FFFF:FFFFFFFFFFFFF:FFFFFFF:FFFFFFFFFFFFF
FFFFFFFFFFFFFFFFFFFFFFFFFFFFFFFFFFFFFFFFFFFFFFFFFFFFFFFFFFFFFFFFFFFF
FFFFFFFFFFFFFFF
@A00155:342:HHGFNDSXY:1:1438:20437:26991 1:N:0:GAACCTAG+TCCGCATA
AGAACCACCTATTATTTAATGTTGTAGATAGTTACATTAGTAAATATTCATATGTAGCATGTTTAAGC
```

ATTAAATCAAATAAGATGAGCAAGATGGAACGTCTGGGTTCCAATTCTGTCAAGACTTATGACATATT
AAATAGATTGGTTAC
+
FFFF:F:FFFFFFFFFFFFFFFFFFF:,F::FFF,FF,F:FFF,FFFF:FFFFF:,::FFFFFF:FF:
FFFFFFFFF,,:F,FFFFFFF,FF:F:F:FFFFFFFFFFFFFF:FFF,:FFFF:F,,FFFFFF:FF,F:
FF,FFFFF::FFFFF
@A00155:342:HHGFNDSXY:1:2518:15655:36307 1:N:0:GAACCTAG+TCCGCATA
AGAACCACCTATTATTTAATGTTGTAGCTAGTTACATTAGTAAATATTCATATGTAGCATGTTTAAGC
ATTAAATCAAATAAGATGAGCAAGATGGAACGTCTGGGTTCCAATTCTGTCAAGACTTATGACATATT
AAATAGATTGGTTAC
+
F:FFFFFFFFFFFFFFFFFFFFFFFFFFFFFFFFFFFFFFFFFFFF:FFFFFFFFFFFFFFFFFFFFFF
FFFFFFFFFFFFFFFFFFFFFFFFFFFFFFFFFFFFFFFFFFFFFFFFFFFFFFFFFFFFFFFFFFFFF
FFFFFFFFFFFFFFF
@A00155:342:HHGFNDSXY:1:2518:15664:36323 1:N:0:GAACCTAG+TCCGCATA
AGAACCACCTATTATTTAATGTTGTAGCTAGTTACATTAGTAAATATTCATATGTAGCATGTTTAAGC
ATTAAATCAAATAAGATGAGCAAGATGGAACGTCTGGGTTCCAATTCTGTCAAGACTTATGACATATT
AAATAGATTGGTTAC
+
FFFFFFFF:FFFFFFFFFFFFFFFFFF:FFFFFFFFFFFFFFFFFFFFFFFFFFFFFFFFFFFFFFFFF
FFFFFFFFFFFFFFFFFFFFFFFFFFFFFFFFFFFFFFFFFFFFFFFFFFFFFFFFFFFFFFFFFFFFF
FFFFFFFFFFFFFFF
@A00155:342:HHGFNDSXY:1:2518:16034:36746 1:N:0:GAACCTAG+TCCGCATA
AGAACCACCTATTATTTAATGTTGTAGCTAGTTACATTAGTAAATATTCATATGTAGCATGTTTAAGC
ATTAAATCAAATAAGATGAGCAAGATGGAACGTCTGGGTTCCAATTCTGTCAAGACTTATGACATATT
AAATAGATTGGTTAC
+
FFFFFFFF:FFFFFFF:FFFFFFFFFFFFFFFFFFFFFFFFFFFFFFFFFFFFFFFFFFFFFFFFFFFF
FFFFFFFFFFFFFFFFFFFFFFFFFFFFFFFFFFFFFFFFFFFFFFFFFFFFFFFFFFFFFFFFFFFFF
FFFFFFFFFFFFFFF
@A00155:342:HHGFNDSXY:1:2526:12491:27226 1:N:0:GAACCTAG+TCCGCATA
CTAACCACCTATTATTTAATGTTGTAGCTAGTTACATTAGTAAATATTCATATGTAGCATGTTTAAGC
ATTAAATCAAATAAGATGAGCAAGATGGAACGTCTGGGTTCCAATTCTGTCAAGACTTATGACATATT
AAATAGATTGGTTAC
+
FFFFFFFFFFFFFF:FFFFFFFFFFFFFFFF:FFFFFFFFFFFFFFFFFFFFFFFFFFFFFFFFFFFF:
FFFFFFFFFFFFFFFFFFFFFFFFFFFFFFFFFFFFFFFFFFFFFFFFFFFFFFFFFFFFFFFFFFFFF
FFFFFFFFFFFFFFF
@A00155:342:HHGFNDSXY:1:2601:12599:3427 1:N:0:GAACCTAG+TCCGCATA
AGAACCACCTATTATTTAATGTTGTAGCTAGTTACATTAGTAAATATTCATATGTAGCATGTTTAAGC
ATTAAATCAAATAAGATGAGCAAGATGGAACGTCTGGGTTCCAATTCTGTCAAGACTTATGACATATT
AAATAGATTGGTTAC
+
FFFFFFFFFFFFFFF:FFFFFFFFFFFFFFFFFFFFFFFFFFFFFFFFFFFFFFFFFFFFFF:FFFFFF
FFFFFFFFFFFFFFFFFFFFFFFFFFFFFFFFFFFFFFFFFFFFFFFFFFFFFFFFFFFFFFFFFFFFF
FFFFFFFFFFFFFFF
@A00155:342:HHGFNDSXY:1:2661:8730:33646 1:N:0:GAACCTAG+TCCGCATA
AGAACCACCTATTATTTAATGTTGTAGCTAGTTACATTAGTAAATATTCATATGTAGCATGTTTAAGC
ATTAAATCAAATAAGATGAGCAAGATGGAACGTCTGGGTTCCAATTCTGTCAAGACTTATGACATATT
AAATAGATTGGTTAC
+
FFFFFFF:FF:FFFFFFFFFFFFFFFFFFFFFFFFFFFFFFFFFFFFFFFFFFFFFFFFFFF:FFFFFF
FFFFFFFFFFFFFFFFFFFFFFFFFFFFFFFFFFFFFFFFFFFFFFFFFFFFFFFFFFFFFFFFFFFFF
FFFFFFFFFFFFFFF

@A00155:342:HHGFNDSXY:1:1150:16016:14763 2:N:0:GAACCTAG+TCCGCATA
AGAACCACCTATTATTTAATGTTGTAGCTAGTTACATTAGTAAATATTCATATGTAGCATGTTTAAGC
ATTAAATCAAATAAGATGAGCAAGATGGAACGTCTGGGTTCCAATTCTGTCAAGACTTATGACATATT
AAATAGATTGGTTAC
+
FFFFFFFFFFFFFFFFFFFFFFFFFFFFFFFFFFFFFFFFFFFFFFFFFFFFFFFFFFFFFFF:F:FFF
FFFFFFFFFFFFFFF:FFFFFFFFFFFFFFFFFFFFFFFFFFFFFFFFFFFF:F:FFFFFFFFFF:FFF
FFFFFFFFFFFF:FF
@A00155:342:HHGFNDSXY:1:1260:20455:35822 2:N:0:GAACCTAG+TCCGCATA
AGAACCACCTATTATTTAATGTTGTAGCTAGTTACATTAGTAAATATTCATATGTAGCATGTTTAAGC
ATTAAATCAAATAAGATGAGCAAGATGGAACGTCTGGGTTCCAATTCTGTCAAGACTTATGACATATT
AAATAGATTGGTTAC
+
FFFFFFFFFFFFFF:FFFF:FFFFFFFFFFFFFFFFFFFFFFFFFFFFFFFFFFFFFFFFFFFFFFFFF
FFFFFFFFFFFFFFFFFFFFFFFFFFFF:FFFFFFFFFFFFFFFFFFFFFFFFFFFFFFFFFFFFFFFF
FF:FFFFF:FFFFFF
@A00155:342:HHGFNDSXY:1:1670:17345:13119 2:N:0:GAACCTAG+TCCGCATA
AGAACCACCTATTATTTAATGTTGTAGCTAGTTACATTAGTAAATATTCATATGTAGCATGTTTAAGC
ATTAAATCAAATAAGATGAGCAAGATGGAACGTCTGGGTTCCAATTCTGTCAAGACTTATGACATATT
AAATAGATTGGTTAC
+
FFFFFFFFFFFFFFFFFFF,F:FFFFFF:FFFFFFFFFFFFFFFFFFFFFFFFFFFFFFFFFFFFFFFF
FFFFF:FFFFFFFFFFFFFFFFFFFFFFFFFFFFFFFFFFFFFFFFFFFF:FFFFFFFFFFFFFFFFFF
FFFFFFFFFFFFFFF
@A00155:342:HHGFNDSXY:1:2316:26142:19398 2:N:0:GAACCTAG+TCCGCATA
AGAACCACCTATTATTTAATGTTGTAGCTAGTTACATTAGTAAATATTCATATGTAGCATGTTTAAGC
ATTAAATCAAATAAGATGAGCAAGATGGAACGTCTGGGTTCCAATTCTGTCAAGACTTATGACATATT
AAATAGATTGGTTAC
+
FFFFFFFFFFFFFFFFFFFFFFFFFFFFFFFFFFFFFFFFFFFFFFFFFFFFFFFFFFFFFFFFFF:FF
FFFFFFFFFFFFFF,FFF:FFFFFFFFFF:FFFFFF:FFFFFFFFFFFFFFFFFFFFFFFFFFFFFFF,
FFFF:FFFFFFFFFF
@A00155:342:HHGFNDSXY:1:1172:2564:27352 1:N:0:GAACCTAG+TCCGCATA
GAACCACCTATTATTTAATGTTGTAGCTAGTTACATTAGTAAATATTCATATGTAGCATGTTTAAGCA
TTAAATCAAATAAGATGAGCAAGATGGAACGTCTGGGTTCCAATTCTGTCAAGACTTATGACATATTA
AATAGATTGGTTACG
+
FFFFFFFFFFFFFFFFFFFFFFFFFFFFFFFFFFFFFFFFFFFFFFFFFFFFFFFFFFFFFFFFFF:FF
FFFFFFFFFFFFFFFFFFFFFFFFFFFFFFFFF,FF:FFFFFFFFFFFFFFFFFFFFFFFFFF:FFFFF
FFFFFFFFFFFFFFF
@A00155:342:HHGFNDSXY:1:1317:20464:17331 1:N:0:GAACCTAG+TCCGCATA
GAACCACCTATTATTTAATGTTGTAGCTAGTTACATTAGTAAATATTCATATGTAGCATGTTTAAGCA
TTAAATCAAATAAGATGAGCAAGATGGAACGTCTGGGTTCCAATTCTGTCAAGACTTATGACATATTA
AATAGATTGGTTACG
+
FFFFFFFFFFFFFFFFFFFFFFFFFFFFFFFFFFFFFFF:FFFFFFFFFFFFFFFFFFFFFFFFFFFFF
FFFFFFFFFFFFFFFFFFFFFFFFFFFFFFFFFFFFFFFFFFFFFFFFFFFFFFFFFFFFFFFFFFFFF
FFFFFFFFFFFFFFF
@A00155:342:HHGFNDSXY:1:1621:29080:11240 1:N:0:GAACCTAG+TCCGCATA
GAACCACCTATTATTTAATGTTGTAGATAGTTACATTAGTAAATATTCATATGTAGCATGTTTAAGCA
TTAAATCAAATAAGATGAGCAAGATGGAACGTCTGGGTTCCAATTCTGTCAAGACTTATGACATATTA
AATAGATTGGTTACG
+
FFFFFFFFFFFFFFFFFFFFFFFFFFF,FFFFFFFFFFFFFFFFFFFFFFFFFFFFFFF,:FFFFFFFFF

FFFFFFFFFFFFFFFFFFFFFFFFFFFFFFFFFFFFFFFFFFFFFFFFFFFFFFFFFFF:FFFFFFFF
FFFFFF:FFFFFFFF
@A00155:342:HHGFNDSXY:1:2278:4670:25770 1:N:0:GAACCTAG+TCCGCATA
GAACCACCTATTATTTAATGTTGTAGCTAGTTACATTAGTAAATATTCATATGTAGCATGTTTAAGCA
TTAAATCAAATAAGATGAGCAAGATGGAACGTCTGGGTTCCAATTCTGTCAAGACTTATGACATATTA
AATAGATTGGTTACG
+
FFFFFF:FFFFFFFFFFFFFFF:FFFFFFFFFFFFFFFFFFFFFFFFFFFFFF,F:F:FFFFFFFFFF
FFFFFFFFFFFFFF,F:FFFFFFFFFFFFFFFFFFFFFFFFFFFFFFFFFFFFFFFFFFFFFFFFFFF
FFFFFFFFFFFFFFF
@A00155:342:HHGFNDSXY:1:2271:10746:12837 1:N:0:GAACCTAG+TCCGCATA
GAACCACCTATTATTTAATGTTGTAGCTAGTTACATTAGTAAATATTCATATGTAGCATGTTTAAGCA
TTAAATCAAATAAGATGAGCAAGATGGAACGTCTGGGTTCCAATTCTGTCAAGACTTATGACATATTA
AATAGATTGGTTACG
+
FFF,FFF:FFFFFFFFFFFFFFFFFFFFFFFFFFFFFFFFF:FFFFFFFFFFFFFFFFFFFFFFFFFF
FFFFFFFFFFFFFFFFFFFFFFFFFFFFFFFFFFFFF,FFFFFFFFFFFFFFFFFFFFFFFFFFFFFF
FFFFFFFFFFFFFFF
@A00155:342:HHGFNDSXY:1:2422:26585:35571 1:N:0:GAACCTAG+TCCGCATA
GAACCACCTATTATTTAATGTTGTAGCTAGTTACATTAGTAAATATTCATATGTAGCATGTTTAAGCA
TTAAATCAAATAAGATGAGCAAGATGGAACGTCTGGGTTCCAATTCTGTCAAGACTTATGACATATTA
AATAGATTGGTTACG
+
FFFFFF:FF:FF:FFF,FFFFFFF,FFF:F:FFF:FFFFFF:FF,FF,FFFFF:F:FFFF:FFFFF:F
FF:FFF:FFFFFFF,FFFFF:FFFFFFFF:FFFFFFFFFFFFF:FFFFFFFFFFFFFFFFFFFFFFFF
FFFFFFFFFFFFFFF
@A00155:342:HHGFNDSXY:1:1628:8829:34788 2:N:0:GAACCTAG+TCCGCATA
GAACCACCTATTATTTAATGTTGTAGCTAGTTACATTAGTAAATATTCATATGTAGCATGTTTAAGCA
TTAAATCAAATAAGATGAGCAAGATGGAACGTCTGGGTTCCAATTCTGTCAAGACTTATGACATATTA
AATAGATTGGTTACG
+
:F,FFFFFF:FFFFFFFFFFFFF:FFF,FFFFFFFFFFFFFFFFFFFFFFF:FFFFFFFFFFFFFFFF
FFFFFFFFFFFFFFFFFFFFF:FFFFFFFFFFFFFFFFFFFFFFFFFFFFF:FFFFF:FFFFFFF,FF
FFFFFFFFFFFFFFF
@A00155:342:HHGFNDSXY:1:2569:22155:2096 2:N:0:GAACCTAG+TCCGCATA
GAACCACCTATTATTTAATGTTGTAGCTAGTTACATTAGTAAATATTCATATGTAGCATGTTTAAGCA
TTAAATCAAATAAGATGAGCAAGATGGAACGTCTGGGTTCCAATTCTGTCAAGACTTATGACATATTA
AATAGATTGGTTACG
+
FFFFFFFFFFFFFFFFFF:FFFFFFFFFFFFFFFFFFFFFFFFFFFFFFFFFFFFFFF:FF:FFFFF:
FFFFF:FFFFFFFFFFFFFFFFFFFFFFFFFFFFFFF,FFFFF:FFFFFFFFFFFFFFFFFFFF:FFF
FFFFFFFFFFFFF:F
@A00155:342:HHGFNDSXY:1:2623:2636:32581 2:N:0:GAACCTAG+TCCGCATA
GAACCACCTATTATTTAATGTTGTAGCTAGTTACATTAGTAAATATTCATATGTAGCATGTTTAAGCA
TTAAATCAAATAAGATGAGCAAGATGGAACGTCTGGGTTCCAATTCTGTCAAGACTTATGACATATTA
AATAGATTGGTTACG
+
FFFFFFFF:FFFFFFFFF,FFFFFFFFFFFFFFFFFFFFFFFFFFFFF:FFFFFFFFFFFFFFFFFFF
FFFFFFFFFF:FFFFFFFFFFFFFFFFFFFFFFFFFFFFFFFF:FFFFFFFFFFFFFFFF:FFFFFFF
FFFFFFFFFFFFFFF
@A00155:342:HHGFNDSXY:1:1115:18521:4539 1:N:0:GAACCTAG+TCCGCATA
AACCACCTATTATTTAATGTTGTAGCTAGTTACATTAGTAAATATTCATATGTAGCATGTTTAAGCAT
TAAATCAAATAAGATGAGCAAGATGGAACGTCTGGGTTCCAATTCTGTCAAGACTTATGACATATTAA
ATAGATTGGTTACGG

+
FFFFFFFFFFFFFFFFFFFFFFFFFFFFFFFFFFFFFFFFFFFFFFFFFFFF:FFFFFFFFFFFFFFF
FFFFFFFFFFFFFFFFFFFFFFFFFFFFFFFFFFFF,FFFFFFFFFFFFFFFFFFFF:FFFF:FFFF:
FFFFFFFFFFFFFFF
@A00155:342:HHGFNDSXY:1:1218:24867:4257 1:N:0:GAACCTAG+TCCGCATA
AACCACCTATTATTTAATGTTGTAGCTAGTTACATTAGTAAATATTCATATGTGGCATGTTTAAGCAT
TAAATCAAATAAGATGAGCAAGATGGAACGTCTGGGTTCCAATTCTGTCAAGACTTATGACATATTAA
ATAGATTGGTTACGG
+
FF,:FF,FFF:FFFFFFFFFFFFFF:FFFFFFFFFFFFFFF:FFFFFFFFF:FFFFFFFFFFFFFFFF
FFF:FF,FFFFFFFFFFFFFFFFFFF,FFFFFFF,FFF:FFFFFFFFF:FFFFFFFFFFFFFFFFFF:
FFFFFFFFFFFFFFF
@A00155:342:HHGFNDSXY:1:1165:1714:8938 1:N:0:GAACCTAG+TCCGCATA
AACCACCTATTATTTAATGTTGTAGCTAGTTACATTAGTAAATATTCATATGTAGCATGTTTAAGCAT
TAAATCAAATAAGATGAGCAAGATGGAACGTCTGGGTTCCAATTCTGTCAAGACTTATGACATATTAA
ATAGATTGGTTACGG
+
FFFFFFFFFFFF::FFFFFFFFFFFFFFFFFFFFFFF,:FFFFFF:FFFFFFFFFFFFFFFFFFFFFF
FFFFFFFFFFFFFFFFFFFFFFFFFFFFFFF:FFFFFF,:FF:F:FFFFFFFFFFFFFFFFFFFFFFF
FFFFFFFFFFFFFFF
@A00155:342:HHGFNDSXY:1:1230:7355:15076 1:N:0:GAACCTAG+TCCGCATA
AACCACCTATTATTTAATGTTGTAGCTAGTTACATTAGTAAATATTCATATGTAGCATGTTTAAGCAT
TAAATCAAATAAGATGAGCAAGATGGAACGTCTGGGTTCCAATTCTGTCAAGACTTATGACATATTAA
ATAGATTGGTTACGG
+
FF:FFFFFFFFFFFFFFFFFF::FF:FFFFFFFFFFFFF:FFFFFFFFFFFFFFF,FFFFFF,FFFFF
FFFFFFFFFFFFFFFFFF:FFFFFFFFFFFFFFFFF:FFF:FFFFFFFFFFFFFFFFFFFFFFFFFFF
FFFFFFF:FFFFFFF
@A00155:342:HHGFNDSXY:1:1255:20265:22968 1:N:0:GAACCTAG+TCCGCATA
CGATCTCTATTATTTAATGTTGTAGCTAGTTACATTAGTAAATATTCATATGTAGCATGTTTAAGCAT
TAAATCAAATAAGATGAGCAAGATGGAACGTCTGGGTTCCAATTCTGTCAAGACTTATGACATATTAA
ATAGATTGGTTACGG
+
:FFFFF:FFFFFFFFFFFFFFF:FFF:FFFFFFFFFFFFFFF:FFFFFFFFFFFFFFFFFFFFFFFFF
:FFFFFFFFFFFFFFFFFFFFFFFFFFFFFFFFFFFFFFFFFFFFFFFFFFFFFFFFFFFFFFFFFFF
FFFFFFFFFFFFFFF
@A00155:342:HHGFNDSXY:1:1272:4679:13886 1:N:0:GAACCTAG+TCCGCATA
AACCACCTATTATTTAATGTTGTAGCTAGTTACATTAGTAAATATTCATATGTAGCATGTTTAAGCAT
TAAATCAAATAAGATGAGCAAGATGGAACGTCTGGGTTCCAATTCTGTCAAGACTTATGACATATTAA
ATAGATTGGTTACGG
+
FFFFF:FFFFFFFFFFFFFFFFFFFFFFFFFFFFF:FFFFFFFFFFFFFFFF,FFFFFF:FFFFFFFF
FFFFFFFFFFFFFFFFFFF:FFFFFFFFFFFFFFFFFFFF:FFFFFFFFFFFFFFFFFFFFFFFFFFF
F:FFFFFFFFFFFFF
@A00155:342:HHGFNDSXY:1:1364:20094:4664 1:N:0:GAACCTAG+TCCGCATA
TCCGATCTATTATTTAATGTTGTAGCTAGTTACATTAGTAAATATTCATATGTAGCATGTTTAAGCAT
TAAATCAAATAAGATGAGCAAGATGGAACGTCTGGGTTCCAATTCTGTCAAGACTTATGACATATTAA
ATAGATTGGTTACGG
+
FFFFFFFFFFFFFFFFFFFFFFFFFFFFFFFFFFFFFFFFFFFFFFFFFFFFFFFFFFFFFFFFFFFF
FFFFFFFFFFFFFFFFFFFFFFFFFFFF:FFFFFFFFFFFFFFFFFFFFFFFFFFFFFFFFFFFFFFF
FFFFFFFFFFFFFFF
@A00155:342:HHGFNDSXY:1:1415:27199:27430 1:N:0:GAACCTAG+TCCGCATA
AACCACCTATTATTTAATGTTGTAGCTAGTTACATTAGTAAATATTCATATGTAGCATGTTTAAGCAT

TAAATCAAATAAGATGAGCAAGATGGAACGTCTGGGTTCCAATTCTGTCAAGACTTATGACATATTAA
ATAGATTGGTTACGG
+
FFFFFFFFFFFFFFFFFFFFFFFFFFFFFFFFFFFFFFFFFFFFFFFFFFF,FFFFFFFFFFFFFFFF
FFFFFFFFFFFFFFFFFFFFFFFFFFFFFFFFFFFFFFFFFFFFFFFFFFFFFFFFFFFFFFFFFFFF
FFFFFFFFFFFFFFF
@A00155:342:HHGFNDSXY:1:1436:5710:2613 1:N:0:GAACCTAG+TCCGCATA
AACCACCTATTATTTAATGTTGTAGCTATTTACATTAGTAAATATTCATATGTAGCATGTTTAAGCAT
TAAATCAAATAAGATGAGCAAGATGGAACTTCTGTGTTCCAATTCTGTCAAGACTTATGACATATTAA
ATAGATTGGTTACGG
+
FFFFF:FFFFFFF,FFFFFFFFFFF,,F,FFF:FF:F,F::FFFFFFFFFF,,FF:FF,FFFFFFFFF
FFFF::FFFFFFFFF:FF:F:FFFFFFFF,FFFF,FF:,FFFFFFFFFF:FFFFFFFFFF:F:FFFF,
FFFFFFFFFFFFFF:
@A00155:342:HHGFNDSXY:1:1519:3414:35994 1:N:0:GAACCTAG+TCCGCATA
AACCACCTATTATTTAATGTTGTAGCTAGTTACATTAGTAAATATTCATATGTAGCATGTTTAAGCAT
TAAATCAAATAAGATGAGCAAGATGGAACGTCTGGGTTCCAATTCTGTCAAGACTTATGACATATTAA
ATAGATTGGTTACGG
+
FFFFFFFFFFFFFFFFFFFFFFFF:FFFFFFFFFFFFFFFFFFFFFFFFFFFFFFFFFFFFFFFFFFF
FFFFFFFFFFFFFFFFFFFFFFFFFFF:FFFFFFFFFFFFFFFFFFFFFFFFFFFFFFFFFFFFFFFF
FFFFFFFFFFFFFFF
@A00155:342:HHGFNDSXY:1:1574:21414:3223 1:N:0:GAACCTAG+TCCGCATA
AACCACCTATTATTTAATGTTGTAGCTAGTTACATTAGTAAATATTCATATGTAGCATGTTTAAGCAT
TAAATCAAATAAGATGAGCAAGATGGAACGTCTGGGTTCCAATTCTGTCAAGACTTATGACATATTAA
ATAGATTGGTCACGG
+
FFF:F:FFFFFFFFF:FFFFFFFFFFF:FFFFFFFFFFFFFFFFFFFFFFFFFFFFFFFFFFFFFFFF
FFFFFFFFFFFFFFFFFFFFFFFFFFFFFFFFFFFFFFFFFFFFFFFFFFFFFFFFFFFFFFFFFFFF
FFFFFFFFFFFFFFF
@A00155:342:HHGFNDSXY:1:2257:15275:11663 1:N:0:GAACCTAG+TCCGCATA
AACCACCTATTATTTAATGTTGTAGCTAGTTACATTAGTAAATATTCATATGTAGCATGTTTAAGCAT
TAAATCAAATAAGATGAGCAAGATGGAACGTCTGGGTTCCAATTCTGTCAAGACTTATGACATATTAA
ATAGATTGGTTACGG
+
FFFFFFFFFFFFFFFFFFFFFFFFFFFFFFFFFFFFFFFFFFFFFFFFFFFFFFFFFFFFFFFFFFFF
FFFFFFFFFFFFFFFFFFFFFFFFFFFFFFFFFFFFFFFFFFFFFFFFFFFFFFFFFFFFFFFFFFFF
FFFFFFFFFFFFFFF
@A00155:342:HHGFNDSXY:1:2338:25952:27054 1:N:0:GAACCTAG+TCCGCATA
AACCACCTATTATTTAATGTTGTAGCTAGTTACATTAGTAAATATTCATATGTAGCATGTTTAAGCAT
TAAATCAAATAAGATGAGCAAGATGGAACGTCTGGGTTCCAATTCTGTCAAGACTTATGACATATTAA
ATAGATTGGTTACGG
+
FFFFFF:FFFFFFFFFFFFFF:FFF:FFFFFFF:FFFFFFFFFFFFFFFFFFFFF,FFFFFFFF:FFF
FFFFFFFFFFFFFFFFFFFFFFFFFFFFFFFFFFFFFFFFFFFFFFFFFFFFFFFFFFF:FFFFFFFF
FFFFFFFFFFFFFFF
@A00155:342:HHGFNDSXY:1:2330:21703:3505 1:N:0:GAACCTAG+TCCGCATA
AACCACCTATTATTTAATGTTGTAGCTAGTTACATTAGTAAATATTCATATGTAGCATGTTTAAGCAT
TAAATCAAATAAGATGAGCAAGATGGAACGTCTGGGTTCCAATTCTGTCAAGACTTATGACATATTAA
ATAGATTGGTTACGG
+
FFFFFFFFFFFFFFFFFFFFFFFFFFFF:FFFFFFFF:FFFFFFFFFFFFFFFFFFFFFFFFFF:FFF
FFFFFFFFFFFFFFFFFFFFFFFFFFFFFFFFFFFFFFFFFFFFFFFFFFFFFFFFFFFFFFFFFFFF
FFFFFFFFFFFFFFF

```
@A00155:342:HHGFNDSXY:1:2453:26232:19867 1:N:0:GAACCTAG+TCCGCATA
AACCACCTATTATTTAATGTTGTAGCTAGTTACATTAGTAAATATTCATATGTAGCATGTTTAAGCAT
TAAATCAAATAAGATGAGCAAGATGGAACGTCTGGGTTCCAATTCTGTCAAGACTTATGACATATTAA
ATAGATTGGTTACGG
+
FF:FFFFFFFFFFFFFFFFF:FFFFFFFFFFFFFFFFFFFFFFFFFFFFFFFFFFFFFFFFFFFFFF:FF
FFFFFFFFFFFFFFFFFFFFFFFFFFFFFFFFFFFFFFFFFFFFFFFFFFFFFFFFFFFFFFFFFFFFFF
FFFFFFFFFFFFFFF
@A00155:342:HHGFNDSXY:1:2527:17002:30029 1:N:0:GAACCTAG+TCCGCATA
AACCACCTATTATTTAATGTTGTAGCTAGTTACATTAGTAAATATTCATATGTAGCATGTTTAAGCAT
TAAATCAAATAAGATGAGCAAGATGGAACGTCTGGGTTCCAATTCTGTCAAGACTTATGACATATTAA
ATAGATTGGTTACGG
+
FFFFFFFFFFFFFFFFFFFFFFFFFFFFFFFFFFFFFFFFFFFFFFFFFFFFFFFFFFFFFFFFFFFFFF
FFFFFFFFFFFFFFFFFFFFFFFFFFFFFFFFFFFFFFFFFFFFFFFFFFFFFFFFFFFFFFFFFFFFFF
FFFFFFFFFFFFFFF
@A00155:342:HHGFNDSXY:1:2537:14290:34475 1:N:0:GAACCTAG+TCCGCATA
AACCACCTATTATTTAATGTTGTAGCTAGTTACATTAGTAAATATTCATATGTAGCATGTTTAAGCAT
TAAATCAAATAAGATGAGCAAGATGGAACGTCTGGGTTCCAATTCTGTCAAGACTTATGACATATTAA
ATAGATTGGTTACGG
+
FFFFFFFFFFFFFF:FFFFFFFFFFFFFFFFFFF:FFFFFFFFFFFFFFFFFF:FFFFFFFFFFFFFFFF
FFFFFFFFFFFFFFFFFFFFFFFFFFFFFFFFFFFFFFFFFFFFFFFFFFFFFFFFFFFFFFFFFFFFFF
FFFFFFFFFFFFFFF
@A00155:342:HHGFNDSXY:1:2665:11966:23813 1:N:0:GAACCTAG+TCCGCATA
AACCACCTATTATTTAATGTTGTAGCTAGTTACATTAGTAAATATTCATATGTAGCATGTTTAAGCAT
TAAATCAAATAAGATGAGCAAGATGGAACGTCTGGGTTCCAATTCTGTCAAGACTTATGACATATTAA
ATAGATTGGTTACGG
+
FFFF:FFFFFFFFFFFFFFFFFFFF:FFFFFFFF:FFFFFFFFFFFFFFFFFFFFFFFFFFFFFFFFFFF
FFFFFFFFFFFFFFFFFFFFFFFFFFFFFFFFFFFFFFFFFFFFFFFFFFFFFFFFFFFFFFFFFFFFFF
FFFFFFFFFFFFFFF
@A00155:342:HHGFNDSXY:1:2526:12491:27226 2:N:0:GAACCTAG+TCCGCATA
AACCACCTATTATTTAATGTTGTAGCTAGTTACATTAGTAAATATTCATATGTAGCATGTTTAAGCAT
TAAATCAAATAAGATGAGCAAGATGGAACGTCTGGGTTCCAATTCTGTCAAGACTTATGACATATTAA
ATAGATTGGTTACAG
+
FFFFFFFFFFFFFFFFFFFFFFFFFFFFFFFFFFFFFFFFFFFF:FFFFFFFFFFFFFFFFFFFFFFFFF
FFFFFFFFFFFFFFFFFFFFFFFFFFFFFFFF:FFFFFFFFFFFFFFFFFFFFFFFFFFFFFFFFFFFFF
FFFFFFFFFFFF:FF
@A00155:342:HHGFNDSXY:1:1155:22363:31548 1:N:0:GAACCTAG+TCCGCATA
ACCACCTATTATTTAATGTTGTAGCTAGTTACATTAGTAAATATTCATATGTAGCATGTTTAAGCATT
AAATCAAATAAGATGAGCAAGATGGAACGTCTGGGTTCCAATTCTGTCAAGACTTATGACATATTAAA
TAGATTGGTTACGGT
+
FFFFFFFFFFFFFFFFFFFFFFFFFFFFFFFFFFFFFFFFFFFFFFFF:FFFFFFFFFFFFFFFFFFFFF
FFFFFFFFFFFFFFFFFFFFFFFFFFFFFFFFFFF:FFFFFFFFFFFFFFFFFFFFFFFFFFFFFFFFF:
FFFFFFFFFFFFFFF
@A00155:342:HHGFNDSXY:1:1174:19217:7717 1:N:0:GAACCTAG+TCCGCATA
ACCACCTATTATTTAATGTTGTAGCTAGTTACATTAGTAAATATTCATATGTAGCATGTTTAAGCATT
AAATCAAATAAGATGAGCAAGATGGAACGTCTGGGTTCCAATTCTGTCAAGACTTATGACATATTAAA
TAGATTGGTTACGGG
+
FFFFFFFFFFFFFFFFFFFFFFFFFFF:FFFFFFFFFFFFFFFFFFFFFFFFF:FFFFFFFFFFFFFFFF
```

FFFFFFFFFFFFFFFFFFFFFFFFFFFFFFFFFFFFFFFFFFFFFFFFFFFFFFFFFFFFFFFFFFFF
FFFFFFFFFFFFFFF
@A00155:342:HHGFNDSXY:1:1350:12689:5556 1:N:0:GAACCTAG+TCCGCATA
ACCACCTATTATTTAATGTTGTAGCTAGTTACATTAGTAAATATTCATATGTAGCATGTTTAAGCATT
AAATCAAATAAGATGAGCAAGATGGAACGTCTGGGTTCCAATTCTGTCAAGACTTATGACATATTAAA
TAGATTGGTTACGGT
+
FFFFFFFFFF:FFFFFFFFFFFFFFFFFFFFFFFFFFFFFFFFFFFFFFFF:FFFFFFFFFFFFFFFF
FFFFFFFF:FFFFFFFFFFFFFFFFF:FFFFFFFFFFFFFF:FFFFFFFFFFFFFFFFFFFFFFFFFF
FFFFFFFFFFFFFFF
@A00155:342:HHGFNDSXY:1:1335:10710:19946 1:N:0:GAACCTAG+TCCGCATA
ACCACCTATTATTTAATGTTGTAGCTAGTTACATTAGTAAATATTCATATGTAGCATGTTTAAGCATT
AAATCAAATAAGATGAGCAAGATGGAACGTCTGGGTTCCAATTCTGTCAAGACTTATGACATATTAAA
TAGATTGGTTACGGT
+
FFFFFFFFFFFFFFFFFFFFFFFFFFFFFFFFFFF:FFFFFFFFFFFFFFFFFFFFFF:FFFFFFFFF
FFFFFFFFFFFFFFFFFFFFFFFFFFFFFFFFFFF:FFFFFFFFFFFFFFFFFFFFFFFFFFFFFFFF
FFFFFFFFFFFFFFF
@A00155:342:HHGFNDSXY:1:1335:11541:36072 1:N:0:GAACCTAG+TCCGCATA
ACCACCTATTATTTAATGTTGTAGCTAGTTACATTAGTAAATATTCATATGTAGCATGTTTAAGCATT
AAATCAAATAAGATGAGCAAGATGGAACGTCTGGGTTCCAATTCTGTCAAGACTTATGACATATTAAA
TAGATTGGTTACGGT
+
FFFFFFFFFFFFFFFFFFFFFFFFFFFFFFFFFFFFFFFFFFFFFF:FFFFFFFFFFFFFFFFFFFFF
FFFFFFFFFFFFFFFFFFFF,FFFFFFFFFFFFFFFFFFFFFFFFFFFFFFFFFFFFFFFFFFFFFF
FFFFFFFFFFFFFFF
@A00155:342:HHGFNDSXY:1:1509:21395:1313 1:N:0:GAACCTAG+TCCGCATA
ACCACCTATTATTTAATGTTGTAGCTAGTTACATTAGTAAATATTCATATGTAGCATGTTTAAGCATT
AAATCAAATAAGATGAGCAAGATGGAACGTCTGGGTTCCAATTCTGTCAAGACTTATGACATATTAAA
TAGATTGGTTACGGT
+
FFFFFFFFFFF:FFFFFFFFFFFFFFFFFFFFFFFFFFFFFFFFFFFFFFFFFFFFFF,FFFF,FFFF
FFFFFFFFFFFFFFFFFFFFFFFFFFFFFFFFFFFFFFFFFFFFFFFFFFFFFFFFFFFFFFFFFFFF
FFFFF,FFFFFFFFF
@A00155:342:HHGFNDSXY:1:1669:28076:29356 1:N:0:GAACCTAG+TCCGCATA
ACCACCTATTATTTAATGTTGTAGCTAGTTACATTAGTAAATATTCATATGTAGCATGTTTAAGCATT
AAATCAAATAAGATGAGCAAGATGGAACGTCTGGGTTCCAATTCTGTCAAGACTTATGACATATTAAA
TAGATTGGTTACGGT
+
FFFFFFFFFFFFFFF:FFFFFFFFFFFFFFFFFFFFFFFFFFFFFFFFFFFFFFFFFFFFFFFFFFFF
FFFFFFFFFFFFFFFFFFFFFFFFFFFFFFFFFFFFFFFFFFFFFFFFFFFFFFFFFFFFFFFFFFFF
FFFFFFFFFFFFFFF
@A00155:342:HHGFNDSXY:1:2113:4146:28494 1:N:0:GAACCTAG+TCCGCATA
ACCACCTATTATTTAATGTTGTAGCTAGTTACATTAGTAAATATTCATATGTAGCATGTTTAAGCATT
AAATCAAATAAGATGAGCAAGATGGAACGTCTGGGTTCCAATTCTGTCAAGACTTATGACATATTAAA
TAGATTGGTTACGGT
+
FFFF:FFFFFFFFF:,FFFFFFFF:F,FFFFFFFFF:FFFFFFFFFFF,FFFFFFFFFFFFFF:FFFF
FFFFFFFFFFFFFFFFFFFFFFFFFFFFFFFFFFFFFFFFFFFFFFFFFFFFFFFFFFFFFFFFFFFF
FFFFFFFFFFFFFFF
@A00155:342:HHGFNDSXY:1:2549:19958:28980 1:N:0:GAACCTAG+TCCGCATA
ACCACCTATTATTTAATGTTGTAGCTAGTTACATTAGTAAATATTCATATGTAGCATGTTTAAGCATT
AAATCAAATAAGATGAGCAAGATGGAACGTCTGGGTTCCAATTCTGTCAAGACTTATGACATATTAAA
TAGATTGGTTACGGT

```
+
FFFFFFFFFFFFFFFFFFFFFFFFFFFFFFFFFFFFFFFFFFFFFFFFFFFFFFFFFFFFFFFFFFFF
FFFFFFFFFFFFFFFFFFFFFFFFFFFFFFFFFFFFFFFFFFFFFFFFFFFFFFFFFFFFFFFFFFFF
FFFFFFFFFFFFFFF
@A00155:342:HHGFNDSXY:1:1407:8883:1720 2:N:0:GAACCTAG+TCCGCATA
ACCACCTATTATTTAATGTTGTAGCTAGTTACATTAGTAAATATTCATATGTAGCATGTTTAAGCATT
AAATCAAATAAGATGAGCAAGATGGAACGTCTGGGTTCCAATTCTGTCAAGACTTATGACATATTAAA
TAGATTGGTTACGGT
+
FFFFFFFFFFFFFFF:FFFFF:FFFFFFFFFFFFFFFFFFFFFFFFFF:FFFFFFFFFFFFFFFFFFF
FFFFFFFFFFFFFFFFFFFFFFFFFFFFFFFFFFFFFFFFFFFFFFFFFFFFFFFFFFFFFFFFFFFF
FFFFFFFFFFFF:FF
@A00155:342:HHGFNDSXY:1:1429:15619:19648 2:N:0:GAACCTAG+TCCGCATA
ACCACCTATTATTTAATGTTGTAGCTAGTTACATTAGTAAATATTCATATGTAGCATGTTTAAGCATT
AAATCAAATAAGATGAGCAAGATGGAACGTCTGGGTTCCAATTCTGTCAAGACTTATGACATATTAAA
TAGATTGGTTACGGT
+
FFFFFFFFFFFFFFFFFFFFFFFFFFFFFFFFFFFFFFFFFFFFFFFFFFFFFFFFFFFFFFFFFFFF
FFFFFFFFFFFF:FFFFFFFFFFF:F:FFFFFF:FFFFFFFFFFFFFFFF:FFFFFFFFFFFFFFF:F
FFFFFF:FFFFFFFF
@A00155:342:HHGFNDSXY:1:1436:9471:16830 2:N:0:GAACCTAG+TCCGCATA
ACCACCTATTATTTAATGTTGTAGCTAGTTACATTAGTAAATATTCATATGTAGCATGTTTAAGCATT
AAATCAAATAAGATGAGCAAGATGGAACGTCTGGGTTCCAATTCTGTCAAGACTTATGACATATTAAA
TAGATTGGTTACGGT
+
FFFFF:FFFFFFFFFFFFFFFFFFFFFFFFFFFFFFFFFFFFFFFFFFFFFFFFFFFFFFFFF:FFFF
FF:FFFF:FFFFFFFFFFFFFFFFFFFFFFFFFFFFFFFFFFFFFFFFFF:FFFFFFFFFFFFFFF,F
FFF:FFFFFFFFFFF
@A00155:342:HHGFNDSXY:1:1431:14498:4742 2:N:0:GAACCTAG+TCCGCATA
ACCACCTATTATTTAATGTTGTAGCTAGTTACATTAGTAAATATTCATATGTAGCATGTTTAAGCATT
AAATCAAATAAGATGAGCAAGATGGAACGTCTGGGTTCCAATTCTGTCAAGACTTATGACATATTAAA
TAGATTGGTTACGGT
+
FFFFFFFFFFFFFFFF:FFFFFFFFFFFFFFFFFFFFFFFFFFFFFFFFFFFFFFFFFFFFFFFFFFF
FFFFFFFFFFFFFFFFFFFF:FFFFF,FFFFFFFFFFFFFFFFFFFFFFFF:FFFFFFFFFFFFFFF:
FF:F,FF:FFFFF,F
@A00155:342:HHGFNDSXY:1:1431:15953:7764 2:N:0:GAACCTAG+TCCGCATA
ACCACCTATTATTTAATGTTGTAGCTAGTTACATTAGTAAATATTCATATGTAGCATGTTTAAGCATT
AAATCAAATAAGATGAGCAAGATGGAACGTCTGGGTTCCAATTCTGTCAAGACTTATGACATATTAAA
TAGATTGGTTACGGT
+
FFFFFFFFFFFFFFFFFFFFFFFFFFFFFFFFFFFFFFFFFFFFFFFFFFFFFFFFFFFFFFFFFFFF
FFFFFFFFFFFFFFFF:FF:FFFFFFFFFFFFFFFFFFFFFFFFFFFFFFFFFFFFFFFFFFFFFFFF
FFFFFFFFFFFFFFF
@A00155:342:HHGFNDSXY:1:2132:13322:11725 2:N:0:GAACCTAG+TCCGCATA
ACCACCTATTATTTAATGTTGTAGCTAGTTACATTAGTAAATATTCATATGTAGCATGTTTAAGCATT
AAATCAAATAAGATGAGCAAGATGGAACGTCTGGGTTCCAATTCTGTCAAGACTTATGACATATTAAA
TAGATTGGTTACGGT
+
FFFFFFFFFFFFFFFFFFFFFFFFFFFFFFFFFFFFFFFFFFFFFFFFFFFFFFFFFFF:FFFFFFFF
FFFFFFFFFFFFFFFFFFFFFFFFFFFFFFFFFFFFFFFFFFFFFFFFFFFFFFF:FFFFFFFFFFFF
FFFFFFFFFFFFFFF
@A00155:342:HHGFNDSXY:1:2277:26919:6151 2:N:0:GAACCTAG+TCCGCATA
ACCACCTATTATTTAATGTTGTAGCTAGTTACATTAGTAAATATTCATATGTAGCATGTTTAAGCATT
```

```
AAATCAAATAAGATGAGCAAGATGGAACGTCTGGGTTCCAATTCTGTCAAGACTTATGACATATTAAA
TAGATTGGTTACGGT
+
FFFFFFFFFFFFFF::FFFFFFFFFFFFFFFFFFFF,FFFFFFFFFFFFFFFFFFFFFFFFFF:FFFFFF
FFFFFFFFFFFFFFFFFFF:FFFF:FFFFFFFFFFFFFFFFFFFFFFFFFFFFFFFFFFFF,FFFFF:
F::FFFF:FFFFF:F
@A00155:342:HHGFNDSXY:1:2349:26910:7263 2:N:0:GAACCTAG+TCCGCATA
ACCACCTATTATTTAATGTTGTAGCTAGTTACATTAGTAAATATTCATATGTAGCATGTTTAAGCATT
AAATCAAATAAGATGAGCAAGATGGAACGTCTGGGTTCCAATTCTGTCAAGACTTATGACATATTAAA
TAGATTGGTTACGGT
+
FFFFFFFFFFFFFFFFFFFFFFFFFFFFFFFFFFFF:FFFFFFFFFFFFFFFFFFFFFFFFFFF:FFFFF
FFFFFFFFFFFFFFFFFFFFFFFFF,FF:FFFFFFFFFFFFFFF,FFFFFF:FFFFF:FFFFFFFFFFF
FFFFFFFFFFFF:FF
@A00155:342:HHGFNDSXY:1:2421:16288:31798 2:N:0:GAACCTAG+TCCGCATA
ACCACCTATTATTTAATGTTGTAGCTAGTTACATTAGTAAATATTCATATGTAGCATGTTTAAGCATT
AAATCAAATAAGATGAGCAAGATGGAACGTCTGGGTTCCAATTCTGTCAAGACTTATGACATATTAAA
TAGATTGGTTACGGT
+
:FFFFFFFFFFFFFFFFFFFFFFFFFFFFFFFFFFFFFFFFFFFFFFFFFFFFFFFFFFFFFFFFFFFF
FFFFFFFFFFFFFFFFFFFFFFFFFFFFFFFFFFFFFFFFFFFFFFFFFFFFFFFFFFFFFFFFFFFFF
FFFFFFFFFFFFFFF
@A00155:342:HHGFNDSXY:1:2429:10628:23844 2:N:0:GAACCTAG+TCCGCATA
ACCACCTATTATTTAATGTTGTAGCTAGTTACATTAGTAAATATTCATATGTAGCATGTTTAAGCATT
AAATCAAATAAGATGAGCAAGATGGAACGTCTGGGTTCCAATTCTGTCAAGACTTATGACATATTAAA
TAGATTGGTTACGGT
+
FFFFFFFFFFFFFFFFFFFFFFFFFFFFFFFFFF:FFFFFFFFFFFFFFFFFFFFFFFFFFFFFFFFFF
FFFFFFFFFFFFFFFFFFFF:FFFFFFFFFFFFFFFFFFFFFFFFFFFFFFFFFF:FFFFFFFFFFFF:F
FFFFFFFFFFFF,FF
@A00155:342:HHGFNDSXY:1:2458:19461:29559 2:N:0:GAACCTAG+TCCGCATA
ACCACCTATTATTTAATGTTGTAGCTAGTTACATTAGTAAATATTCATATGTAGCATGTTTAAGCATT
AAATCAAATAAGATGAGCAAGATGGAACGTCTGGGTTCCAATTCTGTCAAGACTTATGACATATTAAA
TAGATTGGTTACGGT
+
FFFFFFFFFFFFFFFFFFFFFFFFFFFFFFFFFF:FFFFFFFFFFFFFFFFFFFFFFFFFFFFFFFFFF
FFFFFFFFFFFFFFFFFFFFFFFFFFFFFFFFFFFFFFFFFFFFF:FFFFFFFF:FFFFFFFFFFFF,F
FFFFFFFFFFFFFFF
@A00155:342:HHGFNDSXY:1:2551:21124:1501 2:N:0:GAACCTAG+TCCGCATA
ACCACCTATTATTTAATGTTGTAGCTAGTTACATTAGTAAATATTCATATGTAGCATGTTTAAGCATT
AAATCAAATAAGATGAGCAAGATGGAACGTCTGGGTTCCAATTCTGTCAAGACTTATGACATATTAAA
TAGATTGGTTACGGT
+
FFFFFFFFFFFFFFF:FFFFFFFFFFFFFFFFFFFFFFFFFFFFFFFFFFFFFFFFFFFFFFFFFFFFF
FFFFFFFFFFFFFFFFFFFFFFFFFFFFFFFFFFFFFFFFFFFFFFFFFFF:FFFFFFFFFFFFFFFFFF
FFFFFFFFFFFFFFF
@A00155:342:HHGFNDSXY:1:2622:30065:29387 2:N:0:GAACCTAG+TCCGCATA
ACCACCTATTATTTAATGTTGTAGCTAGTTACATTAGTAAATATTCATATGTAGCATGTTTAAGCATT
AAATCAAATAAGATGAGCAAGATGGAACGTCTGGGTTCCAATTCTGTCAAGACTTATGACATATTAAA
TAGATTGGTTACGGT
+
FFFFFFFFFFFFFFFFFFFFFFFFFFFFFFFFFFFFFFFFFFFFFFFFFFFF,FFFFFF:FFFFFFFF
FFFFFFFFFFFFFFF:FFFFFFFFFFFFFFFFFF:FFFFFFFFFF:FFFFFF:FFFFFFFFFFFFFFFFF
FFFFFFFFFFFFFFF
```

@A00155:342:HHGFNDSXY:1:2641:14615:31501 2:N:0:GAACCTAG+TCCGCATA
ACCACCTATTATTTAATGTTGTAGCTAGTTACATTAGTAAATATTCATATGTAGCATGTTTAAGCATT
AAATCAAATAAGATGAGCAAGATGGAACGTCTGGGTTCCAATTCTGTCAAGACTTATGACATATTAAA
TAGATTGGTTACGGT
+
FFFFFFFFFFFFFFFFFFFFFFFFFFFFFFFFFF:FFFFFFFFFFFFFFFFFFFFFFFFFFFFFFFFFF
FFFFFFFFFFFFFFFFFFFFFFFFFFFFFFFFFFFFFFFFFFFFFFFFFFFFFFFFFFFFFFFFFFFF
:FFFFFFFFFFFFFF
@A00155:342:HHGFNDSXY:1:2116:3857:28244 1:N:0:GAACCTAG+TCCGCATA
CCACCTATTATTTAATGTTGTAGCTAGTTACATTAGTAAATATTCATATGTAGCATGTTTAAGCATTA
AATCAAATAAGATGAGCAAGATGGAACGTCTGGGTTCCAATTCTGTCAAGACTTATGACATATTAAAT
AGATTGGTTACGGTA
+
FFFF,FFFFFFFFF:FFFFFFFFFFFFFFFFFFFFFFFFFFFFFFFFFFFFFFFFFFFFFFFFFFFFF
FFFFFFFFFFFFFFFFFFFFFFFFFFFFFFFFFF:FFFFFFFFFFFFFFFFFFFFFFFFFFFFFFFFFF
FFFFFFFFFFFFFFF
@A00155:342:HHGFNDSXY:1:1117:4354:24189 2:N:0:GAACCTAG+TCCGCATA
CACCTATTATTTAATGTTGTAGCTAGTTACATTAGTAAATATTCATATGTAGCATGTTTAAGCATTAA
ATCAAATAAGATGAGCAAGATGGAACGTCTGGGTTCCAATTCTGTCAAGACTTATGACATATTAAATA
GATTGGTTACGGTAA
+
FFFFFFFFFFFFFFFFFFFFFFFFFFFFFFF::FFFFFFFFFFFFFFFFFFFFFFFFFFFFFFFFFFF
FFFFFFFFFFFFFFFFFFF:FFFF,FFFFFFFFFFFFFFFFFFFF,FFFFFFFFFFFFFF:FFFFFFF
FFFFFFFFFFFFFFF
@A00155:342:HHGFNDSXY:1:1223:20898:4586 2:N:0:GAACCTAG+TCCGCATA
CACCTATTATTTAATGTTGTAGCTAGTTACATTAGTAAATATTCATATGTAGCATGTTTAAGCATTAA
ATCAAATAAGATGAGCAAGATGGAACGTCTGGGTTCCAATTCTGTCAAGACTTATGACATATTAAATA
GATTGGTTACGGTAA
+
FFFFFFFFFFFFFFFFFFFFFFFFFFFFFFFFFFFFFFFFFFFFFFFFFFFFFFFFFFFFFFFFFFFF
FFFFFFFFFFFFFFFFFFFFFFFFFFFFFFFFFFFFFFFFFFFFFFFFFFFFFFFFFFFFFF:FFFFF
FFFFFFFFFFFFFFF
@A00155:342:HHGFNDSXY:1:1350:21947:11537 2:N:0:GAACCTAG+TCCGCATA
CACCTATTATTTAATGTTGTAGCTAGTTACATTAGTAAATATTCATATGTAGCATGTTTAAGCATTAA
ATCAAATAAGATGAGCAAGATGGAACGTCTGGGTTCCAATTCTGTCAAGACTTATGACATATTAAATA
GATTGGTTACGGTAA
+
FFFFFFFFFFFFFFFFFFFFFFFFFFFFFFFFFFFFFFFFFFFFFFFFFFFFFFFFFFFFFFFFFFFF
FFFFFFFFFFFFFFFFFFFFFFFFFFFFFFFFFFFFFFFFFFFFFFFFFFFFFFFFFFFFFFFFFFFF
FF:FF:FFFFFFFFF
@A00155:342:HHGFNDSXY:1:2234:19976:22185 2:N:0:GAACCTAG+TCCGCATA
CACCTATTATTTAATGTTGTAGCTAGTTACATTAGTAAATATTCATATGTAGCATGTTTAAGCATTAA
ATCAAATAAGATGAGCAAGATGGAACGTCTGGGTTCCAATTCTGTCAAGACTTATGACATATTAAATA
GATTGGTTACGGTAA
+
FFFFFF:FFFFFFFFFFFFFFF:FFFFFF:FFFFFFFFFFFFFFFFFFF:FFFFF:FFFFFFFFF:FFF
FFFFFF:FF:FFFF:FFFFFFFFFFF:F:FFF:F:F:FFFFFFFFFF:,F:FFFFFFFFFFFFFF::F
:FF:FFFFFF,:FFF
@A00155:342:HHGFNDSXY:1:2264:3459:14779 2:N:0:GAACCTAG+TCCGCATA
CACCTATTATTTAATGTTGTAGCTAGTTACATTAGTAAATATTCATATGTAGCATGTTTAAGCATTAA
ATCAAATAAGATGAGCAAGATGGAACGTCTGGGTTCCAATTCTGTCAAGACTTATGACATATTAAATA
GATTGGTTACGGTAA
+
FFFFFFFFFFFF:FFFFFFFFFFFFFFFFFFFFFF,FFFFFFFFFFFFFFFF:FFFFFFFFFFFFFFFF

FFFFFFFFFFFFFFFFFFFFFFFFFFFFFFFFFFFF,FFFF:FFFFFFFF:FFFFFF:FFFFFFFF::
FFF:F,FFFFF:FFF
@A00155:342:HHGFNDSXY:1:1219:4273:7921 2:N:0:GAACCTAG+TCCGCATA
ACCTATTATTTAATGTTGTAGCTAGTTACATTAGTAAATATTCATATGTAGCATGTTTAAGCATTAAA
TCAAATAAGATGAGCAAGATGGAACGTCTGGGTTCCAATTCTGTCAAGACTTATGACATATTAAATAG
ATTGGTTACGGTAA
+
FFFFFFFFFFFFFFFFFFFFFFFFFFFFFFFFFFFFFFFFFFFFFFFFFFFFFFFFFFFFFFFFFFFF
FFFFFFFFFFF:FFFFF:FFFFFFFFFFFFFFFFFFFFFFFFFFFFF:FFFFFFFFFFFFFFFFFFFF
FFFFFFFFFFFF:F
@A00155:342:HHGFNDSXY:1:2625:7464:7748 2:N:0:GAACCTAG+TCCGCATA
ACCTATTATTTAATGTTGTAGCTAGTTACATTAGTAAATATTCATATGTAGCATGTTTAAGCATTAAA
TCAAATAAGATGAGCAAGATGGAACGTCTGGGTTCCAATTCTGTCAAGACTTATGACATATTAAATAG
ATTGGTTACGGTAA
+
FFFFFFFFFFFFFFFFFFFFFFFFFFFFFFFFFFFFFFFFFFFFFFFFFFFFFFFFFFFFFFFFFFFF
FFFF:FFFFFFFFFFFFFFFFFFFFFFF:FFFFFFFFFFFFFFFFF:FFFFFFFFFFFFFFFFFFFFF
F,FFF:FF:FFF,F
@A00155:342:HHGFNDSXY:1:1547:14461:4366 2:N:0:GAACCTAG+TCCGCATA
ACCTATTATTTAATGTTGTAGCTAGTTACATTAGTAAATATTCATATGTAGCATGTTTAAGCATTAAA
TCAAATAAGATGAGCAAGATGGAACGTCTGGGTTCCAATTCTGTCAAGACTTATGACATATTAAATAG
ATTGGTTACGGTAAA
+
FFFFFFFFFFFFFFFFFF:FFFFFFFFFFFFFFFFFFFFFFFFFFFFFFFFFFFFFFFFFFFFFFFFF
FFFF,FFFFFFFFFFFFFFF,FFFFFFFFFFFFFFFFFFFFFFF:FFFFFFFF,FFFF,FFFFFFFFF
FFFF:FFFF,F,F:F
@A00155:342:HHGFNDSXY:1:1564:30798:31250 2:N:0:GAACCTAG+TCCGCATA
ACCTATTATTTAATGTTGTAGCTAGTTACATTAGTAAATATTCATATGTAGCATGTTTAAGCATTAAA
TCAAATAAGATGAGCAAGATGGAACGTCTGGGTTCCAATTCTGTCAAGACTTATGACATATTAAATAG
ATTGGTTACGGTAAA
+
FFFFFFFFFFFFFFFF:FFFFFFFFFFFFFFFFFFFFFFFFFFFFFF:FFFFFFFF:FFFFFFFFF,F
FFFFFFFFFFFFFFFFFFFFFFFFFFFFFFFFFFFFFFFFFFFFF,FFFF:FFFFFFFFF:FFFFFFF
FFFFF:FFF:FFFF:
@A00155:342:HHGFNDSXY:1:2219:5168:14481 2:N:0:GAACCTAG+TCCGCATA
ACCTATTATTTAATGTTGTAGCTAGTTACATTAGTAAATATTCATATGTAGCATGTTTAAGCATTAAA
TCAAATAAGATGAGCAAGATGGAACGTCTGGGTTCCAATTCTGTCAAGACTTATTACATATTAAATAG
ATTGGTTACGGTAAA
+
FFFFFFFFFFFFFFFFFFFFFFFFFFFFFFFFFFFFFFFFFFFFFFFFFF:FFFFFFFFFFFFFFFF:
FFFFFFFFFFFFFFFFFFFFFFFFFFFFFFF:FFFFFFFFFF,FFFFFF:FFF:,FFFFFFFFFFFF,
FFFF::FFFFF:FFF
@A00155:342:HHGFNDSXY:1:2337:18566:12853 2:N:0:GAACCTAG+TCCGCATA
ACCTATTATTTAATGTTGTAGCTAGTTACATTAGTAAATATTCATATGTAGCATGTTTAAGCATTAAA
TCAAATAAGATGAGCAAGATGGAACGTCTGGGTTCCAATTCTGTCAAGACTTATGACATATTAAATAG
ATTGGTTACGGTAAA
+
FFFFFFFFFFFFFFFFFFFFFFFFFFFFFFFFFFFFFFFFFFFFFFFFFFFFFFFFFFFFFFFFFFFF
FFFFFFFFFFFFFFFFFFFFFFFFFFFFFFFFFFFFFFFFFFFFFFFFFFFFFFFFFFFFFFFFFFFF
FFFFFFFFF:FFFFF
@A00155:342:HHGFNDSXY:1:2436:32579:26005 2:N:0:GAACCTAG+TCCGCATA
ACCTATTATTTAATGTTGTAGCTAGTTACATTAGTAAATATTCATATGTAGCATGTTTAAGCATTAAA
TCAAATAAGATGAGCAAGATGGAACGTCTGGGTTCCAATTCTGTCAAGACTTATGACATATTAAATAG
ATTGGTTACGGTAAA

+
FFFF,FFFFFFFF:,FFFFFFFFFFFF,FF,FFFFFFFFFF:FFFFFFFFFFFFFFFFFFFFFFFFFF
FFFFFFFFFFFFFFFF:FFFFFFFFFFFFFFFFFFFFFFFFFFFF:FFFFFFFFFFFFFFFF:FFFFF
FFFFFF,FFFFFFFF
@A00155:342:HHGFNDSXY:1:2625:7437:7733 2:N:0:GAACCTAG+TCCGCATA
ACCTATTATTTAATGTTGTAGCTAGTTACATTAGTAAATATTCATATGTAGCATGTTTAAGCATTAAA
TCAAATAAGATGAGCAAGATGGAACGTCTGGGTTCCAATTCTGTCAAGACTTATGACATATTAAATAG
ATTGGTTACGGTAAA
+
FFFFFFFFFFF:FF:FFFFFFF:,FFFF,FFFFF:FFFFFFFFFFFFFFFFFFFFF:FFFFFFFFFFF
FFFFF:FFF:FFFFFFFF,FF:FFFFFFFF:FFFFFFFF:FFFFFF,:FFF,FFFFFFF,FF:FFFFF
::FF:F:::FFFF:F
@A00155:342:HHGFNDSXY:1:1361:29035:15922 1:N:0:GAACCTAG+TCCGCATA
TCTATTATTTAATGTTGTAGCTAGTTACATTAGTAAATATTCATATGTAGCATGTTTAAGCATTAAAT
CAAATAAGATGAGCAAGATGGAACGTCTGGGTTCCAATTCTGTCAAGACTTATGACATATTAAATAGA
TTGGTTACGGTAAAA
+
F:FFFFFF:FFFFFFFFFFFF:FFFFFFFFFFFFFFFFFFFFFFFFFFFFFFFFFFFFFFFFFFFFFF
FFFFFFFFFFFFFFFFFFFFFFFFFFFFFFFFFFFFFFF:FFFFFFFFFFFFFFFFFFFFFFFFFFFF
FFFFFFFFFFFFFFF
@A00155:342:HHGFNDSXY:1:1602:3314:4069 2:N:0:GAACCTAG+TCCGCATA
CCTATTATTTAATGTTGTAGCTAGTTACATTAGTAAATATTCATATGTAGCATGTTTAAGCATTAAAT
CAAATAAGATGAGCAAGATGGAACGTCTGGGTTCCAATTCTGTCAAGACTTATGACATATTAAATAGA
TTGGTTAAGGTAAAA
+
FFFFFFFFFFF,FF:F:FF:FF:FFFF,FFFFFFFFFFFFFFFFFF,FFFFFFF:FFFF::FFFFFFF
,FFFFFFFFFFFFFFFFFF,FF:F,:,FFFFFFFFFFFF,:FF,FFFFFFFFFFF,FFFFFFFFFFFF
FFFFF:F,F,:FFF:
@A00155:342:HHGFNDSXY:1:1361:29035:15922 2:N:0:GAACCTAG+TCCGCATA
CTATTATTTAATGTTGTAGCTAGTTACATTAGTAAATATTCATATGTAGCATGTTTAAGCATTAAATC
AAATAAGATGAGCAAGATGGAACGTCTGGGTTCCAATTCTGTCAAGACTTATGACATATTAAATAGAT
TGGTTACGGTAAAA
+
FFFFFFFFFF:FF:FFFFFFFFFFFFFFFFFFFFFFFFFFFFFFFFFFFFF:FFFFFFFFFFFFFFFF
FFFFFFFFFFFFFFFFFFFFFFFFFFFFFFFFFFFFFFFFFFFFFFFFFFFFFFFFFFFFFFFFFFFF
FFF::FF:FFFF,F
@A00155:342:HHGFNDSXY:1:2355:29903:7028 2:N:0:GAACCTAG+TCCGCATA
CTATTATTTAATGTTGTAGCTAGTTACATTAGTAAATATTCATATGTAGCATGTTTAAGCATTAAATC
AAATAAGATGAGCAAGATGGAACGTCTGGGTTCCAATTCTGTCAAGACTTATGACATATTAAATAGAT
TGGTTACGGTAAAA
+
FFFFFFFFFFF::FFFFFFFFFFFFFFFFFFFFFFF:FFFFFFFFFFF:FFFFFFFFFF:F:FFFFFF
FFFFFFFFFFFFF:FFFFFFFFFFFFFFFFFFFFFFFFFFFFFFFFFFFF:FFFFFFFFFFFF:FFF:
FF,FFFFFFFFF:F
@A00155:342:HHGFNDSXY:1:1169:32805:16376 1:N:0:GAACCTAG+TCCGCATA
CTATTATTTAATGTTGTAGCTAGTTACATTAGTAAATATTCATATGTAGCATGTTTAAGCATTAAATC
AAATAAGATGAGCAAGATGGAACGTCTGGGTTCCAATTCTGTCAAGACTTATGACATATTAAATAGAT
TGGTTACGGTAAAAG
+
FFFFFFFFFFFFFFFFFFFFFFFFFFFFFFFFFFFFFFFFFFFFFFFFFFFFFFFFFFFFFFFFFFFF
FFFFFFFFFFFFFFFFFFFFFFFFFFFFFFFFFFFFFFFFFFFFFFFFFFFFFFFFFFFFFFFFFFFF
FFFFFFFFFFFFFFF
@A00155:342:HHGFNDSXY:1:1412:18792:33317 1:N:0:GAACCTAG+TCCGCATA
CTATTATTTAATGTTGTAGCTAGTTACATTAGTAAATATTCATATGTAGCATGTTTAAGCATTAAATC

```
AAATAAGATGAGCAAGATGGAACGTCTGGGTTCCAATTCTGTCAAGACTTATGACATATTAAATAGAT
TGGTTACGGTAAAAG
+
FFFFFFFFFFFFFFFF:FF:FF:FF,FFFFFFFF:FFFFFFFFFFFFFFFFFFFFFFFFFFFFFFFFF
:FFFF:FFFFF,FF:F:FF,FFFFFFF:FFFFFFFFFFFFFFFF:FFFFFFFFF::FFFFFFFFF,:F
F:FF:FFFFFFFFF:
@A00155:342:HHGFNDSXY:1:1502:31955:3035 1:N:0:GAACCTAG+TCCGCATA
CTATTATTTAATGTTGTAGCTAGTTACATTAGTAAATATTCATATGTAGCATGTTTAAGCATTAAATC
AAATAAGATGAGCAAGATGGAACGTCTGGGTTCCAATTCTGTCAAGACTTATGACATATTAAATAGAT
TGGTTACGGTAAAAG
+
FFFFFFFF:FFFFFFFFFFF:FFFFFFFF:FFFFFFFFFFFFF:FFFFFFFFFFFFFFFFFFFFFFFF
FFFFFFFFFFFFFFFFFFFFFFFFFFFFFFFFFFFF,FFFFFFFFFFFFFFFFFFFFFFFFFFFFFFF
FFFFFFFFFFFFFFF
@A00155:342:HHGFNDSXY:1:1636:9543:12320 1:N:0:GAACCTAG+TCCGCATA
CTATTATTTAATGTTGTAGCTAGTTACATTAGTAAATATTCATATGTAGCATGTTTAAGCATTAAATC
AAATAAGATGAGCAAGATGGAACGTCTGGGTTCCAATTCTGTCAAGACTTATGACATATTAAATAGAT
TGGTTACGGTAAAAG
+
:FFFFF:FFFFFFFFFFFFFFFFFFFFFFFFFFFFFFFFFFFFFFFFFFFFFFFFFFFFF:FFFFFF
FFFFFFFFFFFFFFFFFFFFFFFF:FFFFFFFFFFFFFFFFFFFFFFFFFFFFFFFFFFFFFFFFFFF
FFFFFFFFFFFFFFF
@A00155:342:HHGFNDSXY:1:2557:28176:25864 1:N:0:GAACCTAG+TCCGCATA
CTATTATTTAATGTTGTAGCTAGTTACATTAGTAAATATTCATATGTAGCATGTTTAAGCATTAAATC
AAATAAGATGAGCAAGATGGAACGTCTGGGTTCCAATTCTGTCAAGACTTATGACATATTAAATAGAT
TGGTTACGGTAAAAG
+
FFFFFFFFFFFFFFFFFFF:FFFFFFFFF:FFFFFFFFFFFFFFFFFFFFFFFFFFFFFFFFFFFFF:
FFFFFFFFFFFFFFFFFFFFFFFFFFFFFFFFFFFFFFFFFFFFFFFFFFFFFFFFFFFFFFFFFFFF
FFFFFFFFFFFFFFF
@A00155:342:HHGFNDSXY:1:1140:21685:1125 2:N:0:GAACCTAG+TCCGCATA
CTATTATTTAATGTTGTAGCTAGTTACATTAGTAAATATTCATATGTAGCATGTTTAAGCATTAAATC
AAATAAGATGAGCAAGATGGAACGTCTGGGTTCCAATTCTGTCAAGACTTATGACATATTAAATAGAT
TGGTTACGGTAAAAG
+
FFFFFFFFFFFFFFFFFFFFFFFFFFFF:FFFFFFFFFFFFFFFFFFFFFFFFFFFFFFFFFFFFFFF
FFFFFFFFFFFFFFFFFFFFFFFFFFFFFFFFFFFFFFFFFFFFFFFFFFFFFFFFFFFFFFFFFFFF
FFFFFFFFFFFFFFF
@A00155:342:HHGFNDSXY:1:1255:20265:22968 2:N:0:GAACCTAG+TCCGCATA
CTATTATTTAATGTTGTAGCTAGTTACATTAGTAAATATTCATATGTAGCATGTTTAAGCATTAAATC
AAATAAGATGAGCAAGATGGAACGTCTGGGTTCCAATTCTGTCAAGACTTATGACATATTAAATAGAT
TGGTTACGGAGATCG
+
FFFFFFFFFFFFF:FFFFFFFFFFFFFFFFFFFFFFFF,FFFFFFF:FFFFFFFFFFFFFFFFFFFFF
FFFFFFFFFFFFF:FFFFFFFFFFFF,FFFFFFFFFFFFFFFFFFFFFFF:FFFFFFFFFFF:FFFFF
FFFFFFFFFFFFFFF
@A00155:342:HHGFNDSXY:1:1314:13684:8563 2:N:0:GAACCTAG+TCCGCATA
CTATTATTTAATGTTGTAGCTAGTTACATTAGTAAATATTCATATGTAGCATGTTTAAGCATTAAATC
AAATAAGATGAGCAAGATGGAACGTCTGGGTTCCAATTCTGTCAAGACTTATGACATATTAAATAGAT
TGGTTACGGTAAAAG
+
FFFFFFFFFFFFFFFFFFFFFFFFFFFFFFFFFFFFFFFFFFFFFFFFFFFFFFFFFFFFFFFFFFFF
FFFFFFF:FFFFFFFFFFFF:FFFFFFFFFFFFFF:FFFFFF:F,F,FFFFFFFFFFFFFFFFFFFFF
FFFFFFFFFFFF:FF
```

```
@A00155:342:HHGFNDSXY:1:1313:18430:17597 2:N:0:GAACCTAG+TCCGCATA
CTATTATTTAATGTTGTAGCTAGTTACATTAGTAAATATTCATATGTAGCATGTTTAAGCATTAAATC
AAATAAGATGAGCAAGATGGAACGTCTGGGTTCCAATTCTGTCAAGACTTATGACATATTAAATAGAT
TGGTTACGGTAAAAG
+
FFFFFFFFFF:FFFFFFFFFFFFFFFFFFFFFFFFFFFFFFFFFF:FFFFFFFFFFFFFFFFFFFFF:FF
FFFFFFF:FFFFFFFFFFFFFFFF:FFFFFFFFFFFFFFFFFFFFF:FFFFFFFFFFFFFFFFFFFFFFF
F,FFFFFFFF,F:FF
@A00155:342:HHGFNDSXY:1:1335:7609:33207 2:N:0:GAACCTAG+TCCGCATA
CTATTATTTAATGTTGTAGCTAGTTACATTAGTAAATATTCATATGTAGCATGTTTAAGCATTAAATC
AAATAAGATGAGCAAGATGGAACGTCTGGGTTCCAATTCTGTCAAGACTTATGACATATTAAATAGAT
TGGTTACGGTAAAAG
+
FFFFFFFFFFFFF:FFFFFFFFFFFFFFFFFFFFFFFFFF:FFFFF:FFFFFFFFFFFF:FFFFFFFFF
FFFFFFFFFFFFFFFFFFFFFFFFF:FFFFFFFFFFFFFFFFFFFF:FFFFFF,FFFFFFFFF:F:FFF
:FFFFF::FFFFFF:
@A00155:342:HHGFNDSXY:1:1406:24216:19069 2:N:0:GAACCTAG+TCCGCATA
CTATTATTTAATGTTGTAGCTAGTTACATTAGTAAATATTCATATGTAGCATGTTTAAGCATTAAATC
AAATAAGATGAGCAAGATGGAACGTCTGGGTTCAAATTCTGTCAAGACTTATGACATATTAAATAGAT
TGGTTACGGTAAAAG
+
FFFFFFFFFFF,FFFFFF:FFFFFFFFFF:F:FFFF,FFFFF:FF:FFF,FFF:,FFFFFFFFFFFF,
FFF:FF:FFFFFFFFFFF:,FFF::FFF,,FFF,FFFFF:,::F,,FFFFFFF:,FFF:F:FFFFFFF
FF,,FF,:,F:,:FF
@A00155:342:HHGFNDSXY:1:1440:14479:6527 2:N:0:GAACCTAG+TCCGCATA
CTATTATTTAATGTTGTAGCTAGTTACATTAGTAAATATTCATATGTAGAATGTTTAAGCATTAAATC
AAATAAGATGAGCAAGATGGAACGACTGGGTTCCAATTCTGTCAAGACTTATGACATATTAAATAGAT
TGGTTACGGTAAAAG
+
FFFFFFF::,FFFFFFFF:FFFFFFF:FFFFFFFFF:FFF,FFF,:FFF,FFF:,FFFFFFF:FFFFF
FFF,FF,FFFF,,FF,FF,FF::,,:,,,FF,FFF:FF,,FFFFFFFFF,FF:FF::,:FFFFFF:F,
F::,,:,:F,FFFF:
@A00155:342:HHGFNDSXY:1:1461:2175:14152 2:N:0:GAACCTAG+TCCGCATA
CTATTATTTAATGTTGTAGCTAGTTACATTAGTAAATATTCATATGTAGCATGTTTAAGCATTAAATC
AAATAAGATGAGCAAGATGGAACGTCTGGGTTCCAATTCTGTCAAGACTTATGACATATTAAATAGAT
TGGTTACGGTAAAAG
+
FFFFFFFFFFFFFFFFFFFFFFFFFFFFFFFFFFFFFFFFFFFFFFFFFFFFFFFFFFFFFFFFFFFF
FFFFFFFFF:FFFFFFFFFFFFFFFFFFFFFFFFFFFFFFFFFFFFFFFFFFFFFFFFFFFFFFFF:FF
FFFFFF:FFFFF:FF
@A00155:342:HHGFNDSXY:1:1612:7455:22827 2:N:0:GAACCTAG+TCCGCATA
CTATTATTTAATGTTGTAGCTAGTCACATTAGTAAATATTCATATGTAGCATGTTTAAGCATTAAATC
AAATAAGATGAGCAAGATGGAACGTCTGGGTTCCAATTCTGTCAAGACTTATGACATATTAAATAGAT
TGGTTACGGTAAAAG
+
FFFFFFFFFFFFFFFFFFFFFFFFFFFFFFFFFFFFFFFFFFFFFFFFFFFFFFFFFFFFFFFFFFFF
FF:FF:FFFFFFFF:FFFFFF:FFFFFFFFFFFFFFFFFFFFFFFFFFFFFF:FFFFFF,FFFFFFFFFF
FFFFFFFFFFFFFF:
@A00155:342:HHGFNDSXY:1:2172:28330:28291 2:N:0:GAACCTAG+TCCGCATA
CTATTATTTAATGTTGTAGCTAGTTACATTAGTAAATATTCATATGTAGCATGTTTAAGCATTAAATC
AAATAAGATGAGCAAGATGGAACGTCTGGGTTCCAATTCTGTCAAGACTTATGACATATTAAATAGAT
TGGTTACGGTAAAAG
+
FFFFFFFFFF:FFFFFFFFFFFFFFFFFFFFFFFFFFFFFFFFFFFF:FFFFFFFFFFFFFFFFFFFFF
```

FFFFFFFFFFFFFFFFFFFF::FFFFFFFFFFFFFFFFFFFFFFFF:FFFFFFFFFFFFFFFFFFFFFF
FFFFFFFFFF:FFF:
@A00155:342:HHGFNDSXY:1:2274:26332:11397 2:N:0:GAACCTAG+TCCGCATA
CTATTATTTAATGTTGTAGCTAGTTACATTAGTAAATATTCATATGTAGCATGTTTAAGCATTAAATC
AAATAAGATGAGCAAGATGGAACGTCTGGGTTCCAATTCTGTCAAGACTTATGTCATATTAAATAGAT
TGGTTACGGTAAAAG
+
FFFFFFFFFF::FFFFFFFFFFFFFFFFFFFFFFFFFFFFFFFFFFFFFFFFFFFFFFFFFFFFFFFF
FFFFFFFFFFFFFFFFFFFFFFFF:FFFFFFFFFF:FFFFFFFFFFFFFFFFFFFFFFFFFFF,FFFF
FFFFFFF:FFFF,F:
@A00155:342:HHGFNDSXY:1:2254:9661:26960 2:N:0:GAACCTAG+TCCGCATA
CTATTATTTAATGTTGTAGCTAGTTACATTAGTAAATATTCATATGTAGCATGTTTAAGCATTAAATC
AAATAAGATGAGCAAGATGGAACGTCTGGGTTCCTATTCTGTCAAGACTTATGACATATTAAATAGAT
TGGTTACGGTAAAAG
+
FFFFFFFFFFF:FFFFFFFFFFFFFFFFFFFFFFFFF:FFFFFFFFFFFFFFFFFFFFFFFFFFFFFF
FFFFFFFFFFF,FFFFFFFFFFFFFFFFFFFFFFFFFFFFFFFF:FFFFFFFFFF:FFFFFFFFFFFF
FFFFF:FFFFFFF,:
@A00155:342:HHGFNDSXY:1:2278:20184:7670 2:N:0:GAACCTAG+TCCGCATA
CTATTATTTAATGTTGTAGCTAGTTACATTAGTAAATATTCATATGTAGCATGTTTAAGCATTAAATC
AAATAAGATGAGCAAGATGGAACGTCTGGGTTCCAATTCTGTCAAGACTTATGACATATTAAATAGAT
TGGTTACGGTAAAAG
+
FFFFFFFFFFFFFFFFFFFFFFFFFFFFFFFFFFFFFFFFFFFFFFFFFFFFFFFFFFFFFFFFFFFF
FFFFFFFFFFFFFFFFFFFFFFFFFFFFFFFFFFFFFFFFFFFFFFFFFFFFFFFFFF:FFFFFFFF,
FFFFFFFFFF,FF:F
@A00155:342:HHGFNDSXY:1:2405:18023:32362 2:N:0:GAACCTAG+TCCGCATA
CTATTATTTAATGTTGTAGCTAGTTACATTAGTAAATATTCATATGTAGCATGTTTAAGCATTAAATC
AAATAAGATGAGCAAGATGGAACGTCTGGGTTCCAATTCTGTCAAGACTTATGACATATTAAATAGAT
TGGTTACGGTAAAAG
+
FFFFFFFFFFFFFFFFF:FFFFFFFFFFFFFFFFFFFFFFFFFFFFFFFFFFFFFFFFFFFFFFFFFF
FFFFFFFFFFFFFFFFFFFFFF:FFFFFFFFFFFFFFFFFFFFFF:FFFFFFFFFFFFFFFFFFFFFF:FF
FF:FFFF:FFFFF:F
@A00155:342:HHGFNDSXY:1:2419:4227:30201 2:N:0:GAACCTAG+TCCGCATA
CTATTATTTAATGTTGTAGCTAGTTACATTATTAAATATTCATATGTAGCATGTTTAAGCATTAAATC
AAATAAGATGAGCAAGATGGAACGTCTGGGTTCCAATTCTGTCAAGACTTATGACATATTAAATAGAT
TGGTTAAGGTAAAAG
+
:F:FFF,FFFFFFFFF,FF:FF,F:FFFFFF,:FF:FFFF,FFFFFFF,FFF:FF,FFFFFFFFFFF,
FFF:FFFFFFF:FFF,FF,FFFFFFFF::F,FF,FF,FFF,FFFF:FFFFF:,F,F:FFFFFFFF:FF
FF:FFF,F,FFFF:F
@A00155:342:HHGFNDSXY:1:2545:19877:11209 2:N:0:GAACCTAG+TCCGCATA
CTATTATTTAATGTTGTAGCTAGTTACATTAGTAAATATTCATATGTAGCATGTTTAAGCATTAAATC
AAATAAGATGAGCAAGATGGAACGTCTGGGTTCCAATTCTGTCAAGACTTATGACATATTAAATAGAT
TGGTTACGGTAAAAG
+
FFFFFFFFFFFFFFFFFFFFFFFFFFFFFFFFFFFFFFFFFFFFFFFFFFFFFFFFFFFFFFFFFFFF
FFFFFFFFFFFFFFFFFFFFFFFFFFFFFFFFFFFFFFFFFFFFFFFFFFFF:FFFFFFFFFFFFFFF
FFFFFFFFFFFFFFF
@A00155:342:HHGFNDSXY:1:2545:19352:12148 2:N:0:GAACCTAG+TCCGCATA
CTATTATTTAATGTTGTAGCTAGTTACATTAGTAAATATTCATATGTAGCATGTTTAAGCATTAAATC
AAATAAGATGAGCAAGATGGAACGTCTGGGTTCCAATTCTGTCAAGACTTATGACATATTAAATAGAT
TGGTTACGGTAAAAG

```
+
FFFFFFFFFF:FFFFFFFFFFFFFFFFFFFFFFFFFFFFFFFFF,FFFFFFFFFFFFFFFFFFFFFFFF
FFFFFFFFFFFFFFFFFFFFFFFFFFFFFFFFFFFFFFFFFFFFFFFFFFFFFFFFFFFFFF:FFFFFF
FFFFFFF:FFFF:FF
@A00155:342:HHGFNDSXY:1:2559:32588:36667 2:N:0:GAACCTAG+TCCGCATA
CTATTATTTAATGTTGTAGCTAGTTACATTAGTAAATATTCATATGTAGCATGTTTAAGCATTAAATC
AAATAAGATGAGCAAGATGGAACGTCTGGGTTCCAATTCTGTCAAGACTTATGACATATTAAATAGAT
TGGTTACGGTAAAAG
+
FFFFFFFFFFFFF:FFFFFFFFFFFFF:,FFFFFFFFFFFFFFFFFFFFFFFFF::FFFFFFFFFF,F
FFFFFFFFFFFFFFFFFFFFFFFFFFFFFF::FFFFFFFFF:FFFFFFFFFFFF:FFFFFFFF:FFF,
FFFF:FFFF::FFFF
@A00155:342:HHGFNDSXY:1:2641:13810:15389 2:N:0:GAACCTAG+TCCGCATA
CTATTATTTAATGTTGTAGCTAGTTACATTAGTAAATATTCATATGTAGCATGTTTAAGCATTAAATC
AAATAAGATGAGCAAGATGGAACGTCTGGGTTCCAATTCTGTCAAGACTTATGACATATTAAATAGAT
TGGTTACGGTAAAAG
+
FFFFFFF:FFFFF:FFFFFFFFFFFFFFFFFFFFFFFF,FFFFFFFFFFFFFF:FFFFFFFFFFF:FF
FFFFFFFFFFFFFFFFFFFFFFFFFFFFFFFFFFFFFFFFFFFFFF,FFFFFFF::FFF:FFFF:FFFF
FFFFFFFFFFFF:FFF
@A00155:342:HHGFNDSXY:1:2664:13214:10034 2:N:0:GAACCTAG+TCCGCATA
CTATTATTTAATGTTGTAGCTAGTTACATTAGTAAATATTCATATGTAGCATGTTTAAGCATTAAATC
AAATAAGATGAGCAAGATGGAACGTCTGGGTTCCAATTCTGTCAAGACTTATGACATATTAAATAGAT
TGGTTACGGTAAAAG
+
FFFFFFFFFFF:FFFFFFFFFFFFFFFFFFFFFFFFFFFFFFFFFFFFFFFFFFFFFFFFFFFFFFFF
FFFFFFFFFFFFFFFFFFFFFFFF:FFFFFFFFFFFFFFFFFFFFFFFFFFFFFFFFFFFFFFFFF:FF
FFFFFFF:FF:FFFF
@A00155:342:HHGFNDSXY:1:2662:22580:7153 2:N:0:GAACCTAG+TCCGCATA
CTATTATTTAATGTTGTAGCTAGTTACATTAGTAAATATTCATATGTAGCATGTTTAAGCATTAAATC
AAATAAGATGAGCAAGATGGAACGTCTGGGTTCCAATTCTGTCAAGACTTATGACATATTAAATAGAT
TGGTTACGGTAAAAG
+
FFFFFFFFFFFFF,FFFFFFFFFFFFFFFFFFFFFFFFFFFFFFFFFFFFFFFFFFFFFFFFFFFFFF
FFF:FFFFFFFFFFFFFFFFFFFFFFFFFFFFFFFFFFFFFFFFFFFFFFFFF:FFFFFFFFFFFFFFF
FFFFFFFFFFFFFFF
@A00155:342:HHGFNDSXY:1:1636:9408:12336 1:N:0:GAACCTAG+TCCGCATA
TATTATTTAATGTTGTAGCTAGTTACATTAGTAAATATTCATATGTAGCATGTTTAAGCATTAAATCA
AATAAGATGAGCAAGATGGAACGTCTGGGTTCCAATTCTGTCAAGACTTATGACATATTAAATAGATT
GGTTACGGTAAAAG
+
FFFFFF::FFFFFFFFFFFFFFFFFFFFFF:FFFFFFFFFFFFFFFFFFFFFFF:FFFFFFFFFFFFFFF
FFFFFFFFFFFFFFFFFFFFFFFFFFFFFFFFFFFFFFFFFFFFFFFFFFFFFFFFFFFFFFFFFFFFF
FFFFFFFFFFFFFF
@A00155:342:HHGFNDSXY:1:1432:31593:35571 2:N:0:GAACCTAG+TCCGCATA
TATTATTTAATGTTGTAGCTAGTTACATTAGTAAATATTCATATGTAGCATGTTTAAGCATTAAATCA
AATAAGATGAGCAAGATGGAACGTCTGGGTTCCAATTCTGTCAAGACTTATGACATATTAAATAGATT
GGTTACGGTAAAAGA
+
FFF:FFF:FFFFFFFFFFFFF:FFFFFFF:FFF,FFFFFFFFFFFFFFFFFFFFFFFFFF:FF:FFF,F
FFFFFFFFFFFFFFFFFFFF,FFFFFFFFFFFFFFFFFFFFFFFFFFFFFFFFFF:FFFFFFFFFFFFF
FFF::FFFFF,FFFF
@A00155:342:HHGFNDSXY:1:1440:6216:12070 2:N:0:GAACCTAG+TCCGCATA
TATTATTTAATGTTGTAGCTAGTTACATTAGTAAATATTCATATGTAGCATGTTTAAGCATTAAATCA
```

AATAAGATGAGCAAGATGGAACGTCTGGGTTCCAATTCTGTCAAGACTTATGACATATTAAATAGATT
GGTTACGGTAAAAGA
+
:FFFFFFFFFFFFFFFFFFFFFFFFFFFFFFFFFFFFFFFFFFFFFFFFFFFFFFFFFFFFFFFFFFFF
FFFFFFFFFFFFFFFFFFFFFFFFFFFFFFFFFFFFFFFFFFFFF:FFFFFFFFFFFFFFFFFFFFFFF
F:FFFFFF:FFFFFF
@A00155:342:HHGFNDSXY:1:1466:13340:1329 2:N:0:GAACCTAG+TCCGCATA
TATTATTTAATGTTGTAGCTAGTTACATTAGTAAATATTCATATGTAGCATGTTTAAGCATTAAATCA
AATAAGATGAGCAAGATGGAACGTCTGGGTTCCAATTCTGTCAAGACTTATGACATATTAAATAGATT
GGTTACGGTAAAAGA
+
FFFFF:FFFFFFFFFFFFFFFFFFFFFFFFFFFFFFFFFFFF:FFFFFFFFF,FFFFFFFFFFFFFFF
FFFF,FFFFFFFFFFFFFFFFF:FFFFFFFFFFFFFFFF:FFFFFFFFF,FFFFFF,FFF:FFFFFFF
FFFFFFFFF:F::FF
@A00155:342:HHGFNDSXY:1:1176:5801:35837 1:N:0:GAACCTAG+TCCGCATA
ATTATTTAATGTTGTAGCTAGTTACATTAGTAAATATTCATATGTAGCATGTTTAAGCATTAAATCAA
ATAAGATGAGCAAGATGGAACGTCTGGGTTCCAATTCTGTCAAGACTTATGACATATTAAATAGATTG
GTTACGGTAAAAGAC
+
FFFFFFFFFFFFFFFFFFFFFFFFFFFFF:FFFFFFFFFFFFFFFFFFFFFFFFFFFFFFFFFFFFFF
FFFFFFFFFFFFFFFFFFFFFFFFFFFFFFFF:FFF:FFFFFFFFFFFFFFFFFFFFFFFFFFFFFFF
FFFFFFFFFFFFFFF
@A00155:342:HHGFNDSXY:1:1205:13105:1548 1:N:0:GAACCTAG+TCCGCATA
TCTATTTAATGTTGTAGCTAGTTACATTAGTAAATATTCATATGTAGCATGTTTAAGCATTAAATCAA
ATAAGATGAGCAAGATGGAACGTCTGGGTTCCAATTCTGTCAAGACTTATGACATATTAAATAGATTG
GTTACGGTAAAAGAC
+
FFFFFFFFFFFFFF:FFFFFFFFFF:FF:FFFFFFFFFFFFFFFFFFFFFFFFFFFFFFFFFFFFFFF
FFFFFFFFFFFFFFFFFFFFFFFFFFFFFFFFFFFFFFFFFFFFFFFFFFFFFFFFFFFFFFFFFFFF
FFFFFFFFFFFFFFF
@A00155:342:HHGFNDSXY:1:1205:13051:2519 1:N:0:GAACCTAG+TCCGCATA
TCTATTTAATGTTGTAGCTAGTTACATTAGTAAATATTCATATGTAGCATGTTTAAGCATTAAATCAA
ATAAGATGAGCAAGATGGAACGTCTGGGTTCCAATTCTGTCAAGACTTATGACATATTAAATAGATTG
GTTACGGTAAAAGAC
+
FFFF:FFFFFFFFFFFFFFFFFFFFFFFFFFFFFFFFFFFFFFFF:FFFFFFFFFFFFFFFFFFFFFFF
FFF:FFFFFFFFFFFFFFFFFFFFFFFFFFFFFFFFFFFFFFFFFFFFFFFFFFFFFFFFFFFFFFFF
FFFFFFFFFFFFFFF
@A00155:342:HHGFNDSXY:1:1205:13422:4131 1:N:0:GAACCTAG+TCCGCATA
TCTATTTAATGTTGTAGCTAGTTACATTAGTAAATATTCATATGTAGCATGTTTAAGCATTAAATCAA
ATAAGATGAGCAAGATGGAACGTCTGGGTTCCAATTCTGTCAAGACTTATGACATATTAAATAGATTG
GTTACGGTAAAAGAC
+
F,FFFFFFFFFFFFFFFFFFFFFFFFFFFFFFFFFFFFFFFFFFFFFFFFF:FFFFFFFFFFFFFFF
FFFFFFFFFF:FFFFFFFFFFFFFFFFF:FFFFFFFFFFFFFFFFFFFFFFFFFFFFFFFFFFFFF:F
FFFFFFFFFFFFFFF
@A00155:342:HHGFNDSXY:1:1253:22245:30217 1:N:0:GAACCTAG+TCCGCATA
ATTATTTAATGTTGTAGCTAGTTACATTAGTAAATATTCATATGTAGCATGTTTAAGCATTAAATCAA
ATAAGATGAGCAAGATGGAACGTCTGGGTTCCAATTCTGTCAAGACTTATGACATATTAAATAGATTG
GTTACGGTAATAGAC
+
FFFFFFFF:FFFFFFFFFFFFFFFFFF:FFFFFFFFFFFFFFFF,FFFFFFFFFFFFFFFFFFFFFFF
FFFFFFFFFFFFFFFFFFFFFFFFFFFFF:FFFFF:FFFFFFFFFFFFFFFFFFFFFFFFFFFFFFFF
FFFFFFFFFF,FFFF

```
@A00155:342:HHGFNDSXY:1:2141:4770:23062 1:N:0:GAACCTAG+TCCGCATA
TCTATTTAATGTTGTAGCTAGTTACATTAGTAAATATTCATATGTAGCATGTTTAAGCATTAAATCAA
ATAAGATGAGCAAGATGGAACGTCTGGGTTCCAATTCTGTCAAGACTTATGACATATTAAATAGATTG
GTTACGGTAAAAGAC
+
FFFFFFFFFFFFFFFFFFFFFFFF:F,:F:FFFFFFFFF:FFFFFFFFFF:FFFFFF,FFFFFFFFFF
FFFFF:FFFFFF:FFFFFF,F,FFFFFFFFFF:FFFFFFFFFFFFFFFFFFFFFFF:FFFFFFFFFFF
FFFFFFFFFFF:,FF
@A00155:342:HHGFNDSXY:1:2360:27317:17707 1:N:0:GAACCTAG+TCCGCATA
ATTATTTAATGTTGTAGCTAGTTACATTAGTAAATATTCATATGTAGCATGTTTAAGCATTAAATCAA
ATAAGATGAGCAAGATGGAACGTCTGGGTTCCAATTCTGTCAAGACTTATGACATATTAAATAGATTG
GTTACGGTAAAAGAC
+
FFFFFFFF:FFFFFFFFFFFFFFFFFFFFFFFFFFFFFFFFFFFFFF:FFFFFFFFFFFFFFFFFFFF
FFFFFFFFFFFFFFFFFFFFFFFFFFFFFFFFFFFFFFFFFFFFFFFFFFFFFFFFFFFFFFFFFFFF
FFFFFFFFFFFFFFF
@A00155:342:HHGFNDSXY:1:1364:20094:4664 2:N:0:GAACCTAG+TCCGCATA
ATTATTTAATGTTGTAGCTAGTTACATTAGTAAATATTCATATGTAGCATGTTTAAGCATTAAATCAA
ATAAGATGAGCAAGATGGAACGTCTGGGTTCCAATTCTGTCAAGACTTATGACATATTAAATAGATTG
GTTACGGAGATCGGA
+
FFFFFFFFFFFFFFFFFFFFFFFFFFFFFFFFFFFFFFFFFFFFFFFFFFFFFFFFFFFFFFFFFFFF
FFFFFFFFFFFFFFFF:FFFFFFFFFFFFFFFFFFFFFFFFFFFFFFFFFFFFFFFFFFFFFFFFFF
:FFFFFFFFFFFFFF
@A00155:342:HHGFNDSXY:1:1522:3332:9956 2:N:0:GAACCTAG+TCCGCATA
ATTATTTAATGTTGTAGCTAGTTACATTAGTAAATATTCATATGTAGCATGTTTAAGCATTAAATCAA
ATAAGATGAGCAAGATGGAACGTCTGGGTTCCAATTCTGTCAAGACTTATGACATATTAAATAGATTG
GTTACGGTAAAAGAC
+
FFFFFFF:FFFFFFFFFFFFFFFFFFFFFFFFFFFFFFFFFF:FFFFFFFFFFFFFFFFFFFFFFFFF
FFFFFFF:FFFFFFFFFFFFFFFFFFFFFFFFFFFFFFFFFFFFFFFFFFFFFFFFFFFFFF:FFFF
FFFFFFFFFFFFF,F
@A00155:342:HHGFNDSXY:1:1607:28456:13228 2:N:0:GAACCTAG+TCCGCATA
ATTATTTAATGTTGTAGCTAGTTACATTAGTAAATATTCATATGTAGCATGTTTAAGCATTAAATCAA
ATAAGATGAGCAAGATGGAACGTCTGGGTTCCAATTCTGTCAAGACTTATGACATATTAAATAGATTG
GTTACGGTAAAAGAC
+
FFFFFFFFFFFFFFFFFFFFFFFFFFFFFFFFFFFFFFFFFFFFFFFFFFFFFFFFFFFFFFFFFFFF
FFFFFFFFFFFFFFFFFFFFFFFFFFFFFFFFFFFFFFFFFFFFFFFFFFFFFFFFFFFF:FFFFFFF
FFFFF:FF:F::FFF
@A00155:342:HHGFNDSXY:1:1636:9408:12336 2:N:0:GAACCTAG+TCCGCATA
ATTATTTAATGTTGTAGCTAGTTACATTAGTAAATATTCATATGTAGCATGTTTAAGCATTAAATCAA
ATAAGATGAGCAAGATGGAACGTCTGGGTTCCAATTCTGTCAAGACTTATGACATATTAAATAGATTG
GTTACGGTAAAAGAG
+
FFFFFFF,:FFFFFFFFFFFFFFFFFFFFFFFFFFFFFFFFFFFFFFFFFFFFFFFFFFFFFFFFFFF
FFFFFFFF:FFFFFFFFFFFFFFFFFFFFFFFFFFFFFFFFFFF:FFFFFFFFFFFFFFFFFFFFFFF
FFFFFFFFFF:FFFF
@A00155:342:HHGFNDSXY:1:1643:3694:16376 2:N:0:GAACCTAG+TCCGCATA
ATTATTTAATGTTGTAGCTAGTTACATTAGTAAATATTCATATGTAGCATGTTTAAGCATTAAATCAA
ATAAGATGAGCAAGATGGAACGTCTGGGTTCCAATTCTGTCAAGACTTATGACATATTAAATAGATTG
GTTACGGTAAAAGAC
+
FFFFFFFFFFFFFFFFFFFFFFFFFFFFFFFFFFFFFFFFFFFFFFFFFFFFFFFFFFFFFFFFFFFF
```

FFFFFFFFFF:FFFFFFFFF,FFFFFFFFFFFFFFFFF:FFFFFFF:FFFF:FFFFFFFFFF:FFFFFFF
FFFFFFFFF:FFFFF
@A00155:342:HHGFNDSXY:1:1636:9543:12320 2:N:0:GAACCTAG+TCCGCATA
ATTATTTAATGTTGTAGCTAGTTACATTAGTAAATATTCATATGTAGCATGTTTAAGCATTAAATCAA
ATAAGATGAGCAAGATGGAACGTCTGGGTTCCAATTCTGTCAAGACTTATGACATATTAAATAGATTG
GTTACGGTAAAAGAG
+
FFFFFFFF:FFFFFFFFFFFFFFFFFFFFFFFFFFFFFFFFFFFFFFFFFFFFFFFFFFFFFF:FFFF
FFFFFFFFFFFFFF:FFFFFFFFFFFFFFFFFFFFFFFFFFFFFFFFFFFFFFFFFF:FF:F:FFFF:FF
FFFFFFFFFFFFF:F
@A00155:342:HHGFNDSXY:1:2205:26666:34021 2:N:0:GAACCTAG+TCCGCATA
ATTATTTAATGTTGTAGCTAGTTACATTAGTAAATATTCATATGTAGCATGTTTAAGCATTAAATCAA
ATAAGATGAGCAAGATGGAACGTCTGGGTTCCAATTCTGTCAAGACTTATGACATATTAAATAGATTG
GTTACGGTAAAAGAC
+
FFFFFFFFFFFFFFFFFFFFFFFFFFFFFFFFFFFFFFFFFFFFFFFFFFFFFFFFFFFFFFFFF:FFFF
FFFFFFFFFFFFFFFF:FFFFFFFFF:FFF:FFFFFF:F:FFFFFFFFFFFFFF:FFFFFFFFFFFFFF
F:FFFFFFFFFFFFF
@A00155:342:HHGFNDSXY:1:2219:20528:29058 2:N:0:GAACCTAG+TCCGCATA
ATTATTTAATGTTGTAGCTAGTTACATTAGTAAATATTCATATGTAGCATGTTTAAGCATTAAATCAA
ATAAGATGAGCAAGATGGAACGTCTGGGTTCCAATTCTGTCAAGACTTATGACATATTAAATAGATTG
GTTACGGTAAAAGAC
+
:FF:FFFFFFFFFFFFFFFFFFFFFFFFFFFFFFFF:FFFFFFFFFFFFFFFFFFFFFFFFFFFFFFFFF
FF:FFFFFFFFFFFFFFFFFFFFFFFFFFFFFFFFFFFFFFFFFFFFFFFFFFFFFFFFFFFFFFFFFF
FFFFFFFFFFFFFFF
@A00155:342:HHGFNDSXY:1:1150:22146:18771 1:N:0:GAACCTAG+TCCGCATA
TTATTTAATGTTGTAGCTAGTTACATTAGTAAATATTCATATGTAGCATGTTTAAGCATTAAATCAAA
TAAGATGAGCAAGATGGAACGTCTGGGTTCCAATTCTGTCAAGACTTATGACATATTAAATAGATTGG
TTACGGTAAAAGACA
+
FFFFFFFFFFFFFFFFFFFFFFFFFFFFFFFFFFFFFFFFFFFFFFFFFFFFFFFFFFFFFFFFFFFFFF
FFFFFFFFFFFFFFFFFFFFFFFFFFFFFFFFFFFFFFFFFFFFFFFFFFFFFFFFFFFFFFFFFFFFFF
FFFFFFFFFFFFFFF
@A00155:342:HHGFNDSXY:1:1150:25301:30311 1:N:0:GAACCTAG+TCCGCATA
TTATTTAATGTTGTAGCTAGTTACATTAGTAAATATTCATATGTAGCATGTTTAAGCATTAAATCAAA
TAAGATGAGCAAGATGGAACGTCTGGGTTCCAATTCTGTCAAGACTTATGACATATTAAATAGATTGG
TTACGGTAAAAGACA
+
FFFFFFFFFFFFFFFFFFFFFFFFFFFFFFFFFFFFFFFFFFFFFFFFFFFFFFFFFFFFFFFFFFFFFF
FFFFFFFFFFFFFFFFFFFFFFFFFFFFFFFFFFFFFFFFFFFFFFFFFFFFFFFFFFFFFFFFFFFFFF
FFFFFFFFFFFFFFF
@A00155:342:HHGFNDSXY:1:1373:27905:17096 1:N:0:GAACCTAG+TCCGCATA
TTATTTAATGTTGTAGCTAGTTACATTAGTAAATATTCATATGTAGCATGTTTAAGCATTAAATCAAA
TAAGATGAGCAAGATGGAACGTCTGGGTTCCAATTCTGTCAAGACTTATGACATATTAAATAGATTGG
TTACGGTAAAAGACG
+
:FFFFFFFFFFFFFF:FFFFFFFF,FFFFFFFFFFFFFFFFFF:FFFFFFFFFFFF:FF:FF:FFFFFFF
FFFFF:FFF,FFFFFFFFF:FFFFFFFFF:FFFFFFFFFFFFFFFFFFF:FFFFFFFFFF:FFFFFFFFF
FFFFFFFFFFFFFFF
@A00155:342:HHGFNDSXY:1:2321:10719:27821 1:N:0:GAACCTAG+TCCGCATA
TTATTTAATGTTGTAGCTAGTTACATTAGTAAATATTCATATGTAGCATGTTTAAGCATTAAATCAAA
TAAGATGAGCAAGATGGAACGTCTGGGTTCCAATTCTGTCAAGACTTATGACATATTAAATAGATTGG
TTACGGTAAAAGACA

+
F:FFFFFFF:FFFFFFFFFFFFFFFFFFFFFFFFFFFFFFFFFFFFFFFFFFFFFFFFFFFFFFFF
FFFFFFFFFFFFFFFFFFFFFFFFFFFFFFFFFFFFFFFFFFFFFFFFFFFFFFFFFFFFFFFFFFFFFF
FFFFFFFFFFFFFFF
@A00155:342:HHGFNDSXY:1:1213:18611:33630 1:N:0:GAACCTAG+TCCGCATA
TATTTAATGTTGTAGCTAGTTACATTAGTAAATATTCATATGTAGCATGTTTAAGCATTAAATCAAAT
AAGATGAGCAAGATGGAACGTCTGGGTTCCAATTCTGTCAAGACTTATGACATATTAAATAGATTGGT
TACGGTAAAAGACAG
+
FFFF:FFFFFFF,:FFFFFFFF:FFFFFFFF:FFFFFFFFFFFFFFFFFFFFFFFFFFFFFFFFFFFFFF
FFFF:FFFFFFF:FFFFFFFFFFFFFFFFFFFFFFFFFFFFFFFFFFFFFFFFFFFFFFFFFFFFFFFFF
FFFFFFFFFFFFFFF
@A00155:342:HHGFNDSXY:1:1336:8359:32377 1:N:0:GAACCTAG+TCCGCATA
TATTTAATGTTGTAGCTAGTTACATTAGTAAATATTCATATGTAGCATGTTTAAGCATTAAATCAAAT
AAGATGAGCAAGATGGAACGTCTGGGTTCCAATTCTGTCAAGACTTATGACATATTAAATAGATTGGT
TACGGTAAAAGACAA
+
FFFFFFFFFFFFFFFFFFFFFFFFFFF:FFFFFFFFFFFFFFFFFFFFFFF,FFFFFFF:FFFFFFFFFF
FFFFFFFFFFFFFFFFFFFFFFFFFFFFFFFFFFFFFFFFFFFFFFFFFFFFFFFFFFFFFFFFFFFFFF
FFFFFFFFFFFFFFF
@A00155:342:HHGFNDSXY:1:1374:13530:6449 1:N:0:GAACCTAG+TCCGCATA
TATTTAATGTTGTAGCTAGTTACATTAGTAAATATTCATATGTAGCATGTTTAAGCATTAAATCAAAT
AAGATGAGCAAGATGGAACGTCTGGGTTCCAATTCTGTCAAGACTTATGACATATTAAATAGATTGGT
TACGGTAAAAGACTC
+
F:FFFFFFFFFFFFFFFFFFFFFFFFFFFFFFFFFFFFFFFFFFFFFFFFFFFFFFFFFFFFFFFFFFFF
FFFFFFFFFFFFFFFFFFFFFFFFFFFFFFFFFFFFFFFFFFFFFFFFFFFFFFFFFFFFFFFFFFFFFF
FFFFFFFFFFFFFFF
@A00155:342:HHGFNDSXY:1:1511:28944:1799 1:N:0:GAACCTAG+TCCGCATA
TATTTAATGTTGTAGCTAGTTACATTAGTAAATATTCATATGTAGCATGTTTAAGCATTAAATCAAAT
AAGATGAGCAAGATGGAACGTCTGGGTTCCAATTCTGTCAAGACTTATGACATATTAAATAGATTGGT
TACGGTAAAAGACAA
+
FFFFFFFF:FFFFFF::FFFFFFFFFFFFFFFFFFFFF:FFFFFFFFFFFFFFFFFFFFFFFFFFFFFFF
FFFFFFFF:FFFFFFFFFFFFFFFFF,FFFFFFFFFFF:FFFFFFFFFFFFFFFFFFFFFFFFFF,FFFF
FFFFFFFFFFFFFFF
@A00155:342:HHGFNDSXY:1:2107:25690:1454 1:N:0:GAACCTAG+TCCGCATA
TATTTAATGTTGTAGCTAGTTACATTAGTAAATATTCATATGTAGCATGTTTAAGCATTAAATCAAAT
AAGATGAGCAAGATGGAACGTCTGGGTTCCAATTCTGTCAAGACTTATGACATATTAAATAGATTGGT
TACGGTAAAAGGCAA
+
FFFFFFFFFFFFFFF:FFFFFFFFFFFFFFFFFFFFFFFFFFFFFFFFFFF:FFFFFFFFFFFFFFFFFF
FFFFFFFFFFFFFFFFFFFFFFFFFFFFFFFFFF:FFFFFFFFFFFFFFFFFFFFFFFFFFFFFFFFFFF
FFFFFFFFFFFFFFF
@A00155:342:HHGFNDSXY:1:2171:11903:1251 2:N:0:GAACCTAG+TCCGCATA
TATTTAATGTTGTAGCTAGTTACATTAGTAAATATTCATATGTAGCATGTTTAAGCATTAAATCAAAT
AAGATGAGCAAGATGGAACGTCTGGGTTCCAATTCTGTCAAGACTTATGACATATTAAATAGATTGGT
TACGGTAAAAGACAA
+
FFFFFFF:FFFFFFFFFFFFFFFFFFFFFFFFFFFFFFFFFFFFFFFFFFFFFFFFF:FFFFFFFFFF:F
FFFFFFFFFFFFFFF:FFFFFFFFF::FFFFFFFFFFF:F:FFFFFFFF:FFFFFFFFF:FFFF:,FFFF
FFFFFFFF::FFFFF
@A00155:342:HHGFNDSXY:1:2171:11605:1861 2:N:0:GAACCTAG+TCCGCATA
TATTTAATGTTGTAGCTAGTTACATTAGTAAATATTCATATGTAGCATGTTTAAGCATTAAATCAAAT

AAGATGAGCAAGATGGAACGTCTGGGTTCCAATTCTGTCAAGACTTATGACATATTAAATAGATTGGT
TACGGTAAAAGACAA
+
FFFFFFFFFFFFFFFFFFFFFFFFFFFFFFFFFFFFFFFFFFFFFFFFFFFFFFFFFFFFFFFFFFFF,F
FFFFFFFFFFFFFFFFFFFFFFFFFFFFFFFFFFFFFFFFFFFFFFFFFFFFFFFFFFFFFFFFFFFFFF
FFF:FFFFFFFFFFF
@A00155:342:HHGFNDSXY:1:1117:4354:24189 1:N:0:GAACCTAG+TCCGCATA
ATTTAATGTTGTAGCTAGTTACATTAGTAAATATTCATATGTAGCATGTTTAAGCATTAAATCAAATA
AGATGAGCAAGATGGAACGTCTGGGTTCAAATTCTGTCAAGACTTATGACATATTAAATAGATTGGTT
ACGGTAAAAGA
+
FFFFFFF:FFFFFF::FFFFFFFFFFFFFFFFFFFFFFFFFFFFFFFFFF:FFFFFFFFFFFFFFFFFFF
FFFF:FF:FFFFFFFFFF:FFFFFFFFF,FFFFFFFFFF:FFFFFFFF:FFFFF::FFFFFFFFFFFFFF
FFFFFFFFFFF
@A00155:342:HHGFNDSXY:1:1127:1588:22748 1:N:0:GAACCTAG+TCCGCATA
ATTTAATGTTGTAGCTAGTTACATTAGTAAATATTCATATGTAGCATGTTTAAGCATTAAATCAAATA
AGATGAGCAAGATGGAACGTCTGGGTTCCAATTCTGTCAAGACTTATGACATATTAAATAGATTGGTT
ACGGTAAAAGA
+
F:FFFFFFFF:FFFFFF:FFFFF,FF:FFFFFFF:FFFFFFFFF:FFF:,:FFF:FFFFFFFFFFFFFFF
FFFFFFFFFFFFFFFFFF,:FFFFFF:FFFFFF,FF:FFFFFFF:FFFFFFFFFFFFFFFFFFFFF:FFF
FFFFFFFFFFF
@A00155:342:HHGFNDSXY:1:1230:16929:24048 1:N:0:GAACCTAG+TCCGCATA
ATTTAATGTTGTAGCTAGTTACATTAGTAAATATTCATATGTAGCATGTTTAAGCATTAAATCAAATA
AGATGAGCAAGATGGAACGTCTGGGTTCCAATTCTGTCAAGACTTATGACATATTAAATAGATTGGTT
ACGGTAAAAGA
+
FFFFFFFFFFFFFFFFFFFFFFFFF:FFFFFFFFFFFFFFFFFFFFFFFFFFFFFFFFFFFFFFFFFFFF
FFFFFFFFF:FFFFFFFFFFFFF:FFFFFFFFFFFFFFFFFFFFFFFFFFFFFFFFFFFFFFFFFFFFFF
FFFFFFF:FFF
@A00155:342:HHGFNDSXY:1:1273:4408:7341 1:N:0:GAACCTAG+TCCGCATA
ATTTAATGTTGTAGCTAGTTACATTAGTAAATATTCATATGTAGCATGTTTAAGCATTAAATCAAATA
AGATGAGCAAGATGGAACGTCTGGGTTCCAATTCTGTCAAGACTTATGACATATTAAATAGATTGGTT
ACGGTAAAAGA
+
FFFFFFFFFFFFFFFFFFFFFFFFFFFFFFFFFFFFFFF:FFFFFFFFFFFFFFFFFFFFFFFFFFFFFF
FFFFFFFFFFFFFFFFFFFFFFFFFF:FFFFF:FFFFFFFFFFFFFFFFFFFFFFFFFFFFFFFFFFFFF
FFFFFFFFFFF
@A00155:342:HHGFNDSXY:1:1278:24198:32784 1:N:0:GAACCTAG+TCCGCATA
ATTTAATGTTGTAGCTAGTTACATTAGTAAATATTCATATGTAGCATGTTTAAGCATTAAATCAAATA
AGATGAGCAAGATGGAACGTCTGGGTTCCAATTCTGTCAAGACTTATGACATATTAAATAGATTGGTT
ACGGTAAAAGA
+
FFFFFFFFFFFFFFFFFFFFFFFFFFFFFFFFFFFFFFFFFFFFFFFFFFFFFFFFFFFFFFFFFFFFFF
FFFFFFFFFFFFFFFFFFFFFFFFFFFFFFFFFFFFFFFFFFFFFFFFFFFFFFFFFFFFFFFFFFFFFF
FFFFFFFFFFF
@A00155:342:HHGFNDSXY:1:1278:24207:32831 1:N:0:GAACCTAG+TCCGCATA
ATTTAATGTTGTAGCTAGTTACATTAGTAAATATTCATATGTAGCATGTTTAAGCATTAAATCAAATA
AGATGAGCAAGATGGAACGTCTGGGTTCCAATTCTGTCAAGACTTATGACATATTAAATAGATTGGTT
ACGGTAAAAGA
+
FFFFFFFFFFFFFFFFFFFFFFFFFFFFFFFFFFFFFFFFFFFFFFFFFFFFFFFFFFFFFFFFFFFFFF
FFFFFFFFFFFFFFFFFFFFFFFFFFFFFFFFFFFFFFFFFFFFFFFFFFFFFFFFFFFFFFFFFFFFFF
FFFFFFFFFFF

```
@A00155:342:HHGFNDSXY:1:1326:9109:36871 1:N:0:GAACCTAG+TCCGCATA
ATTTAATGTTGTAGCTAGTTACATTAGTAAATATTCATATGTAGCATGTTTAAGCATTAAATCAAATA
AGATGAGCAAGATGGAACGTCTGGGTTCCAATTCTGTCAAGACTTATGACATATTAAATAGATTGGTT
ACGGTAAAAGA
+
FFFFFFFFFF,FFFFFFFFFFF:FFFFF,FFFFFFFFFFFFFFFFFFFFFFFFF,:FFFF:FFFFFFF
FFFFFFF,FFFFFF:::FFFFFF:FFFFF,FFFFFFFFFF:FFFFFFFFFFFFFFFFFFFFFFFFFFF
F:FFFFFFFFF
@A00155:342:HHGFNDSXY:1:1354:30680:22968 1:N:0:GAACCTAG+TCCGCATA
ATTTAATGTTGTAGCTAGTTACATTAGTAAATATTCATATGTAGCATGTTTAAGCATTAAATCAAATA
AGATGAGCAAGATGGAACGTCTGGGTTCCAATTCTGTCAAGACTTATGACATATTAAATAGATTGGTT
ACGGTAAAAGA
+
FFFFFFFFFFF,F:,FFFFFFFFFFFFFFFFF:FFFFFFFFFFFFFFFFFFFFFFFFFFFFFFFFFFF
FFFFFFF:FFFFFFFFFFFF:FFFFFFFFFFFFFFFFFFF:FFFFFFFFFFFFFFFFFFFFFF:FFFF
FFFFFFFFFFF
@A00155:342:HHGFNDSXY:1:1411:3088:13886 1:N:0:GAACCTAG+TCCGCATA
ATTTAATGTTGTAGCTAGTTACATTAGTAAATATTCATATGTAGCATGTTTAAGCATTAAATCAAATA
AGATGAGCAAGATGGAACGTCTGGGTTCCAATTCTGTCAAGACTTATGACATATTAAATAGATTGGTT
ACGGTAAAAGA
+
FFFFFFFFFFFFFFFFFFFFFFFFFFFFFFFFFFFFFFFFFFFFFFF:FFFFFFFFFFFFFFFFFFFF
FFFFFFFFFFFFFFFFFFFFFFFFFFFFFFFFFFFFFFFFFFFFFFFFFFFFFFFFFFFFFFFFFFFF
FFFFFFFFFFF
@A00155:342:HHGFNDSXY:1:1476:6054:32831 1:N:0:GAACCTAG+TCCGCATA
TCTTAATGTTGTAGCTAGTTACATTAGTAAATATTCATATGTAGCATGTTTAAGCATTAAATCAAATA
AGATGAGCAAGATGGAACGTCTGGGTTCCAATTCTGTCAAGACTTATGACATATTAAATAGATTGGTT
ACGGTAAAAGA
+
FFF:FFFFFFFFFFFFFFFFFFFFFFFFFFFFFFFFFFF:FFFFFFFFFFFFFFFFFFFFFFF:FFFF
FFFFFFFFFFFFFFFFFFFFFFFFF:FFFF:FFFFF:FFFFFFFFFFFFFFFFFFFFFFFFFFFFFFF
FFFFFFFFFFF
@A00155:342:HHGFNDSXY:1:1528:32588:11584 1:N:0:GAACCTAG+TCCGCATA
ATTTAATGTTGTAGCTAGTTACATTAGTAAATATTCATATGTAGCATGTTTAAGCATTAAATCAAATA
AGATGAGCAAGATGGAACGTCTGGGTTCCAATTCTGTCAAGACTTATGACATATTAAATAGATTGGTT
ACGGTAAAAGA
+
FFFFFFFFFFFFFFFFFFFFFFFFFFFFFFFFFFFFFFFFFF,FFFFFFFFFFFFFFFFFFFFFFFFF
FFFFFFFFFFFFFFFFFFFFFFFFFFFFFFFFFFFFFFFFFFFFFFFFFFFFFFFFFFFFFFFFFFFF
FFFFFFFFFFF
@A00155:342:HHGFNDSXY:1:1517:27073:7983 1:N:0:GAACCTAG+TCCGCATA
ATTTAATGTTGTAGCTAGTTACATTAGTAAATATTCATATGTAGCATGTTTAAGCATTAAATCAAATA
AGATGAGCAAGATGGAACGTCTGGGTTCCAATTCTGTCAAGACTTATGACATATTAAATAGATTGGTT
ACGGTAAAAGA
+
FFFFFFFFFFFFFF:FFFFFFFFFFFFFFFFF:FFFFFFFFFFFFFFF,FFFFFFFFFFFFFFFFFFF
FFFFFFFFF:FFFFFFFFFFFFFFFFFFFFFFFFFFFFFFFFFFFFFFFFFFFFFFFFFFFFFFFFFF
FFFFFFFFFFF
@A00155:342:HHGFNDSXY:1:1517:27019:8015 1:N:0:GAACCTAG+TCCGCATA
ATTTAATGTTGTAGCTAGTTACATTAGTAAATATTCATATGTAGCATGTTTAAGCATTAAATCAAATA
AGATGAGCAAGATGGAACGTCTGGGTTCCAATTCTGTCAAGACTTATGACATATTAAATAGATTGGTT
ACGGTAAAAGA
+
FFFFFFFFFFFFFFFFFFFFFFFFFFFFFFFFFFFFFFFFFFFFFFF:FFFFFFFFFFFFFFFF:FFF
```

FFFFFFFFFFFFFFFFFFFFFFFFFFFFFFFFFFFFFFFFFFFFFFFFFFFFFFFFFFFFFFFFFFFF
FFFFFFFFFFF
@A00155:342:HHGFNDSXY:1:1670:17345:13119 1:N:0:GAACCTAG+TCCGCATA
ATTTAATGTTGTAGCTAGTTACATTAGTAAATATTCATATGTAGCATGTTTAAGCATTAAATCAAATA
AGATGAGCAAGATGGAACGTCTGGGTTCCAATTCTGTCAAGACTTATGACATATTAAATAGATTGGTT
ACGGTAAAAGA
+
FFFFFFFFFFFFFFFFFFFFFFFFFFFFFFFFFFFFFFFFFFFFFFFFFFFFFFFFFFFFFFFFFFFF
FFFFFFFFFFFFFFFFFFFFFFFFFFFFFFFFFFFFFFFFFFFFFFFFFFFFFFFFFFFFFF:FFFFF
FFFFFFFFFFF
@A00155:342:HHGFNDSXY:1:1675:22896:24612 1:N:0:GAACCTAG+TCCGCATA
ATTTAATGTTGTAGCTAGTTACATTAGTAAATATTCATATGTAGCATGTTTAAGCATTAAATCAAATA
AGATGAGCAAGATGGAACGTCTGGGTTCCAATTCTGTCAAGACTTATGACATATTAAATAGATTGGTT
ACGGTAAAAGA
+
FFFFFFFFF,FFFFFFFFFFFFFFFFFFFFFFFFFFFFFFFFFFFFFFFFFFFFFFFF:FFF,FFFFF
FFFFFFFFFFFFFFFFFFFF:FFFFFFF:FFFFFFFFFFFFFFFFFFFFFFFFFFFFFFFFFFFFFFF
FFFF:FFFFFF
@A00155:342:HHGFNDSXY:1:2152:15619:23594 1:N:0:GAACCTAG+TCCGCATA
ATTTAATGTTGTAGCTAGTTACATTAGTAAATATTCATATGTAGCATGTTTAAGCATTAAATCAAATA
AGATGAGCAAGATGGAACGTCTGGGTTCCAATTCTGTCAAGACTTATGACATATTAAATAGATTGGTT
ACGGTAAAAGA
+
FFFFFFFFFFFFFFFFFFFFFFFFFFFFFFFFFFFFFFFFFFFFFFFFFFFFFFFFFFFFFFFFFFFF
FFFFFFFFFFFFFFFFFFFFFFFFFFFFFFFFFFFFFFFFFFFFFFFFFFFFFFFFFFFFFFFFFFFF
FFFFFFFFFFF
@A00155:342:HHGFNDSXY:1:2243:32732:25426 1:N:0:GAACCTAG+TCCGCATA
ATTTAATGTTGTAGCTAGTTACATTAGTAAATATTCATATGTAGCATGTTTAAGCATTAAATCAAATA
AGATGAGCAAGATGGAACGTCTGGGTTCCAATTCTGTCAAGACTTATGACATATTAAATAGATTGGTT
ACGGTAAAAGA
+
FFFFFFFFFFFFFFFFFFFFFF:F:FFFFFFF:FFFFFFFFFFFFFFFFFFFF,FFFFFFFFFFFFFF
FFFFFFFFFFFFFFFFFFFFFFFFFFFFFFFFFFFFFFFFFFFFFFFFFFFFFFFFFFFFFFFFFFFF
FFFFFFFFFFF
@A00155:342:HHGFNDSXY:1:2318:18249:7701 1:N:0:GAACCTAG+TCCGCATA
ATTTAATGTTGTAGCTAGTTACATTAGTAAATATTCATATGTAGCATGTTTAAGCATTAAATCAAATA
AGATGAGCAAGATGGAACGTCTGGGTTCCAATTCTGTCAAGACTTATGACATATTAAATAGATTGGTT
ACGGTAAAAGA
+
FFFFFFFFFFFFFFFFFFF:FFFFFFFFFFFF:FF:FFFFFFFFFFFFFFFFFFFFFFFFFFFFFFFF
FFFFFFFFFFFFFFFFFFFFFFFFFFFFFFFFFFFFFFFFFFFFFFFFFFFFFFFFF:FFFFFFFFFF
FFFFFFFFFFF
@A00155:342:HHGFNDSXY:1:2420:30626:22905 1:N:0:GAACCTAG+TCCGCATA
TCTTAATGTTGTAGCTAGTTACATTAGTAAATATTCATATGTAGCATGTTTAAGCATTAAATCAAATA
AGATGAGCAAGATGGAACGTCTGGGTTCCAATTCTGTCAAGACTTATGACATATTAAATAGATTGGTT
ACGGTAAAAGA
+
FFFFF:FFFFFFFFFFF:FFFFFFFFFFFFFFFFFFFFFFF:FFFFFFFFFFFFFFFFFFFFF:FFFF
FFFFFFFFFFFFFFFFFFFFFFFFFFFFFFFFFFF:FFFFFFFFFFFFFFFFFFFFFFFFFFFFFF:F
FFFFFFFFFFF
@A00155:342:HHGFNDSXY:1:2405:11812:36511 1:N:0:GAACCTAG+TCCGCATA
TCTTAATGTTGTAGCTAGTTACATTAGTAAATATTCATATGTAGCATGTTTAAGCATTAAATCAAATA
AGATGAGCAAGATGGAACGTCTGGGTTCCAATTCTGTCAAGACTTATGACATATTAAATAGATTGGTT
ACGGTAAAAGA

+
FFFFF:FFFFFFFF:FFF:FFFFFFF:FFFFFFFFFFFFFF:FFFFFFFFFFFFFFFFFFFFF:FFFFFF
FFFFFFFFFFFFFFFFFFFFFFFFFFFFFFFFFFFFFFFFFFFFFFFFFFFFFFFFFFFFFFFFFFFFFF
FFFFFFFFFFF
@A00155:342:HHGFNDSXY:1:2438:4381:8046 1:N:0:GAACCTAG+TCCGCATA
TCTTAATGTTGTAGCTAGTTACATTAGTAAATATTCATATGTAGCATGTTTAAGCATTAAATCAAATA
AGATGAGCAAGATGGAACGTCTGGGTTCCAATTCTGTCAAGACTTATGACATATTAAATAGATTGGTT
ACGGTAAAAGA
+
F:FFFFFFFFFFFF,FFFFFF:FFFF,FFFFFFFFFFFFFFFFFFFFFFFFFF:FFFFFFFFFFFFFFFF
FFFFFFFFFFFFFFFFFFFFFFFFFFFFFFFFFFFFFFFFFFFFF:FFFFFFFFFFFFFFFFFFFFFFFF
FFFFFFFFFFF
@A00155:342:HHGFNDSXY:1:2436:32579:26005 1:N:0:GAACCTAG+TCCGCATA
ATTTAATGTTGTAGCTAGTTACATTAGTAAATATTCATATGTAGCATGTTTAAGCATTAAATCAAATA
AGATGAGCAAGATGGAACGTCTGGGTTCCAATTCTGTCAAGACTTATGACATATTAAATAGATTGGTT
ACGGTAAAAGA
+
FFFFFFFFFFFFFFFFFFFFF:FFFFFFFFFFFFFFFFFFFFFFF,FFFFFFF:FFFFFFFFFFFF:FFF
FFFFFFFFFFFFFF:FF:FFFFFFF,FFFFFFFFFFFFFFFFFFFFFFFFFFFFFFFFFFFFFFFFFF:F
FFFFFFFFFFF
@A00155:342:HHGFNDSXY:1:2478:2392:35070 1:N:0:GAACCTAG+TCCGCATA
TCTTAATGTTGTAGCTAGTTACATTAGTAAATATTCATATGTAGCATGTTTAAGCATTAAATCAAATA
AGATGAGCAAGATGGAACGTCTGGGTTCCAATTCTGTCAAGACTTATGACATATTAAATAGATTGGTT
ACGGTAAAAGA
+
:,FFFFFFF:FFF:FFFFFFFFFFFFFFFFFFFFFFFFFFFFFFFFFFFF:FFFFFFFFFFFFFFFFFFF
FFFFFFFFFFFFFFFFFFFFFFFFFFFFFFFF:FFFFFFFFFFFFFFFFFFFFFFFFFFFFFFFFFFFFF
FFFFFFFFFFF
@A00155:342:HHGFNDSXY:1:2551:21124:1501 1:N:0:GAACCTAG+TCCGCATA
ATTTAATGTTGTAGCTAGTTACATTAGTAAATATTCATATGTAGCATGTTTAAGCATTAAATCAAATA
AGATGAGCAAGATGGAACGTCTGGGTTCCAATTCTGTCAAGACTTATGACATATTAAATAGATTGGTT
ACGGTAAAAGA
+
FFFFFFFFFFFFFFFFFFFFFFFFFFFFFFFFFFFFFFFFFFFFFFFFFFFFFFFFFFFFFFFFFFFFFF
FFFFFFFFFFFFFFFFFFFFFFFFFFFFFFFFFFFFFFFFFFFFFFFFFFFFFFFFFFFFFFFFFFFFFF
FFFFFFFFFFF
@A00155:342:HHGFNDSXY:1:2551:28203:10817 1:N:0:GAACCTAG+TCCGCATA
ATTTAATGTTGTAGCTAGTTACATTAGTAAATATTCATATGTAGCATGTTTAAGCATTAAATCAAATA
AGATGAGCAAGATGGAACGTCTGGGTTCCAATTCTGTCAAGACTTATGACATATTAAATAGATTGGTT
ACGGTAAAAGA
+
FFF:FFFFFFFFFF:FFF:FFFFFFFFFFF:FFFFFFFFFFF:F,FFFFFFFFFFFFFFFFFFFFFFFFF
FFFFFFFFFFFFFFFFFFFFFFFFFFFFFFFFFFFFFFFFFFFFFFFFFFFFFFFFFFFFFFFFFFFFFF
FFFFFFFFFFF
@A00155:342:HHGFNDSXY:1:2575:15194:30123 1:N:0:GAACCTAG+TCCGCATA
ATTTAATGTTGTAGCTAGTTACATTAGTAAATATTCATATGTATCATGTTTAAGCATTAAATCAAATA
AGATGAGCAAGATGGAACGTCTGGGTTCCAATTCTGTCAAGTCTTATGACATATTAAATAGATTGGTT
ACGGTAAAAGA
+
F:FF,FFF:FFF,F,FFFFF:FFFFF:FFFFF:FF,FF,F:FF,FFFFFFFF,FFFFF:,FFFF,:F,
,FFFFF,:F,FF:,F,FFFFFFFFFFFF:,F:FF:FFFF,:F,,FFFFFF::FFFFF,:FF,FFFFFFF
FF:FF,FFFFF
@A00155:342:HHGFNDSXY:1:2662:23366:17347 1:N:0:GAACCTAG+TCCGCATA
ATTTAATGTTGTAGCTAGTTACATTAGTAAATATTCATATGTAGCATGTTTAAGCATTAAATCAAATA

AGATGAGCAAGATGGAACGTCTGGGTTCCAATTCTGTCAAGACTTATGACATATTAAATAGATTGGTT
ACGGTAAAAGA
+
FFFF:FFF:F:FFF:FF:F,F:FFFFFFFFFFFFFFFFFFFF:F,FFFFFFFF,FFF:::,F,FFFFF
FF,F,,FFFFFFFFFFF:FFFFFFF:FFFFFFFFFFFFFFFFFF,FFFF::F,,FF:FFF,FFFFFFF
:,FFFF,FFFF
@A00155:342:HHGFNDSXY:1:2667:13268:32612 1:N:0:GAACCTAG+TCCGCATA
ATTTAATGTTGTAGCTAGTTACATTAGTAAATATTCATATGTAGCATGTTTAAGCATTAAATCAAATA
AGATGAGCAAGATGGAACGTCTGGGTTCCAATTCTGTCAAGACTTATGACATATTAAATAGATTGGTT
ACGGTAAAAGA
+
FFFFF:FFFFFFFFFFFFFFFFFFFFFFFFFFFFFFFFFFFFF:FFFFFFFFFF,FFFFFFFFFFFFF
FFFFFFFFFFFFFFFFFFFFFFFFFFFF:FFFFFFFFFFFFFFFFFFFFFFFFFFFFFFFFFFFFFFF
FFFFFFFFFFF
@A00155:342:HHGFNDSXY:1:2141:4770:23062 2:N:0:GAACCTAG+TCCGCATA
ATTTAATGTTGTAGCTAGTTACATTAGTAAATATTCATATGTAGCATGTTTAAGCATTAAATCAAATA
AGATGAGCAAGATGGAACGTCTGGGTTCCAATTCTGTCAAGACTTATGACATATTAAATAGATTGGTT
ACGGTAAAAGACAG
+
FFFFFFFFFFFFFFFFFFFFFF:FFFFFFFFFFFFFFFFFFFFFFFFFFFFFF,FFFFFFFFFFFFFF
FFFFFFFFFFFFFFFFFFFFFFFFFFFFFFFFF:FFFFFFFF,FF:FFFFFFFFFFFFFFFFFFFF:F
FFFFFFFFFFF::F
@A00155:342:HHGFNDSXY:1:1177:27823:29387 2:N:0:GAACCTAG+TCCGCATA
ATTTAATGTTGTAGCTAGTTACATTAGTAAATATTCATATGTAGCATGTTTAAGCATTAAATCAAATA
AGATGAGCAAGATGGAACGTCTGGGTTCCAATTCTGTCAAGACTTATGACATATTAAATAGATTGGTT
ACGGTAAAAGACAAG
+
FF:FFFFFFFFFFFFFFFFFFFFFFFFFFFFFFFFFFFFFFFFFFFFFFFFFFFFFFFFFFFFFFFFF
FFFFFFFFFFFFFFFFFFFFFFF:FFFFFFFFFFFFFFFFFFFFFFFFFFFFFFFFFFFFFFFFFFFF
FFFFFFFFFFFFFFF
@A00155:342:HHGFNDSXY:1:1205:13105:1548 2:N:0:GAACCTAG+TCCGCATA
ATTTAATGTTGTAGCTAGTTACATTAGTAAATATTCATATGTAGCATGTTTAAGCATTAAATCAAATA
AGATGAGCAAGATGGAACGTCTGGGTTCCAATTCTGTCAAGACTTATGACATATTAAATAGATTGGTT
ACGGTAAAAGACAGA
+
FFFFF:FFFFFFFFFFFFFFFFFFFFFFFFFFFFFFFFFFFFFFFFFFFFFFFFFFFFFFFFFFFFFF
FFFFFFFFFFFFFFFF:FFFFFFFFFFFFFFFFFFFFFFFFFFFFFFFFFFFFFFFFFFFFFFFFFF
FFFFFFFF:F:FFFF
@A00155:342:HHGFNDSXY:1:1205:13051:2519 2:N:0:GAACCTAG+TCCGCATA
ATTTAATGTTGTAGCTAGTTACATTAGTAAATATTCATATGTAGCATGTTTAAGCATTAAATCAAATA
AGATGAGCAAGATGGAACGTCTGGGTTCCAATTCTGTCAAGACTTATGACATATTAAATAGATTGGTT
ACGGTAAAAGACAGA
+
FFFFFF,F:FFFFFFFFFFFFFFFFFFFFFFFFFFFFFFFFFFFFFFFFF:::FFFFFFFFFFFF:FF
FFFFFF::FFFFFFFFFFFFFFFFFFFFFFFFFFFFFFFFFFFFFFFFFFFFFFFF:FFFF,FF
FFFFFFF,FFFFFF:
@A00155:342:HHGFNDSXY:1:1205:13422:4131 2:N:0:GAACCTAG+TCCGCATA
ATTTAATGTTGTAGCTAGTTACATTAGTAAATATTCATATGTAGCATGTTTAAGCATTAAATCAAATA
AGATGAGCAAGATGGAACGTCTGGGTTCCAATTCTGTCAAGACTTATGACATATTAAATAGATTGGTT
ACGGTAAAAGACAGA
+
FFFFFFFFFFFFFFFFFFFFFFFFFFFFFFFFFFFFFFFFFFFFFFFFFFFFFFFFFFFFFFFFFFFF
FFFFFFFFFFFFFFFFFFFFFFFFFFFFFFFFFFFFFFFFFFFFFFFFFFFFFFFFFFFFFFFFFFFF
FFFFFFFFFFFFFFF

```
@A00155:342:HHGFNDSXY:1:1213:18611:33630 2:N:0:GAACCTAG+TCCGCATA
ATTTAATGTTGTAGCTAGTTACATTAGTAAATATTCATATGTAGCATGTTTAAGCATTAAATCAAATA
AGATGAGCAAGATGGAACGTCTGGGTTCCAATTCTGTCAAGACTTATGACATATTAAATAGATTGGTT
ACGGTAAAAGACAGA
+
FFFFFFFF,FFFFFF:FFFFFFFFFFFFFFFFFFFFFFFFFFFFFFFFFFFFFFFFFFFFFFFFF:FF
FFFFFFFFFFFFFF:FFF,FFFFF:FFFFFFFFFFFFFFF:FFFFFFFFFFFFFFFFFFFFFFFFFFF
FFF,FFF:FFFFFFF
@A00155:342:HHGFNDSXY:1:1357:19822:8453 2:N:0:GAACCTAG+TCCGCATA
ATTTAATGTTGTAGCTAGTTACATTAGTAAATATTCATATGTGGCATGTTTAAGCATTAAATCAAATA
AGATGAGCAAGATGGAACGTCTGGGTTCCAATTCTGTCAAGACTTATGACATATTAAATAGATTGGTT
ACGGTATAAGACAAG
+
FFFFFFFFFFF,FFFFFFFFFFFFFFFFFFFFFFFFFFFFFFFFFFFFFFFFFFFFFFFFFFFFFFFF
FFFFFFFFFFFFFFF:FFFFFFFFFFFFFFFFFFFFFFFFFFFFFFFFFFFFFFFFFFFFFFFFFFFF
,F:FFF,FFFFFFFF
@A00155:342:HHGFNDSXY:1:2101:16378:33959 2:N:0:GAACCTAG+TCCGCATA
ATTTAATGTTGTAGCTAGTTACATTAGTAAATATTCATATGTAGCATGTTTAAGCATTAAATCAAATA
AGATGAGCAAGATGGAACGTCTGGGTTCCAATTCTGTCAAGACTTATGACATATTAAATAGATTGGTT
ACGGTAAAAGACAAG
+
FFFF:FFFFFFFFFFFFFFFFFFFFFFFFFFFFFFFFFFFFFFFFFFFFFFFFFFFFF:FFF:FFFF
F,FFFFF:FFFFFF:F:FFFFFFFF:FFFFFFFFFFFFFFFFFFF,FFFFFFFFFFFFFFFFFFFFFF
FFFFFFFFFFFFFFF
@A00155:342:HHGFNDSXY:1:2133:28637:20149 2:N:0:GAACCTAG+TCCGCATA
ATTTAATGTTGTAGCTAGTTACATTAGTAAATATTCATATGTAGCATGTTTAAGCATTAAATCAAATA
AGATGAGCAAGATGGAACGTCTGGGTTCCAATTCTGTCAAGACTTATGACATATTAAATAGATTGGTT
ACGGTAAAAGACAAG
+
FFFFFFFFFFFFFFFFFFFFFFFFFFFFFFFFFFFFFFFFFFFFFFFFFFFFFFFFFFFFFFFFFFFF
FFFFFFFFFFFFFFFFFFFFFFFFFFFFFFFFFFFFFFFFFFFFFFFFFFFFFFF,FFFFFFFFFF:F
FFFFFFFFFFFFFFF
@A00155:342:HHGFNDSXY:1:2172:25238:18615 2:N:0:GAACCTAG+TCCGCATA
ATTTAATGTTGTAGCTAGTTACATTAGTAAATATTCATATGTAGCATGTTTAAGCATTAAATCAAATA
AGATGAGCAAGATGGAACGTCTGGGTTCCAATTCTGTCAAGACTTATGACATATTAAATAGATTGGTT
ACGGTAAAAGACAAG
+
FFFFFFFF:FF:FFF:FFFFFFFFFFFFFFFFFFFFF:FFF:FFFFFFF:FFF::FFFFFFFFFFF:F
FFFF:FFFFFFFFFFFFFF,FFFFFFFFFFFFFFFFFFFFFFFFFFFFFF:FFF:FFF,FFFFFFFFF
FFFFFFFFFFFFFFF
@A00155:342:HHGFNDSXY:1:2250:11270:15092 2:N:0:GAACCTAG+TCCGCATA
ATTTAATGTTGTAGCTAGTTACATTAGTAAATATTCATATGTGGCATGTTTAAGCATTAAATCAAATA
AGATGAGCAAGATGGAACGTCTGGGTTCCAATTCTGTCAAGACTTATGACATATTAAATAGATTGGTT
ACGGTATAAGACAAG
+
FFFFFFFFFFFFFFFFFFFFFFFFFFFFFFFFFFFFFFFFFFFFFFFFFFFFFFFFFFFF:FFFFFFF
FFFFFFFFFFFFFFFFFFFFFFFFFFFFFFFFFFFFFFFFFFFFFFFFFFFFFFFFFFFFFFFFFFFF
FFFFFFFFFFFFFFF
@A00155:342:HHGFNDSXY:1:2528:22544:27821 2:N:0:GAACCTAG+TCCGCATA
ATTTAATGTTGTAGCTAGTTACATTAGTAAATATTCATATGTAGCATGTTTAAGCATTAAATCAAATA
AGATGAGCAAGATGGAACGTCTGGGTTCCAATTCTGTCAAGACTTATGACATATTAAATAGATTGGTT
ACGGTAAAAGACAAG
+
FFFFFFFFFFFFFFFFFFFFFFFFFFFFFFFFFFFFFFFFFFFFFFFFFFFFFFFFFFFFFFFFF:FF
```

```
FFFFFFFFFFFFFFFFFFFFFFFFFFFFFFFFFFFFFFFFFFFFFFFFFFFFFFFFFFFF:F:FF:FF
FFFFFFFFFFFFFFF
@A00155:342:HHGFNDSXY:1:2535:19687:29230 2:N:0:GAACCTAG+TCCGCATA
ATTTAATGTTGTAGCTAGTTACATTAGTAAATATTCATATGTAGCATGTTTAAGCATTAAATCAAATA
AGATGAGCAAGATGGAACGTCTGGGTTCCAATTCTGTCAAGACTTATGACATATTAAATAGATTGGTT
ACGGTAAAAGACAAG
+
:FFFFFFFFFFFFFFFFFFFFFFFFFFFFFFFFFF:FFFFFFFFFFFFFFFFF:FFFFFFFFFFFFFF
FFFFFFF:FFFFFFFFFFF:FFFFFFFFFFFFF,FFFFFF:FFFF:F:FFFFFFFFFFF:::FF:FFF
F:::FFFFF,FFFFF
@A00155:342:HHGFNDSXY:1:1105:2103:14904 1:N:0:GAACCTAG+TCCGCATA
TTAATGTTGTAGCTAGTTACATTAGTAAATATTCATATGTAGCATGTTTAAGCATTAAATCAAATAAG
ATGAGCAAGATGGAACGTCTGGGTTCCAATTCTGTCAAGACTTATGACATATTAAATAGATTGGTTAC
GGTAAAAGACAAGGC
+
F:FFFFFF:FFFFFFFFFFFFFF:,FFFFFFFFFFFFF,FFFFFF,FFFFF,,FFFFFFFFFFFFFFF
FFFFFFF:FFFFFF:FFFFFF:,FFF,FFFFFF::FF:FFFFFFFFFFFFFFFFFFFFFFFFFFFFFF
FFFFFFFFFFFF:FF
@A00155:342:HHGFNDSXY:1:1236:28682:7827 1:N:0:GAACCTAG+TCCGCATA
TTAATGTTGTAGCTAGTTACATTAGTAAATATTCATATGTAGCATGTTTAAGCATTAAATCAAATAAG
ATGAGCAAGATGGAACGTCTGGGTTCCAATTCTGTCAAGACTTATGACATATTAAATAGATTGGTTAC
GGTAAAAGACAAGGC
+
FFFFFFFFFFFFFFFF:FFFFF,FFFFFFFFFFFFFFFFFFFFFFFFFFFFFFFFFFFFFFFFFFFFF
FFFFFFFFFFFFFFFFFFFFFFFFFFFFFFFFFFFFFFFFFFFFFFFFFFFFFFFFFFFFFFFFFFFF
FFFFFFFFFFFFFFF
@A00155:342:HHGFNDSXY:1:1230:5348:22467 1:N:0:GAACCTAG+TCCGCATA
TTAATGTTGTAGCTAGTTACATTAGTAAATATTCATATGTAGCATGTTTAAGCATTAAATCAAATAAG
ATGAGCAAGATGGAACGTCTGGGTTCCAATTCTGTCAAGACTTATGACATATTAAATAGATTGGTTAC
GGTAAAAGACAAGGC
+
FFF:FFFFFFFFFFFFFFFFF:FFFFFFFFFFFFFFFFFFFFFFFFFFFFFFFFFFFFFFFFFFFFFF
FFFFFFFFFFFFFFFFFFFFFFFFFFFFFFFFFFFF:FFFFFFFFFFFFFFFFFFFFFFFFFFFFFFF
FFFFFFFFFFFFFFF
@A00155:342:HHGFNDSXY:1:1329:6298:32941 1:N:0:GAACCTAG+TCCGCATA
TTAATGTTGTAGCTAGTTACATTAGTAAATATTCATATGTAGCATGTTTAAGCATTAAATCAAATAAG
ATGAGCAAGATGGAACGTCTGGGTTCCAATTCTGTCAAGACTTATGACATATTAAATAGATTGGTTAC
GGTAAAAGACAAGGC
+
FFFFFFFFFFFFFFFFFFFFFFFFFFFFFFFFFFFFFFFFFF:FFFFFFFFFFFFFFFFFFFFFFFFF
FFFFFFFFFFFFFFFFFFFFFFFFFFFFFFFFFFFF,:FFFFFFFFFFFFF:FFFFFFFFFFFFFFFF
FFFFFFFFFFFFFFF
@A00155:342:HHGFNDSXY:1:1535:31159:33599 1:N:0:GAACCTAG+TCCGCATA
TTAATGTTGTAGCTAGTTACATTAGTAAATATTCATATGTAGCTTGTTTAAGCATTAAATCAAATAAG
ATGAGCAAGATGGAACGTCTGGGTTCCAATTCTGTCAAGACTTATGACTTATTAAATAGATTGGTTAC
GGTATAAGACAAGGC
+
FF,,F,F:FF:F:FF:F:F,,FFF:F,F,FFFF,F:FF:::F:,:FFFFFF,FFFFFFFF,,F,FFFF
FF:FF:FFF,:,FF,:FF:FFF,FF,:,::FFFFF:FF:,FFF,FFF:,FF:F,FFFFFFFFFFF:,,
FFF:,,FFF:FFFF:
@A00155:342:HHGFNDSXY:1:1563:4038:9017 1:N:0:GAACCTAG+TCCGCATA
TTAATGTTGTAGCTAGTTACATTAGTAAATATTCATATGTAGCATGTTTAAGCATTAAATCAAATAAG
ATGAGCAAGATGGAACGTCTGGGTTCCAATTCTGTCAAGACTTATGACATATTAAATAGATTGGTTAC
GGTAAAAGACAAGGC
```

```
+
FFFF,FFFFFFFFF:FFFFFFF:FFFFFFFFFF:FFFFFF:FFFFF:FFFFFFFFFFFFFFFFFFFFF
FFFFFFFFFFFFFFFFFF:FFFFFFFFFFFF:FFFFFFFFFFFFF:FFFFFFFF:FFFFFFFFF:FFF
FFFFFFFFFFFFFFF
@A00155:342:HHGFNDSXY:1:2154:9824:22701 1:N:0:GAACCTAG+TCCGCATA
TTAATGTTGTAGCTAGTTACATTAGTAAATATTCATATGTAGCATGTTTAAGCATTAAATCAAATAAG
ATGAGCAAGATGGAACGTCTGGGTTCCAATTCTGTCAAGACTTATGACATATTAAATAGATTGGTTAC
GGTAAAAGACAAGGC
+
FFF,FFFFFFFFFFFFFFF:FFFFFFFFFFFF:FFFFFFFFF:FFF::FFFFFFFFFFFFFFFFFFFF
FFFFFFFFFFFFFFFFFFFFFFFFFFFFFFFFFFFFFFFFFFFFFFFFFFFFFFFFFFFFFFFFFFFF
FFFFFFFFFFFFFFF
@A00155:342:HHGFNDSXY:1:2164:6207:35321 1:N:0:GAACCTAG+TCCGCATA
TTAATGTTGTAGCTAGTTACATTAGTAAATATTCATATGTAGCATGTTTAAGCATTAAATCAAATAAG
ATGAGCAAGATGGAACGTCTGGGTTCCAATTCTGTCAAGACTTATGACATATTAAATAGATTGGTTAC
GGTAAAAGACAAGGC
+
FFFFFFFFFFFFFFFFFFFFFFFFFFFFFFFFF,FFFFFFFF:FFFFFFFFFFFFFFFFFFFFFFFFF
FFFFFFFFF:FFFFFFFFFFFFF:FFFFF,FFFFFFFFFFFFFFFFFFFFFFFFFFFFFFFFFFFFFF
FFFFF:FFFFFF:FF
@A00155:342:HHGFNDSXY:1:2172:28330:28291 1:N:0:GAACCTAG+TCCGCATA
TTAATGTTGTAGCTAGTTACATTAGTAAATATTCATATGTAGCATGTTTAAGCATTAAATCAAATAAG
ATGAGCAAGATGGAACGTCTGGGTTCCAATTCTGTCAAGACTTATGACATATTAAATAGATTGGTTAC
GGTAAAAGACAAGGC
+
FFFFFFFFFFF,FFFFFFFFFFFFFFFFFFFFFFFFFFFFFFFFFFFFFFFFFFFFFFFFFFFFFFFF
FFFFFFFFFFFFFFFF:FFFFFFFFFFFFFFFFFFFFFFFFFFFFFFFFFFFFFFFFFFFFFFFFFFF
FFFFFFFFFFFFFFF
@A00155:342:HHGFNDSXY:1:2528:22544:27821 1:N:0:GAACCTAG+TCCGCATA
TTAATGTTGTAGCTAGTTACATTAGTAAATATTCATATGTAGCATGTTTAAGCATTAAATCAAATAAG
ATGAGCAAGATGGAACGTCTGGGTTCCAATTCTGTCAAGACTTATGACATATTAAATAGATTGGTTAC
GGTAAAAGACAAGGC
+
:FFFFFFFFFFFFFFFFFFF:FFFFFFFFFFFFFFFFFFFFFFFFFFFFFFFFFFFFFFFFFFFFFFF
FFFFFFFFFFFFFFFFFFFFFFFFFFFFFFFFFFFFFFFFFFFFFFFFFFFFFFFFFFFFFFFFFFFF
FFFFFFFFFFFFFFF
@A00155:342:HHGFNDSXY:1:2645:32615:1579 1:N:0:GAACCTAG+TCCGCATA
TTAATGTTGTATCTAGTTACATTAGTAAATATTCATATGTAGCATGTTTAAGCATTAAATCAAATAAG
ATGAGCAAGATGGAACGTCTGGGTTCCAATTCTGTCAAGACTTATGACATATTAAATAGATTGGTTAC
GGTAAAAGACAAGGC
+
FFFFFFFFFFFFFFFFFFFFFFFFFFFFFF:FFFFFFFFF:FFFFFFFFFFFFFFFFFFFFFFFFFFF
FFFFFFFFFFFFFFFFFFFFFFFFFFFFFF:FFFFFFFFFFFFFFFFFFFFFFF:FFFFFFFFFFFFFF
FFFFFFFFFFF:F:F
@A00155:342:HHGFNDSXY:1:1476:6054:32831 2:N:0:GAACCTAG+TCCGCATA
TAATGTTGTAGCTAGTTACATTAGTAAATATTCATATGTAGCATGTTTAAGCATTAAATCAAATAAGA
TGAGCAAGATGGAACGTCTGGGTTCCAATTCTGTCAAGACTTATGACATATTAAATAGATTGGTTACG
GTAAAAGACAAGAGA
+
FFFFFFFFFFFFFFFFFFFFFFFFFFFFFFFFFFFFFFFFFFFFFFFFFFFFFFFFFFFFFFFFFFFF
FFFFFFFFFFFFFFFFFFFFFFFFFFFFFFFFFFFFFFFFFFFFFFFFFFFFFFFFFFFFFFFFFFFF
FFFFFFFFFFFFFFF
@A00155:342:HHGFNDSXY:1:2405:11812:36511 2:N:0:GAACCTAG+TCCGCATA
TAATGTTGTAGCTAGTTACATTAGTAAATATTCATATGTAGCATGTTTAAGCATTAAATCAAATAAGA
```

TGAGCAAGATGGAACGTCTGGGTTCCAATTCTGTCAAGACTTATGACATATTAAATAGATTGGTTACG
GTAAAAGACAAGAGA
+
FFFFFFFFFFFFFFFFFFFFFFFFFFFFFFFFFFF:FFFFFFFFFFFFFFFFFFFFFFFFFFFFFFFF
FFFFFFFFFFFFFFFFFFFFFFFFFFFFFFFFFFFFFFFFFFFFFFFFFFFFFFFFFFFFFFFFFFFF
:FFFFFFFFFFFFFF
@A00155:342:HHGFNDSXY:1:2420:30626:22905 2:N:0:GAACCTAG+TCCGCATA
TAATGTTGGAGCTAGTTACATTAGTAAATATTCATATGTAGCATGTTTAAGCATTAAATCAAATAAGA
TGAGCAAGATGGAACGTCTGGGTTCCAATTCTGTCAAGACTTATGACATATTAAATAGATTGGTTACG
GTAAAAGACAAGAGA
+
F:FFF:F:,FFFF:FFF,FF::FFFFFFFFFFFFFFFFFF:FFFFFFFFFFFF:FFFF:FFFF:FFFF
FFFFFFFFFFFFFFFF:FFFFFFFFFFFFFFFFFF:FFFFF:FFFFFFFFFFFFFFFFFFF,:FFFFF
:FF:FFFFFF:FF:F
@A00155:342:HHGFNDSXY:1:2438:4381:8046 2:N:0:GAACCTAG+TCCGCATA
TAATGTTGTAGCTAGTTACATTAGTAAATATTCATATGTAGCATGTTTAAGCATTAAATCAAATAAGA
TGAGCAAGATGGAACGTCTGGGTTCCAATTCTGTCAAGACTTATGACATATTAAATAGATTGGTTACG
GTAAAAGACAAGAGA
+
FFFFFFFFFFFFFFFFFFFFFFFFFFFFFFFFFFFFFFFFFFFFFFFFFFFFFFFFF:FFFFFFFFFF
FFFFFFFFFFFFFFFFFFFFFFFFFFFFFFFFFFFFFFFFFFFFFFFFFFFFF:FFFFFFFFFFFFFF
FFFFFFFFFFFFFFF
@A00155:342:HHGFNDSXY:1:2478:2392:35070 2:N:0:GAACCTAG+TCCGCATA
TAATGTTGTAGCTAGTTACATTAGTAAATATTCATATGTAGCATGTTTAAGCATTAAATCAAATAAGA
TGAGCAAGATGGAACGTCTGGGTTCCAATTCTGTCAAGACTTATGACATATTAAATAGATTGGTTACG
GTAAAAGACAAGAGA
+
FFFFFFFFFFFFFFFFFFFFFFFFFFFFFFFFFFFFFFFFFFFFFFFFF:FFFFFF::FF,FFFF:FF
FFFFFFFFFFFF:FF:FFFFFFFFFFFFFFFFFFFFFFF:FFFF,:FFFFFFF,:FFFFFFFFFFFFF
FFFF,:FF:FFFFFF
@A00155:342:HHGFNDSXY:1:1108:28863:32690 1:N:0:GAACCTAG+TCCGCATA
ATGTTGTAGCTAGTTACATTAGTAAATATTCATATGTAGCATGTTTAAGCATTAAATCAAATAAGATG
AGCAAGATGGAACGTCTGGTTTCCAATTCTGTCAAGACTTATGACATATTAAATAGATTGGTTACGGT
AAAAGACAAGG
+
FFFFFFFFFFFFFFFF::FFFFFFFFF:FFFFFFFFFFFFFF,,FFFFFFFFFFFFFFF:FFFFFFFF
FFFFFFFFFFFFFFFFF:FFFFFFFFFF:FFFFFFFFFFFFFFFFFFFFFF:FFF:FFFFFFFFFFFF
FFFFFFFFFFF
@A00155:342:HHGFNDSXY:1:1149:17418:24925 1:N:0:GAACCTAG+TCCGCATA
ATGTTGTAGCTAGTTACATTAGTAAATATTCATATGTAGCATGTTTAAGCATTAAATCAAATAAGATG
AGCAAGATGGAACGTCTGGGTTCCAATTCTGTCAAGACTTATGACATATTAAATAGATTGGTTACGGT
AAAAGACAAGG
+
FFFFFF:F:FFFFFFFFFFFFFFFFFFFFFFFFFFFFFFFFFFFFFFFFFFFFFFFFFFFFFFFFFFF
FFFF,FFFFFFFFFFFFFFFFFFFFFFFFFFFFFFFFFFFFFFFFFFFFFFFFFFFFFFFFFFFFFFF
FFFFFFFFFFF
@A00155:342:HHGFNDSXY:1:1155:22254:34084 1:N:0:GAACCTAG+TCCGCATA
ATGTTGTAGCTAGTTACATTAGTAAATATTCATATGTAGCATGTTTAAGCATTAAATCAAATAAGATG
AGCAAGATGGAACGTCTGGGTTCCAATTCTGTCAAGACTTATGACATATTAAATAGATTGGTTACGGT
AAAAGACAAGG
+
FFFFFFFFFFFFFFFFFFFFFFFFFFFFFFFFFFFF:FFFFFFFFFFFF:FFFFFFFFFFFFFFFFFF
FFFFFFFFFFFFFFFFFFFFFFFFFFFFFFFFFFFFFFFFFF:FFFFFFFFFFFFFFFFFFFFFFFFF
FFFFFFFFFFF

```
@A00155:342:HHGFNDSXY:1:1454:8160:14904 1:N:0:GAACCTAG+TCCGCATA
ATGTTGTAGCTAGTTACATTAGTAAATATTCATATGTAGCATGTTTAAGCATTAAATCAAATAAGATG
AGCAAGATGGAACGTCTGGGTTCCAATTCTGTCAAGACTTATGACATATTAAATAGATTGGTTACGGT
AAAAGACAAGG
+
FFFFFFFFFFFFFFFFFFFFFFFFFFFFFFFFFFFFFFFFF:FFFFFFFFFFFFFFFFFFFFFFFFFF
FFFFFFFFFFFFFFFFFFFFFFFFFFFFFFFFFFFFFFFFFFFFFFFFFFFF,FFFFFFFFFFFFFFF
FFFFFFFFFFF
@A00155:342:HHGFNDSXY:1:1564:30798:31250 1:N:0:GAACCTAG+TCCGCATA
ATGTTGTAGCTAGTTACATTAGTAAATATTCATATGTAGCATGTTTAAGCATTAAATCAAATAAGATG
AGCAAGATGGAACGTCTGGGTTCCAATTCTGTCAAGACTTATGACATATTAAATAGATTGGTTACGGT
AAAAGACAAGG
+
FFFFFFFFFFFFFFFFFFFFFFFFFFFFFFFFFFFFFFFFFF:FFFFFFFFFFFFFFFFFFFFFFFFF
FFFFFFFFFFFFFFFFFFFFFFFFFFFF,FFFFFFFFFFFFFFFFFFFFFFFFFFFFFFFFFFFFFFF
FFFFFFFFFFF
@A00155:342:HHGFNDSXY:1:1605:17047:35712 1:N:0:GAACCTAG+TCCGCATA
CCGATCTAGCTAGTTACATTAGTAAATATTCATATGTAGCATGTTTAAGCATTAAATCAAATAAGATG
AGCAAGATGGAACGTCTGGGTTCCAATTCTGTCAAGACTTATGACATATTAAATAGATTGGTTACGGT
AAAAGACAAGG
+
::FFFFFFF:F:F:FFFFFFFFFFFFFFFFFFFFF:FFFF:FF:FFFFFFFFFFFFFFFFFFFFFFFF
FFFFFFFFFFFFFF:FFFFFFFFFFF,FFFFFFFFFFFFFF:FFFFFFFF:FFFFFFFFFF:FFFFFF
FFFFF:FFFFF
@A00155:342:HHGFNDSXY:1:1605:17110:35853 1:N:0:GAACCTAG+TCCGCATA
CCGATCTAGCTAGTTACATTAGTAAATATTCATATGTAGCATGTTTAAGCATTAAATCAAATAAGATG
AGCAAGATGGAACGTCTGGGTTCCAATTCTGTCAAGACTTATGACATATTAAATAGATTGGTTACGGT
AAAAGACAAGG
+
F:FFFFFFFFFFFFFFFFFFFFFFFFFFFFFFFFFFFFFFFFFFFFFFFFFFFFFFFFFFFFFFFFFF
FFFFFFFFFFFFFFFFFFFFFFFFFFFFFFFFFFFFFFFFFFFFFFFFFFFFFFFFFFFFFFFFFFFF
FFFFFFFFFFF
@A00155:342:HHGFNDSXY:1:2101:16378:33959 1:N:0:GAACCTAG+TCCGCATA
ATGTTGTAGCTAGTTACATTAGTAAATATTCATATGTAGCATGTTTAAGCATTAAATCAAATAAGATG
AGCAAGATGGAACGTCTGGGTTCCAATTCTGTCAAGACTTATGACATATTAAATAGATTGGTTACGGT
AAAAGACAAGG
+
FFF:FFFFFFFFFFFFFFFFFFFFFFFFFFFFFFFFFFFFFFFFFFFFFFFFFFFFFFFFFF:FFFFF
FFFFFFFFFFFFFFFFFFFFFFFFFFFFFFFFFFFFFFFFFFFFFFFFFFFFFFFFFFFFFFFFFFFF
FFFFFFFFFFF
@A00155:342:HHGFNDSXY:1:2209:14208:11725 1:N:0:GAACCTAG+TCCGCATA
ATGTTGTAGCTAGTTACATTAGTAAATATTCATATGTAGCATGTTTAAGCATTAAATCAAATAAGATG
AGCAAGATGGAACGTCTGGGTTCCAATTCTGTCAAGACTTATGACATATTAAATAGATTGGTTACGGT
AAAAGACAAGG
+
FFFFFFFFFFFFFFFFFFFFFFFFFFFFFF:FFFFFFF:FFFFFFFFFFFFFFFFFFFFFFFFFFFFF
FFFFFFFFFFFFFFFFFFFFFFFFFFFFFFFFFFFFFFFFFFFFFFFFFFFFFFFFFFFFFFFFFFFF
FFFFFFFFF:F
@A00155:342:HHGFNDSXY:1:2622:21079:28166 1:N:0:GAACCTAG+TCCGCATA
ATGTTGTAGCTAGTTACATTAGTAAATATTCATATGTAGCATGTTTAAGCATTAAATCAAATAAGATG
AGCAAGATGGAACGTCTGGGTTCCAATTCTGTCAAGACTTATGACATATTAAATAGATTGGTTACGGT
AAAAGACAAGG
+
FFFFFFFFFFFFFFFFFFFFFFFFFFFFFFFFFFFFFFFFFFFF,FFFFFFFFFFFFFFFFFFFFFFF
```

FFFFFFFFFFFFFFFFFFFFFFFFFFFFFFFFFFFFFFFFFFFFFFFFFFFFFFFFFFFFFFFFFFFFFFFFFFFFFF
FFFFFFFFFFF
@A00155:342:HHGFNDSXY:1:2617:11026:2581 2:N:0:GAACCTAG+TCCGCATA
ATGTTGTAGCTAGTTACATTAGTAAATATTCATATGTAGCATGTTTAAGCATTAAATCAAATAAGATG
AGCAAGATGGAACGTCTGGGTTCCAATTCTGTCAAGACTTATGACATATTAAATAGATTGGTTACGGT
AAAAGACAAGG
+
FFFFFF,FFFFFFFFFFFFF::F:FFF:FFFFFFFFFF:FFF:FFFFFFFFFFFFFFFFFFF:F::F:
F:FFFFFF:FFF,FF:FFFF,FFFFFFFFFFFFFFF:FFF:FFFFFFFFFFFFFFFFFFFFFFFFFFFF
FFFFFFFF,FF
@A00155:342:HHGFNDSXY:1:1503:17418:5321 2:N:0:GAACCTAG+TCCGCATA
ATGTTGTAGCTAGTTACATTAGTAAATATTCATATGTAGCATGTTTAAGCATTAAATCAAATAAGATG
AGCAAGATGGAACGTCTGGGTTCCAATTCTGTCAAGACTTATGACATATTAAATAGATTGGTTACGGT
AAAAGACAAGGCAAG
+
FFFFFFFFFFFFFFFFFFFFFFFFFFFFFFFFFFFFFFFFFFFFFFFFFFFFFFFFFFFFFFFFFFFF:
,FFFFFFFFFFFFFFFFFFFFFFFFF:FFFFFFFFFFFFFF:FFFFFFFFFF:FFFFFFFFFFFFFF,
FFF:FFFFFFFFFFF
@A00155:342:HHGFNDSXY:1:1149:11198:28056 1:N:0:GAACCTAG+TCCGCATA
TGTTGTAGCTAGTTACATTAGTAAATATTCATATGTAGCATGTTTAAGCATTAAATCAAATAAGATGA
GCAAGATGGAACGTCTGGGTTCCAATTCTGTCAAGACTTATGACATATTAAATAGATTGGTTACGGTA
AAAGACAAGGCAAGG
+
FFFFFFFFFFFFFFFFFFFFFFFFFFFFFFFFFFFFFFFFFFFFFFFFFFFFFFFFFFFFFFFFFFFFF
FFFFFFFFFF:FFFFFFFFFFFFFFFFFFFFFFFFFFFFFFFFFFFFFFFFFFFFFFFFFFFFFFFFFF
FFFFFFFFFFFFFFF
@A00155:342:HHGFNDSXY:1:1436:9471:16830 1:N:0:GAACCTAG+TCCGCATA
TGTTGTAGCTAGTTACATTAGTAAATATTCATATGTAGCATGTTTAAGCATTAAATCAAATAAGATGA
GCAAGATGGAACGTCTGGGTTCCAATTCTGTCAAGACTTATGACATATTAAATAGATTGGTTACGGTA
AAAGACAAGGCAAGG
+
FFFFFFFFFFFFFFFFFFFFFFFFFFFFFFFFFFFFFFFFFFFFFFFFFFFFFFFFFFFFFFFFFFFFF
FFFFFFFFFFFFFFFFFFFFFFFFFFFFFFFFFFFFFFFFFFFFFFFFFFFFFFFFFFFFFFFFFFFFF
FFFFFFFFFFFFFFF
@A00155:342:HHGFNDSXY:1:1346:3188:31187 2:N:0:GAACCTAG+TCCGCATA
GTTGTAGCTAGTTACATTAGTAAATATTCATATGTAGCATGTTTAAGCATTAAATCAAATAAGATGAG
CAAGATGGAACGTCTGGGTTCCAATTCTGTCAAGACTTATGACATATTAAATAGATTGGTTACGGTAA
AAGACAAGGCAAGGT
+
::FFFFFFFFFFFFFFFFFFFFFFFFFFFFFFFFFFFFFFFFFFFFFFFFFFFFFFFFFFFFF:FFFFF
FFF:FFFFFF:FFFFFFFFFFFFFFF:FFFFFF,FFFFFFFFFFFFFFFFFFF:FFFFFFFFFFFFFF
FF,F:FF::,FF,F:
@A00155:342:HHGFNDSXY:1:1435:8657:15389 2:N:0:GAACCTAG+TCCGCATA
GTTGTAGCTAGTTACATTAGTAAATATTCATATGTAGCATGTTTAAGCATTAAATCAAATAAGATGAG
CAAGATGGAACGTCTGGGTTCCAATTCTGTCAAGACTTATGACATATTAAATAGATTGGTTACGGTAA
AAGACAAGGCAAGGT
+
F:FFFFFFFFFFFFFFFFFFFFFFFFFFFFFFFFFFFFFFFFFFFFFFFFFFFFF,FFFFFFFFFFFFF
FFFFFFFFFFFFFFFFFFFFFFFFFFFFFFFFFFFFFFFFFFFFFFFFFFFFFFFFFFFFFFFFFFFFF
FFFFFFFFFFF:FFF
@A00155:342:HHGFNDSXY:1:1458:22598:33270 2:N:0:GAACCTAG+TCCGCATA
GTTGTAGCTAGTTACATTAGTAAATATTCATATGTAGCATGTTTAAGCATTAAATCAAATAAGATGAG
CAAGATGGAACGTCTGGGTTCCAATTCTGTCAAGACTTATGACATATTAAATAGATTGGTTACGGTAA
AAGACAAGGCAAGGT

+
FFFFFFFFFFFFFFFFFF:FFFFFF:FFFFFF:FFFFFFFFFFFFFFFFFFFFFFFFFFFFFFFFFFFFF
FFFFFFFFFFFFFFFFFFFFFFFFFFFFFFFFFFFFFFFFFFFFFFFFFFFFFFFFFFFFFFFFFF:FFF
F:FFFFFFFFF:FFF
@A00155:342:HHGFNDSXY:1:1502:12897:7232 2:N:0:GAACCTAG+TCCGCATA
GTTGTAGCTAGTTACATTAGTAAATATTCATATGTAGCATGTTTAAGCATTAAATCAAATAAGATGAG
CAAGATGGAACGTCTGGGTTCCAATTCTGTCAAGACTTATGACATATTAAATAGATTGGTTACGGTAA
AAGACAAGGCAAGGT
+
FFFFFFFFFFFFFFFFFFFFFFFFFFFFFFFFFFFFFFFFFFFFFFFFFFFFFFFFFFFFFFFFFFFFFF
FFFFFFFFFFFFFFFFFFFFFFFFFFFFFFFFFFFFFFFFFFFFFFFFFFFFFFFFFFFFFFFFFFFFFF
F:FFFFFFFFFFFFF
@A00155:342:HHGFNDSXY:1:1545:10936:9157 2:N:0:GAACCTAG+TCCGCATA
GTTGTAGCTAGTTACATTAGTAAATATTCATATGTAGCATGTTTAAGCATTAAATCAAATAAGATGAG
CAAGATGGAACGTCTGGGTTCCAATTCTGTCAAGACTTATGACATATTAAATAGATTGGTTACGGTAA
AAGACAAGGCAAGGT
+
FFFFFFFFFFFFFFFFFFFFFFFFFFFFFFFFFFFFFFFFFFFFFFF:FFFFFFF:FFFF:FFFFFFFFF
FF:FFFFFF:FFFFFFFFFFFFFFFFFFFFFFFFFFFFFFFFFFFFFFFFFFFFFFFFFFFFFFFFFFFF
FFFFFFFF:FFFFFF
@A00155:342:HHGFNDSXY:1:1545:11026:9909 2:N:0:GAACCTAG+TCCGCATA
GTTGTAGCTAGTTACATTAGTAAATATTCATATGTAGCATGTTTAAGCATTAAATCAAATAAGATGAG
CAAGATGGAACGTCTGGGTTCCAATTCTGTCAAGACTTATGACATATTAAATAGATTGGTTACGGTAA
AAGACAAGGCAAGGT
+
FFFFFFFFFFFFF:FFFFFFFFFFFFFFFFFFFFFFFFFFFFFFFFFFFFFFFFF:FFFFFFFFFFFFFF
FFFFFFFFFFFFFFFF:FF:FFFFFFFFFFFFFFFFFFFFFFFFFFFF:FFFFFFFFFF:FFFFFFFFF
FF,FFFF::FFFF,F
@A00155:342:HHGFNDSXY:1:1577:9362:17863 2:N:0:GAACCTAG+TCCGCATA
GTTGTAGCTAGTTACATTAGTAAATATTCATATGTAGCATGTTTAAGCATTAAATCAAATAAGATGAG
CAAGATGGAACGTCTGGGTTCCAATTCTGTCAAGACTTATGACATATTAAATAGATTGGTTACGGTAA
AAGACAAGGCAAGGT
+
FFFFF:FFFFFFFFFFFFFFFFFFFFFFFFFFFFFFFFFFF:FFFFFFFFF:::FFFFFFFFF:FFFF
FFFFFFFFFFFFFFFFFFFFFFFFFFFFFFFFFFFFFFFFFFFFFFFFF:FF:FFFFFFFFFFFFF:FFFF
,FF:FFFFFFFFFFF
@A00155:342:HHGFNDSXY:1:2371:31168:8375 2:N:0:GAACCTAG+TCCGCATA
GTTGTAGCTAGTTACATTAGTAAATATTCATATGTAGCATGTTTAAGCATTAAATCAAATAAGATGAG
CAAGATGGAACGTCTGGGTTCCAATTCTGTCAAGACTTATGACATATTAAATAGATTGGTTACGGTAA
AAGACAAGGCAAGGT
+
FFFFFFFFFFFFFFFFFFFFFFFFFFFFFFFFFFFFFFFFFFFFFFFFFFFFFFFFFFFFFFFFFFFFFF
FFFFFFFFFFFFFFFFFFFFFFFFFFFFFFFFFFFFFFFFFFFFFFFFFFFFFFFFFFFFFFFFFFFFFF
FFFFFFFF:FF,FFF
@A00155:342:HHGFNDSXY:1:2538:21549:6903 2:N:0:GAACCTAG+TCCGCATA
GTTGTAGCTAGTTACATTAGTAAATATTCATATGTAGCATGTTTAAGCATTAAATCAAATAAGATGAG
CAAGATGGAACGTCTGGGTTCCAATTCTGTCAAGACTTATGACATATTAAATAGATTGGTTACGGTAA
AAGACAAGGCAAGGT
+
FFFFFFFFFFFFFFFFFFFFFFFFFFFFFFFFFFFFFFFFFFFFFFFFFFFFFFFFFFFFFFFFFFFFFF
FFFFFFFFFFFFFFFFFFFFFFFFFFFFFFFFFFFFFFFFFFFFFFFFFFFFFFFFFFF:FFFF,FFF::
FF:FFFFFFFF,F:F
@A00155:342:HHGFNDSXY:1:2556:9263:23954 2:N:0:GAACCTAG+TCCGCATA
GTTGTAGCTAGTTACATTAGTAAATATTCATATGTAGCATGTTTAAGCATTAAATCAAATAAGATGAG

CAAGATGGAACGTCTGGGTTCCAATTCTGTCAAGACTTATGACATATTAAATAGATTGGTTACGGTAA
AAGACAAGGCAAGGT
+
FFFFFFFFFFFFFFFFFFFFFFFFFFFFFFFFFFFFFFFF:FFFFFFFFFFFFFFFFFFFFF:FFFF:F
FFFFFFFFF:FFFFFFFFFFFFFFFFFFF:FFFFFFFFF:FFFFFFFFFF:FFFFFFFFFFF:FFFF,:
F:FFFFFFFFF:FFFF
@A00155:342:HHGFNDSXY:1:2633:4797:20134 2:N:0:GAACCTAG+TCCGCATA
GTTGTAGCTAGTTACATTAGTAAATATTCATATGTAGCATGTTTAAGCATTAAATCAAATAAGATGAG
CAAGATGGAACGTCTGGGTTCCAATTCTGTCAAGACTTATGACATATTAAATAGATTGGTTACGGTAA
AAGACAAGGCAAGTT
+
FFFFFFFFFFFFFFFFFFFFFFFFFFFFFFFFFFFFFFFFF:FFFF:FFFFFFFFFFFFFFFFFFFFFF
FFFFFFFFFFFFF:FFFFFFFFFFFFFFFFFFFFFFFFFFFFFFF:FFFFFF,FFFFFFF:FFFFF:FFF:
FFFFFFFFFFFFFF,F
@A00155:342:HHGFNDSXY:1:1605:18231:28933 2:N:0:GAACCTAG+TCCGCATA
GATCTGCTAGTTACATTAGTAAATATTCATATGTAGCATGTTTAAGCATTAAATCAAATAAGATGAGC
AAGATGGAACGTCTGGGTTCCAATTCTGTCAAGACTTATGACATATTAAATAGATTGGTTACGGTAAA
AGACAAGGCAAGGTA
+
F:FFF:FFFFFFFFFFF:FFFFFFFFFFFFFFFFFFFFFFFFFFFFFFFFFFFFFFFFFFFFFF:FFFFF
FFFFFFFFFFFFFFFFFF:FFFFFFFFFFFFFFFFFFFFFFFFFFFFFFFFFFFFFFFFFFFFFFFFFF
FFFFFFFFFFFFFFFF
@A00155:342:HHGFNDSXY:1:1324:18738:12336 2:N:0:GAACCTAG+TCCGCATA
TGTAGCTAGTTACATTAGTAAATATTCATATGTAGCATGTTTAAGCATTAAATCAAATAAGATGAGCA
AGATGGAACGTCTGGGTTCCAATTCTGTCAAGACTTATGACATATTAAATAGATTGGTTACGGTAAAA
GACAAGGCAAGGTAT
+
FFFFFFFFFFFFFF,FFFFFFFFFFFFFFFFFFFFFF:FFFFFFFFF:FFFF:FFFFFFFFFFFFF:FF
FF:FFFFFFFFFFFFFF:FFFFFFFFFFFFFFFFFFFFFFFFFFFFFFFFFFFFF:F:FFFFFFFFFFFFFF
F:FFFFFFF:FFFF:
@A00155:342:HHGFNDSXY:1:2136:3595:35994 2:N:0:GAACCTAG+TCCGCATA
TGTAGCTAGTTACATTAGTAAATATTCATATGTAGCATGTTTAAGCATTAAATCAAATAAGATGAGCA
AGATGGAACGTCTGGGTTCCAATTCTGTCAAGACTTATGACATATTAAATAGATTGGTTACGGTAAAA
GACAAGGCAAGGTAT
+
FFFFFFFFFFFF:FFFFFFFFFF:FF:F:,FFFFF:FFFFFFFF:FFFFFFFFFFFFFFFFFFFFFFFF
FFF:FFFFFF:F,F:FFF:FF,FFFFFFFFFFFFFFFFFFFFFFF:FFFFFFF:FFFFFFFFFFFFFFF
FFFFFFF:F:FFFFF
@A00155:342:HHGFNDSXY:1:2174:32624:7169 2:N:0:GAACCTAG+TCCGCATA
TGTAGCTAGTTACATTAGTAAATATTCATATGTAGCATGTTTAAGCATTAAATCAAATAAGATGAGCA
AGATGGAACGTCTGGGTTCCAATTCTGTCAAGACTTATGACATATTAAATAGATTGGTTACGGTAAAA
GACAAGGCAAGGTAT
+
FFFFFFFFFFFFFFFFFFFFFFFFFFFFFFFFFFFFFFFFFFFFFFFFFFFFFFFFFFFFFFFFFFFFF
FFFFFFFFFFFFFFFFFFFFFFFFFFFFFFFFFFFF:FFFFFFFFFFFFFFFFFFFFFFFFFFFFFFFFF
FFFFFFFF::FFF::
@A00155:342:HHGFNDSXY:1:2258:19117:27899 1:N:0:GAACCTAG+TCCGCATA
CCGATCTGTTACATTAGTAAATATTCATATGTAGCATGTTTAAGCATTAAATCAAATAAGATGAGCAA
GATGGAACGTCTGGGTTCCAATTCTGTCAAGACTTATGACATATTAAATAGATTGGTTACGGTAAAAG
ACAAGGCAAGGTATG
+
FFFFFFFFFFFFFFFFFFFFFFFFFFFFFFFFFFFFFFFF:FFFFFFFFFFFFFFFFFFFFFFFFFFFFF
FFFFFFFFFFFFFFFFFFFFFFFFFFFFFFFFFFFFFFFFFF:FFFFFFFFFFFFFFFFFFFFFFFFFFF
FFFFFFFFFFFFFFF

@A00155:342:HHGFNDSXY:1:2446:24035:7482 1:N:0:GAACCTAG+TCCGCATA
GTAGCTAGTTACATTAGTAAATATTCATATGTAGCATGTTTAAGCATTAAATCAAATAAGATGAGCAA
GATGGAACGTCTGGGTTCCAATTCTGTCAAGACTTATGACATATTAAATAGATTGGTTACGGTAAAAG
ACAAGGCAAGGTATG
+
FFFFFFFFFFF:FFFFFFFFFFFFFFFFFFFFFFFFFFFFFFFFFFFFFFFFFFFFFFFFFFFFFFFF
FFFFFFFFFFFFFFFFFFFFFFFFFFFFFFFFFFFFFFFFFFFFFFFFFFFFFFFFFFFFFFFFFFFF
FFFFFFFF:FFFFFF
@A00155:342:HHGFNDSXY:1:1611:30626:5744 2:N:0:GAACCTAG+TCCGCATA
GTAGCTAGTTACATTAGTAAATATTCATATGTAGCATGTTTAAGCATTAAATCAAATAAGATGAGCAA
GATGGAACGTCTGGGTTCCAATTCTGTCAAGACTTATGACATATTAAATAGATTGGTTACGGTAAAAG
ACAAGGCAAGGTATG
+
FFFFFFFFFFFFFFFFFFFFFFFFFFFFFFFFFFFFFFFFFFFFFFFFFFFFFFFFFFFFFFFFFFFF
F:FFFFFFFFFFFFFFFFFFFFFFFFFFFFFFFFFFFFFFFFFFFFFFFFFFFFFFFFFFFFFFFFFF
FFFFFFFFFFFFFFF
@A00155:342:HHGFNDSXY:1:1611:30011:6057 2:N:0:GAACCTAG+TCCGCATA
GTAGCTAGTTACATTAGTAAATATTCATATGTAGCATGTTTAAGCATTAAATCAAATAAGATGAGCAA
GATGGAACGTCTGGGTTCCAATTCTGTCAAGACTTATGACATATTAAATAGATTGGTTACGGTAAAAG
ACAAGGCAAGGTATG
+
FFFFFFFFFFFFFFFFFFFFFFFFFFFFFFFFFFFFFFFFFFFFFFFFFFFFFFF:FFFFFFFFFFFF
F:FFFFF:FFFFFFFFFFF:FFFFFFFFF:FFFFFFFFF:FFFFFFFFFFFFFFFFFFFFFF:FFFFF
FFFFFFFFFFFFFFF
@A00155:342:HHGFNDSXY:1:2223:1597:13902 2:N:0:GAACCTAG+TCCGCATA
GTAGCTAGTTACATTAGTAAATATTCATATGTAGCATGTTTAAGCATTAAATCAAATAAGATGAGCAA
GATGGAACGTCTGGGTTCCAATTCTGTCAAGACTTATGACATATTAAATAGATTGGTTACGGTAAAAG
ACAAGGCAAGGTATG
+
FFFFFFFFFFFFFFFFFFFFFFFFFFFFFFFFFFFFFFFFFFFFFFFFFFFFFFFFFFFFFFFFFFFF
FFFFFF:FFFFFFFFFFFFFFFFFFFFFFFFFFFFFFFFFFFFFFFFFFFFFFFFFFFFFFF:FFFF
FFFFF:FFFFFFFF:
@A00155:342:HHGFNDSXY:1:2222:4472:21981 2:N:0:GAACCTAG+TCCGCATA
GTAGCTAGTTACATTAGTAAATATTCATATGTAGCATGTTTAAGCATTAAATCAAATAAGATGAGCAA
GATGGAACGTCTGGGTTCCAATTCTGTCAAGACTTATGACATATTAAATAGATTGGTTACGGTAAAAG
ACAAGGCAATGTATG
+
FFFFFF:FFFFFFFFFFFFFFFFFFFFFFFFFFFFFFFFFFFFFFFFFFFFFFFFFFFFFF:FFFFF
FFF:FFFFFFFFFFFFF:FFF:F:FFFFFF:FF,FF:FFFFFFF:FFFFF,FFF,,,FFF,:F:FFF:
FFFF:::FF,,::::
@A00155:342:HHGFNDSXY:1:2248:23258:11240 2:N:0:GAACCTAG+TCCGCATA
GTAGCTAGTTACATTAGTAAATATTCATATGTAGCATGTTTAAGCATTAAATCAAATAAGATGAGCAA
GATGGAACGTCTGGGTTCCAATTCTGTCAAGACTTATGACATATTAAATAGATTTGTTACGGTAAAAG
ACAAGGCAAGGTATG
+
FFFFFFFFFFFFFFFFFFFFFFFFFFFFFFFFFFFFFFFFFFFFFFFFFFFFFFFFFFFFFFFFFFFF
:FFFFFFFFFFFFFFFFFFFFFFFFFFFFFFFFFFFFFFFFFFF:FFFFFFFFFFFFFFFF:FFFFFF
FFFFF,FFFF:FFFF
@A00155:342:HHGFNDSXY:1:2267:25898:31782 2:N:0:GAACCTAG+TCCGCATA
GTAGCTAGTTACATTAGTAAATATTCATATGTAGCATGTTTAAGCATTAAATCAAATAAGATGAGCAA
GATGGAACGTCTGGGTTCCAATTCTGTCAAGACTTATGACATATTAATTAGATTGGTTACGGTAAAAG
ACAAGGCAAGGTATG
+
FFFFFFFFFFFFFFFFFFFFFFFFFFFFFFFFFFFFFFFFFFFFFFFFFFFFFFFFFFFF:FFFFFFF

FFF:FFFFFFFFFFFFFFFFFFFFFFFFFFFFFFFFFFFFFFFFFF,FFFFFFFFFFF:FFFFFFFF
FFFFFFFFFFFFFFF
@A00155:342:HHGFNDSXY:1:2647:18982:2112 2:N:0:GAACCTAG+TCCGCATA
GTAGCTAGTTACATTAGTAAATATTCATATGTAGCATGTTTAAGCATTAAATCAAATAAGATGAGCAA
GATGGAACGTCTGGGTTCCAATTCTGTCAAGACTTATGACATATTAAATAGATTGGTTACGGTAAAAG
ACAAGGCAAGGTATG
+
FFFFFFFFFFFFFFFFFFFFFFFFFFFFFFFFFFFFFFFFFFFFFFFFFFFFFFFFFFFFFFFFFFFF
FFFFFFFFFFF,FFFFFFFFFFFFFFFFFFFFFFFFFFFFF:FFFFFFFFFFFFFFF:FFFFFFF:FF
FFFFF:FFFFF:F:F
@A00155:342:HHGFNDSXY:1:1229:27362:30780 1:N:0:GAACCTAG+TCCGCATA
TAGCTAGTTACATTAGTAAATATTCATATGTAGCATGTTTAAGCATTAAATCAAATAAGATGAGCAAG
ATGGAACGTCTGGGTTCCAATTCTGTCAAGACTTATGACATATTAAATAGATTGGTTACGGTAAAAGA
CAAGGCAAGGTATGG
+
FFFFFF:FFFFFFFFFFFFFFFFFFFFFFFFFFFFFFFFFFFFFFFFFFFFFFFFFFFFFFFFFFFFF
FFFFFFFFFFFFFFFFFFFFFFFFFFFFFFFFFFFFFFFFFFFFFFFFFFFFFFFFFFFFFFFFFFFF
FFFFFFFFFFFFFFF
@A00155:342:HHGFNDSXY:1:2171:11903:1251 1:N:0:GAACCTAG+TCCGCATA
TAGCTAGTTACATTAGTAAATATTCATATGTAGCATGTTTAAGCATTAAATCAAATAAGATGAGCAAG
ATGGAACGTCTGGGTTCCAATTCTGTCAAGACTTATGACATATTAAATAGATTGGTTACGGTAAAAGA
CAAGGCAAGGTATGG
+
FFFFFFFFFFFFFFFFFFFFFFFFFFFFFFFFF,FFFFFFF:FFFFFFFFFFFFFFFFFFFFFFFFFF
FFFFFFF:FFFFFF:FFF:FFFF::FFFFFFFFFFFFFFFFFFFFFFFFF:FFFFFFFFFFFFFFFF
F:FFFFFFFFFFFFF
@A00155:342:HHGFNDSXY:1:2171:11605:1861 1:N:0:GAACCTAG+TCCGCATA
TAGCTAGTTACATTAGTAAATATTCATATGTAGCATGTTTAAGCATTAAATCAAATAAGATGAGCAAG
ATGGAACGTCTGGGTTCCAATTCTGTCAAGACTTATGACATATTAAATAGATTGGTTACGGTAAAAGA
CAAGGCAAGGTATGG
+
FFFFFFFFFFFFFFFFFFFFFFFFFFFFFFFFFFFFFFFFFFFFFFFFFFFFFFFFFFFFFFFFFFFF
FFFFFFFFFFFFFFFFFFFFFFFFFFFFFFFFFFFFFFFFFFFFFFFFFFFFFFFFFFFFFFFFFFFF
FFFFFFFFFFFFFFF
@A00155:342:HHGFNDSXY:1:2208:11351:17832 1:N:0:GAACCTAG+TCCGCATA
TAGCTAGTTACATTAGTAAATATTCATATGTAGCATGTTTAAGCATTAAATCAAATAAGATGAGCAAG
ATGGAACGTCTGGGTTCCAATTCTGTCAAGACTTATGACATATTAAATAGATTGGTTACGGTAAAAGA
CAAGGCAAGGTATGG
+
FFFFFFF:FFFFF:FFFFFFFFFFFFFFFFFFFF:FFFFFFFFFFFFFFFFFFFFFFFFFFFFFFFFF
FFFFFFFFFFFFFFFFFFFFF:FFFFFFFFFFFFFFFFFFFFFFFFFFFFFFFFFFFFFFFFFFFFFF
FFFFFFFFFFFFFFF
@A00155:342:HHGFNDSXY:1:2208:11487:18380 1:N:0:GAACCTAG+TCCGCATA
TAGCTAGTTACATTAGTAAATATTCATATGTAGCATGTTTAAGCATTAAATCAAATAAGATGAGCAAG
ATGGAACGTCTGGGTTCCAATTCTGTCAAGACTTATGACATATTAAATAGATTGGTTACGGTAAAAGA
CAAGGCAGGGTATGG
+
FFFFFF:FFFFFFFFFFFFFFFFFFFFFFFFFFFFF:FFFFFFFFFFFFFFFFFFFFFFFFFFFFFFF
FFFFFFFFFFFFFFFFFFFFFFFFFFFFFFFFFFFFFFFFFFFFFFFFFFFFFFFFFFFFFFFFFFFF
FFFFFFFFFFFFFFF
@A00155:342:HHGFNDSXY:1:2234:19976:22185 1:N:0:GAACCTAG+TCCGCATA
TAGCTAGTTACATTAGTAAATATTCATATGTAGCATGTTTAAGCATTAAATCAAATAAGATGAGCAAG
ATGGAACGTCTGGGTTCCAATTCTGTCAAGACTTATGACATATTAAATAGATTGGTTACGGTAAAAGA
CAAGGCAAGGTATGG

+
FFFFFFFFFFFFFFFFFFFFFFFF,FFFF:FFFFFFF:FFFFFFFFFFFFFFFFFFFFFFFFFFFFFFF
FFFFFFFFFFFFFFFFFFFFFFFFFFFFFFFFFFFFFFFFFFFFFFFFFFFFFFFFFFFFFFFF:FFF
FFFFFFFFFFFFFFF
@A00155:342:HHGFNDSXY:1:2509:7021:21793 1:N:0:GAACCTAG+TCCGCATA
TAGCTAGTTACATTAGTAAATATTCATATGTAGCATGTTTAAGCATTAAATCAAATAAGATGAGCAAG
ATGGAACGTCTGGGTTCCAATTCTGTCAAGACTTATGACATATTAAATAGATTGGTTACGGTAAAAGA
CAAGGCAAGGTATGG
+
FF:FFFFF:FFFFFFFFFFFFFFFFFFFFFFFFFFFFFFFFFF:FFFFFFFFFFFFFFFFFFFFFFFF
FFFFFFFFFFFFFFFFFFFFFFFFFFFFFFFFFFFFFFFFFFFFFFFFFFFFFFFFFFFFFFFFFFFF
FFFFFFFFFFFFFFF
@A00155:342:HHGFNDSXY:1:2559:32588:36667 1:N:0:GAACCTAG+TCCGCATA
TAGCTAGTTACATTAGTAAATATTCATATGTAGCATGTTTAAGCATTAAATCAAATAAGATGAGCAAG
ATGGAACGTCTGGGTTCCAATTCTGTCAAGACTTATGACATATTAAATAGATTGGTTACGGTAAAAGA
CAAGGCAAGGTATGG
+
FFFF,FF,F,,:FFFFFF:,FFFFFFF,FFF,F,,FFF:FFFFF,FF,,FFFF:FF,FF:FFFF,FFF
:FFFFFFFFFFFFF,FFFFFFFFFFFFFF,:FF:FFFF:,FFFFFFFFFF::FFFFFF,F:FF,FFF:
FFFFFFF:FFFFFFF
@A00155:342:HHGFNDSXY:1:1414:32353:12461 1:N:0:GAACCTAG+TCCGCATA
GATCTGTTACATTAGTAAATATTCATATGTAGCATGTTTAAGCATTAAATCAAATAAGATGAGCAAGA
TGGAACGTCTGGGTTCCAATTCTGTCAAGACTTATGACATATTAAATAGATTGGTTACGGTAAAAGAC
AAGGCAAGGTATGGA
+
FFFFFFFFFFFFFFFFFFFFFFFFF:FFFFFFFFFFFFF:FFFFFFFFFFFFFFFF:FFFFFFFFFFF
FFFFFFFFFFFFF:FFFF,FFFFFFFFFFFFF:FFFFFFFFFFFFFFFFFFFFFFFFFFFFFFFFFFF
FFFFFFFFFFFFFFF
@A00155:342:HHGFNDSXY:1:2175:15835:5682 1:N:0:GAACCTAG+TCCGCATA
AGCTAGTTACATTAGTAAATATTCATATGTAGCATGTTTAAGCATTAAATCAAATAAGATGAGCAAGA
TGGAACGTCTGGGTTCCAATTCTGTCAAGACTTATGACATATTAAATAGATTGGTTACGGTAAAAGAC
AAGGCAAGGTATGGA
+
FF:FFF:FFF:FFFFFFFFFFFFFFF:FFFFFFFFFFFFFFFFFFFFFFFFFFFFFFFFFFFFFFFFF
FFFFFFFFFFFFFFFFFFFFFFFFFFFFFFFFFF:FFFFFFFFFFFFFFFFFFFFFFFF:FFFFFFFFFF
FFFFFF:FFFFFFFF
@A00155:342:HHGFNDSXY:1:2274:19135:4288 1:N:0:GAACCTAG+TCCGCATA
AGCTAGTTACATTAGTAAATATTCATATGTAGCATGTTTAAGCATTAAATCAAATAAGATGAGCAAGA
TGGAACGTCTGGGTTCCAATTCTGTCAAGACTTATGACATATTAAATAGATTGGTTACGGTAAAAGAC
AAGGCAAGGTATGGA
+
FF,FFFFFFFFFFFFFFFFFFFFFFFFFFFFFFFFFF:FFFFFFFFFFFFFFFFFFFFFFFFFFFFFFF
FFFFFFFFFFFFFFFFFF:FFFFFFFFFFFFFFFFFFFFFFFFFFFFFFFFFFFFFFFFFFFFFFFFF
FFFFFFFFFFFFFFF
@A00155:342:HHGFNDSXY:1:1605:17047:35712 2:N:0:GAACCTAG+TCCGCATA
AGCTAGTTACATTAGTAAATATTCATATGTAGCATGTTTAAGCATTAAATCAAATAAGATGAGCAAGA
TGGAACGTCTGGGTTCCAATTCTGTCAAGACTTATGACATATTAAATAGATTGGTTACGGTAAAAGAC
AAGGCAAGAGATCGG
+
FFFFFFFFFFFF:FFFFFFFFFFFFFFFFFFFFFFFFFFFFFFFFFFFFFFFFFFFFFFFFFFFFFFF
FFFFFFFFFFFFFFFFFFFFFFFFFFFFFFFFFFFFFFFFFFFFFFFFFFFFFFFFFFFFFF:FFFFFF
FFF:FFFFFFFFFFF
@A00155:342:HHGFNDSXY:1:1605:17110:35853 2:N:0:GAACCTAG+TCCGCATA
AGCTAGTTACATTAGTAAATATTCATATGTAGCATGTTTAAGCATTAAATCAAATAAGATGAGCAAGA

TGGAACGTCTGGGTTCCAATTCTGTCAAGACTTATGACATATTAAATAGATTGGTTACGGTAAAAGAC
AAGGCAAGAGATCGG
+
FFFFFFFFFFFFFFFFFFFFFFFFFFFFFFFFFFFFFFFFFFFFFFFFFFFFFFFFFFFFFFFFFFFF
FFFFFFFFFFFFFFFFFFFFFFFFFFFFFFFFFFFFFFFFF:FFF:FFFFFFFFFFFFFFFFFFF:FF
FFFFFFFFFFF:FFF
@A00155:342:HHGFNDSXY:1:1122:3125:6370 1:N:0:GAACCTAG+TCCGCATA
GCTAGTTACATTAGTAAATATTCATATGTAGCATGTTTAAGCATTAAATCAAATAAGATGAGCAAGAT
GGAACGTCTGGGTTCCAATTCTGTCAAGACTTATGACATATTAAATAGATTGGTTACGGTAAAAGACA
AGGCAAGGTATGGAC
+
FFFFFFFF:FFFFFFFFFFFFFFFFFFFFFFFFFF:FFFFF:FFF:FFFFFFFFFFFFF:FFFFFFFF
FFFFFFFFFFFF:FFFFFFFFFFFFFFFFFFFFFFFFFFFFFFFFFFFFFFFFFFFFFFFFFF:FFF
FFFFFFFFFFFFFFF
@A00155:342:HHGFNDSXY:1:1150:16016:14763 1:N:0:GAACCTAG+TCCGCATA
GCTAGTTACATTAGTAAATATTCATATGTAGCATGTTTAAGCATTAAATCAAATAAGATGAGCAAGAT
GGAACGTCTGGGTTCCAATTCTGTCAAGACTTATGACATATTAAATAGATTGGTTACGGTAAAAGACA
AGGCAAGGTATGGAC
+
FFFFFFFFFFFFFFFFFFFFFFFFFFFFFFFFFFF:FFFFFFFFFFFFFFFFFFFFF:FFFFFFFFFF
FFFFFFFFFFFFFFFFFFFFFFFFFFFFFFFFFFFFFFFFFFFFFFFFFFFFFFFFFFFFFFFFFFFF
FFFFFFFFFFFFFFF
@A00155:342:HHGFNDSXY:1:1243:3613:17707 1:N:0:GAACCTAG+TCCGCATA
GCTAGTTACATTAGTAAATATTCATATGTAGCATGTTTAAGCATTAAATCAAATAAGATGAGCAAGAT
GGAACGTCTGGGTTCCAATTCTGTCAAGACTTATGACATATTAAATAGATTGGTTACGGTAAAAGACA
AGGCAAGGTATGGAC
+
FFFFFFFFFFFFFFFFFFFFFFFFFF,FF:FFFFFFFFFFFFFFFFFFFFFF:FFF:FFFFFFFFFF,
FFFFFFFFFFFFFFFFFF,FFFFFFF:FFFFFFFFFFFFFFFFFFFFFFFFFFFFFFFFFFFFFFFFF
FFFFFFFFFFFFFFF
@A00155:342:HHGFNDSXY:1:1323:31611:1752 1:N:0:GAACCTAG+TCCGCATA
GCTAGTTACATTAGTAAATATTCATATGTAGCATGTTTAAGCATTAAATCAAATAAGATGAGCAAGAT
GGAACGTCTGGGTTCCAATTCTGTCAAGACTTATGACATATTAAATAGATTGGTTACGGTAAAAGACA
AGGCAAGGTATGGAC
+
FFFFF:FFF:FFFFFFFFF,FFFFFFFFFFFF:FFFFFFFFFFFFFFFFFFFF:FFFFFFFFFFFFFF
FFFFFFFFFFFF:FFFFFFFFFFFFF:F::FFF:FFFFFFFFF:FFFFF:FFFFFFFFFFFFFFFFFF
FFFFFFFFFFFFFFF
@A00155:342:HHGFNDSXY:1:1366:19434:32675 1:N:0:GAACCTAG+TCCGCATA
GCTAGTTACATTAGTAAATATTCATATGTAGCATGTTTAAGCATTAAATCAAATAAGATGAGCAAGAT
GGAACGTCTGGGTTCCAATTCTGTCAAGACTTATGACATATTAAATAGATTGGTTACGGTAAAAGACA
AGGCAAGGTATGGAC
+
FFFFFFFFFFFFFFF:FFFFFFFFFFFFFFFFFFFFFFFFFFFFFFFFFFFFFFFFFFFFF,F:FFFF
FFFFFFFFFFFFFFFFFF:FFFFFFFFFFFFFFFFFFFFFFFFFFFFFFFFFFFFFFFFFFFFFFFFF
FFFFFFFFFFFFFFF
@A00155:342:HHGFNDSXY:1:1408:16486:5337 1:N:0:GAACCTAG+TCCGCATA
GCTAGTTACATTAGTAAATATTCATATGTAGCATGTTTAAGCATTAAATCAAATAAGATGAGCAAGAT
GGAACGTCTGGGTTCCAATTCTGTCAAGACTTATGACATATTAAATAGATTGGTTACGGTAAAAGACA
AGGCAAGGTATGGAC
+
FFFFFFFFFFFFFFFFFFFFFFFFFFFFFFF,FF:FFFFFFFFFFFFFFFFFFFFFFFFFFFFFFFFF
:FFFFFFFFF:FFFFFFFFFFFFFFFFFFFFFFFFFFFFFFFFFFFFFFFFFFFFFFFFFFFFFFFFF
FFFFFFFFFFFFFFF

```
@A00155:342:HHGFNDSXY:1:1402:22435:7936 1:N:0:GAACCTAG+TCCGCATA
GCTAGTTACATTAGTAAATATTCATATGTAGCATGTTTAAGCATTAAATCAAATAAGATGAGCAAGAT
GGAACGTCTGGGTTCCAATTCTGTCAAGACTTATGACATATTAAATAGATTGGTTACGGTAAAAGACA
AGGCAAGGTATGGAC
+
:FFFFFFFFFF:FFFFFFFFFFFFFFFFFFFFFFF:FFFFFFFFFFFFFFFFFFFFFFFFFFFFFFFF
FFFFFFFFFFFFFFFFFFFFFFFFFFFFFFFFFFFFFFFFFFFFFFFFFFFFFFFFFFFFFFFFFFFF
FFFFFFFFFFFFFFF
@A00155:342:HHGFNDSXY:1:1424:11008:7780 1:N:0:GAACCTAG+TCCGCATA
GCTAGTTACATTAGTAAATATTCATATGTAGCATGTTTAAGCATTAAATCAAATAAGATGAGCAAGAT
GGAACGTCTGGGTTCCAATTCTGTCAAGACTTATGACATATTAAATAGATTGGTTACGGTAAAAGACA
AGGCAAGGTATGGAC
+
FFFFFFFFF:F,FFFFFFFFFFFFFFFFFFFF:FFFFFFFFFFFFF:FFFFFFFFFF:FFFFFFFFFF
FFFFFFFFFFFF,FFFFFFFFFFFFFFFFFFFFFFFFFFFFFFFFFFFFFFFFFFFFFFFFFFFFFF
FFFFFFFFFFFFFFF
@A00155:342:HHGFNDSXY:1:1432:31593:35571 1:N:0:GAACCTAG+TCCGCATA
GCTAGTTACATTAGTAAATATTCATATGTAGCATGTTTAAGCATTAAATCAAATAAGATGAGCAAGAT
GGAACGTCTGGGTTCCAAGTCTGTCAAGACTTATGACATATTAAATAGATTGGTTACGGTAAAAGACA
AGGCAAGGTATGGAC
+
F:F:,FFFFFFFFFFF:FFFFFF::FFFFFFFFFFFFFF:FFFFFFFFFFFFFF:FFFFFFFF:FFFF
,FFFFFF,F,FFFFFF,F,F:FFF:,F,FFFFFFFFFFFF:FFF:FFF,FF,FFFF,:FFF:FFFFF:
,FF::F:FFFFFF,:
@A00155:342:HHGFNDSXY:1:1448:18412:10989 1:N:0:GAACCTAG+TCCGCATA
GCTAGTTACATTAGTAAATATTCATATGTAGCATGTTTAAGCATTAAATCAAATAAGATGAGCAAGAT
GGAACGTCTGGGTTCCAATTCTGTCAAGACTTATGACATATTAAATAGATTGGTTACGGTAAAAGACA
AGGCAAGGTATGGAC
+
FFFFFFFF:FFFFFFFFFFFFFFFFFFFFFFFFFFFFFFFFF:FFFFFFFFFFFFFFFFFFFF,,:FF
FFFFFFF,FFFFFFFFFFFF:FFFFFFFFFFFFFFFFFFFFFFFFFFF:FFFFFFFFFFFFFFFFFFF
FFFFFFFFFFFFFFF
@A00155:342:HHGFNDSXY:1:1462:9769:21136 1:N:0:GAACCTAG+TCCGCATA
GCTAGTTACATTAGTAAATATTCATATGTAGCATGTTTAAGCATTAAATCAAATAAGATGAGCAAGAT
GGAACGTCTGGGTTCCAATTCTGTCAAGACTTATGACATATTAAATAGATTGGTTACGGTAAAAGACA
AGGCAAGGTATGGAC
+
FFFFFFFFFFFFFFFFFFFFFFFF::FFFFFFFFF:FFFFFFFFFFFFFFFFFFFFFFFFFFFFFFFF
FFFFFFFFFFFFFFFFFF:FFFFFFF:FFFFFFFFFFFFFFFFFFFFFFFFFFFFFFFFFFFFFFFFF
FFFF:FFFFFFFFFF
@A00155:342:HHGFNDSXY:1:1528:30978:6042 1:N:0:GAACCTAG+TCCGCATA
ATCTGTTACATTAGTAAATATTCATATGTAGCATGTTTAAGCATTAAATCAAATAAGATGAGCAAGAT
GGAACGTCTGGGTTCCAATTCTGTCAAGACTTATGACATATTAAATAGATTGGTTACGGTAAAAGACA
AGGCAAGGTATGGAC
+
:FFFFFFFFFFFFFFFFFFFFFFFFFFFFFFFFFFFFFFFFFFFFFFFFFFFFFFFFFFFFFFFFFFF
FFFFFFFFFFFFFFFFFFFFFFFFFFFFFFFFFFFFFFFFFFFFFFFFFFFFFFFFFFFFFFFFFFFF
FFFFFFFFFFFFFFF
@A00155:342:HHGFNDSXY:1:1539:22661:24017 1:N:0:GAACCTAG+TCCGCATA
GCTAGTTACATTAGTAAATATTCATATGTAGCATGTTTAAGCATTAAATCAAATAAGATGAGCAAGAT
GGAACGTCTGGGTTCCAATTCTGTCAAGACTTATGACATATTAAATAGATTGGTTACGGTAAAAGACA
AGGCAAGGTATGGAC
+
FFFFFFFFFFFFFFFFFFFFFFFFFFFFFFFFFFFFFFFFFFFFFFFFFFFFFFFFFFFFFFFFFF:F
```

```
FFFFFFFFFFFFFFFFFFFFFFFFFFFFFFFFFFFFFFFFFFFFFFFFFF:FFFFFFFFFFFFFFFFF
FFFFFFFFFFFFFFF
@A00155:342:HHGFNDSXY:1:1540:5728:37059 1:N:0:GAACCTAG+TCCGCATA
GCTAGTTACATTAGTAAATATTCATATGTAGCATGTTTAAGCATTAAATCAAATAAGATGAGCAAGAT
GGAACGTCTGGGTTCCAATTCTGTCAAGACTTATGACATATTAAATAGATTGGTTACGGTAAAAGACA
AGGCAAGGTATGGAC
+
FFFFFFFFFFF,FFFFFF:FFFFFFFFFFFFFFFF:FFFF:FFFFFFFFFFFFFFFFFFFFFFFFFFFF
FFFFFFFFFFFFFFFFFFFFFFFFF:FFFFFFFFFFFFFFFFFFFFFFFFFFFFFFFFFFFFFFFFFF
FF:FFFFFFFFFFFF
@A00155:342:HHGFNDSXY:1:1558:1741:33599 1:N:0:GAACCTAG+TCCGCATA
GCTAGTTACATTAGTAAATATTCATATGTAGCATGATTAAGCATTAAATCAAATAAGATGAGCAAGAT
GGAACGTCTGGGTTCCAATTCTGTCAAGACTTATGACATATTAAATAGATTGGTTACGGTAAAAGACA
AGTCAAGGTATGGAC
+
F:FFFFFFFFFFFFFFFFFFFFFF::FFFFFFF:F,F:FFF:FFFFFFFFFFFFFFFFFFFFFFFFFFF
FFFFFFFFFFFFFFFFFFFFFFFFFFFFFFFFFFFFFFFFFFFFFFFFFFFFFFFFFFFFFFFFFFFF
FF:FFFFFFFFFFFF
@A00155:342:HHGFNDSXY:1:1605:18231:28933 1:N:0:GAACCTAG+TCCGCATA
GCTAGTTACATTAGTAAATATTCATATGTAGCATGTTTAAGCATTAAATCAAATAAGATGAGCAAGAT
GGAACGTCTGGGTTCCAATTCTGTCAAGACTTATGACATATTAAATAGATTGGTTACGGTAAAAGACA
AGGCAAGGTAAGATC
+
FFFFFFFFFFFFFFFFFFFFFFFFFFFFFFFFFFFFFFFFFFFFFFFFFFFFFFFFFFFFFFFFFFFF
FFFFFFFFFFFFFFFFFFFFFFFFFFFFFFFFFFFFFFFF:FFFFFFFFFFFFFFFFFFFFFFFFFFFF
FFFFF:FFFFFFFFF
@A00155:342:HHGFNDSXY:1:1606:11767:25661 1:N:0:GAACCTAG+TCCGCATA
GCTAGTTACATTAGTAAATATTCATATGTAGCATGTTTAAGCATTAAATCAAATAAGATGAGCAAGAT
GGAACGTCTGGGTTCCAATTCTGTCAAGACTTATGACATATTAAATAGATTGGTTACGGTAAAAGACA
AGGCAAGGTATGGAC
+
FFFFFFFFFFFFFFFFFFFFFFFFFFFFFFFFFFFFFFFFFFFFFFFFFFFFFFFFFFFFFFFFFFFF
FFFFFFFFFFFFFFFFFFFFFFFFFFFFFFFFFFFFFFFFFFFFFFFFFFFFFFFFFFFFFFFFFFFF
FFFFFFFFFFFFFFF
@A00155:342:HHGFNDSXY:1:1671:31937:36573 1:N:0:GAACCTAG+TCCGCATA
GCTAGTTACATTAGTAAATATTCATATGTAGCATGTTTAAGCATTAAATCAAATAAGATGAGCAAGAT
GGAACGTCTGGGTTCCAATTCTGTCAAGACTTATGACATATTAAATAGATTGGTTACGGTAAAAGACA
AGGCAAGGTATGGAC
+
FFFFFFFFFFFFFFFFFFFFFFFFF:FFF:FFFFF:FFFFFFFFFFF::FFFFFFFFFFFFFFFFFFF
FFFFFFFFFFFFFFFFFF:FF:FFFFFFFFFFFFFFFFFFFFFFFFF:FFFFFFFFFFFFFFFFF,FFFF
FFFFFFFFFFFFFFF
@A00155:342:HHGFNDSXY:1:2165:7355:10191 1:N:0:GAACCTAG+TCCGCATA
TCTAGTTACATTAGTAAATATTCATATGTAGCATGTTTAAGCATTAAATCAAATAAGATGAGCAAGAT
GGAACGTCTGGGTTCCAATTCTGTCAAGACTTATGACATATTAAATAGATTGGTTACGGTAAAAGACA
AGGCAAGGTATGGAC
+
FFFFFFFFFFFFF:FFFFFFFF,FFFFF:FFFFFF,FFFFFFFFFFFFFFF:FFFFFFFFFFFFF::F
FFFFFFF:FFFFFFFFFFFFFFFFFFFFFFFFFFFFFFFFFFFFFFFFFFFFFFFFFFFFFFFFFFFF
:FFFFFFFFFFFF:F
@A00155:342:HHGFNDSXY:1:2205:26666:34021 1:N:0:GAACCTAG+TCCGCATA
GCTAGTTACATTAGTAAATATTCATATGTAGCATGTTTAAGCATTAAATCAAATAAGATGAGCAAGAT
GGAACGTCTGGGTTCCAATTCTGTCAAGACTTATGACATATTAAATAGATTGGTTACGGTAAAAGACA
AGGCAAGGTATGGAC
```

+
F:FFFFFFFFFFFFFFFFFFFFFFFFFFFFFFFFF::FFFFFFFFFF:FFFFFFFFFFFFFFFFFFFF
FFFFFFFFFFFFFFFFFFFFFFFFFFFFFFFFFFFFFFFFFFFFFFFFFFFFFFFFFFFFFFFFFFFF
FFFFFFFFFFFFFFF
@A00155:342:HHGFNDSXY:1:2209:15429:33599 1:N:0:GAACCTAG+TCCGCATA
GCTAGTTACATTAGTAAATATTCATATGTAGCATGTTTAAGCATTAAATCAAATAAGATGAGCAAGAT
GGAACGTCTGGGTTCCAATTCTGTCAAGACTTATGACATATTAAATAGATTGGTTACGGTAAAAGACA
AGGCAAGGTATGGAC
+
FFFFFFFFFFFFFFFFFFFFFFFFFFFFFFFFFFFFFFFFFFFFFFFFFFFFFFFFFFFFFFFFFFFF
FFFFFFFFFFFFFFFFFFFFFFFFFFFFFFFFFFFFFFFFFFFFFFFFFFFFFFFFFFFFFFFFFFFF
FFFFFFFFFFFFFFF
@A00155:342:HHGFNDSXY:1:2249:31132:29763 1:N:0:GAACCTAG+TCCGCATA
GCTAGTTACATTAGTAAATATTCATATGTAGCATGTTTAAGCATTAAATCAAATAAGATGAGCAAGAT
GGAACGTCTGGGTTCCAATTCTGTCAAGACTTATGACATATTAAATAGATTGGTTACGGTAAAAGACA
AGGCAAGGTATGGAC
+
FFFFFFFFFFFFFFFFFFFFFFFFFFFFFFFFFFF:FFFFFFFFFFFFFFFFFFFFFFFFFFFFFFFF
FFFFFFFFFFFF,FFFFF:FFFFFFFFFFFFFFFFFFFFFFFFFFFFFFFFFFFFFFFFFFFFFFFFF
FFFFFFFFFFFFFFF
@A00155:342:HHGFNDSXY:1:2361:2501:5979 1:N:0:GAACCTAG+TCCGCATA
GCTAGTTACATTAGTAAATATTCATATGTAGCATGTTTAAGCATTAAATCAAATAAGATGAGCAAGAT
GGAACGTCTGGGTTCCAATTCTGTCAAGACTTATGACATATTAAATAGATTGGTTACGGTAAAAGACA
AGGCAAGGTATGGAC
+
:FFFFFFFFFFFF:FF:FFF::FFFFFFFFF:FF,FFFFFFFFFFFFFFFFFFFFFFFFFF,:FFFFF
FFFFFFFFFF::FFF,FFFFFF:FFFFFFFFFFFFFFFFFFFFFFFFFFFFFFFFFFFFFFFFFFFFF
FFFFFFFFFFFFFFF
@A00155:342:HHGFNDSXY:1:2540:16821:13307 1:N:0:GAACCTAG+TCCGCATA
GCTAGTTACATTAGTAAATATTCATATGTAGCATGTTTAAGCATTAAATCAAATAAGATGAGCAAGAT
GGAACGTCTGGGTTCCAATTCTGTCAAGACTTATGACATATTAAATAGATTGGTTACGGTAAAAGACA
AGGCAAGGTATGGAC
+
:FFFFFFFFFFF:FFFFFFFFFFFFFFFFFFFFFF:FFFFFFFFFFFFFFFFFFFFFFFFFFFFFFFF
FFFFFFFFFFFFFFFFFFFFFFFFFFFFFFFFFFFFFFFFFFFFFFFFFFFFFFFFFFFFFFFFFFFF
FFFFFFFFFFFFFFF
@A00155:342:HHGFNDSXY:1:2557:25283:34601 1:N:0:GAACCTAG+TCCGCATA
GCTAGTTACATTAGTAAATATTCATATGTAGCATGTTTAAGCATTAAATCAAATAAGATGAGCAAGAT
GGAACGTCTGGGTTCCAATTCTGTCAAGACTTATGACATATTAAATAGATTGGTTACGGTAAAAGACA
AGGCAAGGTATGGAC
+
F,FFFFFFF:FFFFFFFFFFFFFFFFFFFFFFFFFFFFFFFFFFFFFFFFFFFFFFFFFFFFFFFFFF
FFFFFFFFFFFFFFFFFFFFFFFFFFFFFFFFFFFFFFFFFFFFFFFFFFFFFFFFFFFFFFFFFFFF
FFFFFFFFFFFFFFF
@A00155:342:HHGFNDSXY:1:2539:20157:22498 1:N:0:GAACCTAG+TCCGCATA
GCTAGTTACATTAGTAAATATTCATATGTAGCATGTTTAAGCATTAAATCAAATAAGATGAGCAAGAT
GGAACGTCTGGGTTCCAATTCTGTCAAGACTTATGACATATTAAATAGATTGGTTACGGTAAAAGACA
AGGCAAGGTATGGAC
+
:FFFFFFFFFFFFFFFFFFFFF:FFFFF:F:FFFFFFFFFFFFFFFFFFFFFFFFFFFFFFFFFFF:F
FFFFFFFFFFFFFFFFFFFFFFFFFFFFFFFFFFFFFFFFFFFFFFFFFFFFFFFFFFFFFFFFFFFF
:FFFFFFFFFFFFFF
@A00155:342:HHGFNDSXY:1:2535:19687:29230 1:N:0:GAACCTAG+TCCGCATA
GCTAGTTACATTAGTAAATATTCATATGTAGCATGTTTAAGCATTAAATCAAATAAGATGAGCAAGAT

```
GGAACGTCTGGGTTCCAATTCTGTCAAGACTTATGACATATTAAATAGATTGGTTACGGTAAAAGACA
AGGCAAGGTATGGAC
+
FFFFFFFFFFFFFFFFFFFFFFFFFFFFFFFFFFF:FFFFFFFFFFFFFFFFFFFFFFFFFFFFFFFF
FFFFFFFFFFFFFFFFFFFFFFFFFFFFFFFFFFFFFFFFFFFFFFFFFFFFFFFFFFFFFFFFFFFF
FFFFFFFFFFFFFFF
@A00155:342:HHGFNDSXY:1:1646:2130:31360 2:N:0:GAACCTAG+TCCGCATA
GCTAGTTACATTAGTAAATATTCATATGTAGCATGTTTAAGCATTAAATCAAATAAGATGAGCAAGAT
GGAACGTCTGGGTTCCAATTCTGTCAAGACTTATGACATATTAAATAGATTGGTTACGGTAAAAGACA
AGGCAAGGTATGGAC
+
FFFFFFFFFFFFFFFFFFFFFFFFFFFFFFFFFFFFFFFFFFFFFFFFFFFFFFFFFFFFFFFFFFFF
FFFFFFFFFFFFFFFF:FFFFFFFFFFFFFFF:FFF:FFFFF:F:FFFFFFFFFFF:F,F:F:FFFFF
FFFFF:FFFFFFFFF
@A00155:342:HHGFNDSXY:1:1646:1651:31720 2:N:0:GAACCTAG+TCCGCATA
GCTAGTTACATTAGTAAATATTCATATGTAGCATGTTTAAGCATTAAATCAAATAAGATGAGCAAGAT
GGAACGTCTGGGTTCCAATTCTGTCAAGACTTATGACATATTAAATAGATTGGTTACGGTAAAAGACA
AGGCAAGGTATGGAC
+
FFFFFFFFFFFFFFFFF:FFFFF:FFFFFFFFFFFFFF,FFFFFFFFFFFFF:FFFFFFF,FFFFFFF
FFFFFFFFFFFFFFFFFFFF,FFFFFF:FFFFFF,:,FFFFFFF,FFF:FFFFFFFFFFFF:,F::FF
:F,FFFFFFFFFFF:
@A00155:342:HHGFNDSXY:1:2228:2555:5509 2:N:0:GAACCTAG+TCCGCATA
GCTAGTTACATTAGTAAATATTCATATGTAGCATGTTTAAGCATTAAATCAAATAAGATGAGCAAGAT
GGAACGTCTGGGTTCCAATTCTGTCAAGACTTATGACATATTAAATAGATTGGTTACGGTAAAAGACA
AGTCAAGGTATGGAC
+
:FFFFFFFFFFFFFFFFFFFFFFFFFFFFFFFFFFFFFFFFFFFFFFFFFFF:FFFFFFFFFFFFFFF
FFFFFFFFFFFFFFFFFFFFF:FF:FFFFFFFFFFFFFFFFFFFFFF:FFFFFFFFFFFFFF,FFFFF
F,,FFF:FFFF:FFF
@A00155:342:HHGFNDSXY:1:2249:31132:29763 2:N:0:GAACCTAG+TCCGCATA
GCTAGTTACATTAGTAAATATTCATATGTAGCATGTTTAAGCATTAAATCAAATAAGATGAGCAAGAT
GGAACGTCTGGGTTCCAATTCTGTCAAGACTTATGACATATTAAATAGATTGGTTACGGTAAAAGACA
AGGCAAGGTATGGAC
+
F:FFFFFFFFFFFFFFFFFFFFFFFFFFFFFFFFFFFFFFFFFFFFFFFFFFFFFFFFFFFFFFFFFF
FFFFFFFFFFFFFFFFFFFFFFFFFFFFFFFFFFFFFFFFFFFFFFFFFFFFFFFFFFFF:FFFFFFF
FFFFFFFFFFFFFFF
@A00155:342:HHGFNDSXY:1:2347:17517:31798 2:N:0:GAACCTAG+TCCGCATA
GCTAGTTACATTAGTAAATATTCATATGTAGCATGTTTAAGCATTAAATCAAATAAGATGAGCAAGAT
GGAACGTCTGGGTTCCAATTCTGTCAAGACTTATGACATATTAAATAGATTGGTTACGGTAAAAGACA
AGGCAAGGTATGGAC
+
FFFFFFFFFFFFFFFFFFFFFFFFF:FFFFFFFFFFFFFFFFFFFFFFFFFFFFFFFFFFFFFFFFFF
FFFFFFFFFFFFFFFFFF:FFFFF:FFFF::FFFFFFFFFFFFFFFFFFFFFFFFFF:FFFF:F:FFF
F:FFF:FFF:FFFFF
@A00155:342:HHGFNDSXY:1:2463:3034:9157 2:N:0:GAACCTAG+TCCGCATA
GCTAGTTACATTAGTAAATATTCATATGTAGCATGTTTAAGCATTAAATCAAATAAGATGAGCAAGAT
GGAACGTCTGGGTTCCAATTCTGTCAAGACTTATGACATATTAAATAGATTGGTTACGGTAAAAGACA
AGGCAAGGTATGGAC
+
F,FFFFFFFFFFFFFFFFFFFFFFFFFFFFFFFFFFFFFFFFFFFFFFF:FFFFFF,FFFFFFFFFFF
FFFFFFFFFFF:FF:FFFFFFFFF:FFFFFFFFF:FFFFFFFFFFFF,FFFF,FFFFF,FFFFF,FFF
F::FFFFFFFF:FFF
```

@A00155:342:HHGFNDSXY:1:2206:10312:21731 1:N:0:GAACCTAG+TCCGCATA
CTAGTTACATTAGTAAATATTCATATGTGGCTTGTTTAAGCATTAAATCTAATAAGATGAGCAAGATG
GAACGTCTGGGTTCCAATTCTTTCAAGACTTATGACATATTAAATAGATTGGTTACGGTAAAAGACAA
GGCAAGGTATGGA
+
FFFFF,,,FFF,FFFFFFFFF,FF:,,FFF,,FFFFFF,F:FFFFF,F:,F,FFFFF:FFF,FF,FFF
F:,FFFFFFFFFF:F::FFFF,FFFFFFFF,FFFFF,F,FF,FFFFFF:FFFFFFFFFFF,FFFFF:F
FFFFFFFFFFFFF
@A00155:342:HHGFNDSXY:1:1646:5511:24471 1:N:0:GAACCTAG+TCCGCATA
CTAGTTACATTAGTAAATATTCATATGTAGCATGTTTAAGCATTAAATCAAATAAGATGAGCAAGATG
GAACGTCTGGGTTCCAATTCTGTCAAGACTTATGACATATTAAATAGATTGGTTACGGTAAAAGACAA
GGCAAGGTATGGAC
+
FFFFFFFFFFFFF:F:FFFFFFFFFFFFFFFFF:,FFF,,FFF:FFFFFFFFFFFFFFFFFFFFFFFF
FF:FFFFFFFFFFFFFFFFFFF:FFFFFFFFFFFFFFFF:FFF:FFFF:FFFFFFFFFFFFFFFFFFF
FFFFFFFFFFFFFF
@A00155:342:HHGFNDSXY:1:2361:2257:11788 1:N:0:GAACCTAG+TCCGCATA
CTAGTTACATTAGTAAATATTCATATGTAGCATGTTTAAGCATTAAATCAAATAAGATGAGCAAGATG
GAACGTCTGGGTTCCAATTCTGTCAAGACTTATGACATATTAAATAGATTGGTTACGGTAAAAGACAA
GGCAAGGTATGGAC
+
FF:,FFF:F,FFFFFF:FFFF,FFFFFFF:F:F:,FFFF:FFFFFFFFFFFFFFF,FFFFFFF,FFFF
,FFFF:FFFFFFFF,FFFFFFFFFFFFFFFFFFFFFFFFFFFFFFFFFFFFFFFFFFFFFFFF,FFFF
FFFFF:,FFFFF:F
@A00155:342:HHGFNDSXY:1:1507:11632:10551 1:N:0:GAACCTAG+TCCGCATA
CTAGTTACATTAGTAAATATTCATATGTAGCATGTTTAAGCATTAAATCAAATAAGATGAGCAAGATG
GAACGTCTGGGTTCCAATTCTGTCAAGACTTATGACATATTAAATAGATTGGTTACGGTAAAAGACAA
GGCAAGGTATGGACC
+
F:FFFFFFFFFFFFFFFFFFFFFFFFFFFFFFFFFFFFFFFFFFFFFFFFFFFFFFFFFFFFFFFFFF
FFFFFFFFFFF:FFFFFFFFFFFFFFFF:FFFFFFF:FFFFF:FFFFFFFFFFFFFFFFFFFFFFFFF
FFFFFFFFFFFFFF:
@A00155:342:HHGFNDSXY:1:1536:7238:32503 1:N:0:GAACCTAG+TCCGCATA
CTAGTTACATTAGTAAATATTCATATGTAGCATGTTTAAGCATTAAATCAAATAAGATGAGCAAGATG
GAACGTCTGGGTTCCAATTCTGTCAAGACTTATGACATATTAAATAGATTGGTTACGGTAAAAGACAA
GGCAAGGTATGGACC
+
FFFFFFFFFFFFFFFFFFFFFFFFFFFFFF:FFFFFFFFFFFFFFFFFFFFFFFFFFFFFFFFFFFFF
FFFFFFFFFFFFFFFFFFFFFFFFFFFFFFFFFFFFFFFFFFFFFFFFFFFFFFFFFFFFFFFFFFFF
FFFFFFFFFFFFFFF
@A00155:342:HHGFNDSXY:1:2165:7355:10191 2:N:0:GAACCTAG+TCCGCATA
CTAGTTACATTAGTAAATATTCATATGTAGCATGTTTAAGCATTAAATCAAATAAGATGAGCAAGATG
GAACGTCTGGGTTCCAATTCTGTCAAGACTTATGACATATTAAATAGATTGGTTACGGTAAAAGACAA
GGCAAGGTATGGACA
+
FFFFFFFFFFFFFFFFFFFFFFFFFFFFFFFFFFFFFFFFFF:FFFFFFFFFFFFFFFFFFFFFFFFF
FFFFFFFFFFFFFFFFFFFFFFFFFFFFFFFFFFFFFFFFFFFFFFFFFFFFFFFFFFFFFFFFFFFF
FFF:FFFF:FFFFFF
@A00155:342:HHGFNDSXY:1:2470:1344:6574 1:N:0:GAACCTAG+TCCGCATA
TAGTTACATTAGTAAATATTCATATGTAGCATGTTTAAGCATTAAATCAAATAAGATGAGCAAGATGG
AACGTCTGGGTTCCAATTCTGTCAAGACTTATGACATATTAAATAGATTGGTTACGGTAAAAGACAAG
GCAAGGTATGGAC
+
FF,,,F:F:FF,:FFFFFF,FF:FF:FF,FF,:,F,FF:,F,:FFF,FFFFFFF:F:FF,:FF,FFFF

FFFF:FFF::F:F:FFF,FFFFFFF,:F:,F:FFFFFFFFFFFFFFFFF:FFFFFF:FFFFFFFFFFF
:FFFFFFFFFFFF
@A00155:342:HHGFNDSXY:1:2219:5168:14481 1:N:0:GAACCTAG+TCCGCATA
TATTTACATTAGTAAATATTCATATGTAGCATGTTTAAGCATTAAATCAAATAAGATGAGCAAGATGG
AACGTCTGGGTTCCAATTCTGTCAAGACTTATGACATATTAAATAGATTGGTTACGGTAAAAGACAAG
GCAAGGTATGGACCC
+
F:,FFF:FFFFFFFFFFFFFFFFFFFFFF:FFFFFFFFFFFFFFFFFFFFFFFF:FFFFFFFFFFFFF
FF,FF:FFFFFFFFFFFFFFFFFFFFFFFFFFFFFFFFFFFFFFFFFFFF,FFFFFFFFFFFFFFFFF
FFFFFFFFFFFFF:F
@A00155:342:HHGFNDSXY:1:1463:24831:19382 1:N:0:GAACCTAG+TCCGCATA
AGTTACATTAGTAAATATTCATATGTAGCATGTTTAAGCATTAAATCAAATAAGATGAGCAAGATGGA
ACGTCTGGGTTCCAATTCTGTCAAGACTTATGACATATTAAATAGATTGGTTACGGTAAAAGACAAGG
CAAGGTATGGACCCC
+
FFFFFFFFFFFFFFFFFFFFFFFFFFFFFFFFFFFFFFFFFFFFFFFFFFFFFFFFFFF:FFFFFFFF
FFFFFFFFFFFFFFFFFFFFFFFFFFFFFFFFFFFFFFFFFFFFFFFFFFFFFFFFFFFFFFFFFFFF
FFFFFFFFFFFFFFF
@A00155:342:HHGFNDSXY:1:2143:30798:1344 1:N:0:GAACCTAG+TCCGCATA
AGTTACATTAGTAAATATTCATATGTAGCATGTTTAAGCATTAAATCAAATAAGATGGGCAAGATGGA
ACGTCTGGGTTCCAATTCTGTCAAGACTTATGACATATTAAATAGATTGGTTACGGTAAAAGACAAGG
CAAGGTATGGACCCG
+
FFFFF::FFFFFF,FFFFFFFFFFFFFFFFFFFFFFF:FFFFFFFFFFFFFFFFFFFFFFFFFFFFFF
FFFFFFFFF:FFFFFFFFFFFFFFFFFFFFFFFFFFFFFFFFFF,FFFFFFFFF:FFFFFF:FFFFFF
FFFFFFFFFFFFFFF
@A00155:342:HHGFNDSXY:1:2607:3106:3490 2:N:0:GAACCTAG+TCCGCATA
AGTTACATTAGTAAATATTCATATGTAGCATGTTTAAGCATTAAATCAAATAAGATGAGCAAGATGGA
ACGTCTGGGTTCCAATTCTGTCAAGACTTATGACATATTAAATAGATTGGTTACGGTAAAAGACAAGG
CAAGGTATGGACCCT
+
FFFFFF:FFFFFFFFFFFFFFFFFFFFFFFFFFFFFF:FFFFFFFFFFFFFFFFFFFFFFFFFFFF:F
:FFFFFF,FFFFFFFFFFFFFFFFF,FFFFFF:FFFFFFFFFFFFF,FFFFFFFFFFFFFFFFFFFF:
FF:FFF,FFFFFFFF
@A00155:342:HHGFNDSXY:1:2507:15637:29168 2:N:0:GAACCTAG+TCCGCATA
GTTACATTAGTAAATATTCATATGTAGCATGTTTAAGCATTAAATCAAATAAGATGAGCAAGATGGAA
CGTCTGGGTTCCAATTCTGTCAAGACTTATGACATATTAAATAGATTGGTTACGGTAAAAGACAAGGC
AAGGTATGGACCCT
+
F:F:F:FF:FFF:FF:FFFFF:FFF:FF:FFFFFFFFF:FFFFFF:FFFF:FFFFFFFFFFFFFFF:F
FFFFFFFFFFFFFFFF:FFFFFFFFFFFFFFFFFFFFFFFF:FFFFFF:FF:FFFFFFFFF:FF:FFF
FFFFF:F:F:FFFF
@A00155:342:HHGFNDSXY:1:1223:9516:21574 2:N:0:GAACCTAG+TCCGCATA
GTTACATTAGTAAATATTCATATGTAGCATGTTTAAGCATTAAATCAAATAAGATGAGCAAGATGGAA
CGTCTGGGTTCCAATTCTGTCAAGACTTATGACATATTAAATAGATTGGTTACGGTAAAAGACAAGGC
AAGGTATGGACCCTT
+
FFFFFFFFFFFFFFFFFFFFFFFFFFFFFFFFFFFFFFFFFF:FFFFFFFFFFFFFFFFFFFFFFFFF
FFFFFFFFFFFFFFFFFFFFFFFFFFFF:FFFFFFFFFFFFFFFFFFFFFFFFFFFFFFFFFFFFFFF
FFFFFFFFF:FFFFF
@A00155:342:HHGFNDSXY:1:1341:29532:26616 2:N:0:GAACCTAG+TCCGCATA
GTTACATTAGTAAATATTCATATGTAGCATGTTTAAGCATTAAATCAAATAAGATGAGCAAGATGGAA
CGTCTGGGTTCCAATTCTGTCAAGACTTATGACATATTAAATAGATTGGTTACGGTAAAAGACAAGGC
AAGGTATGGACCCTT

+
FFFFFFFFFFFFFFFFFFFFFFFFFFFFFFFFFFFF:FFFFFFFFFF:FFFFFFFFFF:FFFFFFFFFFF
FFFFFFFFFFFFFFFFFFFFFFFFFFFFFFFFFFFFFFFFFFFFFFFFFFFFFF:FFFFFFF,FFFFF:F
FFFF:FFFFFFFFFF
@A00155:342:HHGFNDSXY:1:1329:16369:4977 2:N:0:GAACCTAG+TCCGCATA
GTTACATTAGTAAATATTCATATGTAGCATGTTTAAGCATTAAATCAAATAAGATGAGCAAGATGGAA
CGTCTGGGTTCCAATTCTGTCAAGACTTATGACATATTAAATAGATTGGTTACGGTAAAAGACAAGGC
AAGGTATGGACCCTT
+
:FFFFFFFFFFF:FFFFFFFFFFF:FFFFFFFFFFFFFFFFFFFFFFF:FF,FFFFFFFFFFFFFFFFFF
FFFF,FFF:FFFFFF:FFFFFFFFFF:F::,FFFFFFFFF:FFFFF,,FFFFFFFF:FFF,FFFFFFFFF
F:FF:FFFFFFFFFF
@A00155:342:HHGFNDSXY:1:1414:32353:12461 2:N:0:GAACCTAG+TCCGCATA
GTTACATTAGTAAATATTCATATGTAGCATGTTTAAGCATTAAATCAAATAAGATGAGCAAGATGGAA
CGTCTGGGTTCCAATTCTGTCAAGACTTATGACATATTAAATAGATTGGTTACGGTAAAAGACAAGGC
AAGGTATGGAAGATC
+
F:FFFFFFFFFFFFFFFFFFFFFFFFFFFFFFFFFFFFFFFFFFFFFFFFFFFFFFFFFFFFFFFFFFFF
FFFFFFFFFFFFFFFFFFFFFFFFFFFFFFFFFFFFFFFFFFFFFFFFFFFFFFFFFFFFFFFFFFFFFF
FFFFFF:FFFFFFFF
@A00155:342:HHGFNDSXY:1:1435:8585:20525 2:N:0:GAACCTAG+TCCGCATA
GTTACATTAGTAAATATTCATATGTAGCATGTTTAAGCATTAAATCAAATAAGATGAGCAAGATGGAA
CGTCTGGGTTCCAATTCTGTCAAGACTTATGACATATTAAATAGATTGGTTACGGTAAAAGACAAGGC
AAGGTATGGACCCTT
+
FFFFFFFFFFFFFFFFFFFFFFFFFF:FFFFFFFFFFFFFFFFFFFFFFFFFFFFFFFFFFFFFFFFFFF
FFFFFFFFFFFFFFFFFFFFFFFFFFFFFFFFFFFFFFFFFFFFFFFFFFFFFFFFFFFFFF:FFFFFF:
FFFFFFFFFFFFFFF
@A00155:342:HHGFNDSXY:1:1528:30978:6042 2:N:0:GAACCTAG+TCCGCATA
GTTACATTAGTAAATATTCATATGTAGCATGTTTAAGCATTAAATCAAATAAGATGAGCAAGATGGAA
CGTCTGGGTTCCAATTCTGTCAAGACTTATGACATATTAAATAGATTGGTTACGGTAAAAGACAAGGC
AAGGTATGGACAGAT
+
FFFFFFFFFFFFFFFFFFFFFFFFFFFFFFFFFFFFFFFFFFFFFFFFFFFFFFFFFFFFFFFFFFFFFF
FFFFFFFFFFFFFFFFFFFFFFFFFFFFFFFFFFFFFFFFFFFFFFFFFFFFFFFFFFFFFFFFFFFFFF
FF:F:FFFFFFFFFF
@A00155:342:HHGFNDSXY:1:1528:16052:20337 2:N:0:GAACCTAG+TCCGCATA
GTTACATTAGTAAATATTCATATGTAGCATGTTTAAGCATTAAATCAAATAAGATGAGCAAGATGGAA
CGTCTGGGTTCCAATTCTGTCAAGACTTATGACATATTAAATAGATTGGTTACGGTAAAAGACAAGGC
AAGGTATGGACCCTT
+
FFFFFFFFFFFFFFFFFFFFFFF:FFFFFFFFFFFFFFFFFFFFFFFFFFFFFFFFFFFFFFFFFFFFFF
FFFFFFFFFFFFFFFFFFFFFFFFFFFFFFFFFFFFFFFFFFFF,FFFFFFFFFFFFFFFFFFFFFF:FF
FFFFFFFFFFFFFFF
@A00155:342:HHGFNDSXY:1:1660:25328:32800 2:N:0:GAACCTAG+TCCGCATA
GTTACATTAGTAAATATTCATATGTAGCATGTTTAAGCATTAAATCAAATAAGATGAGCAAGATGGAA
CGTCTGGGTTCCAATTCTGTCAAGACTTATGACATATTAAATAGATTGGTTACGGTAAAAGACAAGGC
AAGGTATGGACCCTT
+
FFFFFFFFFFFFFFFFFFFFFFFFFFFFFFFFFFFFFFFFFFFFFFFFF:FFFFFFFFFFFF,FFFFFFF
FFFFFFFFFFFFFFFFFFFFFFFFFFFFFFFFFFFFFFFFFFFFFFFFFFFFFFFFFF:FFFFFFFFFF:
F:FFFFFF,FFFFFF
@A00155:342:HHGFNDSXY:1:2149:3387:7326 2:N:0:GAACCTAG+TCCGCATA
GTTACATTAGTAAATATTCATATGTAGCATGTTTAAGCATTAAATCAAATAAGATGAGCAAGATGGAA

CGTCTGGGTTCCAATTCTGTCAAGACTTATGACATATTAAATAGATTGGTTACGGTAAAAGACAAGGC
AAGGTATGGACCCTT
+
FFFFFFFFFFFFFFFFFFFFFFFFFFFFFFFFFFFFFFFFFFFFFFFFFFFFFFFFFFFFFFFFFFFF
FFFFFFFFFFF,FFFFFFFFFF:FFFFFFFFFFFFFFFFFFFFFFFFFFFFFFFFFFF:FFFFFFFFF
FFFFFFFFFFFFFFF
@A00155:342:HHGFNDSXY:1:2177:4155:33238 2:N:0:GAACCTAG+TCCGCATA
GTTACATTAGTAAATATTCATATGTAGCATGTTTAAGCATTAAATCAAATAAGATGAGCAAGATGGAA
CGTCTGGGTTCCAATTCTGTCAAGACTTATGACATATTAAATAGATTGGTTACGGTAAAAGACAAGGC
AAGGTATGGACCCTT
+
FFFFFFFFFFFFFFFFFFFFFFFFFFFFFFFFFFFFFFFFFFFFFFFFFFFFFFFFFFFFFFFFFFFF
FFFFFFFFFFFFFFFFFFFFFFF:FFFFFFFFFF:FFFFFFFFFF:FFFFFFFFFFFFFFFFFFFFFF
:FFFF,FFFFFFF:F
@A00155:342:HHGFNDSXY:1:2211:11948:9283 2:N:0:GAACCTAG+TCCGCATA
GTTACATTAGTAAATATTCATATGTAGCATGTTTAAGCATTAAATCAAATAAGATGAGCAAGATGGAA
CGTCTGGGTTCCAATTCTGTCAAGACTTATGACATATTAAATAGATTGGTTACGGTAAAAGACAAGGC
AAGGTATGGACCCTT
+
FFFFFFFFFFFFFFFFFFFFFFFFFFFFFFFFFFFFFFFFFFFFFFFFFFFFFFFFFFFFFFFFFFFF
FFFFFFFFFFFFFFFFFFFFFF:FF:FFFFFFFFFFFFFFFFF,:FFFFFFFFFFF:FFFF:FFFFFF
FFFFFFFFFFFFFFF
@A00155:342:HHGFNDSXY:1:2177:3360:36933 2:N:0:GAACCTAG+TCCGCATA
GTTACATTAGTAAATATTCATATGTAGCATGTTTAAGCATTAAATCAAATAAGATGAGCAAGATGGAA
CGTCTGGGTTCCAATTCTGTCAAGACTTATGACATATTAAATAGATTGGTTACGGTAAAAGACAAGGC
AAGGTATGGACCCTT
+
FFFFFFFFFFFFFFFFFFFFFFFFFFFFFFFFFFFFFFFFFFFFFFFFFFFFFFFFFFFFFFFFFFFF
FFFFFFFFFFFFFFFFFFFFFFF:FFFFFFFFFFFFFFF,FF:FFFFFF:FFFFFFFF,FFFFFFFFF
FFFFFFFFFFFFFFF
@A00155:342:HHGFNDSXY:1:2258:19117:27899 2:N:0:GAACCTAG+TCCGCATA
GTTACATTAGTAAATATTCATATGTAGCATGTTTAAGCATTAAATCAAATAAGATGAGCAAGATGGAA
CGTCTGGGTTCCAATTCTGTCAAGACTTATGACATATTAAATAGATTGGTTACGGTAAAAGACAAGGC
AAGGTATGAGATCGG
+
FFFFFF:FFFFFFFFFFFFFFFFFFFFFFFFFFFFFFFFFFFFFFFFFFFFFFFFFFFFFFF:FFFFF
FFFFFF:FFFFFFFFFFFFFFFFFFFFFFFFFFFFFFFFFFFFFFFFFFFFFFFFFFF,FFFFFFFFF
:FFFFF:FFFFFFFF
@A00155:342:HHGFNDSXY:1:2325:32208:17159 2:N:0:GAACCTAG+TCCGCATA
GTTACATTAGTAAATATTCATATGTAGCATGTTTAAGCATTAAATCAAATAAGATGAGCAAGATGGAA
CGTCTGGGTTCCAATTCTGTCAAGACTTATGACATATTAAATAGATTGGTTACGGTAAAAGACAAGGC
AAGGTATGGACCCTT
+
FFFFF:FFFFFFFFFFFFFFFFFF,FFFFFFFFFFFFFFFFFFFFFFFFFFFFFFFFFFFFFFFFFFF
FFFFFFFFFFFFFFFFFFFFFFFFFFFFFFF:FFFFFFFFFFFFFFFFFFFFFFFFFFFFFFFFFFFF
F:FFFFFFFFFFFF:
@A00155:342:HHGFNDSXY:1:2505:29161:36526 2:N:0:GAACCTAG+TCCGCATA
GTTACATTAGTAAATATTCATATGTGGCATGTTTAAGCATTAAATCAAATAAGATGAGCAAGATGGAA
CGTCTGGGTTCCAATTCTGTCAAGACTTATGACATATTAAATAGATTGGTTACGGTAAAAGACAAGGC
AAGGTATGGACCCTT
+
FFFFFFFFFFFFFFFFFFFFFFFFFFFFFFFFFFFFFFFFFFFFFFFFFFFFFFFFFFFFFFFFFFFF
FFFF:FFFFFFFFFFFFFFFFFFFFFFFFFF:FFFFFFFF:FFFFFFFFFFFFFFF:FFFFFFFFFFF
FFFFFFFFFFF:FFF

@A00155:342:HHGFNDSXY:1:2508:4300:33176 2:N:0:GAACCTAG+TCCGCATA
GTTACATTAGTAAATATTCATATGTAGCATGTTTAAGCATTAAATCAAATAAGATGAGCAAGATGGAA
CGTCTGGGTTCCAATTCTGTCAAGACTTATGACATATTAAATAGATTGGTTACGGTAAAAGACAAGGC
AAGGTATGGACCCTT
+
FFFFFFFFFFFFFFFFFFFFFFFFFFFFFFF:FFFFFFFFFFFFFFFFFFFFFFFFFFFFFFFFFFFF
FFFFFFFFFFFFFFFFF:FFFFFFFFFFFFFFFFFFFF:F:FFFFFFFFFFFFFFFFF:FFFFFFFFF
,FFFFFFFFFFFFFF
@A00155:342:HHGFNDSXY:1:2551:22073:17519 2:N:0:GAACCTAG+TCCGCATA
GTTACATTAGTAAATATTCATATGTAGCATGTTTAAGCATTAAATCAAATAAGATGAGCAAGATGGAA
CGTCTGGGTTCCAATTCTGTCAAGACTTATGACATATTAAATAGATTGGTTACGGTAAAAGACAAGGC
AAGGTATGGACCCTT
+
FFFFFFFFFFFFFFFFFFFFFFFFFFFFFFFFFFFFFFFFFFFFFFFFFFFFFFFFFFFFFFFFFFF:
FFFFFFFFFFFFFFFFFFFFFFFFFFFFFFFFFFFFFFFFFFFF:FFFFFFFFFFFFFFFFFFF:FFF
FFFFFFFFFFFFFFF
@A00155:342:HHGFNDSXY:1:2563:25590:28432 2:N:0:GAACCTAG+TCCGCATA
GTTACATTAGTAAATATTCATATGTAGCATGTTTAAGCATTAAATCAAATAAGATGAGCAAGATGGAA
CGTCTGGGTTCCAATTCTGTCAAGACTTATGACATATTAAATAGATTGGTTACGGTAAAAGACAAGGC
AAGGTATGGACCCTT
+
FFFFFF,FFFFFFFFFFFFFF:FFFFFFFFFFFFFFFFFFFFF:FFFFFFFFFFFFFFFFFFFFFFFF
FFFFFFFFFFFFFFF:FFFFFFFFFFFFFFFFFFFFFFFFFFF:FFFFFFFFFFFFFFFFFFFFFFFF
FFFFFFFF:FFFFFF
@A00155:342:HHGFNDSXY:1:2563:25328:29825 2:N:0:GAACCTAG+TCCGCATA
GTTACATTAGTAAATATTCATATGTAGCATGTTTAAGCATTAAATCAAATAAGATGAGCAAGATGGAA
CGTCTGGGTTCCAATTCTGTCAAGACTTATGACATATTAAATAGATTGGTTACGGTAAAAGACAAGGC
AAGGTATGGACCCTT
+
F:FFFFFFFFFFFFFFFFFFF:FFFFFFFFFFFFFFFFFFFFFFFFFFFFFFFFFFFFFFFFFFFFFF
FFFFFFFFFFFFFFFFFFFFFFFFFFFFFFFFFFFF:FFFFFFFFFFFFFFF:FFFFFFFFFFFFFFF
F:FFFFFFFFFFFF:
@A00155:342:HHGFNDSXY:1:1160:12400:4554 2:N:0:GAACCTAG+TCCGCATA
TTACATTAGTAAATATTCATATGTAGCATGTTTAAGCATTAAATCAAATAAGATGAGCAAGATGGAAC
GTCTGGGTTCCAATTCTGTCAAGACTTATGACATATTAAATAGATTGGTTACGGTAAAAGACAAGGCA
AGGTATGGACCCTTA
+
FFFFFFFFFFFFFFFFFFFFFFFFFFFFFFFFFFFFFFFFFFFFFFFFFFFF:FFFFFFFFFFFFFFF
FFFFFFFF:FFFFFFFFFFFFFFFFFFFFFFFFFFFFFFFFFFFFFFFFFF:FFFFF,FF:FFFFFFF
:FFFFFFFFFFFFF:
@A00155:342:HHGFNDSXY:1:1354:3983:21010 2:N:0:GAACCTAG+TCCGCATA
TTACATTAGTAAATATTCATATGTAGCATGTTTAAGCATTAAATCAAATAAGATGAGCAAGATGGAAC
GTCTGGGTTCCAATTCTGTCAAGACTTATGACATATTAAATAGATTGGTTACGGTAAAAGACAAGGCA
AGGTATGGACCCTTA
+
FFFFFFFFFFFFFFFFFFFFFFFFFFFFFFFFFFFFFFFFFFFFFFFFFFFFFFFFFFFFFFFFFFFF
FFFFFFFFFFFFFFFFFFFFFFFFFFFFFFFFFFF:FFF:FFFFFFFFFF,FFFFFFFFF:FFFFFFF
FFFFFFFFFFFFFFF
@A00155:342:HHGFNDSXY:1:1514:27525:1031 2:N:0:GAACCTAG+TCCGCATA
TTACATTAGTAAATATTCATATGTAGCATGTTTAAGCATTAAATCAAATAAGATGAGCAAGATGGAAC
GTCTGGGTTCCAATTCTGTCAAGACTTATGACATATTAAATAGATTGGTTACGGTAAAAGACAAGGCA
AGGTATGGACCCTTA
+
FFFFFFFF:FFFFFFFFFFFFFFFFF:F,:FFFFFFFFFFFFFFFFFFFFFFFFFFFFFFFFFFFFFF

FFFFFFFFFFFF:FFFFFFFFFFFFFF:FFFFFFFFFFFFFFFF,:FFF,FFFFFFF:FFFFFFFF,F
,F,F::FFFFF:FFF
@A00155:342:HHGFNDSXY:1:2505:28257:17895 2:N:0:GAACCTAG+TCCGCATA
TTACATTAGTAAATATTCATATGTAGCATGTTTAAGCATTAAATCAAATAAGATGAGCAAGATGGAAC
GTCTGGGTTCCAATTCTGTCAAGACTTATGACATATTAAATAGATTGGTTACGGTAAAAGACAAGGCA
AGGTATGGACCCTTA
+
FFFFFFFFFFFFFFFFFFFFFFFFFFFF,FFFFFFFFFFFFFFFFFFFFFFFFFFFFFFFFFFFFFFF
FFFFFFFFFFFFFFFFFFFFFFFFFFFF:FFFFFFFFFFFFFFFFFFFFFF:FFFFFFFFFFF::F:F
FFFF::FFFFFF:FF
@A00155:342:HHGFNDSXY:1:2254:9661:26960 1:N:0:GAACCTAG+TCCGCATA
TACATTAGTAAATATTCATATGTAGCATGTTTAAGCATTAAATCAAATAAGATGAGCAAGATGGAACG
TCTGGGTTCCTATTCTGTCAAGACTTATGACATATTAAATAGATTGGTTACGGTAAAAGACAAGGCAA
GGTATGGACCCTTAG
+
FFFFFFFFFFFFFFFFFFFFFFFFFFFF:FFFFF:FFFFFFFFFFFFFFFFFFFFFFFFFFFFFFFFF
FFFFFFFFFFFFFFFFFFFFFFFFFFFF:FFFFFFFFFFFFFFFFFFFFFFFFFFFFFFFFFFFFFFF
FFFFFFFFFFFFFFF
@A00155:342:HHGFNDSXY:1:2333:8594:14403 1:N:0:GAACCTAG+TCCGCATA
TACATTAGTAAATATTCATATGTAGCATGTTTAAGCATTAAATCAAATAAGATGAGCAAGATGGAACG
TCTGGGTTCCAATTCTGTCAAGACTTATGACATATTAAATAGATTGGTTACGGTAAAAGACAAGGCAA
GGTATGGACCCTTAG
+
FFFFFFFFFFFFFFFFFFFFFFFFFFFFFFFFFFFFFFFFFFFFFFFFFFFFFFFFFFFFFFFFF:FF
FFFFFFFFFFFFFFFFFFFFFFFFFFFFFFFFFFFFFFFFFFFFFFFFFFFFFFFFFFFFFFFFFFFF
FFFFFFFFFFFFFFF
@A00155:342:HHGFNDSXY:1:1615:10257:35008 2:N:0:GAACCTAG+TCCGCATA
TACATTAGTAAATATTCATATGTAGCATGTTTAAGCATTAAATCAAATAAGATGAGCAAGATGGAACG
TCTGGGTTCCAATTCTGTCAAGACTTATGACATATTAAATAGATTGGTTACGGTAAAAGACAAGGCAA
GGTATGGACCCTTAG
+
FFFFFFFFFFFFFFFFFFFFFFFFFFFFFFFFFFFFFFFFFFFFFFFFFFFFFFFFFFFFFFFFFFF:
FFFF:FFFFFFFFFFFFFFFF,FFFFFFFFFFFFFFFFFFFFFFFFFFFF:FFFFF:FFFFFFFFFF:
FFFFFFFFFFFFFFF
@A00155:342:HHGFNDSXY:1:2272:26928:16000 2:N:0:GAACCTAG+TCCGCATA
TACATTAGTAAATATTCATATGTAGCATGTTTAAGCATTAAATCAAATAAGATGAGCAAGATGGAACG
TCTGGGTTCCAATTCTGTCAAGACTTATGACATATTAAATAGATTGGTTACGGTAAAAGACAAGGCAA
GGTATGGACCCTTAG
+
FFFFFFFFFFFFFFFFFFFFFFFFFFFFFFFFFFFFFFFFFFFFFFFFFFFFFFFFFFFFFFFFFFFF
FFFFFFFF:FFFFFF:FFFFFFFFFFFFFFFFFFFFFFFFFFFFFFFFFFFFFFF:FFFFFFFFFFFF
FFFF:FFFFFFFFFF
@A00155:342:HHGFNDSXY:1:2124:5981:33301 1:N:0:GAACCTAG+TCCGCATA
ACATTAGTAAATATTCATATGTAGCATGTTTAAGCATTAAATCAAATAAGATGAGCAAGATGGAACGT
CTGGGTTCCAATTCTGTCAAGACTTATGACATATTAAATAGATTGGTTACGGTAAAAGACAAGGAAAG
GTATGGACCCTTAGC
+
FFFFFFFFFFFFFF:FFFFFFFFFFFFFFFFFFFFF:FFFFFFFFFFFFFFFFFFFFFFFFFFFFFFF
FFFFFFFFFFFFFFFFFFFFFFFFFFFFFFFFF:F:FFFFFF,FFFFFFFFFFFF,FFFFFFFFFFFF
FFFFFFFFFFFFFFF
@A00155:342:HHGFNDSXY:1:2260:25464:14998 1:N:0:GAACCTAG+TCCGCATA
ACATTAGTAAATATTCATATGTAGCATGTTTAAGCATTAAATCAAATAAGATGAGCAAGATGGAACGT
CTGGGTTCCAATTCTGTCAAGACTTATGACATATTAAATAGATTGGTTACGGTAAAAGACAAGGAAAG
GTATGGACCCTTAGC

```
+
FFF,FFFFFFFFFFFFFF:FFFFFFFFF:FFFFFFFFFFFFFFFFFFFFFFFF:FFFFFFFFFFFFFF
FFFFFFFFFFFFFFFFFFFFFFFFFFFFFF:F,FFFF:FFFFFFFFF,FFFFF,FFF:FF:FFFFF,F
FFFFFFFFFFFFFFF
@A00155:342:HHGFNDSXY:1:2546:32524:26506 2:N:0:GAACCTAG+TCCGCATA
ACATTAGTAAATATTCATATGTAGCATGTTTAAGCATTAAATCAAATAAGATGAGCAAGATGGAACGT
CTGGGTTCCAATTCTGTCAAGACTTATGACATATTAAATAGATTGGTTAGGGTAAAAGACAAGGCAAG
GTATGGACCCTTAGC
+
FF,,FFFFFFFFFFFFFFFF:FFF::F:FFFFFF,FF:FFFFFFFFFFFFFFFFF,FFFFFFFFFF
FFFFF:FFFFFF:FFFFFFFFFFFFFFF,FF:FF:FF:::FFF,FF,FFFFF:F,FFFFFFFFFF:FF
F:,FFF:FFF:F:FF
@A00155:342:HHGFNDSXY:1:2546:32136:26772 2:N:0:GAACCTAG+TCCGCATA
ACATTAGTAAATATTCATATGTAGCATGTTTAAGCATTAAATCAAATAAGATGAGCAAGATGGAACGT
CTGGGTTCCAATTCTGTCAAGACTTATGACATATTAAATAGATTGGTTACGGTAAAAGACAAGGCAAG
GTATGGACCCTTAGC
+
FFFFFFFFFFFFFFFFFFFFFFFFFFFFFFFFFFFFFFFFFFFFFFFFFFFFFFFFFFFFFFFFFFFF
FFFFFFFFFFFFFFFFFFFFFFFFFFFFFFFFFFFFFFFFF:FFF:FFFF,FFF:FFFFFFFF:FFF,F
FFFFFFFFFFFFFFF
@A00155:342:HHGFNDSXY:1:1512:19416:28855 2:N:0:GAACCTAG+TCCGCATA
CATTAGTAAATATTCATATGTAGCATGTTTAAGCATTAAATCAAATAAGATGAGCAAGATGGAACGTC
TGGGTTCCAATTCTGTCAAGACTTATGACATATTAAATAGATTGGTTACGGTAAAAGACAAGGCAAGG
TATGGACCCTTAGCA
+
FFFFFFFFFFFFFFFFFFFFFFFFFFFFFFFFFFFFFFFFFFFFFFFFFFFFFFFFFFFFFFFFFFFF
FFFFFFFFFFFFF,FFFFFFFFFFFFFFFFFFFFFFFFFFFFFFFFFFFFFFFFFFFFFFFFFFFFFF
FFFFFFFFFFFFFFF
@A00155:342:HHGFNDSXY:1:2525:6424:18693 2:N:0:GAACCTAG+TCCGCATA
CATTAGTAAATATTCATATGTAGCATGTTTAAGCATTAAATCAAATAAGATGAGCAAGATGGAACGTC
TGGGTTCCAATTCTGTCAAGACTTATGACATATTAAATAGATTGGTTACGGTAAAAGACAAGGCAAGG
TATGGACCCTTAGCA
+
FFFFFFFFFFFFFFFFFF:FFFFFFFFFFFFFFFF:FFFFFFFFF:F:FFFFFF:FFFF:FFFFF:FF
FFFFFFFFFFFF,FFFFFFFFFFFFF,F,FF:FFFFFFFFFFFF,FFFFFFFFFFFFF:FFFFFF:FF
F::FF:F,F:FFF:F
@A00155:342:HHGFNDSXY:1:2569:22155:2096 1:N:0:GAACCTAG+TCCGCATA
TAGTAAATATTCATATGTAGCATGTTTAAGCATTAAATCAAATAAGATGAGCAAGATGGAACGTCTGG
GTTCCAATTCTGTCAAGACTTATGACATATTAAATAGATTGGTTACGGTAAAAGACAAGGCAAGGTAT
GGACCCTTAGCAAAG
+
FFFFFFFFFFFFFFFF:FFFFFFF:FFFFF:FFFFFFFFFFFFFFFFFFFFFFFFFFFFFFFFFFFFF
FFFFFFFFFFFFFFFFFFFFFFFFFFFFFFFFFFFFFFFFFFFFFFFFFFFFFFFFFFFFFFFFFFFF
FFFFFFFFFFFFFFF
@A00155:342:HHGFNDSXY:1:1607:28456:13228 1:N:0:GAACCTAG+TCCGCATA
AGTAAATATTCATATGTAGCATGTTTAAGCATTAAATCAAATAAGATGAGCAAGATGGAACGTCTGGG
TTCCAATTCTGTCAAGACTTATGACATATTAAATAGATTGGTTACGGTAAAAGACAAGGCAAGGTATG
GACCCTTAGCAAAGC
+
FFFFFFFFFFFFFFFFFFFFFFFFFFFFFFFFFFFFFFFFFFFFFFFFFFFFFFFFFFFFFFFFFFFF
FFFFFFFFFFFFFFFFFFFFFFFFFFFFFFFFFFFFFFFFFFFFFFFFFFFFFFFFFFFFFFFFFFFF
FFFFFFFFFFFFFFF
@A00155:342:HHGFNDSXY:1:1562:30789:29919 1:N:0:GAACCTAG+TCCGCATA
AGTAAATATTCATATGTAGCATGTTTAAGCATTAAATCAAATAAGATGAGCAAGATGGAACGTCTGGG
```

TTCCAATTCTGTCAAGACTTATGACATATTAAATAGATTGGTTACGGTAAAAGACAAGGCAAGGTATG
GACCCTTAGCAAAGC
+
FFFFFFFFFFFFFFFF:FF,FFF,FFFF::FFFFFFFFFFFFFFFFF:FFFFF,FFFFFFFFFFFFFFFF
FFFFFF,FFFFFFFFFFFFFFFFFFFFFFFFFFFFFFFFFFFFFFFFFFFFFFFF:FFFFFFFFFFF
FFFFFFFFFFFFFFF
@A00155:342:HHGFNDSXY:1:1618:30987:10066 1:N:0:GAACCTAG+TCCGCATA
AGTAAATATTCATATGTAGCATGTTTAAGCATTAAATCAAATAAGATGAGCAAGATGGAACGTCTGGG
TTCCAATTCTGTCAAGACTTATGACATATTAAATAGATTGGTTACGGTAAAAGACAAGGCAAGGTATG
GACCCTTAGCAAAGC
+
:FFFFFFFFFFFFFFFFFFFFFFFFFFF:FFFFFFFFFFFFFFFFFFFFFFFFFFFFFFFFFFFFFFFFF
FFFFFFFFFFFFFF:F:FFFFFFFFFFFFFFFFFFFFFFFFFFFFFFFFFFFFFFFFFFFFFFFFFFFF
FFFFFFFFFFFFFFF
@A00155:342:HHGFNDSXY:1:1634:17499:7122 1:N:0:GAACCTAG+TCCGCATA
AGTAAATATTCATATGTAGCATGTTTAAGCATTAAATCAAATAAGATGAGCAAGATGGAACGTCTGGG
TTCCAATTCTGTCAAGACTTATGACATATTAAATAGATTGGTTACGGTAAAAGACAAGGCAAGGTATG
GACCCTTAGCAAAGC
+
FFFFFFFFFFFFFFFFFFFF:FFFFFFFFFFFFFFFFFF:FFFFFFFFFFFFFFFFFFFFFFFFFFFFFF
FFFFFFFFFFFFFFFFFFFFFFFFFFFFFFFFFFFFFFFFFFFFFFFFFF:FFFFFFFFFFFFFFFFFFF
FFFFFFFFF:FFFFF
@A00155:342:HHGFNDSXY:1:1643:3694:16376 1:N:0:GAACCTAG+TCCGCATA
AGTAAATATTCATATGTAGCATGTTTAAGCATTAAATCAAATAAGATGAGCAAGATGGAACGTCTGGG
TTCCAATTCTGTCAAGACTTATGACATATTAAATAGATTGGTTACGGTAAAAGACAAGGCAAGGTATG
GACCCTTAGCAAAGC
+
FFFFFFFFFFFFFFFFFFFFFFF:FFFFFFFFFFFFFFFFFFFFFFFFFFFFFFFFFFFFFFFFFFFFFF
FFFFFF,:FFFFFFF:FFFFF:FFFFFFFFFFFFFFFFFFF:FFFFFFFFFFFFFFFFFFFFF:FFFFFF
FFFFFFFFFFFFFFF
@A00155:342:HHGFNDSXY:1:2371:31168:8375 1:N:0:GAACCTAG+TCCGCATA
AGTAAATATTCATATGTAGCATGTTTAAGCATTAAATCAAATAAGATGAGCAAGATGGAACGTCTGGG
TTCCAATTCTGTCAAGACTTATGACATATTAAATAGATTGGTTACGGTAAAAGACAAGGCAAGGTATG
GACCCTTAGCAAAGC
+
FFFFFFFFFFFFFFFFFFFFFFFFF:FFFFFFFFFFFFFFFFFFFFFFFFFFFFFFFFFFFFFFFFFFFF
FFFFFFFFFFFFFFFFFFFFFFFFFFFFFFFFFFFFFFFFFFFFFFFFFFFFFFFFFFFFFFFFFFFFFF
FFFFFFFFFFFFFFF
@A00155:342:HHGFNDSXY:1:2563:24713:33739 1:N:0:GAACCTAG+TCCGCATA
AGTAAATATTCATATGTAGCATGTTTAAGCATTAAATCAAATAAGATGAGCAAGATGGAACGTCTGGG
TTCCAATTCTGTCAAGACTTATGACATATTAAATAGATTGGTTACGGTAAAAGACAAGGCAAGGTATG
GACCCTTAGCAAAGC
+
FFFFFFFFFFFFFFFFF:FFFFFFFFFFFFFFFFFFFFFFFFFFFFFFFFFFFFFFFFFFFFFFFFFFFF
FFFFFFFFFFFFFFFFFFFFFFFFFFFFFFFFFFFFFFFFFFFFFFFFFFFFFFFFFFFFFFFFFFFFFF
FFFFFFFFFFFFFFF
@A00155:342:HHGFNDSXY:1:2668:13810:36432 1:N:0:GAACCTAG+TCCGCATA
AGTAAATATTCATATGTAGCATGTTTAAGCATTAAATCAAATAAGATGAGCAAGATGGAACGTCTGGG
TTCCAATTCTGTCAAGACTTATGACATATTAAATAGATTGGTTACGGTAAAAGACAAGGCAAGGTATG
GACCCTTAGCAAAGC
+
FFFFFFFFFFF:FFFFFFFFFFF,FFFFFFFFFFFFFFFFFFFFFFFFFFFFFFFFFFFFFFFFFFFFFF
FFFFFF:FFFFFFFFFFFFFFFFFFFFFFFFFFFFFFFFFFFFFFFFFFFFFFFFFFFFFFFFFFFFFFF
FFFFFFFFFFFFFFF

```
@A00155:342:HHGFNDSXY:1:1321:2284:15812 2:N:0:GAACCTAG+TCCGCATA
AGTAAATATTCATATGTAGCATGTTTAAGCATTAAATCAAATAAGATGAGCAAGATGGAACGTCTGGG
TTCCAATTCTGTCAAGACTTATGACATATTAAATAGATTGGTTACGGTAAAAGACAAGGCAAGGTATG
GACCCTTAGCAAAGC
+
:FFFFFFFFFFFFFFFFFFFFFFFFFFFFFFFFFFFFFFFFFFFFFFFFFFFFFFFFFFFFFFFFFFF
FFFFFFFFFFFFFFFFFFFFFFFFFFFFFFFFFFFFFFFFFFFFFFFFFFFFFFFFFFFFFFFFFFFF
FFF:FFFFF:FF:FF
@A00155:342:HHGFNDSXY:1:1322:4074:12493 2:N:0:GAACCTAG+TCCGCATA
AGTAAATATTCATATGTAGCATGTTTAAGCATTAAATCAAATAAGATGAGCAAGATGGAACGTCTGGG
TTCCAATTCTGTCAAGACTTATGACATATTAAATAGATTGGTTACGGTAAAAGACAAGGCAAGGTATG
GACCCTTAGCAAAGC
+
FFFFFFFFFFFFFFFFFFFFFFFFFFFFFFFFFFF:FFFFFFFFFFFF::FFFFFFFFFFFFFFFFFF
FFFFF:FFF:FFFFFFFFFFFFF:FFFFFFFF:FFFFFFF,:F::FFFFF:F:FFF:FFFFFFF::FF
FFF,FFFFFFF:FFF
@A00155:342:HHGFNDSXY:1:1375:3676:18693 2:N:0:GAACCTAG+TCCGCATA
AGTAAATATTCATATGTAGCATGTTTAAGCATTAAATAAAATAAGATGAGCAAGATGGAACGTCTGGG
TTCCAATTCTGTCAAGACTTATGACATATTAAATAGATTGGTTACGGTAAAAGACAAGGCAAGGTATG
GACCCTTAGCAAAGC
+
FFF:FFFFFFFFFFFFFF:FF::FFFFFFFFFFFFFF,FFFFFFFFFFFFFFFFFFFFFFFFFFFFFF
FFFFFFFFFFFFFFFFFFFFFFFFFFFFF:F:FFFFFFFFF:FFFFFFF::FFFFFFF:FFFFFFFFF
FFFFFFFFFFFFFFF
@A00155:342:HHGFNDSXY:1:1516:1994:1752 2:N:0:GAACCTAG+TCCGCATA
AGTAAATATTCATATGTAGCATGTTTAAGCATTAAATCAAATAAGATGAGCAAGATGGAACGTCTGGG
TTCCAATTCTGTCAAGACTTATGACATATTAAATAGATTGGTTACGGTAAAAGACAAGGCAAGGTATG
GACCCTTAGCAAAGC
+
FFFFFFFFFFFFFFFFFFFFFFF:FFFFFFFFFFF,FFFFFFFFFFFFFFFFF,FFFF:FFFFFFFFFF
FF,FFFFF:FFFFFF,FFFFFFFFFFFFFFFFFFFFFFFFFF,FFFFFFFFFFFFFFFFFFFFFFF:FF
FFFFFFFFF:FFFFF
@A00155:342:HHGFNDSXY:1:1634:17499:7122 2:N:0:GAACCTAG+TCCGCATA
AGTAAATATTCATATGTAGCATGTTTAAGCATTAAATCAAATAAGATGAGCAAGATGGAACGTCTGGG
TTCCAATTCTGTCAAGACTTATGACATATTAAATAGATTGGTTACGGTAAAAGACAAGGCAAGGTATG
GACCCTTAGCAAAGC
+
FFFFFFFFF:FFFFFFFFFFFFFFFFFFFFFFFFFFFFFFFFFFFFFFFFFFFFFFFFF,FFFFFFFF
FFFFFFFFFFFFFFFFFFFFFFFFFFFFFFFFFFFFF,FFFFF:FFFFFFFFFFFFFFFFFFFFF,FF
FFFFFFFFFFFFFFF
@A00155:342:HHGFNDSXY:1:2276:1913:19523 2:N:0:GAACCTAG+TCCGCATA
AGTAAATATTCATATGTAGCATGTTTAAGCATTAAATCAAATAAGATGAGCAAGATGGAACGTCTGGG
TTCCAATTCTGTCAAGACTTATGACATATTAAATAGATTGGTTACGGTAAAAGACAAGGCAAGGTATG
GACCCAGATCGGACG
+
FFFFFFFFF:FFFFFFFFFFF,FFFFFFFFFF,FFF:FFFFFFFFFFFFFF:FF,F::FFFFFFFFFF
F:FFFFF:FF,FFFFFFFFFF:FFFFFFFFFFF,FFFFFF,:FFFF:F,FFFFFFFFFFFFFFFF:F:
FFFF:F:FFF:FF,:
@A00155:342:HHGFNDSXY:1:2261:22833:10473 2:N:0:GAACCTAG+TCCGCATA
AGTAAATATTCATATGTAGCATGTTTAAGCATTAAATCAAATAAGATGAGCAAGATGGAACGTCTGGG
TTCCAATTCTGTCAAGACTTATGACATATTAAATAGATTGGTTACGGTAAAAGACAAGGCAAGGTATG
GACCCTTAGCAAAGC
+
FFFFFFFFFFFFFFFFFFFFFFFFFFFFFFFFFFFFFFFFFFFFFFFFFFFFFFFFFFFFFFFFFFFF
```

```
FFFFFFFFFFFFFFFFFFFFFFFFFFFFFFFFF:FFFFFFFFFFFFFFF:FFFFFFFFFFFFFFFFF:FF
FFFFFFFFFFFFFFF
@A00155:342:HHGFNDSXY:1:2311:23710:33473 2:N:0:GAACCTAG+TCCGCATA
AGTAAATATTCATATGTAGCATGTTTAAGCATTAAATCAAATAAGATGAGCAAGATGGAACGTCTGGG
TTCCAATTCTGTCAAGACTTATGACATATTAAATAGATTGGTTACGGTAAAAGACAAGGCAAGGTATG
GACCCTTAGCAAAGC
+
FFFFFFFFFFFFFFFFFFFFFFFFFFFFF:FFFFFFF:FFFFFFFFFFFFFFFFFFF::FFFFFFFFFFF
FFF,FFFFFFFF:FF:FFFFFFFFFFFFFFFFFFFFF:FFF:FFFFFF:FFFFFFFFFFF:FFF::FFF:
FFFFFFFFFFFFFFF
@A00155:342:HHGFNDSXY:1:2645:16179:24752 2:N:0:GAACCTAG+TCCGCATA
AGTAAATATTCATATGTAGCATGTTTAAGCATTAAATCAAATAAGATGAGCAAGATGGAACGTCTGGG
TTCCAATTCTGTCAAGACTTATGACATATTAAATAGATTGGTTACGGTAAAAGACAAGGCAAGGTATG
GACCCTTAGCAAAGC
+
FFFFFFFFFFFFFF:FFFFFF:FFFFFFFFFFFFFFFFFFFFFFFFFFFFFFFFFFFFFFFFFFFFFFFF
FFFFFFFFFFFFFFF:FFFFFFFFFFFFFFFFFFFFFFFFFFFFFFFFFFFFFFFFFFFFFFFFF:FFFF
FFFFFFFFF:FFFFF
@A00155:342:HHGFNDSXY:1:1349:8739:30373 2:N:0:GAACCTAG+TCCGCATA
GTAAATATTCATATGTAGCATGTTTAAGCATTAAATCAAATAAGATGAGCAAGATGGAACGTCTGGGT
TCCAATTCTGTCAAGACTTATGACATATTAAATAGATTGGTTACGGTAAAAGACAAGGCAAGGTATGG
ACCCTTAGCAAA
+
F::FFFFFFFFFFFFFFFFFFFFFFFFFFFFFFFFFFFFFFFFFFFFFFFFFFFFFFFFFFFFFFFFFFF
F:FFFFFFFFFFFFFFFFFFFFFFFFFFFFFFFFFF:FFF:FFFF:::FFFFF:F:FF:::FF:,FFF:,
F:,,F:F,:FFF
@A00155:342:HHGFNDSXY:1:1520:5656:10504 2:N:0:GAACCTAG+TCCGCATA
GTAAATATTCATATGTAGCATGTTTAAGCATTAAATCAAATAAGATGAGCAAGATGGAACGTCTGGGT
TCCAATTCTGTCAAGACTTATGACATATTAAATAGATTGGTTACGGTAAAAGACAAGGCAAGGTATGG
ACCCTTAGCAAAGC
+
FFFFFFFFFFFFFFFFFFFFFFFFFFFFFFFFFFFFFFFFFFFFFFFFFFFFFFFFFFFFFF:FFFFFFF
FFFFFFFFFFFFFFFFFF::FFFFFFF,FFFFFFFFF:FFFFFFFFFFF:FF:F,FFFFFFFFFFF,FF:
FFFFFFFFFFFFFF
@A00155:342:HHGFNDSXY:1:1124:31096:10817 1:N:0:GAACCTAG+TCCGCATA
GTAAATATTCATATGTAGCATGTTTAAGCATTAAATCAAATAAGATGAGCAAGATGGAACGTCTGGGT
TCCAATTCTGTCAAGACTTATGACATATTAAATAGATTGGTTACGGTAAAAGACAAGGCAAGGTATGG
ACCCTTAGCAAAGCC
+
:FF:FFFFFFFFFFFFFFFFFFFFFFFFFFFFFFFFFFFFFFFFFFFFFFFFFFFFFFFFFFFFFFFFFF
FFFFF:FFFFFFFFFFFFFFFFFFFFFFFFF:FFFFFFFFFFFFFFFFFFFFFFFFFFFFFFFFFFFFFF
FFFFFFFFFFFFFFF
@A00155:342:HHGFNDSXY:1:1160:12400:4554 1:N:0:GAACCTAG+TCCGCATA
GTAAATATTCATATGTAGCATGTTTAAGCATTAAATCAAATAAGATGAGCAAGATGGAACGTCTGGGT
TCCAATTCTGTCAAGACTTATGACATATTAAATAGATTGGTTACGGTAAAAGACAAGGCAAGGTATGG
ACCCTTAGCAAAGCC
+
FFFFFFFFFFFFFFFFFFFFFFFFFFFFFFFFFFFFFFFFFFFFFFFFFFFFFFFFFFFFFFFFFFFFFF
FFFFFFFFFFFFFFFFFFFFFFFFFFFFFFFFFFFFFFFFFFFFFFFFFFFFFFFFFFFFFFFFFFFFFF
FFFFFFFFFFFFFFF
@A00155:342:HHGFNDSXY:1:1231:23312:27868 1:N:0:GAACCTAG+TCCGCATA
GTAAATATTCATATGTAGCATGTTTAAGCATTAAATCAAATAAGATGAGCAAGATGGAACGTCTGGGT
TCCAATTCTGTCAAGACTTATGACATATTAAATAGATTGGTTACGGTAAAAGACAAGGCAAGGTATGG
ACCCTTAGCAAAGCC
```

```
+
FFFFFFFFFFFFFFFFFFFFFFFFFFFFFFFFFFFFFFFFFFFFFFFFFFFFFFFFFFFFFFFFFFFF
FFFFFFFFFFFFFFFFFFFFFFFFFFFFFFFFFFFFFFFFFFFFFFFFFFFFFFFFFFFFFFFFFFFF
FFFFFFFFFFFFFFF
@A00155:342:HHGFNDSXY:1:1231:23122:28072 1:N:0:GAACCTAG+TCCGCATA
GTAAATATTCATATGTAGCATGTTTAAGCATTAAATCAAATAAGATGAGCAAGATGGAACGTCTGGGT
TCCAATTCTGTCAAGACTTATGACATATTAAATAGATTGGTTACGGTAAAAGACAAGGCAAGGTATGG
ACCCTTAGCAAAGCC
+
FFFFFFFFFFFFFFFFFFFFFFFFFFFFFFFFFFFFFFFFFFFFFFFFFFFFFFFFFFFFFF:F:FFF
FFFFFFFFFFFFFFFFFFFFFFFFFFFFFFFFFFFFFFFFFFFFFFFFFFFFFFFFFFFFFFFFFFFF
FFFFFFFFFFFFFFF
@A00155:342:HHGFNDSXY:1:1232:13340:31422 1:N:0:GAACCTAG+TCCGCATA
GTAAATATTCATATGTAGCATGTTTAAGCATTAAATCAAATAAGATGAGCAAGATGGAACGTCTGGGT
TCCAATTCTGTCAAGACTTATGACATATTAAATAGATTGGTTACGGTAAAAGACAAGGCAAGGTATGG
ACCCTTAGCAAAGCC
+
FFFFFFFFFFFFFFFFFFFFFFFFF:FFFFFFFFFFFFFFFFFFFFFFFFFF:FFFFFFFFFFFFFFF
F:FFFFFFFFFFFFFFFFFF:FFFFFFFFFFFFFFFFFFFFFFFFFFFFFFFFFFFFFFFFFFFFFFF
:FFFFFFFFFFFFFF
@A00155:342:HHGFNDSXY:1:1575:4318:19210 1:N:0:GAACCTAG+TCCGCATA
GTAAATATTCATATGTAGCATGTTTAAGCATTAAATCAAATAAGATGAGCAAGATGGAACGTCTGGGT
TCCAATTCTGTCAAGACTTATGACATATTAAATAGATTGGTTACGGTAAAAGACAAGGCAAGGTATGG
ACCCTTAGCAAAGCC
+
FFFFFFFFFFFFFF:FFFFFFF:FFFF:FFF:FFFFFFFFFFFFF:FFFFFFFFFFFFFFFFFFFFFF
FFFFFFFFFF:FFFFFFFFFFFFFFF:FFFFFFFFFFFFFFFFFF,FFFFFFFFFFFFFFFF:FFFFF
FFFFFFFFFFFFFFF
@A00155:342:HHGFNDSXY:1:1615:10257:35008 1:N:0:GAACCTAG+TCCGCATA
GTAAATATTCATATGTAGCATGTTTAAGCATTAAATCAAATAAGATGAGCAAGATGGAACGTCTGGGT
TCCAATTCTGTCAAGACTTATGACATATTAAATAGATTGGTTACGGTAAAAGACAAGGCAAGGTATGG
ACCCTTAGCAAAGCC
+
:FFFFFFFFFFF:FFFFFFFFFFFFFFFFFFFFFFFFFFFFFFFFFFFFFFFFFFFFFFFFFFFFFFF
FFFFFFFFFFFFFFFFFFFFFFFFFFFFFFFFFFFFFFFFFFFFFFFFFFFFFFFFFFFFFFFFFFFF
FFFFFFFFFFFFFFF
@A00155:342:HHGFNDSXY:1:1646:15510:30545 1:N:0:GAACCTAG+TCCGCATA
GTAAATATTCATATGTAGCATGTTTAAGCATTAAATCAAATAAGATGAGCAAGATGGAACGTCAGGGT
TCCAATTCTGTCAAGACTTATGACATATTAAATAGATTGGTTACGGTAAAAGACAAGGCAAGGTATGG
ACCCTTAGCAAAGCC
+
FFFF:::,FFFFFFFF:,:FFFFFFF:FFFFFFFFFFFF:FFF,FFFFFFFFFFFFF,FFF:F,FF:,
FFFFFF,FF:FFFF,FFFFFFFFFFFF:FFFFFF:F,:F:FFFFF:FFFFFFFF:F::F:FFF,FFFF
FFFFFFF,FFF::FF
@A00155:342:HHGFNDSXY:1:1635:30662:16736 1:N:0:GAACCTAG+TCCGCATA
GTAAATATTCATATGTAGCATGTTTAAGCATTAAATCAAATAAGATGAGCAAGATGGAACGTCTGGGT
TCCAATTCTGTCAAGACTTATGACATATTAAATAGATTGGTTACGGTAAAAGACAAGGCAAGGTATGG
ACCCTTAGCAAAGCC
+
FFFFFFFFFFFFFFF:FFFFFFFFFFFFFFFFFFFFFFFFFFFFFFFFFFFFFFFFFFFFFFFFFFFF
FFFFFFFFFFFFFFFFFFFFFFFFFFFFFFFFFFFFFFFFFFFFFFFFFFFFFFFFFFFFFFFFFFFF
FFFFFFFFFFFFFFF
@A00155:342:HHGFNDSXY:1:2140:13096:28995 1:N:0:GAACCTAG+TCCGCATA
GTAAATATTCATATGTAGCATGTTTAAGCATTAAATCAAATAAGATGAGCAAGATGGAACGTCTGGGT
```

TCCAATTCTGTCAAGACTTATGACATATTAAATAGATTGGTTACGGTAAAAGACAAGGCAAGGTATGG
ACCCTTAGCAAAGCC
+
FFFFFFFFF,FFFFFFFFFFFFFFFFFFFFFFFFFFFFFFFFFFFFFFFFFFFFFFFFFFFFFFFFFF
FFFFFFFFFFFFFFFFFFFFFFFFFFFFFFFFFFFFFFFFFFFFFFFFFFFFFFFFFFFFF,FFFFFF
FFFFFFFFFFFFFFF
@A00155:342:HHGFNDSXY:1:2172:25238:18615 1:N:0:GAACCTAG+TCCGCATA
GTAAATATTCATATGTAGCATGTTTAAGCATTAAATCAAATAAGATGAGCAAGATGGAACGTCTGGGT
TCCAATTCTGTCAAGACTTATGACATATTAAATAGATTGGTTACGGTAAAAGACAAGGCAAGGTATGG
ACCCTTAGCAAAGCC
+
:FFFFFFFF:FF:FFFFFFFFFFFF:FFFFFFFFFFFF,:FFFFFFFFFFFFFFFFFFFFFFFFFFF:
F:::F,FFFFFFFFF:FFFFFF,FFFFFFF:FFFFFFF:FFFFFFFFFF:FFFFF:FFFFFFFFFFFF
FFFFFF,FFFFFFFF
@A00155:342:HHGFNDSXY:1:2278:20184:7670 1:N:0:GAACCTAG+TCCGCATA
GTAAATATTCATATGTAGCATGTTTAAGCATTAAATCAAATAAGATGAGCAAGATGGAACGTCTGGGT
TCCAATTCTGTCAAGACTTATGACATATTAAATAGATTGGTTACGGTAAAAGACAAGGCAAGGTATGG
ACCCTTAGCAAAGCC
+
FFFFFFFFFFFFFFFFFFFFFF:FFFFFFFFFFFFFFFFFFFFFFFFFFFFFFFFFFFFFFFFFFFFF
FFFFFFFFFFFFFFFFFFFFFFFFFFFFFFFFFFFFFFFFFFFFFFFFFFFFFF:FFFFFFFFFFFF
FFFFFFFFFFFFFFF
@A00155:342:HHGFNDSXY:1:2471:31910:24001 1:N:0:GAACCTAG+TCCGCATA
GTAAATATTCATATGTAGCATGTTTAAGCATTAAATCAAATAAGATGAGCAAGATGGAACGTCTGGGT
TCCAATTCTGTCAAGACTTATGACATATTAAATAGATTGGTTACGGTAAAAGACAAGGCAAGGTATGG
ACCCTTAGCAAAGCC
+
FFFFFFFFFFFFFFFFF:FFFFFFF:FFFFF:FFFFFFFFFFFFFFFFFFF:FFFFFFFFFFFFFFFF
FFFFFFFFFFFFFFFFFFFFFFFFFFFFFFFFFFFFFFFFFFFFFFF:FFFFFFF:FFFFFFFFFFFF
FFFFFFFFFFF:FFF
@A00155:342:HHGFNDSXY:1:2513:6822:21637 1:N:0:GAACCTAG+TCCGCATA
GTAAATATTCATATGTAGCATGTTTAAGCATTAAATCAAATAAGATGAGCAAGATGGAACGTCTGGGT
TCCAATTCTGTCAAGACTTATGACATATTAAATAGATTGGTTACGGTAAAAGACAAGGCAAGGTATGG
ACCCTTAGCAAAGCC
+
FFFFFFFFF::FF:FFFFFFFFF,FFFFFFFFFFFFFFFFFFFFFFFFFFFFFFFFFFFFFFFFFFFF
FFFFFFFFFFFFFFFF:FFFFFFFFFFFFF:FFFFFFFFFFFFFFFFFFFFFFFFFFFFFFFFFFFFF
FFFFFFF:FFFFFFF
@A00155:342:HHGFNDSXY:1:2548:25012:14622 1:N:0:GAACCTAG+TCCGCATA
GTAAATATTCATATGTAGCATGTTTAAGCATTAAATCAAATAAGATGAGCAAGATGGAACGTCTGGGT
TCCAATTCTGTCAAGACTTATGACATATTAAATAGATTGGTTACGGTAAAAGACAAGGCAAGGTATGG
ACCCTTAGCAAAGCC
+
FFFFFFFFFFFF,FFFFFFFFFFFFFFFFFFFFFFFFFFFFFFFFFFFFFFFFFFFFFFFFFFFFFF
FFFFFFFFFFFFFFFFFFFFFFFFFFFFFFFFFFFFFFFFFFFFFFFFFFFFFFFFFFFFFFFFFFFF
FFFFFFFFFFFFFFF
@A00155:342:HHGFNDSXY:1:2617:17653:1814 1:N:0:GAACCTAG+TCCGCATA
GTAAATATTCATATGTAGCATGTTTAAGCATTAAATCAAATAAGATGAGCAAGATGGAACGTCTGGGT
TCCAATTCTGTCAAGACTTATGACATATTAAATAGATTGGTTACGGTAAAAGACAAGGCAAGGTATGG
ACCCTTAGCAAAGCC
+
FFFFFFFFFFFFFFFFFFFFFF:FFFF:FFF:FFFFFFFFFFFFFFFFFFFFFFFFFFF:FFFFFFFF
FFFFFF:FFFFFFFFFFFFFFFFFFFFFFFFFFFFFFFFFFFFFFFFFFFFFFFFFFFFFFFFFF:FF
FFFFFFFFFFFFFFF

```
@A00155:342:HHGFNDSXY:1:2651:30002:5854 1:N:0:GAACCTAG+TCCGCATA
GTAAATATTCATATGTAGCATGTTTAAGCATTAAATCAAATAAGATGAGCAAGATGGAACGTCTGGGT
TCCAATTCTGTCAAGACTTATGACATATTAAATAGATTGGTTACGGTAAAAGACAAGGCAAGGTATGG
ACCCTTAGCAAAGCC
+
FFFFFFFFFFFFFFFFFFFFFFFFFFFFFFFFFFFFFFFFFFFFFFFFFFFFFFFFFFFFFFFFFFFF
FFFFFFFFFFFFFFFF:FF:FFFFFFFFFFFFFFFFFFFFFFFFFFFFFFFFFFFFFFFFFFFFFFFF
FFFFFFFFFFFFFFF
@A00155:342:HHGFNDSXY:1:2651:29912:5948 1:N:0:GAACCTAG+TCCGCATA
GTAAATATTCATATGTAGCATGTTTAAGCATTAAATCAAATAAGATGAGCAAGATGGAACGTCTGGGT
TCCAATTCTGTCAAGACTTATGACATATTAAATAGATTGGTTACGGTAAAAGACAAGGCAAGGTATGG
ACCCTTAGCAAAGCC
+
FFFFF:FFFFFFFFFFFFFFFF:FFFF::FFFFFFFFFFFFFFFFFFFFFFFFFFFFFFFFFFFFFFF
FFFFFFFFFFFFFFFFFFFFFFFFFFFFFFFFFFFFFFFFFFFFFFFFFFFFFFFFFFFFFFFFFFFF
FFFFFFFFFFFFFFF
@A00155:342:HHGFNDSXY:1:1210:19000:1047 2:N:0:GAACCTAG+TCCGCATA
GTAAATATTCATATGTAGCATGTTTAAGCATTAAATCAAATAAGATGAGCAAGATGGAACGTCTGGGT
TCCAATTCTGTCAAGACTTATGACATATTAAATAGATTGGTTACGGTAAAAGACAAGGCAAGGTATGG
ACCCTTAGCAAAGCC
+
FFFFFFFFFFFF,FFFFFFFFFFFFFFFFFFFFFFFFFFFFFFFFFFFFFFFFFFFFFFFFFFFFFFF
FFFFFFFFFFFFF:FFFFFFFFFFFFFFFFFFFFFF:FFFFFFFFFFFFFFFFFFF:FFFFFFFFFFF
FFFFFFFFFFF,FFF
@A00155:342:HHGFNDSXY:1:1235:5891:13166 2:N:0:GAACCTAG+TCCGCATA
GTAAATATTCATATGTAGCATGTTTAAGCATTAAATCAAATAAGATGAGCAAGATGGAACGTCTGGGT
TCCAATTCTGTCAAGACTTATGACATATTAAATAGATTGGTTACGGTAAAAGACAAGGCAAGGTATGG
ACCCTTAGCAAAGCC
+
FFFFFFFFFFFFFFFFFFFFFFFFFFFFFFFFFFFFFFFFFFFFFFFFFFFFFFFFFFFFFFFFFFFF
FFFFFFFFFFFFFFFFFFFFFFFFFFFFFFFF:FFFFFFFFFFFFFFFFF:FFFFFFFFFFFFFFFFF
FFFFF:FFFF,FFFF
@A00155:342:HHGFNDSXY:1:1444:28366:10598 2:N:0:GAACCTAG+TCCGCATA
GTAAATATTCATATGTAGCATGTTTAAGCATTAAATCAAATAAGATGAGCAAGATGGAACGTCTGGGT
TCCAATTCTGTCAAGACTTATGACATATTAAATAGATTGGTTACGGTAAAAGACAAGGCAAGGTATGG
ACCCTTAGCAAAGCC
+
::FFFFFFFFFFFFFFFFFFFFFFFFFFFFFFFFFFFFFFFFFFFFFFFFFFFFFFFFFFFFFFFFFF
FFFFFFFFFFFFFFFFFFFFFFFFFFFFFFFFFFFFFFFFFFFFFFFFFFFFFFFFFFFFFFFFFFF:
FFFFF:FFFFFFFFF
@A00155:342:HHGFNDSXY:1:1510:21025:16673 2:N:0:GAACCTAG+TCCGCATA
GTAAATATTCATATGTAGCATGTTTAAGCATTAAATCAAATAAGATGAGCAAGATGGAACGTCTGGGT
TCCAATTCTGTCAAGACTTATGACATATTAAATAGATTGGTTACGGTAAAAGACAAGGCAAGGTATGG
ACCCTTAGCAAAGCC
+
FFFFFFFFFFFFFFFFFFFFFFFFFFFFFFFFFFFFFFFFFFFFFFFFFFFFFFFFFFFFFFFFFFFF
FFFFFFFFFFFFFFFFFFFFFFFFFFFFFFFFFFFFFFFFFFFFFFFFFFFFFFFFFFFF:FFFFFFF
FF:FFFFFFFFFFFF
@A00155:342:HHGFNDSXY:1:1572:27552:27038 2:N:0:GAACCTAG+TCCGCATA
GTAAATATTCATATGTAGCATGTTTAAGCATTAAATCAAATAAGATGAGCAAGATGGAACGTCTGGGT
TCCAATTCTGTCAAGACTTATGACATATTAAATAGATTGGTTACGGTAAAAGACAAGGCAAGGTATGG
ACCCTTAGCAAAGCC
+
FFFFFFFFFFF:FFFFFFFFFFFFFFFFFFFFFFFFFFFFFFFFFFFFFFFFFFFFFF:FFFFFFFFF
```

FFFFFFFFFFFFFFFFFFFFFFFFFFFFFFFFFFFF,FFFFFFFFFF:FFFFFFFFFFFFFFFFFFFF
FFFFFFFFFFFFFFF
@A00155:342:HHGFNDSXY:1:2118:14959:27524 2:N:0:GAACCTAG+TCCGCATA
GTAAATATTCATATGTAGCATGTTTAAGCATTAAATCAAATAAGATGAGCAAGATGGAACGTCTGGGT
TCCAATTCTGTCAAGACTTATGACATATTAAATAGATTGGTTACGGTAAAAGACAAGGCAAGGTATGG
ACCCTTAGCAAAGCC
+
FFFFFFFFFFFFFFFFFFFFFFFFFFFFFFFFFFFFFFFFFFFFFFFFFFFFFFFFFFFFF:FFFFFF
FFFFFFFFFFFFFFFFFFF:FFFFFFFF:FF,FFFFFFFFFFFFFFFFFFFFFFFFF:FFFFFF:FFF
FFFFFFFFFFFFFFF
@A00155:342:HHGFNDSXY:1:2140:13096:28995 2:N:0:GAACCTAG+TCCGCATA
GTAAATATTCATATGTAGCATGTTTAAGCATTAAATCAAATAAGATGAGCAAGATGGAACGTCTGGGT
TCCAATTCTGTCAAGACTTATGACATATTAAATAGATTGGTTACGGTAAAAGACAAGGCAAGGTATGG
ACCCTTAGCAAAGCC
+
FFFFFFFFFFFFF,FFFFFFFFFFFFFFFFFFFFFFFFFFFFFFFFFFFFFFFFFFFFFFFFFFFFFF
FFFFFFFFFFFFFFFFFFFFFFFFFFFFFFFFFFFFFFFFFFFFFFFFFFFFF:FFFFFFFFFFFFFF
FFFFFFFFFFFFFFF
@A00155:342:HHGFNDSXY:1:2161:5683:23985 2:N:0:GAACCTAG+TCCGCATA
GTAAATATTCATATGTAGCATGTTTAAGCATTAAATCAAATAAGATGAGCAAGATGGAACGTCTGGGT
TCCAATTCTGTCAAGACTTATGACATATTAAATAGATTGGTTACGGTAAAAGACAAGGCAAGGTATGG
ACCCTTAGCAAAGCC
+
FFFFFFFFFFFFFFFFFFFFFFFFFFFFFFFFFFFFFFFFFFFFFFFFFFFFFFFFFFFFFFFFFFFF
F:FFFFFFFFFFFFFFFFFFF:,FF:FFFFFF:FFFFFFFFF,F:FF:FF,::FFFFFFFFFFFFFFF
FFFFFFFFFFFFF,F
@A00155:342:HHGFNDSXY:1:2120:13756:33458 2:N:0:GAACCTAG+TCCGCATA
GTAAATATTCATATGTAGCATGTTTAAGCATTAAATCAAATAAGATGAGCAAGATGGAACGTCTGGGT
TCCAATTCTGTCAAGACTTATGACATATTAAATAGATTGGTTACGGTAAAAGACAAGGCAAGGTATGG
ACCCTTAGCAAAGCC
+
FFFFFFFFFFFFFFFFFFFFFFFFFFFFFFFFFFFFFFFFFFFFFFFFFFFFFFFFFFFFFFFFFFFF
FFFFFFFFFFFFFFFFFFFFFFFFFFFFFFFFFFFFFFFFFFFFFFFFF:FFFFFFFFFFFFFFFFFF
FFFFFFFFFFFFFFF
@A00155:342:HHGFNDSXY:1:2302:24813:10144 2:N:0:GAACCTAG+TCCGCATA
GTAAATATTCATATGTAGCATGTTTAAGCATTAAATCAAATAAGATGAGCAAGATGGAACGTCTGGGT
TCCAATTCTGTCAAGACTTATGACATATTAAATAGATTGGTTACGGTAAAAGACAAGGCAAGGTATGG
ACCCTTAGCAAAGCC
+
FFFFFFFFFFFFFFF:FFFFFFFFFFFFFFFFFFFFFFFFFFFFFFFFFFFFFFFFFF:FFFFFFFFF
FFFFFFFFFFFFFFFFFFFF:FFFFFFFFFFFFFFFFFFFFFFFFFFFFFFFFFF:FFFFFFFFFFFF
FFFFFFFFFFFFFFF
@A00155:342:HHGFNDSXY:1:2333:6108:16297 2:N:0:GAACCTAG+TCCGCATA
GTAAATATTCATATGTAGCATGTTTAAGCATTAAATCAAATAAGATGAGCAAGATGGAACGTCTGGGT
TCCAATTCTGTCAAGACTTATGACATATTAAATAGATTGGTTACGGTAAAAGACAAGGCAAGGTATGG
ACCCTTAGCAAAGCC
+
FFFFFFFFFFFFF::FFFFFFFFFF:FFFFFFFFFFFFFF:FFFFF:FFFFFFFFFFFFF:FFFFFFF
FF:FFFFF:FFFFF,FFFFFFFFFFF:FFFFFFF:FFF:F:FFFFFFF:FFFF,FFF,:F:,FFFFF:
FF,FFFFFFF,F,FF
@A00155:342:HHGFNDSXY:1:2341:17951:29794 2:N:0:GAACCTAG+TCCGCATA
GTAAATATTCATATGTAGCATGTTTAAGCATTAAATCAAATAAGATGAGCAAGATGGAACGTCTGGGT
TCCAATTCTGTCAAGACTTATGACATATTAAATAGATTGGTTACGGTAAAAGACAAGGCAAGGTATGG
ACCCTTAGCAAAGCC

+
FFFFFF:FFFFFFFFFFFFFFFFFFFFFFFFFFFFFFFFFFFFFFFFFFFFFFFFFFFFFFFFFFFFF
FFFFFFFFFFFFFFFFFFFFFFFFFFFFFFFFFFFFFFFFFFFFFFFF,F:FFFFFFFFFFFFFFFFF
FFFFFFFFFFFFFFF
@A00155:342:HHGFNDSXY:1:2341:18213:36041 2:N:0:GAACCTAG+TCCGCATA
GTAAATATTCATATGTAGCATGTTTAAGCATTAAATCAAATAAGATGAGCAAGATGGAACGTCTGGGT
TCCAATTCTGTCAAGACTTATGACATATTAAATAGATTGGTTACGGTAAAAGACAAGGCACGGTATGG
ACCCTTAGCAAAGCC
+
FFFFFFFFFFFF:FFFFFFFFFFFFFFFFF,FFFFFFFFFFFFFFFFFFFFFFFFFFFFFFFFFFFFF
FFFFFFFFFFFF:F:FFFFFFFFFFFFFFFFFFFFFFFFFFFFF:FF,FFFFFFFFF:FF,FFFFFFF
FFFFFFFFFFFFFFF
@A00155:342:HHGFNDSXY:1:2525:31684:31093 2:N:0:GAACCTAG+TCCGCATA
GTAAATATTCATATGTAGCATGTTTAAGCATTAAATCAAATAAGATGAGCAAGATGGAACGTCTGGGT
TCCAATTCTGTCAAGACTTATGACATATTAAATAGATTGGTTACGGTAAAAGACAAGGCAAGGTATGG
ACCCTTAGCAAAGCC
+
FFFFFFFFFFFFFFFFFFFFFFFFFFFFFFFFFFFFFFFFFFFFFFFFFFFFFFFFFFFFFFFFFFFF
:FFFFFFFFFFFFFFFFFFFFFFFFFFFFFFFFFFFFFFFFFFFFFFFFF:FFFFFFFFFFFFFFFFF
FFFFFFFFFFFFFF:
@A00155:342:HHGFNDSXY:1:2528:13512:12712 2:N:0:GAACCTAG+TCCGCATA
GTAAATATTCATATGTAGCATGTTTAAGCATTAAATCAAATAAGATGAGCAAGATGGAACGTCTGGGT
TCCAATTCTGTCAAGACTTATGACATATTAAATAGATTGGTTACGGTAAAAGACAAGGCAAGGTATGG
ACCCTTAGCAAAGCC
+
FFFFFFFFFFFFFFFFFFFFFFFFFFFFFFFFFFFFFFFFFFFFFFFFFFFFFFFFFFFFFFFFFFFF
FFFFFFFFFFFFFFFFFFFFFFFFFFFFFFF:FFFFFFFFFFFFFFFFF,,FFFFFF:FF,FFFFFFF
FFFFFFFFFFFFFFF
@A00155:342:HHGFNDSXY:1:1551:17635:9048 1:N:0:GAACCTAG+TCCGCATA
TAAATATTCATATGTAGCATGTTTAAGCATTAAATCAAATAAGATGAGCAAGATGGAACGTCTGGGTT
CCAATTCTGTCAAGACTTATGACATATTAAATAGATTGGTTACGGTAAAAGACAAGGCAAGGTATGGA
CCCTTAGCAAAGCC
+
FFFFFFFFFFFFFFFF:FFFFFFFFF:FFFFFFFFFFFFFFFFFFFFFFFFFFFFFFFFFFFFFFFFF
FFFFFFFFFFFFFFFFFFFFFFFFFFFFFFFFFFFFFFFFFFFFFFFFFFFFFFFFFFF:FFFF:FFF
FFFFFFFFFFFFFF
@A00155:342:HHGFNDSXY:1:1575:2347:21496 1:N:0:GAACCTAG+TCCGCATA
TAAATATTCATATGTAGCATGTTTAAGCATTAAATCAAATAAGATGAGCAAGATGGAACGTCTGGGTT
CCAATTCTGTCAAGACTTATGACATATTAAATAGATTGGTTACGGTAAAAGACAAGGCAAGGTATGGA
CCCTTAGCAAAGCC
+
FFFFFFFFFFFFFFFFFFFFF,FFFFFFF:FFFFFFFFFFFFFF:FFFFFFFFFFFFFFF,FFFFFFF
FFFFFFFFFFFFFFFFFFFFFFFFFFFFFFFFF:FFF::FFFFFFFFFFFFFFFFFFFFFFFFFFFFF
FFFFFFFFFFFFFF
@A00155:342:HHGFNDSXY:1:2505:28257:17895 1:N:0:GAACCTAG+TCCGCATA
TAAATATTCATATGTAGCATGTTTAAGCATTAAATCAAATAAGATGAGCAAGATGGAACGTCTGGGTT
CCAATTCTGTCAAGACTTATGACATATTAAATAGATTGGTTACGGTAAAAGACAAGGCAAGGTATGGA
CCCTTAGCAAAGCC
+
FFFFFFFFFFFFFFFFF:FFFFFFFFFFFFFFFFFFFFFFFFFFFFFFFFFFFFFFFFFFFFFFFFFF
FFFFFFFFFFFFFFFFFFFFFFFFFFFFFFFFFFFFFFFFFFFFFFFFFFFFFFFFFFFFFFFFFFFF
FFFFFFFFFFFFFF
@A00155:342:HHGFNDSXY:1:1127:29125:24095 1:N:0:GAACCTAG+TCCGCATA
TAAATATTCATATGTAGCATGTTTAAGCATTAAATCAAATAAGATGAGCAAGATGGAACGTCTGGGTT

```
CCAATTCTGTCAAGACTTATGACATATTAAATAGATTGGTTACGGTAAAAGACAAGGCAAGGTATGGA
CCCTTAGCAAAGCCG
+
FFFFFFFFFFFFFFFF:FFFF:FFFFF:FFFFFFFFFFFFFFFFFFFFFFFFFFFFFF:FFFFFFFFFF
FFFFFFFFFFFFFFFFFFFFFFFFFFFFFFFFFFFFFFFFFF:FFFFFFFFFFFFFFFFFFFFFFFFFF
FFFFFFFFFFFFFFF
@A00155:342:HHGFNDSXY:1:1477:5249:13964 1:N:0:GAACCTAG+TCCGCATA
TAAATATTCATATGTAGCATGTTTAAGCATTAAATCAAATAAGATGAGCAAGATGGAACGTCTGGGTT
CCAATTCTGTCAAGACTTATGACATATTAAATAGATTGGTTACGGTAAAAGACAAGGCAAGGTATGGA
CCCTTAGCAAAGCCG
+
FFFFFFFF:FFFFFFFFFFFFFFFFFFFFFFFFFFFFFFFFFFFFFFFFFFFFFFFFFFFFFFFFFFF:
FFFFFFFFFFFFFFFFFF:FFFFF:FF:FFFFFFF:FFFFFFF:FFFFFFFFFFFFFFFFFFFFFFFFF
FFFFFFFFFFFFFFF
@A00155:342:HHGFNDSXY:1:2625:7437:7733 1:N:0:GAACCTAG+TCCGCATA
TAAATATTCATATGTATCATGTTTAAGCATTAAATCAAATAAGATGAGCAAGATGGAACGTCTGGGTT
CCAATTCTGTCAAGACTTATGACATATTAAATAGATTGGTTACGGTAAAAGACAAGGCAAGGTATGGA
CCCTTAGCAAAGCCA
+
FFFFFFFFFFFFFFF,,FFFFFFFFF,F:FFFFFFFFFFFFFFFFFF::FF:FFFFF:FFFFFFFFFF
FFFFFFFFFFFFFFFFFFFFFFFFFFFFFFFFFFFF,FFFFFF:FFFFFFFFFFFF,FFFFFFFFFFF
FFFFFF,FFF:FFFF
@A00155:342:HHGFNDSXY:1:2625:7464:7748 1:N:0:GAACCTAG+TCCGCATA
TAAATATTCATATGTAGCATGTTTAAGCATTAAATCAAATAAGATGAGCAAGATGGAACGTCTGGGTT
CCAATTCTGTCAAGACTTATGACATATTAAATAGATTGGTTACGGTAAAAGACAAGGCAAGGTATGGA
CCCTTAGCAAAGCCA
+
FFFFFFFFFFFFFFFF:FFF:FFFFFFFFFFFFFFFF:FFFFFFFFFFFFFFFFFFFFFFFFFFFFFFF
FFFFFFFFFFFFF:F:FFFFFFFFFFFFFFFFFFFFFFFFFFFFFFFFFFFFFFFFFFFFFFFFFFFFF
FFFFFFFFFFFFFFF
@A00155:342:HHGFNDSXY:1:1569:11930:33833 2:N:0:GAACCTAG+TCCGCATA
TAAATATTCATATGTAGCATGTTTAAGCATTAAGTCAAATAAGATGAGCAAGATGGAACGTCTGGGTT
CCAATTCTGTCAAGACTTATGACATATTAAATAGATTGGTTACGGTAAAAGACAAGGCAAGGTATGGA
CCCTTAGCAAAGCCA
+
FFFFFFFFFFFFFFFFFFFFFFFFFF:FFFFFFFFFFFFFFFFFFFFFFFFFFFFFF,FFFFFFFFFFF
FFFFFFFFFFFFFFFFFFFFFFFFFFFFF,:FFFFFFFFFFFFFFFFF,FF:FFF:FFFFFFFFFF:F
FFFFFFFFFFFFFFF
@A00155:342:HHGFNDSXY:1:1605:28330:29794 2:N:0:GAACCTAG+TCCGCATA
TAAATATTCATATGTAGCATGTTTAAGCATTAAATCAAATAAGATGAGCAAGATGGAACGTCTGGGTT
CCAATTCTGTCAAGACTTATGACATATTAAATAGATTGGTTACGGTAAAAGACAAGGCAAGGTATGGA
CCCTTAGCAAAGCCA
+
FFFFFFFFFFFFFFFFFFFF:FFFFFFFFFFFFFFFFFFFFFFFFFFFFFFF:FFFFFFFFFFFFFFFF
FFFFFFFFFFFFFFFFFFFFFFFFFFFFFFFFFFFFFF,FFFFFF:F:FFFFFFFFFFFFFFFFFFFF
FFFFFFFFFFFFFFF
@A00155:342:HHGFNDSXY:1:2139:19651:8218 2:N:0:GAACCTAG+TCCGCATA
TAAATATTCATATGTAGCATGTTTAAGCATTAAATCAAATAAGATGAGCAAGATGGAACGTCTGGGTT
CCAATTCTGTCAAGACTTATGACATATTAAATAGATTGGTTACGGTAAAAGACAAGGCAAGGTATGGA
CCCTTAGCAAAGCCA
+
FFFFFFFFFFFFFFFFFFFFFFFFFFFFFFFFFFFFFFFFFFFFFFFFFFFFFFFFFFFFFFFFFFFFF
FFFFFFFFFFFFFFFFFFFFFFFFFFFFFFFFFFFFFFFFFFFFFFFFFF,:FFFFFFFFFFFFFFFFF
FFFFFFFFFFFFFFF
```

```
@A00155:342:HHGFNDSXY:1:2426:7355:17769 2:N:0:GAACCTAG+TCCGCATA
TAAATATTCATATGTAGCATGTTTAAGCATTAAATCAAATAAGATGAGCAAGATGGAACGTCTGGGTT
CCAATTCTGTCAAGACTTATGACATATTAAATAGATTGGTTACGGTAAAAGACAAGGCAAGGTATGGA
CCCTTAGCAAAGCCA
+
FFFFFFFFFFFFFFF:FFFFF:FFFFFFFFFFFFFFFF:FFFFFF::FFFFFFFFFFFFFFFFFFFFF
FFFFFFFFFFFFFFFFFFFFFF,FFFFFF,FFFFFFFFFFFFF:,:,F:F,FFFFFFFFFFFFFFF,F
FFFF:FF,FFFFFFF
@A00155:342:HHGFNDSXY:1:2638:24234:10802 2:N:0:GAACCTAG+TCCGCATA
AAATATTCATATGTAGCATGTTTAAGCATTAAATCAAATAAGATGAGCAAGATGGAACGTCTGGGTTC
CAATTCTGTCAAGACTTATGACATATTAAATAGATTGGTTACGGTAAAAGACAAGGCAAGGTATGGAC
CCTTAGCAAAGCCA
+
FFFFFFFF,FFFFFF:FFFFF:FFFFF:FFFFFFFFFFFFFFFF:FFFFFFFFFFFF:FFFFFFFFFF
FFFFFFFFFFFFFFFFFFFFF,FFFFFFF:FFFFFFFFFFFFFFFF,FFFFFFFFFFFFF:,,FFF:F
:FF,FFFFFFFF:F
@A00155:342:HHGFNDSXY:1:1210:19000:1047 1:N:0:GAACCTAG+TCCGCATA
AAATATTCATATGTAGCATGTTTAAGCATTAAATCAAATAAGATGAGCAAGATGGAACGTCTGGGTTC
CAATTCTGTCAAGACTTATGACATATTAAATAGATTGGTTACGGTAAAAGACAAGGCAAGGTATGGAC
CCTTAGCAAAGCCAG
+
FFFFFFFFFFFFFFFFFFFFFFFFFFFFFFFFFFFFFFFFFFFFFFFF:FFFFFFFFFFFFFFFFFFF
FFFFFFFFFFFFFFFFFFFFFFFFFFFFFFFFFFFFFFFFFFFFFFFFFFFFFFFFFFFFFFFFFFFF
FFFFFFFFFFFFFFF
@A00155:342:HHGFNDSXY:1:1408:26313:23171 1:N:0:GAACCTAG+TCCGCATA
AAATATTCATATGTAGCATGTTTAAGCATTAAATCAAATAAGATGAGCAAGATGGAACGTCTGGGTTC
CAATTCTGTCAAGACTTATGACATATTAAATAGATTGGTTACGGTAAAAGACAAGGCAAGGTATGGAC
CCTTAGCAAAGCCAG
+
FFFFFFFFFFFFFFFF:FFFFFFFFFFFFFFFFFFFFFFFFFFFFFFFFFFF:FFFFFFFFFFFFFFF
FFFFFFFFFFFFFFFFFFFFFFFFFFFFFF,FFFFFFFFFFFFFFFFFFFFFFFFFFFFFFFFFFFFF
FFF:FFFFFFFFFFF
@A00155:342:HHGFNDSXY:1:1459:27019:17002 1:N:0:GAACCTAG+TCCGCATA
AAATATTCATATGTAGCATGTTTAAGCATTAAATCAAATAAGATGAGCAAGATGGAACGTCTGGGTTC
CAATTCTGTCAAGACTTATGACATATTAAATAGATTGGTTACGGTAAAAGACAAGGCAAGGTATGGAC
CCTTAGCAAAGCCAG
+
FFFFFFFFFFFFFFFFFFFFFFFFFFFFFFFFFFFFFFFFFFFFFFFFFFFFFFFFFFFFFFFFFFFF
FFFFFFFFFFFFFFFFFFFFFFFFFFFFFFFFFFFFFFFFFFFFFFFFFFFFFFFFFFFFFFFFFFFF
FFFFFFFFFFFFFFF
@A00155:342:HHGFNDSXY:1:2237:24406:24189 1:N:0:GAACCTAG+TCCGCATA
AAATATTCATATGTAGCATGTTTAAGCATTAAATCAAATAAGATGAGCAAGATGGAACGTCTGGGTTC
CAATTCTGTCAAGACTTATGACATATTAAATAGATTGGTTACGGTAAAAGACAAGGCAAGGTATGGAC
CCTTAGCAAAGCCAG
+
FFFFFFFFFFFFFFFFFFFFFFFFFF,FFFFFFFFFFFFFFFFFFF:FFFF:F:FFF:FFFFFFFFFF
FFFFFFFFFFFFFF:FFFFFF::FFFFFFF:FFF:FFFFFFFFFFFFFFFFFFFFFFFFFFFFFFFFF
FFFFFFFFFFFFFFF
@A00155:342:HHGFNDSXY:1:2230:7563:15123 1:N:0:GAACCTAG+TCCGCATA
AAATATTCATATGTAGCATGTTTAAGCATTAAATCAAATAAGATGAGCAAGATGGAACGTCTGGGTTC
CAATTCTGTCAAGACTTATGACATATTAAATAGATTGGTTACGGTAAAAGACAAGGCAAGGTATGGAC
CCTTAGCAAAGCCAG
+
FF:FFFFFFFFF:FFFFFF,FFFFFFFFFFFFFFFFFFFFFFFFFFFFFFFFFFFFFFFFFFFFFFFF
```

FFFFFFFFFFFFFFFFFFFFFFFFFFFFFFFFFFFFFFFFFFFFFFF:FFFFFFFFFFFFFFFFFFFF
FFFFFFFFFFFFFFF
@A00155:342:HHGFNDSXY:1:2534:10212:12602 1:N:0:GAACCTAG+TCCGCATA
AAATATTCATATGTAGCATGTTTAAGCATTAAATCAAATAAGATGAGCAAGATGGAACGTCTGGGTTC
CAATTCTGTCAAGACTTATGACATATTAAATAGATTGGTTACGGTAAAAGACAAGGCAAGGTATGGAC
CCTTAGCAAAGCCAG
+
FFFFFFF,:FFFFFF:FFFFFF:FFFFFFFFFFFFFFFFFFFFFFFFFFFFFFFFFFFFFFFFFFFFF
FFFFFFFFFFFFFFFFFFFFFFFFFFFFFFFFFFFFFFFFFFFFFFFFFFFFFFFFFFFFFFFFFFFF
FFFFFFFFFFFFFFF
@A00155:342:HHGFNDSXY:1:2607:3106:3490 1:N:0:GAACCTAG+TCCGCATA
AAATATTCATATGTAGCATGTTTAAGCATTAAATCAAATAAGATGAGCAAGATGGAACGTCTGGGTTC
CAATTCTGTCAAGACTTATGACATATTAAATAGATTGGTTACGGTAAAAGACAAGGCAAGGTATGGAC
CCTTAGCAAAGCCAG
+
FFFFFFF:FFFFF:F,FFFF,FFFFFFFFFFFFF:FFF:FFFFF:FFFFFFFFFF:::FFFFFFFFFF
FFFFFFFFFFFFFFFFFFFFFFFFFFFFFFFFFFFFF:FFFFFFFF:FFFFFFFFFFFF:FFFFFFFF
FFFFFFFFFFFFFFF
@A00155:342:HHGFNDSXY:1:2647:18982:2112 1:N:0:GAACCTAG+TCCGCATA
AAATATTCATATGTAGCATGTTTAAGCATTAAATCAAATAAGATGAGCAAGATGGAACGTCTGGGTTC
CAATTCTGTCAAGACTTATGACATATTAAATAGATTGGTTACGGTAAAAGACAAGGCAAGGTATGGAC
CCTTAGCAAAGCCAG
+
FFFFFFFFFFFFFFFF:FFFFFFFFF,FFFFFFFFFFFF,FFF:FFFFFFFFFFFFFFFF:FFFFFFF
FFFFFFFFFFFFFFFFFFFFFFFFFFFFFFFFFFFFFFFFFFFFFFFFFFFFFFFFFFFFFFFFFFFF
FFFFFFFFFFFFFFF
@A00155:342:HHGFNDSXY:1:2158:26612:11600 2:N:0:GAACCTAG+TCCGCATA
AAATATTCATATGTAGCATGTTTAAGCATTAAATCAAATAAGATGAGCAAGATGGAACGTCTGGGTTC
CAATTCTGTCAAGACTTATGACATATTAAATAGATTGGTTACGGTAAAAGACAAGGCAAGGTATGGAC
CCTTAGCAAAGCCAG
+
FFFFFFFFFFFFFFFFFFFFFFFFFFFFFFFFFFFFFFFFFFFFFFFFFFFFFFFFFFFFFFFFFFFF
FFFFFFFFFFFF:FFFFFFFFFFFFFFFFFFFFFFFFFFFFFFFFFFFFFFFFFFFFFFFFFFFFFF
FFFFFF,FFFFFFFF
@A00155:342:HHGFNDSXY:1:2217:8124:25645 2:N:0:GAACCTAG+TCCGCATA
AAATATTCATATGTAGCATGTTTAAGCATTAAATCAAATAAGATGAGCAAGATGGAACGTCTGGGTTC
CAATTCTGTCAAGACTTATGACATATTAAATAGATTGGTTACGGTAAAAGACAAGGCAAGGTATGGAC
CCTTAGCAAAGCCAG
+
FFFF:FFFFFFFFFFFFFFFFFFFF:FFFFFFFFFFFFFFFFFFFFFF::FFFFFFFFFFFFFFFFFF
FFFFFFF:FFF::FFFFFFFFFFFFFFFF:FFFFFFFFFFFFF,,FFF:FFFFFF:F:FFFF,FFFFF
FFFFFFFFFFFFF:F
@A00155:342:HHGFNDSXY:1:2342:1362:29309 2:N:0:GAACCTAG+TCCGCATA
AATATTCATATGTAGCATGTTTAAGCATTAAATCAAATAAGATGAGCAAGATGGAACGTCTGGGTTCC
AATTCTGTCAAGACTTATGACATATTAAATAGATTGGTTACGGTAAAAGACAAGGCAAGGTATGGACC
CTTATCAAA
+
FFF:FFF,F:FFF:FF:FFFFF:,FF:FF::,FF:,,F:::FFFF:FFF::FF:FF:F:FFFFFFFF:
,FFFFFFF:::,,:FFFFF,:,F,FF:,,F:F:FFFFFF::F:FFF,:,,:FFF,FFFFFFFFF:F,FF
,FF:,,:::
@A00155:342:HHGFNDSXY:1:1140:21685:1125 1:N:0:GAACCTAG+TCCGCATA
AATATTCATATGTAGCATGTTTAAGCATTAAATCAAATAAGATGAGCAAGATGGAACGTCTGGGTTCC
AATTCTGTCAAGACTTATGACATATTAAATAGATTGGTTACGGTAAAAGACAAGGCAAGGTATGGACC
CTTAGCAAAGCCAGC

+
FFFFFFFFFFFFFFFFFFFFFF:FFFFFFFFFFFFFFFFFFFFFFFFFFFFFFFFFFF:FFFFFFFFFFF
:FFFFFFFFFFFFFFFFF,F:FFFFFFFFFFFFF:FFFFFFFFFFFFF:FFFFFFFFFFFFFFFFFFFFF
:FFFFFFFFFFFFFF
@A00155:342:HHGFNDSXY:1:1343:4381:2065 1:N:0:GAACCTAG+TCCGCATA
AATATTCATATGTAGCATGTTTAAGCATTAAATCAAATAAGATGAGCAAGATGGAACGTCTGGGTTCC
AATTCTGTCAAGACTTATGACATATTAAATAGATTGGTTACGGTAAAAGACAAGGCAAGGTATGGACC
CTTAGCAAAGCCAGG
+
FFFFFFFFFFFFFFFFFFFFFFFFFFFFFFFFFFFFFFFFFFFF:F:FFFFFFFFFFFFFFFF:FFFF:F
FFFFFFFFFFFFF,FFFFFFFFFFFFFF:FF:FFFFFFFFFFFFFFFFFFFFFFFFFFFFFFFFFFFFFF
FFFFFFFFFFFFFFF
@A00155:342:HHGFNDSXY:1:1354:3983:21010 1:N:0:GAACCTAG+TCCGCATA
AATATTCATATGTAGCATGTTTAAGCATTAAATCAAATAAGATGAGCAAGATGGAACGTCTGGGTTCC
AATTCTGTCAAGACTTATGACATATTAAATAGATTGGTTACGGTAAAAGACAAGGCAAGGTATGGACC
CTTAGCAAAGCCAGT
+
FFFFFFFFFFFFFFFFFF,:FFFFFFFFFFFFFFFFFFFFFFFFFFFFFFFFFFFFFFFFFFFFFFFFFF
,FFFFFFFFFFFFFFFFFFFFFFFFFFFFFFFFFFFFFFF:FFFFFFFFF:FFFFFFFFFFFFFFFFFFF
FFFFFFFFFFFFFFF
@A00155:342:HHGFNDSXY:1:1339:14425:13072 1:N:0:GAACCTAG+TCCGCATA
AATATTCATATGTAGCATGTTTAAGCATTAAATCAAATAAGATGAGCAAGATGGAACGTCTGGGTTCC
AATTCTGTCAAGACTTATGACATATTAAATAGATTGGTTACGGTAAAAGACAAGGCAAGGTATGGACC
CTTAGCAAAGCCAGG
+
FFFFFFFFFFFFFFFFFFFFFFFFFFFFFFFFFFFFFFFFFFFFFFFF:FFFFFFFFFFFFFFFFFFFFF
FFFFFFFFFFFFFFFFFFFFFFFFFFFFFFFFFFFFFFFFFFFFFFFFFFFFFFFFFFFFFFFFFFFFFF
FFFFFFFFFFFFFFF
@A00155:342:HHGFNDSXY:1:1452:17083:32174 1:N:0:GAACCTAG+TCCGCATA
AATATTCATATGTAGCATGTTTAAGCATTAAATCAAATAAGATGAGCAAGATGGAACGTCTGGGTTCC
AATTCTGTCAAGACTTATGACATATTAAATAGATTGGTTACGGTAAAAGACAAGGCAAGGTATGGACC
CTTAGCAAAGCCAGG
+
FFFFFFFFFFFFFFF::FFFFFFFFFFFFFFFFFFFFFFFFFFFFFFFFFFFFFFFFFFFFFFFFFFFFF
FFFFFFFFFFFFFFFFFFFFFFFFFFFFFFFFFFFFFFFFFFFFFFFFFFFFFFFFFFFFFFFFFFFFFF
FFFFFFFFFFFFFFF
@A00155:342:HHGFNDSXY:1:1504:30174:18552 1:N:0:GAACCTAG+TCCGCATA
AATATTCATATGTAGCATGTTTAAGCATTAAATCAAATAAGATGAGCAAGATGGAACGTCTGGGTTCC
AATTCTGTCAAGACTTATGACATATTAAATAGATTGGTTACGGTAAAAGACAAGGCAAGGTATGGACC
CTTAGCAAAGCCAGG
+
FFFFFFFFFFFFFFFFFFFFFFFFFF:FFFFFFFFFFFFFFFFFFFFFFFFFFFFFFFFFFFFFFF:FFF
FFFFFFFFFFFFFFFFFFFFFFFFFFFFFFFFFFFFFFFFFFFFFFFFFFFFF:FFFFFFFFFFFFFFFF
FFFFFFFFFFFFFFF
@A00155:342:HHGFNDSXY:1:1545:10936:9157 1:N:0:GAACCTAG+TCCGCATA
AATATTCATATGTAGCATGTTTAAGCATTAAATCAAATAAGATGAGCAAGATGGAACGTCTGGGTTCC
AATTCTGTCAAGACTTATGACATATTAAATAGATTGGTTACGGTAAAAGACAAGGCAAGGTATGGACC
CTTAGCAAAGCCAGT
+
:F:FFFF:FFFFFFFFFFFFFFFFFFFFFFFFFFFFFFFFFFFFFFFFFFFFFFFFFFFF:FFFFFFFFF
FFFFFFFFFFFFFFFFFFFFFFFFFFFFFFFFFFFFFFFFFFFFFFFFFFFFF:FFFFFFFFFFFFFFFF
FFFFFFFFFFFFFFF
@A00155:342:HHGFNDSXY:1:1545:11026:9909 1:N:0:GAACCTAG+TCCGCATA
AATATTCATATGTAGCATGTTTAAGCATTAAATCAAATAAGATGAGCAAGATGGAACGTCTGGGTTCC

AATTCTGTCAAGACTTATGACATATTAAATAGATTGGTTACGGTAAAAGACAAGGCAAGGTATGGACC
CTTAGCAAAGCCAGT
+
FF:F:FF:FFFFFFF,FFF:FFFFFFFFFFFFFFF,FFF,,:FFF:FFFFFFF,:FF:FFFFFFFFFF
FFFFFFFFF:FFFFFFFFFF::FFFFFFFFFF:FFFFFFFFFFFFFFFFFF,:FFFFF:FFFFF:FFF
FFFFFFFFFFFFFFF
@A00155:342:HHGFNDSXY:1:1664:24505:35383 1:N:0:GAACCTAG+TCCGCATA
AATATTCATATGTAGCATGTTTAAGCATTAAATCAAATAAGATGAGCAAGATGGAACGTCTGGGTTCC
AATTCTGTCAAGACTTATGACATATTAAATAGATTGGTTACGGTAAAAGACAAGGCAAGGTATGGACC
CTTAGCAAAGCCAGA
+
FFF:FFFFFFFFFFF,FFF:FFFFFFFFFFFFFFF,FFFFFFFFFFFFFFFFFFFF:FFFFFFFFFFF
FF:FFFFFFFFFFFFFFFFFFFFFFFFFFFFFFF:FFFFFFFFFFFFFFFFFFFFFFFFFFFFFFFFF
FFFFFF:FFFFFFFF
@A00155:342:HHGFNDSXY:1:2223:1597:13902 1:N:0:GAACCTAG+TCCGCATA
AATATTCATATGTAGCATGTTTAAGCATTAAATCAAATAAGATGAGCAAGATGGAACGTCTGGGTTCC
AATTCTGTCAAGACTTATGACATATTAAATAGATTGGTTACGGTAAAAGACAAGGCAAGGTATGGACC
CTTAGCAAAGCCAGT
+
FF,FFFFFFFFFFFFFFFF:FFFFFFFFFFFFFFFFFFFFF:FFFFFFFFFFFFFFFFFFFFFF,FFF
FF:FFFFFFFFFFFFFFFFFFFF:F:FFFFFFFF:FFFFFFFFFFFFFFFFFFFFFFFFFFF:FFFFF
FFFFFFFFFFFFFFF
@A00155:342:HHGFNDSXY:1:2447:19443:35571 1:N:0:GAACCTAG+TCCGCATA
AATATTCATATGTAGCATGTTTAAGCATTAAATCAAATAAGATGAGCAAGATGGAACGTCTGGGTTCC
AATTCTGTCAAGACTTATGACATATTAAATAGATTGGTTACGGTAAAAGACAAGGCAAGGTATGGACC
CTTAGCAAAGCCAGT
+
FFFF,FFFFFFFFFFFF:FFFFFFFFFFFFFFFFFFFFFFFFFFFFFFFFFFFFFFFFFFFFFFFFFF
FFFFFFFFFFFFFFFFFFFFFFFFFFFFFFFFFFFFFFFFFFFFFFFFFFFFFFFFFFFFFFFFFFFF
FFFFFFFFFFFFFFF
@A00155:342:HHGFNDSXY:1:2665:24288:5729 1:N:0:GAACCTAG+TCCGCATA
AATATTCATATGTAGCATGTTTAAGCATTAAATCAAATAAGATGAGCAAGATGGAACGTCTGGGTTCC
AATTCTGTCAAGACTTATGACATATTAAATAGATTGGTTACGGTAAAAGACAAGGCAAGGTATGGACC
CTTAGCAAAGCCAGA
+
FFFFFFFFFFFFFFFFFFFFFFFFFFFFFFFFFFFFFFFFFFFFFFFFFFFFFFFFFFFFFFFF:FFF
FFFFFFFFFFFFFFFFFFFFFFFFFFFFFFFFFFFFFFFFFFFFFFFF:FFFFFFFFFFFFFFFFFFF
FFFFFFFFFFFFFFF
@A00155:342:HHGFNDSXY:1:2616:29622:17942 2:N:0:GAACCTAG+TCCGCATA
AATATTCATATGTAGCATGTTTAAGCATTAAATCAAATAAGATGAGCAAGATGGAACGTCTGGGTTCC
AATTCTGTCAAGACTTATGACATATTAAATAGATTGGTTACGGTAAAAGACAAGGCAAGGTATGGACC
CTTAGCAAAGCCAGA
+
FFFFFFFFFFFFFFFFFFFFFFFFFFFFFFFFFFFFFFFFFFF:FFFFFFFFFFFFFFFFFF,FFFF:
FFFFFFFFFFFFFFFFFFFFFFFFFFFFFFF,FFFFFFFFFFFFF:FFF:FFFFFFFFF:FFFF:F::
,FFF,FFFFFFFFFF
@A00155:342:HHGFNDSXY:1:1417:6813:25567 1:N:0:GAACCTAG+TCCGCATA
ATTCATATGTAGCATGTTTAAGCATTAAATCAAATAAGATGAGCAAGATGGAACGTCTGGGTTCCAAT
TCTGTCAAGACTTATGACATATTAAATAGATTGGTTACGGTAAAAGACAAGGCAAGGTATGGACCCTT
AGCAAAGCCAGAAAG
+
FFFFFFFFFFFFFFFF:FFFFFFFFFFFFFFFFFFFFFFFFFFFFFFFFFFFFFFFFFFFFFFFFFFF
FFFFFFFFFFFFFFFFFFFFFFFFFFFFFFFFFF:FFFFFFFFFFFFFFFFFFFFFFFFFFFFFFFF:
FFFFFFFFFFFFFFF

```
@A00155:342:HHGFNDSXY:1:1417:6967:25614 1:N:0:GAACCTAG+TCCGCATA
ATTCATATGTAGCATGTTTAAGCATTAAATCAAATAAGATGAGCAAGATGGAACGTCTGGGTTCCAAT
TCTGTCAAGACTTATGACATATTAAATAGATTGGTTACGGTAAAAGACAAGGCAAGGTATGGACCCTT
AGCAAAGCCAGAAAG
+
FFFFFFF:FF:FFFFF:FFF,FFFFFFFFF:FFFFFFF:FFF:F:FFFF:FFFFFFFFFFFFFFFFFF:F
FFFFFFFFFFFFFFFFFFFFFFFFF,FFF,FF:FFFFFF:FFFFF:FFF:,FFFFFFFFFFFFFFFFFFF
FFFFFFFFFFFFFFF
@A00155:342:HHGFNDSXY:1:1536:7627:13761 1:N:0:GAACCTAG+TCCGCATA
ATTCATATGTAGCATGTTTAAGCATTAAATCAAATAAGATGAGCAAGATGGAACGTCTGGGTTCCAAT
TCTGTCAAGACTTATGACATATTAAATAGATTGGTTACGGTAAAAGACAAGGCAAGGTATGGACCCTT
AGCAAAGCCAGAAAG
+
F:FFF:F:FFFFFFFFFFFFFFFFF:FFFFF:FFFFFFFFFFFFFFFFFFFFFFFFFFFFFFFFFFFFFF
FFFFFFFFFFFFFFFFFFFFFFFFFFFFFFFFFFFFFFFFFFFFFFFFFFFFFFFFFFFFFFFFFFFFFF
FFFFFFFFFFFFFFF
@A00155:342:HHGFNDSXY:1:1312:13810:30577 2:N:0:GAACCTAG+TCCGCATA
ATTCATATGTAGCATGTTTAAGCATTAAATCAAATAAGATGAGCAAGATGGAACGTCTGGGTTCCAAT
TCTGTCAAGACTTATGACATATTAAATAGATTGGTTACGGTAAAAGACAAGGCAAGGTATGGACCCTT
AGCAAAGCCAGAAAG
+
FFFFFFFFFFFFFFFFFFFFFFFFFFFFFFFFFFFFFFFFFFFFFFFFFFFFFFFFFFFFFFFFFFFFFF
FF:FFFFFFFFFFFFF:FFFFFFFFFFFFFFFF:FFFFFFFFFFFF:FFFFFFFFFFFFFFFFFFF:FFF
FFFFFFFFFFFFFFF
@A00155:342:HHGFNDSXY:1:1631:14434:18693 2:N:0:GAACCTAG+TCCGCATA
ATTCATATGTAGCATGTTTAAGCATTAAATCAAATAAGATGAGCAAGATGGAACGTCTGGGTTCCAAT
TCTGTCAAGACTTATGACATATTAAATAGATTGGTTACGGTAAAAGACAAGGCAAGGTATGGACCCTT
AGCAAAGCCAGAAAG
+
FFFFFFF:FFFFFFFF:FFFFFFFFFFFFFFFFFFFFFFFFFFFFFFFFFFFFFFFFFFFFFFFFFFFFF
FFFFFFF,FFFFFFFFFFFFFF:FFFFFFFFFFFFFFFFFFFFFF:FFFF:FFFFFFFFFFFF:FFFFFF
FF:FFFFFFFFFFFF
@A00155:342:HHGFNDSXY:1:2134:30273:25895 2:N:0:GAACCTAG+TCCGCATA
ATTCATATGTAGCATGTTTAAGCATTAAATCAAATAAGATGAGCAAGATGGAACGTCTGGGTTCCAAT
TCTGTCAAGACTTATGACATATTAAATAGATTGGTTACGGTAAAAGACAAGGCAAGGTATGGACCCTT
AGCAAAGCCAGAAAG
+
FFFFFFFFFFFFFF:FFFFFFFFFFFFFFFFFFFFFFFF:FFFFFFFFFFFFFFFFFFFFFFFFFFFF:F
FFFFFFFFFFFF:FFFFFFFFFFFFFFFFFFFFF:FFFFFFFFFFFFFFFFFF,FFFFFFFF:FFFFFFF
FFFFFFFFFFFF:FF
@A00155:342:HHGFNDSXY:1:2124:5981:33301 2:N:0:GAACCTAG+TCCGCATA
ATTCATATGTAGCATGTTTAAGCATTAAATCAAATAAGATGAGCAAGATGGAACGTCTGGGTTCCAAT
TCTGTCAAGACTTATGACATATTAAATAGATTGGTTACGGTAAAAGACAAGGAAAGGTATGGACCCTT
AGCAAAGCCAGAAAG
+
FF:FFFFFFFF:,FFFFFFFFFF:FFFFFFFFFFFFFFFF::F,F:FFFFFFFFFFFFFFFFFFFFFFFF
FFFFFFFFFFFFFFFFFFFFFFFFFFFFFFFFFFFFFFF,FFFFFFFFFFFFFFFFFFFFFFFFFFFFFF
FFFFFFFFFFFFFFF
@A00155:342:HHGFNDSXY:1:2260:25464:14998 2:N:0:GAACCTAG+TCCGCATA
ATTCATATGTAGCATGTTTAAGCATTAAATCAAATAAGATGAGCAAGATGGAACGTCTGGGTTCCAAT
TCTGTCAAGACTTATGACATATTAAATAGATTGGTTACGGTAAAAGACAAGGAAAGGTATGGACCCTT
AGCAAAGCCAGAAAG
+
FFFFFFF,FFF:FFFFFFFFFF,FFFFFFFFFFFFFFFFFFFFFFF:FFFFFF:FFFF:FFFFF:FFFFF
```

FFFFFFFFFFFFFFFFFFFFFFFFFFFFFFFFFFFFFFFFFFFFFFFFFFFFFFFFFFFFFFFFFFFFFF
FFFFFFFFFFFFFFF
@A00155:342:HHGFNDSXY:1:2318:3215:4366 2:N:0:GAACCTAG+TCCGCATA
ATTCATATGTAGCATGTTTAAGCATTAAATCAAATAAGATGAGCAAGATGGAACGTCTGGGTTCCAAT
TCTGTCAAGACTTATGACATATTAAATAGATTGGTTACGGTAAAAGACAAGGCAAGGTATGGACCCTT
AGCAAAGCCAGAAAG
+
FFFFFFFFFFFFFFFFFFFFFFFFFFFFFFFFFFFFFFFFFFFFFFFFFFFFFFFF:FFFFFFFFFFFFF
FFF,FFFFFF:FFFFFFFFFFFFFF:FFFFFFFFFFFFFFFFFFF:FFFFFF:FF,FFFFFF,FFFFFF
FFFFF,FFF,FFF:F
@A00155:342:HHGFNDSXY:1:2540:15709:25817 2:N:0:GAACCTAG+TCCGCATA
ATTCATATGTAGCATGTTTAAGCATTAAATCAAATAAGATGAGCAAGATGGAACGTCTGGGTTCCAAT
TCTGTCAAGACTTATGACATATTAAATAGATTGGTTACGGTAAAAGACAAGGCAAGGTATGGACCCTT
AGCAAAGCCAGAAAG
+
FFFFFFF:FFFFFFFFFF:FFFFFFFFFF:FFFFF:FFFFFFFFFFFFF:FFFFFFFFFFFFFFFFFFFF
FFFFFFFFFFFFFFFFFFFFFFFFFFFFFFFFFFFFFFFF:FFFFFFFFFFFFFF:FFFFF,FFFFFFF
:FFFFFFFFFFFFFF
@A00155:342:HHGFNDSXY:1:2570:5367:21778 2:N:0:GAACCTAG+TCCGCATA
ATTCATATGTAGCATGTTTAAGCATTAAATCAAATAAGATGAGCAAGATGGAACGTCTGGGTTCCAAT
TCTGTCAAGACTTATGACATATTAAATAGATTGGTTACGGTAAAAGACAAGGCAAGGTATGGACCCTT
AGCAAAGCCAGAAAG
+
FFFFFFFFFFF:FFFFFFFFFF:F,F::F:FFFFFFFFFFFF::F:FFFFFFFFFFFFF,FFFF:FFF
F:FFFFFF:FFFFFF:FFFFFF:FFFFFFFFFFFF:FFFF:FFFFFF:FFFF:F:FFFFFFFFFFFFF
F:FF,FFF,,F:FFF
@A00155:342:HHGFNDSXY:1:2631:13340:20588 2:N:0:GAACCTAG+TCCGCATA
ATTCATATGTAGCATGTTTAAGCATTAAATCAAATAAGATGAGCAAGATGGAACGTCTGGGTTCCAAT
TCTGTCAAGACTTATGACATATTAAATAGATTGGTTACGGTAAAAGACAAGGCAAGGTATGGACCCTT
AGCAAAGCCAGAAAG
+
FFFFFFFFFFFFFFFFFFFFFFFFFFFFFFFFFFFFFFFFFFFFFFFFFFFFFFFFFFFFFFFFFFFFFF
FFFFFFFFFFFFFFFFFFFFFFFFFFF,F:FFFFFFFFFFFFFFFFFFFFFFFFFF:FFFFFFFFFFFF:
:F:FFFFFFFFFFF:
@A00155:342:HHGFNDSXY:1:1148:17137:17018 2:N:0:GAACCTAG+TCCGCATA
TTCATATGTAGCATGTTTAAGCATTAAATCAAATAAGATGAGCAAGATGGAACGTCTGGGTTCCAATT
CTGTCAAGACTTATGACATATTAAATAGATTGGTTACGGTAAAAGACAAGGCAAGGTATGGACCCTTA
GCAAAGCCAGAAAGA
+
FFFFFF:FFFFFFFFFFFFFFFFFFFF:FFFFFFFFFFFFFFFF:FFFFFFFFFFFFFFFFFF:FFFF
FFFFFFFFFFFFFFFFFFFF:FFFFFFFFFFFFFFFFFFFFFFFFFFFFFFFFF:F,FFFFFF:FFFFFFF
F:FFFFFFFFFF,F:
@A00155:342:HHGFNDSXY:1:1375:1072:36010 2:N:0:GAACCTAG+TCCGCATA
TTCATATGTAGCATGTTTAAGCATTAAATCAAATAAGATGAGCAAGATGGAACGTCTGGGTTCCAATT
CTGTCAAGACTTATGACATATTAAATAGATTGGTTACGGTAAAAGACAAGGCAAGGTATGGACCCTTA
GCAAAGCCAGAAAGA
+
:FFFFFFFFFFFFFFFFFFFFFFFF:F:FF::FFF:FFFFFFFF,FFFFFF:FFFFFFFFFFFFFFF
FFFFFFFFFFFFFFFFFFF:FF:FFFFFFFFFFFF:FFFF,FFFFFF::FFF:,FFF,FFF:FFFFFF
FF:FFFFF:FF:FFF
@A00155:342:HHGFNDSXY:1:1517:18240:31767 2:N:0:GAACCTAG+TCCGCATA
TTCATATGTAGCATGTTTAAGCATTAAATCAAATAAGATGAGCAAGATGGAACGTCTGGGTTCCAATT
CTGTCAAGACTTATGACATATTAAATAGATTGGTTACGGTAAAAGACAAGGCAAGGTATGGACCCTTA
GCAAAGCCAGAAAGA

```
+
FFFFFFFFFFFFFFFFFFFFFFFFFFFFFFFFFFFFFFFFFFFFFFFFFFFFFFFFFFFFFFFFFFFF
FFFFFFFFFFFFFFFFFFFFFFFFFFFFFFFFFFFFFFFFFFFFF:FFFFFFFFFFFFFFFFFFFFFFFFFF
FF:FFFFFFFFFFFF
@A00155:342:HHGFNDSXY:1:1634:13232:11287 2:N:0:GAACCTAG+TCCGCATA
TTCATATGTAGCATGTTTAAGCATTAAATCAAATAAGATGAGCAAGATGGAACGTCTGGGTTCCAATT
CTGTCAAGACTTATGACATATTAAATAGATTGGTTACGGTAAAAGACAAGGCAAGGTATGGACCCTTA
GCAAAGCCAGAAAGA
+
:FFFFFFFFFFFF:FFFFFFFFFFFFFFFFFFFFFFFFFFFFFFFFFFFFFFFFFFFFFFFFFFFF:FF
FFFFFFFFFFFFFFFFFFFFFFFF:FFF,FFFFFFFFFFFFFFFFFFF:FFFFF:FFFFFFFFFFFFFF
FFF,FFFFFFFFFFF
@A00155:342:HHGFNDSXY:1:2213:25355:25770 2:N:0:GAACCTAG+TCCGCATA
TTCATATGTAGCATGTTTAAGCATTAAATCAAATAAGATGAGCAAGATGGAACGTCTGGGTTCCAATT
CTGTCAAGACTTATGACATATTAAATAGATTGGTTACGGTAAAAGACAAGGCAAGGTATGGACCCTTA
GCAAAGCCAGAAAGA
+
FFFFFFFFFF:FFFFFFFFFFFFFFFFFFFFFFFF:FFFFFFF:FFFFFFFFF:FFFFFFFFFFFFFF
FFFFFFFFFFFFFFFFFFFFFFF:FFFFFFFFFFFFFFFFFFFFF,FFFF,F:FFFFFFFFFFFFFFFF
FFFFFF:FFFFFFFF
@A00155:342:HHGFNDSXY:1:2375:2383:34303 2:N:0:GAACCTAG+TCCGCATA
TTCATATGTAGCATGTTTAAGCATTAAATCAAATAAGATGAGCAAGATGGAACGTCTGGGTTCCAATT
CTGTCAAGACTTATGACATATTAAATATATTGGTTACGGTAAAAGACAAGGCAAGGTATGGACCCTTA
GCAAAGCCAGAAAGA
+
FFFFF:FFFFFFFFFFFFF:FF:FFFF:FFFF:FFFFFFFFFFFF:FFFFFFFFFFFFFFFFFFFFFFF
FFFFFFF:FFFFFFFFFFFFFFFF:FF,FFFFFFFFFFFFFFF:FFFFFFFFFFFFFFFFFFFF:FFF
FFFFF:FFFF:FFFF
@A00155:342:HHGFNDSXY:1:2506:30933:32675 2:N:0:GAACCTAG+TCCGCATA
TTCATATGTAGCATGTTTAAGCATTAAATCAAATAAGATGAGCAAGATGGAACGTCTGGGTTCCAATT
CTGTCAAGACTTATGACATATTAAATAGATTGGTTACGGTAAAAGACAAGGCAAGGTATGGACCCTTA
GCAAAGCCAGAAAGA
+
FFFFF:FFFFFFFFFFFFFFFFFFFFFFFFFFFFFFFFFFFFFF:FFFFFFFF:FFFF:FFFF:FFFFFF
FFFFFFFFFFFFFFFFFFFFFFFFFFFFFFFFFFFFFFFFF:FF:FFFFFFFFF:FF:FFF:FFFFF:F
FFFFFFFFFFFFFFF
@A00155:342:HHGFNDSXY:1:2214:4770:27289 2:N:0:GAACCTAG+TCCGCATA
TCATATGTAGCATGTTTAAGCATTAAATCAAATAAGATGAGCAAGATGGAACGTCTGGGTTCCAATTC
TGTCAAGACTTATGACATATTAAATATATTTGTTACTGTAAAAGACAAGGCAAGGTATGGACCCTTAG
AAAAGACAGAAAGAT
+
FFFFFF,FFFFFFFFFFFFFFFFFFFFFFFFFFFF,:FFF:FFFFFFFF:F:FFF:FFF,FFFFF:FFF
FFFFFFFFFFFFFFFFF:FFFFFFFF,FF,,FFFF,,FF:F,:FFFFF,FFFF,FFFFFFFF,FF:FF
,FFFF,FF,F:FF,F
@A00155:342:HHGFNDSXY:1:2271:3007:26428 2:N:0:GAACCTAG+TCCGCATA
TCATATGTAGCATGTTTAAGCATTAAATCAAATAAGATGAGCAAGATGGAACGTCTGGGTTCCAATTC
TGTCAAGACTTATGACATATTAAATAGATTGGTTACGGTAAAAGACAAGGCAAGGTATGGACCCTTAG
CAAAGCCAGAAAGAT
+
FFFFFFFFFFFFFFFFFFFFFFFFFFFFFFFFFFFFFFFFFFFFFFFFFFFFFFFF:FFFFFFFFFFF
FFFFFF:FFFFFFFFFFFFFFFFFFFFFFFFFFFFFFFFFFFFF:FFFFFFFFFFFFFFFFF:FFFFFF
FFFFFFFFFFFF:FF
@A00155:342:HHGFNDSXY:1:1450:11125:29089 1:N:0:GAACCTAG+TCCGCATA
CATATGTAGCATGTTTAAGCATTAAATCAAATAAGATGAGCAAGATGGAACGTCTGGGTTCCAATTCT
```

```
GTCAAGACTTATGACATATTAAATAGATTGGTTACGGTAAAAGACAAGGCAAGGTATGGACCCTTAGC
AAAGCCAGAA
+
FFFFFFFFFFFFFFFF:FFFFFFFFFFFFFFFFFFFFFFFFFFFFFFF:FFFFFFFFFFFFFFFFFFFF
FFFFFFFFFFFFFFFFFFFFF:FFFFFFFFFFFFFFFFFF:FFFFFFFFFFFFFFFFFFFFFFFFFFFF
FFFFFFFFFF
@A00155:342:HHGFNDSXY:1:2248:23258:11240 1:N:0:GAACCTAG+TCCGCATA
CATATGTAGCATGTTTAAGCATTAAATCAAATAAGATGAGCAAGATGGAACGTCTGGGTTCCAATTCT
GTCAAGACTTATGACATATTAAATAGATTTGTTACGGTAAAAGACAAGGCAAGGTATGGACCCTTAGC
AAAGCCAGAA
+
:FFFFFFFF:FFF:FFFFFFFFF:FFFFFFFFFFFFFFFFFFFFFFFFFFFFFFFFFFFFFFFFFFFFF
FFFFFFFFFFFFFFFFFFFFFFFFFFFFFFFFFFFFFFFFFFFFFFFFFFFFFFFFFFFFFFFFFFFFF
FFFFFFFFFF
@A00155:342:HHGFNDSXY:1:2311:21585:11005 1:N:0:GAACCTAG+TCCGCATA
CATATGTAGCATGTTTAAGCATTAAATCAAATAAGATGAGCAAGATGGAACGTCTGGGTTCCAATTCT
GTCAAGACTTATGACATATTAAATAGATTGGTTACGGTAAAAGACAAGGCAAGGTATGGACCCTTAGC
AAAGCCAGAA
+
FFFFFFFFFFFFFFFFFF:FFFFFFFF:FFFF:FFFFFFFFFFFFFFFF:FFFFFFFF:FF:FFFFFF
FFFFFFF:FFFFFFFFF:FFFFFFFFFFFFFFFFFFFFFFFFFFFFFFFFFFFFFFFFFFFFFFFFFFF
FFFFFFFFFF
@A00155:342:HHGFNDSXY:1:2463:3034:9157 1:N:0:GAACCTAG+TCCGCATA
CATATGTAGCATGTTTAAGCATTAAATCAAATAAGATGAGCAAGATGGAACGTCTGGGTTCCAATTCT
GTCAAGACTTATGACATATTAAATAGATTGGTTACGGTAAAAGACAAGGCAAGGTATGGACCCTTAGC
AAAGCCAGAA
+
FFFFFFF:,:FFF:FFFFFFFFFFFFF:FFFFFFFFFFFFFFFFFFFFFFFFFFFFF,FFFF:FFF:FF
FFFFFFF:FFFFFFFFFFFFFFFFFFFFFFFFFFFFFFFFFFFFFFFFFFFFFFFFFFFFFFFFFFFFF
FFFFFFFFFF
@A00155:342:HHGFNDSXY:1:2673:24686:20040 1:N:0:GAACCTAG+TCCGCATA
CATATGTAGCATGTTTAAGCATTAAATCAAATAAGATGAGCAAGATGGAACGTCTGGGTTCCAATTCT
GTCAAGACTTATGACATATTAAATAGATTGGTTACGGTAAAAGACAAGGCAAGGTATGGACCCTTAGC
AAAGCCAGAA
+
FFFFFFFFFFFFFFFFFFFFFFFFFFFF:FFFFFFFFFFFFFFFFFFFFFFFFFFFFFFFFFFFFFFFF
FFFFFFFFFFFFFFFFFFFFFF:FFFFFFFFFFFFFFFFFFFFFFFFFFFFFFFFFF:FFFFFFFFFFFFF
FFFFFFFFFF
@A00155:342:HHGFNDSXY:1:1301:25265:19413 2:N:0:GAACCTAG+TCCGCATA
CATATGTAGCATGTTTAAGCATTAAATCAAATAAGATGAGCAAGATGGAACGTCTGGGTTCCAATTCT
GTCAAGACTTATGACATATTAAATAGATTGGTTACGGTAAAAGACAAGGCAAGGTATGGACCCTTAGC
AAAGCCAGAAAGATC
+
FF:FFFFFFFFFFFFFFFFFFFFFFFFFFFFFFFFFFFFFFFFFFFFF,FFFFFFFFFFFFF:FFFFFFF
FFFFFFFFFFFFFFF:FFFFFFFFFFFFFF:FFFFFFFFFFFFFFFF:FFF:FFFFF:FF:FFFFFF:
FFFF,F::FFFFFFF
@A00155:342:HHGFNDSXY:1:1431:7717:7717 2:N:0:GAACCTAG+TCCGCATA
CATATGTAGCATGTTTAAGCATTAAATCAAATAAGATGAGCAAGATGGAACGTCTGGGTTCCAATTCT
GTCAAGACTTATGACATATTAAATAGATTGGTTACGGTAAAAGACAAGGCAAGGTATGGACCCTTAGC
AAAGCCAGAAAGATC
+
FFFFFFFFFFFFFFFFFFFFFFFFFFFFFFFFFFFFFFFFFFFFFFFFFFFFFFFFFFFFFFFFFFFFF
FFFFFFFFFFFFFFFFFFFFFFFFFFFFFFFFFFF:FFFFFFFFFFFFFFFF,FFFFFFFFFFFFFFFF
F,FFFFFFFFFFFF
```

@A00155:342:HHGFNDSXY:1:1629:11514:36401 2:N:0:GAACCTAG+TCCGCATA
CATATGTAGCATGTTTAAGCATTAAATCAAATAAGATGAGCAAGATGGAACGTCTGGGTTCCAATTCT
GTCAAGACTTATGACATATTAAATAGATTGGTTACGGTAAAAGACAAGGCACGGTATGGACCCTTAGC
AAAGCCAGAAAGATC
+
FFFFFF:FFFFFFFFFFFFFFFFFFFFFFFFFFFFFFFFFFFFFFFFFFFFFFFFFFFFFFFFFFFFF
FFFFFFFFFFFFFFFFFFFFFFFFFFFFFFFFFFFFFFF,FFFFFFFFFFF,FFFFFFFFFFFFFFF:
FFFFFFFFFFFFFFF
@A00155:342:HHGFNDSXY:1:1652:28999:12101 2:N:0:GAACCTAG+TCCGCATA
CATATGTAGCATGTTTAAGCATTAAATCAAATAAGATGAGCAAGATGGAACGTCTGGGTTCCAATTCT
GTCAAGACTTATGACATATTAAATAGATTGGTTACGGTAAAAGACAAGGCAAGGTATGGACCCTTAGC
AAAGCCAGAAAGATC
+
FFFFFFFFFFFFFFFFFFFFFFFFFFFFFFFFFFFFFFF,FFFFFFFFFFFFFF:FFFFFFFFFFFFF
FFFF::FFFFFFFFFF:F,FFFFFFFF:FFF,FF,FFF,FFFFFFFFF:FF:F:FFFFFFFFFFFFFF
F:FFFFFFFFFFFFF
@A00155:342:HHGFNDSXY:1:2601:32687:5588 2:N:0:GAACCTAG+TCCGCATA
CATATGTAGCATGTTTAAGCATTAAATCAAATAAGATGAGCAAGATGGAACGTCTGGGTTCCAATTCT
GTCAAGACTTATGACATATTAAATAGATTGGTTACGGTAAAAGACAAGGCAAGGTATGGACCCTTAGC
AAAGCCAGAAAGATC
+
FFFFFFFFFFFFFFFFFFFFFFFFFFFFFF:FFFFFFFFFFFFF:FFFFFFFF:FFFFFFFF:FFFFF
FFFFFFFFFFFFFFFFFFFFF:FFFFFFFFFFFFFFFFFF:FFFFFFFFFFFFFFFFFFFFFFFFFFF
FFFFFFFFFFFF:FF
@A00155:342:HHGFNDSXY:1:2553:30689:28776 2:N:0:GAACCTAG+TCCGCATA
CGATCTTGCATGTTTAAGCATTAAATCAAATAAGATGAGCAAGATGGAACGTCTGGGTTCCAATTCTG
TCAAGACTTATGACATATTAAATAGATTGGTTACGGTAAAAGACAAGGCAAGGTATGGACCCTTAGCA
AAGCCAGAA
+
FFFFFFF:,FFFFFFFF,FFFFFFFFFFFFFFFFFF:F:FFF:FFFFFFF:FFFFF:FF,FFFF:FFF
FFFF,:FFFFFFFFFFFFFFFF:FFFFFFFFFFFFFFFFFFFFFFFFFFFFFFFFFFFFFFFFFFFF
FFFFFFFFF
@A00155:342:HHGFNDSXY:1:2506:9082:36980 2:N:0:GAACCTAG+TCCGCATA
ATATGTAGCATGTTTAAGCATTAAATCAAATAAGATGAGCAAGATGGAACGTCTGGGTTCCAATTCTG
TCAAGACTTATGACATATTAAATAGATTGGTTACGGTAAAAGACAAGGCAAGGTATGGACCCTTAGCA
AAGCCAGAAAGATC
+
FFFFFFFFF:FFFFFFFFFFFFFFFFFFFFF:FFFFFFFFFFFFFFFF:FFFFFF,FFFFFFFF:FFF
FFFFFFFFFFFFFFFFFFFFFFFFFFFFF,FFF,FF:FF:F:FFFFFFFF:,FFFFFFFFFFF:FFFF
:FFFFFFFFFFFFF
@A00155:342:HHGFNDSXY:1:1260:20455:35822 1:N:0:GAACCTAG+TCCGCATA
ATATGTAGCATGTTTAAGCATTAAATCAAATAAGATGAGCAAGATGGAACGTCTGGGTTCCAATTCTG
TCAAGACTTATGACATATTAAATAGATTGGTTACGGTAAAAGACAAGGCAAGGTATGGACCCTTAGCA
AAGCCAGAAAGATCA
+
FFFFFFFFFFFF:FFFFF:FFFFFFFFFFFFFFFFFFFFFFFFFFFFFFFFFFFFFFFFFFFFFFFFF
FFFFFFFFFFFFFFFFFFFFFFFFFFFFFFFFFFFFFFFFFFFFFFFFFFFFFFFFFFFFFFFFFFFF
FFFFFFFFFFFFFFF
@A00155:342:HHGFNDSXY:1:2347:27670:24612 1:N:0:GAACCTAG+TCCGCATA
ATATGTAGCATGTTTAAGCATTAAATCAAATAAGATGAGCAAGATGGAACGTCTGGGTTCCAATTCTG
TCAAGACTTATGACATATTAAATAGATTGGTTACGGTAAAAGACAAGGCAAGGTATGGACCCTTAGCA
AAGCCAGAAAGATCA
+
FFFFFFFFFFFF:FFFFFFFF:FFFFFFFFFFFFF:F:FFFFFFFFFFFFFFFFFFFFFFFFFFFFFF

```
FFFFFFFFFFFFFFFFFFFFFFFFFFFFFFFFFFFFFFFFFFFFFFFFFFFFF:FFFF:FFFFF:FFF
FFFFFFFFFFFFFFF
@A00155:342:HHGFNDSXY:1:1413:23674:33191 1:N:0:GAACCTAG+TCCGCATA
TATGTAGCATGTTTAAGCATTAAATCAAATAAGATGAGCAAGATGGAACGTCTGGGTTCCAATTCTGT
CAAGACTTATGACATATTAAATAGATTGGTTACGGTAAAAGACAAGGCAAGGTATGGACCCTTAGCAA
AGCCAGAAAGATCAC
+
FFFFFFFFFFF:FFFFFFFFFFFFFFFFFFFFFFFFFFFFFFFFFFFFFFFFFFFFFFFFFFFFFFFF
FFFFFFFFFFFFFFFFFFFFFFFFFFFFFFFFFFFFFFFFFFFFFFFFFFFFFFFFFFFFFFFFFFFF
FFFFFFFFFFFFFFF
@A00155:342:HHGFNDSXY:1:2211:11948:9283 1:N:0:GAACCTAG+TCCGCATA
TATGTAGCATGTTTAAGCATTAAATCAAATAAGATGAGCAAGATGGAACGTCTGGGTTCCAATTCTGT
CAAGACTTATGACATATTAAATAGATTGGTTACGGTAAAAGACAAGGCAAGGTATGGACCCTTAGCAA
AGCCAGAAAGATCAC
+
FFFFFFFFFFF:FFFFFFFF:FFFFFFFFFFFFFFFFFFFFFFFFFFFFFFFFFF:FFFFFFFFFFFF
FFFFFFFFFFFFFFFFFFFFFFFFFFFFFFFFFFFFFFFFFFFFFFFFFFFFFFFFFFFFFFFFFFFF
FFFFFFFFFFFFFFF
@A00155:342:HHGFNDSXY:1:2308:14922:1376 1:N:0:GAACCTAG+TCCGCATA
GATCTAGCACGTTTAAGCATTAAATCAAATAAGATGAGCAAGATGGAACGTCTGGGTTCCAATTCTGT
CAAGACTTATGACATATTAAATAGATTGGTTACGGTAAAAGACAAGGCAAGGTATGGACCCTTAGCAA
AGCCAGAAAGATCAC
+
FFFFFFFFFFFFFFFFFFFFFFFFFFFFFFFFFFFFFFFFFFFFFFFFFFFFFFFFFFFFFFFFFFFF
FFFFFFFFFFFFFFFFFFFFFFFFFFFFFFFFFFFFFFFFFFFFF:FFFFFFFFFFFFFFFFFFFFFF
FFFFFFFFFFFFFFF
@A00155:342:HHGFNDSXY:1:2308:15989:4006 1:N:0:GAACCTAG+TCCGCATA
GATCTAGCATGTTTAAGCATTAAATCAAATAAGATGAGCAAGATGGAACGTCTGGGTTCCAATTCTGT
CAAGACTTATGACATATTAAATAGATTGGTTACGGTAAAAGACAAGGCAAGGTATGGACCCTTAGCAA
AGCCAGAAAGATCAC
+
FFFFFFFFFF:FFFFFFFFFFFFFFFFFFFFFFFFFFFFFFFFFFFFFFFFFFFFFFFFFFFFFFFFF
FFFFFFFFFFFFFFFFFFFFFFFFFFFFFFFFFFFFFFFFFFFFFFFFFFFFFFFFFFFFFFFFFFFF
FFFFFFFFFFFFFFF
@A00155:342:HHGFNDSXY:1:2503:28745:14826 1:N:0:GAACCTAG+TCCGCATA
TATGTAGCATGTTTAAGCATTAAATCAAATAAGATGAGCAAGATGGAACGTCTGGGTTCCAATTCTGT
CAAGACTTATGACATATTAAATAGATTGGTTACGGTAAAAGACAAGGCAAGGTATGGACCCTTAGCAA
AGCCAGAAAGATCAC
+
FFFFFFFFFFF,FFFFFFFFFFFFFFFFFFFFFFFFFFFFFFFFFFFFFFFFFFFFFFFFF:FFFFF
FFFFFFFFFFFFFFFFFFFFFFFFFFFFFFFFFFFFFFFFFFFFFFFFFFFFFFFFFFFFFFFFFFFF
FFFFFFFFFFFFFFF
@A00155:342:HHGFNDSXY:1:2508:4300:33176 1:N:0:GAACCTAG+TCCGCATA
TATGTAGCATGTTTAAGCATTAAATCAAATAAGATGAGCAAGATGGAACGTCTGGGTTCCAATTCTGT
CAAGACTTATGACATATTAAATAGATTGGTTACGGTAAAAGACAAGGCTAGGTATGGACCCTTAGCAA
AGCCAGAAAGATCAC
+
FFFFFFFFFF,FFFFF:FFFFFFFFFFFFFFFFFFFFFFFFFFFFFFF:FFFFFFFFFFFFFFFFFFF
FFFFFFFFFFFFFFFFFFFFFFFFFFFFFFFFFFFFFFFFFFFFFF,FF,FFFFFFFFFFFFFFFF
FFFFFFFFFFFFFFF
@A00155:342:HHGFNDSXY:1:1625:19425:11835 2:N:0:GAACCTAG+TCCGCATA
GATCTAGCATGTTTAAGCATTAAATCAAATAAGATGAGCAAGATGGAACGTCTGGGTTCCAATTCTGT
CAAGACTTATGACATATTAAATAGATTGGTTACGGTAAAAGACAAGGCAAGGTATGGACCCTTAGCAA
AGCCAGAAAGATCAC
```

+
FFFFFFFFFFFFFFFFFFFFFFFFFFFFFFFFFFFFFFFFFFFFFFFFFFFFFFFFFFFFFFFFFFFF
FFFFFFFFFFFFFFFFFFFFFFFFFFFFFFFFFFFFFFFFFFFFFFFFFFFFFFFFFFFFFFFFFFFF
FFFFFFFFFFFFFFF
@A00155:342:HHGFNDSXY:1:1641:9335:8578 2:N:0:GAACCTAG+TCCGCATA
TATGTAGCATGTTTAAGCATTAAATCAAATAAGATGAGCAAGATGGAACGTCTGGGTTCCAATTCTGT
CAAGACTTATGACATATTAAATAGATTGGTTACGGTAAAAGACAAGGCAAGGTATGGACCCTTAGCAA
AGCCAGAAAGATCAC
+
F:FFFF,FFFFFFFFFFFFFFFFFFFFFFFFFFFFFFF:FFFFFFFFFFFFFFFFFFFFFFFFFFFFF
FFFFFFFFFFFFFFFF:FFFFFFFFFFFFFFFFFFFFFFFFFFFFFFFFFF:FFFFFFFFFFFFFFFF
FFFFFFFFFFFFFFF
@A00155:342:HHGFNDSXY:1:1442:24316:18677 2:N:0:GAACCTAG+TCCGCATA
ATGTAGCATGTTTAAGCATTAAATCAAATAAGATGAGCAAGATGGAACGTCTGGGTTCCAATTCTGTC
AAGACTTATGACATATTAAATAGATTGGTTACGGTAAAAGACAAGGCAAGGTATGGACCCTTAGCAAA
GCCAGAAAGATCACC
+
F:FFFFFFFFFFFFFFFFFFFFFFFFFFFFFFFFFFFFFFFFFFFFFFFFFFFFFFFFFFFFFFFFFF
FF:FFFFFFFFFFFFFFFFFFFFFFF,FFFFFFFFFFFFFFF:FFFFFFFFFFFFFFFFFFFF:FFFF
FFFFFFFFFFFFFFF
@A00155:342:HHGFNDSXY:1:1459:21441:10911 2:N:0:GAACCTAG+TCCGCATA
ATTTAGCATGTTTAAGCATTAAATCAAATAAGATGAGCAAGATGGAACGTCTGGGTTCCAATTCTGTC
AAGACTTATGACATATTAAATAGATTGGTTACGGTAAAAGACAAGGCAAGGTATGGACCCTTAGCAAA
GCCAGAAAGATCACC
+
FFFFFFFFFFFFFFFFFFFFFFFFFFFFFFFFFFFFFFFFFFFFFF:FFFFFFFFFFFFFFFFFFFFF
FFFFFFFFFFFFFFFFFFFFFFFFFFFFFFFFFFFF:FFFFFFFFFFFFFFFFFFFFFFFFFF:FFFF
::FFFFFFFFF:FFF
@A00155:342:HHGFNDSXY:1:2603:7066:2331 2:N:0:GAACCTAG+TCCGCATA
ATGTAGCATGTTTAAGCATTAAATCAAATAAGATGAGCAAGATGGAACGTCTGGGTTCCAATTCTGTC
AAGACTTATGACATATTAAATAGATTGGTTACGGTAAAAGACAAGGCAAGGTATGGACCCTTAGCAAA
GCCAGAAAGATCACC
+
FFFFFFFFFFFFFFFFFFFFFFFFFFFFFFFFFFFFFFFFFFFFFFFFFFFFFFFFFFFFFF:FFFFF
F:FFFFFFFFFFFFFFFFFFFFFFFFFFFFFFFFFFFFFFFFFFFFFF:FFFFFFFFFF:FFFFFFF,
FFFFFFFFFFFFFFF
@A00155:342:HHGFNDSXY:1:2271:3586:27492 1:N:0:GAACCTAG+TCCGCATA
TGTAGCATGTTTAAGCATTAAATCAAATAAGATGAGCAAGATGGAACGTCTGGGTTCCAATTCTGTCA
AGACTTATGACATATTAAATAGATTGGTTACGGTAAAAGACAAGGCAAGGTATGGACCCTTAGCAAAG
CCAGAAAGATCACCC
+
FFFFF,FFF:FFFFF::FFFFFFFF,FFFFFFFFFFFFFFFFFFFFFFFFFFFFFF:FFFFFFFFFFF
FF:FFFFFFFFFFFFFFF:FFFFFFFFFFFFFFF:FFFFFFFFFFFFFFFFFFFFFFFFFFFFF:FFF
FFFFF:FFFFFFFFF
@A00155:342:HHGFNDSXY:1:2627:8024:3928 1:N:0:GAACCTAG+TCCGCATA
TGTAGCATGTTTAAGCATTAAATCAAATAAGATGAGCAAGATGGAACGTCTGGGTTCCAATTCTGTCA
AGACTTATGACATATTAAATAGATTGGTTACGGTAAAAGACAAGGCAAGGTATGGACCCTTAGCAAAG
CCAGAAAGATCACCC
+
FFF,FFFFF:F:FF,FFFFFFFFFFFFFFFFFFFFFFFFFFFFFFFFFFFFFFFFFFFFFFFFFFFFF
FFFFFFFFFFFFFFFFFFFFFFFFFFFFFFFFFFFFFFFFFFFFFFFFFFFFFFFFFFFFFFFFFFFF
FFFFFF:FFFFFFFF
@A00155:342:HHGFNDSXY:1:2601:4499:23187 2:N:0:GAACCTAG+TCCGCATA
TCTGTATGTTTAAGCATTAAATCAAATAAGATGAGCAAGATGGAACGTCTGGGTTCCAATTCTGTCAA

```
GACTTATGACATATTAAATAGATTGGTTACGGTAAAAGACAAGGCAAGGTATGGACCCTTAGCAAAGC
CAGAAAGATCAC
+
FFF,FFFFFFFFFF:FFFFFFFFF:FFF:FFFFFFF:F:F:FF:,FFF:FFFFFFF,FFFFFF,F,:F
F:FFFFF:,FFFFFFFFFFF:FFFF,FFFFFFFFFFFFFFFFF:F:F:FFFFFFFF:FFFF,FFFFF:
FFFFFFFFFFFF
@A00155:342:HHGFNDSXY:1:1273:20500:17143 2:N:0:GAACCTAG+TCCGCATA
GTAGCATGTTTAAGCATTAAATCAAATAAGATGAGCAAGATGGAACGTCTGGGTTCCAATTCTGTCAA
GACTTATGACATATTAAATAGATTGGTTACGGTAAAAGACAAGGCAAGGTATGGACCCTTAGCAAAGC
CAGAAAGATCACCTT
+
FFFFFFFFFFFFFFFFFFFFFFFFFFFFFFFFFFFFFFFFFFFFFFFFFFFFFFFFFFFFFFFFFFFF
FFFFFFFFFFFFFFFFFFFFFFFFFFFFFFFFFFFFFFFFFF:FFFFFF:FFFFFFFFFFFFFFFFFF
FFFFFFFFFFFFFFF
@A00155:342:HHGFNDSXY:1:1273:4119:21527 2:N:0:GAACCTAG+TCCGCATA
GTAGCATGTTTAAGCATTAAATCAAATAAGATGAGCAAGATGGAACGTCTGGGTTCCAATTCTGTCAA
GACTTATGACATATTAAATAGATTGGTTACGGTAAAAGACAAGGCAAGGTATGGACCCTTAGCAAAGC
CAGAAAGATCACCTT
+
F:FFFFFFFFFFFFFFFFFFFFFFFFFFFFFFFFFFFFFFFFFFFFFFFFFFFFFFFFFFFFFFFFFF
FFFFFFFFFFFFFFFFFFFFFFFFFFFFFFFFFFFFFFFFFFFFFFFFFFFFFFFFFFFFFFFFFFFF
FFFFFFFFFFFFFFF
@A00155:342:HHGFNDSXY:1:1378:3161:28134 2:N:0:GAACCTAG+TCCGCATA
GTAGCATGTTTAAGCATTAAATCAAATAAGATGAGCAAGATGGAACGTCTGGGTTCCAATTCTGTCAA
GACTTATGACATATTAAATAGATTGGTTACGGTAAAAGACAAGGCAAGGTATGGACCCTTAGCAAAGC
CAGAAAGATCACCTT
+
FFFFFFFFFFFFFFFFFFFFFFFFFFFFFF:FFFFFFFFFFFFFFFFFFFFFFFFFFFFFFFFFFFFF
FFFFFFFFFFFFFFFF,FFFFFFFFFFFFFFFFFFFFFFFFFFFFFFFFFFFFFFFFFFFFFFFFFFF
FFFFFFF:FF:FFFF
@A00155:342:HHGFNDSXY:1:1426:28384:20212 2:N:0:GAACCTAG+TCCGCATA
GTAGCATGTTTAAGCATTAAATCAAATAAGATGAGCAAGATGGAACGTCTGGGTTCCAATTCTGTCAA
GACTTATGACATATTAAATAGATTGGTTACGGTAAAAGACAAGGCAAGGTATGGACCCTTAGCAAAGC
CAGAAAGATCACCTT
+
FFFFFFFFFFFFFFFFFFFFFFFFFFFFFFFFFFFFFFFFFFFFFFFFFFFFFFFFFFFFFFFFFFFF
FFFFFF:FFFFFFFFFFFFFFFFFFFFFFFFFFFFFFFFFFFFFFFFFFFFFFFFFFFFFFFFF:FFF
FFFFFFFFFFFFFFF
@A00155:342:HHGFNDSXY:1:1540:1244:20838 2:N:0:GAACCTAG+TCCGCATA
GTAGCATGTTTAAGCATTAAATCAAATAAGATGAGCAAGATGGAACGTCTGGGTTCCAATTCTGTCAA
GACTTATGACATATTAAATAGATTGGTTACGGTAAAAGACAAGGCAAGGTATGGACCCTTAGCAAAGC
CAGAAAGATCACCTT
+
FFFFFFFFFFFFFFFFFFFFFF:F:FFFFFFFFFFFFFFFFFFF:FFFF,FFFFFFFFFFFFFFFFFF
FFFFF:FFFFFFFFFFFFFFFF:FFF,FFFFF::FFFFFFFFFFFFFFFF,FFF:FFF:FFFF:FFFF
FFFF,FFFFFFFFFF
@A00155:342:HHGFNDSXY:1:2408:16297:2660 2:N:0:GAACCTAG+TCCGCATA
GTAGCATGTTTAAGCATTAAATCAAATAAGATGAGCAAGATGGAACGTCTGGGTTCCAATTCTGTCAA
GACTTATGACATATTAAATAGATTGGTTACGGTAAAAGACAAGGCAAGGTATGGACCCTTAGCAAAGC
CAGAAAGATCACCTT
+
FFFFFFFFFFFFFFFFFFFFFFFFFFFFFFFFFFFFFFFFFFFF:FFFFFFFFFFFFFFFFFFFFFFF
FFFFFFFFFFFFFFFFFFFFFF,FFFFFFFFFFF::FFFFFF,FFFFFFFFFFFFFFFFFFFFFFFFFF
FFFF:FFFFFFFFFF
```

@A00155:342:HHGFNDSXY:1:2408:24921:3161 2:N:0:GAACCTAG+TCCGCATA
GTAGCATGTTTAAGCATTAAATCAAATAAGATGAGCAAGATGGAACGTCTGGGTTCCAATTCTGTCAA
GACTTATGACATATTAAATAGATTGGTTACGGTAAAAGACAAGGCAAGGTATGGACCCTTAGCAAAGC
CAGAAAGATCACCTT
+
FFFFFFFFFFFFFFFFFFFFFFFFFFFFFFFFFFFFFFFFFFFFFFFFFFFFFFFFFFFF:FFFFFFF
FFFFFFFFFFFFFFFFFFFFFF:FFFFFFFFFFFFFFFFFFFF:FFFFFF,FFFFFFFFFFFFFFFFF
FFFFFFFFFFFFFFF
@A00155:342:HHGFNDSXY:1:2650:30734:24189 2:N:0:GAACCTAG+TCCGCATA
TAGCATGTTTAAGCATTAAATCAAATAAGATGAGCAAGATGGAACGTCTGGGTTCCAATTCTGTCAAG
ACTTATGACATATTAAATAGATTGGTTACGGTAAAAGACAAGGCAAGGTATGGACCCTTAGCAAAGCC
AGAAAGATCACCTTG
+
FFFFFFFFFFFFFFFFFFFFFFFFFFFFFFFFFFFFFFFFFFFFFFFFFFFFFFFFFFFFFFFFFFFF
FFFFFFFFFFFFFFFFFFFFFFFFFFFFFFFFFFFFFFFFFFFFFFFFFFFFFFFFFFFFFFFFFFFF
FFF:FFFFFFFFFFF
@A00155:342:HHGFNDSXY:1:1404:17788:15796 2:N:0:GAACCTAG+TCCGCATA
AGCATGTTTAAGCATTAAATCAAATAAGATGAGCAAGATGGAACGTCTGGGTTCCAATTCTGTCAAGA
CTTATGACATATTAAATAGATTGGTTACGGTAAAAGACAAGGCAAGGTATGGACCCTTAGCAAAGCCA
GAAAGATCACCTT
+
:::F::FFFFFFFFFFFFFFFFFFFFFFFFFFFFFFFFFFFFF:FFFFFFFFFFFFFFFFF:FFFFFF
FFFFFFFFFFFFFFFFFFFFFFFFFFFFFFFFFFFFFFFFF:FFFFFF:F,FF:FF:::FFFFFFFFF
FFFFFFFFFFFFF
@A00155:342:HHGFNDSXY:1:2529:8449:13777 2:N:0:GAACCTAG+TCCGCATA
AGCATGTTTAAGCATTAAATCAAATAAGATGAGCAAGATGGAACGTCTGGGTTCCAATTCTGTCAAGA
CTTATGACATATTAAATAGATTGGTTACGGTAAAAGAAAAGGCAAGGTATGGACCCTTAGCAAAGACA
GAAAGATAACCTT
+
:F:FFFFFFFFF:FFFFFFFFFFFFFF:FFF:::FFFFF:FFFFFFFFFFFFFFFFFFF::FFFFFFF
FFFFFFFFFF:F:FFFFF::F:::FFF,,,:FFFF,F,FF,,FFF::F:F,:F:,:FF:,:FFF,,FF
,FFF,F,,F:,FF
@A00155:342:HHGFNDSXY:1:2601:25590:16814 2:N:0:GAACCTAG+TCCGCATA
AGCATGTTTAAGCATTAAATCAAATAAGATGAGCAAGATGGAACGTCTGGGTTCCAATTCTGTCAAGA
CTTATGACATATTAAATAGATTGGTTACGGTAAAAGACAAGTCAAGGTATGGACCCTTAGCAAAGCCA
GAAAGATCACCTTG
+
FF,FFFF:FFFFFFFFFFFFFFFFFFFFFF,FFFFFFFFFFFFF::F:FFF:FFFFF::FFFFF:F,F
FF:FFFF:FFF,FFFFFF:F:F,F,:FF:FFFF:FFFFFFF,FFFFFFFFFFFF,:,FFF,FF,FFFF
F:FFFFFFFFFF,F
@A00155:342:HHGFNDSXY:1:1203:8974:8860 1:N:0:GAACCTAG+TCCGCATA
AGCATGTTTAAGCATTAAATCAAATAAGATGAGCAAGATGGAACGTCTGGGTTCCAATTCTGTCAAGA
CTTATGACATATTAAATAGATTGGTTACGGTAAAAGACAAGGCAAGGTATGGACCCTTAGCAAAGCCA
GAAAGATCACCTTGC
+
FFFFFFFFFFFFFFFFFFFFFFFFFFFFFFFFFFFFFFFFFFFFFFFFFFFFFFFFFFFFFFFFFF:F
FFFFFFFFFFFFFFFFFFFFFFFFFFFFFFFFF:FFFFFFFFFFFFFF:FFFFFFFFFFFFFFFFFFF
FFFFFFFFFFF:FFF
@A00155:342:HHGFNDSXY:1:1349:8739:30373 1:N:0:GAACCTAG+TCCGCATA
AGCATGTTTAAGCATTAAATCAAATAAGATGAGCAAGATGGAACGTCTGGGTTCCAATTCTGTCAAGA
CTTATGACATATTAAATAGATTGGTTACGGTAAAAGACAAGGCAAGGTATGGACCCTTAGCAAAGCCA
GAAAGATCACCTTGC
+
FFFFFFFFFFFFFFFFFFFFFFFFFFFFFFFFFFFFFFFFFFFFFFFFFFFFFFFFFFFFFFFFFFFF

FFFFFFFFFFFFFFFFFFFFFFFFFFFFFFFFFFFFFFFFFFFFFFFFFFFFFFFFFFFFFFFFFFFF
FFFFFFFFFFFFFFF
@A00155:342:HHGFNDSXY:1:1517:18240:31767 1:N:0:GAACCTAG+TCCGCATA
AGCATGTTTAAGCATTAAATCAAATAAGATGAGCAAGATGGAACGTCTGGGTTCCAATTCTGTCAAGA
CTTATGACATATTAAATAGATTGGTTACGGTAAAAGACAAGGCAAGGTATGGACCCTTAGCAAAGCCA
GAAAGATCACCTTGC
+
FFFFFFFFFFFFFFFFFFFFFFFFFFFFFFF:FFFFFFFFFFFFFFFFFFFFFFFFFFFFFFFFFFFF
FFFFFFFFFFFFFFFFFFFFFFFFFFFFFFFFFFFFFFFFFFFFFFFFFFFFFFFFFFFFFFFFFFFF
FFFFFFFFFFFFFFF
@A00155:342:HHGFNDSXY:1:1555:14181:12649 1:N:0:GAACCTAG+TCCGCATA
AGCATGTTTAAGCATTAAATCAAATAAGATGAGCAAGATGGAACGTCTGGGTTCCAATTCTGTCAAGA
CTTATGACATATTAAATAGATTGGTTACGGTAAAAGACAAGGCAAGGTATGGACCCTTAGCAAAGCCA
GAAAGATCACCTTGC
+
FFFFFF:FFFFFFFFFFFFFFFFFFFFFFFFFFFF:FFFFFFFFFFFFFFFF:FFFFFFFFFFFFFFF
FFFFFFFFFFFFFFFFFFFFFFFFFFFFFFFFFFFFFFFFFFFFFFFFFFFFFF:FFFFFFFFFFFFF
FFFFFFFFFFF:FFF
@A00155:342:HHGFNDSXY:1:1625:19425:11835 1:N:0:GAACCTAG+TCCGCATA
AGCATGTTTAAGCATTAAATCAAATAAGATGAGCAAGATGGAACGTCTGGGTTCCAATTCTGTCAAGA
CTTATGACATATTAAATAGATTGGTTACGGTAAAAGACAAGGCAAGGTATGGACCCTTAGCAAAGCCA
GAAAGATCACAGATC
+
FFFFFFFFFFFFFFFFFFFFFFFFFFFFFFFFFFFFFFFFFFFFFFFFFFFFFFFFFFFFFFFFFFFF
FFFFFFFFFFFFFFF:FFFFFFFFFFFFFFFFFFFFFFFFFFFF:FFF:FFFFF:FFFFFFFFFFFFF
FFFFFFFFFFFFF:F
@A00155:342:HHGFNDSXY:1:2506:30933:32675 1:N:0:GAACCTAG+TCCGCATA
AGCATGTTTAAGCATTAAATCAAATAAGATGAGCAAGATGGAACGTCTGGGTTCCAATTCTGTCAAGA
CTTATGACATATTAAATAGATTGGTTACGGTAAAAGACAAGGCAAGGTATGGACCCTTAGCAAAGCCA
GAAAGATCACCTTGC
+
FFFFFFFFFFFFFFFFFFFFFFFFFFFFFFFFFFFFFFFFFFFFFFFFFFFFFFFFFFFF:FF:FFFF
FFFFFFFFFFFFFFFFFFFFFFFFFFFFFFFFFFFFFFFFFFFFFFFFFFFFFFFFFFFFFFFFFFFF
FFFFFFFFFFFFFFF
@A00155:342:HHGFNDSXY:1:2553:30689:28776 1:N:0:GAACCTAG+TCCGCATA
TGCATGTTTAAGCATTAAATCAAATAAGATGAGCAAGATGGAACGTCTGGGTTCCAATTCTGTCAAGA
CTTATGACATATTAAATAGATTGGTTACGGTAAAAGACAAGGCAAGGTATGGACCCTTAGCAAAGCCA
GAAAGATCAGATCGG
+
FFFFFFFFFFFFFFFFFFFFFFFFFFFFFFFFFFFFFFFFFFFFFFFFFFFFFFFFFF:FFFFFFFFF
FFFFF,FFF,FFFFFFFFFFFFFFFFFFFFFFFFFFFFFFFFFFFFFFFFFFF:FFFFFFFFFFF,FF
FFFFFFFFFFFFFFF
@A00155:342:HHGFNDSXY:1:1131:6976:11506 2:N:0:GAACCTAG+TCCGCATA
AGCATGTTTAAGCATTAAATCAAATAAGATGAGCAAGATGGAACGTCTGGGTTCCAATTCTGTCAAGA
CTTATGACATATTAAATAGATTGGTTACGGTAAAAGACAAGGCAAGGTATGGACCCTTAGCAAAGCCA
GAAAGATCACCTTGC
+
FFFFFFFFFFFFFFFFFFFFFFF:FFFFFFFFFFFFFFFFFFFFFFFFFFFFFFFFFFFFFFFFFFFF
FFF:FFFFFFFFFFFFFFFF:FFFFFFFF::FF,FFFFFFFFFFFFFFFFFFFFFFFFFFFFFFFFFF
FFFF:FFFFFFFFFF
@A00155:342:HHGFNDSXY:1:1271:11767:8531 2:N:0:GAACCTAG+TCCGCATA
AGCATGTTTAAGCATTAAATCAAATAAGATGAGCAAGATGGAACGTCTGGGTTCCAATTCTGTCAAGA
CTTATGACATATTAAATAGATTGGTTACGGTAAAAGACAAGGCAAGGTATGGACCCTTAGCAAAGCCA
GAAAGATCACCTTGC

```
+
FFFFFFFFF:FFFFFFFFFFFFFF:FFFFFFFFFFFFFFFFFFFFFFFFFFFFFFFFF:FFFFFFFFF
FFFFFFFFFFFFFFFFFFFFFFFFFFFFFFFFF:FFFFF:FFFFFFFFFFFFFFFFFFFFFFF,FFFF
FFFFFFFFFFFFFFF
@A00155:342:HHGFNDSXY:1:1445:21043:5149 2:N:0:GAACCTAG+TCCGCATA
ACCATGTTTAAGCATTAAATCAAATAAGATGAGCAAGATGGAACGTCTGGGTTCCAATTCTGTCAAGA
CTTATGACATATTAAATAGATTGGTTACGGTAAAAGACAAGGCAAGGTATGGACCCTTAGCAAAGCCA
GAAAGATCACCTTGC
+
FFFF,FFFFFFF:FFFFFFFFFFF,FFFFFFFFFFFFFFFFFFFFFFF:::FFFFFFFFFF:F::FFF
FFFFFFFF:FFFFFFFFFFF:FF:FFFFFF::F:FFFF:FFFFF:FFF:FFFFFFFFFF:,:FFF:FF
FFFFFFFFFFFFFFF
@A00155:342:HHGFNDSXY:1:1573:24668:21386 2:N:0:GAACCTAG+TCCGCATA
AGCATGTTTAAGCATTAAATCAAATAAGATGAGCAAGATGGAACGTCTGGGTTCCAATTCTGTCAAGA
CTTATGACATATTAAATAGATTGGTTACGGTAAAAGACAAGGCAAGGTATGGACCCTTAGCAAAGCCA
GAAAGATCACCTTGC
+
FFFFFFFFFFFFFFFFFFFFFFFFFFFFFFFFFFFFFFFFFFFFFFFFFFFFFFFFFFFFFFFFFFFF
FFFFFFFFFFFFFFFFFFFFFFFFFFFFFFFFF:FFFFFFFFFFFFFFFFFFFFFFFFFFFFFFFFFF
FFFF::FFFFFFFFF
@A00155:342:HHGFNDSXY:1:2146:24623:11694 2:N:0:GAACCTAG+TCCGCATA
AGCATGTTTAAGCATTAAATCAAATAAGATGAGCAAGATGGAACGTCTGGGTTCCAATTCTGTCAAGA
CTTATGACATATTAAATAGATTGGTTACGGTAAAAGACAAGGCAAGGTATGGACCCTTAGCAAAGCCA
GAAAGATCACCTTGC
+
:FFFFFFFFFFFFFFFFFFFFFFFFFFFFFFFFFFFFFFFFFFFFFFFFFFFFFFFFFFFFFFFFFFF
FFFFFFFFFFFFFFFFFFFFFFFFFFFFFFFFFFFFFFFFFFFFFFFFFFFFFFFFFFFFFFFFFFFF
FFFFFFFFFFFFFFF
@A00155:342:HHGFNDSXY:1:2205:16125:15076 2:N:0:GAACCTAG+TCCGCATA
AGCATGTTTAAGCATTAAATCAAATAAGATGAGCAAGATGGAACGTCTGGGTTCCAATTCTGTCAAGA
CTTATGACATATTAAATAGATTGGTTACGGTAAAAGACAAGGCAAGGTATGGACCCTTAGCAAAGCCA
GAAAGATCACCTTGC
+
F:FFFFFFFFF:FFFFFFFFFFFFFFFFFFF:FFFFFFFFFFFFFFFFFFFFFFFFFFFFFFFFFFFF
FFFFFFFFFFFFFFFFFFFFFFFFFFFFFFFFFFFFFFFFFFFFFFFF,FF:FFFFFFFFFFFFFF:F
FFFFFFFFFF:FFFF
@A00155:342:HHGFNDSXY:1:2206:20772:5024 2:N:0:GAACCTAG+TCCGCATA
AGCATGTTTAAGCATTAAATCAAATAAGATGAGCAAGATGGAACGTCTGGGTTCCAATTCTGTCAAGA
CTTATGACATATTAAATAGATTGGTTACGGTAAAAGACAAGGCAAGGTATGGACCCTTAGCAAAGCCA
GAAAGATCACCTTGC
+
FFFFFFFFFFFFFFFFFFFFFFFFFFFFFFFFFFFFFFFFFFFFFFFFFFFFFFFFFFFFFFFFFFFF
FFFFFFFFFFFFFFFFFFFFFFFFFFFFFFFFFFFFFFFFFFFF:FFFFFFFFFFFFFFFFFFFFFFF
FFFFFFFFFFFFFFF
@A00155:342:HHGFNDSXY:1:2266:4752:17550 2:N:0:GAACCTAG+TCCGCATA
AGCATGTTTAAGCATTAAATCAAATAAGATGAGCAAGATGGAACGTCTGGGTTCCAATTCTGTCAAGA
CTTATGACATATTAAATAGATTGGTTACGGTAAAAGACAAGGCAAGGTATGGACCCTTAGCAAAGCCA
GAAAGATCACCTTGC
+
FFFFFFFFFFFFFFFFFFFFFFFFFFFFFFFFFFFFFF:FFF:FFFFFFFFFFFFFFFFFFFFFFFFF
FFFFFFFFFFFFFFFFFFFFFFFFFFFFFFFFFF:FFFFFFFFFFFFFFFFFF:FFFFFFFFFFFFFF
FFFFF,FFFFFFFFF
@A00155:342:HHGFNDSXY:1:2266:4155:18145 2:N:0:GAACCTAG+TCCGCATA
AGCATGTTTAAGCATTAAATCAAATAAGATGAGCAAGATGGAACGTCTGGGTTCCAATTCTGTCAAGA
```

```
CTTATGACATATTAAATAGATTGGTTACGGTAAAAGACAAGGCAAGGTATGGACCCTTAGCAAAGCCA
GAAAGATCACCTTGC
+
FFFFFFFFFFFFFFFFFFFFFFFFFFFFFFFFFFFFFFFFF:FFFFFFFFFFFFFFF:F:FFF:FFF
F:FFFFFFFFFFFFFFFFFFFFFFFFFFFFFFFFF:FFFFFFFFFFFFF:FFFFFFFFFFF:FFFFFFFF
:FFFFFFFFFFF:FF
@A00155:342:HHGFNDSXY:1:2308:14922:1376 2:N:0:GAACCTAG+TCCGCATA
AGCACGTTTAAGCATTAAATCAAATAAGATGAGCAAGATGGAACGTCTGGGTTCCAATTCTGTCAAGA
CTTATGACATATTAAATAGATTGGTTACGGTAAAAGACAAGGCAAGGTATGGACCCTTAGCAAAGCCA
GAAAGATCACAGATC
+
FFF:FFFFFFFFFFFFFFFFFFFFFFFFFFFFFFFFFFFFFFFFFFFFFFFFFFFFFFFFFFFFFFFF
FFFFFFFFFFFFFF:FFFFF:FFFFFFFFFFFFFFFFFFFFFFFFFFFFFFFFFFFFFFFFFFFFFFF
FFFFF:FFFFFFFFF
@A00155:342:HHGFNDSXY:1:2308:15989:4006 2:N:0:GAACCTAG+TCCGCATA
AGCATGTTTAAGCATTAAATCAAATAAGATGAGCAAGATGGAACGTCTGGGTTCCAATTCTGTCAAGA
CTTATGACATATTAAATAGATTGGTTACGGTAAAAGACAAGGCAAGGTATGGACCCTTAGCAAAGCCA
GAAAGATCACAGATC
+
:FF:FFFFFFFFF:FF:FFFFFFFFFFFFFFFFFFFFFFFFFFFFFFFFFFFFFFFFFFFFFFFFFFF
FFFFFFFF:FFFFFFFFFFFFFFFFFFFFFFFFFFFFFFFFFFFFFF:FFF:FFFFFFFFFFFFFFFFFFF
FFFFFFFFFFFFFF:
@A00155:342:HHGFNDSXY:1:2413:12138:21856 2:N:0:GAACCTAG+TCCGCATA
AGCATGTTTAAGCATTAAATCAAATAAGATGAGCAAGATGGAACGTCTGGGTTCCAATTCTGTCAAGA
CTTATGACATATTAAATAGATTGGTTACGGTAAAAGACAAGGCAAGGTATGGACCCTTAGCAAAGCCA
GAAAGATCACCTTGC
+
F:FFFF:FFFFFFFFFFFFFFFF:FF,FFFFFFFFFFFFFFFFFFFFFFFF:FFFFFFFFFFFFFFFF
FFFFFF:FFFFFF,FFFF::FFFF::FFFFFFFFFFFFFFFFF:FFF:FFFFFFFFFFFF,:FFFFFF
FFFFFFFFFFFFFFF
@A00155:342:HHGFNDSXY:1:2449:29884:16924 2:N:0:GAACCTAG+TCCGCATA
AGCATGTTTAAGCATTAAATCAAATAAGATGAGCAAGATGGAACGTCTGGGTTCCAATTCTGTCAAGA
CTTATGACATATTAAATAGATTGGTTACGGTAAAAGACAAGGCAAGGTATGGACCCTTAGCAAAGCCA
GAAAGATCACCTTGC
+
:FFFFFFFFFFFFFFFFFFFFFFFFFFFFFFFFFFFFFFFFFFFFFFFFFFFFFFFFFFFFFFFFFFF
FFFFFFFFFFFFFFFFFFFFFFFFFFFFFFFFFFFFFFFFFFF:FFFFFFFFFFFFFFFFFFFFFFFF
FFFFFFFFFFFFF:F
@A00155:342:HHGFNDSXY:1:2561:25979:8876 2:N:0:GAACCTAG+TCCGCATA
AGCATGTTTAAGCATTAAATCAAATAAGATGAGCAAGATGGAACGTCTGGGTTCCAATTCTGTCAAGA
CTTATGACATATTAAATAGATTGGTTACGGTAAAAGACAAGGCAAGGTATGGACCCTTAGCAAAGCCA
GAAAGATCACCTTGC
+
FFFFFFFFFFFFFFFFFFFFFFFFFFFFFFFFFFFFFFFFFFF,FFFFFFFFFFFFFFFFFFFFFFFFF
FFFFFFFFFFFFFFFFFFFFFFFFFF:FFF:FFF:F:FFFFFFF:FFFFFFFFFFFFFFFFFFFFFFFF
FFF:FFFF:FFFFFF
@A00155:342:HHGFNDSXY:1:2639:31901:6010 2:N:0:GAACCTAG+TCCGCATA
AGCATGTTTAAGCATTAAATCAAATAAGATGAGCAAGATGGAACGTCTGGGTTCCAATTCTGTCAAGA
CTTATGACATATTAAATAGATTGGTTACGGTAAAAGACAAGGCAATGTATGGACCCTTAGCAAAGCCA
GAAAGATCACCTTGC
+
FFFF:FFFFFFF:FFFFFFFFFFFFFFFFFFFFFFFFFFFFFFF:FFFFFFFF:FF:FFFFFFFFF:FFF
F:FFFFFFFFF:FFFFFFFFFFFFFF:FFFFFFF:FFFFFFFFF,,:F,FF:FFF,F:FFFFFFFFFF
FFFF,FFFF,FFFF:
```

```
@A00155:342:HHGFNDSXY:1:1461:2175:14152 1:N:0:GAACCTAG+TCCGCATA
GCATGTTTAAGCATTAAATCAAATAAGATGAGCAAGATGGAACGTCTGGGTTCCAATTCTGTCAAGAC
TTATGACATATTAAATAGATTGGTTACGGTAAAAGACAAGGCAAGGTATGGACCCTTAGCAAAGCCAG
AAAGATCACCTTGC
+
FFFFF:FFFFFFFFFFFFFFFFFFFFFFFFFFFFFFFFFFFFFFFFFFFFFFF:FF:FFFFFFFFFFF
FFFFFFFFFFFFFFFFFFFFFFFFFFFFFFFFFFFFFFFFFFFFFFFFFFFFFFFFFFFFFFFFFFFF
FFFFFFFFFFFFFF
@A00155:342:HHGFNDSXY:1:1243:29803:25927 1:N:0:GAACCTAG+TCCGCATA
GCATGTTTAAGCATTAAATCAAATAAGATGAGCAAGATGGAACGTCTGGGTTCCAATTCTGTCAAGAC
TTATGACATATTAAATAGATTGGTTACGGTAAAAGACAAGGCAAGGTATGGACCCTTAGCAAAGCCAG
AAAGATCACCTTGCG
+
FFFFFFFFFFFFFFFFFFFFFFFF,FFFFFFFFFFFFFFFFFFFFFFFFFFFFFFFFFFFFFFFFFFF
FFFFFFFFFFFFFFFFFFFFFFFFFFFFFFFFFFFFFFFFFFFFFFFFFFFFFFFFFFFFFFFFFFFF
FFFFFFFFFFFFFFF
@A00155:342:HHGFNDSXY:1:1329:16369:4977 1:N:0:GAACCTAG+TCCGCATA
GCATGTTTAAGCATTAAATCAAATAAGATGAGCAAGATGGAACGTCTGGGTTCCAATTCTGTCAAGAC
TTATGACATATTAAATAGATTGGTTACGGTAAAAGACAAGGCAAGGTATGGACCCTTAGCAAAGCCAG
AAAGATCACCTTGCC
+
:,FFFFFFFF,::FFFFFFFFF,F::,FFF:FFF:FFFFFF,FFF,FF,FFFF,:F:FFFFFFFFFFF
F,FF:FF,FFFF,F,F,F,FFFFFF:FFFFF::FFFF:FFFFFFFFFFFF:,FFFFFFFF:FFFFFFF
FFFFFFFFFFFFF:F
@A00155:342:HHGFNDSXY:1:1437:27263:20306 1:N:0:GAACCTAG+TCCGCATA
GCATGTTTAAGCATTAAATCAAATAAGATGAGCAAGATGGAACGTCTGGGTTCCAATTCTGTCAAGAC
TTATGACATATTAAATAGATTGGTTACGGTAAAAGACAAGGCAAGGTATGGACCCTTAGCAAAGCCAG
AAAGATCAGATCGGA
+
FFFFFFFFFFFFFFFFFFFFFFFFFFFFFFFFFFFFFFFFFFFFFFFF:FFFFFFFFFFFFFFFFFFF
FFFFFFFFFFFFFFFFFFFFFFFFFFFFFFFFFFFFFFFFFFF,FF:,FFFFFFFFFFFFFFFFFF:F
FFFFFFFF:FFFFFF
@A00155:342:HHGFNDSXY:1:1573:24668:21386 1:N:0:GAACCTAG+TCCGCATA
GCATGTTTAAGCATTAAATCAAATAAGATGAGCAAGATGGAACGTCTGGGTTCCAATTCTGTCAAGAC
TTATGACATATTAAATAGATTGGTTACGGTAAAAGACAAGGCAAGGTATGGACCCTTAGCAAAGCCAG
AAAGATCACCTTGCC
+
FFFFFFFFFFFFFFFFFFFFFFFFFFFFFFFFFFFFFFFFFFFFFFFFFFFFFFFFFFFFFFFFFFFF
FFFFFFFFFFFFFFFFFFFFFFFFFFFFFFFFFFFFFFFFFFFFFFFFFFFFFFFFFFFFFFFFFFFF
FFFFFFFFFFFFFFF
@A00155:342:HHGFNDSXY:1:1602:3314:4069 1:N:0:GAACCTAG+TCCGCATA
GCATGTTTAAGCATTAAATCAAATAAGATGAGCAAGATGGAACGTCTGGGTTCCAATTCTGTCAAGAC
TTATGACATATTAAATAGATTGGTTACGGTAAAAGACAAGGCAAGGTATGGACCCTTAGCAAAGCCAG
AAAGATCACCTTGCC
+
:FFF,FFFFF:FFFFFFFFFFFFFFF,FFFF,FFF:FFFFFFFFFFFF,FFFFFFFFFFFFFFFFFFF
FFFFFFFFFFFFFFFFF,F:FFFFFFFFFFFFFFFFFF:FFFFFFFFFF:FFFFFF:FFFFFFFFFFF
FFFFFFFFFFFFFFF
@A00155:342:HHGFNDSXY:1:2107:7925:28025 1:N:0:GAACCTAG+TCCGCATA
GCATGTTTAAGCATTAAATCAAATAAGATGAGCAAGATGGAACGTCTGGGTTCCAATTCTGTCAAGAC
TTATGACATATTAAATAGATTGGTTACGGTAAAAGACAAGGCAAGGTATGGACCCTTAGCAAAGCCAG
AAAGATCACCTTGCT
+
FFFFF:FFFFFFFFFFFFF,FFFFFFFFFFFFFFFFFFFFFFFFFFF:FFFFF:FFFFFF,FFFFFFF
```

FFFFFFFFFFFFFFFFFFFFFFFFFFFFFFFFFFFFFFF:FFFFFFFFFFFFFFFFFFFFFFFFFFFF
FFFFFFFFFFFFFFF
@A00155:342:HHGFNDSXY:1:2133:28637:20149 1:N:0:GAACCTAG+TCCGCATA
GCATGTTTAAGCATTAAATCAAATAAGATGAGCAAGATGGAACGTCTGGGTTCCAATTCTGTCAAGAC
TTATGACATATTAAATAGATTGGTTACGGTAAAAGACAAGGCAAGGTATGGACCCTTAGCAAAGCCAG
AAAGATCACCTTGCC
+
FFFFFFFFFFFFFFFFFFFFFFFFFFFFFFFFFFFFFFFFFFFFFFFFFF:FFFFFFFFFFFFFFFFF
FFFFFFFFFFFFFFFFFFFFFFFFFFFFFFFFFFFFFFFFFFFFFFFFFFFFFFFFFFFFFFFFFFFF
FFFFFFFFFFFFFFF
@A00155:342:HHGFNDSXY:1:2142:18683:15562 1:N:0:GAACCTAG+TCCGCATA
GCATGTTTAAGCATTAAATCAAATAAGATGAGCAAGATGGAACGTCTGGGTTCCAATTCTGTCAAGAC
TTATGACATATTAAATAGATTGGTTACGGTAAAAGACAAGGCAAGGTATGGACCCTTAGCAAAGCCAG
AAAGATCACCTTGCC
+
FFFFF:FFFFFFFFFFFFFFFFFFFFFFFFFFFFFFFFFFFFFFFFFFFFFFFFFFFFFFFFFFFFFF
FFFFFFFFFFFFF,FFFFFFFFFFFFFFFFFFFFFFFFFFFFFFFFFFFFFFFFFFFFFFFFFFFFFF
FFFFFFFFFFFFFFF
@A00155:342:HHGFNDSXY:1:2142:18240:15577 1:N:0:GAACCTAG+TCCGCATA
GCATGTTTAAGCATTAAATCAAATAAGATGAGCAAGATGGAACGTCTGGGTTCCAATTCTGTCAAGAC
TTATGACATATTAAATAGATTGGTTACGGTAAAAGACAAGGCAAGGTATGGACCCTTAGCAAAGCCAG
AAAGATCACCTTGCC
+
FFFFFFFFFFFF:FFFFFFFFFFFFFFFFFFFFFFFFFFFFFFFFFFF:F:FFFFFFFFFFFFF:FFF
FFFFFFFFFFFFFFFFFFFFFFFFFFFFFFFFFFFFFFFFFFFFFFFFFFFFFFFFFFFFFFFFFFFF
FFFFFFFFFFFFFFF
@A00155:342:HHGFNDSXY:1:2274:26332:11397 1:N:0:GAACCTAG+TCCGCATA
GCATGTTTAAGCATTAAATCAAATAAGATGAGCAAGATGGAACGTCTGGGTTCCAATTCTGTCAAGAC
TTATGTCATATTAAATAGATTGGTTACGGTAAAAGACAAGGCAAGGTATGGACCCTTAGCAAAGCCAG
AAAGATCACCTTGCC
+
FFFFFFFFFFFFFFFFFFFFFFFFFFFFFFFFFF:FFFFFFFFFF,FFFFFFFF:FFFFFFFFFFFFF
FFFFFFFFFFFFFFFFFFFFFFFFFFFFFFFFFFFFFFFFFFFFFFFFFFFFFFFFF:FFFFFFFFFF
FFFFFFFFFFFFFFF
@A00155:342:HHGFNDSXY:1:2255:14895:36088 1:N:0:GAACCTAG+TCCGCATA
GCATGTTTAAGCATTAAATCAAATAAGATGAGCAAGATGGAACGTCTGGGTTCCAATTCTGTCAAGAC
TTATGACATATTAAATAGATTGGTTACGGTAAAAGACAAGGCAAGGTATGGACCCTTAGCAAAGCCAG
AAAGATCACCTTGCC
+
FFFFFFFFFFFFFFFFFFFFFFFFFFFFFFFFFFFFFFFFFFFFFFFFFFFFFFFF,FFFFFFFFFFF
FFFFFFFFFFFFFFFFFFFFFFFFFFFFFFFFFFFFFFFFFFFFFFFFFFFFFFFFFFFFFFFFFFFF
FFFFFFFFFFFFFFF
@A00155:342:HHGFNDSXY:1:2437:15103:17440 1:N:0:GAACCTAG+TCCGCATA
GCATGTTTAAGCATTAAATCAAATAAGATGAGCAAGATGGAACGTCTGGGTTCCAATTCTGTCAAGAC
TTATGACATATTAAATAGATTGGTTACGGTAAAAGACAAGGCAAGGTATGGACCCTTAGCAAAGCCAG
AAAGATCACCTTGCC
+
:,FFFFFFFFF,FFFF:FF,FFFFFFFFFFFFFFFFFFF:FFFFFFFFFFFF:FF:FFFFFFFFFFFF
FFFFFF,FFFFFFFFFFFFFFF:FF:F:FFFFFFFFFFFF:FFF:FF,F,FF:F:FFF,FFFFFFFFF
FFFFFFFF:FFF:,F
@A00155:342:HHGFNDSXY:1:2449:29884:16924 1:N:0:GAACCTAG+TCCGCATA
GCATGTTTAAGCATTAAATCAAATAAGATGAGCAAGATGGAACGTCTGGGTTCCAATTCTGTCAAGAC
TTATGACATATTAAATAGATTGGTTACGGTAAAAGACAAGGCAAGGTATGGACCCTTAGCAAAGCCAG
AAAGATCACCTTGCC

```
+
FFFFFFFFFFFFFFFFFFFFFFFFFFFFFFFFFFFFFFF:FFFFFFFFFFF:FFFFFFFFFFFFFFFFF
FFFFFFFFFFFFFFFFFFFFFFFFFFFFFFFFFFFFFFFFFFFFFFFFFFFFFFFFFFFFFFFFFFFFF
FFFFFFFFFFFFF:F
@A00155:342:HHGFNDSXY:1:2507:15637:29168 1:N:0:GAACCTAG+TCCGCATA
GCATGTTTAAGCATTAAATCAAATAAGATGAGCAAGATGGAACGTCTGGGTTCCAATTCTGTCAAGAC
TTATGACATATTAAATAGATTGGTTACGGTAAAAGACAAGGCAAGGTATGGACCCTTAGCAAAGCCAG
AAAGATCACCTTGCC
+
FFFF,FFFFFFFFFFFFFFFFFFFFFFFFFFFFFFFFFFFFFFFFFFFFFFFFFFFFFFFFFFFFFFFF
FFFFFFFFFFFFFFFFFFFFFFFFFFFFFFFFFFFFFFFFFFFFFFFFFFFFFFFFFFFFFFFFFFFFF
FFFFFFFFFFFFFFF
@A00155:342:HHGFNDSXY:1:2601:4499:23187 1:N:0:GAACCTAG+TCCGCATA
GTATGTTTAAGCATTAAATCAAATAAGATGAGCAAGATGGAACGTCTGGGTTCCAATTCTGTCAAGAC
TTATGACATATTAAATAGATTGGTTACGGTAAAAGACAAGGCAAGGTATGGACCCTTAGCAAAGCCAG
AAAGATCACAGATCG
+
FFFFFFFFFF,FFFFFFFFFFFFFFF:FFFFFFFFFFFF:FFFFFFFFFFFFFFFFFFFFFFFFFFFFF
FFF,FFFFFFFFFFFFF:FFFF,FF:FFFFFFFFFFFFFF,FFF,:FFFFFF,FF::FF:FFFF:FFF
F,FFFF,FFF:F::F
@A00155:342:HHGFNDSXY:1:2601:4200:27273 1:N:0:GAACCTAG+TCCGCATA
GTATGTTTAAGCATTAAATCAAATAAGATGAGCAAGATGGAACGTCTGGGTTCCAATTCTGTCAAGAC
TTATGACATATTAAATAGATTGGTTACGGTAAAAGACAAGGCAAGGTATGGACCCTTAGCAAAGCCAG
AAAGATCACAGATCG
+
FFFFFF:FFFFFFFFFFFFFFF,FFFFFFFFFFFFFFFFFFFFFFFFFFFFFFFFFFFFFFFFF:FFFF
FFFF,FFFFFFFF:FFFFFFFFFFFFFFFF:FFF:FFF:FFFFFFFF::FFFFF,:FF::FFFFFFFF
FFFFFFFFFFFFF:F
@A00155:342:HHGFNDSXY:1:2564:32398:25974 1:N:0:GAACCTAG+TCCGCATA
GCATGTTTAAGCATTAAATCAAATAAGATGAGCAAGATGGAACGTCTGGGTTCCAATTCTGTCAAGAC
TTATGACATATTAAATAGATTGGTTACGGTAAAAGACAAGGCAAGGTATGGACCCTTAGCAAAGCCAG
AAAGATCACCTTGCC
+
FF::F::FF:FF::FFFFFFFFFFFFFFFF,FFF:FFFFFF:FFFFFFFFFFFFF:FFFFFFFFFFFF
FFFFF:FFFFFFF::,FF:::FFFFFFFFFFFFFFFF,FFFFFFFFFFFFFFFFFF:FFFFFFFFFFF
FFFFFFFFFFFFFFF
@A00155:342:HHGFNDSXY:1:2578:14986:9189 1:N:0:GAACCTAG+TCCGCATA
GCATGTTTAAGCATTAAATCAAATAAGATGAGCAAGATGGAACGTCTGGGTTCCAATTCTGTCAAGAC
TTATGACATATTAAATAGATTGGTTACGGTAAAAGACAAGGCAAGGTATGGACCCTTAGCAAAGCCAG
AAAGATCACCTTGCC
+
F:FF:FFFFFFFFFFFFFFFFFFFFFFFFFF:FFFFFFFFFFFFF,:F:FFFFFFFFFFFFFFF:FFF
FFFF:FFFFF:FFFFF:F,,FF,F,FFFFFFFF:FFFFF:FFF:,FF:FFFFFFFFFF,FFF:FFFFF
FFFF:FFFF:FFFFF
@A00155:342:HHGFNDSXY:1:2639:31901:6010 1:N:0:GAACCTAG+TCCGCATA
GCATGTTTAAGCATTAAATCAAATAAGATGAGCAAGATGGAACGTCTGGGTTCCAATTCTGTCAAGAC
TTATGACATATTAAATAGATTGGTTACGGTTAAAGACAAGGCAAGGTATGGACCCTTAGCAAAGCCAG
AAAGATCACCTTGCC
+
FF,FFFFFFFFFFFFFFFFFFFFFFFFFFFFFFFFFFFFFFF:FFFFF,FF:FFF,FFFFFFFFFFF:F
F:FFFFFFFFFF:FFFFFF,FFFFFFFFFF,FFFFFFF:FF,:FFFFFFFFFFFFFFFFFFF,FFFFFF
FF:FFFF:FF::FFF
@A00155:342:HHGFNDSXY:1:2650:30734:24189 1:N:0:GAACCTAG+TCCGCATA
GCATGTTTAAGCATTAAATCAAATAAGATGAGCAAGATGGAACGTCTGGGTTCCAATTCTGTCAAGAC
```

TTATGACATATTAAATAGATTGGTTACGGTAAAAGACAAGGCAAGGTATGGACCCTTAGCAAAGCCAG
AAAGATCACCTTGCC
+
FFFFFFFFFFF:FFFFFFFFFFFFFFFFFFFFFFFFFFFFFFFFFFFFFFFFFFFFFFFF
FFFFFFFFFFFFFFFFFFFFFFFFFFFFFFFFFFFFFFFFFFFFFFFFFFFFFFFFFFFFFFFFFFFF
FFFFFFFFFFFFFFF
@A00155:342:HHGFNDSXY:1:2645:16179:24752 1:N:0:GAACCTAG+TCCGCATA
GCATGTTTAAGCATTAAATCAAATAAGATGAGCAAGATGGAACGTCTGGGTTCCAATTCTGTCAAGAC
TTATGACATATTAAATAGATTGGTTACGGTAAAAGACAAGGCAAGGTATGGACCCTTAGCAAAGCCAG
AAAGATCACCTTGCC
+
FFFFFFFFFF,FFFFFFFFFFFFFFFFFFFFFFFFFFFFFFFFFFFFFFFFFFFFFFFFFFFFFF
FFFFFFFFFFFFFFFFFFFFFFFFFFFFFFFFFFFFFFFFFFFFFFFFFFFFFFFFFFFFFFFFFFFF
FFFFFFFFFF:FFFF
@A00155:342:HHGFNDSXY:1:2623:2636:32581 1:N:0:GAACCTAG+TCCGCATA
GCATGTTTAAGCATTAAATCAAATAAGATGAGCAAGATGGAACGTCTGGGTTCCAATTCTGTCAAGAC
TTATGACATATTAAATAGATTGGTTACGGTAAAAGACAAGGCAAGGTATGGACCCTTAGCAAAGCCAG
AAAGATCACCTTGCC
+
F::FFFFFFFFFFFFFFFFFFFFFFFFFFFFFFFFF:FFFFFFFF:FFFFFFFFFFFFFFFFFFFFFF
FFFFFFFFFFFFFFFFFFFFFFFFFFFFFFFFFFFFFFFFFFFFFFFFFFFFFFFFFFFFFFFFFFFF
FFFFFFFFFFFFFFF
@A00155:342:HHGFNDSXY:1:2215:10963:6010 2:N:0:GAACCTAG+TCCGCATA
GCATGTTTAAGCATTAAATCAAATAAGATGAGCAAGATGGAACGTCTGGGTTCCAATTCTGTCAAGAC
TTATGACATATTAAATAGATTGGTTACGGTAAAAGACAAGGCAAGGTATGGACCCTTAGCAAAGCCAG
AAAGATCACCTTGCC
+
:F:FFFF:FF:FFF:FFFFFFF:FFFFFFFFFFFFFFFFFFFFFFFFFFFFFFFF:FFFFF:FFFF:F
:FF:FFFFFFFFFFFFF:FFFF:FFFF:FFFFFFFFFFFFF:FFFFFFFF:FFF:FFFFFFFF:FFF,
FFFFFFFFF:F:FFF
@A00155:342:HHGFNDSXY:1:2221:9625:35383 2:N:0:GAACCTAG+TCCGCATA
GCATGTTTAAGCATTAAATCAAATAAGATGAGCAAGATGGAACGTCTGGGTTCCAATTCTGTCAAGAC
TTATGACATATTAAATAGATTGGTTACGGTAAAAGACAAGGCAAGGTATGGACCCTTAGCAAAGCCAG
AAAGATCACCTTGCC
+
FFFFFFFFFFFFFFFF:FFFFFFFFFFFFF:FFFFFFFFFFFFFFFFFFFFFFFFFFFFFFFFFFFFF
FFFFFFFFFFFF,FFFFFFFFFFFFFF:FFFF:,FFFFFFFFF:FFFFFFFFFFFFFFFFFFFFFFFF
FFFFFF:FFFF:FFF
@A00155:342:HHGFNDSXY:1:1350:21947:11537 1:N:0:GAACCTAG+TCCGCATA
CATGTTTAAGCATTAAATCAAATAAGATGAGCAAGATGGAACGTCTGGGTTCCAATTCTGTCAAGACT
TATGACATATTAAATAGATTGGTTACGGTAAAAGACAAGGCAAGGTATGGACCCTTAGCAAAGCCAGA
AAGATCACCTTGCC
+
:FFF,FFFFFFFFFFFFFFFFFF:FFFFFFFF:FFFFFFFFFFFFFFFFFFFFFFFFFFFF:FFFFFF
FFFFFFFFFFFFFFFFFFFFFFFFFFFFFFFFFFFFFFFFFFFFFFFFFFFFFFFFFFFFFFFFFFFF
FFFFFFFFFFFFFF
@A00155:342:HHGFNDSXY:1:2649:8395:9455 1:N:0:GAACCTAG+TCCGCATA
CATGTTTAAGCATTAAATCAAATAAGATGAGCAAGATGGAACGTCTGGGTTCCAATTCTGTCAAGACT
TATGACATATTAAATAGATTGGTTACGGTAAAAGACAAGGCAAGGTATGGACCCTTAGCAAAGCCAGA
AAGATCACCTTGCC
+
FFFF:FFFFFFF:FFFFFFFFFFFFFFFFFFFFFFFFFFFFFFFFFFFFFFFFFFFFFFFFFFFFFFF
FFFFFFFFFFFFFFFFFFFFFFFFFFFF:FFFFFFFFFFFFFFFFFFFFFFFFFFFFFFFFFFFFFFF
FFFFFFFFFFFFFF

```
@A00155:342:HHGFNDSXY:1:1226:29568:27649 1:N:0:GAACCTAG+TCCGCATA
CATGTTTAAGCATTAAATCAAATAAGATGAGCAAGATGGAACGTCTGGGTTCCAATTCTGTCAAGACT
TATGACATATTAAATAGATTGGTTACGGTAAAAGACAAGGCAAGGTATGGACCCTTAGCAAAGCCAGA
AAGATCACCTTGCCC
+
FFFFFFFFFFFFFFFFFFFFFFFFFFFFFFFFFFFFFFFFFFFFFFFFFFFFFFFFFFFFFFFFFFFF
FFFFFFFFFFFFFFFFFFFFFFFFFFFFFFFFFFFFFFFFFFFFFFFFFFFFFFFFFFFFFFFFFFFF
FFFFFFFFFFFFFFF
@A00155:342:HHGFNDSXY:1:1271:11767:8531 1:N:0:GAACCTAG+TCCGCATA
CATGTTTAAGCATTAAATCAAATAAGATGAGCAAGATGGAACGTCTGGGTTCCAATTCTGTCAAGACT
TATGACATATTAAATAGATTGGTTACGGTAAAAGACAAGGCAAGGTATGGACCCTTAGCAAAGCCAGA
AAGATCACCTTGCCC
+
FFFFFFFFFFFFFFFFFFFFFFFFFFFFFFFFFFFFFFFFFFFFFFFFFFFFFF:FFFFFFFFFFFF
FFFFFFFFFFFFFFFF,FFFFFFFFFFFFFFFFFFFFFFFFFFFFFFFFFFFFFF:FFFFFFFFFFFF
FFFFFFFFFFFFFFF
@A00155:342:HHGFNDSXY:1:1324:18738:12336 1:N:0:GAACCTAG+TCCGCATA
CATGTTTAAGCATTAAATCAAATAAGATGAGCAAGATGGAACGTCTGGGTTCCAATTCTGTCAAGACT
TATGACATATTAAATAGATTGGTTACGGTAAAAGACAAGGCAAGGTATGGACCCTTAGCAAAGCCAGA
AAGATCACCTTGCCC
+
FFFFFFFFFFFFFFFFFFFFFFFFFFFFFFFFFFFFFFFFFFFFFFFFFFFFF:FFFFFFFFFFFFFF
FFFFFFFFFFFFFFFFFFFFFFFFFFFFFFFFFFFFFFFFFFFFFFFFFFFFFFFFFFFFFFFFFFFF
FFFFFFFFFFFFFFF
@A00155:342:HHGFNDSXY:1:1526:29812:33583 1:N:0:GAACCTAG+TCCGCATA
CATGTTTAAGCATTAAATCAAATAAGATGAGCAAGATGGAACGTCTGGGTTCCAATTCTGTCAAGACT
TATGACATATTAAATAGATTGGTTACGGTAAAAGACAAGGCAAGGTATGGACCCTTAGCAAAGCCAGA
AAGATCACCTTGCCC
+
FFFF,FFFFFFFFFFFFFFFFFFFFFFFFFFFFFFFFFFFFFFFFFFFFFFFFFFFFFFFFFFFFFF
FFFFFFFFFFFFFFFFFFFFFFFFFFFFFFFFFFFFFFF:FFFFFFFFFFFFFFFFFFFFFFFFFFFF
FFFFFFFFFFFFFFF
@A00155:342:HHGFNDSXY:1:2157:23800:27117 1:N:0:GAACCTAG+TCCGCATA
CATGTTTAAGCATTAAATCAAATAAGATGAGCAAGATGGAACGTCTGGGTTCCAATTCTGTCAAGACT
TATGACATATTAAATAGATTGGTTACGGTAAAAGACAAGGCAAGGTATGGACCCTTAGCAAAGCCAGA
AAGATCACCTTGCCC
+
FFFFFFFFF:FFFFFFFFFFFFFFFFFFFFFFFFFFFFFFFFFFFFFFFFFFFFFFFFFFFFFFFFFF
FFFFFFFFFFFFFFFFFFFFFFFFFFFFFFFFFFFFFFFFFFFFFFFFFFFFFFFFFFFFFFFFFFFF
FFFFFFFFFFFFFFF
@A00155:342:HHGFNDSXY:1:2174:32624:7169 1:N:0:GAACCTAG+TCCGCATA
CATGTTTAAGCATTAAATCAAATAAGATGAGCAAGATGGAACGTCTGGGTTCCAATTCTGTCAAGACT
TATGACATATTAAATAGATTGGTTACGGTAAAAGACAAGGCAAGGTATGGACCCTTAGCAAAGCCAGA
AAGATCACCTTGCCC
+
FFFF:FFFFFFFFFFFFFFFFFFFFFFFFFFFFFFFFFFFFFFFFFFFFFFFFFFFFFFFFFFFFFF
FFFFFFFFFFFFFFFFFFFFFFFF,FFFFFFFFFFF:FFFFFFFFF:FFFFFFFFFFFFFFFFFFFFF
FFFF:FFFF:FFFFF
@A00155:342:HHGFNDSXY:1:2227:26042:3004 1:N:0:GAACCTAG+TCCGCATA
CATGTTTAAGCATTAAATCAAATAAGATGAGCAAGATGGAACGTCTGGGTTCCAATTCTGTCAAGACT
TATGACATATTAAATAGATTGGTTACGGTAAAAGACAAGGCAAGGTATGGACCCTTAGCAAAGCCAGA
AAGATCACCTTGCCC
+
FFFFFFFFFFFFFFFFFFFFFFFFFFFFFFFFFFFFFFFFFFFFFFFFFFFFFFFFFFFFFFFFFFFF
```

FFFFF:FFFFFFFFFFFFFFFFFFFFFFFFFFFFFFFFFFFFFFFFFFFFFFFFFFFFFFFFFFFF
FFFFFFFFFFFFFFF
@A00155:342:HHGFNDSXY:1:2601:31765:11882 1:N:0:GAACCTAG+TCCGCATA
CATGTTTAAGCATTAAATCAAATAAGATGAGCAAGATGGAACGTCTGGGTTCCAATTCTGTCAAGACT
TATGACATATTAAATAGATTGGTTACGGTAAAAGACAAGGCAAGGTATGGACCCTTAGCAAAGCCAGA
AAGATCACCTTGCCC
+
FFFFFFFFFF,FFFFFFFFFFF:FFFFFFFFFFFFFFFFFFFFFFFFFFFFFFFFFFFFFFFFFFFFF
FFFFFFFFFFFFF:FFFFFFFFFFFFFFFFFFFFFFFFFFFFFFFFFFFFFFFFFFFFFFFFFFFFFF
FFFFFFFFFFFFFFF
@A00155:342:HHGFNDSXY:1:1227:8947:21151 2:N:0:GAACCTAG+TCCGCATA
CATGTTTAAGCATTAAATCAAATAAGATGAGCAAGATGGAACGTCTGGGTTCCAATTCTGTCAAGACT
TATGACATATTAAATAGATTGGTTACGGTAAAAGACAAGGCAAGGTATGGACCCTTAGCAAAGCCAGA
AAGATCACCTTGCCC
+
FFFFFFFFFFFFFFF:FFFFFFFFFFFFFFFF:FFFFFFFFFFFFFFFFFFFFFFFFFFFFFF,FFFF
FFFF:FFFF:FFFFFFFFFFFFFFFFFFF,FF:F:FFFF:FFFFFFFFFFFFFFFFFFFF,:FF:FFF
FFFFFFFF,FFFF,F
@A00155:342:HHGFNDSXY:1:1269:5864:14434 2:N:0:GAACCTAG+TCCGCATA
CATGTTTAAGCATTAAATCAAATAAGATGAGCAAGATGGAACGTCTGGGTTCCAATTCTGTCAAGACT
TATGACATATTAAATAGATTGGTTACGGTAAAAGACAAGGCAAGGTATGGACCCTTAGCAAAGCCAGA
AAGATCACCTTGCCC
+
FF,FFFFFFFFFF,FFFFFFFFFFFFFFF:FFFFFFFFFFFFFFFFFFFFFFFFFFFFFFFFFFFFFF
FFFFFFFFFFFFFFFFFFFFFFFFFFFFFFFFFF,FFFFFFFFFFFFFFFFFFFFF:FFFFFF:FF:FF
FFFFFFFFFFFFFF:
@A00155:342:HHGFNDSXY:1:1557:11415:1626 2:N:0:GAACCTAG+TCCGCATA
CATGTTTAAGCATTAAATCAAATAAGATGAGCAAGATGGAACGTCTGGGTTCCAATTCTGTCAAGACT
TATGACATATTAAATAGATTGGTTACGGTAAAAGACAAGGCAAGGTATGGACCCTTAGCAAAGCCAGA
AAGATCACCTTGCCC
+
FFFF:FFFFFFFFFFFFFFFFFFFFFFFFFFFFFFFFF:FFFFFFFFFF:FFFFFFFFFFFFFFFFFF
FFFFFFFFFFFFFFFFFFFFFFFFFFFFFFFF,FFFFFFFFFFFFFFFFFFFFFFFFFFFFFFFFFFF
F:FFFFFFFFFF::F
@A00155:342:HHGFNDSXY:1:2212:6831:26663 2:N:0:GAACCTAG+TCCGCATA
CATGTTTAAGCATTAAATCAAATAAGATGAGCAAGATGGAACGTCTGGGTTCCAATTCTGTCAAGACT
TATGACATATTAAATAGATTGGTTACGGTAAAAGACAAGGCAAGGTATGGACCCTTAGCAAAGCCAGA
AAGATCACCTTGCCC
+
FFFFFFFFFFFFFFFFFFFFFFFF:FFFFFFFFFFFFFFF::FFFFFFFFF:F:F,FFFFFFFFF:FFF
FFFFFFFFFFF:FFFFFFFFFFFFFFFFFF:FFFFFFFFFFFFFFFFFFFFFFFFFFFF:F:F:F,FFFF
FFF:FFFFFFFF,FF
@A00155:342:HHGFNDSXY:1:1273:3703:36683 1:N:0:GAACCTAG+TCCGCATA
ATGTTTAAGCATTAAATCAAATAAGATGAGCAAGATGGAACGTCTGGGTTCCAATTCTGTCAAGACTT
ATGACATATTAAATAGATTGGTTACGGTAAAAGACAAGGCAAGGTATGGACCCTTAGCAAAGCCAGAA
AGATCACCTTGCCCA
+
FFFFFFFFFFFFFFFFFFFFFFFFFFFFFFFFFFFFFFFFFFFFFFFFFFF:FFF:FFFFFFFFFFFF
FF:FFFFFFFFFFFFFFFFFFFFFFFFFFFFFFFFF,:FF:FFFFFFFFFFFFFFFFFFFFFFFFFFFFF
FFFFFFF:FFFFFFF
@A00155:342:HHGFNDSXY:1:2554:7247:2988 2:N:0:GAACCTAG+TCCGCATA
ATGTTTAAGCATTAAATCAAATAAGATGAGCAAGATGGAACGTCTGGGTTCCAATTCTGTCAAGACTT
ATGACATATTAAATAGATTGGTTACGGTAAAAGACAAGGCAAGGTATGGACCCTTAGCAAAGCCAGAA
AGATCACCTTGCCCA

+
:FFFFFFFFFFFFFFFFFFFFFFFFFFFFFFFFFFFFFFFFFFFFFFFFFFFFFFFFFFFFFFFFFFFFFF
FFFFFFFFFFFFFFFFFFFFFFFFFFFFFFFF:FFFFFFFFFFFFFFFFFFFFFFFF:FFFFFFFFFFF
FFFFFFFFF:FFFFF
@A00155:342:HHGFNDSXY:1:1326:8739:11710 2:N:0:GAACCTAG+TCCGCATA
TGTTTAAGCATTAAATCAAATAAGATGAGCAAGATGGAACGTCTGGGTTCCAATTCTGTCAAGACTTA
TGACATATTAAATAGATTGGTTACGGTAAAAGACAAGGCAAGGTATGGACCCTTAGCAAAGCCAGAAA
GATCACCTTGCCCAA
+
FFFFFFFFFFF:FFFFFFFFFFFFFFFFFFFFFFFFFFFFFFFFFFFFFFFFFFFFFFFFFFFFFFFF
FF:FFFFFFFF,:FFFFFFF:FFFFFFFF:FFFFFFFFFFFFFFFFFFFFFFFFFFFFFFFFFFFFFFF
FFF,F:FFF:FFFFF
@A00155:342:HHGFNDSXY:1:1557:32000:25222 2:N:0:GAACCTAG+TCCGCATA
TGTTTAAGCATTAAATCAAATAAGATGAGCAAGATGGAACGTCTGGGTTCCAATTCTGTCAAGACTTA
TGACATATTAAATAGATTGGTTACGGTAAAAGACAAGGCAAGGTATGGACCCTTAGCAAAGCCAGAAA
GATCACCTTGCCCAA
+
FFFFFFFFFFFFFFFFFFFFFFFFFFFFFFFFFFFFFFFFFFFFFFFFFFFFFFFFFFFFFFFFFFFF
FFFFFFFFF:FFFFFFFFFFFFFFFFFF:::FFFFFFFF:FFFFFFFFFFFFFFFF:FFFFF:FFFFF
FFFFFFFFFFFFFFF
@A00155:342:HHGFNDSXY:1:2139:11433:26490 2:N:0:GAACCTAG+TCCGCATA
TGTTTAAGCATTAAATCAAATAAGATGAGCAAGATGGAACGTCTGGGTTCCAATTCTGTCAAGACTTA
TGACATATTAAATAGATTGGTTACGGTAAAAGACAAGGCAAGGTATGGACCCTTAGCAAAGCCAGAAA
GATCACCTTGCCCAA
+
FFFFFFFFFFFFFFFFFFFFFFFFFFFFFFFFFFFFFFFFFFFFFFFFFFFFFFFFFFFFFFFFFFFF
FFFF:FFFFFFFFFFFFFFFFFFFFFFFFFFFFFFFFFFFFFFFFFFFFFFFFFFFFFFFFFFFFFFF
FFFFFFFFFFFFFFF
@A00155:342:HHGFNDSXY:1:2439:16080:13589 2:N:0:GAACCTAG+TCCGCATA
TGTTTAAGCATTAAATCAAATAAGATGAGCAAGATGGAACGTCTGGGTTCCAATTCTGTCAAGACTTA
TGACATATTAAATAGATTGGTTACGGTAAAAGACAAGGCAAGGTATGGACCCTTAGCAAAGCCAGAAA
GATCACCTTGCCCAA
+
FFFFFFFFFFFFFF:FFFF:FF:FFFFFFFFFFFFFFFFFFFFFFFFFFFFFFFFFFFFFFFFFFFFF
FFFFFFFFFFFFFFFFFFFFFFFFFFFFFF,:FFFFFFFFFFFFFFFFFFFFFFFFFFFFFFFFFFFFF
FFFFFFFFFFFFFFF
@A00155:342:HHGFNDSXY:1:2445:28257:8594 2:N:0:GAACCTAG+TCCGCATA
TGTTTAAGCATTAAATCAAATAAGATGAGCAAGATGGAACGTCTGGGTTCCAATTCTGTCAAGACTTA
TGACATATTAAATAGATTGGTTACGGTAAAAGACAAGGCAAGGTATGGACCCTTAGCAAAGCCAGAAA
GATCACCTTGCCCAA
+
:FFFFFFF:FFFFF:FFFFFFFFFFFFFFFFFFFFFFFFF:FF:FFFFFFFFFFF,FFFF:FFFFF:FFF
FFFFFFFFFFFFF:FF,:FFFFFFFFFFFFFFFFFFF:FFFFFF,FFFFFFF:F:FFFFFF,F:F:FF
FFFFFF:FFF:FFFF
@A00155:342:HHGFNDSXY:1:2628:2103:17785 2:N:0:GAACCTAG+TCCGCATA
TGTTTAAGCATTAAATCAAATAAGATGAGCAAGATGGAACGTCTGGGTTCCAATTCTGTCAAGACTTA
TGACATATTAAATAGATTGGTTACGGTAAAAGACAAGGCAAGGTATGGACCCTTAGCAAAGCCAGAAA
GATCAACTTGCCCAA
+
FFFFFFFFFFFFFFFFFFFFFFFFFFFFFFFFFF:FFFFFFFF:FFFFFFFFFFFFFFFFFFFFFFFFFF
FF:FFFFF:FFFFFFF,FFFFF:FFFFF:,:FFFFFFFFF:FFFFFFF:FFF,FFF:FF::F,FFFFF
FFFFF,:::F::F,:
@A00155:342:HHGFNDSXY:1:1551:21124:28682 2:N:0:GAACCTAG+TCCGCATA
GTTTAAGCATTAAATCAAATAAGATGAGCAAGATGGAACGTCTGGGTTCCAATTCTGTCAAGACTTAT

```
GACATATTAAATAGATTGGTTACGGTAAAAGACAAGGCAAGGTATGGACCCTTAGCAAAGCCAGAAAG
ATCACCTTGCCCAA
+
FFFFFFFFFFFFFFFFFFFFFFFFFFFFFFFFFFFFFFFFFFFFFFFFFFFFFFFFFFFFFFFFFFFF
FFFFFFFFFFFFFFF:FFFFFFFFFFF:,FFFFFFFFFFFFFFFFFFFFFFFFFFFFFFFFFFFFFFF
FFFFFFFFFFFFFF
@A00155:342:HHGFNDSXY:1:2515:8359:35039 2:N:0:GAACCTAG+TCCGCATA
GTTTAAGCATTAAATCAAATAAGATGAGCAAGATGGAACGTCTGGGTTCCAATTCTGTCAAGACTTAT
GACATATTAAATAGATTGGTTACGGTAAAAGACAAGGCAAGGTATGGACCCTTAGCAAAGCCAGAAAG
ATCACCTTGCCCAA
+
FFFFFFFFFFFFF,FFFFFFFFF:F:FFFFFFFFFFFFFFFFFFFFFFFFFFFFFFFFFFFFFF:FFF
FFFFFFFFFFFFFFFFFFFFFFF:FFF:F,FFFFFFF:FFF:FFFFFF:FF:FFFFFFFFFFFFFFFF
FF:FFFFFFFFFFF
@A00155:342:HHGFNDSXY:1:1244:9570:17910 2:N:0:GAACCTAG+TCCGCATA
GTTTAAGCATTAAATCAAATAAGATGAGCAAGATGGAACGTCTGGGTTCCAATTCTGTCAAGACTTAT
GACATATTAAATAGATTGGTTACGGTAAAAGACAAGGCAAGGTATGGACCCTTAGCAAAGCCAGAAAG
ATCACCTTGCCCAAA
+
FFFFFFFFFFFFFFFFFFFFFFFFFFFFFFFFFFFFFFFFFFFFFFFFFFFFFFFFFFFFFFFFFFFF
FFFFFFFFFFFFFFFFFFFFFFFFFFFF,,FFFFFFFF:FFFF:FFFFFFFFFFFFFFFFFFFFFFFF
FFFFFFFFFF:F:FF
@A00155:342:HHGFNDSXY:1:1363:20754:32456 2:N:0:GAACCTAG+TCCGCATA
GTTTAAGCATTAAATCAAATAAGATGAGCAAGATGGAACGTCTGGGTTCCAATTCTGTCAAGACTTAT
GACATATTAAATAGATTGGTTACGGTAAAAGACAAGGCAAGGTATGGACCCTTAGCAAAGCCAGAAAG
ATCACCTTGCCCAAA
+
FFFFFFFFFFFFFFFFFFFFFFFFFFFFFFFFFFFFFFFFFFFFFFFFFFFFFFFFFFFFFFFFFFFF
FFFFFFFFFFFFFFFFFFFFFFFFFF:FFFFFFFFFFFFFFFFFFFFFF,FFFFFFFFFFFFFFFFFF
FF:FFFFFFFF:F:F
@A00155:342:HHGFNDSXY:1:1407:4860:2863 2:N:0:GAACCTAG+TCCGCATA
GTTTAAGCATTAAATCAAATAAGATGAGCAAGATGGAACGTCTGGGTTCCAATTCTGTCAAGACTTAT
GACATATTAAATAGATTGGTTACGGTAAAAGACAAGGCAAGGTATGGACCCTTAGCAAAGCCAGAAAG
ATCACCTTGCCCAAA
+
FFFFFFFFFFFFFFFFFFFFFFFFFFFFFFFFFFFFFFFFFFFFFFFFFFFFFFFFFFFFFFFFFFFF
FFFFFFFFFFFFFFFFFFFFFFFFFFFFFFFFFFFFFFFFFFFFFFFFFFFFFFFFF:FFFFFFFFFF
FFFFFFFFFFFFFFF
@A00155:342:HHGFNDSXY:1:1472:7609:11631 2:N:0:GAACCTAG+TCCGCATA
GTTTAAGCATTAAATCAAATAAGATGAGCAAGATGGAACGTCTGGGTTCCAATTCTGTCAAGACTTAT
GACATATTAAATAGATTGGTTACGGTAAAAGACAAGGCAAGGTATGGACCCTTAGCAAAGCCAGAAAG
ATCACCTTGCCCAAA
+
FFFFFFFFFFFFFFFFFFFFFFFFFFF:FFFFFFFFFFFFFFFFFFFFFFFFFFFFF:FFFFFFFFFF
FFFFFFFFF,FFFFFFFF,FFFFFFFFF,FF:FFFFFFFFFFFFFFF:F:F:FFFFFF,FFFFFFFFF
FFFFF:FF:FFFFFF
@A00155:342:HHGFNDSXY:1:1618:2329:12602 2:N:0:GAACCTAG+TCCGCATA
GTTTAAGCATTAAATCAAATAATATGAGCAAGATGGAACGTCTGGGTTCCAATTCTGTCAAGACTTAT
GACATATTAAATAGATTGGTTACGGTAAAAGACAAGGCAAGGTATGGACCCTTAGCAAAGCCAGAAAG
ATCACCTTGCCCAAA
+
FFFFFFFFFFFFFFFFFFFFFF,FFFFFFFFFFFFFFFFFFFF:FF:FFFFFFF:FFFFFFFFFFFFF
FFFFFFFFFFFFF,FFFFFFF:FFFF:F:F,FFFFFF,FFF:FFFF,FFFFFFFFFFFFFFFFFFFFF
:FFFFFFF:F:FFFF
```

```
@A00155:342:HHGFNDSXY:1:1678:9245:9079 2:N:0:GAACCTAG+TCCGCATA
GTTTAAGCATTAAATCAAATAAGATGAGCAAGATGGAACGTCTGGGTTCCAATTCTGTCAAGACTTAT
GACATATTAAATAGATTGGTTACGGTAAAAGACAAGGCAAGGTATGGACCCTTAGCAAAGCCAGAAAG
ATCACCTTGCCCAAA
+
FFFFFFFFFFFFFFFFFFFFFFFFFFFFFFFFFFFFFFFFFFFFFFFFFFFFFFFFFFFFFFFFFFFFFF
FFFFFFFFFFFFFFFFFFFF:FFFFFFFFFFFFFFFFFFFFFFFFF:FFFFFFFFFFFFFFFFFFFFFFF
FFFFFFFFF,FFFFF
@A00155:342:HHGFNDSXY:1:2207:3134:17440 2:N:0:GAACCTAG+TCCGCATA
GTTTAAGCATTAAATCAAATAAGATGAGCAAGATGGAACGTATGGGTTCCAATTCTTTCAAGACTTAT
GACATATTAAATAGATTGGTTACGGTAAAAGACAAGGAAAGGTATTGACCATTAGCAAAGCCAGAAAG
ATCAACTTGCCCAAA
+
FFFFFFFFFF:FF:FFFF:F,FF,FF:FFF,FFFFFF::FF,:FFFFFF:FFFFFFFF,:FF:F:FFFFF
FFFFF:FFF:FFFFFFFF,,FFFFFFFFFFFFFFFFF:,,FFFF,FF,FFFF,FFF,F:F,FFFFFF:FF
,FF,,FF,FFFFF:F
@A00155:342:HHGFNDSXY:1:2203:1868:33129 2:N:0:GAACCTAG+TCCGCATA
GTTTAAGCATTAAATCAAATAAGATGAGCAAGATGGAACGTCTGGGTTCCAATTCTGTCAAGACTTAT
GACATATTAAATAGATTGGTTACGGTAAAAGACAAGGCAAGGTATGGACCCTTAGCAAAGCCAGAAAG
ATCACCTTGCCCAAA
+
FFFFFFFFFFFFFFFF:FFFFFFFFFFFFFF:FFFFFFF:FFFFFFFFFFFFFFFFFFFFFFFFFFF,FF
FFFFFFFFFFFFF:FF:FFFFFFFFFF,FFFFFFF:FFFF:FFFFFFFFFFFFFFFFFFFFFFFFFFF:F
FFFFFFFFFFFFF,:
@A00155:342:HHGFNDSXY:1:2266:12391:5447 2:N:0:GAACCTAG+TCCGCATA
GTTTAAGCATTAAATCAAATAAGATGAGCAAGATGGAACGTCTGGGTTCCAATTCTGTCAAGACTTAT
GACATATTAAATAGATTGGTTACGGTAAAAGACAAGGCAAGGTATGGACCCTTAGCAAAGCCAGAAAG
ATCACCTTGCCCAAA
+
FFFFFFFFFFFFFFFFFFFFFFFFFFF:FFFFFFFFFFFFF,FFFFFFFFFFFFFFFFFFFFFF:F:FFF
FFFFFFFF:FFFFFFFFFFFFFFFFFFFFF:FFFFFFFFFFFFFFFFFFFFFFFFFFFFFFFFFFFFFFF
FFFFFFFFFFFFFFF
@A00155:342:HHGFNDSXY:1:2339:26883:20556 2:N:0:GAACCTAG+TCCGCATA
GTTTAAGCATTAAATCAAATAAGATGAGCAAGATGGAACGTCTGGGTTCCAATTCTGTCAAGACTTAT
GACATATTAAATAGATTGGTTACGGTAAAAGACAAGGCAAGGTATGGACCCTTAGCAAAGCCAGAAAG
ATCACCTTGCCCAAA
+
FFFFFFF:FFFFFFFFFFFFFFFFFFFFFFF:FFFFFFFFFFFFFFFFFFFFFFFFFFFFF:FFFFFFFF
FFFFFFFFFFFFFFFFFFFF:FFFFFFFF:FFFFFFFFFFFFFFFFFFFFFFFFFFFFF,FFFFFFFFFF
FFFFFFFFFFFFFFF
@A00155:342:HHGFNDSXY:1:2368:6144:22529 2:N:0:GAACCTAG+TCCGCATA
GTTTAAGCATTAAATCAAATAAGATGAGCAAGATGGAACGTCTGGGTTCCAATTCTGTCAAGACTTAT
GACATATTAAATAGATTGGTTACGGTAAAAGACAAGGCAAGGTATGGACCCTTAGCAAAGCCAGAAAG
ATCACCTTGCCCAAA
+
FFFFFFFFFFFFFFFFFFFFFFFFFFFFFFFFFFFFFFFFFFF:FFFFFFFFFFFFFF:FFFFFFFFFFF
FFFFFF,:F,FFF:F,FFFFFFFFFFF:F:FFFFFFFFF:FFFFFFFFFFFFFFFFFF:FFFFFFFFFFF
F:FFFFFFFFFFFFF
@A00155:342:HHGFNDSXY:1:2436:10664:20650 2:N:0:GAACCTAG+TCCGCATA
GTTTAAGCATTAAATCAAATAAGATGAGCAAGATGGAACGTCTGGGTTCCAATTCTGTCAAGACTTAT
GACATATTAAATAGATTGGTTACGGTAAAAGACAAGGCAAGGTATGGACCCTTAGCAAAGCCAGAAAG
ATCACCTTGCCCAAA
+
FFFFFFFFFFFFFFFFFFFFFFFFFFFFFFFFFFFFFFFFFFFFFFFFFFFFFFFFFFFFFFFFFFFFFF
```

```
FFFFFFFF,:FFFFFFFFFFFFFFF:F:FFFFFFFFFFFFFFFFFFFFFFFFFFFFFFFFFFFFFFFF
FFFFFFFFFFFF:FF
@A00155:342:HHGFNDSXY:1:2446:5900:4225 2:N:0:GAACCTAG+TCCGCATA
GTTTAAGCATTAAATCAAATAAGATGAGCAAGATGGAACGTCTGGGTTCCAATTCTGTCAAGACTTAT
GACATATTAAATAGATTGGTTACGGTAAAAGACAAGGCAAGGTATGGACCCTTAGCAAAGCCAGAAAG
ATCACCTTGCCCAAA
+
FFFFFFFFFFFFFFFFFFFFFFFFFFFFFFFFFFFFFFFFFFFFFFFFFFFFFFFFFFFFFFFFFFF:
FFFFFFFFFFFFFFF:FFFFFFFFFF:F:,FFFFFFFFFFFFFFFFFFFFFFFFFFFFFFFFFFFFFF
FFFFFFFFFFFFFFF
@A00155:342:HHGFNDSXY:1:1333:29017:14763 1:N:0:GAACCTAG+TCCGCATA
TTTAAGCATTAAATCAAATAAGATGAGCAAGATGGAACGTCTGGGTTCCAATTCTGTCAAGACTTATG
ACATATTAAATAGATTGGTTACGGTAAAAGACAAGGCAAGGTATGGACCCTTAGCAAAGCCAGAAAGA
TCACCTTGCCCAAAG
+
FFFFFFFFFFF:FFFFFFFFFFFFFFF:FFFF:FFFFFFFFFFFF,FFFFFF:FFFFFFFFFFFFF:,
FFFFFFFF:F:FFFFFFFFF:FFFFFFFFFFF,:FFFFFFFFFF:FFFFFFFFFFFFFFFFFFFFFFF
FFF:FFFFF:FFFFF
@A00155:342:HHGFNDSXY:1:1333:28872:15389 1:N:0:GAACCTAG+TCCGCATA
TTTAAGCATTAAATCAAATAAGATGAGCAAGATGGAACGTCTGGGTTCCAATTCTGTCAAGACTTATG
ACATATTAAATAGATTGGTTACGGTAAAAGACAAGGCAAGGTATGGACCCTTAGCAAAGCCAGAAAGA
TCACCTTGCCCAAAG
+
FFFFFFFFFFFFFF:FFFFFFFFFFFFFFFFFFFFFFFFFFFFFFFFFFFFFFFFFFFFFFFFFFFFF
FFFFFFFFFFFFFFFFFFFFFFFF,FFFFFFFFF:FFFFFFFFFFFFFFFFFFFFFFFFFFFFFFFFF
FFFFF::FFFFFFFF
@A00155:342:HHGFNDSXY:1:1333:28131:30953 1:N:0:GAACCTAG+TCCGCATA
TTTAAGCATTAAATCAAATAAGATGAGCAAGATGGAACGTCTGGGTTCCAATTCTGTCAAGACTTATG
ACATATTAAATAGATTGGTTACGGTAAAAGACAAGGCAAGGTATGGACCCTTAGCAAAGCCAGAAAGA
TCACCTTGCCCAAAG
+
FFFFFFFFFFFFFFFFFFFFFFFFFFFFFFFFFFFFFFFFFFFFFFFFFFFFFFFFFFFFFFFFFFFF
FFFFFFFFFFFFFFFFFFFFFFFFFFFFFFFFF,FFFFFFFFFFFFFFFFFFFFFFFFFFFFFFFFFF
FFFFFFFFFFFFFFF
@A00155:342:HHGFNDSXY:1:1410:24813:19570 1:N:0:GAACCTAG+TCCGCATA
TTTAAGCATTAAATCAAATAAGATGAGCAAGATGGAACGTCTGGGTTCCAATTCTGTCAAGACTTATG
ACATATTAAATAGATTGGTTACGGTAAAAGACAAGGCAAGGTATGGACCCTTAGCAAAGCCAGAAAGA
TCACCTTGCCCAAAG
+
FFFFFFFFFFFFFF:FFFFFFFFFFFFFFFFFFFFFFFFFFFFFFFFFFFFFFFFFFFFFFFFFFFFF
FFFFFFFFFFFFFFFFFFFFFFFFFFFFFFFFFFFFFFFFFFFFFFFFFFFFFFFFFFFFFFFFFFFF
FFFFFFFFFFFFFFF
@A00155:342:HHGFNDSXY:1:1545:15465:5228 1:N:0:GAACCTAG+TCCGCATA
TTTAAGCATTAAATCAAATAAGATGAGCAAGATGGAACGTCTGGGTTCCAATTCTGTCAAGACTTATG
ACATATTAAATAGATTGGTTACGGTAAAAGACAAGGCAAGGTATGGACCCTTAGCAAAGCCAGAAAGA
TCACCTTGCCCAAAG
+
FFFFFFFFFFFFFF:FFFFFFFFFFFFFFFFFFFFFFFFFFFFFFFFFFFFFFFFFFFFFFFFFFFFF
FF:FFFFFFFFFFFFFFFFFF:FFFFFFFFFFFFFFFFFFFFFFFFFFFFFFFFFFF:FFFFFFFFFFFF
FFFFFFFFFFFFFFF
@A00155:342:HHGFNDSXY:1:1522:3332:9956 1:N:0:GAACCTAG+TCCGCATA
TTTAAGCATTAAATCAAATAAGATGAGCAAGATGGAACGTCTGGGTTCCAATTCTGTCAAGACTTATG
ACATATTAAATAGATTGGTTACGGTAAAAGACAAGGCAAGGTATGGACCCTTAGCAAAGCCAGAAAGA
TCACCTTGCCCAAAG
```

+
FFFFFFFFFFFFFFFFFFFFFFFFFFFFFFFFFFFFFFFFFFFFFFFFF:FFFFFFFFFFFFFFFFFFFF
FFFFFFFFFFFFFFFFFFFFFFFFFFFFFFFFFFFFFFFFFFFFFFFFFFFFFFFFFFFFFFFFFFFFFF
FFFFFFFFFFFFFFF
@A00155:342:HHGFNDSXY:1:1666:7952:34303 1:N:0:GAACCTAG+TCCGCATA
CCGATCTATTAAATCAAATAAGATGAGCAAGATGGAACGTCTGGGTTCCAATTCTGTCAAGACTTATG
ACATATTAAATAGATTGGTTACGGTAAAAGACAAGGCAAGGTATGGACCCTTAGCAAAGCCAGAAAGA
TCACCTTGCCCAAAG
+
FFFFFFFFFFFFFFFFFFFFFFFFFFFFFFFFFFFFFFFFFFFFFFFFFFFFFF:FFFFFFFFFFFFFFF
FFFFFFFFFFFFFFFFFFFFFFFFFFFF,FFFFF:FFFFFFFFFFFFFFFFFFFFFFFFFFFFFFFFFFF
FFFFFFFFFFFFFFF
@A00155:342:HHGFNDSXY:1:2146:24623:11694 1:N:0:GAACCTAG+TCCGCATA
TTTAAGCATTAAATCAAATAAGATGAGCAAGATGGAACGTCTGGGTTCCAATTCTGTCAAGACTTATG
ACATATTAAATAGATTGGTTACGGTAAAAGACAAGGCAAGGTATGGACCCTTAGCAAAGCCAGAAAGA
TCACCTTGCCCAAAG
+
FFFFFFFFFFFFFFFFFFFFFFFFFFFFFFFFFFFFFFFFFFF:FFFFFFFFFFFFFFFFFFFFFFFFFF
FFFFFFFFFFFFFFFFFFFFFFFFFFFFFFFFFFFFFFFFFFFFFFFFFFFFFFFFFFFFFFFFFFFFFF
FFFFFFFFFFFFFFF
@A00155:342:HHGFNDSXY:1:2158:26612:11600 1:N:0:GAACCTAG+TCCGCATA
TTTAAGCATTAAATCAAATAAGATGAGCAAGATGGAACGTCTGGGTTCCAATTCTGTCAAGACTTATG
ACATATTAAATAGATTGGTTACGGTAAAAGACAAGGCAAGGTATGGACCCTTAGCAAAGCCAGAAAGA
TCACCTTGCCCAAAG
+
:FFFFFFFFFFFFFFFFFFFFFFFFFFFFFFFFFFFFFFFFFFFFFFFFFFFFFFFFFFFFFFFFFFFFF
FFFFFFFFFFFFFFFFFFFFFFFFFFFFFFFFFFFFFFFFFFFFFFFFFFFFFFFFFFFFFFFFFFFFFF
FFFFFFFFFFFFFFF
@A00155:342:HHGFNDSXY:1:2525:31684:31093 1:N:0:GAACCTAG+TCCGCATA
TTTAAGCATTAAATCAAATAAGATGAGCAAGATGGAACGTCTGGGTTCCAATTCTGTCAAGACTTATG
ACATATTAAATAGATTGGTTACGGTAAAAGACAAGGCAAGGTATGGACCCTTAGCAAAGCCAGAAAGA
TCACCTTGCCCAAAG
+
FFFFFFFFFFFFFFFF:FFFFFFFFFFFFFFFFFFFFFFFFFFFFFFFFF:FFFFFFFFFFFFFFFFFFF
FFFFFFFFFFFFFFFFFFFFFFFFFFFFFFFFFFFFFFFFFFFFFFFFFFFFFFFFFFFFFFFFFFFFFF
FF:FFFFFFFFFFFF
@A00155:342:HHGFNDSXY:1:2556:9263:23954 1:N:0:GAACCTAG+TCCGCATA
TTTAAGCATTAAATCAAATAAGATGAGCAAGATGGAACGTCTGGGTTCCAATTCTGTCAAGACTTATG
ACATATTAAATAGATTGGTTACGGTAAAAGACAAGGCAAGGTATGGACCCTTAGCAAAGCCAGAAAGA
TCACCTTGCCCAAAG
+
FFFFFFFFFFFFFFFFFFFFFFFFFFFFFFFFFFFFFFFFFFFFFFF,FFFFFFFFFFFFFFFFFFFFFF
FFFFFFFFFFFFFFFFFFFFFFFFFFFFFFFFFFFFFFFFFFFFFFFFFFFFFFFFFFFFFFFFFFFFFF
FFFFFFFFFFFFFFF
@A00155:342:HHGFNDSXY:1:2603:7066:2331 1:N:0:GAACCTAG+TCCGCATA
TTTAAGCATTAAATCAAATAAGATGAGCAAGATGGAACGTCTGGGTTCCAATTCTGTCAAGACTTATG
ACATATTAAATAGATTGGTTACGGTAAAAGACAAGGCAAGGTATGGACCCTTAGCAAAGCCAGAAAGA
TCACCTTGCCCAAAG
+
:FFFF:FFFFFFFFFFFFFFFFFFFFFFFFFFFFFF:FFFFFFFFFFF,F:FFFF:FFFFFFFFFFFFFF
FFFFFFFFFFFFFFFFFFFFFFFFFFFFFFFFFFFFFFFFFFFFFFFFFFFFFFFFFFFFFFFFFFFFFF
FFFFFFFFFFFFFFF
@A00155:342:HHGFNDSXY:1:2601:25590:16814 1:N:0:GAACCTAG+TCCGCATA
TTTAAGAATTAAATCAAATAAGATGAGCTAGATGGAACGTCTGGGTTCCAATTCTGTCAAGACTTATG

ACATATTAAATAGATTGGTTACGGTAAAAGACAAGGCAAGGTATGGACCCTTAGCAAAGCCAGAAAGA
TCACCTTGCCCAAAG
+
FFF,,F,FFFF:FF,FFF,FFF:FFFF,,FFFF:,FFFFF:F:F::F,FFF,FFFFF:FFFF,FFFFF
F:FF:FFFFF:FFF,FFFFFF:FFFFFFFFF:,:FFF:FF,FFF,FFFFFFFF,FFFFF,:,F:F:FF
F,,F:FFF::,FFFF
@A00155:342:HHGFNDSXY:1:2628:2103:17785 1:N:0:GAACCTAG+TCCGCATA
TTTAAGCATTAAATCAAATAAGATGATCAAGATGGAACGTCTGGGTTCCAATTCTGTCAAGACTTATG
ACATATTAAATAGATTGGTTACGGTAAAAGACAAGGCAAGGTATGGACCCTTAGCAAAGCCAGAAAGA
TCACCTTGCCCAAAG
+
FFFFF,FFF:FFFFFF:F:FF,FFFF,FFFF:FFF:F,FFFFFF,FFF:F:FFFFF,FFF:FFFFFF:
F,FFFFFFFFFFFFFF,FFFFFFFFFFFFFF:FFFFFFF,FF:FFFFFFFFFFFFFFFFFFFFFFFFF
FFFFFFFFFFFFFFF
@A00155:342:HHGFNDSXY:1:2638:24234:10802 1:N:0:GAACCTAG+TCCGCATA
TTTAAGCATTAAATCAAATAAGATGAGCAAGATGGAACGTCTGGGTTCCAATTCTGTCAAGACTTATG
ACATATTAAATAGATTGGTTACGGTAAAAGACAAGGCAAGGTATGGACCCTTAGCAAAGCCAGAAAGA
TCACCTTGCCCAAAG
+
FFFFF,:FF:FFFFFFFFFFFFFFFF:FFFFFFF:FFFFFFF,FF:FFFFFFFFFFFFFFF::FFFF,
F:FFFFF:FFFFFF:FFFFFFFF:FFFFFFFFFFFFFFFFFFFFFFFFFFFFF:FFFFFF:FFFFFFF
F:FFFFF:FFFF:FF
@A00155:342:HHGFNDSXY:1:2245:14073:28463 2:N:0:GAACCTAG+TCCGCATA
TTTAAGCATTAAATCAAATAAGATGAGCAAGATGGAACGTCTGGGTTCCAATTCTGTCAAGACTTATG
ACATATTAAATAGATTGGTTACGGTAAAAGACAAGGCAAGGTATGGACCCTTAGCAAAGCCAGAAAGA
TCACCTTGCCCAAAG
+
FFFF:FFFFFFFFFFFFFFFFFFFFFFFFFFFFFF:FFFFFFFFFFFFFFFFFFFFFFFFFFFFFFF,
FFFFFFFF:FFFFFFFF:FFFF:FF:F:FFFFFFFFFFFFFFFF:FFF:FFFFFFFF:FF:FFFFFFF
FFFF:F:,,FFFFF:
@A00155:342:HHGFNDSXY:1:2578:14986:9189 2:N:0:GAACCTAG+TCCGCATA
TTTAAGCATTAAATCAAATAAGATGAGCAAGATGGAACGTCTGGGTTCCAATTCTGTCAAGACTTATG
ACATATTAAATAGATTGGTTACGGTAAAAGACAAGGCAAGGTATGGACCCTTAGCAAAGCCAGAAAGA
TCACCTTGCCCAAAG
+
:F:FFF::FFFFFFFFFFFFFFF:FF::FFFFFFF:FFFFFFFFFFFFFFFFFFFFFFFFFFFFFFFF
FFFFFFFFFFFFFFFFFFFFFFFFFFFFFFFFFFFF:FFFFFFFFFFFFFFFFFFFF:FFFFFFFFFF
FFFFFF:FFFFFFFF
@A00155:342:HHGFNDSXY:1:2662:22580:7153 1:N:0:GAACCTAG+TCCGCATA
TTAAGCATTAAATCAAATAAGATGAGCAAGATGGAACGTCTGGGTTCCAATTCTGTCAAGACTTATGA
CATATTAAATAGATTGGTTACGGTAAAAGACAAGGCAAGGTATGGACCCTTAGCAAAGCCAGAAAGAT
CACCTTGCCCAAAG
+
FFFFFFFFFFFFFFFFFFFFFFFFFFFFFFFFFFFFFFFFFFFFFFFFFFFFFFFFFFFFFFFFFFFF
FFFFFFFFFFFFFFFFFFFFFFFFFFFFFFFFFFFFFFFFFFFFFFFFFFFFFFFFFFFFFFFFFF:F
FFFFFFFFFFFFFF
@A00155:342:HHGFNDSXY:1:2206:20772:5024 1:N:0:GAACCTAG+TCCGCATA
TTAAGCATTAAATCAAATAAGATGAGCAAGATGGAACGTCTGGGTTCCAATTCTGTCAAGACTTATGA
CATATTAAATAGATTGGTTACGGTAAAAGACAAGGCAAGGTATGGACCCTTAGCAAAGCCAGAAAGAT
CACCTTGCCCAAAGA
+
FFFFFFFFFFFFFFFFFFFFFFFFFFFFFFFF:FFFFFFFFFFF:FFFFFFFFFFFFFFFFFFFFFFF
FFFFFFFFFFFFFFFFFFFFFFFFFFFFFFFFFFFFFFFFFFFFFFFFFFFFFFFFFFFFFFFFFFFF
FFFFFFFFFFFFFFF

```
@A00155:342:HHGFNDSXY:1:1246:6189:11083 2:N:0:GAACCTAG+TCCGCATA
TTAAGCATTAAATCAAATAAGATGAGCAAGATGGAACGTCTGGGTTCCAATTCTGTCAAGACTTATGA
CATATTAAATAGATTGGTTACGGTAAAAGACAAGGCAAGGTATGGACCCTTAGCAAAGCCAGAAAGAT
CACCTTGCCCAAAGA
+
FFFFFFFFFFFFFFFF:FFFFFFFFFFFFFF:FFFFFFFFFFFFFFFFFFFFFFFFFFFFFFFFFFF:F:
FFFFFFFFFFFFFFFFFFFFFFFFFFFFFFFFFFFFFFFFFFFFFFFFFFFFFFFFFFFFFF,FFFFFFF
:FFFFFFFFFFFFFF
@A00155:342:HHGFNDSXY:1:2405:18304:36699 2:N:0:GAACCTAG+TCCGCATA
TAAGCATTAAATCAAATAAGATGAGCAAGATGGAACGTCTGGGTTCCAATTCTGTCAAGACTTATGAC
ATATTAAATAGATTGGTTACGGTAAAAGACAAGGCAAGGTATGGACCCTTAGCAAAGCCAGAAAGATC
ACCTTGCCCAAAGAA
+
FFFFFFFFFFFFFFFFFFFFFFFFFFFFFFFFFFFFFFFFFFFFFFFFFFFFFFFFFFFFFFFFFFFFFF
FFFFFFFF:FFFFFFFF:FFFFFFF:F::FFFFFFFFFFFFFFFFFFFFFFF,FFFFFFFFFFFFFFFFF
FFFFFFFFFFFFFFF
@A00155:342:HHGFNDSXY:1:2342:1362:29309 1:N:0:GAACCTAG+TCCGCATA
AAGCATTAAATCAAATAAGATGAGCAAGATGGAACGTCTGGGTTCCAATTCTGTCAAGACTTATGACA
TATTAAATAGATTGGTTACGGTAAAAGACAAGGCAAGGTATGGACCCTTAGCAAAGCCAGAAAGATCA
CCTTGCCCAAAG
+
FF::F,FFFFFFFFFFFF,F,FF:FFFFFFFFFFFFF,F:F,,F:F::F::F::,FFF:FF,:FFFFFFF
FF:FFFFFF:FFFFF:FFF:::FFFF,FFFF,FFFF::FFF:FFFFF,FFFFFFFF:FFFFFFFFFF,FF
FFFFFFFFFFFF
@A00155:342:HHGFNDSXY:1:1375:1072:36010 1:N:0:GAACCTAG+TCCGCATA
AAGCATTAAATCAAATAAGATGAGCAAGATGGAACGTCTGGGTTCCAATTCTGTCAAGACTTATGACA
TATTAAATAGATTGGTTACGGTAAAAGACAAGGCAAGGTATGGACCCTTAGCAAAGCCAGAAAGATCA
CCTTGCCCAAAGAAG
+
:FFFFFFF:FFFFF,FF,F:FFFFFFFFFF:FFFFFF:FFFFF,,FFFFFFFFFFFFFFFFF,FFFF:FF
FFFFFF::FFFFFFFF,FFFFFF,:,FFFFF,FFFF,FFF:FFFFFFFFFF:FFFFFFFFFFFFFFFFFF
FF,FFFFFFFFF,FF
@A00155:342:HHGFNDSXY:1:1407:8883:1720 1:N:0:GAACCTAG+TCCGCATA
AAGCATTAAATCAAATAAGATGAGCAAGATGGAACGTCTGGGTTCCAATTCTGTCAAGACTTATGACA
TATTAAATAGATTGGTTACGGTAAAAGACAAGGCAAGGTATGGACCCTTAGCAAAGCCAGAAAGATCA
CCTTGCCCAAAGAAG
+
FFFFFFFFFFFFFFFFFFFFFFFFFFFFFFFF:FFFFFFF,FFFF,F:FFFFFFFFFFFFFFFFFFFFFF
FFFFFFFFFFFFFFFFFFFFFFFFFFFFFFFFFFFFFFFFFFFFFFFFFFFFFFFFFFFFFFFFFFFFFF
FFFFFFFFFFFFFFF
@A00155:342:HHGFNDSXY:1:1445:21043:5149 1:N:0:GAACCTAG+TCCGCATA
AAGCATTAAATCAAATAAGATGAGCAAGATGGAACGTCTGGGTTCCAATTCTGTCAAGACTTATGACA
TATTAAATAGATTGGTTACGGTAAAAGACAAGGCAAGGTATGGACCCTTAGCAAAGCCAGAAAGATCA
CCTTGCCCAAAGAAG
+
F:FF,FFF:FF,F,F,F,FFFFFF:FFFFFF:FFFFFF:FFFFF:FFFFFFFFFFFFFFFFFFFFFFFFF
FFFFF:,FFFFF,F:FFFF:FFF:FFFF,:::FFFFF,FFFF:FF:FFFFFFFFFFFFFFFFF::FFFFF
FFFFFFFFFFFFFFF
@A00155:342:HHGFNDSXY:1:1451:18828:3474 1:N:0:GAACCTAG+TCCGCATA
AAGCATTAAATCAAATAAGATGAGCAAGATGGAACGTCTGGGTTCCAATTCTGTCAAGACTTATGACA
TATTAAATAGATTGGTTACGGTAAAAGACAAGGCAAGGTATGGACCCTTAGCAAAGCCAGAAAGATCA
CCTTGCCCAAAGAAG
+
FFFFFFFFFFFFFFFFFFFFFFFF:F:FFFFFFFFFFFFFFFFFFFFFFFFFFFF:FFFFFFFFFFFFFF
```

FFFFFFFFFFFFFFFFFFFFFFFFFFFFFFF:FFFF:FFFFFFFFFFFFFFFFFFFFFFFFFFFFFF:
FFFFFFFFFFFFFFF
@A00155:342:HHGFNDSXY:1:1444:28366:10598 1:N:0:GAACCTAG+TCCGCATA
AAGCATTAAATCAAATAAGATGAGCAAGATGGAACGTCTGGGTTCCAATTCTGTCAAGACTTATGACA
TATTAAATAGATTGGTTACGGTAAAAGACAAGGCAAGGTATGGACCCTTAGCAAAGCCAGAAAGATCA
CCTTGCCCAAAGAAG
+
FFFFFFFFFFFFFFFFFFFFFFFFFFFFFFFFFFFFFFFFFFFFFF:FFFFF:FFFFFFFFFFFFFFF
FFFFFFFFFFFFFFFFFFFFFFFFFFFFFFFFFFFFFFFFFFFFFFFFFFFFFFFFFFFFFFFFFFFF
FFFFFFFFFFFFFFF
@A00155:342:HHGFNDSXY:1:1436:8124:6167 1:N:0:GAACCTAG+TCCGCATA
AAGCATTAAATCAAATAAGATGAGCAAGATGGAACGTCTGGGTTCCAATTCTGTCAAGACTTATGACA
TATTAAATAGATTGGTTACGGTAAAAGACAAGGCAAGGTATGGACCCTTAGCAAAGCCAGAAAGATCA
CCTTGCCCAAAGAAG
+
FFFFFF:FFFFFFFFFFFFFFF:FFFFFFFFFFFFFFFFFFFFFFFFFFFFFFFFFFFFFFFFFFFFF
FFFFFFFFFFFFFFFFFFFFFFFFFFFFFFFFFFFFFFFFFFFFFFFFFFFFFFFFFFFFFFFFFF:
FFFFFFFFFFFFFFF
@A00155:342:HHGFNDSXY:1:1611:30626:5744 1:N:0:GAACCTAG+TCCGCATA
AAGCATTAAATCAAATAAGATGAGCAAGATGGAACGTCTGGGTTCCAATTCTGTCAAGACTTATGACA
TATTAAATAGATTGGTTACGGTAAAAGACAAGGCAAGGTATGGACCCTTAGCAAATCCAGAAAGATCA
CCTTGCCCAAAGAAG
+
FFFFFFFFFFFFFFFFFFFFFFFFFFFFFFFFFFFFFFFFFFFFFF:FFFFFFFFFFFFFFFFFFFFF
FFFFFFFFFFFFFFFFFFFFFFFFFFFFFFFFFFFFFFFFFFFFFFFFFFFFFFFFFFFFFFFFFFFF
FFFFFFFFFFFFFFF
@A00155:342:HHGFNDSXY:1:1611:30011:6057 1:N:0:GAACCTAG+TCCGCATA
AAGCATTAAATCAAATAAGATGAGCAAGATGGAACGTCTGGGTTCCAATTCTGTCAAGACTTATGACA
TATTAAATAGATTGGTTACGGTAAAAGACAAGGCAAGGTATGGACCCTTAGCAAATCCAGAAAGATCA
CCTTGCCCAAAGAAG
+
FFFFFFFFFFFFFFFFFFFFFFFFFFFFFFFFFFFFFFFFFFFFFF:FFFFFFFFFFFFFFFFFFFFF
FFFFFFFFFFFFFFFFFFFFFFFFFFFFFFFFFFFFFFFFFFFF,FFFFFFFFFFFFFFFFFFFFFFF
FFFFFFFFFFFFFFF
@A00155:342:HHGFNDSXY:1:2203:1868:33129 1:N:0:GAACCTAG+TCCGCATA
AAGCATTAAATCAAATAAGATGAGCAAGATGGAACGTCTGGGTTCCAATTCTGTCAAGACTTATGACA
TATTAAATAGATTGGTTACGGTAAAAGACAAGGCAAGGTATGGACCCTTAGCAAAGCCAGAAAGATCA
CCTTGCCCAAAGAAG
+
F:FFFFFFFFFFFFF:FFFF:FF:FFF:,FF:,FF:FF::FF:FFF,F,F::FFF:F:FFFFF:FFFF
FF:FF:FFFFFFFFF:FFFFF:FFFFFFF,:FFF:FFFFFFFFFFFFFFFFFFFFFFFFFFFFFF,FF
FFF,FFFFFFFFFFF
@A00155:342:HHGFNDSXY:1:2302:24813:10144 1:N:0:GAACCTAG+TCCGCATA
AAGCATTAAATCAAATAAGATGAGCAAGATGGAACGTCTGGGTTCCAATTCTGTCAAGACTTATGACA
TATTAAATAGATTGGTTACGGTAAAAGACAAGGCAAGGTATGGACCCTTAGCAAAGCCAGAAAGATCA
CCTTGCCCAAAGAAG
+
FFF:FFFFFFFFFFFFFFFFFFFFFF:FFFFFFFFFFFFFFFFFFFFF,FFFFFFFFFFFFFFFFFF:
FFFFFFFFFFFFFFFFFFFFFFFFFFFFFFFFFFFFFFFFFFFFFFFFFFFFFFFFFFFFFFFFFFFF
FFFFFFFFFFFFFFF
@A00155:342:HHGFNDSXY:1:2375:2383:34303 1:N:0:GAACCTAG+TCCGCATA
AAGCATTAAATCAAATAAGATGAGCAAGATGGAACGTCTGGGTTCCAATTCTGTCAAGACTTATGACA
TATTAAATAGATTGGTTACGGTAAAAGACAAGGCAAGGTATGGACCCTTAGCAAAGCCAGAAAGATCA
CCTTGCCCAAAGAAG

```
+
FFFFFFFFFFFFFFFFFFFFFFFFFFFF:FFFFFFFFFFFFFFFFFFFFFFFFFFFFFFFFFFFFFFF
FFFFFFFFFFFFFFFFFFFFFFFFF:FFFFFFFFFFFFFFFFFFFFFFFFFFFFFFFFFFFFFFFFF:
FFFFFFFFFFFFFFF
@A00155:342:HHGFNDSXY:1:2421:16288:31798 1:N:0:GAACCTAG+TCCGCATA
AAGCATTAAATCAAATAAGATGAGCAAGATGGAACGTCTGGGTTCCAATTCTGTCAAGACTTATGACA
TATTAAATAGATTGGTTACGGTAAAAGACAAGGCAAGGTATGGACCCTTAGCAAAGCCAGAAAGATCA
CCTTGCCCAAAGAAG
+
FF:FFFFFFF:FFFFFFFFFFFFFFFFFFFFFFFFFFFFFFF:FFFFFFFFFFFFFFFF:FFFFFFFF
FFFFFFFFFFFFFFFFFFFFFFFFFFFFFFFFFFFFFFFFFFFFFFFFFFFFFFFFFFFFFFFFFFFF
FFFFFFFFFFFFFFF
@A00155:342:HHGFNDSXY:1:2540:15709:25817 1:N:0:GAACCTAG+TCCGCATA
AAGCATTAAATCAAATAAGATGAGCAAGATGGAACGTCTGGGTTCCAATTCTGTCAAGACTTATGACA
TATTAAATAGATTGGTTACGGTAAAAGACAAGGCAAGGTATGGACCCTTAGCAAAGCCAGAAAGATCA
CCTTGCCCAAAGAAG
+
FFFFFFFFFFFF:FFFFFFFFFFFFFFFFFFFFFFFFFFFFFF:FFFFFFFFFFFFFFFFFFFFFFFF
FFFFFFFFFFFFFFFFFFFFFFFFFFFFFFFFFFFF:FFFFFFFFFFF,FFFFFFFFFFFFFFFFFFF
FFFFFFFFFFFFFFF
@A00155:342:HHGFNDSXY:1:2651:26946:25708 1:N:0:GAACCTAG+TCCGCATA
AAGCATTAAATCAAATAAGATGAGCAAGATGGAACGTCTGGGTTCCAATTCTGTCAAGACTTATGACA
TATTAAATAGATTGGTTACGGTAAAAGACAAGGCAAGGTATGGACCCTTAGCAAAGCCAGAAAGATCA
CCTTGCCCAAAGAAG
+
FFFFFFFFFFFFFFFFFFFFFFFFFFFFFFFFFFFFFFFFFF:FFFFFFFFFFFFFFFFFFFFFFFFF
FFFFFFFFFFFFFFFFFFFFFFFFF:FFFFFFFFFFFFFFFFFFFFFFFFFFFFFFFFFFFFFFFFFF
FFF:FFFFFFFFFFF
@A00155:342:HHGFNDSXY:1:2121:8576:11209 2:N:0:GAACCTAG+TCCGCATA
AAGCATTAAATCAAATAAGATGAGCAAGATGGAACGTCTGGGTTCCAATTCTGTCAAGACTTATGACA
TATTAAATAGATTGGTTACGGTAAAAGACAAGGCAAGGTATGGACCCTTAGCAAAGCCAGAAAGATCA
CCTTGCCCAAAGAAG
+
FFFFFFFFFFFFFFFFFFFFFF:FFFFFFFFFFFFFFFFFFFFFFFFFFFFFFFFFFFFFFFFFFFFF
FFFFFFFFFFFFFFFFFFFFFFFFFFFFFF:FFF:FFFF,FFFFFF:FFFFFFFFFFFFFFFFFFFFF
FFFFFF,FFFFFFFF
@A00155:342:HHGFNDSXY:1:2570:5367:21778 1:N:0:GAACCTAG+TCCGCATA
AGCATTAAATCAAATAAGATGAGCAAGATGGAACGTCTGGGTTCCAATTCTGTCAAGACTTATGACAT
ATTAAATAGATTGGTTACGGTAAAAGACAAGGCAAGGTATGGACCCTTAGCAAAGCCAGAAAGATCAC
CTTGCCCAAAGAAG
+
FF,:FF:FF:F,::FF,FFFFFFFFFFFF:FFFFFF::F,F:FFFFF,FFFFFF:FF,FF:FFFFF:F
FFFFFF,F::F,FFFFFFFFF:FFFF:FFFFFFFFFFFFFFFFFFFF:FFFFFFFFFF,FF:FFFF:F
F:,F,FFF:FFFFF
@A00155:342:HHGFNDSXY:1:1625:8323:16595 1:N:0:GAACCTAG+TCCGCATA
AGCATTAAATCAAATAAGATGAGCAAGATGGAACGTCTGGGTTCCAATTCTGTCAAGACTTATGACAT
ATTAAATAGATTGGTTACGGTAAAAGACAAGGCAAGGTATGGACCCTTAGCAAAGCCAGAAAGATCAC
CTTGCCCAAAGAAGA
+
FFFFFF:FFFFFFFF:FFFFFFFFFFFFFFFFFFFFFFFFFFFFFFFFFFFFFFFFFFFFFFF,F:FF
FFFFFFFFFFFFFFFFFFFFFFFFFFFFFFFFFFFFFFFFFFFFFFFFFFFFFFFFFFFFFFFFFFFF
FFFFFFFFFFFFFFF
@A00155:342:HHGFNDSXY:1:2149:3387:7326 1:N:0:GAACCTAG+TCCGCATA
AGCATTAAATCAAATAAGATGAGCAAGATGGAACGTCTGGGTTCCAATTCTGTCAAGACTTATGACAT
```

ATTAAATAGATTGGTTACGGTAAAAGACAAGGCAAGGTATGGACCCTTAGCAAAGCCAGAAAGATCAC
CTTGCCCAAAGAAGA
+
FFFFFFFFFFFFFFFFFFFFFFFFFFFFFFFFFFFFFFFFFFF,FFFFFFFFF:FFFFFFFFFFFFF
FFFFFFFFFFFFFFFFFFFFFFFFFFFFFFFFFFFFFFFFFFFF:FFFFFFFF:FFFFFFFFFFFFF
FFFFFFFFFFFFFFF
@A00155:342:HHGFNDSXY:1:2173:23999:31751 1:N:0:GAACCTAG+TCCGCATA
AGCATTAAATCAAATAAGATGAGCAAGATGGAACGTCTGGGTTCCAATTCTGTCAAGACTTATGACAT
ATTAAATAGATTGGTTACGGTAAAAGACAAGGCAAGGTATGGACCCTTAGCAAAGCCAGAAAGATCAC
CTTGCCCAAAGAAGA
+
FFFFFFFFFFFFFFFFFFFFFFFFFFFFFFFFFFFFFFFFFFFFFFFFFFFFFFFFFFFFFFFFFFFF
FFFFFFFFFFFFFFFFFFFFFFFFFFFFFFFFFFFFFF:FFFFFFFFFFFFFFFFFFFFFFFFFFFFF
FFFFFFFFFFFFFFF
@A00155:342:HHGFNDSXY:1:2173:23502:32643 1:N:0:GAACCTAG+TCCGCATA
AGCATTAAATCAAATTAGATGAGCAAGATGGAACGTCTGGGTTCCAATTCTGTCTAGACTTATGACAT
ATTAAATAGATTGGTTACGGTAAAAGACAAGGCAAGGTATGGACCCTTAGCAAAGCCAGAAAGATCAC
CTTGCCCAAAGAAGA
+
F::FFF:F:FFFFFF,FFFFFF,F,:,,F:FFFF,,,F:FFF:FFF:FF,F:F,,FF:FFFFF,:FF:
FFFF::FFFFF:::FFFF,FFFFFFFFF,F:FFFF::FF,,FFF,:FFF::F:,:,F:FFF,F:F::,
:FF,:FFF::,:,FF
@A00155:342:HHGFNDSXY:1:1425:9860:3756 2:N:0:GAACCTAG+TCCGCATA
AGCATTAAATCAAATAAGATGAGCAAGATGGAACGTCTGGGTTCCAATTCTGTCAAGACTTATGACAT
ATTAAATAGATTGGTTACGGTAAAAGACAAGGCAAGGTATGGACCCTTAGCAAAGCCAGAAAGATCAC
CTTGCCCAAAGAAGA
+
FFFFFFFFFFFFFFFFFFFFFFFFFFFFFFFFFFF:FFFFFFFFFFFFFFFFFF:FF:FFFFFFFFFF
FFFFFFFFFFFFFFFFFFFFFFFF,:FFFFFFFFF:FFFFFFFFFFFFFFFF:FFFFFFFFFFFFFFFF
FFF:,FFFF:FFFFF
@A00155:342:HHGFNDSXY:1:1177:6867:7247 1:N:0:GAACCTAG+TCCGCATA
GCATTAAATCAAATAAGATGAGCAAGATGGAACGTCTGGGTTCCAATTCTGTCAAGACTTATGACATA
TTAAATAGATTGGTTACGGTAAAAGACAAGGCAAGGTATGGACCCTTAGCAAAGCCAGAAAGATCACC
TTGCC
+
:::FFF:FF,,,FF:,FFF:FFFF,F:FFFFFFFFFFFFFFFFFFFFFFFFFFFFF:FFFFFFFFFFF
FFFFFFFFFF,F,FFFFFFFFFFFF:FFF:,FFFF::FFFFFF:FFFFFFF:FFFF:FFFFFFFF:FF
FF:FF
@A00155:342:HHGFNDSXY:1:1223:20898:4586 1:N:0:GAACCTAG+TCCGCATA
GCATTAAATCAAATAAGATGAGCAAGATGGAACGTCTGGGTTCCAATTCTGTCAAGACTTATGACATA
TTAAATAGATTGGTTACGGTAAAAGACAAGGCAAGGTATGGACCCTTAGCAAAGCCAGAAAGATCACC
TTGCC
+
::FFFF:FFFFFFFFFFFFFFFFFFFFFFFFFFFFFFFFFF:FF:FFFFF:FFFFFFFFFFFFFFFFFF
FFFFFFFFFFFFFFFFFFFFFFFFFFFFFFFFFFFFFFFFFFFFFFFFFFFFFFFFFFFFFFFFFFFF
FFFFF
@A00155:342:HHGFNDSXY:1:1216:32425:25770 1:N:0:GAACCTAG+TCCGCATA
GCATTAAATCAAATAAGATGAGCAAGATGGAACGTCTGGGTTCCAATTCTGTCAAGACTTATGACATA
TTAAATAGATTGGTTACGGTAAAAGACAAGGCAAGGTATGGACCCTTAGCAAAGCCAGAAAGATCACC
TTGCC
+
F:FFFFFFFFFFFFFFFFFFFFFFFFFFFFFFFFFFFFFFFFFFFF:FFFFFFFFFFFFFFFFFFFFF
FFFFFFFFFFFFFFFFFFFFFFFF,FFFFFFFFFFFFFFFFFFFFFFFFFFFFFFFFFFFFF:FFFFFFF
FFFFF

```
@A00155:342:HHGFNDSXY:1:1225:28049:16125 1:N:0:GAACCTAG+TCCGCATA
GCATTAAATCAAATAAGATGAGCAAGATGGAACGTCTGGGTTCCAATTCTGTCAAGACTTATGACATA
TTAAATAGATTGGTTACGGTAAAAGACAAGGCAAGGTATGGACCCTTAGCAAAGCCAGAAAGATCACC
TTGCC
+
::FF:FFFF:F,FF:F:FF,FFFF:FFFFF,FF:FFFFFFFFFF,FFFFFFF,FFFFFFF:FF::FFF
FFFFFFFFFFFFFFFF:,:FFF::FFFFFFF:FF,,FFFFF,FF:FFFF,F,:F,FFFFF::FFFFFF
FFFFF
@A00155:342:HHGFNDSXY:1:1261:3875:17409 1:N:0:GAACCTAG+TCCGCATA
GCATTAAATCAAATAAGATGAGCAAGATGGAACGTCTGGGTTCCAATTCTGTCAAGACTTATGACATA
TTAAATAGATTGGTTACGGTAAAAGACAAGGAAAGGTATGGACCCTTAGCAAAGCCAGAAAGATCACC
TTGCC
+
:FFFFFFFFF:FFFFFFFFFFFFFFFFFFFFFFFFFFFFFFFFF:FF:FFF:FFFFFFFFFFFF,FFF
FFFFF:FFFFFFFFFFFFFFFFFFFFFFFFF,FFFFFFFF:FF:FFFF,FFFFF,FFFFFFFFFFFFF
FFFFF
@A00155:342:HHGFNDSXY:1:1325:29261:3662 1:N:0:GAACCTAG+TCCGCATA
GCATTAAATCAAATAAGATGAGCAAGATGGAACGTCTGGGTTCCAATTCTGTCAAGACTTATGACATA
TTAAATAGATTGGTTACGGTAAAAGACAAGGCAAGGTATGGACCCTTAGCAAAGCCAGAAAGATCACC
TTGCC
+
FFFFFFFFFFFFFFFFFFF,FFFFFFFFFFFF,FFFFFFFFFFFFFFFFFFF,FFFFFFFFFFF:FFF
FFF:FFFFFFFFFFFF,,FFFF:FFFFFFFFFFFFFFFFFFFFF,FFFFFFFFFFFFFFFFFFFFFFF
FFFFF
@A00155:342:HHGFNDSXY:1:1307:2257:30765 1:N:0:GAACCTAG+TCCGCATA
GCATTAAATCAAATAAGATGAGCAAGATGGAACGTCTGGGTTCCAATTCTGTCAAGACTTATGACATA
TTAAATAGATTGGTTACGGTAAAAGACAAGGCAAGGTATGGACCCTTAGCAAAGCCAGAAAGATCACC
TTGCC
+
:FFFF:FFFFFFFFF:FFFFFFFFFFFFFFFFFFFFFFFFFFFFFFFFFFFFFFFFFFFFFFFFFFFF
FFFFFFFFFFFFFFFFFFFFFFFFFF:F,:FFFFFFFFFFFFFFFFFFFFFFFFFFFFFFFFFFFFFF
FFFFF
@A00155:342:HHGFNDSXY:1:1310:12472:17895 1:N:0:GAACCTAG+TCCGCATA
GCATTAAATCAAATAAGATGAGCAAGATGGAACGTCTGGGTTCCAATTCTGTCAAGACTTATGACATA
TTAAATAGATTGGTTACGGTAAAAGACAAGGCAAGGTATGGACCCTTAGCAAAGCCAGAAAGATCACC
TTGCC
+
FFFFFFFFFFFFFFFFFFFFFFFFFFFFFFFFFFFFFFFFFFFFFFFFFFFFFFFFFFFFFFFFFFFF
FFFFFFFFFFFFFFFFFFFFFFFFFFFF:FFFFFFFFFFFFFFFFFFFFFFFFFFFFFFFFFFFFFFFF
FFFFF
@A00155:342:HHGFNDSXY:1:1319:24270:8140 1:N:0:GAACCTAG+TCCGCATA
GCATTAAATAAAATAAGATGAGCAAGATGGAACGTCTGGGTTCCAATTCTGTCAAGACTTATGACATA
TTAAATAGATTGGTTACGGTAAAAGACAAGGCAAGGTATGGACCCTTAGCAAAGCCAGAAAGATCACC
TTGCC
+
FFFFFFFFF,FFFFFFFFFFFFFFFFFFFFFFFFFFFFFFFFFFFFFFFFFFFFFFFFFFFFFFFFFF
FFFFFFFFFFFFFFFFFFFFFFFFFFFFFFFFFFFFFFFFFFFFFFFFFFFFFFFFFFFFFFFFFFFF
FFFFF
@A00155:342:HHGFNDSXY:1:1334:1642:8688 1:N:0:GAACCTAG+TCCGCATA
GCATTAAATCAAATAAGATGAGCAAGATGGAACGTCTGGGTTCCAATTCTGTCAAGACTTATGACATA
TTAAATAGATTGGTTACGGTAAAAGACAAGGCAAGGTATGGACCCTTAGCAAAGCCAGAAAGATCACC
TTGCC
+
F:FFFFFFF,FFF:FF,FFFFFFFFFFFFFFFFFFFFFFFFFFFFFF:FFFFFFFFFFFFFFFFFFFF
```

FFFFFFFFFFFFFF:FFFFFFFFFFFFFFFFFFFFFFFFFFFFFFFFFFFFFFFFFFFFFFFFFFFFF
FFFFF
@A00155:342:HHGFNDSXY:1:1404:6316:28933 1:N:0:GAACCTAG+TCCGCATA
GCATTAAATCAAATAAGATGAGCAAGATGGAACGTCTGGGTTCCAATTCTGTCAAGACTTATGACATA
TTAAATAGATTGGTTACGGTAAAAGACAAGGCAAGGTATGGACCCTTAGCAAAGCCAGAAAGATCACC
TTGCC
+
FFFFFFFFFFFFFFFFFFFFFFFFFFFFFFFFFFFFF:,FFFFFFFFF:F:FFFFFFFFFFFFFFFFF
FFFFFFFFFFFFFFF:FFFFFFFFF:FFFFFFFFFFFFFFFFFFFFFFFFFFFFFFFFFFFFFF,FFF
FFFFF
@A00155:342:HHGFNDSXY:1:1429:15619:19648 1:N:0:GAACCTAG+TCCGCATA
GCATTAAATCAAATAAGATGAGCAAGATGGAACGTCTGGGTTCCAATTCTGTCAAGACTTATGACATA
TTAAATAGATTGGTTACGGTAAAAGACAAGGCAAGGTATGGACCCTTAGCAAAGCCAGAAAGATCACC
TTGCC
+
FFFFFFFFFFFFFFFFFFFFFFFFFFFFFFFFFFFFF:FFFFFFFFFFFFFFFFFFFFFFFFFFFFFF
FFFFFFFFFFFFFFFFFFFFFFFFFFFFFFFFFFFFFFFFFFFFFFFFFFFFFFFFFFFFFFFFFFFF
FFFFF
@A00155:342:HHGFNDSXY:1:1431:14498:4742 1:N:0:GAACCTAG+TCCGCATA
GCATTAAATCAAATAAGATGAGCAAGATGGAACGTCTGGGTTCCAATTCTGTCAAGACTTATGACATA
TTAAATAGATTGGTTACGGTAAAAGACAAGGCAAGGTATGGACCCTTAGCAAAGCCAGAAAGATCACC
TTGCC
+
FFFFFFFFFFFFFFFFFFFFFFFFFFFFFFFFFFFFFFFFFFFF:FFFFFFFFFFFFFFFFFFFFFFF
FFFFFFFFFFFFFFFFFFFFFFFFFFFFFFFFFFFFFFFFFFFFFFFFFFFFFFFFFFFFFFFFFFFF
FFFFF
@A00155:342:HHGFNDSXY:1:1431:7717:7717 1:N:0:GAACCTAG+TCCGCATA
GCATTAAATCAAATAAGATGAGCAAGATGGAACGTCTGGGTTCCAATTCTGTCAAGACTTATGACATA
TTAAATAGATTGGTTACGGTAAAAGACAAGGCAAGGTATGGACCCTTAGCAAAGCCAGAAAGATCACC
TTGCC
+
F:FFFFFFFFFFFFFFFFFFFFFFFFFFFFFFFFFFFFFFFFFFFF:FFFFFFFFFFFFFFFFFFFFF
FFFFFFFFFFFFFFFFFFFFFFFFFFFFFFFFFFFFFFFFFFFFFFFFFFFFFFFFFFFFFFFFFFFF
FFFFF
@A00155:342:HHGFNDSXY:1:1431:15953:7764 1:N:0:GAACCTAG+TCCGCATA
GCATTAAATCAAATAAGATGAGCAAGATGGAACGTCTGGGTTCCAATTCTGTCAAGACTTATGACATA
TTAAATAGATTGGTTACGGTAAAAGACAAGGCAAGGTATGGACCCTTAGCAAAGCCAGAAAGATCACC
TTGCC
+
:FFFFFFFFFFFFFFFFFFFFFFFFFFFFFFFFFFFFFFF:FFFFF:FFFFFFFFFFFFFFFFFFFFF
FFFFFFFFFFFFFFFFFFFFFFFFFFFFFFFFFFFFFFFFFFFFFFFFFFFFFFFFFFFFFFFFFFFF
FFFFF
@A00155:342:HHGFNDSXY:1:1473:12933:28056 1:N:0:GAACCTAG+TCCGCATA
GCATTAAATCAAATAAGATGAGCAAGATGGAACGTCTGGGTTCCAATTCTGTCAAGACTTATGACATA
TTAAATAGATTGGTTACGGTAAAAGACAAGGCAAGGTATGGACCCTTAGCAAAGCCAGAAAGATCACC
TTGCC
+
FF,FF:FFF:FF:F:FFF:FFF,:FFFFF:F,FFF,,:,FFFFF,FFFFFFFFFFFFFFFFFFFF:F:
FFFFFF:FFFFFFFF::F:::FFFFFFFFFFF:F,FFFFFFF,FFFFFFF,FFFFFFF::,FFFFFFF
FFFFF
@A00155:342:HHGFNDSXY:1:1527:18954:25989 1:N:0:GAACCTAG+TCCGCATA
GCATTAAATCAAATAAGATGAGCAAGATGGAACGTCTGGGTTCCAATTCTGTCAAGACTTATGACATA
TTAAATAGATTGGTTACGGTAAAAGACAAGGCAAGGTATGGACCCTTAGCAAAGCCAGAAAGATCACC
TTGCC

```
+
::FFFFFFFFFFFFFFFFFFFFFFFFFFFFFFFFFFFFFFFFFFFFFFFFFFFFFFFFFFFFFFFFFFFF
FFFFFFFFFFFFFFFFFFFFFFFFFFFFFFFFFFFFFFFFFFFFFFFFFFFFFFFFFFFFFFFFFFFFFF
FFFFF
@A00155:342:HHGFNDSXY:1:1527:19741:27696 1:N:0:GAACCTAG+TCCGCATA
GCATTAAATCAAATAAGATGAGCAAGATGGAACGTCTGGGTTCCAATTCTGTCAAGACTTATGACATA
TTAAATAGATTGGTTACGGTAAAAGACAAGGCAAGGTATGGACCCTTAGCAAAGCCAGAAAGATCACC
TTGCC
+
:FFFFFFFFFFFFFFF:FFFFFFFFFFFFFFFFFFFFFFFFFFFFFFFFFFFFFFFFFFFFFFFFFFFFF
FFFFFFFFFFFFFFFFFFFFFFFFFFFFFFFFFFFFFFFFFFFFFFFFFFFFFFFFFFFFFFFFFFFFFF
FFFFF
@A00155:342:HHGFNDSXY:1:1528:6804:23453 1:N:0:GAACCTAG+TCCGCATA
GCATTAAATCAAATAAGATGAGCAAGATGGAACGTCTGGGTTCCAATTCTGTCAAGACTTATGACATA
TTAAATAGATTGGTTACGGTAAAAGACAAGGCAAGGTATGGACCCTTAGCAAAGCCAGAAAGATCACC
TTGCC
+
FFFFFF,FFF:FF:,FFFFF,F:FFFFFFFFFF:FF:FFFFFFFF:FFFFFFFFFFFFFFFFFFFFFF
FFFFF,FFFFFFFFF,FFFF:FFFFF:FF:FFF,:FFFFFFFFF:FFFFF:FFFFFFFFFFFF:FF:FF
F:FFF
@A00155:342:HHGFNDSXY:1:1551:3712:9674 1:N:0:GAACCTAG+TCCGCATA
GCATTAAATCAAATAAGATGAGCAAGATGGAACGTCTGGGTTCCAATTCTGTCAAGACTTATGACATA
TTAAATAGATTGGTTACGGTAAAAGACAAGGCAAGGTATGGACCCTTAGCAAAGCCAGAAAGATCACC
TTGCC
+
FFFFFFFFFFFFFFFFFFFFFFFFFFFFFFFFFFFFFFFFFFFFFFFFFFFFFFFFFFFFFFFFFF:FFF
FFFFFFFFFFFFFFFFFFFFFFFFFFFFF:FFFFF:FFFFFFFF,FFFFFFFFFFFFFFFFFFFFFFFF
F:FFF
@A00155:342:HHGFNDSXY:1:1540:1244:20838 1:N:0:GAACCTAG+TCCGCATA
GCATTAAATCAAATAAGATGAGCAAGATGGAACGTCTGGGTTCCAATTCTGTCAAGACTTATGACATA
TTAAATAGATTGGTTACGGTAAAAGACAAGGCAAGGTATGGACCCTTAGCAAAGCCAGAAAGATCACC
TTGCC
+
FFFFFFFFFFFF,FFFFFFFFFF:FFFFFFFFFFFF:FFF:FFFFFFFFFFFFFFFFFFFF:FFFFFF
FFFFFFFFFFFFF,FFFFFFF:FFFFF:,FFFFFFFFFFFFFFFFF:FFFFFFFFFFFFF:FFFFFFF
,FFFF
@A00155:342:HHGFNDSXY:1:1577:9362:17863 1:N:0:GAACCTAG+TCCGCATA
GCATTAAATCAAATAAGATGAGCAAGATGGAACGTCTGGGTTCCAATTCTGTCAAGACTTATGACATA
TTAAATAGATTGGTTACGGTAAAAGACAAGGCAAGGTATGGACCCTTAGCAAAGCCAGAAAGATCACC
TTGCC
+
:F:FFFFFF:FFFFFFFFFFFFFFFF:FFF:FFF:FFFFFFFFF:FF:FFFF:FFFFFFFFFFF:,FF
FFFFFFFF,FFFFFFFFFFFFFFFFFF:FFF:FFFFFFFFFFFFFFFFFFFFFFFFFFFFFFFFFF:FF
FFFFF
@A00155:342:HHGFNDSXY:1:1604:2076:8844 1:N:0:GAACCTAG+TCCGCATA
GCATTAAATCAAATAAGATGAGCAAGATGGAACGTCTGGGTTCCAATTCTGTCAAGACTTATGACATA
TTAAATAGATTGGTTACGGTAAAAGACAAGGCAAGGTATGGACCCTTAGCAAAGCCAGAAAGATCACC
TTGCC
+
:FFFFFFFFFFFFFFFFFFFFFFFFFFFF,FFFFFFFFFFFFFFFFFFFFFFFFFFFFFFFFFFFFFFFF
FFFFFFFFFFFFFFFFFFFFFFFFFF,FFFFFFFFFFFFFFFFFFFFFFFFFFFFF:FFFFFFFFFFFFF
FFFFF
@A00155:342:HHGFNDSXY:1:1604:2130:8844 1:N:0:GAACCTAG+TCCGCATA
GCATTAAATCAAATAAGATGATCAAGATGGAACGTCTGGGTTCCAATTCTGTCAAGACTTATGAAATA
```

```
TTAAATAGATTGGTTACGGTAAAAGACAAGGAAAGGTATGGACCCTTAGCAAAGCCAGAAAGATCACC
TTGCC
+
FFFFFFFFF,FFF:FFFFFFF,:FFFFFFFFF,FFFFFFFFF:,FFF:FFFF:FFFFFFFFF::,FFF
FFFFFFF,FFF,:FFFFFFFFFFFFF:FF,F,FF,FFFFFFFFFFFFFFFFFFF:,FFFFFFFFFFFF
FFFFF
@A00155:342:HHGFNDSXY:1:1630:2410:25394 1:N:0:GAACCTAG+TCCGCATA
GCATTAAATCAAATAAGATGAGCAAGATGGAACGTCTGGGTTCCAATTCTGTCAAGACTTATGACATA
TTAAATAGATTGGTTACGGTAAAAGACAAGGCAAGGTATGGACCCTTAGCAAAGCCAGAAAGATCACC
TTGCC
+
::FFFFFFF:FFFFFF:FFFFFFFFFFFF,FFF:F:FF,FFFFFFFFFFFFFFFFFF,FFFFFF,FFF
FFFFFFFFFFFFFFFFF,,FFFFFFF,FFFFFFFF,FFFFFFF:FFFFFF:FFFFFFFFFFFFFFFFF
FFFFF
@A00155:342:HHGFNDSXY:1:1631:14434:18693 1:N:0:GAACCTAG+TCCGCATA
GCATTAAATCAAATAAGATGAGCAAGATGGAACGTCTGGGTTCCAATTCTGTCAAGACTTATGACATA
TTAAATAGATTGGTTACGGTAAAAGACAAGGCAAGGTATGGACCCTTAGCAAAGCCAGAAAGATCACC
TTGCC
+
FFFFFFFFFFFFFFFFFFFFFFFFFFFFFFFFFFFFFFFFFFFFFF,FFFFFFFFFFFFFFFFF:FFF
FFFFFFFFFF:FFFFFFFFFFFFFFFFFFFFFFFFFFFFFFFFFFF:FFFFFFFFFFFFFFFFFFFFF
:FFFF
@A00155:342:HHGFNDSXY:1:1657:22896:17848 1:N:0:GAACCTAG+TCCGCATA
GCATTAAATCAAATAAGATGAGCAAGATGGAACGTCTGGGTTCCAATTCTGTCAAGACTTATGACATA
TTAAATAGATTGGTTACGGTAAAAGACAAGGCAAGGTATGGACCCTTAGCAAAGCCAGAAAGATCACC
TTGCC
+
FFFFFFFFFFFFFFFFFFFFFFFFFFFFFFFFFFFFFFFFFFFFFFFFFFFFFFFFFFFFFFFFFFFF
FFFFFFFFFFFFFFFFFFFFFFFFFFFFFFFFFFFFFFFFFFFFFFFFFFFFFFFFFFFFFFFFFFFF
FFFFF
@A00155:342:HHGFNDSXY:1:1660:19904:6057 1:N:0:GAACCTAG+TCCGCATA
GCATTAAATCAAATAAGATGAGCAAGATGGAACGTCTGGGTTCCAATTCTGTCAAGACTTATGACATA
TTAAATAGATTGGTTACGGTAAAAGACAAGGCAAGGTATGGACCCTTAGCAAAGCCAGAAAGATCACC
TTGCC
+
FFFFFFFFFFFFFFFFFFFFFFFFFFFFFFFFFFFFFFFFFFFFFFFFFFFFFFFFFFFFF:FFFFFF
FFFFFFFFFFFFFFFFFFFFFFFFFFFFFFFFFFFFFFFFFFFFFF:FFFFFFFFFFFF:FFFFFFFF
FFFFF
@A00155:342:HHGFNDSXY:1:1660:25328:32800 1:N:0:GAACCTAG+TCCGCATA
GCATTAAATCAAATAAGATGAGCAAGATGGAACGTCTGGGTTCCAATTCTGTCAAGACTTATGACATA
TTAAATAGATTGGTTACGGTAAAAGACAAGGCAAGGTATGGACCCTTAGCAAAGCCAGAAAGATCACC
TTGCC
+
FFFFF:FFFFFFFFF,FFFFFFFFFFFFFFFFF:FFF:FFFFFFFF:FFFFFFFFF:FFFFFFFFFFF
F:FFFFFFFFF:FFFFFFFFFFFFFFFFFFFFFFFFFFFFFFFFFFFFFFFFFFFFFFFFFFFFFFFF
FFFFF
@A00155:342:HHGFNDSXY:1:2132:13322:11725 1:N:0:GAACCTAG+TCCGCATA
GCATTAAATCAAATAAGATGAGCAAGATGGAACGTCTGGGTTCCAATTCTGTCAAGACTTATGACATA
TTAAATAGATTGGTTACGGTAAAAGACAAGGCAAGGTATGGACCCTTAGCAAAGCCAGAAAGATCACC
TTGCC
+
FFFFFFFFFFFFFFFFFFFFFFFFFFFFFFFFFFFFFFFFFFFFFFFFFFFFFFFFFFFFFFFFFFFF
FFFFFFFFFFFFFFFFFFFFFFFFFFFFFFFFFFFFFFFFFFFFFFFFFFFFFFFFFFFFFFFFFFFF
FFFFF
```

@A00155:342:HHGFNDSXY:1:2201:19334:8985 1:N:0:GAACCTAG+TCCGCATA
CTATTAAATCAAATAAGATGAGCAAGATGGAACGTCTGGGTTCCAATTCTGTCAAGACTTATGACATA
TTAAATAGATTGGTTACGGTAAAAGACAAGGCAAGGTATGGACCCTTAGCAAAGCCAGAAAGATCACC
TTGCC
+
FFFFFFFFFFFFFFFFFFFFFFFFFFFFFF:FFFFFFFFF:FFFFF:F,F:FFFFFFFFFFFF:FFFFFF
FFFFFFFFF:FFFFFFFFFFFFFFFFFFFFFFFFFFFFFFFFFFFFFFFFFFFFFF:FFFFFFFFFF:FF
F:FFF
@A00155:342:HHGFNDSXY:1:2165:5159:12179 1:N:0:GAACCTAG+TCCGCATA
GCATTAAATCAAATAAGATGAGCAAGATGGAACGTCTGGGTTCCAATTCTGTCAAGACTTATGACATA
TTAAATAGATTGGTTACGGTAAAAGACAAGGCAAGGTATGGACCCTTAGCAAAGCCAGAAAGATCACC
TTGCC
+
:FFFFFFFFFFFFFFFFFFFFFFFFFFFFFFFFFFFFF:FFFFFFFFFFF:FFFFF:FFFFFFFFFFFFF
FFFFFFFFFFFFFFFFFFFFFFFFFFFFFFFFFFFFFFFFFF:FFFFFFFFFFFFF:FFFFFFFFFFFFF
FFFF:
@A00155:342:HHGFNDSXY:1:2161:5683:23985 1:N:0:GAACCTAG+TCCGCATA
GCATTAAATCAAATAAGATGAGCAAGATGGAACGTCTGGGTTCCAATTCTGTCAAGACTTATGACATA
TTAAATAGATTGGTTACGGTAAAAGACAAGGCAAGGTATGGACCCTTAGCAAAGCCAGAAAGATCACC
TTGCC
+
F:F:FFFFF:FFFFFFFFFFFF:FFFFF:FFFFFFFFFFFFFFFFFFFFFFFFFFFFFF:FFFFFFFFFF
FFFFFFFFFFFFFFFFF:FFFFFF:FFFFF:FFFFFFFFFFFFFFFFFFFFFFFFFFFFFFFFFFFFFFF
FFFFF
@A00155:342:HHGFNDSXY:1:2177:4155:33238 1:N:0:GAACCTAG+TCCGCATA
GCATTAAATCAAATAAGATGAGCAAGATGGAACGTCTGGGTTCCAATTCTGTCAAGACTTATGACATA
TTAAATAGATTGGTTACGGTAAAAGACAAGGCAAGGTATGGACCCTTAGCAAAGCCAGAAAGATCACC
TTGCC
+
FFFFFFFFFFFFFFFFFFFFFFFFFFFFFFFFFFFFFFFFFFFFFFF,FFFFFFFFFFFFFFFFFFFFFF
FFFFFFFFFFFFFFFFFFFFFFFFFFFFFFFFFFFFFFFFFFFFFFFFFFFFFFFFFFFFFFFFFFFFFF
,FFFF
@A00155:342:HHGFNDSXY:1:2177:3360:36933 1:N:0:GAACCTAG+TCCGCATA
GCATTAAATCAAATAAGATGAGCAAGATGGAACGTCTGGGTTCCAATTCTGTCAAGACTTATGACATA
TTAAATAGATTGGTTACGGTAAAAGACAAGGCAAGGTATGGACCCTTAGCAAAGCCAGAAAGATCACC
TTGCC
+
F:FFFFFFFFFFFFFFFFFFFFFFFFFFFFFF:FFFFFFFFFFFFFF:FFFFFFFFFFFFFFFFFFFFFF
FFFFFFFFFFFFFFFFFFFFFFFFFFFFFFFFFFFFFFFFFFFFFFFFFFFFFFFFFFFFFFFFFFFFFF
FFFFF
@A00155:342:HHGFNDSXY:1:2238:14389:12759 1:N:0:GAACCTAG+TCCGCATA
CCGATCTATCAAATAAGATGAGCAAGATGGAACGTCTGGGTTCCAATTCTGTCAAGACTTATGACATA
TTAAATAGATTGGTTACGGTAAAAGACAAGGCAAGGTATGGACCCTTAGCAAAGCCAGAAAGATCACC
TTGCC
+
:,FFF:FFFFFFFFFF::FFFFFF:F:F:FFFFFFFFF:FF:FFF:FFFFFF:FFFFFFFFFF:FFFFFF
FFFFFFFFFFFFFFFFFF:FFFFFFFFFFFFFFF::FFF:FFF:FFFFFFFFFFFFFFFFFFFFFFFFFF
FFFFF
@A00155:342:HHGFNDSXY:1:2264:3459:14779 1:N:0:GAACCTAG+TCCGCATA
GCATTAAATCAAATAAGATGAGCAAGATGGAACGTCTGGGTTCCAATTCTGTCAAGACTTATGACATA
TTAAATAGATTGGTTACGGTAAAAGACAAGGCAAGGTATGGACCCTTAGCAAAGCCAGAAAGATCACC
TTGCC
+
FFFFFFFF:FFFFFFFFFFFFFFFFFFFFFFFFFFF:FF:FFFFFFFFFFFFFFFFFFFFFFFFFFFFFF

FFFFFFFF:FFFFFFFFFFFFFFFFFFFFFFFFFFFFFFFFFFFFFFFFFFFFFFFFFFF
FFFFF
@A00155:342:HHGFNDSXY:1:2231:18511:12414 1:N:0:GAACCTAG+TCCGCATA
GCATTAAATCAAATAAGATGAGCAAGATGGAACGTCTGGGTTCCAATTCTGTCAAGACTTATGACATA
TTAAATAGATTGGTTACGGTAAAAGACAAGGCAAGGTATGGACCCTTAGCAAAGCCAGAAAGATCACC
TTGCC
+
FFFFFFFFFFFFFFFFF:FFFFFFFFFF:FFFFFFFFFFF:FFFFF:FFFFFFFFFFFFFFFFFFFFF
FFFFFFFFFFFFFFFFFFFF:FFFFFFF:FFFFFFFFFFFFFFFFFFFFFFFFFFFFFFFFFFFFFFF
FFFFF
@A00155:342:HHGFNDSXY:1:2231:19560:13573 1:N:0:GAACCTAG+TCCGCATA
GCATTAAATCAAATAAGATGAGCAAGATGGAACGTCTGGGTTCCAATTCTGTCAAGACTTATGACATA
TTAAATAGATTGGTTACGGTAAAAGACAAGGCAAGGTATGGACCCTTAGCAAAGCCAGAAAGATCACC
TTGCC
+
FFF:FF:FF:FFFFFFFFFFFFFFFFFF:FFFFFFFFFFFFFFFFFFFFFFFFFFFFFFFFFFFFFFF
FFFFFFFFFFFFFFFFFFFFFFFFFFFFFFFFF:FFFFFFFFFFFFFFFFFFFFFFFFFFFFFFFFFF
FFFFF
@A00155:342:HHGFNDSXY:1:2266:4752:17550 1:N:0:GAACCTAG+TCCGCATA
GCATTAAATCAAATAAGATGAGCAAGTTGGAACGTCTGGGTTCCAATTCTGTCAAGACTTATGACATA
TTAAATAGATTGGTTACGGTAAAAGACAAGGCAAGGTATGGACCCTTAGCAAAGCCAGAAAGATCACC
TTGCC
+
FFFFFFF:FFFFFFFFFFFFFFFF:F,FFFFFFFFFFFFFFFFFFF:FFFFFF,FFF:FFFFFFFFFF
FFFFFFFFFFFFFFF:FFFFFFFFFFFFFFFFFFFFFFFFF:FFF::FFFFFFFFFF:FFFFFFFFFF
FFFFF
@A00155:342:HHGFNDSXY:1:2266:4155:18145 1:N:0:GAACCTAG+TCCGCATA
GCATTAAATCAAATAAGATGAGCAAGATGGAACGTCTGGGTTCCAATTCTGTCAAGACTTATGACATA
TTAAATAGATTGGTTACGGTAAAAGACAAGGCAAGGTATGGACCCTTAGCAAAGCCAGAAAGATCACC
TTGCC
+
::F:FFFFF,FFFFFF,F:FFF,FFFFFFFFFFFFFFFFFFFFFFFFFFFFFFFFFF:FFFFFFFFFF
FFFFFFFFFFFFFFFFFFFFFFFFFFFFFFFF:FFFFFFFFFFFFFFFFFFFFFFFFFFFFFFFFFFF
:FFFF
@A00155:342:HHGFNDSXY:1:2303:32678:1313 1:N:0:GAACCTAG+TCCGCATA
CTATTAAATCAAATAAGATGAGCAAGATGGAACGTCTGGGTTCCAATTCTGTCAAGACTTATGACATA
TTAAATAGATTGGTTACGGTAAAAGACAAGGCAAGGTATGGACCCTTAGCAAAGCCAGAAAGATCACC
TTGCC
+
:FFFFF:FFFFFFFFFFFFFFFFFFFFFFFFFFFFFFFFFFFFFFF:FF:FFFFFFFFFFFFFFFFFF
FFFFFFFFFFFFFFFFFFFFFFFFFFFFFFFFFFFFFFFFFFFFFFFFFFFFFFFFFFFFFFFFFFFF
FFFFF
@A00155:342:HHGFNDSXY:1:2303:30689:13307 1:N:0:GAACCTAG+TCCGCATA
CTATTAAATCAAATAAGATGAGCAAGATGGAACGTCTGGGTTCCAATTCTGTCAAGACTTATGACATA
TTAAATAGATTGGTTACGGTAAAAGACAAGGCAAGGTATGGACCCTTAGCAAAGCCAGAAAGATCACC
TTGCC
+
FFFFFFFFFFFFFFFFFFFFFFFFFFFFFFFFFFFFFFFFFFFFFFFFFFFFFFFFFFFFFFFFFFFF
FFFFFFFFFFFFFFFFFFFFFFFFFFFFFFFFFFFFFFFFFFFFFFFFFFFFFFFFFFFFFFFFFFFF
FFFFF
@A00155:342:HHGFNDSXY:1:2271:3007:26428 1:N:0:GAACCTAG+TCCGCATA
GCATTAAATCAAATAAGATGAGCAAGATGGAACGTCTGGGTTCCAATTCTGTCAAGACTTATGACATA
TTAAATAGATTGGTTACGGTAAAAGACAAGGCAAGGTATGGACCCTTAGCAAAGCCAGAAAGATCACC
TTGCC

```
+
F,FFFFFFFFFFFFFFFFFFFFFFFFFFFFFFFFFFFFFFFFFFFFFFF:FFFFFFFFFFF:FFFFFFFF
FFFFFFFFFFFFFFFFFFFFFFFFFFFFFFFFFFFFFFFFFFFFFFFFFFFFFFFFFFFFFFFFFFFFFF
F:FFF
@A00155:342:HHGFNDSXY:1:2339:19180:14669 1:N:0:GAACCTAG+TCCGCATA
GATCTAAATCAAATAAGATGAGCAAGATGGAACGTCTGGGTTCCAATTCTGTCAAGACTTATGACATA
TTAAATAGATTGGTTACGGTAAAAGACAAGGCAAGGTATGGACCCTTAGCAAAGCCAGAAAGATCACC
TTGCC
+
FFFFFFFFFFFFFFFFFFFFFFFFFFFFFFFFFFFFFFFFFFFFFFFFFFFFFFFFFFFFFFFFFFFFFF
FFFFFFFFFFFFFFFFFFFFFFFFFFFFFFFFFFFFFFFFFFFFFFFFFFFFFFFFFFFFFFFFFFFFFF
FFFFF
@A00155:342:HHGFNDSXY:1:2329:24912:25003 1:N:0:GAACCTAG+TCCGCATA
CGATCTAATCAAATAAGATGAGCAAGATGGAACGTCTGGGTTCCAATTCTGTCAAGACTTATGACATA
TTAAATAGATTGGTTACGGTAAAAGACAAGGCAAGGTATGGACCCTTAGCAAAGCCAGAAAGATCACC
TTGCC
+
FFFFFFFFFFFFFFFFFFFFFFFFFFFF:FFFFFFFFFFFFFFFFFFFFF:F:FFFFFFFFFFFFFFF
FFFFFFFFFFFFF:FFFFFFFFFFFFFFFFFFF:FFFFFFFFFFFFFFFFFFFFFFFFFFFFFFFFFFFF
FF:FF
@A00155:342:HHGFNDSXY:1:2368:6144:22529 1:N:0:GAACCTAG+TCCGCATA
GCATTAAATCAAATAAGATGAGCAAGATGGAACGTCTGGGTTCCAATTCTGTCAAGACTTATGACATA
TTAAATAGATTGGTTACGGTAAAAGACAAGGCAAGGTATGGACCCTTAGCAAAGCCAGAAAGATCACC
TTGCC
+
FF,FFFFFF:::F:FF:FFFFFFFFF,F:FFFFFFFF:FFFFFF:FFFFFFFFFFF,FFFFFFFF,FF
FFF:FF:FFFFFFFFFFFFFF,FFFFF:FFF:FFFFFFFFF,FFFFFFFFFFFFFFFF:F:FFFFFFF
FFF:F
@A00155:342:HHGFNDSXY:1:2429:10628:23844 1:N:0:GAACCTAG+TCCGCATA
GCATTAAATCAAATAAGATGAGCAAGATGGAACGTCTGGGTTCCAATTCTGTCAAGACTTATGACATA
TTAAATAGATTGGTTACGGTAAAAGACAAGGCAAGGTATGGACCCTTAGCAAAGCCAGAAAGATCACC
TTGCC
+
FFFFFFFFF:FFFFFFFFFFFFFFFFFFFFFFFFFFFFFFFFFFFFFFFFFFFFFFFFFFFFFFFFFF
FFFFFFFFFFFFFFFFFFFFFFFFFFFFFFFFFFFFFFFFFFFFFFFFFFFFFFFFFFFFFFFFFFFFFF
FFFFF
@A00155:342:HHGFNDSXY:1:2413:12138:21856 1:N:0:GAACCTAG+TCCGCATA
GCATTAAATCAAATAAGATGAGCAAGATGGAACGTCTGGGTTCCAATTCTGTCAAGACTTATGACATA
TTAAATAGATTGGTTACGGTAAAAGACAAGGCAAGGTATGGACCCTTAGCAAAGCCAGAAAGATCACC
TTGCC
+
:FF:FFFFFFFFFFFFFFFFFF:FFFFFFFFFFFFFF,FFFFFFFFF,FFFFFFFFFFFFFFFFFFFF
FFFFFFFFFFFFFFFFFFFFFFFFFFFFF:FFFFFFFFFFFFFFFFFFFFFFFFFFFFFFFFFFFFFFFF
FFFFF
@A00155:342:HHGFNDSXY:1:2436:10664:20650 1:N:0:GAACCTAG+TCCGCATA
GCATTAAATCAAATAAGATGAGCAAGATGGAACGTCTGGGTTCCAATTCTGTCAAGACTTATGACATA
TTAAATAGATTGGTTACGGTAAAAGACAAGGCAAGGTATGGACCCTTAGCAAAGCCAGAAAGATCACC
TTGCC
+
FFFFFFFFFFFFFFFFFFFFFFFFFFFFFFFFFFFFFFFF:FFFFFFFFFFFFFFFFFFFFFFFFFFFFF
FFFFFFFFFFFFFFFFFFFFFFFFFFFFFFFFFFFFFFFFFFFFFFFFFFFFFFFFFFFFFFFFFFFFFF
FFFFF
@A00155:342:HHGFNDSXY:1:2532:1551:15139 1:N:0:GAACCTAG+TCCGCATA
TAATTAAATCAAATAAGATGAGCAAGATGGAACGTCTGGGTTCCAATTCTGTCAAGACTTATGACATA
```

```
TTAAATAGATTGGTTACGGTAAAAGACAAGGCAAGGTATGGACCCTTAGCAAAGCCAGAAAGATCACC
TTGCC
+
F,F,FFFFF,F:FF::FFFFFFFFF,:FFFFFFF:FFF:FFF:F:FFF:FFFFFF:FFFFFFFFF:FF
FF:FFFFFFFFFFF:FFFFFFFFFFFFFFFFFFFFFFFFFFFFFFFFFFFFFFFFFF:FFFFFFFFF
FFFFF
@A00155:342:HHGFNDSXY:1:2542:28745:24690 1:N:0:GAACCTAG+TCCGCATA
GCATTAAATCAAATAAGATGAGCAAGATGGAACGTCTGGGTTCCAATTCTGTCAAGACTTATGACATA
TTAAATAGATTGGTTACGGTAAAAGACAAGGCAAGGTATGGACCCTTAGCAAAGCCAGAAAGATCACC
TTGCC
+
::FFFFFFFFFFFFFF:FFFFF:FFF:FFFFFFFFFFFFFFF:FFFFFFFFFFFFFF:FF:F:FFFFF
FFFFFFFF:FF:FFFFFFFFFFFFFFFFFFFFFFFFFFFFFFFFFFFFFFFFFFFFFFFF:FFFFFF
FF:FF
@A00155:342:HHGFNDSXY:1:2561:25979:8876 1:N:0:GAACCTAG+TCCGCATA
GCATTAAATCAAATAAGATGAGCAAGATGGAACGTCTGGGTTCCAATTCTGTCAAGACTTATGACATA
TTAAATAGATTGGTTACGGTAAAAGACAAGGCAAGGTATGGACCCTTAGCAAAGCCAGAAAGATCACC
TTGCC
+
FFFFFFFFFFFFFFFFFFFFFFFFFFFFFFFFFFFFFFFFFF::F:FF,FFFFFFFFFFFFFFFFFFF
FFFFFFFFFFFFFFFFFFFFFFFFFFFFFFFFFFFFFFFFFFFFFFFFFFFFFFFFFFFFFFFFFFFF
FFFFF
@A00155:342:HHGFNDSXY:1:2550:4752:29042 1:N:0:GAACCTAG+TCCGCATA
GCATTAAATCAAATAAGATGAGCAAGATGGAACGTCTGGGTTCCAATTCTGTCAAGACTTATGACATA
TTAAATAGATTGGTTACGGTAAAAGACAAGGCAAGGTATGGACCCTTAGCAAAGCCAGAAAGATCACC
TTGCC
+
FFFFFFFFFFFFFFFF,FFFFF,FFFFFFFFFFFFFFFFFFFFFFFFFFFFFFFFFFFFFFFFFFFFF
FFFF:FFFFFFFFFFFFFFFFFFFFFFFFFFFFFFFFFFFFFFFFFFFFFFFFFFFFFFFFFFFFFFF
FFFFF
@A00155:342:HHGFNDSXY:1:2534:3269:34397 1:N:0:GAACCTAG+TCCGCATA
GCATTAAATCAAATAAGATGAGCAAGATGGAACGTCTGGGTTCCAATTCTGTCAAGACTTATGACATA
TTAAATAGATTGGTTACGGTAAAAGACAAGGCAAGGTATGGACCCTTAGCAAAGCCAGAAAGATCACC
TTGCC
+
:FFFFFFFFFFFFFFFFFFFFFFFFF:FFFFFFFFFF,FFFFFFFF:,FFFFFFFFFFFFFFFFFFF:,
FF:FFFFFFFFFFFFFFFFFFFFFFFFFFFFFFFFFFFFFFFFFFFFFFFFFFFFFFFFFFFFFFFFF
FFFFF
@A00155:342:HHGFNDSXY:1:2535:19144:4836 1:N:0:GAACCTAG+TCCGCATA
CCGATCTATCAAATAAGATGAGCAAGATGGAACGTCTGGGTTCCAATTCTGTCAAGACTTATGACATA
TTAAATAGATTGGTTACGGTAAAAGACAAGGCAAGGTATGGACCCTTAGCAAAGCCAGAAAGATCACC
TTGCC
+
FFFFF:FF,FFFFFFF:FFFFFFFFFFFF:FFF:FFFF::FFFFFFFF:FFFFFFFFFFFFFFFF:FF
FFF,FFFFFFFFFFFFF:FFFFFF:FFFF,FFF,F:FFFFFF:FFFF:FF,:FFFFFFFFFFFFFFFF
,FFFF
@A00155:342:HHGFNDSXY:1:2563:25590:28432 1:N:0:GAACCTAG+TCCGCATA
GCATTAAATCAAATAAGATGAGCAAGATGGAACGTCTGGGTTCCAATTCTGTCAAGACTTATGACATA
TTAAATAGATTGGTTACGGTAAAAGACAAGGCAAGGTATGGACCCTTAGCAAAGCCAGAAAGATCACC
TTGCC
+
FFFFFFFFFFFFFFFFFF:FFFFFFFFFFFFFFFFFFFFFFFFFFFFFFFFFFFFFFFFFFFFFFFFF
FFFFFFFFFFFFFFFFFFFFFFF:FFFFFFFFFFFFFFFFFFFFFFFFFFFFFFFFFFFFFFFFFFFF
FFFFF
```

```
@A00155:342:HHGFNDSXY:1:2563:25328:29825 1:N:0:GAACCTAG+TCCGCATA
GCATTAAATCAAATAAGATGAGCAAGATGGAACGTCTGGGTTCCAATTCTGTCAAGACTTATGACATA
TTAAATAGATTGGTTACGGTAAAAGACAAGGCAAGGTATGGACCCTTAGCAAAGCCAGAAAGATCACC
TTGCC
+
FFFFFFFFFFFFFFFFFFFFFFFFFFFFFFFFFFFFFFFFF:F:FFFFFFFFFFFFFFFFFFFFFFFFFF
FFFFFFFFFFFFFFFFFFFFFFFFFFFFFFFFFFFFFFFFFFFFFFFFFFFFFFFFFFFFFFFFFFFFFF
FFFFF
@A00155:342:HHGFNDSXY:1:2601:32687:5588 1:N:0:GAACCTAG+TCCGCATA
GCATTAAATCAAATAAGATGAGCAAGATGGAACGTCTGGGTTCCAATTCTGTCAAGACTTATGACATA
TTAAATAGATTGGTTACGGTAAAAGACAAGGCAAGGTATGGACCCTTAGCAAAGCCAGAAAGATCACC
TTGCC
+
FFF:FFFF:FFFFFFFFFFFFFFFFFFFFFFFFFFFF:FFF::FF:F:FFFF:FFFFFFFFFFFFFFFFF
FFFFFFFFFFFFFFFFFFFFFFFFFFFFFFFFF:FFFFFFFFFFFFFFFFFFFFFFFFFFFFFFFFFFFF
FFFFF
@A00155:342:HHGFNDSXY:1:2631:13340:20588 1:N:0:GAACCTAG+TCCGCATA
GCATTAAATCAAATAAGATGAGCAAGATGGAACGTCTGGGTTCCAATTCTGTCAAGACTTATGACATA
TTAAATAGATTGGTTACGGTAAAAGACAAGGCAAGGTATGGACCCTTAGCAAAGCCAGAAAGATCACC
TTGCC
+
F:FFFFFFFF:FFFFFFFFFFFFFFFFFFFFFFFFFFFFFF:FFFFFFFFFFFFFFFFFFFFFFFFFFFF
FFFFFFFFFFFFFFFFFFFFFFFFFF:FFFFFFFFFFFFFFFFFFFFFFFFFFFFFFFFFFFFFFFFFFF
FFFFF
@A00155:342:HHGFNDSXY:1:2633:4797:20134 1:N:0:GAACCTAG+TCCGCATA
GCATTAAATCAAATAAGATGAGCAAGATGGAACGTCTGGGTTCCAATTCTGTCAAGACTTATGACATA
TTAAATAGATTGGTTACGGTAAAAGACAAGGCAAGGTATGGACCCTTAGCAAAGCCAGAAAGATCACC
TTGCC
+
F::F:FFFFF:FFFFFFFFFFFF,FFFFFFFFFFF:FF:FFFFFF:FFFFFFFFFFFFFFFFFFFFFF:F
FFFFFFFFFFFFFFFFFFFFFFFFFFFFFFFFF:FFFFFFFFFFFFFFFFFFFFFFFFFFFFFFFFFFFF
FFF:F
@A00155:342:HHGFNDSXY:1:2676:3965:36761 1:N:0:GAACCTAG+TCCGCATA
GCATTAAATCAAATAAGATGAGCAAGATGGAACGTCTGGGTTCCAATTCTGTCAAGACTTATGACATA
TTAAATAGATTGGTTACGGTAAAAGACAAGGCAAGGTATGGACCCTTAGCAAAGCCAGAAAGATCACC
TTGCC
+
:FFFFFFF:FFFFFFFFFFFFFFFFFFFFFFFFFFFFFFFFFFFFFF,FFFFFFFFFFFFFFFFFFFFFF
FFFFFFFFFFFFFFFFFFFFFFFFFFFFFFFFFFFFFFFFFFFFFFFFFFFFFFFFFFFFFFFFFFFFFF
FFFFF
@A00155:342:HHGFNDSXY:1:2670:22978:13322 2:N:0:GAACCTAG+TCCGCATA
GCATTAAATCAAATAAGATGAGCAAGATGGAACGTCTGGGTTCCAATTCTGTCAAGACTTATGACATA
TTAAATAGATTGGTTACGGTAAAAGACAAGGCAAGGTATGGACCCTTAGCAAAGCCAGAAAGATCACC
TTGCCCAAAGAAGAC
+
FFFFFFFFFFFFFFFFFFFFFFFFFFFFFFFFFFFFFFFFFFFFFF:FFFFFFFFFFFFFFFFFFFFFFF
FFFF:FFFFFFFFFFFFFFFFFFFFFFFFF,FFFFFFFFFFFFF:FFFFFFFFFFFFFFFF:FFFFFFFF
FFFF:FFFFFFFFFF
@A00155:342:HHGFNDSXY:1:1163:21576:29058 1:N:0:GAACCTAG+TCCGCATA
CATTAAATCAAATAAGATGAGCAATATGGAACGTCTGGGTTCCAATTCTGTCAAGACTTATGACATAT
TAAATAGATTGGTTACGGTAAAAGACAAGGCAAGGTATGGACCCTTAGCAAAGCCAGAAAGATCACCT
TGCC
+
F:FFFFFFFFFFFFFFFFFF:FFF,FFFFFF:FFFFF:FFFF:FFFFFFFFFFFFFFFFFF:F,F,FFFF
```

```
FFFFFFFFFFFFFF,FFFFFF:FFF:FFFFFF:,FFFFFFFFF:FFFFFF:F:F:FF::F:FFF:F:F
F:FF
@A00155:342:HHGFNDSXY:1:1404:17788:15796 1:N:0:GAACCTAG+TCCGCATA
CATTAAATCAAATAAGATGAGCAAGATGGAACGTCTGGGTTCCAATTCTGTCAAGACTTATGACATAT
TAAATAGATTGGTTACGGTAAAAGACAAGGCAAGGTATGGACCCTTAGCAAAGCCAGAAAGATCACCT
TGCC
+
F:FFFFFF,F,FFF:FFFFFF,F::FFF:FFFFFFF:FF:F,F,FFFFF:FFFFFF:FFFFFFFFFFF
FFFFFFFFFFFFFFFFFFFFFFFF:FFFFFFFFFFFFFFFFFFFFFFFFFFFFFFFFFFFFFFFFFFF
FFF:
@A00155:342:HHGFNDSXY:1:1466:6497:16971 1:N:0:GAACCTAG+TCCGCATA
CATTAAATCAAATAAGATGAGCAAGATGGAACGTCTGGGTTCCAATTCTGTCAAGACTTATGACATAT
TAAATAGATTGGTTACGGTAAAAGACAAGGCAAGGTATGGACCCTTAGCAAAGCCAGAAAGATCACCT
TGCC
+
FFFFFFFFFFFFFFFFFFFFFFFFFFFFFFFFFFFFFFFFFFFFFFFFFFFFFFFFFFFFFFFFFFFF
FFFFFFFFFFFFFFFFFFFFFFFFFFFFF:FFFFFFFFFFFF:FFFFFFFFFFFFFFFFFFFFFFFFF
FFFF
@A00155:342:HHGFNDSXY:1:1516:1994:1752 1:N:0:GAACCTAG+TCCGCATA
CATTAAATCAAATAAGATGAGCAAGATGGAACGTCTGGGTTACAATTCTTTCAAGACTTATGACATAT
TAAATAGATTGGTTACGGTAAAAGACAAGGCAAGGTATGGACCCTTAGCAAAGCCAGAAAGATCACCT
TGCC
+
:,FFF:,F,FFF,FFFFFFFFF,F:FF:F::FFFFFF:::F,F,:,FFF,FF,:FFFFF:FFFFFF,F
:FF,F:FFF::F:FFFF,FF:FFF:,:F,FFFFFFFFFFFFFFFFFFFFFF:FFFFF:FFFFFFFFFF
FFFF
@A00155:342:HHGFNDSXY:1:2139:19651:8218 1:N:0:GAACCTAG+TCCGCATA
CATTAAATCAAATAAGATGAGCAAGATGGAACGTCTGGGTTCCAATTCTGTCAAGACTTATGACATAT
TAAATAGATTGGTTACGGTAAAAGACAAGGCAAGGTATGGACCCTTAGCAAAGCCAGAAAGATCACCT
TGCC
+
FFFFFFFFFFFFFFFFFFFFFFFFFFFFFFFFFFFFFFFF:FFF:F:FFFFFFFFFFFFFFFFFFFFF
FFFF:FFF:FFFFFFFFFFFFFFF:FFFFFFFFFFFFFFFFFFFFFFFFFFFFFFFFFFFFFFFFFFF
FFFF
@A00155:342:HHGFNDSXY:1:2219:20528:29058 1:N:0:GAACCTAG+TCCGCATA
CATTAAATCAAATAAGATGAGCAAGATGGAACGTCTGGGTTCCAATTCTGTCAAGACTTATGACATAT
TAAATAGATTGGTTACGGTAAAAGACAAGGCAAGGTATGGACCCTTAGCAAAGCCAGAAAGATCACCT
TGCC
+
F:FFFFFFFFFFFFFFFFFFFFFFFFFFFFFFFFFFFFFFFFFFFF:FFFFFFFFFFFFFFFFFFFFF
FF:F:FFFFFFFFFFFFFFFFFFFFFFFFFFFFFFFFFFFFFFFFFFFFFFFFFFFFFFFFFFFFFF:
FFFF
@A00155:342:HHGFNDSXY:1:2316:26142:19398 1:N:0:GAACCTAG+TCCGCATA
CATTAAATCAAATAAGATGAGCAAGATGGAACGTCTGGGTTCCAATTCTGTCAAGACTTATGACATAT
TAAATAGATTGGTTACGGTAAAAGACAAGGCAAGGTATGGACCCTTAGCAAAGCCAGAAAGATCACCT
TGCC
+
FFFFFFFFFFFFFFFFFFFFFFFFFFFFFFFFF:FFFFFFFFFFFFFFFFFFFFFFFFFFFFFFFFFF
FFFFFFFFFFFFFFFFFFFF:FFFFFFFFFFFFFFFFFFFFFFFFFFFFFFFFFFFFFFFFFFFFFFF
:FFF
@A00155:342:HHGFNDSXY:1:2408:24921:3161 1:N:0:GAACCTAG+TCCGCATA
CATTAAATCAAATAAGATGAGCAAGATGGAACGTCTGGGTTCCAATTCTGTCAAGACTTATGACATAT
TAAATAGATTGGTTACGGTAAAAGACAAGGCAAGGTATGGACCCTTAGCAAAGCCAGAAAGATCACCT
TGCC
```

```
+
FFFFF:FFFFFFFFFFFFFFFFFFFFFFF:FFFFFFFF:FFFFFFFFFFFFFFFFFFFFFFFFFFFFF
FFFFFFFFFFFFFFFFFFFFFFFFFFFFFFFFFFFFFFFFFFFFFFFFFFFFFFFFFFFFFFFFFFFF
FFFF
@A00155:342:HHGFNDSXY:1:2501:30563:30091 1:N:0:GAACCTAG+TCCGCATA
CATTAAATCAAATAAGATTAGCAAGATGGAACGTCTGGGTTCCAATTCTGTCAAGACTTATGACATAT
TAAATAGATTGGTTACGGTAAAAGACAAGGCAAGGTATGGACCCTTAGCAAAGCCAGAAAGATCACCT
TGCC
+
:FFFFFFFFFFFFFFFFFF,F::FFFFFFFFF,FFFFFFFFFF,FFFF:FFF::FFFFFFFFFFFFFF
FFFFFFFFFF,:FFF::,FFFFFFFFFF::::F:FFFF,FF:F,FFFFFFFFF:FFFFFFFFFFFF:F
FFFF
@A00155:342:HHGFNDSXY:1:2532:2157:15405 1:N:0:GAACCTAG+TCCGCATA
CATTAAATCAAATAAGATGAGCAAGATGGAACGTCTGGGTTCCAATTCTGTCAAGACTTATGACATAT
TAAATAGATTGGTTACGGTAAAAGACAAGGCAAGGTATGGACCCTTAGCAAAGCCAGAAAGATCACCT
TGCC
+
:FFFFFFF,FFFFFFFFFFFF:FF:FFFF,FFFFFFFFFFFF:FFFFFFFFFFFFFFFFFFFFFFFFF
FFFFFFFFFFFFFFFFFFFFFFFFFFFFFFFFFFFFFFFFFFFFFFFFFFFFFFFFFFFFFFFFFFFF
FFFF
@A00155:342:HHGFNDSXY:1:2554:7247:2988 1:N:0:GAACCTAG+TCCGCATA
CATTAAATCAAATAAGATGAGCAAGATGGAACGTCTGGGTTCCAATTCTGTCAAGACTTATGACATAT
TAAATAGATTGGTTACGGTAAAAGACAAGGCAAGGTATGGACCCTTAGCAAAGCCAGAAAGATCACCT
TGCC
+
FFFFFFFFFFFFFFFFFFFFFFFFFFFFFFFFFFFFFFFFF,FFFFFFFFFFFFFFFFFFFFFFFFFF
FFFFFFFFFFFFFFFFFFFFFFFFFFFFFFFFFFFFFFFFFFFFFFFFFFFFFFFFFFFFFFFFFFFF
FFFF
@A00155:342:HHGFNDSXY:1:2601:10384:18693 1:N:0:GAACCTAG+TCCGCATA
CATTAAATCAAATAAGATGAGCAAGATGGAACGTCTGGGTTCCAATTCTGTCAAGACTTATGACATAT
TAAATAGATTGGTTACGGTAAAAGACAAGGCAAGGTATGGACCCTTAGCAAAGCCAGAAAGATCACCT
TGCC
+
FFFFFFFFFFFFFFFFFFFFFFFFFFFFFFFFFFFFF:FFFFFFFFFFFFFFFFF:FFFFFFFFFFFF
FFFFFFFFFFFFFFFFFFFFFFFFFFFFFFFFFFFFFFFFFFFFFFFFFFFFFFFFFFFFFFFFFFFF
FFFF
@A00155:342:HHGFNDSXY:1:2631:14000:13996 1:N:0:GAACCTAG+TCCGCATA
CATTAAATCAAATAAGATGAGCAAGATGGAACGTCTGGGTTCCAATTCTGTCAAGACTTATGACATAT
TAAATAGATTGTTTACGGTAAAAGACAAGGCAAGGTATGGACCCTTAGCAAAGCCAGAAAGATCACCT
TGCC
+
FFFF:FFFFFFFFFF:F,FF,F,,FFFFFFFFFF,FF:FFF:,FFF:FFFFFF,FFF:F:FF,,FFFF
FFFFFFFFF:F,FFFFFFF:,FFF:FFFFFF,FFFFFFF,,F:FFFFF,FFFF:,FFFFFF:FFF,F:
F,FF
@A00155:342:HHGFNDSXY:1:2641:14615:31501 1:N:0:GAACCTAG+TCCGCATA
CATTAAATCAAATAAGATGAGCAAGATGGAACGTCTGGGTTCCAATTCTGTCAAGACTTATGACATAT
TAAATAGATTGGTTACGGTAAAAGACAAGGCAAGGTATGGACCCTTAGCAAAGCCAGAAAGATCACCT
TGCC
+
FFFFFFFFFF:FF:FFFFFF:FFFFFFFFFFFFFFFFF:FFFFFFFFFFFFFFFFFFFFFFFFFFFFF
FFFFFFFFFFFFFFFFFFFFFFFFFFFFFFFFFFFFFFFFFFFFFFFFFFFFFFFFFFFFFFFFFFFF
FFFF
@A00155:342:HHGFNDSXY:1:2245:14073:28463 1:N:0:GAACCTAG+TCCGCATA
CATTAAATCAAATAAGATGAGCAAGATGGAACGTCTGGGTTCCAATTCTGTCAAGACTTATGACATAT
```

```
TAAATAGATTGGTTACGGTAAAAGACAAGGCAAGGTATGGACCCTTAGCAAAGCCAGAAAGATCACCT
TGCCCAAAGAAGAC
+
FFFFF:FF,FF,FFFFFFFF,FF:FFF:F,FF:F:FF,,FFFF:FFFFFFFFFFFFFFFFFFFFFFFF
FFF,F:F,FF,FFFFFF:FFF,FF:FFF:F::FFFFFFFF:F,FFFF:::F:F:FFFFF:FFFF:FFF
F:F::FFFF:,FF:
@A00155:342:HHGFNDSXY:1:2508:22236:33865 1:N:0:GAACCTAG+TCCGCATA
TATTAAATCAAATAAGATGAGCAAGATGGAACGTCTGGGTTCCAATTCTGTCAAGACTTATGACATAT
TAAATAGATTGGTTACGGTAAAAGACAAGGCAAGGTATGGACCCTTAGCAAAGCCAGAAAGATCACCT
TGCCCATAGAAGAC
+
FFFFFFFFFFFF:FFFFFFFFFFFFFFFFFFFFFFFFFF:FFFFFFFFFFFFFFFFFFFFFFFFFF:FF
FFFFFFFFFFFFFFFFFFFFFFFFFFFFFFFFFFFFFFFFFFFFFFFFFFFFFFFFFFFFFFFFFFFF
FFFFFF,FFFFFFF
@A00155:342:HHGFNDSXY:1:2105:14488:20948 1:N:0:GAACCTAG+TCCGCATA
TATTAAATCAAATAAGATGAGCAAGATGGAACGTCTGGGTTCCAATTCTGTCAAGACTTATGACATAT
TAAATAGATTGGTTACGGTAAAAGACAAGGCAAGGTATGGACCCTTAGCAAAGCCAGAAAGATCACCT
TGCCCAAAGAAGACC
+
FFFFFFFF:FFFFFFFFFFFFFFFFFFFFFFFFFFFFFF:FFFFFFFFFFFFFFFFFFFFFFFFFFFF
FFFFFFFFFFFFFFFFFFFFFFFFFFFFFFFFFFFFFFFFFFFFFFFFFFFFFFFFFFFFFFFFFFFF
FFFFFFFFFFFFFFF
@A00155:342:HHGFNDSXY:1:2158:18159:9392 1:N:0:GAACCTAG+TCCGCATA
CATTAAATCAAATAAGATGAGCAAGATGGAACGTCTGGGTTCCAATTCTGTCAAGACTTATGACATAT
TAAATAGATTGGTTACGGTAAAAGACAAGGCAAGGTATGGACCCTTAGCAAAGCCAGAAAGATCACCT
TGCCCAAAGAAGACC
+
FFFFFFF:FFFFFFFFF,FFFFFFFFFFFFFFFFFFFFFFFFF:FFFFFFFFFFFFFFFFFFFFFFFF
FFFFFFFFFFFFFFFFFFFFFFFFFFFFFFFFFFFFFFFFFFFFFFFFFFFFFFFFFFFFFFFFFFFF
FFFFFFFFFFFFFFF
@A00155:342:HHGFNDSXY:1:1622:28854:11350 1:N:0:GAACCTAG+TCCGCATA
TTCCGATCTAATAAGATGAGCAAGATGGAACGTCTGGGTTCCAATTCTGTCAAGACTTATGACATATT
AAATAGATTGGTTACGGTAAAAGACAAGGCAAGGTATGGACCCTTAGCAAAGCCAGAAAGATCACCTT
GCCCAAAGAAGACCA
+
FFFFFFFFFFFFFFFFF:F:FFFFFF:FFFFF:FFFFF:FFFFF,FFFFFFFFFFFFFFFFFFFFFFF
FFFFFFFFFFFFFFFFFFFFFFFFFFFFFFFFFFFFFFFFFFFFFFFFFFFFFFFFFFFFF:FFFFFF
FFFFFF:FFFFFFFF
@A00155:342:HHGFNDSXY:1:2203:18186:35963 1:N:0:GAACCTAG+TCCGCATA
ATTAAATCAAATAAGATGAGCAAGATGGAACGTCTGGGTTCCAATTCTGTCAAGACTTATGACATATT
AAATAGATTGGTTACGGTAAAAGACAAGGCAAGGTATGGACCCTTAGCAAAGCCAGAAAGATCACCTT
GCCCAAAGAAGACCA
+
FFFFFFFFFFFFFFFFFFFFFFFFFFFFFFFFFFFFFFFFFFFFFFFFFFFFFFFFFFFFFFFFFFFF
FFFFFFFFFFFFFFF:FFFFFFFFFFFFFFFFFFFFFFFFFF:FFF:FFFFFFFFFFFFFFFFFFFFF:
:FFFFFFFFFFFFFF
@A00155:342:HHGFNDSXY:1:2267:25898:31782 1:N:0:GAACCTAG+TCCGCATA
ATTAAATCAAATAAGATGAGCAAGATGGAACGTCTGGGTTCCAATTCTGTCAAGACTTATGACATATT
AAATAGATTGGTTACGGTAAAAGACAAGGCAAGGTATGGACCCTTAGCAAAGCCAGAAAGATCACCTT
GCCCAAAGAAGACCA
+
FFFFFFFFFFFFFFFFFFFFFFFFFFFFFFFFF:FFFFFFFFFFFFFFFFFFFFFFFFFFFFFFFFFF
FFFFFFFFFFFFFFFFFFFFFFFFFFFFFFFFFFFFFFFFFFFFFFFFFFFFFFFFFFFFFFFFFFFF
FFFFFFFFFFFFFFF
```

```
@A00155:342:HHGFNDSXY:1:2302:4544:28025 1:N:0:GAACCTAG+TCCGCATA
ATTAAATCAAATAAGATGAGCAAGATGGAACGTCTGGGTTCCAATTCTGTCAAGACTTATGACATATT
AAATAGATTGGTTACGGTAAAAGACAAGGCAAGGTATGGACCCTTAGCAAAGCCAGAAAGATCACCTT
GCCCAAAGAAGACCA
+
F,FFFFFFFFFF:FFFFFFFFFFFFFF:FFFFF:FFF:FF,FFFFFFFFFFFFF:FFFFFFFFFFFFF
FFFFFFFFFFFFFFF:F:FFFFF:F,FFFFFFF:FFFFFFFFFFFF:F:FF:FF:FFFFFFFFFF:FF
:FF,FFFFFFFFFF:
@A00155:342:HHGFNDSXY:1:2439:28637:35900 1:N:0:GAACCTAG+TCCGCATA
ATTAAATCAAATAAGATGAGCAAGATGGAACGTCTGGGTTCCAATTCTGTCAAGACTTATGACATATT
AAATAGATTGGTTACGGTAAAAGACAAGGCAAGGTATGGACCCTTAGCAAAGCCAGAAAGATCACCTT
GCCCAAAGAAGACCC
+
FFFFFFFFFFFFFFFFFFFFFFFFFFFFFFFFFFFFFF:FFFFFFFFFFFFFFFFFFFFFFF:FFFFF
FFFFFFFFFFFFFFFFFFFFFFFFFFFFFFFFFFFFFFFFFFFFFFFFFFFFFFFFFFFFFFFFFFFF
FFFFFFFFFFFFFFF
@A00155:342:HHGFNDSXY:1:2554:1922:8578 1:N:0:GAACCTAG+TCCGCATA
ATTAAATCAAATAAGATGAGCAAGATGGAACGTCTGGGTTCCAATTCTGTCAAGACTTATGACATATT
AAATAGATTGGTTACGGTAAAAGACAAGGCAAGGTATGGACCCTTAGCAAAGCCAGAAAGATCACCTT
GCCCAAAGAAGACCG
+
FFFF:F:FFFFF:FFFFF:FFF,FFFFFFFFFFFFFFFFFFFFF:FFFFFFFFFFFFFFFF:FFFFFF
F:FFFFFFFFFFFFFFFFFFFFFFFFFFFFFFFFFFFFFFF:FFFFFFFFF:FFFF,FFFFF:FFF::
FFFFFF:FFFFFFFF
@A00155:342:HHGFNDSXY:1:1137:26250:4022 2:N:0:GAACCTAG+TCCGCATA
ATTAAATCAAATAAGATGAGCAAGATGGAACGTCTGGGTTCCAATTCTGTCAAGACTTATGACATATT
AAATAGATTGGTTACGGTAAAAGACAAGGCAAGGTATGGACCCTTAGCAAAGCCAGAAAGATCACCTT
GCCCAAAGAAGACCA
+
FFFFFFFFFFFFFFFFFFFFFFFFFFFFFFFFFFFFFFFFFF:FFFFFFFFFFF:FFFFFFFFFFFFF
FFFFFFFFFFFFFFFFFFFF:FFFFFFFFFFFFFFFFFFFFFFFFFF:FFFFFFFFFFFFFFFFFFFF
FFFFFFFFFFFFFFF
@A00155:342:HHGFNDSXY:1:1161:28140:20697 2:N:0:GAACCTAG+TCCGCATA
ATTAAATCAAATAAGATGAGCAAGATGGAACGTCTGGGTTCCAATTCTGTCAAGACTTATGACATATT
AAATAGATTGGTTACGGTAAAAGACAAGGCAAGGTATGGACCCTTAGCAAAGCCAGAAAGATCACCTT
GCCCAAAGAAGACCA
+
FFFF:FFFFFFFFFFFFFFFFFFFFFFFFFFFFFFFFFFFFFFFFFFFFFFFFFFFFFFFFFFFFFF:
FF:FFFFFFFFFFFFFFFF:F:F:FFFFFFFFFFFFFFFFFFFFFFFFFFF:FFFFFFFFFFFFFFFF
FF:FFFFFFFFFFFF
@A00155:342:HHGFNDSXY:1:1133:16640:7921 2:N:0:GAACCTAG+TCCGCATA
ATTAAATCAAATAAGATGAGCAAGATGGAACGTCTGGGTTCCAATTCTGTCAAGACTTATGACATATT
AAATAGATTGGTTACGGTAAAAGACAAGGCAAGGTATGGACCCTTAGCAAAGCCAGAAAGATCACCTT
GCCCAAAGAAGACCA
+
FFFFFFFFFFFFFFFFFFFFFFFFFFFFFFFFFFFFFFFFFFFFFFFFFFFFFFFFFFFFFFFFFFFF
FFFFFFFFFFFFFFFFFFFF:FFFFFFFFFFFFFFFFFFFFFFFFFFFFFFFFFFFFFFFFFFFFFF
FFFFFFFFFFFFFFF
@A00155:342:HHGFNDSXY:1:1172:8965:18270 2:N:0:GAACCTAG+TCCGCATA
ATTAAATCAAATAAGATGAGCAAGATGGAACGTCTGGGTTCCAATTCTGTCAAGACTTATGACATATT
AAATAGATTGGTTACGGTAAAAGACAAGGCAAGGTATGGACCCTTAGCAAAGCCAGAAAGATCACCTT
GCCCAAAGAAGACCA
+
FFFFFFF:FF:FFFF,FF:FFFFFFFFFFFFFFFFFFFFFFFFFFFFFFFFFFFFFFF,FFFFFFFFF
```

FFFFFFFFFFFFFF:FFFFF,FFFFFFFFFFFFFF:FFFFFFFFFFFFFFFFFFFFFFFFFFFFFFFFFF
FFFFFFFFFFFFFFF
@A00155:342:HHGFNDSXY:1:1448:18132:12226 2:N:0:GAACCTAG+TCCGCATA
ATTAAATCAAATAAGATGAGCAAGATGGAACGTCTGGGTTCCAATTCTGTCAAGACTTATGACATATT
AAATAGATTGGTTACGGTAAAAGACAAGGCAAGGTATGGACCCTTAGCAAAGCCAGAAAGATCACCTT
GCCCAAAGAAGACCA
+
FFFFFFFFFFFFFFFFFFFFFF:FFFFFF:FFFFFFFFFFFFFFFFFFFFFFFFFFFFFFFFFFFFFFFF
FFFFFFFFFFFFF:FFFF:FFFFFFFFFFF:FFFF:FFFFFFFFFFFFFFFFFFFFFFFFFFFFFFFFFF
FFFFFFFFFFFFFFF
@A00155:342:HHGFNDSXY:1:1448:18837:12696 2:N:0:GAACCTAG+TCCGCATA
ATTAAATCAAATAAGATGAGCAAGATGGAACGTCTGGGTTCCAATTCTGTCAAGACTTATGACATATT
AAATAGATTGGTTACGGTAAAAGACAAGGCAAGGTATGGACCCTTAGCAAAGCCAGAAAGATCACCTT
GCCCAAAGAAGACCA
+
FFFFFFFFFFFFFFFFFFFFFFFFFFFFFFFFFFFFFFFFFFFFFFFFFFFFFFFFFFFF:FFFFFFFFF
FFFFFFFFFFFFFFFFFFFF,FFFFFFFFFFFFF:FFFFFFFFFFFFFFFFFFFFFFFFFFFFFFFFFFF
FFFFFFFFFFF:FFF
@A00155:342:HHGFNDSXY:1:1505:12933:34319 2:N:0:GAACCTAG+TCCGCATA
ATTAAATCAAATAAGATGAGCAAGATGGAACGTCTGGGTTCCAATTCTGTCAAGACTTATGACATATT
AAATAGATTGGTTACGGTAAAAGACAAGGCAAGGTATGGACCCTTAGCAAAGCCAGAAAGATCACCTT
GCCCAAAGACGACCA
+
FFFFFFFFFFFFFFFFFFFFFFFFFFFFFFFFFFFFFF:FFFFFFFFFFFFFFFFFFFFFF:FFFFF:FF
FFFF,FF:F:FFFFFFFFFF,:FFFFFFFFF:FFF:FFFFFFFFF,FFFFFFFFFFFFF,FFF:FFFFFF
FFFFFFFFF,,FFFF
@A00155:342:HHGFNDSXY:1:1666:7952:34303 2:N:0:GAACCTAG+TCCGCATA
ATTAAATCAAATAAGATGAGCAAGATGGAACGTCTGGGTTCCAATTCTGTCAAGACTTATGACATATT
AAATAGATTGGTTACGGTAAAAGACAAGGCAAGGTATGGACCCTTAGCAAAGCCAGAAAGATCACCTT
GCCCAAAGAGATCGG
+
FFFFFFFFFFFFFFFFFFFFFFFFFFFFFFFFFF:FFFFFFFFFFFFFFFFFFFFFFFFFFFFFFFFFFF
FFFFFFF:FFFFF:FFFFFFFFFFFFFFFFFFFFFFFFFFFFFFFFFFFF:FFFFFFFFFFF,FFFFFFF
FFFFF,FFFFFFFFF
@A00155:342:HHGFNDSXY:1:2105:14488:20948 2:N:0:GAACCTAG+TCCGCATA
ATTAAATCAAATAAGATGAGCAAGATGGAACGTCTGGGTTCCAATTCTGTCAAGACTTATGACATATT
AAATAGATTGGTTACGGTAAAAGACAAGGCAAGGTATGGACCCTTAGCAAAGCCAGAAAGATCACCTT
GCCCAAAGAAGACCA
+
FFFFFFFFFFFFFFFFFFFFFFFFFFFFFFFFFFFFFFFFFFFFFFFFFFFFFFFFFFFFFFFFFFFFFF
F:FFFFFFFFFFFFFFFFFFFFFFFFFFFFFFFFFFFFFFFFFFFFFFFFFFFFFFFFFFFFFFFFFFFF
FFFFFFFFFFF:FFF
@A00155:342:HHGFNDSXY:1:2164:24894:17394 2:N:0:GAACCTAG+TCCGCATA
ATTAAATCAAATAAGATGAGCAAGATGGAACGTCTGGGTTCCAATTCTGTCAAGACTTATGACATATT
AAATAGATTGGTTACGGTAAAAGACAAGGCAAGGTATGGACCCTTAGCAAAGCCAGAAAGATCACCTT
GCCCAAAGAAGACCA
+
FFFFFFFFFFFFFFFFFFFFFFFFFFFFF:FFFFFFFFFFFFFFFFFFFFFFFFFFFFFFFFFFFFFF:F
FFFFFFFFFFFFFFFFFF:FFFFFFFFFFFFFFFFFFFFFFFFFFFFFFFFFFFFFFFFFFFFFFFFFFF
FFFF:FFFFFFFFFF
@A00155:342:HHGFNDSXY:1:2201:19334:8985 2:N:0:GAACCTAG+TCCGCATA
ATTAAATCAAATAAGATGAGCAAGATGGAACGTCTGGGTTCCAATTCTGTCAAGACTTATGACATATT
AAATAGATTGGTTACGGTAAAAGACAAGGCAAGGTATGGACCCTTAGCAAAGCCAGAAAGATCACCTT
GCCCAAAGAAGACAG

```
+
FFFFFFFFFFFFFFFFFFFFFFFFFFFFFFFFFFFFFFFFFFFFFF,FFFFFFFFFFFFF::FFF:FF:F
F:F,FFFF:FFFF:FFFF:FFFFFFFFFFFFFFF:FFFFFFFFFFFFFFFFFFFFFFF,FFFFFFFFFF
FFFFFFFFFFFFFFF
@A00155:342:HHGFNDSXY:1:2303:32678:1313 2:N:0:GAACCTAG+TCCGCATA
ATTAAATCAAATAAGATGAGCAAGATGGAACGTCTGGGTTCCAATTCTGTCAAGACTTATGACATATT
AAATAGATTGGTTACGGTAAAAGACAAGGCAAGGTATGGACCCTTAGCAAAGCCAGAAAGATCACCTT
GCCCAAAGAAGACAG
+
:FFFFFFFFFFFFFFFFFFFFFFFFFFFFFFFFFFFFFFFFFFFFFFFFFFFFFFFFFFFFFFFFFFF
FFFFFFFFFFFFFFFFFFFF:FFFFFFFFFF:FFFFFFFFFFFFFFFFFFFFFFFFFFFFFFFFFFFF
FFFFFFFFFFF:FFF
@A00155:342:HHGFNDSXY:1:2303:30689:13307 2:N:0:GAACCTAG+TCCGCATA
ATTAAATCAAATAAGATGAGCAAGATGGAACGTCTGGGTTCCAATTCTGTCAAGACTTATGACATATT
AAATAGATTGGTTACGGTAAAAGACAAGGCAAGGTATGGACCCTTAGCAAAGCCAGAAAGATCACCTT
GCCCAAAGAAGACAG
+
FFFFFFFFFFFFFFFFFFFFFFFFFFFFFFFF:FFFFFFFFFFFFFFFFFFFFFFFFFFFFFFF:FFFF
FFFFFFFFFFFFFFFFFFFF:FFFFFFFFFFFFFFFF:,FFFFFFFFFFFFFFFFFFFFFFFFFFFFFF
:FFFFF,FFFFFFFF
@A00155:342:HHGFNDSXY:1:2337:2899:33630 2:N:0:GAACCTAG+TCCGCATA
ATTAAATCAAATAAGATGAGCAAGATGGAACGTCTGGGTTCCAATTCTGTCAAGACTTATGACATATT
AAATAGATTGGTTACGGTAAAAGACAAGGCAAGGTATGGACCCTTAGCAAAGCCAGAAAGATCACCTT
GCCCAAAGAAGACCA
+
FFFFFFF,FFFFFFFFFFFFFFFFFFFFFFFFFFFFFFFFFFFFFFFFFF:FF:FFFFFF:FFFFFF
F:FF:FF:FF:FFFFFFFF:,FFFFF:FFFFFFFFFFFFFFFFFFFFFFFF:F,FFFFFFF,FFF,FFF
FF,:FF:FFFF:,FF
@A00155:342:HHGFNDSXY:1:2321:2781:30326 2:N:0:GAACCTAG+TCCGCATA
ATTAAATCAAATAAGATGAGCAAGATGGAACGTCTGGGTTCCAATTCTGTCAAGACTTATGACATATT
AAATAGATTGGTTACGGTAAAAGACAAGGCAAGGTATGGACCCTTAGCAAAGCCAGAAAGATCACCTT
GCCCAAAGAAGACCA
+
FFFFF:FFFF:FFFFFFFFFFF:FFFFFF:FF::FFFFFFFF:FFFFFFFFFFFFFFFFFFFFFFFFFF
FFFFFFFFFFFFFFFFFFFF:,FFFFFFFFFFFFF:FFFF:FFFFFFFFF:F:FFFFFFF:FFFFFFF
FFFF:F:FFFFFFFF
@A00155:342:HHGFNDSXY:1:2362:5746:5212 2:N:0:GAACCTAG+TCCGCATA
ATTAAATCAAATAAGATGAGCAAGATGGAACGTCTGGGTTCCAATTCTGTCAAGACTTATGACATATT
AAATAGATTGGTTACGGTAAAAGACAAGGCAAGGTATGGACCCTTAGCAAAGCCAGAAAGATCACCTT
GCCCAAAGAAGACCA
+
FFFFFFFFFFFFFFF:FFFFFFFFFFFFFFFFFFFFFFFFFFFFFFFFFFFFFFFFFFFFFFFFFFFF
FFFFF:FFFFFFF,:FFFFFFFFFFFFFFFFFFFFFFFFFFFFFFFFFFF:FFFFFFFFFFFFFFFFF
FFFFFF:FFFFFFFF
@A00155:342:HHGFNDSXY:1:2435:11541:28557 2:N:0:GAACCTAG+TCCGCATA
ATTAAATCAAATAAGATGAGCAAGATGGAACGTCTGGGTTCCAATTCTGTCAAGACTTATGACATATT
AAATAGATTGGTTACGGTAAAAGACAAGGCAAGGTATGGACCCTTAGCAAAGCCAGAAAGATCACCTT
GCCCAAAGAAGACCA
+
FFFFFFFFFFFFFFFFFFFFFFFFFFFFFFFFFFFFFFFFFFFFFFFFFFFFFF:FFFFFFFFFFFFF
F:,FFFFFFFFFFFFFFFFF,:FFFFFFFFFFFFFFFFFFFFFFFFFFFFFFFFFFFFFFFFFFFFFF
FFFFFF:FF:FFFFF
@A00155:342:HHGFNDSXY:1:2508:22236:33865 2:N:0:GAACCTAG+TCCGCATA
ATTAAATCAAATAAGATGAGCAAGATGGAACGTCTGGGTTCCAATTCTGTCAAGACTTATGACATATT
```

AAATAGATTGGTTACGGTAAAAGACAAGGCAAGGTATGGACCCTTAGCAAAGCCAGAAAGATCACCTT
GCCCAAAGAAGACAG
+
FFFFFFFFFFFFFFFFFFFFFFFFFFFFFFFFFFFFFFFFFFFFFFFFFFFFFFFFF:FFFFFF,FFF
FFFFFFFFFFFF,FFFFFFF:FF:FF:FFFF:FFFFFFFFFFFFFFFFFFFFFFFFFFFFFFFFFFFF
FFFFF:FFFFFFFFF
@A00155:342:HHGFNDSXY:1:2544:30083:33458 2:N:0:GAACCTAG+TCCGCATA
ATTAAATCAAATAAGATGAGCAAGATGGAACGTCTGGGTTCCAATTCTGTCAAGACTTATGACATATT
AAATAGATTGGTTACGGTAAAAGACAAGGCAAGGTATGGACCCTTAGCAAAGCCAGAAAGATCACCTT
GCCCAAAGAAGACCA
+
FFFFFFFFFFFFFFFFFFFFFFFFFFFFFFFFFFFFFFFFFFFFFFFFFFFFFFFFFFFFFFFFFFFF
FFFFFFFFFFFFFFFFFFFFFFFFFFFFFFFFFFFFFFFFFFFFFFFFFFFFFFFFFFFFFFFFFFFF
FFFFFFFFFFFFFFF
@A00155:342:HHGFNDSXY:1:2544:30382:34006 2:N:0:GAACCTAG+TCCGCATA
ATTAAATCAAATAAGATGAGCAAGATGGAACGTCTGGGTTCCAATTCTGTCAAGACTTATGACATATT
AAATAGATTGGTTACGGTAAAAGACAAGGCAAGGTATGGACCCTTAGCAAAGCCAGAAAGATCACCTT
GCCCAAAGAAGACCA
+
FFFFFFFFFFFFFFFFFFFFFFFFFFFFFFFF:FFFFFFFFFFFFFFFFFFFFFFFFFFFFFFFFFFF
FFFFFFFFFFF:FFFFF,FF:FFFFFFFFFFFFFFFFFFFFFFFFFFFFFFFFFFFFFFFFFFFFFF
FFFFFFFFFFF:FFF
@A00155:342:HHGFNDSXY:1:2561:6144:22780 2:N:0:GAACCTAG+TCCGCATA
ATTAAATCAAATAAGATGAGCAAGATGGAACGTCTGGGTTCCAATTCTGTCAAGACTTATGACATATT
AAATAGATTGGTTACGGTAAAAGACAAGGCAAGGTATGGACCCTTAGCAAAGCCAGAAAGATCACCTT
GCCCAAAGAAGACCA
+
FFFFFFFFFFFFFFFFFFFFFFFFFFFFFFF,FFFFFFFFFFFFF:FFFFFFFF:FFFFFFFFF,FFF
FFF:FFFFF:FFFFFFFF:FFFFFFFFF:FFFFFF,FFFFFFFFFF,FFFFF,FF:FFFFFF,F,FFF
FFFFFFFFFFFFFFF
@A00155:342:HHGFNDSXY:1:2561:6623:23171 2:N:0:GAACCTAG+TCCGCATA
ATTAAATCAAATAAGATGAGCAAGATGGAACGTCTGGGTTCCAATTCTGTCAAGACTTATGACATATT
AAATAGATTGGTTACGGTAAAAGACAAGGCAAGGTCTGGACCCTTAGCAAAGCCAGAAAGATCACCTT
GCCCAAAGAAGACCA
+
FFFFFFFFFFFFFFFFFF:FFFFF:FFF:FFFFFFFFFFFFFFFFFFFFFFFF,FFFF:F,FFF:FFF
:FF,FFFFFFF,FFFFF::,FFFFFFFFFFFFFFF,FFF:FFF,FFFFFFFFFFFFFFFFFFFFFF::
FFF,F::,FF,FFFF
@A00155:342:HHGFNDSXY:1:2651:15329:14669 2:N:0:GAACCTAG+TCCGCATA
ATTAAATCAAATAAGATGAGCAAGATGGAACGTCTGGGTTCCAATTCTGTCAAGACTTATGACATATT
AAATAGATTGGTTACGGTAAAAGACAAGGCAAGGTATGGACCCTTAGCAAAGCCAGAAAGATCACCTT
GCCCAAAGAAGACCA
+
FFFFFFFFFFFFFFFFFFFFFFFFFFFFFFFFFFFFFFFFFFFFFFFFFFFFFFFFFFFFFFFFFFFF
FFFFFFFFFFFFFFFFFFFFFFFFFFFFFFFFFFFFFFFFFFFFFFFFFFFFFFFFFFFFFFFFFFFF
FFFFFFFFFFFFF:F
@A00155:342:HHGFNDSXY:1:2665:15383:8406 2:N:0:GAACCTAG+TCCGCATA
ATTAAATCAAATAAGATGAGCAAGATGGAACGTCTGGGTTCCAATTCTGTCAAGACTTATGACATATT
AAATAGATTGGTTACGGTAAAAGACAAGGCAAGGTATGGACCCTTAGCAAAGCCAGAAAGATCACCTT
GCCCAAAGAAGACCA
+
FFFFFFFFFFFFFFFFFFFFFFFFFFFFFFFFFFFFFFFFFFFFFFFFFFFFFFFFFFFFFFFFFFFF
FFFFFFFFFFFFFFFFF:FFFFFFFFFFFFFFFFFFFFFFFFFFFFFFFFFFFFFFFFFFFFFFFFFF
FFFFFFFFFFFFFFF

```
@A00155:342:HHGFNDSXY:1:2636:7464:4523 2:N:0:GAACCTAG+TCCGCATA
ATTAAATCAAATAAGATGAGCAAGATGGAACGTCTGGGTTCCAATTCTGTCAAGACTCATGACATATT
AAATAGATTGGTTACGGTAAAAGACAAGGCAAGGTATGGACCCTTAGCAAAGCCAGAAAGATCACCTT
GCCCAAAGAAGACCA
+
FF:FFFFFFFFFFFFFFFFFFFFFFFFFFFFFFF:FFFFFFFFFFFFFFFFFFFFFFFF:FFFFFFFF:F
F,FFFFF:FFFFF:FFFFF:F,FFFFFFFFFFFFFFFFFFFFFFFF:FFFFFFF,FF:FFFFFF:FFFF
FF:F,FF:FFFFFFF
@A00155:342:HHGFNDSXY:1:2261:16405:26710 2:N:0:GAACCTAG+TCCGCATA
TTAAATCAAATAAGATGAGCAAGATGGAACGTCTGGGTTCCAATTCTGTCAAGACTTATGACATATTA
AATAGATTGGTTACGGTAAAAGACAAGGCAAGGTATGGACCCTTAGCAAAGCCAGAAAGATCACCTTG
CCCAAAGAAGACCA
+
FF:FFFFFFFFFFFFFFFFFFFFFFFFFFFFFFFFFFFFFFFFFFFF:FFFFFFFF:FFFFFFFFFF:F
FFFFFFF:FFFFFF:FFFFFFFFFFFF:FFFFFF:FFFFFFFFF:FFFFFFFFFFFFFFFF,FF::F:
FF:FFFFFF:FF:F
@A00155:342:HHGFNDSXY:1:1267:6831:32643 1:N:0:GAACCTAG+TCCGCATA
TTAAATCAAATAAGATGAGCAAGATGGAACGTCTGGGTTCCAATTCTGTCAAGACTTATGACATATTA
AATAGATTGGTTACGGTAAAAGACAAGGCAAGGTATGGACCCTTAGAAAAGCCAGAAAGATCACCTTG
CCCAAAGAAGACCCC
+
FFFFFF,FFFFFFFFFFFFFFFFFFFFFFFFFFFFFF:FFFFFFFFF:FFFF,FFFFFFFFFFFFFFF
FFFFFFFFFFFFF,:FFFFF:FFFFF:FF:FFFF:FFFFFFFFFF:,FFFFFFFFF:FFFFFFFF:FF
FFF:FFFFFFFFFFF
@A00155:342:HHGFNDSXY:1:2339:26883:20556 1:N:0:GAACCTAG+TCCGCATA
TTAAATCAAATAAGATGAGCAAGATGGAACGTCTGGGTTCCAATTCTGTCAAGACTTATGACATATTA
AATAGATTGGTTACGGTAAAAGACAAGGCAAGGTATGGACCCTTAGCAAAGCCAGAAAGATCACCTTG
CCCAAAGAAGACCAG
+
FFFFFFFFFFFFFFFFFF:FFFFFFFFFFFFFFFFFFFFFFFFFFFFFFFFFFFFFFFFFFFFFFFFFF
FFFFFFF:FFFFFFFFFFFFFFFFFFFFFFFFFFFFFFFFFFFFFFFFFFFFFFFFFFFFFFFFFFFF
FFFFFFFFFFFFFFF
@A00155:342:HHGFNDSXY:1:2337:2899:33630 1:N:0:GAACCTAG+TCCGCATA
TTAAATCAAATAAGATGAGCAAGATGGAACGTCTGGGTTCCAATTCTGTCAAGACTTATGACATATTA
AATAGATTGGTTACGGTAAAAGACAAGGCAAGGTATGGACCCTTAGCAAAGCCAGAAAGATCACCTTG
CCCAAAGAAGACCAC
+
FFFFFF,F:FFF,FFF:::F,:F,FF::FFFFFFFFFFFF,FFFFFFFFFFFF:FFFFFF:FFFFFFF
FFF:FFFFFFFFF:FFFFFFFF,FFFF:,FFFFFFFFFFFFFFF,FFFFFFFFFFFF:F:FFFFFFFF
FFFFFFF,FF:F:FF
@A00155:342:HHGFNDSXY:1:1273:20500:17143 1:N:0:GAACCTAG+TCCGCATA
TAAATCAAATAAGATGAGCAAGATGGAACGTCTGGGTTCCAATTCTGTCAAGACTTATGACATATTAA
ATAGATTGGTTACGGTAAAAGACAAGGCAAGGTATGGACCCTTAGCAAAGCCAGAAAGATCACCTTGC
CCAAAGAAGACCAAC
+
FFFFFFFFFFFFFFFFFFFFFFFF:FFFFFFFFFFFFFFFFFFFFFFFFFFFFFFFFFFFFFFF:FFFF
FFFFFFFFFFFFFFFFFFFFFFFFFFFFFFFFFFFFFFFFFFFFFFFFFFFFFFFFFFFFFFFFFFFF
FFFFFFFFFFFFFFF
@A00155:342:HHGFNDSXY:1:1276:2022:21120 1:N:0:GAACCTAG+TCCGCATA
TAAATCAAATAAGATGAGCAAGATGGAAAGTCTGGGTTCCAATTCTGTCAAGACTTATGACATATTAA
ATAGATTGGTTACGGTAAAAGACAAGGCAAGGTATGGACCCTTAGCAAAGCCAGAAAGATCACCTTGC
CCAAAGAAGACCAAC
+
FFFFFFFFFFFFFFFFF,FFFFFFFFFF,FF,:::FF,FFFFFF,FFFFFFFFFFF:FFFFFFFFFFF
```

FFFFFFFFFFFFFFFFFFFFFFFFF::FFF:FFFF,F:F,:FFFF,,:FFF,FFFFFFFFFF:FFFF:
F,FFFFF:FFFFF:F
@A00155:342:HHGFNDSXY:1:1435:8657:15389 1:N:0:GAACCTAG+TCCGCATA
TAAAACAAATAAGATGAGCAAGATGGAACGTCTGGGTTCCAATTCTGTCAAGACTTATGACATATTAA
ATAGATTGGTTACGGTAAAAGACAAGGCAAGGTATGGACCCTTAGCAAAGCCAGAAAGATCACCTTGC
CCAAAGAAGACCAAC
+
FFFF:,FFFFFFFFFFFFFFFFFFFFFFFF:FFF:FFF:F:FFFFFFFFFFFFFFFFFFF,FFFFFFF
FFFFFFFFFFFFFFFFFFFFFFFFFFFFFFFFFFFFFFFFFFFFF:FFFFFFFFFFFFFFFFFFFFFF
FFFFFFFFFFFFFFF
@A00155:342:HHGFNDSXY:1:1675:24008:16611 1:N:0:GAACCTAG+TCCGCATA
TAAATCAAATAAGATGAGCAAGATGGAACGTCTGGGTTCCAATTCTGTCAAGACTTATGACATATTAA
ATAGATTGGTTACGGTAAAAGACAAGGCAAGGTATGGACCCTTAGCAAAGCCAGAAAGATCACCTTGC
CCAAAGAAGACCAAC
+
FFFFFFFFFFFFFFFFFFFFFFFFFFFFFFFFFFFFFFFFFFFFFF:FFFFFFFFFF:FFFFFFFFFFF
::FFFFFFFFFFFFFFFFFFFFFFFFFFFFFFFFFFFFFFFFFFFFFFFFFFFFFFFFFFFFFFFFFF
FFFFFFFFFFFFFFF
@A00155:342:HHGFNDSXY:1:2103:2112:6903 1:N:0:GAACCTAG+TCCGCATA
TAAATCAAATAAGATGAGCAAGATGGAACGTCTGGGTTCCAATTCTGTCAAGACTTATGACATATTAA
ATAGATTGGTTACGGTAAAAGACAAGGCAAGGTATGGACCCTTAGCAAAGCCAGAAAGATCACCTTGC
CCAAAGAAGACCAAC
+
FFFFF:FFFFFFFFFFFF,FF:FF,FFFFFF:F:FFF:FF:FFF:FFFF:F:FFFFFFFFFFFFFFF:
FFFFF:FFFFFFFFFFF:FFFF,:FFFFFFFFFFFFFFF,,FFFFFFFFFFFFFFFFFFFFF:FFFFF
FFFFFFFFFFFFFFF
@A00155:342:HHGFNDSXY:1:2120:13756:33458 1:N:0:GAACCTAG+TCCGCATA
TAAATCAAATAAGATGAGCAAGATGGAACGTCTGGGTTCCAATTCTGTCAAGACTTATGACATATTAA
ATAGATTGGTTACGGTAAAAGACAAGGCAAGGTATGGACCCTTAGCAAAGCCAGAAAGATCACCTTGC
CCAAAGAAGACCAAC
+
FFFFFFFFFFFFFFFFFFFFFFFFFFFFFFFFFFFF:FFFFFFFFFFFFFFFFFFFFFFFFFFFFFFF
FFFFFFFFFFFFFFFFFFFFFFFFFFFFFFFFFFFFFFFFFFFFFFFFFFFFFFFFFFFFFFFFFFFF
FFFFFFFFFFFFFFF
@A00155:342:HHGFNDSXY:1:2134:30273:25895 1:N:0:GAACCTAG+TCCGCATA
TAAATCAAATAAGATGAGCAAGATGGAACGTCTGGGTTCCAATTCTGTCAAGACTTATGACATATTAA
ATAGATTGGTTACGGTAAAAGACAAGGCAAGGTATGGACCCTTAGCAAAGCCAGAAAGATCACCTTGC
CCAAAGAAGACCAAC
+
FFFFFFFFFFFFFFFFFFFFFFFFFFFFFFFFFFF:FFFFFF:FFFFFFFFFFFFFFFFFFFFFFFFF
FFFFFFFFFFFFFFFFFFFFFFFFFFFFFFFFFFFFFFFFFFFFFFFFFFFFFFFFFFFFFFFFFFFF
FFFFFFFFFFFFFFF
@A00155:342:HHGFNDSXY:1:2330:5556:5760 1:N:0:GAACCTAG+TCCGCATA
GATCTCAAATAAGATGAGCAAGATGGAACGTCTGGGTTCCAATTTTGTCAAGACTTATGACATATTAA
ATAGATTGGTTACGGTAAAAGACAAGGCAAGGTATGGACCCTTAGCAAAGCCAGAAAGATCACCTTGC
CCAAAGAAGACCAAC
+
FFF:FFFFFFFFFFFFFFF:FFFF:FF,:FF,F:FFFF::FFFFFFF::FFF,,FFFFFF:FFFFFF:
FFFF:F:FFFFFFF:FFFFFFF:,FFFFFFFFF:FFFF:FFFFFFFFFFFFFFFFFFFFFFFF:FFFFF
FF:F,FFF:FFF::F
@A00155:342:HHGFNDSXY:1:2341:17951:29794 1:N:0:GAACCTAG+TCCGCATA
TAAATCAAATAAGATGAGCAAGATGGAACGTCTGGGTTCCAATTCTGTCAAGACTTATGACATATTAA
ATAGATTGGTTACGGTAAAAGACAAGGCAAGGTATGGACCCTTAGCAAAGCCAGAAAGATCACCTTGC
CCAAAGAAGACCAAC

```
+
FFFFFFFFFFFFFFFFFFFFFFFFFFFFFFFFFFFFFFFFFFFFFFFFF:FFFFFFFFFFFFFFFFFF
FFFFFFFFFFFFFFFFFFFFFFFFFFFFFFFFFFFFFFFFFFFFFFFFFFFFFFFFFFFFFFFFFFFF
FFFFFFFFF,FFFFF
@A00155:342:HHGFNDSXY:1:2341:18213:36041 1:N:0:GAACCTAG+TCCGCATA
TAAATCAAATAAGATGAGCAAGATGGAACGTCTGGGTTCCAATTCTGTCAAGACTTATGACATATTAA
ATAGATTGGTTACGGTAAAAGACAAGGCAAGGTATGGACCCTTAGCAAAGCCAGAAAGATCACCTTGC
CCAAAGAAGACCAAC
+
FFFFFFFFFFFFFFFFF:FFFFFFFFFFFFFFFFF::FFFFFFFFFFFFFFFFFFF::FFFFFFFFFF
FFFFFFFFFFFFFFF:FFFFFFFFFFFFFFFFFFFFFFFFFFFFFFFFFFFFFFFFFFFFFFFFFFFF
FFFFFFFFFFFFFFF
@A00155:342:HHGFNDSXY:1:2525:20654:1094 1:N:0:GAACCTAG+TCCGCATA
TAAATCAAATAAGATGAGCAAGATGGAACGTCTGGGTTCCAATTCTGTCAAGACTTATGACATATTAA
ATAGATTGGTTACGGTAAAAGACAAGGCAAGGTATGGACCCTTAGCAAAGCCAGAAAGATCACCTTGC
CCAAAGAAGACCAAC
+
F:FFFFFFFFFFFFFF:FFF:FFFFFFFFFFFFFFFFFFFFFFFFFFFFFFFFFFFFFFFFFFFFFFF
FFFFFFFFFFFFFFF:FFFFFFFFFFFFFFFFFFFFFFFFFFFFFFFFFFFFFFFFFFFFFFFFFFFF
FFFFFFFFFFFFFFF
@A00155:342:HHGFNDSXY:1:2506:8856:14888 1:N:0:GAACCTAG+TCCGCATA
TAAATCAAATAAGATGAGCAAGATGGAACGTCTGGGTTCCAATTCTGTCAAGACTTATGACATATTAA
ATAGATTGGTTACGGTAAAAGACAAGGCAAGGTATGGACCCTTAGCAAAGCCAGAAAGATCACCTTGC
CCAAAGAAGACCAAC
+
FFFFFFFFFFFFFFFFFFFFFFFFFFFFFFFFFFFFFFFFFFFFFFFFFFFFFFFFFFFFFFFFFFFF
FFFFFFFFFFFFF:FFFFFFFFFFFFFFFFFFFFFFFFFFFFFFFFFFFFFFFFFFFFFFFFFFFFFF
FFFFFFFFFFF:FFF
@A00155:342:HHGFNDSXY:1:2573:18005:5274 1:N:0:GAACCTAG+TCCGCATA
GATCTCAAATAAGATGAGCAAGATGGAACGTCTGGGTTCCAATTTTGTCAAGACTTATGACATATTAA
ATAGATTGGTTACGGTAAAAGACAAGGCAAGGTATGGACCCTTAGCAAAGCCAGAAAGATCACCTTGC
CCAAAGAAGACCAAC
+
FFFFFFFFFFFFFFFFFFFFFFFFFFFFFFFFFFFFFFFFFFFFFFFFFFFFFFFFFFFFFFFFFFFF
FFFFFF:FFFFFFFFFFFFFFFFFFFFFFFFFFFFFFFFFFFFFFFFFFFFFFFFFFFFFFFFFF:FF
FFFFFFFFFFFFFFF
@A00155:342:HHGFNDSXY:1:2617:4381:26741 1:N:0:GAACCTAG+TCCGCATA
TAAATCAAATAAGATGAGCAAGATGGAACGTCTGGGTTCCAATTCTGTCAAGACTTATGACATATTAA
ATAGATTGGTTACGGTAAAAGACAAGGCAAGGTATGGACCCTTAGCAAAGCCAGAAAGATCACCTTGC
CCAAAGAAGACCAAC
+
FFFFFF:FFFFFFFFFFFFFFFFFFFF:FFFFFF,FFFFFFFFFFFFF:FFFF:FF:FFF:FFFFFFF
FFFFFFFFFFFFFFFFFFFFFFFFFFFFFFFFFFFFFFF:FFFFFFFFFFFFFFFFFFFFFFFFFFFF
FFFFFFFFFFFFFFF
@A00155:342:HHGFNDSXY:1:2612:7274:9111 1:N:0:GAACCTAG+TCCGCATA
TAAATCAAATAAGATGAGCAAGATGGAACGTCTGGGTTCCAATTCTGTCAAGACTTATGACATATTAA
ATAGATTGGTTACGGTAAAAGACAAGGCAAGGTATGGACCCTTAGCAAAGCCAGAAAGATCACCTTGC
CCAAAGAAGACCAAC
+
FFFFFFFFFFFFFFFF::FFFFFF,FFFFFFFFFF,FF:FF:FFFFFFFFFFFFFFFFFFFFFFFFFF
FFFF:FFFFFFFFFFFFFFFFF:FFFFFFFFFFFF::FF,FFFFFFFFFFFFFFFFFFFFFFF:FFFF
FFFFFFFFFFFF:FF
@A00155:342:HHGFNDSXY:1:2616:29622:17942 1:N:0:GAACCTAG+TCCGCATA
TAAATCAAATAAGATGAGCAAGATGGAACGTCTGGGTTCCAATTCTGTCAAGACTTATGACATATTAA
```

```
ATAGATTGGTTACGGTAAAAGACAAGGCAAGGTATGGACCCTTAGCAAAGCCAGAAAGATCACCTTGC
CCAAAGAAGACCAAC
+
:FFFFFFFFFFFFFFFFFFFFFFFFFFF:FFFFFFFF:FFFF:FFFFFFFFFFF:FFFFFFFFFFFFFF
FFFFFFFFFFFFFFFFFFFFF:FFFFFFFFFFFFFF:FFFFF:FFF:FFFFFFFFFFFFFFFFF:FFF
FFFFFFFFFFFFFFF
@A00155:342:HHGFNDSXY:1:2325:32208:17159 1:N:0:GAACCTAG+TCCGCATA
AAATCAAATAAGATGAGCAAGATGGAACGTCTGGGTTCCAATTCTGTCAAGACTTATGACATATTAAA
TAGATTGGTTACGGTAAAAGACAAGGCAAGGTATGGACCCTTAGCAAAGCCAGAAAGATCACCTTGCC
CAAAGAAGACCAACC
+
::FFFFFFFF:FFF:FFFFFFF:FFFFFFFFFFFF:FFFFFFFFFFFFFFFFFFFFFFFFFFFFFFFF
FFFFFF:FFFFFFFFFFFFFFFFFFFFFFFFFFFFFFFFFFFFFFFFFFFFFFFFFFFFFFFFFFFFF
FFFFFFFFFFFFFFF
@A00155:342:HHGFNDSXY:1:2339:19180:14669 2:N:0:GAACCTAG+TCCGCATA
AAATCAAATAAGATGAGCAAGATGGAACGTCTGGGTTCCAATTCTGTCAAGACTTATGACATATTAAA
TAGATTGGTTACGGTAAAAGACAAGGCAAGGTATGGACCCTTAGCAAAGCCAGAAAGATCACCTTGCC
CAAAGAAGACAGATC
+
FFFFFFFFFFFFFFFFFFFFFFFFFFFFFFFFFFFFFFFFFFFFFFFFFF:FFFFFFFFFFFF:FFFF
FFFFFFFFFFFFFFFFF:FFFFFFFFFFFFFFFFFFFFFFFFFFFFFFFFFFFFFFFFFFFFFFFFFF
FFFFFFFFFFFFFFF
@A00155:342:HHGFNDSXY:1:2405:23050:13542 2:N:0:GAACCTAG+TCCGCATA
AAATCAAATAAGATGAGCAAGATGGAACGTCTGGGTTCCAATTCTGTCAAGACTTATGACATATTAAA
TAGATTGGTTACGGTAAAAGACAAGGCAAGGTATGGACCCTTAGCAAAGCCAGAAAGATCACCTTGCC
CAAAGAAGACCAACA
+
FFF:FFFFFFFFFFFFFFFFFFFFFFFFFFFFFFFF:FFFFFFFFFFFFFFFFFFFFFFFFFFFFFFF
:FFF:FFFFFFFFFFF,:FFFFFFFFFFFFFFFFFFFFFFFFFFFFFFFFFFFFFFFFFFFFFF:FFF
FFFFFFFFFFFFFFF
@A00155:342:HHGFNDSXY:1:2620:15248:30749 2:N:0:GAACCTAG+TCCGCATA
AAATCAAATAAGATGAGCAAGATGGAACGTCTGGGTTCCAATTCTGTCAAGACTTATGACATATTAAA
TAGATTGGTTACGGTAAAAGACAAGGCAAGGTATGGACCCTTAGCAAAGCCAGAAAGATCACCTTGCC
CAAAGAAGACCAACA
+
FFFFFFFFFFFFFFFFFFFFFFFFFFFFFFFFFFFFFFFFFFFFFFFFFF:FFFFFFFFFFFFF:FFF:F
FFFFFFFFFF:FFF:F:::FFFFFFFFFFFFFFFFFFFFFFFFFFFFFFFFFFFFFFF:FFFFFF:FFF
FFFFFFFFFFFFFFF
@A00155:342:HHGFNDSXY:1:2458:19461:29559 1:N:0:GAACCTAG+TCCGCATA
AATCAAATAAGATGAGCAAGATGGAACGTCTGGGTTCCAATTCTGTCAAGACTTATGACATATTAAAT
AGATTGGTTACGGTAAAAGACAAGGCAAGGTATGGACCCTTAGCAAAGCCAGAAAGATCACCTTGCCC
AAAGTAGACCAACG
+
FFFFFF:F:FF,FFFFFFFF:FFFFFFF:FFFFFF::FF:,FFF,F,,:F:FFFFF,,:FF:FF:FFF
:FF:FFF:FFF,FF,F:,FFFF,FFF:FFFFFFFFFFFFFFFFFF:FFFFF:FFFFFFF:FFF,FF,FF
::FF,,,F:FF,:F
@A00155:342:HHGFNDSXY:1:1125:26449:2644 2:N:0:GAACCTAG+TCCGCATA
AATCAAATAAGATGAGCAAGATGGAACGTCTGGGTTCCAATTCTGTCAAGACTTATGACATATTAAAT
AGATTGGTTACGGTAAAAGACAAGGCAAGGTATGGACCCTTAGCAAAGCCAGAAAGATCACCTTGCCC
AAAGAAGACCAACAT
+
FFFFFFFFFFFFFFFFFFFFFFFFFF:FFFFFFFFFFFFFFFFFFFFFFFFFFFFFFFFFFFFFFFFFF
FFFFFFFFFFFFFFFFF:FFFFFFFFFFFFFF:FFFFFFFFFFFFFFFFFFFFFF,FFFFFFFFFF:FFFF
F:FFFFFFFF:FF:F
```

```
@A00155:342:HHGFNDSXY:1:1170:10547:1908 2:N:0:GAACCTAG+TCCGCATA
AATCAAATAAGATGAGCAAGATGGAACGTCTGGGTTCCAATTCTCTCAAGACTTATGACATATTAAAT
AGATTGGTTACGGTAAAAGACAAGGCAAGGTATGGACCCTTAGCAAAGCCAGAAAGATCACCTTGCCC
AAAGAAGACCAACAT
+
FFFFFFFFFFFFFFFFFFFFFFFFFFFFFFFFFFFFFFFF:FFFFFFFFFFFF,FFFFFFFFFFFFFF
FFFFFFFFFFFFFFFFFFFFFFFFFFFFFFFFFFFFFFFFFFFFFF,FFFFFFFFFFFFFFFFFFFF:
FFFFFFFFF:FFFFF
@A00155:342:HHGFNDSXY:1:1612:26196:21308 2:N:0:GAACCTAG+TCCGCATA
AATCAAATAAGATGAGCAAGATGGAACGTCTGGGTTCCAATTCTGTCAAGACTTATGACATATTAAAT
AGATTGGTTACGGTAAAAGACAAGGCAAGGTATGGACCCTTAGCAAAGCCAGAAAGATCACCTTGCCC
AAAGAAGACCAACAT
+
FFFFFFFFFFFFFFFFFFFFFFFFFFFFFFFFFFFFFFFFFFFFFFFFFFFFFFFFFFFFFFFFFFFF
FFFFFFFFFFFFFFFFF:FFFFFFFFFFFFFFFFFFFFFFFFFFFFFFFFFFFFFFFFFFFFFFFFFF
FFFFFFFFFFFFFFF
@A00155:342:HHGFNDSXY:1:2178:27344:18035 2:N:0:GAACCTAG+TCCGCATA
AATCAAATAAGATGAGCAAGATGGAACGTCTGGGTTCCAATTCTGTCAAGACTTATGACATATTAAAT
AGATTGGTTACGGTAAAAGACAAGGCAAGGTATGGACCCTTAGCAAAGCCAGAAAGATCACCTTGCCC
AAAGAAGACCAACAT
+
FFFFFFFFFFFFFF::FFFFFFFFFFFFFFFFFFFFFFFFFFFFFFFFFFFFFFFFFFFFFFFFFFFF
FFFFFFFFFFFFFFFFF:FFFFFFFFFFFFFFFFFFFFFFFFFFFFFFFFFFFFFFFFFFFFFFFFFF
FFFFF:FFFFF:FFF
@A00155:342:HHGFNDSXY:1:2329:24912:25003 2:N:0:GAACCTAG+TCCGCATA
AATCAAATAAGATGAGCAAGATGGAACGTCTGGGTTCCAATTCTGTCAAGACTTATGACATATTAAAT
AGATTGGTTACGGTAAAAGACAAGGCAAGGTATGGACCCTTAGCAAAGCCAGAAAGATCACCTTGCCC
AAAGAAGACAGATCG
+
FFFFFFFFFFFF:FFFFFFFFFFFFFFFFFFFFFFFFFFFFFFFFFFFFFFFFFFFFFFFFFFFF:FF
FFFFFFFFFFFFFFFFF,FFFFFFFFFFFFFFFFFFFFFFFFFFFFFFFFFFFFFFFFFFFFFFFF:F
FFFFFFFFFFFFFFF
@A00155:342:HHGFNDSXY:1:2508:25617:12727 2:N:0:GAACCTAG+TCCGCATA
AATCAAATAAGATGAGCAAGATGGAACGTCTGGGTTCCAATTCTGTCAAGACTTATGACATATTAAAT
AGATTGGTTACGGTAAATGACAAGGCAAGGTATGGACCCTTAGCAAAGCCAGAAAGATCACCTTGCCC
AAAGAAGACCAACAT
+
FFFFFFFFFFFFFFFFFFFFFFFFFFFFFFFFFFFFFFFFFFFFFFFFFFFFFFFFFFFFFFFFFFFF
FFFFFFFFFFFFFFFFF:,FFFFFFFFFFFFFFFFFFFFFFFFFFFFFFFFFFFFFFFFFFFFFFFFF
FFFFFFFFFFFFFFF
@A00155:342:HHGFNDSXY:1:2119:15103:6292 2:N:0:GAACCTAG+TCCGCATA
ATCAAATAAGATGAGCAAGATGGAACGTCTGGGTTCCAATTCTGTCAAGACTTATGACATATTAAATA
GATTGGTTACGGTAAAAGACAAGGCAAGGTATGGACCCTTAGCAAAGCCAGAAAGATCACCTTGCCCA
AAGAAGACCAACATT
+
FFFFFFFFFFFFFFFFFFFFFFFFFFFF,FFFFF:FFFFFFFFFFFFFFFFFFFFF:FFFFFFFFFFFF
FFFFFFFFFFFFF:F,FFFFFFFFFFFFFFFFFFFFFFFFFFFFFFFFFF:FFFFFFFFFFFFFFF:FF
FFFFFFFFF:FFFFF
@A00155:342:HHGFNDSXY:1:2119:14805:7216 2:N:0:GAACCTAG+TCCGCATA
ATCAAATAAGATGAGCAAGATGGAACGTCTGGGTTCCAATTCTGTCAAGACTTATGACATATTAAATA
GATTGGTTACGGTAAAAGACAAGGCAAGGTATGGACCCTTAGCAAAGCCAGAAAGATCACCTTGCCCA
AAGAAGACCAACATT
+
F:FFFFFFFFFFFFFFFFFFFFFFFFFF:FFFFFFFFFFFFFFFFFFFFFFFFFFF:FFFFFFFFFF:FF
```

FFFFFFFFFFFFFF:F:FFFFFFFFFFFF::FFFFFFF,FFFFFFFFFFFFFF:FFFFFFFFFFFFF
FFFFFFFFFFFFFFF
@A00155:342:HHGFNDSXY:1:2238:14389:12759 2:N:0:GAACCTAG+TCCGCATA
ATCAAATAAGATGAGCAAGATGGAACGTCTGGGTTCCAATTCTGTCAAGACTTATGACATATTAAATA
GATTGGTTACGGTAAAAGACAAGGCAAGGTATGGACCCTTAGCAAAGCCAGAAAGATCACCTTGCCCA
AAGAAGACAGATCGG
+
FFFFFFFFFFFFFFFFFFFFFFFFFFFFFFFFFFFFFFFFFFFFFFFFFFFFFFFFFFFFFFFFFFFF
FFFFFFFFFFFFFFF,FFFFFF:FFFFFFFFFFFFFFFFFFFFFFFFFFFFFFFFFFFFFFFFFFFFF
FFFFFFFFFFFFFFF
@A00155:342:HHGFNDSXY:1:2535:19144:4836 2:N:0:GAACCTAG+TCCGCATA
ATCAAATAAGATGAGCAAGATGGAACGTCTGGGTTCCAATTCTGTCAAGACTTATGACATATTAAATA
GATTGGTTACGGTAAAAGACAAGGCAAGGTATGGACCCTTAGCAAAGCCAGAAAGATCACCTTGCCCA
AAGAAGACAGATCGG
+
FFFFFFFFFFFFFFFFFFFFFFFFFFFFFFFFFFFFFFFFFFFFFFFFFFFFFFFFFFFFFFFFFFFF
FFFFFFFFFFFFFFFFFFFFFFFFFFFFFFFFFFFFF:FFFFFFFFFFFFFFFFFFFFFFFFFFFF:FF
FFFFFFFFF:FFFFF
@A00155:342:HHGFNDSXY:1:2610:27832:24549 2:N:0:GAACCTAG+TCCGCATA
ATCAAATAAGATGAGCAAGATGGAACGTCTGGGTTCCAATTCTGTCAAGACTTATGACATATTAAATA
GATTGGTTACGGTAAAAGACAAGGCAAGGTATGGACCCTTAGCAAAGCCAGAAAGATCACCTTGCCCA
AAGAAGACCAACATT
+
FFFFFFFFFF:FFFFFFFFFFFFFFFFFFFFFFFFFFFFFFFFFFFFFFFFFF::FFF::FFFFFFFF
F:FFFFFFFFFFFFFFFFFFFFFFFFFFFF::FFFFFFFFFFFFFFFFFFFFFFFFFFFFFFFFFFFFF
FFFFFFFFFFFFFFF
@A00155:342:HHGFNDSXY:1:1104:26196:15045 2:N:0:GAACCTAG+TCCGCATA
CAAATAAGATGAGCAAGATGGAACGTCTGGGTTCCAATTCTGTCAAGACTTATGACATATTAAATAGA
TTGGTTACGGTAAAAGACAAGGCAAGGTATGGACCCTTAGCAAAGCCAGAAAGATCACCTTGCCCAAA
GAAGACCAACATTTT
+
FFFFFFFFFFFFFFFFFF:FFFFFFFFFFFFFFFFFFFFFFFFFFFFFFFFFFFFFFFFFFFFFFFFFF
FFFFFF,FFFFFF:FFFFFFFFFFFFFFFFFFFFF:FFF:FFFFFFF:FFFFF,FFFFFFFFFFFFFFF
FFF,FFFFFFFFFFF
@A00155:342:HHGFNDSXY:1:2330:5556:5760 2:N:0:GAACCTAG+TCCGCATA
CAAATAAGATGAGCAAGATGGAACGTCTGGGTTCCAATTTTGTCAAGACTTATGACATATTAAATAGA
TTGGTTACGGTAAAAGACAAGGCAAGGTATGGACCCTTAGCAAAGCCAGAAAGATCACCTTGCCCAAA
GAAGACCAACAGATC
+
FFFFFFFFFF:FFFFFFF:FFFFFFFFFFFF:FFFFFFFFFFFFFFFFFFFFFFFFFFFFFFFFFFFFF
FFFF:FFF,FFFFFFFFFFFFFFF:FFF:FFFFFFFFFFFFFFF:FFFFFFFFFFFFFFF:FFFFFFF
,F,,FFFFFF,FFFF
@A00155:342:HHGFNDSXY:1:2573:18005:5274 2:N:0:GAACCTAG+TCCGCATA
CAAATAAGATGAGCAAGATGGAACGTCTGGGTTCCAATTTTGTCAAGACTTATGACATATTAAATAGA
TTGGTTACGGTAAAAGACAAGGCAAGGTATGGACCCTTAGCAAAGCCAGAAAGATCACCTTGCCCAAA
GAAGACCAACAGATC
+
FFFFFFFFFFFFFFFFFFFFFFFFFFFFFFFFFFFFFFFFFFFFFFFFFFFFFFFFFFFFFFFFFFFF
FFFFFFFFFFFFFFFFFFFFFFFFFFFFFFFFFFFFFFFFFFFFFFFFFFFFFFFFFFFFFFFFFFFF
FFFFFFFFFFF:FFF
@A00155:342:HHGFNDSXY:1:1136:26060:32941 1:N:0:GAACCTAG+TCCGCATA
AAATAAGATGAGCAAGATGGAACGTCTGGGTTCCAATTCTGTCAAGACTTATGACATATTAAATAGAT
TGGTTACGGTAAAAGACAAGGCAAGGTATGGACCCTTAGCAAAGCCAGAAAGATCACCTTGCCCAAAG
AAGACCAACATTTT

```
+
FFFFFFFFF:FFFFFFFFFFF:FFFFF:FFFF,FFFFFFFFFFFFFFFFFFFFFFFFFFFFFFFFFFFFF
FFFFFFFFFFFFFFFFFFFFFFFFFFFFFFFFFFFFFFFFFFFFFFFFFFFFFFFFFFFFFFFFFFFFFF
FFFFFFFFFFFFFF
@A00155:342:HHGFNDSXY:1:1125:26449:2644 1:N:0:GAACCTAG+TCCGCATA
AAATAAGATGAGCAAGATGGAACGTCTGGGTTCCAATTCTGTCAAGACTTATGACATATTAAATAGAT
TGGTTACGGTAAAAGACAAGGCAAGGTATGGACCCTTAGCAAAGCCAGAAAGATCACCTTGCCCAAAG
AAGACCAACATTTTC
+
FFFFFFFFFFFFFFFFFF:FFFFFFFF:F::FFFFFFFFFFFFFFFFFFFFFFFFFFFFFFFFFFFFFFF
FFFFFFFFFFFFFFFFFFFFFFFFFFFFFFFFFF:FFFFFFFFFFFFFFFFFFFFFFFFFFFFFFFFFFF
F:FFFFFFFFFFFFF
@A00155:342:HHGFNDSXY:1:1378:3161:28134 1:N:0:GAACCTAG+TCCGCATA
AAATAAGATGAGCAAGATGGAACGTCTGGGTTCCAATTCTGTCAAGACTTATGACATATTAAATAGAT
TGGTTACGGTAAAAGACAAGGCAAGGTATGGACCCTTAGCAAAGCCAGAAAGATCACCTTGCCCAAAG
AAGACCAACATTTTC
+
FFFFFFFFFFFFFFFFFFFFFFFFFFFFFFF:FFFFFFFFFFFFFFFFFFFFFFFFFFFFFFFFFFFFFF
FFFFFFFFFFFFFFFFFFFFFFFFFFFFFFFFFFFFFFFFFFFFFFFFFFFFFFFFFFFFFFFFFFFFFF
FFFFFFFFFFFFFFF
@A00155:342:HHGFNDSXY:1:1365:19244:11083 2:N:0:GAACCTAG+TCCGCATA
AAATAAGATGAGCAAGATGGAACGTCTGGGTTCCAATTCTGTCAAGACTTATGACATATTAAATAGAT
TGGTTACGGTAAAAGACAAGGCAAGGTATGGACCCTTAGCAAAGCCAGAAAGATCACCTTGCCCAAAG
AAGACCAACATTTTC
+
FFFFFFFFFFFFFFFFFFFFFFFFFFFFFF,FFFFFFFFFFFF:FFFFFFFFFFFFFFFFFFFFFFFFFF
FFFFFFFFFFFF:FFFFFFFFFFFFFFFFF:FFFFFFFFFFFFFFFFFFFFFFF:FFFF:F:FFFFFFFF
FFFFFFFFFFFFFFF
@A00155:342:HHGFNDSXY:1:2450:10818:4914 2:N:0:GAACCTAG+TCCGCATA
AAATAAGATGAGCAAGATGGAACGTCTGGGTTCCAATTCTGTCAAGACTTATGACATATTAAATAGAT
TGGTTACGGTAAAAGACAAGGCAAGGTATGGACCCTTAGCAAAGCCAGAAAGATCACCTTGCCCAAAG
AAGACCAACATTTTC
+
FFFFFFFFFFFFFFFFFFFF:FFFFFFFFFFFFFFFFFFFFFFFFFFFFFFFFFFFFFF:FFFF:FFFFF
FFFFFFFFFFFFF:FFFFFFFFFFFFFFFFFFFFFFFFFFF,FFFFFFFFFFFFFFFFFFFFF:FF:FFF
,FF,FFFFFFF,FFF
@A00155:342:HHGFNDSXY:1:2545:3341:17237 2:N:0:GAACCTAG+TCCGCATA
AAATAAGATGAGCAAGATGGAACGTCTGGGTTCCAATTCTGTCAAGACTTATGACATATTAAATAGAT
TGGTTACGGTAAAAGACAAGGCAAGGTATGGACCCTTAGCAAAGCCAGAAAGATCACCTTGCCCAAAG
AAGACCAACATTTTC
+
FFFFFFFFF:FFFFFFFFFFFFFFFFFFFFFFFFFFFFFFFFFFFFFFFFFFFFFFFFFFFFFF:FFFFF
FFFFFFF:FFFF:FFFFFFFFF:FFFFFFFFFF:F:FFFFFFFFFFF::FFFFFFFFF:FFFF:FFFFFF
FFFFF:FF,F:FFFF
@A00155:342:HHGFNDSXY:1:1227:8947:21151 1:N:0:GAACCTAG+TCCGCATA
AATAAGATGAGCAAGATGGAACGTCTGGGTTCCAATTCTGTCAAGACTTATGACATATTAAATAGATT
GGTTACGGTAAAAGACAAGGCAAGGTATGGACCCTTAGCAAAGCCAGAAAGATCACCTTGCCCAAAGA
AGACCAACATTTTCA
+
FFFFFFFFFFFFFFF:FFFFFFFFFFFFFFFFFFF:FFFF:FFFFFFFFFFFFFFFFFFFFFFFFFFFFF
FFFFFFFFFFFFFFFFFFFFFFFFFFFFFFFFFFFFFFFFFFFFFFFFFFFFFFFFFFFFFFFFFFFFFF
FF:FFFFFFFFFFFF
@A00155:342:HHGFNDSXY:1:1357:19822:8453 1:N:0:GAACCTAG+TCCGCATA
AATAAGATGAGCAAGATGGAACGTCTGGGTTCCAATTCTGTCAAGACTTATGACATATTAAATAGATT
```

```
GGTTACGGTATAAGACAAGGCAAGGTATGGACCCTTAGCAAAGCCAGAAAGATCACCTTGCCCAAAGA
AGACCAACATTTTCA
+
FFFFFF:FFFFFFFFFFFFFFFFFFFFFFFFFFF:FFFFFFFFF:FFFFFFFFFFFFFFFFFFFFFFFF
FFFFFFFFFFFF:FFFFFFFFFFFFFFFFFFFF:FFFFFFFFFFFFFFFFFFFFFFFFFFFFFFFFFFF
,FFFFFFFFFFFFFF
@A00155:342:HHGFNDSXY:1:1457:23827:11193 1:N:0:GAACCTAG+TCCGCATA
AATAAGATGAGCAAGATGGAACGTCTGGGTTCCAATTCTGTCAAGACTTATGACATATTAAATAGATT
GGTTACGGTAAAAGACAAGGCAAGGTATGGACCCTTAGCAAAGCCAGAAAGATCACCTTGCCCAAAGA
AGACCAACATTTTCA
+
FFFFFFFFFFFFFFFFFF:FFFFFFFF:FFFFFFFFFFFFFFFFFFFFFFFFFFFFFFFFFFFFFFFFF
FFFFFFFFFFFFFFFFFFFFFFFFFFFFFFFFFFFFFFFFFFFFFFFFFFFFFFFFFFFFFFFFFFFFF
FFFFFFFFFFFFFFF
@A00155:342:HHGFNDSXY:1:2212:6831:26663 1:N:0:GAACCTAG+TCCGCATA
AATAAGATGAGCAAGATGGAACGTCTGGGTTCCAATTCTGTCAAGACTTATGACATATTAAATAGATT
GGTTACGGTAAAAGACAAGGCAAGGTATGGACCCTTAGCAAAGCCAGAAAGATCACCTTGCCCAAAGA
AGACCAACATTTTCA
+
FFFFF:FFFFF,FFFFF:FFFFFFFFFFFF:F:F:FFFFFFFFFFF,FFFFFFFFFFFFFFFFFFFFFF
FFFFFFFFFFFFFFFFFF:,FFFFFFFFFFFFFFFFFFFFFFFFFFFFFFFFFFFF:FFFFFFFFFFFF
FFFFFFFFFFFFFFF
@A00155:342:HHGFNDSXY:1:2250:11270:15092 1:N:0:GAACCTAG+TCCGCATA
AATAAGATGAGCAAGATGGAACGTCTGGGTTCCAATTCTGTCAAGACTTATGACATATTAAATAGATT
GGTTACGGTATAAGACAAGGCAAGGTATGGACCCTTAGCAAAGCCAGAAAGATCACCTTGCCCAAAGA
AGACCAACATTTTCA
+
FFFFFFFFFFFF:FFFFFFFFFFFFFF:FFFFFFFFFFFFFFFFFFFFFFFFFFFFFFFFFFFFFFFFF
FF:FFFFFFFFFFFFFFFFFFFFFFFFFFFFFFFFFFFFFFFFFFFFFFFFFFFFFFFFFFFFFFFFFF
FFFFFFFFFFFFFFF
@A00155:342:HHGFNDSXY:1:1266:17698:4085 2:N:0:GAACCTAG+TCCGCATA
AATAAGATGAGCAAGATGGAACGTCTGGGTTCCAATTCTGTCAAGACTTATGACATATTAAATAGATT
GGTTACGGTAAAAGACAAGGCAAGGTATGGACCCTTAGCAAAGCCAGAAAGATCACCTTGCCCAAAGA
AGACCAACATTTTCA
+
FFFFF:FF:FFFF:FFFFFFFFF:FFFFFFFFFFF,FFFFFF:FFFFFFFFFFFFFFFFFFFFFFFFF:
:FFFFFFFFFF:F:FFFFFF:FFFFFFFFFFFFFFFFFFFFFFFF:FFFFFFFFFFFFFFFF:FFFFFF
F,FFFFFFFFFF:FF
@A00155:342:HHGFNDSXY:1:1316:3215:13792 2:N:0:GAACCTAG+TCCGCATA
AATAAGATGAGCAAGATGGAACGTCTGGGTTCCAATTCTGTCAAGACTTATGACATATTAAATAGATT
GGTTACGGTAAAAGACAAGGCAAGGTATGGACCCTTAGCAAAGCCAGAAAGATCACCTTGCCCAAAGA
AGACCAACATTTTCA
+
FFFFFFFFFFFFFFFFFFFFFFFFFFFFFFFFFFFFFFFFFFFFFFFFFFFFFFFFFFFFFFFFFFFFF
FFFF,FFFFFFFFF:FFFFFF:FFFFFFFFFFFFFFF:FFFFFFFFFFFFFFFFFFFFFFFFFFFFFFF
FFFFFFFFFFFFFFF
@A00155:342:HHGFNDSXY:1:1572:22408:31407 2:N:0:GAACCTAG+TCCGCATA
AATAAGATGAGCAAGATGGGACGTCTGGGTTCCAATTCTGTCAAGACTTATGACATATTAAATAGATT
GGTTACGGTAAAAGACAAGGCAAGGTATGGACCCTTAGCAAAGCCAGAAAGATCACCTTGCCCAAAGA
AGACCAACATTTTCA
+
F:FFFFFFFFFFFFFFFFFFFFFFFFFFFFFFFFFFFFFFFFFFFFFFFFFFFFFFFFFFFFFFFFFFF
FFFFFFFFFFFFFFFFFFFFFFFFFFFFFFFFFFFFFFFFFFFFFFFF:FFFFFFFFFFFFFF:FFFFFF
FFFFFFFFFFFFFFF
```

```
@A00155:342:HHGFNDSXY:1:1622:28854:11350 2:N:0:GAACCTAG+TCCGCATA
AATAAGATGAGCAAGATGGAACGTCTGGGTTCCAATTCTGTCAAGACTTATGACATATTAAATAGATT
GGTTACGGTAAAAGACAAGGCAAGGTATGGACCCTTAGCAAAGCCAGAAAGATCACCTTGCCCAAAGA
AGACCAAGATCGGAA
+
FFFFFFFFFFFFFFFFFFFFFFFFFFFFFFFFFFFFFFFFFFFFFFFFFFFFFFFFFFFFFFFFFFFF
FFFFFFFFFFFFFFFFFFFFFFFFFFFFFFFFFFFFFFFF:FFFFFFFFFFFFFFFFFFFFFFFFFFF
FFFFFFFFFFFFFFF
@A00155:342:HHGFNDSXY:1:2212:14696:14888 2:N:0:GAACCTAG+TCCGCATA
AATAAGATGAGCAAGATGGAACGTCTGGGTTCCAATTCTGTCAAGACTTATGACATATTAAATAGATT
GGTTACGGTAAAAGACAAGGCAAGGTATGGACCCTTAGCAAAGCCAGAAAGATCACCTTGCCCAAAGA
AGACCAACATTTTCA
+
FFFFFFFFFFFFFFFFFFFFFFFFFFFFFFFFFFFFFFFFFFFFFFFFFFFFFFFFFFFFFFFFFFFF
FFF:FFFFFFF:FFFFFFFFFF:FFFFFFFFFFFFFFFFFFFFFFFFFFFFFFFFFFFFFFFFFFFFF
FFFFFFFFFFFFFFF
@A00155:342:HHGFNDSXY:1:2545:5113:22874 2:N:0:GAACCTAG+TCCGCATA
AATAAGATGAGCAAGATGGAACGTCTGGGTTCCAATTCTGTCAAGACTTATGACATATTAAATAGATT
GGTTACGGTAAAAGACAAGGCAAGGTATGGACCCTTAGCAAAGCCAGAAAGATCACCTTGCCCAAAGA
AGACCAACATTTTCA
+
F:FFFFFFFFFFFFFFFFFFFFFFFFFFFFFFFFFFFFFFFFFFFFFFFFFFFFFFFFF:FFFF,FFFF
FFFFFF,FFFF:FFFFFF,FFFFF:FF:FFFF:FFFFFFFFFFFFFFFF,FF,FFFFFFF,FFF,FFF
FFFFFFFFFFFFFF:
@A00155:342:HHGFNDSXY:1:2632:2645:1094 2:N:0:GAACCTAG+TCCGCATA
AATAAGATGAGCAAGATGGAACGTCTGGGTTCCAATTCTGTCAAGACTTATGACATATTAAATAGATT
GGTTACGGTAAAAGACAAGGCAAGGTATGGACCCTTAGCAAAGCCAGAAAGATCACCTTGCCCAAAGA
AGACCAACATTTTCA
+
F:F,FFFFFFFFFFFFFFFFFFFFFFFFFFFFFFFFFFFFFFFF:FFFFFFFFFFFFFFFFFFFFFFFF
:FFFFFF,FFF:FFFFFFFFFFFFFFFFFFFF:FFFFFFFFFFFFFFFFFFFFFFFFFFFFFFFFFFF
FFF:FF,FFFFFFF:
@A00155:342:HHGFNDSXY:1:2632:4417:5447 2:N:0:GAACCTAG+TCCGCATA
AATAAGATGAGCAAGATGGAACGTCTGGGTTCCAATTCTGTCAAGACTTATGACATATTAAATAGATT
GGTTACGGTAAAAGACAAGGCAAGGTATGGACCCTTAGCAAAGCCAGAAAGATCACCTTGCCCAAAGA
AGACCAACATTTTCA
+
FFFFFFFFF:FFFFFFFFFFFFF:FFFFFFFFFFFFFFFFFFFFFFFFFFFFFFF:FFFFFFFF:FF:F
FFFFFFFFFFF::FFFFFFFFFFFFFFFFFFFFFFFFFFFFFFFFFFFFFFFFFFFF:F:FFF,FFF
,FFFFFFFFFFFFFF
@A00155:342:HHGFNDSXY:1:2417:15619:26412 1:N:0:GAACCTAG+TCCGCATA
ATAAGATGAGCAAGATGGAACGTCTGGGTTCCAATTCTGTCAAGACTTATGACATATTAAATAGATTG
GTTACGGTAAAAGACAAGGCAAGGTATGGACCCTTAGCAAAGCCAGAAAGATCACCTTGCCCAAAGAA
GACCAACATTTTCAT
+
FFFFFFFFFFFFFFFFFFFFFFFFFFFFFFFFFFFFFFFFFFFFFFFFFFFFFFFFFFFFFFFFFFFF
FFFFFFFFFFFFFFFFFFFFFFFFFFFFFFFFFFFFFFFFFFFFFFFFFFFFFFFFFFFFFFFFFFFF
FFFFFFFFFFFFFFF
@A00155:342:HHGFNDSXY:1:2164:24894:17394 1:N:0:GAACCTAG+TCCGCATA
TAAGATGAGCAAGATGGAACGTCTGGGTTCCAATTCTGTCAAGACTTATGACATATTAAATAGATTGG
TTACGGTAAAAGACAAGGCAAGGTATGGACCCTTAGCAAAGCCAGAAAGATCACCTTGCCCAAAGAAG
ACCAACATTTTCATA
+
FFFFFFFF:FFF:FF:FFFFFF:FFFF:FFFFFFF:FF:FFFFFFFFFFFFFFFFFFFFFFFFF:FFFF
```

```
FFF,:FFFFFFF:FFFFFFFFFFFFFF:FFFFFFFFFFFFFFFFF:FFFFFFFFF:FFFFFFFFFFFF
FFFFFFFFFFFFFFF
@A00155:342:HHGFNDSXY:1:2227:29812:13730 1:N:0:GAACCTAG+TCCGCATA
TAAGATGAGCAAGATGGAACGTCTGGGTTCCAATTCTGTCAAGACTTATGACATATTAAATAGATTGG
TTACGGTAAAAGACAAGGCAAGGTATGGACCCTTAGCAAAGCCAGAAAGATCACCTTGCCCAAAGAAG
ACCAACATTTTCATA
+
FFFFFFFFFFF:F:FFFF,FFFFFFFFFFFFF:FFFFFFFFFFFFFFFFFFFFFFFFFFFFFFFFFFF
FFFFFFFFFFFFFFFFFFFFFFFFFFFFFFFFFFFFFFFFFFFFFFFFFFFFFFFFFFFFFFFFFFFF
FFFFFFFFFFFFFFF
@A00155:342:HHGFNDSXY:1:2525:31421:8375 1:N:0:GAACCTAG+TCCGCATA
TAAGATGAGCAAGATGGAACGTCTGGGTTCCAATTCTGTCAAGACTTATGACATATTAAATAGATTGG
TTACGGTAAAAGACAAGGCAAGGTATGGACCCTTAGCAAAGCCAGAAAGATCACCTTGCCCAAAGAAG
ACCAACATTTTCATA
+
:FFFFFFFFFFFFFFFFFFFFFFFFFFFFFFFFF:FFFFFFFFFFFFFFFFFFFFFFFF:FFFFFFFFF
FFFFFFFFFFFFFFFFFFFFFFFFFFFFFFFFFFFFFFFFFFFFFFFFFFFFFFF,FFFFFFF:FFFF
FFFFFFFFFFFFFF:
@A00155:342:HHGFNDSXY:1:1244:9570:17910 1:N:0:GAACCTAG+TCCGCATA
AAGATGAGCAAGATGGAACGTCTGGGTTCCAATTCTGTCAAGACTTATGACATATTAAATAGATTGGT
TACGGTAAAAGACAAGGCAAGGTATGGACCCTTAGCAAAGCCAGAAAGATCACCTTGCCCAAAGAAGA
CCAACATTTTCATAC
+
FFFFFFFFFFFFFFFFFFFFFFFFFFFFFFFFFFFFFFFFFFFFFFFFFFFFFFFFFFFFFFFFFFFF
FFFFFFFFFFFFFFFFFFFFFFFFFFFFFFFFFFFFFFFFFFFFFFFFFFFFFFFFFFFFFFFFFFFF
FFFFFFFFFFFFFFF
@A00155:342:HHGFNDSXY:1:2127:22887:5650 1:N:0:GAACCTAG+TCCGCATA
AAGATGAGCAAGATGGAACGTCTGGGTTCCAATTCTGTCAAGACTTATGACATATTAAATAGATTGGT
TACGGTAAAAGACAAGGCAAGGTATGGACCCTTAGCAAAGCCAGAAAGATCACCTTGCCCAAAGAAGA
CCAACATTTTCATAC
+
FFFFFFFFFFFFFFFFFFFFFFFFFFFFFFFFFFFFFFFFFFFFFFFFFFFFFFFFFFFFFFFFFFFF
FFFFFFFFFFFFFFFFFFFFFFFFFFFFFFFFFFFFFFFFFFFFFFFFFFFFFFFFFFFFFFFFFFFF
FFFFFFFFFFFFFFF
@A00155:342:HHGFNDSXY:1:2162:10511:9768 1:N:0:GAACCTAG+TCCGCATA
AAGATGAGCAAGATGGAACGTCTGGGTTCCAATTCTGTCAAGACTTATGACATATTAAATAGATTGGT
TACGGTAAAAGACAAGGCAAGGTATGGACCCTTAGCAAAGCCAGAAAGATCACCTTGCCCAAAGAAGA
CCAACATTTTCATAC
+
FFFFF:FFF:FFFFF:F:FFFFFFFFFFF:FFFFFFFFF,FFFFFFFFFF:FFFFFFFF:FFFFFFFF
FFFFFFFFFFFFFFFFFFFFFFFFFFFFFFFFFFFFFFFFFFFFFFFFFFFFFFFFFFFFFFFFFFF:F
FFF:FFFFFFFFFFF
@A00155:342:HHGFNDSXY:1:2417:16577:34021 1:N:0:GAACCTAG+TCCGCATA
CCGATCTGCAAGATGGAACGTCTGGGTTCCAATTCTGTCAAGACTTATGACATATTAAATAGATTGGT
TACGGTAAAAGACAAGGCAAGGTATGGACCCTTAGCAAAGCCAGAAAGATCACCTTGCCCAAAGAAGA
CCAACATTTTCATAC
+
FFFFFFFFFFFFFFFFFFFFFFFFFFFFFFFFFFFFFFFFFFFFFFFFFFFFFFFFFFFFFFFFFFFF
FFFFFFFFFFFFFF:FFFFFFFFFFFFFFFFFFFFFFFFFFFFFFFFFFFFFFFFFFFFFFFFFFFFF
FFFFFFFFFFFFFFF
@A00155:342:HHGFNDSXY:1:2539:30481:8437 1:N:0:GAACCTAG+TCCGCATA
AAGATGAGCAAGATGGAACGTCTGGGTTCCAATTCTGTCAAGACTTATGACATATTAAATAGATTGGT
TACGGTAAAAGACAAGGCAAGGTATGGACCCTTAGCAAAGCCAGAAAGATCACCTTGCCCAAAGAAGA
CCAACATTTTCATAC
```

+
:FFFFFFFFFFFFFFFFFFFFFFFFFFFFFFFFFFFFFFFFFFFFFFFFFFFFFFFFFFFFFFFFFFF
FFFFFFFFFFFFFFFFFFFFFFFFFFFFFFFFFFFFFFFFFFFFFFFFFFFFFFFFFFFFFFFFFFFF
FFFFFFFFFFFFFFF
@A00155:342:HHGFNDSXY:1:2632:2645:1094 1:N:0:GAACCTAG+TCCGCATA
AAGATGAGCAAGATGGAACGTCTGGGTTCCAATTCTGTCAAGACTTATGACATATTAAATAGATTGGT
TACGGTAAAAGACAAGGCAAGGTATGGACCCTTAGCAAAGCCAGAAAGATCACCTTGCCCAAAGAAGA
CCAACATTTTCATAC
+
FFFFFFFFFFFFFFFFFFFFFFFFF:FF::FFFFFFFFFFFFFFFFFFFFFFFFFFFFFFFFFFFFFF
FFFFFFFFFFF:FFFFFFFFFFFFFF:FFFFFFFFFFFFFFFFFF:FFFF:FFFFFFFFFFFFF:FFF
FFFFFFFFFFFFFFF
@A00155:342:HHGFNDSXY:1:2632:4417:5447 1:N:0:GAACCTAG+TCCGCATA
AAGATGAGCAAGATGGAACGTCTGGGTTCCAATTCTGTCAAGACTTATGACATATTAAATAGATTGGT
TACGGTAAAAGACAAGGCAAGGTATGGACCCTTAGCAAAGCCAGAAAGATCACCTTGCCCAAAGAAGA
CCAACATTTTCATAC
+
FFFFFFFF:FFFFFFFFFFFF:FFFF,F,FFFFFFFFFF:FF:FFFFF:FFFFFFFFFF:FFFFFFFF
FFFFFFFFFFFFFFFFFFFFFFFFFFFFFFFF:FFFFFFFFFFFFFFFFFFFFFF:FFFFFFFFFFFF
FFFFFFFFFFFFFFF
@A00155:342:HHGFNDSXY:1:1359:30499:27853 2:N:0:GAACCTAG+TCCGCATA
AAGATGAGCAAGATGGAACGTCTGGGTTCCAATTCTGTCAAGACTTATGACATATTAAATAGATTGGT
TACGGTAAAAGACAAGGCAAGGTATGGACCCTTAGCAAAGCCAGAAAGATCACCTTGCCCAAAGAAGA
CCAACATTTTCATAC
+
FFFFFFFFFFFFFFFFFFFFFFFFFFFFFFFFFFFFFFFFFFFFFFFFFFFFFF,FFF:FFFF:FFFF
:FFFFFF::FFFFFFFFFF:FF,FFFFFFFF:FFFFF:FFFFFFFFFFFFFFFFFFFFFFFFFFFFFF
FFFFFFFFFFFFFFF
@A00155:342:HHGFNDSXY:1:2456:26838:3192 2:N:0:GAACCTAG+TCCGCATA
AAGATGAGCAAGATGGAACGTCTGGGTTCCAATTCTGTCAAGACTTATGACATATTAAATAGATTGGT
TACGGTAAAAGACAAGGCAAGGTATGGACCCTTAGCAAAGCCAGAAAGATCACCTTGCCCAAAGAAGA
CCAACATTTTCATAC
+
FFFFFFFFFFFFFFFFF:FFFFFFFFFFFFFFFFFFFFFFFFFFFFFFFFFFFFFFFFFFFFF:FFFF
FFFFFFFFFF:FFFFF:FFFFFFFFFFFFFFFFFFFFFFFFFFFFFFFFFFFFFFFFFFFFFFFF:F
FFFFFF:FFFFFFFF
@A00155:342:HHGFNDSXY:1:2663:18801:34397 2:N:0:GAACCTAG+TCCGCATA
AGATGAGCAAGATGGAACGTCTGGGTTCCAATTCTGTCAAGACTTATGACATATTAAATAGATTGGTT
ACGGTAAAAGACAAGGCAAGGTATGGACCCTTAGCAAAGCCAGAAAGATCACCTTGCCCAAAGAAGAC
CAACATTTTCATAC
+
F:FFFFFFFFFFFFFFFF:FFFFFF,FFFFFFF:FFFFFFFF:FFFFFFFFFFF:FFF:F:FFF,:FF
FFFFFFF:FFFFFFF,FFF,FFFFFFFFF:FFF,FFFFFFFFFF:FFFFFFFF:FFFF:FFFFFFFF:
FFFFF,FFFFFFFF
@A00155:342:HHGFNDSXY:1:2246:21531:25347 1:N:0:GAACCTAG+TCCGCATA
AGATGAGCAAGATGGAACGTCTGGGTTCCAATTCTGTCAAGACTTATGACATATTAAATAGATTGGTT
ACGGTAAAAGACAAGGCAAGGTATGGACCCTTAGCAAAGCCAGAAAGATCACCTTGCCCAAAGAAGAC
CAACATTTTCATACA
+
FFFFFFFFFFFFFFFFFFFFFFFFFFFFFFFFFFFFFFFFFFFFFFFFFFFFFFFFFF:FFFFFFFFF
FFFFFFFFFFFFFFFFFFFFFFFFFFFFFFFFFFFFFFFFFFFFFFFFFFFFFFFFFFFFFFFF:F,F
FFFFFFFFFFFFFFF
@A00155:342:HHGFNDSXY:1:2246:21531:25347 2:N:0:GAACCTAG+TCCGCATA
AGATGAGCAAGATGGAACGTCTGGGTTCCAATTCTGTCAAGACTTATGACATATTAAATAGATTGGTT

```
ACGGTAAAAGACAAGGCAAGGTATGGACCCTTAGCAAAGCCAGAAAGATCACCTTGCCCAAAGAAGAC
CAACATTTTCATACA
+
FFFFFFFFFFFFFFFFFFFFFFFFFFFFFFFFFFFFFFFFFFFFFFFFFFFFFFFFFFFFFFFFFFFF
FFFF:FFFFFFFFFFFFFFFFFFFFFFFFFFFFFFFFFFFFFFFFFFFFFFFFFFFFFFFFFFFFFFF
FFFFFFFFFFFFFFF
@A00155:342:HHGFNDSXY:1:2505:21495:7279 2:N:0:GAACCTAG+TCCGCATA
AGATGAGCAAGATGGAACGTCTGGGTTCCAATTCTGTCAAGACTTATGACATATTAAATAGATTGGTT
ACGGTAAAAGACAAGGCAAGGTATGGACCCTTAGCAAAGCCAGAAAGATCACCTTGCCCAAAGAAGAC
CAACATTTTCATACA
+
FFFFFFFFF:F,FFFFFFF,FFFFFFFFFFFFFFFFFFF:FFFFFF:FFFFFF:FFFFFFFFFFFFFF
FFFFF:FFFFFFFFFFFFFFFFFFFFFFFFFFFFFFFFFFFFFFFFFFFFFFFFFFFFFFFFFFFF:F
FFFFFFFFFFFFFFF
@A00155:342:HHGFNDSXY:1:1425:11966:28103 2:N:0:GAACCTAG+TCCGCATA
GATGAGCAAGATGGAACGTCTGGGTTCCAATTCTGTCAAGACTTATGACATATTAAATAGATTGGTTA
CGGTAAAAGACAAGGCAAGGTATGGACCCTTAGCAAAGCCAGAAAGATCACCTTGCCCAAAGAAGACC
AACATTTTCATACAT
+
FFFFFFFFFFFFFFFFFFFFFFFFFFFFFFFFFFFFFFFFFFFFFFFFFFFFFFFFFFFFFFFFFFFF
FFF:FFFFFFFFFFFFFFFFFFFFFFFFFFFFFFFFFFFFFFFFFFFFFFFFFFFFFFFFFFFFFFFF
FFFFFFFFFFFFFFF
@A00155:342:HHGFNDSXY:1:2459:4869:11992 2:N:0:GAACCTAG+TCCGCATA
GATGAGCAAGATGGAACGTCTGGGTTCCAATTCTGTCAAGACTTATGACATATTAAATAGATTGGTTA
CGGTAAAAGACAAGGCAAGGTATGGACCCTTAGCAAAGCCAGAAAGATCACCTTGCCCAAAGAAGACC
AACATTTTCATACAT
+
FFFFFFFFFFFFFFFFFFFFFFFFFFFFFFFFFFFFFFFFFFFFFFFFFFFFFFFFFFFFFF:FFFFFF
FFF:FFFFFFFFFFFFFFFFFFFFFFFFFFFFFFFFFFFFFFFFFFFFFFFFFFFFFFFFFFF:FFFFF
FFFFFFFFFFFFFFF
@A00155:342:HHGFNDSXY:1:1261:19976:10316 1:N:0:GAACCTAG+TCCGCATA
ATGAGCAAGATGGAACGTCTGGGTTCCAATTCTGTCAAGACTTATGACATATTAAATAGATTGGTTAC
GGTAAAAGACAAGGCAAGGTATGGACCCTTAGCAAAGCCAGAAAGATCACCTTGCCCAAAGAAGACCA
ACATTTTCATACATG
+
FFFFFFFFFFFFFFFFFFFFFFFFFFFFFFFFFFFFFFFFFFFFFFFFFFFFFFFFFFFFFFFFFFFF
FFFFFFFFFFFFFFFFFFFFFFFFFFF:FFFFFFFFFFFFFFFFFFFFFFFFFFFFFFFFFFFFFFFF
FFFFFFFFFFFFFFF
@A00155:342:HHGFNDSXY:1:1510:21025:16673 1:N:0:GAACCTAG+TCCGCATA
ATGAGCAAGATGGAACGTCTGGGTTCCAATTCTGTCAAGACTTATGACATATTAAATAGATTGGTTAC
GGTAAAAGACAAGGCAAGGTATGGACCCTTAGCAAAGCCAGAAAGATCACCTTGCCCAAAGAAGACCA
ACATTTTCATACATG
+
FFFFFFFFFFFFFFFFFFFFFFFFFFFFFFFFFFFFFFFFFFFFFFFFFFFFFFFFFFFFFFFFFFFF
FFFFFFFFFFFFFFFFFFFFFFFFFF:FFFFFFFFFFFFFFFFFFFFFFFFFFFFFFFFFFF:FFFFF
FFFFFFFFFFFFFFF
@A00155:342:HHGFNDSXY:1:2272:25409:36949 1:N:0:GAACCTAG+TCCGCATA
ATGAGCAAGATGGAACGTCTGGGTTCCAATTCTGTCAAGACTTATGACATATTAAATAGATTGGTTAC
GGTAAAAGACAAGGCAAGGTATGGACCCTTAGCAAAGCCAGAAAGATCACCTTGCCCAAAGAAGACCA
ACATTTTCATACATG
+
FFFFFFFFFFFFFFFFFFFFFFFFFFFFFFFFFFFFFFFFFFFFFFFFFFFFFFFFFFFFFFFFFFFF
FFFFFFFFFFFFFFFFFFFFFFFFFFFFFFFFFFFFFFFFFF:FFFFFFFFFFFFFFFFFFFFFFFFF
FFFFFFFFFFFFFFF
```

@A00155:342:HHGFNDSXY:1:2261:18041:14074 1:N:0:GAACCTAG+TCCGCATA
ATGAGCAAGATGGAACGTCTGGGTTCCAATTCTGTCAAGACTTATGACATATTAAATAGATTGGTTAC
GGTAAAAGACAAGGCAAGGTATGGACCCTTAGCAAAGCCAGAAAGATCACCTTGCCCAAAGAAGACCA
ACATTTTCATACATG
+
FFFFFFFFFFFFFF:FFFFFFFFFF:FFFFFFFFFFFFFFFFFFFF:FFFFFFFFFFFFFFFFFFFFF
FFFFFFFFFFFFFFFFF:FFFFFFFFFFFFFFFFFFFFFFFFFFFFFFFFFFFFFFFFFFFFFFFF:F
FFFFFFFFFFFFFFF
@A00155:342:HHGFNDSXY:1:2261:16405:26710 1:N:0:GAACCTAG+TCCGCATA
ATGAGCAAGATGGAACGTCTGGGTTCCAATTCTGTCAAGACTTATGACATATTAAATAGATTGGTTAC
GGTAAAAGACAAGGCAAGGTATGGACCCTTAGCAAAGCCAGAAAGATCACCTTGCCCAAAGAAGACCA
ACATTTTCATACATG
+
FFFF:FFFFFF:FFF:FFFF::::F,FFFF:FFFFFFFFF:FFFFFFFFFFFFFFFFFFFFFFFFFFF
FFFFF:FFFFFFFFFFFFFFFFFFFFFFFFFFFFFFFFFFFFFFFFFFF,FFFFFFFFFFFFFFFFFF
FFFFFFFFFFFF,FF
@A00155:342:HHGFNDSXY:1:2325:32515:28463 1:N:0:GAACCTAG+TCCGCATA
ATCTGCAAGATGGAACGTCTGGGTTCCAATTCTGTCAAGACTTATGACATATTAAATAGATTGGTTAC
GGTAAAAGACAAGGCAAGGTATGGACCCTTAGCAAAGCCAGAAAGATCACCTTGCCCAAAGAAGACCA
ACATTTTCATACATG
+
FFFFFFFF:F::FFFFF:FFF:F:FFFFF,FFFFFFFFFFFFFFFFFFFFFFFFFFFFFFFFFFFFFF
FFFFFFFFFFFFFFFFFFFFFFFF:FFFFFFFFFFFFFFFFFFFFFFFFFF,FFFFFFFFFF:FFFFF
FFFFFFFFFFFF,FF
@A00155:342:HHGFNDSXY:1:2321:2781:30326 1:N:0:GAACCTAG+TCCGCATA
ATGAGCAAGATGGAACGTCTGGGTTCCAATTCTGTCAAGACTTATGACATATTAAATAGATTGGTTAC
GGTAAAAGACAAGGCAAGGTATGGACCCTTAGCAAAGCCAGAAAGATCACCTTGCCCAAAGAAGACCA
ACATTTTCATACATG
+
FFFFFFFFFFFFFFFFFFFFFFFFF:FFFFFFFFFFFFFFFFFFFFFFFFFFFFFFFFFFFFFFFFFF
FFFFFFFFFFFFFFFFFFFFFFFFFFFFFFFFFFFFFFFFFFFFFFFFFFFFFFFFFFFFFFFFFFFF
FFFFFFFFFFFFFFF
@A00155:342:HHGFNDSXY:1:2362:5746:5212 1:N:0:GAACCTAG+TCCGCATA
ATGAGCAAGATGGAACGTCTGGGTTCCAATTCTGTCAAGACTTATGACATATTAAATAGATTGGTTAC
GGTAAAAGACAAGGCAAGGTATGGACCCTTAGCAAAGCCAGAAAGATCACCTTGCCCAAAGAAGACCA
ACATTTTCATACATG
+
FFFFFFFFFFF:FFFFFFFFFFFFFFFFFFFFFFFFFFFFFFFFFFFFFFFFFFFFFFFFFFFFFFFF
FFFFFFFFFFFFFFFFFFFFFFFFFFFFFFFFFFFFFFFFFFFFFFFFFFFFFFFFFFFFFFFF:FFF
FFFFFFFFFFFFFFF
@A00155:342:HHGFNDSXY:1:2435:11541:28557 1:N:0:GAACCTAG+TCCGCATA
ATGAGCAAGATGGAACGTCTGGGTTCCAATTCTGTCAAGACTTATGACATATTAAATAGATTGGTTAC
GGTAAAAGACAAGGCAAGGTATGGACCCTTAGCAAAGCCAGAAAGATCACCTTGCCCAAAGAAGACCA
ACATTTTCATACATG
+
FFFF:FFFFFFFFFFFFFFFFFFFFFFFFFFFFFFFFFFFFFFFFFFFFFFFFFFFFFFFFFFFFFFF
FFFFFFFFFFFFFFFFFFFFFFFFFFFFFFFFFFFFFFFFFFFFFFFFFFFFFFFFFFFFFFFFFFFF
FFFFFFFFFFFFFFF
@A00155:342:HHGFNDSXY:1:1678:16034:12352 2:N:0:GAACCTAG+TCCGCATA
ATGAGCAAGATGGAACGTCTTGGTTCCAATTCTGTCAAGACTTATGACATATTAAATAGATTGGTTAC
GGTAAAAGACAAGGCAAGGTATGGACCCTTAGCAAAGCCAGAAAGATCACCTTGCCCAAAGAAGACCA
ACATTTTCATACATG
+
FFFFFFFFFFFFFFFFFFFFFFF:FFFFFFFFFFFFF:FFFFFFFFFFFFFFFFFFFFFFFFFFFFFF

```
FF:FFFFFFFFFFFFF:FFFFFFFFFFFFFFFFFFFFFFFFFFFFFFFFFFFFFFFFFFFF:FFFFF
FFFFFFFFFFFFFFF
@A00155:342:HHGFNDSXY:1:2217:8124:25645 1:N:0:GAACCTAG+TCCGCATA
GAGCAAGATGGAACGTCTGGGTTCCAATTCTGTCAAGACTTATGACATATTAAATAGATTGGTTACGG
TAAAAGACAAGGCAAGGTATGGACCCTTAGCAAAGCCAGAAAGATCACCTTGCCCAAAGAAGACCAAC
ATTTTCATACA
+
FFFFFFFFFFFFFFFFFFFFFFFFFFFFFFFFFFFFFFFFFFFFFFFFFFFFFFFFFFFFFFFFFFFF
FF:FFFFFFFFFFFFFFFFFFFFFFFFFFFFFFFFFFFFFFFFFFFFFFFFFFFFF:FFFF:FFFFF
FFFFFFFFFFF
@A00155:342:HHGFNDSXY:1:1321:2284:15812 1:N:0:GAACCTAG+TCCGCATA
GAGCAAGATGGAACGTCTGGGTTCCAATTCTGTCAAGACTTATGACATATTAAATAGATTGGTTACGG
TAAAAGACAAGGCAAGGTATGGACCCTTAGCAAAGCCAGAAAGATCACCTTGCCCAAAGAAGACCAAC
ATTTTCATACATGAT
+
FFF:FFFFFFFFFFFFFFFFFF:FFFF::FFFFFFFFFFFFFFFFFF,FFFFFFFFFFFFFFFFFFFF
FFFFFFFFFFF:FFFF:FFFFFFFFFFFFFFFFFFFFFFFFFFFFFF:FFFFFFFFFFFF:FFFFFF
FFFFFFFFFFFFFFF
@A00155:342:HHGFNDSXY:1:1426:28384:20212 1:N:0:GAACCTAG+TCCGCATA
GAGCAAGATGGAACGTCTGGGTTCCAATTCTGTCAAGACTTATGACATATTAAATAGATTGGTTACGG
TAAAAGACAAGGCAAGGTATGGACCCTTAGCAAAGCCAGAAAGATCACCTTGCCCAAAGAAGACCAAC
ATTTTCATACATGAT
+
FFF:FFFFFFFFFFFFFFFFFFFFFFFFFFFFFFFFFFFFFFFFFFFFFFFF:FFFFFFFFFFFF
FFFFFFFFFFFFFFFFFFFFFFFFFFFFFFFFFFFFFFFFFFFFFFFFFFFFFFFFFFFFFFFFFFFF
FFFFFFFFFFFFFFF
@A00155:342:HHGFNDSXY:1:1364:4273:25708 2:N:0:GAACCTAG+TCCGCATA
GAGCAAGATGGAACGTCTGGGTTCCAATTCTGTCAAGACTTATGACATATTAAATAGATTGGTTACGG
TAAAAGACAAGGCAAGGTATGGACCCTTAGCAAAGCCAGAAAGATCACCTTGCCCAAAGAAGACCAAC
ATTTTCATACATGAT
+
FFFFFFFFFFFFFFFFFFFFFFFFFFFFFFFFFFFFFFFFFFFFFFFFFFFFFFFFFFFFFFFFFFFF
FFFFFFFFFFFFFFFFFFFFFFFFFFFFFFFFFFF,FFFFFFFFFFFFFFFFFFFFFFFFFFFFFFFF
FFF:FFFFFFFFFFF
@A00155:342:HHGFNDSXY:1:2256:22281:18317 2:N:0:GAACCTAG+TCCGCATA
GAGCAAGATGGAACGTCTGGGTTCCAATTCTGTCAAGACTTATGACATATTAAATAGATTGGTTACGG
TAAAAGACAAGGCAAGGTATGGACCCTTAGCAAAGCCAGAAAGATCACCTTGCCCAAAGAAGACCAAC
ATTTTCATACATGAT
+
FFFFFFFFFFFFFFFFFFFFFFFFFFFFFFFFFFF:FFFFFFFFFFFFFFFFFFFFFFFFFFFFFFFF
FFFFFFFFFFFFFF,FFFFFFFFFFFFFFFFFFFF:FFFFFFFFFFFFFFFFFF,FFFF:FFFF:FFF
FFFF,FFFFFFFFFF
@A00155:342:HHGFNDSXY:1:2256:4101:21214 2:N:0:GAACCTAG+TCCGCATA
GAGCAAGATGGAACGTCTGGGTTCCAATTCTGTCAAGACTTATGACATATTAAATAGATTGGTTACGG
TAAAAGACAAGGCAAGGTATGGACCCTTAGCAAAGCCAGAAAGATCACCTTGCCCAAAGAAGACCAAC
ATTTTCATACATGAT
+
FFFFFFFFFFFFFFFFFFFFFFFFFFFFFFFFFFFFFFFFFFFFFFFFFFFFFFFFFFFFFFFFFFFF
FFFF:FFFFFFFFFFF:FFFFFFFFFFFFFFFF:FFFFFFFFFFFFFFFFFFFFFFFFFFFFFFFF,FF
FFFFFFFFFFF:FFF
@A00155:342:HHGFNDSXY:1:1207:6958:17394 2:N:0:GAACCTAG+TCCGCATA
AGCAAGATGGAACGTCTGGGTTCCAATTCTGTCAAGACTTATGACATATTAAATAGATTGGTTACGGT
AAAAGACAAGGCAAGGTATGGACCCTTAGCAAAGCCAGAAAGATCACCTTGCCCAAAGAAGACCAACA
TTTTCATACATGATG
```

+
FFFFFFFFF,FFFF:FFFF:::FF:FFFFF:FFFFFFFFFFFFFF:F:FFFFFFFFFFFFFF:FFFFF
FFFFFFFFFFFFFF:FFFFFFFFFFFFFFFFFFFF:F:FFFFFF:FFFF,:FF:FFFFFFFFFFFF:FF
,FFFFFFFFF,FFFF
@A00155:342:HHGFNDSXY:1:1560:23050:6245 2:N:0:GAACCTAG+TCCGCATA
AGCAAGATGGAACGTCTGGGTTCCAATTCTGTCAAGACTTATGACATATTAAATAGATTGGTTACGGT
AAAAGACAAGGCAAGGTATGGACCCTTAGCAAAGCCAGAAAGATCACCTTGCCCAAAGAAGACCAACA
TTTTCATACATGATG
+
FFFFFFFFFFFFFFFFFFFFFFFFFFFFFFFFFFFFFFFFFFFFFFFFFFFFFFFFFFFFFFFFFF:FF
F,FFFFFF:FFFFFFFF:FFFFFFFFFFFFFFF:FFFFFFFFF:FFFFFFFFFFFFFFFFFFFFFFFFF
FFFFFFF:FFFFFFF
@A00155:342:HHGFNDSXY:1:2165:4264:6151 2:N:0:GAACCTAG+TCCGCATA
AGCAAGATGGAACGTCTGGGTTCCAATTCTGTCAAGACTTATGACATATTAAATAGATTGGTTACGGT
AAAAGACAAGGCAAGGTATGGACCCTTAGCAAAGCCAGAAAGATCACCTTGCCCAAAGAAGACCAACA
TTTTCATACATGATG
+
FF:FFFFFFFFFFFF:FF:FFFFFFFFFFFFFFFFFFFF:FFFFFFFFFFFF:FFF:FFFFF:FFFF
FF:FFFFFFFFFFFF::FFFFFFFFFFF:FF:FFFFFFFFFFFFFFFF,FFFFF,FF:FFFFFFFFFF
FF:FFFFFFFF,FFF
@A00155:342:HHGFNDSXY:1:2402:25328:29168 2:N:0:GAACCTAG+TCCGCATA
AGCAAGATGGAACGTCTGGGTTCCAATTCTGTCAAGACTTATGACATATTAAATAGATTGGTTACGGT
AAAAGACAAGGCAAGGTATGGACCCTTAGCAAAGCCAGAAAGATCACCTTGCCCAAAGAAGACCAACA
TTTTCATACATGATG
+
FFFFFFFFFFFFFFFFFFFFFFFFFFFFFFFFFFFFFFFF:FFFFFFFFFFFFFFFFFF:F:F:FFFF
FFFF:FFFFFFFF,FFFFFFFFFFFFFFFFF,FFFFFFFFFFFFFFFFFFF,FFFFF:FFFFFFFFFF
FFFFF:FFFFFFF:F
@A00155:342:HHGFNDSXY:1:2611:17345:5040 2:N:0:GAACCTAG+TCCGCATA
AGCAAGATGGAACGTCTGGGTTCCAATTCTGTCAAGACTTATGACATATTAAATAGATTGGTTACGGT
AAAAGACAAGGCAAGGTATGGACCCTTAGCAAAGCCAGAAAGATCACCTTGCCCAAAGAAGACCAACA
TTTTCATACATGATG
+
FFFFFFFF:FFFFFFFFFFFFFFFFFFFFFFFFFFFFFFFFFFFFFFFF:FFFFFFFFFFFFFFF:FFF
FFFF:FFFFFFFFF:FFFFFFFFFFFFFFFFFFFFFFFFFFFFFFFFFFFFFFFFFFFFFFFFFFFFFF
FF:FFFFFFFFFF,F
@A00155:342:HHGFNDSXY:1:2105:1208:27258 2:N:0:GAACCTAG+TCCGCATA
GCAAGATGGAACGTCTGGGTTCCAATTCTGTCAAGACTTATGACATATTAAATAGATTGGTTACGGTA
AATGACAAGGCAAGGTATGGACCCTTAGCAAAGCCAGAAAGATCACCTTGCCCAAAGAAGACCAACAT
TTTCATACAT
+
:F:FF:FFF:FFFF,FFFFFFFF:,FFFFF:::FF:FFFFFF,:FFFFFF,,F,FFFFFFFFF:FFF:
,,,F,FF:FFFFFFFFFFFF,F::FF:FFFF:FFF:F,FFF:F:F:,FFF:FFF,,F,F,F:::FFFF
FFF,:F:,FF
@A00155:342:HHGFNDSXY:1:2325:32515:28463 2:N:0:GAACCTAG+TCCGCATA
GCAAGATGGAACGTCTGGGTTCCAATTCTGTCAAGACTTATGACATATTAAATAGATTGGTTACGGTA
AAAGACAAGGCAAGGTCTGGACCCTTAGCAAAGCCAGAAAGATCACCTTGCCCAAAGAAGACCAACAT
TTTCATACATGAGA
+
F:FFFFFFFF,FF,FFFFFFFFFF::FFFFFF::F:FFFF,FFF,,:FF:FFFFFFFFFFFF,FFF::
FF:FFF:FFFFFFFFF,FFFFFFFFF,FFFFFFFFFF:FFFF:F:FF:FFFFFF:FFFFFFFFFFFFF
FFFFFF,FF,FFF:
@A00155:342:HHGFNDSXY:1:1110:15754:28150 2:N:0:GAACCTAG+TCCGCATA
GCAAGATGGAACGTCTGGGTTCCAATTCTGTCAAGACTTATGACATATTAAATAGATTGGTTACGGTA

AAAGACAAGGCAAGGTATGGACCCTTAGCAAAGCCAGAAAGATCACCTTGCCCAAAGAAGACCAACAT
TTTCATACATGATGA
+
FFFFFFFFFFFFFFFFFFFFFFFFFFFFFFFFFFFFFFFF:FFFFFFFFFF:FFFFFFFFFFFFFFFF:
F:FFF:FFFFFFFFFFFFFFFFFFFFFFFFFFFF,FFFFFFFFFFFFFFFF:FFFFFFFFFFFFFFF
FFFFFFFF:FFFFFF
@A00155:342:HHGFNDSXY:1:1149:3821:33786 2:N:0:GAACCTAG+TCCGCATA
GCAAGATGGAACGTCTGGGTTCCAATTCTGTCAAGACTTATGACATATTAAATAGATTGGTTACGGTA
AAAGACAAGGCAAGGTATGGACCCTTAGCAAAGCCAGAAAGATCACCTTGCCCAAAGAAGACCAACAT
TTTCATACATGATGA
+
FFFFFFFFFFFFFFFFFFFFFFFFFFFFFFFFFFFFFFFFFFFFFFFFFFFFFFFFFFFFFFFFFFFF
FFFFFFFFFFFFFFFF:FFFFFFFFFFFFFFFF:FFFFFF:FFFFFFFFFFFF:FFFFF:FFFFFFFF
FFFFFFFFFFFFFFF
@A00155:342:HHGFNDSXY:1:1550:22815:3897 2:N:0:GAACCTAG+TCCGCATA
GCAAGATGGAACGTCTGGGTTCCAATTCTGTCAAGACTTATGACATATTAAATAGATTGGTTACGGTA
AAAGACAAGGCAAGGTATGGACCCTTAGCAAAGCCAGAAAGATCACCTTGCCCAAAGAAGACCAACAT
TTTCATACATGATGA
+
FFFFFFFFFF:FFFFFFFFFFFFFFFFFFFFFFFFFFFFFFFFFFFFFFFFFFFF:FFFFFF,FFF,
,FFFFFFFFFFFFFFFFFFFFFFFFFFFFFF,FFFFFFFFFFFFFFFFFFFFFFFFFFFFFFFFFFFFF
FF:FFFFFFFFFFFF
@A00155:342:HHGFNDSXY:1:2117:27968:35368 2:N:0:GAACCTAG+TCCGCATA
GCAAGATGGAACGTCTGGGTTCCAATTCTGTCAAGACTTATGACATATTAAATAGATTGGTTACGGTA
AAAGACAAGGCAAGGTATGGACCCTTAGCAAAGCCAGAAAGATCACCTTGCCCAAAGAAGACCAACAT
TTTCATACATGATGA
+
FFFFFFFFFFFFFFFFFFFFFFFFFFFFFFFFFFFFFFFFFFFFFFFFFFFF:FFFFFFFFFFFFFFF
FFFFFFFFFFFFFFFFFFFFFFFFFFFFFFFFFFF:FFFFFFFF:FFFFFFFFFFFFFFFFFFF:FFFFF
FFFFFFFFFFFF:FF
@A00155:342:HHGFNDSXY:1:2134:8386:9627 2:N:0:GAACCTAG+TCCGCATA
GCAAGATGGAACGTCTGGGTTCCAATTCTGTCAAGACTTATGACATATTAAATAGATTGGTTACGGTA
AAAGACAAGGCAAGGTATGGACCCTTAGCAAAGCCAGAAAGATCACCTTGCCCAAAGAAGACCAACAT
TTTCATACATGATGA
+
FFFFFFFFFFFFFFFFFFFFFFFFFFFFFFFFFFFFFFFFFFFFFFFFFFFFFFFFFFFFFFFFFFFF
FFFFFFFFFFFFFFFFF:FFFFFFFFFFFFFFFFFFFFFFFFFFFFFFFFFFFFFFFFFF:FFFFFFF
FFFFFFFFFFFFFFF
@A00155:342:HHGFNDSXY:1:2232:20265:29042 2:N:0:GAACCTAG+TCCGCATA
GCAAGATGGAACGTCTGGGTTCCAATTCTGTCAAGACTTATGACATATTAAATAGATTGGTTACGGTA
AAAGACAAGGCAAGGTATGGACCCTTAGCAAAGCCAGAAAGATCACCTTGCCCAAAGAAGACCAACAT
TTTCATACATGATGA
+
F,::F:FFFF:FFFFFFFFFFFFFFFFFFFFFFFFFFFFFFFFFFFFFFFFFFFFFFFFFFFFFFFFF
FFFFFFFFFFFF:FFF:FFFFFFFFFFFFFFFFFFFFFFFFFFFFFFFFFFFFFFFFFFFFFFFFF:F
FFFFFFFFFFFFFFF
@A00155:342:HHGFNDSXY:1:2417:16577:34021 2:N:0:GAACCTAG+TCCGCATA
GCAAGATGGAACGTCTGGGTTCCAATTCTGTCAAGACTTATGACATATTAAATAGATTGGTTACGGTA
AAAGACAAGGCAAGGTATGGACCCTTAGCAAAGCCAGAAAGATCACCTTGCCCAAAGAAGACCAACAT
TTTCATACAGATCGG
+
FFFFF:FFFFFFFFF:FFF,FFFFFFFF:F:FFFFFFFFF:FFF,FFFFFFFFFFFFFFF:FFFFF:F
FF:FFFFFFF:FFFFFFFFFFFFFFFFFFF,FFFFFFFFF:FF:FFFFFFFFFFFFFFFFF:FFFFFFFF
FFFFFFFFFFFFFFF

@A00155:342:HHGFNDSXY:1:1301:25265:19413 1:N:0:GAACCTAG+TCCGCATA
CAAGATGGAACGTCTGGGTTCCAATTCTGTCAAGACTTATGACATATTAAATAGATTGGTTACGGTAA
AAGACAAGGCAAGGTATGGACCCTTAGCAAAGCCAGAAAGATCACCTTGCCCAAAGAAGACCAACATT
TTCATACATGAT
+
FFF:FF,FFF,:FFFF:FFF:F,:FF:FFFFFFFFFFF:FFF,FFFFF:FFF:,FF::FFF,F,F:,F
F,FFF,FFFFFFFFF:FFF,F:,FFFF:FF,FF:F::F:F,F::FFFF:FFF,,:F:,:F::FF,FFF
FF,:FFFFFF:F
@A00155:342:HHGFNDSXY:1:1554:32154:8703 2:N:0:GAACCTAG+TCCGCATA
CAAGATGGAACGTCTGGGTTCCAATTCTGTCAAGACTTATGACATATTAAATAGATTGGTTACGGTAA
AAGACAAGGCAAGGTATGGACCCTTAGCAAAGCCAGAAAGATCACCTTGCCCAAAGAAGACCAACATT
TTCATACATGATGAA
+
FFFFFFFFFFFFFFFFFFFFFFFFFF:FFFFFFFFFFFFFFFFFFFFFFFFFFFFFFFFFFFFFFFFF
FFFFFFFFFFFFFFFFFFFFFFFFFFFFF,FFFFFF:FFFFFFFFFFFFFFFFF:FFFFFFFFFFFFF
FF:FFFFFFFFFFFF
@A00155:342:HHGFNDSXY:1:1554:31638:9846 2:N:0:GAACCTAG+TCCGCATA
CAAGATGGAACGTCTGGGTTCCAATTCTGTCAAGACTTATGACATATTAAATAGATTGGTTACGGTAA
AAGACAAGGCAAGGTATGGACCCTTAGCAAAGCCAGAAAGATCACCTTGCCCAAAGAAGACCAACATT
TTCATACATGATGAA
+
FFFFFFFFFFFF:FFFFFFFFFFFFFFFFFFFFFFFFFFFFFFFFFFFFFFFFFFFFFFFFFFFFFF
FFFFFFFFFFF:FFFFFFFFFFFFFFFFFFFFFFFFFFFFFFFFFFFFFFFFFFFFFFFFFFFFFFFF
FFFFFFFFFFFFFFF
@A00155:342:HHGFNDSXY:1:1672:19434:26224 2:N:0:GAACCTAG+TCCGCATA
AAGATGGAACGTCTGGGTTCCAATTCTGTCAAGACTTATGACATATTAAATAGATTGGTTACGGTAAA
AGACAAGGCAAGGTATGGACCCTTAGCAAAGCCAGAAAGATCACCTTGCCCAAAGAAGACCAACATTT
TCATACATGATGAA
+
FFFFFFFFFFFFFFFFFFFFFFFFFFFFFFFFFFFFFFFFFFFFFFF:F:FF,FFFFFFFF,FFF,::
:FFFFFFFF:FFF:F,F,FFFFFFFF:FFFFFF,:,FFFFFFFFFFFFFFFF,FFFFFFF:FFFFFFF
FFFFFFFFF::F,F
@A00155:342:HHGFNDSXY:1:2616:3251:33144 2:N:0:GAACCTAG+TCCGCATA
GATGGAACGTCTGGGTTCCAATTCTGTCAAGACTTATGACATATTAAATAGATTGGTTACGGTAAAAG
ACAAGGCAAGGTATGGACCCTTAGCAAAGCCAGAAAGATCACCTTGCCCAAAGAAGACCAACATTTTC
ATACATGAGATCGGA
+
F::FF::FFFFFFFFFFFFFFFFFFFFFFFFFFFFFFFFFFFFFFFFFFFFFFFFFFFFFFFFFFF:F
FFFFFFFF:FFFFFFFFFFFFFFFFFFFFFFFFFFFFFFFFFFFFFFFFFFFFFFFFFFFFFFFFFFF
FFFFFFFFFFFFFFF
@A00155:342:HHGFNDSXY:1:1615:9489:10285 2:N:0:GAACCTAG+TCCGCATA
ATGGAACGTCTGGGTTCCAATTCTGTCAAGACTTATGACATATTAAATAGATTGGTTACGGTAAAAGA
CAAGGCAAGGTATGGACCCTTAGCAAAGCCAGAAAGATCACCTTGCCCAAAGAAGACCAACATTTTCA
TACATGATGAAATT
+
FFFFFFFFFFFFFFFFFFFFFFFFFFFFFFFFFFF,FFFFFFFFFFFFFFFFFFFFFF:FFFFFFFFF
F:FFFFFFFFFFFFFFFFFFFFFFFFFFFFFFFFFF,FFFFFFFFFFFFFF:FFFFFFFF:FFFFFFF
FFFFFFFFFFFFFF
@A00155:342:HHGFNDSXY:1:1501:6090:14512 2:N:0:GAACCTAG+TCCGCATA
ATGGAACGTCTGGGTTCCAATTCTGTCAAGACTTATGACATATTAAATAGATTGGTTACGGTAAAAGA
CAAGGCAAGGTATGGACCCTTAGCAAAGCCAGAAAGATCACCTTGCCCAAAGAAGACCAACATTTTCA
TACATGATGAAATTC
+
:FFFF,FFFFFFFFFFFFFF,FFFFFFF:FFFFFF:FFFFFFFFFFF:FFFFFFFFFFFFFFFF:FFF

FFFFFFFFFF:FFFFFFFFFFFFFF,FFFFFFFFFFFFFFFF,FFFFFFFFFFFFFFFFFFFFF:FFF
FFFF:FFFF:FF,FF
@A00155:342:HHGFNDSXY:1:1533:16577:2206 2:N:0:GAACCTAG+TCCGCATA
ATGGAACGTCTGGGTTCCAATTCTGTCAAGACTTATGACATATTAAATAGATTGGTTACGGTAAAAGA
CAAGGCAAGGTATGGACCCTTAGCAAAGCCAGAAAGATCACCTTGCCCAAAGAAGACCAACATTTTCA
TACATGATGAAATTC
+
FFFFFFFFFFFFFFFFFFFFFFFFFFFFFFFFFFFFF,FFFFFFFFFFFFFFFFFFFFFFF:F,FFFF
FFFFFFFFFFF:FFFFFFFFFFFFFFFFFF,FFFFFFFFFFFFFFFFFFFFFFFFFFFFFFFFF,FFF
FFFFFFFFFFFFFFF
@A00155:342:HHGFNDSXY:1:2201:13955:30170 2:N:0:GAACCTAG+TCCGCATA
ATGGAACGTCTGGGTTCCAATTCTGTCAAGACTTATGACATATTAAATAGATTGGTTACGGTAAAAGA
CAAGGCAAGGTATGGACCCTTAGCAAAGCCAGAAAGATCACCTTGCCCAAAGAAGACCAACATTTTCA
TACATGATGACATTC
+
FFFFFFFFFFFFFFFFFFFFFFFFFFFFFFFFFFFFF:FFFFF:FFFF:FFFFFFFF,FFF,F,,FFF
F:FFFFF,FFFFFFFFFFFFFFFF:FFFF:FFFFFFF:FFFFFFFFFFFFFFFFFFFFFFFFFFFFF:
FFFFFFFFFF,FFFF
@A00155:342:HHGFNDSXY:1:2154:26621:22545 2:N:0:GAACCTAG+TCCGCATA
ATGGAACGTCTGGGTTCCAATTCTGTCAAGACTTATGACATATTAAATAGATTGGTTACGGTAAAAGA
CAAGGCAAGGTATGGACCCTTAGCAAAGCCAGAAAGATCACCTTGCCCAAAGAAGACCAACATTTTCA
TACATGATGAAATTC
+
FFFFFFFFFFFFFFFFFFFFFFFFFFFF:FFFFFFFFFFFFFFFFFFFFFFFFFFFFFF:FFF:FFFF
FFFFFFFFFFFFFF,FFFFF:FFFFFFFFFFFFFFFFFFFFFFFF,FFFFFFF:FFFFFFFFFFFFFF
FFFFFFFFFFFFFFF
@A00155:342:HHGFNDSXY:1:2262:4508:15280 2:N:0:GAACCTAG+TCCGCATA
ATGGAACGTCTGGGTTCCAATTCTGTCAAGACTTATGACATATTAAATAGATTGGTTACGGTAAAAGA
CAAGGCAAGGTATGGACCCTTAGCAAAGCCAGAAAGATCACCTTGCCCAAAGAAGACCAACATTTTCA
TACATGATGAAATTC
+
FFFFFFFFFFFFFFFFFFFFFFFFFFFFFFFFFFFFFFFFFFFFFF:FFFFFFFFFFFFFFFFFF:FF
FFFFFFFFFFFFFFFFFFFFFFFFFFFFFFFFFFFFFFFFFFFFFFFFFFFF:FFFFFFFFFFFFFFF
FFFFFFFFFFF,FFF
@A00155:342:HHGFNDSXY:1:2461:22688:15483 2:N:0:GAACCTAG+TCCGCATA
ATGGAACGTCTGGGTTCCAATTCTGTCAAGACTTATGACATATTAAATAGATTGGTTACGGTAAAAGA
CAAGGCAAGGTATGGACCCTTAGCAAAGCCAGAAAGATCACCTTGCCCAAAGAAGACCAACATTTTCA
TACATGATGAAATTC
+
FFFFFFFFFFFFFFFFFFFFFFFFFFFFFFFFFFFFFFFFFFFFFFFFFFFFFFFFFFFFFFFFFFFF
FFFFFFFFFFFFFFFFFFFFFFFFFFFFFFFFFFFFFFFFFFFFFFFFFFFFFFFFFFFFFFFFFFFF
FFFFFFFFFFFFFFF
@A00155:342:HHGFNDSXY:1:2620:21531:33458 2:N:0:GAACCTAG+TCCGCATA
ATGGAACGTCTGGGTTCCAATTCTGTCAAGACTTATGACATATTAAATAGATTGGTTACGGTAAAAGA
CAAGGCAAGGTATGGACCCTTAGCAAAGCCAGAAAGATCACCTTGCCCAAAGAAGACCAACATTTTCA
TACATGATGAAATTC
+
FFFFFFFFFFFFFFFFFFFFFFFFFFFFFFFFFFFFFFFFFFFFFFFFFFF:FFFFFFFFFFFFFFFF
FFFFFFFFFFFFFFFFFFFFFFFFFFFFFFFFFFFFFFFFFFFFFFFFFFFFFFFFFFFFFFFFFFFF
FFFFFFFFFFF:FFF
@A00155:342:HHGFNDSXY:1:1317:7274:22138 2:N:0:GAACCTAG+TCCGCATA
TGGAACGTCTGGGTTCCAATTCTGTCAAGACTTATGACATATTAAATAGATTGGTTACGGTAAAAGAC
AAGGCAAGGTATGGACCCTTAGCAAAGCCAGAAAGATCACCTTGCCCAAAGAAGACCAACATTTTCAT
ACATGATGAAATTCA

```
+
FFFF:FFFFFFFFFFFFFFFFFFFFFFF:FFFFF:FFFFFFFFFFFFFFFFFFFFFFFFFFFFFFFFFFF
FFFFFFFFFFF:FFFFFFFFFFFFFFFFFFFFFFFFFFFFFFFFFFFFF:FFFF:FFFFFFFFFFFFF
FFFFFFFFFFFFFFF
@A00155:342:HHGFNDSXY:1:1456:28357:16752 2:N:0:GAACCTAG+TCCGCATA
TGGAACGTCTGGGTTCCAATTCTGTCAAGACTTATGACATATTAAATAGATTGGTTACGGTAAAAGAC
AAGGCAAGGTATGGACCCTTAGCAAAGCCAGAAAGATCACCTTGCCCAAAGAAGACCAACATTTTCAT
ACATGATGAAATTCA
+
FFFFFFFFFFFFF:FFFFFFFFFFFF:FFFFFFFFFFFFFF,FFFFFFFFFFFFFFFFFF::FFFFFF
FFFFFFFF,FFFFFFFFFFFFFFFFFFFFFFFFFFFF:FFFFFFFFFFFFF:FFFFFFFFFFFFFFFFF
FFFFFF:F:::FFFF
@A00155:342:HHGFNDSXY:1:1456:28384:16611 2:N:0:GAACCTAG+TCCGCATA
TGGAACGTCTGGGTTCCAATTCTGTCAAGACTTATGACATATTAAATAGATTGGTTACGGTAAAAGAC
AAGGCAAGGTATGGACCCTTAGCAAAGCCAGAAAGATCACCTTGCCCAAAGAAGACCAACATTTTCAT
ACATGATGAAATTCA
+
FFFFFFF,FFFFFFFFFFFFF:FFFFFFFFFF,FFFFFFF:FF:FF:F::F:FFFFFFFFFFF:FFFF
FFFFFFFF:FFFFFFF:F:FFFFFFFFFFFFF:FFFFFFFFFFFF:FFFFFFFFFFF:FFFFFFFFFF
FFFFFFFFF:FFFFF
@A00155:342:HHGFNDSXY:1:2271:7672:24956 2:N:0:GAACCTAG+TCCGCATA
TGGAACGTCTGGGTTCCAATTCTGTCAAGACTTATGACATATTAAATAGATTGGTTACGGTAAAAGAC
AAGGCAAGGTATGGACCCTTAGCAAAGCCAGAAAGATCACCTTGCCCAAAGAAGACCAACATTTTCAT
ACATGATGAAATTCA
+
FFFFFFFFFFFFFFFFFFFFFFFFFFFFFFFFFFFFFFFFFFFFF:FFFFFFFFFFFFFFFFFFFFFFF
FFFFFF:FFF,FFFFFFFFFFFFFFFFFFFFFFFFFFFFFFFFFFFFFFFFF:FFFFFFFFFFFF,
FFFFFFFFFF,FF:F
@A00155:342:HHGFNDSXY:1:2466:18331:22654 2:N:0:GAACCTAG+TCCGCATA
TGGAACGTCTGGGTTCCAATTCTGTCAAGACTTATGACATATTAAATAGATTGGTTACGGTAAAAGAC
AAGGCAAGGTATGGACCCTTAGCAAAGCCAGAAAGATCACCTTGCCCAAAGAAGACCAACATTTTCAT
ACATGATGAAATTCA
+
FFFFFFFFFFFFFFFFFFFFFFFFFFFFFFFFFFFFFFFFFFFFFFFFFFFFFFFFFFFFFFFFF:FF:F
FFFFFFFFFFFFFFFFFFFFFFFFFFFFFFFFFFFFFFFFFFFFFFFFFFFFFFFFFFFFFFFFFFFF
FFFFFFFFFFFFFFF
@A00155:342:HHGFNDSXY:1:2538:31864:10488 2:N:0:GAACCTAG+TCCGCATA
TGGAACGTCTAGGTTCCAATTCTGTCAAGACTTATGACATATTAAATAGATTGGTTACGGTAAAAGAC
AAGGCAAGGTATGGACCCTTAGCAAAGCCAGAAAGATCACCTTGCCCAAAGAAGACCAACATTTTCAT
ACATGATGAACTTCA
+
FFFF:FFFFFFFFFFFFFFFFFFFFF:FFFF:FF,FFFFFFFFF:F,FFF,FFFFF,FFF:,F:FFFF
:FFFF,:FF,FFFFFFFFFFFFFFFFFFF:FFFFFFFFFFFFFFFFFF:FFFFFFFFFFFFF,:FFFF
FFFFFF,FFF,FFFF
@A00155:342:HHGFNDSXY:1:2672:22408:35446 2:N:0:GAACCTAG+TCCGCATA
TGGAACGTCTGGGTTCCAATTCTGTCAAGACTTATGACATATTAAATAGATTGGTTACGGTAAAAGAC
AAGGCAAGGTATGGACCCTTAGCAAAGCCAGAAAGATCACCTTGCCCAAAGAAGACCAACATTTTCAT
ACATGAGGAAATTCA
+
FFFFFFFFFFFFFFFFFFFFFFFFFFFFF,FFFFFFFFFF,FFFFFFF:FFFFFFFFFFFFF,F,FFF
FF:FFFFFFFFFFFFFFFFF,FFF:FFFF:FFFFFF,FFF::FFFFFFFFFF:FFFFFFFFFFFFFF
FFFFFF,FFFFFFFF
@A00155:342:HHGFNDSXY:1:2229:17201:19225 2:N:0:GAACCTAG+TCCGCATA
GGAACGTCTGGGTTCCAATTCTGTCAAGACTTATGACATATTAAATAGATTGGTTACGGTAAAAGACA
```

AGGCAAGGTATGGACCCTTAGCAAAGCCAGAAAGATCACCTTGCCCAAAGAAGACCAACATTTTCATA
CATGATGAAATTC
+
FFFFFFFFFFFFFFFFFFFFFFFFFFFFFFFFFF:FFFFFFFFFF:FFFFFFFF:FFFFFFFFFFFFFF
FFFF:FF::FFFFF:FFFFF:FFF:FFFF:FFF:F::F:,FFF,FFF,F:FFFF:,:FFFFFFF::FF
FFFF,F,::,FFF
@A00155:342:HHGFNDSXY:1:2651:15329:14669 1:N:0:GAACCTAG+TCCGCATA
GGAACGTCTGGGTTCCAATTCTGTCAAGACTTATGACATATTAAATAGATTGGTTACGGTAAAAGACA
AGGCAAGGTATGGACCCTTAGCAAAGCCAGAAAGATCACCTTGCCCAAAGAAGACCAACATTTTCATA
CATGATGAAATTCAT
+
FFFFFFFFFFFF:FFFFFFFFFFFFFFFFFFFFFFFFFFFFFFFFFFFFFFFFFFFFFFFFFFFFFFFF
FFFFFFFFFFFFFFFFFFFFFFFFFFFFFFFFFFFFFFFFFFFFFFFFFFFFFFFFFFFFFFFFFFFFF
FFFFFFFFFFFFFFF
@A00155:342:HHGFNDSXY:1:1114:24361:29559 2:N:0:GAACCTAG+TCCGCATA
GGAACGTCTGGGTTCCAATTCTGTCAAGACTTATGACATATTAAATAGATTGGTTACGGTAAAAGACA
AGGCAAGGTATGGACCCTTAGCAAAGCCAGAAAGATCACCTTGCCCAAAGAAGACCAACATTTTCATA
CATGATGAAATTCAT
+
FFFFFFFFFFFFFFF:FFFFFFFFFFFFFFFFFFFFFFFFFFFFFFFFFFF:FFFFFFFFFFFF:FFFFF
FFFFF,FFFFFFFFFFFFFFFFFFFFFFFFFFFFFFFFFFFFFFFFFFFFFFFFFFFFFFFFFFFFFFF
FFFFFFFFFFFFFFF
@A00155:342:HHGFNDSXY:1:1464:25880:2816 2:N:0:GAACCTAG+TCCGCATA
GGAACGTCTGGGTTCCAATTCTGTCAAGACTTATGACATATTAAATAGATTGGTTACGGTAAAAGACA
AGGCAAGGTATGGACCCTTAGCAAAGCCAGAAAGATCACCTTGCCCAAAGAAGACCAACATTTTCATA
CATGATGAAATTCAT
+
FFFFFFFFFFFFFFFFFFFFFFFFFFFFFFFF,FFFFFFFF,FFFFFFFFF:FFFFFFFFFFFFFFFFF
FFFFFFFFFF:FFFFFFFFFFFFFFFFFFFFFF:FFFFFFFFFFFFFFFFFFFFFFFFFFFFFFFFFFFF
FFFFFFFFF,:FFFF
@A00155:342:HHGFNDSXY:1:1453:20853:9298 2:N:0:GAACCTAG+TCCGCATA
GGAACGTCTGGGTTCCAATTCTGTCAAGACTTATGACATATTAAATAGATTGGTTACGGTAAAAGACA
AGGCAAGGTATGGACCCTTAGCAAAGCCAGAAAGATCACCTTGCCCAAAGAAGACCAACATTTTCATA
CATGATGAAATTCAT
+
FFFFFFFFFFFFFFFFFFFFFFFFFFFFFFFFFFFFFFFFFFFFF:FFFFFFFFFFFFFFFFFFFFFFFF
FFFFF,:FFFFFFFF:FFFFFFFFFFFFFFFFFFFFFFFFFFFFFFFFFFFFFFFFFFFFFFFFFFFFF
FFFFFFFFF,FFFFF
@A00155:342:HHGFNDSXY:1:2114:21423:25285 2:N:0:GAACCTAG+TCCGCATA
GGAACGTCTGGGTTCCAATTCTGTCAAGACTTATGACATATTAAATAGATTGGTTACGGTAAAAGACA
AGGCAAGGTATGGACCCTTAGCAAAGCCAGAAAGATCACCTTGCCCAAAGAAGACCAACATTTTCATA
CATGATGAAATTCAT
+
FFFFFFFFFFFFFFFFFFFFFFFFFF:FFFFFFF:FFFFFFFFFFFFFFFFFFFFFFFFFFFFF:FFFFF
:FFFFFFFFFFFFFFFFFFFFFFFFFFFFFFFFFFFFFFFFFFFFFFFFFF:FFFFFFFFFFFFFF:FF
FF,FFFFF:FFFFFF
@A00155:342:HHGFNDSXY:1:2138:14561:36448 2:N:0:GAACCTAG+TCCGCATA
GGAACGTCTGGGTTCCAATTCTGTCAAGACTTATGACATATTAAATAGATTGGTTACGGTAAAAGACA
AGGCAAGGTATGGACCCTTAGCAAAGCCAGAAAGATCACCTTGCCCAAAGAAGACCAACATTTTCATA
CATGATGAAATTCAT
+
FFF:FFFFFFFFFFFFFFFFFFFFFFFFFFFFFFFFFFFFFFFFFFFFFFFFFFFFFFFFFFFFFFFFF
FF:FFFFFFFFFFFFFF:FFFFFFFFFFFFFFFFFFFFFFFFFFFFF:FFFFFFF:FFFF:FFF:FFFFFF
FFFFFFFFFF:FFFF

```
@A00155:342:HHGFNDSXY:1:2217:27597:30029 2:N:0:GAACCTAG+TCCGCATA
GGAACGTCTGGGGTCCAATTCTGTCAAGACTTATGACATATTAAATAGATTGGTTACGGTAAAAGACA
AGGCAAGGTATGGACCCTTAGCAAAGCCAGAAAGATCACCTTGCCCAAAGAAGACCAACATTTTCATA
CATGATGAAATTCAT
+
FFF::F,FFFFF,FFFFFF,FFFFFF:FFFFFFFF:FFFF,FFFFFFFFFF:FFFFFFFFFFF:FFFF
FFFFFFFFFFFFFF::FFFFFFFFFFFFFFF:F:FFFFFFFFFFFFFFFFFFF:FFFFFFF:FF:FFF
FFFFFFFF,FFFFFF
@A00155:342:HHGFNDSXY:1:2262:30798:18599 2:N:0:GAACCTAG+TCCGCATA
GGAACGTCTGGGTTCCAATTCTGTCAAGACTTATGACATATTAAATAGATTGGTTACGGTAAAAGACA
AGGCAAGGTATGGACCCTTAGCAAAGCCAGAAAGATCACCTTGCCCAAAGAAGACCAACATTTTCATA
CATGATGAAATTCAT
+
FFF:FFFFFFFFFFFFFFFFFFFFFFFFFFFFFFFFFFFFFFFFFFFFFFFFFFFFFFFFFFFFFFFF
FFFFFFFFFFFFFFFFFFFFFFFFFFFFFFFFFFFFFFFFFFFFFFFFFFFFFFFFFFFFFFFFFFFF
FFFFFFFFFFFFFFF
@A00155:342:HHGFNDSXY:1:2308:29261:17754 2:N:0:GAACCTAG+TCCGCATA
GGAACGTCTGGGTTCCAATTCTGTCAAGACTTATGACATATTAAATAGATTGGTTACGGTAAAAGACA
AGGCAAGGTATGGACCCTTAGCAAAGCCAGAAAGATCACCTTGCCCAAAGAAGACCAACATTTTCATA
CATGATGAAATTCAT
+
FFFFFFFFFFFFFFFFFFFFFFFFFFFFFFFFFFFFFFFFFFFFFFFFFFFFFFFFFFFFFF:FFFFF
FFFFF:FFFFFFFFFFFFFFFFFFFFFFFFFFFFFFFFFFFFFFFFFFFFFFFFFFFFFFFFFFFFFF
FFFFFFFFFFFFFFF
@A00155:342:HHGFNDSXY:1:2569:8874:16423 2:N:0:GAACCTAG+TCCGCATA
GGAACGTCTGGGTTCCAATTCTGTCAAGACTTATGACATATTAAATAGATTGGTTACGGTAAAAGACA
AGGCAAGGTATGGACCCTTAGCAAAGCCAGAAAGATCACCTTGCCCAAAGAAGACCAACATTTTCATA
CATGATGAAATTCAT
+
FFFFFFFFFFFFFFFFF:FFF:FFFFFFFFFFFFFFFFFFFFFFFFFFFFFFFFFFFFFFFFFFF:FF
FFFFFFFFFFFFFFFFFFFFFFFF:FFFFFFFFFFFFFFFFFFFFFF:FFFF:FFFFFFFFFFFFFFF
FFFFFFFFFFFFFFF
@A00155:342:HHGFNDSXY:1:2574:25328:12007 2:N:0:GAACCTAG+TCCGCATA
GGAACGTCTGGGTTCCAATTCTGTCAAGACTTATGACATATTAAATAGATTGGTTACGGTAAAAGACA
AGGCAAGGTATGGACCCTTAGCAAAGCCAGAAAGATCACCTTGCCCAAAGAAGACCAACATTTTCATA
CATGATGAAATTCAT
+
FFF:FFFFFFFFFFFFFFFFFFFFFFFFFFFFFFFFFFFFFFFFFFFFFFFFFFFFFFFFFFFFFFFF
FFFFFFFFFFFFFFFFFFFFFFFFFFFFFFFFFFFFFFFFFFFFFFFFFFFFFFFFFFFFFFFFFFFF
FFFFFFFFFFFFFFF
@A00155:342:HHGFNDSXY:1:2602:22390:3286 2:N:0:GAACCTAG+TCCGCATA
GGAACGTCTGGGTTCCAATTCTGTCAAGACTTATGACATATTAAATAGATTGGTTACGGTAAAAGACA
AGGCAAGGTATGGACCCTTAGCAAAGCCAGAAAGATCACCTTGCCCAAAGAAGACCAACATTTTCATA
CATGATGAAATTCAT
+
FFF:FFFFFFFFFFFFFFFFFFFFFFFFFFFFFFFFFFFFFFFFFFFFFFFFFFFFFFFFFFFFFFFF
FFFFF:FFFFFFFFFFFFFFFFFFFFFFFFFFFFFFFFFFFFFFFFFFFFFFFFFFFFFFFF:FFF
FFFFFFFFFFFFFFF
@A00155:342:HHGFNDSXY:1:1431:6153:32690 2:N:0:GAACCTAG+TCCGCATA
CGTCTGGGTTCCAATTCTGTCAAGACTTATGACATATTAAATAGATTGGTTACGGTAAAAGACAAGGC
AAGGTATGGACCCTTAGCAAAGCCAGAAAGATCACCTTGCCCAAAGAAGACCAACATTTTCATACATG
ATGAAATTCATTATT
+
FFFFFFFFFFFFFFFFFFFFFFFFFFFFFFFFFFFFFFFFF:FFFFFFF,F,FFFFFFFFFFFFFFFF
```

```
F,,FF,FFFFF:FF:FFFFFFF:FFF:FFFF:FFFFFFFFF:FFFFFFFFFFFFFFFFFFFFFFFFFF:
:FFFFFFFFF::F:F
@A00155:342:HHGFNDSXY:1:2432:2871:4993 2:N:0:GAACCTAG+TCCGCATA
CGTCTGGGTTCCAATTCTGTCAAGACTTATGACATATTAAATAGATTGGTTACGGTAAAAGACAAGGC
AAGGTATGGACCCTTAGCAAAGCCAGAAAGATCACCTTGCCCAAAGAAGACCAACATTTTCATACATG
ATGAAATTCATTATT
+
FFFFFFFF:FFFFFFFFFFFFFFFFFFFFFFFFFFFFFF:FFFFFFF,FFFFFFFF:FFFFFFFFFFF
FFFFFFFF::FFFFFF:FF:FFF,FFFFFFFF:FFFFFFFFFFFFFFFF:FFFFFFFFFFFFFFFF
FFF::FFF,FFFF:F
@A00155:342:HHGFNDSXY:1:2519:26928:12148 2:N:0:GAACCTAG+TCCGCATA
CGTCTGGGTTCCAATTCTGTCAAGACTTATGACATATTAAATAGATTGGTTACGGTAAAAGACAAGGC
AAGGTATGGACCCTTAGCAAAGCCAGAAAGATCACCTTGCCCAAAGAAGACCAACATTTTCATACATG
ATGAAATTCATTATT
+
FFFFFFFFFFFFFFFFFFFFFFFFFFF:FFFFFFFFFFFFFFFFFFFFFFFFFFFFF:FFFFFFFFFF
FFFFFFFFFFFFF:FFFFFFFFFFFFF:FFFFFFFFFFFFFFFFFFFFFFFFFFFFFFFFFFFFFFFF
FFFF,FFFFFFFFFF
@A00155:342:HHGFNDSXY:1:2450:10818:4914 1:N:0:GAACCTAG+TCCGCATA
GTCTGGGTTCCAATTCTGTCAAGACTTATGACATATTAAATAGATTGGTTACGGTAAAAGACAAGGCA
AGGTATGGACCCTTAGCAAAGCCAGAAAGATCACCTTGCCCAAAGAAGACCAACATTTTCATACATGA
TGAAATTCATTATTG
+
FFFFFFFFFFFFFFFFFFFFFFFFFF:FFFFFF,FFFFFFFFF:,FFFFFFFFFFFFFFFFFFFFFFF
FFFFFFFFFFFFFFFFFFFFFFFFFFFFFFFFFFFFFFFFFFFFF,F:FFFFFFFFFFFFFFFFFFFF
FFFFFFFFFFFFFFF
@A00155:342:HHGFNDSXY:1:1123:17327:24111 2:N:0:GAACCTAG+TCCGCATA
GTCTGGGTTCCAATTCTGTCAAGACTTATGACATATTAAATAGATTGGTTACGGTAAAAGACAAGGCA
AGGTATGGACCCTTAGCAAAGCCAGAAAGATCACCTTGCCCAAAGAAGACCAACATTTTCATACATGA
TGAAATTCATTATTG
+
FFFFFFFFFFFFFFFFFFFFFFFFFFFFFFFFF:FFFFFFFFFFFFFFFFFFFFFFFFFFFFFFFFFF
:FFFFFFFFFFFFFFFFFFFFFFFFFFFFFFFFFFFFFFFFFFFFFFFFFFFFFFFFFFFFFFFFFFF
FFF:FFFFF,FFFFF
@A00155:342:HHGFNDSXY:1:1178:23656:2315 2:N:0:GAACCTAG+TCCGCATA
GTCTGGGTTCCAATTCTGTCAAGACTTATGACATATTAAATAGATTGGTTACGGTAAAAGACAAGGCA
AGGTATGGACCCTTAGCAAAGCCAGAAAGATCACCTTGCCCAAAGAAGACCAACATTTTCATACATGA
TGAAATTCATTATTG
+
FFFFFFFFFFFFFFFFFFFFFFFFFFFFFFFFFFFFFFFFFFFFFFFFFFFFFFFFFF:FFFFFFFFF
FFF:FFFFFFFFFFFFFFFFFFFFFFFFFFFFFFFFFFFFFF:FFFFFFFFFFFFFFFFFF:FFFFFFF
FFF:FFFFF,F:FFF
@A00155:342:HHGFNDSXY:1:1171:11261:9596 2:N:0:GAACCTAG+TCCGCATA
GTCTGGGTTCCAATTCTGTCAAGACTTATGACATATTAAATAGATTGGTTACGGTAAAAGACAAGGCA
AGGTATGGACCCTTAGCAAAGCCAGAAAGATCACCTTGCCCAAAGAAGACCAACATTTTCATACATGA
TGAAATTCATTATTG
+
FFFFFFFFFFFFFFFFFFFFFFFFFFFFFFFFFFFFFFFFFFFFFFFFFFFFFFFFFFFFFFFFFFFF
FFFFFFFFFFF:FFFFFFFFFFFFFFFFFFFFFFFFFFFFFFFFFFFF:FFFFFFFFFFFFFFFFFFF
FFFFFFFFFFFFFFF
@A00155:342:HHGFNDSXY:1:1441:14416:27305 2:N:0:GAACCTAG+TCCGCATA
GTCTGGGTTCCAATTCTGTCAAGACTTATGACATATTAAATAGATTGGTTACGGTAAAAGACAAGGCA
AGGTATGGACCCTTAGCAAAGCCAGAAAGATCACCTTGCCCAAAGAAGACCAACATTTTCATACATGA
TGAAATTCATTATTG
```

+
FFFFFFFFFFFFFFFFFFFFFFFFFFFFFFFFF:FFFFFFFFFFFFFFFFFFFFFFF,FFFFFFFFFF
FFFFFFFFFFFFFFFFFFFFFFFFFFFFFFFFFFFFFFFFFFFFFFFFFFF:FFFFFFFFFFFFFFFF
FFFFFFFFF:FF:FF
@A00155:342:HHGFNDSXY:1:2112:30264:14450 2:N:0:GAACCTAG+TCCGCATA
GTCTGGGTTCCAATTCTGTCAAGACTTATGACATATTAAATAGATTGGTTACGGTAAAAGACAAGGCA
AGGTATGGACCCTTAGCAAAGCCAGAAAGATCACCTTGCCCAAAGAAGACCAACATTTTCATACATGA
TGAAATTCATTATTG
+
FFFFFFFFF:FFFFFFFFFFFFFF:FFFFFFFFFF::FFFFFFFFFFF:FFFFFFFFFFFFFFFFFFF
FFFFFFFFFFFFFFFFFFFFFFFFFFFFFFFFFFFF:FFFFF:FFFFFFF:FFFFF::FFFFFFFFFF
FFFF::FFF,FFFFF
@A00155:342:HHGFNDSXY:1:2211:4996:20760 2:N:0:GAACCTAG+TCCGCATA
GTCTGGGTTCCAATTCTGTCAAGACTTATGACATATTAAATAGATTGGTTACGGTAAAAGACAAGGCA
AGGTATGGACCCTTAGCAAAGCCAGAAAGATCACCTTGCCCAAAGAAGACCAACATTTTCATACATGA
TGAAATTCATTATTG
+
FFFFF:FFFFFFFFFFFFFF:F:FFFFFFFFFFFFFFFF:FF:FF:FFFFFFFFFFFFF:FFFF:FFF
F:FFFFFFF:,FFFFFFFF:FF::FFFFFFFFFFFFF:F,FFFFFFFFFF:FFFFFFFFFFFF:FFFF
FFFF,FF:F,FFFF:
@A00155:342:HHGFNDSXY:1:2674:27977:8265 2:N:0:GAACCTAG+TCCGCATA
GTCTGGGTTCCAATTCTGTCAAGACTTATGACATATTAAATAGATTGGTTACGGTAAAAGACAAGGCA
AGGTATGGACCCTTAGCAAAGCCAGAAAGATCACCTTGCCCAAAGAAGACCAACATTTTCATACATGA
TGAAATTCATTATTG
+
FFFFFFFFFFFFFFFFFFFF:FFFFFFFFFFFFFFFFFFFFFFFFFFFFFFFFFF:FFFF:FFFFFFF
FFFFFFFFFFFFFFFFFFFFFFFFFFFFFFFFFFFFFFFFFFFFFFFFFF,FFFFFFFFFFFFFFFFF
FFF:FFFFFF:FFFF
@A00155:342:HHGFNDSXY:1:1447:28980:6245 2:N:0:GAACCTAG+TCCGCATA
CTGGGTTCCAATTCTGTCAAGACTTATGACATATTAAATAGATTGGTTACGGTAAAAGACAAGGCAAG
GTATGGACCCTTAGCAAAGCCAGAAAGATCACCTTGCCCAAAGAAGACCAACATTTTCATACATGATG
AAATTCATTATTGGT
+
FFFFFFFFFFFFFFFFFFFFFFFFFFFFFFFFFFFFF:FF:FFFFFFFFFFFFFFFFFFFFFFFFF:F
FF::FFFFFFFFFFFFFFFFFFFFFFFFFFFFFFFFFFFFFFFFFFFFFFFFFFFFFFFFFFFFFFFF
FFFFFFF:F::FFFF
@A00155:342:HHGFNDSXY:1:1505:5936:2942 2:N:0:GAACCTAG+TCCGCATA
CTGGGTTCCAATTCTGTCAAGACTTATGACATATTAAATAGATTGGTTACGGTAAAAGACAAGGCAAG
GTATGGACCCTTAGCAAAGCCAGAAAGATCACCTTGCCCAAAGAAGACCAACATTTTCATACATGATG
AAATTCATTATTGTT
+
FFFFFFFF:FFFFFFFFFFF:FFFFFFFFFFFFFFFFF,FFFFFFFFFFFFFFFFFFFFFFFFFF:FF
FF:FFFFFFFFF:FFF,FFFFFFFFFF:FFFFFFFFFFFFFFFFFFFFFFFFFFFFFFFFFFFFFFFF
F,FFFFFFFFFFF,F
@A00155:342:HHGFNDSXY:1:1505:5990:3098 2:N:0:GAACCTAG+TCCGCATA
CTGGGTTCCAATTCTGTCAAGACTTATGACATATTAAATAGATTGGTTACGGTAAAAGACAAGGCAAG
GTATGGACCCTTAGCAAAGCCAGAAAGATCACCTTGCCCAAAGAAGACCAACATTTTCATACATGATG
AAATTCATTATTGGT
+
FFFFFFFFFFFFFFFFFFFFFFFFFFFFFFFFFFFFFFFFFFFFFFFFFFFFFFFFFFFFFFFFFFFF
FFFFFFFFFFFFFFFFFFFFFFFFFFFFFFFFFFFFFFFFFFFFFFFFFFFFFFFFFFFFFFFFFFFF
FFFFFFFFFFFFFFF
@A00155:342:HHGFNDSXY:1:1527:2094:13479 2:N:0:GAACCTAG+TCCGCATA
CTGGGTTCCAATTCTGTCAAGACTTATGACATATTAAATAGATTGGTTACGGTAAAAGACAAGGCAAG

```
GTATGGACCCTTAGCAAAGCCAGAAAGATCACCTTGCCCAAAGAAGACCAACATTTTCATACATGATG
AAATTCATTATTGGT
+
FFFFFFFFFFFFFFFFFFFFFFFFFFF,F:FFFFFF,FFFFFFFFFFFFFFFFFFFFFFFFFF:FFF:
FFFFFFFFFFFFFFFFFFFFFFFFFFFFFFFFFFFFFFFFFFFFFFFFFFFFFFFFFFFFFFFFFFFF
FFFFF:FFFFFFFFF
@A00155:342:HHGFNDSXY:1:1608:10691:14904 2:N:0:GAACCTAG+TCCGCATA
CTGGGTTCCAATTCTGTCAAGACTTATGACATATTAAATAGATTGGTTACGGTAAAAGACAAGGCAAG
GTATGGACCCTTAGCAAAGCCAGAAAGATCACCTTGCCCAAAGAAGACCAACATTTTCATACATGATG
AAATTCATTATTGGT
+
FFFFFFFFFFFFFFFF:FF:FFFFFF:FFFFFFFFF:F:FFFFFFFFF,FFFFF:FFFFFFFFFFFFF
F::FF:FFFFFFFFFFFFFFFFFFFFFFFFFFFFFFFFF:FFFFFFFFF:FFFFFFFFFFFFFFFFFF
F:FFFFFFFFF:FFF
@A00155:342:HHGFNDSXY:1:1626:26214:24846 2:N:0:GAACCTAG+TCCGCATA
CTGGGTTCCAATTCTGTCAAGACTTATGACATATTAAATAGATTGGTTACGGTAAAAGACAAGGCAAG
GTATGGACCCTTAGCAAAGCCAGAAAGATCACCTTGCCCAAAGAAGACCAACATTTTCATACATGATG
AAATTCATTATTGGT
+
FFFFFFFFFFFFFFFFFFFFFFFFFFFFFFFFFFFFFFFFFFFFFFFFFFFFFFF:FFFFFFFFFFFF
FFFFFFFF:FFFFFFFFFFFF:FFFFFFFFFFFFFFFFFFFFFFFFFFF,FFFFFFFFFFFFFFFFFF
FFFFFFFFFFFFFFF
@A00155:342:HHGFNDSXY:1:1638:28320:12524 2:N:0:GAACCTAG+TCCGCATA
CTGGGTTCCAATTCTGTCAAGACTTATGACATATTAAATAGATTGGTTACGGTAAAAGACAAGGCAAG
GTATGGACCCTTAGCAAAGCCAGAAAGATCACCTTGCCCAAAGAAGACCAACATTTTCATACATGATG
AAATTCATTATTGGT
+
FFFFFFFFFFFFFFFFFFFFFFFFF,FFFFFFFFFFFFFFFFFFFFFFFFFFFFFFFFFFFFFFFFFF
FFFFFFFFFFFFFFFFFFFFFFFFFFF:FFFFFFFFFFFFFFFFFFFFFFFFFFFFFFFFFFFFFFFF
FFFFFFFFFF,FFFF
@A00155:342:HHGFNDSXY:1:1641:8865:23797 2:N:0:GAACCTAG+TCCGCATA
CTGGGTTCCAATTCTGTCAAGACTTATGACATATTAAATAGATTGGTTACGGTAAAAGACAAGGCAAG
GTATGGACCCTTAGCAAAGCCAGAAAGATCACCTTGCCCAAAGAAGACCAACATTTTCATACATGATG
AAATTCATTATTGGT
+
FFFFFFFFFFFFFFFFFFFFFFFFFFFFFFFFFFFFFFF:FFFFFFFFFFFFFFFF:FFFFFFFFFFFF
FFFFFFFFFFFFFFFFFFFFFFFFFFFFFFFFFFFFFFFFFFFFFFFFFFFFFFFFFFFFFFFFFFFF
FFFF:FFFFFFFFFF
@A00155:342:HHGFNDSXY:1:1675:18041:26193 2:N:0:GAACCTAG+TCCGCATA
CTGGGTTCCAATTCTGTCAAGACTTATGACATATTAAATAGATTGGTTACGGTAAAAGACAAGGCAAG
GTATGGACCCTTAGCAAAGCCAGAAAGATCACCTTGCCCAAAGAAGACCAACATTTTCATACATGATG
AAATTCATTATTGGT
+
FFFFFFFFFFFFFFFFFFFFFFF:F:FFFFFFFFFFFFFFF,FFFFFFFFFF:F:FFFFFFFFFFFF
FFFFFFFFFFFFFFFFFFFFFFFFFFFFFFFFFFFFFFFF:F:FFFFFFFFFFFFF:FFF:FF:F
F:FF,FFFF,FFFFF
@A00155:342:HHGFNDSXY:1:2109:1949:25598 2:N:0:GAACCTAG+TCCGCATA
CTGGGTTCCAATTCTGTCAAGACTTATGACATATTAAATAGATTGGTTACGGTAAAAGACAAGGCAAG
GTATGGACCCTAAGCAAAGCCAGAAAGATCACCTTGCCCAAAGAAGACCAACATTTTCATACATGATG
AAATTCATTATTGGT
+
FFFFFFFFFFF:FFF,FFFFFFFFF,FFFFFFFF:FFF,FFFF,:F,FFFFF:,FFFFFFFFFFFF,F
FFFFFFFFFFF,:FFFFFF,FF:FF:FFFFFFF::FFFFFFFFF::FFF:FFFFFF,FF,FFFFFFF:
FF::FFF,:,,FF::
```

```
@A00155:342:HHGFNDSXY:1:2234:11243:33270 2:N:0:GAACCTAG+TCCGCATA
CTGGGTTCCAATTCTGTCAAGACTTATGACATATTAAATAGATTGGTTACGGTAAAAGACAAGGCAAG
GTATGGACCCTTAGCAAAGCCAGAAAGATCACCTTGCCCAAAGAAGACCAACATTTTCATACATGATG
AAATTCATTATTGGT
+
FFFFFFFFFFFFFF:FFFFFFFFFFFFFFFFFFFFFFFFFFFFFFFFFFFFFFFF:FFFFFFFF:FFF
FFFFFFFFFFFFFFFFFFFF:FFFFFFFFFFFFFFFFFFFFFFFFFFFFFFFFFFFFFFFFFFFFFFF
:FFFFFFF,FFFFFF
@A00155:342:HHGFNDSXY:1:2249:9046:19100 2:N:0:GAACCTAG+TCCGCATA
CTGGGTTCCAATTCTGTCAAGACTTATGACATATTAAATAGATTGGTTACGGTAAAAGACAAGGCAAG
GTATGGACCCTTAGCAAAGCCAGAAAGATCACCTTGCCCAAAGAAGACCAACATTTTCATACATGATG
AAATTCATTATTGGT
+
FFFFFFFFFFFFFFFFFFFFFFFFFFFFFFFFFFFFFFFFFFFFFFFFFFFFFFFFFFFFFFFFFFFF
FFFFFFFFFFFFFFFFFFFFFFFFFFFFFFFFFFFFFFFFFFFFFFFFFFFFFFFFFFFFFFF:FFFF
FFFFFFFFFFFFFFF
@A00155:342:HHGFNDSXY:1:2304:3830:19429 2:N:0:GAACCTAG+TCCGCATA
CTGGGTTCCAATTCTGTCAAGACTTATGACATATTAAATAGATTGGTTACGGTAAAAGACAAGGCAAG
GTATGGACCCTTAGCAAAGCCAGAAAGATCACCTTGCCCAAAGAAGACCAACATTTTCATACATGATG
AAATTCATTATTGGT
+
FFFFFFF,FFFFFFFFFFFF,FFFFFF,FFFFFFFFFFFFFFFFFFFFF:F:F,FFFFFFFF,F:FFF
F:F:F,FF:FFFFFFFFFF,FFF:FF:FFFFFFFFF:FFFFFFFFF:FFFFFFF:FF:F,FFFFFFF:
:FF,FF:FFFFFF,F
@A00155:342:HHGFNDSXY:1:2359:14163:29872 2:N:0:GAACCTAG+TCCGCATA
CTGGGTTCCAATTCTGTCAAGACTTATGACATATTAAATAGATTGGTTACGGTAAAAGACAAGGCAAG
GTATGGACCCTTAGCAAAGCCAGAAAGATCACCTTGCCCAAAGAAGACCAACATTTTCATACATGATG
AAATTCATTATTGGT
+
FFFFFFFFFFFFFFFFFFFFFFFFFFFFFFFFFFFFFFFFFFFFFFFFFFFFFFFFFFFFFFFFFF:F
FFFFFFFFFFFFFFFFFFFFFFFFFFFFFFFFFFFFFFFFFFFFFFFFFFFFFFFFFFFFFFFFFFFF
FFFFFFFFFFFFFFF
@A00155:342:HHGFNDSXY:1:2372:11623:27132 2:N:0:GAACCTAG+TCCGCATA
CTGGGTTCCAATTCTGTCAAGACTTATGACATATTAAATAGATTGGTTACGGTAAAAGACAAGGCAAG
GTATGGACCCTTAGCAAAGCCAGAAAGATCACCTTGCCCAAAGAAGACCAACATTTTCATACATGATG
AAATTCATTATTGGT
+
F:,:FFFF:FFFFFF:FFFF:FFFFFFFFFFFFF:FFFFFFFFF:FFFFFFFFFFFFFFFFFFFFFF,
,FFFFFFFFFFFFFFFFFF,FF:FFFFFFFFF:F,F,FFFFF,FFFFF,FFFFFFFFFFFFFFFFFF:
F:FFFFFF,FFFFFF
@A00155:342:HHGFNDSXY:1:2423:17029:30577 2:N:0:GAACCTAG+TCCGCATA
CTGGGTTCCAATTCTGTCAAGACTTATGACATATTAAATAGATTGGTTACGGTAAAAGACAAGGCAAG
GTATGGACCCTTAGCAAAGCCAGAAAGATCACCTTGCCCAAAGAAGACCAACATTTTCATACATGATG
AAATTCATTATTGGT
+
FFFFFFFFFFFFFFFFFFFFFFFFFF,FFFF:FFFFFFFFFFFFFFFFFFFFFFF:FFFFFFFFFFFF
FFFFFF:FFFFFFFFFFFFFFFFFFFFFFFFFFFFFFFFFFFFFFFFFFFFFFFFFFFFFFFFFFFF:
FF:FFFFFFFFFFFF
@A00155:342:HHGFNDSXY:1:2447:14850:36448 2:N:0:GAACCTAG+TCCGCATA
CTGGGTTCCAATTCTGTCAAGACTTATGACATATTAAATAGATTGGTTACGGTAAAAGACAAGGCAAG
GTATGGACCCTTAGCAAAGCCAGAAAGATCACCTTGCCCAAAGAAGACCAACATTTTCATACATGATG
AAATTCATTATTGGT
+
FFFFFFFFFFFFFFFFFFFFFFFFFFFFFFFFFFFFFFFFFFFFFFFFFFFFFFFF:FFFFFFFFFFF
```

FFFFFFFFFFFFFFFFFFFFFFFFFFFFFFFFFFFFFFFFFFFFFFFFFFFFFFFFFFFFFFFFFFFFFF
FFFFFFF::FFFFFF
@A00155:342:HHGFNDSXY:1:2501:10339:18364 2:N:0:GAACCTAG+TCCGCATA
CTGGGTTCCAATTCTGTCAAGACTTATGACATATTAAATAGATTGGTTACGGTAAAAGACAAGGCAAG
GTATGGACCCTTAGCAAAGCCAGAAAGATCACCTTGCCCAAAGAAGACCAACATTTTCATACATGATG
AAATTCATTATTGTT
+
:FFFFFFFFFFFFFFFFFF:FFFFFFFFFFFFFFFFFFFFF:FFFFFFF:FFFFFF:FFFFFFFFFFFFF
FFFF:FFFF:FFF,FFFFF,,FFFFFF:FFFFFFFFFFFFFFFFFF:F:FFFFF,:FFFFFFF:F::FF:
FFF,:FFFF:FF:,F
@A00155:342:HHGFNDSXY:1:2663:31530:36933 2:N:0:GAACCTAG+TCCGCATA
CTGGGTTCCAATTCTGTCAAGACTTATGACATATTAAATAGATTGGTTACGGTAAAAGACAAGGCAAG
GTATGGACCCTTAGCAAAGCCAGAAAGATCACCTTGCCCAAAGAAGACCAACATTTTCATACATGATG
AAATTCATTATTGGT
+
FFFFFFFFFFFFFFFFFFFFFFFFFFFFFFFFFFFFFFFFFFFFFFFFFFFFFFFFFFFFFFFFFFFFFF
FFFFF:FFFFFFFFFFFFFFFFFFFFFFFFFFFFFFFFFFFFFFFFFFFFFFFFFFFFFFFFFFFFFFFF
FF:FFFFFFFFFFFF
@A00155:342:HHGFNDSXY:1:1213:1967:4899 2:N:0:GAACCTAG+TCCGCATA
GTTCCAATTCTGTCAAGACTTATGACATATTAAATAGATTGGTTACGGTAAAAGACAAGGCAAGGTAT
GGACCCTTAGCAAAGCCAGAAAGATCACCTTGCCCAAAGAAGACCAACATTTTCATACATGATGAAAT
TCATTATTGGTCTCG
+
FFFFFFFFFFFFFFFFFFFFFFFFFFFFFFFFF:FFFFFFFFFFFFFFFFFFFFFFFFFF:FFFFFFFFF
FFFFFFFFFFFFFFFFFFFFFFFFFFFFFFFFFFFFFFFFFFFFFFFF:FFFFFFFFFFFFFFFFFFFFF
FFFFFFFFFFFFFFF
@A00155:342:HHGFNDSXY:1:2151:14136:14857 1:N:0:GAACCTAG+TCCGCATA
TTCCAATTCTGTCAAGACTTATGACATATTAAATAGATTGGTTACGGTAAAAGACAAGGCAAGGTATG
GACCCTTAGCAAAGCCAGAAAGATCACCTTGCCCAAAGAAGACCAACATTTTCATACATGATGAAATT
CATTATTGGTCTCGC
+
FFFFFF::FFFFFFFFFFFFFFFFFFF:FFFFFFFFFFFFFFFFFFFFFFF:FFFFF:FFFFFFFFFFFF
FFFFFFFFFFFFFFFFFFFFFFFFFFFFF:FFFFFFFFFF,F:FFFFFFFFFFFFFFFFFFFFFFFFFFF
FFFFFFFFFFFFFFF
@A00155:342:HHGFNDSXY:1:2551:22073:17519 1:N:0:GAACCTAG+TCCGCATA
TTCCAATTCTGTCAAGACTTATGACATATTAAATAGATTGGTTACGGTAAAAGACAAGGCAAGGTATG
GACCCTTAGCAAAGCCAGAAAGATCACCTTGCCCAAAGAAGACCAACATTTTCATACATGATGAAATT
CATTATTGGTCTCGC
+
FFF:FFF:FFFFFFFFFFFFFFFFFFFFFFFFFFFFFFFFFFFFFFFFFFFFFFFFFFFFFFFFFFFFFF
FFFFFFFFFFFFFFFFFFFFFFFFFFFFFFFFFFFFFFFFFFFFFFFFFFFFFFFFFFFFFFFFFFFFFF
FFFFFFFFFFFFFFF
@A00155:342:HHGFNDSXY:1:1409:1506:9424 1:N:0:GAACCTAG+TCCGCATA
TTCCGATCTGTCAAGACTTATGACATATTAAATAGATTGGTTACGGTAAAAGACAAGGCAAGGTATGG
ACCCTTAGCAAAGCCAGAAAGATCACCTTGCCCAAAGAAGACCAACATTTTCATACATGATGAAATTC
ATTATTGGTCTCGCG
+
FFFF,F:FFF:FFF,FFFFFF,FFFFF:FFFFFFFFFFFFFFFFFF:FFFFF:FFFFF:FFFF:FFFFFF
FFFFFFFFFFFFFFFFF:FFFFFFFFFFFFFFFFFFFF:FF:FFFFFFFFFF:FFFFFFFFFFFFFFFFF
FFFFFFFFFFFFFFF
@A00155:342:HHGFNDSXY:1:2235:16125:24596 1:N:0:GAACCTAG+TCCGCATA
TTCCGATCTGTCAAGACTTATGACATATTAAATAGATTGGTTACGGTAAAAGACAAGGCAAGGTATGG
ACCCTTAGCAAAGCCAGAAAGATCACCTTGCCCAAAGAAGACCAACATTTTCATACATGATGAAATTC
ATTATTGGTCTCGCG

+
:FFFFFFFFFFFFFFFFFFFFFFFFFFFFF:FF::FFFFFFFFFFFFFF:FFFFFFFFFFFFFFFFFF
FFFFFFFFFFFFFFFFFFFFFFFFFFFFFFFFFFFFFF:FFFFFFFFFFFFFFFFFFFFFFFFFFFFF
FFFFFFFFFFFFFFF
@A00155:342:HHGFNDSXY:1:2535:7328:32377 1:N:0:GAACCTAG+TCCGCATA
TTCCGATCTGTCAAGACTTATGACATATTAAATAGATTGGTTACGGTAAAAGACAAGGCAAGGTATGG
ACCCTTAGCAAAGCCAGAAAGATCACCTTGCCCAAAGAAGACCAACATTTTCATACATGATGAAATTC
ATTATTGGTCTCGCG
+
FFFFFFFFFFFFFFFFFFFFFFFFFFFFFFF:FFFFFFFFFFFFFFFFFFFFFFFFFFFFFFFFFFFF
FFFFFFF:FFFFFFFFFFFFFFFFFFF::FFFFFFFFFF:FFFFFFFFFFFFFFFFFFFFFFFFFFFF
FFFFFFFFFFFFFFF
@A00155:342:HHGFNDSXY:1:2201:2220:13604 2:N:0:GAACCTAG+TCCGCATA
CAATTCTGTCAAGACTTATGACATATTAAATAGATTGGTTACGGTAAAAGACAAGGCAAGGTATGGAC
CCTTAGCAAAGACAGAAAGATCACCTTGCCCAAAGAAGACCAACATTTTCTTACATGATGAAATGAAT
TATTGGTCTCGCGA
+
FF,F,F,FFFF::FFFF:F:FFF,,FFFF::FF:,FFFFF,:FF,,F,F:FFF:FFFF,::F:FFF::
:FFF,F,:F:F,:F:FF:FFFFF:FF:FFFFF,F,FFF:FFFF:,F::F,,F,FFF,FF::,,F,,,F
:,::FFFF:,:FF:
@A00155:342:HHGFNDSXY:1:1447:28980:6245 1:N:0:GAACCTAG+TCCGCATA
CAATTCTGTCAAGACTTATGACATATTAAATAGATTGGTTACGGTAAAAGACAAGGCAAGGTATGGAC
CCTTAGCAAAGCCAGAAAGATCACCTTGCCCAAAGAAGACCAACATTTTCATACATGATGAAATTCAT
TATTGGTCTCGCGAT
+
FFFFFFFFF:FFFFFFFFFFFFFFFFFFF::FFFFFFFFFFFFFFFFFFFFFFFFFFFFFFFFFFFFF
FFFFFFFFFFFFFFFFFFFFFFFFFFFFFFFFFFFFFFFFFFFFFFFFFFFFFFFFFFFFFFFFFFFF
FFFFFFFFFFFFFFF
@A00155:342:HHGFNDSXY:1:1557:11415:1626 1:N:0:GAACCTAG+TCCGCATA
CAATTCTGTCAAGACTTATGACATATTAAATAGATTGGTTACGGTAAAAGACAAGGCAAGGTATGGAC
CCTTAGCAAAGCCAGAAAGATCACCTTGCCCAAAGAAGACCAACATTTTCATACATGATGAAATTCAT
TATTGGTCTCGCGAT
+
FF::FFFFFFFFFFFFFFFFFFFFFFFFFFFF:FFFF:FFFFFFFFFF:FFFF::FFFFFFFFFFFFF
FFFFFFFFFFFFFFFFFF:FFFFFF:FFFFFFF:F:FFFFFFFFFFFFFFFFFFFFFFFFFFFFFFFF
FFFFFFFFFFFFFFF
@A00155:342:HHGFNDSXY:1:1605:28330:29794 1:N:0:GAACCTAG+TCCGCATA
CAATTCTGTCAAGACTTATGACATATTAAATAGATTGGTTACGGTAAAAGACAAGGCAAGGTATGGAC
CCTTAGCAAAGCCAGAAAGATCACCTTGCCCAAAGAAGACCAACATTTTCATACATGATGAAATTCAT
TATTGGTCTCGCGAT
+
FFFFFFFFFFFFFFFFFFFFFFFFFFFFFFFFFFFFFFFFFFFFF:FFFFFFF,FFFFFFFFFFFFFF
FFFFFFFFFFFFFFFFFFFFFFFFFFFFFFFFFFFFFFFFFFFFFFFFFFFFFFFFFFFFFFFFFFFF
FFFFFFFFFFFFFFF
@A00155:342:HHGFNDSXY:1:1641:8865:23797 1:N:0:GAACCTAG+TCCGCATA
CAATTCTGTCAAGACTTATGACATATTAAATAGATTGGTTACGGTAAAAGACAAGGCAAGGTATGGAC
CCTTAGCAAAGCCAGAAAGATCACCTTGCCCAAAGAAGACCAACATTTTCATACATGATGAAATTCAT
TATTGGTCTCGCGAT
+
FFFFFFFFFFFFFFFFFFFFFFFFFFFFFFFFFFFFFFFFFFFFFFFFFFFFFFFFFFFFFFFFFFFF
FFFFFFFFFFFFFFFFFFFFFFFFFFFFFFFFFFFFFF:FFFFFFFFFFFFFFFFFFFFFFFFFFFFF
FFFFFFFFFFFFFFF
@A00155:342:HHGFNDSXY:1:2109:1949:25598 1:N:0:GAACCTAG+TCCGCATA
CATTTCTGTCTAGACTTATGACATATTAAATAGATTGGTTACGGTAAAAGACAAGGCTAGGTATGGAC

```
CCTTAGCAAAGCCAGAAAGATCACCTTGCCCTAAGAAGACCAACATTTTGATTCATGATGAAATTCAT
TATTGGTCTCGCGAT
+
:F,::,FFF:::F,FFF:::,,,FFFFFFF,FFFF:F::F,,FFF:FFFFF,:FFF,,:FFFFF:F::
FFFF:F:FF::FFFF,F,:FF:,F,,F:F,F,:FFFF:::FF,FFFF,F,FF,,:FFFFFFFFFFF:
FF:F:FFF:FF,:,F
@A00155:342:HHGFNDSXY:1:2201:13955:30170 1:N:0:GAACCTAG+TCCGCATA
CAATTCTGTCAAGACTTATGACATATTAAATAGATTGGTTACGGTAAAAGACAAGGCAAGGTATGGAC
CCTTAGCAAAGCCAGAAAGATCACCTTGCCCAAAGAAGACCAACATTTTCATACATGATGAAATTCAT
TATTGGTCTCGCGAT
+
FFF:FFFFFFFFFFFFFFFFFFFFFFFFF:FFFFFFFFFFFFF:F,FFFFF:FFFFFFFFFFFFFFF
FFFFFFFFFFFFFFFFFFFFFFFFFFFFFFFFFF,FF:FFFFFFFFFFFFFFFFFFFFFFFFFFFFF
FFFFFFFFFFFFFFF
@A00155:342:HHGFNDSXY:1:2262:4508:15280 1:N:0:GAACCTAG+TCCGCATA
CAATTCTGTCAAGACTTATGACATATTAAATAGATTGGTTACGGTAAAAGACAAGGCAAGGTATGGAC
CCTTAGCAAAGCCAGAAAGATCACCTTGCCCAAAGAAGACCAACATTTTCATACATGATGAAATTCAT
TATTGGTCTCGCGAT
+
FFFFFFFFFFFFFFFFFFFFFFFFFFFFFFFFFFFFFFFFFFFFFFFFFFFFFFFFFFFFFFFFFFFF
FFFFFFFFF:FFFFFFFFFFFFFFFFFFFFFFFFFF:FF:FFF:FFFFFFFFFFFFFFFFFFFFFFFFF
FFFFFFFFFFFFFF:
@A00155:342:HHGFNDSXY:1:2447:14850:36448 1:N:0:GAACCTAG+TCCGCATA
CAATTCTGTCAAGACTTATGACATATTAAATAGATTGGTTACGGTAAAAGACAAGGCAAGGTATGGAC
CCTTAGCAAAGCCAGAAAGATCACCTTGCCCAAAGAAGACCAACATTTTCATACATGATGAAATTCAT
TATTGGTCTCGCGAT
+
FFFFFFFFFFFFFFFFFFFFFFFFFFFFFFFFFFF:FFFFFFFFFFFFFFFFFFFFFFFFFFFFFFFF
FFFFFFFFFFFFFFFFFFFFFFFFFFF:FFFFFFFFFFFFFFFFFFFFFFFFFFFFFFFFFFFFFFFF
FFFFFFFFFFFFFFF
@A00155:342:HHGFNDSXY:1:2459:4869:11992 1:N:0:GAACCTAG+TCCGCATA
CAATTCTGTCAAGACTTATGACATATTAAATAGATTGGTTACGGTAAAAGACAAGGCAAGGTATGGAC
CCTTAGCAAAGCCAGAAAGATCACCTTGCCCAAAGAAGACCAACATTTTCATACATGATGAAATTCAT
TATTGGTCTCGCGAT
+
FFFFFFFFFFFFFFFFFFFFFFFFFFFFFFFFFFFFFFFFFFFFFFFFFFFF:FFFFFFFFFFFFFFF
FFFFFFFFFFFFFFFFFFFFFFFFFFFFFFFFFFFFFFFF:FFFFFFFFFFFFFFFFFFFFFFFFFFFF
FFFFFFFFFFFFFFF
@A00155:342:HHGFNDSXY:1:2454:22815:21308 1:N:0:GAACCTAG+TCCGCATA
TTCCGATCTCAAGACTTATGACATATTAAATAGATTGGTTACGGTAAAAGACAAGGCAAGGTATGGAC
CCTTAGCAAAGCCAGAAAGATCACCTTGCCCAAAGAAGACCAACATTTTCATACATGATGAAATTCAT
TATTGGTCTCGCGAT
+
FFFFFFFFFFFFFFFFFFFFFFFFFFFFFFFFFFFFFFFFFFFFFFFFFF:FFFFFFFFFFFFFFFFF
FFFFFFFFFFFFFFFFFFFFFFFFFF:FFFFFFF:FF:FFFFFFFFFFFFFFFFFFFFFFFFFFFFFFF
FFFFFFFFFFFFFFF
@A00155:342:HHGFNDSXY:1:1302:8232:6480 2:N:0:GAACCTAG+TCCGCATA
CAATTCTGTCAAGACTTATGACATATTAAATAGATTGGTTACGGTAAAAGACAAGGCAAGGTATGGAC
CCTTAGCAAAGCCAGAAAGATCACCTTGCCCAAAGAAGACCAACATTTTCATACATGATGAAATTCAT
TATTGGTCTCGCGAT
+
FFFFFFFFFFFFFFFFFFFFFFFFFFFF:F:FFFFFFFFFFFFFFFF:,FF:FFFFFFFFFFFFFFFFF
FFFFFFFFF:FFFFFFFFFFFFFFFFFFFFFFFFFFFFFFFFFFFF:FFFFFFFFFF,FFFFFFFFFFFF
F,FFFFFFFFF:FFF
```

```
@A00155:342:HHGFNDSXY:1:1476:29758:12446 2:N:0:GAACCTAG+TCCGCATA
CAATTCTGTCAAGACTTATGACATATTAAATAGATTGGTTACGGTAAAAGACAAGGCAAGGTATGGAC
CCTTAGCAAAGCCAGAAAGATCACCTTGCCCAAAGAAGACCAACATTTTCATACATGATGAAATTCAT
TATTGGTCTCGCGAT
+
FFFFFFFFFFF:FFFFFFFFFFFFFFFFFFFFFFFFFFFFFFFFFF,FFFFFFFFFFFFFFFFFFFFFF
FFFFFFFFFFFFFFFFFFFFFFFFFFFFFFFFFFFFF:FFFFFFFFFFFFFFFFFFFFFFFFFFFFFF
,FFFF:FFFFFFFFF
@A00155:342:HHGFNDSXY:1:1664:12120:9392 1:N:0:GAACCTAG+TCCGCATA
AATTCTGTCAAGACTTATGACATATTAAATAGATTGGTTACGGTAAAAGACAAGGCAAGGTATGGACC
CTTAGCAAAGCCAGAAAGATCACCTTGCCCAAAGAAGACCAACATTTTCATACATGATGAAATTCATT
ATTGGTCTCGCGATC
+
FFFF:FFFFFFFFFFFFFFFFFFFFFFFFFFFFFFFFFFFFFFFFFFFFFFFFFFFFFFFFFFFFFFF
FFFFFFF:FFFFFFFFFFFFFFFFFFFFFFFFFFFFFFFFFFFFFFFFFFFFFFFFFFFFFFFFFF:F
FFFFFFFFFFFFFFF
@A00155:342:HHGFNDSXY:1:1661:1642:15734 1:N:0:GAACCTAG+TCCGCATA
AATTCTGTCAAGACTTATGACATATTAAATAGATTGGTTACGGTAAAAGACAAGGCAAGGTATGGACC
CTTAGCAAAGCCAGAAAGATCACCTTGCCCAAAGAAGACCAACATTTTCATACATGATGAAATTCATT
ATTGGTCTCGCGATC
+
FFF:FFF,FFFFFFFFFFFFFFFFFF,FF:FFFFFFFFFFFFF:FFFFFFFFFF:FFFFFFFF:FFFF
FFFFFFFFFFFFF:FFFFF:FFF:FFFFF,FFFFFFF:FFFFFF::FFFFFF:FFFFFFFFFFFFFFF
FFFFF:FFFFFFFFF
@A00155:342:HHGFNDSXY:1:1659:18701:31970 2:N:0:GAACCTAG+TCCGCATA
AATTCTGTCAAGACTTATGACATATTAAATAGATTGGTTACGGTAAAAGACAAGGCAAGGTATGGACC
CTTAGCAAAGCCAGAAAGATCACCTTGCCCAAAGAAGACCAACATTTTCATACATGATGAAATTCATT
ATTGGTCTCGCGATC
+
FFFFFFFFF:FFFFFFFFFFFFFFFFFFFFFFFFFFFFFFFFFFFFF,FFFFFFFFFFFFFFFFFFFF
FFFFFFFFFFFFFF:FFFFFFFFFFFFFFFFFFFFFFFFFFFFFFFFFFFFFFFFFFFFFFFFFFFFF
F:FFFFFFFFFFFFF
@A00155:342:HHGFNDSXY:1:2367:28302:29653 2:N:0:GAACCTAG+TCCGCATA
AATTCTGTCAAGACTTATGACATATTAAATAGATTGGTTACGGTAAAAGACAAGGCAAGGTATGGACC
CTTAGCAAAGCCAGAAAGATCACCTTGCCCAAAGAAGACCAACATTTTCATACATGATGAAATTCATT
ATTGGTCTCGCGATC
+
FFFFFFFFFFFFFFFFFFFFFFFFFFFFFFFFFFFFFFFFFFFFFFFFFFFFFFFFFFFFFF,FFFFFF
FFFFFFFFFFFFFFFFFFFFFFFFFFFFFFFFFFFFFFFFFFFFFFFFFFFFFFFFFFFFFFFFFF,F
FFFFFFFFFFFFFFF
@A00155:342:HHGFNDSXY:1:2578:32000:23469 2:N:0:GAACCTAG+TCCGCATA
AATTCTGTCAAGACTTATGACATATTAAATAGATTGGTTACGGTAAAAGACAAGGCAAGGTATGGACC
CTTAGCAAAGCCAGAAAGATCACCTTGCCCAAAGAAGACCAACATTTTCATACATGATGAAATTCATT
ATTGGTCTCGCGATC
+
FFFFFFFFFFFFFFFFFFFFFFFFFFFFFFFFFFFFFFFFFFFFFFFFFFFFFFFFFFFFFF:FFFFFF
FFFFFFFFFFFFFFFFFFFFFFFFFFFFFFFFFFF:FFFFFFFFFFFFFFFFFFFFFFFFFFFFFF,F
FFFFFFF:FFFFFFF
@A00155:342:HHGFNDSXY:1:1557:17056:22138 2:N:0:GAACCTAG+TCCGCATA
ATTCTGTCAAGACTTATGACATATTAAATAGATTGGTTACTGTAAAGGACAAGGCAAGGTATGGACCC
TTAGCAAAGACAGAAAGATCACCTTGCCCAAAGAAGACCAACATTTTCATACATGATGAAATTCATTA
TTGGTCTCGCGA
+
FFFF::FFFF:FF,FFFFFFFFFFFFF,FF,F,FFFFFFF,FFFFF,,FFFF,:F:F:FFF,FFFFFF
```

```
::F,FFFF:,FF:FFFFF,FFFFFFF:F:F::,::F:FFFFFFFF,,F:FFFF::FFFF:FF:FF:,F
FFF,:FF,,F,F
@A00155:342:HHGFNDSXY:1:2232:1289:16407 2:N:0:GAACCTAG+TCCGCATA
ATTCTGTCAAGACTTATGACATATTAAATAGATTGGTTACGGTAAAATACAAGGCAAGGTATGGACCC
TTAGCAAAGCCAGAAAGATCACCTTGCCCAAAGAAGACCAACATTTTCATACATGATGAAATTAATTA
TTGTTCTCGCGATC
+
FFF:FFF,,,:F:FF,FF:FFFFFF,:,F:FFFFFFFF,:,FF,F,,,,:,F,:FFFFFFFFFFF,::
F:,F,FF,,F:FF,F,:,FF::FFF,,,::,FF,,,,::F,:FFFF:,:FF:FF:,FF,,:FF,,FFF
FF,,F,FF,,:,F:
@A00155:342:HHGFNDSXY:1:2338:7654:5071 2:N:0:GAACCTAG+TCCGCATA
ATTCTGTCAAGACTTATGACATATTAAATAGATTGGTTACGGTAAAAGACAAGGCAAGGTATGGACCC
TTAGCAAAGCCAGAAAGATCACCTTGCCCAAAGAAGACCAACATTTTCATACATGATGAAATTCATTA
TTGGTCTCGCGATC
+
FFFFFFFFFFFFFFFFFFFFFFFFFFFFFFFFFFFFFFFFFFFFFFFFFFFFFFFFFFFFFFFFFFFF
FFFFFFFFFF:FFFFFFFFFFFFFFFFF:FFFFFFFFF:FFFFFFFFFFFFFFFFFFFFFFFFFFFFF
FF,FF:FFFFFFFF
@A00155:342:HHGFNDSXY:1:2510:30734:36652 2:N:0:GAACCTAG+TCCGCATA
ATTCTGTCAAGACTTATGACATATTAAATAGATTGGTTACGGTCAAAGACAAGGCAAGGTATGGACCC
TTAGCAAAGCCAGAAAGATCACCTTGCCCAAAGAAGACCAACATTTTCATACATGATGAAATTCATTA
TTGGTCTCGCGATC
+
FFFFFFFF:FFFF,FFFFFFFFFFFFFFFFFFFFFFFFFFFFF:,FFFFFFFFFFFF:F:FFFFFFFFF
FFFFFFF:FFFFF,FFFF,F:FFFFFFFFF::FFF:FFFFFFFF:F:F,F,FFFF:FFF,:F,,,:,:
::FFFFFFFFFF:F
@A00155:342:HHGFNDSXY:1:1234:10077:19883 2:N:0:GAACCTAG+TCCGCATA
ATTCTGTCAAGACTTATGACATATTAAATAGATTGGTTACGGTAAAAGACAAGGCAAGGTATGGACCC
TTAGCAAAGCCAGAAAGATCACCTTGCCCAAAGAAGACCAACATTTTCATACATGATGAAATTCATTA
TTGGTCTCGCGATCA
+
FFFFFFFFFFFFFFFFFFFFFFFFFFFFFFFFFFFFFFFFFFFFFFF:F:FFFFFFFFFFFFF:FFFF
FFFFFFFF:FFFFFFFFFFFFFF:FFF:FFFF,:FFFFFFFFFFFFF:FFFFFFFFFFFFFF:FFFFF
FFF,FFFFFF:FFFF
@A00155:342:HHGFNDSXY:1:1234:16441:31407 2:N:0:GAACCTAG+TCCGCATA
ATTCTGTCAAGACTTATGACATATTAAATAGATTGGTTACGGTAAAAGACAAGGCAAGGTATGGACCC
TTAGCAAAGCCAGAAAGATCACCTTGCCCAAAGAAGACCAACATTTTCATACATGATGAAATTCATTA
TTGGTCTCGCGATCA
+
FFFFFFFFFFFFFFFFFFFFFFFF:FFFFFFFFFFFFFFFFFFFFFFFFFFFFFFFFFFFFFFFFFFF
FFFFFFFFFFFFFFFFFFFFFFFFFFFFFFFFFFFFFFFFFFFFFFFFFFFFFFFFFFFFFFFFFFFF
FFFFFFFFF:FFFFF
@A00155:342:HHGFNDSXY:1:1276:14235:10770 2:N:0:GAACCTAG+TCCGCATA
ATTCTGTCAAGACTTATGACATATTAAATAGATTGGTTACGGTAAAAGACAAGGCAAGGTATGGACCC
TTAGCAAAGCCAGAAAGATCACCTTGCCCAAAGAAGACCAACATTTTCATACATGATGAAATTCATTA
TTGGTCTCGCGATCA
+
FFFFFFFF:FFFFFFFFFFFFFFFFFFFFFFFFFFFFFFFFFFFFF:FFFFFFFFFFFFFFFFFFFFF
FFFFFFFFF:FFF:FFFFFFFFFFFFFFFFFFFFF:F:FFFFFFFFFFFFFFFFFFFFFFFFFFFFFF
FFFFFFFFFFFFFFF
@A00155:342:HHGFNDSXY:1:1467:26205:29403 2:N:0:GAACCTAG+TCCGCATA
ATTCTGTCAAGACTTATGACATATTAAATAGATTGGTTACGGTAAAAGACAAGGCAAGGTATGGACCC
TTAGCAAAGCCAGAAAGATCACCTTGCCCAAAGAAGACCAACATTTTCATACATGATGAAATTCATTA
TTGGTCTCGCGATCA
```

+
FFFFFFFFFFFFF:FF:FFFFFFFFFFFFFFFFFFFF:FFFFFFF,FFFFFFFFFFFFF:FFFFFFFF
FFFFFFFFFFFFFFF:FFFFFFFFFFFFFFFFFFFFFFFFFFFF:FFFFF:FFFFFFFFFFFFFFFFF
FFFFF:FFFFFFFFF
@A00155:342:HHGFNDSXY:1:1611:10917:7874 2:N:0:GAACCTAG+TCCGCATA
ATTCTGTCAAGACTTATGACATATTAAATAGATTGGTTACGGTAAAAGACAAGGCAAGGTATGGACCC
TTAGCAAAGCCAGAAAGATCACCTTGCCCAAAGAAGACCAACATTTTCATACATGATGAAATTCATTA
TTGGTCTCGCGATCA
+
FFFFFFFFFFFFFFFFFFFFFFFFFFFFFF:FFFFFFFFFFFFFFFFFFFFFFFFFFFFFFFFFFFFF
FFFFFFFFFFFFFFFFFFFFFFFFFFFFFFFFFFFFFFFFFFFFFFFFFFFF:FFFFFFFFFFFFFFF
FFFFFFFFFFFFFFF
@A00155:342:HHGFNDSXY:1:2173:11559:14246 2:N:0:GAACCTAG+TCCGCATA
ATTCTGTCAAGACTTATGACATATTAAATAGATTGGTTACGGTAAAAGACAAGGCAAGGTATGGACCC
TTAGCAAAGCCAGAAAGATCACCTTGCCCAAAGAAGACCAACATTTTCATACATGATGAAATTCATTA
TTGGTCTCGCGATCA
+
FFFFFFFFF:FFFFFFFFFFFFFFFFFF:FFFFFFFFFFFFFFFFFFFFFFFFFFFFFFFFFFFFFFF
FFFFFFFFFFFFFFFFFF:FFF:FFFFF:FFFFFFF:FFFFFFFFFFFFFFFFFFFFFFFFFFFFF:F
FFFFFFFFFFFFFFF
@A00155:342:HHGFNDSXY:1:2160:25880:2942 2:N:0:GAACCTAG+TCCGCATA
ATTCTGTCAAGACTTATGACATATTAAATAGATTGGTTACGGTAAAAGACAAGGCAAGGTATGGACCC
TTAGCAAAGCCAGAAAGATCACCTTGCCCAAAGAAGACCAACATTTTCATACATGATGAAATTCATTA
TTGGTCTCGCGATCA
+
FFFFFFFFFFFFFFFFFFFFFFFFFFFFFFFFFFFFFFFFFFFFFFFFFFFFFFFFFFFFFFFFFFFF
FFFFFFFFF,FFFF:FFFF,FFFFFFFFFFFF:F:FFFFFFFF::FFFFFFFF:FFF,FFFFFFFFFF
FFFFFFFF,FFFFF:
@A00155:342:HHGFNDSXY:1:2209:23674:30499 2:N:0:GAACCTAG+TCCGCATA
ATTCTGTCAAGACTTATGACATATTAAATAGATTGGTTACGGTAAAAGACAAGGCAAGGTATGGACCC
TTAGCAAAGCCAGAAAGATCACCTTGCCCAAAGAAGACCAACATTTTCATACATGATGAAATTCATTA
TTGGTCTCGCGATCA
+
FFFFFFFF:FFFFFFFFFFFFFF:FFFFFFFFFFFFFFFFFFFFFFFFFFFFFFFF:FFFFFFFFFFF
FFFFFFFFFFFFFFFFFF:FFFFFFFFFFFFFFFFFFFFFFFFFFFFFFFFFFFFF:FFFFFFFFFFF
FFFFFFFFFFFFFFF
@A00155:342:HHGFNDSXY:1:2302:15167:22529 2:N:0:GAACCTAG+TCCGCATA
ATTCTGTCAAGACTTATGACATATTAAATAGATTGGTTACGGTAAAAGACAAGGCAAGGTATGGACCC
TTAGCAAAGCCAGAAAGATCACCTTGCCCAAAGAAGACCAACATTTTCATACATGATGAAATTCATTA
TTGGTCTCGCGATCA
+
FFFFFFFF:FFFF,FFFFFFFFFFFFFFFFFFFFFFFFFFFFF:,:F:FFF:FFFFFFFF::FFFFFF
FFF,:FFFFF,FFFFFFFFFFFFFFF,FFFF,FFF,FFFFFFFFFFFFFFFFFFFFFF,FFFFFFFFF
FFFFFFFFFFFFF:FF
@A00155:342:HHGFNDSXY:1:2356:5367:27508 2:N:0:GAACCTAG+TCCGCATA
ATTCTGTCAAGACTTATGACATATTAAATAGATTGGTTACGCTAAAAGACAAGGCAAGGTATGGACCC
TTAGCAAAGCCAGAAAGATCACCTTGCCCAAAGAAGACCAACATTTTCATACATGATGAAATTCATTA
TTGGTCTCGCGATCA
+
FFFFFFFFFFFFFFFFFFFFFFFFFFFFFFFFFFFFFFFFFFFFF:FFFFFFFFFFFFFFFFFFFFFF
FFFFFFFFFFFFFFFFFFFFFFFFFFFFFFF:,FFFFFFFFFFFFFFFFFFFFFFFFFF,F:FFFFFF
FFFFFFFFFFFFFFF
@A00155:342:HHGFNDSXY:1:2460:23005:4570 2:N:0:GAACCTAG+TCCGCATA
ATTCTGTCAAGACTTATGACATATTAAATAGATTGGTTACGGTAAAAGACAAGGCAAGGTATGGACCC

TTAGCAAAGCCAGAAAGATCACCTTGCCCAAAGAAGACCAACATTTTCATACATGATGAAATTCATTA
TTGGTCTCGCGATCA
+
FFFFFFFFFFFFFFFFFFFFFFFFFFFFFFFFFFFFFFFFFFFFFF:FFFFFFFFFFFFFFFFFFFFF
FFFFFFFFFFFFFFFFFFFFFFFFFFFFFFFFFFFFFF:FFFFFFFF:FFFFFFFFFFFFFFF:FFFF
FFFFFFFF:FFFFFF
@A00155:342:HHGFNDSXY:1:2511:32262:11459 2:N:0:GAACCTAG+TCCGCATA
ATTCTGTCAAGACTTATGACATATTAAATAGATTGGTTACGGTAAAAGACAAGGCAAGGTATGGACCC
TTAGCAAAGCCAGAAAGATCACCTTGCCCAAAGAAGACCAACATTTTCATACATGATGAAATTCATTA
TTGGTCTCGCGATCA
+
FFFFFFFFFFFFFFFFFFFFFFFFFFFFF:FF:FFFFFFFFFFFF:FFFFFFFFF:FFFFFFFFFFFF
FFFFFFFFFFFFFFFFFFFFFFFFFFFFFFFFFFFFFFFFFFFFFFFFFFFFF:FFFFF:FF:FF,FF
FFFFFFFFFFF:,FF
@A00155:342:HHGFNDSXY:1:2644:3839:6950 2:N:0:GAACCTAG+TCCGCATA
ATTCTGTCAAGACTTATGACATATTAAATAGATTGGTTACGGTAAAAGACAAGGCAAGGTATGGACCC
TTAGCAAAGCCAGAAAGATCACCTTGCCCAAAGAAGACCAACATTTTCATACATGATGAAATTCATTA
TTGGTCTCGCGATCA
+
FFFFFFFF::FFFF:,FFFFFFFFFFFFFFFFFFFFFFFFFFF:FFFFF:FFFFFFFFFFFF:FF:FF
FF:,FFFFFFFFFFFF,FFF:FFFFF:FFF:FF:F,FF,FFF:FFFFFFF::FF::FF,,F:,,FFFF
FFFFFFFFF:FF:F:
@A00155:342:HHGFNDSXY:1:2655:15302:20353 2:N:0:GAACCTAG+TCCGCATA
ATTCTGTCAAGACTTATGACATATTAAATAGATTGGTTACGGTAAAAGACAAGGCAAGGTATGGACCC
TTAGCAAAGCCAGAAAGATCACCTTGCCCAAAGAAGACCAACATTTTCATACATGATGAAATTCATTA
TTGGTCTCGCGATCA
+
FFFFFFFFFFFFFFFFFFFFFFFFFFFFFFFFFFFFFFFFFFFFFF:FFFFFFFFFFFFFFFFFFFFF
FFFFFFFFFFFFFFFFFFFFFFFFFFFFFFFFFFFFFFFFFFFFFFFFFFFFF:FFFFFFFFFFFFFF
FFFFFFFF:FFFFFF
@A00155:342:HHGFNDSXY:1:2652:5846:14465 2:N:0:GAACCTAG+TCCGCATA
ATTCTGTCAAGACTTATGACATATTAAATAGATTGGTTACGGTAAAAGACAAGGCAAGGTATGGACCC
TTAGCAAAGCCAGAAAGATCACCTTGCCCAAAGAAGACCAACATTTTCATACATGATGAAATTCATTA
TTGGTCTCGCGATCA
+
FF:FFFFFFFFFFFF:FFFFFFFFFF:FFFFF:FFFFFFFFFF:FFFFFFFFFFFFFFFFFFFFFFFF
FFFFFFF:FFF,FFFFFFFFFFFFFFFFFFFFFFFFFFFFFFFFFFFFFFFFF,FFFFFFFFFFFF,,
,FFFFFFFF,FF:F:
@A00155:342:HHGFNDSXY:1:2652:6614:15013 2:N:0:GAACCTAG+TCCGCATA
ATTCTGTCAAGACTTATGACATATTAAATAGATTGGTTACGGTAAAAGACAAGGCAAGGTATGGACCC
TTAGCAAAGCCAGAAAGATCACCTTGCCCAAAGAAGACCAACATTTTCATACATGATGAAATTCATTA
TTGGTCTCGCGATCA
+
FFFFFFFFFFFFFFFFFFFFFFFFFFFFFFFFFFFFFFFFFFFFFFFFFFFFFFFFFFFFFFFFFFFF
FFFFFFFFFF:FFFFFFFFFFFFFFFFFFFFFFFFFFFFFFFFFFFFFFFFFFFFFFFFFFFFFFFFF
FFFFFFFF,FFFFFF
@A00155:342:HHGFNDSXY:1:2654:3884:1767 2:N:0:GAACCTAG+TCCGCATA
ATTCTGTCAAGACTTATGACATATTAAATAGATTGGTTACGCTAAAAGACAAGGCAAGGTATGGACCC
TTAGCAAAGCCAGAAAGATCACCTTGCCCAAAGAAGACCAACATTTTCATACATGATGAAATTAATTA
TTGGTCTCGCGATCA
+
FFFFFFFFFFFFFFFFFFFFFFFFFFFFFFFFFFFFFFFFFFFFFFFFFFFFFFFFFFFFFF:FFFFF
FFFFFFFFFF:FF:FFFFFFFFFFFFFFFFFFFFFFFFFFFFFFFFFFFFFFFFFFFFF,FFF,FFFF
FFFFFFFF:FFFFFF

```
@A00155:342:HHGFNDSXY:1:1178:23656:2315 1:N:0:GAACCTAG+TCCGCATA
TTCTGTCAAGACTTATGACATATTAAATAGATTGGTTACGGTAAAAGACAAGGCAAGGTATGGACCCT
TAGCAAAGCCAGAAAGATCACCTTGCCCAAAGAAGACCAACATTTTCATACATGATGAAATTCATTAT
TGGTCTCGCGATCAG
+
FFFFFF:FFFFFFFFFFFFFFFFFFFFFFFFFFFFFFFFFFFFFFFFFFFFFFFFFFFFFFFFFFF
FFFFFFFFFFFFFFFFFFFFFFFFFFFFFFFFFFFFFFFFFFFFFFFFFFFFFFFFFFFFFFFFFFFFFF
FFFFFFFFFFFFFFF
@A00155:342:HHGFNDSXY:1:1464:25880:2816 1:N:0:GAACCTAG+TCCGCATA
TTCTGTCAAGACTTATGACATATTAAATAGATTGGTTACGGTAAAAGACAAGGCAAGGTATGGACCCT
TAGCAAAGCCAGAAAGATCACCTTGCCCAAAGAAGACCAACATTTTCATACATGATGAAATTCATTAT
TGGTCTCGCGATCAG
+
F:FFFFFFFFFFFFFFFFFFFFFFF:FFFFFFFFFFFFFFFFFFFFFFFFFFFFFFFFFFFFFFFFFFFF
FFFFFFFFFFFFFFFFFFFFFFFFFFFFFFFFFFFFFFFFFFFFFFFFFFFFFFFFFFFFFFFFFFFFFF
FFFFFFFFFFFFFFF
@A00155:342:HHGFNDSXY:1:2112:29541:26224 1:N:0:GAACCTAG+TCCGCATA
TTCTGTCAAGACTTATGACATATTAAATAGATTGGTTACGGTAAAAGACAAGGCAAGGTATGGACCCT
TAGCAAAGCCAGAAAGATCACCTTGCCCAAAGAAGACCAACATTTTCATACATGATGAAATTCATTAT
TGGTCTCGCGATCAG
+
FFFFF:FFFFFFFFFFFFFFFFFFFFFFFFFFFFFFFFFFFFFFFFFFFFFFF:FFFF:FFFFFFFFFFF
FFFFFFFFFFFFFFFFFFFFFFFFFFFFFFFFFFFFFFFFFFFFFFFFFFFFFFFFFFFFFFFFFFFFFF
FFFFFFFFFFFFFFF
@A00155:342:HHGFNDSXY:1:2211:4996:20760 1:N:0:GAACCTAG+TCCGCATA
TTCTGTCAAGACTTATGACATATTAAATAGATTTGTTACGGTAAAAGACAAGGCAAGGTATGGACCCT
TAGCAAAGCCAGAAAGATCACCTTGCCCAAAGAAGACCAACATTTTCATACATGATGAAATTCATTAT
TGGTCTCGCGATCAG
+
FFFFFFFFFFF,FFFFFFFFFFFFFFFF:F:FFF,FFFF,FFFFFFFFF:FFFFFFFFFFFFFFFFF:FF
FFFFFFFFFFFFFFFFFFFFFFFF,FF::FFFFFFFFFFFFFFFFFF,FFF,FFFFFFFFFFFFFFFFFF
F,FFFFFFFFFFFFF
@A00155:342:HHGFNDSXY:1:1424:27543:10833 2:N:0:GAACCTAG+TCCGCATA
TCTGTCAAGACTTATGACATATTAAATAGATTGGTTACGGTAAAAGACAAGGCAAGGTATGGACCCTT
AGCAAAGCCAGAAAGATCACCTTGCCCAAAGAAGACCAACATTTTCATACATGATGAAATTCATTATT
GGTCTCGCGATCAG
+
FFFFFFFF,FFFFFFF:FFFFFF:,FFF:FFF:FFFF,FFFFFFFFFFFFFFF:FFFFFFFFFFFF:FFF
F:FFFF,FFF,FFFFFFF:FFFF:FFFFFFFFFFF::FF:FFFFFFFFFFFFFFFFFFFFFF:FFFF:FF
FF:FFFF,,FFF:F
@A00155:342:HHGFNDSXY:1:1521:6623:5228 2:N:0:GAACCTAG+TCCGCATA
TCTGTCAAGACTTATGACATATTAAATAGATTGGTTACGGTAAAAGACAAGGCAAGGTATGGACCCTT
AGCAAAGCCAGAAAGATCACCTTGCCCAAAGAAGACCAACATTTTCATACATGATGAAATTCATTATT
GGTCTCGCGATCAGC
+
FFFFFF:FFFFFFFFFFFFFFFFFFFFFFFFFFFFFFFFFFFFFFFFFF:FFF,FF:FFFFFF:FFFFFF
FFFFFFFF,FFFFFFFFFFFFFFFFFFFFFFFFF:F::FFFFFFFFFF,FFFFFFFFFFFFFFFFFFFFF
:FFFFFFFFFFFFFF
@A00155:342:HHGFNDSXY:1:1560:7301:30827 2:N:0:GAACCTAG+TCCGCATA
TCTGTCAAGACTTATGACATATTAAATAGATTGGTTACGGTAAAAGACAAGGCAAGGTATGGACCCTT
AGCAAAGCCAGAAAGATCACCTTGCCCAAAGAAGACCAACATTTTCATACATGATGAAATTCATTATT
GGTCTCGCGATCAGC
+
FFFFFFF:FFFFFFFFFFFFFFFF:FFFFFFFFFFFFFFFFFFFFFFFFFFFFFFFFFFFFFFFFFFFF
```

```
FFFF:FFFFFFFFFFFFFFFFFFFFFFFFFFFFFFFFFFFFFFFFFFFFFFFFFFFFFFFFFFFFFFFFFF
FFFFFFFFFF:,FFF
@A00155:342:HHGFNDSXY:1:1541:21983:12665 1:N:0:GAACCTAG+TCCGCATA
CTGTCAAGACTTATGACATATTAAATAGATTGGTTACGGTAAAAGACAAGGCAAGGTATGGACCCTTA
GCAAAGCCAGAAAGATCACCTTGCCCAAAGAAGACCAACATTTTCATACATGATGAAATTCATTATTG
GTCTCGCGATCAGCG
+
FFFFFFFFFFFFFFFFFFFFFF:FFFFFFF:FFFFFFFFFFFFFFFFFFFFFFFFFFFFFFFFFFFFFF
FFF:FFFFFFFFFFFFFFFFFFFFFFFFF:F,FFFFFFFFFFFFFFFFFFF:FFFFFFFFFFFFFFFFF
FFFFFFFFFFFFFFF
@A00155:342:HHGFNDSXY:1:1565:18692:32080 1:N:0:GAACCTAG+TCCGCATA
CTGTCAAGACTTATGACATATTAAATAGATTGGTTACGGTAAAAGACAAGGCAAGGTATGGACCCTTA
GCAAAGCCAGAAAGATCACCTTGCCCAAAGAAGACCAACATTTTCATACATGATGAAATTCATTATTG
GTCTCGCGATCAGCT
+
FFFFFFFFF:F,FFFFFFFFFFFFFFFFFFFFFFFFFFFFFFFFFFFFFFFFF:FFFFFFFFFFFFF
FFFFFFFFFFFFFFFFFFFFFFFFFFFFFFFFFFFFFFFFFFFFFFFFFFFFFFFFFFFFFFFFFFFFF
FFFFFFFFFFFFFFF
@A00155:342:HHGFNDSXY:1:1557:17056:22138 1:N:0:GAACCTAG+TCCGCATA
CTGTCAAGACTTATGACATATTAAATAGATTGGTTACGTTAAATGACAAGGCAAGGTATGGACCCTTT
GCAAAGCAAGAAAGATCACCTTGCCCAAAGAAGACCTACATTTTCATACTTGATGATATTCATTATTG
GTCTCGCGATCAGCT
+
FFFF::FFF:FF:F:FF:FFFF,FFFF,F:FF:F:,,:,FFFF,:F,:F::,F,:,,F::F,F,FFF,
F::F,,,,:,F,F,:F,F,,FF:F:F,FFFFF:F,,,F,,FFFFFFFF,,:FFF,F,,F::FFFFF:,
,F,FFF:FF:FF,:F
@A00155:342:HHGFNDSXY:1:2118:14959:27524 1:N:0:GAACCTAG+TCCGCATA
CTGTCAAGACTTATGACATATTAAATAGATTGGTTACGGTAAAAGACAAGGCAAGGTATGGACCCTTA
GCAAAGCCAGAAAGATCACCTTGCCCAAAGAAGACCAACATTTTCATACATGATGAAATTCATTATTG
GTCTCGCGATCAGCC
+
FFFF:FFF,FFF:F:FFFFFFFFFFFFFFFFFFFFFFFFF,FFFFFFFF::F:FFFFFFFFF:F:F:FFF
FFFFFFFFFFFFFFFFFFFFFFFFFFFFF:FFFFFFFFFFFFFFFFFFFFFFFFFFFFFFFFFFFFFFFF
FFFFFFFFFFF:FFF
@A00155:342:HHGFNDSXY:1:2232:20265:29042 1:N:0:GAACCTAG+TCCGCATA
CTGTCAAGACTTATGACATATTAAATAGATTGGTTACGGTAAAAGACAAGGCAAGGTATGGACCCTTA
GCAAAGCCAGAAAGATCACCTTGCCCAAAGAAGACCAACATTTTCATACATGATGAAATTCATTATTG
GTCTCGCGATCAGCA
+
FFFFFFFFFFFFFFFFFFFFFFFFFFFFFFFFFFFFFFFFFFFFFFFFFFFFFFFFFFFFFFFFFFFFF
FFFFFFFFFFFFFFFFFFFFFFFFFFFFFFFFFFFFFFFFFFFFFFFFFFFFFFFFFFFFFFFFFFFFF
FFFFFFFFFFFFFFF
@A00155:342:HHGFNDSXY:1:2663:18801:34397 1:N:0:GAACCTAG+TCCGCATA
CTGTCAAGACTTATGACATATTAAATAGATTGGTTACGGTAAAAGACAAGGCAAGGTATGGACCCTTA
GCAAAGCCAGAAAGATCACCTTGCCCAAAGAAGACCAACATTTTCATACATGATGAAATTCATTATTG
GTCTCGCGATCAGCT
+
FFFFFFFFFFF::F:F:FFFFFF,FFF:FFF,FFFFF,FFFF:FFFFFF,FFFFFFFFFFFFFFFFFF
FFFFFFFFFFFFFFFFFFFFFFFFFF:FFFF:F,FFFFF:FFFFF:FFFFFFFFFFFFFFFFFFFFFFFF
FFFFFFFF:FFF:F:
@A00155:342:HHGFNDSXY:1:1649:29333:14278 2:N:0:GAACCTAG+TCCGCATA
CTGTCAAGACTTATGACATATTAAATAGATTGGTTACGGTAAAAGACAAGGCAAGGTATGGACCCTTA
GCAAAGCCAGAAAGATCACCTTGCCCAAAGAAGACCAACATTTTCATACATGATGAAATTCATTATTG
GTCTCGCGATCAGCT
```

+
FFFFFFFFFFFFFFFFFFFFFFFFFFFFFFFFFFFFFFFFFFFFFFFFFFFFFFFFFFFFFFFFFFFF
FFFFFFFFFFFFFFFFFFFFFFFFFFFFFFFFFFFFFFFFFF,F:FFFFF:FFFFF:FFFF,,FFFFF
FFFFFFF:FFFFFFF
@A00155:342:HHGFNDSXY:1:1619:19623:7827 1:N:0:GAACCTAG+TCCGCATA
TGTCAAGACTTATGACATATTAAATAGATTGGTTACGGTAAAAGACAAGGCAAGGTATGGACCCTTAG
CAAAGCCAGAAAGATCACCTTGCCCAAAGAAGACCAACATTTTCATACATGATGAAATTCATTATTGG
TCTCGCGATCAGCTT
+
FFFFFF:FFFFFFFFFFFFFF:FFFFFFFFFFFFFFFFFFFFFFFFFFFFFFFFFFFFFFF:FFFFFF
FFFFFFFFFFFFFFFFFFFFFFFFFF:FFFFFFFFFFFFFFFFFFFFFFFFFFFFFFFFFFFFFFFFF
FFFFFFFFFFFFFFF
@A00155:342:HHGFNDSXY:1:2116:19723:5807 1:N:0:GAACCTAG+TCCGCATA
TGTCAAGACTTATGACATATTAAATAGATTGGTTACGGTAAAAGACAAGGCAAGGTATGGACCCTTAG
CAAAGCCAGAAAGATCACCTTGCCCAAAGAAGACCAACATTTTCATACATGATGAAATTCATTATTGG
TCTCGCGATCAGCTT
+
FFFFFFFFFFFFFFFFFFFFFFFFFFFFFFFFFFFFF:FFFFFFFFFFFFFFFFFFFFFFFFFFFFFF
FFFFFFFFFFFFFFFFFFFFFFFFFFFFFFFFFFFFFFFFFFFFFFFFFFFFFFFFFFFFFFFFFFFF
FFFFFFFFFFFFFFF
@A00155:342:HHGFNDSXY:1:2372:20989:6715 1:N:0:GAACCTAG+TCCGCATA
TCTCAAGACTTATGACATATTAAATAGATTGGTTACGGTAAAAGACAAGGCAAGGTATGGACCCTTAG
CAAAGCCAGAAAGATCACCTTGCCCAAAGAAGACCAACATTTTCATACATGATGAAATTCATTATTGG
TCTCGCGGTCAGCTT
+
FFFFFFFFFFFFFFFFFFFFFFFF:FF:FFFFFFFFFFFFFFFFFFFFFFFFFFFFFFFFFFFFFFFF
FFFFFFFFFFFFFFFFFFFFFFFFFFFFFFFFFFFFFFFFFFFFFFFFFFFFFFFFFFFFFFFFFFFF
FFFFFFF,FFFFFFF
@A00155:342:HHGFNDSXY:1:2606:6081:12117 1:N:0:GAACCTAG+TCCGCATA
TGTCAAGACTTATGACATATTAAATAGATTGGTTACGGTAAAAGACACGGCAAGGTATGGACCCTTAG
CAAAGCCAGAAAGATCACCTTGCCCAAAGAAGACCAACATTTTCATACATGATGAAATTCATTATTGG
TCTCGCGATCAGCTT
+
FFFFFFFFFFFFF:FFFFFFF:FF:F:F:,F:FF,FFFFF:FFFFF:,F,FFF:FFFFFFFFF:F,F:
FFFFF:FFFFFFFFFFFFF,:FFFFFFFFF::F:FFFFFFFFFFFFFFFFFFFFFFFFFFFFFFFFFF
FFFFFFFFFFFFFFF
@A00155:342:HHGFNDSXY:1:2606:6614:11976 1:N:0:GAACCTAG+TCCGCATA
TGTCTAGACTTATGACATATTAAATAGATTGGTTACGTTAAAAGACAAGGCAATGTATGGACCCTTAG
CAAAGCCAGAAAGATCACCTTGCCCAAAGAAGACCAACATTTTCATACATGATGAAATTCATTATTGT
TCTCGCGATCAGCTT
+
FFF,,,F,FF:F,,,FF:,FFF,:F,,,F,,,FF::F,FF,,,FF:,,F::FF,FFFF,:FF,:F:F,
FF:FF:,FFFFFFF,FF,:,FF,,FFFF,F,,FFF,::,F:FFF,:F,,FF:FFF,,FFF:FF::F,,
FFFFFF,:FFFFFFF
@A00155:342:HHGFNDSXY:1:1171:13467:5776 2:N:0:GAACCTAG+TCCGCATA
TGTCAAGACTTATGACATATTAAATAGATTGGTTACGGTAAAAGACAAGGCAAGGTATGGACCCTTAG
CAAAGCCAGAAAGATCACCTTGCCCAAAGAAGACCAACATTTTCATACATGATGAAATTCATTATTGG
TCTCGCGATCAGCTT
+
FFFFFFFFFFFFFFFFFFFFFFFFFFFFFFFFFFFFFFFFFFFFFFFFFFFFFFF:FFFFFFFFFFFF
FFFFFFFFFFFFFFFFFFFFFFFFFFFFFF:,FFFFFFFF:FFFFFFFFFFFFFFFFFFFFFFF:FF,
F:FFFFFFFFFFFFF
@A00155:342:HHGFNDSXY:1:1623:17508:22701 2:N:0:GAACCTAG+TCCGCATA
TGTCAAGACTTATGACATATTAAATAGATTGGTTACGGTAAAAGACAAGGCAAGGTATGGACCCTTAG

CAAAGCCAGAAAGATCACCTTGCCCAAAGAAGACCAACATTTTCATACATGATGAAATTCATTATTGG
TCTCGCGATCAGCTT
+
FFFFFFFFFFFFFFFFFFFFFFFFFFFFFFFFFFFFFFFFFFFFFFFFFFFFFFFFFFFFFFFFFFFF
FFFFFFFFFFFFFFFFFFFFFFFFFFFFFFFFFFFFFFFFFFFFFFFFFFFFFFFFFFFFFFFFFFFF
FFFFFFFFFFFFFFF
@A00155:342:HHGFNDSXY:1:1638:28320:12524 1:N:0:GAACCTAG+TCCGCATA
GTCAAGACTTATGACATATTAAATAGATTGGTTACGGTAAAAGACAAGGCAAGGTATGGACCCTTAGC
AAAGCCAGAAAGATCACCTTGCCCAAAGAAGACCAACATTTTCATACATGATGAAATTCATTATTGGT
CTCGCGATCAGCTTG
+
FFFFFFFFFFFFFFFFFFFFFFF:FF,FFFFFFFFFFFFFFFFFFFFFFFFFFFFFFFFFFFFFFFFF
FFFFFFFFFFFFFFFFFF:FFFFFFFFFFFFFFFFFFFFFFFFFFFFF,FFFFFFFFFFFFFFFFFFFF
FFFFFFFFFFFFFFF
@A00155:342:HHGFNDSXY:1:2330:17110:10676 1:N:0:GAACCTAG+TCCGCATA
GTCAAGACTTATGACATATTAAATAGATTGGTTACGGTAAAAGACAAGGCAAGGTATGGACCCTTAGC
AAAGCCAGAAAGATCACCTTGCCCAAAGAAGACCAACATTTTCATACATGATGAAATTCATTATTGGT
CTCGCGATCAGCTTG
+
FFFFFFFFFFFFFFFFFFFFFFFFFFFFFFFFFFFFFFFFFFFFFFFFFFFFFFFFFFFFFFFFFFFF
FFFFFFFFFFFFFFFFFFFFFFFFFFFF:,FFFFFFFFFFFFFFFFFFFFFFFFFFFFFFFFFFFFFF
FFFFFFFFFFFFFFF
@A00155:342:HHGFNDSXY:1:2538:31864:10488 1:N:0:GAACCTAG+TCCGCATA
GTCAAGACTTATGACATATTAAATAGATTGGTTACGGTAAAAGACAAGGCAAGGTATGGACCCTTAGC
AAAGCCAGAAAGATCACCTTGCCCAAAGAAGACCAACATTTTCATACATGATGAAATTCATTATTGGT
CTCGCGATCAGCTTG
+
FFFFFFFFFFFFFFFFFFFFFFF:FFF:FFFFFFFFF:FFFFFFFFFFFFFFFFFFFFFFFFFFFFFF
FFFFFFFFFFFFFFFFFF:FFFFFFFFF::FFFFFFFFFFFFFFFFFFFFFFFFFFFFFFFFFFFFFF
F:FFFFFFFFFFFFF
@A00155:342:HHGFNDSXY:1:2307:9815:15107 2:N:0:GAACCTAG+TCCGCATA
TCAAGACTTATGACATATTAAATAGATTGGTTACGGTAAAAGACAAGGCAAGGTATGGACCCTTAGCA
AAGCCAGAAAGATCACCTTGCCCAAAGAAGACCAACATTTTCATACATGATGAAATTCATTATTGGTC
TCGCGATCAGCTTGA
+
FFFFFFFFFFFFFFFFFFFFF:FFFFFFFFFFFFFFFFFFFFFFFFF:FFFFFFFFFF:FFF:F:FF:F
FF:F:FFFFFFFFFFFFFF:FFFFF,F:FFFFFFFFFFFFFFFFF,FFF:FFF:FFF:FFFFF:FFFF
FF:FFFFFF:FFFFF
@A00155:342:HHGFNDSXY:1:2606:6614:11976 2:N:0:GAACCTAG+TCCGCATA
CAAGACTTATGACATATTAAATAGATTGTTTACGGTAAAAGACAAGGCAAGGTATGTACCCTTAGCAA
AGCCAGAAAGATCACCATGCCCAAAGAAGACCAACATTTTCATAAATGATGAAAATCATTATTGGTCT
CGCGATCA
+
:FFFF:FFF:FFFFFFF,FFFFFFFFF,,::F,:FFFFFF:F:FF,F:F:F,::F:,FF,FF:FF:FF
:F,:F,FFF,FFFF::,,F:F:F:F,:F:FF,F::F::,F,FFF,F,,F,::FF,,FF,,F,,,::FF
::,:FFF:
@A00155:342:HHGFNDSXY:1:1265:18747:15327 2:N:0:GAACCTAG+TCCGCATA
CAAGACTTATGACATATTAAATAGATTGGTTACGGTAAAAGACAAGGCAAGGTATGGACCCTTAGCAA
AGCCAGAAAGATCACCTTGCCCAAAGAAGACCAACATTTTCATACATGATGAAATTCATTATTGGTCT
CGCGATCAGCTTGA
+
FFFFFFFFF:,FFFF::FFFFFFFFFFFF:FFFFFFFFFFFFFFFFF:FF,F:FFFFFFFFFFFFFFF
F,FFFFFFFFF,:FFFFFFF,:FFFFFFFFF,FF,F:FF:FFFFFFF,FFFFFFFFFFFFFFFFFFFF
FF:FF,,:F:F,FF

```
@A00155:342:HHGFNDSXY:1:2454:22815:21308 2:N:0:GAACCTAG+TCCGCATA
CAAGACTTATGACATATTAAATAGATTGGTTACGGTAAAAGACAAGGCAAGGTATGGACCCTTAGCAA
AGCCAGAAAGATCACCTTGCCCAAAGAAGACCAACATTTTCATACATGATGAAATTCATTATTGGTCT
CGCGATAGATCGGA
+
FFFFFFFFFFFFFFFFFFFFFFFFFFFFFFFFFFFFFFFFFFFFFFFFFFFFFFFFFFFFFFFFFFFF
FFFFFFFFFFFFFFFFFFFFFFFFFFFFFFFFFFFFFFFFFF:FFFFFFFFFFFFFFFFFFFFFFFFF
FFFFFFFFFFFFFF
@A00155:342:HHGFNDSXY:1:1235:5891:13166 1:N:0:GAACCTAG+TCCGCATA
CAAGACTTATGACATATTAAATAGATTGGTTACGGTAAAAGACAAGGCAAGGTATGGACCCTTAGCAA
AGCCAGAAAGATCACCTTGCCCAAAGAAGACCAACATTTTCATACATGATGAAATTCATTATTGGTCT
CGCGATCAGCTTGAG
+
FFFFFFFFFFFFFFFFFFFFFFFFFFFFFFFFFFFFFFFFFFFFFFFFF:FFFFFFFFFFFFFFFFFF
FFFFFFFFFFFFFFFF,FFFFFFFFF:FFFFFFFFFFFFFFFFFFFFFFFFFFFFFFFFFFFFFFFFF
FFFFFFFFFFFFFFF
@A00155:342:HHGFNDSXY:1:1251:8205:32424 1:N:0:GAACCTAG+TCCGCATA
CAAGACTTATGACATATTAAATAGATTGGTTACGGTAAAAGACAAGGCAAGGTATGGACCCTTAGCAA
AGCCAGAAAGATCACCTTGCCCAAAGAAGACCAACATTTTCATACATGATGAAATTCATTATTGGTCT
CGCGATCAGCTTGAG
+
FFF:FFFFFFFF:FFFFFFFFFFFFFFFFFFFFFFFFFFFFFFFFFFFFFFFFFFFFF:FFFFFFFFF
FFFFFFFFFFFFFFFFFFFF:FFFFFFFFFF:FFFFFFFFFFFFFFFFFFFFFFFFFFFFFFFFFFFF
FFFFFFFFFFFFFFF
@A00155:342:HHGFNDSXY:1:1278:25708:12978 1:N:0:GAACCTAG+TCCGCATA
CAAGACTTATGACATATTAAATAGATTGGTTACGGTAAAAGACAAGGCAAGGTATGGACCCTTAGCAA
AGCCAGAAAGATCACCTTGCCCAAAGAAGACCAACATTTTCATACATGATGAAATTCATTATTGGTCT
CGCGATCAGCTTGAG
+
FFFFFFFFFFFFFFFFFFFFFFFFFFFFFFFFF:FFFFFFFFFFFFFFFFF:FF,FFFFFFFFFFFFF
FFFFFFFFFFFFFFFFFFFFFFFFFFFFFFFFFFFFFFFFFFFFFFFFFFFFFFFFFFFFFFFFFFFF
FFFFFFFFFFFFFFF
@A00155:342:HHGFNDSXY:1:1441:14416:27305 1:N:0:GAACCTAG+TCCGCATA
CAAGACTTATGACATATTAAATAGATTGGTTACGGTAAAAGACAAGGCAAGGTATGGACCCTTAGCAA
AGCCAGAAAGATCACCTTGCCCAAAGAAGACCAACATTTTCATACATGATGAAATTCATTATTGGTCT
CGCGATCAGCTTGAG
+
:FFFFFFFFFFFFFFFF:FFF:FFF,:FFFFFFFFFFFFFFFFFFFFFFFFFFFFFFFFFFFFFFFFF
:FFFFFFF:FFFFFFFF,FFFFFFFFFFFFFFF,FFFFFFFFFFFFFFFFFFFFFFFFFFFFFFFFFF
FFFFFFFFFFFFFFF
@A00155:342:HHGFNDSXY:1:1438:30807:25504 1:N:0:GAACCTAG+TCCGCATA
CGATCTTTATGACATATTAAATAGATTGGTTACGGTAAAAGACAAGGCAAGGTATGGACCCTTAGCAA
AGCCAGAAAGATCACCTTGCCCAAAGAAGACCAATATTTTCATACATGATGAAATTCATTATTGGTCT
CGCGATCAGCTTGAG
+
FFFFFFFFFFFFFFFFFFFFF:FFFF:FFFFFFFFFF,,FFFFF,FFFFFFFFFFFFFFFFFFFFFFF
:FFFFFFFFFFFFFFF:FF:FFFFF:FFF,:FFFFFFFFFF:FFFFFFFFFFFFFFFFFFFFFF:FFF
FFFFFFFFFFFFFFF
@A00155:342:HHGFNDSXY:1:1435:8585:20525 1:N:0:GAACCTAG+TCCGCATA
CAAGACTTATGACATATTAAATAGATTGGTTACGGTAAAAGACAAGGCAAGGTATGGACCCTTAGCAA
AGCCAGAAAGATCACCTTGCCCAAAGAAGACCAACATTTTCATACATGATGAAATTCATTATTGGTCT
CGCGATCAGCTTGAG
+
FFFFFFFFFFFFFFFFFFFFFFFFFF:FFFFFFFFFFFFFFFFFFFFFFFFFFFFFFFFFFFFFFFFF
```

```
F:FFFFFFFFFFFFFFFFFFFFFFF:F:FFFFFFFFF:FFFFFFFFFFFFFFFFFFFFFFFFFFFFFFF
FFFFFFFFFFFFFFF
@A00155:342:HHGFNDSXY:1:1502:12897:7232 1:N:0:GAACCTAG+TCCGCATA
CAAGACTTATGACATATTAAATAGATTGGTTACGGTAAAAGACAAGGCAAGGTATGGACCCTTAGCAA
AGCCAGAAAGATCACCTTGCCCAAAGAAGACCAACATTTTCATACATGATGAAATTCATTATTGGTCT
CGCGATCAGCTTGAG
+
FFFFFFFFFFFFFFFFFFFFFFFFFFFFFFFFFFFFFFFFFFFFFFFFFFFFFFFFFFFFFFF,FFFFF
FFFFFFFFFFFFFFFF:FFFFFFFFFF::FFFFFFFFFFFFF:FFFFFFFFFFFFFFFFFFFFFFFFFF
FFFF:FFFFFFFFFF
@A00155:342:HHGFNDSXY:1:2234:11243:33270 1:N:0:GAACCTAG+TCCGCATA
CAAGACTTATGACATATTAAATAGATTGGTTACGGTAAAAGACAAGGCAAGGTATGGACCCTTAGCAA
AGCCAGAAAGATCACCTTGCCCAAAGAAGACCAACATTTTCATACATGATGAAATTCATTATTGGTCT
CGCGATCAGCTTGAG
+
FFFFFFFFFFFF:FFFFFFFFF:FFFFFFFFFF:FFFFFFFFFFFFFFFFFFFFFFFFFFFFFFFFFFF
FFFFFFFFFFFFFFFFFFFFFFFFFFFFFFFFFFFFFFFFFFFFFFFFFFFFFFFFFFFFFFFFFFFFF
FFFFFFFFFFFFFFF
@A00155:342:HHGFNDSXY:1:2664:13214:10034 1:N:0:GAACCTAG+TCCGCATA
CAAGACTTATGACATATTAAATAGATTGGTTACGGTAAAAGACAAGGCAAGGTATGGACCCTTAGCAA
AGCCAGAAAGATCACCTTGCCCAAAGAAGACCAACATTTTCATACATGATGAAATTCATTATTGGTCT
CGCGATCAGCTTGAG
+
FFFFFFFFFFFFFFFFFFFFFFFFFFFFFFFFFFFFFFFFFFFFF:FFFFFFFFFFFFFFFFFFFFFFF
FFFFFFFFFFFFFFFFFFFFFFFFFFFFFFFFFFFFFFFFFFFFFFFFFFFFFFFFFFFFFFFFFFFFF
FFFFFFFFFFFFFFF
@A00155:342:HHGFNDSXY:1:1302:28348:13510 2:N:0:GAACCTAG+TCCGCATA
CAAGACTTATGACATATTAAATAGATTGGTTACGGTAAAAGACAAGGCAAGGTATGGACCCTTAGCAA
AGCCAGAAAGATCACCTTGCCCAAAGAAGACCAACATTTTCATACATGATGAAATTCATTATTGGTCT
CGCGATCAGCTTGAG
+
FFFFFFFFF:FFFFFFFFFFFFFFFFFFFFFFFFFFFFFFFFFFFFFFFF,FFFFFFFFFF:FFFFFFF
FFFFFFFFFFFFFFFFFFFFFFFFFF:FF:FFFFFFFFFFFFFFFFFFFFFFFFFF:F:FFFFFFFFFF
FFFFFFFFFFFFFFF
@A00155:342:HHGFNDSXY:1:2265:18792:28181 2:N:0:GAACCTAG+TCCGCATA
CAAGACTTATGACATATTAAATAGATTGGTTACGGTAAAAGACAAGGCAAGGTATGGACCCTTAGCAA
AGCCAGAAAGATCACCTTGCCCAAAGAAGACCAACATTTTCATACATGATGAAATTCATTATTGGTCT
CGCGATCAGCTTGAG
+
FFF,FFFFF:FFFFFFFFFFFFFFFF:FFFFFFFFF:FFFFFFFFFFFFFFFFFFF,FFFFF:FFFFF
FFFFFFFFFFFFFFFFFFFFFFF,FFFFF:F:FFFFFFFFFFF:FFFFFFFFFFFFFFFFFFFFFFFFFF
FFFFFFFF:FFFFFF
@A00155:342:HHGFNDSXY:1:2372:20989:6715 2:N:0:GAACCTAG+TCCGCATA
CAAGACTTATGACATATTAAATAGATTGGTTACGGTAAAAGACAAGGCAAGGTATGGACCCTTAGCAA
AGCCAGAAAGATCACCTTGCCCAAAGAAGACCAACATTTTCATACATGATGAAATTCATTATTGGTCT
CGCGGTCAGCTTAGA
+
FFFFFFFFFFFFFFFFFFFFFFFFFFFFFFFFFFFFFFFFFFFFFFFFFFFFFF:FFFFFFFFFFFFFF
FFFFFFFFFFFFFFFFFFFFFFFFFFF:FFFFFFFFFFFFFFFFFFFFFFFFF,FFFFF:FFFFFFFFF
FFFF,FFFFFFFFFF
@A00155:342:HHGFNDSXY:1:2672:22408:35446 1:N:0:GAACCTAG+TCCGCATA
AAGACTTATGACATATTAAATAGATTGGTTACGGTAAAAGACAAGGCAAGGTATGGACCCTTAGCAAA
GCCAGAAAGATCACCTTGCCCAAAGAAGACCAACATTTTCATACATGATGAAATTCATTATTGGTCTC
GCGATCAGCTTGAGA
```

```
+
FFFFFFFFFFFFFFFFFFFFFFFFFFFF:FFFFFFFFFFFFFFFFFFFFFFFFFFFFFFFFFFFFFFFF
FFFFFFFFFFFFFFF,FFFFFFFFFFFFFFFFFFFFFFFFFFFFFFFFFFFFFFFFFFFFFFFFFFFFF
FFFFFFFFFFFFFFF
@A00155:342:HHGFNDSXY:1:1339:13729:9330 2:N:0:GAACCTAG+TCCGCATA
AAGACTTATGACATATTAAATAGATTGGTTACGGTAAAAGACAAGGCAAGGTATGGACCCTTAGCAAA
GCCAGAAAGATCACCTTGCCCAAAGAAGACCAACATTTTCATACATGATGAAATTCATTATTGGTCTC
TCGATCAGCTTGAGA
+
FFFFFFFFFFFFFFFFFFFFFFFFFFFFFFFFFFFFFFFFFFFFFFFFFFFFFFFFFFFFFFFFFFFFF
FFFFFFFFFFFFFFFFFFFFFFFFFFFFFFFFFFFFFFFFFFFFFFFFFFF,FFF:FFFFFFFFFFFFF
FFFFFFFFFFFFFFF
@A00155:342:HHGFNDSXY:1:1629:19822:19163 2:N:0:GAACCTAG+TCCGCATA
AAGACTTATGACATATTAAATAGATTGGTTACGGTAAAAGACAAGGCAAGGTATGGACCCTTAGCAAA
GCCAGAAAGATCACCTTGCCCAAAGAAGACCAACATTTTCATACATGATGAAATTCATTATTGGTCTC
TCGATCAGCTTGAGA
+
FFFFFFF,FFFFFFFFFFFFFFFFFFFFFFFFFFFFF:FFFFFFFFFFF:FFFFFFFFFFFFFFFFFFF
FFFFFF:FFFFFFFFFFFF:FFFFFFFFFFFFFFFFFFFFFFFFFFFFFFFFFFFFFFFFFFFFFFFFF
FFFFFFFFFFFFFFF
@A00155:342:HHGFNDSXY:1:1114:10050:23469 2:N:0:GAACCTAG+TCCGCATA
AGACTTATGACATATTAAATAGATTGGTTACGGTAAAAGACAAGGCAAGGTATGGACCCTTAGCAAAG
CCAGAAAGATCACCTTGCCCAAAGAAGACCAACATTTTCATACATGATGAAATTCATTATTGGTCTCG
CGATCAGCTTG
+
FFFFFFFFFFF:FFFFFFFFFFFFFFFFF:FFFFFF:FFFFFFFFFFFFFFFFFFFFFFFFFFFFFFFF
FFFFFF:FFFFFFFFFFFFFFFFFFFFFFFFFFFFFFFFFFFFFFFFFFFFF:FFFFFFFFF,FFFFF
FFFFFFFFFFF
@A00155:342:HHGFNDSXY:1:1227:16613:34804 2:N:0:GAACCTAG+TCCGCATA
AGACTTATGACATATTAAATAGATTGGTTACGGTAAAAGACAAGGCAAGGTATGGACCCTTAGCAAAG
CCAGAAAGATCACCTTGCCCAAAGAAGACCAACATTTTCATACATGATGAAATTCATTATTGGTCTCG
CGATCAGCTTG
+
FFFFFFFFFFFFFFFFFFFFFFFFFFFFFFFFFFFFFFFFFFFFFFFFFFFFFFFFFFFFFFFFFFFFF
FFFFFFFFFFFFFFFFFFFFFFFF:FFFFFFFFFFF:FFFFFFFFFFFFFFFFFFFFFFFFFFFFFFFF
FFFFFFFFFFF
@A00155:342:HHGFNDSXY:1:1258:28140:6417 2:N:0:GAACCTAG+TCCGCATA
AGACTTATGACATATTAAATAGATTGGTTACGGTAAAAGACAAGGCAAGGTATGGACCCTTAGCAAAG
CCAGAAAGATCACCTTGCCCAAAGAAGACCAACATTTTCATACATGATGAAATTCATTATTGGTCTCG
CGATCAGCTTG
+
FFFFFFFFFFFF:FFFFFFFFFFFFFFFFFFFFFFFFFFFFFFFFFFFFFFFFFFFFFFFFFFFFFFFF
FFFFFFFFFFFFFFFFFFFFFFFFFFFFFFFFFFFFFFFFFFFFFFFFF:FF,FFFFFFFFFFFF:FFFF:
FFFFFFFFFFF
@A00155:342:HHGFNDSXY:1:1258:25446:10488 2:N:0:GAACCTAG+TCCGCATA
AGACTTATGACATATTAAATAGATTGGTTACGGTAAAAGACAAGGCAAGGTATGGACCCTTAGCAAAG
CCAGAAAGATCACCTTGCCCAAAGAAGACCAACATTTTCATACATGATGAAATTCATTATTGGTCTCG
CGATCAGCTTG
+
FFFFFFFFFFFFFFFFFFFFFFFFFFFFFFFFFFFFFFFFFFFFFFFFFFFFFFFFFFFFFFFFFFFFF
FFFFFFFFFFFFFFFFFFFFFFFFFF:FFFFFFFFFFFFFFFFFFFFFFFFFFFFFFFFFFFFFFFFFF
FFFFFFFFFFF
@A00155:342:HHGFNDSXY:1:1258:25473:10504 2:N:0:GAACCTAG+TCCGCATA
AGACTTATGACATATTAAATAGATTGGTTACGGTAAAAGACAAGGCAAGGTATGGACCCTTAGCAAAG
```

```
CCAGAAAGATCACCTTGCCCAAAGAAGACCAACATTTTCATACATGATGAAATTCATTATTGGTCTCG
CGATCAGCTTG
+
FFFFFFFFFFFFFFFFFFFFFFFFFF:FFFFFFFFFF:FFFFFFFFFF:FFFFFFFFFFFFFFFFFF:FF
FFFFFFFFFFFFFFFFFFFFFFFFFFFFFFFFFFFFF:FFFFFFFFFFFFFFFFFFFFFFFFFFFFFFFF
FFFFFFFF:F:
@A00155:342:HHGFNDSXY:1:1619:19623:7827 2:N:0:GAACCTAG+TCCGCATA
AGACTTATGACATATTAAATAGATTGGTTACGGTAAAAGACAAGGCAAGGTATGGACCCTTAGCAAAG
CCAGAAAGATCACCTTGCCCAAAGAAGACCAACATTTTCATACATGATGAAATTCATTATTGGTCTCG
CGATCAGCTTA
+
FFFFFFFFFFFFFFFFFFFFFFFFFFFFFFFFFFFFFFFFF:FF:FFFFFFF,FFFFFFFFFFFFFFFFF
FFFFFFFFFF:FFFFFFFFFFF:FFFFFFFFFFFFFFFFFFFFFFFFFFFFFFF,FFFFF:FF:FFFF::
F,FFF:FF:FF
@A00155:342:HHGFNDSXY:1:2152:17996:10300 2:N:0:GAACCTAG+TCCGCATA
AGACTTATGACATATTAAATAGATTGGTTACGGTAAAAGACAAGGCAAGGTATGGACCCTTAGCAAAG
CCAGAAAGATCACCTTGCCCAAAGAAGACCAACATTTTCATACATGATGAAATTCATTATTGGTCTCG
CGATCAGCTTG
+
FFFFFFFFFFFFFFFFFFFFFFFFFFFFFFFFFFFFFFFFFFFFFFFF:FFFFFFFFFFFFFFFFFFFFF
FFFFFFFFFFFFFFFFFFFFFFFFFFFFFF:FFFFFFFFFFFFFFFFFFFFFFFFFFFFFFFFFFFFFFF
FFFFFFFFFFF
@A00155:342:HHGFNDSXY:1:2174:8097:21057 2:N:0:GAACCTAG+TCCGCATA
AGACTTATGACATATTAAATAGATTGGTTACGGTAAAAGACAAGGCAAGGTATGGACCCTTAGCAAAG
CCAGAAAGATCACCTTGCCCAAAGAAGACCAACATTTTCATACATGATGAAATTCATTATTGGTCTCG
CGATCAGCTTG
+
FFFFFF:FFFFFFFFFFF:FFF:FFFFFFFFFFFFFFFFFFFFFFFFFFFFFFFFFFFFFFFFF:FFFFF
FFFFFFFFFFFFFFFFFFFFFFFFFFF,FFFFFFFFFFFFFFFFFFFFFFFFFFFFF::FFFFFFFFFFF
FFFFFFFFFFF
@A00155:342:HHGFNDSXY:1:2302:7283:36558 2:N:0:GAACCTAG+TCCGCATA
AGACTTATGACATATTAAATAGATTGGTTACGGTAAAAGACAAGGCAAGGTATGGACCCTTAGCAAAG
CCAGAAAGATCACCTTGCCCAAAGAAGACCAACATTTTCATACATGATGAAATTCATTATTGGTCTCG
CGATCAGCTTG
+
FFFFFFFFFFFFFFFFFFFFFFFFFFFFFFFFFFFFFFFFFFFFFFFFFFFFFFFFFFFFFFFFFFFFFF
FFFFFFFFFFFFFFFFFFFFFFFFFFF:FFFFFFFFFFFFFFFFFFFFF:FFFFFFFFFFFFFF:FFFFF
FFFFFFFFFFF
@A00155:342:HHGFNDSXY:1:2319:17806:32894 2:N:0:GAACCTAG+TCCGCATA
AGACTTATGACATATTAAATAGATTGGTTACGGTAAAAGACAAGGCAAGGTATGGACCCTTAGCAAAG
CCAGAAAGATCACCTCGCCCAAAGAAGACCAACATTTTCATACATGATGAAATTCATTATTGGTCTCG
CGATCAGCTTG
+
FFFFFFFFFFFFFFFFFFFFFFFFFFFFFFFFFFFFFFFFFFFFFFFFFFFFFFFFFFFFFFFFFFFFFF
FFFFFFFFFFFFFFFFFFFFFFFFFFFFFFFFFFFFFFFFFFFFFFFFFFFFFFFFFFFFFFFFFFFFF:
FFFFFFFFFFF
@A00155:342:HHGFNDSXY:1:2314:22607:13088 2:N:0:GAACCTAG+TCCGCATA
AGACTTATGACATATTAAATAGATTGGTTACGGTAAAAGACAAGGCAAGGTATGGACCCTTAGCAAAG
CCAGAAAGATCACCTTGCCCAAAGAAGACCAACATTTTCATACATGATGAAATTCATTATTGGTCTCG
CGATCAGCTTG
+
FFFFF:FFFFFFFFFFFFFFFFFFFFFFFFFFFFFFFFFFFFFFFFFFFFFFFFFFFFFFFFFFFFFFFF
FFFFFFFFFFFFFFFFFFFFFFFFFF:FFFFFFFFFF:FFFFFFFFFFFFFF:FFFFFFFFFFF:FFFFF
FFF:FFFFFFF
```

```
@A00155:342:HHGFNDSXY:1:2314:22489:13260 2:N:0:GAACCTAG+TCCGCATA
AGACTTATGACATATTAAATAGATTGGTTACGGTAAAAGACAAGGCAAGGTATGGACCCTTAGCAAAG
CCAGAAAGATCACCTTGCCCAAAGAAGACCAACATTTTCATACATGATGAAATTCATTATTGGTCTCG
CGATCAGCTTG
+
FFFFFFF,FFFFFFFFFFFFFFFFFFFFFFFFFFFF:FFFFFFFFFFF:FFFF::FFFFFFFFFFFFFF
:F,FFFFFFFFFFFFFFFFF:FFFFFFF:FFFFFFFF::FFFFFFFFFFFFFF,FFFFF:F:F,F:FF:
::FFFFF:FFF
@A00155:342:HHGFNDSXY:1:2547:9390:5259 2:N:0:GAACCTAG+TCCGCATA
AGACTTATGACATATTAAATAGATTGGTTACGGTAAAAGACAAGGCAAGGTATGGACCCTTAGCAAAG
CCAGAAAGATCACCTTGCCCAAAGAAGACCAACATTTTCATACATGATGAAATTCATTATTGGTCTCT
CGATCAGCTTG
+
FFFFFFFF:FFFFFFFFFFFFFFFFFFFFFFFFFFFFF,FFFFFFFFFFFFFFFFFFFF,FFFFF,FFFF
F,FFFFFFFFFFFFFFF:F,FFFFFFF:FFFFFF:FFF:FFFFFFFFFFFF::FFFF:FF,FFFFFF,
FFF,FFFFFF:
@A00155:342:HHGFNDSXY:1:2606:6081:12117 2:N:0:GAACCTAG+TCCGCATA
AGACTTATGACATATTAAATAGATTGGTTACGGTAAAAGACAAGGCAAGGTATGGACCCTTAGCAAAG
CCAGAAAGATCACCTTGCCCAAAGAAGACCAACATTTTCATACATGATGAAATTCATTATTTGTCTCG
CGATCAGCTTA
+
FFFFFFFFFFFFFFFFFFFFFF,FFFFFFFFFFFFFFFFFF,FFFFFFFFFF:FFFFFFFFFFFFFFFF
FFFFFFFFFFFFFFFFFFFFFFFFFFF:FF,FFFFF:FFFFFFFFFFFFFFFFFF:F,FFF:,FFFFFF
,FFFFFFFF:F
@A00155:342:HHGFNDSXY:1:2616:5701:26616 2:N:0:GAACCTAG+TCCGCATA
AGACTTATGACATATTAAATAGATTGGTTACGGTAAAAGACAAGGCAAGGTATGGACCCTTAGCAAAG
CCAGAAAGATCACCTTGCCCAAAGAATACCAACATTTTCATACATGATTAAATTCATTATTGGTCTCG
CGATCAGCTTGAGA
+
FFFFFF,FFF:FFFF:F,FFFFFFFFFFFFFF,FFFFF,FFFFF:FFF,:FFFFFFFFFF:F,FFFF:
F,F:FFFFFFFFFFFFF,:FFFFFFF,FFFFF,FF:F::FFFFFFFFF,FFF:,,FF,FFFF::FF,F
:FFFFF:FFFFFFF
@A00155:342:HHGFNDSXY:1:1176:13304:16986 1:N:0:GAACCTAG+TCCGCATA
AGACTTATGACATATTAAATAGATTGGTTACGGTAAAAGACAAGGCAAGGTATGGACCCTTAGCAAAG
CCAGAAAGATCACCTTGCCCAAAGAAGACCAACATTTTCATACATGATGAAATTCATTATTGGTCTCG
CGATCAGCTTGAGAC
+
FFF:FFFFFFFFFFFFFFFFFFFFFFFFFFFFFFFFFFFFFFFFFFFFFFFFFFFFFFFFFFFFFFFFF
FFFFFF,FFFFFFFFFFFFFFFFFFFF:FFFFFFFFFFFFFFFFFFFFFFFFFFFFFFFFFFFFFFFFF
FFFFFFFFFFFFFFF
@A00155:342:HHGFNDSXY:1:1213:1967:4899 1:N:0:GAACCTAG+TCCGCATA
AGACTTATGACATATTAAATAGATTGGTTACGGTAAAAGACAAGGCAAGGTATGGACCCTTAGCAAAG
CCAGAAAGATCACCTTGCCCAAAGAAGACCAACATTTTCATACATGATGAAATTCATTATTGGTCTCG
CGATCAGCTTGAGAC
+
FFFFFFFFFFFFFFFFFFFFFFFFFFFFFFFFFFFFFFFFFFFFFFFFFFFFFFFFFFF:FFFFFFFFF
FFFFFFFFFFFFFF:FFFFFFFFFFF:FF:FFFFFFFFFFFFFFFFFFFFFFFFFFFFFFFFFFFFFFF
FFFFFFFFFFFFFFF
@A00155:342:HHGFNDSXY:1:1302:8232:6480 1:N:0:GAACCTAG+TCCGCATA
AGACTTATGACATATTAAATAGATTGGTTACGGTAAAAGACAAGGCAAGGTATGGACCCTTAGCAAAG
CCAGAAAGATCACCTTGCCCAAAGAAGACCAACATTTTCATACATGATGAAATTCATTATTGGTCTCG
CGATCAGCTTGAGAC
+
FFFFFFFFFFFFFFFFFFFFFF,FF:FFFFF,FFFF:FFFFFFFFFFFFFFFFFFFFFFFFFFFFFFFF
```

```
FFFFFFFFFFFFFFFFFFF:FFFFF,:F:FFFFF:FFFFFFFFFFFFFFFFFFFFFFFFFFFFFFFFF
FFFFFFFFFFFFFFF
@A00155:342:HHGFNDSXY:1:1565:7925:28369 1:N:0:GAACCTAG+TCCGCATA
AGACTTATGACATATTAAATAGATTGGTTACGGTAAAAGACAAGGCAAGGTATGGACCCTTAGCAAAG
CCAGAAAGATCACCTTGCCCAAAGAAGACCAACATTTTCATACATGATGAAATTCATTATTGGTCTCG
CGATCAGCTTGAGAC
+
FFFFF:FFFFFFFFFFFFFFFFFFFFF:FFFFFFFFFFFFFFFFFFFFFFFFFFFFFFFFFFFFFFFF
FFFFFFFFFFFFFFFFFFFFFFFFFF,FFFFFFFFFFFFFFFFFFFFFFFFFFFFFFFFFFFFFFFFF
FFFFFFFFFFFFFFF
@A00155:342:HHGFNDSXY:1:1618:2329:12602 1:N:0:GAACCTAG+TCCGCATA
AGACTTATGACATATTAAATAGATTGGTTACGGTAAAAGACAAGGCAAGGTATGGACCCTTAGCAAAG
CCAGAAAGATCACCTTGCCCAAAGAAGACCAACATTTTCATACATGATGAAATTCATTATTGGTCTCG
CGATCAGCTTGAGAC
+
F:FFFFFF:FFFFFFFFFFFF:FFFF:FFF:,,FFFFF:FFFF,:FFFFFFFFFFFF,FFFFFFFFFF
FFFFFFFFFFFFFFFFFFFFFFF,FFF,FFFFFFFFFFFFFFFFFFFFFFFFFFFFFFFFFFFFFFFF
FFFFFFFFFF:FFF:
@A00155:342:HHGFNDSXY:1:1673:14317:35368 1:N:0:GAACCTAG+TCCGCATA
TCCGATCTGACATATTAAATAGATTGGTTACGGTAAAAGACAAGGCAAGGTATGGACCCTTAGCAAAG
CCAGAAAGATCACCTTGCCCAAAGAAGACCAACATTTTCATACATGATGAAATTCATTATTGGTCTCG
CGATCAGCTTGAGAC
+
FFFFFFFFFFFFFFFFFFFFFFFFF:F:FFFFFFFFFFFFFFFFFFFFFFFFFFF:FFFFFFFFFFFFFF
FFFFF:FFFFFFFFFFFFFFFFFFFFFF:FFFFFFFFFFFFFFFFFFFFFFFFFFFFFFFFFFFFFFFF
FFFFFFFFFFFFFFF
@A00155:342:HHGFNDSXY:1:2120:2582:30044 1:N:0:GAACCTAG+TCCGCATA
AGACTTATGACATATTAAATAGATTGGTTACGGTAAAAGACAAGGCAAGGTATGGACCCTTAGCAAAG
CCAGAAAGATCACCTTGCCCAAAGAAGACCAACATTTTCATACATGATGAAATTCATTATTGGTCTCG
CGATCAGCTTGAGAG
+
FFF:FFFFFF:FFFFFFFFF:FFFFFFFFFFFFFFFF:FF:FF,FFFFFFFFFFFFF:FFFFFFFFFFF
FFFFFFFFFF::FFFFF:F:FF,FFFF:F:FFFFFF:FFFFFFFFFFFFFFF:FFFFFFFFFFFFFFF
FFFFFFFFFFFFFFF
@A00155:342:HHGFNDSXY:1:2162:4824:19742 1:N:0:GAACCTAG+TCCGCATA
AGACTTATGACATATTAAATAGATTGGTTACGGTAAAAGACAAGGCAAGGTATGGACCCTTAGCAAAG
CCAGAAAGATCACCTTGCCCAAAGAAGACCAACATTTTCATACATGATGAAATTCATTATTGGTCTCG
CGATCAGCTTGAGAC
+
FFFFFFFFFFFFFFFFFFFFFFFFFFFFFFFFFFFFF:FFFFFFFFFFFFFFFFFFFF,FFFFFFFFF
F:FFFFFFFFFFFFFFFFFFFFFF:F:FFF:FFFFFFFFFFFFFFF:FFFFFFFFFFFFFFFFFFFFFFF
FFFFFFFFFFFFFFF
@A00155:342:HHGFNDSXY:1:2165:4264:6151 1:N:0:GAACCTAG+TCCGCATA
AGACTTATGACATATTAAATAGATTGGTTACGGTAAAAGACAAGGCAAGGTATGGACCCTTAGCAAAG
CCAGAAAGATCACCTTGCCCAAAGAAGACCAACATTTTCATACATGATGAAATTCATTATTGGTCTCG
CGATCAGCTTGAGAC
+
FFFFFFFFFFFFFFFFFFFFFFFFFFFFFFFFFFFFFFFFFFFFFFFFFFFFFFFFFFFFFFFFFFFF
FFFFFFFFFFFFFFFFFFFFFFFFFFFFFFFFFFFFFFFFFFFFFFFFFF:FFFFFFFFFFFFFFFFF
FFFFF,FFFFFFFFF
@A00155:342:HHGFNDSXY:1:2169:20401:29528 1:N:0:GAACCTAG+TCCGCATA
AGACTTATGACATATTAAATAGATTGGTTACGGTAAAAGACAAGGCAAGGTATGGACCCTTAGCAAAG
CCAGAAAGATCACCTTGCCCAAAGAAGACCAACATTTTCATACATGATGAAATTCATTATTGGTCTCG
CGATCAGCTTGAGAC
```

```
+
FFFFFFFFFFFFFFFFF:FFF:FFFFFFFFFFFFFFFFFFFF:FFFFFFFFFFFFFFFFFFFFFFFFFF
FFFFFFFFFFFFFFFFFFFFFFFFFFFFFFFFFFFFFFFFFFFFFFFFFFFFFFFFFFFFFFFFFFFFF
FFFFFFFFFFFFFFF
@A00155:342:HHGFNDSXY:1:2426:7355:17769 1:N:0:GAACCTAG+TCCGCATA
AGACTTATGACATATTAAATAGATTGGTTACGGTAAAAGACAAGGCAAGGTATGGACCCTTAGCAAAG
CCAGAAAGATCACCTTGCCCAAAGAAGACCAACATTTTCATACATGATGAAATTCATTATTGGTCTCG
CGATCAGCTTGAGAC
+
FFFFFFFFFFFFFFFFFFFFFFFFFFFFFFFFFFFFFFFFFF:FFFFFFFFFFFFFFFFFFFFFFFFFF
FFFFFFFFFFFFFFFFFFFFFFF,FF,FFFFFFFFFFFFFFFFFFFFFFFFFFFFFFFFFFFFFFFFFF
FFFFFFFFFFFFFFF
@A00155:342:HHGFNDSXY:1:2445:6605:17409 1:N:0:GAACCTAG+TCCGCATA
AGACTTATGACATATTAAATAGATTGGTTACGGTAAAAGACAAGGCAAGGTATGGACCCTTAGCAAAG
CCAGAAAGATCACCTTGCCCAAAGAAGACCAACATTTTCATACATGATGAAATTCATTATTGGTCTCG
CGATCAGCTTGAGAG
+
FFFFFFFFFFFFFFFFFFFFFFFFFFFFFF:FFFFFFFFFFF:FFFFFFFFFFFFFFFFFFFFF:FF:
FFFFFFFFFFFFFFFFFFFFFFFFFFFFFFFFFFFFFFFFFFFFFFFFFFFFFFFFFFFFFFFFFFFFF
FFFFF,FFFFFFFFF
@A00155:342:HHGFNDSXY:1:2476:19262:29434 1:N:0:GAACCTAG+TCCGCATA
AGACTTATGACATATTAAATAGATTGGTTACGGTAAAAGACAAGGCAAGGTATGGACCCTTAGCAAAG
CCAGAAAGATCACCTTGCCCAAAGAAGACCAACATTTTCATACATGATGAAATTCATTATTGGTCTCG
CGATCAGCTTGAGAC
+
FFFF:FFFFFFFFFFFFFFFFFFFFFFFFFFFFFFFFFFFFF,FFFFFFFFFFFFFFFFFFFFFFFFFF
FFFFFFFF:FFFFFFFFFFFFFFFFFF:FFF:FFFFFFFFFFFFFFFFFFFFFFFFFFFFFFFFFFFFF
FFFFFFFFFFFFFFF
@A00155:342:HHGFNDSXY:1:2627:17598:8891 1:N:0:GAACCTAG+TCCGCATA
AGACTTATGACATATTAAATAGATTGGTTACGGTAAAAGACAAGGCAAGGTATGGACCCTTAGCAAAG
CCAGAAAGATCACCTTGCCCAAAGAAGACCAACATTTTCATACATGATGAAATTCATTATTGGTCTCG
CGATCAGCTTGAGAC
+
FFFFFFFFFFFFFFFFFFFFFFFFFF:FFFFFFFFFFFFFFFF::F:FFFFFFFFFFFFFFFFFFFFFF
F:FFFFFFFFFFFFFFFFFFFFFFF:FFFFFF:FFFFFFFFFFFFF:FFFFFFFFFFFFFFFFFFFFFF
FFFFFFFFFFFFFFF
@A00155:342:HHGFNDSXY:1:2627:18078:9471 1:N:0:GAACCTAG+TCCGCATA
AGACTTATGACATATTAAATAGATTGGTTACGGTAAAAGACAAGGCAAGGTATGGACCCTTAGCAAAG
CCAGAAAGATCACCTTGCCCAAAGAAGACCAACATTTTCATACATGATGAAATTCATTATTGGTCTCG
CGATCAGCTTGAGAC
+
FFFFFFFFFFFFFFFFFFFFFFFFFFFFFFFFFFFFFFFFFFFFFFFFFFFFFFFFFFFFFFFFFFFFF
FFFFFFFFFFFFFFFFFFFFFFFFFFFFF:FFF:FFFFFFFFFFFFFFFFFFFFFFFFFFFFFFFFFFF
FFFFFFFFFFFFFFF
@A00155:342:HHGFNDSXY:1:2634:7970:36620 1:N:0:GAACCTAG+TCCGCATA
AGACTTATGACATATTAAATAGATTGGTTACGGTAAAAGACAAGGCAAGGTATGGACCCTTAGCAAAG
CCAGAAAGATCACCTTGCCCAAAGAAGACCAACATTTTCATACATGATGAAATTCATTATTGGTCTCG
CGATCAGCTTGAGAC
+
FFFFFFFFFFFFFFFFFFFFFFFFFFFFFFFFFF,FFFF:FFFFFFFFFF:FFFFFFFFFFFFFFFFF:
FFFFFFFFFFFFFF:FFFFFFF::FF:FFFFFFFFFFFFFFFFFFFFFFFFFFFFFFFFFFFFFFFFFF
FFFFFFFFFFFFFFF
@A00155:342:HHGFNDSXY:1:2114:11831:21167 2:N:0:GAACCTAG+TCCGCATA
AGACTTATGACATATTAAATAGATTGGTTACGGTAAAAGACAAGGCAAGGTATGGACCCTTAGCAAAG
```

```
CCAGAAAGATCACCTTGCCCAAAGAAGACCAACATTTTCATACATGATGAAATTCATTATTGGTCTCG
CGATCAGCTTGAGAC
+
FFFFFFFFFFFFFFFFFFFFFFFFFFFFFFFFFFFFFFFFFFFFFFFFFFFFFFFFFFFFFFFFFFFF
FFFFFFFFFFFFFFFFFFFFFFFFFFFFFFFFFFFFFF:FFFFFFFFFFFFFFFFFFFFFFF:FFFFF
FFFFFFFFFFFF:FF
@A00155:342:HHGFNDSXY:1:2508:31069:33567 2:N:0:GAACCTAG+TCCGCATA
AGACTTATGACATATTAAATAGATTGGTTACGGTAAAAGACAAGGCAAGGTATGGACCCTTAGCAAAG
CCAGAAAGATCACCTTGCCCAAAGAAGACCAACATTTTCATACATGATGAAATTCATTATTGGTCTCG
CGATCAGCTTGAGAC
+
FFFFFFFFFFFFFFFFFFFFFFFFFFFFFFFFFFFFFFFFFFFFFFF:FFF:FFFFFFFFFFFFF:FF
FFFFFFFFFFFFFFFFFFFFFFFFFFFFFFFFFFFFFFFFFFFFFFFFFFFFFFFFFFFFFFFFFFFF
FFFFFFFFFFFFFFF
@A00155:342:HHGFNDSXY:1:2613:29017:2863 2:N:0:GAACCTAG+TCCGCATA
AGACTTATGACATATTAAATAGATTGGTTACGGTAAAAGACAAGGCAAGGTATGGACCCTTAGCAAAG
CCAGAAAGATCACCTTGCCCAAAGAAGACCAACATTTTCATACATGATGAAATTCATTATTGGTCTCG
CGATCAGCTTGAGAC
+
FFF:FFFFFFFFFFFFFFFFFFFFFFFFFFFFFFFFFFFFFFFFFFFFFFFFF:FFFFFFFFFFFFF:
FFFFFFFFFFFFFFFFFF,FFFFFFFFFFFFFFFFFF::FFFFF:FFFFFFFFFFFFFFF:F,FFFFF
FFFFFFFFFFFFFFF
@A00155:342:HHGFNDSXY:1:1263:5828:36385 2:N:0:GAACCTAG+TCCGCATA
GACTTATGACATATTAAATAGATTGGTTACGGTAAAAGACAAGGCAAGGTATGGACCCTTAGCAAAGC
CAGAAAGATCACCTTGCCCAAAGAAGACCAACATTTTCATACATGATGAAATTCATTATTGGTCTCGC
GATCAGCTTGAGACA
+
FFFFFFFFFFFFFF:FFFFFFFFFFFFFFFFFFFFFFFFFFFFFF:FFF:FFFFFFFFFFFFFFFFFF
FFFFFFFFFFFFFFFFFFFFFFFF:FFFFFFFFFFFFFFFFFFFFFFFFFFFFFFFFFFF:FFFFFFF
FFFF:FFFFFFFFFF
@A00155:342:HHGFNDSXY:1:2555:20030:8469 2:N:0:GAACCTAG+TCCGCATA
GACTTATGACATATTAAATAGATTGGTTACGGTAAAAGACAAGGCAAGGTATGGACCCTTAGCAAAGC
CAGAAAGATCACCTTGCCCAAAGAAGACCAACATTTTCATACATGATGAAATTCATTATTGGTCTCGC
GATCAGCTTGAGACA
+
FFFFFFFFFFFFFFFFFFFFFFFFFFFFFF:FFFFFFFFFFFFFFFFFFFFFF,FFFFFFFFFFFFFF
FFFFFFFFFFFFFFFFFFFFFFFFFF:FFFFFFFFFFFFFFFFFFFFFFFFFFFFFFFFF,FFFFFFF
FFFFFFFFFFFFFFF
@A00155:342:HHGFNDSXY:1:2616:24921:23766 2:N:0:GAACCTAG+TCCGCATA
ACTTATGACATATTAAATAGATTGGTTACGGTAAAAGACAAGGCAAGGTATGGACCCTTAGCAAAGCC
AGAAAGATCACCTTGCCCAAAGAAGACCAACATTTTCATACATGATGAAATTCATTATTGGTCTCGCG
ATCAGCTTGAGACAT
+
FFFFFFFFFFFFFFFFFFFFFFFFFFFFFFFFF:FFFFFF:FFFF:FFFFFFFFFFFFFF:FFFFFFF
FFFFFF:FFFFFFFFFFFFFFFF:FFFFFFFFFFFFFFFFFFFFFFFFFF:FFFFFFFFF,FFFFF:F
FFFFFFF:FFFFF::
@A00155:342:HHGFNDSXY:1:2578:12834:28447 2:N:0:GAACCTAG+TCCGCATA
ACTTATGACATATTAAATAGATTGGTTACGGTAAAAGACAAGGCAAGGTATGGACCCTTAGCAAAGCC
AGAAAGATCACCTTGCCCAAAGAAGACCAACATTTTCATACATGATGAAATTCATTATTGGTCTCGCG
ATCAGCTTGAGACAT
+
FFFFFFFFFFFFFFFFFFFFFFFFFFFFFFFF:FFFFFFFFFFFFFFFFFFFFFFFFFFFFFFF:FFF
FFFFFFFFFFFFFFFFFFFFFF:FF:FFFFFFFFFFFFFFFFFFFFFFFFFFFFFFFFFFFFFFFF:F
FFFFFFF:FFF,FFF
```

```
@A00155:342:HHGFNDSXY:1:1370:6795:26256 2:N:0:GAACCTAG+TCCGCATA
CTTATGACATATTAAATAGATTGGTTACGGTAAAAGACAAGGCAAGGTATGGACCCTTAGCAAAGCCA
GAAAGATCACCTTGCCCAAAGAAGACCAACATTTTCATACATGATGAAATTCATTATTGGTCTCGCGA
TCAGCTTGAGACATT
+
FFFF,FFFFFFFFFFFFFFFFFFFFFFFFFFFFFFFFFFFFFFFFFFFFFFF:FFFFFFFFFFFFFFF
FFFFFFFFFFFFFFFFFFFFF:FFFFFFFFFFFFFFFFFFFFFFFFFFF:FFFFFFFFFFFFFFFFFF
FFFFFFFFFFFFFFF
@A00155:342:HHGFNDSXY:1:1370:6162:26537 2:N:0:GAACCTAG+TCCGCATA
CTTATGACATATTAAATAGATTGGTTACGGTAAAAGACAAGGCAAGGTATGGACCCTTAGCAAAGCCA
GAAAGATCACCTTGCCCAAAGAAGACCAACATTTTCATACATGATGAAATTCATTATTGGTCTCGCGA
TCAGCTTGAGACATT
+
FFFFFFFFFFFFFFFFFFFFFFFFFFFFFFFFFFFFFFFFFFFFFFFFFFFFFFFFFFFFFFFFFFFF
FFFFF:FFFFFFFFFFFFFFFFF,FFFFFFFFFFFFFFFFFFFFFFFFFFFFFFF:FFFFFFFFFFFF
FFFFFFFFFFFFFFF
@A00155:342:HHGFNDSXY:1:1540:3052:20087 2:N:0:GAACCTAG+TCCGCATA
CTTATGACATATTAAATAGATTGGTTACGGTAAAAGACAAGGCAAGGTATGGACCCTTAGCAAAGCCA
GAAAGATCACCTTGCCCAAAGAAGACCAACATTTTCATACATGATGAAATTCATTATTGGTCTCGCGA
TCAGCTTGAGACATT
+
F:FFFFFFFFFFFFFFFFFFFFFFFFFFFFF:F:FFFFFFFFFFFFFFFFFFFFFFFFFFFFFF:FFF
FFFFFFFFFF:FFFFFFFFFFFFFFFFFFFFFFFFFFFFFFFFFFFF,FFFFFFFFF:F:FFFFFFFF
FFFFFFFFFFFFFFF
@A00155:342:HHGFNDSXY:1:2201:10312:25488 2:N:0:GAACCTAG+TCCGCATA
CTTATGACATATTAAATAGATTGGTTACGGTAAAAGACAAGGCAAGGTATGGACCCTTAGCAAAGCCA
GAAAGATCACCTTGCCCAAAGAAGACCAACATTTTCATACATGATGAAATTCATTATTGGTCTCGCGA
TCAGCTTGAGACATT
+
FFFFFFFFFFFFFFFFFFFFFFFFFFFFFFFFF:FFFFFFFFFFFFFFFFFFF:FFFFFFFFFFFFFF
FF:FFFFFFFFFFFFFFFFFFFFFFFFF:FFFFFFFFFFFFFFFFFFFFFFFFFFFFFFFFFFFFFFF
FFFFFFFF:FFFFFF
@A00155:342:HHGFNDSXY:1:2217:21404:30984 2:N:0:GAACCTAG+TCCGCATA
CTTATGACATATTAAATAGATTGGTTACGGTAAAAGACAAGGCAAGGTATGGACCCTTAGCAAAGCCA
GAAAGATCACCTTGCCCAAAGAAGACCAACATTTTCATACATGATGAAATTCATTATTGGTCTCGCGA
TCAGCTTGAGACATT
+
FFFFFFFFFFFFFFFFFFFFFFFFFFFFFFFFFFFFFFFFFFFFFFFFFFFFFFFFFFFFFFFFFFFF
FFFFFFFFFF:FFFFFFFFFFFFFFFFFFFFFFFFFFFFFFFFFFFFFFFFFF:FFFFFFFFFFFFFF
FFFFFFFFFFFFFFF
@A00155:342:HHGFNDSXY:1:2266:7021:32597 2:N:0:GAACCTAG+TCCGCATA
CTTATGACATATTAAATAGATTGGTTACGGTAAAAGACAAGGCAAGGTATGGACCCTTAGCAAAGCCA
GAAAGATCACCTTGCCCAAAGAATACCAACATTTTCATACATGATGAAATTCATTATTGGTCTCGCGA
TCAGCTTGAGACATT
+
F,FFFFFFFFFFFFFFFFFFFFFFFFFFFFFFFFFFFFFFFFFFFFFFFFFFFFFFFFFFFFFFFFFF
FFFFFFFFFFFFF:FFFFFFF:F,FFFFFFFFFFF,FFF,FFFFFFFFFFFFFFFFFFF,:F:FF:FF
FFFFFF:FFFFFFF:
@A00155:342:HHGFNDSXY:1:2266:6958:32675 2:N:0:GAACCTAG+TCCGCATA
CTTATGACATATTAAATAGATTGGTTACGGTAAAAGACAAGGCAAGGTATGGACCCTTAGCAAAGCCA
GAAAGATCACCTTGCCCAAAGAAGACCAACATTTTCATACATGATGAAATTCATTATTGGTCTCGCGA
TCAGCTTGAGACATT
+
FFFFFFFFFFFFFFFFFFFFFFFF,FFFFFFFFF,FFFFFFF:F:FFF:FFFFFFFFFFFFFFFFFFF
```

FFFFFFFFFFF::FFFFFFF,FFFFFFFFFF,FFFFFFFFFFFFFFFF:FFFFFF,F:FFFFFFFFFF
FFFFFFFFFFF,F:F
@A00155:342:HHGFNDSXY:1:2323:8232:13150 2:N:0:GAACCTAG+TCCGCATA
CTTATGACATATTAAATAGATTGGTTACGGTAAAAGACAAGGCAAGGTATGGACCATTAGCAAAGCCA
GAAAGATCACCTTGCCCAAAGAAGACCAACATTTTCAAACATGATGAAATTCATTATTGGTCTCGCGA
TCAGCTTGAGACATT
+
:,FFFFFFFFFFFF:F:FFFFFF::FFFFFFFFF,F:FFFFFFFFFFFF:,FFFF,FFFFFFFFFFFF
,FFF,FFFFFF,FFFFFFFFFFF,FFFFFFFFFF,FF,FFFFFFF,FFFFF:FF:FF,:F,FF:F:FF
FFFF,F,FF:FFFF:
@A00155:342:HHGFNDSXY:1:1151:13910:12023 2:N:0:GAACCTAG+TCCGCATA
TTATGACATATTAAATAGATTGGTTACGGTAAAAGACAAGGCAAGGTATGGACCCTTAGCAAAGCCAG
AAAGATCACCTTGCCCAAAGAAGACCAACATTTTCATACATGATGAAATTCATTATTGGTCTCGCGAT
CAGCTTGAGACATTT
+
FFFFFFFFFFFFFFFF:FFFFFFFF:FFFFFF:FFFFFFFFFFFFFFFFFFFFFFFFFFFFFFFFFFF
FFFFFFFFFFFFFFFFFF:FFFFFFFFFFFFF:FFFFFFFFFFFFFFFFFFFFFFFFFFFFF:F:FFF
FFFFFFFFFFFFFFF
@A00155:342:HHGFNDSXY:1:1347:32913:9298 2:N:0:GAACCTAG+TCCGCATA
TTATGACATATTAAATAGATTGGTTACGGTAAAAGACAAGGCAAGGTATGGACCCTTAGCAAAGCCAG
AAAGATCACCTTGCCCAAAGAAGACCAACATTTTCATACATGATGAAATTCATTATTGGTCTCGCGAT
CAGCTTGAGACATTT
+
FFFFFFFFFFFFFFFFFFFFFFF:FFFFFFFFFFFFFF:FFF:FFFFFFFFFFFFFFFF:FFFF:FFF
FFF:FFFFFFFFFFFFF:FFFFFFFFFFF,FFFFFFFFFFFFFFFFFFFFFFFFFFFF:FFFFFFFFF
FFFFFFFFFFFFFF:
@A00155:342:HHGFNDSXY:1:1438:30807:25504 2:N:0:GAACCTAG+TCCGCATA
TTATGACATATTAAATAGATTGGTTACGGTAAAAGACAAGGCAAGGTATGGACCCTTAGCAAAGCCAG
AAAGATCACCTTGCCCAAAGAAGACCAATATTTTCATACATGATGAAATTCATTATTGGTCTCGCGAT
CAGCTTGAGAGATCG
+
FFFFFFFFFFFFFF:FFFFFFFFFFFFFFFFFFFFFFFFFF:FF:FFFFFFFFFFFFFFF:FFFFFFFF
FFFFFFFFFFFFFFFFFFFFF:FFFFFFFFFFFFF:FFFFFFFFFFF:FFFF:FFFFF:FFFFFFFFF
FFFF:FFFFFFFF::
@A00155:342:HHGFNDSXY:1:1314:13684:8563 1:N:0:GAACCTAG+TCCGCATA
TATGACATATTAAATAGATTGGTTACGGTAAAAGACAAGGCAAGGTATGGACCCTTAGCAAAGCCAGA
AAGATCACCTTGCCCAAAGAAGACCAACATTTTCATACATGATGAAATTCATTATTGGTCTCGCGATC
AGCTTGAGACATTTC
+
FFFFFFFFFFFFFFF:FFFFFF,FFFF:FFFFFFFFFFFFFFFFFFFFFFFFFFFFFFFFFFFFFFFF
FFFFFFFFF:FFFFFFFFFFFF,FFFFFFFFFFFFFFF:FFFFFFFFFFFFFFFFFFFFFFFFFFFFFF
FFFFFFFFFFFFFFF
@A00155:342:HHGFNDSXY:1:1448:18132:12226 1:N:0:GAACCTAG+TCCGCATA
TATGACATATTAAATAGATTGGTTACGGTAAAAGACAAGGCAAGGTATGGACCCTTAGCAAAGCCAGA
AAGATCACCTTGCCCAAAGAAGACCAACATTTTCATACATGATGAAATTCATTATTGGTCTCGCGATC
AGCTTGAGACATTTC
+
F,FFFFFFFFFFF:FFFFFFFFFFFFFFFFFFFFFFFFFFFFFFFFFFFFFFFFFFFFFFF:FFFFFFF
F,F:FFFFFFFFFFFFFFFF:FFFFFFFFFFFFFFFFFFFFFFFFFFFFFFFFFFFFFFFFFFFFFFF
FFFFFFFFFFFFFFF
@A00155:342:HHGFNDSXY:1:1448:18837:12696 1:N:0:GAACCTAG+TCCGCATA
TATGACATATTAAATAGATTGGTTACGGTAAAAGACAAGGCAAGGTATGGACCCTTAGCAAAGCCAGA
AAGATCACCTTGCCCAAAGAAGACCAACATTTTCATACATGATGAAATTCATTATTGGTCTCGCGATC
AGCTTGAGACATTTC

```
+
FFFFFFFFFFFFFFFFFFFFFFFFFFFFFFFFFFFFFFFFFFFFFFFFFFFFFFFFFFFFFFFFFFFF
FFFFFFFFFFFFFFFFFFFFFFFFFFFFFFFFFFFFFF:FFFFFFFFFFFFFFFFFFFFFFFFFFFFF
FFFFFFFFFFFFFFF
@A00155:342:HHGFNDSXY:1:2173:6234:16329 1:N:0:GAACCTAG+TCCGCATA
TCCGATCTATTAAATAGATTGGTTACGGTAAAAGACAAGGCAAGGTATGGACCCTTAGCAAAGCCAGA
AAGATCACCTTGCCCAAAGAAGACCAACATTTTCATACATGATGAAATTCATTATTGGTCTCGCGATC
AGCTTGAGACATTTC
+
FFFFFFFFFFFFFFFFFFFFFFFFFFFFFFFFFFFFFFFFFFFFFFFFFFFFFFFFFFFFFFFFFFFF
FFFFFFFFFFFFFFFFFFFFFFF,FFF:FFFF:FFFF:FFFFFFFFFFFFFFFFFFFFFFFFFFFFFF
FFFFFFFFFFFFFFF
@A00155:342:HHGFNDSXY:1:1222:30201:15749 2:N:0:GAACCTAG+TCCGCATA
ATGACATATTAAATAGATTGGTTACGGTAAAAGACAAGGCAAGGTATGGACCCTTAGCAAAGCCAGAA
AGATCACCTTGCCCAAAGAAGACCAACATTTTCATACATGATGAAATTCATTATTGGTCTCGCGATCA
GCTTGAGACATTTCT
+
FFFFFFFFFFFFFFFFFFFFFFFFFFFF,FFFFFFFFFFFF:FFFFFFFFFFFFFFFFFFFFFFFFFF
FFFFFFF,FFFFFFFFFFFFFFFFFFFFFFFFF:FFFFFFFFFFFFFFFFFFFFFFFFFFFFFFFFFF
FFFFFFFFFFFFFFF
@A00155:342:HHGFNDSXY:1:1410:31069:36229 2:N:0:GAACCTAG+TCCGCATA
ATGACATATTAAATAGATTGGTTACGGTAAAAGACAAGGCAAGGTATGGACCCTTAGCAAAGCCAGAA
AGATCACCTTGCCCAAAGAAGACCAACATTTTCATACATGATGAAATTCATTATTGGTCTCGCGATCA
GCTTGAGACATTTCT
+
FFFFFFFFFFFFFFFFFFFFFFFFFFFFFFFFFFFFFFFFFF:FFFFFFFFFFFFFFFFFFFFFFFFF
FFFFFFFFFFFFFFFFFFFFFFFFFFFFFFFFFFFFFFFFFFFFFFFF:FFFFFFFFFFF:FFFFFFFF
FFFFFFFFFFFFFFF
@A00155:342:HHGFNDSXY:1:1442:20564:4539 2:N:0:GAACCTAG+TCCGCATA
ATGACATATTAAATAGATTGGTTACGGTAAAAGACAAGGCAAGGTATGGACCCTTAGCAAAGCCAGAA
AGATCACCTTGCCCAAAGAAGACCAACATTTTCATACATGATGAAATTCATTATTGGTCTCGCGATCA
GCTTGAGACATTTCT
+
FFFFFFFFFFFFFFFFFFFFFFFFFFFFF:FFFFFFFFFFFFFF:FFFFFFFFFFFFFFF:FFFFFFFF
FFFFFFFFFFFFFFFFFFFFFFFFFFFFFFFFFFFFFFFF:FFFFFFFFFFFFFFFF,FFFFFFFFFFF
FFFFFFFFFFFFFFF
@A00155:342:HHGFNDSXY:1:1455:16134:21386 2:N:0:GAACCTAG+TCCGCATA
ATGACATATTAAATAGATTGGTTACGGTAAAAGACAAGGCAAGGTATGGACCCTTAGCAAAGCCAGAA
AGATCACCTTGCCCAAAGAAGACCAACATTTTCATACATGATGAAATTCATTATTGGTCTCGCGATCA
GCTTGAGACATTTCT
+
FFFFFFFFFFFFFFFFFFFFFFFFFFFFFFF:FFFFFFFFFFFFFFFFFFFFFFFFFF:FFFFFFFFFFF
FFFFFFFFFFFFFFFFFFFFFFFFFFFFFFFFFFFFF:FFFFFFFFFFFFF:FFFFFFFFFFFF:FFF
FFFFFFFFFFFFFFF
@A00155:342:HHGFNDSXY:1:2315:8847:9737 2:N:0:GAACCTAG+TCCGCATA
ATGACATATTAAATAGATTGGTTACGGTAAAAGACAAGGCAAGGTATGGACCCTTAGCAAAGCCAGAA
AGATCACCTTGCCCAAAGAAGACCAACATTTTCATACATGATGAAATTCATTATTGGTCTCGCGATCA
GCTTGAGACATTTCT
+
FFFFFFFFFFFF:FFFFFFFFFFFFFFFFFF,FFFFFFFFFFFFFFFFFFFFFFFFFFFFFFFFFFFFF
FFFFFFFFFFFF,,FF,FFFFFFFFFFFFFFFFFFFFFFFFFFFFFFFFFF:FFFFFFFFFFFFFFFFF
FFFFF:FFFFFFFFF
@A00155:342:HHGFNDSXY:1:2453:31494:11694 2:N:0:GAACCTAG+TCCGCATA
ATGACATATTAAATAGATTGGTTACGGTAAAAGACAAGGCAAGGTATGGACCCTTAGCAAAGCCAGAA
```

AGATCACCTTGCCCAAAGAAGACCAACATTTTCATACATGATGAAATTCATTATTGGTCTCGCGATCA
GCTTGAGACATTTCT
+
FFFFFFFFFFFFFFFFFFFFFFFFFFFF::FFFFFFFFFFFF:FFF:FFFFF:FFFFFFFFFFFFFFFF
FFFFFFFFFFFFFFFFFFFFFFFFFFFFFF:FFFFFFFFFFFFFFFFFFFFFFFFFFFFFFFFFFFFFF
FFFFFFFFF:FFFFF
@A00155:342:HHGFNDSXY:1:1407:3341:9283 1:N:0:GAACCTAG+TCCGCATA
TGACATATTAAATAGATTGGTTACGGTAAAAGACAAGGCAAGGTATGGACCCTTAGCAAAGCCAGAAA
GATCACCTTGCCCAAAGAAGACCAACATTTTCATACATGATGAAATTCATTATTGGTCTCGCGATCAG
CTTGAGACATTTCTC
+
FFFFFFFFFFFFFFFFFFFFFFFFFFFFFFFFFFFFFFFFFFFFFFFFFFFFFFFFFFFFFFFFFFFFF
FFFFFFFFFFFFFFF,FFF:FFFFFFFFFFFFFFFFFFFFFFFFFFFFFFFFFFFFFFFFFFFFFFFFF
FFFFFFFFFFFFFFF
@A00155:342:HHGFNDSXY:1:1458:22598:33270 1:N:0:GAACCTAG+TCCGCATA
TGACATATTAAATAGATTGGTTACGGTAAAAGACAAGGCAAGGTATGGACCCTTAGCAAAGCCAGAAA
GATCACCTTGCCCAAAGAAGACCAACATTTTCATACATGATGAAATTCATTATTGGTCTCGCGATCAG
CTTGAGACATTTCTC
+
FFFFFFFFFFFFFFFFFFFFFFFFFFFFFFFFFFFFFFFFFFFFFFFFFFFFF:FFFFFFFFFFFFF:F
FF:FFFF:FFFFF:FFF,FFFFFFFFFFFFFFFFFFFFFFFFFFFFFFFFFFFFFFFFFFFFFFFFFFF
FFFFFFFFFFFFFFF
@A00155:342:HHGFNDSXY:1:1649:29333:14278 1:N:0:GAACCTAG+TCCGCATA
TGACATATTAAATAGATTGGTTACGGTAAAAGACAAGGCAAGGTATGGACCCTTAGCAAAGCCAGAAA
GATCACCTTGCCCAAAGAAGACCAACATTTTCATACATGATGAAATTCATTATTGGTCTCGCGATCAG
CTTGAGACATTTCTC
+
FFFFFFFFFFFFFFFFFFFFFFFFFFFFFFFFFFFFFFFFFFF:FFFFFFFFFFFFFFFFFFFFFFFFF
FFFFFFFFFFFFFFFFFF:FFFFFFFFFFFFFFFFFFFFFFFFFFFFFFFFFFFFFFFFFFFFFFFFFF
FFFFFFFFFFFFFFF
@A00155:342:HHGFNDSXY:1:2137:28375:5509 1:N:0:GAACCTAG+TCCGCATA
TGACATATTAAATAGATTGGTTACGGTAAAAGACAAGGCAAGGTATGGACCCTTAGCAAAGCCAGAAA
GATCACCTTGCCCAAAGAAGACCAACATTTTCATACATGATGAAATTCATTATTGGTCTCGCGATCAG
CTTGAGACATTTCTC
+
F:FFFFFFFFFFFFFFFFFFFFFFFFFFFFFFFFFF:FFFF:FFFFFFFFFFFFFFFFFFFFFFFFFFF
F:FFFFF:FFFFFFFFFF:,FFFF:FFFFFFFFFFF:FFFFFFFFFFFFFFFFFFFFFFF:FFFFFFFF
FFFFFFFFFFFFFFF
@A00155:342:HHGFNDSXY:1:2235:3748:31845 1:N:0:GAACCTAG+TCCGCATA
TGACATATTAAATAGATTGGTTACGGTAAAAGACAAGGCAAGGTATGGACCCTTAGCAAAGCCAGAAA
GATCACCTTGCCCAAAGAAGACCAACATTTTCATACATGATGAAATTCATTATTGGTCTCGCGATCAG
CTTGAGACATTTCTC
+
FFF,:FFFFFFFFFFFFFFFFFFFFFFFFFFFF:FF:FFFFFFFFFFFFFF:FFFFFFFFFFFFFFFFF
FFFFFFFFFFFFFFFFFFF:F:FFFFFFFFF,FFFFFFF:FFFFFFFFFFFFFFFFFF:FF:FFFFFFF
FFFFFFFFFFFFFFF
@A00155:342:HHGFNDSXY:1:2318:3215:4366 1:N:0:GAACCTAG+TCCGCATA
TGACATATTAAATAGATTGGTTACGGTAAAAGACAAGGCAAGGTATGGACCCTTAGCAAAGCCAGAAA
GATCACCTTGCCCAAAGAAGACCAACATTTTCATACATGATGAAATTCATTATTGGTCTCGCGATCAG
CTTGAGACATTTCTC
+
FFFFFFFFFFFFFFFFFFFFFFFFFF::FFFFFFFFFFFFFFFFFFFFFFFFFFFFFFFFF:FFFFFFF
FFFFFFFFFFF:FFFFFF::FFFF:FFFFFFFFFFFFFFFFFFFFFFFFFFFFFFFFFFFFFFFFFFFF
FFFFFFFFFFFFFFF

```
@A00155:342:HHGFNDSXY:1:2359:25292:13917 2:N:0:GAACCTAG+TCCGCATA
GACATATTAAATAGATTGGTTACGGTAAAAGACAAGGCAAGGTATGGACCCTTAGCAAAGCCAGAAAG
ATCACCTTGCCCAAAGAAGACCAACATTTTCATACATGATGAAATTCATTATTGGTCTCGCGATCAGC
TTGAGACATTTCTC
+
FFFFFFFFFFFFFFFFFFFFFFFFFFF:FFFFFFFFFFFF:FFFFFFFFFFFFFFFFFFFFFFFFFFFFF
FFFFFFFFFFFFFFFFFFFFFFFFFFFFFFFFFFFFFFFFFFFFFFFFFFFFFFFFFFFFF:FFFFFFFF
FFFFFFF:FFFFFF
@A00155:342:HHGFNDSXY:1:1114:24361:29559 1:N:0:GAACCTAG+TCCGCATA
GACATATTAAATAGATTGGTTACGGTAAAAGACAAGGCAAGGTATGGACCCTTAGCAAAGCCAGAAAG
ATCACCTTGCCCAAAGAAGACCAACATTTTCATACATGATGAAATTCATTATTGGTCTCGCGATCAGC
TTGAGACATTTCTCC
+
FFFFFFFFFFFFFFFFFFFFFFFFFFFFFFFFFFFFFFFFFFFFFFFFFFFFFFFFFFFFFFFFFFFFFF
FFFFFFFFFFFFFFFF:FFF,FFFFFFFFFFFFFFFFFFFFFFFFFFFFFFFFFFFFFFFFFFFFFFFFF
FFFFFFFFFFFFFFF
@A00155:342:HHGFNDSXY:1:1316:3215:13792 1:N:0:GAACCTAG+TCCGCATA
GACATATTAAATAGATTGGTTACGGTAAAAGACAAGGCAAGGTATGGACCCTTAGCAAAGCCAGAAAG
ATCACCTTGCCCAAAGAAGACCAACATTTTCATACATGATGAAATTCATTATTGGTCTCGCGATCAGC
TTGAGACATTTCTCC
+
FF::FFFFFFFFFFFFFFFFFFFFF:FFF:FFF:FFFFFFFFFFFFFFFFFFFFFFFFFFF:F,FFFFFF
FFFFFFFFFFFFF:FFFFFFFFF:FFFFFFFFFFFFFFFFFFFFFFFFFFFFFFFFFFFFFFFFFFFFFF
FFFFFFFFFFFFFFF
@A00155:342:HHGFNDSXY:1:1539:6253:15139 1:N:0:GAACCTAG+TCCGCATA
TTCCGATCTAATAGATTGGTTACGGTAAAAGACAAGGCAAGGTATGGACCCTTAGCAAAGCCAGAAAG
ATCACCTTGCCCAAAGAAGACCAACATTTTCATACATGATGAAATTCATTATTGGTCTCGCGATCAGC
TTGAGACATTTCTCC
+
FFFFFFFFFFFFFFFFFFFFFFFFFFFFFFFFFFF:FFFFFFFFFFFFFFFFFFFFFFFFFFFFFFFFFF
FFFFFFFFFFFFFFFFF:FFFFFFFFFFFFFFF:F:FFFFFFFFFFFFFFFFFFFFFFFFFFFFFFFFFF
FFFFFFFFFFFFFFF
@A00155:342:HHGFNDSXY:1:1560:23050:6245 1:N:0:GAACCTAG+TCCGCATA
GACATATTAAATAGATTGGTTACGGTAAAAGACAAGGCAAGGTATGGACCCTTAGCAAAGCCAGAAAG
ATCACCTTGCCCAAAGAAGACCAACATTTTCATACATGATGAAATTCATTATTGGTCTCGCGATCAGC
TTGAGACATTTCTCC
+
FFFF:FFFF:F,FFF,FFFFFFFFFFFF,FFFFFFFFFFFFFFFFFFFFFFFFFFFFFFFFFFFFFFFFF
F:FFFFFFFFFFF,::FFF:FFFFFFFFFFFFFFFFFFFFFFFFFFFFFFFFFFFFFFFFFFFFFFFFFF
FFFFFFFFFFFFFFF
@A00155:342:HHGFNDSXY:1:1572:22408:31407 1:N:0:GAACCTAG+TCCGCATA
GACATATTAAATAGATTGGTTACGGTAAAAGACAAGGCAAGGTATGGACCCTTAGCAAAGCCAGAAAG
ATCACCTTGCCCAAAGAAGACCAACATTTTCATACATGATGAAATTCATTATTGGTCTCGCGATCAGC
TTGAGACATTTCTCC
+
FFFFFFFF:FFFFFFFFFFFFFFFFFFFFFFFFFFFFFFFFFFFFFFFFFFFFFFFFFFFFFFFFFFFFF
FFFFFFFFFFFFFFFFFFFF:FFFFFFFFFFFFFFFFFFFFFFFFFF:FFFFFFFFFFFFFFFFFFFFFF
FFFFFFFFFFFFFFF
@A00155:342:HHGFNDSXY:1:2117:27968:35368 1:N:0:GAACCTAG+TCCGCATA
GACATATTAAATAGATTGGTTACGGTAAAAGACAAGGCAAGGTATGGACCCTTAGCAAAGCCAGAAAG
ATCACCTTGCCCAAAGAAGACCAACATTTTCATACATGATGAAATTCATTATTGGTCTCGCGATCAGC
TTGAGACATTTCTCC
+
FFFFFFFFFFFFFFFFFFFFFFFFFFFFFFFFF:FFFFFFFFFFFFFFFFFFFFFFFFFFFFFFFFFFFF
```

```
FFFFFF:FFFFFFF:FF:F:FFFF:FFFFFFFFFFFFFFFFFFFFFFFFFFFFFFFFFFFFFFFFFFF
FFFFFFFFFFFFFFF
@A00155:342:HHGFNDSXY:1:2114:21423:25285 1:N:0:GAACCTAG+TCCGCATA
GACATATTAAATAGATTGGTTACGGTAAAAGACAAGGCAAGGTATGGACCCTTAGCAAAGCCAGAAAG
ATCACCTTGCCCAAAGAAGACCAACATTTTCATACATGATGAAATTCATTATTGGTCTCGCGATCAGC
TTGAGACATTTCTCC
+
FFFFFFFFFFFFFFFFFFFFFFFFFFFFFFFFFFFFFFFFFFFFFFFFFFFFFFFFFFFFFFFFFFFF
FFFFFFFFFFFFFFFFF,FFFFFFFFFFFFFFFFFFFFFFFFFFFFFFFFFFFFFFFFFFFFFFFFFF
FFFFFFFFFFFFFFF
@A00155:342:HHGFNDSXY:1:2138:14561:36448 1:N:0:GAACCTAG+TCCGCATA
GACATATTAAATAGATTGGTTACGGTAAAAGACAAGGCAAGGTATGGACCCTTAGCAAAGCCAGAAAG
ATCACCTTGCCCAAAGAAGACCAACATTTTCATACATGATGAAATTCATTATTGGTCTCGCGATCAGC
TTGAGACATTTCTCC
+
FFFFFFFFF:FFFFFFFFFFFFFFFFFFFFFFFFFFFFFFFFFFFFFFFFFFFFFFFFFFFFFFFFFF
FFFFFFFFFFF,FFF:FFF::FFFFFFFFFFFFF::FFFFFF:FFFFFFFFFFFFFFFFFFFFFFFFF
FFF:FFFFFFFFFFF
@A00155:342:HHGFNDSXY:1:2209:23674:30499 1:N:0:GAACCTAG+TCCGCATA
GACATATTAAATAGATTGGTTACGGTAAAAGACAAGGCAAGGTATGGACCCTTAGCAAAGCCAGAAAG
ATCACCTTGCCCAAAGAAGACCAACATTTTCATACATGATGAAATTCATTATTGGTCTCGCGATCAGC
TTGAGACATTTCTCC
+
FFFFFFFFFFFFFFFFFFFFFFFFFFFFFFFFFFFFFFFFFFFFFFFFFFFFFFFFFF:FFFFFFFFF
FFFFFFFFFFFFFFFF:FFFFFFFFFFF:FFFFFFFFFFFFFFFFFFFFFFFFFFFFFFFFFFFFFFF
FFFFFFFFFFFFFFF
@A00155:342:HHGFNDSXY:1:2262:30798:18599 1:N:0:GAACCTAG+TCCGCATA
GACATATTAAATAGATTGGTTACGGTAAAAGACAAGGCAAGGTATGGACCCTTAGCAAAGCCAGAAAG
ATCACCTTGCCCAAAGAAGACCAACATTTTCATACATGATGAAATTCATTATTGGTCTCGCGATCAGC
TTGAGACATTTCTCC
+
FFFFFFFFFFFFFFFFFFFFFFFFFFFFFFFFFFFFFFFFFFFFFFFFFFFFFFFFFFFFFFFFFFFF
FFFFFFFFFFFFFFFF:FFFFFFFFFFFFFFFFFFFFFFFFFFFFFFFFFFFFFFFFFFFFFFFFFF
FFFFFFFFFFFFFFF
@A00155:342:HHGFNDSXY:1:2453:31494:11694 1:N:0:GAACCTAG+TCCGCATA
GACATATTAAATAGATTGGTTACGGTAAAAGACAAGGCAAGGTATGGACCCTTAGCAAAGCCAGAAAG
ATCACCTTGCCCAAAGAAGACCAACATTTTCATACATGATGAAATTCATTATTGGTCTCGCGATCAGC
TTGAGACATTTCTCC
+
FFF::FFFFFF:FFFFFFFFFFFFFFFFFFFFFFFFFFFFFFFFFFFFFFFFFFFFFFFFFFFFFFFF
FFFFFFFFFFFFFF,FF:FFFFFFFFFFFFFFFFFFFFFFFFFFFFFFFFFFFFFFFFFFFFFFFFFF
FFFFFFFFFFFFFFF
@A00155:342:HHGFNDSXY:1:2466:18331:22654 1:N:0:GAACCTAG+TCCGCATA
GACATATTAAATAGATTGGTTACGGTAAAAGACAAGGCAAGGTATGGACCCTTAGCAAAGCCAGAAAG
ATCACCTTGCCCAAAGAAGACCAACATTTTCATACATGATGAAATTCATTATTGGTCTCGCGATCAGC
TTGAGACATTTCTCC
+
FFFF:,FFFFFFFFFFFFFFFFFFFFFFFFFFFFFFFFFFFFFFFFFFFFFFFF::FF:,FFFF:F:F
FFFFFFFFFF:FFFFFFFFFFFFFF:FFFFFFFFFFF:FF:FFFFFFFFFFFFFFFFFFFFFFFF:FF
FFFFFFFFFFFFFFF
@A00155:342:HHGFNDSXY:1:2508:25617:12727 1:N:0:GAACCTAG+TCCGCATA
GACATATTAAATAGATTGGTTACGGTAAAAGACAAGGCAAGGTATGGACCCTTAGCAAAGCCAGAAAG
ATCACCTTGCCCAAAGAAGACCAACATTTTCATACATGATGAAATTCATTATTGGTCTCGCGATCAGC
TTGAGACATTTCTCC
```

```
+
FF:FFFFFFFFFFFFFFFFFFFFFFFFFFF,FFFFFFFFFFFFFFFFFFFFFFFFFFFFFFFFFFFFFFF
FFFFFFFFFFFFFFFFFFFFFFFFFFFFFFFFFFFFFFFFFFFFFFFFFFFFFFFFFFFFFFFFFFFFFF
FFFFFFFFFFFFFFF
@A00155:342:HHGFNDSXY:1:2544:30083:33458 1:N:0:GAACCTAG+TCCGCATA
GACATATTAAATAGATTGGTTACGGTAAAAGACAAGGCAAGGTATGGACCCTTAGCAAAGCCAGAAAG
ATCACCTTGCCCAAAGAAGACCAACATTTTCATACATGATGAAATTCATTATTGGTCTCGCGATCAGC
TTGAGACATTTCTCC
+
FFFFFFFFFFFFFFFF:FFFFFFFFFFFFFFFFFFFFFFFFFFFFFFFFFFFFFFFFFFFFFFFFFFFFF
FFFFFFFFFFFFFFFF:FF:FFFFFFFFFFFFFFFFFFFFFFFFFFFFFFFFFFFFFFFFFF:FFFF:FF
FFFFFFFFFFFFFFF
@A00155:342:HHGFNDSXY:1:2544:30382:34006 1:N:0:GAACCTAG+TCCGCATA
GACATATTAAATAGATTGGTTACGGTAAAAGACAAGGCAAGGTATGGACCCTTAGCAAAGCCAGAAAG
ATCACCTTGCCCAAAGAAGACCAACATTTTCATACATGATGAAATTCATTATTGGTCTCGCGATCAGC
TTGAGACATTTCTCC
+
FFFFFFFF:FFFFFFFFFFFFFFFFFFF:FFFFFF,FFFFFFFFFFFFFFFFFFFFFFFFFFFFFFFFFF
FFFF:F:FFFFFFFFFF,F:FFFFFFFFFFFFFFFFFFFFFFFFFFFFFFFFFFFFFFFFFFFFFFFFFF
FFFFFFFFFFFFFFF
@A00155:342:HHGFNDSXY:1:2611:17345:5040 1:N:0:GAACCTAG+TCCGCATA
GACATATTAAATAGATTGGTTACGGTAAAAGACAAGGCAAGGTATGGACCCTTAGCAAAGCCAGAAAG
ATCACCTTGCCCAAAGAAGACCAACATTTTCATACATGATGAAATTCATTATTGGTCTCGCGATCAGC
TTGAGACATTTCTCC
+
FFFFFFFFFFFFFFFFFFFFFFFFFFFFFFFFFFFFFFFFFFFFFFFFFFFFFFF:FFFFFFFFFFFFFF
FFFFFFFFFFFFFFFFFFFFF:F:FFFFFFFFFFFFFFFFFFFFFFFFFFFFFFFFFFFFFFFFFFF:FF
FFFFFFFFFFFFFFF
@A00155:342:HHGFNDSXY:1:1426:19199:25911 2:N:0:GAACCTAG+TCCGCATA
GACATATTAAATAGATTGGTTACGGTAAAAGACAAGGCAAGGTATGGACCCTTAGCAAAGCCAGAAAG
ATCACCTTGCCCAAAGAAGACCAACATTTTCATACATGATGAAATTCATTATTGGTCTCGCGATCAGC
TTGAGACATTTCTCC
+
FFFFFFFFFFFFFFFFFFFFFFFFFFFFFFFFFFFFFFFF:FFFFFFFFFFFFFFFFFFF:FFFFFFFFF
FFFFFFFFFFFFFF:FFFFFFFFFF:FFFFFFFFFFFFFFF:FFFFFFFFFFFFFFFFFF:FFFFFFFFF
FFFF:FFFFFFFFFF
@A00155:342:HHGFNDSXY:1:1563:31150:20964 2:N:0:GAACCTAG+TCCGCATA
GACATATTAAATAGATTGGTTACGGTAAAAGACAAGGCAAGGTATGGACCCTTAGCAAAGCCAGAAAG
ATCACCTTGCCCAAAGAAGACCAACATTTTCATACATGATGAAATTCATTATTGGTCTCGCGATCAGC
TTGAGACATTTCTCC
+
FFFFFFFFFFFFFFFFFFFFFFFFFF:FFFFFFFFFFFF,FFF:FFFFFFFFFFFFF:F:FFFFFFFFFF
FFFFFFFFFFFFFFFFFFFFFFFFFFFFFFFFFF:FFFFFFFFFFFFFFFFFFFFFFFFFFFFFFFFFFF
FFFFFFFFFFFFFFF
@A00155:342:HHGFNDSXY:1:1673:14317:35368 2:N:0:GAACCTAG+TCCGCATA
GACATATTAAATAGATTGGTTACGGTAAAAGACAAGGCAAGGTATGGACCCTTAGCAAAGCCAGAAAG
ATCACCTTGCCCAAAGAAGACCAACATTTTCATACATGATGAAATTCATTATTGGTCTCGCGATCAGC
TTGAGACAGATCGGA
+
FFFFFFFFFFFF:FFFFFFFFFFFFFFFFFFFFFFFFFFFFFFFFFFFFFFFF:FFFFFFFFFFFFFFFF
FFFFFFFFFFFFFFFFFFFFFFFFFFFFFFFFFFFFFFFFFFFFFFFFFF:FFFFFFFFF::FFFFFFFF
FFFF:FFFFFFFFFF
@A00155:342:HHGFNDSXY:1:2137:28375:5509 2:N:0:GAACCTAG+TCCGCATA
GACATATTAAATAGATTGGTTACGGTAAAAGACAAGGCAAGGTATGGACCCTTAGCAAAGCCAGAAAG
```

```
ATCACCTTGCCCAAAGAAGACCAACATTTTCATACATGATGAAATTCATTATTGGTCTCGCGATCAGC
TTGAGACATTTCTCA
+
FFFFFFFFFFFFFFFFFFFFF:FFFFFFFFFFFFFFFFFFFFFFFFFFFFFFFFFFFFFFFFFFFFFF
FFFFFFFFFFFFFFFFFFFFF:FFFFFFFFFFFFFFFFF:FFF:FFFFF:FFFFFFFFFFFFFFFF:F
FFFFFFFFF:FFFFF
@A00155:342:HHGFNDSXY:1:2327:5746:4836 2:N:0:GAACCTAG+TCCGCATA
GACATATTAAATAGATTGGTTACGGTAAAAGACAAGGCAAGGTATGGACCCTTAGCAAAGCCAGAAAG
ATCACCTTGCCCAAAGAAGACCAACATTTTCATACATGATGAAATTCATTATTGGTCTCGCGATCAGC
TTGAGACATTTCTCC
+
FFFFFFFFFFFFFFFFFFFFFFFFFFFFFFFFFFFFFFFF:FFFFFFFFFFFFFFFFFFFFFF:FFFF
FFFFFFFFFFFFFFFFFFFFFFF:FFFFFFFFFFFFFFFFFFFFFFFFFFFFF:FFFF:FFFFFFFF
FFFFF:FFFFFFFF:
@A00155:342:HHGFNDSXY:1:2576:3884:11819 2:N:0:GAACCTAG+TCCGCATA
GACATATTAAATAGATTGGTTACGGTAAAAGACAAGGCAAGGTATGGACCCTTAGCAAAGCCAGAAAG
ATCACCTTGCCCAAAGAAGACCAACATTTTCATACATGATGAAATTCATTATTGGTCTCGCGATCAGC
TTGAGACATTTCTCC
+
FFFFFFFFFFF:FFFFFFFFFFFFFF:F:FFF,FFFFF:FFFFFFFFFFFFFFFFFF::FFFFFFFFF
FFFFFFF:FFFFFFFFFFFFFFFFFFFFFFF:FFFFFF:FFF,FFFFF,FFFF:FFFFFFF:F:::FF
FFFFF,FFFFFFFFF
@A00155:342:HHGFNDSXY:1:2114:4634:25238 2:N:0:GAACCTAG+TCCGCATA
ACATATTAAATAGATTGGTTACGGTAAAAGACAAGGCAAGGTATGGACCCTTAGCAAAGCCAGAAAGA
TCACCTTGCCCAAAGAAGACCAACATTTTCATACATGATGAAATTCATTATTGGTCTCGCGATCAGCT
TGAGACATTTCT
+
F,FFFFFFFFFF:FFFFFFFFF::FFFFFFFFFFF:FFFFF:::F::FF,FFF::FFF,FFF,FFF:F
FFFFF:::FF:FFFFF::FF:FF,FF:,F,F:FFF:FFFFFFFFFFFFFFF:F,F:F,::FFF,F,:F
F,FFFFFFFF::
@A00155:342:HHGFNDSXY:1:1223:9516:21574 1:N:0:GAACCTAG+TCCGCATA
ACATATTAAATAGATTGGTTACGGTAAAAGACAAGGCAAGGTATGGACCCTTAGCAAAGCCAGAAAGA
TCACCTTGCCCAAAGAAGACCAACATTTTCATACATGATGAAATTCATTATTGGTCTCGCGATCAGCT
TGAGACATTTCTCCA
+
FFFFFFFFFFFFFFFFFFFFFFFFFFFFFFFFFFFFFFFFFFFFFFFFFFFFFFFFFFFFFFFFFFFF
FFFFFFFFFFFFFFFFFFFFFFFFFFFFFFFFFFFFFFFFFFFFFFFFFFFFF:FFFFFFFFFFFFFF
FFFFFFFFF:FFFFF
@A00155:342:HHGFNDSXY:1:1528:16052:20337 1:N:0:GAACCTAG+TCCGCATA
ACATATTAAATAGATTGGTTACGGTAAAAGACAAGGCAAGGTATGGACCCTTAGCAAAGCCAGAAAGA
TCACCTTGCCCAAAGAAGACCAACATTTTCATACATGATGAAATTCATTATTGGTCTCGCGATCAGCT
TGAGACATTTCTCCA
+
FFFFFFFFFFFFFFFFFFFFFFFFFFFFFFFFFFFFFFFFFFFFFFFFFFFFFFFFFFFFFFFFFFFF
FFFFFFFFFFFFFFF,FFFFFFFFFFFFFFFFFFFFFFFFFFFFFFFFFFFFFFFFFFFFFFFFFFFF
FFFFFFFFFFFFFFF
@A00155:342:HHGFNDSXY:1:2228:2555:5509 1:N:0:GAACCTAG+TCCGCATA
ACATATTAAATATATTGGTTACGGTAAAAGACAAGGCAAGGTATGGACCCTTAGCAAAGCCAGAAAGA
TCACCTTGCCCAAAGAAGACCAACATTTTCATACATGATGAAATTCATTATTGGTCTCGCGATCAGCT
TGAGACATTTCTCCA
+
F,F:F:F:F,:F,FFFFFFFFFF:FFFFFFFFFFFFFFF:FFFFFFFFFFFFFFFFFFFFFFFFFF:F
:FFFFFFFF::FFFFFF:FFFFFFFFFF:FFFFFFFFFFFFFFFFFFFFFFFFFFFFFFFFFFF:FFF
FFFFFFFFFFFFFFF
```

```
@A00155:342:HHGFNDSXY:1:2347:17517:31798 1:N:0:GAACCTAG+TCCGCATA
ACATATTAAATAGATTGGTTACGGTAAAAGACAAGGCAAGGTATGGACCCTTAGCAAAGCCAGAAAGA
TCACCTTGCCCAAAGAAGACCAACATTTTCATACATGATGAAATTCATTATTGGTCTCGCGATCAGCT
TGAGACATTTCTCCA
+
FFFFFFFFFFFFFFFFFFFFFFFFFFFFFFFFFFFFFFF:FFFFFFFFFFFFFFFFFFFFFFFFFF:F
FFFFF:FFFFFFF:FFFFFFFFFFFFFFFFFFFFFFFFFFFFFFFFFFFFFFFFFFFFFFFFFFFFFF
FFFFFFFFFFFFFFF
@A00155:342:HHGFNDSXY:1:2665:15383:8406 1:N:0:GAACCTAG+TCCGCATA
ACATATTAAATAGATTGGTTACGGTAAAAGACAAGGCAAGGTATGGACCCTTAGCAAAGCCAGAAAGA
TCACCTTGCCCAAAGAAGACCAACATTTTCATACATGATGAAATTCATTATTGGTCTCGCGATCAGCT
TGAGACATTTCTCCA
+
FFFFFFFFFFFFFFFFFFFFFFFFFFFFFFFFFFFFFFFFFFFFFFFFFFFFFFFFFFFFFFFFFFFF
FFFFFFFFFFFFFFFFF:FFFFFFFFFFFFFFFFFFFFFFFFFFFFFFFFFFFFFFFFFFFFFFFFFF
FFFFFFFFFFFFFFF
@A00155:342:HHGFNDSXY:1:1412:9037:21809 2:N:0:GAACCTAG+TCCGCATA
ACATATTAAATAGATTGGTTACGGTAAAAGACAAGGCAAGGTATGGACCCTTAGCAAAGCCAGAAAGA
TCACCTTGCCCAAAGAAGACCAACATTTTCATACATGATGAAATTCATTATTGGTCTCGCGATCAGCT
TGAGACATTTCTCCA
+
FFFFFFFFFFFFFFFFFFFFFFFFFFFFFFFFFFFFFFFFFFFFFFFFFFFFFFFFFFFFFFFFFFFF
FFFFFFFFFFFFFFFFFFFFFFFFFFFFFFFFFFFFFFFFFFFFFFFFFFFFFFFFFFFFFFFFFFFF
FFFFFFFFFFFFFFF
@A00155:342:HHGFNDSXY:1:1572:22191:2973 2:N:0:GAACCTAG+TCCGCATA
ACATATTAAATAGATTGGTTACGGTAAAAGACAAGGCAAGGTATGGACCCTTAGCAAAGCCAGAAAGA
TCACCTTGCCCAAAGAAGACCAACATTTTCATACATGATGAAATTCATTATTGGTCTCGCGATCAGCT
TGAGACATTTCTCCA
+
FFFFFFFFFFFFFFFFFFFFFFFFF:FFFFFFFFFFFFFFFFFFFFFFFFFF:FFFFFFFF,FFFFFF
FFFFFFFFFFFFFFFFFFFFFFFFFFFFFFFFFFFFFFFFFFFFFFFFFFFFFFFFFFFFFFFFFFFF
FFFFFFFFFFFFFFF
@A00155:342:HHGFNDSXY:1:2615:22128:26569 2:N:0:GAACCTAG+TCCGCATA
ACATATTAAATAGATTGGTTACGGTAAAAGACAAGGCAAGGTATGGACCCTTAGCAAAGCCAGAAAGA
TCACCTTGCCCAAAGAAGACCAACATTTTCATACATGATGAAATTCATTATTGGTCTCGCGATCAGCT
TGAGACATTTCTCCA
+
FFFFFFFFFFFFFFFFFFFFFFFFFFFF:FFFFFFFFFFFFFFFFFFFFFFFFFFFFF:FFFFFFFFF
FFF:FF:FFFFFFFFF:FFFFFFFFFFFFFFFFFFFFFFFFF:FFF::FFFF,FFFFFFFFFFFFFFF
FF:FFFFFFFFFFFF
@A00155:342:HHGFNDSXY:1:1151:13910:12023 1:N:0:GAACCTAG+TCCGCATA
CATATTAAATAGATTGGTTACGGTAAAAGACAAGGCAAGGTATGGACCCTTAGCAAAGCCAGAAAGAT
CACCTTGCCCAAAGAAGACCAACATTTTCATACATGATGAAATTCATTATTGGTCTCGCGATCAGCTT
GAGACATTTCTCCAG
+
:FFFFFFF:FFF:FFFFFFFFFFFFFFFFFFFFFFFF:FF:FFFFFFFFFFFFFF:FF:F:F:FFFFF
:,FFFFFFFFF:FF:FFFFFFFFFFFFFFFF:F:FFFFFFFF,FFFFFFFFFFFFF:FFFFFFF:FFF
FFFFFFF:FFFFFFF
@A00155:342:HHGFNDSXY:1:1516:4770:26130 1:N:0:GAACCTAG+TCCGCATA
TCCGATCTATAGATTGGTTACGGTAAAAGACAAGGCAAGGTATGGACCCTTAGCAAAGCCAGAAAGAT
CACCTTGCCCAAAGAAGACCAACATTTTCATACATGATGAAATTCATTATTGGTCTCGCGATCAGCTT
GAGACATTTCTCCAG
+
FFFFFFFFFFFFFFFF:FFFFFFFFF:FFFFFFFFFF:FFFFFFFFFFFF:FFFFFFFFFFFFFFFFF
```

```
FFFFFFFFFFFFF:FFFFFFFFF:F:FFFFFFFFFFFFFFFFFFFFFFFFFFFFFFFFFFFFFFFFFF
FFFFFFFFFFFFFFF
@A00155:342:HHGFNDSXY:1:1547:4282:23563 1:N:0:GAACCTAG+TCCGCATA
TCTATTAAATAGATTGGTTACGGTAAAAGACAAGGCAAGGTATGGACCCTTAGCAAAGCCAGAAAGAT
CACCTTGCCCAAAGAAGACCAACATTTTCATACATGATGAAATTCATTATTGGTCTCGCGATCAGCTT
GAGACATTTCTCCAG
+
FFFFFFFFFFFF:FFFFFFFFFFFFFFFFFFFFFFFFFFFFFFFFFFFFFFFFFFFFFFFFFFFFFF
FFFF:FFFFFFFFFFFF,FFFFFFFFFFFFF:FFFFFFFFFFFFFFFFFFFFFFFFFFFFFFFFFFFF
FFFFFFFFFFFFFFF
@A00155:342:HHGFNDSXY:1:1572:27552:27038 1:N:0:GAACCTAG+TCCGCATA
CATATTAAATAGATTGGTTACGGTAAAAGACAAGGCAAGGTATGGACCCTTAGCAAAGCCAGAAAGAT
CACCTTGCCCAAAGAAGACCAACATTTTCATACATGATGAAATTCATTATTGGTCTCGCGATCAGCTT
GAGACATTTCTCCAG
+
FFFFFFFFFFFFFFFFFFFF:FFFF:FFFFFF,F:FFFFFF:FFFFFFFFFFFFFFFFFFFFFFFFFF
FFFF,:FFFFFFFFF:FFFFFFFFFFFFFFFFF:FFFFFFFFFFFFFFFFFFFFFFFFFFFFFFFFFF
FFFFFFFFFFFFFFF
@A00155:342:HHGFNDSXY:1:2222:4472:21981 1:N:0:GAACCTAG+TCCGCATA
CATATTAAATAGATTGGTTACGGTAAAAGACAAGGCAAGGTATGGACCCTTAGCAAAGCCAGAAAGAT
CACCTTGCCCAAAGAAGACCAACATTTTCATACATGATGAAATTCATTATTGGTCTCGCGATCAGCTT
GAGACATTTCTCCAG
+
:FFFFFFFFFFFFFFFFFFFFFFFF::FFFFF:FFFFFFFFFFFFFFFFFFFFFFFFFFFFFFFFFFF
FFFFFFFFFFFFFFFFFFFFFFFFFFFFFFFFFFFFFFFFFFFFFFFFFFFFFFFFFFFFFFFFFFFF
FFFFFFFFFFFFFFF
@A00155:342:HHGFNDSXY:1:1439:30535:10128 2:N:0:GAACCTAG+TCCGCATA
CATATTAAATAGATTGGTTACGGTAAAAGACAAGGCAAGGTATGGACCCTTAGCAAAGCCAGAAAGAT
CACCTTGCCCAAAGAAGACCAACATTTTCATACATGATGAAATTCATTATTGGTCTCGCGATCAGCTT
GAGACATTTCTCCAG
+
FFFFFFFFFFFFFFFF:FFFFFFFFFFF:FFFFFF:F:FF:FFFFFF:FFFFFFFFFFFFFFFFFFFF
FFFFFFFFFF:FFF:,FFFFFFFFFFFFFFFFFFFFFFFFFFFFFFFFFFFFFFFFFFFFFFFFFFFF
:FFFFFFFFFFFFFF
@A00155:342:HHGFNDSXY:1:2177:10809:15295 2:N:0:GAACCTAG+TCCGCATA
ATATTAAATAGATTGGTTACGGTAAAAGACAAGGCAAGGTATGGACCCTTAGCAAAGCCAGAAAGATC
ACCTTGCCCAAAGAAGACCAACATTTTCATACATGATGAAATTCATTATTGGTCTCGCGATCAGCTTG
AGACATTTCTCCAG
+
:FFFFF,FFFFFFFFFFFFFFFFFFFFFFFFFFFFFFFFFFFFFFFFFFFFFFFFFFFFFFFFFFFFF
FFFFFFFFFFFFFFFF:FFFFFFFFFFFFFFFFFFFFFFFFFFFFFFFFFFFFFFFFFFFFFFFFFFF
FFFFFFFFFFFFFF
@A00155:342:HHGFNDSXY:1:1432:25211:34882 1:N:0:GAACCTAG+TCCGCATA
ATATTATATAGATTGGTTACGGTAAAAGACAAGGCAAGGTATGGACCCTTAGCAAAGCCAGAAAGATC
ACCTTGCCCAAAGATGACCAACATTTTCATACATGATGAAATTCATTATTGGTCTCGCGATCAGCTTG
AGACATTTCTCCAGG
+
:,FFF:,,,FFFF,::FFFF,FFFFFFF:FFFFFFFFFFF,F,FF:F:FFF,FF:FF:FFFFFF:FF:
FFFFFF:FFF:FFF,,:,FFF,FFF:FFFFFFFFFFFF:,::F:FFFF:FFFF:F:FFFFF,F:FFFF
FFF:FFFFFFFF::F
@A00155:342:HHGFNDSXY:1:2546:22978:27445 1:N:0:GAACCTAG+TCCGCATA
ATATTAAATAGATTGGTTACGGTAAAAGACAAGGCAAGGTATGGACCCTTAGCAAAGCCAGAAAGATC
ACCTTGCCCAAAGAAGACCAACATTTTCATACATGATGAAATTCATTATTGGTCTCGCGATCAGCTTG
AGACATTTCTCCAGG
```

+
FFFFFFFFFFFFFF,FFFFF,FFFFFFFFFFFFFFFFFFFFFFFFFFFFFFFFFFFFF:FFFFFFFFF
FFFFFFFFFFFFFFFFFFFF:FFFFFFFFFFFFFFFFFFFFFFFFFFFFFFFFFFFFFFFFFFFFFF
FFFFFFFFFFFFFFF
@A00155:342:HHGFNDSXY:1:2363:17083:19053 2:N:0:GAACCTAG+TCCGCATA
ATATTAAATAGATTGGTTACGGTAAAAGACAAGGCAAGGTATGGACCCTTAGCAAAGCCAGACAGATC
ACCTTGCCCAAAGAAGACCAACATTTTCATACATGATGAACTTCATTATTGGTCTCGCGCTCAGCTTG
AGACATTTCTCCAGG
+
FFFFFFFF:FFFFFFF::FFFFFFFFFFFFFF,FFFFFFF:FFFFFFFFFFFFFF:,F:::F,,:FFF
FFFFF,FFFF:FFFF:FFFF:FFFF,F,FF,:F,:FFFFF,FFFFFF:FFFFF:FF::F,,,FFFFFF
FF::FFF,FFFFFFF
@A00155:342:HHGFNDSXY:1:2221:7627:29794 1:N:0:GAACCTAG+TCCGCATA
TATTAAATAGATTGGTTACGGTAAAAGACAAGGCAAGGTATGGACCCTTAGCAAAGCCAGAAAGATCA
CCTTGCCCAAAGAAGACCAACATTTTCATACATGATGAAATTCATTATTGGTCTCGCGATCAGCTTGA
GACATTTCTCCAGG
+
FFFFFF:FF:FFF,F:F:FFF:FFFFFFFF:FFFFFF,FFFFFFFFFFFFFFFFF:FFFFFFFFFFFF
FFFFFFFFFF,FFFFFFFFFFFFFFFFFFFFFFFFFFFFFFFFFFFFFFFFFFFFFFFFFFFFFFFF
FFFFFFFFFFFFFF
@A00155:342:HHGFNDSXY:1:2612:24623:13416 1:N:0:GAACCTAG+TCCGCATA
TATTAAATAGATTGGTTACGGTAAAAGACAAGGCAAGGTATGGACCCTTAGCAAAGCCAGAAAGATCA
CCTTGCCCAAAGAAGACCAACATTTTCATACATGATGAAATTCATTATTGGTCTCGCGATCAGCTTGA
GACATTTCTCCAGGC
+
FFFF,FFFFFFFF,FFFF:FFFFFFFFFFF,,FFFF:FFFFFFFFF:FFFFFFFFFFFFFFFFFFF:F
FFFFFFFFF:FFF:FFFFFFFFFFFFFFFFFFFFFFFFFFFFFFFFFFFFFFFFFF,FFFFFFFFFFF
FFFFFFFFFFFFFFF
@A00155:342:HHGFNDSXY:1:1111:26657:15060 2:N:0:GAACCTAG+TCCGCATA
ATTAAATAGATTGGTTACGGTAAAAGACAAGGCAAGGTATGGACCCTTAGCAAAGCCAGAAAGATCAC
CTTGCCCAAAGAAGACCAACATTTTCATACATGATGAAATTCATTATTGGTCTCGCGATCAGCTTGAG
ACATTTCTCCAGGTT
+
FFFFFFFF:FFFFFFFFFFFFFFFFFFFFFFFFFFFFFFFFFFFFFFFFFFFFFFFFFFFFFFFFFF
FFFFFFFFFFFF:FFFFFFFFFFFFFFFFFFFFFFFFFFFFFFFFFFFFFFFFFFFFFFFFF:FFFFF
FFFF:FFFFFF:FFF
@A00155:342:HHGFNDSXY:1:1438:16532:33849 2:N:0:GAACCTAG+TCCGCATA
ATTAAATAGATTGGTTACGGTAAAAGACAAGGCAAGGTATGGACCCTTAGCAAAGCCAGAAAGATCAC
CTTGCCCAAAGAAGACCAACATTTTCATACATGATGAAATTCATTATTGGTCTCGCGATCAGCTTGAG
ACATTTCTCCAGGTT
+
FFFFFFFFFFFFFFFFFFFFFFF:FFFFFFFFFFFFFFFFFFFFFFFFFFFFFFFF:FFFFFFFFFFF
FFFFFFFFFFFFFFFFFFFFFFFFFFFFFFFFFFFFFFFFFFFFFFFFF:FFFFFFFFFFFFFFFFFF
FFFFFFFFFFFFFFF
@A00155:342:HHGFNDSXY:1:1532:22851:16830 2:N:0:GAACCTAG+TCCGCATA
ATTAAATAGATTGGTTACGGTAAAAGACAAGGCAAGGTATGGACCCTTAGCAAAGCCAGAAAGATCAC
CTTGCCCAAAGAAGACCAACATTTTCATACATGATGAAATTCATTATTGGTCTCGCGATCAGCTTGAG
ACATTTCTCCAGGTT
+
FFFFFFFFFFFFFFFFFFFFFFFFFFFF:FFFFFFFFFFFFFFFFFFFFFFFFFFFFFFFFFFFFFFF
FFFFFFFFFFFFFFFFFFFFFFFFFFFFFFFFFFFFFFFFFFFFFFFFFFFFFFFFFFFFFFFFFFFF
FF,FFFFFFFFFFFF
@A00155:342:HHGFNDSXY:1:1532:23882:30984 2:N:0:GAACCTAG+TCCGCATA
ATTAAATAGATTGGTTACGGTAAAAGACAAGGCAAGGTATGGACCCTTAGCAAAGCCAGAAAGATCAC

CTTGCCCAAAGAAGACCAACATTTTCATACATGATGAAATTCATTATTGGTCTCGCGATCAGCTTGAG
ACATTTCTCCAGGTT
+
FFFFFFFFFFFFFFFFFFFFFFFFFFFFFFFFFFFFFFFFFFFFFFFFFFFFFFFFFFFFFFFFFFFFFF
FFFFFFFFFFFFFFFFFFFFFFFFF:FFFFFFFFFFFFFFFFFFFFFFFFFFFFFFFFFFFFFFFFFFFF
FFFFFFFFFFFFFFF
@A00155:342:HHGFNDSXY:1:1547:4282:23563 2:N:0:GAACCTAG+TCCGCATA
ATTAAATAGATTGGTTACGGTAAAAGACAAGGCAAGGTATGGACCCTTAGCAAAGCCAGAAAGATCAC
CTTGCCCAAAGAAGACCAACATTTTCATACATGATGAAATTCATTATTGGTCTCGCGATCAGCTTGAG
ACATTTCTCCAGAGA
+
FFFFFFFFFFFFFFFFFFFFFFFFFFFFFFFFFF:FFFFFFFFFFFFFFFFFFFFFFF:FFFFFFFFFFF
FFFFFFFFFFF:FFFFFFFFFFFFFFFFFFFFFFFFFFFFFFFFFFFFFFFFFFFFFFFFFFFFFFFFFF
FFFFFFFFFFFFFFF
@A00155:342:HHGFNDSXY:1:1610:13367:6386 2:N:0:GAACCTAG+TCCGCATA
ATTAAATAGATTGGTTACGGTAAAAGACAAGGCAAGGTATGGACCCTTAGCAAAGCCAGAAAGATCAC
CTTGCCCAAAGAAGACCAACATTTTCATACATGATGAAATTCATTATTGGTCTCGCGATCAGCTTGAG
ACATTTCTCCAGGTT
+
FFFFF,FFFFFFFFFFFFFFFFFFFFFF:FFFFFFFFFFFFFFFFF:FFFF:FFFFFFFFFFFFFFFFFF
FF:FFFFFFFFFFFFF:FFFFFFFFFFFFF:FFFFFFFFFFFFFFFFFFFFFFF,:FFFFFFFFFFF,FF
:FFFFF:F:FF,F:F
@A00155:342:HHGFNDSXY:1:1558:22345:28260 2:N:0:GAACCTAG+TCCGCATA
ATTAAATAGATTGGTTACGGTAAAAGACAAGGCAAGGTATGGACCCTTAGCAAAGCCAGAAAGATCAC
CTTGCCCAAAGAAGACCAACATTTTCATACATGATGAAATTCATTATTGGTCTCGCGATCAGCTTGAG
ACATTTCTCCAGGTT
+
FFFFFFFFFFFFFFFFFFFFFFFFFFFFFFFFFFFFFFFFFFFFFFFFFFFFF:FFFFFFFFFFFFFFFF
FFFFFFFFFFFFFFFFFFFFFFFFFFFFFFFFFFFFFFFF:FFFFFFFF:FFFFFFFFFFFFFFFFFFFF
FFFFFFFFFF,FFFF
@A00155:342:HHGFNDSXY:1:2138:11939:14841 2:N:0:GAACCTAG+TCCGCATA
ATTAAATAGATTGGTTACGGTAAAAGACAAGGCAAGGTATGGACCCTTAGCAAAGCCAGAAAGATCAC
CTTGCCCAAAGAAGACCAACATTTTCATACATGATGAAATTCATTATTGGTCTCGCGATCAGCTTGAG
ACATTTCTCCAGGTT
+
FFFFFFFFFFFFFFFFFFFFFFFFFFFFFFFFFFFFFFFFFFFFFFFFFFFFFFFFFFFFFFFFFFFFFF
FFFFFFFFFFFFFF:FFFFFFFFFFFFFFFFFFFFFFFFFFFFFFFFFFFFFFFFFFFFFFFFFFFFFFF
FFFFFFFFFFFFFFF
@A00155:342:HHGFNDSXY:1:2173:6234:16329 2:N:0:GAACCTAG+TCCGCATA
ATTAAATAGATTGGTTACGGTAAAAGACAAGGCAAGGTATGGACCCTTAGCAAAGCCAGAAAGATCAC
CTTGCCCAAAGAAGACCAACATTTTCATACATGATGAAATTCATTATTGGTCTCGCGATCAGCTTGAG
ACATTTCAGATCGGA
+
:FFFFFFFFFFFFF:FFFFFFFFFFFFFFFFFFFFFFFFFF:FFFFFFFFF:F:FFFFF,FFFFFFFFFF
FFFFFFFFFFF,:,FFFF:FFF:FFFFFFFFF,F:FFFFFFF,FFFFFF,:::F,FF,FFFFFFFF:FFF
FFFFFFFFFFF:FFF
@A00155:342:HHGFNDSXY:1:2413:12671:21245 2:N:0:GAACCTAG+TCCGCATA
ATTAAATAGATTGGTTACGGTAAAAGACAAGGCAAGGTATGGACCCTTAGCAAAGCCAGAAAGATCAC
CTTGCCCAAAGAAGACCAACATTTTCATACATGATGAAATTCATTATTGGTCTCGCGATCAGCTTGAG
ACATTTCTCCAGGTT
+
FF,F:FFFFFFFFFFFFFFFFFFFF,FFFFFFFFF:FF,FFFFFFFFFFFFFFFFF:,FFF:FFFFFFFF
:FFFF:FFFFFFFFFFFFFF,FFF:,::FFFFFFFFF:FFF,,,FFF:F:F:FFFF:FFF,FFFFFFFFF
FFFF,F:F:FF,FFF

```
@A00155:342:HHGFNDSXY:1:2507:29668:26443 2:N:0:GAACCTAG+TCCGCATA
ATTAAATAGATTGGTTACGGTAAAAGACAAGGCAAGGTATGGACCCTTAGCAAAGCCAGAAAGATCAC
CTTGCCCAAAGAAGACCAACATTTTCATACATGATGAAATTCATTATTGGTCTCGCGATCAGCTTGAG
ACATTTCTCCAGGTT
+
FFFFFFFFFFFFFFFFFFFFFFFFFFFFFFFFFF:FFFFFFFFFFFFFFF:FFFFFFFFFFFFFFFFF
FFFFFFFFFFFFF:FF:FFFFFFFFFFFFFFFFFFFFFFFFFFFFFFF:FFFFFFFFFFFFFFFFFFF
FFFFFFFFFFFFFFF
@A00155:342:HHGFNDSXY:1:1164:22083:18599 2:N:0:GAACCTAG+TCCGCATA
TAAATAGATTGGTTACGGTAAAAGACAAGGCAAGGTATGGACCCTTAGCAAAGCCAGAAAGATCACCT
TGCCCAAAGAAGACCAACATTTTCATACATGATGAAATTCATTATTGGTCTCGCGATCAGCTTGAGAC
ATTTCTCCAGGTTTA
+
FFFFFFFFFFFFFFFFFFFFFF::FFF::FFFFF:F:FFFFFFFFFFFFFFFFFFFFFFFFFFFFFFF
FFFFFFFFFF:FFF:FFFFFFFFFFFFFFFFFFFFFFF:FFFFFFFFFFFFFFFFFFFFFFFF:FFFFFF
F:FFFFFFFFFFFFF
@A00155:342:HHGFNDSXY:1:2670:12337:34726 2:N:0:GAACCTAG+TCCGCATA
TAAATAGATTGGTTACGGTAAAAGACAAGGCAAGGTATGGACCCTTAGCAAAGCCAGAAAGATCACCT
TGCCCAAAGAAGACCAACATTTTCATACATGATGAAATTCATTATTGGTCTCGCGATCAGCTTGAGAC
ATTTCTCCAGGTTTA
+
FFF::FFFF::F:FFFFFFFFFFFFFFFFF:FFF:FF,F:FFFFF:FFFFFFFFFFFFFFFFF,FF,,
FFFFFFFFFFFFFFFFFFFFFFFFFFFFFF:F::FFFFFFFFFF:F:,F:FF:FF:FFFFF:FFFFFF
F:,FFF:FFF,FF:F
@A00155:342:HHGFNDSXY:1:1643:4616:2973 1:N:0:GAACCTAG+TCCGCATA
AATAGATTGGTTACGGTAAAAGACAAGGCAAGGTATGGACCCTTAGCAAAGCCAGAAAGATCACCTTG
CCCAAAGAAGACCAACATTTTCATACATGATGAAATTCATTATTGGTCTCGCGATCAGCTTGAGACAT
TTCTCCAGGTTTACA
+
FFFFFF,:F:,:FFF,FFF:FFFFFFF:FFFFFFFFFFFFFFF:FFFFFF:FFFFFFF:FFFFFFFFF
FFFFFF:FF,FFFFF,F,FF:FF,FFFFFFFFFFFF,::FFFFFFF:FFF:F:FFFFF:FFF:FFFFF
FFFFF,FF,:,FFFF
@A00155:342:HHGFNDSXY:1:1128:32832:16078 2:N:0:GAACCTAG+TCCGCATA
AATAGATTGGTTACGGTAAAAGACAAGGCAAGGTATGGACCCTTAGCAAAGCCAGAAAGATCACCTTG
CCCAAAGAAGACCAACATTTTCATACATGATGAAATTCATTATTGGTCTCGCGATCAGCTTGAGACAT
TTCTCCAGGTTTACA
+
FFFFF:FFFFF:FFFFFFFFFFFFFFFFFFF,FFFFFFFFFFFF:FFFFFFFFFFFFFFFFFFFFFFFF
FFFFFFFFFFFFFFFFFFFFFFFFFFFFFFFFFFFFFFFFFF:F,FFFFFFFFFFFFFFFFFFFFFFFF
FFFFFF:FFFFFFFF
@A00155:342:HHGFNDSXY:1:1172:21043:33959 2:N:0:GAACCTAG+TCCGCATA
AATAGATTGGTTACGGTAAAAGACAAGGCAAGGTATGGACCCTTAGCAAAGCCAGAAAGATCACCTTG
CCCAAAGAAGACCAACATTTTCATACATGATGAAATTCATTATTGGTCTCGCGATCAGCTTGAGACAT
TTCTCCAGGTTTACA
+
FFFFFFFFFF:FFFFFFFFFFFFFFFFFFFF:FFFFFFF:FFFFFFFFFFFFFFFFFFFFFFFFFFF::F
FFFFFFFFF:FFFFFFFFFF,FFFFF:FF:FF:FF,FFF:FFF:FFFFFFFFF:FFFFFFFFFF:FFF
FFFFFFFFFFFFFFF
@A00155:342:HHGFNDSXY:1:1253:27416:19413 2:N:0:GAACCTAG+TCCGCATA
AATAGATTGGTTACGGTAAAAGACAAGGCAAGGTATGGACCCTTAGCAAAGCCAGAAAGATCACCTTG
CCCAAAGAAGACCAACATTTTCATACATGATGAAATTCATTATTGGTCTCGCGATCAGCTTGAGACAT
TTCTCCAGGTTTACA
+
FFFFFFFFFFFFFFFFF:FFFFFFFFFFFFFFFFFFFFFFFFFFFFFFFFFFFFFFFFFF:FFFFFFF:
```

FFFFFFFFFFFFFFFFFFFFFFFFFFFFFFFFFFFFFFFFFFFFFFFFFFFFFFFFFFFFFFFFFFFF
FFFFFFFFFFFFFFF
@A00155:342:HHGFNDSXY:1:1438:19533:6856 2:N:0:GAACCTAG+TCCGCATA
AATAGATTGGTTACGGTAAAAGACAAGGCAAGGTATGGACCCTTAGCAAAGCCAGAAAGATCACCTTG
CCCAAAGAAGACCAACATTTTCATACATGATGAAATTCATTATTGGTCTCGCGATCAGCTTGAGACAT
TTCTCCAGGTTTACA
+
FFFFFFFFFFFFFFFFFFFFFFFFFFFFFFFFFFFFFFFFFFFFFFFFFFFFFFFFFFF:FFFFFFFF
FFFFFFFFFFFFFFFFFFFFFFFFFFFFFFFFFFFFFFF:FFFFFFFFFFFFFFFFFFFFFFFFFFFF
FFFFFFFFFFFFFFF
@A00155:342:HHGFNDSXY:1:1440:2311:19460 2:N:0:GAACCTAG+TCCGCATA
AATAGATTGGTTACGGTAAAAGACAAGGCAAGGTATGGACCCTTAGCAAAGCCAGAAAGATCACCTTG
CCCAAAGAAGACCAACATTTTCATACATGATGAAATTCATTATTGGTCTCGCGATCAGCTTGAGACAT
TTCTCCAGGTTTACA
+
FFFFFFFFFFFFFFFFFFFFFFFFFFFFFFFFFFFFFFFFFFFFFFFFFFFFFFFFFFFFFFFFFFFF
FFFF:FF:F:FFFFFFFFFFFFFFFFFFFFFFFFF:FF,FFFFF,FFFFFFFFFFFF:FFFFFFF:FF
FFFFFFFFFFFFFFF
@A00155:342:HHGFNDSXY:1:1453:3224:31407 2:N:0:GAACCTAG+TCCGCATA
AATAGATTGGTTACGGTAAAAGACAAGGCAAGGTATGGACCCTTAGCAAAGCCAGAAAGATCACCTTG
CCCAAAGAAGACCAACATTTTCATACATGATGAAATTCATTATTGGTCTCGCGATCAGCTTGAGACAT
TTCTCCAGGTTTACA
+
FFFFFFFFFFFFFFFFFFF:FFFFFFFFFFFFFFFFFFFFFFFFFFFFFFFFFFFFFFFFFFFFFFFF
FFFFFFFF:,FFFF:FFFFFFFFFFFFFFFFFFFFFFFFFFFFFF:FFFFF:FFFFFF:FFFFFFFFF
FFFFFFFFFFFFFFF
@A00155:342:HHGFNDSXY:1:1454:21549:12602 2:N:0:GAACCTAG+TCCGCATA
AATAGATTGGTTACGGTAAAAGACAAGGCAAGGTATGGACCCTTAGCAAAGCCAGAAAGATCACCTTG
CCCAAAGAAGACCAACATTTTCATACATGATGAAATTCATTATTGGTCTCGCGATCAGCTTGAGACAT
TTCTCCAGGTTTACA
+
FFFFFFFFFFFFFFFFF:FFFFFFFFFFF::FFFFFFFFFFFFFFFFFFFFFFFFFFFFFFFFFFFFF
FFFFFFFFFFFFFFFFFFFFFFFFFFFFFFFFFFF,FFFFFFFFFFFFFFFFFFFFFFFFFFFFFFFF
FFFFF:FFF:FFFFF
@A00155:342:HHGFNDSXY:1:1539:6253:15139 2:N:0:GAACCTAG+TCCGCATA
AATAGATTGGTTACGGTAAAAGACAAGGCAAGGTATGGACCCTTAGCAAAGCCAGAAAGATCACCTTG
CCCAAAGAAGACCAACATTTTCATACATGATGAAATTCATTATTGGTCTCGCGATCAGCTTGAGACAT
TTCTCCAGATCGGAA
+
FFFFFFFFFFFFFFFFFFFFFFFFFFFFFFFFFFFFFFFFFFFFFFFFFFFFFFFFFFFFFFFFFFFF
FFFFFFFFFFFFFFFFFFFFFFFFFFFFFFFF:FFFFFFFFFFFFFFFFFFFFFFFFFFFFFFFFFFF
FFFFFFFFFFFFFFF
@A00155:342:HHGFNDSXY:1:1553:25211:5008 2:N:0:GAACCTAG+TCCGCATA
AATAGATTGTTTACGGTAAAAGACAAGTCAAGGTATGGACCCTTAGCAAAGCCAGAAAGATCACCTTG
CCCAAAGAAGACCAACATTTTCATACATGATGAAATTCATTATTGGTCTCTCGATCAGCTTGAGACAT
TTCTCCAGGTTTACA
+
FFFFFFFFF,FFFF,FFFFF:FFFFF,,:FFF:::F:,FFFFF,F,F:FFFFF,:FFFFFFFF:FFFF
FF:F:F,F:,:FFFFFFFFFF,FFFFFFFFFFF,,:FF::FFFFFF,,F,,FFF,,FF,FF,,F:FFF
FF:FFF,,,,F:F:F
@A00155:342:HHGFNDSXY:1:1655:14009:15702 2:N:0:GAACCTAG+TCCGCATA
AATAGATTGGTTACGGTAAAAGACAAGGCAAGGTATGGACCCTTAGCAAAGCCAGAAAGATCACCTTG
CCCAAAGAAGACCAACATTTTCATACATGATGAAATTCATTATTGGTCTCGCGATCAGCTTGAGACAT
TTCTCCAGGTTTACA

+
FFFFFFFFFFFFFFFFFFF,FFFFFFFF:FFFFFFFFFFFFFFFFFFFFFFFFFFFFFFFFFFFFFFFFF
FFFFFFFFFFFFFFFFFFFFFFFFFFFFFFFFFFFFFFFFF:FFFFFFFFFFFFFFFFFFFFFFFFFFFFF
FFFFFFFFFFFFFFF
@A00155:342:HHGFNDSXY:1:2257:9146:10097 2:N:0:GAACCTAG+TCCGCATA
AATAGATTGGTTACGGTAAAAGACAAGGCAAGGTATGGACCCTTAGCAAAGCCAGAAAGATCACCTTG
CCCAAAGAAGACCAACATTTTCATACATGATGAAATTCATTATTGGTCTCGCGATCAGCTTGAGACAT
TTCTCCAGGTTTACA
+
FFFFFFFFFFFFFF:FFFF:FFFFFFFFFFFFFFFFFFFFFFFF:FFFFFFFFFFFFFFFFF:FFFFFF
FFFF:FFFFFFFFFFFFFFFFFFFFFFFFFFFFFFFFFFFFFFF:FFFFFFFFFFFFFFFFFFFF:FFF
FFFFFFF:FFFFFFF
@A00155:342:HHGFNDSXY:1:2678:20546:13839 2:N:0:GAACCTAG+TCCGCATA
AATAGATTGGTTACGGTAAAAGACAAGGCAAGGTATGGACCCTTAGCAAAGCCAGAAAGATCACCTTG
CCCAAAGAAGAACAACATTTTCATACATGATGAAATTCATTATTGGTCTCGCGATCAGCTTGAGACAT
TTCTCCAGGTTTACA
+
FF,F:FFFF:FFFF:::FFFF:FFFFF,FFFF,:FF::FF,:FFF::FFFFFFF:FFFFFF:FFF,,F
FF,FF:,FF,F,,FF:FF:FFFF:FFFF,FFFFFFFF,FFFFFFF,FFFF,,:FF:F:FFF:F,FFFF
FFFFFFFFFFFFFFF
@A00155:342:HHGFNDSXY:1:1516:4770:26130 2:N:0:GAACCTAG+TCCGCATA
ATAGATTGGTTACGGTAAAAGACAAGGCAAGGTATGGACCCTTAGCAAAGCCAGAAAGATCACCTTGC
CCAAAGAAGACCAACATTTTCATACATGATGAAATTCATTATTGGTCTCGCGATCAGCTTGAGACATT
TCTCCAGAGATCGGA
+
FFFFFFFFFFFFFFFFFFFFFFFFFFFFFFFFFFFFFFFFFFFFFFFFFFFFFFFFFFFFFFFFFFFF
FFFFFFFFFFFFFFFFFFFFFFFFFFFFFFFFFFFFFFFFFFFFFFFFFFFFFFFFFFFFFFFFFFFF
FFFFFFFFFFFF:FF
@A00155:342:HHGFNDSXY:1:1658:9887:26005 2:N:0:GAACCTAG+TCCGCATA
ATAGATTGGTTACGGTAAAAGACAAGGCAAGGTATGGACCCTTAGCAAAGCCAGAAAGATCACCTTGC
CCAAAGAAGACCAACATTTTCATACATGATGAAATTCATTATTGGTCTCGCGATCAGCTTGAGACATT
TCTCCAGGTTTACAA
+
FFFFFFFFFFFFFFFFFFFF:FFFFFFFFFFFFFFFFFFFFFFFFFFFFFFFFFFFFFF:FFFFFFFFF
FFFFFFFFFFFFF:FFFFFFFFFFFFFFFFFFFFFFFFFFFFFFFFFFFFFFFFFFFFFFFFFFFFFF
FFFFFFFFFFF:FFF
@A00155:342:HHGFNDSXY:1:1658:10077:32158 2:N:0:GAACCTAG+TCCGCATA
ATAGATTGGTTACGGTAAAAGACAAGGCAAGGTATGGACCCTTAGCAAAGCCAGAAAGATCACCTTGC
CCAAAGAAGACCAACATTTTCATACATGATGAAATTCATTATTGGTCTCGCGATCAGCTTGAGACATT
TCTCCAGGTTTACAA
+
FFFFFFFFFFFFFFFFFFFFFFFFFFFFFFFFFFFFFFFFFFFFFFFFFFFFFFFFFFFFFFFFFFFF
FFFFFFFFFFFFFFFFFFFFFFFFFFFFFFFFFFFFFFFFFFFFFFFFFFFFFFFFFFFFF:FFFFFF
FFFFFFFFFFFFFFF
@A00155:342:HHGFNDSXY:1:1658:8522:32534 2:N:0:GAACCTAG+TCCGCATA
ATAGATTGGTTACGGTAAAAGACAAGGCAAGGTATGGACCCTTAGCAAAGCCAGAAAGATCACCTTGC
CCAAAGAAGACCAACATTTTCATACATGATGAAATTCATTATTGGTCTCGCGATCAGCTTGAGACATT
TCTCCAGGTTTACAA
+
FFFFFFFFFFFFFFFFFFFFFFFFFFFFFFFFFFFFFFFFFFFFFFFFFFFFFFFFFFFFFFFFFFFF
FFFFFFFFFFFFFFFFFFFFFFFFFFFFFFFFFFFFFFFFFFFFFFFFFFFFFFFFFFFFFFFFFFFF
FFFFFFFFFFFFFFF
@A00155:342:HHGFNDSXY:1:2228:25699:9173 2:N:0:GAACCTAG+TCCGCATA
ATAGATTGGTTACGGTAAAAGACAAGGCAAGGTATGGACCCTTAGCAAAGCCAGAAAGATCACCTTGC

CCAAAGAAGACCAACATTTTCATACATGATGAAATTCATTATTGGTCTCGCGATCAGCTTGAGACATT
TCTCCAGGTTTACAA
+
F:F:FFFFFFFFFFF:FFFFFFFFFFFFF,FFFFFFFF:FFFFFFFFFFFFFFFFFFFFF:FFFFFFF
FFFFFFF:FFFFFFF::FFFFFFFFFFFFFFFFFFFFFFFFFFFFFFFFFFFFFFFFFFFFFFFFFFF
FFFFFFFFFFFFFF:
@A00155:342:HHGFNDSXY:1:2545:24560:29152 2:N:0:GAACCTAG+TCCGCATA
ATAGATTGGTTACGGTAAAAGACAAGGCAAGGTATGGACCCTTAGCAAAGCCAGAAAGATCACCTTGC
CCAAAGAAGACCAACATTTTCATACATGATGAAATTCATTATTGGTCTCGCGATCAGCTTGAGACATT
TCTCCAGGTTTACAA
+
F:FFFFFFFFFFFFFFF:F:FFFFFFFFFFFFFFFFFFFFFFFFFFFFF:FFFFFFFFFFFFF,FFFF
FFFFFFFFFFFFFFFF::,FFFFFFFF:FF:FFFFFFFFFFFFF,FFFFFFFFFFF:FFFFFFFFFFF
FF:FFFF,FFFF:FF
@A00155:342:HHGFNDSXY:1:1667:32624:32972 2:N:0:GAACCTAG+TCCGCATA
TAGATTGGTTACGGTAAAAGACAAGGCAAGGTATGGACCCTTAGCAAAGCCAGAAAGATCACCTTGCC
CAAAGAAGACCAACATTTTCATACATGATGAAATTCATTATTGGTCTCGCGATCAGCTTGAGACATTT
CTCCAGGTTTACAAA
+
F,FFFFFFFFFFFFFFFFFFFFFFFFFFFFFF:FFFFFFFFFFFFFFFFFFFFFFFFFFFFFFFFFFF
FFF:FFFF:FFFFFFFFFFFFFFFFFFFFFFFFFFFF:FFFFFFFFFFFFFFFFF:FFFFFFFFFFFF
FFFFFFFFFFFFFFF
@A00155:342:HHGFNDSXY:1:2424:1353:12195 2:N:0:GAACCTAG+TCCGCATA
AGATTGGTTACGGTAAAAGACAAGGCAAGGTATGGACCCTTAGCAAAGCCAGAAAGATCACCTTGCCC
AAAGAAGTCCAACATTTTCATACATGATGAAATTCATTATTTGTCTCGCGATCAGCTTGAGACATTTC
TCCAGGTTTACAA
+
:F:FFFFFFF:FFFFF:F,F,FFFFFFFFFF,:FFFFFFF:FFFF:FFFFFFFF,F:FFFFFFFFFFF
FFFFFFF,FF,,FFFFF:FFF:F:F:FFFF,FFF:,FF:FF,:FFFFF:F:F,:,FFFF:,,FFFFF,
FF,F:F,F:F:::
@A00155:342:HHGFNDSXY:1:2652:5846:14465 1:N:0:GAACCTAG+TCCGCATA
AGATTGGTTACGGTAAAAGACAAGGCAAGGTATGGACCCTTAGCAAAGCCAGAAAGATCACCTTGCCC
AAAGAAGACCAACATTTTCATACATGATGAAATTCATTATTGGTCTCGCGATCAGCTTGAGACATTTC
TCCAGGTTTACAAAC
+
FFFFF:FFFFF:FFFFFFFFFFFFFFFFFFFFFFFFFFFF:FFFFFFFFFFFFFFFFFFFFFFFFFFF
FFFF:FFFFFFFFFFFFFFFFFFFFFFFFFFFFFFFFFFFFFFFFFFFFFFFFFFFFFFFFFFFFFFF
FFFFFFFFFFFFFFFF
@A00155:342:HHGFNDSXY:1:2652:6614:15013 1:N:0:GAACCTAG+TCCGCATA
AGATTGGTTACGGTAAAAGACAAGGCAAGGTATGGACCCTTAGCAAAGCCAGAAAGATCACCTTGCCC
AAAGAAGACCAACATTTTCATACATGATGAAATTCATTATTGGTCTCGCGATCAGCTTGAGACATTTC
TCCAGGTTTACAAAC
+
FFFFFF:FFFFF,FFFFFFFFFFFFFFFFFFFFFFFFFFFFFFFFFFFFFFFFFFFFFFFFFFFFFFF
FFFFFFFFFFFFFFFFF:FFFFF:FFFFFFFFFFFFFFFFFFFFFFFFFFFFFFFFFFFFFFFFFFFF
FFFFFFFFFFFFFFFF
@A00155:342:HHGFNDSXY:1:1119:8368:6746 2:N:0:GAACCTAG+TCCGCATA
AGATTGGTTACGGTAAAAGACAAGGCAAGGTATGGACCCTTAGCAAAGCCAGAAAGATCACCTTGCCC
AAAGAAGACCAACATTTTCATACATGATGAAATTCATTATTGGTCTCGCGATCAGCTTGAGACATTTC
TCCAGGTTTACAAAC
+
FFFFFFFF:FFFFFFFFFFFFFFFFFF,FFF:FFFFFFFFFFFFFFFFFFFFFFFFFFFFFFFFFFFF
FFFF::FFFFFFFFFFFFFFFFFFFFFFFFFF:FFFFFFFFFFFFFFFFFFFFFFF:FFFFFFFFFFF
FFF:FFFFFFFFFFFF

```
@A00155:342:HHGFNDSXY:1:1165:5204:21559 2:N:0:GAACCTAG+TCCGCATA
AGATTGGTTACGGTAAAAGACAAGGCAAGGTATGGACCCTTAGCAAAGCCAGAAAGATCACCTTGCCC
AAAGAAGACCAACATTTTCATACATGATGAAATTCATTATTGGTCTCGCGATCAGCTTGAGACATTTC
TCCAGGTTTACAAAC
+
FFFFFFFFFFFFFFF:FFFFFFFF,:FFFFFFFFFFFFFFFFFFFFFFFFF::FFF:FFFFFFFFFFF
FFFF:FF:FFFFFFFFFFFFFFFFFFFFFFFFFFFFFFFFFFF,FFFF,FFFFFFF:FFFF:FFFF,FF
FFF:F,FF,FFFFFF
@A00155:342:HHGFNDSXY:1:1275:6171:33129 2:N:0:GAACCTAG+TCCGCATA
AGATTGGTTACGGTAAAAGACAAGGCAAGGTATGGACCCTTAGCAAAGCCAGAAAGATCACCTTGCCC
AAAGAAGACCAACATTTTCATACATGATGAAATTCATTATTGGTCTCGCGATCAGCTTGAGACATTTC
TCCAGGTTTACAGAC
+
FFFFFFFFFFFFFFFFFFFFFFFFFFFFFFFFFFFFFFFFFFFFFFFFFFFFFFFFFFFFFFFFFFFF
FFFFFFFFFFFFFFFFFFFFFFF:FFF:F,FFFFFFFFFFFFFFFFF:FFFFFFFFFFFFFFFFFF:FFF
FFFFFFFFFFFFFFF
@A00155:342:HHGFNDSXY:1:1470:14145:32440 2:N:0:GAACCTAG+TCCGCATA
AGATTGGTTACGGTAAAAGACAAGGCAAGGTATGGACCCTTAGCAAAGCCAGAAAGATCACCTTGCCC
AAAGAAGACCAACATTTTCATACATGATGAAATTCATTATTGGTCTCGCGATCAGCTTGAGACATTTC
TCCAGGTTTACAAAC
+
FFFFFFFFFFFFFFFFFFFFFFF:FFFFFFFFFFF,FFFFFFFFFFFFFFFFF:FFFFFFFFFFFFFF
FFFFFFFFFFFFFFFFFFFFFFFFFFFFFFF:FFFFFFFF,FFFFFFFFFF:FFFFFFF:F:FFFFFF
:FFFFFFFFFFFFFF
@A00155:342:HHGFNDSXY:1:1518:25816:2112 2:N:0:GAACCTAG+TCCGCATA
AGATTGGTTACGGTAAAAGACAAGGCAAGGTATGGACCCTTAGCAAAGCCAGAAAGATCACCTTGCCC
AAAGAAGACCAACATTTTCATACATGATGAAATTCATTATTGGTCTCGCGATCAGCTTGAGACATTTC
TCCAGGTTTACAGAC
+
FFFFFFFFFFFFFFFF,FFFFFFFFFFFFFFFFFFFFFFFFFFFFFF,FFFFFFFFFFFFFFFFFFFFF
FFFFFFFFFFF:FFFFFFFFFFFFFFFFFFFFFFFFFFFF,FFFFFFFFFFFFFFFFF:FFFFFFFF:FF
FFFFFFFFFFFFFFF
@A00155:342:HHGFNDSXY:1:2271:10104:9126 2:N:0:GAACCTAG+TCCGCATA
AGATTGGTTACGGTAAAAGACAAGGCAAGGTATGGACCCTTAGCAAAGCCAGAAAGATCACCTTGCCC
AAAGAAGACCAACATTTTCATACATGATGAAATTCATTATTGGTCTCGCGATCAGCTTGAGACATTTC
TCCAGGTTTACAAAC
+
:FFFFFFFF:FFFF:F:FFFFFFFFFFFFFF:FFFFFFFFFFFFFFFFFFFFFFFFFFFFFFFFFFFF
FFFFFFFFFFFFFFF:F:FFFFFFFFFFFFFFFFFF,FFFFFFFFFFFFFFFFFFFFFFFFFFFFFFF
:FFFFFFFFFFFFFF
@A00155:342:HHGFNDSXY:1:2446:19714:10614 2:N:0:GAACCTAG+TCCGCATA
AGATTGGTTACGGTAAAAGACAAGGCAAGGTATGGACCCTTAGCAAAGCCAGAAAGATCACCTTGCCC
AAAGAAGACCAACATTTTCATACATGATGAAATTCATTATTGGTCTCGCGATCAGCTTGAGACATTTC
TCCAGGTTTAAAACC
+
FFFFFFFFFFFFFFFFFF:FFFFFFFFFFFFFFFFFFFFFFFFFFFFFFFFFFFFFFFFFFFFFFFFF
FFFFFFFFFFFFFFFF:FFFFFFFFFFFFFFFFFFFFF:FFFFFFFFFFFFFFFFFFFFFFFFFF:FFFF
FF:FFFFFFFFFFFF
@A00155:342:HHGFNDSXY:1:2247:1136:27665 1:N:0:GAACCTAG+TCCGCATA
GATTGGTTACGGTAAAAGACAAGGCAAGGTATGGACCCTTAGCAAAGCCAGAAAGATCACCTTGCCCA
AAGAACACCAACATTTTCATACATGATGAAATTCATTATTGGTCTCGCGATCAGCTTGAGACATTTCT
CCAGGTTTACAAACC
+
:F:F:,F:FFF,FFFFF,F:FF:FFFF:FFFFFFFFFF:FFFFFFF:FFFFFFFFFFFFFFF::FFFF
```

```
FF,FF,FFFFFFF,FF:FFFFFF,FFFFFFFFFFFFFFFFFF:FFFFFFFFFF:FF:FFFFFFFFFFF
FFFFFFFFFFFFFFF
@A00155:342:HHGFNDSXY:1:1358:11360:6261 2:N:0:GAACCTAG+TCCGCATA
GATTGGTTACGGTAAAAGACAAGGCAAGGTATGGACCCTTAGCAAAGCCAGAAAGATCACCTTGCCCA
AAGAAGACCAACATTTTCATACATGATGAAATTCATTATTGGTCTCGCGATCAGCTTGAGACATTTCT
CCAGGTTTACAAACC
+
FFFFFFFFFFFFFFF:FFFFFFFFFFFFFFFFFFFFFFFFFFFFFFFFFFFFFFFFFFFFFFFFFFFF
FFFFFFFFFFFFFFFFFFFFFFFFFFFFFFFFFFFFFFFFFFFFFFFFFFFFFFFFFFFFFFFFFFFF
FFFFFFFFFFFFFFF
@A00155:342:HHGFNDSXY:1:1474:30508:11584 2:N:0:GAACCTAG+TCCGCATA
GATTGGTTACGGTAAAAGACAAGGCAAGGTATGGACCCTTAGCAAAGCCAGAAAGATCACCTTGCCCA
AAGAAGACCAACATTTTCATACATGATGAAATTCATTATTGGTCTCGCGATCAGCTTGAGACATTTCT
CCAGGTTTACAAACC
+
FFFF:FF:FFFFFFFFFFFFFFFFFFFFFFFFFFFFFFFFFFFFFFFFFF:FFFFFFFFFFFFF:F
FFFFFFFFFFFFFFFFFFFFF:FFFFFFFF:FFFFFFFFFFFFFFFFFFFFFFFFFFFFFFFFFFFFF
FFFF:FF:FFFFF:F
@A00155:342:HHGFNDSXY:1:1520:1976:23672 2:N:0:GAACCTAG+TCCGCATA
GATTGGTTACGGTAAAAGACAAGGCAAGGTATGGACCCTTAGCAAAGCCAGAAAGATCACCTTGCTCA
AAGAATACCAACATTTTCATACATGATGAAATTCATTATTGGTCTCGCGATCAGCTTGAGACATTTCT
CCAGGTTTACAAACC
+
FFFFFFFFF:FFFF:FFFFFFFFFFFFFFFFFFFFFFFFFFFFFFFFFFFFFFFF,FFFFFFFFFFFF
FFFFF:FFFFFFFFFFFFFFFFFFFFFFF,FFFFFFFFFFFFFFFFFFFFFFFFFFFFFF:FFFFFFFF
FFFFFFFFFFFFFF:
@A00155:342:HHGFNDSXY:1:2103:16541:34835 2:N:0:GAACCTAG+TCCGCATA
GATTGGTTACGGTAAAAGACAAGGCAAGGTATGGACCCTTAGCAAAGCCAGAAAGATCACCTTGCCCA
AAGAAGACCAACATTTTCATACATGATGAAATTCATTATTGGTCTCGCGATCAGCTTGAGACATTTCT
CCAGGTTTACAAACC
+
FFFFFFFFFFFFFFFFFFFFFFFFFFFFFFFFFFFFFFFFF:FFFFFFFFFFFFFFFFFFFFFFFFFF
FFFFFFFFFFFFFFFFFFFFFFFFFFFFFFFFFFFFFFFFFFFFFFFFFFFFFFFFFFFFFFFFFFFF
:F:FFFFFFFFFF,F
@A00155:342:HHGFNDSXY:1:2319:30002:14747 2:N:0:GAACCTAG+TCCGCATA
GATTGGTTACGGTAAAAGACAAGGCAAGGTATGGACCCTTAGCAAAGCCAGAAAGATCACCTTGCCCA
AAGAAGACCAACATTTTCATACATGATGAAATTCATTATTGGTCTCGCGATCAGCTTGAGACATTTCT
CCAGGTTTACAAACC
+
FFFFFFFFFFFFFFFFFFFFFFFFFFFFFFFFFFFF:FFFFFFFFFFFFFFF,FFFFFFFFFFFFF
FFFFFFFFFFFFFFFFFFFFFFFFFFFFFFFFFFFF,FF:FFFFFFFFFFFFFFFFFFFFFFFFFFFFF
FFFFFFFFFFFFFFF
@A00155:342:HHGFNDSXY:1:2509:7102:31548 2:N:0:GAACCTAG+TCCGCATA
GATTGGTTACGGTAAAAGACAAGGCAAGGTATGGACCCTTAGCAAAGCCAGAAAGATCACCTTGCCCA
AAGAAGACCAACATTTTCATACATGATGAAATTCATTATTGGTCTCGCGATCAGCTTGAGACATTTCT
CCAGGTTTACAAACC
+
:F:FFFF:F,FF:FF:FFFFFFFF:FFF:FFFFFFFFF,FFFFFFFFFFFFFFFFFFFFFFFFF,,FF
FFFFFFFFFF,FFF:FF:FF:FFFFFFFFF:,::F:FFFFFFFFFFFFFFFF:FFFFF:FF:FFFFFF
F:FFFFFFF:FFF,F
@A00155:342:HHGFNDSXY:1:1117:25654:36464 2:N:0:GAACCTAG+TCCGCATA
ATTGGTTACGGTAAAAGACAAGGCAAGGTATGGACCCTTAGCAAAGCCAGAAAGATCACCTTGCCCAA
AGAAGACCAACATTTTCATACATGATGAAATTCATTATTGGTCTCGCGATCAGCTTGAGACATTTCTC
CAGGTTTACAAACCT
```

+
FFFFFFFFFFFFFFFF:F:FFFFFFFFFFF:FFFFFF:FFFFFFFFFFFFFFFF,FFFFFFFFFFFFF
FF:FFFFFFFFF:FFFFFFFFF:FFFFFFF:FFFFFFFFFFFFFFFFFFFFFFFFFFFFFFFFFF:FF
FFFFFFFFFFFFFFF
@A00155:342:HHGFNDSXY:1:1122:5186:5995 2:N:0:GAACCTAG+TCCGCATA
ATTGGTTACGGTAAAAGACAAGGCAAGGTATGGACCCTTAGCAAAGCCAGAAAGATCACCTTGCCCAA
AGAAGACCAACATTTTCATACATGATGAAATTCATTATTGGTCTCGCGATCAGCTTGAGACATTTCTC
CAGGTTTACAAACCT
+
FFFFFFFFFFFFFFFFFFFFFFFFF:F:FFFFFFFFFFFFFFFFFFFFFFFFFFFFFFFFFFFFFFFF
FFFFFFFFFFFFFFFFFFFFFFFFFFFFFFFFFFFFFFFF,FFFFFF:FFFFFFFFFF:FFFFFFFFFF
FFFFFFFFFFFF:FF
@A00155:342:HHGFNDSXY:1:1154:11388:16924 2:N:0:GAACCTAG+TCCGCATA
ATTGGTTACGGTAAAAGACAAGGCAAGGTATGGACCCTTAGCAAAGCCAGAAAGATCACCTTGCCCAA
AGAAGACCAACATTTTCATACATGATGAAATTCATTATTGGTCTCGCGATCAGCTTGAGACATTTCTC
CAGGTTTACAAACCT
+
FFFFFFFFFFFFFFF,:FFFFFFFFFFFFFFFFFFFFFFFFFFFFFFFFFFFFFFFFFFFFFFFFFFF
FFFFFFFFFFFFFFFFFFFFFFFFFFFFF:FFFFFFFFFFFFFFF,FFFFFFFFFFFFFFFFFFF:FF
FF,FFFFFFFFFFFF
@A00155:342:HHGFNDSXY:1:1174:18078:19053 2:N:0:GAACCTAG+TCCGCATA
ATTGGTTACGGTAAAAGACAAGGCAAGGTATGGACCCTTAGCAAAGCCAGAAAGATCACCTTGCCCAA
AGAAGACCAACATTTTCATACATGATGAAAGTCATTATTGGTCTCGCGATCAGCTTGAGACATTTCTC
CAGGGTTACAAACCT
+
FFFFFFFFFFFFFFFFFFFFFFFFFFFFFFFFFFFFFFFFFFFFFFFFFFFFFFF:FFFFFFF:FFFF
FF:F::FFFFFFFFFFFFFFFFFFFFF,F:,F:FFFFFFFF:FFFFFFFFFFFFFFF,FFFF:FFFFF
FFFF,F:FFFFFFFF
@A00155:342:HHGFNDSXY:1:1215:31801:14356 2:N:0:GAACCTAG+TCCGCATA
ATTGGTTACGGTAAAAGACAAGGCAAGGTATGGACCCTTAGCAAAGCCAGAAAGATCACCTTGCCCAA
AGAAGACCAACATTTTCATACATGATGAAATTCATTATTGGTCTCGCGATCAGCTTGAGACATTTCTC
CAGGTTTACAAACCT
+
FFFFF:FFFFFF::FFFFFFFFFFF:FFF,FFFFFFFFFFFFFFFFFFFFF:FFFFFFFFFFFFFF:F
FFFFFFFFFFFF,:FFFFFFFFFFFFFFFFF,FFFFFFFFFFFFFFFFFFFFFFFFFFFFFFFFFFFF
FFFFFFFFFFFFFFF
@A00155:342:HHGFNDSXY:1:1261:15718:1157 2:N:0:GAACCTAG+TCCGCATA
ATTGGTTACGGTAAAAGACAAGGCAAGGTATGGACCCTTAGCAAAGCCAGAAAGATCACCTTGCCCAA
AGAAGACCAACATTTTCATACATGATGAAATTCATTATTGGTCTCGCGATCAGCTTGAGACATTTCTC
CAGGTTTACAAACCT
+
FFFFFFFFFFFFFFFFFFF,FFFFFFFFF,FFFFFFFFFFFFFFFFFFFFFFFFFFFFFFFFFFFFFF,
FFFFFFFFFFFFF:FFFFFFFFFFFFFFF:FFFFFFFFFFFFFFFFFFF,FFFFFFFFFFF:FFFFFF
FFFFFFF:FFFFFFF
@A00155:342:HHGFNDSXY:1:1367:30563:17315 2:N:0:GAACCTAG+TCCGCATA
ATTGGTTACGGTAAAAGACAAGGCAAGGTATGGACCCTTAGCAAAGCCAGAAAGATCACCTTGCCCAA
AGAAGACCAACATTTTCATACATGATGAAATTCATTATTGGTCTCGCGATCAGCTTGAGACATTTCTC
CAGGTTTACAAACCT
+
FFFFFFFFFFFFFFFFFFF:FFFFFFFFFFFFFFFFFFFFFFFFFFFFFFFFFFFFFFFFFFFFFFFF
FFFFFFFFFFFFFFFFFFFFFFFFFFFFFFFFFFFFFFFFFFFFFFFFFFFFFFFFFFFFFFFFFFFF
FFFFFFFFFFFFFFF
@A00155:342:HHGFNDSXY:1:1458:26729:12837 2:N:0:GAACCTAG+TCCGCATA
ATTGGTTACGGTAAAAGACAAGGCAAGGTATGGACCCTTAGCAAAGCCAGAAAGATCACCTTGCCCAA

AGAAGACCAACATTTTCATACATGATGAAATTCATTATTGGTCTCGCGATCAGCTTGAGACATTTCTC
CAGGTTTACAAACCT
+
FFFFFFFFFFFFFF:FFFFFF:FFFFFFFFFFFFFFFFFFFF:FFFF:FFFFFFFFFFFFFFFF:FFFFF
FFFFFFFFFFFFFFFFFFFFFFFFFFFFFFFFFFFFFFFFFF:FFFFFFFFFFFFFFFFFFFFFFFFFFF
FFFFFFFF:FFFFFF
@A00155:342:HHGFNDSXY:1:2147:5556:3630 2:N:0:GAACCTAG+TCCGCATA
ATTGGTTACGGTAAAAGACAAGGCAAGGTATGGACCCTTAGCAAAGCCAGAAAGATCACCTTGCCCAA
AGAAGACCAACATTTTCATACATGATGAAATTCATTATTGGTCTCGCGATCAGCTTGAGACATTTCTC
CAGGTTTACAAACCT
+
FFFFFFFFFFFFFFFFFFFFFFFFFFFFFFFFFFFFFFFFFFFFFFFFFFFFFFFFFFFFFFFFFFFFFF
FFFFFFFFFFFFFFFFFFFFFFFFFFFFFFFFFFFFFFFFFFFFFFFFFFFFFFFFFFFFFF:FFFFFFF
FFFFFFFFFFFF:FF
@A00155:342:HHGFNDSXY:1:2214:20745:33160 2:N:0:GAACCTAG+TCCGCATA
ATTGGTTACGGTAAAAGACAAGGCAAGGTATGGACCCTTAGCAAAGCCAGAAAGATCACCTTGCCCAA
AGAAGACCAACATTTTCATACATGATGAAATTCATTATTGGTCTCGCGATCAGCTTGAGACATTTCTC
CAGGTTTACAAACCT
+
FFFFFFFFFFFFFFFFFFFFFFF:FFFFFFFFFFFFFFFFFFFFFFF:FFFFFFFFFFFFFFFF:FFFFF
FFFFFFFFFFFFFFFFFFFFFFFFFFFFFFFFFFFFFFFFFFFFFF:FFFFFFFFFFF:FFFFFFFFFFF
FFFFFFFFFFFFFFF
@A00155:342:HHGFNDSXY:1:2221:19045:24455 2:N:0:GAACCTAG+TCCGCATA
ATTGGTTACGGTAAAAGACAAGGCAAGGTATGGACCCTTAGCAAAGCCAGAAAGATCACCTTGCCCAA
AGAAGACCAACATTTTCATACATGATGAAATTCATTATTGGTCTCGCGATCAGCTTGAGACATTTCTC
CAGGTTTACAAACCT
+
FFFFFFFFFFFFFFFFFFFFFFFFFFFFFFFFFFFFFFFFFFFFFFFFFFFFFFFFFFFFFFFFFFFFFF
FFFFF:FFFFFFFFFFFFFFFFFFFFFFFFFFFFFFFFFFFFFFFFFFFFFFFFFFFFFFFFFFFFFFFF
:FFFFFFFFFFFFFF
@A00155:342:HHGFNDSXY:1:2452:9317:4820 2:N:0:GAACCTAG+TCCGCATA
ATTGGTTACGGTAAAAGACAAGGCAAGGTATGGACCCTTAGCAAAGCCAGAAAGATCACCTTGCCCAA
AGAAGACCAACATTTTCATACATGATGAAATTCATTATTGGTCTCGCGATCAGCTTGAGACATTTCTC
CAGGTTTACAAACCT
+
FFFFFFFFFFFFFFFFFFFFFFFFFFFFFFFFFFFFFFFFFFFFFFFFFFFFFFFF:FFFFFFFFFFFFF
FFFFFFFFFFFFFFFFFFFFFFFFFFFFFFFFFFFFFFFFFFFFFFFFFFFFFFFFFFFFFFFFFFFFFF
:FFFFFFFFFFFFFF
@A00155:342:HHGFNDSXY:1:2545:3052:6652 2:N:0:GAACCTAG+TCCGCATA
ATTGGTTACGGTAAAAGACAAGGCAAGGTATGGACCCTTAGCAAAGCCAGAAAGATCACCTTGCCCAA
AGAAGACCAACATTTTCATACATGATGAAATTCATTATTGGTCTCGCGATCAGCTTGAGACATTTCTC
CAGGTTTACAAACCT
+
FFFFFFFFFFFFFFF:FFFFFFFFFFFFFFFFFFFFFFFFFFFFFFFFFFFFFFFFFFFFFFFFFFFFFF
FFFFFFFF:FFFFFFFFFFFFFFFFFFFFFFFFFFFFFFFFF,FFFFFFF:FFFFFFFF:FFFFFFFFFF
FFFFFFFFFFFFFFF
@A00155:342:HHGFNDSXY:1:2541:14452:30248 2:N:0:GAACCTAG+TCCGCATA
ATTGGTTACGGTAAAAGACAAGGCAAGGTATGGACCCTTAGCAAAGCCAGAAAGATCACCTTGCCCAA
AGAAGACCAACATTTTCATACATGATGAAATTCATTATTGGTCTCGCGATCAGCTTGAGACATTTCTC
CAGGTTTACAAACCT
+
FFFFFFF:FFFFFFFFFFFFFFFFF:FFFFFFFFFFFFFFFFFFFFFFFFFFFFFFFFFFFFFFFFFFFF
FFFFFFFFFFFFFFFFFFFFFFFFFFFFFFFFFFFFFFFFFFFFFFFFFFFFFFFFFFFFFFFFFFFFFF
:FFF:FFFFFFFFFF

```
@A00155:342:HHGFNDSXY:1:2501:26657:3161 2:N:0:GAACCTAG+TCCGCATA
ATTGGTTACGGTAAAAGACAAGGCAAGGTATGGACCCTTAGCAAAGCCAGAAAGATCACCTTGCCCAA
AGAAGACCAACATTTTCATACATGATGAAATTCATTAATGGTCTCGCGATCAGCTTGAGACATTTCTC
CAGGTTTACAAACCT
+
FFFFFFFFFFFFFFFFFFFFFFFFFFFFFFFFFFFFFFFFFFFFFFFFFFFFFFFFFFFFFFFFFFFF
FFFFFFFFFFFFFFFFFFFFFFFFFFFFFFFFFFFF,FFFFFFFFFFFFFFFFFFFFFFFFFFFFFFF
FFFFFFFFFFFFFFF
@A00155:342:HHGFNDSXY:1:1142:10926:16313 2:N:0:GAACCTAG+TCCGCATA
GGTTACGGTAAAAGACAAGGCAAGGTATGGACCCTTAGCAAAGCCAGAAAGATCACCTTGCCCAAAGA
AGACTAACATTTTCATACATGATGAAATTCATTATTGGTCTCGCGATCAGCTTGAGACATTTCTCCAG
GTTTACAAACCTAAA
+
FFFFFFFFFFFFFFFFFFFFFFFFFFFFFFFFFFFFFFFFFFFFFFFFFFFFFFFFFFFFFFFFFFFF
FFFFFFFFFFFFFFFFFFFFFFFFFFFFFFFFFFFFFFFFFFFFFFFFFFFFFFFFFFFFFFFFFFFF
FFFFFFFFFFFFFFF
@A00155:342:HHGFNDSXY:1:2209:4517:15483 2:N:0:GAACCTAG+TCCGCATA
GGTTACGGTAAAAGACAAGGCAAGGTATGGACCCTTAGCAAAGCCAGAAAGATCACCTTGCCCCAAGA
AGACCAACATTTTCATACATGATGAAATTCATTATTGGTCTCGCGATCAGCTTGAGACATTTCTCCAG
GTTTACAAACCTAAA
+
FF,F:FFFFFFFFFFFFFFFFFFFF:FFFFFFF:FFF,FFFFFFFFFFFFFFFFFFFFFFFFFFFF,F
FF:FFFFFF:FFFFFFFFFFFFFFFF:FF:FFFFFFFFFFFF,FFFFFFFFFFFFFFFFFFFFFFFFF
FFFFFFFFFFFFFFF
@A00155:342:HHGFNDSXY:1:2209:4354:15890 2:N:0:GAACCTAG+TCCGCATA
GGTTACGGTAAAAGACAAGGCAAGGTATGGACCCTTAGCAAAGCCAGAAAGATCACCTTGCCCCAAGA
AGACCAACATTTTCATACATGATGAAATTCATTATTGTTCTCGCGATCAGCTTGAGACATTTCTCCAG
GTTTACAAACCTAAA
+
FFFFFFFFF:FFFFFFFFFFFFFFFF::FFFFFFFFFFF::FF:FF::FFF,FFFFFFFFFFFFFFF:
FFFFFFF:FF:FFFFFFFFFFFFFFFFFFFFFF:FFFF,FFFFFFFFFFFFFFFFFFFFFFFFFFFFF
FFFFFFFF:,,FFFF
@A00155:342:HHGFNDSXY:1:2550:22806:14403 2:N:0:GAACCTAG+TCCGCATA
GGTTACGGTAAAAGACAAGGCAAGGTATGGACCCTTAGCAAAGCCAGAAAGATCACCTTGCCCCAAGA
AGACCAACATTTTCATACATGATGAAATTCATTATTGGTCTCGCGATCAGCTTGAGACATTTCTCCAG
GTTTACAAACCTAAA
+
FFFFFFFFFFFFFFFFFFF:FFFFFFFFFFFFFFFFFFFFFFFFFFFFFFFFFFF,FFFFFFFFF:FF,F
FFFFFFFFFFFFFFFFFFFFFFFFFFF,F:FFFFFFFFFFFFFF:FFF:F:FFFFFFFFFFFFFFFFFF
FFF,F:FFFFFFFFF
@A00155:342:HHGFNDSXY:1:2603:1145:11083 2:N:0:GAACCTAG+TCCGCATA
GTTACGGTAAAAGACAAGGCAAGGTATGGACCCTTAGCAAAGCCAGAAAGATCACCTTGCCCAAAGAA
GACCAACATTTTCATACATGATGAAATTCATTATTTGTCTCGCGATCAGCTTGAGACATTTCTCCAGG
TTTACAAACCTA
+
FFF::FFFFF,FFFFFFFFFFF,FFFFF:FFFFF:FFFFF:,FFFFFFFF:FFFF,FFFFF:F,,::F
FF,F:FFF:,:FFFF:FFF:FF:F:FFF,FFFFF::F::FFF,F:FFFFFF:F:F:FFFFF:FFFFFF
::F:,F,,,:FF
@A00155:342:HHGFNDSXY:1:2543:21287:12931 2:N:0:GAACCTAG+TCCGCATA
GTTACGGTAAAAGACAAGGCAAGGTATGGACCCTTAGCAAAGCCAGAAAGATCACCTTGCCCAAAGAA
GACCAACATTTTCATACATGATGAAATTCATAATTGGTCTCGCGATCAGCTTGAGACATTTCACCAGG
TTTACAAACCTAAA
+
:::FFF,FFFFF:FFFFFFFFF,,,FF::FFF,FFFFFFFF,F,FFFFF,FF:F::F::F:FFFF::F
```

```
FFF,FF:F,,:FFFFF:FF,F::FFFFFFF,,F,:,:FFFF,,,:FFFFFF:FFFF:FF,FF,FFFFF
F,FFFFFFF,FFFF
@A00155:342:HHGFNDSXY:1:1141:14805:34679 2:N:0:GAACCTAG+TCCGCATA
GTTACGGTAAAAGACAAGGCAAGGTATGGACCCTTAGCAAAGCCAGAAAGATCACCTTGCCCAAAGAA
GACCAACATTTTCATACATGATGAAATTCATTATTGGTCTCGCGATCAGCTTGAGACATTTCTCCAGG
TTTACAAACCTAAAA
+
FFFFFFFFFFFFFFFFFFFFFFF:FFFFFFFFFFFFFFFFFFFFF:FFFF:FFFFFF:FFFFFF:F:F
FFFFFFFFFFFFFFFFFFFFFFFFFFFFFFFFFFFFFFFFFFFFFFFFFFFFFFFFFFFFFFFF:FFF
FFFFFFFFFFFFFFF
@A00155:342:HHGFNDSXY:1:1167:27444:12226 2:N:0:GAACCTAG+TCCGCATA
GTTACGGTAAAAGACAAGGCAAGGTATGGACCCTTAGCAAAGCCAGAAAGATCACCTTGCCCAAAGAA
GACCAACATTTTCATACATGATGAAATTCATTATTGGTCTCGCGATCAGCTTGAGACATTTCTCCAGG
TTTACAAACCTAAAA
+
FFFFFFFFFFFFFFFFFFFFFFFFFFFFFFFFFFFFFFFFFFFFFFFFFFFFFFFFFFFFFFFFFFFF
FFFFFFFFFFFFFFFFFFFFFFFFFFFFFFFFFFFFFFFFFFFFFFFFFFF,,FFFFFFFFFFFFFFF
FFFFFF:FF:FFFFF
@A00155:342:HHGFNDSXY:1:1333:25319:26647 2:N:0:GAACCTAG+TCCGCATA
GTTACGGTAAAAGACAAGGCAAGGTATGGACCCTTAGCAAAGCCAGAAAGATCACCTTGCCCAAAGAA
GACCAACATTTTCATACATGATGAAATTCATTATTGGTCTCGCGATCAGCTTGAGACATTTCTCCAGG
TTTACAAACCTAAAA
+
FFFFFFFF:FFFFFFFFFFFF:FFFFFFFFFFFFFFFFFFFFFFFFFFFFFFFFFFFFFFFFFFFFFF
FFFFFFFFFFFFFFFFFFFFFFFFF:FFFFFFFFFFFFFFFFFFFFFFFFFFFFFFFFFFFFFFF:FF
FFFFFFFFFFFFF,F
@A00155:342:HHGFNDSXY:1:1268:11496:36652 2:N:0:GAACCTAG+TCCGCATA
GTTACGGTAAAAGACAAGGCAAGGTATGGACCCTTAGCAAAGCCAGAAAGATCACCTTGCCCAAAGAA
GACCAACATTTTCATACATGATGAAATTCATTATTGGTCTCGCGATCAGCTTGAGACATTTCTCCAGG
TTTACAAACCTAAAA
+
FFFFFFFFFFFFFFFFFFFFFFFFFFFFFFFFFFFFFFFFFFFFFF:FFFFFFFFFFFFFFFFFFFFF
FFFFFFFFFFFF:FFFFFFFFFFFF:FFFFFFFFFF:FFFFFFFFFFFFFFFFFFFFFFFFFFFFFFF
FFFFFFFFFFFFFFF
@A00155:342:HHGFNDSXY:1:1464:25545:4460 2:N:0:GAACCTAG+TCCGCATA
GTTACGGTAAAAGACAAGGCAAGGTATGGACCCTTAGCAAAGCCAGAAAGATCACCTTGCCCAAAGAA
GACCAACATTTTCATACATGATGAAATTCATTATTGGTCTCGCGATCAGCTTGAGACATTTCTCCAGG
TTTACAAACCTAAAA
+
FFFFFFFFFFFFFFFFFFFF:FFFFFFFFFFFFFFFFFFFFFFFFFFFFFFFFFFF:FFFFFFFFFFF
FFFFFFFFFFFFFFFFFFFFFFFFFFFFFFFFFFFFFFFFFFFFFFFFF:FFFFFFFFFFFFFFFFFF
FFFFFFFFFFF,FFF
@A00155:342:HHGFNDSXY:1:2178:10682:2957 2:N:0:GAACCTAG+TCCGCATA
GTTACGGTAAAAGACAAGGCAAGGTATGGACCCTTAGCAAAGCCAGAAAGATCACCTTGCCCAAAGAA
GACCAACATTTTCATACATGATGAAATTCATTATTGGTCTCGCGATCAGCTTGAGACATTTCTCCAGG
TTTACAAACCTAAAA
+
FFFFFFFF:FFFFFFFFFFFFFFFF,FFFFFFFFFFFFFFFFFFFFFFFFFFFFFFFFFFFFFFFFFF
FFFFFFFFFFFFFFFFFFFFFFFFFFFFFFFFFFFFFFFFFFFFFFFFFFFFFFFFFFFFFFFFFFFF
:FFFFF:FFFFFFFF
@A00155:342:HHGFNDSXY:1:2539:23710:4006 2:N:0:GAACCTAG+TCCGCATA
GTTACGGTAAAAGACAAGGCAAGGTATGGACCCTTAGCAAAGCCAGAAAGATCACCTTGCCCAAAGAA
GACCAACATTTTCATACATGATGAAATTCATTATTGGTCTCGCGATCAGCTTGAGACATTTCTCCAGG
TTTACAAACCTAAAA
```

+
FFF:FFF,FFFFFFFF:FFFFFFFFFFFFFFFFFFFFFFFFFFFFFFFFFFFFFFFFFFFFFFFFFFF
FFFFFFFFFFFF:FFFFFFFFFFFFFFFFFFFFFFFFFFFFFFFFFFFFFFFFFFFFFFFFFFFFFF
FFFFFFFFFFF:FFF
@A00155:342:HHGFNDSXY:1:1207:20708:2754 2:N:0:GAACCTAG+TCCGCATA
TACGGTAAAAGACAAGGCAAGGTATGGACCCTTAGCAAAGCCAGAAAGATCACCTTGCCCAAAGAAGA
CCAACATTTTCATACATGATGAAATTCATTATTGGTCTCGCGATCAGCTTGAGACATTTCTCCAGGTT
TACAAACCTAAAAAT
+
FFFFFFFF:FFFFFFFFFFFFFFFFFFFFFFFFFFFFFFFFFFFFFFFFFF:FFFFFFFFFF,FFFFF
FFFFF:FF:FFFFFFFFFFFFFF,FFFFF,F:FFFFFFFFFFFFFFFFFFFFFFFFFFFFFFFFFFFF
FFFF:FFFF,FF:FF
@A00155:342:HHGFNDSXY:1:2456:26838:3192 1:N:0:GAACCTAG+TCCGCATA
CGGTAAAAGACAAGGCAAGGTATGGACCCTTAGCAAAGCCAGAAAGATCACCTTGCCCAAAGAAGACC
AACATTTTCATACATGATGAAATTCATTATTGGTCTCGCGATCAGCTTGAGACATTTCTCCAGGTTTA
CAAACCTAAAAATTT
+
FFFFFFFFFFF:FFFFFFFFFFFFFFFFFFFFFFFFFFFFFFFFFFFFFFFFFFFFFFFFFF::FF:F
FFFFFFFFFFFFFFFFFFFFFFFFFFFFFFFFFFFFFFFFFFFFFFFFFFFFFFFFFFFFFFFFFFFF
FFFFFFFFFFFFFFF
@A00155:342:HHGFNDSXY:1:2528:13512:12712 1:N:0:GAACCTAG+TCCGCATA
CGGTAAAAGACAAGGCAAGGTATGGACCCTTAGCAAAGCCAGAAAGATCACCTTGCCCAAAGAAGACC
AACATTTTCATACATGATGAAATTCATTATTGGTCTCGCGATCAGCTTGAGACATTTCTCCAGGTTTA
CAAACCTAAAAATTT
+
F:FFFFFFFFF:FFFFFFFFFFFFFFFFFFFFFFFFFFFFFFFFFFFFFFFFFFFFFFFFFFFF,FFF
FFFFFFFFFFFFFFFFFFFFFFFFFFFFFFFFFFFFFFFFFFFFFFFFFFFFFFFFFF:FFFFFFFFF
FFFFFFFFFFFFFFF
@A00155:342:HHGFNDSXY:1:1362:18394:16000 2:N:0:GAACCTAG+TCCGCATA
CGGTAAAAGACAAGGCAAGGTATGGACCCTTAGCAAAGCCAGAAAGATCACCTTGCCCAAAGAAGACC
AACATTTTCATACATGATGAAATTCATTATTGGTCTCGCGATCAGCTTGAGACATTTCTCCAGGTTTA
CAAACCTAAAAATTT
+
FFFFFFFFFFFFFFFFF:FFFFFFFFFFFFFFFFFFFFFFFFFFFFFFFFFFFFFFFFFFFFFFF:FF
FFFFFFFFFFFFFFFFFFFFFFFFFFFFFFFFFFFFFFFFFFFFFF:FFFFFFFFFFFFFFFFFF:FF
FFFF,FFFFFFFFFF
@A00155:342:HHGFNDSXY:1:1548:20139:8249 2:N:0:GAACCTAG+TCCGCATA
CGGTAAAAGACAAGGCAAGGTATGGACCCTTAGCAAAGCCAGAAAGATCACCTTGCCCAAAGAAGACC
AACATTTTCATACATGATGAAATTCATTATTGGTCTCGCGATCAGCTTGAGACATTTCTCCAGGTTTA
CAAACCTAAAAATTT
+
FFFFFFFFFFFFFFFFFF:FFFFFFFFFFFFFFFFFFFFFFFFFFFFFFFFFFFFFFFFFFFFFFFFF
FFFFFFFFFFFFFFFFFFFFFFFFFFFFFFFFFFFFFFFFFFFFFFFFFFFFFF:FFFFFFFFFFFF
F,FFFFFFFFFFFFF
@A00155:342:HHGFNDSXY:1:2541:27814:28087 2:N:0:GAACCTAG+TCCGCATA
CGGTAAAAGACAAGGCAAGGTATGGACCCTTAGCAAAGCCAGAAAGATCACCTTGCCCAAAGAAGACC
AACATTTTCATACATGATGAAATTCATTATTGGTCTCGCGATCAGCTTGAGACATTTCTCCAGGTTTA
CAAACCTAAAAATTT
+
FFFFF:FFFFFFFFFFFFFFFFFFFFFFFFFFFFFFFFFFFFFFFFFFFFFFFFFFFFFFFFFFFFFF
FFFFFFFFFFFFFFFFFFFFFFFFFFFFFFFFFFFFF:FFFFFFFFFFFFFFFFFFFFFFFFFFFFFF
FFFFFFFFFFFFFFF
@A00155:342:HHGFNDSXY:1:1111:26657:15060 1:N:0:GAACCTAG+TCCGCATA
GGTAAAAGACAAGGCAAGGTATGGACCCTTAGCAAAGCCAGAAAGATCACCTTGCCCAAAGAAGACCA

```
ACATTTTCATACATGATGAAATTCATTATTGGTCTCGCGATCAGCTTGAGACATTTCTCCAGGTTTAC
AAACCTAAAAATTT
+
F,FFFFFFFFFFFFFFFFFFFFFFFFFFFFFFFFFFFFFFFFFFFFFFFFFFFFFFFFFFFFFFFFFF
FFFFFFFFFFFFFFFFFFFFFFFFFFFFFFFFFFFFFFFFFFFFFFFFFFFFFFFFFFFFFFFFFFFF
FFFFFFFFFFFFFF
@A00155:342:HHGFNDSXY:1:1154:2202:2519 2:N:0:GAACCTAG+TCCGCATA
GGTAAAAGACAAGGCAAGGTATGGACCCTTAGCAAAGCCAGAAAGATCACCTTGCCCAAAGAAGACCA
ACATTTTCATACATGATGAAATTCATTATTGGTCTCGCGATCAGCTTGAGACATTTCTCCAGGTTTAC
AAACCTAAAAATTT
+
FFFFFFFFFFFFFFFFFFFFFFFFFFFFFFFFFFFFFFFFFFFFFFFFFFFFFFFFFFFFFFFF:FFF
:FFFFFFFFFFFFFFFFFFF,FFFFFFFFFF:FFFF:FFFFFFFFFFFFFFFFFFFFFFFFF:FFFFFF
FFFFFF,F,FFFFF
@A00155:342:HHGFNDSXY:1:1114:10050:23469 1:N:0:GAACCTAG+TCCGCATA
GGTAAAAGACAAGGCAAGGTATGGACCCTTAGCAAAGCCAGAAAGATCACCTTGCCCAAAGAAGACCA
ACATTTTCATACATGATGAAATTCATTATTGGTCTCGCGATCAGCTTGAGACATTTCTCCAGGTTTAC
AAACCTAAAAATTTG
+
FFFFFFFFFFFFFFFFFFFFFFFFFFFFFFFFFFFFFFFFFFFFFFFFFFFFFFFFFFFFFFFF:FFF
FFFFFFFFFFFFFFFFFFFFFFFFFFF:FFFFFFFFFFFFFFFFFFFFFFFFFFFFFFFFFFFFFFFFF
FFFFFFFFFFFFFFF
@A00155:342:HHGFNDSXY:1:1219:4273:7921 1:N:0:GAACCTAG+TCCGCATA
GGTAAAAGACAAGGCAAGGTATGGACCCTTAGCAAAGCCAGAAAGATCACCTTGCCCAAAGAAGACCA
ACATTTTCATACATGATGAAATTCATTATTGGTCTCGCGATCAGCTTGAGACATTTCTCCAGGTTTAC
AAACCTAAAAATTTG
+
FFFFFFFFFFFFF:FFFFFFFFFFF,FFFFFFFFFFFFFFFFFFFFFFFFFFFFFFFFFFFF:F:FFFF
FFFFFFFFFFF:FFFFFFFFFFFFFFFFFF:FFFFFFFFFFF:FFFFFFFFFFFFFFFFFFFFFFFFFF
FFFFFFFFFFFFFFF
@A00155:342:HHGFNDSXY:1:1273:4119:21527 1:N:0:GAACCTAG+TCCGCATA
GGTAAAAGACAAGGCAAGGTATGGACCCTTAGCAAAGCCAGAAAGATCACCTTGCCCAAAGAAGACCA
ACATTTTCATACATGATGAAATTCATTATTGGTCTCGCGATCAGCTTGAGACATTTCTCCAGGTTTAC
AAACCTAAAAATTTG
+
FFFFFFFFFFFFFFFFFFFFFFFFFFFFFFFFFFFFFFFFFFFFFFFFFFF:FFFFFFFFFF:F:FFF
FFFFFFFFFFFF:FFFFFFFFFFFFFFFFFFFFF,FFFFFFFF:FFFFFFFFFFFFFFFFFFFFFFFFFF
FFFFFFFFFFFFFFF
@A00155:342:HHGFNDSXY:1:1373:27335:31454 1:N:0:GAACCTAG+TCCGCATA
TCTAAAAGACAAGGCAAGGTATGGACCCTTAGCAAAGCCAGAAAGATCACCTTGCCCAAAGAAGACCA
ACATTTTCATACATGATGAAATTCATTATTGGTCTCGCGATCAGCTTGAGACATTTCTCCAGGTTTAC
AAACCTAAAAATTTG
+
FFFFFFFFFF:FF:FFFFFFFFFFFFFFFFFFFFFFFFF:FFFFFFFFFFFFFFFFFFFFFFF:F:FFFF
FFFFFFFFFFFFFFFFFFFFFFFFFFFFFFFFFFFFF:FFFFFFFFFFFFFFFFFFFFFFFFFFFFFFFF
FFFFFFFFFFFFFFF
@A00155:342:HHGFNDSXY:1:1407:31096:10097 1:N:0:GAACCTAG+TCCGCATA
GGTAAAAGACAAGGCAAGGTATGGACCCTTAGCAAAGCCAGAAAGATCACCTTGCCCAAAGAAGACCA
ACATTTTCATACATGATGAAATTCATTATTGGTCTCGCGATCAGCTTGAGACATTTCTCCAGGTTTAC
AAACCTAAAAATTTG
+
FFFFFFFFFFFFFFFFFFFFFFFFFFFFFFFFFFF:FFFFFFFFFFFFFFFFFF:FFFFFFFFFFFFFF
FFFFFFFFFFFFFFFFFFFFFFFFFFFFFFFFFFFFFFFFFFFFFFFFFFFFFFFFFFFFFFFFFFFFF
FFFFF:FFFFF,FFF
```

```
@A00155:342:HHGFNDSXY:1:1436:13874:19820 1:N:0:GAACCTAG+TCCGCATA
CCGATCTGACAAGGCAAGGTATGGACCCTTAGCAAAGCCAGAAAGATCACCTTGCCCAAAGAAGACCA
ACATTTTCATACATGATGAAATTCATTATTGGTCTCGCGATCAGCTTGAGACATTTCTCCAGGTTTAC
AAACCTAAAAATTTG
+
FFFFFFFFFFFFFFFFFFFFFFFFFFFFFFFFFF,FFFFFFFFFFFFFFFFFFFFFFFFFFFFFFFFF
FFFFFFFF,FFFFFFFFFFFFFFFFFFFFFFFFFFFFFFFFFFFFFFFFFFFFFFFFFFFFFFFFFF
FFFFFFFFFFFFFFF
@A00155:342:HHGFNDSXY:1:1529:28420:31735 1:N:0:GAACCTAG+TCCGCATA
GGTAAAAGACAAGGCAAGGTATGGACCCTTAGCAAAGCCAGAAAGATCACCTTGCCCAAAGAAGACCA
ACATTTTCATACATGATGAAATTCATTATTGGTCTCGCGATCAGCTTGAGACATTTCTCCAGGTTTAC
AAACCTAAAAATTTG
+
FFFF:FFFFF:FFFFFF:FFFFFFFF:FFFFFFFFFFFFFFFFFFFFFFFFFFFFFFFFFFFFFFFFF
FFFFFFFFFFFFFFFFFFFFFFFFFFFF:FFFFFFFFFFFFFFFFFFFFFFFF:FFFFFFFFFFFFFF
FFFFFFFFFFFFFFF
@A00155:342:HHGFNDSXY:1:1629:11514:36401 1:N:0:GAACCTAG+TCCGCATA
GGTAAAAGACAAGGCAAGGTATGGACCCTTAGCAAAGCCAGAAAGATCACCTTGCCCAAAGAAGACCA
ACATTTTCATACATGATGAAATTCATTATTGGTCTCGCGATCAGCTTGAGACATTTCTCCAGGTTTAC
AAACCTAAAAATTTG
+
FFFFFFFFFFFFFFFFFFFFFFFFFFFFFFFFFFFFFFFFFFFFFFFFFFFFFFFFFFFFFFFFFFFF
FFFFFFFFFFFFFFFFFFFFFFFFFFFFFFFFFFFFFFF,FFFFFFFFFFFFFFFFFFFFFFFFFFFF
FFFFFFFFFFFFFFF
@A00155:342:HHGFNDSXY:1:2205:16125:15076 1:N:0:GAACCTAG+TCCGCATA
GGTAAAAGACAAGGCAAGGTATGGACCCTTAGCAAAGCCAGAAAGATCACCTTGCCCAAAGAAGACCA
ACATTTTCATACATGATGAAATTCATTATTGGTCTCGCGATCAGCTTGAGACATTTCTCCAGGTTTAC
AAACCTAAAAATTTG
+
F,FFFF:FFFFFFFFFFFFFFFFFFFFFF:FFFF:FFFFFFFF:::FFFFF,FFFFFF:FFF:::FFF
FFFFFFFFFFFFFFFFFFFFFFFFFFFFFFFFFFFFFFFFFF:FFFFFFFFFFFFFFFFFFFFFFFFF
FFFFFFFFFFFFFFF
@A00155:342:HHGFNDSXY:1:2212:14696:14888 1:N:0:GAACCTAG+TCCGCATA
GGTAAAAGACAAGGCAAGGTATGGACCCTTAGCAAAGCCAGAAAGATCACCTTGCCCAAAGAAGACCA
ACATTTTCATACATGATGAAATTCATTATTGGTCTCGCGATCAGCTTGAGACATTTCTCCAGGTTTAC
AAACCTAAAAATTTG
+
FFFFFFFFFFFFFFFFFFFFFFFFFFFFFFFFFFFFFFFFFFFFFFFFFFFFFFFFFFFFFF:FFFFF
FFFFFFFFFFFFFFFFFFFFFFFFFFFFFFFFFFFFFFFFFFFFFFFFFFFFFFFFFFFFFFFFFFFF
FFFFFFFFFFFFFFF
@A00155:342:HHGFNDSXY:1:2266:7021:32597 1:N:0:GAACCTAG+TCCGCATA
GGTAAAAGACAAGGCAAGGTATGGACCCTTAGCAAAGCCAGAAAGATCACCTTGCCCAAAGAAGACCA
ACATTTTCATACATGATGAAATTCATTATTGGTCTCGCGATCAGCTTGAGACATTTCTCCAGGTTTAC
AAACCTAAAAATTTG
+
FFFFFFFFFFFFFFFFFF:FFF:FFFFFFFFFFFFFFFFFFFFFFFFFFFFFFFFFFFFF:FFFFFFF
FFFFFFFFF:FFFFFFFFFF:FFFFFFFFFFFFFFFFF:FFFFFFFFFFFFFFFFFFFFFFFFFFFFF
FFFFFFFFFFFFFFF
@A00155:342:HHGFNDSXY:1:2266:6958:32675 1:N:0:GAACCTAG+TCCGCATA
GGTAAAAGACAAGGCAAGGTATGGACCCTTAGCAAAGCCAGAAAGATCACCTTGCCCAAAGAAGACCA
ACATTTTCATACATGATGAAATTCATTATTGGTCTCGCGATCAGCTTGAGACATTTCTCCAGGTTTAC
AAACCTAAAAATTTG
+
F,FFFF:F:FF,:F,,FFFFFFFFF:FFF:FFFFFFFFFFFFF:FFFFFF:,FF:,FFFFFF,:FFFF
```

```
FFFFFFFFFFFFFFFFFFFFFFF:FFFFFFFFFFFF,FFFFFFFFFFFFFFFFFFFF,F:FFF:F:FFF
FF:FFF,FFF:FFFF
@A00155:342:HHGFNDSXY:1:2249:9046:19100 1:N:0:GAACCTAG+TCCGCATA
GGTAAAAGACAAGGCAAGGTATGGACCCTTAGCAAAGCCAGAAAGATCACCTTGCCCAAAGAAGACCA
ACATTTTCATACATGATGAAATTCATTATTGGTCTCGCGATCAGCTTGAGACATTTCTCCAGGTTTAC
AAACCTAAAAATTTG
+
FFFFFFFFFFFFFFFFFFFFFFFFFFFFFFFFFFFFFFFFFFFFFFFFFFFFFFFFFFFFFFFFFFFF
FFFFFFFFFFFFFFFFFFFFFFFFFFFFFFFFFFFFFFFFFFFFFFFFFFFFFFFFFFFFFFFFFFFF
FFFFFFFFFFFFFFF
@A00155:342:HHGFNDSXY:1:2337:18566:12853 1:N:0:GAACCTAG+TCCGCATA
GGTAAAAGACAAGGCAAGGTATGGACCCTTAGCAAAGCCAGAAAGATCACCTTGCCCAAAGAAGACCA
ACATTTTCATACATGATGAAATTCATTATTGGTCTCGCGATCAGCTTGAGACATTTCTCCAGGTTTAC
AAACCTAAAAATTTG
+
F:FFFFFFFFFFFFFFFFFFFFFFFFFFFFFFFFFFFFFFFFFFFFFFFFFFFFFFFFFF:FF:FFFF
FFFFFFFFFFFFFFFFFFFFFFFFFFFFFFFFFFFFFFFFFFFFFFFFFFFFFFFFFFFFFFFFFFFF
FFFFFFFFFFFFFFF
@A00155:342:HHGFNDSXY:1:1521:19967:1814 2:N:0:GAACCTAG+TCCGCATA
GGTAAAAGACAAGGCAAGGTATGGACCCTTAGCAAAGCCAGAAAGATCACCTTGCCCAAAGAAGACCA
ACATTTTCATACATGATGAAATTCATTATTGGTCTCGCGATCAGCTTGAGACATTTCTCCAGGTTTAC
AAACCTAAAAATTTG
+
FFFFFFFFFFFFFFFFFFFFFFFFFFFFFFFFFFFFFFFFFFFFFFFFFFFFFFFFFFFFFFFFFFFF
FFFFFFFFFFFFFFFFFFFFFFFFFFFFFFFFFFFFFFFFFFFFFFFFF:FFFFFFFFFFFFFFFFFF
FFFFFFFFFFFFFFF
@A00155:342:HHGFNDSXY:1:2142:3459:15562 2:N:0:GAACCTAG+TCCGCATA
TGTAAAAGACAAGGCAAGGTATGGACCCTTAGCAAAGCCAGAAAGATCACCTTGCCCAAAGAAGACCA
ACATTTTCATACATGATGAAATTCATTATTGGTCTCGCGATCAGCTTGAGACATTTCTCCAGGTTTAC
AAACCTAAAAATTTG
+
FFFFFFFFFFFFFFFFFFFFFFFFFFFFF:FFFFFFFF:FFFFFFFFFFFFF::FFFFFFFFFFFFFF
FFFFFFFFFFFFFFFFFFFFFFFFFFFFFFFFFFFFFFFFFFFFFFFFFFFFFFFFFFFFFFFFFFFF
:FFFFFFFFFFFFFF
@A00155:342:HHGFNDSXY:1:2545:5113:22874 1:N:0:GAACCTAG+TCCGCATA
GTAAAAGACAAGGCAAGGTATGGACCCTTAGCAAAGCCAGAAAGATCACCTTGCCCAAAGAAGACCAA
CATTTTCATACATGATGAAATTCATTATTGGTCTCGCGATCAGCTTGAGACATTTCTCCAGGTTTACA
AACCTAAAAATTTG
+
FFF:FFFFFFF,FFFFF,FF,:FFFFFF:FFFFFFFFFFFFFFFFFFFFFF,FFFFFF,F:FF::FFFF
FFFFFFFFFFFFFFFFFFFF,FFFFFFFFFFFFFFFFFFFFFFFFFFFFFFFFFFFFFFFFFFFFFFF
F,FFFFFFFFFFFF
@A00155:342:HHGFNDSXY:1:2539:9923:4554 1:N:0:GAACCTAG+TCCGCATA
CTAAAAGACAAGGCAAGGTATGGACCCTTAGCAAAGCCAGAAAGATCACCTTGCCCAAAGAAGACCAA
CATTTTCATACATGATGAAATTCATTATTGGTCTCGCGATCAGCTTGAGACATTTCTCCAGGTTTACA
AACCTAAAAATTTG
+
FFFFFFFFFFFFFFF:,FFFFFFF:FFFFFFFFFFFFFFFF:FFFFFFFFFFFFFFFFFFFF:FFFF,
FF:FFFFFFFFFFFFFFFFFFFFFFFFFFFFFFFFFFFFFFFFFFFFFFFFFFFFFFFFFFFFFFFFF
F:FFFFFFFFFFFF
@A00155:342:HHGFNDSXY:1:1128:32832:16078 1:N:0:GAACCTAG+TCCGCATA
GTAAAAGACAAGGCAAGGTATGGACCCTTAGCAAAGCCAGAAAGATCACCTTGCCCAAAGAAGACCAA
CATTTTCATACATGATGAAATTCATTATTGGTCTCGCGATCAGCTTGAGACATTTCTCCAGGTTTACA
AACCTAAAAATTTGT
```

+
FFFFFFFFFFFFFFFFF:FFFFFFFFFFFFFFFF:FFFFF:FFFFFFFFF:FFFFFF:,FFFF,FFFF
FFFFFFFFFFFFFFFFF:FFFFFFFFFFFFFFFFFFFFFFF:FFFFFFFFFFFFFFFFFFFFFFFFFF
F:FFFFFFFFFFF:F
@A00155:342:HHGFNDSXY:1:1213:22137:17127 1:N:0:GAACCTAG+TCCGCATA
GTAAAAGACAAGGCAAGGTATGGACCCTTAGCAAAGCCAGAAAGATCACCTTGCCCAAAGAAGACCAA
CATTTTCATACATGATGAAATTCATTATTGGTCTCGCGATCAGCTTGAGACATTTCTCCAGGTTTACA
AACCTAAAAATTTGT
+
FFFFFFFFFFFFFFFFFFF:FFFFFFFFFFFFFFFFFFFFFFFFFFFFFFFFFFFFFFFFFFFFFFFF
FFFFFFFFFFFFFFFFFFFFFFFFFFFFFFFFF:FFFFFFFFFFFFFFFFFFFFFFFFFFFFFFFFFF
FFFFFFFFFFFFFFF
@A00155:342:HHGFNDSXY:1:1236:5611:21355 1:N:0:GAACCTAG+TCCGCATA
GTAAAAGACAAGGCAAGGTATGGACCCTTAGCAAAGCCAGAAAGATCACCTTGCCCAAAGAAGACCAA
CATTTTCATACATGATGAAATTCATTATTGGTCTCGCGATCAGCTTGAGACATTTCTCCAGGTTTACA
AACCTAAAAATTTGT
+
FFFFF:FFFFFFFFFFFFF:FFFFFFFFFFFFFFFFFFFFFFFF:FFFFFFFFFFFFFFFFFFFFFFF
FF:FFFFFFFFF,:FFFFFFFFFFFFFFFFFFFFFFFFFFFFFFFFFFFF,FFFFFFFFFFFFFFFFF
FFFFFFFFFFFFFF:
@A00155:342:HHGFNDSXY:1:1246:6189:11083 1:N:0:GAACCTAG+TCCGCATA
GTAAAAGACAAGGCAAGGTATGGACCCTTAGCAAAGCCAGAAAGATCACCTTGCCCAAAGAAGACCAA
CATTTTCATACATGATGAAATTCATTATTGGTCTCGCGATCAGCTTGAGACATTTCTCCAGGTTTACA
AACCTAAAAATTTGT
+
FFFFFFFFFFFFFFFFFFFFFFFFFFFFFFFFFFFFFFFFFFFFFFFFFFFFFFFFFFFFFFFFFFFF
FFFFFFFFFFFFFFFFFFFFFFFFFFFFFFFFFFFFFFFFFFFFFFFFFFFFFFFFFFFFFFFFFFFF
FFFFFFFFFFFFFFF
@A00155:342:HHGFNDSXY:1:1253:27416:19413 1:N:0:GAACCTAG+TCCGCATA
GTAAAAGACAAGGCAAGGTATGGACCCTTAGCAAAGCCAGAAAGATCACCTTGCCCAAAGAAGACCAA
CATTTTCATACATGATGAAATTCATTATTGGTCTCGCGATCCGCTTGAGACATTTCTCCAGGTTTACA
AACCTAAAAATTTGT
+
FFFFFFFFFFFFFFFFFFFFFFFFFF:FFFFFFFFFFFFFFFFFFFFFFF:FFFFFFF,FFFFFFFFF
FF:FFFFFFFFFFFFFFFF:FFFFFFFFFFFFFFFFFFFFF,FFFFFFFFFFFFFFFFFFFFFFFFFF
FFFFFFFFFFFFFFF
@A00155:342:HHGFNDSXY:1:1412:9037:21809 1:N:0:GAACCTAG+TCCGCATA
GTAAAAGACAAGGCAAGGTATGGACCCTTAGCAAAGCCAGAAAGATCACCTTGCCCAAAGAAGACCAA
CATTTTCATACATGATGAAATTCATTATTGGTCTCGCGATCAGCTTGAGACATTTCTCCAGGTTTACA
AACCTAAAAATTTGT
+
FFFFFFFFFF,FFFFFFFFFFFFFFFFFFFFFFFFFFFFFFFFFFFFFFF:FFFFFFFFFF:FFFFFF
FFFFFFFFFFFFFFFFFFFFFFFFFFFFFFFFFFFFFFFFFFFFFFFFFFFFFFFFFFFFFFFFFFFF
FFFFFFFFFFFFFFF
@A00155:342:HHGFNDSXY:1:1437:26937:27477 1:N:0:GAACCTAG+TCCGCATA
GTAAAAGACAAGGCAAGGTATGGACCCTTAGCAAAGCCAGAAAGATCACCTTGCCCAAAGAAGACCAA
CATTTTCATACATGATGAAATTCATTATTGGTCTCGCGATCAGCTTGAGACATTTCTCCAGGTTTACA
AACCTAAAAATTTGT
+
FFFF:FFFF:FFFFF:FFFFFFFFFFFFFFFFFFFF:FFFFFFFFFFFFFFFFFFFFFFFFFFFFFFF
FFFFFFFFFFFFF:FFFFFFFFFFFFFFFFFFFFFFFFFFFFFFFFFFFFFFFFFFFFFFFFFFFFFF
FFFFFFFFFFFFFFF
@A00155:342:HHGFNDSXY:1:1453:3224:31407 1:N:0:GAACCTAG+TCCGCATA
GTAAAAGACAAGGCAAGGTATGGACCCTTAGCAAAGCCAGAAAGATCACCTTGCCCAAAGAAGACCAA

```
CATTTTCATACATGATGAAATTCATTATTGGTCTCGCGATCAGCTTGAGACATTTCTCCAGGTTTACA
AACCTAAAAATTTGT
+
FFFFFFFFFFF::FFF,FFF,FFFFFFFFFFFFFF:FFFFFFFFFFFFFF::FFFFFF:F:F:,FFFF
FFFFFFFFFFFFFFFF:FFFFFFFFFFFFF:FF:FFFF:FFF,FFF:FFFFFFFFFFFFFFFFFFFFF
FFFFFFFFFFFFFFF
@A00155:342:HHGFNDSXY:1:1521:6623:5228 1:N:0:GAACCTAG+TCCGCATA
GTAAAAGACAAGGCAAGGTATGGACCCTTAGCAAAGCCAGAAAGATCACCTTGCCCAAAGAAGACCAA
CATTTTCATACATGATGAAATTCATTATTGGTCTCGCGATCAGCTTGAGACATTTCTCCAGGTTTACA
AACCTAAAAATTTGT
+
FFFFFFFFFFFFFFFFFFFFFFFFFFFFFFFFFFFFFFFFFFFFFFFFFFFFFFFFFFF:FFFF:FFF
FFFFFFFFFFFFFFFFFFFFFFFFFFFFFFFFFFFFFFFFFFFFFFFFFFFFFFFFFFFFFFF:FFFFF
:FFFFFFFFFFFFFF
@A00155:342:HHGFNDSXY:1:1604:5339:32033 1:N:0:GAACCTAG+TCCGCATA
GTAAAAGACAAGGCAAGGTATGGACCCTTAGCAAAGCCAGAAAGATCACCTTGCCCAAAGAAGACCAA
CATTTTCATACATGATGAAATTCATTATTGGTCTCGCGATCAGCTTGAGACATTTCTCCAGGTTTACA
AACCTAAAAATTTGT
+
FFFFFFFFFFFFFFFF:FFFFFFFFFFF:FFFFFFFFFFFFF:FFFFFFFFFFFFFFF:FF,FFFFF:
F,FFFFFFFFFFFFFFFFFFFFFFFFFFFFFFFFFFFFF,FFFFFF:FFFFFFFFFFFFFFFFFFFFF
FFFFFFFFFFFFFFF
@A00155:342:HHGFNDSXY:1:1572:22191:2973 1:N:0:GAACCTAG+TCCGCATA
GTAAAAGACAAGGCAAGGTATGGACCCTTAGCAAAGCCAGAAAGATCACCTTGCCCAAAGAAGACCAA
CATTTTCATACATGATGAAATTCATTATTGGTCTCGCGATCAGCTTGAGACATTTCTCCAGGTTTACA
AACCTAAAAATTTGT
+
FFFFFFFFFFFFFFFFFFFFFFFFFFFFFFFFFFFFFFFFFFFFFFFFFFFFFFFFFFFFFFF::,FFFF
FFFFFFFFFFFFFFFFFFFFFFFFFFFFFFFFFFFFFFFFFFFFFFFFFFFFFFFFFFFFFFFFFFF:
FFFFFFFFFFFFFFF
@A00155:342:HHGFNDSXY:1:1628:8829:34788 1:N:0:GAACCTAG+TCCGCATA
GTAAAAGACAAGGCAAGGTATGGACCCTTAGCAAAGCCAGAAAGATCACCTTGCCCAAAGAAGACCAA
CATTTTCATACATGATGAAATTCATTATTGGTCTCGCGATCAGCTTGAGACATTTCTCCAGGTTTACA
AACCTAAAAATTTGT
+
FFFFFFFFF,FFFF,,,FF,FFFFFF:FF:FF,FFFF:FFFFFFFFF,FFFFFFFF:F:FFFFF,F::
::FFFFF,FF:,FFFFFF:FF:FF:FFFFFFFFFFFFFFFFFFFFFFFFF:FF:FFFF:FFFFFFFF:F
F::FFFFF:FFFFFF
@A00155:342:HHGFNDSXY:1:1612:26196:21308 1:N:0:GAACCTAG+TCCGCATA
GTAAAAGACAAGGCAAGGTATGGACCCTTAGCAAAGCCAGAAAGATCACCTTGCCCAAAGAAGACCAA
CATTTTCATACATGATGAAATTCATTATTGGTCTCGCGATCAGCTTGAGACATTTCTCCAGGTTTACA
AACCTAAAAATTTGT
+
FFFFFFFFF:FFFFFFFFFFFFFFF,FFFFFFFFFFFFFFFFFFFFFFFFFFFFFFFFFFFFFFFFFFF
FFFFFFFFFFFFFFFFFFFFFFFFFFFFFFFFFFFFFFFFFFFFFFFFFFFFFFFFFFFFFFFFFFFF
FFFFFFFFFFFFFFF
@A00155:342:HHGFNDSXY:1:1678:9245:9079 1:N:0:GAACCTAG+TCCGCATA
GTAAAAGACAAGGCAAGGTATGGACCCTTAGCAAAGCCAGAAAGATCACCTTGCCCAAAGAAGACCAA
CATTTTCATACATGATGAAATTCATTATTGGTCTCGCGATCAGCTTGAGACATTTCTCCAGGTTTACA
AACCTAAAAATTTGT
+
FFFFFFFFFFFFF:FFFFFFF:FFFFFFFFFFF:FFFFFFFFFFFF:FFFFF:FFFFFFFFF,:F:FF
FFFFFFFFFFFFFFFFFF:FFFFFFFFFFFFF:FFFFFFFFFF,FFFFFFFFFFFFFFFFFF:FFFFFFF
FFFFFF:FFFFFFFF
```

@A00155:342:HHGFNDSXY:1:2113:12120:2628 1:N:0:GAACCTAG+TCCGCATA
GTAAAAGACAAGGCAAGGTATGGACCCTTAGCAAAGCCAGAAAGATCACCTTGCCCAAAGAAGACCAA
CATTTTCATACATGATGAAATTCATTATTGGTCTCGCGATCAGCTTGAGACATTTCTCCAGGTTTACA
AACCTAAAAATTTGT
+
FFFFFFFF:FFFF,F:FFF:FFFFFFFFFFFFFFFFFFFFFFFF,FFFFFFFFF:FFFFF:FFFFFFF
FFF:FFFFFFFFF:FFFF:FFFFF:FF:F:FFFFFF,FFFFFFFF:FFFFFFFFFFFF:FFFFFFFFF
FFFFFFFFFFFFF:F
@A00155:342:HHGFNDSXY:1:2142:3459:15562 1:N:0:GAACCTAG+TCCGCATA
GTAAAAGACAAGGCAAGGTATGGACCCTTAGCAAAGCCAGAAAGATCACCTTGCCCAAAGAAGACCAA
CATTTTCATACATGATGAAATTCATTATTGGTCTCGCGATCAGCTTGAGACATTTCTCCAGGTTTACA
AACCTAAAAATTTGA
+
FFFF:FFFFFFFFFFFFFFFFFFFFFFFFFFFFFFFFFFFFFFFFFFFFFFFFFFFFFFFFFF:FFFF
FFFFFFFFFFFFFFFFFFFFFFFFFFFFFFFFFFFFFFFFFFFFFFFFFFFFFFFFFFFFF:FFFFFF
FF:FFFFFFFFFF,F
@A00155:342:HHGFNDSXY:1:2178:27344:18035 1:N:0:GAACCTAG+TCCGCATA
GTAAAAGACAAGGCAAGGTATGGACCCTTAGCAAAGCCAGAAAGATCACCTTGCCCAAAGAAGACCAA
CATTTTCATACATGATGAAATTCATTATTGGTCTCGCGATCAGCTTGAGACATTTCTCCAGGTTTACA
AACCTAAAAATTTGT
+
FFFFFFFFFFFFFFFFFFFFFFFFFFFFFFFFFFFFFFFFFFFFFFFFFFFFFFFFFFFFFFFFFFFF
FFFFFFFFFFFFFFFFFFFFFFFFFFFFFFFFFFFFFFFFFFFFFFFFFFFFFFFFFFFFFFFFFFFF
FFFFFFFFFFFFFFF
@A00155:342:HHGFNDSXY:1:2228:25699:9173 1:N:0:GAACCTAG+TCCGCATA
GTAAAAGACAAGGCAAGGTATGGACCCTTAGCAAAGCCAGAAAGATCACCTTGCCCAAAGAAGACCAA
CATTTTCATACATGATGAAATTCATTATTGGTCTCGCGATCAGCTTGAGACATTTCTCCAGGTTTACA
AACCTAAAAATTTGT
+
FFFFFFFFFFFFFFFFFFF:FFFFFFFFFFFFFFFFFFFFFFFFFFFFFFFFFFFFFFFFFFF:FFFF
FFF:FFFFFFF:FF:FFFFFFFFFFFFFFFFFFFFFFFFF:FFFFFFFFFFFFFFFFFFFFFFFFFFF
FFFFFFFFFFFFFFF
@A00155:342:HHGFNDSXY:1:2256:4101:21214 1:N:0:GAACCTAG+TCCGCATA
GTAAAAGACAAGGCAAGGTATGGACCCTTAGCAAAGCCAGAAAGATCACCTTGCCCAAAGAAGACCAA
CATTTTCATACATGATGAAATTCATTATTGGTCTCGCGATCAGCTTGAGACATTTCTCCAGGTTTACA
AACCTAAAAATTTGT
+
FFFFFFFFFFF:FFFFF:FFFFFFF::FFFFFFFFFFFFFFFFFFFFFFFFFFFFFFFF::FF:FFFF
FFFFFFFFFFFFFFFFFFFFFFFFFFFFFFFFFFFFFFFFFFFFFFFFFFFFFFFFFFFFFFFFFFFF
FFFFFFFFFFFFFFF
@A00155:342:HHGFNDSXY:1:2302:7283:36558 1:N:0:GAACCTAG+TCCGCATA
GTAAAAGACAAGGCAAGGTATGGACCCTTAGCAAAGCCAGAAAGATCACCTTGCCCAAAGAAGACCAA
CATTTTCATACATGATGAAATTCATTATTGGTCTCGCGATCAGCTTGAGACATTTCTCCAGGTTTACA
AACCTAAAAATTTGT
+
FFFF,FFFFFFFFFFFFFFFFFFFFFFFFFFFFFFFFFFFFFFFFFFFFFFFFFFFFF:F:FFFFFFF
FFFFFFFFFFFFFFFFFFFFFFFFFFFFFFFFFFFFFFFFFFFFFFFFFFFFFFFFFFFFFFFFFFFF
FFFFFFFFFFFFFFF
@A00155:342:HHGFNDSXY:1:2372:11623:27132 1:N:0:GAACCTAG+TCCGCATA
GTAAAAGACAAGGCAAGGTATGGACCCTTAGCAAAGCCAGAAAGATCACCTTGCCCAAAGAAGACCAA
CTTTTTCATACATGATGAAATTCATTATTGGTCTCGCGATCAGCTTGAGACATTTCTCCAGGTTTACA
AACCTAAAAATTTGT
+
FFFFF,F:FF:FF,,,FFF:FFF::,FFFFF,FFFFFF,FF,FFFFF,F:FFF,F,FFFF,FFF,FF:

```
:,FFFFFFF,FFFFFFFFF,:F,FFF,F:FFF,FFFFF:FF,:FFFF,FF,FFFFFFFF:FFF:FFFF
,,FFFFFFF:FFFFF
@A00155:342:HHGFNDSXY:1:2515:8359:35039 1:N:0:GAACCTAG+TCCGCATA
GTAAAAGACAAGGCAAGGTATGGACCCTTAGCAAAGCCAGAAAGATCACCTTGCCCAAAGAAGACCAA
CATTTTCATACATGATGAAATTCATTATTGGTCTCGCGATCAGCTTGAGACATTTCTCCAGGTTTACA
AACCTAAAAATTTGT
+
FFFFFFFFFFFF:FFFFFFFFFFFFFFFFFFFFFFFFFFFFFFFFFFFFFFFFFFFFFFFFFFFFFFF
FFFFFFFFFFFFFFFFFFFFFFFFFFFFFFFFFFFFFFFFFFFFFF:FFFFFFFFFFFFFFFFFFFFFFFFFFFFF
FFFFFFFFFFFFFFF
@A00155:342:HHGFNDSXY:1:2506:9082:36980 1:N:0:GAACCTAG+TCCGCATA
GTAAAAGACAAGGCAAGGTATGGACCCTTAGCAAAGCCAGAAAGATCACCTTGCCCAAAGAAGACCAA
CATTTTCATACATGATGAAATTCATTATTGGTCTCGCGATCAGCTTGAGACATTTCTCCAGGTTTACA
AACCTAAAAATTTGT
+
F:FF,F:FFF,F:FFFFFFF:FFFFFFFFFFFFFF:FFFFFFFFFFFFFFFFFFFFFFFFFF,FFFFF:
FFFFFFFFFFFFFFFFFFFFFFFFFFFFFFFFFFFFFFFFFFFFFFFFFFFFFFFFF:FFFFFFFFFF
FFFFFFFFFFFFFFF
@A00155:342:HHGFNDSXY:1:2574:25328:12007 1:N:0:GAACCTAG+TCCGCATA
GTAAAAGACAAGGCAAGGTATGGACCCTTAGCAAAGCCAGAAAGATCACCTTGCCCAAAGAAGACCAA
CATTTTCATACATGATGAAATTCATTATTGGTCTCGCGATCAGCTTGAGACATTTCTCCAGGTTTACA
AACCTAAAAATTTGT
+
FFFFFFFFFFFFFFFFFFFFFFFFFFFFFFFFFFFFFFFFFFFFFFFFFFFFFFFFFFF:FFFFFFFF
FFFFFFFFFFFFFFFFFFFFFFFFFFFFFFFFFFFFFFFFFFFFFFFFFFFFFFFFFFFFFFFFFFFF
FFFFFFFFFFFFFFF
@A00155:342:HHGFNDSXY:1:1110:16405:7232 2:N:0:GAACCTAG+TCCGCATA
GTAAAAGACAAGGCAAGGTATGGACCCTTAGCAAAGCCAGAAAGATCACCTTGCCCAAAGAAGACCAA
CATTTTCATACATGATGAAATTCATTATTGGTCTCGCGATCAGCTTGAGACATTTCTCCAGGTTTACA
AACCTAAAAATTTGT
+
FFFFFFFFFFFFFFFFFFFFFFFFFFFFFFFFFFFFFFFFFFFFFFFFFFFFFFFFFFF:FFFFFFFFF
FFFFFFFFFFFFFFFFFFFFFFFFFFFFFFFFFFFF:FFFFFFFFFFFFFFFFFFFFFFFFFFFFFF:FFF
F:FFFFFFFFFFFFF
@A00155:342:HHGFNDSXY:1:1106:6081:11490 2:N:0:GAACCTAG+TCCGCATA
GTAAAAGACAAGGCAAGGTATGGACCCTTAGCAAAGCCAGAAAGATCACCTTGCCCAAAGAAGACCAA
CATTTTCATACATGATGAAATTCATTATTGGTCTCGCGATCAGCTTGAGACATTTCTCCAGGTTTACA
AACCTAAAAATTTGT
+
F:FFFFFFFFFFFF:FFFFFFFFFFFFFFFF,FFFFFFFFFFFFFFFFFFFFFFFFFFFFFFFFFFF:F
FFFFFFFFFFFFFFFFFFFFFFF,FFFFFFFFFFFFFFF,FFFFFFF:FFFF,F:FFFFFFFFFFFFFF
FFFFFFFFFFFF:FF
@A00155:342:HHGFNDSXY:1:1355:15393:17785 2:N:0:GAACCTAG+TCCGCATA
GTAAAAGACAAGGCAAGGTATGGACCCTTAGCAAAGCCAGAAAGATCACCTTGCCCAAAGAAGACCAA
CATTTTCATACATGATGAAATTCATTATTGGTCTCGCGATCAGCTTGAGACATTTCTCCAGGTTTACA
AACCTAAAAATTTGT
+
FFFFFFFFFFFFFFFFFFFFFFFFFFFFFFFFFFFFFFFFFFFFFFFFFFFFFFFFFFFFFFFFFFFF
FFFFFFFFFFFFFFFFFFFFFFFFFFFFFFFFFFFFFFFFFFFFFFFFFFFFFFFFF:FFFFFFFFFF
FFFFFFFFFFFFFFF
@A00155:342:HHGFNDSXY:1:1357:9362:16203 2:N:0:GAACCTAG+TCCGCATA
GTAAAAGACAAGGCAAGGTATGGACCCTTAGCAAAGCCAGAAAGATCACCTTGCCCAAAGAAGACCAA
CATTTTCATACATGATGAAATTCATTATTGGTCTCGCGATCAGCTTGAGACATTTCTCCAGGTTTACA
AACCTAAAAATTTGT
```

+
FFFFFFFFFFFFFFFFFFFFFFFFFFFFFFFFFFFFFFFFFFFFFFFFFFFFFFFFFFFFFFFFFFFF
FFFFFFFFFFFFFFFFFFFF:FFFFFFFFFFFFFFFFFFFFFFFFFFFFFF:FFFFFFFFFFFFFFFF
FFFFF:FFFFFFFFF
@A00155:342:HHGFNDSXY:1:1415:32316:10207 2:N:0:GAACCTAG+TCCGCATA
GTAAAAGACAAGGCAAGGTATGGACCCTTAGCAAAGCCAGAAAGATCACCTTGCCCAAAGAAGACCAA
CATTTTCATACATGATGAAATTCATTATTGGTCTCGCGATCAGCTTGAGACATTTCTCCAGGTTTACA
AACCTAAAAATTTGT
+
FFFFFFFFFFFFFFFFFF:,FFFFFFFFFFFFF:FFFFFFFFFFFFFFFFFFFFFFFFFFFFFFFFFF
FFFFFFFFFFFFFFFFFFFFFFFFFFFFFFFFFFFFFF:FFFFFFFFFFFFFFFFFF:FFFFFFFFFF
FFFFFFFFFFF,FFF
@A00155:342:HHGFNDSXY:1:1374:14615:34350 2:N:0:GAACCTAG+TCCGCATA
GTAAAAGACAAGGCAAGGTATGGACCCTTAGCAAAGCCAGAAAGATCACCTTGCCCAAAGAAGACCAA
CATTTTCATACATGATGAAATTCATTATTGGTCTCGCGATCAGCTTGAGACATTTCTCCAGGTTTACA
AACCTAAAAATTTGT
+
FFFFFFF:FFFFFFFFFFFFFFFFFFFFFFFFFFFFFFFFFFFF:FFFFFFFFFFFFFFFFFFFFFFF
FFFF:FFFFFFFFFFFFFFFFFFFFFFFFFFFFFFFFFFFFFFF:FFFF:FFFFFFFFFFFFFFFFFF
FFFFFFFFFFFFFFF
@A00155:342:HHGFNDSXY:1:1511:4860:29293 2:N:0:GAACCTAG+TCCGCATA
GTAAAAGACAAGGCAAGGTATGGACCCTTAGCAAAGCCAGAAAGATCACCTTGCCCAAAGAAGACCAA
CATTTTCATACATGATGAAATTCATTATTGGTCTCGCGATCAGCTTGAGACATTTCTCCAGGTTTACA
AACCTAAAAATTTGT
+
FFFFFFFFFFFFFFFFFFFFFFFFFFFFFFFFFF:FFFFFFFFFFFFFFFFFFFFFFFFFFFFFFFFF
FFFFFFFFFFFFFFFFFFFFFFFFFFFFFFFFFFFFFFFFFFFFFFFFFFFFFFFFFFFFFFFFFFFF
FFFFFFFFFFFF:FF
@A00155:342:HHGFNDSXY:1:1537:29288:3396 2:N:0:GAACCTAG+TCCGCATA
GTAAAAGACAAGGCAAGGTATGGACCCTTAGCAAAGCCAGAAAGATCACCTTGCCCAAAGAAGACCAA
CATTTTCATACATGATGAAATTCATTATTGGTCTCGCGATCAGCTTGAGACATTTCTCCAGGTTTACA
AACCTAAAAATTTGT
+
FFFFFFFFFFFFFFFFFFFFFFFFFFFFFFFFFFFFFFFFFFFFFFFFFFFFFFFFFF:FFFFFFFFF
FFFFFFFFFFFFFFFFFFFFFFFFFFFFFFFFFFFFFFFFFFFFFF:FFFFFFFFFFF:FFFFFF:FF
FFFFFFFFFFFF:FF
@A00155:342:HHGFNDSXY:1:1611:21441:6965 2:N:0:GAACCTAG+TCCGCATA
GTAAAAGACAAGGCAAGGTATGGACCCTTAGCAAAGCCAGAAAGATCACCTTGCCCAAAGAAGACCAA
CATTTTCATACATGATGAAATTCATTATTGGTCTCGCGATCAGCTTGAGACATTTCTCCAGGTTTACA
AACCTAAAAATTTGT
+
F:FFFFFFFFFFFFFFFFFFFFFFFFFFFFFFFFFFFFFFFFFFFFFFFFFFFFFFFFFFFF:FFFFF
FF:FFFFFFFFFFFFFFFFFFFFFFFFFFFFFFFFFFFFFFFFFFFFFFFFFFFFFFFFFFFFFFFF
FFFFF:FFFFFFFFF
@A00155:342:HHGFNDSXY:1:1604:5339:32033 2:N:0:GAACCTAG+TCCGCATA
GTAAAAGACAAGGCAAGGTATGGACCCTTAGCAAAGCCAGAAAGATCACCTTGCCCAAAGAAGACCAA
CATTTTCATACATGATGAAATTCATTATTGGTCTCGCGATCAGCTTGAGACATTTCTCCAGGTTTACA
AACCTAAAAATTTGT
+
FFFFFFFFFFFFFFFFFF:FFFFFFFFFFFFFFFFFFFFFFFFFFFFFFFFFFFFFFFFFFFFFFFFF
FFFFFFFFFFFFFFFFFFFFFFFFFFFFFFFFFFFFFFFFFFFFFFFFFFFFFFFFFF,FFFFFFFFF
F,FFFFFFFF:FFFF
@A00155:342:HHGFNDSXY:1:1650:14244:1642 2:N:0:GAACCTAG+TCCGCATA
GTAAAAGACAAGGCAAGGTATGGACCCTTAGCAAAGCCAGAAAGATCACCTTGCCCAAAGAAGACCAA

CATTTTCATACATGATGAAATTCATTATTGGTCTCGCGATCAGCTTGAGACATTTCTCCAGGTTTACA
AACCTAAAAATTTGT
+
FFFFFFFFFFFFFFFFFFFFFFFFFFFFFFFFFFFFFFFFFFFFFFFFF:FFFFFFFFFFFFFFFFFF
FFFFFFFFFFFFFFFFFFFFFFFFFFFFFFFFFFFFFFFFFFFFFFF:FFFFFFFFFFFFFFFFFFFF
FFFFF:FFFFF:FFF
@A00155:342:HHGFNDSXY:1:2133:3604:5228 2:N:0:GAACCTAG+TCCGCATA
GTAAAAGACAAGGCAAGGTATGGACCCTTAGCAAAGCCAGAAAGATCACCTTGCCCAAAGAAGACCAA
CATTTTCATACATGATGAAATTCATTATTGGTCTCGCGATCAGCTTGAGACATTTCTCCAGGTTTACA
AACCTAAAAATTTGT
+
FFFF:FFFFF,FFFFFFFF:FFFFFFFFFFF:FFFFFFFFFFFFFFFFFFFFFFFFFFFFFFFFFFFF
FFFFFFFFFFFFF,FFFFF:FFFFFFFFF:FFFFFFFFFF:FFFFFFFF:FFFFF,FFFFF:FFFFFF
:FF,,FFFFFFFFFF
@A00155:342:HHGFNDSXY:1:2172:28836:6621 2:N:0:GAACCTAG+TCCGCATA
GTAAAAGACAAGGCAAGGTATGGACCCTTAGCAAAGCCAGAAAGATCACCTTGCCCAAAGAAGACCAA
CATTTTCATACATGATGAAATTCATTATTGGTCTCGCGATCAGCTTGAGACATTTCTCCAGGTTTACA
AACCTAAAAATTTGT
+
FFFFFFFFFFFFFFF:FFFFFFFFFFFFFF:FFFFFFFFFFFFFFFFFFFFFFFFFFFF:FFFFFFFF
FFFFFFFFFFFFFFFFFFFF:FFFFFFFFFFFFFFFFFFFFFFFFFFFFFF:FFFFFFFFFFFFFFFF
FF,FFFFFFF:FF:F
@A00155:342:HHGFNDSXY:1:2262:1515:34585 2:N:0:GAACCTAG+TCCGCATA
GTAAAAGACAAGGCAAGGTATGGACCCTTAGCAAAGCCAGAAAGATCACCTTGCCCAAAGAAGACCAA
CATTTTCATACATGATGAAATTCATTATTGGTCTCGCGATCAGCTTGAGACATTTCTCCAGGTTTACA
AACCTAAAAATTTGT
+
FFFF,:FFFFFFFFFFFFFFFFFFFFFFFFFFFF:FFFFF:FFF:FFFFFFFFFFFFF:F,FF:FFFF
F:FFFFFFFFFFFFFFFF,FFF:FFFFFF:FFFF:F:FFFFFF,FFF,F,FFFFF:F,FFFFFFF::F
F,,FFF::::FFF:F
@A00155:342:HHGFNDSXY:1:2315:3893:32503 2:N:0:GAACCTAG+TCCGCATA
GTAAAAGACAAGGCAAGGTATGGACCCTTAGCAAAGCCAGAAAGATCACCTTGCCCAAAGAAGACCAA
CATTTTCATACATGATGAAATTCATTATTGGTCTCGCGATCAGCTTGAGACATTTCTCCAGGTTTACA
AACCTAAAAATTTGT
+
FFFFFFFFFFFFFFFFFFFFFFFFFFFFFFFFFFFFFFFFFFFFFFFFFFFFFFFFFFFFFFFFFFFF
FFFFFFFFFFFFFFFFFFF:FFFFFFFFFFFFFFFFFFFFFFFFFFFFFFFFFFF:FFFFFF:FFFFF
FFF,F:FFFFFFFFF
@A00155:342:HHGFNDSXY:1:2334:15664:4257 2:N:0:GAACCTAG+TCCGCATA
GTAAAAGACAAGGCAAGGTATGGACCCTTAGCAAAGCCAGAAAGATCACCTTGCCCAAAGAAGACCAA
CATTTTCATACATGATGAAATTCATTATTGGTCTCGCGATCAGCTTGAGACATTTCTCCAGGTTTACA
AACCTAAAAATTTGT
+
FFFFFFFFFFFFFFFFFFFFFFFFFFFFFFFFFFFFFFFFFFFFFFFFFFFFFFFFFFFFFFFFFFFF
FFFFFFFFFFFFFFFFFFFFFFFFFFFFFFFFFFFFFFFFFFFFFFFFFFFFFFFFFF:FFFFFFFFF
,F:FFFFFFFFFF,F
@A00155:342:HHGFNDSXY:1:2321:11153:36182 2:N:0:GAACCTAG+TCCGCATA
GTAAAAGACAAGGCAAGGTATGGACCCTTAGCAAAGCCAGAAAGATCACCTTGCCCAAAGAAGACCAA
CATTTTCATACATGATGAAATTCATTATTGGTCTCGCGATCAGCTTGAGACATTTCTCCAGGTTTACA
AACCTAAAAATTTGT
+
FFFFFFFFFFFFFFFFFFFFFFFFFFFFFFFFFFFFFFFFFFFFFFFFFFFFFFFFFFFFFFFFFFFF
FFFFFFFFFFFFFFFFFFFFFFFFFFFFFFFFFFFFFFFFFFFFFFFFFFFFFFFFFF:FFFFF:FFF
F:FFFFFFFFF:FFF

```
@A00155:342:HHGFNDSXY:1:2321:11433:36448 2:N:0:GAACCTAG+TCCGCATA
GTAAAAGACAAGGCAAGGTATGGACCCTTAGCAAAGCCAGAAAGATCACCTTGCCCAAAGAAGACCAA
CATTTTCATACATGATGAAATTCATTATTGGTCTCGCGATCAGCTTGAGACATTTCTCCAGGTTTACA
AACCTAAAAATTTGT
+
FFFFFFFFFFFFFFFFFFFFFFFFFFFFFFFFFFFFFFFFFFFFFFFFFFFFFFFFFFFFFFFFFFFF
FFFFFFFFFFFFFFFFFFFFFFFFF:FFFFFFFFFFFFFFFFFFFFFFFFFFFFFFFFFFFFFFFFFF
:FFFFF:FFFFFFFF
@A00155:342:HHGFNDSXY:1:2356:23339:26412 2:N:0:GAACCTAG+TCCGCATA
GTAAAAGACAAGGCAAGGTATGGACCCTTAGCAAAGCCAGAAAGATCACCTTGCCCAAAGAAGACCAA
CATTTTCATACATGATGAAATTCATTATTGGTCTCGCGATCAGCTTGAGACATTTCTCCAGGTTTACA
AACCTAAAAATTTGT
+
FFFFFFFFFFFFFFFFFFFFFFFFFFFFFFFFFFFFFFFFFFFF:FFFFFFFFFFFFFFFFFFFFFFF
FFFFFFFFFFFFFFFFFFFFFFFFFF:FFFFFFFFFFFFFFFFFFFFFFFFFFFFFFFFFFFFFFFFF
FFFFFFFFFFFFFFF
@A00155:342:HHGFNDSXY:1:2359:13810:26631 2:N:0:GAACCTAG+TCCGCATA
GTAAAAGACAAGGCAAGGTATGGACCCTTAGCAAAGCCAGAAAGATCACCTTGCCCAAAGAAGACCAA
CATTTTCATACATGATGAAATTCATTATTGGTCTCGCGATCAGCTTGAGACAGTTCTCCAGGTTTACA
AACCTAAAAATTTGT
+
FFFFFFF:FFFFFFFFFFFFFFFFFFFFFFFFFFFFFFFFFFFF:FFFFFFFFFFFFFFFFFFFFFFF
FFFFFFFFFFFFFFFFFFFFFFFFFFFFFFFFFFFFFFFFFFFFFFFFF:FF,FFFFFFFFFFFFFFF
:FFFFFFF:FFFFFF
@A00155:342:HHGFNDSXY:1:2428:1976:5165 2:N:0:GAACCTAG+TCCGCATA
GTAAAAGACAAGGCAAGGTATGGACCCTTAGCAAAGCCAGAAAGATCACCTTGCCCAAAGAAGACCAA
CATTTTCATACATGATGAAATTCATTATTGGTCTCGCGATCAGCTTGAGACATTTCTCCAGGTTTACA
AACCTAAAAATTTGT
+
FFFFFFFFFFFFFFFFFFF:FFFFFFFFFFFFFFFFFFFFFFFFFFFFFFFFFFFFFFFFFFFFFFFF
FFFFFFFFFFFFFFFFFFF:FFFF:FFFFFFFFFFFFFFFFFFF:FFFFFFFFFFFFFFFFFFFFFF
,FFFFF,FFFF,F:F
@A00155:342:HHGFNDSXY:1:2428:1515:5431 2:N:0:GAACCTAG+TCCGCATA
GTAAAAGACAAGGCAAGGTATGGACCCTTAGCAAAGCCAGAAAGATCACCTTGCCCAAAGAAGACCAA
CATTTTCATACATGATGAAATTCATTATTGGTCTCGCGATCAGCTTGAGACATTTCTCCAGGTTTACA
AACCTAAAAATTTGT
+
FFFFF:F:FFFFFFFFFFFFFFF:FFFFFFFFFFFF:FFFFFFFFFFFFFFFFFFFFFFFFFFFFFF,
FFFFFFFFFFFFFF:FF:FFFFFFFFFFFFFFFFFF:FFFFFF:FFFFFFFFFFFFFFFFFFFFF,FF
FF:,FF:FFFF:F,F
@A00155:342:HHGFNDSXY:1:2429:25012:23109 2:N:0:GAACCTAG+TCCGCATA
GTAAAAGACAAGGCAAGGTATGGACCCTTAGCAAAGCCAGAAAGATCACCTTGCCCAAAGAAGACCAA
CATTTTCATACATGATGAAATTCATTATTGGTCTCGCGATCAGCTTGAGACATTTCTCCAGGTTTACA
AACCTAAAAATTTGT
+
FFFFFFFFFFFFFFFFFFFFFFFFFFFFFFFFFFFFFFFFFFFFFFFFFFFFFFFFFFFFFFFFFFFF
FFFFFFFFFFFFFFFFFFFFFFFFFFFFFFFFFFFFFFFFFFFFFFFFFFFFFFFFFFFFFFFFFFFF
FFFFFFFFFFFFFFF
@A00155:342:HHGFNDSXY:1:2439:14660:29700 2:N:0:GAACCTAG+TCCGCATA
GTAAAAGACAAGGCAAGGTATGGACCCTTAGCAAAGCCAGAAAGATCACCTTGCCCAAAGAAGACCAA
CATTTTCATACATGATGAAATTCATTATTGGTCTCGCGATCAGCTTGAGACATTTCTCCAGGTTTACA
AACCTAAAAATTTGT
+
F:FF:F,FFFFFFFFFFFFFFFFFFFFFFFFFFFFFFFFFFFFFFFFFFFFFFFFFFFFFFFFFFFFF
```

FFFFFFFFFFFFFFFFFF,FFFFFFFFF:FFFFFFFFF,FFFFF:FFFFFF:FFFFF:FFFFFFFFFF
FFFFF:FFFFFFFFF
@A00155:342:HHGFNDSXY:1:2511:1949:17487 2:N:0:GAACCTAG+TCCGCATA
GTAAAAGACAAGGCAAGGTATGGACCCTTAGCAAAGCCAGAAAGATCACCTTGCCCAAAGAAGACCAA
CATTTTCATACATGATGAAATTCATTATTGGTCTCGCGATCAGCTTGAGACATTTCTCCAGGTTTACA
AACCTAAAAATTTGT
+
FFFFFFFFFFFFFFFFFFFF,FFFFFFFFFFFFFFFFFFFFFFFFFFFFFFFFFFFFFF:FF,FFFFFF
FF,FFFFFFFFFFFFFFFF:FFFFFFFFFFFFFFFFFF:FFFFFFFFFFFFF,FFFFFFF:F:FFFF
FF:F,F:FFF,F:FF
@A00155:342:HHGFNDSXY:1:2543:17119:34147 2:N:0:GAACCTAG+TCCGCATA
GTAAAAGACAAGGCAAGGTATGGACCCTTAGCAAAGCCAGAAAGATCACCTTGCCCAAAGAAGACCAA
CATTTTCATACATGATGAAATTCATTATTGGTCTCGCGATCAGCTTGAGACATTTCTCCAGGTTTACA
AACCTAAAAATTTGT
+
FFFFFFFFFFFFFFF:FFFFFFFFFFFFFFFFFFFFFFFFFFFFFFFFFFFFFFFFFFFFFF:FF:FF
FFFFFFFFFFFFFFFFFFFFFFFFFFFFFFFFFFFFFFF:FFFFFFFFFFFFFF:FFFFFFFFFFFF
FFF:FFFFFFFFFFF
@A00155:342:HHGFNDSXY:1:2618:20292:8954 2:N:0:GAACCTAG+TCCGCATA
GTAAAAGACAAGGCAAGGTATGGACCCTTAGCAAAGCCAGAAAGATCACCTTGCCCAAAGAAGACCAA
CATTTTCATACATGATGAAATTCATTATTGGTCTCGCGATCAGCTTGAGACATTTCTCCAGGTTTACA
AACCTAAAAATTTGT
+
FFFFFFFFFFFFFFFFFFFFFFFFFFFFFFFFFFFFFFFFFFFFFFFFFFFFFFFFFFFFFFFFFFFF
FFFFFFFFFFFFFFFFFFFFFFFFFFFFFFFFFFFFFFFFFFFFFFFFFFFFFFFFFFFFFFFFFFFF
F:FFFFFFFFFFFFF
@A00155:342:HHGFNDSXY:1:2672:25626:4257 2:N:0:GAACCTAG+TCCGCATA
GTAAAAGACAAGGCAAGGTATGGACCCTTAGCAAAGCCAGAAAGATCACCTTGCCCAAAGAAGACCAA
CATTTTCATACATGATGAAATTCATTATTGGTCTCGCGATCAGCTTGAGACATTTCTCCAGGTTTACA
AACCTAAAAATTTGT
+
FFFFFFFFFFFFFFFFFFFFFFFFFFFFFFFFFFFFFFFFFFFFFFFFFFFFFFFFFFFFFFFFFFFF
FFFFFFFFFFFFFFFFFFFF:FFFFFFFFFFFFFFFFFFFFFFFFFFFFFFFFFFFFFFFFFFFFFF
FFFFFFFFFFFFFFF
@A00155:342:HHGFNDSXY:1:1104:26196:15045 1:N:0:GAACCTAG+TCCGCATA
TAAAAGACAAGGCAAGGTATGGACCCTTAGCAAAGCCAGAAAGATCACCTTGCCCAAAGAAGACCAAC
ATTTTCATACATGATGAAATTCATTATTGGTCTCGCGATCAGCTTGAGACATTTCTCCAGGTTTACAA
ACCTAAAAATTTGTG
+
FFFFFFFFFFFFFFFFFFFFFFFFFFFFFFFFFFFFFFFFFFFFFFFFFFFFFFFFFFFF:,FFFFFF
FFFFFFFFFFFFFFFFFFFFFFFFFFFFF:FFFFFFFFFFFFFFFFFFFFFFFFFFFFFFFF:FFFFFF
FFFFFFFFF,FFFFF
@A00155:342:HHGFNDSXY:1:1115:32560:28823 1:N:0:GAACCTAG+TCCGCATA
TAAAAGACAAGGCAAGGTATGGACCCTTAGCAAAGCCAGAAAGATCACCTTGCCCAAAGAAGACCAAC
ATTTTCATACATGATGAAAGTCATTATTGGTCTCGCGATCAGCTTGAGACATTTCTCCAGGTTTACAA
ACCTAAAAATTTGTG
+
FFF:FFFFFFFFFFFFFF:FFFFFFFF,FFFFFFFFFFFF:FFFFFFFF,FFFFFFFFFFFFFFFFFF
FFFFFFFFFFFFFFFF:FF,FFFFFFF:FFFFFFFFFFFF:FF:FFFFFFF:FFF:FFFFFFFFFFFF
:FFFFFFFFFFFFFF
@A00155:342:HHGFNDSXY:1:1313:18430:17597 1:N:0:GAACCTAG+TCCGCATA
TAAAAGACAAGGCAAGGTATGGACCCTTAGCAAAGCCAGAAAGATCACCTTGCCCAAAGAAGACCAAC
ATTTTCATACATGATGAAATTCATTATTGGTCTCGCGATCAGCTTGAGACATTTCTCCAGGTTTACAA
ACCTAAAAATTTGTG

+
FF,:FFFFF,FFFFFFFFFFFFFFFFFFFFFFFFFFFFFFF:FFFFFFFFFFFFFF,:::F,:FFFFF
FFFFFFFFFFFFFFFFFFFFFFFFFFFFFFFFFFFFFFFF:FFFFFFFFFFFFFFFFFFFFFFFFFF:
FFFFFFFFFFFFF:F
@A00155:342:HHGFNDSXY:1:1347:32913:9298 1:N:0:GAACCTAG+TCCGCATA
TAAAAGACAAGGCAAGGTATGGACCCTTAGCAAAGCCAGAAAGATCACCTTGCCCAAAGAAGACCAAC
ATTTTCATACATGATGAAATTCATTATTGGTCTCGCGATCAGCTTGAGACATTTCTCCAGGTTTACAA
ACCTAAAAATTTGTG
+
FFFFFFFF:FFFFFFFFF:FFFFFFFFFFFFFFFFFFFFFFFFFFFFFF:FFFFFFFFF:FF,FFFFF
FFFFFFFFFFFFFFFFFFFFFFFFFFFFFFFFFFFFFFFFFFFFFFFFFFFFFFFFFFFFFFFFFFFF
FFFFFFFFFFFFFFF
@A00155:342:HHGFNDSXY:1:1431:6153:32690 1:N:0:GAACCTAG+TCCGCATA
TAAAAGACAAGGCAAGGTATGGACCCTTAGCAAAGCCAGTAAGATCACCTTGCCCAAAGAAGACCAAC
ATTTTCATACATGATGAAATTCATTATTGGTCTCGCGATCAGCTTGAGACATTTCTCCAGGTTTACAA
ACCTAAAAATTTGGG
+
FF:FFFF:FFFFFFFF:FFFFFFFFFFFF,FFF:FF,,,,FFFFFF:FFFF:F:,,,FFF,,,:FFF:
FFFFF,FFFFFF,FFFFF:FFFFFFFFFF:FFFFFFF:FF,FFFFFF:FFFFFFFFFFFFFFFFF:FF
,F:FF::,,FFFFFF
@A00155:342:HHGFNDSXY:1:1527:2094:13479 1:N:0:GAACCTAG+TCCGCATA
TAAAAGACAAGGCAAGGTATGGACCCTTAGCAAAGCCAGAAAGATCACCTTGCCCAAAGAAGACCAAC
ATTTTCATACATGATGAAATTCATTATTGGTCTCGCGATCAGCTTGAGACATTTCTCCAGGTTTACAA
ACCTAAAAATTTGTG
+
FFFFFFFFFFFF:FFFF:FFFFFFFFFFF:FFFFFFFFFFFFFFFFFFFFFFFFFFFFFFFFFFFFFF
FFFFFFFFFFFFFFFFFFFFFFFFFFFFFF:FFFFFFFFFFFFFFFFFFFFFFFFFFFFFF,FFFFFF
FFFFFFFFFFFFFFF
@A00155:342:HHGFNDSXY:1:1637:30445:9815 1:N:0:GAACCTAG+TCCGCATA
TTCCGATCTAGGCAAGGTATGGACCCTTAGCAAAGCCAGAAAGATCACCTTGCCCAAAGAAGACCAAC
ATTTTCATACATGATGAAATTCATTATTGGTCTCGCGATCAGCTTGAGACATTTCTCCAGGTTTACAA
ACCTAAAAATTTGTG
+
FFFFFFFFFFFFFFFFFFFFFFFFFFFFFFFFFFFFFFFFFFFFFFFFFFFFFFFFFFFFFFFFFFFF
FFFFFFFFFF:FFFFFFFFFFFFFFFFFFFFFFFFFFFFFFFFFFFFFFFFFFFFFFFFFFFFFFFFF
FFFFFFFFFFFFFFF
@A00155:342:HHGFNDSXY:1:1650:14244:1642 1:N:0:GAACCTAG+TCCGCATA
TAAAAGACAAGGCAAGGTATGGACCCTTAGCAAAGCCAGAAAGATCACCTTGCCCAAAGAAGACCAAC
ATTTTCATACATGATGAAATTCATTATTGGTCTCGCGATCAGCTTGAGACATTTCTCCAGGTTTACAA
ACCTAAAAATTTGTG
+
FFFFFFFFFF::FFF:FFFFFFFFFFFFFFFFFFFFFFFFFFFFFFFFFFFFFFFFFFFFF::FFFFF
FFFFFFFFFFFFFFFFFFFFFFFFFFFFFFFFFFFFFFFFFFFFFFFFFFFFFFFFFFFFFFFFFFFF
FFFFFFFFFFFFFFF
@A00155:342:HHGFNDSXY:1:2271:7672:24956 1:N:0:GAACCTAG+TCCGCATA
TAAAAGACAAGGCAAGGTATGGACCCTTAGCAAAGCCAGAAAGATCACCTTGCCCAAAGAAGACCAAC
ATTTTCATACATGATGAAATTCATTATTGGTCTCGCGATCAGCTTGAGACATTTCTCCAGGTTTACAA
ACCTAAAAATTTGTG
+
FFFFFFFFFFFFFFFFFFFFFFFFFFFFFFFFFFFFFFFFFFFFFFFFF:FFFFFFFFFFFFFFFFFF
FFFFFFFFFFFFFFFFFFFFFFFFFFFFFFFFFFFFFFFFFFFFFFFFFFFFFFFFFFFFFFFFFFFF
FFFFFFFFFFFFFFF
@A00155:342:HHGFNDSXY:1:2319:17806:32894 1:N:0:GAACCTAG+TCCGCATA
TAAAAGACAAGGCAAGGTATGGACCCTTAGCAAAGCCAGAAAGATCACCTCGCCCAAAGAAGACCAAC

```
ATTTTCATACATGATGAAATTCATTATTGGTCTCGCGATCAGCTTGAGACATTTCTCCAGGTTTACAA
ACCTAAAAATTTGTG
+
FFFFFFFFFFFFFFFFFFFFFFFFFFFFFFFFFFFFFFFFFFFFFFFFFFFFFFFFFFFFFFFFFFFFFF
FFFFFFFFFFFFFFFFFFFFFFFFFFFFFFFFFFFFFFFFF:FFFFFFFFFFFFFFFFFFFFFFFFFFFF
FFFFFFFFFFFFFFF
@A00155:342:HHGFNDSXY:1:2446:5900:4225 1:N:0:GAACCTAG+TCCGCATA
TAAAAGACAAGGCAAGGTATGGACCCTTAGCAAAGCCAGAAAGATCACCTTGCCCAAAGAAGACCAAC
ATTTTCATACATGATGAAATTCATTATTGGTCTCGCGATCAGCTTGAGACATTTCTCCAGGTTTACAA
ACCTAAAAATTTGTG
+
FFFFFFFFFFFFFFFFFFFFFFFFFFFFFFFFFFFFFFFFFFFFFFFFFFFFFFFFFFFFFFFFFFFFFF
FFFFFFFFFFFFFFFFFFFFFFFFFFFFFFFFFFFFFFFFFFFFFFFFFFFFFFFFFFFFFFFFFFFFFF
FFFFFFFFFFFFFFF
@A00155:342:HHGFNDSXY:1:2432:2871:4993 1:N:0:GAACCTAG+TCCGCATA
TAAAAGACAAGGCAAGGTATGGACCCTTAGCAAAGCCAGAAAGATCACCTTGCCCAAAGAAGACCAAC
ATTTTCATACATGATGAAATTCATTATTGGTCTCGCGATCAGCTTGAGACATTTCTCCAGGTTTACAA
ACCTAAAAATTTGGG
+
FFFFFFF:FF:FFFFFFFFF:FFFFFFFF:FFFFFFFFFFFFFFFFFFFFFFFFFFF:FF:FF:FFFFFF
FFFFFFFFFFFFFFFFFFFFF:FFFFFFFFFFFFFFFFFFFFFFFFFFFFFFFFFFFFFFFFFFFFFFFFFF
FFFFFFFFFFFFFFF
@A00155:342:HHGFNDSXY:1:2519:26928:12148 1:N:0:GAACCTAG+TCCGCATA
TAAAAGACAAGGCAAGGTATGGACCCTTAGCAAAGCCAGAAAGATCACCTTGCCCAAAGAAGACCAAC
ATTTTCATACATGATGAAATTCATTATTGGTCTCGCGATCAGCTTGAGACATTTCTCCAGGTTTACAA
ACCTAAAAATTTGGG
+
FFFFFFFFFFFF:FFFFFFFFFFFFFFFFFFFFFFFFFFFFFFF:FFFFFFFFFFFFFFFFFFFFFFFFF
FFFFFFFFFFFFFFFFFFFFFFFFFFFFFFFFFFFFFFFFFFFFFFFFFFFFFFFFFFFFFFFFFFFFFF
FFFFFFFFFFFFFFF
@A00155:342:HHGFNDSXY:1:2610:27832:24549 1:N:0:GAACCTAG+TCCGCATA
TAAAAGACAAGGCAAGGTATGGACCCTTAGCAAAGCCAGAAAGATCACCTTGCCCAAAGAAGACCAAC
ATTTTCATACATGATGAAATTCATTATTGGTCTCGCGATCAGCTTGAGACATTTCTCCAGGTTTACAA
ACCTAAAAATTTGTG
+
FFFFFFFFFFFFFFFFFFFFFFFFFFFFFFFFFFFFFFFFFFFFFFFFFFFFFFFFFFFFFFFFFFFFFF
FFFFFFFFFFFFFFFFFFFFFFFFFFFFFFFFFFFFFFFFFFFFFFFFFFFFFFFFFFFFFFFFFFFFFF
FFFFFFFFFFFFFFF
@A00155:342:HHGFNDSXY:1:1176:10619:36198 2:N:0:GAACCTAG+TCCGCATA
TAAAAGACAAGGCAAGGTATGGACCCTTAGCAAAGCCAGAAAGATCACCTTGCCCAAAGAAGACCAAC
ATTTTCATACATGATGAAATTCATTATTGGTCTCGCGATCAGCTTGAGACATTTCTCCAGGTTTACAA
ACCTAAAAATTTGTG
+
FFFFFFFFFFFFFFFFFFFFFFFFFFFFFFFFFFFFFFFFF:FFFFFFFFFFFFFFFFFFFFFFFFFFFF
FFFFFFFFFFFFFFFFFFFFFFFFFFFFFFFFFFFFFFFFFFFFFFFFFFFFFFFFFFFFFFFFFFFFFF
FFFFFFF:FFFFFFF
@A00155:342:HHGFNDSXY:1:1525:13539:34397 2:N:0:GAACCTAG+TCCGCATA
TAAAAGACAAGGCAAGGTATGGACCCTTAGCAAAGCCAGAAAGATCACCTTGCCCAAAGAAGACCAAC
ATTTTCATACATGATGAAATTCATTATTGGTCTCGCGATCAGCTTGAGACATTTCTCCAGGTTTACAA
ACCTAAAAATTTGTG
+
FFF,FFFFFFFFFFFFFFFFFFFFFFFFFFFFFFFFFFFFFFFFFFFFFFFFFFFFFFFFFFFFFFFFFF
FFFFFFFFFFFFFFFFF:FFFFFFFFFFFFFFFFFFFFFFFFFFFFFFFFFFFFFFFFFFFFFFFFFFFF
FFFFFFFFFFFFFFF
```

```
@A00155:342:HHGFNDSXY:1:2447:2618:31673 2:N:0:GAACCTAG+TCCGCATA
TAAAAGACAAGGCAAGGTATGGACCCTTAGCAAAGCCAGAAAGATCACCTTGCCCAAAGAAGACCAAC
ATTTTCATACATGATGAAATTCATTATTGGTCTCGCGATCAGCTTGAGACATTTCTCCAGGTTTACAA
ACCTAAAAATTTGTG
+
FFF,F,FFFFFFFF,FFFFFFFFFFFFFFFFFFFFFFFFFFF:FFFFFFFFFF:FF:FFFFFFFFFFF
FFFFFFFFFFFFFFFFFFFFFFFFFFFFFFFFFFF:FFFFF:FFFFFFFFFFFFFF,FFFF,FFFFFF
FFF:FFFFFFFF,FF
@A00155:342:HHGFNDSXY:1:2648:7048:15890 2:N:0:GAACCTAG+TCCGCATA
AAAAGACAAGGCAAGGTATGGACCCTTAGCAAAGCCAGAAAGATCACCTTGCCCAAAGAAGACCAACA
TTTTCATACATGATGAAATTCAATATTTGTATCTCGATCAGCTTGAGACATTTCTCCATGTGTACAAA
CCTAAAAATTTG
+
FFF,:F::F,,FFFFF,:FFFFF,FFFF,,F:,:F,:FFFF:FF,FFFFFFF,FFFF:FF,FF,FFFF
F,:FFF:FFFF,FFF:F,F,:F,FF:,:,,,FF,::F:FF:,:F:FFF,:FFF:F:FF,,F,FF:FF:
F,F,,FFF:,FF
@A00155:342:HHGFNDSXY:1:1520:1976:23672 1:N:0:GAACCTAG+TCCGCATA
AAAAGACAAGGCAAGGTATGGACCCTTAGCAAAGCCAGAAAGATCACCTTGCTCAAAGAATACCAACA
TTTTCATACATGATGAAATTCATTATTGGTCTCGCGATCAGCTTGAGACATTTCTCCAGGTTTACAAA
CCTAAAAATTTGCGG
+
FFFFFFFFFFFFFFF,:FFFFFFFFFFFFFFFFFFFFFFFFFFFFFFFFFFFFFFFFFFFFFFFFFFF
FFF:FFFFFFFFFFFFFFFFFFFFFFFFFFFFFFFFFFFFFFFFFFFFFFFFFFFFFFFFFFFFFFFF
FFFFFFFFFFFFFFF
@A00155:342:HHGFNDSXY:1:1675:18041:26193 1:N:0:GAACCTAG+TCCGCATA
AAAAGACAAGGCAAGGTATGGACCCTTAGCAAAGCCAGAAAGATCACCTTGCCCAAAGAAGACCAACA
TTTTCATACATGATGAAATTCATTATTGGTCTCGCGATCAGCTTGAGACATTTCTCCAGGTTTACAAA
CCTAAAAATTTGTGG
+
FFFFFFFFFFFFFFFFFFFFFFFFFFFFFFFFFFFFFFFFFFFFFFFFF:FFFFFFFFFFFFFFFFFF
FFFFFFFFFFFFFFFFFFFFFFFFFFFFFFFFFFFFFFFFFFFFFFFFFFFFFFFFFFFFFFFFFFFF
FFFFFFFFFFFFFFF
@A00155:342:HHGFNDSXY:1:2340:5448:21637 1:N:0:GAACCTAG+TCCGCATA
TAAAGACAAGGCAAGGTATGGACCCTTAGCAAAGCCAGAAAGATCACCTTGCCCAAAGAAGACCAACA
TTTTCATACATGATGAAATTCATTATTGGTCTCGCGATCAGCTTGAGACATTTCTCCAGGTTTACAAA
CCTAAAAATTTGTGG
+
FFFFFFFFFFFFFFFFFFFFFFFFFFFFFFFFFFFFFFFFFFFFFFFFFFFFFFFF,FF::FFF:FFF
FFFFFFFFF:FFFFFFFFFFFFFFFFFFFFFFFFFFFFFFFFFFFFFFFFFFFFFFFFFFFFFFFFFF
FFFFFFFFFFFFFFF
@A00155:342:HHGFNDSXY:1:2529:8449:13777 1:N:0:GAACCTAG+TCCGCATA
AAAAGACAAGGCAAGGTATGGACCCTTAGCAAAGCCAGAAAGATCACCTTGCCCAAAGAAGACCAACA
TTTTCATACATGATGAAATTCATTATTGGTCTCGCGATCAGCTTGAGACATTTCTCCAGGTTTACAAA
CCTAAAAATTTGTGG
+
FFFFFFFFFFFFFFFFFFFFFFFFFFFFFFFFFFFFFFFFF:FFFFFFFFFFFFFFF:FF,FFFF:FF
FFFFFFFFFFFFF:FFFFFFFFFFFFFFFFFFFFFFFFFFFFFFFFFFFFFFFFFFFFFFFFFFFFFF
FFFFFFFFFFFFFFF
@A00155:342:HHGFNDSXY:1:1149:21920:4225 2:N:0:GAACCTAG+TCCGCATA
AAAAGACAAGGCAAGGTATGGACCCTTAGCAAAGCCAGAAAGATCACCTTGCCCAAAGAAGACCAACA
TTTTCATACATGATGAAATTCATTATTGGTCTCGCGATCAGCTTGAGACATTTCTCCAGGTTTACAAA
CCTAAAAATTTGTGG
+
FFF:FFFFFFFFFFFFFFFFFFFFFFFFFFFFFFFFFFFFFFFFFFFFFFFFFFFFFFFFFFFFFFFF
```

```
FF:FFFFFFFFFFFFFFFFFFFFFFFFFFFFFFFFFFFFFFFFFFFFFFFFFFFFFFFFFFFFFFFFFF
FFFFFFFFFF::FF:
@A00155:342:HHGFNDSXY:1:1245:20356:25003 2:N:0:GAACCTAG+TCCGCATA
AAAAGACAAGGCAAGGTATGGACCCTTAGCAAGGCCAGAAAGATCACCTTGCCCAAAGAAGACCAACA
TTTTCATACATGATGAAATTCATTATTGGTCTCGCGATCAGCTTGAGACATTTCTCCAGGTTTACAAA
CCTAAAAATTTGTGG
+
FFFFFFFFFFFFFFFFFFFFFFFFFFFFFFFF,FFFFFFFFFFFFFFFFFFFFFFFFFFFFFFFFFFF
FFFFFFFFFFFFFFFFFFFF:FFFFFFFFFFFFFFFFFFFFFFFFFFFFFFFFFFFFFFFFFFFFFFF
FFFFFFFFFFFFFFF
@A00155:342:HHGFNDSXY:1:1373:27335:31454 2:N:0:GAACCTAG+TCCGCATA
AAAAGACAAGGCAAGGTATGGACCCTTAGCAAAGCCAGAAAGATCACCTTGCCCAAAGAAGACCAACA
TTTTCATACATGATGAAATTCATTATTGGTCTCGCGATCAGCTTGAGACATTTCTCCAGGTTTACAAA
CCTAAAAATTTGAGA
+
FFFFFFFFFFFFFFFFFFFFFFFFFFFFFFFFFFFFFFFFFFF:FFFFFFFFFFFFFFFFFFFFFFFF
FFFFFFFFFFFFFFFFFFFFFFFFFFFFFFFFFFFFFFFFFFFFFFFFFFFFFFFFFFFFFFFFFFFF
FFFFFFFFFFFFFFF
@A00155:342:HHGFNDSXY:1:1623:25039:14199 2:N:0:GAACCTAG+TCCGCATA
AAAAGACAAGGCAAGGTATGGACCCTTAGCAAAGCCAGAAAGATCACCTTGCCCAAAGAAGACCAACA
TTTTCATACATGATGAAATTCATTATTGGTCTCGCGATCAGCTTGAGACATTTCTCCAGGTTTACAAA
CCTAAAAATTTGTGG
+
FFFFFFFFFFFFF:FFFFFFFFFFFFFF:FFFFFFFFFFFFFFFFFFFFFF:FFFFFFFFFF:FFFFFFF
FFFFFFFFFFFFFFFFFFFF:FFFFFFFFFFFFFFFFFFFFFFFFFFFFFFFFFFFFFFFFFFFFFFF
FFFFFFFFFFFFFFF
@A00155:342:HHGFNDSXY:1:1666:2636:28134 2:N:0:GAACCTAG+TCCGCATA
AAAAGACAAGGCAAGGTATGGACCCTTAGCAAAGCCAGAAAGACCACCTTGCCCAAAGAAGACCAACA
TTTTCATACATGATGAAATTCATTATTGGTCTCGCGATCAGCTTGAGACATTTCTCCAGGTTTACAAA
CCTAAAAATTTGTGG
+
FFFFFFFFFFFFFFFFFFFFFFFFFFFFFFFFFF:FFF:FFFFFFFFF:FFFFFFFFFFFFFFFFFFF
FFFFFFFF:FF:FFFF:FFFFFFFFFFFFFFFFFFFFFFFFFFFFFFFFFF:FFFFFFFFFF:FFFFFF
FFFFFFFFFFFFFFF
@A00155:342:HHGFNDSXY:1:2311:7473:10520 2:N:0:GAACCTAG+TCCGCATA
AAAAGACAAGGCAAGGTATGGACCCTTAGCAAAGCCAGAAAGATCACCTTGCCCAAAGAAGACCAACA
TTTTCATACATGATGAAATTCATTATTGGTCTCGCGATCAGCTTGAGACATTTCTCCAGGTTTACAAA
CCTAAAAATTTGTGG
+
FFFFFFFFFFFFFFFFFFFFFFFF,FFFFFFFFFFFFFFFFFFFFFFFFFFFFF:FFFFFFFFFFFFF
FFFFFFFF:FFFFFFFFFF:FFFFFFFFFFFFFFFFFFFFFFFF:FFFFF:FFFFFFFFFFF:FF,FFF
FFFF:FFFFFFFFF:
@A00155:342:HHGFNDSXY:1:2401:5032:17378 2:N:0:GAACCTAG+TCCGCATA
AAAAGACAAGGCAAGGTATGGACCCTTAGCAAAGCCAGAAAGATCACCTTGCCCAAAGAAGACCAACA
TTTTCATACATGATGAAATTCATTATTGGTCTCGCGATCAGCTTGAGACATTTCTCCAGGTTTACAAA
CCTAAAAATTTGTGG
+
FFFFFFFFFFFFFFFFF:FFFFFFFFFFFFFFFFFFFFFFFFFFFFFFFFFFFFFF,FFFFFFFFFFFF
:FFFFFFFFFFFFFFFFFFF:FFFFFFFFFFFF,FFFFFFFF:FFFFFFFFFFFFFFFFFFFFFFFFF:
FFFF:FF,,FFFFFF
@A00155:342:HHGFNDSXY:1:2539:9923:4554 2:N:0:GAACCTAG+TCCGCATA
AAAAGACAAGGCAAGGTATGGACCCTTAGCAAAGCCAGAAAGATCACCTTGCCCAAAGAAGACCAACA
TTTTCATACATGATGAAATTCATTATTGGTCTCGCGATCAGCTTGAGACATTTCTCCAGGTTTACAAA
CCTAAAAATTTGAGA
```

```
+
FFFFFFFFFFFFFFFFFFFFFFFFFFFFFFFFFFFFFFFFFFFFFFFFFF,FFFFFFFFFFFFFFFFFFFFFFFFFF
FFFFFFFFFFFF:FFFFFFFFF:FFFFFFFFFF:FFFFFFFFFFFFFFFFFFFFFFFFFFFFFFFFFFF
FFFFFFFFFFFFFFF
@A00155:342:HHGFNDSXY:1:2638:13006:35790 2:N:0:GAACCTAG+TCCGCATA
AAAAGACAAGGCAAGGTATGGACCCTTAGCAAAGCCAGAAAGATCACCTTGCCCAAAGAAGACCAACA
TTTTCATACATGATGAAATTCATTATTGGTCTCGCGATCAGCTTGAGACATTTCTCCAGGTTTACAAA
CCTAAAAATTTGTGG
+
FFFFFFFFFFFFFFFFFFFFFFFFFFFFFFFFFFFFFFFFFFFFFFFFFFFFFFFFFFFFFFFFFFFFF
FFFFFFFFFFFFFFFFFFFFFFFFFFFFFFFFFFFFFFFFFFFFFFFFFFFFFFFFFFFFFFF:FFFFFF
FFFF:FFFFFFFFFF
@A00155:342:HHGFNDSXY:1:2667:31891:11130 2:N:0:GAACCTAG+TCCGCATA
AAAAGACAAGGCAAGGTATGGACCCTTAGCAAAGCCAGAAAGATCACCTTGCCCAAAGAAGACCAACA
TTTTCATACATGATGAAATTCATTATTGGTCTCGCGATCAGCTTGAGACATTTCTCCAGGTTTACAAA
CCTAAAAATTTGTGG
+
FFFFFFFFFFFFFFFFFF:FFFFFFFFFFFFFFFFFFF:FFFFFFFFFFFFFFFFFFFFFFFFFFFFFFF
FFFFFFFFFFFFFFFF,,F,FFF,FFFFFFFFFFFFFF,FFFFFFFFFFF,F,FFFFF:FFFFFFFFF,
FFFFFFFFFF,FFFF
@A00155:342:HHGFNDSXY:1:1172:8965:18270 1:N:0:GAACCTAG+TCCGCATA
AAAGACAAGGCAAGGTATGGACCCTTAGCAAAGCCAGAAAGATCACCTTGCCCAAAGAAGACCAACAT
TTTCATACATGATGAAATTCATTATTGGTCTCGCGATCAGCTTGAGACATTTCTCCAGGTTTACAAAC
CTAAAAATTTGTGGG
+
FFFFFFFFFFFFFFFFFFFFFFFFFFFFFFFFFFFFFFFFFFFFFFFFFFFFFFFFFFFFF:FFFFFFFF
FFFFFFFFFFFFFFFFFFFFFFFFFFFFFFFFFFFFFF:FF:FFFFFFFFFFFFFFFFFFFFFFFFFFFFF
FFFFFFFFFFFFFFF
@A00155:342:HHGFNDSXY:1:1326:19605:2378 1:N:0:GAACCTAG+TCCGCATA
AAAGACAAGGCAAGGTATGGACCCTTAGCAAAGCCAGAAAGATCACCTTGCCCAAAGAAGACCAACAT
TTTCATACATGATGAAATTCATTATTGGTCTCGCGATCAGCTTGAGACATTTCTCCAGGTTTACAAAC
CTAAAAATTTGCGGG
+
FFFFFFFFFFFF,F:FFFFFFFFFFFFFFFFFFFFFFFFFFFFFFFFFF:FFFFFFFFFFFFFFFFFFFF
FFFFFFFFFFFFFFFFFFF:FFFFFFFFFFFFFFFFFF,FFFFFFFFFFFFFFFFFFFFFFFFFFFFFF
FFFFFFFFFFFFFFF
@A00155:342:HHGFNDSXY:1:2207:3134:17440 1:N:0:GAACCTAG+TCCGCATA
AAAGACAAGGCAAGGTATGGACCCTTAGCAAAGCCAGAAAGATCACCTTGCCCAAAGAAGACCAACAT
TTTCATACATGATGAAATTCATTATTGGTCTCGCGATCAGCTTGAGACATTTCTCCAGGTTTACAAAC
CTAAAAATTTGTGGG
+
:FFFFFF:F,FFFFFFFFFFFFFFFFFF,FFFFFF:FFFFFFFFFFFF:FFF,,FFF:FF,:FFFFFFF
FFFFFFFFFFFFFFFFFFFFFFFFFFFFFFFFFFFFFFFFF:FFFF,FFFFFFFFFFFFFFFFFFFFFFF
FFFFFFFFFFFF:FF
@A00155:342:HHGFNDSXY:1:2545:3052:6652 1:N:0:GAACCTAG+TCCGCATA
AAAGACAAGGCAAGGTATGGACCCTTAGCAAAGCCAGAAAGATCACCTTGCCCAAAGAAGACCAACAT
TTTCATACATGATGAAATTCATTATTGGTCTCGCGATCAGCTTGAGACATTTCTCCAGGTTTACAAAC
CTAAAAATTTGTGGG
+
FFFFFFFF:F:FFFFFFFFFFFFFFFFFFFFFFFFFFF:FFFFF:FFFFFFFFFFFFFFFF:FFFFFFFF
FFFFFFFFFFFFFFFFFFFFFFFFFFFFFFFFFFFFFFFFFFFFFFFFFFFFFFFFFFFFFFFFFFFFF:F
FFFFFFFFFFFFFFF
@A00155:342:HHGFNDSXY:1:1673:10836:18787 2:N:0:GAACCTAG+TCCGCATA
AAAGACAAGGCAAGGTATGGACCCTTAGCAAAGCCAGAAAGATCACCTTGCCCAAAGAAGACCAACAT
```

TTTCATACATGATGAAATTCATTATTGGTCTCGCGATCAGCTTGAGACATTTCTCCAGGTTTACAAAC
CTAAAAATTTGTGGG
+
FFFFFFFFFFFFFFFFFFFFFFFFFFFFFFFFFFFFFFFFFFFFFFFFFFFFFFFFFFFFFFFFFFFFFF
FFFFFFFFFFFFFFF:FFFFFFFFFFFFFFFFFFFFFFFFFFFFFFFFFFFFFFFFFFFFFFFFFFFFFF
FFFFFFFFFFFFFFF
@A00155:342:HHGFNDSXY:1:1110:16405:7232 1:N:0:GAACCTAG+TCCGCATA
AAGACAAGGCAAGGTATGGACCCTTAGCAAAGCCAGAAAGATCACCTTGCCCAAAGAAGACCAACATT
TTCATACATGATGAAATTCATTATTGGTCTCGCGATCAGCTTGAGACATTTCTCCAGGTTTACAAACC
TAAAAATTTGTGGGC
+
FFFFFFFF:FFFFFFFFFFFFFFFFFFFFFFFFFFFFFFFFFFFFFFFFFFFFFFFFF,FFFFF,FFFFF
FFFFFFF:FFFFFFFFFFFFFFFFFFFFFFFFFFFFFFFFFFFFFFFFFFFFFFFFFFFFFFFFFFFFFF
:FFFFFFFFFFFFFF
@A00155:342:HHGFNDSXY:1:1123:17327:24111 1:N:0:GAACCTAG+TCCGCATA
AAGACAAGGCAAGGTATGGACCCTTAGCAAAGCCAGAAAGATCACCTTGCCCAAAGAAGACCAACATT
TTCATACATGATGAAATTCATTATTGGTCTCGCGATCAGCTTGAGACATTTCTCCAGGTTTACAAACC
TAAAAATTTGTGGGC
+
FFFFFFFFFFFFFFFFFFFFFFFFFFFFFFFFFFFFFFFFFFFFFFFFFFF:FFFFFFFFFFFFFFFFFF
FFFFFFFFF:FFFFFFFFFFFFFFFFFFFFFFFFFFFFFFFFFFFFFFFFFFFF:FFFFFFFFFFFF:FF
FFFFFFFFFFFFFFF
@A00155:342:HHGFNDSXY:1:1148:30382:8140 1:N:0:GAACCTAG+TCCGCATA
CTGACAAGGCAAGGTATGGACCCTTAGCAAAGCCAGAAAGATCACCTTGCCCAAAGAAGACCAACATT
TTCATACATGATGAAATTCATTATTGGTCTCGCGATCAGCTTGAGACATTTCTCCAGGTTTACAAACC
TAAAAATTTGTGGGC
+
:FFF:FFFF:FF:FFFF,FFFFFFFFFFFFFFFFFFFFFFFFFFFFFFFFFFFFFFF::FFFFFFFFFFF
:F:::FFFFFFFFFFFFFFFFFFFFFFFFFFFFFFFFFF,FFFFFFFFFFFFFFFFFF:FFFFFFFFFFF
FFFFFFFFFFFFFFF
@A00155:342:HHGFNDSXY:1:1245:20356:25003 1:N:0:GAACCTAG+TCCGCATA
AAGACAAGGCAAGGTATGGACCCTTAGCAATGCCAGAAAGATCACCTTGCCCAAAGAAGACCAACATT
TTCATACATGATGAAATTCATTATTGGTCTCGCGATCAGCTTGAGACATTTCTCCAGGTTTACAAACC
TAAAAATTTGTGGAC
+
FFFFFFFFFFFFFFFFFFFFFFFFFFFFFFF,FFFFFFFFFFFFFFFF:FFFFFF:FFFFFFFFF:FF:F
FFFFFFFFFFFFFFFFFFFFFFFFFFFFFFFFFFFFFFFFFFFFFFFFFFFFFFFFFFFFFFFFFFFFFF
FFFFFFFFFFFFFFF
@A00155:342:HHGFNDSXY:1:1359:30499:27853 1:N:0:GAACCTAG+TCCGCATA
AAGACAAGGCAAGGTATGGACCCTTAGCAAAGCCAGAAAGATCACCTTGCCCAAAGAAGACCAACATT
TTCATACATGATGAAATTCATTATTGGTCTCGCGATCAGCTTGAGACATTTCTCCAGGTTTACAAACC
TAAAAATTTGTGGGC
+
FFFFFFFFFFFFFFFFFFFFFFFFFFFFFFFFFFFFFFFFFFFFFFFFFFFFFFFFFF:FFFFFFFFFFF
FFFFFFFFFFFFFFFFFFFFFFFFFFFFFFFFFFFFFFFFFFFFFFFFFFFFFFFFFFFFFFFFFFFFFF
FF:FFFFFFFFFFFF
@A00155:342:HHGFNDSXY:1:1413:20763:15718 1:N:0:GAACCTAG+TCCGCATA
AAGACAAGGCAAGGTATGGACCCTTAGCAAAGCCAGAAAGATCACCTTGCCCAAAGAAGACCAACATT
TTCATACATGATGAAATTCATTATTGGTCTCGCGATCAGCTTGAGACATTTCTCCAGGTTTACAAACC
TAAAAATTTGTGGGC
+
FFFFFFFFFFFFFFFFFFFFFFFFFFFFFFFFFFFFFFFF:FFFFFFFFFFFFFFFFF,FFFFFFFFFFF
FFFFFFFFFFFFFFFFFFFFFFFFFFFFFFFFFFFFFFFFFFFFFFFFFFFFFFFFFFFFFFFFFFFFFF
FFFFF:FFFFFFFFF

@A00155:342:HHGFNDSXY:1:1407:4860:2863 1:N:0:GAACCTAG+TCCGCATA
AAGACAAGGCAAGGTATGGACCCTTAGCAAAGCCAGAAAGATCACCTTGCCCAAAGAAGACCAACATT
TTCATACATGATGAAATTCATTATTGGTCTCGCGATCAGCTTGAGACATTTCTCCAGGTTTACAAACC
TAAAAATTTGTGGGC
+
FFFFFFFFFFFFFFFFFFFFFFFFFFFFFFFF,FFFF:FFFFFFFFFFFFFFFFFFFFF,FFFFFFFF
FFFFFFFFFFFFFFFF:FFFFFFFFFFFFFFFFFFFFFFFFFFFFFFFFFFFFFFFFFFFFFFFFFF
FFFFFFFFFFFFFFF
@A00155:342:HHGFNDSXY:1:1439:30535:10128 1:N:0:GAACCTAG+TCCGCATA
AAGACAAGGCAAGGTATGGACCCTTAGCAAAGCCAGAAAGATCACCTTGCCCAAAGAAGACCAACATT
TTCATACATGATGAAATTCATTATTGGTCTCGCGATCAGCTTGAGACATTTCTCCAGGTTTACAAACC
TAAAAATTTGTGGGC
+
FFFFFF:FFFFFFFFFFFFFFF:FF:FFFFFFF:FFFFFFFFFFFF:FFFFFF:FF::F,,FFFFFFF
FFFFFF:FFFFFFFFFFFFFFFFFFFFFFFFFFF::FFFFFFFFFFFFFFFFFFFFFFFFFFFFFFFF
FFFFFFFFFFFFFFF
@A00155:342:HHGFNDSXY:1:1474:30508:11584 1:N:0:GAACCTAG+TCCGCATA
AAGACAAGGCAAGGTATGGACCCTTAGCAAAGCCAGAAAGATCACCTTGCCCAAAGAAGACCAACATT
TTCATACATGATGAAATTCATTATTGGTCTCGCGATCAGCTTGAGACATTTCTCCAGGTTTACAAACC
TAAAAATTTGTGGGC
+
FFFFFFFF:FF::FFFFFFFFFFFFFFFFFFFFFFFFFFFFFFFFF:FFFFFFFF:FFFF:FFFFFFF
FFFFFFFFFFFFFFFFFFFFFFFFFFF:FFFFFFFFFFFFFFFFFFFFFFFFFFFFFFFFFFFF::FF
FFFFFFFFFFFFFFF
@A00155:342:HHGFNDSXY:1:1520:5656:10504 1:N:0:GAACCTAG+TCCGCATA
AAGACAAGGCAAGGTATGGACCCTTAGCAAAGCCAGAAAGATCACCTTGCCCAAAGAAGACCAACATT
TTCATACATGATGAAATTCATTATTGGTCTCGCGATCAGCTTGAGACATTTCTCCAGGTTTACAAACC
TAAAAATTTGTGGAC
+
FFFFFFF:FFFFFFFFF:FFFFFFFFFFF:FF:FFFFFFFFFFFFFFFFFFFFFFFFFFFFFFFFFFF
FFFFFFFFFFFFFFFFFFFFFFFFFFFFFFF:FF:FFFFFFFFFFFFFFFFFFFFFFFFFFFFFFFFF
FFFFFFFFFFFFFFF
@A00155:342:HHGFNDSXY:1:1514:27525:1031 1:N:0:GAACCTAG+TCCGCATA
AAGACAAGGCAAGGTATGGACCCTTAGCAAAGCCAGAAAGATCACCTTGCCCAAAGAAGACCAACATT
TTCATACATGATGAAATTCATTATTGGTCTCGCGATCAGCTTGAGACATTTCTCCAGGTTTACAAACC
TAAAAATTTGTGGGC
+
FFFFFFFFFFFFFFFFFFFFFFFFFFFFFFFF::FFFFFFFFFFFFFFFFFFFFFFFFFFFFFFFFFF
FFFFFFFFFFFFFFFFFFFFFFFFFFFFFF:FFFFFFFFFF:FFFFFFFFFFFFFFFFFFFFFFFFFF
FFFFFFFFFFFFFFF
@A00155:342:HHGFNDSXY:1:1673:10836:18787 1:N:0:GAACCTAG+TCCGCATA
AAGACAAGGCAAGGTATGGACCCTTAGCAAAGCCAGAAAGATCACCTTGCCCAAAGAAGACCAACATT
TTCATACATGATGAAATTCATTATTGGTCTCGCGATCAGCTTGAGACATTTCTCCAGGTTTACAAACC
TAAAAATTTGTGGGC
+
FFFFFFFFFFFFFFF:FFFFFFFFFFFFFFFFFFFFFF:FFFFFFFFFFFFFFFFFFF:FFF,FFFFF
FFFFFFF:FFFFFFFFFFFFFFFFFFFFFFFFFFFFFFFFFFFFFFFFFFFFFFFFFFFFFFFFFFFF
FFFFFFFFFFFFFFF
@A00155:342:HHGFNDSXY:1:2259:13566:33223 1:N:0:GAACCTAG+TCCGCATA
AAGACAAGGCAAGGTATGGACCCTTAGCAAAGCCAGAAAGATCACCTTGCCCAAAGAAGACCAACATT
TTCATACATGATGAAATTCATTATTGGTCTCGCGATCAGCTTGAGACATTTCTCCAGGTTTACAAACC
TAAAAATTTGTGGGC
+
FFFFFFFFFFFFFFFFFFFFFFFFFFFFFFFFFFFFFFFFFFFFFFFFFFFFFFFFFFFFFFFFFFFF

F,FFFFFFFFFFFFFFFFFFFFFFFFFFFFFFFFFFFFFFFFFFFFFFFFFFFFFFFFFFFFFFFFFF
FFFFFFFFFFFFFFF
@A00155:342:HHGFNDSXY:1:2408:16297:2660 1:N:0:GAACCTAG+TCCGCATA
AAGACAAGGCAAGGTATGGACCCTTAGCAAAGCCAGAAAGATCACCTTGCCCAAAGAAGACCAACATT
TTCATACATGATGAAATTCATTATTGGTCTCGCGATCAGCTTGAGACATTTCTCCAGGTTTACAAACC
TAAAAATTTGTGGGC
+
:FFFFFFFFFFFFFFFFFFFFFFFFFFFFFFFFFFFFFFF:FFFFF:FFFFFFFFFFFFFFFFFFFFF
FFFFFFFFFFFFFFFFFFFFFFFF:FFFFFFFFFFFFFFFFFFFFFFFFFFFFFFFFFFFFFFFFFFF
FFFFFFFFFFFFFFF
@A00155:342:HHGFNDSXY:1:2306:12762:1705 2:N:0:GAACCTAG+TCCGCATA
AAGACAAGGCAAGGTATGGACCCTTCGCAAAGCCAGAAAGATCACCTTGCCCAAAGAAGACCAACATT
TTCATACATGATGAAATTCATTATTGGTCTCGCGATCAGCTTGAGACATTTCTCCAGGTTTACAAACC
TAAAAATTTGTGGGC
+
FFFFFFFFFFFFFFFFFFFFFFFFF,FFFFFFFFFFFFFFFFFFFFFFFFFFFFFFFFFFFFFFFFFF
FFFFFFFFFFFFFFFFFFFFFFFFFFFFFFFFFFFFFFFFFFFFFFF:FF:FFFFFFFFFFFFFF:FF
FFFFFFFFFFFFFF:
@A00155:342:HHGFNDSXY:1:1346:3188:31187 1:N:0:GAACCTAG+TCCGCATA
AGACAAGGCAAGGTATGGACCCTTAGCAAAGCCAGAAAGATCACCTTGCCAAAAGAAGACCAACATTT
TCATACATGATGAAATTCATTATTGGTCTCGCGATCAGCTTGAGACATTTCTCCAGGTTTACAAACCT
AAAAATTTGTGGGGC
+
FFFFFFFFFFFFFFFF:FFFFFFFFFFFFFFFFFFFFFFFFFFFFFFFFF,FFFFFF:FFFFFFFFFF
FFFFFFFFFFF:FFFFF:FFFFFFFFFFFFF:FFFFFFFFFFFFFFFFFFFFFFFFFFFFFFFF:FFF
FFFFFFFFFFFFFFF
@A00155:342:HHGFNDSXY:1:2135:23059:15029 1:N:0:GAACCTAG+TCCGCATA
AGACAAGGCAAGGTATGGACCCTTAGCAAAGCCAGAAAGATCACCTTGCCCAAAGAAGACCAACATTT
TCATACATGATGAAATTCATTATTGGTCTCGCGATCAGCTTGAGACATTTCTCCAGGTTTACAAACCT
AAAAATTTGTGGGCG
+
FFFFFFFFFFFFFFFFFFFFFFFFFFFFFFFFFFFFFFFFFFFFFFFFFFFFFFFFF:FFFF:FFFFF
FFFFFFFFFFFFFFFFFFFFFFFFFFFFFFFFFFFFFFFFFFFFFFFFFFFFFFFFFFFFFFFFFFFF
FFFFFFFFFFFFFFF
@A00155:342:HHGFNDSXY:1:2355:29903:7028 1:N:0:GAACCTAG+TCCGCATA
AGACAAGGCAAGGTATGGACCCTTAGCAAAGCCAGAAAGATCACCTTGCCCAAAGAAGACCAACATTT
TCATACATGATGAAATTCATTATTGGTCTCGCGATCAGCTTGAGACATTTCTCCAGGTTTACAAACCT
AAAAATTTGTGGGGC
+
FFFFFFFFFFFFFF:FFFFFFFFFFFFFFFFFFFFFFFFFFFFFFFFFFFFF:FF::FFFFFFFFFFF
FFFFFFFFFFFFFFFFFFFFFFFFFFFFFFFFFFFFFFFFFFFFFFFFFFFFFFFFFFFFFFFFFFFF
FFFFFFFFFFFFFFF
@A00155:342:HHGFNDSXY:1:2359:25292:13917 1:N:0:GAACCTAG+TCCGCATA
AGACAAGGCAAGGTATGGACCCTTAGCAAAGCCAGAAAGATCACCTTGCCCAAAGAAGACCAACATTT
TCATACATGATGAAATTCATTATTGGTCTCGCGATCAGCTTGAGACATTTCTCCAGGTTTACAAACCT
AAAAATTTGTGGGCG
+
FFFFFFFFFFFFFFFFFFFFFFFFFFFFFFFFFFFFFFFFFFFFFFFFFFFFFFFFFFFFFFFFFFFF
FFFFFFFFFF:FFFFFFFFFFFFFFFFFFFFFFFFFFFFFFFFFFFFFFFFFFFFFFFFFFFFFFFFF
FFFFFFFFFFFFFFF
@A00155:342:HHGFNDSXY:1:2505:4806:4773 1:N:0:GAACCTAG+TCCGCATA
AGACAAGGCAAGGTATGGACCCTTAGCAAAGCCAGAAAGATCACCTTGCCCAAAGAAGACCAACATTT
TCATACATGATGAAATTCATTATTGGTCTCGCGATCAGCTTGAGACATTTCTCCAGGTTTACAAACCT
AAAAATTTGTGAGCT

+
F:FF:FFFFFFF:F,F,FF::FFFF:F,FFFFFFFF,FFFFFFFFFFFFFFFFFFFFF:FFFF:FFFF
F:FFF:FFFFF:FFFFFFFFFFFFFFFFFFFFFFFFFFF:FFFFFFFFFFFFFFFFFFFFFFFFFFFFF
FFFF:FFFFFFFFFF
@A00155:342:HHGFNDSXY:1:2538:21549:6903 1:N:0:GAACCTAG+TCCGCATA
AGACAAGGCAAGGTATGGACCCTTAGCAAAGCCAGAAAGATCACCTTGCCCAAAGAAGACCAACATTT
TCATACATGATGAAATTCATTATTGGTCTCGCGATCAGCTTGAGACATTTCTCCAGGTTTACAAACCT
AAAAATTTGTGGGGC
+
FFFFFFF:FFFFFFFFFFFFFFFFFFFFFFFFFFFFFFFFFFFFFFFFFFFFFFFFFFFFFFFFFFFF
FFFFFFFFFFFFFFFFFFFFFFFFFFFFFFFFFFFFFFFFFFFFFFFFFFFFFFFFFFFFFFFFFFFF
FFFFFFFFFFFFFFF
@A00155:342:HHGFNDSXY:1:2569:5104:8046 1:N:0:GAACCTAG+TCCGCATA
GATCTAGGCAAGGTATGGACCCTTAGCAAAGCCAGAAAGATCACCTTGCCCAAAGAAGACCAACATTT
TCATACATGATGAAATTCATTATTGGTCTCGCGATCAGCTTGAGACATTTCTCCAGGTTTACAAACCT
AAAAATTTGTGGGCG
+
FFF:FFFFFF:FFFFFFFFFFFFFFF:FFFFFFFFFFFFFFFFFF:FFFFFFFFFFFFFFFFF,FFF:
FFFFFFFFFFFFFFFFFFFFFFFFFF:FFFFFFFFFFFFFFFFFFFFFFFFFFFF:FFFFFFFFFFFFF
FFFFFFFFFFFFFFF
@A00155:342:HHGFNDSXY:1:1403:29306:6965 2:N:0:GAACCTAG+TCCGCATA
AGACAAGGCAAGGTATGGACCCTTAGCAAAGCCAGAAAGATCACCTTGCCCAAAGAAGACCAACATTT
TCATACATGATGAAATTCATTATTGGTCTCGCGATCAGCTTGAGACATTTCTCCAGGTTTACAAACCT
AAAAATTTGTGGGCT
+
FFFFFFFFFFFFFFFFFFFF:FFFFFFFFFFFFFFFFFFFFFFFFFFFFFFFFFFFFFFFFFFFFFFF
FFFFFFFFFFFFFFF,FFFFFFFFF,FFFFFFFFFFFFFF,:FFFFFF:FFFFF,FF,FFFFFFFFFF
FFFF:F::FFFFFFF
@A00155:342:HHGFNDSXY:1:1503:12319:18693 2:N:0:GAACCTAG+TCCGCATA
AGACAAGGCAAGGTATGGACCCTTAGCAAAGCCAGAAAGATCACCTTGCCCAAAGAAGACCAACATTT
TCATACATGATGAAATTCATTATTGGTCTCGCGATCAGCTTGAGACATTTCTCCAGGTTTACAAACCT
AAAAATTTGTGGGCT
+
FFFFFFFFFF:FF:FFFFFFFFFFFFFFFFFFFFFFFFFFFFFFFFFFFFFFFFFFFFFFFFFFFFFF
FFFFFFFFFFFFFFFFFFF:FFFFFFFFFFFFFFFFFFFFFFFFFFFFFFFFFFFFFFFFFFFF:FFF
FFFFFFFFFFFFFFF
@A00155:342:HHGFNDSXY:1:1571:16568:17566 2:N:0:GAACCTAG+TCCGCATA
AGACAAGGCAAGGTATGGACCCTTAGCAAAGCCAGAAAGATCACCTTGCCCAAAGAAGACCAACATTT
TCATACATGATGAAATTCATTATTGGTCTCGCGATCAGCTTGAGACATTTCTCCAGGTTTACAAACCT
AAAAATTTGTGGGCT
+
FFFFFFFFFFFFFFFFFFFFFFFFFFFFFFFFFFFFFFFFFFFFFFFFFFFFFFFFFFFFFFFFFFFF
FFFFFFFFFFFFFFF,FFFFFFFFFFFFFFFFFFFFFFFFFFF:FFFFFFFFFFFFFFFFFFFFFFFF
FFFFFFFFFFFFFFF
@A00155:342:HHGFNDSXY:1:2125:19470:4178 2:N:0:GAACCTAG+TCCGCATA
AGACAAGGCAAGGTATGGACCCTTAGCAAAGCCAGAAAGATCACCTTGCCCAAAGAAGACCAACATTT
TCATACATGATGAAATTCATTATTGGTCTCGCGATCAGCTTGAGACATTTCTCCAGGTTTACAAACCT
AAAAATTTGTGGGCT
+
FFFFFFFFFFFFFFFFFFFFFFFFFFFFFFFFFFFFFFFFFFFFFFFFFFFFFFFFFFFFFFF,FF:F
FFFFFFFFFFFFFFFFFFFFFFFFFF,FFFFFF:,FFFFFFF:FFFFFFFFFFF,FFFFFFFFFFF:F
FFFFFFFFFFF:FFF
@A00155:342:HHGFNDSXY:1:2208:19741:10817 2:N:0:GAACCTAG+TCCGCATA
AGACAAGGCAAGGTATGGACCCTTAGCAAAGCCAGAAAGATCACCTTGCCCAAAGAAGACCAACATTT

TCATACATGATGAAATTCATTATTGGTCTCGCGATCAGCTTGAGACATTTCTCCAGGTTTACAAACCT
AAAAATTTGTGGGCT
+
FFFFFFFFFFFFFFFFFFFFFFFFFFFFFFFFFFFFFFFFFFFFFFFFFFFFFFFFFFFFFFFFFFFFFF
FFFFFFFFFFFFFFFFFFFFFFFFFFFFFFFFFFFFFFFFFFFFFFFFFFFFFFFFFFFFFFFFFFFFFF
FFFFFFFFFFFFFFF
@A00155:342:HHGFNDSXY:1:2125:23637:1188 2:N:0:GAACCTAG+TCCGCATA
AGACAAGGCAAGGTATGGACCCTTAGCAAAGCCAGAAAGATCACCTTGCCCAAAGAAGACCAACATTT
TCATACATGATGAAATTCATTATTGGTCTCGCGATCAGCTTGAGACATTTCTCCAGGTTTACAAACCT
AAAAATTTGTGGGCT
+
FFFFFFFFFFFFFFFFFFFFFFFFFFFFFFFFFFFFFFFFFFFFFFFFFFFFFFFFFFFFFFFFFFFFFF
FFFFFFFFFFFFFFFFFFF,FF:FF:FFFFFFFFFFFFFFFFFFFFFFFFFFFFFFFFFFFFFFFF:FFF
FFFFFFFFFFFFFFF
@A00155:342:HHGFNDSXY:1:2256:29288:32957 2:N:0:GAACCTAG+TCCGCATA
AGACAAGGCAAGGTATGGACCCTTAGCAAAGCCAGAAAGATCACCTTGCCCAAAGAAGACCAACATTT
TCATACATGATGAAATTCATTATTGGTCTCGCGATCAGCTTGAGACATTTCTCCAGGTTTACAAACCT
AAAAATTTGTGGGCT
+
FFFFFFFFFFFFFFFFFFFFFFFFFFFFFFFFFFFFFFFFFFFFFFFFFFFFFFFFFFFFFFFFFFFFF:
FFFFFFFFFFFFFFFFFFFFFFFFFFFFFFFFFF:FFFFFFFFF:FFFFFFFFFFFFFFFFFFFFFFF:F
FFFFFFFFFFF:FFF
@A00155:342:HHGFNDSXY:1:2403:31367:14606 2:N:0:GAACCTAG+TCCGCATA
AGACAAGGCAAGGTATGGACCCTTAGCAAAGCCAGAAAGATCACCTTGCCCAAAGAAGACCAACATTT
TCATACATGATGAAATTCATTATTGGTCTCGCGATCAGCTTGAGACATTTCTCCAGGTTTACAAACCT
AAAAATTTGTGGGCT
+
FFFF:FFFFFFFFFF:FFFFFFFFFFFFFFFFFF,FFFFFFFFFFFFFFFFFF:FFFF:FFFFFFFFFFF
F:FFFFFFFFFFF:FFFFF:FFFFFFFFFFFFFFFFFFFFFFFFFFFF:FFFFFFFFFFFFFFFFFFFFF
,FFFFFFFFFFFFFF
@A00155:342:HHGFNDSXY:1:2403:32072:15515 2:N:0:GAACCTAG+TCCGCATA
AGACAAGGCAAGGTATGGACCCTTAGCAAAGCCAGAAAGATCACCTTGCCCAAAGAAGACCAACATTT
TCATACATGATGAAATTCATTATTGGTCTCGCGATCAGCTTGAGACATTTCTCCAGGTTTACAAACCT
AAAAATTTGTGGGCT
+
FFFFFFFFFFFFFFF,FFFFFFFFFFFFFFFFFFFFFFFFFFFFFFFFFFFFFFFFFFFFFFFFFFFFFF
FFFFFFFFFFFFFFF:FFFFFFFFFFFFFFFFFFFFFFFF:FFFFFFFFFFF:FFFFFFFFFFFF::FFF
FFFFFFFFFFFFFFF
@A00155:342:HHGFNDSXY:1:2403:31801:15671 2:N:0:GAACCTAG+TCCGCATA
AGACAAGGCAAGGTATGGACCCTTAGCAAAGCCAGAAAGATCACCTTGCCCAAAGAAGACCAACATTT
TCATACATGATGAAATTCATTATTGGTCTCGCGATCAGCTTGAGACATTTCTCCAGGTTTACAAACCT
AAAAATTTGTGGGCT
+
FFFF:FFFF:FFFFFFFFFFFFFFFFFFFFFFFF:FFFFFFFFFFFFFFFFFFFFFFFFFFFFFFFFFFF
FFF,FFFFFFFFF:FFFFF:FFFFFFFFFFFFFFF,FFFF,FFFFFFFFFFFFFFFFFFF:FFFFFFFFF
FFFFF,F:FFFFFFF
@A00155:342:HHGFNDSXY:1:2515:26386:33035 2:N:0:GAACCTAG+TCCGCATA
AGACAAGGCAAGGTATGGACCCTTAGCAAAGCCAGAAAGATCACCTTGCCCAAAGAAGACCAACATTT
TCATACATGATGAAATTCATTATTGGTCTCGCGATCAGCTTGAGACATTTCTCCAGGTTTACAAACCT
AAAAATTTGTGGGCT
+
FFFFFFFFFF:FFFFFFFFFFFFFFFFFFFFFFFFFFFFFFFFFFFFFFFFFFFFFFFFFFFFFFFFFFF
FFFFFFFFFFFFFFFFFFFFFFFFFFFFFFFFFFFFFFFFFFFFFFFFFFFFFFFFFFFFFFFFFFFFFF
FFFFFFFFFFFFFFF

@A00155:342:HHGFNDSXY:1:2629:27968:25254 2:N:0:GAACCTAG+TCCGCATA
AGACAAGGCAAGGTATGGACCCTTAGCAAAGCCAGAAAGATCACCTTGCCCAAAGAAGACCAACATTT
TCATACATGATGAAATTCAGTATTGGTCTCGCGATCAGCTTGAGACATTTCTCCAGGTTTACAAACCT
AAAAATTTGTGGGCT
+
FFFFFFFFFFFFFFFFFFFFFFFFFFFFFFFFF,FF,FF,FFFFFFFFFFFFFFFFFFFFFF,F::FF
FFFFFF:FFFFF:F:,:FF,,FFFFFFFFFFFF:FF,FF,FFF:FF,F,,F,F:,FFF,FFF:,,F,,
F:,F:FF:F,FFFF:
@A00155:342:HHGFNDSXY:1:2344:2483:19727 2:N:0:GAACCTAG+TCCGCATA
GACAAGGCAAGGTATGGACCCTTAGCAAAGCCAGAAAGATCACCTTGCCCAAAGAAGACCAACATTTT
CATACATGATGAAATTCATTATTGGTCTCGCGATCAGCTTGAGACATTTCTCCAGGTTTACAAACCTA
AAAATTTGTGG
+
FFFFFFFFFF,FFFFFFFFF:FFFFFFFFFFFFFFFFFFF:FFFFF::F,FFFFFFFFF:FFFFF:FF
:FFFFFFFFFFFFF,,FFFFFFFF,FFF,:FFFF,FF:FF:FFFFFFFFFF:FFFFFFFFFFF,:FF,
FFFF:,,FFFF
@A00155:342:HHGFNDSXY:1:1335:7609:33207 1:N:0:GAACCTAG+TCCGCATA
GACAAGGCAAGGTATGGACCCTTAGCAAAGCCAGAAAGATCACCTTGCCCAAAGAAGACCAACATTTT
CATACATGATGAAATTCATTATTGGTCTCGCGATCAGCTTGAGACATTTCTCCAGGTTTACAAACCTA
AAAATTTGTGGGCTA
+
:FF:FFFFFF,FFFFFFFFFFFFFFFFFFFFFFFFFFFFFFFFFFFFFFFFFFFFF:FFFFFFFFFFF
F:FFFFFFFFFFFFFFFFFFFFFFF:FF:,FFFFFFFFFFFFFFFFFFFFFFFFFFFFFFFFFFFFFF
FFFFFFFFFFFFFFF
@A00155:342:HHGFNDSXY:1:1363:20754:32456 1:N:0:GAACCTAG+TCCGCATA
GACAAGGCAAGGTATGGACCCTTAGCAAAGCCAGAAAGATCACCTTGCCCAAAGAAGACCAACATTTT
CATACATGATGAAATTCATTATTGGTCTCGCGATCAGCTTGAGACATTTCTCCAGGTTTACAAACCTA
AAAATTTGTGGGCCG
+
FFFFFFFFFFFFFFFFFFFFFFFFFFFFFFFFFFFFFFFFFFFF:FFFFFFFFFFF:FFFFFFFFFFF
FFFFFFFFFFFFFFFFFFFFFFFFFFFFFFFFFFFFFFFFFFFFFFFFFFFFFFFFFFFFFFFFFFFF
FFFFFFFFFFFFFFF
@A00155:342:HHGFNDSXY:1:1454:21549:12602 1:N:0:GAACCTAG+TCCGCATA
GACAAGGCAAGGTATGGACCCTTAGCAAAGCCAGAAAGATCACCTTGCCCAAAGAAGACCAACATTTT
CATACATGATGAAATTCATTATTGGTCTCGCGATCAGCTTGAGACATTTCTCCAGGTTTACAAACCTA
AAAATTTGTGGGCCC
+
FFFFFFFFFFFFFFFFFFFFFFFFFFFFFFFFFFFFFFFFFFFFFFF:FFFFFFFFFFFFFFFFFFFF
FFF:FFFFFFFFFFFFFFFFFFFFFFFFFFFFFFFFFFFFFFFFFFFFFFFFFFFFFFFFFFFFFFFF
FFFFFFFFFFFFFFF
@A00155:342:HHGFNDSXY:1:1551:21124:28682 1:N:0:GAACCTAG+TCCGCATA
GACAAGGCAAGGTATGGACCCTTAGCAAAGCCAGAAAGATCACCTTGCCCAAAGAAGACCAACATTTT
CATACATGATGAAATTCATTATTGGTCTCGCGATCAGCTTGAGACATTTCTCCAGGTTTACAAACCTA
AAAATTTGTGGGCCG
+
FFFFFFFFFFFFFFFFFFFFFFFFFFFFFFFFFFF:FFFFFFFFFFFFFFFFFFFFF:FFFFFFFFFF
FFFFFFFFFFFFFFFFFFFFFFFFFFFFFFFFFFFFFFFFFFFFFF:FFFFFFFFFFFFFFFFFFFFF
FFFFFFFFFFFFFFF
@A00155:342:HHGFNDSXY:1:1560:7301:30827 1:N:0:GAACCTAG+TCCGCATA
GACAAGGCAAGGTATGGACCCTTAGCAAAGCCAGAAAGATCACCTTGCCCAAAGAAGACCAACATTTT
CATACATGATGAAATTCATTATTGGTCTCGCGATCAGCTTGAGACATTTCTCCAGGTTTACAAACCTA
AAAATTTGTGGGCCG
+
FFFFFFFFFFFFFFFFFFFFFFFFFFFFFFFFFFFFFFFFFFFFFFFFFFFFFF:FFFFFFFFFFFFF

```
FFFFFFFFFFFFFFFFFFFFFFFFFFFFFFFF:FF:FFFFFFFFFFFFFFFFFFFFFFFFFFFFFFFF
FFFFFFFFFFFFFFF
@A00155:342:HHGFNDSXY:1:1148:30382:8140 2:N:0:GAACCTAG+TCCGCATA
GACAAGGCAAGGTATGGACCCTTAGCAAAGCCAGAAAGATCACCTTGCCCAAAGAAGACCAACATTTT
CATACATGATGAAATTCATTATTGGTCTCGCGATCAGCTTGAGACATTTCTCCAGGTTTACAAACCTA
AAAATTTGTGGGCAG
+
FFFFFFFFFFFFF:FFFFFFFFFFFFFFFFFFFFFFFFFFFFFFFFFFFFFFFFFFFFFFFFFFFFFFFF
FFFFFFFFFFFFFFFF:F:FFFFFFFFFFFFFFFFFFFFFFFFFFFFFFFFFFFFFFFFFFFFFFFFF
F:FFFFFFFFFFFFF
@A00155:342:HHGFNDSXY:1:1329:15591:14935 2:N:0:GAACCTAG+TCCGCATA
GACAAGGCAAGGTATGGACCCTTAGCAAAGCCAGAAAGATCACCTTGCCCAAAGAAGACCAACATTTT
CATACATGATGAAATTCATTATTGGTCTCGCGATCAGCTTGAGACATTTCTCCAGGTTTACAAACCTA
AAAATTTGTGGGCTA
+
FFFFFFFFF:FFFFFFFFFFFF,FFFFFFFFFFFFFFFFFFFFFFFFFFFFFFFFFFFFFFFFFFFFF
FFFFFFFFFFF:F,FFFFF,FFFF:FFFFFFFFFFFFFFFF,FFFF,FFFFFFFFFFFFFF:FFFFFF
FFFFFFFFFFFFFFF
@A00155:342:HHGFNDSXY:1:1436:13874:19820 2:N:0:GAACCTAG+TCCGCATA
GACAAGGCAAGGTATGGACCCTTAGCAAAGCCAGAAAGATCACCTTGCCCAAAGAAGACCAACATTTT
CATACATGATGAAATTCATTATTGGTCTCGCGATCAGCTTGAGACATTTCTCCAGGTTTACAAACCTA
AAAATTTGAGATCGG
+
FFFFFFFFFFFFFFFFFFFFFFFFFFFF,FFFFFFFFF:FFFFFFFFFFFFFFFFFFFFFFFFFFFFF
FFFFFFFFFFFF:FFFFF:FFFFFFFFFFFFFFFFFFFFFFFFFFFFFFFFFFFFFFFFFFFFFF:FF
FFFFFFFFFFFFFFF
@A00155:342:HHGFNDSXY:1:1522:3432:12696 2:N:0:GAACCTAG+TCCGCATA
GACAAGGCAAGGTATGGACCCTTAGCAAAGCCAGAAAGATCACCTTGCCCAAAGAAGACCAACATTTT
CATACATGATGAAATTCATTATTGGTCTCGCGATCAGCTTGAGACATTTCTCCAGGTTTACAAACCTA
AAAATTTGTGGGCTA
+
FF:FFFFFFFFFFFFFFFFFFFFFFFFFF:F:F:,FFFFFFFFFFFFFFFFFFFF:FFFFF,FFFFFF
FFFFFFFFFFFFFFFFFFFFFFF::F:FFFFFFFF:FFFFFFF,FF:FFFFFFFFFFFFFFFFFF,:,
FFFFFFF,FFFFFFF
@A00155:342:HHGFNDSXY:1:2122:28664:36511 2:N:0:GAACCTAG+TCCGCATA
GACAAGGCAAGGTATGGACCCTTAGCAAAGCCAGAAAGATCACCTTGCCCAAAGAAGACCAACATTTT
CATACATGATGAAATTCATTATTGGTCTCGCGATCAGCTTGAGACATTTCTCCAGGTTTACAAACCTA
AAAATTTGTGGGCTA
+
FFFFFFFFFFFFF,FFFFFFFFFFFFFFFFFFFFFFFFFFFFFFFFFFFFFFFFFFFFFFFFFFFFFF
FFFFFFFFFFFFFFFFFFFFFFFFFFFFFFFFFFFFFFFFFFFFFFFFFFFFFFFFFFFFFFFF:FFF
FFFFF:FFFFFFFFF
@A00155:342:HHGFNDSXY:1:2349:17653:3474 2:N:0:GAACCTAG+TCCGCATA
GACAAGGCAAGGTATGGACCCTTAGCAAAGCCAGAAAGATCACCTTGCCCAAAGAAGACCAACATTTT
CATACATGATGAAATTCATTATTGGTCTCGCGATCAGCTTGAGACATTTCTCCAGGTTTACAAACCTA
AAAATTTGCCCGTTC
+
F:FFFFFF,FFF,FFFFFFFFFFFFFFFFFFFFFFF:FFFFFFFFFFFFFFFFFF:FFFFFFFFFFFF
FFFFFFFFFFFFFFFFFF:FFFFFF:FFFFFFFFFFFFFFFFFFFFFFFF:FFFFFFFFFFFFFFFFF
FFFF:FFFFFFFFFF
@A00155:342:HHGFNDSXY:1:2559:17074:12023 2:N:0:GAACCTAG+TCCGCATA
GACAAGGCAAGGTATGGACCCTTAGCAAAGCCAGAAAGATCACCTTGCCCAAAGAAGACCAACATTTT
CATACATGATGAAATTCATTATTGGTCTCGCGATCAGCTTGAGACATTTCTCCAGGTTTACAAACCTA
AAAATTTGTGGGCTA
```

```
+
FFFFFFFFFFFFFFFFFFFFFFFFFFFFFFFFFFFFFFFFFFFFFFFFFFFFFFFFFFFFFFFFFFFF
FFFFFFFFFFFFFFFFFFFFFFFFFFFFFFFFFFFFFFFFFFFFFFFFFFFFFFFFFFFFFFF,FFFF
FFFFFFFFFFFFFFF
@A00155:342:HHGFNDSXY:1:1177:27823:29387 1:N:0:GAACCTAG+TCCGCATA
ACAAGGCAAGGTATGGACCCTTAGCAAAGCCAGAAAGATCACCTTGCCCAAAGAAGACCAACATTTTC
ATACATGATGAAATTCATTATTGGTCTCGCGATCACCTTGAGACATTTCTCCAGGTTTACAAACCTAA
AAATTTGTGGGCTAC
+
FFFFFFFFFFFFFFFFFFFFFFFFFFFFFFF:FFFFFFFFFFFFFFFFFFFFFFF:FFFFFFFFFFFFF
FFFF:FFFFFFFFFFFFFFFFFFFFFFFFFFFFFFFFFFFFFFFFFFFFFFFFFFFFFFFFFFFFFFF
FFFFFFFFFFFFFFF
@A00155:342:HHGFNDSXY:1:1245:2148:9377 1:N:0:GAACCTAG+TCCGCATA
ACAAGGCAAGGTATGGACCCTTAGCAAAGCCAGAAAGATCACCTTGCCCAAAGAAGACCAACATTTTC
ATACATGATGAAATTCATTATTGGTCTCGCGATCAGCTTGAGACATTTCTCCAGGTTTACAAACCTAA
AAATTTGTGAGCTAC
+
FFFF,FFFF,FFFFFFFFF:FFF:FFFFFFFFFFFFFFFFFFFFFFFFFFFFFF::FFFFFFFFFFFF
FFFFFFFFFFFFFFFFFFFFFFFFFFF:FF:F:FFFF:FFFFFFFFFFFFFFFFFFFFFFFFFFFFFF
FFF:FFFFFFFFFFF
@A00155:342:HHGFNDSXY:1:1312:13810:30577 1:N:0:GAACCTAG+TCCGCATA
ACAAGGCAAGGTATGGACCCTTAGCAAAGCCAGAAAGATCACCTTGCCCAAAGAAGACCAACATTTTC
ATACATGATGAAATTCATTATTGGTCTCGCGATCAGCTTGAGACATTTCTCCAGGTTTACAAACCTAA
AAATTTGTGGGCTAC
+
FFFFFFFFFFFFFFFFFFFFFFFFFFFFFFFFFF:FFFFFFFFFFFFF,FFFFFFFFFFFFFFFFFFF
F,FFFFFFFFFFFFFFFFFFFFFFFFFFFFFFFFFFFFFFFFFFFFFFFFFFFFFFFFFFFFFFFFFF
FFFFFFFFFFFFFFF
@A00155:342:HHGFNDSXY:1:2508:31069:33567 1:N:0:GAACCTAG+TCCGCATA
ACAAGGCAAGGTATGGACCCTTAGCAAAGCCAGAAAGATCACCTTGCCCAAAGAAGACCAACATTTTC
ATACATGATGAAATTCATTATTGGTCTCGCGATCAGCTTGAGACATTTCTCCAGGTTTACAAACCTAA
AAATTTGTGGGCCAC
+
FFFFFFFFFFFFFFFFFFFFFFFFFFFFFFFFFFFFFFFFFFFFFFFFF:FFFFF:FFF:FFFFFFF
FFFFFFF:FFFFFFFFFFFFFFFFFFFFFFFFFFFFFFFFFFFFFFFFFFFFFFFFFFFFFFFFFFFF
FFFFFFFFFFFFFFF
@A00155:342:HHGFNDSXY:1:2505:29161:36526 1:N:0:GAACCTAG+TCCGCATA
ACAAGGCAAGGTATGGACCCTTAGCAAAGCCAGAAAGATCACCTTGCCCAAAGAAGACCAACATTTTC
ATACATGATGAAATTCATTATTGGTCTCGCGATCAGCTTGAGACATTTCTCCAGGTTTACAAACCTAA
AAATTTGTGGGCTAC
+
FFFFFF:FFFFFFFFFFFFFFFFFFFFFFFFFFFFFFFFFFFFFFFFFFFFFFFFFFFFFFFFFFFFF
FFFFFFFFFFFFFFFFFFFFFFFFFFFFFFFFFFFFFFFFFFFFFFFFFFFFFF:FFFFFFFFFFFFF
FFFFFFFFFFFFFFF
@A00155:342:HHGFNDSXY:1:1327:8395:26804 2:N:0:GAACCTAG+TCCGCATA
CAAGGCAAGGTATGGACCCTTAGCAAAGCCAGAAAGATCACCTTGCCCAAAGAAGACCAACATTTTCA
TACATGATGAAATTCATTATTGGTCTCGCGATCAGCTTGAGACATTTCTCCAGGTTTACAAACCTAAA
AATTTGTGGGCTACT
+
FFFFFFFFFFFFFFFFFFFFFFFFFFFFFFFFFF:FFFFFFFFFFFFFFFFFFFFFFFFFFFFFFFFF
FFFFFFFFFFFFFFFFFFFFFFFFFFFFFFFFFFFFFFFFFFFFFFFFFFFFFFFFFFFFFFFFF,FF
FFFFFFFFFFFFFFF
@A00155:342:HHGFNDSXY:1:1168:6334:15718 2:N:0:GAACCTAG+TCCGCATA
AGGCAAGGTATGGACCCTTAGCAAAGCCAGAAAGATCACCTTGCCCAAAGAAGACCAACATTTTCATA
```

CATGATGAAATTCATTATTGGTCTCGCGATCAGCTTGAGACATTTCTCCAGGTTTACAAACCTAAAAA
TTTGTGGGCTACTCT
+
FFFFFFFFFFFFFFFFFFFFFFFFFFFFFFFFFFFFFFFFFFFFFFFFFFF:FF::FFFFFFFFFF
FFFFFFFFFFFFFFFFFFFFFFFFFFFFFFFFFFFFFFFFFFFFFFFFFFFFFFFFF:FFFFFFFF
FFFFFFFFFFFFFFF
@A00155:342:HHGFNDSXY:1:1227:27362:32283 2:N:0:GAACCTAG+TCCGCATA
AGGCAAGGTATGGACCCTTAGCAAAGCCAGAAAGATCACCTTGCCCAAAGAAGACCAACATTTTCATA
CATGATGAAATTCATTATTGGTCTCGCGATCAGCTTGAGACATTTCTCCAGGTTTACAAACCTAAAAA
TTTGTGGGCTACTCT
+
FFFFFFFFFFFFFFFFFFFFFFFFFFFFFFFFFFFFFFFFFFFFFFFFFFFFFFFFFFFFFFFFFF
FFFFFFFF:FFFFFFFFFFF:FFFFFFFFFFFFFFFFFFFFFFFFFFFFFF:FFFFFFFFFFFFFF
FFFFFFFFFFFFFFF
@A00155:342:HHGFNDSXY:1:1234:26341:18490 2:N:0:GAACCTAG+TCCGCATA
AGGCAAGGTATGGACCCTTAGCAAAGCCAGAAAGATCACCTTGCCCAAAGAAGACCAACATTTTCATA
CATGATGAAATTCATTATTGGTCTCGCGATCAGCTTGAGACATTTCTCCAGGTTTACAAACCTAAAAA
TTTGTGGGCTACTCT
+
FFFF:FFFFFFFFFFFFFFFFF:FFFFFFFFFFFFFFFFFFFFFFFFFFFFFFFFFFFFFFFFFFF
FFFFFFFFF::FFF:FFFFFFFFFFFFFFFFFFFFFFFFFFFFFFFFFFFFFFFFFFFFFFFFFFF
FFFFFFFFFFFFFFF
@A00155:342:HHGFNDSXY:1:1637:30445:9815 2:N:0:GAACCTAG+TCCGCATA
AGGCAAGGTATGGACCCTTAGCAAAGCCAGAAAGATCACCTTGCCCAAAGAAGACCAACATTTTCATA
CATGATGAAATTCATTATTGGTCTCGCGATCAGCTTGAGACATTTCTCCAGGTTTACAAACCTAAAAA
TTTGTGAGATCGGAA
+
FFFFFFFF:FFFFFFFFFFFFFFFFFFFFFFFFFFFFFFFFFFFFFFFFFFFFFFFFFFFFFFFFF
FFFFFFF:FFFFFFFFFFFFFFFFFFFFFFFFF:FFFFFFFFFFFFFFFFFFF:FFFFFFFF:FFF
FFFFFFFFF:FFFFF
@A00155:342:HHGFNDSXY:1:2120:32633:31610 2:N:0:GAACCTAG+TCCGCATA
AGGCAAGGTATGGACCCTTAGCAAAGCCAGAAAGATCACCTTGCCCAAAGAAGACCAACATATTCATA
CATGATGAAATTCATTATTGGTCTCGCGATCAGCTTGAGACATTTCTCCAGGTTTACAAACCTAAAAA
TTTGTGGGCTACTCT
+
FFFFFFFFFFFFFFFFFF:FFFFFFFFFFFFFFFFFFFFFFFFFFFFFFFFFFFFFFFF,::FFFF
FFFFFFFFFF:,FFFFFFF,:FFFFFFFFFFFFFFFFFF:FFFFFFFF,FF,FFFF:FFFF,FFFF
F,,FF:F,:F,F,FF
@A00155:342:HHGFNDSXY:1:2471:21712:6183 2:N:0:GAACCTAG+TCCGCATA
AGGCAAGGTATGGACCCTTAGCAAAGCCAGAAAGATCACCTTGCCCAAAGAAGACCAACATTTTCATA
CATGATGAAATTCATTATTGGTCTCGCGATCAGCTTGAGACATTTCTCCAGGTTTACAAACCTAAAAA
TTTGTGGGCTACTCT
+
FFFFFFFFFFFFFFFFFFFFFFFF:FFFFFFFFFFFFFFFFFFFFFFFFFFFFFFFFFFFFFFFFF
FFFFFFFFFFFFFFFFFFFFFFFFFFFFFFFFFFFFFFFFFFFFFFFFFFFFFFFFF,FFFFFFFF
FFF:FFFFFFFFFFF
@A00155:342:HHGFNDSXY:1:2569:5104:8046 2:N:0:GAACCTAG+TCCGCATA
AGGCAAGGTATGGACCCTTAGCAAAGCCAGAAAGATCACCTTGCCCAAAGAAGACCAACATTTTCATA
CATGATGAAATTCATTATTGGTCTCGCGATCAGCTTGAGACATTTCTCCAGGTTTACAAACCTAAAAA
TTTGTGGGCGAGATC
+
FF:FFFFFF:FFFFFFFFFFFFFFFFFFFFFFFFFFFFFFFFFFFFFFFFFFFFFFFFFFFFFFFF
FFFFFFFFFFFF::FFFFFFFFFFFFFFFFFFFFFFFFFF:FFFFFFFFFFFFFFFFFFFFFFFFF
FF,,FFFFFFFFFFF

@A00155:342:HHGFNDSXY:1:1547:19877:20697 1:N:0:GAACCTAG+TCCGCATA
CGATCTGTATGGACCCTTAGCAAAGCCAGAAAGATCACCTTGCCCAAAGAAGACCAACATTTTCATAC
ATGATGAAATTCATTATTGGTCTCGCGATCAGCTTGAGACATTTCTCCAGGTTTACAAACCTAAAAAT
TTGTGGGCTACTCTG
+
FFFFFFFFFFFFFFFFFFFFFFFFFFFFFFFFFFFFFFFFFFFFFFFFFFFFFFFFFFFFFFFFFFFF
:FFFFFFFFFFF:FFFFFFFFFFFFFFFFFFFFFFFFFFFFFFFFFFFFFFFFFFFFFFFFFFFFFFF
FFFFFFFFFFFFFFF
@A00155:342:HHGFNDSXY:1:1120:10700:21621 2:N:0:GAACCTAG+TCCGCATA
GGCAAGGTATGGACCCTTAGCAAAGCCAGAAAGATCACCTTGCCCAAAGAAGACCAACATTTTCATAC
ATGATGAAATTCATTATTGGTCTCGCGATCAGCTTGAGACATTTCTCCAGGTTTACAAACCTAAAAAT
TTGTGGGCTACTCTG
+
FFFFFFFFFFFFFFFFFFFFFFFFFFFFFFFFFFFFFFFFFFFFFFFFFFFFFFFFFFFFFFFFFFFF
:FFFFFFF:FFFFFF:FFFFFFFFFFF:FFFFFFFFFFFFFFFFFFFFFFFF:FFFFF:FFFFFFFFF
FFFFFFFFF:FFFFF
@A00155:342:HHGFNDSXY:1:1626:1425:3959 2:N:0:GAACCTAG+TCCGCATA
GGCAAGGTATGGACCCTTAGCAAAGCCAGAAAGATCACCTTGCCCAAAGAAGACCAACATTTTCATAC
ATGATGAAATTCATTATTGGTCTCGCGATCAGCTTGAGACATTTCTCCAGGTTTACAAACCTAAAAAT
TTGTGGGCTACTCTG
+
FFFFFFFFFFFFFFFFFFFFFFFFFFFFF:FFFFFFFFFFFFFFFF::FFFFFF:F,FFFFFFFFFF:
FFF:FF::FFFFFFFFFFFFF:FFFFF,FF,F:FFFFF,FFFFFFFFFFFFFFFFFFF,,FFFFFF:F
FFFFFFF,FF:FFFF
@A00155:342:HHGFNDSXY:1:1654:3947:10645 2:N:0:GAACCTAG+TCCGCATA
GGCAAGGTATGGACCCTTAGCAAAGCCAGAAAGATCACCTTGCCCAAAGAAGACCAACATTTTCATAC
ATGATGAAATTCATTATTGGTCTCGCGATCAGCTTGAGACATTTCTCCAGGTTTACAAACCTAAAAAT
TTGTGGGCTACTCTG
+
FFFFFFFFFFF:FFFFFFFFFFF:FFFFFFFFFFFFFFFFFFFFFFFFFFFFFFFFFFFFFFFFFFFF
FF:FFFFFFFFFFFFFFFFFFFFFFFFFFFFFFFFFFFFFFFFFFFFFFFFFFFFFFFFFFFFFFFFF
FFFFFFFFFFFFFFF
@A00155:342:HHGFNDSXY:1:2361:2302:34162 2:N:0:GAACCTAG+TCCGCATA
GCAAGGTATGGACCCTTAGCAAAGCCAGAAAGATCACCTTGCCCAAAGAAGACCAACATTTTCATACA
TGATGAAATTCATTATTGGTCTCGCGATCAGCTTGAGACATTTCTCCAGGTTTACAAACCTAAAAATT
TGTGGGCTACTCTG
+
:FFFFFFFFFFFFFFFFFFFFFFFFFFFFF:FFFFFFFFFFFFFFFFFFFFFFFFFFFFFFFFFFFFF
FFFFFFF:FFFFFFFFF:FFFFFFFFFF:FFFFFFFFFFFFFFFFFFF:FFFFF:FF:::FF,FF:FF
FFFFFFFFFFFF:FF
@A00155:342:HHGFNDSXY:1:1153:13928:34225 2:N:0:GAACCTAG+TCCGCATA
GCAAGGTATGGACCCTTAGCAAAGCCAGAAAGATCACCTTGCCCAAAGAAGACCAACATTTTCATACA
TGATGAAATTCATTATTGGTCTCGCGATCAGCTTGAGACATTTCTCCAGGTTTACAAACCTAAAAATT
TGTGGGCTACTCTGG
+
FFFFFFFFFFFFFFFFFFFFF::FFFFFFFFFFFFFFFFFFFFFFFFFFFFFF:FFFFFF:FFFFFFF
FFFFFFFFF:FFFFFF:FFFFFFFFFFFFFFFFFFFFFFFFFFFFFFFFFFFFFFF:FFFFFFFFFFF
FFFFFFFF:FFFFF:
@A00155:342:HHGFNDSXY:1:1438:2103:11490 2:N:0:GAACCTAG+TCCGCATA
GCAAGGTATGGACCCTTAGCAAAGCCAGAAAGATCACCTTGCCCAAAGAAGACCAACATTTTCATACA
TGATGAAATTCATTATTGGTCTCGCGATCAGCTTGAGACATTTCTCCAGGTTTACAAACCTAAAAATT
TGTGGGCTACTCTGG
+
FFFFFFFFFFFFFFFFFFFFFFFFFFFFFFFFFFFFFFFFFFFFFFFFFFFF,FFFFFFFFFFFFFFF

```
FFFFFF::FFFFFFFFFFFFFFF:FFFFFFFFFFFFFFFFFFFFFFFFFFFFFFFFFFFFFF:FFFFFF
FFFFFFFFFFFFFFF
@A00155:342:HHGFNDSXY:1:2128:2537:19507 2:N:0:GAACCTAG+TCCGCATA
GCAAGGTATGGACCCTTAGCAAAGCCAGAAAGATCACCTTGCCCAAAGAAGACCAACATTTTCATACA
TGATGAAATTCATTATTGGTCTCGCGATCAGCTTGAGACATTTCTCCAGGTTTACAAACCTAAAAATT
TGTGGGCTACTCTGG
+
FFFFFFFFFFFFFFFFFFFFFFFFFFFFFFFFFFFFFFFFFFFFFFFFFFFFFFFFFFFFFFFFFFFF
FFFFFFF::FFFFFFFFFFFFFFFFFFFF:FF,FFFFFFFFFFFFFFFFFFFF::FFFFFF:FFFF,F
FFFFFFFFFFFF,::
@A00155:342:HHGFNDSXY:1:1171:11261:9596 1:N:0:GAACCTAG+TCCGCATA
CAAGGTATGGACCCTTAGCAAAGCCAGAAAGATCACCTTGCCCAAAGAAGACCAACATTTTCATACAT
GATGAAATTCATTATTGGTCTCGCGATCAGCTTGAGACATTTCTCCAGGTTTACAAACCTAAAAATTT
GTGGGCTACTCTGGT
+
FFFFFFFFFFFFFFFFFFFFFFFFFFFFFFFFFFFFFFFFFFFFFFFFFFFFFFFFFFFFFFFFFFFF
FFFFFFFFFFFFFFFFFFFFFFFFFFFFFFFFFFFFFFFFFFFFFFFFFFFFFFFFFFFFFFFFFFFF
FFFFFFFFFFFFFFF
@A00155:342:HHGFNDSXY:1:1124:10646:34867 2:N:0:GAACCTAG+TCCGCATA
CAAGGTATGGACCCTTAGCAAAGCCAGAAAGATCACCTTGCCCAAAGAAGACCAACATTTTCATACAT
GATGAAATTCATTATTGGTCTCGCGACCAGCTTGAGACATTTCTCCAGGTTTACAAACCTAAAAATTT
GTGGGCTACTCTGGT
+
FFFFFFFFFFFFFFFFFFFFFFFFFFFFFFFFFFFFFFFFFFFFFFFFFFFFFFFFFFFFFFFFFFFF
FFFFFFFFFFFFF:FFFFFF:F,FFFFFFF:FFFFFFFFFFFFFFFFFFFFFFFFF:FFFFFFFFFFF
FFFF:FFFFFFF:FF
@A00155:342:HHGFNDSXY:1:1274:14416:34037 2:N:0:GAACCTAG+TCCGCATA
CAAGGTATGGACCCTTAGCAAAGCCAGAAAGATCACCTTGCCCAAAGAAGACCAACATTTTCATACAT
GATGAAATTCATTATTGGTCTCGCGATCAGCTTGAGACATTTCTCCAGGTTTACAAACCTAAAAATTT
GTGGGCTACTCTGGT
+
FFFFFF:FFFFFFFFFFFFFFFFFFFFFFFFF,FFFFFFF:FFFFFFFFFFFFFFFFFFFF:FFFFFFF
FF:F:F:FFFFFFFFFFFFFFFFFFF,FFFFFFFFFFFFFFFF,FFFFF::FFF,,FFF,::FFF,F:
:FFFFFF:FFFFFFF
@A00155:342:HHGFNDSXY:1:2543:14651:18630 2:N:0:GAACCTAG+TCCGCATA
CAAGGTATGGACCCTTAGCAAAGCCAGAAAGATCACCTTGCCCAAAGAAGACCAACATTTTCATACAT
GATGAAATTCATTATTGGTCTCGCGATCAGCTTGAGACATTTCTCCAGGTTTACAAACCTAAAAATTT
GTGGGCTACTCTGGT
+
FFFFFFFFFFFFFFFFFFFFFFFFFFFFFFFFFFFFFFFFFFFFFFFFFFFFFFFFFFFFFFF:FFFFF
FFFFFFFFFFFFFFFFFFFFFFFFFFFFFFFFFFFFFFFFFFFFFFFFFFFFF:FFFFFFFFFFFFF
FFFFFFFFFFFFFFF
@A00155:342:HHGFNDSXY:1:2201:5764:5901 1:N:0:GAACCTAG+TCCGCATA
AAGGTATGGACCCTTAGCAAAGCCAGAAAGATCACCTTGCCCAAAGAAGACCAACATTTTCATACATG
ATGAAATTCATTATTGGTCTCGCGATCAGCTTGAGACATTTCTCCAGGTTTACAAACCTAAAAATTTG
TGGGCTACTCTGGGC
+
F,F:FFFFFFFFFFFFFFFFFFFFFFFFF:F:F,F,F,FFFFFFFFFFFFFF,FF:FFFFFFFFFF:FF
FFFFFFFFFFFFFFFFFFFFFFFFFFFFFFFFFFFFFFFF:FFFFFFFFFFFFF:FFFFFFFFFFFFF
FFFFFFFFFFFFFFF
@A00155:342:HHGFNDSXY:1:1123:32226:9674 2:N:0:GAACCTAG+TCCGCATA
AAGGTATGGACCCTTAGCAAAGCCAGAAAGATCACCTTGCCCAAAGAAGACCAACATTTTCATACATG
ATGAACTTCATTATTGGTCTCGCGATCAGCTTGAGACATTTCTCCAGGTTTACAAACCTAAAAATTTG
TGGGCTACTCTGGTT
```

```
+
FFFFFFFFF:FFFFFFFFFFFFFFFFF:FFFFFFFFFFFFFF:FFFFFFFFFFFFFFFFFFFFFF,FF
FFFF:,FFFF:FFFFFFFFFFFFFFFFFFF:FF:FFFFFF:FFFFFFF:FFFFFFFFF:FFFFFF,FF
FFFFFFFFFFF:FFF
@A00155:342:HHGFNDSXY:1:2454:1814:12179 2:N:0:GAACCTAG+TCCGCATA
AAGGTATGGACCCTTAGCAAAGCCAGAAAGATCACCTTGCCCAAAGAAGACCAACATTTTCATACATG
ATGACATTCATTATTGGTCTCGCGATCAGCTTGAGACATTTCTCCAGGTTTACACACCTAAAAATTTG
TGGGCTACTCTGGTT
+
:,FF:,FFF:FFF:F,FFFFFFFF,FFFFFF,FFFF,FFFFF:FFFF,FFFF:FFFFFF::,::F,FF
FFFF,,:FFF,FFFFFFFF,FFFFF:FFFFFFF::FFF:FFFFFF:FF,:FFFF,FFF,F:FF,F:FF
FFFFFF,FF::FF::
@A00155:342:HHGFNDSXY:1:1530:17011:12383 1:N:0:GAACCTAG+TCCGCATA
AGGTATGGACCCTTAGCAAAGCCAGAAAGATCACCTTGCCCAAAGAAGACCAACATTTTCATACATGA
TGAAATTCATTATTGGTCTCGCGATCAGCTTGAGACATTTCTCCAGGTTTACAAACCTAAAAATTTGT
GGGCTACTCTGGTTT
+
FFF:FFFFFFFFFFFFFFFFFFFFFFFFFFFFFFFFFFFFFFFFFFFFFFFFFFFFFFFFFFFFFFFF
FFFFFFFFFFFFFFFFFFFFFFFFFFFFFFFFFFFFFFFFFFFFFFFFFFFFFFFFFFFFFFF:FFFFF
FFFFFF:FFFFFFF:
@A00155:342:HHGFNDSXY:1:1352:10104:28385 2:N:0:GAACCTAG+TCCGCATA
AGGTATGGACCCTTAGCAAAGCCAGAAAGATCACCTTGCCCAAAGAAGACCAACATTTTCATACATGA
TGAAATTCATTATTGGTCTCGCGATCAGCTTGAGACATTTCTCCAGGTTTACAAACCTAAAAATTTGT
GGGCTACTCTGGTTT
+
FFFFFFFFFFFFFFFFFFFFFFFFFFFFFFFFFFFFFFFFFFFFFFFFFFFFFFFFFFFFFFFFFFFF
FFFFFFFFF,FFFFFFFFFFFFFFFFFFFFFFFFFFFF,FFFFFFFFFFFFFFFF:FFFFFFFFFFFFF
FFFFF,FFFFFFFFF
@A00155:342:HHGFNDSXY:1:2326:2474:17519 2:N:0:GAACCTAG+TCCGCATA
AGGTATGGACCCTTAGCAAAGCCAGAAAGATCACCTTGCCCAAAGAAGACCAACATTTTCATACATGA
TGAAATTCATTATTGGTCTCGCGATCAGCTTGAGACATTTCTCCAGGTTTACAAACCTAAAAATTTGT
GGGCTACTCTGGTTT
+
FFFFFFFFFFFFFFFFFFFFFFFFFFFFFFFFFFFFFFFFFFFFFFFFFFFF:FFFFFFFFFFFFFFF
FFFFFFFF:FFFFF:FFFFFFFFFFFFFFFFFFFFFFFFFFFFFFFFFFFFFF:FFFFFFFFF:FFFFFF
FF:FFFFFFFFFFFF
@A00155:342:HHGFNDSXY:1:1351:19063:19038 2:N:0:GAACCTAG+TCCGCATA
GGTATGGACCCTTAGCAAAGCCAGAAAGATCACCTTGCCCAAAGAAGACCAACATTTTCATACATGAT
GAAATTCATTATTGGTCTCGCGATCAGCTTGAGACATTTCTCCAGGTTTACAAACCTAAAAATTTGTG
GGCTACTCTGGTTTT
+
FFFFFFFFFFFFFFFFFFFFFFFFFFFFFFFFFFFFFFFFFFFFFFFFFFFFFFFFFFFFFFFFFFFF
:FFFFFFFF:FFFFFFF:FFFFFFFFFFFFFFFFFFFFFFFFFFFFFFFFFFFFFF:FFFFFFFFFFFF
F:FFFFFFFF:FFFF
@A00155:342:HHGFNDSXY:1:1519:2338:4883 2:N:0:GAACCTAG+TCCGCATA
GGTATGGACCCTTAGCAAAGCCAGAAAGATCACCTTGCCCAAAGAAGACCAACATTTTCATACATGAT
GAAATTCATTATTGGTCTCGCGATCAGCTTGAGACATTTCTCCAGGTTTACAAACCTAAAAATTTGTG
GGCTACTCTGGTTTT
+
FFFFFFFFFFFFFFFFFFFFFFFFFFFFFFFFFFFFFFFFFF:FFFF:FFF:FFFFFFFFFFFFFFFF
FFFFFF:FFFFFFFFFFFFF:FFFFFFFFFFF::FFFFFFFF:FFFFFFFFFF:FFFFF:FFFFFFFF
:FFFFFF:FFFFFFF
@A00155:342:HHGFNDSXY:1:1602:1027:22404 2:N:0:GAACCTAG+TCCGCATA
GGTATGGACCCTTAGCACAGCCAGAAAGATCACCTTGCCCAAAGAAGACCAACATTTTCATACAGGAT
```

GAACTTCATTATTGGTCTCGCGATCAGCTTGAGACATTTCTCCCGGTTTACAAACCTAAAAATTTGTG
GGCTACTCTGGTTTT
+
FF:,FFFFFFFFF:FFF,:FFF:FF:FFFFFFFF:FFFFF:FFFF:FFFFFFFFFF,FFF:FFF,FFF
FFF,FFFF,,:FFFFFFFFFFF:FF:FFFFFFFFFFF,FF:FF,FFFFFFFF:F:FF:FFF,FFFFFF
FFFF,F,F,FF::FF
@A00155:342:HHGFNDSXY:1:1670:25699:21198 2:N:0:GAACCTAG+TCCGCATA
GGTATGGACCCTTAGCAAAGCCAGAAAGATCACCTTGCCCAAAGAAGACCAACATTTTCATACATGAT
GAAATTCATTATTGGTCTCGCGATCAGCTTGAGACATTTCTCCAGGTTTACAAACCTAAAAATTTGTG
GGCTACTCTGGTTTT
+
FFFFFFFFFFFFFFFFFFFFFFFFFFFFFFFFFFFFFFFFFFFFFFFFFFFFFFFFFFFFFFFFFFFF
FFFF,FFFFFFFFFFFFFFFFFFFFFFF:FFFFFFFFFFFFFFFFF::FFFFF:FFF::F:F:FFFFF
FFFFFFFFFFFFFFF
@A00155:342:HHGFNDSXY:1:2341:8359:8923 2:N:0:GAACCTAG+TCCGCATA
GGTATGGACCCTTAGCAAAGCCAGAAAGATCACCTTGCCCAAAGAAGACCAACATTTTCATACATGAT
GAAATTCATTATTGGTCTCGCGATCAGCTTGAGACATTTCTCCAGGTTTACAAACCTAAAAATTTGTG
GGCTACTCTGGTTTT
+
FFFFFFFFFFFFFFFFFFFFFFFFFFFFFFFFFFFFFFFFFFFFFFFFFFFFFFFFFFFFFFFFFFFF
FFFFFFFFFFFFFFFFFFFFFFFFFFFFFFFFFFFFFFFFFFFFFFFFFFFFF:FFFFFFFFFFFFFF
FFFFFFFFFFFFFFF
@A00155:342:HHGFNDSXY:1:2526:15013:18599 2:N:0:GAACCTAG+TCCGCATA
GGTATGGACCCTTAGCAAAGCCAGAAAGATCACCTTGCCCAAAGAAGACCAACATTTTCATACATGAT
GAAATTCATTATTGGTCTCGCGATCAGCTTGAGACATTTCTCCAGGTTTACAAACCTAAAAATTTGTG
GGCTACTCTGGTTTT
+
FFFFFFFFFFFFFFFFFFFFFFFFFFFFFFFFFFFFFFFFFFFFFFFFFFFFFFFFFFFFFFFFFFFF
FFFF,FFF:FF:FFFFFFFFFFFFFFFFFFFFFFF::FFFFFFFFFFFFFFFFFFFFFFFFF:FFFFF
FFFFFFFFFFFFFFF
@A00155:342:HHGFNDSXY:1:1112:28574:12054 2:N:0:GAACCTAG+TCCGCATA
GTATGGACCCTTAGCAAAGCCAGAAAGATCACCTTGCCCAAAGAAGACCAACATTTTCATACATGATG
AAATTCATTATTGGTCTCGCGATCAGCTTGAGACATTTCTCCAGGTTTACAAACCTAAAAATTTGTGG
GCTACTCTGGTTTT
+
FF:FFFFFFFFFFFFFFFFFFFFFFFFFFFFF:FFFFFFFFFFFFFFFFFFFFFFFF:FFFFFFFFFF
FFFFFFF::FFFFF:FFF,FFFFFFFFFF:FFFF:FFFFFFFFFFFFFFFFFFFFF:F:FFF,FFFF:
F:FF:FFF:F,:FF
@A00155:342:HHGFNDSXY:1:1146:30716:11256 2:N:0:GAACCTAG+TCCGCATA
GTATGGACCCTTAGCAAAGCCAGAAAGATCACCTTGCCCAAAGAAGACCAACATTTTCATACATGATG
AAATTCATTATTGGTCTCGCGATCAGCTTGAGACATTTCTCCAGGTTTACAAACCTAAAAATTTGTGG
GCTACTCTGGTTTTT
+
FFFFFFFFFFFFFFFFFFFFFFFFFFFFFFFFFFFFFFFFFFFFFFFFFFFFFFFFFFFFFFFFFFFF
FFFFFFFFFFFFFFFFFFFFFFFFFFFFFFFFFFFFFFFFFFFFFFFFFFFFFFFFFFFFFFFFFFFF
FFFFFFFFFFFFFFF
@A00155:342:HHGFNDSXY:1:1170:30969:2707 2:N:0:GAACCTAG+TCCGCATA
GTATGGACCCTTAGCAAAGCCAGAAAGATCACCTTGCCCAAAGAAGACCAACATTTTCATACATGATG
AAATTCATTATTGGTCTCGCGATCAGCTTGAGACATTTCTCCAGGTTTACAAACCTAAAAATTTGTGG
GCTACTCTGGTTTTT
+
:::FFFFFFFFFFFFFFFFFFFFFFFFFFFFFFFFFFFFFFFFFFFFFFFFFFFFFFFFFFFFFFFFF
FFFFFFFFFFFFFFFFFFFFFFFFFFFFFFFFFFFFFFFFFFFFFFFFFFFFFFFFFFFFFFFFFFFF
FFFFFFFFFFFFFFF

```
@A00155:342:HHGFNDSXY:1:1442:28031:31688 2:N:0:GAACCTAG+TCCGCATA
GTATGGACCCTTAGCAAAGCCAGAAAGATCACCTTGCCCAAAGAAGACCAACATTTTCATACATGATG
AAATTCATTATTGGTCTCGCGATCAGCTTGAGACATTTCTCCAGGTTTACAAACCTAAAAATTTGTGG
GCTACTCTGGTTTTT
+
FFFFFFFFFFFFFFFFFFFFFFFFFFFFFFFFFFFFFFFFFFFFFFFFFFFFFFFFFFFFFFFFFFFF
F:FFFFFFF:FFFFFFFFFFFFFFFFFFFFFFFFFFFFFFFFFFFFFFFFFFFFFFFFFFFFFFFFFF
FFFFFFFFFFFFFFF
@A00155:342:HHGFNDSXY:1:1524:11749:25441 2:N:0:GAACCTAG+TCCGCATA
GTATGGACCCTTAGCAAAGCCAGAAAGATCACCTTGCCCAAAGAAGACCAACATTTTCATACATGATG
AAATTCATTATTGGTCTCTCGATCAGCTTGAGACATTTCTCCAGGTTTACAAACCTAAAAATTTGTGG
GCTACTCTGGTTTTT
+
FFFFFFFFFFFFF:FFFFFF:FFFFFFFFFFFFFFFFFFFFFFFFFFF,,FFF:FFFFFFFFFFFFFF
FFF:F:,FFFFFFFF:FF,FFFFFFFFFFFFFFFF:FFFFFFFFFF:FF,FFFF::FFF:FFF:FFFF
FFFF:::FF:F,FFF
@A00155:342:HHGFNDSXY:1:1508:28212:29684 2:N:0:GAACCTAG+TCCGCATA
GTATGGACCCTTAGCAAAGCCAGAAAGATCACCTTGCCCAAAGAAGACCAACATTTTCATACATGATG
AAATTCATTATTGGTCTCGCGATCAGCTTGAGACATTTCTCCAGGTTTACAAACCTAAAAATTTGTGG
GCTACTCTGGTTTTT
+
FFFFF:FFFFFFFFFFFFFFFFFFFFFFFFFFFFFFFFFFFFFFFFFFFFFFFFFFFFFFFFFFFFFF
FFFFFFFFFFFFFFFFFFFFFFFFFFFFFFFFFFFFFFFFFFFFFFFFF:FFFFFFFFFFFFFF,FFFFFF
FFFFFFFFFFFFFFF
@A00155:342:HHGFNDSXY:1:1547:19877:20697 2:N:0:GAACCTAG+TCCGCATA
GTATGGACCCTTAGCAAAGCCAGAAAGATCACCTTGCCCAAAGAAGACCAACATTTTCATACATGATG
AAATTCATTATTGGTCTCGCGATCAGCTTGAGACATTTCTCCAGGTTTACAAACCTAAAAATTTGTGG
GCTACTCTGAGATCG
+
FFFFFFFFFFFFFFFFFFFFFFFFFFFFFFFFFFFFFFFFFFFFFFFFFFFFFFFFFFFFFFFFFFFF
FFFFFFF:FFFFFFFFFFFFFFFFFFFFFFFFFFFFFFFFFFFFFFFFFFFFF:FFFFFFFFFFFFFF
:FFFFFFFFFFFFFF
@A00155:342:HHGFNDSXY:1:2218:17662:4679 2:N:0:GAACCTAG+TCCGCATA
GTATGGACCCTTAGCAAAGCCAGAAAGATCACCTTGCCCAAAGAAGACCAACATTTTCATACATGATG
AAATTCATTATTGGTCTCGCGATCAGCTTGAGACATTTCTCCAGGTTTACAAACCTAAAAATTTGTGG
GCTACTCTGGTTTTT
+
FFFFFFFFFFFFFFFFFFFFFFFFFFFFFFFFFFFFFFFFFFFFFFFFFFFFFFFFFFFFFFFFFFFF
FFF:FF:FF:FFFFFFFFFFFFFFFFFFFFFFFFFFFFFFFFFFFFFFFFF:FFFFFFFFF:FFFFF
FFF,FFFFFFFFFFF
@A00155:342:HHGFNDSXY:1:2361:4797:14841 2:N:0:GAACCTAG+TCCGCATA
GTATGGACCCTTAGCAAAGCCAGAAAGATCACCTTGCCCAAAGAAGACCAACATTTTCATACATGATG
AAATTCATTATTGGTCTCGCGATCAGCTTGAGACATTTCTCCAGGTTTACAAACCTAAAAATTTGTGG
GCTACTCTGGTTTTT
+
FFFFFFFFFFFFFFFF,FFFFFFFFFFFFFFFFFFFFFFFFFFFFFFFFFFFFFFFFFFFFFFFFFFF
FFFFFFF:FFFFFFFFFFFFFFFFFFFFFFFFFFFFFFFFFFFFFFFFFFFFFFFFFFFFF:FFFFF
FFFFF:FFFFFFFFF
@A00155:342:HHGFNDSXY:1:2549:15691:10097 2:N:0:GAACCTAG+TCCGCATA
GTATGGACCCTTAGCAAAGCCAGAAAGATCACCTTGCCCAAAGAAGACCAACATTTTCATACATGATG
AAATTCATTATTGGTCTCGCGATCAGCTTGAGACATTTCTCCAGGTTTACAAACCTAAAAATTTGTGG
GCTACTCTGGTTTTT
+
FFFFFFFFFFFFFFFFFFFFFFFFFFFFFFFFFFFFFFFFFFFFFFFFFFFFFFFFFFFFFFFFFFFF
```

FF:FFFFFFFFFFFFFFFFFFFFFFFFFFFFFFFFFFFFFFFFFFFFFFFFFFFFFF:FFFFFFFFFF
FFFFFFFFFFFFFFF
@A00155:342:HHGFNDSXY:1:2516:6244:27461 2:N:0:GAACCTAG+TCCGCATA
GTATGGACCCTTAGCAAAGCCAGAAAGATCACCTTGCCCAAAGAAGACCAACATTTTCATACATGATG
AAATTCATTATTGGTCTCGCGATCAGCTTGAGACATTTCTCCAGGTTTACAAACCTAAAAATTTGTGG
GCTACTCTGGTTTTT
+
FFFFFFFFFFFFFFFFFFFFFFFFFFFFFFFFFFFFFFFF:F:FFFFFFFFFFFFFFFFF:FFFF
FFFF:F:FFFFFFFFFFFFFFFFFFFFF:FFFFFF:FFFFFFFFFF:FFFF,FFFFFFF,FF:FFFFF
FFFF::FFFFFFFFF
@A00155:342:HHGFNDSXY:1:1458:28203:18364 2:N:0:GAACCTAG+TCCGCATA
TATGGACCCTTAGCAAAGCCAGAAAGATCACCTTGCCCAAAGAAGACCAACATTTTCATACATGATGA
AATTCATTATTGGTCTCGCGATCAGCTTGAGACATTTCTCCAGGTTTACAAACCTAAAAATTTGTGGG
CTACTCTGGTTTTTC
+
FFFFFFFFFFFFFFFFFFFFFFFFFFFFFFFFFFFFFFFFF:FFFFFFFFFFFFFFFFFFFFFFFFFF
FFFFFFFFFFFFFF:FFFFFFFFFFFFFFFFFFFFFFFFFFFFFFFFFFFFFFFFFFFFFFFFFFFFF
FFFFFFFF,FFF:FF
@A00155:342:HHGFNDSXY:1:1520:19443:35133 2:N:0:GAACCTAG+TCCGCATA
TATGGACCCTTAGCAAAGCCAGAAAGATCACCTTGCCCAAAGAAGACCAACATTTTCATACATGATGA
AATTCATTATTGGTCTCGCGATCAGCTTGAGACATTTCTCCAGGTTTACAAACCTAAAAATTTGTGGG
CTACTCTGGTTTTTC
+
FF,:FFFFFFFFFFFFFFFFFFFFFF,FFFFFFFFFFFFFFFFFFFFFFFFFFFFFFFFFFFFFFF:F
FFF:FFFFFFFFFFFFFFFFFF:FF:FFFFFFFFFFF:FFFFFF:FF:FFFFFFFFFFF:FFFF:FFFF
FF,,F:,FFF:FFFF
@A00155:342:HHGFNDSXY:1:1521:17788:8563 2:N:0:GAACCTAG+TCCGCATA
TATGGACCCTTAGCAAAGCCAGAAAGATCACCTTGCCCAAAGAAGACCAACATTTTCATACATGATGA
AATTCATTATTGGTCTCGCGATCAGCTTGAGACATTTCTCCAGGTTTACAAACCTAAAAATTTGTGGG
CTACTCTGGTTTTTC
+
FFFFFFFFFFFFFFFFFFFFFFFFFFFFFFFFFFFFFFFFF:FFFFFFFFFFFFF:FFFFFFFFFFFF
F:FFFF,FFFFFFFFFFFFFFFFFFFFFFFFFFFFFFFF:FFFFFFFFFFFF:FF:FFF:FF,FF:FFF
FFFFFFFFFFF:F:F
@A00155:342:HHGFNDSXY:1:2118:8576:12806 2:N:0:GAACCTAG+TCCGCATA
TATGGACCCTTAGCAAAGCCAGAAAGATCACCTTGCCCAAAGAAGACCAACATTTTCATACATGATGA
AATTCATTATTGGTCTCGCGATCAGCTTGAGACATTTCTCCAGGTTTACAAACCTAAAAATTTGTGGG
CTACTCTGGTTTTTC
+
FFFFFFFFFFFFFFFFFFFFFFFFFFFFFFFFFFFFFFFFF:FFFF:FFF:FFFFFFFFFFFFFFFFFF
F:FFFF:FF:FFFFFFFFFFFFFFFFFFFFFFFFFFFFFFFFFFFFFFFFFFFFFFFFFFFFFFFFFFF
FFFFFFFFFFFFFFF
@A00155:342:HHGFNDSXY:1:2416:2058:9251 2:N:0:GAACCTAG+TCCGCATA
TATGGACCCTTAGCAAAGCCAGAAAGATCACCTTGCCCAAAGAAGACCAACATTTTCATACATGATGA
AATTCATTATTGGTCTCGCGATCAGCTTGAGACATTTCTCCAGGTTTACAAACCTAAAAATTTGTGGG
CTACTCTGGTTTTTC
+
FFFFFFFFFFFFFFFFFFFFFFFFFFFFFFFFFFFFFF,FFFFFFFFFFFFFFFFFFFFFFFFFFFFF:F
FFFFFF:FFFFFFFFFFFFFF,FFFFFFFFFFFFF:FFFFFFFF,FFFFFF:FFFF:FFFFFF:FF:F
:F::FFFFFFF,FFF
@A00155:342:HHGFNDSXY:1:1635:2564:26412 2:N:0:GAACCTAG+TCCGCATA
ATGGACCCTTAGCAAAGCCAGAAAGATCACCTTGCCCAAAGAAGACCAACATTTTCATACATGATGAA
ATTCATTATTGGTCTCGCGATCAGCTTGAGACATTTCTCCAGGTTTACAAACCTAAAAATTTGTGGGC
TACTCTGGTTTTT

+
FFFFFFFFFFFFFFFFFFFFFFFFFFFFFFFFFFFFFFFFFFFFFFFFFFFFFFFF,FFFFFFFFFFFF
FFFFFFFFFF:FFFFFFFFFFFFFFFFFFFFFFFFFFFFFFFFFFFFFFFFF:FFFFFFFFFF,FF:FF
FF,F:F,FFFFFF
@A00155:342:HHGFNDSXY:1:2512:4363:4194 2:N:0:GAACCTAG+TCCGCATA
ATGGACCCTTAGCAAAGCCAGAAAGATCACCTTGCCCAAAGAAGACCAACATTTTCATACATGATGAA
ATTCATTATTGGTCTCGCGATCAGCTTGAGAAATTTCTCCAGGTTTACAAACCTAAAAATTTGTGGGC
TACTCTGGTTTTTC
+
FF,,FFFFFF:FFFFFFFFF:FFF:FFFFFFFFFFF:FFFFFF,FF,F:FFF::FFFFFFFFFFF:FF
FFFFFFFFFFF,FFFFFF:FFFFFFFFFF:F,F:FFFFFFFFFFFFFFFF:F,:FFFFFF:F:F:,F,
F:FFFFFFFFF:F:
@A00155:342:HHGFNDSXY:1:2545:19696:22013 2:N:0:GAACCTAG+TCCGCATA
ATGGACCCTTAGCAAAGCCAGAAAGATCACCTTGCCCAAAGAAGACCAACATTTTCATACATGATGAA
ATTCATTATTGGTCTCGCGATCAGCTTGAGACATTTCTCCAGGTTTACAAACCTAAAAATTTGTGGGC
TACTCTGGTTTTTC
+
FFFFFFFFFFFFFFFFFFFFFFFFFFFFFFFFFFF::FFFFFFFFFFFFFFFFFFFFFFFFFFF:FF,F
FFF:FFFFFFFFFFFFFFFFFF:FFFFFFFFFFFFFFF:F:FFFFFFFFFFFF::FFFFFFFFFFFFFF
FFFFFFFFFFFFFF
@A00155:342:HHGFNDSXY:1:2628:19895:11177 2:N:0:GAACCTAG+TCCGCATA
ATGGACCCTTAGCAAAGCCAGAAAGATCACCTTGCCCAAAGAAGACCAACATTTTCATACATGATGAA
ATTCATTATTGGTCTCGCGATCAGCTTGAGACATTTCTCCAGGTTTACAAACCTAAAAATTTGTGGGC
TACTCTGGTTTTTC
+
:FFF:FFFFF:FFFFFFFFFFFFFF:FFFFF:FFFFFFFFFFFFFFFFFFFFFFFFFFFFFF:F:FFFF
FF:FFFF:FFFF:FFFFFF:FFFFFFFF:FFFFFFF:FFFFFFFFFFFFFFF::FFFFFF::,FF,F:
FFF,FF:F:FFFF:
@A00155:342:HHGFNDSXY:1:1438:2103:11490 1:N:0:GAACCTAG+TCCGCATA
ATGGACCCTTAGCAAAGCCAGAAAGATCACCTTGCCCAAAGAAGACCAACATTTTCATACATGATGAA
ATTCATTATTGGTCTCGCGATCAGCTTGAGACATTTCTCCAGGTTTACAAACCTAAAAATTTGTGGGC
TACTCTGGTTTTTCC
+
FFFFFFFFFFFFFFFFFFFFFFFFFFFFFFFFFFFFFFFFFF:FFFFFFFFFF:FFFFFFFFFFFFFF
FFFFFFFFFFFFFFFFFFFFFFFFFFFFFFFFFFFFFFFFFFFFF:FFFFFFFFFFFFFFFFFFFFFFFF
FFFFFFFFF:FFFFF
@A00155:342:HHGFNDSXY:1:2177:10809:15295 1:N:0:GAACCTAG+TCCGCATA
ATGGACCCTTAGCAAAGCCAGAAAGATCACCTTGCCCAAAGAAGACCAACATTTTCATACATGATGAA
ATTCATTATTGGTCTCGCGATCAGCTTGAGACATTTCTCCAGGTTTACAAACCTAAAAATTTGTGGGC
TACTCTGGTTTTTCC
+
FFFFFFFFFFFFFFFFFFFFFFFFFFFFFFFFFFFFFFFF:FF::FFFFFFFFFFFFFFFFFFFFFFFF
FFFFFFFFFFFFFFFFFFFFFFFFFFFFFFFFFFFFFFFFFFFFFFFFFFFFFFFFFFFFFFFFFFFFF
FFFFFFFFFFFFFFF
@A00155:342:HHGFNDSXY:1:2361:2302:34162 1:N:0:GAACCTAG+TCCGCATA
ATGGACCCTTAGCAAAGCCAGAAAGATCACCTTGCCCAAAGAAGACCAACATTTTCATACATGATGAA
ATTCATTATTGGTCTCGCGATCAGCTTGAGACATTTCTCCAGGTTTACAAACCTAAAAATTTGTGGGC
TACTCTGGTTTTTCC
+
FFF:F:FF:FFFFFFFFFFF:FFF:FFFFFFFFF,:FFFFFFF,FFFFFFFFFFFFFFFFFFFFFFFFF
FF:FFFFFFFFFFFFFFF:FFFFFFFFFFFFFFFFFFFFFFFFFFFFFFF:FFFFF:FFFFFFFFFFFFF
FFFFFFFFFFFFFFF
@A00155:342:HHGFNDSXY:1:2516:6244:27461 1:N:0:GAACCTAG+TCCGCATA
ATGGACCCTTAGCAAAGCCAGAAAGATCACCTTGCCCAAAGAAGACCAACATTTTCATACATGATGAA

```
ATTCATTATTGGTCTCGCGATCAGCTTGAGACATTTCTCCAGGTTTACAAACCTAAAAATTTGTGGGC
TACTCTGGTTTTTCC
+
FFFFFFFFF:FFFF:,FFFF:FF:FFFFFFFFFFFFFFFF:FFF,FF:FFFF:FFF:F:::F,FFFFFFF
FFFFFFFFFFFFFFFFFFFF:F:FFFFFFFFFF:FFFFFFFFFFFFFFFFFFFFFFFFFFFFFFFFFFFF
FFFFFFFFF::F:FF
@A00155:342:HHGFNDSXY:1:1206:19307:9345 2:N:0:GAACCTAG+TCCGCATA
ATGGACCCTTAGCAAAGCCAGAAAGATCACCTTGCCCAAAGAAGACCAACATTTTCATACATGATGAA
ATTCATTATTGGTCTCGCGATCAGCTTGAGACATTTCTCCAGGTTTACAAACCTAAAAATTTGTGGGC
TACTCTGGTTTTTCC
+
FFFFFFFFFFFFFFFFFFFFFFFFFFFFFFFFFFFFFFFFFFFFFFFFFFFFFFFFFFFFFFFF:FFFFF
FFFFFF:FFFFFFFFFFFFFFFFFFFFFFFFFFFFFFFFFFFFFFFFFFFFFFFFFFFFFFFFFFFFFFF
FFFFFFFFFFFFFFF
@A00155:342:HHGFNDSXY:1:1415:19678:34194 2:N:0:GAACCTAG+TCCGCATA
ATGGACCCTTAGCAAAGCCAGAAAGATCACCTTGCCCAAAGAAGTCCAACATTTTCATACATGATGAA
ATTCATTATTGGTCTCGCGATCAGCTTGAGACATTTCTCCAGGTTTACAAACCTAAAAATTTGTGGGC
TACTCTGGTTTTTCC
+
FFFFFFFFFFFFFFF:FFFFFFFFFFFFFFFFFFFFFFFFF:FFFFFFFFFFFFFFFFFFFFFF:FF:FF
F,:FFF,FFFFFFFFFFFFFF,FF::,FFFFFFFFFFFFFFFFFFFFFFFFFFFFFFF:FFFFF:FFF:F
FFFFFFFFF,:FFFF
@A00155:342:HHGFNDSXY:1:1426:1154:21183 2:N:0:GAACCTAG+TCCGCATA
ATGGACCCTTAGCAAAGCCAGAAAGATCACCTTGCCCAAAGAAGACCAACATTTTCATACATGATGAA
ATTCATTATTGGTCTCGCGATCAGCTTGAGACATTTCTCCAGGTTTACAAACCTAAAAATTTGTGGGC
TACTCTGGTTTTTCC
+
FFFFFFFFFFFF:FFFFFFFFFFFFFFFFFFF:FFFFFFFFF,FFFFFFFFFF:FFFFFFFF:FF:FFFF
F:FFF:FFFFFF:FFFFFFFFFFFF:FFFFFF,,FFFFFFFF,FFF,FFF,FFFF:,:FFF,,FFFFFFF
::F:FFFFF:F::FF
@A00155:342:HHGFNDSXY:1:1568:10212:3396 2:N:0:GAACCTAG+TCCGCATA
ATGGACCCTTAGCAAAGCCAGAAAGATCACCTTGCCCAAAGAAGACCAACATTTTCATACATGATGAA
ATTCATTATTGGTCTCGCGATCAGCTTGAGACATTTCTCCAGGTTTACAAACCTAAAAATTTGTGGGC
TACTCTGGTTTTTCC
+
FFFFFFFFFFFFFFFFFFFFFFFFFFFFFFFFFFFFFFFFFFFFFFFFFFFFFFFFFFFFFFFFFFFFFF
FFFFFFFFFFFFFFFFFFFFFFFFFFFFFFFFFFFFFFFFFFFFFFFFFFFFFFFFFFFF:FFFFFFFFF
FF:FFFFFFFFFF::
@A00155:342:HHGFNDSXY:1:2229:30870:9674 2:N:0:GAACCTAG+TCCGCATA
ATGGACCCTTAGCAAAGCCAGAAAGAACACCTTGCCCAAAGAAGACCAACATTTTCATACATGATGAA
ATTCATTATTGGTCTCGCGATCAGCTTGAGACATTTCTCCAGGTTTACAAACCTAAAAATTTGTGGGC
TACTCTGGTTTTTCC
+
FFFFFFFFFFFFFFFFFFFFFFFFFFFFFFFFFFFFFFFFFFFFFFFFFFFFFFFFFFFFFFFFFFFFFF
FFFFFFFFFFFFFFFFFFFFFFFFFFFFFFFFFF:FFFFFFFFFFFFFFFFFFFFFFFFFFFFFFFFFFF
FFFFFFFFFFFFFF:
@A00155:342:HHGFNDSXY:1:2434:10068:2801 2:N:0:GAACCTAG+TCCGCATA
ATGGACCCTTAGCAAAGCCAGAAAGATCACCTTGCCCAAAGAAGACCAACATTTTCATACATGATGAA
ATTCATTATTGGTCTCGCGATCAGCTTGAGACATTTCTCCAGGTTTACAAACCTAAAAATTTGTGGGC
TACTCTGGTTTTTCC
+
FFFFFFFFFFFFFFFFFFFFFFFFFFFFFFFFFFFFFFFFFFFFFFFFFFFFFFFFFFFFFFFFFFFFFF
FFFFFFFFFFFFFFFFFFFFFFFFFFFFFF:FFFFFFFFF,FFFFFFFFFFFFF:FFFFFFFF:FFF:FF
F:FFFFFFFFFFFFF
```

```
@A00155:342:HHGFNDSXY:1:2504:3622:17065 2:N:0:GAACCTAG+TCCGCATA
ATGGACCCTTAGCAAAGCCAGAAAGATCACCTTGCCCAAAGAAGACCAACATTTTCATACATGATGAA
ATTCATTATTGGTCTCGCGATCAGCTTGAGACATTTCTCCAGGTTTACAAACCTAAAAATTTGTGGGC
TACTCTGGTTTTTCC
+
FFFFFFFFFFFFFFFFFFFFFFFFFFFFFFFFFFFFFFFFFFFFFFFFFFFFFFFFFFFFFFFFFFFF
FFFFFFFFFFFFFFFFFFFFFFFFFF:FFFFFFFFFFFFFFFFFFFFFFFF:FFFFFFFFFFFF,FFFFF
FF:FFFFFFFFFF:F
@A00155:342:HHGFNDSXY:1:2514:14633:26960 2:N:0:GAACCTAG+TCCGCATA
ATGGACCCTTAGCAAAGCCAGAAAGATCACCTTGCCCAAAGAAGACCAACATTTTCATACATGATGAA
ATTCATTATTGGTCTCGCGATCAGCTTGAGACATTTCTCCAGGTTTACAAACCTAAAAATTTGTGGGC
TACTCTGGTTTTTCC
+
FFFFFFFFFFFFFFFFFFFFFFFFFFFFFFFFFFFF:FFFFFFFFFFFFFFFF:FF:FFFFFFFFFFFF
FFFFFFFFFFFFFFFFFFFFFFFFFF:FFFFFFFFFFFFFFFFFFFFFFFFFFFF:FFFFFFF,F:FFFFF
FFFFFFFF:FF:F:F
@A00155:342:HHGFNDSXY:1:2304:28266:35509 2:N:0:GAACCTAG+TCCGCATA
TGGACCCTTAGCAAAGCCAGAAAGATCACCTTGCCCAAAGAAGACCAACATTTTCATACATGATGAAA
TTCATTATTGGTCTCGCGATCAGCTTGAGACATTTCTCCAGGTTTACAAACCTAAAAATTTGTGGGCT
ACTCTGGTTTTTCCA
+
FFFFFFFFFFFFFFFFFFFFFFFFFFFFFFFFFFFFFFFFFFFFFFFFFF:FFFFFFFFFFFFFFFFF:
FFFFFFFFFFFFFFFFFFFFFFFFFFFFFF:FFF,FFFFFFFFFFFFFFFFFFFFFFFFFF:FFFFF:FF
F,,FFF:FFFFFF:F
@A00155:342:HHGFNDSXY:1:2305:24740:35321 2:N:0:GAACCTAG+TCCGCATA
GGACCCTTAGCAAAGCCAGAAAGATCACCTTGACCAAAGAAGACCAACATTTTCATACACGATGAAAT
TCATTATTGGTCTCGCGATCATCTTGAGACATTTCTCCAGGTTTACAAAACTAAAAATTTGTGGGCTA
CTCTGGTTTTTCAA
+
F,FFFFFFFF:FFFFF:F,FFFF:FFF,FF:F,F:FFFF:F:FF,FF:F,::F,F:FFFFFF,:F:::
F,:,FFFF,:FF::F,FF::,,,FF:F,FFF,F:,FFFF,::FFF,:FF,,,FFFFFF,:,,F,,::F
:F:,,,:F,::F,F
@A00155:342:HHGFNDSXY:1:1646:2130:31360 1:N:0:GAACCTAG+TCCGCATA
GGACCCTTAGCAAAGCCAGAAAGATCACCTTGCCCAAAGAAGACCAACATTTTCATACATGATGAAAT
TCATTATTGGTCTCGCGATCAGCTTGAGACATTTCTCCAGGTTTACAAACCTAAAAATTTGTGGGCTA
CTCTGGTTTTTCCAC
+
FFFFFFFFFFFFFFFFFFFFFFFFFFF:,FFFFFFFFFF:FFFFFFFFFFFFFFFFFFFFFF:FFFFFFF
FFFFFFFFFFFFFFFFFFFFFFFFFFFFFFFFFFF:FFFFFFFFFFFFFFFFFFFFFFFFFFFFFFFFFF
FFFFFFFFFFFFFFF
@A00155:342:HHGFNDSXY:1:1646:1651:31720 1:N:0:GAACCTAG+TCCGCATA
GGACCCTTAGCAAAGCCAGAAAGATCACCTTGCCCAAAGAAGACCAACATTTTCATACATGATGAAAT
TCATTATTGGTCTCGCGATCAGCTTGAGACATTTCTCCAGGTTTACAAACCTAAAAATTTGTGGGCTA
CTCTGGTTTTTCCAC
+
FFFFFF:FFFFFFFFFFFFFFFFFFFFFFFFF,FFFFFF,FF,:FFFFFFFFFFFF:FFFFFFFFFFFF
FFFFFFFFFFFFFFFFFFFFFFFFFFFFFFFF:FFF:FFFFF:FFFFFFFFFFFFFFFFFFFFFFFFFFFF
FFFFFF:,FFFFFFF
@A00155:342:HHGFNDSXY:1:2118:8576:12806 1:N:0:GAACCTAG+TCCGCATA
GGACCCTTAGCAAAGCCAGAAAGATCACCTTGCCCAAAGAAGACCAACATTTTCATACATGATGAAAT
TCATTATTGGTCTCGCGATCAGCTTGAGACATTTCTCCAGGTTTACAAACCTAAAAATTTGTGGGCTA
CTCTGGTTTTTCCAC
+
FFFFFFFFFFFFFFFFFFFFFFFFFFFFFFFFFFFFFFFFFFFFFFF:FFF,FFFFFFFFFFFFFFFFFF
```

FFFFFFFFFFFFFFFFFFFFFFFFFFFFFFFFFFFFFFFFFFFFFFFFFFFFFFFFFFFFFFFFFFFF
FFFFFFFFFFFFFFF
@A00155:342:HHGFNDSXY:1:1159:7247:12978 2:N:0:GAACCTAG+TCCGCATA
GGACCCTTAGCAAAGCCAGAAAGATCACCTTGCCCAAAGAAGACCAACATTTTCATACATGATGAAAT
TCATTATTGGTCTCGCGATCAGCTTGAGACATTTCTCCAGGTTTACAAACCTAAAAATTTGTGGGCTA
CTCTGGTTTTTCCAC
+
FFFFFFFFFFFFFFFFFFFFFFFFFFFFFFFFFFFFFFFFFFFFFFFFFFFFFFFFFFFFFFFFFFFF
FFFFFFFFFFFFFFFFFF:FFFFFFFFF:FF:FFFFFFFFFFFFFFFFFFF:FF:FFFFFFFFFFFFF
F:FFFFFFFFFFFFF
@A00155:342:HHGFNDSXY:1:1217:27190:4429 2:N:0:GAACCTAG+TCCGCATA
GGACCCTTAGCAAAGCCAGAAAGATCACCTTGCCCAAAGAAGACCAACATTTTCATACATGATGAAAT
TCATTATTGGTCTCGCGATCAGCTTGAGACATTTCTCCAGGTTTACAAACCTAAAAATTTGTGGGCTA
CTCTGGTTTTTCCAC
+
FF:FFFFFFFFFFFFFFFFFFFFFFFFFFFFFFFFFFFFFFFFFFFFFFFFFFFFFFFFFFFFFFFFF
FFFFFFFFFFFFFFFFFFFFFFFFFFFFFFFFFFFFFFFFFFFFFFFFFFFFFFFFFFFFFFFFFFFF
FFFFFFFFFFFFFFF
@A00155:342:HHGFNDSXY:1:1402:26992:19648 2:N:0:GAACCTAG+TCCGCATA
GGACCCTTAGCAAAGCCAGAAAGATCACCTTGCCCAAAGAAGACCAACATTTTCATACATGATGAAAT
TCATTATTGGTCTCGCGATCAGCTTGAGACATTTCTCCAGGTTTACAAACCTAAAAATTTGTGGGCTA
CTCTGGTTTTTCCAC
+
FFFFFFFFFFFFF:FFFFFFFFFFFFFFFFFFFFFFFFFFFFFFFFFFFFFFFFFFFFFFFFFFFFF:
FFFF,FFFFFFFFFFFFFFFFFFFFFFFFFFFFFFFFFFFFFFFFFFFFFFF:FFFFFFFFFFFFFFF
FFFFFFF,FFFFFFF
@A00155:342:HHGFNDSXY:1:1413:21522:20885 2:N:0:GAACCTAG+TCCGCATA
GGACCCTTAGCAAAGCCAGAAAGATCACCTTGCCCAAAGAAGACCAACATTTTCATACATGATGAAAT
TCATTATTGGTCTCGCGATCAGCTTGAGACATTTCTCCAGGTTTACAAACCTAAAAATTTGTGGGCTA
CTCTGGTTTTTCCAC
+
FFFFFFFFFFFFFFFFFFFFFFFFFFFFFFFFFFFFFFFFFF:FF:FFFFFFFFFFFFFFFFFFF:FF
FFF:FFFFFFFFFFFFFFFFFFFFFFFFFF:FFFFFFFFFFFFFFFFFFFFF,FFFFFFFFF:FFFFF
FFFFFFFFFFFFFFF
@A00155:342:HHGFNDSXY:1:1427:20971:8312 2:N:0:GAACCTAG+TCCGCATA
GGACCCTTAGCAAAGCCAGAAAGATCACCTTGCCCAAAGAAGACCAACATTTTCATACATGATGAAAT
TCATTATTGGTCTCGCGATCAGCTTGAGACATTTCTCCAGGTTTACAAACCTAAAAATTTGTGGGCTA
CTCTGGTTTTTCCAC
+
FFFFFFFFFFFFFFFFFFFFFFFFFFFFFFFFFFFFFFFFFFFFFFFFFFF:FFFFFFFFFFFFF,F:
FFFFF:FFFFFFFFFFFFFFFFF,FFFFFFF::FFFFFFFF:F::FFFFFFFFFFFFFFFFFFFFFFF
FFFFFFFFFFFFFFF
@A00155:342:HHGFNDSXY:1:2217:28754:15154 2:N:0:GAACCTAG+TCCGCATA
GGACCCTTAGCAAAGCCAGAAAGATCACCTTGCCCAAAGAAGACCAACATTTTCATACATGATGAAAT
TCATTATTGGTCTCGCGATCAGCTTGAGACATTTCTCCAGGTTTACAAACCTAAAAATTTGTGGGCTA
CTCTGGTTTTTCCAC
+
FFFFFF:FFFFFFFFFFFFFFFFFFFFFFFFFFFFFFFFFFFFFFFFFFFFFFFFFFFFFFFFFFFFF
FFF,FFFFFFFFFFFFFFFFFFFFFFFFFFF,FFFFFFFFF:FFFFFFFFFFFFFFFFFFFFFFFFFF
FFFFFFF:FFFFFFF
@A00155:342:HHGFNDSXY:1:2219:14027:12915 2:N:0:GAACCTAG+TCCGCATA
GGACCCTTAGCAAAGCCAGAAAGATCACCTTGCCCAAAGAAGACCAACATTTTCATACATGATGAAAT
TCATTATTGGTCTCGCGATCAGCTTGAGACATTTCTCCAGGTTTACAAACCTAAAAATTTGTGGGCTA
CTCTGGTTTTTCCAC

+
FFFFFFFFFFFFFFFFFFFFFFFFFFFFFFFFFFFFFFFFFFFFFFFFFFFFFFFFFFFFFFFFFF
FFFFFFFFFFFFFFFFFFFFFFFFFFFFFFFFFFFFFFFFFFF:FFF,FFFF:FFFFFFFFF:FFF
FFFFFFFFFFFF:FF
@A00155:342:HHGFNDSXY:1:2326:12138:20509 2:N:0:GAACCTAG+TCCGCATA
GGACCCTTAGCAAAGCCAGAAAGATCACCTTGCCCAAAGAAGACCAACATTTTCATACATGATGAAAT
TCATTATTGGTCTCGCGATCAGCTTGAGACATTTCTCCAGGTTTACAAACCTAAAAATTTGTGGGCTA
CTCTGGTTTTTCCAC
+
FFFFFFFFFFFFFFFFFFFFFFFFFFFFFFFFFFFFFFFFFFFFFFFFFFFFFFFFFFFFFFFFFF
FFF:FFFFFFFFFFFFFFFFFFFFFFFFFFFFFFFFFFFFFFFFFFFFFFFFFFFFFFFFFFFFFF
FFFFFFF,FFFFFFF
@A00155:342:HHGFNDSXY:1:2334:8793:28087 2:N:0:GAACCTAG+TCCGCATA
GGACCCTTAGCAAAGCCAGAAAGATCACCTTGCCCAAAGAAGACCAACATTTTCATACATGATGAAAT
TCATTATTGGTCTCGCGATCAGCTTGAGACATTTCTCCAGGTTTACAAACCTAAAAATTTGTGGGCTA
CTCTGGTTTTTCCAC
+
FFFFFFFFFFFFFFFFFFFFFFFFFFFFFFF:FFFFFFFFFFFFFFFFFFFFFFFFFFFFFFFFFF
FFFFFFFFFFFFFFFFFFFFFFFFFFFFFFFFFFFFFFFFFFFFFFF,FFFFFFFFFFFFFFF:FF
FFFFFFFFFFFFFFF
@A00155:342:HHGFNDSXY:1:2643:16776:3771 2:N:0:GAACCTAG+TCCGCATA
GGACCCTTAGCAAAGCCAGAAAGATCACCTTGCCCAAAGAAGACCAACATTTTCATACATGATGAAAT
TCATTATTGGTCTCGCGATCAGCTTGAGACATTTCTCCAGGTTTACAAACCTAAAAATTTGTGGGCTA
CTCTGGTTTTTCCAC
+
FFFFFFFFFFFFFFFFFFFFFFFFFFFFFFFFFFFFFFFFFFFFFF:FFFFFFFFFFFFFFFFFFF
FFFFFFFFFFFFFFFFFFFFFFFFFFFFFF,FFFFFFFFFF:FFFFF:FFF,FFFFFFFFFFFFFF
FFFFFFFFFFFFFFF
@A00155:342:HHGFNDSXY:1:2663:20184:23954 2:N:0:GAACCTAG+TCCGCATA
GGACCCTTAGCAAAGCCAGAAAGATCACCTTGCCCAAAGAAGACCAACATTTTCATACATGATGAAAT
TCATTATTGGTCTCGCGATCAGCTTGAGACATTTCTCCAGGTTTACAAACCTAAAAATTTGTGGGCTA
CTCTGGTTTTTCCAC
+
FFFFFFFFFFFFFFFFFFF:FFFFFFFFFFFFFFFFFFFFFFFFFFFFFFFFFFFFFFFFFFFFF,
FFFFFFFFFFFFFFFFFFFFFFFFFFFFFFFFFFFFFFFFFFFFFFFFFFFFFFFFFFFFFFFFFF
FFFFFFFFFFFF:FF
@A00155:342:HHGFNDSXY:1:1318:17951:3333 2:N:0:GAACCTAG+TCCGCATA
GACCCTTAGCAAAGCCAGAAAGATCACCTTGCCCAAAGAAGACCAACATTTTCATACATGATGAAATT
CATTATTGGTCTCGCGATCAGCTTGAGACATTTCTCCAGGTTTACAAACCTAAAAATTTGTGGGCTAC
TCTGGTTTTTCCAC
+
F:FFFFFFFFFFFFFFFFFFFFFFFFFFFFFFFFFFFFFFFFFFFFFFFFFFFFFFFFFFFFFFFF
FFFFFFFFFFFFFFFFFFFFFFFFFFFFFFFFFFFFFFFFFFFFFFFFFFFFFFFFFFFFFFFFFF
FFFFFFFFFFFFFF
@A00155:342:HHGFNDSXY:1:1141:14805:34679 1:N:0:GAACCTAG+TCCGCATA
GACCCTTAGCAAAGCCAGAAAGATCACCTTGCCCAAAGAAGACCAACATTTTCATACATGATGAAATT
CATTATTGGTCTCGCGATCAGCTTGAGACATTTCTCCAGGTTTACAAACCTAAAAATTTGTGGGCTAC
TCTGGTTTTTCCACC
+
FF:FFFFFFF:FFFFFFFFFF:FFFF:F::FF:FFFFFFF:FFFFFFFFFFFFFF:FFFFFFFFFF
FFFFFFFFFFFFFFF:FFFFF:FFFFFFFFFFFFFF:FFFFFFFFFFFFFFFFFFFFFFFFFFFF:
FFFFFFFF:F:FFFF
@A00155:342:HHGFNDSXY:1:2321:3685:5400 2:N:0:GAACCTAG+TCCGCATA
GACCCTTAGCAAAGCCAGAAAGATCACCTTGCCCAAAGAAGACCAACATTTTCATACATGATGAAATT

```
CATTATTGGTCTCGCGATCAGCTTGAGACATTTCTCCAGGTTTACAAACCTAAAAATTTCTGGGCTAC
TCTGGTTTTTCCACC
+
F:FFFFFFFFFFF,FFFFFFFFF,,FF:FF,FFFFF:FFFF,FFFFFFF:FFFFFFFF:FFFF:FFFF
FF:,FFF,FFFFFFFFF:FFFFFFF:FFFFFFFFFFFFFFFFFFFFF:FFFFF:FF,FFFF,FFFFF,
FF,:FFFFFFFFFFF
@A00155:342:HHGFNDSXY:1:1570:23909:26428 2:N:0:GAACCTAG+TCCGCATA
CCCTTAGCAAAGCCAGAAAGATCACCTTGCCCAAAGAAGACCAACATTTTCATACATGATGAAATTCA
TTATTGGTCTCGCGATCAGCTTGAGACATTTCTCCAGGTTTACAAACCTAAAAATTTGTGGGCTACTC
TGGTTTTTCCACCTG
+
FFFFFFFFFFFFFFFFFFFFFFFFFFFFFFFFF:FFFFFFFFFFFFFFFFFFFFFFFFFFFFFF:FFF
FFF,FFFFFFFFFFFFFFFFFFFFFFFFFFFFFFFFFFFFFFFFFFF:FFFFFFFFFFFFFFFFFFFF
FFFFFFFFFFFFFFF
@A00155:342:HHGFNDSXY:1:2410:29930:6010 2:N:0:GAACCTAG+TCCGCATA
CCCTTAGCAAAGCCAGAAAGATCACCTTGCCCAAAGAAGACCAACATTTTCATACATGATGAAATTCA
TTATTGGTCTCGCGATCAGCTTGAGACATTTCTCCAGGTTTACAAACCTAAAAATTTGTGGGCTACTC
TGGTTTTTCCACCTG
+
FFF:FFF:FFFF:FFFFFF:FFFFFFFFFFFFFFFFFFFFF:F,FFF:FFFFFFFFFFFFFFFF:F:F
FFFFFFFFFFFFFFFFFFFFFFFFFFFF,,FFFFFFFFFFFFFFFFFFF:FFFFFF,FFFF,FFF:FF
F,FF,FFF,FFFF:F
@A00155:342:HHGFNDSXY:1:1270:18394:30217 2:N:0:GAACCTAG+TCCGCATA
CCTTAGCAAAGCCAGAAAGATCACCTTGCCCAAAGAAGACCAACATTTTCATACATGATGAAATTCAT
TATTGGTCTCGCGATCAGCTTGAGACATTTCTCCAGGTTTACAAACCTAAAAATTTGTGGCCTACTCT
GGTTTTTCCACCTGA
+
FFFFFFFFFFFFFFFFFFFFFFFFFFFFFFFFFFFFFFFFFFFFFFFFFFFFFFFFF:FF,FFFFFFF
FFFFFFFFFFFFFFFFFFFFFFFFFFFFFFFFFFFFFFFFFFFFFF::FFFFF,:FFFFFF:FFFFFF
FFFFFFFFFFFFFFF
@A00155:342:HHGFNDSXY:1:2111:28989:16000 2:N:0:GAACCTAG+TCCGCATA
CCTTAGCAAAGCCAGAAAGATCACCTTGCCCAAAGAAGACCAACATTTTCATACATGATGAAATTCAT
TATTGGTCTCGCGATCAGCTTGAGACATTTCTCCAGGTTTACAAACCTAAAAATTTGTGGGCTACTCT
GGTTTTTCCACCTGA
+
FFFFFFFFFFFFFFFFFFFFFFFFFFFFFFFFFFFFFFFFFFFFFFFFFFFFFFFFFFFFFFF:FFFF
FFFFFFFFFFF:FFFFFFFFFFFFFFFFFFFFFFFFFF:FFFF,FFFFFFFFF:FFFF:FFFFFFFFF
FFFFFFFFFFFFFFF
@A00155:342:HHGFNDSXY:1:2403:23719:16454 2:N:0:GAACCTAG+TCCGCATA
CCTTAGCAAAGCCAGAAAGATCACCTTGCCCAAAGAAGACCAACATTTTCATACATGATGAAATTCAT
TATTGGTCTCGCGATCAGCTTGAGACATTTCTCCAGGTTTACAAACCTAAAAATTTGTGGGCTACTCT
GGTTTTTCCACCTGA
+
FFFFFFFFFFFFFFFFFFFFFFFFFFFFFFF:FFFFFF,FFFFFFFFFFFFFFFFFFFFF:FFFFFFF
F:FFFFFFFFFFFFFFFFFFFFFFFFFFFFFFFFFFFFFFFFFFFFFFFFFFFFFFFFFFFFFFFFFF
FFFFFFFFFFFFFFF
@A00155:342:HHGFNDSXY:1:2473:22842:4288 2:N:0:GAACCTAG+TCCGCATA
CCTTAGCAAAGCCAGAAAGATCACCTTGCCCAAAGAAGACCAACATTTTCATACATGATGAAATTCAT
TATTGGTCTCGCGATCAGCTTGAGACATTTCTCCAGGTTTACAAACCTAAAAATTTGTGGGCTACTCT
GGTTTTTCCACCTGA
+
FFFFFFFFFFFFFFFFFFFFFFFFFFFFFFFFFFFFFFFFFFFFFFFFFFFFFFFFFFFFFF:FFFFF
FFFFFFFFFFFFFFFFFFFFFFFFFFFFFFFFFFFFFFFFFFFFFFFF:FFFFFFFFFFFFFFFFFFF
FFFFFFFFFFFFFFF
```

```
@A00155:342:HHGFNDSXY:1:2550:10691:21919 2:N:0:GAACCTAG+TCCGCATA
CCTTAGCAAAGCCAGAAAGATCACCTTGCCCAAAGAAGACCAACATTTTCATACATGATGAAATTCAT
TATTGGTCTCGCGATCAGCTTGAGACATTTCTCCAGGTTTACAAACCTAAAAATTTGTGGGCTACTCT
GGTTTTTCCACCTGA
+
FFFFFFFFFFFFFFFFFFFFFFFFFFFFFFFFFFFFFFFFFFFFFFFFFFFFFFFFFFFFFFFFFFF:
:FFFFFFFFFFFFFFFFFFFFFFFF:FFFFFFFFFFFFFFFFFFFFFFFFFFFFFFFFFFFFFFFFFFF
FFFFFFFFF:FFFFF
@A00155:342:HHGFNDSXY:1:2550:9860:22138 2:N:0:GAACCTAG+TCCGCATA
CCTTAGCAAAGCCAGAAAGATCACCTTGCCCAAAGAAGACCAACATTTTCATACATGATGAAATTCAT
TATTGGTCTCGCGATCAGCTTGAGACATTTCTCCAGGTTTACAAACCTAAAAATTTGTGGGCTACTCT
GGTTTTTACACCTGA
+
:,FFFFFFFF::FFFFFFFFFFF:FFFFFFFFFFFFF:FF,FFFFFFFFFFFF:FF:FFFFFFFFFFF
FFFFFFFFFFF,FFFFF:FFFFFFFFFFFFFFFFFFF:FFFF,FFFF,FFFFFFFFFFFF,FFFF,F:F
FFFFFFF,,F,F:,F
@A00155:342:HHGFNDSXY:1:2405:11406:25629 2:N:0:GAACCTAG+TCCGCATA
CTTAGCAAAGCCAGAAAGATCACCTTGCCCAAAGAAGACCAACATTTTCATACATGATGAAATTCATT
ATTGGTCTCGCGATCAGCTTGAGACATTTCTCCAGGTTTACAAACCTAAAAATTTGTGGGCTACTCTG
GTTTTTCCACCTGA
+
FFFFFFFFFFFFFFFFFFFFFFFFFFFFFFFFFFFFFFFFFFFFFFFFFFFFFFFFFFFFFFFFFFFFF
FFFFFFFFFFFFFFFFFFFFFFFFFFFFFFFFFFFFFFFFFFFFFF:FFFFFFFFFFFFFFFFFFFFFF
FFFFFFFFFFFFFF
@A00155:342:HHGFNDSXY:1:1137:26250:4022 1:N:0:GAACCTAG+TCCGCATA
CTTAGCAAAGCCAGAAAGATCACCTTGCCCAAAGAAGACCAACATTTTCATACATGATGAAATTCATT
ATTGGTCTCGCGATCAGCTTGAGACATTTCTCCAGGTTTACAAACCTAAAAATTTGTGGGCTACTCTG
GTTTTTCCACCTGAG
+
FFFFFFFFFFFFFFFFFFFFFFFFF:FFFFFFF,:F,F:FFFFFFFFFFFFFF,FFFFFFFFFFFFFF
FFFFFFFFFFFFFFFFFFFFFFFFFFFF:FFFFFFFFFFFFFFFFFFFFFFFFFFFFFFFFFFFFFFFF
FFFFFFFFFFFFFFF
@A00155:342:HHGFNDSXY:1:1426:1154:21183 1:N:0:GAACCTAG+TCCGCATA
CTTAGCAAAGCCAGAAAGATCACCTTGCCCAAAGAAGACCAACATTTTCATACATGATGAAATTCATT
ATTGGTCTCGCGATCAGCTTGAGACATTTCTCCAGGTTTACAAACCTAAAAATTTGTGGGCTACTCTG
GTTTTTCCACCTGAG
+
F:,FFFF:FFFFFFF,FFF:FFFF::FFFFF,:FF,F:FF,FF,:FFFFFFFFF:F:FFFFF,FFFF,
:,FFFFFFFFFF::FFFF:FFFFFFF,,FFFFFFFFFFF:FF:,FFFFF:F:F:FFFFFFFF:FFF:F
FFFFFFFFFFFFFFF
@A00155:342:HHGFNDSXY:1:1508:28212:29684 1:N:0:GAACCTAG+TCCGCATA
CTTAGCAAAGCCAGAAAGATCACCTTGCCCAAAGAAGACCAACATTTTCATACATGATGAAATTCATT
ATTGGTCTCGCGATCAGCTTGAGACATTTCTCCAGGTTTACAAACCTAAAAATTTGTGGGCTACTCTG
GTTTTTCCACCTGAG
+
FF:FFFFFFFFFFFFFFFFFFFFFFFFFFFFFFFFFFFFFFFFFFFFFFFFFFFFFFFFFFFFFFFFFF
FFFFFFFFFFFFFFFFFFFFFFFFFFFFFFFFFFFFFFFFFFFFFFFFFFFFFFFFFFFFFFFFFFFFF
FFFFFFFFFFFFFFF
@A00155:342:HHGFNDSXY:1:1611:21441:6965 1:N:0:GAACCTAG+TCCGCATA
CTTAGCAAAGCCAGAAAGATCACCTTGCCCAAAGAAGACCAACATTTTCATACATGATGAAATTCATT
ATTGGTCTCGCGATCAGCTTGAGACATTTCTCCAGGTTTACAAACCTAAAAATTTGTGGGCTACTCTG
GTTTTTCCACCTGAG
+
FFFFFFFFFFFFFFFFFFFFFFFFFFFFFFFFFF:FFF,FF:FFFF::FFFFF:FFFFFFFFFFFFFF
```

FFFFFFFFFFFFFFFFFFFFFFFFFFFFFFFFFFFFFFFFFFFFFFFFFFFFFFFFFFFFFFFFFFFF
FFFFFFFFFFFFFFF
@A00155:342:HHGFNDSXY:1:2173:2049:19382 1:N:0:GAACCTAG+TCCGCATA
CTTAGCAAAGCCAGAAAGATCACCTTGCCCAAAGAAGACCAACATTTTCATACATGATGAAATTCATT
ATTGGTCTCGCGATCAGCTTGAGACATTTCTCCAGGTTTACAAACCTAAAAATTTGTGGGCTACTCTG
GTTTTTCCACCTGAG
+
F:FFFFFFFFFFFFFFFFFFFFFFFFFFF:F:FFFF:FFFF:FFFFFFFFFFFFFFFFFFFFFFFFF,
FFFFFFFFFFFFFFFFFFFFFFFFFFFFFFFFFFFFFFF:FFFFFFFFFFFFFFFFFFFFFFFFFFFF
FFFFFFFFFFFFFFF
@A00155:342:HHGFNDSXY:1:2221:19045:24455 1:N:0:GAACCTAG+TCCGCATA
CTTAGCAAAGCCAGAAAGATCACCTTGCCCAAAGAAGACCAACATTTTCATACATGATGAAATTCATT
ATTGGTCTCGCGATCAGCTTGAGACATTTCTCCAGGTTTACAAACCTAAAAATTTGTGGGCTACTCTG
GTTTTTCCACCTGAG
+
FFF:FFFFFFFFFFFFFFFFFFFFF,FFFFFFFFFFFFFFFFFFFFFFFFFFFFFFFFFFFFFFFFFF
FFFFFFFFFFFFFFFFFFFFFFFFFFFFFFFFFFFF:FFFFFFFFFFFFFFFFFFFFFFFFFFFFFFF
FFFFFFFFFFFFFFF
@A00155:342:HHGFNDSXY:1:2261:22833:10473 1:N:0:GAACCTAG+TCCGCATA
CTTAGCAAAGCCAGAAAGATCACCTTGCCCAAAGAAGACCAACATTTTCATACATGATGAAATTCATT
ATTGGTCTCGCGATCAGCTTGAGACATTTCTCCAGGTTTACAAACCTAAAAATTTGTGGGCTACTCTG
GTTTTTCCACCTGAG
+
FFFFFFFFFFFFFFFFFFFFFFFF:FFFFFFF,FFF:,FFFFFFFFFFFFFFFFFFFFFFFFFFFFFF
FFFFFFFFFFFFFFFFFFFFFFFFFFFFFFFFFFFFFFFFFFFFFFFFFFFFFFFFFFFFFFFFFFFF
FFFFFFFFFFFF:FF
@A00155:342:HHGFNDSXY:1:2504:3622:17065 1:N:0:GAACCTAG+TCCGCATA
CTTAGCAAAGCCAGAAAGATCACCTTGCCCAAAGAAGACCAACATTTTCATACATGATGAAATTCATT
ATTGGTCTCGCGATCAGCTTGAGACATTTCTCCAGGTTTACAAACCTAAAAATTTGTGGGCTACTCTG
GTTTTTCCACCTGAG
+
FF:FFFFFFFFFFF::FFFFFFFFFF:FFFF,,FF:FFF:FFFFFF,FFF,FFFFFFFFFFFFFFFFF
FFFFFFFFFF:FFFF::FFFFF:FF:FFFFFFFFFFFFFFFFFFFFFFFFFF:FFFFFFFFFFFFFFF
FFFFFFFFFFFFFFF
@A00155:342:HHGFNDSXY:1:2602:22390:3286 1:N:0:GAACCTAG+TCCGCATA
CTTAGCAAAGCCAGAAAGATCACCTTGCCCAAAGAAGACCAACATTTTCATACATGATGAAATTCATT
ATTGGTCTCGCGATCAGCTTGAGACATTTCTCCAGGTTTACAAACCTAAAAATTTGTGGGCTACTCTG
GTTTTTCCACCTGAG
+
FFFFFFFFFFFFFFFFFFFFFFFFFFFFFFFFFFFFFFFFFFFFFFFFFFFFFFFFFFFFFFFFFFFF
FFFFFFFFFFFFFFFFFFFFFFFFFFFFFFFFFFFFFFFFFFFFFFFFFFFFFFFFFFFFFFFFFFFF
FFFFFFFFFFFFFFF
@A00155:342:HHGFNDSXY:1:1203:8974:8860 2:N:0:GAACCTAG+TCCGCATA
CTTAGCAAAGCCAGAAAGATCACCTTGCCCAAAGAAGACCAACATTTTCATACATGATGAAATTCATT
ATTGGTCTCGCGATCAGCTTGAGACATTTCTCCAGGTTTACAAACCTAAAAATTTGTGGGCTACTCTG
GTTTTTCCACCTGAG
+
FFF:FFFFFF:FFFF:FFFFFFFFFFF:FFFFFF:F:FFFF,FFFFFFFFFFFFFFFFFFFFFFFFFF
FFFFFFFFFFFFFFFFFFFFFFFFFFFFFFFFFFFFFFFFFF:FFFFFFFFFFFFFFFFFFFFFFFFF
FFFFFFF:FFFFFFF
@A00155:342:HHGFNDSXY:1:1213:22137:17127 2:N:0:GAACCTAG+TCCGCATA
CTTAGCAAAGCCAGAAAGATCACCTTGCCCAAAGAAGACCAACATTTTCATACATGATGAAATTCATT
ATTGGTCTCGCGATCAGCTTGAGACATTTCTCCAGGTTTACAAACCTAAAAATTTGTGGGCTACTCTG
GTTTTTCCACCTGAG

```
+
FFFFFFFF,FFFFFFFFF:FFFFF::FFFFFFFFFFFF,FF:F:FFFFFFFFFFFFFFFFFFFFFFFF
FFFFFFFFFFFFFFFFFFFFFFFFFFFFFFFFFFFFFFFFFFFFFF:FFFFFFFFFFFFFFFFFFFF
FFFFFFFFFFFFFFF
@A00155:342:HHGFNDSXY:1:1217:4408:14293 2:N:0:GAACCTAG+TCCGCATA
CTTAGCAAAGCCAGAAAGATCACCTTGCCCAAAGAAGACCAACATTTTCATACATGATGAAATTCATT
ATTGGTCTCGCGATCAGCTTGAGACATTTCTCCAGGTTTACAAACCTAAAAATTTGTGGGCTACTCTG
GTTTTTCCACCTGAG
+
FFFFFFFFFFFFFFFFFFFFFFFFFFFFFFFFFFFFFFFFFFFFFFFFFFFFFFFFFFFFFFFFFFFF
FFFFFFFFFFFFFFFFFFFFFFFFFFFFFFFFFFFFFFFFFFFFFFFF:FFFF:FFFFFFFFFFFFFF
FFFFFFFF:FFFFFF
@A00155:342:HHGFNDSXY:1:1520:20582:17503 2:N:0:GAACCTAG+TCCGCATA
CTTAGCAAAGCCAGAAAGATCACCTTGCCCAAAGAAGACCAACATTTTCATACATGATGAAATTCATT
ATTGGTCTCGCGATCAGCTTGAGACATTTCTCCAGGTTTACAAACCTAAAAATTTGTGGGCTACTCTG
GTTTTTCCACCTGAG
+
FFFFFFFFFFFFFFFFFF,FFFFFFFFFFFFFFFFFFFFFFFFFFFFFFFFFFF:FFFFFF:FFFFF:
FFFFFFF,FFFFFFFFFFFFFFFFFFFFFFFFFFFFFFFFFFF:FFFFFFFFFF,FFFFFFFFFFFFFF
FFFFFFFFFFFFFFF
@A00155:342:HHGFNDSXY:1:2153:21594:13683 2:N:0:GAACCTAG+TCCGCATA
CTTAGCAAAGCCAGAAAGATCACCTTGCCCAAAGAAGACCAACATTTTCATACATGATGAAATTCATT
ATTGGTCTCGCGATCAGCTTGAGACATTTCTCCAGGTTTACAAACCTAAAAATTTGTGGGCTACTCTG
GTTTTTCCACCTGAG
+
FFFFFFF:FFFFFFFFFFFFF:FFFFFFFFFFFFFFFFFFFFFFFFFFFFFFFFFFFFFFFFF,FF:F:
FFFFFFFFFFFFFFFFFF:FFFFFFFFFFFFFF,FFF:FFFF:FFFFFFF,FFFFFFFFFFFFFFFF
FFFFFFFFFFFFFFF
@A00155:342:HHGFNDSXY:1:2405:9815:27978 2:N:0:GAACCTAG+TCCGCATA
CTTAGCAAAGCCAGAAAGATCACCTTGCCCAAAGAAGACCAACATTTTCATACATGATGAAATTCATT
ATTGGTCTCGCGATCAGCTTGAGACATTTCTCCAGGTTTACAAACCTAAAAATTTGTGGGCTACTCTG
GTTTTTCCACCTGAG
+
FFFFFFFF,FFFFFFFFFFFFFFFFFFFFFFF:FFFFFFFFFFFFFFFFFFFFFFFFFFFFFFFF:FFF
FFFFFFFFFFFFFFFFFFFFFFFF:FFFFFFFFFFFFFFFF:FFFFFFFF:FFFFFFFFFFF,FFFF:
FFFFFFFFFF:FFFF
@A00155:342:HHGFNDSXY:1:2471:25626:36855 2:N:0:GAACCTAG+TCCGCATA
CTTAGCAAAGCCAGAAAGATCACCTTGCCCAAAGAAGACCAACATTTTCATACATGATGAAATTCATT
ATTGGTCTCGCGATCAGCTTGAGACATTTCTCCAGGTTTACAAACCTAAAAATTTGTGGGCTACTCTG
GTTTTTCCACCTGAG
+
FFFFFFFFFFFFFFFFFF:FFFFFFFFFFFFFFFFFFFFFFFFFFFFFFFFFFFFFFF:FFFFFFFFFF
FFFFFFFFFFFFFFFFFFFFFF:FFFFFFFFFFFFFFFFFFFFFFFFFFFFFFFFFFFFFFFFF:FFFF
FFFFFFFFFFFFFFF
@A00155:342:HHGFNDSXY:1:2471:25690:36808 2:N:0:GAACCTAG+TCCGCATA
CTTAGCAAAGCCAGAAAGATCACCTTGCCCAAAGAAGACCAACATTTTCATACATGATGAAATTCATT
ATTGGTCTCGCGATCAGCTTGAGACATTTCTCCAGGTTTACAAACCTAAAAATTTGTGGGCTACTCTG
GTTTTTCCACCTGAG
+
FFFFFFFFFFFFFFFFFFFFFFFFFFFFFFFFFFFFFFFFFFFFFFFFFFFFFFFFFFFFFFFFFF,F
FFFFFFFFFFFFFFFFFFFFFFFFFFFFFFFFFFFFFFFFFFFF:FFFFFFFFFFFFFFFFFFFFFFF
FFFF:FFFFFFFFFF
@A00155:342:HHGFNDSXY:1:2504:6361:16047 2:N:0:GAACCTAG+TCCGCATA
CTTAGCAAAGCCAGAAAGATCACCTTGCCCAAAGAAGACCAACATTTTCATACATGATGAAATTCATT
```

```
ATTGGTCTCGCGATCAGCTTGAGACATTTCTCCAGGTTTACAAACCTAAAAATTTGTGGGCTACTCTG
GTTTTTCCACCTGAG
+
FFFFFFFFFFFFFFFFFFFFFFFFFFFFFFFFFFFFFFFFFFFFFFFFFFFFFFFFFFFFFFFFFFFF
FFFFFFFFFFFF:FFFFF:FFFFFFFFFFFFFFFFFFFFFFF::FFFFFFFFFFFFFFFFFF,FFFFF
FF,FFFFF:F:FFFF
@A00155:342:HHGFNDSXY:1:2477:23882:1110 2:N:0:GAACCTAG+TCCGCATA
CTTAGCAAAGCCAGAAAGATCACCTTGCCCAAAGAAGACCAACATTTTCATACATGATGAAATTCATT
ATTGGTCTCGCGATCAGCTTGAGACATTTCTCCAGGTTTACAAACCTAAAAATTTGTGGGCTACTCTG
GTTTTTCCACCTGAG
+
FFFFFFFFFFFFFFFFFFFFFFFFFFFFFFFFFFFFFFFFFFFFFFFFFFFFFFFFFFFFFFFFFFFF
FFFFFFFFFFFFFFFFFFFFFFF:FFFFFFFFFFFFFFFFFFFFFFFFFFFFFFFFFFFFFFFFFFFF
FFFFFFFFFFFFFFF
@A00155:342:HHGFNDSXY:1:2617:15691:7999 2:N:0:GAACCTAG+TCCGCATA
CTTAGCAAAGCCAGAAAGATCACCTTGCCCAAAGAAGACCAACATTTTCATACATGATGAAATTCATT
ATTGGTCTCGCGATCAGCTTGAGACATTTCTCCAGGTTTACAAACCTAAAAATTTGTGGGCTACTCTG
GTTTTTCCACCTGAG
+
FFFFFFFF,FFFFFFFFF:FFFFFFFFFFFFFFFFFFFFFFFFFFFFFFFFFFFFFFFFFFFFFFFFF
FFFFFFFFFFFFFFFFFFFFFFFFFFFFFFFFFFFFFFFFF,FFFFFFFFFFFFFFFFFFFFFFFFFF
FF,FFFFFFFFFFFF
@A00155:342:HHGFNDSXY:1:2337:31665:1595 2:N:0:GAACCTAG+TCCGCATA
TAGCAAAGCCAGAAAGATCACCTTGCCCAAAGAAGACCAACATTTTCATACATGATGAAATTCATTAT
TGGTCTCGCGATCAGCTTGAGACATTTCTCCAGGTTTACAAACCTAAAAATTTGTGGGCTACTCTGGT
TTTTCCACCTGAGAT
+
FFFFF,FFFFFFFFFFFFFFFFFFFFFFFFFFFFFFFFFFFFFFFFFFFFFFFFFFFF:FFFFFF,
FFFFFFFFFFF::FFFFFFFFFFFFFFFF:FFFFFFFFFF,FFFFFFF:F::FFFFFFFFFFFFFFFF
FFFFFFFFFFFFFFF
@A00155:342:HHGFNDSXY:1:1643:8160:13244 2:N:0:GAACCTAG+TCCGCATA
AGCAAAGCCAGAAAGATCACCTTGCCCAAAGAAGACCAACATTTTCATACATGATGAAATTCATTATT
GGTCTCGCGATCAGCTTGAGACATTTCTCCAGGTTTACAAACCTAAAAATTTGTGGGCTACTCTGGTT
TTTCCACCTGAGATC
+
FFFFFFFFFFFFFFFFFFFFFFFFFFFFFFFFFFFFFFFFFFFFFFFFFFFFFFFFFFFFFFFFFFFF
FFFFFFFFFFFFFFFFFFFFFFFFFFFFFFFFFFFFFFFFFFFFFFFF:FFFFFFFFFFFFFFFFFFF
FFFFFFFFFFFFFF:
@A00155:342:HHGFNDSXY:1:1677:1732:12978 2:N:0:GAACCTAG+TCCGCATA
AGCAAAGCCAGAAAGATCACCTTGCCCAAAGAAGACCAACATTTTCATACATGATGAAATTCATTATT
GGTCTCGCGATCAGCTTGAGACATTTCTCCAGGTTTACAAACCTAAAAATTTGTGGGCTACTCTGGTT
TTTCCACCTGAGATC
+
F:FFFFFFFFFFFFFFFFFFFFFFFFFFFF:FFFFFFFFFFFFFFFFFFFFFFFFFFFFFFF:FFFFF
FFFFFFFFFFFFFFF:FFFF:FFFFFFFFFFFFFFFFFFF:FFFFFFFFFFFFFF:FFFFFFFFFFFF
FFF,F:FFFFFFFFF
@A00155:342:HHGFNDSXY:1:2356:6479:23610 2:N:0:GAACCTAG+TCCGCATA
AGCAAAGCCAGAAAGATCACCTTGCCCAAAGAAGACCAACATTTTCATACATGATGAAATTCATTATT
GGTCTCGCGATCAGCTTGAGACATTTCTCCAGGTTTACAAACCTAAAAATTTGTGGGCTACTCTGGTT
TTTCCACCTGAGATC
+
FFFFFFFFFFFFFFFFFFFFFFFFFFFFFFFFFFFFFF:FFFFFFFFFFFFFFFFFFFFFFFFFFFFF
FFFFFFFFFFFFFFFFFFFFFFFFFFFFFFFFFFFFFFF:FFFFFFFFFFFF::FFFFFF:FFFFFFF
FFF,FFFFFFFFF:F
```

@A00155:342:HHGFNDSXY:1:2416:2058:9251 1:N:0:GAACCTAG+TCCGCATA
GCAAAGCCAGAAAGATCACCTTGCCCAAAGAAGACCAACATTTTCATACATGATGAAATTCATTATTG
GTCTCGCGATCAGCTTGAGACATTTCTCCAGGTTTACAAACCTAAAAATTTGTGGGCTACTCTGGTTT
TTCCACCTGAGATCT
+
:FF,FFFFFFF:FFFFFFFFF,FFFFFFFFFF:FFFFFFFFF,FFF:FF,:FFFFFFF:FFFFFFFFF
FFFFFFFFFFFFFFFFFFFFFFFFFF:FFFFFFFFFFFFFFFFFFFFFFFFFFFFFFFFFFFFFFFFF
FFFFFFFFFFFFFFF
@A00155:342:HHGFNDSXY:1:1437:26937:27477 2:N:0:GAACCTAG+TCCGCATA
GCAAAGCCAGAAAGATCACCTTGCCCAAAGAAGACCAACATTTTCATACATGATGAAATTCATTATTG
GTCTCGCGATCAGCTTGAGACATTTCTCCAGGTTTACAAACCTAAAAATTTGTGGGCTACTCTGGTTT
TTCCACCTGAGATCT
+
FFFFFFFFF:FF:FFFFFFFFFFFFFFF,FFF,FFFF:FFF:FFFF:FFFFFFFFFFFFFFFFFFFFF
FFFFFFFFFFFFFFFFFFFFFFFFFFFFFFFFFFFFFFFFFFFFFFFFFFFF:FFFFFFFFFFFFFFF
FFFFFFFFFFFFFFF
@A00155:342:HHGFNDSXY:1:1662:5113:28322 2:N:0:GAACCTAG+TCCGCATA
GCAAAGCCAGAAAGATCACCTTGCCCAAAGAAGACCAACATTTTCATACATGATGAAATTCATTATTG
GTCTCGCGATCAGCTTGAGACATTTCTCCAGGTTTACAAACCTAAAAATTTGTGGGCTACTCTGGTTT
TTCCACCTGAGATCT
+
FFFFFFFFFFFFFFFFFFFFFFF:F::FFFFFFFFFFFFFFFFFFFFFFFFFFFFFFFF,FFFFFFFFF
FFFFFFFFFFFFFFFFFFFFFFFFFFFFFFFFFFFFFFFFFFFFFFFFFF:FFFFFFFFFFFFFFFFFF
FFFFFFFFFF:FFFF
@A00155:342:HHGFNDSXY:1:2328:1045:15796 2:N:0:GAACCTAG+TCCGCATA
GCAAAGCCAGAAAGATCACCTTGCCCAAAGAAGACCAACATTTTCATACATGATGAAATTCATTATTG
GTCTCGCGATCAGCTTGAGACATTTCTCCAGGTTTACAAACCTAAAAATTTGTGGGCTACTCTGGTTT
TTCCACCTGAGATCT
+
FFFF:FFFFFFF:F:F:FFFFFFFFFF,,F:FF:FF,FF:::FFFFF,FFFFFF:FF,FF,FFFFF::
F:FFFFFFFFFFF:FFF:FFFFFFF,FFF:F:FF::FFF,,FF,F:F,FFFFFFFFFFFFFFFFFFFF
FF::,,FFFFF,F:F
@A00155:342:HHGFNDSXY:1:1402:26992:19648 1:N:0:GAACCTAG+TCCGCATA
CAAAGCCAGAAAGATCACCTTGCCCAAAGAAGACCAACATTTTCATACATGATGAAATTCATTATTGG
TCTCGCGATCAGCTTGAGACATTTCTCCAGGTTTACAAACCTAAAAATTTGTGGGCTACTCTGGTTTT
TCCACCTGAGATCTT
+
FFFFFFFFFFFFFFFFFFFF:FFFFFFFFFFFFFFF,F::FFFFFFFF:FFFFFFFFFFFFFFFFFFF
FFFFFFFF:FFFFFFFFFFFFFFFFFFFF:FFFFFFFF:FFFFFFFFFFFFFFFFFFFFFFFFFFFFF
FFFFFFFFFFFFFFF
@A00155:342:HHGFNDSXY:1:1138:2528:14700 2:N:0:GAACCTAG+TCCGCATA
CAAAGCCAGAAAGATCACCTTGCCCAAAGAAGACCAACATTTTCATACATGATGAAATTCATTATTGG
TCTCGCGATCAGCTTGAGACATTTCCCCAGGTTTACAAACCTAAAAATTTGTGGGCTACTCTGGTTTT
TCCACCTGAGATCTT
+
FFFFFFFFFFFFFFFFFFFFFFFFFFFFFFFFFFFFFFFFFFFFFFFFFFFFFFFFFFFFFFFFFFFF
FFFFFFFFFFFFFFFFFFFFFFFFFF,FFFFFFFFFFFFFFFFFFFFFFFFFFFFFFFFF:FFFFFFFF
FF:FFFFFFFFFFFF
@A00155:342:HHGFNDSXY:1:1161:20157:27727 2:N:0:GAACCTAG+TCCGCATA
CAAAGCCAGAAAGATCACCTTGCCCAAAGAAGACCAACATTTTCATACATGATGAAATTCATTATTGG
TCTCGCGATCAGCTTGAGACATTTCTCCAGGTTTACAAACCTAAAAAGTTGTGGGCTACTCTGGTTTT
TCCACCTGAGATCTT
+
FFFFFFFFFFFFFFFFFFFFFFFFFFFFFFFFFFFFFFFFFFFFFFFFFFFFFFFFF:F,FFFFFFFFF

FFFFFFFFFFFFFFFFFFFFF:FFF:FFFFFF,FFFFFFFFF:FFFF,FFFFFFFFFFFFFFFFFFFF
FFFFFFFFFFFFFFF
@A00155:342:HHGFNDSXY:1:1207:18982:6120 2:N:0:GAACCTAG+TCCGCATA
CAAAGCCAGAAAGATCACCTTGCCCAAAGAAGACCAACATTTTCATACATGATGAAATTCATTATTGG
TCTCGCGATCAGCTTGAGACATTTCTCCAGGTTTACAAACCTAAAAATTTGTGGGCTACTCTGGTTTT
TCCACCTGAGATCTT
+
FFFFFFFFFFFFFFFFFFFFFFFFFFFFFFFFFFFFFFFFFFFFFFFFFFFFFFF:FFFFFFFFFFFF
FFFFFFFFFFFFFFFFFFFFFFFFFFFFFFFFFFFFFF,FFFFFFFFFFFFFFFFFFFFFFFFFFFFF
:FFFFFFFFFFFFFF
@A00155:342:HHGFNDSXY:1:1305:2311:34929 2:N:0:GAACCTAG+TCCGCATA
CAAAGCCAGAAAGATCACCTTGCCCAAAGAAGACCAACATTTTCATACATGATGAAATTCATTATTGG
TCTCGCGATCAGCTTGAGACATTTCTCCAGGTTTACAAACCTAAAAATTTGTGGGCTACTCTGGTTTT
TCCACCTGAGATCTT
+
FFFFF::FFFFFFF:FFFFFFFFF,FFFFFF::FFFFFFFF:FF::FFFF:FF:FF:FFFFFFFFFFF
FFFFFFFFFF:FF:FFF:FFFFFFFFFFF:FFF:FFFF:FFFFF:FFFF,:F,:F,FFFF:,,:,:FF
F:,F,,::F,FF:::
@A00155:342:HHGFNDSXY:1:1662:12861:32597 2:N:0:GAACCTAG+TCCGCATA
CAAAGCCAGAAAGATCACCTTGCCCAAAGAAGACCAACATTTTCATACATGATGAAATTCATTATTGG
TCTCGCGATCAGCTTGAGACATTTCTCCAGGTTTACAAACCTAAAAATTTGTGGGCTACTCTGGTTTT
TCCACCTGAGATCTT
+
FFFFFFFFFFFFFFFFFFFFFFFFFFFFFFFFFFFFFFFFFFFFFFFFFFFFFFFFFFFFFFFFFFFF
FFFFFFFFFFFFFFFFFFFFFFFFFFFFFFFFFFFFFFFFFFFFFFF:FFFFFFFFFFFFFFFFFFFF
FFFFFFFFFFFFFFF
@A00155:342:HHGFNDSXY:1:2207:19135:31093 2:N:0:GAACCTAG+TCCGCATA
CAAAGCCAGAAAGATCACCTTGCCCAAAGAAGACCAACATTTTCATACATGATGAAATTCATTATTGG
TCTCGCGATCAGCTTGAGACATTTCTCCAGGTTTACAAACCTAAAAATTTGTGGGCTACTCTGGTTTT
TCCACCTGAGATCTT
+
FFFFFFFFFFFFFFFFFFFFFFFFFFFFFFFFFFFFFFFFFFFFFFFFFFFFFFFFFFFFFFFFFFFF
FFFFFFFFFFFFFFFFFFFFFFFFFFFFFFFFFFFFFF:FFFFFFFFFFFFFFFFFFFFFFFFFFFFF
FFFFFFFFFFF:FFF
@A00155:342:HHGFNDSXY:1:2215:19533:7764 2:N:0:GAACCTAG+TCCGCATA
CAAAGCCAGAAAGATCACCTTGCCCAAAGAAGACCAACATTTTCATACATGATGAAATTCATTATTGG
TCTCGCGATCAGCTTGAGACATTTCTCCAGGTTTACAAACCTAAAAATTTGTGGGCTACTCTGGTTTT
TCCACCTGAGATCTT
+
FFFFFFFFFFFFFFFFFFFFFFFFFFF:FFFFFFFFFFFFFFFFFFFFFFFFFFFF:FFFFF,FFFFF
FFFFFFFFFFFFFFFFFFFFFFFFFFFFFFFFFFFFFFFFFFFFFFFFFFFFFFFFFFFFFFFFFFFF
FFFFFFFFFFFFFFF
@A00155:342:HHGFNDSXY:1:2216:22399:34491 2:N:0:GAACCTAG+TCCGCATA
CAAAGCCAGAAAGATCACCTTGCCCAAAGAAGACCAACATTTTCATACATGATGAAATTCATTATTGG
TCTCGCGATCAGCTTGAGACATTTCTCCAGGTTTACAAACCTAAAAATTTGTGGGCTACTCTGGTTTT
TCCACCTGAGATCTT
+
FFFFFFFFFFFFFFFFFFFFFFFFFFFFFFFFFFFFFF,FFFFFFFFFFFFFFFFFFFFFFF:FF,FF
FFFFFFFF:FFFFFFFFF:FF,FFFFFFFFFF:FFFFFFFFFFFFF:F:FFFFFFFFFFFFFFFFFFF
FFFFFFFFFFFFF:F
@A00155:342:HHGFNDSXY:1:2262:32560:26663 2:N:0:GAACCTAG+TCCGCATA
CAAAGCCAGAAAGATCACCTTGCCCAAAGAAGACCAACATTTTCATACATGATGAAATTCATTATTGG
TCTCGCGATCAGCTTGAGACATTTCTCCAGGTTTACAAACCTAAAAATTTGTGGGCTACTCTGGTTTT
TCCACCTGAGATCTT

```
+
FFFFFFFFFFFFFFFFFFFFFFFFFFFFFF:FFFFFFFFFF,FFF,FFF:FFFF:F:FFFF,FFFFFF
FFFFFFFFFFFFF:FFFFFFF:F:FFFFFFFFFFFFFFFFFFF:FFF,FFFFFFFFF:F,FFFFFF:F
:FFFFFFFFFFFFFF
@A00155:342:HHGFNDSXY:1:2671:17336:13166 2:N:0:GAACCTAG+TCCGCATA
CAAAGCCAGAAAGATCACCTTGCCCAAAGAAGACCAACATTTTCATACATGATGAAATTCATTATTGG
TCTCGCGATCAGCTTGAGACATTTCTCCAGGTTTACAAACCTAAAAATTTGTGGGCTACTCTGGTTTT
TCCACCTGAGATCTT
+
FFFFFFFFFFFFFFFFFFFFFFFFFFFFFFFFFFFFFFFFFFFFFFFFFFFFFFFFFFFFFFFFFFFF
FFFFFFFFFFFFFFFFFFFFFF::FFFFFFFFFFFFFFF,FFF:FFFFFFFFFFFFFFFFFFFFFFFF
FFFFFFFFFFFFFFF
@A00155:342:HHGFNDSXY:1:1120:10700:21621 1:N:0:GAACCTAG+TCCGCATA
AAGCCAGAAAGATCACCTTGCCCAAAGAAGACCAACATTTTCATACATGATGAAATTCATTATTGGTC
TCGCGATCAGCTTGAGACATTTCTCCAGGTTTACAAACCTAAAAATTTGTGGGCTACTCTGGTTTTTC
CACCTGAGATCTTAG
+
FFFFFFFFFFFFFFFFFFFFFFFFFFFFFFFFFFFFFFFFFFFF:FFFFFFFFFFFFFFFFFFFFFFF
FFFFFFFFFFFFFFFFFFFFFFFFFFFFFFF:FFFFFFFFFFFFFFFFFFFFFFFFFFFFFFFFFFFF
FFFFFFFFFFFFFFF
@A00155:342:HHGFNDSXY:1:1146:30716:11256 1:N:0:GAACCTAG+TCCGCATA
AAGCCAGAAAGATCACCTTGCCCAAAGAAGACCAACATTTTCATACATGATGAAATTCATTATTGGTC
TCGCGATCAGCTTGAGACATTTCTCCAGGTTTACAAACCTAAAAATTTGTGGGCTACTCTGGTTTTTC
CACCTGAGATCTTAG
+
FFFFFFFFFFFFFFFFFFFFFFFFFFFFFFFFFFFFFFFFFFFFFFF:FFFFFFFFFFFFFFFFFFFF
FFFFFFFFFFFFFFFFFFFFFFFFFFFFFFFFFFFFFFFFFFFFFFFFFFFFFFFFFFFFFFFFFFFF
FFFFFFFFFFFFFFF
@A00155:342:HHGFNDSXY:1:1159:7247:12978 1:N:0:GAACCTAG+TCCGCATA
AAGCCAGAAAGATCACCTTGCCCAAAGAAGACCAACATTTTCATACATGATGAAATTCATTATTGGTC
TCGCGATCAGCTTGAGACATTTCTCCAGGTTTACAAACCTAAAAATTTGTGGGCTACTCTGGTTTTTC
CACCTGAGATCTTAG
+
F:FFFFFFFFFFFFFFFFFFFFFFFFFFFFF:FFFFF::F:FFFFFFFFFFFFFFFFFFFFFFFFFF:F
FFFFFFFFFFFFFFFFFFFFFFFFFFF:FFFFFFFFFFFFFFFFFFFFFFFFFFFFFFFFFFFFFFFF
FFFFFFFFFFFFFFF
@A00155:342:HHGFNDSXY:1:1176:10619:36198 1:N:0:GAACCTAG+TCCGCATA
AAGCCAGAAAGATCACCTTGCCCAAAGAAGACCAACATTTTCATACATGATGAAATTCATTATTGGTC
TCGCGATCAGCTTGAGACATTTCTCCAGGTTTACAAACCTAAAAATTTGTGGGCTACTCTGGTTTTTC
CACCTGAGATCTTAG
+
FFFFFFFFFFFFFFFFFFFFFFFFFFFFF:FFFFF,F,FF,FFFFFFFFFFFFFFFFFFFFFFFFFFF
FFFFFFFFFFFFFFFFFFFFFFFFFFFFFFFFFFFFFFFFFFFFFFFFFFFFFFFFFFFFFFFFFFFF
FFFFFFFFFFFFFFF
@A00155:342:HHGFNDSXY:1:1234:10077:19883 1:N:0:GAACCTAG+TCCGCATA
AAGCCAGAAAGATCACCTTGCCCAAAGAAGACCAACATTTTCATACATGATGAAATTCATTATTGGTC
TCGCGATCAGCTTGAGACATTTCTCCAGGTTTACAAACCTAAAAATTTGTGGGCTACTCTGGTTTTTC
CACCTGAGATCTTAG
+
FFFFFFFFFFFFFFFFFFFFFFFFFFFFFF:FF:FFFF,FFFFFFFFF:FFFFFFFFFFFFFFFFFFF
FFFFFFFFFFFFFFFFFFFFFFFFFFFFFFFFFFFFFFFFFFFFFFFFFFFFFFFFFFFFFFFFFFFF
FFFFFFFFFFFFFFF
@A00155:342:HHGFNDSXY:1:1426:19199:25911 1:N:0:GAACCTAG+TCCGCATA
AAGCCAGAAAGATCACCTTGCCCAAAGAAGACCAACATTTTCATACATGATGAAATTCATTATTGGTC
```

```
TCGCGATCAGCTTGAGACATTTCTCCAGGTTTACAAACCTAAAAATTTGTGGGCTACTCTGGTTTTTC
CACCTGAGATCTTAG
+
FFFFFFF:FFFFFFFFFFFFFFFFF:F:,FFFFFFFFFFFFFFFFFFFFFFFFFFFFFFFFFFFFFFFFF
FFFFFFFFFFFFFFFFFFFFFFFFFFFFFFFFFFFFFFFFFFFF:FFFFFFFFFFFFFFFFFFFFFFFFF
FFFFFFFFFFFFFFF
@A00155:342:HHGFNDSXY:1:1427:20971:8312 1:N:0:GAACCTAG+TCCGCATA
AAGCCAGAAAGATCACCTTGCCCAAAGAAGACCAACATTTTCATACATGATGAAATTCATTATTGGTC
TCGCGATCAGCTTGAGACATTTCTCCAGGTTTACAAACCTAAAAATTTGTGGGCTACTCTGGTTTTTC
CACCTGAGATCTTAG
+
FFFFFFFFFFFFFFFFFFFFFFFFFFFFFFFFFFFFFF,FFFFF:FFFFFFFFFFFFFFFFFFFFFFFFF
FFFFFFFFFFFFFFFFFFFFFFFFFFFFFFFFFFFFFFFF:FFFF:FFFFFFFFFFFFFFFFFFFFFFFF
FFFFFFFFFFFFFFF
@A00155:342:HHGFNDSXY:1:1438:16532:33849 1:N:0:GAACCTAG+TCCGCATA
AAGCCAGAAAGATCACCTTGCCCAAAGAAGACCAACATTTTCATACATGATGAAATTCATTATTGGTC
TCGCGATCAGCTTGAGACATTTCTCCAGGTTTACAAACCTAAAAATTTGTGGGCTACTCTGGTTTTTC
CACCTGAGATCTTAG
+
FFFFFFFFFFFFFFFFFF:FFFFFFFFF:,FFFFFFFFFFFFFFFFFFFFFFFFFFFFFFFFFFFFFFFF
F:FFFFFF:FFFFFFFFFFFFFFFFFFFFFFFF:FF,FFFFFFFFFFFFFFFFFFFFFFFFFFFFFFFFF
FFFFFFFFFFFFFFF
@A00155:342:HHGFNDSXY:1:1453:20853:9298 1:N:0:GAACCTAG+TCCGCATA
AAGCCAGAAAGATCACCTTGCCCAAAGAAGACCAACATTTTCATACATGATGAAATTCATTATTGGTC
TCGCGATCAGCTTGAGACATTTCTCCAGGTTTACAAACCTAAAAATTTGTGGGCTACTCTGGTTTTTC
CACCTGAGATCTTAG
+
FF:FFFFFFFFFFFFFFFFFFFFFFFFFFF::FFFFFF:FFFFFFFFFFFFFFFFFFFFFFFFFFFFFFF
FFFFF:FFFFFFFFFFFFFFFFFFFFFFFFFFFFFFFFF:FFFFFFFFFFFFFFFFFFFFFFFFFFFFFF
FFFFFFFFFFFFFFF
@A00155:342:HHGFNDSXY:1:1458:28203:18364 1:N:0:GAACCTAG+TCCGCATA
AAGCCAGAAAGATCACCTTGCCCAAAGAAGACCAACATTTTCATACATGATGAAATTCATTATTGGTC
TCGCGATCAGCTTGAGACATTTCTCCAGGTTTACAAACCTAAAAATTTGTGGGCTACTCTGGTTTTTC
CACCTGAGATCTTAG
+
FFFFFFFFFFFFFFFFFFF:FFFFFFFFFFF:FFFFF:FF:F:FFFFFFFFFFFFFF,FFFFFF:FFFFF
FFFFFFFF:FFFFFFFFFFFFFFFFFFF:FFFFFFFFFFFFFFFFFFFFFFFFFFFFFFFFFFFFFFFFF
FFFFFFFFFFFFFFF
@A00155:342:HHGFNDSXY:1:1541:27543:19382 1:N:0:GAACCTAG+TCCGCATA
CGATCTGAAAGATCACCTTGCCCAAAGAAGACCAACATTTTCATACATGATGAAATTCATTATTGGTC
TCGCGATCAGCTTGAGACATTTCTCCAGGTTTACAAACCTAAAAATTTGTGGGCTACTCTGGTTTTTC
CACCTGAGATCTTAG
+
FFFFFFFFFFFFFFFFFFFFFFFFFFFF:FFFFFFFFFFFFFFFFF:FFFFFFFFFFFFFFFFFFFFFFF
FFFFFFFFFFFFFFFFFFFFFFFFFFFFFFFFFFFFFFFFFFFFFFFFFFFFFFFFFFFFFFFFFFFFFF
FFFFFFFFFFFFFFF
@A00155:342:HHGFNDSXY:1:1615:9489:10285 1:N:0:GAACCTAG+TCCGCATA
AAGCCAGAAAGATCACCTTGCCCAAAGAAGACCAACATTTTCATACATGATGAAATTCATTATTGGTC
TCGCGATCAGCTTGAGACATTTCTCCAGGTTTACAAACCTAAAAATTTGTGGGCTACTCTGGTTTTTC
CACCTGAGATCTTAG
+
FFFFFFFFFFFFFFFFFFFFFFFFFFF:,F::FFFFFFFFFFFFFFFFFFFFFFFFFFFFFFFFFFFFFF
FFFFFFFFFFFFFFFFFFFFFFFFFFFFFFFFFFFFFFFFFFFFFFFFFFFFFFFFFFFFFFFFFFFFFF
FFFFFFFFF:FFFFF
```

```
@A00155:342:HHGFNDSXY:1:2127:13847:22936 1:N:0:GAACCTAG+TCCGCATA
CGATCTGAAAGATCACCTTGCCCAAAGAAGACCAACATTTTCATACATGATGAAATTCATTATTGGTC
TCGCGATCAGCTTGAGACATTTCTCCAGGTTTACAAACCTAAAAATTTGTGGGCTACTCTGGTTTTTC
CACCTGAGATCTTAG
+
FFFFFFFFFFFFFFFFFFFFFFFF,FFFFF,FFF:FFFFFFFFFFFFFFFFFFFFFFFFFFFFFFFFFF
FFFFFFFFFFFFFFFFFFFFFFFFFFFFFFFFFFFFFFFFFFFFFFFFFFFFFFFFFFFFFFFFFFFF
FFFFFFFFFFFFFFF
@A00155:342:HHGFNDSXY:1:2304:28266:35509 1:N:0:GAACCTAG+TCCGCATA
AAGCCAGAAAGATCACCTTGCCCAAAGAAGACCAACATTTTCATACATGATGAAATTCATTATTGGTC
TCGCGATCAGCTTGAGACATTTCTCCAGGTTTACAAACCTAAAAATTTGTGGGCTACTCTGGTTTTTC
CACCTGAGATCTTAG
+
F:FFFFFF::F:FFFFF:FFFFFF:FFFFFFFF::FF:FFFFFFFFF:FFFFFFFFFFFFFFFFFFFF
FFFFFFFFFFFFFFFFFFFFF:FFFFFFFFFFFFFFFFFFFFFFFFFFFFFFFFFFFFFFFFFFFFFF
FFFFFFFFFFFFFFF
@A00155:342:HHGFNDSXY:1:2327:5746:4836 1:N:0:GAACCTAG+TCCGCATA
AAGCCAGAAAGATCACCTTGCCCAAAGAAGACCAACATTTTCATACATGATGAAATTCATTATTGGTC
TCGCGATCAGCTTGAGACATTTCTCCAGGTTTACAAACCTAAAAATTTGTGGGCTACTCTGGTTTTTC
CACCTGAGATCTTAG
+
FFFF:FFFFF:FFFFFFFFFFFFFFFFF:FFFFFFFFFFFFFFFFFFFFFFFFFFFFFFFFFFFFFFF
FFF:FFFFFFFFFFFFFFFFFFFFFFFFFFFFFFFFFFFFFFFFFFFFFFFFFFFFFFFFFFFFFFFF
FFFFFFFFFFFFFFF
@A00155:342:HHGFNDSXY:1:2356:5367:27508 1:N:0:GAACCTAG+TCCGCATA
AAGCCAGAAAGATCACCTTGCCCAAAGAAGACCAACATTTTCATACATGATGAAATTCATTATTGGTC
TCGCGATCAGCTTGAGACATTTCTCCAGGTTTACAAACCTAAAAATTTGTGGGCTACTCTGGTTTTTC
CACCTGAGATCTTAG
+
FFFFFFFFFFFFFFFF:FFFFFFFF:FF,:FFFFFFFFFFFFFFFFFFFFFFFFFFFFFFFFFFFFFF
FFFFFFFFFFFFFFFFFFFFFFFFFFFFFFFFFFFFFFFFFFFFFFFFFFFFFFFFFFFFFFFFFFFF
FFFFFFFFFFFFFFF
@A00155:342:HHGFNDSXY:1:2405:11406:25629 1:N:0:GAACCTAG+TCCGCATA
AAGCCAGAAAGATCACCTTGCCCAAAGAAGACCAACATTTTCATACATGATGAAATTCATTATTGGTC
TCGCGATCAGCTTGAGACATTTCTCCAGGTTTACAAACCTAAAAATTTGTGGGCTACTCTGGTTTTTC
CACCTGAGATCTTAG
+
FFFF,F,FFFFFFFFFF:FF,FFF:FFFFFFFFFF:,FFFFF:FFF:FFFFFFFFFFFFFFFFFFFFF
FFFFFFFFFF:FFFF:F:FFFFF,,FFFF:FFFFFFFF:FFFFFFFFFFFFFFFFF,FFFFFFFFFFF
FF,FFFFFFFFFFFF
@A00155:342:HHGFNDSXY:1:2405:9815:27978 1:N:0:GAACCTAG+TCCGCATA
AAGCCAGAAAGATCACCTTGCCCAAAGAAGACCAACATTTTCATACATGATGAAATTCATTATTGGTC
TCGCGATCAGCTTGAGACATTTCTCCAGGTTTACAAACCTAAAAATTTGTGGGCTACTCTGGTTTTTC
CACCTGAGATCTTAG
+
FF:FFFFFFFFFFFFFF:FFFFFFFFFFFFF:F,FFFFFF:FFFFFFF,FFFFFFFFFFFFFFFFFFF
FFFFFFFFF:FFFFFFFFFF:FFFFFFFFFFFFFFFFFFFFFFFFFFFFFFFFFFFFFFFFFFFFFFF
FFFF:FF:FFFFFFF
@A00155:342:HHGFNDSXY:1:2410:29930:6010 1:N:0:GAACCTAG+TCCGCATA
AAGCCAGAAAGATCACCTTGCCCAAAGAAGACCAACATTTTCATACATGATGAAATTCATTATTGGTC
TCGCGATCAGCTTGAGACATTTCTCCAGGTTTACAAACCTAAAAATTTGTGGGCTACTCTGGTTTTTC
CACCTGAGATCTTAG
+
FF,F:FFFFFFFF,FFFFFFFFFF:FFF:FFFFF:FFFFFFFFF:,:F,FFFFFFFFFFF:,FF:FFF
```

FFFFFFFFFFFFFFFFFFFFFFFFFFF,:FFFFFFFFFFFFFFFFFFFFFF:FF:FFF:FFFFFFFFF
FFFFFFFFFFFFFFF
@A00155:342:HHGFNDSXY:1:2461:22688:15483 1:N:0:GAACCTAG+TCCGCATA
AAGCCAGAAAGATCACCTTGCCCAAAGAAGACCAACATTTTCATACATGATGAAATTCATTATTGGTC
TCGCGATCAGCTTGAGACATTTCTCCAGGTTTACAAACCTAAAAATTTGTGGGCTACTCTGGTTTTTC
CACCTGAGATCTTAG
+
FFFFFFFFFFFFFFFFFF:FFFFFFFFFFFFFFFFFFFFFFFFFFFFFFFFFFFFFFFFFFFFFFFFF
FFFFFFFFFFFFFFFFFFFFFFFFFFFFFFFFFF:FFFFFFFFFFFFFFFFFFFFFFFFFFFFFFFFF
FFFFFFFFFFFFFFF
@A00155:342:HHGFNDSXY:1:2477:23882:1110 1:N:0:GAACCTAG+TCCGCATA
AAGCCAGAAAGATCACCTTGCCCAAAGAAGACCAACATTTTCATACATGATGAAATTCATTATTGGTC
TCGCGATCAGCTTGAGACATTTCTCCAGGTTTACAAACCTAAAAATTTGTGGGCTACTCTGGTTTTTC
CACCTGAGATCTTAG
+
FFFFFFFFFFFFFFFFFFFFFFFFFFFFFFFFF:,FFFFFFFFFFFFFFFFFFFFFFFFFFFFFFFFF
FFFFFFFFFFFFFFFFFF:FFFFFFFFFFFFFFFFFFFFFFFFFFFFFFFFFFFFFFFFFFFFFFFFF
FFFFFFFFFFFFFFF
@A00155:342:HHGFNDSXY:1:2509:7102:31548 1:N:0:GAACCTAG+TCCGCATA
AAGCCAGAAAGATCACCTTGCCCAAAGAAGACCAACATTTTCATACATGATGAAATTCATTATTGGTC
TCGCGATCAGCTTGAGACATTTCTCCAGGTTTACAAACCTAAAAATTTGTGGGCTACTCTGGTTTTTC
CACCTGAGATCTTAG
+
FFFFFF:FFF:FF:FFFFFFFFFFFF,,:FFFFFFFFFFFFFF:FFF::FFFFFFFFFFFFFFF:FFF
FFFFFFFFFFFFFFFFFFFFFFFFFFFF:FFFFFFFFFFFFFFF:FFFFFFFFFFFFF,FFFFFFFFF
FFFFFFFFFFFFFFF
@A00155:342:HHGFNDSXY:1:2522:12002:2581 1:N:0:GAACCTAG+TCCGCATA
CGATCTGAAAGATCACCTTGCCCAAAGAAGACCAACATTTTCATACATGATGAAATTCATTATTGGTC
TCGCGATCAGCTTGAGACATTTCTCCAGGTTTACAAACCTAAAAATTTGTGGGCTACTCTGGTTTTCC
CACCTGAGATCTTAG
+
FFFF,FFFFF:FFFFFFFFFF,FFFFFFFFFFFF:FF,FFFFF,:FF::FF:F,FFFFFFFFFFFFFF
F:FFF:FFFFFFFFFFF:FFFFFFF:,F:FFFFFFFFFFFFF:FF:FFFFFFFFFFFFFFFFFFFFFF
FFFFFFFFFF:FFFF
@A00155:342:HHGFNDSXY:1:2576:3884:11819 1:N:0:GAACCTAG+TCCGCATA
AAGCCAGAAAGATCACCTTGCCCAAAGAAGACCAACATTTTCATACATGATGAAATTCATTATTGGTC
TCGCGATCAGCTTGAGACATTTCTCCAGGTTTACAAACCTAAAAATTTGTGGGCTACTCTGGTTTTTC
CACCTGAGATCTTAG
+
FFFFFFFFF:F:FFFFFFFFFFFFFFF,,:,FFFFFFFFFFFFFFFFFFFFFFFFFFFF:FFFFFF:F
FFFFFFFFFFFFFFFFFFFFFFFFFFFFFFFFFFFF:FFFFFFFFFFFFFFFFFFFFFFFFFFFFFFF
FFFFFFFFFFFFFFF
@A00155:342:HHGFNDSXY:1:2654:3884:1767 1:N:0:GAACCTAG+TCCGCATA
AAGCCAGAAAGATCACCTTGCCCAAAGAAGACCAACATTTTCATACATGATGAAATTCATTATTGGTC
TCGCGATCAGCTTGAGACATTTCTCCAGGTTTACAAACCTAAAAATTTGTGGGCTACTCTGGTTTTTC
CACCTGAGATCTTAG
+
FFFFFF:FFFFFFFFFF:FFFFFFF:F,:F:FFFFFFFF,FFFFFF:FFFFFFFFFFFFFFFFFFFFF
FFFFFFFFFFFFFFFFFFFFFFFFFFF,FFFFFFFFFFFFFFFFFFFFFFFFFFFFFFFFFFFFFFFF
FFFFFFFFFFFFFFF
@A00155:342:HHGFNDSXY:1:2622:30065:29387 1:N:0:GAACCTAG+TCCGCATA
AAGCCAGAAAGATCACCTTGCCCAAAGAAGACCAACATTTTCATACATGATGAAATTCATTATTGGTC
TCGCGATCAGCTTGAGACATTTCTCCAGGTTTACAAACCTAAAAATTTGTGGGCTACTCTGGTTTTTC
CACCTGAGATCTTAG

+
FFFFFFFFFFFFFFFFF,FFFFFFFFFFFF,FFFFFFFFFFFFFFFFFFFFFFFFFFFFFFFFFFFFF
FFFFFFFFFFFFFFFFFFFFFFFFFFFFFFFFFFFFFFFF:FFFFFFFFFFFFFFFFFFFFFFFFFFF
FFFFFFFFFFFFFFF
@A00155:342:HHGFNDSXY:1:2674:27977:8265 1:N:0:GAACCTAG+TCCGCATA
AAGCCAGAAAGATCACCTTGCCCAAAGAAGACCAACATTTTCATACATGATGAAATTCATTATTGGTC
TCGCGATCAGCTTGAGACATTTCTCCAGGTTTACAAACCTAAAAATTTGTGGGCTACTCTGGTTTTTC
CACCTGAGATCTTAG
+
FFFFFFFFFFFFFFFFFFFFFFFFFFFFFFFFFF:FFFFFFFFFFFFFFFFFFFFFFFFFFFFFFFFF
FFFFFFFFFF:FFFFFFFFFFFFFFFFFFFFFFFFFFFFFFFFFFFFFFFFFFFFFFFFFFFFFFFFF
FFFFFFFFFFFFFFF
@A00155:342:HHGFNDSXY:1:1364:21748:27571 2:N:0:GAACCTAG+TCCGCATA
AAGCCAGAAAGATCACCTTGCCCAAAGAAGACCAACATTTTCATACATGATGAAATTCATTATTGGTC
TCGCGATCAGCTTGAGACATTTCTCCAGGTTTACAAACCTAAAAATTTGTGGGCTACTCTGGTTTTTC
CACCTGAGATCTTAG
+
FFFFFFFFFFFFFFFFFFFFFFFFFFFFFFFFFFFFFFFFFFFFFFFFFFFFFFFFFFF:FFFFFFFF
FFFFFFFFFFF:FFFFFFFFFFFFFFFFFFFFFFFFFFFFFFFFFFF:FFFFFFFFFFFFFFFFFFFF
FFFFFFFFFFFFFFF
@A00155:342:HHGFNDSXY:1:1202:11388:33927 2:N:0:GAACCTAG+TCCGCATA
AGCCAGAAAGATCACCTTGCCCAAAGAAGACCAACATTTTCATACATGATGAAATTCATTATTGGTCT
CGCGATCAGCTTGAGACATTTCTCCAGGTTTACAAACCTAAAAATTTGTGGGCTACTCTGGTTTTTCC
ACCTGAGATCTTAG
+
FFFFFFFFFFFFFFFFFFFFFFFFFFFFFFFFFFFFFFFFFFFFFFFFFFFFFFFFFFFFFFFFFFFF
FFFFFFFFFFFFFFFFFFFFFFFFFFFFFFFFFFFFF:FFFFFFFFFFFF:FFFFFFFFFFFFFFFF:
FFFFFFFFFFFFFF
@A00155:342:HHGFNDSXY:1:1131:6976:11506 1:N:0:GAACCTAG+TCCGCATA
AGCCAGAAAGATCACCTTGCCCAAAGAAGACCAACATTTTCATACATGATGAAATTCATTATTGGTCT
CGCGATCAGCTTGAGACATTTCTCCAGGTTTACAAACCTAAAAATTTGTGGGCTACTCTGGTTTTTCC
ACCTGAGATCTTAGC
+
FFF:FFFFF:FFFFFF:FFFFFFFFFFFFFFFFFFFFFFFFFFFFFFFFFFFFFFFFFFFFFFFFFFF
FFFFFFFFFFFFFFFFFFFFFFFFFFFFFFFFFFFFFFFFFFFF:FFFFFFF:FFFFFFF:FFFFFFF
FFFFFFFFFFFFFFF
@A00155:342:HHGFNDSXY:1:1511:4860:29293 1:N:0:GAACCTAG+TCCGCATA
AGCCAGAAAGATCACCTTGCCCAAAGAAGACCAACATTTTCATACATGATGAAATTCATTATTGGTCT
CGCGATCAGCTTGAGACATTTCTCCAGGTTTACAAACCTAAAAATTTGTGGGCTACTCTGGTTTTTCC
ACCTGAGATCTTAGC
+
FFFFFFFF:F:FFFFFFFFFFFFFFFF,:FFFFFFF:FFFFF::FFFFFFFFFFFFFFFFFFFFFFFF
FFFFFFF:FFFFFFFFFFFFFFFFFFFFFFFFFFF,FFFFF,:FFFFFFFFFFFFFFFFFFFFFFFFF
FFFFFFFFFFFFFFF
@A00155:342:HHGFNDSXY:1:1476:29758:12446 1:N:0:GAACCTAG+TCCGCATA
AGCCAGAAAGATCACCTTGCCCAAAGAAGACCAACATTTTCATACATGATGAAATTCATTATTGGTCT
CGCGATCAGCTTGAGACATTTCTCCAGGTTTACAAACCTAAAAATTTGTGGGCTACTCTGGTTTTTCC
ACCTGAGATCTTAGC
+
FFFFFFF:FFFFFFFFFFFFFFFFFFFF:,FFFFFFFFFFFFFFFFFFFFFFFFFFFFFFFFFFFFFF
FFFFFFFFFFFFFFFFFFFFFFFFFFFFFFFFFFFFFFFFFFFFFFFFFFFFFFFFFFFFFFFFFFFF
FFFFFFFFFFFFFFF
@A00155:342:HHGFNDSXY:1:1521:19967:1814 1:N:0:GAACCTAG+TCCGCATA
AGCCAGAAAGATCACCTTGCCCAAAGAAGACCAACATTTTCATACATGATGAAATTCATTATTGGTCT

CGCGATCAGCTTGAGACATTTCTCCAGGTTTACAAACCTAAAAATTTGTGGGCTACTCTGGTTTTTCC
ACCTGAGATCTTAGC
+
FFFFFFFFFFFFFFFFFFFFFFFFFF:FFFFFFFFFFFFFFFFFFFFFFFFFFFFFFFFFFFFFFFFF
FFFFFFFFFFFFFFFFFFFFFFFFFFFFFFFFFFFFFFFFFFFFFFFFFFFFFFFFFFFFFFFFFFFF
FFFFFFFFFFFFFFF
@A00155:342:HHGFNDSXY:1:2112:30264:14450 1:N:0:GAACCTAG+TCCGCATA
AGCCAGAAAGATCACCTTGCCCAAAGAAGACCAACATTTTCATACATGATGAAATTCATTATAGGTCT
CGCGATCAGCTTGAGACATTTCTCCAGGTTTACAAACCTAAAAATTTGTGGGCTACTCTGGTTTTTCC
ACCTGAGATCTTAGC
+
:FFFF:FFFFFFFFFFF:FFFFF,FF:FFFFF:FFFFFFFFFFFFFFFFFFFFFFF:FF:FFFFFFFF
FFFFFF:FFFFFFFFFF:F:FFFFFF:FFFFFFFFFFFFFF:FFFFFFFFFFFFFFFFFFFFFFFF:F
FFFFFFFFFFFFF:F
@A00155:342:HHGFNDSXY:1:2229:17201:19225 1:N:0:GAACCTAG+TCCGCATA
AGCCAGAAAGATCACCTTGCCCAAAGAAGACCAACATTTTCATACATGATGAAATTCATTATTGGTCT
CGCGATCAGCTTGAGACATTTCTCCAGGTTTACAAACCTAAAAATTTGTGGGCTACTCTGGTTTTTCC
ACCTGAGATCTTAGC
+
FFFFFFFFFFF:FFF:,,FF:FF:FFFFFF,FFF:FFFFFFFFFFF:FFFFFFFF:FFFFFFFFFFFF
FFFFFFF:FFFFFFFFFFFFFFFF:FFFFFFFFFFFFFFFFFFFFFFFFFFFFFFFFFFFFFFFFFFF
FF:FFFFFFFFFFFF
@A00155:342:HHGFNDSXY:1:2277:26919:6151 1:N:0:GAACCTAG+TCCGCATA
AGCCAGAAAGATCACCTTGCCCAAAGAAGACCAACATTTTCATACATGATGAAATTCATTATTGGTCT
CGCGATCAGCTTGAGACATTTCTCCAGGTTTACAAACCTAAAAATTTGTGGGCTACTCTGGTTTTTCC
ACCTGAGATCTTAGC
+
FFFFFFFFFFFFFFFFF:FFFFFFFFF:FFFFFFFFFFFFFFFFFFFFFFFFFFFFFFFFFFFFFFFF
FFFFFFFFFFFFFFFFFFFFFFFFFFFFFFFFFFFFFFFFFFFFFFFFFFFFFFFFFFFFFFFFFFFF
FFFFFFFFFFFFFFF
@A00155:342:HHGFNDSXY:1:2337:31665:1595 1:N:0:GAACCTAG+TCCGCATA
AGCCAGAAAGATCACCTTGCCCAAAGAAGACCAACATTTTCATACATGATGAAATTCATTATTGGTCT
CGCGATCAGCTTGAGACATTTCTCCAGGTTTACAAACCTAAAAATTTGTGGGCTACTCTGGTTTTTCC
ACCTGAGATCTTAGC
+
FFFFFFFFFFFFFFFFFFFFFFF,FFF:FFFFFFF:FF:FFFFFFFFFFFFFFFFFFFFFFFFFFFFF
FFFFFFFFFF:FFFFFFFFFFFFFFFFFFFFFFFFFFFFFFF:FFFFFFFFFFFFFFFFFFFFFFFFF
FFFFFFFFFFFFFFF
@A00155:342:HHGFNDSXY:1:2349:26910:7263 1:N:0:GAACCTAG+TCCGCATA
AGCCAGAAAGATCACCTTGCCCAAAGAAGACCAACATTTTCATACATGATGAAATTCATTATTGGTCT
CGCGATCAGCTTGAGACATTTCTCCAGGTTTACAAACCTAAAAATTTGTGGGCTACTCTGGTTTTTCC
ACCTGAGATCTTAGC
+
FFFFFFFFFFFFFFFFF::FFFFFF:FFFFFFFFFFFFFFFFFFFFFFFFFFFFFFFFFFFFFFFFFF
FFFFFFFFFFFFFFFFFFFFFFFFFFFFFFFFFFFFFFFFFFFFFF:FFFFFFFFFFFFFFFFFFFFF
FFFFFFFFFFFFFFF
@A00155:342:HHGFNDSXY:1:2402:25328:29168 1:N:0:GAACCTAG+TCCGCATA
AGCCAGAAAGATCACCTTGCCCAAAGAAGACCAACATTTTCATACATGATGAAATTCATTATTGGTCT
CGCGATCAGCTTGAGACATTTCTCCAGGTTTACAAACCTAAAAATTTGTGGGCTACTCTGGTTTTTCC
ACCTGAGATCTTAGC
+
:FFFFFFF::FFFFFF,FFFFFFF,:F,,FFFFFFF,FFFFFFFF::FFFFFFFFFFFFFFFFFFFFF
FFFFFFF:,FFFFFFFFFFFFFFFFFFFFF,FFFFFFFFFFFFFFFFFFFFFFFFFF:FFF:FFFFFF
FFFFFFFFFFF,:FF

```
@A00155:342:HHGFNDSXY:1:2428:1976:5165 1:N:0:GAACCTAG+TCCGCATA
AGCCAGAAAGATCACCTTGCCCAAAGACGACCAACATTTTCATACATGATGAAATTCATTATTGGTCT
CGCGATCAGCTTGAGACATTTCTCCAGGTTTACAAACCTAAAAATTTGTGGGCTACTCTGGTTTTTCC
ACCTGAGATCTTAGC
+
FF:FFFFF:F,:FFFFF:FF:FFF:FF,:,F:F:FFFFFFFFFFFF:FFFFFFFFFFFFFFFFFFFFF
FF:FFFFFFFFFFFFFFF:FFFFFFFFFFFFFFFFFFFFFFFFFFFFFFFFFFFF:FFFFFFFFFFFF
FFFFFFFFFFF:FFF
@A00155:342:HHGFNDSXY:1:2428:1515:5431 1:N:0:GAACCTAG+TCCGCATA
AGCCAGAAAGATCACCTTGCCCAAAGAAGACCAACATTTTCATACATGATGAAATTCATTATTGGTCT
CGCGATCAGCTTGAGACATTTCTCCAGGTTTACAAACCTAAAAATTTGTGGGCTACTCTGGTTTTTCC
ACCTGAGATCTTAGC
+
F:FFFFFFF:F,FFF::F,FFFFFF:FF,FFFFFFF:F:,FFFF:FFFFFFFFFFFFFFFFFFFF:FF
FFFFFFFFFFFFFFFFFFFFFF,FFFF::FFFFFFFFFFFFFFFF:FFFFFFFFFFFFF:FF,FFFFFF
FFFFFFFF:FFFFFF
@A00155:342:HHGFNDSXY:1:2511:1949:17487 1:N:0:GAACCTAG+TCCGCATA
AGCCAGAAAGATCACCTTGCCCAAAGAAGACCAACATTTTCATACATGATGAAATTCATTATTGGTCT
CGCGATCAGCTTGAGACATTTCTCCAGGTTTACAATCCTAAAAATTTGTGGGCTACTCTGGTTTTTCC
ACCTGAGATCTTAGC
+
::F:FFF:F:F:FFF:FFFFFFFFFFFF:FFFFFF:FF:F:FFFFFFFFFFFFFFF:,FFFFFFFF,:
FF:FFFFFF:FFFFFFFFFFFFFFFFFFFFFFFFFFF,FF::FFF:FFFFFFFFFFFFF:FFFFFFF:FF
FFFFFFFFFFFFFF:
@A00155:342:HHGFNDSXY:1:2541:27814:28087 1:N:0:GAACCTAG+TCCGCATA
AGCCAGAAAGATCACCTTGCCCAAAGAAGACCAACATTTTCATACATGATGAAATTCATTATTGGTCT
CGCGATCAGCTTGAGACATTTCTCCAGGTTTACAAACCTAAAAATTTGTGGGCTACTCTGGTTTTTCC
ACCTGAGATCTTAGC
+
FFFFFFFFFFFFFFF:FFFFFFFFFFF::,FFFFFFFF:FFFFFFFFFFFFFF,FFFFFFFFFFFFF
FFFFFFFFFFFFFFFFFFFFFFFFFFFFFFFFFFFFFFFFFFFFFFFF:FFFFFFFFFFFFFFFFFFF
FFFFFFFFFFFFFFF
@A00155:342:HHGFNDSXY:1:2616:5701:26616 1:N:0:GAACCTAG+TCCGCATA
AGCCAGAAAGATCACCTTGCCCAAAGAAGACCAACATTTTCATACATTATGAAATTCATTATTGGTCT
CGCGATCAGCTTGAGACATTTCTCCAGGTTTACAAACCTAAAAATTTGTGGGCTACTCTGGTTTTTCC
ACCTGAGATCTTAGC
+
F::FFFFFFFF:,F:FFF,F,FFFFFFF,FFFFF:FFFFFFFFFFFF,:FFFFFFFFF:FFFFFFFFF
FF:FFFFFFFF:FFFFFFFFFFFFFFFFFFFF,FFFFFFFFFFFFFFFFFFFFFFFFFFFFFFFFFFFF
FFFFFFFFFFFFFFF
@A00155:342:HHGFNDSXY:1:2569:8874:16423 1:N:0:GAACCTAG+TCCGCATA
AGCCAGAAAGATCACCTTGCCCAAAGAAGACCAACATTTTCATACATGATGAAATTCATTATTGGTCT
CGCGATCAGCTTGAGACATTTCTCCAGGTTTACAAACCTAAAAATTTGTGGGCTACTCTGGTTTTTCC
ACCTGAGATCTTAGC
+
FFFFFFFFFFFFFFFFFFFFFFFFFFFFFFFFFFFFFFFFFFFFFFFFFFFFFFFFFFFFFFFFFF:FF
FFFFFFFFFFFFFFFFFFFFFFFFFFFFFFFFFFFFFFFFFFFFFFFF:FFFFFFFFFFFFFFFFFFF
FFFFFFFFFFFFFFF
@A00155:342:HHGFNDSXY:1:2618:20292:8954 1:N:0:GAACCTAG+TCCGCATA
AGCCAGAAAGATCACCTTGCCCAAAGAAGACCAACATTTTCATACATGATGAAATTCATTATTGGTCT
CGCGATCAGCTTGAGACATTTCTCCAGGTTTACAAACCTAAAAATTTGTGGGCTACTCTGGTTTTTCC
ACCTGAGATCTTAGC
+
:FFFFFF:FFF:FFFF:FFFF:FFF:FFFFFF:FFFFFFFFFFFFFFFFFFFFFFFFFFFFFFFFFFF
```

FFFF::FFFFFFFFFFFFFFFFFFFFFFFFFFFFFFFFFFFFFFFFFFFFFFFFFFFFFFFFFF
FFFFFFFFFFFFFFF
@A00155:342:HHGFNDSXY:1:2233:14714:25817 2:N:0:GAACCTAG+TCCGCATA
AGCCAGAAAGATCACCTTGCCCAAAGAAGACCAACATTTTCATACATGATGAAATTCATTATTGGTCT
CGCGATCAGCTTGAGACATTTCTCCAGGTTTACAAACCTAAAAATTTGTGGGCTACTCTGGTTTTTCC
ACCTGAGAGATCGGA
+
FFFFFFFFFFFFFFFFFFFFFFFFFFFFFFFFFFFFFFFFFFFFFFFFFFFFFFFFFFFFFFFFFFFF
FFFFFFFFFFFFFFFFFFFFFFFF:FFFFFFFFFFFFFFFFFFFFFFFFFFFFFFFFFFFFFFFFFFF
F:FFFFFFFFFFFFF
@A00155:342:HHGFNDSXY:1:2509:6451:24627 2:N:0:GAACCTAG+TCCGCATA
AGCCAGAAAGATCACCTTGCCCAAAGAAGACCAACATTTTCATACATGATGAAATTCATTATTGGTCT
CGCGATCAGCTTGAGACATTTCTCCAGGTTTACAAACCTAAAAATTTGTGGGCTACTCTGGTTTTTCC
ACCTGAGATCTTAGC
+
FFFFFFFFFF,FF:FFFFFFFFFFFFFFFFFFFFFFFFFFFFFFFFFFFFFFFFFFFFFFFFF:FF:F
FF:F:FFFFFFFFFFFFF:FFFFFFFF:FFFFFFF:FFFFFFFF,FF:FFFFFFFFFFFFFF:FFFFF
F,FFFFFFFFFFFFF
@A00155:342:HHGFNDSXY:1:1269:5864:14434 1:N:0:GAACCTAG+TCCGCATA
GCCAGAAAGATCACCTTGCCCAAAGAAGACCAACATTTTCATACATGATGAAATTCATTATTGGTCTC
GCGATCAGCTTGAGACATTTCTCCAGGTTTACAAACCTAAAAATTTGTGGGCTACTCTGGTTTTTCCA
CCTGAGATCTTAGCT
+
FFFFFFFFFFFFFFFFFFFFFFFF:F,F,FFFFFFFFFFFFFFFFFFFFFFFFFFFFFFFFFFFFFFF
FFF,FFFFFFFFFFFFFFFFFFFF:FF,FFFFFF,:F,FFFFFFFFFFFFFFFFFFFFFFFFFFFFFF
FFFFFFFF,FFFFFF
@A00155:342:HHGFNDSXY:1:1450:9534:25269 1:N:0:GAACCTAG+TCCGCATA
GCCAGAAAGATCACCTTGCCCAAAGAAGACCAACATTTTCATACATGATGAAATTCATTATTGGTCTC
GCGATCAGCTTGAGACATTTCTCCAGGTTTACAAACCTAAAAATTTGTGGGCTACTCTGGTTTTTCCA
CCTGAGATCTTAGCT
+
:FFF,FFFF,FFFFFFFFFFFFFF::F:FFFFFFFFFF:FFFFFFFFFFFFFFFF:FFFF:FFFFFFF
FFFFFFFFFFFFFFFFFFFFFFFFFFFFFFFFFFF:FFFFFFFFFFFFFFFFFFFFFFFFFFFFFFFF
FFFFFFFFFFF:FFF
@A00155:342:HHGFNDSXY:1:1450:9995:25285 1:N:0:GAACCTAG+TCCGCATA
GCCAGAAAGATCACCTTGCCCAAAGAAGACCAACATTTTCATACATGATGAAATTCATTATTGGTCTC
GCGATCAGCTTGAGACATTTCTCCAGGTTTACAAACCTAAAAATTTGTGGGCTACTCTGGTTTTTCCA
CCTGAGATCTTAGCT
+
FFFFFFFFFFFFFFFFFFFFFFFFFFF:FFFF:FFFFFFFFFFF:FFFFFFFFFFFFFFFFFFFFFFF
FFFFFFFFFFFFFFFFFFFFFFFFFFFFFFFFFFFFFFFFFFFFFFFFFFFFFFFFFFFFFFFFFFFF
FFFFFFFFFFFFFFF
@A00155:342:HHGFNDSXY:1:1442:24316:18677 1:N:0:GAACCTAG+TCCGCATA
GCCAGAAAGATCACCTTGCCCAAAGAAGACCAACATTTTCATACATGATGAAATTCATTATTGGTCTC
GCGATCAGCTTGAGACATTTCTCCAGGTTTACAAACCTAAAAATTTGTGGGCTACTCTGGTTTTTCCA
CCTGAGATCTTAGCT
+
FFFFFFFFFFFFFFFFFF:FFFFFF:FF:FFFFFFFFFFFFFFFFFFFFFFFFFFFFFFFFFFFFFFF
FFFFFFFFFFFFFFFFFFFFFFFFFFFFFFFFFFF:FFFFF:FFFFFFFFFFFFFFFFFFFFFFFFFF
FFFFFFFFFFFFFFF
@A00155:342:HHGFNDSXY:1:1442:28031:31688 1:N:0:GAACCTAG+TCCGCATA
GCCAGAAAGATCACCTTGCCCAAAGAAGACCAACATTTTCATACATGATGAAATTCATTATTGGTCTC
GCGATCAGCTTGAGACATTTCTCCAGGTTTACAAACCTAAAAATTTGTGGGCTACTCTGGTTTTTCCA
CCTGAGATCTTAGCT

+
FFFFFFFFFFFFFFFFFFFFFFFFFFFFFFFFFFFF:FFFFFFF:FFFFFFFFFFFFFFFFFFFFFFFFF
FFFFFFFFFFFFFFFFFFFFFFFFFF:F:FFFFFFFFFFFFFFFFF:FFFFFFFFFFFFFFFFFFFFFFF
FFFFFFFFFFFFFFF
@A00155:342:HHGFNDSXY:1:1458:26729:12837 1:N:0:GAACCTAG+TCCGCATA
GCCAGAAAGATCACCTTGCCCAAAGAAGACCAACATTTTCATACATGATGAAATTCATTATTGGTCTC
GCGATCAGCTTGAGACATTTCTCCAGGTTTACAAACCTAAAAATTTGTGGGCTACTCTGGTTTTTCCA
CCTGAGATCTTAGCT
+
FFFF:FFFFFFFFFFFFFFFFFFFF:FFFFFFFFFFFFFFFFFFFF:FFFFFFFFFFFFFFFFFFFFFFF
FFFFFFFFFFFFFFFFFFFFFFFFFFFFFFFFFFFFFFFFFFFFFFFFFFFFFFFFFFFFFFFFFFFFFF
FFFFFFFFFFFFFFF
@A00155:342:HHGFNDSXY:1:2159:30237:24455 1:N:0:GAACCTAG+TCCGCATA
TCCGATCTGATCACCTTGCCCAAAGAAGACCAACATTTTCATACATGATGAAATTCATTATTGGTCTC
GCGATCAGCTTGAGACATTTCTCCAGGTTTACAAACCTAAAAATTTGTGGGCTACTCTGGTTTTTCCA
CCTGAGATCTTAGCT
+
FFFFFFFFFFFFFFFFFFFFFFFFFFFFFFFFFFF:FFFFFFFFFFFFFFFFFFFFFFFFFFFFFFFFFF
FFFFFFFFFFFFFFFFFFFFFFFFFFFFFFFFFFFFFFFFFFFFFFFFFFFFFFFFFFFFFFFFFFFFFF
FFFFFFFFFFFFFFF
@A00155:342:HHGFNDSXY:1:2334:15664:4257 1:N:0:GAACCTAG+TCCGCATA
GCCAGAAAGATCACCTTGCCCAAAGAAGACCAACATTTTCATACATGATGAAATTCATTATTGGTCTC
GCGATCAGCTTGAGACATTTCTCCAGGTTTACAAACCTAAAAATTTGTGGGCTACTCTGGTTTTTCCA
CCTGAGATCTTAGCT
+
FFFFFFFFFFFFFFFFFFFFFFFFFFFFF:FFFFFFFF:FFFFFFFFFFFFFFFF:FFFFFFFFFFFFFF
FFFFFFFFFFFFFFFFFFFFFFFFFFFFFFFFFFFFFFFFFFFFFFFFFFFFFFFFFFFFFFFFFFFFFF
FFFFFFFFFFFFFFF
@A00155:342:HHGFNDSXY:1:2344:2483:19727 1:N:0:GAACCTAG+TCCGCATA
GCCAGAAAGATCACCTTGCCCAAAGAAGACCAACATTTTCATACATGATGAAATTCATTATTGGTCTC
GCGATCAGCTTGAGACATTTCTCCAGGTTTACAAACCTAAAAATTTGTGGGCTACTCTGGTTTTTCCA
CCTGAGATCTTAGCT
+
FF,FFFFFFFFFF,FFF:F,FFFF,FFFF,::FFFFFFF,FF::FFFFFF,F:FF:FFFFFFFF,FF,FF
F:FF:FFF,FFFFF::FFFFFFFF:F:FFFFFFFFF::FFFFFFFFFFFFFFFFFFFFFFFFFFFFFF:F
F,FFFFFFFFFFFFF
@A00155:342:HHGFNDSXY:1:2363:17083:19053 1:N:0:GAACCTAG+TCCGCATA
GCCAGAAAGATCACCTTGCACAAAGAAGACCATCATTTTCATACATGATGAAATTCATTATTGGTCTC
GCTATCAGCTTGAGACATTTCTCCTGGTTTACAAACCTAAAACTTTGTGGGCTACTCTGGTTTTTCCA
CCTGAGATCTTAGCT
+
FFFF::,FFFFFFFFFFF,FF,FF:F,FFFFF:,,:,,FF,FFF,F,FFFFF,FFFF:FFFFFFFFFFFF
::,:F,F,FFFF,FFFFF:F,FFF,FFFFF,:FFFFFFFFFFFF,:FFFFFFFFFFFF:FFFFFFFF:FF
,:FFFFFF:FFFF:F
@A00155:342:HHGFNDSXY:1:2549:15691:10097 1:N:0:GAACCTAG+TCCGCATA
GCCAGAAAGATCACCTTGCCCAAAGAAGACCAACATTTTCATACATGATGAAATTCATTATTGGTCTC
GCGATCAGCTTGAGACATTTCTCCAGGTTTACAAACCTAAAAATTTGTGGGCTACTCTGGTTTTTCCA
CCTGAGATCTTAGCT
+
FFFFFFFFFFFFFFFFFFFFFFFF:FFFFFFFFFFFFFFFFFFFFFFFFFFFFFFFFFFFFFFFFFFFF
FFFFFFFFFFFFFFFFFFFFFFFFFFFFFFFFFFFFFFFFF,FF:FFFFFFFFFFFFFFFFFFFFFFFFF
FFFFFFFFFFFFFFF
@A00155:342:HHGFNDSXY:1:2550:10691:21919 1:N:0:GAACCTAG+TCCGCATA
GCCAGAAAGATCACCTTGCCCAAAGAAGACCAACATTTTCATACATGATGAAATTCATTATTGGTCTC

```
GCGATCAGCTTGAGACATTTCTCCAGGTTTACAAACCTAAAAATTTGTGGGCTACTCTGGTTTTTCCA
CCTGAGATCTTAGCT
+
FFFFFFFFFFFFFFFFFFFFFFFFFFFFFFFFFFF:FFFFFF:FFFFFFFFFFFFFFFFFFFFFFFFF
FFFF:FFFFFFFFFFFFFFFFFFFFFFFFFFFFFFFFFFFFFFFFFFFFFFFFFFFFFFFFFFF
FFFFFFFFFFFFFFF
@A00155:342:HHGFNDSXY:1:2550:9860:22138 1:N:0:GAACCTAG+TCCGCATA
GCCAGAAAGATCACCTTGCCCAAAGAAGACCAACATTTTCATACATGATGAAATTCATTATTGGTCTC
GCGATCAGCTTGAGACATTTCTCCAGGTTTACAAACCTAAAAATTTGTGGGCTACTCTGGTTTTTCCA
CCTGAGATCTTAGCT
+
F:FF:FFF:FFFFFFFF:F,FFFF,FFFF::F:FFFFFFFFFF,FFFFFFFFFFF::FFFFFFF:FFF
FFFFFFFFFFFFFFFFFFFFFFFFFFFFFFFFFFFFFFFFFFFFFFFFFFFFFFFFFFF::FFFFFFF
FFFFFFFFFFFFFFF
@A00155:342:HHGFNDSXY:1:2618:15555:36980 1:N:0:GAACCTAG+TCCGCATA
CTCAGAAAGATCACCTTGCCCAAAGAAGACCAACATTTTCATACATGATGAAATTCATTATTGGTCTC
GCGATCAGCTTGAGACATTTCTCCAGGTTTACAAACCTAAAAATTTGTGGGCTACTCTGGTTTTTCCA
CCTGAGATCTTAGCT
+
FFFFFFFFFFFFFFFFFFFFFFFFFF:FFFF:FFFFFFFFFFFFFFFFFFFFFFFFFFFFFFFFFFFF
FFFFFFFFFFFFFFFFFFFFFFFFFFFFFFFFFFFFFFFFFFFFFFFFFFFFFFFFFFFFFFFFFFFF
FFFFFFFFFFFFFFF
@A00155:342:HHGFNDSXY:1:1115:11785:34021 2:N:0:GAACCTAG+TCCGCATA
GCCAGAAAGATCACCTTGCCCAAAGAAGACCAACATTTTCATACATGATGAAATTCATTATTGGTCTC
GCGATCAGCTTGAGACATTTCTCCAGGTTTACAAACCTAAAAATTTGTGGGCTACTCTGGTTTTTCCA
CCTGAGATCTTAGCT
+
F:FFFFFFFFFFFFFFFFFFFF:FF:FFFFFFFFFFFFFFFFFFFFFFFFFFFFFFFFFFFFFFFFFF
FFFFFFFFFFFFFFFFFFFFFFFFF:FFFFFFFF:,FFFFF:FFFFFFFFFFFFFFFFFFFFFFFFF:
:FFFF,FFFFFF,FF
@A00155:342:HHGFNDSXY:1:1108:31331:9189 2:N:0:GAACCTAG+TCCGCATA
GCCAGAAAGATCACCTTGCCCAAAGAAGACCAACATTTTCATACATGATGAAATTCATTATTGGTCTC
GCGATCAGCTTGAGACATTTCTCCAGGTTTACAAACCTAAAAATTTGTGGGCTACTCTGGTTTTTCCA
CCTGAGATCTTAGCT
+
:FFFFFFFFFFFFFFFFFFFFFFFFFFFFFFFFFFFFFFFFFFFFFFFFFFFFFFFFFFFFFFFFFFF
FFFFFFFFFFFFFFFFFFFFFFFFFFFFFFFFFFFFFFFFFFFFFFFFFFFFFFFFFFFFFFFFFFFF
FFFFFFFFFFFFFFF
@A00155:342:HHGFNDSXY:1:1133:29143:17769 2:N:0:GAACCTAG+TCCGCATA
GCCAGAAAGATCACCTTGCCCAAAGAAGACCAACATTTTCATACATGATGAAATTCATTATTGGTCTC
GCGATCAGCTTGAGACATTTCTCCAGGTTTACAAACCTAAAAATTTGTGGGCTACTCTGGTTTTTCCA
CCTGAGATCTTAGCT
+
FFFFFFFFFFFF:FFFFFFFFFFFFFF:FFFFFFFFFFFFFFFFFFFFFFFFFFFFF:F:FFFFFFFF
FFFFFFFFFFFFFFF:FFFFFF,FFFFFFFFFFFFFFFFFFF:FFFFFFFFFFFFFFFFFFF:FFFFF
FFFFFFFFFFFFFFF
@A00155:342:HHGFNDSXY:1:1416:27543:23422 2:N:0:GAACCTAG+TCCGCATA
GCCAGAAAGATCACCTTGCCCAAAGAAGACCAACATTTTCATACATGATGAAATTCATTATTGGTCTC
GCGATCAGCTTGAGACATTTCTCCAGGTTTACAAACCTAAAAATTTGTGGGCTACTCTGGTTTTTCCA
CCTGAGATCTTAGCT
+
FFFFFFFFFFFFFFFFFFFFFFFFFFFFFFFFFFFFFFFFFFFFFFFFFFFFFFFFFFFFFFFFFFFF
FFFFFFFFFFFFFFFFFFF,:FFFFFFFFFFFFFFFFFFFFFFFFFFFFFFFFFFFFFFFF:FFFFFF
:FFFFFFF,FFFFF:
```

@A00155:342:HHGFNDSXY:1:1436:30644:28416 2:N:0:GAACCTAG+TCCGCATA
GCCAGAAAGATCACCTTGCCCAAAGAAGACCAACATTTTCATACATGATGAAATTCATTATTGGTCTC
GCGATCAGCTTGAGACATTTCTCCAGGTTTACAAACCTAAAAATTTGTGGGCTACTCTGGTTTTTCCA
CCTGAGATCTTAGCT
+
FFFFFFFFFFFFFFF:FFFFFFFFFFFFFFFFFFFFFFFFFFFFFFFFFFFFFFFFFF:FFFFFFFFF
FFFFFFFFFFFFFFFFFFFFFFFFFFFFFFFFFFFFFF:FFFFFF:FFFFFFF:FFFFFFFF:,FFF,
FFFFFFFFFFFFFFF
@A00155:342:HHGFNDSXY:1:2472:22327:24283 2:N:0:GAACCTAG+TCCGCATA
GCCAGAAAGATCACCTTGCCCAAAGAAGACCAACATTTTCATACATGATGAAATTCATTATTGGTCTC
GCGATCAGCTTGAGACATTTCTCCAGGTTTACAAACCTAAAAATTTGTGGGCTACTCTGGTTTTTCCA
CCTGAGATCTTAGCT
+
FFFFFFFFFFFFFFFFFFFFFFFFFFFFFFFFFFFFFFFFFFFFFFFFFFF,FFFFFFFFFFFFFFFF
FFFFFFFFFFFFFFFFFFFFFFFFFFFFFFFFFFFFFFFFFFFFFFFF:FFFFFFFFFFFFFFFFFFF
FFFFFFFFFFFFFFF
@A00155:342:HHGFNDSXY:1:1149:3821:33786 1:N:0:GAACCTAG+TCCGCATA
CCAGAAAGATCACCTTGCCCAAAGAAGACCAACATTTTCATACATGATGAAATTCATTATTGGTCTCG
CGATCAGCTTGAGACATTTCTCCAGGTTTACAAACCTAAAAATTTGTGGGCTACTCTGGTTTTTCCAC
CTGAGATCTTAGCTG
+
FFFFFFFFFFFFFFFFFFFFFFF::F:FFFFFFFFFFFFFFFFFFFFFFFFFFFFFFFFFFFFFFFFF
FFFFFFFFFFFFFFFFFFFFFFFFFFFFFFFFFF:FFFFFFFFFFFFFFFFFFFFFFFFFFFFFFFFF
FFFFFFFFFFFFFFF
@A00155:342:HHGFNDSXY:1:1227:16613:34804 1:N:0:GAACCTAG+TCCGCATA
CCAGAAAGATCACCTTGCCCAAAGAAGACCAACATTTTCATACATGATGAAATTCATTATTGGTCTCG
CGATCAGCTTGAGACATTTCTCCAGGTTTACAAACCTAAAAATTTGTGGGCTACTCTGGTTTTTCCAC
CTGAGATCTTAGCTG
+
FFFFFFFFFFFFFFFFFFFFFFFFFFFFFFFFFFFFFFFFFFFFFFFFFFFFFFFFFFFFFFFFFFFF
FFFFFFFFFFFFFFFFFFFFFFFFFFFFFFFFFFFFFFFFFFFFFFFFFFFFFFFFFFFFFFFFFFFF
FFFFFFFFFFFFFFF
@A00155:342:HHGFNDSXY:1:1329:15591:14935 1:N:0:GAACCTAG+TCCGCATA
CCAGAAAGATCACCTTGCCCAAAGAAGACCAACATTTTCATACATGATGAAATTCATTATTGGTCTCG
CGATCAGCTTGAGACATTTCTCCAGGTTTACAAACCTAAAAATTTGTGGGCTACTCTGGTTTTTCCAC
CTGAGATCTTAGCTG
+
FFFFFFFFFFFFFFFFFFFFFFFFFFFFFFFFFFFF:FFFFFFFFFFFFFFFFFFFFFFFFFFFFFFF
FFFFFFFFFFFFFFFFFFFF:FFFFFFFFFFFFFFFFFFFFFFFFFFFFFFFFFFFFFFFFFFFFFF
FFFFFFFF:FFFFFF
@A00155:342:HHGFNDSXY:1:1355:15393:17785 1:N:0:GAACCTAG+TCCGCATA
CCAGAAAGATCACCTTGCCCAAAGAAGACCAACATTTTCATACATGATGAAATTCATTATTGGTCTCG
CGATCAGCTTGAGACATTTCTCCAGGTTTACAAACCTAAAAATTTGTGGGCTACTCTGGTTTTTCCAC
CTGAGATCTTAGCTG
+
FFFFFFFFFFFFFFFFFFFFFFFFFF:FFFFFFFFFFFFFFF:FFFFFFFFFFFFFFFFFFFFFFFFF
FFFFFFFFFFFFFFFFFFFFFFFFFFFFFFFFFFFFFFFFFFFFFFFFFFFFFFFFFFFFFFFFFFFF
FFFFFFFFFFFFFFF
@A00155:342:HHGFNDSXY:1:1370:6795:26256 1:N:0:GAACCTAG+TCCGCATA
CCAGAAAGATCACCTTGCCCAAAGAAGACCAACATTTTCATACATGATGAAATTCATTATTGGTCTCG
CGATCAGCTTGAGACATTTCTCCAGGTTTACAAACCTAAAAATTTGTGGGCTACTCTGGTTTTTCCAC
CTGAGATCTTAGCTG
+
FFFFFFFFFFFFFFFFFFFFFFFFF:,FFFFFFFFFFFFFFFFFFFFFFFFFFFFFFFFFFFFFFFFF

```
FFFFFFFFFFFFFFFFFFFFFFFFFFFFFFFFFFFFFFFFFFFFFFFFFFFFFFFFFFFFFFFFFFFF
FFFFFFFFFFFFFFF
@A00155:342:HHGFNDSXY:1:1370:6162:26537 1:N:0:GAACCTAG+TCCGCATA
CCAGAAAGATCACCTTGCCCAAAGAAGACCAACATTTTCATACATGATGAAATTCATTATTGGTCTCG
CGATCAGCTTGAGACATTTCTCCAGGTTTACAAACCTAAAAATTTGTGGGCTACTCTGGTTTTTCCAC
CTGAGATCTTAGCTG
+
FFFFFFFFFFFFFFFFFFFFF:FFFFFFFFFFFFFFFFFFFFFFFFFFFFFFFFFFFFFFFFFFFFFF
FFFFFFFFFFFFFFFFFFFFFFFFFFFFFFFF:FFFFFFFFFFF:FFFFFFFFFFFFFFFFFFFFFFF
FFFFFFFFFFFFFFF
@A00155:342:HHGFNDSXY:1:1456:28384:16611 1:N:0:GAACCTAG+TCCGCATA
CCAGAAAGATCACCTTGCCCAAAGAAGCCCAACATTTTCATACATGATGAAATTCATTATTGGTCTCG
CGATCAGCTTGAGACATTTCTCCAGGTTTACAAACCTAAAAATTTGTGGGCTACTCTGGTTTTTCCAC
CTGAGATCTTAGCTG
+
FFFFFFFFFFFFFF:FFFFFFF:FF,F,,FFFFFF,FFFFFFFFFFFFFFFFFFFFFFFFFFFFFFFF
FFFFFFFFFFFFFF:FF:FFFFFFFF,FFFFF,:FFFFF,FF:FFFFFFFFFFFFFFFFFFFFFFFFF
FFFFFFFFFFFFFFF
@A00155:342:HHGFNDSXY:1:1456:28357:16752 1:N:0:GAACCTAG+TCCGCATA
CCAGAAAGATCACCTTGCCCAAAGAAGACCAACATTTTCATACATGATGAAATTCATTATTGGTCTCG
CGATCAGCTTGAGACATTTCTCCAGGTTTACAAACCTAAAAATTTGTGGGCTACTCTGGTTTTTCCAC
CTGAGATCTTAGCTG
+
FFFFFFFFFFFFFFFFFFFFFF:F,FFFFFFFFFFFFFFFFFFFFFFFFFFFFFFF:FFFFFFFFFFF
FFF:FFF:FFFFFFFFFFFFFFFFFFFFFF:FFFFFFFFFFFFFFFFFFFFFFFFFFFFFFFFFFFFF
FFFFFFFFFFFFFFF
@A00155:342:HHGFNDSXY:1:1512:19416:28855 1:N:0:GAACCTAG+TCCGCATA
CCAGAAAGATCACCTTGCCCAAAGAAGACCAACATTTTCATACATGATGAAATTCATTATTGGTCTCG
CGATCAGCTTGAGACATTTCTCCAGGTTTACAAACCTAAAAATTTGTGGGCTACTCTGGTTTTTCCAC
CTGAGATCTTAGCTG
+
FFFFFF:FFFFFFFFFFFFFFFFFF,FFFFFFFFFFFFFFFFFFFFFFFFFFFFFF:FF:FFFFFFFF
FF,FFFFFFFFFFFFFFFFFFFFFFFFFFFFFFFFFFFFFFFFFFFFF,FFFFFFFFFFFFFFFFFFF
FFFFFFFFFFFFFFF
@A00155:342:HHGFNDSXY:1:1537:29288:3396 1:N:0:GAACCTAG+TCCGCATA
CCAGAAAGATCACCTTGCCCAAAGAAGACCAACATTTTCATACATGATGAAATTCATTATTGGTCTCG
CGATCAGCTTGAGACATTTCTCCAGGTTTACAAACCTAAAAATTTGTGGGCTACTCTGGTTTTTCCAC
CTGAGATCTTAGCTG
+
FFFFFFFF,FFFF:FFFFFFF:FFF:::FF:FFFFFFFFFFFFFFFFFFFFFFFFFFFFFFFFFFFFF
FFFFFFFFFFFFFFFFFFFFFF:FFFFFFFFF:,FFFFFFFFFFFFFFFFFFFFFFFFFFFFFFFFFF
FFFFFFFFFFFFFFF
@A00155:342:HHGFNDSXY:1:1521:17788:8563 1:N:0:GAACCTAG+TCCGCATA
CCAGAAAGATCACCTTGCCCAAAGAAGACCAACATTTTCATACATGATGAAATTCATTATTGGTCTCG
CGATCAGCTTGAGACATTTCTCCAGGTTTACAAACCTAAAAATTTGTGGGCTACTCTGGTTTTTCCAC
CTGAGATCTTAGCTG
+
FFFFFFFFFFFFFFFFFFFFFFF:FFFFFFFFFFFFFFFFFFFFFFFFFFFFFFFFFFFFFFFFFFFF
FFFFF::FFFFFFFFFFFFFFFFFFFFFFFFFFFFFF:FFFFFFFFFFFF:FFFFFFFFFFFFF:FFF
FFFFFFFFFFFFFFF
@A00155:342:HHGFNDSXY:1:1522:3432:12696 1:N:0:GAACCTAG+TCCGCATA
CCAGAAAGATCACCTTGCCCAAAGAAGACCAACATTTTCATACATGATGAAATTCATTATTGGTCTCG
CGATCAGCTTGAGACATTTCTCCAGGTTTACAAACCTAAAAATTTGTGGGCTACTCTGGTTTTTCCAC
CTGAGATCTTAGCTG
```

+
FFFFFFFFFFFFFF:FFFFFFFFFFFFF,FF,F,FFFFFFFFFFFF:FFFFFFFFFF::FFFFFFF:F
FFFF:FFFFF,FFFF:FFFFFF,F,FFFFFFFFFF:FFFFFFFFFF::FFFFF,FFFFFFFFFFFFFF
FFFFFFF:FF::FFF
@A00155:342:HHGFNDSXY:1:1540:3052:20087 1:N:0:GAACCTAG+TCCGCATA
CCAGAAAGATCACCTTGCCCAAAGAAGACCAACATTTTCATACATGATGAAATTCATTATTGGTCTCG
CGATCAGCTTGAGACATTTCTCCAGGTTTACAAACCTAAAAATTTGTGGGCTACTCTGGTTTTTCCAC
CTGAGATCTTAGCTG
+
FFFFFFFF:FFFFFFFFFFFFFF:FF:FFFFFFFFFFFFFFFFFFFFFFFFFFFFFFFFFFFFFFFFF
FFFFFFFFFFFFFFFFFFFFFFFFF:FFFFFFFFFFFFFFFFFFFFFFFFFFFFFFFFFFFFFFFFFF
FFFFFFFFFFFFFFF
@A00155:342:HHGFNDSXY:1:1611:10917:7874 1:N:0:GAACCTAG+TCCGCATA
CCAGAAAGATCACCTTGCCCAAAGAAGACCAACATTTTCATACATGATGAAATTCATTATTGGTCTCG
CGATCAGCTTGAGACATTTCTCCAGGTTTACAAACCTAAAAATTTGTGGGCTACTCTGGTTTTTCCAC
CTGAGATCTTAGCTG
+
FFFFFFFFFFFFFFFFFFFFFFFF,:,FFFFFFFFFFFFFFFFFFFFFFFFFFFFFFFFFFFFFFFFF
FFFF,:FFFFFFFFFFFFFFFFFFFFFFFFFFFFFFFFFF:FFFFFFFFFFFFFFFFFFFFFFFFFFF
FFFFFFFFFFFFFFF
@A00155:342:HHGFNDSXY:1:2133:2248:24643 1:N:0:GAACCTAG+TCCGCATA
CCAGAAAGATCACCTTGCCCAAATAAGACCAACATTTTCATACATGATGAAATTAATTATTGGTCTCG
CGATCAGCTTGAGACATTTCTCCAGGTTTACAAACCTAAAAATTTGTGGGCTACTCTGGTTTTTCCAC
CTGAGATCTTAGCTG
+
F::,FFFFF::FF:::F,F:FFF,FFFFFFF::FF,FF:F,F:F:,F,,FFFF,,F:FFF:F:FFFF:
FFFFFF:FF:FF,F:F,FFF,F:FFF:FF:,F:F:FFFFFFF,FFFFFF:FFFFF,F,,F:F::FFFF
:FFFFF,FFFFFFFF
@A00155:342:HHGFNDSXY:1:2134:8386:9627 1:N:0:GAACCTAG+TCCGCATA
CCAGAAAGATCACCTTGCCCAAAGAAGACCAACATTTTCATACATGATGAAATTCATTATTGGTCTCG
CGATCAGCTTGAGACATTTCTCCAGGTTTACAAACCTAAAAATTTGTGGGCTACTCTGGTTTTTCCAC
CTGAGATCTTAGCTG
+
FFFFFFFFFFFFFFFFFFFFFF,:,FFFFFFFFFFFFFFFFFFFFFFFFFFFFFFFFFFFFFFFFFFF
FFFFFF:FFFFFFFFFFFFFFFFFFFFFFFFFFFFFFFFFFFFFFFFFFFFFFFFFFFFFFFFFFFFF
FFFFFFFFFFFFFFF
@A00155:342:HHGFNDSXY:1:2143:23737:22091 1:N:0:GAACCTAG+TCCGCATA
CCAGAAAGATCACCTTGCCCAAAGAAGACCAACATTTTCATACATGATGAAATTCATTATTGGTCTCG
CGATCAGCTTGAGACATTTCTCCAGGTTTACAAACCTAAAAATTTGTGGGCTACTCTGGTTTTTCCAC
CTGAGATCTTAGCTG
+
FFFFF:FFFFFFFFFFFFFFFF,FFFFFFFFFFF,FFFFFFFFFFFFFFFFFFFFFFFFFFFFFFFFF
FFFFFFFFFFFFFFFFFFFFFFFFFFFFFFFFFFFFFFFFFFFFFFFFFFFFFFFFFFFFFFFFFFFF
FFFFFFFFFFFFFFF
@A00155:342:HHGFNDSXY:1:2308:29261:17754 1:N:0:GAACCTAG+TCCGCATA
CCAGAAAGATCACCTTGCCCAAAGAAGACCAACATTTTCATACATGATGAAATTCATTATTGGTCTCG
CGATCAGCTTGAGACATTTCTCCAGGTTTACAAACCTAAAAATTTGTGGGCTACTCTGGTTTTTCCAC
CTGAGATCTTAGCTG
+
FFFFFFFFFFFFFFFFFFFFFFFFF:F::FFFFFFFFFFFFFFFFFFFFFFFFFFFFFFFFFFFFFFF
FFFFFFFFFFFFFFFFFFFFFFFFFFFFFFFFFFFFFFFFFFFFFFFFF:FFFFFFFFFFFFFFFFFF
FFFFFFFFFFFFFFF
@A00155:342:HHGFNDSXY:1:2525:6424:18693 1:N:0:GAACCTAG+TCCGCATA
CCAGAAAGATCACCTTGCCCAAAGAAGACCAACATTTTCATACATGATGAAATTCATTATTGGTCTCG

CGATCAGCTTGAGACATTTCTCCAGGTTTACAAACCTAAAAATTTGTGGGCTACTCTGGTTTTTCCAC
CTGAGATCTTAGCTG
+
:FFFFFFFFF:F,FFFFFFFFF,FFF:,FFFFFFFFFFFFFFFF:FFFFFFFFFFFFFFFFFFFFFFF
FF,FFFFFFFFFFFFFFFFFFFFFFFFFFFFFFFFFFFFFFFFFFFFFFFFFFFFFFFFFFFFFFFFF
FFFFFFFFFFFFFFF
@A00155:342:HHGFNDSXY:1:1560:25400:32894 2:N:0:GAACCTAG+TCCGCATA
CCAGAAAGATCACCTTGCCCAAAGAAGACCAACATTTTCATACATGATGAAATTCATTATTGGTCTCG
CGATCAGCTTGAGACATTTCTCCAGGTTTACAAACCTAAAAATTTGTGGGCTACTCTGGTTTTTCCAC
CTGAGATCTTAGCTG
+
FFFFFFFFFFFFFFFFFFFF:F:FFFFFFFFFFFFFFFFFFFFFFFFFFF:FFFFFFFFFFFFFFFFF
FFFFFFFFFFFFFFFFFF:FFFFFFFFFFFFFFFFFFFFFFFFFFFFFFFFFFFFFFFFFFFFFFFFF
FFFFF:FFFFFFFFF
@A00155:342:HHGFNDSXY:1:2666:20880:14544 2:N:0:GAACCTAG+TCCGCATA
CCAGAAAGATCACCTTGCCCAAAGAAGACCAACATTTTCATACATGATGAAATTCATTATTGGTCTCG
CGATCAGCTTGAGACATTTCTCCAGGTTTACAAACCTAAAAATTTGTGGGCTACTCTGGTTTTTCCAC
CTGAGATCTTAGCTG
+
FFFFFFFFFFFFFFFFFFFFFFFFFFFFFFFFFFFFFFFFFFFFFFFFFFFFFFFFFFFFFFFFFFFF
FFFFFFFFFFFFFFFFF:FFFFFFFFFFFFFFF,FFFFFFFFFFFFFFFFFFFFFFFFF:FFFFFFFF
FFFFFFFFF:FFFFF
@A00155:342:HHGFNDSXY:1:1424:27543:10833 1:N:0:GAACCTAG+TCCGCATA
CAGAAAGATCACCTTGCCCAAAGAAGACCAACATTTTCATACATGATGAAATTCATTATTGGTCTCGC
GATCAGCTTGAGACATTTCTCCAGGTTTACAAACCTAAAAATTTGTGGGCTACTCTGGTTTTTCCACC
TGAGATCTTAGCTG
+
FFFFF:FFF:FFFFFFFFFF,FF:F:FF:F:F,FFFFFFF:FFFFFFFFF::FFFFFFFFFFFFFFF:
F:FFFFFFFFFFF:FFFFFFFFFFFFFFFFF,F:FFF::,:FFFFFFFFFFFFFFFFFF,FFF,FF:F
F:F:F:,:FFFFFF
@A00155:342:HHGFNDSXY:1:1520:19443:35133 1:N:0:GAACCTAG+TCCGCATA
CAGAAAGATCACCTTGCCCAAAGAAGACCAACATTTTCATACATGATGAAATTAATTATTGTTCTCGC
GATCAGCTTGAGACATTTCTACTGGTTTACAATCCTAAAAATTTGTGGGCTACTCTGGTTTTTCCACC
TGAGATCTTAGCTG
+
:FF,F,FFFFF,,F,FF,F::FFFFFFF,,FFFFFFFFFFF,,FF,FFFF,FF,FFFFFFF,FFFFFF
F,FF,F:FF:::F,FFFFFF,,,FFFFF:,:F,FFFFFFF,:FFFFFF,:FFFF,FF:FFFFFFF,FF
FF:FFFFFF:FFF:
@A00155:342:HHGFNDSXY:1:1122:5186:5995 1:N:0:GAACCTAG+TCCGCATA
CAGAAAGATCACCTTGCCCAAAGAAGACCAACATTTTCATACATGATGAAATTCATTATTGGTCTCGC
GATCAGCTTGAGACATTTCTCCAGGTTTACAAACCTAAAAATTTGTGGGCTACTCTGGTTTTTCCACC
TGAGATCTTAGCTGG
+
FFFFFFFFFFFFFFFFFFFFFFF,FF,:FFFFFFFFFFFFFFFFFFFFFFFFFFFFFFFFFFFFFFFF
FFFFFFFFFFFFFFFFFFFFFFFFFFFFF:FF:FFFFFF:FFFFFFFFFFFFFFFFFFFFFFF:FF:F
FFFFFF:FFFFFFFF
@A00155:342:HHGFNDSXY:1:1214:21260:10222 1:N:0:GAACCTAG+TCCGCATA
CAGAAAGATCACCTTGCCCAAAGAAGACCAACATTTTCATACATGATGAAATTCATTATTGGTCTCGC
GATCAGCTTGAGACATTTCTCCAGGTTTACAAACCTAAAAATTTGTGGGCTACTCTGGTTTTTCCACC
GGAGATCTTAGCTGG
+
FFFFFFFFFFFFFFFFFFFFFFFFFFFFFFFFFFFFFFFFFFFFFFFFFFFFFFFFFFFFFFFFFFFF
FFFFFFFFFFFFFFFFFFFFFFFFFFFFFFFFFFFFFFFFFFFFFFFFFFFFFFFFFFFFFFFFFFFF
,FFFFFFFFFFFFF:

```
@A00155:342:HHGFNDSXY:1:1215:31801:14356 1:N:0:GAACCTAG+TCCGCATA
CAGAAAGATCACCTTGCCCAACGAAGACCAACATTTTCATACATGATGAAATTCATTATTGGTCTCGC
GATCAGCTTGAGACATTTCTCCAGGTTTACAAACCTAAAAATTTGTGGGCTACTCTGGTTTTTCCACC
TGAGATCTTAGCTGG
+
FFFF,FFFFFFFFFFFFFFFF,F::FFFFFFFFFF:FFFFFF:FFF:FFFFFFFFFFF,,FFFFFFFF
F:FF:FFFFFFFFFFF,FFFFFFFFFFFFFFFFFFFFFFF:FFFFFFFFFFFFFFFFFFFFFFFFFFF
FFFFFFFFFFFFFFF
@A00155:342:HHGFNDSXY:1:1227:27362:32283 1:N:0:GAACCTAG+TCCGCATA
CAGAAAGATCACCTTGCCCAAAGAAGACCAACATTTTCATACATGATGAAATTCATTATTGGTCTCGC
GATCAGCTTGAGACATTTCTCCAGGTTTACAAACCTAAAAATTTGTGGGCTACTCTGGTTTTTCCACC
TGAGATCTTAGCTGG
+
FFFFFFFFFFFFFFFFFFFFF,:FFFFFFFFFFFFF:FFFFFFFFFFFFFFFFFFFFFFF,FFFFFFF
FFFFFFFFFFFFFFFFFFFFFFFFFFFFFFFFF:FFFFFFFFFFFFFFFFFFFFFFFFFFFFFFFFFF
FFFFFFFFFFFFFFF
@A00155:342:HHGFNDSXY:1:1339:13729:9330 1:N:0:GAACCTAG+TCCGCATA
CAGAAAGATCACCTTGCCCAAAGAAGACCAACATTTTCATACATGATGAAATTCATTATTGGTCTCTC
GATCAGCTTGAGACATTTCTCCAGGTTTACAAACCTAAAAATTTGTGGGCTACTCTGGTTTTTCCACC
TGAGATCTTAGCTGG
+
FFFFFFFFFFFFFFFFFFFFFFFFFF:FFFFFFFFFFFFFFFFFFFFFFFFFFFFFFFFFFFFFFFFF
FFFFFFFFFFFFFFFFFFFFFFFFFFFFFFFFFFFFFFFFFFFFFFFFFFFFFFFFFFFFFFFFFFFF
FFFFFFFFFFFFFFF
@A00155:342:HHGFNDSXY:1:1440:2311:19460 1:N:0:GAACCTAG+TCCGCATA
CAGAAAGATCACCGTGCCCAAAGAAGACCAACATTTTCATAAATGATGAAATTCATTATTGGTCTCGC
GATCAGCTTGAGACATTTCTCCAGGTTTACAAACCTAAAAATTTGTGGGCTACTCTGGTTTTTCCACC
TGAGATCTTAGCTGG
+
FF,FFF,FFFFF:,FFF,FF:F:F,,FFFFFFF:FFFFFF:,FF:FFFFFFFFFFFFFFF:FFFFFFF
FFFFF,FFFFFFFFFFFFFFF,FFFFFFFFFFFFFFFFFFFFFFF,FF,F,FFFFFFFF:FFF:FFFFF
:FFFFFFFFFFFFF:
@A00155:342:HHGFNDSXY:1:1629:19822:19163 1:N:0:GAACCTAG+TCCGCATA
CAGAAAGATCACCTTGCCCAAAGAAGACCAACATTTTCATACATGATGAAATTCATTATTGGTCTCTC
GATCAGCTTGAGACATTTCTCCAGGTTTACAAACCTAAAAATTTGTGGGCTACTCTGGTTTTTCCACC
TGAGATCTTAGCTGG
+
FFFFFFFFFFFFFFFFFFFFFFFFFFFFFFFFFFF:FFFFFFFFFFFFFFFFFFFFFFFFFF:FFFFF
FFFFFFFFFFFFFFFFFFFFFFFFFFFFFFFFFFFFFFFFFFFFFFFFFFFFFFFFFFFFFFFFFFFF
FFFFFFFFFFFFFFF
@A00155:342:HHGFNDSXY:1:2122:28664:36511 1:N:0:GAACCTAG+TCCGCATA
CAGAAAGATCACCTTGCCCAAAGAAGACCAACATTTTCATACATGATGAAATTCATTATTGGTCTCGC
GATCAGCTTGAGACATTTCTCCAGGTTTACAAACCTAAAAATTTGTGGGCTACTCTGGTTTTTCCACC
TGAGATCTTAGCTGG
+
FFFFFFFFFFFFFFFFFFFFFFFFFFFFFFFFFFFFFFFFFFFFFFFFFFFFFFFFFFFFFFFFFFFF
FFFFFFFFFFFFFFFFFFFFFFFFFFFFFFFFFFFFFFFFFFFFFFFFFFFFFFFFFFFFFFFFFFFF
FFFFFFFFFFFFFFF
@A00155:342:HHGFNDSXY:1:2217:21404:30984 1:N:0:GAACCTAG+TCCGCATA
CAGAAAGATCACCTTGCCCAAAGAAGACCAACATTTTCATACATGATGAAATTCATTATTGGTCTCGC
GATCAGCTTGAGACATTTCTCCAGGTTTACAAACCTAAAAATTTGTGGGCTACTCTGGTTTTTCCACC
TGAGATCTTAGCTGG
+
FFFFFFF:FFFFFFFFFFFFFFF,F:FFFFFFFFFFFF:FFFFFFFFFFFFFFFFFFFFFFFFFFFFF
```

FFFFFFFFFFFFFFFFFFFFFFFFFFFFFFFFFFFFFFFFFFFFFFFFFFFFFFFFFFFFFFFFFFFF
FFFFFFFFFFFFFFF
@A00155:342:HHGFNDSXY:1:2214:24189:13980 1:N:0:GAACCTAG+TCCGCATA
CAGAAAGATCACCTTGCCCAAAGAAGACCAACATTTTCATACATGATGAAATTCATTATTGGTCTCGC
GATCAGCTTGAGACATTTCTCCAGGTTTACAAACCTAAAAATTTGTGGGCTACTCTGGTTTTTCCACC
GGAGATCTTAGCTGG
+
FFFFFFFFFFFFFFFFFFFFFFFFFFFFFFFFFFFFFFFFFFFFFFFFFFFFFFFFFFFFFFFFFFFF
FFFFFFFFFFFFFFFFFFFFFFFFFFFFFFFFFFFFF::FF:FFFFFFFFFFFFFFFFFFFFFFFFFF
,FFFFFF:FFFFFFF
@A00155:342:HHGFNDSXY:1:2321:11153:36182 1:N:0:GAACCTAG+TCCGCATA
CAGAAAGATCACCTTGCCCAAAGAAGACCAACATTTTCATACATGATGAAATTCATTATTGGTCTCGC
GATCAGCTTGAGACATTTCTCCAGGTTTACAAACCTAAAAATTTGTGGGCTACTCTGGTTTTTCCACC
TGAGATCTTAGCTGG
+
FFFFFFFFFFFFFFFFFFFFFFFFF:FFFFFFFFFFFFFFFFFFFFFFFFFFFFFFFFFFFFFFFFFF
FFFFFFFFFFFFFFFFFFFFFFFFFFFFFFFFFFFFFFFFFFFFFFFFFFFFFFFFFFFFFFFFFFFF
FFFFFFFFFFFFFFF
@A00155:342:HHGFNDSXY:1:2321:11433:36448 1:N:0:GAACCTAG+TCCGCATA
CAGAAAGATCACCTTGCCCAAAGAAGACCAACATTTTCATACATGATGAAATTCATTATTGGTCTCGC
GATCAGCTTGAGACATTTCTCCAGGTTTACAAACCTAAAAATTTGTGGGCTACTCTGGTTTTTCCACC
TGAGATCTTAGCTGG
+
FFFFFFFFFFFFFFFFFFFFFFFFFFFFFFFFFF:FFFFFFFFFFFFFFFF,FFFFFFFFFFFFFFFF
FFFFFFFFFFFFFFFFFFFFFFFFFFFFFFFFFFFFFF:FFFFFFFFFFFFFFFFFFFFFFFFFFFFF
FFFFFFFFFFFFFFF
@A00155:342:HHGFNDSXY:1:2405:18304:36699 1:N:0:GAACCTAG+TCCGCATA
CAGAAAGATCACCTTGCCCAAAGAAGACCAACATTTTCATACATGATGAAATTCATTATTGGTCTCGC
GATCAGCTTGAGACATTTCTCCAGGTTTACAAACCTAAAAATTTGTGGGCTACTCTGGTTTTTCCACC
TGAGATCTTAGCTGG
+
FFFFFFFFFFFFF:FFFFFFFFFFFFFFFFFFFFFFFFFFFFFFFFFFFFFFFFFFFFFFFFFFFFFF
F:FFFFFFFFFFFFFFFFFFFFFFFFFFFFFFFFFFFFFFFFFFFFFFFFFFFFFFFFFFFFFFFFFF
FFFFFFFFFFFFFFF
@A00155:342:HHGFNDSXY:1:2505:21495:7279 1:N:0:GAACCTAG+TCCGCATA
CAGAAAGATCACCTTGCCCAAAGAAGACCAACATTTTCATACATGATGAAATTCATTATTGGTCTCGC
GATCAGCTTGAGACATTTCTCCAGGTTTACAAACCTAAAAATTTGTGGGCTACTCTGGTTTTTCCACC
TGAGATCTTAGCTGG
+
FFFFFFFFFFFFFFFFFFFFFFF:F:FFFFFFFFFFFFFFFFFFFFFFFFFFFFFFFFFFFFFFFFFF
FFFFFF:FFFFFFFFFFFFFFFFFFFFFFFFFFFFFFFFFFFFFFFFFFFFFFFFFFFFFFFFFFFFF
FFFFFFFFFFFFFFF
@A00155:342:HHGFNDSXY:1:2648:7048:15890 1:N:0:GAACCTAG+TCCGCATA
CAGAAAGATCACCTTGCCCAAAGAAGACCCACATTTTCATACATGATGAAATTCATTATTGGTCTCGC
GATCAGCTTGAGACATTTCTCCAGGTTTTCAAACCTAAAAATTTTTGGGCTACTCTGGTTTTTCCACC
TGAGATCTTAGCTGG
+
F,F:F,,FFF:,F:FF,FFFF,:F,,:F:,F:F:F:,::FFF,F,:,FFFFF:,,:F:FFFFF::,FF
:,,FFFFF:FF:FFFFFF:FF:FFFFFF,:,FFF,F,:F:FFFF,FF,F::FFF,:,FFFFFFFFF,F
:F:FF::::FF,::F
@A00155:342:HHGFNDSXY:1:2667:31891:11130 1:N:0:GAACCTAG+TCCGCATA
CAGAAAGATCACCTTGCCCAAAGAAGACCAACATTTTCATACATGATGAAATTCATTATTGGTCTCGC
GATCAGCTTGAGACATTTCTCCAGGTTTACAAACCTAAAAATTTGTGGGCTACTCTGGTTTTTCCACC
TGAGATCTTAGCTGG

```
+
FFFFFFF,FFFFFFFFFFFFF:F::F:FF::FFFFFFFF:FFFFFFFFFFFFFFFFFFFFFFFFFFFF
FFFFFFFFFFFFFFFFFFFFFFFFFFFFFFFFFFFFFFFF,FFFFFFFFF:FFFFFFFFFFFFFFFFF
FFFFFFFFFFFFFFF
@A00155:342:HHGFNDSXY:1:2643:16776:3771 1:N:0:GAACCTAG+TCCGCATA
CAGAAAGATCACCTTGCCCAAAGAAGACCAACATTTTCATACATGATGAAATTCATTATTGGTCTCGC
GATCAGCTTGAGACATTTCTCCAGGTTTACAAACCTAAAAATTTGTGGGCTACTCTGGTTTTTCCACC
TGAGATCTTAGCTGG
+
FFFFFFFFFFFFFFFFF:FFFFFFFFFFFFFFFFFFFFFFFF,FFFFFFFFFFFFFFFFFFFFFFFFF
FFFFFFFFFFFFFFFFFFFFFFFFF:FFFFFFFFFFFFFFFFFFFFFFFFFFFFFFFFFFFFFFFFFF
FFFFFFFFFFFFFFF
@A00155:342:HHGFNDSXY:1:1542:12400:19680 2:N:0:GAACCTAG+TCCGCATA
CAGAAAGATCACCTTGCCCAAAGAAGACCAACATTTTCATACATGATGAAATTCATTATTGGTCTCGC
GATCAGCTTGAGACATTTCTCCAGGTTTACAAACCTAAAAATTTGTGGGCTACTCTGGTTTTTCCACC
TGAGATCTTAGCTGG
+
FFFFFFFFFFFFFF:FFFFFFFFFF::FFFFFFFFFFFFFFFFFFFFFFFFFFFFFFFFFFFF:FFFF
FFFFFFF:FFFF,F:FFFFFFFFFFFFFFFFFFFFFFFFFFFFFFFFFFFFFFFFFF:FFFFFFFFFF
FFFFFFFFFFFFFFF
@A00155:342:HHGFNDSXY:1:2348:14407:22529 2:N:0:GAACCTAG+TCCGCATA
CAGAAAGATCACCTTGCCCAAAGAAGACCAACATTTTCATACATGATGAAATTCATTATTGGTCTCGC
GATCAGCTTGAGACATTTCTCCAGGTTTACAAACCTAAAAATTTGTGGGCTACTCTGGTTTTTCCACC
TGAGATCTTAGCTGG
+
FFFFFFFFFFFFFFFFFFFFFFFFFFFFFFFFFFFFFFFFFFFFFFFFFFFFFFFFFFFFFFFFFFFF
FFFFFFFFFFFFFFFFFFFFFFFFFFFFFFFFFF:FF:FFFFFFFFFFFFF:FFFFFFFFFFFFFFFF
FFFFFFFFFFFFFF:
@A00155:342:HHGFNDSXY:1:2430:26901:20306 2:N:0:GAACCTAG+TCCGCATA
CAGAAAGATCACCTTGCCCAAAGAAGACCAACATTTTCATACATGATGAAATTCATTATTGGTCTCGC
GATCAGCTTGAGACATTTCTCCAGGTTTACAAACCTAAAAATTTGTGGGCTACTCTGGTTTTTCCACC
TGAGATCTTAGCTGG
+
FFFFFFFFFFFFFFFFFFFFFFFFFFFFFFFFFFFFFFFFFFFFFFFFFFFFFFFFFFFFFFFFFFFF
FFFFFFFFFFFFFFFFFFFFFFFFFFFFFFFFFFFFFFFFFFFFFFFFFFFFFFFFFFFFFFFFFFFF
FFFFFFFFFFFFFFF
@A00155:342:HHGFNDSXY:1:2510:1669:32127 2:N:0:GAACCTAG+TCCGCATA
CAGAAAGATCACCTTGCCCAAAGAAGACCAACATTTTCATACATGATGAAATTCATTATTGGTCTCGC
GATCAGCTTGAGACATTTCTCCAGGTTTACAAACCTAAAAATTTGTGGGCTACTCTGGTTTTTCCACC
TGAGATCTTAGCTGG
+
FFFFFFFFFFFFFFFFFFFF,FFFFFFFFFFFFFFFFFF:F::FFF:FF:F,FFF,,FFFFF:FFFFF
FF,F:FF:FFFFFFF,:FFFFFFFFFFFFFFFFFFFFFFF:F,,FF:FFFF::FF:F::FFFFFF::F
FFFFFFFFF,FFFFF
@A00155:342:HHGFNDSXY:1:2543:16676:30655 2:N:0:GAACCTAG+TCCGCATA
CAGAAAGATCACCTTGCCCAAAGAAGACCAACATTTTCATACATGATGAAATTCATTATTGGTCTCGC
GATCAGCTTGAGACATTTCTCCAGGTTTACAAACCTAAAAATTTGTGGGCTACTCTGGTTTTTCCACC
TGAGATCTTAGCTGG
+
FFFFFFFFFFFFFFFFFFFFFFFFFFFFFFFFFFFFFFFFFFFFFFFFFFFFFFFFFF:FFFFFFFFF
FFFFFFFFFFFFFFFFFFFFFFFFFFFFFFFFFFFFFFFFFFFFFFFFFFFFFFFFFFFFFFFFFFFF
FFFFFFFFFFFFFFF
@A00155:342:HHGFNDSXY:1:2618:15555:36980 2:N:0:GAACCTAG+TCCGCATA
CAGAAAGATCACCTTGCCCAAAGAAGACCAACATTTTCATACATGATGAAATTCATTATTGGTCTCGC
```

GATCAGCTTGAGACATTTCTCCAGGTTTACAAACCTAAAAATTTGTGGGCTACTCTGGTTTTTCCACC
TGAGATCTTAGCTAG
+
FFFFFFFFFFFFFFFFFFFFFFFFFFFFFFFFFFFFFFFFFFFFFFFFFFFFFFFFFFFFFFFFFFFF
FFFFFFFFFFFFFFFFFFFFFFFFFFFFFFFFFFFFFFFFFFFFFFFFFFFFFFFFFFFFFFFFFFFF
FFFFFFF:FFFFFFF
@A00155:342:HHGFNDSXY:1:2424:1353:12195 1:N:0:GAACCTAG+TCCGCATA
AGAAAGATCACCTTGCCCAAAGAAGACCAACATTTTCATACATGATGAAATTCATTATTGGTCTCGCG
ATCAGCTTGAGACATTTCTCCAGGTTTACAAACCTAAAAATTTGTGGGCTACTCTGGTTTTTCCACCT
GAGATCTTAGCTG
+
F,FFF,F,FF:FFF:FFFFFF,FF,FF,FFFF,FF,FFFFFFF:FFFFFFFF,FFFFFFFFFF:FFFF
F::FFFFFFFFFFFFFFFFFFF::FFFFFFFF:FFFFFFF,FFFFFFFFFFF:FFFFFFFFFFFFFFF
FFFF:FFFFFFFF
@A00155:342:HHGFNDSXY:1:1167:27444:12226 1:N:0:GAACCTAG+TCCGCATA
AGAAAGATCACCTTGCCCAAAGAAGACCAACATTTTCATACATGATGAAATTCATTATTGGTCTCGCG
ATCAGCTTGAGACATTTCTCCAGGTTTACAAACCTAAAAATTTGTGGGCTACTCTGGTTTTTCCACCT
GAGATCTTAGCTGGT
+
FFFFFFFFFFFFFFFFFFFFFF:FFFFFF:FFFFFFF:FFFFFFFFFFFFFFFFFFF,FFFFFFFFFF
FFFFFFFFFFFFFFFFFFFFFFFFFFFFFFFFFFFFFFF:FFFFFFFFFFFFFFFFFFFFFFFFFFFF
FFFFFFFFFFFFFFF
@A00155:342:HHGFNDSXY:1:1217:27190:4429 1:N:0:GAACCTAG+TCCGCATA
AGAAAGATCACCTTGCCCAAAGAAGACCAACATTTTCATACATGATGAAATTCATTATTGGTCTCGCG
ATCAGCTTGAGACATTTCTCCAGGTTTACAAACCTAAAAATTTGTGGGCTACTCTGGTTTTTCCACCT
GAGATCTTAGCTGGT
+
FFFFFFFFFFFFFFFFFFFFFFFF,FFF,FFFFFFFFFFFFFFFFFFFFFFFFFFFFFFFFFFFFFFF
FFFFFFFF:FFFFFFFFFFFF:FFFFFFFFFFFFFFFFFFFFFFFFFFFFFFFFFFFFFFFFFFFFFF
FFFF::FFFFFFFFF
@A00155:342:HHGFNDSXY:1:1302:28348:13510 1:N:0:GAACCTAG+TCCGCATA
AGAAAGATCACCTTGCCCAAAGAAGACCAACATTTTCATACATGATGAAATTCATTATTGGTCTCGCG
ATCAGCTTGAGACATTTCTCCAGGTTTACAAACCTTAAAATTTGTGGGCTACTCTGGTTTTTCCACCT
GAGATCTTAGCTGGC
+
FFFFFFFFFFFFFFFFFFFFFFFFF:FFFFFFFFFFFFFFFFFFFFFFFFFFFFFFFFFFFFFFFFFF
:FFFFFFFFFFFFFFFFFFFFFFFFFFFFFFFFFFFFFFFFFFFFFFFFFFFFFFFFFFFFFFFFFFF
FFFFFFFFFFFFFFF
@A00155:342:HHGFNDSXY:1:1333:25319:26647 1:N:0:GAACCTAG+TCCGCATA
AGAAAGATCACCTTGCCCAAAGAAGACCAACATTTTCATACATGATGAAATTCATTATTGGTCTCGCG
ATCAGCTTGAGACATTTCTCCAGGTTTACAAACCTAAAAATTTGTGGGCTACTCTGGTTTTTCCACCT
GAGATCTTAGCTGGT
+
FFFFFFFFFFFFFFFFFFFF:FFFFFFFFFFFFFFFFFFFFFFFFFFFFFFFFFFFFFFFFFFFFFFF
FFFFFFFFFFFFFFFFFFFFFFFFFFFFFFF,FFFFFFFFFFFFFFFFFFFFFFFFFFFFFFFFFFFF
FFFFFFFFFFFFFFF
@A00155:342:HHGFNDSXY:1:1503:12319:18693 1:N:0:GAACCTAG+TCCGCATA
AGAAAGATCACCTTGCCCAAAGAAGACCAACATTTTCATACATGATGAAATTCATTATTGGTCTCGCG
ATCAGCTTGAGACATTTCTCCAGGTTTACAAACCTAAAAATTTGTGGGCTACTCTGGTTTTTCCACCT
GAGATCTTAGCTGGT
+
FFFFFFFFFFFFFFFFFFFFFFFFFFFFFFFFFFFFFFFFFFFFFFFFFFFFFFFFFFFFFFFFFFFF
FFFFFFFFFFFFFFFFFFFFF:FFFFFFFFFFFFFFFFFFFFFFFFFFFFFFFFFFFFFFFFFFFFFF
FFFFFFFFFFFFFFF

@A00155:342:HHGFNDSXY:1:1501:6090:14512 1:N:0:GAACCTAG+TCCGCATA
AGAAAGATCACCTTGCCCAAAGAAGACCAACATTTTCATACATGATGAAATTCATTATTGGTCTCGCG
ATCAGCTTGAGACATTTCTCCAGGTTTACAAACCTAAAAATTTGTGGGCTACTCTGGTTTTTCCACCT
GAGATCTTAGCTGGC
+
FFF,FFFFFFFF:FFFFFF:FF,:FFFFFF,F:FFFFFFFFFFFFFFFFFFFFF:FFFFFFFFFFFFF
FFF:FFFFFFFFFFF:FFFFFFFFFFFFFFF,FFFF:FFFFFF:FFFFFFFFFFFFFFFFFFFFFFFF
FFFFFFF,FFFFFFF
@A00155:342:HHGFNDSXY:1:1548:20139:8249 1:N:0:GAACCTAG+TCCGCATA
AGAAAGATCACCTTGCCCAAAGAAGACCAACATTTTCATACATGATGAAATTCATTATTGGTCTCGCG
ATCAGCTTGAGACATTTCTCCAGGTTTACAAACCTAAAAATTTGTGGGCTACTCTGGTTTTTCCACCT
GAGATCTTAGCTGGT
+
FFFFFFFFFFFFFFFFFFF:FFFFF:FFF:FFFFFFFFFFFFFFFFFFFFFFFFFFFFF:FFFFFFFF
FFF:FFFFFFFFFFFFFFF:FFFF:FFFFFFFFFFFFFFFFFFFFFFFFFFFFFFFFFFFFFFFFFFF
FFFF:FFFFFFFFFF
@A00155:342:HHGFNDSXY:1:1525:13539:34397 1:N:0:GAACCTAG+TCCGCATA
AGAAAGATCACCTTGCCCAAAGAAGACCAACATTTTCATACATGATGAAATTCATTATTGGTCTCGCG
ATCAGCTTGAGACATTTCTCCAGGTTTACAAACCTAAAAATTTGTGGGCTACTCTGGTTTTTCCACCT
GAGATCTTAGCTGGT
+
FFFFFFFFFFFFFFFFFFFFFF:FFFFFFFFFFFFFFFFFFFFFFFFFFFFFFFFFFFFFFFFFFFFF
FFFFFFFFFFFFFFFFFFFFFFFFFFFFFFFFFFFFFFFFFFFFFFFFFFFFFFFFFFFFFFFFFFFF
FFFFFFFFFFFFFFF
@A00155:342:HHGFNDSXY:1:2208:19741:10817 1:N:0:GAACCTAG+TCCGCATA
AGAAAGATCACCTTGCCCAAAGAAGACCAACATTTTCATACATGATGAAATTCATTATTGGTCTCGCG
ATCAGCTTGAGACATTTCTCCAGGTTTACAAACCTAAAAATTTGTGGGCTACTCTGGTTTTTCCACCT
GAGATCTTAGCTGGT
+
FFFFFFFFFFFFFFFFFFFFFFF:FFFFFFFF:FFFFFFFFFFFFFFFFFFFFFFFFFFFFFFFFFFF
FFFFFFFFFFFFFFFFFFFFFFFFFFFFFFFFFFFFFFFFFFFFFFFFFFFFFFFFFFFFFFFFFFFF
FFFFFFFFFFFFFFF
@A00155:342:HHGFNDSXY:1:2217:28754:15154 1:N:0:GAACCTAG+TCCGCATA
AGAAAGATCACCTTGCCCAAAGAAGACCAACATTTTCATACATGATGAAATTCATTATTGGTCTCGCG
ATCAGCTTGAGACATTTCTCCAGGTTTACAAACCTAAAAATTTGTGGGCTACTCTGGTTTTTCCACCT
GAGATCTTAGCTGGT
+
FFF:FF:FFFFFFFFFFFFF:FFFF,FFF:FFFFFFFFFFFFFFFFFFFFFFFFF:FFFFFFFFFFFF
FFFFFFFFFFFF:FFFFFFFFFFFFFFFFFFFFFFFFFFFFFFFFFFFFFFFFFFFFFFFFFFFFFFF
FFFFFFFFFFFFFFF
@A00155:342:HHGFNDSXY:1:2620:21531:33458 1:N:0:GAACCTAG+TCCGCATA
AGAAAGATCACCTTGCCCAAAGAAGACCAACATTTTCATACATGATGAAATTCATTATTGGTCTCGCG
ATCAGCTTGAGACATTTCTCCAGGTTTACAAACCTAAAAATTTGTGGGCTACTCTGGTTTTTCCACCT
GAGATCTTAGCTGGC
+
F,FFFFFFFFFFFFFFFFFFFF:FF:FFFFFFFFFFFFFFFFFFFFFFFFFFFFFFFFFFFFFFFFFF
FFFFFFFFFFFFFFFFFFFFFFFFFFFFFFFFFFFFFFFFFFFFFFFFFFFFFFFFFFFFFFFFFFFF
FFFFFFF:FFFFFFF
@A00155:342:HHGFNDSXY:1:1129:29803:31908 2:N:0:GAACCTAG+TCCGCATA
AGAAAGATCACCTTGCCCAAAGAAGACCAACATTTTCATACATGATGAAATTCATTATTGGTCTCGCG
ATCAGCTTGAGACATTTCTCCAGGTTTACAAACCTAAAAATTTGTGGGCTACTCTGGTTTTTCCACCT
GAGATCTTAGCTGGT
+
FFFFFFFFFFFFFFF:FFFFFFFFFFFFFFFFFFFFFFFFFFFFFFF:FFFFFFFFFFFFFFFFFFFF

FFF:FFFF:FFFFF:FFFFFFFFFFFFFFFFFFFFFFFFFFFFFFFFFFFFFFFFFFFFFFFF,FFF:F
FF::FFFFFFFFFFF
@A00155:342:HHGFNDSXY:1:1248:26910:10363 2:N:0:GAACCTAG+TCCGCATA
AGAAAGATCACCTTGCCCAAAGAAGACCAACATTTTCATACATGATGAAATTCATTATTGGTCTCGCG
ATCAGCTTGAGACATTTCTCCAGGTTTACAAACCTAAAAATTTGTGGGCTACTCTGGTTTTTCCACCT
GAGATCTTAGCTGGT
+
FFFFFFFFFFFFFFFFFFFFFFFFFFFFFFFFFFF:FFFFFFFFFFFFFFFFFFFFFFFFFFFFFFFF
F:FFFFFFFFFFFFFFFFFFFFFF:FFFFFFFFFFFFFFFFFFFFFFF:FFF,FFFFFFFFFFFFFF
FFFFF:FFFFFFF:F
@A00155:342:HHGFNDSXY:1:1464:3929:35102 2:N:0:GAACCTAG+TCCGCATA
AGAAAGATCACCTTGCCCAAAGAAGACCAACATTTTCATACATGATGAAATTCATTATTGGTCTCGCG
ATCAGCTTGAGACATTTCTCCAGGTTTACAAACCTAAAAATTTGTGGGCTACTCTGGTTTTTCCACCT
GAGATCTTAGCTGGT
+
FFFFFFFFFFFFFFFFFFFFFFFFFFFFFFFFFFFFFFFFFFFFFFFFFF:FFFFFFFFFFFFFFFFFF
FF:FFFFFFFFFFFFFFFFFFFFFFFFFFFF,FFFFFFFFFFFF::FFFFFFFFFFFFFF:FF:FF
FF:FFFFFFFFFF:F
@A00155:342:HHGFNDSXY:1:2165:25482:1783 2:N:0:GAACCTAG+TCCGCATA
AGAAAGATCACCTTGCCCAAAGAAGACCAACATTTTCATACATGATGAAATTCATTATTGGTCTCGCG
ATCAGCTTGAGACATTTCTCCAGGTTTACAAACCTAAAAATTTGTGGGCTACTCTGGTTTTTCCACCT
GAGATCTTAGCTGGT
+
FFFFFFFFFFFFFFFFFFFFFFFFFFFFFFFFFFFFFFFFFFFFFFFFFFFFFFFF:FFFFFFFFFFF
FFFFFFFFFFFFFFF:FFFFFFFFFFFFFFFFFFFFFFFFFFFFFFFFFFFFFFFFFFFFFFFFFFFF
FFFFFF:FFFFFFFF
@A00155:342:HHGFNDSXY:1:2221:3134:14841 2:N:0:GAACCTAG+TCCGCATA
AGAAAGATCACCTTGCCCAAAGAAGACCAACATTTTCATACATGATGAAATTCATTATTGGTCTCGCG
ATCAGCTTGAGACATTTCTCCAGGTTTACAAACCTAAAAATTTGTGGGCTACTCTGGTTTTTCCACCT
GAGATCTTAGCTGGT
+
FFFFFFFFFFFFFFFFFFFFFFFFFFFFFFFFFFFFFFFFFFFFFFFFFF:FFFFFFFFFFFFFFF:FF
FFFFFFFFFFFFFFFFFFFFFFFFFFFFFF:FFF:FFFF:FFFFFFFFFFFFFFFFFFFFFFFF:FFFFF
FFFFFFFFFFFFF:F
@A00155:342:HHGFNDSXY:1:2316:15845:19069 2:N:0:GAACCTAG+TCCGCATA
AGAAAGATCACCTTGCCCAAAGAAGACCAACATTTTCATACATGATGAAATTCATTATTGGTCTCGCG
ATCAGCTTGAGACATTTCTCCAGGTTTACAAACCTAAAAATTTGTGGGCTACTCTGGTTTTTCCACCT
GAGATCTTAGCTGGT
+
F:FFFFFFFFFF:F:FF:FFFFFF,,F:FFFF:FFFFFFFFFFFFFFFFFFFF::F,FFFFFFFFFFF
F,FFFFFFFF:FFFFFFFFF,FFF:FFFFFF:FFFFFFFF::F,FF:FFFFFFFFF,FF:,F,FF:FF
:F,FFFF,FFFF:,F
@A00155:342:HHGFNDSXY:1:2413:3115:32816 2:N:0:GAACCTAG+TCCGCATA
AGAAAGATCACCTTGCCCAAAGAAGACCAACATTTTCATACATGATGAAATTCATTATTGGTCTCGCG
ATCAGCTTGAGACATTTCTCCAGGTTTACAAACCTAAAAATTTGTGGGCTACTCTGGTTTTTCCACCT
GAGATCTTAGCTGGT
+
FFFFFFFFFFFFFFFFFFFFFFFFFFFFFF:FFFFFFFFFFFFFFFFFFFFFFFFFFFFFFFFFFFFFF
FFFFFFFFF:FFFFFFFFFFFFFFFFF:FFFFFFFFFFFF::FFFFFFFFF:FFFFFFFFFFFFFFFF
F:FFFFFFFFFFFFF
@A00155:342:HHGFNDSXY:1:2543:17300:17237 2:N:0:GAACCTAG+TCCGCATA
AGAAAGATCACCTTGCCCAAAGAAGACCAACATTTTCATACATGATGAAATTCATTATTGGTCTCGCG
ATCAGCTTGAGACATTTCTCCAGGTTTACAAACCTAAAAATTTGTGTGCTACTCTGGTTTTTCCACCT
GAGATCTTAGCTGGT

```
+
FFFFFFFFFFFFFFFFF:FFFFFF:FF,F:FFFFFF:FFFFFFFFFFFFFFF:F:FFFFFFFFFFFFF
FF:FFF::FFFFFFFFFFF:FF,FF:FFF:FF,F:FFFFFFFF:FF,FFFFFF,FF,FFFFFFFF:FF
:FFF:FFFFFFFFFF
@A00155:342:HHGFNDSXY:1:1161:28140:20697 1:N:0:GAACCTAG+TCCGCATA
GAAAGATCACCTTGCCCAAAGAAGACCAACATTTTCATACATGATGAAATTCATTATTGGTCTCGCGA
TCAGCTTGAGACATTTCTCCAGGTTTACAAACCTAAAAATTTGTGGGCTACTCTGGTTTTTCCACCTG
AGATCTTAGCTGGTT
+
FFFFFFFFFFFFFFFFFFFF:F,F:FFFF:FFFFFFFFFFFFFFFFFFFFFFFFFFFFFFFFFFFFFF
FFFFFFFFFFFFFFFFFFFF:FFFFFFFFFFFFFFFFFFF:FFFFFFFFFFFFFFFFFFFFFFFFFFFF
FFFFFFF:FFFFFFF
@A00155:342:HHGFNDSXY:1:1352:10104:28385 1:N:0:GAACCTAG+TCCGCATA
GAAAGATCACCTTGCCCAAAGAAGACCAACATTTTCATACATGATGAAATTCATTATTGGTCTCGCGA
TCAGCTTGAGACATTTCTCCAGGTTTACAAACCTAAAAATTTGTGGGCTACTCTGGTTTTTCCACCTG
AGATCTTAGCTGGTG
+
FFFFFFFFFFFFFFFFFFFFFFFFFFFF:FFFFFFFFFFFFFFFFFFFFFFFFFFFFFFFFFFFFFFF
FFFFFFFFFFFFFFFFFFFFFFFFF:FFFFFFFFFFFFFFFFFFFFFFFFFFFFFFFFFFFFFFFFFF
FFFFFFFFFFFFFFF
@A00155:342:HHGFNDSXY:1:1358:11360:6261 1:N:0:GAACCTAG+TCCGCATA
GAAAGATCACCTTGCCCAAAGAAGACCAACATTTTCATACATGATGAAATTCATTATTGGTCTCGCGA
TCAGCTTGAGACATTTCTCCAGGTTTACAAACCTAAAAATTTGTGGGCTACTCTGGTTTTTCCACCTG
AGATCTTAGCTGGAT
+
:F,FFFFFFFFFFFFFFFFFFFFFFFFFFFFFFFFFFFFFFFFFFFFFFFFFFFFFFFFFFFFFFFFF
FF:FFFFFFFFFFFFFFFFFFFFFFFFFFFFFFFFFFFFF:FFFFFFFFFFFFFFFFFFFFFFFFFFFF
FFFFFFFFFFFFFFF
@A00155:342:HHGFNDSXY:1:1425:19280:4977 1:N:0:GAACCTAG+TCCGCATA
GAAAGATCACCTTGCCCAAAGAAGACCAACATTTTCATACATGATGAAATTCATTATTGGTCTCGCGA
TCAGCTTGAGACATTTCTCCAGGTTTACAAACCTAAAAATTTGTGGGCTACTCTGGTTTTTCCACCTG
AGATCTTAGCTGGTG
+
FFFFFFFFFFFFFFFFFFFF:FFFFFFFFFFFFFFFFFFFFFFFFFFFFFFFFFFFFFFFFFFFFFFF
FFFFFFFFFFFFFFFFFFFFFFFFFFFFFFFFFFFFFFFFFFFFFFFFF:FFFFFFFFFFFFFF:FFF
FFFFFFFFFFFFFFF
@A00155:342:HHGFNDSXY:1:1569:11930:33833 1:N:0:GAACCTAG+TCCGCATA
GAAAGATCACCTTGCCCAAAGAAGACCAACATTTTCATACATGATGAAATTCATTATTGGTCTCGCGA
TCAGCTTGAGACATTTCTCCAGGTTTACAAACCTAAAAATTTGTGGGCTACTCTGGTTTTTCCACCTG
AGATCTTAGCTGGTT
+
FF:FFFFFFFFFFFFFFFFFFFFFFFFFFFFFFFFFFFFFFFFFFFFFFFFFFFFFFFFFFFFFFFFF
FF:FFFFFFFFFFFFFFFFFFFFFFFFFFFFFFFFFFFFFFFFFFFFFFFF:FFFFFFFFFFFFFFFF
FFFFFFFFFFFFFFF
@A00155:342:HHGFNDSXY:1:1670:25699:21198 1:N:0:GAACCTAG+TCCGCATA
GAAAGATCACCTTGCCCAAAGAAGACCAACATTTTCATACATGATGAAATTCATTATTGGTCTCGCGA
TCAGCTTGAGACATTTCTCCAGGTTTACAAACCTAAAAATTTGTGGGCTACTCTGGTTTTTCCACCTG
AGATCTTAGCTGGTA
+
FFFFFFFFFFFFFFFFFFFFFFFFFFFFFFFFFFFFFFFFFFFFFFFFFFFFFFFFFFFFFFFFFFFF
FFFFFFFFFFFFFFFFFFFFFFFFFFFFFFFFFFFFFFFFFFFFFFFFFFFFFFFFFFFFFFFFFFFF
FFFFFFFFFFFFFFF
@A00155:342:HHGFNDSXY:1:2319:30002:14747 1:N:0:GAACCTAG+TCCGCATA
GAAAGATCACCTTGCCCAAAGAAGACCAACATTTTCATACATGATGAAATTCATTATTGGTCTCGCGA
```

TCAGCTTGAGACATTTCTCCAGGTTTACAAACCTAAAAATTTGTGGGCTACTCTGGTTTTTCCACCTG
AGATCTTAGCTGGTT
+
FFFFF:FFFFFFFFFFFFFFFFFFF:FFFFFFFFFFFFFFFFFFFFFFFFFFFFFFFFFFFFFFFFFFFF
FF:FFFFFFFFFFFFFFFFFFFFFFFFFFFFFFFFFFFFFFFFFFFFFFFFFFFFFFFFFFFFFFFFFFF
FFFFFFFFFFFFFFF
@A00155:342:HHGFNDSXY:1:2326:2474:17519 1:N:0:GAACCTAG+TCCGCATA
GAAAGATCACCTTGCCCAAAGAAGACCAACATTTTCATACATGATGAAATTCATTATTGGTCTCGCGA
TCAGCTTGAGACATTTCTCCAGGTTTACAAACCTAAAAATTTGTGGGCTACTCTGGTTTTTCCACCTG
AGATCTTAGCTGGTG
+
:F,FFFFFFFF:FFFFFFFFFFFFFFF::FFFFF:F:FFFFFFFFFFFFFFFFFFFFFFFFFFFFFFFFF
FFFFFFFFFFFFFFFFFFFFFFFFFFFFFFFFFFFFFFFFFFFFFFFFFFFFFFFFFFFFFFFFFFFFFF
FFFFFFFFFFFFFFF
@A00155:342:HHGFNDSXY:1:2348:14407:22529 1:N:0:GAACCTAG+TCCGCATA
GAAAGATCACCTTGCCCAAAGAAGACCAACATTTTCATACATGATGAAATTCATTATTGGTCTCGCGA
TCAGCTTGAGACATTTCTCCAGGTTTACAAACCTAAAAATTTGTGGGCTACTCTGGTTTTTCCACCTG
AGATCTTAGCTGGTT
+
FFFFFFFFFFFFFFFFFFFFFFFFFFFFFFFFFFFFFFF:FFFFFFFFFFFFFFFFFFFFFFFFFFFFFF
FFFFFFFFFFFFFFFFFFFFFFFFFFFFFFFFFFFFFFFFFFFFFFFFFFFFFFFFFF:FFFFFFFFFFF
FFFFFFFFFFFFF:F
@A00155:342:HHGFNDSXY:1:2367:18747:17331 1:N:0:GAACCTAG+TCCGCATA
GAAAGATCACCTTGCCCAAAGAAGACCAACATTTTCATACATGATGAAATTCATTATTGGTCTCGCGA
TCAGCTTGAGACATTTCTCCAGGTTTACAAACCTAAAAATTTGTGGGCTACTCTGGTTTTTCCACCTG
AGATCTTAGCTGGTC
+
FFFFFFFFFFFFFFFFFFF:FF:FFFFFFFFFFFFFFFFFFFFFFFFFFFFFFFFFFFFFFFFFFFFFFF
FFFFFFFFFFFFFFFFFFFFFFFFFFFFFFFFFFFFFFFF:FFFFFFFFFFFFFFFFFFFFFFFFFFFFF
FFFFFFFFFFFFFFF
@A00155:342:HHGFNDSXY:1:2507:29668:26443 1:N:0:GAACCTAG+TCCGCATA
GAAAGATCACCTTGCCCAAAGAAGACCAACATTTTCATACATGATGAAATTCATTATTGGTCTCGCGA
TCAGCTTGAGACATTTCTCCAGGTTTACAAACCTAAAAATTTGTGGGCTACTCTGGTTTTTCCACCTG
AGATCTTAGCTGGTC
+
FFF:FFFFFFFFFFFFFFFFFFF,FFFFF,FFFFFFFFFFFFFFFFFFFFFFFFFFFFFFFFFFFFFFFF
FFFFFFFFFFFFFFFFFFFFFFFFFFFFFFFFFFFFFFFFFFFFFFFFFFFFFFFFFFFFFFFFFFFFFF
FFFFFFFFFFFFFFF
@A00155:342:HHGFNDSXY:1:1309:13349:8453 2:N:0:GAACCTAG+TCCGCATA
GAAAGATCACCTTGCCCAAAGAAGACCAACATTTTCATACATGATGAAATTCATTATTGGTCTCGCGA
TCAGCTTGAGACATTTCTCCAGGTTTACAAACCTAAAAATTTGTGGGCTACTCTGGTTTTTCCACCTG
AGATCTTAGCTGGTT
+
FFFFFFFFFFFFFFFFFFFFFFFFFFFFFFFFFFFFFFFFFFFFFFFFFF:,FFFFFFFFFFFFFFFFFF
FFFFFFFFFFFFFFFFFFFFFFFFFFFFFFF:FFFFFFFFFFFFFFFFF:FFFFFFFFFFFFFFFFFFFF
FFFFFFFFFFFF:FF
@A00155:342:HHGFNDSXY:1:1541:27543:19382 2:N:0:GAACCTAG+TCCGCATA
GAAAGATCACCTTGCCCAAAGAAGACCAACATTTTCATACATGATGAAATTCATTATTGGTCTCGCGA
TCAGCTTGAGACATTTCTCCAGGTTTACAAACCTAAAAATTTGTGGGCTACTCTGGTTTTTCCACCTG
AGATCTTAGAGATCG
+
FFFFFFFFFFFFFFFFFFFFFFFFFFFFFFFFFFFFFFFFFFFFFFFFF::FFFFFFF,FFFFFFFFFFF
,FFFFFFFFFFF::FFFFFFFFFFFFFFFFFFFFFF,FFFFFF::FFFFFFFFFFFFFFFFFFFFFF:FF
FFFFFFFFFFFF:FF

```
@A00155:342:HHGFNDSXY:1:1607:28357:31000 2:N:0:GAACCTAG+TCCGCATA
GAAAGATCACCTTGCCCAAAGAAGACCAAAATTTTCATACATGATGAAATTCATTATTGGTCTCGCGA
TCAGCTTGAGACAGTTCTCCAGTTTTACAAACATAAAAATTTGTGGTCTACTCTGGTTTTTCCACCTG
AGATCTTAGCTGGTT
+
F:FF,FFFFF,FFFF,FFFFFFF,FF,FF,FFFFF:FFFFFFFFFF,FF,F,FFFFFFF,FFFFFFFF
F:::,,:,FFFFF,FF:F,FF,,FF:F,:F:F,FFFFFFFF:,F,,,FFF:FFF,F:::FF::F:,,:
F,:,FF,F:FFF,FF
@A00155:342:HHGFNDSXY:1:1630:7627:27258 2:N:0:GAACCTAG+TCCGCATA
GAAAGATCACCTTGCCCAAAGAAGACCAACATTTTCATACATGATGAAATTCATTATTGGTCTCGCGA
TCAGCTTGAGACATTTCTCCAGGTTTACAAACCTAAAAATTTGTGGGCTACTCTGGTTTTTCCACCTG
AGATCTTAGCTGGTT
+
FFFFFFFFFFFFFFFFFFFFFFFFFFFFFFFFFFFFFFFFFFFFFFFFF:FFFFFFFFFFFFFFFFFFFF
FFFFFFFFFFFFFFFFFFFFFFFFFFFFFFFFFFFFFFFFFFFFFFFFFFFFFFFFFFFFFFFF:FFFFF
FFFFF:FFFFFFFFF
@A00155:342:HHGFNDSXY:1:2127:13847:22936 2:N:0:GAACCTAG+TCCGCATA
GAAAGATCACCTTGCCCAAAGAAGACCAACATTTTCATACATGATGAAATTCATTATTGGTCTCGCGA
TCAGCTTGAGACATTTCTCCAGGTTTACAAACCTAAAAATTTGTGGGCTACTCTGGTTTTTCCACCTG
AGATCTTAGAGATCG
+
F:::FFFFFFFFFFFFFFFFFFFFFFFFFFFFFFFFFFFFFFFFFFFFFFFFFFFFFFFFFFFFFFFFFF
FFFFFFFFFFFFFFFFFFFFFFFFFFFFFFFFFFFFFFFFFFFFFFFFFFFFFFFFFFFFFFFFFFFFFF
FFFFFFFFFFFFFFF
@A00155:342:HHGFNDSXY:1:2308:10511:34287 2:N:0:GAACCTAG+TCCGCATA
GAAAGATCACCTTGCCCAAAGAAGACCAACATTTTCATACATGATGAAATTCATTATTGGTCTCGCGA
TCAGCTTGAGACATTTCTCCAGGTTTACAAACCTAAAAATTTGTGGGCTACTCTGGTTTTTCCACCTG
AGATCTTAGCTGGTT
+
:FFFFFFF:FFFFFFFFFF:FFFFFFFFFFFFFFFFFFFFFFFFFFFFFFFFFF,FFFFFFFFFFFFFF
FF:FFFFFFFFFFFFFFFFFFFFFFFFFFFFFFF:F:FFFFFFF:FFFFFFFFFFFFFFFFFFFFFFFFF
FFFFFFFFFFFFFFF
@A00155:342:HHGFNDSXY:1:2278:22489:36370 2:N:0:GAACCTAG+TCCGCATA
GAAAGATCACCTTGCCCAAAGAAGACCAACATTTTCATACATGATGAAATTCATTATTGGTCTCGCGA
TCAGCTTGAGACATTTCTCCAGGTTTACAAACCTAAAAATTTGTGGGCTACTCTGGTTTTTCCACCTG
AGATCTTAGCTGGTT
+
FFFFFFFFFFFFFFFFFFFFFFFFFFFFFFFFFFFFFFFFFFFFFFFFFFFFFFF,FFFFFFFFFFFFF
FFFFFFFFFFFFF:FFFFFFFFFFFFFFFFFFFFFFFFFFFFFFFFFFFFFF:FFFFFFF:FFFFFFFFF
FFFFFFFFFFFFFFF
@A00155:342:HHGFNDSXY:1:2522:1913:25316 2:N:0:GAACCTAG+TCCGCATA
GAAAGATCACCTTGCCCAAAGAAGACCAACATTTTCATACATGATGAAATTCATTATTGGTCTCGCGA
TCAGCTTGAGACATTTCTCCAGGTTTACAAACCTAAAAATTTGTGGGCTACTCTGGTTTTTCCACCTG
AGATCTTAGCTGGTT
+
FFFFF:FFFFFFFFFFFFF:FF,FFFFFFFFFFFF:FFFFFFF:FFF:FFFFF,,FFFFFFFFFFFFF
FF:FFFFFFFFFFFFFFFFFFFFFF:FFF::FFFFFFFFF,F:FFFFFFF:FFF:FFF:FFFFF,FFFF
FFF:F,FFFF,FFFF
@A00155:342:HHGFNDSXY:1:2522:12002:2581 2:N:0:GAACCTAG+TCCGCATA
GAAAGATCACCTTGCCCAAAGAAGACCAACATTTTCATACATGATGAAATTCATTATTGGTCTCGCGA
TCAGCTTGAGACATTTCTCCAGGTTTACAAACCTAAAAATTTGTGGGCTACTCTGGTTTTCCCACCTG
AGATCTTAGAGATCG
+
FFFFFFFFFFFFFFFFFFFFFFFF:FFFFFFFFFFFFFFFFFFFFFF:FFFFFFFFFFFFFFFFFFFF:F
```

FFFFFFFF,FFFFFFFFFFFFFFFFFF:FFFFFF,FFFF:FFFFFFFFF,FFFFFFFFFFF,FFFFFF
FFFFFFFFFFFFF:F
@A00155:342:HHGFNDSXY:1:2456:4056:20917 2:N:0:GAACCTAG+TCCGCATA
GAAAGATCACCTTGCCCAAAGAAGACCAACATTTTCATACATGATGAAATTCATTATTGGTCTCGCGA
TCAGCTTGAGACATTTCTCCAGGTTTACAAACCTAAAAATTTGTGGGCTACTCTGGTTTTTCCACCTG
AGATCTTAGCTGGTT
+
FFFFFFFFFFFFFFFFFFFFFFFFFFFFFFFFFFFFFFFFFFFFFFFFFF:FFFFFFFFFFFFFF
FFFFFFFFFFFFFFFFFFFFFFFFFFFFFFFF:FFFFFFFFFFFFFFFFFFFFFFFF:FFFFFFFFF:F
FF,FFFFFF:FF:FF
@A00155:342:HHGFNDSXY:1:2601:18792:36824 2:N:0:GAACCTAG+TCCGCATA
GAAAGATCACCTTGCCCAAAGAAGACCAACATTTTCATACATGATGAAATTCATTATTGGTCTCGCGA
TCAGCTTGAGACATTTCTCCAGGTTTACAAACCTAAAAATTTGTGGGCTACTCTGGTTTTTCCACCTG
AGATCTTAGCTGGTT
+
FFFFFFFFFFFFFFFFFFFFFFFFFFFFFFFFFFFFFFFFFFFFFFFF:FFFFFFFFFFFFFFFFF
FFFFFFFFFFFFFFFFFFFFFFFFFFFFFFFFFFFFFFFFFFFFFFFFFFFFFFFF:FFFFFFFFFF
FFFFFF,FFFFFFFF
@A00155:342:HHGFNDSXY:1:2602:18991:4852 2:N:0:GAACCTAG+TCCGCATA
GAAAGATCACCTTGCCCAAAGAAGACCAACATTTTCATACATGATGAAATTCATTATTGGTCTCGCGA
TCAGCTTGAGACATTTCTCCAGGTTTACAAACCTAAAAATTTGTGGGCTACTCTGGTTTTTCCACCTG
AGATCTTAGCTGGTT
+
FFFFFFFFFFFFFFFFFFFFFFFFFFFFFFFFFFFFFFFFFFFFFFFFFFFFFFFFFFFFFFFFFFFF
FFFFFFFFFFFFFFFFFFFFFFFFFFFFFFFFFFFFFFFFFF:FFFFFFFFFFFFFFFFFFFFFFFFF
FFFFFFFFFF:FFFF
@A00155:342:HHGFNDSXY:1:2456:3929:18035 2:N:0:GAACCTAG+TCCGCATA
AAAGATCACCTTGCCCAAAGAAGACCAACATTTTCATACATGATGAAATTCATTATTGGTCTCGCGAT
CAGCTTGAGACATTTCTCCAGGTTTACAAACCTAAAAATTTGTGGGCTACTCTGGTTTTTCCACCTGA
GATCTTAGCTGGTT
+
:FFFFFFFFFFFFFFFFFFFFFFFFFFFFFFFFFFFFFFFFFFFFFFFFFFFFFFFFFFFFFFFFFFF
FFFFFFFFFFFFFFFFFFFFFFFFFFFFFFFFFFFFFFFFFFFFFF:FFFFFFFFFFFFFFFFFFFFFF
FFFFFFFFFFFFFF
@A00155:342:HHGFNDSXY:1:1613:4417:2065 1:N:0:GAACCTAG+TCCGCATA
CTAGATCACCTTGCCCAAAGAAGACCAACATTTTCATACATGATGAAATTCATTATTGGTCTCGCGAT
CAGCTTGAGACATTTCTCCAGGTTTACAAACCTAAAAATTTGTGGGCTACTCGGGTTTTTCCACCTGA
GATCTTAGCTGGTTA
+
FFF,FFFFF:F:FFFFFFF:FF,FFFF:F::FFFF:FFFF:FFFFFFFFFFFFFFFFFFFFFFF:FFFF
F:FFFFFFFFFFFFF::FFFFFFFFFFFFFFFFFFFFFFFFFFFFFFFFFFFFFFF::FFFFFFFF,FF
FFFFFFFFFFFFFFF
@A00155:342:HHGFNDSXY:1:1554:31828:35603 2:N:0:GAACCTAG+TCCGCATA
AAAGATCACCTTGCCCAAAGAAGACCAACATTTTCATACATGATGAAATTCATTATTGGTCTCGCGAT
CAGCTTGAGACATTTCTCCAGGTTTACAAACCTAAAAATTTGTGGGCTACTCTGGTTTTTCCACCTGA
GATCTTAGCTGGTTA
+
FFFFFFFFFFFFFFFFFFFFFFFFFF:FFFFFFFFFFFFFFFFFF:FF:FFFFFF:FFFFFFFFFFFF
FFFFFFFFFFFFFFFFFFFFFFFFFFFF,FFFFFFFFFFFFFFFFFFFFFFFFFFFFFFFF,FFFFFFF
FF:FFFFFFFFFFF:
@A00155:342:HHGFNDSXY:1:2366:5267:9048 2:N:0:GAACCTAG+TCCGCATA
AAAGATCACCTTGCCCAAAGAAGACCAACATTTTCATACATGATGAAATTCATTATTGGTCTCGCGAT
CAGCTTGAGACATTTCTCCAGGTTTACAAACCTAAAAATTTGTGGGCTACTCTGGTTTTTCCACCTGA
GATCTTAGCTGGTTA

+
FFFFFFFFFFFFFFFFFFFFFFFFFFFFFF:FFFFFFFFFFFFFFFFF::FFF::FFFFFFFFFFFFFFF
FFFFFFFFFFFFFFFFFFFFFFFFFFFFFFFFFF:FFFFFFFFFFFFFFFFFFFFFF:FFFF:FFFFFFF
,FFFFFFFFFF,FFF
@A00155:342:HHGFNDSXY:1:2366:5258:9064 2:N:0:GAACCTAG+TCCGCATA
AAAGATCACCTTGCCCAAAGAAGACCAACATTTTCATACATGATGAAATTCATTATTGGTCTCGCGAT
CAGCTTGAGACATTTCTCCAGGTTTACAAACCTAAAAATTTGTGGGCTACTCTGGTTTTTCCACCTGA
GATCTTAGCTGGTTA
+
FFFFFFFFFFFFFFFFFFFFFFFFFFFFFFFFFFF:FFFFFFFFFFFFFFFFFFFFFFFFFFFFFFFFFF
FFFFFFFFFFFFFFFFFFFFFFFFFFFFF,FFFFFFFFFFFFFFF:FFFFFFFFFF,FFFFF:FFFFFFF
FFF,FFFFFFFFF:F
@A00155:342:HHGFNDSXY:1:2473:22842:4288 1:N:0:GAACCTAG+TCCGCATA
AGATCACCTTGCCCAAAGAAGACCAACATTTTCATACATGATGAAATTCATTATTGGTCTCGCGATCA
GCTTGAGACATTTCTCCAGGTTTACAAACCTAAAAATTTGTGGGCTACTCTGGTTTTTCCACCTGAGA
TCTTAGCTGGTTACA
+
FFFFFFFFFFFFFFFFFFFFFFFFFFFFFFFFFFFFFFFFFFFFFFFFFFFFFFFFFFFFFFFFFFFFFF
FFFFFFFFFFFFFFFFFFFFFFFFFFFFFFFFFFFFFFFFFFFFFFFFFFFFFFFFFFFFFFFFFFFFFF
FFFFFFFFFFFFFFF
@A00155:342:HHGFNDSXY:1:1152:9570:15280 2:N:0:GAACCTAG+TCCGCATA
AGATCACCTTGCCCAAAGAAGACCAACATTTTCATACATGATGAAATTCATTATTGGTCTCGCGATCA
GCTTGAGACATTTCTCCAGGTTTACAAACATAAAAATTTGTGGGCAACTCTGGTTTTTCCACCTGAGA
TCGTAGCTGGTTACA
+
FFF:FFFFFFFFFFFF:FFFFFFFFFFFF:FFFFFFFFFFFFFFFFFFF,FF:FFFFFFFFFFFFFFFFF
FFF:::FFFF:F,FFFFFFF:FFFFFFFF,:FFFFFF:FF:::F:,F:,FFF:F,FFFFFFFFFFFFF
FF:FFFF,FF,,FFF
@A00155:342:HHGFNDSXY:1:1613:4417:2065 2:N:0:GAACCTAG+TCCGCATA
AGATCACCTTGCCCAAAGAAGACCAACATTTTCATACATGATGAAATTCATTATTGGTCTCGCGATCA
GCTTGAGACATTTCTCCAGGTTTACAAACCTAAAAATTTGTGGGCTACTCGGGTTTTTCCACCTGAGA
TCTTAGCTGGTTAAG
+
FFFFFFFFFFFF:FFFFFFFFFFFFFFFFFFFFFFFFFFFFFFFF:FFFFFFFFFFFF:FFFFFFFFFFF
FFFFFFFFFFFFFFFFFFFFFFFFFFFFFFFFFFFFFFFFFFF,FFFF:FFFFFFFFFFFFFFFFFFFFF
FFFFFF:FFFFFF:F
@A00155:342:HHGFNDSXY:1:1616:4580:3881 2:N:0:GAACCTAG+TCCGCATA
AGATCACCTTGCCCAAAGAAGACCAACATTTTCATACATGATGAAATTCATTATTGGTCTCGCGATCA
GCTTGAGACATTTCTACAGGTTTAAAAACCTAAAAATTTGTGGGCTACTCTGGTTTTTCAACCTGAGA
TATTATCTGGTTACA
+
F:FFFFFFF,:,FFFFFF,:FF,F:FFFFF:FF,FFF:FFFFFF:FFFFFFF:,FFF:FFFFFF:FFFFF
,F:F,FFFFFF,FF,,FF::FFFF,FFF:,FF:FF::FF,FFFFFF,FF,F,,:FFFF:,F,F::F,F
,,F,F,FF:,FFF:F
@A00155:342:HHGFNDSXY:1:2325:13331:14090 2:N:0:GAACCTAG+TCCGCATA
AGATCACCTTGCCCAAAGAAGACCAACATTTTCATACATGATGAAATTCATTATTGGTCTCGCGATCA
GCTTGAGACATTTCTCCAGGTTTACAAACCTAAAAATTTGTGGGCTACTCTGGTTTTTCCACCTGAGA
TCTTAGCTGGTTACA
+
FFFFFFFFFFFFFFFFFFFFFFFFFFFFFFFFFFFFFFFFFFFFFFFFFFFFFFFFFFFFFFFFFFFFFF
FFFFFFFFFFFFFFFFFFFFFFFFFFFFFFFFFFFFFFFFFFFFFFFFFFFFFFFFFFFFFFFFFFFFFF
FFFFFFFFFFFFFFF
@A00155:342:HHGFNDSXY:1:2603:25554:35008 2:N:0:GAACCTAG+TCCGCATA
AGATCACCTTGCCCAAAGAAGACCAACATTTTCATACATGATGAAATTCATTATTGGTCTCGCGATCA

GCTTGAGACATTTCTCCAGGTTTACAAACCTAAAAATTTGTGGGCTACTCTGGTTTTTCCACCTGAGA
TCTTAGCTGGTTACA
+
FFFFFFFFFFFFFFFFFF:FFFFFFFFFFFFFFFFFFFFFFFFFFFFFFFFFFFFFFFFFFFFFFFFF
FFFFFFFFFFFFFFFFFFFFFFFFFFFFFFFFFFFFFFFFFFFFFFF:FFFFFFFFFFFFFFFFFFFF
FF,FFFFFFFFFFFF
@A00155:342:HHGFNDSXY:1:2603:25138:35477 2:N:0:GAACCTAG+TCCGCATA
AGATCACCTTGCCCAAAGAAGACCAACATTTTCATACATGATGAAATTCATTATTGGTCTCGCGATCA
GCTTGAGACATTTCTCCAGGTTTACAAACCTAAAAATTTGTGGGCTACTCTGGTTTTTCCACCTGAGA
TCTTAGCTGGTTACA
+
FFFFFFFFFFFFFFFFFFFFFFFFFFFFFFFFFFFFFFFFFFFFFFFFFFFFFFFFFFFFFFFFFFFF
FFFFFFFFFFFFFFFFFFFFFFFFFFFFFFFFFFFFFFFFF:FFFFFFFFFFFFFFFFFFFFFFFFFF
F:FFFFFFFFFFFFF
@A00155:342:HHGFNDSXY:1:2631:21875:5744 2:N:0:GAACCTAG+TCCGCATA
AGATCACCTTGCCCAAAGAAGACCAACATTTTCATACATGATGAAATTCATTATTGGTCTCGCGATCA
GCTTGAGACATTTCTCCAGGTTTACAAACCTAAAAATTTGTGGGCTACTCTGGTTTTTCCACCTGAGA
TCTTAGCTGGTTACA
+
FFFFFFFFFFFFFFFFFFFFFFFFFFFFFFFFFFFFFFFFFFFFFFFFFFFFFFFFFFFFFFFFFFFF
FFFFFFFFFFFFFFFFFFFFFFFFFFFFFFFFFFFFFFFFFFFFFF:FFFFFFFFFFFFFFFFFFFFF
FFFFFFFFFFFFFFF
@A00155:342:HHGFNDSXY:1:1569:21450:27242 2:N:0:GAACCTAG+TCCGCATA
GATCACCTTGCCCAAAGAAGACCAACATTTTCATACATGATGAAATTCATTATTGGACTCGCGATCAG
CATGAGACATTTCTCCAGGTTTACAAAACTAAAAATTTGTGGGCTACTCTGGTTTTTCCACCTGAGAT
CTTAGCTGGTTACAA
+
FFFFF,F:FFF:FFFFF,FFF:FF:F::FF,F,:F,:,:FFF:FF,FFF::FF:::,FFFFFFF:FF,
F,,FFFFFF,FFFFFFFF,::FFFFF:,FFFF,FF,:FFF:,:FFFFF:F:F,:F:F:FF,F,FFFFF
:,FFFF:F:F:FFFF
@A00155:342:HHGFNDSXY:1:2134:5394:4131 2:N:0:GAACCTAG+TCCGCATA
GATCACCTTGCCCAAAGAAGACCAACATTTTCATACATGATGAAATTCATTATTGGTCTCGCGATCAG
CTTGAGACATTTCTCCAGGTTTACAAACCTAAAAATTTGTGGGCTACTCTGGTTTTTCCACCTGAGAT
CTTAGCTGGTTACAA
+
FFFFFFFFFFFFFFF:FFFFFFFFFFFFFFFFFFFFFFFFFFFFFFFFFFFFFFFFFFFFFFFFFFFF
FFFFFFFFFFFFFFFFFFFFFFFFFF:FFFFFFFFFFFFFFFFFFFFFFFFF:FFFFFFFFFFFFFFF
FFFF:FF:FFFFFFF
@A00155:342:HHGFNDSXY:1:2165:12970:26522 2:N:0:GAACCTAG+TCCGCATA
GATCACCTTGCCCAAAGAAGACCAACATTTTCATACATGATGAAATTCATTATTGGTCTCGCGATCAG
CTTGAGACATTTCTCCAGGTTTACAAACCTAAAAATTTGTGGGCTACTCTGGTTTTTCCACCTGAGAT
CTTAGCTGGTTACAA
+
FFFFFFFFFFFFFFFFFFFFFFFFFFFFFFFFFFFFFFFFFFFFFFFFFFFFFFFFFFFFFFFFFFFF
FFFFFFFFFFFFFFFFFFFFFFFFFFFFFFFFFFFFFFFFFFFFFFFFFFFFFFFFFFFFFFFFFFFF
FFFFFFFFFFFFFFF
@A00155:342:HHGFNDSXY:1:2159:30237:24455 2:N:0:GAACCTAG+TCCGCATA
GATCACCTTGCCCAAAGAAGACCAACATTTTCATACATGATGAAATTCATTATTGGTCTCGCGATCAG
CTTGAGACATTTCTCCAGGTTTACAAACCTAAAAATTTGTGGGCTACTCTGGTTTTTCCACCTGAGAT
CTTAGCTAGATCGGA
+
FFFFFFFFFFFFFFFFFFFFFFFFFFFFFFFFFFFFFFFFFFFFF:FFFFFFFFFFFFFFFFFFFFFF
FFFFFF:FFFFFFFFFFFFFFFFFFFFFFFFFFFFFFFFFFFFFFFFFFFFFFFFFFFFFFFFFFFFF
FFFFFFFFFFFFFFF

```
@A00155:342:HHGFNDSXY:1:2432:25147:24095 2:N:0:GAACCTAG+TCCGCATA
GATCACCTTGCCCAAAGAAGACCAACATTTTCATACATGATGAAATTCATTATTGGTCTCGCGATCAG
CTTGAGACATTTCTCCAGGTTTACAAACCTAAAAATTTGTGGGCTACTCTGGTTTTTCCACCTGAGAT
CTTAGCTGGTTACAA
+
FFFFFFFFFFFFFFFFFFFFFFFFFFFFFFFFFFFFFFFFFFFFFFFFFF:FFFFFFFFFFFFFFFFFF
F:FFFFFFFFFFFFFFFFFFFFFFFFFFFFFFFFFFFFFFFFFFFFFFFFFFFFFFFFFFFFFFFFFFF
FFFFFFFFFFFFFFF
@A00155:342:HHGFNDSXY:1:2439:19678:36605 2:N:0:GAACCTAG+TCCGCATA
GATCACCTTGCCCAAAGAAGACCAACATTTTCATACATGATGAAATTCATTATTGGTCTCGCGATCAG
CTTGAGACATTTCTCCAGGTTTACAAACCTAAAAATTTGTGGGCTACTCTGGTTTTTCCACCTGAGAT
CTTAGCTGGTTACAA
+
:F:FFFFFFFFFFFFFFFFFFFFFFFFFFFFFFFFFFFFFFFFFFFFFFFFFFFFFFFFFFFF:FFFFF
FFFFFFFFFFFFFFFFFFFF:FFFFFFFFFFFFFFFFFFFFFF:FFFFFFFFFFFFFFFFFFFF:FF:FFF
FFFFF:FFFFFFFFF
@A00155:342:HHGFNDSXY:1:2447:17219:30185 2:N:0:GAACCTAG+TCCGCATA
GATCACCTTGCCCAAAGAAGACCAACATTTTCATACATGATGAAATTCATTATTGGTCTCGCGATCAG
CTTGAGACATTTCTCCAGGTTTACAAACCTAAAAATTTGTGGGCCACTCTGGTTTTTCCACCTGAGAT
CTTAGCTGGTTACAA
+
FFFFFFFFFFFFFFFFFFFFFFFFFFFFFFFFFFFFFFFFFFFFFFFFFFFF:FFFFFFFFFFFFFFFF
FFFFFFFFFFFFFFFFFFFFFFFFFFFFFFFFFFFFFFFFFFFFFFFFFFFFFFFFFFFFFFFFFFF:F
FFFFFFFFFFFFFFF
@A00155:342:HHGFNDSXY:1:2564:26169:29590 2:N:0:GAACCTAG+TCCGCATA
GATCACCTTGCCCAAAGAAGACCAACATTTTCATACATGATGAAATTCATTATTGGTCTCGCGATCAG
CTTGAGACATTTCTCCAGGTTTACAAACCTAAAAATTTGTGGGCTACTCTGGTTTTTCCACCTGAGAT
CTTAGCTGGTTACAA
+
FFFFFFFFFFFFFFFFFF:FFFFFFFFFFFFFFFFFFFFFFFFFFFFFFFFFFFFFFFFFFFFFFFFFF
FFFFFFFFFFFFFFFFFFFFFFFFFFFFFFFFFFFFFFFFFFFFFFFFFFFFFFF:FFFFFFFFFFFFFF
FFFF:FFFFFFFFFF
@A00155:342:HHGFNDSXY:1:1474:2880:29465 2:N:0:GAACCTAG+TCCGCATA
ATCACCTTGCCCAAAGAAGACCAACATTTTCATACATGATGAAATTCATTATTGGTCTCGCGATCAGC
TTGAGACATTTCTCCAGGTTTACAAACCTAAAAATTTGTGGGCTACTCTGGTTTTTCCACCTGAGATC
TTAGCTGGTTACAA
+
FFFFFFFFFFFFFFFFFFFFFFFFFFFFFFFFFFFFFFFFFFFFFFFFF,FFFFFFFFFFFFF:FFFFF
FFFFFFFFFFFFFFFFFFFFFFFFFF,FFFF:FFFFFFFFFFFFFFFFFFFF:FFFFFFFFFFFFFFFFF
FFFFFFFFFFFFFF
@A00155:342:HHGFNDSXY:1:1126:30499:13166 2:N:0:GAACCTAG+TCCGCATA
ATCACCTTGCCCAAAGAAGACCAACATTTTCATACATGATGAAATTCATTATTGGTCTCGCGATCAGC
TTGAGACATTTCTCCAGGTTTACAAACCTAAAAATTTGTGGGCTACTCTGGTTTTTCCACCTGAGATC
TTAGCTGGTTACAAA
+
FFFFFFFF:FFFFFFF:FFFFFFFFFFFFFFFFFFFFFFFFFFFFFFFFFF:FFFFFFFFFFFFFFFFFFF
FFFFFFFFF:FFFFFFFFFFFFFFFFFFFFFFFFFFFFFFFFFFFFFFFFFFFFFFFFFFFFFFFFFFF:
FFFF:FFF:FFFFFF
@A00155:342:HHGFNDSXY:1:1153:1805:13291 2:N:0:GAACCTAG+TCCGCATA
ATCACCTTGCCCAAAGAAGACCAACATTTTCATACATGATGAAATTCATTATTGGTCTCGCGATCAGC
TTGAGACATTTCTCCAGGTTTACAAACCTAAAAATTTGTGGGCTACTCTGGTTTTTCCACCTGAGATC
TTAGCTGGTTACAAA
+
FFFFFFFFFFFFFFFFFFFFFFFFFFFFFFFFFFFFFFFFFFFFFFFFFFFFFFFFFFFFFFFFFFFFF
```

FFFFFFFFFFFFFFFFFFFFFFFFF,FFFFFFF:F,FFFFFFFFFFFFFFFFFFF:FFFFFFFFFFFF
FFFFFFFFFFFFFFF
@A00155:342:HHGFNDSXY:1:1169:22073:31798 2:N:0:GAACCTAG+TCCGCATA
ATCACCTTGCCCAAAGAAGACCAACATTTTCATACATGATGAAATTCATTATTGGTCTCGCGATCAGC
TTGAGACATTTCTCCAGGTTTACAAACCTAAAAATTTGTGGGCTACTCTGGTTTTTCCACCTGAGATC
TTAGCTGGTTACAAA
+
FFFFFFFFFFFFFFFFFFFFFFFFFFFFFFFFFFFFFFFFFFFFFFFFFFFFFFFFFFFFFFFFFFFF
FFFFFFFFFFFFFFFFFFFFFFFFFFFFFFFFFFFFFFFFFFFFFFFFFFFFFFFFFFFFFFFFFFFF
FFFFFFFFFFFFFFF
@A00155:342:HHGFNDSXY:1:1622:12744:9439 2:N:0:GAACCTAG+TCCGCATA
ATCACCTTGCCCAAAGAAGACCAACATTTTCATACATGATGAAATTCATTATTGGTCTCGCGATCAGC
TTGAGACATTTCTCCAGGTTTACAAACCTAAAAATTTGTGGGCTACTCTGGTTTTTCCACCTGAGATC
TTAGCTGGTTACAAA
+
FFFFFFFFFFFFFFFFFFFFFFFFFFFFFFFFFFFFFFFFFFFFFFFFFFFFFFFFFFFFFFFFFFFF
FFFFFFFFFFFFFFFFFFFFFFFFFFFFFFFFFFFFFFFFFFFFFFFFFFFFFFFFFFFFFFFFFFFF
F:FFFFFFFFFFFFF
@A00155:342:HHGFNDSXY:1:1653:17400:14591 2:N:0:GAACCTAG+TCCGCATA
ATCACCTTGCCCAAAGAAGACCAACATTTTCATACATGATGAAATTCATTATTGGTCTCGCGATCAGC
TTGAGACATTTCTCCAGGTTTACAAACCTAAAAATTTGTGGGCTACTCTGGTTTTTCCACCTGAGATC
TTAGCTGGTTACAAA
+
FFFFFFFFFFFFFFFFFFFFFFFFFFFFFFFFFFFFFFFFFFFF,FF:FFFFFFFFFFFFFFFFFFFF
FFFFFFFFFFFFFFFFFFFFFFFFFFFFFFFFFFFFFFFFFFFFFFFFFFFFFFFFFFFFFF:FFFFF
FFFFFFFFFFFFFFF
@A00155:342:HHGFNDSXY:1:2135:18150:2174 2:N:0:GAACCTAG+TCCGCATA
ATCACCTTGCCCAAAGAAGACCAACATTTTCATACATGATGAAATTCATTATTGGTCTCGCGATCAGC
TTGAGACATTTCTCCAGGTTTTCAAACCTAAAAATTTGTGGGCTACTCTGGTTTTTCCACCTGAGATC
TTAGCTGGTTACAAA
+
FFF:FFFFFFFFFFFFFFFFFFFFFFFFFFFFFFFFFFFFFFFFFFFFFFFFFFFFFFFFFFFFFFFF
FFFFFFFF:FFFFFFFFFFFFFFFFFFFFFFFFFFFFFFFFFFF:FFFFFFFFFFFFFFFFFFFFFFF
:,FFF,FFFF,FFFF
@A00155:342:HHGFNDSXY:1:2406:24641:15671 2:N:0:GAACCTAG+TCCGCATA
ATCACCTTGCCCAAAGAAGACCAACATTTTCATACATGATGAAATTCATTATTGGTCTCGCGATCAGC
TTGAGACATTTCTCCAGGTTTACAAACCTAAAAATTTGTGGGCTACTCTGGTTTTTCCACCTGAGATC
TTAGCTGGTTACAAA
+
FFFFFFFFFFFFFFFFFFFFFFFFFFFFFFFFFFFFFFFFFFFF:FFF,FFFFFFFFFFFFFFFFFFF
FFFFFFFFFFFFFFFFFFFFFFF:F:FFFFF:FFFFFFFFFFFFFFFFFFFF,FFFF:FFFFFFFFFF
FFFFFFFFFFFFFFF
@A00155:342:HHGFNDSXY:1:2332:26350:13870 2:N:0:GAACCTAG+TCCGCATA
ATCACCTTGCCCAAAGAAGACCAACATTTTCATACATGATGAAATTCATTATTGGTCTCGCGATCAGC
TTGAGACATTTCTCCAGGTTTTCAAACCTAAAAATTTGTGGGCTACTCTGGTTTTTCCACCTGAGATC
TTAGCTGGTTACAAA
+
FFFFFFFFFFFFFFFFFFFFFFFFFFFFFFFFFFFFFFFFFFFFFFFFFFFFFFFFFFFFFFFFFFFF
FFFFFFFFFFFFFFFFFFFFFFFFFFFF:FFFFFFFFFFFFFFFFFFFFFFFFFFFFFFFFFFFFFFF
FFFFFFFFFFFFFFF
@A00155:342:HHGFNDSXY:1:2436:9760:37059 2:N:0:GAACCTAG+TCCGCATA
ATCACCTTGCCCAAAGAAGACCAACATTTTCATACATGATGAAATTCATTATTGGTCTCGCGATCAGC
TTGAGACATTTCTCCAGGTTTACAAACCTAAAAATTTGTGGGCTACTCTGGTTTTTCCACCTGAGATC
TTAGCTGGTTACAAA

```
+
FFFFFFFFFFFFFFFFFFFFFFFFFFFFFFFFFFFFF:FFFFFFFFFF,:FFFFFFFFFFFFFFFFFF
FFFFFFFFFFFFFFFFFFFFFFFFFFFFFFFF:FFF,FFF:FFFFFFFFFFFFFFFFFFFFFFFFFFFF
FFFFFFFFFFFFFFF
@A00155:342:HHGFNDSXY:1:1655:14009:15702 1:N:0:GAACCTAG+TCCGCATA
TCACCTTGCCCAAAGAAGACCAACATTTTCATACATGATGAAATTCATTATTGGTCTCGCGATCAGCT
TGAGACATTTCTCCAGGTTTACAAACCTAAAAATTTGTGGGCTACTCTGGTTTTTCCACCTGAGATCT
TAGCTGGTTACAAAT
+
:FFFFFFFFFFFFF:FF:FFFFFFFFFFFFFFFFFFFFFFFFFFFFFFFFFFFFFFFFFFFFFFFFFF
FFFFFFFFFFFFFFFFFFFFFFFFFFFFFFFFFFFFFFFFFFFFFFFFFFFFFFFFFFFFFFFFFFFF
FFFFFFFFFFFFFFF
@A00155:342:HHGFNDSXY:1:1420:32018:23030 2:N:0:GAACCTAG+TCCGCATA
CACCTTGCCCAAAGAAGACCAACATTTTCATACATGATGAAATTCATTATTGGTCTCGCGATCAGCTT
GAGACATTTCTCCAGGTTTACAAACCTAAAAATTTGTGGGCTACTCTGGTTTTTCCACCTGAGATCTT
AGCTGGTTACAAATC
+
FFFFFFFFFFFFFFFFFFFFFFFFFFFFFFFFFFFFFFFFFFFF:FFFFFFFFFFFFFFFFFFFFFFFF
FFFFFFFFFFFFFFFFF,FFFFFFFFF:FFFFFFFFFFFFFFFFFFFFFFF:FFFFFFFFFFFFFFFFFF
:FFFFFFFFFFFFFF
@A00155:342:HHGFNDSXY:1:2353:19958:17863 2:N:0:GAACCTAG+TCCGCATA
CACCTTGCCCAAAGAAGACCAACATTTTCATACATGATGAAATTCATTATTGGTCTCGCGATCAGCTT
GAGACATTTCTCCAGGTTTACAAACCTAAAAATTTGTGGGCTACTCTGGTTTTTCCACCTGAGATCTT
AGCTGGTTACAAATC
+
FFFFFFFFFFFFFFFFFFFFFFFFFFFFFFF:FFFFFFFFFFFFFFFFFFFFFFFFFFFFFFFFFFFFF
FFFFFFFFFFFFFFFFFFFFFFFFFFFFFFFFFF:FFFFFFFFFFFFFFFFFFFFFFFFFFFFFFFFFF
FFFFFFFFFFFFFFF
@A00155:342:HHGFNDSXY:1:2134:16297:6136 2:N:0:GAACCTAG+TCCGCATA
ACCTTGCCCAAAGAAGACCAACATTTTCATACATGATGAAATTCATTATTGGTCTCGCGATCAGCTTG
AGACATTTCTCCAGGTTTACAAACCTAAAAATTTGTGGGCTACTCTGGTTTTTCCACCTGAGATCTTA
GCTGGTTACAAATCC
+
F:FFFFFF:FFFFFFFFFFFFFFF:FFFFFFFFF::FFFFFFF,F:FFFFFFFFFF:FFFFFFFFFFF
FFFFFFFF:FFFFFFFFFFFFFFFFFFFFFFFFFFFFFFFFFFFFFFFFFFFFFFF:FFFFFFF,FF
FFFFFFFFFFFFFF:
@A00155:342:HHGFNDSXY:1:2454:1814:12179 1:N:0:GAACCTAG+TCCGCATA
CCTTGCCCAAAGAAGACCAACATTTTCATACATGATGAAATTCATTATTGGTCTCGCGATCAGCTTGA
GACATTTCTCCAGGTTTACAAACCTAAAAATTTGTGGGCTACTCTGGTTTTTCCACCTGAGATCTTAG
CTGGTTACAAATCC
+
FFFFFFFFFF,F:,F:FF:FFFFFFFFFFFF,FF,FFFFFFFFFF:F,:FFFFFFFFF,FF:FF,FF:
FFF::F:FFFF:FF,FFFFFF:FF:F:FFFFFFFFFFFF,FF:FFFFFFF,FFF:FFFFFFFFF:,F:
FFFF,F:FFF:FFF
@A00155:342:HHGFNDSXY:1:1142:10926:16313 1:N:0:GAACCTAG+TCCGCATA
CCTTGCCCAAAGAAGACTAACATTTTCATACATGATGAAATTCATTATTGGTCTCGCGATCAGCTTGA
GACATTTCTCCAGGTTTACAAACCTAAAAATTTGTGGGCTACTCTGGTTTTTCCACCTGAGATCTTAG
CTGGTTACAAATCCT
+
FFFFFFFFFFFFFFFFFFFFFFFFFFFFFFFFFFFFFFFFFFFFFFFFFFFFFFFFFFFFFFFFFFFF
FFFFFFFFFFFFFFFFFFFF:FFFFFFFF:FFFFFFFFFFFFFFFFF:FFFFFFFFFFFFFFFFFFFF
FFFFFFFFFFFFFFF
@A00155:342:HHGFNDSXY:1:1172:21043:33959 1:N:0:GAACCTAG+TCCGCATA
CCTTGCCCAAAGAAGACCAACATTTTCATACATGATGAAATTCATTATTGGTCTCGCGATCAGCTTGA
```

```
GACATTTCTCCAGGTTTACAAACCTAAAAATTTGTGGGCTACTCTGGTTTTTCCACCTGAGATCTTAG
CTGGTTACAAATCCT
+
FFF:FFFFFFFF::,:FFFFFFFFFFFFFFF:FFFFFFFFFFFFFFFFFFFFFFFFFFFFFFFF:FFFF
FFFFF,FFFFFFFFFFFFFFF,:FFFFFFFFFFFFFFFFFFFFFFFFFFFFFFFFFFFFFFFFFFFF
FFFFFFFFFFFFFFF
@A00155:342:HHGFNDSXY:1:1309:13349:8453 1:N:0:GAACCTAG+TCCGCATA
CCTTGCCCAAAGAAGACCAACATTTTCATACATGATGAAATTCATTATTGGTCTCGCGATCAGCTTGA
GACATTTCTCCAGGTTTACAAACCTAAAAATTTGTGGGCTACTCTGGTTTTTCCACCTGAGATCTTAG
CTGGTTACAAATCCC
+
FFFFF:FFFFFFFFFFFFFFFFFFFFFFFFFFFFFFFFFFFFFFFFFFFFFFFFFFFFFFFFFFFFFF
FFFFFFFFFFFFFF:FFFFF:FFFFFFFFFFFFFFFFFFFFFFFFFFFFFFFFFFFFFFFFFFFFFFF
FFFFFFFFFFFFFFF
@A00155:342:HHGFNDSXY:1:1440:14479:6527 1:N:0:GAACCTAG+TCCGCATA
CCTTGCCCAAAGAAGACCAACATTTTCATACATGATGAAATTCATTATTGGTCTCGCGATCAGCTTGA
GACATTTCTCCAGGTTTACAAACCTAAAAATTTGTGGGCTACTCTGGTTTTTCCACCTGAGATCTTAG
CTGGTTACAAATCCT
+
FFFFFFFFFF::F,:F:FF,FFFFFF:FFF::FFFF:::FFFFFFF,,F:FFFFF:FFFFFFFFFFFF
FFFFFFFFFFFFFFFFFFFFFFFFFFFFFFFFFFFFFFFF:FFFFFFFFFFFF,:::FFFFF:FFFFF
FF:FFF,FFFFFF:F
@A00155:342:HHGFNDSXY:1:1654:3947:10645 1:N:0:GAACCTAG+TCCGCATA
CCTTGCCCAAAGAAGACCAACATTTTCATACATGATGAAATTCATTATTGGTCTCGCGATCAGCTTGA
GACATTTCTCCAGGTTTACAAACCTAAAAATTTGTGGGCTACTCTGGTTTTTCCACCTGAGATCTTAG
CTGGTTACAAATCCT
+
FFFFFFFFFFFFFFFFFFFFFFFFFFFFFFFFFFFFFFFFFFFFFFFFFFFFFFFFFFFFFFFFFFFF
FFFFFFFF:FFFFFFFF:FFFFF,FFFFFFFFFFFFFFF:FFFFFFFFFFFFFFFFFFFFFFFFFFFF
FFFFFFFFFFFFFFF
@A00155:342:HHGFNDSXY:1:2125:23637:1188 1:N:0:GAACCTAG+TCCGCATA
CCTTGCCCAAAGAAGACCAACATTTTCATACATGATGAAATTCATTATTGGTCTCGCGATCAGCTTGA
GACATTTCTCCAGGTTTACAAACCTAAAAATTTGTGGGCTACTCTGGTTTTTCCACCTGAGATCTTAG
CTGGTTACAAATCCT
+
FFFFFFFFFFFF:F:FFFFFFFFF:FFFFFFFFFFFFFFFFFFFFFFFFFFFFFFFFFFFFFFFFFFF
FFFFFFFFFFFFFFFFFFFFFFFFF:FFFFFFFFFFFFFFFFFFFFFFFFFFFFFFFFFFFFFFFFFF
FFFFFFFFFFFFFFF
@A00155:342:HHGFNDSXY:1:2125:19470:4178 1:N:0:GAACCTAG+TCCGCATA
CCTTGCCCAAAGAAGACCAACATTTTCATACATGATGAAATTCATTATTGGTCTCGCGATCAGCTTGA
GACATTTCTCCAGGTTTACAAACCTAAAAATTTGTGGGCTACTCTGGTTTTTCCACCTGAGATCTTAG
CTGGTTACAAATCCT
+
FF:FFFFFFFFFFFFFFFFFFFFFFFFFFFFFFFFFFFFFFFFFFFFFFFFFFFFFFFFFFFFFFFFF
FFFFFFFFFFFFFFFFFFFFFFFFFFFFFFFFFFFFFFFFFFFFFFFFFFFFFFFFFFFFFFFFFFFF
FFFFFFFFFFFFFFF
@A00155:342:HHGFNDSXY:1:2515:26386:33035 1:N:0:GAACCTAG+TCCGCATA
CCTTGCCCAAAGAAGACCAACATTTTCATACATGATGAAATTCATTATTGGTCTCGCGATCAGCTTGA
GACATTTCTCCAGGTTTACAAACCTAAAAATTTGTGGGCTACTCTGGTTTTTCCACCTGAGATCTTAG
CTGGTTACAAATCCT
+
FFFFFFFFFFFFFFFFFFFFFFFFFFFFFFFFFFFFFFFFFFFFFFFFFFFFFFFFFFFFFFFFFFFF
FFFFFFFFFFFFFFFFFFFFFFFFFFFFFFFFFFFFFFFFFFFFFFFFFFFFFFFFFFFFFFFFFFFF
FFFFFFFFFFFFFFF
```

@A00155:342:HHGFNDSXY:1:2601:18792:36824 1:N:0:GAACCTAG+TCCGCATA
CCTTGCCCAAAGAAGACCAACATTTTCATACATGATGAAATTCATTATTGGTCTCGCGATCAGCTTGA
GACATTTCTCCAGGTTTACAAACCTAAAAATTTGTGGGCTACTCTGGTTTTTCCACCTGAGATCTTAG
CTGGTTACAAATCCC
+
FFFFFF:FFFFFFFFFFF,FFFFFFFFFFFFFFFFFFFFFFFFFFFFFFFFFFFFFFFFFFFFFFFFF
FFFFFFFFFFFFFFFFFFFFF:FFFFFFFFFFFFFFFFFFFFFFFFFFFFFFFFFFFFFFFFFFFFFF
FFFFFFFFFFFFFFF
@A00155:342:HHGFNDSXY:1:2602:18991:4852 1:N:0:GAACCTAG+TCCGCATA
CCTTGCCCAAAGAAGACCAACATTTTCATACATGATGAAATTCATTATTGGTCTCGCGATCAGCTTGA
GACATTTCTCCAGGTTTACAAACCTAAAAATTTGTGGGCTACTCTGGTTTTTCCACCTGAGATCTTAG
CTGGTTACAAATCCC
+
FFFFFFFFFFFFFFFFFF:FFFFFFFFFFFFFFFFFFFFFFFFFFFFFFFFFFFFFFFFFFFFFFFFF
FFFFFFFFFFFFFFFFFFFFFFFFFFFFFFFFFFFFFFFFFFFFFFFFFFFFFFFFFFFFFFFFFFFF
FFFFFFFFFFFFFFF
@A00155:342:HHGFNDSXY:1:1138:3821:7138 2:N:0:GAACCTAG+TCCGCATA
CCTTGCCCAAAGAAGACCAACATTTTCATACATGATGAAATTCATTATTGGTCTCGCGATCAGCTTGA
GACATTTCTCCAGGTTTACAAACCTAAAAATTTGTGGGCTACTCTGGTTTTTCCACCTGAGATCTTAG
CTGGTTACAAATCCT
+
FFFFFFFFFFFFFFFFFFFFFFFFFFFFFFFFFFFFFFFFFFFFFF:FFFFFFFFFFFFFFFFFFFFF
FFFFFFFFFFFFFF:FFFF:FFFFFFFFFFFFFFFFFFFFFFFFFFFFFFFFFFFFFFFFFFFFFFFF
:FFFFFFFFFFFFFF
@A00155:342:HHGFNDSXY:1:1329:23122:34961 2:N:0:GAACCTAG+TCCGCATA
CCTTGCCCAAAGAAGACCAACATTTTCATACATGATGAAATTCATTATTGGTCTCGCGATCAGCTTGA
GACATTTCTCCAGGTTTACAAACCTAAAAATTTGTGGGCTACTCTGGTTTTTCCACCTGAGATCTTAG
CTGGTTACAAATCCT
+
FFFFFFFFFFFFFFFFFFFFFFF:FFFFFFFFFFFFFFFFFFFFFFFFFFFFFFFFFFFFFFFFFFFF
FFFFFFFFFFFFFFFFFFFFFFFFFFFFFFFFFFFFFFFFFFFFFFFFFFFFFFFFFFFFFFFFFFFF
FFFFFFFFFFFFFFF
@A00155:342:HHGFNDSXY:1:2502:27398:15562 2:N:0:GAACCTAG+TCCGCATA
CCTTGCCCAAAGAAGACCAACATTTTCATACATGATGAAATTCATTATTGGTCTCGCGATCAGCTTGA
GACATTTCTCCAGGTTTACAAACCTAAAAATTTGTGGGCTACTCTGGTTTTTCCACCTGAGATCTTAG
CTGGTTACAAATCCT
+
FFFFFFFFFFFFFFFFFFFFFFFFFFFFFF:FFFFF:F,FFFFFFFFFFFFFFFFFFFFFFFFFFFFF
FFFFF:FFFFFFFFFFFFFFFFFFF:FFFF:F::FFFFFFFFFFFFFF:FFFFFFF:FFFFFFFFFFF
FFFFFFFFFFFFFFF
@A00155:342:HHGFNDSXY:1:2333:6108:16297 1:N:0:GAACCTAG+TCCGCATA
CTTGCCCAAAGAAGACCAACATTTTCATACATGATGAAATTCATTATTGGTCTCGCGATCAGCTTGAG
ACATTTCTCCAGGTTTACAAACCTAAAAATTTGTGGGCTACTCTGGTTTTTCCACCTGAGATCTTAGC
TGGTTACAAATCCT
+
FFFFFFFFFFFFFFFFFFFFFFFFFFFFFFFFFFFFFFFFFFFFFFFFFFFFFFFFFFFFFF:FFFFF
FFFFFFFFFFFFFFFFFFFF:F,FFFFFFFFFFFFFFFFFFFFFFFFFFFFFFFFFFFFFFFFFFFFF
FFFFFFFFFFFFFF
@A00155:342:HHGFNDSXY:1:2670:12337:34726 1:N:0:GAACCTAG+TCCGCATA
CTTGCCCAAAGAAGACCAACATTTTCATACATGATGAAATTCATTATTGGTCTCGCGATCAGATTGAG
ACATTTCTCCAGGTTTACAAACCTAAAAATTTGTGGGCTACTCTGGTTTTTCCACCTGAGATCTTAGC
TGGTTACAAATCCT
+
:FF,:FFFFFFF,,FF,,:F,FFFF,:FF,,FFFFFFF,FFFFFF,FF:FF,F,FFF,F::F,FFFFF

F,,FFF:F:FF,FFFFFF:FF:FFFFFF:FFFFFFF:FF,:F,FFFF:FFF,FF::F:,F:FFFFFFF
FF:FFFFFF:F:FF
@A00155:342:HHGFNDSXY:1:1108:31331:9189 1:N:0:GAACCTAG+TCCGCATA
CTTGCCCAAAGAAGACCAACATTTTCATACATGATGAAATTCATTATTGGTCTCGCGATCAGCTTGAG
ACATTTCTCCAGGTTTACAAACCTAAAAATTTGTGGGCTACTCTGGTTTTTCCACCTGAGATCTTAGC
TGGTTACAAATCCTC
+
FFFFFFFFFFFFFF:FFFFFFFFFFFFFFFFFFFFFFFFFFFFFFFFFFFFFFFFFFFFFFFFFFFFF
FF:FFFFFFFFFFFFFFFFFFFFFFFFFFFFFFFFFFFFFFFFFFFFFFFFFFFFFFFFFFFFFFFFF
FFFFFFFFFFFFFFF
@A00155:342:HHGFNDSXY:1:1425:9860:3756 1:N:0:GAACCTAG+TCCGCATA
CTTGCCCAAAGAAGACCAACATTTTCATACATGATGAAATTCATTATTGGTCTCGCGATCAGCTTGAG
ACATTTCTCCAGGTTTACAAACCTAAAAATTTGTGGGCTACTCTGTTTTTTCCACCTGAGATCTTAGC
TGGTTACAAATCCTC
+
F:FFFFFFFFFFF:FFFFF,FFFFFFFFFFFF:FFFFFFFFFFFFFFFFFFFFFFFFFFFFF:FFFFFF
FFFFFFFFFFFFFFFFFFFFF:FF:FFFFFFFFFFFFFFFFFFFFFFFFFFFFFFFFFFFFFFFFFFF
FFFFFFFFFFFFFFF
@A00155:342:HHGFNDSXY:1:1524:11749:25441 1:N:0:GAACCTAG+TCCGCATA
CTTGCCCAAAGAAGACCAACATTTTCATAAATTATGAAATTCATTATTGGTCTCGCGATCAGCTTGAG
ACATTTCTCCAGGTTTACAAACCTAAAAATTTGTGGGCTACTCTGGTTTTTCCACCTGAGATCTTAGC
TGGTTACAAATCCTC
+
FFF::F::FFF,FFFFFF,,:F:FF:FFF,FF,FF,FFFFF:FFFFFFFFFFFF::,FFF,FFFFFFF
F,:FFFFFFFFFFFFFF,FFFFFFFFFF,FFFFFFFFFFFFFFFFFF:FFF:FFF:FFFFFFFFFF,F
FF:FFFF:FFFFF:F
@A00155:342:HHGFNDSXY:1:2128:2537:19507 1:N:0:GAACCTAG+TCCGCATA
CTTGCCCAAAGAAGACCAACATTTTCATACATGATGAAATTCATTATTGGTCTCGCGATCAGCTTGAG
ACATTTCTCCAGGTTTACAAACCTAAAAATTTGTGGGCTACTCTGGTTTTTCCACCTGAGATCTTAGC
TGGTTACAAATCCTC
+
FFFFFFFFFFFF:FFFFFFFFFFFFFFFFFFFFFFFFFFFFFFFFFFFFFFFF:FFFFFFFFFFFFF:FF
FFFFFFFFFFFF:FFFFFFFFFFF:FFFFFFFFFFFFFFFFFFFFFFFFFFFFFFFFFFFFFFFFFFF
FFFFFFFFFFFFFFF
@A00155:342:HHGFNDSXY:1:2154:26621:22545 1:N:0:GAACCTAG+TCCGCATA
CTTGCCCAAAGAAGACCAACATTTTCATACATGATGAAATTCATTATTGGTCTCGCGATCAGCTTGAG
ACATTTCTCCAGGTTTACAAACCTAAAAATTTGTGGGCTACTCTGGTTTTTCCACCTGAGATCTTAGC
TGGTTACAAATCCTC
+
FFFF:FFFF:FFF:FFFFFFFFFFFFFFFFFFF:FFFFFFFF,FFFFFFFFFFFFFF,FFFF,FFFFFF
FFFFFFFF::FFFFFFFFFFFF,FFFFFFFFFFFF,FFFFFFFFFFFFFFF:FFFFFFFFFF:FF::F
FFFFFFFFFFFFFFF
@A00155:342:HHGFNDSXY:1:2405:23050:13542 1:N:0:GAACCTAG+TCCGCATA
CTTGCCCAAAGAAGACCAACATTTTCATACATGATGAAATTCATTATTGGTCTCGCGATCAGCTTGAG
ACATTTCTCCAGGTTTACAAACCTAAAAATTTGTGGGCTACTCTGGTTTTTCCACCTGAGATCTTAGC
TGGTTACAAATCCTC
+
FFFFFFFFFFFFFFF:FFFFFFFFFFFFFFFFFFFFFFFFFFFFFFFFFFFFFFFFFFFFFFFFFFFFF
FFFFFFFFFFFFFFFFFFFFFFFFFFFFFFFFFFFFFFFFFFFFFFFFFFFFFFFFFFFFFFFFFFFF
FFFFFFFFFFFFFFF
@A00155:342:HHGFNDSXY:1:2650:27633:36511 1:N:0:GAACCTAG+TCCGCATA
GATCTCCAAAGAAGACCAACATTTTCATACATGATGAAATTCATTATTGGTCTCGCGATCAGCTTGAG
ACATTTCTCCAGGTTTACAAACCTAAAAATTTGTGGGCTACTCTGGTTTTTCCACCTGAGATCTTAGC
TGGTTACAAATCCTC

+
FFFFFFF:F:FFFFFFFFFFFFFFFFFFFF:FFFFF,FFFFFFFFFFFFFFFFFFFFFFFF:FFFFFF
FFFFFFFFFFFFFFFFFFFFFFFFFFFFFFFFFFFFF:FFFFFFFFFFFFFFFFFFFFFFFFFFFFF
FFFFFFFFFFFFFFF
@A00155:342:HHGFNDSXY:1:1236:5611:21355 2:N:0:GAACCTAG+TCCGCATA
CTTGCCCAAAGAAGACCAACATTTTCATACATGATGAAATTCATTATTGGTCTCGCGATCAGCTTGAG
ACATTTCTCCAGGTTTACAAACCTAAAAATTTGTGGGCTACTCTGGTTTTTCCACCTGAGATCTTAGC
TGGTTACAAATCCTC
+
FFFFFFFFFFFFFFFFFF:FFFF:FFFFFFFFFFFFFFF,FFFF:FFF:FFFFFFFFFFFFFFFFFFF
FFFFFFFFF:FFFFFFFFFFFFFFFFF::FFFFF:FFFFFFFFFFFFFFFFFFFFFFFFF:FFFFFF:F
FFFFFFFFFFFFFFF
@A00155:342:HHGFNDSXY:1:2113:12120:2628 2:N:0:GAACCTAG+TCCGCATA
CTTGCCCAAAGAAGACCAACATTTTCATACATGATGAAATTCATTATTGGTCTCGCGATCAGCTTGAG
ACATTTCTCCAGGTTTACAAACCTAAAAATTTGTGGGCTACTCTGGTTTTTCCACCTGAGATCTTAGC
TGGTTACAAATCCTC
+
FFFFFFFF,FF,F,,F:F:FF:F,:FF:FF:FFFFF:FFFFF,FFFFFFFFFFFFFFFFFFFFFFFFF
F:F:FFFFFFF:FFFFFFFFFFFFFFFFFFF::F:FFFFFFFFFFFFFFFFFFFFFFFFFFFFFFFFF
FFFFFF:FFFFFFFF
@A00155:342:HHGFNDSXY:1:1138:3821:7138 1:N:0:GAACCTAG+TCCGCATA
TTGCCCAAAGAAGACCAACATTTTCATACATGATGAAATTCATTATTGGTCTCGCGATCAGCTTGAGA
CATTTCTCCAGGTTTACAAACCTAAAAATTTGTGGGCTACTCTGGTTTTTCCACCTGAGATCTTAGCT
GGTTACAAATCCTCC
+
:FFF:FFFFFFFFFFFFFFF:FFFFFFFFFFFFFFFFFFFFFFFFFFFFFFFFFFFFFFFFFFFFFFF
F:FFFFFFFFFFFFFFFFFFFFFFFFFFFFFFFFFFFFFFFFFFFFF:FFFFFFFFFFFFFFFFFFFFF
FFFFFFFFFFFF:FF
@A00155:342:HHGFNDSXY:1:1240:28510:10191 1:N:0:GAACCTAG+TCCGCATA
CCGATCTAAGAAGACCAACATTTTCATACATGATGAAATTCATTATTGGTCTCGCGATCAGCTTGAGA
CATTTCTCCAGGTTTACAAACCTAAAAATTTGTGGGCTACTCTGGTTTTTCCACCTGAGATCTTAGCT
GGTTACAAATCCTCC
+
FFFFFFFFFFFFFFFFFFFFFFFF:FFFF:FFFFFFFFFFFFFFFFFFFFFFFFFFFFFFFFFFFFFFF
FFFFFFFFFFFFFFFFFFFFFFFFFFFFFFFFFFFFFFFFFFFFFFFFFFFFFFFFFFFFFFFFFFFF
FFFFFFFFFFFFFFF
@A00155:342:HHGFNDSXY:1:1416:27543:23422 1:N:0:GAACCTAG+TCCGCATA
TTGCCCAAAGAAGACCAACATTTTCATACATGATGAAATTCATTATTGGTCTCGCGATCAGCTTGAGA
CATTTCTCCAGGTTTACAAACCTAAAAATTTGTGGGCTACTCTGGTTTTTCCACCTGAGATCTTAGCT
GGTTACAAATCCTCC
+
FFFF:F:FFFFFFFFFF:FFF:FFFFFFFFFFFFFFFFFFFFFFFFFFFFFFFFFFFFFFFFFFFFFF
FFFFFFFFFFFFFFFFFFFFFFFFF:FFFFFFFFFFFFFFFFFFFFFFFFFFFFFFFFFFFFFFFFFF
FF:FFFFFFFFFFFF
@A00155:342:HHGFNDSXY:1:1440:6216:12070 1:N:0:GAACCTAG+TCCGCATA
TTGCCCAAAGAAGACCAACATTTTCATACATGATGAAATTCATTATTGGTCTCGCGATCAGCTTGAGA
CATTTCTCCAGGTTTACAAACCTAAAAATTTGTGGGCTACTCTGGTTTTTCCACCTGAGATCTTAGCT
GGTTACAAATCCTCC
+
FFFFFFFFFFFFFFFFFFFFFFFFFFFFFFFFFFFFFFFFFFFFFFFFFFFFFFFFFFFFFFFFFFFF
FFFFFFFFFFFF:FFFFF:FFFFFFFFFFFFFFFFFFFFFFFFFFFF:FFFFFFFFFFFFFFFFF,FFF
FFFFFFFFFFFFFFF
@A00155:342:HHGFNDSXY:1:1466:13340:1329 1:N:0:GAACCTAG+TCCGCATA
TTGCCCAAAGAAGACCAACATTTTCATACATGATGAAATTCATTATTGGTCTCGCGATCAGCTTGAGA

```
CATTTCTCCAGGTTTACAAACCTAAAAATTTGTGGGCTACTCTGGTTTTTCCACCTGAGATCTTAGCT
GGTTACAAATCCTCC
+
F,FFFFFFFF:FFFFFFFFFFF:FFFFFFFFFFFFFFFFFFFFFFFFFFFFFFFFFFFFFFFFFFF
FFFFFFFFFFFFFFFFFFF:FFFFFFFFFFFFFF,FFFFFFFF:FFFFFFFFFFFFF:FFFF:FFF
FFFFFFFFFFFFFFF
@A00155:342:HHGFNDSXY:1:1558:22345:28260 1:N:0:GAACCTAG+TCCGCATA
TTGCCCAAAGAAGACCAACATTTTCATACATGATGAAATTCATTATTGGTCTCGCGATCAGCTTGAGA
CATTTCTCCAGGTTTACAAACCTAAAAATTTGTGGGCTACTCTGGTTTTTCCACCTGAGATCTTAGCT
GGTTACAAATCCTCC
+
FFFFFFFFFFFFFFFFFFFFFFFFFFFFFFFFFFFFFFFFFFFFFFFFFFFFFFFFFFFFFFFFFF
FFFFFFFFFFFFFFFFFFFFFFFFFFFFFFFFFFFFFFFFFFFFFFFFFFFFFFFFFFFFFFFFFF
FFFFFFFFFFFFFFF
@A00155:342:HHGFNDSXY:1:1571:16568:17566 1:N:0:GAACCTAG+TCCGCATA
TTGCCCAAAGAAGACCAACATTTTCATACATGATGAAATTCATTATTGGTCTCGCGATCAGCTTGAGA
CATTTCTCCAGGTTTACAAACCTAAAAATTTGTGGGCTACTCTGGTTTTTCCACCTGAGATCTTAGCT
GGTTACAAATCCTCC
+
FFFFFFFFFFFFFFFFFFFF,FFFFFFFFFFFFFFFFFFFFFFFFFFFFFFFFFFFFFFFFFFFFF
FFFFFFFFFFFFFFFFFFFFFFFFFFFFFFFFFFFFFFFFFFFF:FFFFFFFFFFFFFFFF:FFFF
FFFFFFFFFFFFFFF
@A00155:342:HHGFNDSXY:1:2266:12391:5447 1:N:0:GAACCTAG+TCCGCATA
TTGCCCAAAGAAGACCAACATTTTCATACATGATGAAATTCATTATTGGTCTCGCGATCAGCTTGAGA
CATTTCTCCAGGTTTACAAACCTAAAAATTTGTGGGCTACTCTGGTTTTTCCACCTGAGATCTTAGCT
GGTTACAAATCCTCC
+
FFFFFFFFFFFFF,FFFFFFFFFFFFFFFFFFFFFFFFFFFFFFFFFFFFFFFFFFFFFFFFFFFF
FFFFFFFFFFFFFFFFFFFFFFFFFFFFFFFFFFFFFFFFFFFFFFFFFFFFFFFFFFFFFFFFFF
FFFFFFFFFFFFFFF
@A00155:342:HHGFNDSXY:1:2272:26928:16000 1:N:0:GAACCTAG+TCCGCATA
TTGCCCAAAGAAGACCAACATTTTCATACATGATGAAATTCATTATTGGTCTCGCGATCAGCTTGAGA
CATTTCTCCAGGTTTACAAACCTAAAAATTTGTGGGCTACTCTGGTTTTTCCACCTGAGATCTTAGCT
GGTTACAAATCCTCC
+
FFFFFFFF,FFFFFFFFFFFFFFFFFFFFFFFF:FFFFFFFFFFFFFFFFFFFFFFFF:FFFFFFF
FFFFFFFFFFFFFFFFFFFFFFFFFFFFFFFFFFFFFFFFFFFFFFFFFFFFFFFFFFFFFFFFFF
FFFFFFFFFFFFFFF
@A00155:342:HHGFNDSXY:1:2361:4797:14841 1:N:0:GAACCTAG+TCCGCATA
TTGCCCAAAGAAGACCAACATTTTCATACATGATGAAATTCATTATTGGTCTCGCGATCAGCTTGAGA
CATTTCTCCAGGTTTACAAACCTAAAAATTTGTGGGCTACTCTGGTTTTTCCACCTGAGATCTTAGCT
GGTTACAAATCCTCC
+
FFFFFFFFFFFFFFFFFFFFFFFFFFFFFFFFFFFFFFFFFFFFFFFFFFFFFFFFFFFFFFFFFF
FFFFFFFFFFFFFFFFFFFFFFFFFFFFFFFFFFFFFFFFFFFFFFFFFFFFFFFFFFFFFFFFFF
FFFFFFFFFFFFF:F
@A00155:342:HHGFNDSXY:1:2439:19678:36605 1:N:0:GAACCTAG+TCCGCATA
TTGCCCAAAGAAGACCAACATTTTCATACATGATGAAATTCATTATTGGTCTCGCGATCAGCTTGAGA
CATTTCTCCAGGTTTACAAACCTAAAAATTTGTGGGCTACTCTGGTTTTTCCACCTGAGATCTTAGCT
GGTTACAAATCCTCC
+
FFFFFFFFFFF:FFFFFFFFFFFFFFFFFFFFFFFFF:FFFFFFFFFFFFFFFFFFFFFFFFFFFF
FFFFFFFFFFFFFFFFFFFFFFFFFFFFFFFFFFFFFFFFFFFFFFFFFFFFFFFFFFFFFFFFFF
FFFFFFFFFFFFFFF
```

```
@A00155:342:HHGFNDSXY:1:1436:30644:28416 1:N:0:GAACCTAG+TCCGCATA
TGCCCAAAGAAGACCAACATTTTCATACATGATGAAATTCATTATTGGTCTCGCGATCAGCTTGAGAC
ATTTCTCCAGGTTTACAAACCTAAAAATTTGTGGGCTACTCTGGTTTTTCCACCTGAGATCTTAGCTG
GTTACAAATCCTCC
+
FFFFFFFFFFFFFFFFF:FFFFFF:FFF:FFFFFFFFFFFFFFFFFFFFFFFFFFFFFFFFFFFFFFF
FFFFFFFFFFFFFFF:FFFFFFFFFFFFFFFFFFFFFFFFFFFFFFFFFFFFFFFFFFFFFFFFFFFF
FFFFFFFFFFFFFF
@A00155:342:HHGFNDSXY:1:2105:1208:27258 1:N:0:GAACCTAG+TCCGCATA
TGCCCAAACAACACCAACATTTTCATACATGATGAAATTCATTATTGGTCTCGCGATCAGCTTGAGAC
ATTTCTCCAGGTTTACAAACCTAAAAATTTGTGGGCTACTCTGGTTTTTCCACCTGAGATCTTAGCTG
GTTACAAATCCTCC
+
::,F,F:F,FF,FF,FFFF,:::FFFFFFFFF:,FFFF,FFFFF:FFFFF,:FF,F,FF:FF:FFFFF
FFF::FFFFF::FFFFFFFFFFFFFFFFFFFFFFFFFFFFFFFF:F:FFFFFFFFFFFF:FFFFFF:F
FFFFFFFF:FFF,F
@A00155:342:HHGFNDSXY:1:2262:32560:26663 1:N:0:GAACCTAG+TCCGCATA
TGCCCAAAGAAGACCAACATTTTCATACATGATGAAATTCATTATTGGTCTCGCGATCAGCTTGAGAC
ATTTCTCCAGGTTTACAAACCTAAAAATTTGTGGGCTACTCTGGTTTTTCCACCTGAGATCTTAGCTG
GTTACAAATCCTCC
+
FFFFFFF:F:FFFFFF,F:F:FF,FFFF,FFFFFFFFFFF:FF:FFFFFFFFFFFFFFFFFFFFFFFF
:FFFFFFF:FFFF::FFF:FFF:F:F:FFFFFFFFFFFFF:,FFFFFFFFF:FFFFFFFFFFF:FFFF
FFFFFFFFFFF:FF
@A00155:342:HHGFNDSXY:1:2308:10511:34287 1:N:0:GAACCTAG+TCCGCATA
TGCCCAAAGAAGACCAACATTTTCATACATGATGAAATTCATTATTGGTCTCGCGATCAGCTTGAGAC
ATTTCTCCAGGTTTACAAACCTAAAAATTTGTGGGCTACTCTGGTTTTTCCACCTGAGATCTTAGCTG
GTTACAAATCCTCC
+
FFFFFFFFFFFFFFFFFFF,FFFFFFFFFFFFFFFFFFFFFFF,F:FFFFFFFFFFFFFFFFFFFFFF
FFFFFFFFFFFFFFFFFFFFFFFFFFFFFFFFFFFFFFFFFFFFFFFFFFFFFFFFFFFFFFFFFFFF
FFFFFFFFFFFF:F
@A00155:342:HHGFNDSXY:1:2574:26964:5697 1:N:0:GAACCTAG+TCCGCATA
TGCCCAAAGAAGACCAACATTTTCATACATGATGAAATTCATTATTGGTCTCGCGATCAGCTTGAGAC
ATTTCTCCAGGTTTACAAACCTAAAAATTTGTGGGCTACTCTGGTTTTTCCACCTGAGATCTTAGCTG
GTTACAAATCCTCC
+
FFFF,F:F:FF:F:FFF,FFFFF,FFFF:F:FFFFFFFFFFFFFFFFFFFFFFFFFFFFFFFFFFFFF
FFFFFF:FFFFFFFF:FFFFFFFF:FFFFFFF,FF,FFFFFFFFFFFFFFFFFFFFFFF:FFFF:FFF
FFFFFFFFFFFFFF
@A00155:342:HHGFNDSXY:1:1138:2528:14700 1:N:0:GAACCTAG+TCCGCATA
TGCCCAAAGAAGACCAACATTTTCATACATGATGAAATTCATTATTGGTCTCGCGATCAGCTTGAGAC
ATTTCCCCAGGTTTACAAACCTAAAAATTTGTGGGCTACTCTGGTTTTTCCACCTGAGATCTTAGCTG
GTTACAAATCCTCCG
+
FFF,FFFFFFFFFFFFFFFFFFF,FFF:FFFFFFFFFFFFFFFFFFFF:FFFFFFFFFFFFFFFFFFF
FFFFF,FFFFFFFFFFFFFFFFFFFFFFFFFF:F:FFFFFFFFFFFFFFFFFFFFFFFFFFFFFFFFF
FFFFFFFFFFFFFFF
@A00155:342:HHGFNDSXY:1:1148:17137:17018 1:N:0:GAACCTAG+TCCGCATA
TGCCCAAAGAAGACCAACATTTTCATACATGATGAAATTCATTATTGGTCTCGCGATCAGCTTGAGAC
ATTTCTCCAGGTTTACAAACCTAAAAATTTGTGGGCTACTCTGGTTTTTCCACCTGAGATCTTAGCTG
GTTACAAATCCTCCG
+
FFFFFFFFF:FFFFFFFFFFFFFFFFFF:FFFFFFFFFFFFFFFFFFFFFFFFFFFFFFFFF:FFFFF
```

FFFFFFFFFFFFFFFFFFFFFFFFFFFFFFFFFFFFFFFFFFFFFFFFFFFFFFFFFFFFFFFFFFFF
F:FFFFF:FFFFFFF
@A00155:342:HHGFNDSXY:1:1207:6958:17394 1:N:0:GAACCTAG+TCCGCATA
TGCCCAAAGAAGACCAACATTTTCATACATGATGAAATTCATTATTGGTCTCGCGATCAGCTTGAGAC
ATTTCTCCAGGTTTACAAACCTAAAAATTTGTGGGCTACTCTGGTTTTTCCACCTGAGATCTTAGCTG
GTTACAAATCCTCCG
+
:FF,FFFFFFFFFFFFFFFFFFFFFFFFFFFFFFFFFFFFFFFF:FFFFFFFFFFFF:FFFFFFFFFF
FFFFFFFFFFFFFFFFFFFFFFFFF:FFFFF,FFFF:F,FFFFFFFFFFFFF,FFFFFFFFFFFFFFF
FFFFF:FFFFFFFFF
@A00155:342:HHGFNDSXY:1:1459:21441:10911 1:N:0:GAACCTAG+TCCGCATA
TGCCCAAAGAAGACCAACATTTTCATACATGATGAAATTCATTATTGGTCTCGCGATCAGCTTGAGAC
ATTTCTCCAGGTTTACAAACCTAAAAATTTGTGGGCTACTCTGGTTTTTCCACCTGAGATCTTAGCTG
GTTACAAATCCTCCG
+
FFFFFF:FFFFFFFFFFFFFFFFFFFFFFFFFFFFFFFF,FFFFFFFFFFFFFFFFFFFFFFFFFFFF
FFFFFFFFFFFFFFFFFFFFFFFFFFFFFFFFFFFFFFFFFFFFFFFFFFFFFFFFFFFFFFFFFFFF
FFFFFFFFFFFFFFF
@A00155:342:HHGFNDSXY:1:1505:5936:2942 1:N:0:GAACCTAG+TCCGCATA
TGCCCAAAGAAGACCAACATTTTCATACATGATGAAATTCATTATTGGTCTCGCGATCAGCTTGAGAC
ATTTCTCCAGGTTTACAAACCTAAAAATTTGTTGGCTACTCTGGTTTTTCCACCTGAGATCTTAGCTG
GTTACAAATCCTCCG
+
FFFFFFFFFFFFFFFFFFFFF:FFFFF:FFFFFFFFFFF:FFFFFFFFFFFFFFFFFFFFFFFFFFFF
FFFFFFFFFFFFFFF:FFFFFFFFFFFFFFFF,FF:FFFFFFFFFFFFFFFFFFFFFFF:FFFFFFFF
FFFFFFFFFFFFFFF
@A00155:342:HHGFNDSXY:1:1505:5990:3098 1:N:0:GAACCTAG+TCCGCATA
TGCCCAAAGAAGACCAACATTTTCATACATGATGAAATTCATTATTGGTCTCGCGATCAGCTTGAGAC
ATTTCTCCAGGTTTACAAACCTAAAAATTTGTGGGCTACTCTGGTTTTTCCACCTGAGATCTTAGCTG
GTTACAAATCCTCCG
+
FFFFFFFFFFFFFFFFFFFFFFFFFFFFFFFFFFFFFFFFFFFFFFFFFFFFFFFFFFFFFFFFFFFF
FFFFFFFFFFFFFFFFFFFFFFFFFF,FFFFFFFFFFFFFFFFFFFFF:FFFFFFFFF:FFFFFFFFF
FFFFFFFFFFFFFFF
@A00155:342:HHGFNDSXY:1:1563:31150:20964 1:N:0:GAACCTAG+TCCGCATA
TGCCCAAAGAAGACCAACATTTTCATACATGATGAAATTCATTATTGGTCTCGCGATCAGCTTGAGAC
ATTTCTCCAGGTTTACAAACCTAAAAATTTGTGGGCTACTCTGGTTTTTCCACCTGAGATCTTAGCTG
GTTACAAATCCTCCG
+
FFFFFFFFFFFFFFFFFFFFFFFFFFFF:FFFFFFFFFFFFFFFFFFFFFFF:FFFFFFFFFFFFFFF
FFFFFFFFFFFFFFFFFF:FFFFFFFFFFFFFFFFFFFFFFFFFFFFFFFF:FFFFFFFFFFFFFFFF
FFFFFFF:FFFFFFF
@A00155:342:HHGFNDSXY:1:1634:13232:11287 1:N:0:GAACCTAG+TCCGCATA
TGCCCAAAGAAGACCAACATTTTCATACATGATGAAATTCATTATTGGTCTCGCGATCAGCTTGAGAC
ATTTCTCCAGGTTTACAAACCTAAAAATTTGTGGGCTACTCTGGTTTTTCCACCTGAGATCTTAGCTG
GTTACAAATCCTCCG
+
FFFFFFFFFF,FFFFFFFFFFFFFFFFF:FFFFFFFFFFFFFFFFFFF:FFFFFFFFFFFFFFFFFFF
F:FFFFFFFFFF:FFFFFFFFFFFFFFFFFFFFFFFFFFFFFFFFFFFFFFFFFFFFFFFFFFFFFFF
FFFFFFFFFFFFFFF
@A00155:342:HHGFNDSXY:1:1666:2636:28134 1:N:0:GAACCTAG+TCCGCATA
TGCCCAAAGAAGACCAACATTTTCATACATGATGAAATTCATTATTGGTCTCGCGATCAGCTTGAGAC
ATTTCTCCAGGTTTACAAACCTAAAAATTTGTGGGCTACTCTGGTTTTTCCACCTGAGATCTTAGCTG
GTTACAAATCCTCCG

```
+
FFFFFFFFF::F:FFFFFF:FFFFFF:F,FF:FFF:FFFFFFFFFFFFFFFFFFFFFFFF:FFFFFFFF
FFFFFFFFFFFFFFFFFFFFFFFFF:FFFFFFF:FFF:FFFFFFFFFFFFFFFFFFFFFFFFFFFFFFF
FFFFFFFFFFFFFFF
@A00155:342:HHGFNDSXY:1:2114:4634:25238 1:N:0:GAACCTAG+TCCGCATA
TGCCCAAAGAAGACCAACATTTTCATACATGATGAAATTCATTATTGGTCTCGCGATCAGCTTGAGAC
ATTTCTCCAGGTTTACAAACCTAAAAATTTGTGGGCTACTCTGGTTTTTCCACCTGAGATCTTAGCTG
GTTACAATTCCTCCG
+
FF:FFFFF:F,FF::FF::FFFF,FFF:FFFFFFF,:FFF,FF::FFFFFFFFFF,FFF,FFFF,FF:
,FFF,:FFFFFFFFF:,FFF:FFFFF:FFFFF,FFFFFFF,FFFFFF:F,,FFFFFFFFFFFFFF:FF
FFFFF,F,FFFFFFF
@A00155:342:HHGFNDSXY:1:2152:17996:10300 1:N:0:GAACCTAG+TCCGCATA
TGCCCAAAGAAGACCAACATTTTCATACATGATGAAATTCATTATTGGTCTCGCGATCAGCTTGAGAC
ATTTCTCCAGGTTTACAAACCTAAAAATTTGTGGGCTACTCTGGTTTTTCCACCTGAGATCTTAGCTG
GTTACAAATCCTCCG
+
FFFFFFFFFFFFFFFFFFFFFFFFFFFFFFFFFFFFFFFFFFFFFFFFFFFFFFFFFFFFFFFFFFFF
FFFFFFFFFFFFFFFFFFFFFFFFFF:FFFFFFFFFFFFFFFFFFFFFFFFFFFFFFFFFFFFFFFFF
FFFFFFFFFFFFFFF
@A00155:342:HHGFNDSXY:1:2216:22399:34491 1:N:0:GAACCTAG+TCCGCATA
TGCCCAAAGAAGACCAACATTTTCATACATGATGAAATTCATTATTGGTCTCGCGATCAGCTTGAGAC
ATTTCTCCAGGTTTACAAACCTAAAAATTTGTGGGCTACTCTGGTTTTTCCACCTGAGATCTTAGCTG
GTTACAAATCCTCCG
+
FFFFFFFFFF:FFFFFFFFFFFFFFFFFFFFFFFFFFFFFFFFFFFFFFFFFFFFFFFF:FFFFFFFFF
FFFFFFFFFFFFF:FFFFFFFFFFFFFFFFFFFFFFFFFFFFFFFFFFFFFFFF:FFFFFFF,FFFFFFFF
FFFFFFFFFFFFFFF
@A00155:342:HHGFNDSXY:1:2219:31033:20729 1:N:0:GAACCTAG+TCCGCATA
TGCCCAAAGAAGACCAACATTTTCATACATGATGAAATTCATTATTGGTCTCGCGATCAGCTTGAGAC
ATTTCTCCAGGTTTACAAACCTAAAAATTTGTGGGCTACTCTGGTTTTTCCACCTGAGATCTTAGCTG
GTTACAAATCCTCCG
+
FFFFFFFFFFFFFFFFFFFFFFFFFFFFFFFFFFFFFFFFFFFFFFFFFFFFFFFFFFFFFFFFFFFF
:FFFFFFFFFFFFFFFFFFFFFFFFFFFFFFFFFFFFFFFFFFFFFFFFFFFFFFFFFFFFFFFFFFFF
FFFFFFFFFFFFFFF
@A00155:342:HHGFNDSXY:1:2257:9146:10097 1:N:0:GAACCTAG+TCCGCATA
TGCCCAAAGAAGACCAACATTTTCATACATGATGAAATTCATTATTGGTCTCGCGATCAGCTTGAGAC
ATTTCTCCAGGTTTACAAACCTAAAAATTTGTGGGCTACTCTGGTTTTTCCACCTGAGATCTTAGCTG
GTTACAAATCCTCCG
+
FFFFFFFFFF:FFFFFF:FFFFFFFFFFFFFFFFFFFFFFFFFFFFFFFFFFFFFFFFFFFFFFFFFFF
FFFFFFFFFFFFFFFFFFFFFFFFFFFFFFFFFFFFFFFFFFFFFFFFFFFFFFFFFFFFFFFFFFFF
FFFFFFFFFFFFFFF
@A00155:342:HHGFNDSXY:1:2304:3830:19429 1:N:0:GAACCTAG+TCCGCATA
TGCCCAAATAAGACCAACATTTTCATTCATGATGATATTCATTATTGGTCTCGCGATCAGCTTGAGAC
ATTTCTCCAGGTTTACAAACCTAAAAATTTGTGGGCTACTCTGGTTTTTCCACCTGAGATCTTAGCTG
GTTACAAATCCTCCG
+
FFF,F,,F,FFFFF,F:FFFFFF:FF,F,F,FFF:,,:F:FFF,FFFF:FFFF,FFF:F,FFF:F,,F
:FFF:FF:F,FFFFFFF:FFFFFFF:FFFFFFFFFFFFFFFF:FFFFFF,F:F,FFFFF::FFFF:FF
FFFFFFFFFFFFFFF
@A00155:342:HHGFNDSXY:1:2429:30689:6981 1:N:0:GAACCTAG+TCCGCATA
TCCGATCTGAAGACCAACATTTTCATACATGATGAAATTCATTATTGGTCTCGCGATCAGCTTGAGAC
```

```
ATTTCTCCAGGTTTACAAACCTAAAAATTTGTGGGCTACTCTGGTTTTTCCACCTGAGATCTTAGCTG
GTTACAAATCCTCCG
+
FFFFFFFFFFFFFFFFFFFFFFFFFFFFFFFFFFFFFFFFFFFFFFFFFFFFFFFFFFFFFFFFFFFF
F:FFFFFFFFFFFFFFFFFFFFFFFFFFFFFFFFFFFFFFFFFFFFFFFFFFFFFFFFFFFFFFFFFF
FFFFFFFFFFFFFFF
@A00155:342:HHGFNDSXY:1:2221:3134:14841 1:N:0:GAACCTAG+TCCGCATA
GCCCAAAGAAGACCAACATTTTCATACATGATGAAATTCATTATTGGTCTCGCGATCAGCTTGAGACA
TTTCTCCAGGTTTACAAACCTAAAAATTTGTGGGCTACTCTGGTTTTTCCACCTGAGATCTTAGCTGG
TTACAAATCCTCCGC
+
F:FFFF:,FFFFF:FFFFFFFFFFFFFFFFFFFFFFFFFFFFFFFFFFFFFFFFFFFFFFFFFFFFFF
FFFFFFFFFFFFFFFFFFFFFFFFFFFFFFFFFFFFFFFFFFFFFFFFFFFFFFFFFFFFFFFFFFFF
FFFFFFFFFFFFFFF
@A00155:342:HHGFNDSXY:1:2178:28004:10911 2:N:0:GAACCTAG+TCCGCATA
GCCCAAAGAAGACCAACATTTTCATACATGATGAAATTCATTATTGGTCTCGCGATCAGCTTGAGACA
TTTCTCCAGGTTTACAAACCTAAAAATTTGTGGGCTACTCTGGTTTTTCCACCTGAGATCTTAGCTGG
TTACAAATCCTCCGT
+
FFFFFFFFFFFFFFFFFFFFFFFFFFFFFFFFFFF:FFFFFFFFFFFFFFFFFFFFFFFFFFFFFFFF
FFFFFFFFFFFFFFFFFFFFFFFFFFFFFFFFFFFFFFFFFFFFF:FFFFF:FFFFFFFFFFFFFF:FF
FFFFFFF:FFFFFFF
@A00155:342:HHGFNDSXY:1:1126:16984:35352 2:N:0:GAACCTAG+TCCGCATA
CCCAAAGAAGACCAACATTTTCATACATGATGAAATTCATTATTGGTCTCGCGATCAGCTTGAGACAT
TTCTCCAGGTTTACAAACCTAAAAATTTGTGGGCTACTCTGGTTTTTCCACCTGAGATCTTAGCTGGT
TACAAATCCTC
+
FFFFFFFFFFFFFFFFFFFFFFFFFFFFFFFFFFFFFFFFFFFFFFFFFFFFFFFFFFFFFFFFFFFF
FFFFFFFFFFFFFFFFFFFFFFFFFFFFFFFFFFFFFFFFFFFFFFFFFFFFFFFFFFFFFFFFFFFF
FFFFFFFFFFF
@A00155:342:HHGFNDSXY:1:1171:26829:12571 2:N:0:GAACCTAG+TCCGCATA
CCCAAAGAAGACCAACATTTTCATACATGATGAAATTCATTATTGGTCTCGCGATCAGCTTGAGACAT
TTCTCCAGGTTTACAAACCTAAAAATTTGTGGGCTACTCTGGTTTTTCCACCTGAGATCTTAGCTGGT
TACAAATCCTC
+
FFFFFFFFFFFFFFFFFFFFFFFFFFFFFFFFFFFFFFFFFFFFFFFFFFFFFFFFFFFFFFFFFFFF
FFFFFFFFFFFFFFFFFFFFFF:FFFF,FFFFFFF:FFF:FFFFFFFFFFFFFFFF:F:FFFFFFFF
FFF:FFFFFFF
@A00155:342:HHGFNDSXY:1:1206:6479:15342 2:N:0:GAACCTAG+TCCGCATA
CCCAAAGAAGACCAACATTTTCATACATGATGAAATTCATTATTGGTCTCGCGATCAGCTTGAGACAT
TTCTCCAGGTTTACAAACCTAAAAATTTGTGGGCTACTCTGGGTTTTCCACCTGAGATCTTAGCTGGT
TACAAATCCTC
+
FFFFFFFFFFFFFFFFFFFFFFFFFFFFFFFFFFF,FFFFFFFFF:FFFFFFFFFFFFFFFFFFFFFF
FFFFFFFFFFFFFFFFFFFFF:FFFFFFFFFFFFFFFFFFFFF,FFFFF:FFFFFFFF:FFFF:FFFFF
FFFF:,FF,FF
@A00155:342:HHGFNDSXY:1:1242:10827:27508 2:N:0:GAACCTAG+TCCGCATA
CCCAAAGAAGACCAACATTTTCATACATGATGACATTCATTATTGGTCTCGCGATCAGCTTGAGACAT
TTCTCCAGGTTTACAAACCTAAAAATTTGTGGGCTACTCTGGTTTTTCCACCTGAGATCTTAGCTGGT
TACAAATCCTC
+
FFFFFFFFFFFFF:FFFFFFFFFFFFFFFFFF,,F,FFF,FFFFFFFFFFFFFFFFFFFFFFFFFFFF
:FFFFFFFFFFFFFF::FFFFFFFFFFFFFFFFFFFFFFFFFFFFFFFFFFFFFFFFFFFFFFFFFFF
FFFF:FFFFFF
```

@A00155:342:HHGFNDSXY:1:1242:8431:30091 2:N:0:GAACCTAG+TCCGCATA
CCCAAAGAAGACCAACATTTTCATACATGATGAAATTCATTATTGGTCTCGCGATCAGCTTGAGACAT
TTCTCCAGGTTTACAAACCTAAAAATTTGTGGGCTACTCTGGTTTTTCCACCTGAGATCTTAGCTGGT
TACAAATCCTC
+
FFFF::FFFFFFFFFFFFFFFFFFFFFFFFFFFFF:FFFFFFFFFFFFFFFFFFFFFFFFFFFFFFFF
FFFFFFFFFFFFFFFFFFFFFFFFFFFFFFFFFFFFFFFF:F:FFFFFF:FFFFFF:FFFFFFFFFFF
FFFFFFFFFFF
@A00155:342:HHGFNDSXY:1:1534:30906:35634 2:N:0:GAACCTAG+TCCGCATA
CCCAAAGAAGACCAACATTTTCATACATGATGAAATTCATTATTGGTCTCGCGATCAGCTTGAGACAT
TTCTCCAGGTTTACAAACCTAAAAATTTGTGGGCTACTCTGGTTTTTCCACCTGAGATCTTAGCTGGT
TACAAATCCTC
+
FFFFF:FFF:FFFFFFFFFFFFFFFFFFFFFFFFFFFFFFFFFFFFFFFFFFFFFFFFFFFFFFFFFF
FFFFFFFFFFFFFFFFFFFFFFFFFF,FFFFFFFFFFFFFFFFFFFFFFFFFFFFFFFF:FFFFFFFF
FFFFFFFFFFF
@A00155:342:HHGFNDSXY:1:2219:31033:20729 2:N:0:GAACCTAG+TCCGCATA
CCCAAAGAAGACCAACATTTTCATACATGATGAAATTCATTATTGGTCTCGCGATCAGCTTGAGACAT
TTCTCCAGGTTTACAAACCTAAAAATTTGTGGGCTACTCTGGTTTTTCCACCTGAGATCTTAGCTGGT
TACAAATCCTC
+
FFFFFFFFFFFFFFFFFFFFFFFFFFFFFFFFF,FFFFFFFFFFFFFFFFFFFFFFFFFFFFFFFFF:
FFFFFFFFFFFFFFFFFFFFFFFFFFFFFFFFFFF:FFFFFFFFFFFFFFFFFFFFFFFF:FFFFFFF
FFFF:FFFFFF
@A00155:342:HHGFNDSXY:1:2252:32425:2315 2:N:0:GAACCTAG+TCCGCATA
CCCAAAGAAGACCAACATTTTCATACATGATGAAATTCATTATTGGTCTCGCGATCAGCTTGAGACAT
TTCTCCAGGTTTACAAACCTAAAAATTTGTGGGCTACTCTGGTTTTTCCACCTGAGATCTTAGCTGGT
TACAAATCCTC
+
FFFFFFFFFFFFFFFFFFFFFFF:FFFFFFFFF:F::FF,F:,:FFFFFFFFFF:FFFFFFFFFFF::
FFF,FF:FFF,FFF:FFFFF,F:FF:F:FFFFFF::FFF:FF:,F:FFFFFFFFFFFFFF:FFF:FFF
F,FFFFFFFFF
@A00155:342:HHGFNDSXY:1:2412:4915:5462 2:N:0:GAACCTAG+TCCGCATA
CCCAAAGAAGACCAACATTTTCATACATGATGAAATTCATTATTGGTCTCGCGATCAGCTTGAGACAT
TTCTCCAGGTTTACAAACCTAAAAATTTGTGGGCTACTCTGGTTTTTCCACCTGAGATCTTAGCTGGT
TACAAATCCTC
+
FFFF:FFFFFFFFFFFFFFFFFFFFFFFFFFFFFF:FFF:FFFFFFFFFFFFFFFFFFFFFFFFFFFF
FFFFFFFFFFFFFFF:FFFFFFFFFFFFFF,FFFFFFFFFFFFFFFFFFFFFFFFFFF:FFFFFFFFF
FFFFFFFFFFF
@A00155:342:HHGFNDSXY:1:2433:22444:24925 2:N:0:GAACCTAG+TCCGCATA
CCCAAAGAAGACCAACATTTTCATACATGATGAAATTCATTATTGGTCTCGCGATCAGCTTGAGACAT
TTCTCCAGGTTTACAAACCTAAAAATTTGTGGGCTACTCTGGTTTTTCCACCTGAGATCTTAGCTGGT
TACAAATCCTC
+
FFFFFFFFFFFFFFFFFFFFFFFFFFFFFFFFFFFFFFFFFFFFFFFFFFFFFFFFFFFFFFFFFFFF
FFFFFFFFFFFFFFFFFFFFFFFF:FFFFFFFFFFFFFFFFFFFFFFFFFFFFFFFFFF:FFFFFFFF
FFFFFFFFFFF
@A00155:342:HHGFNDSXY:1:2432:25147:24095 1:N:0:GAACCTAG+TCCGCATA
CCCAAAGAAGACCAACATTTTCATACATGATGAAATTCATTATTGGTCTCGCGATCAGCTTGAGACAT
TTCTCCAGGTTTACAAACCTAAAAATTTGTGGGCTACTCTGGTTTTTCCACCTGAGATCTTAGCTGGT
TACAAATCCTCCGTT
+
FFFFFFFFFFFF,FF:,FFFFFFFFFFFFFFFFFFFFFFFFFFFFFFFFFFFFFFFFFFFFFFFFFFF

FFFFFF::FFFFFFFFFFFFFFFFFFFFFFFFFFFFFFFFFFFFFFFFFFFFFFFFFFFFFFFFFFFF
FFFFFFFFFFFFFFF
@A00155:342:HHGFNDSXY:1:2201:10312:25488 1:N:0:GAACCTAG+TCCGCATA
CCAAAGAAGACCAACATTTTCATACATGATGAAATTCATTATTGGTCTCGCGATCAGCTTGAGACATT
TCTCCAGGTTTACAAACCTAAAAATTTGTGGGCTACTCTGGTTTTTCCACCTGAGATCTTAGCTGGTT
ACAAATCCTCCGTTC
+
FFFFF:FFFFFFFFFFFFFF,FFFFFFFFFFFFFF:FFFFFFFFFFFFFFFFFFFFFFFFFFFFFFFF
FFFFFFFFFFFFFFFFFFFFFFFFFFFFFFFFFFFFFFFFFFFFFFF:FFFFFFFFFFFFFFFFFFFF
FFFFFFFFFFFFFFF
@A00155:342:HHGFNDSXY:1:2613:29017:2863 1:N:0:GAACCTAG+TCCGCATA
CCAAAGAAGACCAACATTTTCATACATGATGAAATTCATTATTGGTCTCGCGATCAGCTTGAGACATT
TCTCCAGGTTTACAAACCTAAAAATTTGTGGGCTACTCTGGTTTTTCCACCTGAGATCTTAGCTGGTT
ACAAATCCTCCGTTC
+
FFFFFFFFFFFFFFFFFFFFFFFFFFFFFFFFFFFFFFFFFFFFFFFFFFFFFFFFFFFFFFFFFFFF
FFFFFFFFFFFFFF:FFFFFFFFFFFFFFFFFFFFFFFFFFFFFFFFFFFFFFFFFFFFFFFFFFFFF
FFFFFFFFFFFF:FF
@A00155:342:HHGFNDSXY:1:2626:31322:6449 1:N:0:GAACCTAG+TCCGCATA
ATCTAGAAGACCAACATTTTCATACATGATGAAATTCATTATTGGTCTCGCGATCAGCTTGAGACATT
TCTCCAGGTTTACAAACCTAAAAATTTGTGGGCTACTCTGGTTTTTCCACCTGAGATCTTAGCTGGTT
ACAAATCCTCCGTTC
+
FFFFFFF:FFFFF:FFFFFFFFFFFFFFFFFFFFFFFFFFFFFFFFFFFFFFFFFFFFFFFFFFFFFF
FFFFFFFFFFFFFFFFFFFFFFFFFFFFFFFFFFFFFFFFFFFFFFFFFFFFFFFFFFFFFFFFFFFF
FFFFFFFFFFFF:FF
@A00155:342:HHGFNDSXY:1:2629:27968:25254 1:N:0:GAACCTAG+TCCGCATA
CCAAAGAAGACCAACATTTTCATACATGATGAAATTCATTATTGGTCTCGCGATCAGCTTGAGACATT
TCTCCAGGTTTACAAACCTAAAAATTTGTGGGCTACTCTGGTTTTTCCACCTGAGATCTTAGCTGGTT
ACAAATCCTCCGTTC
+
FFFF,FFFF,FFF,FF:::,F,:FFF,:FFFFFF:FFFF,FFFFFFFFFFFFF:FFFFF:FFFFFFFF
FFFFFFFF:FFFFFFFFFFFFFF:,FFF:FFFF,FFFFFFFFFFFFFFFFFFFFFFFFF,FFFFFFFF
FFFFFFFFFFFFFFF
@A00155:342:HHGFNDSXY:1:2650:27633:36511 2:N:0:GAACCTAG+TCCGCATA
CCAAAGAAGACCAACATTTTCATACATGATGAAATTCATTATTGGTCTCGCGATCAGCTTGAGACATT
TCTCCAGGTTTACAAACCTAAAAATTTGTGGGCTACTCTGGTTTTTCCACCTGAGATCTTAGCTGGTT
ACAAATCCTCAGATC
+
FFFFFFFFFFFFFFFFFFFFFFFFFFFFFFFFFFFFFFFF:FFFFFFFFFFFFFFFFFFFFFFFF,FF
FFFFFFFFFFFF:F,FFFF,:FFFFFFFFFFFFFFFFFFFFFFFFFFFFF:FFFFFFF:FFFFFFFF
FFF,FFFFFFFFFFF
@A00155:342:HHGFNDSXY:1:1626:1425:3959 1:N:0:GAACCTAG+TCCGCATA
CAAAGAAGACCAACATTTTCATACATGATGAAATTCATTATTGGTCTCGCGATCAGCTTGAGACATTT
CTCCAGGTTTACAAACCTAAAAATTTGTGGGCTACTCTGGTTTTTCCACCTGAGATCTTAGCTGGTTA
CAAATCCTCCGTTCT
+
FFFFFFF:FFFFFFF:F,FFFFFFF:FFFFFFFFFFFFFFFF:FFFFFFFFFFFFFFF:FFFFFFFFF
FFFFF:::FFFFFF:FFFFFFFFFFFFFFF:FFFFFFF:FFFFFFFFFFFFFFFFFFFFFFFFFFFFF
FFFFFFFFFFFF:FF
@A00155:342:HHGFNDSXY:1:1347:27724:34475 2:N:0:GAACCTAG+TCCGCATA
CAAAGAAGACCAACATTTTCATACATGATGAAATTCATTATTGGTCTCGCGATCAGCTTGAGACATTT
CTCCAGGTTTACAAACCTAAAAATTTGTGGGCTACTCTGGTTTTTCCACCTGAGATCTTAGCTGGTTA
CAAATCCTCCGTTCT

+
FFFFFFFFFFFFFFFFFFFFFFFFFFFFFFFFFFFF:FFFF:FFFFFFFFFFFF:FFFFFFFFFFFFF
FFFFFFFFFFFFFF:FFFFFFFFFFFFFFFFFFFFFFFFFFFFFFFF:FFFFFFF:F::FFF:FFFFF
FFFFFFFFFFFFFFF
@A00155:342:HHGFNDSXY:1:1413:5737:27211 2:N:0:GAACCTAG+TCCGCATA
CAAAGAAGACCAACATTTTCATACATGATGAAATTCATTATTGGTCTCGCGATCAGCTTGAGACATTT
CTCCAGGTTTACAAACCTAAAAATTTGTGGGCTACTCTGGTTTTTCCACCTGAGATCTTAGCTGGTTA
CAAATCCTCCGTTCT
+
FFFFFFFFFFFFFFFFFFFFFFFFFFFFFFFFF,FFFFFFFFFFFFFFFFFFFFFFFFFFFFFFFFFF
FFFFFFFFFFFFFFFFFFFF:FFFFFFFFFFFFFFFFFFFFFFFFFFFFFFFFFFFFFF:FFFFFFFF
FFFFFFFFFFFFFFF
@A00155:342:HHGFNDSXY:1:1544:14281:13135 2:N:0:GAACCTAG+TCCGCATA
CAAAGAAGACCAACATTTTCATACATGATGAAATTCATTATTGGTCTCGCGATCAGCTTGAGACATTT
CTCCAGGTTTACAAACCTAAAAATTTGTGGGCTACTCTGGTTTTTCCACCTGAGATCTTAGCTGGTTA
CAAATCCTCCGTTCT
+
FFFFFFFFFFFFFFFFFFFFFFFFFFFFFFF,FFFFFFFFFFFFFFFFFFFFFFFFFFFFFFFFF:,F
FFFFFFFFFFFFFFFFFFFFFFFFFFFFFFFFFFFFFFFFFFFFFFFFFFFFFFFFFFFFFFFFFFFF
FFFFFFFFFFFFFFF
@A00155:342:HHGFNDSXY:1:1641:23610:11913 2:N:0:GAACCTAG+TCCGCATA
CAAAGAAGACCAACATTTTCATACATGATGAAATTCATTATTGGTCTCGCGATCAGCTTGAGACATTT
CTCCAGGTTTACAAACCTAAAAATTTGTGGGCTACTCTGTTTTTTCCACCTGAGATCTTAGCTGGTTA
CAAATCCTCCGTTCT
+
FFF,:FFFFFFFFFFFFFFFFFFFFFFFFFFF:FFFFFFFFFFF:FFFF,FFFFFFFFFFFFFFFFFF
F:FFFFFF:FF:FFFF:FFFFF:FF:FFFFF:FFFFFFF,FFFFFFFF,FFFFFFFFFFF,F:F,FFF
FFFFFF:FFF,FFFF
@A00155:342:HHGFNDSXY:1:1673:6659:26741 2:N:0:GAACCTAG+TCCGCATA
CAAAGAAGACCAACATTTTCATACATGATGAAATTCATTATTGGTCTCGCGATCAGCTTGAGACATTT
CTCCAGGTTTACAAACCTAAAAATTTGTGGGCTACTCTGGTTTTTCCACCTGAGATCTTAGCTGGTTA
CAAATCCTCCGTTCT
+
FFFFFFF:FFFFFFFF:FFFFFFFFFFFFFFFFFF:FFFFFFF,FFF:FFFF,FFFF:FFFFFFFFFF
FFF:FF::FFFFFFF:,FFFFFFFFFFFFFFFFF,FFFF:FFFFFF:FF:FFF:FFFFFF:FFF,FFF
F,FFFF,F,FFFFFF
@A00155:342:HHGFNDSXY:1:2147:17155:15608 2:N:0:GAACCTAG+TCCGCATA
CAAAGAAGACCAACATTTTCATACATGATGAAATTCATTATTGGTCTCGCGATCAGCTTGAGACATTT
CTCCAGGTTTACAAACCTAAAAATTTGTGGGCTACTCTGGTTTTTCCACCTGAGATCTTAGCTGGTTA
CAAATCCTCCGTTCT
+
FFFFFF:FFFFFFFFFFFFFFFFFFFFFFFFFFFFFFF:FFFFFFFFFFFFFFFFFF:FFFFFFFFFF
FFFFFFFFFFFFFF:FFFFFFFF:FFFFFFFF:FFFFFFF:FFF:FFFFFFFFFFFF::FFF,FFFFF
FFFFFFFFFFFFFFF
@A00155:342:HHGFNDSXY:1:2657:30906:4914 2:N:0:GAACCTAG+TCCGCATA
CAAAGAAGACCAACATTTTCATACATGATGAAATTCATTATTGGTCTCGCGATCAGCTTGAGACATTT
CTCCAGGTTTACAAACCTAAAAATTTGTGGGCTACTCTGGTTTTTCCACCTGAGATCTTAGCTGGTTA
CAAATCCACCGTTCT
+
FFFFFFFFFFFFFFFFFFFFFFFFFFFFFFFFF,FFF:FFFFFFFFFFFFFFFFFFFFFFFFFFF:FF
FFFFFFFFFFFFFFFFFFFFFFFFFFFFFFFFFFFFFFFFFFFFFFFFFFFFFFF,F,FFFF,FFFFF
FFFFFFF,FFFFFF:
@A00155:342:HHGFNDSXY:1:1326:8739:11710 1:N:0:GAACCTAG+TCCGCATA
AAAGAAGACCAACATTTTCATACATGATGAAATTCATTATTGGTCTCGCGATCAGCTTGAGACATTTC

```
TCCAGGTTTACAAACCTAAAAATTTGTGGGCTACTCTGGTTTTTCCACCTGAGATCTTAGCTGGTTAC
AAATCCTCCGTTCTG
+
FFFFFF:FFFFFFFFFFFFFFFFFFFFFFFFFFFFFFFFFFFFFFFFFFFF:FFFFFFFFFFFFFF
FFFFFFFFFFFFFFFFFFFFFFFFFF:FFFFFFFFFFFFFFFFFFFFFFF:FFFFFFFFFFFFFFFFFFF
FFFFFFFFFFFFFFF
@A00155:342:HHGFNDSXY:1:1662:5113:28322 1:N:0:GAACCTAG+TCCGCATA
AAAGAAGACCAACATTTTCATACATGATGAAATTCATTATTGGTCTCGCGATCAGCTTGAGACATTTC
TCCAGGTTTACAAACCTAAAAATTTGTGGGCTACTCTGGTTTTTCCACCTGAGATCTTAGCTGGTTAC
AAATCCTCCGTTCTG
+
FFFFFFFFFFF,FFFFFFFFFFFFFFFFFFFFFFFFFFFFFFFFFFFFFFFFFFFFFFFFFFFFFFFF
FFF:FFFFFFFFF:FFFFFF:FFFFFFFFFFFFFFFFFFFFFFFFFFFFFFFFFFFFFFFFFFFFFF
FFFFFFFFFFFFFFF
@A00155:342:HHGFNDSXY:1:2445:28257:8594 1:N:0:GAACCTAG+TCCGCATA
AAAGAAGACCAACATTTTCATACATGATGAAATTCATTATTGGTCTCGCGATCAGCTTGAGACATTTC
TCCAGGTTTACAAACCTAAAAATTTGTGGGCTACTCTGGTTTTTCCACCTGAGATCTTAGCTGGTTAC
AAATCCTCCGTTCTG
+
FF:F,F:FFFFFFF:F:FFFFFFF:FFF:FFFFFFFFFFFFFFFFFFFFFFFFFFFFFFFFFFFFFFF
FFFFF:FFFFFFFFFFFFFFFFFFFFFFFFF:FFFFFFFFFFFFF,FFFFFFFFFFFFFFFFF,FFFFF
FFFFFFFFFFFFFF:
@A00155:342:HHGFNDSXY:1:1347:28528:1799 2:N:0:GAACCTAG+TCCGCATA
AAAGAAGACCAACATTTTCATACATGATGAAATTCATTATTGGTCTCGCGATCAGCTTGAGACATTTC
TCCAGGTTTACAAACCTAAAAATTTGTGGGCTACTCTGGTTTTTCCACCTGAGATCTTAGCTGGTTAC
AAATCCTCCGTTCTG
+
FFFFFFFFFFFFFFFFFFFFFFFFFFFFFFFFF,FFFFFFFFFFFFFFFFFFFFFFFFFFFFFFFFFF
FFFFFFFFFFFFFFFFFFFFFFFFF:FFFFFFFFFFFFFFFFFFFFFFFFFFFFFFFFFFFFFFFFFF
FFFFFFFFFFFFFFF
@A00155:342:HHGFNDSXY:1:1207:20708:2754 1:N:0:GAACCTAG+TCCGCATA
AAGAAGACCAACATTTTCATACATGATGAAATTCATTATTGGTCTCGCGATCAGCTTGAGACATTTCT
CCAGGTTTACAAACCTAAAAATTTGTGGGCTACTCTGGTTTTTCCACCTGAGATCTTAGCTGGTTACA
AATCCTCCGTTCTGC
+
:,FF:FFFF:FF:FFFFFFFFFF:FFFFFFFFFFFFFFFFFFFFFFFFFFFFFFFFFFFFFFFFFFFF
FFFFFFFFFFFFFFFFFFFFFFFFFFFFFFFFFFFFFFFFFFFFFFFFFFFFFFFFFFFFFFFFFFFF
FFFFFFFFFFFFFFF
@A00155:342:HHGFNDSXY:1:1364:4273:25708 1:N:0:GAACCTAG+TCCGCATA
AAGAAGACCAACATTTTCATACATGATGAAATTCATTATTGGTCTCGCGATCAGCTTGAGACATTTCT
CCAGGTTTACAAACCTTAAAATTTGTGGGCTACTCTGGTTTTTCCACCTGAGATCTTAGCTGGTTACA
AATCCTCCGTTCTGC
+
FFFFF:FFFFFFFFFFFFFFFFFFFFFFFFFFFFFFFFFFFFFFFFFFFFFFFFFFFFFFFFFFFFFF
FFFFFFFFFFFFFFFFFFFFFFFFFFFF,FFFF,FFFFFF:FFFFFFFFFFFFFFFF:FFFFFFFFFFF
F:FFFFFFFFFFFFF
@A00155:342:HHGFNDSXY:1:1367:30563:17315 1:N:0:GAACCTAG+TCCGCATA
AAGAAGACCAACATTTTCATACATGATGAAATTCATTATTGGTCTCGCGATCAGCTTGAGACATTTCT
CCAGGTTTACAAACCTAAAAATTTGTGGGCTACTCTGGTTTTTCCACCTGAGATCTTAGCTGGTTACA
AATCCTCCGTTCTGC
+
FFF:FFFFFFFFFFFFFFFFFFFFFFFFFFFFFFFFFFFFFFFFFFFFFF:FFFFFFFFFFFFFFFFFF
FFFFFFFFFFFFFFFFFFFFFFFFFFFFFFFFFFFFFFFFFFFFFFFFFFFFFFFFFFFFFFFFFFFF
FFFFFFFFFFF::FF
```

```
@A00155:342:HHGFNDSXY:1:1474:2880:29465 1:N:0:GAACCTAG+TCCGCATA
AAGAAGACCAACATTTTCATACATGATGAAATTCATTATTGGTCTCGCGATCAGCTTGAGACATTTCT
CCAGGTTTACAAACCTAAAAATTTGTGGGCTACTCTGGTTTTTCCACCTGAGATCTTAGCTGGTTACA
AATCCTCCGTTCTAC
+
FFFFFFFFFFFFF:FFFFFFFFFFFFF:FFFFFFFFFFFFFFFFFFFFFFFFFFFFFFFFFFFFFFFF
FFFFFFFFFFFFFFFFFFF,FFFFFFFF,,FF:FFFFFFFFFFFFFFFFFFFFFFFFFFFFFFFFFFF
FFFFFFFFFFFFFFF
@A00155:342:HHGFNDSXY:1:1547:14461:4366 1:N:0:GAACCTAG+TCCGCATA
AAGAAGACCAACATTTTCATACATGATGAAATTCATTATTGGTCTCGCGATCAGCTTGAGACATTTCT
CCAGGTTTACAAACCTAAAAATTTGTGGGCTACTCTGGTTTTTCCACCTGAGATCTTAGCTGGTTACA
AATCCTCCGTTCTGC
+
FFFFF:FFFFFFFFFFFFFFFFFFFFFF::FFFFFFFFFFFFFFFFFFFFFFFF:FFFFFFFFFFFFFF
FFFFFFFFFFFFFFFFFFF:FFFFFFFFFFFFFFFFFFFFFFFFFFFFFFFFF:FFFFFFFFFFFFFFF
FFFFFFFFFFFFFFF
@A00155:342:HHGFNDSXY:1:2111:28989:16000 1:N:0:GAACCTAG+TCCGCATA
AAGAAGACCAACATTTTCATACATGATGAAATTCATTATTGGTCTCGCGATCAGCTTGAGACATTTCT
CCAGGTTTACAAACCTAAAAATTTGTGGGCTACTCTGGTTTTTCCACCTGAGATCTTAGCTGGTTACA
AATCCTCCGTTCTGC
+
F::FFFFFFFFFFFFFFFFFFFFFFFFFFFFFFFFFFFFFFFFFFFFFFFFFFFFFFFFFFFFFFFFF
FF:FF:FFFFFFFFFFFFFFFFFFFFFFFFFFFFFFFFFFFFFFFFFFFFFFFFFFFFFFFFFFFFFF
FFFFFFFFFFFFFFF
@A00155:342:HHGFNDSXY:1:2119:15103:6292 1:N:0:GAACCTAG+TCCGCATA
AAGAAGACCAACATTTTCATACATGATGAAATTCATTATTGGTCTCGCGATCAGCTTGAGACATTTCT
CCAGGTTTACAAACCTAAAAATTTGTGGGCTACTCTGGTTTTTCCACCTGAGATCTTAGCTGGTTACA
AATCCTCCGTTCTGC
+
:FFFF:,FFFFFFFFFFF:FFFFFFFFFFFFFFFFFFFFFFFFFFFFFFFFFFFFFFFFFFFFFFFFF
FFFFFFFFFFFFFFFFFFFFFFFFFFFFFFFFFFFFFFFFFFFFFFFFFFFFFFFFF:FFFFFFFFFF
FFFFFFFFFFFFFFF
@A00155:342:HHGFNDSXY:1:2119:14805:7216 1:N:0:GAACCTAG+TCCGCATA
AAGAAGACCAACATTTTCATACATGATGAAATTCATTATTGGTCTCGCGATCAGCTTGAGACATTTCT
CCAGGTTTACAAACCTAAAAATTTGTGGGCTACTCTGGTTTTTCCACCTGAGATCTTAGCTGGTTACA
AATCCTCCGTTCTGC
+
FFFFFF:FFFFFFFFFFFF:FFFFFFFFFFFFFFFFFFFFFFFFFFFFFFFFFFFFFFFFFFFFFFFF
FFFFFF:FFFFFFFFFFFF,FF:FFFFFFFFFFFFFFFFFFFFFFFFFFFFFFFFFF::FFFFFFFFF
FFFFFFFFFFFFFFF
@A00155:342:HHGFNDSXY:1:2114:11831:21167 1:N:0:GAACCTAG+TCCGCATA
AAGAAGACCAACATTTTCATACATGATGAAATTCATTATTGGTCTCGCGATCAGCTTGAGACATTTCT
CCAGGTTTACAAACCTAAAAATTTGTGGGCTACTCTGGTTTTTCCACCTGAGATCTTAGCTGGTTACA
AATCCTCCGTTCTGC
+
FFFF::FFFFFFFFFFFFFFFFFFFFFFFFFFFFFFFFFFFFFFFFFFFFFFFFFFFFFFFFFFFFFF
FFFFFFFFFFFFFFFFFFFFFFFFFFFFFFFFFFFFFFFFFFFFFFFFFFFFFFFFFFFFFFFFFFFF
FFFFFFFFFFFFFFF
@A00155:342:HHGFNDSXY:1:2139:11433:26490 1:N:0:GAACCTAG+TCCGCATA
AAGAAGACCAACATTTTCATACATGATGAAATTCATTATTGGTCTCGCGATCAGCTTGAGACATTTCT
CCAGGTTTACAAACCTAAAAATTTGTGGGCTACTCTGGTTTTTCCACCTGAGATCTTAGCTGGTTACA
AATCCTCCGTTCTGC
+
FFFF,FFFFFFFFFFFFFFFFFFFFFFFFFFFFFFF,FFFFFFFFFFFFFFFFFFFFFFFFFFFFFFF
```

FFFFFFFFFFFFFFFFFFFFFFFFFFFFFFFFFFFFFFFFFFFFFFFFFFFFFFFFFFFFFFFFFFFF
FFFFFFFFFFFFFFF
@A00155:342:HHGFNDSXY:1:2165:25482:1783 1:N:0:GAACCTAG+TCCGCATA
AAGAAGACCAACATTTTCATACATGATGAAATTCATTATTGGTCTCGCGATCAGCTTGAGACATTTCT
CCAGGTTTACAAACCTAAAAATTTGTGGGCTACTCTGGTTTTTCCACCTGAGATCTTAGCTGGTTACA
AATCCTCCGTTCTGC
+
FFFF,FFFFFFFFFF,FFFFFFFFFFFFFFFFFFFFF:FFFFFFFFFFFFFFFFFFFFFFFFFFFFFF
FFFFFFFFFFFFFFFFFFFFFFFFFFFFFFFFFFFFFFFFFFFFFFFFFFFFFFFFFFFFFFFFFFFF
FFFFFFFFFFFFFFF
@A00155:342:HHGFNDSXY:1:2217:27597:30029 1:N:0:GAACCTAG+TCCGCATA
AAGAAGACCAACATTTTCATACATGATGAAATTCATTATTGGTCTCGCGATCAGCTTGAGACATTTCT
CCAGGTTTACAAACCTAAAAATTTGTGGGCTACTCTGGTTTTTCCACCTGAGATCTTAGCTGGTTACA
AATCCTCCGTTCTGC
+
FFFF:F:F:FFFFFFFFFFFFFFF::FFFFFF:FFFFFFF:FFFFFFFFFFFFFFFFFFFFFFFFFFF
:FFFFFFF,F:F::FFFFFFFFFFFFFFFFFFFFFFFFFFFFFFFFFFFFFFFFFFFFFFFFFFFFFF
FFFFFFFFFFFFFFF
@A00155:342:HHGFNDSXY:1:2315:8847:9737 1:N:0:GAACCTAG+TCCGCATA
AAGAAGACCAACATTTTCATACATGATGAAATTCATTATTGGTCTCGCGATCAGCTTGAGACATTTCT
CCAGGTTTACAAACCTAAAAATTTGTGGGCTACTCTGGTTTTTCCACCTGAGATCTTAGCTGGTTACA
AATCCTCCGTTCTGC
+
F:FFFFFFFFFFFFFFFFFFFFFFFFFFFFFFFFFFF:FFFFFFFFFFFFFF:FFFFFFFFFFFFFFF
FFFFFFFFFFFFFFFFFFFFFFFFFFFFFFFFFFFFFFFFFFFFFFFFFFFFFFFFF:FFFFFFFFFF
FFFFFFFFF:FFFFF
@A00155:342:HHGFNDSXY:1:2328:1045:15796 1:N:0:GAACCTAG+TCCGCATA
AAGAAGACCAACATTTTCATACATGATGAAATTCATTATTGGTCTCGCGATCAGCTTGAGACATTTCT
CCAGGTTTACAAACCTAAAAATTTGTGGGCTACTCTGGTTTTTCCACCTGAGATCTTAGCTGGTTACA
AATCCTCCGTTCTGC
+
FFFFF:FFFFFFF::F:FFFFFFF:FF:FFFF,FF:FF:::,F:FFF,FFFFFFFFFFFFF:FFFFFF
FFFFF,F:FFFFFFFFFFFFF:FFFFFFF:FFFFFFFF::FFFFFFFFFFFFFFFFFFFFFFFFFFFF
FFFFFFFFFFFFFFF
@A00155:342:HHGFNDSXY:1:2406:24641:15671 1:N:0:GAACCTAG+TCCGCATA
AAGAAGACCAACATTTTCATACATGATGAAATTCATTATTGGTCTCGCGATCAGCTTGAGACATTTCT
CCAGGTTTACAAACCTAAAAATTTGTGGGCTACTCTGGTTTTTCCACCTGAGATCTTAGCTGGTTACA
AATCCTCCGTTCTGC
+
F,FFFFFFFFFFFFFFFFFFFFFFFFFFFFFFFFFFFFFFFFFFFFFFFFF:FFFFFFFFFFFFFFFF
FFFFFFFFFFFFFFFFFFFFFFFFFFFFFFFFFFFFFFFFFFFFFFFFFFFFFFFFFFFFFFFFFFFF
FFFFFFFFFFFFFFF
@A00155:342:HHGFNDSXY:1:2537:7798:32440 1:N:0:GAACCTAG+TCCGCATA
ATCTAGACCAACATTTTCATACATGATGAAATTCATTATTGGTCTCGCGATCAGCTTGAGACATTTCT
CCAGGTTTACAAACCTAAAAATTTGTGGGCTACTCTGGTTTTTCCACCTGAGATCTTAGCTGGTTACA
AATCCTCCGTTCTGC
+
FFFFFFFFFFFFFF:,FFF:FFFFFFFFFFFFFFFFFFFFFFFFFFFFFFFFFFFFFFFFFFFF:FFF
FFFFFFFFFFFFFFFFFFFFFFFFFFFFFFFFFFFFFFFFFFFFFFFFFFFFFFFFFFFFFFFFFFFF
FFFFFFFFFFFFFFF
@A00155:342:HHGFNDSXY:1:2564:26169:29590 1:N:0:GAACCTAG+TCCGCATA
AAGAAGACCAACATTTTCATACATGATGAAATTCATTATTGGTCTCGCGATCAGCTTGAGACATTTCT
CCAGGTTTACAAACCTAAAAATTTGTGGGCTACTCTGGTTTTTCCACCTGAGATCTTAGCTGGTTACA
AATCCTCCGTTCTGC

```
+
FFFFFFFFFFFFFF,FF:FF::,FFFFFFFFFFFFFFFFFFFFFFFFFFFF:FFFFFFFFFFFFFFFF:
FFFFFFFFFFFFFFFFFFFFFFFFFFFFFFFFFFFFFFFFFFFFFFFFFFFFFFFFFFFFFFFFFFFFF
FFFFFFFFFFFFFFF
@A00155:342:HHGFNDSXY:1:2578:12834:28447 1:N:0:GAACCTAG+TCCGCATA
AAGAAGACCAACATTTTCATACATGATGAAATTCATTATTGGTCTCGCGATCAGCTTGAGACATTTCT
CCAGGTTTACAAACCTAAAAATTTGTGGGCTACTCTGGTTTTTCCACCTGAGATCTTAGCTGGTTACA
AATCCTCCGTTCTGC
+
FFFFFFFFFFFFFF:F:FFFFFFFFFFFFFFFFF:FFFFFFFFFFFFFFFFFFFFFFFFFFFFFFFFFF
FFFFFFF,FFFFFFFFFFFFFF:FFFFFFFFFFFFFFFFFFFFFFFFFFFFFFFFFFFFFFFFFFFFFF
FFFFFFFFFFFFFFF
@A00155:342:HHGFNDSXY:1:2641:13810:15389 1:N:0:GAACCTAG+TCCGCATA
AAGAAGACCAACATTTTCATACATGATGAAATTCATTATTGGTCTCGCGATCAGCTTGAGACATTTCT
CCAGGTTTACAAACCTAAAAATTTGTGGGCTACTCTGGTTTTTCCACCTGAGATCTTAGCTGGTTACA
AATCCTCCGTTCTGC
+
FFFF,F:FFFFFFFFFFFFFFFFFF,FFFFFFFFFFFFFFFFFFFFFFFFFFFFFFFFFFFFFFFFFFF
FFFFFFFFFFFFFFFFFFFFFFFFFFFFFFFFFFFFFFFFFFFFFFFFFFFFFFFFFFFFFFFFFFFFF
FFFFFFFFFFFFFFF
@A00155:342:HHGFNDSXY:1:1307:10728:24048 2:N:0:GAACCTAG+TCCGCATA
AAGAAGACCAACATTTTCATACATGATGAAATTCATTATTGGTCTCGCGATCAGCTTGAGACATTTCT
CCAGGTTTACAAACCTAAAAATTTGTGGGCTACTCTGGTTTTTCCACCTGAGATCTTAGCTGGTTACA
AATCCTCCGTTCTGC
+
FFFF:FFFFFFFFFFFFFFFFFFFFFFFFFFF,FFFFFFFFFFFFFFFFFFFFFFFFFFFFFFFFFFFF
FFFFFFFFFFFFFFFFFFFFFF:FFFFFFFFFFFF:FFFFFFFFFFFFFFFFFFFFFFFFFFFFFFF:F
FFFFFFFFFFFFFFF
@A00155:342:HHGFNDSXY:1:1240:28510:10191 2:N:0:GAACCTAG+TCCGCATA
AAGAAGACCAACATTTTCATACATGATGAAATTCATTATTGGTCTCGCGATCAGCTTGAGACATTTCT
CCAGGTTTACAAACCTAAAAATTTGTGGGCTACTCTGGTTTTTCCACCTGAGATCTTAGCTGGTTACA
AATCCTCCAGATCGG
+
FFFFFFFFFFFFFFFFFFFFFFFFFFFFFFFF:FFFFFFFFFFFFFFFFFFFFFFFFFFFFFFFF::FFF
FFFFFFFFFFFFFFFFFFFFFF:FFFFFF:FFFFFFFFFFFFFFFFFFFFFFF:F,,FFFF:FF:FFFF
FFFFFFFFFFFFFFF
@A00155:342:HHGFNDSXY:1:1328:30463:17519 2:N:0:GAACCTAG+TCCGCATA
AAGAAGACCAACATTTTCATACATGATGAAATTCATTATTGGTCTCGCGATCAGCTTGAGACATTTCT
CCAGGTTTACAAACCTAAAAATTTGTGGGCTACTCTGGTTTTTCCACCTGAGATCTTAGCTGGTTACA
AATCCTCCGTTCTGC
+
FFFFFFFFFFFFFFFFFFFFFFFFFFFFFFF:FFFFFFFFFFFFFFFFFFFFFFFFFFFFFFFFFFFFF
FFFFFFFFFFFFFFFFFFFFFFFFFFFFFFFFFFFFFFFFFFFFFFFFFFFFFFFFFFFFFFFFFFFFF
FFF:FFFFFFFFFFF
@A00155:342:HHGFNDSXY:1:1415:26829:16172 2:N:0:GAACCTAG+TCCGCATA
AAGAAGACCAACATTTTCATACATGATGAAATTCATTATTGGTCTCGCGATCAGCTTGAGACATTTCT
CCAGGTTTACAAACCTAAAAATTTGTGGGCTACTCTGGTTTTTCCACCTGAGATCTTAGCTGGTTACA
AATCCTCCGTTCTGC
+
FFFFFFFFFFFF:FFFFFFFFFFFFFFFF:F:,FFFFFFFFFFFFFFFFFFFFFFFFFFFFFFFFFFFF
FFFFFFFFFF:FFFFFFFFFF,FFFFFFFFFFFFFFFFFFFF:FFFFFFFFF:FFF:FFFFFFFFFFF
FFFFFFFFFFFFFFF
@A00155:342:HHGFNDSXY:1:1645:18159:9424 2:N:0:GAACCTAG+TCCGCATA
AAGAAGACCAACATTTTCATACATGATGAAATTCATTATTGGTCTCGCGATCAGCTTGAGACATTTCT
```

```
CCAGGTTTACAAACCTAAAAATTTGTGGGCTACTCTGGTTTTTCCACCTGAGATCTTAGCTGGTTACA
AATCCTCCGTTCTGC
+
FFFFFFFFFFFFFFFFFFFFFFFFFFFFFFFFFFFF,FFFFFFFFFFFFFFFFFFFFFFFFFFFFFFF
FFFFFFFFFFFFFFFFFFFF,FFFFFFFF:FF:FFFFFFFFFFFFF::F:F,FFFF:FFFFFFFFFFF
FFFFFFFFFFFFFFF
@A00155:342:HHGFNDSXY:1:1645:18213:9424 2:N:0:GAACCTAG+TCCGCATA
AAGAAGACCAACATTTTCATACATGATGAAATTCATTATTGGTCTCGCGATCAGCTTGAGACATTTCT
CCAGGTTTACAAACCTAAAAATTTGTGGGCTACTCTGGTTTTTCCACCTGAGATCTTAGCTGGTTACA
AATCCTCCGTTCTGC
+
FFFFFFFFFFFFFFFFFFFFFFFFFFFFFFFFFFFFFFFFFFFFFFFFFF:FFFFFFFFFFFF::FFF
FFFFFFFFFFFF:FFFFFFFFFFFFFFFFFFFFFFFFFFFFFFFFFFFFFFFFFFFFFFFFFFFFFF
FFFFFFFFFF,FFFF
@A00155:342:HHGFNDSXY:1:2165:8377:7670 2:N:0:GAACCTAG+TCCGCATA
AAGAAGACCAACATTTTCATACATGATGAAATTCATTATTGGTCTCGCGATCAGCTTGAGACATTTCT
CCAGGTTTACAAACCTAAAAATTTGTGGGCTACTCTGGTTTTTCCACCTGAGATCTTAGCTGGTTACA
AATCCTCCGTTCTGC
+
FFF:FFFFFFFFFFFFFFFFFFFFFFFFFFF:F:FFFFFFFFFFFFFFFFFFFFFFFFFF:FFFFFFF
FFFFFFFFFFFFFFFFFFFFFFFFFFFFFFF:FFFFFFFFFFFFFFFFFFFFFFFFFFFFFFFFFFFF
F,FFFFFFFFFFFFF
@A00155:342:HHGFNDSXY:1:2523:21992:15029 2:N:0:GAACCTAG+TCCGCATA
AAGAAGACCAACATTTTCATACATGATGAAATTCATTATTGGTCTCGCGATCAGCTTGAGACATTTCT
CCAGGTTTACAAACCTAAAAATTTGTGGGCTACTCTGGTTTTTCCACCTGAGATCTTAGCTGGTTACA
AATCCTCCGTTCTGC
+
FFFFFFFFFFFFFFFFFFFFFFFFFFFFFFF,FFF:FFFFFFFFFFFFFFFFFFFFFFFFFFFF:FFF
FFFFFFFFFFFFFFFFFFFFFFFFFFFFFFFFFFFFFFFFFFFFFFFFFFFFFFFFFFFFFFFFFFFF
FFFFFFFFFFFFFFF
@A00155:342:HHGFNDSXY:1:2654:29695:13651 2:N:0:GAACCTAG+TCCGCATA
AAGAAGACCAACATTTTCATACATGATGAAATTCATTATTGGTCTCGCGATCAGCTTGAGACATTTCT
CCAGGTTTACAATCCTAAAAATTTGTGGGCTACTCTGGTTTTTCCACCTGAGATCTTAGCTGGTTACA
AATCCTCCGTTCTGC
+
FF:FF,FFFFFFFFFFF,FFFFF:FFF:FFFF,FFFFFF:FFFFF:FF:FF,FFFFFFF,FFFFFFFF
::FF:FFFFFFF,:FFF,FF:FFFFFFFFFFFFFFFFF:FFFF:FF:F:FFFFF:,,,FFFFFFF,FF
FFFFFFFFFFFFFFF
@A00155:342:HHGFNDSXY:1:2654:30337:13823 2:N:0:GAACCTAG+TCCGCATA
AAGAAGACCAACATTTTCATACATGATGAAATTCATTATTGGTCTCGCGATCAGCTTGAGACATTTCT
CCAGGTTTACAAACCTAAAAATTTGTGGGCTACTCTGGTTTTTCCACCTGAGATCTTAGCTGGTTACA
AATCCTCCGTTCTGC
+
:FFFFFFFFFFFFFFFFFFFFFFFFFFFFFFFFFFFFFFFFFFFFFFFFFFFFFFFFFFFFFFFFFF
FFFFFFFFFFFFFFFFFFFFFFFFFFFFFFFFFFFFFFFFFFFFFFFFFFFFFFFFFFFFFFFFFFFF
FFFFFFFFFFFFFFF
@A00155:342:HHGFNDSXY:1:2662:9272:11506 2:N:0:GAACCTAG+TCCGCATA
AAGAAGACCAACATTTTCATACATGATGAAATTCATTATTGGTCTCGCGATCAGCTTGAGACATTTCT
CCAGGTTTACAAACCTAAAAATTTGTGGGCTACTCTGGTTTTTCCACCTGAGATCTTAGCTGGTTACA
AATCCTCCGTTCTGC
+
FFFFF,FFFFFFFFFFFFFFF:FFFFF:FFFFF:FF,FFFFFFFFFFFFFF:FFFFFFFF:FFFFFFF
FFFF,F:FFFFFFFFFFFFF:FFFFFF:FFFFFF:FFFFFFFFFFF:,FFFFFFFFFF,,:F:FFF,F
FFFF,F:FFFFFF,F
```

```
@A00155:342:HHGFNDSXY:1:1112:28574:12054 1:N:0:GAACCTAG+TCCGCATA
AGAAGACCAACATTTTCATACATGATGAAATTCATTATTGGTCTCGCGATCAGCTTGAGACATTTCTC
CAGGTTTACAAACCTAAAAATTTGTGGGCTACTCTGGTTTTTCCACCTGAGATCTTAGCTGGTTACAA
ATCCTCCGTTCTGC
+
FFFFF:,F,FF,FFFF:,F:FFFFFFFF,:FFF,:FFFFFFFFFFFFFFFF,:,FFFF,FFFFFF:FF
FFFF,FF:FFF:FFF:FF,FFFFFFFFFFFF:FFFFFFF,FFFFF::FFFFFFFFFFF:F:FFF:,F,
,FFF:F,FFFFFFF
@A00155:342:HHGFNDSXY:1:2147:5556:3630 1:N:0:GAACCTAG+TCCGCATA
AGAAGACCAACATTTTCATACATGATGAAATTCATTATTGGTCTCGCGATCAGCTTGAGACATTTCTC
CAGGTTTACAAACCTAAAAATTTGTGGGCTACTCTGGTTTTTCCACCTGAGATCTTAGCTGGTTACAA
ATCCTCCGTTCTGC
+
FFF::FFFFFFFFFFFFFFFFFFFFFFFFFFFFFFFFFFFFFFFFFFFFFFFFFFFFFFFFFFFFFFF
FFFFFFFFFFFFFFFFFFFFFFFFFFFFFFFFFFFFFFFFFFFFFFFFFFFF:FFFFFFFFFFFFFFF
FFFFFFFFFFFFFF
@A00155:342:HHGFNDSXY:1:2622:6533:25332 2:N:0:GAACCTAG+TCCGCATA
AGAAGACCAACATTTTCATACATGATGAAATTCATTATTGGTCTCGCGATCAGCTTGAGACATTTCTC
CAGGTTTACAAACCTAAAAATTTGTGGGCTACTCTGGTTTTTCCACCTGAGATCTTAGCTGGTTACAA
ATCCTCCGTTCTGC
+
:FFFFFFFFFFFFFFFFFFFFFFFFFFFFFFFFFFF:FFF:FFFFFFFFFFFF,FFFFFFFFFFFFFF
FFF:FFFFFF,:F:FFFFFFFFFFFFF::FFFFFFFFFFFFFFFFFFFFFFFFFFFFFFFFFFFFFFF
FF:F:FF:F,FFF:
@A00155:342:HHGFNDSXY:1:1110:15754:28150 1:N:0:GAACCTAG+TCCGCATA
AGAAGACCAACATTTTCATACATGATGAAATTCATTATTGGTCTCGCGATCAGCTTGAGACATTTCTC
CAGGTTTACAAACCTAAAAATTTGTGGGCTACTCTGGTTTTTCCACCTGAGATCTTAGCTGGTTACAA
ATCCTCCGTTCTACC
+
F:F,FFFFFFFFFFFFFFFFFFFFFFFFFFFFFFFFFFFFFFFFFFFFFFFFFFFFFFFFFFFFFFFF
FFFFFFFFFFFFFFFFFFFFFFFFFFFFF:FFFFFFFFFFFFFFFFFFFFFFFFFFF,FFFFFFFFFF
FFFFFFFFFFFFFFF
@A00155:342:HHGFNDSXY:1:1123:32226:9674 1:N:0:GAACCTAG+TCCGCATA
AGAAGACCAACATTTTCATACATGATGAAATTCATTATTGGTCTCGCGATCAGCTTGAGACATTTCTC
CAGGTTTACAAACCTAAAAATTTGTGGGCTACTCTGGTTTTTCCACCTGAGATCTTAGCTGGTTACAA
ATCCTCCGTTCTGCC
+
:FF,FFFFFFFFFFFFF,FFFF:FFFFFFFFFFFF:FFFFFFFFFFFFFFFFFFFFFFFFFFFF:FFF
F,FFFFFFFFFFFFFF:FFFFFFFFFFFFFFFFFFFFFFFFFFFFFFFFFFFF,FFFFFFFFFF:FFFF
FFFFFFFFFFFFFFF
@A00155:342:HHGFNDSXY:1:1126:16984:35352 1:N:0:GAACCTAG+TCCGCATA
AGAAGACCAACATTTTCATACATGATGAAATTCATTATTGGTCTCGCGATCAGCTTGAGACATTTCTC
CAGGTTTACAAACCTAAAAATTTGTGGGCTACTCTGGTTTTTCCACCTGAGATCTTAGCTGGTTACAA
ATCCTCCGTTCTGCC
+
FFFFFFFFF:FFFFFFFFFFFFFFFFFFFFFFFFFFFF:FFF:FFFFFFFFFFFFFFFFFFFFFFFFFF
FFFFFFFFFFFFFFFFFFFFFFFFFFFFFFFFFFFFFFFFFFFFFFFFFFFFFFFFFFFFFFFFFFFF
FFFFFFFFFFFFFFF
@A00155:342:HHGFNDSXY:1:1129:14823:19053 1:N:0:GAACCTAG+TCCGCATA
TCTAGACCAACATTTTCATACATGATGAAATTCATTATTGGTCTCGCGATCAGCTTGAGACATTTCTC
CAGGTTTACAAACCTAAAAATTTGTGGGCTACTCTGGTTTTTCCACCTGAGATCTTAGCTGGTTACAA
ATCCTCCGTTCTGCC
+
FFFFFFFFFFFFFFFFFFFFFF:FFFFFFFFFFFFF:FFFFFFFFFFFFFFFFFFFFFFFFFFFFFFF
```

```
FFFF:FFFF:FFFFFFFFFFFFFFFFFFFFFFFFFFFF:FFFFFFFFFFFFFFFFFFFFFFFFFFFFFF
FFFFFFFFFFFFFFF
@A00155:342:HHGFNDSXY:1:1171:13467:5776 1:N:0:GAACCTAG+TCCGCATA
AGAAGACCAACATTTTCATACATGATGAAATTCATTATTGGTCTCGCGATCAGCTTGAGACATTTCTC
CAGGTTTACAAACCTAAAAATTTGTGGGCTACTCTGGTTTTTCCACCTGAGATCTTAGCTGGTTACAA
ATCCTCCGTTCTGCC
+
FF,FFFFFFFFFFFFFFFFFFFFFFFFFFFFFFFFFFFFFFFFFFFFFFFFFFFFFFFFFFFF:FFF:
FFFFFFFFFFFFFFFFFFFFFFFFFFFFFFFFFFFFFFFFFFFFFFFFFFFFFFFFFFFFFFFFFFFF
FFFFFFFFFFFFFFF
@A00155:342:HHGFNDSXY:1:1168:6334:15718 1:N:0:GAACCTAG+TCCGCATA
AGAAGACCAACATTTTCATACATGATGAAATTCATTATTGGTCTCGCGATCAGCTTGAGACATTTCTC
CAGGTTTACAAACCTAAAAATTTGTGGGCTACTCTGGTTTTTCCACCTGAGATCTTAGCTGGTTACAA
ATCCTCCGTTCTGCC
+
FFFFFFFFF:FFFFFFFFFF,FF:FFFFFFFFFFFFFFFFFFFFFFFFFFFFFFFFFFFFFFFFFFFF
FFFFFFFFFFF:FFFFFFF:FFFFFFFFFFFFFFFFFF::FF:FFFF:FFFFFFFFFFFFFF:FFFFF
FFFFFFFFFFFFFFF
@A00155:342:HHGFNDSXY:1:1206:6479:15342 1:N:0:GAACCTAG+TCCGCATA
AGAAGACCAACATTTTCATACATGATGAAATTCATTATTGGTCTCGCGATCAGCTTGAGACATTTCTC
CAGGTTTACAAACCTAAAAATTTGTGGGCTACTCTGGTTTTTCCACCTGAGATCTTAGCTGGTTACAA
ATCCTCCGTTCTGCC
+
FFFF:FFFFFFFFFFFFFFFFFFFFFFFFFFFFFFFFFFFFFFFFFFFFFFFFFFFFFFFFFFFFFFF
FFFFFFFFFFFFFFFFFFFF::FFFFFFFFFFFF:FF:FFFFFFFFFFFFFFFFFFFFFFFFFFFFFF
FFFFFFFFFFFFFFF
@A00155:342:HHGFNDSXY:1:1206:6515:20854 1:N:0:GAACCTAG+TCCGCATA
TCCGATCTAACATTTTCATACATGATGAAATTCATTATTGGTCTCGCGATCAGCTTGAGACATTTCTC
CAGGTTTACAAACCTAAAAATTTGTGGGCTACTCTGGTTTTTCCACCTGAGATCTTAGCTGGTTACAA
ATCCTCCGTTCTGCC
+
FFF:FFFFFFFFFF,FFFFFF,FFFFFF,FFFFFFFFFF,FFFFFFFFFFFFFFFFFFFFFFFFFFFF
FFFFFFFFFF:FFFFFF::FFFFFFFFF,FFFFFF:FFFFFFFFFFFFFFFFFFF:FFFFFFFFFFFF
FFFFFFFFFFFFFFF
@A00155:342:HHGFNDSXY:1:1217:4408:14293 1:N:0:GAACCTAG+TCCGCATA
AGAAGACCAACATTTTCATACATGATGAAATTCATTATTGGTCTCGCGATCAGCTTGAGACATTTCTC
CAGGTTTACAAACCTAAAAATTTGTGGGCTACTCTGGTTTTTCCACCTGAGATCTTAGCTGGTTACAA
ATCCTCCGTTCTGCC
+
FFFFFFFFFFFFFF,FFFFF:FFFFFFFFFFFFFFFFFFFFFFFFFFFFFFFFFFFFFFFF:FFFFFFF
FF:FFFFFFFFFFFFFFFFFFFFFFFFFFFFFFFFFFFFFFFFFFFFFFFFFFFFFFFFFFFFFFFFFF
FFFFFFFFFFFFFFF
@A00155:342:HHGFNDSXY:1:1222:30201:15749 1:N:0:GAACCTAG+TCCGCATA
AGAAGACCAACATTTTCATACATGATGAAATTCATTATTGGTCTCGCGATCAGCTTGAGACATTTCTC
CAGGTTTACAAACCTAAAAATTTGTGGGCTACTCTGGTTTTTCCACCTGAGATCTTAGCTGGTTACAA
ATCCTCCGTTCTGCC
+
FFFFFFFFFFFFFF:FFFFFFFFFFFFFFFFFFFFFFFFFFFFFFFFFFFFFFFFFFFFFFFFFFFFF
FFFFFFFFFFFFFFFFFFFFFFFFFFFFFFFFFFFFFFFFFFFFFFFFFFFFFFFFFFFFFFFFFFFF
FFFFFFFFFFFFFFF
@A00155:342:HHGFNDSXY:1:1234:26341:18490 1:N:0:GAACCTAG+TCCGCATA
AGAAGACCAACATTTTCATACATGATGAAATTCATTATTGGTCTCGCGATCAGCTTGAGACATTTCTC
CAGGTTTACAAACCTAAAAATTTGTGGGCTACTCTGGTTTTTCCACCTGAGATCTTAGCTGGTTACAA
ATCCTCCGTTCTGCC
```

```
+
FFFFFFFFFFFFFFFFFFFFFFFFFFFFFFFFFFFFFFFFFFFFFFFFFFFFFFFFFFFFFFFFFFFF
FFFFFFFFFFFFFFFFFFFFFFFFFFFFFFFFFFFFF:FFFFFFFFFFFFFFFFFFFFFFFFFFFFFF
FFFFFFFFF:FFFFF
@A00155:342:HHGFNDSXY:1:1234:16441:31407 1:N:0:GAACCTAG+TCCGCATA
AGAAGACCAACATTTTCATACATGATGAAATTCATTATTGGTCTCGCGATCAGCTTGAGACATTTCTC
CAGGTTTACAAACCTAAAAATTTGTGGGCTACTCTGGTTTTTCCACCTGAGATCTTAGCTGGTTACAA
ATCCTCCGTTCTGCC
+
FFFFFFFFFFFFFFFFFF:FF:FFFFFFFF:FFFFFFFFFFFFFFFFFFFFFFFFFFFFFFFFFFFFF
FFFFFFFFFFFFFFFFFFFFFFFFFFFFFFFFFFFFFFFFFFFFFFFFFFFFFFFF,FFFFFFFFFFF
FFFFFFFFFFFFFFF
@A00155:342:HHGFNDSXY:1:1258:28140:6417 1:N:0:GAACCTAG+TCCGCATA
AGAAGACCAACATTTTCATACATGATGAAATTCATTATTGGTCTCGCGATCAGCTTGAGACATTTCTC
CAGGTTTACAAACCTAAAAATTTGTGGGCTACTCTGGTTTTTCCACCTGAGATCTTAGCTGGTTACAA
ATCCTCCGTTCTGCC
+
FFFFFFFFFFFFFFFFFFFFFFFFFFFFFFFFFFFFFFFFFFFFFFFFFFFFFFFFFFFFFFFFFFFF
FFFFFFFFFFFFFFFFFFFFFFFFFFFFFFFFFFFFFFFFFFFFFFFFFFFFFFF:FFFFFFFFFFFF
FFFFFFFFFFFFFFF
@A00155:342:HHGFNDSXY:1:1258:25446:10488 1:N:0:GAACCTAG+TCCGCATA
AGAAGACCAACATTTTCATACATGATGAAATTCATTATTGGTCTCGCGATCAGCTTGAGACATTTCTC
CAGGTTTACAAACCTAAAAATTTGTGGGCTACTCTGGTTTTTCCACCTGAGATCTTAGCTGGTTACAA
ATCCTCCGTTCTGCC
+
FFFFFFFFFFFFFFFFFFFFFFFFFFFFFFFFFFFFFFFFFFFFFFFFFFFFFFFFFFFFFFFFFFFF
FFFFFFFFFFFFFFFFFFFFFFFFFFFFFFFFFFFFFFFFFFFFFFFFFFFFFFFFFFFFFFFFFFFF
FFFFFFFFFFFFFFF
@A00155:342:HHGFNDSXY:1:1258:25473:10504 1:N:0:GAACCTAG+TCCGCATA
AGAAGACCAACATTTTCATACATGATGAAATTCATTATTGGTCTCGCGATCAGCTTGAGACATTTCTC
CAGGTTTACAAACCTAAAAATTTGTGGGCTACTCTGGTTTTTCCACCTGAGATCTTAGCTGGTTACAA
ATCCTCCGTTCTGCC
+
FFFF:FFFFFFF:FFFFFFFF,FFFFFFFFFFFFFFFFFFFFFFFFFFFFFFFFFFFFFFFF:FFFFF
FFFFFFFFFFFFFFFFFF:F:FFFFFFFFFFFFFF:FFFFFFFFFFFFFFFFFFFFFFFFFFFFFFFF
FFFFFFFFFFFFFFF
@A00155:342:HHGFNDSXY:1:1276:14235:10770 1:N:0:GAACCTAG+TCCGCATA
AGAAGACCAACATTTTCATACATGATGAAATTCATTATTGGTCTCGCGATCAGCTTGAGACATTTCTC
CAGGTTTACAAACCTAAAAATTTGTGGGCTACTCTGGTTTTTCCACCTGAGATCTTAGCTGGTTACAA
ATCCTCCGTTCTGCC
+
FFF,FFFFFFFFF:FFFFFFF:FFFFFFFFFFFFFFFFFFFFFFFFFFFFF:FFFFFFFFFFFFFFFF
FFFFFFF:FFF,FFFFFFFFFFFFFFFFFFFFFFFFFFFFFFFFFFFFFFFFFFFFFFFFFFFFFFFF
FFFFFFFFFFFFFFF
@A00155:342:HHGFNDSXY:1:1274:14416:34037 1:N:0:GAACCTAG+TCCGCATA
AGAAGACCAACATTTTCATACATGATGAAATTCATTATTGGTCTCGCGATCAGCTTGAGACATTTCTC
CAGGTTTACAAACCTAAAAATTTGTGGGCTACTCTGGTTTTTCCACCTGAGATCTTAGCTGGTTACAA
ATCCTCCGTTCTGCC
+
FFF:FFFFFFF:FFFFFFFFFFFFFFFFFFFFFFFFFFFFFFFFFFFFFFFFFFFFFFFFF,FFFFFF
FFFFFFFFFFFFFFFFFFFFFFFFFFFFF:FFFFFFF:FF:FFFFFFFFFFFFFFFFFFFFFFFFFFF
FFFFFFFFFFFFFFF
@A00155:342:HHGFNDSXY:1:1305:11460:35900 1:N:0:GAACCTAG+TCCGCATA
AGAAGACCAACATTTTCATACATGATGAAATTCATTATTGGTCTCGCGATCAGCTTGAGACATTTCTC
```

CAGGTTTACAAACCTAAAAATTTGTGGGCTACTCTGGTTTTTCCACCTGAGATCTTAGCTGGTTACAA
ATCCTCCGTTCTGCC
+
:FFFFFFFFFFFFFFFFFFFFFFFFFFFFFFFFFFFFF,FFF:FFFFFFFFFFFFFFFFFFFFFFFFF
FFFFFFFFFFFFFFFFFFFFFFFFFFFFFFFFFFFFFFFFFFFFFFFFFFFFFFFFFFFFFFFFFFFF
FFFFFFFFFFFFFFF
@A00155:342:HHGFNDSXY:1:1318:17951:3333 1:N:0:GAACCTAG+TCCGCATA
AGAAGACCAACATTTTCATACATGATGAAATTCATTATTGGTCTCGCGATCAGCTTGAGACATTTCTC
CAGGTTTACAAACCTAAAAATTTGTGGGCTACTCTGGTTTTTCCACCTGAGATCTTAGCTGGTTACAA
ATCCTCCGTTCTGCC
+
FFFFFFFFF:FFFFFFFFFFFFFFFFFFFFFFFFFFFFFFFFFF:FFFFFF:FFFFFFFFFFFFFFFF
FFFFFFFFFFF:FFFFFFFF:FFFFFFFFFFFFFFFFFFFFFFFFFFFFFFFFFF:FFFFFFFFFFFF
FFFFFFFFFFFFFFF
@A00155:342:HHGFNDSXY:1:1348:19551:18067 1:N:0:GAACCTAG+TCCGCATA
CTAAGACCAACATTTTCATACATGATGAAATTCATTATTGGTCTCGCGATCAGCTTGAGACATTTCTC
CAGGTTTACAAACCTAAAAATTTGTGGGCTACTCTGGTTTTTCCACCTGAGATCTTAGCTGGTTACAA
ATCCTCCGTTCTGCC
+
:FFFFF:FFFFFFFFFFFF:FF:FFFFFFFFFFFFFFFFFFFFFFFFFFFFFFFFFF:FFFFFFFFFF
F:FFFFFFF:F,FFFFFFFFFFFFFFFFF:FFFFFFFFFFFFFFFFFFF:FFFFFFFFFFFF:FFFFF
FFFFFFFFFFFFFFF
@A00155:342:HHGFNDSXY:1:1357:9362:16203 1:N:0:GAACCTAG+TCCGCATA
AGAAGACCAACATTTTCATACATGATGAAATTCATTATTGGTCTCGCGATCAGCTTGAGACATTTCTC
CAGGTTTACAAACCTAAAAATTTGTGGGCTACTCTGGTTTTTCCACCTGAGATCTTAGCTGGTTACAA
ATCCTCCGTTCTGCC
+
FFFF:FFFFFF:FFFFFFFFFFFFFFFFFFF:FFFFFFFFFFFFFFFFFFFFFFFFFFFFFFFFFFFF
FFFF:FFFFFFFFFFFFF:FFFFFFFFFFFFFFFFFFFFFFFFFFFFFFFFFFFFFFFFFFFFFFFFF
FFFFFFFFFFFFFFF
@A00155:342:HHGFNDSXY:1:1375:3676:18693 1:N:0:GAACCTAG+TCCGCATA
AGAAGCCCAACATTTTCATACATGATGAAATTCATTATTGGTCTCGCGATCAGCTTGAGACATTTCTC
CAGGTTTACAAACCTAAAAATTTGTGGGCTACTCTGGTTTTTCCACCTGAGATCTTAGCTGGTTACAA
ATCCTCCGTTCTGCC
+
FFF::,FFF::FFFFFFFFFFFFFFFFFFFFFFFFFFFFFFFFFFFFFFFFFFFFFFFFFFFFFFFFF
FFFFFFFFFFFFFFFFFFFFF:FFFFFFF:FFFFFFFFFFFFFFFFFFFFFFFFF:FFFFFFFFFFFF
FFFFFFFFFFFFFFF
@A00155:342:HHGFNDSXY:1:1420:32018:23030 1:N:0:GAACCTAG+TCCGCATA
AGAAGACCAACATTTTCATACATGATGAAATTCATTATTGGTCTCGCGATCAGCTTGAGACATTTCTC
CAGGTTTACAAACCTAAAAATTTGTGGGCTACTCTGGTTTTTCCACCTGAGATCTTAGCTGGTTACAA
ATCCTCCGTTCTGCC
+
FFFFFFFFF,F:FFFFFFFFF,FFFFFFFFFFFFFFFFFFFFFFFFFFFFFFFFFFFFFFFFFFFFF:
FFFFFFFFFFFFFFFFFFFFFFFFFFFFFFFFFFFFFFFFFFFFFFFFFFFFFFFFFFFFFFFFFFFF
FFFFFFFFFFFFFFF
@A00155:342:HHGFNDSXY:1:1406:24216:19069 1:N:0:GAACCTAG+TCCGCATA
AGAAGACCAACATTTTCATACATGATGAAATTCATTATTGGTCTCGCGATCAGCTTGAGACATTTCTC
CAGGTTTACAAACCTAAAAATTTGTGGGCTACTCTGGTTTTTCCACCTGAGATCTTAGCTGGTTACAA
ATCCTCCGTTCTGCC
+
FFF::,:FFFFFF:FFFFFFFFFFFFFFFFFFFFFFFFFFFFFFFFFFFFFFFFFFFFFFFFFFFFFF
FFFFFFFFFF:FFFFF,FFFFFFFFFF:FFF:FFFFFFFFFFFFFF:FFFFFFFF,FFFFFFFFFFFF
FFFFFFFFFFFFFFF

```
@A00155:342:HHGFNDSXY:1:1403:29306:6965 1:N:0:GAACCTAG+TCCGCATA
AGAAGACCAACATTTTCATACATGATGAAATTCATTATTGGTCTCGCGATCAGCTTGAGACATTTCTC
CAGGTTTACAAACCTAAAAATTTGTGGGCTACTCTGGTTTTTCCACCTGAGATCTTAGCTGGTTACAA
ATCCTCCGTTCTGCC
+
FFFFFFFFFFFFFFFFFFFFFFFFFFFFFF:FFFFFFFFFFFFFFFFFFFFFFF:FFFFFFFFFFFFFFF
FF:FF:FFFFFFFFFFFFFFFFFFFFFFFF,FFFFFFFFFFFFFFFFFFFFFFFFFFFFFFFFFFFFFFF
FFFFFFFFFFFFFFF
@A00155:342:HHGFNDSXY:1:1410:31069:36229 1:N:0:GAACCTAG+TCCGCATA
AGAAGACCAACATTTTCATACATGATGAAATTCATTATTGGTCTCGCGATCAGCTTGAGACATTTCTC
CAGGTTTACAAACCTAAAAATTTGTGGGCTACTCTGGTTTTTCCACCTGAGATCTTAGCTGGTTACAA
ATCCTCCGTTCTGCC
+
FF:FFFFFFFFFFFFFFFFFFFFFFFFFFFFFFFFFFFFFFFFFFFFFFFFFFFFFFFFFFFFFFFFFFF
FFFFFFFFFFFFFFFFFFFFFFFFFFFFFFFFFFFFFF:FFFFFFFFFFFFFFFFFFFFFFFFFFFFFFF
FFFFFFFFFFFFFFF
@A00155:342:HHGFNDSXY:1:1442:20564:4539 1:N:0:GAACCTAG+TCCGCATA
AGAAGACCAACATTTTCATACATGATGAAATTCATTATTGGTCTCGCGATCAGCTTGAGACATTTCTC
CAGGTTTACAAACCTAAAAATTTGTGGGCTACTCTGGTTTTTCCACCTGAGATCTTAGCTGGTTACAA
ATCCTCCGTTCTGCC
+
FFFFFFFFFFFFFFFFFFFFFF,FFFFFFFFFF:FFFFF:FFFFFFFFFFFFFFFFFFFFFFFFFFFFFF
FFFFFFFFFFFFFFFFFFFFFFFFFFFFFFFFFFFFFFFFFFFFFFFFFFFFFFFFFF,FFFFFFFFFFF
FFFFFFFFFFFFFFF
@A00155:342:HHGFNDSXY:1:1455:16134:21386 1:N:0:GAACCTAG+TCCGCATA
AGAAGACCAACATTTTCATACATGATGAAATTCATTATTGGTCTCGCGATCAGCTTGAGACATTTCTC
CAGGTTTACAAACCTAAAAATTTGTGGGCTACTCTGGTTTTTCCACCTGAGATCTTAGCTGGTTACAA
ATCCTCCGTTCTGCC
+
FF:FFFFFFFFFFF::FFFFFFFFFFFFFFFFFFFFFFFFFFFFFFFFFFFF,FFFFFFFFFFFFFFFFF
FFFFFFFFFFFFFFFFFFFFFFFFFFFFFFFFFFFFFFFFFFF:FFFFFFFFFFFFFFFFFFFFFFFFFF
FFFFFFFFFFFFFFF
@A00155:342:HHGFNDSXY:1:1519:2338:4883 1:N:0:GAACCTAG+TCCGCATA
AGAAGACCAACATTTTCATACATGATGAAATTCATTATTGGTCTCGCGATCAGCTTGAGACATTTCTC
CAGGTTTACAAACCTAAAAATTTGTGGGCTACTCTGGTTTTTCCACCTGAGATCTTAGCTGGTTACAA
ATCCTCCGTTCTGCC
+
FFFF:FFFFFFFFFFFFFFFFFFF:FFFFFFFFFFFFFFFFFFFFFFFFFFFFFFFFFFFFFFFFFFFFF
FFFFFFFFFFFFFFFFFFFFFFFFFFFFFFFF:FFFFFFFFFFFFFFFFFFFFFFFFFFFFFFFFFFFFF
FFFFFFFFFFFFFFF
@A00155:342:HHGFNDSXY:1:1472:7609:11631 1:N:0:GAACCTAG+TCCGCATA
AGAAGACCAACATTTTCATACATGATGAAATTCATTATTGGTCTCGCGATCAGCTTGAGACATTTCTC
CAGGTTTACAAACCTAAAAATTTGTGGGCTACTCTGGTTTTTCCACCTGAGATCTTAGCTGGTTACAA
ATCCTCCGTTCTGCC
+
FF:FFFFFFFFFFFFFFFFFFFFFFFF:FFFFFFFFFFFFFFFFFF:FFFFFFFFFFFFFFFFFFFFFFF
::FF:FFFFFFFFFFFFFFFFFFFFFFFF:FFFFFFFFFFFFFFFF:FFFFFFFFFFF:FFFFFFFFFFF
FFFFFFFFFFFFFFF
@A00155:342:HHGFNDSXY:1:1532:23882:30984 1:N:0:GAACCTAG+TCCGCATA
AGAAGACCAACATTTTCATACATGATGAAATTCATTATTGGTCTCGCGATCAGCTTGAGACATTTCTC
CAGGTTTACAAACCTAAAAATTTGTGGGCTACTCTGGTTTTTCCACCTGAGATCTTAGCTGGTTACAA
ATCCTCCGTTCTGCC
+
FFFFFFFFFFFFFFFFFFFFFFFFFFFFFFFFFFFFFFFFFFFFFFFFFFFFFFFFFFFFFFFFFFFFFF
```

FFFFFFFFFFFFFFFFFFFFFFFFFFFFFFFFFFFFFFFFFFFFFFFFFFFFFFFFFFFFFFFFFFFF
FFFFFFFFFFFFFFF
@A00155:342:HHGFNDSXY:1:1533:16577:2206 1:N:0:GAACCTAG+TCCGCATA
AGAAGACCAACATTTTCATACATGATGAAATTCATTATTGGTCTCGCGATCAGCTTGAGACATTTCTC
CAGGTTTACAAACCTAAAAATTTGTGGGCTACTCTGGTTTTTCCACCTGAGATCTTAGCTGGTTACAA
ATCCTCCGTTCTGCC
+
FF:FFF:FFFFFFFFFFFFFF:FFFFFFFFFFFFF:FFFFFFFFFFFFFFFFFFFFFFFFFFFFFFFF
FFFFFFFFFF,FFFFFFFFFFFFFFFFF:FFFFFFFFFFFFFFFFFFFFFFFFFFFFFFFFFFFFFFF
FFFFFFFFFFFFFFF
@A00155:342:HHGFNDSXY:1:1520:20582:17503 1:N:0:GAACCTAG+TCCGCATA
AGAAGACCAACATTTTCATACATGATGAAATTCATTATTGGTCTCGCGATCAGCTTGAGACATTTCTC
CAGGTTTACAAACCTAAAAATTTGTGGGCTACTCTGGTTTTTCCACCTGAGATCTTAGCTGGTTACAA
ATCCTCCGTTCTGCC
+
FFFFFFFFFFFFFFFFFFFFFFFFFFFFFFFFFFFFFFFFFFFFFFFFFFFFFFFFFFFFFFFFFFFF
FFFFFFFFFFFFFFFFFFFFFFFFFFFFFFFFFFFFFFFFFFFFFFFFFFFFFFFFFFFFFFFFFFFF
FFFFFFFFFFFFFFF
@A00155:342:HHGFNDSXY:1:1520:8558:28181 1:N:0:GAACCTAG+TCCGCATA
CCGATCTCAACATTTTCATACATGATGAAATTCATTATTGGTCTCGCGATCAGCTTGAGACATTTCTC
CAGGTTTACAAACCTAAAAATTTGTGGGCTACTCTGGTTTTTCCACCTGAGATCTTAGCTGGTTACAA
ATCCTCCGTTCTGCC
+
:FFFFFFFF:FFFFFFFFFFFFFFFFFFFFFFFFFFFFFFFFFFFFFFFFFFFFFFFFFFFFFFFFFF
FFFFFFFFFFFFFFFFFFFF:FFFFFFFFFFFFFFFFFFFFFFFFFFFFFFFFFFFFFFFFFFFFFFF
FFFFFFFFFFFFFFF
@A00155:342:HHGFNDSXY:1:1521:26711:34976 1:N:0:GAACCTAG+TCCGCATA
ATCTGACCAACATTTTCATACATGATGAAATTCATTATTGGTCTCGCGATCAGCTTGAGACATTTCTC
CAGGTTTACAAACCTAAAAATTTGTGGGCTACTCTGGTTTTTCCACCTGAGATCTTAGCTGGTTACAA
ATCCTCCGTTCTGCC
+
:FFFFFFFFFFFFFFF:FFFFFFFFFFFFFFFF::FF:FFFFFFFFFFF:FFFFFFFFFFFFFFFFFF
FFFFFFFFF:,FFFFFFFFFFFFFFFF:FFF:FF,FFFFFFFFFFFFFFFFFFFFFFFFFFFFF,FFF
FFFFFFFFFFFFFFF
@A00155:342:HHGFNDSXY:1:1534:30906:35634 1:N:0:GAACCTAG+TCCGCATA
AGAAGACCAACATTTTCATACATGATGAAATTCATTATTGGTCTCGCGATCAGCTTGAGACATTTCTC
CAGGTTTACAAACCTAAAAATTTGTGGGCTACTCTGGTTTTTCCACCTGAGATCTTAGCTGGTTACAA
ATCCTCCGTTCTGCC
+
FFFFFFFFFFFFFFFFFF:FFFFFFFFFFFFFFFFFFFFFFFFFFFFFFFFFFFFFFFFFFFFFFFFF
FFFFFFFFFFFFFFFFFFFFFFFFFFFFFFFFFFFFFFFFFFFFFFFFFFFFFFFFFFFFFFFFFFFF
FFFFFFFFFFFFFFF
@A00155:342:HHGFNDSXY:1:1532:22851:16830 1:N:0:GAACCTAG+TCCGCATA
AGAAGACCAACATTTTCATACATGATGAAATTCATTATTGGTCTCGCGATCAGCTTGAGACATTTCTC
CAGGTTTACAAACCTAAAAATTTGTGGGCTACTCTGGTTTTTCCACCTGAGATCTTAGCTGGTTACAA
ATCCTCCGTTCTGCC
+
FFFFFFFFFFF:FFFFFFFFFFFFFFFFFFFFFFFFFFFFFFFFFFFFFFFFFFFFFFFFFFFFFFFF
FFFFFFFFFFFFFFFFFFFFFFFFFFFFFFFFFFFFFFFFFFFFFFFFFFFFFFFFFFFFFFFFFFFF
FFFFFFFFFFFFFFF
@A00155:342:HHGFNDSXY:1:1557:32000:25222 1:N:0:GAACCTAG+TCCGCATA
AGAAGACCAACATTTTCATACATGATGAAATTCATTATTGGTCTCGCGATCAGCTTGAGACATTTCTC
CAGGTTTACAAACCTAAAAATTTGTGGGCTACTCTGGTTTTTCCACCTGAGATCTTAGCTGGTTACAA
ATCCTCCGTTCTGCC

```
+
FF:FF:FFFFFFFFFFFFFFFFFFFFFFFFFFFFFFF:FFFFFFFFFFFFFFFFFFFFFFFFFFFFFFFF
FFFFFFFFFFFFFFFFFFFFFFFFFFFFFFFFFFFFFFFFFFFFFFFFFFFFFFFFFFFFFFFFFFFFFF
FFFFFFFFFFFFFFF
@A00155:342:HHGFNDSXY:1:1608:19940:31579 1:N:0:GAACCTAG+TCCGCATA
AGAAGACCAACATTTTCATACATGATGAAATTCATTATTGGTCTCGCGATCAGCTTGAGACATTTCTC
CAGGTTTACAAACCTAAAAATTTGTGGGCTACTCTGGTTTTTCCACCTGAGATCTTAGCTGGTTACAA
ATCCTCCGTTCTGCC
+
:FFFFFFFF:FFFFFFFFFFFFFFFFFFFFFFFFFFF:FFFFFFFFFFFFFFFFFFFFFFFFFFFFF:F
FFFFFFFFFF:FFFFFF:FFF:,FFFFFFFFFFFFFFF:FFFFFFFFFFFFFFFFFFFFFFFFFFFFFF
FFFFFFFFFFFFFFF
@A00155:342:HHGFNDSXY:1:1623:17508:22701 1:N:0:GAACCTAG+TCCGCATA
AGAAGACCAACATTTTCATACATGATGAAATTCATTATTGGTCTCGCGATCAGCTTGAGACATTTCTC
CAGGTTTACAAACCTAAAAATTTGTGGGCTACTCTGGTTTTTCCACCTGAGATCTTAGCTGGTTACAA
ATCCTCCGTTCTGCC
+
FFFF:FFFFFFFFFFFFFFFFFFFFFFFFFFFFFFFFFFFFFFFFFFFFFFFFFFFFFFFFFFFFFFFF
FFFFFFFFFFFFFFFFFFFFFFFFFFFFFFFFFFFFF:FFFFFFFFFFFFFFFFFFFFFFFFFFFFFFF
FFFFFFFFFFFFFFF
@A00155:342:HHGFNDSXY:1:1622:12744:9439 1:N:0:GAACCTAG+TCCGCATA
AGAAGACCAACATTTTCATACATGATGAAATTCATTATTGGTCTCGCGATCAGCTTGAGACATTTCTC
CAGGTTTACAAACCTAAAAATTTGTGGGCTACTCTGGTTTTTCCACCTGAGATCTTAGCTGGTTACAA
ATCCTCCGTTCTGCC
+
FFFFFFFFF,FFFFFFFFFFFFFFFFFFFFFFFFFFFFFFFFFFFFFFFFFFFFFFFFFFFFFFFFFFF
FFFFFFFFFFFFFFFFFFFFFFFFFFFFFF:FFFFFFFFFFFFFFFFFFFFFFFFFFFFFFFFFFFFFF
FFFFFFFFFFF:FFF
@A00155:342:HHGFNDSXY:1:1658:9887:26005 1:N:0:GAACCTAG+TCCGCATA
AGAAGACCAACATTTTCATACATGATGAAATTCATTATTGGTCTCGCGATCAGCTTGAGACATTTCTC
CAGGTTTACAAACCTAAAAATTTGTGGGCTACTCTGGTTTTTCCACCTGAGATCTTAGCTGGTTACAA
ATCCTCCGTTCTGCC
+
FFFFF,FFFFFFF:F:FF,FFFFFFFFFFFFFFFFFFFFFFFFFFFFFFFF:FFFFFFFFF:FFFFFF
FFFF:FFFFFF:FFFFFFFFFFFFFFFFFFFFFFFFFFFFFFFFFF:FFFFFF:FFFFFFFFFFFFFF
FFFFFFFFFFFFFFF
@A00155:342:HHGFNDSXY:1:1626:26214:24846 1:N:0:GAACCTAG+TCCGCATA
AGAAGACCAACATTTTCATACATGATGAAATTCATTATTGGTCTCGCGATCAGCTTGAGACATTTCTC
CAGGTTTACAAACCTAAAAATTTGTGGGCTACTCTGGTTTTTCCACCTGAGATCTTAGCTGGTTACAA
ATCCTCCGTTCTGCC
+
FFFF:FFFFFFFFFFFFFFFFFFFFFFFFFFFFFFFFFFFFFFFFFFFFFFFFFFFFFFFFFFFFFFFF
FFFFFFFFFFFFFFF:FFFFFFFFFFFFFFFFFFFFFFFFFFFFFFFFFFFFFFFFFFFFFFFFFFFFF
FFFFFFFFFFFFFFF
@A00155:342:HHGFNDSXY:1:1658:10077:32158 1:N:0:GAACCTAG+TCCGCATA
AGAAGACCAACATTTTCATACATGATGAAATTCATTATTGGTCTCGCGATCAGCTTGAGACATTTCTC
CAGGTTTACAAACCTAAAAATTTGTGGGCTACTCTGGTTTTTCCACCTGAGATCTTAGCTGGTTACAA
ATCCTCCGTTCTGCC
+
FFFFF:FFFFFFFFFFFFFFFFFFFFFFFFFFFFFFFFFFFF:FFFFFFFFFFFFFFFFFFFFFFFFFFF
FFFFFFFFFFFFFFFFFFFFFFFFFFFFFFFFFFFFFFFFFFFFFFFFFFFFFFFFFFFFFFFFFFFFF
FFFFFFFFFFFFFFF
@A00155:342:HHGFNDSXY:1:1658:8522:32534 1:N:0:GAACCTAG+TCCGCATA
AGAAGACCAACATTTTCATACATGATGAAATTCATTATTGGTCTCGCGATCAGCTTGAGACATTTCTC
```

CAGGTTTACAAACCTAAAAATTTGTGGGCTACTCTGGTTTTTCCACCTGAGATCTTAGCTGGTTACAA
ATCCTCCGTTCTGCC
+
F:FFFFFFFFFFFFFFFFFFFFFFFFFFFFFFFFFFFFFFFFFFFFFFFFFFFFFFFFFFFFFFFFFF
FFFFFFFFFFFFFFFFFFFFFFFFFFFFFFFFFFFFFFFFFFFFFFFFFFFFFFFFFFFFFFFFFFFF
FFFFFFFFFFFFFFF
@A00155:342:HHGFNDSXY:1:1662:12861:32597 1:N:0:GAACCTAG+TCCGCATA
AGAAGACCAACATTTTCATACATGATGAAATTCATTATTGGTCTCGCGATCAGCTTGAGACATTTCTC
CAGGTTTACAAACCTAAAAATTTGTGGGCTACTCTGGTTTTTCCACCTGAGATCTTAGCTGGTTACAA
ATCCTCCGTTCTGCC
+
FFFFFFFFFFFFFFFFFF:FFFFFFFFFFFFFFFFFFFFFFFFFFFFFFFFFFFFFFFFFFFFFFFFF
FFFFFFFFFFFFFFFFFFFFFFFFFFFFFFFFFFFFFFFFFFFFFFFFFFFFFFFFFFFFFFFFFFFF
FFFFFFFFFFFFFFF
@A00155:342:HHGFNDSXY:1:1659:18701:31970 1:N:0:GAACCTAG+TCCGCATA
AGAAGACCAACATTTTCATACATGATGAAATTCATTATTGGTCTCGCGATCAGCTTGAGACATTTCTC
CAGGTTTACAAACCTAAAAATTTGTGGGCTACTCTGGTTTTTCCACCTGAGATCTTAGCTGGTTACAA
ATCCTCCGTTCTGCC
+
FFF::FFFFFFFFFFFFFFFFFFFFFFFFFFFFFFFFFFFFFFFFFFFFFFFFFFFFFFFFFFFFFFF
FFFFFFFFFFFFFFFFFFFFFFFFFFFFFFFFFFFFFFFF:FFFFFFFFFFFFFFFFFFFFFFFFFFF
FFFFFFFFFFFFFFF
@A00155:342:HHGFNDSXY:1:1667:32624:32972 1:N:0:GAACCTAG+TCCGCATA
AGAAGACCAACATTTTCATACATGATGAAATTCATTATTGGTCTCGCGATCAGCTTGAGACATTTCTC
CAGGTTTACAAACCTAAAAATTTGTGGGCTACTCTGGTTTTTCCACCTGAGATCTTAGCTGGTTACAA
ATCCTCCGTTCTGCC
+
FF:F:FFFFFFFFFFFFFFFFFFFFFFFFFFFFFFFFFFFFFFFFFFFFFFFFFFFFFFFFFFFFF:F
FFFFF:FFFF:FFFFFFFF:FFFFFFFFFFFFFFFFFFFFFFFFFFFFFFFFFFF:FFFFFFFFFFFF
FFFFFFFFFFFFFFF
@A00155:342:HHGFNDSXY:1:1660:1787:36307 1:N:0:GAACCTAG+TCCGCATA
AGAAGACCAACATTTTCATACATGATGAAATTCATTATTGGTCTCGCGATCAGCTTGAGACATTTCTC
CAGGTTTACAAACCTAAAAATTTGTGGGCTACTCTGGTTTTTCCACCTGAGATCTTAGCTGGTTACAA
ATCCTCCGTTCTGCC
+
F:FFFFFFFFFFFFFFF:FFFFF:,FF,FFFF:FFFFF:F:FFFFFFFFFFFFFFFFFFFFFFFFFFF
,F:FFFFFFF:FFFFFFFFF,FFFFF:FFFFFFFF:FFF:FFFFFFF:FFFFFFF:FFFFFFFFFFFF
FFFFFFFFFFFFFFF
@A00155:342:HHGFNDSXY:1:1664:20220:35227 1:N:0:GAACCTAG+TCCGCATA
AGAAGACCAACATTTTCATACATGATGAAATTCATTATTGGTCTCGCGATCAGCTTGAGACATTTCTC
CAGGTTTACAAACCTAAAAATTTGTGGGCTACTCTGGTTTTTCCACCTGAGATCTTAGCTGGTTACAA
ATCCTCCGTTCTGCC
+
FFFFFFF,FFFFFFFFFFFFFFFFFFFFFFFFFFFFFFFFFFFFFFFFFFFFF:FFFFFFFFFFFFFF
FFFFFFFFFFFFFFFFFFFFFFFF:FFFFFFFFFFFFFFFFFFFFFFFFFFFFFFFFFFFFFFFFFFF
FFFFFFFFFFFFFFF
@A00155:342:HHGFNDSXY:1:1677:1732:12978 1:N:0:GAACCTAG+TCCGCATA
AGAAGACCAACATTTTCATACATGATGAAATTCATTATTGGTCTCGCGATCAGCTTGAGACATTTCTC
CAGGTTTACAAACCTAAAAATTTGTGGGCTACTCTGGTTTTTCCACCTGAGATCTTAGCTGGTTACAA
ATCCTCCGTTCTGCC
+
FFFF:FFFFF:FFF::FF::FFFFFFFFFF,,FF,FFFF:FFFFFFFFFFFFFFFFFF:FFFFFFFFF
,FF,FFFFFFFFFFFFFFFFFFFFFFF::FFF,F:,,,FFFFFFFFF:FFFF:FFFFFFFFFFFFFFF
FFFFFFFFFFFFFFF

```
@A00155:342:HHGFNDSXY:1:2127:2862:22733 1:N:0:GAACCTAG+TCCGCATA
AGAAGACCATCATTTTCATACATGATGAAATTCATTATTGGTCTCGCGATCAGCTTGAGACATTTCTC
CAGGTTTACAAACCTAAAAATTTGTGGGCTACTCTGGTTTTTCCACCTGAGATCTTAGCTGGTTACAA
ATCCTCCGTTCTGCC
+
F:FF,FF,:,FFFF:F,FF::FFFF:FFFFFFFFFFFFFFFFFFFF:FFFFFFF:FF,F:FFFFFFFFFF
::FF:FFF:FFFF:F::F:FFFFFF:FFFFFFFFFFFFFFFF:FF,,FFFFFFFFFFFFFF:F:FFFF::
FF:FF:FFFFFFF,:
@A00155:342:HHGFNDSXY:1:2120:32633:31610 1:N:0:GAACCTAG+TCCGCATA
AGAAGACCAACATTTTCATACATGATGAAATTCATTATTGGTCTCGCGATCAGCTTGAGACATTTCTC
CAGGTTTACAAACCTAAAAATTTGTGGGCTACTCTGGTTTTTCCACCTGAGATCTTAGCTGGTTACAA
ATCCTCCGTTCTGCC
+
FFFFFF,:FFFFFFFFFFFFFF:FFFFFFFFFFFFFF:FFFFFFFFFFFFFFFFF,FFF:FF:,:FF:FF
:FFFF:F,:,:FFFFFFFFF:FFFFFFFFFFFFFFFFFFFFF:F,FFFFFFFFFFFFFF:FFFFF,F:F
:FFFFF:FFFF:FFF
@A00155:342:HHGFNDSXY:1:2133:3604:5228 1:N:0:GAACCTAG+TCCGCATA
AGAAGACCAACATTTTCATACATGATGAAATTCATTATTGGTCTCGCGATCAGCTTGAGACATTTCTC
CAGGTTTACAAACCTAAAAATTTGTGGGCTACTCTGGTTTTTCCACCTGAGATCTTAGCTGGTTACAA
ATCCTCCGTTCTGCC
+
:FFFF:::FFFFF,FFFFFF:FFFFFFFFFFFFFFFFFF,,FFFF::FFFFFF,FFFFFFFFFFF:FFF:
FFFFFFFFFFFF,FFFFFF::FF,FFF,FFFFFFF:FFFFFFFFFFFFFFFFF:FFFFF,:FF,FF::FF
:FF:FFFFFF,FFFF
@A00155:342:HHGFNDSXY:1:2147:17155:15608 1:N:0:GAACCTAG+TCCGCATA
AGAAGACCAACATTTTCATACATGATGAAATTCATTATTGGTCTCGCGATCAGCTTGAGACATTTCTC
CAGGTTTACAAACCTAAAAATTTGTGGGCTACTCTGGTTTTTCCACCTGAGATCTTAGCTGGTTACAA
ATCCTCCGTTCTGCC
+
FFFFFFFFFFFFFFFFFFFFFFFFFFFFFFFFFFFFFFFFFFFFFFFF:FFFFFFFFFFFFFFFFFFFFF
FFFFFFFFFFFFFFFFFFFFFFFFFFFFFFFFFFFFFFFFFFFFFFFFFFFFFFFFFFFFFFFFFFFFFF
FFFFFFFFFFFFFFF
@A00155:342:HHGFNDSXY:1:2160:25880:2942 1:N:0:GAACCTAG+TCCGCATA
AGAAGACCAACATTTTCATACATGATGAAATTCATTATTGGTCTCGCGATCAGCTTGAGACATTTCTC
CAGGTTTACAAACCTAAAAATTTGTGGGCTACTCTGGTTTTTCCACCTGAGATCTTAGCTGGTTACAA
ATCCTCCGTTCTGCC
+
FFFF:FFFFFFFF:FFFFFFFFFFFFFFFFFFFFFFF:FFFFFFFFFFFFFFFFFFFFFFFF:FFFFFFF
FFF:FFFFFFFFFFFFFFFFFFFFFFFFFFFFFFFFFFFFFFFFFFFFFFFFFFFFFFFFFFFFFFFFFF
FFFFFFFFFFFFFFF
@A00155:342:HHGFNDSXY:1:2165:12970:26522 1:N:0:GAACCTAG+TCCGCATA
AGAAGACCAACATTTTCATACATGATGAAATTCATTATTGGTCTCGCGATCAGCTTGAGACATTTCTC
CAGGTTTACAAACCTAAAAATTTGTGGGCTACTCTGGTTTTTCCACCTGAGATCTTAGCTGGTTACAA
ATCCTCCGTTCTGCC
+
FFFFFFFFFFFFFFFFFFFFFFFFFFFFFFFFFFFFFFFFFFFFFFFFFFFFFFFFFFFFFFFFFFFFFF
FFFFFFFFFFFFFFFFFFF:FFFFFFFFFFFFFFFFFFFFFFFFFFFFFFFFFFFFFFFFFFFFFFFFFF
FFFFFFFFFFFFFFF
@A00155:342:HHGFNDSXY:1:2178:28004:10911 1:N:0:GAACCTAG+TCCGCATA
AGAAGACCAACATTTTCATACATGATGAAATTCATTATTGGTCTCGCGATCAGCTTGAGACATTTCTC
CAGGTTTACAAACCTAAAAATTTGTGGGCTACTCTGGTTTTTCCACCTGAGATCTTAGCTGGTTACAA
ATCCTCCGTTCTGCC
+
FFFFFFFFFFFFFF:FFFFFFFFFFFFFFFFFFFFFFFFFFFFFFFFFFFFFFFFFFFFFFFFFFFFFFF
```

FFFFFFFFFFFFFFFFFFFFFFFFFFFFFFFFFFFFFFFFFFFFFFFFFFFFFFFFFFFFFFFFFFFF
FFFFFFFFFFFFFFF
@A00155:342:HHGFNDSXY:1:2232:1289:16407 1:N:0:GAACCTAG+TCCGCATA
AGAAGACCAACATTTTCATACATGATGAAATTCATTATTGGTCTCGCGATCAGCTTGAGACATTTCTC
CAGGTTTACAAACCTAAAAATTTGTGGGCTACTCTGGTTTTTCCACCTGAGATCTTAGCTGGTTACAA
ATCCTCCGTTCTGCC
+
F,FF,F,,FF:F::::FFFFF::F::FFFF,FFFFFFFFF:FFF:FFFFFF:,FFF:FF:FFFFFFF
FFF::FFF:FFFFFFFFFFFFFFFFFFFFFFFFFFFF::F:FFFFFFFFFFF::FFF:FFFFFFFFFF
FFFF:FFFFFF:FF:
@A00155:342:HHGFNDSXY:1:2218:17662:4679 1:N:0:GAACCTAG+TCCGCATA
AGAAGACCAACATTTTCATACATGATGAAATTCATTATTGGTCTCGCGATCAGCTTGAGACATTTCTC
CAGGTTTACAAACCTAAAAATTTGTGGGCTACTCTGGTTTTTCCACCTGAGATCTTAGCTGGTTACAA
ATCCTCCGTTCTGCC
+
FFFFFFFF:FFFFFFFF:FFFFFFFFFFFFFFFFFFFFFFFFFFFFFFFFFFFFFFFFFFFFFFFFFF
FFFFFFFFFFFFFFFFFFFFFFFFFFFFFFFFFFFFFFFFFFFFFFFFFFFFFFFFFFFFFFFFFFFF
FFFFFFFFFFFFFF:
@A00155:342:HHGFNDSXY:1:2219:14027:12915 1:N:0:GAACCTAG+TCCGCATA
AGAAGACCAACATTTTCATACATGATGAAATTCATTATTGGTCTCGCGATCAGCTTGAGACATTTCTC
CAGGTTTACAAACCTAAAAATTTGTGGGCTACTCTGGTTTTTCCACCTGAGATCTTAGCTGGTTACAA
ATCCTCCGTTCTGCC
+
FFFF:FFFFFFFFFFFFFFFFFFFFFFFFFFFFFFFFFFFFFFFFFFFFFFFFFFFFFFFFFFFFFFF
FFFFFFFFFFFFFFFFFFFFFFFFFFFFFFFFFFFFFFFFFFFFFFF:FFFFFFFFFFFFFFFFFFFF
FFFFFFFFFF:FFFF
@A00155:342:HHGFNDSXY:1:2221:9625:35383 1:N:0:GAACCTAG+TCCGCATA
AGAAGACCAACATTTTCATACATGATGAAATTCATTATTGGTCTCGCGATCAGCTTGAGACATTTCTC
CAGGTTTACAAACCTAAAAATTTGTGGGCTACTCTGGTTTTTCCACCTGAGATCTTAGCTGGTTACAA
ATCCTCCGTTCTGCC
+
F:FF:FFFFFFFFF,FFFFFF,FFFFFFFF,FFFFFFFFFFFFFFFFFFFFFFFFFFFFFFFFFFFFF
FFFFFFFFFF:FFFFFFFFFFFFFFFFFFFFFFFFFFFFFFFFFFFFFFFFFFFFFFFFFFFFFFFFF
FFFFFFFFFFFFFFF
@A00155:342:HHGFNDSXY:1:2278:22489:36370 1:N:0:GAACCTAG+TCCGCATA
AGAAGACCAACATTTTCATACATGATGAAATTCATTATTGGTCTCGCGATCAGCTTGAGACATTTCTC
CAGGTTTACAAACCTAAAAATTTGTGGGCTACTCTGGTTTTTCCACCTGAGATCTTAGCTGGTTACAA
ATCCTCCGTTCTGCC
+
FFFFFFFFF:FFFFFFFFFFFFFFFFFFFFFFFFFFFFFFFFFFFFFFFFFFFFFFFFFFFFFFFFFF
FFFFFFFFFFFFFFFFFFFFFFFFFFFFFFFFFFFFFFFFFFFFFFFFFFFFFFFFFFFFFFFFFFFF
FFFFFFFFFFFFFFF
@A00155:342:HHGFNDSXY:1:2262:1515:34585 1:N:0:GAACCTAG+TCCGCATA
AGAAGACCAACATTTTCATACATGATGAAATTCATTATTGGTCTCGCGATCAGCTTGAGACATTTCTC
CAGGTTTACAAACCTAAAAATTTGTGGGCTACTCTGGTTTTTCCACCTGAGATCTTAGCTGGTTACAA
ATCCTCCGTTCTGCC
+
F:FF,FFFFFFF,FFFFFFFFFF:FFFFFFF:FFFFFFFFFFF:FFFFFFFFFFFFFFFFFFFFFFFF
FFFFFFFFFFFFFFFFFFFFFFFFFFFFFFFFFFFFFFFFFFFFFFFFFFFF:FFFFFFFFFFFFFFF
FFFFFFFFFFFFFFF
@A00155:342:HHGFNDSXY:1:2314:22489:13260 1:N:0:GAACCTAG+TCCGCATA
AGAAGACCAACATTTTCATACATGATGAAATTCATTATTGGTCTCGCGATCAGCTTGAGACATTTCTC
CAGGTTTACAAACCTAAAAATTTGTGGGCTACTCTGGTTTTTCCACCTGAGATCTTAGCTGGTTACAA
ATCCTCCGTTCTGCC

```
+
FFF:FF:::FFFFFFFFFFF:FFFFFFFFFFF::F:FFFFFFFF:F:F,FFFFFFFFFFFFFFFFFFF
FF:FFFFFFFF:FFFFFF,FFFFFFFFFFFFFF:F:FFFFFFF:FFFFFFFFFFFFFFFFFFFFFFFFF
F:FFFFFFFFFFFFFF
@A00155:342:HHGFNDSXY:1:2314:22607:13088 1:N:0:GAACCTAG+TCCGCATA
AGAAGACCAACATTTTCATACATGATGAAATTCATTATTGGTCTCGCGATCAGCTTGAGACATTTCTC
CAGGTTTACAAACCTAAAAATTTGTGGGCTACTCTGGTTTTTCCACCTGAGATCTTAGCTGGTTACAA
ATCCTCCGTTCTGCC
+
FFF:FFFFFFFFFFFFFFFFFFFFFFFFFFF:FFFFFFFFFFFFFFFFFFFFFFFFFFFFFFFFFFFFF
FFFFFFFFFFFFFFFFFFFFFFFFFFFFFFFFFFFFFFFFFFFFFFFFFFFFFFFFFFFFFFFFFFFF:
FFFFFFFFFFFFFFF
@A00155:342:HHGFNDSXY:1:2302:15167:22529 1:N:0:GAACCTAG+TCCGCATA
AGAAGACCAACATTTTCATACATGATTAAATTCATTATTGGTCTCGCGATCAGCTTGAGACATTTCTC
CAGGTTTACAAACCTAAAAATTTGTGGGCTACTCTGGTTTTTCCACCTGAGATCTTAGCTGGTTACAA
ATCCTCCGTTCTGCC
+
FFF,:FFFF,FFFFFFFFFF,FFFFF,FF:FFF:FF,FF::FFFFFFFFF:::FFFFFFFFFFFFFFF
:F:FFFFFF:FFFFFFFFF,FFFFFFFF,F:FFFFFFF:FFF:FFFFFFF:FFFFFF:FFFFFFF:F:
FF::FFFFFFFFFFF
@A00155:342:HHGFNDSXY:1:2311:7473:10520 1:N:0:GAACCTAG+TCCGCATA
AGAAGACCAACATTTTCATACATGATGAAATTCATTATTGGTCTCGCGATCAGCTTGAGACATTTCTC
CAGGTTTACAAACCTAAAAATTTGTGGGCTACTCTGGTTTTTCCACCTGAGATCTTAGCTGGTTACAA
ATCCTCCGTTCTGCC
+
FF,FFFFFF,FFFFF:FFFF,FFFFFFFFFFFFFFFFFFFFFFFFFFFFFFFFFFFFFFFFFFFFFFFF
FFFFFFFFFFFFFFFFFFFFFFFFFFFFFFFFFFFFFFFFFFFFFFFFFFFFFFFFFFFFFFF:FFFFF
FFFFFFFFFFFFFFF
@A00155:342:HHGFNDSXY:1:2315:3893:32503 1:N:0:GAACCTAG+TCCGCATA
AGAACACCAACATTTTCATACATGATGAAATTCATTATTGGTCTCGCGATCAGCTTGAGACATTTCTC
CAGGTTTACAAACCTAAAAATTTGTGGGCTACTCTGGTTTTTCCACCTGAGATCTTAGCTGGTTACAA
ATCCTCCGTTCTGCC
+
FFF,,:FFF,FF:FFFFFF:FFFFFFFFFFFFFFFFFFFFFFFFFFFFFFFFFFFFFFFFFFFFFFFFF
FFFFFFFFFFF,FFFFFFFFFFFFFFFFFFFFFFFFFFFFFFFFFFFFFFFFFFFFFFFFFFFFFFFFF
FFFFFFFFFFFFFFF
@A00155:342:HHGFNDSXY:1:2326:12138:20509 1:N:0:GAACCTAG+TCCGCATA
AGAAGACCAACATTTTCATACATGATGAAATTCATTATTGGTCTCGCGATCAGCTTGAGACATTTCTC
CAGGTTTACAAACCTAAAAATTTGTGGGCTACTCTGGTTTTTCCACCTGAGATCTTAGCTGGTTACAA
ATCCTCCGTTCTGCC
+
FFFFFFFFFFFFFFFFFFFFFFFFFFFFFFFFFFFFFFFFFF:FFFFFFFFFFFFFFFFFFFFFFFFFF
FFFFFFFFFFFFFFFFFFFFFFFFFFFFFFFFFFFFFFFFFFFFFFFFFFFFFFFFFFFFFFFFFFFFF
FFFFFFFFFFFFFFF
@A00155:342:HHGFNDSXY:1:2323:8232:13150 1:N:0:GAACCTAG+TCCGCATA
AGAAGACCAACATTTTCATACATGATGAAATTCATTATTGGTCTCGCGATCAGCTTGAGACATTTCTC
CAGGTTTACAAACCTAAAAATTTGTGGGCTACTCTGGTTTTTCCACCTGAGATCTTAGCTGGTTACAA
ATCCTCCGTTCTGCC
+
FF,FFFF,FF::FFFFFFF,F,FFFFFFF,FF:FFFFFF,F::F:,FFFF:FF,FFFF:,FFFFFFFF
F:FFFFF:FFFFF:FFFFFFFFFFF:FFFF:F,FF:F:FFFF::FFFF,,F:FF::F:F::FFFFFF
FFF:FFFFFFF,,FF
@A00155:342:HHGFNDSXY:1:2305:24740:35321 1:N:0:GAACCTAG+TCCGCATA
AGAAGACCAACATTTTCATACACGATGAAATTCATTATTGGTCTCGCGATCAGCTTGAGACATTTCTC
```

CAGGTTTACAAACCTAAAAATTTGTGGGCTACTCTGGTTTTTCCACCTGAGATCTTAGCTGGTTACAA
ATCCTCCGTTCTGCC
+
F:FF,F,:,,,F,FF::FFFF:F:F:,FFFFF::FFFFFF,FFFFFFFF,FFFF:,FFFF:FFFFFF:FF
FF,:,:FF:FF:F:FFFFFFFFFFFF:::F:FFF:FFFFFFFFFFFFF:FFFFFFFF:FFFFFFFFFFFF
FFFFFFFFF:F,:FF
@A00155:342:HHGFNDSXY:1:2311:23710:33473 1:N:0:GAACCTAG+TCCGCATA
AGAAGACCAACATTTTCATACATGATGAAATTCATTATTGGTCTCGCGATCAGCTTGAGACATTTCTC
CAGGTTTACAAACCTAAAAATTTGTGGGCTACTCTGGTTTTTCCACCTGAGATCTTAGCTGGTTACAA
ATCCTCCGTTCTGCC
+
:F:F,FFFF:FFFFF:FF:FFFFFFFFFFFFFFFFFFFFFFFFFFFFFFFFFFF:FFFFFFFFFFFFFFF
FFFFFFFFFFFFFFFFFFFFFFFFFFFFFFFFFFFF:FFFFFFFFFFFFFFFFFFFFFFFFFFFFFFFFF
FFFFFFFFFFFFFFF
@A00155:342:HHGFNDSXY:1:2312:11921:18787 1:N:0:GAACCTAG+TCCGCATA
CCGATCTCAACATTTTCATACATGATGAAATTCATTATTGGTCTCGCGATCAGCTTGAGACATTTCTC
CAGGTTTACAAACCTAAAAATTTGTGGGCTACTCTGGTTTTTCCACCTGAGATCTTAGCTGGTTACAA
ATCCTCCGTTCTGCC
+
FFFFFFFFFFFFFFFFFFFFFFFFFFFFFFFFFFFFFFFFFFFFFFFFFFFFFFFFFF:FFFFFFFFFFF
FFFFFFFFFFFFFFFFFFFFFFFFFFFFFF:FFFFFFFFFFFFFFFFFFFFFFFFFFFFFFFFFFFFFFF
FFFFFFFFFFFFFFF
@A00155:342:HHGFNDSXY:1:2325:13331:14090 1:N:0:GAACCTAG+TCCGCATA
AGAAGACCAACATTTTCATACATGATGAAATTCATTATTGGTCTCGCGATCAGCTTGAGACATTTCTC
CAGGTTTACAAACCTAAAAATTTGTGGGCTACTCTGGTTTTTCCACCTGAGATCTTAGCTGGTTACAA
ATCCTCCGTTCTGCC
+
FFF:FFFFFFFFFFFFFFFFFFFFFFFFFFFFFFFFFFFFFFFFFFFFFFFFFFFFFFFFFFFFFFFFFF
F,FFFFFFFFFFFFF:FFFFFFFFFFFFFFFFFFFFFFFFFFFFFFFFFFFFFFFFFFFFFFFFFFFFFF
FFFFFFFFFFFFFFF
@A00155:342:HHGFNDSXY:1:2353:19958:17863 1:N:0:GAACCTAG+TCCGCATA
AGAAGACCAACATTTTCATACATGATGAAATTCATTATTGGTCTCGCGATCAGCTTGAGACATTTCTC
CAGGTTTACAAACCTAAAAATTTGTGGGCTACTCTGGTTTTTCCACCTGAGATCTTAGCTGGTTACAA
ATCCTCCGTTCTGCC
+
:FF:FFFFFFFFFF,FFFFFFFFFFFFFFFF:FFFFFFFFFFFFFFFFFFFFF:FFFFFFFFFFFFFFFF
F:FFFFFFFFFFFFFFFFFFFFFFFFFFFF:FFFFFFFFFFFFFFFFFFFFFFFFFFFFFFFFFFFFFFF
FFFFFFFFFFFFFFF
@A00155:342:HHGFNDSXY:1:2358:21612:22044 1:N:0:GAACCTAG+TCCGCATA
TCCGATCTAACATTTTCATACATGATGAAATTCATTATTGGTCTCGCGATCAGCTTGAGACATTTCTC
CAGGTTTACAAACCTAAAAATTTGTGGGCTACTCTGGTTTTTCCACCTGAGATCTTAGCTGGTTACAA
ATCCTCCGTTCTGCC
+
FFFF:FFFFFF,FFFFFFFFFF:FFFFFFFFFFFFFF:FFFFFFFFFFFFFFFFFFFFFFFFFFFFFFFF
F:FFFFFFFFFFFFFFFFFFFFFFFFFFFFFFFFFFFFFFFFFFFFFFFFFFFFFFFFFFFFFFFFFFFF
FFFFFFFFFFFFF:F
@A00155:342:HHGFNDSXY:1:2358:20383:23171 1:N:0:GAACCTAG+TCCGCATA
TCCGATCTAACATTTTCATACATGATGAAATTCATTATTGGTCTCGCGATCAGCTTGAGACATTTCTC
CAGGTTTACAAACCTAAAAATTTGTGGGCTACTCTGGTTTTTCCACCTGAGATCTTAGCTGGTTACAA
ATCCTCCGTTCTGCC
+
FFFFFFFFFFFFF:FFFFFFFFFFFFFFFFFFFFFFFFFFFFFFFFFFFFFFFFFFFFFFFFFFFFFFFF
FFFFFFFFFFFFFFFFFFFFFFFFFFFFFFFFFFFFFFFFFFFFFFFFFFFFFFFFFF:FFFFFFFFFFF
FFFFFFFFFFFFFFF

@A00155:342:HHGFNDSXY:1:2401:5032:17378 1:N:0:GAACCTAG+TCCGCATA
ATAAGACCAACATTTTCATACATGATGAAATTCATTATTGGTCTCGCGATCAGCTTGAGACATTTCTC
CAGGTTTACAAACCTAAAAATTTGTGGGCTACTCTGGTTTTTCCACCTGAGATCTTAGCTGGTTACAA
ATCCTCCGTTCTGCC
+
F,:F:FF:FFFFFF,FFFFFFFF,FFFFFFFFFFFFF:FFFFFFFFFFFFFFFF:FFFF:FFFFFFFF
FFFFFFFFFFFFF,FFFFFFFFFFFFFFFFFFFFFFFFFFFFFFFFFFFFFFFFFFFFF:FFFFFFFF
FFFFFFFFFFFFFFF
@A00155:342:HHGFNDSXY:1:2405:18023:32362 1:N:0:GAACCTAG+TCCGCATA
AGAAGACCAACATTTTCATACATGATGAAATTCATTATTGGTCTCGCGATCAGCTTGAGACATTTCTC
CAGGTTTACAAACCTAAAAATTTGTGGGCTACTCTGGTTTTTCCACCTGAGATCTTAGCTGGTTACAA
ATCCTCCGTTCTGCC
+
FFFFFFFFFFFFFFFFFFFFFFFFFFFFFFFFFFFFFFFFFFFFFFFFFFFFFFFFFFFFFFFFFFFF
FFFFFFFFFFFFFFFFFFFFF:FFFFFFFFFFFFFFFFFFFFFFFFFFFFFFFFFFFFFFFFFFFFFF
FFFFFFFFFFFFFFF
@A00155:342:HHGFNDSXY:1:2403:31367:14606 1:N:0:GAACCTAG+TCCGCATA
AGAAGACCAACATTTTCATACATGATGAAATTCATTATTGGTCTCGCGATCAGCTTGAGACATTTCTC
CAGGTTTACAAACCTAAAAATTTGTGGGCTACTCTGGTTTTTCCACCTGAGATCTTAGCTGGTTACAA
ATCCTCCGTTCTGCC
+
FFFFFFFFFFFFFFFFFFFFFFFFFFFFFFFFFFFFFFFFFFFFFFFFFFFFFFFFFFFFFFF:FFFF
FFFFFFFFFFFFFFFFFFFFFFFFFFFFFFFFFFFFFFFFFFFFFFFFFFFFFFFFFFFFFFFFFFFF
FFFFFFFFFFFFFFF
@A00155:342:HHGFNDSXY:1:2403:32072:15515 1:N:0:GAACCTAG+TCCGCATA
AGAAGACCAACATTTTCATACATGATGAAATTCATTATTGGTCTCGCGATCAGCTTGAGACATTTCTC
CAGGTTTACAAACCTAAAAATTTGTGGGCTACTCTGGTTTTTCCACCTGAGATCTTAGCTGGTTACAA
ATCCTCCGTTCTGCC
+
FFFFFFFFFFFFFFFFFFFFFFFFFFFFFFFFFFFFFFFFFFFFFFFFFFFFFFFFFFFFFFFFFFFF
FFFFFFFFFFFFFFFFFFFFFFFFFFFFFFFFFFFFFFFFFFFFFFFFFFFFFFFFFFFFFFFFFFFF
FFFFFFFFFFFFFFF
@A00155:342:HHGFNDSXY:1:2403:31801:15671 1:N:0:GAACCTAG+TCCGCATA
AGAAGACCAACATTTTCATACATGATGAAATTCATTATTGGTCTCGCGATCAGCTTGAGACATTTCTC
CAGGTTTACAAACCTAAAAATTTGTGGGCTACTCTGGTTTTTCCACCTGAGATCTTAGCTGGTTACAA
ATCCTCCGTTCTGCC
+
:FFFFFFFFFFFFF:FFFFFF:FFFFFFFFFFFFFFFFFFFFFFFFFFFFFFFFFFFFFFFFFFFFFF
F:FFFFFFFFF:FFFFFF:FFFFFFFFFFFFFFFFFFFFFFFFFFFFFFFFFFFFFFFFFFFFFFFFF
FFFFFFFFFFFFFFF
@A00155:342:HHGFNDSXY:1:2412:4915:5462 1:N:0:GAACCTAG+TCCGCATA
AGAAGACCAACATTTTCATACATGATGAAATTCATTATTGGTCTCGCGATCAGCTTGAGACATTTCTC
CAGGTTTACAAACCTAAAAATTTGTGGGCTACTCTGGTTTTTCCACCTGAGATCTTAGCTGGTTACAA
ATCCTCCGTTCTGCC
+
F:FFFFF:FFFF:F:FFFFFFFFFFFFFFFFFFFFFFFFFFFFFFFFFFFFFFFFF:FFFFFFFFFFF
FFFFFFFFFF::FFFFFFFFFFFFF:FFFFFFFFFFFFFFFFFFFFFFFFFFFFF:FFF:FFFFFFFF
FFFFFFFFFFFFFFF
@A00155:342:HHGFNDSXY:1:2377:25084:23860 1:N:0:GAACCTAG+TCCGCATA
CGATCTCCAACATTTTCATACATGATGAAATTCATTATTGGTCTCGCGATCAGCTTGAGACATTTCTC
CAGGTTTACAAACCTAAAAATTTGTGGGCTACTCTGGTTTTTCCACCTGAGATCTTAGCTGGTTACAA
ATCCTCCGTTCTGCC
+
FFFFFFFFFFFFFFFFFFFFFFFFFFFFFFFFFFFFFFFFFFFFFFFFFFFFFFFFFFFFFFFFFFFF

```
FFFFFFFFFFFFFFFFFFFFFFFFFFFFFFFFFFFFFFFFFFFFFFFFFFFFFFFFFFFFFFFFFFFFFF
FFFFFFFFFFFFFFF
@A00155:342:HHGFNDSXY:1:2413:3115:32816 1:N:0:GAACCTAG+TCCGCATA
AGAAGACCAACATTTTCATACATGATGAAATTCATTATTGGTCTCGCGATCAGCTTGAGACATTTCTC
CAGGTTTACAAACCTAAAAATTTGTGGGCTACTCTGGTTTTTCCACCTGAGATCTTAGCTGGTTACAA
ATCCTCCGTTCTGCC
+
FFFFFFFFFFFFFFFFFFFFFFFFFFFFFFFFFFFFFFFFFFFFFFFFFFFFFFFFFFFFFFFFFFFFFF
FFFFFFFFFFFFFFFFFFFFFFFFFFFFFFFFFFFFFFFFFFFFFFFFFFFFFFFFFFFFFFFFFFFFFF
FFFFFFFFFFFFFFF
@A00155:342:HHGFNDSXY:1:2439:16080:13589 1:N:0:GAACCTAG+TCCGCATA
AGAAGACCAACATTTTCATACATGATGAAATTCATTATTGGTCTCGCGATCAGCTTGAGACATTTCTC
CAGGTTTACAAACCTAAAAATTTGTGGGCTACTCTGGTTTTTCCACCTGAGATCTTAGCTGGTTACAA
ATCCTCCGTTCTGCC
+
F:FFFFFFFFFFFFF:FFFFFFFFFFFFFFFFFFFFFFFFFFFFFFFFFFFFFFFFFFFFFFFFFFFFFF
FFFFFFFFFFFFFFFFFFF:FFFFFFFFFFFFFFFFFFFFFF:FFFFFFFFFFFFFFFFFFFFFFFFFFF
FFFFFFFFFFFFFFF
@A00155:342:HHGFNDSXY:1:2430:26901:20306 1:N:0:GAACCTAG+TCCGCATA
AGAAGACCAACATTTTCATACATGATGAAATTCATTATTGGTCTCGCGATCAGCTTGAGACATTTCTC
CAGGTTTACAAACCTAAAAATTTGTGGGCTACTCTGGTTTTTCCACCTGAGATCTTAGCTGGTTACAA
ATCCTCCGTTCTGCC
+
FFFFFFFFFFFFFFFFFFFFFFFFFFFFFFFFFFFFFFFFFFFFFFFFFFFFFFFFFFFFFFFFFFFFFF
FFFFFFFFFFFFFFFFFFFFFFFFFFFFFFFFFFFFFFFFFFFFFFFFFFFFFFFFFFFFFFFFFFFFFF
FFFFFFFFFFFFFFF
@A00155:342:HHGFNDSXY:1:2447:2618:31673 1:N:0:GAACCTAG+TCCGCATA
AGAAGACCAACATTTTCATACATGATGAAATTCATTATTGGTCTCGCGATCAGCTTGAGACATTTCTC
CAGGTTTACAAACCTAAAAATTTGTGGGCTACTCTGGTTTTTCCACCTGAGATCTTAGCTGGTTACAA
ATCCTCCGTTCTGCC
+
FFFFFFFFF:FF:FFFFFFFFFFFFFFFFFFFFFFFFFFFFFFFFFFFFFFFFFFFFFFFFFFFFFFFFF
FFFFFFFFFFFFFFFFFFFFFFFFFFFFFFFFFFFFFFFFFFFFFFFFFFFFFFFFFFFFFFFFFFFFFF
FFFFFFFFFFFFFFF
@A00155:342:HHGFNDSXY:1:2429:25012:23109 1:N:0:GAACCTAG+TCCGCATA
AGAAGACCAACATTTTCATACATGATGAAATTCATTATTGGTCTCGCGATCAGCTTGAGACATTTCTC
CAGGTTTACAAACCTAAAAATTTGTGGGCTACTCTGGTTTTTCCACCTGAGATCTTAGCTGGTTACAA
ATCCTCCGTTCTGCC
+
FFFFFFFFFFFFFFFFFFFFFFFFFFFFFFFFFFFFFFFFFFFFFFFFFFFFF:FFFFFFFFFFFFFFFF
FFFFFFFFFFFFFFFFFFFFFFFFFFFFFFFFFFFFFFFFFFFFFFFFFFFFFFFFFFFFFFFFFFFFFF
FFFFFFFFFFFFFFF
@A00155:342:HHGFNDSXY:1:2511:32262:11459 1:N:0:GAACCTAG+TCCGCATA
AGAAGACCAACATTTTCATACATGATGAAATTCATTATTGGTCTCGCGATCAGCTTGAGACATTTCTC
CAGGTTTACAAACCTAAAAATTTGTGGGCTACTCTGGTTTTTCCACCTGAGATCTTAGCTGGTTACAA
ATCCTCCGTTCTGCC
+
FFF:FFFFFFFFFFFFFFFFFFFFFFFFFFFFFFFFFFFFFFFFFFFFFFFFFFFFFFFFFFFFF:FFFF
FFFFFFFFFF:FFFFFFFFFFFFFFFFFFFFFFFFFFFFFFFFFFFFFFFFFFFFFF:,FFFFFFFFFFF
FFFFFFFFFFFFFFF
@A00155:342:HHGFNDSXY:1:2504:6361:16047 1:N:0:GAACCTAG+TCCGCATA
AGAAGACCAACATTTTCATACATGATGAAATTCATTATTGGTCTCGCGATCAGCTTGAGACATTTCTC
CAGGTTTACAAACCTAAAAATTTGTGGGCTACTCTGGTTTTTCCACCTGAGATCTTAGCTGGTTACAA
ATCCTCCGTTCTGCC
```

```
+
FFFFFFFFFFFFF:FFFFFFFFFFFFFFFFFFFFFFFFFFFFFFFFFFFFFFFFFFFFFFFFFFFFFFF
FFFFFFFFFFFFFFFFFFFFFFFFFFFFFFFFFFFFFFFFFFFFFFFFFFFFFF:FFFFFFFFFFFF:
FFFFFFFFFFFFFFF
@A00155:342:HHGFNDSXY:1:2502:27398:15562 1:N:0:GAACCTAG+TCCGCATA
AGAAGACCAACATTTTCATACATGATGAAATTCATTATTGGTCTCGCGATCAGCTTGAGACATTTCTC
CAGGTTTACAAACCTAAAAATTTGTGGGCTACTCTGGTTTTTCCACCTGAGATCTTAGCTGGTTACAA
ATCCTCCGTTCTGCC
+
FFFFFFF,FFFFF:FF,FFFF:FFFFFFFFFFFFFFFFFFFFFFFFFFFFFFFFFFFFFFFFFFFFFF
FFFFFFFFFFFFFFFFFFFFFFFFFFFFFFFFFFFFFFFFFFFFFFFFFFFFFFFFFFFFFFFFFFFF
FFFFFFFFFFFFFFF
@A00155:342:HHGFNDSXY:1:2561:6144:22780 1:N:0:GAACCTAG+TCCGCATA
AGAAGACCAACATTTTCATACATGATGAAATTCATTATTGGTCTCGCGATCAGCTTGAGACATTTCTC
CAGGTTTACAAACCTAAAAATTTGTGGGCTACTCTGGTTTTTCCACCTGAGATCTTAGCTGGTTACAA
ATCCTCCGTTCTGCC
+
F,:F,FFFFFFF:F,FFFFFFFF,FFFFFFFFFFFFFFFFFFFFFFFFFFFFFFFFFFFFFFFFFFFF
FFFFF:FFFFFFFFF:FFFFF:FFFFFFFFFFFFFFF:FFFFFFFFFFFFFF:FFFFFFFFFFFFFFF
FFFFFFFFFFFFFFF
@A00155:342:HHGFNDSXY:1:2561:6623:23171 1:N:0:GAACCTAG+TCCGCATA
AGAAGACCAACATTTTCATACATGATGAAATTCATTATTGGTCTCGCGATCAGCTTGAGACATTTCTC
CAGGTTTACAAACCTAAAAATTTGTGGGCTACTCTGGTTTTTCCACCTGAGATCTTAGCTGGTTACAA
ATCCTCCGTTCTGCC
+
FFF:F:FFFFFFFFFFFFFFFFFFFFFFFFFFFFFFFFFFFFFFFFFFFFFFFFFFFFFFFFFFFFFF
FFFFFFFFFFFFFFFFFFFFFFFFFFFFFFFFFF:FFFFFFFFFFFFFFFFFFFFFFFFFFFFFFFFF
FFF:FFFFFFFFFFF
@A00155:342:HHGFNDSXY:1:2616:24921:23766 1:N:0:GAACCTAG+TCCGCATA
AGAAGACCAACATTTTCATACATGATGAAATTCATTATTGGTCTCGCGATCAGCTTGAGACATTTCTC
CAGGTTTACAAACCTAAAAATTTGTGGGCTACTCTGGTTTTTCCACCTGAGATCTTAGCTGGTTACAA
ATCCTCCGTTCTGCC
+
FFFFF:FFFFFFFFFFFFFFF,FFFFFFFFFFFFFFFFFFFFFFFFFFFFFFFFFFFFFFFFFFFFFF
FFFFFFFFFFFFFF:FFFFFFFFFFFFFFFFFFFFFFFFFFFFFFFFFFFFFFFFF:FFFFFFFFFFF
FFFFFFFFFFFFFF:
@A00155:342:HHGFNDSXY:1:2636:7464:4523 1:N:0:GAACCTAG+TCCGCATA
AGAAGACCAACATTTTCATACATGATGAAATTCATTATTGGTCTCGCGATCAGCTTGAGACATTTCTC
CAGGTTTACAAACCTAAAAATTTGTGGGCTACTCTGGTTTTTCCACCTGAGATCTTAGCTGGTTACAA
ATCCTCCGTTCTGCC
+
FFFF,::FFFFF,FFF:FFFF:FFFF,FFFF:FFF:FFFFFFFFFFFFFFFFFFFFFFF,F,FFFFFFF
FFFFFFFFFFFFFFFFFFFFFFFFFFFFFFFFFFFFFFFFFFFFFFFFFFFFFFFFF:FFFFFFFFFF
FFFFFFFFFFFFF:F
@A00155:342:HHGFNDSXY:1:2615:22128:26569 1:N:0:GAACCTAG+TCCGCATA
AGAAGACCAACATTTTCATACATGATGAAATTCATTATTGGTCTCGCGATCAGCTTGAGACATTTCTC
CAGGTTTACAAACCTAAAAATTTGTGGGCTACTCTGGTTTTTCCACCTGAGATCTTAGCTGGTTACAA
ATCCTCCGTTCTGCC
+
FFFFFFFFFFFFFFFFFFFF:FFFFFFFFFFFFFFFFFFFFFFFFFFFFFFFFFFFFFFFFFFFFFFF
FFFFFFFFFFFFFFFFFFFFFFFFFFFFFFFFFFFFFFFFFFFFFFFFFFFFFFFFFFFFFFFFFFFF
FFFFFFFFFFFFFFF
@A00155:342:HHGFNDSXY:1:2655:28320:30624 1:N:0:GAACCTAG+TCCGCATA
TCCGATCTAACATTTTCATACATGATGAAATTCATTATTGGTCTCGCGATCAGCTTGAGACATTTCTC
```

```
CAGGTTTACAAACCTAAAAATTTGTGGGCTACTCTGGTTTTTCCACCTGAGATCTTAGCTGGTTACAA
ATCCTCCGTTCTGCC
+
FF,FFFFFFF:,F:FFFFFFFFF:FFFF,FFFFFFFFFF,FF:F:F:F,F::FFFF,:F::FFFFFFF
F:FFFFFFFFFFFFF,FF,,FF:FFF:FFF:FF:FFFFFFFF:F::FFFFFFFFF,FF:FFFFFFF:F
FF::FF::FFFFFFF
@A00155:342:HHGFNDSXY:1:2663:20184:23954 1:N:0:GAACCTAG+TCCGCATA
AGAAGACCAACATTTTCATACATGATGAAATTCATTATTGGTCTCGCGATCAGCTTGAGACATTTCTC
CAGGTTTACAAACCTAAAAATTTGTGGGCTACTCTGGTTTTTCCACCTGAGATCTTAGCTGGTTACAA
ATCCTCCGTTCTGCC
+
FFFFFFFFFFFFFFFFFFFFFFFFFFFFFFFFFFFFFFFFFFFFFFFFFFFFFFFFFFFFFFFFFFFF
FFFFFFFFFFFFFFFFFFFFFFFFFFFFF:FFFFFFFFFFFFFFFFFFFFFFFFFFFFFFFFFFFFFF
FFFFFFFFFFFFFFF
@A00155:342:HHGFNDSXY:1:2657:30906:4914 1:N:0:GAACCTAG+TCCGCATA
AGAAGACCAACATTTTCATACATGATGAAATTCATTATTGGTCTCGCGATCAGCTTGAGACATTTCTC
CAGGTTTACAAACCTAAAAATTTGTGGGCTACTCTGGTTTTTCCACCTGAGATCTTAGCTGGTTACAA
ATCCTCCGTTCTGCC
+
FFFFFFFFF:F:FFFFFFFFF:FFFFFFFFFFFFFFFFFFFFFFFFFFF:FFFFFFFFFFFFFFFFFF
FFFFFFFFFFFFFFFFFFFFFFFFFFFFFFFFFFFFFFFFFFFFFFFFFFFFFFFFFFFFFFFFFFFF
FFFFFFFFFFFFFFF
@A00155:342:HHGFNDSXY:1:2672:25626:4257 1:N:0:GAACCTAG+TCCGCATA
AGAAGACCAACATTTTCATACATGATGAAATTCATTATTGGTCTCGCGATCAGCTTGAGACATTTCTC
CAGGTTTACAAACCTAAAAATTTGTGGGCTACTCTGGTTTTTCCACCTGAGATCTTAGCTGGTTACAA
ATCCTCCGTTCTGCC
+
FFF:FFFFFFFFFFFFFFFFFFFFFFFFFFFFFFFFFFFFFFFFFFF:FFFF:FFFFFFFFFFFFFFF
FFFF:FFFFFFFFFFFFFFFFFFFFFFFFFFFF:FFFFFFFFFFFFFFFFFFFFFFFFFFFFFFFFFF
FFFFFFFFFFFFFFF
@A00155:342:HHGFNDSXY:1:2675:29143:33082 1:N:0:GAACCTAG+TCCGCATA
CCGATCTCAACATTTTCATACATGATGAAATTCATTATTGGTCTCGCGATCAGCTTGAGACATTTCTC
CAGGTTTACAAACCTAAAAATTTGTGGGCTACTCTGGTTTTTCCACCTGAGATCTTAGCTGGTTACAA
ATCCTCCGTTCTGCC
+
FFFFFFFF:FFFFFFFFFFFFFFFFFFFFFFFFFFFFFFFFFFFFFFFFFFFFFFFFFFFFFFFFFFF
FFFF:FFFFFFFFFFFFFFFFFFFFFFFFFFFFFFFFFFFFFFFFFFFFFFFFFFFFFFFFFFFFFFF
FFFFFFFFFFFFFFF
@A00155:342:HHGFNDSXY:1:1309:15411:8797 2:N:0:GAACCTAG+TCCGCATA
AGAAGACCAACATTTTCATACATGATGAAATTCATTATTGGTCTCGCGATCAGCTTGAGACATTTCTC
CAGGTTTACAAACCTAAAAATTTGTGGGCTACTCTGGTTTTTCCACCTGAGATCTTAGCTGGTTACAA
ATCCTCCGTTCTGCC
+
FFFFFFFFFFFFFFFFFFFFFFFFFFFFFFFFFFFFFFFFFFFFFFFFFFFFFFFFFFFFFFFFFFFF
FFFFFFFFFFFFFFFFFFFFFFFFFFFFFFFFFFFFFFFFFFFFFFFFFFFFFFFFFFFFFFFFFFFF
FFFFFFFF:FFFFFF
@A00155:342:HHGFNDSXY:1:1315:18132:14732 2:N:0:GAACCTAG+TCCGCATA
AGAAGACCAACATTTTCATACATGATGAAATTCATTATTGGTCTCGCGATCAGCTTGAGACATTTCTC
CAGGTTTACAAACCTAAAAATTTGTGGGCTACTCTGGTTTTTCCACCTGAGATCTTAGCTGGTTACAA
ATCCTCAGATCGGAA
+
FFFFFFFFFFFFFFFFFFFFFFFFFFFFFFFFFFFFFFFFFFFFFFFFFFFFFFFFFFFFFFFFFFFF
FFFFFFFFFFFFFFFFFFFFFFFFFFFFFFF:FFFFFFFFFFFFFFFFFFFF:FFFFFFFFFFFFFFF
FFFFFFFFFFFFFFF
```

```
@A00155:342:HHGFNDSXY:1:1315:17806:23813 2:N:0:GAACCTAG+TCCGCATA
AGAAGACCAACATTTTCATACATGATGAAATTCATTATTGGTCTCGCGATCAGCTTGAGACATTTCTC
CAGGTTTACAAACCTAAAAATTTGTGGGCTACTCTGGTTTTTCCACCTGAGATCTTAGCTGGTTACAA
ATCCTCAGATCGGAA
+
FFFFFFFFFFFFFFFFF,FF:FFFFFF,F:,FF,FF::FFFFFFFFFF:FFFFFFFF,FFF,FFFFFF
FFFF:FFFFFFF:FF,FFF::FFFFFFFFFFFFFFF,FFF:FFF,FFFFFFFFF,FFF:FFF:FFFFF
,FFFFF:FFFFFF:F
@A00155:342:HHGFNDSXY:1:1411:30861:6840 2:N:0:GAACCTAG+TCCGCATA
AGAAGACCAACATTTTCATACATGATGAAATTCATTATTGGTCTCGCGATCAGCTTGAGACATTTCTC
CAGGTTTACAAACCTAAAAATTTGTGGGCTACTCTGGTTTTTCCACCTGAGATCTTAGCTGGTTACAA
ATCCTCCGTTCTGCC
+
FFFFFFFFFFFFFFFFFFFFFFFFFFF:FF:FFFFFFFFFFFFFFFFFFFF:FFFFFFFFFFFFFFFF
FFFFFFFFFFFFFFFF,FFFFFFFFFFFFF:F:FFFFFFFFFFFFFFFFFFFFFFFFF:FFFFFFFF::
FFFFFFFFFFF:FFF
@A00155:342:HHGFNDSXY:1:1411:6325:31829 2:N:0:GAACCTAG+TCCGCATA
AGAAGACCAACATTTTCATACATGATGAAATTCATTATTGGTCTCGCGATCAGCTTGAGACATTTCTC
CAGGTTTACAAACCTAAAAATTTGTGGGCTACTCTGGTTTTTCCACCTGAGATCTTAGCTGGTTACAA
ATCCTCCGTTCTGCC
+
FFFFFFFFFFFFFFFFFFFFFFFFFFFFFFFFFFFFFFFFFFFFFFFFFFFFFFFFFFFFFFFFFFFF
FFFFFFFFFFFFFFFFFFFFFFFFFFFFFFFFFFFFFFFFFFFF:FF,FFFFFFFFFFFFFFFFFFFFF
FFFFFFFFFFFFFFF
@A00155:342:HHGFNDSXY:1:1441:18954:34319 2:N:0:GAACCTAG+TCCGCATA
AGAAGACCAACAATTTCATACATGATGAAATTCATTATTGGTCTCGCGATCAGCTTGAGACATTTCTC
CAGGTTTACAAACCTAAAAATTTGTGGGCTACTCTGGTTTTTCCACCTGAGATCTTAGCTGGTTACAA
ATCCTCCGTTCTGCC
+
FFFFFFFFFFFF,FFF:FFFFFFFFFFFFFFFFF:FFFFFFFFFFFFFFFFFFFFFFFFFFFFFFFFFF
FFFFFFFFFFFF,FFFFFFFFFFFFFFFFFFFFF:FFFF:FFFFF:FFFFFFFFFFFFFFFFFFFFFF
FFFFFF:F:FFFFFF
@A00155:342:HHGFNDSXY:1:1511:26413:6558 2:N:0:GAACCTAG+TCCGCATA
AGAAGACCAACATTTTCATACATGATGAAATTCATTATTGGTCTCGCGATCAGCTTGAGACATTTCTC
CAGGTTTACAAACCTAAAAATTTGTGGGCTACTCTGGTTTTTCCACCTGAGATCTTAGCTGGTTACAA
ATCCTCCGTTCTGCC
+
FFF:FFFFF:FFFFFFFFFFFFFFFFFFFFFFFFFFFFFFFFFFFFFFFFFFFFFFFFFFFFFFFFFF
:FFFFFFFFFFFFFFFFFFFFFFFFFF:FFFFFFFFFFFFFFFFFFFFFFFFFFFFFF:FFFFFFFF:
FFFFFFFFFF:FFFF
@A00155:342:HHGFNDSXY:1:1511:26874:6637 2:N:0:GAACCTAG+TCCGCATA
AGAAGACCAACATTTTCATACATGATGAAATTCATTATTGGTCTCGCGATCAGCTTGAGACATTTCTC
CAGGTTTACAAACCTAAAAATTTGTGGGCTACTCTGGTTTTTCCACCTGAGATCTTAGCTGGTTACAA
ATCCTCCGTTCTGCC
+
FFFFFFFFFFFF:FFFFFFFFFFFFFFF:F:FFFFFFF:FFFFFFFFFFFFFFFFFFFFFFFFFFFFFF
FFFFFFFFFFF,FFFFFFFFF:FFFFFFFF:FFFFFFFF:FFFFFFFFFF:FFFF:FF:FFFFFFFF:
FFFFFFFFFFFFFFF
@A00155:342:HHGFNDSXY:1:1532:18041:21183 2:N:0:GAACCTAG+TCCGCATA
AGAAGACCAACATTTTCATACATGATGAAATTCATTATTGGTCTCGCGATCAGCTTGAGACATTTCTC
CAGGTTTACAAACCTAAAAATTTGTGGGCTACTCTGGTTTTTCCACCTGAGATCTTAGCTGGTTACAA
ATCCTCCGTTCTGCC
+
:FFFFFFFFFFFF:FFFFFFFFFFFFFFFFFFFF:FFFFFFF:FFFFFF:FFFFFFFFFFFFF:FFFF
```

FFF:FFFFFFFFFFFFFFFF:FFFFFFFFFFFFFFFF,F:FFF:FF:FFFFFFFF:FFFFFFFFFFFF
FFFFFFFF:FFFFFF
@A00155:342:HHGFNDSXY:1:1535:12816:27258 2:N:0:GAACCTAG+TCCGCATA
AGAAGACCAACATTTTCATACATGATGAAATTCATTATTGGTCTCGCGATCAGCTTGAGACATTTCTC
CAGGTTTACAAACCTAAAAATTTGTGGGCTACTCTGGTTTTTCCACCTGAGATCTTAGCTGGTTACAA
ATCCTCCGTTCTGCC
+
FFFFFFFFFFFFF:FFFFFFFFFFFFFFF,FFFF:FFFFFFFFFFFFFFFFFFFFFFFFFFFFFFFFF
FFFFFFFFFFFFFFFFFFFFFFFFFFFFFFFFFFFFFFFFFFF,FFFFFFFFFFFFFFFFFFFFFFFF
FFFFFFFFFFFFFFF
@A00155:342:HHGFNDSXY:1:1609:28022:2769 2:N:0:GAACCTAG+TCCGCATA
AGAAGACCAACATTTTCATACATGATGAAATTCATTATTGGTCTCGCGATCAGCTTGAGACATTTCTC
CAGGTTTACAAACCTAAAAATTTGTGGGCTACTCTGGTTTTTCCACCTGAGATCTTAGCTGGTTACAA
ATCCTCCGTTCTGCC
+
F:FFFFFFFFFFFFFFFFFFFFFFFFFF:F:FFFFFFFFFFFFFFFFFFFFFFFFFFFFFFFFFFFFF
FFFFFFFFFFFFFFFFFFFFF:FF:FFFFFF::FFFFFFFFFF:FFFFFFFFFFFFFFFFFF:FFFFF
FFFFFFFFFFFFFFF
@A00155:342:HHGFNDSXY:1:1609:27281:2957 2:N:0:GAACCTAG+TCCGCATA
AGAAGACCAACATTTTCATACATGATGAAATTCATTATTGGTCTCGCGATCAGCTTGAGACATTTCTC
CAGGTTTACAAACCTAAAAATTTGTGGGCTACTCTGGTTTTTCCACCTGAGATCTTAGCTGGTTACAA
ATCCTCCGTTCTGCC
+
FFFFFFFFFFFFFFFFFFFFFFFFFFFF:FFFFFFFFFFFFFFFFFFFFFFFFFFFFFFFFFFFFFFF
FFFFFFFFFFF:FFFFFFF:F,FFFFFFFFFFFFFFFFFFFFFFFF:FFFFFFFF,F:FFFFFFFFFF
FFFFFFFFFFFFFFF
@A00155:342:HHGFNDSXY:1:2155:20772:2957 2:N:0:GAACCTAG+TCCGCATA
AGAAGACCAACATTTTCATACATGATGAAATTCATTATTGGTCTCGCGATCAGCTTGAGACATTTCTC
CAGGTTTACAAACCTAAAAATTTGTGGGCTACTCTGGTTTTTCCACCTGAGATCTTAGCTGGTTACAA
ATCCTCCGTTCTGCC
+
FFF:FFFFFFFFFF:FFFFFFFFFFFFFFFFFFFFFFFFFFFFFFFFFFFFFFFFFFFFFFF:FFFFF
FFFFFFFFFFFFFFF:FFFFFFFFFFFFFFFFFFFFFF:FFFFFFFFFFFFFFFFFFFFFFFFFFFFF
FFFFFFFFFFFFFFF
@A00155:342:HHGFNDSXY:1:2124:29333:10300 2:N:0:GAACCTAG+TCCGCATA
AGAAGACCAACATTTTCATACATGATGAAATTCATTATTGGTCTCGCGATCAGCTTGAGACATTTCTC
CAGGTTTACAAACCTAAAAATTTGTGGGCTACTCTGGTTTTTCCACCTGAGATCTTAGCTGGTTACAA
ATCCTCCGTTCTGCC
+
FFFFFFFFFFFFFFFFFFFFFFFFFFFFFFF:FFFFFFFFFFFFFFFFFFFFFFFFFFFFFF::FFFF
FFFFFFFFFFFFFFFFFFFFFFFFFF:FFF:FFFFFFFF:FFFFFFFFFFFFFFF,FFF:FF,FFFFF
FFF:FFFFFFFFFFF
@A00155:342:HHGFNDSXY:1:2303:16333:14904 2:N:0:GAACCTAG+TCCGCATA
AGAAGACCAACATTTTCATACATGATGAAATTCATTATTGGTCTCGCGATCAGCTTGAGACATTTCTC
CAGGTTTACAAACCTAAAAATTTGTGGGCTACTCTGGTGTTTCCACCTGAGATCTTAGCTGGTTACAA
ATCCTCCGTTCTGCC
+
FFFFFFFFFFFFFF:FFF,FFFFFFFFFFFFFFF:FF:FFFFFFFFFFFFFFFFFFFFFFFFFFFFFF
F:FFFFFFFF:FFFFFFFFFFFFFFFFFFFF:FFFFFF,FFFFFFFFFFFFFFFFFFFFFFFF:FFFF
FFFFFF:FFFFFFFF
@A00155:342:HHGFNDSXY:1:2356:29749:27305 2:N:0:GAACCTAG+TCCGCATA
AGAAGACCAACATTTTCATACATGATGAAATTCATTATTGGTCTCGCGATCAGCTTGAGACATTTCTC
CAGGTTTACAAACCTAAAAATTTGTGGGCTACTCTGGTTTTTCCACCTGAGATCTTAGCTGGTTACAA
ATCCTCCGTTCTGCC

```
+
FFF:FFFFFFFFFFFFFFFFFFFFFFFFF:F::FFFFFFFFFFFFFFFFFFFFFFFFFFFFFFF:FFFFF
FFFFFFFFFFFFFF:FFFFFFF,:F:F:FFFFFFFFFFF::FF,FF:FFF:FFFFFFFF:FF:FFFFF
FFFFFF:F:FFFFFF
@A00155:342:HHGFNDSXY:1:2430:21847:36292 2:N:0:GAACCTAG+TCCGCATA
AGAAGACCAACATTTTCATACATGATGAAATTCATTATTGGTCTCGCGATCAGCTTGAGACATTTCTC
CAGGTTTACAAACCTAAAAATTTGTGGGCTACTCTGGTTTTTCCACCTGAGATCTTAGCTGGTTACAA
ATCCTCCGTTCTGCC
+
FFF:FFFFFFFFFFFFFFFFFFFFFFFFFFFFFFFFFFFFFFFFFFFF:FFFFFFFFFFFF:FFFF
FFFFFFFFFF::FFFFFF:F:FFFFFFFFFF:FFFFF::FFFFFFFFFFFFF:FFFFFF,FFFFFFFF
FFFFFFFFFFFFFF:
@A00155:342:HHGFNDSXY:1:2447:4381:19382 2:N:0:GAACCTAG+TCCGCATA
AGAAGACCAACATTTTCATACATGATGAAATTCATTATTGGTCTCGCGATCAGCTTGAGACATTTCTC
CAGGTTTACAAACCTAAAAATTTGTGGGCTACTCTGGTTTTTCCACCTGAGATCTTAGCTGGTTACAA
ATCCTCCGTTCTGCC
+
FFFFFFFFFFFFFFFFFF:FFFFFFFFFF:FFFFFF:FFFFFFFFFFFFFFFFFFFFFFFF:FFFFFF
FFFFFFFFFFFFFFFFF:FFFFFFFFFFFFF:FFFF:FFFFFFFFF:FFFFFFFFFF,FFFFFFFFF:
FF:FFF:FFFFFF,F
@A00155:342:HHGFNDSXY:1:2532:18331:15640 2:N:0:GAACCTAG+TCCGCATA
AGAAGACCAACATTTTCATACATGATGAAATTCATTATTGGTCTCGCGATCAGCTTGAGACATTTCTC
CAGGTTTACAAACCTAAAAATTTGTGGGCTACTCTGGTTTTTCCACCTGAGATCTTAGCTGGTTACAA
ATCCTCCGTTCTGCC
+
FFFFFFFFFFFFFFFFFFFFFFFFFFFFFFFFFFFFFFFFFFFFFFFFFFFFFFFFFFFFFFFF:FFF
FFFFFFFFFFF:FFFFFFFF:FFFFFFFFFFFFFFFFFFFFFF,FFFFFFFFFFFFFFFFFFFFFFFF
FFFFFFFFFFFFFFF
@A00155:342:HHGFNDSXY:1:2550:9525:7905 2:N:0:GAACCTAG+TCCGCATA
AGAAGACCAACATTTTCATACATGATGAAATTCATTATTGGTCTCGCGATCAGCTTGAGACATTTCTC
CAGGTTTACAAACCTAAAAATTTGTGGGCTACTCTGGTTTTTCCACCTGAGATCTTAGCTGGTTACAA
ATCCTCCGTTCTGCC
+
FFFFFFFFFFFFFFFFFFFFFFFFFFFFFFFFFFFFFFFFFFFFFFFFFFFFFFFFFFFFFFFFFFFF
FFFFFFFFFFFFFFFFFFFFFFFFFFFFFFFFFFFFFFFFFFFFFFFFFFFFFFFFFFFFFFFFFFFF
FFFFFFFFFFFFFFF
@A00155:342:HHGFNDSXY:1:2626:31322:6449 2:N:0:GAACCTAG+TCCGCATA
AGAAGACCAACATTTTCATACATGATGAAATTCATTATTGGTCTCGCGATCAGCTTGAGACATTTCTC
CAGGTTTACAAACCTAAAAATTTGTGGGCTACTCTGGTTTTTCCACCTGAGATCTTAGCTGGTTACAA
ATCCTCCGTTCAGAT
+
FFF:FFFFFFFFFFFFFFFFFFFFFFFFF,FFFFFFFFFFFFFFFFFFFFFFFFFFFFFFFFFFFFFF
FFFFFFFFFFFFFFFFFFFFFFFFFFFFFFF:FFFFFFFFFFFFFFFFFFFFF:F,FFFFFFFFFFFF
FFFFFFFFFFFFFFF
@A00155:342:HHGFNDSXY:1:2662:9435:33959 2:N:0:GAACCTAG+TCCGCATA
AGAAGACCAACATTTTCATACATGATGAAATTCATTATTGGTCTCGCGATCAGCTTGAGACATTTCTC
CAGGTTTACAAACCTAAAAATTTGTGGGCTACTCTGGTTTTTCCACCTGAGATCTTAGCTGGTTACAA
ATCCTCCGTTCTGCC
+
FFFFFFFFFFFFFFFFFFFFFFFFFFFFFFFFFFFFFFFFFF:FFFFFFFFFFFFFFFFFFFFFFFFFF
FFFFFFFFFFFFF:FFFFFFFFFFFFFFFFFFFFFFFFFFFFFF,FFFFFFFFFFFFFFFFFFFFFFFF
FFFFFFFFFFFFF,F
@A00155:342:HHGFNDSXY:1:2635:14118:6120 2:N:0:GAACCTAG+TCCGCATA
AGAAGACCAACATTTTCATACATGATGAAATTCATTATTGGTCTCGCGATCAGCTTGAGACATTTCTC
```

```
CAGGTTTACAAACCTAAAAATTTGTGGGCTACTCTGGTTTTTCCACCTGAGATCTTAGCTGGTTACAA
ATCCTCCGTTCTGCC
+
FFFFFFFFFFFFFFFF:FFFFFFFFFFFFF:FF:FFFFFFFFFFFFFFFFFFFFFFFFFFFF:FFFFF
FFFFFFFFFFFFFFFFFFFFFFFFFFFFFFFFFFFFFFFFFFFFFFFFFFFFFF:FFFFFFFFFFFFF
FFFFFFFFFFFFFFF
@A00155:342:HHGFNDSXY:1:1660:1787:36307 2:N:0:GAACCTAG+TCCGCATA
GAAGACCAACATTTTCATACATGATGAAATTCATTATTGGTCTCGCGATCAGCTTGAGACATTTCTCC
AGGTTTACAAACCTAAAAATTTGTGGGCTACTCTGGTTTTTCCACCTGAGATCTTAGCTGGTTACAAA
TCCTCCGTTCTGC
+
:FFF:F,F:FFFFFF:FFFFFFF,FFFF,FF:FFFFFFFFFFFFFFFFFFFF:FFFFFFFFFFFFFFF
FFFFFFFFFF:F:FFFFFFFFFFFFFFFFF:F:FFFFFFFF:FF:FFFFF:F::FFF:FFFFFFFFF:
FF:FFFFFFFFFF
@A00155:342:HHGFNDSXY:1:1374:14615:34350 1:N:0:GAACCTAG+TCCGCATA
GAAGACCAACATTTTCATACATGATGAAATTCATTATTGGTCTCGCGATCAGCTTGAGACATTTCTCC
AGGTTTACAAACCTAAAAATTTGTGGGCTACTCTGGTTTTTCCACCTGAGATCTTAGCTGGTTACAAA
TCCTCCGTTCTGCC
+
FFF:,FFF:FFFFFFFFFFFFFFFFFFFF:FFFFFFFFFFFFFFFFFFF:FFFFFFFFFFFFFFFFFF
FFFFFFFFFFFFFFFFFFFFFFFFFFFFFFFFFFFFFFFFFFFFFFFFFFF:FFF,FFFFFFFFFFFF
FFFFFFFFFFFFFF
@A00155:342:HHGFNDSXY:1:2149:3378:12383 1:N:0:GAACCTAG+TCCGCATA
GAAGACCAACATTTTCATACATGATGAAATTCATTATTGGTCTCGCGATCAGCTTGAGACATTTCTCC
AGGTTTACAAACCTAAAAATTTGTGGGCTACTCTGGTTTTTCCACCTGAGATCTTAGCTGGTTACAAA
TCCTCCGTTCTGCC
+
FFFFFFFFF:FFFFF,FFFFFFFFFFFFFFFFFFFFFFFFFFFFFFFFFFFFFFFFFFFFFFFFFFFF
FFFFFFFFFFFFFFFFFFFFFFFFFFFFFFFFFFFFFFFFFFFFFFFFFFFFFFFFFFFFFFFFFFFF
FFFFFFFFFFFFFF
@A00155:342:HHGFNDSXY:1:1154:2202:2519 1:N:0:GAACCTAG+TCCGCATA
GAAGACCAACATTTTCATACATGATGAAATTCATTATTGGTCTCGCGATCAGCTTGAGACATTTCTCC
AGGTTTACAAACCTAAAAATTTGTGGGCTACTCTGGTTTTTCCACCTGAGATCTTAGCTGGTTACAAA
TCCTCCGTTCTGCCC
+
:FF:FFFF,FFFFFFFFFFF:FFFFFFFFF:FFF:FFFFFFFFFFFFFFFFFFFFFFFFFFFFFFFFF
FFFFFFFFFFFFFFFFFFFFFFFFFFFFFFFFFFFFFFFFFFFFFFFFFFFFFFFFFFFFFFFFF:FF
FFFFFFFFFFFFF:F
@A00155:342:HHGFNDSXY:1:1505:12933:34319 1:N:0:GAACCTAG+TCCGCATA
GAAGACCAACATTTTCATACATGATGAAATTCATTATTGGTCTCGCGATCAGCTTGAGACATTTCTCC
AGGTTTACAAACCTAAAAATTTGTGGGCTACTCTGGTTTTTCCACCTGAGATCTTAGCTGGTTACAAA
TCCTCCGTTCTGCCG
+
FFF:FFFF,F,FFFFFFFFFFFFFFFFFFFFFFFFFFFFFFFFFFFFFFFFFFFFFFFFFFFFFFFFF
FFFFFFFFFFFFFFFFFFFFFFFFFFFFFFFFFFFFFFFFFFFFFFFFFFFFFFFF,FFFFFFFFFFFF
FFFFFFFFFFFFFFF
@A00155:342:HHGFNDSXY:1:1503:17418:5321 1:N:0:GAACCTAG+TCCGCATA
GAAGCCCAACATTTTCATACATGATGAAATTCATTATTGGTCTCGCGATCAGCTTGAGACATTTCTCC
AGGTTTACAAACCTAAAAATTTGTGGGCTACTCTGGTTTTTCCACCTGAGATCTTAGCTGGTTACAAA
TCCTCCGTTCTGCCC
+
:FFF,FFFFFFFFFFFFFFFFFFFFFFFFFFFFFFFFFFFFFFFFFFFFFFFFFFFFFFFFFFFFFFF
FFFF:FFFFFFFFFFFFFFFFFFFFFFFFFFFFFFFFFFFFFFFFFFFFFFFFFFFFFFFFFFFFFFF
FFFFFFFFFFFFFFF
```

@A00155:342:HHGFNDSXY:1:1535:12816:27258 1:N:0:GAACCTAG+TCCGCATA
GAAGACCAACATTTTCATACATGATGAAATTCATTATTGGTCTCGCGATCAGCTTGAGACATTTCTCC
AGGTTTACAAACCTAAAAATTTGTGGGCTACTCTGGTTTTTCCACCTGAGATCTTAGCTGGTTACAAA
TCCTCCGTTCTGCCC
+
:FFFFFFFFF:::FF,FF:FFFFFFFFFFFFFFFF:FF:FFFFFF:FFFFFFFFFFFFFF:FFFFFFF
:FFFFFFFFFFFFFFFFFFFFFFFFFFFF:FFFFFFFFFFFFFFFFFFFFFFFFFFF::FFFFFFFFF
FFFFFFFFFFFFFFF
@A00155:342:HHGFNDSXY:1:1604:24777:3787 1:N:0:GAACCTAG+TCCGCATA
GAAGACCAACATTTTCATACATGATGAAATTCATTATTGGTCTCGCGATCAGCTTGAGACATTTCTCC
AGGTTTACAAACCTAAAAATTTGTGGGCTACTCTGGTTTTTCCACCTGAGATCTTAGCTGGTTACAAA
TCCTCCGTTCTGCCC
+
:FFF:FFFFFFFFF:FFFFFFFFFFFFFFFFFFFFFFFFFFFF:F:FFFFFFFFFFFFFFFFFFFFFF
F:FFFFFFFFF:FFFFFFFFFFFFFFFFFFFFFFFF,FFFFFFFFFFFFFFFFFFFFFFFFFFFFFFF
FFFFFFFFFFFFFFF
@A00155:342:HHGFNDSXY:1:2172:28836:6621 1:N:0:GAACCTAG+TCCGCATA
GAAGACCAACATTTTCATACATGATGAAATTCATTATTGGTCTCGCGATCAGCTTGAGACATTTCTCC
AGGTTTACAAACCTAAAAATTTGTGGGCTACTCTGGTTTTTCCACCTGAGATCTTAGCTGGTTACAAA
TCCTCCGTTCTGCCC
+
:FFFFFFFFFFFFFF:FFFFFFFFFFFFFFFFFFFFFFFFF:FFFF:FFFFFFFFFFFFFFFFFFFFF
FF:FFFFFFFFFFFFFFFFFFFFF:FFFFFFFFFFFFFF:FFFFFFFFFFFFFFFFFFFFFFFFFFFF
FFFFFFFFFFFFFFF
@A00155:342:HHGFNDSXY:1:1322:29722:15233 2:N:0:GAACCTAG+TCCGCATA
GAAGACCAACATTTTCATACATGATGAAATTCATTATTGGTCTCGCGATCAGCTTGAGACATTTCTCC
AGGTTTACAAACCTAAAAATTTGTGGGCTACTCTGGTTTTTCCACCTGAGATCTTAGCTGGTTACAAA
TCCTCCGTTCTGCCC
+
FFFFFFFFFFFFFFFFFFFFFFFFFFFFF:FFFFFF:FFFFFFFFFFFFFFFFFFFFFFFFFFFFFFF
FFFFFFFFFFFF:FFFFFFFF:FFF,FFFFFFFFFF:FFFFFFFF,FFFFFFF,:FF:FF:FFFFFFF
FFFFF,F:FFFF,F:
@A00155:342:HHGFNDSXY:1:1425:28411:27492 2:N:0:GAACCTAG+TCCGCATA
GAAGACCAACATTTTCATACATGATGAAATTCATTATTGGTCTCGCGATCAGCTTGAGACATTTCTCC
AGGTTTACAAACCTAAAAATTTGTGGGCTACTCTGGTTTTTCCACCTGAGATCTTAGCTGGTTACAAA
TCCTCCGTTCTGCCC
+
FFFFFFFFFFFFFFFF:FFFFFFFFFFFFFFFFFFFFFFFFFFFFFFFFFFFFFFFFFFFFFFFFFF
FFFFFFFFFFFFFFFFFFFFFFFFFFFFF,FFFFFFFFFFFFFFFFFFFFFFF:FFFF:FFFFFFFFF
FFFFFFFFFFFFFFF
@A00155:342:HHGFNDSXY:1:2149:3378:12383 2:N:0:GAACCTAG+TCCGCATA
GAAGACCAACATTTTCATACATGATGAAATTCATTATTGGTCTCGCGATCAGCTTGAGACATTTCTCC
AGGTTTACAAACCTAAAAATTTGTGGGCTACTCTGGTTTTTCCACCTGAGATCTTAGCTGGTTACAAA
TCCTCCGTTCTGCCA
+
FFFFFFFF:FFFFFFFFFFFFFFFFF::FFFFFFFFFFFFFFFFFFFFFFFFFFFFFF,FFFFFFFFF
FFFFFFFFFF:F:FF:F::F:FFFFF:FFFFFFFFFF,FFF:FFFFFFFFFF:FFFFFFFFFFFFFF,
FF,FFFFFFFFFFF:
@A00155:342:HHGFNDSXY:1:2162:27814:17722 2:N:0:GAACCTAG+TCCGCATA
GAAGACCAACATTTTCATACATGATGAAATTCATTATTGGTCTCGCGATCAGCTTGAGACATTTCTCC
AGGTTTACAAACCTAAAAATTTGTGGGCTACTCTGGTTTTTCCACCTGAGATCTTAGCTGGTTACAAA
TCCTCCGTTCTGCCC
+
FFFFFFFFFFFFFFFFFFFFFFFFFFFFF:F,FFFFFFFFFFFFFFFFFFFFF:FFFFFFFFFFFFFF

FFF::FFFFFFFFF:FFFFFFFFFFFFFFFFFFFFFFFFF:FFFFFFFFFFFFFFFFFFFFFFFFFFFF
FFFFF:FFFFFF::F
@A00155:342:HHGFNDSXY:1:2429:30689:6981 2:N:0:GAACCTAG+TCCGCATA
GAAGACCAACATTTTCATACATGATGAAATTCATTATTGGTCTCGCGATCAGCTTGAGACATTTCTCC
AGGTTTACAAACCTAAAAATTTGTGGGCTACTCTGGTTTTTCCACCTGAGATCTTAGCTGGTTACAAA
TCCTCCGAGATCGGA
+
FFFFFFFFFFFFFFFFFFFFFFFFFFFFF:FFFFFFFFFFFFFFFFFFFFFFFFFFFFFFFFFFFFFF
FFFFFFFFFFFFFFFFFFFFFFFFFFFFFFFFFFFFFFFFFFFFFFFFFFFFFFFFF:FFFFFFFFF
FFFFFFFFFFFFFFF
@A00155:342:HHGFNDSXY:1:2537:12283:16783 2:N:0:GAACCTAG+TCCGCATA
GAAGACCAACATTTTCATACATGATGAAATTCATTATTGGTCTCGCGATCAGCTTGAGACATTTCTCC
AGGTTTACAAACCTAAAAATTTGTGGGCTACTCTGGTTTTTCCACCTGAGATCTTAGCTGGTTACAAA
TCCTCCGTTCTGCCC
+
FFFFFFFFFFFFFFFFFFFFFFFFFFFFFFFFFFFFFFFFFFFFFFFFFFFFFFFFFFFFFFFFFFFF
FFFFFFFFFFFFFFFFFFFFFFFFFFFFFFFFFFFFFFFFFFFFFFFFFFFFFFFFFFFFFFFFFFFF
FFFFFFF:FFFFFFF
@A00155:342:HHGFNDSXY:1:2537:10926:19257 2:N:0:GAACCTAG+TCCGCATA
GAAGACCAACATTTTCATACATGATGAAATTCATTATTGGTCTCGCGATCAGCTTGAGACATTTCTCC
AGGTTTACAAACCTAAAAATTTGTGGGCTACTCTGGTTTTTCCACCTGAGATCTTAGCTGGTTACAAA
TCCTCCGTTCTGCCC
+
F,:FFF:FFFFFFFFFFFFFFFFFFFFFFFFFFFFFFFFFFFFFFFFFFFFFFFFFFFFFFFFFFFFF
FFFFFFFFFFFFFFFFFFFFFFFFFFFFFFFFFFFFFFFFFFF:FFFFFFFFFFFFFFFFFFFFFFFF
FFFFFFF:F,FFFFF
@A00155:342:HHGFNDSXY:1:2543:21287:12931 1:N:0:GAACCTAG+TCCGCATA
AAGACCAACATTTTCATACATGATGAAATTCATTATTTGTCTCGCGATCAGCTTGATACATTTCTCCA
GGTTTACAAACCTAAAAATTTGTGGGCTACTCTGGTTTTTCCACATGAGATCTTAGCTGGTTACTAAT
CCTC
+
:,F::,FF::,F:FF,F,FFF,FFF:,FFF,,FF:FF,:F,F:,FFFFF:F:FFFF,F,FFFF,FF,F
FFFFFFFFFF,FFFFFFFFFF,FFF::F,FFFFFFFFFFFF:FF,FF:FFFFFF:F,FFFFF:,,FFF
,FFF
@A00155:342:HHGFNDSXY:1:2456:15013:23891 2:N:0:GAACCTAG+TCCGCATA
AAGACCAACATTTTCATACATGATGAAATTCATTATTGGTCTCGCGATCAGCTTGAGACATTTCTCCA
GGTTTACAAACCTAAAAATTTGTGGGCTACTCTGGTTTTTCCACCTGAGATCTTAGCTGGTTACAAAT
CCTCCGTTCTGCCC
+
FFFFFFFFFFFFFFFFFFFFFFFFFFFFFFFFFFFFFFFFFFFF:FFFFFFFF:FFFFFFF:FFFFFFFF
:FFFFFFF:FFFFFFFFFFFFFFFFFFFF:FFFFFFFFFFFFFFFFFFFFFFFFF:F:FFFFFFFFFFF
FFFFFFFFFF:FF:
@A00155:342:HHGFNDSXY:1:1362:18394:16000 1:N:0:GAACCTAG+TCCGCATA
AAGACCAACATTTTCATACATGATGAAATTCATTATTGGTCTCGCGATCAGCTTGAGACATTTCTCCA
GGTTTACAAACCTAAAAATTTGTGGGCTACTCTGGTTTTTCCACCTGAGATCTTAGCTGGTTACAAAT
CCTCCGTTCTGCCCG
+
FFFFFFFFFFFFFFFFFFFFFFFFFFFFFFFFFFFFFFFFFFFFFFFFFFFFFFFFFFFFFFFFFFFF
FF:FFFFFFFFFFFFFFFFFFFFFFFFFFFFFFFFFFFFFFFFFFFFFFFFFFFFFFFFFFFFFFFFF
FFFFFFFFFFFFFFF
@A00155:342:HHGFNDSXY:1:1425:28411:27492 1:N:0:GAACCTAG+TCCGCATA
AAGACCAACATTTTCATACATGATGAAATTCATTATTGGTCTCGCGATCAGCTTGAGACATTTCTCCA
GGTTTACAAACCTAAAAATTTGTGGGCTACTCTGGTTTTTCCACCTGAGATCTTAGCTGGTTACAAAT
CCTCCGTTCTGCCCA

+
FFFFFFFFFFFFFFF:FFFFFFFFFFFF:FFFFFFFFFFFFFFFFFFFFFF:FFFFFFFFFFFFFF:F
FFFFFFFFFFFFFFFFFFFFFFFFFFFFFFFFFFFFFFFFFFFFFFFFFFFFFFFFFFFFFFFFFFFF
FFFFFFFFFFFFFFF
@A00155:342:HHGFNDSXY:1:2174:8097:21057 1:N:0:GAACCTAG+TCCGCATA
AAGACCAACATTTTCATACATGATGAAATTCATTATTGGTCTCGCGATCAGCTTGAGACATTTCTCCA
GGTTTACAAACCTAAAAATTTGTGGGCTACTCTGGTTTTTCCACCTGAGATCTTAGCTGGTTACAAAT
CCTCCGTTCTGCCCG
+
FFFFFFFFFFFFFFFFFFFFFFFFFFFFFFFFFFFFFFFFFFFFFFFFFFFFFFFFFFFFFFFFFFFF
FFFFFFFFFFFFFFFFFFFFFFFFFFFFFFFFFFFFFFFFFFFFFFFFFFFFFFFFFFFFFFFFFFFF
FFFFFFFFFFFFFFF
@A00155:342:HHGFNDSXY:1:2537:12283:16783 1:N:0:GAACCTAG+TCCGCATA
AAGACCAACATTTTCATACATGATGAAATTCATTATTGGTCTCGCGATCAGCTTGAGACATTTCTCCA
GGTTTACAAACCTAAAAATTTGTGGGCTACTCTGGTTTTTCCACCTGAGATCTTAGCTGGTTACAAAT
CCTCCGTTCTGCCCA
+
FFFFFFFFFFFFFFFFFFFFFFFFFFFFFFFFFFFFFFFFFFFFFFFFFFFFFFFFFFFFFFFFFFFF
FFFFFFFFFFFFFFFFFFFFFFFFFFFFFFFFFFFFFFFFFFFFFFFFFFFFFFFFFFFFFFFFFFFF
FFFFFFFFF:FFFFF
@A00155:342:HHGFNDSXY:1:2537:10926:19257 1:N:0:GAACCTAG+TCCGCATA
AAGACCAACATTTTCATACATGATGAAATTCATTATTGGTCTCGCGATCAGCTTGAGACATTTCTCCA
GGTTTACAAACCTAAAAATTTGTGGGCTACTCTGGTTTTTCCACCTGAGATCTTAGCTGGTTACAAAT
CCTCCGTTCTGCCCA
+
:FFFFFFFF:FFFFFFFFF:FFFFFFFFFFFFFF:FFFFFFFFFFFFFF:FFFFFFFFF:FFFFFFFF
FFFFFFFFFFFFFFFFFFFFFFFFFFFFFFFFFFFFFF:FFF:FFFFFFFFFFFFFFFFFFFFFFFFF
FFFFFFFFFFFFFFF
@A00155:342:HHGFNDSXY:1:1348:19551:18067 2:N:0:GAACCTAG+TCCGCATA
AAGACCAACATTTTCATACATGATGAAATTCATTATTGGTCTCGCGATCAGCTTGAGACATTTCTCCA
GGTTTACAAACCTAAAAATTTGTGGGCTACTCTGGTTTTTCCACCTGAGATCTTAGCTGGTTACAAAT
CCTCCGTTCTGCCAG
+
FFF:FFFFFFFFFFFFFFFFFFFFFFF,FFFF:F:FFFFFFFFFFFFFF::FF:FFFFFFFFFFFFFF
FFFFFFFFFFFFFFFFFFF:FFFFFFFFFFFFFFFF:F,FFF::FFFFFF:F:F:FFFF:FFFFFFFF
:FFFFFFFFFF,FFF
@A00155:342:HHGFNDSXY:1:1609:21178:11929 2:N:0:GAACCTAG+TCCGCATA
AAGACCAACATTTTCATACATGATGAAATTCATTATTGGTCTCGCGATCAGCTTGAGACATTTCTCCA
GGTTTACAAACCTAAAAATTTGTGGGCTACTCTGGTTTTTCCACCTGAGATCTTAGCTGGTTACAAAT
CCTCCGTTCTGCCCT
+
FFFFFFFFFFFFFFFFFFFFFFFFFFFFFFFF:FFFFFFFFFFFFFFFFFFFFFFFFFFFFFFFFFFF
FFFFFFFFF:FFFFFFFFFFFFFFFFFFFFFFFFFFFFFFFFFFFFFFFFF:,FFFFFFFFFFFFFFF
FFFFFFFFFFFF:FF
@A00155:342:HHGFNDSXY:1:2548:32398:20901 2:N:0:GAACCTAG+TCCGCATA
AGACCAACATTTTCATACATGATGAAATTCATTATTGGTCTCGCGATCAGCTTGAGACATTTCTCCAG
GTTTACAAACCTAAAAATTTGTGGGCTACTCTGGTTTTTCCACCTGAGATCTTAGCTGGTTACAAATC
CTCCGTTCTGCCC
+
FFFFFFFFFFFFFFFFFFFFFFFFFF,,FF,:FFFFFFFF:FFFFF,FFFFFFF:FFF::F:FFFFFF
FFFFFFFFFFFFFFFFFFFFFFFFFFFFF:FFFF,FFFFFFFFFFFFF:FF,FFFFFFFFFFFF,FFF
FFFFFFFFFFFFF
@A00155:342:HHGFNDSXY:1:2356:23339:26412 1:N:0:GAACCTAG+TCCGCATA
AGACCAACATTTTCATACATGATGAAATTCATTATTGGTCTCGCGATCAGCTTGAGACATTTCTCCAG

```
GTTTACAAACCTAAAAATTTGTGGGCTACTCTGGTTTTTCCACCTGAGATCTTAGCTGGTTACAAATC
CTCCGTTCTGCCCTT
+
FFFFFFFFFFFFFFFFFFFFFFFFFFFFFFFFFFFFFFFFFFFFFFFFFFFFFFFFFFFFFFFFFFFF
FFFFFFFFFFFFFFFFFFFFFFFFFFFFFFFFFFFFFFFFFFFFFFFFFFFFFFFFFFFFFFFFFFFF
FFFFFFFFFFF:FFF
@A00155:342:HHGFNDSXY:1:1119:31006:5400 2:N:0:GAACCTAG+TCCGCATA
AGACCAACATTTTCATACATGATGAAATTCAGTATTGGTCTCGCGATCAGCTTGAGACATTTCTCCAG
GTTTACAAACCTAAAAATTTGTGGGCTACTCTGGTTTTTCCACCTGAGATCTTAGCTGGTTACAAATC
CTCCGTTCTGCCCTT
+
FFFFFFFFFFFFFFFFFFFFFFFFFFFFFFF,FFFFFFFFFFFFFFFFFFFFFFFFFFFFFFFFF:FF
FFFFFFFF:FFFFF:FFFF:FFFFFFFFFFFFFFFFFFFFFFFFFFFFFFFFFFFFFFFFFFFFFFFF
:FFFFFFFFFF:,FF
@A00155:342:HHGFNDSXY:1:1129:14823:19053 2:N:0:GAACCTAG+TCCGCATA
AGACCAACATTTTCATACATGATGAAATTCATTATTGGTCTCGCGATCAGCTTGAGACATTTCTCCAG
GTTTACAAACCTAAAAATTTGTGGGCTACTCTGGTTTTTCCACCTGAGATCTTAGCTGGTTACAAATC
CTCCGTTCTGCCAGA
+
FFFFFFFFFFFFFFFFFFFFFFFFFFFFFFFFFFFFFFFFFFFFFFF:FFFFFF:FFF:FFFFFFFFF
FFFFFFFFFFFFFFFFFFFFFFFFFFFFFFFFFFFFFFF:FFFFFF:FFFFFFFFFFFFFFFFFFFFF
FFFFFFFFFFFFFF:
@A00155:342:HHGFNDSXY:1:2131:30337:1799 2:N:0:GAACCTAG+TCCGCATA
AGACCAACATTTTCATACATGATGAAATTCATTATTGGTCTCGCGATCAGCTTGAGACATTTCTCCAG
GTTTACAAACCTAAAAATTTGTGGGCTACTCTGGTTTTTCCACCTGAGATCTTAGCTGGTTACAAATC
CTCCGTTCTGCCCTT
+
FFFFFFFFFFFFFFFFFFFFFFFFFFFFFFF:FFFFFFFFF:FFFFFFFFFFFFFFFFFFFFFFFFFF
FFFFFFFFF:FFFFFFFFFFFFFFF:FFFFFFFFF,FFFFFFFFFFFFFFFFFFFFFF:FFFFFFFFF
FFF:FFFFFF:FFF:
@A00155:342:HHGFNDSXY:1:2266:17409:17143 2:N:0:GAACCTAG+TCCGCATA
AGACCAACATTTTCATACATGATGAAATTCATTATTGGTCTCGCGATCAGCTTGAGACATTTCTCCAG
GTTTACAAACCTAAAAATTTGTGGGCTACTCTGGTTTTTCCACCTGAGATCTTAGCTGGTTACAAATC
CTCCGTTCTGCCCTT
+
F:FFFFFFFFFFFFFFFFFFFFFFFFFFFFFFFFFFFF:F,FFFFFFFFFFFFFFFFFFFFFFFFFFF
FFFFFFFFFFFFFFFFFFF:FFFFFFFFFFFFFFFFFFFFFFFFFFFFFFFFFFFF:FFFFFFFFFF:
FFFFFFFFFFF::FF
@A00155:342:HHGFNDSXY:1:2437:4860:32894 2:N:0:GAACCTAG+TCCGCATA
AGACCAACATTTTCATACATGATGAAATTCATTATTGGTCTCGCGATCAGCTTGAGACATTTCTCCAG
GTTTACAAACCTAAAAATTTGTGGGCTACTCTGGTTTTTCCACCTGAGATCTTAGCTGGTTACAAATC
CTCCGTTCTGCCCTT
+
FFFFFFFFFF::FFFFFFFFFFF:F,FFFFF,FFFFF,FFFFFFFFFFFFFFFF:FFFF,FFFFFFFF
FFFF:FF::,FFFF,FFFFFFFFFFFFF::FFFFF,::FFFFFFFF:F,FFFFFFFFFFFFF,,F:FF
FFFFFFFFFF:::FF
@A00155:342:HHGFNDSXY:1:2537:7798:32440 2:N:0:GAACCTAG+TCCGCATA
AGACCAACATTTTCATACATGATGAAATTCATTATTGGTCTCGCGATCAGCTTGAGACATTTCTCCAG
GTTTACAAACCTAAAAATTTGTGGGCTACTCTGGTTTTTCCACCTGAGATCTTAGCTGGTTACAAATC
CTCCGTTCTGCAGAT
+
FFFFFFFFFFFFFFFFFFFFFFFFFF:FFFFFFFFFFFFFFFFFFFFFFFFFFFFFFFFFFFFFFFFF
FFFFFFFFFFFFFFFFFFFFFFFFFFFFFFFFFFFFFFFFFFFFFFFFFFFFFF:FFFFFFFFFFFFF
:FF:F,FFFFFFFFF
```

```
@A00155:342:HHGFNDSXY:1:1162:4969:7028 2:N:0:GAACCTAG+TCCGCATA
GACCAACATTTTCATACATGATGAAATTCATTATTGGTCTCGCGATCAGCTTGAGACATTTCTCCAGG
TTTACAAACCTAAAAATTTGTGGGCTACTCTGGTTTTTCCACCTGAGATCTTAGCTGGTTACAAATCC
TCCGTTCTGCCCTT
+
FFFFFFFFFFFFFFFFFFFFFFFFFFFFFFFFFFFFFFFFFFFFFFFFFFFFFFFFFFFFFFFFFFFF
FFFFFFFFFF:FFFFFFFFFFFFF:FFFFFFFFFFFFFFFFFFFFFF:FFFFFFFFFFFFFFFFFF:F
FFFFFFFFFF:F:F
@A00155:342:HHGFNDSXY:1:2353:23231:16736 2:N:0:GAACCTAG+TCCGCATA
GACCAACATTTTCATACATGATGAAATTCATTATTGGTCTCGCGATCAGCTTGAGACATTTCTCCAGG
TTTACAAACCTAAAAATTTGTGGGCTACTCTGGTTTTTCCACCTGAGATCTTAGCTGGTTACAAATCC
TCCGTTCTGCCCTT
+
FFFFFFFFFFFFFFFFFFFFFFFFFFFFFFFFFFFFFFFFF:FFFFFFFF:FFFFFFFFFFFFFFFFF
,FFFFFFFFFF:FFFFFFFFFFFFFFF:F:FFFFFFFFF,FFFFFFFF:FF:FFFFFFFFFFFFFFFF
FF,FFFFFFFFFF:
@A00155:342:HHGFNDSXY:1:2632:4761:35571 2:N:0:GAACCTAG+TCCGCATA
GACCAACATTTTCATACATGATGAAATTCATTATTGGTCTCGCGATCAGCTTGAGACATTTCTCCAGG
TTTACAAACCTAAAAATTTGTGGGCTAATCTGGTTTTTCCACCTGAGATCTTAGCTGGTTACAAATCA
TCCGTTCTGCAATT
+
FFFFFFFFFFFFFFFFFFFFFFFF:FFFFFFFFFFFFFFFFFFFF,FFFF:FF:FFFFFFFFFFFFFF
FFFFFFFFFF::FFFFFFFFF,FF,FF,FFFFF:FFFFFF:F,FFFFF:FFFF,F,FFFFFFFF:F:,
F,FFFFFF:F,,FF
@A00155:342:HHGFNDSXY:1:1509:10899:9189 2:N:0:GAACCTAG+TCCGCATA
GACCAACATTTTCATACATGATGAAATTCATCATTGGTCTCGCGATCAGCTTGAGACATTTCTCCAGG
TTTACAAACCTAAAAATTTGTGGGCTACTCTGGTTTTTCCACCTGAGATCTTAGCTGGTTACAAATCC
TCCGTTCTGCCCTTC
+
FFFFFFFFFFFF,FFFFFFFFFFFFF:FF:FFFFFFFFFFFFFFFFFFFFFFFFFFFFFFFFFFFFFF
FFFFFFFFFFFFF:FFFFFFFFFFFFFFFFFFFFFF,FFFFFFFFFFFFFFFFFFFFFFFFFFFFFFFF
FFFFFFFFF,FFFF:
@A00155:342:HHGFNDSXY:1:1521:26711:34976 2:N:0:GAACCTAG+TCCGCATA
GACCAACATTTTCATACATGATGAAATTCATTATTGGTCTCGCGATCAGCTTGAGACATTTCTCCAGG
TTTACAAACCTAAAAATTTGTGGGCTACTCTGGTTTTTCCACCTGAGATCTTAGCTGGTTACAAATCC
TCCGTTCTGCCAGAT
+
FF:FFFFFFFFFFFFFFFFFFFFFFF,FFFFFFFFFFFFFFFFFFFFFFFFFFFFFFFFFFFFFFFFFF
FFFFFFF::FFFFFFFFFFFFF:FFFF:FFFFFFFFFFF:FFFFFFFFF:FFFFFFFF:FFFFFFFFF
F:FFFFFFFFFFFFF
@A00155:342:HHGFNDSXY:1:1668:12454:26819 2:N:0:GAACCTAG+TCCGCATA
GACCAACATTTTCATACATGATGAAATTCATTATTGGTCTCGCGATCAGCTTGAGACATTTCTCCAGG
TTTACAAACCTAAAAATTTGTGGGCTACTCTGGTTTTTCCACCTGAGATCTTAGCTGGTTACAAATCC
TCCGTTCTGCCCTTC
+
FFFFF:FFFFFFFFFFFFFFFFFFFFFFFFF:FFFFFFFFFFFFFFFFFFFFFFF:FFFFFFFFFFFFFF
FFFFFFFFFFFFFFF:FFFFFFFFFFFFFFFFFFF,FFFFFFFFFFFFFFFFFFFFFFFFFFFFFFFFFF
FFFFFFFFFFFFFFF
@A00155:342:HHGFNDSXY:1:1350:4942:4445 2:N:0:GAACCTAG+TCCGCATA
CCAACATTTTCATACATGATGAAATTCATTATTGGTCTCGCGATCAGCTTGAGACATTTCTCCAGGTT
TACAAACCTAAAAATTTGTGGGCTACTCTGGTTTTTCCACCTGAGATCTTAGCTGGTTACAAATCCTC
CGTTCTGCCCTTCTT
+
FFFFFFFFFFFFFFFFFFFFFF:FFFFFFFFFFFFFFFFFFFFFFFFFFFFFFFFFFFFFFFFFFFFFF
```

FFF:FFFFFFFFFFFFFFFFFFFFFFFFFFFFFFFFFFFFFFFFFFFFFFFFFFFFFFFFFFFFFFFFFF
FFFFFFFFFFFFF,F
@A00155:342:HHGFNDSXY:1:1441:16712:17785 2:N:0:GAACCTAG+TCCGCATA
CCAACATTTTCATACATGATGAAATTCATTATTGGTCTCGCGATCAGCTTGAGACATTTCTCCAGGTT
TACAAACCTAAAAATTTGTGGGCTACTCTGGTTTTTCCACCTGAGATCTTAGCTGGTTACAAATCCTC
CGTTCTGCCCTTCTT
+
FFFFFFFFFFFFFFFFFFFFFFFFFFFFFFFFFFFFFFFFFFFFFFFFFFFFFFFFFFFFFFFFFFFF
FFFFF:FFFFFFFFFFFFFFFFFFFFFFFFFFFFFFFFFFFFFFFFF,FFFFFFFFFFFFFFFFFFF
FFFFFFFFFF:FFFF
@A00155:342:HHGFNDSXY:1:1675:22372:15875 2:N:0:GAACCTAG+TCCGCATA
CCAACATTTTCATACATGATGAAATTCATTATTGGTCTCGCGATCAGCTTGAGACATTTCTCCAGGTT
TACAAACCTAAAAATTTGTGGGCTACTCTGGTTTTTCCACCTGAGATCTTAGCTGGTTACAAATCCTC
CGTTCTGCCCTTCTT
+
FFFFFFFFFFFFFFFFFFFFFFFFFFFFFFFFFFFFFFFFFFFFFFFF:FFFFFFFFFFFFFFFFFFF
FFFFFFFFFFFFFFF:FFFFFFFFFFFFFFFFFFFFFFFFFFFFFFFFFFFFFFFFFFFFFFFFFFFF
FFFFFFFFF:FFFFF
@A00155:342:HHGFNDSXY:1:2377:25084:23860 2:N:0:GAACCTAG+TCCGCATA
CCAACATTTTCATACATGATGAAATTCATTATTGGTCTCGCGATCAGCTTGAGACATTTCTCCAGGTT
TACAAACCTAAAAATTTGTGGGCTACTCTGGTTTTTCCACCTGAGATCTTAGCTGGTTACAAATCCTC
CGTTCTGCCAGATCG
+
FFFFFFFFFFFFFFFFFFFFFFFFFFFFFFFFFFFFFFFFFFFFFFFFFFFFFFFFFFFFFFFFFFFF
FFFFFFFFFFFFFFFFFFFFFFFFFFFFFFFFFFFFFF:FFFFFFFFFFFFFFFFF:FFFFFFFFFFFF
FFFFFFFFFFFFFFF
@A00155:342:HHGFNDSXY:1:2627:10565:26960 2:N:0:GAACCTAG+TCCGCATA
CCAACATTTTCATACATGATGAAATTCATTATTGGTCTCGCGATCAGCTTGAGACATTTCTCCAGGTT
TACAAACCTAAAAATTTGTGGGCTACTCTGGTTTTTCCACCTGAGATCTTAGCTGGTTACAAATCCTC
CGTTCTGCCCTTCTT
+
FFFFFFFFFFFFFFFFFFFFFFFF:::FFFFFFFFFFFFFFFFFFFFFFFFFFFFFFFFFFFFFFFFF
FFFFFF::FFFFFFF:FFFFFFFF:FFFFFFFFFFFF::FFFFFFF:F:,,FFFFFFFFFFFFF:FFF
FFFFFFF,::F:FFF
@A00155:342:HHGFNDSXY:1:2358:1425:2049 1:N:0:GAACCTAG+TCCGCATA
ATCTATTTTCATACATGATGAAATTCATTATTGGTCTCGCGATCAGCTTGAGACATTTCTCCAGGTTT
ACAAACCTAAAAATTTGTGGGCTACTCTGGTTTTTCCACCTGAGATCTTAGCTGGTTACAAATCCTCC
GTTCTGCC
+
F:::F::F,,F,F,F,:F:,FFFF:FFF:F::F,FFFFFFFF:FFFFF:FFFFFFFF:FFFFF::FFF
F:FFFFFFFFFFF:FFFFFF:FFFF:FFFF:FFFFFFFFFFFFFFFFFFFFFFFF::FFFFFFFFFFFF
FFFFFFF:
@A00155:342:HHGFNDSXY:1:2271:21350:17080 2:N:0:GAACCTAG+TCCGCATA
CAACATTTTCATACATGATGAAATTCATTATTGGTCTCGCGATCAGCTTGAGACATTTCTCCAGGTTT
ACAAACCTAAAAATTTGTGGGCTACTCTGGTTTTTCCACCTGAGATCTTAGCTGGTTACAAATCCTCC
GTTCTGCCC
+
F,FFFFFFFFFFFFFFFFFFFFFFFFFF,FFFFFFFFFFFFFFFFFFFFFFFFFFFFFFFFFFFFFFFF
FFFFFFFFFF:FF:FFFFFFFFFFFFFFFFFFFFFFFF,FFFFFFFFF:FFFFFFF,FFFFF:FFFFFF
F:FFFFF:F
@A00155:342:HHGFNDSXY:1:1373:6885:18051 2:N:0:GAACCTAG+TCCGCATA
CAACATTTTCATACATGATGAACTTCATTATTGGTCTCGCGATCAGCTTGAGACATTTCTCCAGGTTT
ACAAACCTAAAAATTTGTGGGCTACTCTGGTTTTTCCACCTGAGATCTTAGCTGGTTACAAATCCTCC
GTTCTGCCCTTCGT

```
+
FFFFFFFFFFF:,FFFFF:FFF,:F:,F,FFFFFFFFFFFFFFFFFF:FFFFFFF:,:FFFFFFFFFF
FFFFFFF:::F::,:FFFFFFFFFF:F,FF,FFF::FFFFFFFFF:FFFFF:FFFFFFFFFFFFF:,:
FFF:FF:F,FF:,F
@A00155:342:HHGFNDSXY:1:1135:17228:33082 2:N:0:GAACCTAG+TCCGCATA
CAACATTTTCATACATGATGAAATTCATTATTGGTCTCGCGATCAGCTTGAGACATTTCTCCAGGTTT
ACAATCCTAAAAATTTGTGGGCTACTCTGGTTTTTCCACCTGAGATCTTAGCTGGTTACAAATCCTCC
GTTCTGCCCTTCTTA
+
FFFFFFFFFFFFFFFFFFFFFFF,FFFFFFFFFFFFFFFF,FFFFFFFF:F:FFFFFFFFFFFFFF
FFFF,:FFFFFFFF:FFFFFFFF,FFFFFFF:F:FFFFF,FFFFFFFFFFFFFFFFFFFF,FFFFFFF
FF,FFF:FFFF:::F
@A00155:342:HHGFNDSXY:1:1136:14669:23766 2:N:0:GAACCTAG+TCCGCATA
CAACATTTTCATACATGATGAAATTCATTATTGGTCTCGCGATCAGCTTGAGACATTTCTCCAGGTTT
ACAAACCTAAAAATTTGTGGGCTACTCTGGTTTTTCCACCTGAGATCTTAGCTGGTTACAAATCCTCC
GTTCTGCCCTTCTTA
+
FFFFFFFFFF:FFFFFFFFFFFFFFFFFFFFFFFFFFFFFFFFFFFFFFFFFFFFFFFFFFFFFFFFF
FFFFFFFFFFFFF,FFFFFFFFFFFFFFFFFFFFFFFFFFFFFFFFFFFFFFFFFFFFFFFFFFF:FF
FFFFFFFFFFFFFFF
@A00155:342:HHGFNDSXY:1:1358:14172:19993 2:N:0:GAACCTAG+TCCGCATA
CAACATTTTCATACATGATGAAATTCATTATTGGTCTCGCGATCAGCTTGAGACATTTCTCCAGGTTT
ACAAACCTAAAAATTTGTGGGCTACTCTGGTTTTTCCACCTGAGATCTTAGCTGGTTACAAATCCTCC
GTTCTGCCCTTCTTA
+
FFFFFFFFFFFFFFFFFFFFFFFFFFFFFFFFF:FFF:F:FFFFFFFFFFFFFFFFFF:F::F,:::F
F,FFFF,FFFFFFFF:,FF:,:FFFFFF,F:FFFF:FFF:F:FFFF:F,FFFF,:,FF,FFF:,,,::
:,FF:,,FF:::FFF
@A00155:342:HHGFNDSXY:1:1508:7654:1282 2:N:0:GAACCTAG+TCCGCATA
CAACATTTTCATACATGATGAAATTCATTATTGGTCTCGCGATCAGCTTGAGACATTTCTCCAGGTTT
ACAAACCTAAAAATTTGTGGGCTACTCTGGTTTTTCCACCTGAGATCTTAGCTGGTTACAAATCCTCC
GTTCTGCCCTTCTTA
+
FFFFFFFFFFFFFFF:FFFFFF:FFFFFFFFFFFFFFFFFFFFFFFFFFFFFFFFFFFFFFFFFFFFF
FFF:FFFFFFFFFFFFFFFFFFFFFFFFFFFFFFFFFFFFFFFFFFFFFFFFFFFFFFFFFFFFFFFF
FFFFFFFFFFFFFFF
@A00155:342:HHGFNDSXY:1:1432:2691:25880 2:N:0:GAACCTAG+TCCGCATA
CAACATTTTCATACATGATGAAATTCATTATTGGTCTCGCGATCAGCTTGAGACATTTCTCCAGGTTT
ACAAACCTAAAAATTTGTGGGCTACTCTGGTTTTTCCACCTGAGATCTTAGCTGGTTACAAATCCTCC
GTTCTGCCCTTCTTA
+
FFFFFFFFFFFFFFFFFFFFFFFFFFFFFFFFFFFFFFFFFFFFFFFFFFFF,FFFFFF:FFFFFFF
FFFFFFFFFFFFFFFFFFFFFFFFFFFFFFFFFFFFFFFFFFFFFFFF:FFFFFFFFFFFFFFFFFFF
FFFFFFFF:F::FFF
@A00155:342:HHGFNDSXY:1:1520:8558:28181 2:N:0:GAACCTAG+TCCGCATA
CAACATTTTCATACATGATGAAATTCATTATTGGTCTCGCGATCAGCTTGAGACATTTCTCCAGGTTT
ACAAACCTAAAAATTTGTGGGCTACTCTGGTTTTTCCACCTGAGATCTTAGCTGGTTACAAATCCTCC
GTTCTGCCAGATCGG
+
FFFFFFFFFFFFFFFFFFFFFFFFFFFFFFFFFFFFFFFFFFFFFFFFFFFFFFFFFFFFFFFFFFFF
FFFFFFFFFF:FFFFFFFFFFFFFFFFFFFFFFFFFFFFFFFFFFFFFFFFFFFFFFFFFFFFFFFFF
FFFFFFFFFFFFFFF
@A00155:342:HHGFNDSXY:1:2312:11921:18787 2:N:0:GAACCTAG+TCCGCATA
CAACATTTTCATACATGATGAAATTCATTATTGGTCTCGCGATCAGCTTGAGACATTTCTCCAGGTTT
```

ACAAACCTAAAAATTTGTGGGCTACTCTGGTTTTTCCACCTGAGATCTTAGCTGGTTACAAATCCTCC
GTTCTGCCAGATCGG
+
FFFFFFFFFFFFFFFFFFFFFFFFFFFFFFFFFFFFFFFFFFFFFFFFFFFFFFFFFFFFFFFFFFFFFF
FFFF:FFFFFFFFFFFFFFFFFFFF:FFFFFFFFFFFFFFFFFFFFFFFFFFFFFFFFFFFFFFFFFFFF
FFFFFFFFFFFFFFF
@A00155:342:HHGFNDSXY:1:2310:31051:25770 2:N:0:GAACCTAG+TCCGCATA
CAACATTTTCATACATGATGAAATTCATTATTGGTCTCGCGATCAGCTTGAGACATTTCTCCAGGTTT
ACAAACCTAAAAATTTGTGGGCTACTCTGGTTTTTCCACCTGAGATCTTAGCTGGTTACAAATCCTCC
GTTCTGCCCTTCTTA
+
:FFFFFFFFFFFFFFFFFFFFFFFFFFFFFFFFFFFFFFFFFFFFFFFFFFFFFFFFFFFFFFFFFFFFF
FFFFFFFFFFFFFFFFFFFFFFFFFFFFFFFFFFFFFFFFFFFFFFFFFFFFFFFFFFFFFFFFFFFFFF
FFFFFFFFFFFFFFF
@A00155:342:HHGFNDSXY:1:2570:11451:27837 2:N:0:GAACCTAG+TCCGCATA
CAACATTTTCATACATGATGAAATTCATTATTGGTCTCGCGATCAGCTTGAGACATTTCTCCAGGTTT
ACAAACCTAAAAATTTGTCGGCTACTCTGGTTTTTCCACCTGAGATCTTAGCTGGTTACAAATCCTCC
GTTCTGCCCTTCTTA
+
FFFFFFFFFFFFFFFFFFFFFFFFFFFFFFFFFFFFFFFFFFFFFFFFFFFFFFFFFFFFFFFFFFFFFF
FFFFFFFFFFFFFFFFFFFFFFFF:F:FFFFFFFFFFFF::FFF:FFFFFF:FFFFFFFFFF:FF:FF:F
FFFFF:F::FFFFFF
@A00155:342:HHGFNDSXY:1:2675:29143:33082 2:N:0:GAACCTAG+TCCGCATA
CAACATTTTCATACATGATGAAATTCATTATTGGTCTCGCGATCAGCTTGAGACATTTCTCCAGGTTT
ACAAACCTAAAAATTTGTGGGCTACTCTGGTTTTTCCACCTGAGATCTTAGCTGGTTACAAATCCTCC
GTTCTGCCAGATCGG
+
FFFFFFFFFFFFFFFFFFFFFFFFFFF:FFFFFFFFFFFFFF:FFFFFFFFFFFFFFFFFFFFFFFFFFF
FFFFFFFFF:FFFFFFFFFFFFFFFF:FFFFFFFF:FFFFFFFFFFFF,FFFFFFFFFFFFFFFFFFFFF
FFFFFFFFFFFFFFF
@A00155:342:HHGFNDSXY:1:1666:3070:21339 2:N:0:GAACCTAG+TCCGCATA
AACATTTTCATACATGATGAAATTCATTATTGGTCTCGCGATCAGCTTGAGACATTTCTCCAGGTTTA
CAAACCTAAAAATTTGTGGGCTACTCTGGTTTTTCCACCTGAGATCTTAGCTGGTTACAAATCCTCCG
TTCTGCCCTTCTTAT
+
FFFFFFFFFFFFFFF:FFFF,FFFFF:FFFFFFFFFFFFFFFFFFFFFFFFFFFFFFFFFFFFFFFFFFF
FFFFFFFFFFFFFFFFFF:FFFFFFFFFFFF:FFFFFFFFFFFFFFFFFFF:FFFFFFFFFF:FF:FFFF
FFFFFFFFFF::FFF
@A00155:342:HHGFNDSXY:1:1232:19388:10113 2:N:0:GAACCTAG+TCCGCATA
CATTTTCATACATGATGAAATTCATTATTGGTCTCGCGATCAGCTTGAGACATTTCTCCAGGTTTACA
AACCTAAAAATTTGTGGGCTACTCTGGTTTTTCCACCTGAGATCTTAGCTGGTTACAAATCCTCCGTT
CTGCCCTTCTTATAT
+
FFFFFFFFFFFFFFFFFFFFFFFFFFFFFFFFFFFFFFFFFFFFF:FFFFFFFFFFFFFFFFFFFFFFFF
FFFFFFFFFFFFFFFFFFFFFFFFFFFFFFFFFFFFFFFFFFFFFFFFFFFFFFFFFFFFFFFFFFFFFF
FFFF:FFFF:FF,FF
@A00155:342:HHGFNDSXY:1:1671:1976:1251 2:N:0:GAACCTAG+TCCGCATA
CATTTTCATACATGATGAAATTCATTATTGGTCTCGCGATCAGCTTGAGACATTTCTCCAGGTTTACA
AACATAAAAATTTGTGGGCTACTCTGGTTTTTCCACCTGAGATCTTAGCTGGTTACAAATCCTCCGTT
CTGCCCTTCTTATAT
+
FFFFFFFFFFFFFFFFFFFFFFFFFFFFFFF:FFFFFFF:FF,F:FFFF,F:FFFFFFFFF:FFFFFFF
FFF,FFFFF:FFFFFF:FFFFFFFF:F,,FFFFFFFFFFFFFFF:FFFFFFFFFF:FFF:FFFFFFF,FF
FFFFF:FFFFFFFFF

```
@A00155:342:HHGFNDSXY:1:2173:8133:8531 2:N:0:GAACCTAG+TCCGCATA
CATTTTCATACATGATGAAATTCATTATTGGTCTCGCGATCAGCTTGAGACATTTCTCCAGGTTTACA
AACCTAAAAATTTGTGGGCTACTCTGGTTTTTCCACCTGAGATCTTAGCTGGTTACAAATCCTCCGTT
CTGCCCTTCTTATAT
+
FFFFFFFFFFFFFFFFFFFFFFFFFFFFFFFFFFFFFFFFFFFFFFFFFFFFFFFFFFFFFFFFFFFF
FFFFFFFFFFFFFFFFFF,FF:FFFFFFFFFFFFFFFFFFFFFFFFFFFFFFFFFFFFFFFFFFFFFF
:FFF:FFFFFFFFFF
@A00155:342:HHGFNDSXY:1:2338:19506:4773 2:N:0:GAACCTAG+TCCGCATA
CATTTTCATACATGATGAAATTCATTATTGGTCTCGCGATCAGCTTGAGACATTTCTCCAGGTTTACA
AACCTAAAAATTTGTGGGCTACTCTGGTTTTTCCACCTGAGATCTTAGCTGGTTACAAATCCTCCGTT
CTGCCCTTCTTATAT
+
FFFFFFFFFFFFFFFFFFFFFFFFFFFFFFFFFFFFFFFFFFFFFFFFFFFFFFFFFFFFFFFFFFFF
FFFFFFFFFFFFFFFFFFFFFFFFF:FFFFFFFFFFFFFFFFFF:FFFFFFFFFFFFFFFFFFFFFFF
FFFFF:FF:FFFFFF
@A00155:342:HHGFNDSXY:1:2378:24668:16438 2:N:0:GAACCTAG+TCCGCATA
CATTTTCATACATGATGAAATTCATTATTGGTCTCGCGATCAGCTTGAGACATTTCTCCAGGTTTACA
AACCTAAAAATTTGTGGGCTACTCTGGTTTTTCCACCTGAGATCTTAGCTGGTTACAAATCCTCCGTT
CTGCCCTTCTTATAT
+
FFFFFFFFFFFFFFFFFFF:FFFFFFFFFFFFFFFFFFFFFFFFFFFFFFFFFFFFFFFFFFFFFFFF
FFFFFFFFFFFFFFFFFFFFFFFFFFFFFFFFFF:FFFFFFFFFFFFF:FFFFFFFFFFFFF:FFF:F
FFF:FFFFFFFFFFF
@A00155:342:HHGFNDSXY:1:1429:17192:5869 2:N:0:GAACCTAG+TCCGCATA
ATTTTCATACATGATGAAATTCATTATTGGTCTCGCGATCAGCTTGAGACATTTCTCCAGGTTTACAA
ACCTAAAAATTTGTGGGCTACTCTGGTTTTTCCACCTGAGATCTTAGCTGGTTACAAATCCTCCGTTC
TGCCCTTCTTATATC
+
FFFFFFFFFFFFFFFFF:FFFFFF:FFFFFFFFFFFFFFFFFFFFFFFFFFFFFFFFFFFFFFFFFFF
FFFFFFFFFFFFFFFFFFF,FFFFFFFFFFFFFFFFFFFFFFFFFFFFFFFFFFFFFFFFFFFFFFFF
FFFFFFFFFFFFFFF
@A00155:342:HHGFNDSXY:1:1429:17978:5916 2:N:0:GAACCTAG+TCCGCATA
ATTTTCATACATGATGAAATTCATTATTGGTCTCGCGATCAGCTTGAGACATTTCTCCAGGTTTACAA
ACCTAAAAATTTGTGGGCTACTCTGGTTTTTCCACCTGAGATCTTAGCTGGTTACAAATCCTCCGTTC
TGCCCTTCTTATATC
+
FFF:FFFF:FFFFFFFFFFFFFFFFFFFFFFFFFFFFFFFFFFFFFFFFFF:FFFFFFFFFFFFFF:FF
F:FFFFFF:F:FFFFFFFFFFFFFFFF,FFFFFFFFFF:FFFF:FFFF:FF:FFFFFFFFFFFFFFFF
FFF:FF:F,:FF,FF
@A00155:342:HHGFNDSXY:1:1541:24840:23469 2:N:0:GAACCTAG+TCCGCATA
ATTTTCATACATGATGAAATTCATTATTGGTCTCGCGATCAGCTTGAGACATTTCTCCAGGTTTACAA
ACCTAAAAATTTGTGGGCTACTCTGGTTTTTCCACCTGAGATCTTAGATGGTTACAAATCCTCCGTTC
TGCCCTTCTTATATC
+
FFFFF:FFF::FF:FF:FFFFFF:FF:FFFF::F:FFFFFF,F:FFFFFF:,:,F,FFF:FF:FFFFF
FFFFFFFFFFFFF,FF:FFFF:FF:FFFFFFFFF:F::FF:FFF:F:,F:FFFFFFFF::FFFFFFF,
F,FFFF:FFFFF,FF
@A00155:342:HHGFNDSXY:1:1549:5737:2534 2:N:0:GAACCTAG+TCCGCATA
ATTTTCATACATGATGAAATTCATTATTGGTCTCGCGATCAGCTTGAGACATTTCTCCAGGTTTACAA
ACCTAAAAATTTGTGGGCTACTCTGGTTTTTCCACCTGAGATCTTAGCTGGTTACAAATCCTCCGTTC
TGCCCTTCTTATATC
+
FFFFFFFFFFFFFFFFFFFFFFF:FFFFFFFFFFFFFFFFFFFFFFFFFFFFFFFFFFFF:FFFFFFF
```

```
FFFFFFFFF:FFFFFFFFFFFFFFFFFFFFFFFFFFFFFFFFFFFF:FFFFFFFFFFFFFFFFFFFFF
FFFF::FFF:FF:FF
@A00155:342:HHGFNDSXY:1:2332:16089:31203 2:N:0:GAACCTAG+TCCGCATA
ATTTTCATACATGATGAAATTCATTATTGTTCTCGCGATCAGCTTGAGACATTTCTCCAGGTTTACAA
ACCTAAAAATTTGTGGGCTACTCTGGTTTTTCCACATGAGATCTTAGCTGGTTACAAATCCTCCGTTC
TGCCCTTCTTATATC
+
FFFFFFFFFFFFFFFFFFFFF:FFFFFFF,FFFFFFFFFFFFFF:FF:FFFFFF:F:FFFFFFFFFFF
F:FFFF:FFF:FFFF:F:FFF:FFF::FFFF:FFF,FFFFFF:FFFF,F,:FFFFFFFFF,FF:F:FF
F:,,:FF,FFFFFFF
@A00155:342:HHGFNDSXY:1:2527:29677:34601 2:N:0:GAACCTAG+TCCGCATA
ATTTTCATACATGATGAAATTCATTATTGGTCTCGCGATCAGCTTGAGACATTTCTCCAGGTTTACAA
ACCTAAAAATTTGTGGGCTACTCTGGTTTTTCCACCTGAGATCTTAGCTGGTTACAAATCCTCCGTTC
TGCCCTTCTTATATC
+
FFFFFFF:FFFFFFFFFFFFFFFFFFFFFFFFFFFFFFFFFFFFFFFFFFFFFFFFFFFFFFFFFFFF
FFFFFFFFFFFFFFFFFFFFF:FFFFFFFFFFFFFFFFFFFFFFFF:FFFFFFFFFFFFFFFFFFFFF
FFFFF:FF::FFFF:
@A00155:342:HHGFNDSXY:1:2209:1922:8453 2:N:0:GAACCTAG+TCCGCATA
TTTTCATACATGATGAAATTCATTATTGGTCTCGCGATCAGCTTGAGACATTTCTCCAGGTTTACAAA
CCTAAAAATTTGTGGGCTACTCTGGTTTTTCCACCTGAGATCTTAGCTGGTTACAAATCCTCCGTTCT
GCCCTTCTTATATC
+
FFFFFF:FFFFFFFFFFFFFFF:FFFFFFFFFFFFFFFFFFFFFFFFFFFFFFFFFFFFFFFFFFFFF
F,FFFFFFFFFFFFFFFFFFFFFFFFFFF:FFFF:FFFFFFFFFFFFFFFFF,FF:FFFFFFFFFFFF
FF:F,FF,FF:F,F
@A00155:342:HHGFNDSXY:1:1512:20320:23719 2:N:0:GAACCTAG+TCCGCATA
TTTTCATACATGATGAAATTCATTATTGGTCTCGCGATCAGCTTGAGACATTTCTCCAGGTTTACAAA
CCTAAAAATTTGTGGGCTACTCTGGTTTTTCCACCTGAGATCTTAGCTGGTTACAAATCCTCCGTTCT
GCCCTTCTTATATCA
+
FFFFFFFFFFFFFFFFFF,FFFFFFFFFFFFFFFFFFFF:FFFFFFFFFFFFFFFFFFFFFFFFFFFF
FFFFFFFFFFFFFFFFFFFFFFFFFFFFFFFFFFFFFFFFFFF,FFFFFFFFFFFFFFFFFFFFFFFF
FFFFFFFFFFFFFFF
@A00155:342:HHGFNDSXY:1:2658:14082:10692 2:N:0:GAACCTAG+TCCGCATA
TTTTCATACATGATGAAATTCATTATTGGTCTCGCGATCAGCTTGAGACATTTCTCCAGGTTTACAAA
CCTAAAAATTTGTGGGCTACTCTGGTTTTTCCACCTGAGATCTTAGCTGGTTACAAATCCTCCGTTCT
GCCCTTCTTATATCA
+
FFFFFFFFFFFFFFFF:FFFFFFFFFFFFFFFFFFFFFFFFFFFFFFFFFFFFFFFFFFFFFFFFFFF
F:FFFFF:FFFFFFFFFFFFFFFFFFFFFFFFFFFFFFFFFFFFFFFFFFFFFFFFFFFFFFFFFFFF
FFFFFFF:FFFFFFF
@A00155:342:HHGFNDSXY:1:2165:8377:7670 1:N:0:GAACCTAG+TCCGCATA
TCATACATGATGAAATTCATTATTGGTCTCGCGATCAGCTTGAGACATTTCTCCAGGTTTACAAACCT
AAAAATTTGTGGGCTACTCTGGTTTTTCCACCTGAGATCTTAGCTGGTTACAAATCCTCCGTTCTGCC
CTTCTTATATCAATT
+
FFFFFFFFFFFFFFFFFFFFFFFFFFFFFFFFFFFFFFFFFFFFFFFFFFFFFFFFFFFFFFFFFFFF
FFFFFFFFFFFFFFFFFFFFFFFFFFFFFFFFFFFFFFFF,FFFFFFFFFFFFFFFFFFFFFFFFFFF
FFFFFFFFFFFFFFF
@A00155:342:HHGFNDSXY:1:2523:21992:15029 1:N:0:GAACCTAG+TCCGCATA
TCATACATGATGAAATTCATTATTGGTCTCGCGATCAGCTTGAGACATTTCTCCAGGTTTACAAACCT
AAAAATTTGTGGGCTACTCTGGTTTTTCCACCTGAGATCTTAGCTGGTTACAAATCCTCCGTTCTGCC
CTTCTTATATCAATT
```

+
FFFFFFFFFFFFFFF:FFFFFFFFFFFFFFFFFFFFFFFFFFFFFFFFFFFFFFFFFFFFFFFFFFFF
FFFFFF:FFFFFFFFFFFFFFFFFFFFFFFFFFFFFFFFFFFFFFFFFFFFFFFFFFFFFFFFF
FFFFFFFFFFFFFFF
@A00155:342:HHGFNDSXY:1:1358:14172:19993 1:N:0:GAACCTAG+TCCGCATA
CATACATGATGAAATTCATTATTGGTCTCGCGATCAGCTTGAGACATTTCTCCAGGTTTACAAACCTA
AAAATTTGTGGGCTACTCTGGTTTTTCCACCTGAGATCTTAGCTGGTTACAAATCCTCCGTTCTGCCC
TTCTTATATCAATTT
+
FFFFFFFFFF:::FFFFFFFF:FFFFFFFFFFFFFFFFFFFFFF:FFFFFFF:FF:FFFF,:FFFFFF
FF:FFFFFFFFFFFFFFFFFFFFFFFFFFFF,FFFFFFFFFFFFFFFFFFFFFFFFFFF:FFFFFF:F
FF:FFFFFFFFFFFF
@A00155:342:HHGFNDSXY:1:2516:16288:7091 2:N:0:GAACCTAG+TCCGCATA
CATACATGATGAAATTCATTATTGGTCTCGCGATCAGCTTGAGACATTTCTCCAGGTTTACAAACCTA
AAAATTTGTGGGCTACTCTGGTTTTTCCACCTGAGATCTTAGCTGGTTACAAATCCTCCGTTCTGCCC
TTCTTATATCAATTT
+
FFFFFFFFFFFFFFFFFFFFFFFFFFFFFFFFFFFFFFFFFFFFFFFFFFFFFFFFFFFFFFFFFFFF
FFFFFFFFFFFFFFFFFFFFFFFFFFF:FFFFFF:FFFFFFFFFFFFFFFFFFFFFFFFFFFFFFFFF
FFFFFFFFFFFFFFF
@A00155:342:HHGFNDSXY:1:2670:22978:13322 1:N:0:GAACCTAG+TCCGCATA
ATGATGAAATTCATTATTGGTCTCGCGATCAGCTTGAGACATTTCTCCAGGTTTACAAACCTAAAAAT
TTGTGGGCTACTCTGGTTTTTCCACCTGAGATCTTAGCTGGTTACAAATCCTCCGTTCTGCCCTTCTT
ATATCAATTTGAAAT
+
FFFFFFFFFFFFFFFFFFFFFFFFFFFFFFFFFFFFFFFFFFFFFFFFFFFFFFFFFFFF:FFFFFFF
FFFFFFFFFFFFFFFF:FFFFFFFFFFFFFFFFFFFFFFFFFFFFFFFFFFFFFFFFFFFFFFFFFF
FFFFFFFFFFFFFFF
@A00155:342:HHGFNDSXY:1:1626:26151:2284 2:N:0:GAACCTAG+TCCGCATA
TGATGAAATTCATTATTGGTCTCGCGATCAGCTTGAGACATTTCTCCAGGTTTACAAACCTAAAAATT
TGTGGGCTACTCTGGTTTTTCCACCTGAGATCTTAGCTGGTTACAAATCCTCCGTTCTGCCCTTCTTA
TATCAATTTGAAATT
+
FFFFFFFFFFFFFFFFFFFFFFFFFFFFFFFFFFFFFFFFFFFFFFFFFFFFFF:FFFFFFFFFFFFF
FFFFFFFFFFFFFFFFFFFFFFFF:FFFFFF:FFFFFFFF:FF:FFFFF:FFFFFFFFFFFFFFFFFF
FFFFFFFFFFFFFFF
@A00155:342:HHGFNDSXY:1:2233:7889:3067 2:N:0:GAACCTAG+TCCGCATA
TGATGAAATTCATTATTGGTCTCGCGATCAGCTTGAGACATTTCTCCAGGTTTACAAACCTAAAAATT
AGTGGGCTACTCTGGTATTTCCACCTGAGATCTTAGCTGGTTACAAATCCTCCGTTCTGCCCTTCTTA
TATCAAATTGAAATT
+
:FFFFFFF,FFFFFF:FFFFFFFFFFFFFF:FF,FFFFFF:F:FFFFF,F,FFFFFFFFF:FFFFF::
,F,FFFFFFFFFF:F,,FFFF:FFF:FFFF:FFFFFFFFFFFFFFFFFFFFFF,,FFF,,F:,FFFFF
FFFFFF,F,FFFFFF
@A00155:342:HHGFNDSXY:1:1438:10908:6324 2:N:0:GAACCTAG+TCCGCATA
GATGAAATTCATTATTGGTCTCGCGATCAGCTTGAGACATTTCTCCAGGTTTACAAACCTAAAAATTT
GTGGGCTACTCTGGTTTTTCCACCTGAGATCTTAGCTGGTTACAAATCCTCCGTTCTGCCCTTCTTAT
ATCAATTTGAAATTC
+
FFFFFFFFFFFFFFFFFFFFFFFFFFFFFFFF:FFFFFFF:FFFFFFFFFFFFFFFFFFFFF,FF:FF
FFFFFFF,FFFFFFFFFFFFFFFFFFFFFFFFFFFF,FFFFFFFFFFFFFF,FFFFFFFFFFFFFFF:
FFFF:FFFF:FFFFF
@A00155:342:HHGFNDSXY:1:1229:31620:27602 1:N:0:GAACCTAG+TCCGCATA
ATGAAATTCATTATTGGTCTCGCGATCAGCTTGAGACATTTCTCCAGGTTTACAAACCTAAAAATTTG

TGGGCTACTCTGGTTTTTCCACCTGAGATCTTAGCTGGTTACAAATCCTCCGTTCTGCCCTTCTTATA
TCAATTTGAAATTCA
+
FFFFFFFFFFFFFFFFFFFFFFFFFFFFFFFFFFFFFFFFFFFFFFFFFFFFFFFFFFFFFFFFFFF:FF
FFFFFFFFFFFFFFFFFFFFFFFFFFFFFFFFFFFFFFFFFFFFFFFFFFFFFFFFFFFFFFFFFFFFFF
FFFFFFFFFFFFFFF
@A00155:342:HHGFNDSXY:1:1441:16712:17785 1:N:0:GAACCTAG+TCCGCATA
ATGAAATTCATTATTGGTCTCGCGATCAGCTTGAGACATTTCTCCAGGTTTACAAACCTAAAAATTTG
TGGGCTACTCTGGTTTTTCCACCTGAGATCTTAGCTGGTTACAAATCCTCCGTTCTGCCCTTCTTATA
TCAATTTGAAATTCA
+
FFFFFFFFFFFFFFFFFFFFFFFFFFFFFFFFFFFFFFFFFFFFFFFFFFFFFFFFFFFFFFFFFFFFFF
FFFFFFFFFFFFFFFFFFFFFFFFFFFFFFFFFFFFFFFFFFFFFFFFFFFFFFFFFFFFFFFFFFFFFF
FFFFFFFFFFFFFFF
@A00155:342:HHGFNDSXY:1:2516:16288:7091 1:N:0:GAACCTAG+TCCGCATA
ATGAAATTCATTATTGGTCTCGCGATCAGCTTGAGACATTTCTCCAGGTTTACAAACCTAAAAATTTG
TGGGCTACTCTGGTTTTTCCACCTGAGATCTTAGCTGGTTACAAATCCTCCGTTCTGCCCTTCTTATA
TCAATTTGAAATTCA
+
FFFFFFFFFFFFFFFFFFFFFFFFFFFFFFFFFFFFFFFFFFFFFFFFFFFFFFFFFFFFFFFFFFFFFF
FFFFFFFFFFFFFFFFFFFFFFFFFFFFFFFFFFFFFFFFFFFFFFFFFFFFFFFFFFFFFFFFFFFFFF
FFFFFFFFFFFFFFF
@A00155:342:HHGFNDSXY:1:2526:15013:18599 1:N:0:GAACCTAG+TCCGCATA
ATGAAATTCATTATTGGTCTCGCGATCAGCTTGAGACATTTCTCCAGGTTTACAAACCTAAAAATTTG
TGGGCTACTCTGGTTTTTCCACCTGAGATCTTAGCTGGTTACAAATCCTCCGTTCTGCCCTTCTTATA
TCAATTTGAAATTCA
+
FFFFFFFFFFFFFFFFFFFF:FFFFFFFFFFFFFFFFFFFFFFFFFFFFFFFFFFFFFFFFFFFFFFFFF
FFFFFFFFFFFFFFFFFFFFFFFFFFFFFFFFFFFFFFFFFFFFFFFFFFFFFFFFFFFFFFFFFFFFFF
FFFFFFFFFFFFFFF
@A00155:342:HHGFNDSXY:1:1229:31620:27602 2:N:0:GAACCTAG+TCCGCATA
ATGAAATTCATTATTGGTCTCGCGATCAGCTTGAGACATTTCTCCAGGTTTACAAACCTAAAAATTTG
TGGGCTACTCTGGTTTTTCCACCTGAGATCTTAGCTGGTTACAAATCCTCCGTTCTGCCCTTCTTATA
TCAATTTGAAATTCA
+
FFFFFFFFFFFF:FFFFFFFFFFFFFFFFFFFFFFFFFF:FFFFFFFFFFFFFFFFFFFFFFFFFFFF,F
FFFFFFFFFFFFFFFFFFF:FFFFFFFFFF:FFFFFFFFFFFFFFFFFFFFFFFF:FFFFFFFFFFFF,F
FFFFFFFFFFFF:FF
@A00155:342:HHGFNDSXY:1:1573:30879:13542 2:N:0:GAACCTAG+TCCGCATA
ATGAAATTCATTATTGGTCTCGCGATCAGCTTGAGACATTTCTCCAGGTTTACAAACCTAAAAATTTG
TGGGCTACTCTGGTTTTTCCACCTGAGATCTTAGCTGGTTACAAATCCTCCGTTCTGCCCTTCTTATA
TCAATTTGAAATTCA
+
FFFFFF:FFFFFFFFFFFFFFFFFFFFFFFFFFFFFFFFFFFFFFFFFFFFFFFFFFFFFFFFFFFFFFF
FFFFFFFFFFFFFFFFFFFFFFFFFFFFFFFF:FFFFFFFFFFFFFFFFFFFFFFFFFFFFFFFF:FFFF
FFFF:FFFFFFFFFF
@A00155:342:HHGFNDSXY:1:1576:8874:33646 2:N:0:GAACCTAG+TCCGCATA
ATGAAATTCATTATTGGTCTCGCGATCAGCTTGAGACATTTCTCCAGGTTTACAAACCTAAAAATTTG
TGGGCTACTCTGGTTTTTCCACCTGAGATCTTAGCTGGTTACAAATCCTCCGTTCTGCCCTTCTTATA
TCAATTTGAAATTCA
+
FFF:FFFFFFFFFFFFFFFFFFFFFFFFFFFFFFFFFFFFFFFFFFFFFFFFFFFFFFFFFFFFFF::FF
FFFFFFFFFFFFFFFFFFFFFFFFFFFFFFFFFFFFFFFFFFFFFFFFFFFFFFF:FFFFFFFFFFFFFF
FFFFFFFFFFFFFFF

```
@A00155:342:HHGFNDSXY:1:1661:21215:4726 2:N:0:GAACCTAG+TCCGCATA
ATGAAATTCATTATTGGTCTCGCGATCAGCTTGAGACATTTCTCCAGGTTTACAAACCTAAAAATTTG
TGGGCTACTCTGGTTTTTCCACCTGAGATCTTAGCTGGTTACAAATCCTCCGTTCTGCCCTTCTTATA
TCAATTTGAAATTCA
+
F,FFFFFFFFFFFFFFFFFFFFFF:FFFFFFFFFF:FFFFFFFFFFFFFFFFFFF,FFFFFFFFFFFF
FFFF:FFF:FFFFFFFF:FFFFFFFFFFFFFFFFFFFFFFFFFFFFFFFFFFFFFFFFFFFFFFFF,F
FFFF:FFFFFFFFFF
@A00155:342:HHGFNDSXY:1:2222:2618:35524 2:N:0:GAACCTAG+TCCGCATA
ATGAAATTCATTATTGGTCTCGCGATCAGCTTGAGACATTTCTCCAGGTTTACAAACCTAAAAATTTG
TGGGCTACTCTGGTTTTTCCACCTGAGATCTTAGCTGGTTACAAATCCTCCGTTCTGCCCTTCTTATA
TCAATTTGAAATTCA
+
FFFF:FFFFFFFFFFFFFFFFFFFFFFFFFFFFFFFF:FFFFFFFFFFFFFFFFF,FFFFFFFFFFFF
FFFFFFFFFFFFFFFFFFFFFFFFFFFFFF:FFFFFFFFFFFFFFFFFFFFFFF:FFFFFFFFFFFFF
FFFFFFFFFFFFFFF
@A00155:342:HHGFNDSXY:1:2223:28203:4116 2:N:0:GAACCTAG+TCCGCATA
ATGAAATTCATTATTGGTCTCGCGATCAGCTTGAGACATTTCTCCAGGTTTACAAACCTAAAAATTTG
TGGGCTACTCTGGTTTTTCCACCTGAGATCTTAGCTGGTTACAAATCCTCCGTTCTGCCCTTCTTATA
TCAATTTGAAATTCA
+
FFFFFF:FFFFFFFFFFFFFFFFFFFFFFFFFFFFFFFFFFFFFFFFFFFFFFFFFFFFFFFFFFFFF
FFFFFFFFFFFFFFFFFFF:FFFFFFFFFFFFFFFFF:FFFFFFFFFFFFFFFFFFFFFFFFFFF:FF
FFFFF,FFF,FFF:F
@A00155:342:HHGFNDSXY:1:2247:8223:36464 2:N:0:GAACCTAG+TCCGCATA
ATGAAATTCATTATTGGTCTCGCGATCAGCTTGAGACATTTCTCCAGGTTTACAAACCTAAAAATTTG
TGGGCTACTCTGGTTTTTCCACCTGAGATCTTAGCTGGTTACAAATCCTCCGTTCTGCCCTTCTTATA
TCAATTTGAAATTCA
+
FFFFFFFFFFFFFFFFFFFFFFFFFFFFFFFFFFFFFFFFFFFFFFFFFF:FFFFFFFFFFFFFFFFF
F,FFFFFFFFFFFFFFFFFFFFFFFFFFFFFF:F:FFFFFFFFFFFFFFF:FFFFFFFFFFFFFFFFF
FFFFFFFF,FFFF:F
@A00155:342:HHGFNDSXY:1:2325:5954:35978 2:N:0:GAACCTAG+TCCGCATA
ATGAAATTCATTATTGGTCTCGCGATCAGCTTGAGACATTTCTCCAGGTTTACAAACCTAAAAATTTG
TGGGCTACTCTGGTTTTTCCACCTGAGATCTTAGCTGGTTACAAATCCTCCGTTCTGCCCTTCTTATA
TCAATTTGAAATTCA
+
FFFFFFFFFFFFFFFFFFFFFFFFFFFFFFFFFFFFFFFFFFFFFFFFFFFFFFFFFFFFFFFFFFFF
FFFFFFFFFFFFFFFFFFFFFFF:FFFFFFFFFFFFFFFFFFFFFFFFFFFFFFFFFFFFF:FFFFFF
FFFFFFFFFFFFFFF
@A00155:342:HHGFNDSXY:1:2464:15103:4006 2:N:0:GAACCTAG+TCCGCATA
ATGAAATTCATTATTGGTCTCGCGATCAGCTTGAGACATTTCTCCAGGTTTACAAACCTAAAAATTTG
TGGGCTACTCTGGTTTTTCCACCTGAGATCTTAGCTGGTTACAAATCCTCCGTTCTGCCCTTCTTATA
TCAATTTGAAATTCA
+
FFFFFFFFFFFFFFFFFFFF:FFFFFFFFFFFFFFFFFFFFFFFFFFFFFFFFFFFFFFFFFFFFFFF
FFFFFFFFFFFFFFFFFF,FFFFFFFFFFFFFFFFFFFFFFFFFFFFFFFFFFFFFFFFFFFFFFFFF
FFFFFFFFFFFFFFF
@A00155:342:HHGFNDSXY:1:2625:12328:11193 2:N:0:GAACCTAG+TCCGCATA
ATGAAATTCATTATTGGTCTCGCGATCAGCTTGAGACATTTCTCCAGGTTTACAAACCTAAAAATTTG
TGGGCTACTCTGGTTTTTCCACCTGAGATCTTAGCTGGTTACAAATCCTCCGTTCTGCCCTTCTTATA
TCAATTTGAAATTCA
+
FFF:FFFFFFFFFFFFFFFFFFFFFFFFFFFFFFFFFFFFFFFFFFFFFFFFFFFFFFFFFFFFFFFF
```

```
FFFFFFFFFFFFF:FFFFFFFFFFFFFFFFFFFFFFFFFFFFFFFFFFFFFFFFFFFFFFFFFFFFFF
FFFFFFFFFFFFFF:
@A00155:342:HHGFNDSXY:1:2659:26033:24283 2:N:0:GAACCTAG+TCCGCATA
ATGAAATTCATTATTGGTCTCGCGATCAGCTTGAGACATTTCTCCAGGTTTACAAACCTAAAAATTTG
TGGGCTACTCTGGTTTTTCCACCTGAGATCTTAGCTGGTTACAAATCCTCCGTTCTGCCCTTCTTATA
TCAATTTGAAATTCA
+
FFFFFFFFFFFFFFFFFFFFFFFFFFFFFFFFFFFFFFFFFFFFFFFFFFFFFFFFFFFFFFFFFFFF
FFFFFFFFFFFFFFFFFFFFFFFFFFFFFFFFF:FFFFFFFFFFFFFFFFFFFFFFFFFFFFFFFFFF
FFFFFFFFFFFFFFF
@A00155:342:HHGFNDSXY:1:1635:2564:26412 1:N:0:GAACCTAG+TCCGCATA
TGAAATTCATTATTGGTCTCGCGATCAGCTTGAGACATTTCTCCAGGTTTACAAACCTAAAAATTTGT
GGGCTACTCTGGTTTTTCCACCTGAGATCTTAGCTGGTTACAAATCCTCCGTTCTGCCCTTCTTATAT
CAATTTGAAATTC
+
FFFFFFFFFFFFFFFFFFFFFFFFFFFFFFFFFFFFFFFFFFFFF,F:FFFFFFF:FFFFFFFFFFFF
FFFFFFFFFFF:FFFFF:,FFFFFFFFF,FFFF,FFFFFF:FFFF,FFF,FFFFFFFFFFFFFF:FFF
:FFFFFFFF::FF
@A00155:342:HHGFNDSXY:1:1103:26910:30405 1:N:0:GAACCTAG+TCCGCATA
TGAAATTCATTATTGGTCTCGCGATCAGCTTGAGACATTTCTCCAGGTTTACAAACCTAAAAATTTGT
GGGCTACTCTGGTTTTTCCACCTGAGATCTTAGCTGGTTACAAATCCTCCGTTCTGCCCTTCTTATAT
CAATTTGAAATTCAC
+
FFFFFFFFFFFFFFFFFFFFFFFFFFFFFFFFFFFFFFFFFFFFFFFFFFFFFFFF:FFFFFFFFFFF
FFFFFFFFFFFFFFFFFFFFFFFFFFFFFFFFFFFFFFFFFFFFFFFFFFF:FFFFFFFFFFFFFFFF
FFFFFFFFFFFFFFF
@A00155:342:HHGFNDSXY:1:1113:26341:7404 1:N:0:GAACCTAG+TCCGCATA
TGAAATTCATTATTGGTCTCGCGATCAGCTTGAGACATTTCTCCAGGTTTACAAACCTAAAAATTTGT
GGGCTACTCTGGTTTTTCCACCTGAGATCTTAGCTGGTTACAAATCCTCCGTTCTGCCCTTCTTATAT
CAATTTGAAATTCAC
+
FFFFFFFFFFFF:FFFFFF:FFFFFFFFFFFFFFFFFFFFFFFFFFFFFFFFFFFFFFFFFFFFFFFF
FFFFFFFFFFFF:FFFFFFFF:FFFFFFFFFFFFFFFFFFFFFFFFFFFFFFFFFFFFFFFFFFFFFF
FFFFFFFFFFFFFFF
@A00155:342:HHGFNDSXY:1:1121:17852:36260 1:N:0:GAACCTAG+TCCGCATA
TGAAATTCATTATTGGTCTCGCGATCAGCTTGAGACATTTCTCCAGGTTTACAAACCTAAAAATTTGT
GGGCTACTCTGGTTTTTCCACCTGAGATCTTAGCTGGTTACAAATCCTCCGTTCTGCCCTTCTTATAT
CAATTTGAAATTCAC
+
FFF,FFFFFFFFF:FFFFFFFFFFFFFFFFFFFFFFFFFFFF::FF,FFFFFFFFF:FFF:FF:F,FF
FFFFFFFF:FFFFFFFFFFFFFFFFFFFFFFF:FFFFFFFFFFFFFFFFF:FFFFFF,:FFFFFFFFF
FF:FFFFF:F,FFFF
@A00155:342:HHGFNDSXY:1:1129:29803:31908 1:N:0:GAACCTAG+TCCGCATA
TGAAATTCATTATTGGTCTCGCGATCAGCTTGAGACATTTCTCCAGGTTTACAAACCTAAAAATTTGT
GGGCTACTCTGGTTTTTCCACCTGAGATCTTAGCTGGTTACAAATCCTCCGTTCTGCCCTTCTTATAT
CAATTTGAAATTCAC
+
FFFFFFFFFFFFFFFFFFFFFFFFFFFFFFFFFFFFFFFFFFFFFFFFFFFFFFF:FFFFFFFFFFFF
FFFFFFFFFFFFFFFFFF:FFFFFFFFF:FFFFFFFFFFFFFFFFFFFFFFFFFFFFFFFFFFFFFFF
FFFFFFFFFFFFFFF
@A00155:342:HHGFNDSXY:1:1142:6162:29794 1:N:0:GAACCTAG+TCCGCATA
TGAAATTCATTATTGGTCTCGCGATCAGCTTGAGACATTTCTCGTGGTTTACAAACCTAAAAATTTGT
GGGCTACTCTGGTTTTTCCACCTGAGATCTTAGCTGGTTACAAATCCTCCGTTCTGCCCTTCTTATAT
CAATTTGAAATTCAC
```

```
+
FFFFFFFFFFFFFFFFFFFFFFFFFFFFFFFFFFFFFFFFFFFFFF:FFFFFFFFFFFFFFFFFFFF,FF
FFFFFFFFFFFFFFFFFFFFFFFFFFFFFFFFFFFFFFFFFFFFFFFFFFFFFFFFFFFFFFFFFFFFFF
FFFFFFFFFFFFFFF
@A00155:342:HHGFNDSXY:1:1161:20157:27727 1:N:0:GAACCTAG+TCCGCATA
TGAAATTCATTATTGGTCTCGCGATCAGCTTGAGACATTTCTCCAGGTTTACAAACCTAAAAATTTGT
GGGCTACTCTGGTTTTTCCACCTGAGATCTTAGCTGGTTACAAATCCTCCGTTCTGCCCTTCTTATAT
CAATTTGAAATTCAC
+
:FFFFFFFFFFFFFFFFFFFFFFFFFFFFFFFFFFFFFFFFFFFFFFFF:FFFFFFFFFFFFFFFFFFFF
FFFF,FFFFFFFFFFFFFFFFFFFFFFF:FFFFFFFFFFFFFFFFFFFFFFFFFFFFFFFFF:FFFFFFF
FFFFFFFFFFFFFFF
@A00155:342:HHGFNDSXY:1:1169:22073:31798 1:N:0:GAACCTAG+TCCGCATA
TGAAATTCATTATTGGTCTCGCGATCAGCTTGAGACATTTCTCCAGGTTTACAAACCTAAAAATTTGT
GGGCTACTCTGGTTTTTCCACCTGAGATCTTAGCTGGTTACAAATCCTCCGTTCTGCCCTTCTTATAT
CAATTTGAAATTCAC
+
FFFFFFFFFFFFFFFFFFFFFFFFFFFFFFFFFFFFFFFFFFFFFFFFFFFFFFFFFFFFFFFFFFFFFF
FFFFFFFFFFFFFFFFFFFFFFFFFFFFFFFFFFFFFFFFFFFFFFFFFFFFFFFFFFFFFFFFFFFFFF
FFFFFFFFFFFFFFF
@A00155:342:HHGFNDSXY:1:1170:30969:2707 1:N:0:GAACCTAG+TCCGCATA
TGAAATTCATTATTGGTCTCGCGATCAGCTTGAGACATTTCTCCAGGTTTACAAACCTAAAAATTTGT
GGGCTACTCTGGTTTTTCCACCTGAGATCTTAGCTGGTTACAAATCCTCCGTTCTGCCCTTCTTATAT
CAATTTGAAATTCAC
+
FFFFFFFFFFFFFFFFFFFFFFFFFFFFFFFFFFFFFFFFFFFFFFFFFFFFFFFFFFFFFFFF:FFFFF
FFFFFFFFFFFFFFFFFFFFFFFFFFFFFFFFFFFFFFFFFFFFFFFFFFFFFFFFFFFFFFFFFFFFFF
FFFFFFFFFFFFFFF
@A00155:342:HHGFNDSXY:1:1206:19307:9345 1:N:0:GAACCTAG+TCCGCATA
TGAAATTCATTATTGGTCTCGCGATCAGCTTGAGACATTTCTCCAGGTTTACAAACCTAAAAATTTGT
GGGCTACTCTGGTTTTTCCACCTGAGATCTTAGCTGGTTACAAATCCTCCGTTCTGCCCTTCTTATAT
CAATTTGAAATTCAC
+
FFFFFFFFFFFFFFFFFFFFFFFFFF:FFFFFFFFFFFFFFFFFFFFFFFFFFFFFFFFFFFFFFFFFFF
FFFFFFFFFFFFFFFFFFFFFFFFFFFFFFFFFFFFFFFFFFFFFFFFFFFFFFFFFFFFFFFFFFFFFF
FFFFFFFFFFFFFFF
@A00155:342:HHGFNDSXY:1:1174:18078:19053 1:N:0:GAACCTAG+TCCGCATA
TGAAATTCATTATTGGTCTCGCGATCAGCTTGAGACATTTCTCCAGGTTTACAAACCTAAAAATTTGT
GGGCTACTCTGGTTTTTCCACCTGAGATCTTAGCTGGTTACAAATCCTCCGTTCTGCCCTTCTTATAT
CAATTTGAAATTCAC
+
FFFFFFFFFFFFFFFFFFFFFFFFFFFFFFFFFFFFFFFFFFFFFFFFFFFFFFFFFFFFFFFFFFFFFF
FFFFFFFFFFFFFFFFFFFFFFFFFFF:FFFFFFFFFFFFFFFFFFFFFFFFFFFFFFFFFFFFFFFFFF
FFFFFFFFFFFFFFF
@A00155:342:HHGFNDSXY:1:1164:22083:18599 1:N:0:GAACCTAG+TCCGCATA
TGAAATTCATTATTGGTCTCGCGATCAGCTTGAGACATTTCTCCAGGTTTACAAACCTAAAAATTTGT
GGGCTACTCTGGTTTTTCCACCTGAGATCTTAGCTGGTTACAAATCCTCCGTTCTGCCCTTCTTATAT
CAATTTGAAATTCAC
+
F:FFFFFFFFFFFFFFFFFFFFFFFFFF,F:FFFFFFFFFFFFFFFFFFFFFF:FFFFFFFFFFFFFFFF
FFFFFFFFFFFFFFFFFFFFFFFFFFFFFFFFFFFFFFFFFFFFFFFFFFFFFFFFFFFFFFFFFFFFFF
FFFFFFFFFFFFFFF
@A00155:342:HHGFNDSXY:1:1237:3016:4460 1:N:0:GAACCTAG+TCCGCATA
TGAAATTCATTATTGGTCTCGCGATCAGCTTGAGACATTTCTCCAGGTTTACAAACCTAAAAATTTGT
```

```
GGGCTACTCTGGTTTTTCCACCTGAGATCTTAGCTGGTTACAAATCCTCCGTTCTGCCCTTCTTATAT
CAATTTGAAATTCAC
+
FFFFFFFFFFFFFFFFFFFFFFFFFFFFFFFFFFFFFFFFFFF:FFFFFFFFFFF:FFFFFFFFFF:F
FFFFFFFFFFFFF:FFFFF:FFFFF:F:FF:FFFFFFFFFF,FFFF,FFFFFFFFFFFFFFFFFFFFF
FFFFFFFFFFFFFFF
@A00155:342:HHGFNDSXY:1:1248:26910:10363 1:N:0:GAACCTAG+TCCGCATA
TGAAATTCATTATTGGTCTCGCGATCAGCTTGAGACATTTCTCCAGGTTTACAAACCTAAAAATTTGT
GGGCTACTCTGGTTTTTCCACCTGAGATCTTAGCTGGTTACAAATCCTCCGTTCTGCCCTTCTTATAT
CAATTTGAAATTCAC
+
FFFFFFFFFFFFFFFFFFFFFFFFFFFFFFFFFFFFFFFFFFFFFFFFFFFFFFFFFFFFFFFFFFFF
FFFFFFFFFFFFFFFFFFFFFFFFFFFFFFFFFFFFFFFFFFFFFFFFFFFFFFFFFFFFFFFFFFFF
FFFFFFFFFFFFFFF
@A00155:342:HHGFNDSXY:1:1263:20464:11193 1:N:0:GAACCTAG+TCCGCATA
TGAAATTCATTATTGGTCTCGCGATCAGCTTGAGACATTTCTCCAGGTTTACAAACCTAAAAATTTGT
GGGCTACTCTGGTTTTTCCACCTGAGATCTTAGCTGGTTACAAATCCTCCGTTCTGCCCTTCTTATAT
CAATTTGAAATTCAC
+
FFFFF:FFFFFFFFFFFFFFFFFFFFFFFFFFFFFFFFFFFFFFFFFFFFFFFFFFFFFFFFFFFFFF
FFFFFFFFFFFFFFFFFFFFFFFFFFF:FFFFFFFFFFFFFFFFFFFFFFFFFFFFFFFFFFFFFFFF
FF:FFFFFFF:FFFF
@A00155:342:HHGFNDSXY:1:1263:5828:36385 1:N:0:GAACCTAG+TCCGCATA
TGAAATTCATTATTGGTCTCGCGATCAGCTTGAGACATTTCTCCAGGTTTACAAACCTAAAAATTTGT
GGGCTACTCTGGTTTTTCCACCTGAGATCTTAGCTGGTTACAAATCCTCCGTTCTGCCCTTCTTATAT
CAATTTGAAATTCAC
+
FFFFFFFFFFFFFFFFFFFFF:FFFFFFFFFFFFFFFFFFFFFFFFFFFFFFFFFFFFFFFFFFFFFF
FFFFFFFFFFFFFFFFFF:FFFFFFFFFFF:FF:FFFFFFFFF:FFFFFFFFFFFFFFFFFFFFFFFF
FFFFFFFFFFFFFFF
@A00155:342:HHGFNDSXY:1:1269:25183:8844 1:N:0:GAACCTAG+TCCGCATA
TGAAATTCATTATTGGTCTCGCGATCAGCTTGAGACATTTCTCCAGGTTTACAAACCTAAAAATTTGT
GGGCTACTCTGGTTTTTCCACCTGAGATCTTAGCTGGTTACAAATCCTCCGTTCTGCCCTTCTTATAT
CAATTTGAAATTCAC
+
FFFFFFFFFF:FFFFFFFFFFFFFFFFFFFFFFFFFFFFFF::FFFFF:FFFFFFFFFFFFFFFFFFF
FFFFFFFFFFFFFFFFFFFFFFFFFFFFFFFFFFFFFFFFFFFFFFFFFFFFFFFFFFFFFF:FFFFF
FFFFFFFFFFFFFFF
@A00155:342:HHGFNDSXY:1:1341:3613:35430 1:N:0:GAACCTAG+TCCGCATA
TGAAATTCATTATTGGTCTCGCGATCAGCTTGAGACATTTCTCCAGGTTTACAAACCTAAAAATTTGT
GGGCTACTCTGGTTTTTCCACCTGAGATCTTAGCTGGTTACAAATCCTCCGTTCTGCCCTTCTTATAT
CAATTTGAAATTCAC
+
FFFFFFFFF:FFFFFFFFFFFFFFFFFFFFFFFFFFFFFFFFFFF:FFFFFFF:FFFFFFFFFFFFFF
FFF:FFFFFFFFFFFFFFFFFFFFFFFFFFFFF:FFFFFFFFFFFF:FFFFFFFFFFFFFFFFFFFFF
FFFFFFFFFFFFFFF
@A00155:342:HHGFNDSXY:1:1347:28528:1799 1:N:0:GAACCTAG+TCCGCATA
TGAAATTCATTATTGGTCTCGCGATCAGCTTGAGACATTTCTCCAGGTTTACAAACCTAAAAATTTGT
GGGCTACTCTGGTTTTTCCACCTGAGATCTTAGCTGGTTACAAATCCTCCGTTCTGCCCTTCTTATAT
CAATTTGAAATTCAC
+
FFFFFFFFFFFFFFFFFFFFFFFFFFFFFFFFFFFFFF:FFFFFFFFFF:FFFFFFFFFFFFFFFFFF
FFFFFFFFFFFFFFFFFFFFFFFFFFFFFF:FFFFFFFFFFFFFFFFFFFFFFFFFFFFFFFFFFFFF
FFFFFFFFFF,FFFF
```

```
@A00155:342:HHGFNDSXY:1:1365:19244:11083 1:N:0:GAACCTAG+TCCGCATA
TGAAATTCATTATTGGTCTCGCGATCAGCTTGAGACATTTCTCCAGGTTTACAAACCTAAAAATTTGT
GGGCTACTCTGGTTTTTCCACCTGAGATCTTAGCTGGTTACAAATCCTCCGTTCTGCCCTTCTTATAT
CAATTTGAAATTCAC
+
FFFFFFFFFFFFFFFFFFFFFFFFFFFFFFFFFFFFFFFFFFFFFFFFFFFFFFFFFFFFFFF,FFFF
FFFFFFFFFFFFFF:FFFFFFFF:FFFFFFFFFFFFFFFFFFFFFFFFFFFFFFFFFFFFFFFFFFFF
FFFFFFFFFFFFFFF
@A00155:342:HHGFNDSXY:1:1415:14977:18599 1:N:0:GAACCTAG+TCCGCATA
ATCTATTCATTATTGGTCTCGCGATCAGCTTGAGACATTTCTCCAGGTTTACAAACCTAAAAATTTGT
GGGCTACTCTGGTTTTTCCACCTGAGATCTTAGCTGGTTACAAATCCTCCGTTCTGCCCTTCTTATAT
CAATTTGAAATTCAC
+
FFFFFFFFF,FFFFFFFFFFFFFFFFFFFFFFFFFFFFFFFFFFFFFFFFFFFFFFFFFFFFFFFFFF
FFFFFFFFFFFFFFFFFFFFFFFFFFFFFFFFFFFFFFFFFFFFFFFFFFFFF:FFFFFFFFFF:FFF
FFFFFFFFFFFFFFF
@A00155:342:HHGFNDSXY:1:1438:19533:6856 1:N:0:GAACCTAG+TCCGCATA
TGAAATTCATTATTGGTCTCGCGATCAGCTTGAGACATTTCTCCAGGTTTACAAACCTAAAAATTTGT
GGGCTACTCTGGTTTTTCCACCTGAGATCTTAGCTGGTTACAAATCCTCCGTTCTGCCCTTCTTATAT
CAATTTGAAATTCAC
+
FFFF:FFFFFFFFFFFFFFFFFFFFFFFFFFFFFFFFFFFFFFFFFFFFFFFFFFFFFFFFFFFFFFF
FFFFFFFFFFFFFFFFFFFFFFFFFFFFFFFFFFFFFFFFFFFFFFFFFFFFFFFFFFFFFFFFFFFF
FFFFFFFFFFFFFFF
@A00155:342:HHGFNDSXY:1:1464:25545:4460 1:N:0:GAACCTAG+TCCGCATA
TGAAATTCATTATTGGTCTCGCGATCAGCTTGAGACATTTCTCCAGGTTTACAAACCTAAAAATTTGT
GGGCTACTCTGGTTTTTCCACCTGAGATCTTAGCTGGTTACAAATCCTCCGTTCTGCCCTTCTTATAT
CAATTTGAAATTCAC
+
FFFFFF:FFFF:FFFFFFFFFFFFFF::FFFFFFFFFFFFFFFFFFFFFFFFFFFFFFFFFFFFFFFF
FFFFFFFFFFFFFFFFFFFFFFFFFFFFFF:FFFFFFFFFFFFFFFFFFFFFFFFFFFFFFFFFFFFF
FFFFFFFFFFFFFFF
@A00155:342:HHGFNDSXY:1:1508:7654:1282 1:N:0:GAACCTAG+TCCGCATA
TGAAATTCATTATTGGTCTCGCGATCAGCTTGAGACATTTCTCCAGGTTTACAAACCTAAAAATTTGT
GGGCTACTCTGGTTTTTCCACCTGAGATCTTAGCTGGTTACAAATCCTCCGTTCTGCCCTTCTTATAT
CAATTTGAAATTCAC
+
FFFFFFFFFFFFFFFFFFFFFFFFFFFFFFFFFFFFFFFFFFFF:FFFFFFFFFFFFFFFFFFFFFFF
FFFFFFFFFFFFFFFFFFFFFFFFFFFFFFFFFFFFFFFFFF:FFFFFF:FFFFFFFFFFFFFFFFFF
FFFFFFFFFFFFFFF
@A00155:342:HHGFNDSXY:1:1550:22815:3897 1:N:0:GAACCTAG+TCCGCATA
TGAAATTCATTATTGGTCTCGCGATCAGCTTGAGACATTTCTCCAGGTTTACAAACCTAAAAATTTGT
GGGCTACTCTGGTTTTTCCACCTGAGATCTTAGCTGGTTACAAATCCTCCGTTCTGCCCTTCTTATAT
CAATTTGAAATTCAC
+
FFFFF:FFFFFFFFFFFFFFFFFFFF::FFFFFFFFFFFFFFFFFFFFFFFFFFFFFF:FFFFFFFFF
FFFFFFFFFFFFFFFFFFFFFFFFFFFFFFFFFFFFFFFFFFFFFFFFFFFFFFFFFFFFFFFFFFFF
FFFFFFFFFFF,FFF
@A00155:342:HHGFNDSXY:1:1565:14461:21089 1:N:0:GAACCTAG+TCCGCATA
TGAAATTCATTATTGGTCTCGCGATCAGCTTGAGACATTTCTCCAGGTTTACAAACCTAAAAATTTGT
GGGCTACTCTGGTTTTTCCACCTGAGATCTTAGCTGGTTACAAATCCTCCGTTCTGCCCTTCTTATAT
CAATTTGAAATTCAC
+
FFFFFFFFFFFFFFFFFFF:FFFFFFFFFFFFFFFFFF,FFFFFFFFFFFFFFF:FFFFFFFFFFFFF
```

FFFFFFFFFFFFFFFFFFFFFFFFFFFFFFFFFFFFFFFFFFFFFFFFFFFFFFFFFFFF:FFFFFFF
FFFFFFFFFF:FFFF
@A00155:342:HHGFNDSXY:1:1562:4553:15201 1:N:0:GAACCTAG+TCCGCATA
TGAAATTCATTATTGGTCTCGCGATCAGCTTGAGACATTTCTCCAGGTTTACAAACCTAAAAATTTGT
GGGCTACTCTGGTTTTTCCACCTGAGATCTTAGCTGGTTACAAATCCTCCGTTCTGCCCTTCTTATAT
CAATTTGAAATTCAC
+
FFFFFFFF:F,FFFFFFFFFFFFFFFFFFFFFFFFFFF,FFFFFFFFFFFFFFFFFFFFFFFFFFFFF
FFFFFFFFFFFFFFFFFFFFFFFFFFFFFFFFFFFFFFFFF:FFFFFFFFFFF:FFFFFFFFFFFFFF
FFFFF:FFFFFFFFF
@A00155:342:HHGFNDSXY:1:1569:21450:27242 1:N:0:GAACCTAG+TCCGCATA
TGAAATTCATTATTGGTCTCGCGATCAGCTTGAGACATTTCTCCAGGTTTACAAACCTAAAAATTTGT
GGGCTACTCTGGTTTTTCCACCTGAGATCTTAGCTGGTTACATATCCTCCGTTCTGCCCTTCTTATAT
CAATTTGAAATTCAC
+
:FFFFFF:FFFF:F:FFFF:FF:FF,,F:FFFFFFF:FFFFF,:,FFFFFFF:FFF,FFFFFFFFFFF
,FFFFF,FFFFFFFFFFFF:F:FF,FFFFFFFF:F:FF:F,F,FF:FF,FF:F,FF::,FF:FFFF:F
FFFFFFFFFFFFFFF
@A00155:342:HHGFNDSXY:1:1554:31828:35603 1:N:0:GAACCTAG+TCCGCATA
TGAAATTCATTATTGGTCTCGCGATCAGCTTGAGACATTTCTCCAGGTTTACAAACCTAAAAATTTGT
GGGCTACTCTGGTTTTTCCACCTGAGATCTTAGCTGGTTACAAATCCTCCGTTCTGCCCTTCTTATAT
CAATTTGAAATTCAC
+
FFFFFFFFFFFFFF:FFFFFFFFFFFFFFFFFFFFFFFFFFFFFFFFFFFFFFFFFFFFFFFFFFFFF
FFFF:FFFFFFFFFFFFFFFF:FFFFFFFFFFFFFFFFFFFFFFFFFFFFFFFFFFFFFFFFFFF:F
FFFFFFFFFFFFFFF
@A00155:342:HHGFNDSXY:1:1576:8874:33646 1:N:0:GAACCTAG+TCCGCATA
TGAAATTCATTATTGGTCTCGCGATCAGCTTGAGACATTTCTCCAGGTTTACAAACCTAAAAATTTGT
GGGCTACTCTGGTTTTTCCACCTGAGATCTTAGCTGGTTACAAATCCTCCGTTCTGCCCTTCTTATAT
CAATTTGAAATTCAC
+
:FFFFF,FFFFF,FFFFFFFFFFFFF:FFFFFFFFFFFFFFFFFFFFFFFFFFFFFFFFFF:FFFFFF
FFFFFFFFFFFFFFFFFFFFFFFFFFFFFFFFFFFFFFFFFFFFFFFFFFFFFFFFFFFFFFFFFFFF
FFFFFFFFFFFFFFF
@A00155:342:HHGFNDSXY:1:1570:23909:26428 1:N:0:GAACCTAG+TCCGCATA
TGAAATTCATTATTGGTCTCGCGATCAGCTTGAGACATTTCTCCAGGTTTACAAACCTAAAAATTTGT
GGGCTACTCTGGTTTTTCCACCTGAGATCTTAGCTGGTTACAAATCCTCCGTTCTGCCCTTCTTATAT
CAATTTGAAATTCAC
+
FFFFFFFFFFFFF,FFFFFFFFF:FFFFFFFFFFFF:FFFFFFFFFFFFFFFFFFFFFFFFFFFFFFF
FFFFFFFF:FFFFFFFFFFFFFFFFF:FFFFFF:FFFFFFFFFFFFFFFFFFFFFFFFFFFFFFFFFF
FFFFFFFFFFFFFFF
@A00155:342:HHGFNDSXY:1:1609:28022:2769 1:N:0:GAACCTAG+TCCGCATA
TGAAATTCATTATTGGTCTCGCGATCAGCTTGAGACATTTCTCCAGGTTTACAAACCTAAAAATTTGT
GGGCTACTCTGGTTTTTCCACCTGAGATCTTAGCTGGTTACAAATCCTCCGTTCTGCCCTTCTTATAT
CAATTTGAAATTCAC
+
FFFFFFFFFFFFFFFFFFFFFFFFFFFFFFFFFFFFFFFFFFFF:FFFFFFFFFFFFFFFFFFFFFFF
FFFFFFFFFFFF:FFFFFFFFFFFFFFFFFF:FFFFFFFFFFFFFFFFFFFFFFFFFFFFFFFFFFFF
FF:FFFFFFFFFFFF
@A00155:342:HHGFNDSXY:1:1609:27281:2957 1:N:0:GAACCTAG+TCCGCATA
TGAAATTCATTATTGGTCTCGCGATCAGCTTGAGACATTTCTCCAGGTTTACAAACCTAAAAATTTGT
GGGCTACTCTGGTTTTTCCACCTGAGATCTTAGCTGGTTACAAATCCTCCGTTCTGCCCTTCTTATAT
CAATTTGAAATTCAC

+
FFFFFFFFFFF:FFFFFFFFFFFFFFFFFFFFFFFFFFFFFFFFFFFFFFFFFFFFFFFFFFFFFFFF
FFFFFFFFFFFF:FFFFFFFFFFFFFFFFFFFFFFFFFFFFFF:FFFFFFFFF:FFFFFFFFFFFFFF
FFFFFFFFFFFFFFF
@A00155:342:HHGFNDSXY:1:1609:21178:11929 1:N:0:GAACCTAG+TCCGCATA
TGAAATTCATTATTGGTCTCGCGATCAGCTTGAGACATTTCTCCAGGTTTACAAACCTAAAAATTTGT
GGGCTACTCTGGTTTTTCCACCTGAGATCTTAGCTGGTTACAAATCCTCCGTTCTGCCCTTCTTATAT
CAATTTGAAATTCAC
+
FFFFFFFFFFFFFFFFFFFFFFFFFFFFFFFFFFFFFFFFFFFFFFFFFFFFFFFFFFFFFFFFFFFF
FFFFFFFFFFFFFFFFFFFFFFFFFFFFFFFFFFFFFFFFFFFFFFFFFFFFFFFFFFFFFFFFFFFF
FFFFFFFFFFFFFFF
@A00155:342:HHGFNDSXY:1:1653:17400:14591 1:N:0:GAACCTAG+TCCGCATA
TGAAATTCATTATTGGTCTCGCGATCAGCTTGAGACATTTCTCCAGGTTTACAAACCTAAAAATTTGT
GGGCTACTCTGGTTTTTCCACCTGAGATCTTAGCTGGTTACAAATCCTCCGTTCTGCCCTTCTTATAT
CAATTTGAAATTCAC
+
FFFFFFFFFFFFFFFFFFFFFFFFFFFFFFFFFFFFFFFFFFFFFFFFFFFFFFFFFFFFFFFFFFFF
FFFFFFFFFFFFFFFFFFFFFFFFFFFFFFF:FFFFFFFFFFFFFFFFFFFFFFFFFFFFFFFFFFFF
FFFFFFFFFFFFFFF
@A00155:342:HHGFNDSXY:1:1671:1976:1251 1:N:0:GAACCTAG+TCCGCATA
TGAAATTCATTATTGGTCTCGCGATCAGATTGAGACATTTCTCCAGGTTTACAAACCTAAAAATTTGT
GGGCTACTCTGGTTTTTCCACCTGAGATCTTAGCTGGTTACAAATCCTCCGTTCTGCCCTTCTTATAT
CAATTTGAAATTCAC
+
F,FFFFFFFFFFFFFFFFFF,FFFFFF:,FFFFFFFFFFF:FFFFFF:FFFFFFFFFFFFFFFFFFFF
FFFFF,FFFF:FFFFFF:F::FF,FFFFFFFFFF:FFFFFFFFFFFFFFFFFF,FFF:FFFFFFFFFF
FFFFFFFFFFFFFFF
@A00155:342:HHGFNDSXY:1:2103:29930:23296 1:N:0:GAACCTAG+TCCGCATA
TGAAATTCATTATTGGTCTCGCGATCAGCTTGAGACATTTCTCCAGGTTTACAAACCTAAAAATTTGT
GGGCTACTCTGGTTTTTCCACCTGAGATCTTAGCTGGTTACAAATCCTCCGTTCTGCCCTTCTTATAT
CAATTTGAAATTCAC
+
FFF:FFFFFF:FFFFFFFFFFFF:FFFFFFFFFFFFFFFFFFFFFFFFFFFFFFFFFFFFFFFFFFFF
FFFFFFFFFFFFFFFFFFFFFFFFFFFFFFFFFFFFFFFFFFFFFFFFFFFFFFFFFFFFFFFFFFFF
F:FFFFFFFFFFFFF
@A00155:342:HHGFNDSXY:1:1678:16034:12352 1:N:0:GAACCTAG+TCCGCATA
TGAAATTCATTATTGGTCTCGCGATCAGCTTGAGACATTTCTCCAGGTTTACAAACCTAAAAATTTGT
GGGCTACTCTGGTTTTTCCACCTGAGATCTTAGCTGGTTACAAATCCTCCGTTCTGCCCTTCTTATAT
CAATTTGAAATTCAC
+
FFFFFFFFFFFFFFFFFFFFFFFFFF,FFFFFFFFFFFFFFFFFFFFFFFFFFFFFFFFFFFFFFFFF
FFFFFFFFFFFFF:FFFFFFFFFFFFFFFFFFFFFFFFFFFFFFFFFFFFFFFFFFFFFFFFFFFFFF
FFFFFFFFFFFFFFF
@A00155:342:HHGFNDSXY:1:2134:5394:4131 1:N:0:GAACCTAG+TCCGCATA
TGAAATTCATTATTGGTCTCGCGATCAGCTTGAGACATTTCTCCAGGTTTACAAACCTAAAAATTTGT
GGGCTACTCTGGTTTTTCCACCTGAGATCTTAGCTGGTTACAAATCCTCCGTTCTGCCCTTCTTATAT
CAATTTGAAATTCAC
+
FFFFFFFFFFFFFFFFFFFFFFFFFFFFFFFFFFFFFFFFFFFFFFFFFFFFFFFFFFFFFFFFFFFF
FFFFFFFFFFFFFFFFFFFFFFFFFFFFFFFFFFFFFFFFFFFFFFFFFFFFFFFFFFFFFFFFFFFF
FFFFFFFFFFFFFFF
@A00155:342:HHGFNDSXY:1:2135:18150:2174 1:N:0:GAACCTAG+TCCGCATA
TGAAATTCATTATTGGTCTCGCGATCAGCTTGAGACATTTCTCCAGGTTTTCAAACCTAAAAATTTGT

GGGCTACTCTGGTTTTTCCACCTGAGATCTTAGCTGGTTACAAATCCTCCGTTCTGCCCTTCTTATAT
CAATTTGAAATTCAC
+
FFFFFFFFF:FFF:FFFFFFFFFFFFFFFFFFFFFFFFFFFFFFFFFFFFFFFFFFFFFFFFFFFFFFFF
FFFFFFFFFFFFFFFFFFFFFFFFFFFFFFF:FFFFFFFFFFFFFFFFFFFFFFFFFFFFFFFFFFFFFF
FFFFFFFFFFFFFFF
@A00155:342:HHGFNDSXY:1:2155:20772:2957 1:N:0:GAACCTAG+TCCGCATA
TGAAATTCATTATTGGTCTCGCGATCAGCTTGAGACATTTCTCCAGGTTTACAAACCTAAAAATTTGT
GGGCTACTCTGGTTTTTCCACCTGAGATCTTAGCTGGTTACAAATCCTCCGTTCTGCCCTTCTTATAT
CAATTTGAAATTCAC
+
FFFFFFFFFFFFFFFFFFFFFFFFFFFFFFFFFFFFFF:FFFFFFFFFFFFFFFFFFFFFF:FFFFFFFF
FFFFFFFFFFFFFFFFFFFFFFFFFFFFFFFFFFFFFFFFFFFFFFFFFFFFFFFFFFFFFFFFFFFFFF
FFFFFFFFFFFFFFF
@A00155:342:HHGFNDSXY:1:2131:30337:1799 1:N:0:GAACCTAG+TCCGCATA
TGAAATTCATTATTGGTCTCGCGATCAGCTTGAGACATTTCTCCAGGTTTACAAACCTAAAAATTTGT
GGGCTACTCTGGTTTTTCCACCTGAGATCTTAGCTGGTTACAAATCCTCCGTTCTGCCCTTCTTATAT
CAATTTGAAATTCAC
+
FFF:FFFF,FFFFFFFFFFFF:FFFFFFFFF:FFFFFFFFFFFFFFFFFFFFFF:FFFFF::FFFFFFFF
FFFFFFFFFFFFFFFFFFFFFFFFFFFFFFFFFFFFFFFFF,FFF:FFFFFFFFFFFFFFFFFFFFFFFF
FFFFFFFFFFFF:FF
@A00155:342:HHGFNDSXY:1:2162:27814:17722 1:N:0:GAACCTAG+TCCGCATA
TGAAATTCATTATTGGTCTCGCGATCAGCTTGAGACATTTCTCCAGGTTTACAAACCTAAAAATTTGT
GGGCTACTCTGGTTTTTCCACCTGAGATCTTAGCTGGTTACAAATCCTCCGTTCTGCCCTTCTTATAT
CAATTTGAAATTCAC
+
FFFFFFF,FFFFFFFFFFFFFFFFFFFFFFFFFFFFFFFFFFFFFFFFFFFFFFFFFFFFFFFFFFFFFF
FFF:FFFFFFFFFFFFFFFFF:FFFFFF:FF:FFFFFFFFFFFFFFFFFFFFFFFFFFFFFFFFFFFFFF
FFFFFFFFFFFFFFF
@A00155:342:HHGFNDSXY:1:2173:11559:14246 1:N:0:GAACCTAG+TCCGCATA
TGAAATTCATTATTGGTCTCGCGATCAGCTTGAGACATTTCTCCAGGTTTACAAACCTAAAAATTTGT
GGGCTACTCTGGTTTTTCCACCTGAGATCTTAGCTGGTTACAAATCCTCCGTTCTGCCCTTCTTATAT
CAATTTGAAATTCAC
+
FFFFFFFFFFFFFFFF:FFFFFFFFFFFFFFFFFFFFFFFFFFFFFFFFFFFFFFFFFFFFFFFFFFFFF
FFFFFFFFFFFFF:FFFFFFFFFFFFFFFFFFFFFFFFFFFFFFFFFFFFFFFFFFFFFFFFFFFFFFFF
FFFFFFFFFFFFFFF
@A00155:342:HHGFNDSXY:1:2222:2618:35524 1:N:0:GAACCTAG+TCCGCATA
TGAAATTCATTATTGGTCTCGCGATCAGCTTGAGACATTTCTCCAGGTTTACAAACCTAAAAATTTGT
GGGCTACTCTGGTTTTTCCACCTGAGATCTTAGCTGGTTACAAATCCTCCGTTCTGCCCTTCTTATAT
CAATTTGAAATTCAC
+
FFFFFF:FFFFF:FFFFFFFFFFFFFFFFFFFFFFFFFFFFFFFFFFFFFFFFFFFFFFFFFFF:FFFFF
FFFFFFFFFFFFFFFFFFFFFFFFFF:FFFFFFFFFFFFFFFFFFFFFFFFFFFFFFFFFFFFFFFFFF:
FFFFFFFFFF:FFFF
@A00155:342:HHGFNDSXY:1:2252:32425:2315 1:N:0:GAACCTAG+TCCGCATA
TGAAATTCATTATTGGTCTCGCGATCAGCTTGAGACATTTCTCCAGGTTTACAAACCTAAAAATTTGT
GGGCTACTCTGGTTTTTCCACCTGAGATCTTAGCTGGTTACAAATCCTCCGTTCTGCCCTTCTTATAT
CAATTTGAAATTCAC
+
FFFFF::FFFFF:::FFFFF:FF:FFFFF,F:FFFFFFFFFFFFFFFFFFFFFFFFFFFFFFFFFF:FFF
FFFF:FFFFFFFFFFFFFFFFFFFFFFFFFF:FFFFFFFFFFFFFFFFFFFFFFFF:FFFFFFFF:FFFF
FF:FFFFFFF,FF:F

```
@A00155:342:HHGFNDSXY:1:2274:1768:35556 1:N:0:GAACCTAG+TCCGCATA
ATCTATTCATTATTGGTCTCGCGATCAGCTTGAGACATTTCTCCAGGTTTACAAACCTAAAAATTTGT
GGGCTACTCTGGTTTTTCCACCTGAGATCTTAGCTGGTTACAAATCCTCCGTTCTGCCCTTCTTATAT
CAATTTGAAATTCAC
+
FF:FF:FFFFFFF,:FF:FF:FFFFFF:FF:FF:FFF:FFFFFF,FFFF:F:F:FFFFFF:F:F:FFF
FFFFFFFFFFFFFFF:FFFF:FFF,FFF,F,FFFF:FFF,FFFF::FFFFFFFFFF,:F:::FFF::F
F,,FFFFFFFFFF:F
@A00155:342:HHGFNDSXY:1:2310:31051:25770 1:N:0:GAACCTAG+TCCGCATA
TGAAATTCATTATTGGTCTCGCGATCAGCTTGAGACATTTCTCCAGGTTTACAAACCTAAAAATTTGT
GGGCTACTCTGGTTTTTCCACCTGAGATCTTAGCTGGTTACAAATCCTCCGTTCTGCCCTTCTTATAT
CAATTTGAAATTCAC
+
FFFFFFFFFFFFFFFFFFFFFFFFFFFFFFFFFFFFFFFFFFFFFFFFF:FFFFFFFFFFFFFFFFFF
FFFFFFFFFFFFFFFFFFFFFFFFFFFFFFFFFFFFFFFFFFFFFFFFFFFFFFFFFFFFFFFFFFFF
FFFFFFFFFFFFFFF
@A00155:342:HHGFNDSXY:1:2332:26350:13870 1:N:0:GAACCTAG+TCCGCATA
TGAAATTCATTATTGGTCTCGCGATCAGCTTGAGACATTTCTCCAGGTTTTCAAACCTAAAAATTTGT
GGGCTACTCTGGTTTTTCCACCTGAGATCTTAGCTGGTTACAAATCCTCCGTTCTGCCCTTCTTATAT
CAATTTGAAATTCAC
+
FFFFFFFFFFFFFFFFFFFFFFFFFFFFFFFFFFFFFFFFFFFFFFFFFFFFFFFFFFFFFFFFFFFF
FFFFFFFFFFFFFFFFFFFFFFFFFFFFFFFFFFFFFFFFFFFFFFFFFFFFFFFFFFFFFFFFFFFF
FFFFFFFFFFFFFFF
@A00155:342:HHGFNDSXY:1:2356:6479:23610 1:N:0:GAACCTAG+TCCGCATA
TGAAATTCATTATTGGTCTCGCGATCAGCTTGAGACATTTCTCCAGGTTTACAAACCTAAAAATTTGT
GGGCTACTCTGGTTTTTCCACCTGAGATCTTAGCTGGTTACAAATCCTCCGTTCTGCCCTTCTTATAT
CAATTTGAAATTCAC
+
FFFF:FFFFF:FFFFFFFFF:FFFFFFFFFFFFFFFFFFFFF:FFF:FFFFFFFFFFF:FFF:FFFFF
:FFFFFFFFFFFFFFFFFFFF:FFFFFFFFFFF:FFFFFFFFFFFFFFFFFFFF:FFFFFFFFFFFFF
FFFFFFFFFFFFFF:
@A00155:342:HHGFNDSXY:1:2366:5267:9048 1:N:0:GAACCTAG+TCCGCATA
TGAAATTCATTATTGGTCTCGCGATCAGCTTGAGACATTTCTCCAGGTTTACAAACCTAAAAATTTGT
GGGCTACTCTGGTTTTTCCACCTGAGATCTTAGCTGGTTACAAATCCTCCGTTCTGCCCTTCTTATAT
CAATTTGAAATTCAC
+
FFFFFFFF:FFFFFFFFFFF:F,FFFF:FFFFFFFFFFFF:FF:F:FFFFFFFFFFFFFFFFFFFFFF
FFFFFFFF:FFFFFFFFFFFFFFFF,FF:FFFFFFFFFFFFFFFFFFFFFFFFFFFFFFFFFFFFFFF
FFFFFFFFFFFFF:F
@A00155:342:HHGFNDSXY:1:2366:5258:9064 1:N:0:GAACCTAG+TCCGCATA
TGAAATTCATTATTGGTCTCGCGATCAGCTTGAGACATTTCTCCAGGTTTACAAACCTAAAAATTTGT
GGGCTACTCTGGTTTTTCCACCTGAGATCTTAGCTGGTTACAAATCCTCCGTTCTGCCCTTCTTATAT
CAATTTGAAATTCAC
+
FFFFFFFFFFFFFFFFFFF:FF:FFFFFFFFFFFFFFFFFFFFFFFF,FFFFFFF:FFFFFFFFFFFF
FFFFFFFFFFFFFFFFFFFFFFFFFFFFFFFFFFFFFFFFFFFFFF:FFFF,FFFFFFFFFFFFFFFF:F
FFFFFFFFFFFFFFF
@A00155:342:HHGFNDSXY:1:2378:24668:16438 1:N:0:GAACCTAG+TCCGCATA
TGAAATTCATTATTGGTCTCGCGATCAGCTTGAGACATTTCTCCAGGTTTACAAACCTAAAAATTTGT
GGGCTACTCTGGTTTTTCCACCTGAGATCTTAGCTGGTTACAAATCCTCCGTTCTGCCCTTCTTATAT
CAATTTGAAATTCAC
+
FFFFFFFFFFFFFFFFFFFFFFFFFFFFFFFFFFFFFFFFFFFFFFFFFFFFFFFFFFFFFFFFFFFF
```

FFFFFFFFFFFFFFFFFFFFFFFFFFF:FFFFFFFFFFFFFFFFFFFFFFFFFFFFFFFFFFFFFFFF
FFFFFFFFFFFFFFF
@A00155:342:HHGFNDSXY:1:2419:4227:30201 1:N:0:GAACCTAG+TCCGCATA
TGAAATTAATTATTTGTCTCGCGATCAGCTTGAGACATTTCTCCAGTTTTACAAACCTAAAAATTTGT
GGGCTACTCTGGTTTTTCCACCTGAGATCTTAGCTGGTTACAAATCCTCCGTTCTGCCCTTCTTATAT
CAATTTGAAATTCAC
+
FFFF,FF:FFFFFF,FFFFFFFFFFFF:,FF,,FF,:FFFFF:FF:,:FFF:FF:F,FFFFFFFFFFF
F:F,FF,F:FFFFFFFFFF:FFFF:FFF,FF,FFFFF:FF:FFFFFFFFF,FFFFF:,:FF:FFFFFF
FFFFFFF:FFFFFFF
@A00155:342:HHGFNDSXY:1:2436:9760:37059 1:N:0:GAACCTAG+TCCGCATA
TGAAATTCATTATTGGTCTCGCGATCAGCTTGAGACATTTCTCCAGGTTTACAAACCTAAAAATTTGT
GGGCTACTCTGGTTTTTCCACCTGAGATCTTAGCTGGTTACAAATCCTCCGTTCTGCCCTTCTTATAT
CAATTTGAAATTCAC
+
::FFFFFFFFFFFFFFF:F:FFFFF:F:FFFFFFFFFFFFFFFFFF:FFFFFFFFFFFFFFFFFFFFF
FF:FFFFFFFFFFFFFFFFFFFFFFFFFFFFFFFFFFFFFFFFFFFFFFFFFFFFFFFFFFFFFFFFF
FFF:FFFFFFFFFFF
@A00155:342:HHGFNDSXY:1:2456:3929:18035 1:N:0:GAACCTAG+TCCGCATA
TGAAATTCATTATTGGTCTCGCGATCAGCTTGAGACATTTCTCCAGGTTTACAAACCTAAAAATTTGT
GGGCTACTCTGGTTTTTCCACCTGAGATCTTAGCTGGTTACAAATCCTCCGTTCTGCCCTTCTTATAT
CAATTTGAAATTCAC
+
:FFFFFF:FFFFFFFFFFFF:FFFFFFFFFFFFFFFFFFFFFFFFFFFFFFFFFFFFFFFFFFFFFFF
FFFFFFF:FFFFFFFFFFFFFFFFFFFFFFFFFFFFFFFFFFFFFFFFFFFFFFFFFFFFFFFFFFFF
FFFFFFFFFFFFFFF
@A00155:342:HHGFNDSXY:1:2456:4056:20917 1:N:0:GAACCTAG+TCCGCATA
TGAAATTCATTATTGGTCTCGCGATCAGCTTGAGACATTTCTCCAGGTTTACAAACCTAAAAATTTGT
GGGCTACTCTGGTTTTTCCACCTGAGATCTTAGCTGGTTACAAATCCTCCGTTCTGCCCTTCTTATAT
CAATTTGAAATTCAC
+
FFFFFFFFFFFFFFF:FFFFFF:FFFFFFFFFFF::FFFFFFFF:FFFFFFFFFFFFFFFFF:FFFFF
FFFFFFFFFFFFFFFFFFFFFFFFFFF:FFFFFFFFFFFFFFFFFFFFFFFFFFFFFFFFFFFFFFFF
FFFFFFFFFFFFF:F
@A00155:342:HHGFNDSXY:1:2460:23005:4570 1:N:0:GAACCTAG+TCCGCATA
TGAAATTCATTATTGGTCTCGCGATCAGCTTGAGACATTTCTCCAGGTTTACAAACCTAAAAATTTGT
GGGCTACTCTGGTTTTTCCACCTGAGATCTTAGCTGGTTACAAATCCTCCGTTCTGCCCTTCTTATAT
CAATTTGAAATTCAC
+
FFFFFFFFFFFFFFFFFFFFFFFFFFFFFFFFFFFFFFFFFFFFFFFFFFFFFFFFFFFFFFFFFFFF
FFFFFFFFFFFFFFFFFFFFFFFFFFFFFFFFFFFFFFFFFFFFFFFFFFFFFFFFFFFFFFFFFFFF
FFFFFFFFFFFFFFF
@A00155:342:HHGFNDSXY:1:2464:15103:4006 1:N:0:GAACCTAG+TCCGCATA
TGAAATTCATTATTGGTCTCGCGATCAGCTTGAGACATTTCTCCAGGTTTACAAACCTAAAAATTTGT
GGGCTACTCTGGTTTTTCCACCTGAGATCTTAGCTGGTTACAAATCCTCCGTTCTGCCCTTCTTATAT
CAATTTGAAATTCAC
+
FFFFFFFFF:FFFFFFFFFFFFFFFFFFFFFFFFFFFFFFFFFFFFFFFFFFFFFFFFFFFFFFFFFF
FFFFF:FFFFFFFFFFFFFFFFFFFFFFFFFFFFFFFFFFFFFFFFFFFFFFFFFFFFFFFFFFFFFF
FFFFF:FFFFFFFFF
@A00155:342:HHGFNDSXY:1:2472:22327:24283 1:N:0:GAACCTAG+TCCGCATA
TGAAATTCATTATTGGTCTCGCGATCAGCTTGAGACATTTCTCCAGGTTTACAAACCTAAAAATTTGT
GGGCTACTCTGGTTTTTCCACCTGAGATCTTAGCTGGTTACAAATCCTCCGTTCTGCCCTTCTTATAT
CAATTTGAAATTCAC

+
FFFFFFFFFFFFFFFFFFFFFFFFFFFFFFFFFFFFFFFFFFFFFFFFFFFFFFFFFFFFFFFFFFFFFF
FFFFFFFFFFFFFFFFFFFFFFFFFFFFFFFFFFFFFFFFFFFFFFFFFFFFFFFFFFFFFFFFFFFFFF
FFFFFFFFFFFFFFF
@A00155:342:HHGFNDSXY:1:2471:23511:32847 1:N:0:GAACCTAG+TCCGCATA
CCGATCTCATTATTGGTCTCGCGATCAGCTTGAGACATTTCTCCAGGTTTACAAACCTAAAAATTTGT
GGGCTACTCTGGTTTTTCCACCTGAGATCTTAGCTGGTTACAAATCCTCCGTTCTGCCCTTCTTATAT
CAATTTGAAATTCAC
+
FFFFFFFFFF:FFFFFFFFFFFFFFFFFFFFFFFFFFFFFFFFFFFFFFFFFFFFF:FFFFFFFFFFFFF
FFFFFFFFFFFFFFFFFFFFFFFFFFFFFFFFFFFFFFFFFFFFFFFFFFFFFFFFFFFFFFFFFFFFFF
FFFFFFFFFFFFFFF
@A00155:342:HHGFNDSXY:1:2512:4363:4194 1:N:0:GAACCTAG+TCCGCATA
TGAAATTCATTATTGGTCTCGCGATCAGCTTGAGACATTTCTCCAGGTTTACAAACCTAAAAATTTGT
GGGCTACTCTGGTTTTTCCACCTGAGATCTTAGCTGGTTACAAATCCTCCGTTCTGCCCTTCTTATAT
CAATTTGAAATTCAC
+
FFFFFFFF:FF,FF:FFFFFFFF:FFFFFFFFFFFFFFFFFFFFFFFFFFFFFFFFFFFFFFFFFFFFFF
FFFFFFFFFFFFFFFFFFFFFFFFFFFFFFFFFFFFFFFFFFFFFFFFFFFFFFFFFFFFFFF:FFFFFF
FFFFFFFFFFFFFFF
@A00155:342:HHGFNDSXY:1:2527:29677:34601 1:N:0:GAACCTAG+TCCGCATA
TGAAATTCATTATTGGTCTCGCGATCAGCTTGAGACATTTCTCCAGGTTTACAAACCTAAAAATTTGT
GGGCTACTCTGGTTTTTCCACCTGAGATCTTAGCTGGTTACAAATCCTCCGTTCTGCCCTTCTTATAT
CAATTTGAAATTCAC
+
FFFFFFFFFFFFFFFFFFFFFFFFFFFF:FFFFFFFFFFFFFFFFFFFFFFFFFFFFFFFFFFFFF:FFF
FFFFFFFFFFFFFFFFFFFFFFFFFFFFFFFFFFFFFFFFFFFFFFFFFFFFFFFFFFFFFFFFFFFFFF
FFFFFFFFFFFFFFF
@A00155:342:HHGFNDSXY:1:2545:19877:11209 1:N:0:GAACCTAG+TCCGCATA
TGAAATTCATTATTGGTCTCGCGATCAGCTTGAGACATTTCTCCAGGTTTACAAACCTAAAAATTTGT
GGGCTACTCTGGTTTTTCCACCTGAGATCTTAGCTGGTTACAAATCCTCCGTTCTGCCCTTCTTATAT
CAATTTGAAATTCAC
+
FFFFFFFFFFFFFFFFFFFFFFFFFFFFFFFFFFFFFFFFFFFFFFFFFFFFFFFFFFFFFFFFFFFFFF
FFFFFFFFFFFFFFFFFFFFFFFFFFFFFFF,FFFFFFFFFFFFFFFFFFFFFFFFFFFFFFFFFFFFFF
FF:FFFFFFF:FFFF
@A00155:342:HHGFNDSXY:1:2545:19352:12148 1:N:0:GAACCTAG+TCCGCATA
TGAAATTCATTATTGGTCTCGCGATCAGCTTGAGACATTTCTCCAGGTTTACAAACCTAAAAATTTGT
GGGCTACTCTGGTTTTTCCACCTGAGATCTTAGCTGGTTACAAATCCTCCGTTCTGCCCTTCTTATAT
CAATTTGAAATTCAC
+
FFFFFFFFFFFFFFFFFFFFFFFFFFFFFFFFFFFFFFFFFFFFFFFFFFFFFFFFFFFFFFFFFFFFFF
FFFF,FFFFFFFFFFFFFFFFFFFFFFFFFFFFFFFFFFFFFFFFFFFFFFFFFFFFFFFFFFFFFFFFF
FFFFFFFFFFFFFFF
@A00155:342:HHGFNDSXY:1:2543:17300:17237 1:N:0:GAACCTAG+TCCGCATA
TGAAATTCATTATTGGTCTCGCGATCAGCTTGAGACATTTCTCCAGGTTTACAAACCTAAAAATTTGT
GGGCTACTCTGGTTTTTCCACCTGAGATCTTAGCTGGTTACAAATCCTCCGTTCTGCCCTTCTTATAT
CAATTTGAAATTCTC
+
FFF,:FFFFFFFFF:FFFFF,FFFFF,,:FFFFFFF:FFF:FF,FFFFFFFF:FFF,FF:FFFFFFFF:
FF::FF:FF:FFFFFFFFFFFFFFFFF::FFFF,FFF,FFF,FFFF:,FF::FF,F:,:,:FFFF,FFF
FFFFFFFF,FFFF,F
@A00155:342:HHGFNDSXY:1:2603:25554:35008 1:N:0:GAACCTAG+TCCGCATA
TGAAATTCATTATTGGTCTCGCGATCAGCTTGAGACATTTCTCCAGGTTTACAAACCTAAAAATTTGT

GGGCTACTCTGGTTTTTCCACCTGAGATCTTAGCTGGTTACAAATCCTCCGTTCTGCCCTTCTTATAT
CAATTTGAAATTCAC
+
FFFFFFFFFFFFFFFFFFFFFFFFFFFFFFFFFFFFFFFFFFFFF:FFFFFFFFFFFFFFFFFFFFFF
FFFFFFFFFFFFFFFFFFFFFFFFFFFFFFFFFFFFFFFFFFFFFFFFFFFFFFFFFFFFF:FFFFF:
FFFFFFFFFFFFFFF
@A00155:342:HHGFNDSXY:1:2603:25138:35477 1:N:0:GAACCTAG+TCCGCATA
TGAAATTCATTATTGGTCTCGCGATCAGCTTGAGACATTTCTCCAGGTTTACAAACCTAAAAATTTGT
GGGCTACTCTGGTTTTTCCACCTGAGATCTTAGCTGGTTACAAATCCTCCGTTCTGCCCTTCTTATAT
CAATTTGAAATTCAC
+
FFFFFF:FFFFFFFFFFFFFFFFFFFFFFFFFFFFFFFFFFFFFFFFFFFFFFFFFFFFFFFFFFFFF
FFFFFFFFFFFFFFFFFFFFFFFFFFFFFFFFFFFFFFFFFFFFFFFFFFFFFFFFFFFFFFFFFFFF
FFFFFFFFFFFFFFF
@A00155:342:HHGFNDSXY:1:2611:14886:20697 1:N:0:GAACCTAG+TCCGCATA
CCGATCTCATTATTGGTCTCGCGATCAGCTTGAGACATTTCTCCAGGTTTACAAACCTAAAAATTTGT
GGGCTACTCTGGTTTTTCCACCTGAGATCTTAGCTGGTTACAAATCCTCCGTTCTGCCCTTCTTATAT
CAATTTGAAATTCAC
+
FFFFFFFFFFFFFFFFFFFFFFFFFFFFFFFFFFFFFFFFFFFFFFFFFFFFFFFFFFFFFFFFFFFF
FFFFFFFFFFFFFFFFFFFFFFFFFFF:FFFFFFFFFFFFFFFFFFFFFFFFFFFFFFFFFFFFFFFFFFFF
FFFFFFFFFFFFFFF
@A00155:342:HHGFNDSXY:1:2631:21875:5744 1:N:0:GAACCTAG+TCCGCATA
TGAAATTCATTATTGGTCTCGCGATCAGCTTGAGACATTTCTCCAGGTTTACAAACCTAAAAATTTGT
GGGCTACTCTGGTTTTTCCACCTGAGATCTTAGCTGGTTACAAATCCTCCGTTCTGCCCTTCTTATAT
CAATTTGAAATTCAC
+
FFFFF:FFFFFFFFFF:FFF:FFFFFFFFFFFFFFFFFFFFFFFFFFFFFFFFFFFFFFFFFFFFFFF
:FFFFFFFFFFFFFFFFFFFFFFFFFFFFFFFFFFFFFFFFFFFFFFFFFFFFFFFFFFFFFFFFFFF
FFFFFFFFFF:FFFF
@A00155:342:HHGFNDSXY:1:2658:14082:10692 1:N:0:GAACCTAG+TCCGCATA
TGAAATTCATTATTGGTCTCGCGATCAGCTTGAGACATTTCTCCAGGTTTACAAACCTAAAAATTTGT
GGGCTACTCTGGTTTTTCCACCTGAGATCTTAGCTGGTTACAAATCCTCCGTTCTGCCCTTCTTATAT
CAATTTGAAATTCAC
+
:FFFFF:FFFFFFFFFFFFFFFFFFFFFFFFFFFFFFFFFFFFFFF:FFFFFFFFFFFFFFFFFFFFF
FFFFFFFFFFFFFFFFFFFFFFFFFFFFFFFFFFFFFFFFFFFFFFFFFFFFFFFFFFFFFFFFFFFF
FFFFFFFFFFFFFFF
@A00155:342:HHGFNDSXY:1:2666:20880:14544 1:N:0:GAACCTAG+TCCGCATA
TGAAATTCATTATTGGTCTCGCGATCAGCTTGAGACATTTCTCCAGGTTTACAAACCTAAAAATTTGT
GGGCTACTCTGGTTTTTCCACCTGAGATCTTAGCTGGTTACAAATCCTCCGTTCTGCCCTTCTTATAT
CAATTTGAAATTCAC
+
FFFFFFFFFFFFFF:FFFFFFFFFFFFFFFFFFFFFF:FFFFFFFFFFFFFFFFFFFFFFFFFFFFFF
F:FFFFFFFFFFFFFFF:FFFFFF:FFF:FFFFF:FFFF:FFFFFFFFFFFFFFFFFFFFF::FF:FF
FFFFFFFFFFFFFF:
@A00155:342:HHGFNDSXY:1:2671:17336:13166 1:N:0:GAACCTAG+TCCGCATA
TGAAATTCATTATTGGTCTCGCGATCAGCTTGAGACATTTCTCCAGGTTTACAAACCTAAAAATTTGT
GGGCTACTCTGGTTTTTCCACCTGAGATCTTAGCTGGTTACAAATCCTCCGTTCTGCCCTTCTTATAT
CAATTTGAAATTCAC
+
FFFFFFFFFFFFFFFFFFFFFFFFFFFFFFFFFFFFFFFFFFFFFFFFFFFFFFFFFFFFFFFFFFFF
FFFFFFFFFFFFFFFFFFFFFFFFFFFFFFFFFFFFFFFFFFFFFFFFFFFFFFFFFFFFFFFFFFFF
FFFFFFFFFFFFFFF

```
@A00155:342:HHGFNDSXY:1:2678:20546:13839 1:N:0:GAACCTAG+TCCGCATA
TGAAATTCATTATTGGTCTCGCGATCAGCTTGAGACATTTCTCCAGGTTTACAAACCTAAAAATTTGT
GGGATACTCTGGTTTTTCCACCTGAGATCTTAGCTGGTTACAAATCCTCCGTTCTGCACTTCTTATAT
CAATTTGAAATTCAC
+
FFFFFFFFFFFFFF:FFFFFF,FFFFF::F:FF,F::FFFFF:FFFFFFFFFFFF,FF:FFF,FFF,F
,F,,FF,FFF:FFFFFF:,:,FF:,FFF,FF,:FF:FFF,,:FFFF,F,:FFF,FFF,FFF:FFFFFF
FF:FFFFFFFFFFFF
@A00155:342:HHGFNDSXY:1:1114:17544:27179 2:N:0:GAACCTAG+TCCGCATA
TGAAATTCATTATTGGTCTCGCGATCAGCTTGAGACATTTCTCCAGGTTTACAAACCTAAAAATTTGT
GGGCTACTCTGGTTTTTCCACCTGAGATCTTAGCTGGTTACAAATCCTCCGTTCTGCCCTTCTTATAT
CAATTTGAAATTCAC
+
FFFFFFFFFFFFFFFFFFFFFFFFFFFFFF,FFFFFFFFFFFFFFFFFFFFFFFF:FFFFFFFFFFFF
F:FFFFFFFFFFFFFFFFF:FFFFFFFFFFFFFFF:FFFFFFFFFFFFFFFFFF:FFFFFFFFFFFFF
FFFFFFFFFFFFFFF
@A00155:342:HHGFNDSXY:1:1142:6162:29794 2:N:0:GAACCTAG+TCCGCATA
TGAAATTCATTATTGGTCTCGCGATCAGCTTGAGACATTTCTCCAGGTTTACAAACCTAAAAATTTGT
GGGCTACTCTGGTTTTTCCACCTGAGATCTTAGCTGGTTACAAATCCTCCGTTCTGCCCTTCTTATAT
CAATTTGAAATTCAC
+
FFFFFFFFFFFFFFFFFFFFFFFFFFFFFFFFFFFFFFFFFFFFFFFFFFFFFFFFFFFFFFFFFFFF
FFFFFFFFFFFFF,FFFFFFFFFFFFFFFFFFFFFFFFFFFFFFFFFFFFFFFFFFFFFFFFFFFFFF
FFFFFFFFFFFFFFF
@A00155:342:HHGFNDSXY:1:1666:27489:1814 2:N:0:GAACCTAG+TCCGCATA
TGAAATTCATTATTGGTCTCGCGATCAGCTTGAGACATTTCTCCAGGTTTACAAACCTAAAAATTTGT
GGGCTACTCTGGTTTTTCCACCTGAGATCTTAGCTGGTTACAAATCCTCCGTTCTGCCCTTCTTATAT
CAATTTGAAATTCAC
+
FFFFFF:FFFFFFFFFFFFFFFFFFFFFFFFFFFFFFFFFFFF:FFFFFFFFFFFF:FF,FFFF:FFFF
FF,FFFFFFFFFFFFFFFFFFFFFFFFFFF:FFFFFFFFFFFFFFFFFFFFFFFFF:FF:FF:FFF:FFF
FFFFFFFFFFFFF:F
@A00155:342:HHGFNDSXY:1:1670:31955:10770 1:N:0:GAACCTAG+TCCGCATA
GAAATTCATTATTGGTCTCGCGATCAGCTTGAGACATTTCTCCAGGTTTACAAACCTAAAAATTTGTG
GGCTACTCTGGTTTTTCCACCTGAGATCTTAGCTGGTTACAAATCCTCCGTTCTGCCCTTCTTATATC
AATTTGAAATTCACG
+
FFFFFFFFFFFFFFFFFFFFFFFFFFFFF:FFFFFFFFFFFFFFFFFFFFFFFFFFFFFFFFFFFFFFFF
FFFFFFFFFFFFFFFFFFFFFFFFFFFF:FFFFFFFFFFFFFFFFFFFFFFFFFFFFFFFFFFFFFFFF
FFFFFFFFFFFFFFF
@A00155:342:HHGFNDSXY:1:2103:16541:34835 1:N:0:GAACCTAG+TCCGCATA
GAAATTCATTATTGGTCTCGCGATCAGCTTGAGACATTTCTCCAGGTTTACAAACCTAAAAATTTGTG
GGCTACTCTGGTTTTTCCACCTGAGATCTTAGCTGGTTACAAATCCTCCGTTCTGCCCTTCTTATATC
AATTTGAAATTCACG
+
:FFF,FFFF:FFFFFFFFFFFFFFFFFFFFFFFFFFFFFFFFFFFFFFFFFFFFFFFFFFFFFFFFFF
FFFFFFFFFFFFFFFFFFFFFFFFFFFFFFFFFFFFFFFFFFFFFFFFFFFFFFFFFFFFFFFFFFFF
FFFFFFF:FFFFFFF
@A00155:342:HHGFNDSXY:1:2403:23719:16454 1:N:0:GAACCTAG+TCCGCATA
GAAATTCATTATTGGTCTCGCGATCAGCTTGAGACATTTCTCCAGGTTTACAAACCTAAAAATTTGTG
GGCTACTCTGGTTTTTCCACCTGAGATCTTAGCTGGTTACAAATCCTCCGTTCTGCCCTTCTTATATC
AATTTGAAATTCACG
+
:FFFFFFFFFFFFFFFFFFFFFFFFFFFFFFFFFFFFFFFFFFFFFFFFFFFFFFFFFFFFFFFFFFF
```

FFFFFFFFFFFFFFFFFFFFFFFFFFFFFFFFFFFFFFFFFFFFFFFFFFFFFFFFFFFFFFFFF:FF
FFFFFFFFFFFFFFF
@A00155:342:HHGFNDSXY:1:2559:17074:12023 1:N:0:GAACCTAG+TCCGCATA
GAAATTCATTATTGGTCTCGCGATCAGCTTGAGACATTTCTCCAGGTTTACAAACCTAAAAATTTGTG
GGCTACTCTGGTTTTTCCACCTGAGATCTTAGCTGGTTACAAATCCTCCGTTCTGCCCTTCTTATATC
AATTTGAAATTCACG
+
FF:FFFFFFF:FFFFFFFFFFFFFFFFFFFFFFFFFFFFFFFFFFFF:FFFFFFFFFFFFFFFFFFFFF
FFFFFFFFFFF:FFFFFFFFFFFFFFFFFFFFFFFFFFFFFFFFFFFFFFFFFFFFFFFFFFFFFFFF
FF:FFFFFFFFFFFF
@A00155:342:HHGFNDSXY:1:1113:26341:7404 2:N:0:GAACCTAG+TCCGCATA
GAAATTCATTATTGGTCTCGCGATCAGCTTGAGACATTTCTCCAGGTTTACAAACCTAAAAATTTGTG
GGCTACTCTGGTTTTTCCACCTGAGATCTTAGCTGGTTACAAATCCTCCGTTCTGCCCTTCTTATATC
AATTTGAAATTCACA
+
FFFFFFFFFFFFFFFFFFFFFFFFFFFFFFFFFFFFFFFFFFFFFFFFFFFFFFFFFFFFFFFFFFFF
FFFFFFFFFFFFFFFFFFFFFFFFFFFFFFFFFFFFFFFFFFFFFFFFFFFFFFFFFFFFFFFFFFFF
FFFFFFFFFFFFFFF
@A00155:342:HHGFNDSXY:1:1341:3613:35430 2:N:0:GAACCTAG+TCCGCATA
GAAATTCATTATTGGTCTCGCGATCAGCTTGAGACATTTCTCCAGGTTTACAAACCTAAAAATTTGTG
GGCTACTCTGGTTTTTCCACCTGAGATCTTAGCTGGTTACAAATCCTCCGTTCTGCCCTTCTTATATC
AATTTGAAATTCACA
+
FFFFFFFFFFFFFFFFFFFFFFFFFFFFFFFFFFFFFFFFFFFFFFFFFFFFFFFFFFFFFFF:FFFFF
FFFFFFFFFFFFFFFFFFFFFFFFFFFFFFFFFFFFFFFFFFFFFFFFFFFFFFFFFFFFFFFFFFFF
FFFFFFFFFFFFFFF
@A00155:342:HHGFNDSXY:1:1343:3893:21292 2:N:0:GAACCTAG+TCCGCATA
GAAATTCATTATTGGTCTCGCGATCAGCTTGAGACATTTCTCCAGGTTTACAAACCTAAAAATTTGTG
GGCTACTCTGGTTTTTCCACCTGAGATCTTAGCTGGTTACAAATCCTCCGTTCTGCCCTTCTTATATC
AATTTGAAATTCACG
+
FFFFFFFFFFFFFFFFFFFFFFFFFFFFFFFFFFFFFFFFFFFFFFFFFFFFFFFFF:FFFFFFFFFF
FFFFFFFFFFFFFFFFFFFFFFFFFFFFFFFFFFFFFFFFFFFFFFFFFFFFFFFFFFFFFFFFFFFF
FFFFFFFFFFFFFFF
@A00155:342:HHGFNDSXY:1:1646:24089:26115 2:N:0:GAACCTAG+TCCGCATA
GAAATTCATTATTGGTCTCGCGATCAGCTTGAGACATTTCTCCAGGTTTACAAACCTAAAAATTTGTG
GGCTACTCTGGTTTTTCCACCTGAGATCTTAGCTGGTTACAAATCCTCCGTTCTGCCCTTCTTATATC
AATTTGAAATTCACG
+
FFFFFFFFFFFFFFFFFFFFFFFFFFFFFFFFFFFFFFFFFFFFFFFFFFFFFFFFFFFFFFFFFFFF
FFFFFFFFFFFFFFFFFFFFFFFFFFFFFFFFFFFFFFFFFFFFFFFFFFFFFFFFFFF:FFFFFF,F
FFF:FFFFFFFFFFF
@A00155:342:HHGFNDSXY:1:2438:10086:8656 2:N:0:GAACCTAG+TCCGCATA
GAAATTCATTATTGGTCTCGCGATCAGCTTGAGACATTTCTCCAGGTTTACAAACCTAAAAATTTGTG
GGCTACTCTGGTTTTTCCACCTGAGATCTTAGCTGGTTACAAATCCTCCGTTCTGCCCTTCTTATATC
AATTTGAAATTCACG
+
FFFFFFF:FFFFFFFFFFFFFFFF:FFFF:FFFFFFFFFFFFFF:FFF:F:FFFFFF:FFFFFFFFFF
,FFFFFFFFFFFFF:FFFFFFFFFFFFFF:,,FFFFFFFFFFF,F,:FFFF,:::FF:F:FFFF:FFF
FFFFFFF:FF:F,FF
@A00155:342:HHGFNDSXY:1:2525:11957:2440 2:N:0:GAACCTAG+TCCGCATA
GAAATTCATTATTGGTCTCGCGATCAGCTTGAGACATTTCTCCAGGTTTACAAACCTAAAAATTTGTG
GGCTACTCTGGTTTTTCCACCTGAGATCTTAGCTGGTTACAAATCCTCCGTTCTGCCCTTCTTATATC
AATTTGAAATTCACG

```
+
FFFFFFFFFFFFFFFFFFFFFFFFFFFFFFFFFFFFFFF:FFFFFFFFFFFFFFFFFFFFFFFFFFFFFF
FFFFFFFFFFFFFFFFFFFFFFFFFFFFFFFFFFFFFFFFFFFFFFFFFFFFFFFFF:FFFFFFFFFFFF
FFFFFF,FF:FFFFF
@A00155:342:HHGFNDSXY:1:2611:4824:10629 2:N:0:GAACCTAG+TCCGCATA
GAAATTCATTATTGGTCTCGCGATCAGCTTGAGACATTTCTCCAGGTTTACAAACCTAAAAATTTGTG
GGCTACTCTGGTTTTTCCACCTGAGATCTTAGCTGGTTACAAATCCTCCGTTCTGCCCTTCTTATATC
AATTTGAAATTCACG
+
FFFFFFFF:FFFFFFFFFFFFFFFFFFFFFFFFFFFFFFFFFFFFFFFFFFFFFFFFFFFFFFFFFFFFF
:FFFFFFFFFFFFFFFFFFFFFFFFFFFFFFFFFFFFFFFFFF:FFFFFFFFFFFF:FFFFFFF:F:F
FFFFF:FF,FFF:,F
@A00155:342:HHGFNDSXY:1:2646:21793:25081 2:N:0:GAACCTAG+TCCGCATA
GAAATTCATTATTGGTCTCGCGATCAGCTTGAGACATTTCTCCAGGTTTACAAACCTAAAAATTTGTG
GGCTACTCTGGTTTTTCCACCTGAGATCTTAGCTGGTTACAAATCCTCCGTTCTGCCCTTCTTATATC
AATTTGAAATTCACG
+
FFFFFFFFFFFFFFFFFFFFFFFFFF:FFFFFFFFFFFFFFFFFFFFFFFFFFFFFFFFFFFFFFFFFF
FFFFFFFFFFFFFFFFF,FFFFFF:FFFFFFF:FFFFFFFFFFFFFFFFFFFFFF:FFFFFFF,FFFF
FFF:F:FFFFFFFFF
@A00155:342:HHGFNDSXY:1:1106:6081:11490 1:N:0:GAACCTAG+TCCGCATA
AAATTCATTATTGGTCTCGCGATCAGCTTGAGACATTTCTCCAGGTTTACAAACCTAAAAATTTGTGG
GCTACTCTGGTTTTTCCACCTGAGATCTTAGCTGGTTACAAATCCTCCGTTCTGCCCTTCTTATATCA
ATTTGAAATTCACGG
+
FFFFFFFFFFFFFFFFFFFFFFFFFFFFFFFFFFFFFFFFFFFFFFFFFFFF,FFFFFFFFFFFFFFF
FFFFFFFFFFFFFFFF,FF:F,F:FF:FFFFFFF,FFFFFFFFFFFFFFFFFFFFFFFFFFFFFFFFFF
FFFFFFFFFFFFFFF
@A00155:342:HHGFNDSXY:1:1115:11785:34021 1:N:0:GAACCTAG+TCCGCATA
AAATTCATTATTGGTCTCGCGATCAGCTTGAGACATTTCTCCAGGTTTACAAACCTAAAAATTTGTGG
GCTACTCTGGTTTTTCCACCTGAGATCTTAGCTGGTTACAAATCCTCCGTTCTGCCCTTCTTATATCA
ATTTGAAATTCACGG
+
F:FFFFFFFFFFFF:FFF,FFFFFFFFFFFFFFFFFFFFFFFFFFFFFFFFFFFFFFFFFFFFFFFFFF
FFFFFFFFFFFFFFFFF,FFFFFFFFFFFFFFFF:FFFFFFFFF::FFFFFFFFFFFF,FFFFFFFFF
F,FFFFFFFFFFFFF
@A00155:342:HHGFNDSXY:1:1171:26829:12571 1:N:0:GAACCTAG+TCCGCATA
AAATTCATTATTGGTCTCGCGATCAGCTTGAGACATTTCTCCAGGTTTACAAACCTAAAAATTTGTGG
GCTACTCTGGTTTTTCCACCTGAGATCTTAGCTGGTTACAAATCCTCCGTTCTGCCCTTCTTATATCA
ATTTGAAATTCACGG
+
FFFFFFFFFFFFFFFFFFFFF,FFFFFFFFFFFFFFFFFFFFFFFFFFFFFFFFFFFFFFFFF,FFFFFF
FF:FFFFFFF,FFFFFFFFFFFFFFFFFFFFFFFFFFFFFFFFFFFFFFFFFFFFFFFFFFFFFFFFFF
FFFFFFFFFFFFFFF
@A00155:342:HHGFNDSXY:1:1173:10863:3521 1:N:0:GAACCTAG+TCCGCATA
AAATTCATTATTGGTCTCGCGATCAGCTTGAGACATTTCTCCAGGTTTACAAACCTAAAAATTTGTGG
GCTACTCTGGTTTTTCCACCTGAGATCTTAGCTGGTTACAAATCCTCCGTTCTGCCCTTCTTATATCA
ATTTGAAATTCACGG
+
FFFFFFFFFFFFFFFFFFFFFFFFFFFFFFFFFFFFFFFF:FFFFFFF:FFFFFFFFFFFFFFFFF
FFFFFFFF:FFFFFFFFFFFFFFFFF,FFFFFFFFFFFFFFFFFFFFFFFFFFFFFFFFFFFFFFFFFF
FFFFFFFFFFFFFFF
@A00155:342:HHGFNDSXY:1:1341:29532:26616 1:N:0:GAACCTAG+TCCGCATA
AAATTCATTATTGGTCTCGCGATCAGCTTGAGACATTTCTCCAGGTTTACAAACCTAAAAATTTGTGG
```

GCTACTCTGGTTTTTCCACCTGAGATCTTAGCTGGTTACAAATCCTCCGTTCTGCCCTTCTTATATCA
ATTTGAAATTCACGG
+
FFFFFFFFFFFFFFFFFFFFFFFFFFFFFFF:FFFF,FFFFFFFFFFFFFFFFF:FFFFFFFFFFFFFFF
FFFFFFFFFFFFFFFFFFFFFFFFFFFFF:FFFFFFFFFFFFFFFFFFFFFFFFFFFFFFFFFFFFFFFF
FFFFFFFFFFFFFFF
@A00155:342:HHGFNDSXY:1:2209:4517:15483 1:N:0:GAACCTAG+TCCGCATA
AAATTCATTATTGGTCTCGCGATCAGCTTGAGACATTTCTCCAGGTTTACAAACCTAAAAATTTGTGG
GCTACTCTGGTTTTTCCACCTGAGATCTTAGCTGGTTACAAATCCTCCGTTCTGCCCTTCTTATATCA
ATTTGAAATTCACGG
+
FFFFFFFFF:FFFFFFFF:FFFFFFFFFFFFF:FFFFFFFF:FFF:FFFFFFF:FFFFFFFF:FFFFFFF
FFFFFFF:FF:FF:FFF:FFFFFFFFFFFFFFFFFFFFFFFFFFFFF:FFFF:FF,FFFFFFFFFFFF:F
FFF,FFFFFFF:FFF
@A00155:342:HHGFNDSXY:1:2209:4354:15890 1:N:0:GAACCTAG+TCCGCATA
AAATTCATTATTGGTCTCGCGATCAGCTTGAGACATTTCTCCAGGTTTACAAACCTAAAAATTTGTGG
GCTACTCTGGTTTTTCCACCTGAGATCTTAGCTGGTTACAAATCCTCCGTTCTGCCCTTCTTATATCA
ATTTGAAATTCACGG
+
FFFFFFFFFFFF:FFFFF::FFFFFFFFFFF,FFFFFFFFF:F:FFFFFFFFFFFFFFFFFFFFFFFFF:
F,FFFFFF::FFFFFFFFFFFFFFFFFFFFFFFFFFFFFFFFFFFFFFFFFFFFFFFFF:FFFFFFFFFF
FF:FFFFFFFFFFFF
@A00155:342:HHGFNDSXY:1:2550:22806:14403 1:N:0:GAACCTAG+TCCGCATA
AAATTCATTATTGGTCTCGCGATCAGCTTGAGACATTTCTCCAGGTTTACAAACCTAAAAATTTGTGG
GCTACTCTGGTTTTTCCACCTGAGATCTTAGCTGGTTACAAATCCTCCGTTCTGCCCTTCTTATATCA
ATTTGAAATTCACGG
+
:F,FFFFFFFFFFFF:F,FFFFF,FFFFFFFFFFF:FFFF,FF,F,:FFFFFFFFFFF:FFF,FFFFFFF
FFF::F:FFFFFFFFFFF::FFFF:F:FFFFFFFFFFFFF:F:FFFFFF:FFF,F,FF,F::FFFFFF:F
FFFFFFFFFFFFFF:
@A00155:342:HHGFNDSXY:1:2543:16676:30655 1:N:0:GAACCTAG+TCCGCATA
AAATTCATTATTGGTCTCGCGATCAGCTTGAGACATTTCTCCAGGTTTACAAACCTAAAAATTTGTGG
GCTACTCTGGTTTTTCCACCTGAGATCTTAGCTGGTTACAAATCCTCCGTTCTGCCCTTCTTATATCA
ATTTGAAATTCACGG
+
FFFFF:FFFFFFFFFFFFFFFFFFFFFFFFFFFFFFFFFFFFFFFFFFFFFFFFFFFFFFFFFFFFFFFF
FFFFFFFFFFFFFFFFFFFFFFFFFFFFFFFFFFFFFFFFFFFFFFFFFFFFFFFFFFFFFFFFFFFFFF
FFFFFFFFFFFFFFF
@A00155:342:HHGFNDSXY:1:2654:29695:13651 1:N:0:GAACCTAG+TCCGCATA
AAATTCATTATTGGTCTCGCGATCAGCTTGAGACATTTCTCCAGGTTTACAAACCTAAAAATTTGTGG
GCTACTCTGGTTTTTCCACCTGAGATCTTAGCTGGTTACAAATCCTCCGTTCTGCCCTTCTTATATCA
ATTTGAAATTCACGG
+
FFF,F:F,FF::F:FF:FFFFFFFFFF,:FFFF:FFF,F,F:,FFF,FFFFFF,FFFFFFF:FFFFFF:F
,,::FF:FFFFFFFF:F:FFF:FFFFFFF:FFFFFFFFFFFF,FFFFF:FFFFFFFFFF:F:FFFFFFFF
FFFFFFFFFFF:FF:
@A00155:342:HHGFNDSXY:1:2654:30337:13823 1:N:0:GAACCTAG+TCCGCATA
AAATTCATTATTGGTCTCGCGATCAGCTTGAGACATTTCTCCAGGTTTACAAACCTAAAAATTTGTGG
GCTACTCTGGTTTTTCCACCTGAGATCTTAGCTGGTTACAAATCCTCCGTTCTGCCCTTCTTATATCA
ATTTGAAATTCACGG
+
FFFFFFFFFFFFFFFFFFFFFF:FFFFFFFFFFFFFFFFFFFFFFFFFFFFFFFF:FF:FFFFF:FFFFF
FFF:FFFF:FFFFFFFFFFFFFFFFFF:F:FFFFFFFFFFFFFFFFFFFF:FF,FFFFFFFFFFFFFFFF
FFFFFFFFFFFFFFF

```
@A00155:342:HHGFNDSXY:1:2663:31530:36933 1:N:0:GAACCTAG+TCCGCATA
AAATTCATTATTGGTCTCGCGATCAGCTTGAGACATTTCTCCAGGTTTACAAACCTAAAAATTTGTGG
GCTACTCTGGTTTTTCCACCTGAGATCTTAGCTGGTTACAAATCCTCCGTTCTGCCCTTCTTATATCA
ATTTGAAATTCACGG
+
FFFFFFFFFFFFFFFFFFF:FFFFFFFFFFFFFFFFFFFFFFFFFFFFFFFFFFFFFFFFFFFFFFFF
FFFFFFFFFFFFFFFFFFFFFFFFFF:FFFFFFFFFFFFFFFFFFFFFFFFFFFFFFFFFFFFFFFFF
F,F:FFFFFFFFFFF
@A00155:342:HHGFNDSXY:1:2413:17083:11162 1:N:0:GAACCTAG+TCCGCATA
AATTCATTATTGGTCTCGCGATCAGCTTGAGACATTTCTCCAGGTTTACAAACCTAAAAATTTGTGGG
CTACTCTGGTTTTTCCACCTGAGATCTTAGCTGGTTACAAATCCTCCGTTCTGCCCTTCTTATATCAA
TTTGAAATTCACGG
+
F:FFFFFFFFFFFFFFFFFFFFFFFFFFFFFFFFFFFFFFFF:FFFFFFFFFFFFFFF,FFFFFFFFF
FFFFFFFFFFFFFFFFFFFFFFFFFFFFFFFFFFFFFFFFFFFFFFFFFFFFFFFFFFFFFF:FFFFF:
:FFFFFFFFFFFFF
@A00155:342:HHGFNDSXY:1:1678:3775:19116 1:N:0:GAACCTAG+TCCGCATA
AATTCATTATTGGTCTCGCGATCAGCTTGATACATTTCTCCAGGTTTACAAACCTAAAAATTTGTGGG
CTACTCTGGTTTTTCCACCTGAGATCTTAGCTGGTTACAAATCCTCCGTTCTGCCCTTCTTATATCAA
TTTGAAATTCACGGG
+
:FFFFFFFFF:FFFFFFFFFFFFFFFFFFF,FFFF:,F:FFFF:FFFF:FFFF:FFFFF:FFF,,FFF
FF,FFFFFFFFFFFFFFFFFFFFFFFFFFFFFF:FFFFFFFFFFFF,F,FFFFFFFFFFFFFF:FFFFF
,FFFFFFFFFF:FFF
@A00155:342:HHGFNDSXY:1:2256:22281:18317 1:N:0:GAACCTAG+TCCGCATA
AATTCATTATTGGTCTCGCGATCAGCTTGAGACATTTCTCCAGGTTTACAAACCTAAAAATTTGTGGG
CTACTCTGGTTTTTCCACCTGAGATCTTAGCTGGTTACAAATCCTCCGTTCTGCCCTTCTTATATCAA
TTTGAAATTCACGGG
+
FFFFFFFFFFFFFFFFFF:FFFFFFFFFFFFFFFFFFFFFFFFFFFFFFFFFFFFFFFFFFFFFFFFF
FFFFFFFFFFFFFFFFFFFFFFFFFFFFFFFFFFFFFFFFFFFFFFFFFFFFFFFFFFFFFFFFFFFF
:FFFFFFFFFFFFFF
@A00155:342:HHGFNDSXY:1:2638:13006:35790 1:N:0:GAACCTAG+TCCGCATA
AATTCATTATTGGTCTCGCGATCAGCTTGAGACATTTCTCCAGGTTTACAAACCTAAAAATTTGTGGG
CTACTCTGGTTTTTCCACCTGAGATCTTAGCTGGTTACAAATCCTCCGTTCTGCCCTTCTTATATCAA
TTTGAAATTCACGGG
+
FFFFFFFFFFFFFFFFFFFFFFFFFFFFFFFFFFFFFFFFFFFFFFFFFFFFFFFFFFFFFFFFFFFF
FFFFFFFFFFFFFFFFFFFFFFFFFFFFFFFFFFFFFFFFFFFFFFFFFFFFFFFFFFFFFFFFFFFF
FFFFFFFFFFFFFFF
@A00155:342:HHGFNDSXY:1:1478:29984:10050 2:N:0:GAACCTAG+TCCGCATA
AATTCATTATTGGTCTCGCGATCAGCTTGAGACATTTCTCCAGGTTTACAAACCTAAAAATTTGTGGG
CTACTCTGGTTTTTCCACCTGAGATCTTAGCTGGTTACAAATCCTCCGTTCTGCCCTTCTTATATCAA
TTTGAAATTCACGGG
+
FFFFFFFFFFFFF:FFFFFFFFFFFFFFFFFFFFFFFFFFF:FFFFFFFFFFF:FFFFFFFFFFFFFF
FFFFFFFFFFFF,FFFFFFFFFFFFFFF,FFFFFFFFFFFF:FFFFFFFFFFFFFFFFFFFF,FFFFF
F,FF:FFFFFFFFFF
@A00155:342:HHGFNDSXY:1:2512:11388:24001 2:N:0:GAACCTAG+TCCGCATA
AATTCATTATTGGTCTCGCGATCAGCTTGAGACATTTCTCCAGGTTTACAAACCTAAAATTTTGTGGG
CTACTCTGGTTTTTCCACCTGAGATCTTAGCTGGTTACAAATCCTCCGTTCTGCCCTTCTTATATCAA
TTTGAAATTCACGGG
+
FFFFFFFFFFFFFFFFFFFFFFFFFFFFFFFFFFFFFFFFFFFFFFFFFFFFFFFFFFFF,FFFFFFFF
```

```
FFFFFFFFFFFFFFFFFFFFFFFFFFFFFFFFFFFFFFFFFFFFFFFF:FFFFFFFFFFFFF,FFFFFFF
FFFFFFFFFFFFFFF
@A00155:342:HHGFNDSXY:1:1411:30861:6840 1:N:0:GAACCTAG+TCCGCATA
ATTCATTATTGGTCTCGCGATCAGCTTGAGACATTTCTCCAGGTTTACAAACCTAAAAATTTGTGGGC
TACTCTGGTTTTTCCACCTGAGATCTTAGCTGGTTACAAATCCTCCGTTCTGCCCTTCTTATATCAAT
TTGAAATTCACGGAC
+
FFFFFFFFFFFFFFFFFFFFFFFFFFFFFFFFFFFFFFFFFFFFFFFFFFFFFFFFFFF:FFFFFFFFFF
FFF,FFFFFFFFFFFFFFFFFFFFFFFFFFFFFFFFFFFFFFFFFFFFFFFFFFFFFFFFFFFFFFFFFF
FFFFFFFFFFFFFFF
@A00155:342:HHGFNDSXY:1:1129:15465:5603 2:N:0:GAACCTAG+TCCGCATA
ATTCATTATTGGTCTCGCGATCAGCTTGAGACATTTCTCCAGGTTTACAAACCTAAAAATTTGTGGGC
TACTCTGGTTTTTCCACCTGAGATCTTAGCTGGTTACAAATCCTCCGTTCTGCCCTTCTTATATCAAT
TTGAAATTCACGGGA
+
FFFFFFFFFFFFFFFFFFFFFFFFFFFFFFFFFFFFFFFFFFFFFFFFFFF:FFFFFFFFFFFFFFFFFF
FFFFFFFFFFFFFFFFFFFFF:FFF:FFFFFFFFFFFFFFFFFFFFFFFFFFFFF:FF:FFFFFFFFFFF
FFFFFFFFFFFFFFF
@A00155:342:HHGFNDSXY:1:1415:14977:18599 2:N:0:GAACCTAG+TCCGCATA
ATTCATTATTGGTCTCGCGATCAGCTTGAGACATTTCTCCAGGTTTACAAACCTAAAAATTTGTGGGC
TACTCTGGTTTTTCCACCTGAGATCTTAGCTGGTTACAAATCCTCCGTTCTGCCCTTCTTATATCAAT
TTGAAATTCACAGAT
+
FFFFFFFFFFFFFFFFFFFFFFFFFFFFFFFFFFFFFFFFFFFFFFFFFFFFFFFFFFFFFFFFFFFFFF
FFFFFFFFF:FFFFFFFFFFFFFF:FFFF:FFFFFFFFFFFFFFFFFFFFFFFFFFFFFFFFFFFFF,FF
FFFFFFFFFFFFFFF
@A00155:342:HHGFNDSXY:1:1617:9995:31955 2:N:0:GAACCTAG+TCCGCATA
ATTCATTATTGGTCTCGCGATCAGCTTGAGACATTTCTCCAGGTTTACAAACCTAAAAATTTGTGGGC
TACTCTGGTTTTTCCACCTGAGATCTTAGCTGGTTACAAATCCTCCGTTCTGCCCTTCTTATATCAAT
TTGAAATTCACGGGA
+
FFFFFFFFFFFFFFFFFFFFFFFFFFFFFFFFFFFFFFFFFFFFFFFFFFFFFFFFFFFFFFFFFFFFFF
FFFFFFFFFFFFFFFFFFFFFFFFFFFFFFFFFFFFFFFFFFFFFF:FFFFFFFFFFFFFFFFFFFFFFF
FFFFFFFFFFFFFFF
@A00155:342:HHGFNDSXY:1:2244:5584:30232 2:N:0:GAACCTAG+TCCGCATA
ATTCATTATTGGTCTCGCGATCAGCTTGAGACATTTCTCCAGGTTTACAAACCTAAAAATTTGTGGGC
TACTCTGGTTTTTCCACCTGAGATCTTAGCTGGTTACAAATCCTCCGTTCTGCCCTTCTTATATCAAT
TTGAAATTCACGGGA
+
FFFFF:FFFFFFFFFFFFFFFFFFFFFFFFFFFFFFFFFFFFFFF:FFFFFF:FFFFFFFFFFFFF:FFF
FFFFFFFFFFFFFFF:FFFFFFFFFFFFFFFFFFFFFFFFFFFFF:FFFFFFFFFFFFFFFFFFFFFFFF
FFFFFFFFFFFFFFF
@A00155:342:HHGFNDSXY:1:2274:1768:35556 2:N:0:GAACCTAG+TCCGCATA
ATTCATTATTGGTCTCGCGATCAGCTTGAGACATTTCTCCAGGTTTACAAACCTAAAAATTTGTGGGC
TACTCTGGTTTTTCCACCTGAGATCTTAGCTGGTTACAAATCCTCCGTTCTGCCCTTCTTATATCAAT
TTGAAATTCACAGAT
+
::FFFF:FFFFFF:F:FFFFFFFFFFFFFF:FF:FFFFFFFFFFFFFFFFFFFFFFFFFFF:FFFFFFFF
FFFFFFFF,FFFFFFFFFFFFFFFFFF,FFFF:FFFFFF:FFFFFFFFFFFFFFFFFFFF:FFFF:FFF:
,FFFFFFFFFFF::F
@A00155:342:HHGFNDSXY:1:2564:29930:24643 2:N:0:GAACCTAG+TCCGCATA
ATTCATTATTGGTCTCGCGATCAGCTTGAGACATTTCTCCAGGTTTACAAACCTAAAAATTTGTGGGC
TACTCTGGTTTTTCCACCTGAGATCTTAGCTGGTTACAAATCCTCCGTTCTGCCCTTCTTATATCAAT
TTGAAATTCACGGGA
```

+
F:FFFFFFFFF,FFFFFFFFFFFFFFFFFFFFFFFFFFFFFFFFFFFFFFF:FFF:FFFFFFFFFFFF
FFFFFFFFFFFFFFFFFFFFFFFFFFFFFFFFFFFFFFFFFFFFFFFFFFFFFFFFFFFFFF,FFFFFF
FFFFFFFFFFFFFFF
@A00155:342:HHGFNDSXY:1:2471:21712:6183 1:N:0:GAACCTAG+TCCGCATA
TCATTATTGGTCTCGCGATCAGCTTGAGACATTTCTCCAGGTTTACAAACCTAAAAATTTGTGGGCTA
CTCTGGTTTTTCCACCTGAGATCTTAGCTGGTTACAAATCCTCCGTTCTGCCCTTCTTATATCAATTT
GAAATTCACGGGAAG
+
FFFFFFFFFF:FFFFFFFFFFFFFFFFFFFF,FFFFFFFFFFFFFFFFFFFFFFFFFFFFFFFFF:FFF
FFFFFFFFFFFFFFFFFFFFFFFFFFFFFFFFFFFFFFFFFFFFFFFFFFFFFFFFFFFFFFFFFFFF
FFFFFFFFFFFFFFF
@A00155:342:HHGFNDSXY:1:1229:23999:12587 2:N:0:GAACCTAG+TCCGCATA
CATTATTGGTCTCGCGATCAGCTTGAGACATTTCTCCAGGTTTACAAACCTAAAAATTTGTGGGCTAC
TCTGGTTTTTCCACCTGAGATCTTAGCTGGTTACAAATCCTCCGTTCTGCCCTTCTTATATCAATTTG
AAATTCACGGGAAGG
+
FFFFFFFFFFFFFFFFFFFFF:FFFFFFFFFFFFFFFFFFFFFFFFFFFFFFFFFFFFFFFFFFFFFF
FFFFFFFFFFFFFFFFFFFFFFFFFFFFFFFFFFFFFFFFFFFFFFFFFFFFFFFFFFFFFFFFFF:FF
FFFFFF:FFFF:FFF
@A00155:342:HHGFNDSXY:1:1527:32786:22169 2:N:0:GAACCTAG+TCCGCATA
CATTATTGGTCTCGCGATCAGCTTGAGACATTTCTCCAGGTTTACAAACCTAAAAATTTGTGGGCTAC
TCTGGTTTTTCCACCTGAGATCTTAGCTGGTTACAAATCCTCCGTTCTGCCCTTCTTATATCAATTTG
AAATTCACGGGAAGG
+
FFFFFFFFFFFFFFFFFFFFFFFFFFFFFFFFFFFFFFFFFFFFFFFFFFFFF:FFFFFFFFFFFFFF
FFFFFF:FFFFFFFFFFFFFFFFFFFFFFFFFFFFFFFFFFFFFFFFFFFFFFF,F:FFFFFFFFFFFFF
FFFFFFFFFFFFFFF
@A00155:342:HHGFNDSXY:1:2436:6714:22075 2:N:0:GAACCTAG+TCCGCATA
CATTATTGGTCTCGCGATCAGCTTGAGACATTTCTCCAGGTTTACAAACCTAAAAATTTGTGGGCTAC
TCTGGTTTTTCCACCTGAGATCTTAGCTGGTTACAAATCCTCCGTTCTGCCCTTCTTATATCAATTTG
AAATTCACGGGAAGG
+
FFFFFFFFFFFFFFFFFFFFFFFFFFFF:FFFFFFFFFFFFFFFFFFFFFFFFFFFFFFFFFFFFFFFF
FFFFFFFFFFFFFFFFFFFFFFFFFFFFFFFFFFFFFFFFFFF:FFFFFFFFFFFFFFFFFFFFFFFFF
FFFFFF:FFFFFFF:
@A00155:342:HHGFNDSXY:1:2471:23511:32847 2:N:0:GAACCTAG+TCCGCATA
CATTATTGGTCTCGCGATCAGCTTGAGACATTTCTCCAGGTTTACAAACCTAAAAATTTGTGGGCTAC
TCTGGTTTTTCCACCTGAGATCTTAGCTGGTTACAAATCCTCCGTTCTGCCCTTCTTATATCAATTTG
AAATTCACAGATCGG
+
FFFF,FFFFFFFFFFFFFFFFFFFFFFFFF:FFFFFFFFFFFFFFFFFFFFFFFFFFFFFFFFFFFFF
FFFFFFFFFFFFFFFFFFFFFFFFFFFFFFFFFFFFFFFFFFFFFFFFFFFFFFFFFFFFFFFFFFFF
FFFFFFFFFFFFFFF
@A00155:342:HHGFNDSXY:1:2611:14886:20697 2:N:0:GAACCTAG+TCCGCATA
CATTATTGGTCTCGCGATCAGCTTGAGACATTTCTCCAGGTTTACAAACCTAAAAATTTGTGGGCTAC
TCTGGTTTTTCCACCTGAGATCTTAGCTGGTTACAAATCCTCCGTTCTGCCCTTCTTATATCAATTTG
AAATTCACAGATCGG
+
FFFFFFFFFFFFFFFFFFFFFFFFFFFFFFFFFFFFFFFF:FFFFFFFFFFFFFFFFFFFFFFFFFFFF
FFFFFFFFFFFFFFFFFFFFFFFFFFFFFFFFFFFFFFFFFFFFF:FFFFFFFFFFFFFFFFFFFFFF:FF
:FFFFFFFFFFFFFF
@A00155:342:HHGFNDSXY:1:1105:22336:22420 2:N:0:GAACCTAG+TCCGCATA
ATTATTGGTCTCGCGATCAGCTTGAGACATTTCTCCAGGTTTACAAACCTAAAAATTTGTGGGCTACT

CTGGTTTTTCCACCTGAGATCTTAGCTGGTTACAAATCCTCCGTTCTGCCCTTCTTATATCAATTTGA
AATTCACGGGAAGGA
+
:FFFFFFFFFFFFFFFFF:FFFFFFFFFFFFFFFFFFF:FFFFFF:FFFFFFFFFFFFFFFFFFFFFFF
FFFFFFFFFFFFF:FFFFFFFFFFFF:FFFFFFFFFFFFFFFFFFFFFFFFF:FFFFFFFFFFFFFFF
FFFFFFFFFFFFFFF
@A00155:342:HHGFNDSXY:1:1131:29812:16423 2:N:0:GAACCTAG+TCCGCATA
ATTATTGGTCTCGCGATCAGCTTGAGACATTTCTCCAGGTTTACAAACCTAAAAATTTGTGGGCTACT
CTGGTTTTTCCACCTGAGATCTTAGCTGGTTACAAATCCTCCGTTCTGCCCTTCTTATATCAATTTGA
AATTCACGGGAAGGA
+
FFFFFFFFFFFFFFFFFFFFFFFFFFFFFFFFFFFFFFFFFFFFFFFFFFFFFFFFFFFFFFFFFFFF
FFFFFFFFFF:FFFFFFFFFF:FFFFFFFFF:FFFFFFFFFFFFFFFFFFFFFFF,F:FFFFFFFF:F
FFFFFF:F:FFFFFF
@A00155:342:HHGFNDSXY:1:1223:3821:33943 2:N:0:GAACCTAG+TCCGCATA
ATTATTGGTCTCGCGATCAGCTTGAGACATTTCTCCAGGTTTACAAACCTAAAAATTTGTGGGCTACT
CTGGTTTTTCCACCTGAGATCTTAGCTGGTTACAAATCCTCCGTTCTGCCCTTCTTATATCAATTTGA
AATTCACGGGAAGGA
+
FFFFFFFFFFFFFFFFFFFFFFFFFFFFFFFFFFFFFFFFFFFFFFFFFFFFFFFFFFFFF:FFFFFF
FFFFFFFFFFFFFFFFFFFFFFFFFFFFFFFFFFFFFFFFFFFFF:FFFFFF,FFFFF:FFFFFFFFFF
FFFFFFFFFFF,FFF
@A00155:342:HHGFNDSXY:1:1411:31430:11647 2:N:0:GAACCTAG+TCCGCATA
ATTATTGGTCTCGCGATCAGCTTGAGACATTTCTCCAGGTTTACAAACCTAAAAATTTGTGGGCTACT
CTGGTTTTTCCACCTGAGATCTTAGCTGGTTACAAATCCTCCGTTCTGCCCTTCTTATATCAATTTGA
AATTCACGGGAAGGA
+
FFFFFFFFFFFFFFFFFFFFFFFFFFFFFFFFFFFFFFFFFFFFFFFFFFFFFFFFFFFFFFFFFFFF
FFFFFFFFFFFFFFFFFFFFFFFFFFFFFFFFFFFFFFFFFFFFFFFFFFFFFFFFFFFFFFFFFFFF
FFFFFFFFFFFFFFF
@A00155:342:HHGFNDSXY:1:1475:15483:6292 2:N:0:GAACCTAG+TCCGCATA
ATTATTGGTCTCGCGATCAGCTTGAGACATTTCTCCAGGTTTACAAACCTAAAAATTTGTGGGCTACT
CTGGTTTTTCCACCTGAGATCTTAGCTGGTTACAAATCCTCCGTTCTGCCCTTCTTATATCAATTTGA
AATTCACGGGAAGGA
+
FFFFFFFFFFFFFFFFFFFFFFFFFFFFFFFFFFFFFFFFFFFFFFFFFFFFFFFFFFFFFFFFFFFF
FFFFFFFFFFFFFFFFFFFFFFFFFFFFFFFFFFFFFFFFFFFFFFFFFFFF:FFFFFFFFFFFFFFF
FFFFFFFFFFFFFFF
@A00155:342:HHGFNDSXY:1:2141:32226:32816 2:N:0:GAACCTAG+TCCGCATA
ATTATTGGTCTCGCGATCAGCTTGAGACATTTCTCCAGGTTTACAAACCTAAAAATTTGTGGGCTACT
CTGGTTTTTCCACCTGAGATCTTAGCTGGTTACAAATCCTCCGTTCTGCCCTTCTTATATCAATTTGA
AATTCACGGGAAGGA
+
FFFFFFFFFFFFFFFFFFFFFFFFFFFFFFF:FFFFFFFFFFFFFFFFFFFFFFFF:FFFFFFFFFFFF
FFFFFFFFFFFFFFFFFFFFFFFFFFFFFFFFFFFFFFFF:FFFFFFFFFFFFFFF,FFFFFF:FFFF
FFFF:FFFFFFFFFF
@A00155:342:HHGFNDSXY:1:2328:6587:25739 2:N:0:GAACCTAG+TCCGCATA
ATTATTGGTCTCGCGATCAGCTTGAGACATTTCTCCAGGTTTACAAACCTAAAAATTTGTGGGCTACT
CTGGTTTTTCCACCTGAGATCTTAGCTGGTTACAAATCCTCCGTTCTGCCCTTCTTATATCAATTTGA
AATTCACGGGAAGGA
+
FFFFFFFFFFFF:FFFFFFFFFFFFFFFFFFFFFFFFFFFFFFFFFFFFFFFFFFFFFFFFFFFFFFF
FFFFFFFFFFFFFFFFFFFFFFFFFFFFFFFFFFFF:F:FFFFFFFFFFF:FFFFFFFFFFFFFFFFFF
FFFFFF:FFFFFFFF

@A00155:342:HHGFNDSXY:1:1606:4282:12320 2:N:0:GAACCTAG+TCCGCATA
TTATTGGTCTCGCGATCAGCTTGAGACATTTCTCCAGGTTTACAAACATAAAAATTTGTGGGCTACTA
TGGTTTTTCCACCTGAGATCTTAGCTGGTTACAAATCCTCCGTTCTGCCCTTCTTATATCAATTTGAA
ATTCACGGGAAGGAT
+
FFFFFFFFFFFFFFFFFFFFFFFFFFFFFFFFFFFFFFFFFFFFF:,,FFFFFFFFF:FF,,FFF,F,
FFFFFFFFF,F:FF:F,FF:FFF:,F,FFFFFFFFF,:::F:FF,F:,::FFFFFFFFFFFFFFFFFF
FFF,F:F,FFF:FFF
@A00155:342:HHGFNDSXY:1:2607:5267:5102 2:N:0:GAACCTAG+TCCGCATA
TTATTGGTCTCGCGATCAGCTTGAGACATTTCTCCAGGTTTACAAACCTAAAAATTTGTGGGCTACTC
TGGTTTTTCCACCTGAGATCTTAGATGGTTACAAATCCTCCGTTCTTCCCTTCTTATATCAATTTGAA
ATTCACGGGAAGGAT
+
FFFFFFFFFFFFFFFFFFFF:FFF:FFFF:FFFFFFFF,FFFFFFF,FFFF:FF:F,FFFFFFFFFFF
FFFFFFFFF:FFFFFFFFFFFFFF,FFFFFF:FFFFFFFFFFFFFF,::FFFFFFFFF:FFFFFF:FF
FFFFFFF,FFFFF::
@A00155:342:HHGFNDSXY:1:2321:3685:5400 1:N:0:GAACCTAG+TCCGCATA
TATTGGTCTCGCGATCAGATTGAGACATTTCTCCAGGTTTACAAACCTAAAAATTTCTGGGCTACTCT
GGTTTTTCCACCTGAGATCTTAGCTGGTTACAAATCCTCCGTTCTGCCCTTCTTATATCAATTTGAAA
TTCACGGGAAGGAT
+
FFFFF:FFFFFFFFFFFF,F:FFFFF,FFFFFFFF,:FFF:FFFF:FF:FFFFFFFFF:FF:FFFFFF
:FFFFFFFFFFFFFFFFFFF:FFFFFFFFFFF,,FFFFFF,:FFFF:FFFFFFFFFFFFFFFFFFFFF
FFFFFFFFFFFFFF
@A00155:342:HHGFNDSXY:1:1470:19162:30483 2:N:0:GAACCTAG+TCCGCATA
TATTGGTCTCGCGATCAGCTTGAGACATTTCTCCAGGTTTACAAACCTAAAAATTTGTGGGCTACTCT
GGTTTTTCCACCTGAGATCTTAGCTGGTTACAAATCCTCCGTTCTGCCCTCCTTATATCAATTTGAAA
TTCACGGGAAGGATC
+
FFFFFFFFFFFFFFFFFFFFFFFFFFFFFFFFFFFFFFFFFFFFFFFFFFFFFF:FFFFFFFFFFFFF
FFFFFFFFF:FFFFFFFFFFFFFFFFF:F:FFFFFFFFFFFFFFFFFFFFFFF:FFFFFFFFFFF:FFF
FFFFFFFFF:FFFFF
@A00155:342:HHGFNDSXY:1:2141:9127:10755 2:N:0:GAACCTAG+TCCGCATA
TATTGGTCTCGCGATCAGCTTGAGACATTTCTCCAGGTTTACAAACCTAAAAATTTGTGGGCTACTCT
GGTTTTTCCACCTGAGATCTTAGCTGGTTACAAATCCTCCGTTCTGCCCTTCTTATATCAATTTGAAA
TTCACGGGAAGGATC
+
FFFFFFFFFFFFFFFFFFFFFFFFFFFFFFFFFFFFFFFFFFFFFFFFFFFFFFFFFFFFFFFFFFFF
FFFFFFFFFFFFFFFFFFFFFFFFFFFFFFFFFFFFFFFFFFFFFFF:FFFFFFFFFFFFFFFF:FFF
FFFFF:FFFFFFFFF
@A00155:342:HHGFNDSXY:1:2430:24180:31344 2:N:0:GAACCTAG+TCCGCATA
ATTGGTCTCGCGATCAGCTTGAGACATTTCTCCAGGTTTACAAACCTAAAAATTTGTGGGCTACTCTG
GTTTTTCCACCTGAGATCTTAGCTGGTTACAAATCCTCCGTTCTGCCCTTCTTATATCAATTTGAAAT
TCACGGGAAGGATCT
+
FFFFFFFFFFFFFFFFFFFFFFFFFFFFFFFFFFFFFFFFFFFFFFFFFFFFFFFFFFFFFFFFFFFF
FFFFFFFFFFFFFFFFFFFFFFFFFFFFFFFFFFFFFFFFFFFFFFFFFFFFFFFFFFFFFFFFFFFF
:FFFFFFFFFFFFFF
@A00155:342:HHGFNDSXY:1:2514:26115:4726 2:N:0:GAACCTAG+TCCGCATA
ATTGGTCTCGCGATCAGCTTGAGACATTTCTCCAGGTTTACAAACCTAAAAATTTGTGGGCTACTCTG
GTTTTTCCACCTGAGATCTTAGCTGGTTACAAATCCTCCGTTCTGCCCTTCTTATATCAATTTGAAAT
TCACGGGAAGGATCT
+
FFFFFFFFFFFFFFFFFFFFFFFFFFFFFFFF:FFFFFFFFFFFFFFFFFFFFFFFFFFFFFFFFFFF

FFFFFFFFFFFFFFFFFFFFF:FFFFFFFFFFFFFFFFFFFFFFFFFFFFF:F,FFFFFFFFFFFFFF
FFFFFFFF::FF:FF
@A00155:342:HHGFNDSXY:1:2550:9525:7905 1:N:0:GAACCTAG+TCCGCATA
TTGGTCTCGCGATCAGCTTGAGACATTTCTCCAGGTTTACAAACCTAAAAATTTGTGGGCTACTCTGG
TTTTTCCACCTGAGATCTTAGCTGGTTACAAATCCTCCGTTCTGCCCTTCTTATATCAATTTGAAATT
CACGGGAAGGATCTG
+
FFFFFFFFFFFFFFFFFFFFFFFFFFFFFFFFFFFFFFFFFFFFFFFFFFFFFFFFFFFFFFFFFFFF
FFFFFFFFFFFFFFFFFFFFFFFFFFFFFFFFFFFFFFFFFFFFFFFFFFFFFFFFFFFFFFFFFFFF
FFFFFFFFFFFFFFF
@A00155:342:HHGFNDSXY:1:1607:28357:31000 1:N:0:GAACCTAG+TCCGCATA
TGGTCTCGCGATCAGCTTGAGACATTTCTCCAGGTTTACAAACCTAAAAATTTGTGGGCTACTCTGGT
TTTTCCACCTGAGATCTTAGCTGGTTACAAATCCTCCGTTCTGCCCTTCTTATATCAATTTGAAATTC
ACGGGAAGGATCTGG
+
FFFFF:::FFFFFF:FF,:F,FFFFF,,FF:F:,FFFFFF:F:FFFFFFF:FF:F:F,FFFFFFFF:F
FFFFFFFF:FFFFFFF:F,FFF:FFFFFFFFFFFFFFFFFFFFF:FFFFFFFFF,:FF,FFFFFFFF,
FFFFFFFFFFFFFFF
@A00155:342:HHGFNDSXY:1:2353:23231:16736 1:N:0:GAACCTAG+TCCGCATA
TGGTCTCGCGATCAGCTTGAGACATTTCTCCAGGTTTACAAACCTAAAAATTTGTGGGCTACTCTGGT
TTTTCCACCTGAGATCTTAGCTGGTTACAAATCCTCCGTTCTGCCCTTCTTATATCAATTTGAAATTC
ACGGGAAGGATCTGG
+
FFFFFFFFFF:FFFFFFFFFFFFFFFFFFFFFFFFFFFFFFFFFFFFFFFFFFFFFF:FFFFFFFFFF
FFFFFFFFFFFFFFFFFFFF:FFFFFFFFFFFFFFFFFFFFFFFFFFFFFFF:FFFF:FFFFFFFFF
FFFFFFFFFFFFFFF
@A00155:342:HHGFNDSXY:1:2510:1669:32127 1:N:0:GAACCTAG+TCCGCATA
TGGTCTCGCGATCAGCTTGAGACATTTCTCCTGGTTTACACACCTAAAAATGTGTGGGCTACTCTGGT
TTTTCCACCTGAGATCTTAGCTGGTTACAAATCCTCCGTTCTGCCCGTCTTATATCAATTTGAAATTC
ACGGGAAGGATCTGG
+
:FFFFFFFFFFF,F:F,FFF,,F,:FFFF:F,FFF,F::F,,:FF,,FFF:,FF:FFF,:,::,::F:
F:FFFF::F,F:F:FF:::FFFFF:FFF,F,FFFFFFF,F:FF,F:,F:FFF,,FFF,:FFFF:FFF,
FF:FFFFFFF,FFFF
@A00155:342:HHGFNDSXY:1:1270:18394:30217 1:N:0:GAACCTAG+TCCGCATA
GGTCTCGCGATCAGCTTGAGACATTTCTCCAGGTTTACAAACCTAAAAATTTGTGGCCTACTCTGGTT
TTTCCACCTGAGATCTTAGCTGGTTACAAATCCTCCGTTCTGCCCTTCTTATATCAATTTGAAATTCA
CGGGAAGGATCTGGT
+
FFFFFFFFFFFFFFFFFFFFFFFFFFFFFFF:FFFFFFFFFFFFFFFFFFFFFFFFFFFFFFF:FFFF
FFFFFFFFFFFFFFFFFFFFFFFFFFFFFFFFFFFFFFFFFFFFFFFFFFFFFFFFFFFFFFFFFFFF
FFFFFFFFFFFFFFF
@A00155:342:HHGFNDSXY:1:1275:6171:33129 1:N:0:GAACCTAG+TCCGCATA
GGTCTCGCGATCAGCTTGAGACATTTCTCCAGGTTTACAGACCTAAAAATTTGTGGGCTACTCTGGTT
TTTCCACCTGAGATCTTAGCTGGTTACAAATCCTCCGTTCTGCCCTTCTTATATCAATTTGAAATTCA
CGGGAAGGATCTGGT
+
FFFFFFFFFFFFFFFFFFFFFFFFFFFFFFFFFFFFFFFFFFFFFFFFFFFFFFFFFFFFFFFFFFFF
FFFFFFFFFFFFFFFFF:FFFFFFFFFFFFFFFFFFFFFFFFFFFFFFFFFFFFFFFFFFFFFFFFFF
FFFFFFFFFFFFFFF
@A00155:342:HHGFNDSXY:1:1322:29722:15233 1:N:0:GAACCTAG+TCCGCATA
GGTCTCGCGATCAGCTTGAGACATTTCTCCAGGTTTACAAACCTAAAAATTTGTGGGCTACTCTGGTT
TTTCCACCTGAGATCTTAGCTGGTTACAAATCCTCCGTTCTGCCCTTCTTATATCAATTTGAAATTCA
CGGGAAGGATCTGGT

```
+
FFF::FFFFFFFFFFFFFFFF:FFFFFF:FFF,FFFFFFF:FFFFFFFFFFFFFFFFFFFFFFFFFFF
FFFFFFFFFFFFFFFFFFFFFFFFFFFFFFFFFFFFFFF,FFFF:FF:FFFFFFFFFFFFFFFFFFFF
FFFFFF:FFFFFFFF
@A00155:342:HHGFNDSXY:1:1317:7274:22138 1:N:0:GAACCTAG+TCCGCATA
GGTCTCGCGATCAGCTTGAGACATTTCTCCAGGTTTACAAACCTAAAAATTTGTGGGCTACTCTGGTT
TTTCCACCTGAGATCTTAGCTGGTTACAAATCCTCCGTTCTGCCCTTCTTATATCAATTTGAAATTCA
CGGGAAGGATCTGGT
+
FFFFFFFFFFFFFFFFFFFFF:FFFFFFFFFFFFFFFFF:FFFFFFFFFF:FFFFFFFFFFFFFFFFF
FFFFFFFFFFFFFFFFFF,FFFFFFFFFF,:FFFF:FFFFFFFFFFFFFFF:FFFFFFFFFFFFFFFF
FFFFFFFFFFFFFFF
@A00155:342:HHGFNDSXY:1:1329:23122:34961 1:N:0:GAACCTAG+TCCGCATA
GGTCTCGCGATCAGCTTGAGACATTTCTCCAGGTTTACAAACCTAAAAATTTGTGGGCTACTCTGGTT
TTTCCACCTGAGATCTTAGCTGGTTACAAATCCTCCGTTCTGCCCTTCTTATATCAATTTGAAATTCA
CGGGAAGGATCTGGT
+
FFFFFFFFFFFFFFFFFFFFFF:FFFFFFFFFFFFFFFFFFFFFFFFFFFFFFFFFFFFFFFFFFFFF
FFFFFFFFFFFFFFFF:FFFFFFF:FFFFFFFFFFFF:FFFFFFFFFFFFFFFFFFFFFFFFFF:FFF
FFFFFFFFFFFFFFF
@A00155:342:HHGFNDSXY:1:1347:27724:34475 1:N:0:GAACCTAG+TCCGCATA
GGTCTCGCGATCAGCTTGAGACATTTCTCCAGGTTTACAAACCTAAAAATTTGTGGGCTACTCTGGTT
TTTCCACCTGAGATCTTAGCTGGTTACAAATCCTCCGTTCTGCCCTTCTTATATCAATTTGAAATTCA
CGGGAAGGATCTGGT
+
FFFFFFFFFF:FFFFFFFFFFFFFFFFFFFFFFFFFFFFFFFFFFFFFFFFFFFFFFFFFFFFFFFF:
FFFFFFFFFFFFFFFFFFFFFFFFFFFFFFFFFFFFFFFFFFFFFFFFFFFFFFFFFFFFFFFFFFFF
FFFFFFFFFFFFFFF
@A00155:342:HHGFNDSXY:1:1411:31430:11647 1:N:0:GAACCTAG+TCCGCATA
GGTCTCGCGATCAGCTTGAGACATTTCTCCAGGTTTACAAACCTAAAAATTTGTGGGCTACTCTGGTT
TTTCCACCTGAGATCTTAGCTGGTTACAAATCCTCCGTTCTGCCCTTCTTATATCAATTTGAAATTCA
CGGGAAGGATCTGGT
+
FFFFFFFFFFFFFFFFFFFFFFFFFFFFFFFFFFFFFFFFFFFFFFFFFFFFFFFFFFFFFFFFFFFF
FFFFFFFFFFFFFFFFFFFFFFFFFFFFFFFFFFFFFFFFFFFFFFFFFFFFFFFFFFFFFFFFFFFF
FFFFFFFFFFFFFFF
@A00155:342:HHGFNDSXY:1:1518:25816:2112 1:N:0:GAACCTAG+TCCGCATA
GGTCTCGCGATCAGCTTGAGACATTTCTCCAGGTTTACAGACCTAAAAATTTGTGGGCTACTCTGGTT
TTTCCACCTGAGATCTTAGCTGGTTACAAATCCTCCGTTCTGCCCTTCTTATATCAATTTGAAATTCA
CGGGAAGGATCTGGT
+
FFFFFFFFFFFFFFFFFFFFFFFFFFFF,FFFFFFFFFFFFFFFFFFFFFFFFFFFFFFFFFFFFFFF
FFFFFFFFFFFFFFFFFFFFF:FFFFFFFFFFFFFFFFFFFFFFFFFFFFFFFFFF:FFFFFFFFFFF
F:FFFFFFFFFFFFF
@A00155:342:HHGFNDSXY:1:1544:14281:13135 1:N:0:GAACCTAG+TCCGCATA
GGTCTCGCGATCAGCTTGAGACATTTCTCCAGGTTTACAAACCTAAAAATTTGTGGGCTACTCTGGTT
TTTCCACCTGAGATCTTAGCTGGTTACAAATCCTCCGTTCTGCCCTTCTTATATCAATTTGAAATTCA
CGGGAAGGATCTGGT
+
FFFFFFFFFFFFFFFFFFFFFFFFFFFFFF:FFFFFFFFFFFFFFFFFFFFFFFFFFFFFFFFFFFFF
FFFFFFFFFFFFFFFFFFFFFFFFFFFFFFFFFFFFFFFFFFFFFFFFFFFFFFFFFFFFFFFFFFFF
FFFFFFFFFFFFFFF
@A00155:342:HHGFNDSXY:1:1573:30879:13542 1:N:0:GAACCTAG+TCCGCATA
GGTCTCGCGATCAGCTTGAGACATTTCTCCAGGTTTACAAACCTAAAAATTTGTGGGCTACTCTGGTT
```

TTTCCACCTGAGATCTTAGCTGGTTACAAATCCTCCGTTCTGCCCTTCTTATATCAATTTGAAATTCA
CGGGAAGGATCTGGT
+
FFFFFFFFFFFFFFFFFFFFFFFFFFFFFF:FFFFFFFFFFFFFFFFFFFFFFFFFFFFFFFFFFFFF
FFFFFFFF:FFFFFFFFFFFFFFFFFFF:FFFFFFFFFFFFFFFFFFFFFFFFFFFF:FFFFFF:FFF
FFFFFFFFFFFFFFF
@A00155:342:HHGFNDSXY:1:1675:22372:15875 1:N:0:GAACCTAG+TCCGCATA
GGTCTCGCGATCAGCTTGAGACATTTCTCCAGGTTTACAAACCTAAAAATTTGTGGGCTACTCTGGTT
TTTCCACCTGAGATCTTAGCTGGTTACAAATCCTCCGTTCTGCCCTTCTTATATCAATTTGAAATTCA
CGGGAAGGATCTGGT
+
FFFFFFFFFFFFFFFFFFFFFFFFFFFFFFFFFFFFFFFFFFFFFFFFFFFFFFFFFFFFFFFFFFFF
FFFFFFFFFFFFFFFFFFFFFFFFFFFFFFFFFFFFFFFFFFFFFFFFFFFFFFFFF:FFFFFFFFFF
FFFFFFFFFFFFFFF
@A00155:342:HHGFNDSXY:1:2229:30870:9674 1:N:0:GAACCTAG+TCCGCATA
GGTCTCGCGATCAGCTTGAGACATTTCTCCAGGTTTACAAACCTAAAAATTTGTGGGCTACTCTGGTT
TTTCCACCTGAGATCTTAGCTGGTTACAAATCCTCCGTTCTGCCCTTCTTATATCAATTTGAAATTCA
CGGGAAGGATCTGGT
+
FFFFFFFFFFFFFFFFFFFFFFFFFF:FFFFFFFFFFFFFFFFFFFFFFFFFFFFFFFFFFFFFFFFF
FFFFFFFFFFFFFFFFFF:FFFFFFFFFFFFFFFFFFFFFFFFFFFFFFFFFFFFFFFFFFFFFFFFF
FFFFFFFFFFFFFFF
@A00155:342:HHGFNDSXY:1:2325:5954:35978 1:N:0:GAACCTAG+TCCGCATA
GGTCTCGCGATCAGCTTGAGACATTTCTCCAGGTTTACAAACCTAAAAATTTGTGGGCTACTCTGGTT
TTTCCACCTGAGATCTTAGCTGGTTACAAATCCTCCGTTCTGCCCTTCTTATATCAATTTGAAATTCA
CGGGAAGGATCTGGT
+
FFFFFFFFFFFFFFFFFFFFFFFFFFFFFFFFFFFFFFFFFFFFFF:FFFFFFFFFFFFFFFFFFFFF
FFFFFFFFFFFFFFFFFFFFFFFFFFFFFFFFFFFFFFFFFFFFFFFFFFFFFFFFFFFFFFFFFFFF
FFFFFFFFFFFFFFF
@A00155:342:HHGFNDSXY:1:2471:25690:36808 1:N:0:GAACCTAG+TCCGCATA
GGTCTCGCGATCAGCTTGAGACATTTCTCCAGGTTTACAAACCTAAAAATTTGTGGGCTACTCTGGTT
TTTCCACCTGAGATCTTAGCTGGTTACAAATCCTCCGTTCTGCCCTTCTTATATCAATTTGAAATTCA
CGGGAAGGATCTGGC
+
FFFFFFFFFFFFFFFFFFFFFFFFFFFFFFFFFFFFFFFFFFFFFFFFFFFFFFFFF:FFFFFFFFFF
FFFFFFFF:FFFFFFFFFFFFFFFFFFFFFFFFFFFFFFFFFFFFFFFFFFFFFFFFFFFFFFFFFFF
FFFFFFFFFFFFFFF
@A00155:342:HHGFNDSXY:1:2471:25626:36855 1:N:0:GAACCTAG+TCCGCATA
GGTCTCGCGATCAGCTTGAGACATTTCTCCAGGTTTACAAACCTAAAAATTTGTGGGCTACTCTGGTT
TTTCCACCTGAGATCTTAGCTGGTTACAAATCCTCCGTTCTGCCCTTCTTATATCAATTTGAAATTCA
CGGGAAGGATCTGGC
+
FFFFFFFFFFFFFFFFFFFFFFFFFFFFFFFFFFFFFFFFFFFFFFFFFFFFFFFFFFFFFFFFFFFF
FFFFFFFFFFFFF:FFFFFFFFFFFFFFFFFFFFFFFFFFFFFFFFFFFFFFFFFFF:FFFFFFFFFF
FFFFFFFFFFFFFFF
@A00155:342:HHGFNDSXY:1:2514:14633:26960 1:N:0:GAACCTAG+TCCGCATA
GGTCTCGCGATCAGCTTGAGACATTTCTCCAGGTTTACAAACCTAAAAATTTGTGGGCTACTCTGGTT
TTTCCACCTGAGATCTTAGCTGGTTACAAATCCTCCGTTCTGCCCTTCTTATATCAATTTGAAATTCA
CGGGAAGGATCTGGT
+
FFFFFFFFFFFFFFFFFFFFFF:FFFFFFFFFFFFFFFFFFFF:FFFF:FFFFFFFFFFFFFFFFFFF
FFFFFFFFFFFFFFFFFFFFFFFFFFFFFFFFFFFFFFFFFFFFFFFFFFFFFFFFFFF:F:FFFFFF
F:FFFFFFFFFFFFF

```
@A00155:342:HHGFNDSXY:1:2555:20030:8469 1:N:0:GAACCTAG+TCCGCATA
GGTCTCGCGATCAGCTTGAGACATTTCTCCAGGTTTACAAACCTAAAAATTTGTGGGCTACTCTGGTT
TTTCCACCTGAGATCTTAGCTGGTTACAAATCCTCCGTTCTGCCCTTCTTATATCAATTTGAAATTCA
CGGGAAGGATCTGGT
+
FFFFFFFFFFFFFFFFFFFFFFFFFFFFFFFFFFFFFFFFFFFFFFFF:FFFFFFFFFFFFFFFFFFF
FFFFFFFFFFFFFFFFFFFFFFFFFFFFFFFFFFFFFFFFFFFFFFFFFFFFFFFFFFFFFFFFFFFF
F:FFFFFFFFFFFFF
@A00155:342:HHGFNDSXY:1:2617:15691:7999 1:N:0:GAACCTAG+TCCGCATA
GGTCTCGCGATCAGCTTGAGACATTTCTCCAGGTTTACAAACCTAAAAATTTGTGGGCTACTCTGGTT
TTTCCACCTGAGATCTTAGCTGGTTACAAATCCTCCGTTCTGCCCTTCTTATATCAATTTGAAATTCA
CGGGAAGGATCTGGC
+
FFFFFFFFFFFFFFFFFFFFFFFFFFFFFFFFFFFFFFFFFFFFFFFFFFFFFFFFFFFFFFFFFF:F
FFFFFFFFFFFFF:FFFFFFFFFFFFFF:FFFFFFFFFFFFFFFFFFFFFFFFFFFFFFFFFFFFFFF
FFFFFFFFFFFFFFF
@A00155:342:HHGFNDSXY:1:2617:3821:8923 1:N:0:GAACCTAG+TCCGCATA
GGTCTCGCGATCAGCTTGAGACATTTCTCCAGGTTTACAAACCTAAAAATTTGTGGGCTACTCTGGTT
TTTCCACCTGAGATCTTAGCTGGTTACAAATCCTCCGTTCTGCCCTTCTTATATCAATTTGAAATTCA
CGGGAAGGATCTGGT
+
FFFFFFFF,:FFFFFFFFFF::FFFFFFFFF:FFFFFFFFFF:FFFFFFFFFFFFF:FFFFFF:,FFF
FFFFFFFFFFFFFFFFFFFFFFFFFFFFFFFFFFFFFFFFFFFFF:FFFFFFFFFFFFFFFFFFFFFF
F,FFFFFFFFFFFFF
@A00155:342:HHGFNDSXY:1:2625:12328:11193 1:N:0:GAACCTAG+TCCGCATA
GGTCTCGCGATCAGCTTGAGACATTTCTCCAGGTTTACAAACCTAAAAATTTGTGGGCTACTCTGGTT
TTTCCACCTGAGATCTTAGCTGGTTACAAATCCTCCGTTCTGCCCTTCTTATATCAATTTGAAATTCA
CGGGAAGGATCTGGT
+
FFFFFFFFFFFFFFFFFFFFFFFFFFFFFFFFFFFFFFFFFFFFFFFF:FFFFFFFFFFFFFFFFFFF
FFFFFFFFFFFFFFFFFFFFFFFFFFFFFFFFFFFFFFFFFFFFFFFFFFFFF:FFFFFFFFFFFFFF
FFFFFFFFFFFFFFF
@A00155:342:HHGFNDSXY:1:2627:10565:26960 1:N:0:GAACCTAG+TCCGCATA
GGTCTCGCGATCAGCTTGAGACATTTCTCCAGGTTTACAAACCTAAAAATTTGTGGGCTACTCTGGTT
TTTCCACCTGAGATCTTAGCTGGTTACAAATCCTCCGTTCTGCCCTTCTTATATCAATTTGAAATTCA
CGGGAAGGATCTGGT
+
:FFFFFF:FFFFFF:FFFFFF:FFFF:FFF,FFFFFFFFFFF,FFFF,FFFFFFFFFFFF:FFFFFFF
FFF:FFFFFFFFFFFFFFFFFFFFFFFFFFFFFFFFFFF:FFFF:FFFFFFFFFFFFFFFFFFFFFFF
FFFFF:F:FFFFFFF
@A00155:342:HHGFNDSXY:1:2628:19895:11177 1:N:0:GAACCTAG+TCCGCATA
GGTCTCGCGATCAGCTTGAGACATTTCTCCAGGTTTACAAACCTAAAAATTTGTGGGCTACTCTGGTT
TTTCCACCTGAGATCTTAGCTGGTTACAAATCCTCCGTTCTGCCCTTCTTATATCAATTTGAAATTCA
CGGGAAGGATCTGGT
+
FFFFFFFFFFFFFFFFFFFFFFFFFFFFFFFF:FFFFFFF:FFFFFFFFFFFFFFFFFFFFFFFFFFF
FFFFFFFFFFFFF:FFFFFFFFFFFFFFFFFFFFFFFFFFFFFFFFFFFFFFFFFFFFF,FFFFFFFF
FFFFFFFFFFFFFFF
@A00155:342:HHGFNDSXY:1:1214:21260:10222 2:N:0:GAACCTAG+TCCGCATA
GGTCTCGCGATCAGCTTGAGACATTTCTCCAGGTTTACAAACCTAAAAATTTGTGGGCTACTCTGGTT
TTTCCACCTGAGATCTTAGCTGGTTACAAATCCTCCGTTCTGCCCTTCTTATATCAATTTGAAATTCA
CGGGAAGGATCTGGT
+
FFFFFFFFFFFFFFFF:FFFFFFFFFFFFFFFF,FFFFFFFFFFFFFF:FFFFFFFFFFFFFFFFFFF
```

FFFFFFFFFFFFFFFFFFFF:FFFFFFF,FFFFFFFFFFFFFFFF:FFFFFFFFFF:FFFFFFFFFFF
FFFFFFFFFFFFFFF
@A00155:342:HHGFNDSXY:1:2214:24189:13980 2:N:0:GAACCTAG+TCCGCATA
GGTCTCGCGATCAGCTTGAGACATTTCTCCAGGTTTACAAACCTAAAAATTTGTGGGCTACTCTGGTT
TTTCCACCTGAGATCTTAGCTGGTTACAAATCCTCCGTTCTGCCCTTCTTATATCAATTTGAAATTCA
CGGGAAGGATCTGGT
+
FFFFFFFFFF:FFFFFFF:FFFFFFFFFFFFFFFFFFFFFFFFFFFFF:FFFFFFFFFFFFFFFFFFF
FFFFFFFFFFFFFFFFFFFFFFFFFFFFF:FFFFFFFFFFFFFFFFFFFF,FFFFFF:FFFFFFFFFF
FFFFFFFFFFFFFFF
@A00155:342:HHGFNDSXY:1:1125:16342:4836 1:N:0:GAACCTAG+TCCGCATA
CGATCTCGATCAGCTTGAGACATTTCTCCAGGTTTACAAACCTAAAAATTTGTGGGCTACTCTGGTTT
TTCCACCTGAGATCTTAGCTGGTTACAAATCCTCCGTTCTGCCCTTCTTATATCAATTTGAAATTCAC
GGGAAGGATCTGGTC
+
FFFFFFFFFFFFFFFFFFF:FFFFFFFFFFFFFFFFFFFFFFFFFFFFFFFFFFFFFFFFFFFFFFFF
FFFFFFFFFFFFFFFFFFFFFFFFFFFFFFFFFFFFFFFFFFFFFFFFFFFFFFF:FFFFFFFFFFFF
FFFFFFFFFFFFFFF
@A00155:342:HHGFNDSXY:1:1364:21748:27571 1:N:0:GAACCTAG+TCCGCATA
GTCTCGCGATCAGCTTGAGACATTTCTCCAGGTTTACAAACCTAAAAATTTGTGGGCTACTCTGGTTT
TTCCACCTGAGATCTTAGCTGGTTACAAATCCTCCGTTCTGCCCTTCTTATATCAATTTGAAATTCAC
GGGAAGGATCTGGTC
+
FFFFFFFFFFFFFFFFFFFFFFFFFFFFFFFFFFFFFFFFFFFFFFFFFFFFFFFFFFFFFFFFFFFF
FFFFFFFFFFFFFFFFFFFFFFFFFFFFFFFFFFFFFFFFFFFFFFFFFFFFFFFFFFFFFFFFFFFF
:FFFFFFFFFFFFFF
@A00155:342:HHGFNDSXY:1:1415:26829:16172 1:N:0:GAACCTAG+TCCGCATA
GTCTCGCGATCAGCTTGAGACATTTCTCCAGGTTTACAAACCTAAAAATTTGTGGGCTACTCTGGTTT
TTCCACCTGAGATCTTAGCTGGTTACAAATCCTCCGTTCTGCCCTTCTTATATCAATTTGAAATTCAC
GGGAAGGATCTGGTC
+
FFFFFFFFFFFFFFFFFFFFFFFFFFFFF:F,F:FFFFFFFFFFF:FF:FFFFFFFFFFFFFFFFFFF
FFFFFFFFFFFFFFFFFFFFFFFFFFFFFFFFFFFFFFFFFFFFFFFFFFFFFFFFFFFFFFFFFFFF
FFFFFFFFFFFFFFF
@A00155:342:HHGFNDSXY:1:2338:7654:5071 1:N:0:GAACCTAG+TCCGCATA
GTCTCGCGATCAGCTTGAGACATTTCTCCAGGTTTACAAACCTAAAAATTTGTGGGCTACTCTGGTTT
TTCCACCTGAGATCTTAGCTGGTTACAAATCCTCCGTTCTGCCCTTCTTATATCAATTTGAAATTCAC
GGGAAGGATCTGGTC
+
FFFF:FFFFFFFFFFFFFFFFFFFFFFFFFFFFFFFFFFFFFFFFFFFFFFFFFFFFFFFFFFFFFFF
FFFFFFFFFFFFFFFFFFFFFFFFFFFFFFFFFFFFFFFFFFFFFFFFFFFFFFFFFFFFFFFFFFFF
FFFFFFFFFFFFFFF
@A00155:342:HHGFNDSXY:1:2367:28302:29653 1:N:0:GAACCTAG+TCCGCATA
GTCTCGCGATCAGCTTGAGACATTTCTCCAGGTTTACAAACCTAAAAATTTGTGGGCTACTCTGGTTT
TTCCACCTGAGATCTTAGCTGGTTACAAATCCTCCGTTCTGCCCTTCTTATATCAATTTGAAATTCAC
GGGAAGGATCTGGTC
+
FFFFFFFFFFFFFFFFFFFFFFFFFFFFFFFFFFFFFFFFFFFFFFFFFFFFFFFFFFFFFFFFFFFF
FFFFFFFFFFFFFFF:FFFFFFFFFFFFFFFFFFFFFFFFFFFFFFFFFFFFFFFFFFFFFFFFFFFF
FFFFFFFFFFFFFFF
@A00155:342:HHGFNDSXY:1:1139:10628:17018 2:N:0:GAACCTAG+TCCGCATA
GTCTCGCGATCAGCTTGAGACATTTCTCCAGGTTTACAAACCTAAAAATTTGTGGGCTACTCTGGTTT
TTCCACCTGAGATCTTAGCTGGTTACAAATCCTCCGTTCTGCCCTTCTTATATCAATTTGAAATTCAC
GGGAAGGATCTGGTC

```
+
FFFFFFFFFFFFFFFFFFFFFFFFFFFFFFFFFFFFFFFFFFFFFFFFFFFFFFFFFFFFFFFFFFFFFF:F
FFFFFFFFFFF,FFFF:FFFFFFFFFFFFFFFFFF:FFFFFFFFFFFF,FFFFFFFFFFFFFFFFFFF
FFFFFFFFFFFFFFF
@A00155:342:HHGFNDSXY:1:1475:15483:6292 1:N:0:GAACCTAG+TCCGCATA
TCTCGCGATCAGCTTGAGACATTTCTCCAGGTTTACAAACCTAAAAATTTGTGGGCTACTCTGGTTTT
TCCACCTGAGATCTTAGCTGGTTACAAATCCTCCGTTCTGCCCTTCTTATATCAATTTGAAATTCACG
GGAAGGATCTGGTCC
+
FFFFFFFFFFFFFFFFFFFFFFFFFFFFFFFFFFFFFFFFFFFFFFFFFFFFFFFFFFFFFFFFFFFFFFFF
FFFFFFFFFFFFFFFFFFFFFFFFFFFFFFFFFFFFFFFFFFFFFFFFFFFFFFFFFFFFFFFFFFFFFFFF
FFFFFFFFFFFFFFF
@A00155:342:HHGFNDSXY:1:2359:13810:26631 1:N:0:GAACCTAG+TCCGCATA
TCTCGCGATCAGCTTGAGACATTTCTCCAGGTTTACAAACCTAAAAATTTGTGGGCTACTCTGGTTTT
TCCACCTGAGATCTTAGCTGGTTACAAATCCTCCGTTCTGCCCTTCTTATATCAATTTGAAATTCACG
GGAAGGATCTGGTCA
+
FFFFFFFFFFFFFFFFFFFFFFFFFFFFFFFFFFFFFFFFFFFFFFFFFFFFFFFFFFFFFFFFFFFFFFFF
FFFFFFFFFFFFFFFFFFFFFFFFFFFFFFFFFFFFFFFFFFFFFFFFFFFFFFFFFFFFFFFFFFFFFFFF
FFFFFFFFFFFFFFF
@A00155:342:HHGFNDSXY:1:2439:14660:29700 1:N:0:GAACCTAG+TCCGCATA
TCTCGCGATCAGCTTGAGACATTTCTCCAGGTTTACAAACCTAAAAATTTGTGGGCTACTCTGGTTTT
TCCACCTGAGATCTTAGCTGGTTACAAATCCTCCGTTCTGCCCTTCTTATATCAATTTGAAATTCACG
GGAAGGATCTGGTCA
+
FFFFF,F::F:F:FFFFFFF,F:FFFFFFFFFF:FFFF:F:FFFF:FFFFFFFF,F,FFFFF,FF:FF
FFFFFFFFFFFFFFF:FFFFFFFFFF:FFFF,F:FFFFFFF,FFFFFFF,F:FFFF,FFFFFFFFFFF
FFFFFFFFFFFFFFF
@A00155:342:HHGFNDSXY:1:1537:6153:15123 2:N:0:GAACCTAG+TCCGCATA
TCTCGCGATCAGCTTGAGACATTTCTCCAGGTTTACAAACCTAAAAATTTGTGGGCTACTCTGGTTTT
TCCACCTGAGATCTTAGCTGGTTACAAATCCTCCGTTCTGCCCTTCTTATATCAATTTGAAATTCACG
GGAAGGATCTGGTCT
+
FFFFFFF:FFFFFFFFFFFFF:FFF:FFFFFFFFFFFFFFFF:FFFFF,FFFFFFFFFFFFFFFFFFF
FFFFFFFFFFFFFFF,FFFFFFFFFFFFF:FFFFFFFFFFFFFF:FFFF,FFFFFFFFFFFFFFFFF:
FFFFFFFFFFFFFFF
@A00155:342:HHGFNDSXY:1:1537:6207:15280 2:N:0:GAACCTAG+TCCGCATA
TCTCGCGATCAGCTTGAGACATTTCTCCAGGTTTACAAACCTAAAAATTTGTGGGCTACTCTGGTTTT
TCCACCTGAGATCTTAGCTGGTTACAAATCCTCCGTTCTGCCCTTCTTATATCAATTTGAAATTCACG
GGAAGGATCTGGTCT
+
FFFFFFFFFFFFFFFFFFFFFFFFFFFFFFFFFFFFFFFFFFFFFFFFFFFFFFFFFFFFFFFFFFFFFFFF
FFFFFFFFFFFFFFFFFFFFFFFFFFFFFFFFFFFFFFFFFFFFFFFFFFFFFFFFFFFFFFFFFFFFFFF,
FFFFF:FFFFFFFFF
@A00155:342:HHGFNDSXY:1:1133:16640:7921 1:N:0:GAACCTAG+TCCGCATA
TCGCGATCAGCTTGAGACATTTCTCCAGGTTTACAAACCTAAAAATTTGTGGGCTACTCTGGTTTTTC
CACCTGAGATCTTAGCTGGTTACAAATCCTCCGTTCTGCCCTTCTTATATCAATTTGAAATTCACGGG
AAGGATCTGGTCTAC
+
FFFFFFFFFFFFFFFFFFFFFFFFFFFFFFFFFFFFFFFFFFFFFFFFFFFFFFFFFFFFFFFFFFFFFFFF
FFFFFFFFFFFFFFFFFFFFFFFFFFFFFFFFFFFFFFFFFFFFFFFFFFFFFFFFFFFFFFFFFFFFFFFF
FFFFFFFFFFFFFFF
@A00155:342:HHGFNDSXY:1:1122:32949:12618 2:N:0:GAACCTAG+TCCGCATA
CGCGATCAGCTTGAGACATTTCTCCAGGTTTACAAACCTAAAAATTTGTGGGCTACTCTGGTTTTTCC
```

ACCTGAGATCTTAGCTGGTTACAAATCCTCCGTTCTGCCCTTCTTATATCAATTTGAAAATCACGGGA
AGGATCTGGTCTACA
+
FFFFFFFFFFFFFF:FF:FFFFFFFFFFFFF:FFFFFFFFFFFFFFFFFFF:FFFFFFFFFFFFFFFF
FFFFFFFFFFFFFFFFFFFF:FF:FFFFFFFFFFFF:F:FF:F,FF::FFFFFFFFFFF,FFFFFFFF
,FFFFFFFFFFFFFF
@A00155:342:HHGFNDSXY:1:1612:9914:33912 2:N:0:GAACCTAG+TCCGCATA
CGCGATCAGCTTGAGACATTTCTCCAGGTTTACAAACCTAAAAATTTGTGGGCTACTCTGGTTTTTCC
ACCTGAGATCTTAGCTGGTTACAAATCCTCCGTTCTGCCCTTCTTATATCAATTTGAAATTCACGGGA
AGGATCTGGTCTACA
+
FFFFFFFF,FFFFFFFFFF:FFFFFFFF:FFFFFFFFFFFFFFFFFFF,FFFFFFFFFFFFFFFFFFF
FFFFFFFFFFFFFFFFFFFFFFFFFFFFFFFFFFFFFFFFFFFFFFFF::FFFFFFFFFFFFFFFF
FFFFFFFFFFFFFFF
@A00155:342:HHGFNDSXY:1:2223:11623:23030 2:N:0:GAACCTAG+TCCGCATA
CGCGATCAGCTTGAGACATTTCTCCAGGTTTACAAACCTAAAAATTTGTGGGCTACTCTGGTTTTTCC
ACCTGAGATCTTAGCTGGTTACAAATCCTCCGTTCTGCCCTTCTTATATCAATTTGAAATTCACGGGA
AGGATCTGGTCTACA
+
FFFFFFFFFFFFFFFFFFFFFFFFFFFFFFFFFFFFFFFFFFFFFFFFFFFFFFFFFFF:FFFFFF:F:F
FF:FFFFFFFFFFF:FFFFFF:FFFFFFF,:FFFFFF,F:FFFFFFFFFFFFFFF,FFFFFFFFF:FF
FFFFFFFFFFFFFFF
@A00155:342:HHGFNDSXY:1:2443:27570:36996 2:N:0:GAACCTAG+TCCGCATA
CGCGATCAGCTTGAGACATTTCTCCAGGTTTACAAACCTAAAAATTTGTGGGCTACTCTGGTTTTTCC
ACCTGAGATCTTAGCTGGTTACAAATCCTCCGTTCTGCCCTTCTTATATCAATTTGAAATTCACGGGA
AGGATCTGGTCTACA
+
FFFFFFFFFFFFFFFFF:FFFFFFFFFFFFFFFFFFFFFFFFF,FFFFFFFFFFFFF:FFFFFFFFFFF
FFFFFFFFFFFFFF:FFFFFFFFFFFFFFFFFFFFFFFFF:F,FFF:F,FFFFFFFFFFFFFFFFFFFFF
FFFFFF:FFFFFFFF
@A00155:342:HHGFNDSXY:1:1207:18982:6120 1:N:0:GAACCTAG+TCCGCATA
GCGATCAGCTTGAGACATTTCTCCAGGTTTACAAACCTAAAAATTTGTGGGCTACTCTGGTTTTTCCA
CCTGAGATCTTAGCTGGTTACAAATCCTCCGTTCTGCCCTTCTTATATCAATTTGAAATTCACGGGAA
GGATCTGGTCTACAT
+
FFFFFFF:FFFFFFFFFFFFFF:FFFFFFFFFFFFFFFFFFFFFFFFFFFFFFFFFFFFFFFFFFFFF
FFFF:FFFFFFFFFFFFFFFFFFFFFFFFFFFFFFFFFFFFFFFFFFFFFFFFFFFFFFFFFFFFFFFF
FFFFFFFFFFFFFFF
@A00155:342:HHGFNDSXY:1:2207:19135:31093 1:N:0:GAACCTAG+TCCGCATA
GCGATCAGCTTGAGACATTTCTCCAGGTTTACAAACCTAAAAATTTGTGGGCTACTCTGGTTTTTCCA
CCTGAGATCTTAGCTGGTTACAAATCCTCCGTTCTGCCCTTCTTATATCAATTTGAAATTCACGGGAA
GGATCTGGTCTACAT
+
F:FFFFFFFF:FFFFFFFFFFFFFFFF:FFFFF:FFFFFFFFFFFFFFFFFFFFFFFFFFFFFFFFFFF
FFFFFFFFFFFFFFFFFFFFFFFFFFFFFFFFFFFFFFFFFFFFFFFFFFFFFFFFFFFFFFFFFFFF
FFFFFFFFFFFFFFF
@A00155:342:HHGFNDSXY:1:1548:24966:30358 2:N:0:GAACCTAG+TCCGCATA
CGATCAGCTTGAGACATTTCTCCAGGTTTACAAACCTAAAAATTTGTGGGCTACTCTGGTTTTTCCAC
CTGAGATCTTAGCTGGTTACAAATCCTCCGTTCTGCCCTACTTATATCAATTTGAAATTCACGGGAAG
GATCTGTAATACAT
+
:FF:F:F,F,:F,FFF:,:,:F,FF,,:,FFFFF:FFFFFFF:F,:FF,FFFFFF,F:F:,:FFF:F:
FFFF,FFF:FFF,F:,F:FFFFFFFFF,,:,F,,::,F,,:FFF,FFFFFFFF,FFFF::F:,,,FF:
,FFF,,,,,:::FF

```
@A00155:342:HHGFNDSXY:1:1125:16342:4836 2:N:0:GAACCTAG+TCCGCATA
CGATCAGCTTGAGACATTTCTCCAGGTTTACAAACCTAAAAATTTGTGGGCTACTCTGGTTTTTCCAC
CTGAGATCTTAGCTGGTTACAAATCCTCCGTTCTGCCCTTCTTATATCAATTTGAAATTCACGGGAAG
GATCTGGTCAGATCG
+
FFFFFFFFFFFFFFFFFFFFFFFFFFFFFFFFFFFFFFFFFFFFFFFFFFFFFFFFFFFFF:FFFFFFF
FFFFFFFFFFFFFFFFFFFFFFFFFFFFFFFFFFFFFFFFFF:FFFFFF,FFFFFFFFFFFFFFFF
F:FF:FFFFFFFFFF
@A00155:342:HHGFNDSXY:1:2250:24975:26146 2:N:0:GAACCTAG+TCCGCATA
CGATCAGCTTGAGACATTTCTCCAGGTTTACAAACCTAAAAATTTGTGGGCTACTCTGGTTGTTCCAC
CTGAGATCTTTGCTGGTTACAAATCCTCCGTGCTGCCCTTCTTATATCAATTTGAAATTCACGGGAAG
GAGCTGGTCTACCTG
+
FFFFFFFFFFFFFFFFFFFFFFFFFFFFFFFFFFFFFFFFFFFFF,FFFFFFFFFFF:FFFF,FFFFFF
FFFFFFF:FF,:FFFFFFFF:FFFFFFFFF:,FFFF:F:,F,F:F,FFF::FFFFFFFFFFFF:FF,F
F:,F:FFFFFFF,FF
@A00155:342:HHGFNDSXY:1:2672:7075:21198 2:N:0:GAACCTAG+TCCGCATA
CGATCAGCTTGAGACATTTCTCCAGGTTTACAAACCTAAAAATTTGTGGGCTACTCTGGTTTTTCCAC
CTGAGATCTTAGCTGGTTACAAATCCTCCGTTCTGCCCTTCTTATATCAATTTGAAATTCACGGGAAG
GATCTGGTCTACATG
+
FFFFFFFFFFFFFFFFFFFFFFFFFFFFFFFFFFFFFFFFFFFFFFFFF,:FFF:FFFFFFFFFFFFFF
FFFFFFFFFFFFFFF:FFFFFFFFFFFFFFFFFFFFFFFFF:FFFF:FFFFFFFFFFFFF,FFFFFF,F
::FFFFFFFFFFF:F
@A00155:342:HHGFNDSXY:1:1351:19063:19038 1:N:0:GAACCTAG+TCCGCATA
ATCAGCTTGAGACATTTCTCCAGGTTTACAAACCTAAAAATTTGTGGGCTACTCTGGTTTTTCCACCT
GAGATCTTAGCTGGTTACAAATCCTCCGTTCTGCCCTTCTTATATCAATTTGAAATTCACGGGAAGGA
TCTGGTCTACATGCC
+
F,F:FFFFFFFF:FFF,F:FFF:FFFFFFF:,FFFFFFFFFFFFFFFFF:FFFFFFF,FFFFF,,:FF
F:FFFFFFFFFF:FFFFFFFFF,FFFFFFFFFFFFFFFFFFFFFFFFFF:FFFFFFFF:FFFFFFFFFF
FFFFFF:FFFFFFFF
@A00155:342:HHGFNDSXY:1:1350:4942:4445 1:N:0:GAACCTAG+TCCGCATA
ATCAGCTTGAGACATTTCTCCAGGTTTACAAACCTAAAAATTTGTGGGCTACTCTGGTTTTTCCACCT
GAGATCTTAGCTGGTTACAAATCCTCCGTTCTGCCCTTCTTATATCAATTTGAAATTCACGGGAAGGA
TCTGGTCTACATGCC
+
FFFFFF:FFFFFFFFFFFFF:FF:FFFFFFFFFFFFFFFFFFFFFF:FF,FF:FFF:FFFFFFFFFFFF
FFFFFFFFFFFFFFFFFFFFFFFFFFFFFFFF:FFFFFFFFFFFFFFFFFFFFFFFFFFFFFFFFFFFF
FFFFFFFFFFFFFFF
@A00155:342:HHGFNDSXY:1:1641:23610:11913 1:N:0:GAACCTAG+TCCGCATA
ATCAGCTTGAGACATTTCTCCAGGTTTACAAACCTAAAAATTTGTGGGCTACTCTGGTTTTTCCACCT
GAGATCTTAGCTGGTTACAAATCCTCCGTTCTGCCCTTCTTATATCAATTTGAAATTCACGGGAAGGA
TCTGGTCTACATGCC
+
FFFFFFFFFFFF::FFFFFF,::,F:FFFFFFFFF:,FFFF,FFFFFF:FF,F,F::FFFFFFF::FF
::F,FFFFFFFFFFFFF:FFFFFFF:FFFFFFFFFFFFF:F:FFF:FF:FFFF:FFFFF:,:FFFFFF
FFFFF::FFFFFFF:
@A00155:342:HHGFNDSXY:1:2655:15302:20353 1:N:0:GAACCTAG+TCCGCATA
ATCAGCTTGAGACATTTCTCCAGGTTTACAAACCTAAAAATTTGTGGGCTACTCTGGTTTTTCCACCT
GAGATCTTAGCTGGTTACAAATCCTCCGTTCTGCCCTTCTTATATCAATTTGAAATTCACGGGAAGGA
TCTGGTCTACATGCC
+
FFFFFFFFFFFFFFFFFFFFFFFFF:FFFFFFFFFFFFFFFFFFFFFFFFFFFFFFFFFFFFFFFFFFF
```

```
FFFFFFFFFFFFFFFFFFFFFFFFFFFFFFFFFFFFFFFFFFFFFFFFFFFFFFFFFFFF:FFFFFFF
FFFFFFFFFFFFFFF
@A00155:342:HHGFNDSXY:1:1437:22073:16705 2:N:0:GAACCTAG+TCCGCATA
ATCAGCTTGAGACATTTCTCCAGGTTTACAAACCTAAAAATTTGTGGGCTACTCTGGTTTTTCCACCT
GAGATCTTAGCTGGTTACAAATCCTCCGTTCTGCCCTTCTTATATCAATTTGAAATTCACGGGAAGGA
TCTGGTCTACATGCC
+
FFFFF,FFFF:F:FFFF,FFFFFFFFFFFFFFF:F,FFFF:F:FFF:F:F,FFFFFFFF:FFF:FFFF
FFFFFFF:,FFFF,FFFFFFFFFFFF,F:FFFFF,:FFFF,F,FFFFFFFFFFFFFF,:F,:FFFFFF
F,FF::FF::FFFFF
@A00155:342:HHGFNDSXY:1:2430:12391:18035 2:N:0:GAACCTAG+TCCGCATA
ATCAGCTTGAGACATTTCTCCAGGTTTACAAACCTAAAAATTTGTGGGCTACTCTGGTTTTTCCACCT
GAGATCTTAGCTGGTTACAAATCCTCCGTTCTGCCCTTCTTATATCAATTTGAAATTCACGGGAAGGA
TCTGGTCTACATGCC
+
FFFFFFFFFFFFFFFFFFFFFFFFFFFFFFFFFFFFFFFFFFFFFFFFFFFFFFFFFFFFFFFFFFFF
FFFFFFFFFFFFFFFFFFFFFFFFFFFFFFFFF:F:FFFFF:FFFFFFFFFFFFFFFFFFFFFFFFFF
FFFFFFFFFFFFF:F
@A00155:342:HHGFNDSXY:1:2522:9598:2112 2:N:0:GAACCTAG+TCCGCATA
ATCAGCTTGAGACATTTCTCCAGGTTTACAAACCTAAAAATTTGTGGGCTACTCTGGTTTTTCCACCT
GAGATCTTAGCTGGTTACAAATCCTCCGTTCTGCCCTTCTTATATCAATTTGAAATTCACGGGAAGGA
TCTGGTCTACATGCC
+
FFFFFFFFFFFFFFFFFFFFFFFFFFFFFFF:FFFFFFFFFFFFFFFFFFFFFFFFFFFFFFFFFFFF
FFF:FFFFFFFFFFFFFFFFFFFFFFFF:FFFFFF,F:F:F:FFFFFFFFFFFFFFFF:FFFFFFFFFF
FFFFFFFFFFFFFFF
@A00155:342:HHGFNDSXY:1:2546:5493:18677 2:N:0:GAACCTAG+TCCGCATA
ATCAGCTAGAGACATTTCTCCAGGTTTAAAAACCTAAAAATTTGTGGGCTACTCTTGTTTTTCCACCT
GAGATCTTAGATGGTTACAAATAATCCGTTCTGCACTTCTTATATCAATTTGAAATTCACGGGAAGGA
TCTGGTCTACATGCC
+
FFFF:F:,:FFFFF,:FFFFFFFF:FFF,FFFF:FFFFFFF:F,F,::,:F,:FF:FFF,F,F,FF::
:FFFFF:FF,,,:,:FFFFFF:,,:,F,:F:::F,::FFFFFFFF,:FF,FF:FFFFFF,F:,FF:FF
FF,,,:FFFFFF:F:
@A00155:342:HHGFNDSXY:1:2559:11442:31234 2:N:0:GAACCTAG+TCCGCATA
ATCAGCTTGAGACATTTCTCCAGGTTTACAAACCTAAAAATTTGTGGGCTACTCTGGTTTTTCCACCT
GAGATCTTAGCTGGTTACAAATCCTCCGTTCTGCCCTTCTTATATCAATTTGAAATTCACGGGAAGGA
TCTGGTCTACATGCC
+
FF:FFFFFFFFFFFFFFFFFFFFFFFFFFFFFFFFFFFFFF:FFFFFFFFFFFFFFFFFFFFFFFFFFF
FFFFFFFFF,FFFFFFFFFFFFFFF:FFFFFFFF:FFF:FFFFFFFFFFFFFFFFFF:FFFFF:FFFF
FFFFFFFFFFFFF,F
@A00155:342:HHGFNDSXY:1:2644:3839:6950 1:N:0:GAACCTAG+TCCGCATA
TCAGCTTGAGACATTTCTCCAGGTTTACAAACCTAAAAAATTGTGGGCTACTCTGGTTTTTCCACCTG
AGATCTTAGCTGGTTACAAATCCTCCGTTCTGCCCTTCTTATATCAATTTGAAATTCACGGGAAGGAT
CTGGTCTACATGCC
+
FF:,FFFFF:F:FF,,FFF:F::::FFFF,:F:F:,FFF,:FF,FFFF:FFF:F,FFFFFFF,FF:FF
FFFF,FF:FFF:FFFF:FFFFFFFFFFFFFFFFFFFFF,FF,FFFF,FFFFFF,FFFFFFFF::FFFFF
,:FF:FFFFFFF:F
@A00155:342:HHGFNDSXY:1:2650:31593:2910 1:N:0:GAACCTAG+TCCGCATA
GATCTTGAGACATTTCTCCTGGTTTACAAACCTAAAAATTTGTGGGCTACTCTGGTTTTTCCACCTGA
GATCTTAGCTGGTTACATATCCTCCGTTCTGCCCTTCTTATATCAATTTGAAATTCACGGGAAGGATC
TGGTCTACATGCC
```

```
+
:FF:FFFFFFFFFFFFFFF:,FFF,FFFF:F,,FFFF:FFFFFFF,:FF,FFFF:FFFFFFFFFFFF,
FFFFF:FFFFFFFF,F,,FF:FFFFFFFFFFFF:F:FFF:FFFFF:FF:F:FFFFF::F:F,FFFFF:
FFFFF:,:FFFFF
@A00155:342:HHGFNDSXY:1:1154:11388:16924 1:N:0:GAACCTAG+TCCGCATA
CAGCTTGAGACATTTCTCCAGGTTTACAAACCTAAAAATTTGTGGGCTACTCTGGTTTTTCCACCTGA
GATCTTAGCTGGTTACAAATCCTCCGTTCTGCCCTTCTTATATCAATTTGAAATTCACGGGAAGGATC
TGGTCTACATGCCGG
+
FFFFFFFFFFFFFFFFFF:FFFFFFFFFFFFFFFFFFFFFFFFFFFFFFFFFFFFF:FFFFFFFFFFF
FF:FFFFFFFFFFFFFFFFFFFFFFFFFFFFFFFFFFFFFFFFFFF:FFFFFFFFFFFFFFF:FFFFF
FFFFFFFFFFFFFFF
@A00155:342:HHGFNDSXY:1:1230:30626:28322 1:N:0:GAACCTAG+TCCGCATA
TCTCTTGAGACATTTCTCCAGGTTTACAAACCTAAAAATTTGTGGGCTACTCTGGTTTTTCCACCTGA
GATCTTAGCTGGTTACAAATCCTCCGTTCTGCCCTTCTTATATCAATTTGAAATTCACGGGAAGGATC
TGGTCTACATGCCGG
+
FFFFFFFFFFFFFFFFFFFFFFFFFFFFFFFFFFFFFFFFFFFFFFFFFFFFFFFFFFFFFFFFFFFF
FFFFFFFFFFFFFFFFFFFFFFFFFFFFFFFFFFFFFFFFFFFFF::FFFFFFFFFFFFFFFFFFFFF
FFFFFFFFFFFFFFF
@A00155:342:HHGFNDSXY:1:1371:9127:34084 1:N:0:GAACCTAG+TCCGCATA
CTGCTTGAGACATTTCTCCAGGTTTACAAACCTAAAAATTTGTGGGCTACTCTGGTTTTTCCACCTGA
GATCTTAGCTGGTTACAAATCCTCCGTTCTGCCCTTCTTATATCAATTTGAAATTCACGGGAAGGATC
TGGTCTACATGCCGG
+
FFFFFFFFFFFFFFFFFFFFFFFF,FFFFFFFFFFFFFFFFFFFFFFFFFFFFFFFFFFFFFFFFFFF
FFFFFFFFFFFFFFFFFFFFFFFFFFFFFFFFFFFFFFFFFFFFFFFFFFFFFFFFFFFFFFFFF:FF
FFFFFFFFFFFFFF:
@A00155:342:HHGFNDSXY:1:2214:20745:33160 1:N:0:GAACCTAG+TCCGCATA
CAGCTTGAGACATTTCTCCAGGTTTACAAACCTAAAAATTTGTGGGCTACTCTGGTTTTTCCACCTGA
GATCTTAGCTGGTTACAAATCCTCCGTTCTGCCCTTCTTATATCAATTTGAAATTCACGGGAAGGATC
TGGTCTACATGCCGG
+
FFFFFFFFFFFFFFFFFFFFFFFFFFFFFFFFFFFFFFFFFFFFFFFFFFFFFFFFFFFFFFFFFFFF
FF:FFFFFFFFFFFFFFFFFFFFFFFFFFFFFFFFFFFFFFFFFFFFFFFFFFFFFFFFFFFFFFFFF
FFFFFFFFFFFFFFF
@A00155:342:HHGFNDSXY:1:2266:17409:17143 1:N:0:GAACCTAG+TCCGCATA
CAGCTTGAGACATTTCTCCAGGTTTACAAACCTAAAAATTTGTGGGCTACTCTGGTTTTTCCACCTGA
GATCTTAGCTGGTTACAAATCCTCCGTTCTGCCCTTCTTATATCAATTTGAAATTCACGGGAAGGATC
TGGTCTACATGCCGG
+
FF::FFFFFFFFFFFFFFFF:FFFFFF:FFFFFFFFF:FFFFFFFF:FFFF:FFF:FFFFFFFFFFFF
F:FFFFFFFFFFFFFFFFFFFFFFFFFFFFFFFFFFFFFFFFFF:FFFF,,FF:FFFFFFFFF:F:FF
FFFFFFFFFFFFFFF
@A00155:342:HHGFNDSXY:1:2306:12762:1705 1:N:0:GAACCTAG+TCCGCATA
CAGCTTGAGACATTTCTCCAGGTTTACAAACCTAAAAATTTGTGGGCTACTCTGGTTTTTCCACCTGA
GATCTTAGCTGGTTACAAATCCTCCGTTCTGCCCTTCTTATATCAATTTGAAATTCACGGGAAGGATC
TGGTCTACATGCAGG
+
FFFFFFFFFFFFFFFFFFFFFFFFFFFFFFFFFFFFFFFFFFFFFFFFFFFFFFFFFFFFFFFFFFFF
FFFFFFFFFFFFFFFFFFFFFFFFFFFFFFFFFFFFFFFFFFFFF,FFFFFFF:FFFFFFFFFFFFFF
FFFFFFFFFFFFFFF
@A00155:342:HHGFNDSXY:1:2306:16839:22983 1:N:0:GAACCTAG+TCCGCATA
CAGCTTGAGACATTTCTCCAGGTTTACAAACCTAAAAATTTGTGGGCTACTCTGGTTTTTCCACCTGA
```

GATCTTAGCTGGTTACAAATCCTCCGTTCTGCCCTTCTTATATCAATTTGAAATTCACGGGAAGGATC
TGGTCTACATGCCGG
+
FFFFFFFFFFFFFFFFFFFFFFFFFFFFFFFFFFFFFFFFFFF:FFFFFFFFFFFFFFFFFFFFFFFF
FFFFFFFFFFFFFFFFFFFFFFFFFFFFFFFFFFFFFFFFFFFFFFFFFFFFFFFFFFFFFFFFFFFF
FFFFFFFFFFFFFFF
@A00155:342:HHGFNDSXY:1:2344:28429:29340 1:N:0:GAACCTAG+TCCGCATA
CAGCTTGAGACATTTCTCCAGGTTTACAAACCTAAAAATTTGTGGGCTACTCTGGTTTTTCCACCTGA
GATCTTAGCTGGTTACAAATCCTCCGTTCTGCCCTTCTTATATCAATTTGAAATTCACGGGAAGGATC
TGGTCTACATGCCGG
+
FFFFFFFFFFFFFFF:FFFFF:FFFFFFFFFFFFFFFFFFFF:FFFFFFFFFFFFFFFFFFFFFFFFF
:FFFFFFFFFFFFFFFFFFFFFFFFFFFFFFFFFFFFFFFFFFFF:FFFFFFFFFFFFFFFFFFFFF
FFFFFFFFFFFFFFF
@A00155:342:HHGFNDSXY:1:2430:21847:36292 1:N:0:GAACCTAG+TCCGCATA
CAGCTTGAGACATTTCTCCAGGTTTACAAACCTAAAAATTTGTGGGCTACTCTGGTTTTTCCACCTGA
GATCTTAGCTGGTTACAAATCCTCCGTTCTGCCCTTCTTATATCAATTTGAAATTCACGGGAAGGATC
TGGTCTACATGCCGG
+
FFFFFFFFFFFFFFFFFFF,FFFFFFFFFFFFFFFFFFFFFFFFFFFFFFFFFFFFFFFFFFFFFFFF
FFFFFFFFFFFFFFFFFFFFFFFFFFFFFFFFFFFFFFFFFFFFFFFFFFFFFFFFFFFFFFF:FFFFF
FFFFFF:FFFFFFFF
@A00155:342:HHGFNDSXY:1:2452:9317:4820 1:N:0:GAACCTAG+TCCGCATA
CAGCTTGAGACATTTCTCCAGGTTTACAAACCTAAAAATTTGTGGGCTACTCTGGTTTTTCCACCTTA
GATCTTAGCTGGTTACAAATCCTCCGTTCTGCCCTTCTTATATCAATTTGAAATTCACGGGAAGGATC
TGGTCTACATGCCGG
+
FFF:FFFFFFFFFFFFFFFF:F:FFFFFFFFFFFFFFFFFFFFFFFFFFFFFFFFF:FFFFFFFFF,F
FFF:FFFFFFFFFFFFFFFFFFFFFFFFFFFFFFFFFFFFFFFFFFFFFFFFFFFFFFFFFFFFFFFF
FFFFFFFFFFFFFFF
@A00155:342:HHGFNDSXY:1:2501:26657:3161 1:N:0:GAACCTAG+TCCGCATA
CAGCTTGAGACATTTCTCCAGGTTTACAAACCTAAAAATTTGTGGGCTACTCTGGTTTTTCCACCTGA
GATCTTAGCTGGTTACAAATCCTCCGTTCTGCCCTTCTTATATCAATTTGAAATTCACGGGAAGGATC
TGGTCTACATGCCGG
+
FFFFFFFFFFFFFFFFFFFFFFFFFFFFFFFFFFFFFFFFFFFFFFFFFFFFFFFFFFFFFFFFFFFF
FFFFFFFFFFFFFFFFFFFFFFFFFFFFFFFFFFFFFFFFFFFFFFFFFFFFFFFFFFFFFFFFFFFF
FFFFFFFFFFFFFFF
@A00155:342:HHGFNDSXY:1:2512:11388:24001 1:N:0:GAACCTAG+TCCGCATA
CAGCTTGAGACATTTCTCCAGGTTTACAAACCTAAAACTTTGTGGGCTACTCTGGTTTTTCCACCTGA
GATCTTAGCTGGTTACAAATCCTCCGTTCTGCCCTTCTTATATCAATTTGAAATTCACGGGAAGGATC
TGGTCTACATGCCGG
+
FFFFFFFFFFFFFFFFFFFFFFFFFFFFFFFFFFFFF,FFFFFFFFF:FFFF:FFFFFFFFFFFFFFF
FFFFFFFFFFFF:FFFFFFFFFFFFFFFFFFFFFFFFFFFFFFFFF:FFFFFFFFFFFFFFFFFFFFFF
FFFFFFFFFFFFFFF
@A00155:342:HHGFNDSXY:1:2548:32398:20901 1:N:0:GAACCTAG+TCCGCATA
CAGCTTGAGACATTTCTCCAGGTTTACAAACCTAAAAATTTGTGGGCTACTCTGGTTTTTCCACCTGA
GATCTTAGCTGGTTACAAATCCTCCGTTCTGCCCTTCTTATATCAATTTGAAATTCACGGGAAGGATC
TGGTCTACATGCCGG
+
FFFF:FFFFFFFFF:F:FFFFFF,FFFF::FF:FF:F,F:FFFFFFFFFFFF:FFFFF::FF,FFFFF
FFFFF:FFFFFF:FFFFFF:FFFFFFFFFF:FFFFFFFFFFFFFFFFFFFFFFFFFFFFF:FFFFFFFFF
,FFFF,FFFFFFFFF

```
@A00155:342:HHGFNDSXY:1:2202:4047:12947 2:N:0:GAACCTAG+TCCGCATA
AGCTTGAGACATTTCTCCAGGTTTACAAACCTAAAAATTTGTGGGCTACTCTGGTTTTTCCACCTGAG
ATCTTAGCTGGTTACAAATCCTCCGTTCTGCCCTTCTTATATCAATTTGAAATTCACGGGAAGGATCT
GGTCTACATGCCGGA
+
FFFFFFFFFFFFFFFFFFFFFFF:FFFFFFF:FFFFFFFFFFF:FFFFFF:FFF,FFFFFFFFFFFFF
FFF:FFFFFFFFFFFFF:FFFFF:FFFFFFF:F:F,FFF,F:F:FFFF::FFFF:FFFF,::FFFF,,
FFFFF::F:FFFFFF
@A00155:342:HHGFNDSXY:1:1105:22336:22420 1:N:0:GAACCTAG+TCCGCATA
GCTTGAGACATTTCTCCAGGTTTACAAACCTAAAAATTTGTGGGCTACTCTGGTTTTTCCACCTGAGA
TCTTAGCTGGTTACAAATCCTCCGTTCTGCCCTTCTTATATCAATTTGAAATTCACGGGAAGGATCTG
GTCTACATGCCGGAC
+
FFFFFFFFFFFFFFFFFFFFFFFFFFFFFFFFFFFFF:FFFFFFFFFFFFFFFFFFFFFFFFFFFFFF
FFFFFFFFFF:FFFFFFFFFFFFFFFFFFFFFFFFFFFFFFFFFFFFFFFFF:FFFFFFFFFFFFFFF
FFFFFFFFFFFFFFF
@A00155:342:HHGFNDSXY:1:1567:20338:4899 1:N:0:GAACCTAG+TCCGCATA
TTCCGATCTATTTCTCCAGGTTTACAAACCTAAAAATTTGTGGGCTACTCTGGTTTTTCCACCTGAGA
TCTTAGCTGGTTACAAATCCTCCGTTCTGCCCTTCTTATATCAATTTGAAATTCACGGGAAGGATCTG
GTCTACATGCCGGAC
+
FFFFFFFFFFFFFFFFFFFFFFFFFFFFFFFFFFFFFFFFFFFFFFFFFFFFFFFFFFFFFFFFFFFF
:F:FFFFFFFFFFFFFFFFFFFFFFFFFFFFFFFFFFFFFFFFFFFFFFFFFFFFFFF:FFFFFFFF
FFFFFFFFFFFFFFF
@A00155:342:HHGFNDSXY:1:2141:9127:10755 1:N:0:GAACCTAG+TCCGCATA
CTTGAGACATTTCTCCAGGTTTACAAACCTAAAAATTTGTGGGCTACTCTGGTTTTTCCACCTGAGAT
CTTAGCTGGTTACAAATCCTCCGTTCTGCCCTTCTTATATCAATTTGAAATTCACGGGAAGGATCTGG
TCTACATGCCGGACG
+
FFFFFFFFFFFFFFFFFFFFFFFFFFFF:FFFFFFFFFFFFFFFFFFFFFFFFFFFFFFFFFFFFFFF
FFFFFFFFFFFFFFFFFFFFFFFFFFFFFFFFFFFFFFFFFFFFFFFFFFFFFFFFFFFFFFFFFFFF
FFFFFFFFFFFFFFF
@A00155:342:HHGFNDSXY:1:1532:18041:21183 1:N:0:GAACCTAG+TCCGCATA
TTGAGACATTTCTCCAGGTTTACAAACCTAAAAATTTGTGGGCTACTCTGGTTTTTCCACCTGAGTTC
TTAGCTGGTTACAAATCCTCCGTTCTGCCCTTCTTATATCAATTTGAAATTCACGGGAATGATCTGGT
CTACATGCCGGACGG
+
FFFF::,F,FFFFFF:FFF,FF:F,FFFF,FF:FFFFF:F:F,FF:FFF:FFFFFFFF:,F:FFF,FF
FFFFFFFFFFFF,FFF,FF,FF:F,FF::,FFF:FF:,:FF,,FF:,:FFFF,FF:F,:,F,FFFFFF
FFFF,,:::FFFFF:
@A00155:342:HHGFNDSXY:1:2233:7889:3067 1:N:0:GAACCTAG+TCCGCATA
TTGAGACATTTCTCCAGGTTTACAAACCTAAAAATTTGTGGGCTACTCTGGTTTTTCCACCTGAGATC
TTAGCTGGTTACAAATCCTCCGTTCTGCCCTTCTTATATCAATTTGAAATTCACGGGAAGGATCTGGT
CTACATGCCGGACAG
+
FFFFFFFFFFFFF:F::F:FFFFFF:FFFFFFFFFFFFFFFFFFFFFFFFFFFFFFFFFFFFFFFFFF
FFFFFFFFFFFFFFFFFFFFFFFFFFFFFFFFFFFFFFFFF,FFFFFFFFFFFFFFFFFFFFFFFFFF
FFFFFFFFFFFFFFF
@A00155:342:HHGFNDSXY:1:2250:24975:26146 1:N:0:GAACCTAG+TCCGCATA
TTGAGACATTTCTCCAGGTTTACAAACCTAAAAATTTGTGGGCTACTCTGGTTTTTCCACCTGAGATC
TTAGCTGGTTACAAATCCTCCGTTCTGCCCTTCTTATATCAATTTGAAATTCACGGGAAGGATCTGGT
CTACATGCCGGACGG
+
FFFFFFFFFFFFFFFFFFFFFFFFFFFFFFF:FFFFFFFFFFFFFFFFFFFFFFFFFFFFFFFFFFFF
```

```
FFFF:FFFFFFFFFFFFFFFFFFFFFFFFFFFFFFFFFFFFFFFFFFFFFFFFF:FFFFFFFFFFFFF
FFFFFFFFFFFFFFF
@A00155:342:HHGFNDSXY:1:2532:18331:15640 1:N:0:GAACCTAG+TCCGCATA
TTGAGACATTTCTCCAGGTTTACAAACCTAAAAATTTGTGGGCTACTCTGGTTTTTCCACCTGAGATC
TTAGCTGGTTACAAATCCTCCGTTCTGCCCTTCTTATATCAATTTGAAATTCACGGGAAGGATCTGGT
CTACATGCCGGACGG
+
FFFFFFFFFFFFFFF:FF:FFFFF:FFFFFFFFFFFFFFFFFFFFFFFFFFFFFFFFFFFFFFFFFFF
FFFFFFFFFFFFFFFFFFFFFFFFFFFFFFFFFFFFFFFFFFFF:FFFFFFFFFFFFFFFFFFFFFFF
FFFFFFFFFFFFFFF
@A00155:342:HHGFNDSXY:1:2635:14118:6120 1:N:0:GAACCTAG+TCCGCATA
TTGAGACATTTCTCCAGGTTTACAAACCTAAAAATTTGTGGGCTACTCTGGTTTTTCCACCTGAGATC
TTAGCTGGTTACAAATCCTCCGTTCTGCCCTTCTTATATCAATTTGAAATTCACGGGAAGGATCTGGT
CTACATGCCGGACGG
+
FFFFFFFFFFFFFFFFFFFFFFFFFFFFFFFFFFFFFFFFFFFFFFFFFFFFFFFFFFFFFFFFFFFF
FFFFFFFFFFFFFFFFFFFFFFFFFFFFFFFFFFFFFFFFFFFFFFFFFFFFFFFFFFFFFFFFFFFF
FFFFFFFFFFFFFFF
@A00155:342:HHGFNDSXY:1:1108:28384:17456 2:N:0:GAACCTAG+TCCGCATA
TTGAGACATTTCTCCAGGTTTACAAACCTAAAAATTTGTGGGCTACTCTGGTTTTTCCACCTGAGATC
TTAGCTGGTTACAAATCCTCCGTTCTGCCCTTCTTATATCAATTTGAAATTCACGGGAAGGATCTGGT
CTACATGCCGGACGG
+
FFFFFFF:FFFFFFFFFFFFFFFF:FFFFFFFFFFFFFFFFFFFFFFFFFFFFFFFFFFFFFFFFFF:
FFFFFFFFFFFFFFFFFFFFFFFFFFFFF:F:FFF,FFFFFFFFFFFFFFFFFFFFFFFFFFFFFFFF
FFFFFFFFFFFFFFF
@A00155:342:HHGFNDSXY:1:1153:13928:34225 1:N:0:GAACCTAG+TCCGCATA
TGAGACATTTCTCCAGGTTTACAAACCTAAAAATTTGTGGGCTACTCTGGTTTTTCCACCTGAGATCT
TAGCTGGTTACAAATCCTCCGTTCTGCCCTTCTTATATCAATTTGAAATTCACGGGAAGGATCTGGTC
TACATGCCGGACGGG
+
FFFFFFFFFFFFFFF:FFFFFFFFFFFFFFFFFFFFFFFFFFFFFFFFFFFFFFFFFFFFFFFFFFFF
FFF:FFFFFFFFFFFFFFFFFFFFFFFFFFFFFFFFFFFFFFFFFFFFFFFFFFFFF:FFFFFF
FFFFFFFFFFFFFFF
@A00155:342:HHGFNDSXY:1:1328:30463:17519 1:N:0:GAACCTAG+TCCGCATA
TGAGACATTTCTCCAGGTTTACAAACCTAAAAATTTGTGGGCTACTCTGGTTTTTCCACCTGAGATCT
TAGCTGGTTACAAATCCTCCGTTCTGCCCTTCTTATATCAATTTGAAATTCACGGGAAGGATCTGGTC
TACATGCCGGACGGG
+
FFFFFFFFFFFFFFFFFFFFFFFFFFFFFFFFFFFFFFFFFFFFFFFFFFFFFFFFFFFFFFFFFFFF
FFFFFFFFFFFFFFFFFFFFFFFFFFFFFFFFFFFF:FFFFFFFFFFFFFFFFFFFF:FFFFFFFFFF
FFFFFFFFFFFFFFF
@A00155:342:HHGFNDSXY:1:1537:6153:15123 1:N:0:GAACCTAG+TCCGCATA
TGAGACATTTCTCCAGGTTTACAAACCTAATAATTTGTGGGCTACTCTGGTTTTTCCACCTGAGATCT
TAGCTGGTTACTAATCCTCCGTTCTGCCCTTCTTATATCAATTTGAATTTCACGGGAAGGATCTGGTC
TACATGCCGGACGGG
+
FFFF:F,FFFFF,F:FFFFFF:,FF::FF,,F,FFFFFFFFFFFFFF,FFFFFFF:,FFF:F,F,FFF
F,,FFF:FFF:,F,FF,F,:F:FF,,F,,FF,FF,,F,FF,FFFF:F,FF,F:F:FFFFF:,FFFFF,
FFF,FFF:FFF:FFF
@A00155:342:HHGFNDSXY:1:1537:6207:15280 1:N:0:GAACCTAG+TCCGCATA
TGAGACATTTCTCCAGGTTTACAAACCTAAAAATTTGTGGGCTACTCTGGTTTTTCCACCTGAGATCT
TAGCTGGTTACAAATCCTCCGTTCTGCCCGTCTTATATCAATTTGAAATTCACGGGAAGGATCTGGTC
TACATGCCGGACGGG
```

```
+
FFF:FFFFFF:FFFFFFFFFFFFFFFFFFF:FFFFFFFFFFFFFFFFFFF:FFFFFFFFFFFFFFFFF
FFFFFFFFFFFFFFFFFFFFFFFFFFFFF,FFFFFFFFFFFFFFFFFFFFFFFFFFFFFFFFFFFFFF
FFFFFFFFFFFFFFF
@A00155:342:HHGFNDSXY:1:2430:12391:18035 1:N:0:GAACCTAG+TCCGCATA
TGAGACATTTCTCCAGGTTTACAAACCTAAAAATTTGTGGGCTACTCTGGTTTTTCCACCTGAGATCT
TAGCTGGTTACAAATCCTCCGTTCTGCCCTTCTTATATCAATTTGAAATTCACGGGAAGGATCTGGTC
TACATGCCGGACGGG
+
FFFFFFFF:FFF:FFFF:FFFFFFFFFFFFFFFFFFFFFFFFFFFFFFFFFFFFFFFFFFFFFFFFFF
FFFFFFFFFFFFFFFFFFFFFFFFFFFFFFFFFFFFFFFFFFFFFFFFFFFFFFFFFFFFFFFFFFFF
FFFFFFFFFFFFFFF
@A00155:342:HHGFNDSXY:1:2578:32000:23469 1:N:0:GAACCTAG+TCCGCATA
TGAGACATTTCTCCAGGTTTACAAACCTAAAAATTTGTGGGCTACTCTGGTTTTTCCACCTGAGATCT
TAGCTGGTTACAAATCCTCCGTTCTGCCCTTTTTATATCAATTTGAAATTCACGGGAAGGATCTGGTC
TACATGCCGGACGGG
+
FFFFFFFFFFFFFFFFFFFFFFF:FFFFFFFFFFFFFFFFFFFFFFFFFFFFFFFFFFFFFFFFFFFF
F,FFFFFFFFFFFFFFFFFFFFFFFFFFFFFFFFFF,FF:F:FFFFFFFFFFFFFFF:FFFFFFFFFF
FFFFFFFFFFFFFFF
@A00155:342:HHGFNDSXY:1:2650:31593:2910 2:N:0:GAACCTAG+TCCGCATA
TGAGACATTTCTCCAGGTTTACACACCTAAAAATTTGTGGGCTACTCGGGTTTTTCCACCTGAGATCT
TAGCTGGTTACAAATCCTCCGTTCTGCCCTTCTTATATCAAATTGAAATTCACGGGAAGGATCTGGTC
TACATGCCAGATCGG
+
:FFFF,F,FFF,,F,:FFF::FF,:FFFFFFFF:F::FFFF,FFFFF,:FFFFFF:FFFFFFF:FFFF
,FF,:F:FF:FF:F:F,FF::FFF::FF,FFF,,:F,,FFF,FF:FF::FFF:,FFF,:,FFF,FF:F
F,FFFFF,:FFFF,:
@A00155:342:HHGFNDSXY:1:1119:8368:6746 1:N:0:GAACCTAG+TCCGCATA
GAGACATTTCTCCAGGTTTACAAACCTAAAAATTTGTGGGCTACTCTGGTTTTTCCACCTGAGATCTT
AGCTGGTTACAAATCCTCCGTTCTGCCCTTCTTATATCAATTTGAAATTCACGGGAAGGATCTGGTCT
ACATGCCGGACGGGG
+
FFFFFFFFFFFFFFFFFFFFFFFFFFFFFFFFFFFFFFFFFFFFFFFFFFFFFFFFFFFFFFFFFFFF
FFFFFFFFFFFFFFFFFFFFFFFFFFFFFFFFFFFFFFFFFFFFFFF:FFFFFFFFFFFFFFFFFFFF
FFFFFFFFFFFFFFF
@A00155:342:HHGFNDSXY:1:1129:15465:5603 1:N:0:GAACCTAG+TCCGCATA
GAGACATTTCTCCAGGTTTACAAACCTAAAAATTTGTGGGCTACTCTGGTTTTTCCACCTGAGATCTT
AGCTGGTTACAAATCCTCCGTTCTGCCCTTCTTATATCAATTTGAAATTCACGGGAAGGATCTGGTCT
ACATGCCGGACGGGG
+
FFFFFFFFFFFFFFFF:,:,FF:FFFFFFFF::FFF:FFF::FFFFFFFFFFFFFF,FF:FFFF:FFF
FFFFFFFFFFFFFFFFFFFFFFFFFFFFFFFFFFFFFF::FFFFFFFFFFFFFFF,FFFF:F:FFFFF
FFFFFFFFFFFFFFF
@A00155:342:HHGFNDSXY:1:1268:11496:36652 1:N:0:GAACCTAG+TCCGCATA
GAGACATTTCTCCAGGTTTACAAACCTAAAAATTTGTGGGCTACTCTGGTTTTTCCACCTGAGATCTT
AGCTGGTTACAAATCCTCCGTTCTGCCCTTCTTATATCAATTTGAAATTCACGGGAAGGATCTGGTCT
ACATGCCGGACGGGG
+
FFFFFFFFFFFFFFFFFFFFFFFFFFFFFFFFFFFFFFFFFFFFFFFFF:FFFFFFFFFFFFFFFFFF
FFFFFFFFFFFFFFFFFFFFFFFFFFFFFFFFFFFFFFFFFFFFFFFFFFFFFFFFFFFFFFFFFFFF
FFFFFFFFFFFFFFF
@A00155:342:HHGFNDSXY:1:2138:11939:14841 1:N:0:GAACCTAG+TCCGCATA
GAGACATTTCTCCAGGTTTACAAACCTAAAAATTTGTGGGCTACTCTGGTTTTTCCACCTGAGATCTT
```

```
AGCTGGTTACAAATCCTCCGTTCTGCCCTTCTTATATCAATTTGAAATTCACGGGAAGGATCTGGTCT
ACATGCCGGACGGGG
+
FFFFFFF,FFFFFFF:FFFFFFFFFFFFFFFFFFFFFFFFFFFFFFFFFFFFFFFFFFFFFFFFFFFF
FFFFFFFFFFFFFFFFFFFFFFFFFFFFFFFFFFFFFFFFFFFFFFFFFFFFFFFFFFFFFFFFFFFF
FFFFFFFFFFFFFFF
@A00155:342:HHGFNDSXY:1:2244:5584:30232 1:N:0:GAACCTAG+TCCGCATA
GAGACATTTCTCCAGGTTTACAAACCTAAAAATTTGTGGGCTACTCTGGTTTTTCCACCTGAGATCTT
AGCTGGTTACAAATCCTCCGTTCTGCCCTTCTTATATCAATTTGAAATTCACGGGAAGGATCTGGTCT
ACATGCCGGACGGGG
+
FFFFFFFFFFFFFFFFFFFFFFFFFF:FFFFFFFFFFFFFFFFFFFFFFFFFFFFFFFFFFFFFFFFF
FFFFFFFFFFFFFFFFFFFFFFFFFFFFFFFFFFFFFFF:FF:FFFFFFFFFFFF:FFFFFFFFFFFF
FFFFFFFFFFFFFFF
@A00155:342:HHGFNDSXY:1:2413:12671:21245 1:N:0:GAACCTAG+TCCGCATA
GAGACATTTCTCCAGGTTTACAAACCTAAAAATTTGTGGGCTACTCTGGTTTTTCCACCTGAGATCTT
AGCTGGTTACAAATCCTCCGTTCTGCCCTTCTTATATCAATTTGAAATTCACGGGAAGGATCTGGTCT
ACATGCCGGACGGGG
+
FFFFFF,FFFFFFFFFFFFFFFFFFFFFFFFF,FFFFF:FFFFFFFFFFFFFFFFFFFFFFFFFFFFF
::FF:FFFFFFFFFFFFFFFFFFFFFFFFFFFFFFFFFFFFFFFFFFFFFFFFFFFFFFFFFFFFFFF
FFFFFFFFFFFFFFF
@A00155:342:HHGFNDSXY:1:2539:23710:4006 1:N:0:GAACCTAG+TCCGCATA
GAGACATTTCTCCAGGTTTACAAACCTAAAAATTTGTGGGCTACTCTGGTTTTTCCACCTGAGATCTT
AGCTGGTTACAAATCCTCCGTTCTGCCCTTCTTATATCAATTTGAAATTCACGGGAAGGATCTGGTCT
ACATGCCGGACGGGG
+
FFFFFFFFFFFFFF,F:FFFFFFFFFFFFFF,FFFFFFFFFFFFFFFFFFFFFFFFFFFFFFFFFFFF
FFFFFFFFFFFFFFFFFFFFFFFFFFFFFFFFFFFFFFFFFFFFFFFFFFFFFFFFFFFFFFF:FFFFF
FFFFFFFFFFFFFFF
@A00155:342:HHGFNDSXY:1:2602:27670:21793 1:N:0:GAACCTAG+TCCGCATA
CTGACATTTCTCCAGGTTTACAAACCTAAAAATTTGTGGGCTACTCTGGTTTTTCCACCTGAGATCTT
AGCTGGTTACAAATCCTCCGTTCTGCCCTTCTTATATCAATTTGAAATTCACGGGAAGGATCTGGTCT
ACATGCCGGACGGAG
+
FFFFFFFFFFFFFFFFFFFFFFFFFFFFFFFFFFFFFFFFFFFFFFFFFFFFFFFFFFFFFFFFFFFF
FFFFFFFFFFFFFFFFFFFFFFFFFFFFFFFFFFFFFFFFFFFFFFFFFFFFFFFFFFFFFFFFFFFF
FFFFFFFFFFFFFFF
@A00155:342:HHGFNDSXY:1:1406:14678:1078 2:N:0:GAACCTAG+TCCGCATA
GAGACATTTCTCCAGGTTTACAAACCTAAAAATTTGTGGGCTACTCTGGTTTTTCCACCTGAGATCTT
AGCTGGTTACAAATCCTCCGTTCTGCCCTTCTTATATCAATTTGAAATTCACGGGAAGGATCTGGTCT
ACATGCCGGACGGGG
+
FFFFFF:FFFFFFFFFFFFFFF,FF:FFFFFFFF:FFFFFFF,FFFFFFF:FFFFFFFFFFFFFFFF
FFFFFF,F:FFFFFFFFFF:FFFFFFFFFFFFFFFF::F,FF:FF:FFFFFFFFFFFFFFFFFFFFFF
F::FFFF:FFFFFFF
@A00155:342:HHGFNDSXY:1:1529:6081:35477 2:N:0:GAACCTAG+TCCGCATA
GAGACATTTCTCCAGGTTTACAAACCTAAAAATTTGTGGGCTACTCTGGTTTTTCCACCTGAGATCTT
AGCTGGTTACAAATCCTCCGTTCTGCCCTTCTTATATCAATTTGAAATTCACGGGAAGGATCTGGTCT
ACATGCCGGACGGGG
+
FFFFFFFFFFFFFFFFFFFFFFFF,FFFFFF,FFFFFFFFFFFFFFFFFFFFFFFFFFFFFFFFFFFF
FFFFFFFFFFFFFFFFFFFFFFFFFFFF::,FFFF:FFFFFFFFFFFFFFF:FFFFFFFF:FFFFFFFF
FFFFFFFFFFFFFFF
```

```
@A00155:342:HHGFNDSXY:1:1661:14733:19805 2:N:0:GAACCTAG+TCCGCATA
GAGACATTTCTCCAGGTTTACAAACCTAAAAATTTGTGGGCTACTCTGGTTTTTCCACCTGAGATCTT
AGCTGGTTACAAATCCTCCGTTCTGCCCTGCTTATATCAATTTGAAATTCACGGGAAGGATCTGGTCT
ACATGCCGGACGGGG
+
FFFFFF::FFFFFFFFFFFFFFF:FFFFFFFFFFFFFFF:FFFFF:FFF:F:FFFFFFFFFFFFFFFF
FFFFFFFFFFFFFFFFFFFFFFFFFFFFF,FF,:FFFFFFFFF:FFFF::FFFFFFFFFFFFFFFFFF
FFFFFFFFFFF:FFF
@A00155:342:HHGFNDSXY:1:2347:8350:6809 2:N:0:GAACCTAG+TCCGCATA
GAGACATTTCTCCAGGTTTACAAACCTAAAAATTTGTGGGCTACTCTGGTTTTTCCACCTGAGATCTT
AGCTGGTTACAAATCCTCCGTTCTGCCCTTCTTATATCAATTTGAAATTCACGGGAAGGATCTGGTCT
ACATGCCGGACGGGG
+
FFFFFFFFFFFFFFFFFFFFFFFFFFFFFFFF:FFFFFFFFFFFFFFFFFFFFFFFFFFFFFFFFF,FF
FF:FFFFFFFFFFF,FFFFFFFFFFFF,FFFF:FF:FFFFFFFFFFFFFFF:FFFF,FFFFFFFFFFF
FFFFF,FFFFFFF:F
@A00155:342:HHGFNDSXY:1:2518:10041:20071 2:N:0:GAACCTAG+TCCGCATA
GAGACATTTCTCCAGGTTTACAAACCTAAAAATTTGTGGGCTACTCTGGTTTTTCCACCTGAGATCTT
AGCTGGTTACAAATCCTCCGTTCTGCCCTTCTTATATCAATTTGAAATTCACGGGAAGGATCTGGTCT
ACATGCCGGACGGGG
+
F,FFFFFFFFFFFFFFFFFFFF:FFFFFFFFF:FFFFFFFFFFFFFFFFFFFFFFFFFFFFFFFFFFF
FFFFFFFFFFFFFFFFFFFFFFFFFFF:FFFFFFF:FFFFFFFFFFFFFFFF:F,F:FFFFFFFFFFF
FFFFFFFFFFFFFFF
@A00155:342:HHGFNDSXY:1:1146:24686:3724 2:N:0:GAACCTAG+TCCGCATA
AGACATTTCTCCAGGTTTACAAACCTAAAAATTTGTGGGCTACTCTGGTTTTTCCACCTGAGATCTTA
GCTGGTTACAAATCCTCCGTTCTGCCCTTCTTATATCAATTTGAAATTCACGGGAAGGATCTGGTCTA
CATGCCGG
+
FFFFFFFFFFFFFFFFFFFFFFF:FFFFFFFFFFFFFFFFFFFFFFFFFFFFFFFFFFFFFFFFFFFF
FFFFFFFFFFFFFFFFFFFFFFFFFFFFFFFFFFFFFFFFFFFFFFFFFFFF,FFFFFFFFFFFFFFF
FFFFFFFF
@A00155:342:HHGFNDSXY:1:1607:23185:9956 2:N:0:GAACCTAG+TCCGCATA
AGACATTTCTCCAGGTTTACAAACCTAAAAATTTGTGGGCTACTCTGGTTTTTCCACCTGAGATCTTA
GCTGGTTACAAATCCTCCGTTCTGCCCTTCTTATATCAATTTGAAATTCACGGGAAGGATCTGGTCTA
CATGCCGG
+
FFFFFFFFFFFFFFFFFFFFFFFFFFFFFFFFFFFFFFFFFFFF:FFFFFFFFFFFFFFFFFFFFFFFF
FFFFFFFFF:FFFFFFFFFFFFFFFFFFFF:FFF:FFFFFFFFFFFFFFFFFFFFFFFFFFFFFFFFF
FFFFFFFF
@A00155:342:HHGFNDSXY:1:2419:29297:13432 2:N:0:GAACCTAG+TCCGCATA
AGACATTTCTCCAGGTTTACAAACCTAAAAATTTGTGGGCTACTCTGGTTTTTCCACCTGAGATCTTA
GCTGGTTACAAATCATCCGTTCTGCCCTTCTTATATCAATTTGAAATTCACGGGAAGGATCTGGTCTA
CATGCCGG
+
F:FF:FFFFFFFF:F:FFFFFF:FFFFFFFFFFFFFF:FFFFFFFFFF:FFFFFFFFFFFFFFFFFFF
FFFFFFFFFFFFFF,F:FF:,FFFFF:,FFFF:FFFFFFF:,FFFFFFFF:,F:FFF:FFFFFFFFFF
FFFF,FFF
@A00155:342:HHGFNDSXY:1:1371:9127:34084 2:N:0:GAACCTAG+TCCGCATA
AGACATTTCTCCAGGTTTACAAACCTAAAAATTTGTGGGCTACTCTGGTTTTTCCACCTGAGATCTTA
GCTGGTTACAAATCCTCCGTTCTGCCCTTCTTATATCAATTTGAAATTCACGGGAAGGATCTGGTCTA
CATGCCGGAG
+
FFFFF:FFFFFFFFFFFFFFFF:FFFFF,FFFFFFFFFFFFFFFFFFFFFFFFFFFFFFFFFFFF:FF
```

```
FF:FFFFFFFFFFFFFFFF:FFFFF:FF,:,:FFFFFFFFFFFFFFFF:FFFFFFFFFF:FF:FFFF
FFFFFFFFFF
@A00155:342:HHGFNDSXY:1:1351:19081:30968 2:N:0:GAACCTAG+TCCGCATA
AGACATTTCTCCAGGTTTACAAACCTAAAAATTTGTGGGCTACTCTGGTTTTTCCACCTGAGATCTTA
GCTGGTTACAAATCCTCCGTTCTGCCCTTCTTATATCAATTTGAAATTCACGGGAAGGATCTGGTCTA
CATGCCGGAC
+
FFFFFFFFFFFFFFFFFFFFFFFFFFFFFFF,FFFFFFFFFFFFFFFFFFFFFFFFFFFFFFFFFFFF
FFFFFFFFFFFFFFFFFFFFFFFFFFF:FFFFFFFFFFFFFFFFFFFFFFF,FFFFFFFFFFFFFFFF
FFFFFFFFFF
@A00155:342:HHGFNDSXY:1:1408:20103:2456 2:N:0:GAACCTAG+TCCGCATA
AGACATTTCTCCAGGTTTACAAACCTAAAAATTTGTGGGCTACTCTGGTTTTTCCACCTGAGATCTTA
GCTGGTTACAAATCCTCCGTTCTGCCCTTCTTATATCAATTTGAAATTCACGGGAAGGATCTGGTCTA
CATGCCGGAC
+
FFFFFFFFFFFFFFFFFFFFFFFFFFFFFF:FFFFFFFFFFFFFFFFFFFFFFFFFFFFFFFFFFFFF
FFFFFFFFFFFFFFFFFFFFFFFFFFFFFFFF:FFFFFFFFFFFFFFFFFFFFFFFFFFFFFFFFFFF
FFFFFFFFFF
@A00155:342:HHGFNDSXY:1:1506:22489:30107 2:N:0:GAACCTAG+TCCGCATA
AGACATTTCTCCAGGTTTACAAACCTAAAAATTTGTGGGCTACTCTGGTTTTTCCACCTGAGATCTTA
GCTGGTTACAAATCCTCCGTTCTGCCCTTCTTATATCAATTTGAAATTCACGGGAAGGATCTGGTCTA
CATGCCGGAC
+
FFFFFFFFFFFFFFFFFFFFFFFFFFFFFFFFF,FFFFFFFFFFFFFFFFFFFFFFFFFFFFFFFFFF
FFFFFFFFFFFFFFFFFFF,FFFFF,F::FFFFFFFFFFFFFFFFFF:FFFFFFFFF:FFFFFFFFFF
FFFFFFF,FF
@A00155:342:HHGFNDSXY:1:1534:26838:31876 2:N:0:GAACCTAG+TCCGCATA
AGACATTTCTCCAGGTTTACAAACCTAAAAATTTGTGGGCTACTCTGGTTTTTCCACCTGAGATCTTA
GCTGGTTACAAATCCTCCGTTCTGCCCTTCTTATATCAATTTGAAATTCACGGGAAGGATCTGGTCTA
CATGCCGGAC
+
FFFFFFFFFFFFFFFFFFFFFFFFFFFFFFFFFFFFFFFFFFFFFFFFFFFFFFFFFFFFFFFFFFFF
FFFFFFF:FFFFFFFFFFFFFFFFFFFFFFFFFFFFFFFFFFFFFFFFFFFFFFFFFFFFFFFFFFFF
FFFFF:FFFF
@A00155:342:HHGFNDSXY:1:1662:9561:17581 2:N:0:GAACCTAG+TCCGCATA
AGACATTTCTCCAGGTTTACAAACCTAAAAATTTGTGGGCTACTCTGGTTTTTCCACCTGAGATCTTA
GCTGGTTACAAATCCTCCGTTCTGCCCTTCTTATATCAATTTGAAATTCACGGGAAGGATCTGGTCTA
CATGCCGGAC
+
FFFFFFFFFFFFFFFFFFFFFFF:FFFFFFFFFFFFFFF:FFFFFFFFFFFF:FFFFFFFFFFFFFFF
FFFFFFFFFFFFFFFFFFFFFFFFFFFFFFFFFFF::FFFFFFFFFFFFFFFFFFFFFFFFFFFFFFF
FFFFFFFFFF
@A00155:342:HHGFNDSXY:1:2237:32090:10661 2:N:0:GAACCTAG+TCCGCATA
AGACATTTCTCCAGGTTTACAAACCTAAAAATTTGTGGGCTACTCTGGTTTTTCCACCTGAGATCTTA
GCTGGTTACAAATCCTCCGTTCTGCCCTTCTTATATCAATTTGAAATTCACGGGAAGGATCTGGTCTA
CATGCCGGAC
+
FFFFFFFFFFFFFFFFFFFFFFF:FFFFFFFFFFFFFFFFFFFF,FFFFFF:F,FFFFFFFFFFFFFFF
FFFF:FFFFFFFFFFFFFFFFFFFFFF:FFFFFFFFFFFFFFFFFFFFFF,F::FFFFFFF:F:FFFFF
FFFFFF:FF:
@A00155:342:HHGFNDSXY:1:2250:25889:36683 2:N:0:GAACCTAG+TCCGCATA
AGACATTTCTCCAGGTTTACAAACCTAAAAATTTGTGGGCTACTCTGGTTTTTCCACCTGAGATCTTA
GCTGGTTACAAATCCTCCGTTCTGCCCTTTTTATATCAATTTGAAATTCACGGGAAGGATCTGGTCTA
CATGCCGGAC
```

+
FFFFFFFFFFFFFFFF,:FFFFFFFFFFFFFFFFFFFFFFFF:FFFFFFFFFFFF:FFFF:FFFFFF,F
F,:F:,FFFFFFFFFFFF,FFFF::FF:FF,FF,:FFFFFFFFFFFF,FFF,,FFF:FF:FFFFFFFF
FFF:FFFFFF
@A00155:342:HHGFNDSXY:1:2348:32271:11443 2:N:0:GAACCTAG+TCCGCATA
AGACATTTCTCCAGGTTTACACACCTAAAAATTTGTGGGCTACTCTGGTTTTTCCACCTGAGATCTTA
GCTGGTTACAAATCCTCCGTTCTGCCCTTCTTATATCAATTTGAAATTCACGGGAAGGATCTGGTCTA
CATGCCGGAC
+
FFFFFFFFFFFFFFFFFFFFFF,FFFFFFFFF,,FFFFFFFF:FFFFFFFFFF:FFFFFFFFFFFF,FF
FFFFFFFFFFFFFFFFFFFFFFFF:FFFF,F:,::FFFF:FFFFF:FFFFFFFFF:FFFF:FFF:FFF:
FFFFFFFF,F
@A00155:342:HHGFNDSXY:1:2563:12527:5588 2:N:0:GAACCTAG+TCCGCATA
AGACATTTCTCCAGGTTTACAAACCTAAAAATTTGTGGGCTACTCTGGTTTTTCCACCTGAGATCTTA
GCTGGTTACAAATCCTCCGTTCTGCCCTTCTTATATCAATTTGAAATTCACGGGAAGGATCTGGTCTA
CATGCCGGAC
+
FFFFFFFFFFFFFFFFFFFFFFFFFFFFFFFFFFFFFFFFFFFFFFFFFFFFFFFFFFFFFFFFFFFF
FFFFFFFFFFFFFFFFFFFFFFFFFFF,FFFFFF,FFFFFFFFFFFFFFFFFFFFFFFFFFFFFFFFFF
FFFFFFFFFF
@A00155:342:HHGFNDSXY:1:1230:30626:28322 2:N:0:GAACCTAG+TCCGCATA
AGACATTTCTCCAGGTTTACAAACCTAAAAATTTGTGGGCTACTCTGGTTTTTCCACCTGAGATCTTA
GCTGGTTACAAATCCTCCGTTCTGCCCTTCTTATATCAATTTGAAATTCACGGGAAGGATCTGGTCTA
CATGCCGGAGA
+
FFFFFFFFFFFFFFFFFFFFFFFFFFFFFFFFFFFFFFFFFFFFFFFFFFFFFFFFFFFFFFFFFFFF
FFFFFFFFFFFFFFFFFFFFFFFFF:FFFFFFF:FFFFFFFFFFFFFFFFFFFFFFFFFFFFFFFFFF
FFFFF:FFFFF
@A00155:342:HHGFNDSXY:1:2116:9136:16094 2:N:0:GAACCTAG+TCCGCATA
AGACATTTCTCCAGGTTTACAAACCTAAAAATTTGTGGGCTACTCTGGTTTTTCCACCTGAGATCTTA
GCTGGTTACAAATCCTCCGTTCTGCCCTTCTTATATCAATTTGAAATTCACGGGAAGGATCTGGTCTA
CATGCCGGACG
+
FFFFFFFFFFFFFFFFFFFFFFF:FFFFFFFFFFFFFFFFFFFFFFFFFFFFFFFFF:FFFFFFFFFFFF
FF,FFFFFFFFFFFFFFF:FFFFFFF,FFFFFFFFFFFFFFFFFFFFFF:FFFF:FFFF:FFFFFFFF
FFFFFFFFFFF
@A00155:342:HHGFNDSXY:1:2366:14606:18959 2:N:0:GAACCTAG+TCCGCATA
AGACATTTCTCCAGGTTTACAAACCTAAAAATTTGTGGGCTACTCTGGTTTTTCCACCTGAGATCTTA
GCTGGTTACAAATCCTCCGTTCTGCCCTTCTTATATCAATTTGAAATTCACGGGAAGGATCTGGTCTA
CATGCCGGACG
+
FFFFFFFFFFFFFFFFFFFFFFFFFFFFFFFFFFFFFFFFFFFFF:FFFFFFFFFFFFFFFFFFF:FFFF
FFFFFFFFFFFFFFFFFFFF:FFFFFFFFFFFF,FFFFFFFFFFFFFFF:FFFFFF:FF:FFFFFFFF
FFFFFFFFFFF
@A00155:342:HHGFNDSXY:1:2553:27516:24909 2:N:0:GAACCTAG+TCCGCATA
AGACATTTCTCCAGGTTTACAAACCTAAAAATTTGTGGGCTACTCTGGTTTTTCCACCTGAGATCTTA
GCTGGTTACAAATCCTCCGTTCTGCCCTTCTTATATCAATTTGAAATTCACGGGAAGGATCTGGTCTA
CATGCCGGACG
+
FFFFFFFFFFFFFFFFFFFFFFFF:FFFFFFFFFFFFFFFFFFFFFFFFFFFFFFFF:FFFF:FFFF:FFF
FFFFFFFFFFFFFFFFFFFFFFFFFFF:FFFFFFFFFFFFFFFFFFFFFFFFFFFFFFFFFFFFFFFFF
FFFFFF,FFFF
@A00155:342:HHGFNDSXY:1:1553:25211:5008 1:N:0:GAACCTAG+TCCGCATA
AGACATTTCTCCAGGTTTACAAACCTAAAAATTTGTTGGCTACTCTGGTTTTTCCACCTGAGATCTTA

GCTGGTTAAAAATCCTCCGTTCTGCCCTTCTTATATCAATTTGAAATTCAAGGGAAGGATCTGGTCTA
CATGCCGGACGGGG
+
FFF,FFFFFF,FFFFFFF::F:F,F:,FFFFFF:F:,F:,F:FFFF,FFFFFFFF:,:FF:FFFFF,:
:FFFF:FF,F,:FFFFF,:F:,F:F:FFF,F:FF,FFF:FFFF,F,:F:F,:FFFF:FFF::FFFFF,
,FFFFFF:FFF:F:
@A00155:342:HHGFNDSXY:1:2218:31105:29496 2:N:0:GAACCTAG+TCCGCATA
AGACATTTCTCCAGGTTTACAAACCTAAAAATTTGTGGGCTACTCTGGTTTTTCCACCTGAGATCTTA
GCTGGTTACAAATCCTCCGTTCTGCCCTTCTTATATCAATTTGAAATTCACGGGAAGGATCTGGTCTA
CATGCCGGACGGGG
+
FFFF:FFFFFFFFFFFFFFFF:FFFFFFFFFFFFFFFFFFFFFFFFFFF,FFFFFFFFFFFFFFFF:FF
FFFFF:FFFFFFFFFFFFFF:FFFFFFFF:F,::,FFFFFFFFFFFFFFFFFFFFF,FFFFFFFFFFFF
FF:FFFFFFFFFFF
@A00155:342:HHGFNDSXY:1:1305:2311:34929 1:N:0:GAACCTAG+TCCGCATA
AGACATTTCTCCAGGTTTACAAACCTAAAAATTTGTGGTCTACTCTGGTTTTTCCACCTGAGATCTTA
GCTGGTTACAAATCCTCCGTTCTTCCCTTCTTATTTCAATTTGAAATTCACGGGAAGGATCTGGTCTA
CATGCCGGACGGGGT
+
FFF::FF:F:F,F:,FFFF:F,F,FFF:FF:FFFFFF:,,FF:F,F:FFFFFF::F,F,F:FFFFFFF
F:FFFFFFF:F:FF:FFFFFFFF,,::FF:FF:F,FFF,FF::F:FFF:,F:FF,FF,FFFFFF:FFF
FFF,,FFFFF,FFFF
@A00155:342:HHGFNDSXY:1:2250:25889:36683 1:N:0:GAACCTAG+TCCGCATA
AGACATTTCTCCAGGTTTACTAACCTAAAAATTTGTGGGCTACTCTGGTTTTTCCACCTGAGATCTTA
GCTGGTTACAAATCCTCCGTTCTGCCCTTTTTATATCACTTTGAAATTCACGGGAAGGATCTGGTCTA
CATGCCGGACGGGGC
+
FFF,,F:,FFFF,FFFFFFF,F,F:FFFFF:FFFFF:FFFF,FF:F::FFFFFFF:,FFFFFFFFFFF
FFF,FFF:FFFFF,FFFF:FF::FF,,FFF,::F,FF:,F:FFFFFFFF:FFFFFFFFFF:,FFFF,:
FFFF,FFFFFFFFFF
@A00155:342:HHGFNDSXY:1:1114:10131:25363 2:N:0:GAACCTAG+TCCGCATA
AGACATTTCTCCAGGTTTACAAACCTAAAAATTTGTGGGCTACTCTGGTTTTTCCACCTGAGATCTTA
GCTGGTTACAAATCCTCCGTTCTGCCCTTCTTATATCAATTTGAAATTCACGGGAAGGATCTGGTCTA
CATGCCGGACGGGGT
+
FFFFFFFFFFFFFFFFFFFFFFFFFFFFFFFFFFFFFFFFFFFFFFFFFFFFFFFFFFFFFFFFFFFFFF
FFFFFFFFFFFFFFFFFFFFFFFFF:FFFFFFFF:FFFFFFFFFFFF:FFFFFFFFFFFFFFFFFFFFF
FFFFFFFFFFFFFFF
@A00155:342:HHGFNDSXY:1:1343:20347:17942 2:N:0:GAACCTAG+TCCGCATA
AGACATTTCTCCAGGTTTACAAACCTAAAAATTTGTGGGCTACTCTGGTTTTTCCACCTGAGATCTTA
GCTGGTTACAAATCCTCCGTTCTGCCCTTCTTATATCAATTTGAAATTCACGGGAAGGATCTGGTCTA
CATGCCGGACGGGGT
+
FFFFFFFFFFFFFFFFFFFFFFFFFFFFFFFFF:FFFFFFFF,FFFFFFFFFFFFFFFFFFFFFFFFFFF
FFFFFFFFFFFFFFFFFFF,FFFFFFF,FFFFFFFFFFFFFFFFFFFFFFFFFFFF,FFFFFFFFFFFF
FFFF:FFFFFFFFFF
@A00155:342:HHGFNDSXY:1:1138:2121:33630 2:N:0:GAACCTAG+TCCGCATA
GACATTTCTCCAGGTTTACAAACCTAAAAATTTGTGGGCTACTCTGGTTTTTCCACCTGAGATCTTAG
CTGGTTACAAATCCTCCGTTCTGCCCTTCTTATATCAATTTGAAATTCACGGGAAGGATCTGGTCTAC
ATGCCGGACGGGGTG
+
FFFFFFFFFFFFFFFFFFFFFFFFFFFFFFFFFFFFFFFFFFF:FFFFFFFFFFFFFFFFFFFFFFFFFF
FFFFFFF:FFFFFFFFFFFFFFFFFFFFFFFFFFFFFFFFFFFFFFFFFFFFFFFFFFFFFFFFFFFFFF
FFFFFFFFFFFFFFF

@A00155:342:HHGFNDSXY:1:1252:28275:18364 2:N:0:GAACCTAG+TCCGCATA
GACATTTCTCCAGGTTTACAAACCTAAAAATTTGTGGGCTACTCTGGTTTTTCCACCTGAGATCTTAG
CTGGTTACAAATCCTCCGTTCTGCCCTTCTTATATCAATTTGAAATTCACGGGAAGGATCTGGTCTAC
ATGCCGGACGGGGTG
+
FFFFFFFFFFFFFFFFFFFFFFFFFFFFFFFF:FFFFFFFFFFFFFFFFFFFFFFFFFFFFFFF,::F
FFFFFFFFFF:FFFFFFFFFFFFFF:FFFF::FFFFFFFFFFFFFFFFFFFFFFFFFFFF:FFFFFFF
FFFFFFFFFFFFFFF
@A00155:342:HHGFNDSXY:1:1259:10050:18208 2:N:0:GAACCTAG+TCCGCATA
GACATTTCTCCAGGTTTACAAACCTAAAAATTTGTGGGCTACTCTGGTTTTTCCACCTGAGATCTTAG
CTGGTTACAAATCCTCCGTTCTGCCCTTCTTATATCAATTTGAAATTCACGGGAAGGATCTGGTCTAC
ATGCCGGACGGGGTG
+
FFFFFFFFFFF:FFFFFFFFFFFFFFFFFFFFFFFFFFFFFFFFFFFFF:FFFFFFFFFFFFFFFFFF
F:FFFFFFFFFFFFFFFFFFFFFFFFFFFFFFFFFFFFFFFFFFFFFFFFFFFFFFFFFFFFFFFFFF
FFFFFFFFFFFFFFF
@A00155:342:HHGFNDSXY:1:1645:9191:29841 2:N:0:GAACCTAG+TCCGCATA
GACATTTCTCCAGGTTTACAAACCTAAAAATTTGTGGGCTACTCTGGTTTTTCCACCTGAGATCTTAG
CTGGTTACAAATCCTCCGTTCTGCCCTTCTTATATCAATTTGAAATTCACGGGAAGGATCTGGTCTAC
ATGCCGGACGGGGTG
+
FFFFFFFFFFFFFFFFFFFFF:FFFFFFFFFFFFFFFFFFFFFFFFFFFFFFFFFFFFFFFFFFFFFF
FFFFFFFFFFFFFFFFFFFFFFFFF:FFFFFFFFFFFFFFFFFFFFFFFFFFFFFFFFFFFF:FFFFF
FF:FFFFFFFFFFFF
@A00155:342:HHGFNDSXY:1:2602:27670:21793 2:N:0:GAACCTAG+TCCGCATA
GACATTTCTCCAGGTTTACAAACCTAAAAATTTGTGGGCTACTCTGGTTTTTCCACCTGAGATCTTAG
CTGGTTACAAATCCTCCGTTCTGCCCTTCTTATATCAATTTGAAATTCACGGGAAGGATCTGGTCTAC
ATGCCGGACGGAGAG
+
F:FFFFFFFFFFFFFFFFFFFFFFFFFFFFFFFFFFFFFFFFFFFFFFFFFFFFFFFFFFFFFFFFFF
FFFFFFFFFFFFFFFFFFFFFFFFFFFFFFFFFFFFFFFFFFFFFFFFFFFFF:FFFFFFFFFFFFFF
FFFFFFFFFFFFFFF
@A00155:342:HHGFNDSXY:1:1527:32786:22169 1:N:0:GAACCTAG+TCCGCATA
CATTTCTCCAGGTTTACAAACCTAAAAATTTGTGGGCTACTCTGGTTTTTCCACCTGAGATCTTAGCT
GGTTACAAATCCTCCGTTCTGCCCTTCTTATATCAATTTGAAATTCACGGGAAGGATCTGGTCTACAT
GCCGGACGGGGTGAG
+
FFFFFFFFFFFFFFFFFFFFFFFFFFF,FFFFFFFFFFFFFFFFFFFFFFFFFFFFFFFFFFFFFFFF
FFFFFFFFFFFFFFFFFFF,FFFFFFFFFFFFFFF:FFFFFFFFFFFFFFFFFFF:FFFFFFFFFFFF
FFFFFFFFFFFFF,F
@A00155:342:HHGFNDSXY:1:1602:1027:22404 1:N:0:GAACCTAG+TCCGCATA
CATTTCTCCAGGTTTACAAACCTAAAAATTTGTGGGCTACTCTGGTTTTTCCACCTGAGATCTTAGCT
GGTTACAAATCCTCCGTTCTGCCCTTCTTATATCAATTTGAAATTCACGGGAAGGATCTGGTCTACAT
GCCGGACGGAGTGAG
+
F:F:FFFFFFFFFFF,FF:FFFFFFF,,F:,FFFFFFFFFFFFFFFFF,FFF:FFFFFF::FF:FFF:
FFF,:F::,:FFFFFF,FF,FFFFFFF:,F:,,F,,FF,FF,:,FF:FFFF,,FFFFFFFFFF::FF,
FFFFFFFFFFFFFFF
@A00155:342:HHGFNDSXY:1:2121:8576:11209 1:N:0:GAACCTAG+TCCGCATA
CATTTCTCCAGGTTTACAAACCTAAAAATTTGTGGGCTACTCTGGTTTTTCCACCTGAGATCTTAGCT
GGTTACAAATCCTCCGTTCTGCCCTTCTTATATCAATTTGAAATTCACGGGAAGGATCTGGTCTACAT
GCCGGACGGGGTGAG
+
:F:FFFFFFFFFFFFFFFF:F:FFFFFFFFFFFFFFFFFFFFFFFFFFFFFFFFFFFFFFFFFF:FFF

FFFFFFFFFFFFFFFFFFFFFFFFFFFFF:FFFFFF:FFFFFFFFFFFFFFFFFFFFFFFFFFFFFFF
FFFFFFFFFFFFFFF
@A00155:342:HHGFNDSXY:1:1212:32859:17190 2:N:0:GAACCTAG+TCCGCATA
CATTTCTCCAGGTTTACAAACCTAAAAATTTGTGGGCTACTCTGGTTTTTCCACCTGAGATCTTAGCT
GGTTACAAATCCTCCGTTCTGCCCTTCTTATATCAATTTGAAATTCACGGGAAGGATCTGGTCTACAT
GCCGGACGGAGTGAG
+
FFFFFFFFFFFFFFFFFFFFFFFFFFFF:FFFFFFFFFFFFFFFFFFFFFFFFFFFFFFFFF:FFFFF
FFFFFFFFFFFFFFFFFFFFFFF:F,F:FFF:FFFFFFFFFFFFFFFFFFFFFFFFFFFFFFFFFFFF
FFFFFFFFFFFFFFF
@A00155:342:HHGFNDSXY:1:1235:26955:27101 2:N:0:GAACCTAG+TCCGCATA
CATTTCTCCAGGTTTACAAACCTAAAAATTTGTGGGCTACTCTGGTTTTTCCACCTGAGATCTTAGCT
GGTTACAAATCCTCCGTTCTGCCCTTCTTATATCAATTTGAAATTCACGGGAAGGATCTGGTCTACAT
GCCGGACGGGGTGAG
+
FFFFFFFFFFFFFF:FFFFFFFFFFFFF:FFFFF:FFFFFFFFFFFFFFFFFFFFFFFFF::FFFFFF
FFFFFFFFFFFFFFFFFFFFFFF:FFFFFFFFFFFFFFFFFFFFFFFFFFFF,FFFFFFFFFFFFFFF
FFFFFFFFFFFFFFF
@A00155:342:HHGFNDSXY:1:2102:7030:23750 2:N:0:GAACCTAG+TCCGCATA
CATTTCTCCAGGTTTACAAACCTAAAAATTTGTGGGCTACTCTGGTTTTTCCACCTGAGATCTTAGCT
GGTTACAAATCCTCCGTTCTGCCCTTCTTATATCAATTTGAAATTCACGGGAAGGATCTGGTCTACAT
GCCGGACGGGGTGAG
+
FFFFFFFFFFFF:FFFFFFFFFFFFFF:FFFFFFFFFFFFFFFFFFFFFFFFFFFFFFFFFFFFFFFF
FFFFFFFFFFFFFFFFFFFFFF:F:F,FFFFFFFFFFFFFFFFFFF:FFFFFFFFFFFFFFFFFFFFF
FFFFF::FFFFFFFF
@A00155:342:HHGFNDSXY:1:2243:2618:15984 2:N:0:GAACCTAG+TCCGCATA
CATTTCTCCAGGTTTACAAACCTAAAAATTTGTGGGCTACTCTGGTTTTTCCACCTGAGATCTTAGCT
GGTTACAAATCCTCCGTTCTGCCCTTCTTATATCAATTTGAAATTCACGGGAAGGATCTGGTCTACAT
GCCGGACGGAGTGAG
+
:FFFFFFFFFFFFFFFFFFFFFFFFFFFFFFFFFFFFFFFFFFFFFFFFFFFFFFFFFFFFFFFFFFF
FFFFFFFFFFFFFFFFFFFFFFFFFFFFFFFFFFFFFFFFFFFFFFF:FFFFF,FFFFF:FFFFFFFF
:FFFFFFF:FFFFFF
@A00155:342:HHGFNDSXY:1:2634:10230:5368 2:N:0:GAACCTAG+TCCGCATA
CATTTCTCCAGGTTTACAAACCTAAAAATTTGTGGGCTACTCTGGTTTTTCCACCTGAGATCTTAGCT
GGTTACAAATCCTCCGTTCTGCCCTTCTTATATCAATTTGAAATTCACGGGAAGGATCTGGTCTACAT
GCCGGACGGAGTGAG
+
FFFFFFFFFFFFFFFFFFFFFFFFFFFFFFFFFFFFFFFFFFFFFFFFFFFFFFFFFFFFFFFFFFFF
FFFFFFFFFFFFFFFFFFFFFFFFFFFFFFFFFFFFFFFFFFFFFFFFFFFFFFFFFFFFFFFFFFFF
FFFFFFFFFFFFFFF
@A00155:342:HHGFNDSXY:1:2302:29071:9565 2:N:0:GAACCTAG+TCCGCATA
ATTTCTCCAGGTTTACAAACCTAAAAATTTGTGGGCTACTCTGGTTTTTCCACCTGAGATCTTAGCTG
GTTACAAATCCTCCGTTCTGCCCTTCTTATATCAATTTGAAATTCACGGGAAGGATCTGGTCTACATG
CCGGACGGGGTGAG
+
FF,:FFFFFFFFFF:FFFF:FFFF,FF:FFF:FFFFFF,FFF:FFF:FFFFFF:FF:FF::FF,FF:F
F,FFFFFFFFFF:FFFFFFFFFFF:,FF:FFFFFFFFFFFFFFFF:F:FFF,,FFFF::FFF:F:FF,
FFFFFF,FFFFFFF
@A00155:342:HHGFNDSXY:1:1139:10628:17018 1:N:0:GAACCTAG+TCCGCATA
ATTTCTCCAGGTTTACAAACCTAAAAATTTGTGGGCTACTCTGGTTTTTCCACCTGAGATCTTAGCTG
GTTACAAATCCTCCGTTCTGCCCTTCTTATATCAATTTGAAATTCACGGGAAGGATCGGGTCTACATG
CCGGACGGAGTGAGG

+
FFFFFFFFFFFFFFFFFFFFFFFFFFFFFFFFFFFFFFFFFFFFFFFFFFFFFFFFFFFFFFFF::FFFF
FF:FFFFFFFFFFFFFFFFFFFFFFFFFFFF:FFF,:FFFFFF:FFFFFFFFFFFF:F,FFFFFFFFFFF
FFFFFFFFFFFFFFF
@A00155:342:HHGFNDSXY:1:1607:23185:9956 1:N:0:GAACCTAG+TCCGCATA
ATTTCTCCAGGTTTACAAACCTAAAAATTTGTGGGCTACTCTGGTTTTTCCACCTGAGATCTTAGCTG
GTTACAAATCCTCCGTTCTGCCCTTCTTATATCAATTTGAAATTCACGGGAAGGATCTGGTCTACATG
CCGGACGGAGTGAGG
+
FFF:FFFF,FFFFFFFFFFFFFFFFFFFFFFFFFFFFFFFFFFFFFFFFF:FFFFFFFFFFFFFFFFFFF
FFFFFFFFFFFFFFF:FFFFFFFFF::,FFFFFFF:FFFFFFF:FFFFFFFFFFFFFF:FFFFFFFFFFF
FFFFFFFFFFFFFFF
@A00155:342:HHGFNDSXY:1:2202:4047:12947 1:N:0:GAACCTAG+TCCGCATA
ATTTCTCCAGGTTTACAAACCTAAAAATTTGTGGGCTACTCTGGTTTTTCCACCTGAGATCTTAGCTG
GTTACAAATCCTCCGTTCTGCCCTTCTTATATCAATTTGAAATTCACGGGAAGGATCTGGTCTACATG
CCGGACGGGGTGAGG
+
FF,FFF,FF:FFFFFFF:FFFFFF,:FFF:FFFFFFFFFFFFFFFFFFFFFFFFFFFFFFFFFFFFFFFF
FF:FFF,FFFFFFFFFFF:,F:F:F:,FFFFFF::FFF:FFFFFFF:FF:FFFFFFFFFFFFFFFFF:FF
FFFFFFFFFFFFFF:
@A00155:342:HHGFNDSXY:1:1227:11984:30264 2:N:0:GAACCTAG+TCCGCATA
ATTTCTCCAGGTTTACAAACCTAAAAATTTGTGGGCTACTCTGGTTTTTCCACCTGAGATCTTAGCTG
GTTACAAATCCTCCGTTCTGCCCTTCTTATATCAATTTGAAATTCACGGGAAGGATCTGGTCTACATG
CCGGACGGGGTGAGG
+
FFFFFFFFFFFFFFFFFFF:FFFFFFFFFFFFFFFFFFFFFFFFFFFFFFF:FFFFFFFFF:FFFFFFFF
FFFFFFFFFFFFFFFFFFFFFFFFFFF:FFF,FFFFFFFF:FFFFFFFFFFFFFFFFFFFFFFFFFFFFF
FFFFFFFFFFFFFFF
@A00155:342:HHGFNDSXY:1:1567:20338:4899 2:N:0:GAACCTAG+TCCGCATA
ATTTCTCCAGGTTTACAAACCTAAAAATTTGTGGGCTACTCTGGTTTTTCCACCTGAGATCTTAGCTG
GTTACAAATCCTCCGTTCTGCCCTTCTTATATCAATTTGAAATTCACGGGAAGGATCTGGTCTACATG
CCGGACAGATCGGAA
+
FFFFFFFFFFFFFFFFFFFFFFFFFFFFFFFFFFFFFFFFFFFFFFFFFFFFFFFFFFFFFFFFFFFFFF
FFFFFFFFFFFFFFFFFFFFFFFFFFFFFF:FFFFFFFFFFFFFFFFFFFFFFFFFFFFFFFFFFFFFFF
FFFFFFFFFFFFFF:
@A00155:342:HHGFNDSXY:1:1626:6705:28667 2:N:0:GAACCTAG+TCCGCATA
ATTTCTCCAGGTTTACAAACCTAAAAATTTGTGGGCTACTCTGGTTTTTCCACCTGAGATCTTAGCTG
GTTACAAATCCTCCGTTCTGCCCTTCTTATATCAATTTGAAATTCACGGGAAGGATCTGGTCTACATG
CCGGACGGGGTGAGG
+
FFFFFFFFFFFFFFFFFFF::FFFFFFFFFFFFFFFFFFF:FFFF:FFFFFFFFFFFFFFFFF,FFFFFF
FFFFFFFFFFFFFFFFFFFFFFF:FFFFFFF:FFFFFFFFFFFFFFFFFFFFFFFFFFFFFFFFFFFFFF
FFFFFFFFFFFFF,F
@A00155:342:HHGFNDSXY:1:1626:6759:29293 2:N:0:GAACCTAG+TCCGCATA
ATTTCTCCAGGTTTACAAACCTAAAAATTTGTGGGCTACTCTGGTTTTTCCACCTGAGATCTTAGCTG
GTTACAAATCCTCCGTTCTGCCCTTCTTATATCAATTTGAAATTCACGGGAAGGATCTGGTCTACATG
CCGGACGGGGTGAGG
+
FFFFFFFFFFFFFFFFFFF:FFFFFFFFFFFFFFFFFFFFF:FFFFFFFFFFFF:FFFFFFF:FFFF:FF
FFFFFFFFFFFFFFFFFFFFFFFFFFFF:FFFFFFFFFFFFF:FFFFFFFFFFFFFFFFFFFFFFFFFFF
FFFFFFFFF:FFFF:
@A00155:342:HHGFNDSXY:1:1670:24406:34992 2:N:0:GAACCTAG+TCCGCATA
ATTTCTCCAGGTTTACAAACCTAAAAATTTGTGGGCTACTCTGGTTTTTCCACCTGAGATCTTAGCTG

GTTACAAATCCTCCGTTCTGCCCTTCTTATATCAATTTGAAATTCACGGGAAGGATCTGGTCTACATG
TCGGACGGGGTGAGG
+
FFFFFFFFFFFFFFFFFFFFFFFFFFFFFFFFFFFFFFFFFFFFFFF,FFFFFFFFFFFFFF:FFFFF
FFFFFFFFFFFFFFFFFFFFFFFFFFFFFFFFFFFFFF:FFFFFFFFFF:FFFFFFFFFFFFFFFFFF
FF:FFFFFFFF:FFF
@A00155:342:HHGFNDSXY:1:1170:10547:1908 1:N:0:GAACCTAG+TCCGCATA
TTTCTCCAGGTTTACAAACCTAAAAATTTGTGGGCTACTCTGGTTTTTCCACCTGAGATCTTAGCTGG
TTACAAATCCTCCGTTCTGCCCTTCTTATATCAATTTGAAATTCACGGGAAGGATCTGGTCTACATGC
CGGACGGAGTGAGGT
+
FFFFFFFFFFFFFFFFFFFFFFFFF:FFFFFFFFFFFFFFFFFFFFFFFFFFFFFFFFFFFF:FFFFF
FFFFFFFFFFFFFFFFFFFFFFFFFFFFFFFFFFFFFFFFFFFF:FFFFFFFFFFFFFFFFFFFFFFF
FFFFFFF:FFFFFFF
@A00155:342:HHGFNDSXY:1:1410:14271:20885 2:N:0:GAACCTAG+TCCGCATA
TTTCTCCAGGTTTACAAACCTAAAAATTTGTGGGCTACTCTGGTTTTTCCACCTGAGATCTTAGCTGG
TTACAAATCCTCCGTTCTGCCCTTCTTATATCAATTTGAAATTCACGGGAAGGATCTGGTCTACATGC
CGGACGGAGTGAGGT
+
FFFFFFFFFFFFFFFFFFFFFFFFFFFFFFFFFFFFFFFFFFFFFFFFFFFFFFFFFFFFFFFFFFFF
FFFFFFFFFFFFFFFFFFFFFFFFF,:FFFFFFFFFFFFFFFFFFFFFF:FFFFFFFF:FFFFFFFFF
FFFFFFFFFFFFFFF
@A00155:342:HHGFNDSXY:1:2276:13946:23735 2:N:0:GAACCTAG+TCCGCATA
TTTCTCCAGGTTTACAAACCTAAAAATTTGTGGGCTACTCTGGTTTTTCCACCTGAGATCTTAGCTGG
TTACAAATCCTCCGTTCTGCCCTTCTTATATCAATTTGAAATTCACGGGAAGGATCTGGTCTACATGC
CGGACGGGGTGAGGT
+
FFFFFFFFFFFFFFFFFFFFFFFFFFFFFFFFFFFFFF:FFFFFFFFFFFFFFFFFFFFF:FFFFFFF
FFFFFFFFFFFFFFFFFFFFFFFFFFFFFFFFFFF:FFFFFFFF:FFFFFFFFFFFFFFFFFFFFFFF
FFFFFFFFFFFFFFF
@A00155:342:HHGFNDSXY:1:2667:1253:1376 2:N:0:GAACCTAG+TCCGCATA
TTTCTCCAGGTTTACAAACCTAAAAATTTGTGGGCTACTCTGGTTTTTCCACCTGAGATCTTAGCTGG
TTACAAATCCTCCGTTCTGCCCTTCTTATATCAATTTGAAATTCAAGGGAAGGATCTGGTCTACATGC
CGGACGGAGTGAGGT
+
FFF:FFFFFFFFFF:::,,:FFF:,,FFFFFFF:FFF,F,FFFFFFFF:F::F:FFF:F,FF:,:FFF
FF:FFF,FF:F,FFFF,FF:,,FF:FFFF:F,::FFF,,F:F:FF,,,:,:::,F,F::FF:,,FF,:
FFF,::::FFFFF,F
@A00155:342:HHGFNDSXY:1:1242:8431:30091 1:N:0:GAACCTAG+TCCGCATA
TTCTCCAGGTTTACAAACCTAAAAATTTGTGGGCTACTCTGGTTTTTCCACCTGAGATCTTAGCTGGT
TACAAATCCTCCGTTCTGCCCTTCTTATATCAATTTGAAATTCACGGGAAGGATCTGGTCTACATGCC
GGACGGGGTGAGGTC
+
FFFFFFFFFFFFFFFFFFFFFFFFF,FFFFFF:F,FFFFFFFFFFFFFFFFFFFFFFFFFFFFFFFFF
FFFFFFFFFFFFFFFFFFFFFFFFFFFFFFFFF:FFFFFFFFFFFFFFFFFFFFFFFFFFFFFFFFFF
FFFFFFFFFFFFFFF
@A00155:342:HHGFNDSXY:1:1242:10827:27508 1:N:0:GAACCTAG+TCCGCATA
TCTCCAGGTTTACAAACCTAAAAATTTGTGGGCTACTCTGGTTTTTCCACCTGAGATCTTAGCTGGTT
ACAAATCCTCCGTTCTGCCCTTCTTATATCAATTTGAAATTCACGGGAAGGATCTGGTCTACATGCCG
GACGGGGTGAGGTC
+
FFFFFFFFFFFFFFFFFFFFFFFFFFFFFFFFFFFFFFFFFFFFFFFFFFFFFFFFFFFFFFFFFFFF
FFFFFFFFFFFFF:FFFFFFFFFFFFFFFFFFFFFFFFFFFFFFFF:FFFFFFFFFFFFFFFFFFFFF
FFFFFFFFFFFFFF

```
@A00155:342:HHGFNDSXY:1:2153:21594:13683 1:N:0:GAACCTAG+TCCGCATA
CTCCAGGTTTACAAACCTAAAAATTTGTGGGCTACTCTGGTTTTTCCACCTGAGATCTTAGCTGGTTA
CAAATCCTCCGTTCTGCCCTTCTTATATCAATTTGAAATTCACGGGAAGGATCTGGTCTACATGCCGG
ACGGGGTGAGGTCTG
+
FFFFFFFFFFFFFFFFFFFFFFFFFFFFFFFFFFFFFFFFFFFFFFFFFFFFFFFFFFFFFFFFFFFF
FFFFFFFFFFFFFFFFFFFFFFFFFFFFFFF:FFFFFFFFFFFFFFFFFFFFFFFFFFFFFFFFFFFF
FFFFFFFFFFFFFFF
@A00155:342:HHGFNDSXY:1:1203:7817:21480 2:N:0:GAACCTAG+TCCGCATA
CTCCAGGTTTACAAACCTAAAAATTTGTGGGCTACTCTGGTTTTTCCACCTGAGATCTTAGCTGGTTA
CAAATCCTCCGTTCTGCCCTTCTTATATCAATTTGAAATTCACGGGAAGGATCTGGTCTACATGCCGG
ACGGGGTGAGGTCTG
+
FFFFFFFFFFFFF:FFFFFFFFFFFFFFFFFFFFFFFFFFFFFFFFFFFFFFFFFFFFFFFFFFFFFF
FFFFFFFFFFF:FFFFFFFFFFFFFFFF,FFFFFFFFFFFFFF:FFFFFFFFFFFFFFFFFFF,FFFF
FF:FFFFFFFFFFFF
@A00155:342:HHGFNDSXY:1:1357:27462:3803 2:N:0:GAACCTAG+TCCGCATA
CTCCAGGTTTACAAACCTAAAAATTTGTGGGCTACTCTGGTTTTTCCACCTGAGATCTTAGCTGGTTA
CAAATCCTCCGTTCTGCCCTTCTTATATCAATTTGAAATTCACGGGAAGGATCTGGTCTACATGCCGG
ACGGAGTGAGGTCTG
+
FFFFFFFFFFFFFFFFFFFFF:FFFFFFFFFFFFFFFFFFFFFFFFFFFFFFFFFFFFFFFFFFFFFF
FFFFFFF:F:FFFFFFFFFF,FFFFFFFFFFFFFFFFFFFFFFFFFFFFFFFFFFFFF,FFFFFF:FFF
FF,FFFFFFFFFFFF
@A00155:342:HHGFNDSXY:1:1551:7401:3317 2:N:0:GAACCTAG+TCCGCATA
CTCCAGGTTTACAAACCTAAAAATTTGTGGGCTACTCTGGTTTTTCCACCTGAGATCTTAGCTGGTTA
CAAATCCTCCGTTCTGCCCTTCTTATATCAATTTGAAATTCACGGGAAGGATCTGGTCTACATGCCGG
ACGGAGTGAGGTCTG
+
FFFFFFFFFFFFFF:FFFFFFFFFFFFFFFFFFFFFFFFFFFFFFFFFFFFFFFFFFFFFFFFFFFFF
FFFFFFFFFFFFFFFFF:FFFFFFFFFFFFFFFFFFFFFFFFFFFFF:FFFFFFFFFFFFFFFFFFFF
FFFFFFFFFFFFFFF
@A00155:342:HHGFNDSXY:1:2245:18674:16047 2:N:0:GAACCTAG+TCCGCATA
CTCCAGGTTTACAAACCTAAAAATTTGTGGGCTACTCTGGTTTTTCCACCTGAGATCTTAGCTGGTTA
CAAATCCTCCGTTCTGCCCTTCTTATATCAATTTGAAATTCACGGGAAGGATCTGGTCTACATGCCGG
ACGGGGTGAGGTCTG
+
FFFFFFFFFFFFFFFFFFFFFFFFFFFFFFFFFFFFFFFFFF:FFFFFFFFFFFFFFFFFFFFFFFFFF
FFFFFFFFFFFFFFFFF:FF,FFFF,F:FFFFFFFFFFFFFFFFFFFFFFFFFFFFFFF:FFF:FFFFF
FFFFFFFFFFFFFFF
@A00155:342:HHGFNDSXY:1:2444:21847:2159 2:N:0:GAACCTAG+TCCGCATA
CTCCAGGTTTACAAACCTAAAAATTTGTGGGCTACTCTGGTTTTTCCACCTGAGATCTTAGCTGGTTA
CAAATCCTCCGTTCTGCCCTTCTTATATCAATTTGAAATTCACGGGAAGGATCTGGTCTACATGCCGG
ACGGGGTGAGGTCTG
+
FFFFFFFFFFFFFF:FFFFFFFFFFFFFFFFFFFFFFFFFFFFFFFFFFFFFFFFFFFFFFFFFFFFF
FFFFFFFFFFFFFFFFFFFF,FFFFFF,FFFFFFFFFF:FFFFF:FFFFFFFFFFFFFFFFFFFFFFFF
FFFFFFFFFFFFFFF
@A00155:342:HHGFNDSXY:1:2450:17472:1376 2:N:0:GAACCTAG+TCCGCATA
CTCCAGGTTTACAAACCTAAAAATTTGTGGGCTACTCTGGTTTTTCCACCTGAGATCTTAGCTGGTTA
CAAATCCTCCGTTCTGCCCTTCTTATATCAATTTGAAATTCACGGGAAGGATCTGGTCTACATGCCGG
ACGGGGTGAGGTCTG
+
FFFFFFFFFFFFFFFFFFFFFFFFFFFFFFFFFFFFFFFFFFFFFFFFFFFFFFFFFFFFFFFFFFFF
```

FFFFFFFFFFFFFFFFFFFFFFFFFFFFFFFFFFFFFFFFFFFF:FFFFFFFFFFFFFFFFFFFFFFF
FFFFFFFFFFFFFFF
@A00155:342:HHGFNDSXY:1:1470:19162:30483 1:N:0:GAACCTAG+TCCGCATA
CCAGGTTTACAAACCTAAAAATTTGTGGGCTACTCTGGTTTTTCCACCTGAGATCTTAGCTGGTTACA
AATCCTCCGTTCTGCCCTCCTTATATCAATTTGAAATTCACGGGAAGGATCTGGTCTACATGCCGGAC
GGAGTGAGGTC
+
FFFFFFFF:FFFFFFFFFFFFFFFFFFFFFFFFFFFFF,FFFFFFFFFFFFFFFFFFFFFFFFFFFFF
:FFFFFFFFFFFFFFFFFFFFFFFFFFF::FFFFFFFFFFFFFFF:FFFFFFFFFFFFFFFFFFFFFF
FFFFFFFFFFF
@A00155:342:HHGFNDSXY:1:1122:32949:12618 1:N:0:GAACCTAG+TCCGCATA
CCAGGTTTACAAACCTAAAAATTTGTGGGCTACTCTGGTTTTTCCACCTGAGATCTTAGCTGGTTACA
AATCCTCCGTTCTGCCCTTCTTATATCAATTTGAAATTCACGGGAAGGATCTGGTCTACATGCCGGAC
GGGGTGAGGTCTGAG
+
FFFFFFFF:FF:,FFFFFFFFFFFFFFFFFF,F:FFFFF:FFFFFFFFFFFFFFF,F:FFFFFFFFFF
FFFFFFFFF,FFFFFFFFFFFFFFF,FFF,FFFF,F,FFFFFFF::FFFFFFFFFF:,FFFFFFFFFF
FFFFFFFFFFFFFFF
@A00155:342:HHGFNDSXY:1:1162:4969:7028 1:N:0:GAACCTAG+TCCGCATA
CCAGGTTTACAAACCTAAAAATTTGTGGGCTACTCTGGTTTTTCCACCTGAGATCTTAGCTGGTTACA
AATCCTCCGTTCTGCCCTTCTTATATCAATTTGAAATTCACGGGAAGGATCTGGTCTACATGCCGGAT
GGGGTGAGGTCTGAG
+
FFFFFFFFFFFFFFFFFFFFF:FFFFFFFF,FFFFFFFFFFFFFFFFFFFFFFFFFFFFFFFFFFFFF
FFFFFFFFFFFFFFFFFFFFFFFFFFFF,:FFFFFFFFFFFFFFFFFFFFFFFFFFF:FF:F:FFFFF
FFFFFFFFFFFFFFF
@A00155:342:HHGFNDSXY:1:1252:28275:18364 1:N:0:GAACCTAG+TCCGCATA
CCAGGTTTACAAACCTAAAAATTTGTGGGCTACTCTGGTTTTTCCACCTGAGATCTTAGCTGGTTACA
AATCCTCCGTTCTGCCCTTCTTATATCAATTTGAAATTCACGGGAAGGATCTGGTCTACATGCCGGAC
GGGGTGAGGTCTGAG
+
FF:FF:FFFFF::FFFFFF:FFFFF:FFFFFFFFFFFFFFFFFFFFFFFFFFFFFF:FFFFFFFFFFF
FFFFFFFFFFFFFFFFFFFFFFFF:FFFFFFFFFFFFFFFFFFFFFFFF:F,FFFFF:FFFFFFFFFF
FFFFFFFFFFFFFFF
@A00155:342:HHGFNDSXY:1:1551:7401:3317 1:N:0:GAACCTAG+TCCGCATA
CCAGGTTTACAAACCTAAAAATTTGTGGGCTACTCTGGTTTTTCCACCTGAGATCTTAGCTGGTTACA
AATCCTCCGTTCTGCCCTTCTTATATCAATTTGAAATTCACGGGAAGGATCTGGTCTACATGCCGGAC
GGAGTGAGGTCTGAG
+
FFFF:FFFFFFFFFFFFFFFFFFFFFFFFFFFFFFFFFFFFFFFFFFFFFFFFFFFFFFFFFFFFFFF
FFFFFFFFFFFFFFFFFFFFFFFFFFFFF:FFFFFFFFFFFFFFFFFFFFFFFFFFFFFFFFFFFFFF
FFFFFFFFFFFFFFF
@A00155:342:HHGFNDSXY:1:1549:5737:2534 1:N:0:GAACCTAG+TCCGCATA
CCAGGTTTACAAACCTAAAAATTTGTGGGCTACTCTGGTTTTTCCACCTGAGATCTTAGCTGGTTACA
AATCCTCCGTTCTGCCCTTCTTATATCAATTTGAAATTCACGGGAAGGATCTGGTCTACATGCCGGAC
GGAGTGAGGTCTGAG
+
:FFFFFFFFFFFFFFFFFFFFFFFFFFFFFF:FFFFFFFFFFFFFFFFFFFFFFFF:FFFFFFFFFFF
FFFFFFFFFFFFFFFFFFFFFFFFFFFFFFFFFFFFFFFFFFFF,FFFF:FFFFFFFFFFFFFFFFFF
FFFFFFFFFFFFFFF
@A00155:342:HHGFNDSXY:1:1617:9995:31955 1:N:0:GAACCTAG+TCCGCATA
CCAGGTTTACAAACCTAAAAATTTGTGGGCTACTCTGGTTTTTCCACCTGAGATCTTAGCTGGTTACA
AATCCTCCGTTCTGCCCTTCTTATATCAATTTGAAATTCACGGGAAGGATCTGGTCTACATGCCGGAC
GGGGTGAGGTCTGAG

+
FFFFFFFFFFFF:FFFFFFF:FFFFFFFFFFFFFFFFFFFFFFFFFFFFFFFFFF:FFFFFFFFFFF
FFFFFFFFFFFFFFFFFFFFFFFFFFFF:F:FFFF:FFFFFFFFFF:FFFFFFFFFFFFFFFFFFF
FFFFFFFFFFFFFFF
@A00155:342:HHGFNDSXY:1:1662:9561:17581 1:N:0:GAACCTAG+TCCGCATA
CCAGGTTTACAAACCTAAAAATTTGTGGGCTACTCTGGTTTTTCCACCTGAGATCTTAGCTGGTTACA
AATCCTCCGTTCTGCCCTTCTTATATCAATTTGAAATTCACGGGAAGGATCTGGTCTACATGCCGGAC
GGGGTGAGGTCTGAG
+
FFFFF:FFFFFFFFFFFF:FFFFFFFFFFF:FFFFFFFFFFFFFFFFFFFFFFFFFFFFFFFFFFFF
FFFFFFFFFFFFFFFFFFFFFFFFFFFF:F,FFFFFFFFFFFFFFFFFFFFFFFFFFFFFFFFFFFFF
FFFFFFFFFFFFFFF
@A00155:342:HHGFNDSXY:1:2245:18674:16047 1:N:0:GAACCTAG+TCCGCATA
CCAGGTTTACAAACCTAAAAATTTGTGGGCTACTCTGGTTTTTCCACCTGAGATCTTAGCTGGTTACA
AATCCTCCGTTCTGCCCTTCTTATATCAATTTGAAATTCACGGGAAGGATCTGGTCTACATGCCGGAC
GGGGTGAGGTCTGAG
+
FFFFFFFFFFFFFFFFFFFFF:FFFFFFFFFFFFFFFFFFFFFFFFFFFFFFFFFFFFFFFFFFFFFF
:FFFFFFFFFFFFFFFFFFFFFFFFFFFF:FFFFFFFFFFFFFFFFFFFFFFFFFFFFFFFFFFFFFF
FFFFFFFFFFFFFFF
@A00155:342:HHGFNDSXY:1:2271:10104:9126 1:N:0:GAACCTAG+TCCGCATA
CCAGGTTTACAAACCTAAAAATTTGTGGGCTACTCTGGTTTTTCCACCTGAGATCTTAGCTGGTTACA
AATCCTCCGTTCTGCCCTTCTTATATCAATTTGAAATTCACGGGAAGGATCTGGTCTACATGCCGGAC
GGAGTGAGGTCTGAG
+
FFFFFFFFFFFFFFFFF:FFF:FFFFFFFFFFFFFFFFFFFFFFFFFFFFFFFFFFFFFFFFFFFFFF
FFFFFFFFFFFFFFFFFFFFFFFFFFFFFFFFFFFFFFFFFFFFFFFFFFF:FFFFFFFFFFFFFFFF
FFFFFFFFFFFFFFF
@A00155:342:HHGFNDSXY:1:2307:9815:15107 1:N:0:GAACCTAG+TCCGCATA
CCAGGTTTACAAACCTAAAAATTTGTGGGCTACTCTGGTTTTTCCACCTGAGATCTTAGCTGGTTACA
AATCCTCCGTTCTGCCCTTCTTATATCAATTTGAAATTCACGGGAAGGATCTGGTCTACATGCCGGAC
GGGGTGAGGTCTGAG
+
FFFFFFFFFFFFFFFFFFFFFFFFFFFFFFFFFFFFFFFFFFFFFFFFFFFFFFFFFFFFFFFFFFFF
FFFFFFFFFFFFFFFFFFFFFFFFFFFFFFFFFFFFFFFFFFFFFFFF:FFFFFFFFFFFFFFFFFFF
FFFFFFFFFFFFFFF
@A00155:342:HHGFNDSXY:1:2316:15845:19069 1:N:0:GAACCTAG+TCCGCATA
CCAGGTTTACAAACCTAAAAATTTGTGGGCTACTCTGGTTTTTCCACCTGAGATCTTAGCTGGTTACA
AATCCTCCGTTCTGCCCTTCTTATATCAATTTGAAATTCACGGGAAGGATCTGGTCTACATGCCGGAC
GGGGTGAGGTCTGAG
+
:FFFFFFFFFFF:FFFFF:FFFFFFFFFF,FFFFFFFFFFFFFFF:FFFFF:FFFFF:FFFFFFFFFF
:FFFFFFFFFFFFFFFFFFFFFFFFFFFF,FFFFFFFFFFFFFFF:FFFFFFF:FFF:FFFFFFF:FF
FFFF:FFFFFFFFFF
@A00155:342:HHGFNDSXY:1:2443:27570:36996 1:N:0:GAACCTAG+TCCGCATA
CCAGGTTTACAAACCTAAAAATTTGTGGGCTACTCTGGTTTTTCCACCTGAGATCTTAGCTGGTTACA
AATCCTCCGTTCTGCCCTTCTTATATCAATTTGAAATTCACGGGAAGGATCTGGTCTACATGCCGGAC
GGGGTGAGGTCTGAG
+
:FFFFF:FFFFFFFFFFFFFF:FFFFFFF:FFFFFFFFFFFFFFFFFFFFFFFFFFFFFFFFFFFFFF
FFFFFFFFFFFFFFFFFFFFFFFFFFFF:FFFFFFFFFFFFFFFFFFFFFFFFFFFFFFFFFFFFFFF
FFFFFFFFFFFFFFF
@A00155:342:HHGFNDSXY:1:2447:4381:19382 1:N:0:GAACCTAG+TCCGCATA
CCAGGTTTACAAACCTAAAAATTTGTGGGCTACTCTGGTTTTTCCACCTGAGATCTTAGCTGGTTACA

```
AATCCTCCGTTCTGCCCTTCTTATATCAATTTGAAATTCACGGGAAGGATCTGGTCTACATGCCGGAC
GGGGTGAGGTCTGAG
+
FFFFFFFFFFFF,FFFFFFF,FFFFFFFFF:FFFFFFFFFFFFFFFFFFFFFFFFFF:FFFFFFFFFF
F:FFFFF,FFFFFFFFFFFFFFFFF:FFF:FFFFFFFFFFFFFFFFFFF:FFFFFF:FFFFFFFFFFF
FFFFFFFFFFFFFFF
@A00155:342:HHGFNDSXY:1:2545:3341:17237 1:N:0:GAACCTAG+TCCGCATA
CCAGGTTTACAAACCTAAAAATTTGTGGGCTACTCTGGTTTTTCCACCTGAGATCTTAGCTGGTTACA
AATCCTCCGTTCTGCCCTTCTTATATCAATTTGAAATTCACGGGAAGGATCTGGTCTACATGCCGGAC
GGGGTGAGGTCTGAG
+
FFFFFFFFFFF,FFFFFFF:FFFFFFFFFFFFFFFFFFFFFFFFFFFFFFFFFFFF:FFFFFFFFFFF
F:FFFFFFFFFFFFFFF:FFFF:FFFFFF,FFFFFFFFFFFFFFFFFFFFF:FFFFFFFFFFFFFFFF
FFFFFFFFFFFFFFF
@A00155:342:HHGFNDSXY:1:1542:27606:30671 2:N:0:GAACCTAG+TCCGCATA
CCAGGTTTACAAACCTAAAAATTTGTGGGCTACTCTGGTTTTTCCACCTGAGATCTTAGCTGGTTACA
AATCCTCCGTTCTGCCCTTCTTATATCAATTTGAAATTCACGGGAAGGATCTGGTCTACATGCCGGAC
GGGGTGAGGTCTGAG
+
FFFFFFFFFFFFFFFFFFFFFFFFFFFFFFFFFFFFFFFFFFFFFFFFFFFFFFFFFFFFFF:FFFFF
FFFFFFFFFFFFF:FFFFFFFFF:FFFFFFFFFFF:FFFFFFFFFFFFFFFFFFFFFFFFFFF:FFFF
FFFFFFFFFFFFFFF
@A00155:342:HHGFNDSXY:1:2512:13711:35227 2:N:0:GAACCTAG+TCCGCATA
CCAGGTTTACAAACCTAAAAATTTGTGGGCTACTCTGGTTTTTCCACCTGAGATCTTAGCTGGTTACA
AATCCTCCGTTCTGCCCTTCTTATATCAATTTGAAATTCACGGGAAGGATCTGGTCTACATGCCGGAC
GGAGTGAGGTCTGAG
+
FFFFFFFFFFFF,FFFFFFFFFFFFFFFFFFFFFFFFFFFFFFFFFFFFFFFFFFFFFFFF:FFF:FF
FFFFFFFFFFFFFFFF:F,FF,:FFFFFFFF:FFFFFFFFFFFF:FFFFFFFFFFFFFFFFFFFFFFF
FFFFFF:FFFFFFFF
@A00155:342:HHGFNDSXY:1:1304:28221:5838 2:N:0:GAACCTAG+TCCGCATA
CAGGTTTACAAACCTAAAAATTTGTGGGCTACTCTGGTTTTTCCACCTGAGATCTTAGCTGGTTACAA
ATCCTCCGTTCTGCCCTTCTTATATCAATTTGAAATTCACGGGAAGGATCTGGTCTACATGCCGGACG
GGGTGAGGTCTGAGA
+
FFFFFFFFFFFFFFFFFFFFFFFFFFFFFFFFFFFFFFFFFFFFFFFFFFFFFFFFFFFFFF:FFFFF
FFFFFFFFFFFFFFFFFFFFFF:FFFF:FFFFFFFFFFFFFFF,FFFFFFFFFFFFFFFF:FFFFFFF
FFFF:FFFFFFFFFF
@A00155:342:HHGFNDSXY:1:2572:15148:34898 2:N:0:GAACCTAG+TCCGCATA
CAGGTTTACAAACCTAAAAATTTGTGGGCTACTCTGGTTTTTCCACCTGAGATCTTAGCTGGTTACAA
ATCCTCCGTTCTGCCCTTCTTATATCAATTTGAAATTCACGGGAAGGATCTGGTCTACATGCCGGACG
GAGTGAGGTCTGAGA
+
FFFFFFFFFFF:FFFFFFFFFFFFFFFFFFFFFFFFFFFFFFFFFFFFFFFFFFFFFFFFFFFFFFFF
FFFFFFFFFFFFFFFFFF:FFFFFFFFFFFFFFFFFFFFFFFFFFFFFFFFFFFFFFFFFFFFFFFFF
FFFFFFFFFFFF:FF
@A00155:342:HHGFNDSXY:1:2101:1805:7780 2:N:0:GAACCTAG+TCCGCATA
AGGTTTACAAACCTAAAAATTTGTGGGCTACTCTGGTATTTCCACCTGAGATCTTAGCTGGTTACAAA
TCCTCCGTTCTGCCCTTCTTATATCAATTTGAAATTCACGGGAAGGATCTGGTCTACATGCCGGACGG
GGTGAGGTCTGAGAG
+
:FFFFFFFFFFFFFFFFFFF,FF:FFFFFF:FF,:FF,FFFFFFF,FFF:FFF,F,:FF:FFF:FFFF
FFFFF:FFFFFFFFF,FF,F,::FFFFFFF:FF:FF:FFF:FFFFFFF:FF,,F,FFFFFFF,F,FFF
FF:FFFFFFFFFFFF
```

@A00155:342:HHGFNDSXY:1:1310:4562:3505 2:N:0:GAACCTAG+TCCGCATA
GGTTTACAAACCTAAAAATTTGTGGGCTACTCTGGTTTTTCCACCTGAGATCTTAGCTGGTTACAAAT
CCTCCGTTCTGCCCTTCTTATATCAATTTGAAATTCACGGGAAGGATCTGGTCTACATGCCGGACGGG
GTGAGGTCTGAGAGT
+
FF:FFFFFFFFFFFFFFFFFFFFFFFFFFFFFFFFFFFFFFFFFFFFFFFF:FFFFFFFFFFFFFFF
FFFFFFFFFFFFFFFFFFFFFFFFFFFFFFFFFFFFFFFFFFFFFFFFFFFFFFFFFFFFFFFFFFF
FFFFFFFFFFFFFFF
@A00155:342:HHGFNDSXY:1:2152:21558:9737 2:N:0:GAACCTAG+TCCGCATA
GGTTTACAAACCTAAAAATTTGTGGGCTACTCTGGTTTTTCCACCTGAGATCTTAGCTGGTTACAAAT
CCTCCGTTCTGCCCTTCTTATATCAATTTGAAATTCACGGGAAGGATCTGGTCTACATGCCGGACGGG
GTGAGGTCTGAGAGT
+
FFFFFFFF:FFFFFFFFF:FFFFFF:FFFFFFFFFFFFF:FFFFFFFF:FFFFFFFFFFFFFFFFFFF
FFFFFFFFF:FF:FFFFF,F::FFFF:FFFFFFFFFFFF:FFFFFFFFFFF,FFFFF::,F:::FFFF
FFFFF:FFFFFFFFF
@A00155:342:HHGFNDSXY:1:2350:13187:25363 2:N:0:GAACCTAG+TCCGCATA
GGTTTACAAACCTAAAAATTTGTGGGCTACTCTGGTTTTTCCACCTGAGATCTTAGCTGGTTACAAAT
CCTCCGTTCTGCCCTTCTTATATCAATTTGAAATTCACGGGAAGGATCTGGTCTACATGCCGGACGGA
GTGAGGTCTGAGAGT
+
FFFFFFFFFFFFFFFFFFFFFFFFFFFFFFFFFFF,FFFFFFFFFFFFFFFFFFFFFFFFFFFFFFF
FF:FFFFFFFFFFFF,FFF:FFFFFF:,FFFFFFFFFFFFFFFFFFFFFFFFFFFFFFF,F::FFFFF
FFFFFF,FF:FFFF:
@A00155:342:HHGFNDSXY:1:1548:24966:30358 1:N:0:GAACCTAG+TCCGCATA
GTTTACAAACCTAAAAATTTGTGGGCTACTCTGGTTTTTCCACCTGAGATCTTAGCTGGTTACAAATC
CTCCGTTCTGCCCTTCTTATATCAATTTGAAATTCACGGGAAGGATCTGGTCTACATGCCGGACGGGG
TGAGGTCTGA
+
:FFF::,FF,FF::F,:FFF:F,F,::FFF,FF:FFF:FFF,F,F:F:FFF:FFF,FFFFFF:F,FF:
,F,F,FFFFFF,FF,:F:FFFF,F,FFF,F,,FFF,FFF,:FFF:FFFFF:,FF,F:FFF:FF::F,:
FFF:F::F,F
@A00155:342:HHGFNDSXY:1:1343:3893:21292 1:N:0:GAACCTAG+TCCGCATA
TTACAAACCTAAAAATTTGTGGGCTACTCTGGTTTTTCCACCTGAGATCTTAGCTGGTTACAAATCCT
CCGTTCTGCCCTTCTTATATCAATTTGAAATTCACGGGAAGGATCTGGTCTACATGCCGGACGGAGTG
AGGTCTGAGAGTTAC
+
FFFFFFFFFFFFFFFFFFFFFFFF:FFFFFFFF:FFFFFFFFFFFFFFFF:FFFFFFFFFFFFFFF
FFFFFFFFFFFF:FFFFFFFFFF:F:FFFFFFFFFFFFFFFFFFFFFFFFFFFFFFFFFFFFFFFFF
FFFFFFFFFFFFFFF
@A00155:342:HHGFNDSXY:1:1415:19678:34194 1:N:0:GAACCTAG+TCCGCATA
TTACAAACCTAAAAATTTGTGGGCTACTCTGGTTTTTCCACCTGAGATCTTAGCTGGTTACAAATCCT
CCGTTCTGCCCTTCTTATATCAATTTGAAATTCACGGGAAGGATCTGGTCTACATGCCGGACGGAGTG
AGGTCTGAGAGTCAC
+
FFFFFF:FFFFFFFFFFFFF,FFFFFFFFFFFFFFFFFFFFFFFFFFFFFFFFFFF:FFFFFFFFFFF
FFFFFFFFFFFFFFFFFFFF:FF:F:FF:F,FFFFFFF:FFFFFF:FFFFF,FFFFF:FF:F:F:FFF
FFFFFFFFF:FFFF:
@A00155:342:HHGFNDSXY:1:1568:10212:3396 1:N:0:GAACCTAG+TCCGCATA
TTACAAACCTAAAAATTTGTGGGCTACTCTGGTTTTTCCACCTGAGATCTTAGCTGGTTACAAATCCT
CCGTTCTGCCCTTCTTATATCAATTTGAAATTCACGGGAAGGATCTGGTCTACATGCCGGACGGAGTG
AGGTCTGAGAGTCAC
+
FFFFF,FFFFFFFFFFFFFFFFFFFFFFFFFF:FFFFFFFFFFFFFFFFFFFFFFFFFFFFFFFFFFF

```
FFFFFFFFFFFFFFFFFFFFFF:FFFFFFFFFFFFFFFFFFFFFFFFFFFFFFFFFFFFFFFFFFFFFF
FFFFFFFFFFFFFFF
@A00155:342:HHGFNDSXY:1:1626:26151:2284 1:N:0:GAACCTAG+TCCGCATA
TTACAAACCTAAAAATTTGTGGGCTACTCTGGTTTTTCCACCTGAGATCTTAGCTGGTTACAAATCCT
CCGTTCTGCCCTTCTTATATCAATTTGAAATTCACGGGAAGGATCTGGTCTACATGCCGGACGGGGTG
AGGTCTGAGAGTTAC
+
FFFFF,FFFFFFFFFFFFFFFFFFFFFFFFFFFFFFFFFFFFFFFFFFFFFFFFF:FFFFFFFFFF
:FFF:FFFFFFFFFFFFFFFFFFFFFFFFFFFFFFFFFFFFFFFFFFFFFFFFFFFFFFFFFFFFFFF
FFFFFFFFFFFFFFF
@A00155:342:HHGFNDSXY:1:2124:29333:10300 1:N:0:GAACCTAG+TCCGCATA
TTACAAACCTAAAAATTTGTGGGCTACTCTGGTTTTTCCACCTGAGATCTTAGCTGGTTACAAATCCT
CCGTTCTGCCCTTCTTATATCAATTTGAAATTCACGGGAAGGATCTGGTCTACATGCCGGACGGGGTG
AGGTCTGAGAGTTAC
+
FFFFFFFFFFFFFFFFFFFF:FFFFFFFFFFF:FFFFFFFFFFFFFFFFFF:FFFFFFFFFF:FFFFF
FFFFFFFFFFFFFFFFFF:FFFFFFFFFFFFFFFFFFFFFFFFFFFFFFFFFFFFFFFFFFFFFFFFF
FFFFFFFFFFFFFFF
@A00155:342:HHGFNDSXY:1:2213:19262:21981 1:N:0:GAACCTAG+TCCGCATA
TCTCAAACCTAAAAATTTGTGGGCTACTCTGGTTTTTCCACCTGAGATCTTAGCTGGTTACAAATCCT
CCGTTCTGCCCTTCTTATATCAATTTGAAATTCACGGGAAGGATCTGGTCTACATGCCGGACGGGGTG
AGGTCTGAGAGTTAC
+
FFFFF,FFFFFFFFFFFFFFFFFFFFFFFFFFFFFFFFFFFFFFFFFFFFFFFFFFFFFFFFFFFFFF
FFFFFFFFFFFFFFFFFFFFFFFFFFFFFFFFFFFFFFFFFFFFFF,FFFFFFFFFFFFFFFFFFFFFF
FFFFFFFFFFFFFFF
@A00155:342:HHGFNDSXY:1:2434:10068:2801 1:N:0:GAACCTAG+TCCGCATA
TTACAAACCTAAAAATTTGTGGGCTACTCTGGTTTTTCCACCTGAGATCTTAGCTGGTTACAAATCCT
CCGTTCTGCCCTTCTTATATCAATTTGAAATTCACGGGAAGGATCTGGTCTACATGCCGGACGGAGTG
AGGTCTGAGAGTCAC
+
FFFFFFFFFFFFFFFFFFFF,FFFFFFFFFFFFFFFFFFFFFFFFFFFFFFF:FFFFFFFFFFFFFFF
FFFFFFFFFFFFFFFFFF:FFFFFFFFFFFFFFFFFFFFFFFFFFFFFFFFFFFFFFFFFFFFFFFFF
FFFFFFFFFFFFFFF
@A00155:342:HHGFNDSXY:1:2377:27082:10441 1:N:0:GAACCTAG+TCCGCATA
CCGATCTCTAAAAATTTGTGGGCTACTCTGGTTTTTCCACCTGAGATCTTAGCTGGTTACAAATCCTC
CGTTCTGCCCTTCTTATATCAATTTGAAATTCACGGGAAGGATCTGGTCTACATGCCGGACGGAGTGA
GGTCTGAGAGTTACA
+
FFFFFFFFFFFFFFF:FFFFFFFFFFFFFFFFFFFFFFFFFFFFFFFFFFFFFFFFFFFFFFFFFFFF
FFFFFFFFFFFFFFFFFFFFFF,FFFFFFFFFFFFFFFF:FFFFFFFFFFFFFFFFFFFFFFFFFFFFF
FFFFFFFFFFFFFFF
@A00155:342:HHGFNDSXY:1:2563:12527:5588 1:N:0:GAACCTAG+TCCGCATA
TACAAACCTAAAAATTTGTGGGCTACTCTGGTTTTTCCACCTGAGATCTTAGCTGGTTACAAATCCTC
CGTTCTGCCCTTCTTATATCAATTTGAAATTCACGGGAAGGATCTGGTCTACATGCCGGACGGGGTGA
GGTCTGAGAGTTACA
+
FFFFFFFFFFFFFFFFFFFFFFFFFFFFFFFFFFFFFFFFFFFFFFFFFFFFFFFFFFFFFFFFFFFF
FFFFFFFFFFFFFFFFFFFFFFF:FFFF:FFFFFFFFFFFFFFFFFFFFFFFFFFFFFFFFFFFFFFF
FFFFFFFFFFFFFFF
@A00155:342:HHGFNDSXY:1:2135:17779:9580 2:N:0:GAACCTAG+TCCGCATA
TACAAACCTAAAAATTTGTGGGCTACTCTGGTTTTTCCACCTGAGATCTTAGCTGGTTACAAATCCTC
CGTTCTGCCCATCTTATATCAATTTGAAATTCACGGGAAGGATCTGGTCTACATGCCGGACGGGGTGA
GGTCTGAGAGTTACA
```

```
+
FFFFFFFFFFFFFFFF,FF:FFF:FFFFFF:FFF,FFFFFFFFF::,FFFFFFF::FF:FFF,FFF,F
F,F:FFF,FF,FFF,F,FFFFF,FF,FFFF:FFFF:FF:FFFFFF:FFFFFFFFFFFFFFF:F:F::F
FFFFFFFFFFFFFFF
@A00155:342:HHGFNDSXY:1:2268:4119:33019 2:N:0:GAACCTAG+TCCGCATA
TACAAACCTAAAAATTTGTGGGCTACTCTGGTTTTTCCACCTGAGATCTTAGCTGGTTACAAATCCTC
CGTTCTGCCCTTCTTATATCAATTTGAAATTCACGGGAAGGATCTGGTCTACATGCCGGACGGGGTGA
GGTCTGAGAGTTACA
+
FFF,FFFFFFFFFFFF:FFFFFFFFFFFFFFFFFFFFFFFFFFFFFFFFFFFFFFFF,FFFFFFFFFFF
FFFFFFFFFFFFFFFFFFFFFFFFFFFFFFFFF:FFFF,F:F,FFFFF:FFFF:FFF:FFF:F:FFFFFF
FFFFFFFFFFFFFFF
@A00155:342:HHGFNDSXY:1:1320:5014:2284 2:N:0:GAACCTAG+TCCGCATA
ACAAACCTAAAAATTTGTGGGCTACTCTGGTTTTTCCACCTGAGATCTTAGCTGGTTACAAATCCTCC
GTTCTGCCCTTCTTATATCAATTTGAAATTCACGGGAAGGATCTGGTCTACATGCCGGACGGAGTGAG
GTCTGAGAGTTACAC
+
FFFFFFFFF:FFFFFFFFFF,FFF,FFFFFFFFFFFFFFF:FFFFFFFFFFFFFFFFFFFFFFFFFFF
FFFFF:FFFFFFFFFFFFFFFFFFFFFFFF:FFFFFFFFFFFFFFFFFFFF:FFFFFF:FFFFFFFFFF
FFFFFFFFFFFFFFF
@A00155:342:HHGFNDSXY:1:1371:3233:15671 2:N:0:GAACCTAG+TCCGCATA
ACAAACCTAAAAATTTGTGGGCTACTCTGGTTTTTCCACCTGAGATCTTAGCTGGTTACAAATCCTCC
GTTCTGCCCTTCTTATATCAATTTGAAATTCACGGGAAGGATCTGGTCTACATGCCGGACGGGGTGAG
GTCTGAGAGTTACAC
+
FFFFFFFFFFFFFFFFFFFFFFFFFFFFFFFFFFFFFFFFFFFFFFFFFFFFFFFFFFFFFFFFFFFF:
FFFFFFF,FFFFFFFFFFFFFFFFFFFFFFFFFF:FFFFFFFFFFFFFFFFFFFF:F:FFFFFFFFFFF
FFFFF,FFFFF:FFF
@A00155:342:HHGFNDSXY:1:1402:31810:27367 2:N:0:GAACCTAG+TCCGCATA
ACAAACCTAAAAATTTGTGGGCTACTCTGGTTTTTCCACCTGAGATCTTAGCTGGTTACAAATCCTCC
GTTCTGCCCTTCTTATATCAATTTGAAATACACGGGAAGGATCTGGTCTACATGCCGGACGGAGTGAG
GTCTGAGAGTTACAC
+
FFF:FFF:,FFFFFF:,,FF:F:FFF:FFF:FFFFFFFFF:FFFFFFFFFFF:,:FF,FFFF:,,,FF
FFFFFFFFFF:FF,FFF,FFFFFFFFFF:,:FFF,FF:F,FFFF,:,FFFFF,FF:FFFFF:FF:::F
FFFFFF:FFFFFFFF
@A00155:342:HHGFNDSXY:1:1472:24099:9001 2:N:0:GAACCTAG+TCCGCATA
ACAAACCTAAAAATTTGTGGGCTACTCTGGTTTTTCCACCTGAGATCTTAGCTGGTTACAAATCCTCC
GTTCTGCCCTTCTTATATCAATTTGAAATTCACGGGAAGGATCTGGTCTACATGCCGGACGGGGTGAG
GTCTGAGAGTTACAC
+
FFFFFFFFFFFFFFFFFFFFFFFFFFFFFFF:FFFFFFFFFFFFFFF:FFFFFFFFFFFFF:FFFFFF
FFFFFFFFFFFFFFF,FFFFFFFFFFFFFFFFFFFFFFFFFFFFFFFFFFF,FFFFFFFFFFFFFFF
F:FFFFFFFFFFFFF
@A00155:342:HHGFNDSXY:1:2259:12274:17112 2:N:0:GAACCTAG+TCCGCATA
ACAAACCTAAAAATTTGTGGGCTACTCTGGTTTTTCCACCTGAGATCTTAGCTGGTTACAAATCCTCC
GTTCTGCCCTTCTTATATCAATTTGAAATTCACGGGAAGGATCTGGTCTACATGCCGGACGGAGTGAG
GTCTGAGAGTTACAC
+
:FFFFFFFFFFFFFFFFFFFFFFFFFFFFFFFFFFFFFFFFFFFFFFFFFFFFFFFFFFFFFFFFFFF
FFFFFFFFFFFFFFFFFFFFFFFFFFFFFFFFFFFFF:FFFFFFFFFFFFFFFFFFFFFFFFFFFFFF
:FFFFFFFFFFFFFF
@A00155:342:HHGFNDSXY:1:2313:2889:17769 2:N:0:GAACCTAG+TCCGCATA
ACAAACCTAAAAATTTGTGGGCTACTCTGGTTTTTCCACCTGAGATCTTAGCTGGTTACAAATCCTCC
```

```
GTTCTGCCCTTCTTATATCAATTTGAAATTCACGGGAAGGATCTGGTCTACATGCCGGACGGGGTGAG
GTCTGAGAGTTACAC
+
FFFFFFFFFFFFFFFFFFFFFFFFFFFFFFFFFFFFFF:FFFFFFFFFFFFFFFFFFFFFFFFF:FF
FFFFFFFFFFFFFFFFFFFFFF:FFFFFF::FFFFFFFFFFFFFFFFFFF:FFFFFFFFFFFFFFFF
FFFFFFFFFFFFFFF
@A00155:342:HHGFNDSXY:1:2420:14479:21715 2:N:0:GAACCTAG+TCCGCATA
ACAAACCTAAAAATTTGTGGGCTACTCTGGTTTTTCCACCTGAGATCTTAGCTGGTTACAAATCCTCC
GTTCTGCCCTTCTTATATCAATTTGAAATTCACGGGAAGGATCTGGTCTACATGCCGGACGGAGTGAG
GTCTGAGAGTTACAC
+
FFFFFFFFFFFFFFFFFFFFFFFFFFFFFFFFFFFFFFFFFFFFFFFFFFFFFFFFFFFFFFFFFFFF
FFFFFFFF:FFF,FFFFFFFFFFFFFFFFFFFFFFFFFFFFFFFFFFFFFFFFFFFFFFFFFFFFFF
FFFFFFFFFFFFFFF
@A00155:342:HHGFNDSXY:1:1630:7627:27258 1:N:0:GAACCTAG+TCCGCATA
CAAACCTAAAAATTTGTGGGCTACTCTGGTTTTTCCACCTGAGATCTTAGCTGGTTACAAATCCTCCG
TTCTGCCCTTCTTATATCAATTTGAAATTCACGGGAAGGATCTGGTCTACATGCCGGACGGAGTGAGG
TCTGAGAGTT
+
FFFFFFFFFFFFFFFFFFFFFFFFFFFFFFFFFFFFFFFFFFFFFFFFFFFFFFFFFFFFFFFFFFFF
FFFFFFFFFFFFFFFFFFFFFFF:FFFFFFFFFFFFFFFFFFFFFFFFFFFFFFFFFFFFFFFFFFFF
FFFFFFFFFF
@A00155:342:HHGFNDSXY:1:1164:5547:30326 2:N:0:GAACCTAG+TCCGCATA
CAAACCTAAAAATTTGTGGGCTACTCTGGTTTTTCCACCTGAGATCTTAGCTGGTTACAAATCCTCCG
TTCTGCCCTTCTTATCTCAATTTGAAATTCACGGGAAGGATCTGGTCTACATGCCGGACGGGGTGAGG
TCTGAGAGTTACAC
+
FFFFFFFFFFFFFFFFFFFFFFFFFFFFFFFFFFFFF:FFFFFFFFF,FFFFFFFFFFFFFFFFFFFF
FFFFFFFFFFFFFFF,FFFFFFFFFF:FF:FFFFFFF:FFFFFFFFFFFFFFFFFFFF:FF:FFFFFF
FFFFFFFFFFFFFF
@A00155:342:HHGFNDSXY:1:1242:20916:16548 2:N:0:GAACCTAG+TCCGCATA
CAAACCTAAAAATTTGTGGGCTACTCTGGTTTTTCCACCTGAGATCTTAGCTGGTTACAAATCCTCCG
TTCTGCCCTTCTTATATCAATTTGAAATTCACGGGACGGATCTGGTCTACATGCCGGACGGGGTGAGG
TCTGAGAGTTACACT
+
FFFFFFFFFFFFFFFFFFFFFFFFFFFFFFFFFFFFFFFFFFFFFFFFFFFFFFFFFFFFFFFFFFFF
FFFFFFFFFFFFFFFFFFFFFFFFFF:FFFFFFFFFF,FFFFFFFFFFFFFFFFFFFFFFFFFFFFFFF
FFFFFFFFFFFFFFF
@A00155:342:HHGFNDSXY:1:1234:17047:10066 2:N:0:GAACCTAG+TCCGCATA
CAAACCTAAAAATTTGTGGGCTACTCTGGTTTTTCCACCTGAGATCTTAGCTGGTTACAAATCCTCCG
TTCTGCCCTTCTTATATCAATTTGAAATTCACGGGAAGGATCTGGTCTACATGCCGGACGGGGTGAGG
TCTGAGAGTTACACT
+
FFFFFFFFFFF::FFFFFFFFFFFFFFFFFFFFFFFFFFFFFFFFFFFFFFFFFFFFFFFFFFFFFFF
FFFFFFFFFFFFFFFFFFFFFF:FFFFFFFFFFFFFFFFFFFFFFFFFFFFFFFFFFFFFFFFFFFFF
FFFFFFFFFFFFFFF
@A00155:342:HHGFNDSXY:1:1312:22309:19711 2:N:0:GAACCTAG+TCCGCATA
CAAACCTAAAAATTTGTGGGCTACTCTGGTTTTTCCACCTGAGATCTTAGCTGGTTACAAATCCTCCG
TTCTGCCCTTCTTATATCAATTTGAAATTCACGGGAAGGATCTGGTCTACATGCCGGACGGGGTGAGG
TCTGAGAGTTACACT
+
FFFFFFFFFFFFFFFFFFFFFFFFFFFFFFFFFFFFFFFFFFFFFFF:FFFFFFFFFFFFFFFFFFFF
FFFFFFF,FFFFFFFFFFFFFFFFFFFFFFFFFFFFFFFFFFFFFFFFFFFFFFFFFFF,FFFFFFFF
FFFFFFFFFFFFFFF
```

```
@A00155:342:HHGFNDSXY:1:1477:10339:6621 2:N:0:GAACCTAG+TCCGCATA
CAAACCTAAAAATTTGTGGGCTACTCTGGTTTTTCCACCTGAGATCTTAGCTGGTTACAAATCCTCCG
TTCTGCCCTTCTTATATCAATTTGAAATTCACGGGAAGGATCTGGTCTACATGCCGGACGGGGTGAGG
TCTGAGAGTTACACT
+
FFFFFFFFFFFFFFFFFFFFFFFFFFFFFFFFFFFFFFFFFFFFFFFFFFFFFFFFFFFFFFFFFFFF
FFFFFFFFFFF:FFFFFFFFFFF:FFFFFFFFFFFF:FFFFFFFFFFF:FFFFFF:FFFF:FFFFFFF
FFFFFFFFFF:FFF:
@A00155:342:HHGFNDSXY:1:1563:31494:35618 2:N:0:GAACCTAG+TCCGCATA
CAAACCTAAAAATTTGTGGGCTACTCTGGTTTTTCCACCTGAGATCTTAGCTGGTTACAAATCCTCCG
TTCTGCCCTTCTTATATCAATTTGAAATTCACGGGAAGGATCTGGTCTACATGCCGGACGGGGTGAGG
TCTGAGAGTTACACT
+
FFFFFFFFFFFFFFFFFFFFFFFFFFFFFFFFF:FF:FFFFFFFFFFFFFFFFFFFFFFFFFFFFFFF
FFFFFFFFFFFFF::FFFFFFFFFFFFFFFFFFFFFFFFF:FFFFFFFFFFFFFFFFFFFFFFFFFFF
FFFFFFFFFF:FFFF
@A00155:342:HHGFNDSXY:1:1632:3016:20588 2:N:0:GAACCTAG+TCCGCATA
CAAACCTAAAAATTTGTGGGCTACTCTGGTTTTTCCACCTGAGATCTTAGCTGGTTACAAATCCTCCG
TTCTGCCCTTCTTATATCAATTTGAAATTCACGGGAAGGATCTGGTCTACATGCCGGACGGGGTGAGG
TCTGAGAGTTACACT
+
FFFFFFFFFFFFFFFFFFFFFFFFFFFFF:FFFFFFFFFFFFFFFFFFFFFFFFFFFFFFFF:FFF,FF
FFFFFF:FFFFFFFFFFFFFFFFFFFFFFFFFF:FFFFFFFFFFFFFFFFFFFFFFFFFFFFFFFFFFF
FFFFFFFFFFFFFFF
@A00155:342:HHGFNDSXY:1:2213:19262:21981 2:N:0:GAACCTAG+TCCGCATA
CAAACCTAAAAATTTGTGGGCTACTCTGGTTTTTCCACCTGAGATCTTAGCTGGTTACAAATCCTCCG
TTCTGCCCTTCTTATATCAATTTGAAATTCACGGGAAGGATCTGGTCTACATGCCGGACGGGGTGAGG
TCTGAGAGTTACAGA
+
FFFFFFFFFFFFFFFFFFFFFFFFFFFFFFFFFFFFFFFFFFFFFFFFFFFFFFFFFFFFFFFFFFFF
FFFFFFFFFFFFFFFFFFFFFFFFFFFFFFFFFFFFFFFFFFFFFFFFFFFFFFFFFFFFFFFFFFFF
FFFFFFFFFFFFFFF
@A00155:342:HHGFNDSXY:1:2154:26494:35571 2:N:0:GAACCTAG+TCCGCATA
CAAACCTAAAAATTTGTGGGCTACTCTGGTTTTTCCACCTGAGATCTTAGCTGGTTACAAATCCTCCG
TTCTGCCCTTCTTATATCAATTTGAAATTCACGGGAAGGATCTGGTCTACATGCCGGACGGAGTGAGG
TCTGAGAGTTACACT
+
F:FFF,FFFFFFFFFFFFF:,FFFFFFFFFFFFFFFFFFFFFFFFFFFFFFFFF,FFFFFFFF,:FFFF
FFFFFFFFFFFFFFFFFFFFF:FFFFFFFFFFF:FFFF::FFF:FFFFFFFFFFFF:FFF,FF:FF:,
:FF:FFFF:FFFFFF
@A00155:342:HHGFNDSXY:1:2229:12653:8061 2:N:0:GAACCTAG+TCCGCATA
CAAACCTAAAAATTTGTGGGCTACTCTGGTTTTTCCACCTGAGATCTTAGCTGGTTACAAATCCTCCG
TTCTGCCCTTCTTATATCAATTTGAAATTCACGGGAAGGATCTGGTCTACATGCCGGACGGGGTGAGG
TCTGAGAGTTACACT
+
FFFFFFFFFFFFFFFFFFFFFFFFFFFFFFFFFFFFFFFFFFFFFFFFFFFFFFFFFFFFFFFFFFFF
FFFFFFF:FFFFF:F,FFFFFFFFFFFFFFFFFFFFFFFFFFFFFFFFFFFFFF:FFFFFFF:FFFFFF
FFFFFFFFFFFFFFF
@A00155:342:HHGFNDSXY:1:2257:19587:7326 2:N:0:GAACCTAG+TCCGCATA
CAAACCTAAAAATTTGTGGGCTACTCTGGTTTTTCCACCTGAGATCTTAGCTGGTTACAAATCCTCCG
TTCTGCCCTTCTTATATCAATTTGAAATTCACGGGAAGGATCTGGTCTACATGCCGGACGGGGTGAGG
TCTGAGAGTTACACT
+
FFFFFFF:FFFFFFFFFFFFFFFFFFFFFFFFFFFFFFFFFFFFFFFFFFFFFFFFFFFFFFFFFFFF
```

FFFFFFFF:FFFFFFFFFFFFFF:FFFFFFFFFFFFFFFFFFFFFFFFFFFFFFFFFFFFFFFFFFFF
FFFFFFFFFFFFFFF
@A00155:342:HHGFNDSXY:1:2438:25437:10786 2:N:0:GAACCTAG+TCCGCATA
CAAACCTAAAAATTTGTGGGCTACTCTGGTTTTTCCACCTGAGATCTTAGCTGGTTACAAATCCTCCG
TTCTGCCCTTCTTATATCAATTTGAAATTCACGGGAAGGATCTGGTCTACATGCCGGACGGAGTGAGG
TCTGAGAGTTACACT
+
FFFFFFFFFF::FFFFFF:FFFFFFFF:FF,FFFFFFFFFFFFFF:FFF:FFFFF:F:FFFFFFFFFF
FFFFFFF,,::F:F:FFFFFF,FFFFFFFFFFFFFF:FFF,F:F:FFFFFFF:F:FFFFFFFFFFFFF
FFFFFFFF,FFFFFF
@A00155:342:HHGFNDSXY:1:2541:24397:29403 2:N:0:GAACCTAG+TCCGCATA
CAAACCTAAAAATTTGTGGGCTACTCTGGTTTTTCCACCTGAGATCTTAGCTGGTTACAAATCCTCCG
TTCTGCCCTTCTTATATCAATTTGAAATTCACGGGAAGGATCTGGTCTACATGCCGGACGGGGTGAGG
TCTGAGAGTTACACT
+
FFFFFF:FFFFFFFF:FFFFFFFFFFFFFFFFFFFFFFF:FFFFFFFFFFFFFFFFFFFFFFF,:FFF
FF,FFFFFFFFFFFFFFFFFFFFFFF::FFFFFFFFF:FFFFF,FFFFF,FFFFFFFFFFFFFF,FFF
FFFFFFFFFFFF:FF
@A00155:342:HHGFNDSXY:1:2674:9082:30217 2:N:0:GAACCTAG+TCCGCATA
CAAACCTAAAAATTTGTGGGCTACTCTGGTTTTTCCACCTGAGATCTTAGCTGGTTACAAATCCTCCG
TTCTGCCCTTCTTATATCAATTTGATATTCACGGGAAGGATCTGGTCTACATGCCGGACGGGGTGAGG
TCTGAGAGTTACACT
+
FFFFFFFFFFFFFFFFFFFFFFFFFFFFFFFFFFFFFFFFFFFFFFFFFFFFFFFFFFFFFFFF:FFF
FFFFFFFFFFFF:F:FFFFFF:FFF,FFFFFFFFFFFFFFFFFFFFFFFFFFF,FFFFF:FFFFFFFF
FFFFFFFF:FFFFFF
@A00155:342:HHGFNDSXY:1:2353:16785:11835 2:N:0:GAACCTAG+TCCGCATA
AAACCTAAAAATTTGTGGGCTACTCTGGTTTTTCCACCTGAGATCTTAGCTGGTTACAAATCCTCCGT
TCTGCCCTTCTTATATCAATTTGAAATTCACGGGAAGGATCTGGTCTACATGCCGGACGGGGTGAGGT
CTGAGAGTTACACTC
+
FFFFFFFFFFF,:FFFFFFFFFFFFFFFFFFFFFFFFFFFFFFFFFFFFFFFF:FFFFFFFFFFFFF:
FFFFFFF,:F,F:FF,FFFFFFFFFFFFFFFFFFFFFFFFFFFFFFFFFFFFFFFFFFFFFFFFFFFF
FFFFFFFFFFFFFFF
@A00155:342:HHGFNDSXY:1:2460:3305:13698 2:N:0:GAACCTAG+TCCGCATA
AAACCTAAAAATTTGTGGGCTACTCTGGTTTTTCCACCTGAGATCTTAGCTGGTTACAAATCCTCCGT
TCTGCCCTTCTTATATCAATTTGAAATTCACGGGAAGGATCTGGTCTACATGCCGGACGGAGTGAGGT
CTGAGAGTTACACTC
+
FFFFFFFFFFFFFFFFFFFFFFFFFFFFFFFFFFFFFFFFFFFFFFFFFFFFFFFFFFFF:FFFFFFF
FFFFF:FF:F:FFFFFFFFFFFFFFFFFFFFFFFFFFFFFFFFFFFFFFFFFFFFFFFFF:FFFFFFF
FFFFFF:FFFFFFFF
@A00155:342:HHGFNDSXY:1:2676:26684:36652 2:N:0:GAACCTAG+TCCGCATA
AAACCTAAAAATTTGTGGGCTACTCTGGTTTTTCCACCTGAGATCTTAGCTGGTTACAAATCCTCCGT
TCTGCCCTTCTTATATCAATTTGAAATTCACGGGAAGGATCTGGTCTACATGCCGGACGGGGTGAGGT
CTGAGAGTTACACTC
+
FFFFFFFFFFFFFFFFFFFFFFFFFFFFFFFFFFFFFFFFFFFFFFFFFFFFFFFFFFFF:FFFFFFF
FFFFFFFFFFFFFFFFFFFFFFFFFFFFFFFFFFFFFFFFFFFFFFFFFFFFFFFFFFFFFFFFFFFF
FFFFFFFFFFF:FFF
@A00155:342:HHGFNDSXY:1:1307:10728:24048 1:N:0:GAACCTAG+TCCGCATA
CCTAAAAATTTGTGGGCTACTCTGGTTTTTCCACCTGAGATCTTAGCTGGTTACAAATCCTCCGTTCT
GCCCTTCTTATATCAATTTGAAATTCACGGGAAGGATCTGGTCTACATGCCGGACGGGGTGAGGTCTG
AGAGTTACACTCAAC

```
+
FFFFFFFFFFFFFFFFFFFFFFFFFFFFFFFFFFFFFFFFFFFFFFFFFFFFFFFFFFFFFFFFFFFF
FFFFFFFFFFFFFFFFFFFFFFFFFFFFFFFFFFFFFFFFFFFFFFFFFFFFFFFFFFFFFFFFFFFF
FFFFFFFFFFFFFFF
@A00155:342:HHGFNDSXY:1:2110:6280:5885 1:N:0:GAACCTAG+TCCGCATA
CGATCTAATTTGTGGGCTACTCTGGTTTTTCCACCTGAGATCTTAGCTGGTTACAAATCCTCCGTTCT
GCCCTTCTTATATCAATTTGAAATTCACGGGAAGGATCTGGTCTACATGCCGGACGGGGTGAGGTCTG
AGAGTTACACTCAAC
+
FFFFFFFFFFFFFFFFFFFFFFFFFFFFFFFFFFFFFFFFFFFFFFFFFFFFFFFFF:FFFFFFFFFF
FFFFFFFFFFF:FFFFFFFFFFFFFFFFFFFFFFFFFFFFFFFFFFFFFFFFFFFFFFFFFFFFFFFF
FFFFFFFFFFFFFFF
@A00155:342:HHGFNDSXY:1:2209:1922:8453 1:N:0:GAACCTAG+TCCGCATA
CCTAAACATTTGTGGGCTACTCTGGTTTTTCCACCTGAGATCTTAGCTGGTTACAAATCCTCCGTTCT
GCCCTTCTTATATCAATTTGAAATTCACGGGAAGGATCTGGTCTACATGCCGGACGGGGTGAGGTCTG
AGAGTTACACTCAAC
+
FFFFF,,FF,FFFFFFF:FFFFFFFFFFFFFFFFFFF:FFFF:FFF:FF::FFF:FFFFF::FF,FFF
FF:FFFFFFFFFFFFF:FFF,FF:FFFFFFFFFFFFFFFFFFFF:FFFFFFFFFFF:FFFFFFFFFFF
FFFF,FFFFFFFF:F
@A00155:342:HHGFNDSXY:1:1641:20609:14669 2:N:0:GAACCTAG+TCCGCATA
CCTAAAAATTTGTGGGCTACTCTGGTTTTTCCACCTGAGATCTTAGCTGGTTACAAATCCTCCGTTCT
GCCCTTCTTATATCAATTTGAAATTCACGGGAAGGATCTGGTCTACATGCCGGACGGAGTGAGGTCTG
AGAGTTACACTCAAC
+
FFFFFFFFFFFFFFFFFFFFFFFFFFFFFFFFFFFFFFFFFFFFFFFFFFFFFFFFF:FFFFFFFFFF
FFFFFFFFFFFFFFFFFFFFF:FFFFFFFFF:FFFFFFFFFFFFFFFFFFFFFFFFFFFFFFFFFFFF
FFFFFFFFFFFFFFF
@A00155:342:HHGFNDSXY:1:2353:30228:35336 2:N:0:GAACCTAG+TCCGCATA
CCTAAAAATTTGTGGGCTACTCTGGTTTTTCCACCTGAGATCTTAGCTGGTTACAAATCCTCCGTTCT
GCCCTTCTTATATCAATTTGAAATTCACGGGAAGGATCTGGTCTACATGCCGGACGGAGTGAGGTCTG
AGAGTTACACTCAAC
+
FFFFFFFFFFFFFFFFFF:FFFFFFFFFFFFFFFFFFFFFFFFFFFFFFFFFFFFFFFFFFFFFFFFF
FFF:FFFFFFFFFFFFFFFFFFFFFFFFFFFFFFFFFFFFFFFFFFFF:FFFFFFFFFFFFFFFFFFF
FFFFFFFFFFFFFFF
@A00155:342:HHGFNDSXY:1:2417:1678:12571 2:N:0:GAACCTAG+TCCGCATA
CCTAAAAATTTGTGGGCTACTCTGGTTTTTCCACCTGAGATCTTAGCTGGTTACAAATCCTCCGTTCT
GCCCTTCTTATATCAATTTGAAATTCACGGGAAGGATCTGGTCTACATGCCGGACGGGGTGAGGTCTG
AGAGTTACACTCAAC
+
FFFFFFFFF:FFFFFFFFFF:FFFFFF:FFFFFFFFFFFF:FFFFFFFF::FF:FFFFFFFFFFFFFF
FFFFFFF:FFF:F:F:FFFFFFFFFFFFFFFFFFFFFFFFFFFFFFF::FFFFFFFFFFFFFFFFFFF
FFFFFF,FFFFFFFF
@A00155:342:HHGFNDSXY:1:2121:27019:4914 2:N:0:GAACCTAG+TCCGCATA
CTAAAAATTTGTGGGCTACTCTGGTTTTTCCACCTGAGATCTTAGCTGGTTACAAATCCTCCGTTCTG
CCCTTCTTATATCAATTTGAAATTCACGGGAAGGATCTGGTCTACATGCCGGACGGGGTGAGGTCTGA
GAGTTACACTCAAC
+
FFF:FFF:,FFF,,:FFFF,F,F:,,,FFF,FFF:FFFFF,,FF,,FF:::::FFFF,FFF,FFFFFF
,F,,F:F,F,FF,FFF,FFFFFFFF:FF:,FFF,FFF,:FF:FF:F,,,F,FF,,FF,,FFFF,::FF
FF,FFFFFFF,FFF
@A00155:342:HHGFNDSXY:1:1666:3070:21339 1:N:0:GAACCTAG+TCCGCATA
CTAAAAATTTGTGGGCTACTCTGGTTTTTCCACCTGAGATCTTAGCTGGTTACAAATCCTCCGTTCTG
```

CCCTTCTTATATCAATTTGAAATTCACGGGAAGGATCTGGTCTACATGCCGGACGGGGTGAGGTCTGA
GAGTTACACTCAACC
+
:FFFFFFFFFFFFFFFFFFFF,,FFFFFFFFFFF:FFFFFFFFFFFFFFFFFFFFFFFFFF:FFFFFF
FFFF:FFFFFFFFFFFFFFFFFFFFFF,FFFF::FF,FFFF,FFFFFFF:FFFFFFFFFFFFFFFFFF
FFFFFFFFFFFFFFF
@A00155:342:HHGFNDSXY:1:1464:23891:1564 2:N:0:GAACCTAG+TCCGCATA
CTAAAAATTTGTGGGCTACTCTGGTTTTTCCACCTGAGATCTTAGCTGGTTACAAATCCTCCGTTCTG
CCCTTCTTATATCAATTTGAAATTCACGGGAAGGATCTGGTCTACATGCCGGACGGGGTGAGGTCTGA
GAGTTACACTCAACC
+
FFFFFFFFFFFFFFFFFFF:FFFFFFFFFFFFFFFFFFFFF:FFFFFF:FFFFFFFFFFFFFFFFFFF
FFFFFFF,FFFFFFFFFFFFFFFFFFFFFFFFFFFFFFFFFFFFFFFFFFFFFF:FF,FFFFFFFFFF
:FFFFFFFFFFFFF:
@A00155:342:HHGFNDSXY:1:2119:15230:23860 2:N:0:GAACCTAG+TCCGCATA
CTAAAAATTTGTGGGCTACTCTGGTTTTTCCACCTGAGATCTTAGCTGGTTACAAATCCTCCGTTCTG
CCCTTCTTATATCAATTTGAAATTCACGGGAAGGATCTGGTCTACATGCCGGACGGGGTGAGGTCTGA
GAGTTACACTCAACC
+
FFFFFFFFFFFFFFFFFFFFFFFFFFFFFFFFFFFFFFFFFFFFFFFFFFFFFFFFFFFFFFFFFFFF
FFFFFFFFFFFFFFFFFF:FFFFFFFFF:FFFFFFFFFFFFFFFFFFFFFFFFF:FFFFFFF:FFFFF
FFFFFFFF:FFFFFF
@A00155:342:HHGFNDSXY:1:2553:19135:28432 2:N:0:GAACCTAG+TCCGCATA
CTAAAAATTTGTGGGCTACTCTGGTTTTTCCACCTGAGATCTTAGCTGGTTACAAATCCTCCGTTCTG
CCCTTCTTATTTCAATTTGAAATTCACGGGAAGGATCTGGTCTACATGCCGGACGGGGTGAGGTCTGA
GAGTTACACTCAACC
+
FFFFFFF:FFFFFFFFFFFFFFFFFFFFFFFFFFFFFFFFFFFFFFFFFFFFFFFFFFFFFFFFFFFF
FFF:FFFFFFF:FFFFFFFFFFFFFFFFFFFFFFFFFFFFFFFFFFFFFFFFFFFFFFFFFFFFFFFF
FFFFFFFFFFFFFFF
@A00155:342:HHGFNDSXY:1:1276:13955:5462 2:N:0:GAACCTAG+TCCGCATA
TAAAAATTTGTGGGCTACTCTGGTTTTTCCACCTGAGATCTTAGCTGGTTACAAATCCTCCGTTCTGC
CCTTCTTATATCAATTTGAAATTCACGGGAAGGATCTGGTCTACATGCCGGACGGGGTGAGGTCTGAG
AGTTACACTCAACCA
+
FFFFFFFFFFFFFFFFFFFFFFFFFFFFFFFFFFFFFFFFFFFFFFFFFFFFFFFFFFFFFFFFFFFF
FFFFFF:FFFFFFFFFFFFFFFFFFFFFFFFFFFFFFFFFFFFFFFFFFFFFFFFFFFFFFFFFFFFF
FFFFFFFFFFFFFFF
@A00155:342:HHGFNDSXY:1:1367:10149:7388 2:N:0:GAACCTAG+TCCGCATA
TAAAAATTTGTGGGCTACTCTGGTTTTTCCACCTGAGATCTTAGCTGGTTACAAATCCTCCGTTCTGC
CCTTCTTATATCAATTTGAAATTCACGGGAAGGATCTGGTCTACATGCCGGACGGGGTGAGGTCTGAG
AGTTACACTCAACCA
+
FFFFF:FFFFF:FFFFFFFFFFFF:FFFFFFF:::F,FFF::F:FFFFFFFFFFFF:FFFFFFFFFFF
FFFFFFFF:FFFFF:FF:FFFF:FFFFFFFFFFFFFF,FFFF,FFFF:F,FFFF,FFFFFF:FF:F:FF
FFF:FFFF:FFF,FF
@A00155:342:HHGFNDSXY:1:1616:7319:8907 2:N:0:GAACCTAG+TCCGCATA
TAAAAATTTGTGGGCTACTCTGGTTTTTCCACCTGAGATCTTAGCTGGTTACAAATCCTCCGTTCTGC
CCTTCTTATATCAATTTGAAATTCACGGGAAGGATCTGGTCTACATGCCGGACGGGGTGAGGTCTGAG
AGTTACACTCAACCA
+
FFFFFF:F:FFFFFFFFFFFF:FFFFFFFFFFFFFFFFFFFFFF,FFFFF:FFFFFFFFFFFF:FFFF
FFFFFFFFF:FFFF,F:FFFFFFFFFF:FFFFFFFFFFFFFFFFFF:FFFFFFF:FFFFFF:FFFF:F
FFFFF,FFF,FFFFF

@A00155:342:HHGFNDSXY:1:1652:19633:29418 2:N:0:GAACCTAG+TCCGCATA
TAAAAATTTGTGGGCTACTCTGGTTTTTCCACCTGAGATCTTAGCTGGTTACAAATCCTCCGTTCTGC
CCTTCTTATATCAATTTGAAATTCACGGGAAGGATCTGGTCTACATGCCGGACGGGGTGAGGTCTGAG
AGTTACACTCAACCA
+
FFFFFFFFFFFFFFFFFFFFFFFFFFFFFFFFFFFFFFFFFFFFFFFFFFFFFFFFFFFFFFFFFFFF
FFFFFFFFFFFFFFFFFFFFFFFFFFFFFFFFFFFFFFFFFFFFFFFFFFFFFFFFFFFFFFFFFFFF
FFFFFFFFFFFFFFF
@A00155:342:HHGFNDSXY:1:2164:20853:28557 2:N:0:GAACCTAG+TCCGCATA
TAAAAATTTGTGGGCTACTCTGGTTTTTCCACCTGAGATCTTAGCTGGTTACAAATCCTCCGTTCTGC
CCTTCTTATATCAATTTGAAATTCACGGGAAGTATCTGGTCTACATGCCGGACGGGGTGAGGTCTGAG
AGTTACACTCAACCA
+
FFFFFFFFFFFFFFFFFFFFFFFFFFFFFFFFF,FFFFFFF:FFFFFFFFFFFFFFF:FF:FFFFFFF
FFFFFFFF:F:FFFFFFFFFFFFFFFF::FFF,FFFFF,:FFF,FF:FFFFFFF:::FFF,,FFFFFF
F:FFF,FF:FFFFF:
@A00155:342:HHGFNDSXY:1:2378:28375:18944 2:N:0:GAACCTAG+TCCGCATA
TAAAAATTTGTGGGCTACTCTGGTTTTTCCACCTGAGATCTTAGCTGGTTACAAATCCTCCGTTCTGC
CCTTCTTATATCAATTTGAAATTCACGGGAAGGATCTGGTCTACATGCCGGACGGGGTGAGGTCTGAG
AGTTACACTCAACCA
+
:FFFFFFF,FFFFFFFFFFFFFFFFFFFFFFFFF:FFFFFFFFFFFFFFFFFFFF:F:FFFF::F,:F
FFF,FFFFFF:FFF::FF:FFFFFFFFFFFF:FFFF:FF:,::FFFFFFFFFFFF:FFFFFFFFFFFF
FFFFFFF:FFFF:F:
@A00155:342:HHGFNDSXY:1:2653:9308:13823 2:N:0:GAACCTAG+TCCGCATA
TAAAAATTTGTGGGCTACTCTGGTTTTTCCACCTGAGATCTTAGCTGGTTACAAATCCTCCGTTCTGC
CCTTCTTATATCAATTTGAAATTCACGGGAAGGATCTGGTCTACATGCCGGACGGGGTGAGGTCTGAG
AGTTACACTCAACCA
+
FFFFFFFFFFFFFFFFFFFFFFFFFFFFFFFFFFFFFFFFFFFFFFFFFFFFFFFFFFFFFFFFFFFF
F:FFFFFFFFFFFFFFFFFFFFFFFFFFFFFFFFFFFFFFFFFFFFFFFFFFFFFFFFFFFFFFFFFF
FFFFFFFFFFFFFFF
@A00155:342:HHGFNDSXY:1:2663:6768:11240 2:N:0:GAACCTAG+TCCGCATA
TAAAAATTTGTGGGCTACTCTGGTTTTTCCACCTGAGATCTTAGCTGGTTACAAATCCTCCGTTCTGC
CCTTCTTATATCAATTTGAAATTCACGGGAAGGATCTGGTCTACATGCCGGACGGGGTGAGGTCTGAG
AGTTACACTCAACCA
+
FFFFFFFFFFFFFFFFFFFFFFFFFFFFFFFFFFFFFFFFFFFFFFFFFFFFFFFFFFFFFFFFFFFF
FFFFFFFFFFFFFFFFFFFFFFFFFFFFFFFFFFFFFFFFFFFFFFFFFFFFFFFFFFFFFFFFFFFF
FFFFFFFFFFFFFFF
@A00155:342:HHGFNDSXY:1:1569:29441:4288 2:N:0:GAACCTAG+TCCGCATA
AAAAATTTGTGGGCTACTCTGGTTTTTCCACCTGAGATCTTAGCTGGTTACAAATCCTCCGTTCTGCC
CTTCTTATATCAATTTGAAATTCACGGGAAGGATCTGGTCTACATGCCGGACGGAGTGAGGTCTGAGA
GTTACACTCAACCAT
+
FFFFFFFFFFFFFFFFFFFFFFFFFFFFFFFFFFFFFFFFFFFFFFFFFFFFFFFFFFFFFFFFFFFF
FFFFFFFFFFFFFFFFFFFFFFFFFFFFF:FFFFFFFFFFFFFFFFFFFFFFFFFFFFFFFFFFFFFF
FFF:FFFFFFFFFFF
@A00155:342:HHGFNDSXY:1:1678:4779:24612 2:N:0:GAACCTAG+TCCGCATA
AAAAATTTGTGGGCTACTCTGGTTTTTCCACCTGAGATCTTAGCTGGTTACAAATCCTCCGTTCTGCC
CTTCTTATATCAATTTGAAATTCACGGGAAGGATCTGGTCTACATGCCGGACGGGGTGAGGTCTGAGA
GTTACACTCAACCAT
+
FFFFFFFFFFFF:FFFFFFFFFFFFFFFFFFFFFFFFFFFFFFFFFFFFFFFFFFFFFFFFFFFFFFF

FFFFFFFFFFFFFFFFFFFFFFF,FFFFFFFFFFFFFFFFFFFFFFFFFFFFFFFFFFFFFFFFFFFFF
FFFFFFFFFFFFFFF
@A00155:342:HHGFNDSXY:1:2435:21784:12383 2:N:0:GAACCTAG+TCCGCATA
AAAAATTTGTGGGCTACTCTGGTTTTTCCACCTGAGATCTTAGCTGGTTACAAATCCTCCGTTCTGCC
CTTCTTATATCAATTTGAAATTCACGGGAAGGATCTGGTCTACATGCCGGACGGGGTGAGGTCTGAGA
GTTACACTCAACCAT
+
FFFFFFFFFFFFFFFFFFFFFFFFFFFFFFFFFFFFFFFFFFFFFFFFFFFFFFFFFFFFFFFFFFFF
:FFFFFFFFFFFFFFFFFFFFFF:FFF:FFFFFFFFFFFFFFFFFFFFFFFFFFFFFFFFFFFFFFFFF
FFFFFFFFFFFFFFF
@A00155:342:HHGFNDSXY:1:2346:23954:35712 2:N:0:GAACCTAG+TCCGCATA
AAAATTTGTGGGCTACTCTGGTTTTTCCACCTGAGATCTTAGCTGGTTACAAATCCTCCGTTCTGCCC
TTCTTATATCAATTTGAAATTCACGGGAAGGATCTGGTCTACATGCCGGACGGGGTGAGGTCTGAGAG
TTACACTCAACCATT
+
FFFFFFFFFFFFFFFFFFFFFFFFFFFFFFFFFFFFFFFFFFFFFFFFFFFFFFFFFFFFFFFFFFFF
FFFFFFFFFFFFFFFFFFFFFFFFFFFFFFFFFFFFFFFFFFFFFF:FFFFFFFFFFFFFFFFFFFFFF
FFFFFFFFFFFFFFF
@A00155:342:HHGFNDSXY:1:2360:31123:20572 2:N:0:GAACCTAG+TCCGCATA
AAAATTTGTGGGCTACTCTGGTTTTTCCACCTGAGATCTTAGCTGGTTACAAATCCTCCGTTCTGCCC
TTCTTATATCAATTTGAAAATCACGGGAAGGATCTGGTCTACATGCCGGACGGGGTGAGGTCTGAGAG
TTACACTCAACAATT
+
:FFF,FF:FF,FFFFFFF:,:F,FFFFFFFFFFF,FFFFFF,FF:FFFFFFFFFFF:FFF:FFFFFF
F,F:FFFFFFFF,,FFFFF,F:F::::F:,FFFF,,,:,F::F,FF,FFF,,F:F,FF,:,,:F:,F,
,:::F:,:FF:,F::
@A00155:342:HHGFNDSXY:1:2421:9100:13589 2:N:0:GAACCTAG+TCCGCATA
AAAATTTGTGGGCTACTCTGGTTTTTCCACCTGAGATCTTAGCTGGTTACAAATCCTCCGTTCTGCCC
TTCTTATATCAATTTGAAATTCACGGGAAGGATCTGGTCTACATGCCGGACGGGGTGAGGTCTGAGAG
TTACACTCAACCATT
+
FFFFFFFFFFFFFFFFFFFFFFFFFFFFFFFFFFFFFFFFFFFFFFFFFFFFFFFFFFFFFFFFFF:F
:FFFFFFFFFFFFFFFFFFFFFFFFFFFFFFFFFFFFFFFFFFFFF:FFFFFFFFFFFFFFFFFFFFFF
FFFFF,F:FFF,FF:
@A00155:342:HHGFNDSXY:1:2459:7120:18709 2:N:0:GAACCTAG+TCCGCATA
AAAATTTGTGGGCTACTCTGGTTTTTCCACCTGAGATCTTAGCTGGTTACAAATCCTCCGTTCTGCCC
TTCTTATATCAATTTGAAATTCACGGGAAGGATCTGGTCTACATGCCGGACGGGGTGAGGTCTGAGAG
TTACACTCAACCATT
+
FFFFFFFFFFFFFFFFFFFFFFFFFFFFFFFFFFFFFFFFFFFFFFFFFFFFFFFFF:FFFFFFFFFF
FFFF,FFFFFFFFFFFFFFFFFFFFFFFF,FFFFFFFFFFFFFFFFFFFFFFFFFFFFFFFFFFFFFFF
FFFFFFFFFFFF:FF
@A00155:342:HHGFNDSXY:1:1229:23999:12587 1:N:0:GAACCTAG+TCCGCATA
AAATTTGTGGGCTACTCTGGTTTTTCCACCTGAGATCTTAGCTGGTTACAAATCCTCCGTTCTGCCCT
TCTTATATCAATTTGAAATTCACGGGAAGGATCTGGTCTACATGCCGGACGGAGTGAGGTCTGAGAGT
TACACTCAACCATTG
+
FFFFFFFFFFFFFFFFFFFFFFFFFFFFFFFFFFFFFFFFFFFFFFFFFFFFFFFFFFFFFFFFFFFF
FFFFFFFFFFFFFFFFFFFFFFFFFFFFFFFFFFFFFFFFFFFFFFFFFFFFFFFFFFFFFFFFFFFF
FFFFFFFFFFFFFFF
@A00155:342:HHGFNDSXY:1:1343:20347:17942 1:N:0:GAACCTAG+TCCGCATA
AAATTTGTGGGCTACTCTGGTTTTTCCACCTGAGATCTTAGCTGGTTACAAATCCTCCGTTCTGCCCT
TCTTATATCAATTTGAAATTCACGGGAAGGATCTGGTCTACATGCCGGACGGGGTGAGGTCTGAGAGT
TACACTCAACCATTG

+
FFFFFFFFFFFFFFFFFFFFFFFFFFFFFFFFFFFFFF:FFFFFFFFFFFFFFFFFFFFFFFFFFFFF
FFFFFFFFFFFFF,FFFFFFFFFFFFFFFFFFFFFFFFFFFFFFFFFFFFFFFFFFFFFFFFFFFFFF
FFFFFFFFFFFFFFF
@A00155:342:HHGFNDSXY:1:1441:18954:34319 1:N:0:GAACCTAG+TCCGCATA
AAATTTGTGGGCTACTCTGGTTTTTCCACCTGAGATCTTAGCTGGTTACAAATCCTCCGTTCTGCCCT
TCTTATATCAATTTGAAATTCACGGGAAGGATCTGGTCTACATGCCGGACGGAGTGAGGTCTGAGAGT
TACACTCAACCATTG
+
FF,F:FFFFFFFFFFFFFFFFFFFFFFFFFFFFFFFFFFFFFFFFFFFFFFFFFFFFFFFFFFFFFFF
FFFFFFFFFFFFFFFFFFFFFFFFFFFFFFF:FFFFFFFFFFFFFFFFFFFFFFFFFFFFFFFFFFFF
FFFFFFFFFFFFFFF
@A00155:342:HHGFNDSXY:1:2302:29071:9565 1:N:0:GAACCTAG+TCCGCATA
AAATTTGTGGGCTACTCTGGTTTTTCCACCTGAGATCTTAGCTGGTTACAAATCCTCCGTTCTGCCCT
TCTTATATCAATTTGAAATTCACGGGAAGGATCTGGTCTACATGCCGGACGGGGTGAGGTCTGAGAGT
TACACTCAACCATTG
+
FFFFFFFFF,:FFFFF:FFFFFFFFF:FF,FF,F,FFF:FF,FF:FFFF,:FFFFFF,FFFFFF:FFF
F,FFFFFF::::FFFFFFFF,FFFFFF:F,F,FFF:F:FF,FF:::FFFFFF,FFFFFFF:FFF,FFF
FFFFFFFFFFF,FF:
@A00155:342:HHGFNDSXY:1:2313:2889:17769 1:N:0:GAACCTAG+TCCGCATA
AAATTTGTGGGCTACTCTGGTTTTTCCACCTGAGATCTTAGCTGGTTACAAATCCTCCGTTCTGCCCT
TCTTATATCAATTTGAAATTCACGGGAAGGATCTGGTCTACATGCCGGACGGGGTGAGGTCTGAGAGT
TACACTCAACCATTG
+
FFFFFFF:FFFFFFFFFFFF:FFFFFFFFFFFFFFFFFFFFFFFFFFFFFFFFFFFFFFFFFFFFFFF
FFFFFFFFFFF:FFFFFFFFFFFFFFFFFFFFF,FFFFFFFFFFFFFFFFFFFFFFFFFFFFFFFFFF
FFFFFFFFFFFFFFF
@A00155:342:HHGFNDSXY:1:2346:23954:35712 1:N:0:GAACCTAG+TCCGCATA
AAATTTGTGGGCTACTCTGGTTTTTCCACCTGAGATCTTAGCTGGTTACAAATCCTCCGTTCTGCCCT
TCTTATATCAATTTGAAATTCACGGGAAGGATCTGGTCTACATGCCGGACGGGGTGAGGTCTGAGAGT
TACACTCAACCATTG
+
FFFFFFFFFFFFFFFFFFFFFFFFFFFFFFFFFFFFFFFFFFFFFFFFFFFFFFFFFFFFFFFFFFFF
FFFFFFFFFFFFFFFFFFFFFFFFFFFFFFFFFFFFFFFFFFFFFFFFFFFFFFFFFFFFFFFFFFFF
FFFFFFFFFFFFFFF
@A00155:342:HHGFNDSXY:1:2421:9100:13589 1:N:0:GAACCTAG+TCCGCATA
AAATTTGTGGGCTACTCTGGTTTTTCCACCTGAGATCTTAGCTGGTTACAAATCCTCCGTTCTGCCCT
TCTTATATCAATTTGAAATTCACGGGAAGGATCTGGTCTACATGCCGGACGGGGTGAGGTCTGAGAGT
TACACTCAACCATTG
+
FFFFFFFFF:FFFFFFFFFFFFFFFFFFFFFFFFFFFFFFFFFFFFFFFFFFFFFFFFFFFFFFFFFF
FFFFFFFFFFFFFFFFFFFFFFFFFFFFFFFFFFFFFFFFFFFFFFFFFFFFFFFFFFFFFFFFFFFF
FFFFFFFFFFFFFFF
@A00155:342:HHGFNDSXY:1:2459:7120:18709 1:N:0:GAACCTAG+TCCGCATA
AAATTTGTGGGCTACTCTGGTTTTTCCACCTGAGATCTTAGCTGGTTACAAATCCTCCGTTCTGCCCT
TCTTATATCAATTTGAAATTCACGGGAAGGATCTGGTCTACATGCCGGACGGGGTGAGGTCTGAGAGT
TACACTCAACCATTG
+
FFFFFFFFFFFFFFFFFFFFFFF:FFFFFFFFFFFFFFFFFFFFFFFFFFFFFFFFFFFFFFFFFFFF
FFFFFFFFFF:FFFFFFF:FFFFFFFFFFFFFFFFFFFFFFFFFFFFFFFFFFFFFFFFFFFFFFFFF
FFFFFFFFFFFFFFF
@A00155:342:HHGFNDSXY:1:2430:24180:31344 1:N:0:GAACCTAG+TCCGCATA
AAATTTGTGGGCTACTCTGGTTTTTCCACCTGAGATCTTAGCTGGTTACAAATCCTCCGTTCTGCCCT

TCTTATATCAATTTGAAATTCACGGGAAGGATCTGGTCTACATGCCGGACGGAGTGAGGTCTGAGAGT
TACACTCAACCATTG
+
F:FFFFFFFFFFFFFFFFFFFFFFFFFFFFFFFFFFFFFFFFFFFFFFFFFFFFFFFFFFFFFFFFFF
FFFFFFFFFF,FFFFFFF:FFFFFFFFFFFFFFFFFFFFFFFFFFFFFFFFFFFFFFFFFFFFFFFFF
FFFFFFFFFFFFFFF
@A00155:342:HHGFNDSXY:1:2433:22444:24925 1:N:0:GAACCTAG+TCCGCATA
AAATTTGTGGGCTACTCTGGTTTTTCCACCTGAGATCTTAGCTGGTTACAAATCCTCCGTTCTGCCCT
TCTTATATCAATTTGAAATTCACGGGAAGGATCTGGTCTACATGCCGGACGGGGTGAGGTCTGAGAGT
TACACTCAACCATTG
+
FFFFFFFFFFFFFFFFFFFFFFFFFFFFFFFFFFFFFFFFFFFFFFFFFFFFFFFFFFFFFFFFFFFF
FFFFFFFFFFFFFFFFFF:FFFFFFFFFFFFFFFFFFFFFFFFFFFFFFFFFFFFFFFFFFFFFFFFF
FFFFFFFFFFFFFFF
@A00155:342:HHGFNDSXY:1:2541:24397:29403 1:N:0:GAACCTAG+TCCGCATA
AAATTTGTGGGCTACTCTGGTTTTTCCACCTGAGATCTTAGCTGGTTACAAATCCTCCGTTCTGCCCT
TCTTATATCAATTTGAAATTCACGGGAAGGATCTGGTCTACATGCCGGACGGGGTGAGGTCTGAGAGT
TACACTCAACCATTG
+
FFFFFFFFFFF,FFFFFF:FFFFFFFF:FFFFFFFFFFFFF:FFFFFFFFFFFF:FF:FFFFFF:::F
FFFFFFFFFF:FFFFF::FFFFFFFF:FFFFF:,F,FFFFFFFFFFFF:FFFFFFFF,FF:FFFFFFF
FFFFFFFFFFFFFFF
@A00155:342:HHGFNDSXY:1:2514:26115:4726 1:N:0:GAACCTAG+TCCGCATA
AAATTTGTGGGCTACTCTGGTTTTTCCACCTGAGATCTTAGCTGGTTACAAATCCTCCGTTCTGCCCT
TCTTATATCAATTTGAAATTCACGGGAAGGATCTGGTCTACATGCCGGACGGAGTGAGGTCTGAGAGT
TACACTCAACCATTG
+
FFFFFFFFFFFFFFFFFF:FFFFFFFFFFFFFFFFFFFFFFFFFFFFFFFFFFFFFFFFFFFFFFFF:
FFFFFFFFFFF:FFFFFFFFFFFFFFFFFFFFFFFFFFFFFFFFFFFFFFFFFFFFFFFFFFFFFFFF
FFFFFFFFFFFFFFF
@A00155:342:HHGFNDSXY:1:2676:26684:36652 1:N:0:GAACCTAG+TCCGCATA
AAATTTGTGGGCTACTCTGGTTTTTCCACCTGAGATCTTAGCTGGTTACAAATCCTCCGTTCTGCCCT
TCTTATATCAATTTGAAATTCACGGGAAGGATCTGGTCTACATGCCGGACGGGGTGAGGTCTGAGAGT
TACACTCAACCATTG
+
FFFFFFFFFFFFFFFFFFFFFFFFFFFFFFFFFFFFFFFFFFFFFFFFFFFFFFFFFFFFFFFFFFFF
FFFFFFFFFFFFFFFFFFFFFFFFFFFFFFFFFFFFFFFFFFFFFFFFFFFFFFFFFFFFFFFFFFFF
FFFFFFFFFFFFFFF
@A00155:342:HHGFNDSXY:1:1412:29270:4022 2:N:0:GAACCTAG+TCCGCATA
AAATTTGTGGGCTACTCTGGTTTTTCCACCTGAGATCTTAGCTGGTTACAAATCCTCCGTTCTGCCCT
TCTTATATCAATTTGAAATTCACGGGAAGGATCTGGTCTACATGCCGGACGGGGTGAGGTCTGAGAGT
TACACTCAACCATTG
+
FFFFFFFFFFFFFFFFFFFFFFFFFFFFFFFFFFFFFF:FFFFFFFFFFFFFFFFFFFFFFFFFF,FF
F:FFFFFFFFFFFFFFFFFFFFFFFFFFFFFFFFFFFFFFFFFFFFFFFFFFFFFFFFFFFFFFFFFF
FFFFFFFF:FFFFFF
@A00155:342:HHGFNDSXY:1:2559:11442:31234 1:N:0:GAACCTAG+TCCGCATA
AATTTGTGGGCTACTCTGGTTTTTCCACCTGAGATCTTAGCTGGTTACAAATCCTCCGTTCTGCCCTT
CTTATATCAATTTGAAATTCACGGGAAGGATCTGGTCTACATGCCGGACGGGGTGAGGTCTGAGAGTT
ACACTCAACCATTGG
+
FFFFFFFFFFFFFFFFF:FFFFFFFFFFFFFF:FFFFFFFFFF:FFFFFFFFFFFF:FFFFFFFFFFF
FFFFFFFFFF:FFFFFFFFFFF:FFFFFFFFFFFFFFFFFFFFFFFFFFFFFFFFFFFFFFFFFFFFF
FFFFFFFFFFFFFFF

@A00155:342:HHGFNDSXY:1:2110:6280:5885 2:N:0:GAACCTAG+TCCGCATA
AATTTGTGGGCTACTCTGGTTTTTCCACCTGAGATCTTAGCTGGTTACAAATCCTCCGTTCTGCCCTT
CTTATATCAATTTGAAATTCACGGGAAGGATCTGGTCTACATGCCGGACGGGGTGAGGTCTGAGAGTT
ACACTCAACAGATCG
+
F:FFFFFFFFFFFFFFFFFFFFFFFFFFFFFFFFFFFFFFFFFFFFFFFFFFFFFFFFFFFFFFF,FF
FFFFFFFFFFFFFF:FFFFFFFFFFFFFFFFFFFFFFF:FFFFFFFFFFFFFFFFFFFFFFFFFFFFF
FFFFFFFFFFFFF:F
@A00155:342:HHGFNDSXY:1:2152:21558:9737 1:N:0:GAACCTAG+TCCGCATA
TTTGTGGGCTACTCTGGTTTTTCCACCTGAGATCTTAGCTGGTTACAAATCCTCCGTTCTGCCCTTCT
TATATCAATTTGAAATTCACGGGAAGGATCTGGTCTACATGCCGGACGGGGTGAGGTCTGAGAGTTAC
ACTCAACCATT
+
FFFFFF,F:,FFFFF,:FFFFFF:FFFFFFFFF,FFFFFFFFFFFFF,FF:FFFF:FF,FF:FFFF,F
FFF:,FFFFFF,FFFFF:FFFF,F:FF:F:FFFF:FF,,:,FFF:,FF::FFF,FFFFFFFF,FFFFF
FFFFFFFFFFF
@A00155:342:HHGFNDSXY:1:1131:29812:16423 1:N:0:GAACCTAG+TCCGCATA
TTTGTGGGCTACTCTGGTTTTTCCACCTGAGATCTTAGCTGGTTACAAATCCTCCGTTCTGCCCTTCT
TATATCAATTTGAAATTCACGGGAAGGATCTGGTCTACATGCCGGACGGGGTGAGGTCTGAGAGTTAC
ACTCAACCATTGGAG
+
FFFFFFFFFFFFFFFFFFFFFFFFFFFFFFFFFFFFFFFFFFFFFFFFFFFFFFFFFFFFFFFFFFFF
FFFFFFFFFFFFFFFFFFFFFFFFFFFFFFFFFFFFFFFFFFFFFFFFFFFFFFFFFFFFFFFFFFFF
FFFFFFFFFFFFFFF
@A00155:342:HHGFNDSXY:1:1165:5204:21559 1:N:0:GAACCTAG+TCCGCATA
TTTGTGGGCTACTCTGGTTTTTACACCTGAGATCTTAGCTGGTTACAAATCCTCCGTTCTGCCCTTCT
TATATCAATTTGAAATTCACGGGAAGGATCTGGTCTACATGCCGGACGGGGTGAGGTCTGAGAGTTAC
ACTCAACCATTGGAG
+
FFFFFFFFFFFFFFFFFFFFFF,FFFFFFFFFFFFFFFFFFFFFFFFFFFFFFFFFFFFFFFFFFFFF
FFFFFFFFFFFF:FFFFF:FFFFF:FFFFFFFFFFFFFFFFFFFFFFFFFFFFFFFFFFFFFFFFFF
FFFFFFFFFFFFFFF
@A00155:342:HHGFNDSXY:1:1259:10050:18208 1:N:0:GAACCTAG+TCCGCATA
TTTGTGGGCTACTCTGGTTTTTCCACCTGAGATCTTAGCTGGTTACAAATCCTCCGTTCTGCCCTTCT
TATATCAATTTGAAATTCACGGGAAGGATCTGGTCTACATGCCGGACGGGGTGAGGTCTGAGAGTTAC
ACTCAACCATTGGAG
+
FFFFFFFFFFFFFFFFFFFFFFFFFFFFFFFFFFFFFFFFFFFFFFF:FFFFFFFFFFFFFFFFFFFF
FFFFFFFFF:FFFFFFFFFFFFFF:FFFFFFFFFF:FFFFFFFFFFFFFFFFFFFFFFFFFFFFFFFF
FFFFFFFFF:FFFFF
@A00155:342:HHGFNDSXY:1:1309:15411:8797 1:N:0:GAACCTAG+TCCGCATA
TTTGTGGGCTACTCTGGTTTTTCCACCTGAGATCTTAGCTGGTTACAAATCCTCCGTTCTGCCCTTCT
TATATCAATTTGAAATTCACGGGAAGGATCTGGTCTACATGCCGGACGGAGTGAGGTCTGAGAGTTAC
ACTCAACCATTGGAG
+
:FFFFFFFFFFFFFFFFFFFFFFFFFF:FFFFFFFFFFFFFFFFFFFFFFFFFFFFFFFFFFFFFFFF
FFFFFFFFFFFFFFFFFFFFFFFFFFFFFFFFFFFFFFF:FFFFFFFFFFFFFFFFFFFFFF:F,FFF
FFFFFFFFFFFFFFF
@A00155:342:HHGFNDSXY:1:1322:4074:12493 1:N:0:GAACCTAG+TCCGCATA
TTTGTGGGCTACTCTGGTTTTTCCACCTGAGATCTTAGCTGGTTACAAATCCTCCGTTCTGCCCTTCT
TATATCAATTTGAAATTCACGGGAAGGATCTGGTCTACATGCCGGACGGGGTGAGGTCTGAGAGTTAC
ACTCAACCATTGGAG
+
FFFFFFFFFFFFFFFFFFFFFFFFFFFFFFFFFFFFFFFFFFFFFFFFFFFFFFFFFFFFFFFFFF,F

```
FFFFFFFFF:FFFFFFFFFFFFF:FFFFF,F:FFFFFFFFFFFFFFFFFFFFFF:FFFFFFFFFFFFFFF
FFFFFFFFFFFFFFF
@A00155:342:HHGFNDSXY:1:1412:29270:4022 1:N:0:GAACCTAG+TCCGCATA
TTTGTGGGCTACTCTGGTTTTTCCACCTGAGATCTTAGCTGGTTACAAATCCTCCGTTCTGCCCTTCT
TATATCAATTTGAAATTCACGGGAAGGATCTGGTCTACATGCCGGACGGGGTGAGGTCTGAGAGTTAC
ACTCAACCATTGGAG
+
FFFFFFFF:FFFFFFFFFFFFFFFFFFFFFFFFFFFFFFFFFFFFFFFFFFFFFFFFFFFFFFFFFFFFF
FFFFFFFFFFFFFFFFFFFFFFFFFFFFFFFFFFFFFFFFFFFFFFFFFFFFFFFFFFFFFFFFFFFFFF
FFFFFFFFFFFFFFF
@A00155:342:HHGFNDSXY:1:1470:14145:32440 1:N:0:GAACCTAG+TCCGCATA
TTTGTGGGCTACTCTGGTTTTTCCACCTGAGATCTTAGCTGGTTACAAATCCTCCGTTCTGCCCTTCT
TATATCAATTTGAAATTCACGGGAAGGATCTGGTCTACATGCCGGACGGGGTGAGGTCTGAGAGTTAC
ACTCAACCATTGGAG
+
FFFFFFFFFFFFFFFFFFFFFFFFFFFFFFFFFFFFFFFFFFFFFFFFFFFFFFFFFFFFFFFFFFFFFF
FFFFFFFFFFFFFFFFFFFFFFFFFFFFFFFFFFFFFFFFFFFFFFFFFFFFFFFFFFFFFFFFFFFFFF
FFFFFFFFFFFFFFF
@A00155:342:HHGFNDSXY:1:2116:9136:16094 1:N:0:GAACCTAG+TCCGCATA
TTTGTGGGCTACTCTGGTTTTTCCACCTGAGATCTTAGCTGGTTACAAATCCTCCGTTCTGCCCTTCT
TATATCAATTTGAAATTCACGGGAAGGATCTGGTCTACATGCCGGACGGGGTGAGGTCTGAGAGTTAC
ACTCAACCATTGGAG
+
:FFFFFFFFF:FFFFFFFFFFFFFFFFFFFFFFFFFFFFFFFFFFFFFFFFFFFFFFFFFFFFFFFFFFF
FFFFFFFFFFFFFFF,FFFFFFFFFFFFFFFFFFFFFFFFFFFFFFFFFFFFFFFFFFFFFFFFFFFFFF
FFFFFFFFFFFFFFF
@A00155:342:HHGFNDSXY:1:2138:4065:6934 1:N:0:GAACCTAG+TCCGCATA
TTTGTGGGCTACTCTGGTTTTTCCACCTGAGATCTTAGCTGGTTACAAATCCTCCGTTCTGCCCTTCT
TATATCAATTTGAAATTCACGGGAAGGATCTGGTCTACATGCCGGACGGGGTGAGGTCTGAGAGTTAC
ACTCAACCATTGGAG
+
FFFFFFFFF,FFFFFFFFFFFFFFFFFFFFFFFFFF:FFFFFFFFFFFFFFFFFFFFFFFFFFF:FFFFF
FFFFFFFFFFFFFFFFFFFFFFFFFFFFFFFFFFFFFFFFFFFFFF:FFFFFFFFFFFFFFFFFFFFFFF
FFFFFFFFFFFFFFF
@A00155:342:HHGFNDSXY:1:2138:4273:7357 1:N:0:GAACCTAG+TCCGCATA
TTTGTGGGCTACTCTGGTTTTTCCACCTGAGATCTTAGCTGGTTACAAATCCTCCGTTCTGCCCTTCT
TATATCAATTTGAAATTCACGGGAAGGATCTGGTCTACATGCCGGACGGGGTGAGGTCTGAGAGTTAC
ACTCAACCATTGGAG
+
FF:F:::F,FFFFF::FF,:FFFFFFFFFFFFFFFFFFFFFFFFFFFFFFFFFFFFFFFFFFFFFFFFFF
FFFFFFFFFFFFFFF:FFFFFFFFFFFFFFF:FFFFF,:FFFFFFFFFFFFFFFFFFFFFFFFFFFFFFF
FFFFFFFFFFFFFFF
@A00155:342:HHGFNDSXY:1:2257:19587:7326 1:N:0:GAACCTAG+TCCGCATA
TTTGTGGGCTACTCTGGTTTTTCCACCTGAGATCTTAGCTGGTTACAAATCCTCCGTTCTGCCCTTCT
TATATCAATTTGAAATTCACGGGAAGGATCTGGTCTACATGCCGGACGGGGTGAGGTCTGAGAGTTAC
ACTCAACCATTGGAG
+
FFFFFFFFFFFFFFFFFFFFFFFFFFFFFFFFFFFFFFFFFFFFFFFFFFFFFFFFFFFFFFFFFFFFFF
FFFFFFFFFFFFFFFFFFFFFFFFFFFFFFFFFFFFFFFFFFFFFFFFFFFFFFFFFFFFFFFFFFFFFF
FFFFFFFFFFFFFFF
@A00155:342:HHGFNDSXY:1:2436:6714:22075 1:N:0:GAACCTAG+TCCGCATA
TTTGTGGGCTACTCTGGTTTTTCCACCTGAGATCTTAGCTGGTTACAAATCCTCCGTTCTGCCCTTCT
TATATCAATTTGAAATTCACGGGAAGGATCTGGTCTACATGCCGGACGGGGTGAGGTCTGAGAGTTAC
ACTCAACCATTGGAG
```

+
FFFFFFFFFFFFFFFFFFFF:FFFFFFFFFFFFFFFFFFFFFFFFFFFFFFFFFFFFFFFFFFFFFFF
FFFFFFFFFFFFFFFFFFFFFFF,FFFFFFFFFFFFFFFFFFFFFFFFFFFFFFFFFFFFFFFF:FFF
FFFFFFFFFFFFFFF
@A00155:342:HHGFNDSXY:1:2456:15013:23891 1:N:0:GAACCTAG+TCCGCATA
TTTGTGGGCTACTCTGGTTTTTCCACCTGAGATCTTAGCTGGTTACAAATCCTCCGTTCTGCCCTTCT
TATATCAATTTGAAATTCACGGGAAGGATCTGGTCTACATGCCGGACGGGGTGAGGTCTGAGAGTTAC
ACTCAACCATTGGAG
+
:FFFFFFFFFFFFFFFFFFFFFFFFFFFFFFFFFFFFFFFFFFFFFFFFFFFFFFFFFFFFFFFFFFF
FFFFFF:FFFFFFFFFFFFFFFFFFFFFFFFFFFFFFFFFFFFFFFFFFFFFFFFFFFFFFFFFFFFF
FFFFFFFFFFFFFFF
@A00155:342:HHGFNDSXY:1:2622:6533:25332 1:N:0:GAACCTAG+TCCGCATA
TTTGTGGGCTACTCTGGTTTTTCCACCTGAGATCTTAGCTGGTTACAAATCCTCCGTTCTGCCCTTCT
TATATCAATTTGAAATTCACGGGAAGGATCTGGTCTACATGCCGGACGGGGTGAGGTCTGAGAGTTAC
ACTCAACCATTGGAG
+
FFFFFF,FFFFFFFFFFFFFFFFFFFFFFFFFFFFF,FFFFFFFFFFFFFFFFFFFFFFFFFFFFFFF
FFFFFFFF,FFFFFFFFFFFFFFFFFFFFFFFFFFFFFFFF:FFFFFFFFFFFFFFFFFFFFFFFFFF
FFFFFFFFFFFFFFF
@A00155:342:HHGFNDSXY:1:1340:27923:36072 2:N:0:GAACCTAG+TCCGCATA
TTTGTGGGCTACTCTGGTTTTTCCACCTGAGATCTTAGCTGGTTACAAATCCTCCGTTCTGCCCTTCT
TATATCAATTTGAAATTCACGGGAAGGATCTGGTCTACATGCCGGACGGGGTGAGGTCTGAGAGTTAC
ACTCAACCATTGGAG
+
FFFFFFFFFFFFFFFFFFFFFFFFFFFFFFFFFF:FFFFFFFFFFFFFFFFFFFFFFFFFFFFFFFF:
FFFFFFFFFFFFFFFFFFFFFFFFFFFFFFFFF:FFFFFFFFFFFFFFFFFF:FFFFFFFFFFFFFFF
FFFFFFFFFF:FFFF
@A00155:342:HHGFNDSXY:1:1415:32316:10207 1:N:0:GAACCTAG+TCCGCATA
TTGTGGGCTACTCTGGTTTTTCCACCTGAGATCTTAGCTGGTTACAAATCCTCCGTTCTGCCCTTCTT
ATATCAATTTGAAATTCACGGGAAGGATCTGGTCTACATGCCGGACGGGGTGAGGTCTGAGAGTTACA
CTCAACCATTGGAGC
+
:FFFFFFFFFFFFFFFFFFFFFFFFFFFFFFFFFFFFFFFFFFFFFFFFFFFFFFFFFFFFFFFFFFF
FFFFFF,FFFFFFFFFFFF:FFFFFFFFFFFFFFFFFFFFFFFFFFFFFFFFFFFFFFFFFFFFFFFF
FFFF:FFFFFFFFFF
@A00155:342:HHGFNDSXY:1:1612:7455:22827 1:N:0:GAACCTAG+TCCGCATA
TGTGGGCTACTCTGGTTTTTCCACCTGAGATCTTAGCTGGTTACAAATCCTCCGTTCTGCCCTTCTTA
TATCAATTTGAAATTCACGGGAAGGATCTGGTCTACATGCCGGACGGGGTGAGGTCTGAGAGTTACAC
TCAACCATTGGAGAA
+
FFFFFFFFFFFFFFFF:FFFFFFFFFFFFFFFFFFFFFFFFFFFFFFFFFFFF:FFFFFFFFFFFFFF
FFFFFFFFFFFFFFFF,FFFF:FFFFFFFFFFFFFFFFFFFFFFFFFFFFFFFFFFFFFFFFFFFFFF
FFFFFFFFFFFFFFF
@A00155:342:HHGFNDSXY:1:2565:12201:31516 2:N:0:GAACCTAG+TCCGCATA
TGTGGGCTACTCTGGTTTTTCCACCTGAGATCTTAGCTGGTTACAAATCCTCCGTTCTGCCCTTCTTA
TATCAATTTGAAATTCACGGGAAGGATCTGGTCTACATGCCGGACGGGGTGAGGTCTGAGAGTTACAC
TCAACCATTGGAGAA
+
FFFFFFFFFFF:FFFFFFFFFFFFFFFFFFFFFFFFFFFFFFFFFFFFFFFFFFFFFFFFFFFFFFF:
FFFFFFFFFFFFFFFFFFFFFFFFFFFFFFFFFFFFFFFFFFFFFFFFFFFFFFFFFFFFFFFFF:FF
FFFFFFFFFF:FFFF
@A00155:342:HHGFNDSXY:1:1139:21124:23234 1:N:0:GAACCTAG+TCCGCATA
GTGGGCTACTCTGGTTTTTCCACCTGAGATCTTAGCTGGTTACAAATCCTCCGTTCTGCCCTTCTTAT

ATCAATTTGAAATTCACGGGAAGGATCTGGTCTACATGCCGGACGGAGTGAGGTCTGAGAGTTACACT
CAACCATTGGAGAAC
+
FFFFFFFF:FF:FF,FFFFFFFFFFFFFFFFF:FFFFFFFFFFFFF:FFFFFFF,FFFFFFFF:FFFFF
,FFF,,:FFFFFFFF:FFFFFFFFFFFFFFFFFFFFFFFFFFFFFFFFFFFFF:FFFFFFFFFFFFFFF
FFFFFFFFFFFFFF:F
@A00155:342:HHGFNDSXY:1:1603:17110:12994 2:N:0:GAACCTAG+TCCGCATA
GTGGGCTACTCTGGTTTTTCCACCTGAGATCTTAGCTGGTTACAAATCCTCCGTTCTGCCCTTCTTAT
ATCAATTTGAAATTCACGGGAAGGATCTGGTCTACATGCCGGACGGGGTGAGGTCTGAGAGTTACACT
CAACCATTGGAGAAC
+
FFFFFFFFF,FFFFFFFFFFFFFFFFFFFFFFFFFFFFFFFFFFFFFFFFFFFFFFFFFFFFFFF:FFF
FFFFFFFFFFFFFFFFFFFFFFFFFFFFFFFFFFFFFFF:FFFFFFFFFFFFF:FFFFFFFF:FFFFFF
FFFFFFFFFFFFFFF
@A00155:342:HHGFNDSXY:1:1603:17146:13182 2:N:0:GAACCTAG+TCCGCATA
GTGGGCTACTCTGGTTTTTCCACCTGAGATCTTAGCTGGTTACAAATCCTCCGTTCTGCCCTTCTTAT
ATCAATTTGAAATTCACGGGAAGGATCTGGTCTACATGCCGGACGGGGTGAGGTCTGAGAGTTACACT
CAACCATTGGAGAAC
+
FFFFFFFFFFFFFFFFFFFFFFFFFFFFFFFFFFFFFFFFFFFFFFFFFFFFFFFFFFFFF:F,FFFFF
FFFFFFFFFFFFFFFFFFFFFFFFFFFFFFFFFFFFFF:FFFFFFFFFFFFFFFFFFFFFFFFFFFFFF
FFFFFFFFFFFFFFF
@A00155:342:HHGFNDSXY:1:2266:30626:29919 2:N:0:GAACCTAG+TCCGCATA
GTGGGCTACTCTGGTTTTTCCACCTGAGATCTTAGCTGGTTACAAATCCTCCGTTCTGCCCTTCTTAT
ATCAATTTGAAATTCACGGGAAGGATCTGGTCTACATGCCGGACGGAGTGAGGTCTGAGAGTTACACT
CAACCATTGGAGAAC
+
FFFFFFFFFFFFFFFFFFFFFFFFFFFFFFFFFFFFFFFFF:FFFFFFFFFFFFFFFFFFFFFFFFF:F
FFFFFFFFFFFFFFFFFFFFFFFFFFFFFFFFFFFFFFFFFFFFFFFFFFFFFFFFFFFFFFFFFFFFF
FFFFFFFFFFFFFFF
@A00155:342:HHGFNDSXY:1:2603:1145:11083 1:N:0:GAACCTAG+TCCGCATA
TGGGCTACTCTGGTTTTTCCACCTGAGATCTTAGCTGGTTACAAATCCTCCGTTCTGCCCTTCTTATA
TCAATTTGAAATTCACGGGAAGGATCTGGTCTACATGCCGGACGGGGTGAGGTCTGAGAGTTACACTC
AACCATTGGAG
+
F:,:FFFF,F:,:,F:F:FFF:,F:F,F,F:FFFFF:FFF,F,FFFFFF:,FFFFFF,FF:FFFFFFFF
:F:F,FFFFFFF:FF,:F:FFFFFFF:,F:,FFFFFFFFFFFFFFFFFFFF:FFFFFFFFFFFF,FFFF
FFFFFFFFFFF
@A00155:342:HHGFNDSXY:1:1472:24099:9001 1:N:0:GAACCTAG+TCCGCATA
TGGGCTACTCTGGTTTTTCCACCTGAGATCTTAGCTGGTTACAAATCCTCCGTTCTGCCCTTCTTATA
TCAATTTGAAATTCACGGGAAGGATCTGGTCTACATGCCGGACGGGGTGAGGTCTGAGAGTTACACTC
AACCATTGGAGAACG
+
FFFFFFFFFFFFF:FFFFFFFFF:FFFFFFFFFFFFFFFFFFFFFFFFFFFFFFFF:FFFFF,FFFFFF
FFFFFFFFFFFFFFFFFFFFFFFFFFFFFFFFFFFFFFFFFFFFFFFFFFFFFFFFFFFFFFFFFFFFF
FFFFFFFFFFFFFFF
@A00155:342:HHGFNDSXY:1:1529:6081:35477 1:N:0:GAACCTAG+TCCGCATA
TGGGCTACTCTGGTTTTTCCACCTGAGATCTTAGCTGGTTACAAATCCTCCGTTCTGCCCTTCTTATA
TCAATTTGAAATTCACGGGAAGGATCTGGTCTACATGCCGGACGGGGTGAGGTCTGAGAGTTACACTC
AACCATTGGAGAACG
+
FF:F:FFFFFFFFFFFFFFF:FFFFFFFFF,FFFFFFFFFFFFFFFFFFFFFFFFFFFFFFFFFFFF:F
FFF:FFFFFFFFFFFFFFFFFFFFFFFFFFFFFFFFFFFFFFFFFFFFFFFFFFFFFFFFFFFFFFFFF
FFFFFFFFFFFFFFF

@A00155:342:HHGFNDSXY:1:2545:24560:29152 1:N:0:GAACCTAG+TCCGCATA
TGGGCTACTCTGGTTTTTCCACCTGAGATCTTAGCTGGTTACAAATCCTCCGTTCTGCCCTTCTTATA
TCAATTTGAAATTCACGGGAAGGATCTGGTCTACATGCCGGACGGGGTGAGGTCTGAGAGTTACACTC
AACCATTGGAGAACG
+
FFFFFFFFFFFFFFFFFFFFFFFFFFFFFFFFFFFFFFFFFFFFFFFFFFFFFFFFFFFFFFFFFFFF
FFFFFFFFFFFFFFFFFFFFFFFFFFFFFFFFFFFFFFFFFFFFFFFFFFFFFFFFFFFFFFFFFFFF
FFFFFFFFFFFFFFF
@A00155:342:HHGFNDSXY:1:1608:10691:14904 1:N:0:GAACCTAG+TCCGCATA
GGGCTACTCTGGTTTTTCCACCTGAGATCTTAGCTGGTTACAAATCCTCCGTTCTGCCCTTCTTATAT
CAATTTGAAATTCACGAGAAGGATCTGGTCTACATGCCGGACGGGGTGAGGTCTGAGAGTTACACTCA
ACCATTGG
+
FFFFFFFFFFFFFFFFFFFFFFFF:FFFFF:FFFFFFFFFFF:FFFFFFFFFFFFFFFFFFFFFF::F
FF,FF:FFFFFFFFFFFF,FFFFFFFFFFFFFFFFFFFFFFFFFFFFFFFFFFFFFFFFFFFFFFFFF
FFFFFFFF
@A00155:342:HHGFNDSXY:1:2349:25943:10786 1:N:0:GAACCTAG+TCCGCATA
CTGCTACTCTGGTTTTTCCACCTGAGATCTTAGCTGGTTACAAATCCTCCGTTCTGCCCTTCTTATAT
CAATTTGAAATTCACGGGAAGGATCTGGTCTACATGCCGGACGGAGTGAGGTCTGAGAGTTACACTCA
ACCATTGG
+
FFFFFFFFFFFFFFFFFFFFFFFFFFFFFFF:FFFFFFFFFFFFFFFFFFFFFFFFFFFFFFFFFFFF
FFFFFFFFFFFFFFFFFFFFFFFFFFFFFFFFFFFFFFFFFFFFFFFFFFFFFFFFFFFFFFFFFFFF
FFFFFFFF
@A00155:342:HHGFNDSXY:1:2359:14163:29872 1:N:0:GAACCTAG+TCCGCATA
GGGCTACTCTGGTTTTTCCACCTGAGATCTTAGCTGGTTACAAATCCTCCGTTCTGCCCTTCTTATAT
CAATTTGAAATTCACGGGAAGGATCTGGTCTACATGCCGGACGGGGTGAGGTCTGAGAGTTACACTCA
ACCATTGG
+
FFFFFFFFFFFFFFFFFFFFFFFFFFFFFFFFFFFFFFFFFFFFFFFFFFFFFFFFFFFFFFFFFFFF
FFFFFFFF:FFFFFFFFFFFFFFFFFFFFFFFFFFFFFFFFFFFFFFFFFFFFFFFFFFFFFFFFFFF
FFFFFFFF
@A00155:342:HHGFNDSXY:1:2447:17219:30185 1:N:0:GAACCTAG+TCCGCATA
GGGCCACTCTGGTTTTTCCACCTGAGATCTTAGCTGGTTACAAATCCTCCGTTCTGCCCTTCTTATAT
CAATTTGAAATTCACGGGAAGGATCTGGTCTACATGCCGGACGGGGTGAGGTCTGAGAGTTACACTCA
ACCATTGG
+
FFFFFFFFFFFFFFFFFFFFFFFFFFFFFFFFFFFFFFFFFFFFFFFFFFFFFF,FFFFFFFFFFFFF
FFF:FFFFFFFFFFFFFFFFFFFFFFFFFFFFFFFFFFFFFFFFFFFFFFFFFFFFFFFFFFFFFFFF
FFFFFFFF
@A00155:342:HHGFNDSXY:1:2501:10339:18364 1:N:0:GAACCTAG+TCCGCATA
GGGCTACTCTGGTTTTTCCACCTGAGATCTTAGCTGGTTACAAATCCTCCGTTCTGCCCTTCTTATAT
CAATTTGAAATTCACGGGAAGGATCTGGTCTACATGCCGGACGGGGTGAGGTCTGAGAGTTACACTCA
ACCATTGG
+
FFFFFFFFFFFF,FFFFFFFFFFFFFFFFFFFFFFFFFFFFFFFFFFFFFFFFFFFFFFFFFFFFF:F
FFFFFFFFFFFFFFFFFFFFFFFFFFFFFFFFFFFFFFFFFFFFFFFFFFFFFFFFFFFFFFFFFFFF
FFFFFFFF
@A00155:342:HHGFNDSXY:1:2653:9308:13823 1:N:0:GAACCTAG+TCCGCATA
GGGCTACTCTGGTTTTTCCACCTGAGATCTTAGCTGGTTACAAATCCTCCGTTCTGCCCTTCTTATAT
CAATTTGAAATTCACGGGAAGGATCTGGTCTACATGCCGGACGGGGTGAGGTCTGAGAGTTACACTCA
ACCATTGG
+
FFFFFFFFFFFFFFFFFFFFFFFFFFFFFFFFFFFFFFFFFFFF:FFFFFFFFFFFFFFF:FFFF:FF

FFFFFFFFFFFFFFFFFFFFFFFFFFFFFFFFFFFFFFFFFFFFFFFFFFFFFFFFFFFFFFFFFFFF
FFFFFFFF
@A00155:342:HHGFNDSXY:1:2511:30083:10473 1:N:0:GAACCTAG+TCCGCATA
GGGCTACTCTGGTTTTTCCACCTGAGATCTTAGCTGGTTACAAATCCTCCGTTCTGCCCTTCTTATAT
CAATTTGAAATTCACGGGAAGGATCTGGTCTACATGCCGGACGGGGTGAGGTCTGAGAGTTACACTCA
ACCATTGGAGAACGG
+
FFFFFFFFFFFFFFFFFFFFFFF,FFFFFFFFFFFFFFFFFFFFFFFFFFFFFFFFFFFFFFFFFFFF
FFFFFFFFFF:FFFFFFFFFFFFFFFFFFFFFFFFFFFFFFFFFFFFFFFFFFFFFFFFFFFFFFFFF
FFFFFFFFFFFFFFF
@A00155:342:HHGFNDSXY:1:1465:5303:30592 2:N:0:GAACCTAG+TCCGCATA
GGGCTACTCTGGTTTTTCCACCTGAGATCTTAGCTGGTTACAAATCCTCCGTTCTGCCCTTCTTATAT
CAATTTGAAATTCACGGGAAGGATCTGGTTTACATGCCGGACGGGGTGAGGTCTGAGAGTTACACTCA
ACCATTGGAGAACGG
+
FFFFFFFFFFFF,FFFFFFFFFFFFFFFFFFFFFFFFFFFFFFFFFFFFFFFFFFFFFFFFFFFFF:F
FFFFFFFFFFFFFFFFFFFFFFFFFFFFFFFFFFFFFF:FFFFFFFFFFFFFFFFFFFFFF:FFFFFFF
FFFFFFFFFFF:FFF
@A00155:342:HHGFNDSXY:1:2315:3703:10692 2:N:0:GAACCTAG+TCCGCATA
GGCTACTCTGGTTTTTCCACCTGAGATCTTAGCTGGTTACAAATCCTCCGTTCTGCCCTTCTTATATC
AATTTGAAATTCACGGGAAGGATCTGGTCTACATGCCGGACGGGGTGAGGTCTGAGAGTTACACTCAA
CCATTGGAGAACGG
+
FFFFFFFFFFFFFF:F,FFFFFFFFFFF:FFF:FFFFFFFFFF:F,FF,FFFFFFFF:FFFFF:FFF:
FFFFFF:F:FFF,FFF:FFFFFFFFFFFFFFFFFFFF,FFFFF:FFFFFFFFFFFFFFFFFFFFFFFF
FFFFFFFFF:FF:F
@A00155:342:HHGFNDSXY:1:1438:4878:28228 1:N:0:GAACCTAG+TCCGCATA
GGCTACTCTGGTTTTTCCACCTGAGATCTTAGCTGGTTACAAATCCTCCGTTCTGCCCGTCTTATATC
AATTTGAAATTCACGGGAAGGATCTGGTCTACATGCCGGACGGGGTGAGGTCTGAGAGTTACACTCAA
CCATTGGAGAACGGT
+
FFFFFFFF:FFFFFFFFFFFFFFFFFFFFFFFFFFFFFFFFFFFF:FFFFFFFFFFFFF,FFFFFFF:F
FFFFF,FFFFFFFFFFFFFFFFF:FFFFFFFFFFFFFFFF,F:FFFFFFFFFF,F:FFFF,FFFFFFF
FFFFFFFFFFFFFFF
@A00155:342:HHGFNDSXY:1:1512:20320:23719 1:N:0:GAACCTAG+TCCGCATA
GGCTACTCTGGTTTTTCCACCTGAGATCTTAGCTGGTTACAAATCCTCCGTTCTGCCCTTCTTATATC
AATTTGAAATTCACGGGAAGGATCTGGTCTACATGCCGGACGGGGTGAGGTCTGAGAGTTACACTCAA
CCATTGGAGAACGGC
+
FFFFFFFFFFFFFFFFFFFFFFFFFFFFFFFFFFFFFFFFFFFFFFFFFFFFFFFFFFFFFFFFFFFF
FFFFFFFFFFFFFFFFFFFFFFFF:FFFFFFFFFFFFFFFFFFFFFFFFFFFFFFFFFFFFFFFFFFF
FFFFFFFFFFFFFFF
@A00155:342:HHGFNDSXY:1:2223:28203:4116 1:N:0:GAACCTAG+TCCGCATA
GGCTACTCTGGTTTTTCCACCTGAGATCTTAGCTGGTTACAAATCCTCCGTTCTGCCCTTCTTATATC
AATTTGAAATTCACGGGAAGGATCTGGTCTACATGCCGGACGGGGTGAGGTCTGAGAGTTACACTCAA
CCATTGGAGAACGGC
+
FFFFFFFFFFFFFFFFFFFFFFFFFFFFFFFFFFFFFFFFFFFFFFFFFFFFFFFFFFFFFFFFFFFF
F,FFFFFFFFFFFFFFFFFFFFFFFFFFFFFFFFFFFFFFFFFFFFFFFFFFFFFFFFF:FFFFFFFFF
FFFFFFFFFFFFFFF
@A00155:342:HHGFNDSXY:1:2250:11089:18537 1:N:0:GAACCTAG+TCCGCATA
GGCTACTCTGGTTTTTCCACCTGAGATCTTAGCTGGTTACAAATCCTCCGTTCTGCCCTTCTTATATC
AATTTGAAATTCACGGGAAGGATCTGGTCTACATGCCGGACGGGGTGAGGTCTGAGAGTTACACTCAA
CCATTGGAGAACGGT

```
+
FFFFFFFFFFF:FFFFFFFFF:FFFFFFFFFFFFFFFFFFFFFFFFFFFFFFFFFFFFFFFFFFFFFF
FFFFFFFFFFFFFFFFFFFFFFFFFFFFFFFFFFFFFFFFFFFFFFFFFFFFFFFFFFFFFFFFFFFF
FFFFFFFFFFFFFFF
@A00155:342:HHGFNDSXY:1:2425:5556:5791 1:N:0:GAACCTAG+TCCGCATA
CGCTACTCTGGTTTTTCCACCTGAGATCTTAGCTGGTTACAAATCCTCCGTTCTGCCCTTCTTATATC
AATTTGAAATTCACGGGAAGGATCTGGTCTACATGCCGGACGGGGTGAGGTCTGAGAGTTACACTCAA
CCATTGGAGAACGGT
+
FFFFFFFFFFFFFFFFFFFFFFFFFFFFFFFFFFF:FFFFFFFFFFFFFFFFFF:FF,FFFFFFFFFFF
FFFFFFFFFFF,FFFFFFFFFFFFFFFFFFFFFFFFFFFFFFFFFFFFF:FFFFFFFFFFFF:F:FFFF
FFFFFFFFFFFFFFF
@A00155:342:HHGFNDSXY:1:2366:6958:29888 2:N:0:GAACCTAG+TCCGCATA
GGCTACTCTGGTTTTTCCACCTGAGATCTTAGCTGGTTACAAATCCTCCGTTCTGCCCTTCTTATATC
AATTTGAAATTCACGGGAAGGATCTGGTCTACATGCCGGACGGGGTGAGGTCTGAGAGTTACACTCAA
CCATTGGAGAACGGT
+
:FF:FFFFFFF:,FFFFFFF:F:FFFFFFFFFFF,FFFFFF:FFF,FFFF:,F:F,F,FF,:FF:F,:
FFFFFFF,FF:FFFF:FFFFFFF:FF:FF:FFFFFFF:F:FF:::FFFF::,F,FF:F,FFFF,,FFF
FFFF,,,FFFFFF,F
@A00155:342:HHGFNDSXY:1:2414:5710:7560 2:N:0:GAACCTAG+TCCGCATA
GCTACTCTGGTTTTTCCACCTGAGATCTTAGCTGGTTACAAATCCTCCGTTCTGCCCTTCTTATATCA
ATTTGAAATTCACGGGAAGGATCTGGTCTACATGCCGGACGGGGTGAGGTCTGAGAGTTACACTCAAC
CATTGGAGAACGGT
+
FFFFFFFFF,:FFFFFFFFFFFFFFFFFFFFF:FFFFF:FFFFFFFFFFFFFFF,F:FFFF:FFFFFF
FFFFFFFFF:FF,FFFFFFFFFFFFFFFFFFFFF:FFF:FFFF:FFFFFF:FFF,FFFFFFFFFFFFF
FFFFFFFF:FF,FF
@A00155:342:HHGFNDSXY:1:1413:5737:27211 1:N:0:GAACCTAG+TCCGCATA
GCTACTCTGGTTTTTCCACCTGAGATCTTAGCTGGTTACAAATCCTCCGTTCTGCCCTTCTTATATCA
ATTTGAAATTCACGGGAAGGATCTGGTCTACATGCCGGACGGGGTGAGGTCTGAGAGTTACACTCAAC
CATTGGAGAACGGGC
+
FFFFFFFFFFFFFFFFFFF,FFFFFFFFFFFFFFFFFFFFFFFFFFFFFFFFFFFFFFFFFFFFFFFF
F:FFFFFFFFFFFFFFFFFFFFFFFFF:FFFFFFFFFFFFFFFFFFFFFFFFFFFFFFFFFFFFFFFF
FFFFFFFFFFFFFFF
@A00155:342:HHGFNDSXY:1:1510:22354:3067 2:N:0:GAACCTAG+TCCGCATA
GCTACTCTGGTTTTTCCACCTGAGATCTTAGCTGGTTACAAATCCTCCGTTCTGCCCTTCTTATATCA
ATTTGAAATTCACGGGAAGGATCTGGTCTACATGCCGGACGGAGTGAGGTCTTAGAGTTACACTCAAC
CATTGGAGAACGGTT
+
FFFFF,FF,FF::FF:,FFFF,F,FF:FFFFFF,::FFFFFFFF,F,FFFFFFF:FFFFFFFFFFFFF
FFFFFFF,FFFF:FF,FF,FFFFF:FF,F:FFFFF,FFF,FF:FF,F:FF:F:FF:,FFFFFFF,FFF
,F:FFFFFFFF,FFF
@A00155:342:HHGFNDSXY:1:1611:28673:28917 2:N:0:GAACCTAG+TCCGCATA
GCTACTCTGGTTTTTCCACCTGAGATCTTAGCTGGTTACAAATCCTCCGTTCTGCCCTTCTTATATCA
ATTTGAAATTCACGGGAAGGATCTGGTCTACATGCCGGACGGGGTGAGGTCTGAGAGTTACACTCAAC
CATTGGAGAACGGTT
+
FF:FFFF,FFFFFFFFFFFFFFF,FFF::F:FFF:FFFFFFFF,FFFFFFFFFFFF,F:FFFFFFFFF
FFFF:FFFFFF:FFFFFFF:FFFFFFFFFFFFFFFFF:FF:FF,FFFF:FFFFFFF:FFF:FFFFFFF
,F,FFFFFF,F:F:F
@A00155:342:HHGFNDSXY:1:2268:22092:29199 2:N:0:GAACCTAG+TCCGCATA
GCTACTCTGGTTTTTCCACCTGAGATCTTAGCTGGTTACAAATCCTCCGTTCTGCCCTTCTTATATCA
```

ATTTGAAATTCACGGGAAGGATCTGGTCTACATGCCGGACGGGGTGAGGTCTGAGAGTTACACTCAAC
CATTGGAGAACGGTT
+
FFFFFFFFFFFFFFFFFFFFFFFFFFFFFFFFFFFFFFFFFFFFFFFFFFFFFFFFFFFFFFFFFFFFFF
FFFFFFFFFFFFFFFFFFFFFFFFFFFFFFFFFFFFFFFFFFFFFFFFFFF:FFFFFFFF:FFFFFFFFF
FFFFFFFFFFFFFFF
@A00155:342:HHGFNDSXY:1:2349:25943:10786 2:N:0:GAACCTAG+TCCGCATA
GCTACTCTGGTTTTTCCACCTGAGATCTTAGCTGGTTACAAATCCTCCGTTCTGCCCTTCTTATATCA
ATTTGAAATTCACGGGAAGGATCTGGTCTACATGCCGGACGGAGTGAGGTCTGAGAGTTACACTCAAC
CATTGGAGAACGGAG
+
FFFFFFFFFFFFFFFFFFFFFFFFFFFFFFFFFFFFFFFFFFFFFFFFFFFFFFFFFFFFFFFFFFFFFF
FFFFFFFFFFFFFFFFFFFFFFFFFFFFFFFFFFFFFFFFFFFFFFFFFFFFFFFFFFFFFFFFFFFFFF
FFFFFFFFFFFFFF:
@A00155:342:HHGFNDSXY:1:2360:23556:1360 2:N:0:GAACCTAG+TCCGCATA
GCTACTCTGGTTTTTCCACCTGAGATCTTAGCTGGTTACAAATCCTCCGTTCTGCCCTTCTTATATCA
ATTTGAAATTCACGGGAAGGATCTGGTCTACATGCCGGACGGGGTGAGGTCTGAGAGTTACACTCAAC
CATTGGAGAACGGTT
+
FFFFFFFFFFFFFFFFFFFFFFFFFFFFFFFFFFFFFFF:FFFFFFFFFFFFFFFFFFFFFFFFFFFFFF
FFFFF:FFFFFFFF:FFFF:FF:FFFFFFFFFFF:FFFFFF:FFFFFFFFFFFFFFFFFFFFFFFFFFFF
FFFFFFFFFFFFFFF
@A00155:342:HHGFNDSXY:1:2375:27407:18615 2:N:0:GAACCTAG+TCCGCATA
GCTACTCTGGTTTTTCCACCTGAGATCTTAGCTGGTTACAAATCCTCCGTTCTGCCCTTCTTATATCA
ATTTGAAATTCACGGGAAGGATCTGGTCTACATGCCGGACGGAGTGAGGTCTGAGAGTTACACTCAAC
CATTGGAGAACGGTT
+
FFFFFFFFFFFFFFFFFFFFFFFFFFFFFFFFFFFFFFFFFFFFFFFFFFFFFFFFFFFFFF:FFFFFFF
FFFFFFFFFFFFFFFFFFFFFFFFFFFFFFFFFFFFFFFFFFFFFFFFFFFFFFFFFFFFFFFFFFFFFF
FFFFFFFFF:F,FFF
@A00155:342:HHGFNDSXY:1:2375:24650:19727 2:N:0:GAACCTAG+TCCGCATA
GCTACTCTGGTTTTTCCACCTGAGATCTTAGCTGGTTACAAATCCTCCGTTCTGCCCTTCTTATATCA
ATTTGAAATTCACGGGAAGGATCTGGTCTACATGCCGGACGGAGTGAGGTCTGAGAGTTACACTCAAC
CATTGGAGAACGGTT
+
FFFFFFFFFFFFFFFFFFFFFFFFFFFFFFFFFFFFFFFFFFFFFFFFFFFFFFFFFFFFFFFFFFFFFF
FFFFFFFFFFFFFFFFFFFFFFFFFFFFFFFFFFFFFFFFFFFFFFFFFFFFFFFFFFFFFFFFFFFFF:
FFFFFFFFF:FFFFF
@A00155:342:HHGFNDSXY:1:2453:19443:35289 2:N:0:GAACCTAG+TCCGCATA
GCTACTCTGGTTTTTCCACCTGAGATCTTAGCTGGTTACAAATCCTCCGTTCTGCCCTTCTTATATCA
ATTTGAAATTCACGGGAAGGATCTGGTCTACATGCCGGACGGGGTGAGGTCTGAGAGTTACACTCAAC
CATTGGAGAACGGTT
+
FFFFFFFFFFFFFFFFFFFFFFFFFFFFFFFFFFFFFFFFFFFFFFFFFFFFFFFFFF:F,FFFFFFFFF
FFFFFFFFFFFFFFFFFFFFFFFFFFFFFFFFFFFFFFFFFFFFFFFFFFFFFFFFFFFFFFFFFFFFFF
F,FFFFFFFFFFFFF
@A00155:342:HHGFNDSXY:1:2622:28845:34538 2:N:0:GAACCTAG+TCCGCATA
GCTACTCTGGTTTTTCCACCTGAGATCTTAGCTGGTTACAAATCCTCCGTTCTGCCCTTCTTATATCA
ATTTGAAATTCACGGGAAGGATCTGGTCTACATGCCGGACGGAGTGAGGTCTGAGAGTTACACTCAAC
CATTGGAGAACGGTT
+
FFFFFF:FFFFFFFFFFFFFFFFFFFFFFFFFFFFFFFFFFFFFFFFFFFF,FFFFFFFFFFFFFFFFFF
FFFFFF:F,FFFFFFFF:FFFFFFFFFFFFFFFFFFFFFFFFFFFFFFFFFFFFFFFFFFFFFFFFF,F
FFF,FFFFFFFFFFF

@A00155:342:HHGFNDSXY:1:2450:14552:9064 2:N:0:GAACCTAG+TCCGCATA
CTACTCTGGTTTTTCCACCTGAGATCTTAGCTGGTTACAAATCCTCCGTTCTGCCCTTCTTATATCAA
TTTGAAATTCACGGGAAGGATCTGGTCTACATGCCGGACGGGGTGAGGTCTGAGAGTTACACTCAACC
ATTGGAGAACGGTTT
+
FFFFFFFFFFFFFFFFFFFFFFFFFFFFFFFFFFFFFFFFFFFFFFFFFFFFFFFFFFFFFFFFFFFF
FFFFFFFFFFFFFFFFFFFFFFFFFFFFFFFFFFFFFFFFFFFFFFFFFFFFFFFFFFFFFFFFFFF:
FFFFFFFFFFFFFFF
@A00155:342:HHGFNDSXY:1:2511:30083:10473 2:N:0:GAACCTAG+TCCGCATA
ACTCTGGTTTTTCCACCTGAGATCTTAGCTGGTTACAAATCCTCCGTTCTGCCCTTCTTATATCAATT
TGAAATTCACGGGAAGGATCTGGTCTACATGCCGGACGGGGTGAGGTCTGAGAGTTACACTCAACCAT
TGGAGAACGGTTTTC
+
FFFFFFF,FFFFFFFFFFFFFFFFFFFFFFFFFFFFF:FFFFFFFFFFFFFFFFFFFFFFF:FFF:FF
FFFFF:FFFFFFFFFFFFFFFFFFFFFFFFFFFFFFFFFFFFFFFFFFFFFFFFFFFFFFFFFF
FFFFFFFFFFFFFFF
@A00155:342:HHGFNDSXY:1:1230:6036:14638 2:N:0:GAACCTAG+TCCGCATA
CTCTGGTTTTTCCACCTGAGATCTTAGCTGGTTACAAATCCTCCGTTCTGCCCTTCTTATATCAATTT
GAAATTCACGGGAAGGATCTGGTCTACATGCCGGACGGGGTGAGGTCTGAGAGTTACACTCAACCATT
GGAGAACGGTTTTCT
+
FFFFFFFFFFFFFFFFFFFFFFFFFFFFFFFFF:FFFFFFFFFFFFFFFF:FFFF,FFFFFFFFFFFF
FF,FFFFFFFFFF,:FFFFFFFFFFF:FFFFFFFFFFFFFFFFFFFFFFFFFFFFFFFFFFFFFFFFF
FFFFFFF:FFFFFFF
@A00155:342:HHGFNDSXY:1:1409:21974:4351 2:N:0:GAACCTAG+TCCGCATA
CTCTGGTTTTTCCACCTGAGATCTTAGCTGGTTACAAATCCTCCGTTCTGCCCTTCTTATATCAATTT
GAAATTCACGGGAAGGATCTGGTCTACATGCCGGACGGGGTGAGGTCTGAGAGTTACACTCAACCATT
GGAGAACGGTTTTCT
+
FFFFFFFFFFFFFFFFFFFFFFFFFFFFFFFFFFFFFFFFFFFFFFFFFFFFFFFFFFF::FFFFFFF
:FFFFFFFFFFFFFFFFFFFFFFFFFFFFFFFFFFFFFFFFFFFFFFFFFFFFFF:FFFFF,FFF:F
FFFFFFFFFFFFFFF
@A00155:342:HHGFNDSXY:1:2205:7907:11898 2:N:0:GAACCTAG+TCCGCATA
CTCTGGTTTTTCCACCTGAGATCTTAGCTGGTTACAAATCCTCCGTTCTGCCCTTCTTATATCAATTT
GAAATTCACGGGAAGGATCTGGTCTACATGCCGGACGGGGTGAGGTCTGAGAGTTACACTCAACCATT
GGAGAACGGTTTTCT
+
FFF:FFFFFFF,FFF:FFFFFFF:FFFFFF,FFFFFFFFF,FFF:FFFFF,,,FFFFFFFFFFFFFFF
,FFFFFF,FFFFFF,FFFFFFFFFFFFFF:,FFFFFFFFFFFFFFFFFF:FF:FFFFFFFFFF:FFFF
:,FF:,,FFFFFFFF
@A00155:342:HHGFNDSXY:1:2334:4408:19366 2:N:0:GAACCTAG+TCCGCATA
CTCTGGTTTTTCCACCTGAGATCTTAGCTGGTTACAAATCCTCCGTTCTGCCCTTCTTATATCAATTT
GAAATTCACGGGAAGGATCTGGTCTACATGCCGGACGGGGTGAGGTCTGAGAGTTACACTCAACCATT
GGAGAACGGTTTTCT
+
FFFFFFFFFFFFFFFFFFFFFFFFFFFFFFFFFFFFFFFFFFFF:FFFFFF:FFFFFFFFFFFFFFFF
:FFFFFF:FFFFFFF:FFFFFFFFFFFFFFFFFFFFFFFFFFF,FFFFFFFFFFFFFFFFFFFFF,FF
FFF:FFFFFFFFFFF
@A00155:342:HHGFNDSXY:1:2408:2980:17174 2:N:0:GAACCTAG+TCCGCATA
CTCTGGTTTTTCCACCTGAGATCTTAGCTGGTTACAAATCCTCCGTTCTGCCATTCTTATATCAATTT
GAAATTAACGGGAAGGATCTGGTCTACATGCCGGACGGGGTGAGGTCTGAGAGTTAAACTCAACAATT
TGAGAACGGTTTTCT
+
FF,FF:F,:,F,FF,FFFF,F,FF,F,::::FFF:FF,F:,F,:FFF,F,:,,FFFFF:FF:FFFF,F

F,F:FF,F,:,,FFF,FFFF::FF,FF:FF,,,FFFF,FFFF:FFF:FF:FFFF:F,F:F,FFF:F:F
,FFF,FF,,::FF,F
@A00155:342:HHGFNDSXY:1:2432:32940:10066 2:N:0:GAACCTAG+TCCGCATA
CTCTGGTTTTTCCACCTGAGATCTTAGCTGGTTACAAATCCTCCGTTCTGCCCTTCTTATATCAATTT
GAAATTCACGGGAAGGATCTGGTCTACATGCCGGACGGGGTGAGGTCTGAGAGTTACACTCAACCATT
GGAGAACGGTTTTCT
+
FFFFFFFFFFFFFFFFFFFFFFFFFFFFFFFFFFFFFFF:F::FFFFFFFF:FFFFFFFFFFFFFFFF
F,FFFFFFFFFFFFFFFFFFFFFFF:FFFF,FFFFFFFFFFFFFFFFFFFFFFFFF:FFFFFFFFFFF
,FFFFFFF,FFFFFF
@A00155:342:HHGFNDSXY:1:2620:2492:31923 2:N:0:GAACCTAG+TCCGCATA
CTCTGGTTTTTCCACCTGAGATCTTAGCTGGTTACAAATCCTCCGTTCTGCCCTTCTTATATCAATTT
GAAATTCACGGGAAGGATCTGGTCTACATGCCGGACGGGGTGAGGTCTGAGAGTTACACTCAACCATT
GGAGAACGGTTTTCT
+
FFFFFFFFFFFFFFFFFFFFFFFFFFFFFFFFFFFFFFFFFFFFFFFFFFFFFFFFFFFFFFFFFFFF
FFFFFFFFFFFFFFFFFFFFFFFFFFFFF:FFFFFFFFFFFFFFFFFFFFFFFFFFFFFFFFFFFFFF
FFFFFF:FFFFFFFF
@A00155:342:HHGFNDSXY:1:2624:18710:17300 2:N:0:GAACCTAG+TCCGCATA
CTCTGGTTTTTCCACCTGAGATCTTAGCTGGTTACAAATCCTCCGTTCTGCCCTTCTTATATCAATTT
GAAATTCACGGGAAGGATCTGGTCTACATGCCGGACGGGGTGAGGTCTGAGAGTTACACTCAACCATT
GGAGAACGGTTTTCT
+
FFFFFFFFFFFFFFFFFFFFFFFFFFF:FFFFFFFFFFFFFFFFFFFFFFFFFFFFF:FFFFFFFFFF
FFFFFFFFFFFFFFFFFFFFFFFFFFFFFFFFFFFFFFFFFFFFFFFFF:FFFFFFFFFFFFFFFFFF
FFFFFFFFFFF:FFF
@A00155:342:HHGFNDSXY:1:1235:26955:27101 1:N:0:GAACCTAG+TCCGCATA
TCTGGTTTTTCCACCTGAGATCTTAGCTGGTTACAAATCCTCCGTTCTGCCCTTCTTATATCAATTTG
AAATTCACGGGAAGGATCTGGTCTACATGCCGGACGGGGTGAGGTCTGAGAGTTACACTCAACCATTG
GAGAACGGTTTTCTC
+
FFFFFFFFFFFFFFFFFFFFFFFFFFFFFFFFFFFFFFFFFFFFFFFFFFF:FF:FFFFFFFFFFFFF
FFFFFFFFFFFFFFFFFFFFFFFFFFFFFFFFFFFFFFFFFFFFFFFFFFFFFFFFFFFFFFFFFFFF
FFFFFFFFFFFFFFF
@A00155:342:HHGFNDSXY:1:1242:20916:16548 1:N:0:GAACCTAG+TCCGCATA
TCTGGTTTTTCCACCTGAGATCTTAGCTGGTTACAAATCCTCCGTTCTGCCCTTCTTATATCAATTTG
AAATTCACGGGAAGGATCTGGTCTACATGCCGGACGGGGTGAGGTCTGAGAGTTACACTCAACCATTG
GAGAACGGTTTTCTC
+
FFFFFFFFFFFFFFFFFFFFFFFFFFFFFFFFFFFFFFFFFFFF:FFFFFFFFFFFFFFFFFF:FF,F
FFFFFFFFFFF::FFFFFFFFFFF:FFFFFFFFFFFFFFFFFFFFFFFFFFF:FFFFFFFFFFFFFFF
FFFFFFFFFFFFFFF
@A00155:342:HHGFNDSXY:1:1117:4056:21480 2:N:0:GAACCTAG+TCCGCATA
CTGGTTTTTCCACCTGAGATCTTAGCTGGTTACAAATCCTCCGTTCTGCCCTTCTTATATCAATTTGA
AATTCACGGGAAGGATCTGGTCTACATGCCGGACGGAGTGAGGTCTGAGAGTTACACTCAACCATTTG
AGAACGGTTTTCTC
+
FFFFFFFFFFFFFFFFFFFFFFFFF:FF:FFFFFFFFFFFFFFFFFFFFFFFFF:F,FFFFFFFFFFF
FFFFFF:FFFF:FFFFFFFFFFFFFFFFFFF,FFFFF,FFFFFFFFFFFFFFFFFFFFFFFFFFFF,:
F:FFFFFFFFFFFF
@A00155:342:HHGFNDSXY:1:2553:27516:24909 1:N:0:GAACCTAG+TCCGCATA
CTGGTTTTTCCACCTGAGATCTTAGCTGGTTACAAATCCTCCGTTCTGCCCTTCTTATATCAATTTGA
AATTCACGGGAAGGATCTGGTCTACATGCCGGACGGGGTGAGGTCTGAGAGTTACACTCAACCATTGG
AGAACGGTTTTCTCC

+
FFFF:FFFFFFFFFFFFFFFFFFFFFFFFFFFFFFFFFFFFFFFFFFFFFFFFFFFFFFFFFFFFFFF
FFFFFFF:FFFFFFFFFFFFFFFFFFFFFFF:FFFFFFFFFFFFFFFFFFFFFFFFFFFFFFFFFFFFF
FFFFFFFFFFFFFFF
@A00155:342:HHGFNDSXY:1:1357:13440:27962 2:N:0:GAACCTAG+TCCGCATA
CTGGTTTTTCCACCTGAGATCTTAGCTGGTTACAAATCCTCCGTTCTGCCCTTCTTATATCAATTTGA
AATTCACGGGAAGGATCTGGTCTACATGCCTGACGGGGTGAGGTCTGAGAGTTACACTCAACCATTGG
AGAACGGTTTTCTCC
+
FF:F:FFFFFF:FFFFFF:FFFFFFF:F::FFFFFFFFFFFFF::F:FF:,FFFFFF:FF:F:FF:FF
FFF:FFFF::FFFFF:FFFFFFFFFFF::F,FFFFFF,FFF:FFFF,FF:F:FFFFFFFFFFFFFFF,
FFFFFFFFFFFFFFF
@A00155:342:HHGFNDSXY:1:1372:7663:4366 2:N:0:GAACCTAG+TCCGCATA
CTGGTTTTTCCACCTGAGATCTTAGCTGGTTACAAATCCTCCGTTCTGCCCTTCTTATATCAATTTGA
AATTCACGGGAAGGATCTGGTCTACATGCCGGACGGGGTGAGGTCTGAGAGTTACACTCAACCATTGG
AGAACGGTTTTCTCC
+
FFFFFFFFFFFFFFFFFFFFFFFFFFFFFFFFFFFFFFFFFFFFFFFFF:FFF:FFFFFFFFFFFFF
FFFFFFFFFFFFFFFFFFFFFFFFFFFFFFFFFFFFFFFFFFFFFFFFFFFFFFFFFFFFFFFFFFFF
FFFFFFFFFFFFFFF
@A00155:342:HHGFNDSXY:1:1545:21124:32158 2:N:0:GAACCTAG+TCCGCATA
CTGGTTTTTCCACCTGAGATCTTAGCTGGTTACAAATCCTCCGTTCTGCCCTTCTTATATCAATTTGA
AATTCACGGGAAGGATCTGGTCTACATGCCGGACGGGGTGAGGTCTGAGAGTTACACTCAACCAATGG
AGAACGGTTTTCTCC
+
FF:FFFFFFFFFFFFFFFFFFF:FFFFFF,FF:FFFFFFFFFFFFFFFF:,FFFF:FFFFFFFFFFFF
FFFFFF,FFFFFFFFFFFF,F:FFFFF::FFF:FFFFF:FFFFF:FFFFFFFFFFFFF:FFFFF,FFF
FF:F:,FFFFFFFF:
@A00155:342:HHGFNDSXY:1:2520:11478:5650 2:N:0:GAACCTAG+TCCGCATA
CTGGTTTTTCCACCTGAGATCTTAGCTGGTTACAAATCCTCCGTTCTGCCCTTCTTATATCAATTTGA
AATTCACGGGAAGGATCTGGTCTACATGCCGGACGGGGTGAGGTCTGAGAGTTACACTCAACCATTGG
AGAACGGTTTTCTCC
+
FFFFFFFFFFFFFFFFFFFFFFFFFFFFFFFFFFFFFFFFFFFFFFF:FF:FFFFFFFFFFFFFFF:F
FFFFFFFFF:F:FFFFFFFFFFFFFFFFF,FFFFFFFFFFFFFFFFFFFFFFFFFFFFFFF:FFFFFFF
FFFF:F:F:FFFFF:
@A00155:342:HHGFNDSXY:1:2545:20292:26929 2:N:0:GAACCTAG+TCCGCATA
CTGGTTTTTCCACCTGAGATCTTAGCTGGTTACAAATCCTCCGTTCTGCCCTTCTTATATCAATTTGA
AATTCACGGGAAGGATCTGGTCTACATGCCGGACGGGGTGAGGTCTGAGAGTTACACTCAACCATTGG
AGAACGGTTTTCTCC
+
FFFFFFFFFFFFFFFFFFFFFF:FFFFFFFFFFFFFFFFFFFFFFFFFFFFF:F::FFFFFFFFFFFFFFF
FFFFFFFFFFFFFFFFFFFFFFFFFFFFFFFFFFFFFFFFFFFFFFFFFFFFFFFFFFFFFFFFFFFF
FFFFFFFFFFFFFFF
@A00155:342:HHGFNDSXY:1:1124:10646:34867 1:N:0:GAACCTAG+TCCGCATA
TGGTTTTTCCACCTGAGATCTTAGCTGGTTACAAATCCTCCGTTCTGCCCTTCTTATATCAATTTGAA
ATTCACGGGAAGGATCTGGTCTACATGCCGGACGGGGTGAGGTCTGAGAGTTACACTCAACCATTGGA
GAACGGTTTTCTCCT
+
FFFFFFFFFFFFFFFFFF:FFFFF:FFFFFFFF:,FFFFFFFFFFFFFFFFF,:FFFFFFFFF,FFFF
FFFFFFFFF,FFFFFFFFFFFFFFFFFFFFFFFFFFFFFFFFFFFFFFFFFFFFFFFFFFFFFFFFFF
FFFFFFFFFFFFFFF
@A00155:342:HHGFNDSXY:1:1146:24686:3724 1:N:0:GAACCTAG+TCCGCATA
GGTTTTTCCACCTGAGATCTTAGCTGGTTACAAATCCTCCGTTCTGCCCTTCTTATATCAATTTGAAA

```
TTCACGGGAAGGATCTGGTCTACATGCCGGACGGGGTGAGGTCTGAGAGTTACACTCAACCATTGGAG
AACGGTTTTCTCCTC
+
FFFFFFFFFFFFFFFFFFFFFFFFFFFFFFFFFFFFFFFFFFFFFFFFFFFFFFFFFFFFFFFFFFF:
FFFFFFFFFFFFFFFFFFFFFFFFFFFFFFFFFFFFFFFFFFFFFFFFFFFFFFFFFFFFFFFFFFFF
FFFFFFFFFFFFFFF
@A00155:342:HHGFNDSXY:1:1668:12454:26819 1:N:0:GAACCTAG+TCCGCATA
GGTTTTTCCACCTGAGATCTTAGCTGGTTACAAATCCTCCGTTCTGCCCTTCTTATATCAATTTGAAA
TTCACGGGAAGGATCTGGTCTACATGCCGGACGGGGTGAGGTCTGAGAGTTACACTCAACCATTGGAG
AACGGTTTTCTCCTC
+
FFFFFFFFFFFFFFFFFFFFFFFFFFFFFFFFFFFFFFFFFFFFFFFFFFFFFFFFFFFFFFF,FFFF
FFFFFFFFFFFFFFFFFFFFFFFFFFFFFFFFFFFFFFFFFFFFFFFFFFFFFFF:FFFFFFFFFFFF
FFFFFFFFFFFFFFF
@A00155:342:HHGFNDSXY:1:1251:1362:34538 2:N:0:GAACCTAG+TCCGCATA
GGTTTTTCCACCTGAGATCTTAGCTGGTTACAAATCCTCCGTTCTGCCCTTCTTATATCAATTTGAAA
TTAACGGGAAGGATCTGGTCTACATGCCGGACGGAGTGAGGTCTGAGAGTTACACTCAACCATTGGAG
AACGGTTTTCTCCTC
+
FF:F:FF:FF,,:,F,:FFF,FF::FFFFFFFFFFF:F,:FFF::F,FFF:FFFFFFF,FFFFFFFFF
F,,FFF:,FF::FF:F:F,:F::FF:,:F:FFFFFFF:FFFFFF:FFFFFFFF,:FFFFF,FFF,FF:
FFF:FFF,F,FFFFF
@A00155:342:HHGFNDSXY:1:1656:16884:9032 2:N:0:GAACCTAG+TCCGCATA
GGTTTTTCCACCTGAGATCTTAGCTGGTTACAAATCCTCCGTTCTGCCCTTCTTATATCAATTTGAAA
TTCACGGGAAGGATCTGGTCTACATGCCGGACGGAGTGAGGTCTGAGAGTTACACTCAACCATTGGAG
AACGGTTTTCTCCTC
+
FFFFFFFFFFFFFFFFFFFFF:FF:FFFFFFFFFFF:FFFF:FFFFFFFF:FFF,FFFFFFFF:FFFF
FFFFFFFF:FFFFFFFFF:FFFFFFFFFFFFFFFFFFF:FF:FFFFFFFFFFFFFFFFFFFFFFFFFF
F:FFFFFFFFFFFFF
@A00155:342:HHGFNDSXY:1:2234:25129:35305 2:N:0:GAACCTAG+TCCGCATA
GGTTTTTCCACCTGAGATCTTAGCTGGTTACAAATCCTCCGTTCTGCCCTTCTTATATCAATTTGAAA
TTCACGGGAAGGATCTGGTCTACATGCCGGACGGGGTGAGGTCTGAGAGTTACACTCAACCATTGGAG
AACGGTTTTCTCCTC
+
FFFFFFFFFFFFFFFFFFFFFFFFFFFFFFFFFFFFFFFFFFFFFFFFFFFFFFFF:FFFFFFFFFFF
FFFFFFFFFFFFFFFFFFFFFFFFFFFFFFFFFFFFFFFFFFFFFFFFFFFFFFFFFFFFFFFFF:,F
FFFFFFFFFFFFFFF
@A00155:342:HHGFNDSXY:1:1114:17544:27179 1:N:0:GAACCTAG+TCCGCATA
GTTTTTCCACCTGAGATCTTAGCTGGTTACAAATCCTCCGTTCTGCCCTTCTTATATCAATTTGAAAT
TCACGGGAAGGATCTGGTCTACATGCCGGACGGAGTGAGGTCTGAGAGTTACACTCAACCATTGGAGA
ACGGTTTTCTCCTCA
+
FFFFFFFFFFFFFFFFFFFFFFFFFFFFFFF:FFFFFFFFFFFFFFFFFFFFFFFFFFFFFFFF:FFF
FFFFFFFF:FFFFFFFFFFFFFFFFFFFFFFFFFFFFFFFFFFFFFFFFFFFFFFFFFFFFFFFFFF
FFFFFFFFFFFFFFF
@A00155:342:HHGFNDSXY:1:2223:11623:23030 1:N:0:GAACCTAG+TCCGCATA
TTTTTCCACCTGAGATCTTAGCTGGTTACAAATCCTCCGTTCTGCCCTTCTTATATCAATTTGAAATT
CACGGGAAGGATCTGGTCTACATGCCGGACGGGGTGAGGTCTGAGAGTTACACTCAACCATTGGAGAA
CGGTTTTCTCCTCAG
+
FFFFFF:FFFFFFFFFFFF,FFF:FFFFFFFFFFFFFFFFFFFFFFFFFFFFFFFFF:FF:FFFFFFF
FFFFFFFFFFFFFFFFFFFFFFFFFFFFFFFFFFFFFFFFFFFFFFFFFFFFFFFFFFFFFFFFFFFF
FFFFFFFFFFFFFFF
```

```
@A00155:342:HHGFNDSXY:1:2406:19596:20462 2:N:0:GAACCTAG+TCCGCATA
TTTTTCCACCTGAGATCTTAGCTGGTTACAAATCCTCCGTTCTGCCCTTCTTATATCAATTTGAAATT
CACGGGAAGGATCTGGTCTACATGCCGGACGGGGTGAGGTCTGAGAGTTACACTCAACCATTGGAGAA
CGGTTTTCTCCTCAG
+
FFFFFFFFFFFFFFFFFFFFF:FFFFFFFFFFFFFFFFFFFFFFFFFFFF:FFFF:FFFFFFFFFFFFF
FFFFFFFFFFFFFFFFFFFFFFFFFFFFFFFFFFFFFFFFFFFFFFFFFFFFF:FFFFFFFFFFFFF:
FFFFFFFFFFFFFFF
@A00155:342:HHGFNDSXY:1:1357:13440:27962 1:N:0:GAACCTAG+TCCGCATA
TTTTCCACCTGAGATCTTAGCTGGTTACAAATCCTCCGTTCTGCCCTTCTTATATCAATTTGAAATTC
ACGGGAAGGATCTGGTCTACATGCCGGACGGGGTGAGGTCTGAGAGTTACACTCAACCATTGGAGAAC
GGTTTTCTCCT
+
FFFFF:,F,FF,FF:FFFF,:FF,FFFF,,F,,FF:FFFF,FFF,FFF,FF,F,FF,FFFFF:::FF:
F,F,F,FFF,FF,FFFFF,FFFFF:F:FFFF,FFFFFFFFFFFFF,F:FFFFF::F:FFFFFF:F,FF
F,F:FF::F,F
@A00155:342:HHGFNDSXY:1:2406:2040:12038 1:N:0:GAACCTAG+TCCGCATA
TCCGATCTCTGAGATCTTAGCTGGTTACAAATCCTCCGTTCTGCCCTTCTTATATCAATTTGAAATTC
ACGGGAAGGATCTGGTCTACATGCCGGACGGGGTGAGGTCTGAGAGTTACACTCAACCATTGGAGAAC
GGTTTTCTCCTCAG
+
FFFF:FFFFFFFFFFFF:FFFFFFFFFFFFFFFFFFFFFFFFFFFFFFFFFFFFFFFF,FFFFFFFFF
FFFF:FFFFFFFFFFFFFFFFFFF,FFFFFFFFFFFFFFFFFFFFFFFFFFFFFFFFFFFFFFFFF:FF
:FFFFFFFFFFFFF
@A00155:342:HHGFNDSXY:1:2169:4806:3552 1:N:0:GAACCTAG+TCCGCATA
TTCCACCTGAGATCTTAGCTGGTTACAAATCCTCCGTTCTGCCCTTCTTATATCAATTTGAAATTCAC
GGGAAGGATCTGGTCTACATGCCGGACGGGGTGAGGTCTGAGAGTTACACTCAACCATTGGAGAACGG
TTTTCTCCTCAGCTG
+
FFFFFFFFFFFFFFFFFFFFFFFFFFFFFFFFFFFFFFFFFFFFF:FFFFFFFFFFFFFFFFFF:FFFF
FFF:FFFFFFFFFFFFFFFFFFFFFFFFFFFFFFFFFFFFFFFFF:FF::FFFFFFFFFFFFFFFFFFF
FFFFFFFFFFFF:FF
@A00155:342:HHGFNDSXY:1:2662:9435:33959 1:N:0:GAACCTAG+TCCGCATA
TTCCACCTGAGATCTTAGCTGGTTACAAATCCTCCGTTCTGCCCTTCTTATATCAATTTGAAATTCAC
GGGAAGGATCTGGTCTACATGCCGGACGGGGTGAGGTCTGAGAGTTACACTCAACCATTGGAGAACGG
TTTTCTCCTCAGCTC
+
FFFFFFFFFFFF:FFFFFFFFFFFFFFFFFFFFF:FFFFFFFFFFFF:FFFFFFFFFFFFFFFFFFFFF
FFFFFFFFFFFFFFFFFFFFFFFFFFFFFFFFFFFFFFFFFFFFFFFFFFFFFFFFFFFFFFFFFFFFF
FFFFFFFFFFFFFFF
@A00155:342:HHGFNDSXY:1:1309:23213:13072 1:N:0:GAACCTAG+TCCGCATA
TCTCTGAGATCTTAGCTGGTTACAAATCCTCCGTTCTGCCCTTCTTATATCAATTTGAAATTCACGGG
AAGGATCTGGTCTACATGCCGGACGGAGTGAGGTCTGAGAGTTACACTCAACCATTGGAGAACGGTTT
TCTCCTCAGCTCCAG
+
FFFFFFFFFFFF:FFFFFFFFFFF:FFFF:FFFFFFFFFFF,FFFFFFFFFF::FFFFFFFFFFFFFF
FFFFFFFFFFFFFFFFFFFFFFFFFFFFFFFFFFFFFFFFFFFFF:FFFFFFFFFFFFFFFFFFFFFFF
FFFFFFFFFFFFFFF
@A00155:342:HHGFNDSXY:1:1410:14271:20885 1:N:0:GAACCTAG+TCCGCATA
CACCTGAGATCTTAGCTGGTTACAAATCCTCCGTTCTGCCCTTCTTATATCAATTTGAAATTCACGGG
AAGGATCTGGTCTACATGCCGGACGGAGTGAGGTCTGAGAGTTACACTCAACCATTGGAGAACGGTTT
TCTCCTCAGCTCCAG
+
FFFFFFFFFFFFFFFFFFFFFFFFFFFFFFFFFFFFFFFFFFFFFFFFFFFFFFFFFFFFFFFFFFFFF
```

F:FFFFFFFFFFFFFFFFFFFFFFFFFFFFFFFFFFFFFFFFFFFFFFFFFFFFFFFFFFFFFFFFFFFF
FFFFFFFFFFFFFFF
@A00155:342:HHGFNDSXY:1:1531:11695:16329 1:N:0:GAACCTAG+TCCGCATA
TCTCTGAGATCTTAGCTGGTTACAAATCCTCCGTTCTGCCCTTCTTATATCAATTTGAAATTCACGGG
AAGGATCTGGTCTACATGCCGGACGGAGTGAGGTCTGAGAGTTACACTCAACCATTGGAGAACGGTTT
TCTCCTCAGCTCCAG
+
FFFF:FFFFFFF:FFFFFFFFFFFFFFFFFFFFFFFFFFFFFFFFFFF:FFFFF,F:FFFFFFFFFFFF
FFFFFFFFFFFFFFFFFFFFFFFFFFFF:FFFFFFFFFFFFFFFFFFFFFFFFFFFFFFFFFFFFFFFF
FFFFFFFFFFFFFFF
@A00155:342:HHGFNDSXY:1:2366:14606:18959 1:N:0:GAACCTAG+TCCGCATA
CACCTGAGATCTTAGCTGGTTACAAATCCTCCGTTCTGCCCTTCTTATATCAATTTGAAATTCACGGG
AAGGATCTGGTCTACATGCCGGACGGGGTGAGGTCTGAGAGTTACACTCAACCATTGGAGAACGGTTT
TCTCCTCAGCTCCAG
+
FFFFFFFFFFFFFFFFFFFFFFFFFF,FFFFFFFFFFFFFFFFFFFFFFFFFFFFFFFFFFFFFFFFFF
:FFFF,F:FFFFFFFFFFFFFFFFFFFFFFFFFFFFFFFFFFFFFFFFFFFFFFFFFFFFFFFFFFFF
FFFFFFFFFFFFFFF
@A00155:342:HHGFNDSXY:1:2622:28845:34538 1:N:0:GAACCTAG+TCCGCATA
CACCTGAGATCTTAGCTGGTTACAAATCCTCCGTTCTGCCCTTCTTATATCAATTTGAAATTCACGGG
AAGGATCTGGTCTACATGCCGGACGGAGTGAGGTCTGAGAGTTACACTCAACCATTGGAGAACGGTTT
TCTCCTCAGCTCCAG
+
FFFFFFFFFFFFF,FFFFFFFFFFFFFFFFFFFFFFFFFFFFFFFFFF::FFFF:FFFFFFFFFFFFFF
FFFFFFFFFFFFFFFFFFFFFFFFFFFFFFFFFFFFFFFFFFFFFFFFFFFFFFFFFFFFFF:FFFFFF
FFFFFFFFFFFFFFF
@A00155:342:HHGFNDSXY:1:2667:1253:1376 1:N:0:GAACCTAG+TCCGCATA
CACCTGAGATCTTAGCTGGTTACAAATCCTCCGTTCTGCCCTTCTTATATCAATTTGAAATTCACGGG
AAGGATCTGGTCTACATGCCGGACGGAGTGAGGTCTGAGAGTTACACTCAACCATTGGAGAACGGTTT
TCTCCTCAGCTCCAG
+
FFFF,:F,F:F:FFF:F,:F,FFFFFFFF:FFFFF,FFF:FFFFF:FFF,FFF::FFFFFF:FFFFFF
FF,FFFFFF:FFFFFFFFFFFFFFFFFFFFFF:F,FFFFFFFFFFFFFFFFFFFF:FFFFFFFFFFFFF
:FFFFFFFFF:FFFF
@A00155:342:HHGFNDSXY:1:1357:10827:12226 2:N:0:GAACCTAG+TCCGCATA
CACCTGAGATCTTAGCTGGTTACAAATCCTCCGTTCTGCCCTTCTTATATCAATTTGAAATTCACGGG
AAGGATCTGGTCTACATGCCGGACGGGGTGAGGTCTGAGAGTTACACTCAACCATTGGAGAACGGTTT
TCTCCTCAGCTCCAG
+
FFFFFFFFFFFFFFFFFFFFFFFFFFFFFFFFFFFFFFFFFFFFFFFFFFFFFFFFFFFFFFFFFFFFF
FFFFFFFFFFFFFFFFFFFFFFFFFFFFFFFFFFFFFFFFFFFFFFFFFFFFFFFFFFFFFFFFFFFFF
FFFFFFFFFFFFFFF
@A00155:342:HHGFNDSXY:1:1223:3821:33943 1:N:0:GAACCTAG+TCCGCATA
ACCTGAGATCTTAGCTGGTTACAAATCCTCCGTTCTGCCCTTCTTATATCAATTTGAAATTCACGGGA
AGGATCTGGTCTACATGCCGGACGGGGTGAGGTCTGAGAGTTACACTCAACCATTGGAGAACGGTTTT
CTCCTCAGCTCCAGC
+
FFFFF:FFF:F,:FFFF:FFF:FFF:FFFFFFFFFFF:FFF:FFFFFFFFF:FF:FFFFFF:FFFFFF
FFFF:F:,FFFFFFF,FFFFFFF:FFFFFFFFFFFFFF::FFFFFFF:FFFFFFFFFFFFFFFFFFFF
FFFFFFFFFFFFFF:
@A00155:342:HHGFNDSXY:1:1372:7663:4366 1:N:0:GAACCTAG+TCCGCATA
ACCTGAGATCTTAGCTGGTTACAAATCCTCCGTTCTGCCCTTCTTATATCAATTTGAAATTCACGGGA
AGGATCTGGTCTACATGCCGGACGGGGTGAGGTCTGAGAGTTACACTCAACCATTGGAGAACGGTTTT
CTCCTCAGCTCCAGC

+
FFFFFFFF:FFFFFFFFFFFFFFFFFFFFFFFFFFFFFFFFFFFFFFFFFFFF:FFFFFFFFFFFFF:
FFFF:FFFFFFFFFFFFFFFFFFFFFFFFF:FFFFFFFF:FFFFFFFFFFFFFFFFF:FFFFFFFFFF
FFFFFFFFFFFFFFF
@A00155:342:HHGFNDSXY:1:1402:31810:27367 1:N:0:GAACCTAG+TCCGCATA
ACCTGAGATCTTAGCTGGTTACAAATCCTCCGTTCTGCCCTTCTTATATCAATTTGAAATTCACGGGA
AGGATCTGGTCTACATGCCGGACGGAGTGAGGTCTGAGAGTTACACTCAACCATTGGAGAACGGTTTT
CTCCTCAGCTCCAGC
+
FF,FF:FFFFFFFF,FF:F:F,F,F:FFFFFF::FFF,F:FF,FFFFFF,F:FFFF,FFFFFF:FFF,
,:F,F:FFFF::,:,FFF:F:FFFF,FFFFF:FF:F,FF:FF,::F::,FFFFFF,,FFFFFFFF:FF
:F,FF:F:FFFF:,F
@A00155:342:HHGFNDSXY:1:1625:27796:36166 1:N:0:GAACCTAG+TCCGCATA
CTCTGAGATCTTAGCTGGTTACAAATCCTCCGTTCTGCCCTTCTTATATCAATTTGAAATTCACGGGA
AGGATCTGGTCTACATGCCGGACGGGGTGAGGTCTGAGAGTTACACTCAACCATTGGAGAACGGTTTT
CTCCTCAGCTCCAGC
+
FFFFFFFFFFFFF:FFFFFFFFFFFFFFFFFFFFFFFFFFFFFFFF:FFFFFFFFFFFFFFFFFFFFF
FFFFFFFFFFFFFFFFFF:FFFFFF:FFFFFFFFFFFFFFFFFFFFFFFFFFFFFFFFFFFFFFFFFF
FFFFFFFFFFFFFFF
@A00155:342:HHGFNDSXY:1:2141:32226:32816 1:N:0:GAACCTAG+TCCGCATA
ACCTGAGATCTTAGCTGGTTACAAATCCTCCGTTCTGCCCTTCTTATATCAATTTGAAATTCACGGGA
AGGATCTGGTCTACATGCCGGACGGGGTGAGGTCTGAGAGTTACACTCAACCATTGGAGAACGGTTTT
CTCCTCAGCTCCAGC
+
FFFFFFFFFFF:FFFFFFFFFFF:FFFFFF:FFFFFFFFFFFFFFFFFFFF:FFFFF:FFFFFFFFF:
FFFFFFFFFFFFFFFFFFFFFFFFFFFFFFFFFF:FFFFFFFFF:FFFFFFFFFFFFFFFFFFFFFFF
FFF:FFFFFFFFFF:
@A00155:342:HHGFNDSXY:1:2259:12274:17112 1:N:0:GAACCTAG+TCCGCATA
ACCTGAGATCTTAGCTGGTTACAAATCCTCCGTTCTGCCCTTCTTATATCAATTTGAAATTCACGGGA
AGGATCTGGTCTACATGCCGGACGGAGTGAGGTCTGAGAGTTACACTCAACCATTGGAGAACGGTTTT
CTCCTCAGCTCCAGC
+
FFFFFFF:FFFFFFFFFFFFFFFFFFFFFFFFFFFFFFFFFFFFFFF:FFFF:FFFFFFFFFFFFFF:
FFFF:F:FFFFFFFFFFFFFFFFFFFFFFFFFFFFFFFFFFFFFFFFFFFFFFFFFFFFFFFFFFFFF
FFFFFFFFFFFFFFF
@A00155:342:HHGFNDSXY:1:2420:14479:21715 1:N:0:GAACCTAG+TCCGCATA
ACCTGAGATCTTAGCTGGTTACAAATCCTCCGTTCTGCCCTTCTTATATCAATTTGAAATTCACGGGA
AGGATCTGGTCTACATGCCGGACGGAGTGAGGTCTGAGAGTTACACTCAACCATTGGAGAACGGTTTT
CTCCTCAGCTCCAGC
+
FFFFFFFFFFFF,FFFFFFFFFFFFFFFFFFFFFFFFFFFFFFFFFFFFFFFFFFFFFFFFFFFFFF
FFFFFF:FFFFFFFFFFFFFFFFFFFFFFFFFFFFFFFFFFFFFFFFFFFFFFFFFFFFFFFFFFFF
FFFFFFFFFFFFFFF
@A00155:342:HHGFNDSXY:1:2117:12237:32362 2:N:0:GAACCTAG+TCCGCATA
CTCTGAGATCTTAGCTGGTTACAAATCCTCCGTTCTGCCCTTCTTATATCAATTTGAAATTCACGGGA
AGGATCTGGTCTACATGCCGGACGGGGTGAGGTCTGAGAGTTACACTCAACCATTGGAGAACGGTTTT
CTCCTCAGCTCCAGC
+
F:FFFFFFFFFFFFFFFFFFFFFFFFFFFFFFFFFFFFFFFFFFFFFFFFFFFFFFFFFFFFFFFFFF
FFFFFFFFFFFFFFFFFFFFFFFFF:FFFFFFFFFFFFFFFFFFFFFFFFFFFFFFFFFFFFFFFFFF
FFFFFFFFFFFF:FF
@A00155:342:HHGFNDSXY:1:2250:11089:18537 2:N:0:GAACCTAG+TCCGCATA
ACCTGAGATCTTAGCTGGTTACAAATCCTCCGTTCTGCCCTTCTTATATCAATTTGAAATTCACGGGA

AGGATCTGGTCTACATGCCGGACGGGGTGAGGTCTGAGAGTTACACTCAACCATTGGAGAACGGTTTT
CTCCTCAGCTCCAGC
+
FFFFFFFFFFFFFFFFFFFFFFFFFFFFFFFFFFFFFFFFFFFFFFF:FFFFFFFFFFFFFFFFFFFFF
FFFFFFFFFFFFFFFFFFFFFFFFFFFFFFFFFFFFFFFFFFFFFFFFFFFFFFFFFFFFFFFFFFFFF
FFFFFFFFFFFFFFF
@A00155:342:HHGFNDSXY:1:2648:23339:16047 2:N:0:GAACCTAG+TCCGCATA
ACCTGAGATCTTAGCTGGTTACAAATCCTCCGTTCTGCCCTTCTTATATCAATTTGAAATTCACGGGA
AGGATCTGGTCTACATGCCGGACGGGGTGAGGTCTGAGAGTTACACTCAACCATTGGAGAACGGTTTT
CTCCTCAGCTCCAGC
+
FFFFF:FFFF,FFF:F:FF::FFFFF:,FFFF,::FFFF,FFF:FF,FFFFF:FFFFFFFF:FFF:F,,
FF:FF:,F,FFFFFFFFF:,FFFF:FF:FFF:FFFFF:FFFFF:FFFFFFFFFFFFFFFFF:::F,:F,
FF:FFFFFFFF,F::
@A00155:342:HHGFNDSXY:1:2522:1913:25316 1:N:0:GAACCTAG+TCCGCATA
CCTGAGATCTTAGCTGGTTACAAATCCTCCGTTCTGCCCTTCTTATATCAATTTGAAATTCACGGGAA
GGATCTGGTCTACATGCCGGACGGAGTGAGGTCTGAGAGTTACACTCAACCATTGGAGAACGGTTTTC
TCCTCAGCTCCAGCT
+
FFFFFFFFF::,FF:FF:FFFFF::FFFFFFFFFFFFFFFFFFFFFFFFFF,FFFFFFFFFFFFFFFFF
FFFFF,FFFF:FFFFFFFFFFFFFFFFFFFFFFFF:FFFF::FFFFFFFFFFFFFFFFFFFFFFFF:FF
FFFFFFFFFFFFFFF
@A00155:342:HHGFNDSXY:1:1424:13449:18114 2:N:0:GAACCTAG+TCCGCATA
CCTGAGATCTTAGCTGGTTACAAATCCTCCGTTCTGCCCTTCTTATATCAATTTGAAATTCACGGGAA
GGATCTGGTCTACATGCCGGACGGAGTGAGGTCTGAGAGTTACACTCAACCATTGGAGAACGGTTTTC
TCCTCAGCTCCAGCA
+
FFFFFFFFFFFFFFFFFFFF:FFFFFFFFFFFFFFFFFFFFFF:FFFFFFFFFFFFFFFFFFFFFFFFF
FFFFFFFFFFFFFFFFFFFFFFFFFFFFFFFFFFFFFFFFFFFFFFFFFFFFFFFFFFFFFFFFFFFFF
FFFFFFFFFFFFFFF
@A00155:342:HHGFNDSXY:1:1232:19388:10113 1:N:0:GAACCTAG+TCCGCATA
CTGAGATCTTAGCTGGTTACAAATCCTCCGTTCTGCCCTTCTTATATCAATTTGAAATTCACGGGAAG
GATCTGGTCTACATGCCGGACGGGGTGAGGTCTGAGAGTTACACTCAACCATTGGAGAACGGTTTTCT
CCTCAGCT
+
FFFFFFFFFFFFFFFFFFFFFFFFFFFFFFFFFFFFFFFFFFFFFFFFFF:FFFFFFFFFFFFFFFFFF
FFFF:FFFFFFFFFFFFFFFFFFFFFFFFFFFFFFFFFFFFFFFFFFFFFFFFFFFFFFFFFFFFFFFF
FFFFFFFF
@A00155:342:HHGFNDSXY:1:2522:9598:2112 1:N:0:GAACCTAG+TCCGCATA
CTGAGATCTTAGCTGGTTACAAATCCTCCGTTCTGCCCTTCTTATATCAATTTGAAATTCACGGGAAG
GATCTGGTCTACATGCCGGACGGGGTGAGGTCTGAGAGTTACACTCAACCATTGGAGAACGGTTTTCT
CCTCAGCT
+
FFFFFFFFFFFFFFFFF:FFFFFFFFFFFFFFFFFFFFFFFFFFFFFFFFF,FFFFFFFFFFFFFFFFF
FFFFFFFFFFFFFFFFFFFFFFFFFFFFFFFFFFFFFFFFFFFFFFFFFFFFFFFFFFFFFFFFFFFFF
FFFFFFFF
@A00155:342:HHGFNDSXY:1:1170:11659:8656 1:N:0:GAACCTAG+TCCGCATA
CTGAGATCTTAGCTGGTTACAAATCCTCCGTTCTGCCCTTCTTATATCAATTTGAAATTCACGGGAAG
GATCTGGTCTACATGCCGGACGGGGTGAGGTCTGAGAGTTACACTCAACCATTGGAGAACGGTTTTCT
CCTCAGCTCCAGCAG
+
F:FFFFFFFFFFFFFFFFFFFFFFFFFFFFFFFFFFFFFFFFFFFFFFFFFFFFFFFFFFFFFFFFFFF
FFFFFFFFFFFFFFFFFFFFFFFFFFFFFFFFFFFFFFFFFFFFFFFFFFFFFFFFFFFFFFFFFFFFF
FFFFFFFFFFFFFFF

@A00155:342:HHGFNDSXY:1:2117:12237:32362 1:N:0:GAACCTAG+TCCGCATA
CTGAGATCTTAGCTGGTTACAAATCCTCCGTTCTGCCCTTCTTATATCAATTTGAAATTCACGGGAAG
GATCTGGTCTACATGCCGGACGGGGTGAGGTCTGAGAGTTACACTCAACCATTGGAGAACGGTTTTCT
CCTCAGCTCCAGCAG
+
FFFF,FFFFFFFFFFFFFFFFFFFFFFFFFFFFFFFF:FFFFFFFFFFFFF:FFFFFFFFFFFFFFFF
FFFFFFFFFFFFFFFFF:FFFFFFFFFFFFFFFFFFFFFFFFFFFFFFFFF:FFFFFFFFFFFFFF:F
FFFFFFFFFFFF:F:
@A00155:342:HHGFNDSXY:1:2554:26196:12602 1:N:0:GAACCTAG+TCCGCATA
CTGAGATCTTAGCTGGTTACAAATCCTCCGTTCTGCCCTTCTTATATCAATTTGAAATTCACGGGAAG
GATCTGGTCTACATGCCGGACGGGGTGAGGTCTGAGAGTTACACTCAACCATTGGAGAACGGTTTTCT
CCTCAGCTCCAGCAG
+
FFFFFFFFFFFFFFFFFFFFFFFFFFFFFFFFFFFFFFFFFFFFFFFFFFFFFFFFFFFFFFFFFFFF
FFFFFFFFFFFFFFFFFFFFFFFFFFFFFFFFFFFFFFFFFFFFFFFFFFFFFFFFFFFFFFFFFFFF
FFFFFFFFFFFFFFF
@A00155:342:HHGFNDSXY:1:1116:16749:5509 2:N:0:GAACCTAG+TCCGCATA
CTGAGATCTTAGCTGGTTACAAATCCTCCGTTCTGCCCTTCTTATATCAATTTGAAATTCACGGGAAG
GATCTGGTCTACATGCCGGACGGGGTGAGGTCTGAGAGTTACACTCAACCATTGGAGAATGGTTTTCT
CCTCAGCTCCAGCAG
+
F:FFF:FFFFFFFFFFF:FFFFF:FFFFFFF:F:FFFFFFFFFFFFFFFFFFFF:FFFFFFFFFFFFF
FFFFFFFFFFF:FFFFFFFF:FFFFFF:FFFFFFFFFFF:FFFFFFFFFFFFFFFFFFFFFF:F:FFF
FFFFFFFFFFFFFFF
@A00155:342:HHGFNDSXY:1:1309:23213:13072 2:N:0:GAACCTAG+TCCGCATA
CTGAGATCTTAGCTGGTTACAAATCCTCCGTTCTGCCCTTCTTATATCAATTTGAAATTCACGGGACG
GATCTGGTCTACATGCCGGACGGAGTGAGGTCTGAGAGTTACACTCAACCATTGGAGAACGGTTTTCT
CCTCAGCTCCAGAGA
+
FFFFFFFFFFFFFFFFFFFFFFFFFFFFFFFFFFFFFF:FF:FFFFFFFFFFFFFFFFFFFFFFFF,F
FFFFFFFFFFFFFFFFFFFFFFFFFFFFFFFFFFFFFFFFFFFFFFFFFFF:FFFFFFFFFFFFFFFF
FFFFFFFFFFFFFF:
@A00155:342:HHGFNDSXY:1:1361:25482:22357 2:N:0:GAACCTAG+TCCGCATA
CTGAGATCTTAGCTGGTTACAAATCCTCCGTTCTGCCCTTCTTATATCAATTTGAAATTCACGGGAAG
GATCTGGTCTACATGCCGGACGGGGTGAGGTCTGAGAGTTACACTCAACCATTGGAGAACGGTTTTCT
CCTCAGCTCCAGCAG
+
FFF:FFFFFFFF:FFFFFFFFFFFFFFFFFFFFFFFFFFFFFFFF:FFFFFFFFFFFFFFFFFFF:FF
FFFFFFFFFFFFFFFFFFFFFFFFFFFFFFFFFFFFFFFFFFFFFFFFFFF:FFFFFFFFFFFFFFFF
FFFFFFFFFFFFFFF
@A00155:342:HHGFNDSXY:1:1358:23294:23422 2:N:0:GAACCTAG+TCCGCATA
CTGAGATCTTAGCTGGTTACAAATCCTCCGTTCTGCCCTTCTTATATCAATTTGAAATTCACGGGAAG
GATCTGGTCTACATGCCGGACGGGGTGAGGTCTGAGAGTTACACTCAACCATTGGAGAACGGTTTTCT
CCTCAGCTCCAGCAG
+
FFFFFFFFFFFFFFFFFFFFFFFFFFFFFFFFFFFFFFF:FFFFFFFFFFFFFFFFFFFFFFFFFFFF
FFFFFFFFFFFFFFFFFFFFFFFFFFFFFFFFFFFFFFFF:FFFFFFFFFFFFFFFFFFFFFFFFFFF
FFFFFFFFFFFFFFF
@A00155:342:HHGFNDSXY:1:1376:20600:33473 2:N:0:GAACCTAG+TCCGCATA
CTGAGATCTTAGCTGGTTACAAATCCTCCGTTCTGCCCTTCTTATATCAATTTGAAATTCACGGGAAG
GATCTGGTCTACATGCCGGACGGGGTGAGGTCTGAGAGTTACACTCAACCATTGGAGAATGGTTTTCT
CCTCAGCTCCAGCAG
+
FFFFFFFFFFFFFFFFFFFFFFFFFFFFFFFFFFFFFFFFFFFFFFFFFFFFFFFFFFFFFFFFFFFF

```
FFFFFFFFFFFFFFFFFFFFFFFFFFFFFFFFFFFFFFFFFFFFFFFFFFFFFFFFFFFFFFFFFFFF
FFFFFFFFFFFFFFF
@A00155:342:HHGFNDSXY:1:1531:11695:16329 2:N:0:GAACCTAG+TCCGCATA
CTGAGATCTTAGCTGGTTACAAATCCTCCGTTCTGCCCTTCTTATATCAATTTGAAATTCACGGGAAG
GATCTGGTCTACATGCCGGACGGAGTGAGGTCTGAGAGTTACACTCAACCATTGGAGAACGGTTTTCT
CCTCAGCTCCAGAGA
+
FFFFFFFF,FFFFFFFFFFFFFFFFFFFFFFFFFFFFFFFF:F:F:FFFFFFFFFFFFFFFFFFFFFF
FFFFFFFFFFFFFFFFFFFFFFFFFFFFFFFFFFFFFFFFFFFFFFFFFFF:FFFFFFFFFFF:FFFF
:FFFFFFFFFFFFFF
@A00155:342:HHGFNDSXY:1:1625:27796:36166 2:N:0:GAACCTAG+TCCGCATA
CTGAGATCTTAGCTGGTTACAAATCCTCCGTTCTGCCCTTCTTATATCAATTTGAAATTCACGGGAAG
GATCTGGTCTACATGCCGGACGGGGTGAGGTCTGAGAGTTACACTCAACCATTGGAGAACGGTTTTCT
CCTCAGCTCCAGCAG
+
FFFFFFFFFFFFFFFFFFFFFFFFFFFFFFFFFFFFFFFFFFFFFFFFFFFFFFFFFFFFFFFFFFFF
FFFFFFFFFFFFFFFFFFFFFFFFFFFFFFFFFFFFFFFFFFFFFFFFFFFFFFFFFFFFFFFFFFFF
FFFFFFFFFFFFFFF
@A00155:342:HHGFNDSXY:1:2243:10167:9706 2:N:0:GAACCTAG+TCCGCATA
CTGAGATCTTAGCTGGTTACAAATCCTCCGTTCTGCCCTTCTTATATCAATTTGAAATTCACGGGAAG
GATCTGGTCTACATGCCGGACGGGGTGAGGTCTGAGAGTTACACTCAACCATTGGAGAACGGTTTTCT
CCTCAGCTCCAGCAG
+
FFFFFFFFFFF:FFFFFFFFFFFFFFFF:FFFFFFFFFFFFF:FFFFFFFFFFFFFFFFFFFFFFFFF
FFFFFFFFFFFFFFFFFFFFFFFFFFFFFFFFFF:FFFFFFFFFFFFFFFFF:FFFFFFFFFFFFFFF
FFFF:FFFFFFFFFF
@A00155:342:HHGFNDSXY:1:2504:4001:18004 2:N:0:GAACCTAG+TCCGCATA
CTGAGATCTTATCTGGTTACAAATCCTCCGTTCTGCCCTTCTTATATCAATTTGAAATTCACGGGAAG
GATCTGGTCTACATGCCGGACGGAGTGAGGTCTGAGAGTTACACTCAACCATTGGAGAACGGTTTTCT
CCTCAGCTCCAGCAG
+
::FFFFF:FFF,FFFF:FFFFFFFFFFFF,FF:FFF,,:F:FF,FF,FFFF,FFFFFF:F:FF,,FFF
FFF:F:FFFFFFFFF:FFFFFFFF,:FFFFFF::FFFF,FFFFFFFFFF:F:,,F:FFFFFFFFFFFF
FF,FFF:F,F:FFFF
@A00155:342:HHGFNDSXY:1:2617:9742:29042 2:N:0:GAACCTAG+TCCGCATA
CTGAGATCTTAGCGGGTTACAAATCCTCCGTTCTGCCCTTCTTATATCAATTTGAAATTCACGGGAAG
GATCTGGTCTACATGCCGGACGGGGTGAGGTCTGAGAGTTACACTCAACCATTGGAGAACGGTTTTCT
CCTCAGCTCCAGCAG
+
FFFFFFFFFFFFF,FFFFFFFFFFFFFFFFFFFFFFF::FF:FFFFFFFFFFF:FFFFFFFFFFFFFF
FFF:FFFFFFFFFFFFFFFF:FFFFFFFFFFFFFFFFFFF,FFFFFFFFFFF:FFFFFFFFFFFF:FF
FF:FFFFFFFF:F:F
@A00155:342:HHGFNDSXY:1:1117:25654:36464 1:N:0:GAACCTAG+TCCGCATA
TGAGATCTTAGCTGGTTACAAATCCTCCGTTCTGCCCTTCTTATATCAATTTGAAATTCACGGGAAGG
ATCTGGTCTACATGCCGGACGGGGTGAGGTCTGAGAGTTACACTCAACCATTGGAGAACGGTTTTCTC
CTCAGCTCCAGCAGC
+
:FFFF:FFFFFFFFFFFFFFFFFFFFFFFFFFFFFFFFF:FFFFFFFF,,FFFFFFFFFFFFFFF:FF
FFFFFFFFFFFFFFFFFFFFFFFFFFFFFFFFFFFFFFFFFFFFFFFFFFFFFFFFFFFFFFFFFFFF
FFFFFFFFFFFFFFF
@A00155:342:HHGFNDSXY:1:1603:17110:12994 1:N:0:GAACCTAG+TCCGCATA
TGAGATCTTAGCTGGTTACAAATCCTCCGTTCTGCCCTTCTTATATCAATTTGAAATTCACGGGAAGG
ATCTGGTCTACATGCCGGACGGGGTGAGGTCTGAGAGTTACACTCAACCATTGGAGAACGGTTTTCTC
CTCAGCTCCAACAGC
```

```
+
FFFFFF:FFFFFFFFFFFFFFFFFFFFFFFFF:FFFFFFFFFFFFFFFF,FFFFFFFFFFFFFFFF:FF
FFFFFFFFFFFFFFFFFFFFFFFFFFFFFFFFFFFFFFFFFFFFFFFFFFFFFFFFFFFFFFFFFFFFF
FFFFFFFFFFFFFFF
@A00155:342:HHGFNDSXY:1:1603:17146:13182 1:N:0:GAACCTAG+TCCGCATA
TGAGATCTTAGCTGGTTACAAATCCTCCGTTCTGCCCTTCTTATATCAATTTGAAATTCACGGGAAGG
ATCTGGTCTACATGCCGGACGGGGTGAGGTCTGAGAGTTACACTCAACCATTGGAGAACGGTTTTCTC
CTCAGCTCCAACAGC
+
FFFFFFFFFFFFFFFFFFFFFFFFFFFFFFFF,FFFFFFFFFFFFFFFFFFFFFFFFFFFFFFFFFFFF
FFFFFFFFFFFFFFFFFFFFFFFFFFFFFFFFFFFFFFFFFFFFFFFFFFFFFFFFFFFFFFFFFFFFF
FFFFFFFFFFFFFFF
@A00155:342:HHGFNDSXY:1:1645:18159:9424 1:N:0:GAACCTAG+TCCGCATA
TGAGATCTTAGCTGGTTACAAATCCTCCGTTCTGCCCTTCTTATATCAATTTGAAATTCACGGGAAGG
ATCTGGTCTACATGCCGGACGGGGTGAGGTCTGAGAGTTACACTCAACCATTGGAGAACGGTTTTCTC
CTCAGCTCCAGCAGC
+
FFFFFFFFFFFFFFFFFFFFFFFFFFFFFFFFFFF:FF:FFFFFFFF:FFFFFFFFFFF,FFFFF:FF
FFFFFFFFFFFFFFFFFFFFFFFFFFFFFFFFFFFFFFFFFFFFFFFFF:FFFFF:FFFFFFFFFFFFF
FFFFF:FFFFFFFFF
@A00155:342:HHGFNDSXY:1:1645:18213:9424 1:N:0:GAACCTAG+TCCGCATA
TGAGATCTTAGCTGGTTACAAATCCTCCGTTCTGCCCTTCTTATATCAATTTGAAATTCACGGGAAGG
ATCTGGTCTACATGCCGGACGGGGTGAGGTCTGAGAGTTACACTCAACCATTGGAGAACGGTTTTCTC
CTCAGCTCCAGCAGC
+
FFFFFF:FFFFFFFFFFFFFFFFFFFFFFFF:FFFFFFFFFFFFFFFFFFFFFFFFFFFFFFFFFFFFF
FFFFFFFFFFFFFFFFFFFFFFFFFFFFFFFFFFFFFFFFFFFFFFFFFFFFFFFFFFFFFFFFFFFFF
FFFFFFFFFFFFFFF
@A00155:342:HHGFNDSXY:1:1661:21215:4726 1:N:0:GAACCTAG+TCCGCATA
TGAGATCTTAGCTGGTTACAAATCCTCCGTTCTGCCCTTCTTATATCAATTTGAAATTCACGGGAAGG
ATCTGGTCTACATGCCGGACGGGGTGAGGTCTGAGAGTTACACTCAACCATTGGAGAACGGTTTTCTC
CTCAGCTCCAGCAGC
+
FFFFFFFFF,FFFF:FFFFFFFFFFFFFFFFFFFFFFFFFFFFFFFFFFFF,F:FFFFFFFFFFFF::
FFFFFFFFFFFF:FFFFFFFFFFFFFFFFFF:FFFFFFFFFFFFFFFFFFFFFFFFFFFFFFFF:FFFF
FFFFFFFFFFFFFFF
@A00155:342:HHGFNDSXY:1:2541:14452:30248 1:N:0:GAACCTAG+TCCGCATA
TGAGATCTTAGCTGGTTACAAATCCTCCGTTCTGCCCTTCTTATATCAATTTGAAATTCACGGGAAGG
ATCTGGTCTACATGCCGGACGGGGTGAGGTCTGAGAGTTACACTCAACCATTGGAGAACGGTTTTCTC
CTCAGCTCCAGCAGC
+
FFFFF:FFFFFFFFFFFFFFFFFFFFFFFFFFFFFFFFFFFFFFFFFFF:FFFFFFFFFFFFFFFFF
FFFFFFFFFFFFFFFFFFFFFFFFFFFFFFFFFFFFFFFFFFFFFFFFFFFFFFFFFFFFFFFFFFFFF
FFFFFFFFFFFFFFF
@A00155:342:HHGFNDSXY:1:1358:23294:23422 1:N:0:GAACCTAG+TCCGCATA
GAGATCTTAGCTGGTTACAAATCCTCCGTTCTGCCCTTCTTATATCAATTTGAAATTCACGGGAAGGA
TCTGGTCTACATGCCGGACGGGGTGAGGTCTGAGAGTTACACTCAACCATTGGAGAACGGTTTTCTCC
TCAGCTCCAGCAGCG
+
F,FFFFFFFFFFFFFFFFFFFFFFFFFFFFFF,FFFFFFFFFFF:FFFFF:FFFFFFFFFFFFFFFFFF
FFFFFFFFFFFFFFFFFFFFFFFFFFFFFFFFFFF:FFF:FFFFFFFFFFFFFFFFFFFFFFFFFFFFF
FFFFFFFFFFFFFFF
@A00155:342:HHGFNDSXY:1:2134:16297:6136 1:N:0:GAACCTAG+TCCGCATA
GAGATCTTAGCTGGTTACAAATCCTCCGTTCTGCCCTTCTTATATCAATTTGAAATTCACGGGAAGGA
```

TCTGGTCTACATGCCGGACGGAGTGAGGTCTGAGAGTTACACTCAACCATTGGAGAACGGTTTTCTCC
TCAGCTCCAGCAGCA
+
FFFFFFFFFFFFFFFFFFFFFFFFFFFFFFFFFFFFFFFFFFFFFFFFFFFFFFFFFFFFFFFFFFFF
FFFFFFFFFFFFFFFFFFFFFFFFFFFFFFFFFFFFFFFFFFFFFFFFFFFFFFFFFFFFFFFFFFFF
FFFFFFFFFFFFFFF
@A00155:342:HHGFNDSXY:1:2178:10682:2957 1:N:0:GAACCTAG+TCCGCATA
GAGATCTTAGCTGGTTACAAATCCTCCGTTCTGCCCTTCTTATATCAATTTGAAATTCACGGGAAGGA
TCTGGTCTACATGCCGGACGGGGTGAGGTCTGAGAGTTACACTCAACCATTGGAGAACGGTTTTCTCC
TCAGCTCCAGCAGCT
+
FFFFFFFFFFFFFFFFFFF:FFFFFFFFFFFFFFFFFFF:FFFFFFFFFFFFFFFFFFFFFFFFFFFF
FFFFFFFFFFFFFFFFFFFFFFFFFFFFFFFFFFFFFFFFFFFFFFFFFFFFFFFFFFFFFFFFFFFF
FFFFFFFFFFFFFFF
@A00155:342:HHGFNDSXY:1:2271:21350:17080 1:N:0:GAACCTAG+TCCGCATA
GAGATCTTAGCTGGTTACAAATCCTCCGTTCTGCCCTTCTTATATCAATTTGAAATTCACGGGAAGGA
TCTGGTCTACATGCCGGACGGGGTGAGGTCTGAGAGTTACACTCAACCATTGGAGAACGGTTTTCTCC
TCAGCTCCAGCAGCT
+
FFFFFFFFFFFFFFFFFFFFFFFFFFFFFFF,FFFFFFFFFFFFFFFFFFFFFFFFFFFFFFFFFFFF
FFFFFFFFFFFFFFFFFFFFFFFFFFFFFFFFFFFFFFFFFFFFFFFFFFFFFFFFFFFFFFFFFFFF
FFFFFFFFFFFFFFF
@A00155:342:HHGFNDSXY:1:2341:8359:8923 1:N:0:GAACCTAG+TCCGCATA
GAGATCTTAGCTGGTTACAAATCCTCCGTTCTGCCCTTCTTATATCAATTTGAAATTCACGGGAAGGA
TCTGGTCTACATGCCGGACGGGGTGAGGTCTGAGAGTTACACTCAACCATTGGAGAACGGTTTTCTCC
TCAGCTCCAGCAGCC
+
FFFFFFFFFFFFFFFFFFFFFFFFFFFFFFFFFFFFFFFFFFF:FFFFFFFFFFFFFFFFFFFFFFFF
FFFFFFFFFFFFFFFFFFFFFFFFFFFFFFFFFFFFFFFFFFFFFFFFFFFFFFFFFFFFFFFFFFFF
FFFFFFFFFFFFFFF
@A00155:342:HHGFNDSXY:1:2350:13187:25363 1:N:0:GAACCTAG+TCCGCATA
GAGATCTTAGCTGGTTACAAATCCTCCGTTCTGCCCTTCTTATATCAATTTGAAATTCACGGGAAGGA
TCTGGTCTACATGCCGGACGGAGTGAGGTCTGAGAGTTACACTCAACCATTGGAGAACGGTTTTCTCC
TCAGCTCCAGCAGCG
+
FFFFFFF:FFFFFFFFFFFFFFFFFFFFFFFFFFFFFFFFFFFFFFF:FFFFFFFFFFFFFFFFFFFF
FFFFFFFFFFFFFFFFFFFFFFFFFFFFFFFFFFFFFFFFFFFFFFFFFFFFFFFFFFFFFFFFFFFF
FFFFFFFFFFF:FFF
@A00155:342:HHGFNDSXY:1:2620:15248:30749 1:N:0:GAACCTAG+TCCGCATA
GAGATCTTAGCTGGTTACAAATCCTCCGTTCTGCCCTTCTTATATCAATTTGAAATTCACGGGAAGGA
TCTGGTCTACATGCCGGACGGAGTGAGGTCTGAGAGTTACACTCAACCATTGGAGAACGGTTTTCTCC
TCAGCTCCAGCAGCA
+
FFFF:FF,FFFFFFFFFFFFFFFFFFFFFFFFFFFFFFFFFFFFFFF:FFFFFFFFFFFFFFFFFFFF
FF:FFFFFFFFFFFFFFFFFFFFFFFFFFFFFFFFFFFFFFFFFFFFFFFFFFFFFFFFFFFFFFFFF
FFFFFFFFFFFFFFF
@A00155:342:HHGFNDSXY:1:2205:7907:11898 1:N:0:GAACCTAG+TCCGCATA
AGATCTTAGCTGGTTACAAATCCTCCGTTCTGCCCTTCTTATATCAATTTGAAATTCACGGGAAGGAT
CTGGTCTACATGCCGGACGGGGTGAGGTCTGAGAGTTACACTCAACCATTGGAGAACGGTTTTCTCCT
CAGCT
+
F,FFFFFFFFFFF,FFF:F,FFFFFFFFFFFFFFFFFFFFFFFFFFF:FFFFFFFFFFFFFFFFFFFF
FFFFFFFFFFFFFFFFFFFFFFFFFFFFFFFFFFFFFFFFFFFF:FFFFFFFFFFFFFFFFFFFFFFF
FFFFF

@A00155:342:HHGFNDSXY:1:1227:11984:30264 1:N:0:GAACCTAG+TCCGCATA
AGATCTTAGCTGGTTACAAATCCTCCGTTCTGCCCTTCTTATATCAATTTGAAATTCACGGGAAGGAT
CTGGTCTACATGCCGGACGGGGTGAGGTCTGAGAGTTACACTCAACCATTGGAGAACGGTTTTCTCCT
CAGCTCCAGCAGCAC
+
FFFFFFFFFFFFFFFFFF:FFFFFFFFFFFFFFFFFFFFFFFFFFFFFFFFFFFFFFFFFFFFFFFFF
FFFFFF,FFFFFFFFFFFFFFFFFFFFFFFFFFFFFFFFFFFFFFFFFFFFFFFFFFFFFFFFFFFFF
FFFFFFFFFFFFFF:
@A00155:342:HHGFNDSXY:1:1670:24406:34992 1:N:0:GAACCTAG+TCCGCATA
AGATCTTAGCTGGTTACAAATCCTCCGTTCTGCCCTTCTTATATCAATTTGAAATTCACGGGAAGGAT
CTGGTCTACATGTCGGACGGGGTGAGGTCTGAGAGTTACACTCAACCATTGGAGAACGGTTTTCTCCT
CAGCTCCAGCAGCAC
+
FFFFFFFFFFFFFFFFFFFFFFFFFFFFF:FFFFFFFFFFFFFFFFFFFFFFFFFFFFFFFFFFFFFF
FFFFFFFFFFFFFFFFFFFFFFFFFFFFFFFFFFFFFFFFFFFFFFFFFFFFFFFFFFFFFFFFFFFF
FFFFFFFFFFFFFFF
@A00155:342:HHGFNDSXY:1:2451:22019:33552 1:N:0:GAACCTAG+TCCGCATA
ATCTCTTAGCTGGTTACAAATCCTCCGTTCTGCCCTTCTTATATCAATTTGAAATTCACGGGAAGGAT
CTGGTCTACATGCCGGACGGGGTGAGGTCTGAGAGTTACACTCAACCATTGGAGAACGGTTTTCTCCT
CAGCTCCAGCAGCAC
+
FFFFF,FFFFFFFFFFFFFFFFFFFFFFFFFFFFFFFFFFFFFFFFFFFFFFFFFFFFFFFFFFFFFF
FFFFFFFFFFFFFFFFFFFFFFFFFFFFFFFFFFFFFFFFFFFFFFFFFFFFFFFFFFFFFFFFFFFF
FFFFFFFFFFFFF:F
@A00155:342:HHGFNDSXY:1:2553:19135:28432 1:N:0:GAACCTAG+TCCGCATA
AGATCTTAGCTGGTTACAAATCCTCCGTTCTGCCCTTCTTATTTCAATTTGAAATTCACGGGAAGGAT
CTGGTCTACATGCCGGACGGGGTGAGGTCTGAGAGTTACACTCAACCATTGGAGAACGGTTTTCTCCT
CAGCTCCAGCAGCAT
+
FFFFFFFFFFFFFFFFFFFFFFFFFFFFFFFFFFFFFFFFFFFFFFFFFFFFFFFFFFFFFFFFFFFF
FFFFFFFFFFFFFFFFF:FFFFFFFFFFFFFFFFFFFFFFFFFFFFFFFFFFFFFFFFFFFFFFFFFF
FFFFFFFFFFFFFFF
@A00155:342:HHGFNDSXY:1:1167:3631:31328 1:N:0:GAACCTAG+TCCGCATA
CTCTTAGCTGGTTACAAATCCTCCGTTCTGCCCTTCTTATATCAATTTGAAATTCACGGGAAGGATCT
GGTCTACATGCCGGACGGGGTGAGGTCTGAGAGTTACACTCAACCATTGGAGAACGGTTTTCTCCTCA
GCTCCAGCAGCATAC
+
FFFFFFFFFFFFFFFFFFFFFFFFFFFFFFFFFFFFFFFFFFFFF:FFFFFFFFFFFFFFFFFFFFFF
FFFFFFFFFFFFFFFFFFFFFFFFFFFFFFFFFFFFFFFFFFFFFFFFFFFFFFFFFFFFFFFFFFFF
FFFFFFFFFFFFFFF
@A00155:342:HHGFNDSXY:1:1652:19633:29418 1:N:0:GAACCTAG+TCCGCATA
ATCTTAGCTGGTTACAAATCCTCCGTTCTGCCCTTCTTATATCAATTTGAAATTCACGGGAAGGATCT
GGTCTACATGCCGGACGGGGTGAGGTCTGAGAGTTACACTCAACCATTGGAGAACGGTTTTCTCCTCA
GCTCCAGCAGCATAC
+
FFFF:FFFFFFFFFFFFFFFFFFFFFFFFFFFFFFFFFFFFFFFFFFFFFFFFFFFFFFFFFFFFFFF
FFFFFFFFFFFFFFFFFFFFFFFFFFFFFFFFFFFFFFFFFFFFFFFFFFFFFFFFFFFFFFFFFFFF
FFFFFFFFFFFFFFF
@A00155:342:HHGFNDSXY:1:1260:22110:20556 2:N:0:GAACCTAG+TCCGCATA
TCTTAGCTGGTTACAAATCCTCCGTTCTGCCCTTCTTATATCAATTTGAAATTCACGGGAAGGATCTG
GTCTACATGCCGGACGGGGTGAGGTCTGAGAGTTACACTCAACCATTGGAGAACGGTTTTCTCCTCAG
CTCCAGCAGCATACT
+
FFFFFFFFFFFFFFFFFFFFFFFFFFFFFF:FFFF:F:FF,FFFFFFFFFFFFFFFFFFF:FFFFFFF

```
FFFFFFFFFFFFFFF,FFFFFFFFFFFFFFFFFFFFFFFFFFFFFFFFFFFFFFFFFFFFFFFFFFFFFF
FFFFFFFFFFFFFFF
@A00155:342:HHGFNDSXY:1:1570:19307:16078 2:N:0:GAACCTAG+TCCGCATA
CTTAGCTGGTTACAAATCCTCCGTTCTGCCCTTCTTATATCAATTTGAAATTCACGGGAAGGATCTGG
TCTACATGCCGGACGGGGTGAGGTCTGAGAGTTACACTCAACCATTGGAGAACGGTTTTCTCCTCAGC
TCCAGCAGCATACT
+
FFFFFFFFFFFFFFFFFFFFFFFFFFFFFFFFFF,FFFFFFFFFFFFFFFFFFFFFFFFFFFFFFFFFF
FFFFFFFFFFFFFFFFFFFFFFFFFFFFFFFFFFFFFFFFFFFFFFFFFF:,FFFFFFFFFFFFFFFF
FFFFFFFFFFFFFF
@A00155:342:HHGFNDSXY:1:1261:15718:1157 1:N:0:GAACCTAG+TCCGCATA
CTTAGCTGGTTACAAATCCTCCGTTCTGCCCTTCTTATATCAATTTGAAATTCACGGGAAGGATCTGG
TCTACATGCCGGACGGGGTGAGGTCTGAGAGTTACACTCAACCATTGGAGAACGGTTTTCTCCTCAGC
TCCAGCAGCATACTT
+
FFFFFFFFFFFFFFFFFFFFFFFFFFFFFFFF:FFFFFFFFFFFF:FFFF:FFFFFFFFFFFFFFFFFF
FFFFFFFFFFFFFFFFFFFFFFFFFFFFFFFFFFFFFFFFFFFFF:FFFFFF:FFFFFFFFFFFFFFFF
FFFFFFFFFFFFFFF
@A00155:342:HHGFNDSXY:1:1167:3631:31328 2:N:0:GAACCTAG+TCCGCATA
CTTAGCTGGTTACAAATCCTCCGTTCTGCCCTTCTTATATCAATTTGAAATTCACGGGAAGGATCTGG
TCTACATGCCGGACGGGGTGAGGTCTGAGAGTTACACTCAACCATTGGAGAACGGTTTTCTCCTCAGC
TCCAGCAGCATACAG
+
FFFFFFFFFFFFFFFFFFFFFFFFFFFFFFFF:FFF:FFFF:FFFFFFFFFFFFFFFFFFFFFFFFFFF
FFFFFFFFFFFFF:FFFFFFFFFFFFFFFFFFFFFFFFFFFFFFFFFFFFFFF:FFFFFFFFFFFFFFFF
FFFFFFFFFFFFFF:
@A00155:342:HHGFNDSXY:1:1209:22525:17174 2:N:0:GAACCTAG+TCCGCATA
CTTAGCTGGTTACAAATCCTCCGTTCTGCCCTTCTTATATCAATTTGAAATTCACGGGAAGGATCTGG
TCTACATGCCGGACGGGGTGAGGTCTGAGAGTTACACTCAACCATTGGAGAACGGTTTTCTCCTCAGC
TCCAGCAGCATACTT
+
FFFFFFFFFFFFFFFFFFFFFFFFFFFFFFFFFFFFFFFFFFFFFFFFFFFFFFFFFFFFFFFFFFFFF
FFFFFFFFFFFFFFFFFFFFFFFFFFFFFFFFFFFFFFFFFFF:FFFFFFFFFFF:FFFFFFFFFFFF
FFFFFFFFFFFFFFF
@A00155:342:HHGFNDSXY:1:1209:21350:17550 2:N:0:GAACCTAG+TCCGCATA
CTTAGCTGGTTACAAATCCTCCGTTCTGCCCTTCTTATATCAATTTGAAATTCACGGGAAGGATCTGG
TCTACATGCCGGACGGGGTGAGGTCTGAGAGTTACACTCAACCATTGGAGAACGGTTTTCTCCTCAGC
TCCAGCAGCATACTT
+
FFFFFFFFFFFFFFFFFFFFFFFFFFFFFFFFF:FFFF,:FFFFF:FFFFFFFFFFFFFFFFFFFFFFFF
FFFFFFFFFFFFFFFFFFFFFFFFFFFFFFFFFFFFFFFFFFFFFFFFFFF:FFFFFFFFFFFFFFFF
FFFFFFFFF:FFFFF
@A00155:342:HHGFNDSXY:1:1227:24939:32002 2:N:0:GAACCTAG+TCCGCATA
CTTAGCTGGTTACAAATCCTCCGTTCTGCCCTTCTTATATCAATTTGAAATTCACGGGAAGGATCTGG
TCTACATGCCGGACGGGGTGAGGTCTGAGAGTTACACTCAACCATTGGAGAACGGTTTTCTCCTCAGC
TCCAGCAGCATACTT
+
FFFFFFFFFFFFFFFFFFFFFFFFFFFFFFFFFFFFF:F:FFFFFFFFFFFFFFFFFFFFFFFFFFFFFF
FFFFFFFFFFFFFFFFFFFFFFFFFFFFFFFFFFFFFFFFFFFFFFFFFFF:FFFFFFFF:FFFFFFF
FFFFFFFFFFFFFFF
@A00155:342:HHGFNDSXY:1:1228:25102:3223 2:N:0:GAACCTAG+TCCGCATA
CTTAGCTGGTTACAAATCCTCCGTTCTGCCCTTCTTATATCAATTTGAAATTCACGGGAAGGATCTGG
TCTACATGCCGGACGGGGTGAGGTCTGAGAGTTACACTCAACCATTGGAGAACGGTTTTCTCCTCAGC
TCCAGCAGCATACTT
```

```
+
FFF:FFFFFFFFFFFFFFFFFFFFFFFFFFFF,FF:FFFFFFFFFFFFFFFFFFFFFFFFFFFFFFFFFF
FFFFFFFF:FFFFFFFFF:FFFFFFFFFFFFFFFFFFFFFFFFFFFFFFFFFFFFFFF:FFFFFFFFFF
FFFFFFFFFFFFFFF
@A00155:342:HHGFNDSXY:1:1219:14760:7012 2:N:0:GAACCTAG+TCCGCATA
CTTAGCTGGTTACAAATCCTCCGTTCTGCCCTTCTTATATCAATTTGAAATTCACGGGAAGGATCTGG
TCTACATGCCGGACGGGGTGAGGTCTGAGAGTTACACTCAACCATTGGAGAACGGTTTTCTCCTCAGC
TCCAGCAGCATACTT
+
FFFFFFFFFFFFFFFFFFFFFFFFFFFFFFFFF,FFF:FFFFFFFFFFFFFFFFFFFFF:FFFFFFFFF
FFFFFFFFFFFFFFFFFFFFFFFFFFFFFFFFFFFFFFFFFFFF:FFFFFFFFFFFFFFFFFFFFFFFF
FFFFFFFFFFFFFF:
@A00155:342:HHGFNDSXY:1:1271:14082:18208 2:N:0:GAACCTAG+TCCGCATA
CTTAGCTGGTTACAAATCCTCCGTTCTGCCCTTCTTATATCAATTTGAAATTCACGGGAAGGATCTGG
TCTACATGCCGGACGGGGTGAGGTCTGAGAGTTACACTCAACCATTGGAGAACGGTTTTCTCCTCAGC
TCCAGCAGCATACTT
+
FFFFFFFFFFFFFFFFFFFFFFFFFFFFFFFFFFFFFFFFF:FFFFFFFFFFFF:F,FFF:FFFFFFFF
FFF:FF:F,FFFFFFFFFFFFFFFFFFFFFFFFFFFFFFFFFFFFFFFFFFFFFFFFFFFFFFFFFFFF
FFFFFFFFFFFF:F:
@A00155:342:HHGFNDSXY:1:1273:22525:36589 2:N:0:GAACCTAG+TCCGCATA
CTTAGCTGGTTACAAATCCTCCGTTCTGCCCTTCTTATATCAATTTGAAATTCACGGGAAGGATCTGG
TCTACATGCCGGACGGAGTGAGGTCTGAGAGTTACACTCAACCATTGGAGAACGGTTTTCTCCTCAGC
TCCAGCAGCATACTT
+
FFFFFFFFFFFF:FFFFFFFFFFFFFFFFFFF:FFFFFFFFFFFFFFFFFFFFFFFFFFFFFFFFFFFF
FFFFFFFFFFFFFFFFFFFFFFFFFFFFFFFFFFFFFFFFFFFFFFFFFFFFFFFFFFFFFFFFFFFFF
FFFFFFFFFFFFFFF
@A00155:342:HHGFNDSXY:1:1310:5032:23703 2:N:0:GAACCTAG+TCCGCATA
CTTAGCTGGTTACAAATCCTCCGTTCTGCCCTTCTTATATCAATTTGAAATTCACGGGAAGGATCTGG
TCTACATGCCGGACGGGGTGAGGTCTGAGAGTTACACTCAACCATTGGAGAACGGTTTTCTCCTCAGC
TCCAGCAGCATACTT
+
FFFF:FFFFFFFFF:FFFFFFFFFFFFFFFFFF:F,,F,FFFFFFFFFFFF:FFFF,FFFFFFFFFFFF
FFFFFFFFFFFFFFFFFFFFFFFFFFFFFFFFFFFFFFFFFFFF:FFFFFFFF:FFFFFFFFFFFFFFF
FFFFFFFFFFFFFFF
@A00155:342:HHGFNDSXY:1:1351:11116:24095 2:N:0:GAACCTAG+TCCGCATA
CTTAGCTGGTTACAAATCCTCCGTTCTGCCCTTCTTATATCAATTTGAAATTCACGGGAAGGATCTGG
TCTACATGCCGGACGGAGTGAGGTCTGAGAGTTACACTCAACCATTGGAGAACGGTTTTCTCCTCAGC
TCCAGCAGCATACTT
+
:FFFFFFFFFFFFFFFFFFFFFFFFFFFFFFFFFFFFFFFFFFFFFFFFFFFFFFFFFFFFFFFFFFFF
FFFFFFFFFFFFFFFFFFFFFFFFFFFFFFFFFFFFFFFFFFFFFFFFFFFFFFFFFFFFFFFFFFFFF
FFFFFFFFFFFFFFF
@A00155:342:HHGFNDSXY:1:1601:32163:17300 2:N:0:GAACCTAG+TCCGCATA
CTTAGCTGGTTACAAATCCTCCGTTCTGCCCTTCTTATATCAATTTGAAATTCACGGGAAGGATCTGG
TCTACATGCCGGACGGGGTGAGGTCTGAGAGTTACACTCAACCATTGGAGAACGGTTTTCTCCTCAGC
TCCAGCAGCATACTT
+
:F,FFFF,:FFF:FFF,,FF:FFFFFFFF,F,:FF:F,:,FFFF:FFFFFF:FFF,FF:::,FFF:FF
FFFFFFFFFFF:FF,FFFF:FFFFFFF::FFFFFFFF,,FFFFFFFFFFF:FFFFFF::FFFFFFFFF
FF:FFFFFFF:F:F:
@A00155:342:HHGFNDSXY:1:1640:28185:20588 2:N:0:GAACCTAG+TCCGCATA
CTTAGCTGGTTACAAATCCTCCGTTCTGCCCTTCTTATATCAATTTGAAATTCACGGGAAGGATCTGG
```

TCTACATGCCGGACGGGGTGAGGTCTGAGAGTTACACTCAACCATTGGAGAACGGTTTTCTCCTCAGC
TCCAGCAGCATACTT
+
FF:FFFFFFFFFFFFFFFFFFFFFFFFFFFFFFFFFFFFFFFFFFF:F:FFFFFFFFFFFFF:FFFFF
FFFFFFFFFFF:FFFFFFFFFFFFFFFFFFFFFFFFFFFFFFFFF:FFFFFFFFFFFFFFFFFFFFFF
FFFFFFFFFFFFFFF
@A00155:342:HHGFNDSXY:1:1678:24180:18098 2:N:0:GAACCTAG+TCCGCATA
CTTAGCTGGTTACAAATCCTCCGTTCTGCCCTTCTTATATCAATTTGAAATTCACGGGAAGGATCTGG
TCTACATGCCGGACGGGGTGAGGTCTGAGAGTTACACTCAACCATTGGAGAACGGTTTTCTCCTCAGC
TCCAGCAGCATACTT
+
FFFFFFFFFFFFFFFFFFFFFFFFFFFFFF:FFFFFFFFFFFFFFFFFFFFFFFFFFFF:FFFFFFFF
FFFFFFFFFFFFFFFFFFFFFFFFFFFFFFFFF:FFFFFFFFFFFFFFFFF:FFFFFFFFFFFFFFFF
FFFFFFFFFFFFFFF
@A00155:342:HHGFNDSXY:1:2222:29188:25864 2:N:0:GAACCTAG+TCCGCATA
CTTAGCTGGTTACAAATCCTCCGTTCTGCCCTTCTTATATCAATTTGAAATTCACGGGAAGGATCTGG
TCTACATGCCGGACGGGGTGAGGTCTGAGAGTTACACTCAACCATTGGAGAACGGTTTTCTCCTCAGC
TCCAGCAGCATACTT
+
FFFFFFFFFFFFFFFFFFFFFFFFFFFFFFFFFFFFFFFFFFFFFFFF:FFFFFFFFFFFFFFFFF:F
FFFFFFFFFFFFFFFFFFFFFFFFFFFFFFFFFFFFFFFFFFFFFFFFFFFF:FFFFFFFFF:FFF:F
FFFFF:FF:FFFFFF
@A00155:342:HHGFNDSXY:1:2328:5683:13275 2:N:0:GAACCTAG+TCCGCATA
CTTAGCTGGTTACAAATCCTCCGTTCTGCCCTTCTTATATCAATTTGAAATTCACGGGAAGGATCTGG
TCTACATGCCGGACGGGGTGAGGTCTGAGAGTTACACTCAACCATTGGAGAACGGTTTTCTCCTCAGC
TCCAGCAGCATACTT
+
FFFFFFFFFFFFFFFFFFFFFFFFFFFFFFFFFFFFFFFFFFFFFFFFFFFFFFFFFFFFFFFFFFFF
FFFFFFFFFFFFFFFFFFFFFFFFFFFFFFFFFFFFFFFFFFFFFFFFFFFFFFFFFFFFFFFFFFF:
FFFFFFFFFFFFFFF
@A00155:342:HHGFNDSXY:1:2423:15564:26099 2:N:0:GAACCTAG+TCCGCATA
CTTAGCTGGTTACAAATCCTCCGTTCTGCCCTTCTTATATCAATTTGAAATTCACGGGAAGGATCTGG
TCTACATGCCGGACGGGGTGAGGTCTGAGAGTTACACTCAACCATTGGAGAACGGTTTTCTCCTCAGC
TCCAGCAGCATACTT
+
FFFFFF:FFFFFFFFFFFFFFFFFFFFFF:F,FF:F,FFFFFFFFFFFFFFFF,FFFFF:FFF:FFFF
FFFFFFFFFFFFFFFFFFFFFFFFFFFFFFFFFFFFFFFF:FFFFFFFFFFFFFF:F:FFFFFFFFFF
FFFFFFFFFF:FF:F
@A00155:342:HHGFNDSXY:1:2451:22019:33552 2:N:0:GAACCTAG+TCCGCATA
CTTAGCTGGTTACAAATCCTCCGTTCTGCCCTTCTTATATCAATTTGAAATTCACGGGAAGGATCTGG
TCTACATGCCGGACGGGGTGAGGTCTGAGAGTTACACTCAACCATTGGAGAACGGTTTTCTCCTCAGC
TCCAGCAGCACAGAT
+
F,FFFFFFFFFFFFFFFFFFFFFFFFFFFFFFFFFFFFFFFFFFFFFFFFFFFFFFFFFFFFFFFFFF
FFFFFFFFFFFFFFFFFFFFFFFFFFFFFFFFFFFFFFFFFFFFFFFFFFFFFFFFFFFFFFFFFFF:
FFFFFFFFFFFFFFF
@A00155:342:HHGFNDSXY:1:2618:22128:20682 2:N:0:GAACCTAG+TCCGCATA
CTTAGCTGGTTACAAATCCTCCGTTCTGCCCTTCTTATATCAATTTGAAATTCACGGGAAGGATCTGG
TCTACATGCCGGACGGAGTGAGGTCTGAGAGTTACACTCAACCATTGGAGAACGGTTTTCTCCTCAGC
TCCAGCAGCATACTT
+
FFFFFFFFFFFFFFFFFFFFFFFFFFFFFFFFFF::FFFFFFFFFFFFFFFFFFFFFFFFFFFFFFFF
FFFFFFFFFFFFFFFFFFFFFFFFFFFFFFFFFFFFFFFFFFFFFFFFFFFFFFFFFFFFFFFFFFFF
FFFFFFFFFFFFFFF

```
@A00155:342:HHGFNDSXY:1:2641:27344:1720 2:N:0:GAACCTAG+TCCGCATA
CTTAGCTGGTTACAAATCCTCCGTTCTGCCCTTCTTATATCAATTTGAAATTCACGGGAAGGATCTGG
TCTACATGCCGGACGGGGTGAGGTCTGAGAGTTACACTCAACCATTGGAGAACGGTTTTCTCCTCAGC
TCCAGCAGCATACTT
+
FFFFFF:FFFFFFFFFFFFFFFFFFFFFFFFFF:FFF:F:FFFFFFFFFFFFFFFF:FFFFFFFFFFFFF
FFFFFFFFFFFFFFFFFFFFFFFFFFFFFFFFFFFFFFFFFFFFFFFFFFFFFFFFFFFFFF:FFFFFFF
FFFFFFFFFFFFFF:
@A00155:342:HHGFNDSXY:1:2648:18313:29669 2:N:0:GAACCTAG+TCCGCATA
CTTAGCTGGTTACAAATCCTCCGTTCTGCCCTTCTTATATCAATTTGAAATTCACGGGAAGGATCTGG
TCTACATGCCGGACGGGGTGAGGTCTGAGAGTTACACTCAACCATTGGAGAACGGTTTTCTCCTCAGC
TCCAGCAGCATACTT
+
FFFFFFFFFFFFFFFFFFFFFFFFFFFFFFFFFFFFFFFFFFFFFFFFFFFFFFFFFFFFFFFFFFFFFF
FFFFFFFFFFFFFFFFFFFFFFFFFFFFFFFFFFFFFFFFFFFFFFFFFFFFFFFFFFFFFFFFFFFFFF
FFFFF:FFFFFFFFF
@A00155:342:HHGFNDSXY:1:1203:7817:21480 1:N:0:GAACCTAG+TCCGCATA
TTAGCTGGTTACAAATCCTCCGTTCTGCCCTTCTTATATCAATTTGAAATTCACGGGAAGGATCTGGT
CTACATGCCGGACGGGGTGAGGTCTGAGAGTTACACTCAACCATTGGAGAACGGTTTTCTCCTCAGCT
CCAGCAGCATACTTG
+
FFFFFFFFFFFFFFFFFFFFFFFFFFFFFFFFFFFFFFFFF:FFFFFFFFFFFFFFFFFFFFFFFFFFFF
:FFFFFFFFFFFFFFFFFFFFFFFFFFFFFF:FFFFFFFF,FFFFFFFFFFFFFFFF:FFFFFFFFFFFF
FFFFFFFFFFFFFFF
@A00155:342:HHGFNDSXY:1:1408:20103:2456 1:N:0:GAACCTAG+TCCGCATA
TTAGCTGGTTACAAATCCTCCGTTCTGCCCTTCTTATATCAATTTGAAATTCACGGGAAGGATCTGGT
CTACATGCCGGACGGAGTGAGGTCTGAGAGTTACACTCAACCATTGGAGAACGGTTTTCTCCTCAGCT
CCAGCAGCATACTTG
+
FFFFFFFFFFFFFFFFFFFFFFFFFFFFFFFFFFFF:FFFFFFFFFFFFFFFFFFFFFFF,FFFFFFFFF
FFFFFFFFFFFFFFFFFFFFFFFFFFFFFFFFFFFFFFFFFFFFFFFFFFFFFFFFFFFFFFFFFFFFFF
FFFFFFFFFFFFFFF
@A00155:342:HHGFNDSXY:1:2229:12653:8061 1:N:0:GAACCTAG+TCCGCATA
TTAGCTGGTTACAAATCCTCCGTTCTGCCCTTCTTATATCAATTTGAAATTCACGGGAAGGATCTGGT
CTACATGCCGGACGGGGTGAGGTCTGAGAGTTACACTCAACCATTGGAGAACGGTTTTCTCCTCAGCT
CCAGCAGCATACTTG
+
FFFFFFFFFFFFFFF:FFFFFF:F:FFFFFFFFFFFFFF:FF:FFFFFFFFFFFFFFFFFFFFFFFFFFF
FFFF:FFFFFFFFFFFFFFFFFFFFFFFFFFFFFFFFFFFFFFFFFFFFFFFFFFFFFFFFFFFFFFFFF
FFFFFFFFFFFFFFF
@A00155:342:HHGFNDSXY:1:2611:4824:10629 1:N:0:GAACCTAG+TCCGCATA
TTAGCTGGTTACAAATCCTCCGTTCTGCCCTTCTTATATCAATTTGAAATTCACGGGAAGGATCTGGT
CTACATGCCGGACGGAGTGAGGTCTGAGAGTTACACTCAACCATTGGAGAACGGTTTTCTCCTCAGCT
CCAGCAGCATACTTG
+
FFFFFFF:FFF,FFFF:FFFFFFF:FFF:FFFFFFFFFFFFFFFF:F,FFFFFFFFFFFFFFFF:FFFFF
FFFFFFFFFFFFFFFFFFFFFFFFFFFFFFFFFFFFFFFFFFFFFFFFFFFFFFFFFFFFFFFFFF:FFF
FFFFFFFFFFFFFFF
@A00155:342:HHGFNDSXY:1:1303:4300:34303 2:N:0:GAACCTAG+TCCGCATA
TAGCTGGTTACAAATCCTCCGTTCTGCCCTTCTTATATCAATTTGAAATTCACGGGAAGGATCTGGTC
TACATGCCGGACGGAGTGAGGTCTGAGAGTTACACTCAACCATTGGAGAACGGTTTTCTCCTCAGCTC
CAGCAGCAT
+
FFFFF:F,FFFFFFFFF:FFFFFFFFFF,:,FFFFFFFF:,FFFFFFFFFFFFF,FFFF:FFFFFFFFFF
```

```
FFFFFFFFFFFFFFFFFFFFFFFFFFFFFFFFFFFFFFFFFFFF:FF:FFFFF:FFFFFFFFFFFFFF
FF:FFFFFF
@A00155:342:HHGFNDSXY:1:1310:1163:36918 2:N:0:GAACCTAG+TCCGCATA
TAGCTGGTTACAAATCCTCCGTTCTGCCCTTCTTATATCAATTTGAAATTCACGGGAAGGATCTGGTC
TACATGCCGGACGGGGTGAGGTCTGAGAGTTACACTCAACCATTGGAGAACGGTTTTCTCCTCAGCTC
CAGCAGCAT
+
FFFFFFFFF::FFFFFFFFFF:FFFFFFF,:FFF:F,FFFFFFFFF:FFFFFFFFFF:FFFFFFFFFF
FFF,FFFFFFFFFFFFFF:FFFFFF:FFFFF,F:FFFF:FF:::FFFF:FFFFFFFFFFFFFFFF:FF
F:FF:FFFF
@A00155:342:HHGFNDSXY:1:1301:4128:36041 2:N:0:GAACCTAG+TCCGCATA
TAGCTGGATACAAATCCTCCGTTCTGCCCTTCTTATATCAATTTGAAATTCACGGGAAGGATCTGGTC
TACATGCCGGACGGAGTGAGGTCTGAGAGTTACACTCAACCATTGGAGAACGGTTTTCTCCTCAGCTC
CAGCAGCATA
+
FFFFFFF,FFFFFFFFFFFFFFFFFFFFFF:FFF:FF:FFFFFFFFFFFFFFFFFFFFFFFFFFFFFF
FFFFFFFFFFFFFFFFFFFFFFFFFFFFFFFFFFFFFFFFFFFFFFFFFFFFFFFFFFFFFFFFFFFF
FFFFFFFFFF
@A00155:342:HHGFNDSXY:1:1339:14335:20713 2:N:0:GAACCTAG+TCCGCATA
TGGCTGGTTACAAATCCTCCGTTCTGCCCTTCTTATATCAATTTGAAATTCACGGGAAGGATCTGGTC
TACATGCCGGACGGAGTGAGGTCTGAGAGTTACACTCAACCATTGGAGAACGGTTTTCTCCTCAGCTC
CAGCAGCATA
+
FFFFFFFFFFFFFFFFFFFFFFFFFFF:FFFFFFFFFFFFFFFFFFFFFFFFFFFFFFFFFFFFFFFF
FFFFFFFFFFFFFFFFFFFFFFFFFFFFFFFFFFFFFFFFFFFFFFFFFFFFFFFFFFFFFFFFFFFF
FFFFFFFFFF
@A00155:342:HHGFNDSXY:1:1459:24867:21324 2:N:0:GAACCTAG+TCCGCATA
TAGCTGGTTACAAATCCTCCGTTCTGCCCTTCTTATATCAATTTGAAATTCACGGGAAGGATCTGGTC
TACATGCCGGACGGAGTGAGGTCTGAGAGTTACACTCAACCATTGGAGAACGGTTTTCTCCTCAGCTC
CAGCAGCATA
+
FFFFFFFFFFFFFFFFFFFFFFFFFFFFFFFFFFFFFFFFFFFFFFFFFFFFFFFFFFFFFFFFFFFF
FFFFFFFFFFFFF:FFFFFFFFFFFFFFFFFFFFFF:FFFFFFF,:FFFFFFFFFFF:FFFFFFFFFF
FFFFFFFFFF
@A00155:342:HHGFNDSXY:1:1668:27290:23516 2:N:0:GAACCTAG+TCCGCATA
TAGCTGGTTACAAATCCTCCGTTCTGCCCTTCTTATATCAATTTGAAATTCACGGGAAGGATCTGGTC
TACATGCCGGACGGAGTGAGGTCTGAGAGTTACACTCAACCATTGGAGAACGGTTTTCTCCTCAGCTC
CAGCAGCATA
+
FFFFFFFFFFFFFFFFFFFFFFFFFFFFFFFFF,FF:FFFFFFFFFFFFFFFFFFFFFFFF:FFFFFF
FFFFFFFFFFFFFFFFFFFFFFFFFFFFFFFFFFFFFF:FFFFFFFFFFFF,FFFFFFFFFFFFFFFF
FFFFFFFFFF
@A00155:342:HHGFNDSXY:1:2134:11704:36354 2:N:0:GAACCTAG+TCCGCATA
TAGCTGGTTACAAATCCTCCGTTCTGCCCTTCTTATATCAATTTGAAATTCACGGGAAGGATCTGGTC
TACATGCCGGACGGAGTGAGGTCTGAGAGTTACACTCAACCATTGGAGAACGGTTTTCTCCTCAGCTC
CAGCAGCATA
+
FFFFFFFFFFFFFFFFFFFFFFFFFFFFFFFFFFFFFFFFFFFFFFFFFFFFFFFFFFFFFFFFFFFF
FFFFFFFFFFFFFFFFFFFFFFFFFFFFFFFFFFFFFFFFFFFFFFFFFFFFFFFFFFFFFFFFFFFF
FFF:FFFFFF
@A00155:342:HHGFNDSXY:1:2638:20283:36401 2:N:0:GAACCTAG+TCCGCATA
TAGCTGGTTACAAATCCTCCGTTCTGCCCTTCTTATATCAATTTGAAATTCACGGGAAGGATCTGGTC
TACATGCCGGACGGAGTGAGGTCTGAGAGTTACACTCAACCATTGGAGAACGGTTTTCTCCTCAGCTC
CAGCAGCATA
```

+
FFFFFFFFFFFFFFFFFFFFFFFFFFFFFFFFFFFFFFFFFFFFFFFFFFFFFFFFFFFFFFFFFFFF
FFFFFFFFFFFFFFFFFFFFFFFFFFFFFFF:FFFFFFFFFFFFFFFFFFFFFFFFFFFFFFFFFFFF
FFFF:FFFFF
@A00155:342:HHGFNDSXY:1:2546:5493:18677 1:N:0:GAACCTAG+TCCGCATA
TAGCTGGTTACAAATCCTCCGTTCTGCCCTTCTTATATCAATTTGAAATTCACGGGAAGGATCTGGTC
TACATGCCGGACGGGGTGAGGTCTGAGAGTTACACTCAACCATTGGAGAACGGTTTTCTCCTCAGCTC
CAGCAGCATACTTGT
+
FFFFFFFFFFFFFFFFFFFFFFFF,FFFFFFFFFFFFFFFFFFFFFFFFFFFFFFFFFFFFFFFFFFF
FFFFFFFFFFFFFFFFFFFFFFFFFFFFFFFFFFFFFFFFFFFFFFFFFFFFFFFFFFFFFFFFFFFF
FFFFFFFFFFFFFFF
@A00155:342:HHGFNDSXY:1:1135:17228:33082 1:N:0:GAACCTAG+TCCGCATA
AGCTGGTTACAAATCCTCCGTTCTGCCCTTCTTATATCAATTTGAAATTCACGGGAAGGATCTGGTCT
ACATGCCGGACGGGGTGAGGTCTGAGAGTTACACTCAACCATTGGAGAACGGTTTTCTCCTCAGCTCC
AGCAGCATACTTGTC
+
FFFFFFFFFFFFFFFFFFFFFFFFFFFFFFFFFFFFF:F,FF:FFFFFFFFFFFFF,FF:FF:FFFFF
FFFFFFFFFFFFFFFFFFFFFFFFFFFFFFFFFFFFFFFFFFFFFFFFFFFFFFFFFFFFFFF:FFFF
FFFFFFFFFFFFFFF
@A00155:342:HHGFNDSXY:1:1136:14669:23766 1:N:0:GAACCTAG+TCCGCATA
AGCTGGTTACAAATCCTCCGTTCTGCCCTTCTTATATCAATTTGAAATTCACGGGAAGGATCTGGTCT
ACATGCCGGACGGGGTGAGGTCTGAGAGTTACACTCAACCATTGGAGAACGGTTTTCTCCTCAGCTCC
AGCAGCATACTTGTC
+
FFFFFFFFFFFFFFFFFFFFFFFFFFFFFFFFFFFFFFFF:FFFFFFFFFFFFFFFFFFFFF,FFFFF
FFFFFFFFFFFFFFFFFFFFFFFFFFFFFFFFFFFFFFFFFFFFFFFFFFFFFFFFFFFFFFFFFFFF
FFFFFFFFFFFFFFF
@A00155:342:HHGFNDSXY:1:1230:6036:14638 1:N:0:GAACCTAG+TCCGCATA
AGCTGGTTACAAATCCTCCGTTCTGCCCTTCTTATATCAATTTGAAATTCACGGGAAGGATCTGGTCT
ACATGCCGGACGGGGTGAGGTCTGAGAGTTACACTCAACCATTGGAGAACGGTTTTCTCCTCAGCTCC
AGCAGCATACTTGTC
+
FFFFFFF,FFFFFFFFFFFFFFFFFFFFFFFFFFFFFFFFFFFFFFFFFFFFFFF:FFFFF:FFFFFF
FFFFFFFFFFFFFFFF:FFFFFFFFFFF:FFFFFFFFFFFFFFFF:FFFFFFFFFFFFFFFFFFFFFF
FFFFFFFFFFFFFFF
@A00155:342:HHGFNDSXY:1:1351:11116:24095 1:N:0:GAACCTAG+TCCGCATA
AGCTGGTTACAAATCCTCCGTTCTGCCCTTCTTATATCAATTTGAAATTCACGGGAAGGATCTGGTCT
ACATGCCGGACGGAGTGAGGTCTGAGAGTTACACTCAACCATTGGAGAACGGTTTTCTCCTCAGCTCC
AGCAGCATACTTGTC
+
FFFFFFFFFFFFFFFFFFFFFFF,FFFFFFFFFFFFFFFFFFFFFFFFFFFFFFFFFFFFFFFFFFFF
FF:FFFFFFFFFFFFFFFFFFFFFFFFFF:FFFFFFFFFFFFFFFFFFFFFFFFFFFFFFFFFFFFFF
FFFFFFFFFFFFFFF
@A00155:342:HHGFNDSXY:1:1351:19081:30968 1:N:0:GAACCTAG+TCCGCATA
AGCTGGTTACAAATCCTCCGTTCTGCCCTTCTTATATCAATTTGAAATTCACGGGAAGGATCTGGTCT
ACATGCCGGACGGGGTGAGGTCTGAGAGTTACACTCAACCATTGGAGAATGGTTTTCTCCTCAGCTCC
AGCAGCATACTTGTC
+
FFFFFFFFFFFFFFFFFFFFFFFFFFFF,:FFF:FFFFFFFFFFFFFFFFFFFFFFFFFFFFFFFFFF
FFFFFFFFFFFFFFFFFFFFFFFFFFFFFFFFFFFFFFFFFFFFFFFFFFFFFFFFFFFFFFFFFFFF
FFFFFFFFFFFFFFF
@A00155:342:HHGFNDSXY:1:1409:21974:4351 1:N:0:GAACCTAG+TCCGCATA
AGCTGGTTACAAATCCTCCGTTCTGCCCTTCTTATATCAATTTGAAATTCACGGGAAGGATCTGGTCT

ACATGCCGGACGGGGTGAGGTCTGAGAGTTACACTCAACCATTGGAGAACGGTTTTCTCCTCAGCTCC
AGCAGCATACTTGTC
+
FFFFFFFFFFFFFFFFFFFF:FFFFFFFFFFFFFFFFFFF,FFFFFFFFFFFFFFFFFFFFFFFFFFF
FFFFFFFFFFFFFFFFFFFFFFFFFFFFFFFFFFFFF:FFFFFFFFFFFFFFFFFFFFFFFF:FFFFF
FFFFFFFFFFFFFFF
@A00155:342:HHGFNDSXY:1:1560:25400:32894 1:N:0:GAACCTAG+TCCGCATA
AGCTGGTTACAAATCCTCCGTTCTGCCCTTCTTATATCAATTTGAAATTCACGGGAAGGATCTGGTCT
ACATGCCGGACGGGGTGAGGTCTGAGAGTTACACTCAACCATTGGAGAACGGTTTTCTCCTCAGCTCC
AGCAGCATACTTGTC
+
FFFFFFFFFFF:FFFFFFFFFFFFFFF:FFFFFFFFFFFFFFFFFFFFFFFFFFFFFFF:FFFFFFFF
FFFFFFFFFFFFFFFFFFFFFFFFFFFFFFFFFFFFFFFFFFFFF:FFFFFFFFFFFFFFFFFFFFFF
FFFFFFFFFFFFFFF
@A00155:342:HHGFNDSXY:1:2121:27019:4914 1:N:0:GAACCTAG+TCCGCATA
AGCTGGTTACAAATCCTCCTTTCTGCCCTTCTTATATCAATTTGAAATTCACGGGAAGGATCTGGTCT
ACATGCCGGACGGGGTGAGGTCTGAGAGTTACACTCAACCATTGGAGAACGGTTTTCTCCTCAGCTCC
AGCAGCATACTTGTC
+
FF,F,F:F::F:F:,FF,F,,:,FF,FF:,::FFFFF,FFF,FF:FFFFFFFFFF,,FFFFFF:FF,F
,F,FF,FFF::,FF,FFF,FF:FF,FFF:F,,,,F:F:,F,FFF:FF:,F,FFFFF:::FF,,F::F:
FFFFFF:FF,FF,FF
@A00155:342:HHGFNDSXY:1:2366:6958:29888 1:N:0:GAACCTAG+TCCGCATA
AGCTGGTTACAAATCCTCCGTTCTGCCCTTCTTATATCAATTTGAAATTCAAGGGAAGGATCTGGTCT
ACATGCCGGACGGGGTGAGGTCTGAGAGTTACACTCAACCATTGGATAAAGGTGTTCTCCTCAGCTCC
AGCAGCATACTTGTC
+
:F,F:FFFF,::F:FFF:F,FFFFFF,,FFFFFFFFF,F,FF:FFFFFF:,,FFF:,FFFFFF:FF,F
,F:FFFFF::FFF,FFFF,FF,FFFFFFFFFFF,F:FFFFF:FFFF,F:,::F,FF:FF:F,,F,FFF
F,FFFFFFF,FFFFF
@A00155:342:HHGFNDSXY:1:2573:19190:1125 1:N:0:GAACCTAG+TCCGCATA
TTCCGATCTCAAATCCTCCGTTCTGCCCTTCTTATATCAATTTGAAATTCACGGGAAGGATCTGGTCT
ACATGCCGGACGGGGTGAGGTCTGAGAGTTACACTCAACCATTGGAGAACGGTTTTCTCCTCAGCTCC
AGCAGCATACTTGTC
+
:FFFFFFFFF,F:FFFFFFF,FFFFFFFFFFF:FFFFFFF:FFFFFFFFF:FFFFF,FFFFFFFFFFF
FFFFFFFFFFFFFFFFFFFFFFFFFFFFFFFFFFFFFFFFFFFFFFFFFFFFFFFFFFFFFFFFFFFF
FFFFFFFFFFFFFFF
@A00155:342:HHGFNDSXY:1:1122:29523:4899 2:N:0:GAACCTAG+TCCGCATA
AGCTGGTTACAAATCCTCCGTTCTGCCCTTCTTATATCAATTTGAAATTCACGGGAAGGATCTGGTCT
ACATGCCGGACGGAGTGAGGTCTGAGAGTTACACTCAACCATTGGAGAACGGTTTTCTCCTCAGCTCC
AGCAGCATACTTGTC
+
FFFFFFF:FFFFFFFFFFFFFFFFFFFFFFFFF:FFFFFFFFFFFFFFFFFFFFFF:FFFFFFFFFFF
FFFFFFFFFFFFFFFFFFFFFFFFFFFFFFFFFFFFFFFFFFFFFFFFFFFFF:FFFFFFFFFFFFFF
FFFFFFFFFFFFFFF
@A00155:342:HHGFNDSXY:1:1136:27787:9909 2:N:0:GAACCTAG+TCCGCATA
AGCTGGTTACAAATCCTCCGTTCTGCCCTTCTTATATCAATTTGAAATTCACGGGAAGGATCTGGTCT
ACATGCCGGACGGAGTGAGGTCTGAGAGTTACACTCAACCATTGGAGAACGGTTTTCTCCTCAGCTCC
AGCAGCATACTTGTC
+
FFFFFFFFFFFFFFFFFFFFFFFFFFFFF:FFFFFFFFFFFFFFFFFFFFFFF:FFFFFFFFFFFFFF
FFFFFFFFFF:FFFFFFFFFFFFFFFFFFFFFFFFFFFFFFFFFFFFFF:FFFFFFFFFFFFFFFFFF
FFFFFFFFFFFFFFF

```
@A00155:342:HHGFNDSXY:1:2531:16297:35978 2:N:0:GAACCTAG+TCCGCATA
GCTGGTTACAAATCCTCCGTTCTGCCCTTCGGATATCAATTTGAAATTCACGGGAAGGATCTGGTCTA
CATGCCGGACGGGGTGAGTTCTGAGAGTTACACGCAACCATTGGTGAACGGTTTTCTCCTCAGCTCCA
GCAGCATACTTGT
+
FFFF:FFFFFFFFFFF,FFFFFF:,FF,F,,,FFF,FFFFF:FF:F,F:F:FFFFFFFFFF::FFFFF
FF,::FFFF:F,FFFF,:,,F::FF:,FFF,F,,,F:FFF::FF,F,F::,FFFFFF,F:,::FF,:F
FFF::,:F,,:,F
@A00155:342:HHGFNDSXY:1:1313:25491:26694 1:N:0:GAACCTAG+TCCGCATA
GCTGGTTACAAATCCTCCGTTCTGCCCTTCTTATATCAATTTGAAATTCACGGGAAGGATCTGGTCTA
CATGCCGGACGGAGTGAGGTCTGAGAGTTACACTCAACCATTGGAGAACGGTTTTCTCCTCAGCTCCA
GCAGCATACTTGTCA
+
FFFF:FFFFFFFFFFFFF::,FF:FFFFFFF:FFF,FFF,FFFFFFFFFFFF:FFF,FF,,FFF,FF
FFFFFFF:FFFFFFFFFFFFFFF:FFFFFF:FFFFFF,FF:F,FF:FF::FFFFFFFFFFFFF:FFFF
F:F,FFFFF:FFFFF
@A00155:342:HHGFNDSXY:1:1313:25482:32847 1:N:0:GAACCTAG+TCCGCATA
GCTGGTTACAAATCCTCCGTTCTGCCCTTCTTATATCAATTTGAAATTCACGGGAAGGATCTGGTCTA
CATGCCGGACGGAGTGAGGTCTGAGAGTTACACTCAACCATTGGAGAACGGTTTTCTCCTCAGCTCCA
GCAGCATACTTGTCA
+
FFFFFFFFFFFFFFFFFFFFFFFFFFFFFFFFFFFFFFFFFFFFFFFFFFFFFFFFFFFFFFFFFFFF
FFFFFFFFFFFFFFFFFFFFFFFFFFFFFFFFFFFFFFFFFFFFFFFFFFFFFFFFFFFFF:FFFFFF
FFFFFFFFFFF:FFF
@A00155:342:HHGFNDSXY:1:1642:17273:24956 1:N:0:GAACCTAG+TCCGCATA
GCTGGTTACAAATCCTCCGTTCTGCCCTTCTTATATCAATTTGAAATTCACGGGAAGGATCTGGTCTA
CATGCCGGACGGGGTGAGGTCTGAGAGTTACACTCAACCATTGGAGAACGGTTTTCTCCTCAGCTCCA
GCAGCATACTTGTCA
+
FFFFFFFFFFFFFFFFFFFFFFFFFFFFFFFFFFFFFFFFFFFFFFFFFFFFFFFFFFFFFFFFFFFF
FFFFFFFFFFFFFFFFFFFFFFFFFFFFFFFFFFFFFFFFFFFFFFFFFFFFFFFFFFFFFFFFFFFF
FFFFFFFFFFFFFFF
@A00155:342:HHGFNDSXY:1:2234:25129:35305 1:N:0:GAACCTAG+TCCGCATA
GCTGGTTACAAATCCTCCGTTCTGCCCTTCTTATATCAATTTGAAATTCACGGGAAGGATCTGGTCTA
CATGCCGGACGGGGTGAGGTCTGAGAGTTACACTCAACCATTGGAGAACGGTTTTCTCCTCAGCTCCA
GCAGCATACTTGTCA
+
FFFFFFFFFFFFFFFFFFFFFFFFFFFFFFFFFFFFFFFFFFFFFFFFFFFFFFFFFFFFFFFFFFFF
FFFFFFFFFFFFFFFFFFFFFFFFFFFFFFFFFFFFFFFFFFFFFFFFFFFFFFFFFFFFFFFFFFFF
FFFFFFFFFFFFFFF
@A00155:342:HHGFNDSXY:1:1152:9082:20040 2:N:0:GAACCTAG+TCCGCATA
GCTGGTTACAAATCCTCCGTTCTGCCCTTCTTATATCAATTTGAAATTCACGGGAAGGATCTGGTCTA
CATGCCGGACGGGGTGAGGTCTGAGAGTTACACTCAACCATTGGAGAACGGTTTTCTCCTCAGCTCCA
GCAGCATACTTGTCA
+
FFFFFFFFFFFFFFFFFFFFFFFFFFFFFFFFFFFFFFFFFFFFFFFFFFFFFFFFFFFFFFFFFFFF
FFFFFFFFFFFFFFFFFFFFFFFF:FFFFFFFFFFFFFFFFFFFFFFF:FFFFFFFFFFFFFFFFFFF
FFFFF:FFFFFFFFF
@A00155:342:HHGFNDSXY:1:1133:12599:1705 2:N:0:GAACCTAG+TCCGCATA
GCTGGTTACAAATCCTCCGTTCTGCCCTTCTTATATCAATTTGAAATTCACGGGAAGGATCTGGTCTA
CATGCCGGACGGGGTGAGGTCTGAGAGTTACACTCAACCATTGGAGAACGGTTTTCTCCTCAGCTCCA
GCAGCATACTTGTCA
+
F:FFFFFFFFFFFFFFFFF:FFFFFFFFFFFFFFF:FFFFFFFFFFFFFFFFFFF:FFFFFFFFFFFF
```

FFFFFFFFFFFFFFFFFFFFFFFFFFFFFFFFFFFFFFFFFFFFFFFFFFFFFFFFFFFFFFFFFFFF
FFFFFFFFFFFFFFF
@A00155:342:HHGFNDSXY:1:1201:10257:36636 2:N:0:GAACCTAG+TCCGCATA
GCTGGTTACAAATCCTCCGTTCTGCCCTTCTTATATCAATTTGAAATTCACGGGAAGGATCTGGTCTA
CATGCCGGACGGGGTGAGGTCTGAGAGTTACACTCAACCATTGGAGAACGGTTTTCTCCTCAGCTCCA
GCAGCATACTTGTCA
+
:FFFFFFFFFFFFF:FFFF,FFFFF::F:F:FFFFFFFFFFFFFFFFFFFFFFFF:FFFFFFFFFFFF
F:FFFFFFFFFFFF:FFFFFFFFFFFF:FFFFFFFFFFFF:FFFFFFFFFFFFFFFFFFFFFFFFF:F
FFFFFFFFFFFFFFF
@A00155:342:HHGFNDSXY:1:1223:11415:20885 2:N:0:GAACCTAG+TCCGCATA
GCTGGTTACAAATCCTCCGTTCTGCCCTTCTTATATCAATTTGAAATTCACGGGAAGGATCTGGTCTA
CATGCCGGACGGAGTGAGGTCTGAGAGTTACACTCAACCATTGGAGAACGGTTTTCTCCTCAGCTCCA
GCAGCATACTTGTCA
+
FFFFFFFFFFFFFFFFFFFFFFFFFFFFFFFFFFFFFFFFFFFFFFFFFFFFFFFFF:FFFFFFFFFF
FFFFFFFFFFFFFFFFFFFFFFFFFFFFFFFFFFFFFFFFFFFFFFFFFFFFFFFFFFFFFFFFFFFF
FFFFFFFFFFFFFFF
@A00155:342:HHGFNDSXY:1:1239:18041:34021 2:N:0:GAACCTAG+TCCGCATA
GCTGGTTACAAATCCTCCGTTCTGCCCTTCTTATATCAATTTGAAATTCACGGGAAGGATCTGGTCTA
CATGCCGGACGGGGTGAGGTCTGAGAGTTACACTCAACCATTGGAGAACGGTTTTCTCCTCAGCTCCA
GCAGCATACTTGTCA
+
FFFFFFFFFFFFFFFFFFFFFFFFFFFFFF:FFFFFFFFFFFFFFFFFFFFFFFFFFFFFFFFFFFFF
FFFFFFFFFFFFFFFFFFFFFFFFFFFFFFFFFFFFFFFFFFFFFFFFFFFFFFFFFFFFFFFFFFFF
FFFFFFFFFFFFFFF
@A00155:342:HHGFNDSXY:1:1424:32271:29199 2:N:0:GAACCTAG+TCCGCATA
GCTGGTTACAAATCCTCCGTTCTGCCCTTCTTATATCAATTTGAAATTCACGGGAAGGATCTGGTCTA
CATGCCGGACGGGGTGAGGTCTGAGAGTTACACTCAACCAGTGGAGAACGGTTTTCTCCTCAGCTCCA
GCAGCATACTTGTCA
+
FFFF,FFFFFFFFFFFFFFFFFFFFF:,FFF,:,,:FFFFF,FFF:FFFFFFFFFF:FF:F,FFFF:F
FFFFFF:F:FFFFF:FFFFFFFFFFFFFF:FFFFFF,FFF,FFFFFF,F,,F,:FFFFFFFFFFFF:F
FF,,FF,FF,,FFFF
@A00155:342:HHGFNDSXY:1:1516:16830:3239 2:N:0:GAACCTAG+TCCGCATA
GCTGGTTACAAATCCTCCGTTCTGCCCTTCTTATATCAATTTGAAATTCACGGGAAGGATCTGGTCTA
CATGCCGGACGGGGTGAGGTCTGAGAGTTACACTCAACCATTGGAGAACGGTTTTCTCCTCAGCTCCA
GCAGCATACTTGTCA
+
F:FFFFFFFFFFFFFFFFFFFFFFFFFFFFFFFFFFFFFFFFFFFFFFFFFFFFFFFFFFFFFFFFFF
FFFFFFFFFFFFFFFFFFFFFFFFFFFFFFFFFFFFFFFFFFFFFFFFFFFFFFFFFFFFFFFFFFFF
FFFFFFFFFF:FFFF
@A00155:342:HHGFNDSXY:1:1640:4327:21793 2:N:0:GAACCTAG+TCCGCATA
GCTGGTTACAAATCCTCCGTTCTGCCCTTCTTATATCAATTTGAAATTCACGGGAAGGATCTGGTCTA
CATGCCGGACGGAGTGAGGTCTGAGAGTTACACTCAACCATTGGAGAACGGTTTTCTCCTCAGCTCCA
GCAGCATACTTGTCA
+
FFFFFFFFFFFFFFFFFFFFFFFFFFF:FFFF:FFFFFFFFFFFFFFFFFFFFFFFFFFFFFFFFFFF
FFFFFFFFFFFFFFFFFFFFFFFFFFFFFFFFFFFFFF:FFFF,,FFFFFFFFFFFFF:FFFFFFFFF
FFFFFFFFFFFFFFF
@A00155:342:HHGFNDSXY:1:1667:10610:15264 2:N:0:GAACCTAG+TCCGCATA
GCTGGTTACAAATCCTCCGTTCTGCCCTTCTTATATCAATTTGAAATTCACGGGAAGGATCTGGTCTA
CATGCCGGACGGAGTGAGGTCTGAGAGTTACACTCAACCATTGGAGAACGGTTTTCTCCTCAGCTCCA
GCAGCATACTTGTCA

+
FFFFFFFFFFFFFFFFFFF:FFFFFFFFFFF:,FFFFFFFFFFFFFFF,FFFFFFFFFFFFFFFFF
FFF,FFFFFFFFFFFFF:FFFFFFFFFFFFFFFFFFFFFFFFFFFFFFFFFFFFFFFFFFFFFFFF
FFFFFFFFFF,:FFF
@A00155:342:HHGFNDSXY:1:2130:11659:34616 2:N:0:GAACCTAG+TCCGCATA
GCTGGTTACAAATCCTCCGTTCTGCCCTTCTTATATCAATTTGAAATTCACGGGAAGGATCTGGTCTA
CATGCCGGACGGAGTGAGGTCTGAGAGTTACACTCAACCATTGGAGAACGGTTTTCTCCTCAGCTCCA
GCAGCATACTTGTCA
+
:FFFFFFFFFFFFFFFFFFFFFFFFFFFFFFFFFFFFFFFFFFFFFFFFFFFFFFFFFFFFFFFFFFF
FFFFFFFFFFFFFFFFFFFFFFFFFFFFFFFFFFF:FFF::FFFFFFFFFFFFF:FFFFFF:FFFFFF
FFFFFFFFFF:FFFF
@A00155:342:HHGFNDSXY:1:2223:5556:35196 2:N:0:GAACCTAG+TCCGCATA
GCTGGTTACAAATCCTCCGTTCTGCCCTTCTTATATCAATTTGAAATTCACGGGAAGGATCTGGTCTA
CATGCCGGACGGAGTGAGGTCTGAGAGTTACACTCAACCATTGGAGAACGGTTTTCTCCTCAGCTCCA
GCAGCATACTTGTCA
+
F::FFFF:FFFF::FFFFFFFFFFF:F:FFFFFFFF,FFFFFFFFFFFFFFFFFFFFFFFFFFF,FFF
FFFFFFFFFFF:FFFFF:FFFFFFFFFFFFFFFFFFFFFF:F::FF:FF::FF:FFFFFFFFFFFF:FF
FF:::,FFFFF:,FF
@A00155:342:HHGFNDSXY:1:2319:22453:1579 2:N:0:GAACCTAG+TCCGCATA
GCTGGTTACAAATCCTCCGTTCTGCCCTTCTTATATCAATTTGAAATTCACGGGAAGGATCTGGTCTA
CATGCCGGACGGGGTGAGGTCTGAGAGTTACACTCAACCATTGGAGAACGGTTTTCTCCTCAGCTCCA
GCAGCATACTTGTCA
+
FFFFFFFFFFFFFFFFFFFFFFFFFF:FFFFFFFFFFFFFFFFFFFFFFFFFFFFFFFFFFFFFFFFF
FF:FFFFFFFFFFFFFFFFFFFFFFFFFFFFFFFFFFFFFFFFFFFFFFFFFFFFFFFFFFFFFFFFF
FFFFFFFFFFFFFFF
@A00155:342:HHGFNDSXY:1:2354:11125:5071 2:N:0:GAACCTAG+TCCGCATA
GCTGGTTACAAATACTCCGTTCTGCCCTTCTTATATCAATTTGAAATTCACGGGAAGGATCTGGTCTA
CATGCCGGACGGAGTGAGGTCTGAGAGTTACACTCAACCATTGGAGAACGGTTTTCTCCTCAGCTCCA
GCAGCATACTTGTCA
+
FFFFFFFFFFFFFFFFFFFFFFFFFFFFFFFFFFFFFFFFFFFFFFFFFFFFFFFFFFFFFFFFFFFF
FFFFFFFFFFFFFFFFFFFFFFFFFFFFFFFFFFFFFFFFFFFFFFFFFFFFFFFFFFFFFFFFFFFF
FFFFFFFFFFFFFFF
@A00155:342:HHGFNDSXY:1:2473:12563:13542 2:N:0:GAACCTAG+TCCGCATA
GCTGGTTACAAATCCTCCGTTCTGCCCTTCTTATATCAATTTGAAATTCACGGGAAGGATCTGGTCTA
CATGCCGGACGGGGTGAGGTCTGAGAGTTACACTCAACCATTGGAGAACGGTTTTCTCCTCAGCTCCA
GCAGCATACTTGTCA
+
FFFFFFFFFFFFFFFFFFFFFFFFFFFFFFFF::FF:FFFFFFFFFFFFFFFFFFFFFFFFFFFFFFFF
FFFFFFFFFFFFFFFFFFFFFFFFFFFFFFFFFFFFFFFFFFFFFFFFFF:FFFFFFFFFF:FFFFFF
FFFFFFFFFF:FFF:
@A00155:342:HHGFNDSXY:1:1418:5077:28166 1:N:0:GAACCTAG+TCCGCATA
GATCTTACAAATCCTCCGTTCTGCCCTTCTTATATCAATTTGAAATTCACGGGAAGGATCTGGTCTAC
ATGCCGGACGGGGTGAGGTCTGAGAGTTACACTCAACCATTGGAGAACGGTTTTCTCCTCAGCTCCAG
CAGCATACTTGTCAG
+
FFFFFFFFFF:FFFFFFFFFFFFFFFFFFFFFFFFFFFFFFFFFFFFFFFFFFFFFFFFFFFFFFFFF
FFFFFFFFFFFFFFFFFFFFFFFFFFFFFFFFFFFFFFFFFFFFFFFFFFFFFFFFFFFFFFFFFFFF
FFFFFFFFFFFFFFF
@A00155:342:HHGFNDSXY:1:1418:5122:28244 1:N:0:GAACCTAG+TCCGCATA
GATCTTACAAATCCTCCGTTCTGCCCTTCTTATATCAATTTGAAATTCACGGGAAGGATCTGGTCTAC

```
ATGCCGGACGGGGTGAGGTCTGAGAGTTACACTCAACCATTGGAGAACGGTTTTCTCCTCAGCTCCAG
CAGCATACTTGTCAG
+
FFFFFFFFFFFFFFFFFFFFFFFFFFFFFFFFFFFFFFFFF:FFFFFFFFFFFFFFFFFFFFFFFFFFFF
FFFFFFFFFFFFFFFFF:FFFFFFFFFFFFFFFFFFF:FFFFFFFFFFFF:FFFFFFFFFFFFFFFFFFF
FFFFFFFFFFFFFFF
@A00155:342:HHGFNDSXY:1:1509:10899:9189 1:N:0:GAACCTAG+TCCGCATA
CTGGTTACAAATCCTCCGTTCTGCCCTTCTTATATCAATTTGAAATTCACGGGAAGGATCTGGTCTAC
ATGCCGGACGGGGTGAGGTCTGAGAGCTACACTCAACCATTGGAGAACGGTTTTCTCCTCAGCTCCAG
CAGCATACTTGTCAG
+
FFFFFFFFFFFFFFFFFFFFFFFFFFF:,FF:FFFFFFFFFFFFFFFFFFFFFFFFFFFFFFFFFFFFFF
FFFFFFFFFFFFFFFFFFFFFFFFFFF,FFFFFFFFFFFFFFFFFFFFFFFFFFFFFFFFFFFFFFFFFF
FFFFFFFFFFFFFFF
@A00155:342:HHGFNDSXY:1:1563:31494:35618 1:N:0:GAACCTAG+TCCGCATA
CTGGTTACAAATCCTCCGTTCTGCCCTTCTTATATCAATTTGAAATTCACGGGAAGGATCTGGTCTAC
ATGCCGGACGGGGTGAGGTCTGAGAGTTACACTCAACCATTGGAGAACGGTTTTCTCCTCAGCTCCAG
CAGCATACTTGTCAG
+
FFFFFFFFFFFFFFFFFFFFFFFFFFFFFFFFFFFFFFFFFFFFFFF:FFFFFFF,FFFFFFFFFFFFFF
FFFFFFFFFFFFFFFFFFFFFFFFFFFFFFFFFFFFFFFFFFFFFFFF:FFFFFFFFFFFFFFFFFFFFF
FFFFFFFFFFFFFFF
@A00155:342:HHGFNDSXY:1:1678:24180:18098 1:N:0:GAACCTAG+TCCGCATA
CTGGTTACAAATCCTCCGTTCTGCCCTTCTTATATCAATTTGAAATTCACGGGAAGGATCTGGTCTAC
ATGCCGGACGGGGTGAGGTCTGAGAGTTACACTCAACCATTGGAGAACGGTTTTCTCCTCAGCTCCAG
CAGCATACTTGTCAG
+
FFFFFFFF:FFFFFFFFFFFFFFFFFFFF:FFFFFFFF:FFFFFFF:FFFFFFFFFFFFFFFFFFFFFFF
FFFFFFFFFFFFFFFFFFFFFFFFFFFFFFFFFFFFFFFFFFFFFFFFFFFFFFFFFFFFFFFFFFFFFF
FFFFFFFFFFFFFFF
@A00155:342:HHGFNDSXY:1:2332:16089:31203 1:N:0:GAACCTAG+TCCGCATA
CTGGTTACAAATCCTCCGTTCTGCCCTTCTTATATCAATTTGAAATTCACGGGAAGGATCTGGTCTAC
ATGCCGGACGGGGTGAGGTCTGAGAGTTACACTCAACCATTGGAGAACGGTTTTCTCCTCAGCTCCAG
CAGCATACTTGTCAG
+
FFFFFFFFF:FFFFFFFF::FFF:FFFFFFFFFF:FFF:FFFFFFFFFFFFFFFFF:F:FFFFFFFFFFF
FFFFFFFFF:FFFFFF:FFFFFFFFFFFFFFFFFFFFFFFFFFFFFFFFFFFFFFFFFFFFFFFFFFFFF
FFFF:FFFFFFFFFF
@A00155:342:HHGFNDSXY:1:2348:32271:11443 1:N:0:GAACCTAG+TCCGCATA
CTGGTTACAAATCCTCCGTTCTGCCCTTCTTATATCAATTTGAAATTCACGGGAAGGATCTGGTCTAC
ATGCCGGACGGGGTGAGGTCTGAGAGTTACACTCAACCATTGGAGAACGGTTTTCTCCTCAGCTCCAG
CAGCATACTTGTCAG
+
FFFFFF:FF::,FFFF:F,:FFFFFFFFFFFF,F:::,:F,FFFF,:FF:FFFFFFFFFFFFFFFF:F:F
,FFFFFFFFFFFFFFFFFFFFFFFFFFF::FFFFFFFFFFFFFFFFFFFFFFFFFFFFFFFFFFFFFFFF
FFFF,F:FF:FFFFF
@A00155:342:HHGFNDSXY:1:2518:10041:20071 1:N:0:GAACCTAG+TCCGCATA
CTGGTTACAAATCCTCCGTTCTGCCCTTCTTATATCAATTTGAAATTCACGGGAAGGATCTGGTCTAC
ATGCCGGACGGGGTGAGGTCTGAGAGTTACACTCAACCATTGGAGAACGGTTTTCTCCTCAGCTCCAG
CAGCATACTTGTCAG
+
FF:FFFFF:FFFFFFFFFFFFFFFFFFFFFFFFF:FFFFFF:FFFFFFF:FFFF:FFFFFFFFFFFFFFF
FFFFFF:FFFFFFFFFFFFFFFFFFFFFFFFFFFFF,::FFFFFFFFFFFFFFFFFFFFFFFFFFFFFFF
F:FFFFFFFFFFF,F
```

@A00155:342:HHGFNDSXY:1:1438:4878:28228 2:N:0:GAACCTAG+TCCGCATA
CTGGTTACAAATCCTCCGTTCTGCCCCTCTTATATCAATTTGAAATTCACGGGAAGGATCTGGTCTAC
ATGCCGGACGGGGTGAGGTCTGAGAGTTACACTCAACCATTGGAGAACGGTTTTCTCCTCAGCTCCAG
CAGCATACTTGTCAG
+
FFF:FFFFFF:FFFFFFFFFFFFFFF,FFFFF,FFFFFFFFFFFFFFFFFFFFFFFFFFFFFFFFFFF
FFFFFFFFFFFFFFFFFFFFFFFFFFFFFFFFFFFFFFFFFFFFFFFFFFFFFFFFFFFFFFFFFFFF
FFFFFFFFFFFFFFF
@A00155:342:HHGFNDSXY:1:2560:16984:25708 2:N:0:GAACCTAG+TCCGCATA
CTGGTTACAAATCCTCCGTTCTGCCCTTCTTATATCAATTTGAAATTCACGGGAAGGATCTGGTCTAC
ATGCCGGACGGGGTGAGGTCTGAGAGTTACACTCAACCATTGGAGAACGGTTTTCTCCTCAGCTCCAG
CAGCATACTTGTCAG
+
FFFFFFFFFFF:FFFFFFFFFFFFFFFFFFF:FFFFFFFF:FFF:FFFF:FFFF::FFFFFF:F:FFF
FFF:FFFFFF:FFFFF:FFFFFFFFFFFFFFFFFFFF::FF:FFFFFFFFFFFFFFFFFFFFFFFFFF
FFFFFFFFFFFFFFF
@A00155:342:HHGFNDSXY:1:2560:17336:19648 2:N:0:GAACCTAG+TCCGCATA
CTGGTTACAAATCCTCCGTTCTGCCCTTCTTATATCAATTTGAAATTCACGGGAAGGATCTGGTCTAC
ATGCCGGACGGGGTGAGGTCTGAGAGTTACACTCAACCATTGGAGAACGGTTTTCTCCTCAGCTCCAG
CAGCATACTTGTCAG
+
FFFFFFFFFFFFFFFFFFFFFFF:FFFFFFFFFFFFFFFFFFFFFFFFFFFFFFFFFFFFFFFFFFFF
FFFFFFFFFFFFFFFFFFFFFFFFFFFFFFFFFFFFFFFFFFFFFFFFFFFFFFFFFFFFFFFFFFFF
FFFFFFFFFFFFFFF
@A00155:342:HHGFNDSXY:1:1623:21594:20134 2:N:0:GAACCTAG+TCCGCATA
TGGTTACAAATCCTCCGTTCTGCCCTTCTTATATCAATTTGAAATTCACGGGAAGGATCTGGTCTACA
TGCCGGACGGGGTGAGGTCTGAGAGTTACACTCAACCATTGGAGAACGGTTTTCTCCTCAGCTCCAGC
AGCATACTTGTCAGA
+
FFFFFFFFFFFFFFFFFFFFFFFFFFFFFFFFFFFFFFFFFFFFFFFFFFFFFFFFFFFFFFFFFFFF
FFFFFFFFFFFFFFFFF,FFFFFFFF:FFFFFFFFFF:FFFFFFFFFFFFFFFFFFFFFFFFFFFFFF
FFFFFFFFFFFFFFF
@A00155:342:HHGFNDSXY:1:1108:28384:17456 1:N:0:GAACCTAG+TCCGCATA
GTTACAAATCCTCCGTTCTGCCCTTCTTATATCAATTTGAAATTCACGGGAAGGATCTGGTCTACATG
CCGGACGGGGTGAGGTCTGAGAGTTACACTCAACCATTGGAGAACGGTTTTCTCCTCAGCTCCAGCAG
CATACTTGTCAGAAA
+
FFFFFFFFFFFFFFFFFF:FFFFFF:FFFFFFF:FFFFFFFFFFFFFFFFFFFFFFFFFFFFFFFFFF
FFFFFFFFFFFFFFFFFFFFFFFFFFFFFFFFFFFFFFFFFFFFFFFFFFFFFFFFFFFFFFFFFFFF
FFFFFFFFFFFFFFF
@A00155:342:HHGFNDSXY:1:1334:15664:11647 1:N:0:GAACCTAG+TCCGCATA
GTTACAAATCCTCCGTTCTGCCCTTCTTATATCAATTTGAAATTCACGGGAAGGATCTGGTCTACATG
CCGGACGGAGTGAGGTCTGAGAGTTACACTCAACCATTGGAGAACGGTTTTCTCCTCAGCTCCAGCAG
CATACTTGTCAGAAA
+
FFFFFFFFFFFFFFFFFFFFFFFFFFFFFFFFFFFFFFFFFFFFFFFFFFFFFFFFFFFFFFFFFFFF
FFFFFFFFFFFFFFFFFFFFFFFFFFFFFFFFFFFFFFFFFFFFFFFFFFFFFFFFFFFFFFFFFFFF
FFFFFFFFFFFFFFF
@A00155:342:HHGFNDSXY:1:2276:10366:2785 2:N:0:GAACCTAG+TCCGCATA
GTTACAAATCCTCCGTTCTGCCCTTCTTATATCAATTTGAAATTCACGGGAAGGATCTGGTCTACATG
CCGGACGGGGTGAGGTCTGAGAGTTACACTCAACCATTGGAGAACGGTTTTCTCCTCAGCTCCAGCAG
CATACTTGTCAGAAA
+
FFFFFFFFFFFFFFFFFFFFF:FFF:,:FFFFFFFFFFFFFFFFFFFFFFFFFFFFFFFFFFF:FFFF

FFFFFFFFFFFFFFFFFFFFFFFFFFFFFFFFFFFFFFFF:FFFFFFFFFFFFFFFFFFFFFFFFFFF
FFFFFFFFFFFFFFF
@A00155:342:HHGFNDSXY:1:2551:2456:24878 2:N:0:GAACCTAG+TCCGCATA
GTTACAAATCCTCCGTTCTGCCCTTCTTATATCAATTTGAAATTCACGGGAAGGATCTGGTCTACATG
CCGGACGGGGTGAGGTCTGAGAGTTACACTCAACCATTGGAGAACGGTTTTCTCCTCAGCTCCAGCAG
CATACTTGTCAGAAA
+
:FFFFFFFFFFFFFFFFFFFFFFFF,FFFFFFFFFFFFFFFFFFFFFFFFFFF:FFFFFFFFFFFFFFFF
FFFFFFFFFFFFFFFFFFFFFFFFFFFFFFFFFFFFFFFFFFFFFFFFFFFFFFFFFFFFFFFFFFFF
FF::FFFFFFFFF:F
@A00155:342:HHGFNDSXY:1:1136:27787:9909 1:N:0:GAACCTAG+TCCGCATA
TTACAAATCCTCCGTTCTGCCCTTCTTATATCAATTTGAAATTCACGGGAAGGATCTGGTCTACATGC
CGGACGGAGTGAGGTCTGAGAGTTACACTCAACCATTGGAGAACGGTTTTCTCCTCAGCTCCAGCAGC
ATACTTGTCAGAAAT
+
FFFFFFFFFFFFFFFFFFFFFFFFFFFFF:FFFFFFFFFFFFFFFFFFFFFFFFFFFFFFFFFFFFFF
FFFFFFFFFFFFFFFFFFFFFFFFFFFFFFFFFFFFFFFFFFFFFFFFFFFFFFFFFFFFFFFFFFFF
FFFFFFFFFFFFFFF
@A00155:342:HHGFNDSXY:1:1152:9082:20040 1:N:0:GAACCTAG+TCCGCATA
TTACAAATCCTCCGTTCTGCCCTTCTTATATCAATTTGAAATTCACGGGAAGGATCTGGTCTACATGC
CGGACGGGGTGAGGTCTGAGAGTTACACTCAACCATTGGAGAACGGTTTTCTCCTCAGCTCCAGCAGC
ATACTTGTCAGAAAG
+
FFFFFFFFFFFFFFFFFFFFFFFFFFFFFFFFFFFFFFFFFFFFFFFFFFFFFFFFFFFFFFFFFFFF
FFFFFFFFFFFFFFFFFFFFFFFFFFFFFFFFFFFFFFFFFFFFFFFFFFFFFFFFFFFFFFFFFFFF
FFFFFFFFFFFFFFF
@A00155:342:HHGFNDSXY:1:1377:5141:24706 1:N:0:GAACCTAG+TCCGCATA
TCTCAAATCCTCCGTTCTGCCCTTCTTATATCAATTTGAAATTCACGGGAAGGATCTGGTCTACATGC
CGGACGGAGTGAGGTCTGAGAGTTACACTCAACCATTGGAGAACGGTTTTCTCCTCAGCTCCAGCAGC
ATACTTGTCAGAAAT
+
FFFFFFFFFFFFFFFFFFFFFFFFFFFFF:FFFFFFFFFFFFFFFFFFFFFFFFFFFFFFFFFFFFFF
FFFFFFFFFFFFFFF:FFFFFFFFFFFFFFFFFFFFFFFFFFFFFFFFFFFFFFFFFFFFFFFFFFFF
FFFFFFFFFFFFFFF
@A00155:342:HHGFNDSXY:1:2222:29188:25864 1:N:0:GAACCTAG+TCCGCATA
TTACAAATCCTCCGTTCTGCCCTTCTTATATCAATTTGAAATTCACGGGAAGGATCTGGTCTACATGC
CGGACGGGGTGAGGTCTGAGAGTTACACTCAACCATTGGAGAACGGTTTTCTCCTCAGCTCCAGCAGC
ATACTTGTCAGAAAT
+
FFFFFFFFFFFFFFFFFFFFFFFFF:FFFFFFFFFFFFFFFFFFFFFFFFFFFFFFFFF:FFFFFFFFFF
FFFFFFFF:FFF:FFFFFFFFFFFFFFFFFFFFFFFFFFFFFFFFFFFFFFFFFFFFFFFFFFFFFFF
FFFFFFFFFFFFFFF
@A00155:342:HHGFNDSXY:1:2378:28375:18944 1:N:0:GAACCTAG+TCCGCATA
TTACAAATCCTCCGTTCTGCCCTTCTTATATCAATTTGAAATTCACGGGAAGGATCTGGTCTACATGC
CGGACGGGGTGAGGTCTGAGAGTTACACTCAACCATTGGAGAACGGTTTTCTCCTCAGCTCCAGCAGC
ATACTTGTCAGAAAT
+
FFF:FFFFFFFFFF:FFFFFFFFFFFFFFFFFFFFFFFFFFFFFFFFFFFFFF,FFFFFFFFFFFFFFFF
:FFFFFFFFFFFFFFFFFFFFFFFFFFFFF:FFFFFFFFFFFFFFFFFFFFFFFFFFFFFFFFFFFFFF
FFFFFFFFFFFFFFF
@A00155:342:HHGFNDSXY:1:2444:21847:2159 1:N:0:GAACCTAG+TCCGCATA
TTACAAATCCTCCGTTCTGCCCTTCTTATATCAATTTGAAATTCACGGGAAGGATCTGGTCTACATGC
CGGACGGGGTGAGGTCTGAGAGTTACACTCAACCATTGGAGAACGGTTTTCTCCTCAGCTCCAGCAGC
ATACTTGTCAGAAAT

```
+
FFFFFFFFFFFFFFFFFFFFFFFFFFFFFFFFF:FFFFFFF:FFFFFFFF:FFFFFFFFFFFFFFFF
FFFFFFFFFFFFFFFFFFFFFFFFFFFFFFFFFFFFFFFFFFFFFFFFFFFFFFFFFFFFFFFFFFF
FFFFFFFFFFFFFFF
@A00155:342:HHGFNDSXY:1:2510:11840:18584 1:N:0:GAACCTAG+TCCGCATA
TTCCGATCTCTCCGTTCTGCCCTTCTTATATCAATTTGAAATTCACGGGAAGGATCTGGTCTACATGC
CGGACGGGGTGAGGTCTGAGAGTTACACTCAACCATTGGAGAACGGTTTTCTCCTCAGCTCCAGCAGC
ATACTTGTCAGAAAT
+
:FFFFFFFFFFFFFFFFFFFFFFFFFFFFFFFFFFFFFFFFFFFFFFFFFFFFFFFFFFFFFFFFFF
FFFFFFFFFFFFFFFFFFFFFFFFFFFFFFFFFFFFFFFFFFFFFFFFFFFFFFFFFFFFFFFFFFF
FFFFFFFFFFFFFFF
@A00155:342:HHGFNDSXY:1:1310:1163:36918 1:N:0:GAACCTAG+TCCGCATA
TACAAATCCTCCGTTCTGCCCTTCTTATATCAATTTGAAATTCTCGGGAAGGATCTGGTCTACATGCC
GGACGGGGTGAGGTCTGAGAGTTACACTCAACCATTGGAGAACGGTTTTCTCCTCAGCTCCAGCAGCA
TACTTGTCAGAAAT
+
FFFFF,FFFFF:F,FF:FFF:FFF:,F::FF,::,:FFFF,FF:::FFFFFF::F,FF::FFF:FF::
FFF::F:F:FFFFFF,FFFFFFFFFFFFFF:FFF,:FF:FF,FFF:F,FFFFFFFFF:FFFF:F:FFF
FFFF,FFFFFF,FF
@A00155:342:HHGFNDSXY:1:2551:2456:24878 1:N:0:GAACCTAG+TCCGCATA
TACAAATCCTCCGTTCTGCCCTTCTTATATCAATTTGAAATTCACGGGAAGGATCTGGTCTACATGCC
GGACGGGGTGAGGTCTGAGAGTTACACTCAACCATTGGAGAACGGTTTTCTCCTCAGCTCCAGCAGCA
TACTTGTCAGAAATA
+
FF:F:FFFFFFFFFFFFF:,FFFFFFFFFFFFFFFFFFFFFF:FFFF,FFFFFF:FFFFFFFFFFF
FFFFF:FFFFFFFFFFFFFFFFFFFFFFFFFFF,FFFFF:FFFFFFFFFFFFFFFFFFFFFFFFFFFF
FFFFFFFFFF:FFFF
@A00155:342:HHGFNDSXY:1:1418:5077:28166 2:N:0:GAACCTAG+TCCGCATA
TACAAATCCTCCGTTCTGCCCTTCTTATATCAATTTGAAATTCACGGGAAGGATCTGGTCTACATGCC
GGACGGGGTGAGGTCTGAGAGTTACACTCAACCATTGGAGAACGGTTTTCTCCTCAGCTCCAGCAGCA
TACTTGTCAGAGATC
+
FFFFFFFFFFFFFFFFFFFFFFFF,FFFFFFFFFFFFFFFFFFFFFFFFFFFFFFFFFFFFFFFFFF
FFFFFFFFFFFFFFFFFFFFFFFFFFFFFFFFFFFFFFFFFFFFFFFFFFFFFF:FFFFFFFFFFF
FFFF,FFFFFFFFFF
@A00155:342:HHGFNDSXY:1:1418:5122:28244 2:N:0:GAACCTAG+TCCGCATA
TACAAATCCTCCGTTCTGCCCTTCTTATATCAATTTGAAATTCACGGGAAGGATCTGGTCTACATGCC
GGACGGGGTGAGGTCTGAGAGTTACACTCAACCATTGGAGAACGGTTTTCTCCTCAGCTCCAGCAGCA
TACTTGTCAGAGATC
+
FFFFFFFFFFFFFFFFFFFFFFFF:FFFFFFFFFFFFFFFFFFFFFFFFFFFFFFFFFFFFFFFFFF
FFFFFFFFFFFFFFFFFFFFFFFFFFFFFFFFFFFFFFFFFFFFFFFFFFFFFFFFFFFFFFFFFFF
FFFFFFFFFFFFF:F
@A00155:342:HHGFNDSXY:1:1234:17047:10066 1:N:0:GAACCTAG+TCCGCATA
CAAATCCTCCGTTCTGCCCTTCTTATATCAATTTGAAATTCACGGGAAGGATCTGGTCTACATGCCGG
ACGGGGTGAGGTCTGAGAGTTACACTCAACCATTGGAGAACGGTTTTCTCCTCAGCTCCAGCAGCATA
CTTGTCAGAAATAAG
+
FFFFFFFFFFFFFFFFFFFFF:FFFFFFFFFFFFFFFFFFFFFFFFFF:FFF:F,FFFFFFFFFFFFF
FFFFFFFFFFFFFFFFFFFFFFFFFFFFFFFFFFFFFFFFFFFFFFFFFFFFFFFFFFFFFFFFFFFF
F:FFFFFFFFFFFFF
@A00155:342:HHGFNDSXY:1:1310:5032:23703 1:N:0:GAACCTAG+TCCGCATA
CAAATCCTCCGTTCTGCCCTTCTTATATCAATTTGAAATTCACGGGAAGGATCTGGTCTACATGCCGG
```

ACGGGGTGAGGTCTGAGAGTTACACTCAACCATTGGAGAACGGTTTTCTCCTCAGCTCCAGCAGCATA
CTTGTCAGAAATAAG
+
FF:FFFFFFFFFFFFFFFFFF,,FFFFFFFFFF,FFFFFFFFF:,FFFFFFFFFFFFFFFFFFF:FFFFF
FFFFFFFFFFFFFFFFFFFFFFFFFFFFFFFFFFFFF:,FFFFFFFFFFFFFFFFFFFFFFFFFFFFFFF
FFFFFFFFFFFFFFF
@A00155:342:HHGFNDSXY:1:1346:31928:29011 1:N:0:GAACCTAG+TCCGCATA
CGATCTCTCCGTTCTGCCCTTCTTATATCAATTTGAAATTCACGGGAAGGATCTGGTCTACATGCCGG
ACGGGGTGAGGTCTGAGAGTTACACTCAACCATTGGAGAACGGTTTTCTCCTCAGCTCCAGCAGCATA
CTTGTCAGAAATAAG
+
FFFFFFFFFFFFFFFFFFFFFFFF:,FFFFFFFFFFFFFFFFFF:FFF,FFFFF,FFFFFFFFF:FFFFF
FFFFFFFFFFFFFFFFFFFFFFFFFFFFFFFFFFFFFFFFFFFFFFFFF::FFFFFFFFFFFFFFFFFFF
FFFFFFFFFFFFFFF
@A00155:342:HHGFNDSXY:1:1375:24153:31955 1:N:0:GAACCTAG+TCCGCATA
TCTATCCTCCGTTCTGCCCTTCTTATATCAATTTGAAATTCACGGGAAGGATCTGGTCTACATGCCGG
ACGGGGTGAGGTCTGAGAGTTACACTCAACCATTGGAGAACGGTTTTCTCCTCAGCTCCAGCAGCATA
CTTGTCAGAAATAAG
+
FFFFFFFFFFFFFFFFFFFFFFFFFFFFFFFFFFFFFFFFFFFFFFFFFFFFFFFFFFFFFFFFFFFFFF
FFFFFFFFFFFFFFFFFFFFFFFFFFFFFFFFFFFFFFFFFFFFFFFFFFFFFFFFFFFFFFFFFFFFFF
FFFFFFFFFFFFFFF
@A00155:342:HHGFNDSXY:1:1455:29215:31078 1:N:0:GAACCTAG+TCCGCATA
GAACGGCTCCGTTCTGCCCTTCTTATATCAATTTGAAATTCACGGGAAGGATCTGGTCTACATGCCGG
ACGGAGTGAGGTCTGAGAGTTACACTCAACCATTGGAGAACGGTTTTCTCCTCAGCTCCAGCAGCATA
CTTGTCAGAAATAAG
+
FFFFFFFFFFFFFF:F:FFFFFFFFFFFFFFFFFFFFFFFFFFF:FFFFFFFFF:FFFFFFFFFFFFFFF
FFFFFFFFFFFFFFFFFFFFFFFFFFFFFFFFFFFFFFFFFFFFFFFFFFFFFFFFFFFFFFFFFFFFFF
FFFFFFFFFFFFFFF
@A00155:342:HHGFNDSXY:1:1511:26413:6558 1:N:0:GAACCTAG+TCCGCATA
CAAATCCTCCGTTCTGCCCTTCTTATATCAATTTGAAATTCACGGGAAGGATCTGGTCTACATGCCGG
ACGGGGTGAGGTCTGAGAGTTACACTCAACCATTGGAGAACGGTTTTCTCCTCAGCTCCAGCAGCATA
CTTGTCAGAAATAAG
+
FFFFFFFFFFFFFFFFFFFFFFFFFFF,FFFFFFFFFFFFFFFFFFFFFFFFFFFFFFFFFFFFFFFFFF
FFFFFFFFFFFFFFFFFFFFFFFFFFFFFFFFFFFFFFFFFFFFFFFFFFFFFFFFFFFFFFFFFFFFFF
FFFFFFFFFFFFFFF
@A00155:342:HHGFNDSXY:1:1511:26874:6637 1:N:0:GAACCTAG+TCCGCATA
CAAATCCTCCGTTCTGCCCTTCTTATATCAATTTGAAATTCACGGGAAGGATCTGGTCTACATGCCGG
ACGGGGTGAGGTCTGAGAGTTACACTCAACCATTGGAGAACGGTTTTCTCCTCAGCTCCAGCAGCATA
CTTGTCAGAAATAAG
+
FFFFFFFFF:F:FFFFFFFFFFFFF:FFFF:FFFFFFFFFFFFFFFFF:FFFFF:FFFFFFFFFFFFFFF
FFF:FFFFFFFFFFFFFFFFFFFFFFFFFFF:FFF,FFFFFFFFFFFFFFFFFF,FFFFFFFFFFFFFFF
:FFFFFFFFF:FFFF
@A00155:342:HHGFNDSXY:1:1545:21124:32158 1:N:0:GAACCTAG+TCCGCATA
CAAATCCTCCGTTCTGCCCTTCTTATATCAATTTGAAATTCACGGGAAGGATCTGGTCTACATGCCGG
ACGGGGTGAGGTCTGAGAGTTACACTCAACCATTGGAGAACGGTTTTCTCCTCAGCTCCAGCAGCATA
CTTGTCAGAAATAAG
+
FFFFFFFFFFFF,,F:FFFFFFFFFFFFFFF::FF:FFFFFFFFFFFFF:FFF:FFFFFFFFFFFF:FFF
FFFF:FFFFFFF,FFF:FFFFFFFFFFFFFFFFFFFFFFFFFFFFFFFFFFFFFFFFFFFFFFFFFFFFF
FFFFFFFF:FFFFFF

```
@A00155:342:HHGFNDSXY:1:1641:20609:14669 1:N:0:GAACCTAG+TCCGCATA
CAAATCCTCCGTTCTGCCCTTCTTATATCAATTTGAAATTCACGGGAAGGATCTGGTCTACATGCCGG
ACGGAGTGAGGTCTGAGAGTTACACTCAACCATTGGAGAACGGTTTTCTCCTCAGCTCCAGCAGCATA
CTTGTCAGAAATAAG
+
FFFF:FFFFFFFFFFFFFFFFFFFFFFFFFFFFFFFFFFFFFFFFFFFFFFFFFFFFFFFFFFFFFFFFF
FFFFFFFFFFFFFFFFFFFFFFFFFFFFFFFFFFFFFFFFFFFFFFFFFFFFFFF:FFFFFFFFFFFFFF
FFFFFFFFFFFFFFF
@A00155:342:HHGFNDSXY:1:1666:27489:1814 1:N:0:GAACCTAG+TCCGCATA
CAAATCCTCCGTTCTGCCCTTCTTATATCAATTTGAAATTCACGGGAAGGATCTGGTCTACATGCCGG
ACGGGGTGAGGTCTGAGAGTTACACTCAACCATTGGAGAACGGTTTTCTCCTCAGCTCCAGCAGCATA
CTTGTCAGAAATAAG
+
FFF:FFFFFFFFFFFFFFFFFFFFFFFFFFFFFFFFFFFFFFFFFFFFFFFFFFFFFFFFFFFFFFFFFF
FFFFFFFFFFFFFFFFFFFFFFFFFFFFFFFFFFFFFFFFFFFFFF:FFFFFFFFFFFF:FFFFFFFFFF
FFFFFFFFFFFFFFF
@A00155:342:HHGFNDSXY:1:1678:4779:24612 1:N:0:GAACCTAG+TCCGCATA
CAAATCCTCCGTTCTGCCCTTCTTATATCAATTTGAAATTCACGGGAAGGATCTGGTCTACATGCCGG
ACGGGGTGAGGTCTGAGAGTTACACTCAACCATTGGAGAACGGTTTTCTCCTCAGCTCCAGCAGCATA
CTTGTCAGAAATAAG
+
FFFFFF:FFF:FFFFFFFF:FFFFFFFFFFFFFFFFFFF:FFFFFFFFFFFFFFFFFFFFFFFFFFFFFF
FFFFF:FFFFFFFFFFFFF:F:FFFFFFFFFFFFFFFFF,FFFFFF:FFFFFFFFFFFFFFFFFFFFFFF
FFFFFFFFFFFFFFF
@A00155:342:HHGFNDSXY:1:2154:26494:35571 1:N:0:GAACCTAG+TCCGCATA
CAAATCCTCCGTTCTGCCCTTCTTATATCAATTTGAAATTCACGGGAAGGATCTGGTCTACATGCCGG
ACGGAGTGAGGTCTGAGAGTTACACTCAACCATTGGAGAACGGTTTTCTCCTCAGCTCCAGCAGCATA
CTTGTCAGAAATAAG
+
FFFFF,:FF:FFFF:F:F,FF:FFFFFFFFFFFFFF,FFFFFFFFFF:F,FFFFFFFFFFFFFFFFFFFF
FFFFFFFFF:FFFFFFFF:FF:FFFFFFFF,FFFFFF:FFFFFFFFFFFFFFFFFFFFFF:FFFFFFFFF
FFFFFFFFFFFFFFF
@A00155:342:HHGFNDSXY:1:2266:30626:29919 1:N:0:GAACCTAG+TCCGCATA
CAAATCCTCCGTTCTGCCCTTCTTATATCAATTTGAAATTCACGGGAAGGATCTGGTCTACATGCCGG
ACGGAGTGAGGTCTGAGAGTTACACTCAACCATTGGAGAACGGTTTTCTCCTCAGCTCCAGCAGCATA
CTTGTCAGAAATAAG
+
FFFFFFFFFFFFFFFFFFFFFFFFFFF:FFFFFFFFFFFFFFFFFFFFFFFFFFFFFFFFFFFFFFFFFF
FFFFFFFFFFFFFFFFFFFFFFFFFFFFFFFFFFFFFFFFFFFFFFFFFFFFFFFFFFFFFFFFFFFFFF
FFFFFFFFFFFFFFF
@A00155:342:HHGFNDSXY:1:2249:17110:14653 1:N:0:GAACCTAG+TCCGCATA
GATCTCCTCCGTTCTGCCCTTCTTATATCAATTTGAAATTCACGGGAAGGATCTGGTCTACATGCCGG
ACGGAGTGAGGTCTGAGAGTTACACTCAACCATTGGAGAACGGTTTTCTCCTCAGCTCCAGCAGCATA
CTTGTCAGAAATAAG
+
FFFFFFFFFFFFFFFFFF:FFF:FFFFF:FFFFFFFFFFFFFFFFFFFFFFFFFFFFFFFFFFFFFFFFF
FFFFFFFFFFFFFFFFFFFFFFFFFFFFFFFFFFF:FFFFFFFF:FFFFFFFFFFFFFFFFFFFFFFFFF
FFFFFFFFFFFFFFF
@A00155:342:HHGFNDSXY:1:2353:30228:35336 1:N:0:GAACCTAG+TCCGCATA
CAAATCCTCCGTTCTGCCCTTCTTATATCAATTTGAAATTCACGGGAAGGATCTGGTCTACATGCCGG
ACGGAGTGAGGTCTGAGAGTTACACTCAACCATTGGAGAACGGTTTTCTCCTCAGCTCCAGCAGCATA
CTTGTCAGAAATAAG
+
FFFFFFFFFFFFFFFFFFFFFFFFFFF:FFF,FFFFFFFFFFFFFFFF:FFFFFFFFFFFFFFFFFFFFF
```

FFFFFFFFFFFFFFFFFFFFFFFFFFFFFFFFFFFFFFFFFFFFFFFFFFFFFFFFFFFFFFFFFFFFFF
FFFFFFFFFFFFFFF
@A00155:342:HHGFNDSXY:1:2375:2220:10254 1:N:0:GAACCTAG+TCCGCATA
CGATCTCTCCGTTCTGCCCTTCTTATATCAATTTGAAATTCACGGGAAGGATCTGGTCTACATGCCGG
ACGGGGTGAGGTCTGAGAGTTACACTCAACCATTGGAGAACGGTTTTCTCCTCAGCTCCAGCAGCATA
CTTGTCAGAAATAAG
+
FFF,FF:FFFFFFFFFFF:,FFFFFFFFFFFFFFFFFFFFFFFFFFFFFFFFFFFFFFF:FFFFFFFFF
FFF:FFFFFFFFF:FFFFFFFFFFF,FFFF:FFFFFFFFFFFFFFFFFFFFFFFFFFFFFFFFFFFFFF
FFFFFFFFFFFFFFF
@A00155:342:HHGFNDSXY:1:2375:27407:18615 1:N:0:GAACCTAG+TCCGCATA
CAAATCCTCCGTTCTGCCCTTCTTATATCAATTTGAAATTCACGGGAAGGATCTGGTCTACATGCCGG
ACGGAGTGAGGTCTGAGAGTTACACTCAACCATTGGAGAACGGTTTTCTCCTCAGCTCCAGCAGCATA
CTTGTCAGAAATAAG
+
:FFFFFFFFFFFFFFFFFFFFFFFFFFFFFFFFFFFFFFFFFFFFFFFFFFFFFFFFFFFFFFFFF:FF
FFFFFF:FFFFFFFFFFFFFFFFFFFFFFFFFFFFFFFFFFFFFFFFFFFFFFFFFFFFFFFFFFFFFF
FFFFFFFFFFFFFFF
@A00155:342:HHGFNDSXY:1:2375:24650:19727 1:N:0:GAACCTAG+TCCGCATA
CAAATCCTCCGTTCTGCCCTTCTTATATCAATTTGAAATTCACGGGAAGGATCTGGTCTACATGCCGG
ACGGAGTGAGGTCTGAGAGTTACACTCAACCATTGGAGAACGGTTTTCTCCTCAGCTCCAGCAGCATA
CTTGTCAGAAATAAG
+
FFFFFFFFFFFFFFFFFFFFFFFFFFFFFFFFFFFFFFFFFFFFFFFFFFFFFFFFFFFFFFFFFFFFFF
FFFFFFFFFFFFFFFFFFFFFFFFFFFFFFFFFFFFFFFFFFFFFFFFFFFFFFFFFFFFFFFFFFFFFF
FFFFFFFFFFFFFFF
@A00155:342:HHGFNDSXY:1:2545:20292:26929 1:N:0:GAACCTAG+TCCGCATA
CAAATCCTCCGTTCTGCCCTTCTTATATCAATTTGAAATTCACGGGAAGGATCTGGTCTACATGCCGG
ACGGGGTGAGGTCTGAGAGTTACACTCAACCATTGGAGAACGGTTTTCTCCTCAGCTCCAGCAGCATA
CTTGTCAGAAATAAG
+
FFFFFFFFFFFFFFFFFFFFFFFFFFFFFFFFFFFFFFFFFFFFFFFFFFFFFFFFFFFFFFFFFFFFFF
FFFFFFFFFFFFFFFFFFFFFFFFFFFFFFFFFFFFFFFFFFFFFFFFFFFFFFFFFFFFFFFFFFFFFF
FFFFFFFFFFFFFFF
@A00155:342:HHGFNDSXY:1:2672:7075:21198 1:N:0:GAACCTAG+TCCGCATA
CAAATCCTCCGTTCTGCCCTTCTTATATCAATTTGAAATTCACGGGAAGGATCTGGTCTACATGCCGG
ACGGAGTGAGGTCTGAGAGTTACACTCAACCATTGGAGAACGGTTTTCTCCTCAGCTCCAGCAGCATA
CTTGTCAGAAATAAG
+
::FFFFFFFFFFFFFFFFFFFFFFFFFFFFFFFFFFFFFFFFFFFFFFFFFF,F:FFF:FFFFFFFFF
FFFFFFFFFFFF,FFFFFFFFFFFFFFFFFFFFFFFFFFFFFFFFFFFFFFFFFFFFFFFFFFFFFFFF
FFFFFFFFFFFFFFF
@A00155:342:HHGFNDSXY:1:1227:31774:9455 2:N:0:GAACCTAG+TCCGCATA
CAAATCCTCCGTTCTGCCCTTCTTATATCAATTTGAAATTCACGGGAAGGATCTGGTCTACATGCCGG
ACGGGGTGAGGTCTGAGAGTTACACTCAACCATTGGAGAACGGTTTTCTCCTCAGCTCCAGCAGCATA
CTTGTCAGAAATAAG
+
FFFFFFFFFFFFFFFFFFFF:FF,FFF:FFFFFFFFFFFFFFFFFFFFFFFF:FFFFFFFFFFFFFFF
FFFFFFFFFFFFFFFFFFFFFFFFFFFFFFFFFFFFFFFFFFFFFFFFFFFFFFFFFFFFFFFFFFF:FF
FF:F:FFFFFFFF:F
@A00155:342:HHGFNDSXY:1:1328:2193:25989 2:N:0:GAACCTAG+TCCGCATA
CAAATCCTCCGTTCTGCCCTTCTTATATCAATTTGAAATTCACGGGAAGGATCTGGTCTACATGCCGG
ACGGGGTGAGGTCTGAGAGTTACACTCAACCATTGGAGAACGGTTTTCTCCTCAGCTCCAGCAGCATA
CTTGTCAGAAATAAG

```
+
FFFFFFFFFFFFFFFFF:FFFFFFFFFF:FFFFFFFFFFFFF,FFF:FFFFFFFFFFFFFFFFF:FFF
FFFFFFFFF:F:FFFFF:FFFFFFFFFFFF,FFFFFFFFF:FFFFFFFFFFFFFFFFFFFFFFFFFFF
FFFFFFFF:FFFFFF
@A00155:342:HHGFNDSXY:1:1377:5141:24706 2:N:0:GAACCTAG+TCCGCATA
CAAATCCTCCGTTCTGCCCTTCTTATATCAATTTGAAATTCACGGGAAGGATCTGGTCTACATGCCGG
ACGGAGTGAGGTCTGAGAGTTACACTCAACCATTGGAGAACGGTTTTCTCCTCAGCTCCAGCAGCATA
CTTGTCAGAAATAGA
+
FFFFFFFFFFFFFFFFFFFFFFFFFFFFFFFFFFFFFFFFFFFFFFFFFFFFFFFFFFFFFFFFFFFF
FFFFFFFFFFFFFFFFFFFFFFFFFFFFFFFFFFFFFFFFFFFFFFFFFFFFFFFFFFFFFFFFFFFF
FFFFFFFFFF,FFFF
@A00155:342:HHGFNDSXY:1:1467:26630:18803 2:N:0:GAACCTAG+TCCGCATA
CGATCTCTCCGTTCTGCCCTTCTTATATCAATTTGAAATTCACGGGAAGGATCTGGTCTACATGCCGG
ACGGAGTGAGGTCTGAGAGTTACACTCAACCATTGGAGAACGGTTTTCTCCTCAGCTCCAGCAGCATA
CTTGTCAGAAATAAG
+
FFFFFFFFFFFFFFFFFFFFFFFF:FFFFF,FFFF,FF:FFFFFFFFFFFFFFFFFFFFFF:FFFFFF
FFFFFFFFFF:FFFFFFFFFFFFFFFFFFFFFFFFFFFFFFFFFFFFFFFFFFFFFFFFFFFFFFFFF
FFFFF:FFFFFFFFF
@A00155:342:HHGFNDSXY:1:2347:7211:32581 2:N:0:GAACCTAG+TCCGCATA
CAAATCCTCCGTTCTGCCCTTCTTATATCAATTTGAAATTCACGGGAAGGATCTGGTCTACATGCCGG
ACGGAGTGAGGTCTGAGAGTTACACTCAACCATTGGAGAACGGTTTTCTCCTCAGCTCCAGCAGCATA
CTTGTCAGAAATAAG
+
FFFFFFFFFFF:FFFFFF:,FF,FFFFFFFFFFFFFFFFFFFFFFFFFFFFFFFFFFFFFFFFFFFFF
FFFFFFFFFFFFFFFFFFFFFFFFFFFFFFFFFFFFFFFFFFFFF:FFFFFFFFFFFFFFFF:FFFFFFFF
FFFFFFFFFFF:FFF
@A00155:342:HHGFNDSXY:1:2573:19190:1125 2:N:0:GAACCTAG+TCCGCATA
CAAATCCTCCGTTCTGCCCTTCTTATATCAATTTGAAATTCACGGGAAGGATCTGGTCTACATGCCGG
ACGGGGTGAGGTCTGAGAGTTACACTCAACCATTGGAGAACGGTTTTCTCCTCAGCTCCAGCAGCATA
CTTGTCAGATCGGAA
+
FFFFFFFFFFFFFFFFFFFFFFFFFFFFFFFFFFFFFFFFFFFFFFFF:FFFFFFFFFFFFFFFFFFF
FFFFFFFFFFFFFFFFFFFFFFFFFFFFFFFFFFFFFFFFFFFFFFFFFFFFFFFFFFFFF:FFFFFF
FF:FFFFFFFFFFFF
@A00155:342:HHGFNDSXY:1:2642:12852:6715 2:N:0:GAACCTAG+TCCGCATA
CAAATCCTCCGTTCTGCCCTTCTTATATCAATTTGAAATTCACGGGAAGGATCTGGTCTACATGCCGG
ACGGGGTGAGGTCTGAGAGTTACACTCAACCATTGGAGAACGGTTTTCTCCTCAGCTCCAGCAGCATA
CTTGTCAGAAATAAG
+
FFFFFFFFFFFFFFFFFFFFFFFFFFFFFFFFFFFFFFFFFFFFFFFFFFFFFFFFFFFFFFFFFFFF
FFFFFFFFFFFFFFFFFFFFFFFFFFFFFFFFFFFFFFFFFFFFFFFFFFFFFFFFFFFFFFFFFFFF
FFFFFFFFFFFFFFF
@A00155:342:HHGFNDSXY:1:2531:16297:35978 1:N:0:GAACCTAG+TCCGCATA
AAATCCTCCGTTCTGCCCTTCTTATATCAATTTGAAATTCACGGGAAGGATCTGGTCTACATGCCGGA
CGGGGTGAGGTCTGAGAGTTACACTCAACCATTGGAGAACGGTTTTCTCCTCAGCTCCAGCAGCATAC
TTGTCAGAAATAAG
+
FFF:FFF,FFFF,FF::,,F,:FF,FFFF,FF:FFFFFFFFFFFFFFFFFF,FFFFFF,F,FFF,:F:
,FF:FFFFFF:FFFFFFFFFFFF,:,F:,F:FF,FF:F:,FFF,FF,F,:FFFF:F,F,FFFF:,FFF
FFFFFFFFFFFFF:
@A00155:342:HHGFNDSXY:1:1301:4128:36041 1:N:0:GAACCTAG+TCCGCATA
AAATCCTCCGTTCTGCCCTTCTTATATCAATTTGAAATTCACGGGAAGGATCTGGTCTACATGCCGGA
```

CGGAGTGAGGTCTGAGAGTTACACTCAACCATTGGAGAACGGTTTTCTCCTCAGCTCCAGCAGCATAC
TTGTCAGAAATAAGG
+
FF:FFFFFFFFFFFFFFFFFFFFFFF:FFF:FFFFFFFFFFFFFFFF,FFFF:FFFFFFFFFFFFFFFFF
FFFFFFFFFFF:FFFFFF,FFFFFFFFFFFF:FFFFFFFFFFFFFFFFFFFFFFFFFFFFFFFFFFFFFF
FFFFFFFFFFFFFFF
@A00155:342:HHGFNDSXY:1:1465:5303:30592 1:N:0:GAACCTAG+TCCGCATA
AAATCCTCCGTTCTGCCCTTCTTATATCAATTTGAAATTCACGGGAAGGATCTGGTTTACATGCCGGA
CGGGGTGAGGTCTGAGAGTTACACTCAACCATTGGAGAACGGTTTTCTCCTCAGCTCCAGCAGCATAC
TTGTCAGAAATAAGG
+
FFFFFFFFFFFFFFFFFFFF:FFFFF:FFF::FFFFFFFFFFFFFFFFFFFFFFFFFFFFFFFFFFFFFF
FFFFFFFFFFFFFFFFFFFFFFFFFFFFFFFFFFFFFFFFFFFFFFFFFFFFFFFFFFFFFFFFFFFFFF
FFFFFFFFFFFFFFF
@A00155:342:HHGFNDSXY:1:1645:9191:29841 1:N:0:GAACCTAG+TCCGCATA
AAATCCTCCGTTCTGCCCTTCTTATATCAATTTGAAATTCACGGGAAGGATCTGGTCTACATGCCGGA
CGGGGTGAGGTCTGAGAGTTACACTCAACCATTGGAGAACGGTTTTCTCCTCAGCTCCAGCAGCATAC
TTGTCAGAAATAAGG
+
FFFFFFFFFFFFFFFFFFFFF:FFFFFFFFFFFFFFFFFFFFFFFFFFFFFFFF:FFFFFFFFFFFFFFF
FFFFFFFF:FFFFFFFFFFFFFFFFFFFFF:FFFFFFFFFFFFFFFFFFFFFFFFFFFFFFFFFFFFFFF
FFFFFFFFFFFFFFF
@A00155:342:HHGFNDSXY:1:1652:28999:12101 1:N:0:GAACCTAG+TCCGCATA
AAATCCTCCGTTCTGCCCTTCTTATATCAATTTGAAATTCACGGGAAGGATCTGGTCTACATGCCGGA
CGGGGTGAGGTCTGAGAGTTACACTCAACCATTGGAGAACGGTTTTCTCCTCAGCTCCAGCAGCATAC
TTGTCAGAAATAAGG
+
FFFFFFFFFFFFFFFFFFFFFFFFFFFFFFFFFFFFFFFFFFF:FFFFFFFFFFFFFFFFFFFFFFFFFF
FFFFFFFFFFFFFFFFFFFFFFFFFFFFFFFFFFFFFFFFFFFFFFFFFFFFFFFFFFFFFFFFFFFFFF
FFFFFFFFFFFFFFF
@A00155:342:HHGFNDSXY:1:2146:19904:7905 1:N:0:GAACCTAG+TCCGCATA
AAATCCTCCGTTCTGCCCTTCTTATATCAATTTGAAATTCACGGGAAGGATCTGGTCTACATGCCGGA
CGGGGTGAGGTCTGAGAGTTACACTCAACCATTGGAGAACGGTTTTCTCCTCAGCTCCAGCAGCATAC
TTGTCAGAAATAAGG
+
FFFFFFFFFFFFFFFFFF:FFFFF:FFFFFFFFFFFFFFFFFFFFFFFFFFF:FFFFFFFFFFFFFFFFF
FFFFFFFFFFFFFFFFFFFFFFFFFFFFFFFFFFFFFFFFFFFFFFFFFFFFFFFFFFFFFFFFFFFFFF
FFFFFFFFFFFFFFF
@A00155:342:HHGFNDSXY:1:2221:3224:15123 1:N:0:GAACCTAG+TCCGCATA
AAATCCTCCGTTCTGCCCTTCTTATATCAATTTGAAATTCACGGGAAGGATCTGGTCTACATGCCGGA
CGGGGTGAGGTCTGAGAGTTACACTCAACCATTGGAGAACGGTTTTCTCCTCAGCTCCAGCAGCATAC
TTGTCAGAAATAAGG
+
FFFFFFFFFFFF:FFF:FFF:FFFFFFFFFFFFFFFFFFFFFFFFFFFFF:FFF:FFFFFFFFFFFFFFF
FFFFFFFFFFFFFFFFFFFFFFFFFFFFFFFFFFFFFFFFFFFFFFFFFFFFFFFFFFFFFFFFFFFFFF
FFFFFFFFFFFFFFF
@A00155:342:HHGFNDSXY:1:2221:4833:18568 1:N:0:GAACCTAG+TCCGCATA
AAATCCTCCGTTCTGCCCTTCTTATATCAATTTGAAATTCACGGGAAGGATCTGGTCTACATGCCGGA
CGGGGTGAGGTCTGAGAGTTACACTCAACCATTGGAGAACGGTTTTCTCCTCAGCTCCAGCAGCATAC
TTGTCAGAAATAAGG
+
FFFFFFFFFFFFF:FFFF,:F,FFFFFF:FFFFFFFFF,FFFFFFFFFFFF:FF::FF:FFFFFFFFFFF
FFFFFFFFFFFFFFFFFFFFFFFFFFFFFFFFFFFFFFFFFFFFFFFFFFFFFFFFFFFF:FFFFFFFFF
FFFFFFFFFFFFFFF

```
@A00155:342:HHGFNDSXY:1:2221:4842:18834 1:N:0:GAACCTAG+TCCGCATA
AAATCCTCCGTTCTGCCCTTCTTATATCAATTTGAAATTCACGGGAAGGATCTGGTCTACATGCCGGA
CGGGGTGAGGTCTGAGAGTTACACTCAACCATTGGAGAACGGTTTTCTCCTCAGCTCCAGCAGCATAC
TTGTCAGAAATAAGG
+
FFFFFFFFFFFFFF:FFFFFFFFFFFF:F:FFFFFFFFFFFFFFFFFFFFFFFFFFFFFFFF:FFFFFFF
FFFFFFFFFFFFFFFFFFFFFFFFFFFFFFFFFFFFFFFFFFFFFFFFFFFFFFFFFFFFFFFFFFFF:F
FFFFFFFFFFFFFFF
@A00155:342:HHGFNDSXY:1:2570:11451:27837 1:N:0:GAACCTAG+TCCGCATA
AAATCCTCCGTTCTGCCCTTCTTATATCAATTTGAAATTCACGGGAAGGATCTGGTCTACATGCCGGA
CGGGGTGAGGTCTGAGAGTTACACTCAACCATTGGAGAACGGTTTTCTCCTCAGCTCCAGCAGCATAC
TTGTCAGAAATAAGG
+
FFFFFFFFFFFFFFFFFFFFFFFFFFFFFFFFFFFFFFFFFFFFFFFFFFFFFFFFFFFFFFFFFFFFF:
FFFFFFFFFFFFFFFFFFFFFFFFFFFFFFFFFFFFFFFFFFFFFFFFFFFFFFFFFFFFFFFFFFFFFF
FFFFFFFFFFFFFFF
@A00155:342:HHGFNDSXY:1:1519:6885:20932 2:N:0:GAACCTAG+TCCGCATA
AAATCCTCCGTTCTGCCCTTCTTATATCAATTTGAAATTCACGGGAAGGATCTGGTCTACATGCCGGA
CGGGGTGAGGTCTGAGAGTTACACTCAACCATTGGAGAACGGTTTTCTCCTCAGCTCCAGCAGCATAC
TTGTCAGAAATAAGG
+
FFFFFFFFFFFFFFFFFFFFFFFFFFFFFFFFFFFFFFFFFFFFFFF,FFFFFFFFFFFFFFFF:FFFFF
FFFFFFFFFFFFFFFFFFFFFFFFFFFFFFFF:FFFFFFFFFFFFFFFFFFFFFFFFFFFFFFFFFFFFF
F:FFFFFFFF:F:FF
@A00155:342:HHGFNDSXY:1:1535:15465:24267 2:N:0:GAACCTAG+TCCGCATA
AAATCCTCCGTTCTGCCCTTCTTATATCAATTTGAAATTCACGGGAAGGATCTGGTCTACATGCCGGA
CGGGGTGAGGTCTGAGAGTTACACTCAACCATTGGAGAACGGTTTTCTCCTCAGCTCCAGCAGCATAC
TTGTCAGAAATAAGG
+
FFFFFF:FFFFFFF:FFFFFFFFFFFFFFFFFF:::FFFF:FFF,FFFFF,FFFF:F:F:FFF:FFFF,F
FFFFFF:FF,FFFFFFFFFFFFFFFFFFFFFFFFF,F:FFFFFFFFFF:FFFFFFFFFFFFFFFFFF:FF
F:FFFFFFFF:FFFF
@A00155:342:HHGFNDSXY:1:1535:14525:26334 2:N:0:GAACCTAG+TCCGCATA
AAATCCTCCGTTCTGCCCTTCTTATATCAATTTGAAATTCACGGGAAGGATCTGGTCTACATGCCGGA
CGGGGTGAGGTCTGAGAGTTACACTCAACCATTGGAGAACGGTTTTCTCCTCAGCTCCAGCAGCATAC
TTGTCAGAAATAAGG
+
FFFFFFFFFFFFFFFFFFFFFFFFFFFFFFFFFF:FFF:FFFFFFFFFFFFFFFFFFFFFFFFFFFFFFF
FFFFFFFFFFFFFFFFFFFFFFFFFFFFFFFFFFFFFFF,FFFFFFFFFFFFFFFFFFFFFFFFFFFFFF
:FFFFFFFFF:FFFF
@A00155:342:HHGFNDSXY:1:2102:28158:31688 2:N:0:GAACCTAG+TCCGCATA
AAATCCTCCGTTCTGCCCTTCTTATATCAATTTGAAATTCACGGGAAGGATCTGGTCTACATGCCGGA
CGGGGTGAGGTCTGAGAGTTACACTCAACCATTGGAGAACGGTTTTCTCCTCAGCTCCAGCAGCATAC
TTGTCAGAAATAAGG
+
FFFFFFFFFFFFFFFFFFFFFFF:FF:FFFFFFFFFFFFFFFFFFFFFFFFFFF:FFFFFFFFFFFFFFF
FFFFFFFFFFFFFFFFFFFFFF:FFFFFFFFFFFFFFFFFFFFFFFFFF:FFFFFFFFFFFFFFFFFFFF
FFFFFFFFFFFFF,F
@A00155:342:HHGFNDSXY:1:2169:5258:29293 2:N:0:GAACCTAG+TCCGCATA
AAATCCTCCGTTCTGCCCTTCTTATATCAATTTGAAATTCACGGGAAGGATATGGTCTACATGCCGGA
CGGGGTGAGGTCTGAGAGTTACACTCAACCATTGGAGAACGGTTTTCTCATCAGCTCCAGCAGCATAC
TTGTCAGAAATAAGT
+
FFFFFFFF,:FFFFFFFFF:FFFFFFFF,:FFFF:::FFFF:FF::,FF,:FF,FFFFFFF:FF,FFFFF
```

FFFFFFFFFFFFFFF:FFF,FFFF:FFFFFFF:::F,FFF,FF:F,FFF,:FFFFF:FFFFF:FF:FF
:FF,FFFFFFFFF:F
@A00155:342:HHGFNDSXY:1:2317:12554:20228 2:N:0:GAACCTAG+TCCGCATA
AAATCCTCCGTTCTGCCCTTCTTATATCAATTTGAAATTCACGGGAAGGATCTGGTCTACATGCCGGA
CGGGGTGAGGTCTGAGAGTTACACTCAACCATTGGAGAACGGTTTTCTCCTCAGCTCCAGCAGCATAC
TTGTCAGAAATAAGG
+
FFFFFFFFFFFFFFFF:FFFFFFFFFFFFFFFFFFFFFFFFFFFFFFFFFFFFFFFFFFFFFFFFFF:
FFFFFFFFFFFFFFFFFFF:FFFFFFFFFFFFF:FFFFFFFFFFFFFFFFFFFFFFFFFFFFFFFFFF
FFFFFFFFFFFFFF:F
@A00155:342:HHGFNDSXY:1:2378:25057:27571 2:N:0:GAACCTAG+TCCGCATA
AAATCCTCCGTTCTGCCCTTCTTATATCAATTTGAAATTCACGGGAAGGATCTGGTCTACATGCCGGA
CGGGGTGAGGTCTGAGAGTTACACTCAACCATTGGAGAACGGTTTTCTCCTCAGCTCCAGCAGCATAC
TTGTCAGAAATAAGG
+
FFFFFFFFFFFFFFFFFFFFFFFFFFFFFFFFFFFFFFFFFFFFFFFFFFFFFFFFFFFFFFFFFFFF
FFFFFFFFFFFFFFFFFFFF:FFFFFF:FFFFFFFFFF:FFFFFFFFFFFFFFFFFFFFFFFF:FFFF
FFFFFFFF:FFFFFF
@A00155:342:HHGFNDSXY:1:2247:8223:36464 1:N:0:GAACCTAG+TCCGCATA
AATCCTCCGTTCTGCCCTTCTTATATCAATTTGAAATTCACGGGAAGGATCTGGTCTACATGCCGGAC
GGGGTGAGGTCTGAGAGTTACACTCAACCATTGGAGAACGGTTTTCTCCTCAGCTCCAGCAGCATACT
TGTCAGAAATAAGGC
+
FFFFFFFFFFFFFFFF:FFFFFFFFFFFFFFFFFFF,FFFFFFFFFFFFFFFFFFFFFFFFFFFFFFF
FFFFFFFFFFFFFFFFFFFFFFFFFFFFFFFFFFFFF:FFFFFFFFFFFFFFFFFFFFFFFFFFFFFF
FFFFFFFFFFFFFFF
@A00155:342:HHGFNDSXY:1:2211:8241:26005 2:N:0:GAACCTAG+TCCGCATA
AATCCTCCGTTCTGCCCTTCTTATATCAATTTGAAATTCACGGGAAGGATCTGGTCTACATGCCGGAC
GGGGTGAGGTCTGAGAGTTACACTCAACCATTGGAGAACGGTTTTCTCCTCAGCTCCAGCAGCATACT
TGTCAGAAATAAGGT
+
FFFFFFFFFFFFFFFFFFFFFFFFFFFFFFFFFFFFFFFFFFFFFFFFFFFFFFFFFFFFFFFFFFFF
FFFFFFFFFFFFFFFFFFFFFFFFFFFFFFFFFFFFFFFFFFFFFFFFFFFFFFFFFFFFFFFFFFFF
FFFFFFFFFFF:FFF
@A00155:342:HHGFNDSXY:1:1122:29523:4899 1:N:0:GAACCTAG+TCCGCATA
ATCCTCCGTTCTGCCCTTCTTATATCAATTTGAAATTCACGGGAAGGATCTGGTCTACATGCCGGACG
GAGTGAGGTCTGAGAGTTACACTCAACCATTGGAGAACGGTTTTCTCCTCAGCTCCAGCAGCATACTT
GTCAGAAATAAGGGC
+
FFFFFFFFFFFFFFFFFFFFFFFFFFFFFFFFFFFFFFFFFFFF:FFFFFFFFFFFFFFFFFFFFFFF
FFFFFFFFFFFFFFFFFFFFFFFFFFFFFFFFFFFFFFFFFFFF:FFFFFFFFFFFFFFFFFFFFFFF
FFFFFFFFFFFFFFF
@A00155:342:HHGFNDSXY:1:1464:3929:35102 1:N:0:GAACCTAG+TCCGCATA
ATCCTCCGTTCTGCCCTTCTTATATCAATTTGAAATTCACGGGAAGGATCTGGTCTACATGCCGGACG
GGGTGAGGTCTGAGAGTTACACTCAACCATTGGAGAACGGTTTTCTCCTCAGCTCCAGCAGCATACTT
GTCAGAAATAAGGTC
+
FFFFFFFFFFFFFFFF:F:FFFFFFFFFFFFFFFF:FFFFFFFFFFFFFFFFFFFFFFFFFFFFFFFF
F:FFFFFFFFFFFFFFFFF:FFFFFFFFFFFFFFFFFFFFFFFFFFFFFFFFFFFFFFFFFFFFFFFF
FFFFFFFFFFFFFFF
@A00155:342:HHGFNDSXY:1:2450:9959:35336 1:N:0:GAACCTAG+TCCGCATA
GAGCTCCGTTCTGCCCTTCTTATATCAATTTGAAATTCACGGGAAGGATCTGGTCTACATGCCGGACG
GGGTGAGGTCTGAGAGTTACACTCAACCATTGGAGAACGGTTTTCTCCTCAGCTCCAGCAGCATACTT
GTCAGAAATAAGGAC

```
+
FFFFF,F,FFFF:FFFFF,FFFFF:FFF:FFFFFFFFFFFFFFFFFFFFFFFFFFFFFFFFFFFFFFFF
FFFFFFFFFFFFFFFFFFFFFFFFFFFFFFFFFFFFFFFFFFFFFFFFFFFFFFFFFFFFFFFFFFF:F
FFFFFFFFF:FFF:F
@A00155:342:HHGFNDSXY:1:2659:26033:24283 1:N:0:GAACCTAG+TCCGCATA
ATCCTCCGTTCTGCCCTTCTTATATCAATTTGAAATTCACGGGAAGGATCTGGTCTACATGCCGGACG
GAGTGAGGTCTGAGAGTTACACTCAACCATTGGAGAACGGTTTTCTCCTCAGCTCCAGCAGCATACTT
GTCAGAAATAAGGTC
+
FFFFFFFFFFFFFFFFFFFFFFFFFFFFFFFFFFFFFFFFFFFFFFFFFFFFFFFFFFFFFFFFFFFF
FFFFFFFFFFFFFFFFFFFFFFFFFFFFFFFFFFFFFFFFFFFFFFFFFFFFFFFFFFFFFFFFFFFF
FFFFFFFFFFFFFFF
@A00155:342:HHGFNDSXY:1:1375:24153:31955 2:N:0:GAACCTAG+TCCGCATA
ATCCTCCGTTCTGCCCTTCTTATATCAATTTGAAATTCACGGGAAGGATCTGGTCTACATGCCGGACG
GGGTGAGGTCTGAGAGTTACACTCAACCATTGGAGAACGGTTTTCTCCTCAGCTCCAGCAGCATACTT
GTCAGAAATAAGAGA
+
FFFFFFFFFFFFFFFFFFF:FFFFFFFFFFFFFFFFFFFFFFFFFFFFFFFFFFFFFFFFFFFFFFFF
FFFFFFFFFFFFFFFFFFFFFFFFFFFFFFFFFFFFFFFFFFFFFFFFFFFFFFFFFFFFFFFF:FFF
FFFFFFFFFFFFFFF
@A00155:342:HHGFNDSXY:1:2120:13901:27007 2:N:0:GAACCTAG+TCCGCATA
ATCCTCCGTTCTGCCCTTCTTATATCAATTTGAAATTCACGGGAAGGATCTGGTCTACATGCCGGACG
GAGTGAGGTCTGAGAGTTACACTCAACCATTGGAGAACGGTTTTCTCCTCAGCTCCAGCAGCATACTT
GTCAGAAATAAGGTC
+
:FFFFFFFFFFFFFFFFFFFFFFFFFFFFFFFFFFFFFFFFFFFFFFFFFFFFFFFFFFFFFFFFFFF
FFFFFFFFFFFFFFFFFFFFFFFFFFFFFFFFFFFFFFFFFFFFFFFFFFFFFFFFFFFFFFFFFFFF
FFFFFFFFFFFFFFF
@A00155:342:HHGFNDSXY:1:2553:30761:14121 2:N:0:GAACCTAG+TCCGCATA
ATCCTCCGTTCTGCCCTTCTTATATCAATTTGAAATTCACGGGAAGGATCTGGTCTACATGCCGGACG
GAGTGAGGTCTGAGAGTTACACTCAACCATTGGAGAACGGTTTTCTCCTCAGCTCCAGCAGCATACTT
GTCAGAAATAAGGTC
+
FFFFFFFFFFFFFFFFFFFFFFFFF:FFFFFFFFFFFFFFFFFFFFFFFFFFFFFFFFFFFFFFFFFF
FFFFFFFFFFFFFFFFFFFFFFFFFFFFFFFFFFFFFFFFFFFFFFFFFFFFFFF:FFFFFFFFFFFF
FFFFFFFFFFFFFFF
@A00155:342:HHGFNDSXY:1:2559:26530:30906 2:N:0:GAACCTAG+TCCGCATA
ATCCTCCGTTCTGCCCTTCTTATATCAATTTGAAATTCACGGGAAGGATCTGGTCTACATGCCGGACG
GAGTGAGGTCTGAGAGTTACACTCAACCATTGGAGAACGGTTTTCTCCTCAGCTCCAGCAGCATACTT
GTCAGAAATAAGGTC
+
FFFFFFFFFFFFFFFFFFFFFFFFFFFFFFF:FFFFFFFFFFFFFFFFFFFFFFFFFFFFFFF:FFFF:
FFFFFFFF:FFFFFFFFF:FFFFFFFFFFFFF:FFFFF:FFFF:FFFFFFFFFF:FFF,FF:FFFFFF
FFFFFFFFFFFFFF:
@A00155:342:HHGFNDSXY:1:1212:32859:17190 1:N:0:GAACCTAG+TCCGCATA
CCTCCGTTCTGCCCTTCTTATATCAATTTGAAATTCACGGGAAGGATCTGGTCTACATGCCGGACGGA
GTGAGGTCTGAGAGTTACACTCAACCATTGGAGAACGGTTTTCTCCTCAGCTCCAGCAGCATACTTGT
CAGAAATAAGGTCAC
+
FFFFFFFFFFFFFFFFFFFFFFFFFFFFFFFFFFFFFFFFFFFFFFFFFFFFFFFFFFFFFFFFFFFF
FFFFFFFFFFFFFFFFFFFFFFFFFFFFFFFFFFFFFFFFFFFFFFFFFFFFFFFFFFFFFFFFFFFF
FFFFFFFFFFFFFFF
@A00155:342:HHGFNDSXY:1:1312:22309:19711 1:N:0:GAACCTAG+TCCGCATA
CCTCCGTTCTGCCCTTCTTATATCAATTTGAAATTCACGGGAAGGATCTGGTCTACATGCCGGACGGG
```

GTGAGGTCTGAGAGTTACACTCAACCATTGGAGAACGGTTTTCTCCTCAGCTCCAGCAGCATACTTGT
CAGAAATAAGGTCAC
+
FFFFFFFFFFFFFFFF:FFFFFFFFFFFFFF:FFFFFFFFFFFFFFFFFFFFFFFFFFFFFFFFFFFF
FFFFFFFFFFFFFFFFFFFFFFFFFFFFFFFFFFFFFFFF:FFFFFFFFFFFFFFFFFFFFFFFFFFF
FFFFFFFFFFFFFF:
@A00155:342:HHGFNDSXY:1:1361:25482:22357 1:N:0:GAACCTAG+TCCGCATA
CCTCCGTTCTGCCCTTCTTATATCAATTTGAAATTCACGGGAAGGATCTGGTCTACATGCCGGACGGG
GTGAGGTCTGAGAGTTACACTCAACCATTGGAGAACGGTTTTCTCCTCAGCTCCAGCAGCATACTTGT
CAGAAATAAGGTCAC
+
FFFFFFFFFFFFFFFFFFFFFFFFF:FFFFFFFFFFFF:FFFFFFFFFFFFFFFFFFFFFFFFFFFFF
FFFFFFFFFFFFFFFFFFFFFFFFFFFFFFF:FFFFFFFFFFFFFFFFFFFFFFFFFFFFFFFFFFFF
FFFFFFFFFFFFFFF
@A00155:342:HHGFNDSXY:1:1646:24089:26115 1:N:0:GAACCTAG+TCCGCATA
CCTCCGTTCTGCCCTTCTTATATCAATTTGAAATTCACGGGAAGGATCTGGTCTACATGCCGGACGGG
GTGAGGTCTGAGAGTTACACTCAACCATTGGAGAACGGTTTTCTCCTCAGCTCCAGCAGCATACTTGT
CAGAAATAAGGTCAC
+
FFFFFFFFFFFFFFFFFFFFFFFFF:FFFFFFFFFFFFFFFFFFFFFFFFFFFFFFFFFFFFFFFFFF
FFFFFFFFFFFFFFFFFFFFFFFFFFFFFFFFFFFFFFFFFFFFFFFFFFFFFFFFFFFFFFFFFFFF
FFFFFFFFFFFFFFF
@A00155:342:HHGFNDSXY:1:2101:1805:7780 1:N:0:GAACCTAG+TCCGCATA
CCTCCGTTCTGCCCTTCTTATATCAATTTGAAATTCACGGGAAGGATCTGGTCTACATGCCGGACGGG
GTGAGGTCTGAGAGTTACACTCAACCATTGGAGAACGGTTTTCTCCTCAGCTCCAGCAGCATACTTGT
CAGAAATAAGGTCAC
+
FF,FFF::FFFFFF:FFF,::FFFFFFFFFFFF,FF:F:FF::FF::F,FFFF,FFFFFFF:FFF:FF
F:FF:FFF,FFFFF,FFFFFFFFFFFFF,FFF:FFFFFFF,FFFFFFFFFF::FFFFFFFF:FFF,FF
FFFF::FFFFFF:FF
@A00155:342:HHGFNDSXY:1:2130:11659:34616 1:N:0:GAACCTAG+TCCGCATA
CCTCCGTTCTGCCCTTCTTATATCAATTTGAAATTCACGGGAAGGATCTGGTCTACATGCCGGACGGA
GTGAGGTCTGAGAGTTACACTCAACCATTGGAGAACGGTTTTCTCCTCAGCTCCAGCAGCATACTTGT
CAGAAATAAGGTCAC
+
FFFFFFFFF:FFFFFFFFFF:FFFFFFFFFFFFFFFFFFFFFFFFFFFFFFFFFFFFFFFFFFFFFFF
FFFFFFFFFFFFFF:FFFFFFFFFFFFFFFF:FFFFFFFFFFFFFFFFFFFFFFFFFFFFFFFFFFFF
FFFFFFFFFFFFFFF
@A00155:342:HHGFNDSXY:1:2237:32090:10661 1:N:0:GAACCTAG+TCCGCATA
CCTCCGTTCTGCCCTTCTTATATCAATTTGAAATTCACGGGAAGGATCTGGTCTACATGCCGGACGGG
GTGAGGTCTGAGAGTTACACTCAACCATTGGAGAACGGTTTTCTCCTCAGCTCCAGCAGCATACTTGT
CAGAAATAAGGTCAC
+
FFF::FFFFFFFFFFF:FFFFFFF:FFFFFFFFFFFFFFFFFFF:FFF,FF:FFFFFFFFFFFFFFFF
FF:FF:FFFFFFFFF:FFF:FFFFFFFFFFFFFFFF,FFF:FFF,FFFFFFFFFFFFFFFFFF,FFFF
FFFF:FFFFFFFFFF
@A00155:342:HHGFNDSXY:1:2243:10167:9706 1:N:0:GAACCTAG+TCCGCATA
CCTCCGTTCTGCCCTTCTTATATCAATTTGAAATTCACGGGAAGGATCTGGTCTACATGCCGGACGGG
GTGAGGTCTGAGAGTTACACTCAACCATTGGAGAACGGTTTTCTCCTCAGCTCCAGCAGCATACTTGT
CAGAAATAAGGTCAC
+
FFFFF:FFFFFFFF:FFFFFFFFFFFFFFFFFFFFFFFFFFFFFFFFFFFFFFFFFFFFFFFFFFFFF
FFFFFFFFFFFFFFFFFFFFFFFFFFFFFFFFFFFFFFFFFFFFFFFFFFFFFFFFFFFFFFFFFFFF
FFFFFFFFFFFFFFF

@A00155:342:HHGFNDSXY:1:2438:10086:8656 1:N:0:GAACCTAG+TCCGCATA
CCTCCTTTCTGCCCTTCTTATATCAATTTGAAATTCACGGGAAGGATCTGGTCTACATGCCGGACGGG
GTGAGGTCTGAGAGTTACACTCAACCATTGGAGAACGGTTTTCTCCTCAGCTCCAGCAGCATACTTGT
CAGAAATAAGGTCAC
+
:FFF,,:FFFFFF:FFFFFFF:F:F:FFF:F:FFF,FF:FFFFFFFFFFFFFFFFFFFF:FFFFFFFF
FFFFF:FFFFFFFFFFFFFFF,FFFFFFFFFFFFFFFFFFFFFFF:FFFFFFFFFFFFF:FFF,FFFF
FFFFFFFFFF:F:FF
@A00155:342:HHGFNDSXY:1:2432:32940:10066 1:N:0:GAACCTAG+TCCGCATA
CCTCCGTTCTGCCCTTCTTATATCAATTTGAAATTCACGGGAAGGATCTGGTCTACATGCCGGACGGG
GTGAGGTCTGAGAGTTACACTCAACCATTGGAGAACGGTTTTCTCCTCAGCTCCAGCAGCATACTTGT
CAGAAATAAGGTCAC
+
FFFFFFFFFFFFFFFF:FFFFF:FFFFFFFFFFFFFFFFFFFFFFFFFFFFFFFFFFFFFFFFFFFFF
FFFFFFFFFFFFFFFFFFFFFFFFFFFFFFFFFFFFFFFFFFFFFFFFFFFFFFFFFFFFFFFFFF:F
FFFFFFFFFFFFFFF
@A00155:342:HHGFNDSXY:1:2525:11957:2440 1:N:0:GAACCTAG+TCCGCATA
CCTCCGTTCTGCCCTTCTTATATCAATTTGAAATTCACGGGAAGGATCTGGTCTACATGCCGGACGGG
GTGAGGTCTGAGAGTTACACTCAACCATTGGAGAACGGTTTTCTCCTCAGCTCCAGCAGCATACTTGT
CAGAAATAAGGTCAC
+
FFFFFFFFFFFFFFFFFFFFF:FFF:F:FFFFFFFFFFFFFFFFFFFFFFFFFFFFFFFFFFFFFFFF
FFFFFFFFFFFFFFFFFFFFFFFFFFFFFFFFFFFFFFFFFFFFFFFFFFFFFFFFFFFFFFFFFFFF
FFFFFFFFFFFFFFF
@A00155:342:HHGFNDSXY:1:2560:17336:19648 1:N:0:GAACCTAG+TCCGCATA
CCTCCGTTCTGCCCTTCTTATATCAATTTGAAATTCACGGGAAGGATCTGGTCTACATGCCGGACGGG
GTGAGGTCTGAGAGTTACACTCAACCATTGGAGAACGGTTTTCTCCTCAGCTCCAGCAGCATACTTGT
CAGAAATAAGGTCAC
+
FFFFFFFFFFFFFFFF:FFFFFFFFFFFFFFFFFFFFFFFFFF:FFFFFFFFFFFFFFFFFFFFFFFF
FFFFFFFFFFFFFFFFFFFFFFFFFFFFFFFFFF:FFFFFFFFFFFFFFFFFFFFFFFFFFFFFFFFF
FFFFFFFFFFFFFFF
@A00155:342:HHGFNDSXY:1:2564:29930:24643 1:N:0:GAACCTAG+TCCGCATA
CCTCCGTTCTGCCCTTCTTATATCAATTTGAAATTCACGGGAAGGATCTGGTCTACATGCCGGACGGA
GTGAGGTCTGAGAGTTACACTCAACCATTGGAGAACGGTTTTCTCCTCAGCTCCAGCAGCATACTTGT
CAGAAATAAGGTCAT
+
FFFFFFFFFFFFFFFF:FFFFFFFFFFFFFFFFFFFFF:FFFFFFFFFFFFFFFFFFFFFFFFFFFFF
FFFFFFFFFFFFFFFFFFFFFFFFFFFFFFFFFFFFFFFFFFFFFFFFFFFFFFFFFFFFFFFFFFFF
FFFFFFFFFFFFFFF
@A00155:342:HHGFNDSXY:1:2646:21793:25081 1:N:0:GAACCTAG+TCCGCATA
CCTCCGTTCTGCCCTTCTTATATCAATTTGAAATTCACGGGAAGGATCTGGTCTACATGCCGGACGGG
GTGAGGTCTGAGAGTTACACTCAACCATTGGAGAACGGTTTTCTCCTCAGCTCCAGCAGCATACTTGT
CAGAAATAAGGTCAC
+
FFFFFFFFFFFFFFFFFFFFFFFFFFFFFFFFFFFFFF:FF:FFFFFFFFFFFFFFFFFFFFFFFFFF
FFFFFFFFFFFFFFFFFFFFFFFFFFFFFFFFFFFFFFFFFFFFFFFFFFFFF:FFFFFFFFFFFFFF
FFFFFFFFFFFFFFF
@A00155:342:HHGFNDSXY:1:2663:6768:11240 1:N:0:GAACCTAG+TCCGCATA
CCTCCGTTCTGCCCTTCTTATATCAATTTGAAATTCACGGGAAGGATCTGGTCTACATGCCGGACGGG
GTGAGGTCTGAGAGTTACACTCAACCATTGGAGAACGGTTTTCTCCTCAGCTCCAGCAGCATACTTGT
CAGAAATAAGGTCAC
+
FFFFFFFFFFFFFFFFFFFFFFFFF:FFFFFFFFFFFFFFFFFFFFFFFFFFFFFFFFFFFFFFFFFF

```
FFFFFFFFFFFFFFFFFFFFFFFFFFFFFFFFFFFFFFFFFFFFFFFFFFFFFFFFFFFFFFFFFFFF
FFFFFFFFFFFFF:F
@A00155:342:HHGFNDSXY:1:2560:16984:25708 1:N:0:GAACCTAG+TCCGCATA
CTCCGTTCTGCCCTTCTTATATCAATTTGAAATTCACGGGAAGGATCTGGTCTACATGCCGGACGGGG
TGAGGTCTGAGAGTTACACTCAACCATTGGAGAACGGTTTTCTCCTCAGCTCCAGCAGCATACTTGTC
AGAAATAAGGTCAC
+
FFFFFFFFF:::FFF,FFFFFFFFFFFFF,:FFFFFFFFFFFFFFFFFFFF,,FFFFFFFFFFFF:F
FF:FFFFFFFFFF:F:FFFFFFFF:,FF:FFFFFF:FFFFFFFFFFFFFFFFF,:FFF:FFFFFFFFF
FFF::FFFFFFFF:
@A00155:342:HHGFNDSXY:1:1158:5113:36777 2:N:0:GAACCTAG+TCCGCATA
CTCCGTTCTGCCCTTCTTATATCAATTTGAAATTCACGGGAAGGATCTGGTCTACATGCCGGACGGAG
TGAGGTCTGAGAGTTACACTCAACCATTGGAGAACGGTTTTCTCCTCAGCTCCAGCAGCATACTTGTC
AGAAATAAGGTCAC
+
FFFFFFFFFFFFFFFFFFFFFFFFFFFFFFFFFFFFFFFFFFFFFFFFFFFFFFFFFFFFFFFFFFFF
FFFFFFFFFFFFFFFFFFFFFFFFFFFFFFFFFFFFFF:FFFFFFFFFFFFFFFFFFFFFFFFFFFFF
FF,:FFFFF:FFFF
@A00155:342:HHGFNDSXY:1:1202:6334:26365 1:N:0:GAACCTAG+TCCGCATA
CTCCGTTCTGCCCTTCTTATATCAATTTGAAATTCACGGGAAGGATCTGGTCTACATGCCGGACGGAG
TGAGGTCTGAGAGTTACACTCAACCATTGGAGAACGGTTTTCTCCTCAGCTCCAGCAGCATACTTGTC
AGAAATAAGGTCACA
+
FFFFFFFFFFFFFFFFFFFFFFFFFFFFFFFFFFFFFFFFFF:FFFFFFFFFFFFFFF:FFFFFFFFF
FFFFFFFFFFFFFF:FFFFFFFFFFFFFFFFFFFFFFFFFFFFFFFFFFFFFFFFFFFFFFFFFFFFF
FFFF:FFFFFFFFFF
@A00155:342:HHGFNDSXY:1:1202:6497:26365 1:N:0:GAACCTAG+TCCGCATA
CTCCGTTCTGCCCTTCTTATATCAATTTGAAATTCACGGGAAGGATCTGGTCTACATGCCGGACGGAG
TGAGGTCTGAGAGTTACACTCAACCATTGGAGAACGGTTTTCTCCTCAGCTCCAGCAGCATACTTGTC
AGAAATAAGGTCACA
+
FFFFFFFFFFFFFFFFFFFFFFFFFFFFFFFFFFFFFFFFFFFFFFFFFFFFFFFFFFFFFFFFFFFF
FFFFFFFFFFFFFFFFFFFFFFFFFFFFFFFFFFFFFFFFFFFFFFFFFFFFFFFFFFFF:FFFFFFF
:FFFFFFFFFFFFFF
@A00155:342:HHGFNDSXY:1:1467:26630:18803 1:N:0:GAACCTAG+TCCGCATA
CTCCGTTCTGCCCTTCTTATATCAATTTGAAATTCACGGGAAGGATCTGGTCTACATGCCGGACGGAG
TGAGGTCTGAGAGTTACACTCAACCATTGGAGAACGGTTTTCTCCTCAGCTCCAGCAGCATACTTGTC
AGAAATAAGAGATCG
+
FFFFFFFFFFFFFFFFFFFFFFFFFFFFFFFFFFFFFF:FFFFFFFFFFFFFFFFFFFFFFFFFFFFF
FFFFFFFFFFFFFFFFFFFFFFFFFFFFFFFFFFFFFFFFFFFFFFFFF:FFFFFFFFFFFFFFFFFF
FFFFFFFFFFFFFFF
@A00155:342:HHGFNDSXY:1:1467:26205:29403 1:N:0:GAACCTAG+TCCGCATA
CTCCGTTCTGCCCTTCTTATATCAATTTGAAATTCACGGGAAGGATCTGGTCTACATGCCGGACGGGG
TGAGGTCTGAGAGTTACACTCAACCATTGGAGAACGGTTTTCTCCTCAGCTCCAGCAGCATACTTGTC
AGAAATAAGGTCACG
+
FFFFFFFFFFFFFFFFFFFFFFFFFFFF:FFFFFFFFFFFF:FFFFFFFFFFFF,FFFFFFFFFFFFF
FFFFFFFFFFFFFFFFFFFFFFFFFFFFFFFFFFFFFFFFFFFFF:FFFFFFFFFFFFFFFFFFFFFF
FFFFFFFFFFFFFFF
@A00155:342:HHGFNDSXY:1:1516:16830:3239 1:N:0:GAACCTAG+TCCGCATA
CTCCGTTCTGCCCTTCTTATATCAATTTGAAATTCACGGGAAGGATCTGGTCTACATGCCGGACGGGG
TGAGGTCTGAGAGTTACACTCAACCATTGGAGAACGGTTTTCTCCTCAGCTCCAGCAGCATACTTGTC
AGAAATAAGGTCACA
```

```
+
FFFFFFFFFFFFFFFFFFFF:FFFF,FFFFFFFFFFFFFFFFFFFFFFFFFFFFFFFFFFFFFFFFFFF
FFFFFFFFFFFFFFFFFFFFFFFFFFFFFFFFFFFFFFFFFFFFFFFFFFFFFFFFFFFFFFFFFFFFF
FFFFFFFFFFFFFFF
@A00155:342:HHGFNDSXY:1:2243:2618:15984 1:N:0:GAACCTAG+TCCGCATA
CTCCGTTCTGCCCTTCTTATATCAATTTGAAATTCACGGGAAGGATCTGGTCTACATGCCGGACGGAG
TGAGGTCTGAGAGTTACACTCAACCATTGGAGAACGGTTTTCTCCTCAGCTCCAGCAGCATACTTGTC
AGAAATAAGGTCACA
+
FFFFF:FFFFFFFFFFFFFFFFFFFFFFFFFFF:FFFFFFFFFFFFFFFFFFFFFFFFFFFFFFFFFFF
FFFFFFFFFFFFFFFFFFFFFFFFFFFFFFFFFFFFFFFFFFFFFFFFFFFFFFFFFFFFFFFFFFFFF
FFFFFFFFFFFFFFF
@A00155:342:HHGFNDSXY:1:2460:3305:13698 1:N:0:GAACCTAG+TCCGCATA
CTCCGTTCTGCCCTTCTTATATCAATTTGAAATTCACGGGAAGGATCTGGTCTACATGCCGGACGGAG
TGAGGTCTGAGAGTTACACTCAACCATTGGAGAACGGTTTTCTCCTCAGCTCCAGCAGCATACTTGTC
AGAAATAAGGTCACA
+
FFFFFFFFFF:FFFFFFFFFFFFFFFFFFFFFFFFFFFFFFFFFFFFFFFFFFFFFFFFFFFFFFFFFF
FFFFFFFFFFFFFFFFFFFFFFFFFFFFFFFFFFFFFFFFFFFFFFFFFFFFFFFFFFFFFFFFFFFFF
FFFFFFFFFFFFFFF
@A00155:342:HHGFNDSXY:1:2473:12563:13542 1:N:0:GAACCTAG+TCCGCATA
CTCCGTTCTGCCCTTCTTATATCAATTTGAAATTCACGGGAAGGATCTGGTCTACATGCCGGACGGGG
TGAGGTCTGAGAGTTACACTCAACCATTGGAGAACGGTTTTCTCCTCAGCTCCAGCAGCATACTTGTC
AGAAATAAGGTCACA
+
FFFFF:FFFFFFFFFFFFFFFFFFF:FFFFFFFFFFFFFFFFFFFFFFFFFFFFFFFFFFFFFFFFFFF
FFFFFFFFFFFFFFFFFFFF:FFFFFFFFFFFFFFFFFFFFFFFFFFFFFFFFFFFFFFFFFFFFFF:F
FFFFFFFFFFFFFFF
@A00155:342:HHGFNDSXY:1:2510:30734:36652 1:N:0:GAACCTAG+TCCGCATA
CTCCGTTCTGCCCTTCTTATCTCAATTTGAAATTCACGGGAAGGATCTGGTCTACATGCCGGACGGGG
TGAGGTCTGAGAGTTACACTCAACCATTGGAGAACGGTTTTCTCCTCAGCTCCAGCAGCATACTTGTC
AGAAATAAGGTCACG
+
F:FFFFFFFFFFF:F:F,F,FF:F:F:FFFF,FFFFFFFF:FFFFFFFFFFFFF:FFFFFFFFFFFF
FFFFFFFFFFFFFFFFFFFFFFFFFF:FFFFFFFFFFFFFFFFFFFFFFFFFFFFFFFFFFFFFFFFFF
FFFFFFFFFFFFFFF
@A00155:342:HHGFNDSXY:1:1346:31928:29011 2:N:0:GAACCTAG+TCCGCATA
CTCCGTTCTGCCCTTCTTATATCAATTTGAAATTCACGGGAAGGATCTGGTCTACATGCCGGACGGGG
TGAGGTCTGAGAGTTACACTCAACCATTGGAGAACGGTTTTCTCCTCAGCTCCAGCAGCATACTTGTC
AGAAATAAGAGATCG
+
FFFFFFFFFFFFFFFFFFFF:FF:FFFFFF:FFFFFFFFFFFFFFFFFFFF:FFFFFFFFFF:FFFFF
FFFFFFFFFFFFFFFFFFFFFFFFFFFFFFFFFF:F:FFFFFFFFFFFFFFFFFFF:FF:FFFFFFFFF
,FFFFFFFFFFFFFF
@A00155:342:HHGFNDSXY:1:1610:3911:21574 2:N:0:GAACCTAG+TCCGCATA
CTCCGTTCTGCCCTTATTATATCAATTTGAAATTCACGGGAAGGATCTGGTCTACATGCCGGACGGGG
TGAGGTCTGAGATTTACACTCAACCATTGGAGAACGGTTTTCTCCTCAGCTCCAGCAGCATACTTGTC
AGAAATAAGCTCACA
+
:FF,FF:FFF:F::F,FF:FFF:FFFFFFFFF:FFFFFFFF::FF,FF:FFF:FFFF:FF:FFF,FFF
FFFF:FFFFF:F,FFFFFFFFFFFFF:FF:FFFFF:FFF,F::F:FFF,FFFFFF:F:,F:F:,FFFF
FFF:FFFF,,FFF,F
@A00155:342:HHGFNDSXY:1:1614:10520:18552 2:N:0:GAACCTAG+TCCGCATA
CTCCGTTCTGCCCTTCTTATATCAATTTGAAATTCACGGGAAGGATCTGGTCTACATGCCGGACGGGG
```

```
TGAGGTCTGAGAGTTACACTCAACCATTGGAGAACGGTTTTCTCCTCAGCTCCAGCAGCATACTTGTC
AGAAATAAGGTCACA
+
FFFFFFFFFFF,FFFFFFFFFFFFFFFFFFFFFFFFFFFFFFFFFFF:FFFFFFFFFFFFFFFFFFFF
FFFFFFFFFFFFFFFFFFFFFFFFF:FF:F:FFFFFFFFFFFFFF:FFFFFFFFFFFFFFFFFFFFFF
FFFFFFFFF:FFFFF
@A00155:342:HHGFNDSXY:1:2254:23502:8343 2:N:0:GAACCTAG+TCCGCATA
CTCCGTTCTGCCCTTCTTATATCAATTTGAAATTCACGGGAAGGATCTGGTCTACATGCCGGACGGAG
TGAGGTCTGAGAGTTACACTCAACCATTGGAGAACGGTTTTCTCCTCAGCTCCAGCAGCATACTTGTC
AGAAATAAGGTCACA
+
FFFFFFFFFFFFFFFFFFF:FFFFFFFFFFFFFFFFFFFFFFFFFFFFFFFFFFFFFFFFFFFFFFFF
FFFFFFFFFFFFFFFFFFFFFFFFFFF:FFFFFFF:FFFFF,FFFFFFFFFFFF:FF:FFFFFFFFF
FFF:FFFFFFFF:FF
@A00155:342:HHGFNDSXY:1:2357:9959:12383 2:N:0:GAACCTAG+TCCGCATA
CTCCGTTCTGCCCTTCTTATATCAATTTGAAATTCACGGGAAGGATCTGGTCTACATGCCGGACGGGG
TGAGGTCTGAGAGTTACACTCAACCATTGGAGAACGGTTTCCTCCTCAGCTCCAGCAGCATACTTGTC
AGAAATAAGGTCACA
+
FFFFFFFFFF:FFFFFFFFFFFFFFFFFFFFFFFFFFFFFFF,FFFFFFFFFFFFFFFFFFFFFF,FFF
FFFFFFFFFFFFFFFFFFFFF:FFFFFFFFFFFFFFFFFF,FFFFFFFFFFFFFFFFFFFFFFFFFFF
F:FFFFFF:FFFFFF
@A00155:342:HHGFNDSXY:1:2375:2220:10254 2:N:0:GAACCTAG+TCCGCATA
CTCCGTTCTGCCCTTCTTATATCAATTTGAAATTCACGGGAAGGATCTGGTCTACATGCCGGACGGGG
TGAGGTCTGAGAGTTACACTCAACCATTGGAGAACGGTTTTCTCCTCAGCTCCAGCAGCATACTTGTC
AGAAATAAGAGATCG
+
FFFFFFFFFFFFFFFFFFFFFFFFFFFFF:FFFFFFFFFFFFF:FFFFFF:FFFFFFFFFFFFFFFF:
FFFFFF::FFFFF:FFFFFFFF,FFFFFFFFFFFF,FFFFFFFF,FFF:FFFFFFFFFFFFFFFFFFF
FFFF:FFF,FFFFFF
@A00155:342:HHGFNDSXY:1:2439:13123:25692 2:N:0:GAACCTAG+TCCGCATA
CTCCGTTCTGCCCTTCTTATATCAATTTGAAATTCACGGGAAGGATCTGGTCTACATGCCGGACGGGG
TGAGGTCTGAGAGTTACACTCAACCATTGGAGAACGGTTTTCTCCTCAGCTCCAGCAGCATACTTGTC
AGAAATAAGGTCACA
+
:FFFFFF,FFFFFFF:F:FFFF:FFFFFFFFFFF,FFFFFFFF,FFFFF,FFFFFFF:FF:FFFF:,F
FFF,,FFF:FFFF:FF::::FFF:FFF:FFFFFFF,FFFFFFFFFFFFFFFF,F:FFFFFFFF:FFFF
FFFFF,:F,,FFFFF
@A00155:342:HHGFNDSXY:1:2458:10312:22952 2:N:0:GAACCTAG+TCCGCATA
CTCCGTTCTGCCCTTCTTATATCAATTTGAAATTCACGGGAAGGATCTGGTCTACATGCCGGACGGGG
TGAGGTCTGAGAGTTACACTCAACCATTGGAGAACGGTTTTCTCCTCAGCTCCAGCAGCATACTTGTC
AGAAATAAGGTCACA
+
FFFFFFFFFFFFFFFFFFFFFFFFFFFFFFFFFFFFFFFFFFFFFFFFFFFFFFFFFFFFFFF:FFFFF
FFFFFFFFFFFFFFFFFFFFFFFFFFFFFFFFFFFFFFFFFFFFFFFFFFFFFF:FFFFFFFFFFFF
FFFFFFFFFF:FFF:
@A00155:342:HHGFNDSXY:1:2510:11840:18584 2:N:0:GAACCTAG+TCCGCATA
CTCCGTTCTGCCCTTCTTATATCAATTTGAAATTCACGGGAAGGATCTGGTCTACATGCCGGACGGGG
TGAGGTCTGAGAGTTACACTCAACCATTGGAGAACGGTTTTCTCCTCAGCTCCAGCAGCATACTTGTC
AGAAATAGATCGGAA
+
FFFFFFFFFFFFFF:FFFFF:FFFFFFFF:FFFFFFFFFFFFFFFFFFFFFFFFFFFFFFFFFFFFFFF
FFFFFFFFFFFFFFFFFFFFFFFFFFFFFFFFFFFFFFFFFFFF:FFFFFFFFFFFFFFFFFFFFFFFF
FF::FF:FFFFFFFF
```

```
@A00155:342:HHGFNDSXY:1:1132:1561:20760 1:N:0:GAACCTAG+TCCGCATA
TCCGTTCTGCCCTTCTTATATCAATTTGAAATTCACGGGAAGGATCTGGTCTACATGCCGGAAGGGGT
GAGGTCTGAGAGTTACACTCAACCATTGGATAACGGTTTTCTACTCAGCTCAAGAATCATACTTGTCA
GAAATAAGGTCACA
+
FF::FF,,FF:,FFFFF::FFFFFFFFFFFF:F,FF:F,FF,FF:,FF,:FFF,FFF,FFFF,::,FF
FFFF:FF,FFFF:FF:FFFFF:,FFFFF,F,FFF:F:FFFFF,FFFFF:FFFFF,F,FFFFFF:FFFF
FFF,FFFF:F,F,:
@A00155:342:HHGFNDSXY:1:1119:31006:5400 1:N:0:GAACCTAG+TCCGCATA
TCCGTTCTGCCCTTCTTATATCAATTTGAAATTCACGGGAAGGATCTGGTCTACATGCCGGACGGAGT
GAGGTCTGAGAGTTACACTCAACCATTGGAGAACGGTTTTCTCCTCAGCTCCAGCAGCATACTTGTCA
GAAATAAGGTCACAG
+
FFFFFFFFFFFFFFFFFFFF:FFF:FFFFFFFFFFFFFFFFFFFFFFFFFFFFFFFFFFFFFFFFFFFFF
FFFFFFFFFFFFFFFFFFFFFFFFFFFFFFFFFFFFFFFFFFFFFFFFFFFFFFFFFFFFFFFFFFFFFF
FFFFFFFFFFFFFFF
@A00155:342:HHGFNDSXY:1:1227:24939:32002 1:N:0:GAACCTAG+TCCGCATA
TCCGTTCTGCCCTTCTTATATCAATTTGAAATTCACGGGAAGGATCTGGTCTACATGCCGGACGGGGT
GAGGTCTGAGAGTTACACTCAACCATTGGAGAACGGTTTTCTCCTCAGCTCCAGCAGCATACTTGTCA
GAAATAAGGTCACAG
+
FFFFFFFFFFFFFFFFFFFFFFFFFFFFFFFFFFFFFFFFF:FFFFFFFFFFFF:FFFF:FFFFFFFFFF
FFFFFFFFFFFFFFFFFFFFFFFFFFFFFFFFFFFFFFFFFFFFFFFFFFFFFFFFFFFFFFFFFFFFFF
FFFFFFFFFFFFFFF
@A00155:342:HHGFNDSXY:1:1228:25102:3223 1:N:0:GAACCTAG+TCCGCATA
TCCGTTCTGCCCTTCTTATATCAATTTGAAATTCACGGGAAGGATCTGGTCTACATGCCGGACGGGGT
GAGGTCTGAGAGTTACACTCAACCATTGGAGAACGGTTTTCTCCTCAGCTCCAGCAGCATACTTGTCA
GAAATAAGGTCACAG
+
FFFFFFFFFFFFFFFFFFFFFF::FFFFFFFFFFFFFFFFF:F:,FFFFFFFFFFFFFFFFFFFFFFFFF
FFFFFFFFFFFFFFFFFFFFFFFFFFFFFFFFFFFFFFFFF:FFFFFFFFFFFFFFFFFFFFFFFFFFFF
FFFFFFFFFFFFFFF
@A00155:342:HHGFNDSXY:1:1424:13449:18114 1:N:0:GAACCTAG+TCCGCATA
TCCGTTCTGCCCTTCTTATATCAATTTGAAATTCACGGGAAGGATCTGGTCTACATGCCGGACGGAGT
GAGGTCTGAGAGTTACACTCAACCATTGGAGAACGGTTTTCTCCTCAGCTCCAGCAGCATACTTGTCA
GAAATAAGGTCACAG
+
FFFFFFFFFFFFFFFFFFFFFFFF,FFFFFFFFFFFFFFFFFFFFFFFFFFFFFFFFFFFFFFFFFFFFF
FFFFFFFFFFFFFFFFFFFFFFFFFFFFFFFFFFFFFFFFFFFFFFFFFFFFFFFFFFFFFFFFFFFFFF
FFFFFFFFFFFFFFF
@A00155:342:HHGFNDSXY:1:1601:32163:17300 1:N:0:GAACCTAG+TCCGCATA
TCCGTTCTGCCCTTCTTATATCAATTTGAAATTCACGGGAAGGATCTGGTCTACATGCCGGACGGGGT
GAGGTCTGAGAGTTACACTCATCCATTGGAGAACGGTTTTCTCCTCAGCTCCAGCAGCATACTTGTCA
GAAATAAGGTCACAG
+
FF,F:FF:F::FF:FF:FF:FFFFFFF:FFFFF,:FFFFFFFF:FFFFFF,FFF,FF:F:FFFFFFFF
:,FFF:FFFF:FFF,FFFFF:,,F,FFFFF:F,,:FFFFFFFF,F:F,F:F::FFFFF:FFFFFFF,:
F:F:FFFFFF,F::F
@A00155:342:HHGFNDSXY:1:1656:16884:9032 1:N:0:GAACCTAG+TCCGCATA
TCCGTTCTGCCCTTCTTATATCAATTTGAAATTCACGGGAAGGATCTGGTCTACATGCCGGACGGAGT
GAGGTCTGAGAGTTACACTCAACCATTGGAGAACGGTTTTCTCCTCAGCTCCAGCAGCATACTTGTCA
GAAATAAGGTCACAG
+
FFFFFFFFFFFFFFFFFFFF:FFFF:FFFFFFFFFFFFFF:FFFFFFFFFFFFFFFFFFFFFFFFFFFFF
```

```
FFFFFFFFFFFFFFFFFFFFFFFFFFFFFFFFFFFFFFFFFFFFFFFFFFFFFFFFFFFFFFFFFFFFFFF
FFFFFFFFFFFFFFF
@A00155:342:HHGFNDSXY:1:1671:20383:26428 1:N:0:GAACCTAG+TCCGCATA
TCCGATCTGCCCTTCTTATATCAATTTGAAATTCACGGGAAGGATCTGGTCTACATGCCGGACGGGGT
GAGGTCTGAGAGTTACACTCAACCATTGGAGAACGGTTTTCTCCTCAGCTCCAGCAGCATACTTGTCA
GAAATAAGGTCACAG
+
FFFFFFFFFFFFFFFFFFFFFFFFF:FFFFFFFFFFFFFFF,FFFFFFFFF:FFFFFFFFFFFFFFFFF
FFFFFFFFFFFFFFFFFFFFFFFFFFFFFFFFFFFFFFFFFFFFFFFFFFFFFFFFFFFFFFFFFFFFFFF
FFFFFFFFF:FFFFF
@A00155:342:HHGFNDSXY:1:2120:13901:27007 1:N:0:GAACCTAG+TCCGCATA
TCCGTTCTGCCCTTCTTATATCAATTTGAAATTCACGGGAAGGATCTGGTCTACATGCCGGACGGAGT
GAGGTCTGAGAGTTACACTCAACCATTGGAGAACGGTTTTCTCCTCAGCTCCAGCAGCATACTTGTCA
GAAATAAGGTCACAG
+
FFFFFFFFFFFFFFFFFFFFFFFFFFFFFFFFFFFFFFFFFFFFFFFFFFFFFFFFFFFFFFFFFFFFFFF
FFFFFFFFFFFFFFFFFFFFFFFFFFFFFFFFFFFFFFFFFFFFFFFFFFFFFFFFFFFFFFFFFFFFFFF
FFFFFFFFFFFFFFF
@A00155:342:HHGFNDSXY:1:2215:19533:7764 1:N:0:GAACCTAG+TCCGCATA
TCCGTTCTGCCCTTCTTATATCAATTTGAAATTCACGGGAAGGATCTGGTCTACATGCCGGACGGGGT
GAGGTCTGAGAGTTACACTCAACCATTGGAGAACGGTTTTCTCCTCAGCTCCAGCAGCATACTTGTCA
GAAATAAGGTCACAG
+
FFFFFFFFF:FFFFFFFFFFFFFFFFF:FFFFFFFFFFFFFFFFFFFFFFFFFFFFFFFFFFFFFFFFFFF
FFFFFFFFFFFFFFFFFFFFFFFFFFFFFFFFFFFFFFFFFFFFFFFFFFFFFFFFFFFFFFFFFFFFFFF
FF:FFFFFFFFFFFF
@A00155:342:HHGFNDSXY:1:2303:16333:14904 1:N:0:GAACCTAG+TCCGCATA
TCCGTTCTGCCCTTCTTATATCAATTTGAAATTCACGGGAAGGATCTGGTCTACATGCCGGACGGGGT
GAGGTCTGAGAGTTACACTCAACCATTGGAGAACGGTTTTCTCCTCAGCTCCAGCAGCATACTTGTCA
GAAATAAGGTCACAG
+
FFFFFFFFFFFFFFFFF:FFFFFFFFFFFFFFFFFF:FFFFFFFFFFFFFFFFFF:FFFFFFFFFFFF
FFFFFFFFFFFFFFFFFFFFFFFFFFFFFFFFFFFFFFFFFFFFFFFFFFFFFFFFFFFFFFFFFFFFFFF
FFFFFFFFFFFFFFF
@A00155:342:HHGFNDSXY:1:2674:9082:30217 1:N:0:GAACCTAG+TCCGCATA
TCCGTTCTGCCCTTCTTATATCAATTTGATATTCACGGGAAGGATCTGGTCTACATGCCGGACGGGGT
GAGGTCTGAGAGTTACACTCAACCATTGGAGAACGGTTTTCTCCTCAGCTCCAGCAGCATACTTGTCA
GAAATAAGGTCACAG
+
FFF::FFFFF:FFF:FFFFFFFF,:FFFF,FFFF:F:FFFFFFFFFFFFFFFFFFFFFFFFFFFFFFFF
FFFF:FFFFFFFFFFFFF:F:FFFFFFFFFFFFFFFFFFFFFFFFFFFFFFFFFFF,FFFFFFFFFFF
FFF:FFFFFFFFFFF
@A00155:342:HHGFNDSXY:1:1202:11388:33927 1:N:0:GAACCTAG+TCCGCATA
GTTCTGCCCTTCTTATATCAATTTGAAATTCACGGGAAGGATCTGGTCTACATGCCGGACGGGGTGAG
GTCTGAGAGTTACACTCAACCATTGGAGAACGGTTTTCTCCTCAGCTCCAGCAGCATACTTGTCAGAA
ATAAGGTCACAGGAG
+
FFFFFFFF:FFFFFFFFFFFFFFFFFFFFFFFFFFFFFFFFFFFFFFFFFFFFFFFFFFFFFFFFFFFFF
FFFFFFFFFFFFFFFFFFFFFFFFFFFFFFFFFFFFFFFFFFFFFFFFFFFFFFFFFFFFFFFFFFFFFFF
FFFFFFFFFFFFFFF
@A00155:342:HHGFNDSXY:1:1239:18041:34021 1:N:0:GAACCTAG+TCCGCATA
GTTCTGCCCTTCTTATATCAATTTGAAATTCACGGGAAGGATCTGGTCTACATGCCGGACGGGGTGAG
GTCTGAGAGTTACACTCAACCATTGGAGAACGGTTTTCTCCTCAGCTCCAGCAGCATACTTGTCAGAA
ATAAGGTCACAGGAG
```

```
+
::FFFFFFFFFFFFFFFFFFF:FF:FFF:FFFFFFFFFFFFFFFFFFFFFFFFFFFFFFFFFFFFFFFFF
FFFFFFFFFFFFFFFFFFFFFFFFFFFFFFFFFFFFFFFFFFFFFFFFFFFFFFFFFFFFFFFFFFFFFF
FFFFFFFFFFFFFFF
@A00155:342:HHGFNDSXY:1:1477:10339:6621 1:N:0:GAACCTAG+TCCGCATA
GTTCTGCCCTTCTTATATCAATTTGAAATTCACGGGAAGGATCTGGTCTACATGCCGGACGGGGTGAG
GTCTGAGAGTTACACTCAACCATTGGAGAACGGTTTTCTCCTCAGCTCCAGCAGCATACTTGTCAGAA
ATAAGGTCACAGGAG
+
FFFFFFFFFFFFFFFFFFFFFFFFFFFFFFFFFF:FFFFFFFFFFFFFFFFFFFFFFFFFFFFFFFFFFF
FFFFFFFFFFFFFFFFFFFFFFFFFFFFF:FFF:FFFFFFFFFFFFFFFFFFFFFFFFFFFFFFFFFFFF
FFFFFFFFFFFFFFF
@A00155:342:HHGFNDSXY:1:1542:27606:30671 1:N:0:GAACCTAG+TCCGCATA
GTTCTGCCCTTCTTATATCAATTTGAAATTCACGGGAAGGATCTGGTCTACATGCCGGACGGGGTGAG
GTCTGAGAGTTACACTCAACCATTGGAGAACGGTTTTCTCCTCAGCTCCAGCAGCATACTTGTCAGAA
ATAAGGTCACAGGAG
+
F:FFFFFFFFFFFFFFFFFFFFFFFFFFFFFFFFFFFFFFFFFFFFFFFFFFFFFFFFFFFFFFFFFFFF
FFFFFFFFFFFFFFFFFFFFFFFFFFFFFFFFFFFFFFFFFFFFFFFFFFFFFFFFFFFFFFFFFFFFFF
FFFFFFFFFFFFFFF
@A00155:342:HHGFNDSXY:1:2268:22092:29199 1:N:0:GAACCTAG+TCCGCATA
GTTCTGCCCTTCTTATATCAATTTGAAATTCACGGGAAGGATCTGGTCTACATGCCGGACGGGGTGAG
GTCTGAGAGTTACACTCAACCATTGGAGAACGGTTTTCTCCTCAGCTCCAGCAGCATACTTGTCAGAA
ATAAGGTCACAGGAG
+
FFFFFFFFFFFFFFFFFFFFFFFFFFFFF:FFFFFFFFFFFFFF:FFFF:FFFFFFFFFFFFFFFFFFFF
FFFFFFFFFFFFFFFFFFFFFFFFFFFFFFFFFFFFFFFFFFFFFFFFFFFFFFFFFFFFFFFFFFFFFF
FFFFFFFFFFFFFFF
@A00155:342:HHGFNDSXY:1:2347:7211:32581 1:N:0:GAACCTAG+TCCGCATA
GTTCTGCCCTTCTTATATCAATTTGAAATTCACGGGAAGGATCTGGTCTACATGCCGGACGGAGTGAG
GTCTGAGAGTTACACTCAACCATTGGAGAACGGTTTTCTCCTCAGCTCCAGCAGCATACTTGTCAGAA
ATAAGGTCACAGGAG
+
F:FFF:FFFFFFFFFFFFFFFFFFFFFFFFFFFFFFFFFFFFFFFFFFFFFFFFFFFFFFFFFFFFFFFF
FFFFFFFFFFFFFFFFFFFFFFFFFFFFFFFFFFFFFFFFFFFFFFFFFFFFFFFFFFFFFFFFFFFFFF
FFFFFFFFFFFFFFF
@A00155:342:HHGFNDSXY:1:2357:9959:12383 1:N:0:GAACCTAG+TCCGCATA
GTTCTGCCCTTCTTATATCAATTTGAAATTCACGGGAAGGATCTGGTCTACATGCCGGACGGGGTGAG
GTCTGAGAGTTACACTCAACCATTGGAGAACGGTTTCCTCCTCAGCTCCAGCAGCATACTTGTCAGAA
ATAAGGTCACAGGAG
+
FFFFFFFFFFFFFFFFFFFF,FF:FFFFFFFFFFFFFFFFFFFFFFFFFFFFFFFFFFFFF:FFFFFFFF
FFFFFFFFFFFFFFFFFFFFFFFFFFFFFFFFFFFFF:FFFFFFFFFFFFFFFFFFFFFFFFFFFFFFFF
FFFFFFFFFFFFFFF
@A00155:342:HHGFNDSXY:1:2509:6451:24627 1:N:0:GAACCTAG+TCCGCATA
GTTCTGCCCTTCTTATATCAATTTGAAATTCACGGGAAGGATCTGGTCTACATGCCGGACGGGGTGAG
GTCTGAGAGTTACACTCAACCATTGGAGAACGGTTTTCTCCTCAGCTCCAGCAGCATACTTGTCAGAA
ATAAGGTCACAGGAG
+
FFFFFFFFFFFFFFF,FFF:FF:FFFFFFFFFFFFFFFFFFFF:,FFFFFFFFFFFFFFFFFFFFFFFFF
FFFFFFFF:FFFFFFFF:FFFFFFFFFFFFFFFFFFFFFFFFFFFFFFFFFFFFFFFFFFFFFFFFFFF
FFFFFFFFFFFFFFF
@A00155:342:HHGFNDSXY:1:2543:14651:18630 1:N:0:GAACCTAG+TCCGCATA
GTTCTGCCCTTCTTATATCAATTTGAAATTCACGGGAAGGATCTGGTCTACATGCCGGACGGGGTGAG
```

GTCTGAGAGTTACACTCAACCATTGGAGAACGGTTTTCTCCTCAGCTCCAGCAGCATACTTGTCAGAA
ATAAGGTCACAGGAG
+
FFFFFFFFFFFFFFFFFFFFFFFFFFFF:FFFFFFFFFFFFFFFFFFFFFFFFFFFFFFFFFFFFFF
FFFFFFFFFFFFFFFFFFFFFFFFFFFFFFFFFFFFFFFFFFFFFFFFFFFFFFFFFFFFFFFFFFF
FFFFFFFFFFFFFFF
@A00155:342:HHGFNDSXY:1:2618:22128:20682 1:N:0:GAACCTAG+TCCGCATA
GTTCTGCCCTTCTTATATCAATTTGAAATTCACGGGAAGGATCTGGTCTACATGCCGGACGGAGTGAG
GTCTGAGAGTTACACTCAACCATTGGAGAACGGTTTTCTCCTCAGCTCCAGCAGCATACTTGTCAGAA
ATAAGGTCACAGGAG
+
FFFFFFFFFFFFFFFFFFFFFFFFFFFFFFFFFFFFFFFFFFFFFFFFFFFFFFFFFFFFFFFFFFF
FFFFFFFFFFFFFFFFFFFFFFFFFFFFFFFFFFFFFFFFFFFFFFFFFFFFFFFFFFFFFFFFFFF
FFFFFFFFFFFFFFF
@A00155:342:HHGFNDSXY:1:2226:8079:10191 2:N:0:GAACCTAG+TCCGCATA
GTTCTGCCCTTCTTATATCAATTTGAAATTCACGGGAAGGATCTGGTCTACATGCCGGACGGGGTGAG
GTCTGAGAGTTACACTCAACCATTGGAGAACGGTTTTCTCCTCAGCTCCAGCAGCATACTTGTCAGAA
ATAAGGTCACAGGAG
+
FFFFFFFF:FFFFFFFFFFFFFFFFFFF,FFFFFFFFFFFFFFF:FFFFFFF:FFFFFFFF:FFFFF
FFFFFFFFF:FFFFFFFF:FFFFFFFFFFFFF:FFFFFFFFFF,FFF,FFFFFFFFFFFFFFFFFFF
FFFFFFFFFFFFFFF
@A00155:342:HHGFNDSXY:1:1373:6885:18051 1:N:0:GAACCTAG+TCCGCATA
CTGCCCTTCTTATATCAATTTGAAATTCACGGGAAGGATCTGGTCTACATGCCGGACGGGGTGAGGTC
TGAGAGTTACACTCAACCATTGGAGAACGGTTTTCTCCTCAGCTCCAGCAGCATACTTGTCAGAAATA
AGGTCACAGGAGTTG
+
FFFFFFFFF:FFFFF,:F:F,FFFF:FF,FFF::,FFFFF:FFFFFFFFFFFFFFFFFFFFFFFFFF
FFFFFFFFFFFFFFFFFFFFFFFFFFFFFF:,FFFFFFFFFFFFFFFFFFF:F:FFFFFFFFF:FFF
:FFFFFFFFFFFFFF
@A00155:342:HHGFNDSXY:1:2576:31087:36417 2:N:0:GAACCTAG+TCCGCATA
CTGCCCTTCTTATATCAATTTGAAATTCACGGGAAGGATCTGGTCTACATGCCGGACGGGGTGAGGTC
TGAGAGTTACACTCAACCATTGGAGAACGGTTTTCTCCTCAGCTCCAGCAGCATACTTGTCAGAAATA
AGGTCACAGGAGTTG
+
FFFFFFFFFFFFFFFFFFFFFFFFFFFFFFFFFFFFFFFFFFFFFFFFFFFFFFFFFFFFFFFFFFF
FFFFFFFFFF:FFFFFFFFFFFFFFF:FFFFFFFFFFFFFFFFFFFFFFFFFFFFFFFFFFFFFFFF
FFFFFFFFFFFFFFF
@A00155:342:HHGFNDSXY:1:1301:4887:35258 2:N:0:GAACCTAG+TCCGCATA
TGCCCTTCTTATATCAATTTGAAATTCACGGGAAGGATCTGGTCTACATGCCGGACGGAGTGAGGTCT
GAGAGTTACACTCAACCATTGGAGAACGGTTTTCTCCTCAGCTCCAGCAGCATACTTGTCAGAAATAA
GGTCACAGGAGTTGA
+
FFFFFFFFFFFFFFFFFFFFFFFFFFFFFFFFFFFFFFFFFFFFFFFFFFFFFFFFFFFFFFFFFFF
FFFF:FFFFFFFFFFFFFFFFFFFFFFFFF:FFFFFFFFFFFFFFFFFFFFFFFFFFFFFFFFFFFF
FFFFFFFFFF:FFFF
@A00155:342:HHGFNDSXY:1:1227:31774:9455 1:N:0:GAACCTAG+TCCGCATA
GCCCTTCTTATATCAATTTGAAATTCACGGGAAGGATCTGGTCTACATGCCGGACGGGGTGAGGTCTG
AGAGTTACACTCAACCATTGGAGAACGGTTTTCTCCTCAGCTCCAGCAGCATACTTGTCAGAAATAAG
G
+
FFFFFFFFFFFFF:F,F:FFFFFFFFFFFF:FFFFFFFFFFF:FFFFFFFFFFFFFFFFFFFFFFFF
FFFF:FFFFFFFFFFFFFFFFFFFFFFFFFFFFFFFFFFFFFFFFFFFFF:FFFFFFFFFFFFFFFF
F

@A00155:342:HHGFNDSXY:1:1424:32271:29199 1:N:0:GAACCTAG+TCCGCATA
GCCCTTCTTATATCAATTTGAAATTCTCGGGAAGGATCTGGTCTACATGCCGGACGGGGTGAGGTCTG
AGAGTTACACTCAACCATTGGAGAACGGTTTTCTCCTCAGCTCCAGCAGCATACTTGTCAGAAATAAG
G
+
:FFFFFF:FF:FFFFFFFF:,,F,FF,F:FFF,FFFFFF:FFFFF,FFFFF:FFFFFFFFFFFFFFFFFF
FFFFFF:FFFF:FFFFF:FFFFF::FFFFFFFF:::FFFFFFFF,F:FFFF:FFFFFFF,FF:FFF,F
F
@A00155:342:HHGFNDSXY:1:2666:15609:18317 1:N:0:GAACCTAG+TCCGCATA
CTCCTTCTTATATCAATTTGAAATTCACGGGAAGGATCTGGTCTACATGCCGGACGGGGTGAGGTCTG
AGAGTTACACTCAACCATTGGAGAACGGTTTTCTCCTCAGCTCCAGCAGCATACTTGTCAGAAATAAG
G
+
FFFFFFFFFFFFFFFFFFFFFFFFFFFFFFFFFFFFFFFFFFFFFFFFFFFFFFFFFFFFFFFFFFFFFF
FFFFFFFFFFFFFFFFFFFFFFFFFFFFFFFFFFFFFFFFFFFFFFFFFFFFFFFFFFFFFFFFFFFFFF
F
@A00155:342:HHGFNDSXY:1:1220:11478:31923 2:N:0:GAACCTAG+TCCGCATA
GCCCTTCTTATATCAATTTGAAATTCACGGGAAGGATCTGGTCTACATGCCGGACGGGGTGAGGTCTG
AGAGTTACACTCAACCATTGGAGAACGGTTTTCTCCTCAGCTCCAGCAGCATACTTGTCAGAAATAAG
GTCAC
+
FFFFFFFFFFFFF,FFF:FFFFFFFFFFFFFFFFFFFFFFFFFFFFFFFFFFFFFFFFFFFFFFFFFFFF
:FFFFFFFFFFFF:FFFFFFFFF:FFFFFFFFFFFFFFFFFFFFFFFFFFFFFFFFFFFFFFFFFFFFF:
FFFFF
@A00155:342:HHGFNDSXY:1:1244:24496:29074 2:N:0:GAACCTAG+TCCGCATA
GCCCTTCTTATATCAATTTGAAATTCACGGGAAGGATCTGGTCTACATGCCGGACGGGGTGAGGTCTG
AGAGTTACACTCAACCATTGGAGAACGGTTTTCTCCTCAGCTCCAGCAGCATACTTGTCAGAAATAAG
GTCAC
+
FFFF:,FFFFFFFFFFFFFFF::FFFFFFFFFFFFFFFFFF:FFFFFFFFFFFFFFFFFFFFFFFFFFFF
FFFFFFFFFFFFFFFFFFFF:FFFFFFFFFFFFFFFFFFFFFFFFFFFFFFFFFFFFFFFFF:FFFFFFF
:FFFF
@A00155:342:HHGFNDSXY:1:1244:23330:31438 2:N:0:GAACCTAG+TCCGCATA
GCCCTTCTTATATCAATTTGAAATTCACGGGAAGGATCTGGTCTACATGCCGGACGGGGTGAGGTCTG
AGAGTTACACTCAACCATTGGAGAACGGTTTTCTCCTCAGCTCCAGCAGCATACTTGTCAGAAATAAG
GTCAC
+
FFFFFFFFFFFFFFFFFFFFFFFFFFFFFFFF,FFFFFFFFFFFFFFFFFFFFFFFFFFFFFFFFFFFFF
FFFFFFFFFFFFFFFFFFFFFFFFFFFFFFFFFFFFFFFFFFFFFFFFFFFFFFFFF:FFFFFFFFFFFF
FFFFF
@A00155:342:HHGFNDSXY:1:1524:31331:6715 2:N:0:GAACCTAG+TCCGCATA
GCCCTTCTTATATCAATTTGAAATTCACGGGAAGGATCTGGTCTACATGCCGGACGGGGTGAGGTCTG
AGAGTTACACTCAACCATTGGAGAACGGTTTTCTCCTCAGCTCCAGCAGCATACTTGTCAGACATAAG
GTCAC
+
FFFFF:FFF:FFFFFFFFFFFFFFFFFFFFFFFFFFFFFFFFFFFFFFF:FFFFFFFFFFFFFFFFFFFF
FFFFFF:FFFFFFFFFFFFFFFFF:FF:FFFFFFF:FFFFFFF:FFFFF:FFFFFFFFFFFF,FFFFF
,FFFF
@A00155:342:HHGFNDSXY:1:2249:17110:14653 2:N:0:GAACCTAG+TCCGCATA
GCCCTTCTTATATCAATTTGAAATTCACGGGAAGGATCTGGTCTACATGCCGGACGGAGTGAGGTCTG
AGAGTTACACTCAACCATTGGAGAACGGTTTTCTCCTCAGCTCCAGCAGCATACTTGTCAGAAATAAG
AGATC
+
FFFFFFFFFF,FFFFFFFFFFFFFFFFFFFFFFFFFFFFFFFFFFFFFFFFFFFFF:FFFFFFFFFFFFF

FFFFFFFFFFFFFFFFFF:FFFFFFFFFFFFFFFFFFFFFFFF:FF:FFFFFFFFF:FFFFFFFFFFF
FFFFF
@A00155:342:HHGFNDSXY:1:2304:20735:4930 2:N:0:GAACCTAG+TCCGCATA
GCCCTTCGTATATCAATTTGAAATTCACGGGAAGGATCTGGTCTACATGCCGGACGGGGTGAGGTCTG
AGAGTTACACTCAACCATTGGAGAACGGTTTTCTCCTCAGCTCCAGCAGCATACTTGTCAGAAATAAG
GTCAC
+
F:FFF::,FF:FFFFFFFFFFFF:FFFF::FF,:FFFFF:,FFFFFFFF::FFFFF,F:FFFFFFFFF
F:FFFFF,FFFFFF,FFFFFFFFFFFF,F::FFFFF:FF:FFF:FFFFFFFF::FFF:FFFFFFF,FF
FFFFF
@A00155:342:HHGFNDSXY:1:2250:16405:29403 2:N:0:GAACCTAG+TCCGCATA
GCCCTTCTTATATCAATTTGAAATTCACGGGAAGGATCTGGTCTACATGCCGGACGGAGTGAGGTCTG
AGAGTTACACTCAACCATTGGAGAACGGTTTTCTCCTCAGCTCCAGCAGCATACTTGTCAGAAATAAG
GTCAC
+
FFFFFFFFFFFFFFFFFFFFFFFFFFFFFFFFFFFFFFFFFFFFFFFFFFFFFFFFFFFFFFFFFFFF
FFFFFFFFFFFFFFFFFFFFFFFFFFFFFFFFFFFFFFFFFFFFFFFFFFF:FFFFFFFFFFFFFFFF
FFFFF
@A00155:342:HHGFNDSXY:1:1528:4580:6637 2:N:0:GAACCTAG+TCCGCATA
GACCTTCTTATATCAATTTGAAATTCACGTGAAGGATCTGGTATACATGCCGGTCGGGGTGAGGTCTG
AGAGTTACACTCAACCATTGGAGAACGGTTTTCTCCTCAGCTACAGCAGCATACTTGTCAGAAATAAG
GTCACATGAGTTG
+
F,F,FFFF:F,F:FFFF:FFFFFFFF,:,,FFFFFFF,FF,F,FF:FFFFFFF,F,F:FFFF:F::FF
FFFFFFFFFFFFFFFFFFFF:FFFFFFFFFF,F:FFFF:FFF,:FF:FFFFFF::FF,FFFFFFFFFF
,,FFFF,FFFFFF
@A00155:342:HHGFNDSXY:1:1315:18493:26068 2:N:0:GAACCTAG+TCCGCATA
GCCCTTCTTATATCAATTTGAAATTCACGGGAAGGATCTGGTCTACATGCCGGACGGGGTGAGGTCTG
AGAGTTACACTCAACCATTGGAGAACGGTTTTCTCCTCAGCTCCAGCAGCATACTTGTCAGAAATAAG
GTCACAGGAGTTGAG
+
FFFFFFFFFFFFFFFFFFFFFFFFF,:FFFFFFFFFFFFF:FFFFFFFFFFFFFFFF:FFFFFFFFFF
FFFFFF::FFFFFFFFFFFFFFFFFF:FFFFFFFFFF:FFF:F:FF:FFFFFFFFFFFFFFFFFFF:F
FFFFFFFF,FFFFFF
@A00155:342:HHGFNDSXY:1:1361:22571:36010 2:N:0:GAACCTAG+TCCGCATA
GCCCTTCTTATATCAATTTGAAATTCACGGGAAGGATCTGGTCTACATGCCGGACGGGGTGAGGTCTG
AGAGTTACACTCAACCATTGGAGAACGGTTTTCTCCTCAGCTCCAGCAGCATACTTGTCAGAAATAAG
GTCACAGGAGTTGAG
+
FFFFFFFFFFFFFFFFFFFFFFFFFFFFFFFFFFFFF:FFFFFFFFFFFFFFFFFFFFFFFFFFFFFF
:FF:FFFFFFF:FFFFFFFFFFFF:F,FFFFFFFFFFFFFFFFFFF:FFFFFFFF:FFFFFF:FFFFF
:F::FFFFFFF:FFF
@A00155:342:HHGFNDSXY:1:1528:4797:7012 2:N:0:GAACCTAG+TCCGCATA
GCCCTTCTTATATCAATTTGAAATTCACGGGAAGGATCTGGTCTACATGCCGGACGGGGTGAGGTCTG
AGAGTTAAACTCAACAATTGGAGAACGGTTTTCTCCTCAGCTCCAGCAGCATACGTGTCAGAAATAAG
GTCACAGGAGTTGAG
+
FFFF:FF:F:,,FFFFFF:FFFFFFFFF::FFFFFFFFF:FFFFFF:F:FFFFF:FFFFFFFF:FF::
FF:FFF,,F:FFFF,,FFF::F,:F,,,F:::FFF,FFFFFF,FFFF:FFFFF,,:FFFF::,,FFF,
FFFF,FFFFFFF,FF
@A00155:342:HHGFNDSXY:1:1671:20383:26428 2:N:0:GAACCTAG+TCCGCATA
GCCCTTCTTATATCAATTTGAAATTCACGGGAAGGATCTGGTCTACATGCCGGACGGGGTGAGGTCTG
AGAGTTACACTCAACCATTGGAGAACGGTTTTCTCCTCAGCTCCAGCAGCATACTTGTCAGAAATAAG
GTCACAGAGATCGGA

+
FFFFFFFFFFFFFFFFFFFFFFFF:FFFFFFFFFFFFFFFFFFFFFFFFFFFFFFFFFFFFFFFFFFF
FFFFFFFFFFFFFF,FFFFFFFFFFF,FFFFFFF:FFFFFFFFFF:FFFFFFFFFFFFFFFFFFFFFF
FFFFFFFFFFF:FFF
@A00155:342:HHGFNDSXY:1:1675:29758:34240 2:N:0:GAACCTAG+TCCGCATA
GCCCTTCTTATATCAATTTGAAATTCACGGGAAGGATCTGGTCTACATGCCGGACGGGGTGAGGTCTG
AGAGTTACACTCAACCATTGGAGAACGGTTTTCTCCTCAGCTCCAGCAGCATACTTGTCAGAAATAAG
GTCACAGGAGTTGAG
+
FFFFFFFFFFFFFFFF,FFFFFFFFFFFFFFF:FFFFF:FFFFFFFFFFFFFFFFFFFFFFFFFFFFF
FFFFFFFFFFFFFFF,FFFFFF:FFF,F:,FFFFFF:FFFFFFFFFFFFFFFFFFFFFFFFFFFFFFF
F:FFFF:::FFFFFF
@A00155:342:HHGFNDSXY:1:2320:19416:5494 2:N:0:GAACCTAG+TCCGCATA
GCCCTTCTTATATCAATTTGAAATTCACGGGAAGGATCTGGTCTACATGCCGGACGGGGTGAGGTCTG
AGAGTTACACTCAACCATTGGAGAACGGTTTTCTCCTCAGCTCCAGCAGCATACTTGTCAGAAATAAG
GTCACAGGAGTTGAG
+
FFFFFFFFFFFFFFFFFFFFFFFFFFFFFFFFFFFFFFF:FFFFFFFFFFFFFFFFFFFFFFFFFFFF
FFFFFFFFFFFFFFFFFFFFFFFFFFFFFFFFFFFFFFFFFFF:FFFFFFFFFFFF:FFFFFFFFFFF
FFFFFF:FFFFFFFF
@A00155:342:HHGFNDSXY:1:2538:11984:3959 2:N:0:GAACCTAG+TCCGCATA
CCCTTCTTATATCAATTTGAAATTCACGGGAAGGATCTGGTCTACATGCCGGACGGAGTGAGGTCTGA
GAGTTACACTCAACCATTGGAGAACGGTTTTCTCCTCAGCTCCAGCAGCATACTTGTCAGAAATAAGG
TCACAGGAGTTGAGA
+
FFFF:FFFFFFFFFFFFFFFFFFFFFFFFFFFFFFFFFF:FFFFFFFFFFFFFFFFFFFFFFFFFFFF
FFFFFFFFFFFFFFFFFFFFFFFFFFFFFF:F:FFF:FFFFFFFFFFFFFFFF:FFFFFFFFFFFFFFFF
FFFFFF:FFFFFFFF
@A00155:342:HHGFNDSXY:1:1244:24496:29074 1:N:0:GAACCTAG+TCCGCATA
CCTTCTTATATCAATTTGAAATTCACGGGAAGGATCTGGTCTACATGCCGGACGGGGTGAGGTCTGAG
AGTTACACTCAACCATTGGAGAACGGTTTTCTCCTCAGCTCCAGCAGCATACTTGTCAGAAATAAGG
+
FFFFFFFFF,FFFFFFFFFFFFFFFFFFF:FFFFFFFFFFFFFFFFFFFFFFFFFFFFFFFFFFFFFF
FFFFFFFFFFFFFFFFFFFFFFFFFFFFFFFFFFFFFFFFFFFFFFFFFFFFFFFFFFFFFFFFFFF
@A00155:342:HHGFNDSXY:1:1244:23330:31438 1:N:0:GAACCTAG+TCCGCATA
CCTTCTTATATCAATTTGAAATTCACGGGAAGGATCTGGTCTACATGCCGGACGGGGTGAGGTCTGAG
AGTTACACTCAACCATTGGAGAACGGTTTTCTCCTCAGCTCCAGCAGCATACTTGTCAGAAATAAGG
+
FFFFFFF:FFFFFFFFF:FFFFFFFFFFFFFFFFFFFFFFFFFFFFF:FFFFF:FFFFFFFFFFFFFF
FFFFFFFFFFFFFFFFFFFFFFFFFFFFFFFFFFFFFFFFFFFFFFFFFFFFFFFFFFFFFFFFFFF
@A00155:342:HHGFNDSXY:1:1220:11478:31923 1:N:0:GAACCTAG+TCCGCATA
CCTTCTTATATCAATTTGAAATTCACGGGAAGGATCTGGTCTACATGCCGGACGGGGTGAGGTCTGAG
AGTTACACTCAACCATTGGAGAACGGTTTTCTCCTCAGCTCCAGCAGCATACTTGTCAGAAATAAGG
+
FFFFFFF:FFFFFF:FFFFFFFFFFFFFFF,FFFFFFFFFFFFFFFFFFFFFFFFFFFFFFFFFFFFF
FFFFFFFFFFFFFFFFFFFFFFFFFFFFF,FFFFFFFFFFFFFFFFFFFFFFFFFFFFFFFFFFFFF
@A00155:342:HHGFNDSXY:1:1467:18719:20509 2:N:0:GAACCTAG+TCCGCATA
CCTTCTTATATCAATTTGAAATTCACGGGAAGGATCTGGTCTACATGCCGGACGGAGTGAGGTCTGAG
AGTTACACTCAACCATTGGAGAACGGTTTTCTCCTCAGCTCCAGCAGCATACTTGTCAGAAATAAGGT
CACAGGAGTTGAG
+
:,FF,FFF:FFFFFFFFFFFFFFFFFFFFFFFFFFFFFFFFFFFFFFFFFFFFFFFF:FFFFFF:F,
F,FFFFFFFFFFFFFFFF:FFFFFFFFFFFFFFFFFFFFFFFFFF::FFFFFFFFFFFFFFFFFFFFF,FF
FFFFF:FFFFFFF

@A00155:342:HHGFNDSXY:1:1263:14877:22467 2:N:0:GAACCTAG+TCCGCATA
CCTTCTTATATCAATTTGAAATTCACGGGAAGGATCTGGTCTACATGCCGGACGGGGTGAGGTCTGAG
AGTTACACTCAACCATTGGAGAACGGTTTTCTCCTCAGCTCCAGCAGCATACTTGTCAGAAATAAGGT
CACAGGAGTTGAGAT
+
FFFFFFFFFFFFFFFFFFFFFFF:FFFFFFFFFFFFFFFF:FFFFFFFFFFFFFFFFFFFFF::FFFF
FF:FFFFFFFFFFF,FFFF:FFFFFFFFFFFFFFFFFF,:F:FFFFFFFFFFFFFFFFFFFFFFF:FF
FFFFFFFFFFFFFFF
@A00155:342:HHGFNDSXY:1:1263:14850:22482 2:N:0:GAACCTAG+TCCGCATA
CCTTCTTATATCAATTTGAAATTCACGGGAAGGATCTGGTCTACATGCCGGACGGGGTGAGGTCTGAG
AGTTACACTCAACCATTGGAGAACGGTTTTCTCCTCAGCTCCAGCAGCATACTTGTCAGAAATAAGGT
CACAGGAGTTGAGAT
+
FFFFFFFFFFFFFFFFFFFFFFFFFF:FFFFFFFFFFFFFFFF,FFFFFFFFFFFFFFFFFFFFFFFF
FFFFFFFFFFFFFFFFFFFFF:FFFFFFFFFFFFFFFFFFFFFFFFFFFFFFFFFFFFFFFFFFFFFF
FFFFFFFFFFFFFFF
@A00155:342:HHGFNDSXY:1:1270:19379:20055 2:N:0:GAACCTAG+TCCGCATA
CCTTCTTATATCAATTTGAAATTCACGGGAAGGATCTGGTCTACATGCCGGACGGAGTGAGGTCTGAG
AGTTACACTCAACCATTGGAGAACGGTTTTCTCCTCAGCTCCAGCAGCATACTTGTCAGAAATAAGGT
CACAGGAGTTGAGAT
+
FFFFFFFFFF,FFFFFFFFFFFFFFFFFFFFFFFFFFFFFFFFFFFFFFFFFFFFFFFFFFFFF:F:F
FFFFFFFFFFFFF:FFFFFFFFFFFFFFF:FFFFFFFFFFFFFF:FF:FFFFFFFFFFFFFF:FFFFF
FFFFFFFFF:FFFFF
@A00155:342:HHGFNDSXY:1:1506:27299:32456 2:N:0:GAACCTAG+TCCGCATA
CCTTCTTATATCAATTTGAAATTCACGGGAAGGATCTGGTCTACATGCCGGACGGGGTGAGGTCTGAG
AGTTACACTCAACCATTGGAGAACGGTTTTCTCCTCAGCTCCAGCAGCATACTTGTCAGAAATAAGGT
CACAGGAGTTGAGAT
+
FFFFFFFFFFFFFFFFF:FFFFFFFFFFFFFFFFFFFFFFFFFFFFFFFFFFFFFFFFFFFFFFFFFF
FFFFFFFFFFFFFFFFFFFFFFFFFF:FFFFFFFFFFFFFFFFFFFFFFFFFF:FFFFFFFFFFFFFF
FFFFFFFFFFFFFFF
@A00155:342:HHGFNDSXY:1:1562:28646:6417 2:N:0:GAACCTAG+TCCGCATA
CCTTCTTATATCAATTTGAAATTCACGGGAAGGATCTGGTCTACATGCCGGACGGAGTGAGGTCTGAG
AGTTACACTCAACCATTGGAGAACGGTTTTCTCCTCAGCTCCAGCAGCATACTTGTCAGAAATAAGGT
CACAGGAGTTGAGAT
+
FFFFFFFFF:FF:FFFFFF:FFFFFFFFFFF:FFFFFFFFFFFFFFFFFFFFFFFFFFFFFFFFFFFF
FFFFFFF:FFFFFFFFFFFFFFFFFFFFFFFFFFFFFFF:FFFFFFFFFFFFFFFFFFFFFF:FFFFF
FFFFFFFFFFFFFFF
@A00155:342:HHGFNDSXY:1:1655:19054:25567 2:N:0:GAACCTAG+TCCGCATA
CCTTCTTATATCAATTTGAAATTCACGGGAAGGATCTGGTCTACATGCCGGACGGGGTGAGGTCTGAG
AGTTACACTCAACCATTGGAGAACGGTTTTCTCCTCAGCTCCAGCAGCATACTTGTCAGAAATAAGGT
CACAGGAGTTGAGAT
+
F,FFFFFFFFFFFFFFFFFFFFFFFFFFFFFFFFFFFFFFFFFFFFFFFFFFFFFFFFFFFFFFFFFF
FFFFFFFFFFFFFFFFFFFFFFFFFFFFFFFFF:::FFFFFFFF:FFFFFFFFFFFFFFF:FFFFFFF
FFFFFFFFFFFFFFF
@A00155:342:HHGFNDSXY:1:2513:18575:13244 2:N:0:GAACCTAG+TCCGCATA
CCTTCTTATATCAATTTGAAATTCACGGGAAGGATCTGGTCTACATGCCGGACGGGGTGAGGTCTGAG
AGTTACACTCAACCATTGGAGAACGGTTTTCTCCTCAGCTCCAGCAGCATACTTGTCAGAAATAAGGT
CACAGGAGTTGAGAT
+
FFFFFFFFFFFFFFFFFFFFFFFFFFFFFFFFFFFFFFFFFFFFFFFFFFFFFFFFFFFFFFFFFFFF

FFFFFFFFFFFFFFFFFFFFFFFFFFFFF:FFFFF:FFFFFFFF:FF,FFFFFFFFFFFFFFFFFFF,
FFFFFFFFFFFFFFF
@A00155:342:HHGFNDSXY:1:2666:15609:18317 2:N:0:GAACCTAG+TCCGCATA
CCTTCTTATATCAATTTGAAATTCACGGGAAGGATCTGGTCTACATGCCGGACGGGGTGAGGTCTGAG
AGTTACACTCAACCATTGGAGAACGGTTTTCTCCTCAGCTCCAGCAGCATACTTGTCAGAAATAAGGT
CACAGGAGTTGAGAG
+
FFFFFFFFFF:FFFFFFF:FFFFFFFFFFFFFFFFFFFFFFFFFFFFFFFFFFFFFFFFFFFFFFFFF
FFFFFFFFFFFFFFFFFFFFFFFFFFFFFFFFFFFFFFFFFFFFFFFFFFFFFFFFFFFFFFFFFFFF
FFFFFFFFFFFFFFF
@A00155:342:HHGFNDSXY:1:1169:24551:10003 2:N:0:GAACCTAG+TCCGCATA
CTTCTTATATCAATTTGAAATTCACGGGAAGGATCTGGTCTACATGCCGGACGGGGTGAGGTCTGAGA
GTTACACTCAACCATTGGAGAACGGTTTTCTCCTCAGCTCCAGCAGCATACTTGTCAGAAATAAGGTC
ACAGGAGTTGAGATG
+
FFFFFFFFFFFFFFFFFFFFFFFFFFFFFFFFFFFFFFFFFFFFFFFFFFFFFFFF:FFFFFFFFFFF
FFFFFFFFFFFFFF:FFFFFFFFFFFFFFFFFFFFFFFFFFFFFFF:F:FFFFFFFFFFFFFFFFFFF
FFFFF:FFFFFFFFF
@A00155:342:HHGFNDSXY:1:1255:12120:1063 2:N:0:GAACCTAG+TCCGCATA
CTTCTTATATCAATTTGAAATTCACGGGAAGGATCTGGTCTACATGCCGGACGGGGTGAGGTCTGAGA
GTTACACTCAACCATTGGAGAACGGTTTTCTCCTCAGCTCCAGCAGCATACTTGTCAGAAATAAGGTC
ACAGGAGTTGAGATG
+
FFF:FFFFFFFFFFFFFFFFFFFFFFFFFFFFFFFFFFFFFFFFFFFFFFFFFFFFFFF:FFFFF:FF
FFFFFFFFFFFFFFFFFF:FFFFFFFFFFFFFFFFFFFFFFFFFFFFFFFFFFFFFFFFFFFFFFFFF
FFFFFFFFFFFFFFF
@A00155:342:HHGFNDSXY:1:1620:14398:33098 2:N:0:GAACCTAG+TCCGCATA
CTTCTTATATCAATTTGAAATTCACGGGAAGGATCTGGTCTACATGCCGGACGGGGTGAGGTCTGAGA
GTTACACTCAACCATTGGAGAACGGTTTTCTCCTCAGCTCCAGCAGCATACTTGTCAGAAATAAGGTC
ACAGGAGTTGAGATG
+
FFFFFFFFFFFFFFFFFFFFFFFFFFFFFFFFFFFFFFFFFFFFFFFFFFFFFFFFFFFFFFFFFFFF
FFFFFFFFFFFFFFFFFFFFFFFFF,FFFFFFFFFFFFFFFFFF,FFFFFFF:FFFFFFFFFFFFFFF
FFFFFFFFF:FFFFF
@A00155:342:HHGFNDSXY:1:2121:32886:9721 2:N:0:GAACCTAG+TCCGCATA
CTTCTTATATCAATTTGAAATTCACGGGAAGGATCTGGTCTACATGCCGGACGGGGTGAGGTCTGAGA
GTTACACTCAACCATTGGAGAACGGTTTTCTCCTCAGCTCCAGCAGCATACTTGTCAGAAATAAGGTC
ACAGGAGTTGAGATG
+
F::FFFFFFFFFFFF:F:FFFFF:FFFFF:,:FFFFFFFFFFFFFFFFFFF::FFFFFFFFFFFFFFF
FFFFF:FFFFFFFF:F::FFFFFFFF,FFFFFFFFFFFFFF:F:FFFFFFF::FFFFFFFFFFFFF:F
FFFFFFFFF:FFFFF
@A00155:342:HHGFNDSXY:1:2406:20835:9173 2:N:0:GAACCTAG+TCCGCATA
CTTCTTATATCAATTTGAAATTCACGGGAAGGATCTGGTCTACATGCCGGACGGGGTGAGGTCTGAGA
GTTACACTCAACCATTGGAGAACGGTTTTCTCCTCAGCTCCAGCAGCATACTTGTCAGAAATAAGGTC
ACAGGAGTTGAGATG
+
:FFFFFFFFFFFFFFFFFFFFFFFFFFFFFFFFFFFFFFFFFFFFFFFFFFFFFFFFFFFFFFFFFFF
FFFFFFFFFFFFFFFFFFFFFFFFFFFFFFFFFFFFFFFFFFFFFF:FFFFFFFFFFFF::FFFFFFF
FFFFFFFFFFFFFFF
@A00155:342:HHGFNDSXY:1:1332:23583:10582 2:N:0:GAACCTAG+TCCGCATA
TTCTTATATCAATTTGAAATTCACGGGAAGGATCTGGTCTACATGCCGGACGGGGTGAGGTCTGAGAG
TTACACTCAACCATTGGAGAACGGTTTTCTCCTCAGCTCCAGCAGCATACTTGTCAGAAATAAGGTCA
CAGGAGTTGAGATGA

```
+
FFFFFFFFFFFFFFFFFFFFFFFFFFFFFFFFFFFFFFFFFFFFFFFFFFFFFFFFFFFFFFFFFFFFFFF
FFFFFFFFFFFFFFFFFFFFFFFFFFFFFFFFFFFFFFFFFFFFF:FFFFFFFFFFFFFFFFFFFFFFFFF
FFFFFFFFFFFFF,FF
@A00155:342:HHGFNDSXY:1:1201:10257:36636 1:N:0:GAACCTAG+TCCGCATA
TCTTATATCAATTTGAAATTCACGGGAAGGATCTGGTCTACATGCCGGACGGGGTGAGGTCTGAGAGT
TACACTCAACCATTGGAGAACGGTTTTCTCCTCAGCTCCAGCAGCATACTTGTCAGAAATAAGG
+
FFFFFFFFF:FFFFFFFFFFFFFF:FFFFFFFFFFFFFFFFFFFFFFFFFFFFFFFFFFFFFFFFFFFFFF
FFFFFFFFFFFFFFFFFFFFFFFFFFFFFFF:FFFFFFFFFFFFFFFFFFFFFFFFFFFFFFFFFFF
@A00155:342:HHGFNDSXY:1:1623:21594:20134 1:N:0:GAACCTAG+TCCGCATA
TCTTATATCAATTTGAAATTCACGGGAAGGATCTGGTCTACATGCCGGACGGGGTGAGGTCTGAGAGT
TACACTCAACCATTGGAGAACGGTTTTCTCCTCAGCTCCAGCAGCATACTTGTCAGAAATAAGG
+
FFFFFFFFFFFFFFFFFFFFFFFFFFFFFFFFFFFFFFFFFFFFFFFFFFFFFFFFFFFFFFFFFFFFFFF
FFFFFFFFFFFFFFFFFFFFFFFFFFFFFFFFFFFFFFFFFFFFFFFFFFFFFFFFFFFFFFFFFFF
@A00155:342:HHGFNDSXY:1:1667:10610:15264 1:N:0:GAACCTAG+TCCGCATA
TCTTATATCAATTTGAAATTCACGGGAAGGATCTGGTCTACATGCCGGACGGAGTGAGGTCTGAGAGT
TACACTCAACCATTGGAGAACGGTTTTCTCCTCAGCTCCAGCAGCATACTTGTCAGAAATAAGG
+
FF:FFFFFFF:,FFFFFF,FFFFFFFFFFFFFFFFFFFFFFFFFFFFFFFFFFFFFFFFFFFFFFFFFFFF
FFFFFFFFFFFFFFFFFFFFFFFFFFFFFFFFFFFFFFFFFFFFFFFFFFFFFFFFFFFFFFFFFFF
@A00155:342:HHGFNDSXY:1:2102:7030:23750 1:N:0:GAACCTAG+TCCGCATA
TCTTATATCAATTTGAAATTCACGGGAAGGATCGGGTCTACATGCCGGACGGGGTGAGGTCTGAGAGT
TACACTCAACCATTGGAGAACGGTTTTCTCCTCAGCTCCAGCAGCATACTTGTCAGAAATAAGG
+
FFFFFFFF:FF,FFFFFFFFFFFFFFFFFFFFFF,FFFFFFFFFFFFFFFFFFFFFFFFFFFFFFFFFFFF
FFFFFFFFFFFFFFFFFFFFFFFFFFFFFFFFFFFFFFFFFFFFFFFFFFFFFFFFFFFFFFFFFFF
@A00155:342:HHGFNDSXY:1:2354:11125:5071 1:N:0:GAACCTAG+TCCGCATA
TCTTATATCAATTTGAAATTCACGGGAAGGATCTGGTCTACATGCCGGACGGAGTGAGGTCTGAGAGT
TACACTCAACCATTGGAGAACGGTTTTCTCCTCAGCTCCAGCAGCATACTTGTCAGAAATAAGG
+
FFFFFFFFFFFFFFFFFFFFFFFFFFFFFFFFFFFFFFFFFFFFFFFFFFFFFFFFFFFFFFFFFFFFFFF
FFFFFFFFFFFFFFFFFFFFFFFFFFFFFFFFFFFFFFFFFFFFFFFFFFFFFFFFFF:FFFFFFFFF
@A00155:342:HHGFNDSXY:1:2512:13711:35227 1:N:0:GAACCTAG+TCCGCATA
TCTTATATCAATTTGAAATTCACGGGAAGGATCTGGTCTACATGCCGGACGGAGTGAGGTCTGAGAGT
TACACTCAACCATTGGAGAACGGTTTTCTCCTCAGCTCCAGCAGCATACTTGTCAGAAATAAGG
+
FFFFFFFFFFFFFFFFFFFFFFFFFFFFFFFFFFFFFFFFFFFFFFFFFFFFFFFFFFFFFFFFFFFFFFF
FFFFFFFFFFFFFFFFFFFFFF:FFFFFFFFFFFFFFFFFFFFFFFFFFFFF:FFFFFFFFFFFFFF
@A00155:342:HHGFNDSXY:1:2620:2492:31923 1:N:0:GAACCTAG+TCCGCATA
TCTTATATCAATTTGAAATTCACGGGAAGGATCTGGTCTACATGCCGGACGGGGTGAGGTCTGAGAGT
TACACTCAACCATTGGAGAACGGTTTTCTCCTCAGCTCCAGCAGCATACTTGTCAGAAATAAGG
+
FFFFFFFFFFFFFFFFFFFFFFFFFFFFFFFFFFFFFFFFFFFFFFFFFFFFFFFFFFFFFFFFFFFFFFF
FFFFFFFFFFFFFFFFFFFF,FFFFFFFFFFFFFFFFFFFFFFFFFFFFFFFFFFFFFFFFFFFFFF
@A00155:342:HHGFNDSXY:1:1334:15664:11647 2:N:0:GAACCTAG+TCCGCATA
TCTTATATCAATTTGAAATTCACGGGAAGGATCTGGTCTACATGCCGGACGGAGTGAGGTCTGAGAGT
TACACTCAACCATTGGAGAACGGTTTTCTCCTCAGCTCCAGCAGCATACTTGTCAGAAATAAGG
+
FFFFFFFFFFFFFFFFFFFFFFFFFFFFFFFFFFFFFFFFFFFFFFFFFFFFFFFFFFFFFFFFFFFFFFF
FFFFFFFFFFFFFFFFFFFFFFFFFFFFFFFFFFFFFFFFFFFFFFFFFFFFFFFFFFFFFFF:FFF
@A00155:342:HHGFNDSXY:1:2158:12364:23563 2:N:0:GAACCTAG+TCCGCATA
CTTATATCAATTTGAAATTCACGGGAAGGATCTGGTCTACATGCCGGACGGAGTGAGGTCTGAGAGTT
```

ACACTCAACCATTGGAGAACGGTTTTCTCCTCAGCTCCAGCAGCATACTTGTCAGAAATAAGGTCACA
GGAGTTGAGATGAG
+
FFFFFFFFFFFFFFFFFFFFFF:FFFFF:FFFFFFFFFFFF:FFFFFFFFFFFFFFFFF:FFFFFFFFFF
FFFFFFFFFFFFFFFFFFFFFFFFFFFFFFFFFFFFFFFFFFFF:FFFFFFFFFFFFFFFFFFFFFFFFF
FFFFFFFFFFFFFF
@A00155:342:HHGFNDSXY:1:1213:16369:24298 2:N:0:GAACCTAG+TCCGCATA
CTTATATCAATTTGAAATTCACGGGAAGGATCTGGTCTACATGCCGGACGGGGTGAGGTCTGAGAGTT
ACACTCAACCATTGGAGAACGGTTTTCTCCTCAGCTCCAGCAGCATACTTGTCAGAAATAAGGTCACA
GGAGTTGAGATGAGA
+
FFFFFFFFFFFFFFFFFFFFFFFFFFFFFFFFFFFFFFFFFFFFFFFFFFFFFFFFFFFFFFFFFFFFFF
FFFFFFFFFFFFFFFFFFFFFFFFFFFFFFFFFFFFFFFFFFFFFFFFFFFFFFFFFFFFFFFFFFFFFF
FFFFFFFFFFFFFFF
@A00155:342:HHGFNDSXY:1:1303:17119:15076 2:N:0:GAACCTAG+TCCGCATA
CTTATATCAATTTGAAATTCACGGGAAGGATCTGGTCTACATGCCGGACGGGGTGAGGTCTGAGAGTT
ACACTCAACCATTGGAGAACGGTTTTCTCCTCAGCTCCAGCAGCATACTTGTCAGAAATAAGGTCACA
GGAGTTGAGATGAGA
+
FFFFFFFFFFFFFFFFFFFFFFFFFFFFFFFFFFFFFFFFFFFFFFFFFFFFFFFFFFFFFFFFFFFFFF
FFFFFFFFFFFFFFFFFFFFFFFFFFFFFFFFFFFFFFFFFFFFFFFFFFFFFFFFFFFFFFFFFFFFFF
FFFFFFFFFFFFFFF
@A00155:342:HHGFNDSXY:1:1406:18945:10504 2:N:0:GAACCTAG+TCCGCATA
CTTATATCAATTTGAAATTCACGGGAAGGATCTGGTCTACATGCCGGACGGGGTGAGGTCTGAGAGTT
ACACTCAACCATTGGAGAACGGTTTTCTCCTCAGCTCCAGCAGCATACTTGTCAGAAATAAGGTCACA
GGAGTTGAGATGAGA
+
FFFFFFFFFFF:FFFFFFFFFFFFFFFFFFFFFFFFFFFFFFFFFFFFFFFFFFF:FFFFFFFFFFFFFF
FFFFFFFFFFFFFFFFFFFFFFFFF:FFFFFFFFFFFFFFFFFFFFFFFFFFFFFFFFFFFFFFFFFFFF
F,FFFFFFFFFFFFF
@A00155:342:HHGFNDSXY:1:1436:19325:10191 2:N:0:GAACCTAG+TCCGCATA
CTTATATCAATTTGAAATTCACGGGAAGGATCTGGTCTACATGCCGGACGGGGTGAGGTCTGAGAGTT
ACACTCAACCATTGGAGAACGGTTTTCTCCTCAGCTCCAGCAGCATACTTGTCAGAAATAAGGTCACA
GGAGTTGAGATGAGA
+
FFFFFFFFFFFFFFFFFFFFFFFFFFFFFFFFFFFFFFFFFFFFFFFFFFFFFFFFFFFFFFFFFFFFFF
FFFFFFFFFFFFFFFFFFFFFFFFFFFFFFFFFFFFFFFFFFFFFFFFFFFFFFFFFFFFFF:FFFFFF
FFFFFFFFFFFFFFF
@A00155:342:HHGFNDSXY:1:1562:17607:32737 2:N:0:GAACCTAG+TCCGCATA
CTTATATCAATTTGAAATTCACGGGAAGGATCTGGTCTACATGCCGGACGGGGTGAGGTCTGAGAGTT
ACACTCAACCATTGGAGAACGGTTTTCTCCTCAGCTCCAGCAGCATACTTGTCAGAAATAAGGTCACA
GGAGTTGAGATGAGA
+
FFFFFFFFFFFFFFFFFFFFFFFFFF:FFFFFFFFFFFFFFFFFFFFFFFFFFFFFFFFFFFFFFFFFFF
FFFFFFFFFFFFFFFFFFFFFFFFFFFFFFFFFFFFFFFFFFFFFFFFFFFFFFFFFFFFFF:FFFFFFF
FFFFFFFFFFFFFFF
@A00155:342:HHGFNDSXY:1:1608:20139:11036 2:N:0:GAACCTAG+TCCGCATA
CTTATATCAATTTGAAATTCACGGGAAGGATCTGGTCTACATGCCGGACGGGGTGAGGTCTGAGAGTT
ACACTCAACCATTGGAGAACGGTTTTCTCCTCAGCTCCAGCAGCATACTTGTCAGAAATAAGGTCACA
GGAGTTGAGATGAGA
+
FFFFF:FFFFFFFFFFFFFFFFFFFFFFFFFFFFFFFFFFFFFFFFFFFFFFFFFFFFFFFFFFFFFFFF
FFFFFFFFFFFFFFFFFFFFFFFFFFFFFFFFFF:FFF:FFFFFFFFFFFFFFFFFFF:FFFFFFFFFFF
FFFFFFFFFFFFFFF

```
@A00155:342:HHGFNDSXY:1:1633:32443:26177 2:N:0:GAACCTAG+TCCGCATA
CTTATATCAATTTGAAATTCACGGGAAGGATCTGGTCTACATGCCGGACGGGGTGAGGTCTGAGAGTT
ACACTCAACCATTGGAGAACGGTTTTCTCCTCAGCTCCAGCAGCATACTTGTCAGAAATAAGGTCACA
GGAGTTGAGATGAGA
+
FFFFFFFFFFFFFFFFFFFFFFFFFFFFFFFFFFFFFFFFFFFFFFFFFFFFFFFFFFFFFFFFFFFF
FFFFFFFFFFFFFFFFFFFFFFFFFFFFFFFFFFFFFFFFFFFFFFFFFFFFFFFFFFFFFFFFFFFF
FFFFFFFFFF:FFFF
@A00155:342:HHGFNDSXY:1:1675:20518:14481 2:N:0:GAACCTAG+TCCGCATA
CTTATATCAATTTGAAATTCACGGGAAGGATCTGGTCTACATGCCGGACGGGGTGAGGTCTGAGAGTT
ACACTCAACCATTGGAGAACGGTTTTCTCCTCAGCTCCAGCAGCATACTTGTCAGAAATAAGGTCACA
GGAGTTGAGATGAGA
+
FFFFFFFFFFFFFFFFFFFFFFFFFFFFFFFFFFFFFFFFFFFFFFFFFFFFFFFFFFFFFFFFFFFF
FFFFFFFFFFFFFFFFFFFFFFFFFFFFFFFFFFFFFFFF:FFFFFFFFFFFFFFFFFFF:FFFFFFF
FFFFFFFFFFFFF:F
@A00155:342:HHGFNDSXY:1:2113:1163:25989 2:N:0:GAACCTAG+TCCGCATA
CTTATATCAATTTGAAATTCACGGGAAGGATCTGGTCTACATGCCGGACGGGGTGAGGTCTGAGAGTT
ACACTCAACCATTGGAGAACGGTTTTCTCCTCAGCTCCAGCAGCATACTTGTCAGAAATAAGGTCACA
GTAGTTGAGATGAGA
+
FFFFFFF,FFFFFFFF:FF,,,FFFF:FFFFFFFFFFFFF:FFFFFF::F:FFFF,FFFFFF:F:FFF
F:F,FFF:,:FFFFFFFF:FFFFFFF:FF:FFF,FF:FFFFFFF:FF:FF,F::F,F:FF::,F::,F
F,FFFFF:,:FFFFF
@A00155:342:HHGFNDSXY:1:2119:10990:26944 2:N:0:GAACCTAG+TCCGCATA
CTTATATCAATTTGAAATTCACGGGAAGGATCTGGTCTACATGCCGGACGGGGTGAGGTCTGAGAGTT
ACACTCAACCATTGGAGAACGGTTTTCTCCTCAGCTCCAGCAGCATACTTGTCAGAAATAAGGTCACA
GGAGTTGAGATGAGA
+
FFFFFFFFFFFFFFFFFFFFFFFFFFFFFFFFFFFFFFFFFFFFFFFFFFFFFFFFFFFFFFFFFFFF
FFFFFFFFFFFFFFFFFFFFFFFFFFFFFFFF:FFFFFFFFFFFFFFFFFFFFFFFFFFFFF:FFFFF
FFFFFFFFFFFFFFF
@A00155:342:HHGFNDSXY:1:2308:22263:26897 2:N:0:GAACCTAG+TCCGCATA
CTTATATCAATTTGAAATTCACGGGAAGGATCTGGTCTACATGCCGGACGGGGTGAGGTCTGAGAGTT
ACACTCAACCATTGGAGAACGGTTTTCTCCTCAGCTCCAGCAGCATACTTGTCAGAAATAAGGTCACA
GGAGTTGAGATGAGA
+
FFFFFFFFFFFFFFFFFFFFFFFFFFFFFFFFFFFFFFFFFFFFFFFFFFFFFFFFFFFFFFFFFFFF
:FFFFFFFFFFFFFFFFFFFFFFFFFFFFFFFFFFFFFFF::FFFFFFFFFFFFFFFFFFFFFFFFFF
FF:FFFFFFFFFFFF
@A00155:342:HHGFNDSXY:1:2356:27281:11882 2:N:0:GAACCTAG+TCCGCATA
CTTATATCAATTTGAAATTCACGGGAAGGATCTGGTCTACATGCCGGACGGAGTGAGGTCTGAGAGTT
ACACTCAACCATTGGAGAACGGTTTTCTCCTCAGCTCCAGCAGCATACTTGTCAGAAATAAGGTCACA
GGAGTTGAGATGAGA
+
F:FFFFFFFFFFFFFFFFFFFFFFFFFFFFFFFFFFFFFFFFFFFFFFFFFFFFFFFFFFFFFFFFFF
:FFFFFFFFF,:FFFFFFFFFFFFFFFFFFFFFFFFFFFFFFFFFFFFFF:FFFFFF,FFFFFFFF:F
FFFFFFFFFFFFFFF
@A00155:342:HHGFNDSXY:1:2377:17499:16861 2:N:0:GAACCTAG+TCCGCATA
CTTATATCAATTTGAAATTCACGGGAAGGATCTGGTCTACATGCCGGACGGGGTGAGGTCTGAGAGTT
ACACTCAACCATTGGAGAACGGTTTTCTCCTCAGCTCCAGCAGCATACTTGTCAGAAATAAGGTCACA
GGAGTTGAGATGAGA
+
FFFFFFFFFFFFF:FFFFFF:FFFFFFFFFFFFFFFFFFFFFFFFFFFFF:FFFF:FFFFFFFFFFFF
```

FFFFFFFFFFFFFFFFFF:FFFFFFFFFFFFF:FFFFFFFFFFFFFFF:FFFFFF:FF:FFFFF:FF
FFFFFFFFFFFFFFF
@A00155:342:HHGFNDSXY:1:2534:25021:7122 2:N:0:GAACCTAG+TCCGCATA
CTTATATCAATTTGAAATTCACGGGAAGGATCTGGTCTACATGCCGGACGGGGTGAGGTCTGAGAGTT
ACACTCAACCATTGGAGAACGGTTTTCTCCTCAGCTCCAGCAGCATACTTGTCAGAAATAAGGTCACA
GGAGTTGAGATGAGA
+
FFFFFFFFFFFFFFFFFFFFFFFFFFFFFFFFFFFFFFFFFFFFFFFFFFFFFFFFFFFFFFFFFFFF
:FFFFFFFFFF:FFFFFFFFFFFFFFFFFFFFFFFFFFFFFFFFFFFFFF:FFFFF:FFFFFFFFFFF
FFFFFFFFFFFFFFF
@A00155:342:HHGFNDSXY:1:2534:25201:12414 2:N:0:GAACCTAG+TCCGCATA
CTTATATCAATTTGAAATTCACGGGAAGGATCTGGTCTACATGCCGGACGGGGTGAGGTCTGAGAGTT
ACACTCAACCATTGGAGAACGGTTTTCTCCTCAGCTCCAGCAGCATACTTGTCAGAAATAAGGTCACA
GGAGTTGAGATGAGA
+
:FFFFFFFFFFFFFFFFFFFFFFFFFFFFFFFFFFFFFFFFFFFFFFFFFFFFFFFFFFFFFFFFFFF
FFFFFFFFFFFFFFFFFFFFFFFFFFFFFFFFFFFFFFFFFFFFFFFFFFFFFFF:FFFFFFFFFF:
F:FFFFFFFFFFFFF
@A00155:342:HHGFNDSXY:1:2675:20473:30091 2:N:0:GAACCTAG+TCCGCATA
CTTATATCAATTTGAAATTCACGGGAAGGATCTGGTCTACATGCCGGACGGAGTGAGGTCTGAGAGTT
ACACTCAACCATTGGAGAACGGTTTTCTCCTCAGCTCCAGCAGCATACTTGTCAGAAATAAGGTCACA
GGAGTTGAGATGAGA
+
FFFFFFFFFFFFFFFFFFFFFFFFFFFFFFFFFFFFFFFFFFFFFFFFFFFFFFFFFFFFFFFFFFFF
FFFFFFFFFFFFFFFFFFFFFFFFFFFFFFFFFFFFFFFFFFFFFFFFFFFFFFFFFFFFFFFFFFFF
FFFFFFFFFFFFFFF
@A00155:342:HHGFNDSXY:1:1132:1561:20760 2:N:0:GAACCTAG+TCCGCATA
TTATATCAATATGAAATTCACGGGAAGGATCTGGTCTACATGCCGGACGGGGTGAGGTCTGAGAGTTA
CACTCAACCATTGGAGAACGGTTTTCTCCTCAGCTCCAGCAGCATACTTGTCAGAAATAAGG
+
FFF:F,FFFF,FFFFFFFFFF,:FFF:,FFFF,F:FFF:F:FF:,:FFFF,F,FFFFFFF:FFFFFF:
FFFFFFF,:FFFFFFFFFFF:F:FF:FFFFFFFFF:FFFFF:FFFFFFF:,FFFFF:FFF::
@A00155:342:HHGFNDSXY:1:1116:16749:5509 1:N:0:GAACCTAG+TCCGCATA
TTATATCAATTTGAAATTCACGGGAAGGATCTGGTCTACATGCCGGACGGGGTGAGGTCTGAGAGTTA
CACTCAACCATTGGAGAATGGTTTTCTCCTCAGCTCCAGCAGCATACTTGTCAGAAATAAGGTCACAG
GAGTTGAGATGAGAG
+
FF,F:FF,:FF:FF::FFFFFFFF::FFFFFFFFFFFFF:FFFFFFFFFFFFF:FFFFFFFFFFFFF
FFFFFF:FF,FFFFFF::FFF:FFFFFFFFFFFFFFFFFFFFFFFFFFFFFFFFFFFFFF:FFF,F:F
F,FFFFFF:FFFFFF
@A00155:342:HHGFNDSXY:1:1376:20600:33473 1:N:0:GAACCTAG+TCCGCATA
TTATATCAATTTGAAATTCACGGGAAGGATCTGGTCTACATGCCGGACGGGGTGAGGTCTGAGAGTTA
CACTCAACCATTGGAGAATGGTTTTCTCCTCAGCTCCAGCAGCATACTTGTCAGAAATAAGGTCACAG
GAGTTGAGATGAGAG
+
FFFFFFFFF:FFFFFFFFFFFFFFFFFFFFFFFFFFFFFF:FFFFFFFFFFFFFFFFFFFFFFFFFFF
FFFFFFFFFFFFFFFFFFFFFFFFFFFFFFFFFFFFFFFFFFFFFFFFFFFFFFF:FFFFFFFFFFFF
FFFFFFFFFFFFFFF
@A00155:342:HHGFNDSXY:1:2441:25328:17456 2:N:0:GAACCTAG+TCCGCATA
TATCAATTTGAAATTCACGGGAAGGATCTGGTCTACATGCCGGACGGGGTGAGGTCTGAGAGTTACAC
TCAACCATTGGAGAACGGTTTTCTCCTCAGCTCCAGCAGCATACTTGTCAGAAATAAGGTCACAGGAG
TTGAGATGAGATATC
+
FFFFFFFFFFFFFFFFFFFFFFFFFFFFFFFFFFFFFFFFFFFFFFFFFFFFFFFFFFFFFFFFFFFF

FFFFFFFFFFFFFFFFFFFFFFFFFFFFFFFFFFFFFFFFFFFFFFFFFFF:FFFFFFFFFFFFFFFF
FFFFFF,FFFFFFFF
@A00155:342:HHGFNDSXY:1:2439:13123:25692 1:N:0:GAACCTAG+TCCGCATA
ATCAATTTGAAATTCACGGGAAGGATCTGGTCTACATGCCGGACGGGGTGAGGTCTGAGAGTTACACT
CAACCATTGGAGAACGGTTTTCTCCTCAGCTCCAGCAGCATACTTGTCAGAAATAAGGTCACAGGAGT
TGAGATGAGATATCA
+
FFF:,FFFFFF:FFFF,FFF,FFFFFFF:F::FFF,FFFFF:FFF,FFFFFF,FFFFFFFFFFFFFFF
FFF,:FFFFFFFFFF,FF:FFFFFFF,FFFF:F,FFFFFFFFFFFFFFFFFF:FF,:FFFFFFFFFFF
F:FFFFFFFFFFFFF
@A00155:342:HHGFNDSXY:1:1135:22535:26772 2:N:0:GAACCTAG+TCCGCATA
ATCAATTTGAAATTCACGGGAAGGATCTGGTCTACATGCCGGACGGGGTGAGGTCTGAGAGTTACACT
CAACCATTGGAGAACGGTTTTCTCCTCAGCTCCAGCAGCATACTTGTCAGAAATAAGGTCACAGGAGT
TGAGATGAGATATCA
+
FFFFFFFFFFFFFFFFFFFFFFFFFFFFFFFFFFFFFFFFFFFFFFFFFFFFFFFFFFFFFFFFFFFF
FFFFFFFFFFFFFFFFFFFFFFFFFFFFFFFFFFFFFFFFFFFFFFFFFFFFFFFFFFFFFFFFFFFF
FFFFFFFFFFFFFFF
@A00155:342:HHGFNDSXY:1:1147:11984:26475 2:N:0:GAACCTAG+TCCGCATA
ATCAATTTGAAATTCACGGGAAGGATCTGGTCTACATGCCGGACGGGGTGAGGTCTGAGAGTTACACT
CAACCATTGGAGAACGGTTTTCTCCTCAGCTCCAGCAGCATACTTGTCAGAAATAAGGTCACAGGAGT
TGAGATGAGATATCA
+
FF,FFFFFFFFFFFFFFFFFFFFFF,FFFF:FFFFFFFFFFFFFFF:FFFFFF,FFFFFFFFFFFFFF
FFFF:FFFFFFFFFFFFFFFFFF,,:FF::FFFFFFFFFFFFFFF:FFFFFFF:FF:FFFFFFFFFFF
FFFFFF,FFFFFF:F
@A00155:342:HHGFNDSXY:1:1173:3531:31469 2:N:0:GAACCTAG+TCCGCATA
ATCAATTTGAAATTCACGGGAAGGATCTGGTCTACATGCCGGACGGGGTGAGGTCTGAGAGTTACACT
CAACCATTGGAGAACGGTTTTCTCCTCAGCTCCAGCAGCATACTTGTCAGAAATAAGGTCACAGGAGT
TGAGATGAGATATCA
+
FFFFFFFFFFFFFFFFFFFFFFFFFFFFFFFFFFFFFFFFFFFFFFFFFFFFFFFFF:FFFFFFFFFF
FFFFFFFFFFFFFFFFFFFF:FFFFFFFFFFFFFFFFFFFFFFFFFFFFFFFFFFFFFF:FFF:FFF
FFFFFFFFFFFFFFF
@A00155:342:HHGFNDSXY:1:1244:11686:32910 2:N:0:GAACCTAG+TCCGCATA
ATCAATTTGAAATTCACGGGAAGGATCTGGTCTACATGCCGGACGGGGTGAGGTCTGAGAGTTACACT
CAACCATTGGAGAACGGTTTTCTCCTCAGCTCCAGCAGCATACTTGTCAGAAATAAGGTCACAGGAGT
TGAGATGAGATATCA
+
FFFFFFFF:FFFFFFFFFFFFFFFFFFFFFFFFFFF:FFFFFFFFFFFFF:FFFFFFFFFFF:F:FFF
FFFFFFFFFFFFFFFF:FFFFFFFFFFFFFF:FFF:FFFFFFFFFFFFFFFFFFFFFFFFFFF:FFFF
F:FFFFFFFFFFF:F
@A00155:342:HHGFNDSXY:1:1262:12319:1752 2:N:0:GAACCTAG+TCCGCATA
ATCAATTTGAAATTCACGGGAAGGATCTGGTCTACATGCCGGACGGGGTGAGGTCTGAGAGTTACACT
CAACCATTGGAGAACGGTTTTCTCCTCAGCTCCAGCAGCATACTTGTCAGAAATAAGGTCACAGGAGT
TGAGATGAGATATCA
+
FFFFFFFFFFFFFFFFFFFFFFFFFFFFFFFFFFFFFFFFFFFFF:FFFFFFFFFFFFFFF:FFFFFF
FFFFFFFFFFFFFFFFFFFFFFFFFFFFFFFFFFFFFFFFFFFFFFFFFFFFFFF,FFFFFFFFFFFF
FFFFFFFFFFFFFFF
@A00155:342:HHGFNDSXY:1:1260:16369:35665 2:N:0:GAACCTAG+TCCGCATA
ATCAATTTGAAATTCACGGGAAGGATCTGGTCTACATGCCGGACGGGTTGAGGTCTGAGAGTTACACT
CAACCATTGGAGAACGGTTTTCTCCTCAGCTCCAGCAGCATACTTGTCAGAAATAAGGTCACAGGAGT
TGAGATGAGATATCA

+
:FFFFF,:FFFFF:FFFFFFFFF:FFFFFFFFFFFFFFFFFFFF:FFFFFFFFFF:FFFFF,FFFFFF
FFFFFF:F,F:FFFFF:FF:FFFFFFFFFFFFFFFFF,FF:F:F:F,FFFFFFFFFFFFFFFFFFFF,
:FFFF:FFFFFFFFF
@A00155:342:HHGFNDSXY:1:1407:21640:32800 2:N:0:GAACCTAG+TCCGCATA
ATCAATTTGAAATTCACGGGAAGGATCTGGTCTACATGCCGGACGGGGTGAGGTCTGAGAGTTACACT
CAACCATTGGAGAACGGTTTTCTCCTCAGCTCCAGCAGCATACTTGTCAGAAATAAGGTCACAGGAGT
TGAGATGAGATATCA
+
FFFFFFFFFFFFFFFFFFFFFFFFFFFFFFFFFFFFFFFFFFFFFFFFFFFFFFFFFFFFFFFFFFFF
FFFFFFFFFFFFFF:FFFFFFFFFFFFFFFFFFFFFFFFFFFFFFF:FFFFFFFFFFFFFFFFFFFFFF
FFFFFFFFFFFFFFF
@A00155:342:HHGFNDSXY:1:1650:2275:11913 2:N:0:GAACCTAG+TCCGCATA
ATCAATTTGAAATTCACGGGAAGGATCTGGTCTACATGCCGGACGGGGTGAGGTCTGAGAGTTACACT
CAACCATTGGAGAACGGTTTTCTCCTCAGCTCCAGCAGCATACTTGTCAGAAATAAGGTCACAGGAGT
TGAGATGAGATATCA
+
FFFFFFFFFFFFFFFFFFFFFFFFFFFFFFFFFFFF:FFFFFFFFFFFFFFFFFFFFFFFFFFFFFFF
FFFFFFFFFFFFFFFFFFFFFFFFFFFFFFFFFFFFFFFFFFFFFFFFFFFFFFFFFFFFFFFFFFFF
FFFFFFFFFFFFFFF
@A00155:342:HHGFNDSXY:1:1678:4354:31767 2:N:0:GAACCTAG+TCCGCATA
ATCAATTTGAAATTCACGGGAAGGATCTGGTCTACATGCCGGACGGGGTGAGGTCTGAGAGTTACACT
CAACCATTGGAGAACGGTTTTCTCCTCAGCTCCAGCAGCATACTTGTCAGAAATAAGGTCACAGGAGT
TGAGATGAGATATCA
+
FFFFFFFFFFFFFFFFFFFFF,FF:FFFFFFFFFFFFFFFFFFFFFFFFFFFFFFFFFFFFFFFF:FF
FFFFFFFFFFFFFFFFFFFFFFFFFFFFFFFFFFFFFFFFFFFFFFFFFFFFF,FFFFFFFFFFFF:FF
FFFFFFFFFFF,FFF
@A00155:342:HHGFNDSXY:1:2458:3568:19695 2:N:0:GAACCTAG+TCCGCATA
ATCAATTTGAAATTCACGGGAAGGATCTGGTCTACATGCCGGACGGGGTGAGGTCTGAGAGTTACACT
CAACCATTGGAGAACGGTTTTCTCCTCAGCTCCAGCAGCATACTTGTCAGAAATAAGGTCACAGGAGT
TGAGATGAGATATCA
+
FFFFFFFFFFFFFFFFFFFFF:FFFFFFFFFFFFFFFFFFFFFFFFFFFFFFFFFFFFFFFFFFFFFF
F:FFFFFFFFFFFFFFFFFFFFFFFFFFFFFFFF,FFFFFFFFFFFFFFFFF:FFFFFFFFFFFFFFF:
FFFFF,FFFFFF:FF
@A00155:342:HHGFNDSXY:1:2528:25626:13182 2:N:0:GAACCTAG+TCCGCATA
ATCAATTTGAAATTCACGGGAAGGATCTGGTCTACATGCCGGACGGGGTGAGGTCTGAGAGTTACACT
CAACCATTGGAGAACGGTTTTCTCCTCAGCTCCAGCAGCATACTTGTCAGAAATAAGGTCACAGGAGT
TGAGATGAGATATCA
+
FFFFFFFFFFFFFFFFFFFFFFFFFFFFFFFFFFFFFFFFFFFFFFFFFFFFFFFFFFFFFFFFFFFF
FFFFFFFFFFFFFFFFFFFFFFFFFFFFFF:FFFFFFFFFFFFFFFFFFFFFFFFFFFFFFFFFFFFF
FFFFFFFFFFFFFFF
@A00155:342:HHGFNDSXY:1:1310:4562:3505 1:N:0:GAACCTAG+TCCGCATA
TCAATTTGAAATTCACGGGAAGGATCTGGTCTACATGCCGGACGGGGTGAGGTCTGAGAGTTACACTC
AACCATTGGAGAACGGTTTTCTCCTCAGCTCCAGCAGCATACTTGTCAGAAATAAGGTCACAGGAGTT
GAGATGAGATATCAG
+
FFFFFFFFFFF,FFFFFFFFFFFFFFFFFFFFFFFFFFFF:FFFFFFFFFFFFFFFFFFFFFFFFFFF
FFFFFFFFFFFFFFFFF:FFFFFFFFFFFFFFFFFFFFFFFFFFFFFFFFFFFFFFFFFFFFFFFFFF
FFFFFFFFFFFFFFF
@A00155:342:HHGFNDSXY:1:1356:19913:4852 1:N:0:GAACCTAG+TCCGCATA
GATCTTTGAAATTCACGGGAAGGATCTGGTCTACATGCCGGACGGGGTGAGGTCTGAGAGTTACACTC

AACCATTGGAGAACGGTTTTCTCCTCAGCTCCAGCAGCATACTTGTCAGAAATAAGGTCACAGGAGTT
GAGATGAGATATCAG
+
FFFFFFFFFFFFFFFFFFFFFFFFF:FFFFFFFFFFFFFFFFFFFFFFFFFFFFFFFFFFFFFFFFFFFF
FFFFFFFFFFFFFFFF:FFFFFFFFFF:FFFFFFFFFFFFFFFFFFFFFFFFFFFFFFFFFFFFFFFFFF
FFFFFFFFFFFFFFF
@A00155:342:HHGFNDSXY:1:1633:32443:26177 1:N:0:GAACCTAG+TCCGCATA
TCAATTTGAAATTCACGGGAAGGATCTGGTCTACATGCCGGACGGGGTGAGGTCTGAGAGTTACACTC
AACCATTGGAGAACGGTTTTCTCCTCAGCTCCAGCAGCATACTTGTCAGAAATAAGGTCACAGGAGTT
GAGATGAGATATCAG
+
:FF:FFFFFFFFFFFF:FFFFFF:FFFFFFFFFFFFFFFFFFFFFFFFFFFFFFFFFFFFFFFFFFFFFF
FFFFFFFFFFFFFFFFFFFFFFFFFFFFFFFFFFFFFFFFFFFFFFFFFFFFFFFFFFFFFFFFFFFFFF
FFFFFFFFFFFFFFF
@A00155:342:HHGFNDSXY:1:2209:15664:19006 1:N:0:GAACCTAG+TCCGCATA
TCAATTTGAAATTCACGGGAAGGATCTGGTCTACATGCCGGACGGAGTGAGGTCTGAGAGTTACACTC
AACCATTGGAGAACGGTTTTCTCCTCAGCTCCAGCAGCATACTTGTCAGAAATAAGGTCACAGGAGTT
GAGATGAGATATCAG
+
:FFFFFFFFFFFFFFFFFFFFFFFFFFFFFFFFFFFFFFFFFFFFFFFFFFFFFFFFFFFFFFFFFFFFF
FFFFFFFFFFFFFFFFFFFFFFFFFFFFFFFFFFFFFFFFFFFFFFFFFFFFFFFFFFFFFFFFFFFFFF
FFFFFFFFFFFFFFF
@A00155:342:HHGFNDSXY:1:2254:23502:8343 1:N:0:GAACCTAG+TCCGCATA
TCAATTTGAAATTCACGGGAAGGATCTGGTCTACATGCCGGACGGAGTGAGGTCTGAGAGTTACACTC
AACCATTGGAGAACGGTTTTCTCCTCAGCTCCAGCAGCATACTTGTCAGAAATAAGGTCACAGGAGTT
GAGATGAGATATCAG
+
FFFFFFFFFFFFFFFF:FFFFFFFFFFFFFFFFFFFFFFFFFFFFFFFFFFFFFFFFFFFFFFFFFFFFF
FFFFFFFFFFFFFFFFFFFFFFFFFFFFFFFFFFFFFFFFFFFFFFFFFFFFFFFFFFFFFFFFFFFFFF
FFFFFFFFFFFFFFF
@A00155:342:HHGFNDSXY:1:2250:16405:29403 1:N:0:GAACCTAG+TCCGCATA
TCAATTTGAAATTCACGGGAAGGATCTGGTCTACATGCCGGACGGAGTGAGGTCTGAGAGTTACACTC
AACCATTGGAGAACGGTTTTCTCCTCAGCTCCAGCAGCATACTTGTCAGAAATAAGGTCACAGGAGTT
GAGATGAGATATCAG
+
FFFFF:FFFFFFFFFFFFFF:FFFFFFFFFFFFFFFFFFFFFFFFFFFFFFFFFFFFFFFFFFFFFFFFF
FFFFFFFFFFFFFFFFFFFFFFFFFFF:FFFFFFFFFFFFFFFFFFFFFFFFFFFFFFFFFFFFFF:FFF
FFFFFFFFFFFFFFF
@A00155:342:HHGFNDSXY:1:2450:17472:1376 1:N:0:GAACCTAG+TCCGCATA
TCAATTTGAAATTCACGGGAAGGATCTGGTCTACATGCCGGACGGGGTGAGGTCTGAGAGTTACACTC
AACCATTGGAGAACGGTTTTCTCCTCAGCTCCAGCAGCATACTTGTCAGAAATAAGGTCACAGGAGTT
GAGATGAGATATCAG
+
:FFFFFFFFFFFFFFFFFFFFFFFFFFFFFFFFFFFFFFFFFFFFFFFFFFFFFFFFFFFFFFFFFFFFF
FFFFFFFFFFFFFFFFFFFFFFFFFFFFFFFFFFFFFFFFFFFFFFFFFFFFFFFFFFFFFFFFFFFFFF
FFFFFFFFFFFFFFF
@A00155:342:HHGFNDSXY:1:1332:23583:10582 1:N:0:GAACCTAG+TCCGCATA
CAATTTGAAATTCACGGGAAGGATCTGGTCTACATGCCGGACGGGGTGAGGTCTGAGAGTTACACTCA
ACCATTGGAGAACGGTTTTCTCCTCAGCTCCAGCAGCATACTTGTCAGAAATAAGGTCACAGGAGTTG
AGATGAGATATCAGG
+
FF,FFFFFFFFFFFFFFFFFFFFFFFFFFFFFFFFFFFFFFFFFFFFFFFFFFFFFFFFFFFFFFFFFFF
FFFFFFFFFFFFFFFFFFFFFFFFFFFFFFFFFFFFFFFFFFFFFFFFFFFFFFFFFFFFFFFFFFFFFF
FFFFFFFFFFFFFFF

```
@A00155:342:HHGFNDSXY:1:1170:11659:8656 2:N:0:GAACCTAG+TCCGCATA
CAATTTGAAATTCACGGGAAGGATCTGGTCTACATGCCGGACGGGGTGAGGTCTGAGAGTTACACTCA
ACCATTGGAGAACGGTTTTCTCCTCAGCTCCAGCAGCATACTTGTCAGAAATAAGGTCACAGGAGTTG
AGATGAGATATCAGG
+
FFFFFFFFFFFFFFFFFFFFFFFFFFFFFFFFFFFFFFFFFFFFFFFFFFFFFFFFFFFFFFFFFFFF
FFFFFFFFFFFFFFFFFFFFFFFFFFFFFFFFFFFFFFFFFFFFFFFFFFFFFFFFFFFFFFFFFFFF
FFFFFFFFFFFFFFF
@A00155:342:HHGFNDSXY:1:1352:21423:25066 2:N:0:GAACCTAG+TCCGCATA
CAATTTGAAATTCACGGGAAGGATCTGGTCTACATGCCGGACGGGGTGAGGTCTGAGAGTTACACTCA
ACCATTGGAGAACGGTTTTCTCCTCAGCTCCAGCAGCATACTTGTCAGAAATAAGGTCACAGGAGTTG
AGATGAGATATCAGG
+
:FFFFF:FFFFFFFFFFFFFFFFFFFFFFFFFFF:FFFFFFFFFFFFFFFFFFFFFFFFFFFFFFFFFF
FFFFFFFFFFFFFFFFFFFFFFFFFFFFFFFFFFFFFFFFFFFFFF:FFFFFFFFFFFFFFFFFFFF:FF
FF:FFFFFFFFF:F:
@A00155:342:HHGFNDSXY:1:1559:32280:21010 2:N:0:GAACCTAG+TCCGCATA
CAATTTGAAATTCACGGGAAGGATCTGGTCTACATGCCGGACGGAGTGAGGTCTGAGAGTTACACTCA
ACCATTGGAGAACGGTTTTCTCCTCAGCTCCAGCAGCATACTTGTCAGAAATAAGGTCACAGGAGTTG
AGATGAGATATCAGG
+
FFFFFFFFFFFFFFFFFFFFFFFFFFFFFFFFFFFFF:FFFFFFFFFFFFFFFFFFFFFFFFFFFFFF
FFFFFFFF:FFFFFFFFFFFFFFFF:FFFFFFFFFFFFFFFFFFFFFFFFFFFFFFFFF::FFFFFFFF
FFFFFFFFF,FFFFF
@A00155:342:HHGFNDSXY:1:2554:26196:12602 2:N:0:GAACCTAG+TCCGCATA
CAATTTGAAATTCACGGGAAGGATCTGGTCTACATGCCGGACGGGGTGAGGTCTGAGAGTTACACTCA
ACCATTGGAGAACGGTTTTCTCCTCAGCTCCAGCAGCATACTTGTCAGAAATAAGGTCACAGGAGTTG
AGATGAGATATCAGG
+
F:FF,FFFFFFFFFFFFFFFFFFFFFFFFFFFF:FFFFFFFFFFFFFFFFFFFFFFFFFFFFFFFFFFF
FFFFFFFFFFFFFFFF:FFFFFFFFFFFFFFFFFFFFFFFFFFFFFFFFFFFFFFFFFFFFFFFFFFF
FFFFFFFFFFFFFFF
@A00155:342:HHGFNDSXY:1:1273:22525:36589 1:N:0:GAACCTAG+TCCGCATA
AATTTGAAATTCACGGGAAGGATCTGGTCTACATGCCGGACGGAGTGAGGTCTGAGAGTTACACTCAA
CCATTGGAGAACGGTTTTCTCCTCAGCTCCAGCAGCATACTTGTCAGAAATAAGGTCACAGGAGTTGA
GATGAGATATCAGGT
+
F,FFFFFFFFFFFFFFFFFFFFFFFFFFFFFFFFFFFFFFFFFFFFFFFFFFFFFFFFFFFFFFFFFF
FFFFFFFFFFFFFFFFFFFFFFFFFFFFFFFFFFFFFFFFFFFFFFFFFFFFFFFFFFFFFFFFFFFF
FFFFFFFFFFFFFFF
@A00155:342:HHGFNDSXY:1:1438:10908:6324 1:N:0:GAACCTAG+TCCGCATA
AATTTGAAATTCACGGGAAGGATCTGGTCTACATGCCGGACGGAGTGAGGTCTGAGAGTTACACTCAA
CCATTGGAGAACGGTTTTCTCCTCAGCTCCAGCAGCATACTTGTCAGAAATAAGGTCACAGGAGTTGA
GATGAGATATCAGGT
+
FFFFF:FFFFFFFFFFFFFFFFFFFFFFFF,FFFFFFFFF:FFFFFFFFFFFFFFFFFFFFFFFFFFFF
FFFFFFFFFFFFFFFFFFFFFFFFFFFFFFFFFFFFFFFFFFFFFFFFFFFFFFFFFFFFFFFFFFFF
FFFFFFF:FFFFFFF
@A00155:342:HHGFNDSXY:1:1608:20139:11036 1:N:0:GAACCTAG+TCCGCATA
AATTTGAAATTCACGGGAAGGATCTGGTCTACATGCCGGACGGGGTGAGGTCTGAGAGTTACACTCAA
CCATTGGAGAACGGTTTTCTCCTCAGCTCCAGCAGCATACTTGTCAGAAATAAGGTCACAGGAGTTGA
GATGAGATATCAGGT
+
FFFFFFFFFFFFFFFFFFFFFFFFFFFFFFFFFFFFFFFFFFFFFFFFFFFFFFFFFFFFFFFFFFFF
```

```
FFFFFFFFF:FFFFFFFFFFFFFFFFFFFFFFFFFFFFFFFFFFFFFFFFFFFFFFFFFFFFFFFFFFFF
FFFFFFFFFFFFFFF
@A00155:342:HHGFNDSXY:1:1620:14398:33098 1:N:0:GAACCTAG+TCCGCATA
AATTTGAAATTCACGGGAAGGATCTGGTCTACATGCCGGACGGGGTGAGGTCTGAGAGTTACACTCAA
CCATTGGAGAACGGTTTTCTCCTCAGCTCCAGCAGCATACTTGTCAGAAATAAGGTCACAGGAGTTGA
GATGAGATATCAGGT
+
FFFFFFFFFFFFFFFFFFFFFFFFFFFFFFFFFFFFFFFFFFFFFFFFFFFFFFFFFFFFFFFFFFFFFF
FFFFFFFFFFFFFFFFFFFFFFFFFFFFFFFFFFFFFFFFFFFFFFFFFFFFFFFFFFFFFFFFFFFFFF
FFFFFFFFFFFFFFF
@A00155:342:HHGFNDSXY:1:2304:20735:4930 1:N:0:GAACCTAG+TCCGCATA
AATTTGAAATTCACGGGAAGGATCTGGTCTACATGCCGGACGGGGTGAGGTCTGAGAGTTACACTCAA
CCATTGGAGAACGGTTTTCTCCTCAGCTCCAGCAGCATACTTGTCAGAAATAAGGTCACAGGAGTTGA
GATGAGATATCAGGT
+
:,FFF::FF:FFF:FFF,FF:FFFFFFFFFFFF,::::FF:FFFFFF::FFFFFFFFFFFFFFFF:FFFF
FFFFFFFFFFFFFF::FFFFFF:FFFFFFFFFFFFFFFFFFFFFFFFFFFF,F:FFFFFFFFFFFFFFFF
FFFFFFFFFFFFF,F
@A00155:342:HHGFNDSXY:1:2328:6587:25739 1:N:0:GAACCTAG+TCCGCATA
AATTTGAAATTCACGGGAAGGATCTGGTCTACATGCCGGACGGAGTGAGGTCTGAGAGTTACACTCAA
CCATTGGAGAACGGTTTTCTCCTCAGCTCCAGCAGCATACTTGTCAGAAATAAGGTCACAGGAGTTGA
GATGAGATATCAGGT
+
FFFF:FFFFFFFFFFFFFFFFFFFFF,FFFFFFFFFFFFFFFFFFFFFFFFFFFFFFFFFFFFFFFFFFF
FFFFFFFFFFFFFFFFFFFFFFFFFFFFFFFFFFFFFFFFFFFFFFFFFFFFFFFFFFFFFFFFFFFFFF
FFFFFFFFFFFFFFF
@A00155:342:HHGFNDSXY:1:2356:27281:11882 1:N:0:GAACCTAG+TCCGCATA
AATTTGAAATTCACGGGAAGGATCTGGTCTACATGCCGGACGGAGTGAGGTCTGAGAGTTACACTCAA
CCATTGGAGAACGGTTTTCTCCTCAGCTCCAGCAGCATACTTGTCAGAAATAAGGTCACAGGAGTTGA
GATGAGATATCAGGT
+
FFFFFFFFFFFFFFFFFFFFFFFFFFFF:FFFFFFFFFFFFFFFFFFFFFFFFFFFFFFFFFFFFFFFFF
FFFFFFFFFFFFFFFFFFFFFFFFFFFFFFFFFFFFFFFFFFFFFFFFFFFFFFFFFFFFFFFFFFFFFF
FFFFFFFFFFFFFFF
@A00155:342:HHGFNDSXY:1:2406:20835:9173 1:N:0:GAACCTAG+TCCGCATA
AATTTGAAATTCACGGGAAGGATCTGGTCTACATGCCGGACGGGGTGAGGTCTGAGAGTTACACTCAA
CCATTGGAGAACGGTTTTCTCCTCAGCTCCAGCAGCATACTTGTCAGAAATAAGGTCACAGGAGTTGA
GATGAGATATCAGGT
+
FFFFFFFFFFFFFFFFFFFFFFFFFFFFFFFFFFFFFFFFFFFFFFFFFFFFFFFFFFFFFFFFFFFFFF
FFFFFFFFFFFFFFFFFFFFFFFFFFFFFFFFFFFFFFFFFFFFFFFFFFFFFFFFFFFFFFFFFFFFFF
FFFFFFFFFFFFFFF
@A00155:342:HHGFNDSXY:1:2576:31087:36417 1:N:0:GAACCTAG+TCCGCATA
AATTTGAAATTCACGGGAAGGATCTGGTCTACATGCCGGACGGGGTGAGGTCTGAGAGTTACACTCAA
CCATTGGAGAACGGTTTTCTCCTCAGCTCCAGCAGCATACTTGTCAGAAATAAGGTCACAGGAGTTGA
GATGAGATATCAGGC
+
F,FFFFFFFFFFFFFFFFFFFFFFFFFFFFFFFFFFFFFFFFFFFFFFFFFFFFFFFFFFFFFFFFFFFF
FFFFFFFFFFFFFFFFFFFFFFFFFFFFFFFFFFFFFFFFFFFFFFFFFFFFFFFFFFFFFFFFFFFFFF
FFFFFFFFFFFFFFF
@A00155:342:HHGFNDSXY:1:1420:14380:33536 2:N:0:GAACCTAG+TCCGCATA
AATTTGAAATTCACGGGAAGGATCTGGTCTACATGCCGGACGGGGTGAGGTCTGAGAGTTACACTCAA
CCATTGGAGAACGGTTTTCTCCTCAGCTCCAGCAGCATACTTGTCAGAAATAAGGTCACAGGAGTTGA
GATGAGATATCAGGT
```

```
+
FFFFFFFFFFFF,FFFFF,:FFFFF:FFFFFFFFFFF:FFFFFFFFFFFFFF:FFFFFFFFFFFFFFF
FF,FFFFF:FFFFFFFFFFFFFFFFFFF:F:FF,FFFFFFFF:FFFF:FFF:FFF::FFFFFFFF:FFF
FFFFFFFFFFFFFFF
@A00155:342:HHGFNDSXY:1:1630:26006:28870 2:N:0:GAACCTAG+TCCGCATA
AATTTGAAATTCACGGGAAGGATCTGGTCTACATGCCGGACGGGGTGAGGTCTGAGAGTTACACTCAA
CCATTGGAGAACGGTTTTCTCCTCAGCTCCAGCAGCATACTTGTCAGAAATAAGGTCACAGGAGTTGA
GATGAGATATCAGGT
+
FFFFFFFFFFFFFFFFFFFFFFFFFFFFFFFFFFFFFFFFFFFFFFFFFFFFFFFFFFFFFFFFFFFF
FFF:FFFFFFFFFFFFFFFFFFFFFFFFFFFFFFFFFFFFFFFFF:FFFF:FFFFFFFFFFFFFFFFFF
FFFFFFFFFFFFFFF
@A00155:342:HHGFNDSXY:1:2166:11803:31203 2:N:0:GAACCTAG+TCCGCATA
ATTTGAAATTCACGGGAAGGATCTGGTCTACATGCCGGACGGAGTGAGGTCTGAGAGTTACACTCAAC
CATTGGAGAACGGTTTTCTCCTCAGCTCCAGCAGCATACTTGTCAGAAATAAGGTCACAGGAGTTGAG
ATGAGATATCAGGTG
+
FFFFFFFFFFFFFFFFFFFFFFFFFFFFFF:FFFFFFFFFFFFFFFFFFFFFFFFFFFFFFFFFFFF:F
FFFFFFFFFFFFFFFFFFFFFFFFFFFFFFFFFFFFFFFFFFFFFFFFFFFFFFFFFFF:FFFFFFFF
FF,FFFFFFFFFFFF
@A00155:342:HHGFNDSXY:1:2122:24523:21825 2:N:0:GAACCTAG+TCCGCATA
ATTTGAAATTCACGGGAAGGATCTGGTCTACATGCCGGACGGAGTGAGGTCTGAGAGTTACACTCAAC
CATTGGAGAACGGTTTTCTCCTCAGCTCCAGCAGCATACTTGTCAGAAATAAGGTCACAGGAGTTGAG
ATGAGATATCAGGTG
+
FFFFFFFFFFFFFFFFFFFFFFFFFFFFFFFFFFFFFFFFFFFFFFFFFFFFFFFFFFFFFFFFFFFF
FFFFFFFFFFFFFFFFFFFFFFFFFFFFFFFFFFFFFFFFFFFFFFFFFFFFFFFFFFFFFFFFFFFF
FFFFFFFFFFFFFFF
@A00155:342:HHGFNDSXY:1:1530:17011:12383 2:N:0:GAACCTAG+TCCGCATA
TTTGAAATTCACGGGAAGGATCTGGTCTACATGCCGGACGGGGTGAGGTCTGAGAGTTACACTCAACC
ATTGGAGAACGGTTTTCTCCTCAGCTCCAGCAGCATACTTGTCAGAAATAAGGTCACAGGAGTTGAGA
TGAGATATCAGGTGA
+
:FFFFFF,FFFFFFFFFFFFFFFFF,FFFFFFFFFFFFFFFFFFFF:FFFFFFFFFFFFFFFFFFFFF
FFFFFFFFFFFF:FFF:FFFFFFFFFFFFFFFFFFFFFFFFFFFFFFF,FFFFFFFFFFFFFFFFFFF
FFFFFFFFFFFFFFF
@A00155:342:HHGFNDSXY:1:1276:13955:5462 1:N:0:GAACCTAG+TCCGCATA
TTGAAATTCACGGGAAGGATCTGGTCTACATGCCGGACGGGGTGAGGTCTGAGAGTTACACTCAACCA
TTGGAGAACGGTTTTCTCCTCAGCTCCAGCAGCATACTTGTCAGAAATAAGGTCACAGGAGTTGAGAT
GAGATATCAGGTGAG
+
FFFFFFFFFFFFFFFFFFFFFFFFFFFFFFFFFFFFFFFFFFFFFFFFFFFFFFFFFFFFFFFFFFFF
FFFFFFFFFFFFFFFFFFFFFFFFFFFFFFFFFFFFFFFFFFFFFFFFFFFFFFFFFFFFFFFFFFFF
FFFFFFFFFFFFFFF
@A00155:342:HHGFNDSXY:1:1303:17119:15076 1:N:0:GAACCTAG+TCCGCATA
TTGAAATTCACGGGAAGGATCTGGTCTACATGCCGGACGGGGTGAGGTCTGAGAGTTACACTCAACCA
TTGGAGAACGGTTTTCTCCTCAGCTCCAGCAGCATACTTGTCAGAAATAAGGTCACAGGAGTTGAGAT
GAGATATCAGGTGAG
+
FFFFFFFFFFFFFFF,FFFFFFFFFFFFFF:FFFFFFFFFFFFFFFFFFFFFFFFFFFFFFFFFFFFFF
FFFFFFFFFFFFFFFFFFFFFFFFFFF:FFFFFFFFFFFFFFFFFFFFFFFFFFFFFFFFFFFFFFFF
FFFFFFF:FFFFF:F
@A00155:342:HHGFNDSXY:1:1614:10520:18552 1:N:0:GAACCTAG+TCCGCATA
TTGAAATTCACGGGAAGGATCTGGTCTACATGCCGGACGGGGTGAGGTCTGAGAGTTACACTCAACCA
```

TTGGAGAACGGTTTTCTCCTCAGCTCCAGCAGCATACTTGTCAGAAATAAGGTCACAGGAGTTGAGAT
GAGATATCAGGTGAG
+
FFFFFFFFFFFFFFFFFFFFFFFFFFFFFFFFFFFFFFFFFFFFFFFFFFFFFFF,FFFFFFFFFFFF
FFFFFFFFFFFFF:FFFFFFFFFFFFFFFFFFFFFFFFFFFFFFFFFFFFFFFFFFFFFFFFFFFFFF
FFFFFFFFFFFFFFF
@A00155:342:HHGFNDSXY:1:1630:26006:28870 1:N:0:GAACCTAG+TCCGCATA
TTGAAATTCACGGGAAGGATCTGGTCTACATGCCGGACGGGGTGAGGTCTGAGAGTTACACTCAACCA
TTGGAGAACGGTTTTCTCCTCAGCTCCAGCAGCATACTTGTCAGAAATAAGGTCACAGGAGTTGAGAT
GAGATATCAGGTGAG
+
FFFFFFFFFFFFFF,FFFFFFFFFFFFFFFFFFFFFFFFFFFFFFFFFFFFFFFFFFFFFF:FFFFFF
FFFFFFFFFFFFFFFFFFFFFFFFFFFFFFFFFFFFFF:FFFFFFFFFFFFFFFFFFFFFFFFFFFFF
FFFFFFFFFFFFFFF
@A00155:342:HHGFNDSXY:1:1678:4354:31767 1:N:0:GAACCTAG+TCCGCATA
TTGAAATTCACGGGAAGGATCTGGTCTACATGCCGGACGGGGTGAGGTCTGAGAGTTACACTCAACCA
TTGGAGAACGGTTTTCTCCTCAGCTCCAGCAGCATACTTGTCAGAAATAAGGTCACAGGAGTTGAGAT
GAGATATCAGGTGAG
+
FFFFFFFFFFFFFFFFFFFFFFFFFFFFFFFF:FFFFFF:FFFFFFFFFFFFFFFF:FFFFFFFFFFF
FFFFFFFFFFFFFFFFF:F:FFFFFFFFFF:FFFF:FFFFFFFFFFFFFFFFFFFFFFFFFFFFFFFF
FFFFFFFFFFFFFFF
@A00155:342:HHGFNDSXY:1:2276:10366:2785 1:N:0:GAACCTAG+TCCGCATA
TTGAAATTCACGGGAAGGATCTGGTCTACATGCCGGACGGGGTGAGGTCTGAGAGTTACACTCAACCA
TTGGAGAACGGTTTTCTCCTCAGCTCCAGCAGCATACTTGTCAGAAATAAGGTCACAGGAGTTGAGAT
GAGATATCAGGTGAG
+
FFFFFFFFFFFFFFFFFFFFFFFFFFFFFFFFFFFFFFFFFFFFFFFFFFFFFF:FFFFFFFFFFFFF
FFFFFFFFFFFFFFFFFFFFFFFFFFFFFFFFFFFFFFFFFFFFFFFFFFFFFFFFFFFFFFFFFFFF
FFFFFFFFFFFFFFF
@A00155:342:HHGFNDSXY:1:2338:19506:4773 1:N:0:GAACCTAG+TCCGCATA
TTGAAATTCACGGGAAGGATCTGGTCTACATGCCGGACGGGGTGAGGTCTGAGAGTTACACTCAACCA
TTGGAGAACGGTTTTCTCCTCAGCTCCAGCAGCATACTTGTCAGAAATAAGGTCACAGGAGTTGAGAT
GAGATATCAGGTGAG
+
FFFFFFFFFFFFFFFFFFFFF:FFFFFFFFFFFFFFFFFFFFFFFFFFFFFFFFFFFFFFFFFFFFFF
FFFFFFFFFFF:FFFFFFFFFFFFFFFFFFFFF:FFFFFFFFFFFFFFFFF:FFFFFFFFFFFFFFFF
FFFFFFFFFFFFFFF
@A00155:342:HHGFNDSXY:1:1226:31376:8328 2:N:0:GAACCTAG+TCCGCATA
CCGATCTCCACGGGAAGGATCTGGTCTACATGCCGGACGGGGTGAGGTCTGAGAGTTACACTCAACCA
TTGGAGAACGGTTTTCTCCTCAGCTCCAGCAGCATACTTGTCAGAAATAAGGTCACAGGAGTTGAGAT
GAGATATCAGGTGAG
+
FFFFFFFFFFFFFFFFFFFFFFFFFFFFFF:FFFFFFFFFFFFFFFFFFFFFFFFFFFFFFFFFFFFF
FFFFFFFFFFFFFFFFFFFFFFFFFFFFFFFFFFFFFFFFFFFFFFFFFFFFFFFFFFFFFFFFFFFF
FFFFFFFFFFFFFFF
@A00155:342:HHGFNDSXY:1:1240:28248:15749 2:N:0:GAACCTAG+TCCGCATA
CCGATCTCCACGGGAAGGATCTGGTCTACATGCCGGACGGGGTGAGGTCTGAGAGTTACACTCAACCA
TTGGAGAACGGTTTTCTCCTCAGCTCCAGCAGCATACTTGTCAGAAATAAGGTCACAGGAGTTGAGAT
GAGATATCAGGTGAG
+
FFFFFFFFF:FFFFFFFFFFFFFFFFFFFF,FFFFFFFFFFFFFFFF:FFFFFFFFFFFFFFFFFFFF
FFFFFFFFFFFFFFFFFFFFFFFFFFFFFFFFFFFFFFFFFFFFFFFFFFF:FFFFFFFFFFFFFFFF
FFFFFFFFFFFFFFF

@A00155:342:HHGFNDSXY:1:1324:20338:21621 2:N:0:GAACCTAG+TCCGCATA
CCGATCTCCACGGGAAGGATCTGGTCTACATGCCGGACGGGGTGAGGTCTGAGAGTTACACTCAACCA
TTGGAGAACGGTTTTCTCCTCAGCTCCAGCAGCATACTTGTCAGAAATAAGGTCACAGGAGTTGAGAT
GAGATATCAGGTGAG
+
FFFFFFFFFFFFFFFFFFFFFFFFFFFFFF:FFFFFFFFFFFFFFFFFFFFFFFFFFFFFFFFFFFFF
FFFFFFFFFFFFFFFFFFFFFFFFFFFFFFFFFFFFFFFFFFFFFFFFFFFFFFFFFFFFFFFFFFFF
FFFFFFFFFFFFFFF
@A00155:342:HHGFNDSXY:1:1356:19913:4852 2:N:0:GAACCTAG+TCCGCATA
TTGAAATTCACGGGAAGGATCTGGTCTACATGCCGGACGGGGTGAGGTCTGAGAGTTACACTCAACCA
TTGGAGAACGGTTTTCTCCTCAGCTCCAGCAGCATACTTGTCAGAAATAAGGTCACAGGAGTTGAGAT
GAGATATCAGAGATC
+
FFFFFFFFFFFFFFFFFFFFFFFFFFFFFFFFFFFFFFFFFFFFFFFFFFFFFFFFFFFFFFFFFFFF
FFFFFFFFFFFFFFFFFFFFFFFFFFFFFFFFFFFFFFFFFFFFFFFFFFFFFFFFFFFFFFFFFFFF
:FFFFFFFFFFFFFF
@A00155:342:HHGFNDSXY:1:1657:29668:8688 2:N:0:GAACCTAG+TCCGCATA
CCGATCTCCACGGGAAGGATCTGGTCTACATGCCGGACGGGGTGAGGTCTGAGAGTTACACTCAACCA
TTGGAGAACGGTTTTCTCCTCAGCTCCAGCAGCATACTTGTCAGAAATAAGGTCACAGGAGTTGAGAT
GAGATATCAGGTGAG
+
FFFFFFFFFFFFFFFFFFFFFFFFFF:FF:FFFFFFFFFFFFFFFFFFFFFFFFF,FFFFFFFFFFF:
FFFFFFFFFFFFFFFFFFFFFFFFFFFFFFFFFFFFFFFFFFFFF:FFFFFFFFFFFFFFFFFFFFFF
FFFFFFFFFFFFFFF
@A00155:342:HHGFNDSXY:1:2324:22110:16423 2:N:0:GAACCTAG+TCCGCATA
CCGATCTCCACGGGAAGGATCTGGTCTACATGCCGGACGGGGTGAGGTCTGAGAGTTACACTCAACCA
TTGGAGAACGGTTTTCTCCTCAGCTCCAGCAGCATACTTGTCAGAAATAAGGTCACAGGAGTTGAGAT
GAGATATCAGGTGAG
+
FFFFFFFFFFFFFFFFFFFFFFFFFFFFFFFFFFFFFFFFFFFFFFFFFFFFFFFFFFFFFFFFFFFF
FFFFFFFFFFFFFFFFFFFFFFFFFFFFFFFFFFFFFFFFFFFFFFFFFFFFFFFFFFFFFFFFFFFF
FFFFFFFFFFFFFFF
@A00155:342:HHGFNDSXY:1:2310:18059:1485 2:N:0:GAACCTAG+TCCGCATA
TGAAATTCACGGGAAGGATCTGGTCTACATGCCGGACGGGGTGAGGTCTGAGAGTTACACTCAACCAT
TGGAGAACGGTTTTCTCCTCAGCTCCAGCAGCATACTTGTCAGAAATAAGGTCACAGGAGTTGAGATG
AGATATCAGGTGAGT
+
FFFFFFFFFFFFFFF:FFFFFFFFFFFFFFFFFFFFFFFFFFFFFFFFFFFFFFFFFFFFFFFFFFFF
FFFFFFFFFFFFFFFFFFFFFFFFFFFFFFFFFFFFFFFFFFFFF:F,FFFFFFFFFFFFFFFFFF:F
FFFFFFFFFFFFFFF
@A00155:342:HHGFNDSXY:1:1341:16306:1141 2:N:0:GAACCTAG+TCCGCATA
ATTCACGGGAAGGATCTGGTCTACATGCCGGACGGAGTGAGGTCTGAGAGTTACACTCAACCATTGGA
GAACGGTTTTCTCCTCAGCTCCAGCAGCATACTTGTCAGAAATAAGGTCACAGGAGTTGAGATGAGAT
ATCAGGTGAGTTTGA
+
FFFFFFFFFFFFFFFFFFFFFFFFFFFFFFFFFFFFFFFFFFFFFFFFFFFFFFFFFFFFFF:FFFFF
FFFFFFFFFFFFFFFFFFFFFFFFFFFFFFFFFFFFFFFFFFFFFFFFF:FFFFFFFFFFFFFFFFFF
FFFFFFFFFFFFFFF
@A00155:342:HHGFNDSXY:1:2655:11758:12023 2:N:0:GAACCTAG+TCCGCATA
TCACGGGAAGGATCTGGTCTACATGCCGGACGGGGTGAGGTCTGAGAGTTACACTCAACCATTGGAGA
ACGGTTTTCTCCTCAGCTCCAGCAGCATACTTGTCAGAAATAAGGTCACAGGAGTTGAGATGAGATAT
CAGGTGAGTTTGATT
+
FFFFFFFFFFFFFFFFFFFFFFFFFFFFFF:FFFFFFFFFFFFFFFFFFFFFFFFFFFFFFFFFFFFF

```
FFFFFFFFFFFFFFFFFFFFFFFFFFFFFF:FFFFFF:FFFFFFFFFFFFFFFFFFF:FFFFFFFFFF
FFFFFFFFFFFFFFF
@A00155:342:HHGFNDSXY:1:1129:6189:19413 2:N:0:GAACCTAG+TCCGCATA
CACGGGAAGGATCTGGTCTACATGCCGGACGGGGTGAGGTCTGAGAGTTACACTCAACCATTGGAGAA
CGGTTTTCTCCTCAGCTCCAGCAGCATACTTGTCAGAAATAAGGTCACAGGAGTTGAGATGAGATATC
AGGTGAGTTTGATTT
+
FFFFFFFFFFFFFF:FFFFFFFFFFFFFFFFFFFFFFFFFFFFFFFFFFFFFFFFFFFFFFFFFFFFF
FFFFFFFFFFFFF:FFFFFFFFFFFFFFFFFFFFFFFFFFFFFFFFFFFFFFFFF,F:FFFFFFFFFF
:FFFFFFFFFFFFFF
@A00155:342:HHGFNDSXY:1:1305:27525:35227 2:N:0:GAACCTAG+TCCGCATA
CACGGGAAGGATCTGGTCTACATGCCGGACGGGGTGAGGTCTGAGAGTTACACTCAACCATTGGAGAA
CGGTTTTCTCCTCAGCTCCAGCAGCATACTTGTCAGAAATAAGGTCACAGGAGTTGAGATGAGATATC
AGGTGAGTTTGATTT
+
FFFFFFFFFFFFFFFFFFFFFFFFFFFF:FFFFFFFFFFFFFFFFFFFFFFFFFFFFFF::FFFFFFF
FFFFFFFFFFF:FFFFFFFFFF:FFFFFFFFFFFFFFFFFFFFFFFFFFFFF:FFFFFFFFFFFFFFF
FFFFFFFFFFFF:FF
@A00155:342:HHGFNDSXY:1:2139:4933:23062 1:N:0:GAACCTAG+TCCGCATA
ACGGGAAGGATCTGGTCTACATGCCGGACGGGGTGAGGTCTGAGAGTTACACTCAACCATTGGAGAAC
GGTTTTCTCCTCAGCTCCAGCAGCATACTTGTCAGAAATAAGGTCACAGGAGTTGAGATGAGATATCA
GGTGAGTTTGATTTA
+
FFFFFF:FFFFFFFFFFFFFFFFFFFFFFFFFFFFFFFFFFFFFFFFFFFFFFFFFFFFFFFFFFFFF
FFFFFFFFFFFFFFFFFFFFFFFFFFFFFFFFFFFF:FFFFFFFFFFFFFFFFFFFFFFFFFFFFFFF
FFFFFFF:FFFFFFF
@A00155:342:HHGFNDSXY:1:1306:13955:1673 2:N:0:GAACCTAG+TCCGCATA
CGGGAAGGATCTGGTCTACATGCCGGACGGGGTGAGGTCTGAGAGTTACACTCAACCATTGGAGAACG
GTTTTCTCCTCAGCTCCAGCAGCATACTTGTCAGAAATAAGGTCACAGGAGTTGAGATGAGATATCAG
GTGAGTTTGATTTAC
+
FFFFFFFFFFFFFFFFFFFFFFFFFFFFFFFFFFFFFFFFFFFFFFFFFF:FFFFFFFFFFFFFFFFF
FFFFFFFFFFFFFFFFFFFFFFFFFFFFFFFFFF:FFFFFFFFFFFFFFFFFFFFFFFFFFFFFFFFF
FFFFFFFFFFFFFFF
@A00155:342:HHGFNDSXY:1:1350:16866:11851 2:N:0:GAACCTAG+TCCGCATA
CGGGAAGGATCTGGTCTACATGCCGGACGGGGTGAGGTCTGAGAGTTACACTCAACCATTGGAGAACG
GTTTTCTCCTCAGCTCCAGCAGCATACTTGTCAGAAATAAGGTCACAGGAGTTGAGATGAGATATCAG
GTGAGTTTGATTTAC
+
FFFFFFFFFFFFFFFFFFFFFFFFFFFFFFFFFFFFFFFFFFFFFFFFFFFFFFFFFFFFFFFFFFFF
FFFFFFFFFFFFFFFFFFFFFFFFFFFFF,FFFFFFFFFFFFFFFFFFFFFFFFFFFFFFFFFFFFFF
FFFFFFFFFFFFFFF
@A00155:342:HHGFNDSXY:1:1567:20302:25254 2:N:0:GAACCTAG+TCCGCATA
CGGGAAGGATCTGGTCTACATGTCGGACGGGGTGAGGTCTGAGAGTTACACTCAACCATTGGAGAACG
GTTTTCTCCTCAGCTCCAGCAGCATACTTGTCACAAATAAGGTCACAGGAGTTGAGATGAGATATCAG
GTGAGTTTGATTTAC
+
FFFFFFFFFFFFFFFFFFFFFFFFFFFFFFFFFFFFFFFFFFFFFFFFFFFFFFFFFFFFFFFFFFFF
FFFFFFFFFFFFFFFFFFFFFFFFF:FFFFFFFFFF:FFFFFFFFFFFFFFFFFFFFFFFFFFFF:FFF
FFFFFFFFFFFFFFF
@A00155:342:HHGFNDSXY:1:2640:2871:11099 2:N:0:GAACCTAG+TCCGCATA
CGGGAAGGATCTGGTCTACATGCCGGACGGGGTGAGGTCTGAGAGTTACACTCAACCATTGGAGAACG
GTTTTCTCCTCAGCTCCAGCAGCATACTTGTCAGAAATAAGGTCACAGGAGTTGAGATGAGATATCAG
GTGAGTTTGATTTAC
```

+
FFFFFFFFFFFFFFFFFFFFFFFFFFFFFFFFFFFFFFFFFFFFFFFFFFFFFFFFFFFFFFFFFFFF
FFFFFFFFFFFFFFFFFFFFFFFFFFFFFFFFFFFFFFFFF:FFFFFFFFFFFFFF:FFFFFFFFFFF
FFFFFF,FFFFFFFF
@A00155:342:HHGFNDSXY:1:1160:30870:22921 2:N:0:GAACCTAG+TCCGCATA
GGGAAGGATCTGGTCTACATGCCGGACGGGGTGAGGTCTGAGAGTTACACTCAACCATTGGAGAACGG
TTTTCTCCTCAGCTCCAGCAGCATACTTGTCAGAAATAAGGTCACAGGAGTTGAGATGAGATATCAGG
TGAGTTTGATTTACT
+
FFFFFFFFFFFFFFFFFFFFFF:FFFFFF::FFFFFFFFFFFFFFFFFFFFFFFFFF:FFFF:F:FFF
FFFFFFFFFFFF:FFFFFFFFFFFFF:FFFFFFFFFFFFF:FFFFFFFFFFFFFFFFFF,FFFFFFFF
FFFF:FFFFFFFFFF
@A00155:342:HHGFNDSXY:1:1202:6334:26365 2:N:0:GAACCTAG+TCCGCATA
GGGAAGGATCTGGTCTACATGCCGGACGGAGTGAGGTCTGAGAGTTACACTCAACCATTGGAGAACGG
TTTTCTCCTCAGCTCCAGCAGCATACTTGTCAGAAATAAGGTCACAGGAGTTGAGATGAGATATCAGG
TGAGTTTGATTTACT
+
FFFFFFFFFFFFFFFFFFFFFFFFFFFFFFFFFFFFFFFFFFF::FFFFFFFFFFFFFFFFFFFFFFF
FFFFFFFFFFFF:FFFFFFFFFFFFFFFFFFFFFFFFFFFFFFFFFFFFFFFFFFFFFFFFFFFFFF
FFFFFFFFFFF:FFF
@A00155:342:HHGFNDSXY:1:1202:6497:26365 2:N:0:GAACCTAG+TCCGCATA
GGGAAGGATCTGGTCTACATGCCGGACGGAGTGAGGTCTGAGAGTTACACTCAACCATTGGAGAACGG
TTTTCTCCTCAGCTCCAGCAGCATACTTGTCAGAAATAAGGTCACAGGAGTTGAGATGAGATATCAGG
TGAGTTTGATTTACT
+
FFF:FFFFFFFFFFFFFFFFFFFF,F,FFFFFFFFFFFFFFFFFFFFFFFFFFF,FFFFFFFFFFFF:
FFFFFFFFFFFFFFFFFFFFFFFFFFFFFFFFFFFFFFFFFFFFFFFFFFFFFFFFFFFFFFFFFFFF
FFFFFFFFFFFFFFF
@A00155:342:HHGFNDSXY:1:1249:26503:16517 2:N:0:GAACCTAG+TCCGCATA
GGGAAGGATCTGGTCTACATGCCGGACGGGGTGAGGTCTGAGAGTTACACTCAACCATTGGAGAACGG
TTTTCTCCTCAGCTCCAGCAGCATACTTGTCAGAAATAAGGTCACAGGAGTTGAGATGAGATATCAGG
TGAGTTTGATTTACT
+
FFFF:FFFFFFFFFFFFFFFFFFFFFFF:FFFFFFFFFFFFFFFFFFFFFFFFFFFFFFFFFFFFFFF
FFFFFFFFFFFF:FFFFFF:FFFFFFFFFFFFFFFFFFFFFFFFFFFFFFFFFFFFFFF:FFFFFFFF
FFFFFFFFFFFFF:F
@A00155:342:HHGFNDSXY:1:1332:25319:14497 2:N:0:GAACCTAG+TCCGCATA
GGGAAGGATCTGGTCTACATGCCGGACGGGGTGAGGTCTGAGAGTTACACTCAACCATTGGAGAACGG
TTTTCTCCTCAGCTCCAGCAGCATACTTGTCAGAAATAAGGTCACAGGAGTTGAGATGAGATATCAGG
TGAGTTTGATTTACT
+
FFFFFFFFFFFFFFFFFFFFFFFFFFFFFFFFFFFFFFFFFFFFFFFFFFFFFFFFFFFFFFFFFFFF
FFFFFFFFFFFFFFFFFFFFF:FFFFFF:FFFFF:FFFFFFFFFFFFFFFFFFFFFFFFFFFFFFFFF
FFFFF:FFFFFFFFF
@A00155:342:HHGFNDSXY:1:1601:21621:32863 2:N:0:GAACCTAG+TCCGCATA
GGGAAGGATCTGGTCTACATGCCGGACGGGGTGAGGTCTGAGAGTTACACTCAACCATTGGAGAACGG
TTTTCTCCTCAGCTCCAGCAGCATACTTGTCAGAAATAAGGTCACAGGAGTTGAGATGAGATATCAGG
TGAGTTTGATTTACT
+
FFFFFFFFFFFFFFFFFFFFFFFFFFFFFFFFFFFFFFFFFFFFFFFFFFFFFFFFFFFFFFFFFFFF
FFFFFFFFFFFFFFFFFF:FFFFF:FFFFFF:FFF:FFFFFFFFFFFFFF:FFFFFFFFFFFFFFFFF
FFFFF:FFFFFFFFF
@A00155:342:HHGFNDSXY:1:2152:2456:21840 2:N:0:GAACCTAG+TCCGCATA
GGGAAGGATCTGGTCTACATGCCGGACGGGGTGAGGTCTGAGAGTTACACTCAACCATTGGAGAACGG

```
TTTTCTCCTCAGCTCCAGCAGCATACTTGTCAGAAATAAGGTCACAGGAGTTGAGATGAGATATCAGG
TGAGTTTGATTTACT
+
FFFFFFFFFFFFFFFFFFFFFFFFFFFFFFFFFFFFFFFFFFFFFFFFFFFFFFFFFFFFFFFFFFFF
FFFFFFFFFFFFFFFFFFFFFFFFFFFFFFFFFFFFFFFFFFFFFFFFFFFFFFFFFFFFFFFFFFF:
FFFFFFFFFFFFFFF
@A00155:342:HHGFNDSXY:1:2367:28185:30013 2:N:0:GAACCTAG+TCCGCATA
GGGAAGGATCTGGTCTACATGCCGGACGGGGTGAGGTCTGAGAGTTACACTCAACCATTGGAGAACGG
TTTTCTCCTCAGCTCCAGCAGCATACTTGTCAGAAATAAGGTCACAGGAGTTGAGATGAGATATCAGG
TGAGTTTGATTTACT
+
FFFFFFFFFFFFFFFFFFFFFFFFFFFFFFFFFFFFFFFFFFFFFFFFFFFFFFFFFFFFFFFFFFFF
FFFFFFFFFFFFFFFFFFFFFFFFFFFFFFFFFFFFFFFFFFFFFFFFFFFFFFFFFFFFFFFFFFFF
FFFFFFFFFFFFFFF
@A00155:342:HHGFNDSXY:1:2444:28836:27477 2:N:0:GAACCTAG+TCCGCATA
GGGAAGGATCTGGTCTACATGCCGGACGGGGTGAGGTCTGAGAGTTACACTCAACCATTGGAGAACGG
TTTTCTCCTCAGCTCCAGCAGCATACTTGTCAGAAATAAGGTCACAGGAGTTGAGATGAGATATCAGG
TGAGTTTGATTTACT
+
FFFFFFFFFFFFFFFFFFFFFFFFFFFFFFFFFFFFFFFFFFFFFFFFFFFFFFFFFFFFFFFFFFFF
FFFFFFFFFFFFFFFFFFFFFFFF,FFFFFFFFFFFFFFFFFFFFFFFFFFFFFFFFFFFFFFFFFFF
FFFFFFFFFFFFFFF
@A00155:342:HHGFNDSXY:1:2603:1642:1329 2:N:0:GAACCTAG+TCCGCATA
GGGAAGGATCTGGTCTACATGCCGGACGGGGTGAGGTCTGAGAGTTACACTCAACCATTGGAGAACGG
TTTTCTCCTCAGCTCCAGCAGCATACTTGTCAGAACTAAGGTCACACGCGTTGAGATGAGATATCAGG
TGAGTTTGATTTACT
+
F,FFFFF:FF,:FFFFF,FF,:F:,FFF:FFF:FFFFFFFFFFFFFFF:FFFF,FF::F,,FFFFFF:
F,,F::FFFF:F:,:FFFF,:FFF,,F,,FFFFF:,,FFFFFFFFF,,,:FFFFFF:,:F,:,::FF,
FF:FFFFFFFFFFFF
@A00155:342:HHGFNDSXY:1:1608:2501:17848 1:N:0:GAACCTAG+TCCGCATA
GGAAGGATCTGGTCTACATGCCGGACGGGGTGAGGTCTGAGAGTTACACTCAACCATTGGAAAACGGT
TTTCTCCTCAGCTCCAGCAGCATACTTGTCAGAAATAAGGTCACAGGAGTTGAGATGAGATATCAGGT
GAGTTTGATTTACTC
+
FFFFFF:FFFFFFFFFFFFFFFFFFFFFFFFFFFFFFFFFFFFFFFFFFFFFF:FF:FFFFFFFF,F
FFFFFFFFFFFFFFFFF:FFFFFF:FFFFFF:FFFFFFFFFFFFFFFFFFFF:FFFFF:FFFFFFFFF
FFFFFFFFFFFFFFF
@A00155:342:HHGFNDSXY:1:1345:23845:18396 2:N:0:GAACCTAG+TCCGCATA
GGAAGGATCTGGTCTACATGCCGGACGGGGTGAGGTCTGAGAGTTACACTCAACCATTGGAGAACGGT
TTTCTCCTCAGCTCCAGCAGCATACTTGTCAGAAATAAGGTCACAGGAGTTGAGATGAGATATCAGGT
GAGTTTGATTTACTC
+
FFFFFFFFFFFFFFFFFFFFFFFFFFFFFFFFFFFFFFFFFFFFFFFFFFFFFFFFFFFFFFFFFFFF
FFFFFFFFFFFFFFFFFFFFFFFFFFFFFFFFFFFFFFFFFFFFFFFFFFFFFFFFFFFFFFFFFFFF
FFFFFFFF:FFFFFF
@A00155:342:HHGFNDSXY:1:1540:6334:17534 2:N:0:GAACCTAG+TCCGCATA
GGAAGGATCTGGTCTACATGCCGGACGGGGTGAGGTCTGAGAGTTACACTCAACCATTGGAGAACGGT
TTTCTCCTCAGCTCCAGCAGCATACTTGTCAGAAATAAGGTCACAGGAGTTGAGATGAGATATCAGGT
GAGTTTGATTTACTC
+
F:FFFFF:FFFFFFFFFFFFFFFF:FFFFFFFFFFFFFFFFF,FFFFFFF,FFFFF,FFFFFFFF:FF
FFFFFFFFFFFFFFFFFFFFFFFFFFFFFFF:FFFFFFFF:FFFFFFFF:,FFFFFFFFFFFFFFFF:F
FFFFFF:FF:FFFFF
```

```
@A00155:342:HHGFNDSXY:1:1575:20672:27492 2:N:0:GAACCTAG+TCCGCATA
GGAAGGATCTGGTCTACATGCCGGACGGAGTGAGGTCTGAGAGTTACACTCAACCATTGGAGAACGGT
TTTCTCCTCAGCTCCAGCAGCATACTTGTCAGAAATAAGGTCACAGGAGTTGAGATGAGATATCAGGT
GAGTTTGATTTACTC
+
FFFFFFFFFFFFFFFFFFFFFFFFFFFFFFFFFFFFFFFFFFFFFFFFFFFFFFFFFFFFFFFFFFFF
FFFFFFFFFFFFFFFFFFFFFFFFFFFFFFFFFFFFFFFFFFFFFFFFFFFFFFFFFFFFFF:FFFFF
FFFFFFFFFFFFFFF
@A00155:342:HHGFNDSXY:1:1645:30472:7388 2:N:0:GAACCTAG+TCCGCATA
GGAAGGATCTGGTCTACATGCCGGACGGAGTGAGGTCTGAGAGTTACACTCAACCATTGGAGAACGGT
TTTCTCCTCAGCTCCAGCAGCATACTTGTCAGAAATAAGGTCACAGGAGTTGAGATGAGATATCAGGT
GAGTTTGATTTACTC
+
FFFFFFFFFFFFFFFFFFFFFFFFFFFFFFFF:FFFFFFFFFFFFFFFFFFFFFFFFFFFFFFFFFFF
FFFFFFFFFFF:FFFFFFFFFFFFFF:FFFFFFFFFFFFFFFFFFFFFFFFFFFFFFFFFFFFFFFFF
FFF:FFFFFFFFF:F
@A00155:342:HHGFNDSXY:1:2220:28293:3145 2:N:0:GAACCTAG+TCCGCATA
GGAAGGATCTGGTCTACATGCCGGACGGAGTGAGGTCTGAGAGTTACACTCAACCATTGGAGAACGGT
TTTCTCCTCAGCTCCAGCAGCATACTTGTCAGAAATAAGGTCACAGGAGTTGAGATGAGATATCAGGT
GAGTTTGATTTACTC
+
FFFFFFFFFFFFFFFFFFFFFFFFFFFFFFFFFFFFFFFFFFFFFFFFFFFFFFFFFFFFFFFFFFFF
F:FFFFFFFFFFFFFFF:FFFFFFFFFFFFF:FFFFFFFFFFFFFFFFFFFFFFFFFF:FF:FFFFFF
FFFFFFFF,FFFFFF
@A00155:342:HHGFNDSXY:1:2336:21187:31704 2:N:0:GAACCTAG+TCCGCATA
GGAAGGATCTGGTCTACATGCCGGACGGGGTGAGGTCTGAGAGTTACACTCAACCATTGGAGAACGGT
TTTCTCCTCAGCTCCAGCAGCATACTTGTCAGAAATAAGGTCACAGGAGTTGAGATGAGATATCAGGT
GAGTTTGATTTACTC
+
FFFFFFFFFFFFFFFFFFFFFFFFFFFFFFFFFFFFFFFFFFFFFFFFFFFFF:FFFFFFFFFFFFFF
FFFF:FFFFFFFFFFFFFFFFFFFFFFFFFFFFFFFFFFFFFFFFFFFFFFFFFFFFFFFFFFFFFFF
FFFFFFFF:FFFFFF
@A00155:342:HHGFNDSXY:1:2567:21106:34006 2:N:0:GAACCTAG+TCCGCATA
GGAAGGATCTGGTCTACATGCCGGACGGGGTGAGGTCTGAGAGTTACACTCAACCATTGGAGAACGGT
TTTCTCCTCAGCTCCAGCAGCATACTTGTCAGAAATAAGGTCACAGGAGTTGAGATGAGATATCAGGT
GAGTTTGATTTACTC
+
FFFFFFFFFFFFFFFFFFFFFFFFFFFFFFFFFFFFFFFFFFFFFFFFFFFFFFFFFFFFFFFFFFFF
FFFFFFFFFFFFFFFFFFFFFFFFFFFFFFFFFFFFFFFFFFFFFFFFFFFFFFFFFFFFFFFFFFFF
F:FFFFFFFFFFFFF
@A00155:342:HHGFNDSXY:1:2608:4164:29904 2:N:0:GAACCTAG+TCCGCATA
GAAGGATCTGGTCTACATGCCGGACGGGGTGAGGTCTGAGAGTTACACTCAACCATTGGAGAACGGTT
TTCTCCTCAGCTCAAGCAGCATACTTGTCAGAAATAAGGTCACAGGAGTTGAGATGAGATATCAGGTG
AGTTTGATTTACTC
+
FFFFFFFFF,FFFFFF,FFFFFFFFFF,FFFF::FFFFFFFFFFFFFFFFFFFFFFFFFF,FFFFF:F
:F:FF:FFFFFFF,:F:FFFFFF:FFFFFFF::FF:FFFFFF,F:FFF:F:FF:F:FFFF:F,F::,F
FFFF::FF:FF::F
@A00155:342:HHGFNDSXY:1:2623:5565:5149 2:N:0:GAACCTAG+TCCGCATA
GAAGGATCTGGTCTACATGCCGGACGGGGTGAGGTCTGAGAGTTACACTCAACCATTGGAGAACGGTT
TTCTCCTCAGCTCCAGCAGCATACTTGTCAGAAATAAGGTCACAGGAGTTGAGATGAGATATCAGGTG
AGTTTGATTTACTC
+
FFFFFFFFFFFFFFF:FFFFFF:FF:,F:FFFF:F:FFF,FFFFFFFFFFFFFFFFFF:FFFF:FFFF
```

```
FFFFF,FFF:FFF:FFFF:FF:F,F::FFF:FFFFF:FFFFFFFFFF,:F,F:FF,F,FFFFFF:FFF
FF:FF,FFFFFFF:
@A00155:342:HHGFNDSXY:1:1215:16297:27806 2:N:0:GAACCTAG+TCCGCATA
GAAGGATCTGGTCTACATGCCGGACGGAGTGAGGTCTGAGAGTTACACTCAACCATTGGAGAACGGTT
TTCTCCTCAGCTCCAGCAGCATACTTGTCAGAAATAAGGTCACAGGAGTTGAGATGAGATATCAGGTG
AGTTTGATTTACTCT
+
FFFFFFFFFFFFFFFFFFFFFFFFFFFFFFFFFFFFFFFFFFFFFFFFFFFFFFFFFFFFFFFFFFFF
FFFFFFFFFFFFFFFFFFFFFFFFFFFFF:FFFFFFFFFFFFFFFFFFFFFFFFFFFFFFFFFFFFFF
FFFFFFFFFFFFFFF
@A00155:342:HHGFNDSXY:1:1324:24080:8563 2:N:0:GAACCTAG+TCCGCATA
GAAGGATCTGGTCTACATGCCGGACGGGGTGAGGTCTGAGAGTTACACTCAACCATTGGAGAACGGTT
TTCTCCTCAGCTCCAGCAGCATACTTGTCAGAAATAAGGTCACAGGAGTTGAGATGAGATATCAGGTG
AGTTTGATTTACTCT
+
FFFFFF:FFFFFFFFFFFFFFFFFFFFFFF:FFFFFFFFFFFFFFFFFFFFFFFFFFF:::FFF:FFF
FFFFF,FF:FFFFFFFFFFFFF:FFFFFFFFFFFFFFFFF:FFFFFFFFFFFFFFFFFFFFFFFF,FF
FFFFFFF::FFFF,F
@A00155:342:HHGFNDSXY:1:1339:30201:21731 2:N:0:GAACCTAG+TCCGCATA
GAAGGATCTGGTCTACATGCCGGACGGGGTGAGGTCTGAGAGTTACACTCAACCATTGGAGAACGGTT
TTCTCCTCAGCTCCAGCAGCATACTTGTCAGAAATAAGGTCACAGGAGTTGAGATGAGATATCAGGTG
AGTTTGATTTACTCT
+
FFFFFFFFFFFFFFFFFFFFFFFFF:FFF:FFFFFFFFFFFFFFFFFFFFFFFFFFFFFFFFFFFFFF
:FFFFFFFFFFFF:FFFFFFFFFFFFFFFFFFFFFFFFFFFFFFFFFFFFFFFFFFFFFFFFFFFFFF
FFFFFFFFFFFFFFF
@A00155:342:HHGFNDSXY:1:1452:7175:13573 2:N:0:GAACCTAG+TCCGCATA
GAAGGATCTGGTCTACATGCCGGACGGGGTGAGGTCTGAGAGTTACACTCAACCATTGGAGAACGGTT
TTCTCCTCAGCTCCAGCAGCATACTTGTCAGAAATAAGGTCACAGGAGTTGAGATGAGATATCAGGTG
AGTTTGATTTACTCT
+
FFFFFFFFFFFFF:FFFFFFFFFFFFFFFFFFFFFFFFFFFFFFFFFFFFFFFFFFF:FFFFFFFFFF
FFFFFFFFFFFFFFFFFFFFFFFFFFFFFFFFFFFFFFFFFFFFFFFF:FFF,FFF:FFFFFFFFFFF
FFFFFFF:FFFFFFF
@A00155:342:HHGFNDSXY:1:1550:7491:16971 2:N:0:GAACCTAG+TCCGCATA
GAAGGATCTGGTCTACATGCCGGACGGGGTGAGGTCTGAGAGTTACACTCAACCATTGGAGAACGGTT
TTCTCCTCAGCTCCAGCAGCATACTTGTCAGAAATAAGGTCACAGGAGTTGAGATGAGATATCAGGTG
AGTTTGATTTACTCT
+
FFFFFFFFFFFFFFFFFFFFFFFFFFFFFFFFFFFFFFFFFFFFFFFFFFFFFFFFFFFFFFFFFFFF
FFFFFFFFFFFFFFFFFFFFFFFFFFFFFFFFFFFFF:FFFFFFFFFFFFFFFFFFFFFFFFFFFFFF
FFFFFFFFFFF:FFF
@A00155:342:HHGFNDSXY:1:1623:5068:9831 2:N:0:GAACCTAG+TCCGCATA
GAAGGATCTGGTCTACATGCCGGACGGGGTGAGGTCTGAGAGTTACACTCAACCATTGGAGAACGGTT
TTCTCCTCAGCTCCAGCAGCATACTTGTCAGAAATAAGGTCACAGGAGTTGAGATGAGATATCAGGTG
AGTTTGATTTACTCT
+
FFFFFFFFFFFFFFFFFFFFFFFFFFFFFFFFFFFFFFFFFFFFFFFFFFFFFFFFFFFFFFFFFFFF
FFFFFFFFFFFFFFFFFFFFFFFFFFFFFFFFFF,FFFFFFFFFFFFFFFF:FFFFFFFFFFFFFFFF
FFFFFF:FFFFFFFF
@A00155:342:HHGFNDSXY:1:2302:25870:6026 2:N:0:GAACCTAG+TCCGCATA
GAAGGATCTGGTCTACATGCCGGACGGAGTGAGGTCTGAGAGTTACACTCAACCATTGGAGAACGGTT
TTCTCCTCAGCTCCAGCAGCATACTTGTCAGAAATAAGGTCACAGGAGTTGAGATGAGATATCAGGTG
AGTTTGATTTACTCT
```

```
+
FFFFFFFFFFFFFFFFFFFFFFFF:FFF:FFFFFFFFFFFFFFFFFFFFFFFFFFFFFFFFF:F:F:FFFFF
FFFFFFFFFFFFFFFFFFFFFFFFFFFFF:FFFFFFFFFFFFFF:FFFFFFFFFFFFFFFFFFF:FFFFF
FFF:F:FFFFFFFFF
@A00155:342:HHGFNDSXY:1:2334:30987:7341 2:N:0:GAACCTAG+TCCGCATA
GAAGGATCTGGTCTACATGCCGGACGGAGTGAGGTCTGAGAGTTACACTCAACCATTGGAGAACGGTT
TTCTCCTCAGCTCCAGCAGCATACTTGTCAGAAATAAGGTCACAGGAGTTGAGATGAGATATCAGGTG
AGTTTGATTTACTCT
+
FFFFFFFFFFFFFFFFFFFFFFFFFFFFFFFFFFFFFFFFFFFFFFFFFFFFFFFFFFFFFFFFFFF:F
FFFFFFFFFFFFFFFFFFFFFFF:FFFFFFFFFFF,FFFFFFFFFFFFFFFFFFFFFFFFFFFFFFFFFF
FFFFFFF:FFFFFF:
@A00155:342:HHGFNDSXY:1:1457:16405:4476 2:N:0:GAACCTAG+TCCGCATA
AAGGATCTGGTCTACATGCCGGACGGAGTGAGGTCTGAGAGTTACACTCAACCATTGGAGAACGGTTT
TCTCCTCAGCTCCAGCAGCATACTTGTCAGAAATAAGGTCACAGGAGTTGAGATGAGATATCAGGTGA
GTTTGATTTACTCTC
+
FFFFFFFFFFFFFFFFFFFFFFFFFFFFFFFFFFFFFFFFFFFFFFFFFFFFFFFFFFFFFFFFFFFFFF
FFFFFFFFFFFFFFFFFFFFFF:FFFFFFFFFFFFFFF:FFFFFFFFFFFFFFFFFFFFFFFFFF:FFFF
FFFFFF::FFF:FFF
@A00155:342:HHGFNDSXY:1:1457:16369:4601 2:N:0:GAACCTAG+TCCGCATA
AAGGATCTGGTCTACATGCCGGACGGAGTGAGGTCTGAGAGTTACACTCAACCATTGGAGAACGGTTT
TCTCCTCAGCTCCAGCAGCATACTTGTCAGAAATAAGGTCACAGGAGTTGAGATGAGATATCAGGTGA
GTTTGATTTACTCTC
+
FFFFFFFFFFFFFFFFFFFFFFFFFFFFFFFFFFFFFFFFFFFFFFFFFFFFFFFFFFFFFFFFFFFF:FF
FFFFFFFFFFFFFFFFFFFFFFFFFFFFFFFFFFFFFFFFFFFFFFFFFFFFFFFFFFFFFFFFFFFFFF
FFFFFFFFFF:FFFF
@A00155:342:HHGFNDSXY:1:2542:24749:32080 2:N:0:GAACCTAG+TCCGCATA
AAGGATCTGGTCTACATGCCGGACGGGGTGAGGTCTGAGAGTTACACTCAACCATTGGAGAACGGTTT
TCTCCTCAGCTCCAGCAGCATACTGGTCAGAAATAAGGTCACAGGAGTTGAGATGAGATATCAGGTGA
GTTTGATTTACTCTC
+
FF:FFFF:FF:F:FFF:FFFF,FFFFFFFFFFFFF:FFFFFFF:FFFFFFFFFF:,FFFFFFFFFF,F
FFFFFFFF:FFFFFF:FFFFFFFF,FFFFF,FFFFFFFFFFF:F:FFFFFFF:F:FFFF:F:F:FF:F
FFF:FF,,FF,FFFF
@A00155:342:HHGFNDSXY:1:1276:21929:3239 2:N:0:GAACCTAG+TCCGCATA
AGGATCTGGTCTACATGCCGGACGGAGTGAGGTCTGAGAGTTACACTCAACCATTGGAGAACGGTTTT
CTCCTCAGCTCCAGCAGCATACTTGTCAGAAATAAGGTCACAGGAGTTGAGATGAGATATCAGGTGAG
TTTGATTTACTCTCT
+
FFFFFFFFFFFFFF:FFFFFFFFFFFFFFFFFFFFFFFFFFFFFFFFF:FFFFFF,FFFFFFFFFFFF,F
FFFFFFFFFFFFFF:F:FFFFFF:FFFFFFFFFFFFF,F:FFFFFFFFFFFFFFFFFFFFFFFFFFFFF
FFFFFFFFFFFFFFF
@A00155:342:HHGFNDSXY:1:1315:24099:28823 2:N:0:GAACCTAG+TCCGCATA
AGGATCTGGTCTACATGCCGGACGGAGTGAGGTCTGAGAGTTACACTCAACCATTGGAGAACGGTTTT
CTCCTCAGCTCCAGCAGCATACTTGTCAGAAATAAGGTCACAGGAGTTGAGATGAGATATCAGGTGAG
TTTGATTTACTCTCT
+
FFFFFFFFFFFFFFFFFFFFFFFFFFFFFFFFFFFFFFFFFFFFFFFFFFFFFFFFFFFFFFFFFFFFFF
FFFFFFFFFFFFFFFFFFFFFFFFFFFFFFFFFFFFFFFFFFFFFFFFF,FFFFFFFFFFFFFFFFFFFF
FFFFFFFFFFFFFFF
@A00155:342:HHGFNDSXY:1:2378:32723:27884 2:N:0:GAACCTAG+TCCGCATA
AGGATCTGGTCTACATGCCGGACGGGGTGAGGTCTGAGAGTTACACTCAACCATTGGAGAACGGTTTT
```

CTCCTCAGCTCCAGCAGCATACTTGTCAGAAATAAGGTCACAGGAGTTGAGATGAGATATCAGGTGAG
TTTGATTTACTCTCT
+
FFFFFFFFFFFFFFF:FFFFFFFFFFFFFFFFFFFFF:FFFFFFFFFFFFFFFFFFFFFFFFFFFFFF
FFFFFFFFFFFFFFFFFFFFFFFFFFFFF:FFFFFFFFFFFFFFFFFFFFFFFFFFFFFFF:FFFFFF
FFFFF:FFFFFFFFF
@A00155:342:HHGFNDSXY:1:1339:14335:20713 1:N:0:GAACCTAG+TCCGCATA
GGATCTGGTCTACATGCCGGACGGAGTGAGGTCTGAGAGTTACACTCAACCATTGGAGAACGGTTTTC
TCCTCAGCTCCAGCAGCATACTTGTCAGAAATAAGGTCACAGGAGTTGAGATGAGATATCAGGTGAGT
TTGATTT
+
FFFFFFFFFFFFFFFFFFFFFFFFFFFFFFFFFFFFFFFFFFFFFFFFFFFFFFFFFFFFFFFFFFFF
FFFFFFFFFFFFFFFFFFFFFFFFFFFFFFFFFFFFFFFFFFFFFFFFFFFFFFFFFFFFFFFFFFFF
FFFFFFF
@A00155:342:HHGFNDSXY:1:2134:11704:36354 1:N:0:GAACCTAG+TCCGCATA
GGATCTGGTCTACATGCCGGACGGAGTGAGGTCTGAGAGTTACACTCAACCATTGGAGAACGGTTTTC
TCCTCAGCTCCAGCAGCATACTTGTCAGAAATAAGGTCACAGGAGTTGAGATGAGATATCAGGTGAGT
TTGATTT
+
FFFFFFFFFFFFFFFFFFFFFFFFFFFFFFFFFFFFFFFFFFFFFFFFFFFFFFFFFFFFFFFFFFFF
FFFFFFFFFFFFFFFFFFFFFFFFFFFFFFFFFFFFFFFFFFFFFFFFFFFFFFFFFFFFFFFFFFF:
FFFFFFF
@A00155:342:HHGFNDSXY:1:2638:20283:36401 1:N:0:GAACCTAG+TCCGCATA
GGATCTGGTCTACATGCCGGACGGAGTGAGGTCTGAGAGTTACACTCAACCATTGGAGAACGGTTTTC
TCCTCAGCTCCAGCAGCATACTTGTCAGAAATAAGGTCACAGGAGTTGAGATGAGATATCAGGTGAGT
TTGATTT
+
FFFFFFFFFFFFFFFFFFFFFFFFFFFFFFFFFFFFFFFFFFFFFFFFFFFFFFFFFFFFFFFFFFFF
FFFFFFFFFFFFFFFFFFFFFFFFFFFFFFFFFFFFFFFFFFFFFFFFFFFFFFFFFFFFFFFFFFFF
FFFFFFF
@A00155:342:HHGFNDSXY:1:1441:27950:28761 1:N:0:GAACCTAG+TCCGCATA
CGGCTGGTCTACATGCCGGACGGGGTGAGGTCTGAGAGTTACACTCAACCATTGGAGAACGGTTTTCT
CCTCAGCTCCAGCAGCATACTTGTCAGAAATAAGGTCACAGGAGTTGAGATGAGATATCAGGTGAGTT
TGATTTACTCTCTTG
+
FFFFFFFFFFFFFFFFFFFFFFFF,FFFFFFFFFFFFFF:FFFFFFFFFFFFFFFFF:FFFFFFFFFF
FFFFFFFFFFFFFFFFFFFFF:FFFFFFFFFFFFFFF:FFFFFFFFFFFFFFFFFFFFFFFFFFFFFF
FFFFFFFFFFFFFFF
@A00155:342:HHGFNDSXY:1:1630:20564:8234 1:N:0:GAACCTAG+TCCGCATA
GATCTGGTCTACATGCCGGACGGGGTGAGGTCTGAGAGTTACACTCAACCATTGGAGAACGGTTTTCT
CCTCAGCTCCAGCAGCATACTTGTCAGAAATAAGGTCACAGGAGTTGAGATGAGATATCAGGTGAGTT
TGATTTACTCTCTTG
+
FFFFFFFFFFFFFFFFFFFFFFFFFFFFFFFFFFFFFFF:FFFFFFFFFFFFFFFFFFFFFFFFFFFF
FFFFFFFFFFFFFFFFFFFFFFFFFFFFFFFFFFFFFFFFFFFFFFFFFFFFFFFFFFFFFFFFFFFF
FFFFFFFFFFFFFFF
@A00155:342:HHGFNDSXY:1:2445:10710:16783 1:N:0:GAACCTAG+TCCGCATA
GATCTGGTCTACATGCCGGACGGGGTGAGGTCTGAGAGTTACACTCAACCATTGGAGAACGGTTTTCT
CCTCAGCTCCAGCAGCATACTTGTCAGAAATAAGGTCACAGGAGTTGAGATGAGATATCAGGTGAGTT
TGATTTACTCTCTTG
+
FFFFFFFFFFFFFFFFFFFFFFFFFFFFFFFFFFFFFFFFFFFFFFFFFFFFFFFFFFFFFFFFFFFF
FFFFFFFFFFFFFFFFFFFFF:FFFFFFFFFFFFFFFFFFFFFFFFFFFFFFFFFFFFFFFFFFFFFF
FFFFFFFFFFFFFFF

@A00155:342:HHGFNDSXY:1:2505:19678:20509 1:N:0:GAACCTAG+TCCGCATA
TCCGATCTCTACATGCCGGACGGGGTGAGGTCTGAGAGTTACACTCAACCATTGGAGAACGGTTTTCT
CCTCAGCTCCAGCAGCATACTTGTCAGAAATAAGGTCACAGGAGTTGAGATGAGATATCAGGTGAGTT
TGATTTACTCTCTTG
+
FFFFFFFFFFFFFFFFF:F,FFFFFFFF:FFFFF:::FFFF:FFFF:FFFFFFFF::FFFFF:FFFF
:FFFFFFFFF:FFFFFFFFFFFFFF:FF::F:FF:FF,FFFFFFFFFFFFFFFFFFFFFFFF::FF:
FFFFFF:FFFFFFFF
@A00155:342:HHGFNDSXY:1:1560:30309:1031 2:N:0:GAACCTAG+TCCGCATA
TCTGGTCTACATGCCGGACGGGGTGAGGTCTGAGAGTTACACTCAACCATTGGAGAACGGTTTTCTCC
TCAGCTCCAGCAGCATACTTGTCAGAAATAAGGTCACAGGAGTTGAGATGAGATATCAGGTGAGTTTG
ATTTACTCTCTTGG
+
FFFFFFFFFFFFFFFFFFFFFFFFFFFFFFFFFFFFFFFF:FFFFFFFFFFFFFFF:F,FFF:FFFF
FFFFFFFFFF:FFFFFFFFFFFFFFFFFFFFFFFFFFFFFFFF,FFFFFFFFFFFFFFFFFFFFFFF
FF:FFFFFFF:FFF
@A00155:342:HHGFNDSXY:1:2213:25355:25770 1:N:0:GAACCTAG+TCCGCATA
TCTGGTCTACATGCCGGACGGGGTGAGGTCTGAGAGTTACACTCAACCATTGGAGAACGGTTTTCTCC
TCAGCTCCAGCAGCATACTTGTCAGAAATAAGGTCACAGGAGTTGAGATGAGATATCAGGTGAGTTTG
ATTTACTCTCTTGGT
+
FFFFFF,FFFFFFFFFFFFFFFFFFFFFFFFFFFFFFFFFFFFFFFFFFFFFFFFFFFFFFFFFFFF
FFFFFFFFFFFFFFFFFFFFFFFFFFFFFFFFFFFFFFFFFFFFFFFFFFFFFFFFFFFFFFFFFFF
FFFFFFFFFFFFFFF
@A00155:342:HHGFNDSXY:1:2561:15320:5948 1:N:0:GAACCTAG+TCCGCATA
CTGGTCTACATGCCGGACGGGGTGAGGTCTGAGAGTTACACTCAACCATTGGAGAACGGTTTTCTCCT
CAGCTCCAGCAGCATACTTGTCAGAAATAAGGTCACAGGAGTTGAGATGAGATATCAGGTGAGTTTGA
TTTACTCTCTTGGTT
+
:FFF:F:FFFFFFF:FFFFFFFFFFFFFFFFFFFFFFFFFFFFFFFFFF:FF,FF:FFFFFFFFF:F
FFFFF,FFFFFFFFFFFFFFFFFFFFFFFFFF:F::FFF:FFFFFFFFFF:F:F,,FF:,,F,:,,F
FFFF,FF:,:FF:FF
@A00155:342:HHGFNDSXY:1:1263:22842:20854 2:N:0:GAACCTAG+TCCGCATA
CTGGTCTACATGCCGGACGGGGTGAGGTCTGAGAGTTACACTCAACCATTGGAGAACGGTTTTCTCCT
CAGCTCCAGCAGCATACTTGTCAGAAATAAGGTCACAGGAGTTGAGATGAGATATCAGGTGAGTTTGA
TTTACTCTCTTGGTT
+
FFFFFFFFFFFFFFFFFFFFFFFFFFFFFFFFFFFFFFFFFFFFFFFFFFFFFFFFFFFFFFFFFFF
FFFFFFFFFFFFFFFFFFFFFFFFFFFFFFFFFFFFFFFFFFFFFFFFFFFF:FFFFFFFFFFFFFF
:FFFFFFFFFFFFFF
@A00155:342:HHGFNDSXY:1:1250:18367:7435 2:N:0:GAACCTAG+TCCGCATA
CTGGTCTACATGCCGGACGGGGTGAGGTCTGAGAGTTACACTCAACCATTGGAGAACGGTTTTCTCCT
CAGCTCCAGCAGCATACTTGTCAGAAATAAGGTCACAGGAGTTGAGATGAGATATCAGGTGAGTTTGA
TTTACTCTCTTGGTT
+
FFFFFFFFFFFFFFFFFFFFFFFFFFFFFFFFFFFFFFFFFFFFFFFFFFFFFFF:FFFFFFFFFFF
FFFFFFFFFFFFFFFFFFFF:FFFFFFFFFFFFFFFFFFFFFFFFFFFFFFFFFFFFFFFFFFFFFF
:FFFFFFFFFFFFFF
@A00155:342:HHGFNDSXY:1:1442:15076:16955 2:N:0:GAACCTAG+TCCGCATA
CTGGTCTACATGCCGGACGGAGTGAGGTCTGAGAGTTACACTCAACCATTGGAGAACGGTTTTCTCCT
CAGCTCCAGCAGCATACTTGTCAGAAATAAGGTCACAGGAGTTGAGATGAGATATCAGGTGAGTTTGA
TTTACTCTCTTGGTT
+
FFFFFFFFFFFFFFFFFFFFFFFFFFFFFFFFFFFFFFFFFFFFFFFFFFFFFFFFFFFFFFFFFFF

```
FFFFFFFFF:FFFFFFFFFFFFFFFFFF:FFFFFFFFFFFFFFFFFFFFFFFF:FFFFFFFFF:FFFFF
FFFFFFF,FFFFFFF
@A00155:342:HHGFNDSXY:1:1630:4146:30937 2:N:0:GAACCTAG+TCCGCATA
CTGGTCTACATGCCGGACGGGGTGAGGTCTGAGAGTTACACTCAACCATTGGAGAACGGTTTTCTCCT
CAGCTCCAGCAGCATACTTGTCAGAAATAAGGTCACAGGAGTTGAGATGAGATATCAGGTGAGTTTGA
TTTACTCTCTTGGTT
+
FFFFFFFFFFFFFFFFFFFFFFFFFFFFFFFFFFFFFFFFFFFFFFFFFFFFFFFFFFFFFFFFFFFF
FFFFFFFFFFFFFFFFFFFFFFFFFFFFFFFFFFFFFFFFFFFFFFFFFFFFFFFFFFFFFFFFFFFF
FFFFFFFFFFFFFFF
@A00155:342:HHGFNDSXY:1:2118:16423:31782 2:N:0:GAACCTAG+TCCGCATA
CTGGTCTACATGCCGGACGGGGTGAGGTCTGAGAGTTACACTCAACCATTGGAGAACGGTTTTCTCCT
CAGCTCCAGCAGCATACTTGTCAGAAATAAGGTCACAGGAGTTGAGATGAGATATCAGGTGAGTTTGA
TTTACTCTCTTGGTT
+
FFFFFFFF:FFFFFFFF:FFFFFFFFFFFFFFFFFFFFFFFFFFFFFFFFFFFFFF:FFFFFFFFFFFF
FFFFFFFFFFFFFFFFFFFFFFFFFFFFF,FFFFF:FFFFF:FFFF:F:FFF:FFFFFFFFFFF:FFF
FFFFFFF:FFFFFFF
@A00155:342:HHGFNDSXY:1:2147:3206:32847 2:N:0:GAACCTAG+TCCGCATA
CTGGTCTACATGCCGGACGGGGTGAGGTCTGAGAGTTACACTCAACCATTGGAGAACGGTTTTCTCCT
CAGCTCCAGCAGCATACTTGTCAGAAATAAGGTCACAGGAGTTGAGATGAGATATCAGGTGAGTTTGA
TTTACTCTCTTGGTT
+
FFFFFFFFFFFFFFFFFFFFFFFFFFFFFFFFF:FFFFFFFFFFFFFFFFFFFFFFFFFFFFFFFFF:F
:FFFFFFFFFFFFFFFFFFFFFFFFFFFFFFF::FFFFF,FFFFF::FFFF:FF:FFFF,FFFFFFF:F
FFFF,F:FFFF,:FF
@A00155:342:HHGFNDSXY:1:2246:4924:16157 2:N:0:GAACCTAG+TCCGCATA
CTGGTCTACATGCCGGACGGGGTGAGGTCTGAGAGTTACACTCAACCATTGGAGAACGGTTTTCTCCT
CAGCTCCAGCAGCATACTTGTCAGAAATAAGGTCACAGGAGTTGAGATGAGATATCAGGTGAGTTTGA
TTTACTCTCTTGGTT
+
FFFFFFFFFFFFFFFFFFFFFFFFFFFFFFFFFFFFFFFFFFFFFFFFFFFFFFFFFFFFFFFFFFFF
FFFFFFFFFFFFFFFFFFFFFFFFFFFFFFFFFFFFFFFFFFFFFFFFFFFF:,FFFFFFFFFFFFFF
FFFFFF:FFFFFFFF
@A00155:342:HHGFNDSXY:1:2259:28429:28244 2:N:0:GAACCTAG+TCCGCATA
CTGGTCTACATGCCGGACGGGGTGAGGTCTGAGAGTTACACTCAACCATTGGAGAACGGTTTTCTCCT
CAGCTCCAGCAGCATACTTGTCAGAAATAAGGTCACAGGAGTTGAGATGAGATATCAGGTGAGTTTGA
TTTACTCTCTTGGTT
+
FFFFFFFFFFFFFFFFFFFFFFFFFFFFFFFFFFFFFFFFFFFFFFFFFFFFFFFFFFFFFFFFFFFF
FFFFFFFFFFFFFFFFF:FFFFFFFFF:FFFFFFFF,FFFF:FFFFFFFFFFFFFFFFFFFFFFFFFF
FFFFFFFFFFFFF:F
@A00155:342:HHGFNDSXY:1:2307:4399:7545 2:N:0:GAACCTAG+TCCGCATA
CTGGTCTACATGCCGGACGGGGTGAGGTCTGAGAGTTACACTCAACCATTGGAGAACGGTTTTCTCCT
CAGCTCCAGCAGCATACTTGTCAGAAATAAGGTCACAGGAGTTGAGATGAGATATCAGGTGAGTTTGA
TTTACTCTCTTGGTT
+
FFFFFFFFFFFFFFFFFFFFFFFFFFFFFFFFFFFFFFFFFFFFFFFFFFFFFFF:FFFFFFFFFFFF
FFFFFFFFFFFFFFFFFFFFFFFFFFFFFFFFFFFFFFFFFFFFFFFFFFFFFFFFFFFFF:FFFFFF
FFFFFFFFFF,FFFF
@A00155:342:HHGFNDSXY:1:2444:24225:13416 2:N:0:GAACCTAG+TCCGCATA
CTGGTCTACATGCCGGACGGAGTGAGGTCTGAGAGTTACACTCAACCATTGGAGAACGGTTTTCTCCT
CAGCTCCAGCAGCATACTTGTCAGAAATAAGGTCACAGGAGTTGAGATGAGATATCAGGTGAGTTTGA
TTTACTCTCTTGGTT
```

```
+
FFFFFFFFFFFFFFFFFFFFFFFFFFFFFFFFFFFFFFFFFFFFFFFFFFFFFFFFFFFFFFFFFFF:F
FFFFFFFFFFFFFFFFFFFFFFF:FFFFFFFFFFFFFFFFFFFFFFFFFFFFFFFF:FFFFFFFFFFFFF
:FFFFFFFF:FFFFF
@A00155:342:HHGFNDSXY:1:2449:24849:28933 2:N:0:GAACCTAG+TCCGCATA
CTGGTCTACATGCCGGACGGGGTGAGGTCTGAGAGTTACACTCAACCATTGGAGAACGGTTTTCTCCT
CAGCTCCAGCAGCATACTTGTCAGAAATAAGGTCACAGGAGTTGAGATGAGATATCAGGTGAGTTTGA
TTTACTCTCTTGGTT
+
FFFFFFFFFFFFFFFF:FFFFF:FFFFFFFFFFFFFFFFFFF:FFFFF:FFFFFFFFFFFFFFFFFFF
FFFFFFFFFFFF:F:FFF:FF:FFFFF:FFFF:FFFF:FF,FFFFFFFFF:FFF:,FFF::F,:FFFF
FFFFFFFFFFF,FFF
@A00155:342:HHGFNDSXY:1:2623:4128:4413 2:N:0:GAACCTAG+TCCGCATA
CTGGTCTACATGCCGGACGGGGTGAGGTCTGAGAGTTACACTCAACCATTGGAGAACGGTTTTCTCCT
CAGCTCCAGCAGCATACTTGTCAGAAATAAGGTCACAGGAGTTGAGATGAGATATCAGGTGAGTTTGA
TTTACTCTCTTGGTT
+
FFFFFFFFFFFFFFFFFFFFFFFFFFFFFFFFFFFFFFFFFFFFFFFFFFFFFFFFFFFFF:FFFFFFF
FFFFFFFFFFFFF:FFFFFFFFFFFFF::FFFFFFFFF:,FFFFFFFFFFFFFFFFFFFFFFFFFFFFF
FFFFFFFFFFFFFFF
@A00155:342:HHGFNDSXY:1:2669:13467:16203 2:N:0:GAACCTAG+TCCGCATA
CTGGTCTACATGCCGGACGGGGTGAGGTCTGAGAGTTACACTCAACCATTGGAGAACGGTTTTCTCCT
CAGCTCCAGCAGCATACTTGTCAGAAATAAGGTCACAGGAGTTGAGATGAGATATCAGGTGAGTTTGA
TTTACTCTCTTGGTT
+
FFFFFFFFFFFFFFFFFFFFFFFFFFFFFFFFFFFFFFFFFFFFFFFFFFFFFFFFFFFFFFFFFFFF
FFFFFFFFFFFFFFFFFFFFFFFFFFFFFFFFFFFFFFFFFFFFFFFFFFFFFFFFFFFFFFFFFFFF
FFFFFFFFFFFFFFF
@A00155:342:HHGFNDSXY:1:2419:20537:31735 2:N:0:GAACCTAG+TCCGCATA
TGGTCTACATGCCGGACGGAGTGAGGTCTGAGAGTTACACTCAACCATTGGAGAACGGTTTTCTCCTC
AGCTCCAGCAGCATACTTGTCAGAAATAAGGTCACAGGAGTTGAGATGAGATATCAGGTGAGTTTGAT
TTACTCTCTTGGTT
+
F:,FF:F,F,FF,FFF,FFF:,,FF,FFFFFFFFF:FFFF,:FFFFF:,F:F:,FF::FF,:FF:FF:
FFFFF,FFFF:FFFFFF:FFFF:FFFF:FFFF,FFFF:FF,FFFFFF:FFF:FFFFFFF:,F,F,FFF
FFFF:FF,F:,:FF
@A00155:342:HHGFNDSXY:1:1117:4056:21480 1:N:0:GAACCTAG+TCCGCATA
TGGTCTACATGCCGGACGGAGTGAGGTCTGAGAGTTACACTCAACCATTGGAGAACGGTTTTCTCCTC
AGCTCCAGCAGCATACTTGTCAGAAATAAGGTCACAGGAGTTGAGATGATATATCAGGTGAGTTTGAT
TTACTCTCTTGGTTC
+
FFFFFFFFFFFFFFFFFF:FFFFFFFFFFFF,FFFFFFF:FFFFFFFFFFFFFFFFFFFFFFF:FFFFF
FFFFFFF,FFFFFFFFFF,FFFFFFFFFF:FFFFFFF,F,FFFF,FFFF,FFFFFFFFFFFFFFFFFF
FFF:FFFFFFFFFFF
@A00155:342:HHGFNDSXY:1:2530:6144:25755 2:N:0:GAACCTAG+TCCGCATA
TGGTCTACATGCCGGACGGGGTGAGGTCTGAGAGTTACACTCAACCATTGGAGAACGGTTTTCTCCTC
AGCTCCAGCAGCATACTTGTCAGAAATAAGGTCACAGGAGTTGAGATGAGATATCAGGTGAGTTTGAT
TTACTCTCTTGGTTC
+
FFFFFFFFFFFFFFFFFFFFFFFFFFF:FFFFFFFFFFFFFFFFFFFFFFFFFFFFFFFFFFFF,FFFFF
FFFFFFFFFFFFFFF,FFFFFFFFFF:FF,FFFF:FFFFFFF,FFFFFF,FFF:,FFFFFFFFFFFFF
FFFFFFFFFFFFFFF
@A00155:342:HHGFNDSXY:1:2530:6234:30358 2:N:0:GAACCTAG+TCCGCATA
TGGTCTACATGCCGGACGGGGTGAGGTCTGAGAGTTACACTCAACCATTGGAGAACGGTTTTCTCCTC
```

AGCTCCAGCAGCATACTTGTCAGAAATAAGGTCACAGGAGTTGAGATGAGATATCAGGTGAGTTTGAT
TTACTCTCTTGGTTC
+
FFFFFFFFFFFFFFFFFFFFFFFFFFFFFFFFFFFFFFFFFFFFFFFFFFFFFFFFFFFFFFFFFFFF
FFFFFFFFFFFFFFFFFFFFFFFFFFFFFFFFFFFFFFFFFFFFFFFFFFFFFFFFFFFFFFFFFFFF
FFFFFFFFFFFFFFF
@A00155:342:HHGFNDSXY:1:1663:11008:29042 2:N:0:GAACCTAG+TCCGCATA
GGTCTACATGCCGGACGGGGTGAGGTCTGAGAGTTACACTCAACCATTGGAGAACGGTTTTCTCCTCA
GCTCCAGCAGCATACTTGTCAGAAATAAGGTCACAGGAGTTGAGATGAGATATCAGGTGAGTTTGATT
TACTCTCTTG
+
FFFFFFFFFFFFFFFFFFFFFFFFFFFFFFFFFFFFFFFFFFFFFFFFFFFFFFFFFFFFFFFFFFFF
FFFFFFFFFFFFFFFF,FFFFFFFFFFFFFFF:FFFFFFFFFFFFFF::FFFFFFFFFFFFFFFFFFF
FFFFFFFFFF
@A00155:342:HHGFNDSXY:1:2424:20085:4429 2:N:0:GAACCTAG+TCCGCATA
GGTCTACATGCCGGACGGGGTGAGGTCTGAGAGTTACACTCAACCATTGGAGAACGGTTTTCTCCTCA
GCTCCAGCAGCATACTTGTCAGAAATAAGGTCACAGGAGTTGAGATGAGATATCAGGTGAGTTTGATT
TACTCTCTTG
+
FFFFFFFFFFFFFF,FFFFFFFF:FFFFFFFFFFFFFFFFFFFFFFFFFFFFFFFFFFFFFFFFFFFF
FFFFFFFFFFFFFFFFFFFFFFFFFFFFFFFFFFFFFFFFFFFFFFFFFFFFFFFFFFFFFFFFFFFF
FFFFF:F:FF
@A00155:342:HHGFNDSXY:1:1163:31268:12806 2:N:0:GAACCTAG+TCCGCATA
GGTCTACATGCCGGACGGGGTGAGGTCTGAGAGTTACACTCAACCATTGGAGAACGGTTTTCTCCTCA
GCTCCAGCAGCATACTTGTCAGAAATAAGGTCACAGGAGTTGAGATGAGATATCAGGTGAGTTTGATT
TACTCTCTTGG
+
FFFFFFFFFFFFFFFFFFFFFFFFFFFFFFFFFFF:FFFFFFFFFFFFFFFFFFFFFFFFFFFFFFFF
FFFFFFFFFFFFFFFFFFFFFFFFFFFFFFFFFFFFF:FFFFFFFFFFFFFFFFFFFFFFFFFFFFFF
FFFFFFFFFFF
@A00155:342:HHGFNDSXY:1:1272:22616:25692 2:N:0:GAACCTAG+TCCGCATA
GGTCTACATGCCGGACGGAGTGAGGTCTGAGAGTTACACTCAACCATTGGAGAACGGTTTTCTCCTCA
GCTCCAGCAGCATACTTGTCAGAAATAAGGTCACAGGAGTTGAGATGAGATATCAGGTGAGTTTGATT
TACTCTCTTGG
+
FFFFFFFFFFFFFFFFFFFFFFFFFFFFFFFFFFF:FFFFFFFFFFFFFFFFFFFFFFFFFFFFFFFF
FFFFFFFFFFFFFFFFFFFFFFF:FFFFFFFFFFFFFFFF:FFFFFFFFFFFFFFFFFFFFFFFFFFF
FFFFFFF:FF:
@A00155:342:HHGFNDSXY:1:1309:31503:35164 2:N:0:GAACCTAG+TCCGCATA
GGTCTACATGCCGGACGGGGTGAGGTCTGAGAGTTACACTCAACCATTGGAGAACGGTTTTCTCCTCA
GCTCCAGCAGCATACTTGTCAGAAATAAGGTCACAGGAGTTGAGATGAGATATCAGGTGAGTTTGATT
TACTCTCTTGG
+
FFFFFFFFFFFFFFFFFFFFFFFFFFFFFFFFFFFFFFFFFFFFFFFFFFFFFFFFFFFFFFFFFFFF
FFFFFFFFF,FFFFFFFFFFFFFFFFFFFFFFFFFFFFFFFFFFFFFF:FFFFFFFFFFFFFFFFFF:
FFFFF::F:FF
@A00155:342:HHGFNDSXY:1:1409:28194:34569 2:N:0:GAACCTAG+TCCGCATA
GGTCTACATGCCGGACGGGGTGAGGTCTGAGAGTTACACTCAACCATTGGAGAACGGTTTTCTCCTCA
GCTCCAGCAGCATACTTGTCAGAAATAAGGTCACAGGAGTTGAGATGAGATATCAGGTGAGTTTGATT
TACTCTCTTGG
+
FFF:FFFFF:FFFFFFFFF::FFFFFF,FFFFFF,:FFFFF:FF,FFFFF:FFFFFFFF:FF,FF,FF
FF:FFFFFFFFFFFFFFF:FF:FF,FFF,FFFFFFF,FF:F:FFF:FFFFFFFFFFFFFFF,FFFF:F
FFFFFFFF:FF

```
@A00155:342:HHGFNDSXY:1:1415:5593:1501 2:N:0:GAACCTAG+TCCGCATA
GGTCTACATGCCGGACGGAGTGAGGTCTGAGAGTTACACTCAACCATTGGAGAACGGTTTTCTCCTCA
GCTCCAGCAGCATACTTGTCAGAAATAAGGTCACAGGAGTTGAGATGAGATATCAGGTGAGTTTGATT
TACTCTCTTGG
+
FFFFFFFFFFFFFFFFFFFFFFFFFFFFFFFFFFFFFFFFFFFFFFFFFFFFFFFFFFFFFFFFFFFF
FFFFFFFFFFFFFFFFFFFFFF::FFFFFFFFFFFFFFFFFFFFFFFFFFFFFFFFFFFFFFFFFFFF
FFFFFFFFFFF
@A00155:342:HHGFNDSXY:1:1462:14009:9784 2:N:0:GAACCTAG+TCCGCATA
GGTCTACATGCCGGACGGAGTGAGGTCTGAGAGTTACACTCAACCATTGGAGAACGGTTTTCTCCTCA
GCTCCAGCAGCATACTTGTCAGAAATAAGGTCACAGGAGTTGAGATGAGATATCAGTTGAGTTTGATT
TACTCTCTTGG
+
FFFFFFFFFFFFFFFF:FFFFFFFFFFFFFFFFFFFFFFFFFFFFFFFFFFFFFFFFFFFFFFFFFFF
FFFFFFFFFFFFFFFFFFFFFF:FFFFFFFFFFFFFFFFFFFFFFFFFFFFFFFFFFFFFFFFFFFFF
FFFFFFFFFFF
@A00155:342:HHGFNDSXY:1:1376:6903:21778 2:N:0:GAACCTAG+TCCGCATA
GGTCTACATGCCGGACGGAGTGAGGTCTGAGAGTTACACTCAACCATTGGAGAACGGTTTTCTCCTCA
GCTCCAGCAGCATACTTGTCAGAAATAAGGTCACAGGAGTTGAGATGAGATATCAGGTGAGTTTGATT
TACTCTCTTGG
+
FFFFFFFFFFFFFFFFFFFFFFFFFFFFFFFFFFF:FFFFFFFFFFFFFFFFFFFFFFFFFFFFFFFF
FFFFFFFFFFFFFFFFFFFFFFFFFFFFFFFFFFFFFFFFFFF::FFFFFFFFFFFFFFFFFFFFFFF
FFFFFFFF:FF
@A00155:342:HHGFNDSXY:1:1478:20898:18709 2:N:0:GAACCTAG+TCCGCATA
GGTCTACATGCCGGACGGAGTGAGGTCTGAGAGTTACACTCAACCATTGGAGAACGGTTTTCTCCTCA
GCTCCAGCAGCATACTTGTCAGAAATAAGGTCACAGGAGTTGAGATGAGATATCAGGTGAGTTTGATT
TACTCTCTTGG
+
FFFFFFFFFFFFFFFFFFFFFFFFFFFFFFFFFFFFFFFFFFFFFFFFFFFF:FFFFFFFFFFFFFFF
FFFFFFFFFFFFFFFFFFFFFFFFFFFFFFFFFF:FFFFFFFFFFFFFFFFFFFFFFFFFFFFFFFFF
FFFFFFF:FFF
@A00155:342:HHGFNDSXY:1:1537:18141:18004 2:N:0:GAACCTAG+TCCGCATA
GGTCTACATGCCGGACGGGGTGAGGTCTGAGAGTTACACTCAACCATTGGAGAACGGTTTTCTCCTCA
GCTCCAGCAGCATACTTGTCAGAAATAAGGTCACAGGAGTTGAGATGAGATATCAGGTGAGTTTGATT
TACTCTCTTGG
+
FFFFFFFFFFFFFFFFFFFFFFFFFFFFFFFFFFFFFFFFFFFFFFFFFFFFFFFFFFFFFFFFFFFF
FFFFFFFFFFFFFFFFFFFFFFFFFFFFFFFFFFFFFFFFFFFFFFFFFFFFFFFFFFFFFFFFFFFF
FFFFFFFFFFF
@A00155:342:HHGFNDSXY:1:1521:6858:20447 2:N:0:GAACCTAG+TCCGCATA
GGTCTACATGCCGGACGGGGTGAGGTCTGAGAGTTACACTCAACCATTGGAGAACGGTTTTCTCCTCA
GCTCCAGCAGCATACTTGTCAGAAATAAGGTCACAGGAGTTGAGATGAGATATCAGGTGAGTTTGATT
TACTCTCTTGG
+
FFFFFFFFFF:FFFFFF:FFFFFFFFFFFFFFFF:FFFFFFFFFFF:FFFFFFFFF,FFF,FFFF,FF
FFFFFFFFFFFFFFF:FFFFFFFFFFFFFFF::FFFFFFFFFF,:F:FFFFFFFFFFFFFFFFFFFFF
FFFFFFFFFFF
@A00155:342:HHGFNDSXY:1:1539:27706:13620 2:N:0:GAACCTAG+TCCGCATA
GGTCTACATGCCGGACGGAGTGAGGTCTGAGAGTTACACTCAACCATTGGAGAACGGTTTTCTCCTCA
GCTCCAGCAGCATACTTGTCAGAAATAAGGTCACAGGAGTTGAGATGAGATATCAGGTGAGTTTGATT
TACTCTCTTGG
+
FFFFFFFF:FFFFFFFFFFF,FFFF:FFFFF::FFFFFFFFF:FFFFFFFFFFFFFFFFFFF:FFFFF
```

```
FFFFFF:FFFFFFFFFFFFFFF::FFFFFFFFFFFF,FFFFFFFFFFFFFF:FFFF:FFFFFFFFFFF
FF,F,F,FF:F
@A00155:342:HHGFNDSXY:1:1539:27706:13683 2:N:0:GAACCTAG+TCCGCATA
GGTCTACATGCCGGACGGAGTGAGGTCTGAGAGTTACACTCAACCATTGGAGAACGGTTTTCTCCTCA
GCTCCAGCAGCATACTTGTCAGAAATAAGGTCACAGGAGTTGAGATGAGATATCAGGTGAGTTTGATT
TACTCTCTTGG
+
FFFFFFFFFFFFFFFFFFFFFFFFFFFFFFFFFFFFFFFFFFFFFFFFFFFFFFFFFFFFFFFFFFFF
FFFFFFFFFFFFFFFFFFFFFFFFFFFFFFFFFFFFFFFFFFFFFFFFFFFFFFFFFFFFFFFFFFF:
FFF:FFFFFFF
@A00155:342:HHGFNDSXY:1:1543:19397:11757 2:N:0:GAACCTAG+TCCGCATA
GGTCTACATGCCGGACGGGGTGAGGTCTGAGAGTTACACTCAACCATTGGAGAACGGTTTTCTCCTCA
GCTCCAGCAGCATACTTGTCAGAAATAAGGTCACAGGAGTTGAGATGAGATATCAGGTGAGTTTGATT
TACTCTCTTGG
+
FFFFFFFFFFFFFFFFFFFFFFFFFFFFFFFFFFFFFFFFFFFFFFFFFFFFFFFFFFFFFFFFFFFF
FFFFFFFFFFFFFFFFFFFFFFFFFFFFFFFFFFFFFFFFFFFFFFFFFFFFFFFFFFFFFFFFFFFF
FFFFFFFFFFF
@A00155:342:HHGFNDSXY:1:1539:32759:20118 2:N:0:GAACCTAG+TCCGCATA
GGTCTACATGCCGGACGGGGTGAGGTCTGAGAGTTACACTCAACCATTGGAGAACGGTTTTCTCCTCA
GCTCCAGCAGCATACTTGTCAGAAATAAGGTCACAGGAGTTGAGATGAGATATCAGGTGAGTTTGATT
TACTCTCTTGG
+
FFFFFFFFFFFFFFFFFFFFFFFFFFFFFFFFFFFFFFFFFFFFF:FFFFFFFFFFFFFFFFFFFFFF
FFFFFFFFFFFFFFFFFFFFFFF:F,FFFFFFFFFFFFFF:FFFFFFFFF:FFFFFFFFFF:FFFFFF
FFFFFFFFFFF
@A00155:342:HHGFNDSXY:1:1630:31711:17581 2:N:0:GAACCTAG+TCCGCATA
GGTCTACATGCCGGACGGGGTGAGGTCTGAGAGTTACACTCAACCATTGGAGAACGGTTTTCTCCTCA
GCTCCAGCAGCATACTTGTCAGAAATAAGGTCACAGGAGTTGAGATGAGATATCAGGTGAGTTTGATT
TACTCTCTTGG
+
FFFFFFFFFFFFFFFFFFFFFFFFFFFFFFFFFFFFFFFFFFFFFFFFFFFFF:FFFFFFFFFFFFFF
FFFFFFFFFFFFFFFFFFFFFFFFFFFFFFFFFFFFFFFFFFFFFFFFFFF:FFFFFFFFFFFFFFFF
FFFFFFFFFFF
@A00155:342:HHGFNDSXY:1:1666:25690:3709 2:N:0:GAACCTAG+TCCGCATA
GGTCTACATGCCGGACGGGGTGAGGTCTGAGAGTTACACTCAACCATTGGAGAACGGTTTTCTCCTCA
GCTCCAGCAGCATACTTGTCAGAAATAAGGTCACAGGAGTTGAGATGAGATATCAGGTGAGTTTGATT
TACTCTCTTGG
+
FFFFFFFFFFFFFFFFFFFFFFFFFFFFFFFFFFFFFFFFFFFFFFFFFFFFFFFFFFFFFFFFFFFF
FFFFFFFFFFFFFFFF,FFFFFFFFFFFFFFFFFFFFFFFFFFFFFFFFFFFFFFFFFFFFFFFFFFF
FFFFFFFFFFF
@A00155:342:HHGFNDSXY:1:2113:9227:10864 2:N:0:GAACCTAG+TCCGCATA
GGTCTACATGCCGGACGGGGTGAGGTCTGAGAGTTACACTCAACCATTGGAGAACGGTTTTCTCCTCA
GCTCCAGCAGCATACTTGTCAGAAATAAGGTCACAGGAGTTGAGATGAGATATCAGGTGAGTTTGATT
TACTCTCTTGG
+
FFFFFFFFFFFFFFFFFFFFFFFFFFFFFFFFFFFFFFFFFFFFFFFFFFFFFFFFFFF:FFFFFFFF
FFFFFFFFFFFFFFFFFF:F:FFFFFFFFFFFFFFFFFFFFFFFFFFFFFFFFFFFFFFFFF,FFFFF
FFFFFFFFFFF
@A00155:342:HHGFNDSXY:1:2166:8757:20635 2:N:0:GAACCTAG+TCCGCATA
GGTCTACATGCCGGACGGAGTGAGGTCTGAGAGTTACACTCAACCATTGGAGAACGGTTTTCTCCTCA
GCTCCAGCAGCATACTTGTCAGAAATAAGGTCACAGGAGTTGAGATGAGATATCAGGTGAGTTTGATT
TACTCTCTGGG
```

```
+
FFFFFFFFFFFFFFFFFFFFFFFFFFFFFFFFFFFFFFFFFFFFFFFFFFFFFFFFFFFFFFFF:FFFFFF
FFF:FFFFFFF:FFFFFFFFFFFFFFFFFFFFFF:F:FFFFFFFFFFF,FFF:FFFFF,FFFFFFFFFF
FFFF:FFF,FF
@A00155:342:HHGFNDSXY:1:2235:29053:12446 2:N:0:GAACCTAG+TCCGCATA
GGTCTACATGCCGGACGGAGTGAGGTCTGAGAGTTACACTCAACCATTGGAGAACGGTTTTCTCCTCA
GCTCCAGCAGCATACTTGTCAGAAATAAGGTCACAGGAGTTGAGATGAGATATCAGGTGAGTTTGATT
TACTCTCTTGG
+
FFFFFFFFFFFFFFFFFFFFFFFFFFFFFFFFFFFFFFFFFFFFFFFFFFFFFFFFFFFFFFFFFFFFF
FFFFFFFFFFFFFFFFFFFFFFFFFFFFFFFFFFFFFFFFFFFFFFFFFFFFFFFFFFFFFFFFFFFFF
FFFFFFF:FFF
@A00155:342:HHGFNDSXY:1:2338:25780:31172 2:N:0:GAACCTAG+TCCGCATA
GGTCTACATGCCGGACGGAGTGAGGTCTGAGAGTTACACTCAACCATTGGAGAACGGTTTTCTCCTCA
GCTCCAGCAGCATACTTGTCAGAAATAAGGTCACAGGAGTTGAGATGAGATATCAGGTGAGTTTGATT
TACTCTCTTGG
+
FFFFFFFFFFFFFFFF:,FFFFFFFFFFFFF:FFFFFFFFFFFFFFFFFFFFFFFFFFFFFFFFFFFFF
FFFFFFFFFFFFFFFFFFFFFFFFFFFFFFFFFFFFFFFFFFFFFFFFFFFFFFFFFFFFFFFFFFFF:
FFFFFFFFFFF
@A00155:342:HHGFNDSXY:1:2338:25861:31187 2:N:0:GAACCTAG+TCCGCATA
GGTCTACATGCCGGACGGAGTGAGGTCTGAGAGTTACACTCAACCATTGGAGAACGGTTTTCTCCTCA
GCTCCAGCAGCATACTTGTCAGAAATAAGGTCACAGGAGTTGAGATGAGATATCAGGTGAGTTTGATT
TACTCTCTTGG
+
FFFFFFFFFFFFFFFFFFFFFFFFF:FFFFFFFFFFF:F:FFFFFF:FFFFFFFFFFFFFFFFFFFFF
FFFFFFFFFFFFFFFFFFFFFF:FFFFFFFFFFFFFFFFFFFFF:FFFFFFFFFFFFFFF:FFFFFFFF
FFFFFFFFFFF
@A00155:342:HHGFNDSXY:1:2441:9579:33520 2:N:0:GAACCTAG+TCCGCATA
GGTCTACATGCCGGACGGAGTGAGGTCTGAGAGTTACACTCAACCATTGGAGAACGGTTTTCTCCTCA
GCTCCAGCAGCATACTTGTCAGAAATAAGGTCACAGGAGTTGAGATGAGATATCAGGTGAGTTTGATT
TACTCTCTTGG
+
FFFFFFFF:FF:FFFFFFFFFF:FFF,FFF,FFFFFFFFFFFF:FFFFFFFFFFF:FFFFFFFF:FFF
F:F:FFFFFFFFFFF,FFF:FFFFFFFFFFFFFFFFFFFFFF,F:FFFFFFFFFFFFFFFFF:FFFFF:
FFFFFFFFFF:
@A00155:342:HHGFNDSXY:1:2441:9670:34773 2:N:0:GAACCTAG+TCCGCATA
GGTCTACATGCCGGACGGAGTGAGGTCTGAGAGTTACACTCAACCATTGGAGAACGGTTTTCTCCTCA
GCTCCAGCAGCATACTTGTCAGAAATAAGGTCACAGGAGTTGAGATGAGATATCAGGTGAGTTTGATT
TACTCTCTTGG
+
FFFFFFFFFFFFFFFFFFFFFFFFFFFFFFFFFFFFFFFFFFFFFFFFFFFFFFFFFFFFFFFFFFFFF
FFFFFFFFFFFFFFFFFFFFFFFFFFFFFFFFFFFFFFFFFFF:FFFFFFFFFFFFFFF,FFFFFFFF
FFFFFFFFFFF
@A00155:342:HHGFNDSXY:1:2318:22092:33614 2:N:0:GAACCTAG+TCCGCATA
GGTCTACATGCCGGACGGGGTGAGGTCTGAGAGTTACACTCAACCATTGGAGAACGGTTTTCTCCTCA
GCTCCAGCAGCATACTTGTCAGAAATAAGGTCACAGGAGTTGAGATGAGATATCAGGTGAGTTTGATT
TACTCTCTTGG
+
FFFFFFFFFFFFFFFFFFF::FFFFFFFFFFFFFFFFFFFFFFFFFFFFFFFFFFFFFFFFF:F:FFFFF
FFFFFFF:FFFFFFFFFFFFFFFFFFFFFFFFFFFFFFFFFFF:FFFFFFFFFFFFFFFFFFFFFFFFF
FFFFFFFFFFF
@A00155:342:HHGFNDSXY:1:2513:18719:35070 2:N:0:GAACCTAG+TCCGCATA
GGTCTACATGCCGGACGGAGTGAGGTCTGAGAGTTACACTCAACCATTGGAGAACGGTTTTCTCCTCA
```

```
GCTCCAGCAGCATACTTGTCAGAAATAAGGTCACAGGAGTTGAGATGAGATATCAGGTGAGTTTGATT
TACTCTCTTGG
+
FFFFFFFFFFFFF:FFFFFFFFFFFFFFFFFFFFFF:FFFFFFFFFFFFFFFFFFFFFFFFFFFFFFFF
FFFFFFFFFFF:FFF:FFFFFFFFFFFFFFFFFFFFFFFFFFFFFFFFFFFFF:FFFFFFFFFFF,F
F,FFFFF,FFF
@A00155:342:HHGFNDSXY:1:2661:31810:7764 2:N:0:GAACCTAG+TCCGCATA
GGTCTACATGCCGGACGGGGTGAGGTCTGAGAGTTACACTCAACCATTGGAGAACGGTTTTCTCCTCA
GCTCCAGCAGCATACTTGTCAGAAATAAGGTCACAGGAGTTGAGATGAGATATCAGGTGAGTTTGATT
TACTCTCTTGG
+
FFFFFFFFFFFFFFFFFFFFFFFFFFFFFFFFFFFFFFFFFFFFFFFFFFFFFFFFFF:FFFFFFFFF
FFFFFFFFFFFFFFFFFFFFFFF:FFFFFFFFFFFFFFFFFFFFFFFFFFF:F:FFFFFFFFFFFFFF
FFFFFFFFFFF
@A00155:342:HHGFNDSXY:1:2661:32244:8015 2:N:0:GAACCTAG+TCCGCATA
GGTCTACATGCCGGACGGGGTGAGGTCTGAGAGTTACACTCAACCATTGGAGAACGGTTTTCTCCTCA
GCTCCAGCAGCATACTTGTCAGAAATAAGGTCACAGGAGTTGAGATGAGATATCAGGTGAGTTTGATT
TACTCTCTTGG
+
FFFFFFFFFFFFFFFFFFFFFFFFFFFFFFFFFFFF:FFFFFFFFFFFFFFFF:FFFF::F:FFFFFF
FF:FFFFFFFFFFFF:FFFFFFFFFFFFFFF:FFFFFFF,FFFFFFFFFF:,:FFFF:FFFFFFFF,F
FFFFFF:F:FF
@A00155:342:HHGFNDSXY:1:2668:12093:32330 2:N:0:GAACCTAG+TCCGCATA
GGTCTACATGCCGGACGGGGTGAGGTCTGAGAGTTACACTCAACCATTGGAGAACGGTTTTCTCCTCA
GCTCCAGCAGCATACTTGTCAGAAATAAGGTCACAGGAGTTGAGATGAGATATCAGGTGAGTTTGATT
TACTCTCTTGG
+
FFFFFFFFFFFFFFFFFF:FFFFFFFFFFFFFFFFFFFFFFFFFFFFFFFFFFFFFFFFFFFFFFFFF
FFFFFFFFFFFF:FFF:FFFFF:FFFFFFFFFFFFFFFF:FF:FFFFFFFFFFFFFFFFFFFFFFFFF
FFFFFFFFFFF
@A00155:342:HHGFNDSXY:1:1157:20202:16470 2:N:0:GAACCTAG+TCCGCATA
GGTCTACATGCCGGACGGGGTGAGGTCTGAGAGTTACACTCAACCATTGGAGAACGGTTTTCTCCTCA
GCTCCAGCAGCATACTTGTCAGAAATAAGGTCACAGGAGTTGAGATGAGATATCAGGTGAGTTTGATT
TACTCTCTTGGTTCA
+
FFFFFFFFFFFFFFFFFFFFFFFFFFFFFFFFFFF:FFFFFFFFFFFFFFFFFFFFFFFFFFFFFFFF
FFFFFFFFFF:FF:FFFFFFFF:FFFFFFFFFFFFFFFFFFFF:FFFF:F:F:FFFFFFF:FFFFFFF
FF:FF:FFFFFFFFF
@A00155:342:HHGFNDSXY:1:1239:17201:3568 2:N:0:GAACCTAG+TCCGCATA
GGTCTACATGCCGGACGGGGTGAGGTCTGAGAGTTACACTCAACCATTGGAGAACGGTTTTCTCCTCA
GCTCCAGCAGCATACTTGTCAGAAATAAGGTCACAGGAGTTGAGATGAGATATCAGGTGAGTTTGATT
TACTCTCTTGGTTCA
+
FFFFFFFFFFFFFFFFFFFFFFFFFFFFFFFFFFFFFFFFFFFFFFFFFFFFFFFFFFFFFFFFFFFF
FFFFFFFFFFFFFFFFFFFFFFFFFFF:FFFFFFFFFFFFFFFF:FFFFFFFFFFFFFFFFFFFFFFF
FFFFF:FFFFFFFFF
@A00155:342:HHGFNDSXY:1:1239:24487:26052 2:N:0:GAACCTAG+TCCGCATA
GGTCTACATGCCGGACGGGGTGAGGTCTGAGAGTTACACTCAACCATTGGAGAACGGTTTTCTCCTCA
GCTCCAGCAGCATACTTGTCAGAAATAAGGTCACAGGAGTTGAGATGAGATATCAGGTGAGTTTGATT
TACTCTCTTGGTTCA
+
F:F:FFFFFFFFFF:FFFFFFFFFFFFFFFFFFFFFFFFFFFFFFFFFFF:FFFFFFFFFFFFFFFFFF
FFFFFFFFFFFFFFFFFFFFFFFFFF:FFF:FFFFFFFFFFFFFFFF:FFFFFFF:F:FFFF:FFFFFFF
FF:FFFFFFFFFFFF
```

```
@A00155:342:HHGFNDSXY:1:1244:5584:19930 2:N:0:GAACCTAG+TCCGCATA
GGTCTACATGCCGGACGGGGTGAGGTCTGAGAGTTACACTCAACCATTGGAGAACGGTTTTCTCCTCA
GCTCCAGCAGCATACTTGTCAGAAATAAGGTCACAGGAGTTGAGATGAGATATCAGGTGAGTTTGATT
TACTCTCTTGGTTCA
+
F,FFFFFFFFFFFFFFFFFFFFFFFFFF:FFFFFFFFFFFFFFFFFFFFFFFFFFFFFFFFFFFFFFF
FFFFFFFFFFFFFFFFFFFFFFFFFFFFFFFFFFFFFFFFFFFFFFFFFFFFFFF:FFFFFFFFFFFF
FFFFFFFFFFFFFFF
@A00155:342:HHGFNDSXY:1:1315:10926:15749 2:N:0:GAACCTAG+TCCGCATA
GGTCTACATGCCGGACGGGGTGAGGTCTGAGAGTTACACTCAACCATTGGAGAACGGTTTTCTCCTCA
GCTCCAGCAGCATACTTGTCAGAAATAAGGTCACAGGAGTTGAGATGAGATATCAGGTGAGTTTGATT
TACTCTCTTGGTTCA
+
FFFFFFFFFFFFFFFF:FFFFFFFFFFFFFFFFFFFFFFFFFFFFF:FFFFFFFFFFFFFFFFFFFFF
FFFFFFFFFFFFFFFFFFFFFFFFFFFFFFFFFFFFFFFFFF:FFFFFFFFFFFFFFFFFFFFFFFFF
FFFFFFF:FFFFFFF
@A00155:342:HHGFNDSXY:1:1571:16839:6543 2:N:0:GAACCTAG+TCCGCATA
GGTCTACATGCCGGACGGAGTGAGGTCTGAGAGTTACACTCAACCATTGGAGAACGGTTTTCTCCTCA
GCTCCAGCAGCATACTTGTCAGAAATAAGGTCACAGGAGTTGAGATGAGATATCAGGTGAGTTTGATT
TACTCTCTTGGTTCA
+
FFFFFFFFFFFFFFFFFFFFFFFFFFFFFFFFFFFFFFFFFFFFFFFFFFFFFFFFFFFFFFFFFFFF
FFFFFFFFFFFFFFFFFFFFFF:FFFFFFFFFFFFFFFFF:FFFFFFFFFFFFFFFFFFF:FFFFFFF
FFFFFFFFFFFFFFF
@A00155:342:HHGFNDSXY:1:1630:20564:8234 2:N:0:GAACCTAG+TCCGCATA
GGTCTACATGCCGGACGGGGTGAGGTCTGAGAGTTACACTCAACCATTGGAGAACGGTTTTCTCCTCA
GCTCCAGCAGCATACTTGTCAGAAATAAGGTCACAGGAGTTGAGATGAGATATCAGGTGAGTTTGATT
TACTCTCTTGAGATC
+
FFFFFFFF:FFFFFFFFFFFFFFFFFFFFFFFFFFFFFFFFFFFFF,FFFFFFFFFFFFFFFFFFFFF
FFFFFFFF:FFFFFF:FFFFFFF:FF:FFFFFFFFFFFFFFFFFFFFFFFFFFFFFFFFFFFFFFFFF
FFFFFFFFFFFFFFF
@A00155:342:HHGFNDSXY:1:1651:29921:28667 2:N:0:GAACCTAG+TCCGCATA
GGTCTACATGCCGGACGGGGTGAGGTCTGAGAGTTACACTCAACCATTGGAGAACGGTTTTCTCCTCA
GCTCCAGCAGCATACTTGTCAGAAATAAGGTCACAGGAGTTGAGATGAGATATCAGGTGAGTTTGATT
TACTCTCTTGGTTCA
+
FFFFFFFFFFFFFFFFFFFFFFFFFFFFFFFFFFFFFFFFFFFFFF,FFFFFFFFFFFFFFFFFFFFF
FFFFFFFFFFFFFFFFFFFFFFFFFFFFFFFFFFFFFFFFFFFFFFFFFFFFFFFFFFFFFFFFFFFF
FF:FFFFFFFFFFFF
@A00155:342:HHGFNDSXY:1:2307:10176:1642 2:N:0:GAACCTAG+TCCGCATA
GGTCTACATGCCGGACGGAGTGAGGTCTGAGAGTTACACTCAACCATTGGAGAACGGTTTTCTCCTCA
GCTCCAGCAGCATACTTGTCAGAAATAAGGTCACAGGAGTTGAGATGAGATATCAGGTGAGTTTGATT
TACTCTCTTGGTTCA
+
FFFFFFFFFFFFFFFFFFFFFFFFFFFFFFFFFFFFFFFFFFFFFFFFFFFFFFFFFFFFFFFFFFFF
FFFFFFFFFFFFFFFFFFFFFFF:FFFFFFFFFFFFFFFFFFF:FFFFFFFFFFFFFFFFFFFFF:FF
FFFFFFFFFFFFFFF
@A00155:342:HHGFNDSXY:1:2313:20256:14434 2:N:0:GAACCTAG+TCCGCATA
GGTCTACATGCCGGACGGGGTGAGGTCTGAGAGTTACACTCAACCATTGGAGAACGGTTTTCTCCTCA
GCTCCAGCAGCATACTTGTCAGAAATAAGGTCACAGGAGTTGAGATGAGATATCAGGTGAGTTTGATT
TACTCTCTTGGTTCA
+
FFFFFFFFFFFFFFFFFFFF:FFFFFFFFFFFF,FFFFFFFFFFFF,:FFF:F:FFF:FFF,:FFFFF
```

```
FFFFFFFFFF:FFFFFFFFF:FFFFFF:FFF:FFF:FF:F:FFFF,FF:FFFF,F:,FFFFF:FF,F:
FF,FFF:,F:FF:FF
@A00155:342:HHGFNDSXY:1:2349:2618:5963 2:N:0:GAACCTAG+TCCGCATA
GGTCTACATGCCGGACGGGGTGAGGTCTGAGAGTTACACTCAACCATTGGAGAACGGTTTTCTCCTCA
GCTCCAGCAGCATACTTGTCAGAAATAAGGTCACAGGAGTTGAGATGAGATATCAGGTGAGTTTGATT
TACTCTCTTGGTTCA
+
FFFFFFFFFFFFFFFFFFFFFFFFFFFFFFFFFFFFFFFFFFFFFFFFFFFFF:FFFFFFFFFFFFFF
FFFF,FFFFFF:FFFFFFFF:FFFFFFFFFFFFFFFFFFFFFFFFFFFFFF:FFFFFFFFFFFFFF:,
FF:FFF:FFFFFFFF
@A00155:342:HHGFNDSXY:1:2414:9760:5400 2:N:0:GAACCTAG+TCCGCATA
GGTCTACATGCCGGACGGAGTGAGGTCTGAGAGTTACACTCAACCATTGGAGAACGGTTTTCTCCTCA
GCTCCAGCAGCATACTTGTCAGAAATAAGGTCACAGGAGTTGAGATGAGATATCAGGTGAGTTTGATT
TACTCTCTTGGTTCA
+
FFFFFFFFFFFFFFFFFFFFFFFFFFFFFFFFFFFFFFFFFFFFFFFFFFFFFFFFFFFFFFFFFFFF
FFFFFFFFFFFFFFFFFFFFFFFFFFFFFFFFFFFFFFFFFFFFFFFFFFFFFFFFFFFFFFFFFF:F
FF,FFFFFFFFFFFF
@A00155:342:HHGFNDSXY:1:2466:32217:26224 2:N:0:GAACCTAG+TCCGCATA
GGTCTACATGCCGGACGGGGTGAGGTCTGAGAGTTACACTCAACCATTGGAGAACGGTTTTCTCCTCA
GCTCCAGCAGCATACTTGTCAGAAATAAGGTCACAGGAGTTGAGATGAGATATCAGGTGAGTTTGATT
TACTCTCTTGGTTCA
+
FFFFFFFFFFFFFFFFFFFFFFFFFFFFFFFFFFFFFFFFFF:FFFFFFFFFFFFFFFFFFFFFFFFF
FFFFFFFFFFFFFFFFFFFFFFFFFFFFFFFFFFFFFFFFFFFFFFFFFFF:FFFFFFFFFFFFFFFF
FFFFF:FF,FF:FFF
@A00155:342:HHGFNDSXY:1:2445:10710:16783 2:N:0:GAACCTAG+TCCGCATA
GGTCTACATGCCGGACGGGGTGAGGTCTGAGAGTTACACTCAACCATTGGAGAACGGTTTTCTCCTCA
GCTCCAGCAGCATACTTGTCAGAAATAAGGTCACAGGAGTTGAGATGAGATATCAGGTGAGTTTGATT
TACTCTCTTGAGATC
+
FF:FFFFFFFFFFFFFFFFFFFFFFFFFFFFFFFFFFFFFFFFFFFFFFFFFFFFFFFFFFFFFFFFF
FFFFFFFFFFFFFFFFFFFFFFFFFFFFFFFFFFFFFFFFFFFFFFFFFFFFFFFFFFFFFFFFFFFF
FFFFFFFFFFFFFFF
@A00155:342:HHGFNDSXY:1:2603:5077:24377 2:N:0:GAACCTAG+TCCGCATA
GGTCTACATGCCGGACGGGGTGAGGTCTGAGAGTTACACTCAACCATTGGAGAACGGTTTTCTCCTCA
GCTCCAGCAGCATACTTGTCAGAAATAAGGTCACAGGAGTTGAGATGAGATATCATGTGAGTTTGATT
TACTCTCTTGGTTCA
+
F:FFFFFFFFFFFFFFFFF,FFFFFFFFFFFFFFFFFFFFFFFFFFFF:FFF:FFFFF,FFF:FFFFFF
FFFFFF:FFFFFFFFFF:FFFFFFFFFFFFFFFFF:FFFFF,FF:FFFFFFFFFF,FFFF:F,FFFFF
FFFFFFFFFF:FFFF
@A00155:342:HHGFNDSXY:1:1303:4300:34303 1:N:0:GAACCTAG+TCCGCATA
GTCTACATGCCGGACGGAGTGAGGTCTGAGAGTTACACTCAACCATTGGAGAACGGTTTTCTCCTCAG
CTCCAGCAGCATACTTGTCAGAAATAAGGTCACAGGAGTTGAGATGAGATATCAGGTGAGTTTGATTT
ACTCTCTTGGTTCAC
+
FFFFFFFFFFF:FFFFFFFFFF:FFFFFFFFFF:FFFFFFFFFFFFFFFFFF:FF,FFFF:FF,FFFF
FFF:FFFFFFFFFF:F:FFFFFFFFFFFFFFFFFF,FFF:FFFFFFF:FFFF,FFFFFFFFFFFFFFF
FFFFFFFFFFFFFFF
@A00155:342:HHGFNDSXY:1:1432:2691:25880 1:N:0:GAACCTAG+TCCGCATA
GTCTACATGCCGGACGGAGTGAGGTCTGAGAGTTACACTCAACCATTGGAGAACGGTTTTCTCCTCAG
CTCCAGCAGCATACTTGTCAGAAATAAGGTCACAGGAGTTGAGATGAGATATCAGGTGAGTTTGATTT
ACTCTCTTGGTTCAC
```

+
FFFFFFFFFFFFFFFFFFFFFFFFFFFFFFFFFFFFFFFFFFFFFFFFFFFFFFFFFFFFFFFFFF
FFFFFFFFFFFFFFFFFFFFFFFFFF:FFFFFFFFFFFFFFFFFFFFFFFFFFFFFFFFFFFFFFF
FFFFFFFFFFFFFFF
@A00155:342:HHGFNDSXY:1:1459:24867:21324 1:N:0:GAACCTAG+TCCGCATA
GTCTACATGCCGGACGGAGTGAGGTCTGAGAGTTACACTCAACCATTGGAGAACGGTTTTCTCCTCAG
CTCCAGCAGCATACTTGTCAGAAATAAGGTCACAGGAGTTGAGATGAGATATCAGGTGAGTTTGATTT
ACTCTCTTGGTTCAC
+
FFFFFFFFFFFFFFFFFFFFFFFFFFFFFFFFFFFFFFFFFFFFFFFFFFFFFFFFFFFFFFFFFF
FFFFFFFFFFFFFFFFFFFFFFFFFFFFFFFFFFFFFFFFFFFFFFFFFFFFFFFFFFFFFFFFFF
FFFFFFFFFFFFFFF
@A00155:342:HHGFNDSXY:1:2308:25635:4648 1:N:0:GAACCTAG+TCCGCATA
GTCTACATGCCGGACGGGGTGAGGTCTGAGAGTTACACTCAACCATTGGAGAACGGTTTTCTCCTCAG
CTCCAGCAGCATACTTGTCAGAAATAAGGTCACAGGAGTTGAGATGAGATATCAGGTGAGTTTGATTT
ACTCTCTTGGTTCAC
+
FFFFFFFFFFFFFFFFFFFFFFFFFFFFFFFFFFFFFFFFFFFFF:FFFFFFFFFFFFFFFFFFFF
FFFFFFFFFFFFFFFFFFFFFFFFFFFFFFFFFFFFFFFFFFFFFFFFFFFFFFFFFFFFFFFFFF
FFFFFFFFFFFFFFF
@A00155:342:HHGFNDSXY:1:2634:10230:5368 1:N:0:GAACCTAG+TCCGCATA
GTCTACATGCCGGACGGAGTGAGGTCTGAGAGTTACACTCAACCATTGGAGAACGGTTTTCTCCTCAG
CTCCAGCAGCATACTTGTCAGAAATAAGGTCACAGGAGTTGAGATGAGATATCAGGTGAGTTTGATTT
ACTCTCTTGGTTCAC
+
FFFFFFFFFFFFFFFFFFFFFFFFFFFFFFFFFFFFFFFFFFFFFFFFFFFFFFFFFFFFFFFFFF
FFFFFFFFFFFFFFFFFFFFFFFFFFFFFFFFFFFFFFFFFFFFFFFFFFFFFFFFFFFFFFFFFF
FFFFFFFFFFFFFFF
@A00155:342:HHGFNDSXY:1:1217:5665:32002 2:N:0:GAACCTAG+TCCGCATA
GTCTACATGCCGGACGGGGTGAGGTCTGAGAGTTACACTCAACCATTGGAGAACGGTTTTCTCCTCAG
CTCCAGCAGCATACTTGTCAGAAATAAGGTCACAGGAGTTGAGATGAGATATCAGGTGAGTTTGATTT
ACTCTCTTGGTTCAC
+
FFFFFFFFFFFFFFFFFFFFFFFFFFFFFFFFFFFFFFFFFFFFFFFFFFFFFFFFFFFFFFFFFF
FFFFFFFFFFFFFFFFFFFFFFFFFFFFFFFFFFF:FFFFFFFFFFFFFFFFFFFFFFFFFFFFFF
FFFFFFFFF:FFFFF
@A00155:342:HHGFNDSXY:1:1642:17273:24956 2:N:0:GAACCTAG+TCCGCATA
GTCTACATGCCGGACGGGGTGAGGTCTGAGAGTTACACTCAACCATTGGAGAACGGTTTTCTCCTCAG
CTCCAGCAGCATACTTGTCAGAAATAAGGTCACAGGAGTTGAGATGAGATATCAGGTGAGTTTGATTT
ACTCTCTTGGTTCAC
+
FFFFFFFFFFFFFFFFFFFFFFFFFFFFFFFFFFFFFFFFFFFFFFFFFFFFFFFF:FFFFFFFFF
FFFFFFFFFFFFFFFFFFFFFF:FFFFFFFFFFFFFFF:FFFFFFFFFFFFFFFFFFFFFFFFFFF
FFFFFFFFFFFFFFF
@A00155:342:HHGFNDSXY:1:2578:8458:10379 2:N:0:GAACCTAG+TCCGCATA
GTCTACATGCCGGACGGGGTGAGGTCTGAGAGTTACACTCAACCATTGGAGAACGGTTTTCTCCTCAG
CTCCAGCAGCATACTTGTCAGAAATAAGGTCACAGGAGTTGAGATGAGATATCAGGTGAGTTTGATTT
ACTCTCTTGGTTCAC
+
FFFFFFFFFFFFFFFFFFFFFFFFFFFFFFFFFFFFFFFFFFFFFFFFFFFFFFFFFFFFFFFFFF
FFFFFFFFFFFFFFFFFFFFFFFFFFFFFFFFFFFFFFFFFFFFFFFFFFFFFFFFFFFFFF,FFF
FFFFFFFFFFFFF:F
@A00155:342:HHGFNDSXY:1:1616:7319:8907 1:N:0:GAACCTAG+TCCGCATA
TCTACATGCCGGACGGGGTGAGGTCTGAGAGTTACACTCAACCATTGGAGAACGGTTTTCTCCTCAGC

```
TCCAGCAGCATACTTGTCAGAAATAAGGTCACAGGAGTTGAGATGAGATATCAGGTGAGTTTGATTTA
CTCTCTTGGTTCACA
+
FF:FFFFFFFFF,FFFFFFFFFFFFFFFFF:FFFFFF,FFF:F,F::,FF:,FFF:FFFFFFFFFFFFFF
FFFFFFFFFFFF::FFFF:FFFFF:FF:F:FFFFFFFFFF:FFFFF,::F,FFFF,FFFFFFFFFFFFFF
FFFFFFFFFFF:FFF
@A00155:342:HHGFNDSXY:1:2121:15790:8985 2:N:0:GAACCTAG+TCCGCATA
TCTACATGCCGGACGGGGTGAGGTCTGAGAGTTACACTCAACCATTGGAGAACGGTTTTCTCCTCAGC
TCCAGCAGCATACTTGTCAGAAATAAGGTCACAGGAGTTGAGATGAGATATCAGGTGAGTTTGATTTA
CTCTCTTGGTTCACA
+
FFFFFFFFFFFFFFFFFFFFFFFFFFFFFFFFFFFFFFFFFFFFFFFFFFFFFFFFFFFFFFFFFFFFFF
FFFFFFFFFFFFFFFFFFFFFFFFFFFFFFFFFFFFFFFFFFFFFFFFFFFFFFFFFFFFFFFFFFFFFF
FFFFFFFFFFFFFFF
@A00155:342:HHGFNDSXY:1:2269:32036:33364 2:N:0:GAACCTAG+TCCGCATA
TCTACATGCCGGACGGGGTGAGGTCTGAGAGTTACACTCAACCATTGGAGAACGGTTTTCTCCTCAGC
TCCAGCAGCATACTTGTCAGAAATAAGGTCACAGGAGTTGAGATGAGATATCAGGTGAGTTTGATTTA
CTCTCTTGGTTCACA
+
FFFFFFFFFFFFFFFFFFFFFFFFFFFFFFFFFFFFFFFFFFFFFFFFFFFFFFFFFFFFFFFFFFFFFF
FFFFFFFFFFFFFFFFFFFFFFFFFFFFFFF:FFFFFFFFFFFF,FFFFFFFFFF:FFFFFFFFFFFFFF
:FFFF,FFFFFFFFF
@A00155:342:HHGFNDSXY:1:2525:23863:17926 2:N:0:GAACCTAG+TCCGCATA
TCTACATGCCGGACGGAGTGAGGTCTGAGAGTTACACTCAACCATTGGAGAACGGTTTTCTCCTCAGC
TCCAGCAGCATACTTGTCAGAAATAAGGTCACAGGAGTTGAGATGAGATATCAGGTGAGTTTGATTTA
CTCTCTTGGTTCACA
+
FFFFFFFFFFFFFFFFFFFFFFFFFFFFFFFFFFFFFFFFFFFFFFFFFFFFFFFFFFFFFFFFFFFFFF
FFFFFFFFFFFFFFFFFFFFF::FFFFFFFFFFFFFFFFFFFFFFFFFFFFFFFFFFFFFFFFFFFFFFF
FFFFF:FFFFFFFFF
@A00155:342:HHGFNDSXY:1:2525:22706:19805 2:N:0:GAACCTAG+TCCGCATA
TCTACATGCCGGACGGAGTGAGGTCTGAGAGTTACACTCAACCATTGGAGAACGGTTTTCTCCTCAGC
TCCAGCAGCATACTTGTCAGAAATAAGGTCACAGGAGTTGAGATGAGATATCAGGTGAGTTTGATTTA
CTCTCTTGGTTCACA
+
FFFFFFFFFFFFFFFFFFF:FFFFFFFFFFFFFFFFFFFFFFFFFFFFFFFFFFFFFFFFFFFFFFFFFF
FFFFFFFFFFFFFFFFFFFFFFFFFFFFFFFFFFFFFFFFFFFFFFFFFFFFFFFFFFFFFFFFFF:FFF
FFF:FFFFFFFFFFF
@A00155:342:HHGFNDSXY:1:2578:8287:2628 2:N:0:GAACCTAG+TCCGCATA
TCTACATGCCGGACGGGGTGAGGTCTGAGAGTTACACTCAACCATTGGAGAACGGTTTTCTCCTCAGC
TCCAGCAGCATACTTGTCAGAAATAAGGTCACAGGAGTTGAGATGAGATATCAGGTGAGTTTGATTTA
CTCTCTTGGTTCACA
+
FFFFFFFFFFFFFFFFFFFFFFFFFFFFFFFFFFFFFFFFFFFFFFFFFFFFFFFFFFFFFFFFFFFFFF
FFFFFFFFFFFFFFFFFFFFFFFFFFFFFFFFFFFFFFFFFFFFFFFFFFFFFFFFFFFFFFFFFFFFFF
FFFFFFFFFFFFFFF
@A00155:342:HHGFNDSXY:1:2101:25229:21731 2:N:0:GAACCTAG+TCCGCATA
CTACATGCCGGACGGAGTGAGGTCTGAGAGTTACACTCAACCATTGGAGAACGGTTTTCTCCTCAGCT
CCAGCAGCATACTTGTCAGAAATAAGGTCACAGGAGTTGAGATGAGATATCAGGTGAGTTTGATTTAC
TCTCTTGGTTCA
+
:::F:FFFFFF:FFF:F:F:FFFFFF:F:FFF:FFF:FFFFF:FFFF:F:FFFF:FFFF:FF:F:FFF
FF:F::FF:F:F::F:F:F:FFF::FF:FFF:FF:FF:F:F:FF:::F::F:F::F:F:FFF:FFF::
:::FFFF:FF:F
```

```
@A00155:342:HHGFNDSXY:1:1266:13277:21574 2:N:0:GAACCTAG+TCCGCATA
CTACATGCCGGACGGGGTGAGGTCTGAGAGTTACACTCAACCATTGGAGAACGGTTTTCTCCTCAGCT
CCAGCAGCATACTTGTCAGAAATAAGGTCACAGGAGTTGAGATGAGATATCAGGTGAGTTTGATTTAC
TCTCTTGGTTCACAT
+
FFFFFFFFFFFFFF:FF:FFFFFFFFFFFFFFFFFFFFFFFFFFFFFFFFF:FFF,FFFFFFFFFFFF
FFFFFFFFFFFFF:FFFFFF,F:F:F::FFFFFFFFF,FFFFFFFFFFFFFFFFFFFFF:FFF,FFFF
FFFFFFFFFFFFFFF
@A00155:342:HHGFNDSXY:1:1669:1832:20040 1:N:0:GAACCTAG+TCCGCATA
TACATGCCGGACGGGGTGAGGTCTGAGAGTTACACTCAACCATTGGAGAACGGTTTTCTCCTCAGCTC
CAGCAGCATACTTGTCAGAAATAAGGTCACAGGAGTTGAGATGAGATATCAGGTGAGTTTGATTTACT
CTCTTGGTTCACATC
+
FFFFFFFFFFFFFFFFFFFFFFFFFFFFFFFFFFFFFFFFFFFFFFFFFFFFFFFFFFFFFFFFFFFF
FFFFFFFFFFFFFFFFFFFFFFFFFFFFFFF:FFFFFFFFFFFFFFFFFFFFFFFFFFFFFFF:FFFFFF
FFFFFFFFFFFFFFF
@A00155:342:HHGFNDSXY:1:1570:19307:16078 1:N:0:GAACCTAG+TCCGCATA
CATGCCGGACGGGGTGAGGTCTGAGAGTTACACTCAACCATTGGAGAACGGTTTTCTCCTCAGCTCCA
GCAGCATACTTGTCAGAAATAAGGTCACAGGAGTTGAGATGAGATATCAGGTGAGTTTGATTTACTCT
CTTGGTTCACATCAC
+
FFFFFFFF:FFFFFFFFFFFFFFFFFFFFFFFFFFFFFFFFFFFFFFFFFFFFFFFFFFFFFFFFFFF
FFFFFFFFFFFFFFFFFFFFFFFFFFFFFFFFFFFFFFFFFFFFF:FFFFFFFFFFFFFFFFFFFFFF
FFFFFFFFFFFFFFF
@A00155:342:HHGFNDSXY:1:2423:15564:26099 1:N:0:GAACCTAG+TCCGCATA
CATGCCGGACGGGGTGAGGTCTGAGAGTTACACTCAACCATTGGAGAACGGTTTTCTCCTCAGCTCCA
GCAGCATACTTGTCAGAAATAAGGTCACAGGAGTTGAGATGAGATATCAGGTGAGTTTGATTTACTCT
CTTGGTTCACATCAC
+
FFFFFFFFFFFFFFFFFFFFFFFFFFFFFFFFFFFFFFFFFFFFFFFFFFFFFFFFFFFFFFFFFFFF
FFFFFFFFFFFFFFFFFFFFFFFFFFFFFFF:FFFFFFFFFFFFFFFFFFFFFFFFFFFFFFFFFFFF
FFFFFFFFFFFFFFF
@A00155:342:HHGFNDSXY:1:2101:29206:8296 2:N:0:GAACCTAG+TCCGCATA
CATGCCGGACGGGGTGAGGTCTGAGAGTTACACTCAACCATTGGAGAACGGTTTTCTCCTCAGCTCCA
GCAGCATACTTGTCAGAAATAAGGTCACAGGAGTTGAGATGAGATATCAGGTGAGTTTGATTTACTCT
CTTGGTTCACATCAC
+
FFFFFFFFFFFFFFFFFFFFFFFFFFFFFFFFFFFFFFFFFFFFFFFFFFFFFFFFFFFFFFFFFFFF
F:FFFFFFFFFFFFFFFFFFFFFF:FFFFFF:FFFF:FFFFFFFFFFFFFFFFFFF:,FFFFFFFFFF
FFFFF,FFFFFFFFF
@A00155:342:HHGFNDSXY:1:2173:31901:20228 2:N:0:GAACCTAG+TCCGCATA
ATGCCGGACGGAGTGAGGTCTGAGAGTTACACTCAACCATTGGAGAACGGTTTTCTCCTCAGCTCCAG
CAGCATACTTGTCAGAAATAAGGTCACAGGAGTTGAGATGAGATATCAGGTGAGTTTGATTTACTCTC
TTGGTTCACATCACC
+
FFFFFFFFFFFFFFFFFFFFFFFFFFFFFFFFFF:FFFFFFFFFFF:FFFFFFFFFFFFFFFFFFFFF
FFFFF,FF:FFFFFFFFFFF:FFFFFFFFFFF,FFFFF:FFFF:FFFFFFFFFFFFFFFF:FFFFF:F
F::FF:FFFFFFFFF
@A00155:342:HHGFNDSXY:1:2450:12120:29684 2:N:0:GAACCTAG+TCCGCATA
CCGGACGGAGTGAGGTCTGAGAGTTACACTCAACCATTGGAGAACGGTTTTCTCCTCAGCTCCAGCAG
CATACTTGTCAGAAATAAGGTCACAGGAGTTGAGATGAGATATCAGGTGAGTTTGATTTACTCTCTTG
GTTCACATCACCTAT
+
F:FFFFFFFFFFFFFFFFFFFFFFFFFFFFFFFFFFFFFFF:FFFFFFFFFFFFFFFFFFFFFFFFFFFF
```

FFFFFF:F:FFFFFFFFF,:FFFFFFFFFFFFFFFFFFFFFFFFFFFFFFFFFFFFFFFFFF:F,FFF
:FFF:FFFFFFFFFF
@A00155:342:HHGFNDSXY:1:1141:9245:5666 2:N:0:GAACCTAG+TCCGCATA
GGACGGGGTGAGGTCTGAGAGTTACACTCAACCATTGGAGAACGGTTTTCTCCTCAGCTCCAGCAGCA
TACTTGTCAGAAATAAGGTCACAGGAGTTGAGATGAGATATCAGGTGAGTTTGATTTACTCTCTTGGT
TCACATCACCTGTTT
+
FFFFFFFFFFFFFFFFFFFFFFFFFFFFFFFFFFFFFFFFFFFFFFFFFFFFFFFFFFFFFFFFFFFF
FFFFFFFFFFFFFFFFFFFFFFFFFFF:FFFFFFFFFF,FFFFFFFFFFFFFFFFFFFFFFFFFFFFF
FFFFFFFFFFFFFFF
@A00155:342:HHGFNDSXY:1:2139:4933:23062 2:N:0:GAACCTAG+TCCGCATA
GGACGGGGTGAGGTCTGAGAGTTACACTCAACCATTGGAGAACGGTTTTCTCCTCAGCTCCAGCAGCA
TACTTGTCAGAAATAAGGTCACAGGAGTTGAGATGAGATATCAGGTGAGTTTGATTTACTCTCTTGGT
TCACATCACCTGTTT
+
FFFFFFFFFFFFFFFFFFFFFFFFFFFFFFFFFFFFFFFFFFFFFFFFFFFFFFFFFFF:FFFFFFFF
FFFFFFFFFFFFF:FFFFFFFFFFFFFFFFFFFFFFFFFFFFFFFFFFFFFFFFFFFFFFFFFFFFFF
FFFFFFFFFFFFFFF
@A00155:342:HHGFNDSXY:1:1342:21594:12117 2:N:0:GAACCTAG+TCCGCATA
TCTGGGGTGAGGTCTGAGAGTTACACTCAACCATTGGAGAACGGTTTTCTCCTCAGCTCCAGCAGCAT
ACTTGTCAGAAATAAGGTCACAGGAGTTGAGATGAGATATCAGGTGAGTTTGATTTACTCTCTTGGTT
CACATCACCTGTTTC
+
FFFFFFFFFFFFFFFFFFFFFFFFFFFFFFFFFFFFFFFFFFFFFFFFFFFFFFFFFFFFFFFFFFFF
FFFFFFFF:FFFFFFF:FFFFF,FFFFFFFFFFFFFFFFFFFFFFFFFFFFFFFFFFFFFFFFFFFFF
FFFFFFFFFFFFFFF
@A00155:342:HHGFNDSXY:1:2563:25762:20275 2:N:0:GAACCTAG+TCCGCATA
GACGGGGTGAGGTCTGAGAGTTACACTCAACCATTGGAGAACGGTTTTCTCCTCAGCTCCAGCAGCAT
ACTTGTCAGAAATAAGGTCACAGGAGTTGAGATGAGATATCAGGTGAGTTTGATTTACTCTCTTGGTT
CACATCACCTGTTTC
+
FFFFFFFFFFFFFFFFFFFFFFFFFFFFFFFFFFFFFFFFFFFFFFFFFFFFFFFFFFFFFFFFFFFF
FFFFFFFFFFFFF:FFFFFFFFFFFFFFFFFFFFFFFFFFFFFFFFFFFFFF,FFFFFF,FFFFFFF
FFFFFFFFFF:FFFF
@A00155:342:HHGFNDSXY:1:2406:11831:1000 2:N:0:GAACCTAG+TCCGCATA
ACGGAGTGAGGTCTGAGAGTTACACTCAACCATTGGAGAACGGTTTTCTCCTCAGCTCCAGCAGCATA
CTTGTCAGAAATAAGGTCACAGGAGTTGAGATGAGATATCAGGTGAGTTTGATTTACTCTCTTGGTTC
ACATCACCTATTTC
+
FFFFFFFFFFFFFFFFFFFFFFFFFFFFFFFFFFFFFFFF:FFFFFFFFFFFFFFFFFFFFFFFFFFF
FFFFFFFFFF:FFFFFFFFFFFFFFFFFFFFFFFFFFFFFFFFFF:FFFFFFFFFF:FFFFF,FFFFF
FFFFFFFFF:FFFF
@A00155:342:HHGFNDSXY:1:1320:5014:2284 1:N:0:GAACCTAG+TCCGCATA
CGGAGTGAGGTCTGAGAGTTACACTCAACCATTGGAGAACGGTTTTCTCCTCAGCTCCAGCAGCATAC
TTGTCAGAAATAAGGTCACAGGAGTTGAGATGAGATATCAGGTGAGTTTGATTTACTCTCTTGGTTCA
CATCACCTATTTCAC
+
:FFFFFFFFFFFFFFFFFFFFFFFFFFFFF:FFFFFFFFFFFFFFFFFFFFFFFFFFFFFFFFFFFFF
:FFFFFFFFFFFFFFFFFFFFFFFFFFFFFFFFFFFFFFFFFFFFFFFFFF:FFFFFFFFFFFFFFF
FFFFFFFFFFFFFFF
@A00155:342:HHGFNDSXY:1:1630:24180:30749 1:N:0:GAACCTAG+TCCGCATA
ATCTGTGAGGTCTGAGAGTTACACTCAACCATTGGAGAACGGTTTTCTCCTCAGCTCCAGCAGCATAC
TTGTCAGAAATAAGGTCACAGGAGTTGAGATGAGATATCAGGTGAGTTTGATTTACTCTCTTGGTTCA
CATCACCTATTTCAC

```
+
FFFFFFFFFFFFFFFFFFF:FFFFFFFFFFFFFFFFFFFFFFFFFFFFFFFFFFFFFFFFFFFFFFFF
FFFFFFFFFFFFFFFFFFFFF:FFFFFFFF:FFFFFFFFFFFFF,F:FFFFFFFFFFFFFFFFFFFFF
FFFFFFFFFFFFFFF
@A00155:342:HHGFNDSXY:1:2557:13593:28166 1:N:0:GAACCTAG+TCCGCATA
ATCTGTGAGGTCTGAGAGTTACACTCAACCATTGGAGAACGGTTTTCTCCTCAGCTCCAGCAGCATAC
TTGTCAGAAATAAGGTCACAGGAGTTGAGATGAGATATCAGGTGAGTTTGATTTACTCTCTTGGTTCA
CATCACCTATTTCAC
+
FFFFFFFFFFFFFFFFFFFFFFFFFFFFFFFFFFFFFFFFFFFFFFFFFFFFFFFFFFFFFFFFFFFF
F:FFFFFFFFFFFFFFFFFF:FFFFFFFFFFFFFFFFFFFFFFFFFFFFFFFFFFFFFFFFFFFFFFF
FFFFFFFFFFFFFFF
@A00155:342:HHGFNDSXY:1:1518:31647:1313 2:N:0:GAACCTAG+TCCGCATA
CGGGGTGAGGTCTGAGAGTTACACTCAACCATTGGAGAACGGTTTTCTCCTCAGCTCCAGCAGCATAC
TTGTCAGAAATAAGGTCACAGGAGTTGAGATGAGATATCAGGTGAGTTTGATTTACTCTCTTGGTTCA
CATCACCTGTTTCAC
+
FFFFFFFFFFFFFFFFFFFFFFFFFFFFFFFFFFFFFFFFFFFFFFFFFFFFFFFFFFFFFFFFFFFF
,FFFFFFFFF:FFFFFFFFFFFFFF:FFFF:FFFFFFFFFFFFFFFFFFFF,FFFFFFFF:FFFFFFF
FFFFFFFFF:FFFFF
@A00155:342:HHGFNDSXY:1:1648:6958:32142 2:N:0:GAACCTAG+TCCGCATA
CGGAGTGAGGTCTGAGAGTTACACTCAACCATTGGAGAACGGTTTTCTCCTCAGCTCCAGCAGCATAC
TTGTCAGAAATAAGGTCACAGGAGTTGAGATGAGATATCAGGTGAGTTTGATTTACTCTCTTGGTTCA
CATCACCTATTTCAC
+
FFFFFFFFFFFFFFFFFFFFFFFFFFFFFFFFFFFFFFFFFFFFFFFFFFFFFFFFFFFFFFFFFFFF
FFFFFFF,FFF:FFFFFFF:FF,FFFFFF:FFFFFF,FF:FFFFFF:FFFFFFFF,F,FFFFF:FFFF
FFFFFFFF,FFFFFF
@A00155:342:HHGFNDSXY:1:2111:21630:28494 2:N:0:GAACCTAG+TCCGCATA
CGGGGTGAGGTCTGAGAGTTACACTCAACCATTGGAGAACGGTTTTCTCCTCAGCTCCAGCAGCATAC
TTGTCAGAAATAAGGTCACAGGAGTTGAGATGAGATATCAGGTGAGTTTGATTTACTCTCTTGGTTCA
CATCACCTGTTTCAC
+
FFFFFFFFFFFFFFFFFFFFFFFFFFFFF:FFFFFFFFFFFFFFFFFFFFFF,FFFFFFFFFFF:FFF
F,FFFFFFFFFFFFFFFFF:FFFFFFFFFFF:FFFFFFFFFFFFFFFFFFFFFFFFFFFFFF:FFFFF
FFFFFFFFFFFFF:F
@A00155:342:HHGFNDSXY:1:2153:32678:10394 2:N:0:GAACCTAG+TCCGCATA
CGGGGTGAGGTCTGAGAGTTACACTCAACCATTGGAGAACGGTTTTCTCCTCAGCTCCAGCAGCATAC
TTGTCAGAAATAAGGTCACAGGAGTTGAGATGAGATATCAGGTGAGTTTGATTTACTCTCTTGGTTCA
CATCACCTGTTTCAC
+
FFF,FFF:FFFFF:FFFFFFFFFFFFFFFFFFFFFF:FFFF:FFFFFFFFFFFFFFFFFFFFFFFFFF
FFF:FFFFFFFFFFFFFFFFF:FFFFFFFFFFFFFFFFFFFFFFFF,FFFF,FFFFF:FFFFFFFFFF
F:FFFFFFFFFFFFF
@A00155:342:HHGFNDSXY:1:1342:21594:12117 1:N:0:GAACCTAG+TCCGCATA
GGGGTGAGGTCTGAGAGTTACACTCAACCATTGGAGAACGGTTTTCTCCTCAGCTCCAGCAGCATACT
TGTCAGAAATAAGGTCACAGGAGTTGAGATGAGATATCAGGTGAGTTTGATTTACTCTCTTGGTTCAC
ATCACCTGTTTCAGA
+
::FFF,FFFFFFFFFFFFFFFFFF:FFFFFFFFFFFFFFFFFFFFFFFFFFFF:FFFFFFFFFFFFFF
:FFFFFFFFFFFFFFFFFFFFFFFFFFFFFFFFFFFFFFFFFFFFFFFFFFFFFFFFFFFFFFFFFFF
FFFFFFFFFFF:FFF
@A00155:342:HHGFNDSXY:1:1210:20609:34554 2:N:0:GAACCTAG+TCCGCATA
GGGGTGAGGTCTGAGAGTTACACTCAACCATTGGAGAACGGTTTTCTCCTCAGCTCCAGCAGCATACT
```

TGTCAGAAATAAGGTCACAGGAGTTGAGATGAGATATCAGGTGAGTTTGATTTACTCTCTTGGTTCAC
ATCACCTGTTTCACA
+
FFFFFFFFFFFFFFFFFFFFFFFFFFFFFFFFFFFFFFFFFFFFFFFFFFFFFFFFFFFFFFFFFFFF
FFFFFFFFFFFFFFFFFFFFFFFFFFFFFFFFFFFFFFFFFFFFFFFFFFFFF:FFFFF,FFFFFFFF
FFFFFFFFFFFFFFF
@A00155:342:HHGFNDSXY:1:1250:6795:12258 2:N:0:GAACCTAG+TCCGCATA
GGAGTGAGGTCTGAGAGTTACACTCAACCATTGGAGAACGGTTTTCTCCTCAGCTCCAGCAGCATACT
TGTCAGAAATAAGGTCACAGGAGTTGAGATGAGATATCAGGTGAGTTTGATTTACTCTCTTGGTTCAC
ATCACCTATTTCACA
+
FFFFFFFFFFFF:FFFFFFFFFFFFFFFFFFFFFFFFF:FFFFFFFFF,FFFFFFFFFFFFFFFFFFF
:FFFFFFFFFFFFFFFFF:FFF,FFFFFFFFFFFFFFFFFFFFFFFFFFFFFFFFFFFFFFFFFFFFF
FFF:FFFFFFFFF:F
@A00155:342:HHGFNDSXY:1:1313:25491:26694 2:N:0:GAACCTAG+TCCGCATA
GGAGTGAGGTCTGAGAGTTACACTCAACCATTGGAGAACGGTTTTCTCCTCAGCTCCAGCAGCATACT
TGTCAGAAATAAGGTCACAGGAGTTGAGATGAGATATCAGGTGAGTTTGATTTACTCTCTTGGTTCAC
ATCACCTATTTCACA
+
FFFFFFFFFFFFFFFFFF,FF:FFFF:FFFFFFFFFF:FF,FFFFFFFFFFFFFFFFFFFFFFFFFF:
,FFFFFFFFFFFFFFFFFFFFFFFFFFFFF:FFFFFFFFFFFFFFFFFFFFFFF,FFF:FFFF:FFFF
FFFFF:FFFFFF:F:
@A00155:342:HHGFNDSXY:1:1313:25482:32847 2:N:0:GAACCTAG+TCCGCATA
GGAGTGAGGTCTGAGAGTTACACTCAACCATTGGAGAACGGTTTTCTCCTCAGCTCCAGCAGCATACT
TGTCAGAAATAAGGTCACAGGAGTTGAGATGAGATATCAGGTGAGTTTGATTTACTCTCTTGGTTCAC
ATCACCTATTTCACA
+
FFFFFFFFFFFFFFFFFFFFF:FFFFFFFFFF:FFFFFFFFFFFFFFFFFFFF:FFFFFFFFFFFFFF
FFFFFFFFFFFFFFFFFFFFFFFFFFFFFFFFFFFFFFFFFFFFF:FFFFFFFFFFFFFFFFFFFFFF
FFFFFFFFFFFFFFF
@A00155:342:HHGFNDSXY:1:1339:9833:36777 2:N:0:GAACCTAG+TCCGCATA
GGAGTGAGGTCTGAGAGTTACACTCAACCATTGGAGAACGGTTTTCTCCTCAGCTCCAGCAGCATACT
TGTCAGAAATAAGGTCACAGGAGTTGAGATGAGATATCGGGTGAGTTTGATTTACTCTCTTGGTTCAC
ATCACCTATTTCACA
+
FFFF,FFFFFFFFFFFF:FF:FFFFFFF:,:FFF,,FFF:FFFFFFFFFFFFFFFFFFFFFFFFFFF,
FFFFFF:,FFFFFFFFFF:FFFFF:FF:FFFFFFFFFF:,F:FFFFF,FF:,FFF:FFF::FF:,,FF
FFF:FFFFFFFFFFF
@A00155:342:HHGFNDSXY:1:2123:3143:7341 2:N:0:GAACCTAG+TCCGCATA
GGGGTGAGGTCTGAGAGTTACACTCAACCATTGGAGAACGGTTTTCTCCTCAGCTCCAGCAGCATACT
TGTCAGAAATAAGGTCACAGGAGTTGAGATGAGATATCAGGTGAGTTTGATTTACTCTCTTGGTTCAC
ATCACCTGTTTCACA
+
FFFF:FFFFFFFFFFFFFFFFFFFFFFFFFFFFFFF:FFFFFFFFFFFFFFFFFFFFFFFFFFFFFFF
FFFFFFFFFFFFFFFF:FF:FFFFFFFFF:FFFFFFF:FFFFFFFFFFFFFFF:FFFFFFFFFFFFFF
FFFFFFFFFFFFFFF
@A00155:342:HHGFNDSXY:1:1639:3106:33301 2:N:0:GAACCTAG+TCCGCATA
GGGTGAGGTCTGAGAGTTACACTCAACCATTGGAGAACGGTTTTCTCCTCAGCTCCAGCAGCATACTT
GTCAGAAATAAGGTCACAGGAGTTGAGATGAGATATCAGGTGAGTTTGATTTACTCTCTTGGTTCACA
TCACCTGTTTCA
+
FF:FFFFFFFFFFFFFFFFFFFF:FFFFFFFFFFFF,:FF:FFFFFFFFFFFFFFFFFFF:FFFFF:F
F:FFFFFFFFFFFFFFFFFFFFFFFFFFFFFFFFFFFFFFFFFFFFFFFFFFFFFFFFFFFFFFFFFF
F:,:FFFFFFFF

@A00155:342:HHGFNDSXY:1:1571:16839:6543 1:N:0:GAACCTAG+TCCGCATA
GAGTGAGGTCTGAGAGTTACACTCAACCATTGGAGAACGGTTTTCTCCTCAGCTCCAGCAGCATACTT
GTCAGAAATAAGGTCACAGGAGTTGAGATGAGATATCAGGTGAGTTTGATTTACTCTCTTGGTTCACA
TCACCTATTTCACAT
+
FFFFFFFFF:FFFFFFF:FFFFFFFFFFFFFFFFFFFFFFFFFFFFFFFFFFFFFFFFFFF:FFFFFF
FFFFFFFFFFFFFFFFFFFFFFFFFFFFFFFFFFFFFFFFFFFF:FFFFFFFFF:FFFFFFF:FFFFF
FFFFFFFFFFFFFFF
@A00155:342:HHGFNDSXY:1:1123:31846:11209 2:N:0:GAACCTAG+TCCGCATA
GGGTGAGGTCTGAGAGTTACACTCAACCATTGGAGAACGGTTTTCTCCTCAGCTCCAGCAGCATACTT
GTCAGAAATAAGGTCACAGGAGTTGAGATGAGATATCAGGTGAGTTTGATTTACTCTCTTGGTTCACA
TCACCTGTTTCACAT
+
FFFFFFFFFFFFFFFFFFFFFFFFFFFFFFFFFFFFFFFFFFFFFFFFFFFFFFFFFFFFFFFF:F,F
FFFFFF:FFFFFFFFFFFFFFF,:FFFFFFFFF::F:FFFFFFF:FFFF:FF,FFFFFFFFFFFFFFF
FFFFFFF:FFFFFFF
@A00155:342:HHGFNDSXY:1:1443:13919:21026 2:N:0:GAACCTAG+TCCGCATA
GGGTGAGGTCTGAGAGTTACACTCAACCATTGGAGAACGGTTTTCTCCTCAGCTCCAGCAGCATACTT
GTCAGAAATAAGGTCACAGGAGTTGAGATGAGATATCAGGTGAGTTTGATTTACTCTCTTGGTTCACA
TCACCTGTTTCACAT
+
FFFFFFFFFFFFFFFFFFFFFFFFFFFFFFFFFFFFFFFFFFFFFFFFFFFFFFFFFFFFFFFFFFFF
FFFFFFFFFFFFFFFFFFFFFFFFFFFFFFFFFFFFFFFFFFFFFFFFFFFFFFFFFFFFFFFFFFFF
,FFFFFFFFFFFFFF
@A00155:342:HHGFNDSXY:1:1466:5864:32127 2:N:0:GAACCTAG+TCCGCATA
GGGTGAGGTCTGAGAGTTACACTCAACCATTGGAGAACGGTTTTCTCCTCAGCTCCAGCAGCATACTT
GTCAGAAATAAGGTCACAGGAGTTGAGATGAGATATCAGGTGAGTTTGATTTACTCTCTTGGTTCACA
TCACCTGTTTCACAT
+
FFFFFFFFFFFFFFFFFFFFFFFFFFFFFFFF:FFFFF:FF:FFFFFFFF:FFFFFFFFFFFFFFFFF
FFFFFFF:FFFFFFFFFFFFFFF:FFFFFFFFFF:FFFF,:FFFFFFF:FFFFFFF,FF,FFFFFFFF
FFFFFFFFFFFFFFF
@A00155:342:HHGFNDSXY:1:1640:4860:3082 2:N:0:GAACCTAG+TCCGCATA
GGGTGAGGTCTGAGAGTTACACTCAACCATTGGAGAACGGTTTTCTCCTCAGCTCCAGCAGCATACTT
GTCAGAAATAAGGTCACAGGAGTTGAGATGAGATATCAGGTGAGTTTGATTTACTCCCTTGGTTCACA
TCACCTGTTTCACAT
+
FFFFFFFFFFFFFFFFFFFFFFFFFFFFF:FFFFFF,FFFFFFFFFFFFFFFFFFFFFFFFFFFFFFF
FFFFFFFFFFFFFFFFFFFFFFFFFFFFFFFFFFFFFFFFFF:FFFFFFFFFFFFFFF:FFFFFFFFF
FFFFFFFFFFFFFFF
@A00155:342:HHGFNDSXY:1:2132:2799:10097 2:N:0:GAACCTAG+TCCGCATA
GGGTGAGGTCTGAGAGTTACACTCAACCATTGGAGAACGGTTTTCTCCTCAGCTCCAGCAGCATACTT
GTCAGAAATAAGGTCACAGGAGTTGAGATGAGATATCAGGTGAGTTTGATTTACTCTCTTGGTTCACA
TCACCTGTTTCACAT
+
FFFFFFFFFFFFFFFFFFFFFFFFFFFFFFFFFFFFF:FFFFFFFFFFFFFFFFFFFFFF:FFFFFFF
FFFFFFFFFFFFFFFFFFFFFFFFFFFFFFFFFFFFFFFFFFFFFFFFFFFFFFFFFFFFFFFFFFFF
FFFFFFFFFFFFFFF
@A00155:342:HHGFNDSXY:1:2144:5385:30358 2:N:0:GAACCTAG+TCCGCATA
GGGTGAGGTCTGAGAGTTACACTCAACCATTGGAGAACGGTTTTCTCCTCAGCTCCAGCAGCATACTT
GTCAGAAATAAGGTCACAGGAGTTGAGATGAGATATCAGGTGAGTTTGATTTACTCTCTTGGTTCACA
TCACCTGTTTCACAT
+
FFFFFFFFFFFFFFFFFFFFFFFFFFFFFFFFFFFFFFFFFFFFFFFFFFFF:FFFFFFFFFFFFFFF

```
FFFFFFFFFFFFFFFFFFFFFFFFFFFFFFFFFFFFFFFFFFFFFFFFFFFFFF,FFFFFFFFF:FF
FFFFFFFFFFFFFFF
@A00155:342:HHGFNDSXY:1:2202:13630:16047 2:N:0:GAACCTAG+TCCGCATA
GGGTGAGGTCTGAGAGTTACACTCAACCATTGGAGAACGGTTTTCTCCTCAGCTCCAGCAGCATACTT
GTCAGAAATAAGGTCACAGGAGTTGAGATGAGATATCAGGTGAGTTTGATTTACTCTCTTGGTTCACA
TCACCTGTTTCACAT
+
FFFFFFFFFFFF:FFFFFFFFFFFFFFFFFFFFFFFF:FFFFFFFFFFFFFFFFFFFFFFFFFFFFFF
FFFFFFFFFFFFFFFFFFFFFFFFFFFFFFFFFFFFFFFFFFFFFFFF:FFFFF:FFFFFFFFFF
FFFFF:FFFFFFFFF
@A00155:342:HHGFNDSXY:1:2245:13729:2440 2:N:0:GAACCTAG+TCCGCATA
GGGTGAGGTCTGAGAGTTACACTCAACCATTGGAGAACGGTTTTCTCCTCAGCTCCAGCAGCATACTT
GTCAGAAATAAGGTCACAGGAGTTGAGATGAGATATCAGGTGAGTTTGATTTACTCTCTTGGTTCACA
TCACCTGTTTCACAT
+
FFFFFFFFFFFFFFFFFFFFFFFFFFFFF:FFFFFFFFFFFFFFFFFFFFFFFFFFFFFFFFFFFFF:,
FFFFFFFF:,FFFFFFF:FFFFFFF:FFFF:FFFF:FFFFFFFFFFFFFFFFFFFFFFFFFFFFFFFF
FFFFFFFF:FFFFFF
@A00155:342:HHGFNDSXY:1:2261:7572:36495 2:N:0:GAACCTAG+TCCGCATA
GGGTGAGGTCTGAGAGTTACACTCAACCATTGGAGAACGGTTTTCTCCTCAGCTCCAGCAGCATACTT
GTCAGAAATAAGGTCACAGGAGTTGAGATGAGATATCAGGTGAGTTTGATTTACTCTCTTGGTTCACA
TCACCTGTTTCACAT
+
FFFFFFFFFFFFFFFFFFFFFFFFFFFFFFFFFFFFFFFFFFFFFFFFFFFFFFFFFFFFFFFFFFFF
FFFFFFFFFFFFFFFFFFFF:FFFFFFFFFFFFFFFFFFFFFFFFFFFFFFFFFFFFFFFFFFFFFF
FFFFFFFFFFF:FFF
@A00155:342:HHGFNDSXY:1:2431:25111:8531 2:N:0:GAACCTAG+TCCGCATA
GGGTGAGGTCTGAGAGTTACACTCAACCATTGGAGAACGGTTTTCTCCTCAGCTCCAGCAGCATACTT
GTCAGAAATAAGGTCACAGGAGTTGAGATGAGATATCAGGTGAGTTTGATTTACTCTCTTGGTTCACA
TCACCTGTTTCACAT
+
F:FFFFFFFFFFFFFFFFFFFFFFFFFFFFFFFFFFFFFFFFFF:FFF,FFFFFFFFFFFFFFFFFFFF
FFFFFFFFFFFFFFFFFFFFFFFFFFFFFFFFFFFFFFFFFFFFFFFFFFFFFFFFFFFFFFFFFFFF
FFFFFFFFFFFFFFF
@A00155:342:HHGFNDSXY:1:2677:25599:22686 2:N:0:GAACCTAG+TCCGCATA
GGGTGAGGTCTGAGAGTTACACTCAACCATTGGAGAACGGTTTTCTCCTCAGCTCCAGCAGCATACTT
GTCAGAAATAAGGTCACAGGAGTTGAGATGAGATATCAGGTGAGTTTGATTTACTCTCTTGGTTCACA
TCACCTGTTTCACAT
+
FFFFFFFFFFFFFFFFF:FFFFFFFFFFFFFFFFFF:FFFFFFFFFFFFFFFFFFFFFFFFFFFFFFF:
FFFFFFFFFFFFFFFFFFFFFFFFFFFFFFFFFFFFFFFFFFFFFFFFFFFFFFFFFFFFFFFFFFFF
FFFFFFFFFFFFFFF
@A00155:342:HHGFNDSXY:1:1260:7048:26819 1:N:0:GAACCTAG+TCCGCATA
TGTGAGGTCTGAGAGTTACACTCAACCATTGGAGAACGGTTTTCTCCTCAGCTCCAGCAGCATACTTG
TCAGAAATAAGGTCACAGGAGTTGAGATGAGATATCAGGTGAGTTTGATTTACTCTCTTGGTTCACAT
CACCTGTTTCACATT
+
FFFFFFFFFFFFFFFFFFFFFFFFFFFFFFFFFFFFFFFF:FFFFFFFFFFFFFFFFFFFFFFFFFFF
FFFFFFFFFFFFFFFFFFFFFFFFFFF,:F,FF,F,FFFFFFFF,,FFFFFFFFFFFFFFFFFFFFFFF
FFFFFFFFFFFFFFF
@A00155:342:HHGFNDSXY:1:1440:2781:5055 2:N:0:GAACCTAG+TCCGCATA
GGTGAGGTCTGAGAGTTACACTCAACCATTGGAGAACGGTTTTCTCCTCAGCTCCAGCAGCATACTTG
TCAGAAATAAGGTCACAGGAGTTGAGATGAGATATCAGGTGAGTTTGATTTACTCTCTTGGTTCACAT
CACCTGTTTCACATT
```

```
+
FFFFFFF:FFFFFFFFFFFFFFFFFFFFFFFFFFFFFFFFFFFFFFFFFFFFFFFFFFFFFFFFFFFF
:FFFFFFFFFFFFFFFFFFFFFFFF:FFFF:FFFFFFFFFFFFFFFFFFF:FFFFFFFFFFFFFFFFFF
FFFFFFFFFF,FFFF
@A00155:342:HHGFNDSXY:1:2254:16694:17190 2:N:0:GAACCTAG+TCCGCATA
GGTGAGGTCTGAGAGTTACACTCAACCATTGGAGAACGGTTTTCTCCTCAGCTCCAGCAGCATACTTG
TCAGAAATAAGGTCACAGGAGTTGAGATGAGATATCAGGTGAGTTTGATTTACTCTCTTGGTTCACAT
CACCTGTTTCACATT
+
FFFFFFFFFFFFFFFFFFFFFFFFFFFFFFFFFFFFFFFFFFFFFFFFFFFFFFFFFFFFFFFFFFFF
FFFFFFFFFFFFFFFFFFFFFFFFFFF:FFFFFFFF:FFFFFFFFFFFFFFFFFFFF:,FF:FFFFFF
FFFFFF:FFF:FFFF
@A00155:342:HHGFNDSXY:1:2316:12861:11490 2:N:0:GAACCTAG+TCCGCATA
AGTGAGGTCTGAGAGTTACACTCAACCATTGGAGAACGGTTTTCTCCTCAGCTCCAGCAGCATACTTG
TCAGAAATAAGGTCACAGGAGTTGAGATGAGATATCAGGTGAGTTTGATTTACTCTCTTGGTTCACAT
CACCTATTTCACATT
+
FFFFFFFFFFFFFFFFFFFFFFFFFFFFFFFFFFFFFFFFFFFFFFFFFFFFFFFFFFFFFFFFFFFF
FFFF:FFFFFFFFFFF:FFFFFFFFFFFFFFFFFFFFFFFFFFFFFFFFFFFFFFFFFFFFFFFFFFF
FFFFFFFFFFFFFF:
@A00155:342:HHGFNDSXY:1:2333:19470:36182 2:N:0:GAACCTAG+TCCGCATA
GGTGAGGTCTGAGAGTTACACTCAACCATTGGAGAACGGTTTTCTCCTCAGCTCCAGCAGCATACTTG
TCAGAAATAAGGTCACAGGAGTTGAGATGAGATATCAGGTGAGTTTGATTTACTCTCTTGGTTCACAT
CACCTGTTTCACATT
+
FFFFFFFFFFFFFFFFFFFFFFFFFFFFFFFFFFFFFFFFFFFFFFFFFFFFFFFFFFFFFFFFFFFF
FFFFFFFFFFFFFFFFFFFFFFFFFFFFFFFFFFFFFFFFFFFFFFFFFFFFFFFFFFFFFFFFFFFF
FFFFFFF:FFFF:FF
@A00155:342:HHGFNDSXY:1:2365:29460:4476 2:N:0:GAACCTAG+TCCGCATA
GGTGAGGTCTGAGAGTTACACTCAACCATTGGAGAACGGTTTTCTCCTCAGCTCCAGCAGCATACTTG
TCAGAAATAAGGTCACAGGAGTTGAGATGAGATATCAGGTGAGTTTGATTTACTCTCTTGGTTCACAT
CACCTGTTTCACATT
+
FFFFFFFFFFFFFFFFFFFFFFFFFFFFFFFFFFFFFFFFFFFFFFFFFFFFFFFFFFFFFFFFFFFF
FFFFFFFF:FFFFFFFFFFFFFFFFFFFFFFFFFFFFFFFFFFFFFFFFFFFFFFFFFFFFFFFFFFF
FFFFFFFFFFFFFFF
@A00155:342:HHGFNDSXY:1:1550:31376:10520 1:N:0:GAACCTAG+TCCGCATA
GTGAGGTCTGAGAGTTACACTCAACCATTGGAGAACGGTTTTCTCCTCAGCTCCAGCAGCATACTTGT
CAGAAATAAGGTCACAGGAGTTGAGATGAGATATCAGGTGAGTTTGATTTACTCTCTTGGTTCACATC
ACCTGTTTCACATTT
+
FFFFFFFFFFFFFFFFFFFFFFFFFFFFFFFFFFFFFFFFFFFFFFFFFFFFFFFFFFFFFFFFFFFF
FFFFFFFFFFFFFFFFFFFFFFFFFFF:FF:FFFFFFFFFFFF:FFFFFFFFFFFFFFFFFFFFFFFFF
FFFFFFFFFFFFFFF
@A00155:342:HHGFNDSXY:1:2314:14678:35305 1:N:0:GAACCTAG+TCCGCATA
GTGAGGTCTGAGAGTTACACTCAACCATTGGAGAACGGTTTTCTCCTCAGCTCCAGCAGCATACTTGT
CAGAAATAAGGTCACAGGAGTTGAGATGAGATATCAGGTGAGTTTGATTTACTCTCTTGGTTCACATC
ACCTGTTTCACATTT
+
FFFFFFFFFFFFFF:FFFFFFFFFFFFFFFFFFFFFFFFFFFFFFFFFFFFFFFFF:FFFFF:FFFFFFF
FFFFFFFFFFFFFFFFFFFFFFFFFFFFFFFFFFFFFFFFFFFFFFFFFFFFFFFFFFFFFFFFFFFF
FFFFFFFFFFF:FFF
@A00155:342:HHGFNDSXY:1:1145:2266:19664 2:N:0:GAACCTAG+TCCGCATA
GTGAGGTCTGAGAGTTACACTCAACCATTGGAGAACGGTTTTCTCCTCAGCTCCAGCAGCATACTTGT
```

CAGAAATAAGGTCACAGGAGTTGAGATGAGATATCAGGTGAGTTTGATTTACTCTCTTGGTTCACATC
ACCTATTTCACATTT
+
FFFFFFFFFFFFFFFFFFFFFFFFFFFFFFFFFFFF:FFFFFFFF:FFFFFFFFFFFFFFFFFFFFFF
FFFFFFFFFFFFFFFFFFF:FFFFFFFFFFFFFFFF:FFFFFFFFFFFFFFFFFF:FFF,FF:FFFFF
FF:FFFFF:F,FFFF
@A00155:342:HHGFNDSXY:1:1133:6885:10974 2:N:0:GAACCTAG+TCCGCATA
GTGAGGTCTGAGAGTTACACTCAACCATTGGAGAACGGTTTTCTCCTCAGCTCCAGCAGCATACTTGT
CAGAAATAAGGTCACAGGAGTTGAGATGAGATATCAGGTGAGTTTGATTTACTCTCTTGGTTCACATC
ACCTATTTCACATTT
+
FFFFFFFF:FFFFFFFFFFFFFFFFF::FFFFFFFFFFFFFFFFFFFFFFFFFFFFFFFFFFFFFFFF
FFFFFFFFFFFFFFFFFFFF:FFFFFFF,:FFF:FFFFFFFFFFFFF:,FFFFF:FF:FFFFF:FFFF
FFFFFF:FFFFFFFF
@A00155:342:HHGFNDSXY:1:1177:12328:32988 2:N:0:GAACCTAG+TCCGCATA
GTGAGGTCTGAGAGTTACACTCAACCATTGGAGAACGGTTTTCTCCTCAGCTCCAGCAGCATACTTGT
CAGAAATAAGGTCACAGGAGTTGAGATGAGATATCAGGTGAGTTTGATTTACTCTCTTGGTTCACATC
ACCTGTTTCACATTT
+
FFFFFFFFFFFFFFFFFFFFFFFFFFFFFFFFFFFFFFFFFFFFFFFFFFFFFFFFFFFFFFFFF,FF
FFFFFF:FFFFFFFFFFFFFFFF:FFFF:FFFFFFFFFFFFFFFFFFFF:FFFFFFFFFFFFFFFFFF
FFFFFFFFF,FFFFF
@A00155:342:HHGFNDSXY:1:1204:19696:13275 2:N:0:GAACCTAG+TCCGCATA
GTGAGGTCTGAGAGTTACACTCAACCATTGGAGAACGGTTTTCTCCTCAGCTCCAGCAGCATACTTGT
CAGAAATAAGGTCACAGGAGTTGAGATGAGATATCAGGTGAGTTTGATTTACTCTCTTGGTTCACATC
ACCTGTTTCACATTT
+
FFFFFFFF:FFFFFFFFFFFFFFFFFFFFFFFFFFFFFFFFFFFFFFFFFFFFFFFFFFFFFFFFFF
FFFFFFFF,FFFFFFFFFFFFFFFFFFFFFFFFFFFFFFFFFFFFFFFFFFFFFFFFFFFFFFFFFF
FFFFFFFFFFFFFFF
@A00155:342:HHGFNDSXY:1:1204:19714:13275 2:N:0:GAACCTAG+TCCGCATA
GTGAGGTCTGAGAGTTACACTCAACCATTGGAGAACGGTTTTCTCCTCAGCTCCAGCAGCATACTTGT
CAGAAATAAGGTCACAGGAGTTGAGATGAGATATCAGGTGAGTTTGATTTACTCTCTTGGTTCACATC
ACCTGTTTCACATTT
+
FFFFFFFFFFFFFFFFFFFFFFFFFFFFFFFFFFFFFFFFFFFFFFFFFFFFFFFFFFFFFFFFFFFF
FFFFFFFFFFFFFFFFFFFFFFFFFFFFFFFFFFFFFFFFFFF,FFFFFFFFFFFFF:FFFFFFFFFF
FFFFFFFFFFFFFFF
@A00155:342:HHGFNDSXY:1:1301:7654:19351 2:N:0:GAACCTAG+TCCGCATA
GTGAGGTCTGAGAGTTACACTCAACCATTGGAGAACGGTTTTCTCCTCAGCTCCAGCAGCATACTTGT
CAGAAATAAGGTCACAGGAGTTGAGATGAGATATCAGGTGAGTTTGATTTACTCTCTTGGTTCACATC
ACCTGTTTCACATTT
+
FFFFFFFFFFFFFFFFFFFFFF:FFFFFFFFFFFFFFFFFFFFFFFFFFFFFFFFFFFFFFFFFFFFF
FFFFFFFF:FFFFFFFFFFFFFFFFFFFFFF:FFFFFFFFFFFFFFFFFFFFFFFFFFFFFFFFFFFF
FFFFFFFFFFFFFFF
@A00155:342:HHGFNDSXY:1:1260:7048:26819 2:N:0:GAACCTAG+TCCGCATA
GTGAGGTCTGAGAGTTACACTCAACCATTGGAGAACGGTTTTCTCCTCAGCTCCAGCAGCATACTTGT
CAGAAATAAGGTCACAGGAGTTGAGATGAGATATCAGGTGAGTTTGATTTACTCTCTTGGTTCACATC
ACCTGTTTCACATTA
+
F:FFFFFFFFFFFFFFFFFFFFFFFFFFFFFFFFFFFFFFFFFFFFFFFFFFF:FFFFFFFFFFFFFF
FFF:FFFFFFFFFFF:FFFFFFFFFFFFFFF:FFFFFF:FFFFFFFFFFFFFFFFFFFFFFFFFFFFF
FFFFFFFFFFFF:F:

@A00155:342:HHGFNDSXY:1:1344:23493:13307 2:N:0:GAACCTAG+TCCGCATA
GTGAGGTCTGAGAGTTACACTCAACCATTGGAGAACGGTTTTCTCCTCAGCTCCAGCAGCATACTTGT
CAGAAATAAGGTCACAGGAGTTGAGATGAGATATCAGGTGAGTTTGATTTACTCTCTTGGTTCACATC
ACCTGTTTCACATTT
+
FFFFFFFFFFFFFFFFFFFFFFFFFFFFFFFFFFFFFFFFF:FFFFFFFFFFFFFFFFFFFFFFFFFF
FFFFFFFFFFFFFFFFFFFFFFFFFFFFFFFFFFFFFFFFFFFFFFFFFFFFFFFFFFFFFFFFFFFF
FFFFFFFFFFFFFFF
@A00155:342:HHGFNDSXY:1:1514:5213:19727 2:N:0:GAACCTAG+TCCGCATA
GTGAGGTCTGAGAGTTACACTCAACCATTGGAGAACGGTTTTCTCCTCAGCTCCAGCAGCATACTTGT
CAGAAATAAGGTCACAGGAGTTGAGATGAGATATCAGGTGAGTTTGATTTACTCTCTTGGTTCACATC
ACCTGTTTCACATTT
+
FFFFFFFFFFFFFFFFFFFFFFFFFFFFFFFFFFFFFFFFFFFFFFFFFFFFFFFFFFFFFFFFF:FF
FFFFFFFFFFFFFFFFFFFFFFFFFFFFF:FFFFFF:FFFFFFFFFFFFFFFFFFF:FFFFF:FFFFF
FFFFFFFFFFFFFF:
@A00155:342:HHGFNDSXY:1:1550:31376:10520 2:N:0:GAACCTAG+TCCGCATA
GTGAGGTCTGAGAGTTACACTCAACCATTGGAGAACGGTTTTCTCCTCAGCTCCAGCAGCATACTTGT
CAGAAATAAGGTCACAGGAGTTGAGATGAGATATCAGGTGAGTTTGATTTACTCTCTTGGTTCACATC
ACCTGTTTCACATTT
+
FFFFFFFFFFFFFFFFFFFFFFFFFFFFFFFFFFFFFFFFFFFFFFFFFFFFFFFFF:FFFFFFFFFF
FFFFFFFFFFFFFFFFFFFF,FFFFFFFFFFFFFFFFFFFFF::FFFFFFFFFFFFFFFFFFFFFFFF
FFFFFFFFFFFF:FF
@A00155:342:HHGFNDSXY:1:1635:25979:1329 2:N:0:GAACCTAG+TCCGCATA
GTGAGGTCTGAGAGTTACACTCAACCATTGGAGAACGGTTTTCTCCTCAGCTCCAGCAGCATACTTGT
CAGAAATAAGGTCACAGGAGTTGAGATGAGATATCAGGTGAGTTTGATTTACTCTCTTGGTTCACATC
ACCTATTTCACATTT
+
:FFFFFFFFFFFFFFFFFF:FFFF:FFFFFFFFFFFFF:FFFFFFFFFFFFFFFFF:FFFFFFFFFFF
FFFFF:F:FFFFFFF:FFFFFFFFFF:FFFFFFFFFFFFFFFFFFFF,FFFFF,FFF:FF,FFFFFFF
FFFF:FFFFFFF,FF
@A00155:342:HHGFNDSXY:1:1630:24180:30749 2:N:0:GAACCTAG+TCCGCATA
GTGAGGTCTGAGAGTTACACTCAACCATTGGAGAACGGTTTTCTCCTCAGCTCCAGCAGCATACTTGT
CAGAAATAAGGTCACAGGAGTTGAGATGAGATATCAGGTGAGTTTGATTTACTCTCTTGGTTCACATC
ACCTATTTCACAGAT
+
FFFFFFFFFFFFFFFFFFFFFFFFFFFFFFFFFF:FFFFFFFFFFFFFFFFFFFFFFFFFFFFFFFFF
FFFFFFFFFFFFFFFFFFFFFFFFFFFFFFFFFFFFFFFFFFFFFFFFFFFFFFFFFFFFFFFFFFFF
FFFF,FFFFFFFFFF
@A00155:342:HHGFNDSXY:1:1622:8431:11960 2:N:0:GAACCTAG+TCCGCATA
GTGAGGTCTGAGAGTTACACTCAACCATTGGAGAACGGTTTTCTCCTCAGCTCCAGCAGCATACTTGT
CAGAAATAAGGTCACAGGAGTTGAGATGAGATATCAGGTGAGTTTGATTTACTCTCTTGGTTCACATC
ACCTATTTCACATTT
+
FFFFFFFFFFFFFFFFFFFFFFFFFFFFFFFFFFFFFFFFFFFFFFFFFFFFFFFFFFFFFFFFFFFF
F:FFFFFFFFFFFFFFFFFFFFFFFFFFFFFFFFFFF:FFFFFFFFFFFFF:FFFFFFFFFFFFFFFF
FF:FFFFFFFFFFFF
@A00155:342:HHGFNDSXY:1:2210:12346:16642 2:N:0:GAACCTAG+TCCGCATA
GTGAGGTCTGAGAGTTACACTCAACCATTGGAGAACGGTTTTCTCCTCAGCTCCAGCAGCATACTTGT
CAGAAATAAGGTCACAGGAGTTGAGATGAGATATCAGGTGAGTTTGATTTACTCTCTTGGTTCACATC
ACCTGTTTCACATTT
+
FFF:FF::FFFFFFFFFFFFFFFFFFFFFFFFFFFFFFFFFFFFFFFFFFFFFFFFFFFFFFFFFFFF

FFFFFFFFFFFFFFFFFFFFFFFFFFFF:FFFFFFFFFFFFFFFFFFFFFFFFFFFFFFFFFFFFFFFF:
FFFFFFFFF:FFFFF
@A00155:342:HHGFNDSXY:1:2221:24514:29324 2:N:0:GAACCTAG+TCCGCATA
GTGAGGTCTGAGAGTTACACTCAACCATTGGAGAACGGTTTTCTCCTCAGCTCCAGCAGCATACTTGT
CAGAAATAAGGTCACAGGAGTTGAGATGAGATATCAGGTGAGTTTGATTTACTCTCTTGGTTCACATC
ACCTGTTTCACATTT
+
FFFFFFFFFFFFFFFFFFFFFFFFFFFFFFFFFFFFFFFFFFFFFFFFFFFFFFFFFFFFFFFFFFFF
FFFFFFFFFFFFFFFFFFFFFFFFFFFFFFFFFFFFFFFFFFFFFFFFFFFFFFFFFFFFFFFFFFFF
FFFFFFFFFFFFFFF
@A00155:342:HHGFNDSXY:1:2305:18439:25003 2:N:0:GAACCTAG+TCCGCATA
GTGAGGTCTGAGAGTTACACTCAACCATTGGAGAACGGTTTTCTCCTCAGCTCCAGCAGCATACTTGT
CAGAAATAAGGTCACAGGAGTTGAGATGAGATATCAGGTGAGTTTGATTTACTCTCTTGGTTCACATC
ACCTGTTTCACATTT
+
FFFFFFFFFFFFFFFFFFFFFFFFFFFFFFFFFFFFFFFFFFFFFFFFFFFFFFFFFFFFFFFFFFFF
FFFFFFFFFFFFFFFFFFFFFFFFFFFFFFFFFFFFFFFFFFFFFFFFFFFFFFFFFFFFFFFFFFFF
FFFFF:FFFFFFFFF
@A00155:342:HHGFNDSXY:1:2266:17788:11350 2:N:0:GAACCTAG+TCCGCATA
GTGAGGTCTGAGAGTTACACTCAACCATTGGAGAACGGTTTTCTCCTCAGCTCCAGCAGCATACTTGT
CAGAAATAAGGTCACAGGAGTTGAGATGAGATATCAGGTGAGTTTGATTTACTCTCTTGGTTCACATC
ACCTGTTTCACATTT
+
FFFFFFFFFFFFFFFFFFFFFFFFFFFFFFFFFFFFFFFFFFFFFFFFFFFFFFFFFFFFFFFFFFFF
FFFFFFFFFFFFFFFFFFFFFFFFFFFFFFFFFFFFFFFFFFFFFFFFFFFFFFFF:FFFFFFFFFFF
FFFFFFFFFFFFFFF
@A00155:342:HHGFNDSXY:1:2262:13575:12978 2:N:0:GAACCTAG+TCCGCATA
GTGAGGTCTGAGAGTTACACTCAACCATTGGAGAACGGTTTTCTCCTCAGCTCCAGCAGCATACTTGT
CAGAAATAAGGTCACAGGAGTTGAGATGAGATATCAGGTGAGTTTGATTTACTCTCTTGGTTCACATC
ACCTATTTCACATTT
+
FF:FFFFF:FF,FFFFFFF:FFFFF:FFFF:FFFFFFFFFFF,FFFF,F:FFF,FF:FF:FFFFFF:F
FFFFFFFFFFFFFFF::FFFFFFFFFFFFF,,FFFFFFFFF,FFFFFFF:FFF,FFF,,,FF:FFFFF
FFFFFFFF,FFFF:F
@A00155:342:HHGFNDSXY:1:2468:3920:34084 2:N:0:GAACCTAG+TCCGCATA
GTGAGGTCTGAGAGTTACACTCAACCATTGGAGAACGGTTTTCTCCTCAGCTCCAGCAGCATACTTGT
CAGAAATAAGGTCACAGGAGTTGAGATGAGATATCAGGTGAGTTTGATTTACTCTCTTGGTTCACATC
ACCTATTTCACATTT
+
FFFFFFFFFFFFF:FFFFFFFFF:FFFFFFFFFFFFFFFFFFFFFFFFFFFFFFFFFFFFFFFFFFFF
FFFFFFFFFFFFFFFFFFFFFF:FFFFFF,FF:FFFFFFFFFFFFFFFFFFF:,FFFFFFFFFFFFFF
FFFFFFFFFFFFFFF
@A00155:342:HHGFNDSXY:1:2557:13593:28166 2:N:0:GAACCTAG+TCCGCATA
GTGAGGTCTGAGAGTTACACTCAACCATTGGAGAACGGTTTTCTCCTCAGCTCCAGCAGCATACTTGT
CAGAAATAAGGTCACAGGAGTTGAGATGAGATATCAGGTGAGTTTGATTTACTCTCTTGGTTCACATC
ACCTATTTCACAGAT
+
FFFFFFFFFFFFFFFFFFFFFFFFFFFFFFFFFFFFFFFFFFFFFFFFFFFFFFFFFFFFFFFFFFFF
FFFFFFFFFFFFFFFFFFFFFFFFFFFFFFFF:FFFFFFFFFFFFFFFFFFFFFFFFFFFFFF:FFFF
FFFFFFFFFFFFFFF
@A00155:342:HHGFNDSXY:1:2617:32181:32518 2:N:0:GAACCTAG+TCCGCATA
GTGAGGTCTGAGAGTTACACTCAACCATTGGAGAACGGTTTTCTCCTCAGCTCCAGCAGCATACTTGT
CAGAAATAAGGTCACAGGAGTTGAGATGAGATATCAGGTGAGTTTGATTTACTCTCTTGGTTCACATC
ACCTATTTCACATTT

+
F:F:FFFFFFFFFFFFFFFFFFFFFFFF:FFFFFFFFFFFFFFFFFFFFFFFFFFFFFFFFFFFFFFF
FFFFFFFFFFFFFFFFFFFFFFFFFFFFFFFFFFFFFFFFFFFFFFFFFFFFFFFFFFFFFFFFF:FF
FFFFFFFFFFFFFFF
@A00155:342:HHGFNDSXY:1:2618:30228:7404 2:N:0:GAACCTAG+TCCGCATA
GTGAGGTCTGAGAGTTACACTCAACCATTGGAGAACGGTTTTCTCCTCAGCTCCAGCAGCATACTTGT
CAGAAATAAGGTCACAGGAGTTGAGATGAGATATCAGGTGAGTTTGATTTACTCTCTTGGTTCACATC
ACCTATTTCACATTT
+
:FFFFFFFFFFFFFFFFF:FFFFFFFFFFFFFFFFFFFFFFFFFFFFFFFFFFFFFFFFFFFFFFFFF
FFFFFFFFFFFFFFFFFFFFFFFFFFFFFFF:FFFFFFFFFFFFFFFFFFFFFFFF,FFFFFFFFFFF
FFF:FFFFFFFFFFF
@A00155:342:HHGFNDSXY:1:2516:17246:37059 2:N:0:GAACCTAG+TCCGCATA
GTGAGGTCTGAGAGTTACACTCAACCATTGGAGAACGGTTTTCTCCTCAGCTCCAGCAGCATACTTGT
CAGAAATAAGGTCACAGGAGTTGAGATGAGATATCAGGTGAGTTTGATTTACTCTCTTGGTTCACATC
ACCTATTTCACATTT
+
FFFFFFFFFFFFFFFFFFFFFFFFFFFFFFFFFFFFFFFFFFFFFFFFFFFFFFFFFFFFFFFFFFFF
FFFFFFFFF:FFFFFFFFFFFFFFFFFFFFFFFFFFFFFFFFFFFFFFFFFFFFFF,FFFFFF:FFFF
FFFFFFFFFFFFFFF
@A00155:342:HHGFNDSXY:1:2626:16703:27633 2:N:0:GAACCTAG+TCCGCATA
GTGAGGTCTGAGAGTTACACTCAACCATTGGAGAACGGTTTTCTCCTCAGCTCCAGCAGCATACTTGT
CAGAAATAAGGTCACAGGAGTTGAGATGAGATATCAGGTGAGTTTGATTTACTCTCTTGGTTCACATC
ACCTATTTCACATTT
+
FFFFFFFFFFFFFFFFFFFFFFFFFFFFFFFFFFFFFFFFFFFFFFFFFFFFFFFFFFFFFFFFFFFF
FFFF:FFFFFFFFFFFFFFFFFFFFFFFFFFFFFFFFFFFFFFFFFFFFFFFFFFF:FFFFFFFFFF
FFFF:FFFFFF:FFF
@A00155:342:HHGFNDSXY:1:2677:13160:15765 2:N:0:GAACCTAG+TCCGCATA
GTGAGGTCTGAGAGTTACACTCAACCATTGGAGAACGGTTTTCTCCTCAGCTCCAGCAGCATACTTGT
CAGAAATAAGGTCACAGGAGTTGAGATGAGATATCAGGTGAGTTTGATTTACTCTCTTGGTTCACATC
ACCTGTTTCACATTT
+
:FFFFFFFFFFFFFFFFFFFFFFFFFFFFFFFFFFFFFFFFFFFFFFFFFFFFFFFFFFFFFFFFFFF
FFFFFFFFFFFFFFFFFFFFFFFFFFFFFFFFFFFFFFFFFFFFFFFFF:FFFFFFFFFFFFFFFFFF
FFFFFFFFFFFFFFF
@A00155:342:HHGFNDSXY:1:1514:4752:17362 2:N:0:GAACCTAG+TCCGCATA
TGAGGTCTGAGAGTTACACTCAACCATTGGAGAAAGGTTTTCTCCTCATCTCCAGCAGAATACTTGTC
AGAAATAAGGTCACAGGAGTAGAGATGAGATATCAGGTGAGTTTGATTTAATCTCTAGGTTCACATCA
CCTGTTTCACATTT
+
FFF,FFFFFF,FF,FFFFFFFFFF,F,FF,F:FF,F:::FFFFFFFFFFFFFFF,FF,,FF:FFFF:F
FFFFF::FF::FFFF,:F,F,:FFF,:FFFFFFFF:,FFF:FFF:FFFFF:FFF,F,,FFFFF:FF:F
,F:FF::F,FFFFF
@A00155:342:HHGFNDSXY:1:2334:4408:19366 1:N:0:GAACCTAG+TCCGCATA
TGAGGTCTGAGAGTTACACTCAACCATTGGAGAACGGTTTTCTCCTCAGCTCCAGCAGCATACTTGTC
AGAAATAAGGTCACAGGAGTTGAGATGAGATATCAGGTGAGTTTGATTTACTCTCTTGGTTCACATCA
CCTGTTTCACATTTA
+
FFFFFFFFFFFF:FFFFFFFFFF:FFFFFFFFFFFFFF:FFFFFFFFFFFFFFFFFFFFFFFFFFFFF
FFFFFFFFFFFFFFFFFFFFFFFFFFFFFFFFFFFFFFFFFFFFFFFFFFFFFFFFFFFFFFFFFFFF
FFFFFFFFFFFFFFF
@A00155:342:HHGFNDSXY:1:2336:21187:31704 1:N:0:GAACCTAG+TCCGCATA
TGAGGTCTGAGAGTTACACTCAACCATTGGAGAACGGTTTTCTCCTCAGCTCCAGCAGCATACTTGTC

AGAAATAAGGTCACAGGAGTTGAGATGAGATATCAGGTGAGTTTGATTTACTCTCTTGGTTCACATCA
CCTGTTTCACATTTA
+
FFFFFFFFFFFFFFFFFFFFFFFFFFFFFFFFFFFFFFFFFFFFFFFFFFFFFFFFFFFFFFFFFFFF
FFFFFFFFFFFFFFFFFFFFFFFFFFFFFFFFFFFFFFFFFFFFFFFFFFFFFFFFFFFFFFFFFFFF
FFFFFFFFFFFFFFF
@A00155:342:HHGFNDSXY:1:2408:2980:17174 1:N:0:GAACCTAG+TCCGCATA
TGAGGTCTGAGAGTTACACTCAACCATTGGAGAACGGTTTTCTCCTCAGCTCCAGCAGCATACTTGTC
AGAAATAAGGTCACAGGAGTTGAGATGAGATATCAGGTGAGTTTGATTTACTCTCTTGGTTCACATCA
CCTGTTTCACATTTA
+
FFF:F:FFFFFFFFFFFFFFFFFFFFFFFFFFFF:FFFFFFFFFFFFFFFFFFFFFFFFFFFFFFFFF
FFFFFF:FFFFFFFFFFFFFFFFFFFFFFFFFFFFFFFFFFFFFFFFFFFFFFFFFFFFFFFFFFFFF
FFFFFFFFFFFFFFF
@A00155:342:HHGFNDSXY:1:2624:18710:17300 1:N:0:GAACCTAG+TCCGCATA
TGAGGTCTGAGAGTTACACTCAACCATTGGAGAACGGTTTTCTCCTCAGCTCCAGCAGCATACTTGTC
AGAAATAAGGTCACAGGAGTTGAGATGAGATATCAGGTGAGTTTGATTTACTCTCTTGGTTCACATCA
CCTGTTTCACATTTA
+
FFFFFFFFFFFFFFFFFFFFFFFFFFFFFFFFFFFFFFFFFFFFFFFFFFFFFFFFFFFFFFFF:FFF
FFFFFFFFFFFFFFFFFFFFFFFFFFFFFFFFFFFFFFFFFFFFFFFF:FFFFFFFFFFFFFFFFFFF
FFFFFFFFFFF:FFF
@A00155:342:HHGFNDSXY:1:2333:16007:21637 2:N:0:GAACCTAG+TCCGCATA
TGAGGTCTGAGAGTTACACTCAACCATTGGAGAACGGTTTTCTCCTCAGCTCCAGCAGCATACTTGTC
AGAAATAAGGTCACAGGAGTTGAGATGAGATATCAGGTGAGTTTGATTTAATCTCTTGGTTCACATCA
CCTATTTCACATTTA
+
FF:FFFFFF:F:FFFFFFFFFFFFFF:FFF:FFFFFFFFFFFFFFF:FFFFFFFFFFFFFFFFFF:FF
:FFFFFFFF:FFFFFF:::FFF:F:F::F:F::,FF,:F:FFFFF:FF:F,FFF:FF:,FF,F,F::F
,,F:FFF,FFFFFF:
@A00155:342:HHGFNDSXY:1:1118:32705:17018 1:N:0:GAACCTAG+TCCGCATA
GATCTCTGAGAGTTACACTCAACCATTGGAGAACGGTTTTCTCCTCAGCTCCAGCAGCATACTTGTCA
GAAATAAGGTCACAGGAGTTGAGATGAGATATCAGGTGAGTTTGATTTACTCTCTTGGTTCACATCAC
CTATTTCACATTTAC
+
FFFFFFFFFFFFFFFFFFFFFFFFFFFFFFFFFFFFFFFFFFFFFFFFFFFFFFFFFFFFFFFFFFFF
FFFFFFFFFFFFFFFFFFFFFFFFFFFFFFFFFFFFFFFFFFFFFFFFFFFFFFFFFFFFFFFFFFFF
FFFFFFFFFFFFFFF
@A00155:342:HHGFNDSXY:1:1141:9245:5666 1:N:0:GAACCTAG+TCCGCATA
GAGGTCTGAGAGTTACACTCAACCATTGGAGAACGGTTTTCTCCTCAGCTCCAGCAGCATACTTGTCA
GAAATAAGGTCACAGGAGTTGAGATGAGATATCAGGTGAGTTTGATTTACTCTCTTGGTTCACATCAC
CTGTTTCACATTTAC
+
FFFFFFFFFFFFFFFFFFFFFFFFFFFFFFFFFFFFF:FFFFFFFFFFFFFFFFFFFFFFFFFFFFFF
FFFFFFFFFFFFFFFFFFFFFFFF:FFFF,F:FFFFFFFFF:FFFFFFFFFFFFFFFFFFFFFFFFFF
FFFFFFFFFFFFFFF
@A00155:342:HHGFNDSXY:1:1164:26431:2957 1:N:0:GAACCTAG+TCCGCATA
TCTGTCTGAGAGTTACACTCAACCATTGGAGAACGGTTTTCTCCTCAGCTCCAGCAGCATACTTGTCA
GAAATAAGGTCACAGGAGTTGAGATGAGATATCAGGTGAGTTTGATTTACTCTCTTGGTTCACATCAC
CTGTTTCACATTTAC
+
FFFFFFFFFFFFFFFFFFFFFFFFFFFFFFFFFFFFF:FFFFFFFFFFFFFFFFFFFFFFFFFFFFFF
FFFFFFFFFFFFFFFFFFFFFFFFFFFFF:F:FFFFFFFF:FFFFFFFFFFFFFFFFFFFFFFFFFFF
FFFFFFFFFFFFFFF

```
@A00155:342:HHGFNDSXY:1:1219:14760:7012 1:N:0:GAACCTAG+TCCGCATA
GAGGTCTGAGAGTTACACTCAACCATTGGAGAACGGTTTTCTCCTCAGCTCCAGCAGCATACTTGTCA
GAAATAAGGTCACAGGAGTTGAGATGAGATATCAGGTGAGTTTGATTTACTCTCTTGGTTCACATCAC
CTGTTTCACATTTAC
+
FFFFFFFFFFFFFFFFFFFFFFFFFFFFFFFFFFFFFFFFFFFFFFFFFFFFFFFFFFFFFFFFFFFF
FFF:FFFFFFFFFFFFFFFFFFFFFF:FFFFFFFFFFFFFFFFFFFFFFFFFFFFFFFFFFFFFFF:F
FFFFFFFFFFFFFFF
@A00155:342:HHGFNDSXY:1:1339:9833:36777 1:N:0:GAACCTAG+TCCGCATA
GAGGTCTGAGAGTTACACTCAACCATTGGAGAACGGTTTTCTCCTCAGCTCCAGCAGCATACTTGTCA
GAAATAAGGTCACAGGAGTTGAGATGAGATATCAGGTGAGTTTGATTTACTCTCTTGGTTCACATCAC
CTATTTCACATTTAC
+
FFFFFFFFFF:FF,FFFFFFFFFFFFFF,,FF,FFFFFFFFFFF:F:FFFFFFFFFFFFFFF:FF:FF
::FFFFFF:FFFFFF:FF:,FFFF:FFFF,,:FFFFFFF::F:FF:FFFFFF:FFF:,FFFFFFFFFF
FFFFFF,F,F:F:,F
@A00155:342:HHGFNDSXY:1:1362:12472:35869 1:N:0:GAACCTAG+TCCGCATA
GAGGTCTGAGAGTTACACTCAACCATTGGAGAACGGTTTTCTCCTCAGCTCCAGCAGCATACTTGTCA
GAAATAAGGTCACAGGAGTTGAGATGAGATATCAGGTGAGTTTGATTTACTCTCTTGGTTCACATCAC
CTATTTCACATTTAC
+
FFFFFFFFFFFFF:FFFFFFFFFFFFFFFFFFFFFFFFFFFFFFFFFFFFFFFFFFFFFFFFFFFFFF
FFFFFFFFFFFFFFFF:FFFFFFFFFFFFFFFFFFFFFFF:FFFFFFFFFFFFFFFFFFFFFFFFFFF
FFFFFFFFFFFFFFF
@A00155:342:HHGFNDSXY:1:1437:22073:16705 1:N:0:GAACCTAG+TCCGCATA
GAGGTCTGAGAGTTACACTCAACCAGTGGAGAACGGTTTTCTCCTCAGCTCCAGCAGCATACTTGTCA
GAAATAAGGTCACAGGAGTTGAGATGAGATATCAGGTGAGTTTGATTTACTCTCTTGGTTCACATCAC
CTGTTTCACATTTAC
+
FFFFFF:FFFFF:FFFFFFFF:FFF,FFF:FFFFFFFFFFFFFF:FFFFF:FFFFFFFFFFFFF:F:F
FFFFF:FFFFF:FFFFFFFFFFFFFFF:FFFFFFFFFFFFFFFFFF:FFF:,F:FFFF:FFFFFF:FF
FFFFFFF:FFFFFFF
@A00155:342:HHGFNDSXY:1:1442:15076:16955 1:N:0:GAACCTAG+TCCGCATA
GAGGTCTGAGAGTTACACTCAACCATTGGAGAACGGTTTTCTCCTCAGCTCCAGCAGCATACTTGTCA
GAAATAAGGTCACAGGAGTTGAGATGAGATATCAGGTGAGTTTGATTTACTCTCTTGGTTCACATCAC
CTATTTCACATTTAC
+
FFFFFFFFFFFFFFFFFFFFFFFFFFFFFFFFFFFFF,FFFFFFFFFFFFFFFFFFFFFFFFFFFFFF
FFFFFFFFFFFFFFFFFFFFFFFFFFFFF,F:FFFFFFFFFFFFFFFFFFFFFFFFFFFFFFFFFFFF
FFFFFFFFFFFFFF:
@A00155:342:HHGFNDSXY:1:1443:13919:21026 1:N:0:GAACCTAG+TCCGCATA
GAGGTCTGAGAGTTACACTCAACCATTGGAGAACGGTTTTCTCCTCAGCTCCAGCAGCATACTTGTCA
GAAATAAGGTCACAGGAGTTGAGATGAGATATCAGGTGAGTTTGATTTACTCTCTTGGTTCACATCAC
CTGTTTCACATTTAC
+
FFFFFFFFFFFFFFFFFFFFFFFFFFFFFFFF:FFFFFFFFFFFFFFFFFFFFFFFFFFFFFFFFFFF
FFFFFFFFFFFFFFFFFFF:FFFFFF,F:::FFFFFFFFFFFFFFFFFFFFFFFFFFFFFFFFFFFFF
FFFFFFFFFFFFFFF
@A00155:342:HHGFNDSXY:1:1537:18141:18004 1:N:0:GAACCTAG+TCCGCATA
GAGGTCTGAGAGTTACACTCAACCATTGGAGAACGGTTTTCTCCTCAGCTCCAGCAGCATACTTGTCA
GAAATAAGGTCACAGGAGTTGAGATGAGATATCAGGTGAGTTTGATTTACTCTCTTGGTTCACATCAC
CTGTTTCACATTTAC
+
FFFFFFFFFFFFFFFFFFFFFFFFFFFFFFFFFFFFFFFFFFFFFFFFFFFFFFFFFFFFFFFFFFFF
```

FFFFFFFFFFFFFFFFFFFFFFFFFFFFFFFFFFFFFFFFFFFFFFFFFFFFFFFFFFFFFFFFFFFFFF
FFFFFFFFFFFFFFF
@A00155:342:HHGFNDSXY:1:1543:19397:11757 1:N:0:GAACCTAG+TCCGCATA
GAGGTCTGAGAGTTACACTCAACCATTGGAGAACGGTTTTCTCCTCAGCTCCAGCAGCATACTTGTCA
GAAATAAGGTCACAGGAGTTGAGATGAGATATCAGGTGAGTTTGATTTACTCTCTTGGTTCACATCAC
CTGTTTCACATTTAC
+
FFFFFFFFFFFFFFFFFFFFFFFFFFFFFFFFFFFFFFFFFFFFFFFFFFFFFFFFFFFFFFFFFFFFFF
FFFFFFFFFFFFFFFFFFFFFFFFFFFFFFFFFFFFFFFFFFFFFFFFFFFFFFFFFFFFFFFFFFFFFF
FFFFFFFFFFFFFFF
@A00155:342:HHGFNDSXY:1:1569:29441:4288 1:N:0:GAACCTAG+TCCGCATA
GAGGTCTGAGAGTTACACTCAACCATTGGAGAACGGTTTTCTCCTCAGCTCCAGCAGCATACTTGTCA
GAAATAAGGTCACAGGAGTTGAGATGAGATATCAGGTGAGTTTGATTTACTCTCTTGGTTCACATCAC
CTATTTCACATTTAC
+
FFFFFFFFFFFFFFFFFFFFFFFFFFFFFFFFFFFFFFFFFFFFFFFFFFFFFFFFFFFFFFFFFFFFFF
FFFFFFFFFFFFFFFFFFFFFFFFFFFFFFFFFFFFFFFF:FFFFFFFFFFFFFFFFFFFFFFFFFFFFF
FFFFFFFFFFFFFFF
@A00155:342:HHGFNDSXY:1:1623:25039:14199 1:N:0:GAACCTAG+TCCGCATA
GAGGTCTGAGAGTTACACTCAACCATTGGAGAACGGTTTTCTCCTCAGCTCCAGCAGCATACTTGTCA
GAAATAAGGTCACAGGAGTTGAGATGAGATATCAGGTGAGTTTGATTTACTCTCTTGGTTCACATCAC
CTGTTTCACATTTAC
+
FFFFFF:FFFF:FFFFFFFFFFFFFFFFFFFFFFFFFFFFFFFFFFFFFFFFFFFFFFFFFFFFFFFFFF
FFFFFFFFFFFFFFFFFFFFFFFFFFFFFFFFFFFFFFFFFFFFFFFFFFFFFFFFFFFFFFFFFFFFFF
FFFFFFFFFFFFFFF
@A00155:342:HHGFNDSXY:1:1630:31711:17581 1:N:0:GAACCTAG+TCCGCATA
GAGGTCTGAGAGTTACACTCAACCATTGGAGAACGGTTTTCTCCTCAGCTCCAGCAGCATACTTGTCA
GAAATAAGGTCACAGGAGTTGAGATGAGATATCAGGTGAGTTTGATTTACTCTCTTGGTTCACATCAC
CTGTTTCACATTTAC
+
FFFFFFFFFFFFFFFFFFFFFFFFFFFFFFFFF:FFFFFFF:FFFFFFFFFFFFFFFFFFFFFFFFFFFF
FFFFFFFFFFFFFFFFFFFFFFFFFFFFFFFFFFFFFFFFFFFFFFF:FFFFFFFFFFFFFFFFFFFFFF
FFFFFFFFFFFFFFF
@A00155:342:HHGFNDSXY:1:1648:6958:32142 1:N:0:GAACCTAG+TCCGCATA
GAGGTCTGAGAGTTACACTCAACCATTGGAGAACGGTTTTCTCCTCAGCTCCAGCAGCATACTTGTCA
GAAATAAGGTCACAGGAGTTGAGATGAGATATCAGGTGAGTTTGATTTACTCTCTTGGTTCACATCAC
CTATTTCACATTTAC
+
FFFFFFFFFFFFFFFFFFFFFFFFFFFFFFFFFFFF:FFFFFFFFFFFFFFFFFFFFFFFFFFFFFFFFF
FFFFFFFFFF,FFFFFFF,FFFFFFFFFF:FFFFFFF:FF:FFFFFFFFFFFFFFFFFFFFFFFFFFFFF
FFFFFFFFFFFFFFF
@A00155:342:HHGFNDSXY:1:1668:27290:23516 1:N:0:GAACCTAG+TCCGCATA
GAGGTCTGAGAGTTACACTCAACCATTGGAGAACGGTTTTCTCCTCAGCTCCAGCAGCATACTTGTCA
GAAATAAGGTCACAGGAGTTGAGATGAGATATCAGGTGAGTTTGATTTACTCTCTTGGTTCACATCAC
CTATTTCACATTTAC
+
FFFFFFFFFFFFF:FFFFFFFFFFFFFFFFFFFFFFFFFFFFFFFFFFFFFFFFFFFFFFFFFFFFFFFF
FFFFFFFFFFFFFFFFFFFFFFFF:FFFFFFFFFFFFFFFFFFFFFFFFFFFFFFFFFFFFFFFFFFFFF
FFFFFFFFFFFFFFF
@A00155:342:HHGFNDSXY:1:2123:3143:7341 1:N:0:GAACCTAG+TCCGCATA
GAGGTCTGAGAGTTACACTCAACCATTGGAGAACGGTTTTCTCCTCAGCTCCAGCAGCATACTTGTCA
GAAATAAGGTCACAGGAGTTGAGATGAGATATCAGGTGAGTTTGATTTACTCTCTTGGTTCACATCAC
CTGTTTCACATTTAC

```
+
FFFFFFFFFFFFFFFFFFFFFFFFFFFFFFFFFFFFFFFFFFFFFFFFFFFFFFFFFFFFFFFFFFFFFF
:FFFFFFFFFF:FFFFFFFFFFFFFFFFF:FFFFFFFFFFFF:FFFFFFFFFFF:FFFFFFFFFFFFFFF
FFFFFFFFFFF:FFF
@A00155:342:HHGFNDSXY:1:2156:21513:11725 1:N:0:GAACCTAG+TCCGCATA
GATCTCTGAGAGTTACACTCAACCATTGGAGAACGGTTTTCTCCTCAGCTCCAGCAGCATACTTGTCA
GAAATAAGGTCACAGGAGTTGAGATGAGATATCAGGTGAGTTTGATTTACTCTCTTGGTTCACATCAC
CTGTTTCACATTTAC
+
FFFFFFFFFFFFFFFFFFFFFFFFFFFFFFFFFFFFFFFFFFFFFFFFFFFFFFFFFFFFFFFFFFFFFF
FFFFFFFFFFFFFFFFFFFFFFFFFFFFFFFFFFFFFFFFFFFFFFFFFFFFFF:FFFFFFFFFFFFFFF
FFFFFFFFFFFFFFF
@A00155:342:HHGFNDSXY:1:2173:8133:8531 1:N:0:GAACCTAG+TCCGCATA
GAGGTCTGAGAGTTACACTCAACCATTGGAGAACGGTTTTCTCCTCAGCTCCAGCAGCATACTTGTCA
GAAATAAGGTCACAGGAGTTGAGATGAGATATCAGGTGAGTTTGATTTACTCTCTTGGTTCACATCAC
CTATTTCACATTTAC
+
FFFFFFFFFFFFFFFFFFFFFFFFFFFFFFFFFFFFFFFFFFFFFFFFFFFFFFFFFFFFFFFFF:FFFF
FFFFFFFFFFFFFFFFFFFFFFFFFFFFFFFFFFFFFFFFFFFFF:FFFFFFFFFFFFFFFFFFFFFFFF
FFFFFFFFFFFFFFF
@A00155:342:HHGFNDSXY:1:2224:5475:6621 1:N:0:GAACCTAG+TCCGCATA
GATCTCTGAGAGTTACACTCAACCATTGGAGAACGGTTTTCTCCTCAGCTCCAGCAGCATACTTGTCA
GAAATAAGGTCACAGGAGTTGAGATGAGATATCAGGTGAGTTTGATTTACTCTCTTGGTTCACATCAC
CTGTTTCACATTTAC
+
FFFFFFFFFFFFFFFFFFFFFFFFFFF:FFFF:FFFFFFFFFFFFFFFF:FFFFFFFFFFFFFFFFFFFF
FFFFFFFFFFFFFFFFFFFFFFFFFFFFFFF:FFFFFFFFFFFFFFFFFFFFFFFFFFFFFFFFFFFFFF
FFFFFFFFFFFFFFF
@A00155:342:HHGFNDSXY:1:2259:28429:28244 1:N:0:GAACCTAG+TCCGCATA
GAGGTCTGAGAGTTACACTCAACCATTGGAGAACGGTTTTCTCCTCAGCTCCAGCAGCATACTTGTCA
GAAATAAGGTCACAGGAGTTGAGATGAGATATCAGGTGAGTTTGATTTACTCTCTTGGTTCACATCAC
CTGTTTCACATTTAC
+
FFFFFFFFFFFFFFFFFFFFFFFFFFFFFFFFFFFFFFFF:FFFFFFFFFFFFFFFFFFFFFFFFFFFFF
FFFFFFFFFFFFFFFFFFFFFFFFFFFFFFFFFFFFFFFFFFFFFFFFFFFF:FFFFFFF:FFFFFFFFF
FFFFFFFFFFFFFFF
@A00155:342:HHGFNDSXY:1:2254:16694:17190 1:N:0:GAACCTAG+TCCGCATA
GAGGTCTGAGAGTTACACTCAACCATTGGAGAACGGTTTTCTCCTCAGCTCCAGCAGCATACTTGTCA
GAAATAAGGTCACAGGAGTTGAGATGAGATATCAGGTGAGTTTGATTTACTCTCTTGGTTCACATCAC
CTGTTTCACATTTAC
+
FFFFFFFFFFFFFFFFFFFFFFFFFFFFFFFFFFFFFFFFFFFFFFFFFFFFFFFFFFFFFFFFFFFFFF
FFFFFFFFFFFFFFFFFF:FFFFFFF:FFFF:FFFFFFFFFFFFFFFFFFFF:FFFFFFF:FFFFFFFFF
FFFFFFFFFFFFFFF
@A00155:342:HHGFNDSXY:1:2307:10176:1642 1:N:0:GAACCTAG+TCCGCATA
GAGGTCTGAGAGTTACACTCAACCATTGGAGAACGGTTTTCTCCTCAGCTCCAGCAGCATACTTGTCA
GAAATAAGGTCACAGGAGTTGAGATGAGATATCAGGTGAGTTTGATTTACTCTCTTGGTTCACATCAC
CTATTTCACATTTAC
+
FF:FFFFFFFFFFFFFFFFFFFFFFFFFFFFFFFFFFFF:FFFFFFFFFFFFFFFFFFFFFFFFFFFFFF
FFFFFFFFFFFFFFFFFFFFFFFFFFFFFFFFFFFFFFFFFF:FFFFFFFFFFFFFFFFFFFFFFFFFFF
FFFFFFFFFFFFFFF
@A00155:342:HHGFNDSXY:1:2307:4399:7545 1:N:0:GAACCTAG+TCCGCATA
GAGGTCTGAGAGTTACACTCAACCATTGGAGAACGGTTTTCTCCTCAGCTCCAGCAGCATACTTGTCA
```

GAAATAAGGTCACAGGAGTTGAGATGAGATATCAGGTGAGTTTGATTTACTCTCTTGGTTCACATCAC
CTGTTTCACATTTAC
+
FFFFFFFFFFF,,FFFFFFFFFFFFFFFFFFFFFFFFFFFFFFFFFFFFFFFFFFFFFFFFFFFFFFFFF
FFFFFFFFFFFFFFFFFFFFFFFFFFFFFFFFFFFFFFFFFFFFFFFFFFFFFFFFFFFFFFFFFFFFFF
FFFFFFFFFFFFFFF
@A00155:342:HHGFNDSXY:1:2310:18059:1485 1:N:0:GAACCTAG+TCCGCATA
GAGGTCTGAGAGTTACACTCAACCATTGGAGAACGGTTTTCTCCTCAGCTCCAGCAGCATACTTGTCA
GAAATAAGGTCACAGGAGTTGAGATGAGATATCAGGTGAGTTTGATTTACTCTCTTGGTTCACATCAC
CTGTTTCACATTTAC
+
FFFFFFFFFF:FFFFFFFFFFFFFFFFFFFFFF:FFFFFFFFFFFFFFFFFFFFFFFFFFFFFF,:FFFF
FFFFFFFFFFFFFFFFFFFFFFFFFFFFFFFFFFFFFFFFFFFFFFFFFFFFFFFFFFFFFFFFFFFFFF
FFFFFFFFFFFFFFF
@A00155:342:HHGFNDSXY:1:2333:16007:21637 1:N:0:GAACCTAG+TCCGCATA
GAGGTCTGAGAGTTACACTCAACCATTGGAGAACGGTTTTCTCCTCAGCTCCAGCAGCATACTTGTCA
GAAATAAGGTCACAGGAGTTGAGATGAGATATCAGGTGAGTTTGATTTACTCTCTTGGTTCACATCAC
CTATTTCACATTTAC
+
FFFFFFFFFFFFFFFFFFFFFFFFFFFFFFFFFFFFFFFFFFFFFFFFFFFFFFFFFFFFFFFFFFFFFF
FFFFFFFFFFFFFFFFFFFFFFFFFFFFFFFFFFFFFFFFFFFFFFFFFFFFFFFFFFFFFFFFFFFFFF
FFFFFFFFFFFFFFF
@A00155:342:HHGFNDSXY:1:2414:9760:5400 1:N:0:GAACCTAG+TCCGCATA
GAGGTCTGAGAGTTACACTCAACCATTGGAGAACGGTTTTCTCCTCAGCTCCAGCAGCATACTTGTCA
GAAATAAGGTCACAGGAGTTGAGATGAGATATCAGGTGAGTTTGATTTACTCTCTTGGTTCACATCAC
CTATTTCACATTTAC
+
FFFFFFFFFFFFFFFFFFFFFFFFFFFFFFFFFFFFFFFFFFFFFFFFFF:FFFFFFFFFFFFFFFFFFF
[truncated: 482,626 more chars]
